# Supplementary material for: What Is the Relationship between Outdoor Time and Physical Activity, Sedentary Behaviour, and Physical Fitness in Children? A Systematic Review
Source: Int J Environ Res Public Health. 2015 Jun 8;12(6):6455–74. doi: 10.3390/ijerph120606455 (PMC4483711; doi:10.3390/ijerph120606455)
Supplement: Supplementary File 1 [file ijerph-12-06455-s001.pdf]

Supplementary Information

What Is the Relationship between Outdoor Time and Physical Activity, Sedentary Behaviour, and Physical Fitness in Children? A Systematic Review

---

Supplementary File 1. Electronic search strategies

MEDLINE (line 6) PreMEDLINE (line 8) PsyhInfo (line 10) and Embase (line 12)
1. (outside or outdoor* or play ground* or playground* or yard* or green space* or greenspace* or recess or natur* setting* or natur* environment* or natur* landscape* or wilderness).mp. or (out adj2 door).tw.
2. (activ* or exercis* or play*).mp.
3. (time or amount or exposur* or hour* or day* or measur* or pattern* or ratio* or higher or weekend* or recess).mp.
4. 1 and 2 and 3
5. from 4 keep 1-19390
6. limit 5 to ("preschool child (2 to 5 years)" or "child (6 to 12 years)")
7. limit 5 to ("in data review" or in process or "pubmed not medline")
8. 7 and child*.mp.
9. from 4 keep 19391-26344
10. limit 9 to (160 preschool age or 180 school age)
11. from 4 keep 26345-50094
12. limit 11 to (child or preschool child <1 to 6 years> or school child <7 to 12 years>)
13. 6 or 8 or 10 or 12

CINAHL
S1 (outside or outdoor* or play ground* or playground* or yard* or green space* or greenspace* or recess or natur* setting* or natur* environment* or natur* landscape* or wilderness) OR out N2 door*
S2 (activ* or exercis* or play*) AND (time or amount or exposur* or hour* or day* or measur* or pattern* or ratio* or higher or weekend* or recess)
S3 (S1 and S2)
Limiters - Exclude MEDLINE records; Age Groups: Child, Preschool: 2-5 years, Child: 6-12 years

SportDiscus
S1 (outside or outdoor* or play ground* or playground* or yard* or green space* or greenspace* or recess or natur* setting* or natur* environment* or natur* landscape* or wilderness) OR out N2 door*
S2 (activ* or exercis* or play*) AND (time or amount or exposur* or hour* or day* or measur* or pattern* or ratio* or higher or weekend* or recess)
S3 (S1 and S2 and child*)

Supplementary File 2

Table S1. Summary of included studies.

First Author	Year	Country	Study Design	Population (N)	Age Group	Intervention or Exposure	Outcome and Measure
Baranowski	1993	USA	Cross-sectional	191	3–4 years Mean = 4.23 ± 0.65 years	Indoor or outdoor location	Physical Activity Direct observation (CARS)
Boldemann	2006	Sweden	Cross-sectional	197	Range = 4.5–6.5 years (preschoolers)	Parent-reported usual time outdoors on a usual Sunday Direct observation (weekdays during preschool)	Physical activity Pedometer (weekdays during preschool)
Burdette	2004	USA	Cross-sectional	250 White and black children Boys = 57%	Range = 29–52 months (preschoolers)	Parent-reported time (hours, minutes) outdoors in the last month (weekdays and weekends	Physical activity Accelerometer (weekdays & weekend days)
Brown	2009	USA	Cross-sectional (only narrative results reported)	476 Boys = 51%	Range = 3–5 years (preschoolers)	Direct Observation OSRAC—P Momentary time sampling of focus child for 30 min observation period. (% of observation intervals outside)	Physical activity OSRAC-P (% of intervals in each activity intensity)
Cleland	2008	Australia	Prospective 3 year follow-up	548	Ranges = 5–6 years; 10–12 years	Parent-reported usual time (hours, minutes) outdoors (after school & weekends, warmer & cooler months)	Physical Activity Accelerometer—MVPA (weekdays &weekends)
Conrad	2013	Germany	Cross-sectional	1790	Range = 3–14 years	Parent- (younger than 11 years) and self-reported (11+ years old) average time (minutes) outdoors (weekdays and weekends)	Physical Activity Parent (<11 years) and self-report (≥11 years)—Leisure time physical activity (average hours per week)

Table S1. *Cont*.

First Author	Year	Country	Study Design	Population (N)	Age Group	Intervention or Exposure	Outcome and Measure
Cooper	2010	England	Cross-sectional	1010	Mean = 11 (0.4) years	GPS-measured outdoor or indoor time (after school)	Physical Activity Accelerometer—Counts per minute) (after school)
Danner	1991	USA	Cross-sectional	51 children (29 boys, 22 girls)	Mean = 3 years 11 months, Range = 2 years 6 months–5 years 5 months (baseline measures)	Parent-direct observation (dichotomous scores outside or inside at 30 minute intervals) (4 h) report for each hour of 4 h period	Physical Activity Accelerometer–activity counts (after school)
Dolinsky	2011	USA	Cross-sectional	337	Range = 2-5 years; Mean = 3.5 (1.1) years (preschoolers) Children of overweight and obese mothers	Parent-reported outdoor time (hours per day)	Physical Activity Accelerometer—MVPA minutes per day
DuRant	1994	USA	Cross-sectional (only narrative results reported)	191	3–4 years, Mean = 4.23 (0.65) years	Direct observation (CARS) of time outdoors/indoors (min/day)	Physical Activity Direct observation (CARS)
Dunton	2011	USA	Cross-sectional	108	Range = 9–13 years	Self-reported outdoor time (Electronic Ecological Momentary Assessment) (after school and weekends)	Physical Activity Accelerometer 1. Total step-count 2. likelihood of meeting at least 5 min of MVPA during a 30 min pre-EMA period
Gopinath	2011	Australia	Cross-sectional	1426	Mean = 6.7 years	Parent-reported time in outdoor and indoor activities (hours per week)	Blood Pressure Automated Sphygmomanometer
Jones	2009	United Kingdom	Cross-sectional	100, Boys = 47, Girls = 53	Range = 9–10 years	GPS-measured outdoor or indoor time (weekdays and weekends)	Physical Activity Accelerometer—Bouts in each intensity (weekdays and weekends)

Table S1. *Cont*.

First Author	Year	Country	Study Design	Population (N)	Age Group	Intervention or Exposure	Outcome and Measure
Klesges	1990	USA	Cross-sectional	222, Boys = 122 girls = 100 White children	Range = 3–6 years, Mean = 4.44 years (0.5), (Preschoolers)	Direct observation SCAN CATS time sampling survey (1 h) (% time outdoors *vs*. indoors) (late afternoon/ early evening)	Physical Activity Direct observation SCAN CATS (1 h) (stationary, minimal activity, slow movement, rapid movement) (late afternoon/early evening)
Klinker	2014	Denmark	Cross-sectional	170, Boys = 83, Girls = 87	Range = 11–16 years, Mean = 12.8 years (1.1), Children = 129, Mean Age = 12.4 (0.7), Adolescents = 41, Mean Age = 14.2 (0.8)	GPS-measured outdoor and indoor time (weekdays and weekend)	Physical Activity Accelerometer—proportion of MVPA spent outdoors (at least 1 weekday, not weekends)
Ludvigsson	2007	Sweden	Cross-sectional	106	Mean = 6.6 years (SD not reported) Range = 5–7.5 years	Parent-reported “playing outdoors”	Physical Activity Parent-report—physical exercise habits
Loucaides	2006	Cyprus	Cross-sectional	104, Boys = 54, Girls = 50	Grades 5 and 6 Estimated age range = 10–12 years based on age per grade level in Cyprus	Parent-reported outdoor time (hours per day)	Physical Activity Pedometer—Steps
Martin	2008	USA	Cross-sectional	331 black children, Girls = 181	Range = 10–14 years, Mean = 12.1 (1.0) years	Self-reported outdoor time (amount, e.g., a lot) (average school day and weekend day)	Physical Activity Self-report—Leisure time exercise (Godin Leisure-Time Exercise Questionnaire)
McKenzie	1992	USA	Cross-sectional	351 Mexican- and Anglo-American children Girls = 169	Mean = 4.3 to 4.5, SDs = 0.47–0.56) years	Direct observation (BEACHES) during 2 recess and 2 home visits of 30 min each.	Direct observation (BEACHES) during 2 recess and 2 home visits of 30 min each

Table S1. *Cont*.

First Author	Year	Country	Study Design	Population (N)	Age Group	Intervention or Exposure	Outcome and Measure
McKenzie	2008	USA	Cross-sectional	139 Mexican-American children from low income neighbourhoods Girls = 70	Mean = 6.5 (0.98) years	Direct-observation of outdoor time (BEACHES) (After school, before dinner)	Physical Activity Direct observation (BEACHES) (I hour observed before dinner) (1) the proportion of observed in MVPA (2) mean estimated energy rate (kcal/kg per min).
Ortlieb	2013	Germany	Cross-sectional	2809	Mean = 10 years (assessed at 4 weeks before 10th birthday of each child in birth cohort study)	Parent-reported usual time in outdoor activities (average weekday)	Physical Activity Parent report - usual time in LPA, MPA, VPA (average weekday)
Raustorp	2012	Sweden and USA	Cross-sectional	50, Boys = 26, Girls = 24	Range = 40–67 months, Mean = 52 (6) months (preschoolers)	Direct observation of outdoor time (minutes) (preschool day)	Physical activity Accelerometer (5 preschool days)
Saakslahti	1999	Finland	Cross-sectional	105, Boys = 50, Girls = 55	Range 3–4 years, Mean = 3.75 (0.6) years (preschoolers)	Parent-reported indoor and outdoor time (recorded in 5 min intervals) (weekend)	Motor Skills APM inventory Arterial Blood Pressure Dinamap
Sallis	1993	USA	Cross-sectional across 2 years	246 Mexican-American and Anglo-American, Low income	Mean = 4.4 (0.5) years (preschoolers)	Direct observation of indoor and outdoor time (BEACHES) (1 h observed before dinner) (% of all intervals spent outdoors	Physical Activity Direct observation (BEACHES) (1 hour observed before dinner) (kcal/kg/min)
Skala	2012	USA	Cross-sectional	844 Hispanic (62.2%) African-American (22.0%), Caucasian (14.19%). Predominantly low income (76.9%)	Range = 3–11 years	Direct observation of outdoor and indoor time (SOFIT) (indoor PE class compared with outdoor PE class)	Physical Activity Direct observation (SOFIT) (% time and mean difference)

Table S1. *Cont*.

First Author	Year	Country	Study Design	Population (N)	Age Group	Intervention or Exposure	Outcome and Measure
Smith	2009	USA	1 year longitudinal	136, Boys = 62, Girls = 74	Range = 8–10 years Mean = 9.4 (0.6) years	Direct observation of indoor and outdoor time (Indoor and outdoor PE classes of the same format and duration were compared)	Physical Activity Pedometer—Average steps per class (during class time)
Tey	2007	Australia	(only narrative results reported)	84, Boys = 36, Girls = 48	Mean = 5.1 (0.1) years	Children's Light Time-Use Diary Outdoor minutes per day	Children's Light Time-Use Diary Physical activity min/day
Vanderloo	2013	Canada	Cross-sectional	31, Boys = 17, Girls = 14	Mean = 4.1 (0.85) years (preschoolers)	Direct observation of indoor and outdoor time (minutes per hour) (1 day) (childcare centre)	Physical Activity Accelerometer - minutes per hour of total and MVPA (1 day in childcare centre)
Wheeler	2010	England	Cross-sectional	1053	Mean = 10.9 years	GPS-measured indoor and outdoor time (after school on weekdays, weekend) (% time indoors/outdoors)	Physical Activity Accelerometer 1. mean counts per 10 second epoch 2. total MVPA 3. proportion of time in MVPA (weekdays and weekend)
Wickel	2013	USA	Cross-sectional	779, Boys = 390	Range = 9–10 years	Self-reported indoor and outdoor time (3:00 PM–6:00 PM)	Physical Activity Self-reported—% of time in LPA and MVPA (3:00 PM–6:00 PM on a typical school day)

Notes: GPS, Global Positioning System; LPA, light physical activity; MVPA, moderate-to-vigorous physical activity.

Supplementary File 3: Search Results

TY - JOUR
ID - 1
T1 - A81: spectrum of mevalonate kinase deficiency: is colitis more common than we think?
A1 - Biggs,C.
A1 - Dedeoglu,F.
Y1 - 2014/03//
N1 - Biggs, Catherine. Dedeoglu, Fatma
Arthritis & rheumatology (Hoboken, N.J.)
101623795
AIM, IM
Journal Article
English
KW - MEDLINE
RP - NOT IN FILE
SP - S114
JF - Arthritis & Rheumatology
JA - Arthritis rheumatol
VL - 66 Suppl 11
CY - United States
N2 - BACKGROUND/PURPOSE: Mevalonate kinase deficiency (MKD) is an autosomal recessive inflammatory disorder caused by a mutation in the mevalonate kinase (MVK) gene. This leads to decreased enzymatic activity, resulting in impaired cholesterol biosynthesis and increased production of inflammatory mediators such as interleukin 1-beta. There are two known forms of MKD, with Hyperimmunoglobulinemia D Syndrome (HIDS) carrying a milder phenotype and Mevalonic Aciduria (MA) representing a severe form. HIDS is characterized by recurrent febrile episodes accompanied by varying clinical features that include lymphadenopathy, gastrointestinal symptoms, oral ulcers, and arthralgia. A recent report identified early onset colitis as a feature of MKD in two patients (Levy M et al, 2013). Here we report the clinical and laboratory phenotypes of patients cared for at Boston Children's Hospital who were diagnosed with HIDS/MKD. METHODS: We conducted a retrospective chart review of children with HIDS/MKD treated from 2002-2012 at the Rheumatology Clinic at Boston Children's Hospital. Charts were first identified by searching for key words or billing codes related to autoinflammatory syndromes. A manual chart review was then performed to confirm appropriate case identification. RESULTS: Four patients were diagnosed with HIDS/MKD over the 10-year period. Age of symptom onset varied from 2 months to 1 year. Fever duration ranged from 1-5 days, with duration between fevers ranging from 9 days to 12 weeks. Symptoms included sore throat, oral lesions, vomiting, diarrhea, abdominal pain, rash, and arthralgia. Prodromal symptoms were identified in all patients, and included irritability, anorexia, fatigue, geographic tongue, rash, and neck pain. Three of the four patients had a history of bloody stools beginning in infancy/early childhood, and were followed by gastroenterology. All patients had lymphadenopathy on exam, and splenomegaly was detected in two patients by ultrasound. Labs obtained during febrile episodes showed elevated inflammatory markers, however, in two out of three patients evaluated in between fevers, the ESR was persistently elevated. All four patients had elevated IgA when compared to age matched normal values, and two patients had elevated IgD levels. Genetic testing was performed in all patients, with all four having two heterozygous mutations within the MVK gene. In addition, one patient also had single variant mutations of unclear significance in two other genes associated with autoinflammatory syndromes. Two patients were treated with steroids and in each case this aborted febrile episodes, however, in one patient steroid use was also associated with increased fever frequency. One patient had decreased severity of symptoms when treated with colchicine as well as with montelukast, and had rapid resolution of fever and associated symptoms when treated with anakinra. CONCLUSION: The persistence of ESR elevation outside of febrile episodes seen in patients with HIDS/MKD may indicate the presence of chronic subclinical inflammation. Hematochezia was commonly seen, and could indicate the presence of colitis. Of the immunomodulatory medications used in this small patient population, anakinra appeared to be effective.
Copyright 2014 by the American College of Rheumatology
SN - 2326-5205
AD - Boston Children's Hospital, Boston, MA
UR - 24677830

ER -

TY - JOUR
ID - 2
T1 - Fibrous hamartoma of infancy: a clinicopathologic analysis of 60 cases
A1 - Saab,S.T.
A1 - McClain,C.M.
A1 - Coffin,C.M.
Y1 - 2014/03//
N1 - Saab, Shahrazad T. McClain, Colt M. Coffin, Cheryl M
The American journal of surgical pathology
3yv, 7707904
IM
Journal Article. Research Support, Non-U.S. Gov't
English
KW - MEDLINE
RP - NOT IN FILE
SP - 394
EP - 401
JF - American Journal of Surgical Pathology
JA - Am J Surg Pathol
VL - 38
IS - 3
CY - United States
N2 - Fibrous hamartoma of infancy is a benign soft tissue tumor with a characteristic triphasic organoid histologic appearance. It typically occurs within the first 2 years of life. The usual anatomic locations include the upper extremities, axilla, and upper back. Diagnostic challenges occur when this tumor arises in older children, outside of the usual anatomic sites, or when unusual histologic features are encountered. This study reports 60 cases of fibrous hamartoma of infancy from institutional and consultation files. All had a triphasic organoid histologic pattern, but half also displayed an unusual pseudoangiomatous histologic pattern. The male to female ratio was 2.0 (40 boys, 20 girls), with a mean age of 1.5 years (range, 16 d to 8 y) at diagnosis. Tumor size ranged from 0.5 to 9 cm, with a mean of 3.7 cm. Sites included the trunk (40 cases), extremities (17 cases), and head and neck (3 cases). All cases had triphasic elements of mature fibrous tissue, mature adipose tissue, and immature mesenchymal tissue in varying proportions, with the additional pseudoangiomatous pattern in 32 cases. Immunohistochemical analysis demonstrated reactivity for smooth muscle actin and CD34 in the mature fibrous tissue, S100 protein in the mature adipose tissue, and variable CD34 reactivity in immature mesenchymal and pseudoangiomatous foci. Ki-67 proliferative activity was noted in the immature mesenchymal and pseudoangiomatous foci, and Bcl-2 reactivity was restricted to mesenchymal and pseudoangiomatous foci. Follow-up information in 12 cases revealed no evidence of recurrence in 10 patients and local recurrence in 2 patients, each at 3.5 years after primary excision. This study demonstrates an expanded age range (up to 8 y) and anatomic distribution (30 cases outside of the classic locations of the upper extremities, axilla, and upper back) of fibrous hamartoma of infancy. The pseudoangiomatous morphologic variation can lead to challenges in diagnosis and may reflect a maturational phenomenon from the immature mesenchymal component
SN - 1532-0979
AD - Department of Pathology, Microbiology, and Immunology, Vanderbilt University Medical Center, Nashville, TN
UR - 24525510
ER -

TY - JOUR
ID - 3
T1 - Near Work, Outdoor Activity, and their Association with Refractive Error
A1 - Lin,Z.
A1 - Vasudevan,B.
A1 - Jhanji,V.
A1 - Mao,G.Y.
A1 - Gao,T.Y.

A1 - Wang,F.H.
A1 - Rong,S.S.
A1 - Ciuffreda,K.J.
A1 - Liang,Y.B.
Y1 - 2014/04//
N1 - Lin, Zhong. Vasudevan, Balamurali. Jhanji, Vishal. Mao, Guang Yun. Gao, Tie Ying. Wang, Feng Hua. Rong, Shi Song. Ciuffreda, Kenneth J. Liang, Yuan Bo
Optometry and vision science : official publication of the American Academy of Optometry
oiz, 8904931
IM
Journal Article
English
KW - MEDLINE
RP - NOT IN FILE
SP - 376
EP - 382
JF - Optometry & Vision Science
JA - Optom.Vis Sci
VL - 91
IS - 4
CY - United States
N2 - PURPOSE: To assess the relationship between near work, outdoor activity, and refractive error in schoolchildren in Beijing. METHODS: The Beijing Myopia Progression Study is a hospital-based myopia study, in which 386 students from primary (aged 6 to 12 years) and secondary (aged 13 to 17 years) schools in the inner city of Beijing were enrolled. Cycloplegic refraction and a detailed questionnaire probing near, intermediate, and distance visual activities were completed. RESULTS: Three hundred seventy (95.9%) of 386 students with complete cycloplegic autorefraction and myopia questionnaire data were enrolled in this study. Children with more near work time did not exhibit a significantly more myopic refraction in both the primary and secondary school levels after adjusting for the children's gender, outdoor activity time, and average parental refractive error. A significant association between outdoor activity time (in hours per day) and the children's spherical equivalent (in diopters) was found in the primary school students (beta = 0.27, p = 0.03) but not in the secondary school students (beta = 0.04, p = 0.70) after adjusting for similar confounders. The time spent on outdoor sports and outdoor leisure in the primary school students was also significantly associated with the children's spherical equivalent (beta = 0.46, p = 0.04 and beta = 0.31, p = 0.02, respectively). Primary school students with more time outdoors exhibited relatively less myopic refraction than their peers (ptrend = 0.0003), but this relation was not demonstrated in the secondary school children (ptrend = 0.53) after adjusting for similar confounders. CONCLUSIONS: Higher levels of outdoor activity were associated with less myopic refraction in primary school students in the inner city of Beijing. Near work activity was not found to be associated with refraction at either school level
SN - 1538-9235
AD - *MD, PhD +BSOptom, PhD ++MD OD, PhD Clinical & Epidemiological Eye Research Center, The Affiliated Eye Hospital of Wenzhou Medical University, Wenzhou, Zhejiang, China (ZL, GYM, YBL); College of Optometry, Midwestern University, Glendale, Arizona (BV); Department of Ophthalmology and Visual Sciences, The Chinese University of Hong Kong, Hong Kong, China (VJ, SSR); School of Environmental Science and Public Health, Wenzhou Medical University, Wenzhou, Zhejiang, China (GYM); Handan Eye Hospital, Handan, Hebei, China (TYG); Beijing Tongren Eye Center, Beijing Tongren Hospital, Capital Medical University, Beijing Ophthalmology and Visual Science Key Lab, Beijing, China (ZL, FHW, YBL); and Department of Biological and Vision Sciences, SUNY College of Optometry, New York, New York (KJC)
UR - 24637483
ER -

TY - JOUR
ID - 4
T1 - Practical applications to modify and control the development of ametropia
A1 - Sankaridurg,P.R.
A1 - Holden,B.A.
Y1 - 2014/02//

N1 - Sankaridurg, P R. Holden, B A
Eye (London, England)
eye, 8703986
IM
Journal Article. Research Support, Non-U.S. Gov't
English
KW - MEDLINE
RP - NOT IN FILE
SP - 134
EP - 141
JF - Eye
VL - 28
IS - 2
CY - England
N2 - For many individuals, the developmental trend of lessening hyperopia from birth continues past emmetropia towards myopia during childhood. The global pattern for prevalence of refractive errors indicates that the prevalence of hyperopia is low; in contrast, the burden of myopia is on the rise because of rising prevalence and magnitude of myopia. This review highlights the need to lessen the global burden of myopia by intervening with the development and/or slowing the progression of myopia. Further, outcomes from human clinical trials of pharmaceutical, optical, and environmental approaches to control myopia will be summarised. Pharmaceutical treatments are effective in controlling eye growth but are associated with deleterious side effects. Optical strategies that induce myopic defocus at the retina such as peripheral defocus reducing lenses, simultaneous defocus lenses, bifocals, and orthokeratology as well as environmental influences such as increased outdoor activity show promise and provide a substantially risk-free environment in which to control eye growth
SN - 1476-5454
AD - 1] Brien Holden Vision Institute, Sydney, New South Wales, Australia [2] Vision Co-operative Research Centre, Sydney, New South Wales, Australia [3] School of Optometry and Vision Science, University of New South Wales, Sydney, New South Wales, Australia., 1] Brien Holden Vision Institute, Sydney, New South Wales, Australia [2] Vision Co-operative Research Centre, Sydney, New South Wales, Australia [3] School of Optometry and Vision Science, University of New South Wales, Sydney, New South Wales, Australia
UR - 24310242
ER -

TY - JOUR
ID - 5
T1 - Update on human health concerns of recombinant bovine somatotropin use in dairy cows
A1 - Collier,R.J.
A1 - Bauman,D.E.
Y1 - 2014/04//
N1 - Collier, R J. Bauman, D E
Journal of animal science
hc7, 8003002
IM
Journal Article
English
KW - MEDLINE
RP - NOT IN FILE
SP - 1800
EP - 1807
JF - Journal of Animal Science
JA - J Anim Sci
VL - 92
IS - 4
CY - United States
N2 - The 20 yr of commercial use of recombinant bovine somatotropin (rbST) in the United States provide the backdrop for reviewing the outcome of use on human health issues by the upcoming 78th meeting of the Joint

Food and Agriculture Organization of the United Nations (FAO)/World Health Organization (WHO) Expert Committee on Food Additives. These results and further advancements in scientific knowledge indicate there are no new human health issues related to the use of rbST by the dairy industry. Use of rbST has no effect on the micro- and macrocomposition of milk. Also, no evidence exists that rbST use has increased human exposure to antibiotic residues in milk. Concerns that IGF-I present in milk could have biological effects on humans have been allayed by studies showing that oral consumption of IGF-I by humans has little or no biological activity. Additionally, concentrations of IGF-I in digestive tract fluids of humans far exceed any IGF-I consumed when drinking milk. Furthermore, chronic supplementation of cows with rbST does not increase concentrations of milk IGF-I outside the range typically observed for effects of farm, parity, or stage of lactation. Use of rbST has not affected expression of retroviruses in cattle or posed an increased risk to human health from retroviruses in cattle. Furthermore, risk for development of type 1 or type 2 diabetes has not increased in children or adults consuming milk and dairy products from rbST-supplemented cows. Overall, milk and dairy products provide essential nutrients and related benefits in health maintenance and the prevention of chronic diseases
SN - 1525-3163
AD - School of Animal and Comparative Biomedical Sciences University of Arizona, Tucson 85719
UR - 24663163
ER -

TY - JOUR
ID - 6
T1 - Biomass fuel use and the exposure of children to particulate air pollution in southern Nepal
A1 - Devakumar,D.
A1 - Semple,S.
A1 - Osrin,D.
A1 - Yadav,S.K.
A1 - Kurmi,O.P.
A1 - Saville,N.M.
A1 - Shrestha,B.
A1 - Manandhar,D.S.
A1 - Costello,A.
A1 - Ayres,J.G.
Y1 - 2014/05//
N1 - Devakumar, D. Semple, S. Osrin, D. Yadav, S K. Kurmi, O P. Saville, N M. Shrestha, B. Manandhar, D S. Costello, A. Ayres, J G
Environment international
du1, 7807270
IM
Journal Article
English
KW - MEDLINE
RP - NOT IN FILE
SP - 79
EP - 87
JF - Environment International
JA - Environ Int
VL - 66
CY - Netherlands
N2 - The exposure of children to air pollution in low resource settings is believed to be high because of the common use of biomass fuels for cooking. We used microenvironment sampling to estimate the respirable fraction of air pollution (particles with median diameter less than 4mum) to which 7-9year old children in southern Nepal were exposed. Sampling was conducted for a total 2649h in 55 households, 8 schools and 8 outdoor locations of rural Dhanusha. We conducted gravimetric and photometric sampling in a subsample of the children in our study in the locations in which they usually resided (bedroom/living room, kitchen, veranda, in school and outdoors), repeated three times over one year. Using time activity information, a 24-hour time weighted average was modeled for all the children in the study. Approximately two-thirds of homes used biomass fuels, with the remainder mostly using gas. The exposure of children to air pollution was very high. The 24-hour time weighted average over the whole year was 168mug/m(3). The non-kitchen related samples

tended to show approximately double the concentration in winter than spring/autumn, and four times that of the monsoon season. There was no difference between the exposure of boys and girls. Air pollution in rural households was much higher than the World Health Organization and the National Ambient Air Quality Standards for Nepal recommendations for particulate exposure. Copyright 2014 The Authors. Published by Elsevier Ltd.. All rights reserved
SN - 1873-6750
AD - UCL Institute for Global Health, 30 Guilford St., London WC1N 1EH, UK. Electronic address: d.devakumar@ucl.ac.uk., University of Aberdeen Scottish Centre for Indoor Air, Division of Applied Health Sciences, Royal Aberdeen Children's Hospital, Westburn Road, Aberdeen AB25 2ZD, UK., UCL Institute for Global Health, 30 Guilford St., London WC1N 1EH, UK., Mother and Infant Research Activities, Thapathali, PO Box 921, Kathmandu, Nepal., Clinical Trial Services Unit and Epidemiological Studies Unit, University of Oxford, Richard Doll Building, Old Road Campus, Roosevelt Drive, Oxford OX3 7LF, UK., UCL Institute for Global Health, 30 Guilford St., London WC1N 1EH, UK., Mother and Infant Research Activities, Thapathali, PO Box 921, Kathmandu, Nepal., Mother and Infant Research Activities, Thapathali, PO Box 921, Kathmandu, Nepal., UCL Institute for Global Health, 30 Guilford St., London WC1N 1EH, UK., Institute of Occupational and Environmental Medicine, University of Birmingham, Edgbaston, Birmingham B15 2TT, UK
UR - 24533994
ER -

TY - JOUR
ID - 7
T1 - Dopamine transporter deficiency syndrome: phenotypic spectrum from infancy to adulthood
A1 - Ng,J.
A1 - Zhen,J.
A1 - Meyer,E.
A1 - Erreger,K.
A1 - Li,Y.
A1 - Kakar,N.
A1 - Ahmad,J.
A1 - Thiele,H.
A1 - Kubisch,C.
A1 - Rider,N.L.
A1 - Holmes,Morton D.
A1 - Strauss,K.A.
A1 - Puffenberger,E.G.
A1 - D'Agnano,D.
A1 - Anikster,Y.
A1 - Carducci,C.
A1 - Hyland,K.
A1 - Rotstein,M.
A1 - Leuzzi,V.
A1 - Borck,G.
A1 - Reith,M.E.
A1 - Kurian,M.A.
Y1 - 2014/04//
N1 - Ng, Joanne. Zhen, Juan. Meyer, Esther. Erreger, Kevin. Li, Yan. Kakar, Naseebullah. Ahmad, Jamil. Thiele, Holger. Kubisch, Christian. Rider, Nicholas L. Holmes Morton, D. Strauss, Kevin A. Puffenberger, Erik G. D'Agnano, Daniela. Anikster, Yair. Carducci, Claudia. Hyland, Keith. Rotstein, Michael. Leuzzi, Vincenzo. Borck, Guntram. Reith, Maarten E A. Kurian, Manju A
Brain : a journal of neurology
0372537
AIM, IM
Journal Article
English
KW - MEDLINE
RP - NOT IN FILE
SP - 1107

EP - 1119
JF - Brain
VL - 137
IS - Pt 4
CY - England
N2 - Dopamine transporter deficiency syndrome due to SLC6A3 mutations is the first inherited dopamine 'transportopathy' to be described, with a classical presentation of early infantile-onset progressive parkinsonism dystonia. In this study we have identified a new cohort of patients with dopamine transporter deficiency syndrome, including, most significantly, atypical presentation later in childhood with a milder disease course. We report the detailed clinical features, molecular genetic findings and in vitro functional investigations undertaken for adult and paediatric cases. Patients presenting with parkinsonism dystonia or a neurotransmitter profile characteristic of dopamine transporter deficiency syndrome were recruited for study. SLC6A3 mutational analysis was undertaken in all patients. The functional consequences of missense variants on the dopamine transporter were evaluated by determining the effect of mutant dopamine transporter on dopamine uptake, protein expression and amphetamine-mediated dopamine efflux using an in vitro cellular heterologous expression system. We identified eight new patients from five unrelated families with dopamine transporter deficiency syndrome. The median age at diagnosis was 13 years (range 1.5-34 years). Most significantly, the case series included three adolescent males with atypical dopamine transporter deficiency syndrome of juvenile onset (outside infancy) and progressive parkinsonism dystonia. The other five patients in the cohort presented with classical infantile-onset parkinsonism dystonia, with one surviving into adulthood (currently aged 34 years) and labelled as having 'juvenile parkinsonism'. All eight patients harboured homozygous or compound heterozygous mutations in SLC6A3, of which the majority are previously unreported variants. In vitro studies of mutant dopamine transporter demonstrated multifaceted loss of dopamine transporter function. Impaired dopamine uptake was universally present, and more severely impacted in dopamine transporter mutants causing infantile-onset rather than juvenile-onset disease. Dopamine transporter mutants also showed diminished dopamine binding affinity, reduced cell surface transporter, loss of post-translational dopamine transporter glycosylation and failure of amphetamine-mediated dopamine efflux. Our data series expands the clinical phenotypic continuum of dopamine transporter deficiency syndrome and indicates that there is a phenotypic spectrum from infancy (early onset, rapidly progressive disease) to childhood/adolescence and adulthood (later onset, slower disease progression). Genotype-phenotype analysis in this cohort suggests that higher residual dopamine transporter activity is likely to contribute to postponing disease presentation in these later-onset adult cases. Dopamine transporter deficiency syndrome remains under-recognized and our data highlights that dopamine transporter deficiency syndrome should be considered as a differential diagnosis for both infantile- and juvenile-onset movement disorders, including cerebral palsy and juvenile parkinsonism
SN - 1460-2156
AD - 1 Neurosciences Unit, UCL Institute of Child Health, London, UK
UR - 24613933
ER -

TY - JOUR
ID - 8
T1 - Development of HomeSTEAD's physical activity and screen time physical environment inventory
A1 - Hales,D.
A1 - Vaughn,A.E.
A1 - Mazzucca,S.
A1 - Bryant,M.J.
A1 - Tabak,R.G.
A1 - McWilliams,C.
A1 - Stevens,J.
A1 - Ward,D.S.
Y1 - 2013///
N1 - Hales, Derek. Vaughn, Amber E. Mazzucca, Stephanie. Bryant, Maria J. Tabak, Rachel G. McWilliams, Christina. Stevens, June. Ward, Dianne S
The international journal of behavioral nutrition and physical activity
101217089
IM

Journal Article. Research Support, N.I.H., Extramural. Research Support, U.S. Gov't, P.H.S.
English
KW - MEDLINE
RP - NOT IN FILE
SP - 132
JF - International Journal of Behavioral Nutrition & Physical Activity
JA - Int
VL - 10
CY - England
N2 - BACKGROUND: The home environment has a significant influence on children's physical activity, sedentary behavior, dietary intake, and risk for obesity and chronic disease. Our understanding of the most influential factors and how they interact and impact child behavior is limited by current measurement tools, specifically the lack of a comprehensive instrument. HomeSTEAD (the Home Self-administered Tool for Environmental assessment of Activity and Diet) was designed to address this gap. This new tool contains four sections: home physical activity and media equipment inventory, family physical activity and screen time practices, home food inventory, and family food practices. This paper will describe HomeSTEAD's development and present reliability and validity evidence for the first section. METHODS: The ANGELO framework guided instrument development, and systematic literature reviews helped identify existing items or scales for possible inclusion. Refinement of items was based on expert review and cognitive interviews. Parents of children ages 3-12 years (n = 125) completed the HomeSTEAD survey on three separate occasions over 12-18 days (Time 1, 2, and 3). The Time 1 survey also collected demographic information and parent report of child behaviors. Between Time 1 and 2, staff conducted an in-home observation and measured parent and child BMI. Kappa and intra-class correlations were used to examine reliability (test-retest) and validity (criterion and construct). RESULTS: Reliability and validity was strong for most items (97% having ICC > 0.60 and 72% having r > 0.50, respectively). Items with lower reliability generally had low variation between people. Lower validity estimates (r < 0.30) were more common for items that assessed usability and accessibility, with observers generally rating usability and accessibility lower than parents. Small to moderate, but meaningful, correlations between physical environment factors and BMI, outside time, and screen time were observed (e.g., amount of child portable play equipment in good condition and easy to access was significantly associated with child BMI: r = -0.23), providing evidence of construct validity. CONCLUSIONS: The HomeSTEAD instrument represents a clear advancement in the measurement of factors in the home environment related to child weight and weight-related behaviors. HomeSTEAD, in its entirety, represents a useful tool for researchers from which they can draw particular scales of greatest interest and highest relevance to their research questions
SN - 1479-5868
AD - Department of Nutrition, Gillings School of Global Public Health, University of North Carolina at Chapel Hill, 245 Rosenau Hall, CB 7461, Chapel Hill, NC 27599-7461, USA. dsward@email.unc.edu
UR - 24313962
ER -

TY - JOUR
ID - 9
T1 - A qualitative study of interviewer-administered physical activity recalls by children
A1 - Wilson,D.K.
A1 - Baxter,S.D.
A1 - Guinn,C.
A1 - Pate,R.R.
A1 - McIver,K.
Y1 - 2013/08//
N1 - Wilson, Dawn K. Baxter, Suzanne Domel. Guinn, Caroline. Pate, Russell R. McIver, Kerry
Journal of physical activity & health
101189457
IM
Journal Article
English
KW - MEDLINE
RP - NOT IN FILE
SP - 833

EP - 849
JF - Journal of Physical Activity & Health
JA - J Phys Act Health
VL - 10
IS - 6
CY - United States
N2 - BACKGROUND: Qualitative methods were used to better understand how to obtain interviewer-administered recalls of physical activity from children. METHODS: Subjects were 24 third- and fifth-grade children from 1 school in Columbia, South Carolina. Cognitive interviews targeted different retention intervals (about the same or previous school day). Round 1's protocols used an open format and had 4 phases (obtain free recall, review free recall, obtain details, review details). Round 2's protocols used a chronological format and had 3 phases (obtain free recall, obtain details, review details). Trained coders identified discrepancies across interview phases in children's recalls of physical activity at physical education (PE) and recess. Based on the school's schedule, children's reports of PE and recess were classified as omissions (scheduled but unreported) or intrusions (unscheduled but reported). RESULTS: Across interview phases, there were numerous discrepancies for Round 1 (regardless of grade, sex, or retention interval) but few discrepancies for Round 2. For Rounds 1 and 2, respectively, 0% and 0% of children omitted PE, while 33% and 0% intruded PE; 44% and 56% of children omitted recess, while 33% and 0% intruded recess. CONCLUSIONS: Results provide important information for facilitating interviewer-administered recalls of physical activity with elementary-age children
SN - 1543-5474
AD - Dept of Psychology, University of South Carolina, Columbia, SC
UR - 23072783
ER -

TY - JOUR
ID - 10
T1 - Context-Specific Outdoor Time and Physical Activity among School-Children Across Gender and Age: Using Accelerometers and GPS to Advance Methods
A1 - Klinker,C.D.
A1 - Schipperijn,J.
A1 - Kerr,J.
A1 - Ersboll,A.K.
A1 - Troelsen,J.
Y1 - 2014///
N1 - Klinker, Charlotte Demant. Schipperijn, Jasper. Kerr, Jacqueline. Ersboll, Annette Kjaer. Troelsen, Jens
Frontiers in public health
101616579
Journal Article
English
KW - MEDLINE
RP - NOT IN FILE
SP - 20
JF - Frontiers in Public Health
JA - Front
VL - 2
CY - Switzerland
N2 - Introduction: Being outdoors has a positive influence on health among children. Evidence in this area is limited and many studies have used self-reported measures. Objective context-specific assessment of physical activity patterns and correlates, such as outdoor time, may progress this field. Aims: To employ novel objective measures to assess age and gender differences in context-specific outdoor weekday behavior patterns among school-children [outdoor time and outdoor moderate to vigorous physical activity (MVPA)] and to investigate associations between context-specific outdoor time and MVPA. Methods: A total of 170 children had at least one weekday of 9h combined accelerometer and global positioning system data and were included in the analyses. The data were processed using the personal activity and location measurement system (PALMS) and a purpose-built PostgreSQL database resulting in context-specific measures for outdoor time, outdoor MVPA, and overall daily MVPA. In addition, 4 domains (leisure, school, transport, and home) and 11 subdomains (e.g., urban green space and sports facilities) were created and assessed. Multilevel analyses provided results on age

and gender differences and the association between outdoor time and MVPA. Results: Girls compared to boys had fewer outdoor minutes (p<0.05), spent a smaller proportion of their overall daily time outdoors (p<0.05), had fewer outdoor MVPA minutes during the day (p<0.001) and in 11 contexts. Children compared to adolescents had more outdoor minutes (p<0.05). During school and within recess, children compared to adolescents had more outdoor MVPA (p<0.001) and outdoor time (p<0.001). A 1-h increase in outdoor time was associated with 9.9 more minutes of MVPA (p<0.001). Conclusion: A new methodology to assess the context-specific outdoor time and physical activity patterns has been developed and can be expanded to other populations. Different context-specific patterns were found for gender and age, suggesting different strategies may be needed to promote physical activity
SN - 2296-2565
AD - Department of Sports Science and Clinical Biomechanics, University of Southern Denmark , Odense , Denmark., Department of Sports Science and Clinical Biomechanics, University of Southern Denmark , Odense , Denmark., Department of Family and Preventive Medicine, University of California San Diego , San Diego, CA , USA., The National Institute of Public Health, University of Southern Denmark , Copenhagen , Denmark., Department of Sports Science and Clinical Biomechanics, University of Southern Denmark , Odense , Denmark
UR - 24653983
ER -

TY - JOUR
ID - 11
T1 - Sociodemographic factors associated with healthy eating and food security in socio-economically disadvantaged groups in the UK and Victoria, Australia
A1 - Thornton,L.E.
A1 - Pearce,J.R.
A1 - Ball,K.
Y1 - 2014/01//
N1 - Thornton, Lukar E. Pearce, Jamie R. Ball, Kylie
Public health nutrition
9808463, dgy
IM
Journal Article
English
KW - MEDLINE
RP - NOT IN FILE
SP - 20
EP - 30
JF - Public Health Nutrition
JA - Public Health Nutr
VL - 17
IS - 1
CY - England
N2 - OBJECTIVE: To investigate the associations between sociodemographic factors and both diet indicators and food security among socio-economically disadvantaged populations in two different (national) contextual settings. DESIGN: Logistic regression was used to determine cross-sectional associations between nationality, marital status, presence of children in the household, education, employment status and household income (four low income categories) with daily fruit and vegetable consumption, low-fat milk consumption and food security. SETTING: Socio-economically disadvantaged neighbourhoods in the UK and Australia. SUBJECTS: Two samples of low-income women from disadvantaged neighbourhoods: (i) in the UK, the 2003-05 Low Income Diet and Nutrition Survey (LIDNS; n 643); and (ii) in Australia, the 2007-08 Resilience for Eating and Activity Despite Inequality (READI; n 1340). RESULTS: The influence of nationality, marital status and children in the household on the dietary outcomes varied between the two nations. Obtaining greater education qualifications was the most telling factor associated with healthier dietary behaviours. Being employed was positively associated with low-fat milk consumption in both nations and with fruit consumption in the UK, while income was not associated with dietary behaviours in either nation. In Australia, the likelihood of being food secure was higher among those who were born outside Australia, married, employed or had a greater income, while higher income was the only significant factor in the UK. CONCLUSIONS: The identification of factors that differently influence dietary behaviours and food security in socio-economically disadvantaged populations in the UK and

Australia suggests continued efforts need to be made to ensure that interventions and policy responses are informed by the best available local evidence
SN - 1475-2727
AD - 1 Centre for Physical Activity and Nutrition Research, School of Exercise and Nutrition Sciences, Deakin University, 221 Burwood Highway, Burwood, Victoria 3125, Australia., 2 Centre for Research on Environment, Society and Health (CRESH), School of Geosciences, University of Edinburgh, Edinburgh, UK., 1 Centre for Physical Activity and Nutrition Research, School of Exercise and Nutrition Sciences, Deakin University, 221 Burwood Highway, Burwood, Victoria 3125, Australia
UR - 23448943
ER -

TY - JOUR
ID - 12
T1 - Children's objective physical activity by location: why the neighborhood matters
A1 - Kneeshaw-Price,S.
A1 - Saelens,B.E.
A1 - Sallis,J.F.
A1 - Glanz,K.
A1 - Frank,L.D.
A1 - Kerr,J.
A1 - Hannon,P.A.
A1 - Grembowski,D.E.
A1 - Chan,K.CG
A1 - Cain,K.L.
Y1 - 2013/08//
N1 - Kneeshaw-Price, Stephanie. Saelens, Brian E. Sallis, James F. Glanz, Karen. Frank, Lawrence D. Kerr, Jacqueline. Hannon, Peggy A. Grembowski, David E. Chan K, C Gary. Cain, Kelli L
Pediatric exercise science
8909729
IM
Journal Article. Research Support, N.I.H., Extramural
English
KW - MEDLINE
RP - NOT IN FILE
SP - 468
EP - 486
JF - Pediatric Exercise Science
JA - Pediatr Exerc Sci
VL - 25
IS - 3
CY - United States
N2 - Knowledge of where children are active may lead to more informed policies about how and where to intervene and improve physical activity. This study examined where children aged 6-11 were physically active using time-stamped accelerometer data and parent-reported place logs and assessed the association of physical-activity location variation with demographic factors. Children spent most time and did most physical activity at home and school. Although neighborhood time was limited, this time was more proportionally active than time in other locations (e.g., active 42.1% of time in neighborhood vs. 18.1% of time at home). Children with any neighborhood-based physical activity had higher average total physical activity. Policies and environments that encourage children to spend time outdoors in their neighborhoods could result in higher overall physical activity
SN - 1543-2920
AD - University of Washington, Seattle, WA, USA
UR - 23877357
ER -

TY - JOUR
ID - 13
T1 - Assessment of nonzoonotic soil-transmitted helminth levels in soils in Yenagoa Metropolis, Niger Delta

A1 - Bariweni,P.A.
A1 - Ekweozor,I.K.
A1 - Ogbonna,D.N.
Y1 - 2014/01//
N1 - Bariweni, Perekibina A. Ekweozor, Ikem K E. Ogbonna, David N
Journal of environmental health
iac, 0405525
IM
Journal Article
English
KW - MEDLINE
RP - NOT IN FILE
SP - 108
EP - 112
JF - Journal of Environmental Health
JA - J Environ Health
VL - 76
IS - 6
CY - United States
N2 - In order to assess the prevalence of nonzoonotic soil-transmitted helminths in the Yenagoa Metropolis, 480 soil samples were collected from five communities for 12 months. The soil samples were collected along two transects from the waterfront and community playgrounds. Analysis was by standard methods. The results obtained from the study described in this article showed that 44.79% (95% confidence interval [CI] = 40.34%-49.24%) of the soil samples tested positive for nonzoonotic soil-transmitted helminths. Ascaris lumbricoides was the most common helminth with a prevalence rate of 35% (95% CI = 30.73%-39.27%). Mixed occurrence of nonzoonotic soil-transmitted helminths was 10.21%. Although the community playgrounds had a higher prevalence of nonzoonotic soil-transmitted helminths than the waterfront ($p > .05$), more cases of mixed occurrence of nonzoonotic soil-transmitted helminths occurred in the waterfront than the community playgrounds ($p > .05$). The wet season had a higher prevalence rate of nonzoonotic soil-transmitted helminths than the dry season ($p < .05$). The observed high prevalence of nonzoonotic soil-transmitted helminths in soil is considered a potential public health risk to swimmers and children playing outdoors in the Yenagoa metropolis
SN - 0022-0892
UR - 24645421
ER -

TY - JOUR
ID - 14
T1 - Parapapillary beta zone in primary school children in Beijing: associations with outdoor activity
A1 - Guo,Y.
A1 - Liu,L.J.
A1 - Xu,L.
A1 - Lv,Y.Y.
A1 - Tang,P.
A1 - Feng,Y.
A1 - Zhou,J.Q.
A1 - Meng,M.
A1 - Jonas,J.B.
Y1 - 2014/02//
N1 - Guo, Yin. Liu, Li Juan. Xu, Liang. Lv, Yan Yun. Tang, Ping. Feng, Yi. Zhou, Jin Qiong. Meng, Meng. Jonas, Jost B
Investigative ophthalmology & visual science
gwi, 7703701
IM
Journal Article. Research Support, Non-U.S. Gov't
English
KW - MEDLINE
RP - NOT IN FILE

SP - 918
EP - 925
JF - Investigative Ophthalmology & Visual Science
JA - Invest Ophthalmol Vis Sci
VL - 55
IS - 2
CY - United States
N2 - PURPOSE: To investigate prevalence and size of parapapillary alpha zone and beta zone and associations with myopia-related factors in primary school children in Beijing. METHODS: The school-based study included 382 grade-1 children and 299 grade-4 children. The children underwent a comprehensive eye examination and the parents, an interview. The examination was repeated after 1 year. RESULTS: Beta zone (prevalence: 44.5% + 2.1%; mean area: 0.17 + 0.29 mm(2)) was significantly associated with more time spent indoors with studying (P = 0.004; standardized correlation coefficient beta: 0.14; regression coefficient B: 0.05; 95% confidence interval [CI]: 0.02, 0.09) after adjusting for longer axial length (P < 0.001; beta: 0.22; B: 0.07; 95% CI: 0.04, 0.10), more myopic refractive error (P < 0.001; beta: -0.29; B: -0.07; 95% CI: -0.09, -0.04), region of habitation (P = 0.03; beta: 0.11; B: 0.07; 95% CI: 0.01, 0.14), and vertical disc diameter (P = 0.03; beta: 0.10; B: 0.16; 95% CI: 0.02, 0.30). As a corollary, indoors studying time was associated with larger area of beta zone (P = 0.01; beta: 0.11; B: 0.30; 95% CI: 0.07, 0.54) after adjusting for higher axial length/corneal curvature radius ratio (AL/CC; P = 0.006; beta: 0.12; B: 0.94; 95% CI: 0.27, 1.62) and urban region of habitation (P < 0.001; beta: -0.44; B: -0.75; 95% CI: -0.89, -0.61). An increase in AL/CC ratio at 1-year follow-up was associated with more indoors studying time (P = 0.04; beta: 0.10; B: 0.01; 95% CI: 0.00, 0.01) and larger beta zone area (P < 0.001; beta: 0.19; B: 0.04; 95% CI: 0.02, 0.05) after adjusting for axial length (P < 0.001; beta: -0.21; B: -0.01; 95% CI: -0.02, -0.01). CONCLUSIONS: Larger parapapillary beta zone area was associated with more indoors studying time after adjustment for axial length, refractive error, and region of habitation, and reversely, more indoors studying time was associated with larger beta zone in multivariate analysis. The results could indicate that parapapillary beta zone is associated with external factors-dependent development of myopia
SN - 1552-5783
AD - Tongren Eye Care Center, Beijing Tongren Hospital, Capital Medical University, Beijing, China
UR - 24425861
ER -

TY - JOUR
ID - 15
T1 - Shade in urban playgrounds in Sydney and inequities in availability for those living in lower socioeconomic areas
A1 - Anderson,C.
A1 - Jackson,K.
A1 - Egger,S.
A1 - Chapman,K.
A1 - Rock,V.
Y1 - 2014/02//
N1 - Anderson, Caroline. Jackson, Kirsten. Egger, Sam. Chapman, Kathy. Rock, Vanessa
Australian and New Zealand journal of public health
ck2, 9611095
IM
Journal Article
English
KW - MEDLINE
RP - NOT IN FILE
SP - 49
EP - 53
JF - Australian & New Zealand Journal of Public Health
JA - Aust N Z J Public Health
VL - 38
IS - 1
CY - Australia

N2 - OBJECTIVE: This study investigated the differences between the amount of shade covering typical activity areas in playgrounds of higher and lower socioeconomic status (SES) areas within metropolitan Sydney, Australia. METHODS: Using an established audit tool, surveyors visited 139 urban playgrounds and made 1,033 shade observations recording the estimated percentage of an activity area that was covered by shade and the type of shade available. RESULTS: Shade coverage was significantly associated with SES of an area and with type of activity area (p<0.001 for both), but not with time since last upgrade of playground (p=0.116). Activity areas in playgrounds in the lowest SES areas had 34% lower mean shade coverage (95%CI 18-50) than activity areas in playgrounds in the highest SES regions. The mean shade coverage for eating areas was found to be 35% higher (95%CI 27-43) than the main play areas for children. CONCLUSIONS: This study highlights inequities in available shade for those living in lower SES urban areas. Furthermore, the results of this study are concerning from a skin cancer prevention perspective as the playground areas where children spend the majority of their time (the main play equipment and stand-alone equipment) were considerably less shaded than the eating areas. IMPLICATIONS: Local government should ensure that the issue of playground shade is comprehensively addressed in all relevant planning and policy documents so that it becomes an increased priority when developing and upgrading parks and playgrounds. 2014 The Authors. ANZJPH 2014 Public Health Association of Australia
SN - 1753-6405
AD - Cancer Council, New South Wales
UR - 24494946
ER -

TY - JOUR
ID - 16
T1 - Impact of a school-based intervention program on obesity risk factors in Mexican children
A1 - Safdie,M.
A1 - Jennings-Aburto,N.
A1 - Levesque,L.
A1 - Janssen,I.
A1 - Campirano-Nunez,F.
A1 - Lopez-Olmedo,N.
A1 - Aburto,T.
A1 - Rivera,J.A.
Y1 - 2013///
N1 - Safdie, Margarita. Jennings-Aburto, Nancy. Levesque, Lucie. Janssen, Ian. Campirano-Nunez, Fabricio. Lopez-Olmedo, Nancy. Aburto, Tania. Rivera, Juan A
Salud publica de Mexico
0404371, uy1
IM
Journal Article
English
KW - MEDLINE
RP - NOT IN FILE
SP - 374
EP - 387
JF - Salud Publica de Mexico
JA - Salud Publica Mex
VL - 55 Suppl 3
CY - Mexico
N2 - Objective. To evaluate the impact of an 18-month school obesity prevention intervention on the health behaviors of 4th and 5th grade students based on ecological principles and formative research conducted in Mexico. Materials and methods. A Randomized Control Trial (RCT), design was used to assign 27 schools to one of three conditions: basic or plus interventions and control. School environment measures, children's eating and physical activity behaviors, and body mass index were assessed four times over a 2-year period in a sample of 830 students. Results. In the intervention schools, the availability of healthy foods increased with a concomitant decrease in unhealthy food availability. Food intake showed the same trend. In the intervention schools, children did not engage in more moderate to vigorous physical activity (MVPA) in physical education

(PE) class or recess but increased steps taken. Obesity prevalence did not change. Conclusion. The intervention improved the school food environment and child healthy behaviors
SN - 1606-7916
AD - Centro de Investigacion en Nutricion y Salud, Instituto Nacional de Salud Publica, Cuernavaca, Morelos, Mexico., Centro de Investigacion en Nutricion y Salud, Instituto Nacional de Salud Publica, Cuernavaca, Morelos, Mexico., School of Kinesiology and Health Studies, Queen's University, Ontario, Canada., School of Kinesiology and Health Studies, Queen's University, Ontario, Canada., Centro de Investigacion en Nutricion y Salud, Instituto Nacional de Salud Publica, Cuernavaca, Morelos, Mexico., Centro de Investigacion en Nutricion y Salud, Instituto Nacional de Salud Publica, Cuernavaca, Morelos, Mexico., Centro de Investigacion en Nutricion y Salud, Instituto Nacional de Salud Publica, Cuernavaca, Morelos, Mexico., Centro de Investigacion en Nutricion y Salud, Instituto Nacional de Salud Publica, Cuernavaca, Morelos, Mexico
UR - 24643486
ER -

TY - JOUR
ID - 17
T1 - "Pre-schoolers in the playground" an outdoor physical activity intervention for children aged 18 months to 4 years old: study protocol for a pilot cluster randomised controlled trial
A1 - Barber,S.E.
A1 - Jackson,C.
A1 - Akhtar,S.
A1 - Bingham,D.D.
A1 - Ainsworth,H.
A1 - Hewitt,C.
A1 - Richardson,G.
A1 - Summerbell,C.D.
A1 - Pickett,K.E.
A1 - Moore,H.J.
A1 - Routen,A.C.
A1 - O'Malley,C.L.
A1 - Brierley,S.
A1 - Wright,J.
Y1 - 2013///
N1 - Barber, Sally E. Jackson, Cath. Akhtar, Shaheen. Bingham, Daniel D. Ainsworth, Hannah. Hewitt, Catherine. Richardson, Gerry. Summerbell, Carolyn D. Pickett, Kate E. Moore, Helen J. Routen, Ash C. O'Malley, Claire L. Brierley, Shirley. Wright, John
Trials
101263253
IM
Journal Article. Research Support, Non-U.S. Gov't
English
KW - MEDLINE
RP - NOT IN FILE
SP - 326
JF - Trials [Electronic Resource]
JA - Trials
VL - 14
CY - England
N2 - BACKGROUND: The pre-school years are considered critical for establishing healthy lifestyle behaviours such as physical activity. Levels of physical activity track through childhood into adulthood, thus establishing habitual physical activity early in life is vital. Time spent outdoors is associated with greater physical activity and playground interventions have been shown to increase physical activity in school aged children. There are few pre-school, playground-based interventions, and evaluations of these have found mixed results. A recent report published by the UK Chief Medical Officer (CMO) highlighted that new interventions to promote movement in the early years (0-5 years old) are needed. The aim of this study is to undertake a pilot cluster randomised controlled trial (RCT) of an outdoor playground-based physical activity intervention for parents and their children aged 18 months to 4 years old ("Pre-schoolers in the Playground"; PiP) and to assess the

feasibility of conducting a full scale cluster RCT. The PiP intervention is grounded in behavioural theory (Social Cognitive Theory), and is in accordance with the CMO guidance for physical activity in the early years. It is informed by existing literature and data collected from focus groups with parents. METHODS/DESIGN: One hundred and fifty pre-school children affiliated to 10 primary schools will be recruited. Schools will be randomised to either the PiP intervention arm or the control arm (usual practice). Children in the intervention arm will be invited to attend three 30 minute outdoor play sessions per week for 30 weeks (3 school terms) at the school. Feasibility will be assessed by examining recruitment rates, attendance, attrition, acceptability of the trial and of the PiP intervention to parents, fidelity of intervention implementation, capability and capacity for schools to deliver the intervention. Health outcomes and the feasibility of outcome measurement tools will be assessed. These include physical activity via triaxial, accelerometry (Actigraph GT3X+), anthropometry (height, body mass, BMI, waist and upper arm circumference), health related quality of life for child (PedsQL) and parent (EQ5D), parent wellbeing (ComQol-A5), injuries and health service use. A health economic evaluation will also be undertaken. DISCUSSION: It is anticipated that results of this pilot trial will be published in spring 2015. TRIAL REGISTRATION: Current controlled trials: ISRCTN54165860
SN - 1745-6215
AD - Born in Bradford Cohort Study, Bradford Institute for Health Research, Bradford, UK. sally.barber@bthft.nhs.uk
UR - 24107473
ER -

TY - JOUR
ID - 18
T1 - Correlates of light and moderate-to-vigorous objectively measured physical activity in four-year-old children
A1 - van Sluijs,E.M.
A1 - McMinn,A.M.
A1 - Inskip,H.M.
A1 - Ekelund,U.
A1 - Godfrey,K.M.
A1 - Harvey,N.C.
A1 - Griffin,S.J.
Y1 - 2013///
N1 - van Sluijs, Esther M F. McMinn, Alison M. Inskip, Hazel M. Ekelund, Ulf. Godfrey, Keith M. Harvey, Nicholas C. Griffin, Simon J
PloS one
101285081
IM
Journal Article. Research Support, Non-U.S. Gov't
English
KW - MEDLINE
RP - NOT IN FILE
SP - e74934
JF - PLoS ONE [Electronic Resource]
JA - PLoS ONE
VL - 8
IS - 9
CY - United States
N2 - BACKGROUND: Correlates of physical activity (PA) are hypothesized to be context and behaviour specific, but there is limited evidence of this in young children. The aim of the current study is to investigate associations between personal, social and environmental factors and objectively measured light and moderate-to-vigorous PA (LPA and MVPA, respectively) in four-year-old children. METHODS: Cross-sectional data were used from the Southampton Women's Survey, a UK population-based longitudinal study. Four-year old children (n = 487, 47.0% male) had valid PA data assessed using accelerometry (Actiheart) and exposure data collected with a validated maternal questionnaire (including data on child personality, family demographics, maternal behaviour, rules and restrictions, and perceived local environment). Linear regression modelling was used to analyse associations with LPA and MVPA separately, interactions with sex were explored. RESULTS: LPA minutes were greater in children whose mothers reported more PA (vs. inactive: regression

coefficient+standard error: 6.70+2.94 minutes), and without other children in the neighbourhood to play with (-6.33+2.44). MVPA minutes were greater in children with older siblings (vs. none: 5.81+2.80) and those whose mothers used active transport for short trips (vs. inactive: 6.24+2.95). Children accumulated more MVPA in spring (vs. winter: 9.50+4.03) and, in boys only, less MVPA with availability of other children in the neighbourhood (-3.98+1.70). DISCUSSION: Young children's LPA and MVPA have differing associations with a number of social and environmental variables. Interventions targeting PA promotion in young children outside of formal care settings should consider including intensity specific factors
SN - 1932-6203
AD - Medical Research Council Epidemiology Unit, Cambridge, United Kingdom ; UKCRC Centre for Diet and Activity Research (CEDAR), Cambridge, United Kingdom
UR - 24040365
ER -

TY - JOUR
ID - 19
T1 - Characteristics associated with requested and required accelerometer wear in children
A1 - Wells,S.L.
A1 - Kipping,R.R.
A1 - Jago,R.
A1 - Brown,J.
A1 - Hucker,D.
A1 - Blackett,A.
A1 - Lawlor,D.A.
Y1 - 2013///
N1 - Wells, Sian L. Kipping, Ruth R. Jago, Russell. Brown, Judith. Hucker, Daniel. Blackett, Ali. Lawlor, Debbie A
BMJ open
101552874
Journal Article
English
KW - MEDLINE
RP - NOT IN FILE
SP - e003402
JF - BMJ Open
VL - 3
IS - 8
CY - England
N2 - OBJECTIVE: To investigate characteristics associated with wearing an accelerometer for the required and requested time among 8-year-old to 10-year-old children. DESIGN: Cross-sectional. SETTING: 60 Bristol and North Somerset primary schools taking part in the 'Active for Life Year 5' randomised controlled trial (RCT) in 2011. PARTICIPANTS: 2048 children, aged 8-10 years, invited to wear an accelerometer for 5 days of recording. PRIMARY OUTCOME MEASURE: Numbers meeting required wear-time for inclusion in main RCT analysis (>8 h/day >3 days) and numbers meeting requested wear-time (>8 h/day for all 5 days). RESULTS: 817 (40%) of the children wore the accelerometer for the requested time and 1629 (80%) for the required time. In adjusted multivariable analyses the odds of wearing the accelerometer for the required time were greater in females as compared with males (OR 1.76 (1.42-2.18)), those with higher scores for reporting their mother restricted time on sedentary behaviours (1.26 (1.04-1.52) per increase of 1 on a 1-4 scale) and in children from schools with larger year group sizes (1.01 (1.00-1.02) per additional child). Living in a neighbourhood with higher levels of deprivation (0.49 (0.33-0.72) comparing highest to lowest third of the deprivation score) or reporting higher levels of weekday outdoor play (0.97 (0.94, 1.00) per 30 min more) were associated with reduced odds of meeting required time. Results were essentially the same for requested wear-time. Other characteristics, including child body mass index, were not associated with required or requested wear-time. CONCLUSIONS: Only 40% of children wore the accelerometer for the requested time but 80% fulfilled the required criteria to be included in the main study analyses. Knowing which characteristics are associated with accelerometer wear could help target interventions to increase wear-time
SN - 2044-6055
AD - School of Social & Community Medicine, University of Bristol, Bristol, UK

UR - 23975106
ER -

TY - JOUR
ID - 20
T1 - Playground designs to increase physical activity levels during school recess: a systematic review
A1 - Escalante,Y.
A1 - Garcia-Hermoso,A.
A1 - Backx,K.
A1 - Saavedra,J.M.
Y1 - 2014/04//
N1 - Escalante, Yolanda. Garcia-Hermoso, Antonio. Backx, Karianne. Saavedra, Jose M
Health education & behavior : the official publication of the Society for Public Health Education
cql, 9704962
IM
Journal Article
English
KW - MEDLINE
RP - NOT IN FILE
SP - 138
EP - 144
JF - Health Education & Behavior
JA - Health Educ Behav
VL - 41
IS - 2
CY - United States
N2 - School recess provides a major opportunity to increase children's physical activity levels. Various studies have described strategies to increase levels of physical activity. The purpose of this systematic review is therefore to examine the interventions proposed as forms of increasing children's physical activity levels during recess. A systematic search of seven databases was made from the July 1 to July 5, 2012, leading to a final set of eight studies (a total of 2,383 subjects-599 "preschoolers" and 1,784 "schoolchildren") meeting the inclusion criteria. These studies were classified according to the intervention used: playground markings, game equipment, playground markings plus physical structures, and playground markings plus game equipment. The results of these studies indicate that the strategies analyzed do have the potential to increase physical activity levels during recess. The cumulative evidence was (a) that interventions based on playground markings, game equipment, or a combination of the two, do not seem to increase the physical activity of preschoolers and schoolchildren during recess and (ii) that interventions based on playground markings plus physical structures do increase the physical activity of schoolchildren during recess in the short to medium term
SN - 1552-6127
AD - 1Universidad de Extremadura, Caceres, Spain
UR - 23836828
ER -

TY - JOUR
ID - 21
T1 - School-based health promotion and physical activity during and after school hours
A1 - Vander Ploeg,K.A.
A1 - McGavock,J.
A1 - Maximova,K.
A1 - Veugelers,P.J.
Y1 - 2014/02//
N1 - Vander Ploeg, Kerry A. McGavock, Jonathan. Maximova, Katerina. Veugelers, Paul J
Pediatrics
oxv, 0376422
AIM, IM
Journal Article. Research Support, Non-U.S. Gov't
English

KW - MEDLINE
RP - NOT IN FILE
SP - e371
EP - e378
JF - Pediatrics
VL - 133
IS - 2
CY - United States
N2 - OBJECTIVES: Comprehensive school health (CSH) is a multifaceted approach to health promotion. A key objective of CSH is to foster positive health behaviors outside of school. This study examined the 2-year change in physical activity during and after school among students participating in a CSH intervention in Edmonton, Alberta, Canada. METHODS: This was a quasi-experimental, pre-post trial with a parallel, nonequivalent control group. Intervention schools had to be located in socioeconomically disadvantaged neighborhoods. In the spring of 2009 and 2011, pedometer recordings (7 full days) and demographic data were collected from cross-sectional samples of fifth grade students from 10 intervention schools and 20 comparison schools. A total of 1157 students participated in the study. Analyses were adjusted for potential confounders and the clustered design. RESULTS: Relative to 2009, children in 2011 were more active on schools days (1172 steps per day; P < .001) and on weekends (1450 steps per day; P < .001). However, the increase in mean steps between 2009 and 2011 was greater in CSH intervention schools than in comparison schools (school days: 1221 steps per day; P = .009; weekends: 2001 steps per day; P = .005). These increases remained significant after adjusting for gender and overweight status. CONCLUSIONS: These findings provide evidence of the effectiveness of CSH to affect children's physical activity during and outside of school. Results of this study justify broader implementation of effective CSH interventions for physical activity promotion and obesity prevention in the long term
SN - 1098-4275
AD - School of Public Health, University of Alberta, Edmonton, Alberta, Canada; and
UR - 24420806
ER -

TY - JOUR
ID - 22
T1 - Personal exposure of primary school children to BTEX, NO2 and ozone in Eskisehir, Turkey: relationship with indoor/outdoor concentrations and risk assessment
A1 - Demirel,G.
A1 - Ozden,O.
A1 - Dogeroglu,T.
A1 - Gaga,E.O.
Y1 - 2014/03/01/
N1 - Demirel, Gulcin. Ozden, Ozlem. Dogeroglu, Tuncay. Gaga, Eftade O
The Science of the total environment
uj0, 0330500
IM
Journal Article. Research Support, Non-U.S. Gov't
English
KW - MEDLINE
RP - NOT IN FILE
SP - 537
EP - 548
JF - Science of the Total Environment
JA - Sci Total Environ
VL - 473-474
CY - Netherlands
N2 - Personal exposures of 65 primary school children to benzene, toluene, ethyl benzene, xylenes (BTEX), nitrogen dioxide (NO2) and ozone (O3) were measured during 24h by using organic vapor monitors and tailor-made passive samplers. Two schools were selected to represent students living in more polluted (urban) and less polluted (sub-urban) areas in the city of Eskisehir, Turkey. The pollutant concentrations were also measured in indoor and outdoor environments during the personal sampling to investigate the contribution of each micro-

environment on measured personal concentrations. Socio-demographic and personal time-activity data were collected by means of questionnaires and half-hour-time resolution activity diaries. Personal exposure concentrations were found to be correlated with indoor home concentrations. Personal, indoor and outdoor concentrations of all studied pollutants except for ozone were found to be higher for the students living at the urban traffic site. Ozone, on the other hand, had higher concentrations at the sub-urban site for all three types of measurements (personal, indoor and outdoor). Analysis of the questionnaire data pointed out to environmental tobacco smoke, use of solvent based products, and petrol station nearby as factors that affect personal exposure concentrations. Cancer and non-cancer risks were estimated using the personal exposure concentrations. The mean cancer risk for the urban school children (1.7x10(-5)) was found to be higher than the sub-urban school children (0.88x10(-5)). Children living with smoking parents had higher risk levels (1.7x10(-5)) than children living with non-smoking parents (1.08x10(-5)). Overall, the risk levels were <1x10(-4). All hazard quotient values for BTEX for the non-cancer health effects were <1 based on the calculations EPA's Risk Assessment Guidance for Superfund (RAGS) part F. Copyright 2013 Elsevier B.V. All rights reserved
SN - 1879-1026
AD - Anadolu University, Faculty of Engineering, Department of Environmental Engineering, 26555 Eskisehir, Turkey. Electronic address: gulcindemirel7@hotmail.com., Anadolu University, Faculty of Engineering, Department of Environmental Engineering, 26555 Eskisehir, Turkey. Electronic address: oozden@anadolu.edu.tr., Anadolu University, Faculty of Engineering, Department of Environmental Engineering, 26555 Eskisehir, Turkey. Electronic address: tdogeroglu@anadolu.edu.tr., Anadolu University, Faculty of Engineering, Department of Environmental Engineering, 26555 Eskisehir, Turkey. Electronic address: egaga@anadolu.edu.tr
UR - 24388904
ER -

TY - JOUR
ID - 23
T1 - Changes in daily activity patterns of caregivers of children with newly diagnosed epilepsy: a case-controlled design
A1 - Painter,E.
A1 - Rausch,J.R.
A1 - Modi,A.C.
Y1 - 2014/02//
N1 - Painter, Elizabeth. Rausch, Joseph R. Modi, Avani C
Epilepsy & behavior : E&B
100892858
IM
Journal Article
English
KW - MEDLINE
RP - NOT IN FILE
SP - 1
EP - 6
JF - Epilepsy & Behavior
JA - Epilepsy Behav
VL - 31
CY - United States
N2 - Having a child diagnosed with a chronic pediatric illness is a major stressor for families that can alter their daily lives. The primary study aim was to use Daily Phone Diaries (DPDs), a cued-recall procedure to track parents through their activities over a 24-hour period, to assess the activity patterns of a group of caregivers with a child diagnosed with new-onset epilepsy (group with NOE; n=30) and a group of matched comparisons (comparison group; n=29). The time caregivers spent in sleep and recreation was evaluated over the first 5.5months after diagnosis. Caregivers of children with NOE spent significantly more time in recreation inside the home, while the comparison group spent significantly more time in recreation outside the home. These data suggest that families with children with NOE reallocate their time post-diagnosis from recreation outside to inside the home, which raises concerns about the overall adaptation of the family to the diagnosis and presents a critical opportunity for health-care teams to intervene and support families with children with NOE. Copyright 2013 Elsevier Inc. All rights reserved

SN - 1525-5069
AD - Division of Behavioral Medicine and Clinical Psychology, Center for the Promotion of Treatment Adherence and Self-Management, Cincinnati Children's Hospital Medical Center, 3333 Burnet Ave. (MLC 7019), Cincinnati, OH 45229, USA., Department of Pediatrics, University of Cincinnati College of Medicine, Cincinnati, OH 45229, USA; Division of Behavioral Medicine and Clinical Psychology, Cincinnati Children's Hospital Medical Center, Cincinnati, 3333 Burnet Ave. (MLC 3015), OH 45229, USA., Division of Behavioral Medicine and Clinical Psychology, Center for the Promotion of Treatment Adherence and Self-Management, Cincinnati Children's Hospital Medical Center, 3333 Burnet Ave. (MLC 7019), Cincinnati, OH 45229, USA; Department of Pediatrics, University of Cincinnati College of Medicine, Cincinnati, OH 45229, USA. Electronic address: avani.modi@cchmc.org
UR - 24291521
ER -

TY - JOUR
ID - 24
T1 - Moderating influences of baseline activity levels in school physical activity programming for children: the Ready for Recess project
A1 - Saint-Maurice,P.F.
A1 - Welk,G.J.
A1 - Russell,D.W.
A1 - Huberty,J.
Y1 - 2014///
N1 - Saint-Maurice, Pedro F. Welk, Gregory J. Russell, Daniel W. Huberty, Jennifer
BMC public health
100968562
IM
Journal Article
English
KW - MEDLINE
RP - NOT IN FILE
SP - 103
JF - BMC Public Health
VL - 14
IS - 1
CY - England
N2 - BACKGROUND: A limitation of traditional outcome studies from behavioral interventions is the lack of attention given to evaluating the influence of moderating variables. This study examined possible moderation effect of baseline activity levels on physical activity change as a result of the Ready for Recess intervention. METHODS: Ready for Recess (August 2009-September 2010) was a controlled trial with twelve schools randomly assigned to one of four conditions: control group, staff supervision, equipment availability, and the combination of staff supervision and equipment availability. A total of 393 children (181 boys and 212 girls) from grades 3 through 6 (8-11 years old) were asked to wear an Actigraph monitor during school time on 4-5 days of the week. Assessments were conducted at baseline (before intervention) and post intervention (after intervention). RESULTS: Initial MVPA moderated the effect of Staff supervision (beta=-0.47%; p<.05), but not Equipment alone and Staff+Equipment (p>.05). Participants in the Staff condition that were 1 standard deviation (SD) below the mean for baseline MVPA (classified as "low active") had lower MVPA levels at post-intervention when compared with their low active peers in the control condition (Mean diff=-10.8+2.9%; p=.005). High active individuals (+1SD above the mean) in the Equipment treatment also had lower MVPA values at post-intervention when compared with their highly active peers in the control group (Mean diff=-9.5+2.9%; p=.009). CONCLUSIONS: These results indicate that changes in MVPA levels at post-intervention were reduced in highly active participants when recess staff supervision was provided. In this study, initial MVPA moderated the effect of Staff supervision on children's MVPA after 6 months of intervention. Staff training should include how to work with inactive youth but also how to assure that active children remain active
SN - 1471-2458
AD - Iowa State University, Department of Kinesiology, Ames, IA 50011, USA. pedrosm@iastate.edu
UR - 24484545

ER -

TY - JOUR
ID - 25
T1 - Effects of age, season, gender and urban-rural status on time-activity: CanadianHuman Activity Pattern Survey 2 (CHAPS 2)
A1 - Matz,C.J.
A1 - Stieb,D.M.
A1 - Davis,K.
A1 - Egyed,M.
A1 - Rose,A.
A1 - Chou,B.
A1 - Brion,O.
Y1 - 2014/02//
N1 - Matz, Carlyn J. Stieb, David M. Davis, Karelyn. Egyed, Marika. Rose, Andreas. Chou, Benedito. Brion, Orly
International journal of environmental research and public health
101238455
IM
Journal Article. Research Support, Non-U.S. Gov't
English
KW - MEDLINE
RP - NOT IN FILE
SP - 2108
EP - 2124
JF - International Journal of Environmental Research & Public Health [Electronic Resource]
JA - Int J Environ Res Public Health
VL - 11
IS - 2
CY - Switzerland
N2 - Estimation of population exposure is a main component of human health risk assessment for environmental contaminants. Population-level exposure assessments require time-activity pattern distributions in relation to microenvironments where people spend their time. Societal trends may have influenced time-activity patterns since previous Canadian data were collected 15 years ago. The Canadian Human Activity Pattern Survey 2 (CHAPS 2) was a national survey conducted in 2010-2011 to collect time-activity information from Canadians of all ages. Five urban and two rural locations were sampled using telephone surveys. Infants and children, key groups in risk assessment activities, were over-sampled. Survey participants (n = 5,011) provided time-activity information in 24-hour recall diaries and responded to supplemental questionnaires concerning potential exposures to specific pollutants, dwelling characteristics, and socio-economic factors. Results indicated that a majority of the time was spent indoors (88.9%), most of which was indoors at home, with limited time spent outdoors (5.8%) or in a vehicle (5.3%). Season, age, gender and rurality were significant predictors of time activity patterns. Compared to earlier data, adults reported spending more time indoors at home and adolescents reported spending less time outdoors, which could be indicative of broader societal trends. These findings have potentially important implications for assessment of exposure and risk. The CHAPS 2 data also provide much larger sample sizes to allow for improved precision and are more representative of infants, children and rural residents
SN - 1660-4601
AD - Air Health Effects Assessment Division, Health Canada, 269 Laurier Ave West, PL 4903C, Ottawa, ON K1A 0K9, Canada. carlyn.matz@hc-sc.gc.ca., Population Studies Division, Health Canada, 445-757 West Hastings Street-Federal Tower, Vancouver, BC V6C 1A1, Canada. dave.stieb@hc-sc.gc.ca., Population Studies Division, Health Canada, 50 Colombine Driveway, Tunney's Pasture, PL 0801A, Ottawa, ON K1A 0K9, Canada. karelyn.davis@hc-sc.gc.ca., Air Health Effects Assessment Division, Health Canada, 269 Laurier Ave West, PL 4903C, Ottawa, ON K1A 0K9, Canada. marika.egyed@hc-sc.gc.ca., Malatest & Associates, Ltd., 858 Pandora Ave, Victoria, BC V8W 1P4, Canada. a.rose@malatest.com., Malatest & Associates, Ltd., 858 Pandora Ave, Victoria, BC V8W 1P4, Canada. b.chou@malatest.com., Population Studies Division, Health Canada, 50 Colombine Driveway, Tunney's Pasture, PL 0801A, Ottawa, ON K1A 0K9, Canada. orly.brion@hc-sc.gc.ca
UR - 24557523

ER -

TY - JOUR
ID - 26
T1 - Momentary assessment of affect, physical feeling states, and physical activity in children
A1 - Dunton,G.F.
A1 - Huh,J.
A1 - Leventhal,A.M.
A1 - Riggs,N.
A1 - Hedeker,D.
A1 - Spruijt-Metz,D.
A1 - Pentz,M.A.
Y1 - 2014/03//
N1 - Dunton, Genevieve F. Huh, Jimi. Leventhal, Adam M. Riggs, Nathaniel. Hedeker, Donald. Spruijt-Metz, Donna. Pentz, Mary Ann
Health psychology : official journal of the Division of Health Psychology, American Psychological Association
ejl, 8211523
IM
Journal Article
English
KW - MEDLINE
RP - NOT IN FILE
SP - 255
EP - 263
JF - Health Psychology
JA - Health Psychol
VL - 33
IS - 3
CY - United States
N2 - Objective: Most research on the interplay of affective and physical feelings states with physical activity in children has been conducted under laboratory conditions and fails to capture intraindividual covariation. The current study used Ecological Momentary Assessment (EMA) to bidirectionally examine how affective and physical feeling states are related to objectively measured physical activity taking place in naturalistic settings during the course of children's everyday lives. Methods: Children (N = 119, ages 9-13 years, 52% male, 32% Hispanic) completed 8 days of EMA monitoring, which measured positive affect (PA), negative affect (NA), feeling tired, and feeling energetic up to 7 times per day. EMA responses were time-matched to accelerometer assessed moderate-to-vigorous physical activity (MVPA) in the 30 min before and after each EMA survey. Results: Higher ratings of feeling energetic and lower ratings of feeling tired were associated with more MVPA in the 30 min after the EMA prompt. More MVPA in the 30 min before the EMA prompt was associated with higher ratings of PA and feeling energetic and lower ratings of NA. Between-subjects analyses indicated that mean hourly leisure-time MVPA was associated with less intraindividual variability in PA and NA. Conclusions: Physical feeling states predict subsequent physical activity levels, which in turn, predict subsequent affective states in children. Active children demonstrated higher positive and negative emotional stability. Although the strength of these associations were of modest magnitude and their clinical relevance is unclear, understanding the antecedents to and consequences of physical activity may have theoretical and practical implications for the maintenance and promotion of physical activity and psychological well-being in children. (PsycINFO Database Record (c) 2014 APA, all rights reserved)
SN - 1930-7810
AD - Department of Preventive Medicine, University of Southern California., Department of Preventive Medicine, University of Southern California., Department of Preventive Medicine, University of Southern California., Department of Preventive Medicine, University of Southern California., Division of Epidemiology and Biostatistics, University of Illinois., Department of Preventive Medicine, University of Southern California., Department of Preventive Medicine, University of Southern California
UR - 23668846
ER -

TY - JOUR

ID - 27
T1 - Training Mixtec Promotores to Assess Health Concerns in Their Community: A CBPR Pilot Study
A1 - Maxwell,A.E.
A1 - Young,S.
A1 - Rabelo,Vega R.
A1 - Herrmann,A.K.
A1 - See,C.
A1 - Glenn,B.A.
A1 - Mistry,R.
A1 - Bastani,R.
Y1 - 2014/04//
N1 - Maxwell, Annette E. Young, Sandra. Rabelo Vega, Roena. Herrmann, Alison K. See, Cha. Glenn, Beth A. Mistry, Ritesh. Bastani, Roshan
Journal of immigrant and minority health / Center for Minority Public Health
101256527
IM
Journal Article
English
KW - MEDLINE
RP - NOT IN FILE
SP - 310
EP - 313
JF - Journal of Immigrant & Minority Health
JA - J Immigr.Minor.Health
VL - 16
IS - 2
CY - United States
N2 - An academic institution and a community organization partnered for one of the first studies assessing health needs of Mixtecs, indigenous immigrants from Southern Mexico, residing in Ventura County, California. Ten bilingual Spanish- and Mixteco-speaking promotores received a 1-day focus group training, participated in a focus group themselves and conducted 5 focus groups with 42 Mixtec community members. The focus group training is described. Health concerns discussed in the focus groups include outdoor exercise among women viewed as flirtatious; reluctance to ask for governmental assistance due to fear that children will have to pay back later; soda consumption perceived as a symbol of socio-economic status; and unwillingness to obtain mammograms or pap smears because private body parts are to be touched by husbands only. Training promotores to conduct focus groups can increase organizational capacity to identify pressing health needs in under-represented and hard-to-reach population groups
SN - 1557-1920
AD - Center for Cancer Prevention and Control Research, Fielding School of Public Health and Jonsson Comprehensive Cancer Center, University of California Los Angeles, Box 956900, A2-125 CHS, Los Angeles, CA, 90095-6900, USA, amaxwell@ucla.edu
UR - 22940912
ER -

TY - JOUR
ID - 28
T1 - Effects of ambient air pollution on respiratory tract complaints and airway inflammation in primary school children
A1 - Altug,H.
A1 - Gaga,E.O.
A1 - Dogeroglu,T.
A1 - Brunekreef,B.
A1 - Hoek,G.
A1 - Van,Doorn W.
Y1 - 2014/05/01/
N1 - Altug, Hicran. Gaga, Eftade O. Dogeroglu, Tuncay. Brunekreef, Bert. Hoek, Gerard. Van Doorn, Wim
The Science of the total environment

uj0, 0330500
IM
Journal Article
English
KW - MEDLINE
RP - NOT IN FILE
SP - 201
EP - 209
JF - Science of the Total Environment
JA - Sci Total Environ
VL - 479-480
CY - Netherlands
N2 - Respiratory health effects of ambient air pollution were studied in 605 school children 9 to 13years in Eskisehir, Turkey. Each child performed a fractional exhaled nitric oxide (FENO) measurement and a lung function test (LFT). Self-reported respiratory tract complaints (having cold, complaints of throat, runny nose and shortness of breath/wheezing) in the last 7days and on the day of testing were also recorded. As acute health outcomes were investigated, weekly average ambient concentrations of ozone (O3), nitrogen dioxide (NO2) and sulfur dioxide (SO2) were determined by passive sampling in the school playgrounds simultaneously with the health survey. Effects of air pollution on respiratory tract complaints and exhaled NO/lung function were estimated by multivariate logistic regression and multivariate linear mixed effects models, respectively. Upper respiratory tract complaints were significantly (p<0.05) associated with weekly average O3 concentrations during the health survey (adjusted odds ratios (OR) of 1.21 and 1.28 for a 10mugm(-3) increment for having cold and a runny nose on day of testing, respectively). FENO levels were significantly (p<0.05) increased in children with various upper respiratory tract complaints (ratio in FENO varied between 1.16 and 1.40). No significant change in FENO levels was detected in association with any of the measured pollutants (p>0.05). Lung function was not associated with upper respiratory tract complaints and FENO levels. Peak Expiratory Flow (PEF) levels were negatively associated with weekly average O3 levels for children without upper respiratory tract complaints. In summary, elevated levels of air pollutants increased respiratory tract complaints in children. Copyright 2014 Elsevier B.V. All rights reserved
SN - 1879-1026
AD - Department of Environmental Engineering, Anadolu University, Iki Eylul Campus, 26555 Eskisehir, Turkey. Electronic address: hcinar@anadolu.edu.tr., Department of Environmental Engineering, Anadolu University, Iki Eylul Campus, 26555 Eskisehir, Turkey. Electronic address: egaga@anadolu.edu.tr., Department of Environmental Engineering, Anadolu University, Iki Eylul Campus, 26555 Eskisehir, Turkey. Electronic address: tdogeroglu@anadolu.edu.tr., IRAS Institute for Risk Assessment Sciences, Utrecht University, The Netherlands. Electronic address: b.brunekreef@uu.nl., IRAS Institute for Risk Assessment Sciences, Utrecht University, The Netherlands. Electronic address: G.Hoek@uu.nl., Royal Haskoning, Business line Industry and Energy, P.O. Box 151, 6500 AD Nijmegen, The Netherlands. Electronic address: wim.vandoorn@royalhaskoning.com
UR - 24561926
ER -

TY - JOUR
ID - 29
T1 - Contamination and risk assessment (based on bioaccessibility via ingestion and inhalation) of metal(loid)s in outdoor and indoor particles from urban centers of Guangzhou, China
A1 - Huang,M.
A1 - Wang,W.
A1 - Chan,C.Y.
A1 - Cheung,K.C.
A1 - Man,Y.B.
A1 - Wang,X.
A1 - Wong,M.H.
Y1 - 2014/05/01/
N1 - Huang, Minjuan. Wang, Wei. Chan, Chuen Yu. Cheung, Kwai Chung. Man, Yu Bong. Wang, Xuemei. Wong, Ming Hung
The Science of the total environment

uj0, 0330500
IM
Journal Article
English
KW - MEDLINE
RP - NOT IN FILE
SP - 117
EP - 124
JF - Science of the Total Environment
JA - Sci Total Environ
VL - 479-480
CY - Netherlands
N2 - Road dust, household air-conditioning (AC) filter dust and PM2.5 were collected to investigate the contamination of metal(loid)s (Cr, Mn, Ni, Cu, Zn, As, Cd, Sn, Sb, Hg and Pb) in outdoor and indoor urban environments of Guangzhou. Zinc was found to be the most abundant element in road dust and household PM2.5, while the concentration of Pb was the highest in AC filter dust. Enrichment factor (EF) was used to assess the influence of human activity on the contamination of these metal(loid)s. Ingestion and inhalation were the two exposure pathways applied for risk assessment. Physiologically based extraction test (PBET) was used to estimate the oral bioaccessibilities of metal(loid)s in road dust and AC filter dust. Respiratory bioaccessible fraction of metal(loid)s via household PM2.5 was extracted with lung simulating solution. Household AC filter dust was more hazardous to human health than road dust, especially to children. Arsenic was found to be the most risky element based on the risk assessment. Copyright 2014. Published by Elsevier B.V
SN - 1879-1026
AD - Croucher Institute for Environmental Sciences and Department of Biology, Hong Kong Baptist University, Kowloon Tong, Kowloon, Hong Kong, China; Faculty of Environmental Science and Engineering, Sun Yat-Sen University, Guangzhou 510275, Hong Kong, China., Croucher Institute for Environmental Sciences and Department of Biology, Hong Kong Baptist University, Kowloon Tong, Kowloon, Hong Kong, China., Faculty of Environmental Science and Engineering, Sun Yat-Sen University, Guangzhou 510275, Hong Kong, China., Croucher Institute for Environmental Sciences and Department of Biology, Hong Kong Baptist University, Kowloon Tong, Kowloon, Hong Kong, China., Croucher Institute for Environmental Sciences and Department of Biology, Hong Kong Baptist University, Kowloon Tong, Kowloon, Hong Kong, China; Department of Science and Environmental Studies, The Hong Kong Institute of Education, Tai Po, Hong Kong, China., Faculty of Environmental Science and Engineering, Sun Yat-Sen University, Guangzhou 510275, Hong Kong, China., Croucher Institute for Environmental Sciences and Department of Biology, Hong Kong Baptist University, Kowloon Tong, Kowloon, Hong Kong, China; Department of Science and Environmental Studies, The Hong Kong Institute of Education, Tai Po, Hong Kong, China. Electronic address: minghwong@ied.edu.hk
UR - 24561290
ER -

TY - JOUR
ID - 30
T1 - Personal tobacco pack display before and after the introduction of plain packaging with larger pictorial health warnings in Australia: an observational study of outdoor cafe strips
A1 - Zacher,M.
A1 - Bayly,M.
A1 - Brennan,E.
A1 - Dono,J.
A1 - Miller,C.
A1 - Durkin,S.
A1 - Scollo,M.
A1 - Wakefield,M.
Y1 - 2014/04//
N1 - Zacher, Meghan. Bayly, Megan. Brennan, Emily. Dono, Joanne. Miller, Caroline. Durkin, Sarah. Scollo, Michelle. Wakefield, Melanie
Addiction (Abingdon, England)
bm3, 9304118
IM

Journal Article
English
KW - MEDLINE
RP - NOT IN FILE
SP - 653
EP - 662
JF - Addiction
VL - 109
IS - 4
CY - England
N2 - AIMS: We tested whether prevalence of cigarette pack display and smoking at outdoor venues and pack orientation changed following the introduction of plain packaging and larger pictorial health warnings in Australia. METHODS: Between October and April 2011-12 (pre-plain packaging, pre-PP) and 2012-13 (post-plain packaging, post-PP), we counted patrons, smokers and tobacco packs at cafes, restaurants and bars with outdoor seating. Pack type (fully branded, plain or unknown) and orientation were noted. Rates of pack display, smoking and pack orientation were analysed using multi-level Poisson regression. RESULTS: Pack display declined by 15% [adjusted incident rate ratio (IRR)=0.85, 95% confidence interval (CI)=0.79-0.91, P<0.001], driven by a 23% decline in active smoking (IRR=0.77, 95% CI=0.71-0.84, P<0.001) between phases. The decline in pack display coincided with the full implementation of plain packaging from December 2012, was stronger in venues with children present and was limited to mid and high socio-economic status (SES) areas. The proportion of packs orientated face-up declined from 85.4% of fully branded packs pre-PP to 73.6% of plain packs post-PP (IRR=0.87, 95% CI=0.79-0.95, P=0.002). Alternatively, the proportions concealed by telephones, wallets or other items (4.4% of fully branded packs pre-PP and 9.5% of plain packs post-PP; IRR=2.33, 95% CI=1.72-3.17, P<0.001) and in an external case (1.5-3.5% of all packs; IRR=2.79, 95% CI=1.77-4.40, P<0.001) increased. Low SES areas evidenced the greatest increase in pack concealment and the greatest decline in face-up pack orientation. CONCLUSIONS: Following Australia's 2012 policy of plain packaging and larger pictorial health warnings on cigarette and tobacco packs, smoking in outdoor areas of cafes, restaurants and bars and personal pack display (packs clearly visible on tables) declined. Further, a small proportion of smokers took steps to conceal packs that would otherwise be visible. Both are promising outcomes to minimize exposure to tobacco promotion. 2014 Society for the Study of Addiction
SN - 1360-0443
AD - Cancer Council Victoria, Melbourne, Vic., Australia
UR - 24428427
ER -

TY - JOUR
ID - 31
T1 - Bone mineral density of the spine in 11,898 chinese infants and young children: a cross-sectional study
A1 - Xu,H.
A1 - Zhao,Z.
A1 - Wang,H.
A1 - Ding,M.
A1 - Zhou,A.
A1 - Wang,X.
A1 - Zhang,P.
A1 - Duggan,C.
A1 - Hu,F.B.
Y1 - 2013///
N1 - Xu, Haiqing. Zhao, Zhiwei. Wang, Hong. Ding, Ming. Zhou, Aiqin. Wang, Xiaoyan. Zhang, Ping. Duggan, Christopher. Hu, Frank B
PloS one
101285081
IM
Journal Article. Research Support, Non-U.S. Gov't
English
KW - MEDLINE
RP - NOT IN FILE

SP - e82098
JF - PLoS ONE [Electronic Resource]
JA - PLoS ONE
VL - 8
IS - 12
CY - United States
N2 - BACKGROUND: Bone mineral density (BMD) increases progressively during childhood and adolescence and is affected by various genetic and environmental factors. The aim of this study was to establish reference values for lumbar BMD in healthy Chinese infants and young children and investigate its influencing factors. METHODS AND FINDINGS: Healthy children aged 0 to 3 years who underwent regular physical examinations at the Child Health Care Clinic of Hubei Maternal and Child Health Hospital (N = 11,898) were recruited for this study. We also chose 379 preterm infants aged 0 to 1 years to preliminarily explore the development of BMD in this special population. BMD (g/cm(2)) measurements of the lumbar spine (L2-L4) were carried out with dual-energy X-ray absorptiometry and a questionnaire was administered to full-term children's parents to gather information on various nutritional and lifestyle factors as well as mothers' nutritional supplement use during pregnancy. Lumbar BMD significantly increased with age among both boys and girls ($p<0.05$), with fastest growth observed during the first postnatal year. There was no significant difference in lumbar BMD between boys and girls of similar age ($p>0.05$), either among healthy reference children or preterm infants. However, BMD values in preterm infants were significantly lower than those in term infants 3 to 8 months old ($p<0.05$) after adjustment for gestational age. Multivariable linear regression analysis indicated significant positive associations between lumbar BMD of healthy children and the child's age and current weight, mother's weight gain during pregnancy, birth weight, children's outdoor activity duration and children's physical activity duration. CONCLUSION: Our study provides reference values of lumbar BMD for healthy Chinese children aged 0 to 3 years and identifies several influencing factors
SN - 1932-6203
AD - Department of Child Health Care, Hubei Maternal and Child Health Hospital, Wuhan, China
UR - 24324752
ER -

TY - JOUR
ID - 32
T1 - Object exploration in 3- to 7-year-old children
A1 - Sniegulska,M.
A1 - Pisula,W.
Y1 - 2013/10//
N1 - Sniegulska, Magdalena. Pisula, Wojciech
Psychological reports
qf6, 0376475
IM
Journal Article. Research Support, Non-U.S. Gov't
English
KW - MEDLINE
RP - NOT IN FILE
SP - 528
EP - 539
JF - Psychological Reports
JA - Psychol Rep
VL - 113
IS - 2
CY - United States
N2 - The aim of the present study was to analyze free exploration of an unfamiliar, novel object in a semi-natural environment in 189 preschool children aged 3 to 7 years. The study used a purpose-built, novel, and complex stimulus object. Each child was shown the complex object lying on the floor and asked to explore it for 15 minutes. The child's behavior was video recorded. Age-dependent differences were found in the children's exploratory activity. No sex differences were found. Cluster analysis showed four main behavioral patterns that differed in their qualitative and quantitative parameters. Cluster membership was mainly age-dependent and not sex-specific. The results showed individual and developmental differences among the preschoolers with respect

to their exploratory activity. This should be taken into account by school psychologists and teachers when designing the educational tasks and play situations
SN - 0033-2941
AD - Warsaw School of Social Sciences and Humanities., Institute of Psychology, Polish Academy of Sciences
UR - 24597446
ER -

TY - JOUR
ID - 33
T1 - Time Since Immigration and Ethnicity as Predictors of Physical Activity among Canadian Youth: A Cross-Sectional Study
A1 - Kukaswadia,A.
A1 - Pickett,W.
A1 - Janssen,I.
Y1 - 2014///
N1 - Kukaswadia, Atif. Pickett, William. Janssen, Ian
PloS one
101285081
IM
Journal Article
English
KW - MEDLINE
RP - NOT IN FILE
SP - e89509
JF - PLoS ONE [Electronic Resource]
JA - PLoS ONE
VL - 9
IS - 2
CY - United States
N2 - BACKGROUND: Little is known about patterns of physical activity engaged in by youth after they immigrate to a new country. This study aims to investigate relationships between immigrant generation and ethnicity with physical activity, and to determine if the relationship between immigrant generation and physical activity was modified by ethnicity. METHODS: The data sources were Cycle 6 (2009-2010) of the Canadian Health Behaviour in School-Aged Children Study and the 2006 Canada Census of Population. Participants (weighted n = 23,124) were young people from grades 6-10 in 436 schools. Students were asked where they were born, how long ago they moved to Canada, their ethnicity, and how many days a week they accumulated at least 60 minutes of moderate-to-vigorous physical activity (MVPA). RESULTS: Youth born outside of Canada were less likely to be active than peers born in Canada; 11% vs 15% reported 7 days/week of at least 60 minutes of MVPA (p = .001). MVPA increased with time since immigration. Compared to Canadian-born youth, youth who immigrated within the last 1-2 years were less likely to get sufficient MVPA on 4-6 days/week (odds ratio: 0.66, 95% confidence interval: 0.53-0.82) and 7 days/week (0.62; 0.43-0.89). East and South-East Asian youth were less active, regardless of time since immigration: 4-6 days/week (0.67; 0.58-0.79) and 7 days/week (0.37; 0.29-0.48). CONCLUSION: Time since immigration and ethnicity were associated with MVPA among Canadian youth. Mechanisms by which these differences occur need to be uncovered in order to identify barriers to physical activity participation among youth
SN - 1932-6203
AD - Department of Public Health Sciences, Queen's University, Kingston, Ontario, Canada., Department of Public Health Sciences, Queen's University, Kingston, Ontario, Canada ; Clinical Research Centre, Kingston General Hospital, Kingston, Ontario, Canada., Department of Public Health Sciences, Queen's University, Kingston, Ontario, Canada ; School of Kinesiology and Health Studies, Queen's University, Kingston, Ontario, Canada
UR - 24586835
ER -

TY - JOUR
ID - 34

T1 - Long-term effects of 4-year longitudinal school-based physical activity intervention on the physical fitness of children and youth during 7-year followup assessment
A1 - Jurak,G.
A1 - Cooper,A.
A1 - Leskosek,B.
A1 - Kovac,M.
Y1 - 2013/12//
N1 - Jurak, Gregor. Cooper, Ashley. Leskosek, Bojan. Kovac, Marjeta
Central European journal of public health
bo6, 9417324
IM
Journal Article
English
KW - MEDLINE
RP - NOT IN FILE
SP - 190
EP - 195
JF - Central European Journal of Public Health
JA - Cent Eur J Public Health
VL - 21
IS - 4
CY - Czech Republic
N2 - Many school-based physical activity (PA) interventions have been developed, but only a few have assessed their long-term effects. A PA intervention taking place in the first four years of some Slovenian primary schools entails an enhanced physical education (PE) curriculum, including two extra lessons of PE per week, a wider selection of PE content, and additional outdoor education delivered by both a specialist PE teacher and a general teacher. The effects of the intervention on children's physical fitness (motor tasks and anthropometry) were evaluated within a quasi-experimental study. In total, 324 children from nine Slovenian primary schools either received the enhanced curriculum (intervention (n=160)) or standard PE (control (n=164)), and were followed for a four-year intervention period and seven years post intervention. Data from the SLOFIT database were used to compare differences in the physical fitness of children each year. Linear Mixed Models were used to test the influence of the PA intervention. Over an 11-year period, the PA intervention group significantly differed in all motor tasks, but not in anthropometric measures or body mass index, after controlling for year of measurement and sex. Differences between the control and intervention groups decreased with time. This study highlights the importance of tracking the long term effects of PA interventions. PA intervention in the first four years of Slovenian primary school offers the possibility of improving physical performance in children; initiatives aiming to increase their performance (physical fitness, physical activity) and health outcomes are warranted
SN - 1210-7778
AD - Faculty of Sport, University of Ljubljana, Ljubljana, Slovenia. gregor.jurak@fsp.uni-Ij.si, Centre for Exercise, Nutrition and Health Sciences, University of Bristol, Bristol, UK., Faculty of Sport, University of Ljubljana, Ljubljana, Slovenia., Faculty of Sport, University of Ljubljana, Ljubljana, Slovenia
UR - 24592722
ER -

TY - JOUR
ID - 35
T1 - Objectively assessed recess physical activity in girls and boys from high and low socioeconomic backgrounds
A1 - Baquet,G.
A1 - Ridgers,N.D.
A1 - Blaes,A.
A1 - Aucouturier,J.
A1 - Van,Praagh E.
A1 - Berthoin,S.
Y1 - 2014///

N1 - Baquet, Georges. Ridgers, Nicola D. Blaes, Aurelie. Aucouturier, Julien. Van Praagh, Emmanuel. Berthoin, Serge
BMC public health
100968562
IM
Journal Article
English
KW - MEDLINE
RP - NOT IN FILE
SP - 192
JF - BMC Public Health
VL - 14
IS - 1
CY - England
N2 - BACKGROUND: The school environment influences children's opportunities for physical activity participation. The aim of the present study was to assess objectively measured school recess physical activity in children from high and low socioeconomic backgrounds. METHODS: Four hundred and seven children (6-11 years old) from 4 primary schools located in high socioeconomic status (high-SES) and low socioeconomic status (low-SES) areas participated in the study. Children's physical activity was measured using accelerometry during morning and afternoon recess during a 4-day school week. The percentage of time spent in light, moderate, vigorous, very high and in moderate- to very high-intensity physical activity were calculated using age-dependent cut-points. Sedentary time was defined as 100 counts per minute. RESULTS: Boys were significantly (p<0.001) more active than girls. No difference in sedentary time between socioeconomic backgrounds was observed. The low-SES group spent significantly more time in light (p<0.001) and very high (p<0.05) intensity physical activity compared to the high-SES group. High-SES boys and girls spent significantly more time in moderate (p<0.001 and p<0.05, respectively) and vigorous (p<0.001) physical activity than low-SES boys. CONCLUSIONS: Differences were observed in recess physical activity levels according to socioeconomic background and sex. These results indicate that recess interventions should target children in low-SES schools
SN - 1471-2458
AD - University of Lille Nord de France, UDSL, EA 4488 "Physical Activity, Muscle Health", Lille, France. georges.baquet@univ-lille2.fr
UR - 24559254
ER -

TY - JOUR
ID - 36
T1 - Evaluating the effects of the Lunchtime Enjoyment Activity and Play (LEAP) school playground intervention on children's quality of life, enjoyment and participation in physical activity
A1 - Hyndman,B.P.
A1 - Benson,A.C.
A1 - Ullah,S.
A1 - Telford,A.
Y1 - 2014///
N1 - Hyndman, Brendon P. Benson, Amanda C. Ullah, Shahid. Telford, Amanda
BMC public health
100968562
IM
Journal Article
English
KW - MEDLINE
RP - NOT IN FILE
SP - 164
JF - BMC Public Health
VL - 14
IS - 1
CY - England

N2 - BACKGROUND: An emerging public health strategy is to enhance children's opportunities to be physically active during school break periods. The aim of this study was to evaluate the effects of the Lunchtime Enjoyment Activity and Play (LEAP) school playground intervention on primary school children's quality of life (QOL), enjoyment and participation in physical activity (PA). METHODS: This study consisted of a movable/recycled materials intervention that included baseline, a 7-week post-test and an 8-month follow-up data collection phase. Children within an intervention school (n=123) and a matched control school (n=152) aged 5-to-12-years-old were recruited for the study. Children's PA was measured using a combination of pedometers and direct observation (SOPLAY). Quality of life, enjoyment of PA and enjoyment of lunchtime activities were assessed in the 8-12 year children. A multi-level mixed effect linear regression model was applied in STATA (version 12.0) using the xtmixed command to fit linear mixed models to each of the variables to examine whether there was a significant difference (p<0.05) between the intervention and control school at the three time points (pre, post and follow-up). RESULTS: Significant overall interaction effects (group x time) were identified for children's mean steps and distance (pedometers) in the intervention school compared to the control school. Intervention school children also spent significantly higher proportions within specified target areas engaged in higher PA intensities in comparison to the control school at both the 7-week post-test and 8-month follow-up. A short-term treatment effect was revealed after 7-weeks for children's physical health scale QOL, enjoyment of PA and enjoyment of intra-personal play activities. CONCLUSIONS: Examining the effects of this school playground intervention over a school year suggested that the introduction of movable/recycled materials can have a significant, positive long-term intervention effect on children's PA. The implications from this simple, low-cost intervention provide impetus for schools to consider introducing the concept of a movable/recycled materials intervention on a wider scale within primary school settings. TRIAL REGISTRATION: Australian and New Zealand Clinical Trials Registration Number: ACTRN12613001155785
SN - 1471-2458
AD - Discipline of Exercise Sciences, School of Medical Sciences, RMIT University, Melbourne, Australia. brendon.hyndman@rmit.edu.au
UR - 24524375
ER -

TY - JOUR
ID - 37
T1 - Using accelerometers and global positioning system devices to assess gender and age differences in children's school, transport, leisure and home based physical activity
A1 - Klinker,C.D.
A1 - Schipperijn,J.
A1 - Christian,H.
A1 - Kerr,J.
A1 - Ersboll,A.K.
A1 - Troelsen,J.
Y1 - 2014///
N1 - Klinker, Charlotte D. Schipperijn, Jasper. Christian, Hayley. Kerr, Jacqueline. Ersboll, Annette K. Troelsen, Jens
The international journal of behavioral nutrition and physical activity
101217089
IM
Journal Article. Research Support, Non-U.S. Gov't
English
KW - MEDLINE
RP - NOT IN FILE
SP - 8
JF - International Journal of Behavioral Nutrition & Physical Activity
JA - Int
VL - 11
CY - England
N2 - BACKGROUND: Knowledge on domain-specific physical activity (PA) has the potential to advance public health interventions and inform new policies promoting children's PA. The purpose of this study is to identify and assess domains (leisure, school, transport, home) and subdomains (e.g., recess, playgrounds, and urban green space) for week day moderate to vigorous PA (MVPA) using objective measures and investigate

gender and age differences. METHODS: Participants included 367 Danish children and adolescents (11-16 years, 52% girls) with combined accelerometer and Global Positioning System (GPS) data (mean 2.5 days, 12.7 hrs/day). The Personal Activity and Location Measurement System and a purpose-built database assessed data in 15-second epochs to determine PA and assign epochs to 4 domains and 11 subdomains. Frequencies and proportions of time spent in MVPA were determined and differences assessed using multi-level modeling. RESULTS: More than 90% of MVPA was objectively assigned to domains/subdomains. Boys accumulated more MVPA overall, in leisure, school and transport (all p<0.05). Children compared with adolescents accumulated more MVPA, primarily through more school MVPA (p<0.05). Boys spent a large proportion of time accumulating MVPA in playgrounds, active transport, Physical Education, sports facilities, urban green space and school grounds. Girls spent a significant proportion of time accumulating MVPA in active transport and playgrounds. No gender or age differences were found in the home domain. CONCLUSIONS: Large variations were found in PA frequency and intensity across domains/subdomains. Significant gender differences were found, with girls being less active in almost all domains and subdomains. Objectively measured patterns of PA across domains/subdomains can be used to better tailor PA interventions and inform future policies for promoting child PA
SN - 1479-5868
AD - Department of Sports Science and Clinical Biomechanics, University of Southern Denmark, Campusvej 55, 5230 Odense M, Denmark. ckl@sdu.dk
UR - 24457029
ER -

TY - JOUR
ID - 38
T1 - Psychometrics of the preschooler physical activity parenting practices instrument among a Latino sample
A1 - O'Connor,T.M.
A1 - Cerin,E.
A1 - Hughes,S.O.
A1 - Robles,J.
A1 - Thompson,D.I.
A1 - Mendoza,J.A.
A1 - Baranowski,T.
A1 - Lee,R.E.
Y1 - 2014///
N1 - O'Connor, Teresia M. Cerin, Ester. Hughes, Sheryl O. Robles, Jessica. Thompson, Deborah I. Mendoza, Jason A. Baranowski, Tom. Lee, Rebecca E
The international journal of behavioral nutrition and physical activity
101217089
IM
Journal Article. Research Support, N.I.H., Extramural. Research Support, U.S. Gov't, Non-P.H.S.
English
KW - MEDLINE
RP - NOT IN FILE
SP - 3
JF - International Journal of Behavioral Nutrition & Physical Activity
JA - Int
VL - 11
CY - England
N2 - BACKGROUND: Latino preschoolers (3-5 year old children) have among the highest rates of obesity. Low levels of physical activity (PA) are a risk factor for obesity. Characterizing what Latino parents do to encourage or discourage their preschooler to be physically active can help inform interventions to increase their PA. The objective was therefore to develop and assess the psychometrics of a new instrument: the Preschooler Physical Activity Parenting Practices (PPAPP) among a Latino sample, to assess parenting practices used to encourage or discourage PA among preschool-aged children. METHODS: Cross-sectional study of 240 Latino parents who reported the frequency of using PA parenting practices. 95% of respondents were mothers; 42% had more than a high school education. Child mean age was 4.5 (+0.9) years (52% male). Test-retest reliability was assessed in 20%, 2 weeks later. We assessed the fit of a priori models using Confirmatory factor analyses (CFA). In a separate sub-sample (35%), preschool-aged children wore accelerometers to assess associations

with their PA and PPAPP subscales. RESULTS: The a-priori models showed poor fit to the data. A modified factor structure for encouraging PPAPP had one multiple-item scale: engagement (15 items), and two single-items (have outdoor toys; not enroll in sport-reverse coded). The final factor structure for discouraging PPAPP had 4 subscales: promote inactive transport (3 items), promote screen time (3 items), psychological control (4 items) and restricting for safety (4 items). Test-retest reliability (ICC) for the two scales ranged from 0.56-0.85. Cronbach's alphas ranged from 0.5-0.9. Several sub-factors correlated in the expected direction with children's objectively measured PA. CONCLUSION: The final models for encouraging and discouraging PPAPP had moderate to good fit, with moderate to excellent test-retest reliabilities. The PPAPP should be further evaluated to better assess its associations with children's PA and offers a new tool for measuring PPAPP among Latino families with preschool-aged children
SN - 1479-5868
AD - USDA/ARS Children's Nutrition Research Center, Department of Pediatrics, Baylor College of Medicine, 1100 Bates St, Houston, TX 77030, USA. teresiao@bcm.edu
UR - 24428935
ER -

TY - JOUR
ID - 39
T1 - Evaluation of tourists' UV exposure in Paris
A1 - Mahe,E.
A1 - Correa,M.P.
A1 - Godin-Beekmann,S.
A1 - Haeffelin,M.
A1 - Jegou,F.
A1 - Saiag,P.
A1 - Beauchet,A.
Y1 - 2013/03//
N1 - Mahe, E. Correa, M P. Godin-Beekmann, S. Haeffelin, M. Jegou, F. Saiag, P. Beauchet, A
Journal of the European Academy of Dermatology and Venereology : JEADV
c2r, 9216037
IM
Journal Article. Research Support, Non-U.S. Gov't
English
KW - MEDLINE
RP - NOT IN FILE
SP - e294
EP - e304
JF - Journal of the European Academy of Dermatology & Venereology
JA - J Eur Acad Dermatol Venereol
VL - 27
IS - 3
CY - England
N2 - BACKGROUND: Ultraviolet (UV) exposure is one of the most important risk factor for skin cancers. If UV hazard has been evaluated in tropical countries or in some population - children, outdoor activities - little information is available about UV hazard in high latitude towns like Paris, considered as the most 'charismatic city' in the world. OBJECTIVE: To evaluate UV exposure in Paris in spring, in sun and shade, in real life conditions. METHODS: We evaluated erythemal UV exposure, during four sunny days in May-June in eight Paris touristic sites during peak hours (2 days), and during two walks in touristic downtown of Paris. Measures were performed in sun and shade. UV radiation exposure was evaluated with UV index performed with a 'Solarmeter ultraviolet index (UVI)' and UV dose with 'standard erythema dose' (SED) and 'minimal erythema dose' (MED) calculations. RESULTS: Despite 'average' UVI in sunny conditions, a 4-h sun exposure reaches 13-20 SED and 3-10 MED according to phototype. Clouds were inefficient to protect against UV. Shade of places reduces moderately UVI (50-60%) in forecourts. Exposure during 1-h walk reach at least one MED in real life conditions for skin phototypes I-IV. CONCLUSIONS: UV risk for tourist is quite high in spring in Paris. UVI remains high despite high cloud fraction. Shade reduces UVI, but UV protection factor is only 2-3 in large places such as Place Notre Dame and Place Charles de Gaulle. So sun protection campaigns should be proposed, and sun protective strategies could be integrated in urban planning. 2012 The Authors. Journal of the

European Academy of Dermatology and Venereology 2012 European Academy of Dermatology and Venereology
SN - 1468-3083
AD - Research Unit EA 4339 'Skin, cancer, and environment', Ambroise Pare University Hospital, University of Versailles-Saint Quentin en Yvelines, France Department of Dermatology, Ambroise Pare University Hospital, University of Versailles-Saint Quentin en Yvelines, Assistance Publique-Hopitaux de Paris, Boulogne-Billancourt, France Laboratoire Atmospheres, Milieux, Observations Spatiales, Service d'Aeronomie (LATMOS), CNRS, Institut Pierre Simon Laplace, Pierre et Marie Curie University, Paris, France Natural Resources Institute, Federal University of Itajuba, Itajuba, Brazil Site Instrumental de Recherche par Teledetection Atmospherique (SIRTA), Institut Pierre Simon Laplace, Ecole Polytechnique, University of Versailles-Saint Quentin en Yvelines, Palaiseau, France Department of Public Health, Ambroise Pare University Hospital, University of Versailles-Saint Quentin en Yvelines, Assistance Publique-Hopitaux de Paris, Boulogne-Billancourt, France
UR - 22775064
ER -

TY - JOUR
ID - 40
T1 - Mortality and Long-term Virologic Outcomes in Children and Infants Treated With Lopinavir/Ritonavir
A1 - Estripeaut,D.
A1 - Mosser,J.
A1 - Doherty,M.
A1 - Acosta,W.
A1 - Shah,H.
A1 - Castano,E.
A1 - Luciani,K.
A1 - Pascale,J.M.
A1 - Bollinger,R.C.
A1 - Page,K.R.
Y1 - 2013/12//
N1 - Estripeaut, Dora. Mosser, Jon. Doherty, Meg. Acosta, William. Shah, Harita. Castano, Elizabeth. Luciani, Kathia. Pascale, Juan Miguel. Bollinger, Robert C. Page, Kathleen R
The Pediatric infectious disease journal
oxj, 8701858
IM
Journal Article
English
KW - MEDLINE
RP - NOT IN FILE
SP - e466
EP - e472
JF - Pediatric Infectious Disease Journal
JA - Pediatr Infect Dis J
VL - 32
IS - 12
CY - United States
N2 - BACKGROUND: There is scant data on young children receiving protease inhibitor-based therapy in real-life resource-limited settings and on the optimal timing of therapy among children who survive infancy. Our aim was to evaluate outcomes at the Hospital del Nino, Panama, where children have been routinely treated with lopinavir/ritonavir (LPV/r)-based therapy since 2002. METHODS: Retrospective cohort analysis of all HIV-infected children enrolled in care between January 1, 1991, and June 1, 2011. Kaplan-Meier method and Cox proportional hazards regression were used to evaluate death, virologic suppression and virologic rebound. RESULTS: Of 399 children contributing 1944 person-years of follow-up, 254 (63.7%) were treated with LPV/r and 94 (23.6%) were never treated with antiretrovirals (ARVs). Among infants, improved survival was associated with male gender (hazard rate of death[HRdeath] 0.54, 95% confidence interval [CI]: 0.32-0.92) and treatment with highly active antiretroviral therapy (HRdeath 0.32, 95% CI: 0.12-0.83), whereas residence outside of Panama City was associated with poorer survival (HRdeath 1.72, 95% CI: 1.01-2.94). Among

children who survived to 1 year of age without exposure to ARVs, LPV/r-based therapy improved survival (HRdeath 0.07, 95% CI: 0.01-0.33). Virologic suppression was achieved in 42.1%, 70.5% and 85.1% by 12, 24 and 60 months of follow-up among children treated with LPV/r. Virologic suppression was not associated with prior ARV exposure or age at initiation of therapy but was associated with residence outside of Panama City (HR suppression 1.93, 95% CI: 1.19-3.14). Patients with a baseline viral load >100,000 copies/mL were less likely to achieve suppression (HR suppression 0.37, 95% CI: 0.21-0.66). No children who achieved virologic suppression after initiating LPV/r died. CONCLUSIONS: LPV/r-based therapy improved survival not only in infants but also in children over 1 year of age. Age at initiation of LPV/r-based therapy or prior ARVs did not impact virologic outcomes
SN - 1532-0987
AD - From the *Department of Pediatrics, Hospital del Nino, Panama; +Johns Hopkins University School of Medicine; ++Department of Medicine, Johns Hopkins University School of Medicine, Baltimore, MD; and Instituto Conmemorativo Gorgas de Estudios de Salud, Panama
UR - 23799516
ER -

TY - JOUR
ID - 41
T1 - Utilization patterns and perceptions of playground users in new york city
A1 - Silver,D.
A1 - Giorgio,M.
A1 - Mijanovich,T.
Y1 - 2014/04//
N1 - Silver, Diana. Giorgio, Maggie. Mijanovich, Tod
Journal of community health
7600747, hut
IM
Journal Article
English
KW - MEDLINE
RP - NOT IN FILE
SP - 363
EP - 371
JF - Journal of Community Health
JA - J Community Health
VL - 39
IS - 2
CY - Netherlands
N2 - Playgrounds are assumed to be an important resource for physical activity. This study investigates seasonal utilization, user preferences, and perceptions of safety and upkeep of public playgrounds in New York City. A cross-sectional survey was conducted from May 2010 to January 2011 across 10 playgrounds in low/middle income neighborhoods in each of the five boroughs in New York City. A total of 1,396 adults accompanying children were surveyed. Outcomes included playground as main place of outdoor play, and perceptions of playground upkeep and safety. Covariates included socio-demographics and other characteristics of playground users. Multivariable logistic regression with playground/season fixed effects were used. Utilization varied substantially across the four seasons. Blacks had higher odds of reporting the playground as the main place of outdoor play (AOR 1.78, 95 % CI 1.13-2.80, p < .05). High income users had lower odds of reporting the playground as the main place of outdoor play ($60-$80,000: AOR 0.47, 95 % CI 0.29-0.76, p < .01, $80,000+: AOR 0.47, 95 % CI 0.28-0.79, p < .01). Racial differences in perceived upkeep and safety were not significant once playground/season fixed effects were included, highlighting the importance of neighborhood conditions. Women were more likely to report feeling unsafe within playgrounds (AOR 1.51, 95 % CI 1.12-2.02, p < .01). While some playground utilization is driven by individual characteristics, perceptions of public resources influences utilization and cannot be separated from neighborhood conditions. Increasing access to opportunities for physical activity for children requires new strategies beyond playground improvements
SN - 1573-3610

AD - Department of Nutrition, Food Studies and Public Health, Steinhardt School of Culture, Education and Human Development, New York University, 411 Lafayette, 5th Floor, New York, NY, 10003, USA, diana.silver@nyu.edu
UR - 24114313
ER -

TY - JOUR
ID - 42
T1 - Environmental circumstances influencing tic expression in children
A1 - Caurin,B.
A1 - Serrano,M.
A1 - Fernandez-Alvarez,E.
A1 - Campistol,J.
A1 - Perez-Duenas,B.
Y1 - 2014/03//
N1 - Caurin, Belen. Serrano, Mercedes. Fernandez-Alvarez, Emilio. Campistol, Jaume. Perez-Duenas, Belen
European journal of paediatric neurology : EJPN : official journal of the European Paediatric Neurology Society
9715169, dgs
IM
Journal Article
English
KW - MEDLINE
RP - NOT IN FILE
SP - 157
EP - 162
JF - European Journal of Paediatric Neurology
JA - Europ.J Paediatr Neurol
VL - 18
IS - 2
CY - England
N2 - AIM: To assess the clinical features and severity of tics and environmental factors influencing tic expression in a cohort of children with tic disorders. METHODS: We performed a cross-sectional study in a cohort of children and adolescents (N = 92) with tic disorders referred to the outpatient clinic of a tertiary-level paediatric centre in Barcelona. The severity of tics was evaluated using the Yale Global Tic Severity Scale (YGTSS). A questionnaire including a list of environmental factors and common daily activities that might influence tic occurrence was completed for patients greater than 5 years old. RESULTS: Children were classified as having Tourette syndrome (TS) (52 patients), chronic motor or phonic tics (22 patients) and tics of less than 12 months' duration (18 patients). Tics worsened with stressful situations, activities related to school, playing video games and watching TV. A significant proportion of children reported a reduction in tics while they were concentrating on artistic or creative activities or when playing sports and participating in outdoor activities. The YGTSS scores were higher for TS patients ($P < .001$) and correlated positively with the time of evolution of tics ($r = .273$, $P = .026$). Poor school performance was associated with TS ($p = .043$) and higher scores on the YGTSS ($P = .018$), as well as attention deficit/hyperactivity disorder ($P = .007$). CONCLUSIONS: Several activities of daily living were identified as modifying tic severity in children and may be important clues for tic management. In a subgroup of children with TS, tics were associated with significant morbidity and poor academic performance. Our results emphasise the importance of developing specific school programmes and tailored recommendations in patients with TS. Copyright 2013 European Paediatric Neurology Society. Published by Elsevier Ltd. All rights reserved
SN - 1532-2130
AD - Department of Child Neurology, Hospital Sant Joan de Deu, Universitat de Barcelona, Spain., Department of Child Neurology, Hospital Sant Joan de Deu, Universitat de Barcelona, Spain; Centre for Biomedical Research on Rare Diseases (CIBER-ER), Instituto de Salud Carlos III, Barcelona, Spain., Department of Child Neurology, Hospital Sant Joan de Deu, Universitat de Barcelona, Spain., Department of Child Neurology, Hospital Sant Joan de Deu, Universitat de Barcelona, Spain; Centre for Biomedical Research on Rare Diseases (CIBER-ER), Instituto de Salud Carlos III, Barcelona, Spain., Department of Child Neurology, Hospital Sant Joan de Deu, Universitat de Barcelona, Spain; Centre for Biomedical Research on

Rare Diseases (CIBER-ER), Instituto de Salud Carlos III, Barcelona, Spain. Electronic address: bperez@hsjdbcn.org
UR - 24210363
ER -

TY - JOUR
ID - 43
T1 - Assessing human exposure to aluminium, chromium and vanadium through outdoor dust ingestion in the Bassin Minier de Provence, France
A1 - Reis,A.P.
A1 - Patinha,C.
A1 - Noack,Y.
A1 - Robert,S.
A1 - Dias,A.C.
Y1 - 2014/04//
N1 - Reis, A P. Patinha, C. Noack, Y. Robert, S. Dias, A C
Environmental geochemistry and health
8903118
IM
Journal Article
English
KW - MEDLINE
RP - NOT IN FILE
SP - 303
EP - 317
JF - Environmental Geochemistry & Health
JA - Environ Geochem.Health
VL - 36
IS - 2
CY - Netherlands
N2 - The Western part of the "Bassin Minier de Provence", a former coal mining area, is still occupied by old polluting industries such as a coal-fired power plant and an alumina factory. The identified pollution sources that raise more concern in the population are the emission of gases and dusts, as well as the storage of raw and transformed materials. In 2011, a preliminary survey was carried out in the area as the first step to an exposure and health risk-assessment study. This first survey intends to assess human exposure through ingestion and health risk associated with potentially harmful elements (PHEs) in ground-level dusts collected in recreational areas used by children. Dust samples were taken at 19 sites distributed across the study area, depending on the location of public parks, public gardens, playgrounds and schools. Pseudo-total concentrations of 53 elements were determined by ICP-MS. Bioaccessible concentrations were estimated using the unified bioaccessibility method. This study presents the results obtained for Al, V and Cr, which seem to be related with industry and show similar distribution patterns. PHEs presumably related to traffic or other urban pollution sources are not discussed in this study. The highest total concentrations occur in dusts near the alumina plant that have significant amounts of Al mineral phases (gibbsite and alumina). However, in these dusts only small fractions of the elements under study are in bioaccessible forms. The highest bioaccessible fractions occur in dusts collected near the coal-fired power plant. Further investigation is required to assess potential pathways of exposure and health risk in this area
SN - 1573-2983
AD - GEOBIOTEC, University of Aveiro, Campus Universitario de Santiago, 3810-193, Aveiro, Portugal, pmarinho@ua.pt
UR - 23990126
ER -

TY - JOUR
ID - 44
T1 - Sex risk behavior among adolescent and young adult children of opiate addicts: outcomes from the focus on families prevention trial and an examination of childhood and concurrent predictors of sex risk behavior
A1 - Skinner,M.L.

A1 - Fleming,C.B.
A1 - Haggerty,K.P.
A1 - Catalano,R.F.
Y1 - 2014/02//
N1 - Skinner, Martie L. Fleming, Charles B. Haggerty, Kevin P. Catalano, Richard F
Prevention science : the official journal of the Society for Prevention Research
d4q, 100894724
IM
Journal Article
English
KW - MEDLINE
RP - NOT IN FILE
SP - 70
EP - 77
JF - Prevention Science
JA - Prev Sci
VL - 15 Suppl 1
CY - United States
N2 - This study reports on rates and predictors of sex risk behavior among a sample of adolescent and young adult children of parents enrolled in methadone treatment for opiate addiction. Data are from 151 participants (80 males, 71 females) in the Focus on Families (FOF) project, a randomized trial of a family intervention and a study of the development of at-risk children. The study participants are children of parents enrolled in methadone treatment between 1990 and 1993. Participants were interviewed in 2005 when they ranged in age from 15 to 29 years. In the year prior to the follow-up, 79 % of the males and 83 % of females were sexually active, 26 % of males and 10 % of females had more than one partner in the prior year, and 34 % of males and 24 % of females reported having sex outside of a committed relationship. Twenty-four percent of males and 17 % of females met criteria for high-risk sexual behavior, reporting casual or multiple partners in the prior year and inconsistent condom use. Participants in the intervention and control conditions did not differ significantly in terms of any measure of sex risk behavior examined. None of the measures of parent behavior and family processes derived from data at baseline of the FOF study predicted whether participants engaged in high-risk sex. Among measures derived from data collected at long-term follow-up, however, having ever met criteria for substance abuse or dependence predicted greater likelihood of high-risk sexual behavior, and being married or being in a romantic relationship was associated with lower likelihood of high-risk sexual behavior. The findings point to the important role of committed relationships in regulating sex risk behavior among this population, as well as heightened levels of sex risk behavior associated with substance abuse or dependence
SN - 1573-6695
AD - Social Development Research Group, School of Social Work, University of Washington, Seattle, WA, USA, skinnm@u.washington.edu
UR - 23404659
ER -

TY - JOUR
ID - 45
T1 - [Chest computed tomography in children: Indications, efficiency and effective dose]. [French]
A1 - Mordacq,C.
A1 - Deschildre,A.
A1 - Petyt,L.
A1 - Santangelo,T.
A1 - Delvart,C.
A1 - Doan,C.
A1 - Thumerelle,C.
Y1 - 2014/03//
N1 - Mordacq, C. Deschildre, A. Petyt, L. Santangelo, T. Delvart, C. Doan, C. Thumerelle, C
Archives de pediatrie : organe officiel de la Societe francaise de pediatrie
9421356, bwh
IM

English Abstract. Journal Article
French
KW - MEDLINE
RP - NOT IN FILE
SP - 279
EP - 286
JF - Archives de Pediatrie
JA - Arch Pediatr
VL - 21
IS - 3
CY - France
N2 - INTRODUCTION: New multidetector row computed tomography (CT) has made the imaging of younger children more feasible and extending CT indications to a wide range of pediatric respiratory diseases in the last few years. However, CT is a source of radiation exposure. The aim of this study was to evaluate the main indications and the contribution of chest CT in pediatric pulmonology as well as induced radiation. METHODS: This was an observational, prospective study. Children whose chest CTs were analyzed during multidisciplinary meetings (radiologist, pulmonary pediatrician) were included from November 2009 to April 2010. We collected demographic data, CT results, contribution of CT to diagnosis and management, and radiation doses (dose-length product [DLP] and effective dose). Radiation doses were compared according to the CT scans (Lille University Hospital with 128-slice dual-source CT or Lille University Hospital single-source 64-slice CT, or CT performed outside the university hospital). RESULTS: One hundred thirty-five patients were included. The mean age was 6.4 years old. The main indications were analysis of bronchial disease (44%), infectious disease (16%), interstitial disease (14%), or a malformation (9%). The aim of CT was diagnosis (61%) or follow-up of previous lung diseases (39%). Diagnosis chest-CT directly contributed to diagnosis in 48% of cases and to treatment in 24%. Follow-up CT contributed to diagnosis in 38% and treatment in 19% of cases. DLP and effective doses were significantly lower for CT performed in the university hospital, especially with the 128-slice CT compared to the others (P<0.001). The effective doses were: 128-slice CT, 0.61mSv+0.32; 64-slice CT, 1.24mSv+0.97; outside university hospital, 2.56mSv+1.98. CONCLUSION: This study confirms the role played by chest CT in children, which contributes to diagnosis and management of lung diseases. The main concern of CT application, especially in children, is the radiation burden. Children are more susceptible to the effects of radiation than adults and have a longer life expectancy to develop complications. Both radiologists and pediatricians should be aware of a potential risk and have to conjugate their efforts in reducing this risk. The wide range of radiation doses in this study for the same CT procedures underlines the extensive efforts still needed to limit radiation exposure in children. Copyright 2014 Elsevier Masson SAS. All rights reserved
SN - 1769-664X
AD - Service de pneumologie et allergologie pediatriques, hopital Jeanne-de-Flandre, CHRU de Lille, avenue Eugene-Avinee, 59037 Lille cedex, France. Electronic address: clemence.mordacq@chru-lille.fr., Service de pneumologie et allergologie pediatriques, hopital Jeanne-de-Flandre, CHRU de Lille, avenue Eugene-Avinee, 59037 Lille cedex, France., Service de radiologie pediatrique, hopital Jeanne-de-Flandre, CHRU de Lille, avenue Eugene-Avinee, 59037 Lille cedex, France., Service de radiologie thoracique, hopital Calmette, CHRU de Lille, boulevard du Pr-Leclercq, 59037 Lille cedex, France., Service de pneumologie et allergologie pediatriques, hopital Jeanne-de-Flandre, CHRU de Lille, avenue Eugene-Avinee, 59037 Lille cedex, France., Service de pediatrie, hopital Victor-Provo, 37, rue de Barbieu, 59056 Roubaix, France., Service de pneumologie et allergologie pediatriques, hopital Jeanne-de-Flandre, CHRU de Lille, avenue Eugene-Avinee, 59037 Lille cedex, France
UR - 24485863
ER -

TY - JOUR
ID - 46
T1 - Walking and child pedestrian injury: a systematic review of built environment correlates of safe walking
A1 - Rothman,L.
A1 - Buliung,R.
A1 - Macarthur,C.
A1 - To,T.
A1 - Howard,A.
Y1 - 2014/02//

N1 - Rothman, Linda. Buliung, Ron. Macarthur, Colin. To, Teresa. Howard, Andrew
Injury prevention : journal of the International Society for Child and Adolescent Injury Prevention
crz, 9510056
IM
Journal Article. Research Support, Non-U.S. Gov't
English
KW - MEDLINE
RP - NOT IN FILE
SP - 41
EP - 49
JF - Injury Prevention
JA - Inj Prev
VL - 20
IS - 1
CY - England
N2 - BACKGROUND: The child active transportation literature has focused on walking, with little attention to risk associated with increased traffic exposure. This paper reviews the literature related to built environment correlates of walking and pedestrian injury in children together, to broaden the current conceptualization of walkability to include injury prevention. METHODS: Two independent searches were conducted focused on walking in children and child pedestrian injury within nine electronic databases until March, 2012. Studies were included which: 1) were quantitative 2) set in motorized countries 3) were either urban or suburban 4) investigated specific built environment risk factors 5) had outcomes of either walking in children and/or child pedestrian roadway collisions (ages 0-12). Built environment features were categorized according to those related to density, land use diversity or roadway design. Results were cross-tabulated to identify how built environment features associate with walking and injury. RESULTS: Fifty walking and 35 child pedestrian injury studies were identified. Only traffic calming and presence of playgrounds/recreation areas were consistently associated with more walking and less pedestrian injury. Several built environment features were associated with more walking, but with increased injury. Many features had inconsistent results or had not been investigated for either outcome. CONCLUSIONS: The findings emphasise the importance of incorporating safety into the conversation about creating more walkable cities
SN - 1475-5785
AD - Department of Child Health Evaluative Science, The Hospital for Sick Children, , Toronto, Ontario, Canada
UR - 23710061
ER -

TY - JOUR
ID - 47
T1 - Physical activity, leisure-time screen use and depression among children and young adolescents
A1 - Kremer,P.
A1 - Elshaug,C.
A1 - Leslie,E.
A1 - Toumbourou,J.W.
A1 - Patton,G.C.
A1 - Williams,J.
Y1 - 2014/03//
N1 - Kremer, Peter. Elshaug, Christine. Leslie, Eva. Toumbourou, John W. Patton, George C. Williams, Joanne
Journal of science and medicine in sport / Sports Medicine Australia
c54, 9812598
IM
Journal Article
English
KW - MEDLINE
RP - NOT IN FILE
SP - 183
EP - 187
JF - Journal of Science & Medicine in Sport

JA - J Sci Med Sport
VL - 17
IS - 2
CY - Australia
N2 - OBJECTIVES: Adolescent mental disorders remain a relatively neglected area of research, despite evidence that these conditions affect youth disproportionately. We examined associations between physical activity, leisure-time screen use and depressive symptoms among Australian children and adolescents. DESIGN: Large cross-sectional observational study. METHODS: Self-reported physical activity and leisure-time screen behaviours, and depressive symptoms using the Short Mood and Feeling Questionnaire were assessed in 8256 students aged 10-16 years (mean age=11.5 years, SD=0.8). RESULTS: Thirty three percent of the sample reported moderate to high depressive symptoms, with rates higher among females (OR=1.18; 95% CI: 1.02, 1.36; p=0.001). Increased opportunities to be active at school outside class (OR=0.70; 0.58, 0.85; p<0.001), being active in physical education classes (OR=0.77; 0.69, 0.86; p<0.001), greater involvement in sports teams at school (OR=0.77; 0.67, 0.88; p<0.001) and outside of school (OR=0.84; 0.73, 0.96; p=0.01) were all independently associated with lower odds for depressive symptoms. Meeting recommended guidelines for physical activity (OR=0.62; 0.44, 0.88; p=0.007) and, for 12-14 year olds, leisure-time screen use (OR=0.77; 0.59, 0.99; p=0.04) were also independently associated with lower odds for depressive symptoms. CONCLUSIONS: Higher levels of physical activity among children and young adolescents, and lower levels of leisure-time screen use among young adolescents, are associated with lower depressive symptoms. Longitudinal studies are needed to understand the causal relationships between these variables. Crown Copyright 2013. Published by Elsevier Ltd. All rights reserved
SN - 1878-1861
AD - School of Exercise and Nutrition Sciences, Deakin University, Australia. Electronic address: peter.kremer@deakin.edu.au., School of Psychology, Deakin University, Australia., Department of Nutrition and Dietetics, School of Medicine, Flinders University, Australia., School of Psychology and Centre for Mental Health and Wellbeing Research, Deakin University, Australia., Centre for Adolescent Health, Royal Children's Hospital, Australia; Murdoch Children's Research Institute, Australia; Department of Paediatrics, The University of Melbourne, Australia., School of Psychology, Deakin University, Australia; Centre for Adolescent Health, Royal Children's Hospital, Australia; Murdoch Children's Research Institute, Australia; Department of Paediatrics, The University of Melbourne, Australia
UR - 23648221
ER -

TY - JOUR
ID - 48
T1 - Airway effects of traffic-related air pollution on outdoor workers
A1 - Choudhary,H.
A1 - Tarlo,S.M.
Y1 - 2014/04//
N1 - Choudhary, Habiba. Tarlo, Susan M
Current opinion in allergy and clinical immunology
100936359
IM
Journal Article
English
KW - MEDLINE
RP - NOT IN FILE
SP - 106
EP - 112
JF - Current Opinion in Allergy & Clinical Immunology
JA - Curr Opin Allergy Clin Immunol
VL - 14
IS - 2
CY - United States
N2 - PURPOSE OF REVIEW: Traffic-related air pollution has been well documented to be associated with increased risks of airway diseases in the general population. Particularly, susceptible subgroups have included children and endurance athletes exercising outdoors. Relatively little has been published on the risks for outdoor

workers. The purpose of this review is to identify the relative airway risks of outdoor work in areas with increased vehicular air pollution. RECENT FINDINGS: We found a small body of recent literature published on this topic. Most of the relevant studies have focused on traffic police, petrol pump workers, and highway workers, especially in the urban areas of high traffic pollution. These studies suggest increases in respiratory symptoms and reduction in spirometric indices in nonsmoking workers in these occupations when compared with control individuals. Research is needed to investigate the relationship with duration of exposures in these workers, and to determine the duration and reversibility of effects in order to develop standards for safe working exposures. SUMMARY: A significant impact on airway function and respiratory symptoms has been reported from outdoor work with exposure to traffic pollution. Further research is needed to confirm these associations and to develop standards for safe outdoor work in urban settings
SN - 1473-6322
AD - aInstitute of Medical Sciences, University of Toronto bToronto Western Hospital, St Michael's Hospital cDepartment of Medicine, Dalla Lana School of Public Health, University of Toronto, Toronto, Ontario, Canada
UR - 24451913
ER -

TY - JOUR
ID - 49
T1 - Associations between children's independent mobility and physical activity
A1 - Schoeppe,S.
A1 - Duncan,M.J.
A1 - Badland,H.M.
A1 - Oliver,M.
A1 - Browne,M.
Y1 - 2014///
N1 - Schoeppe, Stephanie. Duncan, Mitch J. Badland, Hannah M. Oliver, Melody. Browne, Matthew
BMC public health
100968562
IM
Journal Article
English
KW - MEDLINE
RP - NOT IN FILE
SP - 91
JF - BMC Public Health
VL - 14
IS - 1
CY - England
N2 - BACKGROUND: Independent mobility describes the freedom of children to travel and play in public spaces without adult supervision. The potential benefits for children are significant such as social interactions with peers, spatial and traffic safety skills and increased physical activity. Yet, the health benefits of independent mobility, particularly on physical activity accumulation, are largely unexplored. This study aimed to investigate associations of children's independent mobility with light, moderate-to-vigorous, and total physical activity accumulation. METHODS: In 2011 - 2012, 375 Australian children aged 8-13 years (62% girls) were recruited into a cross-sectional study. Children's independent mobility (i.e. independent travel to school and non-school destinations, independent outdoor play) and socio-demographics were assessed through child and parent surveys. Physical activity intensity was measured objectively through an Actiheart monitor worn on four consecutive days. Associations between independent mobility and physical activity variables were analysed using generalized linear models, accounting for clustered sampling, Actiheart wear time, socio-demographics, and assessing interactions by sex. RESULTS: Independent travel (walking, cycling, public transport) to school and non-school destinations were not associated with light, moderate-to-vigorous and total physical activity. However, sub-analyses revealed a positive association between independent walking and cycling (excluding public transport) to school and total physical but only in boys (b=36.03, p<0.05). Frequent independent outdoor play (three or more days per week) was positively associated with light and total physical activity (b=29.76, p<0.01 and b=32.43, p=0.03, respectively). No significant associations were found between independent outdoor play and moderate-to-vigorous physical activity. When assessing differences by sex, the observed significant associations of independent outdoor play with light and total physical activity remained in

girls but not in boys. All other associations showed no significant differences by sex. CONCLUSIONS: Independent outdoor play may boost children's daily physical activity levels, predominantly at light intensity. Hence, facilitating independent outdoor play could be a viable intervention strategy to enhance physical activity in children, particularly in girls. Associations between independent travel and physical activity are inconsistent overall and require further investigation
SN - 1471-2458
AD - Central Queensland University, School of Human, Health and Social Sciences, Centre for Physical Activity Studies, Building 18, Bruce Highway, Rockhampton QLD 4702, Australia. s.schoeppe@cqu.edu.au
UR - 24476363
ER -

TY - JOUR
ID - 50
T1 - Rationale and design of active play @ home: a parent-led physical activity program for children with and without disability
A1 - Rubin,D.A.
A1 - Wilson,K.S.
A1 - Wiersma,L.D.
A1 - Weiss,J.W.
A1 - Rose,D.J.
Y1 - 2014///
N1 - Rubin, Daniela A. Wilson, Kathleen S. Wiersma, Lenny D. Weiss, Jie W. Rose, Debra J
BMC pediatrics
100967804
IM
Journal Article
English
KW - MEDLINE
RP - NOT IN FILE
SP - 41
JF - BMC Pediatrics
JA - BMC Pediatr
VL - 14
IS - 1
CY - England
N2 - BACKGROUND: Compared to other children, those with disability have additional challenges to being physically active. Prader-Willi Syndrome is a genetic form of childhood obesity that is characterized by hypotonia, growth hormone deficiency, behavioral, and cognitive disability. In children, the low prevalence of this syndrome (1 in 10,000 to 15,000 live births) makes group-based physical activity interventions difficult. In contrast, the home environment presents a natural venue to establish a physical activity routine for this population. This manuscript describes the design of a parent-led physical activity intervention incorporating playground and interactive console-based games to increase physical activity participation in youth with and without Prader-Willi Syndrome. METHODS/DESIGN: The study participants will be 115 youth ages 8-15 y (45 with the syndrome and 70 without the syndrome but categorized as obese). The study will use a parallel design with the control group receiving the intervention after serving as control. Participants will be expected to complete a physical activity curriculum 4 days a week for 6 months including playground games 2 days a week and interactive console games 2 days a week. Parents will be trained at baseline and then provided with a curriculum and equipment to guide their implementation of the program. Tips related to scheduling and coping with barriers to daily program implementation will be provided. Throughout, parents will be contacted by phone once a week (weeks 1-4) and then every other week to receive support in between visits. Measurements of children and parents will be obtained at baseline, 12 weeks, and at the end (week 24) of the intervention. Children main outcomes include physical activity (accelerometry), body composition (dual x-ray absorptiometry), motor proficiency (Bruininks-Oseretsky Test of Motor Proficiency), quality of life and physical activity self-efficacy (questionnaires). Intervention compliance will be monitored using mail-in daily self-report checklists. DISCUSSION: This parent-guided physical activity intervention aims to increase physical activity by using a curriculum that builds physical activity related self-confidence through the development and/or enhancement of motor skill competency. Ultimately, helping children develop these skills as well as joy

in being physically active will translate into sustained behavior change. TRIAL REGISTRATION: Current Controlled Trial: NCT02058342
SN - 1471-2431
AD - Department of Kinesiology, California State University, 800 N, State College Blvd,, KHS-138, Fullerton, CA 92834, USA. drubin@fullerton.edu
UR - 24529259
ER -

TY - JOUR
ID - 51
T1 - Eye exercises of acupoints: their impact on refractive error and visual symptoms in Chinese urban children
A1 - Lin,Z.
A1 - Vasudevan,B.
A1 - Jhanji,V.
A1 - Gao,T.Y.
A1 - Wang,N.L.
A1 - Wang,Q.
A1 - Wang,J.
A1 - Ciuffreda,K.J.
A1 - Liang,Y.B.
Y1 - 2013///
N1 - Lin, Zhong. Vasudevan, Balamurali. Jhanji, Vishal. Gao, Tie Ying. Wang, Ning Li. Wang, Qi. Wang, Ji. Ciuffreda, Kenneth J. Liang, Yuan Bo
BMC complementary and alternative medicine
101088661
IM
Journal Article. Research Support, Non-U.S. Gov't
English
KW - MEDLINE
RP - NOT IN FILE
SP - 306
JF - BMC Complementary & Alternative Medicine
JA - BMC Altern Med
VL - 13
CY - England
N2 - BACKGROUND: Traditional Chinese eye exercises of acupoints involve acupoint self-massage. These have been advocated as a compulsory measure to reduce ocular fatigue, as well as to retard the development of myopia, among Chinese school children. This study evaluated the impact of these eye exercises among Chinese urban children. METHODS: 409 children (195 males, 47.7%), aged 11.1+3.2 (range 6-17) years, from the Beijing Myopia Progression Study (BMPS) were recruited. All had completed the eye exercise questionnaire, the convergence insufficiency symptom survey (CISS), and a cycloplegic autorefraction. Among these, 395 (96.6%) performed the eye exercises of acupoints. Multiple logistic regressions for myopia and multiple linear regressions for the CISS score (after adjusting for age, gender, average parental refractive error, and time spent doing near work and outdoor activity) for the different items of the eye exercises questionnaire were performed. RESULTS: Only the univariate odds ratio (95% confidence interval) for "seriousness of attitude" towards performing the eye exercises of acupoints (0.51, 0.33-0.78) showed a protective effect towards myopia. However, none of the odds ratios were significant after adjusting for the confounding factors. The univariate and multiple beta coefficients for the CISS score were -2.47 (p=0.002) and -1.65 (p=0.039), -3.57 (p=0.002) and -2.35 (p=0.042), and -2.40 (p=0.003) and -2.29 (p=0.004), for attitude, speed of exercise, and acquaintance with acupoints, respectively, which were all significant. CONCLUSIONS: The traditional Chinese eye exercises of acupoints appeared to have a modest effect on relieving near vision symptoms among Chinese urban children aged 6 to 17 years. However, no remarkable effect on reducing myopia was observed
SN - 1472-6882
AD - The Affiliated Eye Hospital, School of Optometry and Ophthalmology, Wenzhou Medical University, 270 West College Road, Wenzhou 325027, Zhejiang, China. yuanboliang@126.com
UR - 24195652
ER -

TY - JOUR
ID - 52
T1 - How to motivate childcare workers to engage preschoolers in physical activity
A1 - Gagne,C.
A1 - Harnois,I.
Y1 - 2014/02//
N1 - Gagne, Camille. Harnois, Isabelle
Journal of physical activity & health
101189457
IM
Journal Article
English
KW - MEDLINE
RP - NOT IN FILE
SP - 364
EP - 374
JF - Journal of Physical Activity & Health
JA - J Phys Act Health
VL - 11
IS - 2
CY - United States
N2 - Background: Data available indicate that numerous childcare workers are not strongly motivated to engage children aged 3-5 in physical activity. Using the theory of planned behavior as the main theoretical framework, this study has 2 objectives: to identify the determinants of the intention of childcare workers to engage preschoolers in physical activity and to identify the variables that could be used to develop an intervention to motivate childcare workers to support preschoolers' physical activity. Methods: 174 childcare workers from 60 childcare centers selected at random in 2 regions of Quebec completed a self-administered questionnaire assessing the constructs of the theory of planned behavior as well as past behavior, descriptive norm and moral norm. Results: Moral norm, perceived behavioral control and subjective norm explained 85% of the variance in intention to engage the children in physical activity. Conclusions: To motivate childcare workers, it is necessary that they perceive that directors, children's parents and coworkers approve of their involvement in children's physical activity. In addition, their ability to overcome perceived barriers (lack of time, loaded schedule, inclement weather) should be developed. Access to a large outdoor yard might also help motivate childcare workers
SN - 1543-5474
AD - Faculty of Nursing, Laval University, Quebec, Prince Edward Island, Canada
UR - 24567488
ER -

TY - JOUR
ID - 53
T1 - Effects of ready for recess, an environmental intervention, on physical activity in third- through sixth-grade children
A1 - Huberty,J.L.
A1 - Beets,M.W.
A1 - Beighle,A.
A1 - Saint-Maurice,P.F.
A1 - Welk,G.
Y1 - 2014/02//
N1 - Huberty, Jennifer L. Beets, Michael W. Beighle, Aaron. Saint-Maurice, Pedro F. Welk, Greg
Journal of physical activity & health
101189457
IM
Journal Article
English
KW - MEDLINE

RP - NOT IN FILE
SP - 384
EP - 395
JF - Journal of Physical Activity & Health
JA - J Phys Act Health
VL - 11
IS - 2
CY - United States
N2 - BACKGROUND: The purpose of this study was to determine the effectiveness of Ready for Recess, an elementary school recess intervention targeting staff training (ST) or providing recreational equipment (EQ) separately, and the combination (EQ+ST) on physical activity (PA). METHODS: Participants were children attending 1 of 12 elementary schools (grades 3rd-6th) included in the study. Separate analytical models were used to evaluate the effects of the intervention conditions on children's accelerometry and direct observation derived PA measures. RESULTS: Boys and girls were measured using accelerometry (n = 667). Boys in EQ+ST increased their MVPA by 14.1% while ST decreased their MVPA by -13.5%. Girls in ST decreased their MVPA by -11.4%. Neither boys nor girls in EQ increased their time spent in MVPA. A total of 523 (boys) and 559 (girls) observations were collected. For boys' and girls' sedentary and vigorous activity there were no significant main effects for treatment condition, time, or treatment condition-by-time effects. CONCLUSIONS: Environmental modifications are only as strong as the staff that implements them. Supervision, if not interactive, may be detrimental to PA participation, especially in girls. Research related to staff training for encouragement and promotion of PA coupled with appropriate use of equipment during recess is warranted
SN - 1543-5474
AD - School Nutrition and Health Promotion, Arizona State University
UR - 23364349
ER -

TY - JOUR
ID - 54
T1 - Pet dog ownership decisions for parents of children with autism spectrum disorder
A1 - Carlisle,G.K.
Y1 - 2014/03//
N1 - Carlisle, Gretchen K
Journal of pediatric nursing
jns, 8607529
IM, N
Journal Article
English
KW - MEDLINE
RP - NOT IN FILE
SP - 114
EP - 123
JF - Journal of Pediatric Nursing
JA - J Pediatr Nurs
VL - 29
IS - 2
CY - United States
N2 - This study aimed to examine the role of pet dogs in families of children with autism. Sixty-seven percent of families owned dogs and 94% reported that their children were bonded to their dogs. Parents described previous experience with dogs and beliefs in their benefits as influential in their dog ownership decision-making process. Children living with dogs interacted with them in play and/or sharing personal space. Sensory issues of the children impacted their interaction with dogs inside and outside the home. Time and cost of care were identified burdens of dog ownership. Benefits were the opportunity to learn responsibility and companionship. 2014 Elsevier Inc. All rights reserved
SN - 1532-8449
AD - University of Missouri, Columbia, MO. Electronic address: carlislegk@missouri.edu
UR - 24183985
ER -

TY - JOUR
ID - 55
T1 - Auditory risk estimates for youth target shooting
A1 - Meinke,D.K.
A1 - Murphy,W.J.
A1 - Finan,D.S.
A1 - Lankford,J.E.
A1 - Flamme,G.A.
A1 - Stewart,M.
A1 - Soendergaard,J.
A1 - Jerome,T.W.
Y1 - 2014/03//
N1 - Meinke, Deanna K. Murphy, William J. Finan, Donald S. Lankford, James E. Flamme, Gregory A. Stewart, Michael. Soendergaard, Jacob. Jerome, Trevor W
International journal of audiology
101140017
IM
Journal Article
English
KW - MEDLINE
RP - NOT IN FILE
SP - S16
EP - S25
JF - International Journal of Audiology
JA - Int J Audiol
VL - 53 Suppl 2
CY - England
N2 - Abstract Objective: To characterize the impulse noise exposure and auditory risk for youth recreational firearm users engaged in outdoor target shooting events. The youth shooting positions are typically standing or sitting at a table, which places the firearm closer to the ground or reflective surface when compared to adult shooters. Design: Acoustic characteristics were examined and the auditory risk estimates were evaluated using contemporary damage-risk criteria for unprotected adult listeners and the 120-dB peak limit suggested by the World Health Organization (1999) for children. Study sample: Impulses were generated by 26 firearm/ammunition configurations representing rifles, shotguns, and pistols used by youth. Measurements were obtained relative to a youth shooter's left ear. Results: All firearms generated peak levels that exceeded the 120 dB peak limit suggested by the WHO for children. In general, shooting from the seated position over a tabletop increases the peak levels, LAeq8 and reduces the unprotected maximum permissible exposures (MPEs) for both rifles and pistols. Pistols pose the greatest auditory risk when fired over a tabletop. Conclusion: Youth should utilize smaller caliber weapons, preferably from the standing position, and always wear hearing protection whenever engaging in shooting activities to reduce the risk for auditory damage
SN - 1708-8186
AD - * Audiology and Speech-Language Sciences, University of Northern Colorado , Greeley , USA
UR - 24564688
ER -

TY - JOUR
ID - 56
T1 - Timeliness of childhood vaccine uptake among children attending a tertiary health service facility-based immunisation clinic in Ghana
A1 - Laryea,D.O.
A1 - Abbeyquaye,Parbie E.
A1 - Frimpong,E.
Y1 - 2014///
N1 - Laryea, Dennis Odai. Abbeyquaye Parbie, Emmanuel. Frimpong, Ebenezer
BMC public health
100968562

IM
Journal Article
English
KW - MEDLINE
RP - NOT IN FILE
SP - 90
JF - BMC Public Health
VL - 14
CY - England
N2 - BACKGROUND: Childhood immunisation is a cost-effective activity in health. Immunisation of children has contributed to reducing child morbidity and mortality. In the last two decades, global deaths from vaccine-preventable illnesses have decreased significantly as a result of immunisation. Similar trends have been observed in Ghana following the introduction of the Expanded Programme on Immunisation. The administration of vaccines is based on the period of highest susceptibility among others. Ghana has long used the proportion of children receiving vaccines and the trends in vaccine preventable illness incidence as performance indicators for immunisation. The addition of timeliness of vaccine uptake as an additional performance indicator has been recommended. This study evaluated the timeliness of vaccine uptake among children immunised at the Komfo Anokye Teaching Hospital, Kumasi, Ghana. METHODS: The study was conducted at the Maternal and Child Health clinic of the hospital between February and March 2012. A representative sample of 259 respondents was selected by simple random sampling. Data collection was by a structured questionnaire and included the examination of Child Health records booklet. Data was entered into a Microsoft Office Access database and analysed using Epi Info Version 3.5.1 2008. RESULTS: The majority of mothers attended antenatal clinics during pregnancy. An overwhelming majority of babies (98.8%) were delivered in a hospital. About 85% of babies were less than 12 months of age. Mean time taken to reach the clinic was 30 minutes. Vaccine uptake was generally timely for initial vaccines. The proportion of children receiving the vaccines later increased with latter vaccines. Overall, 87.3% of babies received vaccines on time with only 5.3% receiving vaccines beyond 28 days of the scheduled date. Children receiving immunisations services in the same facility as they were born were more likely to receive the BCG vaccine on time. CONCLUSIONS: Vaccine uptake is mostly timely among respondents in the study. The BCG vaccine in particular was received on time among children born in the same facility as the immunisation clinic. There is the need to further examine the timeliness of vaccine uptake among children delivered outside health facilities in Ghana
SN - 1471-2458
AD - Public Health Unit, Komfo Anokye Teaching Hospital, P,O, Box 1934, Kumasi, Ghana. dlaryea@kathhsp.org
UR - 24476339
ER -

TY - JOUR
ID - 57
T1 - Promotion of a healthy lifestyle among 5-year-old overweight children: health behavior outcomes of the 'Be active, eat right' study
A1 - van,Grieken A.
A1 - Renders,C.M.
A1 - Veldhuis,L.
A1 - Looman,C.W.
A1 - Hirasing,R.A.
A1 - Raat,H.
Y1 - 2014///
N1 - van Grieken, Amy. Renders, Carry M. Veldhuis, Lydian. Looman, Caspar Wn. Hirasing, Remy A. Raat, Hein
BMC public health
100968562
IM
Journal Article
English
KW - MEDLINE
RP - NOT IN FILE

SP - 59
JF - BMC Public Health
VL - 14
CY - England
N2 - BACKGROUND: This study evaluates the effects of an intervention performed by youth health care professionals on child health behaviors. The intervention consisted of offering healthy lifestyle counseling to parents of overweight (not obese) 5-year-old children. Effects of the intervention on the child having breakfast, drinking sweet beverages, watching television and playing outside were evaluated. METHODS: Data were collected with the 'Be active, eat right' study, a cluster randomized controlled trial among nine youth health care centers in the Netherlands. Parents of overweight children received lifestyle counseling according to the intervention protocol in the intervention condition (n=349) and usual care in the control condition (n=288). Parents completed questionnaires regarding demographic characteristics, health behaviors and the home environment at baseline and at 2-year follow-up. Cluster adjusted regression models were applied; interaction terms were explored. RESULTS: The population for analysis consisted of 38.1% boys; mean age 5.8 [sd 0.4] years; mean BMI SDS 1.9 [sd 0.4]. There were no significant differences in the number of minutes of outside play or television viewing a day between children in the intervention and the control condition. Also, the odds ratio for having breakfast daily or drinking two or less glasses of sweet beverages a day showed no significant differences between the two conditions. Additional analyses showed that the odds ratio for drinking less than two glasses of sweet beverages at follow-up compared with baseline was significantly higher for children in both the intervention (p<0.001) and the control condition (p=0.029). CONCLUSIONS: Comparison of the children in the two conditions showed that the intervention does not contribute to a change in health behaviors. Further studies are needed to investigate opportunities to adjust the intervention protocol, such as integration of elements in the regular well-child visit. The intervention protocol for youth health care may become part of a broader approach to tackle childhood overweight and obesity. TRIAL REGISTRATION: Current Controlled Trials ISRCTN04965410
SN - 1471-2458
AD - Department of Public Health, Erasmus MC University Medical Center Rotterdam, P,O, Box 2040, Rotterdam 3000 CA, the Netherlands. h.raat@erasmusmc.nl
UR - 24447459
ER -

TY - JOUR
ID - 58
T1 - A cross-sectional study of the environment, physical activity, and screen time among young children and their parents
A1 - Carson,V.
A1 - Rosu,A.
A1 - Janssen,I.
Y1 - 2014///
N1 - Carson, Valerie. Rosu, Andrei. Janssen, Ian
BMC public health
100968562
IM
Journal Article. Research Support, Non-U.S. Gov't
English
KW - MEDLINE
RP - NOT IN FILE
SP - 61
JF - BMC Public Health
VL - 14
CY - England
N2 - BACKGROUND: To develop evidence-based interventions promoting healthy active lifestyles among young children and their parents, a greater understanding is needed of the correlates of physical activity and screen time in these dyads. Physical environment features within neighborhoods may have important influences on both children and their parents. The purpose of this study was to examine the associations between several features of the physical environment with physical activity and screen time among 511 young children (<5 years old) and their parents, after adjusting for socio-demographic factors. METHODS: From May to September,

2011, parents of 0-5 year old children from Kingston, Canada completed a questionnaire that assessed socio-demographic characteristics, their physical activity and screen time, and their child's physical activity and screen time. Guided by a previously developed conceptual framework, several physical environment features were assessed using Geographic Information Systems including, function (walkability), safety (road speed), aesthetics (streetscape), and destination (outdoor play/activity space, recreation facilities, distance to closest park, yard at home). Multilevel linear regression analyses were used to examine the relationships while adjusting for several socio-demographic factors. RESULTS: The only independent association observed for the physical environment features was between higher outdoor play/activity space and higher screen time levels among parents. Several associations were observed with socio-demographic variables. For physical activity, child age, child care status, and family socioeconomic status (SES) were independent correlates for children while sex was an independent correlate for parents. For screen time, child age and family SES were independent correlates for children while neighborhood SES was an independent correlate for parents. CONCLUSIONS: The findings suggest that socio-demographic factors, including social environment factors, may be more important targets than features of the physical environment for future interventions aiming to promote healthy active lifestyles in young children and their parents. Given this was one of the first studies to examine these associations in young child-parent dyads, future research should confirm and build on these findings
SN - 1471-2458
AD - Faculty of Physical Education and Recreation, University of Alberta, W1-34 Van Vliet Centre, Edmonton, AB T6G 2H9, Canada. vlcarson@ualberta.ca
UR - 24447532
ER -

TY - JOUR
ID - 59
T1 - Light exposure and physical activity in myopic and emmetropic children
A1 - Read,S.A.
A1 - Collins,M.J.
A1 - Vincent,S.J.
Y1 - 2014/03//
N1 - Read, Scott A. Collins, Michael J. Vincent, Stephen J
Optometry and vision science : official publication of the American Academy of Optometry
oiz, 8904931
IM
Journal Article
English
KW - MEDLINE
RP - NOT IN FILE
SP - 330
EP - 341
JF - Optometry & Vision Science
JA - Optom.Vis Sci
VL - 91
IS - 3
CY - United States
N2 - PURPOSE: To objectively assess daily light exposure and physical activity levels in myopic and emmetropic children. METHODS: One hundred two children (41 myopes and 61 emmetropes) aged 10 to 15 years old had simultaneous objective measures of ambient light exposure and physical activity collected over a 2-week period during school term, using a wrist-worn actigraphy device (Actiwatch 2). Measures of visible light illuminance and physical activity were captured every 30 seconds, 24 hours a day over this period. Mean hourly light exposure and physical activity for weekdays and weekends were examined. To ensure that seasonal variations did not confound comparisons, the light and activity data of the 41 myopes was compared with 41 age- and gender-matched emmetropes who wore the Actiwatch over the same 2-week period. RESULTS: Mean light exposure and physical activity for all 101 children with valid data exhibited significant changes with time of day and day of the week ($p < 0.0001$). On average, greater daily light exposure occurred on weekends compared to weekdays ($p < 0.05$), and greater physical activity occurred on weekdays compared to weekends ($p < 0.01$). Myopic children ($n = 41$, mean daily light exposure 915 + 519 lx) exhibited significantly lower average light exposure compared to 41 age- and gender-matched emmetropic children (1272 + 625 lx, $p < 0.01$). The

amount of daily time spent in bright light conditions (>1000 lx) was also significantly greater in emmetropes (127 + 51 minutes) compared to myopes (91 + 44 minutes, p < 0.001). No significant differences were found between the average daily physical activity levels of myopes and emmetropes (p > 0.05). CONCLUSIONS: Myopic children exhibit significantly lower daily light exposure, but no significant difference in physical activity compared to emmetropic children. This suggests the important factor involved in documented associations between myopia and outdoor activity is likely exposure to bright outdoor light rather than greater physical activity
SN - 1538-9235
AD - *PhD, FAAO +PhD Contact Lens and Visual Optics Laboratory, School of Optometry and Vision Science, Queensland University of Technology, Brisbane, Queensland, Australia
UR - 24413273
ER -

TY - JOUR
ID - 60
T1 - Correlates of Parental Misperception of Their Child's Weight Status: The 'Be Active, Eat Right' Study
A1 - Remmers,T.
A1 - van,Grieken A.
A1 - Renders,C.M.
A1 - Hirasing,R.A.
A1 - Broeren,S.M.
A1 - Raat,H.
Y1 - 2014///
N1 - Remmers, Teun. van Grieken, Amy. Renders, Carry M. Hirasing, Remy A. Broeren, Suzanne M L. Raat, Hein
PloS one
101285081
IM
Journal Article
English
KW - MEDLINE
RP - NOT IN FILE
SP - e88931
JF - PLoS ONE [Electronic Resource]
JA - PLoS ONE
VL - 9
IS - 2
CY - United States
N2 - OBJECTIVE: This study reported on correlates of parental perception of their child's weight status. Associations between parental misperception (i.e., underestimation of the child's weight) and parental intention to improve their child's overweight-related health behaviors and their child meeting guidelines regarding these behaviors were also investigated. METHODS: Baseline data from the population-based 'Be active, eat right study' were used. The population for analysis consisted of 630 overweight and 153 obese five year-old children and their parents. Questionnaires were used to measure parental perception of the child's weight status, correlates of misperception (i.e., child age, child gender, child BMI, parental age, parental gender, parental country of birth, parental educational level and parental weight status), overweight-related health behaviors (i.e., child playing outside, having breakfast, drinking sweet beverages, and watching TV), and parental intention to improve these behaviors. Height and weight were measured using standardized protocols. Multivariable logistic regression analyses were performed. RESULTS: In total, 44.40% of the parents misperceived their child's weight status. Parental misperception was associated with lower child BMI, the parent being the father, a foreign parental country of birth, and a lower parental education level (p<0.05). Parental misperception was not associated with parental intention to improve child overweight-related health behavior, nor with child meeting the guidelines of these behaviors. DISCUSSION: This study showed that almost half of the parents with an overweight or obese child misperceived their child's weight status. A correct parental perception may be a small stepping-stone in improving the health of overweight and obese children
SN - 1932-6203

AD - Department of Public Health, Erasmus MC University Medical Center Rotterdam, Rotterdam, the Netherlands ; Department of Epidemiology, CAPHRI School for Public Health and Primary Care, Maastricht University Medical Center, Maastricht, the Netherlands., Department of Public Health, Erasmus MC University Medical Center Rotterdam, Rotterdam, the Netherlands., Institute of Health Sciences, Faculty of Earth and Life Sciences, VU University Amsterdam, Amsterdam, the Netherlands., Department of Public and Occupational Health, EMGO Institute of Health and Care Research, VU University Medical Center Amsterdam, Amsterdam, the Netherlands., Department of Public Health, Erasmus MC University Medical Center Rotterdam, Rotterdam, the Netherlands., Department of Public Health, Erasmus MC University Medical Center Rotterdam, Rotterdam, the Netherlands
UR - 24551191
ER -

TY - JOUR
ID - 61
T1 - What helps children to move more at school recess and lunchtime? Mid-intervention results from Transform-Us! cluster-randomised controlled trial
A1 - Yildirim,M.
A1 - Arundell,L.
A1 - Cerin,E.
A1 - Carson,V.
A1 - Brown,H.
A1 - Crawford,D.
A1 - Hesketh,K.D.
A1 - Ridgers,N.D.
A1 - Te Velde,S.J.
A1 - Chinapaw,M.J.
A1 - Salmon,J.
Y1 - 2014/02//
N1 - Yildirim, Mine. Arundell, Lauren. Cerin, Ester. Carson, Valerie. Brown, Helen. Crawford, David. Hesketh, Kylie D. Ridgers, Nicola D. Te Velde, Saskia J. Chinapaw, Mai J M. Salmon, Jo
British journal of sports medicine
0432520
IM
Journal Article. Research Support, Non-U.S. Gov't
English
KW - MEDLINE
RP - NOT IN FILE
SP - 271
EP - 277
JF - British Journal of Sports Medicine
JA - BJSM online
VL - 48
IS - 3
CY - England
N2 - BACKGROUND: To investigate the interpersonal and physical environment mediators of the Transform-Us! mid-intervention effects on physical activity (PA) during recess and lunchtime. METHODS: Transform-Us! is a clustered randomised school-based intervention with four groups: sedentary behaviour intervention (SB-I), PA intervention (PA-I), combined PA+SB-I and control group. All children in grade 3 from 20 participating primary schools in Melbourne, Australia were eligible to complete annual evaluation assessments. The outcomes were the proportion of time spent in moderate-to-vigorous PA (MVPA) and light PA (LPA) during recess and lunchtime assessed by accelerometers. Potential mediators included: perceived social support from teachers; perceived availability of line markings; perceived accessibility of sports equipment; and perceived school play environment. Generalised linear models were used and mediation effects were estimated by product-of-coefficients (ab) approach. RESULTS: 268 children (8.2 years, 57% girls at baseline) provided complete data at both time points. A significant intervention effect on MVPA during recess in the SB-I and PA-I groups compared with the control group (proportional difference in MVPA time; 38% (95% CI 21% to 57%) and 40% (95% CI 20% to 62%), respectively) was found. The perceived school play environment was

significantly positively associated with MVPA at recess among girls. An increase in perceived social support from teachers suppressed the PA+SB-I effect on light PA during recess (ab= -0.03, 95% CI -0.06 to -0.00). No significant mediating effects on PA during recess and lunchtime were observed. CONCLUSIONS: A positive perception of the school play environment was associated with higher MVPA during recess among girls. Future studies should conduct mediation analyses to explore underlying mechanisms of PA interventions
SN - 1473-0480
AD - Department of Public and Occupational Health and the EMGO Institute for Health and Care Research, VU University Medical Center, , Amsterdam, The Netherlands
UR - 24124036
ER -

TY - JOUR
ID - 63
T1 - Source contributions of lead in residential floor dust and within-home variability of dust lead loading
A1 - Lucas,J.P.
A1 - Bellanger,L.
A1 - Le,Strat Y.
A1 - Le,Tertre A.
A1 - Glorennec,P.
A1 - Le,Bot B.
A1 - Etchevers,A.
A1 - Mandin,C.
A1 - Sebille,V.
Y1 - 2014/02/01/
N1 - Lucas, Jean-Paul. Bellanger, Lise. Le Strat, Yann. Le Tertre, Alain. Glorennec, Philippe. Le Bot, Barbara. Etchevers, Anne. Mandin, Corinne. Sebille, Veronique
The Science of the total environment
uj0, 0330500
IM
Journal Article. Research Support, Non-U.S. Gov't
English
KW - MEDLINE
RP - NOT IN FILE
SP - 768
EP - 779
JF - Science of the Total Environment
JA - Sci Total Environ
VL - 470-471
CY - Netherlands
N2 - Evidence of the impact of exposure to low levels of lead on children's health is increasing. Residential floor dust is the assumed origin of lead exposure by young children. In this study, we estimate the contribution of different lead sources to household interior floor dust contamination. We also estimate the within-home variability of interior floor dust lead loadings. A multilevel model was developed based on data collected in a French survey in 2008-2009 (484 housing units, 1834 rooms). Missing data were handled by multiple imputation using chained equations. The intra-home correlation between interior floor Log dust lead loadings was approximately 0.6. Dust lead from the landing of an apartment, mostly originating outside the building, was the major contributor to interior floor dust lead. Secondary contributors included the lead-based paint on exterior railings, track-in of the exterior soil of the children's play area into the dwelling, smoking inside the home, demolition of nearby old buildings and sites of pollution in the vicinity. Interior lead-based paint contaminated interior floor dust only in old and non-renovated dwellings. To reduce interior floor dust lead levels in the general population of dwellings, common areas should be maintained, and track-in from the outside should be limited as much as possible. 2013
SN - 1879-1026
AD - Universite Paris Est, CSTB - Centre Scientifique et Technique du Batiment, 84 avenue Jean Jaures, 77447 Marne-la-Vallee Cedex 2, France; Universite de Nantes, EA 4275 Biostatistique, Pharmacoepidemiologie et Mesures Subjectives en Sante, 1 rue Gaston Veil BP 53508, 44035 Nantes Cedex 1, France. Electronic address: jean.paul.lucas@free.fr., UMR CNRS 6629 Laboratoire de Mathematiques Jean Leray, 2 rue de la Houssiniere

BP 92208, F-44322 Nantes Cedex, France., InVS, 12 rue du Val d'Osne, 94415 Saint-Maurice Cedex, France., InVS, 12 rue du Val d'Osne, 94415 Saint-Maurice Cedex, France., EHESP Rennes, Sorbonne Paris Cite, Avenue du Professeur Leon-Bernard, CS 74312, 35043 Rennes Cedex, France; INSERM UMR 1085 Institut de Recherche sur la Sante, l'Environnement et le Travail, Rennes, France., EHESP Rennes, Sorbonne Paris Cite, Avenue du Professeur Leon-Bernard, CS 74312, 35043 Rennes Cedex, France; INSERM UMR 1085 Institut de Recherche sur la Sante, l'Environnement et le Travail, Rennes, France., INSERM UMR 1085 Institut de Recherche sur la Sante, l'Environnement et le Travail, Rennes, France., Universite Paris Est, CSTB - Centre Scientifique et Technique du Batiment, 84 avenue Jean Jaures, 77447 Marne-la-Vallee Cedex 2, France., Universite de Nantes, EA 4275 Biostatistique, Pharmacoepidemiologie et Mesures Subjectives en Sante, 1 rue Gaston Veil BP 53508, 44035 Nantes Cedex 1, France
UR - 24184749
ER -

TY - JOUR
ID - 64
T1 - Soil ingestion rate determination in a rural population of Alberta, Canada practicing a wilderness lifestyle
A1 - Irvine,G.
A1 - Doyle,J.R.
A1 - White,P.A.
A1 - Blais,J.M.
Y1 - 2014/02/01/
N1 - Irvine, G. Doyle, J R. White, P A. Blais, J M
The Science of the total environment
uj0, 0330500
IM
Journal Article. Research Support, Non-U.S. Gov't
English
KW - MEDLINE
RP - NOT IN FILE
SP - 138
EP - 146
JF - Science of the Total Environment
JA - Sci Total Environ
VL - 470-471
CY - Netherlands
N2 - The inadvertent ingestion of contaminated soil can be a major pathway for chemical exposure to humans. Few studies to date have quantified soil ingestion rates to develop exposure estimates for human health risk assessments (HHRA), and almost all of those were for children in suburban/urban environments. Here we employed a quantitative mass balance tracer approach on a rural population practicing outdoor activities to estimate inadvertent soil ingestion. This study followed 9 subjects over a 13 day period in Cold Lake, Alberta, near the largest in situ thermal heavy oil (bitumen) extraction operation in the world. The mean soil ingestion rate in this study using Al Ce, La, and Si tracers was 32 mg d(-1), with a 90th percentile of 152 mg d(-1) and median soil ingestion rate of 18 mg d(-1). These soil ingestion values are greater than the standard recommended soil ingestion rates for HHRA from Health Canada, and are similar to soil ingestion estimates found in the only other study on a rural population. 2013. Published by Elsevier B.V. All rights reserved
SN - 1879-1026
AD - 30 Marie Curie, Pvt., Department of Biology, University of Ottawa, Ottawa, Ontario, Canada, K1N 6N5. Electronic address: graham.irvine@uottawa.ca., 30 Marie Curie, Pvt., Department of Biology, University of Ottawa, Ottawa, Ontario, Canada, K1N 6N5. Electronic address: jamiedoyle@sympatico.ca., 30 Marie Curie, Pvt., Department of Biology, University of Ottawa, Ottawa, Ontario, Canada, K1N 6N5. Electronic address: paul.white@hc-sc.gc.ca., 30 Marie Curie, Pvt., Department of Biology, University of Ottawa, Ottawa, Ontario, Canada, K1N 6N5. Electronic address: jules.blais@uottawa.ca
UR - 24126134
ER -

TY - JOUR
ID - 65

T1 - Risk factors for mercury exposure of children in a rural mining town in northern Chile
A1 - Ohlander,J.
A1 - Huber,S.M.
A1 - Schomaker,M.
A1 - Heumann,C.
A1 - Schierl,R.
A1 - Michalke,B.
A1 - Jenni,O.G.
A1 - Caflisch,J.
A1 - Munoz,D.M.
A1 - von Ehrenstein,O.S.
A1 - Radon,K.
Y1 - 2013///
N1 - Ohlander, Johan. Huber, Stella Maria. Schomaker, Michael. Heumann, Christian. Schierl, Rudolf. Michalke, Bernhard. Jenni, Oskar G. Caflisch, Jon. Munoz, Daniel Moraga. von Ehrenstein, Ondine S. Radon, Katja
PloS one
101285081
IM
Journal Article
English
KW - MEDLINE
RP - NOT IN FILE
SP - e79756
JF - PLoS ONE [Electronic Resource]
JA - PLoS ONE
VL - 8
IS - 11
CY - United States
N2 - OBJECTIVE: Traditional gold mining is associated with mercury exposure. Especially vulnerable to its neurotoxic effects is the developing nervous system of a child. We aimed to investigate risk factors of mercury exposure among children in a rural mining town in Chile. METHODS: Using a validated questionnaire distributed to the parents of the children, a priori mercury risk factors, potential exposure pathways and demographics of the children were obtained. Mercury levels were measured through analyzing fingernail samples. Logistic regression modeling the effect of risk factors on mercury levels above the 75(th) percentile were made, adjusted for potential confounders. RESULTS: The 288 children had a mean age of 9.6 years (SD = 1.9). The mean mercury level in the study population was 0.13 g/g (SD 0.11, median 0.10, range 0.001-0.86 g/g). The strongest risk factor for children's odds of high mercury levels (>75(th) percentile, 0.165 g/g) was to play inside a house where a family member worked with mercury (OR adjusted 3.49 95% CI 1.23-9.89). Additionally, children whose parents worked in industrial gold mining had higher odds of high mercury levels than children whose parents worked in industrial copper mining or outside mining activities. CONCLUSION: Mercury exposure through small-scale gold mining might affect children in their home environments. These results may further help to convince the local population of banning mercury burning inside the households
SN - 1932-6203
AD - Center for International Health Ludwig-Maximilians-University (LMU) at the Institute and Outpatient Clinic for Occupational, Social and Environmental Medicine, University Hospital Munich (LMU), Munich, Germany
UR - 24278170
ER -

TY - JOUR
ID - 66
T1 - Determinants of change in children's sedentary time
A1 - Atkin,A.J.
A1 - Corder,K.
A1 - Ekelund,U.
A1 - Wijndaele,K.

A1 - Griffin,S.J.
A1 - van Sluijs,E.M.
Y1 - 2013///
N1 - Atkin, Andrew J. Corder, Kirsten. Ekelund, Ulf. Wijndaele, Katrien. Griffin, Simon J. van Sluijs, Esther M F
PloS one
101285081
IM
Journal Article. Research Support, Non-U.S. Gov't
English
KW - MEDLINE
RP - NOT IN FILE
SP - e67627
JF - PLoS ONE [Electronic Resource]
JA - PLoS ONE
VL - 8
IS - 6
CY - United States
N2 - BACKGROUND: Understanding the determinants of sedentary time during childhood contributes to the development of effective intervention programmes. PURPOSE: To examine family and home-environmental determinants of 1-year change in objectively measured sedentary time after-school and at the weekend. METHODS: Participants wore accelerometers at baseline and 1 year later. Longitudinal data for after-school and weekend analyses were available for 854 (41.5%male, mean + SD age 10.2 + 0.3 years) and 718 (41.8%male, age 10.2 + 0.3 years) participants. Information on 26 candidate determinants, including socioeconomic status (SES), availability of electronic media and parental rules for sedentary behaviours was self-reported by children or their parents at baseline. Change in the proportion of registered time spent sedentary was used as the outcome variable in multi-level linear regression models, adjusted for age, sex, body mass index and baseline sedentary time. Simple and multiple models were run and interactions with sex explored. RESULTS: Children from higher socioeconomic status families exhibited greater increases in after-school (beta; 95% CI for change in % time spent sedentary 1.02; 0.37, 1.66) and weekend (1.42; 0.65, 2.18) sedentary time. Smaller increases in after-school sedentary time were observed in children with more siblings (-1.00; -1.69, -0.30), greater availability of electronic media (-0.81; -1.29, -0.33) and, for boys, more frequent family visits to the park (-1.89; -3.28, -0.51) and family participation in sport (-1.28; -2.54, -0.02). Greater maternal weekend screen-time (0.45; 0.08, 0.83) and, in girls, greater parental restriction on playing outside (0.91; 0.08, 1.74) were associated with larger increases in weekend sedentary time. The analytical sample was younger, more likely to be female, had lower BMI and was of higher SES than the original baseline sample. CONCLUSIONS: Intervention strategies aimed at reducing parents' weekend screen-time, increasing family participation in sports or recreation (boys) and promoting freedom to play outside (girls) may contribute towards preventing the age-related increase in sedentary time
SN - 1932-6203
AD - UKCRC Centre for Diet and Activity Research, Institute of Public Health, Cambridge, United Kingdom. aa595@medschl.cam.ac.uk
UR - 23840753
ER -

TY - JOUR
ID - 67
T1 - Shoe leather epidemiology: active travel and transport infrastructure in the urban landscape
A1 - Ogilvie,D.
A1 - Mitchell,R.
A1 - Mutrie,N.
A1 - Petticrew,M.
A1 - Platt,S.
Y1 - 2010///
N1 - Ogilvie, David. Mitchell, Richard. Mutrie, Nanette. Petticrew, Mark. Platt, Stephen
The international journal of behavioral nutrition and physical activity
101217089

Journal Article
English
KW - MEDLINE
RP - NOT IN FILE
SP - 43
JF - International Journal of Behavioral Nutrition & Physical Activity
JA - Int
VL - 7
CY - England
N2 - BACKGROUND: Building new transport infrastructure could help to promote changes in patterns of mobility, physical activity, and other determinants of population health such as economic development. However, local residents may not share planners' goals or assumptions about the benefits of such interventions. A particularly contentious example is the construction of major roads close to deprived residential areas. We report the qualitative findings of the baseline phase of a longitudinal mixed-method study of a new urban section of the M74 motorway in Glasgow, Scotland, that aims to combine quantitative epidemiological and spatial data with qualitative interview data from local residents. METHODS: We interviewed 12 residents purposively sampled from a larger study cohort of 1322 to include men and women, different age groups, and people with and without cars, all living within 400 metres of the proposed route of the new motorway. We elicited their views and experiences of the local urban environment and the likely impact of the new motorway using a topic guide based on seven key environmental constructs (aesthetics, green space, convenience of routes, access to amenities, traffic, road danger and personal danger) reflecting an overall ecological model of walking and cycling. RESULTS: Traffic was widely perceived to be heavy despite a low local level of car ownership. Few people cycled, and cycling on the roads was widely perceived to be dangerous for both adults and children. Views about the likely impacts of the new motorway on traffic congestion, pollution and the pleasantness of the local environment were polarised. A new motorway has potential to cause inequitable psychological or physical severance of routes to local amenities, and people may not necessarily use local walking routes or destinations such as parks and shops if these are considered undesirable, unsafe or 'not for us'. Public transport may have the potential to promote or discourage active travel in different socioeconomic contexts. CONCLUSIONS: Altering the urban landscape may influence walking and cycling in ways that vary between individuals, may be inequitable, and may not be predictable from quantitative data alone. A more applied ecological behavioural model may be required to capture these effects
SN - 1479-5868
AD - Medical Research Council Epidemiology Unit and Centre for Diet and Activity Research (CEDAR), Cambridge, UK. david.ogilvie@mrc-epid.cam.ac.uk
UR - 20459803
ER -

TY - JOUR
ID - 68
T1 - Patterns of GPS measured time outdoors after school and objective physical activity in English children: the PEACH project
A1 - Cooper,A.R.
A1 - Page,A.S.
A1 - Wheeler,B.W.
A1 - Hillsdon,M.
A1 - Griew,P.
A1 - Jago,R.
Y1 - 2010///
N1 - Cooper, Ashley R. Page, Angie S. Wheeler, Benedict W. Hillsdon, Melvyn. Griew, Pippa. Jago, Russell
The international journal of behavioral nutrition and physical activity
101217089
Journal Article
English
KW - MEDLINE
RP - NOT IN FILE
SP - 31
JF - International Journal of Behavioral Nutrition & Physical Activity

JA - Int
VL - 7
CY - England
N2 - BACKGROUND: Observational studies have shown a positive association between time outdoors and physical activity in children. Time outdoors may be a feasible intervention target to increase the physical activity of youth, but methods are required to accurately measure time spent outdoors in a range of locations and over a sustained period. The Global Positioning System (GPS) provides precise location data and can be used to identify when an individual is outdoors. The aim of this study was to investigate whether GPS data recorded outdoors were associated with objectively measured physical activity. METHODS: Participants were 1010 children (11.0 +/- 0.4 years) recruited from 23 urban primary schools in South West England, measured between September 2006 and July 2008. Physical activity was measured by accelerometry (Actigraph GT1M) and children wore a GPS receiver (Garmin Foretrex 201) after school on four weekdays to record time outdoors. Accelerometer and GPS data were recorded at 10 second epochs and were combined to describe patterns of physical activity when both a GPS and accelerometer record were present (outdoors) and when there was accelerometer data only (indoors). ANOVA was used to investigate gender and seasonal differences in the patterns of outdoor and indoor physical activity, and linear regression was used to examine the cross-sectional associations between GPS-measured time outdoors and physical activity. RESULTS: GPS-measured time outdoors was a significant independent predictor of children's physical activity after adjustment for potential confounding factors. Physical activity was more than 2.5 fold higher outdoors than indoors (1345.8 +/- 907.3 vs 508.9 +/- 282.9 counts per minute; F = 783.2, p < .001). Overall, children recorded 41.7 +/- 46.1 minutes outdoors between 3.30 pm and 8.30 pm, with more time spent outdoors in the summer months (p < .001). There was no gender difference in time spent outdoors. Physical activity outdoors was higher in the summer than the winter (p < .001), whilst there was no seasonal variation in physical activity indoors. CONCLUSIONS: Duration of GPS recording is positively associated with objectively measured physical activity and is sensitive to seasonal differences. Minute by minute patterning of GPS and physical activity data is feasible and may be a useful tool to investigate environmental influences on children's physical activity and to identify opportunities for intervention
SN - 1479-5868
AD - Department of Exercise, Nutrition and Health Sciences, University of Bristol, Tyndall Avenue, Bristol BS8 1TP, UK. ashley.cooper@bris.ac.uk
UR - 20412582
ER -

TY - JOUR
ID - 69
T1 - Associations between sport and screen-entertainment with mental health problems in 5-year-old children
A1 - Griffiths,L.J.
A1 - Dowda,M.
A1 - Dezateux,C.
A1 - Pate,R.
Y1 - 2010///
N1 - Griffiths, Lucy J. Dowda, Marsha. Dezateux, Carol. Pate, Russell
The international journal of behavioral nutrition and physical activity
101217089
Journal Article
English
KW - MEDLINE
RP - NOT IN FILE
SP - 30
JF - International Journal of Behavioral Nutrition & Physical Activity
JA - Int
VL - 7
CY - England
N2 - BACKGROUND: Few studies have examined the benefits of regular physical activity, and risks of sedentary behaviour, in young children. This study investigated associations between participation in sports and screen-entertainment (as components of physical activity and sedentary behaviour), and emotional and behavioural problems in this population. METHODS: Cross-sectional analysis of data from 13470 children

(50.9% boys) participating in the nationally representative UK Millennium Cohort Study. Time spent participating in sports clubs outside of school, and using screen-entertainment, was reported by the child's mother at child age 5 years, when mental health was also measured using the Strengths and Difficulties Questionnaire. RESULTS: 45% of children did not participate in sport clubs and 61% used screen-entertainment for >/= 2 hours per day. Children who participated in sport had fewer total difficulties; emotional, conduct, hyperactivity-inattention and peer relationship problems; and more prosocial behaviours. These relationships were similar in boys and girls. Boys and girls who used screen-entertainment for any duration, and participated in sport, had fewer emotional and behavioural problems, and more prosocial behaviours, than children who used screen-entertainment for >/= 2 hours per day and did not participate in sport. CONCLUSIONS: Longer durations of screen-entertainment usage are not associated with mental health problems in young children. However, our findings suggest an association between sport and better mental health. Further research based on longitudinal data is required to examine causal pathways in these associations and to determine the potential role of this and other forms of physical activity in preventing mental health disorders
SN - 1479-5868
AD - MRC Centre of Epidemiology for Child Health, UCL Institute of Child Health, London, UK. l.griffiths@ich.ucl.ac.uk
UR - 20409310
ER -

TY - JOUR
ID - 70
T1 - Independent mobility, perceptions of the built environment and children's participation in play, active travel and structured exercise and sport: the PEACH Project
A1 - Page,A.S.
A1 - Cooper,A.R.
A1 - Griew,P.
A1 - Jago,R.
Y1 - 2010///
N1 - Page, Angie S. Cooper, Ashley R. Griew, Pippa. Jago, Russell
The international journal of behavioral nutrition and physical activity
101217089
Journal Article
English
KW - MEDLINE
RP - NOT IN FILE
SP - 17
JF - International Journal of Behavioral Nutrition & Physical Activity
JA - Int
VL - 7
CY - England
N2 - BACKGROUND: Independent mobility (IM) and perceptions of the built environment may relate differentially to children's participation in various physical activity contexts. This cross-sectional study investigated whether independent mobility and perceptions of the built environment in boys and girls were related to physical activity in three different contexts (outdoor play, structured exercise/sport, active commuting). METHODS: Thirteen hundred and seven 10-11 year old boys and girls from 23 schools in a large UK city completed a computerised questionnaire. Independent variables in logistic regression analyses were weekly self-reported frequency of participation in outdoor play, structured exercise/sport and mode of travel home from school. Dependent variables were perceptions of the environment (aesthetics, nuisance, safety, social norm, constraint, play space, accessibility), local and area independent mobility and linear distance from home to school. Analyses were adjusted for body mass index, minutes of daylight after school, level of neighbourhood deprivation and pubertal status. RESULTS: For boys, local independent mobility (Local-IM) was related to an increased likelihood of everyday participation in play (OR 1.58: 95% CI 1.19-2.10), structured exercise/sport (OR 1.42: 1.06-1.89) and active commuting (OR 1.40: 1.07-1.87) but was only related to active commuting for girls (OR1.49: 1.07-2.07). Boys and girls were more likely to report playing out every day if they had higher scores for Social Norm (Boys: OR 1.63 (1.12-2.37); Girls: OR 1.53 (1.01-2.31)) and, for girls only, more positive perceptions of traffic safety (OR 1.63: 1.14-2.34). Easy access to a range of destinations was the dominant predictor for taking part in structured exercise/sport everyday (Boys: OR 1.62 (1.01-2.66); Girls: OR

1.65 (1.07-2.53)). Shorter distance from home to school (OR 0.99: 0.98-0.99) and, for boys only, greater perceived accessibility (OR 1.87: 1.04-3.36) were significantly related to active commuting to school. CONCLUSIONS: Perceptions of the physical environment relate differently to different physical activity contexts and by gender. The only consistent correlate for outdoor play, structured ex/sport and active commuting was higher independent mobility to visit local destinations (Local-IM) for boys. Considering both the physical activity context and its independent correlates should improve the specificity of physical activity interventions in children
SN - 1479-5868
AD - Department of Exercise, Nutrition & Health Sciences, University of Bristol, Bristol, UK. a.s.page@bris.ac.uk
UR - 20170504
ER -

TY - JOUR
ID - 71
T1 - Environmental supportiveness for physical activity in English schoolchildren: a study using Global Positioning Systems
A1 - Jones,A.P.
A1 - Coombes,E.G.
A1 - Griffin,S.J.
A1 - van Sluijs,E.M.
Y1 - 2009///
N1 - Jones, Andrew P. Coombes, Emma G. Griffin, Simon J. van Sluijs, Esther Mf
The international journal of behavioral nutrition and physical activity
101217089
Journal Article
English
KW - MEDLINE
RP - NOT IN FILE
SP - 42
JF - International Journal of Behavioral Nutrition & Physical Activity
JA - Int
VL - 6
CY - England
N2 - BACKGROUND: There is increasing evidence that the environment plays a role in influencing physical activity in children and adults. As children have less autonomy in their behavioural choices, neighbourhood environment supportiveness may be an important determinant of their ability to be active. Yet we know rather little about the types of environment that children use for bouts of physical activity. This study uses accelerometery and global positioning system technologies to identify the charactieristics of environments being used for bouts of continuous moderate to vigorous physical activity (MVPA) in a sample of English schoolchildren. METHODS: The study used a convenience sample of 100 children from SPEEDY (Sport, Physical activity and Eating behaviour: Environmental Determinants in Young people), a cohort of 2064 9-10 year-olds from Norfolk, England, recruited in 2007. Children wore an ActiGraph GT1M accelerometer and a Garmin Forerunner 205 GPS unit over four consecutive days. Accelerometery data points were matched to GPS locations and bouts (5 minutes or more) of MVPA were identified. Bout locations were overlaid with a detailed landcover dataset developed in a GIS to identify the types of environment supporting MVPA. Findings are presented using descriptive statistics. RESULTS: Boys were also more active than girls, spending an average of 20 (SD 23) versus 11 (SD 15) minutes per day in MVPA bouts. Children who spent more time outside the home were more active ($p = 0.002$), especially girls and children living in rural locations (both $p < 0.05$). Children tended to be active close to home, with 63% of all bout time occurring inside neighbourhoods, although boys ($p = 0.05$) and rural children ($p = 0.01$) were more likely to roam outside their neighbourhood. Amongst urban children, gardens (28% of bout time) and the street environment (20%) were the most commonly used environments for MVPA bouts. Amongst rural children farmland (22%) and grassland (18%) were most frequently used. CONCLUSION: The study has developed a new methodology for the identification of environments in which bouts of continuous physical activity are undertaken. The results highlight the importance of the provision of urban gardens and greenspaces, and the maintenance of safe street environments as places for children to be active

SN - 1479-5868
AD - Medical Research Council Epidemiology Unit, Institute of Metabolic Science, Box 285, Addenbrookes Hospital, Hills Road, Cambridge, CB2 0QQ, UK. esther.vansluijs@mrc-epid.cam.ac.uk
UR - 19615073
ER -

TY - JOUR
ID - 72
T1 - School children's personal exposure to ultrafine particles in the urban environment
A1 - Mazaheri,M.
A1 - Clifford,S.
A1 - Jayaratne,R.
A1 - Megat Mokhtar,M.A.
A1 - Fuoco,F.
A1 - Buonanno,G.
A1 - Morawska,L.
Y1 - 2014/01/07/
N1 - Mazaheri, Mandana. Clifford, Sam. Jayaratne, Rohan. Megat Mokhtar, Megat Azman. Fuoco, Fernanda. Buonanno, Giorgio. Morawska, Lidia
Environmental science & technology
dvo, 0213155
IM
Journal Article. Research Support, Non-U.S. Gov't
English
KW - MEDLINE
RP - NOT IN FILE
SP - 113
EP - 120
JF - Environmental Science & Technology
JA - Environ Sci Technol
VL - 48
IS - 1
CY - United States
N2 - There has been considerable scientific interest in personal exposure to ultrafine particles (UFP). In this study, the inhaled particle surface area doses and dose relative intensities in the tracheobronchial and alveolar regions of lungs were calculated using measured 24-h UFP time series of school children personal exposures. Bayesian hierarchical modeling was used to determine mean doses and dose intensities for the various microenvironments. Analysis of measured personal exposures for 137 participating children from 25 schools in the Brisbane Metropolitan Area showed similar trends for all participating children. Bayesian regression modeling was performed to calculate the daily proportion of children's total doses in different microenvironments. The proportion of total daily alveolar doses for home, school, commuting, and other were 55.3%, 35.3%, 4.5%, and 5.0%, respectively, with the home microenvironment contributing a majority of children's total daily dose. Children's mean indoor dose was never higher than the outdoor's at any of the schools, indicating there were no persistent indoor particle sources in the classrooms during the measurements. Outdoor activities, eating/cooking at home, and commuting were the three activities with the highest dose intensities. Children's exposure during school hours was more strongly influenced by urban background particles than traffic near the school
SN - 1520-5851
AD - International Laboratory for Air Quality and Health, Institute of Health and Biomedical Innovation, Queensland University of Technology , 2 George Sreet, Brisbane 4001, Australia
UR - 24274338
ER -

TY - JOUR
ID - 73
T1 - Trends and patterns of under-5 vaccination in Nigeria, 1990-2008: what manner of progress?
A1 - Ushie,B.A.

A1 - Fayehun,O.A.
A1 - Ugal,D.B.
Y1 - 2014/03//
N1 - Ushie, B A. Fayehun, O A. Ugal, D B
Child: care, health and development
7602632, c9x
IM
Journal Article
English
KW - MEDLINE
RP - NOT IN FILE
SP - 267
EP - 274
JF - Child: Care, Health & Development
JA - Child Care Health Dev
VL - 40
IS - 2
CY - England
N2 - BACKGROUND: Despite efforts towards reducing childhood morbidity and mortality, Nigeria ranks among countries with the highest rates of vaccine-preventable diseases including tuberculosis, poliomyelitis, measles, diphtheria, pertussis and tetanus. These efforts include regular rounds of immunization days and routine exercises. The government of Nigeria periodically undertakes National Demographic and Health (NDH) surveys, which tap information on various health indices including vaccination coverage. Limited studies have used the NDHS data to examine the trends in vaccination coverage for the assessment of successes or failures of the immunization efforts. METHODS: This study used four NDH Surveys datasets between 1990 and 2008, which generated child health information including the proportion that had had any or all basic childhood vaccines. A combined total of 44071 (weighted) children were involved in the study. The trend and pattern of vaccination over 18 years were examined while selected factors were regressed to obtain predictors of child vaccinations in Nigeria. RESULTS: The most recent survey (2008) reported more complete vaccination apart from 1990, which was said to be inaccurate. In all surveys, children from mothers with higher education, who were delivered in hospitals, lived in urban areas, and whose mothers work outside the home had significantly higher proportions of completed basic vaccination. A lower level of childhood vaccination is observed in the northern parts, while higher rates are observed in the southern parts. More complete vaccination coverage was reported in the 1990 survey, followed by 2008, 1999 and 2003. In addition, children from mothers with higher levels of education, who were delivered in hospitals, who lived in urban areas, and whose mothers work outside the home had significantly higher proportions of completed basic vaccination. CONCLUSION: Much more work needs to be done if more children are to be covered and thus reduce vaccine-preventable diseases. 2013 John Wiley & Sons Ltd
SN - 1365-2214
AD - Institute of Child Health, College of Medicine, University of Ibadan, Ibadan, Nigeria
UR - 23573874
ER -

TY - JOUR
ID - 74
T1 - Family planning and pregnancy issues for women with systemic inflammatory diseases: patient and physician perspectives
A1 - Chakravarty,E.
A1 - Clowse,M.E.
A1 - Pushparajah,D.S.
A1 - Mertens,S.
A1 - Gordon,C.
Y1 - 2014///
N1 - Chakravarty, Eliza. Clowse, Megan E B. Pushparajah, Daphnee S. Mertens, Sarah. Gordon, Caroline
BMJ open
101552874

Journal Article
English
KW - MEDLINE
RP - NOT IN FILE
SP - e004081
JF - BMJ Open
VL - 4
IS - 2
CY - England
N2 - OBJECTIVES: To identify family planning and pregnancy (FPP) issues for female patients of childbearing age living with a chronic inflammatory disease and to assess whether current clinical practice routinely provides adequate support to alleviate these concerns. SETTING: Multinational survey and an analysis of online patient activity. PARTICIPANTS: Premenopausal women (aged 20-45 years; N=969) were surveyed in the USA, the UK, Germany, France, Italy and Spain. Rheumatologists were surveyed in Germany (N=50), France (N=50), Italy (N=50) and the USA (N=100), and gastroenterologists were also surveyed in the USA (N=100). PRIMARY AND SECONDARY OUTCOME MEASURES: Two online surveys were undertaken to identify FPP issues for physicians and patients. The surveys examined the frequency of dialogue on these topics between physicians and patients, alongside assessment of patient satisfaction regarding these conversations. Online analysis identified key themes for patient discussion outside their doctors' office/clinic/surgery. RESULTS: 32-56% of physicians spontaneously reported having talked about FPP with their female patients of childbearing age. When prompted, the majority of rheumatologists (74-92%) and gastroenterologists (74%) reported having discussed conception/pregnancy with female patients; however, less than half reported consulting their patient's treating general practitioner/gynaecologist about these topics. The majority of patients reported their FPP-related concerns are not adequately addressed/settled during their medical appointments. Furthermore, only 30-40% of patients considered advice/information to be consistent across multiple healthcare professionals. Key online FPP-related patient discussions included disease state, adverse effects, treatment, switch behaviour and wash-out requirements. CONCLUSIONS: Female patients who live with chronic inflammatory disease have important FPP concerns. The majority of patients, however, do not feel that their FPP concerns are adequately addressed in current clinical practice and report that they receive inconsistent advice from the various healthcare professionals who manage different aspects of their care. There is a clear need for provision of up-to-date and consistent information/support to female patients
SN - 2044-6055
AD - Oklahoma Medical Research Foundation, Oklahoma City, Oklahoma, USA
UR - 24500612
ER -

TY - JOUR
ID - 75
T1 - Increasing the athletic group play of children with autism
A1 - Miltenberger,C.A.
A1 - Charlop,M.H.
Y1 - 2014/01//
N1 - Miltenberger, Catherine A. Charlop, Marjorie H
Journal of autism and developmental disorders
7904301, hgw
IM
Journal Article
English
KW - MEDLINE
RP - NOT IN FILE
SP - 41
EP - 54
JF - Journal of Autism & Developmental Disorders
JA - J Autism Dev Disord
VL - 44
IS - 1
CY - United States

N2 - A multiple baseline design across three children with autism and within child across activity was used to assess the effects of interventions designed to teach children with autism to play two common athletic group games, handball and 4-square. Treatment consisted of two phases. In Phase I, athletic skills training, the children participated in sessions designed facilitate their acquisition of the athletic skills required by the targeted games. During Phase II, rules training, the children were instructed on the rules of the targeted games. Mastering the athletic skills and participating in rules training resulted in increased athletic group play and concomitant increases in speech. These gains were maintained at 8-16 weeks follow-up. However, generalization to participation in school recess activities did not occur
SN - 1573-3432
AD - Claremont Graduate University, 850 Columbia Ave, Claremont, CA, 91711, USA
UR - 23700189
ER -

TY - JOUR
ID - 76
T1 - School reform: the role of physical education policy in physical activity of elementary school children in Alabama's Black Belt Region
A1 - Robinson,L.E.
A1 - Wadsworth,D.D.
A1 - Webster,E.K.
A1 - Bassett,D.R.,Jr.
Y1 - 2014/01//
N1 - Robinson, Leah E. Wadsworth, Danielle D. Webster, E Kipling. Bassett, David R Jr
American journal of health promotion : AJHP
amj, 8701680
T
Journal Article. Research Support, Non-U.S. Gov't
English
KW - MEDLINE
RP - NOT IN FILE
SP - S72
EP - S76
JF - American Journal of Health Promotion
JA - Am J Health Promot
VL - 28
IS - 3 Suppl
CY - United States
N2 - PURPOSE: To investigate the implementation of state- and school-mandated physical education (PE) and physical activity policies on students' school-day physical activity behaviors. DESIGN: Observational, cross-sectional study. SETTING: Five elementary schools in rural Alabama. SUBJECTS: Six hundred and eighty-three school-age children. MEASURES: School-day physical activity behaviors were assessed with pedometer step count, the System for Observing Fitness Instruction Time (SOFIT), and the System for Observing Play and Leisure Activity in Youth (SOPLAY). ANALYSIS: Descriptive statistics (means and standard deviations) were used to describe the current health and in-school physical activity behaviors of students. RESULTS: Students accumulated a daily average of 4079.57 (+ 1678.1) and 3473.44 (+ 1073.37) steps for boys and girls, respectively, throughout the school day. SOFIT findings indicate that students spent an average of 23.80 + 5.33 min/d in PE and that 14.33 + 6.10 min/d were spent in moderate to vigorous physical activity (MVPA). This equates to 59.38% of PE time spent in MVPA. SOPLAY findings showed that recess and before/after school activity was not provided. CONCLUSIONS: It appears that schools were in compliance with the state-mandated PE policies on paper but not in actuality. Students did not attend PE for 30 min/d. The implementation of PE as the sole physical activity opportunity provides inadequate amounts of physical activity during the school day
SN - 0890-1171
UR - 24380469
ER -

TY - JOUR
ID - 77

T1 - Impact of the Boston Active School Day policy to promote physical activity among children
A1 - Cradock,A.L.
A1 - Barrett,J.L.
A1 - Carter,J.
A1 - McHugh,A.
A1 - Sproul,J.
A1 - Russo,E.T.
A1 - Dao-Tran,P.
A1 - Gortmaker,S.L.
Y1 - 2014/01//
N1 - Cradock, Angie L. Barrett, Jessica L. Carter, Jill. McHugh, Anne. Sproul, Jonathan. Russo, Elizabeth T. Dao-Tran, Patricia. Gortmaker, Steven L
American journal of health promotion : AJHP
amj, 8701680
T
Journal Article. Research Support, N.I.H., Extramural. Research Support, Non-U.S. Gov't. Research Support, U.S. Gov't, P.H.S.
English
KW - MEDLINE
RP - NOT IN FILE
SP - S54
EP - S64
JF - American Journal of Health Promotion
JA - Am J Health Promot
VL - 28
IS - 3 Suppl
CY - United States
N2 - PURPOSE: To test effectiveness of Active School Day policy implementation on physical activity outcomes and estimate school-level implementation costs. DESIGN: The design of the study was quasi-experimental (pretest-posttest matched controls). SETTING: The study took place in six elementary schools with three matched pairs in Boston, Massachusetts, February to June 2011. SUBJECTS: Subjects were 455 consenting fourth- and fifth-grade students among 467 eligible. INTERVENTION: Active School Day policy implementation provided equipment, curricular materials, and training to physical educators and school wellness champions to promote 150 weekly minutes of quality physical education, recess, and physical activity integrated into classrooms. MEASURES: Accelerometer assessments of accumulated minutes and bouts of moderate, vigorous, and sedentary physical activity on 5 school days before and after implementation were used. Implementation costs were collected by record review and reported resource utilization. ANALYSIS: Analysis was conducted using multivariate mixed models estimated with repeated measures of daily physical activity, adjusted for student demographics and other confounding and design/clustering variables. RESULTS: Accelerometer data were provided by 201 intervention and 192 comparison students for an average of 4 days per period (84% response). During school time, students in intervention schools demonstrated greater increases in minutes per day of moderate-to-vigorous physical activity (3.9, 95% confidence interval [CI] 1.8-6.0; $p < .001$) and vigorous physical activity (1.8, 95% CI .7-3.0; $p < .001$), and greater decreases in minutes per day of sedentary time (-10.6, 95% CI -15.3- -5.8; $p < .001$) than controls. Ongoing annual implementation costs totaled $4,523/school ($14/student). CONCLUSION: Active School Day implementation increased student moderate-to-vigorous physical activity levels by 24% and decreased sedentary time during school at modest cost
SN - 0890-1171
UR - 24380467
ER -

TY - JOUR
ID - 78
T1 - Childcare outdoor renovation as a built environment health promotion strategy: evaluating the preventing obesity by design intervention
A1 - Cosco,N.G.
A1 - Moore,R.C.
A1 - Smith,W.R.

Y1 - 2014/01//
N1 - Cosco, Nilda G. Moore, Robin C. Smith, William R
American journal of health promotion : AJHP
amj, 8701680
T
Journal Article. Research Support, Non-U.S. Gov't
English
KW - MEDLINE
RP - NOT IN FILE
SP - S27
EP - S32
JF - American Journal of Health Promotion
JA - Am J Health Promot
VL - 28
IS - 3 Suppl
CY - United States
N2 - PURPOSE: To evaluate the effectiveness of Preventing Obesity by Design (POD), a childcare center outdoor renovation intervention. DESIGN: Pre-post intervention evaluation. SETTING: North Carolina licensed childcare centers (N = 27). SUBJECTS: Preschool children. INTERVENTION: Outdoor renovation, teacher training. MEASURES: Behavior mapping, Preschool Outdoor Environment Measurement Scale (POEMS), center director interview. ANALYSIS: Descriptive statistics, ordinary least squares and logistic regressions calculated to assess levels of association between environmental change, children's physical activity (PA), social behaviors, and environmental quality. Qualitative interview data analyzed to help understand intervention impact. RESULTS: Behavior mapping showed that site layout attributes, such as the form (i.e., "single loop" and "double loop") of pathways (functioning as circulation routes and wheeled toy settings), are associated with higher levels of PA. Teacher interaction was associated with decreased children's PA. Absence of teacher or lack of child/child interaction was associated with increased PA. POEMS assessment of environmental quality was higher after renovation. POEMS domains (Physical Space and Teacher/Caregiver Roles) were positively associated with PA. After renovation, 68% of center directors reported positive changes in children's behavior and 40% mentioned edible plant installations as greatest success. CONCLUSION: Built environment renovation of childcare center outdoors, including looped pathways installation, coupled with teacher training, may support increased PA. Renovation, including food gardens, may be a key to success for preschool health promotion and support change in childcare policy
SN - 0890-1171
UR - 24380462
ER -

TY - JOUR
ID - 79
T1 - The potential for pocket parks to increase physical activity
A1 - Cohen,D.A.
A1 - Marsh,T.
A1 - Williamson,S.
A1 - Han,B.
A1 - Derose,K.P.
A1 - Golinelli,D.
A1 - McKenzie,T.L.
Y1 - 2014/01//
N1 - Cohen, Deborah A. Marsh, Terry. Williamson, Stephanie. Han, Bing. Derose, Kathryn Pitkin. Golinelli, Daniella. McKenzie, Thomas L
American journal of health promotion : AJHP
amj, 8701680
T
Journal Article. Research Support, N.I.H., Extramural
English
KW - MEDLINE
RP - NOT IN FILE

SP - S19
EP - S26
JF - American Journal of Health Promotion
JA - Am J Health Promot
VL - 28
IS - 3 Suppl
CY - United States
N2 - PURPOSE: To assess the use of new pocket parks in low-income neighborhoods. DESIGN: The design of the study was a quasi-experimental post-test only comparison. SETTING: Los Angeles, California, was the setting for the study. SUBJECTS: Subjects were park users and residents living within .5 mile of three pocket parks and 15 neighborhood parks. INTERVENTION: The creation of pocket parks. MEASURES: We used the System of Observing Play and Recreation in Communities (SOPARC) tool to measure park use and park-based physical activity, and then surveyed park users and residents about their park use. ANALYSIS: We surveyed 392 and 432 household members within .5 mile of the three pocket parks before and after park construction, respectively, as well as 71 pocket park users, and compared them to 992 neighborhood park users and 342 residents living within .5 mile of other neighborhood parks. We compared pocket park use to playground area use in the larger neighborhood parks. We used descriptive statistics and generalized estimating equations for the analysis. RESULTS: Overall, pocket park use compared favorably in promoting moderate-to-vigorous physical activity with that of existing playground space in nearby parks, and they were cost-effective at $0.73/MET hour (metabolic equivalent hour) gained. Pocket park visitors walked an average of .25 miles to get to a park. CONCLUSIONS: Pocket parks, when perceived as attractive and safe destinations, may increase physical activity by encouraging families with children to walk there. Additional strategies and programs may be needed to encourage more residents to use these parks
SN - 0890-1171
UR - 24380461
ER -

TY - JOUR
ID - 80
T1 - Impact of recess interventions on children's physical activity--a meta-analysis
A1 - Erwin,H.E.
A1 - Ickes,M.
A1 - Ahn,S.
A1 - Fedewa,A.
Y1 - 2014/01//
N1 - Erwin, Heather E. Ickes, Melinda. Ahn, Soyeon. Fedewa, Alicia
American journal of health promotion : AJHP
amj, 8701680
T
Journal Article
English
KW - MEDLINE
RP - NOT IN FILE
SP - 159
EP - 167
JF - American Journal of Health Promotion
JA - Am J Health Promot
VL - 28
IS - 3
CY - United States
N2 - OBJECTIVE: The purpose of this paper was to explore the effect of recess interventions on children's physical activity (PA) levels and to examine which specific interventions/characteristics have more influence on children's PA. DATA SOURCE: Academic Search Premier, CINAHL, ERIC, MEDLINE, ProQuest, SPORT Discus. STUDY INCLUSION AND EXCLUSION CRITERIA: Papers published between 1986 and 2012, published in the English language, and evaluating any recess intervention with PA as an outcome. DATA EXTRACTION: Study outcomes were distilled into seven domains related to PA: age, gender, intervention type, intervention duration, duration of PA per intervention session, outcome measures, and study region.

DATA SYNTHESIS: Effect sizes were pooled within and across studies for each domain separately. Moderator analyses were performed using gender, age, and total length of intervention. Twenty-three independent samples were provided from 13 studies. RESULTS: The estimated overall effect size for time in PA was .56 (SE .07), suggesting a positive and significant mean difference between pretest and posttest PA time after implementing a recess intervention. Differences in PA levels were moderated by age, duration and type of intervention, and type of outcome measures. CONCLUSION. The limited evidence suggests recess interventions can improve youth PA levels. Implications reinforce the importance of schools in advocating for recess during the school day. Strategies shown to increase student PA during recess should be implemented
SN - 0890-1171
UR - 23875990
ER -

TY - JOUR
ID - 81
T1 - Work-life balance of female versus male surgeons in Hong Kong based on findings of a questionnaire designed by a Japanese surgeon
A1 - Kwong,A.
A1 - Chau,W.W.
A1 - Kawase,K.
Y1 - 2014/01//
N1 - Kwong, Ava. Chau, Wai Wang. Kawase, K
Surgery today
bfy, 9204360
IM
Journal Article
English
KW - MEDLINE
RP - NOT IN FILE
SP - 62
EP - 72
JF - Surgery Today
JA - SURG
VL - 44
IS - 1
CY - Japan
N2 - PURPOSE: We evaluated the attitudes of female and male surgeons in Hong Kong to work, personal life, and work-life balance, through a questionnaire survey. METHODS: We sent an online questionnaire survey designed by a female Japanese surgeon to 142 female surgeons practicing in Hong Kong, while its customized version was sent to 953 of their male counterparts. RESULTS: "Home life" and "Work" were the first and second priorities for both the women and the men. More of the female surgeons reported that they could not find enough time to participate in community activities ($p = 0.038$) or rest ($p = 0.024$). Both reported moderate satisfaction at work ($p = 0.114$) and in their life outside work ($p = 0.346$), as well as equality at home ($p = 0.548$) and at work ($p = 0.177$). Of the men, 89 % agreed with the necessity for granting paternity leave and reported that they would like to work part-time during the child-rearing years ($p = 0.013$). CONCLUSIONS: The number of female surgeons involved in key roles is increasing in parallel with the increasing number of female medical students. The introduction of paternity leave for male surgeons is an important concern. The issues of work-life balance may need to be addressed to attract more talent into an important specialty in Hong Kong and this is likely to apply to other parts of Asia as well
SN - 1436-2813
AD - Division of Breast Surgery, Department of Surgery, The University of Hong Kong, Hong Kong, Hong Kong, akwong@asiabreastregistry.com
UR - 23595786
ER -

TY - JOUR
ID - 82

T1 - Studying the effects of smell and taste experience in the pediatric population using functional near infrared spectroscopy: a hypothesis
A1 - Rohlfs-Dominguez,P.
Y1 - 2014/01//
N1 - Rohlfs-Dominguez, Paloma
Medical hypotheses
m0m, 7505668
IM
Journal Article
English
KW - MEDLINE
RP - NOT IN FILE
SP - 89
EP - 93
JF - Medical Hypotheses
JA - Med Hypotheses
VL - 82
IS - 1
CY - United States
N2 - There are different postnatal sensitive periods throughout the development course of sensory functions. During sensitive periods, there is a biological display of an extreme neural sensitivity to the storage of experience-driven sensory information that is not present outside these developmental stages. This neural property is reflected in subjects' reported preferences for sensory stimuli, such as odors and tastes. The human brain mapping approach (HBA) has demonstrated that disease-free human postnatal and later development of any sensory function parallels morphological and functional development of the CNS and that this development correlates with signal changes that have been acquired by means of neuroimaging techniques. Whether experience with tastes and/or odors has a stronger effect on the perception of gustatory and/or olfactory stimuli the earlier subjects are exposed to certain odors and tastes is still unknown. It is also unknown, whether as well as how this effect is reflected in brain activation patterns and whether we are currently able to identify sensitive periods of gustatory and olfactory development from the imaging signals. To answer these research questions, repeated exposure to tastes and/or odors should be applied in children of different age ranges in order to induce different age-related degrees of olfactory/gustatory preferences as well as different aged-related patterns of oxyhemoglobin (OH) and deoxyhemoglobin (DOH) changes that should be measured by means of the functional near-infrared spectroscopy (fNIRS) technique. Copyright 2013 Elsevier Ltd. All rights reserved
SN - 1532-2777
AD - Department of Psychology and Anthropology of the University of Extremadura, Spain. Electronic address: prohlfsdomnguez@yahoo.es
UR - 24315448
ER -

TY - JOUR
ID - 83
T1 - Effectiveness and feasibility of lowering playground density during recess to promote physical activity and decrease sedentary time at primary school
A1 - D'Haese,S.
A1 - Van,Dyck D.
A1 - De,Bourdeaudhuij,I
A1 - Cardon,G.
Y1 - 2013///
N1 - D'Haese, Sara. Van Dyck, Delfien. De Bourdeaudhuij, Ilse. Cardon, Greet
BMC public health
100968562
IM
Journal Article. Research Support, Non-U.S. Gov't
English
KW - MEDLINE
RP - NOT IN FILE

SP - 1154
JF - BMC Public Health
VL - 13
CY - England
N2 - BACKGROUND: This pilot study aimed at investigating the effectiveness of lowering playground density on increasing children's physical activity and decreasing sedentary time. Also the feasibility of this intervention was tested. METHODS: Data were collected in September and October 2012 in three Belgian schools in 187, 9-12 year old children. During the intervention, playground density was decreased by splitting up recesses and decreasing the number of children sharing the playground. A within-subject design was used. Children wore accelerometers during the study week. Three-level (class - participant - measurement (baseline or intervention)) linear regression models were used to determine intervention effects. After the intervention week the school principals filled out a questionnaire concerning the feasibility of the intervention. RESULTS: The available play space was 12.18 + 4.19 m2/child at baseline and increased to 24.24 + 8.51 m2/child during intervention. During the intervention sedentary time decreased (-0.58 min/recess; -3.21%/recess) and moderate-to-vigorous physical activity (+1.04 min/recess; +5.9%/recess) increased during recess and during the entire school day (sedentary time: -3.29%/school day; moderate-to-vigorous physical activity +1.16%/school day). All principals agreed that children enjoyed the intervention; but some difficulties were reported. CONCLUSIONS: Lowering playground density can be an effective intervention for decreasing children's sedentary time and increasing their physical activity levels during recess; especially in least active children
SN - 1471-2458
AD - Faculty of Medicine and Health Sciences, Department of Movement and Sports Sciences, Ghent University, Watersportlaan 2, Ghent 9000, Belgium. sara.dhaese@ugent.be
UR - 24325655
ER -

TY - JOUR
ID - 84
T1 - Energy consumption, body composition and physical activity levels in 11- to 13-year-old Spanish children
A1 - Cordova,A.
A1 - Villa,G.
A1 - Sureda,A.
A1 - Rodriguez-Marroyo,J.A.
A1 - Martinez-Castaneda,R.
A1 - Sanchez-Collado,M.P.
Y1 - 2013///
N1 - Cordova, Alfredo. Villa, Gerardo. Sureda, Antoni. Rodriguez-Marroyo, Jose A. Martinez-Castaneda, Raul. Sanchez-Collado, Maria P
Annals of nutrition & metabolism
58e, 8105511
IM
Journal Article. Research Support, Non-U.S. Gov't
English
KW - MEDLINE
RP - NOT IN FILE
SP - 223
EP - 228
JF - Annals of Nutrition & Metabolism
JA - Ann Nutr Metab
VL - 63
IS - 3
CY - Switzerland
N2 - BACKGROUND/AIMS: The prevalence of overweight and obesity in childhood is increasing markedly. The aim of this study was to evaluate the relationship between physical activity, energy consumption and weight status in a cohort of Spanish children. METHODS: A total of 137 children (11-13 years old) participated in the study voluntarily (with paternal consent). Children were classified into 3 groups according to their physical activity, i.e. sedentary, active and sporty groups. Body composition measures, physical fitness and total caloric and macronutrient intake were determined. RESULTS: Weight, body mass index, waist circumference, skinfold

thickness measures and body fat percentage of children were lower in the active children, whereas body water content increased with activity. Nutritional habits were similar in the 3 studied groups. No significant differences in total energy intake or percentage of carbohydrates, fat and proteins were found. All parameters related to caloric expenditure were higher in children carrying out more physical activity. CONCLUSIONS: Children with higher levels of physical activity presented more favorable anthropometric profiles, but there were no differences in respect to their dietary habits. An increase in weekly energy expenditure through physical activity outside school seems essential to prevent overweight and the risk of childhood obesity
SN - 1421-9697
AD - Departamento de Bioquimica, Biologia Molecular y Fisiologia, Facultad de Fisioterapia, Universidad de Valladolid, Soria, Spain
UR - 24192533
ER -

TY - JOUR
ID - 85
T1 - Primary prevention of overweight in preschool children, the BeeBOFT study (breastfeeding, breakfast daily, outside playing, few sweet drinks, less TV viewing): design of a cluster randomized controlled trial
A1 - Raat,H.
A1 - Struijk,M.K.
A1 - Remmers,T.
A1 - Vlasblom,E.
A1 - van,Grieken A.
A1 - Broeren,S.M.
A1 - Te Velde,S.J.
A1 - Beltman,M.
A1 - Boere-Boonekamp,M.M.
A1 - L'Hoir,M.P.
Y1 - 2013///
N1 - Raat, Hein. Struijk, Mirjam K. Remmers, Teun. Vlasblom, Eline. van Grieken, Amy. Broeren, Suzanne M L. te Velde, Saskia J. Beltman, Maaike. Boere-Boonekamp, Magda M. L'Hoir, Monique P
BMC public health
100968562
IM
Journal Article. Research Support, Non-U.S. Gov't
English
KW - MEDLINE
RP - NOT IN FILE
SP - 974
JF - BMC Public Health
VL - 13
CY - England
N2 - BACKGROUND: Two overweight prevention interventions were developed to be offered by preventive Youth Health Care (YHC) in addition to the currently applied overweight prevention protocol to parents of 0-3 year old children. The two interventions aim to support parents of preschool children to realize healthy child nutrition and activity behaviors of their young child. The aim of this study is to assess the effects of the two overweight prevention interventions with regard to child health behaviors and child Body Mass Index. METHODS/DESIGN: A cluster randomized controlled trial was conducted among parents and their preschool children who attend one of 51 participating YHC teams. The teams were randomly allocated to one of the two intervention groups, or to the control group (care as usual).The 'BBOFT+' intervention focuses on effective child rearing by parents from birth onwards by enlarging parental skills concerning healthy behavioural life-style habits. Parents who are allocated to the 'E-health4Uth Healthy toddler' intervention group, at the child age of circa 18 and 24 months old, are invited to complete an online E-health module providing tailored health education regarding healthy child nutrition and activity behaviors. The E-health messages are discussed and reinforced during the subsequent regularly scheduled visits by YHC professionals, and were repeated after 4 weeks.The primary outcome measures at child age 3 years are: overweight inducing/reducing behaviors, (for 'BBOFT+' only) healthy sleep, Body Mass Index and prevalence of overweight and obesity. Secondary outcome measures are attitudes and other cognitive characteristics of the parents regarding the overweight-related

behaviors of their child, parenting styles and practices, and health-related quality of life of the children. DISCUSSION: We hypothesize that the use of the additional interventions will result in a healthier lifestyle of preschool children and an improved BMI and less development of overweight and obesity compared to usual care. TRIAL REGISTRATION: Nederlands Trial Register NTR1831
SN - 1471-2458
AD - Department of Public Health, Erasmus MC - University Medical Center Rotterdam, Dr, Molewaterplein 50, PO Box 2040, 3000 CA Rotterdam, the Netherlands. h.raat@erasmusmc.nl
UR - 24138805
ER -

TY - JOUR
ID - 86
T1 - Evaluation of policies and practices to prevent mother to child transmission of hepatitis B virus in China: results from China GAVI project final evaluation
A1 - Cui,F.
A1 - Luo,H.
A1 - Wang,F.
A1 - Zheng,H.
A1 - Gong,X.
A1 - Chen,Y.
A1 - Wu,Z.
A1 - Miao,N.
A1 - Kane,M.
A1 - Hennessey,K.
A1 - Hadler,S.C.
A1 - Hutin,Y.J.
A1 - Liang,X.
A1 - Yang,W.
Y1 - 2013/12/27/
N1 - Cui, Fuqiang. Luo, Huiming. Wang, Fuzhen. Zheng, Hui. Gong, Xiaohong. Chen, Yuansheng. Wu, Zhenhua. Miao, Ning. Kane, Mark. Hennessey, Karen. Hadler, Stephen C. Hutin, Yvan J. Liang, Xiaofeng. Yang, Weizhong
Vaccine
x6o, 8406899
IM
Journal Article
English
KW - MEDLINE
RP - NOT IN FILE
SP - J36
EP - J42
JF - Vaccine
VL - 31 Suppl 9
CY - Netherlands
N2 - BACKGROUND: Mother to Child Transmission (MTCT) has remained a leading cause of HBV infection in China, accounting for 40% of total infections. Providing hepatitis B vaccine (HepB) to all infants within 24h of birth (Timely Birth Dose, TBD), and subsequent completion of at least 3 vaccine doses is key to preventing perinatal HBV infection. In 2002, with the financial support of the Global Alliance on Vaccine and Immunization (GAVI) targeted to Western region and 223 poverty-affected counties in Central region, hepatitis B vaccine was provided for free. In 2010, we evaluated the China GAVI project in terms of its activities to prevent perinatal infections. OBJECTIVE: The objectives of the evaluation were to (1) measure achievements in the China GAVI project in terms of TBD coverage, and (2) describe practices for HBsAg screening of pregnant women and HBIG use outside the GAVI China project. METHODS: We used the methods recommended by WHO to select a cluster sample of health care facilities for the purpose of an injection safety assessment. We stratified China into three regions based on economic criteria, and selected eight counties with a probability proportional to population size in each region. In each selected county, we selected (a) 10 townships at random among the list of townships of the county and (b) the one county level hospital. In each hospital, we abstracted

2002 through 2009 records to collect information regarding birth cohorts, hospitals deliveries, vaccine management, hepatitis B vaccination delivery, HBsAg screening practices and results, and HBIG administration. In addition, in all hospitals, we abstracted records regarding the delivery of TBD. RESULTS: We visited 244 facilities in the three regions, including 24 county hospitals and 220 township hospitals. We reviewed 837,409 birth summary records, 699,249 for infants born at county or township hospitals. Hospital delivery rates increased from 58% in 2002 to 93% in 2009. Surveyed TBD coverage increased from 60% in 2002 to 91% in 2009 (+31%). Surveyed TBD coverage among children born in hospitals increased from 73% in 2002 to 98% in 2009. Between 2002 and 2009, the proportion of pregnant women screened for HBsAg increased from 64% in 2002 to 85% in 2009. In 2009, the proportion of infants born to women screened and found to be HBsAg positive who did not receive any immunization within 24h after birth ranged from 0% to 0.7% across regions. CONCLUSIONS: Increased availability of hepatitis B vaccine, along with efforts to improve hospital deliveries, increased TBD coverage in China. This decreased perinatal HBV transmission and will reduce disease burden in the future. Screening for HBsAg to guide HBIG administration has begun, but with heterogeneous immuno-prophylaxis practices and a poor system for follow up. Copyright 2012 Elsevier Ltd. All rights reserved
SN - 1873-2518
AD - Chinese Center for Disease Control and Prevention, Beijing, China., Chinese Center for Disease Control and Prevention, Beijing, China., Chinese Center for Disease Control and Prevention, Beijing, China., Chinese Center for Disease Control and Prevention, Beijing, China., Chinese Center for Disease Control and Prevention, Beijing, China., Chinese Center for Disease Control and Prevention, Beijing, China., Chinese Center for Disease Control and Prevention, Beijing, China., Chinese Center for Disease Control and Prevention, Beijing, China., Mercer Island, WA, USA., World Health Organization, West Pacific Region, Manila, Philippines., Centers for Disease Control and Prevention, Atlanta, USA., World Health Organization office in China, Beijing, China., Chinese Center for Disease Control and Prevention, Beijing, China., Chinese Center for Disease Control and Prevention, Beijing, China. Electronic address: Yangwz@chinacdc.cn
UR - 24331019
ER -

TY - JOUR
ID - 87
T1 - Detecting indoor and outdoor environments using the ActiGraph GT3X+ light sensor in children
A1 - Flynn,J.I.
A1 - Coe,D.P.
A1 - Larsen,C.A.
A1 - Rider,B.C.
A1 - Conger,S.A.
A1 - Bassett,D.R.,Jr.
Y1 - 2014/01//
N1 - Flynn, Jennifer I. Coe, Dawn P. Larsen, Chelsea A. Rider, Brian C. Conger, Scott A. Bassett, David R Jr
Medicine and science in sports and exercise
8005433, mg8
IM, S
Journal Article
English
KW - MEDLINE
RP - NOT IN FILE
SP - 201
EP - 206
JF - Medicine & Science in Sports & Exercise
JA - Med Sci Sports Exerc
VL - 46
IS - 1
CY - United States
N2 - INTRODUCTION: Experts recommend children spend more time playing outdoors. The ambient light sensor of the ActiGraph GT3X+ provides lux measurements. A lux is the International System's unit of illumination, equivalent to 1 lmm. Few studies have established a lux threshold for determining whether a child is indoors or outdoors. PURPOSE: This study aimed 1) to assess the reliability of the ActiGraph GT3X+

ambient light sensor, 2) to identify a lux threshold to accurately discriminate between indoor and outdoor activities in children, and 3) to test the accuracy of the lux threshold in a free-living environment. METHODS: In part 1, a series of reliability tests were performed using 20 ActiGraph GT3X+ monitors under different environmental conditions. Cronbach's alpha was used to determine interinstrument reliability. In part 2, 18 children performed 11 different activities (five indoors and six outdoors) for 6 min each. The optimal threshold for detecting indoor/outdoor activity was determined using a receiver operator characteristic curve analysis. In part 3, 18 children at a preschool wore the monitor during a school day. Percent accuracy was determined for all conditions. RESULTS: In part 1, the devices had Cronbach's alpha values of 0.992 and 1.000 for indoor and outdoor conditions, respectively, indicating high interinstrument reliability. In part 2, the optimal lux threshold was determined to be 240 lux (sensitivity = 0.92, specificity = 0.88, area under the curve = 0.96, 95% CI = 0.951-0.970). In part 3, results of the school-day validation demonstrated the monitor was 97.0% accurate for overall detection of indoor and outdoor conditions (outdoor = 88.9%, indoor = 99.1%). CONCLUSIONS: The results demonstrate that an ActiGraph GT3X+ lux threshold of 240 can accurately assess indoor and outdoor conditions of preschool children in a free-living environment
SN - 1530-0315
AD - Department of Kinesiology, Recreation, and Sport Studies, University of Tennessee, Knoxville, TN
UR - 23846164
ER -

TY - JOUR
ID - 88
T1 - Vitamin C for asthma and exercise-induced bronchoconstriction
A1 - Milan,S.J.
A1 - Hart,A.
A1 - Wilkinson,M.
Y1 - 2013///
N1 - Milan, Stephen J. Hart, Anna. Wilkinson, Mark
The Cochrane database of systematic reviews
100909747
IM
Journal Article. Research Support, Non-U.S. Gov't
English
KW - MEDLINE
RP - NOT IN FILE
SP - CD010391
JF - Cochrane Database of Systematic Reviews
JA - Cochrane Database Syst Rev
VL - 10
CY - England
N2 - BACKGROUND: Dietary antioxidants, such as vitamin C, in the epithelial lining and lining fluids of the lung may be beneficial in the reduction of oxidative damage (Arab 2002). They may therefore be of benefit in reducing symptoms of inflammatory airway conditions such as asthma, and may also be beneficial in reducing exercise-induced bronchoconstriction, which is a well-recognised feature of asthma and is considered a marker of airways inflammation. However, the association between dietary antioxidants and asthma severity or exercise-induced bronchoconstriction is not fully understood. OBJECTIVES: To examine the effects of vitamin C supplementation on exacerbations and health-related quality of life (HRQL) in adults and children with asthma or exercise-induced bronchoconstriction compared to placebo or no vitamin C. SEARCH METHODS: We identified trials from the Cochrane Airways Group's Specialised Register (CAGR). The Register contains trial reports identified through systematic searches of a number of bibliographic databases, and handsearching of journals and meeting abstracts. We also searched trial registry websites. The searches were conducted in December 2012. SELECTION CRITERIA: We included randomised controlled trials (RCTs). We included both adults and children with a diagnosis of asthma. In separate analyses we considered trials with a diagnosis of exercise-induced bronchoconstriction (or exercise-induced asthma). We included trials comparing vitamin C supplementation with placebo, or vitamin C supplementation with no supplementation. We included trials where the asthma management of both treatment and control groups provided similar background therapy. The primary focus of the review is on daily vitamin C supplementation to prevent exacerbations and improve HRQL. The short-term use of vitamin C at the time of exacerbations or for cold symptoms in people with asthma are outside

the scope of this review. DATA COLLECTION AND ANALYSIS: Two review authors independently screened the titles and abstracts of potential studies, and subsequently screened full text study reports for inclusion. We used standard methods expected by The Cochrane Collaboration. MAIN RESULTS: A total of 11 trials with 419 participants met our inclusion criteria. In 10 studies the participants were adults and only one was in children. Reporting of study design was inadequate to determine risk of bias for most of the studies and poor availability of data for our key outcomes may indicate some selective outcome reporting. Four studies were parallel-group and the remainder were cross-over studies. Eight studies included people with asthma and three studies included 40 participants with exercise-induced asthma. Five studies reported results using single-dose regimes prior to bronchial challenges or exercise tests. There was marked heterogeneity in vitamin C dosage regimes used in the selected studies, compounding the difficulties in carrying out meaningful analyses.One study on 201 adults with asthma reported no significant difference in our primary outcome, health-related quality of life (HRQL), and overall the quality of this evidence was low. There were no data available to evaluate the effects of vitamin C supplementation on our other primary outcome, exacerbations in adults. One small study reported data on asthma exacerbations in children and there were no exacerbations in either the vitamin C or placebo groups (very low quality evidence). In another study conducted in 41 adults, exacerbations were not defined according to our criteria and the data were not available in a format suitable for evaluation by our methods. Lung function and symptoms data were contributed by single studies. We rated the quality of this evidence as moderate, but further research is required to assess any clinical implications that may be related to the changes in these parameters. In each of these outcomes there was no significant difference between vitamin C and placebo. No adverse events at all were reported; again this is very low quality evidence.Studies in exercise-induced bronchoconstriction suggested some improvement in lung function measures with vitamin C supplementation, but theses studies were few and very small, with limited data and we judged the quality of the evidence to be low. AUTHORS' CONCLUSIONS: Currently, evidence is not available to provide a robust assessment on the use of vitamin C in the management of asthma or exercise-induced bronchoconstriction. Further research is very likely to have an important impact on our confidence in the estimates of effect and is likely to change the estimates. There is no indication currently that vitamin C can be recommended as a therapeutic agent in asthma. There was some indication that vitamin C was helpful in exercise-induced breathlessness in terms of lung function and symptoms; however, as these findings were provided only by small studies they are inconclusive. Most published studies to date are too small and inconsistent to provide guidance. Well-designed trials with good quality clinical endpoints, such as exacerbation rates and health-related quality of life scores, are required
SN - 1469-493X
AD - Population Health Sciences and Education, St George's University of London, London, UK
UR - 24154977
ER -

TY - JOUR
ID - 89
T1 - Barriers and facilitators to the implementation of lay health worker programmes to improve access to maternal and child health: qualitative evidence synthesis
A1 - Glenton,C.
A1 - Colvin,C.J.
A1 - Carlsen,B.
A1 - Swartz,A.
A1 - Lewin,S.
A1 - Noyes,J.
A1 - Rashidian,A.
Y1 - 2013///
N1 - Glenton, Claire. Colvin, Christopher J. Carlsen, Benedicte. Swartz, Alison. Lewin, Simon. Noyes, Jane. Rashidian, Arash
The Cochrane database of systematic reviews
100909747
IM
Journal Article. Research Support, Non-U.S. Gov't
English
KW - MEDLINE
RP - NOT IN FILE

SP - CD010414
JF - Cochrane Database of Systematic Reviews
JA - Cochrane Database Syst Rev
VL - 10
CY - England
N2 - BACKGROUND: Lay health workers (LHWs) perform functions related to healthcare delivery, receive some level of training, but have no formal professional or paraprofessional certificate or tertiary education degree. They provide care for a range of issues, including maternal and child health. For LHW programmes to be effective, we need a better understanding of the factors that influence their success and sustainability. This review addresses these issues through a synthesis of qualitative evidence and was carried out alongside the Cochrane review of the effectiveness of LHWs for maternal and child health. OBJECTIVES: The overall aim of the review is to explore factors affecting the implementation of LHW programmes for maternal and child health. SEARCH METHODS: We searched MEDLINE, OvidSP (searched 21 December 2011); MEDLINE Ovid In-Process & Other Non-Indexed Citations, OvidSP (searched 21 December 2011); CINAHL, EBSCO (searched 21 December 2011); British Nursing Index and Archive, OvidSP (searched 13 May 2011). We searched reference lists of included studies, contacted experts in the field, and included studies that were carried out alongside the trials from the LHW effectiveness review. SELECTION CRITERIA: Studies that used qualitative methods for data collection and analysis and that focused on the experiences and attitudes of stakeholders regarding LHW programmes for maternal or child health in a primary or community healthcare setting. DATA COLLECTION AND ANALYSIS: We identified barriers and facilitators to LHW programme implementation using the framework thematic synthesis approach. Two review authors independently assessed study quality using a standard tool. We assessed the certainty of the review findings using the CerQual approach, an approach that we developed alongside this and related qualitative syntheses. We integrated our findings with the outcome measures included in the review of LHW programme effectiveness in a logic model. Finally, we identified hypotheses for subgroup analyses in future updates of the review of effectiveness. MAIN RESULTS: We included 53 studies primarily describing the experiences of LHWs, programme recipients, and other health workers. LHWs in high income countries mainly offered promotion, counselling and support. In low and middle income countries, LHWs offered similar services but sometimes also distributed supplements, contraceptives and other products, and diagnosed and treated children with common childhood diseases. Some LHWs were trained to manage uncomplicated labour and to refer women with pregnancy or labour complications.Many of the findings were based on studies from multiple settings, but with some methodological limitations. These findings were assessed as being of moderate certainty. Some findings were based on one or two studies and had some methodological limitations. These were assessed have low certainty.Barriers and facilitators were mainly tied to programme acceptability, appropriateness and credibility; and health system constraints. Programme recipients were generally positive to the programmes, appreciating the LHWs' skills and the similarities they saw between themselves and the LHWs. However, some recipients were concerned about confidentiality when receiving home visits. Others saw LHW services as not relevant or not sufficient, particularly when LHWs only offered promotional services. LHWs and recipients emphasised the importance of trust, respect, kindness and empathy. However, LHWs sometimes found it difficult to manage emotional relationships and boundaries with recipients. Some LHWs feared blame if care was not successful. Others felt demotivated when their services were not appreciated. Support from health systems and community leaders could give LHWs credibility, at least if the health systems and community leaders had authority and respect. Active support from family members was also important.Health professionals often appreciated the LHWs' contributions in reducing their workload and for their communication skills and commitment. However, some health professionals thought that LHWs added to their workload and feared a loss of authority.LHWs were motivated by factors including altruism, social recognition, knowledge gain and career development. Some unsalaried LHWs wanted regular payment, while others were concerned that payment might threaten their social status or lead recipients to question their motives. Some salaried LHWs were dissatisfied with their pay levels. Others were frustrated when payment differed across regions or institutions. Some LHWs stated that they had few opportunities to voice complaints. LHWs described insufficient, poor quality, irrelevant and inflexible training programmes, calling for more training in counselling and communication and in topics outside their current role, including common health problems and domestic problems. LHWs and supervisors complained about supervisors' lack of skills, time and transportation. Some LHWs appreciated the opportunity to share experiences with fellow LHWs.In some studies, LHWs were traditional birth attendants who had received additional training. Some health professionals were concerned that these LHWs were over-confident about their ability to manage danger signs. LHWs and recipients pointed to other problems, including women's reluctance to be referred after bad experiences with health professionals, fear of caesarean sections, lack of transport, and cost. Some LHWs were reluctant to refer

women on because of poor co-operation with health professionals.We organised these findings and the outcome measures included in the review of LHW programme effectiveness in a logic model. Here we proposed six chains of events where specific programme components lead to specific intermediate or long-term outcomes, and where specific moderators positively or negatively affect this process. We suggest how future updates of the LHW effectiveness review could explore whether the presence of these components influences programme success. AUTHORS' CONCLUSIONS: Rather than being seen as a lesser trained health worker, LHWs may represent a different and sometimes preferred type of health worker. The close relationship between LHWs and recipients is a programme strength. However, programme planners must consider how to achieve the benefits of closeness while minimizing the potential drawbacks. Other important facilitators may include the development of services that recipients perceive as relevant; regular and visible support from the health system and the community; and appropriate training, supervision and incentives
SN - 1469-493X
AD - Global Health Unit, Norwegian Knowledge Centre for the Health Services, PO Box 7004 St Olavs plass, Oslo, Norway, N-0130
UR - 24101553
ER -

TY - JOUR
ID - 90
T1 - Elementary school practices and children's objectively measured physical activity during school
A1 - Carlson,J.A.
A1 - Sallis,J.F.
A1 - Norman,G.J.
A1 - McKenzie,T.L.
A1 - Kerr,J.
A1 - Arredondo,E.M.
A1 - Madanat,H.
A1 - Mignano,A.M.
A1 - Cain,K.L.
A1 - Elder,J.P.
A1 - Saelens,B.E.
Y1 - 2013/11//
N1 - Carlson, Jordan A. Sallis, James F. Norman, Gregory J. McKenzie, Thomas L. Kerr, Jacqueline. Arredondo, Elva M. Madanat, Hala. Mignano, Alexandra M. Cain, Kelli L. Elder, John P. Saelens, Brian E
Preventive medicine
pm4, 0322116
IM
Journal Article. Research Support, N.I.H., Extramural. Research Support, Non-U.S. Gov't
English
KW - MEDLINE
RP - NOT IN FILE
SP - 591
EP - 595
JF - Preventive Medicine
JA - Prev Med
VL - 57
IS - 5
CY - United States
N2 - OBJECTIVE: To examine the relation of physical activity practices covering physical education (PE), recess, and classroom time in elementary schools to children's objectively measured physical activity during school. METHODS: Participants were 172 children from 97 elementary schools in the San Diego, CA and Seattle, WA USA regions recruited in 2009-2010. Children's moderate-to-vigorous physical activity (MVPA) during school was assessed via accelerometry, and school practices were assessed via survey of school informants. Multivariate linear mixed models were adjusted for participant demographics and unstandardized regression coefficients are reported. The 5 practices with the strongest associations with physical activity were combined into an index to investigate additive effects of these practices on children's MVPA. RESULTS: Providing > 100 min/week of PE (B=6.7 more min/day; p=.049), having < 75 students/supervisor in recess

(B=6.4 fewer min/day; p=.031), and having a PE teacher (B=5.8 more min/day; p=.089) were related to children's MVPA during school. Children at schools with 4 of the 5 practices in the index had 20 more min/day of MVPA during school than children at schools with 0 or 1 of the 5 practices (p<.001). CONCLUSIONS: The presence of multiple school physical activity practices doubled children's physical activity during school. 2013
SN - 1096-0260
AD - University of California, San Diego, 9500 Gilman Dr., La Jolla, CA 92093 USA. Electronic address: jacarlson@ucsd.edu
UR - 23948107
ER -

TY - JOUR
ID - 91
T1 - Epidemiological and clinical features of paediatric open globe injuries in southwestern Turkey
A1 - Ilhan,H.D.
A1 - Bilgin,A.B.
A1 - Cetinkaya,A.
A1 - Unal,M.
A1 - Yucel,I.
Y1 - 2013///
N1 - Ilhan, Hatice Deniz. Bilgin, Ahmet Burak. Cetinkaya, Asli. Unal, Mustafa. Yucel, Iclal
International journal of ophthalmology
101553860
Journal Article
English
KW - MEDLINE
RP - NOT IN FILE
SP - 855
EP - 860
JF - International Journal of Ophthalmology
VL - 6
IS - 6
CY - China
N2 - AIM: To evaluate the epidemiologic, anatomic, and clinical features of open globe injuries in children. METHODS: The medical files of patients under the age of 16 who had been operated for an open globe injury at Akdeniz University Hospital's Department of Ophthalmology were retrospectively evaluated. RESULTS: A total of 90 patients were evaluated in this study. Among these patients, 26 (28.9%) were female and 64 (71.1%) were male. The mean age of the patients was 7.7+4.2 years. The male/female ratio was observed to increase with increasing age (P=0.006, r=7.48). Injuries were most likely to occur in spring and autumn (P=0.028). The time interval between the injury and the surgical repair was 9.36+27.4h. Forty (44.4%) of the injuries occurred in the home, 27 (30%) occurred in the yard, and 21 (23.3%) happened while playing in the street. The most common causes of injury were sharp metal objects (P<0.001). Injury to the cornea occurred in 47 (52.2%) of the patients (P<0.001). The most common complication to occur was cataract formation. Additional operations were necessary for 37 (41.1%) of the patients. The final visual acuity was correlated with both the initial visual acuity of the wounded eye prior to surgery and the length of the wound (P<0.001, r=0.502 and P<0.001, r=-0.442, respectively). CONCLUSION: Open globe injuries that are suffered in childhood generally occur either at home, in the yard, or on the street, with sharp metal objects being the most common cause of injury. The initial visual acuity and the length of the wound are the most important determinants of the final visual acuity
SN - 2222-3959
AD - Department of Ophthalmology, Akdeniz University, Antalya 07059, Turkey., Department of Ophthalmology, Akdeniz University, Antalya 07059, Turkey., Department of Ophthalmology, Akdeniz University, Antalya 07059, Turkey., Department of Ophthalmology, Akdeniz University, Antalya 07059, Turkey., Department of Ophthalmology, Akdeniz University, Antalya 07059, Turkey
UR - 24392337
ER -

TY - JOUR
ID - 92

T1 - Do preschools differ in promoting children's physical activity? An instrument for the assessment of preschool physical activity programmes
A1 - Sterdt,E.
A1 - Pape,N.
A1 - Kramer,S.
A1 - Urban,M.
A1 - Werning,R.
A1 - Walter,U.
Y1 - 2013///
N1 - Sterdt, Elena. Pape, Natalie. Kramer, Silke. Urban, Michael. Werning, Rolf. Walter, Ulla
BMC public health
100968562
IM
Journal Article. Research Support, Non-U.S. Gov't
English
KW - MEDLINE
RP - NOT IN FILE
SP - 795
JF - BMC Public Health
VL - 13
CY - England
N2 - BACKGROUND: Preschools offer high potential for preventive interventions. However, little is known about the structure of preschool programmes to promote physical activity (PA) in preschoolers although almost all children aged three to six years spend one third of the day at preschool. The aim of this study was to determine whether and to what extent preschools implement systematic PA promotion measures using an instrument specifically developed to assess and systematize preschool PA programmes. METHODS: In the cross-sectional study a baseline survey of preschool education policies was conducted to identify and assess the type and extent of PA programmes and opportunities in preschools in the State of Lower Saxony, Germany. An assessment instrument was developed to identify preschools with systematic PA programmes (type 1) and those without PA programmes (type 2) based on the following quality criteria: A) written PA policy, B) structured weekly PA offerings for all children; C) at least one qualified physical education teacher; D) PA-friendly indoor and outdoor facilities (exercise room, situational PA opportunities, outdoor areas, play equipment etc.), and E) structured PA promotion in place for at least two years. A third type of preschool that promotes PA in children to some extent (i.e., that meets the criteria partially but not completely) was classified as "preschools with limited PA programmes". RESULTS: 2415 preschools participated in the survey (response rate: 59%). The results show that 26% (n=554) have a systematic PA programme while 3% (n=64) have no PA programme. Most (71%, n=1514) were classified as limited PA programme preschools. All three types of preschools differed significantly (p=.000) from each other in terms of size (small vs. large). Most of the preschools without PA programmes are small half-day preschools. CONCLUSIONS: The study investigated an assessment-instrument providing extensive insight into the nature, extent and routine practical implementation of PA promotion in preschools. The criteria used to evaluate preschool PA programmes are well-suited to identify the different preschool PA programme types and target areas in the field of PA promotion in which specific measures (teacher education, structured PA offerings, etc.) can be implemented in future interventions
SN - 1471-2458
AD - Institute for Epidemiology, Social Medicine and Health Systems Research, Hannover Medical School, Carl-Neuberg-Str,1, D-30625 Hanover, Germany. sterdt.elena@mh-hannover.de
UR - 24005037
ER -

TY - JOUR
ID - 93
T1 - Myopic shift and outdoor activity among primary school children: one-year follow-up study in Beijing
A1 - Guo,Y.
A1 - Liu,L.J.
A1 - Xu,L.
A1 - Tang,P.
A1 - Lv,Y.Y.

A1 - Feng,Y.
A1 - Meng,M.
A1 - Jonas,J.B.
Y1 - 2013///
N1 - Guo, Yin. Liu, Li Juan. Xu, Liang. Tang, Ping. Lv, Yan Yun. Feng, Yi. Meng, Meng. Jonas, Jost B
PloS one
101285081
IM
Journal Article. Research Support, Non-U.S. Gov't
English
KW - MEDLINE
RP - NOT IN FILE
SP - e75260
JF - PLoS ONE [Electronic Resource]
JA - PLoS ONE
VL - 8
IS - 9
CY - United States
N2 - PURPOSE: To assess whether a change in myopia related oculometric parameters of primary school children in Beijing was associated with indoors and outdoors activity. METHODS: The longitudinal school-based study included school children who were examined in 2011 and who were re-examined in 2012. The children underwent a comprehensive eye examination including ocular biometry by optical low-coherence reflectometry and non-cycloplegic refractometry. Parents and children had a detailed interview including questions on time spent indoors and outdoors. RESULTS: Out of 681 students examined at baseline, 643 (94.4%) returned for follow-up examination. Within the one-year period, mean time spent daily outdoors increased by 0.4+0.9 hours, mean axial length by 0.26+0.49 mm, the ratio of axial length divided by anterior corneal curvature (AL/CC) by 0.03+0.06, and myopic refractive error by -0.06+0.89 diopters. In multivariate analysis, elongation of axial length was significantly associated with less total time spent outdoors (P = 0.02; standardized coefficient beta -0.12) and more time spent indoors with studying (P = 0.007; beta: 0.14) after adjustment for maternal myopia (P = 0.02; beta: 0.12). An increase in AL/CC was significantly associated with less time spent outdoors (P = 0.01; beta:-0.12) after adjustment for paternal myopia (P = 0.003; beta: 0.15) and if region of habitation was excludedors for leisure (P = 0.006; beta:-0.13), with less total time spent outdoors (P = 0.04; beta:-0.10), or with more time spent i. An increase in myopic refractive error, after adjustment for age, was significantly associated with less time spent outdo ndoors with studying (P = 0.005; beta: 0.13). CONCLUSIONS: A change in oculometric parameters indicating an increase in myopia was significantly associated with less time spent outdoors and more time spent indoors in school children in Greater Beijing within a study period of one year. Our study provides additional information on the potentially helpful role of outdoors activity in the prevention of myopia. Public health care measures such as school agendas may potentially take it into account
SN - 1932-6203
AD - Tongren Eye Care Center, Beijing Tongren Hospital, Capital Medical University, Beijing, China
UR - 24086484
ER -

TY - JOUR
ID - 94
T1 - What would my avatar do? Gaming, pathology, and risky decision making
A1 - Bailey,K.
A1 - West,R.
A1 - Kuffel,J.
Y1 - 2013///
N1 - Bailey, Kira. West, Robert. Kuffel, Judson
Frontiers in psychology
101550902
Journal Article
English
KW - MEDLINE

RP - NOT IN FILE
SP - 609
JF - Frontiers in Psychology
JA - Front Psychol
VL - 4
CY - Switzerland
N2 - Recent work has revealed a relationship between pathological video game use and increased impulsivity among children and adolescents. A few studies have also demonstrated increased risk-taking outside of the video game environment following game play, but this work has largely focused on one genre of video games (i.e., racing). Motivated by these findings, the aim of the current study was to examine the relationship between pathological and non-pathological video game use, impulsivity, and risky decision making. The current study also investigated the relationship between experience with two of the most popular genres of video games [i.e., first-person shooter (FPS) and strategy] and risky decision making. Consistent with previous work, ~7% of the current sample of college-aged adults met criteria for pathological video game use. The number of hours spent gaming per week was associated with increased impulsivity on a self-report measure and on the temporal discounting (TD) task. This relationship was sensitive to the genre of video game; specifically, experience with FPS games was positively correlated with impulsivity, while experience with strategy games was negatively correlated with impulsivity. Hours per week and pathological symptoms predicted greater risk-taking in the risk task and the Iowa Gambling task, accompanied by worse overall performance, indicating that even when risky choices did not pay off, individuals who spent more time gaming and endorsed more symptoms of pathological gaming continued to make these choices. Based on these data, we suggest that the presence of pathological symptoms and the genre of video game (e.g., FPS, strategy) may be important factors in determining how the amount of game experience relates to impulsivity and risky-decision making
SN - 1664-1078
AD - Social Cognitive Neuroscience Laboratory, Department of Psychological Sciences, University of Missouri Columbia, MO, USA
UR - 24058356
ER -

TY - JOUR
ID - 95
T1 - Promoting smoke-free homes: a novel behavioral intervention using real-time audio-visual feedback on airborne particle levels
A1 - Klepeis,N.E.
A1 - Hughes,S.C.
A1 - Edwards,R.D.
A1 - Allen,T.
A1 - Johnson,M.
A1 - Chowdhury,Z.
A1 - Smith,K.R.
A1 - Boman-Davis,M.
A1 - Bellettiere,J.
A1 - Hovell,M.F.
Y1 - 2013///
N1 - Klepeis, Neil E. Hughes, Suzanne C. Edwards, Rufus D. Allen, Tracy. Johnson, Michael. Chowdhury, Zohir. Smith, Kirk R. Boman-Davis, Marie. Bellettiere, John. Hovell, Melbourne F
PloS one
101285081
IM
Journal Article. Research Support, N.I.H., Extramural
English
KW - MEDLINE
RP - NOT IN FILE
SP - e73251
JF - PLoS ONE [Electronic Resource]
JA - PLoS ONE
VL - 8

IS - 8
CY - United States
N2 - Interventions are needed to protect the health of children who live with smokers. We pilot-tested a real-time intervention for promoting behavior change in homes that reduces second hand tobacco smoke (SHS) levels. The intervention uses a monitor and feedback system to provide immediate auditory and visual signals triggered at defined thresholds of fine particle concentration. Dynamic graphs of real-time particle levels are also shown on a computer screen. We experimentally evaluated the system, field-tested it in homes with smokers, and conducted focus groups to obtain general opinions. Laboratory tests of the monitor demonstrated SHS sensitivity, stability, precision equivalent to at least 1 g/m(3), and low noise. A linear relationship (R(2) = 0.98) was observed between the monitor and average SHS mass concentrations up to 150 g/m(3). Focus groups and interviews with intervention participants showed in-home use to be acceptable and feasible. The intervention was evaluated in 3 homes with combined baseline and intervention periods lasting 9 to 15 full days. Two families modified their behavior by opening windows or doors, smoking outdoors, or smoking less. We observed evidence of lower SHS levels in these homes. The remaining household voiced reluctance to changing their smoking activity and did not exhibit lower SHS levels in main smoking areas or clear behavior change; however, family members expressed receptivity to smoking outdoors. This study established the feasibility of the real-time intervention, laying the groundwork for controlled trials with larger sample sizes. Visual and auditory cues may prompt family members to take immediate action to reduce SHS levels. Dynamic graphs of SHS levels may help families make decisions about specific mitigation approaches
SN - 1932-6203
AD - Center for Behavioral Epidemiology and Community Health (C-BEACH), Graduate School of Public Health, SDSU Research Foundation, San Diego State University, San Diego, California, USA. nklepeis@projects.sdsu.edu
UR - 24009742
ER -

TY - JOUR
ID - 96
T1 - Playground characteristics to encourage children to visit and play
A1 - Nasar,J.L.
A1 - Holloman,C.H.
Y1 - 2013/11//
N1 - Nasar, Jack L. Holloman, Christopher H
Journal of physical activity & health
101189457
IM
Journal Article. Research Support, Non-U.S. Gov't
English
KW - MEDLINE
RP - NOT IN FILE
SP - 1201
EP - 1208
JF - Journal of Physical Activity & Health
JA - J Phys Act Health
VL - 10
IS - 8
CY - United States
N2 - BACKGROUND: The research sought to find the salient perceived characteristics of playgrounds for African-American children and their parents, and to test effects of changes in those characteristics on playground choice. METHODS: Thirty-one African-American children and their parents sorted 15 photographs of playgrounds for similarity. Nonmetric multidimensional scaling on the similarity scores and correlations between the resulting dimensions and judged characteristics of each playground revealed salient perceived characteristics. Study 2 had 40 African-American children and their parents view pairs of photographs, manipulated on the salient characteristics, and pick the one to play on (child question) or for the child to play on (parent question). A third study inventoried and observed children's activities in 14 playgrounds. RESULTS: Study 1 found seats, fence, playground type, and softness of surface as salient perceived characteristics of the playground. Study 2 found that participants were more likely to pick playgrounds with equipment and

playgrounds with a softer surface. Study 3 found higher levels of physical activity for playground settings with equipment. CONCLUSIONS: The findings confirm correlational findings on the desirability of equipment and safety. Communities need to test the effects of changes in playgrounds
SN - 1543-5474
AD - Dept of City and Regional Planning, The Ohio State University, Columbus, OH
UR - 23220790
ER -

TY - JOUR
ID - 97
T1 - Inertial sensing algorithms for long-term foot angle monitoring for assessment of idiopathic toe-walking
A1 - Chalmers,E.
A1 - Le,J.
A1 - Sukhdeep,D.
A1 - Watt,J.
A1 - Andersen,J.
A1 - Lou,E.
Y1 - 2014/01//
N1 - Chalmers, Eric. Le, Jonathan. Sukhdeep, Dulai. Watt, Joe. Andersen, John. Lou, Edmond
Gait & posture
9416830, dcm
IM
Journal Article
English
KW - MEDLINE
RP - NOT IN FILE
SP - 485
EP - 489
JF - Gait & Posture
JA - Gait Posture
VL - 39
IS - 1
CY - England
N2 - When children walk on their toes for no known reason, the condition is called Idiopathic Toe Walking (ITW). Assessing the true severity of ITW can be difficult because children can alter their gait while under observation in clinic. The ability to monitor the foot angle during daily life outside of clinic may improve the assessment of ITW. A foot-worn, battery-powered inertial sensing device has been designed to monitor patients' foot angle during daily activities. The monitor includes a 3-axis accelerometer, 2-axis gyroscope, and a low-power microcontroller. The device is necessarily small, with limited battery capacity and processing power. Therefore a high-accuracy but low-complexity inertial sensing algorithm is needed. This paper compares several low-complexity algorithms' aptitude for foot-angle measurement: accelerometer-only measurement, finite impulse response (FIR) and infinite impulse response (IIR) complementary filtering, and a new dynamic predict-correct style algorithm developed using fuzzy c-means clustering. A total of 11 subjects each walked 20 m with the inertial sensing device fixed to one foot; 10 m with normal gait and 10 m simulating toe walking. A cross-validation scheme was used to obtain a low-bias estimate of each algorithm's angle measurement accuracy. The new predict-correct algorithm achieved the lowest angle measurement error: <5degree mean error during normal and toe walking. The IIR complementary filtering algorithm achieved almost-as good accuracy with less computational complexity. These two algorithms seem to have good aptitude for the foot-angle measurement problem, and would be good candidates for use in a long-term monitoring device for toe-walking assessment. Copyright 2013 Elsevier B.V. All rights reserved
SN - 1879-2219
AD - Department of Electrical and Computer Engineering, University of Alberta, Edmonton, AB, Canada T6G 2V4
UR - 24050952
ER -

TY - JOUR

ID - 98
T1 - Cortisol responsivity differences in children with autism spectrum disorders during free and cooperative play
A1 - Schupp,C.W.
A1 - Simon,D.
A1 - Corbett,B.A.
Y1 - 2013/10//
N1 - Schupp, Clayton W. Simon, David. Corbett, Blythe A
Journal of autism and developmental disorders
7904301, hgw
IM
Journal Article. Research Support, N.I.H., Extramural
English
KW - MEDLINE
RP - NOT IN FILE
SP - 2405
EP - 2417
JF - Journal of Autism & Developmental Disorders
JA - J Autism Dev Disord
VL - 43
IS - 10
CY - United States
N2 - Children with autism spectrum disorder (ASD) demonstrate significant heterogeneity in their profiles of social interaction and stress responsivity. We evaluated behavior and stress response in 52 male children ages 8-12 in a naturalistic playground interaction paradigm involving a child with ASD, a typically developing peer, and a same-age confederate. Younger children in the ASD group engaged in 5.8 times more approach behavior and showed a lower cortisol response than their older peers. Those that verbally initiated with their peers also showed a higher cortisol response. Older children with ASD exhibited the highest stress responsivity, while younger children with ASD showed more willingness to approach others without apparent stress. Intervening early and often may contribute to improvements in social engagement in youth with ASD
SN - 1573-3432
AD - Cancer Prevention Institute of California, Fremont, CA, USA
UR - 23430177
ER -

TY - JOUR
ID - 99
T1 - Cytokine status in Ukrainian children with irritable bowel syndrome residing in a radioactive contaminated area
A1 - Sheikh Sajjadieh,M.R.
A1 - Kuznetsova,L.
A1 - Bojenko,V.
Y1 - 2012/12//
N1 - Sheikh Sajjadieh, Mohammad Reza. Kuznetsova, Larisa. Bojenko, Vadim
Iranian journal of immunology : IJI
101282932
IM
Journal Article. Research Support, Non-U.S. Gov't
English
KW - MEDLINE
RP - NOT IN FILE
SP - 248
EP - 253
JF - Iranian Journal Of Immunology: IJI
JA - Iran J Immunol
VL - 9
IS - 4

CY - Iran
N2 - BACKGROUND: The effect of low dose radiation on immune system is shown. Ionizing radiation can affect cytokine production and polarization of T helper cells. OBJECTIVE: The current study focused on ionizing radiation in Ukrainian children residing in a contaminated area with clinical irritable bowel syndrome. METHOD: Our study included 75 rural children population aged 4-18 yrs, who lived in a contaminated area exposed to natural environmental radiation with clinical irritable bowel syndrome (categorized in three groups) and 20 rural children participants aged 5-15 yrs who were living in areas with similar levels of radioactive contamination without clinical irritable bowel syndrome as control group. Internal radiation activity was measured by gamma-ray spectrometry. Serum levels of IL-4 and IFN- were measured by enzyme linked immunosorbent assay. RESULTS: A trend towards increased levels of IL-4 was observed in children with clinical irritable bowel syndrome. In these children, IFN- levels were lower than that of the control group. CONCLUSION: The IBS symptoms in Ukrainian children residing in a contaminated area may have stemmed from Th1 to Th2 immune deviation and differential expression of IL-4 and IFN-
SN - 1735-367X
AD - Department of Clinical Immunology and Allergology, National Medical Academy for Post Graduate Education, Kiev, Ukraine, e-mail: mohammad_esfahan@yahoo.com
UR - 23268291
ER -

TY - JOUR
ID - 100
T1 - Neighborhood perceptions moderate the association between the family environment and children's objectively assessed physical activity
A1 - D'Haese,S.
A1 - Timperio,A.
A1 - Veitch,J.
A1 - Cardon,G.
A1 - Van,Dyck D.
A1 - Salmon,J.
Y1 - 2013/11//
N1 - D'Haese, Sara. Timperio, Anna. Veitch, Jenny. Cardon, Greet. Van Dyck, Delfien. Salmon, Jo
Health & place
din, 9510067
T
Journal Article. Research Support, Non-U.S. Gov't
English
KW - MEDLINE
RP - NOT IN FILE
SP - 203
EP - 209
JF - Health & Place
JA - Health Place
VL - 24
CY - England
N2 - This study aimed to investigate whether parents' perceptions of the neighborhood environment moderate associations between the family environment and children's moderate- to vigorous-intensity physical activity (MVPA) outside of school hours. In total, 929 parents of 10-12 year-old children completed a questionnaire concerning the family environment, MVPA levels, and the neighborhood environment. Children wore an Actigraph (AM7164-2.2C) accelerometer. Compared with neighborhood environment factors, the family environment was more frequently associated with children's MVPA. Parental MVPA was positively associated with children's MVPA, but only among children whose parents reported a high presence of sporting venues. Having more restrictive physical activity rules was negatively associated with children's weekday MVPA in neighborhoods with high perceived stranger danger. 2013 Published by Elsevier Ltd
SN - 1873-2054
AD - Faculty of Medicine and Health Sciences, Department of Movement and Sports Sciences, Ghent University, Watersportlaan 2, 9000 Ghent, Belgium; Research Foundation Flanders (FWO), Egmontstraat 5, 1000 Brussels, Belgium. Electronic address: Sara.DHaese@UGent.be

UR - 24148202
ER -

TY - JOUR
ID - 101
T1 - Measuring the physical activity practices used by parents of preschool children
A1 - Vaughn,A.E.
A1 - Hales,D.
A1 - Ward,D.S.
Y1 - 2013/12//
N1 - Vaughn, Amber E. Hales, Derek. Ward, Dianne S
Medicine and science in sports and exercise
8005433, mg8
IM, S
Journal Article. Research Support, N.I.H., Extramural. Research Support, U.S. Gov't, P.H.S.
English
KW - MEDLINE
RP - NOT IN FILE
SP - 2369
EP - 2377
JF - Medicine & Science in Sports & Exercise
JA - Med Sci Sports Exerc
VL - 45
IS - 12
CY - United States
N2 - INTRODUCTION: Parents play a critical role in shaping children's attitudes, beliefs, and behaviors, including those around physical activity and inactivity. Our ability to identify which practices effectively promote children's physical activity and limit inactivity is limited by existing measurement instruments. This project will present a newly developed physical activity parenting practices survey, the psychometric properties of this survey's scales, and their association with child physical activity and screen time behaviors. METHODS: A sample of 324 parents with 2- to 5-yr-old children from central North Carolina completed a series of questionnaires, including this newly developed survey of physical activity parenting practices. Child physical activity was assessed by ActiGraph GT3X accelerometers and parent report. Exploratory factor analysis was used to identify physical activity parenting practice constructs, and Pearson correlation was used to explore relations between constructs and child physical activity. RESULTS: Fourteen parent practices used to either control or support children's physical activity or screen time were identified. Limits on screen time (r = -0.44), use of screen time to control behavior (r = 0.23), exposure to TV (r = 0.33), and parent modeling of physical activity (r = 0.37) were all significantly associated with children's TV viewing. Use of physical activity to control child behavior was significantly associated with time spent outside (r = 0.15) and minutes of moderate or vigorous physical activity (r = 0.16). Several supportive practices were associated with time outside (+) and TV time (-). CONCLUSIONS: Results provide support for this new measure of physical activity parenting practices and identify several practices that are clearly associated with child physical activity
SN - 1530-0315
AD - 1Center for Health Promotion and Disease Prevention, University of North Carolina at Chapel Hill, Chapel Hill, NC; and 2Department of Nutrition, Gillings School of Global Public Health, University of North Carolina at Chapel Hill, Chapel Hill, NC
UR - 23715430
ER -

TY - JOUR
ID - 102
T1 - Patient-provider communication about gestational weight gain among nulliparous women: a qualitative study of the views of obstetricians and first-time pregnant women
A1 - Duthie,E.A.
A1 - Drew,E.M.
A1 - Flynn,K.E.
Y1 - 2013///

N1 - Duthie, Elizabeth A. Drew, Elaine M. Flynn, Kathryn E
BMC pregnancy and childbirth
100967799
IM
Journal Article. Research Support, Non-U.S. Gov't. Research Support, U.S. Gov't, P.H.S.
English
KW - MEDLINE
RP - NOT IN FILE
SP - 231
JF - BMC Pregnancy & Childbirth
JA - BMC Pregnancy Childbirth
VL - 13
CY - England
N2 - BACKGROUND: In 2009 the Institute of Medicine updated its guidelines for weight gain during pregnancy, in part because women of childbearing age now weigh more pre-pregnancy and tend to gain more weight during pregnancy than women did when the previous set of guidelines were released in 1990. Women who begin pregnancy overweight or obese and women who gain weight outside IOM recommendations are at risk for poor maternal and fetal health outcomes. With these concerns in mind, we examined what obstetricians communicate about gestational weight gain to their pregnant patients and how nulliparous patients perceive weight-related counseling from their obstetricians. METHODS: We conducted one-on-one, semi-structured interviews with 19 nulliparous women and 7 obstetricians recruited from a single clinic at a large academic medical center in the United States. Interviews were transcribed verbatim and analyzed inductively using thematic analysis. RESULTS: We identified 4 major themes: 1) Discussions about the amount and pace of gestational weight gain: obstetricians reported variation in the frequency and timing of weight-related discussions with patients while most patients said that weight was not emphasized by their obstetricians; 2) The content of communication about nutrition and physical activity: obstetricians said they discuss nutrition and activity with all patients while most patients reported that their obstetrician either discussed these topics in general terms or not at all; 3) Communication about postpartum weight loss: obstetricians said that they do not typically address postpartum weight loss with patients during prenatal visits while patients had concerns about postpartum weight; and 4) Patient feelings about obstetrician advice: most patients said that their obstetrician does not tend to offer "unsolicited advice", instead offering information in response to patient questions or concerns. Women were divided about whether they desired more advice from their obstetrician on weight gain, nutrition, and activity. CONCLUSIONS: Our analysis revealed discrepancies between obstetricians' and patients' perceptions of their weight-related clinical interactions. Our findings suggest that there is a missed opportunity to use prenatal visits as opportunities to discuss healthy eating and exercise during pregnancy, the postpartum period, and beyond. Additional research on the design, implementation, and testing of interventions to address prenatal nutrition and physical activity is warranted
SN - 1471-2393
AD - Center for Patient Care and Outcomes Research, Medical College of Wisconsin, 8701 Watertown Plank Rd,, Milwaukee, WI, USA. elduth@mcw.edu
UR - 24330402
ER -

TY - JOUR
ID - 103
T1 - Effects of body size and gender on the population pharmacokinetics of artesunate and its active metabolite dihydroartemisinin in pediatric malaria patients
A1 - Morris,C.A.
A1 - Tan,B.
A1 - Duparc,S.
A1 - Borghini-Fuhrer,I.
A1 - Jung,D.
A1 - Shin,C.S.
A1 - Fleckenstein,L.
Y1 - 2013/12//
N1 - Morris, Carrie A. Tan, Beesan. Duparc, Stephan. Borghini-Fuhrer, Isabelle. Jung, Donald. Shin, Chang-Sik. Fleckenstein, Lawrence

Antimicrobial agents and chemotherapy
6hk, c98, 0315061, 0116415
IM
Journal Article. Research Support, Non-U.S. Gov't
English
KW - MEDLINE
RP - NOT IN FILE
SP - 5889
EP - 5900
JF - Antimicrobial Agents & Chemotherapy
JA - Antimicrob.Agents Chemother
VL - 57
IS - 12
CY - United States
N2 - Despite the important role of the antimalarial artesunate and its active metabolite dihydroartemisinin (DHA) in malaria treatment efforts, there are limited data on the pharmacokinetics of these agents in pediatric patients. This study evaluated the effects of body size and gender on the pharmacokinetics of artesunate-DHA using data from pediatric and adult malaria patients. Nonlinear mixed-effects modeling was used to obtain a base model consisting of first-order artesunate absorption and one-compartment models for artesunate and for DHA. Various methods of incorporating effects of body size descriptors on clearance and volume parameters were tested. An allometric scaling model for weight and a linear body surface area (BSA) model were deemed optimal. The apparent clearance and volume of distribution of DHA obtained with the allometric scaling model, normalized to a 38-kg patient, were 63.5 liters/h and 65.1 liters, respectively. Estimates for the linear BSA model were similar. The 95% confidence intervals for the estimated gender effects on clearance and volume parameters for artesunate fell outside the predefined no-relevant-clinical-effect interval of 0.75 to 1.25. However, the effect of gender on apparent DHA clearance was almost entirely contained within this interval, suggesting a lack of an influence of gender on this parameter. Overall, the pharmacokinetics of artesunate and DHA following oral artesunate administration can be described for pediatric patients using either an allometric scaling or linear BSA model. Both models predict that, for a given artesunate dose in mg/kg of body weight, younger children are expected to have lower DHA exposure than older children or adults
SN - 1098-6596
AD - College of Pharmacy, University of Iowa, Iowa City, Iowa, USA
UR - 24041884
ER -

TY - JOUR
ID - 104
T1 - Environmental risk factors and lung diseases in children: from guidelines to health effects
A1 - La,Grutta S.
A1 - Indinnimeo,L.
A1 - di,Coste A.
A1 - Ferrante,G.
A1 - Landi,M.
A1 - Pelosi,U.
A1 - Rusconi,F.
Y1 - 2013/10//
N1 - La Grutta, Stefania. Indinnimeo, Luciana. di Coste, Annalisa. Ferrante, Giuliana. Landi, Massimo. Pelosi, Umberto. Rusconi, Franca
Early human development
edh, 7708381
IM
Journal Article
English
KW - MEDLINE
RP - NOT IN FILE
SP - S59
EP - S62

JF - Early Human Development
JA - Early Hum Dev
VL - 89 Suppl 3
CY - Ireland
N2 - During the last decades research all over the world has highlighted the deleterious effects of outdoor and indoor pollution on respiratory health of adults and children. The World Health Organization (WHO) "Air quality guidelines for Europe" played a fundamental role in providing information and guidance to authorities involved in the air pollution field and they are considered the key source on which the European Commission's directive on air quality is based. Children appear to be most vulnerable to the harmful effects of outdoor pollutants, which can cause both acute exacerbations, as well as chronic respiratory symptoms and diseases. Possible mechanisms include the induction of oxidative stress, and/or allergic sensitization, as well as increased susceptibility to infections. Cigarette smoke is one of the environmental pollutant influencing morbidity and death rate in childhood as responsible for adverse health effects in both prenatal and postnatal. There is growing epidemiological evidence that indoor allergen exposure may contribute to the development of allergic respiratory symptoms. In Italy the housing and social situation, with regard to the aspects related to exposure to secondhand smoke or the presence of fungal spores, moisture linked to household vapor and poor ventilation of the rooms are problems still not completely resolved. From a medical point of view the field of pediatrics has certainly made great strides in promoting the health of children and pediatricians to have a central role for pursuing this objective. 2013
SN - 1872-6232
AD - Institute of Biomedicine and Molecular Immunology, IBIM, National Research Council, CNR, Palermo, Italy. Electronic address: stefania.lagrutta@ibim.cnr.it
UR - 23972292
ER -

TY - JOUR
ID - 105
T1 - Parental obesity as a predictor of childhood overweight/obesity in Australian migrant children
A1 - Li,Y.
A1 - Brock,K.
A1 - Cant,R.
A1 - Ke,L.
A1 - Morrell,S.
Y1 - 2008/09//
N1 - Li, Yang. Brock, Kaye. Cant, Rosemary. Ke, Liang. Morrell, Stephen
Obesity research & clinical practice
101303911
Journal Article
English
KW - MEDLINE
RP - NOT IN FILE
SP - I
EP - II
JF - Obesity Research & Clinical Practice
JA - Obes Res Clin Pract
VL - 2
IS - 3
CY - Netherlands
N2 - Summary: Obesity levels are increasing disproportionately in immigrant children worldwide. We investigated predictors of immigrant children's obesity status in a well-documented 3-year follow-up study of children at 9 years (n = 1232) and 12 years (n = 628) of age living in inner city areas of Sydney (Australia). The major immigrant groups in this sample were from Europe, the Middle East and SE Asia. Having an obese parent and having either parent or child not being born in Australia and not playing organised sport were predictors of childhood obesity. If either parent was obese compared to non-obese, then the odds ratio for a 9-year-old child of being obese compared to normal weight was 4.9 (95% CI: 3.0-8.0); for 12-year olds the odds ratio was 8.0 (95% CI: 3.6-18). For the survey of 9-years old, a parent born outside Australia or if the child himself was born outside Australia was associated with an almost twofold chance of being obese (OR = 1.8, 95% CI: 1.1-3.0), and

not participating in organised sports was significantly associated with childhood obesity. Nine-year olds who did not participate in organised sports outside of school hours were almost twice as likely to be obese (OR = 1.9, 95% CI: 1.2-2.9). When stratified by ethnicity, participation in sports was not related to lower obesity status in children of SE Asian origin. One reason for this could be because SE Asian had very limited participation in sports compared to their counterparts. Accordingly, we believe that health promotion messages to avoid obesity need to be targeted ethnic-specifically.: A 2008 Asian Oceanian Association for the Study of Obesity . Published by Elsevier Ltd. All rights reserved
SN - 1871-403X
AD - School of Public Health, Faculty of Medicine, University of Sydney, NSW, Australia., School of Public Health, Faculty of Medicine, University of Sydney, NSW, Australia. Electronic address:k.brock@usyd.edu.au., School of Public Health, Faculty of Medicine, University of Sydney, NSW, Australia., School of Public Health, Faculty of Medicine, University of Sydney, NSW, Australia., School of Public Health, Faculty of Medicine, University of Sydney, NSW, Australia
UR - 24351775
ER -

TY - JOUR
ID - 106
T1 - Changes in community readiness among key school stakeholders after Ready for Recess
A1 - Ehlers,D.K.
A1 - Huberty,J.L.
A1 - Beseler,C.L.
Y1 - 2013/12//
N1 - Ehlers, Diane K. Huberty, Jennifer L. Beseler, Cheryl L
Health education research
bqp, 8608459
T
Journal Article. Research Support, Non-U.S. Gov't
English
KW - MEDLINE
RP - NOT IN FILE
SP - 943
EP - 953
JF - Health Education Research
JA - Health Educ Res
VL - 28
IS - 6
CY - England
N2 - School community readiness (CR) for health promotion efforts may be critical to the effectiveness of school-based interventions aimed at promoting youth physical activity and reducing childhood obesity. The purpose of this study was to: (i) identify key informants who scored highest on school CR at baseline and (ii) determine the effects of Ready for Recess on changes in CR among school key informants from baseline to post-intervention. Key informants (N = 98) across 17 schools participated in CR interviews. Interview questions focused on school CR for physical activity and childhood obesity efforts across six dimensions. At baseline, principals scored higher than teachers in overall readiness and knowledge of the issue and higher than recess staff and nurses in leadership. Leadership readiness decreased across key informants at post-intervention and principals demonstrated greater decreases when compared with recess staff. Baseline disparities between principals and other key informants suggest principals may have overestimated the readiness of staff implementing the intervention. Declines among principals indicate that they may not have been prepared to deliver adequate support to successfully implement the intervention. These results illuminate the importance of assessing/improving school readiness prior to interventions. The CR model may provide an opportunity to improve school-based physical activity interventions
SN - 1465-3648
AD - Department of Exercise and Wellness, Arizona State University, 500 N 3rd Street, Phoenix, AZ 85004-0698, USA and Department of Psychology, Colorado State University, 1879 Campus Delivery, Fort Collins, CO 80523-1879, USA
UR - 24045411

ER -

TY - JOUR
ID - 107
T1 - Analysis of energy flow during playground surface impacts
A1 - Davidson,P.L.
A1 - Wilson,S.J.
A1 - Chalmers,D.J.
A1 - Wilson,B.D.
A1 - Eager,D.
A1 - McIntosh,A.S.
Y1 - 2013/10//
N1 - Davidson, Peter L. Wilson, Suzanne J. Chalmers, David J. Wilson, Barry D. Eager, David. McIntosh, Andrew S
Journal of applied biomechanics
9315240
IM
Journal Article. Research Support, Non-U.S. Gov't
English
KW - MEDLINE
RP - NOT IN FILE
SP - 628
EP - 633
JF - Journal of Applied Biomechanics
JA - J
VL - 29
IS - 5
CY - United States
N2 - The amount of energy dissipated away from or returned to a child falling onto a surface will influence fracture risk but is not considered in current standards for playground impact-attenuating surfaces. A two-mass rheological computer simulation was used to model energy flow within the wrist and surface during hand impact with playground surfaces, and the potential of this approach to provide insights into such impacts and predict injury risk examined. Acceleration data collected on-site from typical playground surfaces and previously obtained data from children performing an exercise involving freefalling with a fully extended arm provided input. The model identified differences in energy flow properties between playground surfaces and two potentially harmful surface characteristics: more energy was absorbed by (work done on) the wrist during both impact and rebound on rubber surfaces than on bark, and rubber surfaces started to rebound (return energy to the wrist) while the upper limb was still moving downward. Energy flow analysis thus provides information on playground surface characteristics and the impact process, and has the potential to identify fracture risks, inform the development of safer impact-attenuating surfaces, and contribute to development of new energy-based arm fracture injury criteria and tests for use in conjunction with current methods
SN - 1065-8483
AD - Injury Prevention Research Unit, University of Otago, Dunedin, New Zealand
UR - 23182916
ER -

TY - JOUR
ID - 108
T1 - Unhealthy and healthy food consumption inside and outside of the school by pre-school and elementary school Mexican children in Tijuana, Mexico
A1 - Vargas,L.
A1 - Jimenez-Cruz,A.
A1 - Bacardi-Gascon,M.
Y1 - 2013/12//
N1 - Vargas, Lilian. Jimenez-Cruz, Arturo. Bacardi-Gascon, Montserrat
Journal of community health
7600747, hut

IM
Journal Article
English
KW - MEDLINE
RP - NOT IN FILE
SP - 1166
EP - 1174
JF - Journal of Community Health
JA - J Community Health
VL - 38
IS - 6
CY - Netherlands
N2 - Food from lunch packs (LP) or food available inside and outside of school can play an important role in the development of obesity. The purpose of this study was to evaluate the LP of elementary school (ES) and preschool children (PS) in Tijuana, and the foods available to them inside and outside of school. Eight public schools participated in the study. A random sample of all the groups from a school district was conducted. A questionnaire was administered to children in first through sixth grade (ES) and to the parents of PS. LP and food available inside and outside of the school were classified as healthy, unhealthy, and adequate according to the guidelines set forth by the Secretariat of Health. A total of 2,716 questionnaires were administered and the content of 648 LP was assessed. It was observed that 99% of PS had LP prepared at home, a higher percentage than ES. None of the LP of the ES was classified as healthy, and 1% was classified as adequate. Among PS, 21% of the LP were classified as healthy and 6% as adequate. More than half of the children recognized the brand name of foods high in fat, salt, and added sugar available inside and outside of school grounds. Most of the LP of ES and PS and the foods available inside and outside of school were unhealthy and inadequate. A strategy to prevent the availability of unhealthy and inadequate food in LP and foods available inside and outside schools is recommended
SN - 1573-3610
AD - Facultad de Medicina y Psicologia, Universidad Autonoma de Baja California, Calzada Tecnologico 14418, Mesa de Otay, Unidad Universitaria, 22390, Tijuana, Baja California, Mexico, lilian.vargas05@yahoo.com.mx
UR - 23864428
ER -

TY - JOUR
ID - 109
T1 - Epidemiology of paediatric injury in low income environment: value of hospital based data prior to the institution of a formal registration system
A1 - Chichom-Mefire,A.
A1 - Fokou,M.
Y1 - 2013/07//
N1 - Chichom-Mefire, Alain. Fokou, Marcus
African journal of paediatric surgery : AJPS
101255062
IM
Journal Article
English
KW - MEDLINE
RP - NOT IN FILE
SP - 265
EP - 270
JF - African Journal of Paediatric Surgery:AJPS
JA - Afr J Paediatr Surg
VL - 10
IS - 3
CY - Nigeria
N2 - Background: Little attention is generally paid to paediatric injuries, especially in low income settings. The aim of this study is to provide an overview of the epidemiology of hospital-based paediatric injuries in a semi-

urban area in Cameroon prior to the initiation of a formal registration system. Patients and Methods: A sixteen items data collection sheet derived from a newly instituted trauma registry is used to retrospectively gather hospital-based basic information about epidemiology of injuries in patients aged 15 years or below in a low income setting. Results: Two hundred and seventy seven cases representing 16% of all injury cases could be analysed. The frequency of injuries significantly increased with age with a peak between 11 and 15 years ($P < 0.001$). Children in school playgrounds carried a significantly higher risk of sustaining an injury ($P < 0.001$). Falls and interpersonal violence were the most frequent mechanisms. The face and locomotor systems were the most commonly involved. More than 60% of patients were discharged back home. Conclusions: The data from the present registration system seem to indicate a higher injury rate in pre-adolescent children and in the school playground. The institution of a formal registration system is likely to improve the quality of data recording system
SN - 0974-5998
AD - Department of General Thoracic Surgery, Faculty of Health Sciences, University of Buea and Regional Hospital Limbe, Yaounde, Cameroon
UR - 24192473
ER -

TY - JOUR
ID - 110
T1 - Prevention of prejudice in elementary school students
A1 - Rooney-Rebeck,P.
A1 - Jason,L.
Y1 - 1986/12//
N1 - Rooney-Rebeck, P. Jason, L
The journal of primary prevention
8213457
Journal Article
English
KW - MEDLINE
RP - NOT IN FILE
SP - 63
EP - 73
JF - Journal of Primary Prevention
JA - J Prim Prev
VL - 7
IS - 2
CY - Netherlands
N2 - This study investigated the effects of cooperative group peer tutoring on the inter-ethnic relations of elementary school age children. Direct observations of social interactions on the playground and sociometric indices were used to measure inter-ethnic associations before and after the eight-week program. For first graders, inter-ethnic interactions and sociometric choices increased and improvements were found in arithmetic and reading grades. However, no significant changes were found among the third grade program children in either inter-ethnic associations or academic performance. These findings suggest that a cooperative peer tutoring classroom structure may improve the inter-ethnic relationships of first grade children who have experienced only a short history of competitive academic exercises and whose overt ethnic prejudice may be less ingrained
SN - 0278-095X
AD - ,
UR - 24271761
ER -

TY - JOUR
ID - 111
T1 - Estimated distributions of personal exposure to respirable particles
A1 - Letz,R.
A1 - Ryan,P.B.
A1 - Spengler,J.D.
Y1 - 1984/12//

N1 - Letz, R. Ryan, P B. Spengler, J D
Environmental monitoring and assessment
dvk, 8508350
Journal Article
English
KW - MEDLINE
RP - NOT IN FILE
SP - 351
EP - 359
JF - Environmental Monitoring & Assessment
JA - Environ Monit Assess
VL - 4
IS - 4
CY - Netherlands
N2 - A method of estimating distributions of exposure to respirable particles is presented. Using pollutant monitoring data from outdoors and indoors, time-activity data and a time-weighted exposure model, means and variances for exposure distributions are generated. Variances are estimated using Gauss' law of error propagation. The model is calibrated using data from a personal monitoring study. Estimated distributions of exposure to respirable particles for children in six cities living in homes with and without smokers are presented. The implications of these estimates for air pollution epidemiology and needs for further research are discussed
SN - 0167-6369
AD - Department of Environmental Science and Physiology, Harvard School of Public Health, 677 Huntington Avenue, 02115, Boston, MA, USA
UR - 24257862
ER -

TY - JOUR
ID - 112
T1 - Associations of polymorphisms in non-HLA loci with autoantibodies at the diagnosis of type 1 diabetes: INS and IKZF4 associate with insulin autoantibodies
A1 - Lempainen,J.
A1 - Harkonen,T.
A1 - Laine,A.
A1 - Knip,M.
A1 - Ilonen,J.
A1 - Finnish Pediatric,Diabetes Register
Y1 - 2013/11//
N1 - Lempainen, J. Harkonen, T. Laine, Ap. Knip, M. Ilonen, J. Finnish Pediatric Diabetes Register
Pediatric diabetes
100939345
IM
Journal Article
English
KW - MEDLINE
RP - NOT IN FILE
SP - 490
EP - 496
JF - Pediatric Diabetes
JA - Pediatr Diabetes
VL - 14
IS - 7
CY - Denmark
N2 - OBJECTIVE: More than 50 loci outside the human leukocyte antigen (HLA) region have been confirmed to affect type 1 diabetes (T1D) risk but their effect on beta-cell autoimmunity is poorly defined. We analyzed the association of 35 single nucleotide polymorphism (SNP) markers previously associated with T1D with the presence of disease-predictive autoantibodies at the time of T1D diagnosis. SUBJECTS AND METHODS: The study cohort comprised 1554 children diagnosed with T1D before the age of 15 yr. The associations between

various genotypes and positivity for antibodies against islet cells [islet cell antibodies (ICA)], insulin [insulin autoantibodies (IAA)], glutamic acid decarboxylase (GADA), islet antigen 2 (IA2A), and zinc transporter 8 (ZnT8A) were analyzed. RESULTS: INS gene polymorphism rs689 and IKZF4 polymorphism (rs1701704) were strongly associated with IAA positivity at the time of T1D diagnosis (p = 0.000004 and 0.00044, respectively). The presence of the T1D-risk conferring INS AA genotype was associated with IAA. In contrast, the presence of the susceptible C allele of the IKZF4 marker was inversely associated with IAA. The INS and IKZF4 polymorphisms were not significantly associated with ICA, GADA, IA2A, or ZnT8A positivity. CONCLUSIONS: Both INS and IKZF4 polymorphisms modified the probability of IAA positivity at time of T1D onset but the inverse association of IKZF4 risk allele with IAA suggests that the IKZF4 polymorphism is involved in a pathway of beta-cell autoimmunity alternate to the route characterized by IAA and development of T1D in early childhood. The IKZF4 gene encodes Eos, which is implicated to play an important role in Treg programming where this gene might exert its influence on T1D risk. 2013 John Wiley & Sons A/S. Published by John Wiley & Sons Ltd
SN - 1399-5448
AD - Immunogenetics Laboratory, University of Turku, Turku, Finland; Department of Pediatrics, Turku University Hospital, University of Turku, Turku, Finland
UR - 23721563
ER -

TY - JOUR
ID - 113
T1 - Seasonal variation, weather and behavior in day-care children: a multilevel approach
A1 - Ciucci,E.
A1 - Calussi,P.
A1 - Menesini,E.
A1 - Mattei,A.
A1 - Petralli,M.
A1 - Orlandini,S.
Y1 - 2013/11//
N1 - Ciucci, Enrica. Calussi, Pamela. Menesini, Ersilia. Mattei, Alessandra. Petralli, Martina. Orlandini, Simone
International journal of biometeorology
gqs, 0374716
IM
Journal Article. Research Support, Non-U.S. Gov't
English
KW - MEDLINE
RP - NOT IN FILE
SP - 845
EP - 856
JF - International Journal of Biometeorology
JA - Int J Biometeorol.
VL - 57
IS - 6
CY - United States
N2 - This study analyzes the effect of weather variables, such as solar radiation, indoor and outdoor air temperature, relative humidity and time spent outdoor, on the behavior of 2-year-old children and their affects across different seasons: winter, spring and summer. Participants were a group of 61 children (33 males and 28 females) attending four day-care centers in Florence (Central Italy). Mean age of children at the beginning of the study was 24.1 months (SD=3.6). We used multilevel linear analyses to account for the hierarchical structure of our data. The study analyzed the following behavioral variables: Activity Level, Attentional Focusing, Frustration, and Aggression. Results showed a different impact of some weather variables on children's behavior across seasons, indicating that the weather variable that affects children's behavior is usually the one that shows extreme values during the studied seasons, such as air temperature and relative humidity in winter and summer. Studying children and their reactions to weather conditions could have potentially wide-reaching implications for parenting and teaching practices, as well as for researchers studying social relationships development
SN - 1432-1254

AD - Department of Psychology, University of Florence, Via di San Salvi 12 Complesso di San Salvi Padiglione 26, 50135, Florence, Italy, enrica.ciucci@unifi.it
UR - 23238531
ER -

TY - JOUR
ID - 114
T1 - Obesity-related behaviors of US- and non-US-born parents and children in low-income households
A1 - Cespedes,E.M.
A1 - McDonald,J.
A1 - Haines,J.
A1 - Bottino,C.J.
A1 - Schmidt,M.E.
A1 - Taveras,E.M.
Y1 - 2013/10//
N1 - Cespedes, Elizabeth M. McDonald, Julia. Haines, Jess. Bottino, Clement J. Schmidt, Marie Evans. Taveras, Elsie M
Journal of developmental and behavioral pediatrics : JDBP
htf, 8006933
IM
Journal Article. Research Support, U.S. Gov't, P.H.S.
English
KW - MEDLINE
RP - NOT IN FILE
SP - 541
EP - 548
JF - Journal of Developmental & Behavioral Pediatrics
JA - J Dev Behav Pediatr
VL - 34
IS - 8
CY - United States
N2 - OBJECTIVE: To examine differences in obesity-related behaviors by parental US-born status among low-income, minority families participating in Healthy Habits, Happy Homes, an intervention trial to improve household routines for childhood obesity prevention. Evidence suggests lower obesity risk among adult immigrants, but research is inconclusive regarding the influence of having a non-US-born parent on childhood obesity. METHOD: We sampled 57 US-born and 64 non-US-born families of children aged 2 to 5.9 years living in the Boston area. At baseline, parents reported their own screen time, physical activity, diet, and sleep as well as their children's behaviors. We used linear and logistic regression to examine the association of parental US-born status with obesity-related behaviors. RESULTS: Mean (SD) body mass index z score was 0.94 (1.16), and it did not differ between the groups. After adjusting for parental education and child race/ethnicity, children of non-US-born (vs US-born) parents had later bedtimes (0.81 hours later; 95% confidence interval [CI], 0.37-1.25) and wake-up times (0.56 hours later; 95% CI, 0.16-0.95) and engaged in less active play (0.15 fewer hr/d; 95% CI, -0.28 to -0.01). Non-US-born (vs US-born) parents had less screen exposure. CONCLUSION: In this cross-section of low-income, urban families, having a parent born outside the United States was associated with a profile of risk and protective behavior; adjustment for education and race/ethnicity removed the protective associations of parental nativity with child behavior. Obesity-related differences in behaviors and home environments should be considered when designing interventions targeting low-income communities with a high proportion of non-US-born participants
SN - 1536-7312
AD - *Obesity Prevention Program, Department of Population Medicine, Harvard Medical School and Harvard Pilgrim Health Care Institute, Boston, MA; +Departments of Nutrition and Epidemiology, Harvard School of Public Health, Boston, MA; ++Department of Family Relations and Applied Nutrition, University of Guelph, Guelph, Ontario, Canada; Department of Medicine, Boston Children's Hospital, Boston, MA; |Division of General Pediatrics, MassGeneral Hospital for Children, Boston, MA
UR - 24131876
ER -

TY - JOUR
ID - 115
T1 - Longitudinal influences of neighbourhood built and social environment on children's weight status
A1 - Gose,M.
A1 - Plachta-Danielzik,S.
A1 - Willie,B.
A1 - Johannsen,M.
A1 - Landsberg,B.
A1 - Muller,M.J.
Y1 - 2013/10//
N1 - Gose, Maria. Plachta-Danielzik, Sandra. Willie, Bianca. Johannsen, Maike. Landsberg, Beate. Muller, Manfred J
International journal of environmental research and public health
101238455
IM
Journal Article. Research Support, Non-U.S. Gov't
English
KW - MEDLINE
RP - NOT IN FILE
SP - 5083
EP - 5096
JF - International Journal of Environmental Research & Public Health [Electronic Resource]
JA - Int J Environ Res Public Health
VL - 10
IS - 10
CY - Switzerland
N2 - The objective was to examine longitudinal 4-year-relationships between neighbourhood social environment and children's body mass index-standard deviation score (BMI-SDS) taking into account the built environment. Furthermore, we have analysed the influence of potential interactions between the social environment and family/social data on children's BMI-SDS. Between 2006-2008 and 2010-2012, anthropometric measurements were conducted among 485 children (age at baseline: 6.1 (5.8-6.4)). Socio-demographic characteristics and perception of residential environment were reported by parents. Geographic Information Systems were used to examine street length, number of food outlets and distance to the nearest playground and park/green space within an 800 m Euclidian buffer of each participant address point. Additional data on neighbourhood characteristics (e.g., traffic density, walkability, crime rates) were obtained from the State Capital of Kiel, Germany. In a multivariate model, walkability, street type, socioeconomic status of the district and perceived frequency of passing trucks/buses were associated with BMI-SDS over 4 years, but only neighbourhood SES had an effect on change in BMI-SDS. However, familial/social factors rather than neighbourhood environment (especially social environment) had an impact on children's BMI-SDS over 4 years. Thus, social inequalities in childhood overweight are only partially explained by social neighbourhood environment
SN - 1660-4601
AD - Institute of Human Nutrition and Food Science, Christian-Albrechts University Kiel, Dusternbrooker Weg 17, Kiel 24105, Germany. mmueller@nutrfoodsc.uni-kiel.de
UR - 24132135
ER -

TY - JOUR
ID - 116
T1 - Effects of a playground marking intervention on school recess physical activity in French children
A1 - Blaes,A.
A1 - Ridgers,N.D.
A1 - Aucouturier,J.
A1 - Van,Praagh E.
A1 - Berthoin,S.
A1 - Baquet,G.
Y1 - 2013/11//

N1 - Blaes, Aurelie. Ridgers, Nicola D. Aucouturier, Julien. Van Praagh, Emmanuel. Berthoin, Serge. Baquet, Georges
Preventive medicine
pm4, 0322116
IM
Journal Article. Research Support, Non-U.S. Gov't
English
KW - MEDLINE
RP - NOT IN FILE
SP - 580
EP - 584
JF - Preventive Medicine
JA - Prev Med
VL - 57
IS - 5
CY - United States
N2 - OBJECTIVE: Playground interventions offer an opportunity to enhance school recess physical activity. We aimed to assess the effects of playground marking on objectively measured school recess physical activity in French children. METHODS: Participants were four hundred and twenty children (6-11years old) from 4 primary schools in Nord-Pas de Calais, France. Children's physical activity (PA) was measured with a uniaxial accelerometer twice a day (morning and afternoon recess) during a 4-day school week in April and May 2009. Two experimental schools (EG) received a recess-based intervention (playground markings) and two others served as controls (CG). Percentage of time spent on the following intensities of physical activity during school recess was measured before and after intervention: sedentary (SED), light physical activity (LPA), moderate physical activity (MPA), vigorous physical activity (VPA), very high physical activity (VHPA) and moderate-to-vigorous physical activity (MVPA). RESULTS: At baseline, school recess PA among children from CG was significantly ($p<0.001$) higher than that among EG children. No interaction was observed between the recess-based intervention and gender. After the intervention, the EG spent significantly ($p<0.05$) more time in MPA, VPA and MVPA with a concomitant significant decrease in SED ($p<0.05$) compared to baseline, while the PA in CG remained unchanged. CONCLUSION: Painted playground markings had a positive short-term effect on school recess physical activity levels. 2013
SN - 1096-0260
AD - University of Lille Nord de France, UDSL, EA 4488 "Physical Activity, Muscle, Health", Faculty of Sport Sciences and Physical Education, 9 Rue de l'Universite, 59790 Ronchin, France
UR - 23933267
ER -

TY - JOUR
ID - 117
T1 - Erythemal ultraviolet solar radiation doses received by young skiers
A1 - Serrano,M.A.
A1 - Canada,J.
A1 - Moreno,J.C.
A1 - Members Of The Valencia Solar Radiation Research Group.
Y1 - 2013/11//
N1 - Serrano, Maria-Antonia. Canada, Javier. Moreno, Juan Carlos. Members Of The Valencia Solar Radiation Research Group
Photochemical & photobiological sciences : Official journal of the European Photochemistry Association and the European Society for Photobiology
101124451
IM
Journal Article. Research Support, Non-U.S. Gov't
English
KW - MEDLINE
RP - NOT IN FILE
SP - 1976
EP - 1983

JF - Photochemical & Photobiological Sciences
JA - Photochem Photobiol Sci
VL - 12
IS - 11
CY - England
N2 - Children are a special group since epidemiological evidence indicates that excessive exposure to sunlight at an early age increases the risk of skin cancer in later life. The purpose of this study is to quantify children's UV exposure when skiing, using dosimeters (VioSpor) placed on the shoulders of 10 participants. The children received a median daily Standard Erythema Dose of 2.1 within a range of 4.9-0.71, this being approximately 35% of the calculated 24 h ambient UV radiation on the horizontal plane. According to the results obtained, young skiers are exposed to UV radiation that can potentially cause skin damage and erythema and increase the risk of skin cancer in the course of a lifetime. These findings emphasise the need for adequate protective measures against solar radiation when skiing. The results also suggest that sun-protection campaigns should be undertaken aimed at children engaged in outdoor sports, including winter activities
SN - 1474-9092
AD - Instituto de Ingenieria Energetica, Universitat Politecnica de Valencia, Camino de Vera s/n, 46022 Valencia, Spain. mserranj@fis.upv.es
UR - 24057156
ER -

TY - JOUR
ID - 118
T1 - Heterosexual gender relations in and around childhood risk and safety
A1 - Brussoni,M.
A1 - Olsen,L.L.
A1 - Creighton,G.
A1 - Oliffe,J.L.
Y1 - 2013/10//
N1 - Brussoni, Mariana. Olsen, Lise L. Creighton, Genevieve. Oliffe, John L
Qualitative health research
9202144, bqc
T
Journal Article. Research Support, N.I.H., Extramural. Research Support, Non-U.S. Gov't
English
KW - MEDLINE
RP - NOT IN FILE
SP - 1388
EP - 1398
JF - Qualitative Health Research
JA - Qual Health Res
VL - 23
IS - 10
CY - United States
N2 - Injuries are a leading cause of child death, and safety interventions frequently target mothers. Fathers are largely ignored despite their increasing childcare involvement. In our qualitative study with 18 Canadian heterosexual couples parenting children 2 to 7 years old, we examined dyadic decision making and negotiations related to child safety and risk engagement in recreational activities. Parents viewed recreation as an important component of men's childcare, but women remained burdened with mundane tasks. Most couples perceived men as being more comfortable with risk than women, and three negotiation patterns emerged: fathers as risk experts; mothers countering fathers' risk; and fathers acknowledging mothers' safety concerns but persisting in risk activities. Our findings suggest that contemporary involved fathering practices privilege men in the outdoors and can erode women's control for protecting children from unintentional injury. We recommend promoting involved fathering that empowers both parents and developing injury-prevention strategies incorporating both fathers' and mothers' perspectives
SN - 1049-7323
AD - 1University of British Columbia, Vancouver, British Columbia, Canada
UR - 24043348

ER -

TY - JOUR
ID - 119
T1 - Patterns of participation in school-related activities and settings in children with spina bifida
A1 - Peny-Dahlstrand,M.
A1 - Krumlinde-Sundholm,L.
A1 - Gosman-Hedstrom,G.
Y1 - 2013/10//
N1 - Peny-Dahlstrand, M. Krumlinde-Sundholm, L. Gosman-Hedstrom, G
Disability and rehabilitation
9207179, a8i
IM
Journal Article. Research Support, Non-U.S. Gov't
English
KW - MEDLINE
RP - NOT IN FILE
SP - 1821
EP - 1827
JF - Disability & Rehabilitation
JA - Disabil Rehabil
VL - 35
IS - 21
CY - England
N2 - PURPOSE: To evaluate how children with spina bifida (SB) participate in school-related activities and to explore if their motor and process skills in task performance were related to their level of active participation in school. METHOD: Fifty children from a geographical cohort of children with SB (aged 6-14 years) and their teachers rated the children's frequency of participation in school-related activities using a Swedish adaptation of the Availability and Participation Scale. The teachers also rated each child's level of active participation with the School Function Assessment, part one. Each child's motor and process skills were evaluated with the Assessment of Motor and Process Skills. The relation between levels of active participation and motor and process skills was subjected to binary logistic regression analysis. RESULTS: The children participated very frequently in school activities, but their level of active participation was restricted, particularly in the recess/playground setting. There was a highly significant relation between full active participation in most school settings and the children's motor and process skills. CONCLUSION: Children with SB need support to become more actively involved, particularly in unstructured peer activities. The school staff need to be informed that not only the motor skills but also the process skills have an impact on the children's active participation. IMPLICATIONS FOR REHABILITATION: The children with spina bifida (SB) showed a low level of active participation and involvement in school settings even though their frequency of participation was high. It is of highest importance to enable children with SB to be actively involved in peer-related and unstructured activities and to encourage them to participate in both academic and societal learning. To achieve active participation, it is important to inform the school staff about how this issue is affected not only by the level of the child's motor skills but also, and probably even more so, by the level of his/her process skills. From this knowledge, individual strategies can be worked out
SN - 1464-5165
AD - Institute of Neuroscience and Physiology at the Sahlgrenska Academy, University of Gothenburg , Sweden
UR - 23350762
ER -

TY - JOUR
ID - 120
T1 - Test balloons? Small signs of big events: a qualitative study on circumstances facilitating adults' awareness of children's first signs of sexual abuse
A1 - Flam,A.M.
A1 - Haugstvedt,E.
Y1 - 2013/09//

N1 - Flam, Anna Margrete. Haugstvedt, Eli
Child abuse & neglect
can, 7801702
IM
Journal Article
English
KW - MEDLINE
RP - NOT IN FILE
SP - 633
EP - 642
JF - Child Abuse & Neglect
JA - Child Abuse Negl
VL - 37
IS - 9
CY - England
N2 - This research examined caregivers' awareness of children's first signs of sexual abuse. The aim was to explore circumstances that facilitate adults' awareness of first signs in everyday natural settings. Data were obtained from a Norwegian university hospital's outpatient specialty mental health clinic. Included were all cases (N=20) referred during a two-year period for treatment after the disclosure of sexual abuse that was reported to the police and child protective service. Nonabusing caregivers' awareness of first signs were recollected in hindsight as part of therapy. Qualitative analysis was conducted to capture caregivers' experiences. As identified by caregivers, all children gave signs. Thereafter, children either stopped, delayed, or immediately disclosed sexual abuse. At first signs, each child had time and attention from trusted adults, connection to the abuser, and exhibited signs of reservation against that person or related activities. Then, if met with closed answers, first signs were rebuffed as once-occurring events. If met with open answers and follow-up questions, children continued to tell. Unambiguous messages were prompted only in settings with intimate bodily activity or sexual abuse related content. In sum, when trusted adults provided door-openings, children used them; when carefully prompted, children talked; when thoughtfully asked, children told. The study suggests that children's signs of sexual abuse can be understood as "test balloons" to explore understanding and whether anything is to be done. A disclosing continuation hinges on the trusted adult's dialogical attunement and supplementary door-openings. Divergent from an idea of behavioural markers, or purposeful versus accidental disclosures, this study calls for a broader attention: Moments of first signs are embedded in dialogue. A uniqueness at moments of first signs appears: Both to form such moments and to transform them into moments of meeting for joint exploration and telling, hinge upon how trusted caregivers scaffold opportunities for the child to disclose. Subsequently, support offers need to be addressed not only to strengthen children to tell, but also for caregivers and professionals to take into account the necessity of a dialogically oriented sensitivity towards children, both for telling to occur and for hearing to take place. Copyright 2013 The Authors. Published by Elsevier Ltd.. All rights reserved
SN - 1873-7757
AD - University Hospital of Northern Norway, Outpatient Specialty Mental Health Service for Children and Adolescents, Post Box 19, UNN, N-9038 Tromso, Norway
UR - 23899535
ER -

TY - JOUR
ID - 121
T1 - Physical activity among preschoolers during indoor and outdoor childcare play periods
A1 - Vanderloo,L.M.
A1 - Tucker,P.
A1 - Johnson,A.M.
A1 - Holmes,J.D.
Y1 - 2013/11//
N1 - Vanderloo, Leigh M. Tucker, Patricia. Johnson, Andrew M. Holmes, Jeffrey D
Applied physiology, nutrition, and metabolism = Physiologie appliquee, nutrition et metabolisme
101264333
IM

Journal Article
English
KW - MEDLINE
RP - NOT IN FILE
SP - 1173
EP - 1175
JF - Applied Physiology, Nutrition, & Metabolism = Physiologie Appliquee, Nutrition et Metabolisme
JA - Appl Physiol Nutr Metab
VL - 38
IS - 11
CY - Canada
N2 - Preschoolers' (n = 31) physical activity (PA) levels during indoor and outdoor childcare hours were explored using accelerometers. Participants engaged in 0.54 min/h (SD = 0.59) of moderate-to-vigorous PA (MVPA) and 14.42 min/h (SD = 6.78) of total PA (TPA) indoors compared with 5.03 min/h (SD = 4.92) of MVPA and 31.68 min/h (SD = 0.83) of TPA outdoors. Boys and girls engaged in significantly more TPA outdoors; however, only boys demonstrated a significant increase in MVPA outdoors
SN - 1715-5320
AD - a Health and Rehabilitation Sciences, Faculty of Health Sciences, University of Western Ontario, London, ON H6G 1H1, Canada
UR - 24053528
ER -

TY - JOUR
ID - 122
T1 - The role of specialisation in the promotion of young football talents: a person-oriented study
A1 - Zibung,M.
A1 - Conzelmann,A.
Y1 - 2013///
N1 - Zibung, Marc. Conzelmann, Achim
European journal of sport science
101146739
IM
Journal Article
English
KW - MEDLINE
RP - NOT IN FILE
SP - 452
EP - 460
JF - European Journal of Sport Science EJSS : Official Journal of the European College of Sport Science
JA - EJSS (Champaign.)
VL - 13
IS - 5
CY - England
N2 - This paper investigates the controversial question whether it is more effective to promote specialisation in a specific sport at the beginning of a career or whether to encourage a broad range of sports when promoting competitive sports talents in order for them to achieve a high level of performance in adulthood. The issue of promoting talents depends on human developmental processes and therefore raises developmental scientific questions. Based on recent, dynamic-interactionist concepts of development, we assume a person-oriented approach focussing on the person as a whole rather than individual features. Theoretical considerations lead to four interacting factors being summarised to form a subsystem: childhood training. The relative weights of these factors lead to patterns. By relating these to a performance criterion at the age of peak performance, particularly promising developmental patterns may be identified. One hundred fifty-nine former Swiss football talents were retrospectively interviewed about their career and the data analysed using the LICUR method. Two early career patterns were identified as having a favourable influence on adult performance. Both are characterised by an above-average amount of in-club training. One pattern also exhibits an above-average amount of informal football played outside the club, the other above-average scores for activity in other sports. Hence, comprehensive training and practice inside and outside the club form the basis for subsequent football expertise

SN - 1536-7290
AD - a Institut fur Sportwissenschaft , Universitat Bern , Bern , Switzerland
UR - 24050461
ER -

TY - JOUR
ID - 123
T1 - Smart growth community design and physical activity in children
A1 - Jerrett,M.
A1 - Almanza,E.
A1 - Davies,M.
A1 - Wolch,J.
A1 - Dunton,G.
A1 - Spruitj-Metz,D.
A1 - Ann,Pentz M.
Y1 - 2013/10//
N1 - Jerrett, Michael. Almanza, Estela. Davies, Molly. Wolch, Jennifer. Dunton, Genevieve. Spruitj-Metz, Donna. Ann Pentz, Mary
American journal of preventive medicine
8704773, apl
IM
Journal Article. Research Support, N.I.H., Extramural
English
KW - MEDLINE
RP - NOT IN FILE
SP - 386
EP - 392
JF - American Journal of Preventive Medicine
JA - Am J Prev Med
VL - 45
IS - 4
CY - Netherlands
N2 - BACKGROUND: Physical inactivity is a leading cause of death and disease globally. Research suggests physical inactivity might be linked to community designs that discourage active living. A "smart growth" community contains features likely to promote active living (walkability, green space, mixed land use), but objective evidence on the potential benefits of smart growth communities is limited. PURPOSE: To assess whether living in a smart growth community was associated with increased neighborhood-centered leisure-time physical activity in children aged 8-14 years, compared to residing in a conventional community (i.e., one not designed according to smart growth principles). METHODS: Participants were recruited from a smart growth community, "The Preserve," located in Chino, California, and eight conventional communities within a 30-minute drive of The Preserve. The analytic sample included 147 children. During 2009-2010, each child carried an accelerometer and a GPS for 7 days to ascertain physical activity and location information. Negative binomial models were used to assess the association between residence in the smart growth community and physical activity. Analyses were conducted in 2012. RESULTS: Smart growth community residence was associated with a 46% increase in the proportion of neighborhood moderate-to-vigorous physical activity (MVPA) as compared to conventional community residence. This analysis included neighborhood activity data collected during the school season and outside of school hours and home. Counterfactual simulations with model parameters suggested that smart growth community residence could add 10 minutes per day of neighborhood MVPA. CONCLUSIONS: Living in a smart growth community may increase local physical activity in children as compared to residence in conventionally designed communities. 2013 American Journal of Preventive Medicine
SN - 1873-2607
AD - School of Public Health, Division of Environmental Health Sciences (Jerrett, Almanza), University of Southern California, Los Angeles, California. Electronic address: jerrett@berkeley.edu
UR - 24050413
ER -

TY - JOUR
ID - 124
T1 - Safe play areas on farms in New South Wales
A1 - Depczynski,J.
A1 - Herde,E.
A1 - Fragar,L.
A1 - Lower,T.
Y1 - 2013/08//
N1 - Depczynski, Julie. Herde, Emily. Fragar, Lyn. Lower, Tony
The Australian journal of rural health
9305903, c0i
N
Journal Article. Research Support, Non-U.S. Gov't
English
KW - MEDLINE
RP - NOT IN FILE
SP - 220
EP - 224
JF - Australian Journal of Rural Health
JA - Aust J Rural Health
VL - 21
IS - 4
CY - Australia
N2 - OBJECTIVE: To assess the prevalence and security of fenced house yards on NSW farms and rural properties with a view to providing information to increase the development of safe play areas on farms. DESIGN: A cross-sectional stratified study using computer-assisted telephone interviewing. SETTING: Interviews were conducted in the four rural Area Health Services throughout February to December 2008. PARTICIPANTS: Randomly selected sample of 1117 adults living on a farm or rural property in the study areas. MAIN OUTCOME MEASURES: Self-reported issues involving fenced house yards or safe play areas for children. RESULTS: Overall, 79.8% of farm respondents reported that they had a fenced house yard or safe play area. For those respondents with a fenced house yard, 66.6% reported that it was secure enough to prevent /make it difficult for a young child to wander away unsupervised. Based on these figures, it is estimated that only 53.1% of all farms or rural properties have a secure fenced house yard or safe play area. There were statistically significant variations between geographic locations, with the North Coast (37.7%) being lower. CONCLUSIONS: This study illustrates a need across rural NSW to further promote, install or upgrade secure fenced house yards or safe play areas. While all geographic regions of the state could improve provision to protect children, there may also be some that are in need of more intensive intervention programs to enhance compliance. Such a proposition may also be relevant on a national level. 2013 The Authors. Australian Journal of Rural Health National Rural Health Alliance Inc
SN - 1440-1584
AD - Australian Centre for Agricultural Health and Safety, Moree, New South Wales, Australia
UR - 24033523
ER -

TY - JOUR
ID - 125
T1 - Physical activity during recess in low-income third-grade Texas students
A1 - Springer,A.E.
A1 - Tanguturi,Y.
A1 - Ranjit,N.
A1 - Skala,K.A.
A1 - Kelder,S.H.
Y1 - 2013/05//
N1 - Springer, Andrew E. Tanguturi, Yasas. Ranjit, Nalini. Skala, Katherine A. Kelder, Steven H
American journal of health behavior
9602338, dz5
IM

Journal Article. Research Support, N.I.H., Extramural. Research Support, Non-U.S. Gov't
English
KW - MEDLINE
RP - NOT IN FILE
SP - 318
EP - 324
JF - American Journal of Health Behavior
JA - Am J Health Behav
VL - 37
IS - 3
CY - United States
N2 - OBJECTIVES: To estimate the prevalence of recess-based moderate and vigorous physical activity (MVPA) and vigorous physical activity (VPA) among third-grade students attending low-income, urban schools in Texas. METHODS: Structured observations (N = 77 class and 616 student observations) using SOFIT were conducted over 3 months in 8 elementary schools with majority Hispanic students. RESULTS: Students engaged in 66.4% and 19.2% of their ~20-minute recess time in MVPA and VPA, respectively. Boys engaged in more MVPA and VPA ($p < .007$). Contextual differences in activity (setting and activity type) were identified. CONCLUSIONS: Findings underscore the importance of scheduled recess time for children's physical activity
SN - 1945-7359
AD - Michael & Susan Dell Center for Healthy Living, University of Texas School of Public Health-Austin Regional Campus, Austin, TX, USA. Andrew.E.Springer@uth.tmc.edu
UR - 23985178
ER -

TY - JOUR
ID - 126
T1 - How physically active are children attending summer day camps?
A1 - Beets,M.W.
A1 - Weaver,R.G.
A1 - Beighle,A.
A1 - Webster,C.
A1 - Pate,R.R.
Y1 - 2013/08//
N1 - Beets, Michael W. Weaver, Robert G. Beighle, Aaron. Webster, Collin. Pate, Russell R
Journal of physical activity & health
101189457
IM
Journal Article
English
KW - MEDLINE
RP - NOT IN FILE
SP - 850
EP - 855
JF - Journal of Physical Activity & Health
JA - J Phys Act Health
VL - 10
IS - 6
CY - United States
N2 - BACKGROUND: Summer day camps (SDC) represent one of the largest settings, outside the academic school year, where children can engage in safe, enjoyable physical activity (PA). Yet, little is known about this setting and how active children are while attending. METHODS: System for Observing Play and Leisure Activity in Youth was used to categorize PA of boys/girls as Sedentary/Walking/Vigorous across multiple days (8 AM to 6 PM) in 4 large-scale community-based SDCs. Contextual characteristics of type of activity, activity management, equipment, and in/outdoors were collected simultaneously. Mixed-model regression analyses examined associations between PA categories and contextual characteristics. RESULTS: A total of 4649 scans of 2462 children were made across 27 days in the SDCs. Physical activity opportunities represented 38% of the

daily schedule. Overall, 74%-79%, 13%-16%, and 7%-9% of children were observed Sedentary, Walking, or Vigorous during the SDC, and this changed to 62%-67%, 18%-19%, and 15%-18% observed Sedentary, Walking, or Vigorous during PA opportunities. Water-based PA, equipment, and free-play were related to increased PA. Children waiting-in-line for turns, staff instructing, and organized PA were related to increased sedentary. CONCLUSIONS: These findings provide evidence of modifiable characteristics of SDCs associated with PA. Improving staff skills related to facilitating active environments is a viable avenue to increase PA accumulated within SDCs
SN - 1543-5474
AD - Dept of Exercise Science, University of South Carolina, Columbia, SC
UR - 23070923
ER -

TY - JOUR
ID - 127
T1 - The beach as a setting for families' health promotion: a qualitative study with parents and children living in coastal regions in Southwest England
A1 - Ashbullby,K.J.
A1 - Pahl,S.
A1 - Webley,P.
A1 - White,M.P.
Y1 - 2013/09//
N1 - Ashbullby, Katherine J. Pahl, Sabine. Webley, Paul. White, Mathew P
Health & place
din, 9510067
T
Journal Article. Research Support, Non-U.S. Gov't
English
KW - MEDLINE
RP - NOT IN FILE
SP - 138
EP - 147
JF - Health & Place
JA - Health Place
VL - 23
CY - England
N2 - This study explores the neglected issue of how families engage with beach environments in their local areas and use them in health promoting ways. Fifteen families with children aged 8-11 years living in coastal regions in Southwest England participated in individual semi-structured interviews. The findings indicate that beaches encouraged families to be physically active. Although families valued the opportunities for physical activity and active play afforded by beaches, the key health benefits emphasised were psychological, including experiencing fun, stress relief and engagement with nature. Increased social and family interaction was also highlighted as benefits. Despite perceiving health benefits, not all families regularly visited the beach. Barriers to visits included parents having limited time, cost of parking, lack of car access and cold weather. Parents played a key role in enabling visits by choosing to share these environments with their children. The social dimension of visits also encouraged families to make regular trips. The findings support the use of beach environments to promote families' health and wellbeing and positive relationships with nature. Copyright 2013 Elsevier Ltd. All rights reserved
SN - 1873-2054
AD - School of Psychology, Faculty of Science and Technology, Plymouth University, Drake Circus, Plymouth PL4 8AA, United Kingdom. katherine.ashbullby@pcmd.ac.uk
UR - 23906586
ER -

TY - JOUR
ID - 128
T1 - Childhood and adult secondhand smoke and type 2 diabetes in women
A1 - Lajous,M.

A1 - Tondeur,L.
A1 - Fagherazzi,G.
A1 - de Lauzon-Guillain,B.
A1 - Boutron-Ruaualt,M.C.
A1 - Clavel-Chapelon,F.
Y1 - 2013/09//
N1 - Lajous, Martin. Tondeur, Laura. Fagherazzi, Guy. de Lauzon-Guillain, Blandine. Boutron-Ruaualt, Marie-Christine. Clavel-Chapelon, Francoise
Diabetes care
eag, 7805975
IM
Journal Article. Research Support, Non-U.S. Gov't
English
KW - MEDLINE
RP - NOT IN FILE
SP - 2720
EP - 2725
JF - Diabetes Care
VL - 36
IS - 9
CY - United States
N2 - OBJECTIVE: The objective of this study was to evaluate the relationship between childhood and adult secondhand smoke and type 2 diabetes. RESEARCH DESIGN AND METHODS: We conducted a prospective cohort study among 37,343 French women from the E3N-EPIC (Etude Epidemiologique aupres des femmes de la Mutuelle Generale de l'Education Nationale-European Prospective Investigation into Cancer and Nutrition) who never smoked and who were free of type 2 diabetes, cancer, or cardiovascular disease at baseline in 1992. Self-reported childhood secondhand smoke exposure was defined as having at least one parent who smoked. Adult secondhand smoke was defined as the sum of self-reported hours recorded at baseline of exposure to tobacco smoke from a spouse who smoked (or domestic close contact) and from outside the home. RESULTS: Between 1992 and 2007, 795 cases of incident type 2 diabetes were identified and validated through a drug reimbursement dataset and a specific questionnaire. Women with at least one parent who smoked appeared to have an 18% higher rate of type 2 diabetes than women with parents who did not smoke (age-adjusted hazard ratio 1.18 [95% CI 1.02-1.36]). Adult secondhand smoke exposure (no exposure versus > 4 h/day) was associated with an increased rate of type 2 diabetes (1.36 [1.05-1.77], P = 0.002 for trend) after adjusting for parental history of diabetes, education, body silhouette at age 8, childhood secondhand smoke exposure, physical activity, body mass index, hypertension, hypercholesterolemia, menopausal status and hormone use, alcohol intake, and processed red meat and coffee consumption. CONCLUSIONS: This prospective analysis suggests that secondhand smoke exposure in childhood and adulthood are associated with a higher rate of type 2 diabetes
SN - 1935-5548
AD - Center for Research on Population Health, National Institute of Public Health, Mexico City, Mexico
UR - 23757428
ER -

TY - JOUR
ID - 129
T1 - Neighborhood safety and green space as predictors of obesity among preschool children from low-income families in New York City
A1 - Lovasi,G.S.
A1 - Schwartz-Soicher,O.
A1 - Quinn,J.W.
A1 - Berger,D.K.
A1 - Neckerman,K.M.
A1 - Jaslow,R.
A1 - Lee,K.K.
A1 - Rundle,A.
Y1 - 2013/09//

N1 - Lovasi, Gina S. Schwartz-Soicher, Ofira. Quinn, James W. Berger, Diana K. Neckerman, Kathryn M. Jaslow, Risa. Lee, Karen K. Rundle, Andrew
Preventive medicine
pm4, 0322116
IM
Journal Article. Research Support, N.I.H., Extramural. Research Support, Non-U.S. Gov't
English
KW - MEDLINE
RP - NOT IN FILE
SP - 189
EP - 193
JF - Preventive Medicine
JA - Prev Med
VL - 57
IS - 3
CY - United States
N2 - BACKGROUND: Neighborhood safety, green space, walkability, and sociodemographics may influence physical activity and childhood obesity. METHODS: Data on measured height and weight, demographic characteristics, and home ZIP code were collected from year 2004 enrollees in a means-tested preschool program in New York City. Each ZIP code was surrounded by a 400-m buffer and characterized using data from the US census, local government departments, New York Times website, and Transportation Alternatives. Linear and Poisson models were constructed using cluster robust standard errors and adjusting for child's sex, race, ethnicity, age, and neighborhood characteristics. RESULTS: Analyses included 11,562 children ages 3-5 years living in 160 residential ZIP codes. A higher homicide rate (at the 75th vs 25th percentile) was associated with a 22% higher prevalence of obesity (95% CI for the prevalence ratio (PR): 1.05 to 1.41). A higher density of street trees (at the 75th vs 25th percentile) was associated with 12% lower prevalence of obesity (95% CI for the PR: 0.79 to 0.99). Other neighborhood characteristics did not have significant associations with childhood obesity. CONCLUSIONS: Among preschool children from low-income families, neighborhood homicide rate was associated with more obesity and street tree density was associated with less obesity. Copyright 2013 Elsevier Inc. All rights reserved
SN - 1096-0260
AD - Columbia University, Mailman School of Public Health, Department of Epidemiology, New York, NY, USA. glovasi@columbia.edu
UR - 23732240
ER -

TY - JOUR
ID - 130
T1 - Behavioural determinants for obesity: a cross-sectional study among Urban Adolescents in India
A1 - Rani,M.A.
A1 - Sathiyasekaran,B.W.
Y1 - 2013/07//
N1 - Rani, M Anitha. Sathiyasekaran, B W C
Journal of preventive medicine and public health = Yebang Uihakhoe chi
101242972
IM
Journal Article. Research Support, Non-U.S. Gov't
English
KW - MEDLINE
RP - NOT IN FILE
SP - 192
EP - 200
JF - Journal of Preventive Medicine & Public Health / Yebang Uihakhoe Chi
JA - J Prev Med Pub Health
VL - 46
IS - 4
CY - Korea (South)

N2 - OBJECTIVES: To measure the prevalence of behavioural risk factors for obesity among urban adolescent school children in Chennai, India. METHODS: This study was performed as a cross-sectional study using a World Health Organization-designed Global School-based Student Health Survey questionnaire (modified for India) among adolescent school children studying in 30 randomly selected secondary and higher secondary schools in Chennai city. 1842 adolescents studying in the VIII to XII standards were randomly selected for the study. RESULTS: In the present study, 40.7% of the students ate fruit one or more times per day and 74.5% of the students ate vegetables one or more times per day. Nearly 20% of the students ate fast food items on 4 to 7 days during the previous week. Among the students, 30.4% watched television for more than two hours per day. Nearly 68% of the girls and 22% of the boys did not participate in outdoor sports activities. When the pattern of physical activity of the students was assessed, it was observed that 15.6% were inactive, 43.4% were minimally active, and the remaining 41.0% belonged to the category of health enhancing physical activity. Among the students, 6.2% were overweight and 5.2% were obese. CONCLUSIONS: The prevalence of risk factors for obesity was quite high among the adolescents. This study also showed that a great proportion of overweight/obese adolescents had a correct perception of their body weight and they were making efforts to modify risk factors such as television viewing, computer use, a sedentary lifestyle, and unhealthy dietary habits
SN - 2233-4521
AD - Department of Community Medicine, Sri Ramachandra Medical College and Research Institute, Sri Ramachandra University, Chennai, India. anithajayaraju@yahoo.co.in
UR - 23946877
ER -

TY - JOUR
ID - 131
T1 - The changing pattern of pediatric both-bone forearm shaft fractures among 86,000 children from 1997 to 2009
A1 - Sinikumpu,J.J.
A1 - Pokka,T.
A1 - Serlo,W.
Y1 - 2013/08//
N1 - Sinikumpu, Juha-Jaakko. Pokka, Tytti. Serlo, Willy
European journal of pediatric surgery : official journal of Austrian Association of Pediatric Surgery ... [et al] = Zeitschrift fur Kinderchirurgie
azo, 9105263
IM
Journal Article
English
KW - MEDLINE
RP - NOT IN FILE
SP - 289
EP - 296
JF - European Journal of Pediatric Surgery
JA - Eur J Pediatr Surg
VL - 23
IS - 4
CY - Germany
N2 - INTRODUCTION: Forearm fractures are common among children. Unlike most pediatric fractures, there is a risk of unsatisfactory results in forearm shaft fractures. The healing of a tubular bone is most unlikely in the diaphysis far away from the metaphyseal zones. The treatment of forearm shaft fractures is evolving. The purpose of the study was to analyze the pattern of forearm shaft fractures and their treatment in a population of children in recent years. MATERIALS AND METHODS: All the children (from 0 to 16 years) with a both-bone forearm shaft fracture (AO-segment 22-D) during the years 1997 to 2009 in a catchment area of about 86,000 children were included in this population-based study. There were 291 fractures in all. The age-related annual incidences, background factors, seasonal variation, injury types, treatment, reoperations, and short-term outcome were determined. RESULTS: The incidence of all forearm shaft fractures increased threefold in 1997 to 2009 ($p < 0.001$). Boys predominated (70%) over the girls ($p < 0.001$). The mean age of the patients was 8.7 years (SD, +/- 3.8) among both genders. The fractures were most common in August and most uncommon in December ($p < 0.001$). A fall in the playground was the most common type of injury (34%). Ninety-two percent

of all patients (N = 269) were treated in the operating room and 44% (N = 119) were invasively stabilized. The incidence of invasive surgery held stable. However, elastic stable intramedullary nailing (ESIN) increased from 10% in 1998-2000 to 30% in 2007-2009 (p = 0.043). There were 77 reoperations (26%); reoperation rate was 5% (6/119) and 41% (N = 71/172) in the invasive and non-invasive treatment groups, respectively. Twenty-three percent of the patients referred did not have better than "good" short-term results. The "excellent" short-term outcome was increasing (p = 0.031). CONCLUSIONS: We found two mega-trends in pediatric forearm shaft fractures in the period from 1997 to 2009: First, the number of fractures was increasing significantly. Second, the forearm shaft fractures were increasingly treated by an invasive intramedullary method. We also found a significant seasonal variation, but the reason for it is not clear. The findings are based on valuable population-based data and the results are certain. Pediatric trauma centres should prepare themselves for on-going changes in forearm shaft fractures. Georg Thieme Verlag KG Stuttgart New York
SN - 1439-359X
AD - Clinic of Pediatric Surgery and Orthopedics, Department of Children and Adolescents, Oulu University Hospital, Oulu, Finland. juha-jaakko.sinikumpu@ppshp.fi
UR - 23444075
ER -

TY - JOUR
ID - 132
T1 - Should they play outside? Cardiorespiratory fitness and air pollution among schoolchildren in Bogota
A1 - Ramirez,A.
A1 - Sarmiento,O.L.
A1 - Duperly,J.
A1 - Wai,Wong T.
A1 - Rojas,N.
A1 - Arango,C.M.
A1 - Maldonado,A.
A1 - Aristizabal,G.
A1 - Perez,L.
A1 - Lobelo,F.
Y1 - 2012/08//
N1 - Ramirez, Andrea. Sarmiento, Olga L. Duperly, John. Wai Wong, Tze. Rojas, Nestor. Arango, Carlos M. Maldonado, Andrea. Aristizabal, Gustavo. Perez, Ligia. Lobelo, Felipe
Revista de salud publica (Bogota, Colombia)
100936348
IM
Journal Article. Research Support, Non-U.S. Gov't
English
KW - MEDLINE
RP - NOT IN FILE
SP - 570
EP - 583
JF - Revista de Salud Publica
JA - Rev Salud Publica (Bogota)
VL - 14
IS - 4
CY - Colombia
N2 - OBJECTIVE: This study was aimed at comparing cardiorespiratory fitness (CF), measured as VO2 max, amongst school children exposed to varying levels of particulate matter (PM10), and air pollution in Bogota, Colombia. METHODS: This was a cross-sectional study; it involved 1,045 children aged 7-12 attending four public schools served by different public transit routes and systems. Three schools were classified as being highly polluted (HP) and one slightly polluted (SP). The children and their parents were surveyed to collect data regarding their socio-demographic characteristics, physical activity habits and respiratory disease background. Objective measurements of weight and height were used to calculate the body mass index. VO2max was estimated using the 20-meter shuttle-run test, previously validated for Bogota. Spirometry was performed on 435 children. RESULTS: After adjustment for covariates, no difference was found in VO2max between children attending SP or HP schools (girls SP 45.8 ml/kg/min vs HP 44.6 ml/kg/min, p=0.11; boys SP 47.2 ml/kg/min cf

HP 48.2 ml/kg/min, p=0.41). CONCLUSIONS: VO2max levels did not differ amongst children attending schools exposed to high compared to low levels of air pollution and PM. A longitudinal study assessing children's VO2max levels in relation to exposure to highly-polluted areas is warranted
SN - 0124-0064
AD - Department of Public Health, School of Medicine, Universidad de los Andes, Bogota, Colombia. osarmien@uniandes.edu.co
UR - 23912511
ER -

TY - JOUR
ID - 133
T1 - Only kids who are fools would do that!: peer social norms influence children's risk-taking decisions
A1 - Morrongiello,B.A.
A1 - McArthur,B.A.
A1 - Kane,A.
A1 - Fleury,R.
Y1 - 2013/08//
N1 - Morrongiello, Barbara A. McArthur, Brae Anne. Kane, Alexa. Fleury, Reba
Journal of pediatric psychology
jl8, 7801773
IM
Journal Article. Research Support, Non-U.S. Gov't
English
KW - MEDLINE
RP - NOT IN FILE
SP - 744
EP - 755
JF - Journal of Pediatric Psychology
JA - J Pediatr Psychol
VL - 38
IS - 7
CY - United States
N2 - OBJECTIVES: The current research examined the impact of peer social norms on the physical risk-taking decisions of elementary-school children. METHOD: Children 6-12 years of age completed a novel video-viewing decision task in which they observed risk and non-risk child behaviors on a playground and, after each behavior, indicated their willingness to model each of the behaviors in their own video, both before and after exposure to peer-communicated social norms (encouragement, discouragement). RESULTS: Exposure to peer social norms resulted in significant changes in risk taking, with changes predicted from ratings of perceived social norms and appraisals of injury vulnerability and severity. CONCLUSION: Exposure to peer social norms can provide another means by which injury prevention programs can aim to reduce injury-risk behaviors among school-age children
SN - 1465-735X
AD - Psychology Department, University of Guelph, Guelph, ON N1G 2W1, Canada. bmorrong@uoguelph.ca
UR - 23629147
ER -

TY - JOUR
ID - 134
T1 - Sources and perceptions of indoor and ambient air pollution in rural Alaska
A1 - Ware,D.
A1 - Lewis,J.
A1 - Hopkins,S.
A1 - Boyer,B.
A1 - Noonan,C.
A1 - Ward,T.
Y1 - 2013/08//

N1 - Ware, Desirae. Lewis, Johnnye. Hopkins, Scarlett. Boyer, Bert. Noonan, Curtis. Ward, Tony
Journal of community health
7600747, hut
IM
Journal Article. Research Support, N.I.H., Extramural
English
KW - MEDLINE
RP - NOT IN FILE
SP - 773
EP - 780
JF - Journal of Community Health
JA - J Community Health
VL - 38
IS - 4
CY - Netherlands
N2 - Even though Alaska is the largest state in the United States, much of the population resides in rural and underserved areas with documented disparities in respiratory health. This is especially true in the Yukon-Kuskokwim (southwest) and Ahtna (southcentral) Regions of Alaska. In working with community members, the goal of this study was to identify the air pollution issues (both indoors and outdoors) of concern within these two regions. Over a two-year period, 328 air quality surveys were disseminated within seven communities in rural Alaska. The surveys focused on understanding the demographics, home heating practices, indoor activities, community/outdoor activities, and air quality perceptions within each community. Results from these surveys showed that there is elevated potential for PM10/PM2.5 exposures in rural Alaska communities. Top indoor air quality concerns included mold, lack of ventilation or fresh air, and dust. Top outdoor air pollution concerns identified were open burning/smoke, road dust, and vehicle exhaust (e.g., snow machines, ATVs, etc.). These data can now be used to seek additional funding for interventions, implementing long-term, sustainable solutions to the identified problems. Further research is needed to assess exposures to PM10/PM2.5 and the associated impacts on respiratory health, particularly among susceptible populations such as young children
SN - 1573-3610
AD - Center for Environmental Health Sciences, The University of Montana, 32 Campus Drive, Missoula, MT 59812, USA
UR - 23526077
ER -

TY - JOUR
ID - 135
T1 - Exercise using the Wii Fit plus with a child with primary raynaud's disease and obesity: a case report
A1 - Qualls,K.K.
A1 - Arnold,S.H.
A1 - McEwen,I.R.
A1 - Jeffries,L.M.
Y1 - 2013/08//
N1 - Qualls, Kathlene K. Arnold, Sandra H. McEwen, Irene R. Jeffries, Lynn M
Physical & occupational therapy in pediatrics
8109120, d0u
IM
Journal Article. Research Support, U.S. Gov't, Non-P.H.S.
English
KW - MEDLINE
RP - NOT IN FILE
SP - 327
EP - 341
JF - Physical & Occupational Therapy in Pediatrics
JA - Phys Occup Ther Pediatr
VL - 33
IS - 3
CY - England

N2 - The purpose of this case report was to describe the use of the Nintendo Wii Fit Plus as an alternative exercise for an 11-year-old child with primary Raynaud's disease (PRD) and obesity who was not involved in organized sports and had limited outdoor physical activity and exercise. The Wii Fit Plus exercise parameters are described as well as outcomes measured at baseline, 12 weeks, and 24 weeks. Specifically, we evaluated changes in body mass index (BMI), cardiorespiratory fitness, and health related quality of life (HRQL). Following the 24-week exercise program, the child's BMI decreased, cardiorespiratory fitness increased, and HRQL increased and were comparable to values in healthy children. These findings suggest that the Wii Fit Plus may have been an effective exercise strategy for this child
SN - 1541-3144
AD - Matanuska-Susitna Borough School District, Palmer, Alaska, USA. Kathlene.qualls@matsuk12.us
UR - 23414388
ER -

TY - JOUR
ID - 136
T1 - Pacing strategies of inexperienced children during repeated 800 m individual time-trials and simulated competition
A1 - Lambrick,D.
A1 - Rowlands,A.
A1 - Rowland,T.
A1 - Eston,R.
Y1 - 2013/05//
N1 - Lambrick, Danielle. Rowlands, Alex. Rowland, Thomas. Eston, Roger
Pediatric exercise science
8909729
IM
Journal Article
English
KW - MEDLINE
RP - NOT IN FILE
SP - 198
EP - 211
JF - Pediatric Exercise Science
JA - Pediatr Exerc Sci
VL - 25
IS - 2
CY - United States
N2 - Prior experience of fatiguing tasks is considered essential to establishing an optimal pacing strategy. This study examined the pacing behavior of inexperienced children during self-paced, 800 m running, both individually and within a competitive environment. Thirteen children (aged 9-11 y) completed a graded-exercise test to volitional exhaustion on a treadmill (laboratory trial), followed by three self-paced, individual 800 m time-trials (Trials 1-3) and one self-paced, competitive 800 m time-trial (Trial 4) on an outdoor athletics track. Ratings of perceived exertion (RPE) and heart rate (HR) were measured throughout all trials. Overall performance time improved from Trial 1-3 (250.1 + 50.4 s & 242.4 + 51.5 s, respectively, p < .017). The difference in overall performance time between Trials 3 and 4 (260.5 + 54.2 s) was approaching significance (p = .06). The pacing strategy employed from the outset was consistent across all trials. These findings dispute the notion that an optimal pacing strategy is learned with exercise experience or training
SN - 1543-2920
AD - Institute of Food, Nutrition and Human Health, Massey University, Wellington, New Zealand
UR - 23504805
ER -

TY - JOUR
ID - 137
T1 - Variables associated with active and inactive behavior during the after-school period
A1 - Wickel,E.
Y1 - 2013/05//

N1 - Wickel, Eric
Pediatric exercise science
8909729
IM
Journal Article. Research Support, N.I.H., Extramural
English
KW - MEDLINE
RP - NOT IN FILE
SP - 288
EP - 299
JF - Pediatric Exercise Science
JA - Pediatr Exerc Sci
VL - 25
IS - 2
CY - United States
N2 - This study analyzed time-use interviews to report levels of active and inactive behavior during the after-school period (3-6 pm). Interviews were conducted on random days from three separate seasons during third and fourth grade. Youth with at least two interviews during third (356 completed 2 interviews; 506 completed 3 interviews [9 yrs; 50% boys]) and fourth (186 completed 2 interviews; 768 completed 3 interviews [10 yrs; 50% boys]) grade were included to report levels of moderate-to-vigorous physical activity (MVPA), light physical activity, inactive screen time, inactive nonscreen time, and travel by location and who the activity was undertaken with. Reporting time outside the home and with peers (single or group) was related to higher levels of MVPA. While inside the home, screen and nonscreen proportions were comparable (38% and 40%, respectively), despite unique patterns (screen: boys > girls; nonscreen: girls > boys). Reporting time with both parents was associated with more nonscreen time; whereas reporting time with peer groups was associated with lower screen time. Understanding active and inactive patterns of children's behavior outside of school hours can be very important in contributing toward the development of innovative interventions for increasing physical activity
SN - 1543-2920
AD - Dept. of Exercise and Sports Science, University of Tulsa, Tulsa, OK, USA
UR - 23502279
ER -

TY - JOUR
ID - 138
T1 - A prevalence study on outdoor air pollution and respiratory diseases in children in Zasavje, Slovenia, as a lever to trigger evidence-based environmental health activities
A1 - Kukec,A.
A1 - Farkas,J.
A1 - Erzen,I.
A1 - Zaletel-Kragelj,L.
Y1 - 2013///
N1 - Kukec, Andreja. Farkas, Jerneja. Erzen, Ivan. Zaletel-Kragelj, Lijana
Arhiv za higijenu rada i toksikologiju
8mk, 0373100
IM
Journal Article. Research Support, Non-U.S. Gov't
English
KW - MEDLINE
RP - NOT IN FILE
SP - 9
EP - 22
JF - Arhiv Za Higijenu Rada i Toksikologiju
JA - Arh Hig Rada Toksikol
VL - 64
IS - 1
CY - Croatia

N2 - The aim of this study was to estimate the population burden of respiratory diseases in the Zasavje region of Slovenia that can be attributed to outdoor air pollution in order to gain relevant grounds for evidence based public health activities. In 2008, 981 schoolchildren (age 6 to 12 years) were observed in a prevalence study. The prevalence of chronic respiratory diseases (CRD) and frequent acute respiratory symptoms (FARS) was related to the level of outdoor air pollution in the local environment (low, moderate and high pollution areas). Logistic regression was used as a method for statistical analysis. The prevalence of CRD was 3.0 % in low pollution areas, 7.5 % in moderate pollution areas, and 9.7 % in high pollution areas (p=0.005). After adjustment for the effects of confounders, 2.91-times higher odds for CRD were registered in high pollution areas in comparison to low pollution areas (p=0.017). The prevalence of FARS was: 7.8 % in low pollution areas, 13.3 % in moderate pollution areas and 15.9 % in high pollution areas (p=0.010). After adjustment for the effects of confounders, 2.02-times higher odds for FARS were registered in high pollution areas in comparison to low pollution areas (p=0.023). The study confirmed a significantly higher prevalence of CRD and FARS in children living in high pollution areas of Zasavje. These results at least partially prompted mutual understanding and cross-sectoral cooperation - prerequisites for solving complex problems involving the impact of air pollution on health
SN - 0004-1254
AD - Public Health Centre, Faculty of Medicine, University of Ljubljana, Ljubljana, Slovenia
UR - 23585197
ER -

TY - JOUR
ID - 139
T1 - Pediatric near-drowning and drowning
A1 - Jan,M.M.
Y1 - 2013/02//
N1 - Jan, Mohammed M
Saudi medical journal
dyw, 7909441
IM
Journal Article
English
KW - MEDLINE
RP - NOT IN FILE
SP - 119
EP - 122
JF - Saudi Medical Journal
JA - Saudi Med J
VL - 34
IS - 2
CY - Saudi Arabia
N2 - Childhood water submersion remains a major public health problem. The aim of this article is to present an updated overview of the topic with data from our region and special attention to prevention strategies. Children less than 5 years and males are particularly at risk. Infants are at higher risk of drowning in bathtubs, toilets, or washing machines, while most drowning in older children occurs in swimming pools. Poor supervision by inexperienced caregivers is a common factor and contributes to most deaths. Adolescents drown more often during outdoor water activity. Acute management should start as soon as possible in order to prevent unfavorable neurological outcome. Cardio-pulmonary resuscitation should be started at the scene. Most children who were rescued quickly will recover neurologically intact. Restricting access to water and close adult supervision are paramount in preventing near-drowning and drowning. High-risk groups, such as new or young parents, and lower socioeconomic families, should be targeted by such prevention programs
SN - 0379-5284
AD - Department of Pediatrics, Faculty of Medicine, King Abdulaziz University, PO Box 80215, Jeddah 21589, Kingdom of Saudi Arabia. mmjan@kau.edu.sa
UR - 23396456
ER -

TY - JOUR

ID - 140
T1 - Female sexual health consensus clinical guidelines
A1 - Lamont,J.
Y1 - 2012/08//
N1 - Lamont, John
Journal of obstetrics and gynaecology Canada : JOGC = Journal d'obstetrique et gynecologie du Canada : JOGC
101126664
IM
Journal Article
English
KW - MEDLINE
RP - NOT IN FILE
SP - 769
EP - 775
JF - Journal of Obstetrics & Gynaecology Canada: JOGC
JA - J Obstet Gynaecol Can
VL - 34
IS - 8
CY - Canada
N2 - Objective: To establish national guidelines for the assessment of women's sexual health concerns and the provision of sexual heath care for women. Evidence: Published literature was retrieved through searches of PubMed, CINAHL, and the Cochrane Library from May to October 2010, using appropriate controlled vocabulary (e.g., sexuality, "sexual dysfunction," "physiological," dyspareunia) and key words (e.g., sexual dysfunction, sex therapy, anorgasmia). Results were restricted, where possible, to systematic reviews, randomized control trials/controlled clinical trials, and observational studies. There were no language restrictions. Searches were updated on a regular basis and incorporated in the guideline to December 2010. Grey (unpublished) literature was identified through searching the websites of health technology assessment and health technology assessment-related agencies, clinical practice guideline collections, clinical trial registries, and national and international medical specialty societies. Each article was screened for relevance and the full text acquired if determined to be relevant. The evidence obtained was reviewed and evaluated by the members of the Expert Workgroup established by the Society of Obstetricians and Gynaecologists of Canada. Values: The quality of evidence was evaluated and recommendations made using the use of criteria described by the Canadian Task Force on Preventive Health Care (Table). Summary Statements and Recommendations Introduction Summary Statements 1. Sexual concerns are prevalent in the population. (II-1) 2. Many women have to look outside medicine for solutions to their sexual concerns. (II-1) 3. Many health care providers have the ability to deal with sexual health issues. (II-3) 4. Health care providers need a better understanding of female sexual issues/problems. (II-3) Chapter 1: Sexuality Across the Lifespan Summary Statements 5. Children are sexual from birth. Expression of sexuality is a developmental process. (II-2) 6. Most discourse on adolescent sexuality focuses on the potential for adverse consequences such as exploitation, sexual assault, unwanted pregnancy, and sexually transmitted infections, and has generally neglected to communicate to girls that expression of sexuality and sexual experimentation are normal and healthy. (II-2) 7. Age-appropriate sexual expression is a positive part of the development of adolescent girls. Negative, coercive, and discriminatory experiences can detrimentally affect sexual well-being. (II-2) 8. Variations exist in same-sex and opposite-sex sexual behaviour; same-sex and opposite-sex sexual behaviour is not equivalent to self-definition as heterosexual or lesbian or bisexual. Some women who have sex with women may be reluctant to define themselves as lesbian because women who identify themselves or who are identified by others as lesbian or bisexual may experience social discrimination. (II-2) 9. Women express their sexuality in a variety of ways and in a variety of situations, including with a partner and through masturbation. (II-2) 10. Masturbation and self-pleasuring can be important for self-knowledge and as a sexual outlet in themselves for women who have and those who do not have a partner. (III) 11. Relationship factors have a major influence on a woman's sexual well-being. (II-2) 12. Pregnancy and breastfeeding, as well as experience with infertility, can affect sexual functioning. (II-2) 13. Decline in frequency of sexual activity at menopause does not alter women's potential for desire, arousal, orgasm, sexual pleasure, or sexual satisfaction. (II-2) 14. Psychological, relationship, social, cultural, and biological factors affect women's sexual well-being as they age and experience menopause. (II-2) 15. Most women with a partner continue to engage in sexual activity. Women often cease sexual activity not because of lack of interest but because they do not have a partner. (II-2) 16. Women's sexuality may be affected by biological events (e.g., puberty, childbirth, menopause, and aging), by their own psychology/psychological

health, by their ethnicity and culture, and by their sexual orientation. (III) 17. Whether or not women's sexual desire and activity continue through periods of pregnancy, childrearing, menopause, and aging may dependent on the presence of a partner, a partner's sexual function, the quality of the relationship, and both partners' general health. (III) 18. There is considerable variation in the patterns of girls' and women's sexual expression and experience. (II-2) Recommendations 1. Health care providers should encourage adolescents to use condoms consistently, and to take other steps to promote sexual health and prevent sexually transmitted infections (e.g., human papillomavirus vaccination), even while they are in a relationship. (II-3A) 2. Health care providers should be well informed about the variability of normal patterns of sexual development before evaluating sexual concerns that pertain to children and adolescents. (II-3A) 3. Health care providers should balance concern about adverse sexual consequences for girls with positive messages about adolescent girls' expression of their sexuality. (II-3A) 4. Health care providers should consider the effect of the relationship when assessing a woman's sexual well-being. (III-A) 5. Health care providers should strive to make their offices open and welcoming environments for women of all sexual preferences and practices (III-A) 6. Health care providers should discuss sexuality at the early prenatal visit, before discharge from the hospital postpartum, and at the postnatal check-up. (III-A) 7. Health care providers should communicate that they are open to discussing sexual concerns; educate patients about normal fluctuations in sexual interest and frequency; discuss the range of non-coital sexual activities if intercourse is difficult, painful, or prohibited for medical reasons; and emphasize the importance of the quality of lovemaking rather than coital frequency to sexual satisfaction. (III-A) 8. Health care providers should provide advice to support sexual adjustment and deal with challenges to sexual function during pregnancy and childbirth (e.g., suggest adapting coital position to accommodate changing body shape, suggest topical lubricant to reduce dyspareunia postpartum). (III-A) 9. Health care providers should help women deal with their concerns related to breastfeeding and sexual activity. This should include providing reassurance about the hormonal causes of erotic feelings during breastfeeding and informing women that if they are distressed by milk ejection during orgasm, this can be reduced by emptying the breast before sexual activity. (III-A) 10. The health care provider should enquire about both the woman's functioning and her partner's functioning in assessing changes to sexual activity with menopause and aging. (II-1A) 11. Changes in sexual functioning should be treated only if the woman expresses distress about these changes. (II-3B) 12. Health care providers should recommend the use of a lubricant or estrogen (local or systemic) for problems arising from vaginal dryness. (II-1A) 13. Health care providers should discuss safer sex, particularly with newly single women. (II-2A) 14. Health care providers should understand that all women are sexual and acknowledge that women have sexual needs. (III-A) 15. Health care providers should have an understanding of and respect for diverse individual patterns of sexual behaviour and orientation across the lifespan. (III-A) 16. Couples should be encouraged to include sexual pleasuring without penetration in their activities if penetration is impossible. (III-A) 17. Health care providers should recognize the need for sensitivity to a woman's life stage, to her individual situation, and to her sexual orientation when they assess sexual health concerns. (III-A) Chapter 2: Classification, Causes, and Assessment of Women's Sexual Dysfunction Recommendations 18. Health care providers should regard the identification and management of a woman's sexual health issues as important and legitimate elements of her clinical care (II-2A) 19. Health care providers should ensure they have and apply the skills and knowledge necessary to assess and manage a woman's sexual health problems. (III-A) 20. Health care providers should provide a clinical environment in which women feel they can discuss their sexual concerns. (III-A) 21. Health care providers should establish a list of clinical sexual health resources in the community for referral when necessary. (III-A) Chapter 3: Management of Sexual Concerns Summary Statements 19. Effective management of sexual concerns requires a biopsychosocial approach that includes both medical and counselling skills. (II-3) 20. A limited problem-focused approach, sometimes called the 20-minute hour, can be used to assess and manage sexual concerns effectively without disruption of the office schedule. (II-3) 21. The PLISSIT (permission, limited information, specific suggestions, intensive therapy) approach can be used to determine the level of intervention required. (II-3) 22. Involvement of the partner can often enhance outcomes in managing sexual health concerns. (II-3) Recommendations 22. All health care providers should include screening questions regarding sexual well-being as a standard of practice. (II-3A) 23. Health care providers who lack confidence in taking a biopsychosocial approach to counselling on sexual health concerns should seek additional training. (III-B) 24. Health care providers should involve the woman's partner in the assessment and treatment of sexual health concerns when it is appropriate and safe to do so. (III-A) Chapter 4: Health Concerns that Affect Female Sexuality Summary Statements 23. (ABSTRACT TRUNCATED)

SN - 1701-2163

AD - Hamilton ON

UR - 22947409

ER -

TY - JOUR
ID - 141
T1 - Natural products as starting points for future anti-malarial therapies: going back to our roots?. [Review]
A1 - Wells,T.N.
Y1 - 2011///
N1 - Wells, Timothy N C
Malaria journal
101139802
IM
Journal Article. Review
English
KW - MEDLINE
RP - NOT IN FILE
SP - S3
JF - Malaria Journal
JA - Malar.J
VL - 10 Suppl 1
CY - England
N2 - BACKGROUND: The discovery and development of new anti-malarials are at a crossroads. Fixed dose artemisinin combination therapy is now being used to treat a hundred million children each year, with a cost as low as 30 cents per child, with cure rates of over 95%. However, as with all anti-infective strategies, this triumph brings with it the seeds of its own downfall, the emergence of resistance. It takes ten years to develop a new medicine. New classes of medicines to combat malaria, as a result of infection by Plasmodium falciparum and Plasmodium vivax are urgently needed. RESULTS: Natural product scaffolds have been the basis of the majority of current anti-malarial medicines. Molecules such as quinine, lapachol and artemisinin were originally isolated from herbal medicinal products. After improvement with medicinal chemistry and formulation technologies, and combination with other active ingredients, they now make up the current armamentarium of medicines. In recent years advances in screening technologies have allowed testing of millions of compounds from pharmaceutical diversity for anti-malarial activity in cellular assays. These initiatives have resulted in thousands of new sub-micromolar active compounds - starting points for new drug discovery programmes. Against this backdrop, the paucity of potent natural products identified has been disappointing. Now is a good time to reflect on the current approach to screening herbal medicinal products and suggest revisions. Nearly sixty years ago, the Chinese doctor Chen Guofu, suggested natural products should be approached by dao-xing-ni-shi or 'acting in the reversed order', starting with observational clinical studies. Natural products based on herbal remedies are in use in the community, and have the potential unique advantage that clinical observational data exist, or can be generated. The first step should be the confirmation and definition of the clinical activity of herbal medicinal products already used by the community. This first step forms a solid basis of observations, before moving to in vivo pharmacological characterization and ultimately identifying the active ingredient. A large part of the population uses herbal medicinal products despite limited numbers of well-controlled clinical studies. Increased awareness by the regulators and public health bodies of the need for safety information on herbal medicinal products also lends support to obtaining more clinical data on such products.
CONCLUSIONS: The relative paucity of new herbal medicinal product scaffolds active against malaria results discovered in recent years suggest it is time to re-evaluate the 'smash and grab' approach of randomly testing purified natural products and replace it with a patient-data led approach. This will require a change of perspective form many in the field. It will require an investment in standardisation in several areas, including: the ethnopharmacology and design and reporting of clinical observation studies, systems for characterizing anti-malarial activity of patient plasma samples ex vivo followed by chemical and pharmacological characterisation of extracts from promising sources. Such work falls outside of the core mandate of the product development partnerships, such as MMV, and so will require additional support. This call is timely, given the strong interest from researchers in disease endemic countries to support the research arm of a malaria eradication agenda. Para-national institutions such as the African Network for Drugs and Diagnostics Innovation (ANDi) will play a major role in facilitating the development of their natural products patrimony and possibly clinical best practice to bring forward new therapeutics. As in the past, with quinine, lapinone and artemisinin, once the activity of herbal medicinal products in humans is characterised, it can be used to identify new molecular scaffolds which will form the basis of the next generation of anti-malarial therapies
SN - 1475-2875

AD - Medicines for Malaria Venture, 20 rte de Pre-Bois, CH-1215 Geneva, Switzerland. wellst@mmv.org
UR - 21411014
ER -

TY - JOUR
ID - 142
T1 - Applying motor-control theory to physical therapy practice: a case report
A1 - Kenyon,L.K.
A1 - Blackinton,M.T.
Y1 - 2011///
N1 - Kenyon, Lisa K. Blackinton, Mary T
Physiotherapy Canada. Physiotherapie Canada
0346574, pcp
H
Journal Article
English
KW - MEDLINE
RP - NOT IN FILE
SP - 345
EP - 354
JF - Physiotherapy Canada
JA - Physiother Can
VL - 63
IS - 3
CY - Canada
N2 - Purpose: This case report describes the application of an integrated, systems-based theory of motor control to physical therapy practice.Client Description: The patient was a 5-year-old boy with spastic hemiplegic cerebral palsy who was entering kindergarten. Parent concerns related to the child's safety in playground activities such as playing on the slide.Interventions: Motor-control theory, including factors related to the task, the environment, and the individual, was used to guide and direct physical therapy management related to the patient goal of safely and effectively climbing the ladder to the playground slide.Patient Outcomes: When the child entered kindergarten, he was able to safely ascend the ladder to the playground slide, using a modified movement pattern, when distractions were minimized. However, attentional issues continued to affect task execution when other children were present.Implications: This case report demonstrates a means by which current knowledge and theory can be integrated into clinical practice.Future Directions: Applying motor-control theory to this case led to the development of clinical questions for future research
SN - 0300-0508
AD - Lisa K. Kenyon, PT, PhD, PCS: Assistant Professor, Physical Therapy Program, Grand Valley State University, Grand Rapids, Michigan, USA
UR - 22654241
ER -

TY - JOUR
ID - 143
T1 - Relationship between occlusion and lisping in children with cleft lip and palate
A1 - Whitaker,M.E.
A1 - de Souza Freitas,J.A.
A1 - Pegoraro-Krook,M.I.
A1 - Ozawa,T.O.
A1 - de Cassia Moura Carvalho Lauris
A1 - Lauris,J.R.
A1 - Dutka,J.C.
Y1 - 2012/01//
N1 - Whitaker, Melina Evangelista. de Souza Freitas, Jose Alberto. Pegoraro-Krook, Maria Ines. Ozawa, Terumi Okada. de Cassia Moura Carvalho Lauris, Rita. Lauris, Jose Roberto Pereira. Dutka, Jeniffer Cassia Rillo
The Cleft palate-craniofacial journal : official publication of the American Cleft Palate-Craniofacial Association

a0r, 9102566
D, IM
Journal Article
English
KW - MEDLINE
RP - NOT IN FILE
SP - 96
EP - 103
JF - Cleft Palate-Craniofacial Journal
JA - Cleft Palate Craniofac J
VL - 49
IS - 1
CY - United States
N2 - Introduction : The literature suggests that individuals with history of cleft lip and palate who present with midfacial growth deficiency are at higher risk of presenting lisping. The relationship between distortions during production of linguoalveolar fricative sounds and the severity of malocclusion, however, has not been established for the population with cleft. Objective : To study the association between lisping and dental arch relationship. Methodology : Speech samples and dental arch casts were obtained from 106 children with operated unilateral cleft lip and palate (UCLP) during the stage of mixed dentition and before orthodontic treatment. Videotaped productions of the phrase /u saci saiw sedu/ were rated by speech-language pathologists for the identification of lisping during [s]. Dental arch casts were rated by orthodontists using the Goslon Yardstick and the Five-Year Index to establish dental arch relationship. Results : Multiple logistic regression showed no significant association between lisping and dento-occlusal index (p = .802) and age (p = .662). Substantial interjudge agreement during auditory-perceptual ratings was found (kappa = .63). Almost perfect agreement was found between orthodontists while establishing the dental arch relationship (kappa = .81). Discussion : This study failed to reveal an association between lisping and dental arch relationship in children with operated UCLP. Multiple variables may play a role in determining occurrence of lisping, warranting further investigation
SN - 1545-1569
UR - 21740184
ER -

TY - JOUR
ID - 144
T1 - The significance of the subplate for evolution and developmental plasticity of the human brain
A1 - Judas,M.
A1 - Sedmak,G.
A1 - Kostovic,I.
Y1 - 2013///
N1 - Judas, Milos. Sedmak, Goran. Kostovic, Ivica
Frontiers in human neuroscience
101477954
Journal Article
English
KW - MEDLINE
RP - NOT IN FILE
SP - 423
JF - Frontiers in Human Neuroscience
JA - Front Hum Neurosci
VL - 7
CY - Switzerland
N2 - The human life-history is characterized by long development and introduction of new developmental stages, such as childhood and adolescence. The developing brain had important role in these life-history changes because it is expensive tissue which uses up to 80% of resting metabolic rate (RMR) in the newborn and continues to use almost 50% of it during the first 5 postnatal years. Our hominid ancestors managed to lift-up metabolic constraints to increase in brain size by several interrelated ecological, behavioral and social adaptations, such as dietary change, invention of cooking, creation of family-bonded reproductive units, and

life-history changes. This opened new vistas for the developing brain, because it became possible to metabolically support transient patterns of brain organization as well as developmental brain plasticity for much longer period and with much greater number of neurons and connectivity combinations in comparison to apes. This included the shaping of cortical connections through the interaction with infant's social environment, which probably enhanced typically human evolution of language, cognition and self-awareness. In this review, we propose that the transient subplate zone and its postnatal remnant (interstitial neurons of the gyral white matter) probably served as the main playground for evolution of these developmental shifts, and describe various features that makes human subplate uniquely positioned to have such a role in comparison with other primates
SN - 1662-5161
AD - Section of Developmental Neuroscience, Department of Neuroscience, Croatian Institute for Brain Research, University of Zagreb School of Medicine Zagreb, Croatia
UR - 23935575
ER -

TY - JOUR
ID - 145
T1 - Rising Prevalence and Neighborhood, Social, and Behavioral Determinants of Sleep Problems in US Children and Adolescents, 2003-2012
A1 - Singh,G.K.
A1 - Kenney,M.K.
Y1 - 2013///
N1 - Singh, Gopal K. Kenney, Mary Kay
Sleep disorders
101600304
Journal Article
English
KW - MEDLINE
RP - NOT IN FILE
SP - 394320
JF - Sleep Disorders Print
JA - sleep disord
VL - 2013
CY - United States
N2 - We examined trends and neighborhood and sociobehavioral determinants of sleep problems in US children aged 6-17 between 2003 and 2012. The 2003, 2007, and 2011-2012 rounds of the National Survey of Children's Health were used to estimate trends and differentials in sleep problems using logistic regression. Prevalence of sleep problems increased significantly over time. The proportion of children with <7 days/week of adequate sleep increased from 31.2% in 2003 to 41.9% in 2011-2012, whereas the prevalence of adequate sleep <5 days/week rose from 12.6% in 2003 to 13.6% in 2011-2012. Prevalence of sleep problems varied in relation to neighborhood socioeconomic and built-environmental characteristics (e.g., safety concerns, poor housing, garbage/litter, vandalism, sidewalks, and parks/playgrounds). Approximately 10% of children in neighborhoods with the most-favorable social environment had serious sleep problems, compared with 16.2% of children in neighborhoods with the least-favorable social environment. Children in neighborhoods with the fewest health-promoting amenities or the greatest social disadvantage had 37%-43% higher adjusted odds of serious sleep problems than children in the most-favorable neighborhoods. Higher levels of screen time, physical inactivity, and secondhand smoke exposure were associated with 20%-47% higher adjusted odds of sleep problems. Neighborhood conditions and behavioral factors are important determinants of sleep problems in children
SN - 2090-3545
AD - US Department of Health and Human Services, Health Resources and Services Administration, Maternal and Child Health Bureau, 5600 Fishers Lane, Room 18-41, Rockville, MD 20857, USA
UR - 23819057
ER -

TY - JOUR
ID - 146
T1 - Determinants of physical activity in primary school students using the health belief model
A1 - Ar-Yuwat,S.

A1 - Clark,M.J.
A1 - Hunter,A.
A1 - James,K.S.
Y1 - 2013///
N1 - Ar-Yuwat, Sireewat. Clark, Mary Jo. Hunter, Anita. James, Kathy S
Journal of multidisciplinary healthcare
101512691
Journal Article
English
KW - MEDLINE
RP - NOT IN FILE
SP - 119
EP - 126
JF - Journal of multidisciplinary healthcare
JA - J Multidiscip.Healthc
VL - 6
CY - New Zealand
N2 - BACKGROUND: Thailand is a middle-income country in which two-thirds of children demonstrate an insufficient level of physical activity. Physical inactivity is a major risk factor for obesity and many other health-related consequences in children. Thus, it is important to understand how primary school children perceive things in their daily life as determinants of physical activity. The purpose of this study was to investigate the impact of cues, perceived benefits, and perceived barriers on the level of physical activity among primary school students. METHODS: A cross-sectional study was conducted in Phitsanulok Province, Thailand, in 2011. Multistage sampling selected a total of 123 primary school students. The Physical Activity Questionnaire for Older Children and the Cues, Perceived Benefits, and Barriers to Physical Activity Questionnaire were used to assess the student levels of physical activity, as well as the perceived benefits, barriers, and cues to action. The association between these factors and the level of physical activity was determined by correlation statistics and confirmed by robust regression. Multivariate analysis of variance compared health belief model determinants: perceived benefits, perceived barriers, and cues to action on physical activity between male and female students. Self-administered questionnaires were validated and tested in a pilot study. RESULTS: The level of activity among primary school children was significantly influenced by perceived barriers, such as fear of strangers when playing outdoors, bad weather, and too much homework. However, activity was not influenced by cues to action or perceived benefits. Perceived benefits, barriers, and cues to physical activity did not differ by gender. CONCLUSION: A safe environment and fewer barriers, such as amount of homework, could enhance physical activity in primary school children
SN - 1178-2390
AD - Boromarajonani College of Nursing Nakhon Lampang (BCNLP), Lampang, Thailand ; Hahn School of Nursing and Health Science, University of San Diego, San Diego, CA
UR - 23569383
ER -

TY - JOUR
ID - 147
T1 - The effect of surface and season on playground injury rates
A1 - Branson,L.J.
A1 - Latter,J.
A1 - Currie,G.R.
A1 - Nettel-Aguirre,A.
A1 - Embree,T.
A1 - Hagel,B.E.
Y1 - 2012/11//
N1 - Branson, Lara Joan. Latter, John. Currie, Gillian R. Nettel-Aguirre, Alberto. Embree, Tania. Hagel, Brent Edward
Paediatrics & child health
9815960
Journal Article
English

KW - MEDLINE
RP - NOT IN FILE
SP - 485
EP - 489
JF - Paediatrics & Child Health
JA - Paediatr child health
VL - 17
IS - 9
CY - Canada
N2 - OBJECTIVE: To examine the effect of season on playground surface injury rates. METHODS: Injuries were identified through student incident report forms used in school districts in Calgary (Alberta) and the surrounding area. Playground surface exposure data were estimated based on school enrollment. RESULTS: A total of 539 injuries were reported during the 2007/2008 school year. Abrasions, bruises and inflammation were the most frequently reported injuries. The head, neck or face were most commonly injured. Injury rates per 1000 student days ranged between 0.018 (rubber crumb in spring) and 0.08 (poured-in-place and natural rock in the fall). Rubber crumb surfacing, compared with natural rock, had a significantly lower rate of injury in the spring, but no other season-surface comparisons were statistically significant. CONCLUSIONS: Rates of injury were similar for natural rock, poured-in-place, and crushed rock in the fall and winter. There was some evidence of a lower rate of injury on rubber crumb surfaces in the spring
SN - 1205-7088
AD - Department of Paediatrics;
UR - 24179416
ER -

TY - JOUR
ID - 148
T1 - Juvenile myopia progression, risk factors and interventions
A1 - Myrowitz,E.H.
Y1 - 2012/07//
N1 - Myrowitz, Elliott H
Saudi journal of ophthalmology : official journal of the Saudi Ophthalmological Society
9425601
Journal Article
English
KW - MEDLINE
RP - NOT IN FILE
SP - 293
EP - 297
JF - Saudi Journal of Ophthalmology
JA - Saudi j
VL - 26
IS - 3
CY - Saudi Arabia
N2 - The development and progression of early onset myopia is actively being investigated. While myopia is often considered a benign condition it should be considered a public health problem for its visual, quality of life, and economic consequences. Nearly half of the visually impaired population in the world has uncorrected refractive errors, with myopia a high percent of that group. Uncorrected visual acuity should be screened for and treated in order to improve academic performance, career opportunities and socio-economic status. Genetic and environmental factors contribute to the onset and progression of myopia. Twin studies have supported genetic factors and research continues to identify myopia genetic loci. While multiple myopia genetic loci have been identified establishing myopia as a common complex disorder, there is not yet a genetic model explaining myopia progression in populations. Environmental factors include near work, education levels, urban compared to rural location, and time spent outdoors. In this field of study where there continues to be etiology controversies, there is recent agreement that children who spend more time outdoors are less likely to become myopic. Worldwide population studies, some completed and some in progress, with a common protocol are gathering both genetic and environmental cohort data of great value. There have been rapid population changes in prevalence rates supporting an environmental influence. Interventions to prevent juvenile myopia progression

include pharmacologic agents, glasses and contact lenses. Pharmacological interventions over 1-2 year trials have shown benefits. Peripheral vision defocus has been found to affect the emmetropization process and may be affected by wearing glasses or contacts. Accommodation accuracy also has been implicated in myopia progression. Further research will aim to assess both the role and interaction of environmental influences and genetic factors
SN - 1319-4534
AD - The Wilmer Eye Institute, The Johns Hopkins University, Baltimore, MD, USA
UR - 23961008
ER -

TY - JOUR
ID - 149
T1 - Epidemiology of paediatric burns in Iran
A1 - Karimi,H.
A1 - Montevalian,A.
A1 - Motabar,A.R.
A1 - Safari,R.
A1 - Parvas,M.S.
A1 - Vasigh,M.
Y1 - 2012/09/30/
N1 - Karimi, H. Montevalian, A. Motabar, A R. Safari, R. Parvas, M S. Vasigh, M
Annals of burns and fire disasters
101251186
Journal Article
English
KW - MEDLINE
RP - NOT IN FILE
SP - 115
EP - 120
JF - Annals of Burns & Fire Disasters
JA - Ann
VL - 25
IS - 3
CY - Italy
N2 - We surveyed the epidemiology of the patients in a tertiary burn care centre (the Motahari Burn Hospital) in Tehran in the 4-yr period 2005-2009. Scalding was the major cause of burn injury for patients under the age of 6, while there were many more flame and electrical burns in late childhood. Males were mainly affected (male to female ratio, 1.7:1). Most burns occurred in the summer, probably due to older children's increased outdoor activities during school vacations. Most of the injuries took place in the kitchen. Age was directly related to the higher total body surface area and mortality rate. Explosion of propane gas at home had a high incidence. Length of hospital stay increased in relation to the burn surface area. Infants were found to be at greatest risk for burn injuries, while older children were at higher risk for severe burns. Before arriving at the hospital, 22 patients had received traditional therapy in the home which was not effective and caused some problems. Pre-hospital care by emergency medicine service personnel was complete and effective. 374 patients had positive results for wound culture (42.9%). The most frequent bacteria found in burn wound cultures was coagulase-negative Staphylococcus (66.8%). Blood culture was positive in 12% of the patients with positive burn wound culture and the most frequent bacteria in blood culture was Pseudomonas aeruginosa. The overall mortality rate was 10.6%. Treatment and prevention programmes should target high risk groups. Important criteria include older age, flame burn, presence of inhalation injury, total body surface area burned above 40%, and sepsis
SN - 1592-9558
AD - Department of Plastic and Reconstructive Surgery
UR - 23466950
ER -

TY - JOUR
ID - 150

T1 - Feasibility of a novel web-based physical activity questionnaire for young children
A1 - Bonn,S.E.
A1 - Surkan,P.J.
A1 - Lagerros,Y.T.
A1 - Balter,K.
Y1 - 2012/12/06/
N1 - Bonn, Stephanie E. Surkan, Pamela J. Lagerros, Ylva Trolle. Balter, Katarina
Pediatric reports
101551542
Journal Article
English
KW - MEDLINE
RP - NOT IN FILE
SP - e37
JF - Pediatric Reports
JA - Pediatric rep
VL - 4
IS - 4
CY - Italy
N2 - Accurate assessment of children's physical activity is critical in determining associations between childhood physical activity and health later in life. We have developed a web-based questionnaire, KidActive-Q, to assess physical activity in early childhood. The feasibility and reproducibility of KidActive-Q were assessed in a sample of 20 children aged 2 to 6 years. The questionnaire was distributed to parents via e-mail on two occasions. The median time required to complete KidActive-Q was 2.5 minutes. All the parents reported that the questionnaire was easy or very easy to fill. The intraclass correlations for test-retest reliability were 0.60 (95% confidence interval: 0.31-0.88) for daily time spent outdoors, and 0.85 (95% confidence interval: 0.72-0.97) for daily time watching television. Our results suggest that KidActive-Q is a user-friendly tool for assessing physical activity in early childhood. To our knowledge, this is the first web-based questionnaire specifically developed for young children
SN - 2036-749X
AD - Department of Medical Epidemiology and Biostatistics, Karolinska Institutet, Sweden;
UR - 23355937
ER -

TY - JOUR
ID - 151
T1 - Injuries presented at a primary care setting in oman
A1 - Al-Balushi,H.
A1 - Al-Kalbani,A.
A1 - Al-Khwaldi,T.
A1 - Al-Suqri,S.
A1 - Al-Maniri,A.
A1 - Alazri,M.
A1 - Al-Hinai,M.
Y1 - 2012/11//
N1 - Al-Balushi, Hassan. Al-Kalbani, Ahmed. Al-Khwaldi, Talal. Al-Suqri, Salim. Al-Maniri, Abdullah. Alazri, Mohamed. Al-Hinai, Mustafa
Oman medical journal
101526350
Journal Article
English
KW - MEDLINE
RP - NOT IN FILE
SP - 486
EP - 490
JF - Oman Medical Journal
JA - Oman med

VL - 27
IS - 6
CY - Oman
N2 - OBJECTIVE: This study aims to measure the rate, types, and causes of injuries presented at a primary care setting in the Muscat governorate. METHODS: A cross-sectional study was carried out where all patients who attended the Al Moabelah Health Center from the period of 1(st) January 2010 to the 31(st) December 2010 were identified from the electronic medical records. Patients identified with any type of injury were then selected for further data collection with their age and gender recorded. Details such as the type, cause and nature of the injury at the clinic were gathered from the clinical notes. RESULTS: A total number of 86,554 visits were recorded for the year 2010. The rate of injury visits was 24 per 1000 visits. In this study, the rate of injury visits was found to be four times more common in males (44.4 per 1000) than in females (11.5 per 1000), p<0.001. While children aged under 12 years accounted for 41.4%, and elderly patients aged over 60 years accounted for 3.5% of the total visits emanating from injuries. The results show that home was the most common place where the majority of injuries occurred (42%), followed by playgrounds (10.2%). The most common causes of injury visits were falls, followed by cutting/piercing by sharp objects and sports injuries, which accounted for 40%, 13.4%, and 12%, respectively. CONCLUSION: Injuries in a primary care setting with close proximity to emergency departments of secondary and tertiary hospitals may only account to less than 3% of the total visits
SN - 1999-768X
AD - Ministry of Health, Muscat, Sultanate of Oman
UR - 23226821
ER -

TY - JOUR
ID - 152
T1 - Service-learning in Higher Education Relevant to the Promotion of Physical Activity, Healthful Eating, and Prevention of Obesity
A1 - Rosenkranz,R.R.
Y1 - 2012/10//
N1 - Rosenkranz, Richard R
International journal of preventive medicine
101535380
Journal Article
English
KW - MEDLINE
RP - NOT IN FILE
SP - 672
EP - 681
JF - International Journal of Preventive Medicine
JA - Int J Prev Med
VL - 3
IS - 10
CY - Iran
N2 - Service-learning is a type of experiential teaching and learning strategy combining classroom instruction and meaningful community service and guided activities for reflection. This educational approach has been used frequently in higher education settings, including an array of disciplines such as medicine, theology, public health, physical education, nutrition, psychology, anthropology, and sociology. The purpose of the present review paper was to provide guidance on the use of service-learning within higher education, relevant to the preventive medicine and public health topics of healthful eating, physical activity, and obesity prevention. In service-learning, coursework is structured to address community needs, and to benefit students through the real-world application of knowledge. The benefits for students include positive impacts on social skills, empathy, awareness, understanding, and concern regarding community issues, plus greater confidence and skills to work with diverse populations, increased awareness of community resources, improved motivation, and enhanced knowledge. Educational institutions may also benefit through improved "town and gown" relations, as strong ties, partnerships, and mutually beneficial activities take place. The present literature review describes several service-learning applications such as nutrition education for kids, dietary improvement for seniors, foodservice recipe modification on a college campus, an intergenerational physical activity program for nursing home residents, motor skill development in kindergarteners, organized elementary school recess physical activities,

health education, and obesity prevention in children. From this review, service-learning appears to have great potential as a flexible component of academic coursework in the areas of preventive medicine and public health
SN - 2008-8213
AD - School of Science and Health, University of Western Sydney, and Department of Human Nutrition Kansas State University, Manhattan, KS 66506
UR - 23112892
ER -

TY - JOUR
ID - 153
T1 - Heat illness surveillance in schoolboys participating in physical education class in tropical climate: an analytical prospective descriptive study
A1 - Somboonwong,J.
A1 - Sanguanrungsirikul,S.
A1 - Pitayanon,C.
Y1 - 2012///
N1 - Somboonwong, Juraiporn. Sanguanrungsirikul, Sompol. Pitayanon, Chatchatchai
BMJ open
101552874
Journal Article
English
KW - MEDLINE
RP - NOT IN FILE
JF - BMJ Open
VL - 2
IS - 4
CY - England
N2 - OBJECTIVES: This study aimed to determine thermoregulatory and cardiovascular responses as well as the occurrence of heat illness in children exercising outdoors in physical education class under hot and humid climate. Little information regarding this issue under real-life situation is available, especially in the Southeast Asia. DESIGN: Analytical, prospective descriptive study. SETTING: A primary school in Bangkok, Thailand. PARTICIPANTS: A total of 457 schoolboys (aged 5.5-12 years) were observed while exercising outdoors during their physical education classes throughout the academic year of 2009, including semester 1 (between July and September 2009) and semester 2 (between November 2009 and February 2010). PRIMARY AND SECONDARY OUTCOME MEASURES: Primary outcome measure was tympanic temperature. Secondary outcome measures included blood pressure, heart rate, hydration status and the occurrence of heat-related illness. RESULTS: Outdoor physical activity consisted of skill practice (duration 24.11+11.04 min, intensity <3 metabolic equivalent of tasks) and playing sports (duration 11.48+5.53 min, intensity 2.6-8.8 metabolic equivalent of tasks). After exercise, tympanic temperature increased by 0.66+0.41degreeC. There were 20 (4.4%) students whose ear temperature exceeded 38degreeC, 18 of whom did not consume water. The RR of increasing body temperature up to 38degreeC in overweight students was 2.1-fold higher than normal-weight students. The per cent change in mean arterial pressure and heart rate increased by 20.16+15.34% and 23.94+19.78%, respectively. Sweat and dehydration rates were 391.16+186.75 ml/h and 0.63+0.26%, respectively. No evidence of heat illness was found. Wet bulb globe temperatures of semesters 1 and 2 were 29.95+1.87degreeC and 28.32+2.39degreeC, respectively. CONCLUSIONS: There is an increased risk for heat illness during outdoor activities in physical education class in primary school children, especially those who are overweight and have poor hydration status
SN - 2044-6055
AD - Department of Physiology, Faculty of Medicine, Chulalongkorn University, Bangkok, Thailand
UR - 22773535
ER -

TY - JOUR
ID - 154
T1 - The role of the lateral amygdala in the retrieval and maintenance of fear-memories formed by repeated probabilistic reinforcement
A1 - Erlich,J.C.

A1 - Bush,D.E.
A1 - Ledoux,J.E.
Y1 - 2012///
N1 - Erlich, Jeffrey C. Bush, David E A. Ledoux, Joseph E
Frontiers in behavioral neuroscience
101477952
Journal Article
English
KW - MEDLINE
RP - NOT IN FILE
SP - 16
JF - Frontiers in Behavioral Neuroscience
JA - Front Behav Neurosci
VL - 6
CY - Switzerland
N2 - The lateral nucleus of the amygdala (LA) is a key element in the neural circuit subserving Pavlovian fear-conditioning, an animal model of fear and anxiety. Most studies have focused on the role of the LA in fear acquisition and extinction, i.e., how neural plasticity results from changing contingencies between a neutral conditioned stimulus (CS) (e.g., a tone) and an aversive unconditioned stimulus (US) (e.g., a shock). However, outside of the lab, fear-memories are often the result of repeated and unpredictable experiences. Examples include domestic violence, child abuse or combat. To better understand the role of the LA in the expression of fear resulting from repeated and uncertain reinforcement, rats experienced a 30% partial reinforcement (PR) fear-conditioning schedule four days a week for four weeks. Rats reached asymptotic levels of conditioned-fear expression after the first week. We then manipulated LA activity with drug (or vehicle) (VEH) infusions once a week, for the next three weeks, before the training session. LA infusions of muscimol (MUSC), a GABA-A agonist that inhibits neural activity, reduced CS evoked fear-behavior to pre-conditioning levels. LA infusions of pentagastrin (PENT), a cholecystokinin-2 (CCK) agonist that increases neural excitability, resulted in CS-evoked fear-behavior that continued past the offset of the CS. This suggests that neural activity in the LA is required for the retrieval of fear memories that stem from repeated and uncertain reinforcement, and that CCK signaling in the LA plays a role in the recovery from fear after the removal of the fear-evoking stimulus
SN - 1662-5153
AD - Department of Molecular Biology, Broby Lab, Princeton University, Princeton NJ, USA
UR - 22514524
ER -

TY - JOUR
ID - 155
T1 - Environmental effects on fractional exhaled nitric oxide in allergic children
A1 - La,Grutta S.
A1 - Ferrante,G.
A1 - Malizia,V.
A1 - Cibella,F.
A1 - Viegi,G.
Y1 - 2012///
N1 - La Grutta, Stefania. Ferrante, Giuliana. Malizia, Velia. Cibella, Fabio. Viegi, Giovanni
Journal of allergy
101535174
Journal Article
English
KW - MEDLINE
RP - NOT IN FILE
SP - 916926
JF - Journal Of Allergy
JA - J Allergy (Cairo)
VL - 2012
CY - United States

N2 - Fractional exhaled nitric oxide (FeNO) is a non-invasive marker of airway inflammation in asthma and respiratory allergy. Environmental factors, especially indoor and outdoor air quality, may play an important role in triggering acute exacerbations of respiratory symptoms. The authors have reviewed the literature reporting effects of outdoor and indoor pollutants on FeNO in children. Although the findings are not consistent, urban and industrial pollution-mainly particles (PM(2.5) and PM(10)), nitrogen dioxide (NO(2)), and sulfur dioxide (SO(2))-as well as formaldehyde and electric baseboard heating have been shown to increase FeNO, whilst ozone (O(3)) tends to decrease it. Among children exposed to Environmental Tobacco Smoke (ETS) with a genetic polymorphisms in nitric oxide synthase genes (NOS), a higher nicotine exposure was associated with lower FeNO levels. Finally, although more studies are needed in order to better investigate the effect of gene and environment interactions which may affect the interpretation of FeNO values in the management of children with asthma, clinicians are recommended to consider environmental exposures when taking medical histories for asthma and respiratory allergy. Further research is also needed to assess the effects of remedial interventions aimed at reducing/abating environmental exposures in asthmatic/allergic patients
SN - 1687-9791
AD - Health and Environment Unit, Regional Agency for Environment Protection/ARPA, Sicilia, Corso Calatafimi 217, 90129 Palermo, Italy
UR - 22162708
ER -

TY - JOUR
ID - 156
T1 - Activity preferences and participation of school-age children living in urban and rural environments
A1 - Brown,T.
A1 - O'Keefe,S.
A1 - Stagnitti,K.
Y1 - 2011/10//
N1 - Brown, Ted. O'Keefe, Sophie. Stagnitti, Karen
Occupational therapy in health care
8309883
Journal Article
English
KW - MEDLINE
RP - NOT IN FILE
SP - 225
EP - 239
JF - Occupational Therapy in Health Care
JA - Occup Ther Health Care
VL - 25
IS - 4
CY - England
N2 - ABSTRACT It is important for therapists to be knowledgeable about the impact of the environment on children's participation patterns and activity preferences. This study investigated the activity preference and participation among school-age children living in urban and rural locations. The participation patterns and preferences for activities of 58 typically developing children (32 males and 26 females; response rate of 38.7%) aged 8-12 years were assessed across both urban (n = 24) and rural (n = 34) regions of southwest Victoria, Australia. The participation patterns and preferences for activities were assessed using the Children's Assessment of Participation and Enjoyment and Preferences for Activities of Children (CAPE/PAC). An independent samples t-test was used to determine whether significant differences existed for the CAPE/PAC scores for urban and rurally based children as well as boys and girls. Significant differences were found between the scores of children living in urban and rural areas on the following subscales: CAPE Diversity, CAPE Intensity, CAPE Whom, CAPE Where, PAC Physical Preference, and PAC Social Preference. A significant difference for rural and urban groups was found on the following CAPE activity types: Recreation Diversity, Recreation Intensity, Social Diversity, Social Intensity, Self-Improvement Diversity, and Self-Improvement Intensity. Rurally based children were engaged in a broader range of activities and did so more frequently than urban children. Differences in gender were identified with girls preferring to participate in social and skill-based activities and being more likely to participate with friends or people outside their home. However, there were no significant differences in the participation patterns of boys and girls. Physical, social, and structural aspects of

the location where a child lives impact the frequency, type of activities, and whom a child participates with most frequently in out-of-school activities. The activity participation of boys and girls in Australia has become quite similar
SN - 1541-3098
AD - 1Department of Occupational Therapy, School of Primary Health Care, Faculty of Medicine, Nursing and Health Sciences, Monash University, Frankston, Victoria, Australia
UR - 23899077
ER -

TY - JOUR
ID - 157
T1 - Vitamin D concentrations among healthy children in Calgary, Alberta
A1 - Stoian,C.A.
A1 - Lyon,M.
A1 - Cox,R.G.
A1 - Stephure,D.K.
A1 - Mah,J.K.
Y1 - 2011/02//
N1 - Stoian, Cristina A. Lyon, Martha. Cox, Robin G. Stephure, David K. Mah, Jean K
Paediatrics & child health
9815960
Journal Article
English
KW - MEDLINE
RP - NOT IN FILE
SP - 82
EP - 86
JF - Paediatrics & Child Health
JA - Paediatr child health
VL - 16
IS - 2
CY - Canada
N2 - OBJECTIVE: To examine the relationship between serum vitamin D concentrations, dietary intake and body mass index among healthy children living in Calgary, Alberta. METHODS: The present cross-sectional study included healthy children two to 13 years of age who presented to the Alberta Children's Hospital for elective surgery during a 12-month period. Data including the child's weight, height, age, sex, ethnicity, dietary intake, use of vitamin supplements, physical activity and time spent outdoors were collected. Serum concentrations of 25-hydroxyvitamin D (25[OH]D) were measured using commercial immunoradiometric assay kits. RESULTS: Serum 25(OH)D concentrations were available for 1442 of 1862 participants, of whom 862 (59.8%) were boys. The mean (+ SD) serum 25(OH)D concentration was 86.1+35.1 nmol/L (range 10 nmol/L to 323 nmol/L). Five hundred thirty-nine (37.4%) participants had insufficient vitamin D status (25[OH]D between 25 nmol/L and lower than 75 nmol/L), and vitamin D deficiency (25[OH]D 25 nmol/L or lower) was present in 29 subjects (2.0%). Children in the older age group (nine to 13 years) were more likely to have suboptimal vitamin D (P<0.001). Other risk factors significantly associated with suboptimal vitamin D status included overweight or obesity, nonwhite ethnicity, winter months, dietary vitamin D intake of less than 200 IU/day and less time spent outdoors. CONCLUSION: A high rate of suboptimal vitamin D concentrations was observed among the participants. Beyond promoting a vitamin D-enriched diet, physicians should also consider the body mass index and other risk factors to determine the optimal vitamin D intake for children living in the area studied
SN - 1918-1485
AD - Department of Pediatrics;
UR - 22294867
ER -

TY - JOUR
ID - 158

T1 - Health risks assessment in children for phthalate exposure associated with childcare facilities and indoor playgrounds.[Erratum appears in Environ Health Toxicol. 2013 Feb;28:e2013004]
A1 - Kim,H.H.
A1 - Yang,J.Y.
A1 - Kim,S.D.
A1 - Yang,S.H.
A1 - Lee,C.S.
A1 - Shin,D.C.
A1 - Lim,Y.W.
Y1 - 2011///
N1 - Kim, Ho-Hyun. Yang, Ji-Yeon. Kim, Sun-Duk. Yang, Su-Hee. Lee, Chung-Soo. Shin, Dong-Chun. Lim, Young-Wook
Environmental health and toxicology
101570455
Journal Article
English
KW - MEDLINE
RP - NOT IN FILE
SP - e2011008
JF - Environmental Health and Toxicology
JA - Environ Health Toxicol
VL - 26
CY - Korea (South)
N2 - OBJECTIVES: This study assessed the health risks for children exposed to phthalate through several pathways including house dust, surface wipes and hand wipes in child facilities and indoor playgrounds. METHODS: The indoor samples were collected from various children's facilities (40 playrooms, 42 daycare centers, 44 kindergartens, and 42 indoor-playgrounds) in both summer (Jul-Sep, 2007) and winter (Jan-Feb, 2008). Hazard index (HI) was estimated for the non-carcinogens and the examined phthalates were diethylhexyl phthalate (DEHP), diethyl phthalate (DEP), dibutyl-n-butyl phthalate (DnBP), and butylbenzyl phthalate (BBzP). The present study examined these four kinds of samples, i.e., indoor dust, surface wipes of product and hand wipes. RESULTS: Among the phthalates, the detection rates of DEHP were 98% in dust samples, 100% in surface wipe samples, and 95% in hand wipe samples. In this study, phthalate levels obtained from floor dust, product surface and children's hand wipe samples were similar to or slightly less compared to previous studies. The 50(th) and 95(th) percentile value of child-sensitive materials did not exceed 1 (HI) for all subjects in all facilities. CONCLUSIONS: For DEHP, DnBP and BBzP their detection rates through multi-routes were high and their risk based on health risk assessment was also observed to be acceptable. This study suggested that ingestion and dermal exposure could be the most important pathway of phthalates besides digestion through food
SN - 2233-6567
AD - Institute for Environmental Research, Yonsei University College of Medicine, Seoul, Korea
UR - 22125769
ER -

TY - JOUR
ID - 159
T1 - Overweight and obesity among school-going children of Lucknow city
A1 - Vohra,R.
A1 - Bhardwaj,P.
A1 - Srivastava,J.P.
A1 - Srivastava,S.
A1 - Vohra,A.
Y1 - 2011/05//
N1 - Vohra, Rajaat. Bhardwaj, Pankaj. Srivastava, Jyoti P. Srivastava, Shekhar. Vohra, Anusha
Journal of family & community medicine
101553144, 100911100
Journal Article
English

KW - MEDLINE
RP - NOT IN FILE
SP - 59
EP - 62
JF - Journal of Family and Community Medicine
JA - J Family Community Med
VL - 18
IS - 2
CY - India
N2 - BACKGROUND: Childhood obesity is increasingly being observed with changing lifestyles of families. The magnitude of overweight ranges from 9% to 27.5% and obesity ranges from 1% to 12.9% among Indian children. OBJECTIVES: The present study was undertaken to study the magnitude of overweight/obesity and its determinants among children in Lucknow city. MATERIALS AND METHODS: A list of government and private school was procured from Office of Basic Shiksha Adhikari. Three government and three private schools were selected by Simple Random Sampling. Students of 5th to 12th grades available at the time of study were included as study unit. Predesigned and pretested questionnaire was used to elicit the information on family characteristics and individual characteristics. Height and weight were measured and BMI was calculated. Children with BMI of 25 and above were considered overweight and children with BMI more than 30 were considered obese. RESULTS: Overweight and obesity was found to be 4.17% and 0.73%, respectively; they together constitute 4.91% for overweight/obesity. The study revealed that the important correlates of overweight/obesity were father's education, father's occupation, class, children playing outdoor games for less than 30 min, and those consuming fast foods. CONCLUSIONS: Children of higher classes (above 8th standard) belonging to higher socioeconomic group with less outdoor activities and consuming fast foods were more predisposed to overweight/obesity. As a preventive strategy, there is a need to apply health and nutritional education programs for inculcating healthy life styles, and incorporating more outdoor activities in Physical Education Department of school curriculum
SN - 2229-340X
AD - Department of Community Medicine, Era's Lucknow Medical College and Hospital, Lucknow, India
UR - 21897912
ER -

TY - JOUR
ID - 160
T1 - Changes in time-use and drug use by young adults in poor neighbourhoods of Greater Buenos Aires, Argentina, after the political transitions of 2001-2002: Results of a survey
A1 - Rossi,D.
A1 - Zunino,Singh D.
A1 - Pawlowicz,M.P.
A1 - Touze,G.
A1 - Bolyard,M.
A1 - Mateu-Gelabert,P.
A1 - Sandoval,M.
A1 - Friedman,S.R.
Y1 - 2011///
N1 - Rossi, Diana. Zunino Singh, Dhan. Pawlowicz, Maria Pia. Touze, Graciela. Bolyard, Melissa. Mateu-Gelabert, Pedro. Sandoval, Milagros. Friedman, Samuel R
Harm reduction journal
101153624
Journal Article
English
KW - MEDLINE
RP - NOT IN FILE
SP - 2
JF - Harm Reduction Journal
JA - Harm Reduct.J
VL - 8
IS - 1

CY - England
N2 - BACKGROUND: In some countries, "Big Events" like crises and transitions have been followed by large increases in drug use, drug injection and HIV/AIDS. Argentina experienced an economic crisis and political transition in 2001/2002 that affected how people use their time. This paper studies how time use changes between years 2001 and 2004, subsequent to these events, were associated with drug consumption in poor neighbourhoods of Greater Buenos Aires. METHODS: In 2003-2004, 68 current injecting drug users (IDUs) and 235 young non-IDUs, aged 21-35, who lived in impoverished drug-impacted neighbourhoods in Greater Buenos Aires, were asked about time use then and in 2001. Data on weekly hours spent working or looking for work, doing housework/childcare, consuming drugs, being with friends, and hanging out in the neighbourhood, were studied in relation to time spent using drugs. Field observations and focus groups were also conducted. RESULTS: After 2001, among both IDUs and non-IDUs, mean weekly time spent working declined significantly (especially among IDUs); time spent looking for work increased, and time spent with friends and hanging out in the neighbourhood decreased.We found no increase in injecting or non-injecting drug consumption after 2001. Subjects most affected by the way the crises led to decreased work time and/or to increased time looking for work--and by the associated increase in time spent in one's neighbourhood--were most likely to increase their time using drugs. CONCLUSIONS: Time use methods are useful to study changes in drug use and their relationships to every day life activities. In these previously-drug-impacted neighbourhoods, the Argentinean crisis did not lead to an increase in drug use, which somewhat contradicts our initial expectations. Nevertheless, those for whom the crises led to decreased work time, increased time looking for work, and increased time spent in indoor or outdoor neighbourhood environments, were likely to spend more time using drugs. These data suggest that young adults in traditionally less-impoverished neighbourhoods may be more vulnerable to Big Events than those in previously drug-impacted impoverished neighbourhoods. Since Big Events will continue to occur, research on the pathways that determine their sequelae is needed
SN - 1477-7517
AD - Intercambios Civil Association, Av, Corrientes 2548 Piso 2 Dto, D - C1046AAP - Ciudad de Buenos Aires - Argentina. drossi@intercambios.org.ar
UR - 21251290
ER -

TY - JOUR
ID - 161
T1 - Has the Alberta daily physical activity initiative been successfully implemented in Calgary schools?
A1 - Kennedy,C.D.
A1 - Cantell,M.
A1 - Dewey,D.
Y1 - 2010/09//
N1 - Kennedy, Christine Diane. Cantell, Marja. Dewey, Deborah
Paediatrics & child health
9815960
Journal Article
English
KW - MEDLINE
RP - NOT IN FILE
SP - e19
EP - e24
JF - Paediatrics & Child Health
JA - Paediatr child health
VL - 15
IS - 7
CY - Canada
N2 - INTRODUCTION: In September 2005, the Alberta government introduced the daily physical activity (DPA) initiative, which requires that students from grades 1 to 9 be physically active in school for a minimum of 30 min per day. OBJECTIVE: To obtain information on whether and how the DPA initiative has been implemented in Calgary schools. METHODS: Information was obtained through a descriptive survey. Principals and vice-principals from elementary schools participated in an interview, in which they were asked questions about the DPA initiative, their definition of physical activity, the types of activities that fulfilled the DPA requirement, and barriers to increasing physical activity and physical education. RESULTS: 98.2% of

respondents reported being aware of the DPA initiative; 100% of respondents reported it being successfully implemented. The leading responses to the question, "How do you define physical activity?" were "moving/movement" (43.5%), "increasing the heart rate" (32.7%) and "being active" (29%). 78.2% of participants responded that physical education was the only type of activity that fulfilled the DPA requirement; the other participants reported that recess, intramurals and DPA periods organized by the teacher also counted. 69.1% and 61.1% of respondents, respectively, stated that there were barriers to increasing physical education and physical activity. A lack of time in the curriculum, a lack of space and a lack of funding were the most frequently reported barriers. CONCLUSION: According to principal and vice-principal reports, the DPA initiative has been successfully implemented in elementary schools in Calgary. This suggests that government initiatives directed at increasing physical activity at school could result in increasing the actual amount of physical activity that children participate in. However, prospective longitudinal research directly measuring the amount of physical activity that children engage in is needed to directly assess the impact of such initiatives
SN - 1918-1485
AD - Department of Pediatrics;
UR - 21886440
ER -

TY - JOUR
ID - 162
T1 - Variables associated with children's physical activity levels during recess: the A-CLASS project
A1 - Ridgers,N.D.
A1 - Fairclough,S.J.
A1 - Stratton,G.
Y1 - 2010///
N1 - Ridgers, Nicola D. Fairclough, Stuart J. Stratton, Gareth
The international journal of behavioral nutrition and physical activity
101217089
Journal Article
English
KW - MEDLINE
RP - NOT IN FILE
SP - 74
JF - International Journal of Behavioral Nutrition & Physical Activity
JA - Int
VL - 7
CY - England
N2 - BACKGROUND: School recess provides a daily opportunity for children to engage in physically active behaviours. However, few studies have investigated what factors may influence children's physical activity levels in this context. Such information may be important in the development and implementation of recess interventions. The aim of this study was to investigate the association between a range of recess variables and children's sedentary, moderate and vigorous physical activity in this context. METHODS: One hundred and twenty-eight children (39% boys) aged 9-10 years old from 8 elementary schools had their physical activity levels observed during school recess using the System for Observing Children's Activity and Relationships during Play (SOCARP). Playground variables data were also collected at this time. Multilevel prediction models identified variables that were significantly associated with children's sedentary, moderate and vigorous physical activity during recess. RESULTS: Girls engaged in 13.8% more sedentary activity and 8.2% less vigorous activity than boys during recess. Children with no equipment provision during recess engaged in more sedentary activity and less moderate activity than children provided with equipment. In addition, as play space per child increased, sedentary activity decreased and vigorous activity increased. Temperature was a significant negatively associated with vigorous activity. CONCLUSIONS: Modifiable and unmodifiable factors were associated with children's sedentary, moderate and vigorous physical activity during recess. Providing portable equipment and specifying areas for activities that dominate the elementary school playground during recess may be two approaches to increase recess physical activity levels, though further research is needed to evaluate the short and long-term impact of such strategies
SN - 1479-5868
AD - Centre for Physical Activity and Nutrition Research, Deakin University, 221 Burwood Highway, Burwood, Victoria 3125, Australia. nicky.ridgers@deakin.edu.au

UR - 20937142
ER -

TY - JOUR
ID - 163
T1 - Elevated cortisol during play is associated with age and social engagement in children with autism
A1 - Corbett,B.A.
A1 - Schupp,C.W.
A1 - Simon,D.
A1 - Ryan,N.
A1 - Mendoza,S.
Y1 - 2010///
N1 - Corbett, Blythe A. Schupp, Clayton W. Simon, David. Ryan, Niles. Mendoza, Sally
Molecular autism
101534222
Journal Article
English
KW - MEDLINE
RP - NOT IN FILE
SP - 13
JF - Molecular Autism
JA - Mol Autism
VL - 1
IS - 1
CY - England
N2 - BACKGROUND: The hallmark characteristic of autism is impaired reciprocal social interaction. While children find social interaction stress-reducing, many children with autism may find social interaction stress-inducing. The current study was designed to examine stress responsivity as measured by cortisol by comparing children with autism to neurotypical peers during an ecologically valid 20-minute playground paradigm. METHODS: The experiment involved sets of three children: a child with autism, a neurotypical child, and a confederate. Participants included 45 prepubescent males between 8 and 12 years of age (21 with autism and 24 neurotypical children). RESULTS: Children with autism showed fewer initiations (chi2(1) = 4.03, P = 0.044), rejected initiations from others more (chi2(1) = 7.10, P = 0.008) and spent less time interacting during motor (F(1,43) = 16.7, P = 0.0002) and cooperative (F(1,43) = 14.78, P = 0.0004) play. Repeated measures analysis of the cortisol values revealed a significant model (chi2(4) = 22.76, P < 0.0005) that included time of measurement, diagnosis and age as main effects and an interaction between diagnosis and age. Thus, as age increased among children with autism, they experienced enhanced cortisol levels while age did not modify expected cortisol levels for typical children. Stress responsivity was associated with more peripheral equipment play for motor (chi2(3) = 12.3, P = 0.006) and cooperative (chi2(3) = 8.24, P = 0.04) play as well as reduced nonverbal social skills during motor (chi2(1) = 5.52, P = 0.018) and cooperative play (chi2(1) = 4.53, P = 0.033). CONCLUSIONS: Overall, children with autism engaged in fewer social overtures and spent less time interacting than typically developing peers during play. The peer interaction paradigm resulted in significantly higher levels of cortisol in many children with autism. Distinct patterns emerged within the autism group based on developmental (older), biological (cortisol responder) and behavioral patterns (peripheral group interaction). The enhanced cortisol response was observed in children who voluntarily engaged in interaction; thus, it does not support the notion of a response to social threat. Rather, it appears to reflect attendant metabolic preparedness and enhanced arousal from engaging socially. The data suggest that many children with autism activate hypothalamic-pituitary-adrenal responses in relatively benign social situations, which appears to be a function of age and level of social engagement. The findings support the need to teach coping strategies in addition to fundamental social skills to youth with autism
SN - 2040-2392
AD - Department of Psychiatry, Vanderbilt University, 1601 23rd Avenue South, Nashville, TN 37212, USA. blythe.corbett@vanderbilt.edu
UR - 20875126
ER -

TY - JOUR

ID - 164
T1 - Correlates of sedentary behaviours in preschool children: a review
A1 - Hinkley,T.
A1 - Salmon,J.
A1 - Okely,A.D.
A1 - Trost,S.G.
Y1 - 2010///
N1 - Hinkley, Trina. Salmon, Jo. Okely, Anthony D. Trost, Stewart G
The international journal of behavioral nutrition and physical activity
101217089
Journal Article
English
KW - MEDLINE
RP - NOT IN FILE
SP - 66
JF - International Journal of Behavioral Nutrition & Physical Activity
JA - Int
VL - 7
CY - England
N2 - BACKGROUND: Sedentary behaviour has been linked with a number of health outcomes. Preschool-aged children spend significant proportions of their day engaged in sedentary behaviours. Research into the correlates of sedentary behaviours in the preschool population is an emerging field, with most research being published since 2002. Reviews on correlates of sedentary behaviours which include preschool children have previously been published; however, none have reported results specific to the preschool population. This paper reviews articles reporting on correlates of sedentary behaviour in preschool children published between 1993 and 2009. METHODS: A literature search was undertaken to identify articles which examined correlates of sedentary behaviours in preschool children. Articles were retrieved and evaluated in 2008 and 2009. RESULTS: Twenty-nine studies were identified which met the inclusion criteria. From those studies, 63 potential correlates were identified. Television viewing was the most commonly examined sedentary behaviour. Findings from the review suggest that child's sex was not associated with television viewing and had an indeterminate association with sedentary behaviour as measured by accelerometry. Age, body mass index, parental education and race had an indeterminate association with television viewing, and outdoor playtime had no association with television viewing. The remaining 57 potential correlates had been investigated too infrequently to be able to draw robust conclusions about associations. CONCLUSIONS: The correlates of preschool children's sedentary behaviours are multi-dimensional and not well established. Further research is required to provide a more comprehensive understanding of the influences on preschool children's sedentary behaviours to better inform the development of interventions
SN - 1479-5868
AD - Centre for Physical Activity and Nutrition Research, Deakin University, 221 Burwood Hwy, Burwood, Vic, 3125 Australia. trina.hinkley@deakin.edu.au
UR - 20825682
ER -

TY - JOUR
ID - 165
T1 - Diet, Screen Time, Physical Activity, and Childhood Overweight in the General Population and in High Risk Subgroups: Prospective Analyses in the PIAMA Birth Cohort
A1 - Wijga,A.H.
A1 - Scholtens,S.
A1 - Bemelmans,W.J.
A1 - Kerkhof,M.
A1 - Koppelman,G.H.
A1 - Brunekreef,B.
A1 - Smit,H.A.
Y1 - 2010///
N1 - Wijga, Alet H. Scholtens, Salome. Bemelmans, Wanda J E. Kerkhof, Marjan. Koppelman, Gerard H. Brunekreef, Bert. Smit, Henriette A

Journal of obesity
101526295
Journal Article
English
KW - MEDLINE
RP - NOT IN FILE
JF - Journal of Obesity
JA - J Obes
VL - 2010, 2010.
CY - Egypt
N2 - Objective. To prospectively identify behavioral risk factors for childhood overweight and to assess their relevance in high risk sub groups (children of mothers with overweight or low education). Methods. In the PIAMA birth cohort (n = 3963), questionnaire data were obtained at ages 5 and 7 on "screen time", walking or cycling to school, playing outside, sports club membership, fast food consumption, snack consumption and soft drink consumption. Weight and height were measured at age 8 years. Results. Screen time, but none of the other hypothesized behavioral factors, was associated with overweight (aOR 1.4 (CI: 1.2-1.6)). The adjusted population attributable risk fraction for screen time > 1 hr/day was 10% in the high risk and 17% in the low risk sub groups. Conclusion. Reduction of screen time to < 1 hr/day could result in a reduction of overweight prevalence in the order of 2 percentage points in both high and low risks sub groups
SN - 2090-0716
AD - Centre for Prevention and Health Services Research, National Institute for Public Health and the Environment, P.O. Box 1, 3720 BA Bilthoven, The Netherlands
UR - 20721356
ER -

TY - JOUR
ID - 166
T1 - Neighborhood and physical activities of Portuguese adolescents
A1 - Loureiro,N.
A1 - Matos,M.G.
A1 - Santos,M.M.
A1 - Mota,J.
A1 - Diniz,J.A.
Y1 - 2010///
N1 - Loureiro, Nuno. Matos, Margarida G. Santos, Maria M. Mota, Jorge. Diniz, Jose A
The international journal of behavioral nutrition and physical activity
101217089
Journal Article
English
KW - MEDLINE
RP - NOT IN FILE
SP - 33
JF - International Journal of Behavioral Nutrition & Physical Activity
JA - Int
VL - 7
CY - England
N2 - BACKGROUND: This study examines associations between perceptions of neighbourhood environment and physical activity and sports within Portuguese adolescents. METHODS: The sample consisted of 4,877 individuals of both genders, with an average age of 14 years. The instrument used was the Health Behavior School-aged Children questionnaire. RESULTS: Perceptions of the neighbourhood being unsafe for children to play and having no place to spend leisure time were associated with lower levels of exercise among adolescents. The perceptions of the neighbourhood being unsafe for children to play (OR = 1.3, p < .005) and the fact of not having a place to spend leisure time (OR = 1.3, p < .005) p < .005) were associated with lower levels of exercise among adolescents. The perception of these variables is associated to a lower probability of exercising. The neighbourhood characteristics are more important to the practice of outdoor sports than of indoor sports. CONCLUSION: The perceptions of the neighbourhood may influence adolescent's physical activity and sports, in different ways

SN - 1479-5868
AD - Faculdade de Motricidade Humana, Cruz Quebrada, Portugal. margaridagaspar@netcabo.pt
UR - 20444283
ER -

TY - JOUR
ID - 167
T1 - Chronic hip pain in a boy with mental retardation and cerebral palsy.[Reprint of J Dev Behav Pediatr. 2005 Apr;26(2):152-7; PMID: 15827468]
A1 - Stein,M.T.
A1 - Gottsegen,D.
A1 - Blasco,P.A.
A1 - Wolraich,M.
A1 - Hennessy,M.J.
Y1 - 2010/04//
N1 - Stein, Martin T. Gottsegen, David. Blasco, Peter A. Wolraich, Mark. Hennessy, Michael J
Journal of developmental and behavioral pediatrics : JDBP
htf, 8006933
Journal Article
English
KW - MEDLINE
RP - NOT IN FILE
SP - S86
EP - S91
JF - Journal of Developmental & Behavioral Pediatrics
JA - J Dev Behav Pediatr
VL - 31
IS - 3 Suppl
CY - United States
N2 - CASE: Jonny is a 13 year old boy with spastic quadriparesis and severe mental retardation following Haemophilus influenza type B (HIB) meningitis at 2-months of age. Signs of meningitis started on the evening of his 2-month immunizations that included the HIB vaccine. He presented to his pediatrician with left hip pain that occurred intermittently for a few years and more frequently in the past six months. His parents initially attributed the pain to whizzing around the back yard in a motorized wheelchair. An earlier evaluation of hip pain led to bilateral femoral osteotomies for hip dysplasia. Obesity, associated with inactivity and a tendency to consume fatty foods, complicates Jonny's disabilities. His only activity is a modest amount of physical therapy at school and "floor time" for about one hour each day at home. In the office of his pediatrician, Jonny is friendly, smiling, and verbalizing a few words with his limited expressive vocabulary. He is resistant to a hip examination and grimaces with manipulation of his left hip. Spasticity of the left leg appears increased compared to previous examinations. He has nonpitting edema of his lower legs and feet, a cryptorchid left testicle, and a somewhat tender left inguinal area. Jonny lives with his mother and father in a small house on a busy street less than one-half mile from the pediatrician's office. Jonny's pediatrician often sees him in his wheelchair, accompanied by his mother or grandmother, and waves or stops to chat. He has van services to school, and there is a Hoyer lift in the home, but his parents do not own a van. Recently, Jonny's father finds it more difficult to lift him. The family has also been challenged by the mental health problems of Jonny's two older brothers, and a serious eye injury suffered by his middle brother in a motor-vehicle accident. Jonny's pediatrician has cared for him and his two brothers since birth. Although the parents continue to believe that the HIB vaccine caused his catastrophic illness, they remain with the pediatrician. In general, they are satisfied with the individualized educational plan at a local public school. When he was 6.5 years old, Jonny's school aid reported that he attempted to touch her in the genital area. The pediatrician attended the meeting to review this incident and successfully advocated for Jonny by pointing out that this was an isolated incident; it did not occur again. At 6 years old Jonny functioned in the 1.5-2.5 year old range with motor skills in the 6-12 month level according to the Bayley Scales of Infant Development and the Vineland Adaptive Behavior Scales. In the past a neurologist and a physiatrist saw Jonny, but both of these individuals moved from the community. He had prior evaluations at a children's orthopedic clinic at a small community hospital and at the local Shriner's hospital. He had a tonsillectomy and adenoidectomy at 7 years old. He is currently treated for constipation and receives dental care at a clinic for people with disabilities. His pediatrician has always respected the parents for their care

and obvious love for their disabled child. However, parental resistance to addressing major issues such as obesity has frustrated his pediatrician. When the pediatrician suggested that Jonny was eligible for the state's managed care program, which would convert Medicaid coverage to a state sponsored program with more extensive services and case management, Jonny's mother repeatedly said that she would "think it over." At the current visit, the pediatrician recommended an adjustment of Jonny's wheelchair, a hip x-ray, a referral to Shriner's Hospital, and an appointment with a pediatric surgeon to address the undescended testicle and possible hernia. Jonny's mother mentioned that he had been to Shriner's Hospital for hip pain two years earlier but was told nothing could be done "because nothing was wrong with his bone." The hip x-ray was normal as well as a complete blood count and a C-reactive protein. The pediatric surgeon did not find a hernia and deferred treatment of the cryptorchid testicle. His parents contacted the wheelchair company to arrange adjustments. The pediatrician called the medical director at the Shriner's Hospital to discuss Jonny's case, but 2 months after the initial visit, the parents had not arranged for an appointment at the Shriner's Hospital. Jonny's hip pain persisted. The pediatrician now wonders how he can more effectively address Jonny's current problems and improve overall care for him and his family
SN - 1536-7312
AD - University of California San Diego, Children's Hospital San Diego, CA, USA
UR - 20414086
ER -

TY - JOUR
ID - 168
T1 - Chronic constipation, atypical eating pattern, weight loss, and anxiety in a 19-year old youth.[Reprint of J Dev Behav Pediatr. 2006 Aug;27(4):338-40; PMID: 16906011]
A1 - Richards,M.M.
A1 - Banez,G.A.
A1 - Dohil,R.
A1 - Stein,M.T.
Y1 - 2010/04//
N1 - Richards, Margaret M. Banez, Gerard A. Dohil, Ranjan. Stein, Martin T
Journal of developmental and behavioral pediatrics : JDBP
htf, 8006933
Journal Article
English
KW - MEDLINE
RP - NOT IN FILE
SP - S83
EP - S85
JF - Journal of Developmental & Behavioral Pediatrics
JA - J Dev Behav Pediatr
VL - 31
IS - 3 Suppl
CY - United States
N2 - CASE: A 19-year-old female was admitted to a children's hospital weighing 75 pounds. She lost 75 pounds over 2 years and did not menstruate for a year. Despite her dissatisfaction with her thin appearance and desire to weigh more, her inpatient treatment focused on "disordered eating." Adolescent medicine, nutrition, and gastroenterology specialists were consulted. A pediatric psychology consultation was initiated to address anxiety associated with eating as well as abnormal toileting behaviors. The patient reported that she had chronic constipation since the age 3 years with a large, hard, and painful bowel movement once every 1-3 weeks. She had numerous medical and nutritional interventions to improve her bowel functioning, including extensive laxative use and a diet high in fiber (mostly fruits and vegetables). Additionally, an extensive medical evaluation to look for physiological causes for chronic constipation, including Hirschsprung's disease, was not diagnostic. The patient described purposeful stool withholding due to her concern over painful bowel movements. She also avoided peer activities because she did not want to use toilets outside her home. The patient acknowledged that her disordered eating stemmed largely from her toileting difficulties. She described altering her eating habits to avoid bowel movements (e.g., eating small meals, not eating at school) and anxious thoughts while defecating. Defecation anxiety was now affecting other areas of her life. For example, she frequently used copious amounts of toilet paper to ensure thorough cleaning. The patient's parents confirmed her need to thoroughly clean after

toilet use, using "almost half a roll of toilet paper." They described other concerning "routines," including refusing to use towels after showering. She dried herself with a fan because of fear that her towel may be dirty. They indicated that during early adolescence, she frequently washed her face to prevent acne breakouts. According to parents, the patient followed doctors' instructions "as though they (were) gospel," deviating from suggestions only with extreme resistance
SN - 1536-7312
AD - Children's Hospital, Cleveland Clinic, Cleveland, Ohio, USA
UR - 20414085
ER -

TY - JOUR
ID - 169
T1 - Disruptive and oppositional behavior in an 11-year old boy.[Reprint of J Dev Behav Pediatr. 2007 Oct;28(5):406-8; PMID: 18049326]
A1 - Garland,A.
A1 - Augustyn,M.
A1 - Stein,M.T.
Y1 - 2010/04//
N1 - Garland, Ann. Augustyn, Marilyn. Stein, Martin T
Journal of developmental and behavioral pediatrics : JDBP
htf, 8006933
Journal Article
English
KW - MEDLINE
RP - NOT IN FILE
SP - S21
EP - S23
JF - Journal of Developmental & Behavioral Pediatrics
JA - J Dev Behav Pediatr
VL - 31
IS - 3 Suppl
CY - United States
N2 - CASE: Tony is an 11-year old boy in the fifth grade whose mother describes him as "really a good kid who is bright and tries to be friendly. But he's always doing things that get him in trouble at school and sometimes at home." Tony was diagnosed with ADHD (combined type) 2 years ago. Stimulant therapy improved his attention and concentration during school, decreased hyperactivity in the classroom and improved educational achievements. However, Tony is oppositional and disruptive on the playground, during team sports and at home. His teacher observed that he wants to fit in, but he quickly gets in arguments with other children. He has difficulty sustaining friendships because he typically annoys others with unreasonable demands. He often has temper tantrums when things do not go his way; the tantrums are not prolonged but frequent. At home, on several occasions Tony hit his younger sister, and he once threw a dinner plate against the wall during a family meal. Although his mother describes these behaviors as present for many years, they seem to be escalating. Tony lives with both parents and his younger sister. There is no history of marital discord or major life event change in the past year. Standardized achievement tests demonstrate average to above average achievement scores. He continues to get mostly B grades and an occasional C. Tony's parents have tried to limit television time as a punishment for disruptive behaviors without any apparent effect. His mother reports that she yelled at him on several occasions when he refused to carry out household chores. "He gets angry at the simplest request for help." After meeting with Tony and his mother and completing a normal physical examination, the pediatrician referred Tony to a child psychologist for behavioral therapy
SN - 1536-7312
AD - University of California San Diego, CA, USA
UR - 20414068
ER -

TY - JOUR
ID - 170

T1 - Individual, social and physical environmental correlates of children's active free-play: a cross-sectional study
A1 - Veitch,J.
A1 - Salmon,J.
A1 - Ball,K.
Y1 - 2010///
N1 - Veitch, Jenny. Salmon, Jo. Ball, Kylie
The international journal of behavioral nutrition and physical activity
101217089
Journal Article
English
KW - MEDLINE
RP - NOT IN FILE
SP - 11
JF - International Journal of Behavioral Nutrition & Physical Activity
JA - Int
VL - 7
CY - England
N2 - BACKGROUND: Children's unstructured outdoor free-play (or active free-play) has the potential to make an important contribution to children's overall physical activity levels. Limited research has, however, examined physical activity in this domain. This study examined associations between individual, social and physical environmental factors and the frequency with which children play in particular outdoor locations outside school hours. This study also investigated whether the frequency of playing in outdoor locations was associated with children's overall physical activity levels. METHODS: Participants including 8-9 year old children and their parents (n = 187) were recruited from a selection of primary schools of varying socioeconomic status across metropolitan Melbourne, Australia. Parents completed a survey and children's overall physical activity levels were measured by accelerometry. Regression models examined the odds of children playing in various outdoor settings according to particular correlates. RESULTS: Inverse associations were found between preference for activities not involving physical activity, and the likelihood of children playing in the yard at home on the weekend (OR = 0.65; CI = 0.45,0.95). Positive correlates of children playing in their own street included: parental perceptions that it was safe for their child to play in their street (weekdays [OR = 6.46; CI = 2.84,14.71], weekend days [OR = 6.01; CI = 2.68,13.47]); children having many friends in their neighbourhood (OR = 2.63; CI = 1.21,5.76); and living in a cul-de-sac (weekdays [OR = 3.99; CI = 1.65,9.66], weekend days [OR = 3.49; CI = 1.49,8.16]). Positive correlates of more frequent play in the park/playground on weekdays included family going to the park together on a weekly basis on weekdays (OR = 6.8; CI = 3.4,13.6); and on weekend days (OR = 7.36; CI = 3.6,15.0). No differences in mean mins/day of moderate-vigorous physical activity were found between children in the highest and lowest tertiles for frequency of playing in particular outdoor locations. CONCLUSION: The presence of friends, safety issues and aspects of the built environment were reported by parents to be associated with children's active free-play in outdoor locations. Future research needs to further examine associations with time spent in active free-play and objectively-measured overall physical activity levels. It is also important to investigate strategies for developing a supportive social and physical environment that provides opportunities for children to engage in active free-play
SN - 1479-5868
AD - Centre for Physical Activity and Nutrition Research, Deakin University, 221 Burwood Hwy, Burwood, Victoria, 3125, Australia. jenny.veitch@deakin.edu.au
UR - 20181061
ER -

TY - JOUR
ID - 171
T1 - Injury-related unsafe behavior among households from different socioeconomic strata in pune city
A1 - Mirkazemi,R.
A1 - Kar,A.
Y1 - 2009/10//
N1 - Mirkazemi, Roksana. Kar, Anita
Indian journal of community medicine : official publication of Indian Association of Preventive & Social Medicine

9315574
Journal Article
English
KW - MEDLINE
RP - NOT IN FILE
SP - 301
EP - 305
JF - Indian Journal of Community Medicine
JA - Indian J
VL - 34
IS - 4
CY - India
N2 - INTRODUCTION: Behavior pattern influences the risk of unintentional injuries. This study was conducted to identify the pattern of household unsafe behavior in different socioeconomic strata, in Pune city, India. MATERIALS AND METHOD: Population-based, cross-sectional study. Behaviors influencing the risk of burn, poisoning, drowning, and road traffic injuries were questioned from 200 randomly selected households. RESULTS: Nearly 28% of the households did not have a separate kitchen, 37.5% cooked at the ground level, 33.5% used a kerosene pressure stove, 12% used unprotected open fire as a source of warmth in winter, and 34.5% stored inflammable substances at home. Ninety one percent of the households reported storing poisonous chemicals in places that could not be locked. In 68.3% of the households with children below five years, these chemicals were kept in places accessible to children. Nearly 21% of the individuals, who could swim, did so in unsafe places and 25.2% of them were not trained in swimming. In 35.5% of the households, children used streets as playgrounds. Among all two-wheeled vehicle riders, 35.6% reported not having a helmet and 57.7% of those who had a helmet did not use it regularly. Socioeconomic status was strongly associated with the unsafe behaviors related to burns, drowning, and road traffic injuries. CONCLUSION: The study identifies the sociocultural and behavioral factors leading to unsafe behaviors, placing individuals at risk of unintentional injuries, which can be used as a first step toward prevention
SN - 1998-3581
AD - Interdisciplinary School of Health Sciences, University of Pune, Pune 411 007, India
UR - 20165622
ER -

TY - JOUR
ID - 172
T1 - Is there an association between spatial access to parks/green space and childhood overweight/obesity in Calgary, Canada?
A1 - Potestio,M.L.
A1 - Patel,A.B.
A1 - Powell,C.D.
A1 - McNeil,D.A.
A1 - Jacobson,R.D.
A1 - McLaren,L.
Y1 - 2009///
N1 - Potestio, Melissa L. Patel, Alka B. Powell, Christopher D. McNeil, Deborah A. Jacobson, R Daniel. McLaren, Lindsay
The international journal of behavioral nutrition and physical activity
101217089
Journal Article
English
KW - MEDLINE
RP - NOT IN FILE
SP - 77
JF - International Journal of Behavioral Nutrition & Physical Activity
JA - Int
VL - 6
CY - England

N2 - BACKGROUND: The recent increase in childhood obesity is expected to add significantly to the prevalence of chronic diseases. We used multivariate multilevel analysis to examine associations between parks/green space and childhood overweight/obesity across communities in Calgary, Canada, a city characterized by intensified urban sprawl and high car use. METHODS: Body Mass Index was calculated from measured height and weight data obtained from 6,772 children (mean age = 4.95 years) attending public health clinics for pre-school vaccinations. Each child's home postal code was geocoded using ESRI ArcGIS 9.2. We examined four measures of spatial access to parks/green space (based on Geographic Information Systems): 1) the number of parks/green spaces per 10,000 residents, 2) the area of parks/green space as a proportion of the total area within a community, 3) average distance to a park/green space, and 4) the proportion of parks/green space service area as a proportion of the total area within a community. Analyses were adjusted for dissemination area median family income (as a proxy for an individual child's family income) community-level education, and community-level proportion of visible minorities. RESULTS: In general, parks/green space at the community level was not associated with overweight/obesity in Calgary, with the exception of a marginally significant effect whereby a moderate number of parks/green spaces per 10,000 residents was associated with lower odds of overweight/obesity. This effect was non-significant in adjusted analyses. CONCLUSION: Our null findings may reflect the popularity of car travel in Calgary, Canada and suggest that the role built environment characteristics play in explaining health outcomes may differ depending on the type of urban environment being studied
SN - 1479-5868
AD - University of Calgary, Department of Community Health Sciences, Faculty of Medicine, 3330 Hospital Drive NW Calgary, Alberta, T2N 4N1, Canada. mlpotest@ucalgary.ca
UR - 19930567
ER -

TY - JOUR
ID - 173
T1 - Task control signals in pediatric tourette syndrome show evidence of immature and anomalous functional activity
A1 - Church,J.A.
A1 - Wenger,K.K.
A1 - Dosenbach,N.U.
A1 - Miezin,F.M.
A1 - Petersen,S.E.
A1 - Schlaggar,B.L.
Y1 - 2009///
N1 - Church, Jessica A. Wenger, Kristin K. Dosenbach, Nico U F. Miezin, Francis M. Petersen, Steven E. Schlaggar, Bradley L
Frontiers in human neuroscience
101477954
Journal Article
English
KW - MEDLINE
RP - NOT IN FILE
SP - 38
JF - Frontiers in Human Neuroscience
JA - Front Hum Neurosci
VL - 3
CY - Switzerland
N2 - Tourette Syndrome (TS) is a pediatric movement disorder that may affect control signaling in the brain. Previous work has proposed a dual-networks architecture of control processing involving a task-maintenance network and an adaptive control network (Dosenbach et al., 2008). A prior resting-state functional connectivity MRI (rs-fcMRI) analysis in TS has revealed functional immaturity in both putative control networks, with "anomalous" correlations (i.e., correlations outside the typical developmental range) limited to the adaptive control network (Church et al., 2009). The present study used functional MRI (fMRI) to study brain activity related to adaptive control (by studying start-cues signals), and to task-maintenance (by studying signals sustained across a task set). Two hypotheses from the previous rs-fcMRI results were tested. First, adaptive control (i.e., start-cue) activity will be altered in TS, including activity inconsistent with typical development

("anomalous"). Second, group differences found in task-maintenance (i.e., sustained) activity will be consistent with functional immaturity in TS. We examined regions found through a direct comparison of adolescents with and without TS, as well as regions derived from a previous investigation that showed differences between unaffected children and adults. The TS group showed decreased start-cue signal magnitude in regions where start-cue activity is unchanged over typical development, consistent with anomalous adaptive control. The TS group also had higher magnitude sustained signals in frontal cortex regions that overlapped with regions showing differences over typical development, consistent with immature task-maintenance in TS. The results demonstrate task-related fMRI signal differences anticipated by the atypical functional connectivity found previously in adolescents with TS, strengthening the evidence for functional immaturity and anomalous signaling in control networks in adolescents with TS
SN - 1662-5161
AD - Department of Neurology, Washington University School of Medicine St. Louis, MO 63110, USA. jchurch@wustl.edu
UR - 19949483
ER -

TY - JOUR
ID - 174
T1 - Flip flops, dress clothes, and no coat: clothing barriers to children's physical activity in child-care centers identified from a qualitative study
A1 - Copeland,K.A.
A1 - Sherman,S.N.
A1 - Kendeigh,C.A.
A1 - Saelens,B.E.
A1 - Kalkwarf,H.J.
Y1 - 2009///
N1 - Copeland, Kristen A. Sherman, Susan N. Kendeigh, Cassandra A. Saelens, Brian E. Kalkwarf, Heidi J
The international journal of behavioral nutrition and physical activity
101217089
Journal Article
English
KW - MEDLINE
RP - NOT IN FILE
SP - 74
JF - International Journal of Behavioral Nutrition & Physical Activity
JA - Int
VL - 6
CY - England
N2 - BACKGROUND: Three-quarters of 3-6 year-old children in the U.S. spend time in childcare; many spend most of their waking hours in these settings. Daily physical activity offers numerous health benefits, but activity levels vary widely across centers. This study was undertaken to explore reasons why physical activity levels may vary. The purpose of this paper is to summarize an unexpected finding that child-care providers cited was a key barrier to children's physical activity. METHODS: Nine focus groups with 49 child-care providers (55% black) from 34 centers (including inner-city, suburban, Head Start and Montessori) were conducted in Cincinnati, OH. Three independent raters analyzed verbatim transcripts for themes. Several techniques were used to increase credibility of findings, including interviews with 13 caregivers. RESULTS: Two major themes about clothing were: 1) children's clothing was a barrier to children's physical activity in child-care, and 2) clothing choices were a significant source of conflict between parents and child-care providers. Inappropriate clothing items included: no coat/hat/gloves in the wintertime, flip flops or sandals, dress/expensive clothes, jewelry, and clothes that were either too loose or too tight. Child-care providers explained that unless there were enough extra coats at the center, a single child without a coat could prevent the entire class from going outside. Caregivers suggested several reasons why parents may dress their child inappropriately, including forgetfulness, a rushed morning routine, limited income to buy clothes, a child's preference for a favorite item, and parents not understanding the importance of outdoor play. Several child-care providers favored specific policies prohibiting inappropriate clothing, as many reported limited success with verbal or written reminders to bring appropriate clothing. CONCLUSION: Inappropriate clothing may be an important barrier to children's physical activity in child-care settings, particularly if the clothing of a few children preclude physical activity for the remaining

children. Center directors and policy makers should consider devising clear and specific policies for the types of clothing that will be permitted in these settings so that children's active play opportunities are not curtailed. To enhance compliance, parents may need education about the importance and benefits of active play for children's development
SN - 1479-5868
AD - Division of General and Community Pediatrics, Cincinnati Children's Hospital Medical Center, Cincinnati, OH, USA. kristen.copeland@cchmc.org
UR - 19895677
ER -

TY - JOUR
ID - 175
T1 - Objectively measured physical activity, physical activity related personality and body mass index in 6- to 10-yr-old children: a cross-sectional study
A1 - Deforche,B.
A1 - De,Bourdeaudhuij,I
A1 - D'hondt,E.
A1 - Cardon,G.
Y1 - 2009///
N1 - Deforche, Benedicte. De Bourdeaudhuij, Ilse. D'hondt, Eva. Cardon, Greet
The international journal of behavioral nutrition and physical activity
101217089
Journal Article
English
KW - MEDLINE
RP - NOT IN FILE
SP - 25
JF - International Journal of Behavioral Nutrition & Physical Activity
JA - Int
VL - 6
CY - England
N2 - BACKGROUND: The prevalence and level of overweight in childhood is rapidly increasing. One potential contributor to the rise in overweight is a decline in physical activity (PA). The purpose of this study was to compare levels and patterns of PA and PA related personality in normal-weight (NW) and overweight (OW) 6- to 10-yr-old children. METHODS: Subjects were grouped into OW (N = 59, BMI = 24.2 +/- 4.8 kg/m2) or NW (N = 61, BMI = 15.7 +/- 1.5 kg/m2) according to International Obesity Task Force cut-offs. PA was assessed by accelerometry. Parents filled in a questionnaire on PA and sedentary behaviour and PA related personality of their child (born tired, moves slowly, is often tired, lacks energy, avoids physical efforts, prefers watching playing children instead of joining them, is always active, needs to let himself/herself go, has a lot of energy). RESULTS: NW children spent on average 77 min/day in MVPA, whereas OW children only 57 min/day (p = .001). OW children had fewer 5, 10 and 20 min bouts of MVPA (p = .01). OW and NW children showed identical PA patterns on both week days and weekends, although at different levels. According to parents' report, a greater percentage of OW children was not engaged in any sport (46% versus 23%, chi2 = 6.3, p = .01). OW children had a less active personality (p < .001), watched more TV during weekend (p < .01), but no differences were found in outside play or non-active play. BMI of mother and father explained 29% of the variance in children's BMI z-score (p < .001). PA related personality, screen behaviour during weekend and MVPA explained an additional 12% (p < 0.01). CONCLUSION: The results of this study demonstrate that NW children spent on average 20 min per day more in MVPA. PA patterns were similar in NW versus OW children, although at different levels. Greatest differences in PA according to weight status were found in the afternoon during after school hours. This is the first study to show distinct PA related personality traits in OW children compared to NW peers
SN - 1479-5868
AD - Department of Movement and Sports Sciences, Ghent University, Watersportlaan 2, 9000 Gent, Belgium. Benedicte.Deforche@UGent.be
UR - 19442293
ER -

TY - JOUR
ID - 176
T1 - Time spent playing outdoors after school and its relationship with independent mobility: a cross-sectional survey of children aged 10-12 years in Sydney, Australia
A1 - Wen,L.M.
A1 - Kite,J.
A1 - Merom,D.
A1 - Rissel,C.
Y1 - 2009///
N1 - Wen, Li Ming. Kite, James. Merom, Dafna. Rissel, Chris
The international journal of behavioral nutrition and physical activity
101217089
Journal Article
English
KW - MEDLINE
RP - NOT IN FILE
SP - 15
JF - International Journal of Behavioral Nutrition & Physical Activity
JA - Int
VL - 6
CY - England
N2 - BACKGROUND: Time spent outdoors is positively associated with physical activity and has been suggested as a proxy for physical activity of children. The role of children's independence in physical activity and time spent outdoors is less understood. This study aimed to assess how much time children spent playing outdoors after school, and to explore the relationship between outdoor play and independence among children aged 10-12 years. METHOD: Children recorded how much time they spent playing outdoors or watching TV/videos or playing computer games after school using a five-day diary, and also reported whether they were allowed to walk on their own in their neighbourhood as an indicator of their independent mobility. Parents were surveyed on family demographics and perception of neighbourhood safety. The surveys were conducted in late 2006 as part of the Central Sydney Walk to School program which involved 1975 children and their parents from 24 primary schools. Factors associated with time spent playing outdoors were determined by logistic regression modelling. RESULTS: Thirty-seven per cent of children spent less than half an hour a day playing outdoors after school, and 43% spent more than 2 hours a day watching TV, videos or playing computer games. Forty-eight per cent of children were allowed to walk on their own near where they lived. Children's independent mobility was significantly associated with outdoor play after adjusting for other confounders. Compared with those who were never allowed to walk on their own near where they lived, students who were allowed to walk on their own were significantly more likely to spend more than half an hour a day playing outdoors after school with an adjusted odds ratio of 2.6, 95% CI 1.84-3.58, P < 0.001. CONCLUSION: The findings that a significant proportion of children spend less than half an hour a day playing outdoors after school and have excessive screen time have important implications for physical activity promotion and obesity prevention. The study also suggests that children's independent mobility should be considered in research and evaluation into children's play and physical activity. Environments that promote greater independent mobility in children may increase their physical activity levels and hence reduce their risk of overweight/obesity
SN - 1479-5868
AD - Health Promotion Service, Sydney South West Area Health Service, Syndey, Australia. lmwen@email.cs.nsw.gov.au
UR - 19291324
ER -

TY - JOUR
ID - 177
T1 - Hand dominance and gender in forearm fractures in children
A1 - Hassan,F.O.
Y1 - 2008/12//
N1 - Hassan, Freih Odeh Abu
Strategies in trauma and limb reconstruction (Online)
101299515

Journal Article
English
KW - MEDLINE
RP - NOT IN FILE
SP - 101
EP - 103
JF - Strategies in Trauma & Limb Reconstruction
JA - Strategies Trauma Limb Reconstr
VL - 3
IS - 3
CY - Italy
N2 - No published studies have addressed the role of hand dominance in various types of forearm fractures. The present study aims to investigate the effects of the dominant hand and gender in forearm fractures in children and adolescents. In a prospective study, 181 children aged 2-15 years presenting with unilateral forearm fracture were examined over a 6-year period, investigating the role of the dominant hand, fractured side, fractured site, and gender in different types of forearm fractures. Forearm fractures occur more often in boys and are more common on the left side (P = 0.001, 0.029, respectively). Isolated distal radius fracture is more common than distal radius and ulna fracture in right-handed children (P = 0.008). Increases in the number of middle forearm fractures in the dominant hand in left-handed children (P = 0.0056) may be due to mechanisms of injury other than a simple indirect fall or severe injury preventing the use of the dominant hand as a preventive measure. The mean age for boys and girls at the time of forearm fractures was 8.97 and 5.98 years, respectively, which may be attributed to older girls tending not to do as many outside-the-home activities as boys at this age. Overall, forearm fractures are more common in the non-dominant hand, in boys, and in both distal forearm bones
SN - 1828-8936
AD - Department of Orthopedics Surgery, Jordan University Hospital, Amman, Jordan, freih@ju.edu.jo
UR - 19030948
ER -

TY - JOUR
ID - 178
T1 - Physical environmental characteristics and individual interests as correlates of physical activity in Norwegian secondary schools: the health behaviour in school-aged children study
A1 - Haug,E.
A1 - Torsheim,T.
A1 - Samdal,O.
Y1 - 2008///
N1 - Haug, Ellen. Torsheim, Torbjorn. Samdal, Oddrun
The international journal of behavioral nutrition and physical activity
101217089
Journal Article
English
KW - MEDLINE
RP - NOT IN FILE
SP - 47
JF - International Journal of Behavioral Nutrition & Physical Activity
JA - Int
VL - 5
CY - England
N2 - BACKGROUND: The school has been identified as a key arena for physical activity promotion for young people. Effective change of physical activity behaviour requires identification of consistent and modifiable correlates. The study explores students' interests in school physical activity and facilities in the school environment and examines their associations with students' participation in physical activity during recess and their cross-level interaction effect. METHODS: This cross-sectional study was based on a national representative sample of Norwegian secondary schools and grade 8 students who participated in the Health Behaviour in School-aged Children (HBSC) 2005/06 study. The final sample comprised 68 schools and 1347 students. Physical environment characteristics were assessed through questionnaires completed by the principals, and students' physical activity and interests in physical activity were assessed through student self-

completion questionnaires. RESULTS: Most students were interested in more opportunities for physical activity in school. Multilevel logistic regression models demonstrated that students attending schools with many facilities had 4.49 times (95% Confidence Interval (CI) = 1.93-10.44) higher odds of being physically active compared to students in schools with fewer facilities when adjusting for socio-economic status, sex and interests in school physical activity. Also open fields (Odds Ratio (OR) = 4.31, 95% CI = 1.65-11.28), outdoor obstacle course (OR = 1.78, 95% CI = 1.32-2.40), playground equipment (OR = 1.73, 95% CI = 1.24-2.42) and room with cardio and weightlifting equipment (OR = 1.58, 95%CI = 1.18-2.10) were associated with increased participation in physical activity. Both students' overall interests and the physical facilitation of the school environment significantly contributed to the prediction of recess physical activity. The interaction term demonstrated that students' interests might moderate the effect of facilities on recess physical activity. CONCLUSION: The findings support the use of an ecological approach and multilevel analyses in the investigation of correlates of physical activity that allows for a broader understanding of the influence of and interaction between factors at multiple levels on physical activity behaviour. In the promotion of physical activity in lower secondary schools, the study suggests that programmes should include a focus on environmental facilitation and incorporate strategies to increase students' interests for school physical activity
SN - 1479-5868
AD - Research Centre for Health Promotion, Faculty of Psychology, University of Bergen, Christiesgt, 13, N-5020, Bergen, Norway. ellen.haug@psyhp.uib.no
UR - 18823545
ER -

TY - JOUR
ID - 179
T1 - Relationships between the home environment and physical activity and dietary patterns of preschool children: a cross-sectional study
A1 - Spurrier,N.J.
A1 - Magarey,A.A.
A1 - Golley,R.
A1 - Curnow,F.
A1 - Sawyer,M.G.
Y1 - 2008///
N1 - Spurrier, Nicola J. Magarey, Anthea A. Golley, Rebecca. Curnow, Fiona. Sawyer, Michael G
The international journal of behavioral nutrition and physical activity
101217089
Journal Article
English
KW - MEDLINE
RP - NOT IN FILE
SP - 31
JF - International Journal of Behavioral Nutrition & Physical Activity
JA - Int
VL - 5
CY - England
N2 - OBJECTIVE: To assess relationships between characteristics of the home environment and preschool children's physical activity and dietary patterns. METHODS: Homes of 280 preschool children were visited and information obtained by direct observation and parent interview regarding physical and nutritional characteristics of the home environment. Children's physical activity, sedentary behaviour and dietary patterns were measured using standardised parent-report questionnaires. Associations were analysed using analysis of variance and correlation. RESULTS: Parental physical activity ($p = 0.03$-0.008), size of backyard ($p = 0.001$) and amount of outdoor play equipment ($p = 0.003$) were associated with more outdoor play. Fewer rules about television viewing ($p < 0.001$) and presence of playstation ($p = 0.02$) were associated with more indoor sedentary time. Higher fruit and vegetable intake was associated with restricting children's access to fruit juice ($p = 0.02$) and restricting high fat/sugar snacks ($p = 0.009$). Lower intake of non-core foods was associated with restricting children's access to fruit juice ($p = 0.007$), cordial/carbonated drinks ($p < 0.001$) and high fat/sugar snacks ($p = 0.003$). Lower fruit and vegetable intake was associated with reminding child to 'eat up' ($p = 0.007$) and offering food rewards to eat main meal ($p = 0.04$). Higher intake of non-core foods was associated with giving food 'treats' ($p = 0.03$) and offering food rewards to eat main meal ($p = 0.04$). The availability of food

groups in the home was associated with children's intake of these foods (fruit and vegetables, p < 0.001; fat in dairy, p = <0.001; sweetened beverages, p = 0.004-<0.001; non-core foods, p = 0.01-<0.001). CONCLUSION: Physical attributes of the home environment and parental behaviours are associated with preschool children's physical activity, sedentary behaviour and dietary patterns. Many of these variables are modifiable and could be targeted in childhood obesity prevention and management
SN - 1479-5868
AD - Department of Paediatrics and Child Health, Flinders University, Bedford Park, South Australia 5042, Australia. nicola.spurrier@flinders.edu.au
UR - 18513416
ER -

TY - JOUR
ID - 180
T1 - Crime rates and sedentary behavior among 4th grade Texas school children
A1 - Brown,H.S.,III
A1 - Perez,A.
A1 - Mirchandani,G.G.
A1 - Hoelscher,D.M.
A1 - Kelder,S.H.
Y1 - 2008///
N1 - Brown, H Shelton 3rd. Perez, Adriana. Mirchandani, Gita G. Hoelscher, Deanna M. Kelder, Steven H
The international journal of behavioral nutrition and physical activity
101217089
Journal Article
English
KW - MEDLINE
RP - NOT IN FILE
SP - 28
JF - International Journal of Behavioral Nutrition & Physical Activity
JA - Int
VL - 5
CY - England
N2 - INTRODUCTION: Although per capita crime has generally fallen over the period which coincides with the obesity epidemic, it has not fallen uniformly across communities. It also has not fallen enough to allay fears on the part of parents. Over the past 30 years, technological changes have made the indoor alternatives to playing outside, where children are more vulnerable to criminal activity, more enjoyable (cable TV, video games, and the internet) and comfortable (the spread of air conditioning to low income neighborhoods). We determined whether indoor sedentary behavior patterns are associated with community crime statistics. 4th graders in the U.S. are typically 9 or 10 years old. METHODS: We used data from the 2004-2005 Texas School Physical Activity and Nutrition (SPAN) survey linked with U.S. Department of Justice, Office of Justice Programs, Bureau of Justice Statistics data for the years 2000 through 2005 and Texas State data on sexual offenders. The probability-based sample included a total of 7,907 children in grade four. Multistage probability sampling weights were used. The dependent variables included were hours of TV watching, video game playing, computer use and total indoor sedentary behavior after school. Incremental Relative Rates were computed for community crime rates including robberies, all violent crimes, murders, assaults, property crimes, rapes, burglaries, larcenies and motor vehicle thefts as well as for sexual offenders living in the neighborhood. The neighborhood refers to the areas where the students at each school live. In the case of sexual offenders, sexual offenders per capita are estimated using the per capita rate in the zip code of the school attended; all other crime statistics are estimated by the crimes per capita in the police department jurisdiction covering the school attended. After controlling for sex, age, and African-American and Hispanic, cross-sectional associations were determined using multivariate Poisson regression. RESULTS: 4th grade boys were more likely to play video games in communities with increased per 100 population rates of larceny and burglary as well as in communities with increased per capita sexual offenders; 4th grade girls were more likely to watch television in communities with increased per capita sexual offenders. While 4th grade girls were more likely to watch TV in communities with increased per capita sex offenders, they were less likely to use computers. Per capita sexual offenders were negatively related to computer use amongst 4th grade girls. CONCLUSION: By combining community crime and cross-sectional individual level data on indoor sedentary behavior, we found that there is

an association between community crimes/sex offender rates and certain types of indoor sedentary behavior. The development of technologies in recent decades which makes supervising children easier indoors, where children are much less vulnerable to crime, may be contributing to the epidemic of childhood obesity
SN - 1479-5868
AD - Michael & Susan Dell Center for Advancement of Healthy Living, Division of Management, Policy and Community Health, University of Texas School of Public Health, 313 E, 12th Street, Suite 220, Austin, TX 78701, USA. h.shelton.brown@uth.tmc.edu
UR - 18479521
ER -

TY - JOUR
ID - 181
T1 - Active commuting to school: how far is too far?
A1 - Nelson,N.M.
A1 - Foley,E.
A1 - O'Gorman,D.J.
A1 - Moyna,N.M.
A1 - Woods,C.B.
Y1 - 2008///
N1 - Nelson, Norah M. Foley, Eimear. O'Gorman, Donal J. Moyna, Niall M. Woods, Catherine B
The international journal of behavioral nutrition and physical activity
101217089
Journal Article
English
KW - MEDLINE
RP - NOT IN FILE
SP - 1
JF - International Journal of Behavioral Nutrition & Physical Activity
JA - Int
VL - 5
CY - England
N2 - BACKGROUND: Walking and cycling to school provide a convenient opportunity to incorporate physical activity into an adolescent's daily routine. School proximity to residential homes has been identified as an important determinant of active commuting among children. The purpose of this study is to identify if distance is a barrier to active commuting among adolescents, and if there is a criterion distance above which adolescents choose not to walk or cycle. METHODS: Data was collected in 2003-05 from a cross-sectional cohort of 15-17 yr old adolescents in 61 post primary schools in Ireland. Participants self-reported distance, mode of transport to school and barriers to active commuting. Trained researchers took physical measurements of height and weight. The relation between mode of transport, gender and population density was examined. Distance was entered into a bivariate logistic regression model to predict mode choice, controlling for gender, population density socio-economic status and school clusters. RESULTS: Of the 4013 adolescents who participated (48.1% female, mean age 16.02 +/- 0.661), one third walked or cycled to school. A higher proportion of males than females commuted actively (41.0 vs. 33.8%, chi2 (1) = 22.21, p < 0.001, r = -0.074). Adolescents living in more densely populated areas had greater odds of active commuting than those in the most sparsely populated areas (chi2 (df = 3) = 839.64, p < 0.001). In each density category, active commuters travelled shorter distances to school. After controlling for gender and population density, a 1-mile increase in distance decreased the odds of active commuting by 71% (chi2 (df = 1) = 2591.86, p < 0.001). The majority of walkers lived within 1.5 miles and cyclists within 2.5 miles. Over 90% of adolescents who perceived distance as a barrier to active commuting lived further than 2.5 miles from school. CONCLUSION: Distance is an important perceived barrier to active commuting and a predictor of mode choice among adolescents. Distances within 2.5 miles are achievable for adolescent walkers and cyclists. Alternative strategies for increasing physical activity are required for individuals living outside of this criterion
SN - 1479-5868
AD - Department of Sport, Culture and the Arts, University of Strathclyde, Glasgow, Scotland. norah.nelson@strath.ac.uk
UR - 18182102
ER -

TY - JOUR
ID - 182
T1 - The contribution of preschool playground factors in explaining children's physical activity during recess
A1 - Cardon,G.
A1 - Van,Cauwenberghe E.
A1 - Labarque,V.
A1 - Haerens,L.
A1 - De,Bourdeaudhuij,I
Y1 - 2008///
N1 - Cardon, Greet. Van Cauwenberghe, Eveline. Labarque, Valery. Haerens, Leen. De Bourdeaudhuij, Ilse
The international journal of behavioral nutrition and physical activity
101217089
Journal Article
English
KW - MEDLINE
RP - NOT IN FILE
SP - 11
JF - International Journal of Behavioral Nutrition & Physical Activity
JA - Int
VL - 5
CY - England
N2 - BACKGROUND: Low levels of physical activity are characteristic in preschoolers. To effectively promote physical activity, it is necessary to understand factors that influence young children's physical activity. The present study aimed to investigate how physical activity levels are influenced by environmental factors during recess in preschool. METHODS: Preschool playground observations and pedometry during recess were carried out in 39 randomly selected preschools (415 boys and 368 girls; 5.3 +/- 0.4 years old). In order to examine the contribution of playground variables to physical activity levels, taking adjustment for clustering of subjects within preschools into account, multilevel analyses were conducted. RESULTS: During recess boys took significantly more steps per minute than girls (65 +/- 36 versus 54 +/- 28 steps/min). In both genders higher step counts per minute were significantly associated with less children per m2 and with shorter recess times. Only in boys a hard playground surface was a borderline significant predictor for higher physical activity levels. In girls higher step counts were associated with the presence of less supervising teachers. Playground markings, access to toys, the number of playing or aiming equipment pieces and the presence of vegetation or height differences were not significant physical activity predictors in both genders. CONCLUSION: In preschool children physical activity during outdoor play is associated with modifiable playground factors. Further study is recommended to evaluate if the provision of more play space, the promotion of continued activity by supervisors and the modification of playground characteristics can increase physical activity levels in preschoolers
SN - 1479-5868
AD - Department of Movement and Sports Sciences, Ghent University, Watersportlaan 2, 9000 Ghent, Belgium. greet.cardon@ugent.be
UR - 18302741
ER -

TY - JOUR
ID - 183
T1 - How should activity guidelines for young people be operationalised?
A1 - Olds,T.
A1 - Ridley,K.
A1 - Wake,M.
A1 - Hesketh,K.
A1 - Waters,E.
A1 - Patton,G.
A1 - Williams,J.
Y1 - 2007///

N1 - Olds, Tim. Ridley, Kate. Wake, Melissa. Hesketh, Kylie. Waters, Elizabeth. Patton, George. Williams, Joanne
The international journal of behavioral nutrition and physical activity
101217089
Journal Article
English
KW - MEDLINE
RP - NOT IN FILE
SP - 43
JF - International Journal of Behavioral Nutrition & Physical Activity
JA - Int
VL - 4
CY - England
N2 - BACKGROUND: If guidelines regarding recommended activity levels for young people are to be meaningful and comparable, it should be clear how they are operationalised. It is usually open to interpretation whether young people are required to meet activity and screen time targets (1) all days of the week, (2) on most days of the week, (3) on average across all days, or (4) whether compliance should be understood as the probability that a randomly selected young person meets the guidelines on a randomly selected day. This paper studies this question using data drawn from the Australian Health of Young Victorians study. METHODS: The subjects for this study were 885 13-19 year olds who recalled four days of activities using a computerised use-of-time instrument, the Multimedia Activity Recall for Children and Adolescents (MARCA). Daily minutes of moderate-to-vigorous physical activity (MVPA) and screen time were calculated. The prevalence of compliance to Australian guidelines (> or = 60 min/day of MVPA and < or = 120 min/day of screen time outside of school hours) was calculated using the four methods. RESULTS: The four methods resulted in significantly different prevalence estimates for compliance to the MVPA guideline (20-68%), screen guideline (12-42%) and both guidelines (2-26%). Furthermore, different individuals were identified as compliant by the different methods. CONCLUSION: Clarification of how compliance to guidelines should be operationalised would assist in comparisons between studies, and in consistency in determining correlates of compliance
SN - 1479-5868
AD - Nutritional Physiology Research Centre, University of South Australia, Adelaide, Australia. timothy.olds@unisa.edu.au
UR - 17883875
ER -

TY - JOUR
ID - 184
T1 - Children's physical activity levels during school recess: a quasi-experimental intervention study
A1 - Ridgers,N.D.
A1 - Stratton,G.
A1 - Fairclough,S.J.
A1 - Twisk,J.W.
Y1 - 2007///
N1 - Ridgers, Nicola D. Stratton, Gareth. Fairclough, Stuart J. Twisk, Jos W R
The international journal of behavioral nutrition and physical activity
101217089
Journal Article
English
KW - MEDLINE
RP - NOT IN FILE
SP - 19
JF - International Journal of Behavioral Nutrition & Physical Activity
JA - Int
VL - 4
CY - England
N2 - BACKGROUND: Recess provides a daily opportunity for children to engage in moderate-to-vigorous (MVPA) and vigorous physical activity (VPA). Limited research has investigated the effects of recess-based interventions on physical activity using large sample sizes whilst investigating variables that may influence the

intervention effect. The aim of the study was to investigate the short-term effects of a playground markings and physical structures intervention on recess physical activity. A secondary aim was to investigate the effects of covariates on the intervention. METHODS: 150 boys and 147 girls were randomly selected from 26 elementary schools to wear uni-axial accelerometers that quantified physical activity every 5 seconds during recess. Fifteen schools located in deprived areas in one large urban city in England received funding through a national initiative to redesign the playground environment. Eleven schools served as matched socioeconomic controls. Data were collected at baseline and 6-weeks following playground intervention. Recess MVPA and VPA levels adjusted for pupil- and school-level covariates (baseline physical activity, age, gender, recess length, body mass index) were analysed using multilevel analyses. RESULTS: Positive but non-significant intervention effects were found for MVPA and VPA when confounding variables were added to the model. Gender was a significant predictor of recess physical activity, with boys engaging in more MVPA and VPA than girls. Significant interactions for MVPA revealed that the intervention effect was stronger for younger elementary aged school children compared to older children, and the intervention effect increased as daily recess duration increased. CONCLUSION: The playground redesign intervention resulted in small but non-significant increases in children's recess physical activity when school and pupil level variables were added to the analyses. Changing the playground environment produced a stronger intervention effect for younger children, and longer daily recess duration enabled children to engage in more MVPA following the intervention. This study concludes that the process of increasing recess physical activity is complex when school and pupil-level covariates are considered, though they should be taken into account when investigating the effects of playground intervention studies on children's physical activity during recess
SN - 1479-5868
AD - Research Institute for Sport and Exercise Sciences, Liverpool John Moores University, Henry Cotton Campus, 15-21 Webster Street, Liverpool L3 2ET, UK. n.ridgers@ljmu.ac.uk
UR - 17517136
ER -

TY - JOUR
ID - 185
T1 - Children`s falls from heights in Ahwaz, Iran
A1 - Soori,H.
Y1 - 2003/10//
N1 - Soori, Hamid
Neurosciences (Riyadh, Saudi Arabia)
101252453
Journal Article
English
KW - MEDLINE
RP - NOT IN FILE
SP - 237
EP - 240
JF - Neurosciences
VL - 8
IS - 4
CY - Saudi Arabia
N2 - OBJECTIVE: To describe the epidemiology of children`s falls in Ahwaz, Iran and to suggest its possible causes and preventive measures. METHODS: A one-year study was carried out in Ahwaz, Iran in 1999. All children aged 0-15 years who were taken to the accident and emergency (A&E) departments of all urban hospitals because of a vertical fall from heights were investigated. All parents of children were interviewed by using a questionnaire that included demographic and fall characteristic information. RESULTS: During the year of study, 2403 fall injuries were admitted to A&E departments, comprising 10.1% of all children`s injuries. There were 64.5% boys and 35.5% girls. Patients ranged in age from 5 months to 15 years (mean 5.9 years), and were distributed equally between Fars and Arabs. The most frequent falls originated from windows and roofs (32.6%), from stairs (24.6%), and from playground equipment (9.3%). Many fall injuries took place at or near the home (77.5%), and involved the head (71.7%). CONCLUSION: A combination of targeted information and safety regulations could be an essential effort for reducing the incidence of children`s falls from heights. This requires educating the parents to the hazards of falls and to mandate safer housing
SN - 1319-6138

AD - The Medical School, Ahwaz University of Medical Sciences, Ahwaz, Iran. Tel. +98 (611) 3363312. Fax. +98 (611) 3361544/3335200. E-mail: hsoori@yahoo.com
UR - 23648887
ER -

TY - JOUR
ID - 186
T1 - Immunizations: recommendations and resources for active patients
A1 - Strikas,R.A.
A1 - Schmidt,J.V.
A1 - Weaver,D.L.
A1 - Wolfe,C.R.
Y1 - 2001/10//
N1 - Strikas, R A. Schmidt, J V. Weaver, D L. Wolfe, C R
The Physician and sportsmedicine
0427461
Journal Article
English
KW - MEDLINE
RP - NOT IN FILE
SP - 33
EP - 48
JF - Physician & Sportsmedicine
JA - Phys Sportsmed.
VL - 29
IS - 10
CY - United States
N2 - Although childhood vaccination rates are at an all-time high, those for adolescents and adults are suboptimal. All adolescents and adults should be immunized against measles, mumps, rubella, varicella, tetanus, and diphtheria, and many should also receive hepatitis A, hepatitis B, influenza, and pneumococcal vaccines. In addition, active patients who engage in outdoor activities may benefit from vaccination against Lyme and meningococcal disease. Regular, strenuous exercise and foreign travel may increase the risk of some infectious diseases. Athletes often see a physician only for sports physical exams and injuries, so it is important for providers to take the opportunity to vaccinate patients during these visits
SN - 0091-3847
AD - National Immunization Program, Center for Disease Control and Prevention, Atlanta, GA, 30333, USA
UR - 20086549
ER -

TY - JOUR
ID - 187
T1 - Clinical research in paediatric oncology and the role of the research nurse in the UK
A1 - Coulson,S.
A1 - Phelan,L.
Y1 - 2000/09//
N1 - Coulson, S. Phelan, L
European journal of oncology nursing : the official journal of European Oncology Nursing Society
100885136
Journal Article
English
KW - MEDLINE
RP - NOT IN FILE
SP - 154
EP - 161
JF - European Journal of Oncology Nursing
JA - EUR J ONCOL NURS
VL - 4

IS - 3
CY - Scotland
N2 - Survival rates for childhood cancer have improved considerably as new drugs, treatment protocols and supportive therapies have developed through clinical trials. Such studies take a considerable amount of time and organization and alongside scientific and medical staff the research nurse plays a vital role. The facets of this role will be outlined in this paper. Involvement begins at an early stage of study development and includes applying for Research Ethics Committee approval of the project. Once the study has opened the research nurse is then responsible for patient recruitment, monitoring and follow-up. The research nurse works within a team but also with a degree of autonomy ensuring that standards of patient care are maintained by adhering to guidelines for clinical research in general and those aimed at children specifically. Providing detailed information and support to the child and family, staff and outside agencies are other notable aspects of the post. The role of the research nurse continues to develop, as clinical trials maintain a significant role in improving the treatment for childhood cancer
SN - 1462-3889
AD - Paediatric Oncology, St. James's University Hospital, Leeds, UK
UR - 12849646
ER -

TY - JOUR
ID - 188
T1 - Comparison of methods of sampling for Toxocara species and fecal coliforms in an outdoor day care environment
A1 - Carabin,H.
A1 - Gyorkos,T.W.
A1 - Kokoskin,E.
A1 - Payment,P.
A1 - Joseph,L.
A1 - Soto,J.
Y1 - 1998/05//
N1 - Carabin, H. Gyorkos, T W. Kokoskin, E. Payment, P. Joseph, L. Soto, J
The Canadian journal of infectious diseases = Journal canadien des maladies infectieuses
9425856
Journal Article
English
KW - MEDLINE
RP - NOT IN FILE
SP - 149
EP - 156
JF - Canadian Journal of Infectious Diseases
JA - Can
VL - 9
IS - 3
CY - Canada
N2 - OBJECTIVE: To compare three sampling methods and to pretest methods for the determination of fecal coliform (FC) counts and Toxocara species from sand in the day care outdoor environment. DESIGN: The sand samples were obtained from the play area and the sandbox of a day care centre and examined for the presence of FC and Toxocara species, the common roundworm of dogs and cats. The sampling methods included random selection and two types of judgement methods. The latter included one method where domestic animals were judged to be likely to defecate and the other where children would be likely to be playing. In addition, to obtain a global estimate of contamination, the entire areas of both the sandbox and the play area were sampled on the last day. SETTING: Outdoor day care environment. MAIN RESULTS: The most representative levels of bacterial contamination and Toxocara species originated from the combined sample of the entire surface areas rather than from any separate random or judgement method of sampling. FCs were found in all sampled areas of the sandbox (median 910 FCs/g of sand) and of the play area (median 350 FCs/g of sand). Toxocara species were recovered from a number of areas in both the sandbox and the play area. CONCLUSIONS: Research on environmental microbial contamination of outdoor day care settings would benefit from the application of standardized and validated sampling and laboratory methods

SN - 1180-2332
AD - Department of Epidemiology and Biostatistics, McGill University, Montreal, Quebec
UR - 22346537
ER -

TY - JOUR
ID - 189
T1 - Influence of Behavioral and Environmental Factors on the Development of Obesity in Three-year-old Children -A Case-Control Study Based on Toyama Study-
A1 - Sugimori,H.
A1 - Yoshida,K.
A1 - Miyakawa,M.
A1 - Izuno,T.
A1 - Kishimoto,T.
A1 - Takahashi,E.
A1 - Yamagami,T.
A1 - Kagamimori,S.
Y1 - 1997/07//
N1 - Sugimori, H. Yoshida, K. Miyakawa, M. Izuno, T. Kishimoto, T. Takahashi, E. Yamagami, T. Kagamimori, S
Environmental health and preventive medicine
9609642
Journal Article
English
KW - MEDLINE
RP - NOT IN FILE
SP - 74
EP - 78
JF - Environmental Health & Preventive Medicine
JA - Environ
VL - 2
IS - 2
CY - Japan
N2 - Obesity in children seems to be a risk factor for chronic diseases in adulthood. From the viewpoint of preventive medicine, factors influencing the development of obese children should be removed early in life.The purpose of this study was to the elucidate relationship between obesity in 3-year-old children and both behavioral and environmental factors by conducting a case-control study. Subjects were selected from the Toyama study. Matched-pair comparisons were performed between obese children whose Kaup's index was 18 or more (N=117) and control children (N=234) . Multivariate stepwised logistic regression analysis also applied to assess influence of confounding factors.The results indicated that the following 6 factors significantly influenced the development of obese 3-year-old children in exact Fisher's method analysis (p<0.05): person other than the mother responsible for taking care of the child, short sleep duration (9 hours or less), physical inactivity, eating snacks irregularly, overweight father (BMI>24), and overweight mother (BMI>24). For both sexes, after adjusting for confounders by multivariate stepwise logistic analysis, overweight mother (OR 2.54, 95 % CI 1.64-3.95), birth overweight (birth weight>3,500g; OR 1.76, 95 % CI 1.15-2.69), the mother not responsible for taking care of the child (OR 1.65, 95% CI 1.10-2.48), overweight father (OR 1.62, 95%CI 1.09-2.40), eating snacks irregularly (OR 1.56, 95% CI 1.04-2.33), and gender (female;OR 0.51, 95% CI 0.34-0.77) had significant relationships with obesity in childhood. For boys, overweight mother (OR 2.53, 95 % CI 1.47-4.35), birth overweight (OR 2.03, 95%CI 1.22-3.39), eating snacks irregularly (OR 1.94, 95 %CI 1.19-3.18), and birth month (36-41 months; OR 0.47, 95 % CI 0.23-0.96) had significant relationships. For girls, overweight mother (OR 2.62, 1.28-5.35), and short sleep duration (OR 2.24, 1.11-4.52) had significant relationships. In neither Fisher's exact method nor multivariate logistic models, time to wake up, bedtime, duration of playing outdoors, regularity of meals, care about salty food, or frequency of eating snacks had significant relations with obesity in 3-year-old children (p<0.05)
SN - 1342-078X
AD - Depart. of Hygiene & Preventive Medicine, Showa University, School of Medicine, 1-5-8 Hatanodai, Shinagawa-ku, 142, Tokyo, Japan

UR - 21432456
ER -

TY - JOUR
ID - 190
T1 - Environmental health in minority and other underserved populations: Benign methods for identifying lead hazards at day care centres of New Orleans
A1 - Viverette,L.
A1 - Mielke,H.W.
A1 - Brisco,M.
A1 - Dixon,A.
A1 - Schaefer,J.
A1 - Pierre,K.
Y1 - 1996/03//
N1 - Viverette, L. Mielke, H W. Brisco, M. Dixon, A. Schaefer, J. Pierre, K
Environmental geochemistry and health
8903118
Journal Article
English
KW - MEDLINE
RP - NOT IN FILE
SP - 41
EP - 45
JF - Environmental Geochemistry & Health
JA - Environ Geochem.Health
VL - 18
IS - 1
CY - Netherlands
N2 - This study tests the hypothesis that exterior sources of lead dust are more important than interior sources in the route of exposure of children. Benign field methods were used to distinguish between potential and actual lead exposure problems. Utilising hand wipe and surface wipe techniques, hand and environmental samples were obtained from selected day care centres at different locations within New Orleans. Previous research has shown that soil lead is determined by location within the city. Private and public day care centres were selected from inner and outer city areas to estimate the extent of hand lead exposure. To measure and identify the extent of environmental lead exposure, hand wipes were taken before and after playing outdoors. Results of preliminary findings show that outdoor lead dust is a more potent contaminant of hands than indoor lead dust. An association was found between the amount of lead on children's hands after playing outdoors and the lead content in the exterior dust and soil. Although two girls out of forty children had exceptionally high hand lead quantities after playing outdoors, in general, boys have higher hand lead levels than girls. The private inner-city day care centre had a severe contamination problem in its outdoor play area. By contrast, the outdoor play area of the public inner city day care centre is of such a high quality that the quantity of lead dust is independent of location in the city
SN - 0269-4042
AD - Xavier Institute of Bioenvironmental Toxicology, Xavier University of Louisiana, College of Pharmacy, 70125, New Orleans, LA, USA
UR - 24194368
ER -

TY - JOUR
ID - 191
T1 - Petrol lead in a small island environment
A1 - Sammut,M.
A1 - Savona-Ventura,C.
Y1 - 1996///
N1 - Sammut, M. Savona-Ventura, C
The International journal of risk & safety in medicine
9100907

Journal Article
English
KW - MEDLINE
RP - NOT IN FILE
SP - 33
EP - 40
JF - International Journal of Risk & Safety in Medicine
VL - 9
IS - 1
CY - Netherlands
N2 - The Maltese population has been repeatedly shown to have high mean blood lead levels irrespective of age. This study confirms that these higher levels also occur in Maltese children. A definite correlation is shown between high blood lead levels and the environment of the subjects studied, being statistically higher in individuals living on the urbanized larger island. A statistical correlation has been shown between hand-wipe specimens taken from children playing in a playground and the traffic density of the area. A similar statistical correlation between playground, street and household dust to traffic density has also been identified
SN - 0924-6479
AD - Department of Toxicology, Malta Medical School, Gwardamangia, Malta, Tel.: 0356 223 035/221 019/239 783; Fax: 0356 235 638
UR - 23512022
ER -

TY - JOUR
ID - 192
T1 - Polio Eradication by the year 2000
A1 - Tukei,P.M.
Y1 - 1996/08//
N1 - Tukei, P M
African journal of health sciences
9439497
Editorial
English
KW - MEDLINE
RP - NOT IN FILE
SP - 65
JF - African Journal of Health Sciences
JA - Afr J Health Sci
VL - 3
IS - 3
CY - Kenya
N2 - In May l988, the Annual Meeting of the World Health Assembly (WHA) committed WHO to the exciting challenge of Global Eradication of Poliomyelitis by the year 2000. The World accepted this challenge based on a number of scientific factors that had already been witnessed and certain other basic epidemiological factors concerning the transmission of the wild polio virus: 1. Smallpox had been eradicated and the global machinery and commitment for repeating such a feat could still be mobilised on She same lines to tackle another global menace; 2. The Pan American Health Organisation (PAHO) had already by May 1985 committed the American region to polio eradication by the year 1990. Although many people were initially sceptical of this initiative, it was quickly realised and recorded that the programme mounted by PAHO was achieving a high level of success and the goal of eradication was achievable; 3. Epidemiological! transmission factors that are persuasive to science for mounting eradication initiatives include: a) The wild polio virus moves from man to man only and has no wild animal reservoir to maintain it. b) An infected individual either dies, is crippled or fully recovers and remains immune without retaining or carrying the virus for many years (no carrier state) c) Available vaccine, particularly the oral (OPV) is not only capable of inducing long standing immunity in an individual but can, by multiplying in the gut, exclude or interrupt the circulation of the wild strain. The basic concepts and strategies for polio eradication recommended by WHO and now adapted globally are quite simple: 1. For each national expanded programme on immunisation (EPI) to raise the primary polio coverage rate with OPV to beyond 80% as a routine in children under one year. Under such conditions of immunisation in every locality in

every district and province, the incidence of paralytic polio is quickly reduced to very low levels. Most countries in the world have already achieved and exceeded this level of coverage. 2. For each country to conduct National Immunisation Days (NIDs) daring which 2 doses of OPV, one month apart, are administered to ALL children under 5 years of age irrespective of their previous vaccination status. This strategy boosts the immunity in (he children already vaccinated and catches those missed by routine services. The wild polio virus cannot live for long periods outside the human body, hence the NIDs effectively remove the wild polio virus from circulation. China was able to vaccinate over SO million children under 5ysars with OPV within two days. Currently the practice is for many neighbouring countries (even upto 15 at a time) to simultaneously operationalise their NIDs on 2 to 3 days. 3. For each country to establish and operate an extremely sensitive surveillance system capable of detecting any new case of acute flaccid paralysis (AFP), Since paralysis can be produced by other conditions, it is necessary to back up the field surveillance with a reliable laboratory service capable of isolating poliovirus from the stool samples of paralytic cases. An isolated poliovirus would then have to be typed as wild or vaccine type strain. The strategies described above have all beers activated in all countries of the world and the world is already1 witnessing a dramatic disappearance of new cases of AFF. The International Certification Commission on Polio Eradication has established formal criteria by which countries can be certified polio-free. A polio-free status has to be maintained for at least 3 years in the countries of a region for that region to be certified as having eradicated the wild poliovirus. Global eradication will have been achieved if and when all regions in the world have been certified. The world is, no doubt, most grateful to all those international organisations, such as the Rotary International, WHO, UNICEF, national governments of the developed world through! donor agencies such as J1CA, US AID, D ANID A, etc., for the enormous resources that have been mobilised to operationalise EPI programmes for polio eradication. This gratitude is also a tribute in the late Dr. Albert Sabin, the discoverer of the oral poliomyelitis vaccine, which has been the major biological tool making it possible to eradicate the wild type poliovirus
SN - 1022-9272
AD - Kenya Medical Research Institute
UR - 17451302
ER -

TY - JOUR
ID - 193
T1 - Good behavior game: effects of individual contingencies for group consequences on disruptive behavior in a classroom
A1 - Barrish,H.H.
A1 - Saunders,M.
A1 - Wolf,M.M.
Y1 - 1969///
N1 - Barrish, H H. Saunders, M. Wolf, M M
Journal of applied behavior analysis
0174763, hdr
Journal Article
English
KW - MEDLINE
RP - NOT IN FILE
SP - 119
EP - 124
JF - Journal of Applied Behavior Analysis
JA - J Appl Behav Anal
VL - 2
IS - 2
CY - United States
N2 - Out-of-seat and talking-out behaviors were studied in a regular fourth-grade class that included several "problem children". After baseline rates of the inappropriate behaviors were obtained, the class was divided into two teams "to play a game". Each out-of-seat and talking-out response by an individual child resulted in a mark being placed on the chalkboard, which meant a possible loss of privileges by all members of the student's team. In this manner a contingency was arranged for the inappropriate behavior of each child while the consequence (possible loss of privileges) of the child's behavior was shared by all members of this team as a group. The privileges were events which are available in almost every classroom, such as extra recess, first to line up for

lunch, time for special projects, stars and name tags, as well as winning the game. The individual contingencies for the group consequences were successfully applied first during math period and then during reading period. The experimental analysis involved elements of both reversal and multiple baseline designs
SN - 0021-8855
AD - University of Kansas
UR - 16795208
ER -

TY - JOUR
ID - 194
T1 - Mobility, non-familial activity, and fertility
A1 - Tien,H.Y.
Y1 - 1967/03//
N1 - Tien, H Y
Demography
0226703, e0h
Journal Article
English
KW - MEDLINE
RP - NOT IN FILE
SP - 218
EP - 227
JF - Demography
VL - 4
IS - 1
CY - United States
N2 - In this analysis of fertility data from a sample of non-Catholic faculty couples in an American university, temporal patterns and variations in education, employment, marriage, and parenthood of the husbands and wives are discussed in reference to (1) the social mobility-fertility hypothesis and (2) a non-familial activity-fertility hypothesis.The couples are divided into four groups on the basis of family size and mobility status: (1) mobile-small, (2) non-mobile-small, (3) mobile-large, and (4) non-mobile-large. Whatever their mobility status, the four groups of husbands successfully completed requirements for the doctoral degree at about the same age and became established at about the same time in life and within the profession. Whatever their husbands' social origins, the wives also differ little with respect to educational attainment and in their work experiences in prematrimonial days. However, a different pattern is found in the work experiences of the wives since marriage. Those with two children are more likely to be employed after marriage and parenthood.On the other hand, a good many more wives with four or more children not only never worked before marriage but also remained outside the labor force after marriage (in the earlier years of marriage as well as after the tenth anniversary). The present data thus seem to support an analytically useful distinction between the "working wives" and the "working mothers."
SN - 0070-3370
AD - University of Illinois, Illinois, USA
UR - 21279774
ER -

TY - JOUR
ID - 195
T1 - Maternal Mortality and its Relation to the Shape of the Female Pelvis
A1 - Vaughan,K.
Y1 - 1929/12//
N1 - Vaughan, K
Proceedings of the Royal Society of Medicine
pxm, 7505890
Journal Article
English
KW - MEDLINE
RP - NOT IN FILE

SP - 191
EP - 196
JF - Proceedings of the Royal Society of Medicine
JA - Proc R Soc Med
VL - 23
IS - 2
CY - England
N2 - Both in the modern and the ancient world, among women who are out-of-door workers and among those who live in natural and primitive conditions, childbirth is easy and safe.The inlet of the female pelvis approaches a circle. The circular form of inlet allows a larger foetal head to pass than the same measure disposed in any other form.In the circular pelvis the sacro-iliac joints are well developed by constant use and are freely movable.In contrast to this, civilization with its confinement of children indoors, and its absence of sunlight on the skin necessary for the proper calcification of growing bone, and the disuse of the sacro-iliac joints, alters the normal shape of the pelvis and makes childbirth difficult and dangerous. This change in pelvic shape leads to an undue proportion of undersized children surviving the ordeal of birth as the children with the larger heads who would easily pass the circular inlet are born dead.The last report on infant mortality tells us that two-thirds of all the stillbirths and infant deaths are due to prematurity and obstetric trauma, and the New Statistical Review issued in 1927 tells us that 20% more of the stillbirths are males.The natural proportion of males to females is thus upset, only the smaller children are born alive, hence perhaps the eventual decay of all civilizations, past and present.I venture to suggest that the solution of our maternal mortality problem does not consist so much in elaborate provision for abnormal childbirth as in trying to regain the lost heritage of natural and easy birth. We must begin with the newborn female child and her development, and return to nature as regards light, food and exercise, and thus we shall ensure proper development of the pelvis so that childbirth becomes again easy and safe
SN - 0035-9157
UR - 19987262
ER -

TY - JOUR
ID - 196
T1 - [Relations between aggressive behavior and family factors among preschool children in Shanghai]. [Chinese]
A1 - Jia,S.M.
A1 - Wang,L.
A1 - Shi,Y.J.
A1 - Li,P.
Y1 - 2011/12//
N1 - Jia, Shou-mei. Wang, Ling. Shi, Ying-juan. Li, Ping
Zhonghua liu xing bing xue za zhi = Zhonghua liuxingbingxue zazhi
cqg, 8208604
IM
English Abstract. Journal Article. Research Support, Non-U.S. Gov't
Chinese
KW - MEDLINE
KW - Aggression
KW - Child
KW - Child Behavior Disorders/et [Etiology]
KW - Preschool
KW - Family Relations
KW - Female
KW - Humans
KW - Male
KW - Parenting
KW - Questionnaires
RP - NOT IN FILE
SP - 1216
EP - 1220

JF - Chung-Hua Liu Hsing Ping Hsueh Tsa Chih Chinese Journal of Epidemiology
JA - Chung Hua Liu Hsing Ping Hsueh Tsa Chih
VL - 32
IS - 12
CY - China
N2 - OBJECTIVE: To describe the prevalence of aggressive behavior among preschool children and its related family factors. METHODS: 1234 preschool children in ten kindergartens were rated on their aggressive behavior by their parents, using Child Behavior Checklist (CBCI), Parent Behavior Inventory (PBI) and a general questionnaire. RESULTS: The overall prevalence of aggressive behavior among preschool children was 12.9% (95%CI: 11.0 - 15.0) according to the CBCL assessment, with the rate being slight higher (13.7%, 93/680) in boys than in girls (11.9%, 66/554). Data from logistic regression analysis showed that parents' hostile/coercive parenting style (OR = 2.396, 95%CI: 1.636 - 3.510) and inconsistent parenting attitude between parents and grandparents (OR = 1.867, 95%CI: 1.287 - 2.710) would lead to more aggressive behaviors in preschool children. Compared with children without difficulty in falling asleep, those who often (OR = 3.415, 95%CI: 1.901 - 6.135) or sometimes (OR = 2.147, 95%CI: 1.256 - 3.671) had problem falling asleep at night had more aggressive behaviors. On the other hand, factors as: watching TV less than 1 hour each day (OR = 0.252, 95%CI: 0.136 - 0.467), father in older age (OR = 0.703, 95%CI: 0.503 - 0.983) and participating in regular outdoor activities (OR = 0.617, 95%CI: 0.399 - 0.955) were protective factors to the aggressive behaviors of the children. CONCLUSION: The prevalence of aggressive behavior in preschool children was high which called for more attention. Intervention programs targeting the family should consider the influencing factors as ways of parenting, consistent attitude on parenting in the family etc. to reduce the occurrence of aggressive behavior among preschool children
SN - 0254-6450
AD - Fudan University, Shanghai, China
UR - 22336603
ER -

TY - JOUR
ID - 197
T1 - Differential methylation of genes in the medial prefrontal cortex of developing and adult rats following exposure to maltreatment or nurturing care during infancy
A1 - Blaze,J.
A1 - Scheuing,L.
A1 - Roth,T.L.
Y1 - 2013///
N1 - Blaze, Jennifer. Scheuing, Lisa. Roth, Tania L
Developmental neuroscience
ec5, 7809375
IM
Journal Article. Research Support, N.I.H., Extramural. Research Support, Non-U.S. Gov't
English
KW - MEDLINE
KW - Animals
KW - Newborn
KW - Behavior
KW - Animal/ph [Physiology]
KW - Brain-Derived Neurotrophic Factor/bi [Biosynthesis]
KW - Brain-Derived Neurotrophic Factor/ge [Genetics]
KW - Cell Adhesion Molecules
KW - Neuronal/bi [Biosynthesis]
KW - Neuronal/ge [Genetics]
KW - Child
KW - Child Abuse
KW - DNA Methylation/ge [Genetics]
KW - Epigenesis
KW - Genetic/ph [Physiology]
KW - Extracellular Matrix Proteins/bi [Biosynthesis]

KW - Extracellular Matrix Proteins/ge [Genetics]
KW - Female
KW - Gene Expression
KW - Humans
KW - Male
KW - Maternal Behavior
KW - Nerve Tissue Proteins/bi [Biosynthesis]
KW - Nerve Tissue Proteins/ge [Genetics]
KW - Neuronal Plasticity/ge [Genetics]
KW - Neuronal Plasticity/ph [Physiology]
KW - Prefrontal Cortex/gd [Growth & Development]
KW - Prefrontal Cortex/me [Metabolism]
KW - Pregnancy
KW - Rats
KW - Long-Evans
KW - Serine Endopeptidases/bi [Biosynthesis]
KW - Serine Endopeptidases/ge [Genetics]
KW - Sex Characteristics
RP - NOT IN FILE
SP - 306
EP - 316
JF - Developmental Neuroscience
JA - Dev Neurosci
VL - 35
IS - 4
CY - Switzerland
N2 - Quality of maternal care in infancy is an important contributing factor in the development of behavior and psychopathology. One way maternal care could affect behavioral trajectories is through environmentally induced epigenetic alterations within brain regions known to play prominent roles in cognition, emotion regulation, and stress responsivity. Whereas such research has largely focused on the hippocampus or hypothalamus, the prefrontal cortex (PFC) has only just begun to receive attention. The current study was designed to determine whether exposure to maltreatment or nurturing care is associated with differential methylation of candidate gene loci (bdnf and reelin) within the medial PFC (mPFC) of developing and adult rats. Using a within-litter design, infant male and female rats were exposed to an adverse or nurturing caregiving environment outside their home cage for 30 min per day during the first postnatal week. Additional littermates remained with their biological caregiver within the home cage during the manipulations. We observed that infant rats subjected to caregiver maltreatment emitted more audible and ultrasonic vocalizations than littermates subjected to nurturing care either within or outside of the home cage. While we found no maltreatment-induced changes in bdnf DNA methylation present in infancy, sex-specific alterations were present in the mPFC of adolescents and adults that had been exposed to maltreatment. Furthermore, while maltreated females showed differences in reelin DNA methylation that were transient, males exposed to maltreatment and both males and females exposed to nurturing care outside the home cage showed differences in reelin methylation that emerged by adulthood. Our results demonstrate the ability of infant-caregiver interactions to epigenetically mark genes known to play a prominent role in cognition and psychiatric disorders within the mPFC. Furthermore, our data indicate the remarkable complexity of alterations that can occur, with both transient and later emerging DNA methylation differences that could shape developmental trajectories and underlie gender differences in outcomes. Copyright 2013 S. Karger AG, Basel
SN - 1421-9859
AD - Department of Psychology, University of Delaware, Newark, DE 19716, USA
UR - 23751776
ER -

TY - JOUR
ID - 198
T1 - Can topical insect repellents reduce malaria? A cluster-randomised controlled trial of the insect repellent N,N-diethyl-m-toluamide (DEET) in Lao PDR
A1 - Chen-Hussey,V.

A1 - Carneiro,I.
A1 - Keomanila,H.
A1 - Gray,R.
A1 - Bannavong,S.
A1 - Phanalasy,S.
A1 - Lindsay,S.W.
Y1 - 2013///
N1 - Chen-Hussey, Vanessa. Carneiro, Ilona. Keomanila, Hongkham. Gray, Rob. Bannavong, Sihamano. Phanalasy, Saysana. Lindsay, Steven W
PloS one
101285081
IM
Journal Article. Randomized Controlled Trial. Research Support, N.I.H., Extramural. Research Support, U.S. Gov't, Non-P.H.S.
English
KW - MEDLINE
KW - Administration
KW - Topical
KW - Adolescent
KW - Adult
KW - Animals
KW - Child
KW - Culicidae/de [Drug Effects]
KW - DEET/ad [Administration & Dosage]
KW - DEET/pd [Pharmacology]
KW - Female
KW - Humans
KW - Insect Bites and Stings/pc [Prevention & Control]
KW - Insect Repellents/ad [Administration & Dosage]
KW - Insect Repellents/pd [Pharmacology]
KW - Insect Vectors/de [Drug Effects]
KW - Intention to Treat Analysis
KW - Laos
KW - Malaria/pc [Prevention & Control]
KW - Malaria/tm [Transmission]
KW - Male
KW - Middle Aged
KW - Patient Compliance/sn [Statistics & Numerical Data]
KW - Young Adult
RP - NOT IN FILE
SP - e70664
JF - PLoS ONE [Electronic Resource]
JA - PLoS ONE
VL - 8
IS - 8
CY - United States
N2 - BACKGROUND: Mosquito vectors of malaria in Southeast Asia readily feed outdoors making malaria control through indoor insecticides such as long-lasting insecticidal nets (LLINs) and indoor residual spraying more difficult. Topical insect repellents may be able to protect users from outdoor biting, thereby providing additional protection above the current best practice of LLINs. METHODS AND FINDINGS: A double blind, household randomised, placebo-controlled trial of insect repellent to reduce malaria was carried out in southern Lao PDR to determine whether the use of repellent and long-lasting insecticidal nets (LLINs) could reduce malaria more than LLINs alone. A total of 1,597 households, including 7,979 participants, were recruited in June 2009 and April 2010. Equal group allocation, stratified by village, was used to randomise 795 households to a 15% DEET lotion and the remainder were given a placebo lotion. Participants, field staff and data analysts were blinded to the group assignment until data analysis had been completed. All households received new LLINs. Participants were asked to apply their lotion to exposed skin every evening and sleep under the LLINs

each night. Plasmodium falciparum and P. vivax cases were actively identified by monthly rapid diagnostic tests. Intention to treat analysis found no effect from the use of repellent on malaria incidence (hazard ratio: 1.00, 95% CI: 0.99-1.01, p = 0.868). A higher socio-economic score was found to significantly decrease malaria risk (hazard ratio: 0.72, 95% CI: 0.58-0.90, p = 0.004). Women were also found to have a reduced risk of infection (hazard ratio: 0.59, 95% CI: 0.37-0.92, p = 0.020). According to protocol analysis which excluded participants using the lotions less than 90% of the time found similar results with no effect from the use of repellent. CONCLUSIONS: This randomised controlled trial suggests that topical repellents are not a suitable intervention in addition to LLINs against malaria amongst agricultural populations in southern Lao PDR. These results are also likely to be applicable to much of the Greater Mekong Sub-region. TRIAL REGISTRATION: This trial is registered with number NCT00938379
SN - 1932-6203
AD - Faculty of Infectious and Tropical Diseases, London School of Hygiene and Tropical Medicine, London, United Kingdom. Vanessa.Chen-Hussey@lshtm.ac.uk
UR - 23967083
ER -

TY - JOUR
ID - 199
T1 - Relationship between media viewing and obesity in school-aged children in Taipei, Taiwan
A1 - Huang,H.M.
A1 - Chien,L.Y.
A1 - Yeh,T.C.
A1 - Lee,P.H.
A1 - Chang,P.C.
Y1 - 2013/09//
N1 - Huang, Hsiu-Mei. Chien, Li-Yin. Yeh, Ting-Chi. Lee, Pi-Hsia. Chang, Pi-Chen
The journal of nursing research : JNR
101128757
IM, N
Journal Article
English
KW - MEDLINE
KW - Case-Control Studies
KW - Child
KW - Female
KW - Humans
KW - Male
KW - Mass Media
KW - Obesity/ep [Epidemiology]
KW - Taiwan/ep [Epidemiology]
RP - NOT IN FILE
SP - 195
EP - 203
JF - Journal of Nursing Research
JA - J Nurs Res
VL - 21
IS - 3
CY - China (Republic : 1949-)
N2 - BACKGROUND: Increased media viewing such as watching television and videos and playing on/using computers has been associated with childhood obesity. Guidelines from the American Academy of Pediatrics recommend limiting children's total media time to no more than 2 hours per day. Information about media usage and school-aged obesity in Taiwan is lacking. PURPOSE: The purpose of this study was to investigate whether media viewing after school is associated with the total and centralobesity in school-aged children in Taipei, Taiwan's capital and largest city. METHODS: A case-control study was designed, and a control approach was used to recruit participants. Two hundred seventy-five obese and 275 normal-weight children currently in the fourth grade were enrolled from 29 elementary schools in Taipei City. Media viewing after school was measured using a 3-day self-reported physical activity log and the daily sedentary activity component of the

National Health Interview Survey in Taiwan. The latter was completed by the participants' parents. RESULTS: Participants with total obesity had significantly more television watching time (60.24 minutes vs. 43.50 minutes, p < .05) and total media watching time (73.61 minutes vs. 52.67 minutes, p < .001) than normal-weight participants. Similar results were found for participants with central obesity. Total television and total media watching durations greater than 2 hours a day correlated significantly with total and central obesity. Time spent using computers did not differ significantly between obese and normal-weight control participants on weekdays. CONCLUSIONS/IMPLICATIONS FOR PRACTICE: Childhood obesity may be multifactorial in origin. Excessive sedentary activity such as watching television may have a variety of consequences beyond the putative effect on body habitus. School nurses should promote health programs targeted to prevent excessive weight and obesity in children, recommend reducing media viewing, and encourage patient participation in extracurricular outdoor activities
SN - 1948-965X
AD - National Taipei University of Nursing and Health Science
UR - 23958609
ER -

TY - JOUR
ID - 200
T1 - Influencing factors of sedentary behavior in European preschool settings: an exploration through focus groups with teachers
A1 - De,Decker E.
A1 - De,Craemer M.
A1 - De,Bourdeaudhuij,I
A1 - Wijndaele,K.
A1 - Duvinage,K.
A1 - Androutsos,O.
A1 - Iotova,V.
A1 - Lateva,M.
A1 - Alvira,J.M.
A1 - Zych,K.
A1 - Manios,Y.
A1 - Cardon,G.
Y1 - 2013/09//
N1 - De Decker, Ellen. De Craemer, Marieke. De Bourdeaudhuij, Ilse. Wijndaele, Katrien. Duvinage, Kristin. Androutsos, Odysseas. Iotova, Violeta. Lateva, Mina. Alvira, Juan Miguel Fernandez. Zych, Kamila. Manios, Yannis. Cardon, Greet
The Journal of school health
k13, 0376370
IM, N
Journal Article. Research Support, Non-U.S. Gov't
English
KW - MEDLINE
KW - Adult
KW - Attitude to Health
KW - Child
KW - Preschool
KW - Europe
KW - Faculty/sn [Statistics & Numerical Data]
KW - Female
KW - Focus Groups
KW - Health Behavior
KW - Health Education/mt [Methods]
KW - Health Promotion/mt [Methods]
KW - Humans
KW - Male
KW - Obesity/pc [Prevention & Control]
KW - Schools/og [Organization & Administration]

KW - Sedentary Lifestyle
KW - Young Adult
RP - NOT IN FILE
SP - 654
EP - 661
JF - Journal of School Health
JA - J Sch Health
VL - 83
IS - 9
CY - United States
N2 - BACKGROUND: Sedentary behavior refers to activities involving sitting down and reclining (eg, watching TV, using the computer) and has been associated with different health outcomes. In preschool, children are sedentary for 50% to 80% of the time, in the classroom as well as during recess. Because of the absence of qualitative studies examining influencing factors of preschoolers' sedentary behavior in preschool settings, this study explored teachers' opinions on potentially influencing factors of this behavior. METHODS: Eighty-seven teachers of 4- to 6-year-old preschoolers from 6 European countries participated in a total of 18 focus groups between October 2010 and January 2011. Key findings were reported separately by country, and were independently analyzed by 2 researchers using qualitative content analysis. RESULTS: Teachers perceive the lack of play space and small classroom size as being influential factors on preschoolers' sedentary behavior; increasing play equipment and using teachers' prompts are mentioned as ways to stimulate children to be less sedentary on the playground. Computer use is reported to be more common in preschool than watching TV. CONCLUSION: Interventions should focus on increasing teachers' awareness of how sedentary preschoolers are during the preschool day. Teachers also should be informed about strategies to decrease sedentariness in the classroom and on the playground. 2013, American School Health Association
SN - 1746-1561
AD - Department of Movement and Sport Sciences, Ghent University, Watersportlaan 2, 9000 Gent, Belgium. ellen.dedecker@ugent.be
UR - 23879785
ER -

TY - JOUR
ID - 201
T1 - Development of a unique multi-contaminant air sampling device for a childhood asthma cohort in an agricultural environment
A1 - Armstrong,J.L.
A1 - Fitzpatrick,C.F.
A1 - Loftus,C.T.
A1 - Yost,M.G.
A1 - Tchong-French,M.
A1 - Karr,C.J.
Y1 - 2013/09//
N1 - Armstrong, Jenna L. Fitzpatrick, Cole F. Loftus, Christine T. Yost, Michael G. Tchong-French, Maria. Karr, Catherine J
Environmental science. Processes & impacts
101601576
IM
Journal Article. Research Support, N.I.H., Extramural. Research Support, U.S. Gov't, P.H.S.
English
KW - MEDLINE
KW - Agriculture
KW - Air Pollutants/ae [Adverse Effects]
KW - Air Pollutants/an [Analysis]
KW - Asthma/et [Etiology]
KW - Child
KW - Cohort Studies
KW - Environment
KW - Environmental Monitoring/is [Instrumentation]

KW - Equipment Design
KW - Humans
RP - NOT IN FILE
SP - 1760
EP - 1767
JF - Environmental Science
JA - Environ Sci Process Impacts.
VL - Processes & Impacts. 15
IS - 9
CY - England
N2 - This research describes the design, deployment, performance, and acceptability of a novel outdoor active air sampler to provide simultaneous measurements of multiple contaminants at timed intervals for the Aggravating Factors of Asthma in Rural Environment (AFARE) study-a longitudinal cohort of 50 children in Yakima Valley, Washington. The sampler was constructed of multiple sampling media connected to individual critical orifices and a rotary vane vacuum pump. It was connected to a timed control valve system to collect 24 hours samples every six days over 18 months. We describe a spatially representative approach with both quantitative and qualitative location criteria to deploy a network of 14 devices at participant residences in a rural region (20 x 60 km). Overall the sampler performed well, as the concurrent mean sample flow rates were within or above the ranges of recommended sampling rates for each exposure metric of interest. Acceptability was high among the study population of Hispanic farmworker participant households. The sampler design may prove useful for future urban and rural community-based studies with aims at collecting multiple contaminant data during specific time periods
SN - 2050-7895
AD - Department of Environmental and Occupational Health Sciences, University of Washington School of Public Health, Health Sciences Building, 1959 NE Pacific Street, Box 357234, Seattle, WA 98195, USA. jennaa@uw.edu
UR - 23896655
ER -

TY - JOUR
ID - 202
T1 - A large community outbreak of blastomycosis in Wisconsin with geographic and ethnic clustering
A1 - Roy,M.
A1 - Benedict,K.
A1 - Deak,E.
A1 - Kirby,M.A.
A1 - McNiel,J.T.
A1 - Sickler,C.J.
A1 - Eckardt,E.
A1 - Marx,R.K.
A1 - Heffernan,R.T.
A1 - Meece,J.K.
A1 - Klein,B.S.
A1 - Archer,J.R.
A1 - Theurer,J.
A1 - Davis,J.P.
A1 - Park,B.J.
Y1 - 2013/09//
N1 - Roy, Monika. Benedict, Kaitlin. Deak, Eszter. Kirby, Miles A. McNiel, Jena T. Sickler, Carrie J. Eckardt, Eileen. Marx, Ruth K. Heffernan, Richard T. Meece, Jennifer K. Klein, Bruce S. Archer, John R. Theurer, Joan. Davis, Jeffrey P. Park, Benjamin J
Clinical infectious diseases : an official publication of the Infectious Diseases Society of America
a4j, 9203213
IM
Journal Article. Research Support, U.S. Gov't, P.H.S.
English
KW - MEDLINE

KW - Adolescent
KW - Adult
KW - Aged
KW - 80 and over
KW - Blastomyces/ip [Isolation & Purification]
KW - Blastomycosis/ep [Epidemiology]
KW - Blastomycosis/mi [Microbiology]
KW - Case-Control Studies
KW - Child
KW - Preschool
KW - Cluster Analysis
KW - Community-Acquired Infections/ep [Epidemiology]
KW - Community-Acquired Infections/mi [Microbiology]
KW - Disease Outbreaks
KW - Ethnic Groups
KW - Female
KW - Geography
KW - Humans
KW - Male
KW - Middle Aged
KW - Wisconsin/ep [Epidemiology]
KW - Young Adult
RP - NOT IN FILE
SP - 655
EP - 662
JF - Clinical Infectious Diseases
JA - Clin Infect Dis
VL - 57
IS - 5
CY - United States
N2 - BACKGROUND: Blastomycosis is a potentially life-threatening infection caused by the soil-based dimorphic fungus Blastomyces dermatitidis, which is endemic throughout much of the Midwestern United States. We investigated an increase in reported cases of blastomycosis that occurred during 2009-2010 in Marathon County, Wisconsin. METHODS: Case detection was conducted using the Wisconsin Electronic Disease Surveillance System (WEDSS). WEDSS data were used to compare demographic, clinical, and exposure characteristics between outbreak-related and historical case patients, and to calculate blastomycosis incidence rates. Because initial mapping of outbreak case patients' homes and recreational sites demonstrated unusual neighborhood and household case clustering, we conducted a 1:3 matched case-control study to identify factors associated with being in a geographic cluster. RESULTS: Among the 55 patients with outbreak-related cases, 33 (70%) were hospitalized, 2 (5%) died, 30 (55%) had cluster-related cases, and 20 (45%) were Hmong. The overall incidence increased significantly since 2005 (average 11% increase per year, P < .001), and incidence during 2005-2010 was significantly higher among Asians than non-Asians (2010 incidence: 168 vs 13 per 100 000 population). Thirty of the outbreak cases grouped into 5 residential clusters. Outdoor activities were not risk factors for blastomycosis among cluster case patients or when comparing outbreak cases to historical cases. CONCLUSIONS: This outbreak of blastomycosis, the largest ever reported, was characterized by unique household and neighborhood clustering likely related to multifocal environmental sources. The reasons for the large number of Hmong affected are unclear, but may involve genetic predisposition
SN - 1537-6591
AD - Epidemic Intelligence Service, Scientific Education and Professional Development Program Office, Centers for Disease Control and Prevention, 1600 Clifton Rd NE, MS C-09, Atlanta, GA 30307, USA. ige6@cdc.gov
UR - 23735332
ER -

TY - JOUR
ID - 203
T1 - Maternal depressive symptoms and child obesity in low-income urban families

A1 - Gross,R.S.
A1 - Velazco,N.K.
A1 - Briggs,R.D.
A1 - Racine,A.D.
Y1 - 2013/07//
N1 - Gross, Rachel S. Velazco, Nerissa K. Briggs, Rahil D. Racine, Andrew D
Academic pediatrics
101499145
IM
Journal Article. Research Support, Non-U.S. Gov't. Research Support, U.S. Gov't, P.H.S.
English
KW - MEDLINE
KW - Adult
KW - Child
KW - Preschool
KW - Cross-Sectional Studies
KW - Depression/ep [Epidemiology]
KW - Depression/px [Psychology]
KW - Feeding Behavior/px [Psychology]
KW - Female
KW - Humans
KW - Male
KW - Mother-Child Relations
KW - Mothers/px [Psychology]
KW - Overweight/ep [Epidemiology]
KW - Overweight/px [Psychology]
KW - Pediatric Obesity/ep [Epidemiology]
KW - Poverty/px [Psychology]
KW - Poverty/sn [Statistics & Numerical Data]
KW - Urban Population/sn [Statistics & Numerical Data]
RP - NOT IN FILE
SP - 356
EP - 363
JF - Academic pediatrics
JA - Acad Pediatr
VL - 13
IS - 4
CY - United States
N2 - OBJECTIVE: To characterize the relationship between maternal depressive symptoms and child weight status, obesity-promoting feeding practices, and activity-related behaviors in low-income urban families. METHODS: We conducted a cross-sectional survey of mothers with 5-year-old children receiving pediatric care at a federally qualified community health center. We used regression analyses to examine the relationship between maternal depressive symptoms (trichotomized: none, mild, moderate to severe) and 1) child weight status; 2) obesity-promoting feeding practices, including mealtime practices and feeding styles; and 3) activity-related behaviors, including sleep time, screen time, and outdoor playtime. RESULTS: The sample included 401 mother-child pairs (78.3% response rate), with 23.4% of mothers reporting depressive symptoms (15.7% mild, 7.7% moderate to severe). Mothers with moderate to severe depressive symptoms were more likely to have overweight and obese children than mothers without depressive symptoms (adjusted odds ratio 2.62; 95% confidence interval 1.02-6.70). Children of mildly depressed mothers were more likely to consume sweetened drinks and to eat out at restaurants and were less likely to eat breakfast than children of nondepressed mothers. Mothers with depressive symptoms were less likely to set limits, to use food as a reward, to restrict their child's intake, and to model healthy eating than nondepressed mothers. Children with depressed mothers had less sleep and outdoor playtime per day than children of nondepressed mothers. CONCLUSIONS: Maternal depressive symptoms are associated with child overweight and obese status and with several obesity-promoting practices. These results support the need for maternal depression screening in pediatric obesity prevention programs. Further research should explore how to incorporate needed mental health support. Copyright 2013 Academic Pediatric Association. Published by Elsevier Inc. All rights reserved

SN - 1876-2867
AD - Department of Pediatrics, Albert Einstein College of Medicine, Children's Hospital at Montefiore, Bronx, NY 10467, USA. rgross@montefiore.org
UR - 23830021
ER -

TY - JOUR
ID - 204
T1 - Therapy in pediatric stroke. [Review]
A1 - Simma,B.
A1 - Holiner,I.
A1 - Luetschg,J.
Y1 - 2013/07//
N1 - Simma, B. Holiner, I. Luetschg, J
European journal of pediatrics
end, 7603873
IM
Journal Article. Review
English
KW - MEDLINE
KW - Adolescent
KW - Anticoagulants/tu [Therapeutic Use]
KW - Aspirin/tu [Therapeutic Use]
KW - Child
KW - Fibrinolytic Agents/tu [Therapeutic Use]
KW - Humans
KW - Practice Guidelines as Topic
KW - Risk Factors
KW - Stroke/dt [Drug Therapy]
KW - Stroke/pa [Pathology]
KW - Stroke/pc [Prevention & Control]
RP - NOT IN FILE
SP - 867
EP - 875
JF - European Journal of Pediatrics
JA - Eur J Pediatr
VL - 172
IS - 7
CY - Germany
N2 - The aim of this review is to summarize the existing literature on therapy and management of cerebrovascular insults in children and adolescents. As data sources, studies were identified by MEDLINE, PubMed, Cochrane Library, and relevant bibliographies for the topic "pediatric stroke." We also reviewed guidelines for "stroke in adults." As a result, pediatric stroke is underestimated. The annual incidence for all stroke entities (cerebral venous thrombosis and hemorrhagic and arterial ischemic stroke) is as high as for pediatric brain tumors, 3-15/100.000 children per year. A distinct etiology can be determined only in a minority of them. Underlying risk factors are multiple, mainly vasculopathies, congential heart diseases, coagulopathies, lipometabolic disorders, and sickle cell anemia. Current recommendations for therapy are based on adult studies, are preliminary, and discussed controversially. Antithrombotic therapy is uniformly recommended for the acute stage of pediatric stroke; no consensus exists on antiplatelet therapy with acetylsalicylic acid (ASA, aspirin) (5 mg/d), with ultra-fractionated or low-molecular-weight heparin. Thrombolysis using recombinant tissue plasminogen activator is not advised, despite the fact that current practice takes a different approach. None of the guidelines specify the duration of ASA for secondary prevention. Additional supportive therapy measures are osmotherapy and decompressive craniectomy. Oxygen in the absence of hypoxemia, intensive insulin therapy, antiepileptic drugs in the absence of clinical or electrographic seizures, corticosteroids, and GP-IIb/IIIa-receptor antagonists should not be used outside clinical trials. In conclusions, current therapeutic guidelines for pediatric stoke are still based on consensus and expert and society opinions and differ between countries. Consensus prevails on the need for acute anticoagulation using either antiplatelets or heparin. Long-

term treatment with acetylsalicylic acid in all or only high-risk patients and for how long remains the subject of debate. Lifelong secondary prevention has never been investigated in children or adults. All guidelines agree that there is no indication for thrombolysis in children outside clinical trials, although clinical practice in large centers differs
SN - 1432-1076
AD - Department of Pediatrics, Academic Teaching Hospital, Landeskrankenhaus Feldkirch, Feldkirch 6800, Austria. burkhard.simma@lkhf.at
UR - 23129344
ER -

TY - JOUR
ID - 205
T1 - Intracranial EEG evaluation of relationship within a resting state network
A1 - Duncan,D.
A1 - Duckrow,R.B.
A1 - Pincus,S.M.
A1 - Goncharova,I.
A1 - Hirsch,L.J.
A1 - Spencer,D.D.
A1 - Coifman,R.R.
A1 - Zaveri,H.P.
Y1 - 2013/10//
N1 - Duncan, Dominique. Duckrow, Robert B. Pincus, Steven M. Goncharova, Irina. Hirsch, Lawrence J. Spencer, Dennis D. Coifman, Ronald R. Zaveri, Hitten P
Clinical neurophysiology : official journal of the International Federation of Clinical Neurophysiology
db4, 100883319
IM
Journal Article
English
KW - MEDLINE
KW - Adolescent
KW - Adult
KW - Brain Mapping/mt [Methods]
KW - Child
KW - Electroencephalography/mt [Methods]
KW - Epilepsy
KW - Frontal Lobe/di [Diagnosis]
KW - Frontal Lobe/pp [Physiopathology]
KW - Female
KW - Gyrus Cinguli/pp [Physiopathology]
KW - Humans
KW - Magnetic Resonance Imaging
KW - Male
KW - Nerve Net/pp [Physiopathology]
KW - Parietal Lobe/pp [Physiopathology]
KW - Young Adult
RP - NOT IN FILE
SP - 1943
EP - 1951
JF - Clinical Neurophysiology
JA - Clin Neurophysiol
VL - 124
IS - 10
CY - Netherlands
N2 - OBJECTIVE: We tested if a relationship between distant parts of the default mode network (DMN), a resting state network defined by fMRI studies, can be observed with intracranial EEG recorded from patients with localization-related epilepsy. METHODS: Magnitude squared coherence, mutual information, cross-

approximate entropy, and the coherence of the gamma power time-series were estimated, for one hour intracranial EEG recordings of background activity from 9 patients, to evaluate the relationship between two test areas which were within the DMN (anterior cingulate and orbital frontal, denoted as T1 and posterior cingulate and mesial parietal, denoted as T2), and one control area (denoted as C), which was outside the DMN. We tested if the relationship between T1 and T2 was stronger than the relationship between each of these areas and C. RESULTS: A low level of relationship was observed among the 3 areas tested. The relationships among T1, T2 and C did not demonstrate support for the DMN. CONCLUSIONS: This study suggests a lack of intracranial EEG support for the fMRI defined default mode network. SIGNIFICANCE: The results obtained underscore the considerable difference between electrophysiological and hemodynamic measurements of brain activity and possibly suggest a lack of neuronal involvement in the DMN. Copyright 2013 International Federation of Clinical Neurophysiology. Published by Elsevier Ireland Ltd. All rights reserved
SN - 1872-8952
AD - Department of Electrical Engineering, Yale University, New Haven, CT 06511, USA. dominique.duncan@yale.edu
UR - 23790525
ER -

TY - JOUR
ID - 206
T1 - Achieving timely adoption. [Review]
A1 - Carnochan,S.
A1 - Moore,M.
A1 - Austin,M.J.
Y1 - 2013///
N1 - Carnochan, Sarah. Moore, Megan. Austin, Michael J
Journal of evidence-based social work
101197676
IM
Journal Article. Review
English
KW - MEDLINE
KW - Adoption
KW - Age Factors
KW - Child
KW - Child Welfare
KW - Foster Home Care/og [Organization & Administration]
KW - Government Agencies/og [Organization & Administration]
KW - Humans
KW - Parents/ed [Education]
KW - Sex Factors
KW - Social Work/og [Organization & Administration]
KW - Socioeconomic Factors
KW - Time Factors
RP - NOT IN FILE
SP - 210
EP - 219
JF - Journal of Evidence-Based Social Work
JA - J Evid Based Soc Work
VL - 10
IS - 3
CY - United States
N2 - While family reunification is the primary permanency objective for children who must be placed temporarily outside of their homes, reunification is not possible for all children. For those children who do not return to their parents and cannot find permanent homes with other family members, adoption is the favored outcome. This review examines the composite measure in the federal Child and Family Services Review that measures agency performance related to the timeliness of adoptions of foster children. It summarizes the multiple factors that research has found to be associated with increased risk for adoption delay and disruption.

These include child characteristics, family of origin and adoptive family characteristics, and features of child welfare services and systems. Practices that have been broadly linked to adoption timeliness or address risk factors associated with delays in adoption are described, including social worker activities and agency or system-wide practice
SN - 1543-3722
AD - Bay Area Social Services Consortium, School of Social Welfare, University of California, Berkeley, California 94720-7400, USA. scarnochan@berkeley.edu
UR - 23705651
ER -

TY - JOUR
ID - 207
T1 - Acute injury risk and severity in indoor climbing-a prospective analysis of 515,337 indoor climbing wall visits in 5 years
A1 - Schoffl,V.R.
A1 - Hoffmann,G.
A1 - Kupper,T.
Y1 - 2013/09//
N1 - Schoffl, Volker R. Hoffmann, Georg. Kupper, Thomas
Wilderness & environmental medicine
dbe, 9505185
IM
Journal Article
English
KW - MEDLINE
KW - Accidental Falls/sn [Statistics & Numerical Data]
KW - Adolescent
KW - Adult
KW - Aged
KW - 80 and over
KW - Athletic Injuries/ep [Epidemiology]
KW - Child
KW - Female
KW - Germany/ep [Epidemiology]
KW - Humans
KW - Lower Extremity/in [Injuries]
KW - Male
KW - Middle Aged
KW - Mountaineering/in [Injuries]
KW - Population Surveillance
KW - Sports
KW - Upper Extremity/in [Injuries]
KW - Young Adult
RP - NOT IN FILE
SP - 187
EP - 194
JF - Wilderness & Environmental Medicine
JA - Wilderness Environ Med
VL - 24
IS - 3
CY - United States
N2 - OBJECTIVE: Rock climbing's popularity continues to rise, with people of all ages regularly participating in the sport. Climbing literature suggests climbers get injured mostly in their upper extremities. Most studies on climbing injury analysis are conducted retrospectively, with all the inherent problems of a retrospective setup (no exact time collection, biased injury perception, etc). Prospective data are still missing. METHODS: We prospectively evaluated all attendees of a major German indoor climbing gym in Stuttgart, Germany, with bouldering and lead climbing facilities. Attendee's age, sex, and time spent climbing were electronically

recorded on each visit. All acute injuries were graded using the Medical Commission of the Union Internationale des Associations d'Alpinisme Score. Injury cause, belayers' and climbers' experience, and outcome were additionally analyzed. RESULTS: During a 5-year period (2007-2011), 515,337 visits to the climbing wall were registered, of which 63.6% were by male visitors, 36.4% female, within an age of 8-80 years (median, 34 years). The average time of climbing was 2 hours 47 minutes. Thirty climbing injuries were recorded, 22 were in male and 8 in female climbers with a total mean age of 27.5 + 10.6 years. Acute injuries happened in 6 cases while bouldering, in 16 cases while lead climbing, in 7 cases while top roping, and in 1 case as a third person (not climbing or belaying) while watching another climber. Bouldering injuries were mostly the result of falls onto the mat, whereas in lead and top rope climbing various scenarios happened. Fifteen (50%) injuries were Medical Commission of the Union Internationale des Associations d'Alpinisme grade 2, 13 (43%) were grade 3, and 2 (7%) were grade 4, with no fatalities. The overall injury rate was 0.02 injuries per 1000 hours of climbing activities. CONCLUSIONS: This was the first study to accurately record time spent indoor climbing digitally and evaluate the acute injuries prospectively in a large cohort. There were few injuries sustained, suggesting indoor climbing has a low risk of acute injury per 1000 hours of participation. The injuries were of minor to moderate injury severity, and no fatalities occurred. Several injuries could have been avoided, and further injury-prevention concepts should be developed. Wilderness Medical Society
SN - 1545-1534
AD - Department of Sports Orthopedics-Sportsmedicine, Klinikum Bamberg (Dr Schoffl); Department of Traumasurgery, Friedrich Alexander University Erlangen-Nuremberg (Dr Schoffl); Medical Commission of the Union Internationale des Associations d'Alpinisme (Drs Schoffl and Kupper); Medical Commission of the International Federation of Sport Climbing (Dr Schoffl). Electronic address: volker.schoeffl@me.com
UR - 23877045
ER -

TY - JOUR
ID - 208
T1 - Understanding family motivations and barriers to participation in community-based programs for overweight youth: one program model does not fit all
A1 - Grow,H.M.
A1 - Hsu,C.
A1 - Liu,L.L.
A1 - Briner,L.
A1 - Jessen-Fiddick,T.
A1 - Lozano,P.
A1 - Saelens,B.E.
Y1 - 2013/07//
N1 - Grow, H Mollie Greves. Hsu, Clarissa. Liu, Lenna L. Briner, Leslie. Jessen-Fiddick, Tricia. Lozano, Paula. Saelens, Brian E
Journal of public health management and practice : JPHMP
c0l, 9505213
T
Journal Article. Research Support, N.I.H., Extramural
English
KW - MEDLINE
KW - Adolescent
KW - Adult
KW - Child
KW - Community Health Services/og [Organization & Administration]
KW - Family/px [Psychology]
KW - Female
KW - Health Promotion/og [Organization & Administration]
KW - Humans
KW - Interviews as Topic
KW - Male
KW - Middle Aged
KW - Models
KW - Organizational

KW - Motivation
KW - Overweight/th [Therapy]
KW - Parents/px [Psychology]
KW - Patient Acceptance of Health Care/px [Psychology]
KW - Patient Acceptance of Health Care/sn [Statistics & Numerical Data]
KW - Risk Reduction Behavior
KW - Washington
RP - NOT IN FILE
SP - E1
EP - E10
JF - Journal of Public Health Management & Practice
JA - J Public Health Manag Pract
VL - 19
IS - 4
CY - United States
N2 - CONTEXT: Successful obesity intervention efforts depend on effective recruitment and retention, an ongoing challenge for community-based programs. OBJECTIVE: We sought to provide insights into the most salient factors affecting family enrollment and retention in community-based programs for overweight youth and their families. We especially sought to understand potentially modifiable program factors affecting participation. DESIGN: : We conducted semistructured, in-depth, face-to-face interviews with parents of overweight children within 1 year of referral to a public health grant-funded community-based healthy lifestyle promotion program. Purposeful sampling was used to select participants across program sites, by level of program completion, and child age and sex. Transcribed interviews were coded independently by 2 staff with a structured codebook and then analyzed by themes through an iterative process using Atlas.ti. The Integrative Model of Behavior served as an orienting theoretical framework. SETTING: Community-based child obesity intervention program in King County, Washington. PARTICIPANTS: Twenty-three parents from diverse socioeconomic backgrounds were interviewed, of which 10 completed the program, 9 did not complete, and 4 did not enroll. MAIN OUTCOME MEASURE(S): Parent-reported factors related to enrollment and retention. RESULTS: Key parent reasons for program enrollment included: (a) addressing both eating and activity, (b) concern about child's weight, (c) seeking help outside the family, and (d) structured parent-child time. Parents perceived a lack of child motivation to enroll; some youth initially opposed attending, which was overcome through positive program experience. All families described barriers to attending, and some identified specific strategies or skills they used to overcome barriers. No single program design emerged to address every family's needs. Instead, using the themes of accessibility and accountability, we present parent- recommended design options. CONCLUSIONS: To meet different families' needs, public health and health care agencies offering youth health promotion programs should consider providing program options that vary intensity level and weight loss emphasis
SN - 1550-5022
AD - Seattle Children's Research Institute, Department of Pediatrics, University of Washington, Seattle, WA 98145, USA. mollie.grow@seattlechildrens.org
UR - 23328502
ER -

TY - JOUR
ID - 209
T1 - Paediatric sunburn: the experience of an Australian paediatric burns unit
A1 - Mah,L.
A1 - Di,Giovine P.
A1 - Quinn,L.
A1 - Sparnon,A.
Y1 - 2013/08//
N1 - Mah, Latifa. Di Giovine, Paul. Quinn, Linda. Sparnon, Anthony
Journal of paediatrics and child health
arp, 9005421
IM
Journal Article
English

KW - MEDLINE
KW - Adolescent
KW - Burn Units
KW - Child
KW - Preschool
KW - Female
KW - Humans
KW - Infant
KW - Male
KW - Retrospective Studies
KW - Sex Distribution
KW - South Australia/ep [Epidemiology]
KW - Sunburn/ep [Epidemiology]
KW - Sunburn/pc [Prevention & Control]
KW - Sunscreening Agents
KW - Ultraviolet Rays
RP - NOT IN FILE
SP - 654
EP - 657
JF - Journal of Paediatrics & Child Health
JA - J Paediatr Child Health
VL - 49
IS - 8
CY - Australia
N2 - AIM: The number of hospital presentations and admissions for treatment of sunburn remains significant, despite efforts to educate the public regarding sun protection. Current literature chiefly examines public health campaigns and sun protection behaviours and attitudes. There are very few articles that explore paediatric sunburn requiring hospital presentation. This study was therefore undertaken to provide a snapshot of this issue and to identify patterns and causative factors in the development of severe sunburn requiring hospital presentation. METHODS: Data were collected for retrospective analysis from case records of patients who presented with sunburn and were registered on the Burns Service database at the Women's and Children's Hospital in South Australia. This study includes patients who presented during the period of October 2006 to March 2011. RESULTS: There were 81 cases identified over the period of 2006-2011 from the Burns database that had sufficient information for the purpose of this study. Factors such as outdoor activity and water sports were predictably apparent, with patients being burned on days with extremely high ultraviolet ratings. Key patterns that emerged were location of sunburn and sun protection use, which were gender and age specific. CONCLUSION: Larger-scale studies are warranted to further delineate the contributing factors and to identify the specific populations of children at risk of sunburn. Future educational programmes can therefore target these subgroups and behaviours for effective prevention of sunburn. Tailored campaigns that address these factors may be of greater impact in reducing hospital presentations and admissions of significant sunburn. 2013 The Authors. Journal of Paediatrics and Child Health 2013 Paediatrics and Child Health Division (Royal Australasian College of Physicians)
SN - 1440-1754
AD - Burns Service, Women's and Children's Hospital, North Adelaide, South Australia, Australia. latifa.mah@health.wa.gov.au
UR - 23869600
ER -

TY - JOUR
ID - 210
T1 - Associations of children's independent mobility and active travel with physical activity, sedentary behaviour and weight status: a systematic review. [Review]
A1 - Schoeppe,S.
A1 - Duncan,M.J.
A1 - Badland,H.
A1 - Oliver,M.
A1 - Curtis,C.

Y1 - 2013/07//
N1 - Schoeppe, Stephanie. Duncan, Mitch J. Badland, Hannah. Oliver, Melody. Curtis, Carey
Journal of science and medicine in sport / Sports Medicine Australia
c54, 9812598
IM
Case Reports. Journal Article. Research Support, Non-U.S. Gov't. Review
English
KW - MEDLINE
KW - Body Weight
KW - Child
KW - Exercise
KW - Humans
KW - Independent Living
KW - Randomized Controlled Trials as Topic
KW - Sedentary Lifestyle
KW - Travel
RP - NOT IN FILE
SP - 312
EP - 319
JF - Journal of Science & Medicine in Sport
JA - J Sci Med Sport
VL - 16
IS - 4
CY - Australia
N2 - UNLABELLED: Health benefits from children's independent mobility and active travel beyond school travel are largely unexplored. OBJECTIVES: This review synthesized the evidence for associations of independent mobility and active travel to various destinations with physical activity, sedentary behaviour and weight status. DESIGN: Systematic review. METHODS: A systematic search in six databases (PubMed, Scopus, CINAHL, SportDiscus, PsychInfo, TRIS) for papers published between January 1990 and March 2012 was undertaken, focussing on children aged 3-18 years. Study inclusion and methodological quality were independently assessed by two reviewers. RESULTS: 52 studies were included. Most studies focussed solely on active travel to and/or from school, and showed significant positive associations with physical activity. The same relationship was detected for active travel to leisure-related places and independent mobility with physical activity. An inverse relationship between active travel to school and weight status was evident but findings were inconsistent. Few studies examined correlations between active travel to school and self-reported screen-time or objectively measured sedentary behaviour, and findings were unclear. CONCLUSIONS: Studies on independent mobility suggested that children who have the freedom to play outdoors and travel actively without adult supervision accumulate more physical activity than those who do not. Further investigation of children's active travel to leisure-related destinations, measurement of diverse sedentary behaviour beyond simply screen-based activities, and consistent thresholds for objectively measured sedentary behaviour in children will clarify the inconsistent evidence base on associations of active travel with sedentary behaviour and weight status.
Copyright 2012 Sports Medicine Australia. Published by Elsevier Ltd. All rights reserved
SN - 1878-1861
AD - Central Queensland University, Institute for Health and Social Science Research, Centre for Physical Activity Studies, Rockhampton, QLD, Australia. s.schoeppe@cqu.edu.au
UR - 23219100
ER -

TY - JOUR
ID - 211
T1 - Physical activity for children in special school environment
A1 - Sit,C.H.
A1 - McKenzie,T.L.
A1 - Cerin,E.
A1 - McManus,A.
A1 - Lian,J.
Y1 - 2013/06//

N1 - Sit, C H P. McKenzie, T L. Cerin, E. McManus, A. Lian, J
Hong Kong medical journal = Xianggang yi xue za zhi / Hong Kong Academy of Medicine
dnz, 9512509
IM
Journal Article. Multicenter Study. Observational Study. Randomized Controlled Trial
English
KW - MEDLINE
KW - Child
KW - Disabled Children
KW - Female
KW - Health Promotion/mt [Methods]
KW - Humans
KW - Male
KW - Motor Activity/ph [Physiology]
KW - Physical Education and Training/mt [Methods]
KW - Play and Playthings
KW - Schools
RP - NOT IN FILE
SP - 42
EP - 44
JF - Hong Kong Medical Journal
JA - HONG KONG MED
VL - 19 Suppl 4
CY - China
N2 - 1. We assessed children's physical activity (PA) in structured (physical education) and unstructured (recess, lunch, before and after school) periods in special schools and examined its association with modifiable area contextual characteristics. 2. Children with disabilities were not highly active, but were more active during recess and lunch periods than at other times including physical education classes. 3. Areas were often not accessible during unstructured settings. Children were more active in areas when supervision and organised activities were provided. 4. Providing an interactive game during free play did not significantly increase group's PA. 5. Children's PA accrual is influenced by contextual characteristics of the school environment. There is a need to make areas more accessible and to use social marketing and programming to attract more users. School and health professionals should modify contextual characteristics by providing more direct supervision and organised activities during free play
SN - 1024-2708
AD - Institute of Human Performance, The University of Hong Kong, Pokfulam Road, Hong Kong SAR, China. sithp@hku.hk
UR - 23775187
ER -

TY - JOUR
ID - 212
T1 - School physical activity policy assessment
A1 - Lounsbery,M.A.
A1 - McKenzie,T.L.
A1 - Morrow,J.R.,Jr.
A1 - Holt,K.A.
A1 - Budnar,R.G.
Y1 - 2013/05//
N1 - Lounsbery, Monica A F. McKenzie, Thomas L. Morrow, James R Jr. Holt, Kathryn A. Budnar, Ronald G
Journal of physical activity & health
101189457
IM
Journal Article. Research Support, Non-U.S. Gov't
English
KW - MEDLINE
KW - Checklist

KW - Child
KW - Health Policy
KW - Humans
KW - Motor Activity
KW - Physical Education and Training/mt [Methods]
KW - Physical Education and Training/og [Organization & Administration]
KW - Program Evaluation
KW - School Health Services/og [Organization & Administration]
RP - NOT IN FILE
SP - 496
EP - 503
JF - Journal of Physical Activity & Health
JA - J Phys Act Health
VL - 10
IS - 4
CY - United States
N2 - BACKGROUND: Physical activity (PA) levels in schools vary widely, and there is interest in studying how student PA accrual relates to school policy and environmental conditions. School PA policy research, however, is in its infancy and generalizable measurement tools do not exist. We developed and assessed reliability of items on the School Physical Activity Policy Assessment (S-PAPA), an instrument designed to assess school PA policy related to physical education (PE), recess, and other opportunities. METHODS: To develop items, we perused associated literature, examined existing instruments, and consulted school policy makers. For test-retest reliability assessment, 31 elementary school PE teachers completed the survey twice, 14 days apart. RESULTS: S-PAPA uses open-ended, dichotomous, multichotomous, and checklist formatting and has 3 modules: 1) Physical Education (47 items), 2) Recess (27 items), and 3) Other Before, During, and After School Programs (15 items). Responses to more than 95% of items were highly related between Times 1 and 2. Generally, physical education and recess items had fair to substantial levels of agreement, and items about other school PA programs had fair to perfect agreement. CONCLUSIONS: Test-retest results suggest S-PAPA items are reliable and useful in assessing PA policies in elementary schools
SN - 1543-5474
AD - Dept. of Kinesiology and Nutrition Sciences, University of Nevada-Las Vegas, USA
UR - 22975809
ER -

TY - JOUR
ID - 213
T1 - Lead, allergen, and pesticide levels in licensed child care centers in the United States
A1 - Viet,S.M.
A1 - Rogers,J.
A1 - Marker,D.
A1 - Fraser,A.
A1 - Friedman,W.
A1 - Jacobs,D.
A1 - Zhou,J.
A1 - Tulve,N.
Y1 - 2013/12//
N1 - Viet, Susan Marie. Rogers, John. Marker, David. Fraser, Alexa. Friedman, Warren. Jacobs, David. Zhou, J. Tulve, Nicolle
Journal of environmental health
iac, 0405525
IM
Journal Article. Research Support, U.S. Gov't, Non-P.H.S.
English
KW - MEDLINE
KW - Allergens/an [Analysis]
KW - Child Day Care Centers
KW - Child

KW - Preschool
KW - Environmental Exposure
KW - Environmental Health
KW - Environmental Monitoring
KW - Environmental Pollutants/an [Analysis]
KW - Health Surveys
KW - Humans
KW - Infant
KW - Lead/an [Analysis]
KW - Pesticides/an [Analysis]
KW - Questionnaires
KW - Spectrometry
KW - X-Ray Emission
KW - United States
RP - NOT IN FILE
SP - 8
EP - 14
JF - Journal of Environmental Health
JA - J Environ Health
VL - 76
IS - 5
CY - United States
N2 - The First National Environmental Health Survey of Child Care Centers was conducted to provide information about lead, allergen, and pesticide levels in licensed U.S. child care centers. Lead levels were measured in settled dust, paint, and play area soil; indoor allergen levels were measured in settled dust; and pesticide residues were measured on indoor surfaces and in play area soil. Fourteen percent of centers had significant lead hazards, suggesting that an estimated 470,000 children under age six (approximately 10% of all children in licensed centers) attend centers with significant lead hazards. Approximately 5% of centers had levels of allergens associated with asthma and allergic conditions. Three-quarters of centers had pesticides applied (either indoors or outdoors) during the previous year. Although most centers did not appear to present risks from lead and allergens, some centers did have unsafe levels of these contaminants. These conclusions cannot be generalized to unlicensed child care arrangements
SN - 0022-0892
AD - Westat, Rockville, MD 20850, USA. susanviet@westat.com, Westat, Rockville, MD 20850, USA., Westat, Rockville, MD 20850, USA., Westat, Rockville, MD 20850, USA., Department of Housing and Urban Development, USA., National Center for Healthy Housing, USA., Department of Energy, USA., U.S. Environmental Protection Agency, USA
UR - 24437044
ER -

TY - JOUR
ID - 214
T1 - [Factors associated with early sexual activity among Chilean adolescents]. [Spanish]
A1 - Gonzalez,A.E.
A1 - Molina,G.T.
A1 - Montero,V.A.
A1 - Martinez,N.,V
Y1 - 2013/03//
N1 - Gonzalez A, Electra. Molina G, Temistocles. Montero V, Adela. Martinez N, Vania
Revista medica de Chile
shd, 0404312
IM
English Abstract. Journal Article
Spanish
KW - MEDLINE
KW - Adolescent
KW - Adolescent Behavior/px [Psychology]

KW - Age Factors
KW - Child
KW - Chile/ep [Epidemiology]
KW - Coitus/px [Psychology]
KW - Cross-Sectional Studies
KW - Family Relations
KW - Female
KW - Humans
KW - Male
KW - Risk Factors
KW - Socioeconomic Factors
KW - Young Adult
RP - NOT IN FILE
SP - 313
EP - 319
JF - Revista Medica de Chile
JA - Rev Med Chil.
VL - 141
IS - 3
CY - Chile
N2 - BACKGROUND: Nowadays, adolescent population begins sexual activity earlier, a behavior with negative psychological ana social consequences. AIM: To determine the association between family factors and early sexual activity in adolescents of low and middle socioeconomic level. MATERIAL AND METHODS: A sample of 3,210 adolescents, who confidentially requested care in a sexual and reproductive health university center, between 2000 and 2007, was analyzed. Adolescents who started sexual activity before 15 years of age, and those who started sexual activity after 15 years of age, were compared. Data was collected from structured interviews conducted at the adolescente first visit. Logistic regression was used to identify family factors associated with early sexual initiating in both, men and women. RESULTS: Family factors associated with early sexual activity start were not being raised by both parents, a poor parent-child relationship, a poor family communication, mothers with a history of adolescent motherhood, mothers employed outside the home and family dysfunction. Among women, the variables associated with a higher risk of early sexual debut were not married parents and history of adolescent fatherhood record among parents. Among men, these variables were permissions without restriction during the week and punishment when family rules were broken. CONCLUSIONS: Family factors must be considered in the design of strategies to prevent early sexual activity
SN - 0717-6163
AD - Centro de Medicina Reproductiva y Desarrollo Integral de la Adolescencia, Facultad de Medicina, Universidad de Chile, Chile. evgonzalez@med.uchile.cl
UR - 23900321
ER -

TY - JOUR
ID - 215
T1 - Perceptions and acceptability of HPV vaccination among parents of young adolescents: a multicenter national survey in China
A1 - Zhang,S.K.
A1 - Pan,X.F.
A1 - Wang,S.M.
A1 - Yang,C.X.
A1 - Gao,X.H.
A1 - Wang,Z.Z.
A1 - Li,M.
A1 - Ren,Z.F.
A1 - Zhao,F.H.
A1 - Qiao,Y.L.
Y1 - 2013/07/11/
N1 - Zhang, Shao-Kai. Pan, Xiong-Fei. Wang, Shao-Ming. Yang, Chun-Xia. Gao, Xiao-Hong. Wang, Zeng-Zhen. Li, Man. Ren, Ze-Fang. Zhao, Fang-Hui. Qiao, You-Lin

Vaccine
x6o, 8406899
IM
Journal Article. Multicenter Study. Research Support, Non-U.S. Gov't
English
KW - MEDLINE
KW - Adolescent
KW - Adult
KW - Child
KW - China
KW - Condylomata Acuminata/pc [Prevention & Control]
KW - Condylomata Acuminata/vi [Virology]
KW - Female
KW - Health Knowledge
KW - Attitudes
KW - Practice
KW - Humans
KW - Male
KW - Papillomavirus Infections/pc [Prevention & Control]
KW - Papillomavirus Vaccines/tu [Therapeutic Use]
KW - Parents/px [Psychology]
KW - Patient Acceptance of Health Care/sn [Statistics & Numerical Data]
KW - Uterine Cervical Neoplasms/pc [Prevention & Control]
KW - Uterine Cervical Neoplasms/vi [Virology]
KW - Vaccination/px [Psychology]
RP - NOT IN FILE
SP - 3244
EP - 3249
JF - Vaccine
VL - 31
IS - 32
CY - Netherlands
N2 - Prophylactic HPV vaccines target young adolescents to prevent related cervical lesions and even genital warts prior to onset of sexual activity. Parental consent is often essential for success of vaccination program for this age group. We conducted a national multicenter study to explore the acceptability of HPV vaccination among parents of young adolescents and associated factors in relevant parent decision making in China. A total of 2899 parents of young adolescents (11-17 years) participated in the survey between November 28, 2011 and May 9, 2012, but four were excluded from analysis because of inconsistencies in their given information in the questionnaire. Mothers accounted for 62.8% of the parent participants. The mean age of the parents was 40.40 (standard deviation, 4.68) years. Only 36.2% of the parents accepted the vaccine for their children. Knowledge about HPV and HPV vaccine was a positive correlate with HPV vaccination acceptability (Ptrend=0.003). Grade of child (Ptrend=0.015), prior vaccination experience outside the National Expanded Program on Immunization (OR: 1.43; 95%CI: 1.19-1.72), fear of cervical cancer and/or genital warts (OR: 2.47; 95%CI: 2.00-3.05), and prior consultation regarding HPV vaccine information (OR: 2.35; 95%CI: 1.57-3.52) were also positively associated with higher HPV vaccine acceptability. The acceptability was lower in mothers (OR: 0.45; 95%CI: 0.37-0.54) and who had better education (Ptrend=0.009). 57.3% of the parents agreed that the most appropriate venue for HPV vaccination was the local center for disease prevention and control. In conclusion, our study indicates a low acceptability of HPV vaccination among parents of young adolescents in China. We understand there are many challenges in implementing HPV vaccination program. Our findings will serve as valuable references for future HPV vaccination policies and campaigns after HPV vaccines are approved in China. Copyright 2013 Elsevier Ltd. All rights reserved
SN - 1873-2518
AD - Department of Cancer Epidemiology, Cancer Institute of Chinese Academy of Medical Sciences and Peking Union Medical College, Beijing 100021, China
UR - 23707446
ER -

TY - JOUR
ID - 216
T1 - District and school physical education policies: implications for physical education and recess time
A1 - Lounsbery,M.A.
A1 - McKenzie,T.L.
A1 - Morrow,J.R.,Jr.
A1 - Monnat,S.M.
A1 - Holt,K.A.
Y1 - 2013/02//
N1 - Lounsbery, Monica A F. McKenzie, Thomas L. Morrow, James R Jr. Monnat, Shannon M. Holt, Kathryn A
Annals of behavioral medicine : a publication of the Society of Behavioral Medicine
8510246, cm5
IM
Journal Article. Research Support, Non-U.S. Gov't
English
KW - MEDLINE
KW - Child
KW - Guideline Adherence
KW - Health Promotion
KW - Humans
KW - Models
KW - Educational
KW - Motor Activity
KW - Physical Education and Training/mt [Methods]
KW - Policy
KW - Program Evaluation
KW - Schools
KW - Time Factors
KW - United States
RP - NOT IN FILE
SP - S131
EP - S141
JF - Annals of Behavioral Medicine
JA - Ann Behav Med
VL - 45 Suppl 1
CY - United States
N2 - BACKGROUND: The relationship between physical education (PE) policies and children's PE and recess time is not well understood. PURPOSE: The purpose of this study is to assess the association of district and school PE policies, the PE environment, and PE and recess time. METHODS: Key informants in 65 schools from 9 states completed instruments assessing district and school PE policies, the school PE environment, and time in PE and recess. RESULTS: Few significant associations were found between PE policies and PE or recess minutes; no policies were associated with both. A number of PE environmental variables were associated with both policies and time in PE and recess. CONCLUSIONS: PE policies, their implementation, and PE environmental variables can have important implications for recess time. Some school PE environment measures designed to improve PE may result in PE time limitations. Deficiencies in PE and recess time are not likely to be effectively addressed through policy adoption alone
SN - 1532-4796
AD - University of Nevada, Las Vegas, Las Vegas, NV, USA. monica.lounsbery@unlv.edu
UR - 23334759
ER -

TY - JOUR
ID - 217
T1 - Strength training and physical activity in boys: a randomized trial
A1 - Meinhardt,U.
A1 - Witassek,F.

A1 - Petro,R.
A1 - Fritz,C.
A1 - Eiholzer,U.
Y1 - 2013/12//
N1 - Meinhardt, Udo. Witassek, Fabienne. Petro, Renato. Fritz, Chris. Eiholzer, Urs
Pediatrics
oxv, 0376422
AIM, IM
Comparative Study. Journal Article. Randomized Controlled Trial
English
KW - MEDLINE
KW - Absorptiometry
KW - Photon
KW - Accelerometry
KW - Adolescent
KW - Body Composition
KW - Child
KW - Child Behavior/ph [Physiology]
KW - Energy Metabolism
KW - Female
KW - Follow-Up Studies
KW - Humans
KW - Leisure Activities
KW - Male
KW - Motor Activity/ph [Physiology]
KW - Muscle Strength
KW - Physical Education and Training
KW - Regression Analysis
KW - Resistance Training/mt [Methods]
KW - Schools
KW - Sex Factors
KW - Switzerland
RP - NOT IN FILE
SP - 1105
EP - 1111
JF - Pediatrics
VL - 132
IS - 6
CY - United States
N2 - BACKGROUND: In developed societies levels of daily physical activity (PA) among school-age children are decreasing. This implies risk factors for cardiovascular and metabolic diseases. Specific strategies to improve levels of PA are needed. In prepubertal boys there is evidence that strength training increases spontaneous PA outside of training. METHODS: A total of 102 schoolchildren (age 10-14 years) in Switzerland were randomly assigned to physical education classes or to participate twice weekly at a guided strength training program for 19 weeks. Spontaneous PA energy expenditure (PAEE; 3axial accelerometry for 7 days), leg and arm strength, and body composition (dual energy radiograph absorptiometry) were measured at baseline, after 19 weeks of training intervention, and after 3 months of washout. RESULTS: There were no significant differences between the groups at baseline. In the intervention group, PAEE increased by 10% from baseline to end of training in boys (P = .02), but not in girls. Leg and arm strength were increased owing to training intervention in both boys and girls. All other variables were unchanged. Baseline PAEE was significantly negatively correlated with changes of PAEE. CONCLUSIONS: Targeted strength training significantly increases daily spontaneous PA behavior in boys. The less active children showed the greatest increase in spontaneous PAEE. Girls showed a similar increase in strength, but not in spontaneous PAEE. This may be explained by their earlier pubertal development. Strength training may be a promising strategy in schools to counteract decreasing levels of PA
SN - 1098-4275

AD - Head of the Center for Pediatric Endocrinology Zurich, Moehrlistrasse 69, CH-8006 Zurich, Switzerland. urs.eiholzer@pezz.ch
UR - 24190687
ER -

TY - JOUR
ID - 218
T1 - Disentangling neighborhood contextual associations with child body mass index, diet, and physical activity: the role of built, socioeconomic, and social environments
A1 - Carroll-Scott,A.
A1 - Gilstad-Hayden,K.
A1 - Rosenthal,L.
A1 - Peters,S.M.
A1 - McCaslin,C.
A1 - Joyce,R.
A1 - Ickovics,J.R.
Y1 - 2013/10//
N1 - Carroll-Scott, Amy. Gilstad-Hayden, Kathryn. Rosenthal, Lisa. Peters, Susan M. McCaslin, Catherine. Joyce, Rebecca. Ickovics, Jeannette R
Social science & medicine (1982)
ut9, 8303205
IM
Journal Article. Research Support, Non-U.S. Gov't
English
KW - MEDLINE
KW - Body Mass Index
KW - Child
KW - Connecticut/ep [Epidemiology]
KW - Diet/sn [Statistics & Numerical Data]
KW - Environment Design/sn [Statistics & Numerical Data]
KW - Female
KW - Health Status Disparities
KW - Humans
KW - Male
KW - Motor Activity
KW - Pediatric Obesity/ep [Epidemiology]
KW - Residence Characteristics/sn [Statistics & Numerical Data]
KW - Social Determinants of Health
KW - Social Environment
KW - Socioeconomic Factors
RP - NOT IN FILE
SP - 106
EP - 114
JF - Social Science & Medicine
JA - Soc Sci Med
VL - 95
CY - England
N2 - Obesity prevalence among US children and adolescents has tripled in the past three decades. Consequently, dramatic increases in chronic disease incidence are expected, particularly among populations already experiencing health disparities. Recent evidence identifies characteristics of "obesogenic" neighborhood environments that affect weight and weight-related behaviors. This study aimed to examine associations between built, socioeconomic, and social characteristics of a child's residential environment on body mass index (BMI), diet, and physical activity. We focused on pre-adolescent children living in New Haven, Connecticut to better understand neighborhood environments' contribution to persistent health disparities. Participants were 1048 fifth and sixth grade students who completed school-based health surveys and physical measures in fall 2009. Student data were linked to US Census, parks, retailer, and crime data. Analyses were conducted using multilevel modeling. Property crimes and living further from a grocery store were associated with higher BMI.

Students living within a 5-min walk of a fast food outlet had higher BMI, and those living in a tract with higher density of fast food outlets reported less frequent healthy eating and more frequent unhealthy eating. Students' reported perceptions of access to parks, playgrounds, and gyms were associated with more frequent healthy eating and exercise. Students living in more affluent neighborhoods reported more frequent healthy eating, less unhealthy eating, and less screen time. Neighborhood social ties were positively associated with frequency of exercise. In conclusion, distinct domains of neighborhood environment characteristics were independently related to children's BMI and health behaviors. Findings link healthy behaviors with built, social, and socioeconomic environment assets (access to parks, social ties, affluence), and unhealthy behaviors with built environment inhibitors (access to fast food outlets), suggesting neighborhood environments are an important level at which to intervene to prevent childhood obesity and its adverse consequences. Copyright 2013 Elsevier Ltd. All rights reserved
SN - 1873-5347
AD - CARE: Community Alliance for Research and Engagement, Yale School of Public Health, USA. ac3343@drexel.edu
UR - 23642646
ER -

TY - JOUR
ID - 219
T1 - Does goal setting in activity-focused interventions for children with cerebral palsy influence treatment outcome?. [Review]
A1 - Brogren,Carlberg E.
A1 - Lowing,K.
Y1 - 2013/11//
N1 - Brogren Carlberg, Eva. Lowing, Kristina
Developmental medicine and child neurology
0006761, e83
IM
Journal Article. Review
English
KW - MEDLINE
KW - Adolescent
KW - Cerebral Palsy/pp [Physiopathology]
KW - Cerebral Palsy/px [Psychology]
KW - Cerebral Palsy/th [Therapy]
KW - Child
KW - Preschool
KW - Goals
KW - Humans
KW - Infant
KW - Psychomotor Performance/ph [Physiology]
KW - Treatment Outcome
RP - NOT IN FILE
SP - 47
EP - 54
JF - Developmental Medicine & Child Neurology
JA - Dev Med Child Neurol
VL - 55 Suppl 4
CY - England
N2 - Today, treatment for children with cerebral palsy predominantly aims at improving the children's possibilities to perform everyday activities in their natural environment. The activities in focus for intervention are often expressed as specific goals, frequently defined in a collaborative goal-setting process between professionals and parents. The role of goal setting to improve the outcome of the intervention has not been shown in the literature so far. Thus, the aim of this systematic review was to explore if goal setting has an impact on treatment outcome assessed by standardized measures. CINAHL and MEDLINE were searched from January 2000 to October 2012, resulting in a final selection of 13 articles, six of which were randomized controlled trials. Methodological quality was assessed and study characteristics were analysed descriptively.

Subject characteristics, type of intervention/s, frequency, and intensity of therapy varied largely. Outcome was assessed by standardized outcome measures as well as evaluated through aspects of goal attainment. Most studies showed robust within-group changes according to study-appropriate standardized measures, whereas the between-group comparisons exhibited less consistent differences in outcome. The review does not provide support for a positive effect of goal setting per se on treatment outcome. Studies that specifically measure the effect of goal setting on treatment outcome are needed. The Authors. Developmental Medicine & Child Neurology 2013 Mac Keith Press
SN - 1469-8749
AD - Department of Women's and Children's Health, Karolinska Institutet, Karolinska Universitetssjukhuset, Stockholm, Sweden
UR - 24237280
ER -

TY - JOUR
ID - 220
T1 - Study of Health and Activity in Preschool Environments (SHAPES): study protocol for a randomized trial evaluating a multi-component physical activity intervention in preschool children
A1 - Pfeiffer,K.A.
A1 - Saunders,R.P.
A1 - Brown,W.H.
A1 - Dowda,M.
A1 - Addy,C.L.
A1 - Pate,R.R.
Y1 - 2013///
N1 - Pfeiffer, Karin A. Saunders, Ruth P. Brown, William H. Dowda, Marsha. Addy, Cheryl L. Pate, Russell R
BMC public health
100968562
IM
Journal Article. Randomized Controlled Trial. Research Support, N.I.H., Extramural
English
KW - MEDLINE
KW - Accelerometry/sn [Statistics & Numerical Data]
KW - Child
KW - Preschool
KW - Energy Metabolism
KW - Humans
KW - Models
KW - Psychological
KW - Motor Activity
KW - Multilevel Analysis
KW - Program Evaluation
KW - Research Design
KW - School Health Services/og [Organization & Administration]
KW - Sedentary Lifestyle
KW - Social Environment
RP - NOT IN FILE
SP - 728
JF - BMC Public Health
VL - 13
CY - England
N2 - BACKGROUND: Physical inactivity is a recognized public health concern. Inadequate proportions of children in the U.S, including those of preschool age, are meeting physical activity recommendations. In response to low numbers of preschool children attaining appropriate physical activity levels, combined with the large number of young children who attend preschool, researchers have identified the need to devise interventions to increase physical activity at preschools. However, few multi-component interventions to increase physical activity in preschool children exist. The aims of this study were to observe the effects of a multi-component intervention on physical activity, sedentary behavior, and physical activity energy expenditure

in 3-5 year-old children; identify factors that associate with change in those variables; and evaluate the process of implementing the multi-component intervention. The purpose of this manuscript is to describe the study design and intervention protocol. METHODS/DESIGN: The overall design of the Study of Health and Activity in Preschool Environments (SHAPES) was a two-year randomized trial (nested cohort design), with two conditions, two measurement occasions, and preschool serving as the unit of analysis. Sixteen schools (eight intervention and eight control) were enrolled. The intervention protocol was based on the social ecological model and included four main components: (a) indoor physical activity ("move inside"), (b) recess ("move outside"), (c) daily lessons ("move to learn"), and (d) social environment. Components were implemented using teacher and administrator trainings and workshops, site support visits, newsletters, and self-monitoring methods. Outcomes included accelerometer assessment of physical activity, sedentary behavior, and physical activity energy expenditure; weight status; and demographic factors; family/home social and physical environment; and parental characteristics. An extensive process evaluation battery was also used to monitor dose delivered by interventionists, completeness of intervention component delivery by teachers, and fidelity of teachers' implementation. DISCUSSION: The study will address important gaps relative to increasing physical activity in preschool children. Few studies to date have incorporated a multi-component approach, rigorous measurement protocol, and thorough evaluation of intervention implementation
SN - 1471-2458
AD - Department of Kinesiology, Michigan State University, 308 West Circle Drive, 27R Intramural Rec Sports- Circle, East Lansing, MI 48824, USA. kap@msu.edu
UR - 23919808
ER -

TY - JOUR
ID - 221
T1 - National burden of invasive methicillin-resistant Staphylococcus aureus infections, United States, 2011
A1 - Dantes,R.
A1 - Mu,Y.
A1 - Belflower,R.
A1 - Aragon,D.
A1 - Dumyati,G.
A1 - Harrison,L.H.
A1 - Lessa,F.C.
A1 - Lynfield,R.
A1 - Nadle,J.
A1 - Petit,S.
A1 - Ray,S.M.
A1 - Schaffner,W.
A1 - Townes,J.
A1 - Fridkin,S.
A1 - Emerging Infections Program-
Y1 - 2013/11/25/
N1 - Dantes, Raymund. Mu, Yi. Belflower, Ruth. Aragon, Deborah. Dumyati, Ghinwa. Harrison, Lee H. Lessa, Fernanda C. Lynfield, Ruth. Nadle, Joelle. Petit, Susan. Ray, Susan M. Schaffner, William. Townes, John. Fridkin, Scott. Emerging Infections Program-Active Bacterial Core Surveillance MRSA Surveillance Investigators
JAMA internal medicine
101589534
AIM, IM
Journal Article. Research Support, Non-U.S. Gov't. Research Support, U.S. Gov't, P.H.S.
English
KW - MEDLINE
KW - Adolescent
KW - Adult
KW - Aged
KW - Child
KW - Preschool
KW - Community-Acquired Infections/ep [Epidemiology]

KW - Community-Acquired Infections/mi [Microbiology]
KW - Cost of Illness
KW - Cross Infection/ep [Epidemiology]
KW - Cross Infection/mi [Microbiology]
KW - Female
KW - Hospitalization
KW - Humans
KW - Infant
KW - Long-Term Care
KW - Male
KW - Methicillin-Resistant Staphylococcus aureus
KW - Middle Aged
KW - Nursing Homes
KW - Renal Dialysis
KW - Risk Factors
KW - Staphylococcal Infections/ep [Epidemiology]
KW - Staphylococcal Infections/mi [Microbiology]
KW - United States/ep [Epidemiology]
RP - NOT IN FILE
SP - 1970
EP - 1978
JF - JAMA Internal Medicine
JA - JAMA Intern Med
VL - 173
IS - 21
CY - United States
N2 - IMPORTANCE: Estimating the US burden of methicillin-resistant Staphylococcus aureus (MRSA) infections is important for planning and tracking success of prevention strategies. OBJECTIVE: To describe updated national estimates and characteristics of health care- and community-associated invasive methicillin-resistant Staphylococcus aureus (MRSA) infections in 2011. DESIGN, SETTING, AND PARTICIPANTS: Active laboratory-based case finding identified MRSA cultures in 9 US metropolitan areas from 2005 through 2011. Invasive infections (MRSA cultured from normally sterile body sites) were classified as health care-associated community-onset (HACO) infections (cultured < 3 days after admission and/or prior year dialysis, hospitalization, surgery, long-term care residence, or central vascular catheter presence < 2 days before culture); hospital-onset infections (cultured >3 days after admission); or community-associated infections if no other criteria were met. National estimates were adjusted using US census and US Renal Data System data. MAIN OUTCOMES AND MEASURES: National estimates of invasive HACO, hospital-onset, and community-associated MRSA infections using US census and US Renal Data System data as the denominator. RESULTS: An estimated 80,461 (95% CI, 69,515-93,914) invasive MRSA infections occurred nationally in 2011. Of these, 48,353 (95% CI, 40,195-58,642) were HACO infections; 14,156 (95% CI, 10,096-20,440) were hospital-onset infections; and 16,560 (95% CI, 12,806-21,811) were community-associated infections. Since 2005, adjusted national estimated incidence rates decreased among HACO infections by 27.7% and hospital-onset infections decreased by 54.2%; community-associated infections decreased by only 5.0%. Among recently hospitalized community-onset (nondialysis) infections, 64% occurred 3 months or less after discharge, and 32% of these were admitted from long-term care facilities. CONCLUSIONS AND RELEVANCE: An estimated 30,800 fewer invasive MRSA infections occurred in the United States in 2011 compared with 2005; in 2011 fewer infections occurred among patients during hospitalization than among persons in the community without recent health care exposures. Effective strategies for preventing infections outside acute care settings will have the greatest impact on further reducing invasive MRSA infections nationally
SN - 2168-6114
AD - Epidemic Intelligence Service, Scientific Education and Professional Development Program Office, Centers for Disease Control and Prevention, Atlanta, Georgia2Division of Healthcare Quality Promotion, Centers for Disease Control and Prevention, Atlanta, Georgia
UR - 24043270
ER -

TY - JOUR

ID - 222
T1 - Traumatic dental injuries at a German university clinic 2004-2008
A1 - Bucher,K.
A1 - Neumann,C.
A1 - Hickel,R.
A1 - Kuhnisch,J.
Y1 - 2013/04//
N1 - Bucher, Katharina. Neumann, Claudia. Hickel, Reinhard. Kuhnisch, Jan
Dental traumatology : official publication of International Association for Dental Traumatology
101091305, d2p
D
Journal Article
English
KW - MEDLINE
KW - Academic Medical Centers
KW - Adolescent
KW - Adult
KW - Age Distribution
KW - Analysis of Variance
KW - Child
KW - Preschool
KW - Female
KW - Germany/ep [Epidemiology]
KW - Guidelines as Topic
KW - Humans
KW - Infant
KW - Male
KW - Middle Aged
KW - Retrospective Studies
KW - Sex Distribution
KW - Tooth Injuries/ep [Epidemiology]
KW - Tooth Injuries/et [Etiology]
KW - Tooth Injuries/th [Therapy]
RP - NOT IN FILE
SP - 127
EP - 133
JF - Dental Traumatology
JA - Dent Traumatol
VL - 29
IS - 2
CY - Denmark
N2 - AIM: This study was designed to analyse the distribution and treatment management of patients who presented at a German university dental emergency department for trauma in a 5-year period. MATERIALS/METHODS: Clinical and radiographical baseline data and recall of patients presenting from 2004 to 2008 for dental trauma were evaluated with regard to age, gender, type of injury and treatment strategies. In addition, trauma management as performed was compared with the guidelines of the International Association of Dental Traumatology (IADT). RESULTS: A total of 361 teeth in 219 patients from 1 to 68 years were treated with 1.7 injured teeth per incident and patient. Over 75% of the patients were < 14 years. In 23% of all incidents, primary teeth were affected with a male-to-female ratio of 2.1:1. Luxation injuries were predominant in the deciduous dentition. The domestic environment was the most common trauma location (44%), followed by nursery schools (36%) and outdoors during recreational activities (20%) mainly caused by falls (72%). In 76% of all dental injuries, permanent teeth were involved; 65% of patients were men; and 35%, women (1.8:1). With permanent teeth, enamel-dentine fracture without pulp involvement was most common. Falls (38%), accidental contacts (21%) and sports accidents (18%) caused the most injuries. The majority of teeth were treated in concordance with the guidelines (89%). CONCLUSIONS: The distribution of traumatic injuries is similar to other national and international data with regard to gender, age and in relation to injury type, location and causes. International and national healthcare associations and teachers in under- and

postgraduate education should put effort on increasing the awareness of the guidelines. 2012 John Wiley & Sons A/S
SN - 1600-9657
AD - Department of Conservative Dentistry, Ludwig Maximilian University, Munich, Germany. kbuecher@dent.med.uni-muenchen.de
UR - 22613081
ER -

TY - JOUR
ID - 223
T1 - Evaluation of 99mTc-succimer dosing in pediatric patients
A1 - Galbraith,W.
A1 - Nguyen,A.
A1 - Harrison,D.L.
A1 - Chen,X.
A1 - Talley,K.
Y1 - 2013/06//
N1 - Galbraith, Wendy. Nguyen, Anthony. Harrison, Donald L. Chen, Xinlian. Talley, Katie
Journal of nuclear medicine technology
cwf, 0430303
IM
Journal Article
English
KW - MEDLINE
KW - Adolescent
KW - Body Surface Area
KW - Child
KW - Preschool
KW - Environmental Exposure/st [Standards]
KW - Female
KW - Humans
KW - Infant
KW - Male
KW - Nuclear Medicine/mt [Methods]
KW - Nuclear Medicine/st [Standards]
KW - Practice Guidelines as Topic
KW - Radiation Dosage
KW - Retrospective Studies
KW - Societies
KW - Medical/st [Standards]
KW - Syringes
KW - Technetium Tc 99m Dimercaptosuccinic Acid/du [Diagnostic Use]
KW - Time Factors
RP - NOT IN FILE
SP - 81
EP - 84
JF - Journal of Nuclear Medicine Technology
JA - J Nucl Med Technol
VL - 41
IS - 2
CY - United States
N2 - UNLABELLED: Balancing image quality with radiation dose is a goal with every diagnostic procedure requiring radiation. Our institution compared the dosing of (99m)Tc-labeled succimer, commonly referred to as dimercaptosuccinic acid ((99m)Tc-DMSA), to pediatric patients using 2 methods of calculation, body surface area (BSA, the method we used from 2009 to 2010) and body weight (BW, the method we used in 2011). METHODS: A retrospective study was conducted in a 230-bed inpatient, tertiary-care academic pediatric hospital to obtain objective data on patients under the age of 17 y who received a renal nuclear medicine

procedure with (99m)Tc-DMSA using a 300,000-count parallel image and four 150,000-count pinhole images. Data collection included patient age, sex, height, weight, calculated activity, assayed activity, administered activity, residual syringe activity, imaging time, and notable patient or equipment factors affecting the procedure. RESULTS: Calculated activities based on BSA were higher than calculated activities based on BW. (99m)Tc-DMSA adsorption to the plastic syringes was significant, with a range of 3%-82%. Because of the adsorption, an average of 23.7 MBq (SD, +31 MBq) was added to the patients' calculated dose when the order was placed. Therefore, assayed activities were significantly higher than calculated activities in both groups. Administered activity correlations to BSA and BW calculations were 0.75 and 0.83, respectively. Administered activities from BSA and BW groups were outside the American College of Radiology (ACR)-recommended guidelines 59% and 45% of the time, respectively. Overall, children less than 2 y old were above the ACR recommendations 80% of the time. There was a poor correlation between administered activity and total imaging time (r = 0.23). Average imaging time overall for 5 planar views was 14.8 min (+7.1 min). Patients receiving less than the ACR-recommended administered activities (<1.85 MBq/kg) had an average increase in imaging time of 4.5 min (+3.4 min). CONCLUSION: The activity administered to patients was significantly affected by the amount of (99m)Tc-DMSA activity adsorbed to the syringe. Syringe residual should be considered when standardizing (99m)Tc-DMSA imaging protocols and calculating patient dose. Although (99m)Tc-DMSA adsorption was variable, the administered activities correlated with calculated activities. In all but one of our patients, the total imaging time was far less than recommended by the ACR and European Association of Nuclear Medicine guidelines. The study indicates that using the BW calculation of 3.7 MBq/kg resulted in a range of administered activity of 1.85-2.59 MBq/kg. (99m)Tc-DMSA dosing of 3.7 MBq/kg for pinhole imaging should be appropriate for most studies
SN - 1535-5675
AD - University of Oklahoma College of Pharmacy, Oklahoma City, OK, USA. wendy-galbraith@ouhsc.edu
UR - 23608072
ER -

TY - JOUR
ID - 224
T1 - Physical activity in children attending preschools
A1 - Olesen,L.G.
A1 - Kristensen,P.L.
A1 - Korsholm,L.
A1 - Froberg,K.
Y1 - 2013/11//
N1 - Olesen, Line Groenholt. Kristensen, Peter Lund. Korsholm, Lars. Froberg, Karsten
Pediatrics
oxv, 0376422
AIM, IM
Journal Article. Randomized Controlled Trial. Research Support, Non-U.S. Gov't
English
KW - MEDLINE
KW - Child
KW - Child Behavior/ph [Physiology]
KW - Preschool
KW - Cross-Sectional Studies
KW - Denmark/ep [Epidemiology]
KW - Female
KW - Humans
KW - Male
KW - Motor Activity/ph [Physiology]
KW - Schools/sn [Statistics & Numerical Data]
KW - Schools
RP - NOT IN FILE
SP - e1310
EP - e1318
JF - Pediatrics
VL - 132

IS - 5
CY - United States
N2 - OBJECTIVE: To identify correlates of objectively measured moderate and vigorous physical activity (MVPA) in children during preschool attendance. METHODS: This cross-sectional study included data from 426 apparently healthy Danish children (49.5% boys), 5 to 6 years of age enrolled in 42 randomly selected preschools. The percentage of time spent in MVPA (> 574 counts/15 second) during preschool attendance was measured using ActiGraph accelerometers over 4.3 preschool days in May and June in 2009. Thirty-seven potential correlates across the child, preschool staff, and preschool environment domains were tested for associations with MVPA. RESULTS: The final multivariate model identified 9 significant correlates of MVPA. Preterm birth, vegetation on the playground, and rainy days were negatively associated with MVPA, whereas child motor coordination, location of preschool building on the playground, gender (boys), percentage afternoon hours, and size of indoor area per child were positively associated with MVPA. The direction of the significant association with the parental mean education level was unclear. CONCLUSIONS: We identified a number of new modifiable correlates of MVPA during preschool attendance. The positive association with size of indoor area per child and location of the preschool building on the playground seem important correlates to be targeted in future studies
SN - 1098-4275
AD - Centre of Research in Childhood Health, Department of Sports Science and Clinical Biomechanics, University of Southern Denmark, 55 Campusvej, Odense M, 5230, Denmark. lgoelsen@health.sdu.dk
UR - 24127470
ER -

TY - JOUR
ID - 225
T1 - Vitamin D in the healthy European paediatric population. [Review]
A1 - Braegger,C.
A1 - Campoy,C.
A1 - Colomb,V.
A1 - Decsi,T.
A1 - Domellof,M.
A1 - Fewtrell,M.
A1 - Hojsak,I.
A1 - Mihatsch,W.
A1 - Molgaard,C.
A1 - Shamir,R.
A1 - Turck,D.
A1 - van,Goudoever J.
A1 - ESPGHAN Committee on Nutrition.
Y1 - 2013/06//
N1 - Braegger, Christian. Campoy, Cristina. Colomb, Virginie. Decsi, Tamas. Domellof, Magnus. Fewtrell, Mary. Hojsak, Iva. Mihatsch, Walter. Molgaard, Christian. Shamir, Raanan. Turck, Dominique. van Goudoever, Johannes. ESPGHAN Committee on Nutrition
Journal of pediatric gastroenterology and nutrition
jl6, 8211545
IM
Journal Article. Review
English
KW - MEDLINE
KW - Adolescent
KW - Adolescent Development
KW - Bone Development
KW - Child
KW - Child Development
KW - Preschool
KW - Diet/ae [Adverse Effects]
KW - Dietary Supplements/ae [Adverse Effects]
KW - Europe/ep [Epidemiology]

KW - Food
KW - Fortified/ae [Adverse Effects]
KW - Health Policy
KW - Health Promotion
KW - Humans
KW - Infant
KW - Practice Guidelines as Topic
KW - Prevalence
KW - Societies
KW - Scientific
KW - Sunlight/ae [Adverse Effects]
KW - Vitamin D/ad [Administration & Dosage]
KW - Vitamin D/ae [Adverse Effects]
KW - Vitamin D/tu [Therapeutic Use]
KW - Vitamin D Deficiency/dh [Diet Therapy]
KW - Vitamin D Deficiency/ep [Epidemiology]
KW - Vitamin D Deficiency/pc [Prevention & Control]
RP - NOT IN FILE
SP - 692
EP - 701
JF - Journal of Pediatric Gastroenterology & Nutrition
JA - J Pediatr Gastroenterol Nutr
VL - 56
IS - 6
CY - United States
N2 - In recent years, reports suggesting a resurgence of vitamin D deficiency in the Western world, combined with various proposed health benefits for vitamin D supplementation, have resulted in increased interest from health care professionals, the media, and the public. The aim of this position paper is to summarise the published data on vitamin D intake and prevalence of vitamin D deficiency in the healthy European paediatric population, to discuss the health benefits of vitamin D and to provide recommendations for the prevention of vitamin D deficiency in this population. Vitamin D plays a key role in calcium and phosphate metabolism and is essential for bone health. There is insufficient evidence from interventional studies to support vitamin D supplementation for other health benefits in infants, children, and adolescents. The pragmatic use of a serum concentration >50 nmol/L to indicate sufficiency and a serum concentration <25 nmol/L to indicate severe deficiency is recommended. Vitamin D deficiency occurs commonly among healthy European infants, children, and adolescents, especially in certain risk groups, including breast-fed infants, not adhering to the present recommendation for vitamin D supplementation, children and adolescents with dark skin living in northern countries, children and adolescents without adequate sun exposure, and obese children. Infants should receive an oral supplementation of 400 IU/day of vitamin D. The implementation should be promoted and supervised by paediatricians and other health care professionals. Healthy children and adolescents should be encouraged to follow a healthy lifestyle associated with a normal body mass index, including a varied diet with vitamin D-containing foods (fish, eggs, dairy products) and adequate outdoor activities with associated sun exposure. For children in risk groups identified above, an oral supplementation of vitamin D must be considered beyond 1 year of age. National authorities should adopt policies aimed at improving vitamin D status using measures such as dietary recommendations, food fortification, vitamin D supplementation, and judicious sun exposure, depending on local circumstances
SN - 1536-4801
AD - Division of Gastroenterology and Nutrition, University Children's Hospital Zurich, Zurich, Switzerland. Christian.Braegger@kispi.uzh.ch
UR - 23708639
ER -

TY - JOUR
ID - 226
T1 - [A survey on the agricultural profession-related injuries among 11 902 rural residents in Shandong province]. [Chinese]
A1 - Li,Z.H.

A1 - Yin,W.Q.
A1 - Ma,H.
A1 - Liu,W.L.
A1 - Li,S.S.
A1 - Zhang,M.L.
A1 - Chu,W.J.
A1 - An,B.
Y1 - 2011/08//
N1 - Li, Zhi-hua. Yin, Wei-qing. Ma, Hong. Liu, Wei-liang. Li, Sha-sha. Zhang, Meng-lin. Chu, Wen-jie. An, Bang
Zhonghua liu xing bing xue za zhi = Zhonghua liuxingbingxue zazhi
cqg, 8208604
IM
English Abstract. Journal Article
Chinese
KW - MEDLINE
KW - Accidents
KW - Occupational/sn [Statistics & Numerical Data]
KW - Adolescent
KW - Adult
KW - Aged
KW - Agriculture
KW - Child
KW - Preschool
KW - China/ep [Epidemiology]
KW - Female
KW - Humans
KW - Incidence
KW - Infant
KW - Male
KW - Middle Aged
KW - Questionnaires
KW - Risk Factors
KW - Rural Population
KW - Wounds and Injuries/ep [Epidemiology]
KW - Young Adult
RP - NOT IN FILE
SP - 768
EP - 772
JF - Chung-Hua Liu Hsing Ping Hsueh Tsa Chih Chinese Journal of Epidemiology
JA - Chung Hua Liu Hsing Ping Hsueh Tsa Chih
VL - 32
IS - 8
CY - China
N2 - OBJECTIVE: To understand the prevalence and risk factors of agricultural activities related injuries among rural residents in Shandong province. METHODS: A retrospective investigation was conducted among agricultural profession-related workers in 20 villages with multistage cluster sampling method in Shandong province. Four times face-to-face interview were conducted by trained interviewers, including 32 students and local medical personnel under constructed questionnaires. Accidental injuries occurred in the activities or in the agricultural profession-related jobs were recorded, from May 1(st)2009 to April 30(st) 2010. Data was input and analyzed by SPSS 13.0 statistical software. RESULTS: A total of 837 cases reported at least 1 job-related injury out of the 11 902 people who had been surveyed in one year. The crude incidence rate was 7.03% and the standardized incidence rate was 7.36%, higher in males (9.01%) than in females (4.10%), chi2 = 105.53, P = 0.000. Children and adolescents (< 14 aged) had the higher incidence rate (9.50%), chi2 = 9.70, P = 0.008. People working in the area of commercial service related to agricultural products had the highest incidence rate (12.94%). In particular, those occupations that related to agricultural construction or materials appeared to have had higher incidence rates as 16.80% and 15.59% respectively, than other kinds of jobs (chi2 = 167.30, P =

0.000). There were higher proportion of injuries occurred in the roads (28.79%), in the fields (28.08%)during labor work (38.00%) transportation (27.97%), respectively. The seasonality of agricultural injures mostly occurred between June and August, accounted for 47.43%. Major external causes related to injuries were instruments or tools (31.42%) being used, transportation (24.13%) and falls (20.19%). Wounds on limbs took the majority (56.39%). The accidental self-inflicted injury occupied 76.82%, while accidents to passive injuries occupied 11.47%, other kinds accounted for 11.71%. Most of the accidents caused mild or moderate damage, accounted for 60.22% and 30.34% respectively. 7 cases died of injuries, with the fatality rate as 0.84% and the mortality rate was 58.81 per one hundred thousand. 72 cases ended up in disability caused by injuries, with the morbidity rate as 6.05 per thousand. Answers to the major internal causes of injuries appeared to be: "did not know how to protect oneself" (29.87%) with females (45.41%) in particular, followed by"over fatigue" and "inappropriate treatment" which took the 2(nd) place in males. Major natural environmental factors of injuries would relate to "high air temperature and humidity but lower wind velocity" (14.93%), "unclear signs on the country road" (12.19%), "the sky was cloudy or dark" (10.87%), "slippery road caused by rain or snow" (10.51%), "kids were unintended" (10.27%) etc. Most of the wounded received treatment clinically (50.18%), with the percentile (P50) of medical cost as 182.76 RMB (Yuan). The proportion of inpatient was 27.72% and with percentile of time as P(50) = 7.57 day and cost as P(50) = 2840.00 RMB Yuan. The wounded had a rest of P50 = 5.9 days, with an indirect cost as P(50) = 233.16 RMB Yuan. CONCLUSION: The results of this study indicated that the incidence of agricultural profession-related injuries was high, with serious harm. Behavioral intervention and awareness of injuries should be enhanced, together with the improvement of environmental condition
SN - 0254-6450
AD - Department of Epidemiology, School of Public Health, Weifang Medical College, China.
lizhihua@wfmc.edu.cn
UR - 22093464
ER -

TY - JOUR
ID - 227
T1 - Differences in movement-related cortical activation patterns underlying motor performance in children with and without developmental coordination disorder
A1 - Pangelinan,M.M.
A1 - Hatfield,B.D.
A1 - Clark,J.E.
Y1 - 2013/06//
N1 - Pangelinan, Melissa M. Hatfield, Bradley D. Clark, Jane E
Journal of neurophysiology
jc7, 0375404
IM
Journal Article. Research Support, N.I.H., Extramural. Research Support, Non-U.S. Gov't
English
KW - MEDLINE
KW - Age Factors
KW - Biomechanical Phenomena
KW - Brain Waves
KW - Case-Control Studies
KW - Child
KW - Female
KW - Humans
KW - Male
KW - Motor Cortex/gd [Growth & Development]
KW - Motor Cortex/pp [Physiopathology]
KW - Motor Skills
KW - Motor Skills Disorders/pp [Physiopathology]
KW - Movement/ph [Physiology]
KW - Task Performance and Analysis
RP - NOT IN FILE
SP - 3041

EP - 3050
JF - Journal of Neurophysiology
JA - J Neurophysiol
VL - 109
IS - 12
CY - United States
N2 - Behavioral deficits in visuomotor planning and control exhibited by children with developmental coordination disorder (DCD) have been extensively reported. Although these functional impairments are thought to result from "atypical brain development," very few studies to date have identified potential neurological mechanisms. To address this knowledge gap, electroencephalography (EEG) was recorded from 6- to 12-yr-old children with and without DCD (n = 14 and 20, respectively) during the performance of a visuomotor drawing task. With respect to motor performance, typically developing (TD) children exhibited age-related improvements in key aspects of motor planning and control. Although some children with DCD performed outside this TD landscape (i.e., age-related changes within the TD group), the group developmental trajectory of the children with DCD was similar to that of the TD children. Despite overall similarities in performance, engagement of cortical resources in the children with DCD was markedly different from that in their TD counterparts. While the patterns of activation are stable in TD children across the age range, the young children with DCD exhibited less engagement of motor cortical brain areas and the older children with DCD exhibited greater engagement of motor cortical brain areas than their TD peers. These results suggest that older children with DCD may employ a compensatory strategy in which increased engagement of relevant motor resources allows these children to perform comparably to their TD peers. Moreover, the magnitude of activation was related to several kinematic measures, particularly in children with DCD, suggesting that greater engagement in motor resources may underlie better behavioral performance
SN - 1522-1598
AD - Neuroscience and Cognitive Science Program and Department of Kinesiology, University of Maryland, College Park, MD, USA. mpangelinan@research.baycrest.org
UR - 23536712
ER -

TY - JOUR
ID - 228
T1 - Learning from the experts: exploring playground experience and activities using a write and draw technique
A1 - Knowles,Z.R.
A1 - Parnell,D.
A1 - Stratton,G.
A1 - Ridgers,N.D.
Y1 - 2013/03//
N1 - Knowles, Zoe Rebecca. Parnell, Daniel. Stratton, Gareth. Ridgers, Nicola Diane
Journal of physical activity & health
101189457
IM
Journal Article
English
KW - MEDLINE
KW - Child
KW - Child Behavior
KW - England
KW - Female
KW - Humans
KW - Male
KW - Motor Activity
KW - Play and Playthings/px [Psychology]
KW - Qualitative Research
KW - Schools
KW - Writing
RP - NOT IN FILE

SP - 406
EP - 415
JF - Journal of Physical Activity & Health
JA - J Phys Act Health
VL - 10
IS - 3
CY - United States
N2 - BACKGROUND: Qualitative research into the effect of school recess on children's physical activity is currently limited. This study used a write and draw technique to explore children's perceptions of physical activity opportunities during recess. METHODS: 299 children age 7-11 years from 3 primary schools were enlisted. Children were grouped into Years 3 & 4 and Years 5 & 6 and completed a write and draw task focusing on likes and dislikes. Pen profiles were used to analyze the data. RESULTS: Results indicated 'likes' focused on play, positive social interaction, and games across both age groups but showed an increasing dominance of games with an appreciation for being outdoors with age. 'Dislikes' focused on dysfunctional interactions linked with bullying, membership, equipment, and conflict for playground space. Football was a dominant feature across both age groups and 'likes/dislikes' that caused conflict and dominated the physically active games undertaken. CONCLUSION: Recess was important for the development of conflict management and social skills and contributed to physical activity engagement. The findings contradict suggestions that time spent in recess should be reduced because of behavioral issues
SN - 1543-5474
AD - School of Sport and Exercise Science, Liverpool John Moores University, Liverpool, UK
UR - 22820462
ER -

TY - JOUR
ID - 229
T1 - Ethnic differences in the home environment and physical activity behaviors among low-income, minority preschoolers in Texas
A1 - Chuang,R.J.
A1 - Sharma,S.
A1 - Skala,K.
A1 - Evans,A.
Y1 - 2013/03//
N1 - Chuang, Ru-Jye. Sharma, Shreela. Skala, Katie. Evans, Alexandra
American journal of health promotion : AJHP
amj, 8701680
T
Comparative Study. Journal Article. Research Support, Non-U.S. Gov't
English
KW - MEDLINE
KW - African Americans
KW - Child
KW - Preschool
KW - Confidence Intervals
KW - Cross-Sectional Studies
KW - Hispanic Americans
KW - Housing
KW - Humans
KW - Logistic Models
KW - Minority Groups
KW - Motor Activity
KW - Odds Ratio
KW - Play and Playthings
KW - Poverty/eh [Ethnology]
KW - Questionnaires
KW - Television/ut [Utilization]
KW - Texas

RP - NOT IN FILE
SP - 270
EP - 278
JF - American Journal of Health Promotion
JA - Am J Health Promot
VL - 27
IS - 4
CY - United States
N2 - PURPOSE: To evaluate the ethnic differences in the home physical activity and screen time environment of preschoolers enrolled in Head Start. DESIGN: Cross-sectional. SETTING: Sixteen Harris County Department of Education (HCDE) Head Start centers, Texas. SUBJECTS: Seven hundred and six preschoolers enrolled in HCDE Head Start centers and their parents. MEASURES. Items from the parent-reported Healthy Home Survey evaluating the home physical activity environment and the Nutrition Screening form evaluating screen time. ANALYSIS: Mixed model linear and logistic regression analysis. RESULTS: Of the parents, 75.06% were overweight or obese. Hispanic parents were more likely to have a yard or open space (p = .014) and usable play equipment in the yard (p = .019) for their children to play in compared to African-Americans. Conversely, more African-American parents reported having a television (TV) in their preschooler's bedroom (p = .032) and were less likely to cut down their child's TV viewing time (p = .036). African-American preschoolers were reportedly more likely to eat while watching TV (p = .009), play video games for > 2 hours on weekdays (p = .018) and have computer time for > 2 hours on weekends (p = .007), compared to Hispanics. CONCLUSION: The Hispanic participants of this study had a healthier home environment for physical activity and screen time as compared with their African-American counterparts. Future interventions targeting lifestyle change need to address these factors in an ethnic-specific manner
SN - 0890-1171
AD - The University of Texas Health Sciences Center at Houston, School of Public Health, 1200 Herman Pressler, RAS E-603, Houston, TX 77030, USA
UR - 23448417
ER -

TY - JOUR
ID - 230
T1 - Fatalities of pedestrians, bicycle riders, and motorists due to distracted driving motor vehicle crashes in the U.S., 2005-2010
A1 - Stimpson,J.P.
A1 - Wilson,F.A.
A1 - Muelleman,R.L.
Y1 - 2013/11//
N1 - Stimpson, Jim P. Wilson, Fernando A. Muelleman, Robert L
Public health reports (Washington, D.C. : 1974)
9716844, qja
AIM, IM
Journal Article. Research Support, Non-U.S. Gov't
English
KW - MEDLINE
KW - Accidents
KW - Traffic/mo [Mortality]
KW - Traffic/td [Trends]
KW - Adolescent
KW - Adult
KW - Aged
KW - Automobile Driving/sn [Statistics & Numerical Data]
KW - Bicycling/sn [Statistics & Numerical Data]
KW - Child
KW - Preschool
KW - Databases
KW - Factual
KW - Female

KW - Humans
KW - Infant
KW - Newborn
KW - Male
KW - Middle Aged
KW - United States
KW - Walking/sn [Statistics & Numerical Data]
KW - Young Adult
RP - NOT IN FILE
SP - 436
EP - 442
JF - Public Health Reports
JA - Public Health Rep
VL - 128
IS - 6
CY - United States
N2 - OBJECTIVE: Distracted driving is an increasingly deadly threat to road safety. This study documents trends in and characteristics of pedestrian, bicycle rider, and other victim deaths caused by distracted drivers on U.S. public roads. METHODS: We obtained data from the Fatality Analysis Reporting System database from 2005 to 2010 on every crash that resulted in at least one fatality within 30 days occurring on public roads in the U.S. Following the definition used by the National Highway Traffic Safety Administration, we identified distracted driving based on whether police investigators determined that a driver had been using a technological device, including a cell phone, onboard navigation system, computer, fax machine, two-way radio, or head-up display, or had been engaged in inattentive or careless activities. RESULTS: The rate of fatalities per 10 billion vehicle miles traveled increased from 116.1 in 2005 to 168.6 in 2010 for pedestrians and from 18.7 in 2005 to 24.6 in 2010 for bicyclists. Pedestrian victims of distracted driving crashes were disproportionately male, 25-64 years of age, and non-Hispanic white. They were also more likely to die at nighttime, be struck by a distracted driver outside of a marked crosswalk, and be in a metro location. Bicycling victims of distracted crashes were disproportionately male, non-Hispanic white, and struck by a distracted driver outside of a crosswalk. Compared with pedestrians, bicyclists were less likely to be hit in early morning. CONCLUSIONS: Distracted drivers are the cause of an increasing share of fatalities found among pedestrians and bicycle riders. Policies are needed to protect pedestrians and bicycle riders as they cross intersections or travel on roadways
SN - 1468-2877
AD - University of Nebraska Medical Center, Department of Health Services Research and Administration, Omaha, NE
UR - 24179255
ER -

TY - JOUR
ID - 231
T1 - Endothelial damage in long-term survivors of childhood cancer
A1 - Brouwer,C.A.
A1 - Postma,A.
A1 - Hooimeijer,H.L.
A1 - Smit,A.J.
A1 - Vonk,J.M.
A1 - van Roon,A.M.
A1 - van den Berg,M.P.
A1 - Dolsma,W.V.
A1 - Lefrandt,J.D.
A1 - Bink-Boelkens,M.T.
A1 - Zwart,N.
A1 - de Vries,E.G.
A1 - Tissing,W.J.
A1 - Gietema,J.A.
Y1 - 2013/11/01/

N1 - Brouwer, Cornelia A J. Postma, Aleida. Hooimeijer, H Louise H. Smit, Andries J. Vonk, Judith M. van Roon, Arie M. van den Berg, Maarten P. Dolsma, Wil V. Lefrandt, Joop D. Bink-Boelkens, Margreet T E. Zwart, Nynke. de Vries, Elisabeth G E. Tissing, Wim J E. Gietema, Jourik A
Journal of clinical oncology : official journal of the American Society of Clinical Oncology
jco, 8309333
IM
Journal Article. Research Support, Non-U.S. Gov't
English
KW - MEDLINE
KW - Adolescent
KW - Adult
KW - Age of Onset
KW - Antineoplastic Agents/ae [Adverse Effects]
KW - Cardiovascular Diseases/ep [Epidemiology]
KW - Carotid Intima-Media Thickness
KW - Child
KW - Preschool
KW - Endothelium/de [Drug Effects]
KW - Endothelium/pa [Pathology]
KW - Endothelium/re [Radiation Effects]
KW - Female
KW - Humans
KW - Infant
KW - Newborn
KW - Male
KW - Middle Aged
KW - Neoplasms/co [Complications]
KW - Radiotherapy/ae [Adverse Effects]
KW - Survivors/sn [Statistics & Numerical Data]
KW - Young Adult
RP - NOT IN FILE
SP - 3906
EP - 3913
JF - Journal of Clinical Oncology
JA - J Clin Oncol
VL - 31
IS - 31
CY - United States
N2 - PURPOSE: To evaluate the presence of vascular damage in long-term childhood cancer survivors (CCS) and sibling controls, and to evaluate the association between vascular damage parameters and cancer treatment and influence of cardiovascular risk factors. PATIENTS AND METHODS: Vascular assessment was performed in 277 adult CCSs (median age at diagnosis, 9 years; range, 0 to 20 years; median current age, 28 years; range, 18 to 48 years) treated with potentially cardiovascular toxic anticancer treatment (ie, anthracyclines, platinum, and/or radiotherapy [RT]). Measurements included carotid- and femoral-wall intima-media thickness (IMT), flow-mediated vasodilatation of the brachial artery by ultrasound, assessment of endothelial and inflammatory marker proteins (including tissue-type plasminogen activator [t-PA], plasminogen activator inhibitor type 1 [PAI-I]), and cardiovascular risk factors. CCS assessments were compared with those of 130 sibling controls (median age, 26 years; range, 18 to 51 years). RESULTS: At a median of 18 years (range, 5 to 31 years) after treatment, carotid and femoral IMTs in CCSs were not different from those of controls. However, CCSs who received RT as part of their treatment regimen had increased carotid and femoral IMTs and higher t-PA and PAI-I levels, indicating vascular damage and persistent endothelial activation. Patients treated with RT to the neck or chest also had greater femoral IMT. Greater IMT was associated with presence of cardiovascular risk factors (eg, hypertension and overweight). CONCLUSION: After potentially cardiovascular toxic anticancer treatment, CCSs who received RT showed signs of endothelial damage and an unfavorable cardiovascular risk profile compared with controls. CCSs treated with localized RT had increased IMT outside the primary irradiation field. These abnormalities are probably involved in the pathogenesis of cardiovascular morbidity in CCSs

SN - 1527-7755
AD - Cornelia A.J. Brouwer, Aleida Postma, H. Louise H. Hooimeijer, Andries J. Smit, Judith M. Vonk, Arie M. van Roon, Maarten P. van den Berg, Wil V. Dolsma, Joop D. Lefrandt, Margreet T.E. Bink-Boelkens, Nynke Zwart, Wim J.E. Tissing, Elisabeth G.E. de Vries, and Jourik A. Gietema, University Medical Center Groningen, University of Groningen, Groningen, the Netherlands
UR - 24062395
ER -

TY - JOUR
ID - 232
T1 - School participation, supports and barriers of students with and without disabilities
A1 - Coster,W.
A1 - Law,M.
A1 - Bedell,G.
A1 - Liljenquist,K.
A1 - Kao,Y.C.
A1 - Khetani,M.
A1 - Teplicky,R.
Y1 - 2013/07//
N1 - Coster, W. Law, M. Bedell, G. Liljenquist, K. Kao, Y-C. Khetani, M. Teplicky, R
Child: care, health and development
7602632, c9x
IM
Comparative Study. Journal Article. Research Support, U.S. Gov't, Non-P.H.S.
English
KW - MEDLINE
KW - Adolescent
KW - Architectural Accessibility
KW - Canada
KW - Child
KW - Child Development
KW - Preschool
KW - Disabled Children/px [Psychology]
KW - Disabled Children/rh [Rehabilitation]
KW - Environment
KW - Female
KW - Health Resources/st [Standards]
KW - Humans
KW - Male
KW - Parents/px [Psychology]
KW - Questionnaires
KW - Schools
KW - Social Participation/px [Psychology]
KW - Students
KW - United States
RP - NOT IN FILE
SP - 535
EP - 543
JF - Child: Care, Health & Development
JA - Child Care Health Dev
VL - 39
IS - 4
CY - England
N2 - BACKGROUND: We compared school participation patterns of students ages 5-17 with and without disabilities and examined whether features of the school environment were perceived to help or hinder their participation. METHODS: Parents (n = 576) residing in the USA and Canada completed the Participation and Environment Measure for Children and Youth (PEM-CY) via the internet. RESULTS: Parents of students with

disabilities reported that their children participated less frequently in school clubs and organizations and getting together with peers outside the classroom and that they were less involved in all school activities. Parents of students with disabilities also were significantly more likely to report that features of the environment hindered school participation and that resources needed to support their child's participation were not adequate. CONCLUSIONS: Parents of students with disabilities report that their children are participating less in important school-related activities. Barriers limiting school participation include features of the physical and social environment as well as limited resources. 2013 John Wiley & Sons Ltd
SN - 1365-2214
AD - Department of Occupational Therapy, Boston University, 635 Commonwealth Avenue, Boston, MA 02215, USA. wjcoster@bu.edu
UR - 23763254
ER -

TY - JOUR
ID - 233
T1 - Is school community readiness related to physical activity before and after the Ready for Recess intervention?
A1 - Ehlers,D.K.
A1 - Huberty,J.L.
A1 - Beseler,C.L.
Y1 - 2013/04//
N1 - Ehlers, Diane K. Huberty, Jennifer L. Beseler, Cheryl L
Health education research
bqp, 8608459
T
Journal Article. Research Support, Non-U.S. Gov't
English
KW - MEDLINE
KW - Child
KW - Female
KW - Humans
KW - Interviews as Topic
KW - Male
KW - Motor Activity
KW - Obesity/pc [Prevention & Control]
KW - Play and Playthings
KW - Residence Characteristics
KW - School Health Services
KW - Schools/og [Organization & Administration]
KW - Schools
RP - NOT IN FILE
SP - 192
EP - 204
JF - Health Education Research
JA - Health Educ Res
VL - 28
IS - 2
CY - England
N2 - The purpose of this study was to determine: (i) the effect of schools' baseline community readiness (CR) on youth physical activity (PA) at recess prior to the Ready for Recess intervention; (ii) if changes in PA due to the intervention were explained by baseline CR and (iii) if specific components of the intervention altered an association between baseline CR and changes in youth PA. Methods that were employed included: six informants from each of 17 schools participated in CR interviews at baseline (N = 101). CR scores were calculated based on the CR model's nine stages. Direct observation was used to measure PA. Poisson models evaluated the association between baseline CR and PA. Results were that seven schools were in denial, eight in vague awareness and two in pre-planning stages. CR marginally predicted pre-intervention PA. When compared with youth at schools in denial, youth at schools in vague awareness and pre-planning demonstrated significant

increases in moderate/vigorous PA. Ready for Recess strengthened this association. This is the first study to demonstrate that school CR may explain changes in PA at recess after a school-based PA intervention. Low CR levels may contraindicate interventions. Efforts to increase school CR a priori may be critical to increasing PA among youth
SN - 1465-3648
AD - Department of Health, Physical Education, and Recreation, University of Nebraska, Omaha, NE 68182, USA. dehlers@unomaha.edu
UR - 23107932
ER -

TY - JOUR
ID - 234
T1 - Rifamycins (rifampicin, rifabutin and rifapentine) compared to isoniazid for preventing tuberculosis in HIV-negative people at risk of active TB. [Review]
A1 - Sharma,S.K.
A1 - Sharma,A.
A1 - Kadhiravan,T.
A1 - Tharyan,P.
Y1 - 2013///
N1 - Sharma, Surendra K. Sharma, Anju. Kadhiravan, Tamilarasu. Tharyan, Prathap
The Cochrane database of systematic reviews
100909747
IM
Journal Article. Meta-Analysis. Research Support, Non-U.S. Gov't. Review
English
KW - MEDLINE
KW - Adult
KW - Antibiotics
KW - Antitubercular/tu [Therapeutic Use]
KW - Child
KW - Directly Observed Therapy
KW - Drug Administration Schedule
KW - HIV Seronegativity
KW - Humans
KW - Isoniazid/tu [Therapeutic Use]
KW - Latent Tuberculosis/dt [Drug Therapy]
KW - Randomized Controlled Trials as Topic
KW - Rifabutin/tu [Therapeutic Use]
KW - Rifampin/aa [Analogs & Derivatives]
KW - Rifampin/tu [Therapeutic Use]
KW - Tuberculosis
KW - Pulmonary/pc [Prevention & Control]
RP - NOT IN FILE
SP - CD007545
JF - Cochrane Database of Systematic Reviews
JA - Cochrane Database Syst Rev
VL - 7
CY - England
N2 - BACKGROUND: Preventing active tuberculosis (TB) from developing in people with latent tuberculosis infection (LTBI) is important for global TB control. Isoniazid (INH) for six to nine months has 60% to 90% protective efficacy, but the treatment period is long, liver toxicity is a problem, and completion rates outside trials are only around 50%. Rifampicin or rifamycin-combination treatments are shorter and may result in higher completion rates. OBJECTIVES: To compare the effects of rifampicin monotherapy or rifamycin-combination therapy versus INH monotherapy for preventing active TB in HIV-negative people at risk of developing active TB. SEARCH METHODS: We searched the Cochrane Infectious Disease Group Specialized Register; Cochrane Central Register of Controlled Trials (CENTRAL); MEDLINE; EMBASE; LILACS; clinical trials registries; regional databases; conference proceedings; and references, without language restrictions to

December 2012; and contacted experts for relevant published, unpublished and ongoing trials. SELECTION CRITERIA: Randomized controlled trials (RCTs) of HIV-negative adults and children at risk of active TB treated with rifampicin, or rifamycin-combination therapy with or without INH (any dose or duration), compared with INH for six to nine months. DATA COLLECTION AND ANALYSIS: At least two authors independently screened and selected trials, assessed risk of bias, and extracted data. We sought clarifications from trial authors. We pooled relative risks (RRs) with their 95% confidence intervals (CIs), using a random-effects model if heterogeneity was significant. We assessed overall evidence quality using the GRADE approach. MAIN RESULTS: Ten trials are included, enrolling 10,717 adults and children, mostly HIV-negative (2% HIV-positive), with a follow-up period ranging from two to five years. Rifampicin (three/four months) vs. INH (six months)Five trials published between 1992 to 2012 compared these regimens, and one small 1992 trial in adults with silicosis did not detect a difference in the occurrence of TB over five years of follow up (one trial, 312 participants; very low quality evidence). However, more people in these trials completed the shorter course (RR 1.19, 95% CI 1.01 to 1.30; five trials, 1768 participants; moderate quality evidence). Treatment-limiting adverse events were not significantly different (four trials, 1674 participants; very low quality evidence), but rifampicin caused less hepatotoxicity (RR 0.12, 95% CI 0.05 to 0.30; four trials, 1674 participants; moderate quality evidence). Rifampicin plus INH (three months) vs. INH (six months)The 1992 silicosis trial did not detect a difference between people receiving rifampicin plus INH compared to INH alone for occurrence of active TB (one trial, 328 participants; very low quality evidence). Adherence was similar in this and a 1998 trial in people without silicosis (two trials, 524 participants; high quality evidence). No difference was detected for treatment-limiting adverse events (two trials, 536 participants; low quality evidence), or hepatotoxicity (two trials, 536 participants; low quality evidence). Rifampicin plus pyrazinamide (two months) vs. INH (six months)Three small trials published in 1994, 2003, and 2005 compared these two regimens, and two reported a low occurrence of active TB, with no statistically significant differences between treatment regimens (two trials, 176 participants; very low quality evidence) though, apart from one child from the 1994 trial, these data on active TB were from the 2003 trial in adults with silicosis. Adherence with both regimens was low with no statistically significant differences (four trials, 700 participants; very low quality evidence). However, people receiving rifampicin plus pyrazinamide had more treatment-limiting adverse events (RR 3.61, 95% CI 1.82 to 7.19; two trials, 368 participants; high quality evidence), and hepatotoxicity (RR 4.59, 95% 2.14 to 9.85; three trials, 540 participants; moderate quality evidence). Weekly, directly-observed rifapentine plus INH (three months) vs. daily, self-administered INH (nine months)A large trial conducted from 2001 to 2008 among close contacts of TB in the USA, Canada, Brazil and Spain found directly observed weekly treatment to be non-inferior to nine months self-administered INH for the incidence of active TB (0.2% vs 0.4%, RR 0.44, 95% CI 0.18 to 1.07, one trial, 7731 participants; moderate quality evidence). The directly-observed, shorter regimen had higher treatment completion (82% vs 69%, RR 1.19, 95% CI 1.16 to 1.22, moderate quality evidence), and less hepatotoxicity (0.4% versus 2.4%; RR 0.16, 95% CI 0.10 to 0.27; high quality evidence), though treatment-limiting adverse events were more frequent (4.9% versus 3.7%; RR 1.32, 95% CI 1.07 to 1.64 moderate quality evidence) AUTHORS' CONCLUSIONS: Trials to date of shortened prophylactic regimens using rifampicin alone have not demonstrated higher rates of active TB when compared to longer regimens with INH. Treatment completion is probably higher and adverse events may be fewer with shorter rifampicin regimens. Shortened regimens of rifampicin with INH may offer no advantage over longer INH regimens. Rifampicin combined with pyrazinamide is associated with more adverse events. A weekly regimen of rifapentine plus INH has higher completion rates, and less liver toxicity, though treatment discontinuation due to adverse events is probably more likely than with INH
SN - 1469-493X
AD - Department of Medicine, All India Institute of Medical Sciences, New Delhi, India. sksharma.aiims@gmail.com
UR - 23828580
ER -

TY - JOUR
ID - 235
T1 - An epidemiological study of cutaneous leishmaniasis in Al-jabal Al-gharbi, Libya
A1 - Abdellatif,M.Z.
A1 - El-Mabrouk,K.
A1 - Ewis,A.A.
Y1 - 2013/02//

N1 - Abdellatif, Manal Z M. El-Mabrouk, Khamis. Ewis, Ashraf A
The Korean journal of parasitology
b3d, 9435800
IM
Journal Article
English
KW - MEDLINE
KW - Adolescent
KW - Adult
KW - Age Factors
KW - Animal Husbandry
KW - Animals
KW - Child
KW - Preschool
KW - Female
KW - Humans
KW - Leishmaniasis
KW - Cutaneous/ep [Epidemiology]
KW - Libya/ep [Epidemiology]
KW - Male
KW - Occupational Exposure
KW - Risk Factors
KW - Sex Factors
RP - NOT IN FILE
SP - 75
EP - 84
JF - Korean Journal of Parasitology
JA - Korean J Parasitol
VL - 51
IS - 1
CY - Korea (South)
N2 - Cutaneous leishmaniasis (CL) is an endemic parasitic infection in the Mediterranean region, including Libya and its Al-jabal Al-gharbi province. We aimed at studying the occupational relevance as well as other epidemiological aspects of CL. We investigated 140 CL cases who attended at Gharyan outpatient polyclinic during a period of 6 months in 2009. CL infection was clinically diagnosed and confirmed by demonstration of Leishmania parasites on smears from lesions. Our findings showed that males were more affected than females (P=0.04), and people above 10-years were more affected than younger ones (P=0.0001). A significant percent of CL cases belonged to Al-Kawasem subprovince (P=0.0001). Farm-related activities were the most frequent occupations among CL cases (P=0.04). In addition to farm workers, housewives and students are at risk groups since they are engaged at farm activities. Moreover, those who have occupations that require staying outdoors for a part of night, e.g., policemen, are also at risk. Compared to children, adult CL patients had multiple lesions (P=0.001) that were more prevalent in their upper and lower extremities than the face (P=0.0001). We conclude that CL is a major health problem in Al-jabal Al-gharbi province of Libya. The presence of rodents and sandflies makes it a suitable environment for Leishmania to spread in an endemic epidemiological pattern. Being engaged in farming activities or outdoor occupations increases the risk of infection. Various clinical patterns of CL suggest the presence of more than 1 species of Leishmania at Al-jabal Al-gharbi province. We propose that the 2 species responsible for CL in this area are L. major and L. tropica. Further investigations to identify the leishmanial species responsible for CL at Al-jabal Al-gharbi together with adoption of preventive and control programs are needed
SN - 1738-0006
AD - Department of Parasitology, Faculty of Medicine, Al-jabal Al-gharbi University, Gharyan, Libya
UR - 23467624
ER -

TY - JOUR
ID - 236

T1 - Does playground improvement increase physical activity among children? A quasi-experimental study of a natural experiment
A1 - Bohn-Goldbaum,E.E.
A1 - Phongsavan,P.
A1 - Merom,D.
A1 - Rogers,K.
A1 - Kamalesh,V.
A1 - Bauman,A.E.
Y1 - 2013///
N1 - Bohn-Goldbaum, Erika E. Phongsavan, Philayrath. Merom, Dafna. Rogers, Kris. Kamalesh, Venugopal. Bauman, Adrian E
Journal of environmental and public health
101516361
IM
Journal Article. Research Support, Non-U.S. Gov't
English
KW - MEDLINE
KW - Child
KW - Preschool
KW - Cities
KW - Cross-Sectional Studies
KW - Environment
KW - Equipment and Supplies
KW - Female
KW - Humans
KW - Male
KW - Motor Activity
KW - New South Wales
KW - Play and Playthings
KW - Questionnaires
KW - Socioeconomic Factors
RP - NOT IN FILE
SP - 109841
JF - Journal Of Environmental & Public Health
JA - J Environ Public Health
VL - 2013
CY - United States
N2 - Outdoor recreational spaces have the potential to increase physical activity. This study used a quasi-experimental evaluation design to determine how a playground renovation impacts usage and physical activity of children and whether the visitations correlate with children's physical activity levels and parental impressions of the playground. Observational data and intercept interviews were collected simultaneously on park use and park-based activity among playground visitors at pre- and postrenovation at an intervention and a comparison park during three 2-hour periods each day over two weeks. No detectable difference in use between parks was observed at followup. In the intervention park, attendance increased among boys, but decreased among girls although this (nonsignificant) decline was less marked than in the comparison park. Following renovation, there was no detectable difference between parks in the number of children engaged in MVPA (interaction between park and time: P = 0.73). At the intervention park, there was a significant decline in girls engaging in MVPA at followup (P = 0.04). Usage was correlated with parental/carer perceptions of playground features but not with physical activity levels. Renovations have limited the potential to increase physical activity until factors influencing usage and physical activity behavior are better understood
SN - 1687-9813
AD - Prevention Research Collaboration, School of Public Health, The University of Sydney, NSW 2006, Australia. erika.goldbaum@sydney.edu.au
UR - 23840227
ER -

TY - JOUR

ID - 237
T1 - Formative research on hygiene behaviors and geophagy among infants and young children and implications of exposure to fecal bacteria
A1 - Ngure,F.M.
A1 - Humphrey,J.H.
A1 - Mbuya,M.N.
A1 - Majo,F.
A1 - Mutasa,K.
A1 - Govha,M.
A1 - Mazarura,E.
A1 - Chasekwa,B.
A1 - Prendergast,A.J.
A1 - Curtis,V.
A1 - Boor,K.J.
A1 - Stoltzfus,R.J.
Y1 - 2013/10//
N1 - Ngure, Francis M. Humphrey, Jean H. Mbuya, Mduduzi N N. Majo, Florence. Mutasa, Kuda. Govha, Margaret. Mazarura, Exevia. Chasekwa, Bernard. Prendergast, Andrew J. Curtis, Valerie. Boor, Kathyrn J. Stoltzfus, Rebecca J
The American journal of tropical medicine and hygiene
3zq, 0370507
AIM, IM
Journal Article. Research Support, Non-U.S. Gov't
English
KW - MEDLINE
KW - Adult
KW - Animals
KW - Chickens
KW - Child
KW - Preschool
KW - Environmental Exposure
KW - Environmental Microbiology
KW - Feces/mi [Microbiology]
KW - Female
KW - Humans
KW - Hygiene/st [Standards]
KW - Infant
KW - Male
KW - Risk Factors
KW - Soil
KW - Soil Microbiology
KW - Young Adult
KW - Zimbabwe
RP - NOT IN FILE
SP - 709
EP - 716
JF - American Journal of Tropical Medicine & Hygiene
JA - Am J Trop Med Hyg
VL - 89
IS - 4
CY - United States
N2 - We conducted direct observation of 23 caregiver-infant pairs for 130 hours and recorded wash-related behaviors to identify pathways of fecal-oral transmission of bacteria among infants. In addition to testing fingers, food, and drinking water of infants, three infants actively ingested 11.3 + 9.2 (mean + SD) handfuls of soil and two ingested chicken feces 2 + 1.4 times in 6 hours. Hand washing with soap was not common and drinking water was contaminated with Escherichia coli in half (12 of 22) of the households. A one-year-old infant ingesting 1 gram of chicken feces in a day and 20 grams of soil from a laundry area of the kitchen yard

would consume 4,700,000-23,000,000 and 440-4,240 E. coli, respectively, from these sources. Besides standard wash and nutrition interventions, infants in low-income communities should be protected from exploratory ingestion of chicken feces, soil, and geophagia for optimal child health and growth
SN - 1476-1645
AD - Division of Nutritional Sciences, Cornell University, Ithaca, New York; Department of International Health, Johns Hopkins Bloomberg School of Public Health, Johns, Baltimore, Maryland; Zvitambo Project, Harare, Zimbabwe; Centre for Pediatrics, Queen Mary University of London, London, United Kingdom; The Hygiene Centre, London School of Hygiene and Tropical Medicine, London, United Kingdom
UR - 24002485
ER -

TY - JOUR
ID - 238
T1 - Nonmedical vaccine exemptions and pertussis in California, 2010
A1 - Atwell,J.E.
A1 - Van,Otterloo J.
A1 - Zipprich,J.
A1 - Winter,K.
A1 - Harriman,K.
A1 - Salmon,D.A.
A1 - Halsey,N.A.
A1 - Omer,S.B.
Y1 - 2013/10//
N1 - Atwell, Jessica E. Van Otterloo, Josh. Zipprich, Jennifer. Winter, Kathleen. Harriman, Kathleen. Salmon, Daniel A. Halsey, Neal A. Omer, Saad B
Pediatrics
oxv, 0376422
AIM, IM
Journal Article. Research Support, U.S. Gov't, P.H.S.
English
KW - MEDLINE
KW - California/ep [Epidemiology]
KW - Child
KW - Preschool
KW - Cluster Analysis
KW - Culture
KW - Female
KW - Humans
KW - Male
KW - Pertussis Vaccine/tu [Therapeutic Use]
KW - Vaccination/es [Ethics]
KW - Vaccination
KW - Whooping Cough/di [Diagnosis]
KW - Whooping Cough/ep [Epidemiology]
KW - Whooping Cough/pc [Prevention & Control]
RP - NOT IN FILE
SP - 624
EP - 630
JF - Pediatrics
VL - 132
IS - 4
CY - United States
N2 - BACKGROUND: In 2010, 9120 cases of pertussis were reported in California, more than any year since 1947. Although this resurgence has been widely attributed to waning immunity of the acellular vaccine, the role of vaccine refusal has not been explored in the published literature. Many factors likely contributed to the outbreak, including the cyclical nature of pertussis, improved diagnosis, and waning immunity; however, it is important to understand if clustering of unvaccinated individuals also played a role. METHODS: We analyzed

nonmedical exemptions (NMEs) for children entering kindergarten from 2005 through 2010 and pertussis cases with onset in 2010 in California to determine if NMEs increased in that period, if children obtaining NMEs clustered spatially, if pertussis cases clustered spatially and temporally, and if there was statistically significant overlap between clusters of NMEs and cases. RESULTS: Kulldorff's scan statistics identified 39 statistically significant clusters of high NME rates and 2 statistically significant clusters of pertussis cases in this time period. Census tracts within an exemptions cluster were 2.5 times more likely to be in a pertussis cluster (odds ratio = 2.47, 95% confidence interval: 2.22-2.75). More cases occurred within as compared with outside exemptions clusters (incident rate ratios = 1.20, 95% confidence interval: 1.10-1.30). The association remained significant after adjustment for demographic factors. NMEs clustered spatially and were associated with clusters of pertussis cases. CONCLUSIONS: Our data suggest clustering of NMEs may have been 1 of several factors in the 2010 California pertussis resurgence
SN - 1098-4275
AD - MBBS, 1518 Clifton Rd, NE, Room 7017, Atlanta, GA 30322. somer@emory.edu
UR - 24082000
ER -

TY - JOUR
ID - 239
T1 - Reference levels in the context of Fukushima and related lessons learned
A1 - Sakai,K.
Y1 - 2013/11//
N1 - Sakai, Kazuo
Health physics
g2h, 2985093r
IM
Journal Article
English
KW - MEDLINE
KW - Child
KW - Emergencies
KW - Expert Testimony
KW - Female
KW - Fukushima Nuclear Accident
KW - Health Knowledge
KW - Attitudes
KW - Practice
KW - Humans
KW - Pregnancy
KW - Radiation Protection
KW - Radiometry/st [Standards]
KW - Reference Standards
RP - NOT IN FILE
SP - 466
EP - 468
JF - Health Physics
JA - Health Phys
VL - 105
IS - 5
CY - United States
N2 - About 1 mo after the Fukushima Dai-ichi nuclear power plant accident, which was caused by the Great East Japan earthquake and tsunami on 11 March 2011, Japanese authorities set a dose criterion for the use of school playgrounds in Fukushima at 20 mSv y- based on the International Commission on Radiological Protection recommendation for the reference level for the public under the existing exposure situation. This dose criterion was intended as a start line for reducing the dose to children; however, it caused much confusion among the public due to the misunderstanding of the concept of optimization and the application of reference level. Also, concerns were caused by the lack of precise but understandable information on radiation effects. This situation highlighted the importance of an understanding of radiation protection concepts by members of

the general public and the outreach activities of radiation protection experts, both of which are essential for Fukushima recovery
SN - 1538-5159
AD - *National Institute of Radiological Sciences, Research Center for Radiation Protection, 4-9-1 Anagawa, Inage-ku, Chiba 263-8555, Japan
UR - 24077049
ER -

TY - JOUR
ID - 240
T1 - Can the reduction of pediatric injury rates be sustained using a community-based approach?
A1 - Pomerantz,W.J.
A1 - Gittelman,M.A.
A1 - Ho,M.
A1 - Hornung,R.W.
Y1 - 2013/10//
N1 - Pomerantz, Wendy J. Gittelman, Michael A. Ho, Mona. Hornung, Richard W
The journal of trauma and acute care surgery
101570622
AIM, IM
Journal Article
English
KW - MEDLINE
KW - Accident Prevention
KW - Adolescent
KW - Age Factors
KW - Case-Control Studies
KW - Child
KW - Preschool
KW - Cohort Studies
KW - Female
KW - Humans
KW - Infant
KW - Newborn
KW - Male
KW - Ohio
KW - Preventive Health Services/og [Organization & Administration]
KW - Program Evaluation
KW - Time Factors
KW - Wounds and Injuries/ep [Epidemiology]
KW - Wounds and Injuries/pc [Prevention & Control]
KW - Young Adult
RP - NOT IN FILE
SP - S276
EP - S280
JF - The Journal of Trauma and Acute Care Surgery
JA - J Trauma Acute Care Surg
VL - 75
IS - 4 Suppl 3
CY - United States
N2 - BACKGROUND: Injuries are the leading cause of morbidity and mortality in US residents aged 1 to 44 years. Community-based interventions are effective in reducing injuries. Using this approach, investigators significantly reduced injuries in Avondale, Ohio, between 1999 and 2004 compared with three control communities (42 vs. 15%, respectively). The objective of this study was to determine if injury reduction was sustained through the 5 years after initial implementation of injury prevention (IP) efforts in Avondale compared with the same three control communities. DESIGN/METHODS: Injury prevention interventions implemented in Avondale, Ohio, during previous study years were sustained. Two new playgrounds were built,

but no other new interventions were introduced. Control communities had no programs introduced by the team during the study period. Data were obtained from the Hamilton County Injury Surveillance System from 2005 to 2009 for deaths, hospitalizations, and emergency department visits due to injury for children 0 to 19 years old. Data from the study community were compared with those of the same three control communities as in our previous work, with similar demographics and socioeconomic characteristics, as well as with data obtained previously from 1999 to 2004. Census data based on annual estimates were used to calculate injury rates. RESULTS: The injury rate in Avondale decreased from 17,073 to 11,284 injuries per 100,000 children per year during the 11-year period (33.9% reduction). The injury rate in the control communities decreased from 14,436 to 12,381 injuries per 100,000 children per year in the same period (14.2% reduction). The difference in the injury rate decrease between the intervention and control communities was statistically significant, $p < 0.001$. From 2005 to 2009, the lower injury rate was sustained in Avondale, $p = 0.58$. CONCLUSIONS: Community-based strategies to prevent injuries to children in high-risk communities can be successful in reducing overall injury rates. These efforts can result in sustained injury reduction over time despite no new interventions being introduced
SN - 2163-0763
UR - 23702627
ER -

TY - JOUR
ID - 241
T1 - Physical activity, adiposity and urbanization level in children: results for the Italian cohort of the IDEFICS study
A1 - Donatiello,E.
A1 - Dello,Russo M.
A1 - Formisano,A.
A1 - Lauria,F.
A1 - Nappo,A.
A1 - Reineke,A.
A1 - Sparano,S.
A1 - Barba,G.
A1 - Russo,P.
A1 - Siani,A.
Y1 - 2013/08//
N1 - Donatiello, E. Dello Russo, M. Formisano, A. Lauria, F. Nappo, A. Reineke, A. Sparano, S. Barba, G. Russo, P. Siani, A
Public health
qi7, 0376507
IM
Journal Article. Research Support, Non-U.S. Gov't
English
KW - MEDLINE
KW - Adiposity
KW - Child
KW - Preschool
KW - Cross-Sectional Studies
KW - Female
KW - Humans
KW - Italy/ep [Epidemiology]
KW - Male
KW - Motor Activity
KW - Pediatric Obesity/ep [Epidemiology]
KW - Questionnaires
KW - Rural Health/sn [Statistics & Numerical Data]
KW - Skinfold Thickness
KW - Urban Health/sn [Statistics & Numerical Data]
KW - Urbanization
RP - NOT IN FILE

SP - 761
EP - 765
JF - Public Health
VL - 127
IS - 8
CY - Netherlands
N2 - OBJECTIVES: While there is extensive evidence about the influence of environmental factors on adult obesity, fewer studies have assessed how the environment influences body fat in children. This cross-sectional study investigated the distribution of adiposity indices according to urbanization level and patterns of physical activity among children in the Italian cohort of the IDEFICS study. METHODS: The sample included 1673 preschool and school-aged children (mean age 6.1 years, standard deviation 1.7) living in rural (n = 579), suburban (n = 442) and urban (n = 652) areas. Anthropometric measures were taken and questionnaires were used to assess children's lifestyles, including patterns of physical activity. RESULTS: Children who lived in rural areas spent significantly more time in outdoor activities but participated in less structured physical activity compared with children living in suburban and urban areas. Adiposity estimated by the sum of skinfold thickness increased linearly from rural to urban areas, with results for suburban areas showing intermediate values. CONCLUSIONS: The data show that geographical environmental factors influence patterns of physical activity and body fat in children. In particular, the results suggest an association between the time spent in unstructured outdoor activities and the degree of adiposity in schoolchildren. These results may have implications for public health, including efforts to increase freely available playgrounds as an effective measure to counteract the obesity epidemic in children. Copyright 2013 The Royal Society for Public Health. Published by Elsevier Ltd. All rights reserved
SN - 1476-5616
AD - Epidemiology and Population Genetics, Institute of Food Sciences, CNR, Avellino, Italy
UR - 23876300
ER -

TY - JOUR
ID - 242
T1 - Seasonal and locational variations in children's play: implications for wellbeing
A1 - Ergler,C.R.
A1 - Kearns,R.A.
A1 - Witten,K.
Y1 - 2013/08//
N1 - Ergler, Christina R. Kearns, Robin A. Witten, Karen
Social science & medicine (1982)
ut9, 8303205
IM
Journal Article. Research Support, Non-U.S. Gov't
English
KW - MEDLINE
KW - Child
KW - Child Welfare
KW - Female
KW - Geography
KW - Medical
KW - Humans
KW - Male
KW - New Zealand
KW - Play and Playthings/px [Psychology]
KW - Play and Playthings
KW - Residence Characteristics/sn [Statistics & Numerical Data]
KW - Seasons
RP - NOT IN FILE
SP - 178
EP - 185
JF - Social Science & Medicine

JA - Soc Sci Med
VL - 91
CY - England
N2 - Physical activity, through independent outdoor play, has come to the fore as a way to improve children's health through it fostering healthy mental and social as well as physiological development. However, in many high-income countries children's autonomous play opportunities have diminished due to urban intensification and declining parental license. Regardless of this trend, children's play varies across countries, cities, cultures and seasons. This paper offers new insights into the complexities of play as a vital aspect of children's wellbeing. Within the context of New Zealand - whose citizens generally regard themselves as outdoor people - this paper explores why 'play' might resonate differently across localities and seasons. We contrast the play affordances provided by Auckland's central city (dominated by apartment living) with Beach Haven, a suburban area. We employed a multi-method approach and included 20 children and their parents who were recruited through school and summer holiday programs embracing different gender and ethnicities to reflect the general cultural mix of the respective neighbourhoods. We advance two arguments. First, we suggest that the rarity of children playing outdoors unsupervised normalises supervised indoor play and reduces children's opportunities to see outdoor play as an alternative to interior or supervised pastimes. Second, we follow Bourdieu's theory of practice to argue that the regard parents and children have towards outdoor play reflects locally constituted beliefs about what is seasonally 'appropriate' children's activity. We found that extra-curricular activities and supervised excursions are undertaken in the central city all year around and only vary between social groups by the type of destination. In the suburb, independent outdoor play in summer represents children's main business after school in ways that enhance their environmental literacy and potential future health gain. For others these symbolic values were replaced by safety concerns. In contrast, it seems that even in a relatively mild climate winter is the time to relax and stay indoors unless children have an outdoor habitus. We find that the determinants of seasonal outdoor play transcend modifiable barriers such as traffic and unsuitable play spaces as well as the inevitable issue of inclement weather. Copyright 2012 Elsevier Ltd. All rights reserved
SN - 1873-5347
AD - The University of Auckland, School of Environment, Private Bag 90219, Auckland 1142, New Zealand. c.ergler@auckland.ac.nz
UR - 23312793
ER -

TY - JOUR
ID - 243
T1 - Assessment of human health risks from heavy metals in outdoor dust samples in a coal mining area
A1 - Rout,T.K.
A1 - Masto,R.E.
A1 - Ram,L.C.
A1 - George,J.
A1 - Padhy,P.K.
Y1 - 2013/06//
N1 - Rout, Tofan Kumar. Masto, R E. Ram, L C. George, Joshy. Padhy, Pratap Kumar
Environmental geochemistry and health
8903118
IM
Journal Article. Research Support, Non-U.S. Gov't
English
KW - MEDLINE
KW - Adolescent
KW - Adult
KW - Age Factors
KW - Aged
KW - Child
KW - Preschool
KW - Coal Mining
KW - Dust/an [Analysis]
KW - Environmental Exposure/ae [Adverse Effects]
KW - Environmental Monitoring/mt [Methods]

KW - Environmental Pollutants/an [Analysis]
KW - Environmental Pollutants/to [Toxicity]
KW - Environmental Pollution/ae [Adverse Effects]
KW - Environmental Pollution/an [Analysis]
KW - Humans
KW - India
KW - Infant
KW - Metals
KW - Heavy/an [Analysis]
KW - Heavy/to [Toxicity]
KW - Middle Aged
KW - Risk Assessment
KW - Young Adult
RP - NOT IN FILE
SP - 347
EP - 356
JF - Environmental Geochemistry & Health
JA - Environ Geochem.Health
VL - 35
IS - 3
CY - Netherlands
N2 - Jharia (India) a coal mining town has been affected by the consequences of mining and associated activities. Samples of outdoor fallen dust were collected at different locations of Jharia covering four different zones: commercial, petrol pump, high traffic, and residential areas. The dust samples were analysed for different trace elements (As, Cd, Co, Cr, Cu, Mn, Ni, Pb, Se, and Zn). The highest concentration of the elements in the dust samples are Mn (658 mg/kg), Zn (163.6 mg/kg), Cr (75.4 mg/kg), Pb (67.8 mg/kg), Ni (66 mg/kg), Cu (56.8 mg/kg), Co (16.9 mg/kg), As (4.1 mg/kg), and Cd (0.78 mg/kg). The concentration of selenium was below detection limit. Except Cd, contents of all the other elements in the dust samples were significantly lower in the residential area. High amount of Ni (145 mg/kg) and Pb (102 mg/kg) was observed in the high traffic and petrol pump areas, respectively. The exposure risk assessment strategies are helpful in predicting the potential health risk of the trace elements in the street dust. Selected receptors for risk assessment were infants, toddlers, children, teens, and adults. The calculated hazard quotient (HQ) for lifetime exposure was <1.0 for all the elements studied, indicating no risks from these elements for adults Among the receptors, toddlers were found to be more vulnerable, with HQ for Co, Cr, and Pb > 0.1. The finding predicts potential health risk to toddlers and children
SN - 1573-2983
AD - Environmental Management Division, CSIR-Central Institute of Mining and Fuel Research (Digwadih Campus), Dhanbad, 828108, India
UR - 23129348
ER -

TY - JOUR
ID - 244
T1 - A preliminary randomized controlled study on the effectiveness of vestibular-specific neuromuscular training in children with hearing impairment
A1 - Rajendran,V.
A1 - Roy,F.G.
A1 - Jeevanantham,D.
Y1 - 2013/05//
N1 - Rajendran, Venkadesan. Roy, Finita Glory. Jeevanantham, Deepa
Clinical rehabilitation
cr7, 8802181
IM
Journal Article. Randomized Controlled Trial
English
KW - MEDLINE
KW - Child

KW - Exercise Therapy/mt [Methods]
KW - Hearing Loss/pp [Physiopathology]
KW - Hearing Loss/rh [Rehabilitation]
KW - Humans
KW - Motor Skills/ph [Physiology]
KW - Postural Balance/ph [Physiology]
KW - Quality of Life
KW - Sickness Impact Profile
KW - Vestibule
KW - Labyrinth/pp [Physiopathology]
RP - NOT IN FILE
SP - 459
EP - 467
JF - Clinical Rehabilitation
JA - Clin Rehabil
VL - 27
IS - 5
CY - England
N2 - OBJECTIVE: To determine the effectiveness of vestibular-specific neuromuscular training on motor skills, balance and health-related quality of life in children with hearing impairment. DESIGN: Controlled, randomized, preliminary study. SETTING: Rehabilitation school for children with hearing impairment. SUBJECTS AND INTERVENTION: Twenty-three children with mean age of 7.5-8.1 years with hearing impairment were randomized to either the intervention or the control group. Children in the experimental group (n = 11) participated in vestibular-specific neuromuscular training for six weeks and the children in the control group continued their regular activities followed at school. OUTCOME MEASURES: Measurement of motor skills (Test of Gross Motor Development-2), postural control (Pediatric Reach Test, One Leg Standing Balance Test and postural sway meter) and health-related quality of life (PedsQL Generic Core Scale). RESULTS: Following intervention, the scores of motor skills (Test of Gross Motor Development P = 0.02; throw for distance P = 0.042; kick for distance P = 0.08; jump for distance P = 0.001; 15-yard dash P = 0.001), postural control measures (Pediatric Reach Test P = 0.001; One Leg Standing Test P = 0.03; and anteroposterior sway (eyes open P = 0.007, eyes closed P = 0.03); mediolateral sway (eyes open P = 0.014, eyes closed P = 0.017) and health-related quality of life (P = 0.01) improved significantly in the experimental group and not in the control group. CONCLUSION: The findings of the study suggest that vestibular-specific neuromuscular training programme may improve the motor skills, balance and health-related quality of life in children with hearing impairment
SN - 1477-0873
AD - Institute of Rehabilitation Science, Holy Cross College affiliated to Bharathidasan University, Trichy, Tamilnadu, India. venkadphysio@gmail.com
UR - 23076993
ER -

TY - JOUR
ID - 245
T1 - Associations between neighborhood resources and physical activity in inner-city minority children
A1 - Galvez,M.P.
A1 - McGovern,K.
A1 - Knuff,C.
A1 - Resnick,S.
A1 - Brenner,B.
A1 - Teitelbaum,S.L.
A1 - Wolff,M.S.
Y1 - 2013/01//
N1 - Galvez, Maida P. McGovern, Kathleen. Knuff, Catherine. Resnick, Susan. Brenner, Barbara. Teitelbaum, Susan L. Wolff, Mary S
Academic pediatrics
101499145
IM

Journal Article. Research Support, N.I.H., Extramural. Research Support, Non-U.S. Gov't. Research Support, U.S. Gov't, P.H.S.
English
KW - MEDLINE
KW - African Americans/sn [Statistics & Numerical Data]
KW - Child
KW - Female
KW - Health Resources/sd [Supply & Distribution]
KW - Hispanic Americans/sn [Statistics & Numerical Data]
KW - Humans
KW - Male
KW - Minority Groups
KW - Minority Health
KW - Motor Activity
KW - New York City
KW - Odds Ratio
KW - Pediatric Obesity/pc [Prevention & Control]
KW - Questionnaires
KW - Residence Characteristics/sn [Statistics & Numerical Data]
KW - Urban Population
RP - NOT IN FILE
SP - 20
EP - 26
JF - Academic pediatrics
JA - Acad Pediatr
VL - 13
IS - 1
CY - United States
N2 - OBJECTIVE: The role of neighborhood physical activity resources on childhood physical activity level is increasingly examined in pediatric obesity research. We describe how availability of physical activity resources varies by individual and block characteristics and then examine its associations with physical activity levels of Latino and black children in East Harlem, New York City. METHODS: Physical activity resource availability by individual and block characteristics were assessed in 324 children. Availability was measured against 4 physical activity measures: average weekly hours of outdoor unscheduled physical activity, average weekly metabolic hours of scheduled physical activity, daily hours of sedentary behavior, and daily steps. RESULTS: Physical activity resource availability differed by race/ethnicity, caregiver education, and income. Presence of one or more playgrounds on a child's block was positively associated with outdoor unscheduled physical activity (odds ratio [OR] = 1.95, 95% confidence interval [CI] 1.11-3.43). Presence of an after-school program on a child's block was associated with increased hours of scheduled physical activity (OR = 3.25, 95% CI 1.41-7.50) and decreased sedentary behavior (OR = 3.24, 95% CI 1.30-8.07). The more resources a child had available, the greater the level of outdoor unscheduled physical activity (P for linear trend = .026). CONCLUSIONS: Neighborhood physical activity resource availability differs by demographic factors, potentially placing certain groups at risk for low physical activity level. Availability of select physical activity resources was associated with reported physical activity levels of East Harlem children but not with objective measures of physical activity. Copyright 2013 Academic Pediatric Association. Published by Elsevier Inc. All rights reserved
SN - 1876-2867
AD - Department of Preventive Medicine, Mount Sinai School of Medicine, New York, NY 10023, USA. maida.galvez@mssm.edu
UR - 23312856
ER -

TY - JOUR
ID - 246
T1 - Amount and environmental predictors of outdoor playtime at home and school: a cross-sectional analysis of a national sample of preschool-aged children attending Head Start
A1 - Marino,A.J.
A1 - Fletcher,E.N.

A1 - Whitaker,R.C.
A1 - Anderson,S.E.
Y1 - 2012/11//
N1 - Marino, Alexis J. Fletcher, Erica N. Whitaker, Robert C. Anderson, Sarah E
Health & place
din, 9510067
T
Journal Article. Research Support, N.I.H., Extramural. Research Support, Non-U.S. Gov't
English
KW - MEDLINE
KW - Child
KW - Preschool
KW - Continental Population Groups/sn [Statistics & Numerical Data]
KW - Cross-Sectional Studies
KW - Early Intervention (Education)/sn [Statistics & Numerical Data]
KW - Educational Status
KW - Environment
KW - Family Characteristics
KW - Female
KW - Humans
KW - Male
KW - Play and Playthings
KW - Schools/sn [Statistics & Numerical Data]
KW - Time Factors
KW - United States
RP - NOT IN FILE
SP - 1224
EP - 1230
JF - Health & Place
JA - Health Place
VL - 18
IS - 6
CY - England
N2 - Outdoor play is an important contributor to children's physical activity and the prevalence, correlates, and environmental predictors of it among young children are not well characterized. This study aims to estimate the amount of time preschool-aged children attending Head Start spend playing outdoors at home and school, and whether aspects of the home and school environment are associated with greater outdoor play. We analyzed data (n=2529) collected in spring 2007 in the Head Start Family and Child Experiences Survey (FACES). Overall, 37.5% of children played outside at home >2h per weekday. Children who had a yard near home to play in or who had visited a park or playground or gone on a picnic with a family member in the last month were more likely to have >2h per weekday outdoor play at home, but having a playground within walking distance of the home was not related to home outdoor playtime. On average children played outdoors at Head Start for 36 min per day. The amount of time children played outdoors at home was not related to school outdoor time.
Copyright 2012 Elsevier Ltd. All rights reserved
SN - 1873-2054
AD - Division of Epidemiology, The Ohio State University College of Public Health, 336 Cunz Hall, 1841 Neil Avenue, Columbus, OH 43210, USA. amarino@cph.osu.edu
UR - 23000895
ER -

TY - JOUR
ID - 247
T1 - The role of schools in children's physical activity participation: staff perceptions
A1 - Huberty,J.
A1 - Dinkel,D.
A1 - Coleman,J.
A1 - Beighle,A.

A1 - Apenteng,B.
Y1 - 2012/12//
N1 - Huberty, J. Dinkel, D. Coleman, J. Beighle, A. Apenteng, B
Health education research
bqp, 8608459
T
Journal Article. Research Support, Non-U.S. Gov't
English
KW - MEDLINE
KW - Child
KW - Faculty
KW - Female
KW - Focus Groups
KW - Health Knowledge
KW - Attitudes
KW - Practice
KW - Humans
KW - Male
KW - Midwestern United States
KW - Physical Education and Training
KW - Qualitative Research
KW - Schools
RP - NOT IN FILE
SP - 986
EP - 995
JF - Health Education Research
JA - Health Educ Res
VL - 27
IS - 6
CY - England
N2 - The school setting provides a promising environment to increase children's physical activity (PA), however, staff often impact the success of PA within schools. The purpose of this article was to describe the knowledge of elementary school staff related to PA and their perception of the importance of the school environment being conducive to PA prior to the implementation of a recess intervention. Qualitative focus groups were conducted in 12 elementary schools in the Midwest. Grounded theory was used to explore participants' knowledge and perceptions. Participants felt PA was important but believed several factors impacted children's ability to be more active: (i) lack of time due to increasing academic demands, (ii) peer pressure (especially in girls) not to be active and (iii) lack of space and equipment. When discussing recess, staff felt that their encouragement of or active participation in PA with children resulted in more activity. Furthermore, even though participants were aware of PA benefits, they noticed eliminating recess was often used as a punishment for misbehavior. School-based PA promotion and PA opportunities hold great promise for increasing PA in children. However, to maximize these efforts school polices related to training staff about PA are needed
SN - 1465-3648
AD - Health, Physical Education, and Recreation, University of Nebraska Omaha, Omaha, NE 68182, USA. jenniferwhite@unomaha.edu
UR - 22717940
ER -

TY - JOUR
ID - 248
T1 - Participation in community-originated interventions is associated with positive changes in weight status and health behaviors in youth
A1 - Whetstone,L.M.
A1 - Kolasa,K.M.
A1 - Collier,D.N.
Y1 - 2012/09//

N1 - Whetstone, Lauren MacKenzie. Kolasa, Kathryn M. Collier, David N
American journal of health promotion : AJHP
amj, 8701680
T
Journal Article. Research Support, Non-U.S. Gov't
English
KW - MEDLINE
KW - Adolescent
KW - Body Mass Index
KW - Body Weight
KW - Child
KW - Preschool
KW - Community Health Services/mt [Methods]
KW - Community Health Services/sn [Statistics & Numerical Data]
KW - Diet/sn [Statistics & Numerical Data]
KW - Female
KW - Food Preferences/px [Psychology]
KW - Health Behavior
KW - Health Promotion/mt [Methods]
KW - Health Promotion/sn [Statistics & Numerical Data]
KW - Humans
KW - Male
KW - Motor Activity
KW - Nutritional Status
KW - Overweight/pc [Prevention & Control]
KW - Prospective Studies
KW - Weight Loss
RP - NOT IN FILE
SP - 10
EP - 16
JF - American Journal of Health Promotion
JA - Am J Health Promot
VL - 27
IS - 1
CY - United States
N2 - PURPOSE: To assess changes in children's health behaviors and weight status after participation in community-originated interventions. DESIGN: Prospective cohort study following body mass index (BMI) z-score trajectory over time. SETTING: Schools and community settings in 19 locations in North Carolina. SUBJECTS: A total of 1144 children, with an average age of 9.5 years, participating in community-originated physical activity and nutrition interventions, such as active recess and healthy cafeteria offerings, and environment and policy changes designed to prevent and reduce the prevalence of childhood obesity. Retention from baseline to final collection was 54%. Measures . Self-reported physical activity and eating behaviors; measured height and weight were collected at baseline and after interventions (average of 20 months). Z-score was compared with expected growth without intervention. ANALYSIS: Descriptive statistics, chi(2) tests and t-tests, and ANOVA analyses of variance to assess changes in health behaviors and weight status. RESULTS: More than 11% of children improved their weight status, and 86% of children who began at a healthy weight remained there. The average BMI for children who were overweight at enrollment was significantly lower after intervention (1.1 kg/m(2) lower for boys and 0.88 kg/m(2) lower for girls). Overweight and obese children who made improvements in key health behaviors showed greater decreases in BMI z-score than those who did not improve those behaviors (increased fruit [-.2207 vs. -.0793] and vegetable [-.2215 vs. -.0855] intake, and drinking less soda [-.1985 vs. -.0912]). There were no significant increases in physical activity, and changes in physical activity were not related to decreases in BMI z-score in this study. CONCLUSION: Community-originated interventions to raise awareness about food choices and to change policies and environments may improve BMI z-scores
SN - 0890-1171
AD - Department of Family Medicine, East Carolina University, 101 Heart Drive, Mail Stop 654, Greenville, NC 27834, USA. whetstonel@ecu.edu

UR - 22950920
ER -

TY - JOUR
ID - 249
T1 - Risk factors for incident myopia in Australian schoolchildren: the Sydney adolescent vascular and eye study
A1 - French,A.N.
A1 - Morgan,I.G.
A1 - Mitchell,P.
A1 - Rose,K.A.
Y1 - 2013/10//
N1 - French, Amanda N. Morgan, Ian G. Mitchell, Paul. Rose, Kathryn A
Ophthalmology
7802443
IM
Journal Article. Research Support, Non-U.S. Gov't
English
KW - MEDLINE
KW - Australia/ep [Epidemiology]
KW - Child
KW - Cross-Sectional Studies
KW - Female
KW - Humans
KW - Incidence
KW - Leisure Activities
KW - Logistic Models
KW - Longitudinal Studies
KW - Male
KW - Myopia/ep [Epidemiology]
KW - Questionnaires
KW - Risk Factors
RP - NOT IN FILE
SP - 2100
EP - 2108
JF - Ophthalmology
VL - 120
IS - 10
CY - United States
N2 - PURPOSE: To examine the risk factors for incident myopia in Australian schoolchildren. DESIGN: Population-based, longitudinal cohort study. PARTICIPANTS: The Sydney Adolescent Vascular and Eye Study (SAVES) was a 5- to 6-year follow-up of the Sydney Myopia Study (SMS). At follow-up, 2103 children were reexamined: 892 (50.5%) from the younger cohort and 1211 (51.5%) from the older cohort. Of these, 863 in the younger cohort and 1196 in the older cohort had complete refraction data. METHODS: Cycloplegic autorefraction (cyclopentolate 1%; Canon RK-F1; Canon, Tokyo, Japan) was measured at baseline and follow-up. Myopia was defined as a spherical equivalent refraction of <-0.50 diopters (D). Children were classified as having incident myopia if they were nonmyopic at baseline and myopic in either eye at follow-up. A comprehensive questionnaire determined the amount of time children spent outdoors and doing near work per week at baseline, as well as ethnicity, parental myopia, and socioeconomic status. MAIN OUTCOME MEASURES: Incident myopia. RESULTS: Children who became myopic spent less time outdoors compared with children who remained nonmyopic (younger cohort, 16.3 vs. 21.0 hours, respectively, P<0.0001; older cohort, 17.2 vs. 19.6 hours, respectively, P=0.001). Children who became myopic performed significantly more near work (19.4 vs. 17.6 hours; P=0.02) in the younger cohort, but not in the older cohort (P=0.06). Children with 1 or 2 parents who were myopic had greater odds of incident myopia (1 parent: odds ratio [OR], 3.2, 95% confidence interval [CI], 1.9-5.2; both parents: OR, 3.3, 95% CI, 1.6-6.8) in the younger but not the older cohort. Children of East Asian ethnicity had a higher incidence of myopia compared with children of European Caucasian ethnicity (both P<0.0001) and spent less time outdoors (both P<0.0001). A less hyperopic refraction

at baseline was the most significant predictor of incident myopia. The addition of time outdoors, near work, parental myopia, and ethnicity to the model significantly improved the predictive power (P<0.0001) in the younger cohort but had little effect in the older cohort. CONCLUSIONS: Time spent outdoors was negatively associated with incident myopia in both age cohorts. Near work and parental myopia were additional significant risk factors for myopia only in the younger cohort. FINANCIAL DISCLOSURE(S): The author(s) have no proprietary or commercial interest in any materials discussed in this article. Copyright 2013 American Academy of Ophthalmology. Published by Elsevier Inc. All rights reserved
SN - 1549-4713
AD - Discipline of Orthoptics, Faculty of Health Sciences, University of Sydney, Sydney, NSW, Australia
UR - 23672971
ER -

TY - JOUR
ID - 250
T1 - Effects of a short-term coordination exercise program during school recess: agility of seven- to eight-year-old elementary school children
A1 - Yasumitsu,T.
A1 - Nogawa,H.
Y1 - 2013/04//
N1 - Yasumitsu, Tatsuo. Nogawa, Haruo
Perceptual and motor skills
ozb, 0401131
IM
Clinical Trial. Journal Article
English
KW - MEDLINE
KW - Athletic Performance/ph [Physiology]
KW - Child
KW - Exercise Test/mt [Methods]
KW - Exercise Therapy/mt [Methods]
KW - Female
KW - Humans
KW - Japan
KW - Male
KW - Schools/og [Organization & Administration]
KW - Treatment Outcome
RP - NOT IN FILE
SP - 598
EP - 610
JF - Perceptual & Motor Skills
JA - Percept.Mot.Skills
VL - 116
IS - 2
CY - United States
N2 - This study tested the effect of a short-term coordination program focused on improving the agility of school children ages 7 to 8 years. Healthy Japanese children were placed in an experimental group of 26 children (10 girls, 16 boys) and a control group of 20 children (9 girls, 11 boys). The experimental group participated in 10 coordination program sessions during recesses; each session was performed 1 to 3 times per week, for 26 days. No differences were observed between the groups in repeated side-step scores prior to the program, although an interaction was observed after the completion of the treatment. The short-term coordination program was effective in increasing the agility of elementary school children aged 7 to 8 years
SN - 0031-5125
AD - PCY, Ltd, Japan. sahara0808@yahoo.co.jp
UR - 24032333
ER -

TY - JOUR

ID - 251
T1 - [Activities after school time and obesity in children. Influence of family and neighborhood environment]. [Spanish]
A1 - Yamamoto-Kimura,L.T.
A1 - vear-Galindo,M.G.
A1 - Moran-Alvarez,C.
A1 - cuna-Sanchez,M.E.
A1 - Torres-Duran,P.V.
A1 - Juarez-Oropeza,M.A.
A1 - Ferreira-Hermosillo,A.
A1 - Solis-Diaz,M.G.
Y1 - 2013/07//
N1 - Yamamoto-Kimura, Liria T. Alvear-Galindo, Ma Guadalupe. Moran-Alvarez, Cristina. Acuna-Sanchez, Ma Eugenia. Torres-Duran, Patricia V. Juarez-Oropeza, Marco Antonio. Ferreira-Hermosillo, Aldo. Solis-Diaz, Ma Guadalupe
Revista medica del Instituto Mexicano del Seguro Social
101243727
IM
English Abstract. Journal Article
Spanish
KW - MEDLINE
KW - Child
KW - Cross-Sectional Studies
KW - Family Characteristics
KW - Female
KW - Humans
KW - Leisure Activities
KW - Male
KW - Obesity/ep [Epidemiology]
KW - Residence Characteristics
RP - NOT IN FILE
SP - 378
EP - 383
JF - Revista Medica del Instituto Mexicano del Seguro Social
JA - REV
VL - 51
IS - 4
CY - Mexico
N2 - Background: elementary school children spend little time at the school in Mexico. The aim was to investigate the activities after school time in children and to determine the presence of overweight and obesity. Methods: one hundred and seventy two children of both sexes were included. They belonged to the first and second grade of an official primary school from a marginalized zone. Somatometry was obtained and the type of physical practice and motifs for no practice physical activity were investigated. Descriptive statistics were applied. Results: the frequency of underweight was 12.1 %, overweight 19 %, and obesity 18.5 %. Sedentary activities like watching television and playing video games were the most common registered, whereas sports were the lowest physical activity practiced, especially in girls. The parents' lack of time, low family income, and the scarcity of safe spaces where children can play and practice physical activity or sports were the most related factors. Conclusions: poverty and insecurity of the neighborhoods influence the outside activities in after school time. The high frequency of underweight, overweight and obesity in school children were associated to the lack of physical activity
SN - 0443-5117
AD - Departamento de Salud Publica, Universidad Nacional Autonoma de Mexico, Distrito Federal, Mexico. liriayk@unam.mx
UR - 24021065
ER -

TY - JOUR

ID - 252
T1 - Health and safety of child care centers: an analysis of licensing specialists' reports of routine, unannounced inspections
A1 - Crowley,A.A.
A1 - Jeon,S.
A1 - Rosenthal,M.S.
Y1 - 2013/10//
N1 - Crowley, Angela A. Jeon, Sangchoon. Rosenthal, Marjorie S
American journal of public health
1254074, 3xw
AIM, IM
Journal Article. Research Support, Non-U.S. Gov't
English
KW - MEDLINE
KW - Child Day Care Centers/lj [Legislation & Jurisprudence]
KW - Child Day Care Centers/st [Standards]
KW - Child
KW - Preschool
KW - Confidence Intervals
KW - Connecticut
KW - Guideline Adherence
KW - Humans
KW - Licensure
KW - Research Report/st [Standards]
KW - Safety Management/st [Standards]
KW - Specialization
RP - NOT IN FILE
SP - e52
EP - e58
JF - American Journal of Public Health
JA - Am J Public Health
VL - 103
IS - 10
CY - United States
N2 - OBJECTIVES: We assessed the prevalence of regulatory noncompliance of licensed child care centers and identified factors associated with improved compliance. METHODS: We analyzed 676 routine, unannounced reports of child care centers collected by the Connecticut Department of Public Health licensing specialists over a 2-year time period, included characteristics of centers, and created categories of regulations. RESULTS: The sample included 41% of licensed child care centers. Of the 13 categories of regulations in the analyses, 7 categories (outdoor safety, indoor safety, indoor health, child and staff documentation, emergency preparedness, infant-toddler indoor health, and infant-toddler indoor safety) had regulations with center noncompliance greater than 10%. Playground hazard-free was the regulation with the highest frequency (48.4%) of noncompliance. Compliance with the regulation for 20 hours of continuing education per year for child care providers was the characteristic most frequently associated with regulations compliance. CONCLUSIONS: Efforts to support continuing education of child care providers are essential to improve and sustain healthy and safe early-care and education programs. Analyses of state child care licensing inspection reports provide valuable data and findings for strategic planning efforts
SN - 1541-0048
AD - Angela A. Crowley and Sangchoon Jeon are with Yale University School of Nursing, Yale University, New Haven, CT. Marjorie S. Rosenthal is with the Department of Pediatrics, Yale University School of Medicine, and the Robert Wood Johnson Clinical Scholars Program, New Haven
UR - 23948016
ER -

TY - JOUR
ID - 253
T1 - [The differential classification of school avoidance- a cluster-analytic investigation]. [German]

A1 - Knollmann,M.
A1 - Reissner,V.
A1 - Kiessling,S.
A1 - Hebebrand,J.
Y1 - 2013/09//
N1 - Knollmann, Martin. Reissner, Volker. Kiessling, Susanne. Hebebrand, Johannes
Zeitschrift fur Kinder- und Jugendpsychiatrie und Psychotherapie
9801717, c0w
IM
English Abstract. Journal Article
German
KW - MEDLINE
KW - Absenteeism
KW - Adolescent
KW - Antisocial Personality Disorder/cl [Classification]
KW - Antisocial Personality Disorder/di [Diagnosis]
KW - Antisocial Personality Disorder/ep [Epidemiology]
KW - Antisocial Personality Disorder/px [Psychology]
KW - Anxiety Disorders/cl [Classification]
KW - Anxiety Disorders/di [Diagnosis]
KW - Anxiety Disorders/ep [Epidemiology]
KW - Causality
KW - Child
KW - Child Behavior Disorders/cl [Classification]
KW - Child Behavior Disorders/di [Diagnosis]
KW - Child Behavior Disorders/ep [Epidemiology]
KW - Child Behavior Disorders/px [Psychology]
KW - Cluster Analysis
KW - Cross-Sectional Studies
KW - Depressive Disorder/cl [Classification]
KW - Depressive Disorder/di [Diagnosis]
KW - Depressive Disorder/ep [Epidemiology]
KW - Depressive Disorder/px [Psychology]
KW - Female
KW - Humans
KW - Internal-External Control
KW - Male
KW - Personality Assessment/sn [Statistics & Numerical Data]
KW - Personality Inventory/sn [Statistics & Numerical Data]
KW - Phobic Disorders/cl [Classification]
KW - Phobic Disorders/di [Diagnosis]
KW - Phobic Disorders/ep [Epidemiology]
KW - Phobic Disorders/px [Psychology]
KW - Psychometrics/sn [Statistics & Numerical Data]
RP - NOT IN FILE
SP - 335
EP - 345
JF - Zeitschrift fur Kinder-und Jugendpsychiatrie und Psychotherapie
JA - Z Kinder Jugendpsychiatr.Psychother
VL - 41
IS - 5
CY - Switzerland
N2 - OBJECTIVE: We investigated subtypes of school-avoiding children and adolescents in a German sample using cluster analysis. METHOD: 169 outpatients of a specialized outpatient unit for children and adolescents with school-avoiding behavior were subjected to cluster analysis using factor scores (principal factoring, varimax-rotation: measures of internalizing and externalizing symptoms, e.g., CBCL, YSR, and variables indicating the quality of school avoiding behavior, such as initial parental knowledge of school absence,

activities during school absence). The resulting clusters were compared to other variables such as parental mental health problems, parental divorce, bullying in school, class repetition, and IQ. RESULTS: Three groups were identified: "school refusers" with low externalizing symptoms who mainly stayed at home with their parents and were informed about their school absence from the beginning. Second, the "truants" with strong externalizing symptoms who spent school time alone or together with peers outside their home and were absent in school without initial parental knowledge. In a third cluster, children showed more externalizing problems than the school refusers and less externalizing problems than the truants. Most of their parents were informed about the school absence from the beginning, although the dominant activity was staying at home alone. Internalizing symptoms had no impact on cluster formation. Comparisons of the three groups, however, showed higher internalizing problems for school refusers and the third cluster, which was then labeled "school avoidance with mixed symptoms." RESULTS AND CONCLUSIONS: In most cases, significant differences regarding potential stressors at home (e.g., parental mental health problems) or in school (e.g., bullying, class repetition) were not obtained
SN - 1422-4917
AD - Klinik fur Psychiatrie, Psychosomatik und Psychotherapie des Kindes- und Jugendalters, LVR-Klinikum Essen, Kliniken und Institut der Universitat Duisburg-Essen
UR - 23988835
ER -

TY - JOUR
ID - 254
T1 - A place for play? The influence of the home physical environment on children's physical activity and sedentary behaviour. [Review]
A1 - Maitland,C.
A1 - Stratton,G.
A1 - Foster,S.
A1 - Braham,R.
A1 - Rosenberg,M.
Y1 - 2013///
N1 - Maitland, Clover. Stratton, Gareth. Foster, Sarah. Braham, Rebecca. Rosenberg, Michael
The international journal of behavioral nutrition and physical activity
101217089
IM
Journal Article. Research Support, Non-U.S. Gov't. Review
English
KW - MEDLINE
KW - Child
KW - Child Behavior
KW - Environment
KW - Exercise
KW - Health Behavior
KW - Humans
KW - Parent-Child Relations
KW - Parenting
KW - Parents
KW - Play and Playthings
KW - Sedentary Lifestyle
KW - Social Environment
KW - Television
KW - Video Games
RP - NOT IN FILE
SP - 99
JF - International Journal of Behavioral Nutrition & Physical Activity
JA - Int
VL - 10
CY - England

N2 - The home environment is an important influence on the sedentary behaviour and physical activity of children, who have limited independent mobility and spend much of their time at home. This article reviews the current evidence regarding the influence of the home physical environment on the sedentary behaviour and physical activity of children aged 8-14 years. A literature search of peer reviewed articles published between 2005 and 2011 resulted in 38 observational studies (21 with activity outcomes, 23 with sedentary outcomes) and 11 experimental studies included in the review. The most commonly investigated behavioural outcomes were television watching and moderate to vigorous physical activity. Media equipment in the home and to a lesser extent the bedroom were positively associated with children's sedentary behaviour. Physical activity equipment and the house and yard were not associated with physical activity, although environmental measures were exclusively self-reported. On the other hand, physical activity equipment was inversely associated with sedentary behaviours in half of studies. Observational studies that investigated the influence of the physical and social environment within the home space, found that the social environment, particularly the role of parents, was important. Experimental studies that changed the home physical environment by introducing a television limiting device successfully decreased television viewing, whereas the influence of introducing an active video game on activity outcomes was inconsistent. Results highlight that the home environment is an important influence on children's sedentary behaviour and physical activity, about which much is still unknown. While changing or controlling the home physical environment shows promise for reducing screen based sedentary behaviour, further interventions are needed to understand the broader impact of these changes. Future studies should prioritise investigating the influence of the home physical environment, and its interaction with the social environment, on objectively measured sedentary time and home context specific behaviours, ideally including technologies that allow objective measures of the home space
SN - 1479-5868
AD - School of Sport Science, Exercise and Health, University of Western Australia, 35 Stirling Highway, Crawley, WA 6009, Australia. clover.maitland@uwa.edu.au
UR - 23958282
ER -

TY - JOUR
ID - 255
T1 - Transmission of multidrug-resistant tuberculosis in the USA: a cross-sectional study
A1 - Moonan,P.K.
A1 - Teeter,L.D.
A1 - Salcedo,K.
A1 - Ghosh,S.
A1 - Ahuja,S.D.
A1 - Flood,J.
A1 - Graviss,E.A.
Y1 - 2013/09//
N1 - Moonan, Patrick K. Teeter, Larry D. Salcedo, Katya. Ghosh, Smita. Ahuja, Shama D. Flood, Jennifer. Graviss, Edward A
The Lancet infectious diseases
101130150
IM
Journal Article. Multicenter Study. Research Support, U.S. Gov't, P.H.S.
English
KW - MEDLINE
KW - Adolescent
KW - Adult
KW - Aged
KW - Antitubercular Agents/pd [Pharmacology]
KW - Child
KW - Preschool
KW - Cross-Sectional Studies
KW - Ethnic Groups
KW - Female
KW - Genotype
KW - Humans

KW - Infant
KW - Male
KW - Medical Records
KW - Middle Aged
KW - Minisatellite Repeats
KW - Mycobacterium tuberculosis/de [Drug Effects]
KW - Mycobacterium tuberculosis/ge [Genetics]
KW - Mycobacterium tuberculosis/ip [Isolation & Purification]
KW - Questionnaires
KW - Recurrence
KW - Time Factors
KW - Travel
KW - Tuberculosis
KW - Multidrug-Resistant/ep [Epidemiology]
KW - Multidrug-Resistant/tm [Transmission]
KW - United States/ep [Epidemiology]
KW - Young Adult
RP - NOT IN FILE
SP - 777
EP - 784
JF - The Lancet Infectious Diseases
JA - Lancet Infect Dis
VL - 13
IS - 9
CY - United States
N2 - BACKGROUND: Multidrug-resistant (MDR) tuberculosis is a potential threat to tuberculosis elimination, but the extent of MDR tuberculosis disease in the USA that is attributable to transmission within the country is unknown. We assessed transmission of MDR tuberculosis and potential contributing factors in the USA. METHODS: In a cross-sectional study, clinical, demographic, epidemiological, and Mycobacterium tuberculosis genotype data were obtained during routine surveillance of all verified cases of MDR tuberculosis reported from eight states in the USA (California from Jan 1, 2007, to Dec 31, 2009; Texas from Jan 1, 2007, to March 31, 2009; and the states of Colorado, Maryland, Massachusetts, New York, Tennessee, and Washington from Jan 1, 2007 to Dec 31, 2008). In-depth interviews and health-record abstraction were done for all who consented to ascertain potential interpersonal connections. FINDINGS: 168 cases of MDR tuberculosis were reported in the eight states during our study period. 92 individuals (55%) consented to in-depth interview. 20 (22%) of these individuals developed MDR tuberculosis as a result of transmission in the USA; a source case was identified for eight of them (9%). 20 individuals (22%) had imported active tuberculosis (ie, culture-confirmed disease within 3 months of entry into the USA). 38 (41%) were deemed to have reactivation of disease, of whom 14 (15%) had a known previous episode of tuberculosis outside the USA. Five individuals (5%) had documented treatment of a previous episode in the USA, and so were deemed to have relapsed. For nine cases (10%), insufficient evidence was available to definitively classify reason for presentation. INTERPRETATION: About a fifth of cases of MDR tuberculosis in the USA can be linked to transmission within the country. Many individuals acquire MDR tuberculosis before entry into the USA. MDR tuberculosis needs to be diagnosed rapidly to reduce potential infectious periods, and clinicians should consider latent tuberculosis infection treatment-tailored to the results of drug susceptibility testing of the putative source case-for exposed individuals. FUNDING: Centers for Disease Control and Prevention. Copyright 2013 Elsevier Ltd. All rights reserved
SN - 1474-4457
AD - Division of Tuberculosis Elimination, Centers for Disease Control and Prevention, Atlanta, GA 30333, USA. pmoonan@cdc.gov
UR - 23759447
ER -

TY - JOUR
ID - 256
T1 - Schoolyard physical activity of 6-11 year old children assessed by GPS and accelerometry
A1 - Dessing,D.

A1 - Pierik,F.H.
A1 - Sterkenburg,R.P.
A1 - van,Dommelen P.
A1 - Maas,J.
A1 - de Vries,S.I.
Y1 - 2013///
N1 - Dessing, Dirk. Pierik, Frank H. Sterkenburg, Reinier P. van Dommelen, Paula. Maas, Jolanda. de Vries, Sanne I
The international journal of behavioral nutrition and physical activity
101217089
IM
Journal Article
English
KW - MEDLINE
KW - Actigraphy/mt [Methods]
KW - Child
KW - Child Behavior
KW - Exercise
KW - Female
KW - Geographic Information Systems
KW - Health Behavior
KW - Humans
KW - Logistic Models
KW - Male
KW - Netherlands
KW - Odds Ratio
KW - Physical Exertion
KW - Recreation
KW - Schools
KW - Sex Factors
RP - NOT IN FILE
SP - 97
JF - International Journal of Behavioral Nutrition & Physical Activity
JA - Int
VL - 10
CY - England
N2 - BACKGROUND: Children's current physical activity levels are disturbingly low when compared to recommended levels. This may be changed by intervening in the school environment. However, at present, it is unclear to what extent schoolyard physical activity contributes towards reaching the daily physical activity guideline. The aim of this study was to examine how long and at what intensity children are physically active at the schoolyard during different time segments of the day. Moreover, the contribution of schoolyard physical activity towards achieving the recommended guideline for daily physical activity was investigated. METHODS: Children (n=76) between the age of 6-11 years were recruited in six different schools in five cities (>70.000 residents) in the Netherlands. During the weekdays of a regular school week, childrens' physical activity and location were measured with ActiGraph accelerometers and Travelrecorder GPS receivers. Data was collected from December 2008 to April 2009. From the data, the amount of moderate to vigorous physical activity (MVPA) on and outside the schoolyard was established. Moreover, the percentage of MVPA on the schoolyard was compared between the following segments of the day: pre-school, school, school recess, lunch break and post-school. Differences between boys and girls were compared using linear and logistic mixed-effects models. RESULTS: On average, children spent 40.1 minutes/day on the schoolyard. During this time, boys were more active on the schoolyard, with 27.3% of their time spent as MVPA compared to 16.7% among girls (OR=2.11 [95% CI 1.54 - 2.90]). The children were most active on the schoolyard during school recess, during which boys recorded 39.5% and girls recorded 23.4% of the time as MVPA (OR=2.55 [95% CI: 1.69 - 3.85]). Although children were only present at the schoolyard for 6.1% of the total reported time, this time contributed towards 17.5% and 16.8% of boys' and girls' minutes of MVPA. CONCLUSIONS: On the schoolyard, children's physical activity levels are higher than on average over the whole day. Physical activity levels are particularly high during school recess. The school environment seems to be an important setting for improving children's

physical activity levels. Further research on the facilitators of these high activity levels may provide targets for further promotion of physical activity among children
SN - 1479-5868
AD - TNO, Department of Urban Environment and Safety, PO Box 80015, 3508 TA Utrecht, The Netherlands
UR - 23945145
ER -

TY - JOUR
ID - 257
T1 - An updated view on the role of dopamine in myopia. [Review]
A1 - Feldkaemper,M.
A1 - Schaeffel,F.
Y1 - 2013/09//
N1 - Feldkaemper, Marita. Schaeffel, Frank
Experimental eye research
epl, 0370707
IM
Journal Article. Review
English
KW - MEDLINE
KW - Animals
KW - Axial Length
KW - Eye/me [Metabolism]
KW - Child
KW - Dopamine/me [Metabolism]
KW - Eye/gd [Growth & Development]
KW - Humans
KW - Light
KW - Myopia/me [Metabolism]
KW - Myopia/pc [Prevention & Control]
KW - Retina/me [Metabolism]
KW - Retina/re [Radiation Effects]
RP - NOT IN FILE
SP - 106
EP - 119
JF - Experimental Eye Research
JA - Exp Eye Res
VL - 114
CY - England
N2 - A large body of data is available to support the hypothesis that dopamine (DA) is one of the retinal neurotransmitters involved in the signaling cascade that controls eye growth by vision. Initially, reduced retinal DA levels were observed in eyes deprived of sharp vision by either diffusers ("deprivation myopia", DM) or negative lenses ("lens induced myopia", LIM). Simulating high retinal DA levels by intravitreal application of a DA agonist can suppress the development of both DM and LIM. Also more recent studies using knock-out mouse models of DA receptors support the idea of an association between decreased DA levels and DM. There seem to be differences in the magnitude of the effects of DA on DM and LIM, with larger changes in DM but the degrees of image degradation by both treatments need to be matched to support this conclusion. Although a number of studies have shown that the inhibitory effects of dopamine agonists on DM and LIM are mediated through stimulation of the D2-receptor, there is also recent evidence that the balance of D2- and D1-receptor activation is important. Inhibition of D2-receptors can also slow the development of spontaneous myopia in albino guinea pigs. Retinal DA content displays a distinct endogenous diurnal, and partially circadian rhythm. In addition, retinal DA is regulated by a number of visual stimuli like retinal illuminance, spatial frequency content of the image, temporal contrast and, in chicks, by the light input from the pineal organ. A close interaction was found between muscarinergic and dopaminergic systems, and between nitric oxide and dopaminergic pathways, and there is evidence for crosstalk between the different pathways, perhaps multiple binding of the ligands to different receptors. It was shown that DA agonists interact with the immediate early signaling molecule ZENK which triggers the first steps in eye growth regulation. However, since long treatment periods were often needed

to induce significant changes in retinal dopamine synthesis and release, the role of dopamine in the early steps is unclear. The wide spatial distribution of dopaminergic amacrine cells in the retina and the observation that changes in dopamine levels can be locally induced by local retinal deprivation is in line with the assumption that dopaminergic mechanisms control both central and peripheral eye growth. The protective effect of outdoor activity on myopia development in children seems to be partly mediated by the stimulatory effect of light on retinal dopamine production and release. However, the dose-response function linking light exposure to dopamine and to the suppression of myopia is not known and requires further studies. Copyright 2013 Elsevier Ltd. All rights reserved
SN - 1096-0007
AD - Centre for Ophthalmology, Institute for Ophthalmic Research, Section of Neurobiology of the Eye, Calwerstrase 7/1, 72076 Tuebingen, Germany. marita.feldkaemper@uni-tuebingen.de
UR - 23434455
ER -

TY - JOUR
ID - 258
T1 - Psychological predictors of children' s recess physical activity motivation and behavior
A1 - Stellino,M.B.
A1 - Sinclair,C.D.
Y1 - 2013/06//
N1 - Stellino, Megan Babkes. Sinclair, Christina D
Research quarterly for exercise and sport
r6y, 8006373
IM
Journal Article
English
KW - MEDLINE
KW - Accelerometry/is [Instrumentation]
KW - Body Weight
KW - Child
KW - Child Behavior/px [Psychology]
KW - Female
KW - Humans
KW - Male
KW - Motivation
KW - Motor Activity
KW - Overweight/px [Psychology]
KW - Personal Autonomy
KW - Regression Analysis
KW - Sex Factors
RP - NOT IN FILE
SP - 167
EP - 176
JF - Research Quarterly for Exercise & Sport
JA - Res Q Exerc Sport
VL - 84
IS - 2
CY - United States
N2 - PURPOSE: This study explored the relationship between children's basic psychological needs satisfaction at recess, level of recess physical activity motivation (RPAM), and recess physical activity (RPA). METHOD: Fifth-grade children (N = 203; 50.2% boys; 71.7% healthy-weight) completed measures of age, gender, basic psychological need satisfaction, and level of self-determined motivation for RPA. Children also wore pedometers during six consecutive 30-min mid-school-day recesses. RESULTS: Multiple regression analyses indicated unique significant predictors of RPAM and RPA according to gender and weight status. RPAM was significantly predicted by all three basic psychological needs for boys and only competence need satisfaction for girls and healthy-weight children. RPA was predicted by RPAM for girls, competence need satisfaction for overweight children, and autonomy need satisfaction for boys and healthy-weight children. CONCLUSIONS:

Findings support self-determination theory and provide important insight into the variations in psychological predictors of motivation for RPA and actual physical activity behavior based on gender and weight status
SN - 0270-1367
AD - School of Sport and Exercise Science, University of Northern Colorado, Gunter Hall, Mailbox 39, Greeley, CO 80639, USA. megan.stellino@unco.edu
UR - 23930542
ER -

TY - JOUR
ID - 259
T1 - The ONE step initiative: quality improvement in a pediatric clinic for secondhand smoke reduction
A1 - Bunik,M.
A1 - Cavanaugh,K.L.
A1 - Herrick,D.
A1 - Mehner,L.
A1 - Venugopalakrishnan,J.
A1 - Crane,L.A.
A1 - Puma,J.
Y1 - 2013/08//
N1 - Bunik, Maya. Cavanaugh, Keith L. Herrick, Diane. Mehner, Lauren. Venugopalakrishnan, Janani. Crane, Lori A. Puma, Jini
Pediatrics
oxv, 0376422
AIM, IM
Journal Article. Research Support, Non-U.S. Gov't
English
KW - MEDLINE
KW - Adult
KW - Child
KW - Colorado
KW - Cooperative Behavior
KW - Documentation/st [Standards]
KW - Electronic Health Records
KW - Female
KW - Follow-Up Studies
KW - Hospitals
KW - Teaching
KW - Urban
KW - Humans
KW - Interdisciplinary Communication
KW - Male
KW - Outpatient Clinics
KW - Hospital
KW - Patient Education as Topic/mt [Methods]
KW - Physician Assistants
KW - Program Evaluation
KW - Quality Improvement
KW - Referral and Consultation
KW - Smoking Cessation/mt [Methods]
KW - Tobacco Smoke Pollution/pc [Prevention & Control]
RP - NOT IN FILE
SP - e502
EP - e511
JF - Pediatrics
VL - 132
IS - 2
CY - United States

N2 - BACKGROUND AND OBJECTIVE: Although comprehensive smoking counseling to limit secondhand smoke (SHS) is widely endorsed, it is often not done. Published evaluations of brief and practical systems that improve screening and counseling to reduce SHS are limited. Our objective was to determine if a quality improvement activity around smoking counseling leads to changes in (1) medical assistant and pediatric provider assessment of smoking history and (2) smoking or other behaviors affecting children's SHS exposure. METHODS: In a large urban teaching clinic we assessed the ONE Step intervention, which included the following: (1) "Ask" (medical assistant asking whether caregivers smoke); (2) "Advise" (providers advising smoking outside and quitting if ready); (3) "Refer" (providers referring to the Colorado telephone QuitLine); and (4) electronic medical record prompts and required documentation regarding smoking. Medical assistant and provider assessments of smoking were evaluated with a chart review by using a pre-/posttest design. Caregiver behavior change was evaluated with a time-series survey that included assessment at baseline and follow-up via telephone at 6 and 12 months from study entry. RESULTS: ONE Step was associated with a statistically significant increase in Ask, Advise, and Refer documentation. Caregiver surveys showed that 97% found discussions of SHS with providers acceptable. Six- and 12-month follow-ups, respectively, showed that 14% and 13% of smokers reported quitting and that 63% and 70% of current smokers reported reduced SHS exposure. CONCLUSIONS: ONE Step was feasible to deliver in a busy outpatient setting, acceptable to families, and appears to have resulted in decreased exposure to SHS in our pediatric population
SN - 1098-4275
AD - Department of Pediatrics, Colorado School of Public Health, University of Colorado Anschutz Medical Campus, Aurora, Colorado 80045, USA. maya.bunik@childrenscolorado.org
UR - 23858424
ER -

TY - JOUR
ID - 260
T1 - High frequency oscillations mirror disease activity in patients with focal cortical dysplasia
A1 - Kerber,K.
A1 - LeVan,P.
A1 - Dumpelmann,M.
A1 - Fauser,S.
A1 - Korinthenberg,R.
A1 - Schulze-Bonhage,A.
A1 - Jacobs,J.
Y1 - 2013/08//
N1 - Kerber, Karolin. LeVan, Pierre. Dumpelmann, Matthias. Fauser, Susanne. Korinthenberg, Rudolf. Schulze-Bonhage, Andreas. Jacobs, Julia
Epilepsia
eix, 2983306r
IM
Journal Article. Research Support, Non-U.S. Gov't
English
KW - MEDLINE
KW - Adolescent
KW - Adult
KW - Brain Diseases/cl [Classification]
KW - Brain Diseases/pp [Physiopathology]
KW - Brain Diseases/su [Surgery]
KW - Brain Mapping
KW - Brain Waves/ph [Physiology]
KW - Child
KW - Electrodes
KW - Implanted
KW - Electroencephalography
KW - Electromyography
KW - Epilepsy/pp [Physiopathology]
KW - Epilepsy/su [Surgery]
KW - Female

KW - Humans
KW - Male
KW - Malformations of Cortical Development/cl [Classification]
KW - Malformations of Cortical Development/pp [Physiopathology]
KW - Malformations of Cortical Development/su [Surgery]
KW - Middle Aged
KW - Outcome Assessment (Health Care)
KW - Signal Processing
KW - Computer-Assisted
KW - Statistics as Topic
KW - Young Adult
RP - NOT IN FILE
SP - 1428
EP - 1436
JF - Epilepsia
VL - 54
IS - 8
CY - United States
N2 - PURPOSE: The study analyzes the occurrence of high frequency oscillations in different types of focal cortical dysplasia in 22 patients with refractory epilepsy. High frequency oscillations are biomarkers for epileptic tissue, but it is unknown whether they can reflect increasingly dysplastic tissue changes as well as epileptic disease activity. METHODS: High frequency oscillations (80-450 Hz) were visually marked by two independent reviewers in all channels of intracranial implanted grid, strips, and depth electrodes in patients with focal cortical dysplasia and refractory epilepsy. Rates of high frequency oscillations in patients with pathologically confirmed focal cortical dysplasia of Palmini type 1a and b were compared with those in type 2a and b. KEY FINDINGS: Patients with focal cortical dysplasia type 2 had significantly more seizures than those with type 1 ($p < 0.001$). Rates of high frequency oscillations were significantly higher in patients with focal cortical dysplasia type 2 versus type 1 ($p < 0.001$). In addition, it could be confirmed that rates of high frequency oscillations were significantly higher in presumed epileptogenic areas than outside ($p < 0.001$). SIGNIFICANCE: Activity of high frequency oscillations mirrors the higher epileptogenicity of focal cortical dysplasia type 2 lesions compared to type 1 lesions. Therefore, rates of high frequency oscillations can reflect disease activity of a lesion. This has implications for the use of high frequency oscillations as biomarkers for epileptogenic areas, because a detailed analysis of their rates may be necessary to use high frequency oscillations as a predictive tool in epilepsy surgery. Wiley Periodicals, Inc. 2013 International League Against Epilepsy
SN - 1528-1167
AD - Department of Neuropediatrics and Muscular Diseases, University of Freiburg, Freiburg, Germany
UR - 23899121
ER -

TY - JOUR
ID - 261
T1 - School-day and overall physical activity among youth
A1 - Long,M.W.
A1 - Sobol,A.M.
A1 - Cradock,A.L.
A1 - Subramanian,S.V.
A1 - Blendon,R.J.
A1 - Gortmaker,S.L.
Y1 - 2013/08//
N1 - Long, Michael W. Sobol, Arthur M. Cradock, Angie L. Subramanian, S V. Blendon, Robert J. Gortmaker, Steven L
American journal of preventive medicine
8704773, apl
IM
Journal Article. Research Support, U.S. Gov't, P.H.S.
English

KW - MEDLINE
KW - Accelerometry/mt [Methods]
KW - Accelerometry/sn [Statistics & Numerical Data]
KW - Adolescent
KW - Child
KW - Child Welfare
KW - Health Policy
KW - Health Promotion/mt [Methods]
KW - Humans
KW - Leisure Activities
KW - Linear Models
KW - Motor Activity
KW - Nutrition Surveys/sn [Statistics & Numerical Data]
KW - Obesity/pc [Prevention & Control]
KW - Physical Fitness
KW - School Health Services/og [Organization & Administration]
KW - Schools
KW - Socioeconomic Factors
KW - Time Factors
KW - United States
RP - NOT IN FILE
SP - 150
EP - 157
JF - American Journal of Preventive Medicine
JA - Am J Prev Med
VL - 45
IS - 2
CY - Netherlands
N2 - BACKGROUND: Increasing school-day physical activity through policy and programs is commonly suggested to prevent obesity and improve overall child health. However, strategies that focus on school-day physical activity may not increase total physical activity if youth compensate by reducing physical activity outside of school. PURPOSE: Objectively measured, nationally representative physical activity data were used to test the hypothesis that higher school-day physical activity is associated with higher overall daily physical activity in youth. METHODS: Accelerometer data from 2003-2004/2005-2006 National Health and Nutrition Examination Surveys were analyzed in 2012 to estimate physical activity levels during the school day (8AM-3PM) among youth aged 6-19 years (n=2548). Fixed-effects regressions were used to estimate the impact of changes in school-day minutes of moderate-to-vigorous physical activity (MVPA) on changes in total daily MVPA. RESULTS: Each additional minute of school-day MVPA was associated with an additional 1.14 minutes (95% CI=1.04, 1.24; p<0.001) of total daily MVPA, or 0.14 additional minutes (95% CI=0.04, 0.24; p=0.008) outside the school day, controlling for total daily accelerometer wear time and age, gender, race/ethnicity, and other non-time varying covariates. There were no differences in the effect of school-day MVPA on total MVPA by age group, gender, race/ethnicity, poverty status, or degree of change in MVPA. CONCLUSIONS: Higher school-day MVPA was associated with higher daily MVPA among U.S. youth with no evidence for same-day "compensation." Increasing school-based physical activity is a promising approach that can improve total daily physical activity levels of youth. Copyright 2013 American Journal of Preventive Medicine. Published by Elsevier Inc. All rights reserved
SN - 1873-2607
AD - Department of Social and Behavioral Sciences, Harvard School of Public Health, Boston, MA, USA. michael.long@mail.harvard.edu
UR - 23867021
ER -

TY - JOUR
ID - 262
T1 - Family child care providers' compliance with state physical activity regulations, Delaware Child Care Provider Survey, 2011
A1 - Leng,S.W.

A1 - Lessard,L.
Y1 - 2013///
N1 - Leng, Sarah Williams. Lessard, Laura
Preventing chronic disease
101205018
IM
Journal Article. Research Support, Non-U.S. Gov't
English
KW - MEDLINE
KW - Child
KW - Child Day Care Centers/lj [Legislation & Jurisprudence]
KW - Child Day Care Centers/st [Standards]
KW - Delaware
KW - Environment Design
KW - Facility Regulation and Control/lj [Legislation & Jurisprudence]
KW - Female
KW - Government Regulation
KW - Guideline Adherence
KW - Health Care Surveys
KW - Health Knowledge
KW - Attitudes
KW - Practice
KW - Humans
KW - Logistic Models
KW - Male
KW - Motor Activity
RP - NOT IN FILE
SP - E114
JF - Preventing Chronic Disease
JA - Prev Chronic Dis
VL - 10
CY - United States
N2 - INTRODUCTION: Delaware is one state that has implemented comprehensive child care regulations to foster healthy dietary and physical activity behaviors of young children. This study describes the Delaware family child care environment and providers' knowledge of and compliance with physical activity regulations. We analyzed the data to determine characteristics associated with predictors of knowledge of and compliance with these regulations. METHODS: A random stratified sample of 663 licensed Delaware family child care providers was mailed a survey on family child care characteristics and providers' awareness and practices of the child care regulations. Three logistic regression models were used to explore the association between provider characteristics and their knowledge of and compliance with the regulations. RESULTS: Ultimately, 313 of the 663 eligible family child care providers participated in the survey (47.2% response rate). Controlling for covariates, we found that family child care providers' education level was significantly associated with knowledge of the physical activity regulation. Another model showed that family child care providers with larger amounts of outdoor space were more likely to report compliance with the recommendation for unstructured physical activity than those without this described space (odds ratio, 2.45). A third model showed a significant association between available indoor space for all activities including running and reported greater compliance with the recommendation for structured physical activity than was reported by caregivers with less indoor space (odds ratio, 11.2). CONCLUSION: To provide the recommended levels of physical activity for children in child care, the available physical space environment is an important area of focus for advocates of physical activity recommendations within the family child care environment
SN - 1545-1151
AD - Nemours/Alfred I. duPont Hospital for Children-Nemours Health and Prevention Services, Wilmington, Delaware, USA
UR - 23845175
ER -

TY - JOUR

ID - 263
T1 - Health care-seeking behavior for childhood diarrhea in Mirzapur, rural Bangladesh
A1 - Das,S.K.
A1 - Nasrin,D.
A1 - Ahmed,S.
A1 - Wu,Y.
A1 - Ferdous,F.
A1 - Farzana,F.D.
A1 - Khan,S.H.
A1 - Malek,M.A.
A1 - El,Arifeen S.
A1 - Levine,M.M.
A1 - Kotloff,K.L.
A1 - Faruque,A.S.
Y1 - 2013/07//
N1 - Das, Sumon K. Nasrin, Dilruba. Ahmed, Shahnawaz. Wu, Yukun. Ferdous, Farzana. Farzana, Fahmida Dil. Khan, Soroar Hossain. Malek, Mohammad Abdul. El Arifeen, Shames. Levine, Myron M. Kotloff, Karen L. Faruque, Abu S G
The American journal of tropical medicine and hygiene
3zq, 0370507
AIM, IM
Journal Article
English
KW - MEDLINE
KW - Adult
KW - Bangladesh/ep [Epidemiology]
KW - Caregivers/px [Psychology]
KW - Child
KW - Preschool
KW - Diarrhea
KW - Infantile/ec [Economics]
KW - Infantile/ep [Epidemiology]
KW - Infantile/pp [Physiopathology]
KW - Infantile/px [Psychology]
KW - Female
KW - Health Knowledge
KW - Attitudes
KW - Practice
KW - Health Surveys
KW - Humans
KW - Infant
KW - Newborn
KW - Male
KW - Patient Acceptance of Health Care/px [Psychology]
KW - Patient Acceptance of Health Care/sn [Statistics & Numerical Data]
KW - Prevalence
KW - Rural Population
KW - Severity of Illness Index
RP - NOT IN FILE
SP - 62
EP - 68
JF - American Journal of Tropical Medicine & Hygiene
JA - Am J Trop Med Hyg
VL - 89
IS - 1 Suppl
CY - United States

N2 - We evaluated patterns of health care use for diarrhea among children 0-59 months of age residing in Mirzapur, Bangladesh, using a baseline survey conducted during May-June 2007 to inform the design of a planned diarrheal etiology case/control study. Caretakers of 7.4% of 1,128 children reported a diarrheal illness in the preceding 14 days; among 95 children with diarrhea, 24.2% had blood in the stool, 12.2% received oral rehydration solution, 27.6% received homemade fluids, and none received zinc at home. Caretakers of 87.9% sought care outside the home; 49.9% from a pharmacy, and 22.1% from a hospital or health center. The primary reasons for not seeking care were maternal perception that the illness was not serious enough (74.0%) and the high cost of treatment (21.9%). To improve management of childhood diarrhea in Mirzapur, Bangladesh, it will be important to address knowledge gaps in caretakers' assessment of illness severity, appropriate home management, and when to seek care in the formal sector. In addition, consideration should be given to inclusion of the diverse care-giving settings in clinical training activities for diarrheal disease management
SN - 1476-1645
AD - International Center for Diarrheal Disease Research, Bangladesh (icddr,b), Dhaka 1212, Bangladesh
UR - 23629937
ER -

TY - JOUR
ID - 264
T1 - Maximum hand-rung coupling forces in children: the effects of handhold diameter
A1 - Ehrlich,P.F.
A1 - Young,J.G.
A1 - Ulin,S.
A1 - Woolley,C.
A1 - Armstrong,T.J.
A1 - Galecki,A.
A1 - Chen,S.
A1 - shton-Miller,J.A.
Y1 - 2013/06//
N1 - Ehrlich, Peter F. Young, Justin G. Ulin, Sheryl. Woolley, Charles. Armstrong, Thomas J. Galecki, Andrzej. Chen, Shu. Ashton-Miller, James A
Human factors
ge5, 0374660
IM, S
Journal Article. Randomized Controlled Trial. Research Support, Non-U.S. Gov't
English
KW - MEDLINE
KW - Accidental Falls/pc [Prevention & Control]
KW - Body Weight
KW - Child
KW - Preschool
KW - Equipment Design
KW - Female
KW - Hand Strength
KW - Humans
KW - Male
KW - Muscle Strength Dynamometer
KW - Play and Playthings
RP - NOT IN FILE
SP - 545
EP - 556
JF - Human Factors
JA - Hum Factors
VL - 55
IS - 3
CY - United States
N2 - OBJECTIVE: To quantify the effect of handhold size (diameter) on the maximum breakaway strength between a hand and handhold for children. BACKGROUND: Falls from playground equipment are a major

cause of childhood injury and death. It is unclear if recommendations for handholds on playground equipment are too broad. METHODS: Breakaway strength was defined as the maximum quasistatic force that can be exerted on a grasped object before the object is forcibly pulled from the grasp. Hand anthropometry, grip, and breakaway strengths were measured for 397 children between the ages of 6 and 11 years. Three cylindrical handhold diameters were tested. RESULTS: Breakaway strength was significantly affected by handhold size, gender, and hand dominance. Significant covariate predictors for breakaway strength included grip strength, age, and hand breadth. Breakaway strength was reduced for the largest diameter (3.81 cm) for children of all ages. CONCLUSION: Handhold design factors significantly affect the breakaway strength of children. APPLICATION: The results can be used as a basis for design recommendations for hand rungs used by children to reliably support their bodyweight
SN - 0018-7208
AD - University of Michigan Medical School, CS Mott Children's Hospital 4-495, 1540 E. Hospital Drive, Ann Arbor, MI 48109-4211, USA. pehrlich@med.umich.edu
UR - 23829029
ER -

TY - JOUR
ID - 265
T1 - A cluster randomized controlled trial of an incentive-based outdoor physical activity program
A1 - Finkelstein,E.A.
A1 - Tan,Y.T.
A1 - Malhotra,R.
A1 - Lee,C.F.
A1 - Goh,S.S.
A1 - Saw,S.M.
Y1 - 2013/07//
N1 - Finkelstein, Eric A. Tan, Yock-Theng. Malhotra, Rahul. Lee, Chun-Fan. Goh, Shao-Sheng. Saw, Seang-Mei
The Journal of pediatrics
jlz, 0375410
AIM, IM
Journal Article. Randomized Controlled Trial. Research Support, Non-U.S. Gov't
English
KW - MEDLINE
KW - Child
KW - Cluster Analysis
KW - Exercise
KW - Female
KW - Humans
KW - Male
KW - Motivation
KW - Motor Activity
KW - Physical Fitness
RP - NOT IN FILE
SP - 167
EP - 172
JF - Journal of Pediatrics
JA - J Pediatr
VL - 163
IS - 1
CY - United States
N2 - OBJECTIVE: To evaluate an incentive-based physical activity intervention to increase physical activity and fitness among children in a 9-month cluster randomized controlled trial. STUDY DESIGN: Children aged 6-12 years were randomized to control (n = 138 from 106 families) or intervention arm (n = 147 from 106 families). The intervention included incentives for meeting step targets as measured by pedometers and structured weekend outdoor activities. Outcomes included trends in activity for the intervention group and between-group differences in pedometer steps, 6-minute walk test, body mass index, and parent-reported

Pediatric Quality of Life Inventory. RESULTS: At follow-up, children in the intervention group recorded significantly more pedometer steps than controls over the entire week (8660 vs 7767; P = .010), on weekdays (8646 vs 7826; P = .041), and on weekends (8779 vs 7684; P = .018). Three different trajectory classes were identified. The first group increased activity but was not sustained, the second group met the target step levels, and the third group significantly surpassed the step goals. The intervention group showed trends toward longer 6-minute walk test times and higher Pediatric Quality of Life Inventory scores, but the differences were not statistically significant. CONCLUSION: Incentives for increased step activity were effective in producing greater steps and showed a (nonsignificant) trend toward improvements in other health outcomes. Thus, future incentive trials should be incorporate greater step targets and longer follow-up periods to provide evidence of the long-term effect of these incentives on children's health. Copyright 2013 Mosby, Inc. All rights reserved
SN - 1097-6833
AD - Health Services and Systems Research, Duke-National University of Singapore Graduate Medical School, Singapore. eric.finkelstein@duke-nus.edu.sg
UR - 23415616
ER -

TY - JOUR
ID - 266
T1 - Neuroinvasive West Nile virus disease in Canada. The Saskatchewan experience
A1 - Tellez-Zenteno,J.F.
A1 - Hunter,G.
A1 - Hernandez-Ronquillo,L.
A1 - Haghir,E.
Y1 - 2013/07//
N1 - Tellez-Zenteno, Jose F. Hunter, Gary. Hernandez-Ronquillo, Lizbeth. Haghir, Edrish
The Canadian journal of neurological sciences. Le journal canadien des sciences neurologiques
cj9, 0415227
IM
Journal Article. Research Support, Non-U.S. Gov't
English
KW - MEDLINE
KW - Adolescent
KW - Adult
KW - Aged
KW - 80 and over
KW - Child
KW - Female
KW - Humans
KW - Male
KW - Middle Aged
KW - Saskatchewan/ep [Epidemiology]
KW - Time Factors
KW - West Nile Fever/co [Complications]
KW - West Nile Fever/ep [Epidemiology]
KW - Young Adult
RP - NOT IN FILE
SP - 580
EP - 584
JF - Canadian Journal of Neurological Sciences
JA - Can J Neurol Sci
VL - 40
IS - 4
CY - Canada
N2 - BACKGROUND: West Nile virus (WNV) is a virus of the family Flaviviridae. The main route of human infection is through the bite of an infected mosquito. Approximately 90% of WNV infections in humans are asymptomatic, but neurologic manifestations can be severe. METHODS: This study reviews the clinical profile of cases with neuroinvasive West Nile infection (NWNI) reported by the Surveillance program of the

government of Saskatchewan in the Saskatoon Health Region (SHR). In 2007, 1456 cases of human West Nile cases were reported by the government of Saskatchewan in the whole province. One hundred and thirteen cases had severe symptoms of NWNI (8%), 1172 (80%) cases had mild symptoms of WNI and 171 (12%) had asymptomatic disease. Three hundred and fifty six cases were reported in the SHR, where 57 (16%) fulfilled criteria for NWNI. RESULTS: From the 57 cases, 39 (68%) were females. Nine (16%) patients had a history of recent camping, two (4%) reported outdoor sports and four (8%) reported outdoor activities not otherwise specified. Twenty five patients had headache (43.9%), 25 confusion (42.1%), 23 meningitis (40.4%), 17 encephalitis (29.8%), 14 encephalopathy (24.6%), 11 meningoencephalitis (19.3%), 10 tremor (17.5%), acute flaccid paralysis 10 (17.5%), myoclonus 1 (1.8%), nystagmus 2 (3.5%), diplopia 2 (3.5%), dizziness 2 (3.5%). Three patients died related with comorbidities during admission. CONCLUSION: During a year of high occurrence of WNI in Saskatchewan, 16% of cases developed NWNI. The recognition of neurological complications associated with WNI is important to improve their referral to tertiary centers
SN - 0317-1671
AD - Division of Neurology, Department of Medicine, University of Saskatchewan, Saskatoon, Saskatchewan, Canada. jftellez@yahoo.com
UR - 23786744
ER -

TY - JOUR
ID - 267
T1 - Bone health in children and adolescents with perinatal HIV infection. [Review]
A1 - Puthanakit,T.
A1 - Siberry,G.K.
Y1 - 2013///
N1 - Puthanakit, Thanyawee. Siberry, George K
Journal of the International AIDS Society
101478566
IM, X
Journal Article. Research Support, Non-U.S. Gov't. Review
English
KW - MEDLINE
KW - Adenine/ae [Adverse Effects]
KW - Adenine/aa [Analogs & Derivatives]
KW - Adenine/tu [Therapeutic Use]
KW - Adolescent
KW - Anti-HIV Agents/ae [Adverse Effects]
KW - Anti-HIV Agents/tu [Therapeutic Use]
KW - Bone Density/de [Drug Effects]
KW - Bone Density/ph [Physiology]
KW - Bone Diseases/ci [Chemically Induced]
KW - Bone Diseases/ep [Epidemiology]
KW - Child
KW - HIV Infections/co [Complications]
KW - HIV Infections/dt [Drug Therapy]
KW - Humans
KW - Organophosphonates/ae [Adverse Effects]
KW - Organophosphonates/tu [Therapeutic Use]
KW - Prevalence
RP - NOT IN FILE
SP - 18575
JF - Journal of the International AIDS Society
JA - J Int AIDS Soc
VL - 16
CY - Switzerland
N2 - The long-term impact on bone health of lifelong HIV infection and prolonged ART in growing and developing children is not yet known. Measures of bone health in youth must be interpreted in the context of expected developmental and physiologic changes in bone mass, size, density and strength that occur from fetal

through adult life. Low bone mineral density (BMD) appears to be common in perinatally HIV-infected youth, especially outside of high-income settings, but data are limited and interpretation complicated by the need for better pediatric norms. The potential negative effects of tenofovir on BMD and bone mass accrual are of particular concern as this drug may be used more widely in younger children. Emphasizing good nutrition, calcium and vitamin D sufficiency, weight-bearing exercise and avoidance of alcohol and smoking are effective and available approaches to maintain and improve bone health in all settings. More data are needed to inform therapies and monitoring for HIV-infected youth with proven bone fragility. While very limited data suggest lack of marked increase in fracture risk for youth with perinatal HIV infection, the looming concern for these children is that they may fail to attain their expected peak bone mass in early adulthood which could increase their risk for fractures and osteoporosis later in adulthood
SN - 1758-2652
AD - Department of Pediatrics, Faculty of Medicine, Chulalongkorn University, Bangkok, Thailand
UR - 23782476
ER -

TY - JOUR
ID - 268
T1 - Validity of a self-report survey tool measuring the nutrition and physical activity environment of primary schools
A1 - Nathan,N.
A1 - Wolfenden,L.
A1 - Morgan,P.J.
A1 - Bell,A.C.
A1 - Barker,D.
A1 - Wiggers,J.
Y1 - 2013///
N1 - Nathan, Nicole. Wolfenden, Luke. Morgan, Philip J. Bell, Andrew C. Barker, Daniel. Wiggers, John
The international journal of behavioral nutrition and physical activity
101217089
IM
Journal Article. Research Support, Non-U.S. Gov't. Validation Studies
English
KW - MEDLINE
KW - Administrative Personnel
KW - Child
KW - Data Collection
KW - Diet
KW - Environment Design
KW - Exercise
KW - Faculty
KW - Food Dispensers
KW - Automatic
KW - Food Services
KW - Fund Raising
KW - Humans
KW - Interviews as Topic
KW - Lunch
KW - New South Wales
KW - Questionnaires/st [Standards]
KW - Recreation
KW - Reproducibility of Results
KW - Schools
KW - Self Report
KW - Sports
RP - NOT IN FILE
SP - 75
JF - International Journal of Behavioral Nutrition & Physical Activity

JA - Int
VL - 10
CY - England
N2 - BACKGROUND: Valid tools measuring characteristics of the school environment associated with the physical activity and dietary behaviours of children are needed to accurately evaluate the impact of initiatives to improve school environments. The aim of this study was to assess the validity of Principal self-report of primary school healthy eating and physical activity environments. METHODS: Primary school Principals (n=42) in New South Wales, Australia were invited to complete a telephone survey of the school environment; the School Environment Assessment Tool - SEAT. Equivalent observational data were collected by pre-service teachers located within the school. The SEAT, involved 65 items that assessed food availability via canteens, vending machines and fundraisers and the presence of physical activity facilities, equipment and organised physical activities. Kappa statistics were used to assess agreement between the two measures. RESULTS: Almost 70% of the survey demonstrated moderate to almost perfect agreement. Substantial agreement was found for 10 of 13 items assessing foods sold for fundraising, 3 of 6 items assessing physical activity facilities of the school, and both items assessing organised physical activities that occurred at recess and lunch and school sport. Limited agreement was found for items assessing foods sold through canteens and access to small screen recreation. CONCLUSIONS: The SEAT provides researchers and policy makers with a valid tool for assessing aspects of the school food and physical activity environment
SN - 1479-5868
AD - Hunter New England Population Health, Hunter New England Area Health Service, Newcastle, NSW, Australia. Nicole.Nathan@hnehealth.nsw.gov.au
UR - 23758936
ER -

TY - JOUR
ID - 269
T1 - Feasibility and safety of a novel in vivo model to assess playground falls in children
A1 - Ehrlich,P.F.
A1 - Young,J.G.
A1 - Ulin,S.
A1 - Wooley,C.
A1 - Armstrong,T.J.
A1 - Buschmann,B.
A1 - Galecki,A.
A1 - shton-Miller,J.A.
Y1 - 2013/07//
N1 - Ehrlich, Peter F. Young, Justin G. Ulin, Sheryl. Wooley, Charles. Armstrong, Thomas J. Buschmann, Bethany. Galecki, Andrzej. Ashton-Miller, James A
The journal of trauma and acute care surgery
101570622
AIM, IM
Comparative Study. Journal Article. Research Support, Non-U.S. Gov't
English
KW - MEDLINE
KW - Accident Prevention/mt [Methods]
KW - Accidental Falls/pc [Prevention & Control]
KW - Anthropometry
KW - Biomedical Engineering
KW - Child
KW - Preschool
KW - Equipment Design
KW - Equipment Safety
KW - Feasibility Studies
KW - Female
KW - Hand Strength
KW - Humans
KW - Male

KW - Models
KW - Theoretical
KW - Play and Playthings
KW - Sensitivity and Specificity
RP - NOT IN FILE
SP - 54
EP - 59
JF - The Journal of Trauma and Acute Care Surgery
JA - J Trauma Acute Care Surg
VL - 75
IS - 1
CY - United States
N2 - BACKGROUND: Falls are the leading cause of nonfatal unintentional injuries among hospitalized children with playground equipment accounting for more than 50%. National standards for playground rung and rail design exist, but there a lack of in vivo models available to test these standards. We developed a novel in vivo model to test rung and rail design. We report the feasibility and safety of the model. METHODS: A device was built to simulate children hanging onto a playground bar until their hand slips off. This was defined as breakaway strength. The handle unit was mounted on a vertical cable that was mechanically raised and lowered using a linear actuator controlled by the experimenter. The unit was padded and contained a video camera that recorded the posture of the hand during each trial. Breakaway force and torque were recorded as they held onto the handle by LabView software. In addition, standard anthropometrics and grip strength were recorded. RESULTS: Biomedical engineering approved the device. There were 425 eligible students aged 5 years to 11 years. Of these, 93% (397) participated (212 males and 185 females). Ninety-nine percent (396 of 397) completed all three experimental stations, one declined because of fear. There were no injuries and no falls. Average time to complete the study was 22 + 0.5 minutes. Ninety-one percent of participants were right handed; the ethnicity was representative of the local area with 79% being white. Mean + SD height, weight, and body mass index for the 397 participants were 1.28 + 0.11 m, 28.0 + 8.12 kg, and 16.31 + 2.59 kg/m2, respectively. Hand size, grip strength, and maximum breakaway force increased with age. CONCLUSION: This model is safe and feasible and maybe a viable method to assess rung and rail design for playgrounds
SN - 2163-0763
AD - Section of Pediatric Surgery, Department of Surgery, College of Engineering, University of Michigan, Ann Arbor, Michigan, USA. pehrlich@med.umich.edu
UR - 23778439
ER -

TY - JOUR
ID - 270
T1 - Mineralization of dense collagen hydrogel scaffolds by human pulp cells
A1 - Coyac,B.R.
A1 - Chicatun,F.
A1 - Hoac,B.
A1 - Nelea,V.
A1 - Chaussain,C.
A1 - Nazhat,S.N.
A1 - McKee,M.D.
Y1 - 2013/07//
N1 - Coyac, B R. Chicatun, F. Hoac, B. Nelea, V. Chaussain, C. Nazhat, S N. McKee, M D
Journal of dental research
hyv, 0354343
D, IM
Journal Article. Research Support, Non-U.S. Gov't
English
KW - MEDLINE
KW - Alkaline Phosphatase/an [Analysis]
KW - Apatites/an [Analysis]
KW - Biological Markers/an [Analysis]
KW - Calcification

KW - Physiologic/ph [Physiology]
KW - Cell Culture Techniques
KW - Cell Differentiation/ph [Physiology]
KW - Cell Shape/ph [Physiology]
KW - Cell Survival/ph [Physiology]
KW - Child
KW - Preschool
KW - Dental Pulp/cy [Cytology]
KW - Extracellular Matrix/ch [Chemistry]
KW - Extracellular Matrix Proteins/an [Analysis]
KW - Fibrillar Collagens/ch [Chemistry]
KW - Fibrillar Collagens
KW - Gels
KW - Humans
KW - Hydrogel/ch [Chemistry]
KW - Hydrogel
KW - Odontogenesis/ph [Physiology]
KW - Osteogenesis/ph [Physiology]
KW - Osteopontin/an [Analysis]
KW - Phosphoproteins/an [Analysis]
KW - Pressure
KW - Stem Cells/ph [Physiology]
KW - Time Factors
KW - Tissue Engineering/is [Instrumentation]
KW - Tissue Scaffolds/ch [Chemistry]
KW - Tissue Scaffolds
KW - Tooth
KW - Deciduous/cy [Cytology]
RP - NOT IN FILE
SP - 648
EP - 654
JF - Journal of Dental Research
JA - J Dent Res
VL - 92
IS - 7
CY - United States
N2 - While advances in biomineralization have been made in recent years, unanswered questions persist on bone- and tooth-cell differentiation, on outside-in signaling from the extracellular matrix, and on the link between protein expression and mineral deposition. In the present study, we validate the use of a bioengineered three-dimensional (3D) dense collagen hydrogel scaffold as a cell-culture model to explore these questions. Dental pulp progenitor/stem cells from human exfoliated deciduous teeth (SHEDs) were seeded into an extracellular matrix-like collagen gel whose fibrillar density was increased through plastic compression. SHED viability, morphology, and metabolic activity, as well as scaffold mineralization, were investigated over 24 days in culture. Additionally, measurements of alkaline phosphatase enzymatic activity, together with immunoblotting for mineralized tissue cell markers ALPL (tissue-non-specific alkaline phosphatase), DMP1 (dentin matrix protein 1), and OPN (osteopontin), demonstrated osteo/odontogenic cell differentiation in the dense collagen scaffolds coincident with mineralization. Analyses of the mineral phase by electron microscopy, including electron diffraction and energy-dispersive x-ray spectroscopy, combined with Fourier-transform infrared spectroscopy and biochemical analyses, were consistent with the formation of apatitic mineral that was frequently aligned along collagen fibrils. In conclusion, use of a 3D dense collagen scaffold promoted SHED osteo/odontogenic cell differentiation and mineralization
SN - 1544-0591
AD - Faculty of Dentistry, McGill University, Montreal, QC, Canada
UR - 23632809
ER -

TY - JOUR

ID - 271
T1 - A new community-based outdoor intervention to increase physical activity in Singapore children: findings from focus groups
A1 - Drury,V.B.
A1 - Saw,S.M.
A1 - Finkelstein,E.
A1 - Wong,T.Y.
A1 - Tay,P.K.
Y1 - 2013/05//
N1 - Drury, Vicki B. Saw, Seang Mei. Finkelstein, Eric. Wong, Tien Yin. Tay, Peter Kc
Annals of the Academy of Medicine, Singapore
53f, 7503289
IM
Journal Article. Research Support, Non-U.S. Gov't
English
KW - MEDLINE
KW - Attitude to Health
KW - Child
KW - Preschool
KW - Consumer Participation
KW - Counseling
KW - Feasibility Studies
KW - Focus Groups
KW - Health Behavior
KW - Health Education
KW - Health Knowledge
KW - Attitudes
KW - Practice
KW - Humans
KW - Intervention Studies
KW - Jogging/ph [Physiology]
KW - Medical Records
KW - Motivation
KW - Motor Activity
KW - Myopia/pc [Prevention & Control]
KW - Parents/px [Psychology]
KW - Program Development
KW - Reinforcement (Psychology)
KW - Running/ph [Physiology]
KW - Singapore
KW - Walking/ph [Physiology]
RP - NOT IN FILE
SP - 225
EP - 231
JF - Annals of the Academy of Medicine, Singapore
JA - Ann Acad Med Singapore
VL - 42
IS - 5
CY - Singapore
N2 - INTRODUCTION: Myopia is a significant public health problem in Singapore with estimates that more than 50% of the population is affected by it by the time of adulthood. Childhood obesity is also increasing and has been linked to long-term health problems. Recent studies have found that Singaporean children in Primary 1 spend less than 3 hours a day outdoors which is less than children in other countries. Physical activity has been shown to be protective against obesity and recently, there has been some evidence to suggest that time spent outdoors may reduce the prevalence and severity of myopia. This study aims to explore the barriers and enablers to children in Singapore participating in outdoor activities. MATERIALS AND METHODS: Qualitative data, gathered from focus group discussions was thematically analysed against the PRECEDE component of the

PRECEDE-PROCEED model which provided a conceptual framework for examining factors relevant to children participating in an outdoor activity intervention. A total of 31 people participated in 4 focus groups held over a 6-month period. RESULTS: This feasibility study was exploratory in nature but provided valuable information concerning barriers and enablers to participation. Data informed the development of a larger study. CONCLUSION: Results indicated that families preferred structured activities such as orienteering and a choice of weekend attendance days and times
SN - 0304-4602
AD - Faculty of Health Sciences, School of Nursing and Midwifery University, Australia. v.drury@curtin.edu
UR - 23771109
ER -

TY - JOUR
ID - 272
T1 - Full correction and Undercorrection of Myopia Evaluation Trial: design and baseline data of a randomized, controlled, double-blind trial
A1 - Li,S.M.
A1 - Li,S.Y.
A1 - Liu,L.R.
A1 - Guo,J.Y.
A1 - Chen,W.
A1 - Wang,N.L.
A1 - Millodot,M.
Y1 - 2013/05//
N1 - Li, Shi-Ming. Li, Si-Yuan. Liu, Luo-Ru. Guo, Ji-Yuan. Chen, Wei. Wang, Ning-Li. Millodot, Michel
Clinical & experimental ophthalmology
100896531, dq3
IM
Journal Article. Randomized Controlled Trial. Research Support, Non-U.S. Gov't
English
KW - MEDLINE
KW - Accommodation
KW - Ocular/ph [Physiology]
KW - Adolescent
KW - Axial Length
KW - Eye/pp [Physiopathology]
KW - Biometry
KW - Child
KW - Disease Progression
KW - Double-Blind Method
KW - Eyeglasses
KW - Female
KW - Humans
KW - Male
KW - Myopia/di [Diagnosis]
KW - Myopia/pp [Physiopathology]
KW - Myopia/th [Therapy]
KW - Questionnaires
KW - Refraction
KW - Research Design
KW - Treatment Outcome
KW - Visual Acuity/ph [Physiology]
RP - NOT IN FILE
SP - 329
EP - 338
JF - Clinical & Experimental Ophthalmology
JA - Clin Experiment.Ophthalmol
VL - 41

IS - 4
CY - Australia
N2 - BACKGROUND: To determine the difference in the rate of myopic progression between children wearing single vision lenses with undercorrection of +0.50 D and children whose myopia is fully corrected, and to explore the factors that may influence the process. DESIGN: Randomized, controlled, double-blind trial. PARTICIPANTS: Two hundred children aged 7-15 years with low-to-moderate myopia (-1.5 D to -6.0 D), astigmatism <-1.5 D and anisometropia <1.0 D. METHODS: The children were randomly allocated to wear single vision lenses with full correction or undercorrection by +0.50 D. Ocular examinations and questionnaire surveys for myopia-related factors will be performed every 6 months. MAIN OUTCOME MEASURES: Cycloplegic autorefraction and axial length. RESULTS: Of 200 children, 100 (50%) were girls, 41 (21%) esophoric and 82 (42%) exophoric at near. The characteristics of gender, age, age of myopia onset, phoria, eye dominance, parental myopia, refractive error, axial length, corneal curvature, mean time spent in near work and outdoor activities between the two groups were not significantly different. The accommodative responses at 33 cm, the accommodative demands and lags at infinity were significantly different in the two groups as they were measured with full correction in one group and undercorrection in the other. CONCLUSION: Full correction and Undercorrection of Myopia Evaluation Trial is a clinical trial designed to determine the effectiveness of undercorrection of myopia by +0.5 D on myopic progression in a population of school-aged children known to be susceptible to myopia and to identify the factors influencing the process. 2012 The Authors. Clinical and Experimental Ophthalmology 2012 Royal Australian and New Zealand College of Ophthalmologists
SN - 1442-9071
AD - Beijing Tongren Eye Center, Beijing Tongren Hospital, Capital Medical University, Beijing, China
UR - 23009037
ER -

TY - JOUR
ID - 273
T1 - Periodic fever, aphthous stomatitis, pharyngitis, cervical adenitis syndrome is linked to dysregulated monocyte IL-1beta production
A1 - Kolly,L.
A1 - Busso,N.
A1 - von Scheven-Gete,A.
A1 - Bagnoud,N.
A1 - Moix,I.
A1 - Holzinger,D.
A1 - Simon,G.
A1 - Ives,A.
A1 - Guarda,G.
A1 - So,A.
A1 - Morris,M.A.
A1 - Hofer,M.
Y1 - 2013/06//
N1 - Kolly, Laeticia. Busso, Nathalie. von Scheven-Gete, Annette. Bagnoud, Nathaliane. Moix, Isabelle. Holzinger, Dirk. Simon, Gregoire. Ives, Annette. Guarda, Greta. So, Alexander. Morris, Michael A. Hofer, Michael
The Journal of allergy and clinical immunology
h53, 1275002
AIM, IM
Journal Article. Research Support, Non-U.S. Gov't
English
KW - MEDLINE
KW - Adolescent
KW - Adult
KW - Aged
KW - Child
KW - Female
KW - Fever/ge [Genetics]
KW - Fever/im [Immunology]

KW - Fever/me [Metabolism]
KW - Genetic Variation
KW - Humans
KW - Inflammation/im [Immunology]
KW - Inflammation/me [Metabolism]
KW - Inflammation Mediators/bl [Blood]
KW - Interleukin 1 Receptor Antagonist Protein/me [Metabolism]
KW - Interleukin-1beta/bi [Biosynthesis]
KW - Leukocyte Count
KW - Leukocytes
KW - Mononuclear/im [Immunology]
KW - Mononuclear/me [Metabolism]
KW - Lipopolysaccharides/im [Immunology]
KW - Lymphadenitis/ge [Genetics]
KW - Lymphadenitis/im [Immunology]
KW - Lymphadenitis/me [Metabolism]
KW - Male
KW - Middle Aged
KW - Monocytes/im [Immunology]
KW - Monocytes/me [Metabolism]
KW - Neutrophils
KW - Pharyngitis/ge [Genetics]
KW - Pharyngitis/im [Immunology]
KW - Pharyngitis/me [Metabolism]
KW - Stomatitis
KW - Aphthous/ge [Genetics]
KW - Aphthous/im [Immunology]
KW - Aphthous/me [Metabolism]
KW - Syndrome
KW - Young Adult
RP - NOT IN FILE
SP - 1635
EP - 1643
JF - Journal of Allergy & Clinical Immunology
JA - J Allergy Clin Immunol
VL - 131
IS - 6
CY - United States
N2 - BACKGROUND: The exact pathogenesis of the pediatric disorder periodic fever, aphthous stomatitis, pharyngitis, cervical adenitis (PFAPA) syndrome is unknown. OBJECTIVES: We hypothesized that PFAPA might be due to dysregulated monocyte IL-1beta production linked to genetic variants in proinflammatory genes. METHODS: Fifteen patients with PFAPA syndrome were studied during and outside a febrile episode. Hematologic profile, inflammatory markers, and cytokine levels were measured in the blood. The capacity of LPS-stimulated PBMCs and monocytes to secrete IL-1beta was assessed by using ELISA, and active IL-1beta secretion was visualized by means of Western blotting. Real-time quantitative PCR was performed to assess cytokine gene expression. DNA was screened for variants of the MEFV, TNFRSF1A, MVK, and NLRP3 genes in a total of 57 patients with PFAPA syndrome. RESULTS: During a febrile attack, patients with PFAPA syndrome revealed significantly increased neutrophil counts, erythrocyte sedimentation rates, and C-reactive protein, serum amyloid A, myeloid-related protein 8/14, and S100A12 levels compared with those seen outside attacks. Stimulated PBMCs secreted significantly more IL-1beta during an attack (during a febrile episode, 575 + 88 pg/mL; outside a febrile episode, 235 + 56 pg/mL; P < .001), and this was in the mature active p17 form. IL-1beta secretion was inhibited by ZYVAD, a caspase inhibitor. Similar results were found for stimulated monocytes (during a febrile episode, 743 + 183 pg/mL; outside a febrile episode, 227 + 92 pg/mL; P < .05). Genotyping identified variants in 15 of 57 patients, with 12 NLRP3 variants, 1 TNFRSF1A variant, 4 MEFV variants, and 1 MVK variant. CONCLUSION: Our data strongly suggest that IL-1beta monocyte production is dysregulated in patients with PFAPA syndrome. Approximately 20% of them were found to have NLRP3 variants, suggesting that inflammasome-related genes might be involved in this autoinflammatory syndrome.

Copyright 2012 American Academy of Allergy, Asthma & Immunology. Published by Mosby, Inc. All rights reserved
SN - 1097-6825
AD - Rheumatology Service, DAL, Lausanne University Hospital, University of Lausanne, Lausanne, Switzerland
UR - 23006543
ER -

TY - JOUR
ID - 274
T1 - Increasing physical activity in under-resourced communities through school-based, joint-use agreements, Los Angeles County, 2010-2012
A1 - Lafleur,M.
A1 - Gonzalez,E.
A1 - Schwarte,L.
A1 - Banthia,R.
A1 - Kuo,T.
A1 - Verderber,J.
A1 - Simon,P.
Y1 - 2013///
N1 - Lafleur, Mariah. Gonzalez, Eloisa. Schwarte, Liz. Banthia, Rajni. Kuo, Tony. Verderber, Joanie. Simon, Paul
Preventing chronic disease
101205018
IM
Journal Article. Research Support, U.S. Gov't, P.H.S.
English
KW - MEDLINE
KW - Adult
KW - Child
KW - Community-Institutional Relations
KW - Cross-Sectional Studies
KW - Female
KW - Health Promotion
KW - Humans
KW - Los Angeles/ep [Epidemiology]
KW - Male
KW - Motor Activity
KW - Obesity/pc [Prevention & Control]
KW - Poverty
KW - Public Facilities/sn [Statistics & Numerical Data]
KW - School Health Services/og [Organization & Administration]
KW - School Health Services/sn [Statistics & Numerical Data]
KW - Schools/sn [Statistics & Numerical Data]
RP - NOT IN FILE
SP - E89
JF - Preventing Chronic Disease
JA - Prev Chronic Dis
VL - 10
CY - United States
N2 - INTRODUCTION: Few studies have examined how joint-use agreements between schools and communities affect use of school facilities after hours for physical activity in under-resourced communities. The objective of this study was to assess whether these agreements can increase community member use of these opened spaces outside of school hours. METHODS: Trained observers conducted school site observations after joint-use agreements were implemented in 7 Los Angeles County school districts. All 7 districts had disproportionately high adult and child obesity rates, and all had executed a joint-use agreement between schools and community or government entities from January 2010 through December 2012. To assess use, we

adapted the System for Observing Play and Recreation in Communities (SOPARC) instrument to record the number, demographic characteristics, and physical activity levels of community members who used the joint-use school sites. To supplement observations, we collected contextual information for each location, including the existence of physical activity programs at the site and the condition of exercise equipment. RESULTS: We completed 172 SOPARC observations and related environmental assessments for 12 school sites. Observations made on 1,669 site users showed that most of them were Hispanic and nearly half were adults; three-quarters engaged in moderate to vigorous physical activity. Community member use of school sites was 16 times higher in joint-use schools that had physical activity programs than in schools without such programs. CONCLUSION: Joint-use agreements are a promising strategy for increasing moderate to vigorous physical activity among adults and children in under-resourced communities. Providing physical activity programs may substantially increase after-hours use of school facilities by community members
SN - 1545-1151
AD - The Sarah Samuels Center for Public Health Research and Evaluation, Oakland, CA 94612, USA. mariah@samuelscenter.com
UR - 23721790
ER -

TY - JOUR
ID - 275
T1 - Attentional threat avoidance and familial risk are independently associated with childhood anxiety disorders
A1 - Brown,H.M.
A1 - McAdams,T.A.
A1 - Lester,K.J.
A1 - Goodman,R.
A1 - Clark,D.M.
A1 - Eley,T.C.
Y1 - 2013/06//
N1 - Brown, Hannah M. McAdams, Tom A. Lester, Kathryn J. Goodman, Robert. Clark, David M. Eley, Thalia C
Journal of child psychology and psychiatry, and allied disciplines
hp3, 0375361
IM
Journal Article. Research Support, Non-U.S. Gov't. Twin Study
English
KW - MEDLINE
KW - Anxiety Disorders/di [Diagnosis]
KW - Anxiety Disorders/ge [Genetics]
KW - Anxiety Disorders/px [Psychology]
KW - Attention
KW - Child
KW - Color Perception
KW - Diseases in Twins/di [Diagnosis]
KW - Diseases in Twins/ge [Genetics]
KW - Diseases in Twins/px [Psychology]
KW - Emotions
KW - Facial Expression
KW - Female
KW - Field Dependence-Independence
KW - Gene-Environment Interaction
KW - Genetic Predisposition to Disease/ge [Genetics]
KW - Genetic Predisposition to Disease/px [Psychology]
KW - Harm Reduction
KW - Humans
KW - Male
KW - Orientation
KW - Pattern Recognition

KW - Visual
KW - Reaction Time
RP - NOT IN FILE
SP - 678
EP - 685
JF - Journal of Child Psychology & Psychiatry & Allied Disciplines
JA - J Child Psychol Psychiatry
VL - 54
IS - 6
CY - England
N2 - BACKGROUND: Twin studies in children reveal that familial aggregation of anxiety disorders is due to both genetic and environmental factors. Cognitive biases for threat information are considered a robust characteristic of childhood anxiety. However, little is known regarding the underlying aetiology of such biases and their role in anxiety disorders. METHOD: A face version of the dot-probe task measuring attentional biases for negative (anger, fear, sad, disgust) and positive (happy) facial expressions was completed by 600, 8-year-old twins; the largest study of its kind. Twin correlations for attentional bias scores were compared to estimate genetic and environmental effects. Parent-report diagnostic interviews identified children with an anxiety disorder. Indices of inferred genetic and familial risk for anxiety disorders were created for each child. Data were analysed using a series of logistic regressions. RESULTS: Anxious children showed greater attentional avoidance of negative faces than nonanxious children; t (548) = 2.55, p < .05. Attentional avoidance was not under genetic or shared environmental influence. Risk for anxiety disorders was predicted by familial factors. Both attentional avoidance and inferred familial risk were significant but independent predictors of anxiety disorders (ORs = .65 and 3.64, respectively). CONCLUSIONS: Anxiety-related attentional biases and familial risk play important but independent roles in childhood anxiety disorders. If replicated, these findings indicate that links between genetic risk and anxiety disorders lie outside the domain of attentional processes. 2012 The Authors. Journal of Child Psychology and Psychiatry 2012 Association for Child and Adolescent Mental Health
SN - 1469-7610
AD - Social, Genetic and Developmental Psychiatry Centre, Institute of Psychiatry, King's College London, London, UK. hannah.brown@kcl.ac.uk
UR - 23176633
ER -

TY - JOUR
ID - 276
T1 - Environmental lead exposure risks associated with children's outdoor playgrounds
A1 - Taylor,M.P.
A1 - Camenzuli,D.
A1 - Kristensen,L.J.
A1 - Forbes,M.
A1 - Zahran,S.
Y1 - 2013/07//
N1 - Taylor, Mark Patrick. Camenzuli, Danielle. Kristensen, Louise Jane. Forbes, Miriam. Zahran, Sammy
Environmental pollution (Barking, Essex : 1987)
dvl, 8804476
IM
Journal Article. Research Support, Non-U.S. Gov't
English
KW - MEDLINE
KW - Air Pollutants/an [Analysis]
KW - Child
KW - Child Care
KW - Environmental Exposure/an [Analysis]
KW - Environmental Exposure/sn [Statistics & Numerical Data]
KW - Environmental Pollution/sn [Statistics & Numerical Data]
KW - Humans
KW - Lead/an [Analysis]
KW - Lead Poisoning/ep [Epidemiology]

KW - Metallurgy
KW - Play and Playthings
KW - Risk Assessment
KW - Soil Pollutants/an [Analysis]
KW - South Australia
RP - NOT IN FILE
SP - 447
EP - 454
JF - Environmental Pollution
JA - Environ Pollut.
VL - 178
CY - England
N2 - This study examines exposure risks associated with lead smelter emissions at children's public playgrounds in Port Pirie, South Australia. Lead and other metal values were measured in air, soil, surface dust and on pre- and post-play hand wipes. Playgrounds closest to the smelter were significantly more lead contaminated compared to those further away (t(27.545) = 3.76; p = .001). Port Pirie post-play hand wipes contained significantly higher lead loadings (maximum hand lead value of 49,432 mug/m(2)) than pre-play hand wipes (t(27) = 3.57, p = .001). A 1% increase in air lead (mug/m(3)) was related to a 0.713% increase in lead dust on play surfaces (95% CI, 0.253-1.174), and a 0.612% increase in post-play wipe lead (95% CI, 0.257-0.970). Contaminated dust from smelter emissions is determined as the source and cause of childhood lead poisoning at a rate of approximately one child every third day. Copyright 2013 Elsevier Ltd. All rights reserved
SN - 1873-6424
AD - Environmental Science, Department of Environment and Geography, Faculty of Science, Macquarie University, North Ryde, Sydney, NSW 2109, Australia. mark.taylor@mq.edu.au
UR - 23643852
ER -

TY - JOUR
ID - 277
T1 - Child outdoor physical activity is reduced by prevalence of the Asian tiger mosquito, Aedes albopictus
A1 - Worobey,J.
A1 - Fonseca,D.M.
A1 - Espinosa,C.
A1 - Healy,S.
A1 - Gaugler,R.
Y1 - 2013/03//
N1 - Worobey, John. Fonseca, Dina M. Espinosa, Carolina. Healy, Sean. Gaugler, Randy
Journal of the American Mosquito Control Association
jao, 8511299
IM
Comparative Study. Journal Article. Research Support, Non-U.S. Gov't. Research Support, U.S. Gov't, Non-P.H.S.
English
KW - MEDLINE
KW - Aedes
KW - Animals
KW - Child
KW - Exercise
KW - Humans
KW - Mosquito Control
KW - Obesity
KW - Urban Population
RP - NOT IN FILE
SP - 78
EP - 80
JF - Journal of the American Mosquito Control Association
JA - J Am Mosq.Control Assoc

VL - 29
IS - 1
CY - United States
N2 - We tested the hypothesis that day-biting mosquitoes contribute to child obesity by reducing opportunities for summer outdoor play. The influence of Asian tiger mosquito (Aedes albopictus) prevalence on child outdoor physical activity was compared in 2 matched urban communities, one treated for mosquito abatement and one untreated. More time was spent outdoors by children where abatement took place
SN - 8756-971X
AD - Department of Nutritional Sciences, Rutgers University, New Brunswick, NJ 08901, USA
UR - 23687862
ER -

TY - JOUR
ID - 278
T1 - [Risk factors for asthma in children in Hefei, China]. [Chinese]
A1 - Xiong,M.
A1 - Ni,C.
A1 - Pan,J.H.
A1 - Wang,Q.
A1 - Zheng,L.L.
Y1 - 2013/05//
N1 - Xiong, Mei. Ni, Chen. Pan, Jia-Hua. Wang, Qiang. Zheng, Li-Lin
Zhongguo dang dai er ke za zhi = Chinese journal of contemporary pediatrics
100909956
IM
English Abstract. Journal Article
Chinese
KW - MEDLINE
KW - Adolescent
KW - Asthma/et [Etiology]
KW - Asthma/pc [Prevention & Control]
KW - Case-Control Studies
KW - Child
KW - Preschool
KW - China
KW - Female
KW - Humans
KW - Infant
KW - Newborn
KW - Logistic Models
KW - Male
KW - Risk Factors
RP - NOT IN FILE
SP - 364
EP - 367
JF - Zhongguo Dangdai Erke Zazhi
JA - Zhongguo Dang.Dai.Er Ke Za Zhi
VL - 15
IS - 5
CY - China
N2 - OBJECTIVE: To investigate the risk factors for asthma in children in Hefei, China and to provide a strategy for asthma control in this region. METHODS: A total of 400 children with a confirmed diagnosis of asthma, as well as 400 children of comparable age, sex, living environment, and family background, who had no respiratory diseases, were selected for a case-control study. A survey questionnaire survey was completed for all children. The obtained data were subjected to univariate and multivariate logistic regression analysis to determine the risk factors for asthma. RESULTS: The logistic regression analysis showed that a family history of allergy, allergic rhinitis, infantile eczema, no breastfeeding, air-conditioning and passive smoking were the

risk factors for asthma in children, with odds ratios of 9.63, 7.56, 4.58, 2.16, 1.73, and 1.55 respectively. CONCLUSIONS: In order to reduce the incidence of asthma, we should advocate breast feeding, promote outdoor activities, keep ventilation natural, prevent passive smoking and cure allergic rhinitis
SN - 1008-8830
AD - Department of Pediatrics, Provincial Hospital Affiliated to Anhui Medical University, Hefei 231000, China
UR - 23676939
ER -

TY - JOUR
ID - 279
T1 - Modifiable lifestyle behavior patterns, sedentary time and physical activity contexts: a cluster analysis among middle school boys and girls in the SALTA study
A1 - Marques,E.A.
A1 - Pizarro,A.N.
A1 - Figueiredo,P.
A1 - Mota,J.
A1 - Santos,M.P.
Y1 - 2013/06//
N1 - Marques, Elisa A. Pizarro, Andreia N. Figueiredo, Pedro. Mota, Jorge. Santos, Maria P
Preventive medicine
pm4, 0322116
IM
Journal Article. Research Support, Non-U.S. Gov't
English
KW - MEDLINE
KW - Body Mass Index
KW - Child
KW - Cholesterol
KW - HDL/bl [Blood]
KW - Cluster Analysis
KW - Exercise Test
KW - Female
KW - Health Behavior
KW - Humans
KW - Male
KW - Motor Activity
KW - Physical Fitness
KW - Portugal
KW - Recreation
KW - Risk Assessment
KW - Risk Factors
KW - Sedentary Lifestyle
KW - Students/sn [Statistics & Numerical Data]
KW - Travel
RP - NOT IN FILE
SP - 413
EP - 415
JF - Preventive Medicine
JA - Prev Med
VL - 56
IS - 6
CY - United States
N2 - OBJECTIVE: To analyze how modifiable health-related variables are clustered and associated with children's participation in play, active travel and structured exercise and sport among boys and girls. METHODS: Data were collected from 9 middle-schools in Porto (Portugal) area. A total of 636 children in the 6th grade (340 girls and 296 boys) with a mean age of 11.64 years old participated in the study. Cluster analyses

were used to identify patterns of lifestyle and healthy/unhealthy behaviors. Multinomial logistic regression analysis was used to estimate associations between cluster allocation, sedentary time and participation in three different physical activity (PA) contexts: play, active travel, and structured exercise/sport. RESULTS: Four distinct clusters were identified based on four lifestyle risk factors. The most disadvantaged cluster was characterized by high body mass index, low high-density lipoprotein cholesterol and cardiorespiratory fitness and a moderate level of moderate to vigorous PA. Everyday outdoor play (OR=1.85, 95%CI 0.318-0.915) and structured exercise/sport (OR=1.85, 95%CI 0.291-0.990) were associated with healthier lifestyle patterns. There were no significant associations between health patterns and sedentary time or travel mode. CONCLUSION: Outdoor play and sport/exercise participation seem more important than active travel from school in influencing children's healthy cluster profiles. Copyright 2013 Elsevier Inc. All rights reserved
SN - 1096-0260
AD - Research Centre in Physical Activity, Health and Leisure (CIAFEL), Faculty of Sport Science, University of Porto, Porto, Portugal. emarques@fade.up.pt
UR - 23567246
ER -

TY - JOUR
ID - 280
T1 - Prenatal and postnatal hormone effects on the human brain and cognition. [Review]
A1 - Auyeung,B.
A1 - Lombardo,M.V.
A1 - Baron-Cohen,S.
Y1 - 2013/05//
N1 - Auyeung, Bonnie. Lombardo, Michael V. Baron-Cohen, Simon
Pflugers Archiv : European journal of physiology
ozx, 0154720
IM
Journal Article. Research Support, Non-U.S. Gov't. Review
English
KW - MEDLINE
KW - Brain/em [Embryology]
KW - Brain/gd [Growth & Development]
KW - Brain/me [Metabolism]
KW - Brain/ph [Physiology]
KW - Child
KW - Child Development
KW - Cognition
KW - Female
KW - Humans
KW - Male
KW - Oxytocin/me [Metabolism]
KW - Sex Characteristics
KW - Testosterone/me [Metabolism]
KW - Young Adult
RP - NOT IN FILE
SP - 557
EP - 571
JF - Pflugers Archiv - European Journal of Physiology
JA - Pflugers Arch
VL - 465
IS - 5
CY - Germany
N2 - This review examines the role of hormones in the development of social and nonsocial cognition and the brain. Research findings from human studies designed to elucidate the effects of both prenatal and postnatal exposure to hormones in children and young adults are summarized. Effects are found to be both time and dose dependent, with exposure to abnormal hormone levels having a limited impact outside the "critical window" in development. Particular attention is given to the role of prenatal hormone exposure, which appears to be vital

for early organization of the brain. In later life, measurements of circulating hormone levels and the administration of testosterone and oxytocin are found to predict behavior, but the effect is thought to be one of "activation" or "fine-tuning" of the early organization of the brain. Possible directions for valuable future research are discussed
SN - 1432-2013
AD - Autism Research Centre, Department of Psychiatry, University of Cambridge, Douglas House, 18b Trumpington Road, Cambridge, CB2 8AH, UK. ba251@cam.ac.uk
UR - 23588379
ER -

TY - JOUR
ID - 281
T1 - Patterns of myopigenic activities with age, gender and ethnicity in Sydney schoolchildren
A1 - French,A.N.
A1 - Morgan,I.G.
A1 - Mitchell,P.
A1 - Rose,K.A.
Y1 - 2013/05//
N1 - French, Amanda N. Morgan, Ian G. Mitchell, Paul. Rose, Kathryn A
Ophthalmic & physiological optics : the journal of the British College of Ophthalmic Opticians (Optometrists)
onk, 8208839
IM
Journal Article. Research Support, Non-U.S. Gov't
English
KW - MEDLINE
KW - Age Factors
KW - Analysis of Variance
KW - Asian Continental Ancestry Group
KW - Child
KW - Cohort Studies
KW - European Continental Ancestry Group
KW - Female
KW - Follow-Up Studies
KW - Humans
KW - Leisure Activities
KW - Male
KW - Myopia/eh [Ethnology]
KW - Myopia/et [Etiology]
KW - New South Wales
KW - Questionnaires
KW - Reading
KW - Sex Factors
RP - NOT IN FILE
SP - 318
EP - 328
JF - Ophthalmic & Physiological Optics
JA - Ophthalmic Physiol Opt.
VL - 33
IS - 3
CY - England
N2 - PURPOSE: To examine the patterns of myopigenic activity (high near work, low time outdoors) in children growing up in Sydney, Australia, by age, ethnicity and gender. METHODS: The Sydney Adolescent Vascular and Eye Study (SAVES) re-examined children from the two age cohorts (6 and 12 years at baseline) from the Sydney Myopia Study (SMS). At 5-6 year follow-up, 863 in the younger cohort and 1196 in the older cohort had complete refraction data. Cycloplegic autorefraction (cyclopentolate 1%; Canon RK-F1) was measured at baseline and follow-up. Children who became myopic (<-0.50 dioptres spherical equivalent refraction) were those classified as non-myopic at baseline and myopic at follow-up. A detailed questionnaire

was administered to measure weekly activities, including time spent outdoors and near work at both baseline and follow-up examination. RESULTS: Overall, 128 (14.8%) children in the younger cohort and 210 (17.6%) in the older cohort became myopic. At follow-up, for both cohorts, children had significantly reduced the amount of time spent outdoors (younger cohort, p = 0.001, older cohort, p < 0.0001) and increased near work time (younger cohort, p < 0.0001, older cohort, p = 0.006). Children of East Asian ethnicity spent significantly less time outdoors by more than 7 h per week (both cohorts at baseline and follow-up, all p < 0.0001) and more time in near work activities by close to 3 h compared to European Caucasian children at all ages examined (both cohorts at baseline and follow-up all, p < 0.03). The average pattern of activity for girls differed from that of boys in a similar way (both cohorts at baseline and follow-up all, p < 0.0001). The two independent samples of 12 year-old children provided by follow-up in the younger cohort and baseline in the older cohort gave very similar answers to the questionnaire, with significant differences only evident for computer use (p = 0.001) and books read (p < 0.0001). CONCLUSIONS: Answers to the activity questionnaire were very similar in the two cohorts of 12 year-olds, suggesting that the questionnaire gives reproducible answers. However, further work is required for validation. Children's pattern of activities become more myopigenic with age, and differed by gender and by ethnicity at all ages, with girls having a more myopigenic activity pattern than boys, and children of East Asian ancestry having a more myopigenic activity pattern than European Caucasian children.
Ophthalmic & Physiological Optics 2013 The College of Optometrists
SN - 1475-1313
AD - Discipline of Orthoptics, Faculty of Health Sciences, University of Sydney, Sydney, Australia
UR - 23452023
ER -

TY - JOUR
ID - 282
T1 - Standardizing operational vector sampling techniques for measuring malaria transmission intensity: evaluation of six mosquito collection methods in western Kenya
A1 - Wong,J.
A1 - Bayoh,N.
A1 - Olang,G.
A1 - Killeen,G.F.
A1 - Hamel,M.J.
A1 - Vulule,J.M.
A1 - Gimnig,J.E.
Y1 - 2013///
N1 - Wong, Jacklyn. Bayoh, Nabie. Olang, George. Killeen, Gerry F. Hamel, Mary J. Vulule, John M. Gimnig, John E
Malaria journal
101139802
IM
Comparative Study. Evaluation Studies. Journal Article. Research Support, Non-U.S. Gov't
English
KW - MEDLINE
KW - Adult
KW - Animals
KW - Child
KW - Preschool
KW - Culicidae/cl [Classification]
KW - Culicidae/gd [Growth & Development]
KW - Culicidae/ps [Parasitology]
KW - Disease Vectors
KW - Entomology/mt [Methods]
KW - Entomology/st [Standards]
KW - Female
KW - Humans
KW - Infant
KW - Kenya
KW - Malaria/tm [Transmission]

KW - Male
KW - Population Density
RP - NOT IN FILE
SP - 143
JF - Malaria Journal
JA - Malar.J
VL - 12
CY - England
N2 - BACKGROUND: Operational vector sampling methods lack standardization, making quantitative comparisons of malaria transmission across different settings difficult. Human landing catch (HLC) is considered the research gold standard for measuring human-mosquito contact, but is unsuitable for large-scale sampling. This study assessed mosquito catch rates of CDC light trap (CDC-LT), Ifakara tent trap (ITT), window exit trap (WET), pot resting trap (PRT), and box resting trap (BRT) relative to HLC in western Kenya to 1) identify appropriate methods for operational sampling in this region, and 2) contribute to a larger, overarching project comparing standardized evaluations of vector trapping methods across multiple countries. METHODS: Mosquitoes were collected from June to July 2009 in four districts: Rarieda, Kisumu West, Nyando, and Rachuonyo. In each district, all trapping methods were rotated 10 times through three houses in a 3 x 3 Latin Square design. Anophelines were identified by morphology and females classified as fed or non-fed. Anopheles gambiae s.l. were further identified as Anopheles gambiae s.s. or Anopheles arabiensis by PCR. Relative catch rates were estimated by negative binomial regression. RESULTS: When data were pooled across all four districts, catch rates (relative to HLC indoor) for An. gambiae s.l (95.6% An. arabiensis, 4.4% An. gambiae s.s) were high for HLC outdoor (RR = 1.01), CDC-LT (RR = 1.18), and ITT (RR = 1.39); moderate for WET (RR = 0.52) and PRT outdoor (RR = 0.32); and low for all remaining types of resting traps (PRT indoor, BRT indoor, and BRT outdoor; RR < 0.08 for all). For Anopheles funestus, relative catch rates were high for ITT (RR = 1.21); moderate for HLC outdoor (RR = 0.47), CDC-LT (RR = 0.69), and WET (RR = 0.49); and low for all resting traps (RR < 0.02 for all). At finer geographic scales, however, efficacy of each trap type varied from district to district. CONCLUSIONS: ITT, CDC-LT, and WET appear to be effective methods for large-scale vector sampling in western Kenya. Ultimately, choice of collection method for operational surveillance should be driven by trap efficacy and scalability, rather than fine-scale precision with respect to HLC. When compared with recent, similar trap evaluations in Tanzania and Zambia, these data suggest that traps which actively lure host-seeking females will be most useful for surveillance in the face of declining vector densities
SN - 1475-2875
AD - Centers for Disease Control and Prevention, Division of Parasitic Diseases and Malaria, Atlanta, GA, USA. vsm0@cdc.gov
UR - 23631641
ER -

TY - JOUR
ID - 283
T1 - Outdoor activity during class recess reduces myopia onset and progression in school children
A1 - Wu,P.C.
A1 - Tsai,C.L.
A1 - Wu,H.L.
A1 - Yang,Y.H.
A1 - Kuo,H.K.
Y1 - 2013/05//
N1 - Wu, Pei-Chang. Tsai, Chia-Ling. Wu, Hsiang-Lin. Yang, Yi-Hsin. Kuo, Hsi-Kung
Ophthalmology
7802443
IM
Journal Article. Research Support, Non-U.S. Gov't
English
KW - MEDLINE
KW - Child
KW - Disease Progression
KW - Female

KW - Humans
KW - Incidence
KW - Leisure Activities
KW - Male
KW - Myopia/ep [Epidemiology]
KW - Myopia/et [Etiology]
KW - Myopia/pc [Prevention & Control]
KW - Prospective Studies
KW - Regression Analysis
KW - Schools
KW - Taiwan/ep [Epidemiology]
KW - Urban Population/sn [Statistics & Numerical Data]
RP - NOT IN FILE
SP - 1080
EP - 1085
JF - Ophthalmology
VL - 120
IS - 5
CY - United States
N2 - PURPOSE: The aim of this study was to investigate the effect of outdoor activity during class recess on myopia changes among elementary school students in a suburban area of Taiwan. DESIGN: Prospective, comparative, consecutive, interventional study. PARTICIPANTS: Elementary school students 7 to 11 years of age recruited from 2 nearby schools located in a suburban area of southern Taiwan. INTERVENTION: The children of one school participated in the interventions, whereas those from the other school served as the control group. The interventions consisted of performing a recess outside the classroom (ROC) program that encouraged children to go outside for outdoor activities during recess. The control school did not have any special programs during recess. MAIN OUTCOME MEASURES: Data were obtained by means of a parent questionnaire and ocular evaluations that included axial length and cycloplegic autorefraction at the beginning and after 1 year. RESULTS: Five hundred seventy-one students were recruited for this study, of whom 333 students participated in the interventional program, and 238 students were in the control school. At the beginning of the study, there were no significant differences between these 2 schools with regard to age, gender, baseline refraction, and myopia prevalence (47.75% vs. 49.16%). After 1 year, new onset of myopia was significantly lower in the ROC group than in the control group (8.41% vs. 17.65%; P<0.001). There was also significantly lower myopic shift in the ROC group compared with the control group (-0.25 diopter [D]/year vs. -0.38 D/year; P = 0.029). The multivariate analysis demonstrated that the variables of intervention of the ROC program and higher school year proved to be a protective factor against myopia shift in nonmyopic subjects (P = 0.020 and P = 0.017, respectively). For myopic subjects, school year was the only variable significantly associated with myopia progression (P = 0.006). CONCLUSIONS: Outdoor activities during class recess in school have a significant effect on myopia onset and myopic shift. Such activities have a prominent effect on the control of myopia shift, especially in nonmyopic children. FINANCIAL DISCLOSURE(S): The author(s) have no proprietary or commercial interest in any materials discussed in this article. Copyright 2013 American Academy of Ophthalmology. Published by Elsevier Inc. All rights reserved
SN - 1549-4713
AD - Department of Ophthalmology, Kaohsiung Chang Gung Memorial Hospital, and Chang Gung University College of Medicine, Kaohsiung, Taiwan
UR - 23462271
ER -

TY - JOUR
ID - 284
T1 - Effects of the Start For Life treatment on physical activity in primarily African American preschool children of ages 3-5 years
A1 - Annesi,J.J.
A1 - Smith,A.E.
A1 - Tennant,G.A.
Y1 - 2013///

N1 - Annesi, James J. Smith, Alice E. Tennant, Gisele A
Psychology, health & medicine
9604099
IM
Journal Article. Randomized Controlled Trial. Research Support, Non-U.S. Gov't
English
KW - MEDLINE
KW - Accelerometry
KW - African Americans/ed [Education]
KW - African Americans/sn [Statistics & Numerical Data]
KW - Analysis of Variance
KW - Child
KW - Preschool
KW - Early Intervention (Education)/mt [Methods]
KW - Exercise/ph [Physiology]
KW - Female
KW - Humans
KW - Male
KW - Motor Activity/ph [Physiology]
KW - Overweight/ep [Epidemiology]
KW - Overweight/pc [Prevention & Control]
KW - Program Evaluation
KW - Psychological Theory
KW - Self Efficacy
KW - Treatment Outcome
KW - United States/ep [Epidemiology]
RP - NOT IN FILE
SP - 300
EP - 309
JF - Psychology Health & Medicine
JA - PSYCHOL HEALTH MED
VL - 18
IS - 3
CY - England
N2 - In U.S. children of ages 2-5 years, combined overweight and obesity has increased to 21%, with African American children of this age range highest at 26%. Lack of physical activity is highly predictive of overweight and obesity in children. Preschools may be a useful point for intervention. An innovative preschool physical activity treatment (Start For Life) was developed based on principles of social cognitive and self-efficacy theory. It incorporated 30 minutes daily of highly structured physical activity with behavioral and self-regulatory skills training (e.g. goal setting, self-monitoring, productive self-talk) interspersed. Data obtained from accelerometry was used to contrast physical activity outputs during the preschool day in the Start For Life condition (n = 202) with a usual-care control condition (n = 136). After controlling for age and sex of the primarily African American participants (M age = 4.7 years), changes over eight weeks in moderate-to-vigorous and vigorous physical activity were significant, and significantly more favorable in the Start For Life group; F(1, 344) = 4.98, p = .026 and F(1, 344) = 3.60, p = .058, respectively. Start For Life was associated with a weekly increase in moderate-to-vigorous physical activity of approximately 40 minutes. After sufficient replications that better account for different sample types, parental effects and physical activity outside of the school day, and long-term effects, widespread dissemination may be considered
SN - 1465-3966
AD - YMCA of Metropolitan Atlanta, Atlanta, GA, USA. jamesa@ymcaatlanta.org
UR - 22882084
ER -

TY - JOUR
ID - 285
T1 - Physical activity and its correlates in children: a cross-sectional study (the GINIplus & LISAplus studies)
A1 - Ortlieb,S.

A1 - Schneider,G.
A1 - Koletzko,S.
A1 - Berdel,D.
A1 - von,Berg A.
A1 - Bauer,C.P.
A1 - Schaaf,B.
A1 - Herbarth,O.
A1 - Lehmann,I.
A1 - Hoffmann,B.
A1 - Heinrich,J.
A1 - Schulz,H.
A1 - GINIplus and LISAplus Study Groups.
Y1 - 2013///
N1 - Ortlieb, Sandra. Schneider, Gabriel. Koletzko, Sibylle. Berdel, Dietrich. von Berg, Andrea. Bauer, Carl-Peter. Schaaf, Beate. Herbarth, Olf. Lehmann, Irina. Hoffmann, Barbara. Heinrich, Joachim. Schulz, Holger. GINIplus and LISAplus Study Groups
BMC public health
100968562
IM
Journal Article. Research Support, Non-U.S. Gov't
English
KW - MEDLINE
KW - Adult
KW - Body Mass Index
KW - Child
KW - Cohort Studies
KW - Cross-Sectional Studies
KW - Environment Design
KW - Female
KW - Follow-Up Studies
KW - Germany/ep [Epidemiology]
KW - Humans
KW - Life Style
KW - Male
KW - Mothers/ed [Education]
KW - Mothers/sn [Statistics & Numerical Data]
KW - Motor Activity
KW - Parent-Child Relations
KW - Questionnaires
KW - Seasons
KW - Sedentary Lifestyle
KW - Socioeconomic Factors
RP - NOT IN FILE
SP - 349
JF - BMC Public Health
VL - 13
CY - England
N2 - BACKGROUND: Physical inactivity among children is an increasing problem that adversely affects children's health. A better understanding of factors which affect physical activity (PA) will help create effective interventions aimed at raising the activity levels of children. This cross-sectional study examined the associations of PA with individual (biological, social, behavioral, psychological) and environmental (East vs. West Germany, rural vs. urban regions) characteristics in children. METHODS: Information on PA and potential correlates was collected from 1843 girls and 1997 boys using questionnaires during the 10-year follow-up of two prospective birth cohort studies (GINIplus and LISAplus). Study regions represent urban and rural sites as well as East and West of Germany. Logistic regression modeling was applied to examine cross-sectional associations between individual as well as environmental factors and PA levels. RESULTS: Five of fourteen variables were significantly associated with PA. Among children aged 10, girls tended to be less active

than boys, especially with respect to vigorous PA (OR=0.72 for summer). Children who were not a member of a sports club showed a substantially reduced amount of PA in winter (OR=0.15). Rural environments promote moderate PA, particularly in winter (OR=1.88), whereas an increased time outdoors primarily promotes moderate PA in summer (OR=12.41). Children with abnormal emotional symptoms exhibited reduced physical activity, particularly in winter (OR=0.60). BMI, puberty, parental BMI, parental education, household income, siblings, TV/PC consumption, and method of arriving school, were not associated with PA. CONCLUSIONS: When considering correlates of PA from several domains simultaneously, only few factors (sex, sports club membership, physical environment, time outdoors, and emotional symptoms) appear to be relevant. Although the causality needs to be ascertained in longitudinal studies, variables which cannot be modified should be used to identify risk groups while modifiable variables, such as sports club activities, may be addressed in intervention programs
SN - 1471-2458
AD - Institute of Epidemiology I, Helmholtz Zentrum Munchen, German Research Center for Environmental Health, Ingolstadter Landstrasse 1, Neuherberg 85764, Germany
UR - 23587274
ER -

TY - JOUR
ID - 286
T1 - Alone is a crowd: social motivations, social withdrawal, and socioemotional functioning in later childhood
A1 - Coplan,R.J.
A1 - Rose-Krasnor,L.
A1 - Weeks,M.
A1 - Kingsbury,A.
A1 - Kingsbury,M.
A1 - Bullock,A.
Y1 - 2013/05//
N1 - Coplan, Robert J. Rose-Krasnor, Linda. Weeks, Murray. Kingsbury, Adam. Kingsbury, Mila. Bullock, Amanda
Developmental psychology
0260564, cn8
IM
Journal Article. Research Support, Non-U.S. Gov't
English
KW - MEDLINE
KW - Analysis of Variance
KW - Child
KW - Child Behavior/ph [Physiology]
KW - Female
KW - Humans
KW - Interpersonal Relations
KW - Male
KW - Models
KW - Theoretical
KW - Motivation/ph [Physiology]
KW - Personality
KW - Questionnaires
KW - Self Concept
KW - Shyness
KW - Social Behavior
KW - Statistics as Topic
RP - NOT IN FILE
SP - 861
EP - 875
JF - Developmental Psychology
JA - Dev Psychol
VL - 49

IS - 5
CY - United States
N2 - The primary goals of this study were to test a conceptual model linking social approach and avoidance motivations, socially withdrawn behaviors, and peer difficulties in later childhood and to compare the socioemotional functioning of different subtypes of withdrawn children (shy, unsociable, avoidant). Participants were 367 children, aged 9-12 years. Measures included assessments of social motivations (i.e., self-reported shyness and preference for solitude) and social withdrawal (observations of solitary behaviors in the schoolyard and self-reports of solitary activities outside of school), as well as self- and parent-reported peer difficulties and internalizing problems. Among the results, both shyness and preference for solitude were associated with socially withdrawn behaviors, which in turn predicted peer difficulties. However, only shyness (but not preference for solitude) also displayed a direct path to peer difficulties. As well, results from person-oriented analyses indicated that different subtypes of socially withdrawn children displayed decidedly different profiles with regard to indices of internalizing problems. For example, whereas unsociable children did not differ from their nonwithdrawn peers on indices of internalizing problems, socially avoidant (i.e., high in both shyness and unsociability) children reported the most pervasive socioemotional difficulties. Findings are discussed in terms of the implications of different forms of social withdrawal for socioemotional functioning in later childhood
SN - 1939-0599
AD - Department of Psychology, Carleton University, 1125 Colonel By Drive, Ottawa, Ontario K1S 5B6, Canada. robert_coplan@carleton.ca
UR - 22686178
ER -

TY - JOUR
ID - 287
T1 - Convergent validity of four accelerometer cutpoints with direct observation of preschool children's outdoor physical activity
A1 - Kahan,D.
A1 - Nicaise,V.
A1 - Reuben,K.
Y1 - 2013/03//
N1 - Kahan, David. Nicaise, Virginie. Reuben, Karen
Research quarterly for exercise and sport
r6y, 8006373
IM
Journal Article. Research Support, Non-U.S. Gov't. Validation Studies
English
KW - MEDLINE
KW - Acceleration
KW - Child
KW - Preschool
KW - Female
KW - Humans
KW - Male
KW - Monitoring
KW - Ambulatory
KW - Motor Activity
KW - Observation
KW - Play and Playthings
KW - Sedentary Lifestyle
KW - United States
RP - NOT IN FILE
SP - 59
EP - 67
JF - Research Quarterly for Exercise & Sport
JA - Res Q Exerc Sport
VL - 84
IS - 1

CY - United States
N2 - PURPOSE: More than one fifth of American preschool-aged children are classified as overweight/obese. Increasing physical activity is one means of slowing/reversing progression to overweight or obesity. Measurement of physical activity in this age group relies heavily on motion sensors such as accelerometers. Output is typically interpreted through application of one of several cutpoints to quantify the amount of time spent at various physical activity levels. Cutpoint nonequivalence has resulted in widely varying estimates of physical activity. Thus, we sought to ascertain the convergent validity of four ActiGraph accelerometer cutpoints with the Observation System for Recording Activity in Children-Preschool (OSRAC-P). METHOD: Two studies were conducted with sixty-nine 4- and 5-year-olds during unstructured outdoor free play at a single university campus preschool in Southern California. In Study 1 (N = 57), percentages of time spent in sedentary behavior (SED) and moderate-to-vigorous physical activity (MVPA) were compared at the aggregate level using the two-proportion z-test and Bland Altman plots. In Study 2 (N = 12), time-matched 5-s intervals between accelerometery and the OSRAC-P were compared using percent agreement, kappa, and classification functions. RESULTS: In Study 1, cutpoints by Sirard, Trost, Pfeiffer, Dowda, and Pate (2005) best converged with the OSRAC-P estimates of SED and MVPA. In Study 2, Sirard et al. cutpoints and those by Pate, Almeida, McIver, Pfeiffer, and Dowda (2006) were more sensitive in detecting SED and MVPA, respectively. CONCLUSIONS: Choosing a cutpoint in this age group should be based on the exposure of interest, epoch/observation interval duration, and required degree of stringency for classifying physical activity level
SN - 0270-1367
AD - School of Exercise & Nutritional Sciences, San Diego State University, 5500 Campanile Drive, San Diego, CA 92182-7251, USA. dkahan@mail.sdsu.edu
UR - 23611009
ER -

TY - JOUR
ID - 288
T1 - Increasing physical activity in young primary school children--it's child's play: a cluster randomised controlled trial
A1 - Engelen,L.
A1 - Bundy,A.C.
A1 - Naughton,G.
A1 - Simpson,J.M.
A1 - Bauman,A.
A1 - Ragen,J.
A1 - Baur,L.
A1 - Wyver,S.
A1 - Tranter,P.
A1 - Niehues,A.
A1 - Schiller,W.
A1 - Perry,G.
A1 - Jessup,G.
A1 - van der Ploeg,H.P.
Y1 - 2013/05//
N1 - Engelen, Lina. Bundy, Anita C. Naughton, Geraldine. Simpson, Judy M. Bauman, Adrian. Ragen, Jo. Baur, Louise. Wyver, Shirley. Tranter, Paul. Niehues, Anita. Schiller, Wendy. Perry, Gabrielle. Jessup, Glenda. van der Ploeg, Hidde P
Preventive medicine
pm4, 0322116
IM
Journal Article. Randomized Controlled Trial. Research Support, Non-U.S. Gov't
English
KW - MEDLINE
KW - Accelerometry
KW - Adult
KW - Child
KW - Preschool
KW - Early Intervention (Education)

KW - Female
KW - Health Promotion/mt [Methods]
KW - Humans
KW - Male
KW - Motor Activity
KW - Play and Playthings
KW - School Health Services
KW - Students/sn [Statistics & Numerical Data]
RP - NOT IN FILE
SP - 319
EP - 325
JF - Preventive Medicine
JA - Prev Med
VL - 56
IS - 5
CY - United States
N2 - OBJECTIVE: To explore the effects of an innovative school-based intervention for increasing physical activity. METHODS: 226 children (5-7 years old) randomly selected from 12 Australian primary schools were recruited to a cluster randomised trial with schools randomly allocated to intervention or control conditions. The 13-week intervention comprised: (1) altering the school playground by introducing loose materials and (2) a teacher-parent intervention exploring perceptions of risk associated with children's free play. The primary outcomes were total accelerometer counts and moderate-vigorous physical activity during break times. Testing took place in Sydney, 2009-2010. RESULTS: 221 participants were tested at baseline. Mixed-effect multilevel regression revealed a small but significant increase from the intervention on total counts (9400 counts, 95% CI 3.5-15.2, p=0.002) and minutes of MVPA (1.8 min, 95% CI 0.5-3.1, p=0.006); and a decrease in sedentary activity (2.1 min, 95% CI 0.5-3.8, p=0.01) during break times. We retested children in one intervention school after 2 years; they maintained the gains. CONCLUSIONS: Capturing children's intrinsic motivations to play while simultaneously helping adults reconsider views of free play as risky provided increases in physical activity during break times. Using accelerometry as the sole measure of physical activity may underestimate the effect. Trial registration: ACTRN12611000089932. Copyright 2013 Elsevier Inc. All rights reserved
SN - 1096-0260
AD - Faculty of Health Sciences, University of Sydney, Lidcombe, Australia. lina.engelen@sydney.edu.au
UR - 23462477
ER -

TY - JOUR
ID - 289
T1 - The effect of school recess interventions on physical activity : a systematic review. [Review]
A1 - Parrish,A.M.
A1 - Okely,A.D.
A1 - Stanley,R.M.
A1 - Ridgers,N.D.
Y1 - 2013/04//
N1 - Parrish, Anne-Maree. Okely, Anthony D. Stanley, Rebecca M. Ridgers, Nicola D
Sports medicine (Auckland, N.Z.)
8412297, spo
IM
Journal Article. Research Support, Non-U.S. Gov't. Review
English
KW - MEDLINE
KW - Adolescent
KW - Child
KW - Exercise
KW - Humans
KW - Motor Activity
KW - School Health Services
KW - Schools

RP - NOT IN FILE
SP - 287
EP - 299
JF - Sports Medicine
JA - Sports Med
VL - 43
IS - 4
CY - New Zealand
N2 - BACKGROUND: The benefits of physical activity to maintain optimal health and well-being in children and adolescents are undisputed. The school environment offers opportunities for children to be physically active. OBJECTIVE: The aim of this review is to systematically examine the effects of recess-based interventions on the physical activity (PA) levels of school-aged children and adolescents. DATA SOURCES: A systematic literature search was conducted to identify papers reporting interventions to promote PA during school recess and/or lunchtime periods. The search was conducted in six databases (PubMed, SPORTDiscus, Web of Science, Proquest, Cochrane and Scopus) for papers published between January 2000 and April 2011. STUDY SELECTION: Articles were included in the review if (i) they reported the findings of an intervention targeting PA levels of children and/or adolescents during school recess and/or lunchtime; (ii) have a measure of PA as an outcome variable; (iii) participants were aged between 5 and 18 years; and (iv) were published in English. METHODS: Two authors independently searched the literature using the same search strategies to identify papers reporting interventions that promote PA during school recess and lunchtime periods. Methodological quality was assessed using an adapted eight item assessment scale. The effects of the interventions were assessed with a rating system used in a recent review of interventions in youth. RESULTS: The search originally retrieved 2,265 articles. Nine published peer-reviewed journal articles met the inclusion criteria for this review. Eight studies used randomized controlled trials and one was a controlled trial. Three studies demonstrated high methodological quality (33%). None of the studies adequately reported the randomization procedure or used power calculations. Few studies reported potential confounders and three studies had less than a 6 week follow-up. Five studies demonstrated a positive intervention effect on children's PA levels, with four reporting statistically significant increases and two reporting significant decreases in recess PA. The summary of the levels of evidence for intervention effects found inconclusive results for all intervention types, though promising strategies that require further investigation were identified. LIMITATIONS: Whilst every effort was made to ensure that this review was as encompassing as possible, it may be limited by its search terms especially if there were studies with unclear titles or abstracts. In addition, only manuscripts published in English were considered, eliminating any possible studies published in other languages. CONCLUSIONS: All of the studies used an objective measure to assess PA outcomes, although several criteria were consistently absent from the studies. The levels of evidence were not sufficient to establish conclusive intervention effects on children's recess PA. This could be due to the small number of published studies. There is a need for higher-quality intervention research to strengthen published findings to inform recess PA interventions. Intervention research is needed in adolescents due to the absence of school recess intervention research in this population
SN - 1179-2035
AD - Faculty of Health and Behavioural Sciences, University of Wollongong, Wollongong, NSW, Australia. aparrish@uow.edu.au
UR - 23512170
ER -

TY - JOUR
ID - 290
T1 - Malaria epidemiology in Lihir Island, Papua New Guinea
A1 - Mitja,O.
A1 - Paru,R.
A1 - Selve,B.
A1 - Betuela,I.
A1 - Siba,P.
A1 - De Lazzari,E.
A1 - Bassat,Q.
Y1 - 2013///

N1 - Mitja, Oriol. Paru, Raymond. Selve, Billy. Betuela, Inoni. Siba, Peter. De Lazzari, Elisa. Bassat, Quique
Malaria journal
101139802
IM
Journal Article. Research Support, Non-U.S. Gov't
English
KW - MEDLINE
KW - Adolescent
KW - Adult
KW - Child
KW - Preschool
KW - Cross-Sectional Studies
KW - Female
KW - Humans
KW - Incidence
KW - Malaria
KW - Falciparum/ep [Epidemiology]
KW - Falciparum/pc [Prevention & Control]
KW - Vivax/ep [Epidemiology]
KW - Vivax/pc [Prevention & Control]
KW - Male
KW - Mosquito Control/mt [Methods]
KW - Papua New Guinea/ep [Epidemiology]
KW - Prevalence
KW - Topography
KW - Medical
KW - Young Adult
RP - NOT IN FILE
SP - 98
JF - Malaria Journal
JA - Malar.J
VL - 12
CY - England
N2 - BACKGROUND: Plasmodium vivax and Plasmodium falciparum malaria remain highly endemic in the Pacific Islands including Lihir Island, Papua New Guinea. Lihir Gold Limited is conducting mining activities and funded an integrated vector control intervention within the villages surrounding the mine. The aim of this study was to assess the impact of such programme by comparing the epidemiological trends of malaria in different parts of the island. METHODS: Two cross-sectional surveys were conducted before and after the intervention (2006-2010) to determine malaria prevalence in mine-impact (MI) and non-MI areas. Incidence of malaria was estimated for the Lihir Medical Centre catchment area using island population denominators and a health-centre passive case detection ongoing from 2006-2011. RESULTS: A total of 2,264 and 1,653 children<15 were surveyed in the cross-sectional studies. The prevalence of any malaria parasitaemia initially was 31.5% in MI areas and, 34.9% in non-MI (POR 1.17; 95 CI 0.97 - 1.39). After four years there was a significant reduction in prevalence in the MI areas (5.8%; POR 0.13, 95 CI 0.09-0.20), but reduction was less marked in non-MI areas (26.9%; POR 0.69, 95 CI 0.58-0.81).28,747 patients were included in the evaluation of incidence trends and overall malaria in local Lihirian population in MI areas declined over time, while it remained at similar high levels among migrants. The age-incidence analysis showed that for each higher age range the malaria incidence declines compared to that of the previous stratum. CONCLUSIONS: There was a substantial reduction in prevalence and incidence rates of both P. vivax and P. falciparum in the mining area following implementation of a malaria control intervention, which was not seen in the area outside the mining activities
SN - 1475-2875
AD - Department of Medicine, Lihir Medical Center, PO Box 34, Lihir Island, NIP, Papua New Guinea. oriolmitja@hotmail.com
UR - 23497296
ER -

TY - JOUR
ID - 291
T1 - Children's physical activity levels during indoor recess dance videos
A1 - Erwin,H.
A1 - Koufoudakis,R.
A1 - Beighle,A.
Y1 - 2013/05//
N1 - Erwin, Heather. Koufoudakis, Ryann. Beighle, Aaron
The Journal of school health
k13, 0376370
IM, N
Journal Article. Research Support, Non-U.S. Gov't
English
KW - MEDLINE
KW - Actigraphy/is [Instrumentation]
KW - Child
KW - Dancing
KW - Exercise
KW - Factor Analysis
KW - Statistical
KW - Female
KW - Humans
KW - Male
KW - Schools
KW - Southeastern United States
KW - Video Recording
RP - NOT IN FILE
SP - 322
EP - 327
JF - Journal of School Health
JA - J Sch Health
VL - 83
IS - 5
CY - United States
N2 - BACKGROUND: Children's physical activity (PA) levels remain low, and schools are being asked to assume a leadership role in PA promotion. Research suggests outdoor recess contributes to children's overall PA levels. However, similar research is not available for indoor recess, which occurs frequently due to a variety of factors. The purpose of this study was to describe PA levels and intensity during indoor recess using PA dance videos. METHODS: Fifty-four children (27 girls) wore accelerometers for 5days during indoor recess. Factorial analyses of variance were conducted to examine the differences in PA between sexes and across grades. RESULTS: Participants averaged 17.66 (SD=2.82) minutes in indoor recess. On average, 22.22% (SD=12.72) was spent in moderate-to-vigorous physical activity (MVPA), and 45.70% (SD=11.29) of time was spent in low-intensity physical activity (LPA) totaling 67.92% (SD=16.54) of total recess time. Boys accumulated a higher percentage of MVPA and total PA compared to girls. CONCLUSIONS: Students engaged in PA 68% of indoor recess time, equaling almost 12minutes of PA during recess. Results suggest PA dance videos are 1 effective method for increasing elementary children's PA during indoor recess as an alternative to sedentary activities. 2013, American School Health Association
SN - 1746-1561
AD - Department of Kinesiology and Health Promotion, University of Kentucky, College of Education, 115 Seaton Bldg., Lexington, KY 40506-0219, USA. heather.erwin@uky.edu
UR - 23516999
ER -

TY - JOUR
ID - 292
T1 - Physical activity behaviours of highly active preschoolers
A1 - Howie,E.K.

A1 - Brown,W.H.
A1 - Dowda,M.
A1 - McIver,K.L.
A1 - Pate,R.R.
Y1 - 2013/04//
N1 - Howie, E K. Brown, W H. Dowda, M. McIver, K L. Pate, R R
Pediatric obesity
101572033
IM
Comparative Study. Journal Article. Multicenter Study. Research Support, N.I.H., Extramural
English
KW - MEDLINE
KW - Accelerometry
KW - Child Behavior
KW - Child
KW - Preschool
KW - Exercise
KW - Female
KW - Health Promotion
KW - Humans
KW - Male
KW - Motor Activity
KW - Obesity/ep [Epidemiology]
KW - Obesity/pc [Prevention & Control]
KW - Play and Playthings
KW - Schools
KW - Nursery/sn [Statistics & Numerical Data]
KW - United States/ep [Epidemiology]
RP - NOT IN FILE
SP - 142
EP - 149
JF - Pediatric Obesity
JA - Pediatr Obes
VL - 8
IS - 2
CY - England
N2 - BACKGROUND: Understanding the physical activity behaviour of young children who are highly active may provide important guidance for promoting physical activity in preschools. OBJECTIVES: The objective of this study was to describe the movement characteristics of high-active (HA) children during attendance at preschools. METHODS: Children in 20 preschools (n=231) wore accelerometers and were classified into tertiles of moderate-to-vigorous physical activity. Children's movement characteristics were observed using the Observational System for Recording Physical Activity in Children-Preschool Version. Mixed-model analyses compared movement types between HA children and lower-active (LA) children during the total school day. RESULTS: HA (n=77) children were observed to be more active than LA children (n=154) indoors (P<0.001), but no differences were observed outdoors. HA children were more frequently observed running, crawling, climbing, jumping, skipping, swinging and throwing across the total school day than LA children. Outdoors, HA children participated in more swinging and throwing and less jumping or skipping than LA children. Indoors, HA children spent more time pulling, pushing and running, and less time walking than LA children. CONCLUSIONS: HA children have unique activity patterns. Further interventions to increase physical activity of all preschoolers should increase the time spent outside and include varied activity types throughout the entire school day. 2012 The Authors. Pediatric Obesity 2012 International Association for the Study of Obesity
SN - 2047-6310
AD - Department of Exercise Science, University of South Carolina, Columbia, SC, USA. howieek@email.sc.edu
UR - 23042629
ER -

TY - JOUR
ID - 293
T1 - [Contextual study of Airsoft gun related ocular injuries]. [French]
A1 - Rambaud,C.
A1 - Tabary,A.
A1 - Contraires,G.
A1 - El,Hassan F.
A1 - Labalette,P.
Y1 - 2013/03//
N1 - Rambaud, C. Tabary, A. Contraires, G. El Hassan, F. Labalette, P
Journal francais d'ophtalmologie
iae, 7804128
IM
English Abstract. Journal Article
French
KW - MEDLINE
KW - Adolescent
KW - Athletic Injuries/ep [Epidemiology]
KW - Athletic Injuries/et [Etiology]
KW - Athletic Injuries/pc [Prevention & Control]
KW - Child
KW - Contact Lenses/sn [Statistics & Numerical Data]
KW - Cornea/in [Injuries]
KW - Equipment Design
KW - Eye Foreign Bodies/et [Etiology]
KW - Eye Injuries/ep [Epidemiology]
KW - Eye Injuries/et [Etiology]
KW - Eye Injuries/pc [Prevention & Control]
KW - Eye Protective Devices/ut [Utilization]
KW - Eyeglasses/sn [Statistics & Numerical Data]
KW - Female
KW - Firearms/lj [Legislation & Jurisprudence]
KW - Firearms
KW - Forensic Ballistics/sn [Statistics & Numerical Data]
KW - France/ep [Epidemiology]
KW - Humans
KW - Iris/in [Injuries]
KW - Male
KW - Plastics
KW - Retrospective Studies
KW - Vitreous Hemorrhage/ep [Epidemiology]
KW - Vitreous Hemorrhage/et [Etiology]
KW - Wounds
KW - Gunshot/ep [Epidemiology]
KW - Gunshot/et [Etiology]
KW - Gunshot/pc [Prevention & Control]
RP - NOT IN FILE
SP - 236
EP - 241
JF - Journal Francais d Opthalmologie
JA - J Fr Ophtalmol.
VL - 36
IS - 3
CY - France
N2 - INTRODUCTION: Airsoft guns have become more common in France since the Decree of March 24, 1999, allowing people over 18 years of age to use weapons with an energy level below 2J. The present study aimed to assess the relationship between the context of Airsoft gun pellet related ocular injuries and their

clinical consequences, in order to determine an effective prevention strategy. METHODS: A retrospective analysis of ocular injuries due to Airsoft gun pellets was conducted in two hospitals in northern France between September 2009 and August 2010. RESULTS: Six patients with a mean age of 12.6 + 3.2 years were included. The most frequent injuries were corneal abrasion, hyphema and iris trauma. Functional sequelae were observed in 50% of cases and surgical intervention was necessary in one third of cases. A direct shot had occurred in all cases. In 83% of cases, the accident took place while playing in or around the house. In one out of every two cases, the gun had been won at a booth in a fair or had been purchased in a big-box store and in no cases did the patients own safety glasses. CONCLUSION: The strong appeal of Airsoft guns to minors leads us to propose several practical measures to help protect them, with the main one being to forbid selling these guns outside of specialized stores. Copyright 2012 Elsevier Masson SAS. All rights reserved
SN - 1773-0597
AD - Service d'ophtalmologie, hopital Huriez, centre hospitalier regional et universitaire de Lille, 1, place de Verdun, 59037 Lille cedex, France. rambaud.camille@gmail.com
UR - 22986026
ER -

TY - JOUR
ID - 294
T1 - Exploring effective strategies for increasing the amount of moderate-to-vigorous physical activity children accumulate during recess: a quasi-experimental intervention study
A1 - Efrat,M.W.
Y1 - 2013/04//
N1 - Efrat, Merav W
The Journal of school health
k13, 0376370
IM, N
Comparative Study. Journal Article. Randomized Controlled Trial. Research Support, N.I.H., Extramural. Research Support, Non-U.S. Gov't
English
KW - MEDLINE
KW - Child
KW - Female
KW - Health Promotion/mt [Methods]
KW - Humans
KW - Male
KW - Motivation
KW - Motor Activity
KW - Obesity/pc [Prevention & Control]
KW - Play and Playthings
KW - Schools
KW - Social Facilitation
RP - NOT IN FILE
SP - 265
EP - 272
JF - Journal of School Health
JA - J Sch Health
VL - 83
IS - 4
CY - United States
N2 - BACKGROUND: Less than half of elementary children meet the physical activity recommendations of 30 to 60 minutes of moderate-to-vigorous physical activity (MVPA) on a daily basis. Recess provides the single biggest opportunity for children to accumulate MVPA. This study explored whether a teacher's social prompting to be active during recess alone and modeling of active recess-time games alone may increase the amount of MVPA children accumulate during recess. METHODS: Three demographically matched schools were randomly assigned to receive a social prompting intervention, modeling intervention, or to serve as a comparison group. Pre- and postintervention physical activity data was collected utilizing accelerometers. RESULTS: Compared with preintervention, MVPA during recess increased significantly in the social prompting group and decreased

significantly in the modeling group. There were no significant changes in the comparison group. There was a significant interaction between time and intervention: pre- to postintervention change between interventions was significant for modeling versus comparison and modeling versus social prompting, but not for social prompting versus comparison. CONCLUSIONS: Modeling was not an effective strategy for increasing MVPA during recess. Social prompting may be an effective strategy for increasing MVPA during recess. However, larger studies are needed to determine whether social prompting increases MVPA during recess. In light of evidence suggesting that MVPA is linked to lower obesity rates, and a substantial number of health and academic benefits, social prompting may be a useful strategy to further explore to address the childhood obesity epidemic, and improve students' health and academic outcomes. 2013, American School Health Association
SN - 1746-1561
AD - Department of Health Science, College of Health and Human Development, California State University, 18111 Nordhoff Street, Northridge, CA 91330-8285, USA. merav.efrat@csun.edu
UR - 23488887
ER -

TY - JOUR
ID - 295
T1 - Severity of playground-related fractures: more than just playground factors?
A1 - Lam,K.Y.
A1 - Sumanth,Kumar G.
A1 - Mahadev,A.
Y1 - 2013/04//
N1 - Lam, Kai Yet. Sumanth Kumar, Gera. Mahadev, Arjandas
Journal of pediatric orthopedics
hsw, 8109053
IM
Journal Article
English
KW - MEDLINE
KW - Child
KW - Preschool
KW - Female
KW - Fractures
KW - Bone/ep [Epidemiology]
KW - Bone/et [Etiology]
KW - Humans
KW - Infant
KW - Injury Severity Score
KW - Male
KW - Play and Playthings/in [Injuries]
KW - Prospective Studies
KW - Risk Factors
RP - NOT IN FILE
SP - 221
EP - 226
JF - Journal of Pediatric Orthopedics
JA - J Pediatr Orthop
VL - 33
IS - 3
CY - United States
N2 - INTRODUCTION: Despite the establishment of playground safety standards, playground-related injuries are still a significant cause of extremity fractures in Singapore. This prospective study evaluates the dimensions and characteristics of our playgrounds, and their effect on fracture severity in an Asian population. We aim to correlate various playground risk factors with severity of the fractures and give recommendations on future safety standards. Our data also allows us to compare the demographics of patients in our study group with that collected in our earlier study in 2004. METHODS: From June 2005 to 2006, children who presented with extremity fractures to KK Women's and Children's Hospital after a playground injury were enrolled. Their

clinical data were collected prospectively. Relevant playground details were collected on-site independently by another investigator. For analysis of severity, fractures were "major" if they required reduction or operative fixation and were "minor" if they did not. RESULTS: Supervision at time of injury, especially from the child's parents or siblings, resulted in a lower likelihood of "major" fractures (P=0.002, likelihood ratio=1.97). Conversely, supervision from grandparents or maids was found to result in a higher likelihood of "major" fractures. Increased weight of patients was directly related to severity of fractures (P=0.000), and a body mass index (BMI) of less than 19.8 kg/m resulted in lower likelihood of "major" fractures (P=0.010, likelihood ratio=2.22). Height of equipments and other playground-related factors were not linked to severity of fractures. CONCLUSIONS: Supervision at the playground, preferably from the child's parents or siblings, and keeping a child's BMI within limits as guided by the BMI charts, may potentially reduce the occurrence of severe fractures. LEVEL OF EVIDENCE: Level I-prognostic study
SN - 1539-2570
AD - Department of Orthopaedic Surgery, KK Women's and Childrens Hospital (KKH), Singapore.
kaiyet@yahoo.com
UR - 23482255
ER -

TY - JOUR
ID - 296
T1 - Programming of respiratory health in childhood: influence of outdoor air pollution. [Review]
A1 - Wright,R.J.
A1 - Brunst,K.J.
Y1 - 2013/04//
N1 - Wright, Rosalind J. Brunst, Kelly J
Current opinion in pediatrics
but, 9000850
IM
Journal Article. Research Support, N.I.H., Extramural. Review
English
KW - MEDLINE
KW - Air Pollutants/to [Toxicity]
KW - Air Pollution/ae [Adverse Effects]
KW - Asthma/et [Etiology]
KW - Asthma/pp [Physiopathology]
KW - Autonomic Nervous System/pp [Physiopathology]
KW - Child
KW - Humans
KW - Metabolic Detoxication
KW - Drug
KW - Mitochondrial Diseases/co [Complications]
KW - Neurosecretory Systems/pp [Physiopathology]
KW - Respiration Disorders/et [Etiology]
KW - Respiration Disorders/pp [Physiopathology]
KW - Respiratory Hypersensitivity/et [Etiology]
KW - Respiratory Hypersensitivity/pp [Physiopathology]
KW - Respiratory System/gd [Growth & Development]
KW - Xenobiotics/pk [Pharmacokinetics]
RP - NOT IN FILE
SP - 232
EP - 239
JF - Current Opinion in Pediatrics
JA - Curr Opin Pediatr
VL - 25
IS - 2
CY - United States
N2 - PURPOSE OF REVIEW: This overview highlights recent experimental and epidemiological evidence for the programming effects of outdoor air pollution exposures during early development on lung function and

chronic respiratory disorders, such as asthma and related allergic disorders. RECENT FINDINGS: Air pollutants may impact anatomy and/or physiological functioning of the lung and interrelated systems. Programming effects may result from pollutant-induced shifts in a number of molecular, cellular, and physiological states and their interacting systems. Specific key regulatory systems susceptible to programming may influence lung development and vulnerability to respiratory diseases, including both central and peripheral components of neuroendocrine pathways and autonomic nervous system (ANS) functioning which, in turn, influence the immune system. Starting in utero, environmental factors, including air pollutants, may permanently organize these systems toward trajectories of enhanced pediatric (e.g., asthma, allergy) as well as adult disease risk (e.g., chronic obstructive pulmonary disease). Evidence supports a central role of oxidative stress in the toxic effects of air pollution. Additional research suggests xenobiotic metabolism and subcellular components, such as mitochondria are targets of ambient air pollution and play a role in asthma and allergy programming. Mechanisms operating at the level of the placenta are being elucidated. Epigenetic mechanisms may be at the roots of adaptive developmental programming. SUMMARY: Optimal coordinated functioning of many complex processes and their networks of interaction are necessary for normal lung development and the maintenance of respiratory health. Outdoor air pollution may play an important role in early programming of respiratory health and is potentially amenable to intervention
SN - 1531-698X
AD - Department of Pediatrics, Division of Pulmonary Medicine, Icahn School of Medicine at Mount Sinai, New York, New York, USA. rosalind.wright@mssm.edu
UR - 23422354
ER -

TY - JOUR
ID - 297
T1 - Traumatic injuries in the primary teeth of 4- to 6-year-old school children in gulbarga city, India. A prevalence study
A1 - Bhayya,D.P.
A1 - Shyagali,T.R.
Y1 - 2013/03//
N1 - Bhayya, Deepak P. Shyagali, Tarulatha R
Oral health and dental management
101568098
D, IM
Comparative Study. Journal Article
English
KW - MEDLINE
KW - Accidental Falls/sn [Statistics & Numerical Data]
KW - Age Factors
KW - Child
KW - Preschool
KW - Cohort Studies
KW - Cross-Sectional Studies
KW - Cuspid/in [Injuries]
KW - Dental Enamel/in [Injuries]
KW - Dental Pulp/in [Injuries]
KW - Dentin/in [Injuries]
KW - Female
KW - Humans
KW - Incisor/in [Injuries]
KW - India/ep [Epidemiology]
KW - Male
KW - Play and Playthings/in [Injuries]
KW - Prevalence
KW - Sex Factors
KW - Tooth Crown/in [Injuries]
KW - Tooth Discoloration/ep [Epidemiology]
KW - Tooth Fractures/ep [Epidemiology]

KW - Tooth Mobility/ep [Epidemiology]
KW - Tooth
KW - Deciduous/in [Injuries]
RP - NOT IN FILE
SP - 17
EP - 23
JF - Oral Helath & Dental Management
JA - Oral Helath Dent
VL - 12
IS - 1
CY - Romania
N2 - AIMS: The aims of the study were: to assess the prevalence of traumatic injuries to the teeth of 4- to 6-year-old children living in Gulbarga City, India, to determine prevalence of such dental traumatic injuries at the ages of 4, 5, and 6 years and to compare the prevalence of these injuries between male and female children. METHODS: A cross-sectional survey was performed. It consisted of a clinical examination of upper and lower deciduous anterior teeth by one examiner and an interview using a questionnaire with a sample of 1500 children aged 4 to 6 years who attended kindergarten and primary schools in Gulbarga city. Garcia-Godoy's (1981) classification was used to classify the traumatic injuries. Intra-examiner consistency was assessed by kappa values on tooth-by-tooth basis. The chisquare test was used to analyse any gender and age differences. RESULTS: The prevalence of traumatic dental injuries was 76.13%, of which crown fracture with enamel involvement of teeth was the most prevalent, followed by crown fracture with both enamel and dentine involvement. Significant and highly significant differences were found between boys and girls for discoloration of teeth ($P<0.05$), crown fracture involving enamel ($P<0.001$) and crown fracture involving both enamel and dentine ($P<0.001$). The prevalence of traumatic dental injuries in the 5-year-old children was higher than that in the 4- and 6-year-olds. The commonest cause of injury was due to a fall (60%) and in 40% of cases of traumatic injury, they occurred in a field/playground. CONCLUSIONS: The prevalence of traumatic injuries to the anterior teeth of the 4- to 6-year-olds who took part in this study was very high. There is a need to run educational programmes to increase parents' awareness of the risks of dental trauma
SN - 2247-2452
AD - Darshan Dental College and Hospital Campus, Loyara, Udaipur, India
UR - 23474577
ER -

TY - JOUR
ID - 298
T1 - Surgery for temporal lobe epilepsy in children: relevance of presurgical evaluation and analysis of outcome
A1 - Miserocchi,A.
A1 - Cascardo,B.
A1 - Piroddi,C.
A1 - Fuschillo,D.
A1 - Cardinale,F.
A1 - Nobili,L.
A1 - Francione,S.
A1 - Russo,G.L.
A1 - Cossu,M.
Y1 - 2013/03//
N1 - Miserocchi, Anna. Cascardo, Beatrice. Piroddi, Chiara. Fuschillo, Dalila. Cardinale, Francesco. Nobili, Lino. Francione, Stefano. Russo, Giorgio Lo. Cossu, Massimo
Journal of neurosurgery. Pediatrics
101463759
IM
Journal Article
English
KW - MEDLINE
KW - Adolescent
KW - Age of Onset

KW - Child
KW - Preschool
KW - Electroencephalography
KW - Epilepsy
KW - Temporal Lobe/di [Diagnosis]
KW - Temporal Lobe/px [Psychology]
KW - Temporal Lobe/su [Surgery]
KW - Female
KW - Humans
KW - Infant
KW - Magnetic Resonance Imaging
KW - Male
KW - Neuroimaging
KW - Neuropsychological Tests
KW - Neurosurgical Procedures/mt [Methods]
KW - Postoperative Care
KW - Preoperative Care
KW - Treatment Outcome
RP - NOT IN FILE
SP - 256
EP - 267
JF - Journal of Neurosurgery
JA - J Neurosurg Pediatrics
VL - Pediatrics.. 11
IS - 3
CY - United States
N2 - OBJECT: The authors' goal in this paper was to retrospectively evaluate the relevance of the presurgical workup and the postoperative outcome in children (< 15 years) who undergo surgery for temporal lobe epilepsy (TLE). METHODS: The authors performed a retrospective analysis of 68 patients (43 boys and 25 girls) who underwent resection for TLE between 2001 and 2010 at a single center and had a minimum postoperative follow-up of 12 months. Presurgical investigations included full clinical evaluation, interictal electroencephalography (EEG), and MRI in all cases; cognitive evaluation in patients older than 5 years; scalp video-EEG in 46 patients; and invasive EEG in 3 patients. Clinical evaluation included a careful assessment of ictal semiology (based on anamnestic reports or video-EEG review), with particular attention to early signs and/or symptoms suggestive of temporal lobe origin of the seizure. Microsurgical resections were performed within the anatomical limits of the temporal lobe, and surgical specimens were processed for histological examination. Postoperative assessment of seizure outcome (Engel classification system) and cognitive performance was conducted at regular intervals. The effect on postoperative seizure outcome (good = Engel Class I; poor = Engel Classes II-IV) of several presurgical and surgical variables was investigated by bivariate statistical analysis. RESULTS: All patients had at least 1 early sign or symptom suggesting a temporal lobe origin of their seizures. Lateralized interictal or ictal EEG abnormalities were seen in all patients, and they were localized to the temporal lobe in 45 patients. In all cases MRI demonstrated a structural abnormality. Surgery consisted of a tailored anterior temporal lobectomy in 64 patients and a neocortical lesionectomy in 4 patients. Postoperatively, 58 patients (85%) were in Engel Class I. Variables significantly associated with a poor outcome were preoperative sensory motor deficit (p = 0.019), mental retardation (p = 0.003), MRI abnormalities extending outside the temporal lobe (p = 0.0018), history of generalized seizures (p = 0.01) or status epilepticus (p = 0.008), unremarkable histology (p = 0.001), seizures immediately postoperatively (p = 0.00001), and ipsilateral epileptiform activity on postoperative EEG (p = 0.005). At postoperative neuropsychological assessment, the percentage of patients with a pathological score at the final visit invariably decreased compared with that at the preoperative evaluation in all considered cognitive domains. CONCLUSIONS: Among the study population, a surgical selection based on a noninvasive evaluation was possible in most patients. The invaluable information resulting from the rigorous noninvasive electroclinical and neuroimaging evaluation can lead to excellent surgical results without the use of invasive, time-consuming, and expensive diagnostic tools. The potential reduction of invasiveness-related risks, complexity, and costs of presurgical investigations should hopefully allow for an increase in the number of children with TLE who will receive surgery, particularly in centers with limited technological resources
SN - 1933-0715

AD - C. Munari Epilepsy Surgery Centre, Department of Neuroscience, Niguarda Hospital, Milan, Italy
UR - 23311387
ER -

TY - JOUR
ID - 299
T1 - Off-label drug prescribing in a Portuguese paediatric emergency unit
A1 - Ribeiro,M.
A1 - Jorge,A.
A1 - Macedo,A.F.
Y1 - 2013/02//
N1 - Ribeiro, Marina. Jorge, Arminda. Macedo, Ana Filipa
International journal of clinical pharmacy
101554912
IM
Journal Article
English
KW - MEDLINE
KW - Adolescent
KW - Child
KW - Preschool
KW - Emergency Service
KW - Hospital
KW - Female
KW - Humans
KW - Infant
KW - Newborn
KW - Male
KW - Off-Label Use/sn [Statistics & Numerical Data]
KW - Pediatrics
KW - Portugal
RP - NOT IN FILE
SP - 30
EP - 36
JF - International Journal of Clinical Pharmacy
JA - Int J Clin Pharm
VL - 35
IS - 1
CY - Netherlands
N2 - BACKGROUND: Many drugs are prescribed outside the terms of the marketing authorization ("off-label"). Several studies have shown that this is a common practice in various European healthcare settings. OBJECTIVE: This study aimed to quantify and characterize off-label drug prescribing in children admitted to a Portuguese Paediatric Unit (PEU). SETTING: This study was conducted in the Paediatric Unit of the university teaching hospital of Cova da Beira Hospital Centre (CHCB), Covilha, located in the Eastern Central Region of Portugal. METHOD: A descriptive study was conducted, including a sample of 700 children, randomly selected from those admitted between January to October 2010. Drug prescription was assessed by retrospective review of clinical files. MAIN OUTCOME MEASURE: Off-label prescribing was defined as the utilization of a drug at an indication, age, dosage, frequency or route of administration different from those recommended in the Summary of Product Characteristics (SPC). For purposes of this study only the medicines prescribed to be used after discharge from the hospital were studied. RESULTS: 364 boys and 336 girls, aged from 4 days to 18 years, were included in this study. Of the 724 medicines prescribed, 32.2 % were off-label. At least one drug was used off-label in 28.1 % of the studied population, corresponding to 46.1 % of the 427 children that received prescriptions. "Alteration in dosage" was the commonest reason for off-label prescribing (28.2 %). The off-label prescriptions comprised mainly drugs acting on the "Respiratory System" and "Anti-infectious agents for systemic use". Amoxicillin/clavulanic acid, paracetamol, amoxicillin, ibuprofen and salbutamol were the five active principles most frequently prescribed off-label. CONCLUSION: The prevalence of off-label drug

prescribing in the Portuguese PEU of CHCB is high, the use in a dose or for an age group not approved in the SPC being the most common reasons for off-label prescription
SN - 2210-7711
AD - Faculty of Health Sciences, University of Beira Interior, Av. Infante D.Henrique, 6200-506, Covilha, Portugal. marinaa.ribeiro@hotmail.com
UR - 23054136
ER -

TY - JOUR
ID - 300
T1 - Birth weight and time spent in outdoor physical activity during adolescence
A1 - Gopinath,B.
A1 - Hardy,L.L.
A1 - Baur,L.A.
A1 - Burlutsky,G.
A1 - Mitchell,P.
Y1 - 2013/03//
N1 - Gopinath, Bamini. Hardy, Louise L. Baur, Louise A. Burlutsky, George. Mitchell, Paul
Medicine and science in sports and exercise
8005433, mg8
IM, S
Journal Article. Research Support, Non-U.S. Gov't
English
KW - MEDLINE
KW - Adolescent
KW - Birth Weight
KW - Body Height
KW - Child
KW - Computers
KW - Female
KW - Head/ah [Anatomy & Histology]
KW - Humans
KW - Infant
KW - Newborn
KW - Linear Models
KW - Longitudinal Studies
KW - Male
KW - Motor Activity
KW - Questionnaires
KW - Sports
KW - Television
KW - Time Factors
KW - Video Games
RP - NOT IN FILE
SP - 475
EP - 480
JF - Medicine & Science in Sports & Exercise
JA - Med Sci Sports Exerc
VL - 45
IS - 3
CY - United States
N2 - PURPOSE: We investigated the association between birth parameters (weight, length, and head circumference) and time spent in physical activity (outdoor and indoor) and screen time (TV viewing, computer, and videogame usage) among adolescents. METHODS: A longitudinal cohort study surveyed 1794 children in 2004-2005 (median age = 12.7 yr), and 752 were resurveyed 5 yr later in 2009-2010 (age = 17-18 yr). Adolescents completed detailed activity questionnaires. Parents extracted birth parameter data from their child's health record booklet. RESULTS: After adjusting for age, sex, ethnicity, gestational age, parental education,

home ownership, exposure to passive smoking, and body mass index, 12-yr-old children in the highest compared with the lowest quartile of birth weight spent on average approximately 56 and 62 min more in total (Ptrend = 0.02) and outdoor physical activity (Ptrend = 0.02) per week, respectively. Similarly, 12-yr-old children in the high (>4000 g) versus very low (<2000 g) birth weight group spent approximately 1.3 hwk more in outdoor activity (Ptrend = 0.02). Among those age 17-18 yr, increasing birth weight (lowest to highest quartile) was associated with greater time spent in outdoor physical activity (~1 hwk-1, Ptrend = 0.04). Significant associations were not observed between all birth parameters and recreational screen time. Also, no associations were observed between head circumference or birth length with physical activity and screen time. CONCLUSIONS: Birth weight could be a potential determinant of physical activity, but not of screen time during adolescence. Hence, this could be part of the underlying mechanism between prenatal influences and future disease risk and could have possible clinical implications
SN - 1530-0315
AD - Centre for Vision Research, Department of Ophthalmology and Westmead Millennium Institute, University of Sydney, NSW, Australia. bamini.gopinath@sydney.edu.au
UR - 23059858
ER -

TY - JOUR
ID - 301
T1 - Lethal brain edema, shock, and coagulopathy after scorpion envenomation
A1 - Cavari,Y.
A1 - Lazar,I.
A1 - Shelef,I.
A1 - Sofer,S.
Y1 - 2013/03//
N1 - Cavari, Yuval. Lazar, Isaac. Shelef, Ilan. Sofer, Shaul
Wilderness & environmental medicine
dbe, 9505185
IM
Case Reports. Journal Article
English
KW - MEDLINE
KW - Animals
KW - Antivenins/tu [Therapeutic Use]
KW - Child
KW - Preschool
KW - Fatal Outcome
KW - Humans
KW - Male
KW - Multiple Organ Failure
KW - Scorpion Stings/mo [Mortality]
KW - Scorpion Stings/th [Therapy]
KW - Scorpion Venoms/ai [Antagonists & Inhibitors]
KW - Scorpions
KW - Time Factors
RP - NOT IN FILE
SP - 23
EP - 27
JF - Wilderness & Environmental Medicine
JA - Wilderness Environ Med
VL - 24
IS - 1
CY - United States
N2 - We report the case of a 2-year-old Bedouin boy in whom developed severe and unusual complications after being stung, most probably, by the yellow scorpion Leiurus quinquestriatus hebraeus. Five hours after arrival to the emergency department, the boy had multisystem organ failure involving the central nervous system (seizure activity followed by coma with dilated, nonreactive pupils, and severe brain edema), shock

(noncardiogenic), disseminated intravascular coagulation, renal failure, hepatic failure, and watery diarrhea, causing his death. In view of the relevant literature, we discuss the pathophysiologic events ultimately leading to his death. Copyright 2013 Wilderness Medical Society. Published by Elsevier Inc. All rights reserved
SN - 1545-1534
AD - Pediatric Intensive Care Unit, Soroka University Medical Center, Faculty of Health Sciences, Ben Gurion University-Negev, Beer Sheva, Israel
UR - 23280335
ER -

TY - JOUR
ID - 302
T1 - What factors are associated with adolescents' school break time physical activity and sedentary time?
A1 - Ridgers,N.D.
A1 - Timperio,A.
A1 - Crawford,D.
A1 - Salmon,J.
Y1 - 2013///
N1 - Ridgers, Nicola D. Timperio, Anna. Crawford, David. Salmon, Jo
PloS one
101285081
IM
Journal Article. Research Support, Non-U.S. Gov't
English
KW - MEDLINE
KW - Adolescent
KW - Age Factors
KW - Child
KW - Exercise/ph [Physiology]
KW - Female
KW - Humans
KW - Male
KW - Multivariate Analysis
KW - Questionnaires
KW - Schools
KW - Sedentary Lifestyle
KW - Time Factors
RP - NOT IN FILE
SP - e56838
JF - PLoS ONE [Electronic Resource]
JA - PLoS ONE
VL - 8
IS - 2
CY - United States
N2 - PURPOSE: Adolescents' physical activity levels during school break time are low and understanding correlates of physical activity and sedentary time in this context is important. This study investigated cross-sectional and longitudinal associations between a range of individual, behavioural, social and policy/organisational correlates and objectively measured school break time physical activity and sedentary time. METHODS: In 2006, 146 adolescents (50% males; mean age = 14.1+0.6 years) completed a questionnaire and wore an accelerometer for >3 school days. Time spent engaged in sedentary, light (LPA) and moderate-to-vigorous physical activity (MVPA) during school break times (recess and lunchtime) were calculated using existing cut-points. Measures were repeated in 2008 among 111 adolescents. Multilevel models examined cross-sectional and longitudinal associations. RESULTS: Bringing in equipment was cross-sectionally associated with 3.2% more MVPA during break times. Females engaged in 5.1% more sedentary time than males, whilst older adolescents engaged in less MVPA than younger adolescents. Few longitudinal associations were observed. Adolescents who brought sports equipment to school engaged in 7.2% less LPA during break times two years later compared to those who did not bring equipment to school. CONCLUSION: These data suggest that

providing equipment and reducing restrictions on bringing in sports equipment to school may promote physical activity during school recess. Strategies targeting females' and older adolescents', in particular, are warranted
SN - 1932-6203
AD - Centre for Physical Activity and Nutrition Research (C-PAN), Deakin University, Melbourne, Australia. nicky.ridgers@deakin.edu.au
UR - 23418606
ER -

TY - JOUR
ID - 303
T1 - Design and methods for evaluating an early childhood obesity prevention program in the childcare center setting
A1 - Natale,R.
A1 - Scott,S.H.
A1 - Messiah,S.E.
A1 - Schrack,M.M.
A1 - Uhlhorn,S.B.
A1 - Delamater,A.
Y1 - 2013///
N1 - Natale, Ruby. Scott, Stephanie Hapeman. Messiah, Sarah E. Schrack, Maria Mesa. Uhlhorn, Susan B. Delamater, Alan
BMC public health
100968562
IM
Journal Article. Randomized Controlled Trial. Research Support, U.S. Gov't, Non-P.H.S.
English
KW - MEDLINE
KW - Body Mass Index
KW - Child
KW - Child Day Care Centers/og [Organization & Administration]
KW - Preschool
KW - Diet
KW - Financing
KW - Government
KW - Health Promotion/mt [Methods]
KW - Humans
KW - Motor Activity
KW - Obesity/pc [Prevention & Control]
KW - Organizational Policy
KW - Program Evaluation/mt [Methods]
KW - Research Design
KW - School Health Services/ec [Economics]
KW - School Health Services/og [Organization & Administration]
KW - United States
RP - NOT IN FILE
SP - 78
JF - BMC Public Health
VL - 13
CY - England
N2 - BACKGROUND: Many unhealthy dietary and physical activity habits that foster the development of obesity are established by the age of five. Presently, approximately 70 percent of children in the United States are currently enrolled in early childcare facilities, making this an ideal setting to implement and evaluate childhood obesity prevention efforts. We describe here the methods for conducting an obesity prevention randomized trial in the child care setting. METHODS/DESIGN: A randomized, controlled obesity prevention trial is currently being conducted over a three year period (2010-present). The sample consists of 28 low-income, ethnically diverse child care centers with 1105 children (sample is 60% Hispanic, 15% Haitian, 12% Black, 2% non-Hispanic White and 71% of caregivers were born outside of the US). The purpose is to test the

efficacy of a parent and teacher role-modeling intervention on children's nutrition and physical activity behaviors. . The Healthy Caregivers-Healthy Children (HC2) intervention arm schools received a combination of (1) implementing a daily curricula for teachers/parents (the nutritional gatekeepers); (2) implementing a daily curricula for children; (3) technical assistance with meal and snack menu modifications such as including more fresh and less canned produce; and (4) creation of a center policy for dietary requirements for meals and snacks, physical activity and screen time. Control arm schools received an attention control safety curriculum. Major outcome measures include pre-post changes in child body mass index percentile and z score, fruit and vegetable and other nutritious food intake, amount of physical activity, and parental nutrition and physical activity knowledge, attitudes, and beliefs, defined by intentions and behaviors. All measures were administered at the beginning and end of the school year for year one and year two of the study for a total of 4 longitudinal time points for assessment. DISCUSSION: Although few attempts have been made to prevent obesity during the first years of life, this period may represent the best opportunity for obesity prevention. Findings from this investigation will inform both the fields of childhood obesity prevention and early childhood research about the effects of an obesity prevention program housed in the childcare setting. TRIAL REGISTRATION: Trial registration number: NCT01722032
SN - 1471-2458
AD - Division of Education, University of Miami Miller School of Medicine, Miami, USA. rnatale@med.miami.edu
UR - 23356862
ER -

TY - JOUR
ID - 304
T1 - Parents' perceptions of children's physical activity compared on two electronic diaries
A1 - Lau,J.
A1 - Engelen,L.
A1 - Bundy,A.
Y1 - 2013/02//
N1 - Lau, Jamie. Engelen, Lina. Bundy, Anita
Pediatric exercise science
8909729
IM
Comparative Study. Journal Article. Research Support, Non-U.S. Gov't
English
KW - MEDLINE
KW - Accelerometry
KW - Analysis of Variance
KW - Child
KW - Preschool
KW - Data Collection/is [Instrumentation]
KW - Female
KW - Humans
KW - Leisure Activities
KW - Male
KW - Motor Activity
KW - Parents/px [Psychology]
KW - Perception
KW - Questionnaires
KW - Sex Factors
RP - NOT IN FILE
SP - 124
EP - 137
JF - Pediatric Exercise Science
JA - Pediatr Exerc Sci
VL - 25
IS - 1
CY - United States

N2 - BACKGROUND: After-school hours provide an opportunity for physical activity (PA). Parental perceptions influence children's PA. The aims were to: compare parents' perceptions of children's PA with objectively measured PA; shed light on PA during after-school hours; and compare two electronic devices for collecting data. METHODS: Twenty parent-child dyads participated. Children (5-7 years, mean 6.25 + 0.64) wore Actical accelerometers; their parents responded to activity diaries on electronic devices. Data were collected twice for 4 consecutive weekday afternoons (15.30-19.00). RESULTS: While parents perceived their children to be quite active, children were, in fact, largely inactive. Parents' responses compared with accelerometer data yielded moderate correlations (r = .44, p < .01). Two thirds of parents' responses were overestimations. Boys were physically more active than girls and had higher PA outdoors than indoors. Girls' PA remained similar indoors and outdoors but parents did not perceive the similarity. Both electronic devices produced similar results and compliance rates. CONCLUSION: Parents consistently over-reported their children's PA. Findings have implications for initiatives to increase PA. If parents perceive their children as very active, they may lack motivation to promote PA. Parents' limitations as proxy reporters aside, the similarity of results yielded by the two electronic devices suggests that the choice is a matter of researcher preference
SN - 1543-2920
AD - Vancouver, British Columbia, Canada
UR - 23406701
ER -

TY - JOUR
ID - 305
T1 - Lower head temperature does not affect children's self-paced running velocity
A1 - Ferreira,Junior J.
A1 - Martini,A.
A1 - Borba,D.
A1 - Gomes,L.
A1 - Pinto,J.
A1 - Oliveira,B.
A1 - Coelho,D.
A1 - Prado,L.
A1 - Rodrigues,L.
Y1 - 2013/02//
N1 - Ferreira Junior, Joao. Martini, Angelo. Borba, Diego. Gomes, Leonardo. Pinto, Joao. Oliveira, Bernardo. Coelho, Daniel. Prado, Luciano. Rodrigues, Luiz
Pediatric exercise science
8909729
IM
Journal Article. Randomized Controlled Trial
English
KW - MEDLINE
KW - Analysis of Variance
KW - Brazil
KW - Child
KW - Environmental Exposure/pc [Prevention & Control]
KW - Exercise Test
KW - Female
KW - Head
KW - Heart Rate
KW - Heat Stress Disorders/pc [Prevention & Control]
KW - Hot Temperature
KW - Humans
KW - Male
KW - Physical Exertion
KW - Protective Clothing
KW - Running/ph [Physiology]
KW - Skin Temperature/ph [Physiology]
KW - Statistics

KW - Nonparametric
KW - Sunlight
RP - NOT IN FILE
SP - 114
EP - 123
JF - Pediatric Exercise Science
JA - Pediatr Exerc Sci
VL - 25
IS - 1
CY - United States
N2 - To test if the use of a peaked cap protects children against sun radiation, allowing increased exercise performance, nineteen healthy children (10.3 + 0.8 years old, 146.2 + 6.9 cm, 36.8 + 5.5 kg, 1.2 + 0.1 m2 and 44.1 + 2.8 mL.kg-1.min-1) took part in 4 experimental situations: 2 initial familiarization runs and 2 self-paced 6km runs (4 x 1.5 km exercise bouts with 3min rest intervals) one of them wearing a peaked cap (CAP) and another situation without the cap (NOCAP). The CAP and NOCAP situations were randomized. Exercise was performed outdoors 3-7 days apart. Environmental variables were measured every 10min, and physiological variables were measured before and after each run and during the rest intervals. Running velocity did not differ between CAP and NOCAP situations. The mean head temperature was reduced by 1.1 degreeC in the CAP situation (p < .05). Average skin temperature, mean heart rate, rate of perceived exertion and wet bulb and globe temperature did not differ between CAP and NOCAP. The decrease in the mean head temperature was not sufficient to alter running velocity
SN - 1543-2920
AD - Federal University of Minas Gerais, Belo Horizonte, Minas Gerais, Brazil
UR - 23406699
ER -

TY - JOUR
ID - 306
T1 - Neighbourhood safety and leisure-time physical activity among Dutch adults: a multilevel perspective
A1 - Kramer,D.
A1 - Maas,J.
A1 - Wingen,M.
A1 - Kunst,A.E.
Y1 - 2013///
N1 - Kramer, Danielle. Maas, Jolanda. Wingen, Marleen. Kunst, Anton E
The international journal of behavioral nutrition and physical activity
101217089
IM
Journal Article. Research Support, Non-U.S. Gov't
English
KW - MEDLINE
KW - Adolescent
KW - Adult
KW - Age Factors
KW - Aged
KW - Automobiles
KW - Bicycling
KW - Child
KW - Crime
KW - Educational Status
KW - Environment
KW - Fear
KW - Female
KW - Health Behavior
KW - Humans
KW - Leisure Activities
KW - Logistic Models

KW - Male
KW - Middle Aged
KW - Netherlands
KW - Residence Characteristics
KW - Safety
KW - Sex Factors
KW - Walking
KW - Young Adult
RP - NOT IN FILE
SP - 11
JF - International Journal of Behavioral Nutrition & Physical Activity
JA - Int
VL - 10
CY - England
N2 - BACKGROUND: Several neighbourhood elements have been found to be related to leisure-time walking and cycling. However, the association with neighbourhood safety remains unclear. This study aimed to assess the association of neighbourhood-level safety with leisure-time walking and cycling among Dutch adults. METHODS: Data were derived from the national health survey (POLS) 2006-2009, with valid data on 20046 respondents residing in 2127 neighbourhoods. Multilevel logistic regression models were used to examine the association between neighbourhood-level safety (general safety and specific safety components: physical disorder, social disorder, crime-related fear, traffic safety) and residents' engagement in outdoor leisure-time walking and cycling for at least 30 minutes per week. RESULTS: An increase in neighbourhood safety (both general safety and each of the safety components) was significantly associated with an increase in leisure-time cycling participation. Associations were strongest for general safety and among older women. In the general population, neighbourhood safety was not significantly associated with leisure-time walking. However, among younger and older adult men and lower educated individuals, an increase in general safety was associated with a decrease in leisure-time walking participation. CONCLUSIONS: In the Netherlands, neighbourhood safety appears to be related to leisure-time cycling but not to walking. Leisure-time cycling may best be encouraged by improving different safety components at once, rather than focusing on one safety aspect such as traffic safety. Special attention is needed for older women
SN - 1479-5868
AD - Department of Public Health, Academic Medical Centre, University of Amsterdam, Amsterdam, The Netherlands. d.kramer@amc.uva.nl
UR - 23356476
ER -

TY - JOUR
ID - 307
T1 - Changes in screen time activity in Norwegian children from 2001 to 2008: two cross sectional studies
A1 - Overby,N.C.
A1 - Klepp,K.I.
A1 - Bere,E.
Y1 - 2013///
N1 - Overby, Nina C. Klepp, Knut-Inge. Bere, Elling
BMC public health
100968562
IM
Journal Article. Research Support, Non-U.S. Gov't
English
KW - MEDLINE
KW - Child
KW - Child Behavior
KW - Computers/ut [Utilization]
KW - Cross-Sectional Studies
KW - Educational Status
KW - Female
KW - Humans

KW - Leisure Activities/px [Psychology]
KW - Male
KW - Norway
KW - Parents
KW - Questionnaires
KW - Sex Factors
KW - Television/ut [Utilization]
KW - Time Factors
RP - NOT IN FILE
SP - 80
JF - BMC Public Health
VL - 13
CY - England
N2 - BACKGROUND: There has been an increase in screen-based communication, leading to concerns about the negative health effects of screen-based activities in children and adolescents. The present study aimed to (1) analyze changes in screen time activity in Norwegian children from 2001 to 2008, and (2) to analyze associations between the changes in screen time activity over time and sex, grade level and parental educational level. METHODS: Within the project Fruits and Vegetables Make the Marks (FVMM), 1488 6th and 7th grade pupils from 27 Norwegian elementary schools completed a questionnaire including a question about time spent on television viewing and personal computer use in 2001 and 1339 pupils from the same schools completed the same questionnaire in 2008. Data were analyzed by multilevel linear mixed models. RESULTS: The proportions of 6th and 7th grade pupils at the 27 schools that reported screen time activity outside school of 2 hours/day or more decreased from 55% to 45% (p<0.001) from 2001 to 2008 when adjusting for sex, grade level and parental education. The decrease was most evident in 6th graders (51% to 37%) and in children with highly educated parents (54% to 39%). CONCLUSION: The present study shows that there has been a marked reduction in screen time activity outside school in this group of Norwegian 10-12 year olds from 2001 to 2008
SN - 1471-2458
AD - Department of Public Health, Sport and Nutrition, Faculty of Health and Sport, University of Agder, Kristiansand, 4604, Norway. nina.c.overby@uia.no
UR - 23356930
ER -

TY - JOUR
ID - 308
T1 - Correlation between the Child Health Questionnaire and the International Knee Documentation Committee score in pediatric and adolescent patients with an anterior cruciate ligament tear
A1 - Boykin,R.E.
A1 - McFeely,E.D.
A1 - Shearer,D.
A1 - Frank,J.S.
A1 - Harrod,C.C.
A1 - Nasreddine,A.Y.
A1 - Kocher,M.S.
Y1 - 2013/03//
N1 - Boykin, Robert E. McFeely, Eric D. Shearer, Dave. Frank, Jeremy S. Harrod, Christopher C. Nasreddine, Adam Y. Kocher, Mininder S
Journal of pediatric orthopedics
hsw, 8109053
IM
Comparative Study. Journal Article
English
KW - MEDLINE
KW - Adolescent
KW - Anterior Cruciate Ligament/in [Injuries]
KW - Anterior Cruciate Ligament/su [Surgery]
KW - Anterior Cruciate Ligament Reconstruction
KW - Child

KW - Cross-Sectional Studies
KW - Female
KW - Follow-Up Studies
KW - Humans
KW - Knee Injuries/pa [Pathology]
KW - Knee Injuries/px [Psychology]
KW - Knee Injuries/su [Surgery]
KW - Male
KW - Prospective Studies
KW - Quality of Life
KW - Questionnaires
KW - Registries
RP - NOT IN FILE
SP - 216
EP - 220
JF - Journal of Pediatric Orthopedics
JA - J Pediatr Orthop
VL - 33
IS - 2
CY - United States
N2 - BACKGROUND: The modified International Knee Documentation Committee (Pedi-IKDC) Subjective Knee Evaluation Form has recently been shown to be valid, reliable, and responsive in a pediatric and adolescent population. The correlations between the Pedi-IKDC and quality-of-life-related health measures have not been studied in depth to determine how a knee injury affects patients in this age group. The purpose of this study was to examine the association between the Pedi-IKDC score and the Child Health Questionnaire (CHQ) in a group of pediatric patients with an anterior cruciate ligament (ACL) injury. METHODS: A prospectively collected registry of patients with ACL injuries was searched to indentify all patients who had completed both the Pedi-IKDC and CHQ (CHQ-CF87) questionnaires. These were analyzed to determine significant correlations between domains of the CHQ and the Pedi-IKDC. RESULTS: A total of 135 patients were included (80 male, 55 female) with a median age of 15.3 years (range, 13.1 to 17.2 y). The cohort included patients treated both operatively (120) and nonoperatively (15). The total Pedi-IKDC score was found to correlate with the majority of the CHQ including expected domains such as physical function (correlation coefficient = 0.64), bodily pain (0.7), and family activities (0.41), in addition to emotional role (0.45), mental health (0.46), self-esteem (0.45), and social limitations--physical (0.38) (P < 0.001 for all correlations). CONCLUSIONS: Seven of the 12 domains on the CHQ are significantly correlated with the IKDC in adolescent patients with an ACL tear. Self-esteem, mental health, emotional role, and social limitations categories are significantly correlated with knee function suggesting that quality-of-life in this population is affected in domains outside of physical function and pain. A greater understanding of the psychosocial impact of injury may be of utility in these patients. STUDY DESIGN: Level III cross-sectional study
SN - 1539-2570
AD - Division of Sports Medicine, Children's Hospital, Boston, MA 02115, USA
UR - 23389579
ER -

TY - JOUR
ID - 309
T1 - Logistic regression analyses for predicting clinically important differences in motor capacity, motor performance, and functional independence after constraint-induced therapy in children with cerebral palsy
A1 - Wang,T.N.
A1 - Wu,C.Y.
A1 - Chen,C.L.
A1 - Shieh,J.Y.
A1 - Lu,L.
A1 - Lin,K.C.
Y1 - 2013/03//
N1 - Wang, Tien-ni. Wu, Ching-yi. Chen, Chia-ling. Shieh, Jeng-yi. Lu, Lu. Lin, Keh-chung
Research in developmental disabilities

8709782, rid
IM
Clinical Trial. Journal Article. Multicenter Study
English
KW - MEDLINE
KW - Activities of Daily Living
KW - Age Factors
KW - Cerebral Palsy/rh [Rehabilitation]
KW - Child
KW - Preschool
KW - Exercise Movement Techniques/sn [Statistics & Numerical Data]
KW - Female
KW - Humans
KW - Logistic Models
KW - Male
KW - Patient Selection
KW - Recovery of Function
KW - Restraint
KW - Physical/sn [Statistics & Numerical Data]
KW - Time Factors
KW - Treatment Outcome
KW - Upper Extremity
RP - NOT IN FILE
SP - 1044
EP - 1051
JF - Research in Developmental Disabilities
JA - Res Dev Disabil
VL - 34
IS - 3
CY - United States
N2 - Given the growing evidence for the effects of constraint-induced therapy (CIT) in children with cerebral palsy (CP), there is a need for investigating the characteristics of potential participants who may benefit most from this intervention. This study aimed to establish predictive models for the effects of pediatric CIT on motor and functional outcomes. Therapists administered CIT to 49 children (aged 3-11 years) with CP. Sessions were 1-3.5h a day, twice a week, for 3-4 weeks. Parents were asked to document the number of restraint hours outside of the therapy sessions. Domains of treatment outcomes included motor capacity (measured by the Peabody Developmental Motor Scales II), motor performance (measured by the Pediatric Motor Activity Log), and functional independence (measured by the Pediatric Functional Independence Measure). Potential predictors included age, affected side, compliance (measured by time of restraint), and the initial level of motor impairment severity. Tests were administered before, immediately after, and 3 months after the intervention. Logistic regression analyses showed that total amount of restraint time was the only significant predictor for improved motor capacity immediately after CIT. Younger children who restrained the less affected arm for a longer time had a greater chance to achieve clinically significant improvements in motor performance. For outcomes of functional independence in daily life, younger age was associated with clinically meaningful improvement in the self-care domain. Baseline motor abilities were significantly predictive of better improvement in mobility and cognition. Significant predictors varied according to the aspects of motor outcomes after 3 months of follow-up. The potential predictors identified in this study allow clinicians to target those children who may benefit most from CIT. Copyright 2012 Elsevier Ltd. All rights reserved
SN - 1873-3379
AD - College of Medicine, National Taiwan University, Taipei, Taiwan
UR - 23291522
ER -

TY - JOUR
ID - 310
T1 - Playground safety and quality in chicago
A1 - Allen,E.M.

A1 - Hill,A.L.
A1 - Tranter,E.
A1 - Sheehan,K.M.
Y1 - 2013/02//
N1 - Allen, Erin M. Hill, Amy L. Tranter, Erma. Sheehan, Karen M
Pediatrics
oxv, 0376422
AIM, IM
Journal Article. Research Support, Non-U.S. Gov't
English
KW - MEDLINE
KW - Accidental Falls/pc [Prevention & Control]
KW - Chicago
KW - Child
KW - Preschool
KW - Cross-Sectional Studies
KW - Equipment Design/st [Standards]
KW - Female
KW - Health Surveys
KW - Humans
KW - Infant
KW - Newborn
KW - Male
KW - Motor Activity
KW - Play and Playthings/in [Injuries]
KW - Public Sector
KW - Safety/st [Standards]
KW - Social Environment
KW - Socioeconomic Factors
KW - Wounds and Injuries/ep [Epidemiology]
KW - Wounds and Injuries/pc [Prevention & Control]
RP - NOT IN FILE
SP - 233
EP - 241
JF - Pediatrics
VL - 131
IS - 2
CY - United States
N2 - OBJECTIVES: To assess playground safety and quality in Chicago, Illinois, identify disparities in access, and use the data to inform collaborative improvement. METHODS: A cross-sectional survey of public park playgrounds in Chicago, Illinois, was conducted in 2009, 2010, and 2011 by using the National Program for Playground Safety Standardized Survey. All playgrounds were surveyed in 2009 and 2010; those that failed in 2010 were resurveyed in 2011. Playgrounds were assessed in 4 main categories: age-appropriate design, fall surfacing, equipment maintenance, and physical environment. Safety scores were generated from the assessment. Geographic information system mapping provided a visual description of the playground pass/fail rate based on neighborhood, child population, race/ethnicity, and poverty level. RESULTS: Of the ~500 playgrounds, 467 were assessed in 2009, and 459 were assessed in 2010. In 2009, half of all playgrounds (55%) and in 2010, nearly two-thirds (61%) earned scores consistent with safe playgrounds (P < .001). Playgrounds scored poorest in fall surfacing and equipment maintenance. Geographic information system mapping showed neighborhoods with a higher percentage of children and impoverished families had fewer playgrounds and more failing playgrounds. In 2011, 154 (85%) of the playgrounds that failed in 2010 were surveyed. The mean playground score among failing playgrounds improved significantly between 2010 (61%) and 2011 (67%, P < .001). CONCLUSIONS: Since the playground improvement initiative began in 2009, considerable progress has been made in the safety scores, although access to high-quality playgrounds varies by neighborhood. Many failing playgrounds can be brought up to standard with improvement in fall surfacing and equipment maintenance
SN - 1098-4275

AD - Baylor College of Medicine, 3925 Fairmont Parkway, Pasadena, TX 77504, USA. emallen@bcm.edu
UR - 23339221
ER -

TY - JOUR
ID - 311
T1 - Outdoor activity and myopia among primary students in rural and urban regions of Beijing
A1 - Guo,Y.
A1 - Liu,L.J.
A1 - Xu,L.
A1 - Lv,Y.Y.
A1 - Tang,P.
A1 - Feng,Y.
A1 - Meng,M.
A1 - Jonas,J.B.
Y1 - 2013/02//
N1 - Guo, Yin. Liu, Li Juan. Xu, Liang. Lv, Yan Yun. Tang, Ping. Feng, Yi. Meng, Meng. Jonas, Jost B
Ophthalmology
7802443
IM
Journal Article. Research Support, Non-U.S. Gov't
English
KW - MEDLINE
KW - Adolescent
KW - Axial Length
KW - Eye/pa [Pathology]
KW - Biometry
KW - Child
KW - Preschool
KW - China/ep [Epidemiology]
KW - Cross-Sectional Studies
KW - Female
KW - Humans
KW - Leisure Activities
KW - Male
KW - Myopia/ep [Epidemiology]
KW - Questionnaires
KW - Refraction
KW - Ocular/ph [Physiology]
KW - Rural Population/sn [Statistics & Numerical Data]
KW - Students
KW - Tomography
KW - Optical Coherence
KW - Urban Population/sn [Statistics & Numerical Data]
KW - Visual Acuity/ph [Physiology]
RP - NOT IN FILE
SP - 277
EP - 283
JF - Ophthalmology
VL - 120
IS - 2
CY - United States
N2 - OBJECTIVE: To assess associations among outdoor activity, ocular biometric parameters, and myopia among grade 1 and grade 4 primary students in Beijing. DESIGN: School-based, cross-sectional study. PARTICIPANTS: A total of 382 grade 1 and 299 grade 4 children participated in the study. METHODS: The children underwent a comprehensive eye examination, including ocular biometry by optical low-coherence reflectometry and noncycloplegic refractometry. Parents and children participated in a detailed interview,

including questions on time spent indoors and outdoors. MAIN OUTCOME MEASURES: Factors associated with myopia. RESULTS: The study included 681 children, with 382 (56.1%) students from grade 1 (mean age, 6.3 + 0.5 years; range, 5-8 years) and 299 students from grade 4 (mean age, 9.4 + 0.7 years; range, 8-13 years); 370 students (54.3%) lived in the urban region. The mean daily time spent outdoors was 1.6 + 0.8 hours (range, 0.5-5.1 hours). In multivariate analysis, axial length was significantly associated with older age (P<0.001; standardized beta coefficient, 0.28), taller body height (P = 0.001; beta, 0.18), maternal myopia (P = 0.03; beta, 0.09), and urban region of habitation (P<0.001; beta, -0.21), or alternatively to the region of habitation, with less time spent outdoors (P = 0.001; beta, -0.16) and more time spent indoors studying (P = 0.02; beta, 0.10). The axial length-to-corneal curvature radius ratio was associated with older age, urban region of habitation, maternal and paternal myopia, and paternal level of education. Presence of myopia (defined as refractive error <-1 diopters in the right eye) was associated with older age (P<0.001; odds ratio [OR], 1.45; 95% confidence interval [CI], 1.24-1.69), maternal myopia (P<0.001; OR, 2.99; 95% CI, 1.94-5.35), and urban region of habitation (P<0.001; OR, 0.17; 95% CI, 0.11-0.26), or alternatively to the region of habitation, with less time spent outdoors (P<0.001; OR, 0.32; 95% CI, 0.21-0.48) and more time spent indoors studying (P<0.001; OR, 1.38; 95% CI, 1.09-1.75). CONCLUSIONS: Less outdoor activity, more indoor studying, older age, maternal myopia, and urban region of habitation were associated with longer ocular axial length and myopia in grade 1 and grade 4 primary school children in Greater Beijing. Remaining outdoors more (e.g., during school) may reduce the high prevalence of myopia in the young generation in Beijing. Copyright 2013 American Academy of Ophthalmology. Published by Elsevier Inc. All rights reserved
SN - 1549-4713
AD - Beijing Institute of Ophthalmology, Beijing Tongren Hospital, Capital Medical University, Beijing, China
UR - 23098368
ER -

TY - JOUR
ID - 312
T1 - Household levels of nitrogen dioxide and pediatric asthma severity
A1 - Belanger,K.
A1 - Holford,T.R.
A1 - Gent,J.F.
A1 - Hill,M.E.
A1 - Kezik,J.M.
A1 - Leaderer,B.P.
Y1 - 2013/03//
N1 - Belanger, Kathleen. Holford, Theodore R. Gent, Janneane F. Hill, Melissa E. Kezik, Julie M. Leaderer, Brian P
Epidemiology (Cambridge, Mass.)
a2t, 9009644
IM
Journal Article. Research Support, N.I.H., Extramural
English
KW - MEDLINE
KW - Air Pollution
KW - Indoor/ae [Adverse Effects]
KW - Indoor/an [Analysis]
KW - Asthma/ci [Chemically Induced]
KW - Asthma/pp [Physiopathology]
KW - Child
KW - Preschool
KW - Environmental Exposure/ae [Adverse Effects]
KW - Environmental Exposure/an [Analysis]
KW - Environmental Monitoring/mt [Methods]
KW - Family Characteristics
KW - Female
KW - Humans
KW - Hypersensitivity/di [Diagnosis]
KW - Logistic Models

KW - Male
KW - Nitrogen Dioxide/ae [Adverse Effects]
KW - Prospective Studies
KW - Seasons
KW - Severity of Illness Index
RP - NOT IN FILE
SP - 320
EP - 330
JF - Epidemiology
VL - 24
IS - 2
CY - United States
N2 - BACKGROUND: Adverse respiratory effects in children with asthma are associated with exposures to nitrogen dioxide (NO2). Levels indoors can be much higher than outdoors. Primary indoor sources of NO2 are gas stoves, which are used for cooking by one-third of U.S. households. We investigated the effects of indoor NO2 exposure on asthma severity among an ethnically and economically diverse sample of children, controlling for season and indoor allergen exposure. METHODS: Children 5-10 years of age with active asthma (n = 1,342) were recruited through schools in urban and suburban Connecticut and Massachusetts (2006-2009) for a prospective, year-long study with seasonal measurements of NO2 and asthma severity. Exposure to NO2 was measured passively for four, month-long, periods with Palmes tubes. Asthma morbidity was concurrently measured by a severity score and frequency of wheeze, night symptoms, and use of rescue medication. We used adjusted, hierarchical ordered logistic regression models to examine associations between household NO2 exposure and health outcomes. RESULTS: Every 5-fold increase in NO2 exposure above a threshold of 6 ppb was associated with a dose-dependent increase in risk of higher asthma severity score (odds ratio = 1.37 [95% confidence interval = 1.01-1.89]), wheeze (1.49 [1.09-2.03]), night symptoms (1.52 [1.16-2.00]), and rescue medication use (1.78 [1.33-2.38]). CONCLUSIONS: Asthmatic children exposed to NO2 indoors, at levels well below the U.S. Environmental Protection Agency outdoor standard (53 ppb), are at risk for increased asthma morbidity. Risks are not confined to inner city children, but occur at NO2 concentrations common in urban and suburban homes
SN - 1531-5487
AD - Center for Perinatal, Pediatric and Environmental Epidemiology, Yale University School of Public Health, New Haven, CT 06510, USA
UR - 23337243
ER -

TY - JOUR
ID - 313
T1 - Lifestyle behaviors of obese children following parental weight loss surgery
A1 - Watowicz,R.P.
A1 - Taylor,C.A.
A1 - Eneli,I.U.
Y1 - 2013/02//
N1 - Watowicz, Rosanna P. Taylor, Christopher A. Eneli, Ihuoma U
Obesity surgery
c0v, 9106714
IM
Comparative Study. Journal Article
English
KW - MEDLINE
KW - Case-Control Studies
KW - Child
KW - Child Nutritional Physiological Phenomena
KW - Child of Impaired Parents/px [Psychology]
KW - Diet
KW - Exercise/px [Psychology]
KW - Family Health
KW - Feeding Behavior/px [Psychology]

KW - Female
KW - Gastroplasty/px [Psychology]
KW - Health Knowledge
KW - Attitudes
KW - Practice
KW - Humans
KW - Life Style
KW - Male
KW - Motivation
KW - Obesity
KW - Morbid/px [Psychology]
KW - Morbid/su [Surgery]
KW - Parents/px [Psychology]
KW - Questionnaires
KW - Retrospective Studies
KW - Social Environment
KW - Weight Loss
RP - NOT IN FILE
SP - 173
EP - 178
JF - Obesity Surgery
JA - Obes Surg
VL - 23
IS - 2
CY - United States
N2 - BACKGROUND: Following weight loss surgery (WLS), patients are expected to make diet and lifestyle changes which may lead to children mimicking the changing behaviors of their parents. The purpose of the study was to identify the differences in diet and lifestyle behaviors between obese children with and without a parent who received WLS. METHODS: Medical records of 45 children whose parents had undergone WLS and 90 age- and gender-matched control children were reviewed from a weight loss program in a large Midwest children's hospital. Differences in dietary choices and behaviors, perceived barriers, and sedentary behaviors were examined between both groups. RESULTS: The mean age for the sample was 12.8 years. Children in the parental weight loss surgery (PWLS) group were more likely to eat two or more helpings of food at each sitting (p=0.02) and less likely to play outdoors for more than an hour each day (p=0.01). Compared to the control group, the PWLS group more frequently reported eating fast food on most days (45.2 vs. 27.0 %), soda consumption several times a week (48.6 vs. 29.4 %), and no vegetable intake (9.5 vs. 1.1 %). The top three barriers to exercise for both groups were lack of self-discipline, lack of interest, and lack of energy. CONCLUSIONS: Obese children who live with a parent that had undergone WLS reported several unhealthy lifestyle behaviors, in some cases worse than the children who live with parents who had not had WLS. Being cognizant of these findings will help obesity providers focus their counseling and expectations appropriately
SN - 1708-0428
AD - School of Health and Rehabilitation Sciences, The Ohio State University, 435 W. 10th Avenue, 306 Atwell Hall, Columbus, OH 43210, USA. Rosanna.Watowicz@osumc.edu
UR - 22941333
ER -

TY - JOUR
ID - 314
T1 - Implementation of a national reference laboratory for Buruli ulcer disease in Togo
A1 - Beissner,M.
A1 - Huber,K.L.
A1 - Badziklou,K.
A1 - Halatoko,W.A.
A1 - Maman,I.
A1 - Vogel,F.
A1 - Bidjada,B.
A1 - Awoussi,K.S.

A1 - Piten,E.
A1 - Helfrich,K.
A1 - Mengele,C.
A1 - Nitschke,J.
A1 - Amekuse,K.
A1 - Wiedemann,F.X.
A1 - Diefenhardt,A.
A1 - Kobara,B.
A1 - Herbinger,K.H.
A1 - Kere,A.B.
A1 - Prince-David,M.
A1 - Loscher,T.
A1 - Bretzel,G.
Y1 - 2013///
N1 - Beissner, Marcus. Huber, Kristina Lydia. Badziklou, Kossi. Halatoko, Wemboo Afiwa. Maman, Issaka. Vogel, Felix. Bidjada, Bawimodom. Awoussi, Koffi Somenou. Piten, Ebekalisai. Helfrich, Kerstin. Mengele, Carolin. Nitschke, Jorg. Amekuse, Komi. Wiedemann, Franz Xaver. Diefenhardt, Adolf. Kobara, Basile. Herbinger, Karl-Heinz. Kere, Abiba Banla. Prince-David, Mireille. Loscher, Thomas. Bretzel, Gisela
PLoS neglected tropical diseases
101291488
IM
Journal Article. Research Support, Non-U.S. Gov't
English
KW - MEDLINE
KW - Adolescent
KW - Adult
KW - Aged
KW - Buruli Ulcer/di [Diagnosis]
KW - Buruli Ulcer/ep [Epidemiology]
KW - Child
KW - Preschool
KW - Clinical Laboratory Techniques/mt [Methods]
KW - Clinical Laboratory Techniques/st [Standards]
KW - Female
KW - Humans
KW - Male
KW - Microscopy/mt [Methods]
KW - Microscopy/st [Standards]
KW - Middle Aged
KW - Polymerase Chain Reaction/mt [Methods]
KW - Polymerase Chain Reaction/st [Standards]
KW - Quality Assurance
KW - Health Care
KW - Reference Standards
KW - Togo/ep [Epidemiology]
KW - Young Adult
RP - NOT IN FILE
SP - e2011
JF - PLoS Neglected Tropical Diseases [electronic resource]
JA - PLoS Negl Trop Dis
VL - 7
IS - 1
CY - United States
N2 - BACKGROUND: In a previous study PCR analysis of clinical samples from suspected cases of Buruli ulcer disease (BUD) from Togo and external quality assurance (EQA) for local microscopy were conducted at an external reference laboratory in Germany. The relatively poor performance of local microscopy as well as effort and time associated with shipment of PCR samples necessitated the implementation of stringent EQA

measures and availability of local laboratory capacity. This study describes the approach to implementation of a national BUD reference laboratory in Togo. METHODOLOGY: Large scale outreach activities accompanied by regular training programs for health care professionals were conducted in the regions "Maritime" and "Central," standard operating procedures defined all processes in participating laboratories (regional, national and external reference laboratories) as well as the interaction between laboratories and partners in the field. Microscopy was conducted at regional level and slides were subjected to EQA at national and external reference laboratories. For PCR analysis, sample pairs were collected and subjected to a dry-reagent-based IS2404-PCR (DRB-PCR) at national level and standard IS2404 PCR followed by IS2404 qPCR analysis of negative samples at the external reference laboratory. PRINCIPAL FINDINGS: The inter-laboratory concordance rates for microscopy ranged from 89% to 94%; overall, microscopy confirmed 50% of all suspected BUD cases. The inter-laboratory concordance rate for PCR was 96% with an overall PCR case confirmation rate of 78%. Compared to a previous study, the rate of BUD patients with non-ulcerative lesions increased from 37% to 50%, the mean duration of disease before clinical diagnosis decreased significantly from 182.6 to 82.1 days among patients with ulcerative lesions, and the percentage of category III lesions decreased from 30.3% to 19.2%. CONCLUSIONS: High inter-laboratory concordance rates as well as case confirmation rates of 50% (microscopy), 71% (PCR at national level), and 78% (including qPCR confirmation at external reference laboratory) suggest high standards of BUD diagnostics. The increase of non-ulcerative lesions, as well as the decrease in diagnostic delay and category III lesions, prove the effect of comprehensive EQA and training measures involving also procedures outside the laboratory
SN - 1935-2735
AD - Department of Infectious Diseases and Tropical Medicine, University Hospital, Ludwig-Maximilians-University, Munich, Germany. beissner@lrz.uni-muenchen.de
UR - 23359828
ER -

TY - JOUR
ID - 315
T1 - [Guideline 'Overweight' for child health care]. [Review] [Dutch]
A1 - Kist-van Holthe,J.E.
A1 - Bulk-Bunschoten,A.M.
A1 - Renders,C.M.
A1 - L'Hoir,M.
A1 - Kuijpers,T.
A1 - Hirasing,R.A.
Y1 - 2013///
N1 - Kist-van Holthe, Joana E. Bulk-Bunschoten, Anneke M W. Renders, Carry M. L'Hoir, Monique. Kuijpers, Ton. HiraSing, Remy A
Nederlands tijdschrift voor geneeskunde
nuk, 0400770
IM
English Abstract. Journal Article. Research Support, Non-U.S. Gov't. Review
Dutch
KW - MEDLINE
KW - Child
KW - Diet
KW - Reducing
KW - Exercise/ph [Physiology]
KW - Humans
KW - Overweight/di [Diagnosis]
KW - Overweight/th [Therapy]
KW - Pediatrics/st [Standards]
KW - Practice Guidelines as Topic
RP - NOT IN FILE
SP - A4718
JF - Nederlands Tijdschrift voor Geneeskunde
JA - Ned Tijdschr Geneeskd
VL - 157

IS - 4
CY - Netherlands
N2 - The '5th National Growth Study' indicates that the percentage of overweight children in the Netherlands has risen from 9-12% in 1997 to 13-15% in 2009. Child Health Care is a unique setting for promotion of development, growth and behaviour of children, in which tailored prevention can be offered. Detection of overweight in children and intervention by Child Health Care takes place in a multidisciplinary setting linking general practitioners, paediatricians, dieticians, teachers, physiotherapists, pedagogues and psychologists. For overweight children, a change plan is created based on exercise, playing outside, having breakfast every day, as little as possible sweetened beverages and fast-food, and less time spent in front of the television or computer, with fewer energy-rich snacks. As recommended in the Dutch CBO guideline 'Obesity', obese children are referred to a general practitioner or paediatrician
SN - 1876-8784
AD - VUmc/EMGO+-Instituut, afd. Sociale Geneeskunde, Amsterdam, the Netherlands. j.kist@vumc.nl
UR - 23343730
ER -

TY - JOUR
ID - 316
T1 - Do features of public open spaces vary between urban and rural areas?
A1 - Veitch,J.
A1 - Salmon,J.
A1 - Ball,K.
A1 - Crawford,D.
A1 - Timperio,A.
Y1 - 2013/02//
N1 - Veitch, Jenny. Salmon, Jo. Ball, Kylie. Crawford, David. Timperio, Anna
Preventive medicine
pm4, 0322116
IM
Comparative Study. Journal Article. Research Support, Non-U.S. Gov't
English
KW - MEDLINE
KW - Adult
KW - Australia
KW - Child
KW - Environment Design
KW - Health Promotion/st [Standards]
KW - Humans
KW - Lighting
KW - Motor Activity
KW - Obesity/pc [Prevention & Control]
KW - Play and Playthings
KW - Public Facilities
KW - Rural Population/sn [Statistics & Numerical Data]
KW - Safety
KW - Social Planning
KW - Urban Population/sn [Statistics & Numerical Data]
KW - Urban Renewal/st [Standards]
RP - NOT IN FILE
SP - 107
EP - 111
JF - Preventive Medicine
JA - Prev Med
VL - 56
IS - 2
CY - United States

N2 - OBJECTIVE: Parks are an important setting for physical activity and specific park features have been shown to be associated with park visitation and physical activity. Most park-based research has been conducted in urban settings with few studies examining rural parks. This study examined differences in features of parks in urban compared with rural areas. METHODS: In 2009/10 a tool was developed to audit 433 urban and 195 rural parks located in disadvantaged areas of Victoria, Australia. Features assessed included: access; lighting/safety; aesthetics; amenities; paths; outdoor courts/ovals; informal play spaces; and playgrounds (number, diversity, age appropriateness and safety of play equipment). RESULTS: Rural parks scored higher for aesthetics compared with urban parks (5.08 vs 4.44). Urban parks scored higher for access (4.64 vs 3.89), lighting/safety (2.01 vs 1.76), and diversity of play equipment (7.37 vs 6.24), and were more likely to have paths suitable for walking/cycling (58.8% vs 40.9%) and play equipment for older children (68.2% vs 17.1%). CONCLUSION: Although the findings cannot be generalized to all urban and rural parks, the results may be used to inform advocacy for park development in rural areas to create parks that are more supportive of physical activity for children and adults. Copyright 2012 Elsevier Inc. All rights reserved
SN - 1096-0260
AD - Centre for Physical Activity and Nutrition Research, Deakin University, Burwood, Victoria 3125, Australia. jenny.veitch@deakin.edu.au
UR - 23201001
ER -

TY - JOUR
ID - 317
T1 - Estimated energy expenditures for school-based policies and active living. [Review]
A1 - Bassett,D.R.
A1 - Fitzhugh,E.C.
A1 - Heath,G.W.
A1 - Erwin,P.C.
A1 - Frederick,G.M.
A1 - Wolff,D.L.
A1 - Welch,W.A.
A1 - Stout,A.B.
Y1 - 2013/02//
N1 - Bassett, David R. Fitzhugh, Eugene C. Heath, Gregory W. Erwin, Paul C. Frederick, Ginny M. Wolff, Dana L. Welch, Whitney A. Stout, Aaron B
American journal of preventive medicine
8704773, apl
IM
Journal Article. Research Support, Non-U.S. Gov't. Review
English
KW - MEDLINE
KW - Adolescent
KW - Child
KW - Energy Metabolism/ph [Physiology]
KW - Health Behavior
KW - Health Policy
KW - Health Promotion/mt [Methods]
KW - Humans
KW - Motor Activity/ph [Physiology]
KW - Organizational Policy
KW - Policy Making
KW - Residence Characteristics
KW - Schools
RP - NOT IN FILE
SP - 108
EP - 113
JF - American Journal of Preventive Medicine
JA - Am J Prev Med
VL - 44

IS - 2
CY - Netherlands
N2 - BACKGROUND: Despite overwhelming evidence of the health benefits of physical activity, most American youth are not meeting the 60 minutes per day recommendation for moderate- to vigorous-intensity physical activity (MVPA). Policy changes have the potential to bring about substantial increases in physical activity in youth, within school and community settings. PURPOSE: The purpose of this study was to quantify the increase in energy expenditure for school-based policies and built environment changes. METHODS: Scientific literature reviews were consulted, and more than 300 published studies (1995-2011) in English were identified based on titles and abstracts. After an initial screening, 85 articles were included. Study quality was assessed, and the impact of various strategies for increasing physical activity in youth was estimated from objective measurements/direct observation. RESULTS: Within school settings, the average minutes of MVPA gained per school day for studies in each intervention category were as follows: mandatory physical education (23 minutes); classroom activity breaks (19 minutes); afterschool activity programs (10 minutes); standardized physical education curricula (6 minutes more than traditional physical education); modified playgrounds (6 minutes); and modified recess (5 minutes more than traditional recess). Within community settings, significant MVPA was associated with active commuting (16 minutes) and park renovations (12 minutes), but proximity to parks had a small effect (1 minute). No conclusions could be drawn regarding joint-use agreements, because of a lack of studies quantifying their impact on energy expenditure. CONCLUSIONS: Of the various policies and built environment changes examined, the largest effects were seen with mandatory physical education, classroom activity breaks, and active commuting to school. Policymakers can use this information along with estimates of the cost, feasibility, and population reach, to identify the best options for increasing physical activity in youth. Copyright 2013 American Journal of Preventive Medicine. Published by Elsevier Inc. All rights reserved
SN - 1873-2607
AD - Department of Kinesiology, Recreation, & Sport Studies, University of Tennessee, Knoxville, 37996, USA. dbassett@utk.edu
UR - 23332325
ER -

TY - JOUR
ID - 318
T1 - Health impact modelling of active travel visions for England and Wales using an Integrated Transport and Health Impact Modelling Tool (ITHIM)
A1 - Woodcock,J.
A1 - Givoni,M.
A1 - Morgan,A.S.
Y1 - 2013///
N1 - Woodcock, James. Givoni, Moshe. Morgan, Andrei Scott
PloS one
101285081
IM
Journal Article. Research Support, Non-U.S. Gov't
English
KW - MEDLINE
KW - Adolescent
KW - Adult
KW - Aged
KW - 80 and over
KW - Air Pollutants/me [Metabolism]
KW - Air Pollution/pc [Prevention & Control]
KW - Automobiles
KW - Bicycling
KW - Carbon Dioxide/me [Metabolism]
KW - Child
KW - Preschool
KW - England
KW - Humans

KW - Infant
KW - Newborn
KW - Middle Aged
KW - Models
KW - Theoretical
KW - Public Health/mt [Methods]
KW - Public Health/sn [Statistics & Numerical Data]
KW - Public Health/td [Trends]
KW - Transportation/mt [Methods]
KW - Transportation/sn [Statistics & Numerical Data]
KW - Wales
KW - Walking
RP - NOT IN FILE
SP - e51462
JF - PLoS ONE [Electronic Resource]
JA - PLoS ONE
VL - 8
IS - 1
CY - United States
N2 - BACKGROUND: Achieving health benefits while reducing greenhouse gas emissions from transport offers a potential policy win-win; the magnitude of potential benefits, however, is likely to vary. This study uses an Integrated Transport and Health Impact Modelling tool (ITHIM) to evaluate the health and environmental impacts of high walking and cycling transport scenarios for English and Welsh urban areas outside London. METHODS: Three scenarios with increased walking and cycling and lower car use were generated based upon the Visions 2030 Walking and Cycling project. Changes to carbon dioxide emissions were estimated by environmental modelling. Health impact assessment modelling was used to estimate changes in Disability Adjusted Life Years (DALYs) resulting from changes in exposure to air pollution, road traffic injury risk, and physical activity. We compare the findings of the model with results generated using the World Health Organization's Health Economic Assessment of Transport (HEAT) tools. RESULTS: This study found considerable reductions in disease burden under all three scenarios, with the largest health benefits attributed to reductions in ischemic heart disease. The pathways that produced the largest benefits were, in order, physical activity, road traffic injuries, and air pollution. The choice of dose response relationship for physical activity had a large impact on the size of the benefits. Modelling the impact on all-cause mortality rather than through individual diseases suggested larger benefits. Using the best available evidence we found fewer road traffic injuries for all scenarios compared with baseline but alternative assumptions suggested potential increases. CONCLUSIONS: Methods to estimate the health impacts from transport related physical activity and injury risk are in their infancy; this study has demonstrated an integration of transport and health impact modelling approaches. The findings add to the case for a move from car transport to walking and cycling, and have implications for empirical and modelling research
SN - 1932-6203
AD - UKCRC Centre for Diet and Activity Research (CEDAR), Institute of Public Health, University of Cambridge, Cambridge, United Kingdom. jw745@medschl.cam.ac.uk
UR - 23326315
ER -

TY - JOUR
ID - 319
T1 - Daily physical activity patterns of children living in an American Indian community
A1 - Brusseau,T.A.
A1 - Kulinna,P.H.
A1 - Tudor-Locke,C.
A1 - Ferry,M.
Y1 - 2013/01//
N1 - Brusseau, Timothy A. Kulinna, Pamela H. Tudor-Locke, Catrine. Ferry, Matthew
Journal of physical activity & health
101189457
IM

Journal Article
English
KW - MEDLINE
KW - Child
KW - Exercise
KW - Female
KW - Humans
KW - Indians
KW - North American/sn [Statistics & Numerical Data]
KW - Male
KW - Physical Education and Training/sn [Statistics & Numerical Data]
KW - Sex Factors
RP - NOT IN FILE
SP - 48
EP - 53
JF - Journal of Physical Activity & Health
JA - J Phys Act Health
VL - 10
IS - 1
CY - United States
N2 - BACKGROUND: Embracing a physically active lifestyle is especially important for American Indian (AI) children who are at a greater risk for hypokinetic diseases, particularly Type 2 diabetes. The purpose of this study was to describe AI children's pedometer-determined physical activity (PA) segmented into prominent daily activity patterns. METHODS: Participants included 5th- and 6th-grade children (N = 77) attending school from 1 Southwestern US AI community. Children wore a pedometer (Yamax Digiwalker SW-200) for 7 consecutive days. RESULTS: Boys accumulated 12,621 (+ 5385) steps/weekday and girls accumulated 11,640 (+ 3695) steps/weekday of which 38% (4,779 + 1271) and 35% (4,027 + 1285) were accumulated at school for boys and girls, respectively. Physical education (PE) provided the single largest source of PA during school for both boys (25% or 3117 steps/day) and girls (23% or 2638 steps/day). Lunchtime recess provided 1612 (13%) and 1241 (11%) steps/day for boys and girls, respectively. Children were significantly less active on weekend days, accumulating 8066 + 1959 (boys) and 6676 + 1884 (girls). CONCLUSIONS: Although children accumulate a majority of their steps outside of school, this study highlights the important contribution of PE to the overall PA accumulation of children living in AI communities. Further, PA programming during the weekend appears to be important for this population
SN - 1543-5474
AD - Department of Exercise and Sport Science, University of Utah, Salt Lake City, UT, USA
UR - 23324486
ER -

TY - JOUR
ID - 320
T1 - Social development conditions and related factors for children in Japanese kindergarten in Shanghai, China
A1 - Onishi,S.
A1 - Okawa,N.
A1 - Utsumi,M.
A1 - Yamada,K.
A1 - Morioka,I.
Y1 - 2013/01//
N1 - Onishi, Shuhei. Okawa, Naoko. Utsumi, Miyoko. Yamada, Kazuko. Morioka, Ikuharu
Environmental health and preventive medicine
9609642
IM
Journal Article. Research Support, Non-U.S. Gov't
English
KW - MEDLINE
KW - Child

KW - Child Development
KW - Preschool
KW - China
KW - Female
KW - Humans
KW - Japan/eh [Ethnology]
KW - Male
KW - Questionnaires
KW - Schools
KW - Social Behavior
RP - NOT IN FILE
SP - 40
EP - 48
JF - Environmental Health & Preventive Medicine
JA - Environ
VL - 18
IS - 1
CY - Japan
N2 - OBJECTIVE: The aim of this study is to clarify the social development conditions and related factors for children in Japanese kindergarten in Shanghai, China. METHODS: The target schools were three Japanese kindergartens in Shanghai, three in Wakayama, Japan, and three in Osaka, Japan. The target kindergarten children were all 5- or 6-year-olds. We used an anonymous questionnaire to the guardian of the child. The question items related to social development, lifestyle and daily activities of the child, and rearing awareness and conditions of the guardian. Social development was scored by Strength and Difficulties Questionnaires (SDQ). RESULTS: The number of respondents was 246 (Shanghai group 72, Wakayama group 86, Osaka group 88). The median value of the score of total difficulties and prosocial behavior, SDQ subscale, and the proportion of children judged as low need showed no significant difference. Results of multiple logistic regression analysis showed that the frequency of eating fast-foods and the rearing condition "I become anxious or lose enthusiasm for childrearing when the child is recalcitrant" were the factors relevant to prosocial behavior in the Shanghai group. In the Wakayama group, the hours spent daily watching TV was designated as the relevant factor. In the Osaka group, hours spent daily playing outdoors was found to be the relevant factor. CONCLUSIONS: Social development of children in Japanese kindergarten in Shanghai was the same as that of those living in Japan. The factors relevant to prosocial behavior in the Shanghai group were different from those in the two cities in Japan. This study may show the important points to keep in mind for childrearing abroad
SN - 1347-4715
AD - Graduate School of Health and Nursing Science, Wakayama Medical University, Mikazura 580, Wakayama, 641-0011, Japan
UR - 22797971
ER -

TY - JOUR
ID - 321
T1 - Published reports of delayed hemolytic anemia after treatment with artesunate for severe malaria--worldwide, 2010-2012
A1 - Centers for Disease Control and Prevention (CDC)
Y1 - 2013/01/11/
N1 - Centers for Disease Control and Prevention (CDC)
MMWR. Morbidity and mortality weekly report
ne8, 7802429
IM
Journal Article
English
KW - MEDLINE
KW - Adolescent
KW - Adult
KW - Aged
KW - Anemia

KW - Hemolytic/ci [Chemically Induced]
KW - Hemolytic/ep [Epidemiology]
KW - Antimalarials/ae [Adverse Effects]
KW - Antimalarials/tu [Therapeutic Use]
KW - Artemisinins/ae [Adverse Effects]
KW - Artemisinins/tu [Therapeutic Use]
KW - Child
KW - Preschool
KW - Female
KW - Humans
KW - Malaria/dt [Drug Therapy]
KW - Male
KW - Middle Aged
KW - Retrospective Studies
KW - Severity of Illness Index
KW - Time Factors
RP - NOT IN FILE
SP - 5
EP - 8
JF - MMWR - Morbidity & Mortality Weekly Report
JA - MMWR Morb Mortal Wkly Rep
VL - 62
IS - 1
CY - United States
N2 - Artesunate has been recommended by the World Health Organization (WHO) as the first-line treatment for severe malaria since 2010. It is not licensed in the United States but is available from CDC under an investigational new drug (IND) protocol. During 2010-2012, a total of 19 cases of delayed hemolytic anemia after treatment of severe malaria with artesunate were published in the peer-reviewed medical literature, but no such cases have been reported in the United States. CDC Malaria Branch staff reviewed each published report of delayed hemolysis after artesunate use. Based on the pathogenesis of malaria, the hemolysis likely is a result of severe malaria and not the treatment itself. However, artesunate used in the United States is produced by the U.S. Army Medical Materiel Development Activity, and artesunate used outside of the United States is not. An unrecognized difference might exist between the U.S. artesunate and the artesunate used elsewhere. Alternatively, cases of artesunate-associated hemolysis might have occurred in the United States but were not reported. To better assess these possibilities, CDC has amended the artesunate IND protocol and now recommends that persons treated for severe malaria with artesunate be followed for 4 weeks after treatment and evaluated for hemolytic anemia
SN - 1545-861X
UR - 23302816
ER -

TY - JOUR
ID - 322
T1 - Demographic variations in observed energy expenditure across park activity areas
A1 - Besenyi,G.M.
A1 - Kaczynski,A.T.
A1 - Wilhelm Stanis,S.A.
A1 - Vaughan,K.B.
Y1 - 2013/01//
N1 - Besenyi, Gina M. Kaczynski, Andrew T. Wilhelm Stanis, Sonja A. Vaughan, Katherine B
Preventive medicine
pm4, 0322116
IM
Journal Article
English
KW - MEDLINE
KW - Adolescent

KW - Child
KW - Preschool
KW - Energy Metabolism/ph [Physiology]
KW - Environment Design
KW - Female
KW - Humans
KW - Male
KW - Missouri
KW - Observation
KW - Public Facilities
KW - Qualitative Research
KW - Recreation/ph [Physiology]
KW - Young Adult
RP - NOT IN FILE
SP - 79
EP - 81
JF - Preventive Medicine
JA - Prev Med
VL - 56
IS - 1
CY - United States
N2 - OBJECTIVE: Parks are important physical activity (PA) settings, but few studies have examined PA differences between park areas according to multiple user demographic characteristics. This study explored variations in adult/senior and child/teen PA intensity across park settings by gender, age, and race/ethnicity. METHODS: In July-August 2009, the System for Observing Play and Recreation in Communities (SOPARC) was used in four parks in Kansas City, Missouri to record park users' PA behavior within specific park areas. A total of 8855 observations were recorded. ANCOVAs compared mean energy expenditure (EE) for various demographic groups across the five most-used park target areas for adults/seniors and children/teens. RESULTS: Among adults/seniors, all sub-samples exhibited higher EE on paved trails (0.086-0.093 kcal/kg/min) and tennis courts (0.086-0.089 kcal/kg/min), than in open spaces, playgrounds, or picnic shelters. Among children/teens, the child, White, and all child/teen sub-samples showed greater EE on playgrounds (0.088-0.089 kcal/kg/min) than in picnic shelters. CONCLUSION: Park planning and design efforts should consider environmental and programming initiatives to increase intergenerational PA levels within park areas such as playgrounds, open spaces, and picnic shelters. Copyright 2012 Elsevier Inc. All rights reserved
SN - 1096-0260
AD - Department of Health Promotion, Education, and Behavior, Arnold School of Public Health, University of South Carolina, Columbia, SC 29208, USA. besenyi@email.sc.edu
UR - 23068023
ER -

TY - JOUR
ID - 323
T1 - Factors predicting severe childhood obesity in kindergarteners.[Erratum appears in Int J Obes (Lond). 2013 May;37(5):758]
A1 - Flores,G.
A1 - Lin,H.
Y1 - 2013/01//
N1 - Flores, G. Lin, H
International journal of obesity (2005)
101256108
IM
Journal Article
English
KW - MEDLINE
KW - Birth Weight
KW - Body Mass Index
KW - Child

KW - Preschool
KW - Diet
KW - Female
KW - Humans
KW - Infant
KW - Logistic Models
KW - Longitudinal Studies
KW - Male
KW - Mothers
KW - Multivariate Analysis
KW - Obesity
KW - Morbid/ep [Epidemiology]
KW - Morbid/eh [Ethnology]
KW - Morbid/pc [Prevention & Control]
KW - Odds Ratio
KW - Predictive Value of Tests
KW - Prevalence
KW - Risk Factors
KW - Sleep
KW - United States/ep [Epidemiology]
RP - NOT IN FILE
SP - 31
EP - 39
JF - International Journal of Obesity
JA - Int J Obes (Lond)
VL - 37
IS - 1
CY - England
N2 - BACKGROUND: Severe obesity has increased >300% in US children since 1976, and is associated with multiple cardiovascular risk factors and high adult obesity rates. OBJECTIVE: The objective of this study was to identify predictors of severe obesity in kindergarteners. METHODS: Multivariable logistic regression and recursive partitioning analysis (RPA) were used to identify prenatal/pregnancy, infant, and early childhood predictors of severe kindergarten obesity (body mass index (BMI) 99th percentile) in the Early Childhood Longitudinal Study Birth Cohort, a nationally representative longitudinal study that followed children from birth through kindergarten. RESULTS: For the 6800 children, the severe kindergarten obesity prevalence was 5.7%, with higher adjusted odds for crossing the 85th percentile of BMI at 2 years old (odds ratio (OR), 8.0; 95% confidence interval (CI), 4.1-15.7), preschool age (OR, 7.9; 95% CI, 4.9-12.8) and 9 months old (OR, 1.8; 95% CI, 1.2-2.6); maternal severe obesity (OR, 3.4; 95% CI, 1.9-5.8) and gestational diabetes (OR, 2.9; 95% CI, 1.5-5.5); drinking tea or coffee between meals/before bedtime at 2 years old (OR, 3.3; 95% CI, 1.3-8.5); Latino (OR, 2.3; 95% CI, 1.4-3.7) and multiracial (OR, 2.3; 95% CI, 1.1-4.8) race/ethnicity; and drinking sugary beverages at kindergarten age at least weekly (OR, 2.3; 95% CI, 1.4-3.7). Ever-attending center-based daycare (OR, 0.3; 95% CI, 0.1-0.9), eating fruit at least weekly at kindergarten age (OR, 0.3; 95% CI, 0.1-0.7), and maternal history of a prior newborn birth weight 4000g (OR, 0.1; 95% CI, 0.02-0.6) were associated with reduced odds of severe obesity. RPA identified low severe obesity prevalence (1.9%) for non-85th BMI-percentile preschool crossers and high severe obesity (56-80%) for predictor clusters which included crossing the 85th BMI percentile at earlier ages, low parental education, specific maternal age cutoffs, preschooler bedtime rules, and outside walking/play frequency for 9-month-olds. CONCLUSIONS: Certain parental, prenatal/pregnancy, infant, and early childhood factors, both alone and in combination, are potent predictors of severe obesity in kindergarteners
SN - 1476-5497
AD - Division of General Pediatrics, Department of Pediatrics, UT Southwestern Medical Center, Dallas, TX 75390, USA. glenn.flores@utsouthwestern.edu
UR - 23147114
ER -

TY - JOUR
ID - 324

T1 - The crucial role of recess in school. [Review]
A1 - Murray,R.
A1 - Ramstetter,C.
A1 - Council on School Health
A1 - American Academy of Pediatrics.
Y1 - 2013/01//
N1 - Murray, Robert. Ramstetter, Catherine. Council on School Health. American Academy of Pediatrics
Pediatrics
oxv, 0376422
AIM, IM
Journal Article. Review
English
KW - MEDLINE
KW - Child
KW - Child Development/ph [Physiology]
KW - Cognition/ph [Physiology]
KW - Educational Status
KW - Humans
KW - Motor Activity/ph [Physiology]
KW - Physical Education and Training/mt [Methods]
KW - Schools
KW - Students/px [Psychology]
KW - Students
RP - NOT IN FILE
SP - 183
EP - 188
JF - Pediatrics
VL - 131
IS - 1
CY - United States
N2 - Recess is at the heart of a vigorous debate over the role of schools in promoting the optimal development of the whole child. A growing trend toward reallocating time in school to accentuate the more academic subjects has put this important facet of a child's school day at risk. Recess serves as a necessary break from the rigors of concentrated, academic challenges in the classroom. But equally important is the fact that safe and well-supervised recess offers cognitive, social, emotional, and physical benefits that may not be fully appreciated when a decision is made to diminish it. Recess is unique from, and a complement to, physical education--not a substitute for it. The American Academy of Pediatrics believes that recess is a crucial and necessary component of a child's development and, as such, it should not be withheld for punitive or academic reasons
SN - 1098-4275
UR - 23277311
ER -

TY - JOUR
ID - 325
T1 - Physical activity during school in urban minority kindergarten and first-grade students
A1 - Reznik,M.
A1 - Wylie-Rosett,J.
A1 - Kim,M.
A1 - Ozuah,P.O.
Y1 - 2013/01//
N1 - Reznik, Marina. Wylie-Rosett, Judith. Kim, Mimi. Ozuah, Philip O
Pediatrics
oxv, 0376422
AIM, IM
Journal Article. Research Support, Non-U.S. Gov't
English
KW - MEDLINE

KW - Child
KW - Preschool
KW - Cross-Sectional Studies
KW - Female
KW - Humans
KW - Male
KW - Minority Groups/ed [Education]
KW - Minority Groups
KW - Motor Activity/ph [Physiology]
KW - New York City/eh [Ethnology]
KW - Physical Education and Training/mt [Methods]
KW - Schools
KW - Students
KW - Urban Population
RP - NOT IN FILE
SP - e81
EP - e87
JF - Pediatrics
VL - 131
IS - 1
CY - United States
N2 - OBJECTIVES: To determine if physical activity (PA) during the school day varied by school, grade level, gender, weight status, and physical education (PE) and recess participation among urban kindergarten and first-grade students. METHODS: Cross-sectional study at 4 Bronx, NY, schools. Student PA was measured by a Yamax Digi-Walker pedometer, an objective and validated measure of PA in children. Each student wore a sealed pedometer during school for 5 consecutive days. Hierarchical models were fit to identify variables predictive of PA. RESULTS: A total of 916 had valid pedometer data (53% male, 70% Hispanic, mean age 5.98 years [SD 0.66], 45% overweight). PA varied by school (P < .0001). First-grade students took a significantly greater mean number of steps during school than kindergarten students. Overall, students took an average of 2479.7 (SD 961.8) steps/school day. PA did not differ by BMI status. Students took on average 724 more steps on PE days than on non-PE days and 490 more steps on days with outdoor recess than nonrecess days (P < .05 for both). Fewer than 1% of all students achieved lower cut points for previously published mean range of steps/school day for boys and girls. Multivariable analysis revealed higher grade level, participation in PE class, and outdoor recess as independent predictors of PA. CONCLUSIONS: PA levels were low in kindergarten and first-grade students. Future interventions incorporating classroom-based PA and outdoor recess may increase PA in resource-poor urban schools with limited PE facilities
SN - 1098-4275
AD - Department of Pediatrics, Children's Hospital at Montefiore, Albert Einstein College of Medicine, Bronx, NY 10467, USA mreznik@montefiore.org
UR - 23209101
ER -

TY - JOUR
ID - 326
T1 - Indoor versus outdoor time in preschoolers at child care
A1 - Tandon,P.S.
A1 - Saelens,B.E.
A1 - Zhou,C.
A1 - Kerr,J.
A1 - Christakis,D.A.
Y1 - 2013/01//
N1 - Tandon, Pooja S. Saelens, Brian E. Zhou, Chuan. Kerr, Jacqueline. Christakis, Dimitri A
American journal of preventive medicine
8704773, apl
IM
Journal Article. Research Support, N.I.H., Extramural. Research Support, Non-U.S. Gov't. Validation Studies
English

KW - MEDLINE
KW - Accelerometry
KW - Child Day Care Centers/mt [Methods]
KW - Child
KW - Preschool
KW - Feasibility Studies
KW - Female
KW - Geographic Information Systems
KW - Humans
KW - Light
KW - Male
KW - Motor Activity/ph [Physiology]
KW - Play and Playthings
KW - ROC Curve
KW - Reproducibility of Results
KW - Time Factors
KW - Weather
RP - NOT IN FILE
SP - 85
EP - 88
JF - American Journal of Preventive Medicine
JA - Am J Prev Med
VL - 44
IS - 1
CY - Netherlands
N2 - BACKGROUND: Being outdoors may have health benefits including being more physically active. Understanding the relationship between outdoor time and health is hampered by the difficulty of measuring outdoor time. PURPOSE: To examine the accuracy and validity of light-sensor and GPS methods for quantifying outdoor time among those aged 3-5 years at child care. METHODS: A total of 45 children (mean age 4.5 years, 64% boys) from five child care centers wore portable accelerometers with built-in light sensors and a separate GPS device around their waists during child care, providing 80,648 episodes (15 seconds each) for analysis. Direct observation (gold standard) of children being outdoors versus indoors was conducted for 2 days at each center. GPS signal-to-noise ratios, processed through the Personal Activity and Location Measurement System were used to define indoor versus outdoor locations. Receiver operating characteristic (ROC) analyses were used to determine thresholds for defining being indoors versus outdoors. Data were collected in Fall 2011, analyzed in 2012. RESULTS: Mean observed outdoor time was 63 [+44; range: 18-152] minutes/day. Mean light-sensor levels were significantly higher outdoors. The area under the ROC curve for location based on light sensor for all weather conditions was 0.82 (range: 0.70 on partly cloudy days to 0.97 on sunny days); for GPS, it was 0.89. The light sensor had a sensitivity of 74% and specificity of 86%. GPS had a sensitivity of 82% and specificity of 88%. CONCLUSIONS: A light sensor and a GPS device both distinguish indoor from outdoor time for preschoolers with moderate to high levels of accuracy. These devices can increase the feasibility and lower the cost of measuring outdoor time in studies of preschool children. Copyright 2013 American Journal of Preventive Medicine. Published by Elsevier Inc. All rights reserved
SN - 1873-2607
AD - Center for Child Health, Behavior, and Development, Seattle Children's Research Institute, Seattle, WA, USA. pooja@uw.edu
UR - 23253655
ER -

TY - JOUR
ID - 327
T1 - Baby unplugged: a novel, market-based approach to reducing screen time and promoting healthy alternatives
A1 - Hutton,J.S.
Y1 - 2013/01//
N1 - Hutton, John S
Clinical pediatrics

dhe, 0372606, 8407647
AIM, IM
Journal Article
English
KW - MEDLINE
KW - Books
KW - Child
KW - Child Development
KW - Health Promotion
KW - Humans
KW - Intelligence
KW - Leisure Activities/px [Psychology]
KW - Leisure Activities
KW - Marketing
KW - Television/ut [Utilization]
KW - Television
RP - NOT IN FILE
SP - 62
EP - 65
JF - Clinical Pediatrics
JA - Clin Pediatr (Phila)
VL - 52
IS - 1
CY - United States
N2 - The issue of electronic media use by young children is increasingly important in pediatrics, a major risk factor for numerous chronic conditions. Despite guidelines in place since 1999, screen time is on the rise, aided by new formats removing practically all barriers of use. Key drivers are technological allure, confusion about developmental readiness, and perception of educational value, fueled by potent marketing. This article describes the development of Baby Unplugged, a series of children's board books celebrating "old-school," screen-free childhood. Written by a pediatrician who also owns a children's bookstore, the books were inspired and informed by advocacy projects in the areas of media use and early literacy as a pediatric resident. They reinforce AAP Electronic Media Guidelines, notably discouraging screen-based media under 2 years old, largely by encouraging healthy, fun alternatives. Examples include Pets, Book, and Yard. Multi-sensorial exploration and parent-child engagement are emphasized in a non-prescriptive way, featuring gender and ethnic diversity and activities that are accessible and inexpensive. The author describes challenges faced by pediatricians providing anticipatory guidance for media use, given limited time and resources and the perception that we are out of touch. This is heightened by oft-deceptive marketing of screen-based products more likely to be perceived as "cool." Reach Out and Read is cited as an example of a successful, "cool" intervention, though limited to select populations. Baby Unplugged takes advocacy to the marketplace, where the screen time battle is being lost
SN - 1938-2707
AD - Cincinnati Children's Hospital Medical Center, Cincinnati, OH 45209, USA.
JohnSandy@bluemanateebooks.com
UR - 23172903
ER -

TY - JOUR
ID - 328
T1 - Elevated blood lead levels and sources of exposure in the population of Kinshasa, the capital of the Democratic Republic of Congo
A1 - Tuakuila,J.
A1 - Lison,D.
A1 - Mbuyi,F.
A1 - Haufroid,V.
A1 - Hoet,P.
Y1 - 2013/01//
N1 - Tuakuila, Joel. Lison, Dominique. Mbuyi, Francois. Haufroid, Vincent. Hoet, Perrine
Journal of exposure science & environmental epidemiology

101262796
IM
Journal Article. Research Support, Non-U.S. Gov't
English
KW - MEDLINE
KW - Adolescent
KW - Adult
KW - Aged
KW - Child
KW - Preschool
KW - Cross-Sectional Studies
KW - Democratic Republic of the Congo
KW - Environmental Exposure
KW - Female
KW - Humans
KW - Infant
KW - Lead/bl [Blood]
KW - Male
KW - Middle Aged
KW - Rural Population
KW - Urban Population
KW - Young Adult
RP - NOT IN FILE
SP - 81
EP - 87
JF - Journal of Exposure Science & Environmental Epidemiology
JA - J Expo Sci Environ Epidemiol
VL - 23
IS - 1
CY - United States
N2 - The objective of this study was to determine blood lead levels (BLLs) and the possible sources of exposure in the population of Kinshasa, the capital of the Democratic Republic of Congo. A cross-sectional survey was carried out from January to May 2008 in a representative sample of the Kinshasan population. BLL was measured in 275 individuals (53.4% women) aged 1-70 years in the urban area of Kinshasa and from 60 additional subjects in the rural area. Pb was also determined in environmental specimens (air and soil, indoor and outdoor). BLL in the study population ranged from 2.9 to 49.3mug/dl (median, 9.9mug/dl). The median BLL among children aged <6 years was 11.5mug/dl (range: 3.0-37.8mug/dl). Of these children, 71% had elevated BLL (>10mug/dl) and 22% had BLL >20mug/dl. The proportion of elevated BLL (>10mug/dl) was higher for children aged <3 years than for children aged 3 to 5 years (97% vs 56%). A higher prevalence of elevated BLL was observed in urban compared with rural children (71% vs 20%). Significantly higher BLLs were also found in children whose mother consumed fired clay during pregnancy. Residential informal activities in the recycling of car batteries also contributed to elevated BLL in children. The elevated background of Pb exposure in the Kinshasan population indicates a public health issue that requires corrective actions. Pb-contaminated dust and air in children's home is an issue of public health concern. The use of leaded gasoline and the activities of car battery recycling in certain residences appear to constitute the main sources of exposure in the city of Kinshasa. The traditional use of fired clay for the treatment of gastritis by pregnant women is another significant contributor for elevated BLL in children
SN - 1559-064X
AD - Environmental Chemistry, Faculty of Sciences, Universite de Kinshasa, Kinshasa, Democratic Republic of Congo
UR - 22617721
ER -

TY - JOUR
ID - 329
T1 - The quality of the outdoor environment influences childrens health -- a cross-sectional study of preschools
A1 - Soderstrom,M.

A1 - Boldemann,C.
A1 - Sahlin,U.
A1 - Martensson,F.
A1 - Raustorp,A.
A1 - Blennow,M.
Y1 - 2013/01//
N1 - Soderstrom, M. Boldemann, C. Sahlin, U. Martensson, F. Raustorp, A. Blennow, M
Acta paediatrica (Oslo, Norway : 1992)
bgc, 9205968
IM
Journal Article. Research Support, Non-U.S. Gov't
English
KW - MEDLINE
KW - Child Day Care Centers
KW - Child Welfare
KW - Child
KW - Preschool
KW - Cross-Sectional Studies
KW - Environment
KW - Female
KW - Health Status
KW - Humans
KW - Male
RP - NOT IN FILE
SP - 83
EP - 91
JF - Acta Paediatrica
JA - Acta Paediatr
VL - 102
IS - 1
CY - Norway
N2 - AIM: To test how the quality of the outdoor environment of child day care centres (DCCs) influences children's health. METHODS: The environment was assessed using the Outdoor Play Environmental Categories (OPEC) tool, time spent outdoors and physical activity as measured by pedometer. 172/253 (68%) of children aged 3.0-5.9 from nine DCCs participated in Southern Sweden. Health data collected were body mass index, waist circumference, saliva cortisol, length of night sleep during study, and symptoms and well-being which were scored (1-week diary - 121 parent responders). Also, parent-rated well-being and health of their child were scored (questionnaire, 132 parent responders). MANOVA, ANOVA and principal component analyses were performed to identify impacts of the outdoor environment on health. RESULTS: High-quality outdoor environment at DCCs is associated with several health aspects in children such as leaner body, longer night sleep, better well-being and higher mid-morning saliva cortisol levels. CONCLUSION: The quality of the outdoor environment at DCCs influenced the health and well-being of preschool children and should be given more attention among health care professionals and community planners. 2012 The Author(s)/Acta Paediatrica 2012 Foundation Acta Paediatrica
SN - 1651-2227
AD - The Section of General Practice, Department of Public Health and The research Unit for General Practice, University of Copenhagen, Copenhagen, Denmark. masod@sund.ku.dk
UR - 23035750
ER -

TY - JOUR
ID - 330
T1 - The effects of a Special Olympics Unified Sports Soccer training program on anthropometry, physical fitness and skilled performance in Special Olympics soccer athletes and non-disabled partners
A1 - Baran,F.
A1 - Aktop,A.
A1 - Ozer,D.

A1 - Nalbant,S.
A1 - Aglamis,E.
A1 - Barak,S.
A1 - Hutzler,Y.
Y1 - 2013/01//
N1 - Baran, Funda. Aktop, Abdurrahman. Ozer, Dilara. Nalbant, Sibel. Aglamis, Ece. Barak, Sharon. Hutzler, Yeshayahu
Research in developmental disabilities
8709782, rid
IM
Journal Article. Randomized Controlled Trial
English
KW - MEDLINE
KW - Adolescent
KW - Anthropometry
KW - Child
KW - Disabled Children/px [Psychology]
KW - Education of Intellectually Disabled/og [Organization & Administration]
KW - Exercise
KW - Humans
KW - Intellectual Disability/px [Psychology]
KW - Intellectual Disability/rh [Rehabilitation]
KW - Male
KW - Motivation
KW - Motor Skills
KW - Physical Education and Training/og [Organization & Administration]
KW - Physical Fitness
KW - Program Evaluation
KW - Soccer
KW - Sports
RP - NOT IN FILE
SP - 695
EP - 709
JF - Research in Developmental Disabilities
JA - Res Dev Disabil
VL - 34
IS - 1
CY - United States
N2 - The study investigated the effects of a Special Olympics (SO) Unified Sport (UNS) soccer program on anthropometry, physical fitness and soccer skills of male youth athletes with and without intellectual disabilities (ID) who participated in a training group (TRG) and in a comparison group (CG) without specific training. Youth with ID (WID) were randomly selected out of all the students between the ages 12 and 15, with a diagnosis of educable mental retardation and no secondary disabilities, who were attending a special education school. Participants without ID (WoID) were randomly selected from a regular secondary school out of the same age groups of male students. All participants were given permission by their parents or guardians to participate in the study. Participants in the TRG included 23 youth WID and 23 youth WoID. Mean ages were = 14.1 (SD = 1.1) and 13.2 (SD = 0.79) respectively. Fifteen WID, and 15 WoID comprised the CG. Mean ages were 14.51 (SD = 0.81) and 13.78 (SD = 0.49) respectively. Prior to and following the program measurements were conducted, and data were collected on students' anthropometric and fitness components of the Brockport physical fitness test as well as a soccer skill performance based on the SO soccer skill test. Participants in the TRG trained 8 weeks, 1.5h per session, three times per week, in an after-school soccer program. CG did not participate in any sports program outside of the school physical education class. Dependent t tests and effect size calculations revealed that SO athletes and non-disabled partners scored significantly higher with regard to physical fitness and football skills in most variables compared with their CG. This Unified Program was successful in increasing fitness and soccer skill performance of youth WID as well as of those WoID. Copyright 2012 Elsevier Ltd. All rights reserved
SN - 1873-3379

AD - Akdeniz University, School of Physical Education and Sport, Antalya, Turkey
UR - 23134807
ER -

TY - JOUR
ID - 331
T1 - Patterns and predictors of participation in leisure activities outside of school in children and adolescents with Cerebral Palsy
A1 - Longo,E.
A1 - Badia,M.
A1 - Orgaz,B.M.
Y1 - 2013/01//
N1 - Longo, Egmar. Badia, Marta. Orgaz, Begona M
Research in developmental disabilities
8709782, rid
IM
Journal Article. Research Support, Non-U.S. Gov't
English
KW - MEDLINE
KW - Adolescent
KW - Cerebral Palsy/px [Psychology]
KW - Cerebral Palsy/rh [Rehabilitation]
KW - Child
KW - Cross-Sectional Studies
KW - Education
KW - Special/sn [Statistics & Numerical Data]
KW - Environment
KW - Family/px [Psychology]
KW - Female
KW - Humans
KW - Leisure Activities/px [Psychology]
KW - Mainstreaming (Education)/sn [Statistics & Numerical Data]
KW - Male
KW - Motor Activity
KW - Personal Autonomy
KW - Predictive Value of Tests
KW - Questionnaires
KW - Regression Analysis
KW - Social Behavior
KW - Spain
RP - NOT IN FILE
SP - 266
EP - 275
JF - Research in Developmental Disabilities
JA - Res Dev Disabil
VL - 34
IS - 1
CY - United States
N2 - This study analyzed the patterns and predictors of participation in leisure activities outside of school of Spanish children and adolescents with Cerebral Palsy (CP). Children and adolescents with CP (n = 199; 113 males and 86 females) participated in this cross-sectional study. Their mean age was 12.11 years (SD = 3.02; range 8-18 years), and they were evaluated using the Spanish version of the Children's Assessment of Participation and Enjoyment (CAPE). Means, standard deviations and percentages were used to characterize the profile of participation, and linear regression analyses were employed to assess associations between the variables (child, family and environmental factors) and the diversity, intensity and enjoyment of participation. Children and adolescents with CP reported low diversity and intensity of participation and high levels of

enjoyment. Participation in leisure activities outside of school was determined more by child and environmental factors than by family ones. Copyright 2012 Elsevier Ltd. All rights reserved
SN - 1873-3379
AD - INICO, Faculty of Psychology, University of Salamanca, Spanish Agency of International Cooperation - MAEC - AECID, Avda de la Merced 109-131, 37005 Salamanca, Spain
UR - 22989575
ER -

TY - JOUR
ID - 332
T1 - The German Environmental Survey for Children (GerES IV): reference values and distributions for time-location patterns of German children
A1 - Conrad,A.
A1 - Seiwert,M.
A1 - Hunken,A.
A1 - Quarcoo,D.
A1 - Schlaud,M.
A1 - Groneberg,D.
Y1 - 2013/01//
N1 - Conrad, Andre. Seiwert, Margarete. Hunken, Andreas. Quarcoo, David. Schlaud, Martin. Groneberg, David
International journal of hygiene and environmental health
do6, 100898843
IM
Journal Article. Research Support, Non-U.S. Gov't
English
KW - MEDLINE
KW - Adolescent
KW - Body Weight
KW - Child
KW - Preschool
KW - Cross-Sectional Studies
KW - Emigrants and Immigrants/sn [Statistics & Numerical Data]
KW - Environment
KW - Environmental Exposure
KW - Exercise
KW - Female
KW - Germany
KW - Humans
KW - Male
KW - Reference Values
KW - Socioeconomic Factors
KW - Time Factors
RP - NOT IN FILE
SP - 25
EP - 34
JF - International Journal of Hygiene & Environmental Health
JA - Int J Hyg Environ Health
VL - 216
IS - 1
CY - Germany
N2 - Children's time-location patterns are important determinants of environmental exposure and other health-relevant factors. Building on data of the German Environmental Survey for Children (GerES IV), our study aimed at deriving reference values and distributions for time-location patterns of 3-14-year-old German children. We also investigated if GerES IV data are appropriate for evaluating associations with children's health determinants by linking them to data of the National Health Interview and Examination Survey for Children and Adolescents (KiGGS). Parents reported on the time their children usually spend at home, in other indoor

environments, and outdoors. This information was characterized by statistical parameters, which were also calculated for different strata concerning socio-demography and the residential environment. Consequently, group differences were evaluated by t-tests and univariate ANOVA. Reference distributions were fitted to the time-location data by a Maximum Likelihood approach to make them also useable in probabilistic exposure modeling. Finally, associations between data on the children's physical activity as well as body weight and their outdoor time were investigated by bivariate correlation analysis and cross tabulation. On daily average, German children spend 15 h and 31 min at home, 4 h and 46 min in other indoor environments, and 3 h and 43 min outdoors. Time spent at home and outdoors decreases with age while time spent in other indoor environments increases. Differences in time-location patterns were also observed for the socio-economic status (SES) and immigration status. E.g., children with a high SES spend 24 min less outdoors than low SES children. Immigrants spend on daily average 20 min more at home and 15 min less outdoors than non-immigrant children. Outdoor time was associated with parameters of the residential environment like the building development. Children living in 1- or 2-family houses spend more time outdoors than children living in building blocks (3 h 48 min vs. 3 h 29 min). Physical activity correlates with outdoor time. For children with diminished age-specific outdoor time, a higher prevalence of obesity was observed (odds ratio: 3.2, 95% CI: 1.5-7.1). GerES IV provides a compilation of current time-location reference values and distributions on German children. This data hint to substantial differences in time-location patterns within the population to be considered in environmental health risk assessment. Copyright 2012 Elsevier GmbH. All rights reserved
SN - 1618-131X
AD - Charite--University Medicine Berlin, Institute of Occupational Medicine, Germany. andre.conrad@uba.de
UR - 22410199
ER -

TY - JOUR
ID - 333
T1 - Walkable for whom? Examining the role of the built environment on the neighbourhood-based physical activity of children
A1 - Loptson,K.
A1 - Muhajarine,N.
A1 - Ridalls,T.
A1 - Smart Cities,Healthy Kids Research Team.
Y1 - 2012///
N1 - Loptson, Kristjana. Muhajarine, Nazeem. Ridalls, Tracy. Smart Cities, Healthy Kids Research Team
Canadian journal of public health = Revue canadienne de sante publique
ck6, 0372714
IM
Journal Article. Research Support, Non-U.S. Gov't
English
KW - MEDLINE
KW - Adolescent
KW - Canada
KW - Child
KW - Child Behavior/px [Psychology]
KW - Environment Design/sn [Statistics & Numerical Data]
KW - Female
KW - Humans
KW - Male
KW - Motor Activity
KW - Parents/px [Psychology]
KW - Qualitative Research
KW - Residence Characteristics/sn [Statistics & Numerical Data]
KW - Safety
KW - Social Environment
KW - Socioeconomic Factors
KW - Walking/px [Psychology]
RP - NOT IN FILE
SP - eS29

EP - eS34
JF - Canadian Journal of Public Health
JA - Can J Public Health
VL - Revue Canadienne de Sante Publique. 103
IS - 9 Suppl 3
CY - Canada
N2 - OBJECTIVES: To date, only a few studies have attempted to study the processes by which community design and the built and social environments affect individual physical activity, especially in children. Qualitative enquiry is useful for exploring perceptions and decision-making, and to understand the processes involved in how people interact with their environments. This study used qualitative methods to gain insight into the pathways linking the neighbourhood environment with children's activity patterns. METHODS: Data were collected in semi-structured interviews with 24 child-parent dyads (children aged 10-14 years). Families lived in neighbourhoods ranging from lowest to highest median income and representing the three main design types found in Saskatoon - urban, semi-suburban and suburban. RESULTS: Parents and children underscored the importance of safe environments for children's physical activity: streets or paths they can cycle on without feeling threatened, parks and green spaces free of criminal activity, and neighbourhoods where people know each other and children have friends to play with. Although grid-pattern urban neighbourhoods with a high density of destinations may in principle promote active transportation, the higher levels of crime and traffic danger that tend to exist in these areas may hinder physical activity in children.CONOCLUSION: Understanding what facilitates activity in children is a complex endeavour. It requires understanding the barriers to physical activity present at the neighbourhood level as well as social and perceptual factors that act in interdependent ways to either promote or hinder children's physical activity
SN - 1920-7476
AD - Saskatchewan Population Health and Evaluation Research Unit, Department of Community Health and Epidemiology, University of Saskatchewan, Saskatoon, SK
UR - 23618085
ER -

TY - JOUR
ID - 334
T1 - The implementation of a pilot playground markings project in four Australian primary schools
A1 - Kelly,A.
A1 - Arjunan,P.
A1 - van der Ploeg,H.P.
A1 - Rissel,C.
A1 - Borg,J.
A1 - Wen,L.M.
Y1 - 2012/12//
N1 - Kelly, Angela. Arjunan, Punitha. van der Ploeg, Hidde P. Rissel, Chris. Borg, Janelle. Wen, Li Ming
Health promotion journal of Australia : official journal of Australian Association of Health Promotion Professionals
9710936
IM
Journal Article
English
KW - MEDLINE
KW - Accelerometry
KW - Australia
KW - Child
KW - Exercise
KW - Female
KW - Health Promotion/og [Organization & Administration]
KW - Humans
KW - Male
KW - Overweight/pc [Prevention & Control]
KW - Play and Playthings
KW - Schools/og [Organization & Administration]

RP - NOT IN FILE
SP - 183
EP - 187
JF - Health Promotion Journal of Australia
JA - HEALTH PROMOT J AUST
VL - 23
IS - 3
CY - Australia
N2 - ISSUE ADDRESSED: Increasing childhood overweight and obesity rates need strategies that promote and engage children in more physical activity and school is one setting for this. This pilot project aimed to determine if coloured playground markings are an effective strategy, in the Australian context, to increase students' physical activity during school break times in primary schools. METHODS: Four schools participated, two as intervention schools and two as control schools. A combination of data collection methods were used, including accelerometers on students, playground observations and a teacher survey. RESULTS: The accelerometer and observation data did not show an improvement in the physical activity levels of the intervention students compared to the control students. The teacher survey was positive in all aspects of the project implementation. Observations suggest that most students were active in large grass areas rather than around the playground markings. CONCLUSION: Based on our data, it is unclear whether playground markings are an effective intervention to increase physical activity in the school setting. The amount of playing space available appears to influence the use of the playground markings
SN - 1036-1073
AD - South Western Sydney & Sydney Local Health Districts, NSW Health, Australia
UR - 23540317
ER -

TY - JOUR
ID - 335
T1 - The prevalence of neurocranium injury in children in Brod-Posavina County
A1 - Mihic,J.
A1 - Rotim,K.
A1 - Marcikic,M.
A1 - Smiljanic,D.
A1 - Dikanovic,M.
A1 - Jurjevic,M.
A1 - Matic,I.
Y1 - 2012/12//
N1 - Mihic, Josip. Rotim, Kresimir. Marcikic, Marcel. Smiljanic, Danko. Dikanovic, Marinko. Jurjevic, Matija. Matic, Ivo
Acta clinica Croatica
9425483
IM
Journal Article
English
KW - MEDLINE
KW - Adolescent
KW - Brain Injuries/ep [Epidemiology]
KW - Brain Injuries/et [Etiology]
KW - Child
KW - Preschool
KW - Craniocerebral Trauma/ep [Epidemiology]
KW - Craniocerebral Trauma/et [Etiology]
KW - Croatia/ep [Epidemiology]
KW - Female
KW - Humans
KW - Infant
KW - Male
KW - Prevalence

RP - NOT IN FILE
SP - 615
EP - 622
JF - Acta Clinica Croatica
JA - ACTA CLIN
VL - 51
IS - 4
CY - Croatia
N2 - Head injuries are very common in children and are the most frequent cause of disability and death among children. This retrospective study included 350 children hospitalized for injury of neurocranium over a 5-year period at Dr Josip Bencevic General Hospital in Slavonski Brod. Boys were more commonly injured (63.4%) than girls. The most common injuries were recorded in children aged 7-14 (47.1%), followed by those aged 1-6 (33.8%) years. The injuries occurred slightly more often in urban (50.9%) than in rural (46.6%) setting. Children were more commonly injured in the street or on the road (38.6%), followed by injuries sustained at home (35.2%), at school (9.3%) and on playgrounds (5.7%). They were most commonly injured by fall (50%), followed by traffic injuries (33.5%). Statistically significant differences were found in the following age groups: all children younger than one year were injured by fall; children aged 1-14 were mostly injured by fall (less in traffic, and due to hitting), and those aged 15-18 mostly in traffic (less by fall and due to hitting). Children were mostly injured in the street or on the road (in traffic accidents), followed by injuries at home (mostly by fall), at school and around the house or in the yard (mostly by fall); on the playground (due to hitting) and on the road (in traffic accidents) (statistically significant difference). Most of them had head contusion and cerebral commotion combined (46.8%), followed by head contusion alone (12.5%) and skull fractures (10.5%). Hemorrhages and hematomas were rare (epidural, subdural, subarachnoid hemorrhage), found in 3.2% of cases. We hope that our results will prove helpful in planning preventive measures and treatment of injured children
SN - 0353-9466
AD - Department of Surgery, Dr Josip Bencevic General Hospital, Slavonski Brod, Croatia. j_mihic@yahoo.com
UR - 23540170
ER -

TY - JOUR
ID - 336
T1 - The role of indoor pollution in the development and maintenance of chronic airway inflammation in children. [Review]
A1 - Packeu,A.
A1 - Chasseur,C.
A1 - Bladt,S.
A1 - Detandt,M.
Y1 - 2012///
N1 - Packeu, A. Chasseur, C. Bladt, S. Detandt, M
B-ENT
101247842
IM
Journal Article. Review
English
KW - MEDLINE
KW - Air Pollution
KW - Indoor/ae [Adverse Effects]
KW - Allergens/ae [Adverse Effects]
KW - Child
KW - Chronic Disease
KW - Humans
KW - Inflammation/ci [Chemically Induced]
KW - Respiratory Tract Diseases/ci [Chemically Induced]
RP - NOT IN FILE
SP - 73
EP - 79

JF - B-ENT
VL - 8 Suppl 19
CY - Belgium
N2 - Air pollution is one of the great problems of this century and it plays an important role in the increasing prevalence of chronic inflammatory problems in the upper airway in children. Since their lungs and immune system are not fully developed when exposure begins, newborns and children appear to be more sensitive to the effects of both outdoor and indoor air pollution. Furthermore, children spend most of their time indoors and are exposed more often to pollutants in indoor air. The link between health problems, chemical products and allergens (the latter mainly from cats and mites) has been extensively studied. Other important indoor contaminants are fungi, which are often present in damp buildings and can cause severe respiratory disease by producing spores, allergens, volatile irritant compounds and toxins. A proper identification of mould contamination of this kind is vital for correct diagnosis, treatment and the prevention of health problems, and improvements have been observed after the removal or cleaning of the contaminated materials and improvements to the ventilation of buildings. While a possible association between respiratory symptoms, such as rhinitis, and the presence of fungi in the indoor environment has been documented by several authors, other studies have observed no significant relationship. The development of standardised sampling, detection and diagnostic tests will be essential to understand the proper role of fungi in the indoor atmosphere and their impact on public health
SN - 1781-782X
AD - Scientific Service of Mycology and Aerobiology, Scientific Institute of Public Health, Brussels, Belgium. ann.packeu@wiv-isp.be
UR - 23431612
ER -

TY - JOUR
ID - 337
T1 - Differences between 9-11 year old British Pakistani and White British girls in physical activity and behavior during school recess
A1 - Pollard,T.M.
A1 - Hornby-Turner,Y.C.
A1 - Ghurbhurrun,A.
A1 - Ridgers,N.D.
Y1 - 2012///
N1 - Pollard, Tessa M. Hornby-Turner, Yvonne C. Ghurbhurrun, Adarshini. Ridgers, Nicola D
BMC public health
100968562
IM
Journal Article. Research Support, Non-U.S. Gov't
English
KW - MEDLINE
KW - Child
KW - Cross-Cultural Comparison
KW - European Continental Ancestry Group/px [Psychology]
KW - Female
KW - Great Britain
KW - Humans
KW - Motor Activity
KW - Pakistan/eh [Ethnology]
KW - Schools
KW - Students/px [Psychology]
KW - Students/sn [Statistics & Numerical Data]
RP - NOT IN FILE
SP - 1087
JF - BMC Public Health
VL - 12
CY - England

N2 - BACKGROUND: School recess provides an important opportunity for children to engage in physical activity. Previous studies indicate that children and adults of South Asian origin are less active than other ethnic groups in the United Kingdom, but have not investigated whether activity differs within the shared school environment. The aim of this study was to test the hypothesis that British Pakistani girls aged 9-11 years are less active during recess than White British girls. METHODS: In Study One, the proportion of recess spent by 137 White British (N = 70) and British Pakistani (N = 67) girls in sedentary behavior, moderate-to-vigorous activity (MVPA) and vigorous activity (VPA) was determined using accelerometry. In Study Two, 86 White British (N = 48) and British Pakistani (N = 38) girls were observed on the playground using the System for Observing Children's Activity and Relationships during Play (SOCARP). Accelerometry data were collected during observations to allow identification of activities contributing to objectively measured physical activity. RESULTS: Accelerometry data indicated that British Pakistani girls spent 2.2% (95% CI: 0.2, 4.3) less of their total recess time in MVPA and 1.3% (95% CI: 0.2, 2.4) less in VPA than White British girls. Direct observation showed that British Pakistani girls spent 12.0% (95% CI: 2.9, 21.1) less playground time being very active, and 12.3% (95% CI: 1.7, 23.0) less time playing games. Time spent being very active according to direct observation data correlated significantly with accelerometer-assessed time spent in MVPA and VPA, and time spent playing games correlated significantly with accelerometer-assessed time spent in VPA, suggesting that differences in behavior observed in Study Two may have contributed to the differences in time spent in MVPA and VPA in Study One. CONCLUSIONS: British Pakistani girls were less active than White British girls during school recess. Recess has been identified as a potentially important target for the delivery of physical activity interventions; such interventions should consider ways in which the activity levels of British Pakistani girls could be increased
SN - 1471-2458
AD - Physical Activity Laboratory, Department of Anthropology, Durham University, Dawson Building, South Road, Durham, DH1 3LE, UK. t.m.pollard@durham.ac.uk
UR - 23249170
ER -

TY - JOUR
ID - 338
T1 - Teaching children with autism spectrum disorder to mand for information using "which?"
A1 - Marion,C.
A1 - Martin,G.L.
A1 - Yu,C.T.
A1 - Buhler,C.
A1 - Kerr,D.
A1 - Claeys,A.
Y1 - 2012///
N1 - Marion, Carole. Martin, Garry L. Yu, C T. Buhler, Charissa. Kerr, Danni. Claeys, Amanda
Journal of applied behavior analysis
0174763, hdr
IM
Journal Article
English
KW - MEDLINE
KW - Behavior Therapy/mt [Methods]
KW - Child
KW - Child Development Disorders
KW - Pervasive/px [Psychology]
KW - Pervasive/th [Therapy]
KW - Preschool
KW - Conditioning
KW - Operant
KW - Generalization (Psychology)
KW - Humans
KW - Male
KW - Verbal Behavior
RP - NOT IN FILE

SP - 865
EP - 870
JF - Journal of Applied Behavior Analysis
JA - J Appl Behav Anal
VL - 45
IS - 4
CY - United States
N2 - We examined a procedure consisting of a preference assessment, prompting, contrived conditioned establishing operations, and consequences for correct and incorrect responses for teaching children with autism to mand "which?" We used a modified multiple baseline design across 3 participants. All the children learned to mand "which?" Generalization occurred to the natural environment, to a novel activity, and to a novel container; the results were maintained over time
SN - 1938-3703
AD - University of Manitoba and St. Amant Research Centre, Winnipeg, Manitoba R2M 3Z9, Canada. cmarion@stamant.mb.ca
UR - 23322944
ER -

TY - JOUR
ID - 339
T1 - Multicity study of air pollution and mortality in Latin America (the ESCALA study)
A1 - Romieu,I.
A1 - Gouveia,N.
A1 - Cifuentes,L.A.
A1 - de Leon,A.P.
A1 - Junger,W.
A1 - Vera,J.
A1 - Strappa,V.
A1 - Hurtado-Diaz,M.
A1 - Miranda-Soberanis,V.
A1 - Rojas-Bracho,L.
A1 - Carbajal-Arroyo,L.
A1 - Tzintzun-Cervantes,G.
A1 - HEI Health Review Committee.
Y1 - 2012/10//
N1 - Romieu, Isabelle. Gouveia, Nelson. Cifuentes, Luis A. de Leon, Antonio Ponce. Junger, Washington. Vera, Jeanette. Strappa, Valentina. Hurtado-Diaz, Magali. Miranda-Soberanis, Victor. Rojas-Bracho, Leonora. Carbajal-Arroyo, Luz. Tzintzun-Cervantes, Guadalupe. HEI Health Review Committee
Research report (Health Effects Institute)
8812230, ah8, 8812230
IM
Journal Article. Research Support, Non-U.S. Gov't
English
KW - MEDLINE
KW - Adolescent
KW - Adult
KW - Age Distribution
KW - Air Pollutants/ae [Adverse Effects]
KW - Air Pollutants/an [Analysis]
KW - Air Pollution/ae [Adverse Effects]
KW - Air Pollution/an [Analysis]
KW - Air Pollution/sn [Statistics & Numerical Data]
KW - Cardiovascular Diseases/ci [Chemically Induced]
KW - Cardiovascular Diseases/mo [Mortality]
KW - Child
KW - Preschool
KW - Climate

KW - Environmental Exposure/sn [Statistics & Numerical Data]
KW - Female
KW - Humans
KW - Infant
KW - Latin America/ep [Epidemiology]
KW - Male
KW - Middle Aged
KW - Ozone/ae [Adverse Effects]
KW - Ozone/an [Analysis]
KW - Particulate Matter/ae [Adverse Effects]
KW - Particulate Matter/an [Analysis]
KW - Respiratory Tract Diseases/ci [Chemically Induced]
KW - Respiratory Tract Diseases/mo [Mortality]
KW - Socioeconomic Factors
KW - Time Factors
KW - Urban Health/sn [Statistics & Numerical Data]
KW - Young Adult
RP - NOT IN FILE
SP - 5
EP - 86
JF - Research Report - Health Effects Institute
JA - Res Rep Health Eff.Inst
IS - 171
CY - United States
N2 - INTRODUCTION: The ESCALA* project (Estudio de Salud y Contaminacion del Aire en Latinoamerica) is an HEI-funded study that aims to examine the association between exposure to outdoor air pollution and mortality in nine Latin American cities, using a common analytic framework to obtain comparable and updated information on the effects of air pollution on several causes of death in different age groups. This report summarizes the work conducted between 2006 and 2009, describes the methodologic issues addressed during project development, and presents city-specific results of meta-analyses and meta-regression analyses. METHODS: The ESCALA project involved three teams of investigators responsible for collection and analysis of city-specific air pollution and mortality data from three different countries. The teams designed five different protocols to standardize the methods of data collection and analysis that would be used to evaluate the effects of air pollution on mortality (see Appendices B-F). By following the same protocols, the investigators could directly compare the results among cities. The analysis was conducted in two stages. The first stage included analyses of all-natural-cause and cause-specific mortality related to particulate matter < or = 10 pm in aerodynamic diameter (PM10) and to ozone (O3) in cities of Brazil, Chile, and Mexico. Analyses for PM10 and O3 were also stratified by age group and O3 analyses were stratified by season. Generalized linear models (GLM) in Poisson regression were used to fit the time-series data. Time trends and seasonality were modeled using natural splines with 3, 6, 9, or 12 degrees of freedom (df) per year. Temperature and humidity were also modeled using natural splines, initially with 3 or 6 df, and then with degrees of freedom chosen on the basis of residual diagnostics (i.e., partial autocorrelation function [PACF], periodograms, and a Q-Q plot) (Appendix H, available on the HEI Web site). Indicator variables for day-of-week and holidays were used to account for short-term cyclic fluctuations. To assess the association between exposure to air pollution and risk of death, the PM10 and O3 data were fit using distributed lag models (DLMs). These models are based on findings indicating that the health effects associated with air pollutant concentrations on a given day may accumulate over several subsequent days. Each DLM measured the cumulative effect of a pollutant concentration on a given day (day 0) and that day's contribution to the effect of that pollutant on multiple subsequent (lagged) days. For this study, exposure lags of up to 3, 5, and 10 days were explored. However, only the results of the DLMs using a 3-day lag (DLM 0-3) are presented in this report because we found a decreasing association with mortality in various age-cause groups for increasing lag effects from 3 to 5 days for both PM10 and O3. The potential modifying effect of socioeconomic status (SES) on the association of PM10 or O3 concentration and mortality was also explored in four cities: Mexico City, Rio de Janeiro, Sao Paulo, and Santiago. The methodology for developing a common SES index is presented in the report. The second stage included meta-analyses and metaregression. During this stage, the associations between mortality and air pollution were compared among cities to evaluate the presence of heterogeneity and to explore city-level variables that might explain this heterogeneity. Meta-analyses were conducted to combine mortality effect estimates across cities and to evaluate the presence of

heterogeneity among city results, whereas meta-regression models were used to explore variables that might explain the heterogeneity among cities in mortality risks associated with exposures to PM10 (but not to O3). RESULTS: The results of the mortality analyses are presented as risk percent changes (RPC) with a 95% confidence interval (CI). RPC is the increase in mortality risk associated with an increase of 10 microg/m3 in the 24-hour average concentration of PM10 or in the daily maximum 8-hour moving average concentration of O3. Most of the results for PM10 were positive and statistically significant, showing an increased risk of mortality with increased ambient concentrations. Results for O3 also showed a statistically significant increase in mortality in the cities with available data. With the distributed lag model, DLM 0-3, PM10 ambient concentrations were associated with an increased risk of mortality in all cities except Concepci6n and Temuco. In Mexico City and Santiago the RPC and 95% CIs were 1.02% (0.87 to 1.17) and 0.48% (0.35 to 0.61), respectively. PM10 was also significantly associated with increased mortality from cardiopulmonary, respiratory, cardiovascular, cerebrovascular-stroke, and chronic obstructive lung diseases (COPD) in most cities. The few nonsignificant effects generally were observed in the smallest cities (Concepcion, Temuco, and Toluca). The percentage increases in mortality associated with ambient O3 concentrations were smaller than for those associated with PM10. All-natural-cause mortality was significantly related to O3 in Mexico City, Monterrey, Sao Paulo and Rio de Janeiro. Increased mortality risks for some specific causes were also observed in these cities and in Santiago. In the analyses stratified by season, different patterns in mortality and O3 were observed for cold and warm seasons. Risk estimates for the warm season were larger and significant for several causes of death in Sao Paulo and Rio de Janeiro. Risk estimates for the cold season were larger and significant for some causes of death in Mexico City, Monterrey, and Toluca. In an analysis stratified by SES, the all-natural-cause mortality risk in Mexico City was larger for people with a medium SES; however we observed that the risk of mortality related to respiratory causes was larger among people with a low SES, while the risk of mortality related to cardiovascular and cerebrovascular-stroke causes was larger among people with medium or high SES. In Sao Paulo, the all-natural-cause mortality risk was larger in people with a high SES, while in Rio de Janeiro the all-natural-cause mortality risk was larger in people with a low SES. In both Brazilian cities, the risks of mortality were larger for respiratory causes, especially for the low- and high-SES groups. In Santiago, all-natural-cause mortality risk did not vary with level of SES; however, people with a low SES had a higher respiratory mortality risk, particularly for COPD. People with a medium SES had larger risks of mortality from cardiovascular and cerebrovascular-stroke disease. The effect of ambient PM10 concentrations on infant and child mortality from respiratory causes and lower respiratory infection (LRI) was studied only for Mexico City, Santiago, and Sao Paulo. Significant increased mortality risk from these causes was observed in both Santiago (in infants and older children) and Mexico City (only in infants). For O3, an increased mortality risk was observed in Mexico City (in infants and older children) and in Sao Paulo (only in infants during the warm season). The results of the meta-analyses confirmed the positive and statistically significant association between PM10 and all-natural-cause mortality (RPC = 0.77% [95% CI: 0.60 to 1.00]) using the random-effects model. For mortality from specific causes, the percentage increase in mortality ranged from 0.72% (0.54 to 0.89) for cardiovascular disease to 2.44% (1.36 to 3.59) for COPD, also using the random-effects model. For O3, significant positive associations were observed using the random-effects model for some causes, but not for all natural causes or for respiratory diseases in people 65 years or older (> or = 65 years), and not for COPD and cerebrovascular-stroke in the all-age and the > or = 65 age groups. The percentage increase in all-natural-cause mortality was 0.16% (-0.02 to 0.33). In the meta-regression analyses, variables that best explained heterogeneity in mortality risks among cities were the mean average of temperature in the warm season, population percentage of infants (< 1 year), population percentage of children at least 1 year old but < 5 years (i.e., 1-4 years), population percentage of people > or = 65 years, geographic density of PM10 monitors, annual average concentrations of PM10, and mortality rates for lung cancer. CONCLUSIONS: The ESCALA project was undertaken to obtain information for assessing the effects of air pollutants on mortality in Latin America, where large populations are exposed to relatively high levels of ambient air pollution. An important goal was to provide evidence that could inform policies for controlling air pollution in Latin America. This project included the development of standardized protocols for data collection and for statistical analyses as well as statistical analytic programs (routines developed in R by the ESCALA team) to insure comparability of results. The analytic approach and statistical programming developed within this project should be of value for researchers carrying out single-city analyses and should facilitate the inclusion of additional Latin American cities within the ESCALA multicity project. Our analyses confirm what has been observed in other parts of the world regarding the effects of ambient PM10 and 03 concentrations on daily mortality. They also suggest that SES plays a role in the susceptibility of a population to air pollution; people with a lower SES appeared to have an increased risk of death from respiratory causes, particularly COPD. Compared with the general population,

infants and young children appeared to be more susceptible to both PM10 and O3, although an increased risk of mortality was not observed in these age groups in all cities. (ABSTRACT TRUNCATED)
SN - 1041-5505
AD - Instituto Nacional de Salud Publica, Cuernavaca, Morelos, Mexico
UR - 23311234
ER -

TY - JOUR
ID - 340
T1 - Mediators of the effect of the JUMP-in intervention on physical activity and sedentary behavior in Dutch primary schoolchildren from disadvantaged neighborhoods
A1 - van Stralen,M.M.
A1 - de,Meij J.
A1 - Te Velde,S.J.
A1 - van der Wal,M.F.
A1 - van,Mechelen W.
A1 - Knol,D.L.
A1 - Chinapaw,M.J.
Y1 - 2012///
N1 - van Stralen, Maartje M. de Meij, Judith. Te Velde, Saskia J. van der Wal, Marcel F. van Mechelen, Willem. Knol, Dirk L. Chinapaw, Mai Jm
The international journal of behavioral nutrition and physical activity
101217089
IM
Journal Article. Multicenter Study. Research Support, Non-U.S. Gov't
English
KW - MEDLINE
KW - Child
KW - Exercise/px [Psychology]
KW - Female
KW - Follow-Up Studies
KW - Health Behavior
KW - Humans
KW - Intervention Studies
KW - Male
KW - Motor Activity
KW - Netherlands
KW - Overweight/pc [Prevention & Control]
KW - Overweight/px [Psychology]
KW - Residence Characteristics
KW - Schools
KW - Sedentary Lifestyle
KW - Self Report
KW - Social Support
KW - Sports
RP - NOT IN FILE
SP - 131
JF - International Journal of Behavioral Nutrition & Physical Activity
JA - Int
VL - 9
CY - England
N2 - BACKGROUND: Important health benefits can be achieved when physical activity in children from low socio-economic status is promoted and sedentariness is limited. By specifying the mediating mechanisms of existing interventions one can improve future physical activity interventions. This study explored potential mediators of the long-term effect of the school-based multicomponent JUMP-in intervention on sport participation, outdoor play and screen time in Dutch primary schoolchildren from disadvantaged neighborhoods. METHODS: In total, 600 primary schoolchildren (aged 9.8 + 0.7, 51% girls, 13% Dutch ethnicity, 35%

overweight) from 9 intervention and 10 control schools were included in the analyses. JUMP-in was developed using Intervention Mapping, and targeted psychological and environmental determinants of physical activity. Outcome behaviors were self-reported sport participation, outdoor play, TV-viewing behavior and computer use. Potential mediators were self-reported psychological, social and physical environmental factors. RESULTS: JUMP-in was effective in improving sport participation after 20 months, but not in improving outdoor play, or reducing TV-viewing or computer time. JUMP-in was not effective in changing hypothesized mediators so no significant mediated effects could be identified. However, changes in self-efficacy, social support and habit strength were positively associated with changes in sport participation, and changes in social support, self-efficacy, perceived planning skills, enjoyment and habit strength were positively associated with changes in outdoor play. Changes in enjoyment was positively associated with changes in TV-viewing while parental rules were negatively associated. Having a computer in the bedroom and enjoyment were positively associated with changes in computer use, while changes in parental rules were negatively associated. CONCLUSIONS: Besides a significant positive effect on sports participation, no significant intervention effect on outdoor play, screen time or any of the potential mediators was found. This suggest that other (unmeasured) factors operated as mediating mechanisms of the intervention, that we used unsuccessful intervention strategies, that the strategies were inappropriately implemented, or that children are unable to accurately recall past activities and cognitions. Additionally, the school setting might not be the sole channel to influence leisure time activities. Still, several personal and environmental constructs were found to be relevant in predicting change in sport participation, outdoor play and screen behavior and seem to be potential mediators. Future interventions are recommended including more effective strategies targeting these relevant constructs, addressing different constructs (e.g. pedagogic skills of parents), and focusing on different implementation settings. TRAIL REGISTRATION: ISRCTN17489378
SN - 1479-5868
AD - EMGO Institute for Health and Care Research and the Department of Public and Occupational Health, VU University Medical Center, Amsterdam, The Netherlands
UR - 23130806
ER -

TY - JOUR
ID - 341
T1 - Determinants of vitamin D status in young children: results from the Belgian arm of the IDEFICS (Identification and Prevention of Dietary- and Lifestyle-Induced Health Effects in Children and Infants) Study
A1 - Sioen,I.
A1 - Mouratidou,T.
A1 - Kaufman,J.M.
A1 - Bammann,K.
A1 - Michels,N.
A1 - Pigeot,I.
A1 - Vanaelst,B.
A1 - Vyncke,K.
A1 - De,Henauw S.
A1 - IDEFICS consortium.
Y1 - 2012/06//
N1 - Sioen, Isabelle. Mouratidou, Theodora. Kaufman, Jean-Marc. Bammann, Karin. Michels, Nathalie. Pigeot, Iris. Vanaelst, Barbara. Vyncke, Krishna. De Henauw, Stefaan. IDEFICS consortium
Public health nutrition
9808463, dgy
IM
Journal Article. Research Support, Non-U.S. Gov't
English
KW - MEDLINE
KW - Adipose Tissue
KW - Belgium/ep [Epidemiology]
KW - Body Constitution
KW - Body Mass Index
KW - Child
KW - Cross-Sectional Studies

KW - Female
KW - Health Status
KW - Humans
KW - Male
KW - Obesity
KW - Abdominal/co [Complications]
KW - Play and Playthings
KW - Prevalence
KW - Reference Values
KW - Regression Analysis
KW - Risk Factors
KW - Seasons
KW - Sunlight
KW - Vitamin D/aa [Analogs & Derivatives]
KW - Vitamin D/bl [Blood]
KW - Vitamin D Deficiency/bl [Blood]
KW - Vitamin D Deficiency/ep [Epidemiology]
KW - Vitamin D Deficiency/et [Etiology]
RP - NOT IN FILE
SP - 1093
EP - 1099
JF - Public Health Nutrition
JA - Public Health Nutr
VL - 15
IS - 6
CY - England
N2 - OBJECTIVE: To describe the vitamin D status of Belgian children and examine the influence of non-nutritional determinants, in particular of anthropometric variables. DESIGN: Cross-sectional data of Belgian participants of the EU 6th Framework Programme IDEFICS (Identification and Prevention of Dietary- and Lifestyle-Induced Health Effects in Children and Infants) Study. SETTING: 25-Hydroxyvitamin D (25(OH)D) was measured using RIA. Vitamin D status was categorized as deficient (<25 nmol/l), insufficient (25-50 nmol/l), sufficient (50-75 nmol/l) and optimal (>75 nmol/l). Anthropometric measurements included height, weight, waist and hip circumferences and triceps and subscapular skinfold thicknesses. SUBJECTS: Children (n 357) aged 4-11 years. RESULTS: Serum 25(OH)D ranged from 136 to 1235 nmol/l (mean 472 (sd 146) nmol/l); with 5 % deficient, 53 % insufficient, 40 % sufficient and 2 % optimal. No significant differences were found by age and gender. Significant differences in 25(OH)D were observed for month of sampling (P < 0001), number of hours playing outside per week (r = 0140), weight (r = -0121), triceps (r = -0112) and subscapular (r = -0119) skinfold thickness, sum of two skinfold thicknesses (r = -0125) and waist circumference (r = -0108). Linear regression analysis of 25(OH)D adjusted for age, month of sampling and hours playing outside per week suggested that (i) weight, (ii) BMI Z-score, (iii) waist circumference and (iv) triceps and subscapular skinfold thickness (as well as the sum of both) independently influenced 25(OH)D. CONCLUSIONS: The majority of Belgian children had a suboptimal vitamin D status, with more than half having an insufficient status in winter and spring. Month of the year, weekly number of hours playing outside and body composition - both central and abdominal obesity - were identified as important determinants of vitamin D status in Belgian children
SN - 1475-2727
AD - Department of Public Health, Ghent University, UZ 2 Blok A, De Pintelaan 185, B-9000 Ghent, Belgium. Isabelle.Sioen@UGent.be
UR - 22152637
ER -

TY - JOUR
ID - 342
T1 - Geographic correlation between tapeworm carriers and heavily infected cysticercotic pigs
A1 - O'Neal,S.E.
A1 - Moyano,L.M.
A1 - Ayvar,V.
A1 - Gonzalvez,G.

A1 - Diaz,A.
A1 - Rodriguez,S.
A1 - Wilkins,P.P.
A1 - Tsang,V.C.
A1 - Gilman,R.H.
A1 - Garcia,H.H.
A1 - Gonzalez,A.E.
A1 - Cysticercosis Working Group
Y1 - 2012///
N1 - O'Neal, Seth E. Moyano, Luz M. Ayvar, Viterbo. Gonzalvez, Guillermo. Diaz, Andre. Rodriguez, Silvia. Wilkins, Patricia P. Tsang, Victor C W. Gilman, Robert H. Garcia, Hector H. Gonzalez, Armando E. Cysticercosis Working Group in Peru
PLoS neglected tropical diseases
101291488
IM
Journal Article. Research Support, N.I.H., Extramural. Research Support, Non-U.S. Gov't
English
KW - MEDLINE
KW - Adolescent
KW - Adult
KW - Aged
KW - 80 and over
KW - Animals
KW - Antibodies
KW - Helminth/bl [Blood]
KW - Cestode Infections/ep [Epidemiology]
KW - Cestode Infections/ve [Veterinary]
KW - Child
KW - Preschool
KW - Feces/ps [Parasitology]
KW - Female
KW - Humans
KW - Infant
KW - Newborn
KW - Male
KW - Middle Aged
KW - Peru/ep [Epidemiology]
KW - Rural Population
KW - Swine
KW - Swine Diseases/ep [Epidemiology]
KW - Swine Diseases/ps [Parasitology]
KW - Taenia solium/ip [Isolation & Purification]
KW - Tongue/pa [Pathology]
KW - Topography
KW - Medical
KW - Young Adult
RP - NOT IN FILE
SP - e1953
JF - PLoS Neglected Tropical Diseases [electronic resource]
JA - PLoS Negl Trop Dis
VL - 6
IS - 12
CY - United States
N2 - BACKGROUND: Neurocysticercosis is a leading cause of preventable epilepsy in the developing world. Sustainable community-based interventions are urgently needed to control transmission of the causative parasite, Taenia solium. We examined the geospatial relationship between live pigs with visible cysticercotic cysts on their tongues and humans with adult intestinal tapeworm infection (taeniasis) in a rural village in

northern Peru. The objective was to determine whether tongue-positive pigs could indicate high-risk geographic foci for taeniasis to guide targeted screening efforts. This approach could offer significant benefit compared to mass intervention. METHODS: We recorded geographic coordinates of all village houses, collected stool samples from all consenting villagers, and collected blood and examined tongues of all village pigs. Stool samples were processed by enzyme-linked immunosorbent assay (ELISA) for presence of Taenia sp. coproantigens indicative of active taeniasis; serum was processed by enzyme-linked immunoelectrotransfer blot for antibodies against T. solium cysticercosis (EITB LLGP) and T. solium taeniasis (EITB rES33). FINDINGS: Of 548 pigs, 256 (46.7%) were positive for antibodies against cysticercosis on EITB LLGP. Of 402 fecal samples, 6 (1.5%) were positive for the presence of Taenia sp. coproantigens. The proportion of coproantigen-positive individuals differed significantly between residents living within 100-meters of a tongue-positive pig (4/79, 5.1%) and residents living >100 meters from a tongue-positive pig (2/323, 0.6%) (p = 0.02). The prevalence of taeniasis was >8 times higher among residents living within 100 meters of a tongue-positive pig compared to residents living outside this range (adjusted PR 8.1, 95% CI 1.4-47.0). CONCLUSIONS: Tongue-positive pigs in endemic communities can indicate geospatial foci in which the risk for taeniasis is increased. Targeted screening or presumptive treatment for taeniasis within these high-risk foci may be an effective and practical control intervention for rural endemic areas
SN - 1935-2735
AD - Department of Public Health and Preventive Medicine, Oregon Health and Science University, Portland, Oregon, United States of America. oneals@ohsu.edu
UR - 23285305
ER -

TY - JOUR
ID - 343
T1 - Human recombinant protein C for severe sepsis and septic shock in adult and paediatric patients. [Review][Update of Cochrane Database Syst Rev. 2012;3:CD004388; PMID: 22419295]
A1 - Marti-Carvajal,A.J.
A1 - Sola,I.
A1 - Gluud,C.
A1 - Lathyris,D.
A1 - Cardona,A.F.
Y1 - 2012///
N1 - Marti-Carvajal, Arturo J. Sola, Ivan. Gluud, Christian. Lathyris, Dimitrios. Cardona, Andres Felipe
The Cochrane database of systematic reviews
100909747
IM
Journal Article. Meta-Analysis. Research Support, Non-U.S. Gov't. Review
English
KW - MEDLINE
KW - Adult
KW - Age Factors
KW - Anti-Infective Agents/ae [Adverse Effects]
KW - Anti-Infective Agents/tu [Therapeutic Use]
KW - Cause of Death
KW - Child
KW - Drug Recalls
KW - Early Termination of Clinical Trials
KW - Hospital Mortality
KW - Humans
KW - Protein C/ae [Adverse Effects]
KW - Protein C/tu [Therapeutic Use]
KW - Randomized Controlled Trials as Topic
KW - Recombinant Proteins/ae [Adverse Effects]
KW - Recombinant Proteins/tu [Therapeutic Use]
KW - Sepsis/dt [Drug Therapy]
KW - Sepsis/mo [Mortality]
KW - Shock

KW - Septic/dt [Drug Therapy]
KW - Septic/mo [Mortality]
RP - NOT IN FILE
SP - CD004388
JF - Cochrane Database of Systematic Reviews
JA - Cochrane Database Syst Rev
VL - 12
CY - England
N2 - BACKGROUND: Sepsis is a common and frequently fatal condition. Human recombinant activated protein C (APC) has been introduced to reduce the high risk of death associated with severe sepsis or septic shock. This systematic review is an update of a Cochrane review originally published in 2007. OBJECTIVES: We assessed the benefits and harms of APC for patients with severe sepsis or septic shock. SEARCH METHODS: We searched CENTRAL (The Cochrane Library 2012, Issue 6); MEDLINE (2010 to June 2012); EMBASE (2010 to June 2012); BIOSIS (1965 to June 2012); CINAHL (1982 to June 2012) and LILACS (1982 to June 2012). There was no language restriction. SELECTION CRITERIA: We included randomized clinical trials assessing the effects of APC for severe sepsis or septic shock in adults and children. We excluded studies on neonates. We considered all-cause mortality at day 28 and at the end of study follow up, and hospital mortality as the primary outcomes. DATA COLLECTION AND ANALYSIS: We independently performed trial selection, risk of bias assessment, and data extraction in duplicate. We estimated relative risks (RR) for dichotomous outcomes. We measured statistical heterogeneity using the I(2) statistic. We used a random-effects model. MAIN RESULTS: We identified one new randomized clinical trial in this update which includes six randomized clinical trials involving 6781 participants in total, five randomized clinical trials in adult (N = 6307) and one randomized clinical trial in paediatric (N = 474) participants. All trials had high risk of bias and were sponsored by the pharmaceutical industry. APC compared with placebo did not significantly affect all-cause mortality at day 28 compared with placebo (780/3435 (22.7%) versus 767/3346 (22.9%); RR 1.00, 95% confidence interval (CI) 0.86 to 1.16; I(2) = 56%). APC did not significantly affect in-hospital mortality (393/1767 (22.2%) versus 379/1710 (22.1%); RR 1.01, 95% CI 0.87 to 1.16; I(2) = 20%). APC was associated with an increased risk of serious bleeding (113/3424 (3.3%) versus 74/3343 (2.2%); RR 1.45, 95% CI 1.08 to 1.94; I(2) = 0%). APC did not significantly affect serious adverse events (463/3334 (13.9%) versus 439/3302 (13.2%); RR 1.04, 95% CI 0.92 to 1.18; I(2) = 0%). Trial sequential analyses showed that more trials do not seem to be needed for reliable conclusions regarding these outcomes. AUTHORS' CONCLUSIONS: This updated review found no evidence suggesting that APC should be used for treating patients with severe sepsis or septic shock. APC seems to be associated with a higher risk of bleeding. The drug company behind APC, Eli Lilly, has announced the discontinuation of all ongoing clinical trials using this drug for treating patients with severe sepsis or septic shock. APC should not be used for sepsis or septic shock outside randomized clinical trials
SN - 1469-493X
AD - Facultad de Ciencias de la Salud Eugenio Espejo, Universidad Tecnologica Equinoccial, Quito, Ecuador. arturo.marti.carvajal@gmail.com
UR - 23235609
ER -

TY - JOUR
ID - 344
T1 - Physical activity among Canadian children on school days and nonschool days
A1 - Vander Ploeg,K.A.
A1 - Wu,B.
A1 - McGavock,J.
A1 - Veugelers,P.J.
Y1 - 2012/11//
N1 - Vander Ploeg, Kerry Ann. Wu, Biao. McGavock, Jon. Veugelers, Paul J
Journal of physical activity & health
101189457
IM
Journal Article. Research Support, Non-U.S. Gov't
English
KW - MEDLINE

KW - Actigraphy/is [Instrumentation]
KW - Alberta
KW - Child
KW - Cross-Sectional Studies
KW - Female
KW - Humans
KW - Male
KW - Motor Activity/ph [Physiology]
KW - Schools
KW - Sex Distribution
KW - Walking/sn [Statistics & Numerical Data]
RP - NOT IN FILE
SP - 1138
EP - 1145
JF - Journal of Physical Activity & Health
JA - J Phys Act Health
VL - 9
IS - 8
CY - United States
N2 - BACKGROUND: Schools are frequently cited as a favorable venue to promote physical activity (PA), however little data exist describing times when students are least active. Our objective was to overcome this limitation and describe time periods when students are least active. METHODS: We used a cross-sectional design to assess patterns of PA in 923 grade 5 students [mean age: 10.9 (+ 0.4) years] from 30 schools in Alberta, Canada. Students wore time-stamped pedometers for 9 consecutive days, providing 7 full days of data. We compared step counts adjusted for nonwear time between school days and nonschool days as well as during school hours and after school hours. RESULTS: 689 (75%) students provided complete data. The average daily step count was higher on school days (boys 13,476 + 4123 step/day; girls 11,436 + 3158 steps/day) than nonschool days (boys 11,009 + 5542 steps/day; girls 10,256 + 5206 steps/day). More steps were also taken during school hours than nonschool hours (boys +206 + 420 steps/hour, P < .001; girls 210 + 347, P < .001 steps/hour). CONCLUSIONS: PA levels of children are below Canadian recommended levels for optimal growth and health. Health promotion should emphasize PA particularly among girls, outside school hours, and weekends
SN - 1543-5474
AD - School of Public Health Science, University of Alberta, Edmonton, Alberta, Canada
UR - 22207077
ER -

TY - JOUR
ID - 345
T1 - Adolescents' daily activities and the restorative niches that support them
A1 - Roe,J.J.
A1 - Aspinall,P.A.
Y1 - 2012/09//
N1 - Roe, Jenny J. Aspinall, Peter A
International journal of environmental research and public health
101238455
IM
Journal Article. Research Support, Non-U.S. Gov't
English
KW - MEDLINE
KW - Adolescent
KW - Child
KW - Cities
KW - Cluster Analysis
KW - Cross-Sectional Studies
KW - Environment
KW - Female

KW - Humans
KW - Male
KW - Models
KW - Biological
KW - Personal Satisfaction
KW - Scotland
KW - Social Environment
KW - Social Participation
KW - Social Support
RP - NOT IN FILE
SP - 3227
EP - 3244
JF - International Journal of Environmental Research & Public Health [Electronic Resource]
JA - Int J Environ Res Public Health
VL - 9
IS - 9
CY - Switzerland
N2 - This paper explores wellbeing from the perspective of the psychological dynamics underlying adolescents' relationship with place. It uses a dynamic model of wellbeing called personal project analysis (PPA) which captures the concept of 'flourishing', defined as functioning well in your activities, strivings and interactions with the world [1]. Using PPA methods we identified adolescents' daily activities and the 'restorative niches' that best support them. A series of settings (including home, urban and natural outdoor places) were explored using PPA with 45 young people (aged 11-13) living in Edinburgh, Central Scotland. Participants were asked to think of eight projects of current importance to them, to say where the project took place and to rate each project against a series of core wellbeing dimensions measuring project meaning, manageability, support and affect (how much fun, stress etc.). Latent class analysis was carried out to explore clusters-or sub-groups-in the data and to identify the significant discriminators between clusters. A three-cluster model produced the best fit with project type, project place and wellbeing indicators (fun and stress) significantly discriminating between the three clusters. The three clusters were labeled by their dominant environmental context, 'faraway' (e.g., beach, national parks, hills), 'everyday' (e.g., home, school, local streets) and 'citywide' (e.g., sport settings, urban town context). 'Faraway' and 'citywide' clusters had a significantly higher wellbeing content, especially for fun and stress; the 'everyday' cluster indicated local environs remain a dominant project place for this age group, but are associated with greater stress. We compare findings with adults and suggest that outdoor settings further afield from home have greater significance within adolescent project systems, but that support is needed to facilitate access to these places
SN - 1660-4601
AD - School of the Built Environment, Heriot-Watt University, Edinburgh EH14 4AS, Scotland, UK. j.roe@hw.ac.uk
UR - 23202680
ER -

TY - JOUR
ID - 346
T1 - Evaluation of a redesigned outdoor space on preschool children's physical activity during recess
A1 - Nicaise,V.
A1 - Kahan,D.
A1 - Reuben,K.
A1 - Sallis,J.F.
Y1 - 2012/11//
N1 - Nicaise, Virginie. Kahan, David. Reuben, Karen. Sallis, James F
Pediatric exercise science
8909729
IM
Comparative Study. Evaluation Studies. Journal Article
English
KW - MEDLINE
KW - Acceleration

KW - Anthropometry
KW - Chi-Square Distribution
KW - Child Welfare
KW - Child
KW - Preschool
KW - Cross-Sectional Studies
KW - Environment Design
KW - Exercise/ph [Physiology]
KW - Female
KW - Humans
KW - Intervention Studies
KW - Male
KW - Motor Activity/ph [Physiology]
KW - Play and Playthings
KW - School Health Services
RP - NOT IN FILE
SP - 507
EP - 518
JF - Pediatric Exercise Science
JA - Pediatr Exerc Sci
VL - 24
IS - 4
CY - United States
N2 - This study investigated the impact of renovation and redesign of a university preschool's outdoor space on children's sedentary behavior, light activity, and moderate-to-vigorous-physical-activity (MVPA) during unstructured recess. Physical activity was measured by accelerometry and direct observation in two independent samples of 50 (baseline) and 57 (postintervention) children (Mage = 4.4 yrs + 0.5). Controlling for gender, age, BMI and recess length, observational data, but not accelerometry, revealed a significant decrease in intervals spent sedentary (-26.5%) and increases in light physical activity (+11.6%) and MVPA (+14.9%). Higher levels of MVPA were associated with specific environmental changes (new looping cycle path, OR = 2.18; increased playground open space, OR = 7.62; and new grass hill, OR = 3.27). Decreased sedentary behavior and increased light activity and MVPA may be realized with environmental changes that promote continuous and novel movement experiences in more expansive spaces
SN - 1543-2920
AD - School of Exercise and Nutritional Sciences, San Diego State University, San Diego, CA, USA
UR - 23196760
ER -

TY - JOUR
ID - 347
T1 - Accelerometry-assessed sedentary behaviour and physical activity levels during the segmented school day in 10-14-year-old children: the HAPPY study
A1 - Bailey,D.P.
A1 - Fairclough,S.J.
A1 - Savory,L.A.
A1 - Denton,S.J.
A1 - Pang,D.
A1 - Deane,C.S.
A1 - Kerr,C.J.
Y1 - 2012/12//
N1 - Bailey, Daniel P. Fairclough, Stuart J. Savory, Louise A. Denton, Sarah J. Pang, Dong. Deane, Colleen S. Kerr, Catherine J
European journal of pediatrics
end, 7603873
IM
Comparative Study. Journal Article. Research Support, Non-U.S. Gov't
English

KW - MEDLINE
KW - Accelerometry/mt [Methods]
KW - Accelerometry
KW - Adolescent
KW - Algorithms
KW - Analysis of Variance
KW - Body Mass Index
KW - Child
KW - Female
KW - Humans
KW - Male
KW - Motor Activity
KW - Obesity/pc [Prevention & Control]
KW - Risk Factors
KW - Sampling Studies
KW - Schools
KW - Sedentary Lifestyle
KW - Sex Factors
KW - Time Factors
RP - NOT IN FILE
SP - 1805
EP - 1813
JF - European Journal of Pediatrics
JA - Eur J Pediatr
VL - 171
IS - 12
CY - Germany
N2 - The school day offers several different time periods that provide varying opportunities for sedentary time (SED) and engagement in physical activity (PA), yet little is known about the PA and sedentary behaviour patterns of boys and girls during these times. The volume, intensity and temporal distributions of SED and PA undertaken by 135 schoolchildren aged 10-14 years, during different segments of the school day: (a) school transport, (b) morning recess, (c) lunch break, (d) class time and (e) after school, were explored using triaxial accelerometry. PA was categorised into SED, light PA (LPA), moderate PA (MPA) and vigorous PA (VPA). Girls engaged in significantly more SED and LPA than boys during recess and lunch break (p<0.05), while boys engaged in significantly higher levels of VPA during recess (p<0.001) and MPA and VPA during lunch break (p<0.001). PA engagement was similar between sexes during other segments of the day. Conclusion: PA patterns appear more beneficial for health in boys during less structured school-based time periods and interventions may therefore target opportunities for girls to be physically active during these times to overcome this observed sex deficit
SN - 1432-1076
AD - Institute for Sport and Physical Activity Research, University of Bedfordshire, Polhill Avenue, Bedford, Bedfordshire, MK41 9EA, UK. daniel.bailey@beds.ac.uk
UR - 22983026
ER -

TY - JOUR
ID - 348
T1 - Does school-based physical activity decrease overweight and obesity in children aged 6-9 years? A two-year non-randomized longitudinal intervention study in the Czech Republic
A1 - Sigmund,E.
A1 - El,Ansari W.
A1 - Sigmundova,D.
Y1 - 2012///
N1 - Sigmund, Erik. El Ansari, Walid. Sigmundova, Dagmar
BMC public health
100968562
IM

Controlled Clinical Trial. Journal Article. Research Support, Non-U.S. Gov't
English
KW - MEDLINE
KW - Child
KW - Czech Republic
KW - Female
KW - Humans
KW - Longitudinal Studies
KW - Male
KW - Motor Activity
KW - Obesity/pc [Prevention & Control]
KW - Overweight/pc [Prevention & Control]
KW - Program Evaluation
KW - School Health Services
RP - NOT IN FILE
SP - 570
JF - BMC Public Health
VL - 12
CY - England
N2 - BACKGROUND: Globally, efforts aimed at the prevention of childhood obesity have led to the implementation of a range of school-based interventions. This study assessed whether augmenting physical activity (PA) within the school setting resulted in increased daily PA and decreased overweight/obesity levels in 6-9-year-old children. METHODS: Across the first to third primary school years, PA of 84 girls and 92 boys was objectively monitored five times (each for seven successive days) using Yamax pedometer (step counts) and Caltrac accelerometer (activity energy expenditure AEE - kcal/kg per day). Four schools were selected to participate in the research (2 intervention, 2 controls), comprising intervention (43 girls, 45 boys) and control children (41 girls, 47 boys). The study was non-randomized and the intervention schools were selected on the basis of existing PA-conducive environment. Analyses of variance (ANOVA) for repeated measures examined the PA programme and gender effects on the step counts and AEE. Logistic regression (Enter method) determined the obesity and overweight occurrence prospect over the course of implementation of the PA intervention. RESULTS: There was a significant increase of school-based PA during schooldays in intervention children (from1718 to3247 steps per day; and from 2.1 to3.6 Kcal/Kg per day) in comparison with the control children. Increased school-based PA of intervention children during schooldays contributed to them achieving >10,500 steps and >10.5 Kcal/Kg per school day across the 2 years of the study, and resulted in a stop of the decline in PA levels that is known to be associated with the increasing age of children. Increased school-based PA had also positive impact on leisure time PA of schooldays and on PA at weekends of intervention children. One year after the start of the PA intervention, the odds of being overweight or obese in the intervention children was almost three times lower than that of control children (p<0.005), and these odds steadily decreased with the duration of the intervention. CONCLUSIONS: The findings suggest that school-based PA (Physical Education lessons, PA during short breaks and longer recesses, PA at after-school nursery) in compatible active environments (child-friendly gym and school playground, corridors with movement and playing around corners and for games) has a vital role in obesity and overweight reduction among younger pupils
SN - 1471-2458
AD - Center for Kinanthropology Research, Institute of Active Lifestyle, Palacky University in Olomouc, Olomouc, Czech Republic. erik.sigmund@upol.cz
UR - 22892226
ER -

TY - JOUR
ID - 349
T1 - [Patellar osteochondral injury as onset of patellar instability]. [Spanish]
A1 - Cepero-Campa,S.
A1 - Ullot-Font,R.
A1 - Perez-Lopez,L.M.
Y1 - 2012/01//
N1 - Cepero-Campa, S. Ullot-Font, R. Perez-Lopez, L M
Revista espanola de cirugia ortopedica y traumatologia

101477399
IM
Case Reports. English Abstract. Journal Article
Spanish
KW - MEDLINE
KW - Cartilage
KW - Articular/in [Injuries]
KW - Child
KW - Female
KW - Fracture Fixation
KW - Internal/mt [Methods]
KW - Fractures
KW - Bone/co [Complications]
KW - Bone/su [Surgery]
KW - Humans
KW - Joint Instability/et [Etiology]
KW - Joint Instability/su [Surgery]
KW - Patella/in [Injuries]
RP - NOT IN FILE
SP - 63
EP - 66
JF - Revista Espanola de Cirugia Ortopedica y Traumatologia
VL - 56
IS - 1
CY - Spain
N2 - Patellar osteochondral fractures with no dislocation are uncommon and usually affect the centromedial facet of the patella. We present the case of a 10 year-old, overweight, female patient. She was seen in the emergency room after suffering an accidental fall, assessed as an osteochondral fracture-dislocation of the right patella with upper-outer displaced free fragments. By patient interview, she referred to no previous episodes of patellar dislocation. To complete the study, we performed an MRI which showed a medial facet patellar fracture, with two osteochondral fragments located in the sub-quadricipital recess, associated with other lesions suggesting patellar subluxation. We considered that the best treatment was surgery, so the following was performed: an open reduction and internal fixation with absorbable bars, lateral patellar release (Ficat technique), patellar coverage by medial portion of quadriceps (Insall technique) and internal moving of the lateral half of the patellar tendon (Goldwaith technique). The injury was checked one year later using arthroscopy. It confirmed a good reconstruction of the articular surface, and right patellar centering. At follow-up, during the physiotherapy period, the patient began to have repeated episodes of instability in the contralateral patella. The CT scan confirmed the patellar lateralisation (TAGT 17). Centering surgery was indicated due to the occurrence of multiple dislocation episodes. The patient currently carries out normal physical activity and she has a complete range of movement. Patellar osteochondral fracture is an injury frequently associated with patellar instability, which may onset in the first episode. The medial location of the lesions and the involvement of the system of medial knee stability is a fundamental finding. This fact reinforces the diagnosis of pre-fracture patellar dislocation. This is not a fracture-dislocation, but a dislocation-fracture. We may, therefore, treat the injury and its cause. Copyright 2011 SECOT. Published by Elsevier Espana. All rights reserved
SN - 1988-8856
AD - Servicio de Ortopedia y Traumatologia Pediatricas, Hospital Sant Joan de Deu, Barcelona, Espana
UR - 23177946
ER -

TY - JOUR
ID - 350
T1 - "HealthKick": formative assessment of the health environment in low-resource primary schools in the Western Cape Province of South Africa
A1 - de,Villiers A.
A1 - Steyn,N.P.
A1 - Draper,C.E.

A1 - Fourie,J.M.
A1 - Barkhuizen,G.
A1 - Lombard,C.J.
A1 - Dalais,L.
A1 - Abrahams,Z.
A1 - Lambert,E.V.
Y1 - 2012///
N1 - de Villiers, Anniza. Steyn, Nelia P. Draper, Catherine E. Fourie, Jean M. Barkhuizen, Gerhard. Lombard, Carl J. Dalais, Lucinda. Abrahams, Zulfa. Lambert, Estelle V
BMC public health
100968562
IM
Journal Article. Research Support, Non-U.S. Gov't
English
KW - MEDLINE
KW - Child
KW - Diet
KW - Humans
KW - Motor Activity
KW - Needs Assessment
KW - Organizational Policy
KW - Poverty
KW - Program Evaluation
KW - Qualitative Research
KW - School Health Services
KW - Schools/og [Organization & Administration]
KW - Social Environment
KW - South Africa
KW - Tobacco Use Disorder/pc [Prevention & Control]
RP - NOT IN FILE
SP - 794
JF - BMC Public Health
VL - 12
CY - England
N2 - BACKGROUND: This study evaluated the primary school environment in terms of being conducive to good nutrition practices, sufficient physical activity and prevention of nicotine use, with the view of planning a school-based health intervention. METHODS: A sample of 100 urban and rural disadvantaged schools was randomly selected from two education districts of the Western Cape Education Department, South Africa. A situation analysis, which comprised an interview with the school principal and completion of an observation schedule of the school environment, was done at all schools. RESULTS: Schools, on average, had 560 learners and 16 educators. Principals perceived the top health priorities for learners to be an unhealthy diet (50%) and to far lesser degree, lack of physical activity (24%) and underweight (16%). They cited lack of physical activity (33%) and non-communicable diseases (NCDs; 24%) as the main health priorities for educators, while substance abuse (66%) and tobacco use (31%) were prioritised for parents. Main barriers to health promotion programmes included lack of financial resources and too little time in the time table. The most common items sold at the school tuck shops were crisps (100%), and then sweets (96%), while vendors mainly sold sweets (92%), crisps (89%), and ice lollies (38%). Very few schools (8%) had policies governing the type of food items sold at school. Twenty-six of the 100 schools that were visited had vegetable gardens. All schools reported having physical activity and physical education in their time tables, however, not all of them offered this activity outside the class room. Extramural sport offered at schools mainly included athletics, netball, and rugby, with cricket and soccer being offered less frequently. CONCLUSION: The formative findings of this study contribute to the knowledge of key environmental and policy determinants that may play a role in the health behaviour of learners, their parents and their educators. Evidently, these show that school environments are not always conducive to healthy lifestyles. To address the identified determinants relating to learners it is necessary to intervene on the various levels of influence, i.e. parents, educators, and the support systems for the school environment including the curriculum, food available at school, resources for physical activity as well as appropriate policies in this regard

SN - 1471-2458
AD - Chronic Diseases of Lifestyle Unit, Medical Research Council (MRC), Tygerberg, South Africa. anniza.de.villiers@mrc.ac.za
UR - 22985326
ER -

TY - JOUR
ID - 351
T1 - Introduction of 2009 pandemic influenza A virus subtype H1N1 into South Africa: clinical presentation, epidemiology, and transmissibility of the first 100 cases
A1 - Archer,B.N.
A1 - Timothy,G.A.
A1 - Cohen,C.
A1 - Tempia,S.
A1 - Huma,M.
A1 - Blumberg,L.
A1 - Naidoo,D.
A1 - Cengimbo,A.
A1 - Schoub,B.D.
Y1 - 2012/12/15/
N1 - Archer, Brett N. Timothy, Geraldine A. Cohen, Cheryl. Tempia, Stefano. Huma, Mmampedi. Blumberg, Lucille. Naidoo, Dhamari. Cengimbo, Ayanda. Schoub, Barry D
The Journal of infectious diseases
ih3, 0413675
AIM, IM
Journal Article. Research Support, N.I.H., Extramural
English
KW - MEDLINE
KW - Adolescent
KW - Adult
KW - Age Distribution
KW - Aged
KW - Child
KW - Preschool
KW - Family Health
KW - Female
KW - Humans
KW - Influenza A Virus
KW - H1N1 Subtype/ip [Isolation & Purification]
KW - Influenza
KW - Human/ep [Epidemiology]
KW - Human/pa [Pathology]
KW - Human/tm [Transmission]
KW - Male
KW - Middle Aged
KW - Prospective Studies
KW - South Africa/ep [Epidemiology]
KW - Travel
KW - Young Adult
RP - NOT IN FILE
SP - S148
EP - S153
JF - Journal of Infectious Diseases
JA - J Infect Dis
VL - 206 Suppl 1
CY - United States

N2 - BACKGROUND: We documented the introduction of 2009 pandemic influenza A virus subtype H1N1 (A[H1N1]pdm09) into South Africa and describe its clinical presentation, epidemiology, and transmissibility. METHODS: We conducted a prospective descriptive study of the first 100 laboratory-confirmed cases of A(H1N1)pdm09 infections identified through active case finding and surveillance. Infected patients and the attending clinicians were interviewed, and close contacts were followed up to investigate household transmission. FINDINGS: The first case was confirmed on 14 June 2009, and by 15 July 2009, 100 cases were diagnosed. Forty-two percent of patients reported international travel within 7 days prior to onset of illness. Patients ranged in age from 4 to 70 years (median age, 21.5 years). Seventeen percent of household contacts developed influenza-like illness, and 10% of household contacts had laboratory-confirmed A(H1N1)pdm09 infection. We found a mean serial interval (+ SD) of 2.3 + 1.3 days (range, 1-5 days) between successive laboratory-confirmed cases in the transmission chain. CONCLUSIONS: A(H1N1)pdm09 established itself rapidly in South Africa. Transmissibility of the virus was comparable to observations from outside of Africa and to seasonal influenza virus strains
SN - 1537-6613
AD - National Institute for Communicable Diseases, National Health Laboratory Service, University of Witwatersrand, Johannesburg, South Africa. bretta@nicd.ac.za
UR - 23169962
ER -

TY - JOUR
ID - 352
T1 - Predictors of physical activity change during adolescence: a 3.5-year follow-up
A1 - Dumith,S.C.
A1 - Gigante,D.P.
A1 - Domingues,M.R.
A1 - Hallal,P.C.
A1 - Menezes,A.M.
A1 - Kohl,H.W.,III
Y1 - 2012/12//
N1 - Dumith, Samuel C. Gigante, Denise P. Domingues, Marlos R. Hallal, Pedro C. Menezes, Ana M B. Kohl, Harold W 3rd
Public health nutrition
9808463, dgy
IM
Journal Article. Research Support, Non-U.S. Gov't
English
KW - MEDLINE
KW - Adolescent
KW - Adolescent Behavior
KW - Brazil
KW - Child
KW - Environment
KW - Exercise
KW - Fear
KW - Female
KW - Follow-Up Studies
KW - Growth
KW - Health Behavior
KW - Health Surveys
KW - Humans
KW - Male
KW - Menarche
KW - Mothers
KW - Overweight
KW - Prospective Studies
KW - Questionnaires
KW - Reference Values

KW - Residence Characteristics
KW - Sedentary Lifestyle
KW - Sex Factors
KW - Social Class
RP - NOT IN FILE
SP - 2237
EP - 2245
JF - Public Health Nutrition
JA - Public Health Nutr
VL - 15
IS - 12
CY - England
N2 - OBJECTIVE: To investigate the predictors of change in physical activity (PA) from early to mid adolescence in a cohort of adolescents. DESIGN: Prospective, population-based birth cohort study. PA level was evaluated by means of questionnaire, and was analysed in continuous form (min/week) and as a trajectory (inactive-inactive, inactive-active, active-inactive, active-active) based on the cut-off point of 300 min/week. SETTING: Pelotas, a city of 340 000 inhabitants in southern Brazil. SUBJECTS: Adolescents (n 4120) followed from 11 to 15 years of age. RESULTS: Maternal PA change and more exposure to outdoors were directly associated with a positive change in PA level (min/week) for both genders. Higher maturation status (among boys) and later menarche were also associated with positive PA change in min/week. Predictors to remain inactive were: maternal PA change (inverse association), more exposure to outdoors, higher socio-economic level, fear of living in the neighbourhood and non-overweight girls. Predictors to become inactive were higher socio-economic level among boys and increase in screen time among girls. CONCLUSIONS: The study demonstrates that social, family, biological, behavioural and environmental factors exert an important role in the PA change among youngsters as they move into adolescence. These findings may be relevant to the design of policies and intervention programmes aimed at promoting PA in teenagers
SN - 1475-2727
AD - Postgraduate Program in Epidemiology, Federal University of Pelotas, Pelotas, Brazil. scdumith@yahoo.com.br
UR - 22464063
ER -

TY - JOUR
ID - 353
T1 - A personal digital assistant for improving independent transitioning in adolescents with high-functioning autism spectrum disorder
A1 - Palmen,A.
A1 - Didden,R.
A1 - Verhoeven,L.
Y1 - 2012///
N1 - Palmen, Annemiek. Didden, Robert. Verhoeven, Lisette
Developmental neurorehabilitation
101304394
IM
Journal Article. Research Support, Non-U.S. Gov't
English
KW - MEDLINE
KW - Adolescent
KW - Child
KW - Child Development Disorders
KW - Pervasive/rh [Rehabilitation]
KW - Computers
KW - Handheld
KW - Female
KW - Humans
KW - Male
KW - Young Adult

RP - NOT IN FILE
SP - 401
EP - 413
JF - Developmental neurorehabilitation
JA - Dev Neurorehabil
VL - 15
IS - 6
CY - England
N2 - OBJECTIVE: This study evaluated the effectiveness of a personal digital assistant (PDA) on independent transitioning between activities in a day treatment centre for youth with high-functioning ASD. METHODS: Within a multiple baseline design across four participants, data were collected on participant's transitioning and staff's prompting behaviour. Intervention by staff consisted of one technical instruction session on use of the PDA and non-specific instruction following incorrect transitions while not using the PDA, in the natural setting. RESULTS: Analysis revealed a significant increase in percentage independent daily transitions, which resulted from the independent use of the PDA. The change in staff's prompt use during intervention was mainly the result of a significant decrease in the use of non-specific prompts in correcting participant's transition behaviour. CONCLUSION: A brief intervention was effective in improving independent transitioning using a PDA. Findings are evaluated in light of their clinical implications and suggestions for future research are discussed
SN - 1751-8431
AD - Dr Leo Kannerhuis Doorwerth, Centre for Autism, Nijmegen, The Netherlands. a.palmen@pwo.ru.nl
UR - 23030628
ER -

TY - JOUR
ID - 354
T1 - The frequency of outdoor play for preschool age children cared for at home-based child care settings
A1 - Tandon,P.S.
A1 - Zhou,C.
A1 - Christakis,D.A.
Y1 - 2012/11//
N1 - Tandon, Pooja S. Zhou, Chuan. Christakis, Dimitri A
Academic pediatrics
101499145
IM
Journal Article. Research Support, Non-U.S. Gov't
English
KW - MEDLINE
KW - Child Day Care Centers/sn [Statistics & Numerical Data]
KW - Child
KW - Preschool
KW - Cross-Sectional Studies
KW - Female
KW - Humans
KW - Male
KW - Motor Activity
KW - Obesity/pc [Prevention & Control]
KW - Play and Playthings
RP - NOT IN FILE
SP - 475
EP - 480
JF - Academic pediatrics
JA - Acad Pediatr
VL - 12
IS - 6
CY - United States
N2 - OBJECTIVE: Given that more than 34% of U.S. children are cared for in home-based child care settings and outdoor play is associated with physical activity and other health benefits, we sought to characterize the

outdoor play frequency of preschoolers cared for at home-based child care settings and factors associated with outdoor play. METHODS: Cross-sectional study of 1900 preschoolers (representing approximately 862,800 children) cared for in home-based child care settings (including relative and nonrelative care) using the nationally representative Early Childhood Longitudinal Study, Birth Cohort. RESULTS: Only 50% of home-based child care providers reported taking the child outside to walk or play at least once/day. More than one-third of all children did not go outside to play daily with either their parent(s) or home-based child care provider. There were increased odds of going outside daily for children cared for by nonrelatives in the child's home compared with care from a relative. Children with >3 regular playmates had greater odds of being taken outdoors by either the parents or child care provider. We did not find statistically significant associations between other child level (age, sex, screen-time), family level (highest education in household, mother's race, employment, exercise frequency), and child care level (hours in care, provider's educational attainment, perception of neighborhood safety) factors and frequency of outdoor play. CONCLUSIONS: At a national level, the frequency of outdoor play for preschoolers cared for in home-based child care settings is suboptimal. Further study and efforts to increase outdoor playtime for children in home-based child care settings are needed. Copyright 2012 Academic Pediatric Association. Published by Elsevier Inc. All rights reserved
SN - 1876-2867
AD - Center for Child Health, Behavior, and Development, Seattle Children's Research Institute, Seattle, WA, USA. pooja@uw.edu
UR - 22980727
ER -

TY - JOUR
ID - 355
T1 - DNA hypomethylation affects cancer-related biological functions and genes relevant in neuroblastoma pathogenesis
A1 - Mayol,G.
A1 - Martin-Subero,J.I.
A1 - Rios,J.
A1 - Queiros,A.
A1 - Kulis,M.
A1 - Sunol,M.
A1 - Esteller,M.
A1 - Gomez,S.
A1 - Garcia,I.
A1 - de,Torres C.
A1 - Rodriguez,E.
A1 - Galvan,P.
A1 - Mora,J.
A1 - Lavarino,C.
Y1 - 2012///
N1 - Mayol, Gemma. Martin-Subero, Jose I. Rios, Jose. Queiros, Ana. Kulis, Marta. Sunol, Mariona. Esteller, Manel. Gomez, Soledad. Garcia, Idoia. de Torres, Carmen. Rodriguez, Eva. Galvan, Patricia. Mora, Jaume. Lavarino, Cinzia
PloS one
101285081
IM
Journal Article. Research Support, Non-U.S. Gov't
English
KW - MEDLINE
KW - Child
KW - Preschool
KW - Cyclin D1/ge [Genetics]
KW - Cyclin D1/me [Metabolism]
KW - DNA Methylation/ge [Genetics]
KW - Gene Expression Profiling
KW - Gene Expression Regulation
KW - Neoplastic

KW - Genes
KW - Neoplasm/ge [Genetics]
KW - Humans
KW - Infant
KW - Newborn
KW - Neuroblastoma/ge [Genetics]
KW - Reproducibility of Results
KW - Sequence Analysis
KW - DNA
KW - Temperature
RP - NOT IN FILE
SP - e48401
JF - PLoS ONE [Electronic Resource]
JA - PLoS ONE
VL - 7
IS - 11
CY - United States
N2 - Neuroblastoma (NB) pathogenesis has been reported to be closely associated with numerous genetic alterations. However, underlying DNA methylation patterns have not been extensively studied in this developmental malignancy. Here, we generated microarray-based DNA methylation profiles of primary neuroblastic tumors. Stringent supervised differential methylation analyses allowed us to identify epigenetic changes characteristic for NB tumors as well as for clinical and biological subtypes of NB. We observed that gene-specific loss of DNA methylation is more prevalent than promoter hypermethylation. Remarkably, such hypomethylation affected cancer-related biological functions and genes relevant to NB pathogenesis such as CCND1, SPRR3, BTC, EGF and FGF6. In particular, differential methylation in CCND1 affected mostly an evolutionary conserved functionally relevant 3' untranslated region, suggesting that hypomethylation outside promoter regions may play a role in NB pathogenesis. Hypermethylation targeted genes involved in cell development and proliferation such as RASSF1A, POU2F2 or HOXD3, among others. The results derived from this study provide new candidate epigenetic biomarkers associated with NB as well as insights into the molecular pathogenesis of this tumor, which involves a marked gene-specific hypomethylation
SN - 1932-6203
AD - Developmental Tumor Biology Laboratory, Hospital Sant Joan de Deu, Fundacion Sant Joan de Deu, Barcelona, Spain
UR - 23144874
ER -

TY - JOUR
ID - 356
T1 - A controlled, class-based multicomponent intervention to promote healthy lifestyle and to reduce the burden of childhood obesity
A1 - Centis,E.
A1 - Marzocchi,R.
A1 - Di,Luzio R.
A1 - Moscatiello,S.
A1 - Salardi,S.
A1 - Villanova,N.
A1 - Marchesini,G.
A1 - Croce School of Health Promotion,G.C.
Y1 - 2012/12//
N1 - Centis, E. Marzocchi, R. Di Luzio, R. Moscatiello, S. Salardi, S. Villanova, N. Marchesini, G. G. C. Croce School of Health Promotion
Pediatric obesity
101572033
IM
Journal Article. Research Support, Non-U.S. Gov't
English
KW - MEDLINE

KW - Behavior Therapy
KW - Body Mass Index
KW - Child
KW - Counseling
KW - Exercise
KW - Faculty
KW - Female
KW - Health Promotion/mt [Methods]
KW - Humans
KW - Italy
KW - Life Style
KW - Male
KW - Motivation
KW - Obesity/pc [Prevention & Control]
KW - Parents
KW - Television
KW - Time Factors
KW - Treatment Outcome
RP - NOT IN FILE
SP - 436
EP - 445
JF - Pediatric Obesity
JA - Pediatr Obes
VL - 7
IS - 6
CY - England
N2 - BACKGROUND: Overweight and obesity prevention in childhood and adolescence represent a priority for public health; school is a privileged place for health promotion interventions. OBJECTIVES: The study aimed to test the effectiveness of a multicomponent 5-month intervention on the habits of primary school children, making the families aware of the importance of healthy choices. METHODS: Two hundred nine children attending the fourth class of primary school, divided into interventional (n=103) and control arm (n=106) were included in the study. In the intervention group, parents and teachers received more intense lifestyle counseling, associated with weekly motivational telephone calls to families to motivate further their lifestyle changes. Standard deviation score (SDS) body mass index (BMI) was the primary outcome measure; on open-air games and TV watching were secondary outcomes. RESULTS: At baseline, no differences were observed between groups. At 8-month follow-up, mean SDS BMI had decreased by 0.06 units in the intervention arm and increased by 0.12 in controls (timextreatment anova, P<0.002). Outdoor activities increased from 6.23h week(-1) to 9.93 in the intervention group (P<0.001), not in controls. This change was associated differences in TV watching from baseline (intervention, -0.96h week(-1); P=0.037; controls, +1.33h week(-1); P=0.031). CONCLUSION: A multicomponent school-based intervention addressing the needs of children, teachers and families produced a significant and favourable short-term effect on overweight/obese schoolchildren. 2012 The Authors. Pediatric Obesity 2012 International Association for the Study of Obesity
SN - 2047-6310
AD - Unit of Metabolic Diseases & Clinical Dietetics, Alma Mater Studiorum University, Bologna, Italy
UR - 22911919
ER -

TY - JOUR
ID - 357
T1 - Estimating youth locomotion ground reaction forces using an accelerometer-based activity monitor
A1 - Neugebauer,J.M.
A1 - Hawkins,D.A.
A1 - Beckett,L.
Y1 - 2012///
N1 - Neugebauer, Jennifer M. Hawkins, David A. Beckett, Laurel
PloS one
101285081

IM
Journal Article. Research Support, N.I.H., Extramural. Research Support, Non-U.S. Gov't
English
KW - MEDLINE
KW - Acceleration
KW - Adolescent
KW - Child
KW - Female
KW - Humans
KW - Locomotion
KW - Male
KW - Models
KW - Statistical
KW - Monitoring
KW - Physiologic
KW - Movement
KW - Regression Analysis
KW - Reproducibility of Results
KW - Running
KW - Walking
RP - NOT IN FILE
SP - e48182
JF - PLoS ONE [Electronic Resource]
JA - PLoS ONE
VL - 7
IS - 10
CY - United States
N2 - To address a variety of questions pertaining to the interactions between physical activity, musculoskeletal loading and musculoskeletal health/injury/adaptation, simple methods are needed to quantify, outside a laboratory setting, the forces acting on the human body during daily activities. The purpose of this study was to develop a statistically based model to estimate peak vertical ground reaction force (pVGRF) during youth gait. 20 girls (10.9 + 0.9 years) and 15 boys (12.5 + 0.6 years) wore a Biotrainer AM over their right hip. Six walking and six running trials were completed after a standard warm-up. Average AM intensity (g) and pVGRF (N) during stance were determined. Repeated measures mixed effects regression models to estimate pVGRF from Biotrainer activity monitor acceleration in youth (girls 10-12, boys 12-14 years) while walking and running were developed. Log transformed pVGRF had a statistically significant relationship with activity monitor acceleration, centered mass, sex (girl), type of locomotion (run), and locomotion type-acceleration interaction controlling for subject as a random effect. A generalized regression model without subject specific random effects was also developed. The average absolute differences between the actual and predicted pVGRF were 5.2% (1.6% standard deviation) and 9% (4.2% standard deviation) using the mixed and generalized models, respectively. The results of this study support the use of estimating pVGRF from hip acceleration using a mixed model regression equation
SN - 1932-6203
AD - Biomedical Engineering Graduate Group, University of California Davis, Davis, California, United States of America
UR - 23133564
ER -

TY - JOUR
ID - 358
T1 - Activated partial thromboplastin time is a better trending tool in pediatric extracorporeal membrane oxygenation
A1 - Maul,T.M.
A1 - Wolff,E.L.
A1 - Kuch,B.A.
A1 - Rosendorff,A.
A1 - Morell,V.O.

A1 - Wearden,P.D.
Y1 - 2012/11//
N1 - Maul, Timothy M. Wolff, Erin L. Kuch, Bradley A. Rosendorff, Adam. Morell, Victor O. Wearden, Peter D
Pediatric critical care medicine : a journal of the Society of Critical Care Medicine and the World Federation of Pediatric Intensive and Critical Care Societies
100954653
IM
Comparative Study. Journal Article. Research Support, Non-U.S. Gov't
English
KW - MEDLINE
KW - Adolescent
KW - Anticoagulants/ad [Administration & Dosage]
KW - Child
KW - Preschool
KW - Extracorporeal Membrane Oxygenation/ae [Adverse Effects]
KW - Extracorporeal Membrane Oxygenation
KW - Heart Failure/th [Therapy]
KW - Hemorrhage/et [Etiology]
KW - Hemorrhage/pc [Prevention & Control]
KW - Heparin/ad [Administration & Dosage]
KW - Humans
KW - Infant
KW - Partial Thromboplastin Time
KW - Point-of-Care Systems
KW - Respiratory Insufficiency/th [Therapy]
KW - Retrospective Studies
KW - Risk Assessment
KW - Statistics
KW - Nonparametric
KW - Whole Blood Coagulation Time
RP - NOT IN FILE
SP - e363
EP - e371
JF - Pediatric Critical Care Medicine
JA - Pediatr Crit Care Med
VL - 13
IS - 6
CY - United States
N2 - OBJECTIVES: To determine whether activated partial thromboplastin times are a better heparin management tool than activated clotting times in pediatric extracorporeal membrane oxygenation. DESIGN: A single-center retrospective analysis of perfusion and patient records. SETTING: Academic pediatric tertiary care center. PATIENTS: Pediatric patients (<21 yrs old) requiring extracorporeal membrane oxygenation support initiated at Children's Hospital of Pittsburgh. INTERVENTIONS: None. MEASUREMENTS AND MAIN RESULTS: Point-of-care activated clotting time and activated partial thromboplastin time values, clinical laboratory activated partial thromboplastin time values, weight-normalized heparin administration (units/kg/hr), and reported outcomes were collected for pediatric patients treated for cardiac and/or respiratory failure with extracorporeal membrane oxygenation. Spearman's ranked correlations were performed for each coagulation test compared to heparin dosage. The Bland-Altman test was used to determine the validity of the point-of-care activated partial thromboplastin time. Hazard analysis was conducted for outcomes and complications for patients whose heparin management was based on the clinical laboratory activated partial thromboplastin time or the activated clotting time. Only the clinical laboratory activated partial thromboplastin time showed a correlation (p = 0.40 vs. p = -0.04 for activated clotting time) with the heparin administration (units/kg/hr). Point-of-care activated partial thromboplastin time and activated partial thromboplastin time values correlated well (p = 0.76), with <5% of samples showing a difference outside 2 SDs, but differences in their absolute values (activated partial thromboplastin time = 100 secs) preclude them from being interchangeable measures. Furthermore, despite no effective change in the mean activated clotting time, cardiac patients showed a

significantly improved correlation to heparin dose for all coagulation tests (e.g., point-of-care activated partial thromboplastin time p = 0.60). Management of patients with the clinical laboratory activated partial thromboplastin time did not significantly affect patient survival rates but did significantly reduce bleeding complications and significantly increased clotting in the extracorporeal membrane oxygenation circuit. A hazard analysis demonstrated that bleeding complications were associated with an increased risk of mortality, whereas clotting complications in the extracorporeal membrane oxygenation circuit were not. CONCLUSIONS: The activated clotting time is not an accurate monitoring tool for heparin management in pediatricextracorporeal membrane oxygenation. The point-of-care activated partial thromboplastin time correlates well with the clinical laboratory activated partial thromboplastin time but cannot be substituted for the clinical laboratory activated partial thromboplastin time values. Management of pediatric extracorporeal membrane oxygenation patients with the clinical laboratory activated partial thromboplastin time reduced bleeding complications which are associated with increases in mortality
SN - 1529-7535
AD - Department of Cardiothoracic Surgery, Children's Hospital of Pittsburgh of University of Pittsburgh Medical Center, Pittsburgh, PA, USA
UR - 22940857
ER -

TY - JOUR
ID - 359
T1 - Leading causes of injury hospitalisation in children aged 0-4 years in New South Wales by injury submechanism: a brief profile by age and sex
A1 - Schmertmann,M.
A1 - Williamson,A.
A1 - Black,D.
Y1 - 2012/11//
N1 - Schmertmann, Marcia. Williamson, Ann. Black, Deborah
Journal of paediatrics and child health
arp, 9005421
IM
Journal Article. Research Support, Non-U.S. Gov't
English
KW - MEDLINE
KW - Accidents
KW - Child
KW - Preschool
KW - Epidemiologic Studies
KW - Female
KW - Hospitalization
KW - Humans
KW - Infant
KW - Male
KW - New South Wales
KW - Risk Assessment/mt [Methods]
KW - Sex Distribution
KW - Wounds and Injuries/et [Etiology]
RP - NOT IN FILE
SP - 978
EP - 984
JF - Journal of Paediatrics & Child Health
JA - J Paediatr Child Health
VL - 48
IS - 11
CY - Australia
N2 - AIM: To identify the leading causes of injury in children aged 0-4 years by single year of age using injury submechanisms and present a brief epidemiologic profile of each cause. METHODS: Hospitalisation data for New South Wales from 1999 to 2009 were used to identify the leading causes of injury for children aged 0-4

years by single year of age. For each leading cause, rates over time and by sex were calculated by single year of age. Associated age and sex risk ratios were estimated. RESULTS: The leading causes of injury for children aged <1, 1 and 2 years were falls while being carried, burns by hot non-aqueous substances and poisoning by other and unspecified pharmaceutical substances, respectively. Falls involving playground equipment ranked first for children aged 3-4 years. Each leading injury cause exhibited an age pattern that remained stable over time and by sex. Age predicted falls while being carried and both age and sex predicted the remaining leading injury causes, with age and sex interacting to predict burns by hot non-aqueous substances. CONCLUSIONS: Epidemiologic analysis using single-year age intervals and injury submechanisms results in a clearer picture of injury risk for young children. The findings of this study provide detailed information regarding the leading causes of hospitalised injury in young children by age and sex. Child health-care providers can use this information to focus discussions of child development and injury risk with families of young children and suggest appropriate prevention measures in terms of a child's age and sex. 2012 The Authors. Journal of Paediatrics and Child Health 2012 Paediatrics and Child Health Division (Royal Australasian College of Physicians)
SN - 1440-1754
AD - University of New South Wales Faculty of Health Sciences, Sydney, New South Wales, Australia. mschmertmann@optusnet.com.au
UR - 23078174
ER -

TY - JOUR
ID - 360
T1 - Lessons from the hepatoblastoma-familial polyposis connection
A1 - Moore,S.W.
A1 - Tshifularo,N.
A1 - Grobbelaar,J.
Y1 - 2012/11//
N1 - Moore, S W. Tshifularo, N. Grobbelaar, J
South African medical journal = Suid-Afrikaanse tydskrif vir geneeskunde
0404520
IM
Case Reports. Journal Article
English
KW - MEDLINE
KW - Adenomatous Polyposis Coli/ep [Epidemiology]
KW - Adenomatous Polyposis Coli/ge [Genetics]
KW - Child
KW - Preschool
KW - Comorbidity
KW - Genetic Association Studies
KW - Hepatoblastoma/ep [Epidemiology]
KW - Hepatoblastoma/ge [Genetics]
KW - Hepatoblastoma/ra [Radiography]
KW - Humans
KW - Infant
KW - Liver Neoplasms/ep [Epidemiology]
KW - Liver Neoplasms/ge [Genetics]
KW - Liver Neoplasms/ra [Radiography]
KW - Male
KW - Retrospective Studies
KW - Tomography
KW - X-Ray Computed
RP - NOT IN FILE
SP - 888
EP - 889
JF - South African Medical Journal
JA - SAMJ, S

VL - Suid-Afrikaanse Tydskrif Vir Geneeskunde. 102
IS - 11 Pt 2
CY - South Africa
N2 - BACKGROUND: Approximately one-third of hepatoblastoma (HB) patients have associated congenital abnormalities, but familial recurrence is rare, except in association with familial adenomatous polyposis (FAP). This correlation may be missed if not actively sought, with implications for long-term outcome and management. METHODS: We retrospectively investigated 3 families with an HB-familial polyposis connection, from a cohort of 113 FAP families (1989 - 2010). Data were analysed to assess clinical problem, treatment, complications and management. Long-term morbidity and functional outcome were analysed to identify management difficulties. RESULTS: Three FAP families (2.65%) had an HB association. In one case, undiagnosed FAP at the time of HB diagnosis was only detected 5 years later, when the mother presented with advanced colorectal carcinoma. A chromosome 5 APC gene mutation (exon 15 codon 793 C->T) was then identified. In a second case, a non-related male child presented with a stage 4 multifocal HB with lung metastases. Genetic studies identified an APC gene mutation (exon 6 codon 232 C->T). Further family investigation showed >20 related FAP patients. A third HB-FAP association was identified in a known FAP family early in the study, prior to the availability of genetic testing. CONCLUSION: Although a rare association, a family history of FAP in HB patients is an important 'hidden connection'. Germline variation may be outside the usual FAP gene site. Identifying families with unknown HB/FAP is important due to long-term management implications and follow-up
SN - 0256-9574
AD - Stellenbosch University, South Africa. swm@sun.ac.za
UR - 23116752
ER -

TY - JOUR
ID - 361
T1 - The relationship between soil geochemistry and the bioaccessibility of trace elements in playground soil
A1 - De,Miguel E.
A1 - Mingot,J.
A1 - Chacon,E.
A1 - Charlesworth,S.
Y1 - 2012/12//
N1 - De Miguel, Eduardo. Mingot, Juan. Chacon, Enrique. Charlesworth, Susanne
Environmental geochemistry and health
8903118
IM
Comparative Study. Journal Article
English
KW - MEDLINE
KW - Arsenic/an [Analysis]
KW - Arsenic/me [Metabolism]
KW - Child
KW - Cities
KW - Environmental Exposure
KW - Environmental Monitoring/mt [Methods]
KW - Humans
KW - Metals/an [Analysis]
KW - Metals/me [Metabolism]
KW - Play and Playthings
KW - Risk Assessment
KW - Soil/ch [Chemistry]
KW - Soil Pollutants/an [Analysis]
KW - Soil Pollutants/me [Metabolism]
KW - Spain
KW - Trace Elements/an [Analysis]
KW - Trace Elements/me [Metabolism]
RP - NOT IN FILE

SP - 677
EP - 687
JF - Environmental Geochemistry & Health
JA - Environ Geochem.Health
VL - 34
IS - 6
CY - Netherlands
N2 - A total of 32 samples of surficial soil were collected from 16 playground areas in Madrid (Spain), in order to investigate the importance of the geochemistry of the soil on subsequent bioaccessibility of trace elements. The in vitro bioaccessibility of As, Co, Cr, Cu, Ni, Pb and Zn was evaluated by means of two extraction processes that simulate the gastric environment and one that reproduces a gastric + intestinal digestion sequence. The results of the in vitro bioaccessibility were compared against aqua regia extractions ("total" concentration), and it was found that total concentrations of As, Cu, Pb and Zn were double those of bioaccessible values, whilst that of Cr was ten times higher. Whereas the results of the gastric + intestinal extraction were affected by a high uncertainty, both gastric methods offered very similar and consistent results, with bioaccessibilities following the order: As = Cu = Pb = Zn > Co > Ni > Cr, and ranging from 63 to 7 %. Selected soil properties including pH, organic matter, Fe and CaCO(3) content were determined to assess their influence on trace element bioaccessibility, and it was found that Cu, Pb and Zn were predominantly bound to organic matter and, to a lesser extent, Fe oxides. The former fraction was readily accessible in the gastric solution, whereas Fe oxides seemed to recapture negatively charged chloride complexes of these elements in the gastric solution, lowering their bioaccessibility. The homogeneous pH of the playground soils included in the study does not influence trace element bioaccessibility to any significant extent except for Cr, where the very low gastric accessibility seems to be related to the strongly pH-dependent formation of complexes with organic matter. The results for As, which have been previously described and discussed in detail in Mingot et al. (Chemosphere 84: 1386-1391, 2011), indicate a high gastric bioaccessibility for this element as a consequence of its strong association with calcium carbonate and the ease with which these bonds are broken in the gastric solution. The calculation of risk assessments are therefore dependant on the methodology used and the specific environment they address. This has impacts on management strategies formulated to ensure that the most vulnerable of society, children, can live and play without adverse consequences to their health
SN - 1573-2983
AD - Environmental Geochemistry Research and Engineering Laboratory, Universidad Politecnica de Madrid, Alenza 4, 28003, Madrid, Spain. eduardo.demiguel@upm.es
UR - 23053927
ER -

TY - JOUR
ID - 362
T1 - Allergic airway diseases in childhood - marching from epidemiology to novel concepts of prevention. [Review]
A1 - Hatzler,L.
A1 - Hofmaier,S.
A1 - Papadopoulos,N.G.
Y1 - 2012/11//
N1 - Hatzler, Laura. Hofmaier, Stephanie. Papadopoulos, Nikolaos G
Pediatric allergy and immunology : official publication of the European Society of Pediatric Allergy and Immunology
bu6, 9106718
IM
Journal Article. Review
English
KW - MEDLINE
KW - Air Pollution/ae [Adverse Effects]
KW - Animals
KW - Child
KW - Child Nutrition Disorders/ep [Epidemiology]
KW - Developing Countries
KW - Humans

KW - Hygiene
KW - Infection/ep [Epidemiology]
KW - Prevalence
KW - Respiratory Hypersensitivity/ep [Epidemiology]
KW - Respiratory Hypersensitivity/pc [Prevention & Control]
KW - Risk Factors
KW - Socioeconomic Factors
RP - NOT IN FILE
SP - 616
EP - 622
JF - Pediatric Allergy & Immunology
JA - Pediatr Allergy Immunol
VL - 23
IS - 7
CY - England
N2 - In the past years, a wide range of epidemiological, clinical, and experimental studies have produced remarkable advances in the field of respiratory allergies in childhood. By the recent investigations on epidemiological trends, risk factors, and prevention of asthma and allergic rhinitis, various exiting concepts have been challenged, and novel innovative approaches have been developed. Pediatric Allergy and Immunology (PAI), with a number of highly relevant contributions between 2010 and 2012, has become an important forum in this area. The prevalence of asthma in some developed countries may have reached a plateau, while in developing countries, where the prevalence was previously low, allergic diseases are still on the increase. A wide array of risk and protective factors, including hygiene, infections, outdoor and indoor air pollution, allergen exposure, breast-feeding practices, nutrition, and obesity, play a multifaceted role in shaping the observed worldwide trends of respiratory allergies. Under the guidance of recent research, prediction and prevention strategies in the clinical practice are progressively changing, the focus moving away from avoidance of allergen exposure and toward tolerance induction. 2012 John Wiley & Sons A/S. Published by Blackwell Publishing Ltd
SN - 1399-3038
AD - Department of Paediatric Pneumology and Immunology, Charite University Medical Centre, Berlin, Germany
UR - 23106446
ER -

TY - JOUR
ID - 363
T1 - Young and free? A study of independent mobility among urban and rural dwelling Australian children
A1 - Carver,A.
A1 - Timperio,A.F.
A1 - Crawford,D.A.
Y1 - 2012/11//
N1 - Carver, Alison. Timperio, Anna F. Crawford, David A
Journal of science and medicine in sport / Sports Medicine Australia
c54, 9812598
IM
Journal Article. Research Support, Non-U.S. Gov't
English
KW - MEDLINE
KW - Adolescent
KW - Adult
KW - Australia/ep [Epidemiology]
KW - Bicycling/sn [Statistics & Numerical Data]
KW - Cellular Phone/sn [Statistics & Numerical Data]
KW - Child
KW - Cross-Sectional Studies
KW - Data Collection/mt [Methods]
KW - Female

KW - Humans
KW - Male
KW - Middle Aged
KW - Personal Autonomy
KW - Rural Population/sn [Statistics & Numerical Data]
KW - Schools/sn [Statistics & Numerical Data]
KW - Sex Factors
KW - Social Environment
KW - Urban Population/sn [Statistics & Numerical Data]
KW - Walking/sn [Statistics & Numerical Data]
RP - NOT IN FILE
SP - 505
EP - 510
JF - Journal of Science & Medicine in Sport
JA - J Sci Med Sport
VL - 15
IS - 6
CY - Australia
N2 - OBJECTIVES: To examine urban/rural differences in children's independent mobility; associations between mobility licences and children's independent mobility; and potential correlates of mobility licences. DESIGN: Cross-sectional study of 430 primary (48% boys; 72% urban) and 258 secondary schoolchildren (52% boys; 51.6% urban) and their parents. METHODS: Parents survey reported the mobility licences they granted to their children (e.g. allowing them to cross main roads on their own), access to outdoor playspaces and mobile phone ownership. Children survey reported their independent mobility on school journeys and on weekends. Differences were examined in mobility licences and independent mobility by sex, urban/rural setting and age-group. Regression analyses examined associations between mobility licences and independent mobility; and how access to playspaces, and mobile phone ownership, were associated with mobility licences. RESULTS: Overall, on average, boys were granted more mobility licences, than were girls, but there were no significant differences by urban/rural location. Variations in independent mobility by urban/rural locality were identified on the school journey but not on weekends. Boys attending urban primary schools had highest rates (44%) of walking/cycling independently to school; those attending rural secondary schools had the lowest (14%). Among urban boys and rural primary school-aged girls access to outdoor playspaces was associated with mobility licences. Mobile phone ownership was associated with mobility licences only among boys attending urban primary schools. CONCLUSIONS: Many Australian children in urban and rural areas lack independent mobility. Further research should examine social/physical environmental influences on parental restrictions, to inform interventions that aim to promote children's independent mobility. Copyright 2012 Sports Medicine Australia. Published by Elsevier Ltd. All rights reserved
SN - 1878-1861
AD - Centre for Physical Activity and Nutrition Research, School of Exercise and Nutrition Sciences, Deakin University, Australia. alison.carver@deakin.edu.au
UR - 22497720
ER -

TY - JOUR
ID - 364
T1 - Levels and indoor-outdoor relationships of size-specific particulate matter in naturally ventilated Portuguese schools
A1 - Madureira,J.
A1 - Paciencia,I.
A1 - Fernandes,Ede O.
Y1 - 2012///
N1 - Madureira, Joana. Paciencia, Ines. Fernandes, Eduardo de Oliveira
Journal of toxicology and environmental health. Part A
ds5, 100960995
IM
Comparative Study. Journal Article. Research Support, Non-U.S. Gov't
English

KW - MEDLINE
KW - Air Pollutants/an [Analysis]
KW - Air Pollution/pc [Prevention & Control]
KW - Air Pollution
KW - Indoor/pc [Prevention & Control]
KW - Indoor
KW - Carbon Dioxide/an [Analysis]
KW - Child
KW - Environmental Monitoring
KW - Humans
KW - Humidity
KW - Particle Size
KW - Particulate Matter/an [Analysis]
KW - Particulate Matter/ch [Chemistry]
KW - Portugal
KW - Schools
KW - Seasons
KW - Temperature
KW - Time Factors
KW - Urban Health
KW - Ventilation
RP - NOT IN FILE
SP - 1423
EP - 1436
JF - Journal of Toxicology & Environmental Health Part A
JA - J Toxicol Environ Health A
VL - 75
IS - 22-23
CY - England
N2 - Indoor exposure to particulate matter (PM) has received great interest due to the epidemiological evidence of its health impact, particularly in susceptible populations such as children. The present study investigated indoor concentrations of three PM size fractions in 11 naturally ventilated schools with static heating systems, and the relationship between indoor and outdoor PM concentrations. The study was performed in Porto, Portugal, during winter and included school buildings and individual classrooms with walk-through surveys, as well as indoor and outdoor air monitoring. Mean 12-h indoor daytime concentrations PM10, PM(2.5), and PM1 were 140, 95, and 91 mug/m, respectively. During the day, PM(2.5) and PM1 concentrations were lower indoors than outdoors (indoor/outdoor ratios of 0.83 and 0.8, respectively), whereas PM10 showed the opposite trend. Concentrations decreased significantly during the night, 49% for PM10 and 27% for PM(2.5) and PM1. These findings reflect the significant contribution from the activities of occupants inside classrooms to higher indoor levels of PM10 levels, whereas the fine fraction of PM(2.5) and PM1 is primarily influenced by outdoor concentrations. This study provides a link between size-specific PM in Portuguese schools with contribution of outdoor versus indoor air. Our results suggest that exposure to PM is high and highlights the need for strategies that provide healthier school environments
SN - 1528-7394
AD - Institute of Mechanical Engineering of Faculty of Engineering of University of Porto, Porto, Portugal. jvm@fe.up.pt
UR - 23095161
ER -

TY - JOUR
ID - 365
T1 - Lipids in Australian children: cause for concern? 2005-2007 Busselton Health Study
A1 - Bell,L.
A1 - Davis,E.
A1 - Knuiman,M.
A1 - Divitini,M.
A1 - Beilby,J.

A1 - Hunter,M.
A1 - Hung,J.
Y1 - 2012/10//
N1 - Bell, Lana. Davis, Elizabeth. Knuiman, Matthew. Divitini, Mark. Beilby, John. Hunter, Michael. Hung, Joseph
Journal of paediatrics and child health
arp, 9005421
IM
Comparative Study. Journal Article. Research Support, Non-U.S. Gov't
English
KW - MEDLINE
KW - Adolescent
KW - Biological Markers/bl [Blood]
KW - Body Mass Index
KW - Child
KW - Cholesterol/bl [Blood]
KW - Cross-Sectional Studies
KW - Dyslipidemias/bl [Blood]
KW - Dyslipidemias/di [Diagnosis]
KW - Dyslipidemias/ep [Epidemiology]
KW - Female
KW - Health Surveys
KW - Humans
KW - Male
KW - New South Wales/ep [Epidemiology]
KW - Triglycerides/bl [Blood]
KW - United States/ep [Epidemiology]
KW - Western Australia/ep [Epidemiology]
RP - NOT IN FILE
SP - E172
EP - E177
JF - Journal of Paediatrics & Child Health
JA - J Paediatr Child Health
VL - 48
IS - 10
CY - Australia
N2 - AIM: To report the current lipid status of Australian school children from a population-based sample and compare this to international and Australian data. METHODS: A cross section of school children aged 6 to 16.9 years in Busselton, Western Australia (WA) between 2005 and 2007 had fasting lipids tested. The first analysis compares the Busselton sample to data recommended by the American Academy of Paediatrics (AAP) 2008. The second analysis compares the Busselton sample to data from the Schools Physical Activity and Nutrition Survey (SPANS) study, New South Wales (NSW), Australia, 2004 and the 1985 Australian Health and Fitness Survey (AHFS). The third analysis applies laboratory-reported cut-points in WA to report percentages over 'healthy desirable norms'. RESULTS: Analysis 1: higher levels of total cholesterol and triglycerides in Busselton children compared to AAP data source. Boys had higher low-density lipoprotein (LDL) levels. Analysis 2: comparable rates of dyslipidaemia to SPANS 2004 but lower rates compared to the AHFS, 1985. Analysis 3: total and LDL-cholesterol above recommended range in 32.7% and 19.4% of boys and 38.2% and 24.6% of girls. CONCLUSION: In a large population-based sample of Australian school children, we found a higher frequency of abnormal lipid profiles when compared to American data. In addition, many children have levels outside reported healthy norms for Australian children. Research tracking lipid profiles of Australian children into adulthood is needed to understand the association of these levels with future cardiovascular risk. 2012 The Authors. Journal of Paediatrics and Child Health 2012 Paediatrics and Child Health Division (Royal Australasian College of Physicians)
SN - 1440-1754
AD - Department of Endocrinology, Princess Margaret Hospital, Australia
UR - 22998088
ER -

TY - JOUR
ID - 366
T1 - Outdoor play among children in relation to neighborhood characteristics: a cross-sectional neighborhood observation study
A1 - Aarts,M.J.
A1 - de Vries,S.I.
A1 - van Oers,H.A.
A1 - Schuit,A.J.
Y1 - 2012///
N1 - Aarts, Marie-Jeanne. de Vries, Sanne I. van Oers, Hans Am. Schuit, Albertine J
The international journal of behavioral nutrition and physical activity
101217089
IM
Journal Article. Research Support, Non-U.S. Gov't
English
KW - MEDLINE
KW - Child
KW - Preschool
KW - Cross-Sectional Studies
KW - Environment Design
KW - Evaluation Studies as Topic
KW - Female
KW - Humans
KW - Male
KW - Motor Activity
KW - Multivariate Analysis
KW - Netherlands
KW - Parents
KW - Play and Playthings
KW - Questionnaires
KW - Residence Characteristics
KW - Safety
RP - NOT IN FILE
SP - 98
JF - International Journal of Behavioral Nutrition & Physical Activity
JA - Int
VL - 9
CY - England
N2 - BACKGROUND: Although environmental characteristics as perceived by parents are known to be related to children's outdoor play behavior, less is known about the relation between independently measured neighborhood characteristics and outdoor play among children. The purpose of this study was to identify quantitative as well as qualitative neighborhood characteristics related to outdoor play by means of neighborhood observations. METHODS: Questionnaires including questions on outdoor play behavior of the child were distributed among 3,651 parents of primary school children (aged 4-12 years). Furthermore, neighborhood observations were conducted in 33 Dutch neighborhoods to map neighborhood characteristics such as buildings, formal outdoor play facilities, public space, street pattern, traffic safety, social neighborhood characteristics, and general impression. Data of the questionnaires and the neighborhood observations were coupled via postal code of the respondents. Multilevel GEE analyses were performed to quantify the correlation between outdoor play and independently measured neighborhood characteristics. RESULTS: Parental education was negatively associated with outdoor play among children. Neither the presence nor the overall quality of formal outdoor play facilities were (positively) related to outdoor play among children in this study. Rather, informal play areas such as the presence of sidewalks were related to children's outdoor play. Also, traffic safety was an important characteristic associated with outdoor play. CONCLUSIONS: This study showed that, apart from individual factors such as parental education level, certain modifiable characteristics in the neighborhood environment (as measured by neighborhood observations) were associated with outdoor play among boys and

girls of different age groups in The Netherlands. Local policy makers from different sectors can use these research findings in creating more activity-friendly neighborhoods for children
SN - 1479-5868
AD - Department Tranzo, Scientific Center for Care and Welfare, Tilburg University, Tilburg, The Netherlands. mj.aarts@maastrichtuniversity.nl
UR - 22901102
ER -

TY - JOUR
ID - 367
T1 - Reference levels in the context of Fukushima - lessons learned and a challenge for the radiation protection system
A1 - Sakai,K.
Y1 - 2012/10//
N1 - Sakai, K
Annals of the ICRP
5a5, 7708044
IM
Journal Article
English
KW - MEDLINE
KW - Adult
KW - Attitude to Health
KW - Child
KW - Fukushima Nuclear Accident
KW - Health Communication
KW - Humans
KW - Japan
KW - Radiation Dosage
KW - Radiation Protection/st [Standards]
KW - Reference Values
KW - Risk Assessment
RP - NOT IN FILE
SP - 282
EP - 285
JF - Annals of the ICRP
JA - Ann ICRP
VL - 41
IS - 3-4
CY - England
N2 - A number of dose criteria were set after the accident in Fukushima, including a criterion regarding the use of school playgrounds in Fukushima. Considering the band of 1-20 mSv/year recommended by the International Commission on Radiological Protection (ICRP) for public exposure under existing exposure situations, Japanese authorities set 20 mSv/year as a 'start line' for reducing the dose to school children. However, this led to considerable confusion among the general public and some experts. They thought that the dose limit was increased to 20 mSv/year (20 times as high as before), and that school children could be exposed to 20 mSv in 1 year. This is just an example of confusion caused by inadequate comprehension of radiation effects, misunderstanding of radiation protection concepts, or both. Another issue was raised regarding the higher radiosensitivity of children compared with adults. In the 2007 ICRP Recommendations, a higher risk coefficient is given to the whole population than the adult population, because the whole population includes children; a subpopulation with higher radiosensitivity and a longer life span. The point of argument was whether a lower reference level should be set for children alone. Radiation protection experts should continue to collect scientific information to improve the radiation protection system. In addition, it is the role of these experts to explain the framework of radiation protection to the general public in plain language. Copyright 2012. Published by Elsevier Ltd
SN - 1872-969X

AD - Research Centre for Radiation Protection, National Institute of Radiological Sciences, 4-9-1 Anagawa, Inage-ku, Chiba 263-8555, Japan. kazsakai@nirs.go.jp
UR - 23089027
ER -

TY - JOUR
ID - 368
T1 - Is he being bad? Social and language brain networks during social judgment in children with autism
A1 - Carter,E.J.
A1 - Williams,D.L.
A1 - Minshew,N.J.
A1 - Lehman,J.F.
Y1 - 2012///
N1 - Carter, Elizabeth J. Williams, Diane L. Minshew, Nancy J. Lehman, Jill F
PloS one
101285081
IM
Journal Article. Research Support, N.I.H., Extramural
English
KW - MEDLINE
KW - Adolescent
KW - Autistic Disorder/pp [Physiopathology]
KW - Brain/pp [Physiopathology]
KW - Brain Mapping
KW - Case-Control Studies
KW - Child
KW - Demography
KW - Eye Movements/ph [Physiology]
KW - Female
KW - Humans
KW - Judgment
KW - Language
KW - Magnetic Resonance Imaging
KW - Male
KW - Nerve Net/pp [Physiopathology]
KW - Social Behavior
RP - NOT IN FILE
SP - e47241
JF - PLoS ONE [Electronic Resource]
JA - PLoS ONE
VL - 7
IS - 10
CY - United States
N2 - Individuals with autism often violate social rules and have lower accuracy in identifying and explaining inappropriate social behavior. Twelve children with autism (AD) and thirteen children with typical development (TD) participated in this fMRI study of the neurofunctional basis of social judgment. Participants indicated in which of two pictures a boy was being bad (Social condition) or which of two pictures was outdoors (Physical condition). In the within-group Social-Physical comparison, TD children used components of mentalizing and language networks [bilateral inferior frontal gyrus (IFG), bilateral medial prefrontal cortex (mPFC), and bilateral posterior superior temporal sulcus (pSTS)], whereas AD children used a network that was primarily right IFG and bilateral pSTS, suggesting reduced use of social and language networks during this social judgment task. A direct group comparison on the Social-Physical contrast showed that the TD group had greater mPFC, bilateral IFG, and left superior temporal pole activity than the AD group. No regions were more active in the AD group than in the group with TD in this comparison. Both groups successfully performed the task, which required minimal language. The groups also performed similarly on eyetracking measures, indicating that the activation results probably reflect the use of a more basic strategy by the autism group rather than performance disparities. Even though language was unnecessary, the children with TD recruited language areas during the

social task, suggesting automatic encoding of their knowledge into language; however, this was not the case for the children with autism. These findings support behavioral research indicating that, whereas children with autism may recognize socially inappropriate behavior, they have difficulty using spoken language to explain why it is inappropriate. The fMRI results indicate that AD children may not automatically use language to encode their social understanding, making expression and generalization of this knowledge more difficult
SN - 1932-6203
AD - Robotics Institute, Carnegie Mellon University, Pittsburgh, Pennsylvania, USA. ejcarter@gmail.com
UR - 23082151
ER -

TY - JOUR
ID - 369
T1 - The amount and value of work time of community medicine distributors in community case management of malaria among children under five years in the Ejisu-Juaben District of Ghana
A1 - gyei-Baffour,P.
A1 - Hansen,K.S.
A1 - Browne,E.N.
A1 - Magnussen,P.
Y1 - 2012///
N1 - Agyei-Baffour, Peter. Hansen, Kristian S. Browne, Edmund N L. Magnussen, Pascal
Malaria journal
101139802
IM
Journal Article. Research Support, Non-U.S. Gov't
English
KW - MEDLINE
KW - Adult
KW - Child
KW - Preschool
KW - Community Health Workers/sn [Statistics & Numerical Data]
KW - Costs and Cost Analysis
KW - Female
KW - Ghana
KW - Health Services Research
KW - Humans
KW - Infant
KW - Longitudinal Studies
KW - Malaria/di [Diagnosis]
KW - Malaria/dt [Drug Therapy]
KW - Male
KW - Middle Aged
KW - Motivation
KW - Rural Population
KW - Time Factors
KW - Workload/sn [Statistics & Numerical Data]
RP - NOT IN FILE
SP - 277
JF - Malaria Journal
JA - Malar.J
VL - 11
CY - England
N2 - BACKGROUND: The contribution of community medicine distributors (CMD) to prompt health service delivery in areas described as "hard-to-reach" is important but the value of their work time remains unknown and thus makes it difficult to design appropriate regular financial incentives to motivate them. This makes CMDs feel their efforts are not recognized. An attempt to estimate the value of 54 CMDs' work time involved in community case management of malaria (CCMm) in a rural district in Ghana is presented. METHODS: Time spent by CMDs on CCMm activities were recorded for a period of 12 months to determine the work-time value.

Cost analysis was performed in Microsoft Excel with data from CMD records and at 2007 market price in Ghana. RESULTS: A CMD spent 4.8 hours, [95% CI: 3.9; 5.3] on all CCMm-related activities per day. The time value of CMD work ranged from GH 2.04 (US$ 2.24) to GH 4.1 [US$ 4.6] per week and GH 19.2 - 86.4 (US$ 21.10-94.95) per month. The gross wage outside CCMm as reported by CMD was GH 58.4 [US$ 64.69] and value of foregone income of GH 86.40 (US$ 94.95) per month, about 14-times higher than the monthly incentives of GH 6.0 given by the CCMm programme. CONCLUSION: The value of work time and the foregone income of CMDs in CCMm are high and yet there are no regular and sustainable incentives provided for them. The results are significant to policy in designing incentives to motivate CMDs in large-scale implementation of CCMm
SN - 1475-2875
AD - Department of Community Health, School of Medical Sciences, College of Health Sciences, Kwame Nkrumah University of Science and Technology, UPO, PMB, KNUST, Kumasi, Ghana. agyeibaffour@yahoo.co.uk
UR - 22898097
ER -

TY - JOUR
ID - 370
T1 - Efficacy and feasibility of lowering playground density to promote physical activity and to discourage sedentary time during recess at preschool: a pilot study
A1 - Van,Cauwenberghe E.
A1 - De,Bourdeaudhuij,I
A1 - Maes,L.
A1 - Cardon,G.
Y1 - 2012/10//
N1 - Van Cauwenberghe, Eveline. De Bourdeaudhuij, Ilse. Maes, Lea. Cardon, Greet
Preventive medicine
pm4, 0322116
IM
Journal Article. Research Support, Non-U.S. Gov't
English
KW - MEDLINE
KW - Accelerometry
KW - Administrative Personnel/px [Psychology]
KW - Belgium
KW - Child
KW - Preschool
KW - Environment Design
KW - Exercise
KW - Feasibility Studies
KW - Humans
KW - Linear Models
KW - Male
KW - Pilot Projects
KW - Play and Playthings
KW - Questionnaires
KW - Recreation/ph [Physiology]
KW - School Health Services
KW - Sedentary Lifestyle
KW - Time Factors
RP - NOT IN FILE
SP - 319
EP - 321
JF - Preventive Medicine
JA - Prev Med
VL - 55
IS - 4

CY - United States
N2 - OBJECTIVE: This pilot study examined if lowering playground density is feasible and effective in improving preschoolers' activity and sedentary levels during recess. METHOD: Between November and December 2011, a within-subject design was used to study preschoolers' activity via accelerometry during recesses in the usual conditions (baseline) and with lower playground density (intervention). During the intervention, preschools scheduled extra recesses so that the number of classes usually sharing the playground was halved. Effects were investigated, using two-level linear regression models, in 128 4- to 6-year-old children (69 boys) from 4 preschools in Ghent, Belgium. The four preschool principals filled in a questionnaire after the intervention. RESULTS: At baseline, available play space was on average 7.4 (SD=1.7) m(2)/child; during the intervention this increased to 16.7 (SD=5.1) m(2)/child. The intervention was effective in decreasing sedentary time (-1 min; -5.1%) and increasing light-to-vigorous (+1 min; +5.1%) and moderate-to-vigorous physical activity (+0.8 min; +4.8%) during recess. None found it difficult to implement the intervention. All agreed it was possible to use this strategy in the future. CONCLUSIONS: Although it was feasible to implement this intervention, improvements in preschoolers' activity levels were small. This intervention could especially be important for preschools with higher playground density. Copyright 2012 Elsevier Inc. All rights reserved
SN - 1096-0260
AD - Department of Movement and Sport Sciences, Ghent University, Watersportlaan 2, Ghent, Belgium. Eveline.VanCauwenberghe@UGent.be
UR - 22846504
ER -

TY - JOUR
ID - 371
T1 - Time outdoors, visual activity, and myopia progression in juvenile-onset myopes
A1 - Jones-Jordan,L.A.
A1 - Sinnott,L.T.
A1 - Cotter,S.A.
A1 - Kleinstein,R.N.
A1 - Manny,R.E.
A1 - Mutti,D.O.
A1 - Twelker,J.D.
A1 - Zadnik,K.
A1 - CLEERE Study Group.
Y1 - 2012/10//
N1 - Jones-Jordan, Lisa A. Sinnott, Loraine T. Cotter, Susan A. Kleinstein, Robert N. Manny, Ruth E. Mutti, Donald O. Twelker, J Daniel. Zadnik, Karla. CLEERE Study Group
Investigative ophthalmology & visual science
gwi, 7703701
IM
Journal Article. Research Support, N.I.H., Extramural. Research Support, Non-U.S. Gov't
English
KW - MEDLINE
KW - Adolescent
KW - Age of Onset
KW - Child
KW - Disease Progression
KW - Female
KW - Health Surveys
KW - Humans
KW - Leisure Activities
KW - Longitudinal Studies
KW - Male
KW - Motor Activity/ph [Physiology]
KW - Myopia/ep [Epidemiology]
KW - Myopia/pp [Physiopathology]
KW - Reading
KW - Risk Factors

KW - Sports/ph [Physiology]
KW - Time Factors
KW - Vision
KW - Ocular/ph [Physiology]
RP - NOT IN FILE
SP - 7169
EP - 7175
JF - Investigative Ophthalmology & Visual Science
JA - Invest Ophthalmol Vis Sci
VL - 53
IS - 11
CY - United States
N2 - PURPOSE: To investigate the association between myopia progression and time spent outdoors and in various visual activities. METHODS: Subjects were 835 myopes (both principal meridians -0.75 diopters [D] or more myopia by cycloplegic autorefraction) in the Collaborative Longitudinal Evaluation of Ethnicity and Refractive Error (CLEERE) Study with both progression data and at least one measure of activity associated with a progression interval. Activity data were collected by parental survey. Average activity level (mean of the activity at the beginning and the end of a 1-year progression interval) was the primary predictor in a repeated-measures mixed model. The model controlled for age, sex, ethnicity, refractive error at the beginning of the progression interval, clinic site, and type of autorefractor used. Effects were scaled based on performing an additional 10 hours per week of an activity. RESULTS: In the multivariate model, the number of hours of reading for pleasure per week was not significantly associated with annual myopia progression at an a priori level of $P < 0.01$, nor were the other near activities, the near-work composite variable diopter-hours, or outdoor/sports activity. The magnitude of effects was clinically small. For example, the largest multivariate effect was that each additional 10 hours of reading for pleasure per week at the end of a progression interval was associated with an increase in average annual progression by -0.08 D. CONCLUSIONS: Despite protective associations previously reported for time outdoors reducing the risk of myopia onset, outdoor/sports activity was not associated with less myopia progression following onset. Near work also had little meaningful effect on the rate of myopia progression
SN - 1552-5783
AD - The Ohio State University College of Optometry, Columbus, Ohio 43210, USA.
ljordan@optometry.osu.edu
UR - 22977132
ER -

TY - JOUR
ID - 372
T1 - Miranos! Look at us, we are healthy! An environmental approach to early childhood obesity prevention
A1 - Yin,Z.
A1 - Parra-Medina,D.
A1 - Cordova,A.
A1 - He,M.
A1 - Trummer,V.
A1 - Sosa,E.
A1 - Gallion,K.J.
A1 - Sintes-Yallen,A.
A1 - Huang,Y.
A1 - Wu,X.
A1 - Acosta,D.
A1 - Kibbe,D.
A1 - Ramirez,A.
Y1 - 2012/10//
N1 - Yin, Zenong. Parra-Medina, Deborah. Cordova, Alberto. He, Meizi. Trummer, Virginia. Sosa, Erica. Gallion, Kipling J. Sintes-Yallen, Amanda. Huang, Yaling. Wu, Xuelian. Acosta, Desiree. Kibbe, Debra. Ramirez, Amelie
Childhood obesity (Print)
101542497

IM
Journal Article. Research Support, Non-U.S. Gov't
English
KW - MEDLINE
KW - Body Mass Index
KW - Chi-Square Distribution
KW - Child
KW - Preschool
KW - Cultural Competency
KW - Faculty
KW - Female
KW - Food Habits
KW - Health Promotion/mt [Methods]
KW - Humans
KW - Male
KW - Mexican Americans/eh [Ethnology]
KW - Mexican Americans
KW - Motor Activity/ph [Physiology]
KW - Obesity/eh [Ethnology]
KW - Obesity/pc [Prevention & Control]
KW - Parents/ed [Education]
KW - Poverty/eh [Ethnology]
KW - Program Evaluation
KW - Texas/eh [Ethnology]
KW - Weight Loss
RP - NOT IN FILE
SP - 429
EP - 439
JF - Childhood Obesity
JA - Child
VL - 8
IS - 5
CY - United States
N2 - BACKGROUND: Obesity prevention research is sparse in young children at risk for obesity. This study tested the effectiveness of a culturally tailored, multicomponent prevention intervention to promote healthy weight gain and gross motor development in low-income preschool age children. METHODS: Study participants were predominantly Mexican-American children (n = 423; mean age = 4.1; 62% in normal weight range) enrolled in Head Start. The study was conducted using a quasi-experimental pretest/posttest design with two treatment groups and a comparison group. A center-based intervention included an age-appropriate gross motor program with structured outdoor play, supplemental classroom activities, and staff development. A combined center- and home-based intervention added peer-led parent education to create a broad supportive environment in the center and at home. Primary outcomes were weight-based z-scores and raw scores of gross motor skills of the Learning Achievement Profile Version 3. RESULTS: Favorable changes occurred in z-scores for weight (one-tailed $p < 0.04$) for age and gender among children in the combined center- and home-based intervention compared to comparison children at posttest. Higher gains of gross motor skills were found in children in the combined center- and home-based ($p < 0.001$) and the center-based intervention ($p < 0.01$). Children in both intervention groups showed increases in outdoor physical activity and consumption of healthy food. Process evaluation data showed high levels of protocol implementation fidelity and program participation of children, Head Start staff, and parents. CONCLUSION: The study demonstrated great promise in creating a health-conducive environment that positively impacts weight and gross motor skill development in children at risk for obesity. Program efficacy should be tested in a randomized trial
SN - 2153-2176
AD - Department of Health and Kinesiology, The University of Texas at San Antonio, USA.
zenong.yin@utsa.edu
UR - 23061498
ER -

TY - JOUR
ID - 373
T1 - The incidence of accidents in child care centers of the local government organizations
A1 - Intraratsamee,J.
A1 - Ratanasiri,A.
A1 - Kanato,M.
Y1 - 2012/08//
N1 - Intraratsamee, Jariya. Ratanasiri, Amornrat. Kanato, Manop
Journal of the Medical Association of Thailand = Chotmaihet thangphaet
izr, 7507216
IM
Journal Article. Research Support, Non-U.S. Gov't
English
KW - MEDLINE
KW - Accidents/sn [Statistics & Numerical Data]
KW - Child
KW - Child Day Care Centers
KW - Female
KW - Humans
KW - Incidence
KW - Male
KW - Safety
KW - Thailand/ep [Epidemiology]
KW - Wounds and Injuries/ep [Epidemiology]
RP - NOT IN FILE
SP - 1098
EP - 1104
JF - Journal of the Medical Association of Thailand
JA - J Med Assoc Thai
VL - 95
IS - 8
CY - Thailand
N2 - OBJECTIVE: To study the incidence of accidents and the environment around the building of child care centers of the local government organizations under the Health Promoting Center 6. MATERIAL AND METHOD: This was a descriptive study carried out among 1,871 child care centers of the local government organizations under the Health Promoting Center 6 within eight Northeastern provinces. The self administered questionnaires were handed out in the process of this study and data were collected between January 1 and February 28, 2011. RESULTS: One thousand eight hundred seventy one child care centers answered the questionnaires. The ratio of children per caregiver was found to be 17:1. In 2010 the incidence of injury in children was 7.17 per 100 persons. Ratio of injury between boys and girls was 1.9:1. The places mostly reported for the accidents were outside the building, the playground instruments, and inside the classrooms. Most child care centers were located on the street side and had no fence near the water sources (pool, pond) and trees. Only 22.9% had children water closets and those with adult water closet provided no support for holding. Most (93.3%) of the toilets had water containers inside. CONCLUSION: Environment around the child care center building may be the cause of many accidents. If the child care centers comply the convention instructions, children will be safe during they stay
SN - 0125-2208
AD - Department of Community Medicine, Faculty of Medicine, Khon Kaen University, Khon Kaen, Thailand
UR - 23061316
ER -

TY - JOUR
ID - 374
T1 - Representative contact diaries for modeling the spread of infectious diseases in Taiwan
A1 - Fu,Y.C.
A1 - Wang,D.W.
A1 - Chuang,J.H.

Y1 - 2012///
N1 - Fu, Yang-chih. Wang, Da-Wei. Chuang, Jen-Hsiang
PloS one
101285081
IM
Journal Article. Research Support, Non-U.S. Gov't
English
KW - MEDLINE
KW - Adolescent
KW - Adult
KW - Age Factors
KW - Aged
KW - 80 and over
KW - Child
KW - Preschool
KW - Communicable Diseases/ep [Epidemiology]
KW - Communicable Diseases/tm [Transmission]
KW - Computer Simulation
KW - Health Surveys/mt [Methods]
KW - Health Surveys/sn [Statistics & Numerical Data]
KW - Humans
KW - Infant
KW - Newborn
KW - Middle Aged
KW - Models
KW - Biological
KW - Rural Population/sn [Statistics & Numerical Data]
KW - Social Behavior
KW - Taiwan/ep [Epidemiology]
KW - Time Factors
KW - Urban Population/sn [Statistics & Numerical Data]
KW - Young Adult
RP - NOT IN FILE
SP - e45113
JF - PLoS ONE [Electronic Resource]
JA - PLoS ONE
VL - 7
IS - 10
CY - United States
N2 - Recent studies of infectious diseases have attempted to construct more realistic parameters of interpersonal contact patterns from diary-approach surveys. To ensure that such diary-based contact patterns provide accurate baseline data for policy implementation in densely populated Taiwan, we collected contact diaries from a national sample, using 3-stage systematic probability sampling and rigorous in-person interviews. A representative sample of 1,943 contact diaries recorded a total of 24,265 wide-range, face-to-face interpersonal contacts during a 24-hour period. Nearly 70% of the contacts occurred outside of respondents' households. The most active age group was schoolchildren (ages 5-14), who averaged around 16-18 daily contacts, about 2-3 times as many as the least active age groups. We show how such parameters of contact patterns help modify a sophisticated national simulation system that has been used for years to model the spread of pandemic diseases in Taiwan. Based on such actual and representative data that enable researchers to infer findings to the whole population, our analyses aim to facilitate implementing more appropriate and effective strategies for controlling an emerging or pandemic disease infection
SN - 1932-6203
AD - Institute of Sociology, Academia Sinica, Taipei, Taiwan, Republic of China. fuyc@sinica.edu.tw
UR - 23056193
ER -

TY - JOUR

ID - 375
T1 - The association between time spent outdoors and myopia in children and adolescents: a systematic review and meta-analysis. [Review]
A1 - Sherwin,J.C.
A1 - Reacher,M.H.
A1 - Keogh,R.H.
A1 - Khawaja,A.P.
A1 - Mackey,D.A.
A1 - Foster,P.J.
Y1 - 2012/10//
N1 - Sherwin, Justin C. Reacher, Mark H. Keogh, Ruth H. Khawaja, Anthony P. Mackey, David A. Foster, Paul J
Ophthalmology
7802443
IM
Journal Article. Meta-Analysis. Research Support, Non-U.S. Gov't. Review
English
KW - MEDLINE
KW - Adolescent
KW - Child
KW - Cross-Sectional Studies
KW - Disease Progression
KW - Environment
KW - Exercise
KW - Humans
KW - Incidence
KW - Leisure Activities
KW - Myopia/ep [Epidemiology]
KW - Myopia/pc [Prevention & Control]
KW - Odds Ratio
KW - Prevalence
KW - Prospective Studies
KW - Time Factors
KW - Young Adult
RP - NOT IN FILE
SP - 2141
EP - 2151
JF - Ophthalmology
VL - 119
IS - 10
CY - United States
N2 - OBJECTIVE: To summarize relevant evidence investigating the association between time spent outdoors and myopia in children and adolescents (up to 20 years). DESIGN: Systematic review and meta-analysis. PARTICIPANTS: Results from 7 cross-sectional studies were pooled in a meta-analysis. A further 16 studies (8 cross-sectional not meeting criteria for meta-analysis; 7 prospective cohort studies; 1 randomized, controlled trial [RCT]) were reported in the systematic review. METHODS: The literature search included 4 databases (Medline, Embase, Web of Science, and Cochrane Central Register of Controlled Trials [CENTRAL]), and reference lists of retrieved studies. Estimates of association were pooled using random effects meta-analysis. We summarized data examining the association between time spent outdoors and prevalent myopia, incident myopia, and myopic progression. MAIN OUTCOME MEASURES: Pooled odds ratios (ORs) and 95% confidence intervals (CIs) for myopia for each additional hour spent outdoors per week from a meta-analysis. RESULTS: The pooled OR for myopia indicated a 2% reduced odds of myopia per additional hour of time spent outdoors per week, after adjustment for covariates (OR, 0.981; 95% CI, 0.973-0.990; P<0.001; I(2), 44.3%). This is equivalent to an OR of 0.87 for an additional hour of time spent outdoors each day. Three prospective cohort studies provided estimates of risk of incident myopia according to time spent outdoors, adjusted for possible confounders, although estimates could not be pooled, and the quality of studies and length of follow-up times varied. Three studies (2 prospective cohort and 1 RCT) investigated time spent outdoors and myopic

progression and found increasing time spent outdoors significantly reduced myopic progression. CONCLUSIONS: The overall findings indicate that increasing time spent outdoors may be a simple strategy by which to reduce the risk of developing myopia and its progression in children and adolescents. Therefore, further RCTs are warranted to investigate the efficacy of increasing time outdoors as a possible intervention to prevent myopia and its progression. Copyright 2012 American Academy of Ophthalmology. Published by Elsevier Inc. All rights reserved
SN - 1549-4713
AD - Department of Public Health and Primary Care, University of Cambridge, Cambridge, UK. justincsherwin@gmail.com
UR - 22809757
ER -

TY - JOUR
ID - 376
T1 - Epileptic high-frequency oscillations in intraoperative electrocorticography: the effect of propofol
A1 - Zijlmans,M.
A1 - Huiskamp,G.M.
A1 - Cremer,O.L.
A1 - Ferrier,C.H.
A1 - van Huffelen,A.C.
A1 - Leijten,F.S.
Y1 - 2012/10//
N1 - Zijlmans, Maeike. Huiskamp, Geertjan M. Cremer, Olaf L. Ferrier, Cyrille H. van Huffelen, Alexander C. Leijten, Frans S S
Epilepsia
eix, 2983306r
IM
Journal Article. Research Support, Non-U.S. Gov't
English
KW - MEDLINE
KW - Adolescent
KW - Adult
KW - Anesthetics
KW - Intravenous/du [Diagnostic Use]
KW - Intravenous/pd [Pharmacology]
KW - Brain Mapping
KW - Brain Waves/de [Drug Effects]
KW - Child
KW - Electroencephalography
KW - Epilepsy/pp [Physiopathology]
KW - Epilepsy/su [Surgery]
KW - Female
KW - Humans
KW - Intraoperative Period
KW - Male
KW - Middle Aged
KW - Propofol/du [Diagnostic Use]
KW - Propofol/pd [Pharmacology]
KW - Young Adult
RP - NOT IN FILE
SP - 1799
EP - 1809
JF - Epilepsia
VL - 53
IS - 10
CY - United States

N2 - PURPOSE: Epileptic high-frequency oscillations (HFOs; 80-500 Hz) may be used to guide neurosurgeons during epilepsy surgery to identify epileptogenic tissue. We studied the effect of the anesthetic agent propofol on the occurrence of HFOs in intraoperative electrocorticography (ECoG). METHODS: We selected patients who were undergoing surgery for temporal lobe epilepsy with a standardized electrode grid placement. Intraoperative ECoG was recorded at 2,048 Hz following cessation of propofol. The number and distribution of interictal spikes, ripples (R [80-250 Hz]), and fast ripples (FRs; 250-500 Hz) were analyzed. The amount of events on mesiotemporal channels and lateral neocortical channels were compared between patients with a suspected mesiotemporal and lateral epileptogenic area (Student's t-test), and HFOs were compared with the irritative zone, using correlation between amounts of events per channel, to provide evidence for the epileptic nature of the HFOs. Next, the amount of events within the first minute and the last minute were compared to each other and the change in events over the entire epochs was analyzed using correlation analyses of 10 epochs during the emergence periods (Spearman rank test). We studied whether the duration of HFOs changed over time. The change in events within presumed epileptogenic area was compared to the change outside this area (Student's t-test). Periods of burst suppression and continuous background activity were compared between and within patients (t-test). KEY FINDINGS: Twelve patients were included: five with suspected mesiotemporal epileptogenic area and three with suspected lateral epileptogenic area (and four were "other"). Spikes, ripples, and FRs were related to the suspected epileptogenic areas, and HFO zones were related to the irritative zones. Ripples and FRs increased during emergence from propofol anesthesia (mean number of ripples from first minute-last minute: 61.5-73.0, $R = 0.46$, $p < 0.01$; FRs: 3.1-5.7, $R = 0.30$, $p < 0.01$) and spikes remained unchanged (80.1-79.9, $R = -0.05$, $p = 0.59$). There was a decrease in number of channels with spikes ($R = -0.18$, $p = 0.05$), but no change in ripples ($R = -0.13$, $p = 0.16$) or FRs ($R = 0.11$, $p = 0.45$). There was no change in the durations of HFOs. The amount of HFOs in the presumed epileptogenic areas did not change more than the amount outside the presumed epileptogenic area, whereas spikes paradoxically decreased more within the suspected epileptogenic area. Six patients showing burst-suppression had lower rates of ripples than six other patients with continuous background activity ($p = 0.02$). No significant difference was found between burst suppression and continuous background activity in four patients, but there was a trend toward showing more ripples during continuous background activity ($p = 0.16$). SIGNIFICANCE: Propofol, known for its antiepileptic effects, reduces the number of epileptic HFOs, but has no effect on spikes. This enforces the hypothesis that, in epilepsy, HFOs mirror the disease activity and HFOs might be useful for monitoring antiepileptic drug treatment. It is feasible to record HFOs during surgery, but propofol infusion should be interrupted for some minutes to improve detection. Wiley Periodicals, Inc. 2012 International League Against Epilepsy
SN - 1528-1167
AD - Department of Neurology, Rudolf Magnus Institute, University Medical Center Utrecht, Utrecht, The Netherlands. g.j.m.zijlmans@umcutrecht.nl
UR - 22984839
ER -

TY - JOUR
ID - 377
T1 - Frequency of parent-supervised outdoor play of US preschool-aged children
A1 - Tandon,P.S.
A1 - Zhou,C.
A1 - Christakis,D.A.
Y1 - 2012/08//
N1 - Tandon, Pooja S. Zhou, Chuan. Christakis, Dimitri A
Archives of pediatrics & adolescent medicine
9422751, bwf
AIM, IM
Journal Article. Research Support, Non-U.S. Gov't
English
KW - MEDLINE
KW - Child
KW - Preschool
KW - Cross-Sectional Studies
KW - Exercise
KW - Female

KW - Humans
KW - Longitudinal Studies
KW - Male
KW - Parents
KW - Play and Playthings
KW - Residence Characteristics
KW - Socioeconomic Factors
KW - United States
RP - NOT IN FILE
SP - 707
EP - 712
JF - Archives of Pediatrics & Adolescent Medicine
JA - Arch Pediatr Adolesc Med
VL - 166
IS - 8
CY - United States
N2 - OBJECTIVE: To characterize preschoolers' daily parent-supervised outdoor play frequency and associated factors. DESIGN: Cross-sectional using data from the Early Childhood Longitudinal Study-Birth Cohort. SETTING: Nationally representative US sample. PARTICIPANTS: Preschool-aged children. MAIN OUTCOME MEASURE: Parent-reported outdoor play frequency. RESULTS: The sample size of 8950 represented approximately 4 million children. Sixty percent of mothers worked outside the home, 79% exercised 0 to 3 days per week, and 93% perceived their neighborhood to be safe. Forty-four percent of mothers and 24% of fathers reported taking their child outside to play at least once per day. Fifty-one percent of children were reported to go outside to play at least once per day with either parent. Fifty-eight percent of children who were not in child care went outside daily. A child's odds of going outside daily were associated with sex (odds ratio [OR] for girls, 0.85;95% CI, 0.75-0.95), having more regular playmates (OR for > 3 playmates, 2.03; 95% CI, 1.72-2.38), mother's race/ethnicity (OR for Asian, 0.51, 95% CI, 0.43-0.61; black,0.59, 95% CI, 0.49-0.70; Hispanic, 0.80, 95% CI, 0.67-0.95), mother's employment (OR for full time, 0.70; 95%CI, 0.62-0.81), and parent's exercise frequency of 4 days or more per week (OR, 1.50; 95% CI, 1.28-1.75). We did not find significant association of outdoor play with child's time spent watching television, household income, mother's marital status, or parent's perceptions of neighborhood safety. CONCLUSIONS: About half the preschoolers in this sample did not have even 1 parent-supervised outdoor play opportunity per day. Efforts to increase active outdoor play should especially target children who are girls and nonwhite. Outdoor play opportunities at child care are critical for children of parents who work outside the home
SN - 1538-3628
AD - Center for Child Health, Behavior, and Development, Seattle, WA 98145, USA. pooja@uw.edu
UR - 22473885
ER -

TY - JOUR
ID - 378
T1 - Factors associated with Leishmania asymptomatic infection: results from a cross-sectional survey in highland northern Ethiopia
A1 - Custodio,E.
A1 - Gadisa,E.
A1 - Sordo,L.
A1 - Cruz,I.
A1 - Moreno,J.
A1 - Nieto,J.
A1 - Chicharro,C.
A1 - Aseffa,A.
A1 - Abraham,Z.
A1 - Hailu,T.
A1 - Canavate,C.
Y1 - 2012///
N1 - Custodio, Estefania. Gadisa, Endalamaw. Sordo, Luis. Cruz, Israel. Moreno, Javier. Nieto, Javier. Chicharro, Carmen. Aseffa, Abraham. Abraham, Zelalem. Hailu, Tsegaye. Canavate, Carmen

PLoS neglected tropical diseases
101291488
IM
Journal Article. Research Support, Non-U.S. Gov't
English
KW - MEDLINE
KW - Adolescent
KW - Animals
KW - Antibodies
KW - Protozoan/bl [Blood]
KW - Asymptomatic Infections
KW - Child
KW - Preschool
KW - Cross-Sectional Studies
KW - Ethiopia/ep [Epidemiology]
KW - Female
KW - Humans
KW - Leishmania/im [Immunology]
KW - Leishmaniasis/ep [Epidemiology]
KW - Leishmaniasis/ps [Parasitology]
KW - Male
KW - Questionnaires
KW - Risk Factors
RP - NOT IN FILE
SP - e1813
JF - PLoS Neglected Tropical Diseases [electronic resource]
JA - PLoS Negl Trop Dis
VL - 6
IS - 9
CY - United States
N2 - BACKGROUND: In northern Ethiopia the prevalence of visceral leishmaniasis is steadily rising posing an increasing public health concern. In order to develop effective control strategies on the transmission of the disease it is important to generate knowledge on the epidemiological determinants of the infection. METHODOLOGY/PRINCIPAL FINDINGS: We conducted a cross-sectional survey on children 4-15 years of age using a multi staged stratified cluster sampling on high incidence sub-districts of Amhara regional state, Ethiopia. The survey included a socio-demographic, health and dietary questionnaire, and anthropometric measurements. We performed rK39-ICT and DAT serological tests in order to detect anti-Leishmania antibodies and carried out Leishmanin Skin Test (LST) using L.major antigen. Logistic regression models were used. Of the 565 children surveyed 56 children were positive to infection (9.9%). The individual variables that showed a positive association with infection were increasing age, being male and sleeping outside [adjusted odds ratios (95% CI): 1.15 (1.03, 1.29), 2.56 (1.19, 5.48) and 2.21 (1.03, 4.71) respectively] and in relation to the household: past history of VL in the family, living in a straw roofed house and if the family owned sheep [adjusted OR (95% CI): 2.92 (1.25, 6.81), 2.71 (1.21, 6.07) and 4.16 (1.41, 12.31) respectively]. CONCLUSIONS/SIGNIFICANCE: A behavioural pattern like sleeping outside is determinant in the transmission of the infection in this area. Protective measures should be implemented against this identified risk activity. Results also suggest a geographical clustering and a household focalization of the infection. The behaviour of the vector in the area needs to be clarified in order to establish the role of domestic animals and house materials in the transmission of the infection
SN - 1935-2735
AD - Centro Nacional de Medicina Tropical, Instituto de Salud Carlos III, Madrid, Spain. ecustodio@isciii.es
UR - 23029576
ER -

TY - JOUR
ID - 379
T1 - School and individual-level characteristics are associated with children's moderate to vigorous-intensity physical activity during school recess

A1 - Martin,K.
A1 - Bremner,A.
A1 - Salmon,J.
A1 - Rosenberg,M.
A1 - Giles-Corti,B.
Y1 - 2012/10//
N1 - Martin, Karen. Bremner, Alexandra. Salmon, Jo. Rosenberg, Michael. Giles-Corti, Billie
Australian and New Zealand journal of public health
ck2, 9611095
IM
Journal Article. Research Support, Non-U.S. Gov't
English
KW - MEDLINE
KW - Accelerometry
KW - Body Mass Index
KW - Child
KW - Exercise
KW - Female
KW - Health Behavior
KW - Health Promotion/mt [Methods]
KW - Humans
KW - Leisure Activities
KW - Male
KW - Motor Activity
KW - Play and Playthings
KW - Qualitative Research
KW - Regression Analysis
KW - Schools
KW - Social Environment
KW - Socioeconomic Factors
KW - Urban Population
KW - Western Australia
RP - NOT IN FILE
SP - 469
EP - 477
JF - Australian & New Zealand Journal of Public Health
JA - Aust N Z J Public Health
VL - 36
IS - 5
CY - Australia
N2 - OBJECTIVE: The objective of this study was to identify school environmental characteristics associated with moderate to vigorous physical activity during school recess, including morning and lunch breaks. METHODS: Accelerometry data, child-level characteristics and school physical activity, policy and socio-cultural data were collected from 408 sixth grade children (mean age 11 years) attending 27 metropolitan primary schools in Perth, Western Australia. Hierarchical modelling identified key characteristics associated with children's recess moderate to vigorous physical activity (RMVPA). RESULTS: Nearly 40% of variability in children's RMVPA was explained by school environment and individual characteristics identified in this study. Children's higher daily RMVPA was associated with newer schools, schools with a higher number of grassed surfaces per child and fewer shaded grassed surfaces, and the physical education coordinator meeting Australian physical activity guidelines. CONCLUSIONS: Characteristics of the school physical and social environments are strongly correlated with children's MPVA during recess. IMPLICATIONS: The school environment is an ideal target for maximising children's physical activity during recess. Future research could examine the impact of modifying these environmental characteristics on children's school physical activity. 2012 The Authors. ANZJPH 2012 Public Health Association of Australia
SN - 1753-6405
AD - School of Population Health, University of Western Australia, Crawley, WA, Australia. karen.martin@uwa.edu.au

UR - 23025370
ER -

TY - JOUR
ID - 380
T1 - [Development of a didactic questionnaire to measure vegetable and fruit intake during the school recess. Pilot test]. [Spanish]
A1 - Medina,C.
A1 - Villanueva-Borbolla,M.
A1 - Barquera,S.
Y1 - 2012/10//
N1 - Medina, Catalina. Villanueva-Borbolla, Montserrat. Barquera, Simon
Salud publica de Mexico
0404371, uy1
IM
English Abstract. Journal Article
Spanish
KW - MEDLINE
KW - Child
KW - Female
KW - Food Habits
KW - Fruit
KW - Government Programs
KW - Humans
KW - Male
KW - Mexico
KW - Nutritional Sciences/ed [Education]
KW - Pilot Projects
KW - Questionnaires
KW - Sampling Studies
KW - School Health Services/og [Organization & Administration]
KW - Schools
KW - Self Report
KW - Vegetables
RP - NOT IN FILE
SP - 479
EP - 486
JF - Salud Publica de Mexico
JA - Salud Publica Mex
VL - 54
IS - 5
CY - Mexico
N2 - OBJECTIVE: To develop an open, self-reported questionnaire, focused in understanding intake and intention to increase vegetable and fruit consumption during the Mexican children's lunchtime. MATERIALS AND METHODS: After assessing pilot tests, the self-reported questionnaire was applied as a classroom exercise to measure children's consumptions of fruit and vegetables. RESULTS: A total of 486 children completed the questionnaire (49.8% males and 50.2% females). The reported food consumption indicates that 24.5% of children do not consume vegetables or fruits, and among consumers the most common portion size is one. Girls consume more vegetables and fruits and they can recognize more benefits. A total of 9.7% of the participants that do not consume VF, expressed less intention of including vegetables or fruits in their lunch. These results were similar to those reported by other authors. CONCLUSION: This study is useful to identify strengths and limitations during the design and implementation of this type of instrument to understand children's food habits during school recess
SN - 1606-7916
AD - Instituto Nacional de Salud Publica, Cuernavaca, Morelos, Mexico
UR - 23011499
ER -

TY - JOUR
ID - 381
T1 - High prevalence of vitamin D insufficiency or deficiency in young adolescents in Korea
A1 - Shin,Y.H.
A1 - Kim,K.E.
A1 - Lee,C.
A1 - Shin,H.J.
A1 - Kang,M.S.
A1 - Lee,H.R.
A1 - Lee,Y.J.
Y1 - 2012/10//
N1 - Shin, Youn Ho. Kim, Ki Eun. Lee, Choae. Shin, Hye Jung. Kang, Myung Suh. Lee, Hye-Ree. Lee, Yong-Jae
European journal of pediatrics
end, 7603873
IM
Journal Article
English
KW - MEDLINE
KW - Adolescent
KW - Body Mass Index
KW - Child
KW - Cross-Sectional Studies
KW - Female
KW - Humans
KW - Insulin Resistance
KW - Linear Models
KW - Lipids/bl [Blood]
KW - Male
KW - Prevalence
KW - Republic of Korea/ep [Epidemiology]
KW - Sex Distribution
KW - Vitamin D/bl [Blood]
KW - Vitamin D Deficiency/di [Diagnosis]
KW - Vitamin D Deficiency/ep [Epidemiology]
RP - NOT IN FILE
SP - 1475
EP - 1480
JF - European Journal of Pediatrics
JA - Eur J Pediatr
VL - 171
IS - 10
CY - Germany
N2 - Recent studies suggest that vitamin D insufficiency or deficiency is not only a problem of older generations anymore but also an important health concern among younger generations. However, comprehensive data are lacking in Korean adolescents. We investigated the vitamin D (25-hydroxy vitamin D [25(OH)D]) status, the prevalence of vitamin D insufficiency or deficiency, and the association between vitamin D levels and insulin resistance and lipid profiles in a sample of 188 Korean adolescents aged 12-13 years who participated in a general health check-up at a tertiary hospital. Vitamin D deficiency was considered as serum concentrations <20 ng/mL (50 nmol/L); a level of 21-29 ng/mL (52-72 nmol/L) was considered to indicate vitamin D insufficiency, whereas a level of 30 ng/mL or greater (>75 nmol/L) was considered sufficient or optimum. In this cross-sectional study, vitamin D insufficiency or deficiency was found in 98.9 % of boys and 100 % of girls, whereas only 1.1 % of boys and 0 % of girls had a serum 25(OH)D level of greater than 30 ng/mL. In multivariate linear regression analysis, HOMA-IR, triglyceride, and LDL cholesterol were inversely associated with 25(OH)D concentrations. We found that vitamin D insufficiency or deficiency is a very common health problem in Korean adolescents, particularly in girls, and that serum 25(OH)D levels are

inversely associated with insulin resistance and lipid profiles. These results suggest that more time spent in outdoor activity for sunlight exposure and higher vitamin D intake may be needed in younger adolescents in South Korea
SN - 1432-1076
AD - Department of Pediatrics, CHA University School of Medicine, Seoul, South Korea
UR - 22562162
ER -

TY - JOUR
ID - 382
T1 - Identity of the fungal species present in the homes of asthmatic children
A1 - Meng,J.
A1 - Barnes,C.S.
A1 - Rosenwasser,L.J.
A1 - Children's Mercy Center for Environmental Health.
Y1 - 2012/10//
N1 - Meng, J. Barnes, C S. Rosenwasser, L J. Children's Mercy Center for Environmental Health
Clinical and experimental allergy : journal of the British Society for Allergy and Clinical Immunology
ceb, 8906443
IM
Journal Article. Research Support, Non-U.S. Gov't
English
KW - MEDLINE
KW - Adolescent
KW - Air Microbiology
KW - Air Pollution
KW - Indoor
KW - Allergens
KW - Asthma/im [Immunology]
KW - Child
KW - Preschool
KW - Colony Count
KW - Microbial
KW - Culture Media
KW - Environmental Monitoring
KW - Female
KW - Fungi/cl [Classification]
KW - Fungi/im [Immunology]
KW - Fungi/ip [Isolation & Purification]
KW - Housing
KW - Humans
KW - Male
KW - Species Specificity
KW - Spores
KW - Fungal/ip [Isolation & Purification]
RP - NOT IN FILE
SP - 1448
EP - 1458
JF - Clinical & Experimental Allergy
JA - Clin Exp Allergy
VL - 42
IS - 10
CY - England
N2 - BACKGROUND: Fungal exposures are believed to play an important role in the development of asthma and atopy, accounting for increased asthmatic symptoms and severe asthma exacerbation. Indoor fungal species vary both in taxa and concentration in different residences and in different regions. OBJECTIVES: We explored the fungal species spectrum in 88 homes with at least one asthmatic child in the Middle West region of the

United States mostly during late spring and fall season in comparison with 85 homes that did not contain an asthmatic child during flu season. METHODS: The average fungal spore counts per cubic metre of air in the bedroom of the enrolled child, the main living spaces and outdoor environments, and the culturable fungal colony-forming units per cubic metre of air samples in the main living space from each home were measured. RESULTS: The results indicated that Cladosporium, Penicillium, Aspergillus, Basidiospores, Epicoccum and Pithomyces were found in more asthmatic homes than in homes without an asthmatic child or existed in higher concentration in asthmatic homes than in homes without an asthmatic child even after adjusting outdoor spore concentration. The results for culturable fungal species confirmed most of these findings even after adjusting for seasonal factors. Although Alternaria was commonly found in both kinds of homes, there was no significant difference in detection rate or concentration of Alternaria between asthmatic homes and homes without an asthmatic child by either spore counting or culturable airborne detection. CONCLUSION AND CLINICAL RELEVANCE: Since many allergens have been identified in these fungal species, identifying and controlling these fungal species in asthmatic homes might be expected to improve asthma care and benefit asthmatic children. 2012 Blackwell Publishing Ltd
SN - 1365-2222
AD - Department of Pediatric Immunology Research, The Children's Mercy Hospital, Kansas City, MO 64108, USA. jmeng@cmh.edu
UR - 22994342
ER -

TY - JOUR
ID - 383
T1 - Benefits and environmental determinants of physical activity in children and adolescents. [Review]
A1 - Loprinzi,P.D.
A1 - Cardinal,B.J.
A1 - Loprinzi,K.L.
A1 - Lee,H.
Y1 - 2012///
N1 - Loprinzi, Paul D. Cardinal, Bradley J. Loprinzi, Kristina L. Lee, Hyo
Obesity facts
101469429
IM
Journal Article. Review
English
KW - MEDLINE
KW - Adiposity
KW - Adolescent
KW - Bone Density
KW - Child
KW - Environment
KW - Exercise
KW - Guidelines as Topic
KW - Health
KW - Health Behavior
KW - Health Promotion
KW - Humans
KW - Motor Skills
KW - Physical Fitness
KW - Sedentary Lifestyle
KW - Social Environment
RP - NOT IN FILE
SP - 597
EP - 610
JF - Obesity Facts
JA - Obes Facts
VL - 5
IS - 4

CY - Switzerland
N2 - In this review, we identify the health benefits associated with physical activity (PA); address the physical activity and sedentary guidelines issued by public health scientists as well as children's compliance to these guidelines; discuss the importance of motor skill acquisition during early childhood; and identify different settings that contribute to physical activity participation and strategies for improving PA in these settings. Results show that regular participation in PA during childhood has numerous immediate benefits, including positive changes in adiposity, skeletal health, psychological health, and cardiorespiratory fitness. Additionally, motor skill development during early childhood may have immediate health benefits as well as long-lasting effects in adulthood. Furthermore, the benefits of PA during childhood also appear to positively influence adult health outcomes, such as increased bone mineral density. Key environmental settings that have been shown to influence children's PA behavior include child care, active commuting to and from school, school recess, school physical education, after-school programs, churches, medical settings, and the home environment. Recommendations for practitioners and researchers are discussed. Copyright 2012 S. Karger AG, Basel
SN - 1662-4033
AD - Lansing School of Nursing and Health Sciences, Department of Exercise Science, Bellarmine University, Louisville, KY, USA. loprinzp@onid.orst.edu
UR - 22986648
ER -

TY - JOUR
ID - 384
T1 - Knowledge, attitude and practice about rabies among children receiving formal and informal education in Samaru, Zaria, Nigeria
A1 - Dzikwi,A.A.
A1 - Ibrahim,A.S.
A1 - Umoh,J.U.
Y1 - 2012/09//
N1 - Dzikwi, Asabe Adamu. Ibrahim, Ayuba Sini. Umoh, Jarlath Udoudo
Global journal of health science
101519495
IM
Journal Article. Randomized Controlled Trial
English
KW - MEDLINE
KW - Adolescent
KW - Adult
KW - Animals
KW - Bites and Stings/co [Complications]
KW - Bites and Stings/vi [Virology]
KW - Child
KW - Preschool
KW - Dog Diseases/pc [Prevention & Control]
KW - Dog Diseases/vi [Virology]
KW - Dogs
KW - Female
KW - Health Education/og [Organization & Administration]
KW - Health Knowledge
KW - Attitudes
KW - Practice
KW - Humans
KW - Male
KW - Nigeria
KW - Pets/vi [Virology]
KW - Rabies/pc [Prevention & Control]
KW - Rabies/th [Therapy]
KW - Rabies/tm [Transmission]
KW - Rabies Vaccines/ad [Administration & Dosage]

RP - NOT IN FILE
SP - 132
EP - 139
JF - Global Journal of Health Science
JA - Glob J Health Sci
VL - 4
IS - 5
CY - Canada
N2 - Every year, about 50,000 people die of rabies of which about 55% of the mortalities occur in Asia and over 40% in Africa. Children are victims of up to 50% of these mortalities. The figure is alarming and immediate action is required to stop this scourge. This study was carried out to assess the knowledge, attitude and practice about rabies among children attending primary schools located in the Ahmadu Bello University (ABU) premises and those outside the university as well as those receiving informal education. The participants for this study were children drawn by random selection from the schools chosen by purposive sampling. With the aid of questionnaires, information was obtained from a total of 477 children with 400 from formal educational settings among 3 schools, and 77 from quaranic schools (almajiris) in the informal setting. More children receiving formal education were aware about the disease (50.8%) than those receiving informal education (32.5%), likewise those residing within ABU quarters (71%) were better informed than those residing outside ABU quarters (43.3%). Among children in the formal schools, 25.9% obtained information from friends and at school (25.9%), while in the informal setting, 56% obtained information from friends and only 16% from school. With regards to attitude and practice, 75.5% of children receiving formal education came from homes where dogs were vaccinated against rabies and 23.3% of them play with dogs they know, while 11.1% of those receiving informal education vaccinate their dogs and fewer of them (14.3%) play with dogs known to them. Many children (65.7%) of those in formal schools know the role of dogs in rabies transmission, compared to only 8% in the informal schools. However, only 9.7% of children in formal schools associate both signs of furious and dumb form of rabies with the disease, compared with 28% in informal schools. Among children bitten by dogs, 87.5% of those receiving informal education received hospital treatment compared to 63.7% of those going to formal schools. About 13% in each of the two categories received traditional treatment. It is, therefore, important for children to be properly educated about rabies so that they can avoid dogs, recognise potential exposures, report to a responsible adult and pass on the knowledge to their peers
SN - 1916-9736
AD - Department of Veterinary Public Health and Preventive Medicine, Ahmadu Bello University, Zaria, Nigeria. asabezik@yahoo.com
UR - 22980386
ER -

TY - JOUR
ID - 385
T1 - Disengagement and inhibition of visual-spatial attention are differently impaired in children with rolandic epilepsy and Panayiotopoulos syndrome
A1 - Bedoin,N.
A1 - Ciumas,C.
A1 - Lopez,C.
A1 - Redsand,G.
A1 - Herbillon,V.
A1 - Laurent,A.
A1 - Ryvlin,P.
Y1 - 2012/09//
N1 - Bedoin, Nathalie. Ciumas, Carolina. Lopez, Celine. Redsand, Gregory. Herbillon, Vania. Laurent, Agathe. Ryvlin, Philippe
Epilepsy & behavior : E&B
100892858
IM
Journal Article. Research Support, Non-U.S. Gov't
English
KW - MEDLINE
KW - Adolescent

KW - Age Factors
KW - Attention Deficit Disorder with Hyperactivity/et [Etiology]
KW - Autonomic Nervous System Diseases/co [Complications]
KW - Cerebral Cortex/pp [Physiopathology]
KW - Child
KW - Electroencephalography
KW - Epilepsy/co [Complications]
KW - Epilepsy
KW - Rolandic/co [Complications]
KW - Female
KW - Humans
KW - Inhibition (Psychology)
KW - Male
KW - Photic Stimulation
KW - Reaction Time
KW - Space Perception/ph [Physiology]
KW - Young Adult
RP - NOT IN FILE
SP - 81
EP - 91
JF - Epilepsy & Behavior
JA - Epilepsy Behav
VL - 25
IS - 1
CY - United States
N2 - We assessed voluntary orientation and reorientation of visuospatial attention in 313 healthy 6- to 22-year-old participants, 30 children suffering from benign epilepsy with centrotemporal spikes (BECTS) and 13 children with Panayiotopoulos syndrome (PS). The developmental section highlights the late development of reorienting skills. Only children with BECTS-R showed a strong tendency toward a rightward bias in attentional orientation. Additionally, a unilateral deficit of disengagement characterizes the patients with BECTS-R and comorbid ADHD. Right rolandic spikes seem to aggravate subclinical reorienting difficulties. Finally, children with PS failed to diffuse inhibition, except in the nearest area outside the attentional focus. This deficit could be attributed to the typical occipital-to-frontal spreading of the spikes in PS. By showing distinct attentional deficiencies according to the epileptic syndrome and the epileptic focus lateralization in BECTS, the results provide new evidence for alterations of attentional mechanisms by interictal epileptic activity, which probably contribute to learning difficulties. Copyright 2012 Elsevier Inc. All rights reserved
SN - 1525-5069
AD - Laboratoire Dynamique du Langage, CNRS UMR 5596, Lyon, France. Nathalie.bedoin@univ-lyon2.fr
UR - 22796339
ER -

TY - JOUR
ID - 386
T1 - Correlates of children's time-specific physical activity: a review of the literature. [Review]
A1 - Stanley,R.M.
A1 - Ridley,K.
A1 - Dollman,J.
Y1 - 2012///
N1 - Stanley, Rebecca M. Ridley, Kate. Dollman, James
The international journal of behavioral nutrition and physical activity
101217089
IM
Journal Article. Research Support, Non-U.S. Gov't. Review
English
KW - MEDLINE
KW - Adolescent
KW - Child

KW - Female
KW - Health Behavior
KW - Humans
KW - Male
KW - Motor Activity
KW - Play and Playthings
KW - Schools
KW - Time Factors
RP - NOT IN FILE
SP - 50
JF - International Journal of Behavioral Nutrition & Physical Activity
JA - Int
VL - 9
CY - England
N2 - Assessment of correlates of physical activity occurring at different times of the day, locations and contexts, is imperative to understanding children's physical activity behaviour. The purpose of this review was to identify the correlates of children's physical activity (aged 8-14 years) occurring during the school break time and after-school periods. A review was conducted of the peer-reviewed literature, published between 1990 and January 2011. A total of 22 studies (12 school break time studies, 10 after-school studies) were included in the review. Across the 22 studies, 17 studies were cross-sectional and five studies were interventions. In the school break time studies, 39 potential correlates were identified, of which gender and age were consistently associated with school break time physical activity in two or more studies, and family affluence, access to a gym, access to four or more physical activity programs and the condition of a playing field were all associated with school break time physical activity in only one study. Access to loose and fixed equipment, playground markings, size of and access to play space and the length of school break time were all positively associated with changes in school break time physical activity in intervention studies. Thirty-six potential correlates of after-school physical activity were identified. Gender (with boys more active), younger age, lower body mass index (for females), lower TV viewing/playing video games, and greater access to facilities were associated with higher levels of after-school physical activity in two or more studies. Parent supervision was negatively associated with females' after-school physical activity in one study. This review has revealed a relatively small number of studies investigating the school break time and after-school periods in the specified age range and only a few correlates have demonstrated a consistent association with physical activity. This highlights the infancy of this area and a need for further investigation into time-specific physical activity behaviour so that interventions designed for these specific periods can target the important correlates
SN - 1479-5868
AD - Health and Use of Time Group, School of Health Sciences, University of South Australia, GPO Box 2471, Adelaide, South Australia, 5001, Australia. starm002@mymail.unisa.edu.au
UR - 22546218
ER -

TY - JOUR
ID - 387
T1 - Walk as directed! Adolescents' adherence to pedometer intervention protocol
A1 - Kahan,D.
A1 - Nicaise,V.
Y1 - 2012/09//
N1 - Kahan, David. Nicaise, Virginie
Journal of physical activity & health
101189457
IM
Journal Article
English
KW - MEDLINE
KW - Adolescent
KW - Body Mass Index
KW - Child
KW - Female

KW - Humans
KW - Male
KW - Monitoring
KW - Ambulatory/mt [Methods]
KW - Ambulatory/px [Psychology]
KW - Patient Compliance/px [Psychology]
KW - Religion
KW - Self Report
KW - Walking
RP - NOT IN FILE
SP - 962
EP - 969
JF - Journal of Physical Activity & Health
JA - J Phys Act Health
VL - 9
IS - 7
CY - United States
N2 - BACKGROUND: Despite frequent use of pedometers in interventions targeting youth PA, there is no literature that addresses the prevalence and reasons for protocol nonadherence. METHODS: Adherence behaviors of early adolescents (n = 43; Meanage = 12.3 + 1.0) in an 8-week, faith-based intervention were monitored/recorded. Students provided reasons for various aspects of protocol breach, which were used to develop a post intervention questionnaire. Analyses included calculations of frequency/percentage as well as cross tabulations/chi square to detect gender/age differences. RESULTS: Over the intervention, recording PA in logs decreased by 85% and was attributed to forgetfulness and lack of time. For pedometers, highest-frequency events included error codes (n = 501), incorrect wear (37%, > 1 day), and shaking (58%-69%, > 1 time). Top reasons for shaking were to make up for lost step opportunities and get further along the route. Of permissible stepping strategies, males used ambulatory activity on the playground and stair usage more, while 6th graders used speed stepping in place more than their respective counterparts. CONCLUSIONS: Students admit to basal levels of nonadherence, which should be taken into consideration when designing/implementing interventions. Integrating intervention tasks into the regular curriculum and providing sufficient opportunities to perform them may alleviate some barriers to adherence. Future research should attempt to confirm results in other school types/levels as well as quantify these behaviors in free-living or unstructured settings
SN - 1543-5474
AD - School of Exercise and Nutritional Sciences, San Diego State University, San Diego, CA, USA
UR - 21975599
ER -

TY - JOUR
ID - 388
T1 - Use of community-level data in the National Children's Study to establish the representativeness of segment selection in the Queens Vanguard Site
A1 - Rundle,A.
A1 - Rauh,V.A.
A1 - Quinn,J.
A1 - Lovasi,G.
A1 - Trasande,L.
A1 - Susser,E.
A1 - Andrews,H.F.
Y1 - 2012///
N1 - Rundle, Andrew. Rauh, Virginia A. Quinn, James. Lovasi, Gina. Trasande, Leonardo. Susser, Ezra. Andrews, Howard F
International journal of health geographics
101152198
IM
Journal Article. Research Support, N.I.H., Extramural. Research Support, U.S. Gov't, Non-P.H.S.
English
KW - MEDLINE

KW - Child
KW - Child Welfare/sn [Statistics & Numerical Data]
KW - Cohort Studies
KW - Emigrants and Immigrants/sn [Statistics & Numerical Data]
KW - Geography
KW - Humans
KW - Infant
KW - Low Birth Weight
KW - Newborn
KW - New York City/ep [Epidemiology]
KW - Residence Characteristics/sn [Statistics & Numerical Data]
KW - Social Environment
KW - Urban Population/sn [Statistics & Numerical Data]
RP - NOT IN FILE
SP - 18
JF - International Journal of Health Geographics [Electronic Resource]
JA - Int J Health Geogr
VL - 11
CY - England
N2 - BACKGROUND: The WHO Multiple Exposures Multiple Effects (MEME) framework identifies community contextual variables as central to the study of childhood health. Here we identify multiple domains of neighborhood context, and key variables describing the dimensions of these domains, for use in the National Children's Study (NCS) site in Queens. We test whether the neighborhoods selected for NCS recruitment, are representative of the whole of Queens County, and whether there is sufficient variability across neighborhoods for meaningful studies of contextual variables. METHODS: Nine domains (demographic, socioeconomic, households, birth rated, transit, playground/greenspace, safety and social disorder, land use, and pollution sources) and 53 indicator measures of the domains were identified. Geographic information systems were used to create community-level indicators for US Census tracts containing the 18 study neighborhoods in Queens selected for recruitment, using US Census, New York City Vital Statistics, and other sources of community-level information. Mean and inter-quartile range values for each indicator were compared for Tracts in recruitment and non-recruitment neighborhoods in Queens. RESULTS: Across the nine domains, except in a very few instances, the NCS segment-containing tracts (N=43) were not statistically different from those 597 populated tracts in Queens not containing portions of NCS segments; variability in most indicators was comparable in tracts containing and not containing segments. CONCLUSIONS: In a diverse urban setting, the NCS segment selection process succeeded in identifying recruitment areas that are, as a whole, representative of Queens County, for a broad range of community-level variables
SN - 1476-072X
AD - Department of Epidemiology, Mailman School of Public Health, 722 West 168th Street, New York, NY 10032, USA. Agr3@columbia.edu
UR - 22668454
ER -

TY - JOUR
ID - 389
T1 - An assessment of swinger techniques for the playground swing oscillatory motion
A1 - Linge,S.O.
Y1 - 2012///
N1 - Linge, Svein O
Computer methods in biomechanics and biomedical engineering
9802899, d1e
IM
Journal Article
English
KW - MEDLINE
KW - Biological Clocks/ph [Physiology]
KW - Child
KW - Computer Simulation

KW - Energy Transfer/ph [Physiology]
KW - Humans
KW - Models
KW - Biological
KW - Movement/ph [Physiology]
KW - Oscillometry/mt [Methods]
KW - Play and Playthings
KW - Psychomotor Performance/ph [Physiology]
RP - NOT IN FILE
SP - 1103
EP - 1109
JF - Computer Methods in Biomechanics & Biomedical Engineering
JA - Comput Methods Biomech.Biomed Engin.
VL - 15
IS - 10
CY - England
N2 - Much attention has been devoted to how playground swing amplitudes are built up by swinger techniques, i.e. body actions. However, very little attention has been given to the requirements that such swinger techniques place on the swinger himself. The purpose of this study was to find out whether different swinger techniques yield significantly different maximum torques, endurance and coordinative skills, and also to identify preferable techniques. We modelled the seated swinger as a rigid dumbbell and compared three different techniques. A series of computer simulations were run with each technique, testing the performance with different body rotational speeds, delayed onset of body rotation and different body mass distributions, as swing amplitudes were brought up towards 90degree. One technique was found to be extremely sensitive to the timing of body actions, limiting swing amplitudes to 50degree and 8degree when body action was delayed by 0.03 and 0.3 s, respectively. Two other more robust techniques reached 90degree even with the largest of these delays, although more time (and endurance) was needed. However, these two methods also differed with respect to maximum torque and endurance, and none was preferable in both these aspects, being dependent on the swinger goals and abilities
SN - 1476-8259
AD - Telemark University College, P.O. Box 203, N-3901 Porsgrunn, Norway. s-ohm@online.no
UR - 21604220
ER -

TY - JOUR
ID - 390
T1 - The value of EEG-fMRI and EEG source analysis in the presurgical setup of children with refractory focal epilepsy
A1 - Elshoff,L.
A1 - Groening,K.
A1 - Grouiller,F.
A1 - Wiegand,G.
A1 - Wolff,S.
A1 - Michel,C.
A1 - Stephani,U.
A1 - Siniatchkin,M.
Y1 - 2012/09//
N1 - Elshoff, Lydia. Groening, Kristina. Grouiller, Frederic. Wiegand, Gert. Wolff, Stephan. Michel, Christoph. Stephani, Ulrich. Siniatchkin, Michael
Epilepsia
eix, 2983306r
IM
Journal Article. Research Support, Non-U.S. Gov't
English
KW - MEDLINE
KW - Adolescent
KW - Brain/pa [Pathology]

KW - Brain/pp [Physiopathology]
KW - Child
KW - Preschool
KW - Electroencephalography/st [Standards]
KW - Epilepsies
KW - Partial/di [Diagnosis]
KW - Partial/pp [Physiopathology]
KW - Female
KW - Follow-Up Studies
KW - Humans
KW - Infant
KW - Magnetic Resonance Imaging/st [Standards]
KW - Male
KW - Postoperative Period
KW - Preoperative Care/st [Standards]
RP - NOT IN FILE
SP - 1597
EP - 1606
JF - Epilepsia
VL - 53
IS - 9
CY - United States
N2 - PURPOSE: In the presurgical evaluation of children and juvenile patients with refractory focal epilepsy, the main challenge is to localize the point of seizure onset as precisely as possible. We compared results of the conventional electroencephalography-functional magnetic resonance imaging (EEG-fMRI) analysis with those obtained with a newly developed method using voltage maps of average interictal epileptiform discharges (IEDs) recorded during clinical long-term monitoring and with the results of the electric source imaging (ESI). METHODS: Simultaneous EEG-fMRI was recorded in nine patients (ages 1.5-17.5 years) undergoing presurgical evaluation. The postoperative outcome and resected area were compared with the following: the localizations of blood oxygen-level dependent (BOLD) signal changes associated with IEDs, which were identified by visual inspection changes using SPM5 software (Analysis I); BOLD signal changes related to IED topography, which was characterized using spike-specific voltage maps of average IED recorded outside the MR scanner during clinical long-term monitoring (Analysis II); as well as results of EEG source analysis based on the distributed linear local autoregressive average (LAURA) algorithm using the Cartool software by Denis Brunet (Analysis III). KEY FINDINGS: All nine patients had postoperative outcome Engel class I-IIb (postoperative time 6-26 months). The analysis I revealed an IED-related area of activation within the resection area in 3 (33%) of 9 patients, analysis II was able to reliably localize the source of epileptic activity in 4 (44%) of 9 patients, and analysis III rendered results concordant with the postoperative resection site in all nine patients. CONCLUSIONS: The localization of seizure onset based on EEG-fMRI may be a useful adjunct in the preoperative evaluation but also has some deficits that impair the reliability of results. In contrast, EEG source analysis is clearly a more credible method for epileptic focus localization in children with refractory epilepsies. It seems likely that the analysis based on IED topography (Analysis II) may increase sensitivity and reliability of EEG-fMRI in some patients. However, the benefit from this innovative method in children is rather limited compared with adults. Wiley Periodicals, Inc. 2012 International League Against Epilepsy
SN - 1528-1167
AD - Neuropediatric Department, Christian-Albrechts-University, Kiel, Germany. lydia.elshoff@web.de
UR - 22779700
ER -

TY - JOUR
ID - 391
T1 - School day segmented physical activity patterns of high and low active children
A1 - Fairclough,S.J.
A1 - Beighle,A.
A1 - Erwin,H.
A1 - Ridgers,N.D.
Y1 - 2012///

N1 - Fairclough, Stuart J. Beighle, Aaron. Erwin, Heather. Ridgers, Nicola D
BMC public health
100968562
IM
Journal Article. Research Support, Non-U.S. Gov't
English
KW - MEDLINE
KW - Accelerometry
KW - Child
KW - Cross-Sectional Studies
KW - England
KW - Female
KW - Humans
KW - Male
KW - Motor Activity/ph [Physiology]
KW - Schools
KW - Social Environment
KW - Time Factors
RP - NOT IN FILE
SP - 406
JF - BMC Public Health
VL - 12
CY - England
N2 - BACKGROUND: Variability exists in children's activity patterns due to the association with environmental, social, demographic, and inter-individual factors. This study described accelerometer assessed physical activity patterns of high and low active children during segmented school week days whilst controlling for potential correlates. METHODS: Two hundred and twenty-three children (mean age: 10.7+0.3 yrs, 55.6% girls, 18.9% overweight/obese) from 8 north-west England primary schools wore ActiGraph GT1M accelerometers for 7 consecutive days during autumn of 2009. ActiGraph counts were converted to minutes of moderate (MPA), vigorous (VPA) and moderate-to-vigorous (MVPA) physical activity. Children were classified as high active (HIGH) or low active (LOW) depending on the percentage of week days they accumulated at least 60 minutes of MVPA. Minutes spent in MPA and VPA were calculated for school time and non-school time and for five discrete school day segments (before-school, class time, recess, lunchtime, and after-school). Data were analysed using multi-level modelling. RESULTS: The HIGH group spent significantly longer in MPA and/or VPA before-school, during class time, lunchtime, and after-school (P<.05), independent of child and school level factors. The greatest differences occurred after-school (MPA=5.5 minutes, VPA=3.8 minutes, P<0.001). MPA and VPA were also associated with gender, BMI z-score, number of enrolled children, playground area per student, and temperature, depending on the segment analysed.The additive effect of the segment differences was that the HIGH group accumulated 12.5 minutes per day more MVPA than the LOW group. CONCLUSIONS: HIGH active children achieved significantly more MPA and VPA than LOW active during four of the five segments of the school day when analyses were adjusted for potential correlates. Physical activity promotion strategies targeting low active children during discretionary physical activity segments of the day, and particularly via structured afterschool physical activity programs may be beneficial
SN - 1471-2458
AD - Research Institute for Sport and Exercise Sciences, Liverpool John Moores University, Tom Reilly Building, Liverpool, UK. s.j.fairclough@ljmu.ac.uk
UR - 22672654
ER -

TY - JOUR
ID - 392
T1 - Bone mineral density and vitamin D status among African American children with forearm fractures
A1 - Ryan,L.M.
A1 - Teach,S.J.
A1 - Singer,S.A.
A1 - Wood,R.
A1 - Freishtat,R.

A1 - Wright,J.L.
A1 - McCarter,R.
A1 - Tosi,L.
A1 - Chamberlain,J.M.
Y1 - 2012/09//
N1 - Ryan, Leticia Manning. Teach, Stephen J. Singer, Steven A. Wood, Rachel. Freishtat, Robert. Wright, Joseph L. McCarter, Robert. Tosi, Laura. Chamberlain, James M
Pediatrics
oxv, 0376422
AIM, IM
Journal Article. Research Support, N.I.H., Extramural. Research Support, U.S. Gov't, P.H.S.
English
KW - MEDLINE
KW - Absorptiometry
KW - Photon
KW - African Americans
KW - Bone Density
KW - Calcium
KW - Dietary/ad [Administration & Dosage]
KW - Case-Control Studies
KW - Child
KW - Preschool
KW - Energy Intake
KW - Female
KW - Humans
KW - Male
KW - Radius Fractures/bl [Blood]
KW - Radius Fractures/eh [Ethnology]
KW - Radius Fractures/pa [Pathology]
KW - Ulna Fractures/bl [Blood]
KW - Ulna Fractures/eh [Ethnology]
KW - Ulna Fractures/pa [Pathology]
KW - Vitamin D/aa [Analogs & Derivatives]
KW - Vitamin D/bl [Blood]
RP - NOT IN FILE
SP - e553
EP - e560
JF - Pediatrics
VL - 130
IS - 3
CY - United States
N2 - OBJECTIVE: To determine whether African American children with forearm fractures have decreased bone mineral density and an increased prevalence of vitamin D deficiency (serum 25-hydroxyvitamin D level < 20 ng/mL) compared with fracture-free control patients. METHODS: This case-control study in African American children, aged 5 to 9 years, included case patients with forearm fracture and control patients without fracture. Evaluation included measurement of bone mineral density and serum 25-hydroxyvitamin D level. Univariable and multivariable analyses were used to test for associations between fracture status and 2 measures of bone health (bone mineral density and 25-hydroxyvitamin D level) while controlling for other potential confounders. RESULTS: The final sample included 76 case and 74 control patients. There were no significant differences between case and control patients in age, gender, parental education level, enrollment season, outdoor play time, height, or mean dietary calcium nutrient density. Cases were more likely than control patients to be overweight (49.3% vs 31.4%, P = .03). Compared with control patients, case patients had lower whole body z scores for bone mineral density (0.62 + 0.96 vs 0.98 + 1.09; adjusted odds ratio 0.38 [0.20-0.72]) and were more likely to be vitamin D deficient (47.1% vs 40.8%; adjusted odds ratio 3.46 [1.09-10.94]). CONCLUSIONS: These data support an association of lower bone mineral density and vitamin D deficiency with increased odds of forearm fracture among African American children. Because suboptimal childhood bone

health also negatively impacts adult bone health, interventions to increase bone mineral density and correct vitamin D deficiency are indicated in this population to provide short-term and long-term benefits
SN - 1098-4275
AD - Children's National Medical Center, Division of Emergency Medicine, 111 Michigan Ave NW, Washington, DC 20010, USA. lryan@childrensnational.org
UR - 22926174
ER -

TY - JOUR
ID - 393
T1 - Can social interventions prevent tuberculosis?: the Papworth experiment (1918-1943) revisited
A1 - Bhargava,A.
A1 - Pai,M.
A1 - Bhargava,M.
A1 - Marais,B.J.
A1 - Menzies,D.
Y1 - 2012/09/01/
N1 - Bhargava, Anurag. Pai, Madhukar. Bhargava, Madhavi. Marais, Ben J. Menzies, Dick
American journal of respiratory and critical care medicine
9421642, bzs
AIM, IM
Evaluation Studies. Journal Article. Research Support, Non-U.S. Gov't
English
KW - MEDLINE
KW - Adolescent
KW - Adult
KW - Child
KW - Preschool
KW - Cohort Studies
KW - Employment
KW - England/ep [Epidemiology]
KW - Female
KW - Follow-Up Studies
KW - Food Services
KW - Health Surveys
KW - Housing
KW - Humans
KW - Incidence
KW - Infant
KW - Newborn
KW - Infectious Disease Transmission
KW - Vertical/pc [Prevention & Control]
KW - Male
KW - Preventive Health Services/mt [Methods]
KW - Preventive Health Services/og [Organization & Administration]
KW - Social Welfare
KW - Tuberculosis/ep [Epidemiology]
KW - Tuberculosis/pc [Prevention & Control]
KW - Tuberculosis/tm [Transmission]
KW - Young Adult
RP - NOT IN FILE
SP - 442
EP - 449
JF - American Journal of Respiratory & Critical Care Medicine
JA - Am J Respir Crit Care Med
VL - 186
IS - 5

CY - United States
N2 - RATIONALE: There is consensus on the need to address social determinants of tuberculosis (TB) to achieve TB control, but evidence based on interventions is lacking. OBJECTIVES: We reanalyzed data from the sociomedical experiment performed at the Papworth Village Settlement in England, where the impact of stable employment and adequate housing and nutrition on the incidence of TB infection and disease in children living with parents with active TB was documented during 1918-1943. METHODS: Information on 315 children of patients, who lived at Papworth, was abstracted from a published monograph. Overall and age-specific occurrence of TB infection, disease, and deaths among children born in the settlement (village-born cohort) were compared with those of children born outside and admitted later (admitted cohort) to Papworth. MEASUREMENTS AND MAIN RESULTS: The annual risks of infection in the village-born and admitted cohorts were 20 and 24%, respectively. Of 24 children who developed TB disease, only one was village-born. Among children 5 years of age or less, there was zero incidence of TB in the village-born, compared with five cases (1,217/100,000 person-years) among children born outside Papworth. In the admitted cohort, among children 13 years of age and older, the incidence of TB before admission to Papworth was 5,263/100,000 person-years, whereas it was 341/100,000 person-years while living in Papworth. CONCLUSIONS: At Papworth social interventions including adequate nutrition did not reduce TB transmission but did reduce the incidence of TB disease in children living with parents with active TB. These results are relevant today for prevention of TB in children of patients with active TB, particularly with multidrug-resistant TB in high-burden settings
SN - 1535-4970
AD - Department of Epidemiology, Biostatistics, and Occupational Health, McGill University, Montreal, Canada
UR - 22773730
ER -

TY - JOUR
ID - 394
T1 - Parents' perceptions of skin cancer threat and children's physical activity
A1 - Tran,A.D.
A1 - Aalborg,J.
A1 - Asdigian,N.L.
A1 - Morelli,J.G.
A1 - Mokrohisky,S.T.
A1 - Dellavalle,R.P.
A1 - Berwick,M.
A1 - Box,N.F.
A1 - Crane,L.A.
Y1 - 2012/08//
N1 - Tran, Alexander D. Aalborg, Jenny. Asdigian, Nancy L. Morelli, Joseph G. Mokrohisky, Stefan T. Dellavalle, Robert P. Berwick, Marianne. Box, Neil F. Crane, Lori A
Preventing chronic disease
101205018
IM
Journal Article. Randomized Controlled Trial. Research Support, N.I.H., Extramural. Research Support, Non-U.S. Gov't
English
KW - MEDLINE
KW - Adult
KW - Behavioral Risk Factor Surveillance System
KW - Body Mass Index
KW - Child
KW - Cohort Studies
KW - Colorado
KW - Cross-Sectional Studies
KW - Environmental Exposure/ae [Adverse Effects]
KW - Female
KW - Health Behavior/eh [Ethnology]

KW - Humans
KW - Male
KW - Motor Activity
KW - Nevus
KW - Pigmented
KW - Parents/px [Psychology]
KW - Parents
KW - Physical Examination
KW - Questionnaires
KW - Recreation
KW - Regression Analysis
KW - Severity of Illness Index
KW - Skin Neoplasms/co [Complications]
KW - Skin Neoplasms/pc [Prevention & Control]
KW - Skin Neoplasms/px [Psychology]
KW - Skin Pigmentation
KW - Social Class
KW - Sunlight/ae [Adverse Effects]
KW - Time Factors
RP - NOT IN FILE
SP - E143
JF - Preventing Chronic Disease
JA - Prev Chronic Dis
VL - 9
CY - United States
N2 - INTRODUCTION: Sun exposure is a major risk factor for skin cancer, but without physical activity, children are at risk of childhood obesity. The objective of this study was to explore relationships between parental perceptions of skin cancer threat, sun protection behaviors, physical activity, and body mass index (BMI) in children. METHODS: This is a cross-sectional analysis nested within the Colorado Kids Sun Care Program sun safety intervention trial. In summer 2007, parent telephone interviews provided data on demographics, perceptions of skin cancer threat, sun protection behaviors, and physical activity. Physical examinations provided data on phenotype, freckling, and BMI. Data from 999 Colorado children born in 1998 were included in analysis. We used analysis of variance, Spearman's rho (p) correlation, and multivariable linear regression analysis to evaluate relationships with total amount of outdoor physical activity. RESULTS: After controlling for sex, race/ethnicity, skin color, and sun protection, regression analysis showed that each unit increase in perceived severity of nonmelanoma skin cancer was associated with a 30% increase in hours of outdoor physical activity (P = .005). Hours of outdoor physical activity were not related to perceived severity of melanoma or perceived susceptibility to skin cancer. BMI-for-age was not significantly correlated with perceptions of skin cancer threat, use of sun protection, or level of physical activity. CONCLUSION: The promotion of sun safety is not likely to inhibit physical activity. Skin cancer prevention programs should continue to promote midday sun avoidance and sun protection during outdoor activities
SN - 1545-1151
AD - Colorado School of Public Health, University of Colorado Anschutz Medical Campus, Aurora, Colorado, USA
UR - 22935145
ER -

TY - JOUR
ID - 395
T1 - The epidemiology of caving injuries in the United States
A1 - Stella-Watts,A.C.
A1 - Holstege,C.P.
A1 - Lee,J.K.
A1 - Charlton,N.P.
Y1 - 2012/09//
N1 - Stella-Watts, Alejandro C. Holstege, Christopher P. Lee, Jae K. Charlton, Nathan P
Wilderness & environmental medicine

dbe, 9505185
IM
Journal Article
English
KW - MEDLINE
KW - Accidental Falls/mo [Mortality]
KW - Accidental Falls/sn [Statistics & Numerical Data]
KW - Adolescent
KW - Adult
KW - Aged
KW - Athletic Injuries/ep [Epidemiology]
KW - Cause of Death
KW - Caves
KW - Child
KW - Preschool
KW - Craniocerebral Trauma/ep [Epidemiology]
KW - Female
KW - Fractures
KW - Bone/ep [Epidemiology]
KW - Humans
KW - Lacerations/ep [Epidemiology]
KW - Leisure Activities
KW - Male
KW - Middle Aged
KW - Recreation
KW - Retrospective Studies
KW - Seasons
KW - United States/ep [Epidemiology]
KW - Wounds and Injuries/ep [Epidemiology]
KW - Young Adult
RP - NOT IN FILE
SP - 215
EP - 222
JF - Wilderness & Environmental Medicine
JA - Wilderness Environ Med
VL - 23
IS - 3
CY - United States
N2 - OBJECTIVE: Caving is a demanding sport practiced throughout the world. Currently, there are no collective data analyzing injury mechanism or type in these austere environments. This study is a retrospective analysis of caving incidents documented by the National Speleological Society (NSS)-American Caving Accidents (ACA) annual publication. METHODS: This study retrospectively analyzes 877 incident reports collected between 1980 and 2008 by NSS-ACA. For each victim, the month, year, location, age, gender, incident type, injury zone of the body, injury type, the result of the incident, and time intervals for rescue were extracted. RESULTS: A total of 1356 victims were identified; 83% of victims were male, 17% were female. Ages ranged from 2 to 69 years old, with an average of 27 years. The greatest number of events occurred in summer months, peaking in July. The most common incident leading to traumatic injury was a caver fall (74%), also contributing to 30% of caver fatalities. Lower extremities were most commonly injured (29%), followed by the upper extremities and head (21% and 15%, respectively). Fractures comprised 41% of injuries, followed by lacerations (13%), bruise, hematoma, and abrasions (12%), and sprains and strains (7%). CONCLUSIONS: The majority of injuries were not life threatening; however, over the course of 28 years there were 81 documented fatalities. Similar to other studies of wilderness injuries, fractures, soft tissue injuries, and lacerations were prominent in this study. In general, the overall precipitating event leading to injuries is falling, leading to orthopedic trauma. To better prepare cave rescue teams we have attempted to describe the characteristics of caving injuries in the United States. Copyright 2012 Wilderness Medical Society. Published by Elsevier Inc. All rights reserved
SN - 1545-1534

AD - Department of Emergence Medicine, University of Rochester, Rochester, NY, USA
UR - 22704081
ER -

TY - JOUR
ID - 396
T1 - Daily inhalation rate and time-activity/location pattern in Japanese preschool children
A1 - Kawahara,J.
A1 - Tanaka,S.
A1 - Tanaka,C.
A1 - Aoki,Y.
A1 - Yonemoto,J.
Y1 - 2012/09//
N1 - Kawahara, Junko. Tanaka, Shigeho. Tanaka, Chiaki. Aoki, Yasunobu. Yonemoto, Junzo
Risk analysis : an official publication of the Society for Risk Analysis
8109978, ria
IM
Journal Article
English
KW - MEDLINE
KW - Air Pollution
KW - Child
KW - Preschool
KW - Female
KW - Humans
KW - Inhalation Exposure
KW - Japan
KW - Male
KW - Motor Activity
RP - NOT IN FILE
SP - 1595
EP - 1604
JF - Risk Analysis
JA - Risk Anal
VL - 32
IS - 9
CY - United States
N2 - Lack of data on daily inhalation rate and activity of children has been an issue in health risk assessment of air pollutants. This study aimed to obtain the daily inhalation rate and intensity and frequency of physical activity in relation to the environment in Japanese preschool children. Children aged four-six years (n= 138) in the suburbs of Tokyo participated in this study, which involved three days' continuous monitoring of physical activity using a tri-axial accelerometer and parent's completion of a time/location diary during daily life. The estimated three-day mean daily inhalation rate (body temperature, pressure, saturated with water vapor) was 9.9 + 1.6 m(3) /day (0.52 + 0.09 m(3) /kg/day). The current daily inhalation rate value of 0.580 m(3) /kg/day proposed for use in health risk assessment in Japan is confirmed to be valid to calculate central value of inhaled dose of air pollutants in five- to six-year-old children. However, the 95th percentile daily inhalation rate of 0.83 m(3) /kg/day based on measurement for five-year-old children is recommended to be used to provide an upper bound estimate of exposure that ensure the protection of all five- to six-year-old children from the health risk of air pollutants. Children spent the majority of their time in sedentary and light level of physical activity (LPA) when indoors, while 85% of their time when outdoors was spent in LPA and moderate-to-vigorous physical activity. The results suggest the need to consider variability of minute respiratory ventilation rate according to the environment for more refined short-term health risk assessment. 2012 Society for Risk Analysis
SN - 1539-6924
AD - Research Center for Environmental Risks, National Institute for Environmental Studies, Tsukuba, Japan. jkawa@nies.go.jp
UR - 22384860
ER -

TY - JOUR
ID - 397
T1 - Accelerometer measured level of physical activity indoors and outdoors during preschool time in Sweden and the United States
A1 - Raustorp,A.
A1 - Pagels,P.
A1 - Boldemann,C.
A1 - Cosco,N.
A1 - Soderstrom,M.
A1 - Martensson,F.
Y1 - 2012/08//
N1 - Raustorp, Anders. Pagels, Peter. Boldemann, Cecilia. Cosco, Nilda. Soderstrom, Margareta. Martensson, Fredrika
Journal of physical activity & health
101189457
IM
Journal Article. Multicenter Study. Research Support, Non-U.S. Gov't
English
KW - MEDLINE
KW - Accelerometry/sn [Statistics & Numerical Data]
KW - Body Mass Index
KW - Body Weights and Measures
KW - Child
KW - Preschool
KW - Cross-Cultural Comparison
KW - Exercise
KW - Female
KW - Humans
KW - Male
KW - North Carolina/ep [Epidemiology]
KW - Sex Factors
KW - Sweden/ep [Epidemiology]
KW - Time Factors
KW - United States/ep [Epidemiology]
RP - NOT IN FILE
SP - 801
EP - 808
JF - Journal of Physical Activity & Health
JA - J Phys Act Health
VL - 9
IS - 6
CY - United States
N2 - BACKGROUND: It is important to understand the correlates of physical activity (PA) to influence policy and create environments that promote PA among preschool children. We compared preschoolers' PA in Swedish and in US settings and objectively examined differences boys' and girls' indoor and outdoor PA regarding different intensity levels and sedentary behavior. METHODS: Accelerometer determined PA in 50 children with mean age 52 months, (range 40-67) was recorded during preschool time for 5 consecutive weekdays at 4 sites. The children wore an Actigraph GTIM Monitor. RESULTS: Raleigh preschool children, opposite to Malmo preschoolers spent significantly more time indoors than outdoors (P<.001). Significantly more moderate-to-vigorous intensity physical activity (MVPA) was observed outdoors (P<.001) in both settings. Malmo children accumulated significantly more counts/min indoors (P<.001). The percent of MVPA during outdoor time did not differ between children at Raleigh and Malmo. CONCLUSION: Physical activity counts/minutes was significantly higher outdoors vs. indoors in both Malmo and Raleigh. Malmo preschoolers spent 47% of attendance time outdoors compared with 18% for Raleigh preschoolers which could have influenced the difference in preschool activity between the 2 countries. Time spent in MVPA at preschool was very limited and predominantly adopted outdoors

SN - 1543-5474
AD - School of Sport Sciences, Linnaeus University, Kalmar, Sweden
UR - 21952100
ER -

TY - JOUR
ID - 398
T1 - Physical activity during school recess: a systematic review. [Review]
A1 - Ridgers,N.D.
A1 - Salmon,J.
A1 - Parrish,A.M.
A1 - Stanley,R.M.
A1 - Okely,A.D.
Y1 - 2012/09//
N1 - Ridgers, Nicola D. Salmon, Jo. Parrish, Anne-Maree. Stanley, Rebecca M. Okely, Anthony D
American journal of preventive medicine
8704773, apl
IM
Journal Article. Research Support, Non-U.S. Gov't. Review
English
KW - MEDLINE
KW - Adolescent
KW - Child
KW - Female
KW - Health Promotion/mt [Methods]
KW - Humans
KW - Leisure Activities
KW - Male
KW - Motor Activity/ph [Physiology]
KW - Play and Playthings
KW - Recreation/px [Psychology]
KW - Schools
KW - Sex Factors
RP - NOT IN FILE
SP - 320
EP - 328
JF - American Journal of Preventive Medicine
JA - Am J Prev Med
VL - 43
IS - 3
CY - Netherlands
N2 - CONTEXT: Interest has increased in examining the physical activity levels of young people during school recess. Identifying correlates of their recess physical activity behaviors is timely, and would inform school-based physical activity programming and intervention development. The review examined the correlates of children's and adolescent's physical activity during school recess periods. EVIDENCE ACQUISITION: A systematic search of six electronic databases, reference lists, and personal archives identified 53 studies (47 focused on children) published between January 1990 and April 2011 that met the inclusion criteria. Data were analyzed in 2011. Correlates were categorized using the social-ecological framework. EVIDENCE SYNTHESIS: Forty-four variables were identified across the four levels of the social-ecological framework, although few correlates were studied repeatedly at each level. Positive associations were found of overall facility provision, unfixed equipment, and perceived encouragement with recess physical activity. Results revealed that boys were more active than girls. CONCLUSIONS: Providing access to school facilities, providing unfixed equipment, and identifying ways to promote encouragement for physical activity have the potential to inform strategies to increase physical activity levels during recess periods. Copyright 2012 American Journal of Preventive Medicine. Published by Elsevier Inc. All rights reserved
SN - 1873-2607

AD - Centre for Physical Activity and Nutrition Research, Deakin University, Melbourne, Australia. nicky.ridgers@deakin.edu.au
UR - 22898126
ER -

TY - JOUR
ID - 399
T1 - Cross-sectional study of motor development among children after the Fontan procedure
A1 - Longmuir,P.E.
A1 - Banks,L.
A1 - McCrindle,B.W.
Y1 - 2012/08//
N1 - Longmuir, Patricia E. Banks, Laura. McCrindle, Brian W
Cardiology in the young
9200019, C4T
IM
Journal Article. Research Support, Non-U.S. Gov't
English
KW - MEDLINE
KW - Accelerometry
KW - Child
KW - Child Development
KW - Preschool
KW - Cross-Sectional Studies
KW - Developmental Disabilities/pp [Physiopathology]
KW - Female
KW - Fontan Procedure
KW - Heart Defects
KW - Congenital/su [Surgery]
KW - Humans
KW - Locomotion
KW - Male
KW - Motor Activity
KW - Motor Skills
KW - Sedentary Lifestyle
RP - NOT IN FILE
SP - 443
EP - 450
JF - Cardiology in the Young
JA - Cardiol Young
VL - 22
IS - 4
CY - England
N2 - OBJECTIVES: To determine the gross motor skills of school-aged children after the Fontan procedure and compare the locomotor and object control skills with normative data. Study design This study followed a cross-sectional design. SETTING: This study was based on hospital outpatient visit, with accelerometry conducted at home. Patients This study included 55 patients, including 22 girls in the age group of 6-10 years, 5.1 years after Fontan. Main outcome measures Test of Gross Motor Development - Version 2, daily activity by accelerometer, medical history review, child and parent perceptions of activity. RESULTS: Being involved in active team sports increased locomotor percentile score by 10.3 points (CI: 4.4, 16.1). Preference for weekend outdoor activities (6.9, CI: 2.0, 11.8), performing at least 30 minutes of moderate-to-vigorous physical activity daily (24.5, CI: 7.3, 41.8), and reporting that parents seldom criticise the child's physical activity (21.8, CI: 8.9, 34.8) were also associated with higher locomotor percentile scores ($p < 0.01$). Object control percentile scores were higher ($p < 0.03$) with involvement in formal instruction (5.9, CI: 1.1, 10.6) and being restricted to "activities within comfortable limits" (27.6, CI: 7.7, 47.5). Older chronological age ($r = 0.28$), a more complicated medical history ($r = 0.36$), and older age at Fontan ($r = 0.28$) were associated with greater skill delay ($p < 0.04$). CONCLUSIONS: Children after Fontan attain basic motor skills at a later age than their peers,

and deficits continue for more complex skills as age increases, suggesting a need for longitudinal monitoring of gross motor skill development through the elementary school years. Future research might investigate whether a gross motor skill rehabilitation programme can provide these children with the motor skills needed to successfully participate in a physically active lifestyle with peers
SN - 1467-1107
AD - Labatt Family Heart Centre, The Hospital for Sick Children, University of Toronto, Ontario, Canada. plongmuir@cheo.on.ca
UR - 22269013
ER -

TY - JOUR
ID - 400
T1 - Design and baseline characteristics of the Short bouTs of Exercise for Preschoolers (STEP) study
A1 - Alhassan,S.
A1 - Nwaokelemeh,O.
A1 - Mendoza,A.
A1 - Shitole,S.
A1 - Whitt-Glover,M.C.
A1 - Yancey,A.K.
Y1 - 2012///
N1 - Alhassan, Sofiya. Nwaokelemeh, Ogechi. Mendoza, Albert. Shitole, Sanyog. Whitt-Glover, Melicia C. Yancey, Antronette K
BMC public health
100968562
IM
Journal Article. Randomized Controlled Trial. Research Support, Non-U.S. Gov't
English
KW - MEDLINE
KW - Child Day Care Centers/mt [Methods]
KW - Child
KW - Preschool
KW - Cluster Analysis
KW - Exercise/ph [Physiology]
KW - Female
KW - Humans
KW - Male
KW - Play and Playthings
KW - Program Evaluation
KW - Research Design
KW - Time Factors
RP - NOT IN FILE
SP - 582
JF - BMC Public Health
VL - 12
CY - England
N2 - BACKGROUND: Most preschool centers provide two 30-min sessions of gross-motor/outdoor playtime per preschool day. Within this time frame, children accumulate most of their activity within the first 10min. This paper describes the design and baseline participant characteristics of the Short bouTs of Exercise for Preschoolers (STEP) study. The STEP study is a cluster randomized controlled study designed to examine the effects of short bouts of structured physical activity (SBS-PA) implemented within the classroom setting as part of designated gross-motor playtime on during-school physical activity (PA) in preschoolers.
METHODS/DESIGN: Ten preschool centers serving low-income families were randomized into SBS-PA versus unstructured PA (UPA). SBS-PA schools were asked to implement age-appropriate 10min structured PA routines within the classroom setting, twice daily, followed by 20min of usual unstructured playtime. UPA intervention consisted of 30min of supervised unstructured free playtime twice daily. Interventions were implemented during the morning and afternoon designated gross-motor playtime for 30min/session, five days/week for six months. Outcome measures were between group difference in during-preschool PA

(accelerometers and direct observation) over six-months. Ten preschool centers, representing 34 classrooms and 315 children, enrolled in the study. The average age and BMI percentile for the participants was 4.1 +0.8years and 69th percentile, respectively. Participants spent 74% and 6% of their preschool day engaged in sedentary and MVPA, respectively. DISCUSSION: Results from the STEP intervention could provide evidence that a PA policy that exposes preschoolers to shorter bouts of structured PA throughout the preschool day could potentially increase preschoolers' PA levels
SN - 1471-2458
AD - Department of Kinesiology, University of Massachusetts Amherst, Amherst, MA 01003-9258, USA. alhassan@kin.umass.edu
UR - 22853642
ER -

TY - JOUR
ID - 401
T1 - Use of actigraphy for assessment in pediatric sleep research. [Review]
A1 - Meltzer,L.J.
A1 - Montgomery-Downs,H.E.
A1 - Insana,S.P.
A1 - Walsh,C.M.
Y1 - 2012/10//
N1 - Meltzer, Lisa J. Montgomery-Downs, Hawley E. Insana, Salvatore P. Walsh, Colleen M
Sleep medicine reviews
9804678
IM
Journal Article. Research Support, N.I.H., Extramural. Review
English
KW - MEDLINE
KW - Actigraphy
KW - Child
KW - Preschool
KW - Humans
KW - Infant
KW - Reproducibility of Results
KW - Sleep/ph [Physiology]
KW - Sleep Medicine Specialty/is [Instrumentation]
KW - Sleep Medicine Specialty/mt [Methods]
RP - NOT IN FILE
SP - 463
EP - 475
JF - Sleep Medicine Reviews
JA - Sleep Med Rev
VL - 16
IS - 5
CY - England
N2 - The use of actigraphs, or ambulatory devices that estimate sleep-wake patterns from activity levels, has become common in pediatric research. Actigraphy provides a more objective measure than parent-report, and has gained popularity due to its ability to measure sleep-wake patterns for extended periods of time in the child's natural environment. The purpose of this review is: 1) to provide comprehensive information on the historic and current uses of actigraphy in pediatric sleep research; 2) to review how actigraphy has been validated among pediatric populations; and 3) offer recommendations for methodological areas that should be included in all studies that utilize actigraphy, including the definition and scoring of variables commonly reported. The poor specificity to detect wake after sleep onset was consistently noted across devices and age groups, thus raising concerns about what is an "acceptable" level of specificity for actigraphy. Other notable findings from this review include the lack of standard scoring rules or variable definitions. Suggestions for the use and reporting of actigraphy in pediatric research are provided. Copyright 2011 Elsevier Ltd. All rights reserved
SN - 1532-2955
AD - National Jewish Health, 1400 Jackson Street, G311, Denver, CO 80206, USA. meltzerL@njhealth.org

UR - 22424706
ER -

TY - JOUR
ID - 402
T1 - Getting kids active by participating in sport and doing it more often: focusing on what matters
A1 - Mandic,S.
A1 - Bengoechea,E.G.
A1 - Stevens,E.
A1 - de la Barra,S.L.
A1 - Skidmore,P.
Y1 - 2012///
N1 - Mandic, Sandra. Bengoechea, Enrique Garcia. Stevens, Emily. de la Barra, Sophia Leon. Skidmore, Paula
The international journal of behavioral nutrition and physical activity
101217089
IM
Journal Article. Research Support, Non-U.S. Gov't
English
KW - MEDLINE
KW - Adolescent
KW - Child
KW - Cross-Sectional Studies
KW - Female
KW - Health Policy
KW - Health Promotion
KW - Health Surveys
KW - Humans
KW - Life Style
KW - Logistic Models
KW - Male
KW - Motor Activity
KW - Multilevel Analysis
KW - Multivariate Analysis
KW - New Zealand
KW - Physical Education and Training
KW - Questionnaires
KW - Residence Characteristics
KW - Schools
KW - Socioeconomic Factors
KW - Sports
RP - NOT IN FILE
SP - 86
JF - International Journal of Behavioral Nutrition & Physical Activity
JA - Int
VL - 9
CY - England
N2 - BACKGROUND: Reduced time dedicated to physical education and free play in recent decades emphasizes the need to promote opportunities for sport participation in adolescents in order to increase physical activity levels. The purpose of this study was to examine the association of sociodemographic and biological characteristics, behavioural patterns, and school-related and sport-specific variables with time spent participating in sport. METHODS: A total of 1837 secondary school students (age: 14.6 + 1.2 years; 50.9 % boys) from 19 of 23 schools in the Otago Region (New Zealand) completed an online sport survey and Youth Physical Activity Questionnaire in 2009. Using multilevel modeling, we examined the association of individual-, school- and sport-related variables on sport participation and the amount of time spent in sports. RESULTS: Higher rates of sport participation were associated with lower neighbourhood deprivation scores (OR (95%CI): 0.75 (0.49-1.14), 0.57 (0.38-0.86), 0.48 (0.28-0.81)), higher quintiles of physical activity (2.89 (2.10-3.96), 2.81 (1.68-4.70), 3.54 (2.24-5.57), 3.97 (1.99-7.95)), highest quintiles of screen time (1.58 (0.94-2.65), 1.99 (1.42-

2.80), 2.17 (1.43-3.30), 1.88 (1.37-2.57)) and boys only school status (2.21 (1.57-3.10)). Greater amount of time spent in sports was associated with male gender (0.56 (0.43-0.74), lower neighbourhood deprivation scores (0.72 (0.59-0.93), 0.78 (0.58-1.04), 0.62 (0.39-1.00)), higher quintiles of physical activity (3.18 (2.29-4.41), 4.25 (2.91-6.20), 8.33 (5.58-12.44), 6.58 (4.07-10.64)), highest quintile of screen time (1.83 (1.31-2.56), greater availability of sports outside school (1.68 (1.22-2.32)), better sport management (2.57 (1.63-4.07)) and provision of sport courts at school (0.57 (0.40-0.81)). Conversely, obesity was associated with less time spent participating in sport (0.50 (0.31-0.80)). CONCLUSION: Results support the use of sport participation as an effective strategy to increase physical activity levels and identify target groups and areas for interventions, program design and policy development. Interventions should focus on improving accessibility to sport programs for all adolescents, providing adequate sport grounds at school, and promoting good sport management practices. Programs and policies encouraging sport participation should address in particular the needs of adolescents living in deprived neighborhoods, those attending coeducational and girls-only schools, and those who are obese
SN - 1479-5868
AD - School of Physical Education, University of Otago, PO Box 56, Dunedin, New Zealand. sandra.mandic@otago.ac.nz
UR - 22788577
ER -

TY - JOUR
ID - 403
T1 - Associations among social capital, parenting for active lifestyles, and youth physical activity in rural families living in upstate New York
A1 - Davison,K.K.
A1 - Nishi,A.
A1 - Kranz,S.
A1 - Wyckoff,L.
A1 - May,J.J.
A1 - Earle-Richardson,G.B.
A1 - Strogatz,D.S.
A1 - Jenkins,P.L.
Y1 - 2012/10//
N1 - Davison, Kirsten K. Nishi, Akihiro. Kranz, Sibylle. Wyckoff, Lynae. May, John J. Earle-Richardson, Giulia B. Strogatz, David S. Jenkins, Paul L
Social science & medicine (1982)
ut9, 8303205
IM
Journal Article
English
KW - MEDLINE
KW - Adolescent
KW - Child
KW - Cross-Sectional Studies
KW - Female
KW - Health Surveys
KW - Humans
KW - Life Style
KW - Male
KW - Motor Activity
KW - New York
KW - Parenting/px [Psychology]
KW - Rural Population
KW - Social Support
KW - Young Adult
RP - NOT IN FILE
SP - 1488
EP - 1496

JF - Social Science & Medicine
JA - Soc Sci Med
VL - 75
IS - 8
CY - England
N2 - While emerging research supports a positive relationship between social capital and youth physical activity (PA), few studies have examined possible mechanisms explaining this relationship and no studies have focused on rural youth. In this study, we examined parents' support of children's PA as an intermediary factor linking social capital and youth PA in a largely rural cross sectional sample of American children aged 6- to 19-years and their parents/guardians (N=767 families) living in upstate New York. Parents completed a self-administered survey assessing demographic factors, perceived social capital, support for children's PA, and children's PA including time spent outdoors and days per week of sufficient PA. Structural equation modeling was used to test the hypothesis that higher social capital is linked with higher parental support for PA and, in turn, higher PA in children. Analyses were conducted separately for younger (6-12 years) and older (13-19 years) children and controlled for demographic factors (child age, household education, participation in a food assistance program) and perceived neighborhood safety. Anticipated relationships among social capital, parents' activity-related support, and children's PA were identified for older, but not younger children. Findings suggest that parent support for children's PA is one possible mechanism linking social capital and youth PA and the parents of adolescents may rely more heavily on cues from their social environment to shape their approaches to supporting their children's PA than parents of younger children. Copyright 2012 Elsevier Ltd. All rights reserved
SN - 1873-5347
AD - Department of Nutrition, Harvard School of Public Health, Boston, MA, USA. kdavison@hsph.harvard.edu
UR - 22818486
ER -

TY - JOUR
ID - 404
T1 - Indignity, exclusion, pain and hunger: the impact of musculoskeletal impairments in the lives of children in Malawi
A1 - Alavi,Y.
A1 - Jumbe,V.
A1 - Hartley,S.
A1 - Smith,S.
A1 - Lamping,D.
A1 - Muhit,M.
A1 - Masiye,F.
A1 - Lavy,C.
Y1 - 2012///
N1 - Alavi, Yasmene. Jumbe, Vincent. Hartley, Sally. Smith, Sarah. Lamping, Donna. Muhit, Mohammad. Masiye, Francis. Lavy, Chris
Disability and rehabilitation
9207179, a8i
IM
Journal Article. Research Support, Non-U.S. Gov't
English
KW - MEDLINE
KW - Adolescent
KW - Adult
KW - Child
KW - Preschool
KW - Disabled Children/px [Psychology]
KW - Female
KW - Focus Groups
KW - Humans
KW - Hunger

KW - Interviews as Topic
KW - Malawi
KW - Male
KW - Models
KW - Psychological
KW - Musculoskeletal Diseases/px [Psychology]
KW - Pain
KW - Personhood
KW - Poverty
KW - Qualitative Research
KW - Questionnaires
KW - Sickness Impact Profile
KW - Social Environment
KW - Social Isolation
KW - Stress
KW - Psychological/et [Etiology]
KW - Young Adult
RP - NOT IN FILE
SP - 1736
EP - 1746
JF - Disability & Rehabilitation
JA - Disabil Rehabil
VL - 34
IS - 20
CY - England
N2 - PURPOSE: To develop a conceptual model representing the impact of musculoskeletal impairments (MSIs) in the lives of children in Malawi. METHOD: A total of 169 children with MSIs (CMSIs), family and other community members participated in 57 interviews, focus groups and observations. An inductive approach to data analysis was used to conceptualise the impact of MSIs in children's day-to-day lives. RESULTS: The main themes that emerged were Indignity, Exclusion, Pain and Hunger. Indignity represents various affronts to children's sense of inherent equal worth as human beings, for example when bullied by peers. Exclusion refers to CMSIs being excluded from three core daily activities: school, play and household chores. Some CMSIs experienced Pain, for example as an outcome of striving to participate. Children with severe mobility impairments were at increased risk of Hunger, having less access to food outside the home and placing a burden of care on the family that could restrict household productivity. Household Poverty was therefore included in the model, as this household impact was inseparable from the impact on CMSIs. CONCLUSION: It is recommended that rehabilitation interventions are planned and evaluated with consideration to their impact on Exclusion, Indignity, Pain, Hunger and Household Poverty using multi-faceted partnerships
SN - 1464-5165
AD - London School of Hygiene & Tropical Medicine, Faculty of Infectious & Tropical Diseases, London, UK. yasmene.alavi@lshtm.ac.uk
UR - 22409227
ER -

TY - JOUR
ID - 405
T1 - Five-year changes in school recess and lunchtime and the contribution to children's daily physical activity
A1 - Ridgers,N.D.
A1 - Timperio,A.
A1 - Crawford,D.
A1 - Salmon,J.
Y1 - 2012/08//
N1 - Ridgers, Nicola D. Timperio, Anna. Crawford, David. Salmon, Jo
British journal of sports medicine
0432520
IM

Journal Article. Research Support, Non-U.S. Gov't
English
KW - MEDLINE
KW - Child
KW - Preschool
KW - Exercise/ph [Physiology]
KW - Female
KW - Humans
KW - Lunch/ph [Physiology]
KW - Male
KW - Sex Factors
KW - Snacks/ph [Physiology]
KW - Victoria
RP - NOT IN FILE
SP - 741
EP - 746
JF - British Journal of Sports Medicine
JA - BJSM online
VL - 46
IS - 10
CY - England
N2 - OBJECTIVE: To investigate the longitudinal changes in children's recess and lunchtime physical activity levels and in the contribution of recess and lunchtime to daily physical activity levels over 5 years among 5-6- and 10-12-year olds. METHODS: Data were drawn from two longitudinal studies that were conducted in metropolitan Melbourne, Australia. Boys and girls (n=2782) aged 5-6 years and 10-12 years participated in baseline (T0) measures. Physical activity (n=2490) was measured every 60 s for eight consecutive days using hip-mounted accelerometry. Subsequent measurements were taken at 3-year (T1; n=773) and 5-year (T2; n=634) follow-up. Physical activity intensities were derived using age-adjusted cut-points. Sedentary time was defined as 100 counts/min. Longitudinal data were analysed using three-level (time, child, school) multilevel analyses, stratified by sex and cohort, and adjusted for potential confounding variables. RESULTS: Significant decreases in recess and lunchtime moderate and vigorous physical activity were observed (p<0.001), with larger decreases occurring in the older cohort. Associated increases were observed in sedentary time over time (p<0.01). Although the contribution of recess to daily moderate intensity physical activity increased in the younger cohort over time (p<0.001), significant decreases were observed in the older cohort (p<0.001). CONCLUSION: Physical activity levels during recess and lunchtime decreased in both cohorts over time. Decreases in the contribution of recess and lunchtime to older children's daily physical activity were also observed. Interventions are needed in both primary and secondary schools to promote physical activity levels during recess and lunchtime, particularly during the early years of secondary school
SN - 1473-0480
AD - Centre for Physical Activity and Nutrition Research, Deakin University, 221 Burwood Highway, Burwood, Victoria 3125, Australia. nicky.ridgers@deakin.edu.au
UR - 21596716
ER -

TY - JOUR
ID - 406
T1 - Organized physical activity in young school children and subsequent 4-year change in body mass index
A1 - Dunton,G.
A1 - McConnell,R.
A1 - Jerrett,M.
A1 - Wolch,J.
A1 - Lam,C.
A1 - Gilliland,F.
A1 - Berhane,K.
Y1 - 2012/08//
N1 - Dunton, Genevieve. McConnell, Rob. Jerrett, Michael. Wolch, Jennifer. Lam, Claudia. Gilliland, Frank. Berhane, Kiros

Archives of pediatrics & adolescent medicine
9422751, bwf
AIM, IM
Journal Article. Research Support, N.I.H., Extramural. Research Support, Non-U.S. Gov't. Research Support, U.S. Gov't, Non-P.H.S.
English
KW - MEDLINE
KW - Body Composition
KW - Body Height
KW - Body Mass Index
KW - Body Weight
KW - California
KW - Child
KW - Preschool
KW - Female
KW - Humans
KW - Longitudinal Studies
KW - Male
KW - Overweight/ep [Epidemiology]
KW - Questionnaires
KW - Schools
KW - Sports/ph [Physiology]
RP - NOT IN FILE
SP - 713
EP - 718
JF - Archives of Pediatrics & Adolescent Medicine
JA - Arch Pediatr Adolesc Med
VL - 166
IS - 8
CY - United States
N2 - OBJECTIVE: To determine whether participation in organized outdoor team sports and structured indoor nonschool activity programs in kindergarten and first grade predicted subsequent 4-year change in body mass index (BMI; calculated as weight in kilograms divided by height in meters squared) during the adiposity rebound period of childhood. DESIGN: Longitudinal cohort study. SETTING: Forty-five schools in 13 communities across Southern California. PARTICIPANTS: Largely Hispanic and non-Hispanic white children (N = 4550) with a mean (SD) age at study entry of 6.60 (0.65) years. MAIN EXPOSURES: Parents completed questionnaires assessing physical activity, demographic characteristics, and other relevant covariates at baseline. Data on built and social environmental variables were linked to the neighborhood around children's homes using geographical information systems. MAIN OUTCOME MEASURES: Each child's height and weight were measured annually during 4 years of follow-up. RESULTS: After adjusting for several confounders, BMI increased at a rate 0.05 unit/year slower for children who participated in outdoor organized team sports at least twice per week compared with children who did not. For participation in each additional indoor nonschool structured activity class, lesson, and program, BMI increased at a rate 0.05 unit/year slower, and the attained BMI level at age 10 years was 0.48 units lower. CONCLUSION: Engagement in organized sports and activity programs as early as kindergarten and the first grade may result in smaller increases in BMI during the adiposity rebound period of childhood
SN - 1538-3628
AD - Department of Preventive Medicine, Keck School of Medicine, University of Southern California, Los Angeles 90033-9045, USA. dunton@usc.edu
UR - 22869403
ER -

TY - JOUR
ID - 407
T1 - 'Trying to make it all come together': structuration and employed mothers' experience of family food provisioning in Canada
A1 - Slater,J.

A1 - Sevenhuysen,G.
A1 - Edginton,B.
A1 - O'neil,J.
Y1 - 2012/09//
N1 - Slater, Joyce. Sevenhuysen, Gustaaf. Edginton, Barry. O'neil, John
Health promotion international
9008939, d00
IM
Journal Article. Research Support, Non-U.S. Gov't
English
KW - MEDLINE
KW - Adult
KW - Canada
KW - Child
KW - Preschool
KW - Choice Behavior
KW - Cooking
KW - Diet/px [Psychology]
KW - Employment/px [Psychology]
KW - Family/px [Psychology]
KW - Female
KW - Food Habits/px [Psychology]
KW - Food Habits
KW - Humans
KW - Interviews as Topic
KW - Mothers/px [Psychology]
KW - Time Factors
RP - NOT IN FILE
SP - 405
EP - 415
JF - Health Promotion International
JA - Health Promot Internation.
VL - 27
IS - 3
CY - England
N2 - This research examined the aetiology of employed mothers' food choice and food provisioning decisions using a qualitative, grounded theory methodology. Semi-structured interviews using the Food Choice Map were conducted with eleven middle-income employed mothers of elementary school-age children. Results demonstrated that the women exhibited conflicting identities with respect to food choice and provisioning. As 'good mothers' they were the primary food and nutrition caregivers for the family, desiring to provide healthy, homemade foods their families preferred at shared family meals. They also sought to be independent selves, working outside the home, within the context of a busy modern family. Increased food autonomy of children, and lack of time due to working outside the home and children's involvement in extracurricular activities, were significant influences on their food choice and provisioning. This resulted in frequently being unable to live up to their expectations of consistently providing healthy homemade foods and having shared family meals. To cope, the women frequently relied on processed convenience and fast foods despite their acknowledged inferior nutritional status. Using Giddens' structuration theory, the dynamic relationships between the women's food choice and provisioning actions, their identities and larger structures including socio-cultural norms, conditions of work and the industrial food system were explored. The ensuing dietary pattern of the women and their families increases the risk of poor health outcomes, including obesity. These results have implications for public health responses to improve population health by shifting the focus from individual-level maternal influences to structural influences on diet
SN - 1460-2245
AD - Department of Human Nutritional Sciences, University of Manitoba, Winnipeg, Manitoba, Canada R3T 2N2. slater@cc.umanitoba.ca
UR - 21693474
ER -

TY - JOUR
ID - 408
T1 - Risk factors predisposing to pedestrian road traffic injury in children living in Lima, Peru: a case-control study
A1 - Pernica,J.M.
A1 - LeBlanc,J.C.
A1 - Soto-Castellares,G.
A1 - Donroe,J.
A1 - Carhuancho-Meza,B.A.
A1 - Rainham,D.G.
A1 - Gilman,R.H.
Y1 - 2012/08//
N1 - Pernica, Jeffrey M. LeBlanc, John C. Soto-Castellares, Giselle. Donroe, Joseph. Carhuancho-Meza, Bristan A. Rainham, Daniel G C. Gilman, Robert H
Archives of disease in childhood
6xg, 0372434
AIM, IM
Journal Article. Research Support, Non-U.S. Gov't
English
KW - MEDLINE
KW - Accidents
KW - Traffic/sn [Statistics & Numerical Data]
KW - Case-Control Studies
KW - Child
KW - Family
KW - Female
KW - Humans
KW - Male
KW - Peru/ep [Epidemiology]
KW - Recreation
KW - Risk Factors
KW - Schools
KW - Walking/in [Injuries]
KW - Wounds and Injuries/ep [Epidemiology]
RP - NOT IN FILE
SP - 709
EP - 713
JF - Archives of Disease in Childhood
JA - Arch Dis Child
VL - 97
IS - 8
CY - England
N2 - OBJECTIVE: To describe the epidemiology of pedestrian road traffic injury in Lima and to identify associated child-level, family-level, and school travel-related variables. DESIGN: Case-control study. SETTING: The Instituto Nacional de Salud del Nino, the largest paediatric hospital in the city. PARTICIPANTS: Cases were children who presented because of pedestrian road traffic injury. Controls presented with other diagnoses and were matched on age, sex and severity of injury. RESULTS: Low socioeconomic status, low paternal education, traffic exposure during the trip to school, lack of supervision during outside play, and duration of outside play were all statistically significantly associated with case-control status. In multivariate logistic regression, a model combining the lack of supervision during outside play and the number of the streets crossed walking to school best predicted case-control status (p<0.001). CONCLUSIONS: These results emphasise that an assessment of children's play behaviours and school locations should be considered and integrated into any plan for an intervention designed to reduce pedestrian road traffic injury. A child-centred approach will ensure that children derive maximum benefit from sorely needed public health interventions
SN - 1468-2044

AD - Department of Pediatrics, McMaster University, Hamilton, Canada
UR - 22685050
ER -

TY - JOUR
ID - 409
T1 - Behavioral risk factors for overweight in early childhood; the 'Be active, eat right' study
A1 - Veldhuis,L.
A1 - Vogel,I.
A1 - Renders,C.M.
A1 - van,Rossem L.
A1 - Oenema,A.
A1 - Hirasing,R.A.
A1 - Raat,H.
Y1 - 2012///
N1 - Veldhuis, Lydian. Vogel, Ineke. Renders, Carry M. van Rossem, Lenie. Oenema, Anke. HiraSing, Remy A. Raat, Hein
The international journal of behavioral nutrition and physical activity
101217089
IM
Journal Article. Research Support, Non-U.S. Gov't
English
KW - MEDLINE
KW - Beverages
KW - Child Behavior
KW - Child
KW - Preschool
KW - Confidence Intervals
KW - Cross-Sectional Studies
KW - Diet
KW - Dietary Sucrose/ad [Administration & Dosage]
KW - Exercise
KW - Feeding Behavior
KW - Female
KW - Health Behavior
KW - Humans
KW - Male
KW - Obesity/et [Etiology]
KW - Odds Ratio
KW - Overweight
KW - Play and Playthings
KW - Risk Factors
KW - Risk-Taking
KW - Sedentary Lifestyle
KW - Television
RP - NOT IN FILE
SP - 74
JF - International Journal of Behavioral Nutrition & Physical Activity
JA - Int
VL - 9
CY - England
N2 - BACKGROUND: The lifestyle-related behaviors having breakfast, drinking sweet beverages, playing outside and watching TV have been indicated to have an association with childhood overweight, but research among young children (below 6 years old) is limited. The aim of the present study was to assess the associations between these four behaviors and overweight among young children. METHODS: This cross-sectional study used baseline data on 5-year-old children (n = 7505) collected for the study 'Be active, eat right'. Age and sex-specific cut-off points for body mass index of the International Obesity Task Force were used to assess

overweight/obesity. Multivariable logistic regression analyses were applied. RESULTS: For children whom had breakfast <7 days/week and watched TV >2 hours/day, the odds ratio (OR) for having overweight (obesity included) was, respectively, 1.49 (95% confidence interval (CI): 1.13-1.95), and 1.25 (95% CI: 1.03-1.51). There was a positive association between the number of risk behaviors present and the risk for having overweight. For children with 3 or all of the risk behaviors having breakfast <7 days/week, drinking sweet beverages >2 glasses/day, playing outside <1 h/day, watching TV >2 hs/day, the OR for overweight was 1.73 (95% CI: 1.11-2.71) (all models adjusted for children's sex and sociodemographic characteristics). CONCLUSION: Given the positive association between the number of behavioral risk factors and overweight, further studies are needed to evaluate the effectiveness of behavioral counseling of parents of toddlers in preventing childhood overweight. In the meantime we recommend physicians to target all four behaviors for counseling during well-child visits
SN - 1479-5868
AD - Department of Public Health, Erasmus MC-University Medical Centre Rotterdam, Rotterdam, The Netherlands. l.veldhuis@erasmusmc.nl
UR - 22704042
ER -

TY - JOUR
ID - 410
T1 - The transient effect of interictal spikes from a frontal focus on language-related gamma activity
A1 - Brown,E.C.
A1 - Matsuzaki,N.
A1 - Asano,E.
Y1 - 2012/08//
N1 - Brown, Erik C. Matsuzaki, Naoyuki. Asano, Eishi
Epilepsy & behavior : E&B
100892858
IM
Journal Article. Research Support, N.I.H., Extramural
English
KW - MEDLINE
KW - Adolescent
KW - Brain Mapping
KW - Brain Waves/ph [Physiology]
KW - Child
KW - Electroencephalography
KW - Epilepsy/co [Complications]
KW - Epilepsy/pa [Pathology]
KW - Female
KW - Frontal Lobe/pp [Physiopathology]
KW - Humans
KW - Language
KW - Male
KW - Neuropsychological Tests
KW - Retrospective Studies
KW - Time Factors
KW - Young Adult
RP - NOT IN FILE
SP - 497
EP - 502
JF - Epilepsy & Behavior
JA - Epilepsy Behav
VL - 24
IS - 4
CY - United States
N2 - Interictal spike activity arising from an epileptic focus may cause transient subclinical changes in language function. We retrospectively studied four patients with a seizure focus of the left frontal lobe who underwent

language mapping via electrocorticography. In three patients, we could group language task trials into 'spike' and 'non-spike' trials, based upon occurrence of spikes arising from the seizure onset zone during presentation of question stimuli. In one patient, we demonstrated a reduction in language-related gamma activity (80-100Hz) at one dorsal superior frontal site outside the seizure onset zone; reduction in mean peak amplitude of 58.4% of baseline reference (95% C.I.: 31.6% to 85.1%). This site was located near the seizure onset zone and was associated with the greatest spike rate among sites of similar function. This is the first preliminary study to show an effect of interictal spikes upon language-related gamma activity of the lateral frontal lobe. Copyright 2012 Elsevier Inc. All rights reserved
SN - 1525-5069
AD - School of Medicine, Wayne State University, Detroit, MI 48201, USA
UR - 22749027
ER -

TY - JOUR
ID - 411
T1 - Effect of physical activity and sun exposure on vitamin D status of Saudi children and adolescents
A1 - Al-Othman,A.
A1 - Al-Musharaf,S.
A1 - Al-Daghri,N.M.
A1 - Krishnaswamy,S.
A1 - Yusuf,D.S.
A1 - Alkharfy,K.M.
A1 - Al-Saleh,Y.
A1 - Al-Attas,O.S.
A1 - Alokail,M.S.
A1 - Moharram,O.
A1 - Sabico,S.
A1 - Chrousos,G.P.
Y1 - 2012///
N1 - Al-Othman, Abdulaziz. Al-Musharaf, Sara. Al-Daghri, Nasser M. Krishnaswamy, Soundararajan. Yusuf, Deqa S. Alkharfy, Khalid M. Al-Saleh, Yousef. Al-Attas, Omar S. Alokail, Majed S. Moharram, Osama. Sabico, Shaun. Chrousos, George P
BMC pediatrics
100967804
IM
Journal Article. Research Support, Non-U.S. Gov't
English
KW - MEDLINE
KW - Adolescent
KW - Age Factors
KW - Biological Markers/bl [Blood]
KW - Calcifediol/bl [Blood]
KW - Calcifediol/df [Deficiency]
KW - Child
KW - Cross-Sectional Studies
KW - Exercise
KW - Female
KW - Health Surveys
KW - Humans
KW - Linear Models
KW - Male
KW - Prevalence
KW - Questionnaires
KW - Saudi Arabia/ep [Epidemiology]
KW - Sunlight
KW - Vitamin D Deficiency/bl [Blood]
KW - Vitamin D Deficiency/di [Diagnosis]

KW - Vitamin D Deficiency/ep [Epidemiology]
KW - Vitamin D Deficiency/et [Etiology]
RP - NOT IN FILE
SP - 92
JF - BMC Pediatrics
JA - BMC Pediatr
VL - 12
CY - England
N2 - BACKGROUND: Accumulating evidence suggests an increased prevalence of vitamin D deficiency in the Middle East. In this context, we aimed to determine whether the prevalence of vitamin D deficiency is related to degree of physical activity and sun exposure among apparently healthy Saudi children and adolescents, a little studied population. METHODS: A total of 331 Saudi children aged 6-17 years (153 boys and 178 girls) were included in this cross sectional study. Levels of physical activity and sun exposure were determined using a standard questionnaire. Anthropometry, serum calcium and 25-(OH) vitamin D were analyzed. RESULTS: All subjects were vitamin D deficient, the majority being moderately deficient (71.6%). Age was the single most significant predictor affecting 25 (OH) Vitamin D levels, explaining 21% of the variance perceived (p = 1.68 x 10-14). Age-matched comparisons revealed that for groups having the same amount of sun exposure, those with moderate or are physically active will have higher levels of vitamin D status, though levels in across groups remained deficient. CONCLUSION: Vitamin D deficiency is common among Saudi children and adolescents, and is influenced by both sun exposure and physical activity. Promotion of an active outdoor lifestyle among Saudi children in both homes and schools may counteract the vitamin D deficiency epidemic in this vulnerable population. Vitamin D supplementation is suggested in all groups, including those with the highest sun exposure and physical activity
SN - 1471-2431
AD - Prince Mutaib Chair for Biomarkers of Osteoporosis, King Saud University, Riyadh, Kingdom of Saudi Arabia
UR - 22759399
ER -

TY - JOUR
ID - 412
T1 - Does the effect of a school recess intervention on physical activity vary by gender or race? Results from the Ready for Recess pilot study
A1 - Siahpush,M.
A1 - Huberty,J.L.
A1 - Beighle,A.
Y1 - 2012/09//
N1 - Siahpush, Mohammad. Huberty, Jennifer L. Beighle, Aaron
Journal of public health management and practice : JPHMP
c0l, 9505213
T
Journal Article. Research Support, Non-U.S. Gov't
English
KW - MEDLINE
KW - Acceleration
KW - Actigraphy
KW - Body Mass Index
KW - Child
KW - Exercise/ph [Physiology]
KW - Female
KW - Health Behavior/eh [Ethnology]
KW - Health Promotion/mt [Methods]
KW - Humans
KW - Leisure Activities
KW - Male
KW - Midwestern United States
KW - Physical Fitness

KW - Pilot Projects
KW - Recreation
KW - Regression Analysis
KW - School Health Services
KW - Schools/sn [Statistics & Numerical Data]
KW - Sex Distribution
KW - Sex Factors
KW - Students/sn [Statistics & Numerical Data]
KW - Time Factors
RP - NOT IN FILE
SP - 416
EP - 422
JF - Journal of Public Health Management & Practice
JA - J Public Health Manag Pract
VL - 18
IS - 5
CY - United States
N2 - BACKGROUND AND OBJECTIVES: The recess environment in schools has been identified as an integral part of school-based programs to enhance physical activity (PA). The purpose of this study was to report pilot findings on the extent to which the Ready for Recess intervention was associated with a different amount of increase in moderate to vigorous PA (MPVA) during recess and the rest of the school day between girls and boys, and between nonwhites and whites. METHODS: The Ready for Recess intervention modified the recess environment of schools by providing staff training and recreational equipment. The MPVA levels of 3rd, 4th, and 5th grade students (n = 93) at 2 schools were measured pre- and post-intervention using ActiGraph accelerometers. Multiple regression models with robust variance were utilized to test for the interaction of intervention with gender and race/ethnicity. RESULTS: The intervention was associated with an adjusted increase of 4.7 minutes (P <.001) in moderate/vigorous PA during recess. There was no evidence that this effect varied by gender (P = .944) or race (P = .731). The intervention was also associated with an adjusted increase of 29.6 minutes (P < .001) in moderate/vigorous PA during rest of the school day. While this effect did not vary by gender, there was some evidence (P = .034) that nonwhites benefited more from the intervention than whites. CONCLUSION: Simple strategies such as staff training and recreational equipment may be an effective way to increase PA in children (despite gender or ethnicity) during recess time as well as during the rest of the school day
SN - 1550-5022
AD - Department of Health Promotion, Social and Behavioral Health, College of Public Health, University of Nebraska Medical Center and Health, University of Nebraska Omaha, Omaha, Nebraska 68198, USA. msiahpush@unmc.edu
UR - 22836532
ER -

TY - JOUR
ID - 413
T1 - Assessment of food, nutrition, and physical activity practices in Oklahoma child-care centers
A1 - Sisson,S.B.
A1 - Campbell,J.E.
A1 - May,K.B.
A1 - Brittain,D.R.
A1 - Monroe,L.A.
A1 - Guss,S.H.
A1 - Ladner,J.L.
Y1 - 2012/08//
N1 - Sisson, Susan B. Campbell, Janis E. May, Kellie B. Brittain, Danielle R. Monroe, Lisa A. Guss, Shannon H. Ladner, Jennifer L
Journal of the Academy of Nutrition and Dietetics
101573920
AIM, IM

Comparative Study. Journal Article. Multicenter Study
English
KW - MEDLINE
KW - Caregivers/px [Psychology]
KW - Chi-Square Distribution
KW - Child Day Care Centers/st [Standards]
KW - Child Day Care Centers/sn [Statistics & Numerical Data]
KW - Child Day Care Centers
KW - Child Nutritional Physiological Phenomena/ph [Physiology]
KW - Child
KW - Preschool
KW - Cross-Sectional Studies
KW - Diet/st [Standards]
KW - Exercise/ph [Physiology]
KW - Feeding Behavior
KW - Female
KW - Food Preferences
KW - Food Services/st [Standards]
KW - Food Services/sn [Statistics & Numerical Data]
KW - Health Behavior
KW - Humans
KW - Male
KW - Nutrition Assessment
KW - Nutrition Policy
KW - Obesity/ep [Epidemiology]
KW - Obesity/pc [Prevention & Control]
KW - Oklahoma/ep [Epidemiology]
KW - Play and Playthings
KW - Social Environment
RP - NOT IN FILE
SP - 1230
EP - 1240
JF - Journal of the Academy of Nutrition & Dietetics
JA - J Acad Nutr Diet
VL - 112
IS - 8
CY - United States
N2 - The purpose of the current study was to determine the obesogenic practices in all-day child-care centers caring for preschool-aged children. This study used a cross-sectional, self-reported survey mailed to centers across Oklahoma (n=314). Frequency of responses and chi(2) were calculated comparing region and star rating. Items where the majority of centers frequently report best practices include: daily fruits served (76%), daily nonfried vegetables served (71%), rarely/never served sugary drinks (92%), rarely/never used food to encourage good behaviors (88%), staff join children at table most of the time (81%), staff rarely eat different foods in view of children (69%), visible self-serve or request availability of water (93%), regular informal communication about healthy eating (86%), opportunities for outdoor play (95%), not withholding activity for punishment (91%), accessible play equipment (59% to 80% for different types of equipment), and minimization of extended sitting time (78%). Practices where centers can improve include increasing variety of vegetables (18%), reducing frequency of high-fat meats served (74% serve more than once per week), increasing high-fiber and whole-grain foods (35% offer daily), serving style of "seconds" (28% help kids determine whether they are still hungry), nonfood holiday celebrations (44% use nonfood treats), having toys and books that encourage healthy eating (27%) and physical activity (25%) in all rooms in the center, a standard nutrition (21%) and physical education (50%) curriculum, and following a written physical activity policy (43%). Practitioners can use these data to develop benchmarks and interventions, as this was the first study to assess statewide obesogenic practices in child care. Copyright 2012 Academy of Nutrition and Dietetics. Published by Elsevier Inc. All rights reserved
SN - 2212-2672

AD - Behavioral Nutrition and Physical Activity Laboratory, Department of NutritionalSciences, University of Oklahoma Health Sciences Center, 1200 N Stonewall Ave, Oklahoma City, OK 73117-1215, USA. susan-sisson@ouhsc.edu
UR - 22818731
ER -

TY - JOUR
ID - 414
T1 - Exercise deficit disorder in youth: an emergent health concern for school nurses
A1 - Faigenbaum,A.D.
A1 - Gipson-Jones,T.L.
A1 - Myer,G.D.
Y1 - 2012/08//
N1 - Faigenbaum, Avery D. Gipson-Jones, Trina L. Myer, Gregory D
The Journal of school nursing : the official publication of the National Association of School Nurses
bgs, 9206498
N
Journal Article
English
KW - MEDLINE
KW - Adolescent
KW - Age Factors
KW - Child
KW - Child Welfare
KW - Exercise/ph [Physiology]
KW - Health Behavior
KW - Humans
KW - Motor Activity/ph [Physiology]
KW - Nurse's Role
KW - Obesity/ep [Epidemiology]
KW - Obesity/et [Etiology]
KW - Obesity/pc [Prevention & Control]
KW - School Nursing
KW - Sedentary Lifestyle
KW - United States/ep [Epidemiology]
RP - NOT IN FILE
SP - 252
EP - 255
JF - Journal of School Nursing
JA - J Sch Nurs
VL - 28
IS - 4
CY - United States
N2 - Although the benefits of regular physical activity are widely acknowledged, recent findings indicate that a growing number of youth are not as active as they should be. The impact of a sedentary lifestyle during childhood on lifelong pathological processes and associated health care costs have created a need for immediate action to manage, if not prevent, unhealthy behaviors during this vulnerable period of life. The concept of identifying children with exercise deficit disorder early in life and prescribing effective interventions to prevent the cascade of adverse health outcomes later in life is needed to raise public awareness, focus on primary prevention, and impact the collective behaviors of health care providers and public health agencies. School nurses are in a unique position to take advantage of well-child visits as an ideal opportunity to assess physical activity habits and encourage daily participation in play, recess, sports, planned exercise, and physical education
SN - 1546-8364
AD - The College of New Jersey, School of Nursing, Health and Exercise Science, Ewing, NJ 08628, USA. faigenba@tcnj.edu
UR - 22427317
ER -

TY - JOUR
ID - 415
T1 - Play equipment, physical activity opportunities, and children's activity levels at childcare
A1 - Gubbels,J.S.
A1 - Van Kann,D.H.
A1 - Jansen,M.W.
Y1 - 2012///
N1 - Gubbels, Jessica S. Van Kann, Dave H H. Jansen, Maria W J
Journal of environmental and public health
101516361
IM
Journal Article. Multicenter Study. Research Support, Non-U.S. Gov't
English
KW - MEDLINE
KW - Child Day Care Centers/og [Organization & Administration]
KW - Child
KW - Preschool
KW - Environment
KW - Female
KW - Humans
KW - Male
KW - Motor Activity/ph [Physiology]
KW - Netherlands/ep [Epidemiology]
KW - Play and Playthings
KW - Social Environment
RP - NOT IN FILE
SP - 326520
JF - Journal Of Environmental & Public Health
JA - J Environ Public Health
VL - 2012
CY - United States
N2 - This study investigated the association between physical activity facilities at childcare (e.g., play equipment) and physical activity of 2- and 3-year olds. Observations of physical activity intensity were performed among 175 children at 9 childcare centers in The Netherlands, using the OSRAC-P. The physical activity facilities were assessed for indoors and outdoors separately, using the EPAO instrument. Regular (single-level) multivariate and multilevel linear regression analyses examined the association of the facilities and child characteristics (age and sex) with children's activity levels. Various physical activity facilities were available in all childcare centers (e.g., balls). Riding toys and a small playing area were associated with lower indoor physical activity levels. Outdoor physical activity levels were positively associated with the availability of portable jumping equipment and the presence of a structured track on the playground. Portable slides, fixed swinging equipment, and sandboxes were negatively associated with outdoor activity levels. In addition, the 3-year old children were more active outdoors than the 2-year olds. In conclusion, not all physical activity facilities at childcare were indeed positively associated with children's activity levels. The current findings provide concrete leads for childcare providers regarding which factors they can improve in the physical environment to facilitate children's physical activity
SN - 1687-9813
AD - Department of Health Promotion, Maastricht University, PO Box 616, 6200 MD Maastricht, The Netherlands
UR - 22811736
ER -

TY - JOUR
ID - 416
T1 - Bronchial and alveolar nitric oxide in exercise-induced bronchoconstriction in asthmatic children
A1 - Chinellato,I.
A1 - Piazza,M.

A1 - Peroni,D.
A1 - Sandri,M.
A1 - Chiorazzo,F.
A1 - Boner,A.L.
A1 - Piacentini,G.
Y1 - 2012/08//
N1 - Chinellato, I. Piazza, M. Peroni, D. Sandri, M. Chiorazzo, F. Boner, A L. Piacentini, G
Clinical and experimental allergy : journal of the British Society for Allergy and Clinical Immunology
ceb, 8906443
IM
Clinical Trial. Journal Article
English
KW - MEDLINE
KW - Asthma
KW - Exercise-Induced/me [Metabolism]
KW - Exercise-Induced/pp [Physiopathology]
KW - Bronchi/me [Metabolism]
KW - Bronchi/pp [Physiopathology]
KW - Bronchoconstriction
KW - Child
KW - Exercise Test
KW - Exhalation
KW - Female
KW - Humans
KW - Male
KW - Nitric Oxide/me [Metabolism]
KW - Nitric Oxide
KW - Pulmonary Alveoli/me [Metabolism]
KW - Pulmonary Alveoli/pp [Physiopathology]
KW - Spirometry
RP - NOT IN FILE
SP - 1190
EP - 1196
JF - Clinical & Experimental Allergy
JA - Clin Exp Allergy
VL - 42
IS - 8
CY - England
N2 - BACKGROUND: Epidemiological studies have shown an association between the severity of exercise-induced bronchoconstriction (EIB) and fractional exhaled nitric oxide at the flow of 50 mL/s (FeNO(50)). However, no study has assessed the correlation between alveolar production (C(alv)) and bronchial flux (J(NO)) of nitric oxide (NO) and EIB in asthmatic children. OBJECTIVE: To identify the relationship between severity of EIB and bronchial or alveolar nitric oxide. METHODS: Our group included 36 allergic children with intermittent asthma. The EIB was determined by a standard exercise challenge and the severity was expressed as the maximum change in percentage from the baseline value of lung function (FEV(1)%, FEF(25-75)%) after exercising. A chemiluminescence analyser at multiple flows was used to calculate FeNO(50), J(NO) and C(alv,) which reflect large airways, J(NO) and alveolar concentration of NO respectively. RESULTS: Sixteen (44.4%) children presented a FEV(1) > 10%, eight (22.2%) had FEV(1) > 15% and nine (25%) children had a FEF(25-75) > 26%. A significant correlation was observed between severity of EIB and FeNO(50) , J(NO) and C(alv.) EIB was significantly more severe in children sensitive to indoor allergens compared with outdoor allergens only (P = 0.014); those children showed also higher levels of C(alv) (P = 0.003) and of J(NO) (P = 0.044). CONCLUSIONS AND CLINICAL RELEVANCE: Our results suggest that inflammation is present in the central and peripheral airways and that it is associated with the severity of EIB. Clinicaltrials.gov NCT00952835. 2012 Blackwell Publishing Ltd
SN - 1365-2222
AD - Department of Pediatrics, University of Verona, Verona, Italy
UR - 22805466

ER -

TY - JOUR
ID - 417
T1 - Where are youth physically active? A descriptive examination of 45 parks in a southeastern community
A1 - Reed,J.A.
A1 - Hooker,S.P.
Y1 - 2012/04//
N1 - Reed, Julian A. Hooker, Steven P
Childhood obesity (Print)
101542497
IM
Journal Article. Research Support, Non-U.S. Gov't
English
KW - MEDLINE
KW - Adolescent
KW - Child
KW - European Continental Ancestry Group/sn [Statistics & Numerical Data]
KW - Female
KW - Humans
KW - Male
KW - Minority Groups/sn [Statistics & Numerical Data]
KW - Motor Activity
KW - Physical Exertion
KW - Public Facilities/sn [Statistics & Numerical Data]
KW - Sedentary Lifestyle
KW - Sex Distribution
KW - Sex Factors
KW - Sports/sn [Statistics & Numerical Data]
RP - NOT IN FILE
SP - 124
EP - 131
JF - Childhood Obesity
JA - Child
VL - 8
IS - 2
CY - United States
N2 - BACKGROUND: The purpose of this study was to identify the activity settings used and physical activity (PA) intensity achieved by boys and girls in 45 parks in a southeastern community. METHODS: PA and sedentary behaviors were assessed at all activity settings by gender, race, and PA intensity. Eight activity settings were identified in 45 parks. The System for Observing Play and Recreation in Communities (SOPARC) instrument was used to assess PA during the summers of 2004 through 2008. RESULTS: More boys (n = 1,668; 58%) were observed at the 45 parks than girls (n = 1,184, 42%). Playgrounds were the most frequently used activity setting by both boys and girls. The vast majority of PA observed on the playgrounds was vigorous PA (n = 584; 41%). Playing fields were the second most frequently used activity setting by male and female youth, and 292 (10.3%) of all boys and girls were observed using this activity setting. However, only 18 of the 45 parks (40%) had a playing field. More males (n = 164; 56%) used the playing fields for PA than girls (n = 128; 44%). Most boys and girls were observed participating in vigorous PA (n = 254; 87%) at this activity setting. Observations of seven of the eight activity settings in the 45 parks indicated a greater frequency and percentage of white youth observed in comparison to minorities. CONCLUSIONS: Identifying the patterns of children and teens in community parks will provide investigators with specific objective data to develop user profiles that can perhaps lead to effective PA interventions in these environmental settings
SN - 2153-2176
AD - Department of Health Sciences, Furman University, Greenville, SC 29613, USA.
Julian.reed@furman.edu
UR - 22799512
ER -

TY - JOUR
ID - 418
T1 - Comparison of measurements of time outdoors and light levels as risk factors for myopia in young Singapore children
A1 - Dharani,R.
A1 - Lee,C.F.
A1 - Theng,Z.X.
A1 - Drury,V.B.
A1 - Ngo,C.
A1 - Sandar,M.
A1 - Wong,T.Y.
A1 - Finkelstein,E.A.
A1 - Saw,S.M.
Y1 - 2012/07//
N1 - Dharani, R. Lee, C-F. Theng, Z X. Drury, V B. Ngo, C. Sandar, M. Wong, T-Y. Finkelstein, E A. Saw, S-M
Eye (London, England)
eye, 8703986
IM
Journal Article. Research Support, Non-U.S. Gov't
English
KW - MEDLINE
KW - Child
KW - Data Collection/mt [Methods]
KW - Female
KW - Humans
KW - Leisure Activities
KW - Male
KW - Myopia/et [Etiology]
KW - Myopia/pc [Prevention & Control]
KW - Risk Factors
KW - Singapore
KW - Sunlight/ae [Adverse Effects]
KW - Time Factors
RP - NOT IN FILE
SP - 911
EP - 918
JF - Eye
VL - 26
IS - 7
CY - England
N2 - AIMS: To compare methods to measure time outdoor and light levels, two possible predictors of myopia, in Singapore children. METHODS: Outdoor time from a diary and portable light meter over a 1-week period was compared in 117 Singapore children aged 6-12 years with and without myopia. All children wore a (HOBO Pendant temp/light Part # UA-002-64) light meter for 1 week and the parents filled the 7-day outdoor diary to track the outdoor activity. RESULTS: Mean outdoor time from diary and time with light levels was 5.44hours per week and 7.91hours per week, respectively, during school term and school holidays. Time spent with light levels of >1000Lux from the light meter were 7.08h per week and 9.81h per week, respectively, during school term and school holidays. The intraclass correlation coefficients were 0.21 and 0.28 for outdoor time from the diary and light meter (1000Lux cut-off) during the school term and holidays, respectively. The correlation coefficient was 0.34 (95% CI 0.05, 0.58) for a weekday during school holidays, 0.17 (-0.14, 0.45) for a weekday during school term, 0.07 (-0.16, 0.29) for a weekday during school term, and 0.25 (0.02, 0.46) for a weekend during school term. CONCLUSIONS: The agreement between the light meter and 1-week diary was poor to fair. Both instruments measure different parameters, time outdoors and light intensity, and could therefore capture different aspects of risk in future myopia studies
SN - 1476-5454

AD - Saw Swee Hock School of Public Health, National University of Singapore, 16 Medical Drive, Singapore
UR - 22562184
ER -

TY - JOUR
ID - 419
T1 - Ecological assessment of cognitive functions in children with acquired brain injury: a systematic review. [Review]
A1 - Chevignard,M.P.
A1 - Soo,C.
A1 - Galvin,J.
A1 - Catroppa,C.
A1 - Eren,S.
Y1 - 2012///
N1 - Chevignard, Mathilde P. Soo, Cheryl. Galvin, Jane. Catroppa, Cathy. Eren, Senem
Brain injury : [BI]
bra, 8710358
IM
Journal Article. Research Support, Non-U.S. Gov't. Review
English
KW - MEDLINE
KW - Activities of Daily Living
KW - Adolescent
KW - Brain Injuries/di [Diagnosis]
KW - Brain Injuries/pp [Physiopathology]
KW - Brain Injuries/px [Psychology]
KW - Child
KW - Cognition Disorders/di [Diagnosis]
KW - Cognition Disorders/pp [Physiopathology]
KW - Cognition Disorders/px [Psychology]
KW - Disability Evaluation
KW - Executive Function
KW - Female
KW - Humans
KW - Male
KW - Neuropsychological Tests/sn [Statistics & Numerical Data]
KW - Reproducibility of Results
RP - NOT IN FILE
SP - 1033
EP - 1057
JF - Brain Injury
JA - Brain Inj
VL - 26
IS - 9
CY - England
N2 - BACKGROUND: Childhood acquired brain injury (ABI) often leads to impairment in cognitive functioning, resulting in disabilities in both the home and school environment. Assessing the impact of these cognitive deficits in everyday life using traditional neuropsychological tests has been challenging. This study systematically reviewed ecological measures of cognitive abilities available for children with ABI. METHOD: Eight databases were searched (until October 2011) for scales: (1) focused on ecological assessment of cognitive functioning; (2) with published data in an ABI population; (3) applicable to children up to 17;11 years of age; and (4) in English. The title and abstract of all papers were reviewed independently by two reviewers. RESULTS: Database searches yielded a total of 12 504 references, of which 17 scales met the inclusion criteria for the review, focusing on executive functions (n=9), memory (n=3), general cognitive abilities (n=2), visuo-spatial skills (n=2) and attention (n=1). Four tasks used observation of actual performance in a natural environment, five were proxy-reports and six were functional paper and pencil type tasks, performed in an office. CONCLUSION: Overall, few measures were found; eight were still experimental tasks which did not

provide norms. Executive functions were better represented in ecological assessment, with relatively more standardized scales available
SN - 1362-301X
AD - Rehabilitation Department for Children with Acquired Brain Injury (INR-A), Hopitaux de Saint Maurice, Saint Maurice, France. m.chevignard@hopitaux-st-maurice.fr
UR - 22715895
ER -

TY - JOUR
ID - 420
T1 - The effect of an unstructured, moderate to vigorous, before-school physical activity program in elementary school children on academics, behavior, and health
A1 - Tompkins,C.L.
A1 - Hopkins,J.
A1 - Goddard,L.
A1 - Brock,D.W.
Y1 - 2012///
N1 - Tompkins, Connie L. Hopkins, Jacob. Goddard, Lauren. Brock, David W
BMC public health
100968562
IM
Journal Article. Randomized Controlled Trial
English
KW - MEDLINE
KW - Child
KW - Child Behavior
KW - Education
KW - Exercise/ph [Physiology]
KW - Exercise/px [Psychology]
KW - Female
KW - Health Status
KW - Humans
KW - Male
KW - Questionnaires
RP - NOT IN FILE
SP - 300
JF - BMC Public Health
VL - 12
CY - England
N2 - BACKGROUND: Physical inactivity has been deemed a significant, contributing factor to childhood overweight and obesity. In recent years, many school systems removed recess and/or physical education from their curriculum due to growing pressure to increase academic scores. With the vast majority of children's time spent in school, alternative strategies to re-introduce physical activity back into schools are necessary. A creative yet underutilized solution to engage children in physical activity may be in before-school programs. The objective of the proposed study is to examine the effect of an unstructured, moderate to vigorous, before-school physical activity program on academic performance, classroom behavior, emotions, and other health related measures. METHODS/DESIGN: Children in 3rd-5th grade will participate in a before-school (7:30-8:15 a.m.), physical activity program for 12 weeks, 3 days a week. Children will be able to choose their preferred activity and asked to sustain physical activity of moderate to vigorous intensity with individual heart rate monitored during each session. DISCUSSION: The proposed study explores an innovative method of engaging and increasing physical activity in children. The results of this study will provide evidence to support the feasibility of an unstructured, moderate to vigorous, before-school physical activity program in children and provide insight regarding the ideal physical activity intensity and duration necessary to achieve a positive increase in academic performance. TRIAL REGISTRATION: ClinicalTrials.gov Identifier: NCT01505244
SN - 1471-2458
AD - Department of Rehabilitation & Movement Science, University of Vermont, 106 Carrigan Drive, 310D Rowell, Burlington, VT 05405-0068, USA. Connie.Tompkins@uvm.edu

UR - 22537028
ER -

TY - JOUR
ID - 421
T1 - Screen time and physical activity behaviours are associated with health-related quality of life in Australian adolescents
A1 - Lacy,K.E.
A1 - Allender,S.E.
A1 - Kremer,P.J.
A1 - de Silva-Sanigorski,A.M.
A1 - Millar,L.M.
A1 - Moodie,M.L.
A1 - Mathews,L.B.
A1 - Malakellis,M.
A1 - Swinburn,B.A.
Y1 - 2012/08//
N1 - Lacy, Kathleen E. Allender, Steven E. Kremer, Peter J. de Silva-Sanigorski, Andrea M. Millar, Lynne M. Moodie, Marjory L. Mathews, Louise B. Malakellis, Mary. Swinburn, Boyd A
Quality of life research : an international journal of quality of life aspects of treatment, care and rehabilitation
9210257, bqm
IM
Journal Article. Research Support, Non-U.S. Gov't
English
KW - MEDLINE
KW - Adolescent
KW - Australia
KW - Child
KW - Computers/ut [Utilization]
KW - Cross-Sectional Studies
KW - Exercise
KW - Female
KW - Health Status
KW - Humans
KW - Male
KW - Obesity
KW - Quality of Life
KW - Questionnaires
KW - Sedentary Lifestyle
KW - Television/ut [Utilization]
KW - Video Games/sn [Statistics & Numerical Data]
RP - NOT IN FILE
SP - 1085
EP - 1099
JF - Quality of Life Research
JA - Qual Life Res
VL - 21
IS - 6
CY - Netherlands
N2 - PURPOSE: To explore the cross-sectional relationships between health-related quality of life (HRQoL) and physical activity (PA) behaviours and screen-based media (SBM) use among a sample of Australian adolescents. METHODS: Data came from baseline measures collected for the It's Your Move! community-based obesity prevention intervention. Questionnaire data on sociodemographics, PA, SBM and HRQoL were collected from 3,040 students (56% boys) aged 11-18 years in grade levels 7-11 in 12 secondary schools. Anthropometric data were measured. RESULTS: The highest level of PA at recess, lunchtime and after school was associated with higher HRQoL scores (boys, by 5.3, 8.1, 6.3 points; girls, by 4.2, 6.1, 8.2 points) compared with not being active during these periods. Exceeding 2 h of SBM use each day was associated with

significantly lower HRQoL scores (boys, by 3.2 points; girls, by 4.0 points). Adolescents who were physically active and low SBM users on school days had higher HRQoL scores (boys, by 6.6 points; girls, by 7.8 points) compared with those who were not physically active every school day and high SBM users on school days. CONCLUSIONS: Several of the relationships between low PA and high SBM use and HRQoL were comparable to those previously observed between chronic disease conditions and HRQoL, indicating that these behaviours deserve substantial attention
SN - 1573-2649
AD - WHO Collaborating Centre for Obesity Prevention, Deakin University, Geelong Waterfront Campus, 1 Gheringhap Street, Geelong, VIC, 3220, Australia. katie.lacy@deakin.edu.au
UR - 21932139
ER -

TY - JOUR
ID - 422
T1 - Physical activity and sedentary behaviors and health-related quality of life in adolescents
A1 - Gopinath,B.
A1 - Hardy,L.L.
A1 - Baur,L.A.
A1 - Burlutsky,G.
A1 - Mitchell,P.
Y1 - 2012/07//
N1 - Gopinath, Bamini. Hardy, Louise L. Baur, Louise A. Burlutsky, George. Mitchell, Paul
Pediatrics
oxv, 0376422
AIM, IM
Journal Article. Research Support, Non-U.S. Gov't
English
KW - MEDLINE
KW - Adolescent
KW - Adolescent Behavior
KW - Analysis of Variance
KW - Child
KW - Cross-Sectional Studies
KW - Dancing
KW - Exercise
KW - Female
KW - Health Surveys
KW - Humans
KW - Linear Models
KW - Longitudinal Studies
KW - Male
KW - Multivariate Analysis
KW - Quality of Life
KW - Sedentary Lifestyle
KW - Self Report
KW - Sports
KW - Television
KW - Video Games
RP - NOT IN FILE
SP - e167
EP - e174
JF - Pediatrics
VL - 130
IS - 1
CY - United States
N2 - OBJECTIVE: Our goal was to assess cross-sectional and longitudinal associations between physical activity and sedentary behaviors (television viewing, computer and video-game usage, and reading) with health-

related QoL. METHODS: Of 2353 children surveyed (median age: 12.7 years), 1216 were resurveyed 5 years later, and 475 were newly recruited into the study (N = 1691). Children completed detailed activity questionnaires. Health-related QoL was assessed by using the Pediatric Quality of Life Inventory (PedsQL). RESULTS: Cross-sectionally, after multivariable adjustment, adolescents in the highest versus lowest tertile of time spent in outdoor physical activity and television viewing had a higher (P(trend) = .001) and lower (P(trend) = .0003) total PedsQL score, respectively. Adolescents who remained in the highest tertiles compared with those in the lowest tertiles of total physical activity over the 5 years had significantly higher scores in the following areas: total (P(trend) = .04), physical summary (P(trend) = .0001), and social (P(trend) = .02) domains. Conversely, those in the highest versus lowest tertile of screen-viewing time during follow-up reported significantly lower values in the following areas: total score (6.34-unit difference), physical summary (4.86-unit difference), psychosocial summary (7.09-unit difference), and emotional (8.33-unit difference) and school (9.78-unit difference) domains. CONCLUSIONS: Regular physical activity over the long-term was associated with higher perceived health-related QoL among adolescents. Conversely, lower PedsQL scores were observed among those who spent the most time in screen-viewing activities. Improved understanding of these relationships could help in developing interventions to promote general well-being among adolescents
SN - 1098-4275
AD - Centre for Vision Research, Department of Ophthalmology and Westmead Millennium Institute, University of Sydney, Sydney, New South Wales, Australia
UR - 22689863
ER -

TY - JOUR
ID - 423
T1 - Physical activity and beverages in home- and center-based child care programs
A1 - Tandon,P.S.
A1 - Garrison,M.M.
A1 - Christakis,D.A.
Y1 - 2012/07//
N1 - Tandon, Pooja S. Garrison, Michelle M. Christakis, Dimitri A
Journal of nutrition education and behavior
101132622
IM
Journal Article. Research Support, Non-U.S. Gov't
English
KW - MEDLINE
KW - Adult
KW - Beverages
KW - Caregivers
KW - Child Day Care Centers
KW - Child
KW - Preschool
KW - Humans
KW - Interviews as Topic
KW - Motor Activity
KW - Nutrition Surveys
KW - Play and Playthings
RP - NOT IN FILE
SP - 355
EP - 359
JF - Journal of Nutrition Education & Behavior
JA - J Nutr Educ Behav
VL - 44
IS - 4
CY - United States
N2 - OBJECTIVE: To describe and compare obesity prevention practices related to physical activity and beverages in home- and center-based child care programs. METHODS: A telephone survey of licensed home- and center-based child care programs in Florida, Massachusetts, Michigan, and Washington between October

and December 2008. RESULTS: Most programs reported almost universal access to outdoor play areas and staff who frequently engage in and lead physical activities. About half of the programs reported < 60 minutes of outdoor play time per day for preschoolers. Fewer home-based programs offered a variety of fixed and portable play equipment. Over a third of the programs did not have adequate indoor space for all physical activities. Over two thirds of programs offered juice 3 or 4 times per week or more. CONCLUSIONS AND IMPLICATIONS: Many child care programs could increase active opportunities (by increasing outdoor time and providing better spaces for indoor physical activity) and decrease juice provision. Copyright 2012 Society for Nutrition Education and Behavior. Published by Elsevier Inc. All rights reserved
SN - 1878-2620
AD - Center for Child Health, Behavior, and Development, Seattle Children's Research Institute, Seattle, WA 98145-5005, USA
UR - 22564855
ER -

TY - JOUR
ID - 424
T1 - Comparing different accelerometer cut-points for sedentary time in children
A1 - Fischer,C.
A1 - Yildirim,M.
A1 - Salmon,J.
A1 - Chinapaw,M.JM
Y1 - 2012/05//
N1 - Fischer, Claudia. Yildirim, Mine. Salmon, Jo. Chinapaw M, J M
Pediatric exercise science
8909729
IM
Comparative Study. Journal Article. Research Support, Non-U.S. Gov't
English
KW - MEDLINE
KW - Acceleration
KW - Actigraphy/mt [Methods]
KW - Age Factors
KW - Child
KW - Preschool
KW - Female
KW - Health Behavior
KW - Humans
KW - Leisure Activities
KW - Male
KW - Motor Activity/ph [Physiology]
KW - Sedentary Lifestyle
KW - Time
RP - NOT IN FILE
SP - 220
EP - 228
JF - Pediatric Exercise Science
JA - Pediatr Exerc Sci
VL - 24
IS - 2
CY - United States
N2 - Actigraph accelerometers are hypothesized to be valid measurements for assessing children's sedentary time. However, there is considerable variation in accelerometer cut-points used. Therefore, we compared the most common accelerometer sedentary cut-points of children performing sedentary behaviors. Actigraph Actitrainer uniaxial accelerometers were used to measure children's activity intensity (29 children, 5-11 years old) during different activities, namely playing computer games, nonelectronic sedentary games, watching television and playing outdoors. A structured protocol was the criterion for assessing the validity of four common cut-points (100, 300, 800, 1100 counts/minute). The median counts during all sedentary behaviors

were below the lowest comparison cut-point of 100 cpm. The 75th percentile values for the sedentary behaviors were always below the cut-point of 300 cpm. Our results suggest that the cut-point of <100 cpm is the most appropriate
SN - 1543-2920
AD - EMGO Institute for Health and Care Research and Dept of Public and Occupational Health, VU University Medical Center, Amsterdam, The Netherlands
UR - 22728414
ER -

TY - JOUR
ID - 425
T1 - Pediatric trauma in the Austrian Alps: the epidemiology of sport-related injuries in helicopter emergency medical service
A1 - Selig,H.F.
A1 - Hupfl,M.
A1 - Trimmel,H.
A1 - Voelckel,W.G.
A1 - Nagele,P.
Y1 - 2012/06//
N1 - Selig, Harald F. Hupfl, Michael. Trimmel, Helmut. Voelckel, Wolfgang G. Nagele, Peter
High altitude medicine & biology
d04, 100901183
IM, S
Journal Article
English
KW - MEDLINE
KW - Adolescent
KW - Air Ambulances/sn [Statistics & Numerical Data]
KW - Austria/ep [Epidemiology]
KW - Bicycling/in [Injuries]
KW - Brain Injuries/ep [Epidemiology]
KW - Child
KW - Preschool
KW - Contusions/ep [Epidemiology]
KW - Facial Injuries/ep [Epidemiology]
KW - Female
KW - Fractures
KW - Bone/ep [Epidemiology]
KW - Humans
KW - Infant
KW - Lower Extremity/in [Injuries]
KW - Male
KW - Mountaineering/in [Injuries]
KW - Rescue Work
KW - Retrospective Studies
KW - Skiing/in [Injuries]
KW - Snow Sports/in [Injuries]
KW - Spinal Injuries/ep [Epidemiology]
KW - Upper Extremity/in [Injuries]
RP - NOT IN FILE
SP - 112
EP - 117
JF - High Altitude Medicine & Biology
JA - High Alt.Med Biol
VL - 13
IS - 2
CY - United States

N2 - BACKGROUND/PURPOSE: We aimed to examine the epidemiological characteristics and injury patterns of pediatric trauma in helicopter emergency medical service (HEMS) caused by sports/outdoor activities in alpine environment. METHODS: This retrospective cohort study analyzed 912 primary HEMS missions for pediatric trauma (0-14 years of age) in Austrian Alps between 1 January 2006 and 30 June 2007. Children were stratified by age into toddlers (3-5 years), children in early (6-9 years), and late school age (10-14 years). RESULTS: The majority of pediatric sports-related trauma in alpine environment was caused by skiing (82.1%; n=749). Pediatric patients were predominately in late school age and boys (72.8%, n=664 and 61.0%, n=556, respectively) and a minority (16.0%, n=146) was severely injured. Overall, fracture (47.0%, n=429), contusion (17.9%, n=163), and traumatic brain injury (17.0%, n=155) were the most common prehospital diagnoses. The most frequent pattern of injury was related to the head/face and spine (36.3%, n=331). CONCLUSIONS: The knowledge about epidemiological characteristics of HEMS use for injured children in alpine environment may be essential for training requirements of HEMS crews and operational considerations of HEMS providers. The incidence of head and spinal injuries requires support for initiatives to promote helmet wear and appropriate risk behavior amongst skiers and snowboarders
SN - 1557-8682
AD - Department of Anesthesiology and General Intensive Care and Pain Therapy, Medical University of Vienna, Vienna, Austria. harald.se@gmx.at
UR - 22724614
ER -

TY - JOUR
ID - 426
T1 - The association between leisure-time physical activities and asthma symptoms among 10- to 12-year-old children: the effect of living environment in the PANACEA study
A1 - Kosti,R.I.
A1 - Priftis,K.N.
A1 - Anthracopoulos,M.B.
A1 - Papadimitriou,A.
A1 - Grigoropoulou,D.
A1 - Lentzas,Y.
A1 - Yfanti,K.
A1 - Panagiotakos,D.B.
Y1 - 2012/05//
N1 - Kosti, Rena I. Priftis, Kostas N. Anthracopoulos, Michael B. Papadimitriou, Anastasios. Grigoropoulou, Dimitra. Lentzas, Yiannis. Yfanti, Konstantina. Panagiotakos, Demosthenes B
The Journal of asthma : official journal of the Association for the Care of Asthma
hf7, 8106454
IM
Journal Article
English
KW - MEDLINE
KW - Asthma/ep [Epidemiology]
KW - Asthma/pp [Physiopathology]
KW - Body Weights and Measures
KW - Child
KW - Diet
KW - Environment
KW - Exercise
KW - Female
KW - Health Behavior
KW - Health Surveys
KW - Humans
KW - Leisure Activities
KW - Life Style
KW - Male
KW - Residence Characteristics
KW - Rural Population

KW - Socioeconomic Factors
KW - Urban Population
RP - NOT IN FILE
SP - 342
EP - 348
JF - Journal of Asthma
JA - J Asthma
VL - 49
IS - 4
CY - England
N2 - OBJECTIVE: This study evaluated the interrelationships of living environment, physical activity, lifestyle/dietary habits, and nutritional status on the prevalence of childhood asthma. METHODS: In a cross-sectional survey 1125 children (529 boys), 10 to 12 years old, were selected from 18 schools located in an urban environment (Athens, n = 700) and from 10 schools located in rural areas (n = 425) in Greece. RESULTS: Children living in Athens had higher likelihood of "ever had" asthma compared with children living in rural areas (odds ratio (OR) = 1.78, 95% confidence interval (CI) = 1.25-2.54), after adjusting for age and sex. After adjusting for age, sex, body mass index, and adherence to the Mediterranean diet (KIDMED score), leisure-time physical activity was inversely associated with "ever had" asthma. When stratifying by county of residence, a trend toward reduced asthma symptoms among children engaged in outdoor physical activities during their leisure time who reside in rural (but not urban) environment was observed (OR = 0.88, 95% CI = 0.77-1.01). CONCLUSIONS: The inverse relationship between asthma symptoms and leisure-time physical activity in the rural environment and the lack of an association between asthma symptoms and organized sports-related activities should draw the attention of public healthcare authorities. Their efforts should focus on the planning of a sustainable natural environment, which will promote the physical health of children and reduce the burden of childhood asthma
SN - 1532-4303
AD - Department of Nutrition and Dietetics, Harokopio University, Athens, Greece
UR - 22300140
ER -

TY - JOUR
ID - 427
T1 - Improving generalization of peer socialization gains in inclusive school settings using initiations training
A1 - Koegel,L.K.
A1 - Kuriakose,S.
A1 - Singh,A.K.
A1 - Koegel,R.L.
Y1 - 2012/05//
N1 - Koegel, Lynn K. Kuriakose, Sarah. Singh, Anjileen K. Koegel, Robert L
Behavior modification
9l5, 7803043
IM
Journal Article. Research Support, N.I.H., Extramural. Research Support, Non-U.S. Gov't
English
KW - MEDLINE
KW - Behavior Therapy/mt [Methods]
KW - Child
KW - Child Development Disorders
KW - Pervasive/px [Psychology]
KW - Preschool
KW - Female
KW - Generalization (Psychology)
KW - Humans
KW - Interpersonal Relations
KW - Mainstreaming (Education)
KW - Male
KW - Peer Group

KW - Social Behavior
KW - Social Participation/px [Psychology]
RP - NOT IN FILE
SP - 361
EP - 377
JF - Behavior Modification
JA - Behav Modif
VL - 36
IS - 3
CY - United States
N2 - Social engagement by children with autism spectrum disorder (ASD) in unstructured school settings generally occurs at very low levels, if at all. Although many interventions improve peer socialization, generalization and maintenance of such gains when interventions are faded are typically low. The present study employed a multiple baseline design across participants to target generalization in the absence of interventionists in elementary school children with ASD at recess. Teaching initiations has been suggested as one method to increase generalization. The results of the present study showed that when initiations were targeted during intervention for social play, the participants demonstrated generalized peer social engagement, increases in unprompted peer-directed initiations, and more positive affect during peer interactions. Results are discussed in terms of theoretical and applied implications of incorporating initiations training into social interventions
SN - 1552-4167
AD - University of California, Santa Barbara, 93106-9490, USA. lynnk@education.ucsb.edu
UR - 22645399
ER -

TY - JOUR
ID - 428
T1 - Fresh Fruit and Vegetable Program participation in elementary schools in the United States and availability of fruits and vegetables in school lunch meals
A1 - Ohri-Vachaspati,P.
A1 - Turner,L.
A1 - Chaloupka,F.J.
Y1 - 2012/06//
N1 - Ohri-Vachaspati, Punam. Turner, Lindsey. Chaloupka, Frank J
Journal of the Academy of Nutrition and Dietetics
101573920
AIM, IM
Journal Article. Research Support, Non-U.S. Gov't
English
KW - MEDLINE
KW - Child
KW - Child Nutritional Physiological Phenomena
KW - Consumer Participation
KW - Cross-Sectional Studies
KW - Female
KW - Food Preferences/px [Psychology]
KW - Food Services/st [Standards]
KW - Food Services/sn [Statistics & Numerical Data]
KW - Food Supply/st [Standards]
KW - Food Supply/sn [Statistics & Numerical Data]
KW - Fruit/sd [Supply & Distribution]
KW - Fruit
KW - Humans
KW - Male
KW - Nutrition Policy
KW - Obesity/ep [Epidemiology]
KW - Obesity/pc [Prevention & Control]

KW - Poverty
KW - Schools
KW - Students/px [Psychology]
KW - United States
KW - Vegetables/sd [Supply & Distribution]
KW - Vegetables
RP - NOT IN FILE
SP - 921
EP - 926
JF - Journal of the Academy of Nutrition & Dietetics
JA - J Acad Nutr Diet
VL - 112
IS - 6
CY - United States
N2 - Dietary intake among children in the United States falls short of national recommendations. Schools can play an important role in improving children's preferences and food consumption patterns. The US Department of Agriculture's Fresh Fruit and Vegetable Program (FFVP) aims to improve children's nutrient intake patterns by offering fresh fruits and vegetables as snacks outside the reimbursable meals programs in elementary schools that serve large numbers of low-income children. Using a nationally representative sample of public elementary schools, this cross-sectional study investigated FFVP participation patterns among schools by demographic and school characteristics. Further, the study investigated the association between FFVP participation and availability of fresh fruits, salads, and vegetables at lunch as reported by school administrators and foodservice staff. Data collected via a mail-back survey from 620 public elementary schools participating in the National School Lunch Program during 2009-2010 were analyzed. Almost 70% of the FFVP-participating schools had a majority of students (>50%) eligible for free and reduced-cost meals. Participating in US Department of Agriculture Team Nutrition Program and having a registered dietitian or a nutritionist on staff were significantly associated with FFVP participation. Based on the results from logistic regression analyses schools participating in the FFVP were significantly more likely (odds ratio 2.07; 95% CI 1.12 to 3.53) to serve fresh fruit during lunch meals. Slightly >25% of public elementary schools across the United States participated in the FFVP, and participation was associated with healthier food availability in school lunches. Copyright 2012 Academy of Nutrition and Dietetics. Published by Elsevier Inc. All rights reserved
SN - 2212-2672
AD - School of Nutrition and Health Promotion, Arizona State University, 500 N 3rd St, Phoenix, AZ 85004, USA. pohrivac@asu.edu
UR - 22709817
ER -

TY - JOUR
ID - 429
T1 - [Study of a population-wide epidemic outbreak of mumps virus G1 in Jerez de la Frontera (Spain)]. [Spanish]
A1 - Gonzalez,P.P.
A1 - Barrios,J.A.
A1 - Morales Serna,J.C.
Y1 - 2012/06//
N1 - Gonzalez, Pascasio Pena. Barrios, Jose Almenara. Morales Serna, Juan Carlos
Atencion primaria / Sociedad Espanola de Medicina de Familia y Comunitaria
9111075, a5f
IM
English Abstract. Journal Article
Spanish
KW - MEDLINE
KW - Adolescent
KW - Adult
KW - Child
KW - Preschool
KW - Cross-Sectional Studies

KW - Disease Outbreaks
KW - Humans
KW - Infant
KW - Mumps/ep [Epidemiology]
KW - Mumps/vi [Virology]
KW - Mumps virus/cl [Classification]
KW - Spain/ep [Epidemiology]
KW - Young Adult
RP - NOT IN FILE
SP - 320
EP - 327
JF - Atencion Primaria
JA - Aten.Primaria
VL - 44
IS - 6
CY - Spain
N2 - OBJECTIVES: To characterize the mumps outbreak, assess the effectiveness of vaccines and discuss prevention and control measures. DESIGN: Observational cross-sectional study of cases by person, place, and time. LOCATION: City Jerez de la Frontera and 8 Primary Care centres. PARTICIPANTS: A total of 116 cases of mumps were reported throughout seven months, with the last case in June 2008. MEASUREMENTS: Main outcomes: age, sex, place of study or work, symptoms start date, clinical description, complications, and genotype of virus isolation, history and vaccination compositions. Calculation of total vaccine effectiveness. Active case finding and contact studies were performed on the families, work places and leisure areas. RESULTS: The mean age of the cases was 16.24 years(SD 10.6). The majority (68.96%) of the cases were children at school and 31.03% arose from family and work areas. Mumps vaccine coverage rates were above 90%. The complete vaccine effectiveness (2 doses); for those under 20 years-old it was: 99.84% (95% CI=99.77 - 99.89), being lower in schools with the highest attack rate, immunised with current vaccine from Jeryl Lynn strain: 71.01% (95% CI=55.85 80.97). Two doses of this strain were used in 13.79% of the cases. Susceptible populations were vaccinated at the start of the outbreak: 11,381 doses used in schools and 93 outside schools. CONCLUSIONS: An outbreak of mumps virus G 1 population with high rates of mumps vaccine coverage, with areas with low vaccine effectiveness with current vaccine. Mass vaccination of population has contributed to the resolution of the outbreak. Copyright 2011 Elsevier Espana, S.L. All rights reserved
SN - 1578-1275
UR - 22019067
ER -

TY - JOUR
ID - 430
T1 - Prospective study of the participation patterns of Grade 6 and Year 8 students in Victoria, Australia in activities outside of school
A1 - McMullan,S.
A1 - Chin,R.
A1 - Froude,E.
A1 - Imms,C.
Y1 - 2012/06//
N1 - McMullan, Sarah. Chin, Rachel. Froude, Elspeth. Imms, Christine
Australian occupational therapy journal
15420200r
IM
Journal Article
English
KW - MEDLINE
KW - Adolescent
KW - Child
KW - Confidence Intervals
KW - Cross-Sectional Studies
KW - Female

KW - Health Care Surveys
KW - Hobbies/px [Psychology]
KW - Humans
KW - Leisure Activities/px [Psychology]
KW - Male
KW - Prospective Studies
KW - Psychometrics
KW - Questionnaires
KW - Recreation/px [Psychology]
KW - Self Report
KW - Sex Factors
KW - Social Participation/px [Psychology]
KW - Victoria
RP - NOT IN FILE
SP - 197
EP - 208
JF - Australian Occupational Therapy Journal
JA - Aust Occup Ther J
VL - 59
IS - 3
CY - Australia
N2 - BACKGROUND: Positive participation outcomes are deemed the ultimate goal of health care and specifically of occupational therapy. Knowledge of the typical participation patterns of children in Australia will provide essential information to support our understanding of participation and the goal of maximising children's engagement. AIMS: This study investigated the participation of Grade 6 and Year 8 Victorian students in activities outside school and explored differences between genders and between students in different year levels. Secondarily, we began to establish Australian normative data on the Children's Assessment of Participation and Enjoyment and Preferences for Activities of Children. METHODS: This cross sectional survey methods study recruited students from a random selection of public schools. Participation was measured using the Children's Assessment of Participation and Enjoyment and Preferences for Activities of Children questionnaires. RESULTS: Participants included 84 (37 female, 47 male) students in Grade 6 (n = 43) and Year 8 (n = 41). Differences between year levels were only evident for participation in Recreational and Active Physical activities. Grade 6 students did more activities, more intensely than Year 8 students, but with no difference in enjoyment. The mean number of Recreational activities done by Grade 6 students was 8.5 (95%CI: 7.9-9.1) compared to Year 8 students 6.9 (95%CI: 6.1-7.7; P = 0.001). Gender differences were evident in the participation patterns within Social, Skill-Based and Self-Improvement activities. CONCLUSION: The findings suggested that gender was a more important influence on participation patterns than a 2-year age gap, with participation patterns being relatively stable between Grade 6 and Year 8. 2012 The Authors Australian Occupational Therapy Journal 2012 Occupational Therapy Australia
SN - 1440-1630
AD - School of Occupational Therapy, La Trobe University, Melbourne, Victoria, Australia
UR - 22690770
ER -

TY - JOUR
ID - 431
T1 - Formaldehyde personal exposure measurements and time weighted exposure estimates in children
A1 - Lazenby,V.
A1 - Hinwood,A.
A1 - Callan,A.
A1 - Franklin,P.
Y1 - 2012/08//
N1 - Lazenby, Victoria. Hinwood, Andrea. Callan, Anna. Franklin, Peter
Chemosphere
b4d, 0320657
IM

Journal Article. Research Support, Non-U.S. Gov't
English
KW - MEDLINE
KW - Air Pollution
KW - Indoor/an [Analysis]
KW - Behavior
KW - Child
KW - Environmental Exposure
KW - Female
KW - Formaldehyde/an [Analysis]
KW - Humans
KW - Life Style
KW - Male
KW - Questionnaires
KW - Seasons
RP - NOT IN FILE
SP - 966
EP - 973
JF - Chemosphere
VL - 88
IS - 8
CY - England
N2 - Residential concentrations of formaldehyde have been associated with poor respiratory health in children, where formaldehyde has been measured using stationary monitors inside homes. Although children spend most of their time indoors at home, there are few studies of children's personal exposure to formaldehyde. The aim of this study was to investigate the relationship between personal exposure formaldehyde concentrations, microenvironmental concentrations and time weighted exposure estimates in children. Forty-one primary school children (aged between 9 and 12 years) wore a personal passive sampler over two 24h periods in two seasons and completed 24h daily activity diaries and a questionnaire about lifestyle and behaviour. Samplers were co located indoors at home, outdoors at centralised locations and indoors at school for the corresponding period. Personal exposure formaldehyde concentrations in this group of children were generally low with a geometric mean concentration of 9.1 ppb (range <detection limit to 27.3 ppb). There were strong correlations between personal exposure concentrations and both domestic indoor (r(s)=.779, p<0.001) and time weighted estimated (r(s)=.802, p<0.001) concentrations. The time weighted model did not improve the estimate of personal exposure compared with stationary indoor concentrations. Indoor air concentration measured with a single stationary monitor was a suitable surrogate for personal exposure. Copyright 2012 Elsevier Ltd. All rights reserved
SN - 1879-1298
AD - Edith Cowan University, Centre for Ecosystem Management, 270 Joondalup Drive, Joondalup, WA 6027, Australia
UR - 22516208
ER -

TY - JOUR
ID - 432
T1 - Look who's walking: social and environmental correlates of children's walking in London
A1 - Steinbach,R.
A1 - Green,J.
A1 - Edwards,P.
Y1 - 2012/07//
N1 - Steinbach, Rebecca. Green, Judith. Edwards, Phil
Health & place
din, 9510067
T
Journal Article. Research Support, Non-U.S. Gov't
English
KW - MEDLINE

KW - Adolescent
KW - Asian Continental Ancestry Group/sn [Statistics & Numerical Data]
KW - Automobiles/ec [Economics]
KW - Child
KW - Data Collection
KW - Environment Design
KW - Female
KW - Health Behavior/eh [Ethnology]
KW - Health Behavior
KW - Humans
KW - London
KW - Male
KW - Ownership
KW - Schools
KW - Social Class
KW - Travel
KW - Walking
RP - NOT IN FILE
SP - 917
EP - 927
JF - Health & Place
JA - Health Place
VL - 18
IS - 4
CY - England
N2 - A substantial literature examines the social and environmental correlates of walking to school but less addresses walking outside the school commute. Using travel diary data from London, we examined social and environmental correlates of walking: to school; outside the school commute during term time; and during the summer and weekends. Living in a household without a car was associated with all journey types; 'Asian' ethnicity was negatively associated with walking for non-school travel; environmental factors were associated with non-school journeys, but not the school commute. Interventions aiming to increase children's active travel need to take account of the range of journeys they make. Copyright 2012 Elsevier Ltd. All rights reserved
SN - 1873-2054
AD - Faculty of Public Health & Policy, London School of Hygiene & Tropical Medicine, 15-17 Tavistock Place, London WC1H 9SH, United Kingdom. rebecca.steinbach@lshtm.ac.uk
UR - 22464978
ER -

TY - JOUR
ID - 433
T1 - The legacy of early interpersonal experience. [Review]
A1 - Roisman,G.I.
A1 - Fraley,R.C.
Y1 - 2012///
N1 - Roisman, Glenn I. Fraley, R Chris
Advances in child development and behavior
2k7, 0370417
IM
Journal Article. Review
English
KW - MEDLINE
KW - Achievement
KW - Adolescent
KW - Child
KW - Preschool
KW - Emotional Intelligence
KW - Gene-Environment Interaction

KW - Humans
KW - Infant
KW - Internal-External Control
KW - Interpersonal Relations
KW - Object Attachment
KW - Parent-Child Relations
KW - Personality Assessment
KW - Personality Development
KW - Socialization
KW - Temperament
KW - Young Adult
RP - NOT IN FILE
SP - 79
EP - 112
JF - Advances in Child Development & Behavior
JA - Adv Child Dev Behav
VL - 42
CY - United States
N2 - Despite decades of research on social development, the question of whether early interpersonal experiences play an important role in human development is just as controversial today as it was decades ago. In this chapter, we argue that there are at least three reasons why this debate has remained unsettled. First, outside of a few landmark longitudinal studies, relatively few investigations have prospectively charted the long-term correlates of experiences with primary caregivers in the first 3 years of life. Second, most analyses of datasets focused on the legacy of early experience have emphasized two-wave test-retest analyses. We contend that, to distinguish between enduring versus merely transient effects of early experience, it is instead necessary to study the pattern of associations that are observed across multiple assessment occasions. Third, despite decades of stinging critiques by behavior-genetic scholars, not enough attention has been paid in social developmental research to determining whether the robust correlations identified between parental behavior and child outcomes are genetically mediated. In this chapter, we discuss each of these issues, in turn, and review recent conceptual models and empirical data that help to address them
SN - 0065-2407
AD - Department of Psychology, University of Illinois at Urbana-Champaign, champaign, Illinois, USA
UR - 22675904
ER -

TY - JOUR
ID - 434
T1 - Geographic variations in elementary school-based physical activity practices
A1 - Turner,L.
A1 - Chaloupka,F.J.
A1 - Slater,S.J.
Y1 - 2012/07//
N1 - Turner, Lindsey. Chaloupka, Frank J. Slater, Sandy J
The Journal of school health
k13, 0376370
IM, N
Journal Article
English
KW - MEDLINE
KW - Child
KW - Data Collection
KW - Geography
KW - Health Promotion/mt [Methods]
KW - Health Promotion/sn [Statistics & Numerical Data]
KW - Humans
KW - Illinois
KW - Logistic Models

KW - Male
KW - Motor Activity/ph [Physiology]
KW - Physical Education and Training/sn [Statistics & Numerical Data]
KW - Play and Playthings
KW - Risk Factors
KW - School Health Services
KW - Schools
KW - Students
RP - NOT IN FILE
SP - 307
EP - 310
JF - Journal of School Health
JA - J Sch Health
VL - 82
IS - 7
CY - United States
N2 - BACKGROUND: Physical activity (PA) is associated with health and academic benefits among children, but many schools do not meet national recommendations. This study examined school-based PA practices in nationally representative samples of public elementary schools, and geographic variations in those practices. METHODS: Mail-back surveys were used to gather data from 578 public elementary schools during the 2006-2007 school year and 680 public elementary schools during the 2009-2010 school year. RESULTS: Nationwide, only 1 in 5 schools met the national recommendation of at least 150 minutes of physical education (PE) per week, although PE was more prevalent in the South. Nearly three-fourths of schools nationwide met recommendations for 20 or more minutes of recess daily, and this was more prevalent in the Midwest but less so in the South. Approximately 40% of schools offered sports and approximately 40% offered other opportunities for PA during the school day. The only practice that increased over time was formal classroom instruction on PA. CONCLUSIONS: There has been little change in elementary school-based PA practices nationwide in recent years. With the upcoming renewal of the Elementary and Secondary Education Act, policymakers have an important opportunity to promote increased PA in schools. 2012, American School Health Association
SN - 1746-1561
AD - Institute for Health Research and Policy, University of Illinois at Chicago, 1747 W. Roosevelt Road, M/C 275, Room 558, Chicago, IL 60608, USA. lindseyt@uic.edu
UR - 22671946
ER -

TY - JOUR
ID - 435
T1 - Sun exposure and risk of lymphoid neoplasms in Singapore
A1 - Wong,K.Y.
A1 - Tai,B.C.
A1 - Chia,S.E.
A1 - Kuperan,P.
A1 - Lee,K.M.
A1 - Lim,S.T.
A1 - Loong,S.
A1 - Mow,B.
A1 - Ng,S.B.
A1 - Tan,L.
A1 - Tan,S.Y.
A1 - Tan,S.H.
A1 - Tao,M.
A1 - Wong,A.
A1 - Wong,G.C.
A1 - Seow,A.
Y1 - 2012/07//
N1 - Wong, Kin-Yoke. Tai, Bee-Choo. Chia, Sin-Eng. Kuperan, Ponnudurai. Lee, Khai-Mun. Lim, Soon-Thye. Loong, Susan. Mow, Benjamin. Ng, Siok-Bian. Tan, Leonard. Tan, Soo-Yong. Tan, Suat-Hoon. Tao, Miriam.

Wong, Alvin. Wong, Gee-Chuan. Seow, Adeline
Cancer causes & control : CCC
a5r, 9100846
IM
Journal Article. Research Support, Non-U.S. Gov't
English
KW - MEDLINE
KW - Adolescent
KW - Adult
KW - Age Factors
KW - Aged
KW - 80 and over
KW - Case-Control Studies
KW - Child
KW - Female
KW - Humans
KW - Logistic Models
KW - Lymphoma/cl [Classification]
KW - Lymphoma/ep [Epidemiology]
KW - Lymphoma
KW - Non-Hodgkin/ep [Epidemiology]
KW - Male
KW - Middle Aged
KW - Risk Factors
KW - Singapore/ep [Epidemiology]
KW - Sunlight
KW - Time Factors
KW - Young Adult
RP - NOT IN FILE
SP - 1055
EP - 1064
JF - Cancer Causes & Control
JA - Cancer Causes Control
VL - 23
IS - 7
CY - Netherlands
N2 - BACKGROUND: Epidemiologic studies have reported an inverse association between sun exposure and non-Hodgkin lymphoma (NHL), but these have been almost exclusively conducted in Western populations residing in temperate locations. We evaluated the association between personal outdoor sun exposure and risk of malignant lymphomas in Singapore. METHODS: A hospital-based case-control study of 541 incident cases of lymphoid neoplasms and 830 controls were recruited during 2004-2008. Participants were interviewed regarding recreational or occupational outdoor activities during childhood and in adulthood. Basic demographics and potential confounders were also collected. Odds ratios (OR) and 95 % confidence intervals (CI) were calculated using unconditional logistic regression analysis. RESULTS: Compared with individuals who did not have regular sun exposure, a lower risk of NHL was observed for those who reported regular exposure on non-school days during childhood [OR, 0.62; 95 % CI, 0.46-0.83] and non-working days in adulthood [OR, 0.70; 95 % CI, 0.51-0.97]. The protective effect was more evident among women. CONCLUSION: Our findings support an inverse relationship between intermittent sun exposure and the risk of NHL. These findings are consistent with the growing evidence from various countries, but further studies, especially prospective studies, are needed in Asian populations
SN - 1573-7225
AD - Saw Swee Hock School of Public Health, National University of Singapore, Singapore
UR - 22543543
ER -

TY - JOUR
ID - 436

T1 - Lead contamination in French children's homes and environment
A1 - Lucas,J.P.
A1 - Le,Bot B.
A1 - Glorennec,P.
A1 - Etchevers,A.
A1 - Bretin,P.
A1 - Douay,F.
A1 - Sebille,V.
A1 - Bellanger,L.
A1 - Mandin,C.
Y1 - 2012/07//
N1 - Lucas, Jean-Paul. Le Bot, Barbara. Glorennec, Philippe. Etchevers, Anne. Bretin, Philippe. Douay, Francis. Sebille, Veronique. Bellanger, Lise. Mandin, Corinne
Environmental research
ei2, 0147621
IM
Journal Article. Research Support, Non-U.S. Gov't
English
KW - MEDLINE
KW - Air Pollution
KW - Indoor/an [Analysis]
KW - Child
KW - Dust/an [Analysis]
KW - Environmental Exposure/an [Analysis]
KW - Environmental Monitoring/mt [Methods]
KW - France
KW - Housing/st [Standards]
KW - Humans
KW - Lead/an [Analysis]
KW - Paint/an [Analysis]
KW - Soil Pollutants/an [Analysis]
KW - Water Pollutants
KW - Chemical/an [Analysis]
RP - NOT IN FILE
SP - 58
EP - 65
JF - Environmental Research
JA - Environ Res
VL - 116
CY - United States
N2 - Lead in homes is a well-known source of childhood lead exposure, which is still of concern due to the health effects of low lead doses. This study aims to describe lead contamination in the homes of children aged 6 months to 6 years in France (without overseas). Between October 2008 and August 2009, 484 housing units were investigated. Lead in tap water and total and leachable lead levels from floor dust, outdoor soils and paint chips were measured. X-ray fluorescence measurements were carried out on non-metallic and metallic substrates. Nationwide results are provided. The indoor floor dust lead (PbD) geometric mean (GM) was 8.8 mug/m2 (0.8 mug/ft2) and 6.8 mug/m2 (0.6 mug/ft2) for total and leachable lead respectively; 0.21% of homes had an indoor PbD loading above 430.5 mug/m2 (40 mug/ft2). The outdoor play area concentration GM was 33.5 mg/kg and 21.7 mg/kg in total and leachable lead respectively; 1.4% of concentrations were higher than or equal to 400 mg/kg. Outdoor floor PbD GM was 44.4 mug/m2 (4.1 mug/ft2) that was approximately 3.2 times higher than the GM of indoor PbD. Lead-based paint (LBP) was present in 25% of dwellings, LBP on only non-metallic substrates was present in 19% of homes and on metallic substrates in 10% of dwellings. The GM of lead concentrations in tap water was below 1 mug/L; 58% of concentrations were lower than 1 mug/L and 2.9% were higher than or equal to 10 mug/L. The age cut-off for homes with lead would be 1974 for paint and 1993 for indoor floor dust. This study provides, for the first time, a look at the state of lead contamination to which children are exposed in French housing. Moreover, it provides policy makers an estimate of the number of

French dwellings sheltering children where abatement should be conducted. Copyright 2012 Elsevier Inc. All rights reserved
SN - 1096-0953
AD - Universite Paris Est, CSTB-Centre Scientifique et Technique du Batiment, ESE/Sante, Marne-la-Vallee, France. jean-paul.lucas@cstb.fr
UR - 22551852
ER -

TY - JOUR
ID - 437
T1 - French children's exposure to metals via ingestion of indoor dust, outdoor playground dust and soil: contamination data
A1 - Glorennec,P.
A1 - Lucas,J.P.
A1 - Mandin,C.
A1 - Le,Bot B.
Y1 - 2012/09/15/
N1 - Glorennec, Philippe. Lucas, Jean-Paul. Mandin, Corinne. Le Bot, Barbara
Environment international
du1, 7807270
IM
Journal Article. Research Support, Non-U.S. Gov't
English
KW - MEDLINE
KW - Air Pollutants/an [Analysis]
KW - Air Pollution
KW - Indoor/sn [Statistics & Numerical Data]
KW - Child
KW - Child Day Care Centers/sn [Statistics & Numerical Data]
KW - Preschool
KW - Dust/an [Analysis]
KW - Environmental Exposure/sn [Statistics & Numerical Data]
KW - Female
KW - France
KW - Humans
KW - Infant
KW - Male
KW - Metals/an [Analysis]
KW - Soil/ch [Chemistry]
KW - Soil Pollutants/an [Analysis]
RP - NOT IN FILE
SP - 129
EP - 134
JF - Environment International
JA - Environ Int
VL - 45
CY - Netherlands
N2 - In addition to dietary exposure, children are exposed to metals via ingestion of soils and indoor dust, contaminated by natural or anthropogenic outdoor and indoor sources. The objective of this nationwide study was to assess metal contamination of soils and dust which young French children are exposed to. A sample of 484 children (6 months to 6 years) was constituted in order to obtain representative results for young French children. In each home indoor settled dust was sampled by a wipe in up to five rooms. Outdoor playgrounds were sampled with a soil sample ring (n=315) or with a wipe in case of hard surfaces (n=53). As, Cd, Cr, Cu, Mn, Pb, Sb, Sr, and V were measured because of their potential health concern due to soil and dust ingestion. The samples were digested with hydrochloric acid, and afterwards aqua regia in order to determine both leachable and total metal concentrations and loadings by mass spectrometry with a quadrupole ICP-MS. In indoor settled dust most (total) loadings were below the Limit of Quantification (LOQ), except for Pb and Sr,

whose median loadings were respectively 9 and 10 mug/m2. The 95th percentile of loadings were 2 mug/m2 for As, <0.8 for Cd, 18 for Cr, 49 for Cu, <64 for Mn, 63 for Pb, 2 for Sb, 56 for Sr, and <8 for V. Median/95th percentile of loadings in settled dust on outdoor playgrounds were 2/16, <0.8/1.3, 17/53, 49/330, 99/424, 32/393, 2/13, 86/661 and 10/37 mug/m2 for As, Cd, Cr, Cu, Mn, Pb, Sb, Sr, and V respectively. In outdoor playground soil median/95th percentile of concentrations (mug/g) were 8/26, <0.65/1, 25/52, <26/53,391/956, 27/254, 0.7/4, 54/295, 23/57 for As, Cd, Cr, Cu, Mn, Pb, Sb, Sr, and V respectively. These results are comparable with those observed in other countries. Because of their representative nature, we can assess children's exposures to these metals via soil and dust and the associated risks in urban and rural environments. Ratios of leachable/total concentrations and loadings, calculated on >LOQ measurements, differed among metals. To a lesser extent, they were also affected by type of matrix, with (except for Cd) a greater leachability of dust (especially indoor) compared to soils. Copyright 2012 Elsevier Ltd. All rights reserved
SN - 1873-6750
AD - EHESP - School of Public Health, Sorbonne Paris Cite, Rennes, France. philippe.glorennec@ehesp.fr
UR - 22613504
ER -

TY - JOUR
ID - 438
T1 - Combining global positioning system and accelerometer data to determine the locations of physical activity in children
A1 - Oreskovic,N.M.
A1 - Blossom,J.
A1 - Field,A.E.
A1 - Chiang,S.R.
A1 - Winickoff,J.P.
A1 - Kleinman,R.E.
Y1 - 2012/05//
N1 - Oreskovic, Nicolas M. Blossom, Jeff. Field, Alison E. Chiang, Sylvia R. Winickoff, Jonathan P. Kleinman, Ronald E
Geospatial health
101302943
IM
Journal Article. Research Support, Non-U.S. Gov't
English
KW - MEDLINE
KW - Acceleration
KW - Adolescent
KW - Child
KW - Child Welfare/sn [Statistics & Numerical Data]
KW - Data Collection
KW - Data Interpretation
KW - Statistical
KW - Female
KW - Geographic Information Systems/sn [Statistics & Numerical Data]
KW - Humans
KW - Male
KW - Massachusetts
KW - Motor Activity/ph [Physiology]
KW - Public Health/is [Instrumentation]
KW - Public Health/mt [Methods]
KW - Social Environment
KW - Software
RP - NOT IN FILE
SP - 263
EP - 272
JF - Geospatial Health
JA - Geospat.Health

VL - 6
IS - 2
CY - Italy
N2 - National trends indicate that children and adolescents are not achieving sufficient levels of physical activity. Combining global positioning system (GPS) technology with accelerometers has the potential to provide an objective determination in locations where youth engage in physical activity. The aim of this study was to identify the optimal methods for collecting combined accelerometer and GPS data in youth, to best locate where children spend time and are physically active. A convenience sample of 24 mid-school children in Massachusetts was included. Accelerometers and GPS units were used to quantify and locate childhood physical activity over 5 weekdays and 2 weekend days. Accelerometer and GPS data were joined by time and mapped with a geographical information system (GIS) using ArcGIS software. Data were collected in winter, spring, summer in 2009-2010, collecting a total of 26,406 matched datapoints overall. Matched data yield was low (19.1% total), regardless of season (winter, 12.8%; spring, 30.1%; summer, 14.3%). Teacher-provided, pre-charged equipment yielded the most matched (30.1%; range: 10.1-52.3%) and greatest average days (6.1 days) of data. Across all seasons, children spent most of their time at home. Outdoor use patterns appeared to vary by season, with street use increasing in spring, and park and playground use increasing in summer. Children spent equal amounts of physical activity time at home and walking in the streets. Overall, the various methods for combining GPS and accelerometer data provided similarly low amounts of combined data. No combined GPS and accelerometer data collection method proved superior in every data return category, but use of GIS to map joined accelerometer and GPS data can demarcate childhood physical activity locations
SN - 1970-7096
AD - MGH Center for Child and Adolescent Health Research and Policy, Boston, MA 02114, USA.
noreskovic@partners.org
UR - 22639128
ER -

TY - JOUR
ID - 439
T1 - Parent emotional expressiveness and children's self-regulation: associations with abused children's school functioning
A1 - Haskett,M.E.
A1 - Stelter,R.
A1 - Proffit,K.
A1 - Nice,R.
Y1 - 2012/04//
N1 - Haskett, Mary E. Stelter, Rebecca. Proffit, Katie. Nice, Rachel
Child abuse & neglect
can, 7801702
IM
Journal Article. Research Support, N.I.H., Extramural
English
KW - MEDLINE
KW - Adaptation
KW - Psychological
KW - Aggression/px [Psychology]
KW - Child
KW - Child Abuse/px [Psychology]
KW - Preschool
KW - Educational Status
KW - Emotions
KW - Female
KW - Humans
KW - Interpersonal Relations
KW - Male
KW - Parent-Child Relations
KW - Parents/px [Psychology]
KW - Social Control

KW - Informal
RP - NOT IN FILE
SP - 296
EP - 307
JF - Child Abuse & Neglect
JA - Child Abuse Negl
VL - 36
IS - 4
CY - England
N2 - OBJECTIVE: Identifying factors associated with school functioning of abused children is important in prevention of long-term negative outcomes associated with school failure. The purpose of this study was to examine the degree to which parent emotional expressiveness and children's self-regulation predicted early school behavior of abused children. METHODS: The sample included 92 physically abused children ages 4-7 and one of their parents (95.7% mothers). Parents completed a measure of their own emotional expressiveness, and parents and teachers provided reports of children's self-regulatory skills. Children's school functioning was measured by observations of playground aggression and teacher reports of aggression and classroom behavior. RESULTS: Parents' expression of positive and negative emotions was associated with various aspects of children's self-regulation and functioning in the school setting. Links between self-regulation and children's school adjustment were robust; poor self-regulation was associated with higher aggression and lower cooperation and self-directed behavior in the classroom. There was minimal support for a mediating role of children's self-regulation in links between parent expressiveness and children's behavior. PRACTICE IMPLICATIONS: Findings point to the relevance of parent emotional expressivity and children's self-regulatory processes in understanding physically abused children's functioning at the transition to school. Although further research is needed, findings indicate that increasing parental expression of positive emotion should be a focus in treatment along with reduction in negativity of abusive parents. Further, addressing children's self-regulation could be important in efforts to reduce aggression and enhance children's classroom competence. Copyright 2012 Elsevier Ltd. All rights reserved
SN - 1873-7757
AD - North Carolina State University, Department of Psychology, Raleigh, NC 27695, USA
UR - 22565040
ER -

TY - JOUR
ID - 440
T1 - Toilet training in daycare centers in Flanders, Belgium
A1 - Kaerts,N.
A1 - Van,Hal G.
A1 - Vermandel,A.
A1 - Wyndaele,J.J.
A1 - participants of the learning study group.
Y1 - 2012/06//
N1 - Kaerts, Nore. Van Hal, Guido. Vermandel, Alexandra. Wyndaele, Jean-Jacques. participants of the learning study group
European journal of pediatrics
end, 7603873
IM
Journal Article
English
KW - MEDLINE
KW - Age Factors
KW - Attitude to Health
KW - Belgium
KW - Child Care/mt [Methods]
KW - Child Care/st [Standards]
KW - Child Care/sn [Statistics & Numerical Data]
KW - Child Day Care Centers/st [Standards]
KW - Child Day Care Centers/sn [Statistics & Numerical Data]

KW - Child Day Care Centers
KW - Child Development
KW - Child
KW - Preschool
KW - Female
KW - Humans
KW - Interpersonal Relations
KW - Male
KW - Questionnaires
KW - Toilet Training
RP - NOT IN FILE
SP - 955
EP - 961
JF - European Journal of Pediatrics
JA - Eur J Pediatr
VL - 171
IS - 6
CY - Germany
N2 - Several changes have occurred in the toilet training (TT) process in recent decades. There has been an increase in the use of daycare as both parents now often work outside the home. Most children attend daycare centers during the period TT usually takes place, and daytime training has shifted from home to daycare. This study is the first to evaluate the way TT is done in daycare centers. A questionnaire was sent to 1,500 daycare centers, of which 429 replied (response rate of 28.6%). The results show that half of the childcare workers base the decision to start TT on readiness signs. The combination of age and readiness signs is used by 44.5%. The majority of the respondents spent more than 1 h per day on TT (81.8%); 79.8% considered that daycare and parents should play an equal role in TT. However, more than a third of the respondents thought that parents pass the responsibility for TT on to daycare and that not enough attention was paid to TT in the home. Most respondents stated that daycare centers had the facilities and means to be able to give the children proper TT (88.5%). Other research indicated that the outcome of TT is not that good overall and that there is room for improvement in the methods used. More research is needed on the role division and on the interaction between daycare and parents and their combined influence on the process and outcome of TT
SN - 1432-1076
AD - Department of Urology, Faculty of Medicine, University of Antwerp, Antwerp, Belgium
UR - 22207492
ER -

TY - JOUR
ID - 441
T1 - Assessment of personal exposure to manganese in children living near a ferromanganese refinery
A1 - Haynes,E.N.
A1 - Ryan,P.
A1 - Chen,A.
A1 - Brown,D.
A1 - Roda,S.
A1 - Kuhnell,P.
A1 - Wittberg,D.
A1 - Terrell,M.
A1 - Reponen,T.
Y1 - 2012/06/15/
N1 - Haynes, Erin N. Ryan, Pat. Chen, Aimin. Brown, David. Roda, Sandy. Kuhnell, Pierce. Wittberg, Dawn. Terrell, Matthew. Reponen, Tiina
The Science of the total environment
uj0, 0330500
IM
Evaluation Studies. Journal Article
English
KW - MEDLINE

KW - Air Pollutants/an [Analysis]
KW - Air Pollutants/bl [Blood]
KW - Biological Markers/an [Analysis]
KW - Biological Markers/bl [Blood]
KW - Child
KW - Cohort Studies
KW - Community-Based Participatory Research
KW - Environmental Exposure
KW - Environmental Monitoring/mt [Methods]
KW - Female
KW - Hair/ch [Chemistry]
KW - Humans
KW - Male
KW - Manganese/an [Analysis]
KW - Manganese/bl [Blood]
KW - Mass Spectrometry
KW - Ohio
KW - Questionnaires
KW - Spectrophotometry
KW - Atomic
RP - NOT IN FILE
SP - 19
EP - 25
JF - Science of the Total Environment
JA - Sci Total Environ
VL - 427-428
CY - Netherlands
N2 - Airborne exposure to manganese (Mn) can result in neurologic effects. Stationary air sampling is the traditional technique to assess Mn exposure for communities, yet may not accurately reflect children's personal exposure. The goal of the study was to characterize personal exposure to Mn and PM(2.5) in a cohort of children ages 7-9 years residing near a ferromanganese refinery. A subset of children living in non-smoking households ages 7-9 enrolled in the Marietta Community Actively Researching Exposure Study during March-June 2009 and 2010 were invited to participate. Blood and hair were collected and analyzed for Mn. Participants wore a PM(2.5) sampler (Personal Modular Impactor) for 48 h. TWD was based on time spent at home and school and the distance of each from the refinery. Stationary outdoor air sampling was conducted 8 km from the refinery using a Harvard-type PM(2.5) impactor. The relationship between personal Mn exposure and TWD was examined by multiple regression adjusting for stationary air Mn concentration, wind speed and direction, and precipitation. Complete personal air sampling data were collected on 38 children. TWD ranged from 4.7 km to 28.5 km with a mean distance of 11.1 (4.7 sd) km. Mn concentration in personal air samples ranged from 1.5 ng/m3 to 54.5 ng/m3 (geometric mean, 8.1 ng/m3). TWD was a significant predictor of natural log personal air Mn concentration (lnMn) with an associated decrease of 0.075 lnMn for each km TWD ($p<0.05$, 95% CI -0.13 to -0.01). Personal Mn exposures were positively associated with stationary air Mn levels and inversely associated with wind speed. A child's location (home and school) relative to the refinery is a significant predictor of personal Mn exposure. Wind speed is also an important contributor to personal Mn exposure. Copyright 2012 Elsevier B.V. All rights reserved
SN - 1879-1026
AD - College of Medicine, Department of Environmental Health, University of Cincinnati, Cincinnati, OH 45267, USA. erin.haynes@uc.edu
UR - 22551936
ER -

TY - JOUR
ID - 442
T1 - Physical activity opportunities in Canadian childcare facilities: a provincial/territorial review of legislation
A1 - Vanderloo,L.M.
A1 - Tucker,P.
A1 - Ismail,A.

A1 - van Zandvroort,M.M.
Y1 - 2012/05//
N1 - Vanderloo, Leigh M. Tucker, Patricia. Ismail, Ali. van Zandvroort, Melissa M
Journal of physical activity & health
101189457
IM
Journal Article
English
KW - MEDLINE
KW - Canada
KW - Child Care/lj [Legislation & Jurisprudence]
KW - Child Day Care Centers/lj [Legislation & Jurisprudence]
KW - Child Welfare/lj [Legislation & Jurisprudence]
KW - Child
KW - Preschool
KW - Female
KW - Government Regulation
KW - Health Behavior
KW - Health Policy/lj [Legislation & Jurisprudence]
KW - Health Promotion/mt [Methods]
KW - Humans
KW - Male
KW - Motor Activity
KW - Play and Playthings
KW - Practice Guidelines as Topic
KW - Social Marketing
RP - NOT IN FILE
SP - 461
EP - 472
JF - Journal of Physical Activity & Health
JA - J Phys Act Health
VL - 9
IS - 4
CY - United States
N2 - BACKGROUND: Preschoolers spend a substantial portion of their day in childcare; therefore, these centers are an ideal venue to encourage healthy active behaviors. It is important that provinces'/territories' childcare legislation encourage physical activity (PA) opportunities. The purpose of this study was to review Canadian provincial/territorial childcare legislation regarding PA participation. Specifically, this review sought to 1) appraise each provincial/territorial childcare regulation for PA requirements, 2) compare such regulations with the NASPE PA guidelines, and 3) appraise these regulations regarding PA infrastructure. METHODS: A review of all provincial/territorial childcare legislation was performed. Each document was reviewed separately by 2 researchers, and the PA regulations were coded and summarized. The specific provincial/territorial PA requirements (eg, type/frequency of activity) were compared with the NASPE guidelines. RESULTS: PA legislation for Canadian childcare facilities varies greatly. Eight of the thirteen provinces/territories provide PA recommendations; however, none provided specific time requirements for daily PA. All provinces/territories did require access to an outdoor play space. CONCLUSION: All Canadian provinces/territories lack specific PA guidelines for childcare facilities. The development, implementation, and enforcement of national PA legislation for childcare facilities may aid in tackling the childhood obesity epidemic and assist childcare staff in supporting and encouraging PA participation
SN - 1543-5474
AD - School of Health & Rehabilitation Sciences, University of Western Ontario, London, Ontario, Canada
UR - 21934169
ER -

TY - JOUR
ID - 443

T1 - Environmental characteristics and student physical activity in PE class: findings from two large urban areas of Texas
A1 - Skala,K.A.
A1 - Springer,A.E.
A1 - Sharma,S.V.
A1 - Hoelscher,D.M.
A1 - Kelder,S.H.
Y1 - 2012/05//
N1 - Skala, Katherine A. Springer, Andrew E. Sharma, Shreela V. Hoelscher, Deanna M. Kelder, Steven H
Journal of physical activity & health
101189457
IM
Journal Article. Research Support, N.I.H., Extramural. Research Support, Non-U.S. Gov't
English
KW - MEDLINE
KW - Age Factors
KW - Child
KW - Curriculum
KW - Environment
KW - Environment Design
KW - Female
KW - Health Behavior
KW - Health Promotion/mt [Methods]
KW - Humans
KW - Linear Models
KW - Male
KW - Motor Activity/ph [Physiology]
KW - Physical Education and Training/mt [Methods]
KW - Public Health
KW - Schools
KW - Sex Factors
KW - Social Marketing
KW - Statistics as Topic
KW - Students/px [Psychology]
KW - Students/sn [Statistics & Numerical Data]
KW - Texas
KW - Urban Population/sn [Statistics & Numerical Data]
RP - NOT IN FILE
SP - 481
EP - 491
JF - Journal of Physical Activity & Health
JA - J Phys Act Health
VL - 9
IS - 4
CY - United States
N2 - BACKGROUND: Physical education (PE) classes provide opportunities for children to be active. This study examined the associations between specific environmental characteristics (teacher characteristics; class size, duration and location; and lesson context) and elementary school-aged children's moderate-to-vigorous activity (MVPA) during PE. METHODS: Environmental characteristics and student activity levels were measured in 211 third-, fourth-, and fifth-grade PE classes in 74 Texas public schools using SOFIT direct observation. RESULTS: Students engaged in less than half their PE class time in MVPA (38%), while approximately 25% of class time was spent in classroom management. Percent time in MVPA was significantly higher in outdoor classes compared with indoors (41.4% vs. 36.1%, P = .037). Larger (P = .044) and longer (P = .001) classes were negatively associated with percentage of MVPA and positively correlated with time spent in management (P < .001). CONCLUSIONS: Findings suggest that children's activity may be influenced by environmental factors such as class size, location, and lesson contexts. These findings hold important policy

implications for PE class organization and the need for strategies that maximize children's MVPA. Further research is needed to test the causal association of these factors with student MVPA
SN - 1543-5474
AD - Michael & Susan Dell Center for Advancement of Healthy Living, School of Public Health, The University of Texas, Houston, TX, USA
UR - 21934165
ER -

TY - JOUR
ID - 444
T1 - [Correlation between acute schistosomiasis japonica and serum interleukin-33 levels]. [Chinese]
A1 - Chen,R.Q.
A1 - Zhou,Y.B.
A1 - Jiang,L.F.
A1 - Song,X.X.
A1 - Zhang,Z.J.
A1 - Ju,L.W.
A1 - Jiang,Q.W.
Y1 - 1939/02/20/
N1 - Chen, Ruo-Qing. Zhou, Yi-Biao. Jiang, Lu-Fang. Song, Xiu-Xia. Zhang, Zhi-Jie. Ju, Li-Wen. Jiang, Qing-Wu
Zhongguo xue xi chong bing fang zhi za zhi = Chinese journal of schistosomiasis control
101144973
IM
English Abstract. Journal Article
Chinese
KW - MEDLINE
KW - Acute Disease
KW - Adolescent
KW - Adult
KW - Child
KW - Female
KW - Humans
KW - Interleukins/bl [Blood]
KW - Interleukins/im [Immunology]
KW - Male
KW - Middle Aged
KW - Schistosomiasis japonica/bl [Blood]
KW - Schistosomiasis japonica/di [Diagnosis]
KW - Schistosomiasis japonica/im [Immunology]
KW - Young Adult
RP - NOT IN FILE
SP - 32
EP - 34
JF - Chinese Journal of Schistosomiasis Control
JA - Zhongguo Xue Xi Chong.Bing Fang Zhi Za Zhi
VL - 24
IS - 1
CY - China
N2 - OBJECTIVE: To study the function of interleukin-33 (IL-33) in the development of human acute schistosomiasis japonica by determining the serum IL-33 levels in acute schistosomiasis japonica patients. METHODS: Four patients with acute schistosomiasis japonica were recruited from schistosomiasis endemic lake areas, and 15 controls were recruited outside the schistosomiasis endemic areas. The demographic data and venous blood were collected from all the subjects. The serum IL-33 levels of all the subjects were tested by using enzyme-linked immunosorbent assay. All the results were statistically analyzed with Stata 10.0 software. RESULTS: The serum IL-33 levels of the patients with acute schistosomiasis japonica [517.33 (334.65, 1 056.88) pg/ml] were significantly higher than those of the controls [1.66(1.66, 6.35) pg/ml] (Z = -3.207, P =

0.001). The correlation coefficients between serum IL-33 levels and numbers of eosinophils, serum IL-33 levels and duration of infection were both 0.8 (P = 0.2). CONCLUSIONS: The serum IL-33 level is significantly elevated in the patients with acute schistosomiasis japonica, indicating that IL-33 may play a pro-inflammatory role in the acute stage of schistosomiasis japonica and participate in initiating the Th2 type immune responses between 7 and 9 weeks after the infection
SN - 1005-6661
AD - Department of Epidemiology, School of Public Health, Fudan University, Key laboratory on Public Health Safety, Ministry of Education, Shanghai 200032, China
UR - 22590860
ER -

TY - JOUR
ID - 445
T1 - Time outdoors and physical activity as predictors of incident myopia in childhood: a prospective cohort study
A1 - Guggenheim,J.A.
A1 - Northstone,K.
A1 - McMahon,G.
A1 - Ness,A.R.
A1 - Deere,K.
A1 - Mattocks,C.
A1 - Pourcain,B.S.
A1 - Williams,C.
Y1 - 2012/05//
N1 - Guggenheim, Jeremy A. Northstone, Kate. McMahon, George. Ness, Andy R. Deere, Kevin. Mattocks, Calum. Pourcain, Beate St. Williams, Cathy
Investigative ophthalmology & visual science
gwi, 7703701
IM
Journal Article. Research Support, Non-U.S. Gov't
English
KW - MEDLINE
KW - Adolescent
KW - Child
KW - England/ep [Epidemiology]
KW - Exercise
KW - Female
KW - Humans
KW - Incidence
KW - Kaplan-Meier Estimate
KW - Leisure Activities
KW - Male
KW - Myopia/ep [Epidemiology]
KW - Prospective Studies
KW - Questionnaires
KW - Regression Analysis
KW - Risk Factors
KW - Time Factors
RP - NOT IN FILE
SP - 2856
EP - 2865
JF - Investigative Ophthalmology & Visual Science
JA - Invest Ophthalmol Vis Sci
VL - 53
IS - 6
CY - United States

N2 - PURPOSE: Time spent in "sports/outdoor activity" has shown a negative association with incident myopia during childhood. We investigated the association of incident myopia with time spent outdoors and physical activity separately. METHODS: Participants in the Avon Longitudinal Study of Parents and Children (ALSPAC) were assessed by noncycloplegic autorefraction at ages 7, 10, 11, 12, and 15 years, and classified as myopic (<-1 diopters) or as emmetropic/hyperopic (>-0.25 diopters) at each visit (N = 4,837-7,747). Physical activity at age 11 years was measured objectively using an accelerometer, worn for 1 week. Time spent outdoors was assessed via a parental questionnaire administered when children were aged 8-9 years. Variables associated with incident myopia were examined using Cox regression. RESULTS: In analyses using all available data, both time spent outdoors and physical activity were associated with incident myopia, with time outdoors having the larger effect. The results were similar for analyses restricted to children classified as either nonmyopic or emmetropic/hyperopic at age 11 years. Thus, for children nonmyopic at age 11, the hazard ratio (95% confidence interval, CI) for incident myopia was 0.66 (0.47-0.93) for a high versus low amount of time spent outdoors, and 0.87 (0.76-0.99) per unit standard deviation above average increase in moderate/vigorous physical activity. CONCLUSION: Time spent outdoors was predictive of incident myopia independently of physical activity level. The greater association observed for time outdoors suggests that the previously reported link between "sports/outdoor activity" and incident myopia is due mainly to its capture of information relating to time outdoors rather than physical activity
SN - 1552-5783
AD - School of Optometry and Vision Sciences, Cardiff University, Cardiff, Wales, United Kingdom. guggenheim@cardiff.ac.uk
UR - 22491403
ER -

TY - JOUR
ID - 446
T1 - Argininosuccinate lyase deficiency. [Review]
A1 - Nagamani,S.C.
A1 - Erez,A.
A1 - Lee,B.
Y1 - 2012/05//
N1 - Nagamani, Sandesh C S. Erez, Ayelet. Lee, Brendan
Genetics in medicine : official journal of the American College of Medical Genetics
d0z, 9815831
IM
Journal Article. Research Support, N.I.H., Extramural. Research Support, Non-U.S. Gov't. Review
English
KW - MEDLINE
KW - Arginine/me [Metabolism]
KW - Arginine/tu [Therapeutic Use]
KW - Argininosuccinate Lyase/ge [Genetics]
KW - Argininosuccinic Acid/bl [Blood]
KW - Argininosuccinic Acid/me [Metabolism]
KW - Argininosuccinic Acid/ur [Urine]
KW - Argininosuccinic Aciduria/di [Diagnosis]
KW - Argininosuccinic Aciduria/ge [Genetics]
KW - Argininosuccinic Aciduria/th [Therapy]
KW - Child
KW - Preschool
KW - Citrulline/bl [Blood]
KW - Cognition Disorders/en [Enzymology]
KW - Cognition Disorders/ge [Genetics]
KW - Diet
KW - Protein-Restricted
KW - Fumarates/me [Metabolism]
KW - Genetic Testing
KW - Glucose/tu [Therapeutic Use]
KW - Humans

KW - Hyperammonemia/en [Enzymology]
KW - Hyperammonemia/ge [Genetics]
KW - Hypertension/en [Enzymology]
KW - Hypertension/ge [Genetics]
KW - Infant
KW - Newborn
KW - Lipids/tu [Therapeutic Use]
KW - Liver Diseases/en [Enzymology]
KW - Liver Diseases/ge [Genetics]
KW - Liver Transplantation
KW - Neonatal Screening
KW - Phenylbutyrates/tu [Therapeutic Use]
KW - Sodium Benzoate/tu [Therapeutic Use]
RP - NOT IN FILE
SP - 501
EP - 507
JF - Genetics in Medicine
JA - Genet Med
VL - 14
IS - 5
CY - United States
N2 - The urea cycle consists of six consecutive enzymatic reactions that convert waste nitrogen into urea. Deficiencies of any of these enzymes of the cycle result in urea cycle disorders (UCDs), a group of inborn errors of hepatic metabolism that often result in life-threatening hyperammonemia. Argininosuccinate lyase (ASL) catalyzes the fourth reaction in this cycle, resulting in the breakdown of argininosuccinic acid to arginine and fumarate. ASL deficiency (ASLD) is the second most common UCD, with a prevalence of ~1 in 70,000 live births. ASLD can manifest as either a severe neonatal-onset form with hyperammonemia within the first few days after birth or as a late-onset form with episodic hyperammonemia and/or long-term complications that include liver dysfunction, neurocognitive deficits, and hypertension. These long-term complications can occur in the absence of hyperammonemic episodes, implying that ASL has functions outside of its role in ureagenesis and the tissue-specific lack of ASL may be responsible for these manifestations. The biochemical diagnosis of ASLD is typically established with elevation of plasma citrulline together with elevated argininosuccinic acid in the plasma or urine. Molecular genetic testing of ASL and assay of ASL enzyme activity are helpful when the biochemical findings are equivocal. However, there is no correlation between the genotype or enzyme activity and clinical outcome. Treatment of acute metabolic decompensations with hyperammonemia involves discontinuing oral protein intake, supplementing oral intake with intravenous lipids and/or glucose, and use of intravenous arginine and nitrogen-scavenging therapy. Dietary restriction of protein and dietary supplementation with arginine are the mainstays in long-term management. Orthotopic liver transplantation (OLT) is best considered only in patients with recurrent hyperammonemia or metabolic decompensations resistant to conventional medical therapy
SN - 1530-0366
AD - Department of Molecular and Human Genetics, Baylor College of Medicine, Houston, Texas, USA. Sandesh C.S. Nagamani
UR - 22241104
ER -

TY - JOUR
ID - 447
T1 - Organophosphate pesticide environmental exposure: analysis of salivary cholinesterase and carboxilesterase activities in preschool children and their mothers
A1 - Bulgaroni,V.
A1 - Rovedatti,M.G.
A1 - Sabino,G.
A1 - Magnarelli,G.
Y1 - 2012/05//
N1 - Bulgaroni, Vanina. Rovedatti, Maria Gabriela. Sabino, Guillermo. Magnarelli, Gladis
Environmental monitoring and assessment

dvk, 8508350
IM
Journal Article. Research Support, Non-U.S. Gov't
English
KW - MEDLINE
KW - Adult
KW - Argentina
KW - Carboxylesterase/me [Metabolism]
KW - Child
KW - Preschool
KW - Cholinesterases/me [Metabolism]
KW - Environmental Exposure/an [Analysis]
KW - Environmental Exposure/sn [Statistics & Numerical Data]
KW - Environmental Pollutants/me [Metabolism]
KW - Environmental Pollutants/to [Toxicity]
KW - Female
KW - Humans
KW - Male
KW - Organophosphorus Compounds/me [Metabolism]
KW - Organophosphorus Compounds/to [Toxicity]
KW - Pesticides/me [Metabolism]
KW - Pesticides/to [Toxicity]
KW - Saliva/me [Metabolism]
KW - Young Adult
RP - NOT IN FILE
SP - 3307
EP - 3314
JF - Environmental Monitoring & Assessment
JA - Environ Monit Assess
VL - 184
IS - 5
CY - Netherlands
N2 - A pilot study was conducted to evaluate the usefulness of salivary cholinesterase and carboxylesterase as biomarkers of exposure to environmental organophosphate pesticides. Ninety samples were obtained from women and 62 samples from their preschool-aged children who live near an agricultural area of the Upper Valley of the Negro River (Patagonia, Argentina) where pesticides are applied 6 months a year. Each participant donated two samples under similar conditions: one in the pre-exposure period and another during the pulverization period. Demographic information, potential confounders, and risk behaviors were registered. Active or passive smoking had no effect on these enzyme activities in either group. During the pulverization period, cholinesterase activity was not detectable in 76% of the children's samples and 23% of the mothers' samples. Comparing samples collected during the pulverization period with respect to the pre-pulverization period, the average mother and child cholinesterase activity decreased by 65.7% ($p<0.001$) and 85.8% ($p<0.001$), respectively. Also, mother and child carboxylesterase activity decreased by 27.5% ($p<0.001$) and 41.9% ($p<0.01$), respectively. Child carboxylesterase activity in the pulverization period was associated to the habit of eating dust outdoors ($p<0.01$). The most frequent inhibition levels observed for cholinesterase and carboxylesterase activity were between 70-100% and 0-29%, respectively, in both groups studied. This shows that at the same level of exposure, cholinesterase was more sensitive to inhibition than carboxylesterase. Therefore, carboxylesterase might more properly reflect the degree of environmental organophosphate exposure and may have potential as a novel tool for biomonitoring
SN - 1573-2959
AD - LIBIQUIMA (Laboratory of Biochemistry, Chemistry and Environmental Research)-IDEPA (CONICET), National University of Comahue (UNCo), Buenos Aires, 1400 (8300), Neuquen, Argentina
UR - 21739279
ER -

TY - JOUR
ID - 448

T1 - Activity pattern of a selected group of school occupants and their family members in Helsinki-Finland
A1 - Hussein,T.
A1 - Paasonen,P.
A1 - Kulmala,M.
Y1 - 2012/05/15/
N1 - Hussein, Tareq. Paasonen, Pauli. Kulmala, Markku
The Science of the total environment
uj0, 0330500
IM
Journal Article
English
KW - MEDLINE
KW - Adolescent
KW - Adult
KW - Aged
KW - 80 and over
KW - Air Pollutants
KW - Child
KW - Preschool
KW - Environmental Exposure
KW - Family
KW - Female
KW - Finland
KW - Human Activities
KW - Humans
KW - Male
KW - Middle Aged
KW - Questionnaires
KW - Schools
KW - Time Factors
KW - Weather
KW - Young Adult
RP - NOT IN FILE
SP - 289
EP - 292
JF - Science of the Total Environment
JA - Sci Total Environ
VL - 425
CY - Netherlands
N2 - The daily time-spent in different environments is an important factor in calculation of personal exposure to air pollutants. Despite this importance, the amount of research done on this topic is rather limited, especially in North Europe where the climate is rather cold. In this study, we gathered detailed information via a standard questionnaire to report the residence time and place of 167 subjects (between 2 and 93 years old) lived in Helsinki during three time periods in winter and spring 2009. Subjects spent 81%-92% of their time indoors and up to ~15% of their time outdoors. The daily time-spent in different environments was affected by several factors: ambient temperature, type of day (workday or weekend/holiday), gender, and age. Therefore, the differences occur individually and can be explained by these factors. For example, subjects spent more time at home on weekends than workdays because obviously the majority of our subjects did not have work on weekends. The time-spent at kindergarten/school/work increased with age until retirement. Females spent more time at home than males. After all, the activity pattern found in this study is rather similar to those previously reported in Germany and North America. Copyright 2012 Elsevier B.V. All rights reserved
SN - 1879-1026
AD - University of Helsinki, Department of Physics, Division of Atmospheric Sciences, P. O. Box 48, FI-00014 UHEL, Helsinki, Finland. tareq.hussein@helsinki.fi
UR - 22464956
ER -

TY - JOUR
ID - 449
T1 - The neighborhood social environment and body mass index among youth: a mediation analysis
A1 - Veitch,J.
A1 - van Stralen,M.M.
A1 - Chinapaw,M.J.
A1 - Te Velde,S.J.
A1 - Crawford,D.
A1 - Salmon,J.
A1 - Timperio,A.
Y1 - 2012///
N1 - Veitch, Jenny. van Stralen, Maartje M. Chinapaw, Mai J M. te Velde, Saskia J. Crawford, David. Salmon, Jo. Timperio, Anna
The international journal of behavioral nutrition and physical activity
101217089
IM
Journal Article. Research Support, Non-U.S. Gov't
English
KW - MEDLINE
KW - Adolescent
KW - Australia
KW - Body Mass Index
KW - Body Weight
KW - Child
KW - Cross-Sectional Studies
KW - Energy Intake
KW - Female
KW - Follow-Up Studies
KW - Health Behavior
KW - Humans
KW - Leisure Activities/px [Psychology]
KW - Linear Models
KW - Male
KW - Multivariate Analysis
KW - Obesity/pc [Prevention & Control]
KW - Parents
KW - Prospective Studies
KW - Questionnaires
KW - Residence Characteristics
KW - Schools
KW - Social Environment
KW - Socioeconomic Factors
KW - Television
KW - Walking/px [Psychology]
RP - NOT IN FILE
SP - 31
JF - International Journal of Behavioral Nutrition & Physical Activity
JA - Int
VL - 9
CY - England
N2 - BACKGROUND: This study aimed to examine associations between aspects of the neighborhood social environment and body mass index (BMI) in youth both cross-sectionally and prospectively; and whether this association was mediated by physical activity, screen-time and sedentary time. METHODS: Data were collected in 2004 and 2006 in high and low socio-economic areas of Melbourne, Australia. In 2004, 185 children aged 8-9 years (47% boys) and 359 children aged 13-15 years (45% boys) participated. Parents reported their perceptions of aspects of the social environment (i.e. social networks and social trust/cohesion), and physical activity (i.e. time spent outdoors by their children; and their younger children's walking and cycling trips) and screen-time

(i.e. TV viewing, computer use). The older children self-reported their walking and cycling trips and their screen-time. All children wore an accelerometer to objectively assess outside-school hours moderate- to-vigorous physical activity and sedentary time. BMI was calculated from height and weight measured in 2004 and 2006. Multilevel linear regression analyses were conducted to examine associations between the social environment and BMI. Mediation analyses using the products of coefficient method were conducted to determine whether associations between the social environment and BMI were mediated by the time spent in a range of physical activity and sedentary behaviors. RESULTS: Cross-sectional and prospective regression analyses showed that a more positive social network and higher social trust/cohesion was related to lower BMI among children. There was no evidence that time spent in physical activity or sedentary behaviors mediated this relation, despite significant associations between social networks and screen-time and between screen-time and BMI. CONCLUSIONS: The findings suggest that the neighborhood social environment may be important for preventing overweight and obesity in children. Further research investigating the mechanisms through which the neighborhood social environment exerts its effect on BMI is needed. 2012 Veitch et al; licensee BioMed Central Ltd
SN - 1479-5868
AD - Centre for Physical Activity and Nutrition Research, Deakin University, 221 Burwood Highway, Burwood, 3125, Australia. jenny.veitch@deakin.edu.au
UR - 22429957
ER -

TY - JOUR
ID - 450
T1 - Spatial classification of youth physical activity patterns
A1 - Rainham,D.G.
A1 - Bates,C.J.
A1 - Blanchard,C.M.
A1 - Dummer,T.J.
A1 - Kirk,S.F.
A1 - Shearer,C.L.
Y1 - 2012/05//
N1 - Rainham, Daniel G. Bates, Christopher J. Blanchard, Chris M. Dummer, Trevor J. Kirk, Sara F. Shearer, Cindy L
American journal of preventive medicine
8704773, apl
IM
Journal Article. Research Support, Non-U.S. Gov't
English
KW - MEDLINE
KW - Adolescent
KW - Adolescent Behavior
KW - Child
KW - Cities/sn [Statistics & Numerical Data]
KW - Exercise
KW - Female
KW - Food
KW - Geographic Information Systems/sn [Statistics & Numerical Data]
KW - Health Behavior
KW - Humans
KW - Male
KW - Residence Characteristics/sn [Statistics & Numerical Data]
KW - Social Environment
KW - Socioeconomic Factors
KW - Transportation/sn [Statistics & Numerical Data]
RP - NOT IN FILE
SP - e87
EP - e96
JF - American Journal of Preventive Medicine

JA - Am J Prev Med
VL - 42
IS - 5
CY - Netherlands
N2 - BACKGROUND: Physical activity is an essential element in reducing the prevalence of obesity, but much is unknown about the intensity and location of physical activity among youth-this is important because adolescent health behaviors are predictive of behaviors in adults. PURPOSE: This study aims to identify the locations where youth moderate-to-vigorous physical activity (MVPA) occurs, and to examine how MVPA varies according to urbanicity (urban, suburban, rural). METHODS: Participants included adolescent students (N=380, aged 12-16 years) from Halifax, Nova Scotia. Locations of MVPA were measured using accelerometers and GPS data loggers for up to 7 days. Specialized software was developed to integrate and process the data. Frequencies of MVPA by location were determined, and differences in MVPA were assessed for association with urbanicity. RESULTS: Active commuting accounted for the largest proportion of time in MVPA among urban and suburban students. Rural students achieved most MVPA at school. Other residential locations, shopping centers, and green spaces accounted for a majority of the remaining MVPA. Minutes in MVPA varied significantly overall (196.6 + 163.8, 84.9 + 103.2, 81.7 + 98.2); at school (45.7 + 45.2, 18.6 + 28.0, 29.8 + 39.7); while commuting (110.3 + 107.1, 31.5 + 55.2, 19.5 + 39.7); and at other activity locations (19.7 + 27.1, 14.8 + 26.8, 12.0 + 22.1) and by urbanicity. CONCLUSIONS: Findings reveal that the journeys between locations are as important as home and school settings in contributing to greater MVPA in adolescent youth. The relative importance of context as a contributor to MVPA varies with urbanicity. Combining actimetry and GPS data provides a precise link between physical activity measurements and contexts of the built environment. Copyright 2012 American Journal of Preventive Medicine. Published by Elsevier Inc. All rights reserved
SN - 1873-2607
AD - Environmental Science Program, Dalhousie University, Halifax, Nova Scotia, Canada. daniel.rainham@dal.ca
UR - 22516507
ER -

TY - JOUR
ID - 451
T1 - Physical activity and physical fitness: standardizing assessment with the PhenX Toolkit
A1 - Haskell,W.L.
A1 - Troiano,R.P.
A1 - Hammond,J.A.
A1 - Phillips,M.J.
A1 - Strader,L.C.
A1 - Marquez,D.X.
A1 - Grant,S.F.
A1 - Ramos,E.
Y1 - 2012/05//
N1 - Haskell, William L. Troiano, Richard P. Hammond, Jane A. Phillips, Michael J. Strader, Lisa C. Marquez, David X. Grant, Struan F. Ramos, Erin
American journal of preventive medicine
8704773, apl
IM
Journal Article. Research Support, N.I.H., Extramural
English
KW - MEDLINE
KW - Adolescent
KW - Adult
KW - Age Factors
KW - Aged
KW - Child
KW - Preschool
KW - Data Collection/mt [Methods]
KW - Data Collection/st [Standards]

KW - Exercise
KW - Female
KW - Health Status Indicators
KW - Humans
KW - Male
KW - Middle Aged
KW - Phenotype
KW - Physical Fitness
KW - Young Adult
RP - NOT IN FILE
SP - 486
EP - 492
JF - American Journal of Preventive Medicine
JA - Am J Prev Med
VL - 42
IS - 5
CY - Netherlands
N2 - The focus of the PhenX (Phenotypes and eXposures) Toolkit is to provide researchers whose expertise lies outside a particular area with key measures identified by experts for uniform use in large-scale genetic studies and other extensive epidemiologic efforts going forward. The current paper specifically addresses the PhenX Toolkit research domain of physical activity and physical fitness (PA/PF), which are often associated with health outcomes. A Working Group (WG) of content experts completed a 6-month consensus process in which they identified a set of 14 high-priority, low-burden, and scientifically supported measures. During this process, the WG considered self-reported and objective measures that included the latest technology (e.g., accelerometers, pedometers, and heart-rate monitors). They also sought the input of measurement experts and other members of the research community during their deliberations. A majority of the measures include protocols for children (or adolescents), adults, and older adults or are applicable to all ages. Measures from the PA/PF domain and 20 other domains are publicly available and found at the PhenX Toolkit website, www.phenxtoolkit.org. The use of common measures and protocols across large studies enhances the capacity to combine or compare data across studies, benefiting both PA/PF experts and non-experts. Use of these common measures by the research community should increase statistical power and enhance the ability to answer scientific questions that previously might have gone unanswered. Copyright 2012 American Journal of Preventive Medicine. All rights reserved
SN - 1873-2607
AD - Prevention Research Center, Department of Medicine, Stanford University, Palo Alto, California 94304, USA. whaskell@stanford.edu
UR - 22516489
ER -

TY - JOUR
ID - 452
T1 - Environmental justice at school: understanding research, policy, and practice to improve our children's health. [Review]
A1 - Sampson,N.
Y1 - 2012/05//
N1 - Sampson, Natalie
The Journal of school health
k13, 0376370
IM, N
Journal Article. Review
English
KW - MEDLINE
KW - Air Pollution
KW - Indoor/pc [Prevention & Control]
KW - Child
KW - Environmental Exposure/pc [Prevention & Control]
KW - Health Policy

KW - Humans
KW - Research
KW - Schools
KW - United States
RP - NOT IN FILE
SP - 246
EP - 252
JF - Journal of School Health
JA - J Sch Health
VL - 82
IS - 5
CY - United States
N2 - BACKGROUND: No overarching federal agencies or policies are responsible for ensuring environmental health at schools in the United States, potentially allowing many inequities for low-income and minority communities to persist. This article examines emergent research, policy, and practice-based efforts that may be used to identify and address environmental justice at school. METHODS: A brief literature review was conducted to understand (1) major mental, behavioral, and physical outcomes associated with environmental risk for school-aged children and (2) current research methods for assessing these relationships. Documents prepared by government agencies, school districts, and advocacy groups were also collected and synthesized to improve understanding of the state of planning and policies for maintaining or improving school environments. RESULTS: Environmental risk can manifest in diverse ways such as mold, poor air quality, poor community design, or contaminated playgrounds. Deeply rooted in national and state structures of school funding and planning, such risks can lead to outcomes including respiratory illness, poor performance in school, and reduced levels of physical activity. With growing attention to these concerns, methods for measuring environmental risks and underlying disparities have advanced tremendously in the last few decades, yet development of innovative research approaches may be necessary to further advance and evaluate appropriate interventions. CONCLUSIONS: Environmental injustice is generally unacknowledged during decision-making in US school districts. Continued dialogue reflective of translatable science is necessary to support school districts with limited funding in ensuring safe, healthy environments for learning. 2012, American School Health Association
SN - 1746-1561
AD - Department of Health Behavior and Health Education, University of Michigan, School of Public Health, Ann Arbor, MI 48109, USA. nsampson@umich.edu
UR - 22494096
ER -

TY - JOUR
ID - 453
T1 - Risk factors for variation in 25-hydroxyvitamin D3 and D2 concentrations and vitamin D deficiency in children
A1 - Tolppanen,A.M.
A1 - Fraser,A.
A1 - Fraser,W.D.
A1 - Lawlor,D.A.
Y1 - 2012/04//
N1 - Tolppanen, Anna-Maija. Fraser, Abigail. Fraser, William D. Lawlor, Debbie A
The Journal of clinical endocrinology and metabolism
hrb, 0375362
AIM, IM
Journal Article. Research Support, Non-U.S. Gov't
English
KW - MEDLINE
KW - 25-Hydroxyvitamin D 2/bl [Blood]
KW - Calcifediol/bl [Blood]
KW - Child
KW - Preschool
KW - Cohort Studies
KW - England/ep [Epidemiology]

KW - Female
KW - Humans
KW - Longitudinal Studies
KW - Male
KW - Prevalence
KW - Prospective Studies
KW - Puberty
KW - Risk Factors
KW - Seasons
KW - Severity of Illness Index
KW - Sex Factors
KW - Socioeconomic Factors
KW - Sunlight
KW - Vitamin D Deficiency/bl [Blood]
KW - Vitamin D Deficiency/ep [Epidemiology]
KW - Vitamin D Deficiency/eh [Ethnology]
RP - NOT IN FILE
SP - 1202
EP - 1210
JF - Journal of Clinical Endocrinology & Metabolism
JA - J Clin Endocrinol Metab
VL - 97
IS - 4
CY - United States
N2 - CONTEXT: Vitamin D status is believed to be best indicated by serum 25-hydroxyvitamin D [25(OH)D; consisting of 25(OH)D3 and 25(OH)D2] that are obtained from different sources. Suboptimal vitamin D status is common and associated with adverse health outcomes. OBJECTIVES: The objectives were to report the prevalence and risk factors of vitamin D deficiency and determine associations of characteristics that have been shown to relate to total 25(OH)D with 25(OH)D3 and 25(OH)D2 concentrations. DESIGN AND SETTING: The Avon Longitudinal Study of Parents and Children is a population-based contemporary birth cohort (children born in 1991-1992) from southwest England. PARTICIPANTS AND OUTCOME MEASURES: Seven thousand five hundred sixty children with serum 25(OH)D3 and 25(OH)D2 concentrations measured at the mean age of 9.9 yr participated in the study. RESULTS: Vitamin D deficiency [total 25(OH)D concentration <20 ng/ml] was common (29%). The main risk factors were winter season, less time spent outdoors, low socioeconomic position, nonwhite ethnicity, older age, more advanced puberty stage, and female gender. Although there were some common risk factors for lower 25(OH)D3 and 25(OH)D2 concentrations (age, gender, puberty stage, body mass index, physical activity, household income, maternal education), several characteristics were associated with 25(OH)D3 only (ethnicity, vitamin D intake, time spent outdoors, and UVB protection score) and others with 25(OH)D2 only (protein and carbohydrate intake, parent's social class, and housing tenure). CONCLUSIONS: Vitamin D deficiency was common in this contemporary U.K. cohort. Despite some overlap, there are differences in potential confounding structures for associations of 25(OH)D3 and 25(OH)D2 with health outcomes. These should be accounted for in future studies
SN - 1945-7197
AD - Medical Research Council, Centre for Causal Analysis in Translational Epidemiology, School of Social and Community Medicine, University of Bristol, Oakfield House, 15-23 Oakfield Grove, Clifton, Bristol BS8 2BN, United Kingdom
UR - 22278429
ER -

TY - JOUR
ID - 454
T1 - Attachment security and HPA axis reactivity to positive and challenging emotional situations in child-mother dyads in naturalistic settings
A1 - Roque,L.
A1 - Verissimo,M.
A1 - Oliveira,T.F.
A1 - Oliveira,R.F.

Y1 - 2012/05//
N1 - Roque, Lisa. Verissimo, Manuela. Oliveira, Tania F. Oliveira, Rui F
Developmental psychobiology
e8a, 0164074
IM
Journal Article
English
KW - MEDLINE
KW - Adult
KW - Child Development/ph [Physiology]
KW - Child
KW - Preschool
KW - Emotions/ph [Physiology]
KW - Fear/ph [Physiology]
KW - Female
KW - Humans
KW - Hydrocortisone/an [Analysis]
KW - Hypothalamo-Hypophyseal System/ph [Physiology]
KW - Infant
KW - Male
KW - Mother-Child Relations
KW - Object Attachment
KW - Pituitary-Adrenal System/ph [Physiology]
KW - Saliva/ch [Chemistry]
RP - NOT IN FILE
SP - 401
EP - 411
JF - Developmental Psychobiology
JA - Dev Psychobiol.
VL - 54
IS - 4
CY - United States
N2 - This study investigated adrenocortical activity in response to different challenging and positive affect emotional contexts in child-mother dyads, as function of attachment security (children's secure base behaviors and mothers' attachment representations). Fifty-one children ranging in age from 18 to 26 months and their mothers participated in this study. Secure children showed significant increases in their cortisol levels after fear episodes and significant decreases, after positive affect ones. No significant changes were found for frustration/anger episodes. Insecure children did not show significant differences in cortisol levels in any of the episodes, which suggests that insecure attachment may be related to hypothalamic-pituitary-adrenal axis suppression in response to challenging and positive contexts. Mothers of insecure children showed significantly higher cortisol concentrations in pre- and post-session samples, than mothers of secure children. Mothers' personal attachment representations influenced their own cortisol responses, as well as their children's (in a marginal significant way). Copyright 2011 Wiley Periodicals, Inc
SN - 1098-2302
AD - UIPCDE, ISPA-Instituto Universitario, Lisboa, Portugal. lisa.roque.psychologist@gmail.com
UR - 22487942
ER -

TY - JOUR
ID - 455
T1 - Development of a pragmatic measure for evaluating and optimizing rapid response systems
A1 - Bonafide,C.P.
A1 - Roberts,K.E.
A1 - Priestley,M.A.
A1 - Tibbetts,K.M.
A1 - Huang,E.
A1 - Nadkarni,V.M.

A1 - Keren,R.
Y1 - 2012/04//
N1 - Bonafide, Christopher P. Roberts, Kathryn E. Priestley, Margaret A. Tibbetts, Kathleen M. Huang, Emily. Nadkarni, Vinay M. Keren, Ron
Pediatrics
oxv, 0376422
AIM, IM
Comparative Study. Evaluation Studies. Journal Article
English
KW - MEDLINE
KW - Cardiopulmonary Resuscitation/td [Trends]
KW - Child
KW - Preschool
KW - Female
KW - Heart Arrest/mo [Mortality]
KW - Heart Arrest/th [Therapy]
KW - Hospital Mortality/td [Trends]
KW - Hospital Rapid Response Team/st [Standards]
KW - Hospitals
KW - Pediatric/og [Organization & Administration]
KW - Humans
KW - Infant
KW - Intensive Care Units
KW - Male
KW - Monitoring
KW - Physiologic/st [Standards]
KW - Reproducibility of Results
KW - Retrospective Studies
KW - United States/ep [Epidemiology]
RP - NOT IN FILE
SP - e874
EP - e881
JF - Pediatrics
VL - 129
IS - 4
CY - United States
N2 - OBJECTIVES: Standard metrics for evaluating rapid response systems (RRSs) include cardiac and respiratory arrest rates. These events are rare in children; therefore, years of data are needed to evaluate the impact of RRSs with sufficient statistical power. We aimed to develop a valid, pragmatic measure for evaluating and optimizing RRSs over shorter periods of time. METHODS: We reviewed 724 medical emergency team and 56 code-blue team activations in a children's hospital between February 2010 and February 2011. We defined events resulting in ICU transfer and noninvasive ventilation, intubation, or vasopressor infusion within 12 hours as "critical deterioration." By using in-hospital mortality as the gold standard, we evaluated the test characteristics and validity of this proximate outcome metric compared with a national benchmark for cardiac and respiratory arrest rates, the Child Health Corporation of America Codes Outside the ICU Whole System Measure. RESULTS: Critical deterioration (1.52 per 1000 non-ICU patient-days) was more than eightfold more common than the Child Health Corporation of America measure of cardiac and respiratory arrests (0.18 per 1000 non-ICU patient-days) and was associated with >13-fold increased risk of in-hospital death. The critical deterioration metric demonstrated both criterion and construct validity. CONCLUSIONS: The critical deterioration rate is a valid, pragmatic proximate outcome associated with in-hospital mortality. It has great potential for complementing existing patient safety measures for evaluating RRS performance
SN - 1098-4275
AD - Division of General Pediatrics, The Children's Hospital of Philadelphia, Philadelphia, Pennsylvania 19104, USA. bonafide@email.chop.edu
UR - 22392182
ER -

TY - JOUR
ID - 456
T1 - Influence of activity patterns in fitness during youth
A1 - Aires,L.
A1 - Silva,G.
A1 - Martins,C.
A1 - Santos,M.P.
A1 - Ribeiro,J.C.
A1 - Mota,J.
Y1 - 2012/04//
N1 - Aires, L. Silva, G. Martins, C. Santos, M P. Ribeiro, J C. Mota, J
International journal of sports medicine
8008349, grk
IM
Journal Article. Research Support, Non-U.S. Gov't
English
KW - MEDLINE
KW - Adolescent
KW - Adolescent Behavior/ph [Physiology]
KW - Body Mass Index
KW - Cardiovascular Physiological Phenomena
KW - Child
KW - Exercise/ph [Physiology]
KW - Female
KW - Humans
KW - Longitudinal Studies
KW - Male
KW - Physical Fitness/ph [Physiology]
KW - Portugal
KW - Respiratory Physiological Phenomena
KW - Schools
KW - Sports/ph [Physiology]
RP - NOT IN FILE
SP - 325
EP - 329
JF - International Journal of Sports Medicine
JA - Int J Sports Med
VL - 33
IS - 4
CY - Germany
N2 - The aim of this study was to analyze longitudinal associations between cardiorespiratory fitness, physical activity and body mass index in a 4-year longitudinal study. 170 students (97 girls and 73 boys) aged at baseline from 11 to 17 years were followed. Students performed 20-m-SR; physical activity patterns and parents' education were evaluated using a standard questionnaire. Body mass index was categorized according to established cut points. In a multilevel analysis using MLwIN, 2 level structures were defined: first for individuals and second for time observations. In a longitudinal 2 level analysis, cardiorespiratory fitness was -negatively associated with body mass index for girls and boys, respectively (p<0.05; R2=0.63; 0.62), especially with obesity category (p<0.01; R2=0.58; 0.60). In girls, independent associations were observed between CRF and PA categories regarding participation "almost every day" in organized (p<0.05; R2=0.50) and non-organized sports outside school (p<0.05; R2=0.52) and participation in sports competitions (p<0.05; R2=0.51). In boys, associations were found only with participation in sports competitions (p<0.05; R2=0.50). The results highlight the importance of youth participation in organized activities and competitive sports over time to achieve health-related fitness benefits. Georg Thieme Verlag KG Stuttgart New York
SN - 1439-3964
AD - Faculty of Sports, Research Centre in Physical Activity, Health and Leisure, University of Porto, Portugal. luisa.aires@gmail.com
UR - 22377948

ER -

TY - JOUR
ID - 457
T1 - The impact of state laws and district policies on physical education and recess practices in a nationally representative sample of US public elementary schools
A1 - Slater,S.J.
A1 - Nicholson,L.
A1 - Chriqui,J.
A1 - Turner,L.
A1 - Chaloupka,F.
Y1 - 2012/04//
N1 - Slater, Sandy J. Nicholson, Lisa. Chriqui, Jamie. Turner, Lindsey. Chaloupka, Frank
Archives of pediatrics & adolescent medicine
9422751, bwf
AIM, IM
Comparative Study. Journal Article. Multicenter Study. Research Support, N.I.H., Extramural. Research Support, Non-U.S. Gov't
English
KW - MEDLINE
KW - Child
KW - Cross-Sectional Studies
KW - Female
KW - Health Policy/lj [Legislation & Jurisprudence]
KW - Health Promotion/lj [Legislation & Jurisprudence]
KW - Humans
KW - Incidence
KW - Male
KW - Obesity/ep [Epidemiology]
KW - Obesity/pc [Prevention & Control]
KW - Organizational Policy
KW - Physical Education and Training/og [Organization & Administration]
KW - Program Evaluation
KW - Retrospective Studies
KW - Schools/og [Organization & Administration]
KW - United States/ep [Epidemiology]
RP - NOT IN FILE
SP - 311
EP - 316
JF - Archives of Pediatrics & Adolescent Medicine
JA - Arch Pediatr Adolesc Med
VL - 166
IS - 4
CY - United States
N2 - OBJECTIVE: To examine the impact of state- and school district-level policies on the prevalence of physical education (PE) and recess in a nationally representative sample of US public elementary schools. DESIGN: Analyses from annual, nationally representative, cross-sectional surveys of school administrators in the United States. SETTING: Data were collected through surveys conducted between February and June during the 2006-2007 through 2008-2009 school years. State laws and district policies were compiled annually by researchers at the University of Illinois at Chicago using established legal research techniques. PARTICIPANTS: The sample size was 47 states, 690 districts, and 1761 schools. MAIN EXPOSURES: State- and school district-level PE and recess-related laws. MAIN OUTCOME MEASURES: Twenty minutes of daily recess and 150 min/wk of PE. RESULTS: The odds of schools having 150 min/wk of PE increased if they were located in states (odds ratio [OR], 2.8; 95% CI, 1.3-5.7) or school districts (OR, 2.4; 95% CI, 1.3-4.3) having a law or policy requiring 150 min/wk of PE. Schools located in states with laws encouraging daily recess were significantly more likely to have 20 minutes of recess daily (OR, 1.8; 95% CI, 1.2-2.8). District policies were not significantly associated with school-level recess practices. Adequate PE time was inversely associated with

recess and vice versa, suggesting that schools are substituting one form of physical activity for another rather than providing the recommended amount of both recess and PE. CONCLUSION: By mandating PE or recess, policy makers can effectively increase school-based physical activity opportunities for youth
SN - 1538-3628
AD - Institute for Health Research and Policy and Department of Health Policy & Administration, School of Public Health, University of Illinois at Chicago, 1747 W Roosevelt Rd, Chicago, IL 60608, USA. sslater@uic.edu
UR - 22147763
ER -

TY - JOUR
ID - 458
T1 - What can global positioning systems tell us about the contribution of different types of urban greenspace to children's physical activity?
A1 - Lachowycz,K.
A1 - Jones,A.P.
A1 - Page,A.S.
A1 - Wheeler,B.W.
A1 - Cooper,A.R.
Y1 - 2012/05//
N1 - Lachowycz, Kate. Jones, Andy P. Page, Angie S. Wheeler, Benedict W. Cooper, Ashley R
Health & place
din, 9510067
T
Journal Article
English
KW - MEDLINE
KW - Actigraphy/is [Instrumentation]
KW - Child
KW - England
KW - Environment Design
KW - Female
KW - Geographic Information Systems
KW - Humans
KW - Male
KW - Motivation
KW - Motor Activity
RP - NOT IN FILE
SP - 586
EP - 594
JF - Health & Place
JA - Health Place
VL - 18
IS - 3
CY - England
N2 - Urban greenspace is hypothesised to be an important location for physical activity in children, but their actual use of the resource to be active is not well known. In this study, global positioning systems (GPS) and accelerometers were used to measure activity within green environments for 902 English children aged 11-12. We summarised activity intensities in different types of greenspace on weekday evenings, weekend days and by season. Around half of outdoor moderate-vigorous activity took place in greenspace at the weekend and use was consistent across seasons. The findings suggest the importance of certain types of greenspace to children's physical activity. Copyright 2012 Elsevier Ltd. All rights reserved
SN - 1873-2054
AD - School of Environmental Sciences, University of East Anglia, Norwich NR4 7JT, UK. k.lachowycz@uea.ac.uk
UR - 22365385
ER -

TY - JOUR
ID - 459
T1 - Effect of stimulant medication use by children with ADHD on heart rate and perceived exertion
A1 - Mahon,A.D.
A1 - Woodruff,M.E.
A1 - Horn,M.P.
A1 - Marjerrison,A.D.
A1 - Cole,A.S.
Y1 - 2012/04//
N1 - Mahon, Anthony D. Woodruff, Megan E. Horn, Mary P. Marjerrison, Andrea D. Cole, Andrew S
Adapted physical activity quarterly : APAQ
8701671
IM
Comparative Study. Journal Article. Research Support, N.I.H., Extramural
English
KW - MEDLINE
KW - Attention Deficit Disorder with Hyperactivity/di [Diagnosis]
KW - Attention Deficit Disorder with Hyperactivity/dt [Drug Therapy]
KW - Attention Deficit Disorder with Hyperactivity/pp [Physiopathology]
KW - Case-Control Studies
KW - Central Nervous System Stimulants/ad [Administration & Dosage]
KW - Child
KW - Confidence Intervals
KW - Dose-Response Relationship
KW - Drug
KW - Exercise Test/mt [Methods]
KW - Female
KW - Heart Rate/de [Drug Effects]
KW - Heart Rate/ph [Physiology]
KW - Humans
KW - Linear Models
KW - Male
KW - Perception/ph [Physiology]
KW - Physical Exertion/de [Drug Effects]
KW - Physical Exertion/ph [Physiology]
KW - Reference Values
KW - Sensitivity and Specificity
KW - Severity of Illness Index
RP - NOT IN FILE
SP - 151
EP - 160
JF - Adapted Physical Activity Quarterly
JA - Adapt Phys Act Q
VL - 29
IS - 2
CY - United States
N2 - The effect of stimulant medication use by children with attention deficit/hyperactivity disorder (ADHD) on the rating of perceived exertion (RPE)-heart rate (HR) relationship was examined. Children with ADHD (n=20; 11.3+1.8 yrs) and children without ADHD (n=25; 11.2+2.1 yrs) were studied. Children with ADHD were examined while on their usual dose of medication on the day of study. HR and RPE, using the OMNI RPE scale, were assessed during a graded exercise to peak voluntary effort. The RPE-HR relationship was determined individually and the intercept and slope responses were compared between groups. The intercept was 132.4+19.5 bpm for children with ADHD and 120.6+15.7 bpm for children without ADHD. The slope was 7.3+1.9 bpm/RPE for the children with ADHD and 8.1+1.6 bpm/ RPE for the children without ADHD. For the group with ADHD the intercept and slope values fell outside of the 95% CI observed in the control group. The

altered relationship between RPE and HR with stimulant medication use in children with ADHD has practical implications with respect to the use of HR and RPE to monitor exercise intensity
SN - 1543-2777
AD - Human Performance Laboratory, Ball State University, Muncie, IN, USA
UR - 22467834
ER -

TY - JOUR
ID - 460
T1 - The contribution of recess to children's school-day physical activity
A1 - Erwin,H.
A1 - Abel,M.
A1 - Beighle,A.
A1 - Noland,M.P.
A1 - Worley,B.
A1 - Riggs,R.
Y1 - 2012/03//
N1 - Erwin, Heather. Abel, Mark. Beighle, Aaron. Noland, Melody P. Worley, Brooke. Riggs, Richard
Journal of physical activity & health
101189457
IM
Comparative Study. Journal Article. Research Support, Non-U.S. Gov't
English
KW - MEDLINE
KW - Analysis of Variance
KW - Body Mass Index
KW - Child
KW - Child Welfare
KW - Female
KW - Humans
KW - Male
KW - Motor Activity/ph [Physiology]
KW - Play and Playthings
KW - Recreation/ph [Physiology]
KW - Schools
KW - Students
KW - Time Factors
KW - Walking/ph [Physiology]
RP - NOT IN FILE
SP - 442
EP - 448
JF - Journal of Physical Activity & Health
JA - J Phys Act Health
VL - 9
IS - 3
CY - United States
N2 - BACKGROUND: Recess is an important component of a Comprehensive School Physical Activity Program, providing approximately 1268 and 914 steps for boys and girls, respectively, within a 15-minute time period. The purpose of this study was to determine the contribution of unstructured recess on children's school-day physical activity levels and to examine if recess and school-day physical activity levels varied by BMI, gender, and grade level. METHODS: One-hundred sixty third- to fifth-grade students from 2 elementary schools wore pedometers during 4 recess periods. RESULTS: Recess accounted for 17% to 44% of school-day step counts. There was a significant main effect for grade level, but not for BMI or gender, on the percentage of school-day steps accumulated during recess. CONCLUSIONS: A 15-minute recess makes a valuable contribution to children's school-day physical activity, especially for the least active children. More research is warranted to determine environmental influences on children's recess physical activity
SN - 1543-5474

AD - Dept of Kinesiology and Health Promotion, University of Kentucky, Lexington, KY, USA
UR - 21934153
ER -

TY - JOUR
ID - 461
T1 - A comparison of patterns of sun protection during beach holidays and everyday outdoor activities in a population sample of young German children
A1 - Li,J.
A1 - Uter,W.
A1 - Pfahlberg,A.
A1 - Gefeller,O.
Y1 - 2012/04//
N1 - Li, J. Uter, W. Pfahlberg, A. Gefeller, O
The British journal of dermatology
aw0, 0004041
IM
Comparative Study. Journal Article
English
KW - MEDLINE
KW - Activities of Daily Living
KW - Adult
KW - Aged
KW - Bathing Beaches
KW - Child
KW - Preschool
KW - Fathers/px [Psychology]
KW - Female
KW - Germany
KW - Health Knowledge
KW - Attitudes
KW - Practice
KW - Holidays
KW - Humans
KW - Male
KW - Middle Aged
KW - Mothers/px [Psychology]
KW - Parenting/px [Psychology]
KW - Protective Clothing/sn [Statistics & Numerical Data]
KW - Questionnaires
KW - Seasons
KW - Skin Neoplasms/pc [Prevention & Control]
KW - Sunburn/pc [Prevention & Control]
KW - Sunscreening Agents/tu [Therapeutic Use]
KW - Ultraviolet Rays/ae [Adverse Effects]
RP - NOT IN FILE
SP - 803
EP - 810
JF - British Journal of Dermatology
JA - Br J Dermatol
VL - 166
IS - 4
CY - England
N2 - BACKGROUND: Reducing exposure to ultraviolet (UV) radiation is the main effective measure for preventing skin cancer. Educational campaigns targeting sun protection have been focused either on behaviour on the beach during the summer holiday alone, or during everyday outdoor activities of the children. Little is known about the comparison between these different settings. OBJECTIVES: To analyse whether parents apply

similar protective measures to reduce UV exposure for their young children in different outdoor environments. METHODS: Families (n = 2619) with children aged 3-6 years (response: 647%) were enrolled in a population-based survey in the German city of Erlangen and its surrounding rural county. Using a self-administered standardized questionnaire parents gave information about demographic and photosensitivity data of their children, their knowledge about risk factors for skin cancer and their typical instructions given to their children when these played outside on a summer day in different outdoor environments. RESULTS: Significant discrepancies regarding the four UV protective measures (clothes, shade, sunhat, sunscreen) for children between an everyday outdoor setting and a holiday setting on the beach were observed. A high level of parental risk factor knowledge was significantly associated with a better protection for children in all four measures only on the beach. Photosensitivity and demographic characteristics had some impact on protective behaviour, too. Measures of sun protection were reduced with children's increasing age. CONCLUSIONS: Skin cancer prevention campaigns should target the encouragement of sun protection for children also in outdoor activities of daily living, not only during a summer holiday on the beach. 2012 The Authors. BJD 2012 British Association of Dermatologists 2012
SN - 1365-2133
AD - Department of Medical Informatics, Biometry and Epidemiology, Friedrich Alexander University of Erlangen-Nuremberg, 91054 Erlangen, Germany
UR - 22229912
ER -

TY - JOUR
ID - 462
T1 - Playground equipment injuries at home versus those in public settings: differences in severity
A1 - Keays,G.
A1 - Skinner,R.
Y1 - 2012/04//
N1 - Keays, Glenn. Skinner, Robin
Injury prevention : journal of the International Society for Child and Adolescent Injury Prevention
crz, 9510056
IM
Journal Article
English
KW - MEDLINE
KW - Accidental Falls/sn [Statistics & Numerical Data]
KW - Accidents
KW - Home/sn [Statistics & Numerical Data]
KW - Canada/ep [Epidemiology]
KW - Case-Control Studies
KW - Child
KW - Preschool
KW - Female
KW - Humans
KW - Injury Severity Score
KW - Male
KW - Odds Ratio
KW - Play and Playthings/in [Injuries]
KW - Wounds and Injuries/di [Diagnosis]
KW - Wounds and Injuries/ep [Epidemiology]
RP - NOT IN FILE
SP - 138
EP - 141
JF - Injury Prevention
JA - Inj Prev
VL - 18
IS - 2
CY - England

N2 - The objective of the present research was to compare the severity of playground-related injuries in backyards of home with those occurring in public settings. This case-control study used emergency-based surveillance data from Canada regarding children, 3-11 years old, who were injured after falling from playground equipment (PGE). Cases were those whose injuries occurred at home (backyards), and controls were those whose injuries occurred in parks, schools or daycare centres. Of the 39,730 subjects selected, 84% happened in public and 16% at home. Children falling from a home PGE had greater odds of severe injuries (OR=1.30; 95% CI 1.23 to 1.37) and fractures (OR=1.47; 95% CI 1.39 to 1.55) than those from public PGE. Children aged 3-5 years falling off slides at home, compared to slides in public settings, had the greatest odds of severe injuries (OR=1.72; 95% CI 1.41 to 2.09) and fractures (OR=2.17; 95% CI 1.79 to 2.64.) When setting up PGE at home, parents should be diligent in using proper landing surfaces, such as those found in public playgrounds
SN - 1475-5785
AD - Department of Trauma, Montreal Children's Hospital, Montreal, Quebec, Canada. glenn@keays.ca
UR - 22345155
ER -

TY - JOUR
ID - 463
T1 - Preventing unintentional injuries to children under 15 years in the outdoors: a systematic review of the effectiveness of educational programs. [Review]
A1 - Pearson,M.
A1 - Hunt,H.
A1 - Garside,R.
A1 - Moxham,T.
A1 - Peters,J.
A1 - Anderson,R.
Y1 - 2012/04//
N1 - Pearson, Mark. Hunt, Harriet. Garside, Ruth. Moxham, Tiffany. Peters, Jaime. Anderson, Rob
Injury prevention : journal of the International Society for Child and Adolescent Injury Prevention
crz, 9510056
IM
Journal Article. Research Support, Non-U.S. Gov't. Review
English
KW - MEDLINE
KW - Accident Prevention/mt [Methods]
KW - Adolescent
KW - Child
KW - Preschool
KW - Female
KW - Health Education/st [Standards]
KW - Humans
KW - Infant
KW - Leisure Activities
KW - Male
KW - Wounds and Injuries/pc [Prevention & Control]
RP - NOT IN FILE
SP - 113
EP - 123
JF - Injury Prevention
JA - Inj Prev
VL - 18
IS - 2
CY - England
N2 - INTRODUCTION: Unintentional injuries to children in the outdoors have a significant impact on child mortality, development and healthcare costs. This paper presents the findings of a systematic review about the effectiveness of programs that provided information, advice or education about the prevention of unintentional injuries to children under 15 years during outdoor play and leisure. METHODS: A structured search strategy

was conducted in a range of databases. All report titles and abstracts were screened using pre-defined criteria. Included reports were quality appraised using a modified Graphical Appraisal Tool for Epidemiological studies (GATE) tool. All quality appraisals and data extraction were checked by a second reviewer. If not provided in the original reports, ORs and mean differences were calculated, where sufficient data were available. RESULTS: Twenty-three studies met the inclusion criteria. There was a paucity of robust study designs. The majority of studies only reported a short-term follow-up of intermediate outcome measures. Only two studies measured injury rates; both reported a reduction, but both studies also had considerable methodological weaknesses. The five studies that measured the use of protective equipment reported mixed results, although there is some evidence that suggests that more extensive educational programs (such as health fairs and media campaigns) increase their use. The 20 studies that measured behaviour, attitude or knowledge outcomes reported highly mixed results. DISCUSSION: Methodological weaknesses of the included studies limit support for a particular course of action. To better inform policy and practice, future research should (1) use robust study designs and (2) not rely on short-term proxy outcome measures
SN - 1475-5785
AD - Peninsula Technology Assessment Group (PenTAG), Peninsula Medical School, University of Exeter, Exeter, UK. mark.pearson@pms.ac.uk
UR - 21890579
ER -

TY - JOUR
ID - 464
T1 - Intensity and duration of physical efforts in Physical Education classes
A1 - Kremer,M.M.
A1 - Reichert,F.F.
A1 - Hallal,P.C.
Y1 - 2012/04//
N1 - Kremer, Marina Marques. Reichert, Felipe Fossati. Hallal, Pedro Curi
Revista de saude publica
0135043, t5x
IM
Journal Article
English Portuguese
KW - MEDLINE
KW - Adolescent
KW - Brazil
KW - Child
KW - Cross-Sectional Studies
KW - Female
KW - Humans
KW - Male
KW - Motor Activity/ph [Physiology]
KW - Physical Education and Training
KW - Physical Exertion/ph [Physiology]
KW - Schools
KW - Sex Factors
KW - Students
KW - Time Factors
RP - NOT IN FILE
SP - 320
EP - 326
JF - Revista de Saude Publica
JA - Rev Saude Publica
VL - 46
IS - 2
CY - Brazil
N2 - OBJECTIVE: To evaluate the intensity and duration of physical efforts in Physical Education classes in primary and secondary school. METHODS: School-based cross-sectional study carried out by means of the

observation of 218 Physical Education classes, including a total of 272 students (each one of the students was evaluated three times). The study was carried out in the city of Pelotas (Southern Brazil), between August and December 2009. In order to evaluate the intensity of the efforts, accelerometers were used and the following cut-off points were adopted (in counts per minute): sedentary activities (0-100), light activities (101-2,000), moderate (2,001-4,999), vigorous (5,000-7,999), and very vigorous activities (>8000). RESULTS: The mean duration of the classes was 35.6 minutes (SD 6.0). The mean proportion of time spent in moderate to vigorous physical activity was 32.7% (SD 25.2). Boys (44.1%) were involved significantly more in moderate to vigorous physical activity as compared to girls (21.0%; p<0.01). Students who practice physical activities outside the classes participated more in moderate to vigorous physical activity in the Physical Education classes. CONCLUSIONS: Besides the fact that Physical Education classes have a short duration, students practice moderate to vigorous physical activity during one third of the class, with a poor significant contribution to students' level of physical activity
SN - 1518-8787
AD - Instituto Federal Sul-Rio-Grandense, Pelotas, Brasil
UR - 22331179
ER -

TY - JOUR
ID - 465
T1 - A recess intervention to promote moderate-to-vigorous physical activity
A1 - Howe,C.A.
A1 - Freedson,P.S.
A1 - Alhassan,S.
A1 - Feldman,H.A.
A1 - Osganian,S.K.
Y1 - 2012/02//
N1 - Howe, C A. Freedson, P S. Alhassan, S. Feldman, H A. Osganian, S K
Pediatric obesity
101572033
IM
Journal Article. Research Support, Non-U.S. Gov't
English
KW - MEDLINE
KW - Body Mass Index
KW - Child
KW - Exercise/ph [Physiology]
KW - Female
KW - Health Promotion
KW - Health Status Indicators
KW - Humans
KW - Male
KW - Obesity/pc [Prevention & Control]
KW - Recreation/ph [Physiology]
KW - Running/ph [Physiology]
KW - Schools
RP - NOT IN FILE
SP - 82
EP - 88
JF - Pediatric Obesity
JA - Pediatr Obes
VL - 7
IS - 1
CY - England
N2 - BACKGROUND: Schools provide a prime environment for interventions that attempt to increase physical activity and prevent obesity. OBJECTIVE: This study examined the effect of a 30-min, structured recess using 22 games of known energy expenditure on moderate-to-vigorous physical activity when compared to free play implemented with third graders from two elementary schools over 9 weeks. METHODS: Moderate-to-vigorous

physical activity and other cardiovascular risk factors were assessed pre- and post-intervention in 27 children. RESULTS: Moderate-to-vigorous physical activity during recess increased significantly in intervention school children from 6.9+0.8 to 14.9+0.9min pre- and post-intervention, respectively (adjusted mean change 8.0+1.1; P<0.0001), with no differences by gender or body mass index (BMI). In-school, moderate-to-vigorous physical activity also increased significantly more for intervention compared to control children (adjusted mean change 14+4min vs. 3+3min; P=0.014, respectively). CONCLUSION: There was no significant difference in BMI and cardiovascular risk factors. A structured recess is feasible to implement and can significantly increase moderate-to-vigorous physical activity. 2012 The Authors Pediatric Obesity 2012 International Association for the Study of Obesity
SN - 2047-6310
AD - Department of Kinesiology, University of Massachusetts, Amherst, MA, USA
UR - 22434742
ER -

TY - JOUR
ID - 466
T1 - Prevalence of overweight, obesity, and associated risk factors among school children and adolescents in Tianjin, China
A1 - Andegiorgish,A.K.
A1 - Wang,J.
A1 - Zhang,X.
A1 - Liu,X.
A1 - Zhu,H.
Y1 - 2012/04//
N1 - Andegiorgish, Amanuel Kidane. Wang, Jianhua. Zhang, Xin. Liu, Xinmin. Zhu, Hong
European journal of pediatrics
end, 7603873
IM
Journal Article. Research Support, Non-U.S. Gov't
English
KW - MEDLINE
KW - Adolescent
KW - Birth Weight
KW - Body Mass Index
KW - Child
KW - China/ep [Epidemiology]
KW - Cross-Sectional Studies
KW - Female
KW - Humans
KW - Life Style
KW - Male
KW - Obesity/ep [Epidemiology]
KW - Overweight/ep [Epidemiology]
KW - Prevalence
KW - Risk Factors
KW - Social Class
RP - NOT IN FILE
SP - 697
EP - 703
JF - European Journal of Pediatrics
JA - Eur J Pediatr
VL - 171
IS - 4
CY - Germany
N2 - UNLABELLED: To estimate the prevalence and determinants of overweight and obesity among school children and adolescents (7-18 years), a cross-sectional study was conducted in Tianjin City of Northeast China. Five primary and middle schools were selected using a multistage random cluster sampling. Anthropometric

measurements were taken by the research team. Then, standardized questionnaires on socioeconomic status, diet and lifestyle, parental weight and height were sent to the parents to be completed together with their child. Overall, 3,140 students were screened for overweight and associated risk factors. Body mass index classification reference proposed by the Working Group on Obesity in China was used as a screening reference to calculate the prevalence of overweight in these groups. The prevalence of overweight and obesity was found to be 12.5% and 15.7%, respectively. Logistic regression analysis (overweight compared with normal weight) showed that, overweight in children was significantly associated with male gender, parental obesity, parental educational level, mother's history of gestational diabetes, high birth weight, less hours of physical activity per day, urban residence, motorized transportation, and eating food not prepared at home. After adjusting for parental obesity, the odds ratio of overweight children was increased by urban residence (rural as a reference, 2.68, 2.16-3.32), positive gestational diabetes (2.76, 1.37-4.50), and fast-food/restaurant food service (2.03, 1.34-3.07). Children who walked to school and participated in outdoor activities were 54% and 63% less likely to be overweight respectively. CONCLUSIONS: The prevalence of overweight and obesity is high among school children and adolescents in Tianjin. Changes in lifestyle, high socioeconomic status, parental obesity, mother's history of gestational diabetes, and high birth weight are among the highly associated risk factors of overweight. An educational approach about maintaining a healthy weight should be introduced much earlier to high-risk children, parents, and women of childbearing age
SN - 1432-1076
AD - Epidemiology Department, College of Public Health, Tianjin Medical University, Tianjin, 300070, Tianjin Province, People's Republic of China
UR - 22160003
ER -

TY - JOUR
ID - 467
T1 - The pain experiences of powered wheelchair users
A1 - Frank,A.O.
A1 - De Souza,L.H.
A1 - Frank,J.L.
A1 - Neophytou,C.
Y1 - 2012///
N1 - Frank, Andrew O. De Souza, Lorraine H. Frank, Julia L. Neophytou, Claudius
Disability and rehabilitation
9207179, a8i
IM
Journal Article. Research Support, Non-U.S. Gov't
English
KW - MEDLINE
KW - Activities of Daily Living
KW - Adolescent
KW - Adult
KW - Aged
KW - 80 and over
KW - Child
KW - Disabled Persons/rh [Rehabilitation]
KW - Electricity
KW - Equipment Design
KW - Female
KW - Humans
KW - Interviews as Topic
KW - Male
KW - Middle Aged
KW - Pain/di [Diagnosis]
KW - Pain/et [Etiology]
KW - Pain Measurement/mt [Methods]
KW - Patient Satisfaction
KW - Qualitative Research

KW - Questionnaires
KW - Wheelchairs/ae [Adverse Effects]
KW - Young Adult
RP - NOT IN FILE
SP - 770
EP - 778
JF - Disability & Rehabilitation
JA - Disabil Rehabil
VL - 34
IS - 9
CY - England
N2 - PURPOSE: To explore the experience of pain and discomfort in users of electric-powered indoor/outdoor wheelchairs (EPIOCs) provided by a National Health Service. METHODS: EPIOC users receiving their chair between February and November 2002 (N=74) were invited to participate in a telephone questionnaire/interview and 64 (aged 10-81 years) agreed. Both specific and open-ended questions examined the presence of pain/discomfort, its severity, minimizing and aggravating factors, particularly in relation to the EPIOC and its use. RESULTS: Most EPIOC users described experiences of pain with 17% reporting severe pain. Over half felt their pain was influenced by the wheelchair and few (25%) considered their chair eased their symptoms. The most common strategy for pain relief was taking medication. Other self-help strategies included changing position, exercise and complementary therapies. Respondents emphasized the provision of backrests, armrests, footrests and cushions which might alleviate or exacerbate pain, highlighting the importance of appropriate assessment for this high dependency group. CONCLUSIONS: Users related pain to their underlying medical condition, their wheelchair or a combination of the two. User feedback is essential to ensure that the EPIOC meets health needs with minimal pain. This becomes more important as the health condition of users changes over time
SN - 1464-5165
AD - Stanmore Specialist Wheelchair Service, Royal National Orthopaedic Hospital, Brockley Hill, Stanmore, United Kingdom. andrew.frank1@btinternet.com
UR - 22013954
ER -

TY - JOUR
ID - 468
T1 - [Children of single mothers: health risks and environmental stress]. [German]
A1 - Scharte,M.
A1 - Bolte,G.
Y1 - 2012/03//
N1 - Scharte, M. Bolte, G
Gesundheitswesen (Bundesverband der Arzte des Offentlichen Gesundheitsdienstes (Germany))
bfd, 9204210
IM
Comparative Study. English Abstract. Journal Article
German
KW - MEDLINE
KW - Affective Symptoms/ep [Epidemiology]
KW - Attention Deficit Disorder with Hyperactivity/ep [Epidemiology]
KW - Child
KW - Child Behavior Disorders/ep [Epidemiology]
KW - Child Rearing
KW - Child Welfare
KW - Preschool
KW - Conduct Disorder/ep [Epidemiology]
KW - Cross-Sectional Studies
KW - Environmental Exposure
KW - Female
KW - Germany
KW - Health Status Indicators

KW - Health Surveys
KW - Humans
KW - Male
KW - Mothers
KW - Obesity/ep [Epidemiology]
KW - Poverty
KW - Psychosocial Deprivation
KW - Residence Characteristics
KW - Sex Factors
KW - Single Parent
KW - Social Environment
KW - Socialization
RP - NOT IN FILE
SP - 123
EP - 131
JF - Gesundheitswesen
VL - 74
IS - 3
CY - Germany
N2 - BACKGROUND AND AIM: In Germany the risk for relative poverty has increased profoundly during the last 15 years, especially among single parent families. As poverty is often associated with bad health we examined the physical and mental health as well as health-related behaviour, housing and environmental conditions in children with lone mothers versus children in couple families. STUDY POPULATION AND METHODS: In 3 cross-sectional surveys conducted during 2004-2007 in 3 cities and 3 rural areas in Bavaria data on 19 039 pre-school children (47% female) were collected. Health, behaviour and exposure assessment was based on parental reports. The 18 327 cases with complete information on family status were analysed. 10% of the children grew up with single mothers. RESULTS: Single mothers evaluated the general state of health of their children more often as moderate to very poor than couple parents (OR [95% CI]: male: 1.37 [1.07-1.77], female 1.77 [1.33-2.35]). Sons with single mothers were more often obese (1.44 [1.09-1.90]). They scored significantly higher in the SDQ total difficulties score (1.94 [1.44-2.62]), on the emotional problem scale (1.91 [1.40-2.59]) as well as on the hyperactivity scale (1.82 [1.35-2.47]) compared to boys from couple families. No difference was found in prosocial behaviour. Girls with single mothers revealed more often conduct problems 1.36 [1.02-1.81] compared to those from couple families. They also showed a significantly higher prevalence of asthma (2.06 [1.29-3.30]). Children living with their single mothers were less often members of sports clubs and the boys were less physical active. No difference was found regarding the consumption of fruits and vegetables. Single mother family was associated with children's environmental tobacco smoke exposure at home (2.03 [1.79-2.29]). Single mothers perceived higher environmental exposures to noise and air pollution, suffered more often from a lack of accessible green spaces in the neighbourhood and reported a higher traffic load on the residential street. CONCLUSION: Children with single mothers reveal increased health risks and live under significantly poorer housing and environmental conditions. Further analyses are necessary to identify the mediating factors. Georg Thieme Verlag KG Stuttgart New York
SN - 1439-4421
AD - Bayerisches Landesamt fur Gesundheit und Lebensmittelsicherheit, Sachbereich Arbeits- und Umweltepidemiologie, Munchen, Germany
UR - 21267816
ER -

TY - JOUR
ID - 469
T1 - Assessing Indiana's Health and Safety in early care and education programs: identifying areas for improvement
A1 - Alkon,A.
A1 - Cole,P.S.
Y1 - 2012/04//
N1 - Alkon, Abbey. Cole, Patricia S
Maternal and child health journal
9715672, di8

IM
Journal Article. Research Support, Non-U.S. Gov't
English
KW - MEDLINE
KW - Adolescent
KW - Checklist
KW - Child
KW - Child Care
KW - Child Day Care Centers/og [Organization & Administration]
KW - Child Health Services/og [Organization & Administration]
KW - Child Welfare
KW - Preschool
KW - Cross-Sectional Studies
KW - Early Intervention (Education)
KW - Female
KW - Humans
KW - Indiana
KW - Male
KW - Program Evaluation
KW - Reproducibility of Results
KW - Safety
RP - NOT IN FILE
SP - 555
EP - 563
JF - Maternal & Child Health Journal
JA - Matern Child Health J
VL - 16
IS - 3
CY - United States
N2 - The objective of this study is to describe the key National Health and Safety Standards (NHS) met in early care and education (ECE) programs in Indiana and to establish reliability for the Indiana Health and Safety Checklist. The Indiana Health and Safety Checklist, modified from the California Childcare Health Program's Health and Safety Checklist, includes 87 items and 11 subscales. It was completed in 2 h by trained child care health consultants in 82 ECE programs to assess the number of key NHS met. The 82 ECE programs met the majority of NHS items. Thirteen items were not met in more than 50% of the programs. The items with the lowest compliance were emergency food supplies, children's hand washing, special health care plans, cleaning and sanitizing counters, and impact surfaces under playground equipment. The Indiana Health and Safety Checklist had moderate internal reliability for 6 of the 9 subscales analyzed. ECE programs strive to provide high quality care and the programs met the majority of NHS on the checklist. The Checklist is a reliable instrument that helped identify health and safety gaps and thus, identified the targeted interventions for child care health consultation
SN - 1573-6628
AD - Department of Family Health Care Nursing, University of California, San Francisco School of Nursing, 2 Koret Way, San Francisco, CA 94143-0606, USA. abbey.alkon@nursing.ucsf.edu
UR - 21509431
ER -

TY - JOUR
ID - 470
T1 - Using interviews and peer pairs to better understand how school environments affect young children's playground physical activity levels: a qualitative study
A1 - Parrish,A.M.
A1 - Yeatman,H.
A1 - Iverson,D.
A1 - Russell,K.
Y1 - 2012/04//

N1 - Parrish, Anne-Maree. Yeatman, Heather. Iverson, Don. Russell, Ken
Health education research
bqp, 8608459
T
Journal Article
English
KW - MEDLINE
KW - Child
KW - Faculty
KW - Female
KW - Humans
KW - Interviews as Topic
KW - Male
KW - Motor Activity
KW - Organizational Policy
KW - Peer Group
KW - Play and Playthings
KW - Schools
RP - NOT IN FILE
SP - 269
EP - 280
JF - Health Education Research
JA - Health Educ Res
VL - 27
IS - 2
CY - England
N2 - School break times provide a daily opportunity for children to be active; however, research indicates this time is underutilized. Reasons for low children's playground activity levels have primarily focused on physical barriers. This research aimed to contribute to physical environmental findings affecting children's playground physical activity levels by identifying additional variables through the interview process. Thirteen public schools were included in the sample (total 2946 children). Physical activity and environmental data were collected over 3 days. Environmental variables were manually assessed at each of the 13 schools. Observational data were used to determine which three schools were the most and least active. The principal, three teachers and 20 students in Grades 4-6 from these six schools (four lower and two average socioeconomic status) were invited to participate in the interview process. Student interviews involved the paired interview technique. The main themes generated from the school interviews included the effect of non-fixed equipment (including balls), playground markings, playground aesthetics, activity preference, clothing, the amount of break time available for play, teacher playground involvement, gender, bullying, school policies, student confidence in break-time activity and fundamental movement skills. The effect of bullying on playground physical activity levels was concerning
SN - 1465-3648
AD - School of Health Sciences, Faculty of Health and Behavioural Sciences, University of Wollongong, Wollongong, New South Wales 2522, Australia. aparrish@uow.edu.au
UR - 21712499
ER -

TY - JOUR
ID - 471
T1 - Toxicity and elemental composition of particulate matter from outdoor and indoor air of elementary schools in Munich, Germany
A1 - Oeder,S.
A1 - Dietrich,S.
A1 - Weichenmeier,I.
A1 - Schober,W.
A1 - Pusch,G.
A1 - Jorres,R.A.
A1 - Schierl,R.

A1 - Nowak,D.
A1 - Fromme,H.
A1 - Behrendt,H.
A1 - Buters,J.T.
Y1 - 2012/04//
N1 - Oeder, S. Dietrich, S. Weichenmeier, I. Schober, W. Pusch, G. Jorres, R A. Schierl, R. Nowak, D. Fromme, H. Behrendt, H. Buters, J T M
Indoor air
9423515, da3
IM
Journal Article. Research Support, Non-U.S. Gov't
English
KW - MEDLINE
KW - Air Pollution
KW - Indoor/ae [Adverse Effects]
KW - Indoor/an [Analysis]
KW - Aryl Hydrocarbon Hydroxylases/me [Metabolism]
KW - Biotransformation
KW - Calcium Carbonate/an [Analysis]
KW - Calcium Carbonate/to [Toxicity]
KW - Cell Line
KW - Cells
KW - Cultured
KW - Child
KW - Cytochrome P-450 CYP3A/me [Metabolism]
KW - Germany
KW - Housing
KW - Humans
KW - Keratinocytes/de [Drug Effects]
KW - Keratinocytes/me [Metabolism]
KW - Microscopy
KW - Electron
KW - Scanning
KW - Oxidoreductases
KW - N-Demethylating/me [Metabolism]
KW - Particle Size
KW - Particulate Matter/an [Analysis]
KW - Particulate Matter/to [Toxicity]
KW - Schools
KW - Silicon/an [Analysis]
KW - Silicon/to [Toxicity]
KW - Sulfur/an [Analysis]
KW - Sulfur/to [Toxicity]
RP - NOT IN FILE
SP - 148
EP - 158
JF - Indoor Air
VL - 22
IS - 2
CY - Denmark
N2 - UNLABELLED: Outdoor particulate matter (PM(10)) is associated with detrimental health effects. However, individual PM(10) exposure occurs mostly indoors. We therefore compared the toxic effects of classroom, outdoor, and residential PM(10). Indoor and outdoor PM(10) was collected from six schools in Munich during teaching hours and in six homes. Particles were analyzed by scanning electron microscopy and X-ray spectroscopy (EDX). Toxicity was evaluated in human primary keratinocytes, lung epithelial cells and after metabolic activation by several human cytochromes P450. We found that PM(10) concentrations during teaching hours were 5.6-times higher than outdoors (117 + 48 mug/m(3) vs. 21 + 15 mug/m(3), P < 0.001).

Compared to outdoors, indoor PM contained more silicate (36% of particle number), organic (29%, probably originating from human skin), and Ca-carbonate particles (12%, probably originating from paper). Outdoor PM contained more Ca-sulfate particles (38%). Indoor PM at 6 mug/cm(2) (10 mug/ml) caused toxicity in keratinocytes and in cells expressing CYP2B6 and CYP3A4. Toxicity by CYP2B6 was abolished with the reactive oxygen species scavenger N-acetylcysteine. We concluded that outdoor PM(10) and indoor PM(10) from homes were devoid of toxicity. Indoor PM(10) was elevated, chemically different and toxicologically more active than outdoor PM(10). Whether the effects translate into a significant health risk needs to be determined. Until then, we suggest better ventilation as a sensible option. PRACTICAL IMPLICATIONS: Indoor air PM(10) on an equal weight base is toxicologically more active than outdoor PM(10). In addition, indoor PM(10) concentrations are about six times higher than outdoor air. Thus, ventilation of classrooms with outdoor air will improve air quality and is likely to provide a health benefit. It is also easier than cleaning PM(10) from indoor air, which has proven to be tedious. 2011 John Wiley & Sons A/S
SN - 1600-0668
AD - ZAUM-Center of Allergy and Environment, Helmholtz Zentrum Munchen/Technische Universitat Munchen, Munich, Germany
UR - 21913995
ER -

TY - JOUR
ID - 472
T1 - Age-related differences in the brain areas outside the classical language areas among adults using category decision task
A1 - Cho,Y.W.
A1 - Song,H.J.
A1 - Lee,J.J.
A1 - Lee,J.H.
A1 - Lee,H.J.
A1 - Yi,S.D.
A1 - Chang,H.W.
A1 - Berl,M.M.
A1 - Gaillard,W.D.
A1 - Chang,Y.
Y1 - 2012/03//
N1 - Cho, Yong Won. Song, Hui-Jin. Lee, Jae Jun. Lee, Joo Hwa. Lee, Hui Joong. Yi, Sang Doe. Chang, Hyuk Won. Berl, Madison M. Gaillard, William D. Chang, Yongmin
Brain and language
7506220, b5h
IM
Journal Article. Research Support, Non-U.S. Gov't
English
KW - MEDLINE
KW - Adolescent
KW - Adult
KW - Aged
KW - Aging/ph [Physiology]
KW - Brain/ph [Physiology]
KW - Brain Mapping
KW - Child
KW - Decision Making/ph [Physiology]
KW - Dominance
KW - Cerebral/ph [Physiology]
KW - Female
KW - Frontal Lobe/ph [Physiology]
KW - Gyrus Cinguli/ph [Physiology]
KW - Hippocampus/ph [Physiology]
KW - Humans
KW - Magnetic Resonance Imaging

KW - Male
KW - Middle Aged
KW - Parietal Lobe/ph [Physiology]
KW - Semantics
KW - Temporal Lobe/ph [Physiology]
KW - Young Adult
RP - NOT IN FILE
SP - 372
EP - 380
JF - Brain & Language
JA - Brain Lang
VL - 120
IS - 3
CY - United States
N2 - Older adults perform much like younger adults on language. This similar level of performance, however, may come about through different underlying brain processes. In the present study, we evaluated age-related differences in the brain areas outside the typical language areas among adults using a category decision task. Our results showed that similar activation patterns were found in classical language processing areas across the three age groups although regional lateralization indices in Broca's and Wernicke's areas decreased with age. The greatest differences, however, among the three groups were found primarily in the brain areas not associated with core language functioning including the hippocampus, middle frontal gyrus, ventromedial frontal cortex, medial superior parietal cortex and posterior cingulate cortex. Therefore, the non-classical language areas may exhibit an age-related difference between three age groups while the subjects show a similar activation pattern in the core, primary language processing during a semantic decision task. Copyright A 2012 Elsevier Inc. All rights reserved
SN - 1090-2155
AD - Department of Neurology, Keimyung University, Dongsan Medical Center, Daegu, Republic of Korea
UR - 22305968
ER -

TY - JOUR
ID - 473
T1 - The daily patterns of time use for parents of children with complex needs: a systematic review. [Review]
A1 - McCann,D.
A1 - Bull,R.
A1 - Winzenberg,T.
Y1 - 2012/03//
N1 - McCann, Damhnat. Bull, Rosalind. Winzenberg, Tania
Journal of child health care : for professionals working with children in the hospital and community
9806360, dcl
N
Journal Article. Review
English
KW - MEDLINE
KW - Activities of Daily Living
KW - Caregivers
KW - Child
KW - Disabled Children
KW - Humans
KW - Parent-Child Relations
KW - Time Factors
RP - NOT IN FILE
SP - 26
EP - 52
JF - Journal of Child Health Care
JA - J Child Health Care
VL - 16

IS - 1
CY - England
N2 - The aim of this systematic review was to critically examine the research that quantifies and describes the daily patterns of time use by parents of children with complex needs. Four electronic databases were searched. A total of 32 studies (30 peer-reviewed journal articles and two theses) met the inclusion criteria. Three key findings emerged from the review: (a) Parents of children with complex needs carry a significant caregiving burden that often does not reduce as the age of the child increases, (b) supervision or 'vigilance' is a category of childcare that carries a particular time requirement for these parents and (c) parents of children with complex needs spend (sometimes considerable) time undertaking health care-related tasks outside the 'normal' parenting role. The time demands placed on parents caring for a child or children with complex needs at home should be considered when health professionals are negotiating essential and/or additional therapies or treatments to be included in a child's home care regime
SN - 1741-2889
AD - School of Nursing and Midwifery, University of Tasmania, Launceston, Tasmania, Australia.
Damhnat.Mccann@utas.edu.au
UR - 22308543
ER -

TY - JOUR
ID - 474
T1 - Fidelity of after-school program implementation targeting adolescent youth: identifying successful curricular and programmatic characteristics
A1 - Rajan,S.
A1 - Basch,C.E.
Y1 - 2012/04//
N1 - Rajan, Sonali. Basch, Charles E
The Journal of school health
k13, 0376370
IM, N
Journal Article. Research Support, N.I.H., Extramural. Research Support, Non-U.S. Gov't
English
KW - MEDLINE
KW - Adolescent
KW - Adolescent Behavior
KW - Canada
KW - Child
KW - Curriculum
KW - Decision Making
KW - Evaluation Studies as Topic
KW - Female
KW - Health Education/mt [Methods]
KW - Health Education/og [Organization & Administration]
KW - Humans
KW - Motor Activity
KW - Program Development
KW - Program Evaluation
KW - Qualitative Research
KW - School Health Services/og [Organization & Administration]
KW - School Health Services/st [Standards]
KW - United States
RP - NOT IN FILE
SP - 159
EP - 165
JF - Journal of School Health
JA - J Sch Health
VL - 82
IS - 4

CY - United States
N2 - BACKGROUND: This study consisted of a formative evaluation of an after-school health education program designed for adolescent females, entitled Girls on Track. Evidence-based after-school programs have potential to supplement the traditional school day, encourage social and emotional skill development, improve the quality of student health, and contribute to a healthier school environment. Implementing comprehensive programs outside of the classroom, however, is challenging and gaps exist in the literature regarding implementation fidelity. METHODS: The national program was in a cohort of adolescent females from 11 Girls on Track locations in fall 2008 and 10 locations in spring 2009. Mixed-method analyses evaluated fidelity of lesson implementation, described curricular and programmatic characteristics associated with implementation fidelity, and assessed coach perceptions toward program quality and feasibility. RESULTS: Specific lesson characteristics associated with higher rates of implementation fidelity and favorable coach perceptions toward program feasibility included improved lesson clarity, more time to process health topics, teaching and learning objectives that were well aligned with lesson activities, incorporation of alternative activities for less-motivated participants, and provision of resources on key health topics for coaches. CONCLUSION: Girls on Track is currently being implemented across the United States and Canada, reaching over 5000 adolescent females annually. Identifying and incorporating specific curricular and programmatic characteristics associated with high levels of implementation fidelity can enhance the quality and benefits of after-school programs. 2012, American School Health Association
SN - 1746-1561
AD - National Development and Research Institutes and Public Health Solutions, 71 West 23rd Street, 8th Floor, New York, NY 10010, USA. sr2345@columbia.edu
UR - 22385088
ER -

TY - JOUR
ID - 475
T1 - Impact of an active video game on healthy children's physical activity
A1 - Baranowski,T.
A1 - Abdelsamad,D.
A1 - Baranowski,J.
A1 - O'Connor,T.M.
A1 - Thompson,D.
A1 - Barnett,A.
A1 - Cerin,E.
A1 - Chen,T.A.
Y1 - 2012/03//
N1 - Baranowski, Tom. Abdelsamad, Dina. Baranowski, Janice. O'Connor, Teresia Margareta. Thompson, Debbe. Barnett, Anthony. Cerin, Ester. Chen, Tzu-An
Pediatrics
oxv, 0376422
AIM, IM
Comparative Study. Journal Article. Randomized Controlled Trial. Research Support, N.I.H., Extramural. Research Support, U.S. Gov't, Non-P.H.S.
English
KW - MEDLINE
KW - Anthropometry
KW - Body Mass Index
KW - Child
KW - Child Welfare
KW - Female
KW - Humans
KW - Male
KW - Motor Activity/ph [Physiology]
KW - Obesity/ep [Epidemiology]
KW - Obesity/et [Etiology]
KW - Obesity/pc [Prevention & Control]
KW - Reference Values

KW - Risk Assessment
KW - Time Factors
KW - United States
KW - Video Games/ae [Adverse Effects]
KW - Video Games/cl [Classification]
KW - Video Games/sn [Statistics & Numerical Data]
RP - NOT IN FILE
SP - e636
EP - e642
JF - Pediatrics
VL - 129
IS - 3
CY - United States
N2 - OBJECTIVE: This naturalistic study tests whether children receiving a new (to them) active video game spontaneously engage in more physical activity than those receiving an inactive video game, and whether the effect would be greater among children in unsafe neighborhoods, who might not be allowed to play outside. METHODS: Participants were children 9 to 12 years of age, with a BMI >50th percentile, but <99th percentile; none of these children a medical condition that would preclude physical activity or playing video games. A randomized clinical trial assigned children to receiving 2 active or 2 inactive video games, the peripherals necessary to run the games, and a Wii console. Physical activity was monitored by using accelerometers for 5 weeks over the course of a 13-week experiment. Neighborhood safety was assessed with a 12 item validated questionnaire. RESULTS: There was no evidence that children receiving the active video games were more active in general, or at anytime, than children receiving the inactive video games. The outcomes were not moderated by parent perceived neighborhood safety, child BMI z score, or other demographic characteristics. CONCLUSIONS: These results provide no reason to believe that simply acquiring an active video game under naturalistic circumstances provides a public health benefit to children
SN - 1098-4275
AD - Children's Nutrition Research Center, Department of Pediatrics, Baylor College of Medicine, Houston, TX 77030, USA. tbaranow@bcm.edu
UR - 22371457
ER -

TY - JOUR
ID - 476
T1 - Requirement of longitudinal synchrony of epileptiform discharges in the hippocampus for seizure generation: a pilot study
A1 - Umeoka,S.C.
A1 - Luders,H.O.
A1 - Turnbull,J.P.
A1 - Koubeissi,M.Z.
A1 - Maciunas,R.J.
Y1 - 2012/03//
N1 - Umeoka, Shuichi C. Luders, Hans O. Turnbull, John P. Koubeissi, Mohamad Z. Maciunas, Robert J
Journal of neurosurgery
jd3, 0253357
AIM, IM
Journal Article
English
KW - MEDLINE
KW - Adolescent
KW - Adult
KW - Amygdala/pp [Physiopathology]
KW - Anterior Temporal Lobectomy/mt [Methods]
KW - Child
KW - Electrodes
KW - Implanted
KW - Electroencephalography/mt [Methods]

KW - Epilepsy
KW - Temporal Lobe/pp [Physiopathology]
KW - Temporal Lobe/su [Surgery]
KW - Female
KW - Functional Laterality
KW - Hippocampus/pp [Physiopathology]
KW - Hippocampus/su [Surgery]
KW - Humans
KW - Male
KW - Middle Aged
KW - Parahippocampal Gyrus/pp [Physiopathology]
KW - Pilot Projects
KW - Seizures/pp [Physiopathology]
RP - NOT IN FILE
SP - 513
EP - 524
JF - Journal of Neurosurgery
JA - J Neurosurg Pediatrics
VL - 116
IS - 3
CY - United States
N2 - OBJECT: The goal in this study was to assess the role of longitudinal hippocampal circuits in the generation of interictal and ictal activity in temporal lobe epilepsy (TLE) and to evaluate the effects of multiple hippocampal transections (MHT). METHODS: In 6 patients with TLE, the authors evaluated the synchrony of hippocampal interictal and ictal epileptiform discharges by using a cross-correlation analysis, and the effect of MHT on hippocampal interictal spikes was studied. Five of the 6 patients were studied with depth electrodes, and epilepsy surgery was performed in 4 patients (anterior temporal lobectomy in 1 and MHT in 3). RESULTS: Four hundred eighty-two (95.1%) of 507 hippocampal spikes showed an anterior-to-posterior propagation within the hippocampus, with a fixed peak-to-peak interval. During seizures, a significant increase of synchronization between different hippocampal regions and between the hippocampus and the ipsilateral anterior parahippocampal gyrus was observed in all seizures. An ictal increase in synchronization between the hippocampus and ipsilateral amygdala was seen in only 24.1% of the seizures. No changes in synchronization were noticed during seizures between the hippocampi and the amygdala on either side. The structure leading the epileptic seizures varied over time during a given seizure and also from one seizure to another. Spike analysis during MHT demonstrated that there were two spike populations that reacted differently to this procedure--namely, 1) spikes that showed maximum amplitude at the head of the hippocampus (type H); and 2) spikes that showed the highest amplitude at the hippocampal body (type B). A striking decrease in amplitude and frequency of type B spikes was noticed in all 3 patients after transections at the head or anterior portion of the hippocampal body. Type H spikes were seen in 2 cases and did not change in amplitude and frequency throughout MHT. Type B spikes showed constantly high cross-correlation values in different derivations and a relatively fixed peak-to-peak interval before MHT. This fixed interpeak delay disappeared after the first transection, although high cross-correlation values persisted unchanged. All patients who underwent MHT remained seizure free for more than 2 years. CONCLUSIONS: These data suggest that synchronized discharges involving the complete anterior-posterior axis of the hippocampal/parahippocampal (H/P) formation underlie the spread of epileptiform discharges outside the H/P structures and, therefore, for the generation of epileptic seizures originating in the H/P structures. This conclusion is supported by the following observations. 1) Hippocampal spikes are consistently synchronized in the whole hippocampal structures, with a fixed delay between the different hippocampal areas. 2) One or two transections between the head and body of the hippocampal formation are sufficient to abolish hippocampal spikes that are synchronized along the anterior-posterior axis of the hippocampus. 3) Treatment with MHT leads to seizure freedom in patients with H/P epilepsy
SN - 1933-0693
AD - Epilepsy Center, University Hospitals Neurological Institute, Case Medical Center, 11100 Euclid Avenue, Cleveland, Ohio 44106-5040, USA
UR - 22175726
ER -

TY - JOUR

ID - 477
T1 - The effectiveness of a community playground intervention
A1 - Quigg,R.
A1 - Reeder,A.I.
A1 - Gray,A.
A1 - Holt,A.
A1 - Waters,D.
Y1 - 2012/02//
N1 - Quigg, Robin. Reeder, Anthony Ivor. Gray, Andrew. Holt, Alec. Waters, Debra
Journal of urban health : bulletin of the New York Academy of Medicine
c5l, 9809909
IM
Evaluation Studies. Journal Article. Research Support, Non-U.S. Gov't
English
KW - MEDLINE
KW - Actigraphy/is [Instrumentation]
KW - Child
KW - Preschool
KW - Exercise
KW - Female
KW - Follow-Up Studies
KW - Health Promotion
KW - Humans
KW - Male
KW - New Zealand
KW - Play and Playthings
KW - Public Facilities
KW - Questionnaires
KW - Residence Characteristics
RP - NOT IN FILE
SP - 171
EP - 184
JF - Journal of Urban Health
JA - J Urban Health
VL - 89
IS - 1
CY - United States
N2 - This study assessed whether an upgrade of playgrounds in a community was associated with changes in the physical activity of local children. The study used a natural experiment design with a local authority project to upgrade two community playgrounds as the intervention and a matched control community. Children's physical activity was measured by an Actigraph GT1M accelerometer worn for 8 days, enabling up to 6 days of data to be analyzed. A self-administered parent/guardian questionnaire was used to collect additional data, including perceptions of the neighborhood, school-travel modes, days involved in extracurricular activities, ethnicity, caregiver age, caregiver sex, household vehicle access, and household income. At baseline, 184 children (5-10 years old) participated. Of these, 156 completed the 1-year follow-up assessment (20% lost to follow-up). There was statistically significant evidence that change in mean total daily physical activity was associated with on an interaction between participant's body mass index (BMI) z-score and her or his community of residence (p=0.006), with the intervention being associated with higher levels of activity for children with lower BMIs but lower levels for children with higher BMIs. Physical activity is not the only focus of local authority playground provision as playgrounds also have benefits for social development and fundamental movement skills. However, making sure that physical activity is always included in the design rationale and that playgrounds are designed to encourage and sustain physical activity could be a useful population health intervention. The effects of such interventions on different subgroups are of importance, especially if the effects differ over levels of BMI
SN - 1468-2869

AD - Cancer Society of New Zealand Social and Behavioural Research Unit, Department of Preventive and Social Medicine, Dunedin School of Medicine, University of Otago, P.O. Box 913, Dunedin, 9054, New Zealand. robin.quigg@otago.ac.nz
UR - 21959697
ER -

TY - JOUR
ID - 478
T1 - Solar UV exposure of children in a summer school in Valencia, Spain
A1 - Serrano,M.A.
A1 - Canada,J.
A1 - Moreno,J.C.
Y1 - 2012/03//
N1 - Serrano, Maria-Antonia. Canada, Javier. Moreno, Juan Carlos
International journal of biometeorology
gqs, 0374716
IM
Journal Article. Research Support, Non-U.S. Gov't
English
KW - MEDLINE
KW - Child
KW - Humans
KW - Radiation Dosage
KW - Radiation Monitoring/is [Instrumentation]
KW - Schools
KW - Seasons
KW - Spain
KW - Sunlight
KW - Ultraviolet Rays
RP - NOT IN FILE
SP - 371
EP - 377
JF - International Journal of Biometeorology
JA - Int J Biometeorol.
VL - 56
IS - 2
CY - United States
N2 - Ultraviolet (UV) exposure is the major environmental factor involved in the development of skin cancers and occurs mainly during outdoor activities. During summer schools, children receive regular and significant solar ultraviolet erythemal radiation (UVER) while practising outdoor activities. Personal dosimeters (VioSpor) were attached to the shoulders of schoolchildren and used to quantify their exposure to UVER. The study took place in Valencia, Spain, during July 2008, with three age groups (7-8, 9-10 and 11-12 years old) and involved about 15 schoolchildren. The median (25, 75 percentiles) twice-daily UV exposure values for all groups was 5.49 (3.59, 8.00) standard erythemal doses (SEDs), where 1 SED is defined as effective 100 Jm(-2) when weighted with the CIE erythemal response function. Exposure ratio (ER) is defined as the ratio between the personal dose on a selected body site and the corresponding ambient dose received on a horizontal plane during the same exposure period. The median (25, 75 percentiles) ER value for all groups in the study was 5.9% (4.1, 8.7)
SN - 1432-1254
AD - Department of Applied Physics, Universidad Politecnica de Valencia, Camino de Vera s/n, 46022, Valencia, Spain. mserranj@fis.upv.es
UR - 21560029
ER -

TY - JOUR
ID - 479
T1 - Profile of pediatric ocular trauma in mid western hilly region of Nepal

A1 - Dulal,S.
A1 - Ale,J.B.
A1 - Sapkota,Y.D.
Y1 - 2012/01//
N1 - Dulal, S. Ale, J B. Sapkota, Y D
Nepalese journal of ophthalmology : a biannual peer-reviewed academic journal of the Nepal Ophthalmic Society : NEPJOPH
101505288
IM
Comparative Study. Journal Article
English
KW - MEDLINE
KW - Adolescent
KW - Age Distribution
KW - Child
KW - Preschool
KW - Eye Injuries/ep [Epidemiology]
KW - Female
KW - Follow-Up Studies
KW - Hospitalization/sn [Statistics & Numerical Data]
KW - Humans
KW - Incidence
KW - Male
KW - Nepal/ep [Epidemiology]
KW - Retrospective Studies
KW - Risk Factors
KW - Sex Distribution
KW - Time Factors
RP - NOT IN FILE
SP - 134
EP - 137
JF - Nepalese Journal of Ophthalmology : A Biannual Peer-reviewed Academic Journal of the Nepal Ophthalmic Society : NEPJOPH
JA - Nepal J Ophthalmol
VL - 4
IS - 1
CY - Nepal
N2 - INTRODUCTION: Majority of blinding ocular injuries can be prevented. OBJECTIVE: To describe the epidemiology of ocular trauma in children. MATERIAL AND METHODS: A retrospective review of medical records of the patients aged below 16 years who attended the Himalaya Eye Hospital, Pokhara with history of ocular injury. RESULTS: Of 6,829 pediatric patients, 554 (8.1 %) had ocular trauma. The ocular trauma was more prevalent (38.1 %) in the age group of 5 - 10 years followed by 10-15 years (16.6 %). The boys (62 % vs 38 %) were more prone to ocular trauma than girls (RR = 1.7 and 95 % CI = 1.41 - 2.02). Of 554 ocular injuries, 32 (5.8 %) were open globe injuries. Sub-conjunctival hemorrhage was the commonest presenting finding in 96 (17.3 %) subjects. Forty-seven (8.5 %) of them attended the hospital after 15 days of injury. Home was the most common place for trauma (n =204, 36.8%), followed by playground (n = 140, 25.3 %). CONCLUSION: Children between the ages of 5-10 years are most vulnerable to ocular trauma.Home is the commonest place for ocular injury followed by playground. By adopting some common safety factors or by reducing the ocular injury risks factor, ocular trauma can be greatly reduced. NEPjOPH
SN - 2072-6805
AD - Nepal Netra Jyoti Sangh Central Office, Kathmandu, Nepal. sunudulal@yahoo.com
UR - 22344011
ER -

TY - JOUR
ID - 480

T1 - The economics of inpatient on-demand treatment for haemophilia with high-responding inhibitors: a US retrospective data analysis
A1 - Pokras,S.M.
A1 - Petrilla,A.A.
A1 - Weatherall,J.
A1 - Lee,W.C.
Y1 - 2012/03//
N1 - Pokras, S M. Petrilla, A A. Weatherall, J. Lee, W C
Haemophilia : the official journal of the World Federation of Hemophilia
c8f, 9442916
IM
Journal Article. Research Support, Non-U.S. Gov't
English
KW - MEDLINE
KW - Adolescent
KW - Adult
KW - Aged
KW - Analgesics
KW - Opioid/tu [Therapeutic Use]
KW - Blood Coagulation Factor Inhibitors/bl [Blood]
KW - Child
KW - Preschool
KW - Factor VIII/ad [Administration & Dosage]
KW - Factor VIII/ec [Economics]
KW - Factor VIII/im [Immunology]
KW - Factor VIIa/ad [Administration & Dosage]
KW - Factor VIIa/ec [Economics]
KW - Health Care Costs
KW - Hemophilia A/dt [Drug Therapy]
KW - Hemophilia A/ec [Economics]
KW - Hemorrhage/dt [Drug Therapy]
KW - Hemorrhage/ec [Economics]
KW - Humans
KW - Length of Stay
KW - Male
KW - Middle Aged
KW - Multivariate Analysis
KW - Recombinant Proteins/ad [Administration & Dosage]
KW - Recombinant Proteins/ec [Economics]
KW - Retrospective Studies
KW - United States
KW - Young Adult
RP - NOT IN FILE
SP - 284
EP - 290
JF - Haemophilia
VL - 18
IS - 2
CY - England
N2 - Inpatient costs comprise >50% of annual healthcare costs for haemophilia patients with inhibitors but no reports exist on inpatient resource use and costs at a US national level. To quantify inpatient resource use and costs for on-demand treatment of bleeds of US haemophilia patients with inhibitors and compare costs and treatment duration between Factor VIII bypassing agents (BAs). Stays with haemophilia A from 2003-2008 were identified from inpatient billing records. Presence of inhibitors was inferred through use of BA; recombinant activated Factor VII and plasma-derived activated prothrombin complex concentrate. Duration and number of infusions of BA, length of stay, use of opioid-containing analgesics and costs were assessed and compared. Among 1322 stays mean BA treatment duration was 4.6 days with 4.9 infusions, 6.1 nights spent in

hospital, and 58% administered opioid-containing analgesics. In unadjusted analyses there were significant differences in the above mentioned outcomes by BA use, reflecting underlying differences between the two patient populations. Average inpatient costs were $82,911. In adjusted analyses, African-American race, greater disease severity, hospital region outside the southern US and older age (cost model only) were significant predictors of longer BA treatment duration and higher costs. The economic burden of inpatient on-demand treatment of haemophilia with inhibitors is substantial and is associated with lengthy stays, high costs and inadequate pain relief. Availability of more effective BAs could reduce the need for re-treatment, reducing treatment costs and other medical costs, while improving health related quality of life. 2011 Blackwell Publishing Ltd
SN - 1365-2516
AD - Health Economics & Outcomes Research, IMS Consulting Group, Alexandria, VA 22314, USA
UR - 21812862
ER -

TY - JOUR
ID - 481
T1 - Making the connection: randomized controlled trial of social skills at school for children with autism spectrum disorders
A1 - Kasari,C.
A1 - Rotheram-Fuller,E.
A1 - Locke,J.
A1 - Gulsrud,A.
Y1 - 2012/04//
N1 - Kasari, Connie. Rotheram-Fuller, Erin. Locke, Jill. Gulsrud, Amanda
Journal of child psychology and psychiatry, and allied disciplines
hp3, 0375361
IM
Journal Article. Randomized Controlled Trial. Research Support, N.I.H., Extramural
English
KW - MEDLINE
KW - Analysis of Variance
KW - Behavior Therapy/mt [Methods]
KW - Child
KW - Child Behavior/px [Psychology]
KW - Child Development Disorders
KW - Pervasive/px [Psychology]
KW - Female
KW - Follow-Up Studies
KW - Friends/px [Psychology]
KW - Humans
KW - Interpersonal Relations
KW - Male
KW - Peer Group
KW - Play and Playthings/px [Psychology]
KW - Program Evaluation/mt [Methods]
KW - Schools
KW - Social Behavior
KW - Socialization
KW - Students/px [Psychology]
KW - Treatment Outcome
RP - NOT IN FILE
SP - 431
EP - 439
JF - Journal of Child Psychology & Psychiatry & Allied Disciplines
JA - J Child Psychol Psychiatry
VL - 53
IS - 4

CY - England
N2 - BACKGROUND: This study compared two interventions for improving the social skills of high functioning children with autism spectrum disorders in general education classrooms. One intervention involved a peer-mediated approach (PEER) and the other involved a child-assisted approach (CHILD). METHOD: The two interventions were crossed in a 2 x 2 factorial design yielding control, PEER, CHILD, and both PEER and CHILD conditions. Sixty children participated from 56 classrooms in 30 schools. Interventions involved 12 sessions over 6 weeks, with a 3-month follow-up. Outcome measures included self, peer and teacher reports of social skills and independent weekly observations of children on their school playground over the course of the intervention. RESULTS: Significant improvements were found in social network salience, number of friendship nominations, teacher report of social skills in the classroom, and decreased isolation on the playground for children who received PEER interventions. Changes obtained at the end of the treatment persisted to the 3-month follow-up. CONCLUSIONS: These data suggest that significant improvements can be made in peer social connections for children with autism spectrum disorders in general education classrooms with a brief intervention, and that these gains persist over time. 2011 The Authors. Journal of Child Psychology and Psychiatry 2011 Association for Child and Adolescent Mental Health
SN - 1469-7610
AD - Center for Autism Research and Treatment, Department of Psychiatry and Biobehavioral Sciences, University of California, 760 Westwood Plaza, Los Angeles, CA 90024, USA. kasari@gseis.ucla.edu
UR - 22118062
ER -

TY - JOUR
ID - 482
T1 - To TST or not to TST: is tuberculin skin testing necessary before BCG immunisation in children?. [Review]
A1 - Ritz,N.
A1 - Tebruegge,M.
A1 - Camacho-Badilla,K.
A1 - Haeusler,G.M.
A1 - Connell,T.G.
A1 - Curtis,N.
Y1 - 2012/02/14/
N1 - Ritz, Nicole. Tebruegge, Marc. Camacho-Badilla, Kattia. Haeusler, Gabrielle M. Connell, Tom G. Curtis, Nigel
Vaccine
x6o, 8406899
IM
Journal Article. Research Support, Non-U.S. Gov't. Review
English
KW - MEDLINE
KW - BCG Vaccine/ad [Administration & Dosage]
KW - Child
KW - Preschool
KW - Guidelines as Topic
KW - Humans
KW - Infant
KW - Latent Tuberculosis/di [Diagnosis]
KW - Latent Tuberculosis/pc [Prevention & Control]
KW - Tuberculin Test/ut [Utilization]
RP - NOT IN FILE
SP - 1434
EP - 1436
JF - Vaccine
VL - 30
IS - 8
CY - Netherlands

N2 - Bacille Calmette-Guerin (BCG) vaccine is one of the most commonly administered vaccines worldwide. In countries with high tuberculosis (TB) prevalence, it is generally given shortly after birth. In a number of low TB prevalence countries, BCG is used as a travel vaccine, typically given to children outside the neonatal period prior to visiting regions where TB is common. In this setting, it is recommended that latent TB infection (LTBI) resulting from prior exposure to Mycobacterium tuberculosis is excluded by a tuberculin skin test (TST) before BCG immunisation. This is to avoid the risk of an accelerated local reaction that is more common in individuals who have LTBI. In addition, BCG immunisation in individuals with LTBI is unnecessary, as it does not provide protection against progression to active TB disease. We review and discuss current international guidelines and recommendations on the need to screen children for LTBI prior to BCG immunisation. Guidelines vary significantly regarding age-related cut-offs and additional selection criteria. This variation primarily reflects the lack of evidence on which to base recommendations. We suggest an alternative strategy using a risk assessment questionnaire to identify children who should have a TST before BCG immunisation. This targeted approach will reduce the number of children unnecessarily screened, whilst allowing the identification of those with LTBI, who need further evaluation and treatment. Copyright 2011 Elsevier Ltd. All rights reserved
SN - 1873-2518
AD - Department of Paediatrics, The University of Melbourne, Royal Children's Hospital Melbourne, Parkville, Australia. nicole.ritz@ukbb.ch
UR - 22155145
ER -

TY - JOUR
ID - 483
T1 - Critical narrative review to identify educational strategies promoting physical activity in preschool. [Review]
A1 - Kreichauf,S.
A1 - Wildgruber,A.
A1 - Krombholz,H.
A1 - Gibson,E.L.
A1 - Vogele,C.
A1 - Nixon,C.A.
A1 - Douthwaite,W.
A1 - Moore,H.J.
A1 - Manios,Y.
A1 - Summerbell,C.D.
A1 - ToyBox-study group.
Y1 - 2012/03//
N1 - Kreichauf, S. Wildgruber, A. Krombholz, H. Gibson, E L. Vogele, C. Nixon, C A. Douthwaite, W. Moore, H J. Manios, Y. Summerbell, C D. ToyBox-study group
Obesity reviews : an official journal of the International Association for the Study of Obesity
100897395
IM
Journal Article. Research Support, Non-U.S. Gov't. Review
English
KW - MEDLINE
KW - Child
KW - Child Nutrition Sciences/ed [Education]
KW - Child Nutritional Physiological Phenomena/ph [Physiology]
KW - Preschool
KW - Exercise/ph [Physiology]
KW - Female
KW - Health Education/mt [Methods]
KW - Health Education/st [Standards]
KW - Health Promotion/mt [Methods]
KW - Health Promotion/st [Standards]
KW - Humans
KW - Male
KW - Obesity/pc [Prevention & Control]

KW - Play and Playthings
RP - NOT IN FILE
SP - 96
EP - 105
JF - Obesity Reviews
JA - Obes Rev
VL - 13 Suppl 1
CY - England
N2 - The aim of this narrative review is critically to evaluate educational strategies promoting physical activity that are used in the preschool setting in the context of obesity prevention programmes. Literature search was conducted between April and August 2010 in English and German databases (PubMED, PsychINFO, PSYNDEX, ERIC, FIS Bildung). Outcomes considered were time and intensity of physical activity, motor skills or measures of body composition. A total of 19 studies were included. Ten studies added physical activity lessons into their curriculum, one study provided more time for free play, eight studies focused on the social and play environment. Studies reporting positive outcomes implemented physical activity sessions that lasted at least 30 min d(-1). Several studies showed that children are most active in the first 10-15 min. The existence or installation of playground markings or fixed play equipment had no effect, whereas the presence or addition of portable play equipment was positively correlated with moderate-to-vigorous physical activity. Teacher training may be a key element for successful interventions. To overcome time constraints, a suggested solution is to integrate physical activity into daily routines and other areas of the preschool curriculum. 2012 The Authors. obesity reviews 2012 International Association for the Study of Obesity
SN - 1467-789X
AD - State Institute of Early Childhood Research, Munich, Germany. susanne.kreichauf@ifp.bayern.de
UR - 22309068
ER -

TY - JOUR
ID - 484
T1 - Factors associated with physical activity in children attending family child care homes
A1 - Gunter,K.B.
A1 - Rice,K.R.
A1 - Ward,D.S.
A1 - Trost,S.G.
Y1 - 2012/02//
N1 - Gunter, Katherine B. Rice, Kelly R. Ward, Dianne S. Trost, Stewart G
Preventive medicine
pm4, 0322116
IM
Journal Article
English
KW - MEDLINE
KW - Acceleration
KW - Adult
KW - Analysis of Variance
KW - Caregivers/px [Psychology]
KW - Child Care
KW - Child Welfare/px [Psychology]
KW - Child Welfare/sn [Statistics & Numerical Data]
KW - Child
KW - Preschool
KW - Cross-Sectional Studies
KW - Female
KW - Health Behavior
KW - Health Promotion/mt [Methods]
KW - Health Status Indicators
KW - Humans
KW - Male

KW - Motor Activity/ph [Physiology]
KW - Play and Playthings/px [Psychology]
KW - Psychometrics
KW - Self Report
KW - Time Factors
RP - NOT IN FILE
SP - 131
EP - 133
JF - Preventive Medicine
JA - Prev Med
VL - 54
IS - 2
CY - United States
N2 - OBJECTIVE: To determine the relationship between family child care home (FCCH) practices and characteristics, and objectively measured physical activity (PA) among children attending FCCHs. METHODS: FCCH practices and characteristics were assessed in 45 FCCHs in Oregon (USA) in 2010-2011 using the Nutrition and Physical Activity Self-Assessment for Child Care Instrument. Within the 45 FCCHs, 136 children between ages 2 and 5 years wore an accelerometer during child care attendance over a one-week period. Time spent in light, moderate, and vigorous PA per hour was calculated using intensity-related cut-points (Pate et al., 2006). RESULTS: FCCH characteristics and practices associated with higher levels of PA (min/h; p<0.05) included provision of sufficient outdoor active play [32.2 (1.0) vs. 28.6 (1.3)], active play using portable play equipment [31.7 (1.0) vs. 29.3 (1.4)], the presence of a variety of fixed play equipment [32.2 (1.0) vs. 28.9 (1.3)], and suitable indoor play space [32.2 (1.0) vs. 28.6 (1.3)], engaging in active play with children [32.1 (1.1) vs. 29.6 (1.2)], and receiving activity-related training [33.1 (1.2) vs. 30.3 (1.1)]. CONCLUSIONS: This is the first study to identify practices and characteristics of FCCHs that influence children's PA. These data should be considered when developing programs and policies to promote PA in FCCHs. Copyright 2011 Elsevier Inc. All rights reserved
SN - 1096-0260
AD - College of Public Health and Human Sciences, Oregon State University, Corvallis, OR 97331, USA. Kathy.gunter@oregonstate.edu
UR - 22178820
ER -

TY - JOUR
ID - 485
T1 - Evaluation of infection control advice for patients at risk of chemotherapy-induced neutropenia in 2 pediatric oncology centers: Cape Town, South Africa, and Newcastle-Upon-Tyne, UK
A1 - Woolner,A.F.
A1 - Davidson,A.
A1 - Skinner,R.
A1 - King,D.
Y1 - 2012/02//
N1 - Woolner, Andrea Forman. Davidson, Alan. Skinner, Rod. King, Derek
Pediatric hematology and oncology
avq, 8700164
IM
Clinical Trial. Comparative Study. Journal Article. Multicenter Study
English
KW - MEDLINE
KW - Adolescent
KW - Antineoplastic Agents/ad [Administration & Dosage]
KW - Antineoplastic Agents/ae [Adverse Effects]
KW - Cancer Care Facilities
KW - Child
KW - Preschool
KW - Cross-Sectional Studies
KW - Female

KW - Great Britain
KW - Hospitals
KW - Pediatric
KW - Humans
KW - Infection/ci [Chemically Induced]
KW - Infection/ep [Epidemiology]
KW - Infection Control
KW - Male
KW - Neoplasms/dt [Drug Therapy]
KW - Neoplasms/ep [Epidemiology]
KW - Neutropenia/ci [Chemically Induced]
KW - Neutropenia/ep [Epidemiology]
KW - Questionnaires
KW - Risk Factors
KW - South Africa
RP - NOT IN FILE
SP - 73
EP - 84
JF - Pediatric Hematology & Oncology
JA - Pediatr Hematol Oncol
VL - 29
IS - 1
CY - England
N2 - Childhood cancers are treated with myelotoxic chemotherapy. Resultant neutropenia can lead to life-threatening infections. There is no consistent guidance on infection control precautions for neutropenic patients who are not yet febrile or infected. Although it is not possible to eradicate infection risk, it is conceivable that the risk could be reduced by effective infection prevention. This study compared infection control measures advised to pediatric and adolescent oncology patients receiving chemotherapy in 2 centers (Cape Town, South Africa, and Newcastle, UK). Prospective, observational, cross-sectional surveys of staff and patients/parents were undertaken using standardized, study-specific questionnaires. Seventy-eight staff and 56 patients/parents participated. Precautions advised in Newcastle were significantly different to Cape Town (all P < .05), except both agreed inpatient isolation was unnecessary. Over 40% of patients/parents felt isolation was important (P < .01). In Cape Town, staff and patients had similar views. In Newcastle, patients/parents had stricter opinions on particular precautions than staff, for example, attending school, playing outside and avoiding busy places (P < .01). Patient/parent responses were similar between centers. Over 90% of staff felt advising patients/parents about hand washing was important. Currently infection prevention advice is inconsistent. Further research is needed to elucidate effective guidance for infection prevention in pediatric neutropenic patients
SN - 1521-0669
AD - School of Medicine and Dentistry, Division of Applied Medicine, University of Aberdeen, Foresterhill, Aberdeen, UK. andrea.woolner.10@aberdeen.ac.uk
UR - 22304013
ER -

TY - JOUR
ID - 486
T1 - Global malaria mortality between 1980 and 2010: a systematic analysis
A1 - Murray,C.J.
A1 - Rosenfeld,L.C.
A1 - Lim,S.S.
A1 - Andrews,K.G.
A1 - Foreman,K.J.
A1 - Haring,D.
A1 - Fullman,N.
A1 - Naghavi,M.
A1 - Lozano,R.
A1 - Lopez,A.D.
Y1 - 2012/02/04/

N1 - Murray, Christopher J L. Rosenfeld, Lisa C. Lim, Stephen S. Andrews, Kathryn G. Foreman, Kyle J. Haring, Diana. Fullman, Nancy. Naghavi, Mohsen. Lozano, Rafael. Lopez, Alan D
Lancet
2985213r, l0s, 0053266
AIM, IM
Journal Article. Research Support, Non-U.S. Gov't
English
KW - MEDLINE
KW - Adolescent
KW - Adult
KW - Aged
KW - Child
KW - Preschool
KW - Female
KW - Humans
KW - Malaria/mo [Mortality]
KW - Malaria/tm [Transmission]
KW - Male
KW - Middle Aged
KW - World Health
KW - Young Adult
RP - NOT IN FILE
SP - 413
EP - 431
JF - Lancet
VL - 379
IS - 9814
CY - England
N2 - BACKGROUND: During the past decade, renewed global and national efforts to combat malaria have led to ambitious goals. We aimed to provide an accurate assessment of the levels and time trends in malaria mortality to aid assessment of progress towards these goals and the focusing of future efforts. METHODS: We systematically collected all available data for malaria mortality for the period 1980-2010, correcting for misclassification bias. We developed a range of predictive models, including ensemble models, to estimate malaria mortality with uncertainty by age, sex, country, and year. We used key predictors of malaria mortality such as Plasmodium falciparum parasite prevalence, first-line antimalarial drug resistance, and vector control. We used out-of-sample predictive validity to select the final model. FINDINGS: Global malaria deaths increased from 995,000 (95% uncertainty interval 711,000-1,412,000) in 1980 to a peak of 1,817,000 (1,430,000-2,366,000) in 2004, decreasing to 1,238,000 (929,000-1,685,000) in 2010. In Africa, malaria deaths increased from 493,000 (290,000-747,000) in 1980 to 1,613,000 (1,243,000-2,145,000) in 2004, decreasing by about 30% to 1,133,000 (848,000-1,591,000) in 2010. Outside of Africa, malaria deaths have steadily decreased from 502,000 (322,000-833,000) in 1980 to 104,000 (45,000-191,000) in 2010. We estimated more deaths in individuals aged 5 years or older than has been estimated in previous studies: 435,000 (307,000-658,000) deaths in Africa and 89,000 (33,000-177,000) deaths outside of Africa in 2010. INTERPRETATION: Our findings show that the malaria mortality burden is larger than previously estimated, especially in adults. There has been a rapid decrease in malaria mortality in Africa because of the scaling up of control activities supported by international donors. Donor support, however, needs to be increased if malaria elimination and eradication and broader health and development goals are to be met. FUNDING: The Bill & Melinda Gates Foundation. Copyright 2012 Elsevier Ltd. All rights reserved
SN - 1474-547X
AD - Institute for Health Metrics and Evaluation, University of Washington, Seattle, WA 98121, USA
UR - 22305225
ER -

TY - JOUR
ID - 487
T1 - Societal values and policies may curtail preschool children's physical activity in child care centers
A1 - Copeland,K.A.

A1 - Sherman,S.N.
A1 - Kendeigh,C.A.
A1 - Kalkwarf,H.J.
A1 - Saelens,B.E.
Y1 - 2012/02//
N1 - Copeland, Kristen A. Sherman, Susan N. Kendeigh, Cassandra A. Kalkwarf, Heidi J. Saelens, Brian E
Pediatrics
oxv, 0376422
AIM, IM
Journal Article. Research Support, N.I.H., Extramural. Research Support, Non-U.S. Gov't
English
KW - MEDLINE
KW - Child Day Care Centers
KW - Child
KW - Preschool
KW - Female
KW - Focus Groups
KW - Health Surveys
KW - Humans
KW - Male
KW - Motor Activity
KW - Ohio
KW - Play and Playthings
KW - Public Policy
KW - Safety Management
KW - Sedentary Lifestyle
KW - Social Values
KW - Wounds and Injuries/et [Etiology]
KW - Wounds and Injuries/pc [Prevention & Control]
RP - NOT IN FILE
SP - 265
EP - 274
JF - Pediatrics
VL - 129
IS - 2
CY - United States
N2 - BACKGROUND AND OBJECTIVES: Three-fourths of US preschool-age children are in child care centers. Children are primarily sedentary in these settings, and are not meeting recommended levels of physical activity. Our objective was to identify potential barriers to children's physical activity in child care centers. METHODS: Nine focus groups with 49 child care providers (55% African American) were assembled from 34 centers (inner-city, suburban, Head Start, and Montessori) in Cincinnati, Ohio. Three coders independently analyzed verbatim transcripts for themes. Data analysis and interpretation of findings were verified through triangulation of methods. RESULTS: We identified 3 main barriers to children's physical activity in child care: (1) injury concerns, (2) financial, and (3) a focus on "academics." Stricter licensing codes intended to reduce children's injuries on playgrounds rendered playgrounds less physically challenging and interesting. In addition, some parents concerned about potential injury, requested staff to restrict playground participation for their children. Small operating margins of most child care centers limited their ability to install abundant playground equipment. Child care providers felt pressure from state mandates and parents to focus on academics at the expense of gross motor play. Because children spend long hours in care and many lack a safe place to play near their home, these barriers may limit children's only opportunity to engage in physical activity. CONCLUSIONS: Societal priorities for young children--safety and school readiness--may be hindering children's physical development. In designing environments that optimally promote children's health and development, child advocates should think holistically about potential unintended consequences of policies
SN - 1098-4275
AD - Division of General and Community Pediatrics, Cincinnati Children's Hospital Medical Center, Cincinnati, OH 45229, USA. kristen.copeland@cchmc.org
UR - 22218842

ER -

TY - JOUR
ID - 488
T1 - The reason for recess. Children who are more physically active may do better in school
A1 - Park,A.
Y1 - 2012/01/16/
N1 - Park, Alice
Time
9877130, bqn
K
News
English
KW - MEDLINE
KW - Child
KW - Educational Measurement
KW - Exercise
KW - Humans
KW - Physical Education and Training
KW - United States
RP - NOT IN FILE
SP - 19
JF - Time
VL - 179
IS - 2
CY - United States
SN - 0040-781X
UR - 22299334
ER -

TY - JOUR
ID - 489
T1 - Quantification and localization of EEG interictal spike activity in patients with surgically removed epileptogenic foci
A1 - Coutin-Churchman,P.E.
A1 - Wu,J.Y.
A1 - Chen,L.L.
A1 - Shattuck,K.
A1 - Dewar,S.
A1 - Nuwer,M.R.
Y1 - 2012/03//
N1 - Coutin-Churchman, Pedro E. Wu, Joyce Y. Chen, Leo L K. Shattuck, Kirk. Dewar, Sandy. Nuwer, Marc R
Clinical neurophysiology : official journal of the International Federation of Clinical Neurophysiology
db4, 100883319
IM
Comparative Study. Evaluation Studies. Journal Article
English
KW - MEDLINE
KW - Action Potentials/ph [Physiology]
KW - Adolescent
KW - Adult
KW - Brain Mapping/mt [Methods]
KW - Child
KW - Preschool
KW - Electroencephalography/mt [Methods]
KW - Epilepsy/pa [Pathology]

KW - Epilepsy/pp [Physiopathology]
KW - Epilepsy/su [Surgery]
KW - Female
KW - Follow-Up Studies
KW - Frontal Lobe/pa [Pathology]
KW - Frontal Lobe/pp [Physiopathology]
KW - Frontal Lobe/su [Surgery]
KW - Humans
KW - Image Processing
KW - Computer-Assisted/mt [Methods]
KW - Infant
KW - Magnetic Resonance Imaging
KW - Male
KW - Neuroimaging/mt [Methods]
KW - Neurosurgical Procedures
KW - Retrospective Studies
KW - Sensitivity and Specificity
KW - Temporal Lobe/pa [Pathology]
KW - Temporal Lobe/pp [Physiopathology]
KW - Temporal Lobe/su [Surgery]
KW - Time Factors
KW - Treatment Outcome
KW - Young Adult
RP - NOT IN FILE
SP - 471
EP - 485
JF - Clinical Neurophysiology
JA - Clin Neurophysiol
VL - 123
IS - 3
CY - Netherlands
N2 - OBJECTIVE: To demonstrate if interictal spike activity was localized within the resected area in surgically treated epilepsy patients; and if there is correspondence between the degree of localization and improvement after surgery. METHODS: We analyzed long-term EEGs from 34 patients. Interictal spikes were grouped in clusters and averaged according to morphology and topography. The relative contribution of each cluster to the total number of spikes in each patient was estimated. Dipole and distributed EEG source localization of each cluster was made. The percentage of spike activity localized within the site of resection (SR) during the onset was estimated. The relationship between the percentage of activity within SR and the surgery outcome was assessed. RESULTS: Fourteen patients had 90-100% of spikes within SR, 9 had 50-89%. Most patients with more than 50% of activity localized within SR were seizure free, while the 5 patients who had all activity localized outside SR were not seizure free. CONCLUSIONS: Localization of clusters containing the largest quantity of interictal spikes during onset generally corresponded to the likely location of the epileptogenic cortex. SIGNIFICANCE: Computer-assisted EEG source localization with our methodology can be a useful adjunct for the evaluation of candidates for epilepsy surgery. Copyright 2011 International Federation of Clinical Neurophysiology. All rights reserved
SN - 1872-8952
AD - Department of Clinical Neurophysiology, Ronald Reagan UCLA Medical Center, Los Angeles, CA 90095, USA. pchurchman@mednet.ucla.edu
UR - 21903463
ER -

TY - JOUR
ID - 490
T1 - Assessment of DDT and DDE levels in soil, dust, and blood samples from Chihuahua, Mexico
A1 - Martinez,F.D.
A1 - Trejo-Acevedo,A.
A1 - Betanzos,A.F.

A1 - Espinosa-Reyes,G.
A1 - egria-Torres,J.A.
A1 - Maldonado,I.N.
Y1 - 2012/02//
N1 - Martinez, Fernando Diaz-Barriga. Trejo-Acevedo, Antonio. Betanzos, Angel F. Espinosa-Reyes, Guillermo. Alegria-Torres, Jorge Alejandro. Maldonado, Ivan Nelinho Perez
Archives of environmental contamination and toxicology
6yd, 0357245
IM
Journal Article. Research Support, Non-U.S. Gov't
English
KW - MEDLINE
KW - Child
KW - Preschool
KW - DDT/bl [Blood]
KW - Dichlorodiphenyl Dichloroethylene/bl [Blood]
KW - Dust/an [Analysis]
KW - Environmental Monitoring/mt [Methods]
KW - Humans
KW - Insecticides/bl [Blood]
KW - Malaria/pc [Prevention & Control]
KW - Mexico
KW - Mosquito Control/mt [Methods]
KW - Soil/an [Analysis]
KW - Soil/ch [Chemistry]
RP - NOT IN FILE
SP - 351
EP - 358
JF - Archives of Environmental Contamination & Toxicology
JA - Arch Environ Contam Toxicol
VL - 62
IS - 2
CY - United States
N2 - The aim of this study was to assess levels of DDT and DDE in two environmental matrices (soil and dust) and to investigate the blood levels of these insecticides in exposed children living in a north Mexican state (Chihuahua) where DDT was sprayed several years ago during (1) health campaigns for the control of malaria and (2) agricultural activities. DDT and DDE were analyzed by gas chromatography/mass spectrometry. In general, lower levels were found in household outdoor samples. The levels in outdoor samples ranged from 0.001 to 0.788 mg/kg for DDT and from 0.001 to 0.642 mg/kg for DDE. The levels in indoor samples ranged from 0.001 to 15.47 mg/kg for DDT and from 0.001 to 1.063 mg/kg for DDE. Similar results to those found in indoor soil were found in dust, in which the levels ranged from 0.001 to 95.87 mg/kg for DDT and from 0.001 to 0.797 mg/kg for DDE. Moreover, blood levels showed that all of the communities studied had been exposed to DDT and/or DDE, indicating a general past or present exposure to DDT. It is important to note that the quotient DDT/DDE in all matrices was always >1. Whether the people living in our study area are at risk is an issue that deserves further analysis. However, applying precautionary principles, it is important to initiate a risk-reduction program to decrease exposure to DDT and its metabolites in people living in this area
SN - 1432-0703
AD - Departamento Toxicologia Ambiental, Facultad de Medicina, Universidad Autonoma de San Luis Potosi, Avenida Venustiano Carranza No. 2405, Col Lomas los Filtros, San Luis Potosi 78210, SLP, Mexico
UR - 21822982
ER -

TY - JOUR
ID - 491
T1 - Acute effects of walking environment and GSTM1 variants in children with asthma
A1 - Newcomb,P.
A1 - Hunt,A.

A1 - Rast,P.
A1 - Cauble,D.
A1 - Rowe,N.
A1 - Li,J.
Y1 - 2012/01//
N1 - Newcomb, Patricia. Hunt, Andrew. Rast, Pamela. Cauble, Denise. Rowe, Nancy. Li, Jianling
Biological research for nursing
9815758, dw1
IM, N
Journal Article. Research Support, Non-U.S. Gov't
English
KW - MEDLINE
KW - Air Pollutants/to [Toxicity]
KW - Asthma/en [Enzymology]
KW - Asthma/ge [Genetics]
KW - Asthma/pp [Physiopathology]
KW - Child
KW - Preschool
KW - Cross-Over Studies
KW - Female
KW - Glutathione Transferase/ge [Genetics]
KW - Humans
KW - Male
KW - Pilot Projects
KW - Respiratory Function Tests
KW - Vehicle Emissions
KW - Walking
RP - NOT IN FILE
SP - 55
EP - 64
JF - Biological Research for Nursing
JA - Biol Res Nurs
VL - 14
IS - 1
CY - United States
N2 - BACKGROUND: Exercise in air polluted by traffic emissions may aggravate airway inflammation in children with asthma, particularly those who produce decreased glutathione-S transferase (GST) as a result of GSTM1 gene deletion. OBJECTIVES: This pilot crossover study investigated whether children with asthma experience more airway changes when exercising outdoors near roadways than when exercising indoors. It also examined differences in risk between children with and without GSTM1 deletion. METHOD: Children between the ages of 5 and 12 years were assigned to groups and walked daily for 1 week in each exercise condition. Airway inflammation indicated by exhaled nitric oxide (eNO) and pulmonary functions measured as forced expiratory volume in 1 s (FEV1) and mid-expiratory flow rate were measured at baseline and at three intervals during the walking program. Independent variables of interest included walking condition (place), time of walking, genotype, and particulate matter (PM) exposure. RESULTS: A linear mixed models approach was used to investigate the contributions of targeted variables to respiratory outcomes. Results indicated that walking location and ambient level of ultrafine particulates during walking influenced function of small airways. Absence of one or both alleles for the GSTM1 gene did not influence airway function acutely. DISCUSSION: Mid-expiratory flow (FEF(25-75)) may be more informative than FEV1 in studies of acute lung function changes in children with asthma. Further study of the effects of varied environmental conditions on lower airway function of children is needed to optimize exercise experiences for urban children with asthma
SN - 1552-4175
AD - College of Nursing, University of Texas at Arlington, Arlington, TX 76019, USA. pnewcomb@uta.edu
UR - 21196426
ER -

TY - JOUR

ID - 492
T1 - Associations of birth weight and physical activity with sex steroids in preschool Japanese children
A1 - Wada,K.
A1 - Nakamura,K.
A1 - Tamai,Y.
A1 - Tsuji,M.
A1 - Masue,T.
A1 - Ando,K.
A1 - Nagata,C.
Y1 - 2012/02//
N1 - Wada, Keiko. Nakamura, Kozue. Tamai, Yuya. Tsuji, Michiko. Masue, Takako. Ando, Kyoko. Nagata, Chisato
Cancer causes & control : CCC
a5r, 9100846
IM
Journal Article. Research Support, Non-U.S. Gov't
English
KW - MEDLINE
KW - Birth Weight
KW - Body Mass Index
KW - Child
KW - Preschool
KW - Cross-Sectional Studies
KW - Female
KW - Gonadal Steroid Hormones/ur [Urine]
KW - Humans
KW - Japan
KW - Male
KW - Motor Activity
RP - NOT IN FILE
SP - 231
EP - 238
JF - Cancer Causes & Control
JA - Cancer Causes Control
VL - 23
IS - 2
CY - Netherlands
N2 - PURPOSE: We aimed to assess the association of body size at birth or physical activity with sex steroid levels, independent of body mass among young children. METHODS: The cross-sectional study was conducted in 2006. Subjects were 230 boys and 198 girls, aged 3-6 years. Birth weight was based on parents' reports. Questions about physical activity focused on outdoor playtime. Urinary estrone, estradiol, testosterone, and 5-androstene-3beta,17alpha diol (3beta,17alpha-AED) levels were measured by liquid chromatography-electrospray ionization tandem mass spectrometry. Urinary dehydroepiandrosterone level was measured with a radioimmunoassay. RESULTS: After adjustments for age and BMI, girls with lower birth weight had higher testosterone (trend p = 0.038) and 3beta,17alpha-AED (trend p = 0.028). Girls with low birth weight and high birth weight had higher estrone (p = 0.014) and estradiol (p = 0.074) than those who had middle birth weight. Boys who were physically active had lower testosterone (p = 0.028) and 3beta,17alpha-AED (p = 0.003) than those who were not active. Girls who were physically active had lower estrone (p = 0.015). CONCLUSIONS: Sex steroid levels in childhood might be affected by body size at birth or by physical activity during childhood. These effects might differ by sex
SN - 1573-7225
AD - Department of Epidemiology and Preventive Medicine, Gifu University Graduate School of Medicine, Gifu 501-1194, Japan. dr_keiko@gifu-u.ac.jp
UR - 22080278
ER -

TY - JOUR

ID - 493
T1 - Influence of parental history of hypertension on screen time and physical activity in young offspring
A1 - Gopinath,B.
A1 - Hardy,L.L.
A1 - Baur,L.A.
A1 - Teber,E.
A1 - Mitchell,P.
Y1 - 2012/02//
N1 - Gopinath, Bamini. Hardy, Louise L. Baur, Louise A. Teber, Erdahl. Mitchell, Paul
Journal of hypertension
iew, 8306882
IM
Journal Article. Research Support, Non-U.S. Gov't
English
KW - MEDLINE
KW - Child
KW - Female
KW - Humans
KW - Hypertension/pp [Physiopathology]
KW - Male
KW - Motor Activity
KW - New South Wales
KW - Parents
KW - Sedentary Lifestyle
RP - NOT IN FILE
SP - 336
EP - 341
JF - Journal of Hypertension
JA - J Hypertens
VL - 30
IS - 2
CY - England
N2 - OBJECTIVE: Epidemiological data on the relationship between parental hypertension and lifestyle factors among children are limited. We assessed the associations between positive parental history of hypertension with a range of indicators of sedentariness (television viewing, computer and videogame usage) and physical activity (outdoor and indoor activity) among prepubertal children. METHODS: Six-year-old (1765 of 2238 eligible) students from a random cluster sample of 34 Sydney schools were examined. Parents completed questionnaires about their medical conditions and the child's activities. If the biological mother and/or father had hypertension, then this was classified as positive parental history of hypertension. RESULTS: Parents of 160 (9.2%) children gave a positive family history of hypertension. After adjusting for age, sex, ethnicity, parental education and employment and exposure to passive smoking, children with, compared with those without, a family history of hypertension were more likely to spend above the median time (hours/day) watching television and playing videogames [odds ratio (OR) 1.41, 95% confidence interval (CI) 1.02-1.96; OR 1.52, CI 1.12-2.06, respectively]. The offspring of hypertensive parents were more likely to spend above the median time in total physical activity (OR 1.67, 95% CI 1.23-2.27). Maternal (but not paternal) hypertension was associated with increased time spent watching television (P = 0.03) and in outdoor activity (P = 0.01). CONCLUSION: Parental hypertension influences the time that prepubertal offspring spend in both active and sedentary pursuits. These findings highlight potential factors that could be addressed in the development of cardiovascular disease-preventive measures starting early in life among the offspring of hypertensive parents
SN - 1473-5598
AD - Centre for Vision Research, Department of Ophthalmology and Westmead Millennium Institute, University of Sydney, Sydney, Australia
UR - 22179085
ER -

TY - JOUR
ID - 494

T1 - Impact and cost-effectiveness of family Fitness Zones: a natural experiment in urban public parks
A1 - Cohen,D.A.
A1 - Marsh,T.
A1 - Williamson,S.
A1 - Golinelli,D.
A1 - McKenzie,T.L.
Y1 - 2012/01//
N1 - Cohen, Deborah A. Marsh, Terry. Williamson, Stephanie. Golinelli, Daniela. McKenzie, Thomas L
Health & place
din, 9510067
T
Journal Article. Research Support, N.I.H., Extramural. Research Support, Non-U.S. Gov't
English
KW - MEDLINE
KW - Adolescent
KW - Adult
KW - Aged
KW - Child
KW - Cost-Benefit Analysis
KW - Environment Design/ec [Economics]
KW - Environment Design/sn [Statistics & Numerical Data]
KW - Family
KW - Female
KW - Humans
KW - Male
KW - Middle Aged
KW - Motor Activity
KW - Physical Fitness
KW - Population Density
KW - Propensity Score
KW - Recreation
KW - Residence Characteristics
KW - Urban Population
KW - Young Adult
RP - NOT IN FILE
SP - 39
EP - 45
JF - Health & Place
JA - Health Place
VL - 18
IS - 1
CY - England
N2 - We evaluated the impact of outdoor exercise equipment (FZ, Fitness Zones) in 12 parks serving diverse populations. We used the System for Observing Play and Recreation in Communities (SOPARC) to assess use and estimate energy expenditure prior to and twice after FZ installation. Park use increased more in FZ parks than in 10 control parks that did not get equipment, but the difference was not statistically significant. However, self-reports of being a new park user increased more in FZ parks, and estimated energy expenditure in FZ parks was higher at both follow-ups than at baseline. Installing Fitness Zones appears to be cost-effective (10.5 cents/MET increase) and most successful in parks in densely populated areas with limited facilities. Longer-term follow-up measures are needed to determine if the early increases in physical activity associated with the Fitness Zone installations are sustained. Copyright 2011 Elsevier Ltd. All rights reserved
SN - 1873-2054
AD - RAND Corporation, 1776 Main Street, Santa Monica, CA 90407, USA. dcohen@rand.org
UR - 22243905
ER -

TY - JOUR

ID - 495
T1 - Physical activity in child-care centers: do teachers hold the key to the playground?
A1 - Copeland,K.A.
A1 - Kendeigh,C.A.
A1 - Saelens,B.E.
A1 - Kalkwarf,H.J.
A1 - Sherman,S.N.
Y1 - 2012/02//
N1 - Copeland, Kristen A. Kendeigh, Cassandra A. Saelens, Brian E. Kalkwarf, Heidi J. Sherman, Susan N
Health education research
bqp, 8608459
T
Journal Article. Research Support, N.I.H., Extramural. Research Support, Non-U.S. Gov't
English
KW - MEDLINE
KW - Attitude
KW - Child Day Care Centers/og [Organization & Administration]
KW - Child Day Care Centers
KW - Child
KW - Preschool
KW - Faculty
KW - Focus Groups
KW - Humans
KW - Interviews as Topic
KW - Motor Activity
KW - Play and Playthings
RP - NOT IN FILE
SP - 81
EP - 100
JF - Health Education Research
JA - Health Educ Res
VL - 27
IS - 1
CY - England
N2 - Many (56%) US children aged 3-5 years are in center-based childcare and are not obtaining recommended levels of physical activity. In order to determine what child-care teachers/providers perceived as benefits and barriers to children's physical activity in child-care centers, we conducted nine focus groups and 13 one-on-one interviews with 49 child-care teachers/providers in Cincinnati, OH. Participants noted physical and socio-emotional benefits of physical activity particular to preschoolers (e.g. gross motor skill development, self-confidence after mastery of new skills and improved mood, attention and napping after exercise) but also noted several barriers including their own personal attitudes (e.g. low self-efficacy) and preferences to avoid the outdoors (e.g. don't like hot/cold weather, getting dirty, chaos of playground). Because individual teachers determine daily schedules and ultimately make the decision whether to take the children outdoors, they serve as gatekeepers to the playground. Participants discussed a spectrum of roles on the playground, from facilitator to chaperone to physical activity inhibitor. These findings suggest that children could have very different gross motor experiences even within the same facility (with presumably the same environment and policies), based on the beliefs, creativity and level of engagement of their teacher
SN - 1465-3648
AD - Department of Pediatrics, University of Cincinnati College of Medicine, Cincinnati, OH 45229, USA. kristen.copeland@cchmc.org
UR - 21804083
ER -

TY - JOUR
ID - 496
T1 - An observational study on socio-economic and ethnic differences in indicators of sedentary behavior and physical activity in preschool children

A1 - van,Rossem L.
A1 - Vogel,I.
A1 - Moll,H.A.
A1 - Jaddoe,V.W.
A1 - Hofman,A.
A1 - Mackenbach,J.P.
A1 - Raat,H.
Y1 - 2012/01//
N1 - van Rossem, Lenie. Vogel, Ineke. Moll, Henriette A. Jaddoe, Vincent W. Hofman, Albert. Mackenbach, Johan P. Raat, Hein
Preventive medicine
pm4, 0322116
IM
Journal Article. Research Support, Non-U.S. Gov't
English
KW - MEDLINE
KW - Child
KW - Preschool
KW - Exercise
KW - Female
KW - Humans
KW - Logistic Models
KW - Male
KW - Netherlands
KW - Observation
KW - Questionnaires
KW - Sedentary Lifestyle/eh [Ethnology]
KW - Social Class
RP - NOT IN FILE
SP - 55
EP - 60
JF - Preventive Medicine
JA - Prev Med
VL - 54
IS - 1
CY - United States
N2 - OBJECTIVE: We studied associations between social disadvantage and indicators of sedentary behavior and physical activity at preschool age. METHODS: Data from 4688 children enrolled in a birth cohort in Rotterdam, the Netherlands, between 2002 and 2006 were analyzed. Indicators of sedentary behavior (watching television > 2 h/day and sitting in a buggy > 0.5 h/day) and physical inactivity (playing outside < 3 h/day) were measured by a parent-reported questionnaire at age 3. Adjustments were made for social circumstances and indicators of health behaviors. Logistic regression was used to obtain odds ratios (OR) and 95% confidence intervals (CI). RESULTS: Children with low-educated mothers (OR: 3.27, 95% CI 2.12-5.05) and non-Dutch children (OR(nonWestern): 2.67, 95% CI 2.04-3.49, OR(Western): 2.09, 95% CI 1.42-3.0) were more likely to watch television for at least 2 h/day. Similar results were seen for sitting in a buggy for at least 0.5h/day. Non western children were more likely to play outside for less than 3 h/day (OR: 1.95, 95% CI: 1.39-2.73) than native Dutch children, while no differences were seen for other western children or children from mothers with a low educational level. CONCLUSIONS: Socio-economic status and ethnicity are already associated with indicators of sedentary lifestyles at preschool age. Copyright 2011. Published by Elsevier Inc
SN - 1096-0260
AD - The Generation R Study Group, Erasmus MC-University Medical Centre Rotterdam, PO Box 2040, 3000 CA Rotterdam, The Netherlands. l.vanrossem@umcutrecht.nl
UR - 22064316
ER -

TY - JOUR
ID - 497

T1 - Correlates of children's moderate and vigorous physical activity during weekdays and weekends
A1 - Fairclough,S.J.
A1 - Ridgers,N.D.
A1 - Welk,G.
Y1 - 2012/01//
N1 - Fairclough, Stuart J. Ridgers, Nicola D. Welk, Gregory
Journal of physical activity & health
101189457
IM
Comparative Study. Journal Article
English
KW - MEDLINE
KW - Acceleration
KW - Actigraphy/mt [Methods]
KW - Age Factors
KW - Child
KW - Child Welfare
KW - Cross-Sectional Studies
KW - England
KW - Epidemiologic Factors
KW - Female
KW - Health Status Indicators
KW - Humans
KW - Male
KW - Motor Activity/ph [Physiology]
KW - Multivariate Analysis
KW - Psychometrics
KW - Sedentary Lifestyle
KW - Sex Factors
KW - Statistics
KW - Nonparametric
KW - Time Factors
RP - NOT IN FILE
SP - 129
EP - 137
JF - Journal of Physical Activity & Health
JA - J Phys Act Health
VL - 9
IS - 1
CY - United States
N2 - BACKGROUND: Vigorous-intensity physical activity (VPA) may confer superior health benefits for children compared to moderate-intensity physical activity (MPA), but the correlates of MPA and VPA may differ. The study purpose was to investigate associations between selected enabling, predisposing, and demographic physical activity correlates, and MPA and VPA during weekdays and at weekends. METHODS: Data were gathered from 175 children (aged 10 to 11 years). MPA and VPA were assessed using accelerometers. Correlates were measured at child and school levels. Multilevel analyses identified correlates that significantly predicted MPA and VPA. RESULTS: Gender significantly predicted weekday MPA ($P < .001$), and weekend MPA ($P = .022$) and VPA ($P = .035$). Weekday VPA was predicted by gender ($P < .001$), indices of multiple deprivation score ($P < .003$), BMI ($P = .018$), and school playground area ($P = .046$). CONCLUSIONS: Gender was the most significant correlate of MPA and VPA. Children most likely to engage in weekday VPA were boys with lower deprivation scores and BMI values, with access to larger playground areas
SN - 1543-5474
AD - REACH Group and Faculty of Education, Community, and Leisure, Liverpool John Moores University, Liverpool, UK
UR - 22232499
ER -

TY - JOUR
ID - 498
T1 - School playground facilities as a determinant of children's daily activity: a cross-sectional study of Danish primary school children
A1 - Nielsen,G.
A1 - Bugge,A.
A1 - Hermansen,B.
A1 - Svensson,J.
A1 - Andersen,L.B.
Y1 - 2012/01//
N1 - Nielsen, Glen. Bugge, Anna. Hermansen, Bianca. Svensson, Jesper. Andersen, Lars Bo
Journal of physical activity & health
101189457
IM
Journal Article
English
KW - MEDLINE
KW - Acceleration
KW - Body Mass Index
KW - Child
KW - Child Welfare/px [Psychology]
KW - Child Welfare/sn [Statistics & Numerical Data]
KW - Confidence Intervals
KW - Cross-Sectional Studies
KW - Denmark
KW - Equipment Design
KW - Female
KW - Humans
KW - Linear Models
KW - Male
KW - Motor Activity/ph [Physiology]
KW - Odds Ratio
KW - Play and Playthings
KW - Schools/sn [Statistics & Numerical Data]
KW - Statistics as Topic
KW - Students/sn [Statistics & Numerical Data]
KW - Time Factors
RP - NOT IN FILE
SP - 104
EP - 114
JF - Journal of Physical Activity & Health
JA - J Phys Act Health
VL - 9
IS - 1
CY - United States
N2 - BACKGROUND: This study investigates the influence of school playground facilities on children's daily physical activity. METHODS: Participants were 594 school children measured at preschool (age 6 to 7 years) and 3 years later in third grade (518 children age 9 to 10 years) from 18 schools in 2 suburban municipalities in Denmark. Physical activity data were obtained using accelerometers. These were related to the number of permanent play facilities in school grounds and the school playground area (m2). RESULTS: The number of play facilities in the school grounds was positively associated with all measures of children's activity. In preschool every 10 additional play facilities the children had access to was associated with an increase in the average accelerometer counts of 14% (r = .273, P < .001) in school time and 6.9% (r = .195, P < .001) overall. For the children in third grade, access to 10 additional play facilities was associated with an increase in school time activity level of 26% (r = .364, P < .001) and an increase in overall activity level of 9.4% (r = .211, P < .001). School playground area did not affect activity levels independently of the number of permanent play

facilities. CONCLUSION: Increasing the number of play facilities in primary school playgrounds may increase the level of children's daily physical activity
SN - 1543-5474
AD - Dept of Exercise and Sport Sciences, University of Copenhagen, Copenhagen, Denmark
UR - 22232496
ER -

TY - JOUR
ID - 499
T1 - Inflammatory disease of the bile ducts-cholangiopathies: liver biopsy challenge and clinicopathological correlation. [Review]
A1 - Portmann,B.
A1 - Zen,Y.
Y1 - 2012/01//
N1 - Portmann, Bernard. Zen, Yoh
Histopathology
gb4, 7704136
IM
Journal Article. Review
English
KW - MEDLINE
KW - Adolescent
KW - Adult
KW - Biopsy
KW - Child
KW - Preschool
KW - Cholangitis/im [Immunology]
KW - Cholangitis/pa [Pathology]
KW - Cholangitis
KW - Sclerosing/pa [Pathology]
KW - Female
KW - Humans
KW - Immunoglobulin G/me [Metabolism]
KW - Infant
KW - Liver/pa [Pathology]
KW - Liver Cirrhosis
KW - Biliary/pa [Pathology]
KW - Male
KW - Middle Aged
KW - Young Adult
RP - NOT IN FILE
SP - 236
EP - 248
JF - Histopathology
VL - 60
IS - 2
CY - England
N2 - Liver biopsy challenge and clinicopathological correlation Liver biopsy interpretation in inflammatory diseases of the bile ducts or chronic cholangiopathies may be challenging, especially for pathologists working outside referral centres, where there is a limited exposure to relatively uncommon conditions. In view of the importance of sampling errors resulting from the patchy distribution of pathognomonic bile duct injuries and the misleading absence of cholestasis in the early stages, there is a need to recognize surrogate markers and subtle changes, in particular the early periportal deposition of copper and mild biliary interface activity. Such findings may either constitute the first indication of a primarily biliary disorder or be supportive of a clinically suspected diagnosis. Histological changes common to chronic cholangiopathies are reviewed at the variable stages of development that patients may first present to clinicians. As awareness of the protean clinical manifestations is essential for histological interpretation, the major and distinctive anatomoclinical features of primary biliary

cirrhosis and primary and acquired sclerosing cholangitis are revisited, together with so-called overlapping syndromes and less common variants and associations, including more recently documented conditions, such as IgG4-related disease and the rarer multidrug resistance 3 deficiency. The review stresses the importance of evaluating histological changes in conjunction with clinical information. 2011 Blackwell Publishing Limited
SN - 1365-2559
AD - Institute of Liver Studies, King's College Hospital, London, UK. bernard.portmann@kcl.ac.uk
UR - 21668470
ER -

TY - JOUR
ID - 500
T1 - Is fear of strangers related to physical activity among youth?
A1 - Ding,D.
A1 - Bracy,N.L.
A1 - Sallis,J.F.
A1 - Saelens,B.E.
A1 - Norman,G.J.
A1 - Harris,S.K.
A1 - Durant,N.
A1 - Rosenberg,D.
A1 - Kerr,J.
Y1 - 2012/01//
N1 - Ding, Ding. Bracy, Nicole L. Sallis, James F. Saelens, Brian E. Norman, Gregory J. Harris, Sion Kim. Durant, Nefertiti. Rosenberg, Dori. Kerr, Jacqueline
American journal of health promotion : AJHP
amj, 8701680
T
Journal Article. Research Support, Non-U.S. Gov't. Validation Studies
English
KW - MEDLINE
KW - Adolescent
KW - California
KW - Child
KW - Continental Population Groups/px [Psychology]
KW - Crime/px [Psychology]
KW - Cross-Sectional Studies
KW - Fear/px [Psychology]
KW - Female
KW - Health Behavior
KW - Humans
KW - Interpersonal Relations
KW - Male
KW - Massachusetts
KW - Motor Activity
KW - Ohio
KW - Psychometrics
KW - Reproducibility of Results
KW - Residence Characteristics
KW - Safety
KW - Social Environment
RP - NOT IN FILE
SP - 189
EP - 195
JF - American Journal of Health Promotion
JA - Am J Health Promot
VL - 26
IS - 3

CY - United States
N2 - PURPOSE: To (1) assess the reliability of the newly developed Fears of Stranger Danger (FSD) scale, (2) examine measurement invariance and identify demographic variation in FSD, and (3) examine associations of FSD with physical activity, screen time, and body mass index (BMI) z score. DESIGN: Cross-sectional survey with test-retest. SETTING: Neighborhoods with various socioeconomic characteristics and walkability in San Diego, Boston, and Cincinnati. PARTICIPANTS: Parent-adolescent pairs (n = 171), and parents of children (n = 116). Response rate was 47% for Survey 1, and 69% were retained for Survey 2. METHODS: Data analyses included test-retest reliability and internal consistency for FSD, tests of differential functioning for measurement invariance, t-test for associations between FSD and demographic variables, and partial correlation for associations of FSD with physical activity, screen time, and BMI z score. RESULTS: The FSD scale had moderate to substantial test-retest reliability (intraclass correlation coefficient = .65-.85) and excellent internal consistency (Cronbach alpha = .88-.94). Measurement invariance was established across gender, race/ethnicity, and income. FSD was higher regarding younger children, females, nonwhites, and lower-income youth. FSD was positively associated with restrictive parental rules for playing outside (partial r = .28-.33), and negatively associated with children's outdoor physical activity in the neighborhood (partial r = -.27), but not associated with other measures of physical activity, screen time, or BMI z score. CONCLUSION: The new measure of FSD had good evidence of reliability and measurement invariance, but there were inconsistent associations of FSD with youth physical activity
SN - 0890-1171
AD - Joint Doctoral Program in Public Health, Department of Psychology, San Diego State University, 3900 5th Avenue, San Diego, CA 92103, USA
UR - 22208419
ER -

TY - JOUR
ID - 501
T1 - Assessing the social and physical contexts of children's leisure-time physical activity: an ecological momentary assessment study
A1 - Dunton,G.F.
A1 - Kawabata,K.
A1 - Intille,S.
A1 - Wolch,J.
A1 - Pentz,M.A.
Y1 - 2012/01//
N1 - Dunton, Genevieve Fridlund. Kawabata, Keito. Intille, Stephen. Wolch, Jennifer. Pentz, Mary Ann
American journal of health promotion : AJHP
amj, 8701680
T
Journal Article. Research Support, N.I.H., Extramural. Research Support, Non-U.S. Gov't
English
KW - MEDLINE
KW - Adolescent
KW - California
KW - Cellular Phone
KW - Child
KW - Exercise/ph [Physiology]
KW - Exercise/px [Psychology]
KW - Female
KW - Health Surveys
KW - Humans
KW - Leisure Activities/px [Psychology]
KW - Linear Models
KW - Male
KW - Motor Activity/ph [Physiology]
KW - Social Environment
KW - Time Factors
RP - NOT IN FILE

SP - 135
EP - 142
JF - American Journal of Health Promotion
JA - Am J Health Promot
VL - 26
IS - 3
CY - United States
N2 - PURPOSE: To use Ecological Momentary Assessment with mobile phones to describe where and with whom children's leisure-time physical activity occurs. DESIGN: Repeated assessments across 4 days (Friday-Monday) during nonschool time (20 total). SETTING: Chino, California, and surrounding communities. SUBJECTS: Primarily low to middle income children (N =121; aged 9-13 years; x=11.0 years, SD =1.2 years; 52% male, 38% Hispanic/Latino). MEASURES: Electronic surveys measured current activity (e.g., active play/sports/exercise, watching TV/movies), social company (e.g., family, friends, alone), physical location (e.g., home, outdoors, school), and other perceived contextual features (e.g., safety, traffic, vegetation, distance from home). Analysis . Multilevel linear and multinomial logistic regression. RESULTS: Most of children's physical activity occurred outdoors (away from home) (42%), followed by at home (indoors) (30%), front/backyard (at home) (8%), someone else's house (8%), at a gym/recreation center (3%), and other locations (9%). Children's physical activity took place most often with multiple categories of people together (e.g., friends and family) (39%), followed by family members only (32%), alone (15%), and with friends only (13%). Age, weight status, income, and racial/ethnic differences in physical activity contexts were observed. CONCLUSIONS: The most frequently reported contexts for children's leisure time physical activity were outdoors and with family members and friends together
SN - 0890-1171
AD - Department of Preventive Medicine, University of Southern California, 1000 S. Fremont Avenue, Alhambra, CA 91803, USA. dunton@usc.edu
UR - 22208410
ER -

TY - JOUR
ID - 502
T1 - Quantified outdoor micro-activity data for children aged 7-12-years old
A1 - Beamer,P.I.
A1 - Luik,C.E.
A1 - Canales,R.A.
A1 - Leckie,J.O.
Y1 - 2012/01//
N1 - Beamer, Paloma I. Luik, Catherine E. Canales, Robert A. Leckie, James O
Journal of exposure science & environmental epidemiology
101262796
IM
Evaluation Studies. Journal Article. Research Support, Non-U.S. Gov't
English
KW - MEDLINE
KW - Age Factors
KW - Child
KW - Child Behavior
KW - Preschool
KW - Environmental Exposure/an [Analysis]
KW - Environmental Monitoring/mt [Methods]
KW - Environmental Pollutants
KW - Female
KW - Humans
KW - Male
KW - Play and Playthings
KW - Quality Control
KW - Sex Factors
KW - Time Factors

KW - Videotape Recording
RP - NOT IN FILE
SP - 82
EP - 92
JF - Journal of Exposure Science & Environmental Epidemiology
JA - J Expo Sci Environ Epidemiol
VL - 22
IS - 1
CY - United States
N2 - Estimation of aggregate exposure and risk requires detailed information regarding dermal contact and mouthing activity. We analyzed micro-level activity time series (MLATS) of children aged 7-12 years to quantify these contact behaviors and evaluate differences by age and gender. In all, 18 children, aged 7-12 years, were videotaped while playing outdoors. Video footage was transcribed via Virtual Timing Device (VTD) software. We calculated the hand and mouth contact frequency, hourly duration and median duration of contact with 16 object categories. Median mouthing frequencies were 12.6 events/h and 2.6 events/h for hands and non-dietary objects, respectively. Median hourly mouthing duration was 0.4 min/h and 0.1 min/h with hands and objects. Median mouthing contact duration was 1 s and 1.5 s with hands and objects, respectively. The median object contact frequency for both the hands combined was 537.3 events/h with an hourly contact duration of 81.8 min/h and a median contact duration of 3 s. There were no significant differences in the mouthing activity between genders or age groups. Female children had longer and more frequent hand contacts with several surface types. Age was negatively correlated with hand contacts of floor and wood surfaces. Contact frequencies in this study are higher than current regulatory recommendations for this age group
SN - 1559-064X
AD - Environmental Health Sciences, Mel and Enid Zuckerman College of Public Health, University of Arizona, Tucson, Arizona, USA. pbeamer@email.arizona.edu
UR - 21989500
ER -

TY - JOUR
ID - 503
T1 - 'The overall quality of my life as a sibling is all right, but of course, it could always be better'. Quality of life of siblings of children with intellectual disability: the siblings' perspectives
A1 - Moyson,T.
A1 - Roeyers,H.
Y1 - 2012/01//
N1 - Moyson, T. Roeyers, H
Journal of intellectual disability research : JIDR
9206090, bga
IM
Journal Article
English
KW - MEDLINE
KW - Adolescent
KW - Adult
KW - Birth Order
KW - Caregivers/px [Psychology]
KW - Child
KW - Cost of Illness
KW - Disabled Children
KW - Family Health
KW - Female
KW - Health Surveys
KW - Humans
KW - Intellectual Disability/px [Psychology]
KW - Male
KW - Middle Aged
KW - Psychometrics/is [Instrumentation]

KW - Psychometrics/mt [Methods]
KW - Quality of Life/px [Psychology]
KW - Questionnaires
KW - Siblings/px [Psychology]
KW - Social Support
RP - NOT IN FILE
SP - 87
EP - 101
JF - Journal of Intellectual Disability Research
JA - J Intellect Disabil Res
VL - 56
IS - 1
CY - England
N2 - BACKGROUND: The concept of family quality of life is becoming increasingly important in family support programmes. This concept describes the quality of life of all family members and the family system as a whole, but only the opinion of the parents has been included. The opinion of the siblings has been incorporated in the opinions of the parents, although research has shown that there is discordance between parents' and siblings' reports. The principal goal of this study is to investigate how young siblings of children with intellectual disability define their quality of life as a sibling. METHOD: As we were more concerned with understanding the experience of being a sibling from the siblings' own frame of reference, we opted for a qualitative research design and more specifically used in-depth, phenomenology-based interviews. Data were sorted by means of a process of continuously comparing the codes according to the principles of grounded theory. RESULTS: Siblings described the following nine domains as domains of sibling quality of life: joint activities, mutual understanding, private time, acceptance, forbearance, trust in well-being, exchanging experiences, social support and dealing with the outside world. CONCLUSIONS: This study shows not only that siblings can define their quality of life, but also that this definition of sibling quality of life differs from the family quality of life concept. Therefore, it may be not only a valuable addition to the family quality of life concept but also an appropriate concept to describe siblings' experience. 2011 The Authors. Journal of Intellectual Disability Research 2011 Blackwell Publishing Ltd
SN - 1365-2788
AD - Research Group Developmental Disorders, Department of Experimental Clinical and Health Psychology, Ghent University, Ghent, Belgium. tinneke.moyson@ugent.be
UR - 21366753
ER -

TY - JOUR
ID - 504
T1 - Comparison of thromboelastometry (ROTEM) with standard plasmatic coagulation testing in paediatric surgery
A1 - Haas,T.
A1 - Spielmann,N.
A1 - Mauch,J.
A1 - Madjdpour,C.
A1 - Speer,O.
A1 - Schmugge,M.
A1 - Weiss,M.
Y1 - 2012/01//
N1 - Haas, T. Spielmann, N. Mauch, J. Madjdpour, C. Speer, O. Schmugge, M. Weiss, M
British journal of anaesthesia
0372541
IM
Comparative Study. Journal Article. Research Support, Non-U.S. Gov't
English
KW - MEDLINE
KW - Blood Coagulation Tests/mt [Methods]
KW - Blood Coagulation Tests/st [Standards]
KW - Child

KW - Preschool
KW - Data Interpretation
KW - Statistical
KW - Female
KW - Fibrinogen/an [Analysis]
KW - Guidelines as Topic
KW - Hospitals
KW - Pediatric
KW - Humans
KW - Infant
KW - Male
KW - Partial Thromboplastin Time
KW - Pediatrics/mt [Methods]
KW - Prospective Studies
KW - Prothrombin Time
KW - Quality Control
KW - Reference Standards
KW - Surgical Procedures
KW - Operative/mt [Methods]
KW - Thrombelastography/mt [Methods]
KW - Thrombelastography/st [Standards]
RP - NOT IN FILE
SP - 36
EP - 41
JF - British Journal of Anaesthesia
JA - Br J Anaesth
VL - 108
IS - 1
CY - England
N2 - BACKGROUND: Thromboelastometry (ROTEM()) might be useful to detect intraoperative coagulation disorders early in major paediatric surgery. This observational trial compares this technique to standard coagulation tests. METHODS: Intraoperative blood sampling was obtained in children undergoing elective major surgery. At each time point, standard coagulation tests [activated partial thromboplastin time (aPTT), prothrombin time (PT), and fibrinogen level] and ROTEM() analyses (InTEM, ExTEM, and FibTEM) were performed simultaneously by trained hospital laboratory staff. RESULTS: A total of 288 blood samples from 50 subjects were analysed. While there was a poor correlation between PT and aPTT to ExTEM clotting time (CT) and InTEM CT, respectively, a good correlation was detected between PT and aPTT to clot formation time, and a very good correlation between fibrinogen level and FibTEM assay (r=0.882, P<0.001). Notably, 64% of PT and 94% of aPTT measurements were outside the reference range, while impaired CT was observed in 13% and 6.3%, respectively. Standard coagulation test results were available after a median of 53 min [inter-quartile range (IQR): 45-63 min], whereas 10 min values of ROTEM() results were available online after 23 min (IQR: 21-24 min). CONCLUSIONS: PT and aPTT cannot be interchangeably used with ROTEM() CT. Based on the results of ROTEM(), recommended thresholds for PT and aPTT might overestimate the need for coagulation therapy. A good correlation was found between the fibrinogen level and the FibTEM assay. In addition, ROTEM() offered faster turnaround times
SN - 1471-6771
AD - Department of Anaesthesia, University Children's Hospital Zurich, Steinwiesstrasse 75, Zurich 8032, Switzerland. thorsten.haas@kispi.uzh.ch
UR - 22086509
ER -

TY - JOUR
ID - 505
T1 - Overweight does not increase asthma risk but may decrease allergy risk at school age after infantile bronchiolitis
A1 - Sidoroff,V.
A1 - Hyvarinen,M.K.

A1 - Piippo-Savolainen,E.
A1 - Korppi,M.
Y1 - 2012/01//
N1 - Sidoroff, Virpi. Hyvarinen, Mari K. Piippo-Savolainen, Eija. Korppi, Matti
Acta paediatrica (Oslo, Norway : 1992)
bgc, 9205968
IM
Journal Article. Research Support, Non-U.S. Gov't
English
KW - MEDLINE
KW - Asthma/et [Etiology]
KW - Asthma/pp [Physiopathology]
KW - Bronchial Hyperreactivity/et [Etiology]
KW - Bronchiolitis/th [Therapy]
KW - Child
KW - Female
KW - Follow-Up Studies
KW - Humans
KW - Hypersensitivity/et [Etiology]
KW - Hypersensitivity
KW - Immediate/et [Etiology]
KW - Infant
KW - Male
KW - Obesity/co [Complications]
KW - Overweight/co [Complications]
KW - Risk Factors
RP - NOT IN FILE
SP - 43
EP - 47
JF - Acta Paediatrica
JA - Acta Paediatr
VL - 101
IS - 1
CY - Norway
N2 - AIM: Increasing evidence suggests that overweight children are at increased risk of asthma. The association between weight gain and allergy is more complex. The aim was to evaluate the association between overweight or obesity and asthma, allergy, bronchial reactivity or atopic sensitization at school age in children with bronchiolitis in infancy. SUBJECTS AND METHODS: Eighty-one children hospitalized for bronchiolitis at <24 months of age attended control visits at 7.2 and 12.3 years of ages. The visits consisted of medical examinations, weight and height measurements, body mass index (BMI) calculations, skin prick tests and exercise challenge tests. BMI >1.3 SD from age- and gender-specific references meant overweight and BMI >2.0 SD obesity. RESULTS: Current or previous overweight or obesity did not increase the risk of asthma, allergy, bronchial reactivity or atopic sensitization at 7.2 or 12.3 years of age. Previous and current obesity decreased the risk of atopic dermatitis, and current overweight and obesity decreased the risk of sensitization to outdoor allergens at 12.3 years of age. CONCLUSION: Previous or current overweight does not increase asthma or allergy risk but current obesity may decrease allergy risk at school age after bronchiolitis in infancy.
2011 The Author(s)/Acta Paediatrica 2011 Foundation Acta Paediatrica
SN - 1651-2227
AD - Department of Pediatrics, University of Eastern Finland, Kuopio, Finland
UR - 21824194
ER -

TY - JOUR
ID - 506
T1 - Do distinct atypical cortical networks process biological motion information in adults with Autism Spectrum Disorders?
A1 - McKay,L.S.

A1 - Simmons,D.R.
A1 - McAleer,P.
A1 - Marjoram,D.
A1 - Piggot,J.
A1 - Pollick,F.E.
Y1 - 2012/01/16/
N1 - McKay, Lawrie S. Simmons, David R. McAleer, Phil. Marjoram, Dominic. Piggot, Judith. Pollick, Frank E
NeuroImage
cpp, 9215515
IM
Journal Article. Research Support, Non-U.S. Gov't
English
KW - MEDLINE
KW - Adolescent
KW - Adult
KW - Cerebral Cortex/pp [Physiopathology]
KW - Child
KW - Child Development Disorders
KW - Pervasive/pp [Physiopathology]
KW - Female
KW - Humans
KW - Magnetic Resonance Imaging
KW - Male
KW - Motion Perception
KW - Nerve Net/pp [Physiopathology]
KW - Young Adult
RP - NOT IN FILE
SP - 1524
EP - 1533
JF - Neuroimage
VL - 59
IS - 2
CY - United States
N2 - Whether people with Autism Spectrum Disorders (ASDs) have a specific deficit when processing biological motion has been a topic of much debate. We used psychophysical methods to determine individual behavioural thresholds in a point-light direction discrimination paradigm for a small but carefully matched groups of adults (N=10 per group) with and without ASDs. These thresholds were used to derive individual stimulus levels in an identical fMRI task, with the purpose of equalising task performance across all participants whilst inside the scanner. The results of this investigation show that despite comparable behavioural performance both inside and outside the scanner, the group with ASDs shows a different pattern of BOLD activation from the TD group in response to the same stimulus levels. Furthermore, connectivity analysis suggests that the main differences between the groups are that the TD group utilise a unitary network with information passing from temporal to parietal regions, whilst the ASD group utilise two distinct networks; one utilising motion sensitive areas and another utilising form selective areas. Furthermore, a temporal-parietal link that is present in the TD group is missing in the ASD group. We tentatively propose that these differences may occur due to early dysfunctional connectivity in the brains of people with ASDs, which to some extent is compensated for by rewiring in high functioning adults. Copyright 2011 Elsevier Inc. All rights reserved
SN - 1095-9572
AD - School of Psychology, University of Glasgow, Glasgow G12 8QB, UK. l.mckay@nin.knaw.nl
UR - 21888982
ER -

TY - JOUR
ID - 507
T1 - [Biological pollution and its relationship with respiratory symptoms indicative of asthma, Bucaramanga, Colombia]. [Spanish]

A1 - Herrera,A.B.
A1 - Rodriguez,L.A.
A1 - Niederbacher,J.
Y1 - 2011/07//
N1 - Herrera, Astrid B. Rodriguez, Laura A. Niederbacher, Jurg
Biomedica : revista del Instituto Nacional de Salud
8205605
IM
Comparative Study. English Abstract. Journal Article. Research Support, Non-U.S. Gov't
Spanish
KW - MEDLINE
KW - Air Microbiology
KW - Air Pollution
KW - Indoor/ae [Adverse Effects]
KW - Animals
KW - Antigens
KW - Dermatophagoides/to [Toxicity]
KW - Asthma/ep [Epidemiology]
KW - Asthma/et [Etiology]
KW - Asthma/ge [Genetics]
KW - Child
KW - Preschool
KW - Colombia
KW - Comorbidity
KW - Family Health
KW - Female
KW - Fungi/ip [Isolation & Purification]
KW - Humans
KW - Infant
KW - Newborn
KW - Premature
KW - Male
KW - Pets
KW - Rhinitis
KW - Allergic
KW - Perennial/ep [Epidemiology]
KW - Perennial/et [Etiology]
KW - Socioeconomic Factors
KW - Spores
KW - Fungal
KW - Tobacco Smoke Pollution
KW - Urban Population
RP - NOT IN FILE
SP - 357
EP - 371
JF - Biomedica
JA - Biomedica (Bogota)
VL - 31
IS - 3
CY - Colombia
N2 - INTRODUCTION: Indoorair pollution may play an important role in development and exacerbation of asthma in children. OBJECTIVE: The association between the presence of indoor biological contaminants and respiratory symptoms related to asthma was assessed in preschool children. Materials and methods. This cross-sectional study was undertaken in Bucaramanga, Colombia, and included children <7 years of age living in two urban areas of with different levels of outdoor air pollution. The 678 children were an average of 3.5 years of age. Respiratory symptoms indicative of asthma and indoor air pollutants were assessed by previously validated questionnaires.. Biological samples potentially containing mites and fungi were collected by standardized

laboratory methods. The log binomial regression model was used for multivariate analysis, using adjusted prevalence ratios (PR). RESULTS: The prevalence of asthmatic respiratory symptoms was 8.0%; (95% C.I: 5.6-9.6), without significant differences between the two areas. Binomial model analysis showed that asthma symptoms were associated with mites (PR 1.78; 95% C.I. 1.0-3.0), Acremonium sp (PR 6.24; 95 C.I.: 3.8-10.0) and a history of child pneumonia (PR 4.0; 95% C.I. 2.5-6.4), allergic rhinitis (PR 1.9; 95% C.I.: 1.2-3.1), prematurity (PR 3.4; 95% C.I. 1.8-6.5), parents with asthma (PR 2.6; 95% C.I. 1.4-5.0) and pet ownership (PR 0.4; 95% C.I. 0.2-0.9). CONCLUSIONS: The indoor exposure to biological contaminants (dust mites and fungi), history of prematurity, pneumonia, rhinitis and family history of asthma increased the occurence of symptoms suggestive of asthma in young children
SN - 0120-4157
AD - Departamento de Salud Publica, Escuela de Medicina, Universidad Industrial de Santander, Bucaramanga, Colombia. aherrera_lopes@hotmail.com
UR - 22674312
ER -

TY - JOUR
ID - 508
T1 - Enhanced right amygdala activity in adolescents during encoding of positively valenced pictures
A1 - Vasa,R.A.
A1 - Pine,D.S.
A1 - Thorn,J.M.
A1 - Nelson,T.E.
A1 - Spinelli,S.
A1 - Nelson,E.
A1 - Maheu,F.S.
A1 - Ernst,M.
A1 - Bruck,M.
A1 - Mostofsky,S.H.
Y1 - 2011/01//
N1 - Vasa, Roma A. Pine, Daniel S. Thorn, Julia M. Nelson, Tess E. Spinelli, Simona. Nelson, Eric. Maheu, Francoise S. Ernst, Monique. Bruck, Maggie. Mostofsky, Stewart H
Developmental cognitive neuroscience
101541838
IM
Journal Article. Randomized Controlled Trial. Research Support, N.I.H., Extramural
English
KW - MEDLINE
KW - Adolescent
KW - Adult
KW - Amygdala/ph [Physiology]
KW - Child
KW - Female
KW - Humans
KW - Magnetic Resonance Imaging/mt [Methods]
KW - Male
KW - Photic Stimulation/mt [Methods]
KW - Psychomotor Performance/ph [Physiology]
KW - Time Factors
KW - Young Adult
RP - NOT IN FILE
SP - 88
EP - 99
JF - Developmental Cognitive Neuroscience
JA - Dev Cogn Neurosci
VL - 1
IS - 1
CY - Netherlands

N2 - While studies among adults implicate the amygdala and interconnecting brain regions in encoding emotional stimuli, few studies have examined whether developmental changes occur within this emotional-memory network during adolescence. The present study examined whether adolescents and adults differentially engaged the amygdala and hippocampus during successful encoding of emotional pictures, with either positive or negative valence. Eighteen adults and twelve adolescents underwent event-related fMRI while encoding emotional pictures. Approximately 30 min later, outside the scanner, subjects were asked to recall the pictures seen during the scan. Age group differences in brain activity in the amygdala and hippocampus during encoding of the pictures that were later successfully and unsuccessfully recalled were separately compared for the positive and negative pictures. Adolescents, relative to adults, demonstrated enhanced activity in the right amygdala during encoding of positive pictures that were later recalled compared to not recalled. There were no age group differences in amygdala or hippocampal activity during successful encoding of negative pictures. The findings of preferential activity within the adolescent right amygdala during successful encoding of positive pictures may have implications for the increased reward and novelty seeking behavior, as well as elevated rates of psychopathology, observed during this distinct developmental period
SN - 1878-9307
AD - Department of Psychiatry, Kennedy Krieger Institute, 716 North Broadway, Baltimore, MD 21205, United States. vasa@kennedykrieger.org
UR - 21127721
ER -

TY - JOUR
ID - 509
T1 - Relationship between impulsiveness and deviant behavior among adolescents in the classroom: age and sex differences
A1 - Esteban,A.
A1 - Tabernero,C.
Y1 - 2011/12//
N1 - Esteban, Angeles. Tabernero, Carmen
Psychological reports
qf6, 0376475
IM
Journal Article. Research Support, Non-U.S. Gov't
English
KW - MEDLINE
KW - Adolescent
KW - Adolescent Behavior/px [Psychology]
KW - Age Factors
KW - Attention Deficit and Disruptive Behavior Disorders/px [Psychology]
KW - Child
KW - Diagnostic Self Evaluation
KW - Female
KW - Humans
KW - Impulsive Behavior/px [Psychology]
KW - Male
KW - Questionnaires
KW - Schools
KW - Self-Assessment
KW - Sex Factors
KW - Students/px [Psychology]
RP - NOT IN FILE
SP - 703
EP - 717
JF - Psychological Reports
JA - Psychol Rep
VL - 109
IS - 3
CY - United States

N2 - To assess the relationship between impulsiveness and deviant behavior among 103 adolescents, taking into account their sociodemographic characteristics, the Barratt Impulsiveness Scale and a self-assessment measure with regard to disruptive and deviant behaviors which had occurred in the last 90 days were used. The results show that impulsiveness and disruptive behavior in the classroom were related to deviant behavior outside of the classroom. Therefore, age and sex explained the relationship between impulsiveness and behavior. The older adolescents and the girls showed less disruptive behaviors than the younger participants and the boys; both variables showed an interactive effect on disruptive behavior. The age at which sexual activity commenced and the number of sexual partners were also significantly related to impulsiveness and disruptive and deviant behavior. Similarly, impulsiveness was shown to have a significant relationship with disruptive and deviant behavior, and disruptive behavior was shown to have a significant relationship with deviant behavior
SN - 0033-2941
AD - University of Cordoba, Spain
UR - 22420105
ER -

TY - JOUR
ID - 510
T1 - Profile of vernal keratoconjunctivitis in Nepal: a hospital based study
A1 - Kansakar,I.
Y1 - 2011/06//
N1 - Kansakar, I
Nepal Medical College journal : NMCJ
101129937
IM
Journal Article
English
KW - MEDLINE
KW - Adolescent
KW - Adult
KW - Child
KW - Preschool
KW - Conjunctivitis
KW - Allergic/ep [Epidemiology]
KW - Cross-Sectional Studies
KW - Female
KW - Humans
KW - Male
KW - Nepal/ep [Epidemiology]
KW - Retinoscopy
KW - Seasons
KW - Young Adult
RP - NOT IN FILE
SP - 92
EP - 95
JF - Nepal Medical College Journal: NMCJ
JA - Nepal Med Coll J
VL - 13
IS - 2
CY - Nepal
N2 - Vernal keratoconjunctivitis (VKC) or Spring Catarrh is recurrent, chronic, allergic conjunctivitis occurring in prepubertal age-group during spring and summer season. Though this is a self-limiting disorder, it often leads to visual impairment due to secondary corneal complications if not treated during active stage of the illness. Apart from this, the illness is known to affect the productivity and quality of life in school going children due to absenteeism and limitations to outdoor activities. Knowing the clinical profile of disease would provide the evidence for developing, treatment protocol in coming years. Therefore a hospital based study was designed to see the profile of VKC. Total of 220 patients were enrolled in this study. Male to female ratio with VKC was found to be 4:1. Clinical presentation showed seasonal variation; 78.2% during the spring and summer season.

Bilateral involvement was seen in 100% cases, out of which 44.5% were limbal type. The best spectacle corrected visual status of better than 6/9 was in 96.4%, while 2.3% had vision less than 6/12. In 9.5% corneal involvement was seen as superficial keratopathy, while others, such as Shield ulcer, pannus, corneal scar, and keratoconus consisted of 8.3%. The disease is predominantly seen in male child with seasonal variation. Visual impairment is mainly due to involvement of cornea
AD - Shree Rana Ambika Shah Eye Hospital, Lumbini Eye Institute, Bhairahawa, Nepal.
irinakans17@hotmail.com
UR - 22364089
ER -

TY - JOUR
ID - 511
T1 - [Analysis of the risk factors for pesticide poisoning among children in countryside of Guigang city]. [Chinese]
A1 - Li,H.
A1 - Yang,L.
A1 - Feng,Q.M.
A1 - Li,C.L.
Y1 - 2011/12//
N1 - Li, Hai. Yang, Li. Feng, Qi-ming. Li, Chun-ling
Zhonghua yu fang yi xue za zhi [Chinese journal of preventive medicine]
d74, 7904962
IM
English Abstract. Journal Article. Research Support, U.S. Gov't, Non-P.H.S.
Chinese
KW - MEDLINE
KW - Adolescent
KW - Case-Control Studies
KW - Child
KW - Preschool
KW - China/ep [Epidemiology]
KW - Female
KW - Humans
KW - Infant
KW - Male
KW - Pesticides/po [Poisoning]
KW - Poisoning/ep [Epidemiology]
KW - Risk Factors
KW - Rural Population
RP - NOT IN FILE
SP - 1103
EP - 1107
JF - Chung-Hua Yu Fang i Hsueh Tsa Chih [Chinese Journal of Preventive Medicine]
JA - Chung Hua Yu Fang I Hsueh Tsa Chih
VL - 45
IS - 12
CY - China
N2 - OBJECTIVE: This study was to investigate the risk factors for pesticide poisoning among rural children in Guigang. METHODS: A 1:4 matched case-control study was conducted. A total of 78 rural children who were hospitalized or visited the out-patient clinic due to pesticide poisoning in Guigang from January to December in 2009 were recruited as cases, and 312 matched controls were recruited during the same time. The children's parents or guardians were surveyed with a questionnaire. The questionnaire including general information and 21 possible risk factors concerned in family structure, guardian status, educational level of parents, average annual family income, family and school health education and dangerous behavior in children. The data were analyzed by conditional logistic regression analysis. RESULTS: Three risk factors and five protective factors were identified significantly associated with pesticide poisoning in rural children. The risk factors included inappropriate deposit of hydrocomion and contaminated working clothes (OR = 3.529, 95%CI: 1.408 - 8.848),

playing outside frequently (OR = 2.846, 95%CI: 1.513 - 5.352), grandparents being children's guardian (OR = 2.187, 95%CI: 1.187 - 4.029). The protective factors included high frequency of guardianship (OR = 0.408, 95%CI: 0.205 - 0.811), knowledge for poisoning prevention (OR = 0.412, 95%CI: 0.224 - 0.758), washing working clothes in time (OR = 0.435, 95%CI: 0.212 - 0.893), taking health educational courses in school (OR = 0.448, 95%CI: 0.232 - 0.867) and teaching children non-access to toxic agents regularly (OR = 0.462, 95%CI: 0.227 - 0.939). CONCLUSION: Childhood pesticide poisoning accidence in countryside of Guigang was caused by multiple factors including children's risk behaviors, family factors, environmental factors and health education
SN - 0253-9624
AD - School of Public Health, Guangxi Medical University, Nanning 530021, China
UR - 22336346
ER -

TY - JOUR
ID - 512
T1 - Where they live, how they play: neighborhood greenness and outdoor physical activity among preschoolers
A1 - Grigsby-Toussaint,D.S.
A1 - Chi,S.H.
A1 - Fiese,B.H.
A1 - STRONG Kids Project Writing Group.
Y1 - 2011///
N1 - Grigsby-Toussaint, Diana S. Chi, Sang-Hyun. Fiese, Barbara H. STRONG Kids Project Writing Group
International journal of health geographics
101152198
IM
Journal Article. Research Support, Non-U.S. Gov't
English
KW - MEDLINE
KW - Child Behavior
KW - Child Welfare
KW - Child
KW - Preschool
KW - Cohort Studies
KW - Environment
KW - Exercise/ph [Physiology]
KW - Female
KW - Humans
KW - Linear Models
KW - Male
KW - Motor Activity/ph [Physiology]
KW - Multivariate Analysis
KW - Parent-Child Relations
KW - Play and Playthings
KW - Residence Characteristics
KW - Risk Assessment
KW - Time Factors
KW - United States
KW - Urban Population
RP - NOT IN FILE
SP - 66
JF - International Journal of Health Geographics [Electronic Resource]
JA - Int J Health Geogr
VL - 10
CY - England
N2 - BACKGROUND: Emerging empirical evidence suggests exposure to "green" environments may encourage higher levels of physical activity among children. Few studies, however, have explored this

association exclusively in pre-school aged children in the United States. We examined whether residing in neighborhoods with higher levels of greenness was associated with higher levels of outdoor physical activity among preschoolers. In addition, we also explored whether outdoor playing behaviors (e.g., active vs. quiet) were influenced by levels of neighborhood greenness independent of demographic and parental support factors. RESULTS: Higher levels of neighborhood greenness as measured by the Normalized Difference Vegetation Index (NDVI) was associated with higher levels of outdoor playing time among preschool-aged children in our sample. Specifically, a one unit increase in neighborhood greenness increased a child's outdoor playing time by approximately 3 minutes. A dose-response relationship was observed between increasing levels of parental support for physical activity (e.g., time spent playing with children) and child outdoor physical activity ($p < 0.01$). CONCLUSIONS: Consistent with previous studies, neighborhood greenness influences physical activity behavior. However, for preschoolers, parental involvement may be more critical for improving physical activity levels
SN - 1476-072X
AD - Department of Kinesiology and Community Health, Division of Nutritional Sciences, University of Illinois at Urbana Champaign, USA. dgrigs1@illinois.edu
UR - 22165919
ER -

TY - JOUR
ID - 513
T1 - Effects of a multi-pronged intervention on children's activity levels at recess: the Aventuras para Ninos study
A1 - Elder,J.P.
A1 - McKenzie,T.L.
A1 - Arredondo,E.M.
A1 - Crespo,N.C.
A1 - Ayala,G.X.
Y1 - 2011/03//
N1 - Elder, John P. McKenzie, Thomas L. Arredondo, Elva M. Crespo, Noe C. Ayala, Guadalupe X
Advances in nutrition (Bethesda, Md.)
101540874
IM
Journal Article. Randomized Controlled Trial. Research Support, N.I.H., Extramural. Research Support, Non-U.S. Gov't. Research Support, U.S. Gov't, P.H.S.
English
KW - MEDLINE
KW - Child
KW - Preschool
KW - Exercise
KW - Female
KW - Hispanic Americans
KW - Humans
KW - Leisure Activities
KW - Male
KW - Play and Playthings
KW - Schools
KW - Sedentary Lifestyle
KW - Social Environment
KW - United States
RP - NOT IN FILE
SP - 171S
EP - 176S
JF - Advances in Nutrition
JA - Adv Nutr (Bethesda)
VL - 2
IS - 2
CY - United States

N2 - Latino children spend more time in sedentary activities than other American children, and only ~1 in 5 Latino children in public elementary and middle schools meet all 6 fitness standards in statewide fitness testing. Schools that facilitate physical activity (PA) by maintaining playgrounds and providing physical education classes have children who are more active and less overweight. The aims of the present study were to examine the extent to which several social and physical environmental changes in school settings resulted in observed changes in area characteristics and children's activity levels during recess. Thirteen elementary schools serving predominantly Mexican American children were randomized into control or activity and nutrition environmental intervention conditions. Playgrounds and activities were restructured in 6 intervention schools to promote more PA. After 1 y, there were no overall statistical differences between treatment groups in PA or sedentary behavior in these settings and results did not differ by gender. Changing the social and physical environments to promote children's moderate-to-vigorous PA is important to the design of active and healthy recess environments. The present results are not conclusive as to the link between these interventions and actual behavior, but show sufficient promise for further population and setting specific research
SN - 2156-5376
AD - Graduate School of Public Health, and Institute for Behavioral and Community Health, San Diego State University Research Foundation, San Diego, CA 9245, USA.
UR - 22332049
ER -

TY - JOUR
ID - 514
T1 - A family based tailored counselling to increase non-exercise physical activity in adults with a sedentary job and physical activity in their young children: design and methods of a year-long randomized controlled trial
A1 - Finni,T.
A1 - Saakslahti,A.
A1 - Laukkanen,A.
A1 - Pesola,A.
A1 - Sipila,S.
Y1 - 2011///
N1 - Finni, Taija. Saakslahti, Arja. Laukkanen, Arto. Pesola, Arto. Sipila, Sarianna
BMC public health
100968562
IM
Journal Article. Randomized Controlled Trial. Research Support, Non-U.S. Gov't
English
KW - MEDLINE
KW - Actigraphy/is [Instrumentation]
KW - Child
KW - Preschool
KW - Counseling/mt [Methods]
KW - Electromyography
KW - Female
KW - Finland
KW - Humans
KW - Male
KW - Motor Activity/ph [Physiology]
KW - Questionnaires
KW - Research Design
KW - Sedentary Lifestyle
RP - NOT IN FILE
SP - 944
JF - BMC Public Health
VL - 11
CY - England
N2 - BACKGROUND: Epidemiological evidence suggests that decrease in sedentary behaviour is beneficial for health. This family based randomized controlled trial examines whether face-to-face delivered counselling is effective in reducing sedentary time and improving health in adults and increasing moderate-to-vigorous

activities in children. METHODS: The families are randomized after balancing socioeconomic and environmental factors in the Jyvaskyla region, Finland. Inclusion criteria are: healthy men and women with children 3-8 years old, and having an occupation where they self-reportedly sit more than 50% of their work time and children in all-day day-care in kindergarten or in the first grade in primary school. Exclusion criteria are: body mass index > 35 kg/m2, self-reported chronic, long-term diseases, families with pregnant mother at baseline and children with disorders delaying motor development.From both adults and children accelerometer data is collected five times a year in one week periods. In addition, fasting blood samples for whole blood count and serum metabonomics, and diurnal heart rate variability for 3 days are assessed at baseline, 3, 6, 9, and 12 months follow-up from adults. Quadriceps and hamstring muscle activities providing detailed information on muscle inactivity will be used to realize the maximum potential effect of the intervention. Fundamental motor skills from children and body composition from adults will be measured at baseline, and at 6 and 12 months follow-up. Questionnaires of family-influence-model, health and physical activity, and dietary records are assessed. After the baseline measurements the intervention group will receive tailored counselling targeted to decrease sitting time by focusing on commute and work time. The counselling regarding leisure time is especially targeted to encourage toward family physical activities such as visiting playgrounds and non-built environments, where children can get diversified stimulation for play and practice fundamental of motor skills. The counselling will be reinforced during the first 6 months followed by a 6-month maintenance period. DISCUSSION: If shown to be effective, this unique family based intervention to improve lifestyle behaviours in both adults and children can provide translational model for community use. This study can also provide knowledge whether the lifestyle changes are transformed into relevant biomarkers and self-reported health. TRIAL REGISTRATION NUMBER: ISRCTN: ISRCTN28668090
SN - 1471-2458
AD - Neuromuscular Research Center, Department of Biology of Physical Activity, University of Jyvaskyla, Jyvaskyla, Finland. taija.finni@jyu.fi
UR - 22185647
ER -

TY - JOUR
ID - 515
T1 - The relationship between brown adipose tissue activity and neoplastic status: an (18)F-FDG PET/CT study in the tropics
A1 - Huang,Y.C.
A1 - Chen,T.B.
A1 - Hsu,C.C.
A1 - Li,S.H.
A1 - Wang,P.W.
A1 - Lee,B.F.
A1 - Kuo,C.Y.
A1 - Chiu,N.T.
Y1 - 2011///
N1 - Huang, Yung-Cheng. Chen, Tai-Been. Hsu, Chien-Chin. Li, Shau-Hsuan. Wang, Pei-Wen. Lee, Bi-Fang. Kuo, Ching-Yuan. Chiu, Nan-Tsing
Lipids in health and disease
101147696
IM
Journal Article. Research Support, Non-U.S. Gov't
English
KW - MEDLINE
KW - Adipose Tissue
KW - Brown/me [Metabolism]
KW - Adolescent
KW - Adult
KW - Aged
KW - Body Fat Distribution
KW - Child
KW - Female
KW - Fluorodeoxyglucose F18/du [Diagnostic Use]

KW - Humans
KW - Male
KW - Middle Aged
KW - Multimodal Imaging
KW - Multivariate Analysis
KW - Neoplasms/me [Metabolism]
KW - Neoplasms/ri [Radionuclide Imaging]
KW - Positron-Emission Tomography
KW - Retrospective Studies
KW - Taiwan
KW - Tomography
KW - X-Ray Computed
KW - Tropical Climate
KW - Young Adult
RP - NOT IN FILE
SP - 238
JF - Lipids in Health & Disease
JA - Lipids health dis
VL - 10
CY - England
N2 - BACKGROUND: Brown adipose tissue (BAT) has thermogenic potential. For its activation, cold exposure is considered a critical factor though other determinants have also been reported. The purpose of this study was to assess the relationship between neoplastic status and BAT activity by 2-deoxy-2-[(18)F]fluoro-D-glucose (18F-FDG) positron emission tomography/computed tomography (PET/CT) in people living in the tropics, where the influence of outdoor temperature was low. METHODS: (18)F-FDG PET/CT scans were reviewed and the total metabolic activity (TMA) of identified activated BAT quantified. The distribution and TMA of activated BAT were compared between patients with and without a cancer history. The neoplastic status of patients was scored according to their cancer history and (18)F-FDG PET/CT findings. We evaluated the relationships between the TMA of BAT and neoplastic status along with other factors: age, body mass index, fasting blood sugar, gender, and outdoor temperature. RESULTS: Thirty of 1740 patients had activated BAT. Those with a cancer history had wider BAT distribution (p = 0.043) and a higher TMA (p = 0.028) than those without. A higher neoplastic status score was associated with a higher average TMA. Multivariate analyses showed that neoplastic status was the only factor significantly associated with the TMA of activated BAT (p = 0.016). CONCLUSIONS: Neoplastic status is a critical determinant of BAT activity in patients living in the tropics. More active neoplastic status was associated with more vigorous TMA of BAT
SN - 1476-511X
AD - Department of Nuclear Medicine, Kaohsiung Chang Gung Memorial Hospital and Chang Gung University College of Medicine, Kaohsiung, Taiwan
UR - 22182284
ER -

TY - JOUR
ID - 516
T1 - Exercise-induced bronchospasm in children with asthma in the United States: results from the Exercise-Induced Bronchospasm Landmark Survey
A1 - Ostrom,N.K.
A1 - Eid,N.S.
A1 - Craig,T.J.
A1 - Colice,G.L.
A1 - Hayden,M.L.
A1 - Parsons,J.P.
A1 - Stoloff,S.W.
Y1 - 2011/11//
N1 - Ostrom, Nancy K. Eid, Nemr S. Craig, Timothy J. Colice, Gene L. Hayden, Mary Lou. Parsons, Jonathan P. Stoloff, Stuart W
Allergy and asthma proceedings : the official journal of regional and state allergy societies
ci9, 9603640

IM
Journal Article. Research Support, Non-U.S. Gov't
English
KW - MEDLINE
KW - Adolescent
KW - Asthma
KW - Exercise-Induced/ep [Epidemiology]
KW - Child
KW - Preschool
KW - Female
KW - Humans
KW - Male
KW - Questionnaires
KW - United States/ep [Epidemiology]
RP - NOT IN FILE
SP - 425
EP - 430
JF - Allergy & Asthma Proceedings
JA - Allergy Asthma Proc
VL - 32
IS - 6
CY - United States
N2 - Despite the availability of effective therapies, uncontrolled asthma remains a common problem. Previous large surveys suggest that exercise-related respiratory symptoms may be a significant element of uncontrolled asthma. The Exercise-Induced Bronchospasm (EIB) Landmark Survey is the first comprehensive, national survey evaluating EIB awareness and impact among the general public, asthma patients, and health care providers. This study was designed to evaluate the prevalence and impact of exercise-related respiratory symptoms in children (aged 4-17 years) with asthma. A national survey was conducted with parents of 516 children diagnosed with asthma or taking medications for asthma in the prior year. The majority of parents reported that their child experienced one or more exercise-related respiratory symptom and almost one-half (47.4%) experienced four or more symptoms. Most commonly reported symptoms were coughing, shortness of breath, and wheezing. Respondents reported that asthma limited their child's ability to participate either "a lot" or "some" in sports (30%), other outdoor activities (26.3%), and normal physical exertion (20.9%). Only 23.1% of children with exercise-related respiratory symptoms were reported to take short-acting beta-agonists such as albuterol "always" or "most of the time" before exercising. Exercise-related respiratory symptoms among pediatric asthma patients are common and substantially limit the ability of children to participate normally and perform optimally in physical activities. Such symptoms may reflect uncontrolled underlying asthma that should be evaluated and treated with appropriate controller medications. Despite the availability of preventative therapy, many children do not use short-acting bronchodilators before exercise as recommended in national guidelines
SN - 1539-6304
AD - Allergy and Asthma Medical Group and Research Center, San Diego, California 92123, USA. nancyostrom@allergyandasthma.com
UR - 22221436
ER -

TY - JOUR
ID - 517
T1 - Protection against solar ultraviolet radiation in childhood. [Review]
A1 - Pustisek,N.
A1 - Situm,M.
Y1 - 2011/09//
N1 - Pustisek, Nives. Situm, Mirna
Collegium antropologicum
8003354
IM

Journal Article. Review
English
KW - MEDLINE
KW - Child
KW - Environmental Exposure/pc [Prevention & Control]
KW - Health Promotion/mt [Methods]
KW - Humans
KW - Skin Neoplasms/pc [Prevention & Control]
KW - Sunlight/ae [Adverse Effects]
KW - Ultraviolet Rays/ae [Adverse Effects]
RP - NOT IN FILE
SP - 343
EP - 346
JF - Collegium Antropologicum
JA - Coll Antropol
VL - 35 Suppl 2
CY - Croatia
N2 - In the last decade, awareness of the harmful effects of solar ultraviolet radiation has increased. Modern lifestyles, outdoor occupations, sports and other activities make total sun avoidance impossible. Children spend more time outdoors than adults and there is compelling evidence that childhood is a particularly vulnerable time for the photocarcinogenic effects of the sun. Sun exposure among infants and pre-school age children is largely depend on the discretion of adult care providers. It is important to learn safe habits about sun-safety behaviours during the childhood. Children deserve to live and play in safe environments, and it is the responsibility of every adult to help children stay safe. Protecting children from excessive sun exposure is protection from sunburn today and other forms of sun damages, especially skin cancers, in the future
SN - 0350-6134
AD - Children's Hospital Klaiceva, "Sestre milosrdnice" University Hospital Center, Zagreb, Croatia. nives.pustisek@kbcsm.hr
UR - 22220468
ER -

TY - JOUR
ID - 518
T1 - Relationship between daily physical activity, recess physical activity, age and sex in scholar of primary school, Spain
A1 - Escalante,Y.
A1 - Backx,K.
A1 - Saavedra,J.M.
A1 - Garcia-Hermoso,A.
A1 - Dominguez,A.M.
Y1 - 2011/10//
N1 - Escalante, Yolanda. Backx, Karianne. Saavedra, Jose M. Garcia-Hermoso, Antonio. Dominguez, Ana M
Revista espanola de salud publica
cia, 9600212
IM
Journal Article. Research Support, Non-U.S. Gov't
English Spanish
KW - MEDLINE
KW - Age Factors
KW - Child
KW - Cross-Sectional Studies
KW - Female
KW - Health Surveys
KW - Humans
KW - Male
KW - Motor Activity
KW - Play and Playthings

KW - Questionnaires
KW - Schools
KW - Sedentary Lifestyle
KW - Sex Factors
KW - Spain
RP - NOT IN FILE
SP - 481
EP - 489
JF - Revista Espanola de Salud Publica
JA - Rev Esp Salud Publica
VL - 85
IS - 5
CY - Spain
N2 - BACKGROUND: Sedentary behavior has negative effects on health. School recess playgrounds provide important settings and opportunities for children to engage in physical activity. The aim of this study was to describe the daily physical activity (PA) and physical activity during recess of Primary School children and the relationship with sex and age. METHOD: 783 children (379 boys and 359 girls; age=8.5+1.7 years; range 6 to 11) participated in the study. Daily PA of each child was measured using a validated questionnaire that was completed by the parents of each child, whilst playground recess PA was measured using accelerometry. An ANOVA was used to determine differences by sex in each age group with confidence intervals and effect sizes. Also MANOVA was used to analyse the main and interaction effects of age and sex on variables. The Scheffe post-hoc test was used for comparisons. RESULTS: Boys reported higher daily PA levels than girls across all age groups. The differences was bigger in seven (47,6+6,5 vs 42,6+6,4 MET/day; p<0,001), eight (48,5+8,6 vs 41,9+4,6MET/day; p<0,001) and nine years old (49,8+8,3 vs 44,1+5,9 MET/day; p<0,001). Measured recess PA was higher for boys compared to girls at age nine (28,4+12,0 vs 23.7+11,8 motion counts; p<0,039), ten (28,5+10,8 vs 23,7+10,0 motion counts; p<0,014) and eleven years old (24,6+12,2 vs 20,7+9,3 motion counts; p<0,047). CONCLUSION: Daily PA is higher in boys compared to girls across all age groups (six to eleven years old). On the other hand, recess PA was higher only in boys between nine and eleven years
SN - 1135-5727
AD - Grupo de Investigacion AFIDES, Facultad de Ciencias del Deporte, Universidad de Extremadura, Caceres, Espana. yescgon@unex.es
UR - 22212835
ER -

TY - JOUR
ID - 519
T1 - Food insecurity among Australian children: potential determinants, health and developmental consequences
A1 - Ramsey,R.
A1 - Giskes,K.
A1 - Turrell,G.
A1 - Gallegos,D.
Y1 - 2011/12//
N1 - Ramsey, Rebecca. Giskes, Katrina. Turrell, Gavin. Gallegos, Danielle
Journal of child health care : for professionals working with children in the hospital and community
9806360, dcl
N
Journal Article
English
KW - MEDLINE
KW - Adolescent
KW - Adult
KW - Child
KW - Child Development
KW - Preschool
KW - Female
KW - Food Supply

KW - Health Status Disparities
KW - Health Surveys
KW - Humans
KW - Male
KW - Poverty
KW - Queensland
KW - Urban Population
RP - NOT IN FILE
SP - 401
EP - 416
JF - Journal of Child Health Care
JA - J Child Health Care
VL - 15
IS - 4
CY - England
N2 - Children in food-insecure households may be at risk of poor health, developmental or behavioural problems. This study investigated the associations between food insecurity, potential determinants and health and developmental outcomes among children. Data on household food security, socio-demographic characteristics and children's weight, health and behaviour were collected from households with children aged 3-17 years in socioeconomically disadvantaged suburbs by mail survey using proxy-parental reports (185 households). Data were analysed using logistic regression. Approximately one-in-three households (34%) were food insecure. Low household income was associated with an increased risk of food insecurity [odds ratio (OR), 16.20; 95% confidence interval (CI), 3.52-74.47]. Children with a parent born outside of Australia were less likely to experience food insecurity (OR, 0.42; 95% CI, 0.19-0.93). Children in food-insecure households were more likely to miss days from school or activities (OR, 3.52; 95% CI, 1.46-8.54) and were more likely to have borderline or atypical emotional symptoms (OR, 2.44; 95% CI, 1.11-5.38) or behavioural difficulties (OR, 2.35; 95% CI, 1.04-5.33). Food insecurity may be prevalent among socioeconomically disadvantaged households with children. The potential developmental consequences of food insecurity during childhood may result in serious adverse health and social implications
SN - 1741-2889
AD - Queensland University of Technology, School of Public Health, Brisbane, Australia.
rebecca.ramsey@qut.edu.au
UR - 22199175
ER -

TY - JOUR
ID - 520
T1 - Improving health-related fitness in children: the Fit-4-Fun randomized controlled trial study protocol
A1 - Eather,N.
A1 - Morgan,P.J.
A1 - Lubans,D.R.
Y1 - 2011///
N1 - Eather, Narelle. Morgan, Philip J. Lubans, David R
BMC public health
100968562
IM
Journal Article. Randomized Controlled Trial. Research Support, Non-U.S. Gov't
English
KW - MEDLINE
KW - Adolescent
KW - Child
KW - Exercise/ph [Physiology]
KW - Follow-Up Studies
KW - Health Promotion/mt [Methods]
KW - Humans
KW - New South Wales
KW - Physical Education and Training

KW - Physical Fitness/ph [Physiology]
KW - Program Evaluation
KW - School Health Services/og [Organization & Administration]
RP - NOT IN FILE
SP - 902
JF - BMC Public Health
VL - 11
CY - England
N2 - BACKGROUND: Declining levels of physical fitness in children are linked to an increased risk of developing poor physical and mental health. Physical activity programs for children that involve regular high intensity physical activity, along with muscle and bone strengthening activities, have been identified by the World Health Organisation as a key strategy to reduce the escalating burden of ill health caused by non-communicable diseases. This paper reports the rationale and methods for a school-based intervention designed to improve physical fitness and physical activity levels of Grades 5 and 6 primary school children. METHODS/DESIGN: Fit-4-Fun is an 8-week multi-component school-based health-related fitness education intervention and will be evaluated using a group randomized controlled trial. Primary schools from the Hunter Region in NSW, Australia, will be invited to participate in the program in 2011 with a target sample size of 128 primary schools children (age 10-13). The Fit-4-Fun program is theoretically grounded and will be implemented applying the Health Promoting Schools framework. Students will participate in weekly curriculum-based health and physical education lessons, daily break-time physical activities during recess and lunch, and will complete an 8-week (3 x per week) home activity program with their parents and/or family members. A battery of six health-related fitness assessments, four days of pedometery-assessed physical activity and a questionnaire, will be administered at baseline, immediate post-intervention (2-months) and at 6-months (from baseline) to determine intervention effects. Details of the methodological aspects of recruitment, inclusion criteria, randomization, intervention program, assessments, process evaluation and statistical analyses are described. DISCUSSION: The Fit-4-Fun program is an innovative school-based intervention targeting fitness improvements in primary school children. The program will involve a range of evidence-based behaviour change strategies to promote and support physical activity of adequate intensity, duration and type, needed to improve health-related fitness. TRIAL REGISTRATION NO: Australia and New Zealand Clinical Trials Register (ANZCTR): ACTRN12611000976987
SN - 1471-2458
AD - Priority Research Centre in Physical Activity and Nutrition, School of Education, University of Newcastle, Callaghan Campus, Newcastle, Australia. narelle.eather@newcastle.edu.au
UR - 22142435
ER -

TY - JOUR
ID - 521
T1 - [Asthmatic exacerbations: specific features in children]. [French]
A1 - Carsin,A.
A1 - Pham-Thi,N.
Y1 - 2011/12//
N1 - Carsin, A. Pham-Thi, N
Revue des maladies respiratoires
rz9, 8408032
IM
English Abstract. Journal Article
French
KW - MEDLINE
KW - Age Factors
KW - Air Pollution/ae [Adverse Effects]
KW - Air Pollution
KW - Indoor/ae [Adverse Effects]
KW - Allergens/ae [Adverse Effects]
KW - Anti-Asthmatic Agents/tu [Therapeutic Use]
KW - Asthma/di [Diagnosis]
KW - Asthma/pp [Physiopathology]

KW - Asthma/pc [Prevention & Control]
KW - Biological Markers
KW - Breath Tests
KW - Child
KW - Cross Infection/co [Complications]
KW - Humans
KW - Nitric Oxide/an [Analysis]
KW - Nutritional Status
KW - Patient Education as Topic
KW - Respiratory Hypersensitivity/co [Complications]
KW - Respiratory Tract Infections/co [Complications]
KW - Risk Factors
KW - Sputum/ch [Chemistry]
KW - Tobacco Smoke Pollution/ae [Adverse Effects]
KW - Virus Diseases/co [Complications]
RP - NOT IN FILE
SP - 1322
EP - 1328
JF - Revue des Maladies Respiratoires
JA - Rev Mal Respir
VL - 28
IS - 10
CY - France
N2 - Asthma concerns more than 10% of 10-year-old children. Despite the similarities between adult and childhood asthma, the pediatric population presents some specific characteristics, notably in relation to exacerbations. Asthma in the newborn infant is a specific entity, the definition of which has recently been officially recognized. In exacerbations, the most important trigger factors are respiratory virus infections, the strain having prognostic importance. The indoor and outdoor environments are risk factors, particularly high levels of atmospheric pollution. Nutrients seem to play a prognostic role through vitamin D or food allergy. Measurement of exhaled nitric oxide and examination of induced sputum may help in diagnosis and adjustment of treatment but these tools are not yet effective as predictive factors in asthma exacerbations. Prevention, early management and continued education of children and their families remain the best methods to improve asthma control. Copyright 2011 Elsevier Masson SAS. All rights reserved
SN - 1776-2588
AD - Unite de pneumologie pediatrique, CHU Timone-Enfants, 264 rue Saint-Pierre, Marseille cedex 05, France
UR - 22152939
ER -

TY - JOUR
ID - 522
T1 - Is the environment near home and school associated with physical activity and adiposity of urban preschool children?
A1 - Lovasi,G.S.
A1 - Jacobson,J.S.
A1 - Quinn,J.W.
A1 - Neckerman,K.M.
A1 - shby-Thompson,M.N.
A1 - Rundle,A.
Y1 - 2011/12//
N1 - Lovasi, Gina S. Jacobson, Judith S. Quinn, James W. Neckerman, Kathryn M. Ashby-Thompson, Maxine N. Rundle, Andrew
Journal of urban health : bulletin of the New York Academy of Medicine
c5l, 9809909
IM
Journal Article. Research Support, N.I.H., Extramural. Research Support, Non-U.S. Gov't
English

KW - MEDLINE
KW - Accidents
KW - Traffic/sn [Statistics & Numerical Data]
KW - Adiposity
KW - Body Weights and Measures
KW - Child
KW - Preschool
KW - Cross-Sectional Studies
KW - Environment
KW - Environment Design
KW - Exercise
KW - Female
KW - Humans
KW - Male
KW - New York City/ep [Epidemiology]
KW - Overweight/ep [Epidemiology]
KW - Poverty Areas
KW - Residence Characteristics/sn [Statistics & Numerical Data]
KW - Schools/sn [Statistics & Numerical Data]
KW - Socioeconomic Factors
KW - Urban Population/sn [Statistics & Numerical Data]
KW - Walking/sn [Statistics & Numerical Data]
RP - NOT IN FILE
SP - 1143
EP - 1157
JF - Journal of Urban Health
JA - J Urban Health
VL - 88
IS - 6
CY - United States
N2 - Preventing sedentary behavior and adiposity in childhood has become a public health priority. We examined urban social and built environment characteristics as correlates of physical activity and anthropometry among 428 preschool children from low-income families in New York City. We measured the children's height, weight, skinfold thicknesses, physical activity by accelerometer, and covariates. We geocoded home and Head Start center addresses and estimated the following for an area within 0.5 km of those two locations using a detailed geographic database: neighborhood composition, walkability, crime and traffic safety, and aesthetic characteristics. Generalized estimating equations were used to examine the associations of area characteristics with physical activity or adiposity, adjusted for characteristics of the child, mother, and home. Participants were 2-5 years old, 53% female, 83% Hispanic, and 43% either overweight or obese. Of the walkability indicators, land use mix was associated with physical activity (26 more activity counts/minute per standard deviation increase in mixed land use, p=0.015) and subway stop density was associated with adiposity (1.2 mm smaller sums of skinfold thicknesses sum per standard deviation increase in subway stop density, p=0.001). The pedestrian-auto injury rate, an indicator of traffic safety problems, was associated with physical activity and adiposity (16 fewer activity counts/minute, p=0.033, and 1.0 mm greater skinfold thickness per standard deviation increase in pedestrian-auto injuries, p=0.018). Children living in areas with more street trees were more physically active and those living in areas with more park access had smaller skinfolds. However, many of the tested associations were not statistically significant and some trends were not in the hypothesized direction. Efforts to enhance walkability, safety, and green spaces in the local environment may be relevant to physical activity and adiposity, and therefore to the health of preschool-aged children from low-income families
SN - 1468-2869
AD - Department of Epidemiology, Mailman School of Public Health, Columbia University, New York, NY, USA. gl2225@columbia.edu
UR - 21826583
ER -

TY - JOUR
ID - 523

T1 - Pattern of childhood falls in a low-income setting: a cross-sectional study in Dar es Salaam
A1 - Kamala,B.
A1 - Wilson,M.L.
A1 - Hasselberg,M.
Y1 - 2011/12//
N1 - Kamala, Benjamin. Wilson, Michael L. Hasselberg, Marie
International journal of injury control and safety promotion
101247254
IM
Journal Article. Research Support, Non-U.S. Gov't
English
KW - MEDLINE
KW - Accidental Falls/sn [Statistics & Numerical Data]
KW - Adolescent
KW - Africa South of the Sahara/ep [Epidemiology]
KW - Child
KW - Child Welfare/sn [Statistics & Numerical Data]
KW - Preschool
KW - Cross-Sectional Studies
KW - Female
KW - Health Surveys
KW - Humans
KW - Incidence
KW - Infant
KW - Newborn
KW - Logistic Models
KW - Male
KW - Poverty/sn [Statistics & Numerical Data]
KW - Public Health
KW - Questionnaires
KW - Residence Characteristics/sn [Statistics & Numerical Data]
KW - Risk Assessment
KW - Risk Factors
KW - Sex Factors
KW - Socioeconomic Factors
KW - Sudan/ep [Epidemiology]
RP - NOT IN FILE
SP - 305
EP - 311
JF - International Journal of Injury Control & Safety Promotion
JA - Int J Inj Contr Saf Promot
VL - 18
IS - 4
CY - England
N2 - The objective was to determine patterns and circumstances of childhood falls in a low-income setting in Dar es Salaam, Tanzania. This cross sectional study is based on a household survey conducted in July 2009. A total of 3927 children up to age 18 from 1928 households in 15 sampled wards were surveyed through a structured questionnaire. The current study includes information regarding fall occurrence, socio-demographic and economic factors. Data were analysed using chi-square, t-test and logistic regression. Male children had 42% higher odds of falls compared to females, and rural residents had more than two times higher odds compared to urban residents. Falls occurred three times more among age group 1-4 and two times more among age group 5-9 compared to those between 15 and 18 years. Most falls occurred outdoors (62%) while playing (51%) with boys being over-represented. Females and children aged 1-4 years fell more from stairs whereas most infants fell from furniture. Male gender, younger age groups and rural residence were significant factors for fall injuries. The circumstances in which these falls occur also differ significantly. Intervention efforts should emphasise these patterns
SN - 1745-7319

AD - Department of Public Health, Division of Global Health/IHCAR, Karolinska Institute, Stockholm, Sweden. benjamin.kamala.182@student.ki.se
UR - 21660795
ER -

TY - JOUR
ID - 524
T1 - Sociocultural and psychological determinants in migrants for noncompliance with occlusion therapy for amblyopia
A1 - Tjiam,A.M.
A1 - Akcan,H.
A1 - Ziylan,F.
A1 - Vukovic,E.
A1 - Loudon,S.E.
A1 - Looman,C.W.
A1 - Passchier,J.
A1 - Simonsz,H.J.
Y1 - 2011/12//
N1 - Tjiam, Angela M. Akcan, Hilal. Ziylan, Fatma. Vukovic, Elizabet. Loudon, Sjoukje E. Looman, Caspar W N. Passchier, Jan. Simonsz, Huibert J
Graefe's archive for clinical and experimental ophthalmology = Albrecht von Graefes Archiv fur klinische und experimentelle Ophthalmologie
fpr, 8205248
IM
Journal Article. Research Support, Non-U.S. Gov't
English
KW - MEDLINE
KW - Amblyopia/eh [Ethnology]
KW - Amblyopia/th [Therapy]
KW - Child
KW - Preschool
KW - Cultural Characteristics
KW - Emigrants and Immigrants/px [Psychology]
KW - Ethnic Groups
KW - Health Services/ut [Utilization]
KW - Humans
KW - Language
KW - Netherlands
KW - Orthoptics/mt [Methods]
KW - Parents/px [Psychology]
KW - Patient Compliance/px [Psychology]
KW - Questionnaires
KW - Sensory Deprivation
KW - Social Class
RP - NOT IN FILE
SP - 1893
EP - 1899
JF - Graefes Archive for Clinical & Experimental Ophthalmology
JA - Graefes Arch Clin Exp Ophthalmol
VL - 249
IS - 12
CY - Germany
N2 - BACKGROUND: Compliance with occlusion therapy for amblyopia in children is low when their parents have a low level of education, speak Dutch poorly, or originate from another country. We determined how sociocultural and psychological determinants affect compliance. METHODS: Included were amblyopic children between the ages of 3 and 6, living in low socio-economic status (SES) areas. Compliance with occlusion therapy was measured electronically. Their parents completed an oral questionnaire, based on the "Social

Position & Use of Social Services by Migrants and Natives" questionnaire that included demographics and questions on issues like education, employment, religion and social contacts. Parental fluency in Dutch was rated on a five-point scale. Regression analysis was used to describe the relationship between the level of compliance and sociocultural and psychological determinants. RESULTS: Data from 45 children and their parents were analyzed. Mean electronically measured compliance was 56+44 percent. Children whose parents had close contact with their neighbors or who were highly dependent on their family demonstrated low levels of compliance. Children of parents who were members of a club and who had positive conceptualizations of Dutch society showed high levels of compliance. Poor compliance was also associated with low income, depression, and when patching interfered with the child's outdoor activity. Religion was not associated with compliance. CONCLUSIONS: Poor compliance with occlusion therapy seems correlated with indicators of social cohesion. High social cohesion at micro level, i.e., family, neighbors and friends, and low social cohesion on macro level, i.e., Dutch society, are associated with noncompliance. However, such parents tend to speak Dutch poorly, so it is difficult to determine its actual cause
SN - 1435-702X
AD - Department of Ophthalmology, Room EE-1607, Erasmus MC, University Medical Center Rotterdam, PO Box 2040, 3000 CA, Rotterdam, The Netherlands. amtjiam@gmail.com
UR - 21399941
ER -

TY - JOUR
ID - 525
T1 - [Seasonal variation in the occurrence of rhegmatogenous retinal detachment at the beginning of the 21st century. Study results and literature review]. [Review] [German]
A1 - Bertelmann,T.
A1 - Cronauer,M.
A1 - Stoffelns,B.
A1 - Sekundo,W.
Y1 - 2011/12//
N1 - Bertelmann, T. Cronauer, M. Stoffelns, B. Sekundo, W
Der Ophthalmologe : Zeitschrift der Deutschen Ophthalmologischen Gesellschaft
bgv, 9206148
IM
English Abstract. Journal Article. Meta-Analysis. Review
German
KW - MEDLINE
KW - Adolescent
KW - Adult
KW - Aged
KW - 80 and over
KW - Child
KW - Female
KW - Germany/ep [Epidemiology]
KW - Humans
KW - Life Style
KW - Male
KW - Middle Aged
KW - Prevalence
KW - Retinal Detachment/ep [Epidemiology]
KW - Risk Assessment
KW - Risk Factors
KW - Seasons
KW - Young Adult
RP - NOT IN FILE
SP - 1155
EP - 1163
JF - Ophthalmologe
VL - 108

IS - 12
CY - Germany
N2 - AIM: The present study aims to ascertain whether there are seasonal variations in the occurrence of rhegmatogenous retinal detachment at the beginning of the 21st century, compare these results with seasonal variations observed in the literature for the 20th century, and determine whether current general lifestyle changes in terms of the increased use of visual media (TV, PC) and a decrease in outdoor activity influence seasonal patterns of rhegmatogenous retinal detachment. METHOD: A quantitative retrospective analysis of 2605 surgical protocols of retinal detachment repair procedures performed at the eye clinic of the Johannes Gutenberg University, Mainz, Germany, between 1998 and 2008 was carried out. RESULTS: Rhegmatogenous retinal detachment was diagnosed in 1490 cases and, as such, was the most frequently occurring type of retinal detachment during the selected period of time (p=0.001). A seasonal distribution was detected with statistically significant maximum and minimum incidences in July and October, respectively, (p=0.008). These results are consistent with the findings of large studies conducted in the 20(th) century that observed a maximum in the summer months and a minimum in the winter months. Furthermore, in the period between 2003 and 2008 significantly more rhegmatogenous retinal detachments were observed than from 1998 to 2002 (p=0.001). During school holiday periods 1.43% fewer rhegmatogenous retinal detachments were observed than had been estimated statistically. The increased number of hours of sunlight and increased average monthly temperatures had no significant impact on the occurrence of rhegmatogenous retinal detachment. CONCLUSION: Current changes in lifestyle in terms of the increased use of visual media and a decrease in outdoor activity do not appear to have influenced the seasonal variation in rhegmatogenous retinal detachment. Factors other than light intensity and average temperature that have not yet been evaluated appear to be the cause of these seasonal variations
SN - 1433-0423
AD - Augenklinik des UKGM GmbH, Standort Marburg, Baldingerstr., 35041, Marburg, Deutschland. thomas.bertelmann@staff.uni-marburg.de
UR - 22130684
ER -

TY - JOUR
ID - 526
T1 - Promoting fundamental movement skill development and physical activity in early childhood settings: a cluster randomized controlled trial
A1 - Jones,R.A.
A1 - Riethmuller,A.
A1 - Hesketh,K.
A1 - Trezise,J.
A1 - Batterham,M.
A1 - Okely,A.D.
Y1 - 2011/11//
N1 - Jones, Rachel A. Riethmuller, Annaleise. Hesketh, Kylie. Trezise, Jillian. Batterham, Marijka. Okely, Anthony D
Pediatric exercise science
8909729
IM
Journal Article. Randomized Controlled Trial. Research Support, Non-U.S. Gov't
English
KW - MEDLINE
KW - Body Mass Index
KW - Child
KW - Preschool
KW - Faculty
KW - Feasibility Studies
KW - Female
KW - Health Promotion/mt [Methods]
KW - Humans
KW - Male
KW - Motor Activity

KW - Motor Skills
KW - Physical Education and Training/mt [Methods]
KW - Pilot Projects
KW - Videotape Recording
RP - NOT IN FILE
SP - 600
EP - 615
JF - Pediatric Exercise Science
JA - Pediatr Exerc Sci
VL - 23
IS - 4
CY - United States
N2 - The aim of this study was to assess the feasibility, acceptability and potential efficacy of a physical activity program for preschool children. A 20-week, 2-arm parallel cluster randomized controlled pilot trial was conducted. The intervention comprised structured activities for children and professional development for staff. The control group participated in usual care activities, which included designated inside and outside playtime. Primary outcomes were movement skill development and objectively measured physical activity. At follow-up, compared with children in the control group, children in the intervention group showed greater improvements in movement skill proficiency, with this improvement statically significant for overall movement skill development (adjust diff. = 2.08, 95% CI 0.76, 3.40; Cohen's d = 0.47) and significantly greater increases in objectively measured physical activity (counts per minute) during the preschool day (adjust diff. = 110.5, 95% CI 33.6, 187.3; Cohen's d = 0.46). This study demonstrates that a physical activity program implemented by staff within a preschool setting is feasible, acceptable and potentially efficacious
SN - 1543-2920
AD - University of Wollongong, Wollongong, NSW, Australia
UR - 22109783
ER -

TY - JOUR
ID - 527
T1 - Assessing children's physical activity behaviors at recess: a multi-method approach
A1 - Saint-Maurice,P.F.
A1 - Welk,G.J.
A1 - Silva,P.
A1 - Siahpush,M.
A1 - Huberty,J.
Y1 - 2011/11//
N1 - Saint-Maurice, Pedro F. Welk, Gregory J. Silva, Pedro. Siahpush, Mohammad. Huberty, Jennifer
Pediatric exercise science
8909729
IM
Journal Article
English
KW - MEDLINE
KW - Analysis of Variance
KW - Child
KW - Environment Design
KW - Female
KW - Health Behavior
KW - Humans
KW - Male
KW - Monitoring
KW - Ambulatory
KW - Motor Activity
KW - Observation
KW - Play and Playthings
KW - Schools

KW - Seasons
KW - Sex Factors
RP - NOT IN FILE
SP - 585
EP - 599
JF - Pediatric Exercise Science
JA - Pediatr Exerc Sci
VL - 23
IS - 4
CY - United States
N2 - To better understand and promote youth physical activity (PA) it is important to determine settings and characteristics that promote or influence behavior. This study evaluated the utility of a multi-method approach (accelerometers plus direct observation) to better understand youth PA at recess. A total of 100 third through fifth grade children (52 males and 48 females) wore an Actigraph accelerometer during school recess for five consecutive days in both Fall and Spring. Trained observers coded PA behaviors at the same recess periods using the System for Observing Play and Leisure Activities (SOPLAY). Overall, gender comparisons based on both instruments indicated that boys were more active than girls. MVPA levels were higher during climbing/sliding activities (40-50%) and when the activity setting was supervised and equipped (30%). Both assessments indicated that boys were more active but the contextual data from the SOPLAY indicate that differences may vary according to the environmental context
SN - 1543-2920
AD - Dept. of Kinesiology, Iowa State University, Ames, IA, USA
UR - 22109782
ER -

TY - JOUR
ID - 528
T1 - Personal and ambient exposures to air toxics in Camden, New Jersey
A1 - Lioy,P.J.
A1 - Fan,Z.
A1 - Zhang,J.
A1 - Georgopoulos,P.
A1 - Wang,S.W.
A1 - Ohman-Strickland,P.
A1 - Wu,X.
A1 - Zhu,X.
A1 - Harrington,J.
A1 - Tang,X.
A1 - Meng,Q.
A1 - Jung,K.H.
A1 - Kwon,J.
A1 - Hernandez,M.
A1 - Bonnano,L.
A1 - Held,J.
A1 - Neal,J.
A1 - HEI Health Review Committee.
Y1 - 2011/08//
N1 - Lioy, Paul J. Fan, Zhihua. Zhang, Junfeng. Georgopoulos, Panos. Wang, Sheng-Wei. Ohman-Strickland, Pamela. Wu, Xiangmei. Zhu, Xianlei. Harrington, Jason. Tang, Xiaogang. Meng, Qingyu. Jung, Kyung Hwa. Kwon, Jaymin. Hernandez, Marta. Bonnano, Linda. Held, Joann. Neal, John. HEI Health Review Committee
Research report (Health Effects Institute)
8812230, ah8, 8812230
IM
Journal Article. Research Support, Non-U.S. Gov't. Research Support, U.S. Gov't, Non-P.H.S.
English
KW - MEDLINE
KW - Adolescent

KW - Adult
KW - Aged
KW - 80 and over
KW - Air Pollutants/an [Analysis]
KW - Aldehydes/an [Analysis]
KW - Case-Control Studies
KW - Child
KW - Environmental Exposure/an [Analysis]
KW - Environmental Monitoring/mt [Methods]
KW - Female
KW - Hazardous Substances/an [Analysis]
KW - Health Surveys
KW - Humans
KW - Linear Models
KW - Male
KW - Middle Aged
KW - New Jersey
KW - Particulate Matter/an [Analysis]
KW - Polycyclic Hydrocarbons
KW - Aromatic/an [Analysis]
KW - Poverty Areas
KW - Small-Area Analysis
KW - Volatile Organic Compounds/an [Analysis]
RP - NOT IN FILE
SP - 3
EP - 127
JF - Research Report - Health Effects Institute
JA - Res Rep Health Eff.Inst
IS - 160
CY - United States
N2 - Personal exposures and ambient concentrations of air toxics were characterized in a pollution "hot spot" and an urban reference site, both in Camden, New Jersey. The hot spot was the city's Waterfront South neighborhood; the reference site was a neighborhood, about 1 km to the east, around the intersection of Copewood and Davis streets. Using personal exposure measurements, residential ambient air measurements, statistical analyses, and exposure modeling, we examined the impact of local industrial and mobile pollution sources, particularly diesel trucks, on personal exposures and ambient concentrations in the two neighborhoods. Presented in the report are details of our study design, sample and data collection methods, data- and model-analysis approaches, and results and key findings of the study. In summary, 107 participants were recruited from nonsmoking households, including 54 from Waterfront South and 53 from the Copewood-Davis area. Personal air samples were collected for 24 hr and measured for 32 target compounds--11 volatile organic compounds (VOCs*), four aldehydes, 16 polycyclic aromatic hydrocarbons (PAHs), and particulate matter (PM) with an aerodynamic diameter < or = 2.5 microm (PM2.5). Simultaneously with the personal monitoring, ambient concentrations of the target compounds were measured at two fixed monitoring sites, one each in the Waterfront South and Copewood-Davis neighborhoods. To understand the potential impact of local sources of air toxics on personal exposures caused by temporal (weekdays versus weekend days) and seasonal (summer versus winter) variations in source intensities of the air toxics, four measurements were made of each subject, two in summer and two in winter. Within each season, one measurement was made on a weekday and the other on a weekend day. A baseline questionnaire and a time diary with an activity questionnaire were administered to each participant in order to obtain information that could be used to understand personal exposure to specific air toxics measured during each sampling period. Given the number of emission sources of air toxics in Waterfront South, a spatial variation study consisting of three saturation-sampling campaigns was conducted to characterize the spatial distribution of VOCs and aldehydes in the two neighborhoods. Passive samplers were used to collect VOC and aldehyde samples for 24- and 48-hr sampling periods simultaneously at 22 and 16 grid-based sampling sites in Waterfront South and Copewood-Davis, respectively. Results showed that measured ambient concentrations of some target pollutants (mean +/- standard deviation [SD]), such as PM2.5 (31.3 +/- 12.5 microg/m3), toluene (4.24 +/- 5.23 microg/m3), and benzo[a]pyrene (0.36 +/- 0.45 ng/m3), were significantly higher (P < 0.05) in Waterfront South than in Copewood-Davis, where the concentrations of PM2.5, toluene,

and benzo[a]pyrene were 25.3 +/- 11.9 microg/m3, 2.46 +/- 3.19 microg/m3, and 0.21 +/- 0.26 ng/m3, respectively. High concentrations of specific air toxics, such as 60 microg/m3 for toluene and 159 microg/m3 for methyl tert-butyl ether (MTBE), were also found in areas close to local stationary sources in Waterfront South during the saturation-sampling campaigns. Greater spatial variation in benzene, toluene, ethylbenzene, and xylenes (known collectively as BTEX) as well as of MTBE was observed in Waterfront South than in Copewood-Davis during days with low wind speed. These observations indicated the significant impact of local emission sources of these pollutants and possibly of other pollutants emitted by individual source types on air pollution in Waterfront South. (Waterfront South is a known hot spot for these pollutants.) There were no significant differences between Waterfront South and Copewood-Davis in mean concentrations of benzene or MTBE, although some stationary sources of the two compounds have been reported in Waterfront South. Further, a good correlation (R > 0.6) was found between benzene and MTBE in both locations. These results suggest that automobile exhausts were the main contributors to benzene and MTBE air pollution in both neighborhoods. Formaldehyde and acetaldehyde concentrations were found to be high in both neighborhoods. Mean (+/- SD) concentrations of formaldehyde were 20.2 +/- 19.5 microg/m3 in Waterfront South and 24.8 +/- 20.8 microg/m3 in Copewood-Davis. A similar trend was observed for the two compounds during the saturation-sampling campaigns. The results indicate that mobile sources (i.e., diesel trucks) had a large impact on formaldehyde and acetaldehyde concentrations in both neighborhoods and that both are aldehyde hot spots. The study also showed that PM2.5, aldehydes, BTEX, and MTBE concentrations in both Waterfront South and Copewood-Davis were higher than ambient background concentrations in New Jersey and than national average concentrations, indicating that both neighborhoods are in fact hot spots for these pollutants. Higher concentrations were observed on weekdays than on weekend days for several compounds, including toluene, ethylbenzene, and xylenes (known collectively as TEX) as well as PAHs and PM2.5. These observations showed the impact on ambient air pollution of higher traffic volumes and more active industrial and commercial operations in the study areas on weekdays. Seasonal variations differed by species. Concentrations of TEX, for example, were found to be higher in winter than in summer in both locations, possibly because of higher emission rates from automobiles and reduced photochemical reactivity in winter. In contrast, concentrations of MTBE were found to be significantly higher in summer than in winter in both locations, possibly because of higher evaporation rates from gasoline in summer. Similarly, concentrations of heavier PAHs, such as benzo[a]pyrene, were found to be higher in winter in both locations, possibly because of higher emission rates from mobile sources, the use of home heating, and the reduced photochemical reactivity of benzo[a]pyrene in winter. In contrast, concentrations of lighter PAHs were found to be higher in summer in both locations, possibly because of volatilization of these compounds from various surfaces in summer. In addition, higher concentrations of formaldehyde were observed in summer than in winter, possibly because of significant contributions from photochemical reactions to formaldehyde air pollution in summer. Personal concentrations of toluene (25.4 +/- 13.5 microg/m3) and acrolein (1.78 +/- 3.7 microg/m3) in Waterfront South were found to be higher than those in the Copewood-Davis neighborhood (13.1 +/- 15.3 microg/m3 for toluene and 1.27 +/- 2.36 microg/m3 for acrolein). However, personal concentrations for most of the other compounds measured in Waterfront South were found to be similar to or lower than those than in Copewood-Davis. (For example, mean +/- SD concentrations were 4.58 +/- 17.3 microg/m3 for benzene, 4.06 +/- 5.32 microg/m3 for MTBE, 16.8 +/- 15.5 microg/m3 for formaldehyde, and 0.40 +/- 0.94 ng/m3 for benzo[a]pyrene in Waterfront South and 9.19 +/- 34.0 microg/m3 for benzene, 6.22 +/- 19.0 microg/m3 for MTBE, 16.0 +/- 16.7 microg/m3 for formaldehyde, and 0.42 +/- 1.08 ng/m3 for benzo[a]pyrene in Copewood-Davis.) This was probably because many of the target compounds had both outdoor and indoor sources. The higher personal concentrations of these compounds in Copewood-Davis might have resulted in part from higher exposure to environmental tobacco smoke (ETS) of subjects from Copewood-Davis. The Spearman correlation coefficient (R) was found to be high for pollutants with significant outdoor sources. The R's for MTBE and carbon tetrachloride, for example, were > 0.65 in both Waterfront South and Copewood-Davis. The R's were moderate or low (0.3-0.6) for compounds with both outdoor and indoor sources, such as BTEX and formaldehyde. A weaker association (R < 0.5) was found for compounds with significant indoor sources, such as BTEX, formaldehyde, PAHs, and PM2.5. The correlations between personal and ambient concentrations of MTBE and BTEX were found to be stronger in Waterfront South than in Copewood-Davis, reflecting the significant impact of local air pollution sources on personal exposure to these pollutants in Waterfront South. Emission-based ambient concentrations of benzene, toluene, and formaldehyde and contributions of ambient exposure to personal concentrations of these three compounds were modeled using atmospheric dispersion modeling and Individual Based Exposure Modeling (IBEM) software, respectively, which were coupled for analysis in the Modeling Environment for Total Risk (MENTOR) system. The compounds were associated with the three types of dominant sources in the two neighborhoods: industrial sources (toluene), exhaust from gasoline-powered motor vehicles (benzene), and

exhaust from diesel-powered motor vehicles (formaldehyde). Subsequently, both the calculated and measured ambient concentrations of each of the three compounds were separately combined with the time diaries and activity questionnaires completed by the subjects as inputs to IBEM-MENTOR for estimating personal exposures from ambient sources. Modeled ambient concentrations of benzene and toluene were generally in agreement with the measured ambient concentrations within a factor of two, but the values were underestimated at the high-end percentiles. The major local (neighborhood) contributors to ambient benzene concentrations were from mobile sources in the study areas; both mobile and stationary (point and area) sources contributed to the ambient toluene concentrations. This finding can be used as guidance for developing better emission inventories to characterize, through modeling, the ambient concentrations of air toxics in the study areas. (ABSTRACT TRUNCATED)
SN - 1041-5505
AD - Environmental and Occupational Health Sciences Institute, Robert Wood Johnson Medical School, Piscataway, New Jersey 08854, USA
UR - 22097188
ER -

TY - JOUR
ID - 529
T1 - Monte Carlo simulations of the electric field close to the body in realistic environments for application in personal radiofrequency dosimetry
A1 - Iskra,S.
A1 - McKenzie,R.
A1 - Cosic,I.
Y1 - 2011/11//
N1 - Iskra, S. McKenzie, R. Cosic, I
Radiation protection dosimetry
d4z, 8109958
IM
Comparative Study. Journal Article
English
KW - MEDLINE
KW - Adult
KW - Body Burden
KW - Child
KW - Computer Simulation
KW - Electromagnetic Fields
KW - Environmental Exposure/an [Analysis]
KW - Humans
KW - Models
KW - Biological
KW - Monte Carlo Method
KW - Radiation Monitoring/is [Instrumentation]
KW - Radiation Monitoring/mt [Methods]
KW - Relative Biological Effectiveness
KW - Uncertainty
RP - NOT IN FILE
SP - 517
EP - 527
JF - Radiation Protection Dosimetry
JA - Radiat Prot.Dosimetry
VL - 147
IS - 4
CY - England
N2 - Personal dosemeters can play an important role in epidemiological studies and in radiofrequency safety programmes. In this study, a Monte Carlo approach is used in conjunction with the finite difference time domain method to obtain distributions of the electric field strength close to a human body model in simulated realistic environments. The field is a proxy for the response of an ideal body-worn electric field dosemeter. A set of eight

environments were modelled based on the statistics of Rayleigh, Rice and log-normal fading to simulate outdoor and indoor multipath exposures at 450, 900 and 2100 MHz. Results indicate that a dosemeter mounted randomly within 10-50 mm of the adult or child body model (torso region) will on average underestimate the spatially averaged value of the incident electric field strength by a factor of 0.52 to 0.74 over the frequencies of 450, 900 and 2100 MHz. The uncertainty in results, assessed at the 95 % confidence level (between the 2.5th and 97.5th percentiles) was largest at 2100 MHz and smallest at 450 MHz
SN - 1742-3406
AD - School of Electrical and Computer Engineering, RMIT University, 3000 Melbourne, Australia. S9609898@student.rmit.edu.au
UR - 21242165
ER -

TY - JOUR
ID - 530
T1 - Desperately seeking diarrhoea: outbreak of haemolytic uraemic syndrome caused by emerging sorbitol-fermenting shiga toxin-producing Escherichia coli O157:H-, Germany, 2009
A1 - Nielsen,S.
A1 - Frank,C.
A1 - Fruth,A.
A1 - Spode,A.
A1 - Prager,R.
A1 - Graff,A.
A1 - Plenge-Bonig,A.
A1 - Loos,S.
A1 - Lutgehetmann,M.
A1 - Kemper,M.J.
A1 - Muller-Wiefel,D.E.
A1 - Werber,D.
Y1 - 2011/12//
N1 - Nielsen, S. Frank, C. Fruth, A. Spode, A. Prager, R. Graff, A. Plenge-Bonig, A. Loos, S. Lutgehetmann, M. Kemper, M J. Muller-Wiefel, D E. Werber, D
Zoonoses and public health
101300786
IM
Journal Article
English
KW - MEDLINE
KW - Child
KW - Preschool
KW - Diarrhea/co [Complications]
KW - Diarrhea/ep [Epidemiology]
KW - Diarrhea/mi [Microbiology]
KW - Disease Outbreaks
KW - Environmental Exposure/ae [Adverse Effects]
KW - Escherichia coli Infections/co [Complications]
KW - Escherichia coli Infections/ep [Epidemiology]
KW - Escherichia coli O157/ip [Isolation & Purification]
KW - Escherichia coli O157/py [Pathogenicity]
KW - Escherichia coli O157
KW - Feces/mi [Microbiology]
KW - Female
KW - Germany/ep [Epidemiology]
KW - Hemolytic-Uremic Syndrome/co [Complications]
KW - Hemolytic-Uremic Syndrome/ep [Epidemiology]
KW - Hemolytic-Uremic Syndrome/mi [Microbiology]
KW - Humans
KW - Infant

KW - Interviews as Topic
KW - Male
KW - Play and Playthings
KW - Risk Factors
KW - Shiga-Toxigenic Escherichia coli
KW - Sorbitol/me [Metabolism]
RP - NOT IN FILE
SP - 567
EP - 572
JF - Zoonoses & Public Health
JA - Zoonoses Public Health
VL - 58
IS - 8
CY - Germany
N2 - Infection with sorbitol-fermenting Shiga toxin-producing Escherichia coli O157:H- (sf STEC O157:H-) is rare, but emerging in Europe. The pathogen is typically isolated from paediatric patients with life-threatening haemolytic uraemic syndrome (HUS). It is unclear whether this observation primarily reflects the pathogen's virulence or its complex laboratory diagnosis, not routinely conducted in diarrhoeal patients. In summer 2009, four boys living in the same suburb in Germany developed diarrhoea-associated HUS: three were infected by sf STEC O157:H- and one died. We conducted two analytical epidemiological studies, an extensive search for diarrhoeal cases in potentially exposed groups, and an environmental investigation. Outbreak cases were residents of the suburb diagnosed with HUS, sf STEC O157:H- infection, or both between 24 July 2009 and 25 August 2009. Overall, we ascertained eight cases with a median age of 4 years (range: from 8 months to 9 years). Stool screening of 220 persons led to the identification of only four additional cases: two asymptomatic carriers and two diarrhoeal cases. HUS was strongly associated with visiting a local playground in July, particularly on 16th July (odds ratio = 42.7, P = 0.002). No other commonality, including food, was identified, and all environmental samples (n = 24) were negative. In this localized non-foodborne outbreak, the place of likely infection was a local playground. Sf STEC O157:H- infection apparently limits itself rarely to diarrhoeal illness and progresses frequently to HUS. Therefore, detection of and response to this hypervirulent pathogen primarily relies on HUS surveillance. 2011 Blackwell Verlag GmbH
SN - 1863-2378
AD - European Programme for Intervention Epidemiology Training (EPIET), European Centre for Disease Prevention and Control (ECDC), Stockholm, Sweden. nielsens@rki.de
UR - 21824358
ER -

TY - JOUR
ID - 531
T1 - Psychometric properties of a Chinese version of the Home Environment Measure for Motor Development
A1 - Hsieh,Y.H.
A1 - Hwang,A.W.
A1 - Liao,H.F.
A1 - Chen,P.C.
A1 - Hsieh,W.S.
A1 - Chu,P.Y.
Y1 - 2011///
N1 - Hsieh, Yu-Hsin. Hwang, Ai-Wen. Liao, Hua-Fang. Chen, Pau-Chung. Hsieh, Wu-Shiun. Chu, Pei-Yi
Disability and rehabilitation
9207179, a8i
IM
Journal Article. Research Support, Non-U.S. Gov't
English
KW - MEDLINE
KW - Child Development
KW - Child
KW - Preschool
KW - China

KW - Developmental Disabilities/rh [Rehabilitation]
KW - Environment
KW - Female
KW - Housing
KW - Humans
KW - Infant
KW - Male
KW - Motor Skills
KW - Play and Playthings
KW - Psychometrics
KW - Reproducibility of Results
RP - NOT IN FILE
SP - 2454
EP - 2463
JF - Disability & Rehabilitation
JA - Disabil Rehabil
VL - 33
IS - 25-26
CY - England
N2 - PURPOSE: This study examined the psychometric properties of the Chinese version of the Affordance in the Home Environment for Motor Development - Toddler version (AHEMD-Toddler-C) for children developing typically (DT) or having motor delays (MD). METHODS: This was a methodology study. Parent-child dyads with DT (n = 106, mean age of 27.9 months) and with MD (n = 45, 23.6 months) were enrolled. For test-retest reliability, parents completed AHEMD-Toddler-C twice within 2 weeks. For convergent validity, correlations were analysed between AHEMD-Toddler-C and Home Observation for Measurement of the Environment Inventory (HOME), and between AHEMD-Toddler-C and family variables. RESULTS: Test-retest reliabilities for AHEMD-Toddler-C were adequate except for Variety of Stimulation (VS) subscale. For convergent validity, the correlation coefficients between AHEMD and HOME were 0.44 (p <0.05). Two subscales of motor toys of AHEMD demonstrated convergent validity with Learning Material subscale of HOME and some family variables in children with MD. Inside Space subscale of AHEMD correlated with family variables. Outside Space (OS) subscale of AHEMD was not significantly correlated with HOME or family variables. CONCLUSION: AHEMD-Toddler-C is a new measure option to explore the relationships between home environment and motor development in Chinese-speaking countries. Nevertheless, VS and OS subscales should be used cautiously
SN - 1464-5165
AD - Taitung Center of Chinese Association of Early Intervention Program for Children with Developmental Delays, Taitung county, Taiwan
UR - 21529124
ER -

TY - JOUR
ID - 532
T1 - Instant Recess: a practical tool for increasing physical activity during the school day
A1 - Whitt-Glover,M.C.
A1 - Ham,S.A.
A1 - Yancey,A.K.
Y1 - 2011///
N1 - Whitt-Glover, Melicia C. Ham, Sandra A. Yancey, Antronette K
Progress in community health partnerships : research, education, and action
101273946
IM
Journal Article. Randomized Controlled Trial
English
KW - MEDLINE
KW - Child
KW - Female
KW - Health Policy

KW - Humans
KW - Inservice Training/ec [Economics]
KW - Inservice Training/mt [Methods]
KW - Male
KW - Motor Activity/ph [Physiology]
KW - North Carolina
KW - Obesity/pc [Prevention & Control]
KW - Observation
KW - Organizational Policy
KW - Pilot Projects
KW - Public-Private Sector Partnerships
KW - Schools/og [Organization & Administration]
KW - Schools/td [Trends]
KW - Teaching/mt [Methods]
KW - Training Support
RP - NOT IN FILE
SP - 289
EP - 297
JF - Progress in Community Health Partnerships
JA - Prog
VL - 5
IS - 3
CY - United States
N2 - BACKGROUND: An increased prevalence of overweight/obesity among children has led to school district level policies to increase physical activity (PA) among elementary school students. Interventions are needed that increase activity levels without sacrificing time spent in academics. OBJECTIVES: We evaluated a policy implementation intervention for to increase in-school PA in elementary schools in Forsyth County, North Carolina, in a randomized study with a delayed intervention control group. METHODS: The study included third- through fifth-grade classrooms in eight elementary schools. Instant Recess was used to introduce 10-minute PA breaks in classrooms on schedules determined by teachers. Direct observation was used to measure activity levels, other student behaviors, and teacher behaviors related to PA in the classrooms. RESULTS: Twenty-eight visits to schools were made during the spring and fall semesters of 2009. At baseline 11% to 44% of intervention and control schools were engaged in classroom-based PA. PA increased from baseline to spring follow-up in intervention schools and was maintained the following fall. Control schools decreased PA from baseline to spring and increased PA once they began the intervention. Students in classrooms engaged in Instant Recess exhibited statistically significant increases in light (51%) and moderate-intensity (16%) PA and increases in time spent in on-task behavior (11%). Control schools experienced similar benefits after they began implementing Instant Recess. CONCLUSIONS: Instant Recess is useful for increasing PA and improving behavior among elementary school children. Additional research may be needed to understand how to create policies supporting classroom activity breaks and how to assess policy adherence
SN - 1557-0541
AD - Gramercy Research Group, Los Angeles School of Public Health, USA
UR - 22080777
ER -

TY - JOUR
ID - 533
T1 - Improving sensitivity of EEG-fMRI studies in epilepsy: the role of sleep-specific activity
A1 - Moehring,J.
A1 - Coropceanu,D.
A1 - Galka,A.
A1 - Moeller,F.
A1 - Wolff,S.
A1 - Boor,R.
A1 - Jansen,O.
A1 - Stephani,U.
A1 - Siniatchkin,M.

Y1 - 2011/11/14/
N1 - Moehring, Jan. Coropceanu, Diana. Galka, Andreas. Moeller, Friederike. Wolff, Stephan. Boor, Rainer. Jansen, Olav. Stephani, Ulrich. Siniatchkin, Michael
Neuroscience letters
n7n, 7600130
IM
Journal Article. Research Support, Non-U.S. Gov't
English
KW - MEDLINE
KW - Alpha Rhythm/ph [Physiology]
KW - Brain Mapping/mt [Methods]
KW - Brain Waves/ph [Physiology]
KW - Cerebral Cortex/ph [Physiology]
KW - Cerebral Cortex/pp [Physiopathology]
KW - Child
KW - Electroencephalography/mt [Methods]
KW - Epilepsy/di [Diagnosis]
KW - Epilepsy/pp [Physiopathology]
KW - Female
KW - Humans
KW - Image Processing
KW - Computer-Assisted/mt [Methods]
KW - Magnetic Resonance Imaging/mt [Methods]
KW - Male
KW - Predictive Value of Tests
KW - Sensitivity and Specificity
KW - Sleep/ph [Physiology]
KW - Sleep Disorders/di [Diagnosis]
KW - Sleep Disorders/pp [Physiopathology]
KW - Sleep Stages/ph [Physiology]
RP - NOT IN FILE
SP - 211
EP - 215
JF - Neuroscience Letters
JA - Neurosci Lett
VL - 505
IS - 2
CY - Ireland
N2 - Using simultaneous recordings of EEG and functional MRI (EEG-fMRI) in patients with focal epilepsy, recent studies have revealed insufficient sensitivity and a lack of correspondence between epileptic EEG foci and activation patterns in some patients. In this study of children with focal epilepsy, we explore whether sleep-specific activity (sleep spindles, k-complexes and vertex sharp waves) may increase the sensitivity of EEG-fMRI of interictal epileptiform discharges (IED). When considering the sleep-specific activity in a statistical model, it was possible to increase the statistical significance of the activated voxels inside of the expected source of the IED and to reduce the number of activated voxels outside of it. According to this study, it could be worthwhile to include sleep-specific activity into the model by analyzing EEG-fMRI data in epilepsy. Copyright 2011 Elsevier Ireland Ltd. All rights reserved
SN - 1872-7972
AD - Department of Neuropediatrics, Christian-Albrechts-University, Kiel, Germany. jan.moehring@uk-sh.de
UR - 22027175
ER -

TY - JOUR
ID - 534
T1 - Pediatric brown adipose tissue: detection, epidemiology, and differences from adults
A1 - Drubach,L.A.
A1 - Palmer,E.L.,III

A1 - Connolly,L.P.
A1 - Baker,A.
A1 - Zurakowski,D.
A1 - Cypess,A.M.
Y1 - 2011/12//
N1 - Drubach, Laura A. Palmer, Edwin L 3rd. Connolly, Leonard P. Baker, Amanda. Zurakowski, David. Cypess, Aaron M
The Journal of pediatrics
jlz, 0375410
AIM, IM
Comparative Study. Journal Article. Research Support, N.I.H., Extramural. Research Support, Non-U.S. Gov't
English
KW - MEDLINE
KW - Adipose Tissue
KW - Brown/ri [Radionuclide Imaging]
KW - Adolescent
KW - Adult
KW - Age Factors
KW - Child
KW - Preschool
KW - Female
KW - Fluorodeoxyglucose F18/du [Diagnostic Use]
KW - Humans
KW - Infant
KW - Male
KW - Positron-Emission Tomography
KW - Radiopharmaceuticals/du [Diagnostic Use]
KW - Young Adult
RP - NOT IN FILE
SP - 939
EP - 944
JF - Journal of Pediatrics
JA - J Pediatr
VL - 159
IS - 6
CY - United States
N2 - OBJECTIVES: To evaluate the prevalence and factors affecting the detection of active brown adipose tissue (BAT) in children and adolescents using (18)F-fluorodeoxyglucose positron emission tomography. STUDY DESIGN: A total of 385 positron emission tomography scans performed for various oncologic indications in 172 patients aged 5-21 years were reviewed. BAT activity was detected by visual inspection as present or absent in the neck, thorax, and abdomen based on its well-characterized and typical appearance and then quantified by comparing the (18)F-fluorodeoxyglucose activity in the cervical-supraclavicular depots with that measured in the liver. Clinical indices were recorded. RESULTS: The BAT detection rate was not significantly different between boys and girls (43.3% vs 45.3%). BAT activity was found most often in the cervical-supraclavicular depots. The highest percentage of patients with detectable BAT and the highest BAT/liver activity were in the 13- to 14.99-year age group in both males and females (P = .005). Body mass index percentile correlated inversely with BAT activity (P = .012). BAT activity did not correlate with outdoor temperature or clinical diagnosis. CONCLUSION: Under typical clinical imaging conditions, BAT is detected more frequently in children than in adults. BAT activity increases from childhood into adolescence, when it is detected in almost half of patients, and it correlates inversely with obesity, suggesting that BAT may play a prominent role in pediatric metabolism. Copyright 2011 Mosby, Inc. All rights reserved
SN - 1097-6833
AD - Department of Radiology, Children's Hospital Boston, Boston, MA 02466, USA. laura.drubach@childrens.harvard.edu
UR - 21839465
ER -

TY - JOUR
ID - 535
T1 - Temporal variation in childhood injury from common recreational activities
A1 - Loder,R.T.
A1 - Abrams,S.
Y1 - 2011/09//
N1 - Loder, Randall T. Abrams, Sam
Injury
0226040, gon
IM
Journal Article. Research Support, Non-U.S. Gov't
English
KW - MEDLINE
KW - Accident Prevention/mt [Methods]
KW - Athletic Injuries/cl [Classification]
KW - Athletic Injuries/ep [Epidemiology]
KW - Athletic Injuries/pc [Prevention & Control]
KW - Child
KW - Data Interpretation
KW - Statistical
KW - Emergency Service
KW - Hospital/sn [Statistics & Numerical Data]
KW - Equipment Safety/st [Standards]
KW - Female
KW - Hospitals/sn [Statistics & Numerical Data]
KW - Humans
KW - Male
KW - Play and Playthings/in [Injuries]
KW - Seasons
KW - Sex Distribution
KW - Software
KW - Sports Equipment/st [Standards]
KW - Time Factors
KW - United States/ep [Epidemiology]
RP - NOT IN FILE
SP - 945
EP - 957
JF - Injury
VL - 42
IS - 9
CY - Netherlands
N2 - BACKGROUND/PURPOSE: To investigate the month and day of the week of injury in common childhood activities using the National Electronic Injury Surveillance System database. METHODS: All emergency department visits 2002-2006 from bicycles/tricycles, scooters, playground equipment, swimming/water activities, skiing/snowboarding, trampolines, and skating were analysed. The NEISS weighted and stratified data set was analysed using SUDAAN software. Weekday and month of injury, gender, race, anatomical location of the injury, geographical location of injury, and disposition were tabulated. Simple variation by month or weekday was analysed using cosinor analysis; combined variation for both month and weekday was analysed by topographical analysis. RESULTS: There were an estimated 4.61 million emergency department visits for injuries from these activities in children in the United States. The average age was 9.5 years; there were 1.65 million girls (35.9%) and 2.97 boys (64.1%). Cosinor analysis demonstrated significant single peaks for month of injury for snow activities (January 27), trampolines (June 10), scooters (June 24), cycling (July 6), and water (July 12) activities. Double cosinor peaks were noted for skating (April 13 and September 12) and playground (April 22 and September 21) activities. Cosinor analyses demonstrated that the peak week days of injury were Monday for trampoline and snow activities, Saturday/Sunday for skating activities, Sunday for cycling, and Wednesday for playground equipment. There was no peak injury day for scooter or water activities. Topographical representation of paediatric injuries demonstrated that injuries from

slides most frequently occurred April-May on Wednesdays and Saturdays, and those on swings April-May all days except Tuesday. Monkeybar injuries were bimodal, with the spring peak on Wednesday-Thursday in April/May and the fall peak Tuesday-Friday in September. Rollerblade injuries occurred Saturday-Sunday from March/April; rollerskates on Saturday-Sunday in January-April, and skateboards Saturday-Monday in August-October and Sundays in April. CONCLUSIONS: These findings can be used to further guide childhood injury prevention programmes/campaigns and especially track improvements after targeted prevention programmes. Public parks and schools should check/correct the status of playground landing surfaces just before the bimodal peaks. Education campaigns reinforcing the need for bicycle helmets could be concentrated immediately before the increase in cycling activity - March on weekends and April/May for weekdays
SN - 1879-0267
AD - Department of Orthopaedic Surgery, School of Medicine, Indiana University, Indianapolis, IN, USA. rloder@iupui.edu
UR - 22081824
ER -

TY - JOUR
ID - 536
T1 - Correlates of moderate-to-vigorous physical activity among preschoolers during unstructured outdoor play periods
A1 - Nicaise,V.
A1 - Kahan,D.
A1 - Sallis,J.F.
Y1 - 2011/10//
N1 - Nicaise, Virginie. Kahan, David. Sallis, James F
Preventive medicine
pm4, 0322116
IM
Journal Article. Research Support, Non-U.S. Gov't
English
KW - MEDLINE
KW - Body Mass Index
KW - California
KW - Child
KW - Preschool
KW - Cross-Sectional Studies
KW - Environment Design
KW - Exercise/ph [Physiology]
KW - Female
KW - Humans
KW - Male
KW - Observation
KW - Play and Playthings
KW - Sex Factors
KW - Statistics as Topic
KW - Time Factors
RP - NOT IN FILE
SP - 309
EP - 315
JF - Preventive Medicine
JA - Prev Med
VL - 53
IS - 4-5
CY - United States
N2 - OBJECTIVE: Quantify moderate-to-vigorous physical activity (MVPA) and its correlates in preschool children during outdoor unstructured play periods using direct observation. METHODS: Cross-sectional data consisting of 204 observation periods collected from 51 four- and five-year-old children using the Observation System for Recording Physical Activity in Children - Preschool (OSRAC-P) at a preschool in southern

California, autumn and spring 2009-2010. Gender and BMI classification and OSRAC-P environmental codes were related to observed MVPA in multiple logistic regression models. RESULTS: Less than 21% of intervals were spent in MVPA overall. Boys and normal weight children engaged in higher intensity levels than their respective counterparts. More MVPA was associated with normal weight (OR=2.49-3.25, R(2)=3%), location (grass, playground, looping cycle path; OR=3.21-4.90, R(2)=4-12%), play context (ball/objects, wheel, open space; OR=2.78-8.51, R(2)=9%), and group composition (solitary, one-on-one; OR=1.34-2.08, R(2)=1%). CONCLUSION: Open spaces located in playgrounds and grass fields, and activity-genic portable equipment, manipulative objects, and riding vehicles are some design and equipment features that appear to foster MVPA. Lowering play space density and engaging children through teacher prompts and teacher-arranged activities may further increase MVPA on playgrounds. Copyright A 2011 Elsevier Inc. All rights reserved
SN - 1096-0260
AD - School of Exercise and Nutritional Sciences, San Diego State University, San Diego, CA 92182, USA. virginienicaise@yahoo.fr
UR - 21878351
ER -

TY - JOUR
ID - 537
T1 - Reirradiation and concomitant metronomic temozolomide: an efficient combination for local control in medulloblastoma disease?
A1 - Padovani,L.
A1 - Andre,N.
A1 - Gentet,J.C.
A1 - Figarella,Branger D.
A1 - Scavarda,D.
A1 - Verschuur,A.
A1 - Chinot,O.
A1 - Cowen,D.
A1 - Muracciole,X.
Y1 - 2011/12//
N1 - Padovani, Laetitia. Andre, Nicolas. Gentet, Jean Claude. Figarella Branger, Dominique. Scavarda, Didier. Verschuur, Arnaud. Chinot, Olivier. Cowen, Didier. Muracciole, Xavier
Journal of pediatric hematology/oncology
9505928, b57
IM
Case Reports. Journal Article
English
KW - MEDLINE
KW - Administration
KW - Metronomic
KW - Adolescent
KW - Adult
KW - Antineoplastic Agents
KW - Alkylating/ad [Administration & Dosage]
KW - Brain Neoplasms/dt [Drug Therapy]
KW - Brain Neoplasms/rt [Radiotherapy]
KW - Chemoradiotherapy/mt [Methods]
KW - Child
KW - Dacarbazine/ad [Administration & Dosage]
KW - Dacarbazine/aa [Analogs & Derivatives]
KW - Fatal Outcome
KW - Female
KW - Humans
KW - Male
KW - Medulloblastoma/dt [Drug Therapy]
KW - Medulloblastoma/rt [Radiotherapy]
KW - Radiation Dosage

KW - Retreatment/mt [Methods]
KW - Young Adult
RP - NOT IN FILE
SP - 600
EP - 604
JF - Journal of Pediatric Hematology/Oncology
JA - J Pediatr Hematol Oncol
VL - 33
IS - 8
CY - United States
N2 - Medulloblastoma (MB) is the most common malignant pediatric brain tumor and a rare adulthood tumor. Twenty percent to 30% of patients relapses and displays a poor prognosis. The management of recurrent disease represents a medical challenge as salvage therapy with high-dose chemotherapy is disappointing. We report a pilot study of reirradiation and concomitant metronomic temozolomide of MB focal recurrence. Five patients from 10 to 27 years old at time of first diagnosis were treated initially with upfront radiation therapy at full dose. They relapsed focally and progressed under chemotherapy with a time recurrence ranged from 2 to 15 years after initial diagnosis. Patients were then treated with 3-dimensional conformal reirradiation focused on the relapsed disease with a median dose of 28 Gy (1.8 Gy per fraction) and concomitant temozolomide (75 mg/m/d) alone or as part of a multidrug metronomic regimen. Five complete responses were obtained at the end of metronomic radiochemotherapy. The median follow-up was 28 months. At last follow-up, 3 patients progressed outside radiation field under maintenance chemotherapy, and 1 is free of disease. Only 1 patient relapsed in the reirradiation field. No neurological toxicity was observed. These results indicate a possible radiosensitizing effect of concomitant metronomic temozolomide with radiation therapy. This association could play a role in the management of high-risk MB patient with oligometastasis disease to increase local control
SN - 1536-3678
AD - Radiation Oncology Department, CHU La Timone, Assistance Publique de Marseille, France. laetitia.padovani@ap-hm.fr
UR - 22042276
ER -

TY - JOUR
ID - 538
T1 - Environmental influences on children's physical activity and eating habits in a rural Oregon County
A1 - Findholt,N.E.
A1 - Michael,Y.L.
A1 - Jerofke,L.J.
A1 - Brogoitti,V.W.
Y1 - 2011/11//
N1 - Findholt, Nancy E. Michael, Yvonne L. Jerofke, Linda J. Brogoitti, Victoria W
American journal of health promotion : AJHP
amj, 8701680
T
Journal Article. Research Support, N.I.H., Extramural. Research Support, Non-U.S. Gov't
English
KW - MEDLINE
KW - Adolescent
KW - Adult
KW - Age Factors
KW - Child
KW - Child Nutritional Physiological Phenomena
KW - Child Welfare/px [Psychology]
KW - Community-Based Participatory Research
KW - Female
KW - Focus Groups
KW - Food Habits/px [Psychology]
KW - Humans
KW - Male

KW - Motor Activity/ph [Physiology]
KW - Obesity
KW - Oregon
KW - Qualitative Research
KW - Recreation
KW - Risk Factors
KW - Rural Population/sn [Statistics & Numerical Data]
KW - Social Environment
KW - Young Adult
RP - NOT IN FILE
SP - e74
EP - e85
JF - American Journal of Health Promotion
JA - Am J Health Promot
VL - 26
IS - 2
CY - United States
N2 - PURPOSE. To identify environmental barriers and facilitators of children's physical activity and healthy eating in a rural county. DESIGN. Community-based participatory research using mixed methods, primarily qualitative. SETTING. A rural Oregon county. SUBJECTS. Ninety-five adults, 6 high school students, and 41 fifth-grade students. MEASURES. In-depth interviews, focus groups, Photovoice, and structured observations using the Physical Activity Resource Assessment, System for Observing Play and Leisure Activity, Community Food Security Assessment Toolkit, and School Food and Beverage Marketing Assessment Tool. ANALYSIS. Qualitative data were coded by investigators; observational data were analyzed using descriptive statistics. The findings were triangulated to produce a composite of environmental barriers and assets. RESULTS. Limited recreational resources, street-related hazards, fear of strangers, inadequate physical education, and denial of recess hindered physical activity, whereas popularity of youth sports and proximity to natural areas promoted physical activity. Limited availability and high cost of healthy food, busy lifestyles, convenience stores near schools, few healthy meal choices at school, children's being permitted to bring snacks to school, candy used as incentives, and teachers' modeling unhealthy eating habits hindered healthy eating, whereas the agricultural setting and popularity of gardening promoted healthy eating. CONCLUSIONS. This study provides data on a neglected area of research, namely environmental determinants of rural childhood obesity, and points to the need for multifaceted and multilevel environmental change interventions
SN - 0890-1171
AD - Oregon Health & Science University, Schoolof Nursing-La Grande Campus, La Grande, USA. findholt@ohsu.edu
UR - 22040399
ER -

TY - JOUR
ID - 539
T1 - Circumstances surrounding the community needle-stick injuries in Georgia
A1 - Butsashvili,M.
A1 - Kamkamidze,G.
A1 - Kajaia,M.
A1 - Kandelaki,G.
A1 - Zhorzholadze,N.
Y1 - 2011/12//
N1 - Butsashvili, Maia. Kamkamidze, George. Kajaia, Maia. Kandelaki, George. Zhorzholadze, Nana
Journal of community health
7600747, hut
IM
Journal Article
English
KW - MEDLINE
KW - Accidents
KW - Home/sn [Statistics & Numerical Data]

KW - Adolescent
KW - Adult
KW - Ambulatory Care Facilities/sn [Statistics & Numerical Data]
KW - Blood-Borne Pathogens/ip [Isolation & Purification]
KW - Child
KW - Preschool
KW - Community-Acquired Infections/co [Complications]
KW - Community-Acquired Infections/et [Etiology]
KW - Female
KW - Georgia
KW - HIV Seropositivity/bl [Blood]
KW - Hepacivirus/im [Immunology]
KW - Hepatitis B Antibodies/bl [Blood]
KW - Hepatitis B virus/im [Immunology]
KW - Humans
KW - Male
KW - Middle Aged
KW - Needlestick Injuries/co [Complications]
KW - Needlestick Injuries/et [Etiology]
KW - Needlestick Injuries/pc [Prevention & Control]
KW - Seroepidemiologic Studies
KW - Young Adult
RP - NOT IN FILE
SP - 1050
EP - 1052
JF - Journal of Community Health
JA - J Community Health
VL - 36
IS - 6
CY - Netherlands
N2 - Community needle-stick injuries are important public health problem due to concern of blood-borne pathogen transmission. Purpose of this study was to describe circumstances related to non-occupational needle-stick injuries in Georgia. Data were collected from one outpatient clinic in Tbilisi. Medical records from 2002 to 2007 were reviewed. Blood tests were performed on HBV, HCV and HIV at first visit and 6 months after exposure. 25 (54.4%) study subjects were children playing in street/yard and being accidentally stuck by used needle. Most frequent circumstances related to needle stick among adult individuals were recapping or discarding used needle while taking care of family member needed home injections (12 cases). Eight participants (17.4%) reported accidentally stepping on used needle at sea shore. No infection with HIV and HCV were documented. Only one case of HBV infection occurred in female patient taking care of mother with chronic HBV infection. Study suggests that seroconversion for blood-borne infections after community needle-stick injuries is very low. Family members of patients receiving home injections should be informed about potential risks and advised using infection-control measures. Parents/teachers should be educated about the circumstances related to exposure to used needles among children
SN - 1573-3610
AD - Maternal and Child Care Union, 8 Nutsubidze st, Tbilisi, 0177, Georgia
UR - 21533886
ER -

TY - JOUR
ID - 540
T1 - SPACE for physical activity--a multicomponent intervention study: study design and baseline findings from a cluster randomized controlled trial
A1 - Toftager,M.
A1 - Christiansen,L.B.
A1 - Kristensen,P.L.
A1 - Troelsen,J.
Y1 - 2011///

N1 - Toftager, Mette. Christiansen, Lars B. Kristensen, Peter L. Troelsen, Jens
BMC public health
100968562
IM
Journal Article. Multicenter Study. Randomized Controlled Trial
English
KW - MEDLINE
KW - Adolescent
KW - Anthropometry
KW - Child
KW - Cluster Analysis
KW - Data Collection
KW - Denmark
KW - Environment Design
KW - Exercise/ph [Physiology]
KW - Female
KW - Health Promotion/og [Organization & Administration]
KW - Health Promotion
KW - Humans
KW - Intervention Studies
KW - Male
KW - Outcome Assessment (Health Care)
KW - Physical Fitness/ph [Physiology]
KW - Questionnaires
KW - Research Design
KW - Schools
RP - NOT IN FILE
SP - 777
JF - BMC Public Health
VL - 11
CY - England
N2 - BACKGROUND: The aim of the School site, Play Spot, Active transport, Club fitness and Environment (SPACE) Study was to develop, document, and assess a comprehensive intervention in local school districts that promote everyday physical activity (PA) among 11-15-year-old adolescents. The study is based on a social ecological framework, and is designed to implement organizational and structural changes in the physical environment. METHODS/DESIGN: The SPACE Study used a cluster randomized controlled study design. Twenty-one eligible schools in the Region of Southern Denmark were matched and randomized in seven pairs according to eight matching variables summarized in an audit tool (crow-fly distance from residence to school for 5-6th graders; area household income; area education level; area ethnicity distribution; school district urbanity; condition and characteristics of school outdoor areas; school health policy; and active transport in the local area). Baseline measurements with accelerometers, questionnaires, diaries, and physical fitness tests were obtained in Spring 2010 in 5-6th grade in 7 intervention and 7 control schools, with follow-up measurements to be taken in Spring 2012 in 7-8th grade. The primary outcome measure is objective average daily physical activity and will be supported by analyses of time spent in moderate to vigorous activity and time spent sedentary. Other secondary outcome measures will be obtained, such as, overweight, physical fitness, active commuting to/from school and physical activity in recess periods. DISCUSSION: A total of 1348 adolescents in 5-6th grade in the Region of Southern Denmark participated at baseline (n = 14 schools). The response rate was high in all type of measurements (72.6-97.4%). There were no significant differences between intervention and control groups at baseline according to selected background variables and outcome measures: gender (p = .54), age (p = .17), BMI (p = .59), waist circumference (p = .17), physical fitness (p = .93), and physical activity (accelerometer) (p = .09). The randomization and matched pair design produced equivalent groups according to central outcome measures and background variables. The SPACE for physical activity Study will provide new insights on the effectiveness of multicomponent interventions to improve adolescents' physical activity level. TRIAL REGISTRATION: Current Controlled Trials ISRCTN79122411
SN - 1471-2458
AD - Institute of Sports Science and Clinical Biomechanics, University of Southern Denmark, Campusvej 55, 5230 Odense M, Denmark. mto@sdu.dk

UR - 21985278
ER -

TY - JOUR
ID - 541
T1 - Trends by age in youth physical activity: Youth Media Campaign Longitudinal Survey
A1 - Wall,M.I.
A1 - Carlson,S.A.
A1 - Stein,A.D.
A1 - Lee,S.M.
A1 - Fulton,J.E.
Y1 - 2011/11//
N1 - Wall, Megan I. Carlson, Susan A. Stein, Aryeh D. Lee, Sarah M. Fulton, Janet E
Medicine and science in sports and exercise
8005433, mg8
IM, S
Journal Article
English
KW - MEDLINE
KW - Adolescent
KW - Child
KW - Female
KW - Health Surveys
KW - Humans
KW - Leisure Activities
KW - Longitudinal Studies
KW - Male
KW - Motor Activity/ph [Physiology]
KW - Sports
KW - United States
RP - NOT IN FILE
SP - 2140
EP - 2147
JF - Medicine & Science in Sports & Exercise
JA - Med Sci Sports Exerc
VL - 43
IS - 11
CY - United States
N2 - PURPOSE: This study aimed to characterize longitudinal age trajectories across 5 yr in the prevalence of free-time and organized physical activity participation among US youth by sex, race, and parental education. METHODS: Study participants were a nationally representative sample of youth, 9-13 yr old in 2002, who participated in the Centers for Disease Control and Prevention's Youth Media Campaign Longitudinal Survey. Baseline data were collected in 2002. Attrition for the next 4 yr resulted in an overall response rate of 23% by 2006 (n = 1623). The survey collected information concerning respondents' frequency of participation in free-time and organized physical activities outside school. Organized activities were defined as activities involving a coach, instructor, or other leader. Orthogonal polynomial contrasts were used to test for linear and quadratic trends in respondents' participation free-time and organized physical activity sessions during the previous 7 d over ages 9-17. Pairwise t-tests were used to determine whether age-specific estimates of participation rates differed significantly by sex, race, and parental education level. RESULTS: Free-time physical activity participation prevalence declined linearly from ages 9 to 17 in both sexes but also demonstrated a quadratic trajectory in boys, peaking at age 13. Organized physical activity demonstrated a quadratic trajectory and declined most notably after age 14 in both sexes. Free-time physical activity participation was lower in girls compared with boys between ages 12 and 16 (difference range = 12-17 percentage points). Both non-white youth and those with less educated parents had lower organized physical activity participation at most ages (difference range = 15-29 percentage points). CONCLUSIONS: Free-time and organized physical activity exhibit different trajectories between ages 9 and 17 and are subject to dissimilar demographic level variation
SN - 1530-0315

AD - National Center for Chronic Disease Prevention and Health Promotion, Centers for Disease Control and Prevention, Atlanta, GA 30345, USA. megan.i.wall@gmail.com
UR - 21502886
ER -

TY - JOUR
ID - 542
T1 - Number of mutations within CTL-defined epitopes of the hepatitis B Virus (HBV) core region is associated with HBV disease progression
A1 - Kim,D.
A1 - Lyoo,K.S.
A1 - Smith,D.
A1 - Hur,W.
A1 - Hong,S.W.
A1 - Sung,P.S.
A1 - Yoon,S.K.
A1 - Mehta,S.
Y1 - 2011/12//
N1 - Kim, Daniel. Lyoo, Kwang Soo. Smith, Davey. Hur, Wonhee. Hong, Sung Woo. Sung, Pil Soo. Yoon, Seung Kew. Mehta, Sanjay
Journal of medical virology
i9n, 7705876
IM
Journal Article. Research Support, N.I.H., Extramural. Research Support, Non-U.S. Gov't
English
KW - MEDLINE
KW - Adolescent
KW - Adult
KW - Aged
KW - 80 and over
KW - Carcinoma
KW - Hepatocellular/co [Complications]
KW - Hepatocellular/pa [Pathology]
KW - Hepatocellular/vi [Virology]
KW - Child
KW - Disease Progression
KW - Epitopes
KW - T-Lymphocyte/ge [Genetics]
KW - T-Lymphocyte/im [Immunology]
KW - Female
KW - Hepatitis B/co [Complications]
KW - Hepatitis B/pa [Pathology]
KW - Hepatitis B/vi [Virology]
KW - Hepatitis B Core Antigens/ge [Genetics]
KW - Hepatitis B Core Antigens/im [Immunology]
KW - Hepatitis B virus/ge [Genetics]
KW - Hepatitis B virus/im [Immunology]
KW - Hepatitis B virus/py [Pathogenicity]
KW - Humans
KW - Liver Cirrhosis/co [Complications]
KW - Liver Cirrhosis/pa [Pathology]
KW - Liver Cirrhosis/vi [Virology]
KW - Liver Neoplasms/co [Complications]
KW - Liver Neoplasms/pa [Pathology]
KW - Liver Neoplasms/vi [Virology]
KW - Male
KW - Middle Aged

KW - Mutation
KW - Missense
KW - Republic of Korea
KW - T-Lymphocytes
KW - Cytotoxic/im [Immunology]
KW - Young Adult
RP - NOT IN FILE
SP - 2082
EP - 2087
JF - Journal of Medical Virology
JA - J Med Virol
VL - 83
IS - 12
CY - United States
N2 - The virologic determinants of progressive liver disease associated with hepatitis B virus (HBV) remain unclear. Previous investigations have associated HBV disease with specific mutations but this association may be confounded by HBV genotype, HLA haplotype of the infected individual or both. The association between non-synonymous mutations located within putative cytotoxic T-lymphocyte directed epitopes (CDE) of the HBV core region and disease states was investigated. Subjects infected with HBV were enrolled from a clinical cohort in Seoul, Korea, and HBV core gene sequences were analyzed for mutational patterns inside and outside of CDE with respect to subject demographics and HBV-related disease states. No specific mutation or pattern of mutations were associated with progressive disease states; however, individuals with cirrhosis and hepatocellular carcinoma had greater numbers of non-synonymous mutations within CDE when compared to those with chronic HBV infection who were HBeAg positive (P=0.007 and 0.026, respectively). In conclusion, this study demonstrates that HBV disease progression is associated with viral escape mutations that are a marker of CTL activity. These data suggest that the number of non-synonymous mutations in the HBV core region may predict HBV disease progression better than any single mutation or pattern of mutations. Copyright 2011 Wiley Periodicals, Inc
SN - 1096-9071
AD - University of California, La Jolla, CA, USA
UR - 22012714
ER -

TY - JOUR
ID - 543
T1 - Correlates of sedentary time and physical activity among preschool-aged children
A1 - Dolinsky,D.H.
A1 - Brouwer,R.J.
A1 - Evenson,K.R.
A1 - Siega-Riz,A.M.
A1 - Ostbye,T.
Y1 - 2011/11//
N1 - Dolinsky, Diana H. Brouwer, Rebecca J Namenek. Evenson, Kelly R. Siega-Riz, Anna Maria. Ostbye, Truls
Preventing chronic disease
101205018
IM
Journal Article. Multicenter Study. Randomized Controlled Trial. Research Support, N.I.H., Extramural. Research Support, Non-U.S. Gov't
English
KW - MEDLINE
KW - Actigraphy/mt [Methods]
KW - Body Mass Index
KW - Child Behavior
KW - Child
KW - Preschool
KW - Cross-Sectional Studies

KW - Female
KW - Health Behavior
KW - Humans
KW - Male
KW - Motor Activity
KW - Obesity/ep [Epidemiology]
KW - Overweight/ep [Epidemiology]
KW - Prevalence
KW - Sedentary Lifestyle
KW - World Health
RP - NOT IN FILE
SP - A131
JF - Preventing Chronic Disease
JA - Prev Chronic Dis
VL - 8
IS - 6
CY - United States
N2 - INTRODUCTION: Few studies have examined the correlates of objectively measured amounts of sedentary time and physical activity in young children. We evaluated the demographic, biological, behavioral, social, and environmental correlates of the amount of sedentary time and moderate-to-vigorous physical activity (MVPA) as measured by accelerometry in preschool-aged children. METHODS: We obtained baseline measurements of physical activity by using an Actical accelerometer among 337 preschool-aged children (aged 2-5) of overweight or obese mothers. For children, we defined sedentary time as less than 12 counts per 15 seconds and MVPA as 715 or more counts per 15 seconds. Body mass index of the mother and child (calculated from measured height and weight) and maternal physical activity as measured by accelerometer were included as potential correlates. Mothers self-reported all other potential correlates. We used multivariable linear regression analyses to examine correlates of the amount of sedentary time and MVPA. RESULTS: Children had an average of 6.1 hours per day of sedentary time and 14.9 minutes per day of MVPA. In multivariable analysis, boys (P <.001) had fewer minutes per day of sedentary time, whereas older children (P <.001), boys (P <.001), children in high-income households (>$60,000/y [P = .005]), and children who spent more time outdoors (P = .001) had more MVPA. CONCLUSION: Both modifiable and nonmodifiable factors were correlated with preschool children's amount of MVPA, which can be helpful when designing interventions for this age group. The lack of correlates for sedentary time indicates the need for further investigation into this behavior
SN - 1545-1151
AD - Duke University Medical Center, Durham, North Carolina, USA. dolin004@mc.duke.edu
UR - 22005624
ER -

TY - JOUR
ID - 544
T1 - The important health impact of where a child lives: neighborhood characteristics and the burden of lead poisoning
A1 - Vivier,P.M.
A1 - Hauptman,M.
A1 - Weitzen,S.H.
A1 - Bell,S.
A1 - Quilliam,D.N.
A1 - Logan,J.R.
Y1 - 2011/11//
N1 - Vivier, Patrick M. Hauptman, Marissa. Weitzen, Sherry H. Bell, Scott. Quilliam, Daniela N. Logan, John R
Maternal and child health journal
9715672, di8
IM
Journal Article. Research Support, Non-U.S. Gov't
English
KW - MEDLINE

KW - Child
KW - Preschool
KW - Cost of Illness
KW - Female
KW - Geographic Information Systems
KW - Humans
KW - Infant
KW - Lead Poisoning/di [Diagnosis]
KW - Lead Poisoning/ep [Epidemiology]
KW - Male
KW - Population Surveillance
KW - Residence Characteristics
KW - Rhode Island/ep [Epidemiology]
RP - NOT IN FILE
SP - 1195
EP - 1202
JF - Maternal & Child Health Journal
JA - Matern Child Health J
VL - 15
IS - 8
CY - United States
N2 - Toxins and other health threats can cause health problems, whether they are present in the child's own home, other neighborhood homes where the child spends time, or common areas such as playgrounds. We assess the impact of where a child lives on the burden of lead poisoning. Statewide lead screening data was obtained from the Rhode Island Department of Health. Block group level indicators of old housing and poverty were obtained from the US Census. Of the 204,746 study children, 35,416 (17.3%) had a blood lead level>10 mug/dL. The proportion of study children who were lead poisoned in each block group ranged from 0.0 to 48.6%. The proportion of study children with an elevated blood lead level increased from 8% among children living in block groups in the lowest quintile of poverty to 31% for those in the highest quintile for poverty. Old housing also had an important impact on the risk of lead poisoning. The proportion of children with an elevated blood lead level increased from 7% among children living in block groups in the lowest quintile for pre-1950 housing to 27% for those in the highest quintile for pre-1950 housing. The adjusted odds ratio was 1.64 for the highest quintile of poverty and 1.77 for the highest quintile of pre-1950 housing. The findings of this large, statewide study demonstrate the powerful impact of where children live on the risk of lead poisoning. The findings have important implications for understanding the problem of lead poisoning and for planning primary prevention programs
SN - 1573-6628
AD - Department of Community Health, Brown University, Box G-S121, 121 South Main St, Providence, RI 02912, USA. Patrick_Vivier@Brown.edu
UR - 20972613
ER -

TY - JOUR
ID - 545
T1 - "My child and I are a package deal": balancing adult and child concerns in repartnering after divorce
A1 - Anderson,E.R.
A1 - Greene,S.M.
Y1 - 2011/10//
N1 - Anderson, Edward R. Greene, Shannon M
Journal of family psychology : JFP : journal of the Division of Family Psychology of the American Psychological Association (Division 43)
8802265, dlv
IM
Journal Article. Research Support, N.I.H., Extramural
English
KW - MEDLINE
KW - Adult

KW - Child
KW - Divorce/px [Psychology]
KW - Female
KW - Humans
KW - Individuality
KW - Interpersonal Relations
KW - Interview
KW - Psychological
KW - Longitudinal Studies
KW - Male
KW - Marriage/px [Psychology]
KW - Middle Aged
KW - Mother-Child Relations
KW - Mothers/px [Psychology]
KW - Parenting/px [Psychology]
KW - Questionnaires
KW - Young Adult
RP - NOT IN FILE
SP - 741
EP - 750
JF - Journal of Family Psychology
JA - J Fam Psychol
VL - 25
IS - 5
CY - United States
N2 - Parents who repartner after divorce must decide how to balance the potentially competing demands of their own desire for adult companionship and romance with the needs of their children for parental attention and affection. In this study, the authors assessed individual differences in divorced custodial mothers' orientation toward repartnering, characterizing it as a continuum, ranging from more child focused to more adult focused. Mothers who are more adult focused tend to be older, more educated, more likely to be employed outside the home, and exiting marriages of longer duration. In addition, using longitudinal data from in-home interviews, mothers who are more adult focused reported having lower rapport with their children, spending less time in joint activities with their children, and their children in turn reported lower rapport with their mothers. Levels of adult-focused orientation are relatively stable over time but increase when mothers become involved or interested in new partners. Using longitudinal diary data over a 2-year period, the authors demonstrated that mothers who are more child focused engage in more active management of emergent relationships in repartnered families and that adult-focused and child-focused mothers respond to different concerns. Whereas all mothers become more active in managing emergent relationships when both partner and child are resisting one another, mothers with greater child focus respond more to concerns of the child, and mothers with greater adult focus respond more to the concerns of the partner. Implications for intervention with divorced families are discussed
SN - 1939-1293
AD - Department of Human Development and Family Sciences, University of Texas at Austin, Austin, TX 78712, USA. edward.anderson@mail.utexas.edu
UR - 21744961
ER -

TY - JOUR
ID - 546
T1 - Quantifying peer interactions for research and clinical use: the Manchester Inventory for Playground Observation
A1 - Gibson,J.
A1 - Hussain,J.
A1 - Holsgrove,S.
A1 - Adams,C.
A1 - Green,J.
Y1 - 2011/11//

N1 - Gibson, Jenny. Hussain, Jamilla. Holsgrove, Samina. Adams, Catherine. Green, Jonathan
Research in developmental disabilities
8709782, rid
IM
Journal Article. Validation Studies
English
KW - MEDLINE
KW - Anxiety/di [Diagnosis]
KW - Anxiety/px [Psychology]
KW - Attention Deficit Disorder with Hyperactivity/di [Diagnosis]
KW - Attention Deficit Disorder with Hyperactivity/px [Psychology]
KW - Attention Deficit and Disruptive Behavior Disorders/di [Diagnosis]
KW - Attention Deficit and Disruptive Behavior Disorders/px [Psychology]
KW - Child
KW - Child Behavior/cl [Classification]
KW - Child Behavior/px [Psychology]
KW - Child Development Disorders
KW - Pervasive/di [Diagnosis]
KW - Pervasive/px [Psychology]
KW - Child Psychology/mt [Methods]
KW - Conduct Disorder/di [Diagnosis]
KW - Conduct Disorder/px [Psychology]
KW - Depression/di [Diagnosis]
KW - Depression/px [Psychology]
KW - Disability Evaluation
KW - Great Britain
KW - Humans
KW - Mental Disorders/di [Diagnosis]
KW - Mental Disorders/px [Psychology]
KW - Observer Variation
KW - Peer Group
KW - Personality Inventory/st [Standards]
KW - Personality Inventory/sn [Statistics & Numerical Data]
KW - Play and Playthings/px [Psychology]
KW - ROC Curve
KW - Sensitivity and Specificity
KW - Social Behavior
RP - NOT IN FILE
SP - 2458
EP - 2466
JF - Research in Developmental Disabilities
JA - Res Dev Disabil
VL - 32
IS - 6
CY - United States
N2 - Direct observation of peer relating is potentially a sensitive and ecologically valid measure of child social functioning, but there has been a lack of standardised methods. The Manchester Inventory for Playground Observation (MIPO) was developed as a practical yet rigorous assessment of this kind for 5-11 year olds. We report on the initial reliability and validity of the MIPO and its ability to distinguish social impairments within different psychopathologies. We observed 144 clinically referred children aged 5;00-11;11 (mean 8.8) years with Externalising (n = 44), Internalising (n = 19), Autism Spectrum Disorders (n = 39) or Specific Language Impairment (n = 42), and 44 class-controls, in naturalistic playground interaction. Observers, blind to clinical diagnosis, completed the MIPO and the teacher checklist from the Social Skills Rating System (SSRS). MIPO items showed high internal consistency (alpha = .924; all 'alpha if item deleted' values>.91), inter-observer reliability (mean (w) = .77) and test-retest stability (over 2 weeks; mean (w) = .58). MIPO totals showed convergence with SSRS (n = 68, r(s) = .78, p<.01) and excellent discrimination between case and control (sensitivity = 0.75 and specificity = 0.88, AUC = .897). Externalising, Autistic Spectrum and Language

Impaired groups showed distinct profiles of MIPO impairment consistent with theory:Internalising disorders less so. 65.3% of clinical cases were classified accurately for primary diagnosis. The MIPO shows reliability and validity as a measure of children's social functioning relevant in developmental research and as a clinical tool to aid differential diagnosis and intervention planning. Copyright 2011 Elsevier Ltd. All rights reserved
SN - 1873-3379
AD - Dept Human Communication and Deafness, Ellen Wilkinson Building, University of Manchester, Oxford Road, Manchester M13 9PL, UK. jenny.l.gibson@postrgad.manchester.ac.uk
UR - 21831591
ER -

TY - JOUR
ID - 547
T1 - The Sydney playground project: popping the bubblewrap--unleashing the power of play: a cluster randomized controlled trial of a primary school playground-based intervention aiming to increase children's physical activity and social skills
A1 - Bundy,A.C.
A1 - Naughton,G.
A1 - Tranter,P.
A1 - Wyver,S.
A1 - Baur,L.
A1 - Schiller,W.
A1 - Bauman,A.
A1 - Engelen,L.
A1 - Ragen,J.
A1 - Luckett,T.
A1 - Niehues,A.
A1 - Stewart,G.
A1 - Jessup,G.
A1 - Brentnall,J.
Y1 - 2011///
N1 - Bundy, Anita C. Naughton, Geraldine. Tranter, Paul. Wyver, Shirley. Baur, Louise. Schiller, Wendy. Bauman, Adrian. Engelen, Lina. Ragen, Jo. Luckett, Tim. Niehues, Anita. Stewart, Gabrielle. Jessup, Glenda. Brentnall, Jennie
BMC public health
100968562
IM
Journal Article. Randomized Controlled Trial. Research Support, Non-U.S. Gov't
English
KW - MEDLINE
KW - Bullying
KW - Child
KW - Preschool
KW - Cluster Analysis
KW - Exercise
KW - Female
KW - Health Promotion/mt [Methods]
KW - Humans
KW - Male
KW - Mental Health
KW - New South Wales
KW - Overweight/pc [Prevention & Control]
KW - Play and Playthings/px [Psychology]
KW - Schools
KW - Socialization
RP - NOT IN FILE
SP - 680
JF - BMC Public Health

VL - 11
CY - England
N2 - BACKGROUND: In the Westernised world, numerous children are overweight and have problems with bullying and mental health. One of the underlying causes for all three is postulated to be a decrease in outdoor free play. The aim of the Sydney Playground Project is to demonstrate the effectiveness of two simple interventions aimed to increase children's physical activity and social skills. METHODS/DESIGN: This study protocol describes the design of a 3-year cluster randomised controlled trial (CRCT), in which schools are the clusters. The study consists of a 13-week intervention and 1 week each of pre-and post-testing. We are recruiting 12 schools (6 control; 6 intervention), with 18 randomly chosen participants aged 5 to 7 years in each school. The two intervention strategies are: (1) Child-based intervention: Unstructured materials with no obvious play value introduced to the playground; and (2) Adult-based intervention: Risk reframing sessions held with parents and teachers with the aim of exploring the benefits of allowing children to engage in activities with uncertain outcomes. The primary outcome of the study, physical activity as measured by accelerometer counts, is assessed at baseline and post-intervention. Additional assessments include social skills and interactions, self-concept, after school time use and anthropometric data. Qualitative data (i.e., transcriptions of audio recordings from the risk reframing sessions and of interviews with selected teacher and parent volunteers) are analysed to understand their perceptions of risk in play. The control schools have recess as usual. In addition to outcome evaluation, regular process evaluation sessions are held to monitor fidelity to the treatment. DISCUSSION: These simple interventions, which could be adopted in every primary school, have the potential of initiating a self-sustaining cycle of prevention for childhood obesity, bullying and mental ill health. TRIAL REGISTRATION: Australian and New Zealand Clinical Trials Registration Number ACTRN12611000089932
SN - 1471-2458
AD - Faculty of Health Sciences, University of Sydney, Lidcombe, Australia. anita.bundy@sydney.edu.au
UR - 21884603
ER -

TY - JOUR
ID - 548
T1 - With or without spikes: localization of focal epileptic activity by simultaneous electroencephalography and functional magnetic resonance imaging
A1 - Grouiller,F.
A1 - Thornton,R.C.
A1 - Groening,K.
A1 - Spinelli,L.
A1 - Duncan,J.S.
A1 - Schaller,K.
A1 - Siniatchkin,M.
A1 - Lemieux,L.
A1 - Seeck,M.
A1 - Michel,C.M.
A1 - Vulliemoz,S.
Y1 - 2011/10//
N1 - Grouiller, Frederic. Thornton, Rachel C. Groening, Kristina. Spinelli, Laurent. Duncan, John S. Schaller, Karl. Siniatchkin, Michael. Lemieux, Louis. Seeck, Margitta. Michel, Christoph M. Vulliemoz, Serge
Brain : a journal of neurology
0372537
AIM, IM
Journal Article. Research Support, Non-U.S. Gov't
English
KW - MEDLINE
KW - Adolescent
KW - Adult
KW - Brain Mapping/mt [Methods]
KW - Cerebral Cortex/pp [Physiopathology]
KW - Child
KW - Preschool
KW - Electroencephalography/mt [Methods]

KW - Epilepsy/pp [Physiopathology]
KW - Female
KW - Humans
KW - Magnetic Resonance Imaging/mt [Methods]
KW - Male
KW - Middle Aged
RP - NOT IN FILE
SP - 2867
EP - 2886
JF - Brain
VL - 134
IS - Pt 10
CY - England
N2 - In patients with medically refractory focal epilepsy who are candidates for epilepsy surgery, concordant non-invasive neuroimaging data are useful to guide invasive electroencephalographic recordings or surgical resection. Simultaneous electroencephalography and functional magnetic resonance imaging recordings can reveal regions of haemodynamic fluctuations related to epileptic activity and help localize its generators. However, many of these studies (40-70%) remain inconclusive, principally due to the absence of interictal epileptiform discharges during simultaneous recordings, or lack of haemodynamic changes correlated to interictal epileptiform discharges. We investigated whether the presence of epilepsy-specific voltage maps on scalp electroencephalography correlated with haemodynamic changes and could help localize the epileptic focus. In 23 patients with focal epilepsy, we built epilepsy-specific electroencephalographic voltage maps using averaged interictal epileptiform discharges recorded during long-term clinical monitoring outside the scanner and computed the correlation of this map with the electroencephalographic recordings in the scanner for each time frame. The time course of this correlation coefficient was used as a regressor for functional magnetic resonance imaging analysis to map haemodynamic changes related to these epilepsy-specific maps (topography-related haemodynamic changes). The method was first validated in five patients with significant haemodynamic changes correlated to interictal epileptiform discharges on conventional analysis. We then applied the method to 18 patients who had inconclusive simultaneous electroencephalography and functional magnetic resonance imaging studies due to the absence of interictal epileptiform discharges or absence of significant correlated haemodynamic changes. The concordance of the results with subsequent intracranial electroencephalography and/or resection area in patients who were seizure free after surgery was assessed. In the validation group, haemodynamic changes correlated to voltage maps were similar to those obtained with conventional analysis in 5/5 patients. In 14/18 patients (78%) with previously inconclusive studies, scalp maps related to epileptic activity had haemodynamic correlates even when no interictal epileptiform discharges were detected during simultaneous recordings. Haemodynamic changes correlated to voltage maps were spatially concordant with intracranial electroencephalography or with the resection area. We found better concordance in patients with lateral temporal and extratemporal neocortical epilepsy compared to medial/polar temporal lobe epilepsy, probably due to the fact that electroencephalographic voltage maps specific to lateral temporal and extratemporal epileptic activity are more dissimilar to maps of physiological activity. Our approach significantly increases the yield of simultaneous electroencephalography and functional magnetic resonance imaging to localize the epileptic focus non-invasively, allowing better targeting for surgical resection or implantation of intracranial electrode arrays
SN - 1460-2156
AD - Presurgical Epilepsy Evaluation Unit and Functional Brain Mapping Laboratory, Neurology Department, University Hospital, University of Geneva, 1 Geneva, Switzerland
UR - 21752790
ER -

TY - JOUR
ID - 549
T1 - Parents seek early intervention services for a two-year-old without autism
A1 - Reddy,A.
A1 - Graves,C.
A1 - Augustyn,M.
Y1 - 2011/10//

N1 - Reddy, Arathi. Graves, Crista. Augustyn, Marilyn
Journal of developmental and behavioral pediatrics : JDBP
htf, 8006933
IM
Case Reports. Journal Article
English
KW - MEDLINE
KW - Autistic Disorder/di [Diagnosis]
KW - Autistic Disorder/px [Psychology]
KW - Autistic Disorder/th [Therapy]
KW - Behavior Therapy/mt [Methods]
KW - Behavior Therapy
KW - Child Development
KW - Child
KW - Preschool
KW - Diagnosis
KW - Differential
KW - Early Intervention (Education)/mt [Methods]
KW - Humans
KW - Male
KW - Parents
KW - Sleep
KW - Treatment Outcome
KW - Walking/px [Psychology]
RP - NOT IN FILE
SP - 616
EP - 618
JF - Journal of Developmental & Behavioral Pediatrics
JA - J Dev Behav Pediatr
VL - 32
IS - 8
CY - United States
N2 - Sam is a 27-month-old boy who you have followed since birth. He lives with his parents in a small resort town approximately 90 miles outside a major city. Both his parents are professionals in their late 30s and have been highly involved in his care since birth. At the 12-month visit, they were concerned about his difficulty regulating. He was not sleeping through the night and had significant difficulty with baths. His physical examination and growth were normal. His eye contact was good, although it was difficult to see him smile. He had 1 or 2 words and was beginning to walk independently.At the 15-month checkup, they continued to be concerned about his poor regulation. He napped sporadically, and he was very difficult to take out on errands as he did not like his car seat. He now had approximately 10 single words, was using his fingers to point, and very clearly waved "bye bye" as soon as you entered the room.At the 18-month checkup, they state that he has not yet learned the word "no." He will follow a 1-step command when he wants to but now has 15 single words without any combinations. He points for his needs and to show them something. He has become increasingly "shy" around strangers and prefers to play with one other child as opposed to a larger group. He does not like loud noises and prefers to go barefoot constantly. His physical examination was again normal as was his growth. He is referred for a full hearing evaluation, which is also normal. The family was referred to early intervention, and he began receiving speech and language therapy and occupational therapy for his sensory challenges as well as a play group.At the 24-month checkup, his language continued to consist of single words-now approximately 30. When the parents do not understand what he wants, he will often tantrum and has started banging his head on the floor when frustrated. He has no repetitive behaviors and is starting to demonstrate imaginative play. Bath time has becoming increasingly challenging because he does not like the sensation of soap and the water temperature must be "just right." You refer the child to a Developmental and Behavioral Pediatrician for evaluation and at 28 months he is seen. During his testing visit, he had decreased eye contact and followed his own agenda but improved significantly as testing progressed. As he got more comfortable, he began making good eye contact, social referenced, and exhibited joint attention with his parents and the examiner. He did not meet criteria for an autism spectrum disorder or specifically pervasive developmental disorder-not otherwise specified (PDD-NOS). He was given a diagnosis of mixed receptive and expressive language delay and

disruptive behavior disorder with sensory processing problems.The parents come to you a month after their evaluation visit asking you to give him a "listed diagnosis of PDD-NOS" that could be removed when he turns 3 years so that he may qualify for increased hours of services-up to 15 hours per week-as well as applied behavioral analysis therapy. A behavioral therapist through early intervention has told the family that he would benefit from this increased intervention, specifically applied behavioral analysis but the only way he can receive it is with a "medical diagnosis" on the autism spectrum. What do you do next?
SN - 1536-7312
AD - Boston University School of Medicine for Reddy, Boston, MA, USA
UR - 21904210
ER -

TY - JOUR
ID - 550
T1 - Geographic disparities in state and district policies targeting youth obesity
A1 - Taber,D.R.
A1 - Chriqui,J.F.
A1 - Chaloupka,F.J.
Y1 - 2011/10//
N1 - Taber, Daniel R. Chriqui, Jamie F. Chaloupka, Frank J
American journal of preventive medicine
8704773, apl
IM
Journal Article. Research Support, Non-U.S. Gov't
English
KW - MEDLINE
KW - Adolescent
KW - Body Mass Index
KW - Child
KW - Exercise
KW - Food Services/lj [Legislation & Jurisprudence]
KW - Food Services/og [Organization & Administration]
KW - Food Services
KW - Health Promotion
KW - Health Status Disparities
KW - Healthcare Disparities
KW - Humans
KW - Local Government
KW - Logistic Models
KW - Nutrition Policy
KW - Obesity/ep [Epidemiology]
KW - Obesity/pc [Prevention & Control]
KW - Policy Making
KW - Prevalence
KW - Schools
KW - State Government
KW - United States/ep [Epidemiology]
RP - NOT IN FILE
SP - 407
EP - 414
JF - American Journal of Preventive Medicine
JA - Am J Prev Med
VL - 41
IS - 4
CY - Netherlands
N2 - BACKGROUND: States and school districts nationwide have enacted policies targeting youth obesity, but many policies lack specific requirements or enforcement language. Geographic disparities in youth obesity could worsen if policies are weaker in areas with higher obesity prevalence. PURPOSE: To determine if state or

district policy strength varies with youth obesity prevalence across Census divisions. METHODS: Policies in five domains related to nutrition and physical activity in schools were obtained from all states and nationally representative samples of 578 and 592 public school districts in the 2006-2007 and 2008-2009 school years, respectively. Policy language strength was rated on a 0-100 scale on both the state and district level. Regression models were used to determine if mean 2006-2007 strength scores, and changes in mean scores from 2006-2007 to 2008-2009, were associated with youth obesity prevalence across Census divisions. Analyses were conducted in 2010. RESULTS: State and district policies governing foods sold outside of school meal programs ("competitive foods") were stronger in 2006-2007 in the two divisions with the highest youth obesity prevalence (East South Central, West South Central). Furthermore, mean competitive food policy strength increased the most from 2006-2007 to 2008-2009 in these divisions. The West South Central had the weakest district physical education policies in 2006-2007, however, and was the only division in which average strength of district school meal policies decreased. CONCLUSIONS: State and districts in Census divisions with the highest youth obesity prevalence are taking steps to restrict competitive foods in schools, but many have not targeted other policy domains as aggressively. Copyright 2011 American Journal of Preventive Medicine. Published by Elsevier Inc. All rights reserved
SN - 1873-2607
AD - Health Policy Center, Institute for Health Research and Policy, University of Illinois at Chicago, USA. dtaber@uic.edu
UR - 21961468
ER -

TY - JOUR
ID - 551
T1 - The influence of physical education on physical activity levels of urban elementary students
A1 - Dauenhauer,B.D.
A1 - Keating,X.D.
Y1 - 2011/09//
N1 - Dauenhauer, Brian D. Keating, Xiaofen D
Research quarterly for exercise and sport
r6y, 8006373
IM
Journal Article
English
KW - MEDLINE
KW - African Continental Ancestry Group
KW - Analysis of Variance
KW - Child
KW - Female
KW - Health Behavior
KW - Hispanic Americans
KW - Humans
KW - Male
KW - Monitoring
KW - Ambulatory/is [Instrumentation]
KW - Motor Activity
KW - Physical Education and Training
KW - Time Factors
KW - Urban Population
RP - NOT IN FILE
SP - 512
EP - 520
JF - Research Quarterly for Exercise & Sport
JA - Res Q Exerc Sport
VL - 82
IS - 3
CY - United States

N2 - The purpose of this study was to examine the role of physical education in shaping physical activity patterns. Seventy-one Hispanic and African American elementary students participated in the study. Students attended one 30- and one 60-min physical education class weekly. Pedometer steps were used to estimate physical activity. Data suggest that students did not engage in enough physical activity on a daily basis to incur health benefits. There were significant step differences in 0-, 30-, and 60-min physical education days, with the most steps occurring on 60-min days. Results from the study suggest physical education may be an important source of physical activity for Hispanic and African American students, especially girls, and may influence participation in physical activity outside of class
SN - 0270-1367
AD - Department of Curriculum and Instruction, College of Education, The University of Texas-Austin, TX 78712, USA. dauenhauer@mail.utexas.edu
UR - 21957710
ER -

TY - JOUR
ID - 552
T1 - Modeling the bullying prevention program design recommendations of students from grades five to eight: a discrete choice conjoint experiment
A1 - Cunningham,C.E.
A1 - Vaillancourt,T.
A1 - Cunningham,L.J.
A1 - Chen,Y.
A1 - Ratcliffe,J.
Y1 - 2011/11//
N1 - Cunningham, Charles E. Vaillancourt, Tracy. Cunningham, Lesley J. Chen, Yvonne. Ratcliffe, Jenna
Aggressive behavior
7502265
IM
Journal Article. Research Support, Non-U.S. Gov't
English
KW - MEDLINE
KW - Adolescent
KW - Bullying/px [Psychology]
KW - Bullying
KW - Child
KW - Humans
KW - Models
KW - Theoretical
KW - Questionnaires
KW - Schools/og [Organization & Administration]
KW - Students/px [Psychology]
RP - NOT IN FILE
SP - 521
EP - 537
JF - Aggressive Behavior
JA - Aggress.Behav
VL - 37
IS - 6
CY - United States
N2 - We used a discrete choice conjoint experiment to model the bullying prevention recommendations of 845 students from grades 5 to 8 (aged 9-14). Students made choices between experimentally varied combinations of 14 four-level prevention program attributes. Latent class analysis yielded three segments. The high impact segment (27.1%) recommended uniforms, mandatory recess activities, four playground supervisors, surveillance cameras, and 4-day suspensions when students bully. The moderate impact segment (49.5%) recommended discretionary uniforms and recess activities, four playground supervisors, and 3-day suspensions. Involvement as a bully or bully-victim was associated with membership in a low impact segment (23.4%) that rejected uniforms and surveillance cameras. They recommended fewer anti-bullying activities, discretionary recess

activities, fewer playground supervisors, and the 2-day suspensions. Simulations predicted most students would recommend a program maximizing student involvement combining prevention with moderate consequences. The simulated introduction of mandatory uniforms, surveillance cameras, and long suspensions reduced overall support for a comprehensive program, particularly among students involved as bullies or bully-victims. 2011 Wiley Periodicals, Inc
SN - 1098-2337
AD - Department of Psychiatry and Behavioural Neurosciences, McMaster University, Hamilton, Ontario, Canada. cunnic@hhsc.ca
UR - 21866555
ER -

TY - JOUR
ID - 553
T1 - Characteristics of microsporidial keratoconjunctivitis in an eastern Indian cohort: a case series
A1 - Sengupta,J.
A1 - Saha,S.
A1 - Khetan,A.
A1 - Pal,D.
A1 - Gangopadhyay,N.
A1 - Banerjee,D.
Y1 - 2011/07//
N1 - Sengupta, Jayangshu. Saha, Suman. Khetan, Archana. Pal, Dipanjan. Gangopadhyay, Nibaran. Banerjee, Debdulal
Indian journal of pathology & microbiology
gkk, 7605904
IM
Journal Article
English
KW - MEDLINE
KW - Adolescent
KW - Adult
KW - Age Distribution
KW - Child
KW - Cohort Studies
KW - Environmental Exposure
KW - Female
KW - Humans
KW - India/ep [Epidemiology]
KW - Keratoconjunctivitis/di [Diagnosis]
KW - Keratoconjunctivitis/ep [Epidemiology]
KW - Keratoconjunctivitis/mi [Microbiology]
KW - Keratoconjunctivitis/pa [Pathology]
KW - Male
KW - Microbiological Techniques/mt [Methods]
KW - Microsporidia/ip [Isolation & Purification]
KW - Microsporidiosis/di [Diagnosis]
KW - Microsporidiosis/ep [Epidemiology]
KW - Microsporidiosis/mi [Microbiology]
KW - Microsporidiosis/pa [Pathology]
KW - Retrospective Studies
KW - Risk Factors
KW - Young Adult
RP - NOT IN FILE
SP - 565
EP - 568
JF - Indian Journal of Pathology & Microbiology
JA - Indian J Pathol Microbiol

VL - 54
IS - 3
CY - India
N2 - BACKGROUND: Microsporidia are intracellular parasites responsible for human infections. Recently, there has been an increase in the incidence of microsporidial keratoconjunctivitis (MKC) affecting normal individuals worldwide. AIM: To determine the characteristics of MKC in an Indian cohort. MATERIALS AND METHODS: This is a retrospective, noncomparative, observational case series, involving patients with MKC between June and September 2009. Of the 24 patients identified, microbiological confirmation in direct smear was obtained in 22 cases and selected. Standard microbiological workup was performed in all the cases. We studied the demographics, predisposing conditions, antecedent treatment received before presentation, clinical characteristics, treatment offered, and resolution time with sequel. The management consisted of simple debridement and application of chloramphenicol ointment (1%) two times a day. RESULTS: Mean age of onset was 18.7 years (95% CI, 15.7-21.7; range, 11-36 s years). All patients gave history of prior outdoor activity and exposure to rain water/mud. Antecedent treatment comprised of Acyclovir eye ointment (45.4%) and antibiotic eye drop (27.3%) most commonly. Microsporidia were identified in Gram stain (81.8%), 10% potassium hydroxide mount (72.7%), modified Ziehl-Neelsen staining (36.4%), and Giemsa (18.2%). Majority presented as unilateral superficial keratoconjunctivitis with punctate epithelial keratitis. Mean resolution time was 9 days (95%CI, 7.9-10.2). CONCLUSIONS: MKC can occur in normal patients with exposure to rain and mud, related to outdoor activity often misdiagnosed as viral ocular infections. Strong clinical suspicion with proper microbiological evaluation helps to diagnose this commonly misdiagnosed condition
SN - 0974-5130
AD - Corneal and Ocular surface disease clinic, Priyamvada Birla Aravind Eye Hospital, Kolkata, India. jayansu@hotmail.com
UR - 21934222
ER -

TY - JOUR
ID - 554
T1 - Physical activity and the achievement gap among urban minority youth. [Review]
A1 - Basch,C.E.
Y1 - 2011/10//
N1 - Basch, Charles E
The Journal of school health
k13, 0376370
IM, N
Journal Article. Review
English
KW - MEDLINE
KW - Absenteeism
KW - Achievement
KW - Adolescent
KW - Adolescent Behavior
KW - Age Factors
KW - Attention
KW - Child
KW - Child Welfare
KW - Cognition
KW - Curriculum
KW - Health Promotion
KW - Health Status Disparities
KW - Humans
KW - Learning
KW - Minority Groups/px [Psychology]
KW - Minority Groups/sn [Statistics & Numerical Data]
KW - Motor Activity
KW - Physical Fitness
KW - Prevalence

KW - Risk Factors
KW - School Health Services
KW - Schools
KW - Socioeconomic Factors
KW - Students
KW - United States
KW - Urban Population/sn [Statistics & Numerical Data]
RP - NOT IN FILE
SP - 626
EP - 634
JF - Journal of School Health
JA - J Sch Health
VL - 81
IS - 10
CY - United States
N2 - OBJECTIVES: To outline the prevalence and disparities of physical activity among school-aged urban minority youth, causal pathways through which low levels of physical activity and fitness adversely affects academic achievement, and proven or promising approaches for schools to increase physical activity and physical fitness among youth. METHODS: Literature review. RESULTS: A large proportion of youth is insufficiently physically active. Estimates of population-wide levels of physical activity indicate that Black and Hispanic youth are less physically active than White youth, with disparities particularly evident for females. The population segments of youth with lowest levels of physical activity and fitness also have least access to school-based physical activity opportunities and resources. Physical activity affects metabolism and all major body systems, exerting powerful positive influences on the brain and spinal cord and, consequently, on emotional stability, physical health, and motivation and ability to learn. The cornerstone of school-based physical activity programs should be a high-quality physical education program based on national standards. Such programs are strongly recommended by the Task Force on Community Preventive Services as a way to increase physical activity and physical fitness among youth. CONCLUSIONS: Physical inactivity is highly and disproportionately prevalent among school-aged urban minority youth, has a negative impact on academic achievement through its effects on cognition, and effective practices are available for schools to address this problem. Increasing students' physical activity and physical fitness can best be achieved through a comprehensive approach that includes physical education, wise use of recess and after-school times, co-curricular physical activity opportunities, and bicycling or walking to and from school. 2011, American School Health Association
SN - 1746-1561
AD - Department of Health and Behavior Studies, Teachers College, Columbia University, 525 West 120th Street, New York, NY 10027, USA. ceb35@columbia.edu
UR - 21923875
ER -

TY - JOUR
ID - 555
T1 - Children's play: a tool for public health interventions
A1 - Vitale,A.
Y1 - 2011/10//
N1 - Vitale, Anna
International journal of pediatric obesity : IJPO : an official journal of the International Association for the Study of Obesity
101256330
IM
Journal Article
English
KW - MEDLINE
KW - Child
KW - Preschool
KW - Faculty
KW - Humans
KW - Italy

KW - Parents
KW - Play and Playthings
KW - Public Health Practice
KW - Social Support
RP - NOT IN FILE
SP - 57
EP - 59
JF - International Journal of Pediatric Obesity
JA - Int J Pediatr Obes
VL - 6 Suppl 2
CY - England
N2 - All children need to spend some time playing outdoors. In Northern European countries, schools are equipped with outdoor facilities where children can play during breaks between lessons. The Italian school system, oriented to a mnemonic learning approach, has attached a poor importance to the role of playing for kindergarten age children, and Italian kindergartens are not so well equipped for children's active play. Furthermore, Italian teachers and parents worried that while playing outdoors, children might catch a cold or hurt themselves, and discourage active play outside. The equipment of kindergartens' open spaces with wooden games, where children can play in the morning or after school, resulted in children's increased time of playing outdoors. These playgrounds are also used during vacation day time by children to play and in the evenings, for theatrical and animation events. Finally, watching over their children when playing, "forced" parents to talk to each other creating a social network
SN - 1747-7174
AD - Town Councillor in charge of Social and Educational Policies, Municipality of Ceglie Messapica, Brindisi, Italy. anna_vital@libero.it
UR - 21923299
ER -

TY - JOUR
ID - 556
T1 - Physical activity and play in kindergarten age children
A1 - Caroli,M.
A1 - Malecka-Tendera,E.
A1 - Epifani,S.
A1 - Rollo,R.
A1 - Sansolios,S.
A1 - Matusik,P.
A1 - Mikkelsen,B.E.
Y1 - 2011/10//
N1 - Caroli, Margherita. Malecka-Tendera, Ewa. Epifani, Susi. Rollo, Rodolfo. Sansolios, Sanne. Matusik, Pawel. Mikkelsen, Bent E
International journal of pediatric obesity : IJPO : an official journal of the International Association for the Study of Obesity
101256330
IM
Comparative Study. Journal Article. Research Support, Non-U.S. Gov't
English
KW - MEDLINE
KW - Child
KW - Preschool
KW - Denmark/ep [Epidemiology]
KW - Female
KW - Habits
KW - Humans
KW - Italy/ep [Epidemiology]
KW - Male
KW - Motor Activity
KW - Nutritional Status

KW - Obesity/ep [Epidemiology]
KW - Obesity/eh [Ethnology]
KW - Overweight/ep [Epidemiology]
KW - Overweight/eh [Ethnology]
KW - Play and Playthings
KW - Poland/ep [Epidemiology]
KW - Prevalence
KW - Questionnaires
KW - Sports
KW - Transportation
RP - NOT IN FILE
SP - 47
EP - 53
JF - International Journal of Pediatric Obesity
JA - Int J Pediatr Obes
VL - 6 Suppl 2
CY - England
N2 - PERISCOPE project assesses factors promoting or preventing obesity development in early age. A specific aim is to assess preschool children's physical activity habits in three different European countries. PERISCOPE has been implemented in 1094 children attending kindergartens in Denmark, Italy and Poland. The parents' and children's physical activity habits and attitudes assessed by a questionnaire filled by the parents. Overweight and obesity assessed by Cole's BMI cut-off points. Statistical analysis performed by chi(2) test and the test of proportion. Denmark shows the lowest rate (14.6 %) of overweight, followed by Poland (17.1%), while Italy shows the highest (21.2 %) ($p < 0.0001$). The Polish families show the highest rate of walking from home to kindergarten and back, followed by the Italians and, lastly, the Danish ones ($p < 0.001$). Almost all the Danish and Polish children, but only the 50.1 % of the Italians play outside ($p < 0.001$). During the weekdays, 34.9 % of Polish children, 22.2 % of Italians and 19.8 % of the Danish play outside more than one hour a day ($p < 0.0001$). During the weekend, 91.1 % of Polish children, 86.7 % of Danish children, but only 54.4 % of Italians play outside more than one hour ($p < 0.0001$). 53.5 % of Danish children, 31.9 % of Polish children, and 18.2 % of Italian ones practice sport ($p < 0.0001$). Danish children are the most active, the Polish are in the middle and the Italians are the least active. The difference in infrastructures (safety of walking streets, access to playgrounds/parks, etc.) can play an important role, in addition to cultural and social family characteristics, to the development of overweight
SN - 1747-7174
AD - Nutrition Unit, Department of Prevention, Azienda Sanitaria Locale Brindisi, Brindisi, Italy. mcaroli@libero.it
UR - 21923297
ER -

TY - JOUR
ID - 557
T1 - Development and results of a new methodology to perform focus group with preschool children on their beliefs and attitudes on physical activity
A1 - Cammisa,M.
A1 - Montrone,R.
A1 - Caroli,M.
Y1 - 2011/10//
N1 - Cammisa, Maria. Montrone, Rosa. Caroli, Margherita
International journal of pediatric obesity : IJPO : an official journal of the International Association for the Study of Obesity
101256330
IM
Journal Article. Research Support, Non-U.S. Gov't
English
KW - MEDLINE
KW - Art
KW - Child

KW - Preschool
KW - Environment
KW - Faculty
KW - Female
KW - Focus Groups/mt [Methods]
KW - Health Behavior
KW - Health Knowledge
KW - Attitudes
KW - Practice
KW - Humans
KW - Male
KW - Motor Activity
KW - Parents
KW - Program Development/mt [Methods]
KW - Questionnaires
RP - NOT IN FILE
SP - 22
EP - 27
JF - International Journal of Pediatric Obesity
JA - Int J Pediatr Obes
VL - 6 Suppl 2
CY - England
N2 - The aim of this study is to develop and test a method to perform focus groups (FGs) and to elicit the subjective views of preschool-age children on physical activity and perceived kindergarten barriers to practice it. FGs have been held in three different kindergarten classes with 49 children who were 4-5 years old. Children were asked to draw themselves in their preferred way of playing and were asked few questions about their drawings to understand their behaviours and ideas. In class A and B, 67% and 75% of the children, respectively, drew sedentary plays (table and impersonation games). Children referred that the main obstacle to perform active games outside home/kindergarten was the parents' and teachers' perceived risk that they could be hurt or catch a cold. The children would like to have more table games in the kindergarten. 81% of children in class C drew active group games. All these children were well satisfied with their kindergarten environment and did not refer to any adults' fear regarding active play. This class teacher spent a lot of time to develop children's motor abilities through active games and often used the garden to let the children to play freely. The use of drawings to understand children's habits on physical activity has turned out to be a reliable and easy tool in preschool children. The different results obtained in the two children groups show the need to change the beliefs and the behaviours of teachers and parents who seem to be non-architectural "invisible" barriers to be knocked down. This protocol has been developed by ASL Brindisi within the framework of PERISCOPE's objective to develop new methodologies
SN - 1747-7174
AD - Nutrition Unit, Department of Prevention, Azienda Sanitaria Locale Brindisi, Brindisi, Italy
UR - 21923291
ER -

TY - JOUR
ID - 558
T1 - Target-motion prediction for robotic search and rescue in wilderness environments
A1 - Macwan,A.
A1 - Nejat,G.
A1 - Benhabib,B.
Y1 - 2011/10//
N1 - Macwan, Ashish. Nejat, Goldie. Benhabib, Beno
IEEE transactions on systems, man, and cybernetics. Part B, Cybernetics : a publication of the IEEE Systems, Man, and Cybernetics Society
9890044
IM
Journal Article. Research Support, Non-U.S. Gov't
English

KW - MEDLINE
KW - Child
KW - Preschool
KW - Computer Simulation
KW - Cybernetics
KW - Humans
KW - Models
KW - Biological
KW - Motor Activity/ph [Physiology]
KW - Rescue Work/mt [Methods]
KW - Robotics/is [Instrumentation]
KW - Wilderness
RP - NOT IN FILE
SP - 1287
EP - 1298
JF - IEEE Transactions on Systems, Man, & Cybernetics, Part B: Cybernetics
JA - IEEE Trans Syst Man Cybern.B
VL - 41
IS - 5
CY - United States
N2 - This paper presents a novel modular methodology for predicting a lost person's (motion) behavior for autonomous coordinated multirobot wilderness search and rescue. The new concept of isoprobability curves is introduced and developed, which represents a unique mechanism for identifying the target's probable location at any given time within the search area while accounting for influences such as terrain topology, target physiology and psychology, clues found, etc. The isoprobability curves are propagated over time and space. The significant tangible benefit of the proposed target-motion prediction methodology is demonstrated through a comparison to a nonprobabilistic approach, as well as through a simulated realistic wilderness search scenario
SN - 1941-0492
AD - Department of Mechanical and Industrial Engineering, University of Toronto, Toronto, ON, Canada. a.macwan@utoronto.ca
UR - 21571612
ER -

TY - JOUR
ID - 559
T1 - Environmental modifications to increase physical activity during recess: preliminary findings from ready for recess
A1 - Huberty,J.L.
A1 - Beets,M.W.
A1 - Beighle,A.
A1 - Welk,G.
Y1 - 2011/09//
N1 - Huberty, Jennifer L. Beets, Michael W. Beighle, Aaron. Welk, Greg
Journal of physical activity & health
101189457
IM
Comparative Study. Journal Article. Research Support, Non-U.S. Gov't
English
KW - MEDLINE
KW - Acceleration
KW - Child
KW - Confidence Intervals
KW - Environment Design
KW - Health Promotion/mt [Methods]
KW - Humans
KW - Motor Activity/ph [Physiology]
KW - Obesity/pc [Prevention & Control]

KW - Program Development
KW - Program Evaluation
KW - Recreation/ph [Physiology]
KW - Schools
KW - Social Marketing
KW - Time Factors
RP - NOT IN FILE
SP - S249
EP - S256
JF - Journal of Physical Activity & Health
JA - J Phys Act Health
VL - 8 Suppl 2
CY - United States
N2 - BACKGROUND: The purpose of this study was to determine the effectiveness of Ready for Recess: an elementary school recess intervention targeting staff training (ST) and providing recreational equipment (EQ). METHODS: Ready for Recess had 4 intervention schools: 1) EQ+ST, 2) EQ, 3) ST, and 4) control. Moderate-to-vigorous physical activity (MVPA) was assessed with accelerometers at the four schools in 257 3rd- to 6th-grade children. Random intercept models for overweight/obese (OWOB) and healthy weight (HW) for boys and girls separately, examined change in percentage of time spent in MVPA during recess across EQ+ST, EQ, and ST compared with the control from baseline to postintervention. RESULTS: HW boys receiving EQ+ST increased MVPA by 19.4%, OWOB boys receiving ST increased MVPA by 4.5%, OWOB girls receiving EQ-ST increased MVPA by 6.0%, while HW girls receiving EQ decreased MVPA by 13.6% in comparison with the control. CONCLUSIONS: Ready for Recess represents a possible means to increase MVPA in OWOB girls/boys, populations least likely to meet MVPA recommendations. However, the effect of the intervention was not uniform across all subgroups
SN - 1543-5474
AD - Dept of Health, Physical Education, and Recreation, University of Nebraska at Omaha, USA
UR - 21918239
ER -

TY - JOUR
ID - 560
T1 - Early pubertal timing and girls' problem behavior: integrating two hypotheses
A1 - Stattin,H.
A1 - Kerr,M.
A1 - Skoog,T.
Y1 - 2011/10//
N1 - Stattin, Hakan. Kerr, Margaret. Skoog, Therese
Journal of youth and adolescence
0333507
IM
Journal Article. Research Support, Non-U.S. Gov't
English
KW - MEDLINE
KW - Adolescent
KW - Adolescent Behavior/ph [Physiology]
KW - Adolescent Behavior
KW - Age Factors
KW - Alcoholic Intoxication
KW - Child
KW - Cross-Sectional Studies
KW - Female
KW - Friends
KW - Humans
KW - Interpersonal Relations
KW - Juvenile Delinquency
KW - Leisure Activities

KW - Longitudinal Studies
KW - Menarche
KW - Peer Group
KW - Puberty/px [Psychology]
KW - Schools
KW - Self Report
KW - Socialization
KW - Sweden
RP - NOT IN FILE
SP - 1271
EP - 1287
JF - Journal of Youth & Adolescence
JA - J Youth Adolesc
VL - 40
IS - 10
CY - United States
N2 - Girls' early pubertal timing has been linked in many studies to behavioral problems such as delinquency and substance use. The theoretical explanations for these links have often involved the girls' peer relationships, but contexts have also been considered important in some explanations. By integrating two theoretical models, the peer-socialization and the contextual-amplification hypotheses, we propose a contextual framework for explaining the link between early pubertal timing and external problem behavior in girls. We hypothesize that early developing girls engage in unhealthy, dangerous, and risky behavior under contextual conditions that promote access to older friends and opposite-sex relationships. Under other conditions it is less likely. We tested this integrated hypothesis in two studies conducted in Sweden. The first was a cross-sectional study with information about school and free-time friends in a community sample (N = 284). Early pubertal timing was linked to having older, more normbreaking friends outside of school, but not in school, thus suggesting that the school context interferes early-developing girls' selection of older peers. The second study involved both a longitudinal (N = 434) and a cross-sectional sample of girls (N = 634), where we examined a leisure setting that is known to attract delinquent youth. Results showed that early pubertal timing was most strongly linked to delinquency for girls who spent time in this context and were heavily involved with boys and peers. In sum, results from both studies supported our predictions that certain contexts would amplify the peer-socialization effect. Overall, we conclude that the integrated peer-socialization/contextual-amplification model satisfactorily explains the link between pubertal timing and external problem behavior
SN - 1573-6601
AD - Center for Developmental Research, Academy of Law, Psychology, and Social Work, JPS: Psychology, Orebro University, 701 82, Orebro, Sweden. hakan.stattin@oru.se
UR - 21769611
ER -

TY - JOUR
ID - 561
T1 - Street connectivity is negatively associated with physical activity in Canadian youth
A1 - Mecredy,G.
A1 - Pickett,W.
A1 - Janssen,I.
Y1 - 2011/08//
N1 - Mecredy, Graham. Pickett, William. Janssen, Ian
International journal of environmental research and public health
101238455
IM
Journal Article. Research Support, Non-U.S. Gov't
English
KW - MEDLINE
KW - Adolescent
KW - Adolescent Behavior
KW - Canada
KW - Child

KW - Child Behavior
KW - Cross-Sectional Studies
KW - Exercise
KW - Female
KW - Geographic Information Systems
KW - Health Surveys
KW - Humans
KW - Male
KW - Questionnaires
KW - Regression Analysis
KW - Residence Characteristics/cl [Classification]
KW - Risk
KW - Schools
KW - Students
KW - Transportation
KW - Walking
RP - NOT IN FILE
SP - 3333
EP - 3350
JF - International Journal of Environmental Research & Public Health [Electronic Resource]
JA - Int J Environ Res Public Health
VL - 8
IS - 8
CY - Switzerland
N2 - Street connectivity, defined as how well streets connect to one and other and the density of intersections, is positively associated with active transportation in adults. Our objective was to study the relation between street connectivity and physical activity in youth. Study participants consisted of 8,535 students in grades 6-10 from 180 schools across Canada who completed the 2006 Health Behaviour in School-aged Children (HBSC) survey. Street connectivity was measured in a 5 km circular buffer around these schools using established geographic information system measures. Physical activity performed outside of school hours was assessed by questionnaire, and multi-level regression analyses were used to estimate associations with street connectivity after controlling for several covariates. Compared to students living in the highest street connectivity quartile, those in the second (relative risk = 1.22, 95% confidence interval = 1.10-1.35), third (1.25, 1.13-1.37), and fourth (1.21, 1.09-1.34) quartiles were more likely to be physically active outside of school. In conclusion, youth in neighbourhoods with the most highly connected streets reported less physical activity outside of school than youth from neighbourhoods with less connected streets. Relationships between street connectivity and physical activity reported in this national study are in the opposite direction to those previously observed for active transportation in adult populations
SN - 1660-4601
AD - Department of Community Health and Epidemiology, Queen's University, 99 University Avenue, Kingston, ON K7L 3N6, Canada. grahammecredy@gmail.com
UR - 21909310
ER -

TY - JOUR
ID - 562
T1 - Exploring the impact of once-daily OROS methylphenidate (MPH) on symptoms and quality of life in children and adolescents with ADHD transitioning from immediate-release MPH
A1 - Kordon,A.
A1 - Stollhoff,K.
A1 - Niederkirchner,K.
A1 - Mattejat,F.
A1 - Rettig,K.
A1 - Schauble,B.
Y1 - 2011/09//
N1 - Kordon, Andreas. Stollhoff, Kirsten. Niederkirchner, Kate. Mattejat, Fritz. Rettig, Klaus. Schauble, Barbara

Postgraduate medicine
0401147, pfk
AIM, IM
Clinical Trial. Journal Article
English
KW - MEDLINE
KW - Activities of Daily Living/px [Psychology]
KW - Adolescent
KW - Attention Deficit Disorder with Hyperactivity/dt [Drug Therapy]
KW - Attention Deficit Disorder with Hyperactivity/px [Psychology]
KW - Central Nervous System Stimulants/ad [Administration & Dosage]
KW - Central Nervous System Stimulants/tu [Therapeutic Use]
KW - Child
KW - Delayed-Action Preparations
KW - Female
KW - Humans
KW - Male
KW - Methylphenidate/ad [Administration & Dosage]
KW - Methylphenidate/tu [Therapeutic Use]
KW - Prospective Studies
KW - Psychiatric Status Rating Scales
KW - Quality of Life/px [Psychology]
RP - NOT IN FILE
SP - 27
EP - 38
JF - Postgraduate Medicine
JA - Postgrad Med
VL - 123
IS - 5
CY - United States
N2 - OBJECTIVES: To explore the clinical and health-related quality of life (HRQoL) outcomes in children/adolescents with attention-deficit/hyperactivity disorder (ADHD) who required a therapy switch from immediate-release (IR) methylphenidate (MPH) and were initiated on Osmotic Release Oral System (OROS()) MPH. METHODS: Prospective, noninterventional study including patients (aged 6-18 years) with a confirmed diagnosis of ADHD who transitioned from IR MPH to OROS() MPH based on medical needs. Patients were transitioned to OROS() MPH and were followed for 12 weeks. Attention-deficit/hyperactivity disorder symptoms, functional outcomes, HRQoL, and tolerability were assessed throughout the study. RESULTS: 598 patients entered the intention-to-treat analysis. The mean OROS() MPH starting dose was 29.5 + 12.0 mg/day, increasing slightly to 33.5 + 13.2 mg/day at final visit. Compared with baseline, there were significant (all P < 0.0001) symptomatic, functional, and HRQoL improvements after transitioning from IR MPH to OROS() MPH as assessed by the Conners' Parent Rating Scale (from 29.0 + 10.5 to 19.5 + 11.1), Children's Global Assessment Scale (by 11.0 + 13.3), and Inventory for Assessing Quality of Life (ILC) LQ0-28 scores (parents' rating from 17.2 + 3.9 to 19.4 + 4.0; patients' rating from 18.7 + 4.0 to 20.5 + 3.9). Overall, no significant changes in quality of sleep or appetite were observed. More than 70% of parents and physicians rated the effectiveness of OROS() MPH as at least "good" and were at least "satisfied" with OROS() MPH. The most common treatment-emergent adverse events were insomnia and anorexia. No clinically relevant changes in body weight or vital signs were observed. CONCLUSIONS: In this naturalistic setting, transitioning from IR MPH to OROS() MPH, in patients who showed previously insufficient response and/or poor tolerability, was successful. Patients' and parents' HRQoL as well as burden of disease showed a clinically relevant improvement. OROS() MPH was generally safe and well tolerated
SN - 1941-9260
AD - Klinik fur Psychiatrie und Psychotherapie, Lubeck, Germany
UR - 21904084
ER -

TY - JOUR
ID - 563

T1 - Widespread cortical thinning in children with frontal lobe epilepsy
A1 - Widjaja,E.
A1 - Mahmoodabadi,S.Z.
A1 - Snead,O.C.,III
A1 - Almehdar,A.
A1 - Smith,M.L.
Y1 - 2011/09//
N1 - Widjaja, Elysa. Mahmoodabadi, Sina Zarei. Snead, O Carter 3rd. Almehdar, Abeer. Smith, Mary Lou
Epilepsia
eix, 2983306r
IM
Journal Article. Research Support, Non-U.S. Gov't
English
KW - MEDLINE
KW - Adolescent
KW - Brain Mapping
KW - Cerebral Cortex/pa [Pathology]
KW - Child
KW - Epilepsy
KW - Frontal Lobe/pa [Pathology]
KW - Female
KW - Functional Laterality
KW - Humans
KW - Magnetic Resonance Imaging
KW - Male
KW - Young Adult
RP - NOT IN FILE
SP - 1685
EP - 1691
JF - Epilepsia
VL - 52
IS - 9
CY - United States
N2 - PURPOSE: Spread of seizure activity outside the frontal lobe due to cortico-cortical connections can result in alteration in the cortex beyond the frontal lobe in children with intractable frontal lobe epilepsy (FLE). The aim of this study was to identify regions of reduced cortical thickness in children with intractable FLE. METHODS: High-resolution volumetric T(1)-weighted imaging was performed on 17 children with FLE, who were being evaluated for epilepsy surgery, and 26 age-matched healthy controls. The cortical thickness of 12 patients with left FLE and 5 patients with right FLE was compared to controls. The clusters of cortical thinning were regressed against age of seizure onset, duration of epilepsy, seizure frequency, and number of medications. KEY FINDINGS: In children with left FLE, cortical thinning was present in the left superior frontal, paracentral, precuneus, cingulate, inferior parietal, supramarginal, postcentral, and superior temporal gyri, as well as in the right superior and middle frontal, medial orbitofrontal, supramarginal, postcentral, banks of superior temporal sulcus, and parahippocampal gyri. In children with right FLE, cortical thinning was present in the right precentral, postcentral, transverse temporal, parahippocampal, lingual, and lateral occipital gyri, as well as in the left superior frontal, inferior parietal, postcentral, superior temporal, posterior cingulate, and lingual gyri. In children with left FLE, following exclusion of one outlier, there was no significant association between age at seizure onset, duration of epilepsy, seizure frequency and number of medications with clusters of cortical thinning. In children with right FLE, age at seizure onset, duration of epilepsy, frequency of seizures, and number of medications were not associated with clusters of cortical thinning within the right and left hemispheres. SIGNIFICANCE: Cortical changes were present in the frontal and extrafrontal cortex in children with intractable FLE. These changes may be related to spread of seizure activity, large epileptogenic zones involving both frontal and extrafrontal lobes, and development of secondary epileptogenic zones that over time lead to cortical abnormality. Further studies correlating cortical changes with neurocognitive measures are needed to determine if the cortical changes relate to cognitive function. Wiley Periodicals, Inc. 2011 International League Against Epilepsy
SN - 1528-1167

AD - Diagnostic Imaging, Hospital for Sick Children, Toronto, Ontario, Canada. Elysa.Widjaja@sickkids.ca
UR - 21627647
ER -

TY - JOUR
ID - 564
T1 - Association between school- and nonschool-based activity programs and physical activity in adolescent girls
A1 - Taber,D.R.
A1 - Stevens,J.
A1 - Lytle,L.A.
A1 - Foreman,R.D.
A1 - Moody,J.
A1 - Parra-Medina,D.
A1 - Pratt,C.A.
Y1 - 2011/09//
N1 - Taber, Daniel R. Stevens, June. Lytle, Leslie A. Foreman, Robyn D. Moody, Jamie. Parra-Medina, Deborah. Pratt, Charlotte A
Journal of physical activity & health
101189457
IM
Journal Article. Research Support, N.I.H., Extramural
English
KW - MEDLINE
KW - Adolescent
KW - Age Factors
KW - Body Mass Index
KW - Child
KW - Cross-Sectional Studies
KW - Exercise
KW - Female
KW - Humans
KW - Schools/og [Organization & Administration]
KW - Socioeconomic Factors
RP - NOT IN FILE
SP - 971
EP - 977
JF - Journal of Physical Activity & Health
JA - J Phys Act Health
VL - 8
IS - 7
CY - United States
N2 - BACKGROUND: Some researchers have questioned if activity programs would be more effective if based outside school (eg, community leagues) rather than within schools. This study compared participation in activity programs based within and outside of school, and estimated the associations between participation and moderate-vigorous physical activity (MVPA) among adolescent girls. METHODS: Within the Trial of Activity for Adolescent Girls, independent samples of 1559 6th-grade girls (age 11 to 12) and 3282 8th-grade girls (age 13 to 14) reported program participation using questionnaires. MVPA was measured using accelerometers. Linear mixed models accounted for school and site clustering. RESULTS: Sixth-grade girls reported 5 times as many programs outside school as within school (4.1 vs. 0.8); daily MVPA was 0.29 minutes higher (1.2% of the mean) for each additional program outside school. Compared with 6th-grade girls, 8th-grade girls participated in 1.3 fewer programs outside school, while programs' association with MVPA was unchanged. Conversely, school programs' association with MVPA was greater in 8th grade. Daily MVPA was 1.33 minutes higher per school program, and participation declined 0.13. CONCLUSION: Programs within and outside schools can both increase activity among adolescent girls. Intervention research should focus on increasing participation in school programs, and increasing movement during programs outside school
SN - 1543-5474

AD - Dept of Epidemiology, University of North Carolina at Chapel Hill, USA
UR - 21885888
ER -

TY - JOUR
ID - 565
T1 - [Epidemiological description of rabies reservoir in bats in the Metropolitan Region: Chile. 2000-2009]. [Spanish]
A1 - Favi,C.M.
A1 - Bassaletti,C.A.
A1 - Lopez,D.J.
A1 - Rodriguez,A.L.
A1 - Yung,P.,V
Y1 - 2011/06//
N1 - Favi C, Myriam. Bassaletti C, Angela. Lopez D, Javier. Rodriguez A, Luis. Yung P, Veronica
Revista chilena de infectologia : organo oficial de la Sociedad Chilena de Infectologia
9305754
IM
English Abstract. Journal Article
Spanish
KW - MEDLINE
KW - Adult
KW - Animals
KW - Child
KW - Chile/ep [Epidemiology]
KW - Chiroptera/vi [Virology]
KW - Disease Reservoirs/ve [Veterinary]
KW - Female
KW - Humans
KW - Male
KW - Middle Aged
KW - Rabies/ep [Epidemiology]
KW - Rabies/tm [Transmission]
KW - Rabies/ve [Veterinary]
KW - Rabies virus/ip [Isolation & Purification]
KW - Urban Population
RP - NOT IN FILE
SP - 223
EP - 228
JF - Revista Chilena de Infectologia
VL - 28
IS - 3
CY - Chile
N2 - In Chile, in 1985 rabies was detected in insectivorous bats. Since then the epidemiological surveillance activities was extended to these species and the epidemiological pattern was characterized as endemic in bats. In this study we analyzed positive rabies cases in the Metropolitan Region between 2000 and 2009. We identified 325 cases of rabies in 11.472 analyzed samples. We determined an increase of positivity cases in bats between the years of the study. Places with the highest number of positives cases were Las Condes, Santiago, Providencia, Puente Alto and Nunoa. In 147 cases we identify the circumstances in which the bat was found: 89 inside, 45 outside the building and 13 in public spaces. In 39 cases there was contact with humans or animals. These results reinforce the relevance of educating the population against the contact with bats and allow health authorities to take early surveillance and control measures
SN - 0716-1018
AD - Instituto de Salud Publica de Chile
UR - 21879147
ER -

TY - JOUR
ID - 566
T1 - Investigating a pediatric hospital's response to an inpatient census surge during the 2009 H1N1 influenza pandemic
A1 - Van Cleve,W.C.
A1 - Hagan,P.
A1 - Lozano,P.
A1 - Mangione-Smith,R.
Y1 - 2011/08//
N1 - Van Cleve, William C. Hagan, Pat. Lozano, Paula. Mangione-Smith, Rita
Joint Commission journal on quality and patient safety / Joint Commission Resources
101238023
IM
Journal Article
English
KW - MEDLINE
KW - Child
KW - Disease Outbreaks
KW - Hospital Records
KW - Hospitals
KW - Pediatric/og [Organization & Administration]
KW - Pediatric/sn [Statistics & Numerical Data]
KW - Humans
KW - Influenza A Virus
KW - H1N1 Subtype
KW - Influenza
KW - Human/ep [Epidemiology]
KW - Interviews as Topic
KW - Organizational Case Studies
KW - Patient Discharge/st [Standards]
KW - Patient Discharge/sn [Statistics & Numerical Data]
KW - Program Evaluation
KW - Surge Capacity/og [Organization & Administration]
KW - Surge Capacity/sn [Statistics & Numerical Data]
KW - Triage/mt [Methods]
KW - Triage/st [Standards]
KW - Washington
RP - NOT IN FILE
SP - 376
EP - 382
JF - Joint Commission Journal on Quality & Patient Safety
JA - Jt.Comm J Qual Patient Saf
VL - 37
IS - 8
CY - United States
N2 - BACKGROUND: On November 4, 2009, the 250-bed Seattle Children's Hospital (SCH) identified a surge in its census--245 inpatients, well above the average midnight census of 207. In response, SCH activated its pandemic influenza surge plan in an effort to decrease the inpatient census. Within 16 hours, 51 patients (20.4% of total bed capacity) had been discharged, and inpatient census at SCH decreased to 222 patients. METHODS: As part of a quality improvement project, SCH's response to the surge was investigated, with data drawn from interviews, a review of records created in the course of the surge plan implementation, an e-mail survey of attending physicians responsible for patient discharges, and models examining predictors of hospital discharges. FINDINGS: Analysis of three years of hospital data (2007-2009) indicated that the high census on November 4 was an uncommon but not unprecedented occurrence. In addition, there was a clear positive association between an evening's census and the number of discharges during the following 24 hours. SCH discharged essentially the same number of patients on November 4 as on previous high-census days when the surge plan was not activated, suggesting that the surge plan did not succeed in creating excess discharges. CONCLUSIONS:

Increasingly, evidence indicates that care quality depends on the degree to which hospital resources are sufficient to meet demand. Reverse triage, at least as implemented by SCH on November 4, 2009, is unlikely to represent an effective solution to surge outside of a disaster setting because of its requirement for centralized decision making. SCH has incorporated the results of this review into the way that it collects and analyzes data, manages flow, and responds to inpatient surges
SN - 1553-7250
AD - Department of Pediatrics, University of Washington and Center for Child Health, Behavior and Development, Seattle Children's Research Institute, USA. vancleve@uw.edu
UR - 21874973
ER -

TY - JOUR
ID - 567
T1 - Personal endotoxin exposure in a panel study of school children with asthma
A1 - Delfino,R.J.
A1 - Staimer,N.
A1 - Tjoa,T.
Y1 - 2011///
N1 - Delfino, Ralph J. Staimer, Norbert. Tjoa, Thomas
Environmental health : a global access science source
101147645
IM
Journal Article. Research Support, N.I.H., Extramural. Research Support, Non-U.S. Gov't
English
KW - MEDLINE
KW - Adolescent
KW - Air Pollutants/ae [Adverse Effects]
KW - Air Pollutants/an [Analysis]
KW - Animals
KW - Asthma/ci [Chemically Induced]
KW - Asthma/ep [Epidemiology]
KW - California/ep [Epidemiology]
KW - Carbon/an [Analysis]
KW - Carbon/to [Toxicity]
KW - Cats
KW - Child
KW - Dogs
KW - Endotoxins/an [Analysis]
KW - Endotoxins/to [Toxicity]
KW - Environmental Monitoring
KW - Epidemiological Monitoring
KW - Female
KW - Humans
KW - Inhalation Exposure/ae [Adverse Effects]
KW - Inhalation Exposure/an [Analysis]
KW - Linear Models
KW - Longitudinal Studies
KW - Male
KW - Nitrogen Dioxide/an [Analysis]
KW - Nitrogen Dioxide/to [Toxicity]
KW - Particulate Matter/an [Analysis]
KW - Particulate Matter/to [Toxicity]
KW - Pets
KW - Reproducibility of Results
KW - Residence Characteristics
KW - Seasons
KW - Statistics

KW - Nonparametric
KW - Time Factors
RP - NOT IN FILE
SP - 69
JF - Environmental Health: A Global Access Science Source
JA - Environ Health
VL - 10
CY - England
N2 - BACKGROUND: Endotoxin exposure has been associated with asthma exacerbations and increased asthma prevalence. However, there is little data regarding personal exposure to endotoxin in children at risk, or the relation of personal endotoxin exposure to residential or ambient airborne endotoxin. The relation between personal endotoxin and personal air pollution exposures is also unknown. METHODS: We characterized personal endotoxin exposures in 45 school children with asthma ages 9-18 years using 376 repeated measurements from a PM2.5 active personal exposure monitor. We also assayed endotoxin in PM2.5 samples collected from ambient regional sites (N = 97 days) and from a subset of 12 indoor and outdoor subject home sites (N = 109 and 111 days, respectively) in Riverside and Whittier, California. Endotoxin was measured using the Limulus Amoebocyte Lysate kinetic chromogenic assay. At the same time, we measured personal, home and ambient exposure to PM2.5 mass, elemental carbon (EC), and organic carbon (OC). To assess exposure relations we used both rank correlations and mixed linear regression models, adjusted for personal temperature and relative humidity. RESULTS: We found small positive correlations of personal endotoxin with personal PM2.5 EC and OC, but not personal PM2.5 mass or stationary site air pollutant measurements. Outdoor home, indoor home and ambient endotoxin were moderately to strongly correlated with each other. However, in mixed models, personal endotoxin was not associated with indoor home or outdoor home endotoxin, but was associated with ambient endotoxin. Dog and cat ownership were significantly associated with increased personal but not indoor endotoxin. CONCLUSIONS: Daily fixed site measurements of endotoxin in the home environment may not predict daily personal exposure, although a larger sample size may be needed to assess this. This conclusion is relevant to short-term exposures involved in the acute exacerbation of asthma
SN - 1476-069X
AD - Department of Epidemiology, School of Medicine, University of California, Irvine, Irvine, CA 92617-7555, USA. rdelfino@uci.edu
UR - 21810249
ER -

TY - JOUR
ID - 568
T1 - Cleft palate, retrognathia and congenital heart disease in velo-cardio-facial syndrome: a phenotype correlation study
A1 - Friedman,M.A.
A1 - Miletta,N.
A1 - Roe,C.
A1 - Wang,D.
A1 - Morrow,B.E.
A1 - Kates,W.R.
A1 - Higgins,A.M.
A1 - Shprintzen,R.J.
Y1 - 2011/09//
N1 - Friedman, Marcia A. Miletta, Nathanial. Roe, Cheryl. Wang, Dongliang. Morrow, Bernice E. Kates, Wendy R. Higgins, Anne Marie. Shprintzen, Robert J
International journal of pediatric otorhinolaryngology
gs2, 8003603
IM
Journal Article. Research Support, N.I.H., Extramural. Research Support, Non-U.S. Gov't
English
KW - MEDLINE
KW - Abnormalities
KW - Multiple/ep [Epidemiology]
KW - Multiple/ge [Genetics]

KW - Adult
KW - Aged
KW - Child
KW - Preschool
KW - Chromosome Aberrations
KW - Chromosomes
KW - Human
KW - Pair 21/ge [Genetics]
KW - Cleft Palate/ep [Epidemiology]
KW - Cleft Palate/ge [Genetics]
KW - Cohort Studies
KW - DiGeorge Syndrome/ep [Epidemiology]
KW - DiGeorge Syndrome/ge [Genetics]
KW - Female
KW - Genetic Predisposition to Disease/ep [Epidemiology]
KW - Heart Defects
KW - Congenital/ep [Epidemiology]
KW - Congenital/ge [Genetics]
KW - Humans
KW - Infant
KW - Newborn
KW - Male
KW - Middle Aged
KW - Phenotype
KW - Prevalence
KW - Retrognathia/ep [Epidemiology]
KW - Retrognathia/ge [Genetics]
KW - Retrospective Studies
KW - Severity of Illness Index
KW - United States/ep [Epidemiology]
KW - Young Adult
RP - NOT IN FILE
SP - 1167
EP - 1172
JF - International Journal of Pediatric Otorhinolaryngology
JA - Int J Pediatr Otorhinolaryngol
VL - 75
IS - 9
CY - Ireland
N2 - OBJECTIVE: Velo-cardio-facial syndrome (VCFS) is caused by a microdeletion of approximately 40 genes from one copy of chromosome 22. Expression of the syndrome is a variable combination of over 190 phenotypic characteristics. As of yet, little is known about how these phenotypes correlate with one another or whether there are predictable patterns of expression. Two of the most common phenotypic categories, congenital heart disease and cleft palate, have been proposed to have a common genetic relationship to the deleted T-box 1 gene (TBX1). The purpose of this study is to determine if congenital heart disease and cleft palate are correlated in a large cohort of human subjects with VCFS. METHODS: This study is a retrospective chart review including 316 Caucasian non-Hispanic subjects with FISH or CGH microarray confirmed chromosome 22q11.2 deletions. All subjects were evaluated by the interdisciplinary team at the Velo-Cardio-Facial Syndrome International Center at Upstate Medical University, Syracuse, NY. Each combination of congenital heart disease, cleft palates, and retrognathia was analyzed by Chi square or Fisher exact test. RESULTS: For all categories of congenital heart disease and cleft palate or retrognathia no significant associations were found, with the exception of submucous cleft palate and retrognathia (nominal p=0.0325) and occult submucous cleft palate and retrognathia (nominal p=0.000013). CONCLUSIONS: Congenital heart disease and cleft palate do not appear to be correlated in human subjects with VCFS despite earlier suggestions from animal models. Possible explanations include modification of the effect of TBX1 by genes outside of the 22q11.2 region that may further influence the formation of the palate or heart, or the presence of epigenetic factors that may effect genes within the deleted region, modifying genes elsewhere, or polymorphisms on the

normal copy of chromosome 22. Lastly, it is possible that TBX1 plays a role in palate formation in some species, but not in humans. In VCFS, retrognathia is caused by an obtuse angulation of the skull base. It is unknown if the correlation between retrognathia and cleft palate in VCFS indicates a developmental sequence related to skull morphology, or direct gene effects of both anomalies. Much work remains to be done to fully understand the complex relationships between phenotypic characteristics in VCFS. Copyright 2011 Elsevier Ireland Ltd. All rights reserved
SN - 1872-8464
AD - Velo-Cardio-Facial Syndrome International Center, Department of Otolaryngology and Communication Sciences, SUNY Upstate Medical University, 725 Irving Avenue, Suite 406, Syracuse, NY 13210, United States
UR - 21763005
ER -

TY - JOUR
ID - 569
T1 - Report of the 2010 society of thoracic surgeons congenital heart surgery practice and manpower survey
A1 - Jacobs,M.L.
A1 - Daniel,M.
A1 - Mavroudis,C.
A1 - Morales,D.L.
A1 - Jacobs,J.P.
A1 - Fraser,C.D.,Jr.
A1 - Turek,J.W.
A1 - Mayer,J.E.
A1 - Tchervenkov,C.
A1 - Conte,J.
Y1 - 2011/08//
N1 - Jacobs, Marshall L. Daniel, Megan. Mavroudis, Constantine. Morales, David L S. Jacobs, Jeffrey P. Fraser, Charles D Jr. Turek, Joseph W. Mayer, John E. Tchervenkov, Christo. Conte, John
The Annals of thoracic surgery
15030100R
AIM, IM
Journal Article
English
KW - MEDLINE
KW - Adult
KW - Aged
KW - Canada
KW - Certification/sn [Statistics & Numerical Data]
KW - Child
KW - Data Collection
KW - Education
KW - Medical
KW - Graduate
KW - Female
KW - Heart Defects
KW - Congenital/su [Surgery]
KW - Humans
KW - Job Satisfaction
KW - Male
KW - Middle Aged
KW - Retirement/sn [Statistics & Numerical Data]
KW - Societies
KW - Specialization
KW - Thoracic Surgery/ed [Education]
KW - Thoracic Surgery/ma [Manpower]
KW - United States
RP - NOT IN FILE

SP - 762
EP - 768
JF - Annals of Thoracic Surgery
JA - Ann Thorac Surg
VL - 92
IS - 2
CY - Netherlands
N2 - BACKGROUND: The Society of Thoracic Surgeons (STS) Workforce on Congenital Heart Surgery undertook a second subspecialty Practice and Manpower Survey (2005, 2010) to obtain contemporary data. METHODS: Preliminary research suggested a potential target group of 273 congenital heart surgeons, including 258 at 125 US centers and 15 at 8 Canadian centers. The web-based survey was sent to these surgeons, plus all individuals listing pediatric cardiac surgery on CTSNet. RESULTS: Two hundred forty-six responders included 213 active congenital heart surgeons, 16 retired congenital heart surgeons, and 17 surgeons in training. Retirement age was 63.5+7.5 years. Two hundred thirteen active congenital heart surgeon responders represent 78% of the original estimate. Their responses generated the following data: The mean age was 49.2+8.5 (range, 35 to 75 years). American medical school graduates included 159 of 201 respondents (79%). Years of postgraduate training was 9.7+1.7. One hundred ninety-seven (92%) respondents were certified in thoracic surgery by the American Board of Thoracic Surgery (ABTS). Twenty-eight of 200 (14%) received their congenital heart surgery training outside the United States or Canada. One hundred forty-three of 190 respondents (75%) perform exclusively congenital heart operations; 31 (16%) perform exclusively pediatric heart operations. Of 186 respondents, 54 (29%) perform fewer than 100 major congenital heart operations per year, 78 (42%) perform 100 to 199 procedures, and 54 (29%) perform 200 procedures or more. Active congenital heart surgeons have been in their current positions for 9.3+8.6 years. Eight respondents are in their first year of practice. For 203 respondents, mean anticipated years to retirement is 16.1+7.6. Twenty-eight anticipate retirement within 5 years; 31 in 6 to 10 years. CONCLUSIONS: These data should help facilitate rational plans to meet workforce needs for an expanding patient population. Copyright 2011. Published by Elsevier Inc. All rights reserved
SN - 1552-6259
AD - Department of Pediatric and Congenital Heart Surgery, Cleveland Clinic, Cleveland, Ohio, USA. marshall.jacobs@comcast.net
UR - 21865107
ER -

TY - JOUR
ID - 570
T1 - Multi-city assessment of lifetime pregnancy involvement among street youth, Ukraine
A1 - Zapata,L.B.
A1 - Kissin,D.M.
A1 - Robbins,C.L.
A1 - Finnerty,E.
A1 - Skipalska,H.
A1 - Yorick,R.V.
A1 - Jamieson,D.J.
A1 - Marchbanks,P.A.
A1 - Hillis,S.D.
Y1 - 2011/08//
N1 - Zapata, Lauren B. Kissin, Dmitry M. Robbins, Cheryl L. Finnerty, Erin. Skipalska, Halyna. Yorick, Roman V. Jamieson, Denise J. Marchbanks, Polly A. Hillis, Susan D
Journal of urban health : bulletin of the New York Academy of Medicine
c5l, 9809909
IM
Journal Article
English
KW - MEDLINE
KW - Adolescent
KW - Adolescent Behavior
KW - Chi-Square Distribution

KW - Child
KW - Female
KW - Geography
KW - Health Behavior
KW - Health Surveys
KW - Homeless Persons/px [Psychology]
KW - Homeless Persons/sn [Statistics & Numerical Data]
KW - Humans
KW - Logistic Models
KW - Male
KW - Multivariate Analysis
KW - Pregnancy
KW - Unplanned
KW - Residence Characteristics
KW - Risk Assessment
KW - Risk Factors
KW - Risk-Taking
KW - Sexuality/px [Psychology]
KW - Sexuality/sn [Statistics & Numerical Data]
KW - Ukraine
KW - Urban Population/sn [Statistics & Numerical Data]
KW - Young Adult
RP - NOT IN FILE
SP - 779
EP - 792
JF - Journal of Urban Health
JA - J Urban Health
VL - 88
IS - 4
CY - United States
N2 - Although street youth are at increased risk of lifetime pregnancy involvement (LPI), or ever becoming or getting someone pregnant, no reports to date describe the epidemiology of LPI among systematically sampled street youth from multiple cities outside of North America. The purpose of our assessment was to describe the prevalence of and risk factors associated with LPI among street youth from three Ukrainian cities. We used modified time-location sampling to conduct a cross-sectional assessment in Odesa, Kyiv, and Donetsk that included citywide mapping of 91 public venue locations frequented by street youth, random selection of 74 sites, and interviewing all eligible and consenting street youth aged 15-24 years found at sampled sites (n = 929). Characteristics of youth and prevalence of LPI overall and by demographic, social, sexual, and substance use risk factors, were estimated separately for males and females. Adjusted odds ratios (AORs) were calculated with multivariable logistic regression and effect modification by gender was examined. Most (96.6%) eligible youth consented to participate. LPI was reported for 41.7% of females (93/223) and 23.5% of males (166/706). For females, LPI was significantly elevated and highest (>70%) among those initiating sexual activity at <12 years and for those reporting lifetime anal sex and exchanging sex for goods. For males, LPI was significantly elevated and highest (>40%) among those who reported lifetime anal sex and history of a sexually transmitted infection. Overall, risk factors associated with LPI were similar for females and males. Among the total sample (females and males combined), significant independent risk factors with AORs >2.5 included female gender, being aged 20-24 years, having five to six total adverse childhood experiences, initiating sex at age <12 or 13-14 years, lifetime anal sex, most recent sex act unprotected, and lifetime exchange of sex for goods. Among street youth with LPI (n = 259), the most recent LPI event was reported to be unintended by 63.3% and to have ended in abortion by 43.2%. In conclusion, our assessment documented high rates of LPI among Ukrainian street youth who, given the potential for negative outcomes and the challenges of raising a child on the streets, are in need of community-based pregnancy prevention programs and services. Promising preventive strategies are discussed, which are likely applicable to other urban populations of street-based youth as well
SN - 1468-2869
AD - Division of Reproductive Health, Centers for Disease Control and Prevention, National Center for Chronic Disease Prevention and Health Promotion, Atlanta, GA, USA. lzapata@cdc.gov
UR - 21779933

ER -

TY - JOUR
ID - 571
T1 - Influence of degree of specific allergic sensitivity on severity of rhinitis and asthma in Chinese allergic patients
A1 - Li,J.
A1 - Huang,Y.
A1 - Lin,X.
A1 - Zhao,D.
A1 - Tan,G.
A1 - Wu,J.
A1 - Zhao,C.
A1 - Zhao,J.
A1 - Spangfort,M.D.
A1 - Zhong,N.
A1 - China Alliance of Research on Respiratory Allergic Disease (CARRAD)
Y1 - 2011///
N1 - Li, Jing. Huang, Ying. Lin, Xiaoping. Zhao, Deyu. Tan, Guolin. Wu, Jinzhun. Zhao, Changqing. Zhao, Jing. Spangfort, Michael D. Zhong, Nanshan. China Alliance of Research on Respiratory Allergic Disease (CARRAD)
Respiratory research
101090633
IM
Journal Article. Multicenter Study. Research Support, Non-U.S. Gov't
English
KW - MEDLINE
KW - Adolescent
KW - Adult
KW - Aged
KW - Allergens/im [Immunology]
KW - Asthma/di [Diagnosis]
KW - Asthma/ep [Epidemiology]
KW - Asthma/im [Immunology]
KW - Chi-Square Distribution
KW - Child
KW - Preschool
KW - China/ep [Epidemiology]
KW - Cross-Sectional Studies
KW - Humans
KW - Immunoglobulin E/bl [Blood]
KW - Inhalation Exposure
KW - Intradermal Tests
KW - Logistic Models
KW - Middle Aged
KW - Odds Ratio
KW - Questionnaires
KW - Rhinitis
KW - Allergic
KW - Perennial/di [Diagnosis]
KW - Perennial/ep [Epidemiology]
KW - Perennial/im [Immunology]
KW - Seasonal/di [Diagnosis]
KW - Seasonal/ep [Epidemiology]
KW - Seasonal/im [Immunology]
KW - Severity of Illness Index
KW - Young Adult

RP - NOT IN FILE
SP - 95
JF - Respiratory Research
JA - Respir Res
VL - 12
CY - England
N2 - BACKGROUND: The association between sensitizations and severity of allergic diseases is controversial. OBJECTIVE: This study was to investigate the association between severity of asthma and rhinitis and degree of specific allergic sensitization in allergic patients in China. METHOD: A cross-sectional survey was performed in 6304 patients with asthma and/or rhinitis from 4 regions of China. Patients completed a standardized questionnaire documenting their respiratory and allergic symptoms, their impact on sleep, daily activities, school and work. They also underwent skin prick tests with 13 common aeroallergens. Among the recruited subjects, 2268 provided blood samples for serum measurement of specific IgE (sIgE) against 16 common aeroallergens. RESULTS: Significantly higher percentage of patients with moderate-severe intermittent rhinitis were sensitized to outdoor allergens while percentage of patients sensitized to indoor allergens was increased with increasing severity of asthma. Moderate-severe intermittent rhinitis was associated with the skin wheal size and the level of sIgE to Artemisia vulgaris and Ambrosia artemisifolia ($p < 0.001$). Moderate-severe asthma was associated with increasing wheal size and sIgE response to Dermatophagoides (D.) pteronyssinus and D. farinae ($p < 0.001$). Moderate-severe rhinitis and asthma were also associated with increase in number of positive skin prick test and sIgE. CONCLUSIONS: Artemisia vulgaris and Ambrosia artemisifolia sensitizations are associated with the severity of intermittent rhinitis and D. pteronyssinus and D. farinae sensitizations are associated with increasing severity of asthma in China. Increase in number of allergens the patients are sensitized to may also increase the severity of rhinitis and asthma
SN - 1465-993X
AD - State Key Laboratory of Respiratory Disease, The First Affiliated Hospital, Guangzhou Medical College, Guangzhou, Guangdong, China
UR - 21831329
ER -

TY - JOUR
ID - 572
T1 - Assessment of oral bioaccessibility of arsenic in playground soil in Madrid (Spain): a three-method comparison and implications for risk assessment
A1 - Mingot,J.
A1 - De,Miguel E.
A1 - Chacon,E.
Y1 - 2011/09//
N1 - Mingot, Juan. De Miguel, Eduardo. Chacon, Enrique
Chemosphere
b4d, 0320657
IM
Comparative Study. Journal Article
English
KW - MEDLINE
KW - Arsenates/an [Analysis]
KW - Arsenates/me [Metabolism]
KW - Arsenic/an [Analysis]
KW - Arsenic/me [Metabolism]
KW - Calcium Compounds/an [Analysis]
KW - Calcium Compounds/me [Metabolism]
KW - Child
KW - Environmental Exposure/an [Analysis]
KW - Environmental Exposure/sn [Statistics & Numerical Data]
KW - Humans
KW - Hydrogen-Ion Concentration
KW - Mouth/me [Metabolism]
KW - Play and Playthings

KW - Risk Assessment
KW - Soil/ch [Chemistry]
KW - Soil Pollutants/an [Analysis]
KW - Soil Pollutants/me [Metabolism]
KW - Spain
RP - NOT IN FILE
SP - 1386
EP - 1391
JF - Chemosphere
VL - 84
IS - 10
CY - England
N2 - Three methodologies to assess As bioaccessibility were evaluated using playground soil collected from 16 playgrounds in Madrid, Spain: two (Simplified Bioaccessibility Extraction Test: SBET, and hydrochloric acid-extraction: HCl) assess gastric-only bioaccessibility and the third (Physiologically Based Extraction Test: PBET) evaluates mouth-gastric-intestinal bioaccessibility. Aqua regia-extractable (pseudo total) As contents, which are routinely employed in risk assessments, were used as the reference to establish the following percentages of bioaccessibility: SBET-63.1; HCl-51.8; PBET-41.6, the highest values associated with the gastric-only extractions. For Madrid playground soils--characterised by a very uniform, weakly alkaline pH, and low Fe oxide and organic matter contents--the statistical analysis of the results indicates that, in contrast with other studies, the highest percentage of As in the samples was bound to carbonates and/or present as calcium arsenate. As opposed to the As bound to Fe oxides, this As is readily released in the gastric environment as the carbonate matrix is decomposed and calcium arsenate is dissolved, but some of it is subsequently sequestered in unavailable forms as the pH is raised to 5.5 to mimic intestinal conditions. The HCl extraction can be used as a simple and reliable (i.e. low residual standard error) proxy for the more expensive, time consuming, and error-prone PBET methodology. The HCl method would essentially halve the estimate of carcinogenic risk for children playing in Madrid playground soils, providing a more representative value of associated risk than the pseudo-total concentrations used at present. Copyright 2011 Elsevier Ltd. All rights reserved
SN - 1879-1298
AD - Environmental Geochemistry Research and Engineering Laboratory, Universidad Politecnica de Madrid, Alenza 4, E-28003 Madrid, Spain
UR - 21601908
ER -

TY - JOUR
ID - 573
T1 - Effect of school-based interventions on physical activity and fitness in children and adolescents: a review of reviews and systematic update. [Review]
A1 - Kriemler,S.
A1 - Meyer,U.
A1 - Martin,E.
A1 - van Sluijs,E.M.
A1 - Andersen,L.B.
A1 - Martin,B.W.
Y1 - 2011/09//
N1 - Kriemler, S. Meyer, U. Martin, E. van Sluijs, E M F. Andersen, L B. Martin, B W
British journal of sports medicine
0432520
IM
Journal Article. Review
English
KW - MEDLINE
KW - Adolescent
KW - Child
KW - Exercise/ph [Physiology]
KW - Health Promotion/og [Organization & Administration]
KW - Health Status

KW - Humans
KW - Motor Skills/ph [Physiology]
KW - Physical Fitness/ph [Physiology]
KW - Prognosis
KW - Randomized Controlled Trials as Topic
KW - Review Literature as Topic
KW - School Health Services/og [Organization & Administration]
RP - NOT IN FILE
SP - 923
EP - 930
JF - British Journal of Sports Medicine
JA - BJSM online
VL - 45
IS - 11
CY - England
N2 - BACKGROUND: School-based interventions are thought to be the most universally applicable and effective way to counteract low physical activity (PA) and fitness although there is controversy about the optimal strategy to intervene. OBJECTIVES: The objective of this review was to summarise recent reviews that aimed to increase PA or fitness in youth and carry out a systematic review of new intervention studies. METHODS: Relevant systematic reviews and original controlled and randomised controlled school-based trials with a PA or fitness outcome measure, a duration of >12 weeks, a sufficient quality and involvement of a healthy population aged 6-18 years that were published from 2007 to 2010 were included. Results In these reviews, 47-65% of trials were found to be effective. The effect was mostly seen in school-related PA while effects outside school were often not observed or assessed. CONCLUSIONS: The school-based application of multicomponent intervention strategies was the most consistent, promising strategy, while controversy existed regarding the effectiveness of family involvement, focus on healthy populations at increased risk or duration and intensity of the intervention. All 20 trials in the review update showed a positive effect on in-school, out-of-school or overall PA, and 6 of 11 studies showed an increase in fitness. Taking into consideration both assessment quality and public health relevance, multicomponent approaches in children including family components showed the highest level of evidence for increasing overall PA. This review confirms the public health potential of high quality, school-based PA interventions for increasing PA and possibly fitness in healthy youth
SN - 1473-0480
AD - Swiss Tropical and Public Health Institute, University of Basel, Socinstr 59, 4002 Basel, Switzerland. susi.kriemler@unibas.ch
UR - 21836176
ER -

TY - JOUR
ID - 574
T1 - Differences in physical activity during school recess
A1 - Ridgers,N.D.
A1 - Saint-Maurice,P.F.
A1 - Welk,G.J.
A1 - Siahpush,M.
A1 - Huberty,J.
Y1 - 2011/09//
N1 - Ridgers, Nicola D. Saint-Maurice, Pedro F. Welk, Gregory J. Siahpush, Mohammad. Huberty, Jennifer
The Journal of school health
k13, 0376370
IM, N
Journal Article
English
KW - MEDLINE
KW - Body Mass Index
KW - Child
KW - Child Welfare

KW - Ethnic Groups
KW - Female
KW - Health Status Indicators
KW - Humans
KW - Male
KW - Motor Activity/ph [Physiology]
KW - Recreation/ph [Physiology]
KW - Running
KW - Schools
KW - Students
RP - NOT IN FILE
SP - 545
EP - 551
JF - Journal of School Health
JA - J Sch Health
VL - 81
IS - 9
CY - United States
N2 - BACKGROUND: School recess provides a daily opportunity for physical activity engagement. The purpose of this study was to examine physical activity levels during recess by gender, ethnicity, and grade, and establish the contribution of recess to daily school physical activity levels. METHODS: Two hundred and ten children (45% boys) from grades 3 to 6 in 4 elementary schools had their physical activity during school quantified using uni-axial accelerometry every 5 seconds for 5 consecutive school days. Data were collected in fall 2009. The proportion of time spent engaged in physical activity during daily school recess was determined using existing age-appropriate cutpoints. The relative contribution of recess to school day physical activity was also determined. RESULTS: Boys were more active than girls during recess. Girls engaged in more sedentary activity than boys. No main effects for ethnicity were observed. Children in grades 3 and 5 were more active than children in grades 4 and 6. Recess contributed 17.9% and 15.5% toward boys' and girls' school day moderate-to-vigorous physical activity, respectively. CONCLUSION: Children engaged in physical activity during recess, though interventions may be needed to increase the intensity of activity in this context. 2011, American School Health Association
SN - 1746-1561
AD - Alfred Deakin Postdoctoral Research Fellow, Centre for Physical Activity and Nutrition Research, Deakin University, Burwood, Victoria, Australia
UR - 21831067
ER -

TY - JOUR
ID - 575
T1 - Kids in the city study: research design and methodology
A1 - Oliver,M.
A1 - Witten,K.
A1 - Kearns,R.A.
A1 - Mavoa,S.
A1 - Badland,H.M.
A1 - Carroll,P.
A1 - Drumheller,C.
A1 - Tavae,N.
A1 - Asiasiga,L.
A1 - Jelley,S.
A1 - Kaiwai,H.
A1 - Opit,S.
A1 - Lin,E.Y.
A1 - Sweetsur,P.
A1 - Barnes,H.M.
A1 - Mason,N.
A1 - Ergler,C.

Y1 - 2011///
N1 - Oliver, Melody. Witten, Karen. Kearns, Robin A. Mavoa, Suzanne. Badland, Hannah M. Carroll, Penelope. Drumheller, Chelsea. Tavae, Nicola. Asiasiga, Lanuola. Jelley, Su. Kaiwai, Hector. Opit, Simon. Lin, En-Yi Judy. Sweetsur, Paul. Barnes, Helen Moewaka. Mason, Nic. Ergler, Christina
BMC public health
100968562
IM
Journal Article. Research Support, Non-U.S. Gov't
English
KW - MEDLINE
KW - Actigraphy/is [Instrumentation]
KW - Actigraphy/mt [Methods]
KW - Body Size
KW - Child
KW - Cities
KW - Cross-Sectional Studies
KW - Environment Design
KW - Exercise
KW - Female
KW - Focus Groups
KW - Humans
KW - Interviews as Topic
KW - Male
KW - New Zealand
KW - Research Design
KW - Sedentary Lifestyle
RP - NOT IN FILE
SP - 587
JF - BMC Public Health
VL - 11
CY - England
N2 - BACKGROUND: Physical activity is essential for optimal physical and psychological health but substantial declines in children's activity levels have occurred in New Zealand and internationally. Children's independent mobility (i.e., outdoor play and traveling to destinations unsupervised), an integral component of physical activity in childhood, has also declined radically in recent decades. Safety-conscious parenting practices, car reliance and auto-centric urban design have converged to produce children living increasingly sedentary lives. This research investigates how urban neighborhood environments can support or enable or restrict children's independent mobility, thereby influencing physical activity accumulation and participation in daily life. METHODS/DESIGN: The study is located in six Auckland, New Zealand neighborhoods, diverse in terms of urban design attributes, particularly residential density. Participants comprise 160 children aged 9-11 years and their parents/caregivers. Objective measures (global positioning systems, accelerometers, geographical information systems, observational audits) assessed children's independent mobility and physical activity, neighborhood infrastructure, and streetscape attributes. Parent and child neighborhood perceptions and experiences were assessed using qualitative research methods. DISCUSSION: This study is one of the first internationally to examine the association of specific urban design attributes with child independent mobility. Using robust, appropriate, and best practice objective measures, this study provides robust epidemiological information regarding the relationships between the built environment and health outcomes for this population
SN - 1471-2458
AD - School of Environment, The University of Auckland, Auckland, NZ. r.kearns@auckland.ac.nz
UR - 21781341
ER -

TY - JOUR
ID - 576
T1 - Lean analysis of a pediatric intensive care unit physician group rounding process to identify inefficiencies and opportunities for improvement
A1 - Vats,A.

A1 - Goin,K.H.
A1 - Fortenberry,J.D.
Y1 - 2011/07//
N1 - Vats, Atul. Goin, Kristin H. Fortenberry, James D
Pediatric critical care medicine : a journal of the Society of Critical Care Medicine and the World Federation of Pediatric Intensive and Critical Care Societies
100954653
IM
Journal Article
English
KW - MEDLINE
KW - Child
KW - Efficiency
KW - Organizational
KW - Humans
KW - Intensive Care Units
KW - Pediatric
KW - Internship and Residency
KW - Interprofessional Relations
KW - Medical Staff
KW - Hospital
KW - Process Assessment (Health Care)
KW - Quality Improvement
KW - Teaching Rounds/og [Organization & Administration]
KW - Time Factors
KW - Workflow
RP - NOT IN FILE
SP - 415
EP - 421
JF - Pediatric Critical Care Medicine
JA - Pediatr Crit Care Med
VL - 12
IS - 4
CY - United States
N2 - OBJECTIVE: A physician group in a pediatric intensive care unit faced challenges when moving to a larger unit. Challenges included increased time for rounds, nonbillable attending physician hours, poor communication with pediatric intensive care unit staff, and meeting resident physician duty hours and teaching requirements. The purpose of this analysis was to identify waste and opportunities for improvement to improve physician efficiency. DESIGN: Human factor (observational data collection) techniques were used to capture >60 hrs of rounding data. Twelve attending physicians and their rounding teams were shadowed to capture rounds on 130 pediatric intensive care unit patients. Rounding events, times, and patient interactions were recorded. Lean methods and scenario analysis were used to analyze the data and identify opportunities for improvement. Rounding events were categorized to determine value-added and nonvalue-added activities. Value-added activities were subclassified as essential or nonessential to morning rounds. SETTING: Thirty-bed pediatric intensive care unit in a children's hospital with academic affiliation. PATIENTS OR SUBJECTS: Eight attending pediatric intensivists and their physician rounding teams. INTERVENTIONS: Eight attending physician-led rounding teams were observed for 12 rounding events and a total of 130 patient contacts. MEASUREMENTS AND MAIN RESULTS: Large variation existed in the rounding process. Nonessential activities was highly correlated with physician preference and created a wide range in rounding time per patient. Essential activities showed the least variation and represents a "lean process." Scenario analysis was used to determine the impact of removing waste and reallocating the nonessential activities outside of rounds. Results of the analysis indicated that rounds could be reduced by 42% and that plan of care completion would be timelier (decreased from a mean of 157 to 82 mins). CONCLUSIONS: In a large physician group, essential activities showed the least variation. Practice variation focused on minimizing nonessential activities could have dramatic impacts on standardizing practice. Further study is indicated to determine whether standardizing rounds to focus on essential activities can lead to more effective processes that require fewer resources while improving outcomes for all stakeholders

SN - 1529-7535
AD - Children's Healthcare of Atlanta, Atlanta, GA, USA. atul.vats@choa.org
UR - 20975612
ER -

TY - JOUR
ID - 577
T1 - Prevalence and predictors of vitamin D insufficiency in children: a Great Britain population based study
A1 - Absoud,M.
A1 - Cummins,C.
A1 - Lim,M.J.
A1 - Wassmer,E.
A1 - Shaw,N.
Y1 - 2011///
N1 - Absoud, Michael. Cummins, Carole. Lim, Ming J. Wassmer, Evangeline. Shaw, Nick
PloS one
101285081
IM
Journal Article. Research Support, Non-U.S. Gov't
English
KW - MEDLINE
KW - Adolescent
KW - Child
KW - Preschool
KW - Cross-Sectional Studies
KW - Female
KW - Great Britain/ep [Epidemiology]
KW - Humans
KW - Logistic Models
KW - Male
KW - Nutrition Surveys
KW - Prognosis
KW - Vitamin D Deficiency/di [Diagnosis]
KW - Vitamin D Deficiency/ep [Epidemiology]
RP - NOT IN FILE
SP - e22179
JF - PLoS ONE [Electronic Resource]
JA - PLoS ONE
VL - 6
IS - 7
CY - United States
N2 - OBJECTIVES: To evaluate the prevalence and predictors of vitamin D insufficiency (VDI) in children in Great Britain. DESIGN: A nationally representative cross-sectional study survey of children (1102) aged 4-18 years (999 white, 570 male) living in private households (January 1997-1998). Interventions provided information about dietary habits, physical activity, socio-demographics, and blood sample. Outcome measures were vitamin D insufficiency (<50 nmol/L). RESULTS: Vitamin D levels (mean = 62.1 nmol/L, 95%CI 60.4-63.7) were insufficient in 35%, and decreased with age in both sexes (p<0.001). Young People living between 53-59 degrees latitude had lower levels (compared with 50-53 degrees, p = 0.045). Dietary intake and gender had no effect on vitamin D status. A logistic regression model showed increased risk of VDI in the following: adolescents (14-18 years old), odds ratio (OR) = 3.6 (95%CI 1.8-7.2) compared with younger children (4-8 years); non white children (OR = 37 [95%CI 15-90]); blood levels taken December-May (OR = 6.5 [95%CI 4.3-10.1]); on income support (OR = 2.2 [95%CI 1.3-3.9]); not taking vitamin D supplementation (OR = 3.7 [95%CI 1.4-9.8]); being overweight (OR 1.6 [95%CI 1.0-2.5]); <1/2 hour outdoor exercise/day/week (OR = 1.5 [95%CI 1.0-2.3]); watched >2.5 hours of TV/day/week (OR = 1.6[95%CI 1.0-2.4]). CONCLUSION: We confirm a previously under-recognised risk of VDI in adolescents. The marked higher risk for VDI in non-white children suggests they should be targeted in any preventative strategies. The association of higher risk of VDI among children who exercised less outdoors, watched more TV and were overweight highlights potentially

modifiable risk factors. Clearer guidelines and an increased awareness especially in adolescents are needed, as there are no recommendations for vitamin D supplementation in older children
SN - 1932-6203
AD - School of Health and Population Sciences, University of Birmingham, Birmingham, United Kingdom. michaelabsoud@childdemyelination.org.uk
UR - 21799790
ER -

TY - JOUR
ID - 578
T1 - Human health risk assessment of synthetic turf fields based upon investigation of five fields in Connecticut
A1 - Ginsberg,G.
A1 - Toal,B.
A1 - Simcox,N.
A1 - Bracker,A.
A1 - Golembiewski,B.
A1 - Kurland,T.
A1 - Hedman,C.
Y1 - 2011///
N1 - Ginsberg, Gary. Toal, Brian. Simcox, Nancy. Bracker, Anne. Golembiewski, Brian. Kurland, Tara. Hedman, Curtis
Journal of toxicology and environmental health. Part A
ds5, 100960995
IM
Comparative Study. Journal Article. Research Support, Non-U.S. Gov't
English
KW - MEDLINE
KW - Adult
KW - Age Factors
KW - Air Pollutants/st [Standards]
KW - Air Pollutants/to [Toxicity]
KW - Air Pollution
KW - Indoor/ae [Adverse Effects]
KW - Athletes
KW - Benzothiazoles/ad [Administration & Dosage]
KW - Benzothiazoles/an [Analysis]
KW - Benzothiazoles/to [Toxicity]
KW - Carcinogens
KW - Environmental/ad [Administration & Dosage]
KW - Environmental/st [Standards]
KW - Environmental/to [Toxicity]
KW - Child
KW - Connecticut/ep [Epidemiology]
KW - Elastomers/ch [Chemistry]
KW - Elastomers/ec [Economics]
KW - Elastomers/to [Toxicity]
KW - Environmental Exposure/ae [Adverse Effects]
KW - Environmental Exposure/st [Standards]
KW - Facility Design and Construction/ec [Economics]
KW - Guidelines as Topic
KW - Humans
KW - Neoplasms/ep [Epidemiology]
KW - Play and Playthings
KW - Public Facilities/ec [Economics]
KW - Public Facilities
KW - Recycling
KW - Respiratory Rate

KW - Risk Assessment
KW - Risk Factors
KW - Seasons
KW - Sports Equipment/ae [Adverse Effects]
KW - Sports Equipment/ec [Economics]
RP - NOT IN FILE
SP - 1150
EP - 1174
JF - Journal of Toxicology & Environmental Health Part A
JA - J Toxicol Environ Health A
VL - 74
IS - 17
CY - England
N2 - Questions have been raised regarding possible exposures when playing sports on synthetic turf fields cushioned with crumb rubber. Rubber is a complex mixture with some components possessing toxic and carcinogenic properties. Exposure is possible via inhalation, given that chemicals emitted from rubber might end up in the breathing zone of players and these players have high ventilation rates. Previous studies provide useful data but are limited with respect to the variety of fields and scenarios evaluated. The State of Connecticut investigated emissions associated with four outdoor and one indoor synthetic turf field under summer conditions. On-field and background locations were sampled using a variety of stationary and personal samplers. More than 20 chemicals of potential concern (COPC) were found to be above background and possibly field-related on both indoor and outdoor fields. These COPC were entered into separate risk assessments (1) for outdoor and indoor fields and (2) for children and adults. Exposure concentrations were prorated for time spent away from the fields and inhalation rates were adjusted for play activity and for children's greater ventilation than adults. Cancer and noncancer risk levels were at or below de minimis levels of concern. The scenario with the highest exposure was children playing on the indoor field. The acute hazard index (HI) for this scenario approached unity, suggesting a potential concern, although there was great uncertainty with this estimate. The main contributor was benzothiazole, a rubber-related semivolatile organic chemical (SVOC) that was 14-fold higher indoors than outdoors. Based upon these findings, outdoor and indoor synthetic turf fields are not associated with elevated adverse health risks. However, it would be prudent for building operators to provide adequate ventilation to prevent a buildup of rubber-related volatile organic chemicals (VOC) and SVOC at indoor fields. The current results are generally consistent with the findings from studies conducted by New York City, New York State, the U.S. Environmental Protection Agency (EPA), and Norway, which tested different kinds of fields and under a variety of weather conditions
SN - 1528-7394
AD - Connecticut Dept of Public Health, Hartford, Connecticut 06106, USA. gary.ginsberg@ct.go
UR - 21797769
ER -

TY - JOUR
ID - 579
T1 - Safety and immunogenicity of the 10-valent pneumococcal nontypeable Haemophilus influenzae protein D conjugate vaccine (PHiD-CV) in Chilean children
A1 - Lagos,R.E.
A1 - Munoz,A.E.
A1 - Levine,M.M.
A1 - Lepetic,A.
A1 - Francois,N.
A1 - Yarzabal,J.P.
A1 - Schuerman,L.
Y1 - 2011/05//
N1 - Lagos, Rosanna E. Munoz, Alma E. Levine, Myron M. Lepetic, Alejandro. Francois, Nancy. Yarzabal, Juan Pablo. Schuerman, Lode
Human vaccines
101265291
IM

Journal Article. Randomized Controlled Trial. Research Support, Non-U.S. Gov't
English
KW - MEDLINE
KW - Antibodies
KW - Bacterial/bl [Blood]
KW - Child
KW - Preschool
KW - Chile/ep [Epidemiology]
KW - Female
KW - Haemophilus Infections/ep [Epidemiology]
KW - Haemophilus Infections/pc [Prevention & Control]
KW - Humans
KW - Immunization
KW - Secondary/mt [Methods]
KW - Infant
KW - Male
KW - Pneumococcal Infections/ep [Epidemiology]
KW - Pneumococcal Infections/pc [Prevention & Control]
KW - Pneumococcal Vaccines/ad [Administration & Dosage]
KW - Pneumococcal Vaccines/ae [Adverse Effects]
KW - Pneumococcal Vaccines/im [Immunology]
KW - Vaccination/mt [Methods]
RP - NOT IN FILE
SP - 511
EP - 522
JF - Human Vaccines
JA - Hum
VL - 7
IS - 5
CY - United States
N2 - The safety and immunogenicity of the 10-valent pneumococcal nontypeable Haemophilus influenzae protein D conjugate vaccine (PHiD-CV, Synflorix) were assessed in 240 healthy Chilean children randomized to receive 3 doses of PHiD-CV (PHiD-CV group) or hepatitis A vaccine (HAV control group) at 2-4-6 months of age. All were offered 1 HAV dose at 12 months (outside study). The PHiD-CV group received a second HAV dose at 18-21 months and PHiD-CV booster at 20-23 months. The HAV control group received 2 PHiD-CV catch-up doses at 18-21 and 20-23 months. Adverse events were recorded and pneumococcal antibody responses and opsonophagocytic activity (OPA) were measured. Both PHiD-CV vaccination schedules were well tolerated and immunogenic against the pneumococcal vaccine serotypes and protein D. The reactogenicity of PHiD-CV primary, booster and catch-up doses was in line with previous PHiD-CV studies, although generally higher than with HAV. For each vaccine serotype, the percentage of subjects with antibody concentrations >0.2 g/ml (GSK's 22F-inhibition ELISA) was at least 93.2% following 3 PHiD-CV primary doses and at least 97.4% post-booster; percentages with OPA titers >8 were at least 91.7% post-booster. After 2-dose catch-up, at least 94.3% of children had antibody concentrations >0.2 g/ml against each serotype except 6B (84.3%); at least 95.2% had OPA titers >8 except against serotypes 1, 5 and 6B. In conclusion, the safety profiles of 2 PHiD-CV vaccination schedules (3-dose primary plus booster and 2-dose catch-up) were in line with previous studies and PHiD-CV was immunogenic for all 10 vaccine serotypes and protein D
SN - 1554-8619
AD - Centro para Vacunas en Desarrollo-Chile, Santiago, Chile
UR - 21441782
ER -

TY - JOUR
ID - 580
T1 - Outdoor-based play and reconnection to nature: a neglected pathway to positive youth development
A1 - Mainella,F.P.
A1 - Agate,J.R.
A1 - Clark,B.S.

Y1 - 2011///
N1 - Mainella, Fran P. Agate, Joel R. Clark, Brianna S
New directions for youth development
101090644
IM
Journal Article
English
KW - MEDLINE
KW - Adaptation
KW - Psychological
KW - Adolescent
KW - Adolescent Development
KW - Attitude
KW - Child
KW - Child Development
KW - Cognition
KW - Humans
KW - Interpersonal Relations
KW - Mental Health
KW - Motor Activity
KW - Nature
KW - Physical Fitness
KW - Play and Playthings/px [Psychology]
KW - Recreation/px [Psychology]
KW - Social Support
RP - NOT IN FILE
SP - 89
EP - 104
JF - New Directions for Youth Development
JA - New Dir.Youth Dev
VL - 2011
IS - 130
CY - United States
N2 - Throughout history, outdoor-based play and the connection to nature have been recognized as important contributors to a happy life and healthy development. At times, however, play and nature have been neglected and viewed as frivolous and wasteful. In the early twentieth century, the first play movement took place to get children out of the factories and back outdoors to play. Now, a century later, factors including twenty-four-hour media,stranger danger, and overscheduling of children's time have resulted in a level of play deprivation that is contributing to a host of social, emotional, and physical problems. This article draws on recent research that shows that as many as 40 percent of America's schools have eliminated or significantly reduced recess. Also, children and adolescents are spending an average of fifty-three hours each week in front of screen media, and only 31 percent of mothers report that their children play outside as frequently as they did as children. The deficits in outdoor-based play that arise from these lifestyles have resulted in significant increases in emotional and psychological disorders, decreased capacity to deal with stressors, and decreased physical fitness. This article describes specific organizations and programs that address the problem of play deprivation and reconnection to the outdoors. Copyright 2011 Wiley Periodicals, Inc., A Wiley Company
SN - 1537-5781
AD - Clemson University, USA
UR - 21786412
ER -

TY - JOUR
ID - 581
T1 - Outdoor sports and risk of ultraviolet radiation-related skin lesions in children: evaluation of risks and prevention
A1 - Mahe,E.
A1 - Beauchet,A.

A1 - de Paula,Correa M.
A1 - Godin-Beekmann,S.
A1 - Haeffelin,M.
A1 - Bruant,S.
A1 - Fay-Chatelard,F.
A1 - Jegou,F.
A1 - Saiag,P.
A1 - Aegerter,P.
Y1 - 2011/08//
N1 - Mahe, E. Beauchet, A. de Paula Correa, M. Godin-Beekmann, S. Haeffelin, M. Bruant, S. Fay-Chatelard, F. Jegou, F. Saiag, P. Aegerter, P
The British journal of dermatology
aw0, 0004041
IM
Journal Article. Randomized Controlled Trial. Research Support, Non-U.S. Gov't
English
KW - MEDLINE
KW - Adult
KW - Child
KW - Cluster Analysis
KW - Environmental Exposure/ae [Adverse Effects]
KW - Environmental Exposure/an [Analysis]
KW - Environmental Exposure/pc [Prevention & Control]
KW - Erythema/et [Etiology]
KW - Health Promotion
KW - Humans
KW - Nevus
KW - Pigmented/ep [Epidemiology]
KW - Pigmented/et [Etiology]
KW - Paris/ep [Epidemiology]
KW - Radiation Dosage
KW - Risk Assessment
KW - School Health Services
KW - Soccer
KW - Sports
KW - Sunlight/ae [Adverse Effects]
KW - Sunscreening Agents/tu [Therapeutic Use]
KW - Ultraviolet Rays/ae [Adverse Effects]
KW - Weather
RP - NOT IN FILE
SP - 360
EP - 367
JF - British Journal of Dermatology
JA - Br J Dermatol
VL - 165
IS - 2
CY - England
N2 - BACKGROUND: Excessive ultraviolet (UV) radiation exposure can cause skin cancers, skin photoageing and cataracts. Children are targeted by sun-protection campaigns because high sun exposure and sunburn in childhood increase the risk of melanoma in adulthood. Little information is available about UV radiation risk and exposure in children who take part in outdoor sports. OBJECTIVE: To evaluate the risk of developing UV radiation-induced skin lesions run by children who practise outdoor sports, and UV radiation exposure and sun-protection measures during a soccer tournament. METHODS: Firstly, we evaluated the relationship between melanocytic naevus - a skin lesion linked with exposure to UV radiation - and outdoor sports in 660 11-year-old children. Secondly, we used the occasion of a 1-day soccer tournament held in the spring to evaluate UV radiation-protective measures used by soccer players and the public. We also evaluated the UV radiation index and cloud cover during the tournament, and calculated the UV radiation dose and minimal erythema dose

depending on skin phototype. RESULTS: The naevus count and acquired naevus count measured over the 2 years of the study were higher in the 344 children who practised outdoor sports. Sun-protective measures were insufficient for soccer players and the public. CONCLUSIONS: This study shows that outdoor sports increase the risk of developing UV radiation-induced skin lesions in childhood. During a 1-day soccer tournament held in the spring, children and their parents were inadequately protected against the sun. These results suggest that sun-protection campaigns should be aimed at children who practise popular outdoor sports. 2011 The Authors. BJD 2011 British Association of Dermatologists 2011
SN - 1365-2133
AD - Department of Dermatology, Ambroise Pare University Hospital, University of Versailles-Saint Quentin en Yvelines, Assistance Publique-Hopitaux de Paris, Boulogne-Billancourt, France. emmanuel.mahe@ch-argenteuil.fr
UR - 21574980
ER -

TY - JOUR
ID - 582
T1 - Examining children's physical activity and play behaviors during school playtime over time
A1 - Ridgers,N.D.
A1 - Carter,L.M.
A1 - Stratton,G.
A1 - McKenzie,T.L.
Y1 - 2011/08//
N1 - Ridgers, Nicola D. Carter, Laura M. Stratton, Gareth. McKenzie, Thomas L
Health education research
bqp, 8608459
T
Journal Article. Randomized Controlled Trial. Research Support, Non-U.S. Gov't
English
KW - MEDLINE
KW - Body Mass Index
KW - Body Weights and Measures
KW - Child
KW - Exercise
KW - Female
KW - Humans
KW - Male
KW - Play and Playthings
KW - Schools/sn [Statistics & Numerical Data]
KW - Social Isolation
KW - Socioeconomic Factors
RP - NOT IN FILE
SP - 586
EP - 595
JF - Health Education Research
JA - Health Educ Res
VL - 26
IS - 4
CY - England
N2 - School playtime (recess) provides children an opportunity to engage in a range of active and sedentary play behaviors on a daily basis. However, little data have investigated changes in playtime behaviors over time. The aim of this study was to investigate how children's physical activity levels, the size of their social group, play behaviors and social interactions changed over one academic year (Study 1), and during the transition from Year 5 (aged 9-10 years) to Year 6 (aged 10-11 years; Study 2). Primary school children were directly observed during school playtime using the System for Observing Children's Activity and Relationships during Play. Results revealed children engaged in moderate-to-vigorous physical activity for at least half of the intervals observed. Incidents of physical antisocial behavior significantly decreased, while incidents of verbal antisocial behavior and time spent alone increased across Study 1. Incidents of verbal antisocial behavior, time spent alone

and engagement in playground games significantly decreased across Study 2. The data suggest that children are highly physically active during school playtime, and while their play behaviors and activity levels fluctuate, these fluctuations were generally small over one academic year and in the transition between Year 5 and Year 6
SN - 1465-3648
AD - Centre for Physical Activity and Nutrition Research, Deakin University, 221 Burwood Highway, Burwood, Victoria 3125, Australia. nicky.ridgers@deakin.edu.au
UR - 21422119
ER -

TY - JOUR
ID - 583
T1 - Results of the "Active by Choice Today" (ACT) randomized trial for increasing physical activity in low-income and minority adolescents
A1 - Wilson,D.K.
A1 - Van Horn,M.L.
A1 - Kitzman-Ulrich,H.
A1 - Saunders,R.
A1 - Pate,R.
A1 - Lawman,H.G.
A1 - Hutto,B.
A1 - Griffin,S.
A1 - Zarrett,N.
A1 - Addy,C.L.
A1 - Mansard,L.
A1 - Mixon,G.
A1 - Brown,P.V.
Y1 - 2011/07//
N1 - Wilson, Dawn K. Van Horn, M Lee. Kitzman-Ulrich, Heather. Saunders, Ruth. Pate, Russell. Lawman, Hannah G. Hutto, Brent. Griffin, Sarah. Zarrett, Nicole. Addy, Cheryl L. Mansard, Leslie. Mixon, Gary. Brown, Porschia V
Health psychology : official journal of the Division of Health Psychology, American Psychological Association
ejl, 8211523
IM
Journal Article. Randomized Controlled Trial. Research Support, N.I.H., Extramural
English
KW - MEDLINE
KW - Behavior Therapy
KW - Child
KW - Choice Behavior
KW - Exercise/px [Psychology]
KW - Female
KW - Humans
KW - Male
KW - Minority Groups/px [Psychology]
KW - Motivation
KW - Motor Activity
KW - Poverty/px [Psychology]
KW - Social Environment
RP - NOT IN FILE
SP - 463
EP - 471
JF - Health Psychology
JA - Health Psychol
VL - 30
IS - 4
CY - United States

N2 - OBJECTIVE: This study reports the results of the "Active by Choice Today" (ACT) trial for increasing moderate-to-vigorous physical activity (MVPA) in low-income and minority adolescents. DESIGN: The ACT program was a randomized controlled school-based trial testing the efficacy of a motivational plus behavioral skills intervention on increasing MVPA in underserved adolescents. Twenty-four middle schools were matched on school size, percentage minorities, percentage free or reduce lunch, and urban or rural setting before randomization. A total of 1,563 6th grade students (mean age, 11.3 years, 73% African American, 71% free or reduced lunch, 55% female) participated in either a 17-week (over one academic year) intervention or comparison after-school program. MAIN OUTCOME MEASURE: The primary outcome measure was MVPA based on 7-day accelerometry estimates at 2-weeks postintervention and an intermediate outcome was MVPA at midintervention. RESULTS: At midintervention students in the intervention condition engaged in 4.87 greater minutes of MVPA per day (95% CI: 1.18 to 8.57) than control students. Students in intervention schools engaged in 9.11 min (95% CI: 5.73 to 12.48) more of MVPA per day than those in control schools during the program time periods; indicating a 27 min per week increase in MVPA. No significant effect of the ACT intervention was found outside of school times or for MVPA at 2-weeks postintervention. CONCLUSIONS: Motivational and behavioral skills programs are effective at increasing MVPA in low-income and minority adolescents during program hours, but further research is needed to address home barriers to youth MVPA
SN - 1930-7810
AD - Department of Psychology, University of South Carolina, Columbia, SC 29208, USA.
wilsondk@mailbox.sc.edu
UR - 21534677
ER -

TY - JOUR
ID - 584
T1 - Effect of a low-cost, teacher-directed classroom intervention on elementary students' physical activity
A1 - Erwin,H.E.
A1 - Beighle,A.
A1 - Morgan,C.F.
A1 - Noland,M.
Y1 - 2011/08//
N1 - Erwin, Heather E. Beighle, Aaron. Morgan, Charles F. Noland, Melody
The Journal of school health
k13, 0376370
IM, N
Journal Article
English
KW - MEDLINE
KW - Actigraphy/mt [Methods]
KW - Analysis of Variance
KW - Child
KW - Exercise
KW - Faculty
KW - Female
KW - Health Promotion/mt [Methods]
KW - Humans
KW - Male
KW - Motor Activity
KW - Recreation
KW - Schools
KW - Students
KW - Walking/sn [Statistics & Numerical Data]
KW - Walking
RP - NOT IN FILE
SP - 455
EP - 461
JF - Journal of School Health
JA - J Sch Health

VL - 81
IS - 8
CY - United States
N2 - BACKGROUND: Effective physical activity (PA) interventions are warranted for youth, and schools have been identified as logical locations for such involvement. Experts and professionals in the field promote comprehensive school PA programs, including classroom PA. The purpose of this study was to determine the effect of a low-cost, teacher-directed classroom-based intervention on the school PA of elementary children. METHODS: Nine classroom teachers were provided inexpensive curricula and trained to implement and instruct PA breaks (2 x 30 minute training sessions). The teachers were encouraged to lead 1 activity break per day after the training. One hundred and six elementary students wore pedometers up to 12 days over 3 monitoring periods during the school year (baseline, follow-up, post follow-up) to assess the effectiveness and the sustainability of the intervention. The teachers self-reported the frequency of activity breaks instructed. RESULTS: The teachers (n = 5) who complied with the recommended 1 activity break per day had students who accrued ~33% more mean school steps/day at follow-up (~1100) and post follow-up (~1350) compared to controls. Teachers (n = 4) in the intervention who did not comply with the 1 activity break per day recommendation had students accrue similar mean school steps/day as controls. CONCLUSION: Inexpensive, teacher-directed classroom-based PA interventions can be effective in improving children's PA levels if teachers implement 1 activity break per school day. We recommend promoting the notion of 1 activity break per day in the classroom as part of a comprehensive school PA program that includes quality physical education, recess, and before/after school programs. 2011, American School Health Association
SN - 1746-1561
AD - Department of Kinesiology and Health Promotion, University of Kentucky, Lexington, USA. heather.erwin@uky.edu
UR - 21740430
ER -

TY - JOUR
ID - 585
T1 - The challenge of low physical activity during the school day: at recess, lunch and in physical education.[Erratum appears in Br J Sports Med. 2011 Aug;45(10):819]
A1 - Nettlefold,L.
A1 - McKay,H.A.
A1 - Warburton,D.E.
A1 - McGuire,K.A.
A1 - Bredin,S.S.
A1 - Naylor,P.J.
Y1 - 2011/08//
N1 - Nettlefold, L. McKay, H A. Warburton, D E R. McGuire, K A. Bredin, S S D. Naylor, P J
British journal of sports medicine
0432520
IM
Journal Article. Randomized Controlled Trial. Research Support, Non-U.S. Gov't
English
KW - MEDLINE
KW - Acceleration
KW - Body Mass Index
KW - British Columbia
KW - Child
KW - Exercise/ph [Physiology]
KW - Female
KW - Humans
KW - Leisure Activities
KW - Male
KW - Monitoring
KW - Ambulatory/is [Instrumentation]
KW - Physical Education and Training/og [Organization & Administration]
KW - School Health Services/og [Organization & Administration]

KW - Sedentary Lifestyle
KW - Sex Distribution
KW - Time Factors
RP - NOT IN FILE
SP - 813
EP - 819
JF - British Journal of Sports Medicine
JA - BJSM online
VL - 45
IS - 10
CY - England
N2 - PURPOSE: To describe physical activity (PA) intensity across a school day and assess the percentage of girls and boys achieving recommended guidelines. METHODS: The authors measured PA via accelerometry in 380 children (8-11 years) and examined data representing (1) the whole school day, (2) regular class time, (3) recess, (4) lunch and (5) scheduled physical education (PE). Activity was categorised as sedentary (SED), light physical activity (LPA) or moderate to vigorous physical activity (MVPA) using age-specific thresholds. They examined sex differences across PA intensities during each time period and compliance with recommended guidelines. RESULTS: Girls accumulated less MVPA and more SED than boys throughout the school day (MVPA -10.6 min; SED +13.9 min) recess (MVPA -1.6 min; SED +1.7 min) and lunch (MVPA -3.1 min; SED +2.9 min). Girls accumulated less MVPA (-6.2 min), less LPA (-2.5 min) and more SED (+9.4 min) than boys during regular class time. Fewer girls than boys achieved PA guidelines during school (90.9% vs 96.2%), recess (15.7% vs 34.1%) and lunch (16.7% vs 37.4%). During PE, only 1.8% of girls and 2.9% of boys achieved the PA guidelines. Girls and boys accumulated similar amounts of MVPA, LPA and SED. CONCLUSION: The MVPA deficit in girls was due to their sedentary behaviour as opposed to LPA. Physical activity strategies that target girls are essential to overcome this deficit. Only a very small percentage of children met physical activity guidelines during PE. There is a great need for additional training and emphasis on PA during PE. In addition schools should complement PE with PA models that increase PA opportunities across the school day
SN - 1473-0480
AD - University of British Columbia, Vancouver, British Columbia, Canada
UR - 20215489
ER -

TY - JOUR
ID - 586
T1 - [Comparison of physical activity and favourite ways of spending free time in preschool girls and boys from the Mazowsze region]. [Polish]
A1 - Merkiel,S.
A1 - Chalcarz,W.
A1 - Deptula,M.
Y1 - 2011///
N1 - Merkiel, Sylwia. Chalcarz, Wojciech. Deptula, Monika
Roczniki Panstwowego Zakladu Higieny
txt, 0414756
IM
Comparative Study. English Abstract. Journal Article
Polish
KW - MEDLINE
KW - Birth Weight
KW - Child
KW - Preschool
KW - Female
KW - Humans
KW - Leisure Activities
KW - Life Style
KW - Male
KW - Motor Activity
KW - Poland

KW - Population Surveillance
KW - Questionnaires
KW - Sex Distribution
RP - NOT IN FILE
SP - 93
EP - 99
JF - Roczniki Panstwowego Zakladu Higieny
JA - Rocz.Panstw.Zakl.Hig
VL - 62
IS - 1
CY - Poland
N2 - The aim of this study was to compare physical activity of preschool girls and boys from the Mazowsze region and their favourite ways of spending free time. The studied population included 131 children aged 3 to 7 years. Parents filled in questionnaires about their children's physical activity and favourite ways of spending free time, as well as about general information on the children and their families. Gender had statistically significant influence on the children's birth weight, attending karate outside the preschool and on riding a bicycle, playing with a ball and playing with a dog as favourite ways of spending free time during sunny weather as well as playing on a computer and playing with dolls as favourite ways of spending free time during rainy weather. Gender had little influence on the studied preschoolers' physical activity. However favourite ways of spending free time turned out to be significantly different according to gender. Physical activity level in both girls and boys was low, similarly to their peers from the previous studies
SN - 0035-7715
AD - Zaklad Zywnosci i Zywienia, Akademia Wychowania Fizycznego w Poznaniu.
sylwiamerkiel@awf.poznan.pl
UR - 21735986
ER -

TY - JOUR
ID - 587
T1 - Parental perspective on sun protection for young children in Bavaria
A1 - Li,J.
A1 - Uter,W.
A1 - Pfahlberg,A.
A1 - Gefeller,O.
Y1 - 2011/08//
N1 - Li, Jiang. Uter, Wolfgang. Pfahlberg, Annette. Gefeller, Olaf
Photodermatology, photoimmunology & photomedicine
awp, 9013641
IM
Comparative Study. Journal Article. Research Support, Non-U.S. Gov't
English
KW - MEDLINE
KW - Adult
KW - Child
KW - Preschool
KW - Female
KW - Humans
KW - Male
KW - Parents
KW - Self Report
KW - Sex Factors
KW - Sunbathing
KW - Sunburn/pc [Prevention & Control]
KW - Sunlight/ae [Adverse Effects]
KW - Ultraviolet Rays/ae [Adverse Effects]
RP - NOT IN FILE
SP - 196

EP - 202
JF - Photodermatology, Photoimmunology & Photomedicine
JA - Photodermatol.Photoimmunol.Photomed.
VL - 27
IS - 4
CY - Denmark
N2 - BACKGROUND: Exposure to ultraviolet (UV) radiation is the most important modifiable risk factor for skin cancer. Parents are responsible for sun protection of their young children and thus their knowledge and attitudes should be assessed. METHODS: A survey that included 2619 children aged 3-6 years (response: 64.7%) was performed in Erlangen during 2001/2002. Parents' attitude, knowledge and behavior regarding sun protection were ascertained by using a self-administered questionnaire. Demographic and photosensitivity data of the children were recorded, along with the UV radiation-related outcomes. RESULTS: Most parents correctly recognized the risk factors of skin cancer, except the intermittent and intensive sun exposure. Nevertheless, most parents also agreed with statements regarding sun- and tanning related beauty and well-being. The overwhelming majority of parents stressed the necessity of sun protection for their children; however, 50% of the children experienced some form of solar radiation injury during the preceding summer. Children eagerly pursued outdoor activities, especially boys compared with girls (P<0.01). A gender difference was also apparent, with boys showing more willingness to wear hats than girls (P<0.01). CONCLUSION: The relatively high level of knowledge was in contrast to a prevalent attitude of regarding sunlight as enhancing health and well-being. This may hamper UV radiation avoidance strategies. Thus, future campaigns should not only address preventive knowledge but also focus on associated attitudes to effectively modify the behavior. 2011 John Wiley & Sons A/S
SN - 1600-0781
AD - Department of Medical Informatics, Biometry and Epidemiology, Friedrich Alexander University of Erlangen-Nuremberg, Erlangen, Germany
UR - 21729168
ER -

TY - JOUR
ID - 588
T1 - Physical fitness of normal, stunted and overweight children 6-13 years in Oaxaca, Mexico
A1 - Malina,R.M.
A1 - Pena Reyes,M.E.
A1 - Tan,S.K.
A1 - Little,B.B.
Y1 - 2011/07//
N1 - Malina, R M. Pena Reyes, M E. Tan, S K. Little, B B
European journal of clinical nutrition
ejc, 8804070
IM
Journal Article. Research Support, U.S. Gov't, Non-P.H.S.
English
KW - MEDLINE
KW - Adolescent
KW - Age Factors
KW - Anthropometry
KW - Body Composition
KW - Body Mass Index
KW - Body Weights and Measures
KW - Child
KW - Cross-Sectional Studies
KW - Female
KW - Growth Disorders/ep [Epidemiology]
KW - Growth Disorders/eh [Ethnology]
KW - Growth Disorders/et [Etiology]
KW - Growth Disorders/pp [Physiopathology]
KW - Humans

KW - Indians
KW - North American
KW - Male
KW - Malnutrition/ep [Epidemiology]
KW - Malnutrition/eh [Ethnology]
KW - Malnutrition/pp [Physiopathology]
KW - Mexico/ep [Epidemiology]
KW - Motor Activity
KW - Muscle Strength
KW - Overweight/ep [Epidemiology]
KW - Overweight/eh [Ethnology]
KW - Overweight/pp [Physiopathology]
KW - Physical Endurance
KW - Physical Fitness
KW - Prevalence
KW - Rural Health
RP - NOT IN FILE
SP - 826
EP - 834
JF - European Journal of Clinical Nutrition
JA - Eur J Clin Nutr
VL - 65
IS - 7
CY - England
N2 - BACKGROUND/OBJECTIVE: The objective of this study is to compare the growth and physical fitness of normal, stunted and overweight/obese (owt/ob) Oaxaca children 6-13 years. SUBJECTS/METHODS: This study was a cross-sectional, included 688 school children (grades 1-3, 4-6), aged 6-13 years, from an indigenous rural community (n=361) and colonia popular (n=327) in Oaxaca, southern Mexico. Measurements: Anthropometry-weight, height, sitting height, limb circumferences, skinfolds. Derived-body mass index, sitting height/height ratio, leg and step lengths, limb muscle areas, sum of skinfolds. Physical fitness-sit and reach, sit-ups, distance run, grip strength, standing long jump, 35 yard dash. Physical activity-steps to and from school, household chores, sports participation. Analysis: Normal-not stunted, not owt/ob; stunted-not owt/ob; and owt/ob-not stunted were compared with multivariate analysis of covariance controlling for age. Two children were stunted and owt/ob, and were excluded. RESULTS: Age-adjusted means for body size, muscularity, adiposity and grip strength showed a gradient, owt/ob>normal>stunted in both sexes and grade levels (P<0.001). Relative position of stunted and owt/ob children was reversed for strength per unit mass. Stunted and normal children ran a greater distance than owt/ob children (P<0.05). Normal, stunted and owt/ob children did not differ consistently in other fitness items and indicators of activity and inactivity. CONCLUSION: Size, muscularity, fatness and strength differed significantly, owt/ob>normal>stunted, but owt/ob children had less strength per unit mass and poorer endurance. Normal and stunted children did not differ consistently in fitness. Physical activity and television time did not differ among the three groups
SN - 1476-5640
AD - Department of Kinesiology and Health Education, University of Texas at Austin, Austin, TX, USA. rmalina@1skyconnect.net
UR - 21448221
ER -

TY - JOUR
ID - 589
T1 - Childhood and adolescent predictors of leisure time physical activity during the transition from adolescence to adulthood: a population based cohort study
A1 - Jose,K.A.
A1 - Blizzard,L.
A1 - Dwyer,T.
A1 - McKercher,C.
A1 - Venn,A.J.
Y1 - 2011///

N1 - Jose, Kim A. Blizzard, Leigh. Dwyer, Terry. McKercher, Charlotte. Venn, Alison J
The international journal of behavioral nutrition and physical activity
101217089
IM
Journal Article. Research Support, Non-U.S. Gov't
English
KW - MEDLINE
KW - Adolescent
KW - Adolescent Behavior
KW - Adult
KW - Age Factors
KW - Australia
KW - Child
KW - Data Interpretation
KW - Statistical
KW - Educational Status
KW - Exercise
KW - Female
KW - Follow-Up Studies
KW - Health Knowledge
KW - Attitudes
KW - Practice
KW - Humans
KW - Leisure Activities
KW - Male
KW - Motor Activity
KW - Parents
KW - Questionnaires
KW - Retrospective Studies
KW - Social Class
KW - Sports/sn [Statistics & Numerical Data]
KW - Young Adult
RP - NOT IN FILE
SP - 54
JF - International Journal of Behavioral Nutrition & Physical Activity
JA - Int
VL - 8
CY - England
N2 - BACKGROUND: Few studies have investigated factors that influence physical activity behavior during the transition from adolescence to adulthood. This study explores the associations of sociodemographic, behavioral, sociocultural, attitudinal and physical factors measured in childhood and adolescence with physical activity behavior during the transition from adolescence to adulthood. METHODS: Childhood and adolescent data (at ages 7-15 years) were collected as part of the 1985 Australian Health and Fitness Survey and subdivided into sociodemographics (socioeconomic status, parental education), behavioral (smoking, alcohol, sports diversity, outside school sports), sociocultural (active father, active mother, any older siblings, any younger siblings, language spoken at home), attitudinal (sports/recreational competency, self-rated health, enjoyment physical education/physical activity, not enjoying school sports) and physical (BMI, time taken to run 1.6 km, long jump) factors. Physical activity between the ages 15 and 29 years was reported retrospectively using the Historical Leisure Activity Questionnaire at follow-up in 2004-2006 by 2,048 participants in the Childhood Determinants of Adult Health Study (CDAH). Australia's physical activity recommendations for children and adults were used to categorize participants as persistently active, variably active or persistently inactive during the transition from adolescence to adulthood. RESULTS: For females, perceived sports competency in childhood and adolescence was significantly associated with being persistently active (RR=1.88, 95% CI=1.39, 2.55). Smoking (RR=0.31 CI=0.12, 0.82) and having younger siblings (RR=0.69 CI=0.52, 0.93) were inversely associated with being persistently active after taking physical and attitudinal factors into account. For males, playing sport outside school (RR=1.47 CI=1.05, 2.08), having active fathers (RR=1.25 CI=1.01, 1.54) and not enjoying school sport (RR=4.07 CI=2.31, 7.17) were associated with being persistently active into adulthood.

Time taken to complete the 1.6 km run was inversely associated with being persistently active into adulthood (RR=0.85 CI=0.78, 0.93) after adjusting for recreational competency. CONCLUSIONS: Perceived sports competency (females) and cardiorespiratory fitness, playing sport outside school and having active fathers (males) in childhood and adolescence were positively associated with being persistently active during the transition from adolescence to adulthood
SN - 1479-5868
AD - Menzies Research Institute Tasmania, University of Tasmania, Private Bag 23, Hobart, Tasmania, Australia. Kim.jose@utas.edu.au
UR - 21631921
ER -

TY - JOUR
ID - 590
T1 - Association between physical activity and blood pressure in prepubertal children
A1 - Gopinath,B.
A1 - Hardy,L.L.
A1 - Teber,E.
A1 - Mitchell,P.
Y1 - 2011/07//
N1 - Gopinath, Bamini. Hardy, Louise L. Teber, Erdahl. Mitchell, Paul
Hypertension research : official journal of the Japanese Society of Hypertension
9307690
IM
Journal Article. Research Support, Non-U.S. Gov't
English
KW - MEDLINE
KW - Australia
KW - Blood Pressure/ph [Physiology]
KW - Body Mass Index
KW - Body Weight/ph [Physiology]
KW - Child
KW - Female
KW - Humans
KW - Hypertension
KW - Life Style
KW - Male
KW - Motor Activity/ph [Physiology]
KW - Obesity
RP - NOT IN FILE
SP - 851
EP - 855
JF - Hypertension Research - Clinical & Experimental
JA - Hypertens Res
VL - 34
IS - 7
CY - England
N2 - Elevated blood pressure (BP) during childhood and adolescence increases the risk of hypertension in later life. Although physical activity is known to positively moderate BP, data regarding this relationship are limited in prepubertal children. We aimed to assess the association between a range of physical activities (including indoor and outdoor activity) and BP in a large community-based sample of prepubertal schoolchildren. Eligible year-1 schoolchildren (n=1765; mean age 6.7 + 0.4 years) from a random cluster sample of 34 schools in Sydney, Australia, were examined. Parents completed detailed questionnaires about their child's activity. Height and weight were measured, and body mass index (BMI) was calculated. BP was measured using a standard protocol, and elevated BP was defined using published guidelines. Physical activity was classified as low, medium or high (that is, as tertiles). After adjusting for age, sex, ethnicity, height, BMI, parental qualifications and family history of hypertension and/or cardiovascular disease, children in the highest tertile of outdoor and indoor activities had significantly lower diastolic BP (~1.5 mm Hg; P(trend)=0.01) and systolic BP (~1.3 mm

Hg; P(trend)=0.03), respectively, compared with those in the lowest tertile (reference). Linearly, time spent in indoor activities (each hour per day) was associated with ~2.4 mm Hg decrease in diastolic BP (P=0.001). Physical activity was independently associated with lower BP in this sample of prepubertal children. The findings emphasize the importance of ensuring regular physical activity programs in primary schools to potentially reduce the risk of elevated BP in childhood and in later life
SN - 1348-4214
AD - 1] Centre for Vision Research, Department of Ophthalmology and Westmead Millennium Institute, University of Sydney, Westmead Hospital, Sydney, New South Wales, Australia
UR - 21525949
ER -

TY - JOUR
ID - 591
T1 - Effectiveness of a primary school-based intervention to reduce overweight
A1 - Jansen,W.
A1 - Borsboom,G.
A1 - Meima,A.
A1 - Zwanenburg,E.J.
A1 - Mackenbach,J.P.
A1 - Raat,H.
A1 - Brug,J.
Y1 - 2011/06//
N1 - Jansen, Wilma. Borsboom, Gerard. Meima, Abraham. Zwanenburg, Evelien Joosten-Van. Mackenbach, Johan P. Raat, Hein. Brug, Johannes
International journal of pediatric obesity : IJPO : an official journal of the International Association for the Study of Obesity
101256330
IM
Journal Article. Randomized Controlled Trial
English
KW - MEDLINE
KW - Body Mass Index
KW - Chi-Square Distribution
KW - Child
KW - Child Behavior
KW - Female
KW - Health Behavior
KW - Health Promotion
KW - Humans
KW - Linear Models
KW - Logistic Models
KW - Male
KW - Netherlands
KW - Overweight/pp [Physiopathology]
KW - Overweight/th [Therapy]
KW - Physical Education and Training
KW - Physical Fitness
KW - School Health Services
KW - Schools
KW - Time Factors
KW - Treatment Outcome
KW - Waist Circumference
KW - Weight Loss
RP - NOT IN FILE
SP - e70
EP - e77
JF - International Journal of Pediatric Obesity

JA - Int J Pediatr Obes
VL - 6
IS - 2-2
CY - England
N2 - OBJECTIVES: The purpose of this study was to evaluate the effect of a school-based intervention program to reduce overweight and improve fitness in primary school children. METHODS: A cluster randomized controlled design was used over one school year with schools as unit of randomization. In total 20 schools and 2,622 children aged 6-12 years (grades 3-8) from multi-ethnic, low income inner-city neighbourhoods in Rotterdam, Netherlands, participated. The intervention, named Lekker Fit! (Enjoy being fit!) was a multi-component intervention based on behavioural and ecological models. Main components of the intervention are the implementation of three physical education (PE) sessions a week by a professional PE teacher, additional sport and play activities outside school hours and an educational program. Main primary outcome measures were weight status, body mass index (BMI), waist circumference and fitness (20 m shuttle run). RESULTS: Significant positive intervention effects were found for percentage overweight children (OR 0.53; 95% CI 0.36-0.78), waist circumference (-1.29 cm; 95% CI -2.16 to -0.42 cm) and 20 m shuttle run (0.57 laps; 95% CI 0.13-1.01 laps) among pupils of grades 3-5 (6-9-year olds). The prevalence of overweight in grades 3-5 increased by 4.3% in the control group and by 1.3% in the intervention group. No significant effects were found for BMI or for grades 6-8 (9-12-year olds). CONCLUSIONS: Our results provide evidence for the effectiveness of the multi-component intervention Lekker Fit! among pupils of grades 3-5 and adds to the growing body of evidence that school-based programs with a focus on PA are most effective in reducing childhood obesity. [ISRCTN84383524]
SN - 1747-7174
AD - Department of Public Health, Erasmus MC, Rotterdam, The Netherlands. w.jansen@ggd.rotterdam.nl
UR - 21609245
ER -

TY - JOUR
ID - 592
T1 - Randomized controlled trial of a good practice approach to treatment of childhood obesity in Malaysia: Malaysian Childhood Obesity Treatment Trial (MASCOT)
A1 - Wafa,S.W.
A1 - Talib,R.A.
A1 - Hamzaid,N.H.
A1 - McColl,J.H.
A1 - Rajikan,R.
A1 - Ng,L.O.
A1 - Ramli,A.H.
A1 - Reilly,J.J.
Y1 - 2011/06//
N1 - Wafa, Sharifah W. Talib, Ruzita A. Hamzaid, Nur H. McColl, John H. Rajikan, Roslee. Ng, Lai O. Ramli, Ayiesah H. Reilly, John J
International journal of pediatric obesity : IJPO : an official journal of the International Association for the Study of Obesity
101256330
IM
Journal Article. Randomized Controlled Trial. Research Support, Non-U.S. Gov't
English
KW - MEDLINE
KW - Actigraphy
KW - Body Mass Index
KW - Child
KW - Child Behavior
KW - Counseling
KW - Diet
KW - Exercise
KW - Female
KW - Habits

KW - Health Behavior
KW - Health Promotion
KW - Humans
KW - Malaysia
KW - Male
KW - Obesity/pp [Physiopathology]
KW - Obesity/px [Psychology]
KW - Obesity/th [Therapy]
KW - Quality of Life
KW - Questionnaires
KW - Risk Reduction Behavior
KW - Time Factors
KW - Treatment Outcome
KW - Weight Loss
RP - NOT IN FILE
SP - e62
EP - e69
JF - International Journal of Pediatric Obesity
JA - Int J Pediatr Obes
VL - 6
IS - 2-2
CY - England
N2 - CONTEXT: Few randomized controlled trials (RCTs) of interventions for the treatment of childhood obesity have taken place outside the Western world. AIM: To test whether a good practice intervention for the treatment of childhood obesity would have a greater impact on weight status and other outcomes than a control condition in Kuala Lumpur, Malaysia. METHODS: Assessor-blinded RCT of a treatment intervention in 107 obese 7- to 11-year olds. The intervention was relatively low intensity (8 hours contact over 26 weeks, group based), aiming to change child sedentary behavior, physical activity, and diet using behavior change counselling. Outcomes were measured at baseline and six months after the start of the intervention. Primary outcome was BMI z-score, other outcomes were weight change, health-related quality of life (Peds QL), objectively measured physical activity and sedentary behavior (Actigraph accelerometry over 5 days). RESULTS: The intervention had no significant effect on BMI z score relative to control. Weight gain was reduced significantly in the intervention group compared to the control group (+1.5 kg vs. +3.5 kg, respectively, t-test $p < 0.01$). Changes in health-related quality of life and objectively measured physical activity and sedentary behavior favored the intervention group. CONCLUSIONS: Treatment was associated with reduced rate of weight gain, and improvements in physical activity and quality of life. More substantial benefits may require longer term and more intensive interventions which aim for more substantive lifestyle changes
SN - 1747-7174
AD - University Kebangsaan Malaysia, Kuala Lumpur, Malaysia
UR - 21604965
ER -

TY - JOUR
ID - 593
T1 - The impact of rainfall and school break time policies on physical activity in 9-10 year old British children: a repeated measures study
A1 - Harrison,F.
A1 - Jones,A.P.
A1 - Bentham,G.
A1 - van Sluijs,E.M.
A1 - Cassidy,A.
A1 - Griffin,S.J.
Y1 - 2011///
N1 - Harrison, Flo. Jones, Andrew P. Bentham, Graham. van Sluijs, Esther M F. Cassidy, Aedin. Griffin, Simon J
The international journal of behavioral nutrition and physical activity
101217089

IM
Journal Article. Research Support, Non-U.S. Gov't
English
KW - MEDLINE
KW - Actigraphy
KW - Child
KW - Female
KW - Great Britain
KW - Humans
KW - Male
KW - Motor Activity
KW - Multilevel Analysis
KW - Policy
KW - Questionnaires
KW - Rain
KW - Regression Analysis
KW - Schools
KW - Sedentary Lifestyle
KW - Time Factors
RP - NOT IN FILE
SP - 47
JF - International Journal of Behavioral Nutrition & Physical Activity
JA - Int
VL - 8
CY - England
N2 - BACKGROUND: The weather may be a driver of seasonal patterns in children's physical activity (PA). A better understanding of the relationships between weather and PA may help increase children's PA. This study aims to examine the association between PA and rainfall in 9-10 year old children, and how it may be modified by school policies. METHODS: 1794 participants in the SPEEDY study in Norfolk, UK recorded PA using ActiGraph accelerometers over up to six days in the summer term of 2007. Multilevel regression models were used to determine the day-by-day association between rainfall and minutes spent sedentary, in moderate-to-vigorous PA (MVPA), and average counts per minute (cpm) over the whole day (07:00-21:00) and the lunchtime period (12:00-14:00). School policies for break times in bad weather were fitted as interaction terms with rainfall. RESULTS: Relative to days with no rain, children spent 9.4 minutes (95%CI 7.0 to 11.9) fewer in MVPA, were sedentary for 13.6 minutes (8.8 to 18.4) more, and accumulated 85.9 cpm (66.2 to 105.5) fewer over the whole day on the wettest days. Children allowed to play outside in wet weather showed the lowest lunchtime PA levels on the wettest days, undertaking 9.8 minutes (6.2 to 13.5) fewer MVPA, 16.1 minutes (10.3 to 21.9) more sedentary, and accumulating 408.0 cpm (250.9 to 565.1) fewer than those allowed to be active indoors. CONCLUSIONS: Rainfall is negatively associated with PA in primary school children, but providing indoor physical activities in wet weather may help children maintain physical activity levels irrespective of rainfall
SN - 1479-5868
AD - School of Environmental Sciences, University of East Anglia, Norwich NR4 7JT, UK. flo.harrison@uea.ac.uk
UR - 21609441
ER -

TY - JOUR
ID - 594
T1 - Trauma and hyperactivity: differentiating symptoms and cause. [Review]
A1 - Arunyanart,W.
A1 - Weitzman,C.
A1 - Groves,B.
A1 - Augustyn,M.
Y1 - 2011/07//
N1 - Arunyanart, Wirongrong. Weitzman, Carol. Groves, Betsy. Augustyn, Marilyn
Journal of developmental and behavioral pediatrics : JDBP

htf, 8006933
IM
Case Reports. Journal Article. Review
English
KW - MEDLINE
KW - Adolescent
KW - Attention Deficit and Disruptive Behavior Disorders/di [Diagnosis]
KW - Attention Deficit and Disruptive Behavior Disorders/et [Etiology]
KW - Child
KW - Child Development/ph [Physiology]
KW - Female
KW - Foster Home Care/px [Psychology]
KW - Humans
KW - Male
KW - Mothers
KW - Pregnancy
KW - Prenatal Exposure Delayed Effects/pp [Physiopathology]
KW - Prenatal Exposure Delayed Effects/px [Psychology]
RP - NOT IN FILE
SP - 482
EP - 484
JF - Journal of Developmental & Behavioral Pediatrics
JA - J Dev Behav Pediatr
VL - 32
IS - 6
CY - United States
N2 - CASE: Quintin is a 7-and-a-half-year-old male who presents to you with his long-term foster parents stating that the school "wants to put him in a special behavior class." You have cared for the child since he went into the foster parents/biological aunt and uncle's care at 14 months of age.Quintin has been healthy and is on no medication; he has no chronic diseases or other medical problems. He has never had physical trauma, but when you first met him at 14 months of age, his aunt and uncle could give no history about his infancy. The aunt's sister who is the biological mother is currently incarcerated for drug trafficking, and his biological father is an unknown.His toddler years were fairly uneventful. The aunt and uncle have 2 children of their own who are currently 12 and 15 years of age-at the time Quintin joined the family they were 5 and 8 years of age and so the initial adjustment was stressful for the family. The aunt worked full time throughout Quintin's toddlerhood and often in childcare he would have difficulty with drop-off.When he began at head start, he was often described as "too busy" and "trouble on the playground," but he was highly verbal and intellectually curious, had many friends, and did well. Kindergarten was fairly uneventful in a full-day program, although he began to be more provocative at home, often getting into his cousins video games and once breaking his cousin's smart phone.He did well academically at the beginning of first grade. He was a solid reader by January of the year, but his activity and oppositionality were increasing. He was suspended 4 times between January and March for "unsafe behavior" including bolting from the playground during recess following the dare of an older student. His aunt and uncle present at primary care at their wits end. They state that at home he is increasingly angry. He responds much better to his uncle but can be very provocative with his aunt stating "I don't have to listen to you because you are not my mother." His aunt expressed concern when she was told by his teacher that "Either he starts medication or he needs to go to a special classroom." Where do you go from there?
SN - 1536-7312
AD - Phramongkutklao Hospital, Bangkok, Thailand
UR - 21654333
ER -

TY - JOUR
ID - 595
T1 - Residential insecticide usage in northern California homes with young children
A1 - Wu,X.M.
A1 - Bennett,D.H.
A1 - Ritz,B.

A1 - Frost,J.
A1 - Cassady,D.
A1 - Lee,K.
A1 - Hertz-Picciotto,I.
Y1 - 2011/07//
N1 - Wu, Xiangmei May. Bennett, Deborah H. Ritz, Beate. Frost, Joshua. Cassady, Diana. Lee, Kiyoung. Hertz-Picciotto, Irva
Journal of exposure science & environmental epidemiology
101262796
IM
Journal Article. Research Support, U.S. Gov't, Non-P.H.S.
English
KW - MEDLINE
KW - Adult
KW - Air Pollution
KW - Indoor/an [Analysis]
KW - Indoor/sn [Statistics & Numerical Data]
KW - Animals
KW - California/ep [Epidemiology]
KW - Child
KW - Preschool
KW - Environmental Exposure/an [Analysis]
KW - Environmental Exposure/sn [Statistics & Numerical Data]
KW - Housing
KW - Humans
KW - Insecticides/an [Analysis]
KW - Insecticides/to [Toxicity]
KW - Pets
KW - Risk Assessment/mt [Methods]
KW - Risk Assessment/sn [Statistics & Numerical Data]
KW - Siphonaptera
KW - Temperature
KW - Time Factors
RP - NOT IN FILE
SP - 427
EP - 436
JF - Journal of Exposure Science & Environmental Epidemiology
JA - J Expo Sci Environ Epidemiol
VL - 21
IS - 4
CY - United States
N2 - Residential insecticide usage and actual application details were collected in a population-based sample of 477 households residing within 22 counties in northern California with at least one child of age < 5 years between January 2006 and August 2008. Structured telephone interviews were conducted collecting information on residential use of insecticides, including outdoor sprays, indoor sprays, indoor foggers, applications by professionals, and pet flea/tick control during the previous year. Interviews also covered post-treatment behaviors, which influence post-application exposure levels. Altogether, 80% of the households applied some type of insecticide in the previous year, with half of this population using two or more application methods. Of the households using insecticides, half reported applying insecticides relatively infrequently (<4 times per year), whereas 11-13% reported high frequency of use (>24 times per year). Application frequency was temperature dependent, with significantly more applications during the warmer months from May through October. Spot treatments appeared to be the most prevalent application pattern for sprays. For one out of three of the indoor applications, children played in the treated rooms on the day of the application, and for 40% of the outdoor applications, pets played in the treated area on the day of the application. These findings describing the intensity of insecticide use and accompanying behaviors in families with young children may inform future insecticide exposure modeling efforts, and ultimately, risk assessments
SN - 1559-064X

AD - Department of Public Health Sciences, University of California, Davis, CA 95616, USA
UR - 20588323
ER -

TY - JOUR
ID - 596
T1 - [Indoor air quality in schools]. [Review] [French]
A1 - Cartieaux,E.
A1 - Rzepka,M.A.
A1 - Cuny,D.
Y1 - 2011/07//
N1 - Cartieaux, E. Rzepka, M-A. Cuny, D
Archives de pediatrie : organe officiel de la Societe francaise de pediatrie
9421356, bwh
IM
English Abstract. Journal Article. Review
French
KW - MEDLINE
KW - Air Pollutants/ae [Adverse Effects]
KW - Air Pollutants/an [Analysis]
KW - Air Pollution
KW - Indoor/ae [Adverse Effects]
KW - Indoor/an [Analysis]
KW - Asthma/di [Diagnosis]
KW - Asthma/ep [Epidemiology]
KW - Asthma/et [Etiology]
KW - Child
KW - Preschool
KW - Cross-Sectional Studies
KW - Diagnosis
KW - Differential
KW - France
KW - Humans
KW - Respiratory Hypersensitivity/di [Diagnosis]
KW - Respiratory Hypersensitivity/ep [Epidemiology]
KW - Respiratory Hypersensitivity/et [Etiology]
KW - Risk Factors
KW - Schools
KW - Statistics as Topic
KW - Ventilation
RP - NOT IN FILE
SP - 789
EP - 796
JF - Archives de Pediatrie
JA - Arch Pediatr
VL - 18
IS - 7
CY - France
N2 - Indoor air quality in schools has received particular attention over the past several years. Children are considered as one of the most sensitive groups to atmospheric pollution because their bodies are actively growing and they breathe higher volumes of air relative to their body weights than adults do. They also spend more time in school or group structures (preschools, day nurseries) than in any indoor environments other than the home. The analysis of children's exposure to air pollution at school requires the identification of the main pollutant sources present in these educational institutions. Both a strong contribution of outdoor pollution and a very specific pollution bound to school activities such as the use of paints, markers, glues, and manufactured ink eraser pens, exist. The ventilation in school buildings also plays an important role in air quality. A higher air exchange may improve thermal comfort and air quality. The cause of indoor air pollution is a combinatory

effect of physical, chemical, and biological factors, and the adequacy of ventilation in the environment. Several pollutants have been reported to exist in classrooms such as bacteria, molds, volatile organic compounds, persistent organic pollutants and microparticles. There is a correlation between the concentrations of the pollutants and onset of health problems in schoolchildren. We observe predominantly respiratory symptoms as well as a prevalence of respiratory diseases such as asthma and allergies. This study shows that poor indoor air quality affects children's health. Copyright 2011 Elsevier Masson SAS. All rights reserved
SN - 1769-664X
AD - Institut lillois d'ingenierie de la sante, 42, rue Ambroise-Pare, 59120 Loos, France
UR - 21621987
ER -

TY - JOUR
ID - 597
T1 - Rickets: concerns over the worldwide increase
A1 - Lowdon,J.
Y1 - 2011/03//
N1 - Lowdon, Jacqui
The journal of family health care
101142028
N
Journal Article
English
KW - MEDLINE
KW - Child
KW - Preschool
KW - Dietary Supplements
KW - Great Britain/ep [Epidemiology]
KW - Humans
KW - Infant
KW - Prevalence
KW - Rickets/di [Diagnosis]
KW - Rickets/dt [Drug Therapy]
KW - Rickets/ep [Epidemiology]
KW - Rickets/pc [Prevention & Control]
KW - Risk Factors
KW - Vitamin D/tu [Therapeutic Use]
KW - World Health
RP - NOT IN FILE
SP - 25
EP - 29
JF - Journal of Family Health Care
JA - J Fam Health Care
VL - 21
IS - 2
CY - England
N2 - Rickets is a childhood disease that causes a softening of the bones, potentially leading to fractures and deformity. Eighty years ago it was thought to have largely been eradicated from the U.K. However a recent increase in cases of rickets, not just in Britain but around the world, has proven this isn't the case. Today the disease affects children from all types of socio-economic backgrounds, not just the poorer ones, and it is primarily caused by low levels of vitamin D and certain foods. In January 2011 the government's chief medical officer Dame Sally Davies recommended all children aged six months to five should be given vitamin D supplements, particularly during winter months when natural sunshine is limited. The irony is that the advice in recent years for children to wear a high factor sunscreen and remain covered up while playing outdoors are partly felt to be behind the reason for its re-emergence. Parents and health professionals alike were shocked when it was revealed that a school girl living on the Isle of Wight developed rickets precisely because of her mother's vigilance at following sun safety rules. NICE, in their latest report (Jan 2011) stated that: "Exposure to the sun has a number of benefits. For example, it increases people's sense of wellbeing, allows them to

synthesise vitamin D and provides opportunities for physical activity". A tendency for children to stay indoors and watch TV or play on computer games, rather than play outside when the sun is shining, is arguably also another contributing factor
SN - 1474-9114
AD - Royal Manchester Children's Hospital
UR - 21678784
ER -

TY - JOUR
ID - 598
T1 - Playground renovations and quality at public parks in Boston,Massachusetts, 1996-2007
A1 - Barrett,J.L.
A1 - Hannon,C.
A1 - Keefe,L.
A1 - Gortmaker,S.L.
A1 - Cradock,A.L.
Y1 - 2011/07//
N1 - Barrett, Jessica L. Hannon, Cynthia. Keefe, Linda. Gortmaker, Steven L. Cradock, Angie L
Preventing chronic disease
101205018
IM
Journal Article. Research Support, U.S. Gov't, P.H.S.
English
KW - MEDLINE
KW - Activities of Daily Living
KW - Adolescent
KW - Architectural Accessibility/st [Standards]
KW - Boston
KW - Child
KW - Humans
KW - Play and Playthings
KW - Retrospective Studies
KW - Safety Management/st [Standards]
RP - NOT IN FILE
SP - A72
JF - Preventing Chronic Disease
JA - Prev Chronic Dis
VL - 8
IS - 4
CY - United States
N2 - INTRODUCTION: Recreational and transportation infrastructure can promote physical activity among children and adolescents. The Play Across Boston community-based research project sought to estimate and compare playground renovation rates across Boston areas before and after a playground quality assessment, to describe changes in playground quality among a subset of parks, and to document features of local transportation infrastructure around parks. METHODS: We used an observational pretest-posttest design to estimate playground renovation rates among 103 city-operated parks. Renovation rates were calculated on the basis of annual city Parks Department capital budgets from fiscal years 1996 through 2007. We used the same design to describe changes between a 2000 to 2001 baseline assessment of playground quality and a 2007 follow-up measured via observation of a subsample of 18 low-scoring parks in disadvantaged areas. We used chi(2) analysis to compare percentages of playgrounds renovated across city areas before and after baseline assessment, logistic regression analysis to calculate odds ratios comparing renovation rates after baseline by city area, and paired t tests to compare playground quality at baseline and follow-up. RESULTS: Overall playground renovation rates before (29%) and after (34%) baseline assessment were similar. Parks scoring low on playground quality at baseline were renovated after baseline at a higher rate than high-scoring playgrounds. After accounting for baseline playground quality, parks in disadvantaged areas were renovated at a rate similar to those in other areas. Playground quality scores improved between baseline (mean, 38.3; 95% confidence interval, 35.3-41.3) and 2007 in a subsample of previously low-scoring parks in disadvantaged areas.

CONCLUSION: The findings of the 2007 follow-up assessment indicate an equitable rate of playground renovation across city areas according to need
SN - 1545-1151
AD - Department of Society, Human Development, and Health, Harvard School of Public Health, Landmark Center, 401 Park Drive, Boston, MA 02215, USA. jbarrett@hsph.harvard.edu
UR - 21672396
ER -

TY - JOUR
ID - 599
T1 - Soil intervention as a strategy for lead exposure prevention: the New Orleans lead-safe childcare playground project
A1 - Mielke,H.W.
A1 - Covington,T.P.
A1 - Mielke,P.W.,Jr.
A1 - Wolman,F.J.
A1 - Powell,E.T.
A1 - Gonzales,C.R.
Y1 - 2011/08//
N1 - Mielke, Howard W. Covington, Tina P. Mielke, Paul W Jr. Wolman, Fredericka J. Powell, Eric T. Gonzales, Chris R
Environmental pollution (Barking, Essex : 1987)
dvl, 8804476
IM
Evaluation Studies. Journal Article. Research Support, Non-U.S. Gov't. Research Support, U.S. Gov't, Non-P.H.S.
English
KW - MEDLINE
KW - Child
KW - Child Care/sn [Statistics & Numerical Data]
KW - Conservation of Natural Resources/mt [Methods]
KW - Environmental Exposure/pc [Prevention & Control]
KW - Environmental Exposure/sn [Statistics & Numerical Data]
KW - Environmental Monitoring
KW - Environmental Policy
KW - Environmental Remediation/ec [Economics]
KW - Environmental Remediation/mt [Methods]
KW - Government Programs
KW - Humans
KW - Lead/an [Analysis]
KW - New Orleans
KW - Play and Playthings
KW - Soil/ch [Chemistry]
KW - Soil Pollutants/an [Analysis]
RP - NOT IN FILE
SP - 2071
EP - 2077
JF - Environmental Pollution
JA - Environ Pollut.
VL - 159
IS - 8-9
CY - England
N2 - The feasibility of reducing children's exposure to lead (Pb) polluted soil in New Orleans is tested. Childcare centers (median = 48 children) are often located in former residences. The extent of soil Pb was determined by selecting centers in both the core and outlying areas. The initial 558 mg/kg median soil Pb (range 14-3692 mg/kg) decreased to median 4.1 mg/kg (range 2.2-26.1 mg/kg) after intervention with geotextile covered by 15 cm of river alluvium. Pb loading decreased from a median of 4887 mug/m(2) (454 mug/ft(2))

range 603-56650 mug/m(2) (56-5263 mug/ft(2)) to a median of 398 mug/m(2) (37 mug/ft(2)) range 86-980 mug/m(2) (8-91 mug/ft(2)). Multi-Response Permutation Procedures indicate similar (P-values = 0.160-0.231) soil Pb at childcare centers compared to soil Pb of nearby residential communities. At ~$100 per child, soil Pb and surface loading were reduced within hours, advancing an upstream intervention conceptualization about Pb exposure prevention. Copyright 2010 Elsevier Ltd. All rights reserved
SN - 1873-6424
AD - Department of Chemistry, Tulane University, New Orleans, LA 70118, USA. howard.mielke@gmail.com
UR - 21131114
ER -

TY - JOUR
ID - 600
T1 - School playgrounds and physical activity policies as predictors of school and home time activity
A1 - Taylor,R.W.
A1 - Farmer,V.L.
A1 - Cameron,S.L.
A1 - Meredith-Jones,K.
A1 - Williams,S.M.
A1 - Mann,J.I.
Y1 - 2011///
N1 - Taylor, Rachael W. Farmer, Victoria L. Cameron, Sonya L. Meredith-Jones, Kim. Williams, Sheila M. Mann, Jim I
The international journal of behavioral nutrition and physical activity
101217089
IM
Journal Article. Research Support, Non-U.S. Gov't
English
KW - MEDLINE
KW - Child
KW - Female
KW - Humans
KW - Male
KW - Motor Activity
KW - New Zealand
KW - Play and Playthings
KW - Policy
KW - Questionnaires
KW - Schools/sn [Statistics & Numerical Data]
KW - Sports
RP - NOT IN FILE
SP - 38
JF - International Journal of Behavioral Nutrition & Physical Activity
JA - Int
VL - 8
CY - England
N2 - BACKGROUND: Previous work has suggested that the number of permanent play facilities in school playgrounds and school-based policies on physical activity can influence physical activity in children. However, few comparable studies have used objective measures of physical activity or have had little adjustment for multiple confounders. METHODS: Physical activity was measured by accelerometry over 5 recess periods and 3 full school days in 441 children from 16 primary schools in Dunedin, New Zealand. The number of permanent play facilities (swing, fort, slide, obstacle course, climbing wall etc) in each school playground was counted on three occasions by three researchers following a standardized protocol. Information on school policies pertaining to physical activity and participation in organized sport was collected by questionnaire. RESULTS: Measurement of school playgrounds proved to be reliable (ICC 0.89) and consistent over time. Boys were significantly more active than girls (P < 0.001), but little time overall was spent in moderate-vigorous physical activity (MVPA). Boys engaged in MVPA for 32 (SD 17) minutes each day of which 17 (10) took place at school compared with 23 (14) and 11 (7) minutes respectively in girls. Each additional 10-unit increase in play

facilities was associated with 3.2% (95% CI 0.0-6.4%) more total activity and 8.3% (0.8-16.3%) more MVPA during recess. By contrast, school policy score was not associated with physical activity in children. CONCLUSION: The number of permanent play facilities in school playgrounds is associated with higher physical activity in children, whereas no relationship was observed for school policies relating to physical activity. Increasing the number of permanent play facilities may offer a cost-effective long-term approach to increasing activity levels in children
SN - 1479-5868
AD - Edgar National Centre for Diabetes and Obesity Research, Department of Medical and Surgical Sciences, University of Otago, PO Box 913, Dunedin 9054, New Zealand. rachael.taylor@otago.ac.nz
UR - 21521530
ER -

TY - JOUR
ID - 601
T1 - Active children use more locations for physical activity
A1 - Corder,K.
A1 - Sallis,J.F.
A1 - Crespo,N.C.
A1 - Elder,J.P.
Y1 - 2011/07//
N1 - Corder, Kirsten. Sallis, James F. Crespo, Noe C. Elder, John P
Health & place
din, 9510067
T
Journal Article. Research Support, N.I.H., Extramural. Research Support, Non-U.S. Gov't
English
KW - MEDLINE
KW - California
KW - Child
KW - Preschool
KW - Environment Design
KW - Female
KW - Humans
KW - Male
KW - Motor Activity
KW - Questionnaires
KW - Randomized Controlled Trials as Topic
KW - Residence Characteristics
RP - NOT IN FILE
SP - 911
EP - 919
JF - Health & Place
JA - Health Place
VL - 17
IS - 4
CY - England
N2 - We examined frequency of use of 11 physical activity (PA) locations among 539 San Diego children (45.0% males, 41.2% Latinos; mean + SD age: 6.6 + 0.7 years) and explored associations between location use, PA and potential correlates. Parents reported child's use (visits/week) of 11 locations. Child PA was assessed by accelerometry (subsample n = 178). The most frequently used locations (mean + SD times/week) were homes (3.2 + 2.3) and parks/playground (1.6 + 1.3). Children used 4.0 + 2.0 locations in a typical week, and made a total of 12.5 + 6.8 visits/week to all locations. Latinos used fewer locations regularly (3.6 + 2.1 vs. 4.3 + 1.9 locations; p < 0.001) and had fewer visits to all locations (11.4 + 7.4 vs. 13.2 + 6.4 visits/week; p = 0.003) than non-Latinos. Accelerometry-assessed vigorous PA (VPA) was positively associated with the number of locations regularly used (s = 0.04, p = 0.03) and total visits to all locations among Latinos (s = 0.09, p = 0.005). Parental PA support was positively associated with locations used (s = 0.64, p < 0.001) and visits to all locations

(s = 2.56, p < 0.001). Children using a greater variety of locations did more VPA. Latinos making more total visits to all locations had higher VPA. Copyright 2011 Elsevier Ltd. All rights reserved
SN - 1873-2054
AD - Centre for Diet and Activity Research, Institute of Public Health, University of Cambridge, Cambridge , UK. klc29@medschl.cam.ac.uk
UR - 21550836
ER -

TY - JOUR
ID - 602
T1 - Impact of short bouts of physical activity on attention-to-task in elementary school children. [Review]
A1 - Mahar,M.T.
Y1 - 2011/06//
N1 - Mahar, Matthew T
Preventive medicine
pm4, 0322116
IM
Journal Article. Review
English
KW - MEDLINE
KW - Attention
KW - Child
KW - Cognition
KW - Curriculum
KW - Exercise
KW - Humans
KW - Leisure Activities/px [Psychology]
KW - Motor Activity
KW - Physical Education and Training/mt [Methods]
KW - Reproducibility of Results
KW - Schools
KW - Students
KW - Teaching/mt [Methods]
RP - NOT IN FILE
SP - S60
EP - S64
JF - Preventive Medicine
JA - Prev Med
VL - 52 Suppl 1
CY - United States
N2 - OBJECTIVE: The study's objective was to describe the measurement of on-task behavior and review the research on the effects of short physical activity breaks on attention-to-task in elementary school students, with a particular focus on a promising approach to improving on-task behavior with short bouts of physical activity in the classroom setting. METHODS: Procedures to directly observe attention-to-task were detailed. Published studies that measured attention-to-task in elementary school students following physical activity breaks were reviewed. RESULTS: Direct measurement of attention-to-task is intensive and demanding on observers. Previous research on attention-to-task following physical activity breaks is sparse. The limited evidence suggests a small to moderate improvement on attention-to-task following physical activity breaks (Effect Sizes typically ranged from 0.13 to 0.60). Teachers can be trained in a relatively short time to effectively lead classroom-based physical activities. Students who participated in classroom-based physical activities that incorporate academic concepts demonstrated significantly better improvements (+8.3%) in attention-to-task than control group participants (-3.1%). CONCLUSIONS: Attention-to-task is a variable that directly relates to concerns of classroom teachers. The limited available research has demonstrated moderate to good evidence that physical activity during the school day improves attention-to-task in elementary school students. Because of the positive effects of physical activity on attention-to-task, it is recommended that elementary school teachers consider implementing physical activity sessions throughout the school day in the form of recess and classroom-based physical activities. Copyright 2011 Elsevier Inc. All rights reserved

SN - 1096-0260
AD - Activity Promotion Laboratory, Department of Exercise and Sport Science, East Carolina University, Greenville, NC, USA
UR - 21281665
ER -

TY - JOUR
ID - 603
T1 - Pattern of injuries among children and adolescents in Riyadh, Saudi Arabia: a household survey
A1 - Gad,A.
A1 - AL-Eid,R.
A1 - Al-Ansary,S.
A1 - bin,Saeed A.
A1 - Kabbash,A.
Y1 - 2011/06//
N1 - Gad, Ashry. AL-Eid, Rasheed. Al-Ansary, Saleh. bin Saeed, Abdulaziz. Kabbash, Abdulmoty
Journal of tropical pediatrics
kaw, 8010948
IM
Journal Article. Research Support, Non-U.S. Gov't
English
KW - MEDLINE
KW - Accidental Falls/sn [Statistics & Numerical Data]
KW - Accidents
KW - Traffic/sn [Statistics & Numerical Data]
KW - Adolescent
KW - Child
KW - Preschool
KW - Cross-Sectional Studies
KW - Female
KW - Foodborne Diseases/ep [Epidemiology]
KW - Humans
KW - Male
KW - Questionnaires
KW - Risk Factors
KW - Saudi Arabia/ep [Epidemiology]
KW - Sex Distribution
KW - Wounds and Injuries/ep [Epidemiology]
KW - Wounds and Injuries/et [Etiology]
RP - NOT IN FILE
SP - 179
EP - 184
JF - Journal of Tropical Pediatrics
JA - J Trop Pediatr
VL - 57
IS - 3
CY - England
N2 - OBJECTIVES: To determine the incidence and pattern of injuries among children and adolescents <18 years old in Riyadh city and to identify associated factors. MATERIAL AND METHODS: A cross sectional household survey included children and adolescents <18 years. A two-stage cluster sample was used to recruit the participants from thirty clusters each consisted OF 23 households. A questionnaire was used for data collection by interview. RESULTS: The study included 1650 children and adolescents. Of them, 22.2% reported having had injuries in the previous 12 months. The most common injuries were falls (40.4%), Road Traffic Accidents (RTA) (15%), food intoxication (8.8%). Males were more affected by injuries than females (26% vs. 18%). Males living near playgrounds or public gardens, playing in the street are independent risk factors for occurrence of both falls and RTA injuries. RECOMMENDATIONS: school safety education and environmental modification should be applied in Riyadh

SN - 1465-3664
AD - Research Chair of Epidemiology, Public Health and Environment, Family & Community Medicine Department, College of Medicine, King Saud University, Riyadh, KSA. ashry59@yahoo.co.uk
UR - 20724436
ER -

TY - JOUR
ID - 604
T1 - Adapting an established measure of supervision for beach settings: is the parent supervision attributes profile questionnaire reliable?
A1 - Petrass,L.A.
A1 - Blitvich,J.D.
A1 - Finch,C.F.
Y1 - 2011/06//
N1 - Petrass, Lauren A. Blitvich, Jennifer D. Finch, Caroline F
International journal of injury control and safety promotion
101247254
IM
Comparative Study. Journal Article. Research Support, Non-U.S. Gov't. Validation Studies
English
KW - MEDLINE
KW - Australia
KW - Bathing Beaches/sn [Statistics & Numerical Data]
KW - Child
KW - Preschool
KW - Health Knowledge
KW - Attitudes
KW - Practice
KW - Humans
KW - Infant
KW - Parents
KW - Questionnaires
KW - Reproducibility of Results
KW - Safety/sn [Statistics & Numerical Data]
KW - Swimming/sn [Statistics & Numerical Data]
RP - NOT IN FILE
SP - 113
EP - 117
JF - International Journal of Injury Control & Safety Promotion
JA - Int J Inj Contr Saf Promot
VL - 18
IS - 2
CY - England
N2 - The Parent Supervision Attributes Profile Questionnaire (PSAPQ), developed to measure aspects of caregiver supervision and protectiveness and previously applied within playgrounds and in the home, was modified for implementation in a beach setting. To assess the test-retest reliability of the PSAPQ beach modification (PSAPQ-BEACH), 20 caregivers completed the PSAPQ-BEACH twice, over a mean interval of 18 days (range 10-24). The test-retest reliability and internal consistency of the PSAPQ-BEACH scores were compared to those of the PSAPQ. All scores on the PSAPQ-BEACH were higher than the PSAPQ, providing evidence that the questionnaire remains reliable after its adaptation to beach settings
SN - 1745-7319
AD - School of Human Movement and Sport Sciences, University of Ballarat, Victoria, Australia. l.petrass@ballarat.edu.au
UR - 21302168
ER -

TY - JOUR

ID - 605
T1 - Influence of physical inactivity on associations between single nucleotide polymorphisms and genetic predisposition to childhood obesity
A1 - Xi,B.
A1 - Wang,C.
A1 - Wu,L.
A1 - Zhang,M.
A1 - Shen,Y.
A1 - Zhao,X.
A1 - Wang,X.
A1 - Mi,J.
Y1 - 2011/06/01/
N1 - Xi, Bo. Wang, Chunyu. Wu, Lijun. Zhang, Meixian. Shen, Yue. Zhao, Xiaoyuan. Wang, Xingyu. Mi, Jie
American journal of epidemiology
3h3, 7910653
IM
Journal Article. Research Support, Non-U.S. Gov't
English
KW - MEDLINE
KW - Adiposity/ge [Genetics]
KW - Adolescent
KW - Apoptosis Regulatory Proteins/ge [Genetics]
KW - Body Mass Index
KW - Brain-Derived Neurotrophic Factor/ge [Genetics]
KW - Carrier Proteins/ge [Genetics]
KW - Child
KW - China
KW - Exercise
KW - Female
KW - Gene Frequency
KW - Genetic Association Studies
KW - Genetic Predisposition to Disease
KW - Genotype
KW - Humans
KW - Life Style
KW - Male
KW - Membrane Glycoproteins/ge [Genetics]
KW - Membrane Proteins/ge [Genetics]
KW - Obesity/ge [Genetics]
KW - Polymorphism
KW - Single Nucleotide
KW - Proteins/ge [Genetics]
KW - Receptor
KW - Melanocortin
KW - Type 4/ge [Genetics]
RP - NOT IN FILE
SP - 1256
EP - 1262
JF - American Journal of Epidemiology
JA - Am J Epidemiol
VL - 173
IS - 11
CY - United States
N2 - Childhood obesity is a complex disease that is influenced by both genetic and environmental factors. The authors' aim was to determine whether sedentary behavior and physical activity modulate the association between single nucleotide polymorphisms (SNPs) and obesity risk in Chinese children. A population-based study was carried out in 2,848 children (6-18 years of age) in Beijing, China, in 2004. It included 1,229 obese

cases and 1,619 normal-weight controls. Lifestyle information was collected through the use of a validated questionnaire, and 6 SNPs were genotyped. The association between the 6 SNPs and obesity risk was modulated by sedentary behavior and physical activity. A higher risk of obesity was observed in children who carried the high-risk alleles of the 6 SNPs and engaged in sedentary behavior >2 hours/day outside of school or participated in low or moderate physical activity. Most notably, the association between 5 SNPs (Fas apoptotic inhibitory molecule 2 rs7138803, Niemann-Pick disease, type C1 rs1805081, fat mass- and obesity-associated gene rs6499640, melanocortin 4 receptor gene rs17782313, and brain-derived neurotrophic factor rs6265) and obesity risk was only observed in children who had moderate-to-low physical activity levels or engaged in sedentary behavior, regardless of which risk alleles they carried. The results indicated that encouraging less sedentary behavior and higher levels of physical activity could alleviate the influence of risk alleles on genetic predisposition to childhood obesity, thereby serving as a promising prevention strategy
SN - 1476-6256
AD - Department of Epidemiology, Capital Institute of Pediatrics, 2 Ya Bao Road, Beijing, China
UR - 21527513
ER -

TY - JOUR
ID - 606
T1 - PLAYgrounds: effect of a PE playground program in primary schools on PA levels during recess in 6 to 12 year old children. Design of a prospective controlled trial
A1 - Janssen,M.
A1 - Toussaint,H.M.
A1 - Van,Willem M.
A1 - Verhagen,E.A.
Y1 - 2011///
N1 - Janssen, Mirka. Toussaint, Huub M. Van Willem, Mechelen. Verhagen, Evert A L M
BMC public health
100968562
IM
Journal Article
English
KW - MEDLINE
KW - Child
KW - Controlled Clinical Trials as Topic
KW - Female
KW - Humans
KW - Male
KW - Motor Activity
KW - Netherlands
KW - Physical Education and Training/og [Organization & Administration]
KW - Play and Playthings
KW - Program Evaluation
KW - Prospective Studies
KW - Questionnaires
KW - Schools
RP - NOT IN FILE
SP - 282
JF - BMC Public Health
VL - 11
CY - England
N2 - BACKGROUND: The relative number of children meeting the minimal required dose of daily physical activity remains execrably low. It has been estimated that in 2015 one out of five children will be overweight. Therefore, low levels of physical activity during early childhood may compromise the current and future health and well-being of the population, and promoting physical activity in younger children is a major public health priority. This study is to gain insight into effects of a Physical Education based playground program on the PA levels during recess in primary school children aged 6-12. METHODS/DESIGN: The effectiveness of the intervention program will be evaluated using a prospective controlled trial design in which schools will be

matched, with a follow-up of one school year. The research population will consist of 6-12 year old primary school children. The intervention program will be aimed at improving physical activity levels and will consist of a multi-component alteration of the schools' playground. In addition, playground usage will be increased through altered time management of recess times, as well as a modification of the Physical Education content. DISCUSSION: The effects of the intervention on physical activity levels during recess (primary outcome measure), overall daily physical activity and changes in physical fitness (secondary outcome measures) will be assessed. Results of this study could possibly lead to changes in the current playground system of primary schools and provide structured health promotion for future public health. TRIAL REGISTRATION: Netherlands Trial Register (NTR): NTR2386
SN - 1471-2458
AD - Academy of Physical Education, Technical University of Applied Sciences of Amsterdam, The Netherlands. m.janssen@hva.nl
UR - 21548998
ER -

TY - JOUR
ID - 607
T1 - Is the neighbourhood environment associated with sedentary behaviour outside of school hours among children?
A1 - Veitch,J.
A1 - Timperio,A.
A1 - Crawford,D.
A1 - Abbott,G.
A1 - Giles-Corti,B.
A1 - Salmon,J.
Y1 - 2011/06//
N1 - Veitch, Jenny. Timperio, Anna. Crawford, David. Abbott, Gavin. Giles-Corti, Billie. Salmon, Jo
Annals of behavioral medicine : a publication of the Society of Behavioral Medicine
8510246, cm5
IM
Journal Article. Research Support, Non-U.S. Gov't
English
KW - MEDLINE
KW - Adult
KW - Child
KW - Environment
KW - Female
KW - Humans
KW - Leisure Activities/px [Psychology]
KW - Male
KW - Parents/px [Psychology]
KW - Perception
KW - Residence Characteristics
KW - Sedentary Lifestyle
RP - NOT IN FILE
SP - 333
EP - 341
JF - Annals of Behavioral Medicine
JA - Ann Behav Med
VL - 41
IS - 3
CY - United States
N2 - BACKGROUND: Little is known about neighbourhood environments and children's sedentary behaviour outside school hours. PURPOSE: This study aims to examine the associations between public open spaces (POS), parent perceptions of the neighbourhood and children's sedentary behaviours. METHODS: Parents reported their child's television viewing and computer/electronic game time and their perceptions of the physical and social neighbourhood. Children's sedentary time was objectively assessed. The closest POS was audited.

RESULTS: Cross-sectionally, living near a POS with a water feature and greater parental satisfaction with POS quality were negatively associated with computer/e-games; greater POS area was negatively associated with TV viewing. Longitudinally, living in a cul-de-sac and greater satisfaction with POS quality were negatively associated with computer/e-games and TV viewing, respectively. A walking path in the POS was positively associated with computer/e-games. CONCLUSION: Neighbourhood features appear to positively and negatively influence children's sedentary behaviours, highlighting the complexity of urban planning on behaviour. Further age- and context-specific studies are required
SN - 1532-4796
AD - Centre for Physical Activity and Nutrition Research, Burwood, Australia. jenny.veitch@deakin.edu.au
UR - 21336879
ER -

TY - JOUR
ID - 608
T1 - The cost of sustaining playground related extremity fractures in Singapore
A1 - Leung,K.W.
A1 - Mahadev,A.
Y1 - 2011/04//
N1 - Leung, Kenneth Wong Pak. Mahadev, Arjandas
Injury
0226040, gon
IM
Journal Article
English
KW - MEDLINE
KW - Absenteeism
KW - Accidental Falls/ec [Economics]
KW - Accidental Falls/sn [Statistics & Numerical Data]
KW - Adolescent
KW - Age Distribution
KW - Arm Injuries/ec [Economics]
KW - Arm Injuries/ep [Epidemiology]
KW - Arm Injuries/px [Psychology]
KW - Child
KW - Preschool
KW - Cost of Illness
KW - Equipment Safety
KW - Female
KW - Fractures
KW - Bone/ec [Economics]
KW - Bone/ep [Epidemiology]
KW - Bone/px [Psychology]
KW - Humans
KW - Leg Injuries/ec [Economics]
KW - Leg Injuries/ep [Epidemiology]
KW - Leg Injuries/px [Psychology]
KW - Male
KW - Play and Playthings/in [Injuries]
KW - Prospective Studies
KW - Risk Factors
KW - Sex Distribution
KW - Singapore/ep [Epidemiology]
RP - NOT IN FILE
SP - 352
EP - 355
JF - Injury
VL - 42

IS - 4
CY - Netherlands
N2 - PURPOSE: To study the cost of sustaining a fracture of the extremity caused by playground equipment. These costs include financial, psychological, clinical and others like loss of school days. METHOD: This is a prospective study of 226 children seen at the Paediatric Orthopaedic Department for a1-year period starting June 2005. Once confirmed to have a playground related fracture by the Orthopaedic specialist, three sets of data are collected. First is the clinical data. Second is the playground related data. This is done on site independently by another investigator. Third is the radiological data,which is assessed independently by an Orthopaedic surgeon to ensure consistency. RESULTS: Out of the 226 children seen, two-thirds were boys. The average age was 7.5 years. 35.8% were forearm fractures and 28.8% were supracondylar fractures with the rest being mostly fractures in the upper limb. 64.2% were treated with immobilisation only whilst 24.2% required closed manipulation and reduction. 1.2% required admission for treatment of their fractures. The average period of cast immobilisation was 34 days. The average number of consults, including that at the emergency department, was 4 with an average length of follow up of 67.2 days. Based on the above, in our institution, the average cost for outpatient treatment would be about S$680.00 (US$485.71) (US$1.00 = S$1.40; 1/1/2010) per injury. The inpatient cost for non-surgical treatment cost would be S$1000.00 and for surgical stabilisation the average cost would be S$3300.00 (US$2357.14). CONCLUSION: Playgrounds are meant for children to play safely. Yet, there appears to be a significant number of injuries sustained. Whilst these fractures are relatively minor, requiring outpatient treatment,there are costs. Besides the financial costs, there are hidden psychological costs of loss of school days and inability to participate in sports. Finally, as with any injury, there can be long-term complications which present further clinical costs. SIGNIFICANCE: Most studies on playground injuries tend to concentrate on the equipment and very superficially cover the clinical aspects, less so the costs. This paper looks at the clinical aspects in greater depth and emphasises that there are significant costs, beyond financial, when a child sustains a playground injury. The next thing to do is to examine what playgrounds factors significantly contribute to these injuries and remedy them when planning the construction of playgrounds. 2010 Elsevier Ltd. All rights reserved
SN - 1879-0267
AD - Department of Orthopaedic Surgery, KK Women's and Children's Hospital, 100 Bukit Timah Road, Singapore 229899, Singapore
UR - 21598457
ER -

TY - JOUR
ID - 609
T1 - Features and amenities of school playgrounds: a direct observation study of utilization and physical activity levels outside of school time
A1 - Colabianchi,N.
A1 - Maslow,A.L.
A1 - Swayampakala,K.
Y1 - 2011///
N1 - Colabianchi, Natalie. Maslow, Andrea L. Swayampakala, Kamala
The international journal of behavioral nutrition and physical activity
101217089
IM
Journal Article. Research Support, N.I.H., Extramural. Research Support, Non-U.S. Gov't
English
KW - MEDLINE
KW - Adolescent
KW - Adolescent Behavior
KW - Adult
KW - Child
KW - Child Behavior
KW - Equipment Safety
KW - Facility Design and Construction
KW - Female
KW - Humans
KW - Leisure Activities

KW - Male
KW - Motor Activity
KW - Ohio
KW - Play and Playthings
KW - Residence Characteristics
KW - Schools
KW - Urban Population
RP - NOT IN FILE
SP - 32
JF - International Journal of Behavioral Nutrition & Physical Activity
JA - Int
VL - 8
CY - England
N2 - BACKGROUND: A significant amount of research has examined whether park or playground availability is associated with physical activity. However, little research has examined whether specific features or amenities of parks or playgrounds, such as the number of unique types of playground equipment or the safety of the equipment is associated with utilization of the facility or physical activity levels while at the facility. There are no studies that use direct observation and a detailed park assessment to examine these associations. METHODS: Twenty urban schoolyards in the Midwest, ten of which were renovated, were included in this study. Using a detailed environmental assessment tool (i.e., Environmental Assessment of Public Recreation Spaces), information on a variety of playground attributes was collected. Using direct observation (i.e., System for Observing Play and Leisure Activity in Youth), the number of adults, girls and boys attending each schoolyard and their physical activity levels were recorded. Each schoolyard was observed ten times for 90 minutes each time outside of school hours. Clustered multivariable negative binomial regressions and linear regressions were completed to examine the association between playground attributes and utilization of the schoolyard and the proportion active on the playground, respectively. Effect modification by renovation status was also examined. RESULTS: At renovated schoolyards, the total number of play features was significantly associated with greater utilization in adults and girls; overall cleanliness was significantly associated with less utilization in girls and boys; and coverage/shade for resting features was significantly associated with greater utilization in adults and boys. At unrenovated schoolyards, overall safety was significantly associated with greater utilization in boys. No playground attribute was associated with the proportion active on the playground after adjusting for all other significant playground attributes. CONCLUSIONS: Having a large quantity of play features and shade at renovated playgrounds were positively associated with utilization of the schoolyard. Modifying playgrounds to have these features may increase the utilization of these facilities outside of school time. Additional research should explore what features and amenities are associated with increased physical activity levels of children and adults who utilize the facilities. 2011 Colabianchi et al; licensee BioMed Central Ltd
SN - 1479-5868
AD - Department of Epidemiology and Biostatistics, Arnold School of Public Health, University of School Carolina, 800 Sumter Street, Columbia, SC 29208, USA. colabianchi@sc.edu
UR - 21492455
ER -

TY - JOUR
ID - 610
T1 - An assessment of schoolyard renovation strategies to encourage children's physical activity
A1 - Anthamatten,P.
A1 - Brink,L.
A1 - Lampe,S.
A1 - Greenwood,E.
A1 - Kingston,B.
A1 - Nigg,C.
Y1 - 2011///
N1 - Anthamatten, Peter. Brink, Lois. Lampe, Sarah. Greenwood, Emily. Kingston, Beverly. Nigg, Claudio
The international journal of behavioral nutrition and physical activity
101217089
IM

Evaluation Studies. Journal Article. Research Support, Non-U.S. Gov't
English
KW - MEDLINE
KW - Child
KW - Child Behavior/px [Psychology]
KW - Child Behavior
KW - Colorado
KW - Facility Design and Construction
KW - Female
KW - Health Behavior
KW - Health Promotion/mt [Methods]
KW - Humans
KW - Male
KW - Motor Activity
KW - Overweight/pc [Prevention & Control]
KW - Play and Playthings/px [Psychology]
KW - Play and Playthings
KW - Schools
RP - NOT IN FILE
SP - 27
JF - International Journal of Behavioral Nutrition & Physical Activity
JA - Int
VL - 8
CY - England
N2 - BACKGROUND: Children in poor and minority neighborhoods often lack adequate environmental support for healthy physical development and community interventions designed to improve physical activity resources serve as an important approach to addressing obesity. In Denver, the Learning Landscapes (LL) program has constructed over 98 culturally-tailored schoolyard play spaces at elementary schools with the goal to encourage utilization of play spaces and physical activity. In spite of enthusiasm about such projects to improve urban environments, little work has evaluated their impact or success in achieving their stated objectives. This study evaluates the impacts of LL construction and recency of renovation on schoolyard utilization and the physical activity rates of children, both during and outside of school, using an observational study design. METHODS: This study employs a quantitative method for evaluating levels of physical activity of individuals and associated environmental characteristics in play and leisure environments. Schools were selected on the basis of their participation in the LL program, the recency of schoolyard renovation, the size of the school, and the social and demographic characteristics of the school population. Activity in the schoolyards was measured using the System for Observing Play and Leisure Activity (SOPLAY), a validated quantitative method for evaluating levels of physical activity of individuals in play and leisure environments. Trained observers collected measurements before school, during school recess, after school, and on weekends. Overall utilization (the total number of children observed on the grounds) and the rate of activity (the percentage of children observed who were physically active) were analyzed. Observations were compared using t-tests and the data were stratified by gender for further analysis. In order to assess the impacts of LL renovation, recently-constructed LL schoolyards were compared to LL schoolyards with older construction, as well as un-renovated schoolyards. RESULTS: Overall utilization was significantly higher at LL schools than at un-renovated schools for most observation periods. Notably, LL renovation had no impact on girl's utilization on the weekends, although differences were observed for all other periods. There were no differences in rates of activity for any comparison. With the exception of the number of boys observed, there was no statistically significant difference in activity when recently-constructed LL schools are compared to LL schools with older construction dates and there was no difference observed in comparisons of older LL with unrenovated sites. CONCLUSIONS: While we observed greater utilization and physical activity in schools with LL, the impact of specific features of LL renovation is not clear. However, schoolyard renovation and programs to encourage schoolyard use before and after school may offer a means to encourage greater physical activity among children, and girls in particular. Additional study of schoolyard renovation may shed light on the specific reasons for these findings or suggest effective policies to improve the physical activity resources of poor and minority neighborhoods. 2011 Anthamatten et al; licensee BioMed Central Ltd
SN - 1479-5868

AD - Department of Geography and Environmental Sciences, University of Colorado Denver, Denver, CO, USA. peter.anthamatten@ucdenver.edu
UR - 21477325
ER -

TY - JOUR
ID - 611
T1 - A cluster randomised school-based lifestyle intervention programme for the prevention of childhood obesity and related early cardiovascular disease (JuvenTUM 3)
A1 - Siegrist,M.
A1 - Hanssen,H.
A1 - Lammel,C.
A1 - Haller,B.
A1 - Halle,M.
Y1 - 2011///
N1 - Siegrist, Monika. Hanssen, Henner. Lammel, Christoph. Haller, Bernhard. Halle, Martin
BMC public health
100968562
IM
Journal Article. Randomized Controlled Trial. Research Support, Non-U.S. Gov't
English
KW - MEDLINE
KW - Adolescent
KW - Cardiovascular Diseases/pc [Prevention & Control]
KW - Child
KW - Feasibility Studies
KW - Feeding Behavior
KW - Female
KW - Germany
KW - Health Promotion/mt [Methods]
KW - Humans
KW - Life Style
KW - Male
KW - Motor Activity
KW - Obesity/pc [Prevention & Control]
KW - Parent-Child Relations
KW - Risk Factors
KW - School Health Services/og [Organization & Administration]
RP - NOT IN FILE
SP - 258
JF - BMC Public Health
VL - 11
CY - England
N2 - BACKGROUND: Childhood obesity is not only associated with adult obesity but also with increased risk of adult onset of type 2 diabetes and subsequent coronary heart disease. The potential effects of school-based health intervention programmes on cardiovascular risk and surrogate markers are unclear, as only few studies have attempted to investigate a complete risk profile including a detailed laboratory analysis or micro- and macrovascular function. In this study a comprehensive school-based randomized intervention programme will be investigated in 10-14-year old children addressing the influence of lifestyle intervention on inactivity, cardiometabolic risk factors and early signs of vascular disease. METHODS/DESIGN: 15 secondary schools in Southern Germany are randomly assigned to intervention or control schools. Children in the fifth grade (10-11 years) will be observed over four years. The study combines a school-based with a home-based approach, aiming at children, teachers and parents. The main components are weekly lifestyle-lessons for children, taught by regular classroom teachers to increase physical activity in- and outside of school, to improve eating patterns at school and at home, to reduce media consumption and to amplify well-being. In 4-6 annual meetings, teachers receive information about health-related topics with worksheets for children and supporting equipment, accounting for school-specific needs and strategies. Parents' trainings are provided on a regular basis.All

examinations are performed at the beginning and at the end of every school year. Anthropometry includes measurements of BMI, waist and upper arm circumferences, skinfold thickness as well as peripheral blood pressure. Blood sampling includes lipid parameters, insulin, glucose, hsCRP, adiponectin, and IL-6 as well as testosteron and estrogen to determine maturation status. Vascular function is non-invasively assessed by measuring arterial stiffness in large arteries using a sphygmograph and by analysing arteriolar and venular diameters in the retinal microcirculation using a non-mydriatric vessel analyser. A questionnaire is filled out to determine daily physical activity, motivational factors, dietary habits, quality of life (KINDL-R) and socio-economic data. Physical fitness is assessed by a six-item test battery. DISCUSSION: Our study aims to provide a feasible long-term intervention strategy to re-establish childhood health and to prevent obesity-related cardiovascular dysfunction in children. TRIAL REGISTRATION: NCT00988754
SN - 1471-2458
AD - Department of Prevention, Rehabilitation and Sports Medicine, Technische Universitaet Muenchen, Klinikum rechts der Isar, Munich, Germany. siegrist@sport.med.tum.de
UR - 21513530
ER -

TY - JOUR
ID - 612
T1 - Orofacial and dental trauma of young children in Dunedin, New Zealand
A1 - Chan,Y.M.
A1 - Williams,S.
A1 - Davidson,L.E.
A1 - Drummond,B.K.
Y1 - 2011/06//
N1 - Chan, Yin Man. Williams, Sheila. Davidson, Lesley E. Drummond, Bernadette K
Dental traumatology : official publication of International Association for Dental Traumatology
101091305, d2p
D
Journal Article. Research Support, Non-U.S. Gov't
English
KW - MEDLINE
KW - Accidental Falls/sn [Statistics & Numerical Data]
KW - Accidents
KW - Home/sn [Statistics & Numerical Data]
KW - Animals
KW - Athletic Injuries/ep [Epidemiology]
KW - Bites and Stings/ep [Epidemiology]
KW - Child
KW - Preschool
KW - Dogs
KW - Facial Injuries/ep [Epidemiology]
KW - Female
KW - Humans
KW - Incisor/in [Injuries]
KW - Infant
KW - Lip/in [Injuries]
KW - Male
KW - Mouth/in [Injuries]
KW - New Zealand/ep [Epidemiology]
KW - Play and Playthings/in [Injuries]
KW - Retrospective Studies
KW - Tooth Avulsion/ep [Epidemiology]
KW - Tooth Fractures/ep [Epidemiology]
KW - Tooth Injuries/ep [Epidemiology]
KW - Tooth
KW - Deciduous/in [Injuries]
RP - NOT IN FILE

SP - 199
EP - 202
JF - Dental Traumatology
JA - Dent Traumatol
VL - 27
IS - 3
CY - Denmark
N2 - AIM: The aims were to identify the predominant causes and types of orofacial injury in young children attending clinics at a University Dental School in Dunedin, New Zealand. MATERIAL AND METHODS: A retrospective analysis of data from the records of all children aged 0-10years who had been seen for orofacial trauma in 1999 and 2000 was undertaken. RESULTS AND CONCLUSIONS: Three hundred traumatic incidents in 288 children were analysed; 86.6% had causes noted. In very young children, most injuries were because of falls, while collisions, falling and sports were responsible for more injuries in school-aged children. Playground equipment and ride-on vehicles played a role particularly in the older children. There were no incidents of trauma as a result of road traffic accidents. Ten injuries were caused by animals, mainly dogs. Location was recorded for two-thirds of accidents: the predominant place was at home, followed by school. No seasonal variation was apparent. There were 228 non-dental injuries, of which the majority were to the lips. The predominant dental injuries in both dentitions were concussions and subluxations with a significantly higher occurrence of both in the primary dentition ($P<0.001$). Upper central incisors were most often involved. The age distribution for boys and girls was similar. In conclusion, the causes and types of orofacial trauma in this group of young New Zealand school children attending a university dental school were similar to other studies, except for the high proportion of concussions recorded in both dentitions. While the injuries were well described, not all records noted the cause or location. This has resulted in changes to the standard recording form to provide consistency in data capture. Information from this study will also be used to support child injury prevention strategies in New Zealand. 2011 John Wiley & Sons A/S
SN - 1600-9657
AD - Faculty of Dentistry, University of Otago, Dunedin, New Zealand
UR - 21457187
ER -

TY - JOUR
ID - 613
T1 - [Yersinia pestis infection in the State of Bahia: effective control or epidemiological silence?]. [Portuguese]
A1 - Saavedra,Rda C.
A1 - Dias,J.P.
Y1 - 2011/03//
N1 - Saavedra, Ramon da Costa. Dias, Juarez Pereira
Revista da Sociedade Brasileira de Medicina Tropical
ret, 7507456
IM
English Abstract. Journal Article. Research Support, Non-U.S. Gov't
Portuguese
KW - MEDLINE
KW - Adolescent
KW - Adult
KW - Animals
KW - Antibodies
KW - Bacterial/bl [Blood]
KW - Brazil/ep [Epidemiology]
KW - Child
KW - Disease Vectors
KW - Dogs
KW - Female
KW - Humans
KW - Male
KW - Middle Aged
KW - Plague/ep [Epidemiology]

KW - Plague/pc [Prevention & Control]
KW - Rats
KW - Risk Factors
KW - Rodentia/mi [Microbiology]
KW - Seroepidemiologic Studies
KW - Siphonaptera/mi [Microbiology]
KW - Socioeconomic Factors
KW - Yersinia pestis/im [Immunology]
KW - Young Adult
RP - NOT IN FILE
SP - 223
EP - 227
JF - Revista Da Sociedade Brasileira de Medicina Tropical
JA - Rev Soc Bras Med Trop
VL - 44
IS - 2
CY - Brazil
N2 - INTRODUCTION: From an epidemiological point of view, the plague is still being considered of great importance, because of its high epidemic potential. Despite the absence of cases of human plague in Brazil, its etiologic agent, the bacteria Yersinia pestis, is still deep rooted in its natural environment. The occurrence of positive serology for plague in domestic carnivores in plague areas in Bahia in the past few years implies the need for a more rigorous evaluation in order to verify whether the bacillus of the plague is still active in these areas. METHODS: In this study, the presence of infection caused by Y. pestis was analyzed by seroprevalence tests on humans, dogs and rodents and by the detection of the bacteria in rodents and fleas. A structured questionnaire was used to analyze the association between environmental, socioeconomic and biological factors and seroprevalence in humans. RESULTS: Of the 630 serum samples examined (88 from humans, 480 from dogs and 62 from rodents), all were nonreactive for plague and bacteriological analyses performed on 14 rodents and 2 flea lots showed no signs of the bacteria. CONCLUSIONS: These results cannot confirm the eradication of the disease in the entire State, since the cyclic nature of the plague indicates that it can go silent for long periods and then resurge, affecting large numbers of people. Thus, maintenance of active, permanent surveillance is required for early detection and the development of adequate control measures
SN - 1678-9849
AD - Secretaria de Saude da Bahia, Diretoria de Vigilancia Epidemiologica, Salvador, BA.
saavedra@saude.ba.gov.br
UR - 21468476
ER -

TY - JOUR
ID - 614
T1 - Participation patterns of children with acquired brain injury
A1 - Law,M.
A1 - Anaby,D.
A1 - DeMatteo,C.
A1 - Hanna,S.
Y1 - 2011///
N1 - Law, Mary. Anaby, Dana. DeMatteo, Carol. Hanna, Steven
Brain injury : [BI]
bra, 8710358
IM
Journal Article. Research Support, Non-U.S. Gov't
English
KW - MEDLINE
KW - Activities of Daily Living/px [Psychology]
KW - Adolescent
KW - Analysis of Variance
KW - Brain Injuries/px [Psychology]
KW - Brain Injuries/rh [Rehabilitation]

KW - Child
KW - Preschool
KW - Continuity of Patient Care
KW - Disability Evaluation
KW - Female
KW - Health Surveys
KW - Humans
KW - Injury Severity Score
KW - Interpersonal Relations
KW - Male
KW - Peer Group
KW - Prognosis
KW - Quality of Life/px [Psychology]
KW - Young Adult
RP - NOT IN FILE
SP - 587
EP - 595
JF - Brain Injury
JA - Brain Inj
VL - 25
IS - 6
CY - England
N2 - OBJECTIVE: To describe the participation patterns of children and youth with Acquired Brain Injury (ABI) and to compare these patterns with typically-developing peers. METHODS: One hundred and thirty-five children with ABI completed the Children Assessment of Participation and Enjoyment (CAPE) to measure their participation diversity and intensity in outside-of-school activities (i.e. recreational, physical, social, self-improvement and skill-based activities). Results were compared to 354 typically-developing peers. ANOVA analyses were performed while controlling for age and gender. RESULTS: Similar to typically-developing children, individuals with ABI proportionally participated mostly in social and recreational activities and were less likely to engage in skill-based activities. However, level of intensity and diversity within each activity type was different between the two groups. Children with ABI participated in fewer activities and were less frequently involved in all the CAPE's activity types except for intensity in social activities. These differences, characterized by small-to-medium effect sizes, were not dependent on the child's age and gender. CONCLUSIONS: Participation of children with ABI is restricted in comparison to their typically-developing peers even in a sample where minor injury is predominant. Future studies might address additional factors that potentially affect participation, e.g. child's preferences and family function
SN - 1362-301X
AD - School of Rehabilitation Science, McMaster University, Hamilton, ON, Canada
UR - 21534736
ER -

TY - JOUR
ID - 615
T1 - Wide variability in physical activity environments and weather-related outdoor play policies in child care centers within a single county of Ohio
A1 - Copeland,K.A.
A1 - Sherman,S.N.
A1 - Khoury,J.C.
A1 - Foster,K.E.
A1 - Saelens,B.E.
A1 - Kalkwarf,H.J.
Y1 - 2011/05//
N1 - Copeland, Kristen A. Sherman, Susan N. Khoury, Jane C. Foster, Karla E. Saelens, Brian E. Kalkwarf, Heidi J
Archives of pediatrics & adolescent medicine
9422751, bwf
AIM, IM

Journal Article. Research Support, N.I.H., Extramural. Research Support, Non-U.S. Gov't
English
KW - MEDLINE
KW - Child Day Care Centers/st [Standards]
KW - Child Day Care Centers/td [Trends]
KW - Child Welfare
KW - Child
KW - Preschool
KW - Cross-Sectional Studies
KW - Environment
KW - Exercise/ph [Physiology]
KW - Female
KW - Humans
KW - Infant
KW - Male
KW - Motor Activity/ph [Physiology]
KW - Ohio
KW - Play and Playthings
KW - Policy Making
KW - Weather
RP - NOT IN FILE
SP - 435
EP - 442
JF - Archives of Pediatrics & Adolescent Medicine
JA - Arch Pediatr Adolesc Med
VL - 165
IS - 5
CY - United States
N2 - OBJECTIVES: To examine the variability of physical activity environments and outdoor play policies in child care centers and to determine whether this variability is associated with the demographic characteristics of the child care centers surveyed. DESIGN: Early Learning Environments Physical Activity and Nutrition Telephone Survey. SETTING: Child care centers in Hamilton County (greater Cincinnati area), Ohio, during the period from 2008 to 2009. PARTICIPANTS: Directors of all 185 licensed full-time child care centers in Hamilton County. OUTCOME MEASURES: Descriptive measures of playground and indoor physical activity environments and weather-related outdoor play policies. RESULTS: Of 185 eligible child care centers, 162 (88%) responded to our survey. Of the 162 centers that responded, 151 (93%) reported an on-site playground, but slightly more than half reported that their playgrounds were large, that they were at least one-third covered in shade, or that they had a variety of portable play equipment. Only half reported having a dedicated indoor gross motor room where children could be active during inclement weather. Only 32 centers (20%) allowed children to go outside in temperatures below 32degreeF (0degreeC), and 70 centers (43%) reported allowing children outdoors during light rain. A higher percentage of children receiving tuition assistance was associated with lower quality physical activity facilities and stricter weather-related practices. National accreditation was associated with more physical activity-promoting practices. CONCLUSION: We found considerable variability in the indoor and outdoor physical activity environments offered by child care centers within a single county of Ohio. Depending on the outdoor play policy and options for indoor physical activity of a child care center, children's opportunities for physical activity can be curtailed as a result of subfreezing temperatures or light rain. Policy changes and education of parents and teachers may be needed to ensure that children have ample opportunity for daily physical activity
SN - 1538-3628
AD - General and Community Pediatrics, Cincinnati Children's Hospital Medical Center, 3333 Burnet Avenue, Cincinnati, OH 45229, USA. kristen.copeland@cchmc.org
UR - 21199969
ER -

TY - JOUR
ID - 616

T1 - Correlates of park-based physical activity among children in diverse communities: results from an observational study in two cities
A1 - Spengler,J.O.
A1 - Floyd,M.F.
A1 - Maddock,J.E.
A1 - Gobster,P.H.
A1 - Suau,L.J.
A1 - Norman,G.J.
Y1 - 2011/05//
N1 - Spengler, John O. Floyd, Myron F. Maddock, Jason E. Gobster, Paul H. Suau, Luis J. Norman, Gregory J
American journal of health promotion : AJHP
amj, 8701680
T
Journal Article. Research Support, Non-U.S. Gov't
English
KW - MEDLINE
KW - Chicago
KW - Child
KW - Environment Design
KW - Female
KW - Florida
KW - Health Behavior/eh [Ethnology]
KW - Humans
KW - Income
KW - Logistic Models
KW - Male
KW - Motor Activity
KW - Observation
KW - Public Facilities
KW - Recreation
KW - Residence Characteristics
KW - Sex Factors
RP - NOT IN FILE
SP - e1
EP - e9
JF - American Journal of Health Promotion
JA - Am J Health Promot
VL - 25
IS - 5
CY - United States
N2 - PURPOSE: This study examined correlates of park-based physical activity (PA) among children in neighborhood parks. DESIGN: Direct observation was used to assess PA among children. SETTING: Public parks in Tampa, Florida (n=10), and Chicago, Illinois (n=18), from low income and high income and racially/ethnically diverse communities. Subjects. Children (n=3410), coded as anyone who appeared to be 10 years or younger, observed at parks in Tampa and Chicago. MEASURES: Physical activity was measured by a modified version of the System for Observing Play and Leisure Among Youth (SOPLAY). ANALYSIS: Descriptive statistics and multilevel regression models were used in data analysis. RESULTS: At the activity observation level, children's PA was positively related to temperature and unstructured activities in Tampa and Chicago parks. Among park activity area predictors, type of activity area was significantly related to PA. In Tampa, more PA was observed on courts, and less PA was observed in shelter areas compared with open space areas. In Chicago, less PA was observed on courts and fields compared with open space areas. Neighborhood income was associated with lower PA in Tampa parks. Neighborhood race/ethnicity was not a significant predictor of children's PA in either city. CONCLUSIONS: Children's PA was linked to modifiable social and environmental features within parks. Strategies to increase PA among children in parks should promote courts, playgrounds, informal activities, and free play. Copyright 2011 by American Journal of Health Promotion, Inc
SN - 0890-1171

AD - College of Health and Human Performance, University of Florida, PO Box 118208, Gainesville, FL 32611-8208, USA. spengler@hhp.ufl.edu
UR - 21534825
ER -

TY - JOUR
ID - 617
T1 - Ready for recess: a pilot study to increase physical activity in elementary school children
A1 - Huberty,J.L.
A1 - Siahpush,M.
A1 - Beighle,A.
A1 - Fuhrmeister,E.
A1 - Silva,P.
A1 - Welk,G.
Y1 - 2011/05//
N1 - Huberty, Jennifer L. Siahpush, Mohammad. Beighle, Aaron. Fuhrmeister, Erin. Silva, Pedro. Welk, Greg
The Journal of school health
k13, 0376370
IM, N
Comparative Study. Journal Article
English
KW - MEDLINE
KW - Actigraphy
KW - Body Mass Index
KW - Child
KW - Exercise/ph [Physiology]
KW - Female
KW - Health Behavior
KW - Health Promotion/mt [Methods]
KW - Humans
KW - Leisure Activities
KW - Male
KW - Midwestern United States
KW - Motor Activity/ph [Physiology]
KW - Physical Fitness
KW - Pilot Projects
KW - Regression Analysis
KW - Schools
KW - Students
RP - NOT IN FILE
SP - 251
EP - 257
JF - Journal of School Health
JA - J Sch Health
VL - 81
IS - 5
CY - United States
N2 - BACKGROUND: Creating an optimal environment at recess may be necessary to maximize physical activity (PA) participation in youth. The purpose of this study was to determine the initial effectiveness of an elementary school recess intervention on the amount of moderate PA (MPA) and vigorous PA (VPA) during recess and the school day. METHODS: This school-based intervention included staff training, activity zones, and playground equipment. The PA levels of third, fourth, and fifth grade students (n = 93) at two schools were measured at baseline and post-intervention using ActiGraph accelerometers. Paired t tests were used to compare percentage of time spent during recess in MPA and VPA. Multiple regressions were utilized to model the effect of intervention, age, sex, race, body mass index, and school on minutes spent in MPA and VPA. RESULTS: The multiple regression results demonstrated increases of 2.5 minutes of MPA ($p < .001$) and 2.2 minutes of VPA ($p < .001$) at recess and an increase of 18.7 minutes of MPA ($p < .001$) and 4.7 minutes of VPA ($p < .001$)

during the school day. These represent respective increases of 51.2% and 112.2% in the adjusted means of MPA and VPA during recess and respective increases of 92.2% and 71.6% in the adjusted mean of MPA and VPA during school day. CONCLUSION: Staff training, recreational equipment, and playground markings are inexpensive, simple ways to increase PA during recess so that children can accumulate minutes of PA to meet the recommended guidelines of 60 minutes per day. 2011, American School Health Association
SN - 1746-1561
AD - Physical Activity in Health Promotion, Health, Physical Education and Recreation Department, University of Nebraska Omaha, 6001 Dodge St., Omaha, NE 68182, USA. jenniferwhite@mail.unomaha.edu
UR - 21517864
ER -

TY - JOUR
ID - 618
T1 - Epidemiological and geographical aspects of leishmaniasis in Tigray, northern Ethiopia: a retrospective analysis of medical records, 2005-2008
A1 - Morrone,A.
A1 - Pitidis,A.
A1 - Pajno,M.C.
A1 - Dassoni,F.
A1 - Latini,O.
A1 - Barnabas,G.A.
A1 - Padovese,V.
Y1 - 2011/05//
N1 - Morrone, Aldo. Pitidis, Alessio. Pajno, Maria Chiara. Dassoni, Federica. Latini, Ottavio. Barnabas, Gebre Ab. Padovese, Valeska
Transactions of the Royal Society of Tropical Medicine and Hygiene
wbu, 7506129
IM
Journal Article
English
KW - MEDLINE
KW - Adolescent
KW - Adult
KW - Aged
KW - Child
KW - Preschool
KW - Disease Outbreaks/sn [Statistics & Numerical Data]
KW - Ethiopia/ep [Epidemiology]
KW - Female
KW - Humans
KW - Infant
KW - Leishmaniasis
KW - Cutaneous/ep [Epidemiology]
KW - Mucocutaneous/ep [Epidemiology]
KW - Male
KW - Medical Records
KW - Middle Aged
KW - Neglected Diseases/ep [Epidemiology]
KW - Prevalence
KW - Risk Factors
KW - Young Adult
RP - NOT IN FILE
SP - 273
EP - 280
JF - Transactions of the Royal Society of Tropical Medicine & Hygiene
JA - Trans R Soc Trop Med Hyg
VL - 105

IS - 5
CY - England
N2 - Leishmaniasis is one of the most neglected tropical diseases and epidemic outbreaks often occur worldwide. This paper reports some epidemiological features of the disease in Tigray, northern Ethiopia, with the aim of studying the disease distribution and the environmental factors that may have influenced it. Medical records from patients with Leishmania attending the Italian Dermatological Centre of Mekele in the period 2005-2008 were retrospectively reviewed. Age and gender distribution, clinical types, occupation, co-morbidity, urban/rural origin, altitude and rainfall were investigated. The result was 471 patients affected and the prevalent clinical form was cutaneous leishmaniasis (86%). Five main risk areas were identified in the Tigray highlands and only isolated cases were reported at altitudes below 1700m. The variables related to a higher risk of catching leishmaniasis were male gender, age over-14, poor education, outdoor activities and living at high altitudes. Climatic and environmental changes occurring in this region and land degradation are discussed as factors influencing leishmaniasis distribution. Further research including field missions and geomapping is needed to quantify the actual disease burden in the region. Copyright 2011 Royal Society of Tropical Medicine and Hygiene. Published by Elsevier Ltd. All rights reserved
SN - 1878-3503
AD - National Institute for Health, Migration and Poverty, via di San Gallicano 25/A, 00153 Rome, Italy
UR - 21439603
ER -

TY - JOUR
ID - 619
T1 - Characterization of coarse particulate matter in school gyms
A1 - Branis,M.
A1 - Safranek,J.
Y1 - 2011/05//
N1 - Branis, Martin. Safranek, Jiri
Environmental research
ei2, 0147621
IM
Journal Article. Research Support, Non-U.S. Gov't
English
KW - MEDLINE
KW - Air Pollutants/an [Analysis]
KW - Air Pollutants/ch [Chemistry]
KW - Air Pollution
KW - Indoor/sn [Statistics & Numerical Data]
KW - Child
KW - Czech Republic
KW - Environmental Monitoring
KW - Gymnastics
KW - Humans
KW - Inhalation Exposure/sn [Statistics & Numerical Data]
KW - Particle Size
KW - Particulate Matter/an [Analysis]
KW - Particulate Matter/ch [Chemistry]
KW - Schools/sn [Statistics & Numerical Data]
RP - NOT IN FILE
SP - 485
EP - 491
JF - Environmental Research
JA - Environ Res
VL - 111
IS - 4
CY - United States
N2 - We investigated the mass concentration, mineral composition and morphology of particles resuspended by children during scheduled physical education in urban, suburban and rural elementary school gyms in Prague

(Czech Republic). Cascade impactors were deployed to sample the particulate matter. Two fractions of coarse particulate matter (PM(10-2.5) and PM(2.5-1.0)) were characterized by gravimetry, energy dispersive X-ray spectrometry and scanning electron microscopy. Two indicators of human activity, the number of exercising children and the number of physical education hours, were also recorded. Lower mass concentrations of coarse particulate matter were recorded outdoors (average PM(10-2.5) 4.1-7.4 mug m(-3) and PM(2.5-1.0) 2.0-3.3 mug m(-3)) than indoors (average PM(10-2.5) 13.6-26.7 mug m(-3) and PM(2.5-1.0) 3.7-7.4 mug m(-3)). The indoor concentrations of coarse aerosol were elevated during days with scheduled physical education with an average indoor-outdoor (I/O) ratio of 2.5-16.3 for the PM(10-2.5) and 1.4-4.8 for the PM(2.5-1.0) values. Under extreme conditions, the I/O ratios reached 180 (PM(10-2.5)) and 19.1 (PM(2.5-1.0)). The multiple regression analysis based on the number of students and outdoor coarse PM as independent variables showed that the main predictor of the indoor coarse PM concentrations is the number of students in the gym. The effect of outdoor coarse PM was weak and inconsistent. The regression models for the three schools explained 60-70% of the particular dataset variability. X-ray spectrometry revealed 6 main groups of minerals contributing to resuspended indoor dust. The most abundant particles were those of crustal origin composed of Si, Al, O and Ca. Scanning electron microscopy showed that, in addition to numerous inorganic particles, various types of fibers and particularly skin scales make up the main part of the resuspended dust in the gyms. In conclusion, school gyms were found to be indoor microenvironments with high concentrations of coarse particulate matter, which can contribute to increased short-term inhalation exposure of exercising children. Copyright 2011 Elsevier Inc. All rights reserved
SN - 1096-0953
AD - Charles University in Prague, Faculty of Science, Institute for Environmental Studies, Prague, Czech Republic. branis@natur.cuni.cz
UR - 21458792
ER -

TY - JOUR
ID - 620
T1 - Inertial-magnetic sensors for assessing spatial cognition in infants
A1 - Campolo,D.
A1 - Taffoni,F.
A1 - Formica,D.
A1 - Schiavone,G.
A1 - Keller,F.
A1 - Guglielmelli,E.
Y1 - 2011/05//
N1 - Campolo, Domenico. Taffoni, Fabrizio. Formica, Domenico. Schiavone, Giuseppina. Keller, Flavio. Guglielmelli, Eugenio
IEEE transactions on bio-medical engineering
gfx, 0012737
IM
Journal Article. Research Support, Non-U.S. Gov't
English
KW - MEDLINE
KW - Child
KW - Preschool
KW - Cognition/ph [Physiology]
KW - Humans
KW - Image Processing
KW - Computer-Assisted
KW - Infant
KW - Magnetics
KW - Monitoring
KW - Physiologic/is [Instrumentation]
KW - Physiologic/mt [Methods]
KW - Play and Playthings
KW - Reproducibility of Results
KW - Space Perception/ph [Physiology]

RP - NOT IN FILE
SP - 1499
EP - 1503
JF - IEEE Transactions on Biomedical Engineering
JA - IEEE Trans Biomed Eng
VL - 58
IS - 5
CY - United States
N2 - This letter describes a novel approach to the assessment of spatial cognition in children. In particular, we present a wireless instrumented toy embedding magneto-inertial sensors for orientation tracking, specifically developed to assess the ability to insert objects into holes. To be used in naturalistic environments (e.g., day cares), we also describe an in-field calibration procedure based on a sequence of manual rotations, not relying on accurate motions or sophisticated equipment. The final accuracy of the proposed system, after the mentioned calibration procedure, is derived by direct comparison with a gold-standard motion tracking device. In particular, both systems are subjected to a sequence of ten single-axis rotations (approximately 90degree, back and forth), about three different axes. The rms of the angular error between the two measurements (gold-standard versus proposed systems) was evaluated for each trial. In particular, the average rms error is under 2degree. This study indicates that a technological approach to ecological assessment of spatial cognition in infants is indeed feasible. As a consequence, prevention through screening of large number of infants is at reach.
2011 IEEE
SN - 1558-2531
AD - School of Mechanical and Aerospace Engineering, Nanyang Technological University, Singapore 639798. d.campolo@ntu.edu.sg
UR - 21233040
ER -

TY - JOUR
ID - 621
T1 - Brain stem responses evoked by stimulation of the mature cochlear nucleus with an auditory brain stem implant
A1 - O'Driscoll,M.
A1 - El-Deredy,W.
A1 - Ramsden,R.T.
Y1 - 2011/05//
N1 - O'Driscoll, Martin. El-Deredy, Wael. Ramsden, Richard T
Ear and hearing
e9g, 8005585
IM
Clinical Trial. Journal Article. Research Support, Non-U.S. Gov't
English
KW - MEDLINE
KW - Acoustic Stimulation
KW - Adolescent
KW - Adult
KW - Auditory Threshold/ph [Physiology]
KW - Child
KW - Cochlear Implantation/is [Instrumentation]
KW - Cochlear Implantation/mt [Methods]
KW - Cochlear Nucleus/ph [Physiology]
KW - Cochlear Nucleus/su [Surgery]
KW - Electrodes
KW - Implanted
KW - Evoked Potentials
KW - Auditory
KW - Brain Stem
KW - Female
KW - Hearing Loss

KW - Sensorineural/et [Etiology]
KW - Sensorineural/pp [Physiopathology]
KW - Sensorineural/rh [Rehabilitation]
KW - Humans
KW - Loudness Perception/ph [Physiology]
KW - Male
KW - Middle Aged
KW - Monitoring
KW - Intraoperative/is [Instrumentation]
KW - Intraoperative/mt [Methods]
KW - Neurofibromatosis 2/co [Complications]
KW - Young Adult
RP - NOT IN FILE
SP - 286
EP - 299
JF - Ear & Hearing
JA - Ear Hear
VL - 32
IS - 3
CY - United States
N2 - OBJECTIVES: The Nucleus auditory brain stem implant (ABI) has been used in the hearing rehabilitation of totally deaf individuals for whom a cochlear implant is not an option such as in the case of neurofibromatosis type 2 (NF2). Intraoperative electrically evoked auditory brain stem responses (EABRs) are recorded to assist in the placement of the electrode array over the dorsal and ventral cochlear nuclei in the lateral recess of the IVth ventricle of the brain stem. This study had four objectives: (1) to characterize EABRs evoked by stimulation with an ABI in adolescents and adults with NF2, (2) to evaluate how the EABR morphology relates to auditory sensations elicited from stimulation by an ABI, (3) to establish whether there is evidence of morphology changes in the EABR with site of stimulation by the ABI, and (4) to investigate how the threshold of the EABR relates to behavioral threshold and comfortably loud sensations measured at initial device activation. DESIGN: Intraoperative EABRs were recorded from 34 subjects with ABIs: 19 male and 15 female, mean age 27 yrs (range 12 to 52 yrs). ABI stimulation was applied at seven different sites using either wide bipolar stimulation across the array or in subsections of the array from medial to lateral and inferior to superior. The EABRs were analyzed with respect to morphology, peak latency, and changes in these characteristics with the site of stimulation. In a subset of eight subjects, additional narrow bipolar sites were stimulated to compare the intraoperative EABR threshold levels with the behavioral threshold (T) and comfortably loud (C) levels of stimulation required at initial device activation. RESULTS: EABRs were elicited from 91% of subjects. Morphology varied from one to four vertex-positive peaks with mean latencies of 0.76, 1.53, 2.51, and 3.64 msecs, respectively. The presence of an EABR from stimulation by electrodes across the whole array had a high predictive value for the presence of auditory electrodes at initial device activation. When examining subsections of the array, the absence of an EABR was a poor predictor for the absence of auditory electrodes. The morphology of the EABRs varied with site of stimulation in 16 cases, but there was no consistent pattern of change with stimulation site. There was a trend for more auditory electrodes to be present in stimulation sites that evoked EABRs with a higher number of peaks in the waveform. The EABR threshold was closer to the behavioral C level than the T level, but there was no overall correlation between the intraoperative EABR threshold level and the behavioral T and C levels. CONCLUSIONS: The presence of an intraoperative EABR corresponded well to the presence of auditory electrodes. The absence of an EABR from stimulating subsections of the array was not; however, a good indicator for the absence of auditory electrodes and the EABR from such stimulation would not be of assistance in identifying the nonauditory sections of the array to exclude in behavioral fitting of the device. The morphology of the EABR did not relate to site of stimulation. More peaks in the EABR was associated with a greater number of electrodes with auditory sensations, suggesting that correct positioning of the ABI activated more auditory subsystems within the cochlear nucleus. The intraoperative EABR thresholds did not correlate with the behavioral T and C levels and could not be used to assist in device fitting
SN - 1538-4667
AD - Auditory Implant Programme, University of Manchester, United Kingdom. martin.odriscoll@manchester.ac.uk
UR - 21157353

ER -

TY - JOUR
ID - 622
T1 - ITKids part I: children's occupations and use of information and communication technologies
A1 - Ciccarelli,M.
A1 - Straker,L.
A1 - Mathiassen,S.E.
A1 - Pollock,C.
Y1 - 2011///
N1 - Ciccarelli, Marina. Straker, Leon. Mathiassen, Svend Erik. Pollock, Clare
Work (Reading, Mass.)
9204382
IM
Journal Article
English
KW - MEDLINE
KW - Child
KW - Computers/ut [Utilization]
KW - Female
KW - Humans
KW - Leisure Activities
KW - Male
KW - Reproducibility of Results
KW - Self Report
KW - Telecommunications/ut [Utilization]
KW - Work
RP - NOT IN FILE
SP - 401
EP - 412
JF - Work
VL - 38
IS - 4
CY - Netherlands
N2 - OBJECTIVE: School children use information and communication technology (ICT) on a regular basis for a variety of purposes. The purpose of this study was to document how school children spend their time and the different types of ICT they use. METHODS: Nine Australian primary school children were observed in their school and away-from-school environments during one school day to record their ICT usage, comparing self-report exposures with direct observations. Self-reported discomfort scores were obtained throughout the day. RESULTS: Paper-based ICT (Old ICT) was mostly used for productive occupations at school, while electronic-based ICT (New ICT) was mostly used during leisure in away-from-school locations. Tasks involving no ICT (Non-ICT) accounted for the largest proportion of time in both locations during self-care, leisure and instrumental occupations. End-of-day self-reported time performing different occupations was consistent with data from independent observations. Self reported time using Old ICT and New ICT was marginally over-estimated, and time spent using Non-ICT was marginally under-estimated. CONCLUSION: The children in this study used a variety of ICT in the performance of daily occupations in their natural environments. New ICT use was primarily for leisure, but time spent was less than reported in other studies. Discomfort reports among the participants were low. Participants' self-reports of occupations performed and ICT use was reliable and could be useful as an exposure assessment metric
SN - 1875-9270
AD - School of Occupational Therapy & Social Work, Curtin Health Innovation Research Institute, Curtin University, Perth, WA, Australia. m.ciccarelli@curtin.edu.au
UR - 21508529
ER -

TY - JOUR
ID - 623

T1 - Indoor and outdoor sources of size-resolved mass concentration of particulate matter in a school gym-implications for exposure of exercising children
A1 - Branis,M.
A1 - Safranek,J.
A1 - Hytychova,A.
Y1 - 2011/05//
N1 - Branis, Martin. Safranek, Jiri. Hytychova, Adela
Environmental science and pollution research international
9441769, dvn
IM
Journal Article. Research Support, Non-U.S. Gov't
English
KW - MEDLINE
KW - Air Pollutants/an [Analysis]
KW - Air Pollution
KW - Indoor/an [Analysis]
KW - Indoor/sn [Statistics & Numerical Data]
KW - Child
KW - Environmental Monitoring
KW - Female
KW - Gymnastics/sn [Statistics & Numerical Data]
KW - Humans
KW - Inhalation Exposure/sn [Statistics & Numerical Data]
KW - Male
KW - Particle Size
KW - Particulate Matter/an [Analysis]
KW - Physical Education and Training/sn [Statistics & Numerical Data]
KW - Schools/sn [Statistics & Numerical Data]
RP - NOT IN FILE
SP - 598
EP - 609
JF - Environmental Science & Pollution Research
JA - Environ Sci Pollut.Res Int
VL - 18
IS - 4
CY - Germany
N2 - INTRODUCTION: It has been noticed many times that schools are buildings with high levels of particulate matter concentrations. Several authors documented that concentrations of particulate matter in indoor school microenvironments exceed limits recommended by WHO namely when school buildings are situated near major roads with high traffic densities. In addition, exercise under conditions of high particulate concentrations may increase the adverse health effects, as the total particle deposition increases in proportion to minute ventilation, and the deposition fraction nearly doubles from rest to intense exercise. SITE AND METHODS: Mass concentrations of size-segregated aerosol were measured simultaneously in an elementary school gym and an adjacent outdoor site in the central part of Prague by two pairs of collocated aerosol monitors-a fast responding photometer DusTrak and a five stage cascade impactor. To encompass seasonal and annual differences, 89 days of measurements were performed during ten campaigns between 2005 and 2009. RESULTS AND DISCUSSION: The average (all campaigns) outdoor concentration of PM(2.5) (28.3 mug m(-3)) measured by the cascade impactors was higher than the indoor value (22.3 mug m(-3)) and the corresponding average from the nearest fixed site monitor (23.6 mug m(-3)). Indoor and outdoor PM(2.5) concentrations exceeded the WHO recommended 24-h limit in 42% and 49% of the days measured, respectively. The correlation coefficient (r) between corresponding outdoor and indoor aerosol sizes increased with decreasing aerodynamic diameter of the collected particles (r=0.32-0.87), suggesting a higher infiltration rate of fine and quasi-ultrafine particles. Principal component analysis revealed five factors explaining more than 82% of the data variability. The first two factors reflected a close association between outdoor and indoor fine and quasi-ultrafine particles confirming the hypothesis of high infiltration rate of particles from outdoors. The third factor indicated that human activity is the main source of indoor emission of coarse particles. The fourth factor involved only outdoor variables showing the resuspension of coarse ambient aerosol on dry and

warm days without its seeming effect on the indoor coarse PM levels. Having in mind that high concentrations of both fine and coarse aerosol were frequently observed in the studied space, our results suggest that indoor exercise in polluted urbanized areas may increase the overall exposure and thus represent a potential health risk to young individuals during physical education at schools
SN - 1614-7499
AD - Faculty of Science, Institute for Environmental Studies, Charles University in Prague, Albertov 6, 128 43, Prague 2, Czech Republic. branis@natur.cuni.cz
UR - 20972889
ER -

TY - JOUR
ID - 624
T1 - Influence of physical activity and screen time on the retinal microvasculature in young children
A1 - Gopinath,B.
A1 - Baur,L.A.
A1 - Wang,J.J.
A1 - Hardy,L.L.
A1 - Teber,E.
A1 - Kifley,A.
A1 - Wong,T.Y.
A1 - Mitchell,P.
Y1 - 2011/05//
N1 - Gopinath, Bamini. Baur, Louise A. Wang, Jie Jin. Hardy, Louise L. Teber, Erdahl. Kifley, Annette. Wong, Tien Y. Mitchell, Paul
Arteriosclerosis, thrombosis, and vascular biology
9505803, B89
IM
Journal Article. Research Support, Non-U.S. Gov't
English
KW - MEDLINE
KW - Blood Pressure
KW - Child
KW - Child Behavior
KW - Computers
KW - Exercise
KW - Female
KW - Fluorescein Angiography
KW - Humans
KW - Linear Models
KW - Male
KW - Microcirculation
KW - Microvessels/ah [Anatomy & Histology]
KW - Microvessels/ph [Physiology]
KW - New South Wales
KW - Questionnaires
KW - Retinal Vessels/ah [Anatomy & Histology]
KW - Retinal Vessels/ph [Physiology]
KW - Risk Assessment
KW - Risk Factors
KW - Sedentary Lifestyle
KW - Television
KW - Time Factors
KW - Video Games
RP - NOT IN FILE
SP - 1233
EP - 1239
JF - Arteriosclerosis, Thrombosis & Vascular Biology

JA - Arterioscler.Thromb.Vasc Biol
VL - 31
IS - 5
CY - United States
N2 - OBJECTIVE: It is not clear whether physical activity and sedentary behavior affect retinal microvascular caliber. We investigated associations among physical activity (outdoor and indoor sporting activities), sedentary behaviors (including screen time, television [TV] viewing, and computer and videogame usage), and retinal microvascular caliber in schoolchildren. METHODS AND RESULTS: Six-year-old students (1765/2238) from a random cluster sample of 34 Sydney schools were examined. Parents completed questionnaires about physical and sedentary activities. Retinal images were taken, and retinal vessel caliber was quantified. After adjusting for age, sex, ethnicity, eye color, axial length, body mass index, birth weight, and mean arterial blood pressure, children who spent more time in outdoor sporting activities (in the highest tertile of activity) had 2.2 mum (95% CI 0.65 to 3.71) wider mean retinal arteriolar caliber than those in the lowest tertile (Ptrend=0.004). Increasing quartiles of time spent watching TV were associated with narrower mean retinal arteriolar caliber2.3 mum (95% CI 0.73 to 3.92), Ptrend=0.003. CONCLUSIONS: These data suggest that physical activity could have a beneficial influence, whereas screen time has a potential adverse influence on retinal microvascular structure. The magnitude of arteriolar narrowing associated with each hour daily of TV viewing is similar to that associated with a 10-mm Hg increase in systolic blood pressure in children
SN - 1524-4636
AD - Centre for Vision Research, Department of Ophthalmology, University of Sydney, Westmead Hospital, Hawkesbury Rd, Westmead, New South Wales 2145, Australia
UR - 21508347
ER -

TY - JOUR
ID - 625
T1 - Mothers' reports of play dates and observation of school playground behavior of children having high-functioning autism spectrum disorders
A1 - Frankel,F.D.
A1 - Gorospe,C.M.
A1 - Chang,Y.C.
A1 - Sugar,C.A.
Y1 - 2011/05//
N1 - Frankel, Frederick D. Gorospe, Clarissa M. Chang, Ya-Chih. Sugar, Catherine A
Journal of child psychology and psychiatry, and allied disciplines
hp3, 0375361
IM
Journal Article. Research Support, N.I.H., Extramural. Research Support, Non-U.S. Gov't
English
KW - MEDLINE
KW - Adaptation
KW - Psychological
KW - Child
KW - Child Development Disorders
KW - Pervasive/px [Psychology]
KW - Family
KW - Female
KW - Humans
KW - Intelligence
KW - Interpersonal Relations
KW - Male
KW - Mothers
KW - Observation
KW - Peer Group
KW - Play and Playthings/px [Psychology]
KW - Schools
KW - Social Environment

RP - NOT IN FILE
SP - 571
EP - 579
JF - Journal of Child Psychology & Psychiatry & Allied Disciplines
JA - J Child Psychol Psychiatry
VL - 52
IS - 5
CY - England
N2 - BACKGROUND: Children with high-functioning autism spectrum disorders (ASD) are generally included with typically developing peers at school. They have difficulties interacting with peers on the school playground. Previous literature suggests that having play dates in the home may be related to better peer acceptance at school. METHODS: This study examines the relationship between mother-reported play date frequency and amount of conflict, and peer interaction observed on the school playground for a sample of 27 boys and 4 girls meeting structured interview and observation criteria for ASD. Measures of intellectual functioning, adaptive behavior, and social skills were included in a stepwise regression analysis to account for their impact on relationships between maternal play date reports, general peer acceptance at school (as rated by the child's teacher) and observations of school playground behavior. RESULTS: Results revealed that children with autism spectrum disorders who had more play dates in their home tended to spend a greater amount of time engaged in behaviors such as mutual offering of objects, conversing and other turn-taking activities with peers on the school playground. They also received more positive responses to their overtures from peers. These relationships remained highly significant even after accounting for other demographic, general social, and cognitive variables. CONCLUSIONS: The present results suggest that play date frequency is strongly related to school playground behavior. Owing to the design of this study, future research must assess whether play dates in the home promote better peer relationships on the playground or the reverse. In either case, the assessment of play dates, as well as observation of spontaneous unsupervised social interactions, are important outcome measures to consider in social skills interventions for children with high-functioning ASD. 2010 The Authors. Journal of Child Psychology and Psychiatry 2010 Association for Child and Adolescent Mental Health
SN - 1469-7610
AD - UCLA Semel Institute for Neuroscience and Human Behavior, Los Angeles, CA, USA. ffrankel@ucla.edu
UR - 20860756
ER -

TY - JOUR
ID - 626
T1 - Age-related increase in inferior frontal gyrus activity and social functioning in autism spectrum disorder
A1 - Bastiaansen,J.A.
A1 - Thioux,M.
A1 - Nanetti,L.
A1 - van der,Gaag C.
A1 - Ketelaars,C.
A1 - Minderaa,R.
A1 - Keysers,C.
Y1 - 2011/05/01/
N1 - Bastiaansen, Jojanneke A. Thioux, Marc. Nanetti, Luca. van der Gaag, Christiaan. Ketelaars, Cees. Minderaa, Ruud. Keysers, Christian
Biological psychiatry
a3s, 0213264
IM
Journal Article. Research Support, Non-U.S. Gov't
English
KW - MEDLINE
KW - Adolescent
KW - Adult
KW - Age Factors
KW - Aging/ph [Physiology]
KW - Attention/ph [Physiology]

KW - Brain Mapping
KW - Child
KW - Child Development Disorders
KW - Pervasive/pp [Physiopathology]
KW - Cross-Sectional Studies
KW - Emotions/ph [Physiology]
KW - Facial Expression
KW - Frontal Lobe/pp [Physiopathology]
KW - Humans
KW - Image Processing
KW - Computer-Assisted
KW - Magnetic Resonance Imaging
KW - Male
KW - Middle Aged
KW - Photic Stimulation
KW - Social Adjustment
KW - Social Perception
RP - NOT IN FILE
SP - 832
EP - 838
JF - Biological Psychiatry
JA - Biol Psychiatry
VL - 69
IS - 9
CY - United States
N2 - BACKGROUND: Hypoactivation of the inferior frontal gyrus during the perception of facial expressions has been interpreted as evidence for a deficit of the mirror neuron system in children with autism. We examined whether this dysfunction persists in adulthood, and how brain activity in the mirror neuron system relates to social functioning outside the laboratory. METHODS: Twenty-one adult males with autism spectrum disorders and 21 typically developing subjects matched for age, sex, and IQ were scanned in three conditions: observing short movies showing facial expressions, performing a facial movement, and experiencing a disgusting taste. Symptom severity and level of social adjustment were measured with the Autism Diagnostic Observation Schedule and the Social Functioning Scale. RESULTS: Inferior frontal gyrus activity during the observation of facial expressions increased with age in subjects with autism, but not in control subjects. The age-related increase in activity was associated with changes in gaze behavior and improvements in social functioning. These age-related neurocognitive improvements were not found in a group of individuals with schizophrenia, who had comparable levels of social functioning. CONCLUSIONS: The results of this cross-sectional study suggest that mirror neuron system activity augments with age in autism and that this is accompanied by changes in gaze behavior and improved social functioning. It is the first demonstration of an age-related neurocognitive improvement in autism. Increased motor simulation may contribute to the amelioration in social functioning documented in adolescence and adulthood. This finding should encourage the development of new therapeutic interventions directed at emotion simulation. Copyright 2011 Society of Biological Psychiatry. Published by Elsevier Inc. All rights reserved
SN - 1873-2402
AD - Social Brain Laboratory, Department of Neuroscience, University Medical Center Groningen, Groningen, the Netherlands
UR - 21310395
ER -

TY - JOUR
ID - 627
T1 - Unintentional childhood injury patterns, odds, and outcomes in Kampala City: an analysis of surveillance data from the National Pediatric Emergency Unit
A1 - Mutto,M.
A1 - Lawoko,S.
A1 - Nansamba,C.
A1 - Ovuga,E.

A1 - Svanstrom,L.
Y1 - 2011/01//
N1 - Mutto, Milton. Lawoko, Stephen. Nansamba, Catherine. Ovuga, Emilio. Svanstrom, Leif
Journal of injury & violence research
101535747
IM
Journal Article. Research Support, Non-U.S. Gov't
English
KW - MEDLINE
KW - Accidents/sn [Statistics & Numerical Data]
KW - Accidents
KW - Home/sn [Statistics & Numerical Data]
KW - Traffic/sn [Statistics & Numerical Data]
KW - Burns/ep [Epidemiology]
KW - Child
KW - Preschool
KW - Cross-Sectional Studies
KW - Emergency Service
KW - Hospital/sn [Statistics & Numerical Data]
KW - Female
KW - Humans
KW - Incidence
KW - Male
KW - Population Surveillance
KW - Uganda/ep [Epidemiology]
KW - Wounds and Injuries/ep [Epidemiology]
RP - NOT IN FILE
SP - 13
EP - 18
JF - Journal of Injury & Violence Research
JA - J Inj Violence Res
VL - 3
IS - 1
CY - Iran
N2 - BACKGROUND: Unintentional Childhood Injuries pose a major public health challenge in Africa and Uganda. Previous estimates of the problem may have underestimated the childhood problem. We set to determine unintentional childhood injury pattern, odds, and outcomes at the National Paediatric Emergency unit in Kampala city using surveillance data. METHODS: Incident proportions, odds and proportional rates were calculated and used to determine unintentional injury patterns across childhood (1-12 years). RESULTS: A total of 556 cases recorded between January and May 2008 were analyzed: majority had been transported to hospital by mothers using mini-buses, private cars, and motorcycles. Median distance from injury location to hospital was 5 km. Homes, roads, and schools were leading injury locations. Males constituted 60% of the cases. Play and daily living activities were commonest injury time activities. Falls, burns and traffic accounted for 70.5% of unintentional childhood injuries. Burns, open wounds, fractures were commonest injury types. Motorcycles, buses and passenger-cars caused most crashes. Play grounds, furniture, stairs and trees were commonest source of falls. Most burn injuries were caused by liquids, fires and hot objects. 43.8% of cases were admitted. 30% were discharged without disability; 10%, were disabled; 1%, died. Injury odds and proportional incidence rates varied with age, place and cause. Poisoning and drowning were rare. Local pediatric injury priorities should include home, road and school safety. CONCLUSIONS: Unintentional injuries are common causes of hospital visit by children under 13 years especially boys. Homes, roads and educational facilities are commonest unintentional injury sites. Significant age and gender differences exist in intentional injury causation, characteristics and outcomes. In its current form, our surveillance system seems inefficient in capturing poisoning and drowning. The local prevention priorities could include home, road and school safety; especially dissemination and uptake of proven interventions. Burns should be focus of domestic injury prevention among under-fives. Commercial passenger motorcycles require better regulation and control
SN - 2008-4072

AD - Pincer Group International Ltd, Karolinska Institutet, Department of Public Health Sciences, Social Medicine, Sweden. mmutto@pincergroup.org
UR - 21483209
ER -

TY - JOUR
ID - 628
T1 - Feasibility of increasing childhood outdoor play and decreasing television viewing through a family-based intervention in WIC, New York State, 2007-2008
A1 - Davison,K.K.
A1 - Edmunds,L.S.
A1 - Wyker,B.A.
A1 - Young,L.M.
A1 - Sarfoh,V.S.
A1 - Sekhobo,J.P.
Y1 - 2011/05//
N1 - Davison, Kirsten K. Edmunds, Lynn S. Wyker, Brett A. Young, Laurie M. Sarfoh, Vanessa S. Sekhobo, Jackson P
Preventing chronic disease
101205018
IM
Comparative Study. Journal Article. Research Support, U.S. Gov't, Non-P.H.S.
English
KW - MEDLINE
KW - Adult
KW - Child
KW - Preschool
KW - Exercise
KW - Feasibility Studies
KW - Female
KW - Follow-Up Studies
KW - Health Promotion
KW - Humans
KW - Male
KW - Maternal-Child Health Centers/sn [Statistics & Numerical Data]
KW - New York
KW - Nutrition Policy
KW - Nutritional Physiological Phenomena
KW - Play and Playthings
KW - Television/sn [Statistics & Numerical Data]
KW - Young Adult
RP - NOT IN FILE
SP - A54
JF - Preventing Chronic Disease
JA - Prev Chronic Dis
VL - 8
IS - 3
CY - United States
N2 - INTRODUCTION: Active Families is a program developed to increase outdoor play and decrease television viewing among preschool-aged children enrolled in the Special Supplemental Nutrition Program for Women, Infants, and Children (WIC). Our objective was to assess its feasibility and efficacy. METHODS: We implemented Active Families in a large WIC clinic in New York State for 1 year. To this end, we incorporated into WIC nutrition counseling sessions a community resource guide with maps showing recreational venues. Outcome measures were children's television viewing and time playing outdoors and parents' behaviors (television viewing, physical activity), self-efficacy to influence children's behaviors, and parenting practices specific to television viewing. We used a nonpaired pretest and posttest design to evaluate the intervention, drawing on comparison data from 3 matched WIC agencies. RESULTS: Compared with the children at

baseline, the children at follow-up were more likely to watch television less than 2 hours per day and play outdoors for at least 60 minutes per day. Additionally, parents reported higher self-efficacy to limit children's television viewing and were more likely to meet physical activity recommendations and watch television less than 2 hours per day. CONCLUSION: Results suggest that it is feasible to foster increased outdoor play and reduced television viewing among WIC-enrolled children by incorporating a community resource guide into WIC nutrition counseling sessions. Future research should test the intervention with a stronger evaluation design in multiple settings, with more diverse WIC populations, and by using more objective outcome measures of child behaviors
SN - 1545-1151
AD - University at Albany, State University of New York, Albany, New York, USA
UR - 21477494
ER -

TY - JOUR
ID - 629
T1 - Surveillance for laboratory-confirmed sporadic cases of cyclosporiasis--United States, 1997-2008
A1 - Hall,R.L.
A1 - Jones,J.L.
A1 - Herwaldt,B.L.
Y1 - 2011/04/08/
N1 - Hall, Rebecca L. Jones, Jeffrey L. Herwaldt, Barbara L
Morbidity and mortality weekly report. Surveillance summaries (Washington, D.C. : 2002)
nnb, 101142015, 8400155
IM
Journal Article
English
KW - MEDLINE
KW - Adolescent
KW - Adult
KW - Aged
KW - 80 and over
KW - Child
KW - Preschool
KW - Cyclospora/ip [Isolation & Purification]
KW - Cyclosporiasis/co [Complications]
KW - Cyclosporiasis/di [Diagnosis]
KW - Cyclosporiasis/ep [Epidemiology]
KW - Diarrhea/et [Etiology]
KW - Disease Notification
KW - Female
KW - Humans
KW - Incidence
KW - Infant
KW - Male
KW - Middle Aged
KW - Population Surveillance
KW - Seasons
KW - Travel
KW - United States/ep [Epidemiology]
RP - NOT IN FILE
SP - 1
EP - 11
JF - Morbidity & Mortality Weekly Report
JA - Morb Mortal Wkly Rep Surveill.Summ.
VL - Surveillance Summaries. 60
IS - 2
CY - United States

N2 - PROBLEM/CONDITION: Cyclosporiasis is an enteric disease caused by the parasite Cyclospora cayetanensis. Cyclosporiasis is reported most commonly in tropical and subtropical regions. In the United States, outbreaks of cyclosporiasis associated with various types of imported fresh produce have been documented and described since the mid-1990s. No molecular tools are available for linking C. cayetanensis cases. National data regarding laboratory-confirmed sporadic cases (i.e., cases not linked to documented outbreaks) have not been summarized previously. REPORTING PERIOD: This summary includes laboratory-confirmed sporadic cases that occurred during 1997-2008 and were reported to CDC by 2009. DESCRIPTION OF SYSTEM: In January 1999, cyclosporiasis became a nationally notifiable disease, and, as of 2008, it was a reportable condition in 37 states, New York City (NYC), and the District of Columbia. For 1997-2008, CDC was notified of laboratory-confirmed cases via two active surveillance systems (the Cyclospora Sentinel Surveillance Network and the Foodborne Diseases Active Surveillance Network), two passive systems (the National Notifiable Diseases Surveillance System and the Public Health Laboratory Information System), and informal mechanisms (e.g., electronic mail). RESULTS: CDC was notified of 1,110 laboratory-confirmed sporadic cases of cyclosporiasis that occurred during 1997-2008. The overall population-adjusted incidence rates ranged from a low of 0.01 cases per 100,000 persons in 1997 to a high of 0.07 in 2002. Of the 1,110 cases, 849 (76.5%) were reported by seven states: 498 (44.9%) occurred in residents of Florida (228 cases), NYC (200 cases), and elsewhere in New York state (70 cases); and >50 cases were reported by each of five other states (Connecticut, Georgia, Massachusetts, New Jersey, and Pennsylvania). Overall, the case-patients' median age was 44 years (range: 3 months-96 years); 50.5% were female, 47.2% were male, and the sex was unknown for 2.3%. A total of 372 case-patients (33.5%) had a documented history of international travel during the 2-week period before symptom onset or diagnosis, 398 (35.9%) reported no international travel, and 340 (30.6%) had an unknown travel history. Some details about the travel were available for 317 (85.2%) of the case-patients with a known history of international travel; 142 (44.8%) had traveled to Mexico (60 persons), Guatemala (44 persons), or Peru (38 persons). Among the 398 case-patients classified as having domestically acquired cases, 124 persons (31.2%) lived in Florida, and 64 persons (16.1%) lived either in NYC (49 persons) or elsewhere in New York state (15 persons). The majority (278 [69.8%]) of onset or diagnosis dates for domestically acquired cases occurred during April-August. INTERPRETATION: Approximately one third of cases occurred in persons with a known history of international travel who might have become infected while traveling outside the continental United States. Domestically acquired cases were concentrated in time (spring and summer) and place (eastern and southeastern states): some of these cases probably were outbreak associated but were not linked to other cases, in part because of a lack of molecular tools. PUBLIC HEALTH ACTION: Surveillance for cases of cyclosporiasis and research to develop molecular methods for linking seemingly sporadic cases should remain U.S. public health priorities, in part to facilitate identification and investigation of outbreaks and to increase understanding of the biology of Cyclospora and the epidemiology of cyclosporiasis. Unidentified, uninvestigated cases and outbreaks represent missed opportunities to identify vehicles of infection, modes of contamination, and preventive measures. Travelers to known areas of endemicity should be advised that food and water precautions for Cyclospora are similar to those for other enteric pathogens, except that this parasite is unlikely to be killed by routine chemical disinfection or sanitizing methods. The diagnosis of cyclosporiasis should be considered for persons with persistent or remitting-relapsing diarrheal illness, and testing for Cyclospora should be requested explicitly
SN - 1545-8636
AD - Center for Global Health, CDC, 4770 Buford Highway N.E., MS F-22, Atlanta, GA 30341, USA
UR - 21471951
ER -

TY - JOUR
ID - 630
T1 - Transitioning to OROS() methylphenidate from atomoxetine is effective in children and adolescents with ADHD
A1 - Niederkirchner,K.
A1 - Slawik,L.
A1 - Wermelskirchen,D.
A1 - Rettig,K.
A1 - Schauble,B.
Y1 - 2011/04//
N1 - Niederkirchner, Kathe. Slawik, Lara. Wermelskirchen, Detlef. Rettig, Klaus. Schauble, Barbara
Expert review of neurotherapeutics

101129944
IM
Journal Article. Research Support, Non-U.S. Gov't
English
KW - MEDLINE
KW - Adolescent
KW - Adrenergic Uptake Inhibitors/ae [Adverse Effects]
KW - Adrenergic Uptake Inhibitors/tu [Therapeutic Use]
KW - Attention Deficit Disorder with Hyperactivity/dt [Drug Therapy]
KW - Central Nervous System Stimulants/ad [Administration & Dosage]
KW - Central Nervous System Stimulants/ae [Adverse Effects]
KW - Central Nervous System Stimulants/tu [Therapeutic Use]
KW - Child
KW - Delayed-Action Preparations
KW - Drug Administration Schedule
KW - Female
KW - Humans
KW - Male
KW - Methylphenidate/ad [Administration & Dosage]
KW - Methylphenidate/ae [Adverse Effects]
KW - Methylphenidate/tu [Therapeutic Use]
KW - Propylamines/ae [Adverse Effects]
KW - Propylamines/tu [Therapeutic Use]
KW - Prospective Studies
KW - Psychiatric Status Rating Scales
KW - Quality of Life
KW - Treatment Outcome
RP - NOT IN FILE
SP - 499
EP - 508
JF - Expert Review of Neurotherapeutics
JA - Expert rev
VL - 11
IS - 4
CY - England
N2 - OBJECTIVE: To explore the clinical outcomes of children/adolescents with attention-deficit/hyperactivity disorder (ADHD) who required a therapy switch from atomoxetine to OROS() methylphenidate (MPH). METHODS: This prospective, noninterventional study involved patients aged 6-18 years with a confirmed diagnosis of ADHD who experienced insufficient clinical response and/or poor tolerability during atomoxetine treatment. Patients were transitioned to OROS MPH and followed for 12 weeks. ADHD symptoms, functional outcomes, health-related quality of life (HRQoL) and tolerability were assessed throughout the study. RESULTS: 42 patients (intention-to-treat) transitioned from atomoxetine 43.2 plus 14.7 mg onto OROS MPH 33.0 plus 17.7 mg (mean daily starting dose), increasing to 38.6 plus 17.6 mg at the final visit. Median treatment duration was 85 days (range: 3-155). Compared with baseline, symptoms, functional outcome and HRQoL improved after transitioning to OROS MPH as assessed by the Conners' Parent Rating Scale (mean change from baseline: -10.1 + 11.6; $p < 0.0001$), Children's Global Assessment Scale (8.7 + 16.2; $p = 0.0015$) and ILC-LQ0-28 scores (parents' rating from 14.9 + 3.6 [baseline] to 17.5 + 4.8 [study end]; $p = 0.0002$; patients' rating from 16.9 + 3.9 [baseline] to 19.3 + 4.4 [study end]; $p = 0.0003$). Social interactions and late afternoon tasks (playing with other children, household chores, school homework and behavior towards visitors/at visits) improved ($p < 0.001$). Approximately 62% expressed satisfaction ('very good' or `good') with OROS MPH therapy compared with prior atomoxetine with respect to symptom control in the late afternoon. The most common treatment-emergent adverse events after switching were involuntary muscle contractions (tics; 16.7%), insomnia (14.3%), abdominal pain (9.5%) and headache (9.5%). No clinically relevant changes in body weight or vital signs were observed. CONCLUSION: In this naturalistic setting, transitioning from atomoxetine to OROS MPH was associated with improved ADHD symptoms and impacted positively on patients' and parents' HRQoL and disease burden in ADHD children who demonstrated an insufficient response and/or poor tolerability to atomoxetine

SN - 1744-8360
AD - Janssen-Cilag GmbH, Raiffeisenstrasse 8, D-41470 Neuss, Germany
UR - 21469923
ER -

TY - JOUR
ID - 631
T1 - Obesity prevention in the family day care setting: impact of the Romp & Chomp intervention on opportunities for children's physical activity and healthy eating
A1 - de Silva-Sanigorski,A.
A1 - Elea,D.
A1 - Bell,C.
A1 - Kremer,P.
A1 - Carpenter,L.
A1 - Nichols,M.
A1 - Smith,M.
A1 - Sharp,S.
A1 - Boak,R.
A1 - Swinburn,B.
Y1 - 2011/05//
N1 - de Silva-Sanigorski, A. Elea, D. Bell, C. Kremer, P. Carpenter, L. Nichols, M. Smith, M. Sharp, S. Boak, R. Swinburn, B
Child: care, health and development
7602632, c9x
IM
Journal Article. Research Support, Non-U.S. Gov't
English
KW - MEDLINE
KW - Australia
KW - Capacity Building
KW - Child Care
KW - Child
KW - Preschool
KW - Community Health Services/og [Organization & Administration]
KW - Cross-Sectional Studies
KW - Family
KW - Female
KW - Health Behavior
KW - Health Promotion
KW - Humans
KW - Infant
KW - Newborn
KW - Male
KW - Motor Activity
KW - Obesity/ep [Epidemiology]
KW - Obesity/pc [Prevention & Control]
KW - Program Evaluation
KW - Social Environment
KW - Victoria
RP - NOT IN FILE
SP - 385
EP - 393
JF - Child: Care, Health & Development
JA - Child Care Health Dev
VL - 37
IS - 3
CY - England

N2 - BACKGROUND: The Romp & Chomp intervention reduced the prevalence of overweight/obesity in pre-school children in Geelong, Victoria, Australia through an intervention promoting healthy eating and active play in early childhood settings. This study aims to determine if the intervention successfully created more health promoting family day care (FDC) environments. METHODS: The evaluation had a cross-sectional, quasi-experimental design with the intervention FDC service in Geelong and a comparison sample from 17 FDC services across Victoria. A 45-item questionnaire capturing nutrition- and physical activity-related aspects of the policy, socio-cultural and physical environments of the FDC service was completed by FDC care providers (in 2008) in the intervention (n= 28) and comparison (n= 223) samples. RESULTS: Select results showed intervention children spent less time in screen-based activities (P= 0.03), organized active play (P < 0.001) and free inside play (P= 0.03) than comparison children. There were more rules related to healthy eating (P < 0.001), more care provider practices that supported children's positive meal experiences (P < 0.001), fewer unhealthy food items allowed (P= 0.05), higher odds of staff being trained in nutrition (P= 0.04) and physical activity (P < 0.001), lower odds of having set minimum times for outside (P < 0.001) and organized (P= 0.01) active play, and of rewarding children with food (P < 0.001). CONCLUSIONS: Romp & Chomp improved the FDC service to one that discourages sedentary behaviours and promotes opportunities for children to eat nutritious foods. Ongoing investment to increase children's physical activity within the setting and improving the capacity and health literacy of care providers is required to extend and sustain the improvements. 2011 Blackwell Publishing Ltd
SN - 1365-2214
AD - WHO Collaborating Centre for Obesity Prevention, Deakin University and McCaughey Centre and Melbourne School of Population Health, The University of Melbourne, Melbourne, Vic. Australia. andreams@unimelb.edu.au
UR - 21276039
ER -

TY - JOUR
ID - 632
T1 - The substance and sources of young children's healthy eating and physical activity knowledge: implications for obesity prevention efforts
A1 - Lanigan,J.D.
Y1 - 2011/05//
N1 - Lanigan, J D
Child: care, health and development
7602632, c9x
IM
Journal Article
English
KW - MEDLINE
KW - Child Development
KW - Child Welfare
KW - Child
KW - Preschool
KW - Drinking
KW - Eating/px [Psychology]
KW - Exercise/px [Psychology]
KW - Female
KW - Health Knowledge
KW - Attitudes
KW - Practice
KW - Humans
KW - Information Dissemination/mt [Methods]
KW - Male
KW - Motor Activity
KW - Obesity/ep [Epidemiology]
KW - Obesity/pc [Prevention & Control]
KW - Preventive Health Services
KW - Qualitative Research

RP - NOT IN FILE
SP - 368
EP - 376
JF - Child: Care, Health & Development
JA - Child Care Health Dev
VL - 37
IS - 3
CY - England
N2 - BACKGROUND: The prevalence of overweight among young children is increasing at an alarming rate. Global efforts to address the issue can benefit from understanding how young children's experiences across multiple contexts shape their perspectives of healthy weight. METHODS: This qualitative study examines the substance and sources of young American children's knowledge related to healthy eating, physical activity and media practices. Role play and semi-structured interviews were conducted in child-care settings with 81 children aged 3-5 who represented diverse socio-economic statuses and ethnic backgrounds. RESULTS: Children demonstrated better understanding of the benefits of healthy eating compared with physical activity. Snacks and beverages consumed outside mealtime were less likely to be healthy even among the 40% of children who demonstrated an understanding of healthy nutrition. The majority of children's leisure activity selections involved media and minimally active pursuits. Three quarters of the children were unable to articulate reasons for healthy choices or identify the sources of their health understandings. The media was listed as source of health information more frequently than adults. CONCLUSION: Obesity prevention efforts targeting young children need to use consistent messaging across all contexts in which children develop in order to increase their understanding that physical activity and eating choices support health. Efforts need to counter inaccurate information and address the rationale for health practices. Key gaps in young children's understanding include: the importance of drinking water, that snacks are part of nutritional intake and the benefits of engaging in physical activities. 2011 Blackwell Publishing Ltd
SN - 1365-2214
AD - Department of Human Development, Washington State University Vancouver, Vancouver, WA 98686 9600, USA. jlanigan@vancouver.wsu.edu
UR - 21276032
ER -

TY - JOUR
ID - 633
T1 - The influence of ICT on the activity patterns of children with physical disabilities outside school
A1 - Lidstrom,H.
A1 - Ahlsten,G.
A1 - Hemmingsson,H.
Y1 - 2011/05//
N1 - Lidstrom, H. Ahlsten, G. Hemmingsson, H
Child: care, health and development
7602632, c9x
IM
Comparative Study. Journal Article. Research Support, Non-U.S. Gov't
English
KW - MEDLINE
KW - Adolescent
KW - Child
KW - Communication
KW - Computers/ut [Utilization]
KW - Cross-Sectional Studies
KW - Disabled Children
KW - Educational Technology
KW - Female
KW - Humans
KW - Interpersonal Relations
KW - Male
KW - Motor Activity

KW - Peer Group
KW - Questionnaires
KW - Sex Distribution
KW - Social Environment
RP - NOT IN FILE
SP - 313
EP - 321
JF - Child: Care, Health & Development
JA - Child Care Health Dev
VL - 37
IS - 3
CY - England
N2 - AIM: To investigate the outside school activity patterns of children with physical disabilities, and specifically their information and communication technology (ICT) usage compared with that of non-disabled children. In addition, the aim was to investigate the children's opinions on computer use and the associations between their use of the Internet and their interaction with peers. METHODS: Questionnaire on activities outside school, answered by 215 children and youths with physical disabilities, mean age 12 years 10 months, attending mainstream schools. For group comparisons with non-disabled children, data from the survey 'Kids and Media' were used. RESULTS: In the analysis, two sets of activity patterns were identified, depending on whether the child was disabled or not and on the gender of the child. A higher proportion of children with physical disabilities were engaged in ICT activities, while non-disabled children tended to be engaged in a broader range of activities outside school. The activity pattern was more uniform for boys and girls with disabilities than for their non-disabled peers. Use of the Internet was positively associated with peer interaction. CONCLUSION: Outside school, the activity patterns of children and youths with physical disabilities seem to be characterized by a focus on ICT activities, which enable children to compensate for their impairment because it suits all. In addition, digital skills developed outside school engage children with physical disabilities, giving them increased access to society and for educational purposes. 2010 Blackwell Publishing Ltd
SN - 1365-2214
AD - Department of Neurobiology, Care Sciences and Society, Karolinska Institutet, Stockholm, Sweden. helene.lidstrom@ki.se
UR - 21143263
ER -

TY - JOUR
ID - 634
T1 - Gross motor ability of native Greek, Roma, and Roma immigrant school-age children in Greece
A1 - Tsimaras,V.
A1 - Arzoglou,D.
A1 - Fotiadou,E.
A1 - Kokaridas,D.
A1 - Kotzamanidou,M.
A1 - Angelopoulou,N.
A1 - Bassa,E.
Y1 - 2011/02//
N1 - Tsimaras, Vasilios. Arzoglou, Despina. Fotiadou, Eleni. Kokaridas, Dimitrios. Kotzamanidou, Marianna. Angelopoulou, Nikoletta. Bassa, Eleni
Perceptual and motor skills
ozb, 0401131
IM
Journal Article
English
KW - MEDLINE
KW - Analysis of Variance
KW - Child
KW - Child Development/ph [Physiology]
KW - Emigrants and Immigrants
KW - Female

KW - Greece/eh [Ethnology]
KW - Gypsies
KW - Humans
KW - Male
KW - Motor Skills/ph [Physiology]
RP - NOT IN FILE
SP - 279
EP - 288
JF - Perceptual & Motor Skills
JA - Percept.Mot.Skills
VL - 112
IS - 1
CY - United States
N2 - The purpose of this study was to estimate and compare gross motor ability of children aged 7 to 10 years, all from Roma minority families (Romas, Roma immigrants) and families of indigenous Greeks. The sample consisted of 180 hildren (60 natives, 60 Romas, 60 Roma immigrants) studying in Greek public primary schools. The Test of Gross Motor Development scores showed that the group of indigenous Greek children had significantly higher performance in terms of locomotion skills, handling skills, and general motor ability compared to the groups of Roma and Roma immigrant children. No statistically significant differences were observed between the two other groups. These findings might be attributed to less participation of minority children in organized physical activities in and outside school, as well as to the reduced parental encouragement for attending related activities
SN - 0031-5125
AD - Laboratory of Developmental Pediatrics and Special Education, Department of Physical Education and Sport Science, Aristotle University of Thessaloniki, Greece. tsimaras@phed.auth.gr
UR - 21466101
ER -

TY - JOUR
ID - 635
T1 - An overview analysis of the time people spend outdoors
A1 - Diffey,B.L.
Y1 - 2011/04//
N1 - Diffey, B L
The British journal of dermatology
aw0, 0004041
IM
Journal Article
English
KW - MEDLINE
KW - Activities of Daily Living
KW - Adolescent
KW - Adult
KW - Child
KW - Preschool
KW - Cohort Studies
KW - Female
KW - Humans
KW - Leisure Activities
KW - Male
KW - Middle Aged
KW - Questionnaires
KW - Seasons
KW - Sunlight
KW - Time Factors
KW - Young Adult
RP - NOT IN FILE

SP - 848
EP - 854
JF - British Journal of Dermatology
JA - Br J Dermatol
VL - 164
IS - 4
CY - England
N2 - BACKGROUND: An important factor in determining our exposure to sunlight, and the consequent impact on skin health and vitamin D status, is the time we spend outdoors. OBJECTIVES: To determine estimates of the typical times per day spent outdoors during weekdays, weekends and holidays during a summer season. METHODS: A number of published studies giving data on the time per day spent outdoors by people were reviewed and a meta-analysis performed. From these data summary estimates of the average time per day outdoors were extracted. RESULTS: Time spent per day outdoors during weekdays and weekends is positively skewed, with a normal distribution of times outdoors during holidays. The median times per day outdoors during weekdays and weekends gave pooled estimates of 104 and 164 h, respectively. Corresponding values for the pooled estimates of mean times outdoors during these two periods were 143 and 238 h. The mean time per day outdoors during holiday exposure is 5-6 h. CONCLUSIONS: Summer-long distribution of times spent outdoors on a daily basis exhibits a highly skewed nature that highlights the difference between our adventitious and recreational exposure. Over the course of a summer season, when people are outside, they spend on average of 1-2 h per day outdoors. 2011 The Author. BJD 2011 British Association of Dermatologists
SN - 1365-2133
AD - Dermatological Sciences, Institute of Cellular Medicine, University of Newcastle, Newcastle upon Tyne NE2 4HH, UK. b.l.diffey@ncl.ac.uk
UR - 21128911
ER -

TY - JOUR
ID - 636
T1 - Visual activity before and after the onset of juvenile myopia
A1 - Jones-Jordan,L.A.
A1 - Mitchell,G.L.
A1 - Cotter,S.A.
A1 - Kleinstein,R.N.
A1 - Manny,R.E.
A1 - Mutti,D.O.
A1 - Twelker,J.D.
A1 - Sims,J.R.
A1 - Zadnik,K.
A1 - CLEERE Study Group.
Y1 - 2011/03//
N1 - Jones-Jordan, Lisa A. Mitchell, G Lynn. Cotter, Susan A. Kleinstein, Robert N. Manny, Ruth E. Mutti, Donald O. Twelker, J Daniel. Sims, Janene R. Zadnik, Karla. CLEERE Study Group
Investigative ophthalmology & visual science
gwi, 7703701
IM
Journal Article. Research Support, N.I.H., Extramural. Research Support, Non-U.S. Gov't
English
KW - MEDLINE
KW - Activities of Daily Living
KW - Adolescent
KW - Child
KW - Ethnic Groups
KW - Female
KW - Humans
KW - Leisure Activities
KW - Male
KW - Motor Activity

KW - Myopia/eh [Ethnology]
KW - Myopia/pp [Physiopathology]
KW - Refraction
KW - Ocular/ph [Physiology]
KW - Sports
KW - Visual Acuity/ph [Physiology]
RP - NOT IN FILE
SP - 1841
EP - 1850
JF - Investigative Ophthalmology & Visual Science
JA - Invest Ophthalmol Vis Sci
VL - 52
IS - 3
CY - United States
N2 - PURPOSE: To investigate visual activities before and after the onset of juvenile myopia. METHODS: The subjects were 731 incident myopes (-0.75 D or more myopia on cycloplegic autorefraction in both meridians) and 587 emmetropes (between -0.25 and +1.00 D) in the Collaborative Longitudinal Evaluation of Ethnicity and Refractive Error (CLEERE) Study. Parents supplied visual activity data annually. Data from myopic children 5 years before through 5 years after myopia onset were compared to data from age-, sex-, and ethnicity-matched models of children who remained emmetropic. RESULTS: Hours per week spent reading or using a computer/playing video games did not differ between the groups before myopia onset; however, hours per week for both activities were significantly greater in myopes than in emmetropes at onset and in 4 of the 5 years after onset by 0.7 to 1.6 hours per week. Hours per week spent in outdoor/sports activities were significantly fewer for children who became myopic 3 years before onset through 4 years after onset by 1.1 to 1.8 hours per week. Studying and TV watching were not significantly different before myopia onset. CONCLUSIONS: Before myopia onset, near work activities of future myopic children did not differ from those of emmetropes. Those who became myopic had fewer outdoor/sports activity hours than the emmetropes before, at, and after myopia onset. Myopia onset may influence children's near work behavior, but the lack of difference before onset argues against a major causative role for near work. Less outdoor/sports activity before myopia onset may exert a stronger influence on development than near work
SN - 1552-5783
AD - The Ohio State University College of Optometry, Columbus, Ohio 43210, USA
UR - 20926821
ER -

TY - JOUR
ID - 637
T1 - Windsor, Ontario exposure assessment study: design and methods validation of personal, indoor, and outdoor air pollution monitoring
A1 - Wheeler,A.J.
A1 - Xu,X.
A1 - Kulka,R.
A1 - You,H.
A1 - Wallace,L.
A1 - Mallach,G.
A1 - Van,Ryswyk K.
A1 - MacNeill,M.
A1 - Kearney,J.
A1 - Rasmussen,P.E.
A1 - bek-Zlotorzynska,E.
A1 - Wang,D.
A1 - Poon,R.
A1 - Williams,R.
A1 - Stocco,C.
A1 - Anastassopoulos,A.
A1 - Miller,J.D.
A1 - Dales,R.

A1 - Brook,J.R.
Y1 - 2011/03//
N1 - Wheeler, Amanda J. Xu, Xiaohong. Kulka, Ryan. You, Hongyu. Wallace, Lance. Mallach, Gary. Van Ryswyk, Keith. MacNeill, Morgan. Kearney, Jill. Rasmussen, Pat E. Dabek-Zlotorzynska, Ewa. Wang, Daniel. Poon, Raymond. Williams, Ron. Stocco, Corinne. Anastassopoulos, Angelos. Miller, J David. Dales, Robert. Brook, Jeffrey R
Journal of the Air & Waste Management Association (1995)
9503111, cbi
IM
Journal Article. Validation Studies
English
KW - MEDLINE
KW - Adult
KW - Air Pollutants/an [Analysis]
KW - Air Pollution
KW - Indoor/an [Analysis]
KW - Asthma/pp [Physiopathology]
KW - Child
KW - Environmental Monitoring/mt [Methods]
KW - Female
KW - Humans
KW - Male
KW - Nitrogen Dioxide/an [Analysis]
KW - Ontario
KW - Ozone/an [Analysis]
KW - Particulate Matter/an [Analysis]
KW - Patient Selection
KW - Quality Control
KW - Questionnaires
KW - Research Design
KW - Vital Capacity
KW - Volatile Organic Compounds/an [Analysis]
RP - NOT IN FILE
SP - 324
EP - 338
JF - Journal of the Air & Waste Management Association
JA - J Air Waste Manag Assoc
VL - 61
IS - 3
CY - United States
N2 - The Windsor, Ontario Exposure Assessment Study evaluated the contribution of ambient air pollutants to personal and indoor exposures of adults and asthmatic children living in Windsor, Ontario, Canada. In addition, the role of personal, indoor, and outdoor air pollution exposures upon asthmatic children's respiratory health was assessed. Several active and passive sampling methods were applied, or adapted, for personal, indoor, and outdoor residential monitoring of nitrogen dioxide, volatile organic compounds, particulate matter (PM; PM-2.5 pm [PM2.5] and < or =10 microm [PM10] in aerodynamic diameter), elemental carbon, ultrafine particles, ozone, air exchange rates, allergens in settled dust, and particulate-associated metals. Participants completed five consecutive days of monitoring during the winter and summer of 2005 and 2006. During 2006, in addition to undertaking the air pollution measurements, asthmatic children completed respiratory health measurements (including peak flow meter tests and exhaled breath condensate) and tracked respiratory symptoms in a diary. Extensive quality assurance and quality control steps were implemented, including the collocation of instruments at the National Air Pollution Surveillance site operated by Environment Canada and at the Michigan Department of Environmental Quality site in Allen Park, Detroit, MI. During field sampling, duplicate and blank samples were also completed and these data are reported. In total, 50 adults and 51 asthmatic children were recruited to participate, resulting in 922 participant days of data. When comparing the methods used in the study with standard reference methods, field blanks were low and bias was acceptable, with most methods being within 20% of reference methods. Duplicates were typically within less than 10% of each other, indicating that

study results can be used with confidence. This paper covers study design, recruitment, methodology, time activity diary, surveys, and quality assurance and control results for the different methods used
SN - 1096-2247
AD - Air Health Science Division, Health Canada, Ottawa, Ontario, Canada. amanda.wheeler@hc-sc.gc.ca
UR - 21416760
ER -

TY - JOUR
ID - 638
T1 - Pedometer-determined segmented physical activity patterns of fourth- and fifth-grade children
A1 - Brusseau,T.A.
A1 - Kulinna,P.H.
A1 - Tudor-Locke,C.
A1 - Ferry,M.
A1 - van der,Mars H.
A1 - Darst,P.W.
Y1 - 2011/02//
N1 - Brusseau, Timothy A. Kulinna, Pamela H. Tudor-Locke, Catrine. Ferry, Matthew. van der Mars, Hans. Darst, Paul W
Journal of physical activity & health
101189457
IM
Journal Article
English
KW - MEDLINE
KW - Age Factors
KW - Body Mass Index
KW - Child
KW - Exercise
KW - Female
KW - Humans
KW - Male
KW - Sex Factors
KW - Socioeconomic Factors
KW - Walking
RP - NOT IN FILE
SP - 279
EP - 286
JF - Journal of Physical Activity & Health
JA - J Phys Act Health
VL - 8
IS - 2
CY - United States
N2 - BACKGROUND: The need to understand where and how much physical activity (PA) children accumulate has become important in assisting the development, implementation, and evaluation of PA interventions. The purpose of this study was to describe the daily PA patterns of children during the segmented school-week. METHODS: 829 children participated by wearing pedometers (Yamax-Digiwalker SW-200) for 5 consecutive days. Students recorded their steps at arrival/departure from school, Physical Education (PE), recess, and lunchtime. RESULTS: Boys took significantly more steps/day than girls during most PA opportunities; recess, t(440)=8.80, P<.01; lunch, t(811)=14.57, P<.01; outside of school, t(763)=5.34, P<.01; school, t(811)=10.61, P<.01; and total day, t(782)=7.69, P<.01. Boys and girls accumulated a similar number of steps t(711) .69, P=.09 during PE. For boys, lunchtime represented the largest single source of PA (13.4%) at school, followed by PE (12.7%) and recess (9.5%). For girls, PE was the largest (14.3%), followed by lunchtime (11.7%) and recess (8.3%). CONCLUSION: An understanding of the contributions of the in-school segments can serve as baseline measures for practitioners and researchers to use in school-based PA interventions
SN - 1543-3080

AD - Department of Kinesiology, Sport Studies, and Physical Education, College of Brockport, Brockport, NY, USA
UR - 21415455
ER -

TY - JOUR
ID - 639
T1 - The role of school physical activity programs in child body mass trajectory
A1 - Fernandes,M.M.
A1 - Sturm,R.
Y1 - 2011/02//
N1 - Fernandes, Meenakshi Maria. Sturm, Roland
Journal of physical activity & health
101189457
IM
Journal Article. Research Support, N.I.H., Extramural
English
KW - MEDLINE
KW - Body Mass Index
KW - Child
KW - Cohort Studies
KW - Exercise
KW - Female
KW - Health Behavior
KW - Humans
KW - Male
KW - Physical Education and Training/sn [Statistics & Numerical Data]
KW - Schools/sn [Statistics & Numerical Data]
KW - Socioeconomic Factors
RP - NOT IN FILE
SP - 174
EP - 181
JF - Journal of Physical Activity & Health
JA - J Phys Act Health
VL - 8
IS - 2
CY - United States
N2 - BACKGROUND: Physical activity at school can support obesity prevention among youth. This paper assesses the role of existing school physical activity programs for a national cohort from first grade to fifth grade. METHODS: We analyzed a cohort from the Early Childhood Longitudinal Survey-Kindergarten Cohort which included 8246 children in 970 schools across the country. Growth curve models estimate the effect of physical education (PE) and recess on individual child body mass trajectories controlling for child and school characteristics. Hierarchical models allow for unobserved school and child effects. RESULTS: Among first graders, 7.0% met the National Association of Sport and Physical Education (NASPE) recommended time for PE and 70.7% met the recommended time for recess in the previous week. Boys experienced a greater increase in body mass than girls. Meeting the NASPE recommended time for recess was associated with a 0.74 unit decrease in BMI (body mass index) percentile for children overall. Meeting the NASPE recommendation for physical education was associated with 1.56 unit decrease in BMI percentile among boys but not girls. CONCLUSIONS: We find evidence that meeting the national recommendations for PE and recess is effective in mitigating body mass increase among children
SN - 1543-3080
AD - Abt Associates Inc., Cambridge, MA, USA
UR - 21415444
ER -

TY - JOUR
ID - 640

T1 - What is the meaning and nature of active play for today's children in the UK?
A1 - Brockman,R.
A1 - Fox,K.R.
A1 - Jago,R.
Y1 - 2011///
N1 - Brockman, Rowan. Fox, Kenneth R. Jago, Russell
The international journal of behavioral nutrition and physical activity
101217089
IM
Journal Article. Research Support, Non-U.S. Gov't
English
KW - MEDLINE
KW - Bicycling
KW - Child
KW - Exercise
KW - Family
KW - Female
KW - Focus Groups
KW - Friends
KW - Great Britain
KW - Humans
KW - Leisure Activities
KW - Male
KW - Perception
KW - Play and Playthings
KW - Sedentary Lifestyle
KW - Sex Factors
KW - Social Values
RP - NOT IN FILE
SP - 15
JF - International Journal of Behavioral Nutrition & Physical Activity
JA - Int
VL - 8
CY - England
N2 - BACKGROUND: Preventing the decline in physical activity which occurs around 10-11 years of age is a public health priority. Physically active play can make unique contributions to children's development which cannot be obtained from more structured forms of physical activity. Encouraging active play in children's leisure time has potential to increase physical activity levels while promoting optimal child development. Aspired wisdom states that contemporary British children no longer play outdoors, but systematic evidence for this is lacking. We need to build a more informed picture of contemporary children's play before we consider interventions to increase it. METHODS: Eleven focus groups were conducted with 77, 10-11 year old children from four primary schools in Bristol, UK. Focus groups examined: 1) children's perceptions of 'play'; 2) how much of their play is active play; and 3) contexts of children's active play. All focus groups were audio-taped and transcribed verbatim. Data were analysed using a thematic approach. RESULTS: Children's perceptions of play were broad and included both physically active and sedentary behaviours. Children reported that they frequently engaged in active play and valued both the physical and social benefits it provided. Whereas boys frequently reported having a 'kick about' or riding bikes as their preferred forms of active play, girls were less likely to report a specific activity. Additionally, boys reported greater independent mobility in their active play compared to girls. Finally, boys were more likely to report playing with neighbourhood friends but girls more frequently reported playing with family members. CONCLUSIONS: Promoting active play in children's leisure time may increase the physical activity of children, but interventions may need to be tailored according to gender
SN - 1479-5868
AD - Centre for Exercise, Nutrition & Health Sciences, School for Policy Studies, University of Bristol, Bristol, UK. Rowan.Brockman@bristol.ac.uk
UR - 21385336
ER -

TY - JOUR
ID - 641
T1 - Analysis of nonsexual injuries of the male genitals in children and adolescents
A1 - Widni,E.E.
A1 - Hollwarth,M.E.
A1 - Saxena,A.K.
Y1 - 2011/04//
N1 - Widni, E E. Hollwarth, M E. Saxena, A K
Acta paediatrica (Oslo, Norway : 1992)
bgc, 9205968
IM
Journal Article
English
KW - MEDLINE
KW - Accidental Falls
KW - Accidents
KW - Adolescent
KW - Athletic Injuries/et [Etiology]
KW - Athletic Injuries/th [Therapy]
KW - Child
KW - Preschool
KW - Humans
KW - Length of Stay/sn [Statistics & Numerical Data]
KW - Male
KW - Penis/in [Injuries]
KW - Retrospective Studies
KW - Scrotum/in [Injuries]
KW - Treatment Outcome
RP - NOT IN FILE
SP - 590
EP - 593
JF - Acta Paediatrica
JA - Acta Paediatr
VL - 100
IS - 4
CY - Norway
N2 - AIM: This study analysed nonsexual male genital injuries in boys with regards to aetiology, age, management and outcome. METHODS: Patients were identified from the hospital database (between 1999 and 2009) were analysed retrospectively. RESULTS: Seventy-four patients (2-17 years) were identified which included 24 toddlers and small children (2-5 years), 36 school age children (6-12 years) and 14 adolescents (13-17 years). Traumas were related to falls (n=14), kicks (n=11), sport (n=10), toilet seats (n=9), motor vehicles (n=8), bicycle (n=4), outdoor activities (n=4) and others causes (n=14). The leading cause of injuries in (i) toddler and small children was related to toilet seat (n=7) and falls (n=7), (ii) school age children to sport (n=8), kicks (n=7) and falls (n=6), and (iii) adolescents to motorcycle accidents (n=5). There were 45 scrotal and 29 penile injuries ranging from lacerations to contusions. Sixty-eight patients required hospital stay (0.5-36 days, =2.4 days). Follow-up examinations were uneventful, except for one minor wound infection. CONCLUSION: Nonsexual traumas to the male external genitalia occur mostly in 6- to 12-year-old boys, with sport accidents, kicks and falls being the major causes. Scrotal and penile lacerations and testicular contusions are the most common injuries. Complications after injuries to the male genitals in children are rare. 2010 The Author(s)/Acta Paediatrica 2010 Foundation Acta Paediatrica
SN - 1651-2227
AD - Department for Pediatric- and Adolescent Surgery, Medical University of Graz, Graz, Austria
UR - 21087313
ER -

TY - JOUR

ID - 642
T1 - Japanese encephalitis in two children--United States, 2010
A1 - Centers for Disease Control and Prevention (CDC)
Y1 - 2011/03/11/
N1 - Centers for Disease Control and Prevention (CDC)
MMWR. Morbidity and mortality weekly report
ne8, 7802429
IM
Case Reports. Journal Article
English
KW - MEDLINE
KW - Animals
KW - Child
KW - Culicidae
KW - DNA
KW - Viral/an [Analysis]
KW - Encephalitis
KW - Japanese/di [Diagnosis]
KW - Japanese/dt [Drug Therapy]
KW - Japanese/tm [Transmission]
KW - Japanese
KW - Fatal Outcome
KW - Female
KW - Humans
KW - Immunoglobulin M/an [Analysis]
KW - Insect Bites and Stings
KW - Male
KW - Neurocysticercosis/di [Diagnosis]
KW - Nevada
KW - Philippines
KW - Refugees
KW - Risk
KW - Texas
KW - Thailand/eh [Ethnology]
KW - Travel
RP - NOT IN FILE
SP - 276
EP - 278
JF - MMWR - Morbidity & Mortality Weekly Report
JA - MMWR Morb Mortal Wkly Rep
VL - 60
IS - 9
CY - United States
N2 - Japanese encephalitis virus (JEV) is the leading cause of vaccine-preventable encephalitis in Asia and the western Pacific. JEV is maintained in an enzootic cycle involving mosquitoes and amplifying vertebrate hosts, mainly pigs and wading birds. The virus is transmitted to humans primarily by Culex mosquitoes, which breed in flooded rice fields and pools of stagnant water and most often feed outdoors during the evening and night. JEV transmission occurs mainly in rural agricultural areas, but occasional human cases occur in urban areas. Japanese encephalitis (JE) in persons who have traveled or lived overseas is diagnosed infrequently in the United States, with only four cases identified from 1992 (when a JE vaccine was first licensed in the United States) to 2008. This report describes the only cases diagnosed in the United States and reported to CDC since then. The first was a fatal case in a U.S. child who had visited relatives in the Philippines. The other occurred in a refugee who became ill while traveling from Thailand to the United States and whose diagnosis was complicated by concurrent neurocysticercosis. JE should be considered in the differential diagnosis for any patient with an acute neurologic infection who recently has been in a JE-endemic country. Travelers to JE-endemic countries should be advised of the risk for JE and the importance of personal protective measures to

prevent mosquito bites. JE vaccine should be considered for travelers who might be at greater risk based on the season, location, and duration of their visit and their planned activities
SN - 1545-861X
UR - 21389931
ER -

TY - JOUR
ID - 643
T1 - Review of unintentional non-fire-related carbon monoxide poisoning morbidity and mortality in Florida, 1999-2007. [Review]
A1 - Harduar-Morano,L.
A1 - Watkins,S.
Y1 - 2011/03//
N1 - Harduar-Morano, Laurel. Watkins, Sharon
Public health reports (Washington, D.C. : 1974)
9716844, qja
AIM, IM
Journal Article. Review
English
KW - MEDLINE
KW - Accidents/mo [Mortality]
KW - Accidents/sn [Statistics & Numerical Data]
KW - Adolescent
KW - Adult
KW - Age Distribution
KW - Aged
KW - 80 and over
KW - Carbon Monoxide Poisoning/ep [Epidemiology]
KW - Carbon Monoxide Poisoning/eh [Ethnology]
KW - Carbon Monoxide Poisoning/mo [Mortality]
KW - Child
KW - Preschool
KW - Continental Population Groups
KW - Female
KW - Florida/ep [Epidemiology]
KW - Humans
KW - Infant
KW - Newborn
KW - Male
KW - Middle Aged
KW - Prevalence
KW - Risk Factors
KW - Sex Distribution
KW - Young Adult
RP - NOT IN FILE
SP - 240
EP - 250
JF - Public Health Reports
JA - Public Health Rep
VL - 126
IS - 2
CY - United States
N2 - OBJECTIVE: Previous Florida evaluations of carbon monoxide (CO) poisoning have been disaster focused. The majority of prevention messages and risk-factor identification efforts have revolved around hurricane season (June-November). We evaluated the prevalence, risk factors, and causes of CO poisoning within Florida throughout the year to produce a more complete presentation of the burden of CO poisoning. METHODS: We obtained data from death certificates, hospital discharge records, and emergency department

records. We limited our analysis to unintentional poisonings, calculating rates for age, gender, race, and ethnicity, and reviewing poisoning chronology, location, and exposure situation. RESULTS: From 1999 to 2007, 493 people were hospitalized, and 230 individuals died as a result of non-fire-related CO poisoning. From 2005 to 2007, 781 people visited emergency departments for non-fire-related CO poisoning. Rates of severe poisoning resulting in hospitalization or death were highest among the elderly (0.56 visits and 0.63 deaths per 100,000 Floridians). Acute poisoning rates were highest among people 25-34 years of age (2.48 visits per 100,000 Floridians). Poisonings were primarily due to motor vehicle exhaust (21%-69%) and generator exposure (12%-33%), and the majority (50%-70%) occurred within the home. A large number of poisonings (25%-29%) occurred during the winter months, outside of hurricane season. CONCLUSION: The findings of this study indicate a need for additional prevention strategies in conjunction with current activities to more effectively reduce the number of CO poisonings in Florida. Prevention activities should be conducted year-round, and additional strategies should include public awareness of the hazards of motor vehicle exhaust
SN - 0033-3549
AD - Division of Environmental Health, Florida Department of Health, Tallahassee, FL 32399, USA
UR - 21387954
ER -

TY - JOUR
ID - 645
T1 - Are child occupants a significant source of driving distraction?
A1 - Koppel,S.
A1 - Charlton,J.
A1 - Kopinathan,C.
A1 - Taranto,D.
Y1 - 2011/05//
N1 - Koppel, Sjaan. Charlton, Judith. Kopinathan, Chelvi. Taranto, David
Accident; analysis and prevention
acs, 1254476
IM
Journal Article. Research Support, Non-U.S. Gov't
English
KW - MEDLINE
KW - Accidents
KW - Traffic/pc [Prevention & Control]
KW - Traffic/px [Psychology]
KW - Adult
KW - Attention
KW - Automobile Driving/px [Psychology]
KW - Automobile Driving/sn [Statistics & Numerical Data]
KW - Child
KW - Child Behavior
KW - Preschool
KW - Data Collection/sn [Statistics & Numerical Data]
KW - Female
KW - Humans
KW - Infant
KW - Male
KW - Parenting/px [Psychology]
KW - Safety
KW - Video Recording
RP - NOT IN FILE
SP - 1236
EP - 1244
JF - Accident Analysis & Prevention
JA - Accid Anal Prev
VL - 43
IS - 3

CY - England
N2 - Driver distraction represents a well-documented and growing contribution to the road safety problem. This study used a naturalistic, observational approach to examine if children in vehicles are a significant source of driving distraction. Families with children aged between 1 and 8 years drove an instrumented "study vehicle" on their regular trips for 3 weeks. A discrete video recording system in the vehicle provided images of the driver and front seat passenger, the rear seat child passengers and the traffic ahead. The video-recordings inside and outside the vehicle were analysed to identify potential distracting activities, where 'distraction' was broadly defined as any activity that distracted the driver or competed for their attention while driving. In addition, all potentially distracting activities that involved the driver looking away from the forward roadway for more than 2s while the vehicle was in motion were also coded. Video-recordings were analysed for 92 driving journeys undertaken by 12 families including 25 children and 19 drivers. The mean journey duration was approximately 16 min (range: 2 min-3h 34 min). Most journeys were undertaken during the day (89%), with the mother driving (65%) and without a front seat passenger (64%). Driving journeys were predominantly undertaken in urban areas (97%), on suburban roads/streets (94%), and under low complexity traffic conditions (91%). Most journeys involved some source of potential driver distraction (98%), with drivers distracted for 18% of the driving journey. The most frequent types of distracting activities that drivers engaged in included: touching their head or their face (35%), interacting with child passengers in the rear seat (12%), and engaging with the front seat passenger (9%). Almost three-quarters of these potentially distracting activities were engaged in by the driver while the study vehicle was in motion (72%) and 14% of all potentially distracting activities involved the driver's eyes off the roadway for greater than 2s while the vehicle was in motion, thereby potentially doubling their crash risk. The most frequent child-related activities that drivers engaged in included: turning to look at the rear seated occupants or viewing the children using the rear-view mirror (76.4%), engaging in conversation with their children (16%), assisting their children (e.g., passing food and drink [7%]) and playing with their children (1%). Drivers spent significantly longer periods of time engaged in non-child occupant-related activities compared with child occupant-related activities and were significantly more likely to have their eyes off the forward roadway for greater than 2s while engaged in non-child occupant-related activities (14%) compared to child occupant-related activities (10%). The results suggest that drivers need to be educated about the potential crash and injury risks associated with both child occupant-related and non-child occupant-related activities while driving their vehicle. Copyright 2011 Elsevier Ltd. All rights reserved
SN - 1879-2057
AD - Monash University Accident Research Centre, Building 70, Clayton, VIC 3800, Australia. Sjaanie.Koppel@muarc.monash.edu.au
UR - 21376923
ER -

TY - JOUR
ID - 646
T1 - The paediatric wrist revisited: redefining MR findings in healthy children
A1 - Muller,L.S.
A1 - Avenarius,D.
A1 - Damasio,B.
A1 - Eldevik,O.P.
A1 - Malattia,C.
A1 - Lambot-Juhan,K.
A1 - Tanturri,L.
A1 - Owens,C.M.
A1 - Rosendahl,K.
Y1 - 2011/04//
N1 - Muller, Lil-Sofie Ording. Avenarius, D. Damasio, B. Eldevik, O P. Malattia, C. Lambot-Juhan, K. Tanturri, L. Owens, C M. Rosendahl, K
Annals of the rheumatic diseases
0372355, 62w
IM
Journal Article
English
KW - MEDLINE
KW - Adolescent

KW - Aging/pa [Pathology]
KW - Bone Marrow/ah [Anatomy & Histology]
KW - Child
KW - Preschool
KW - Female
KW - Humans
KW - Magnetic Resonance Imaging/mt [Methods]
KW - Male
KW - Prospective Studies
KW - Reference Values
KW - Synovial Fluid/cy [Cytology]
KW - Wrist Joint/ah [Anatomy & Histology]
RP - NOT IN FILE
SP - 605
EP - 610
JF - Annals of the Rheumatic Diseases
JA - Ann Rheum Dis
VL - 70
IS - 4
CY - England
N2 - OBJECTIVES: During a multicentre study on juvenile idiopathic arthritis, wide variations were observed in bone shape, signal intensity and volume of joint fluid as shown by MRI which in part appeared to be unrelated to disease activity. A study was undertaken to examine these features in a cohort of healthy children. METHODS: 88 children of mean age 9.8 years (range 5-15) underwent MRI imaging (T1-weighted Spin Echo and Spectral Selection Attenuated Inversion Recovery (SPAIR)) of the left wrist. The number of bony depressions, distribution and amount of joint fluid and the presence of bone marrow changes were assessed. RESULTS: Bony depressions were present in all children, increasing with age from a mean of 4.0 in children aged 4-6 years to 9.2 in those aged 12-15 years (p<0.001)). 45 of 84 children (53.6%) had a high signal on SPAIR with a corresponding low signal on T1 in at least one bone. No associations were seen between bone marrow change (present or not) and sex (p=0.827) or sports club membership (p=0.616). All children had visible joint fluid in at least one of the joints assessed. No associations were seen between the presence of joint fluid and age group, except for the radius/scaphoid and capitate-scaphoid joints and a recess lateral to the hamate. CONCLUSIONS: It is important to be aware of the high prevalence of bony depressions, signal changes suggestive of bone marrow oedema and the volume of joint fluid seen in normal children. Such findings must be interpreted with care in children with suspected disease such as juvenile arthritis
SN - 1468-2060
AD - Department of Radiology, University Hospital North Norway, Tromso, Norway. lil-sofie.ording.muller@unn.no
UR - 21173014
ER -

TY - JOUR
ID - 647
T1 - Ultraviolet radiation: a hazard to children and adolescents. [Review]
A1 - Council on Environmental Health
A1 - Section on Dermatology
A1 - Balk,S.J.
Y1 - 2011/03//
N1 - Council on Environmental Health. Section on Dermatology. Balk, Sophie J
Pediatrics
oxv, 0376422
AIM, IM
Journal Article. Review
English
KW - MEDLINE
KW - Adolescent
KW - Child

KW - Environmental Exposure/ae [Adverse Effects]
KW - Humans
KW - Practice Guidelines as Topic
KW - Skin/re [Radiation Effects]
KW - Skin Neoplasms/pc [Prevention & Control]
KW - Sunburn/pc [Prevention & Control]
KW - Ultraviolet Rays/ae [Adverse Effects]
RP - NOT IN FILE
SP - 588
EP - 597
JF - Pediatrics
VL - 127
IS - 3
CY - United States
N2 - Ultraviolet radiation (UVR) causes the 3 major forms of skin cancer: basal cell carcinoma; squamous cell carcinoma; and cutaneous malignant melanoma. Public awareness of the risk is not optimal, overall compliance with sun protection is inconsistent, and melanoma rates continue to rise. The risk of skin cancer increases when people overexpose themselves to sun and intentionally expose themselves to artificial sources of UVR. Yet, people continue to sunburn, and teenagers and adults alike remain frequent visitors to tanning parlors. Pediatricians should provide advice about UVR exposure during health-supervision visits and at other relevant times. Advice includes avoiding sunburning, wearing clothing and hats, timing activities (when possible) before or after periods of peak sun exposure, wearing protective sunglasses, and applying and reapplying sunscreen. Advice should be framed in the context of promoting outdoor physical activity. Adolescents should be strongly discouraged from visiting tanning parlors. Sun exposure and vitamin D status are intertwined. Cutaneous vitamin D production requires sunlight exposure, and many factors, such as skin pigmentation, season, and time of day, complicate efficiency of cutaneous vitamin D production that results from sun exposure. Adequate vitamin D is needed for bone health. Accumulating information suggests a beneficial influence of vitamin D on many health conditions. Although vitamin D is available through the diet, supplements, and incidental sun exposure, many children have low vitamin D concentrations. Ensuring vitamin D adequacy while promoting sun-protection strategies will require renewed attention to children's use of dietary and supplemental vitamin D
SN - 1098-4275
UR - 21357336
ER -

TY - JOUR
ID - 648
T1 - Parenthood and factors that influence outdoor recreational physical activity from a gender perspective
A1 - Sjogren,K.
A1 - Hansson,E.E.
A1 - Stjernberg,L.
Y1 - 2011///
N1 - Sjogren, Katarina. Hansson, Eva Ekvall. Stjernberg, Louise
BMC public health
100968562
IM
Journal Article. Research Support, Non-U.S. Gov't
English
KW - MEDLINE
KW - Adult
KW - Child
KW - Preschool
KW - Exercise
KW - Family Characteristics
KW - Female
KW - Humans
KW - Infant
KW - Newborn

KW - Life Style
KW - Male
KW - Parents
KW - Questionnaires
KW - Recreation
KW - Sex Factors
KW - Sweden
RP - NOT IN FILE
SP - 93
JF - BMC Public Health
VL - 11
CY - England
N2 - BACKGROUND: A physically active life promotes both physical and mental health, increasing well-being and quality of life. Physical activity (PA) performed outdoors has been found to be particularly good for promoting well-being. However, participation in PA can change during the course of a lifetime. Parenthood has been found to be a life event associated with decreased PA, especially among women, although studies in the field are sparse. The aim of this study was to investigate participation in outdoor recreational PA, and factors influencing participation among parents-to-be, with and without previous children, from a gender perspective. METHODS: This study included baseline data from parents-to-be, 224 women and 208 men, from the municipality of Karlskrona in south-east Sweden. Data collection was carried out during 2008-2009. We measured the self-reported amount of outdoor recreational PA undertaken during the last year and analysed the probability of participating in this PA using 25 variables covering individual and socioeconomic factors. RESULTS: Seventy-six per cent of the women and 65% of the men had participated in outdoor recreational PA, varying from several times per month to every day, over a 12-month period prior to one month before pregnancy. Participation in PA indoors and owning a dog or a horse emerged as the most important factors associated with the probability of participation in outdoor recreational PA. Men were affected by a greater number of factors than women, for example men who had a family situation that permitted outdoor recreational PA participated in activities to a greater extent than men without such a family situation. The physical aspect, i.e. improved physical condition, staying power and vigour, also played a significant role with regard to participation among men. CONCLUSIONS: Becoming a parent is a life-changing event that affects participation in PA. By offering family-oriented PA choices that involve both parents and children, midwives and health promoters can encourage parents to be active and to support each other. The promotion of outdoor recreational PA, which also has restorative effects on well-being, needs to focus on activities which are attractive and affordable for the majority of both women and men
SN - 1471-2458
AD - Faculty of Medicine, Department of Clinical Sciences in Malmo, General Practice, Lund University, Malmo University Hospital, 205 02 Malmo, Sweden. katarina.sjogren@med.lu.se
UR - 21310038
ER -

TY - JOUR
ID - 649
T1 - Young children in urban areas: links among neighborhood characteristics, weight status, outdoor play, and television watching
A1 - Kimbro,R.T.
A1 - Brooks-Gunn,J.
A1 - McLanahan,S.
Y1 - 2011/03//
N1 - Kimbro, Rachel Tolbert. Brooks-Gunn, Jeanne. McLanahan, Sara
Social science & medicine (1982)
ut9, 8303205
IM
Journal Article. Research Support, N.I.H., Extramural. Research Support, Non-U.S. Gov't
English
KW - MEDLINE
KW - Adult
KW - Body Mass Index

KW - Child
KW - Preschool
KW - Cohort Studies
KW - Female
KW - Humans
KW - Male
KW - Mother-Child Relations
KW - Mothers/px [Psychology]
KW - Mothers/sn [Statistics & Numerical Data]
KW - Motor Activity
KW - Obesity/ep [Epidemiology]
KW - Overweight/ep [Epidemiology]
KW - Play and Playthings
KW - Residence Characteristics/sn [Statistics & Numerical Data]
KW - Social Class
KW - Television/ut [Utilization]
KW - United States
KW - Urban Health
KW - Urban Population/sn [Statistics & Numerical Data]
RP - NOT IN FILE
SP - 668
EP - 676
JF - Social Science & Medicine
JA - Soc Sci Med
VL - 72
IS - 5
CY - England
N2 - Although research consistently demonstrates a link between residential context and physical activity for adults and adolescents, less is known about young children's physical activity. Using data from the U.S. Fragile Families and Child Wellbeing Study (N=1822, 51% male), we explored whether outdoor play and television watching were associated with children's body mass indexes (BMIs) at age five using OLS regression models, controlling for a wide array of potential confounders, including maternal BMI. We also tested whether subjective and objective neighborhood measures - socioeconomic status (from U.S. Census tract data), type of dwelling, perceived collective efficacy, and interviewer-assessed physical disorder of the immediate environment outside the home - were associated with children's activities, using negative binomial regression models. Overall, 19% of the sample were overweight (between the 85th and 95th percentiles), and 16% were obese (> 95th percentile). Hours of outdoor play were negatively associated with BMI, and hours of television were positively associated with BMI. Moreover, a ratio of outdoor play to television time was a significant predictor of BMI. Higher maternal perceptions of neighborhood collective efficacy were associated with more hours of outdoor play, fewer hours of television viewing, and more trips to a park or playground. In addition, we found that neighborhood physical disorder was associated with both more outdoor play and more television watching. Finally, contrary to expectations, we found that children living in public housing had significantly more hours of outdoor play and watched more television, than other children. We hypothesize that poorer children may have more unstructured time, which they fill with television time but also with outdoor play time; and that children in public housing may be likely to have access to play areas on the grounds of their housing facilities. Copyright 2011 Elsevier Ltd. All rights reserved
SN - 1873-5347
AD - Department of Sociology, Rice University, 6100 Main St., MS-28, Houston, TX 77005, USA. rtkimbro@rice.edu
UR - 21324574
ER -

TY - JOUR
ID - 650
T1 - Associations between the home and school environments and child body mass index
A1 - Miller,D.P.
Y1 - 2011/03//

N1 - Miller, Daniel P
Social science & medicine (1982)
ut9, 8303205
IM
Journal Article. Research Support, U.S. Gov't, P.H.S.
English
KW - MEDLINE
KW - Body Mass Index
KW - Child
KW - Female
KW - Humans
KW - Longitudinal Studies
KW - Male
KW - Obesity/ep [Epidemiology]
KW - Overweight/ep [Epidemiology]
KW - Residence Characteristics/sn [Statistics & Numerical Data]
KW - Schools/sn [Statistics & Numerical Data]
KW - Social Environment
KW - United States/ep [Epidemiology]
RP - NOT IN FILE
SP - 677
EP - 684
JF - Social Science & Medicine
JA - Soc Sci Med
VL - 72
IS - 5
CY - England
N2 - This paper examined associations between various aspects of home and school environments and child body mass index (BMI) in the Early Childhood Longitudinal Study - Kindergarten cohort, a panel dataset of US children collected from 1998 to 2004. Using three-level growth curve modeling with a sample of approximately 11,400 children, it assessed whether these aspects were related to initial BMI and to the rate of growth of BMI over the period from kindergarten to fifth grade, independent of a large number of controls. A number of home and school factors were associated with initial BMI and the growth of BMI. Greater hours of sleep by children, more lunches eaten at school, and the adequacy of their school cafeterias and the adequacy of their school gymnasiums were all significantly associated with lower initial levels of BMI. More breakfasts typically eaten per week with their families and greater minutes of recess (free time for activity at school) were each associated with decreases in the rate of BMI growth over time, while more television watched, greater average hours of weekly maternal employment, more school lunches and school breakfasts eaten, and the adequacy of children's gymnasiums were associated with faster rates of BMI growth over the study period. The study adds to the existing literature on environmental influences on child BMI by illustrating the utility and necessity of examining multiple influences within a single analytic framework. Further research and policy efforts should continue to acknowledge the multi-etiological manner by which the environment can affect rates of child obesity. Copyright 2010 Elsevier Ltd. All rights reserved
SN - 1873-5347
AD - Boston University School of Social Work, 264 Bay State Road, Boston, MA 02215, United States. dpmiller@bu.edu
UR - 21227558
ER -

TY - JOUR
ID - 651
T1 - Vitamin D status in Norwegian children and adolescents with excess body weight
A1 - Lagunova,Z.
A1 - Porojnicu,A.C.
A1 - Lindberg,F.A.
A1 - Aksnes,L.
A1 - Moan,J.

Y1 - 2011/03//
N1 - Lagunova, Zoya. Porojnicu, Alina C. Lindberg, Fedon A. Aksnes, Lage. Moan, Johan
Pediatric diabetes
100939345
IM
Journal Article. Research Support, Non-U.S. Gov't
English
KW - MEDLINE
KW - Adolescent
KW - Body Composition/ph [Physiology]
KW - Body Mass Index
KW - Body Weight/ph [Physiology]
KW - Child
KW - Female
KW - Humans
KW - Male
KW - Norway/ep [Epidemiology]
KW - Nutritional Status/ph [Physiology]
KW - Overweight/bl [Blood]
KW - Overweight/ep [Epidemiology]
KW - Seasons
KW - Vitamin D/bl [Blood]
KW - Young Adult
RP - NOT IN FILE
SP - 120
EP - 126
JF - Pediatric Diabetes
JA - Pediatr Diabetes
VL - 12
IS - 2
CY - Denmark
N2 - OBJECTIVES: The prevalence of childhood and adolescent obesity has increased during the past decades. A high body mass index (BMI) is associated with a low vitamin D status. The purpose of this study was to determine the prevalence of vitamin D deficiency and insufficiency in Norwegian children and adolescents with excess body weight. METHODS: Vitamin D status and seasonal variations of 25(OH)D and 1,25(OH)(2)D were analyzed in 102 children and adolescents (70 girls and 32 boys), 8-19 yr of age, with overweight and obesity. RESULTS: Overall, 50% of the children and adolescents included in the study had a low vitamin D status (25(OH)D <75 nmol/L) and 19% had vitamin D deficiency (25(OH)D <50 nmol/L). This was most prevalent in adolescents. Only 42% of teenagers had 25(OH)D levels >75 nmol/L vs. 72% of preteens. Both 25(OH)D and 1,25(OH)(2)D showed seasonal variations. A peak in serum 25(OH)D concentrations was observed during the summer while the lowest values were seen during the spring. In contrast, serum 1,25(OH)(2)D had a peak during the spring and the lowest concentrations during the winter. CONCLUSIONS: The prevalence of vitamin D deficiency and insufficiency is higher in obese and overweight adolescents than in overweight children. This might be related to low outdoor activities and low vitamin D intake in teenagers. Seasonal variations of both the vitamin D metabolites were observed. 2010 John Wiley & Sons A/S
SN - 1399-5448
AD - Department of Radiation Biology, Oslo University Hospital, The Norwegian Radium Hospital, 0310 Oslo, Norway. zoya.lagunova@rr-research.no
UR - 21352427
ER -

TY - JOUR
ID - 652
T1 - Multisystem Langerhans cell histiocytosis in children: current treatment and future directions. [Review]
A1 - Minkov,M.
Y1 - 2011/04/01/

N1 - Minkov, Milen
Paediatric drugs
dse, 100883685
IM
Journal Article. Review
English
KW - MEDLINE
KW - Adolescent
KW - Child
KW - Preschool
KW - Clinical Trials as Topic
KW - Histiocytosis
KW - Langerhans-Cell/dt [Drug Therapy]
KW - Langerhans-Cell/th [Therapy]
KW - Humans
KW - Infant
KW - Newborn
KW - Societies
KW - Medical
RP - NOT IN FILE
SP - 75
EP - 86
JF - Paediatric Drugs
JA - Paediatr Drugs
VL - 13
IS - 2
CY - New Zealand
N2 - Langerhans cell histiocytosis (LCH) is a rare (about 3-5 cases per million children aged 0-14 years), non-malignant disease characterized by proliferation and accumulation of clonal dendritic cells, extreme clinical heterogeneity, and an unpredictable course. Three large-scale, international, prospective therapeutic studies (LCH-I to III) for multisystem LCH (MS-LCH) have been conducted by the Histiocyte Society since 1991. The cumulative lessons from these studies are summarized in this review. Patients with MS-LCH represent a heterogeneous group with respect to disease severity and outcome, therefore treatment stratification and risk-tailored treatment are mandatory. The risk for mortality can be predicted based on involvement of 'risk organs' (e.g. hematopoietic system, liver, and/or spleen) at diagnosis and on response to initial therapy (assessed after 6-12 weeks of treatment). Thus, patients without involvement of risk organs (low-risk group) are not at risk for mortality but need systemic therapy in order to control the disease activity and avoid reactivations and permanent consequences. Patients with risk organ involvement (risk group) are at risk for mortality, and lack of therapy response defines a subgroup with a particularly dismal prognosis (high-risk group). Those patients in the risk group who respond to therapy and survive are at risk for reactivations and permanent consequences. The LCH-I study compared the efficacy of vinblastine and etoposide, and concluded that they are equivalent single-agent treatments for children with MS-LCH. However, the results of this trial were inferior with respect to response rate at week 6, disease reactivation rate, and sequelae, when compared with historical trials using more intensive regimens. The combination of prednisolone and vinblastine was established as a standard first-line treatment through the LCH-II and LCH-III studies. The regimen consists of one to two 6-week courses (continuous oral corticosteroids 40mg/m2/day for 4 weeks, tapered over 2 weeks plus weekly vinblastine intravenous push) of initial therapy, followed by a continuation phase (three weekly pulses of oral prednisolone 40mg/m2/day for 5 days plus a vinblastine injection). The addition of a third drug to the standard combination (etoposide in LCH-II and methotrexate in LCH-III) failed to significantly improve survival in the risk group. The remaining mortality in the risk group is about 20%, and up to 40% in the high-risk group. Concerning low-risk MS-LCH, comparison of results of the LCH-II study with historical data suggested that the remaining reactivation rate of about 50% (and possibly permanent consequences) could be reduced by prolongation of the total treatment duration. To study this hypothesis, in the low-risk group of the LCH-III study standard maintenance therapy was randomly given for a total treatment duration of 6 and 12 months. Unpublished preliminary data from this recently closed trial suggested that prolongation of the treatment duration may significantly improve reactivation-free survival. In summary, several studies have shown that systemic therapy is indicated for all patients with MS-LCH. A standard two-drug regimen consisting of an initial 'intensive' phase

for 6-12 weeks, followed by a less intensive 'maintenance phase' for a total treatment duration of at least 12 months is recommended for patients treated outside of clinical trials. Non-responders, particularly those with progressive disease in risk organs, are eligible for experimental salvage approaches. Remaining questions will be addressed in the upcoming LCH-IV trial, which is in the process of intensive preparation
SN - 1179-2019
AD - Department of Outpatient Hematology/Oncology, St Anna Children's Hospital, Kinderspitalgasse 6, Vienna, Austria. milen.minkov@stanna.at
UR - 21351807
ER -

TY - JOUR
ID - 653
T1 - Physical and social contextual influences on children's leisure-time physical activity: an ecological momentary assessment study
A1 - Dunton,G.F.
A1 - Liao,Y.
A1 - Intille,S.
A1 - Wolch,J.
A1 - Pentz,M.A.
Y1 - 2011/01//
N1 - Dunton, Genevieve F. Liao, Yue. Intille, Stephen. Wolch, Jennifer. Pentz, Mary Ann
Journal of physical activity & health
101189457
IM
Journal Article. Research Support, N.I.H., Extramural. Research Support, Non-U.S. Gov't
English
KW - MEDLINE
KW - Child
KW - Female
KW - Humans
KW - Leisure Activities
KW - Male
KW - Motor Activity
KW - Questionnaires
KW - Regression Analysis
KW - Social Environment
RP - NOT IN FILE
SP - S103
EP - S108
JF - Journal of Physical Activity & Health
JA - J Phys Act Health
VL - 8 Suppl 1
CY - United States
N2 - BACKGROUND: This study used real-time electronic surveys delivered through mobile phones, known as Ecological Momentary Assessment (EMA), to determine whether level and experience of leisure-time physical activity differ across children's physical and social contexts. METHODS: Children (N = 121; ages 9 to 13 years; 52% male, 32% Hispanic/Latino) participated in 4 days (Fri.-Mon.) of EMA during nonschool time. Electronic surveys (20 total) assessed primary activity (eg, active play/sports/exercise), physical location (eg, home, outdoors), social context (eg, friends, alone), current mood (positive and negative affect), and enjoyment. Responses were time-matched to the number of steps and minutes of moderate-to-vigorous physical activity (MVPA; measured by accelerometer) in the 30 minutes before each survey. RESULTS: Mean steps and MVPA were greater outdoors than at home or at someone else's house (all $P < .05$). Steps were greater with multiple categories of company (eg, friends and family together) than with family members only or alone (all $P < .05$). Enjoyment was greater outdoors than at home or someone else's house (all $P < .05$). Negative affect was greater when alone and with family only than friends only (all $P < .05$). CONCLUSION: Results describing the value of outdoor and social settings could inform context-specific interventions in this age group
SN - 1543-3080

AD - Dept of Preventive Medicine, University of Southern California, Alhambra, CA, USA
UR - 21350250
ER -

TY - JOUR
ID - 654
T1 - [Environmental approaches in the prevention of obesity in children and adolescents]. [Review] [German]
A1 - Loss,J.
A1 - Leitzmann,M.
Y1 - 2011/03//
N1 - Loss, J. Leitzmann, M
Bundesgesundheitsblatt, Gesundheitsforschung, Gesundheitsschutz
101181368
IM
English Abstract. Journal Article. Review
German
KW - MEDLINE
KW - Adolescent
KW - Child
KW - Female
KW - Health Behavior
KW - Health Promotion/mt [Methods]
KW - Humans
KW - Male
KW - Obesity/pc [Prevention & Control]
KW - Prevalence
KW - Primary Prevention/mt [Methods]
KW - Risk Reduction Behavior
KW - Treatment Outcome
RP - NOT IN FILE
SP - 281
EP - 289
JF - Bundesgesundheitsblatt, Gesundheitsforschung, Gesundheitsschutz
JA - Bundesgesundheitsblatt Gesundheitsforschung Gesundheitsschutz
VL - 54
IS - 3
CY - Germany
N2 - This article aims to provide a structured review of how to create settings and environments that prevent the development of childhood overweight and obesity. It also describes which institutions and levels are responsible for environmental (global) approaches in obesity prevention and which evidence exists in terms of process and outcomes of different preventive measures. Environmental approaches in disease prevention deal with social and technical-material conditions of daily living, as those conditions significantly influence health behavior. Strategies that focus on the obesogenic environment are considered increasingly important in the prevention of obesity in children and adolescents. They can be applied at different levels (e.g., schools, communities). These interventions should aim to improve the availability of healthy foods and physical activity facilities, e.g., by provision of healthy meals and foods in schools, restaurants, and stores and by price reductions of healthy foods. Physical activity can be supported by creating attractive green spaces and playgrounds in schools and cities, improving sidewalk networks and a supportive pedestrian environment, and implementing walk-to-school projects. On a national level, policies and legislation can support changes in the social and situational environments, e.g., relating to catering in schools or TV advertisement. The practice of environmental approaches is complex, because many stakeholders from different sectors have to be involved. This may account for the observation that environmental approaches are currently underrepresented in obesity prevention
SN - 1437-1588
AD - Medizinische Soziologie, Institut fur Epidemiologie und Praventivmedizin, Fakultat fur Medizin, Universitat Regensburg, Franz-Josef-Straus-Allee 11, Regensburg,Germany. julika.loss@klinik.uni-regensburg.de
UR - 21347760

ER -

TY - JOUR
ID - 655
T1 - Indoor air quality differences between urban and rural preschools in Korea
A1 - Yoon,C.
A1 - Lee,K.
A1 - Park,D.
Y1 - 2011/03//
N1 - Yoon, Chungsik. Lee, Kiyoung. Park, Donguk
Environmental science and pollution research international
9441769, dvn
IM
Comparative Study. Journal Article
English
KW - MEDLINE
KW - Air Pollutants/an [Analysis]
KW - Air Pollution
KW - Indoor/an [Analysis]
KW - Indoor/sn [Statistics & Numerical Data]
KW - Asbestos/an [Analysis]
KW - Carbon Dioxide/an [Analysis]
KW - Child
KW - Preschool
KW - Cities
KW - Environmental Monitoring/mt [Methods]
KW - Formaldehyde/an [Analysis]
KW - Humans
KW - Inhalation Exposure/an [Analysis]
KW - Inhalation Exposure/sn [Statistics & Numerical Data]
KW - Lead/an [Analysis]
KW - Particulate Matter/an [Analysis]
KW - Republic of Korea
KW - Rural Population
KW - Schools/sn [Statistics & Numerical Data]
KW - Urban Population
KW - Volatile Organic Compounds/an [Analysis]
RP - NOT IN FILE
SP - 333
EP - 345
JF - Environmental Science & Pollution Research
JA - Environ Sci Pollut.Res Int
VL - 18
IS - 3
CY - Germany
N2 - BACKGROUND, AIMS, AND SCOPE: Preschool indoor air quality (IAQ) is believed to be different from elementary school or higher school IAQ and preschool is the first place for social activity. Younger children are more susceptible than higher-grade children and spend more time indoors. The purpose of this study was to compare the indoor air quality by investigating the concentrations of airborne particulates and gaseous materials at preschools in urban and rural locations in Korea. METHODS: We investigated the concentrations of airborne particulates and gaseous materials in 71 classrooms at 17 Korean preschools. For comparison, outdoor air was sampled simultaneously with indoor air samples. Airborne concentrations of total suspended particulates, respirable particulates, lead, asbestos, total volatile organic compounds and components, formaldehyde, and CO(2) were measured with National Institute for Occupational Safety and Health and/or Environmental Protection Agency analytical methods. RESULTS: The concentration profiles of the investigated pollutants in indoor and urban settings were higher than those in outdoor and rural areas, respectively. The ratios of indoor/outdoor concentrations (I/O) of particulates and gaseous pollutants were characterized in urban and

rural preschools. Total dust concentration was highest in urban indoor settings followed by urban outdoor, rural indoor, and rural outdoor locations with an I/O ratio of 1.37 in urban and 1.35 in rural areas. Although I/O ratios of lead were close to 1, lead concentrations were much higher in urban than in rural areas. The I/O ratio of total VOCs was 2.29 in urban and 2.52 in rural areas, with the highest level in urban indoor settings. The I/O ratio of formaldehyde concentrations was higher in rural than in urban areas because the outdoor rural level was much lower than the urban concentration. Since an I/O ratio higher than 1 implies the presence of indoor sources, we concluded that there are many indoor sources in preschools. CONCLUSIONS: We confirmed that pollutants in indoor and urban settings were higher than those in outdoor and rural areas, respectively. Preschool children are expected to spend more time inside preschool facilities and therefore to be more exposed to pollutants. As far as we know, preschool IAQ is different from elementary school or higher school IAQ. Also, they are more vulnerable than higher-grade children. We found that the indoor and urban concentration profiles of the studied pollutants in preschools were higher than those in outdoor and rural areas. We believe that our findings may be useful for understanding the potential health effects of exposure and intervention studies in preschools
SN - 1614-7499
AD - Department of Environmental Health, Seoul National University, 599 Gwanak-ro, Gwanak-gu, Seoul, 151-742, South Korea. csyoon@snu.ac.kr
UR - 20668959
ER -

TY - JOUR
ID - 656
T1 - Role of social networks in shaping disease transmission during a community outbreak of 2009 H1N1 pandemic influenza
A1 - Cauchemez,S.
A1 - Bhattarai,A.
A1 - Marchbanks,T.L.
A1 - Fagan,R.P.
A1 - Ostroff,S.
A1 - Ferguson,N.M.
A1 - Swerdlow,D.
A1 - Pennsylvania,H.
Y1 - 2011/02/15/
N1 - Cauchemez, Simon. Bhattarai, Achuyt. Marchbanks, Tiffany L. Fagan, Ryan P. Ostroff, Stephen. Ferguson, Neil M. Swerdlow, David. Pennsylvania H1N1 working group
Proceedings of the National Academy of Sciences of the United States of America
pv3, 7505876
IM
Historical Article. Journal Article. Research Support, N.I.H., Extramural. Research Support, Non-U.S. Gov't. Research Support, U.S. Gov't, P.H.S.
English
KW - MEDLINE
KW - Child
KW - Female
KW - History
KW - 21st Century
KW - Humans
KW - Influenza A Virus
KW - H1N1 Subtype
KW - Influenza
KW - Human/ep [Epidemiology]
KW - Human/tm [Transmission]
KW - Interviews as Topic
KW - Male
KW - Markov Chains
KW - Models
KW - Theoretical
KW - Monte Carlo Method

KW - Pandemics/hi [History]
KW - Pennsylvania
KW - Sex Factors
KW - Social Support
KW - Students
RP - NOT IN FILE
SP - 2825
EP - 2830
JF - Proceedings of the National Academy of Sciences of the United States of America
JA - Proc Natl Acad Sci U S A
VL - 108
IS - 7
CY - United States
N2 - Evaluating the impact of different social networks on the spread of respiratory diseases has been limited by a lack of detailed data on transmission outside the household setting as well as appropriate statistical methods. Here, from data collected during a H1N1 pandemic (pdm) influenza outbreak that started in an elementary school and spread in a semirural community in Pennsylvania, we quantify how transmission of influenza is affected by social networks. We set up a transmission model for which parameters are estimated from the data via Markov chain Monte Carlo sampling. Sitting next to a case or being the playmate of a case did not significantly increase the risk of infection; but the structuring of the school into classes and grades strongly affected spread. There was evidence that boys were more likely to transmit influenza to other boys than to girls (and vice versa), which mimicked the observed assortative mixing among playmates. We also investigated the presence of abnormally high transmission occurring on specific days of the outbreak. Late closure of the school (i.e., when 27% of students already had symptoms) had no significant impact on spread. School-aged individuals (6-18 y) facilitated the introduction and spread of influenza in households, but only about one in five cases aged >18 y was infected by a school-aged household member. This analysis shows the extent to which clearly defined social networks affect influenza transmission, revealing strong between-place interactions with back-and-forth waves of transmission between the school, the community, and the household
SN - 1091-6490
AD - Medical Research Council Centre for Outbreak Analysis and Modelling, Department of Infectious Disease Epidemiology, School of Public Health, Imperial College London, London W2 1PG, United Kingdom. s.cauchemez@imperial.ac.uk
UR - 21282645
ER -

TY - JOUR
ID - 657
T1 - Factors associated with the physical activity level of children who have the Fontan procedure
A1 - Longmuir,P.E.
A1 - Russell,J.L.
A1 - Corey,M.
A1 - Faulkner,G.
A1 - McCrindle,B.W.
Y1 - 2011/02//
N1 - Longmuir, Patricia E. Russell, Jennifer L. Corey, Mary. Faulkner, Guy. McCrindle, Brian W
American heart journal
0370465
AIM, IM
Journal Article
English
KW - MEDLINE
KW - Child
KW - Preschool
KW - Cross-Sectional Studies
KW - Exercise
KW - Female
KW - Fontan Procedure

KW - Humans
KW - Male
KW - Motor Activity
RP - NOT IN FILE
SP - 411
EP - 417
JF - American Heart Journal
JA - Am Heart J
VL - 161
IS - 2
CY - United States
N2 - BACKGROUND: Children with complex heart defects are sedentary, with activity level unrelated to exercise capacity. We sought to identify factors associated with physical activity level for children who have the Fontan procedure. METHODS: We used a cross-sectional study, 64 children (25 female, 5-11 years) after Fontan. Measurements were weekly minutes of moderate-to-vigorous physical activity, cardiac status, resting/exercise cardiopulmonary capacity, gross motor skill, health-related endurance/strength/body composition, and parent/child activity perceptions. RESULTS: Participants performed 361 + 137 minutes per week of moderate-to-vigorous physical activity. Increased activity related to antithrombotic medication use (86 min/wk), lower resting heart rate (3 min/wk), higher weekday outdoor time (0.7 minutes per outside minute), lower family income (13 minutes per $10,000), and higher parent rating of child's activity relative to peers (36 min/wk). Factors related to decreased activity were winter season (-84 min/wk), history of arrhythmia (-96 min/wk), and greater child confidence in own ability to be active (-113 min/wk). CONCLUSIONS: Physical activity after the Fontan procedure is primarily associated with factors unrelated to cardiac status. Interventions that impact these modifiable factors would be expected to enable these children to achieve the recommended activity levels associated with optimal health. Copyright 2011 Mosby, Inc. All rights reserved
SN - 1097-6744
AD - Labatt Family Heart Centre, The Hospital for Sick Children, Toronto, Canada; Department of Physical Therapy, Faculty of Medicine, University of Toronto, Toronto, Canada
UR - 21315227
ER -

TY - JOUR
ID - 658
T1 - Boys and girls on the playground: sex differences in social development are not stable across early childhood
A1 - Barbu,S.
A1 - Cabanes,G.
A1 - Le Maner-Idrissi,G.
Y1 - 2011///
N1 - Barbu, Stephanie. Cabanes, Guenael. Le Maner-Idrissi, Gaid
PloS one
101285081
IM
Journal Article. Research Support, Non-U.S. Gov't
English
KW - MEDLINE
KW - Age Factors
KW - Child
KW - Cross-Sectional Studies
KW - Female
KW - Gender Identity
KW - Humans
KW - Infant
KW - Male
KW - Play and Playthings/px [Psychology]
KW - Schools
KW - Social Behavior

RP - NOT IN FILE
SP - e16407
JF - PLoS ONE [Electronic Resource]
JA - PLoS ONE
VL - 6
IS - 1
CY - United States
N2 - Sex differences in human social behaviors and abilities have long been a question of public and scientific interest. Females are usually assumed to be more socially oriented and skillful than males. However, despite an extensive literature, the very existence of sex differences remains a matter of discussion while some studies found no sex differences whereas others reported differences that were either congruent or not with gender stereotypes. Moreover, the magnitude, consistency and stability across time of the differences remain an open question, especially during childhood. As play provides an excellent window into children's social development, we investigated whether and how sex differences change in social play across early childhood. Following a cross-sectional design, 164 children aged from 2 to 6 years old, divided into four age groups, were observed during outdoor free play at nursery school. We showed that sex differences are not stable over time evidencing a developmental gap between girls and boys. Social and structured forms of play emerge systematically earlier in girls than in boys leading to subsequent sex differences in favor of girls at some ages, successively in associative play at 3-4 years, cooperative play at 4-5 years, and social interactions with peers at 5-6 years. Preschool boys also display more solitary play than preschool girls, especially when young. Nevertheless, while boys catch up and girls move on towards more complex play, sex differences in social play patterns are reversed in favor of boys at the following ages, such as in associative play at 4-5 years and cooperative play at 5-6 years. This developmental perspective contributes to resolve apparent discrepancies between single-snapshot studies. A better understanding of the dynamics of sex differences in typical social development should also provide insights into atypical social developments which exhibit sex differences in prevalence, such as autism
SN - 1932-6203
AD - Laboratoire EthoS-Ethologie animale et humaine, UMR 6552-CNRS, Universite de Rennes 1, Rennes, France. stephanie.barbu@univ-rennes1.fr
UR - 21297987
ER -

TY - JOUR
ID - 659
T1 - Barriers to adolescent girls' participation in physical activity defined by physical activity levels
A1 - Zaragoza,J.
A1 - Generelo,E.
A1 - Julian,J.A.
A1 - barca-Sos,A.
Y1 - 2011/03//
N1 - Zaragoza, J. Generelo, E. Julian, J A. Abarca-Sos, A
The Journal of sports medicine and physical fitness
0376337, k6v
IM
Journal Article. Research Support, Non-U.S. Gov't
English
KW - MEDLINE
KW - Adolescent
KW - Adolescent Behavior/ph [Physiology]
KW - Adolescent Behavior/px [Psychology]
KW - Attitude to Health
KW - Child
KW - Environment
KW - Exercise/px [Psychology]
KW - Female
KW - Health Behavior
KW - Humans
KW - Questionnaires

KW - Safety
KW - Spain
KW - Time Factors
RP - NOT IN FILE
SP - 128
EP - 135
JF - Journal of Sports Medicine & Physical Fitness
JA - J Sports Med Phys Fitness
VL - 51
IS - 1
CY - Italy
N2 - AIM: Perceived barriers were the single most powerful predictors of a change in health behaviours and the most consistent negative correlate of children's actual physical activity (PA). The purpose of this study was to compare the perceived barriers of physical activity in 714 adolescent girls defined by physical activity levels. METHODS: The final sample consisted of 714 adolescent, student girls. The age of these students ranged from 12 to 15 years, with a mean age of 13.5 + 0.7 years. Perceived physical activity and perceived barriers to physical activity were measured using the Physical Self-Description Questionnaire and a Scale of Barriers that includes 17 units of barriers divided into two categories: intrapersonal barriers and environmental/contextual barriers. RESULTS: In our study, girls with a low activity level perceived a greater number of barriers than those with a high activity level, more specifically three intrapersonal barriers and two environmental/contextual barriers. Girls with a low activity level reported greater barriers to physical activity than high-active girls for these barriers: don't like physical activity (F=23.405, p=0.001), not good at physical activity and sports (F=24.160, p=0.001), I'm too lazy to do physical activity (F=8.415, P=0.001), it is not safe to do physical activity outdoors (F=11.39, P<0.001) and there is no-one to do physical activity with (F=15.22 P<0.001). CONCLUSION: This study shows the need to learn and effectively use time management strategies that emphasize prioritising and better scheduling of physical activities
SN - 0022-4707
AD - Faculty of Social Sciences and Education, University of Zaragoza, Zaragoza, Spain. zaragoza@unizar.es
UR - 21297572
ER -

TY - JOUR
ID - 660
T1 - An assessment of indoor air concentrations and health risks of volatile organic compounds in three primary schools
A1 - Sofuoglu,S.C.
A1 - Aslan,G.
A1 - Inal,F.
A1 - Sofuoglu,A.
Y1 - 2011/01//
N1 - Sofuoglu, Sait C. Aslan, Guler. Inal, Fikret. Sofuoglu, Aysun
International journal of hygiene and environmental health
do6, 100898843
IM
Journal Article. Research Support, Non-U.S. Gov't
English
KW - MEDLINE
KW - Adolescent
KW - Air Pollutants/an [Analysis]
KW - Air Pollutants/to [Toxicity]
KW - Air Pollution
KW - Indoor/ae [Adverse Effects]
KW - Indoor/an [Analysis]
KW - Benzene/an [Analysis]
KW - Benzene/to [Toxicity]
KW - Carcinogens
KW - Environmental/an [Analysis]

KW - Environmental/to [Toxicity]
KW - Child
KW - Formaldehyde/an [Analysis]
KW - Formaldehyde/to [Toxicity]
KW - Humans
KW - Irritants/an [Analysis]
KW - Irritants/to [Toxicity]
KW - Naphthalenes/an [Analysis]
KW - Naphthalenes/to [Toxicity]
KW - Odors/an [Analysis]
KW - Risk Assessment
KW - Schools
KW - Seasons
KW - Suburban Health
KW - Toluene/an [Analysis]
KW - Toluene/to [Toxicity]
KW - Turkey
KW - Urban Health
KW - Volatile Organic Compounds/an [Analysis]
KW - Volatile Organic Compounds/to [Toxicity]
RP - NOT IN FILE
SP - 36
EP - 46
JF - International Journal of Hygiene & Environmental Health
JA - Int J Hyg Environ Health
VL - 214
IS - 1
CY - Germany
N2 - Concentrations of volatile organic compounds (VOCs), including formaldehyde, in classrooms, kindergartens, and outdoor playgrounds of three primary schools were measured in spring, winter, and fall terms in Izmir, Turkey. A health-risk assessment was conducted for odor detection, sensory irritation, chronic toxic effects, and cancer. Active sampling was applied for VOCs and formaldehyde on Tenax TA and DNPH tubes, respectively. VOCs were analyzed in a thermal desorption-GC-MS system. Formaldehyde analysis was performed using an HPLC instrument. Benzene, toluene, and formaldehyde were the most abundant compounds with 95th percentile indoor air concentrations of 29, 87, and 106 mug/m(3), respectively. Naphthalene and xylenes followed them with an order of magnitude lower concentrations. Two isomers of dichlorobenzene (1,3 and 1,4) were the other notable compounds. The concentrations were utilized to classify the indoor air pollutants with respect to potential health effects. In addition, carcinogenic and chronic toxic risks were estimated using Monte-Carlo simulation. Formaldehyde appears to be the most concerning pollutant with high chronic toxic and carcinogenic risk levels according to the health assessment followed by naphthalene, benzene, and toluene due to their chronic effects. Copyright 2010 Elsevier GmbH. All rights reserved
SN - 1618-131X
AD - Izmir Institute of Technology, Department of Chemical Engineering and Environmental Research Center, Gulbahce, Urla 35430 Izmir, Turkey
UR - 20833585
ER -

TY - JOUR
ID - 661
T1 - Epidemiology of acute soccer injuries in canadian children and youth
A1 - Giannotti,M.
A1 - Al-Sahab,B.
A1 - McFaull,S.
A1 - Tamim,H.
Y1 - 2011/02//
N1 - Giannotti, Maria. Al-Sahab, Ban. McFaull, Steve. Tamim, Hala
Pediatric emergency care

pau, 8507560
IM
Comparative Study. Journal Article
English
KW - MEDLINE
KW - Acute Disease
KW - Adolescent
KW - Age Distribution
KW - Athletic Injuries/di [Diagnosis]
KW - Athletic Injuries/ep [Epidemiology]
KW - Athletic Injuries/th [Therapy]
KW - Canada/ep [Epidemiology]
KW - Child
KW - Preschool
KW - Cohort Studies
KW - Confidence Intervals
KW - Databases
KW - Factual
KW - Emergency Service
KW - Hospital/ut [Utilization]
KW - Female
KW - Fractures
KW - Bone/ep [Epidemiology]
KW - Bone/et [Etiology]
KW - Bone/th [Therapy]
KW - Humans
KW - Incidence
KW - Injury Severity Score
KW - Logistic Models
KW - Male
KW - Patient Admission/sn [Statistics & Numerical Data]
KW - Risk Assessment
KW - Sex Distribution
KW - Soccer/in [Injuries]
KW - Soft Tissue Injuries/ep [Epidemiology]
KW - Soft Tissue Injuries/et [Etiology]
KW - Soft Tissue Injuries/th [Therapy]
KW - Sprains and Strains/ep [Epidemiology]
KW - Sprains and Strains/et [Etiology]
KW - Sprains and Strains/th [Therapy]
KW - Young Adult
RP - NOT IN FILE
SP - 81
EP - 85
JF - Pediatric Emergency Care
JA - Pediatr Emerg Care
VL - 27
IS - 2
CY - United States
N2 - OBJECTIVES: To describe acute injury characteristics in children and youth soccer players and to identify the characteristics of patients who required hospital admission. METHODS: The analysis of the study was based on the Canadian Hospitals Injury Reporting and Prevention Program. A total of 32,149 patients (aged 5-19 years) with soccer-related injuries presenting to 16 participating hospital emergency departments from 1994 to 2004 were included in the analysis. RESULTS: Males had the highest proportion of injuries (62%). The leading injuries were sprains/strains (38%), followed by fractures/dislocations (31%) and superficial injuries (23%). A total of 896 cases (3%) required hospital admission. Based on logistic regression analysis, being a male, playing unorganized soccer, having multiple body injuries, playing soccer outside school premises, and

playing during the summer/fall increased the likelihood of hospital admission. Moreover, having a head/face/neck injury (Odds ratio [OR], 1.3; 95% confidence interval [95% CI], 1.1-1.7) and trunk injury (OR, 1.7; 95% CI, 1.2-2.4) as compared with an upper extremity injury and having injuries from contact with structures/surfaces (OR, 3.1; 95% CI, 2.2-4.3) and with other players (OR, 2.5; 95% CI, 1.8-3.5) as compared with ball contact had the highest odds of hospital admission. CONCLUSIONS: Soccer accounted for a significant proportion of injuries presented to Canadian Hospitals Injury Reporting and Prevention Program emergency departments during 1994-2004. Further studies investigating potential interventional programs and techniques among this population are highly warranted
SN - 1535-1815
AD - School of Kinesiology and Health Science, York University, Toronto, Canada
UR - 21252813
ER -

TY - JOUR
ID - 662
T1 - [Sun protection questionnaire in Buenos Aires adolescent athletes]. [Spanish]
A1 - Laffargue,J.A.
A1 - Merediz,J.
A1 - Bujan,M.M.
A1 - Pierini,A.M.
Y1 - 2011/02//
N1 - Laffargue, Jorge A. Merediz, Javier. Bujan, Maria M. Pierini, Adrian M
Archivos argentinos de pediatria
6q4, 0372460
IM
English Abstract. Journal Article
Spanish
KW - MEDLINE
KW - Adolescent
KW - Argentina
KW - Athletes
KW - Attitude to Health
KW - Child
KW - Cross-Sectional Studies
KW - Female
KW - Humans
KW - Male
KW - Questionnaires
KW - Sunburn/pc [Prevention & Control]
KW - Sunscreening Agents
RP - NOT IN FILE
SP - 30
EP - 35
JF - Archivos Argentinos de Pediatria
JA - Arch Argent.Pediatr
VL - 109
IS - 1
CY - Argentina
N2 - INTRODUCTION: Excessive sun exposure is the major environmental factor in the development of skin cancer. Adolescents who play outdoor sports are highly exposed to ultraviolet rays, which will result in cumulative photodamage with a higher risk of developing skin cancer in adulthood. OBJECTIVES: 1) To evaluate sun protection habits in Buenos Aires adolescent athletes. 2) To identify the characteristics of those adolescents who have developed sunburns. PATIENTS AND METHODS: An observational descriptive cross-sectional study was carried out using a voluntary anonymous written and self-reported survey addressed to adolescent athletes aged 11 to 18 years, who participated in a competitive sporting activity. RESULTS: The study included 554 adolescents. The 26% reported to have taken sunscreen to the competition. The 5.2% reported to have used it on a routine basis during training, and the 6.1% while doing sports. The 45% of the

adolescents referred that sun protection factor rating was not relevant to them. Males most frequently inclined to believe that a good suntan was synonymous of health and women believed that suntan was synonymous of beauty. Most adolescents (73%) had developed at least one sunburn in the previous summer. CONCLUSIONS: In this study, a low compliance with the use of sunscreen was observed in adolescent athletes, despite the high rates of sunburns they had had recently. Nowadays, sun protection campaigns addressed to this age group are urgently needed
SN - 1668-3501
AD - Hospital de Ninos "Pedro de Elizalde", Argentina
UR - 21283941
ER -

TY - JOUR
ID - 663
T1 - Obese adolescents are less active than their normal-weight peers, but wherein lies the difference?
A1 - Olds,T.S.
A1 - Ferrar,K.E.
A1 - Schranz,N.K.
A1 - Maher,C.A.
Y1 - 2011/02//
N1 - Olds, Tim S. Ferrar, Katia E. Schranz, Natasha K. Maher, Carol A
The Journal of adolescent health : official publication of the Society for Adolescent Medicine
a0j, 9102136
IM
Journal Article. Research Support, Non-U.S. Gov't
English
KW - MEDLINE
KW - Adolescent
KW - Anthropometry
KW - Australia
KW - Body Mass Index
KW - Child
KW - Energy Metabolism
KW - Female
KW - Humans
KW - Male
KW - Monitoring
KW - Physiologic/is [Instrumentation]
KW - Physiologic/mt [Methods]
KW - Motor Activity/ph [Physiology]
KW - Obesity/me [Metabolism]
KW - Obesity/th [Therapy]
KW - Sports/sn [Statistics & Numerical Data]
RP - NOT IN FILE
SP - 189
EP - 195
JF - Journal of Adolescent Health
JA - J Adolesc Health
VL - 48
IS - 2
CY - United States
N2 - BACKGROUND: Obese adolescents achieve less moderate to vigorous physical activity (MVPA) than normal-weight adolescents, but the nature and extent of the deficit is unclear. This study aimed to describe differences in MVPA across weight status categories by domain of activity (sport, play, and active transport) and specific activity-sets (e.g. team sports, playground games, and chores) using minutes of activity, estimated intensity, and estimated energy expenditure as metrics of MVPA. METHOD: Anthropometric, use-of-time, and pedometer data were collected on a random sample of 2,200 Australian children aged 9-16 years. Minutes of activity, intensity metabolic equivalents of tasks (METs), and energy expenditure (MET.min) within each

domain were estimated using an energy cost compendium. RESULTS: Obese adolescents reported 174 MET.min/d lower MVPA energy expenditure than normal-weight peers (p < .0001), more than accounting for the entire difference in total daily energy expenditure (154 MET.min/d). Of this difference, 121 MET.min/d was associated with lower sports participation (p < .0001) and 45 MET.min/d with less free play (p = .03). There were no differences in minutes of active transport or in reported activity intensity across weight status categories in sport, free play, or active transport. The differences in MVPA participation between obese and normal-weight adolescents were largely because of different levels of participation in team sports, cycling, partner sports (boys), and dancing (girls). CONCLUSION: More than two-thirds of the difference in energy expenditure between obese and normal-weight Australian adolescents was because of lower participation in sport. Strategies for engaging obese adolescents in sport may help to redress deficits in energy expenditure. Copyright 2011 Society for Adolescent Health and Medicine. Published by Elsevier Inc. All rights reserved
SN - 1879-1972
AD - Health and Use of Time Group, School of Health Sciences, University of South Australia, Adelaide, Australia
UR - 21257119
ER -

TY - JOUR
ID - 664
T1 - [Influence of near-work and outdoor activities on myopia progression in school children]. [Chinese]
A1 - Yi,J.H.
A1 - Li,R.R.
Y1 - 2011/01//
N1 - Yi, Jun-Hui. Li, Rong-Rong
Zhongguo dang dai er ke za zhi = Chinese journal of contemporary pediatrics
100909956
IM
English Abstract. Journal Article. Randomized Controlled Trial
Chinese
KW - MEDLINE
KW - Child
KW - Disease Progression
KW - Female
KW - Humans
KW - Leisure Activities
KW - Male
KW - Myopia/et [Etiology]
KW - Questionnaires
KW - Refraction
KW - Ocular
RP - NOT IN FILE
SP - 32
EP - 35
JF - Zhongguo Dangdai Erke Zazhi
JA - Zhongguo Dang.Dai.Er Ke Za Zhi
VL - 13
IS - 1
CY - China
N2 - OBJECTIVE: To study the influence of near-work and outdoor activities on myopia progression in school children. METHODS: Eighty 7-11-year-old school children with myopia were randomly assigned into an intervention group (n=41) and a control group (n=39). The children in the intervention group did near- and middle-vision activities less than 30 hrs per week and more outdoor activities than 14-15 hrs per week. Myopia progression was observed regularly over 2 years after which ophthalmologists administered questionnaires regarding near-vision work (reading, writing and using computer), middle-vision work (watching TV and extracurricular learning activities), outdoor activities, using nature light, wearing glasses, etc. RESULTS: The annual mean myopia progression (0.38 + 0.15 D) in the intervention group was significantly lower than that in the control group (0.52 + 0.19 D; P<0.01). The children in the two groups spent similar amounts of time in near-

vision activities, but the children in the intervention group spent less time in middle-vision activities (P<0.01) and more outdoor activities (13.7 + 2.4 vs 6.2 + 1.6 hrs/wk; P<0.01). When considering all children in the study, there were 4 factors that significantly correlated with less myopia progression: more outdoor activities, more time spent wearing glasses, more time spent in natural light and less time using a computer. When analyzing the intervention group separately, more outdoor activity was inversely correlated with myopia progression (t=-2.510, P<0.05). Separate analysis of the control group indicated that more time wearing glasses was correlated with less myopia progression (t=-3.115, P<0.05). CONCLUSIONS: Myopia progression in school children may be slowed by more outdoor activities, more time spent in natural light and more time wearing corrective glasses
SN - 1008-8830
AD - Department of Ophthalmology, Third Xiangya Hospital, Central South University, Changsha 410013, China. yijunhui@hotmail.com
UR - 21251384
ER -

TY - JOUR
ID - 665
T1 - Effect of cesium radioisotope on humoral immune status in Ukrainian children with clinical symptoms of irritable bowel syndrome related to Chernobyl disaster
A1 - Sheikh Sajjadieh,M.R.
A1 - Kuznetsova,L.V.
A1 - Bojenko,V.B.
Y1 - 2011/02//
N1 - Sheikh Sajjadieh, M R. Kuznetsova, L V. Bojenko, V B
Toxicology and industrial health
vws, 8602702
IM
Journal Article
English
KW - MEDLINE
KW - Adolescent
KW - B-Lymphocytes/me [Metabolism]
KW - Cesium Radioisotopes/to [Toxicity]
KW - Chernobyl Nuclear Accident
KW - Child
KW - Preschool
KW - Enzyme-Linked Immunosorbent Assay
KW - Humans
KW - Immunity
KW - Humoral/re [Radiation Effects]
KW - Immunoglobulin A/bl [Blood]
KW - Immunoglobulin G/bl [Blood]
KW - Immunoglobulin M/bl [Blood]
KW - Irritable Bowel Syndrome/et [Etiology]
KW - Irritable Bowel Syndrome/im [Immunology]
KW - Linear Models
KW - T-Lymphocytes/me [Metabolism]
KW - Ukraine/ep [Epidemiology]
RP - NOT IN FILE
SP - 51
EP - 56
JF - Toxicology & Industrial Health
JA - Toxicol Ind Health
VL - 27
IS - 1
CY - England
N2 - UNLABELLED: The aim of this study is to determine humoral immune status in Ukrainian children with clinical symptoms of irritable bowel syndrome 23 years after the Chernobyl disaster. Method and material: The

test population consisted of 95 participants: 75 rural patients aged 4-18, who lived in a contaminated area exposed to natural environmental radiation (falling under three groups) and 20 healthy urban participants from Kiev aged 5-15 as a control group. Internal radiation activity has been measured by gamma-ray spectrometry. B-lymphocytes population was analyzed with monoclonal antibody against antigen CD22(+). Serum immunoglobulins were evaluated by enzyme-linked immunosorbent assay (ELISA) method. $p < 0.05$ was considered significant. Result: The percentage of CD22(+) in study groups is increased significantly in comparison to control group at $p < 0.05$. Reduced serum immunoglobulins levels have developed in the majority of the participants. CONCLUSION: Humoral immune status of study groups with clinical symptom of irritable bowel syndrome residing in a contaminated area has changed
SN - 1477-0393
AD - Department of Clinical Immunology and Allergology, National Medical Academy for Post Graduate Education, Kiev, Ukraine. mohammad_esfahan@yahoo.com
UR - 20826551
ER -

TY - JOUR
ID - 666
T1 - Vitamin D receptor gene polymorphism and bone mineral density in 0-6-year-old Han children
A1 - Yu,X.D.
A1 - Shen,X.M.
A1 - Xue,M.B.
A1 - Yan,C.H.
Y1 - 2011/01//
N1 - Yu, Xiao-Dan. Shen, Xiao-Ming. Xue, Ming-Bao. Yan, Chong-Huai
Journal of bone and mineral metabolism
da5, 9436705
IM
Journal Article. Research Support, Non-U.S. Gov't
English
KW - MEDLINE
KW - Bone Density/ge [Genetics]
KW - Child
KW - Preschool
KW - Female
KW - Genotype
KW - Humans
KW - Infant
KW - Newborn
KW - Male
KW - Polymorphism
KW - Genetic/ge [Genetics]
KW - Restriction Fragment Length/ge [Genetics]
KW - Receptors
KW - Calcitriol/ge [Genetics]
RP - NOT IN FILE
SP - 54
EP - 61
JF - Journal of Bone & Mineral Metabolism
JA - J Bone Miner.Metab
VL - 29
IS - 1
CY - Japan
N2 - This study aims to investigate the four vitamin D receptor (VDR) gene single nucleotide polymorphisms and their possible relationship with bone mineral density (BMD) in Chinese 0-6-year-old Han children. Two hundred four 0-6-year-old Han children without metabolic bone disease were randomly recruited in Shanghai, China. The BMD of the middle tibia was measured by an ultrasonic bone density instrument. VDR genotypes were determined by polymerase chain reaction restriction fragment length polymorphism using endonuclease

ApaI, BsmI, TaqI and FokI. The alleles of a, T, b and F and the genotypes of aa, TT, bb and Ff were predominant. The frequency alleles of a, T, b and F were, respectively, 70.6, 95.8, 95.3 and 57.6%. When the influences of confounders such as serum 25(OH)D, serum zinc and outdoor activities on BMD were removed, the genotypes of BsmI and FokI were found apparently to be related to BMD. The BMD of the Bb carrier was much lower than that of the bb carrier (22.00 + 27.84 and 43.14 + 31.98, P < 0.05). The BMD of the ff carrier was lower than that of the Ff or FF carrier (26.97 + 34.22 and 37.95 + 29.70 and 53.52 + 30.35, P < 0.001), while the genotypes of ApaI and TaqI have no relation with BMD in 0-6-year-old Han children. These findings show that the Bb and ff genotypes of the VDR BsmI and FokI variants are significantly associated with a decreased BMD in Chinese Han children aged 0-6 years, while the VDR ApaI and TaqI polymorphisms are not significantly associated with it
SN - 1435-5604
AD - Shanghai Institute of Pediatric Research, Xinhua Hospital, Medical School of Shanghai Jiaotong University, Shanghai, China
UR - 20458603
ER -

TY - JOUR
ID - 667
T1 - Wideband energy reflectance findings in presence of normal tympanogram in children with Down's syndrome
A1 - Kaf,W.A.
Y1 - 2011/02//
N1 - Kaf, Wafaa A
International journal of pediatric otorhinolaryngology
gs2, 8003603
IM
Comparative Study. Journal Article. Research Support, Non-U.S. Gov't
English
KW - MEDLINE
KW - Acoustic Impedance Tests
KW - Case-Control Studies
KW - Causality
KW - Child
KW - Preschool
KW - Down Syndrome/di [Diagnosis]
KW - Down Syndrome/ep [Epidemiology]
KW - Ear Canal/pp [Physiopathology]
KW - Female
KW - Humans
KW - Incidence
KW - Male
KW - Otitis Media/di [Diagnosis]
KW - Otitis Media/ep [Epidemiology]
KW - Otoscopy/mt [Methods]
KW - Reference Values
KW - Risk Assessment
KW - Sensitivity and Specificity
KW - Tympanic Membrane/pp [Physiopathology]
RP - NOT IN FILE
SP - 219
EP - 226
JF - International Journal of Pediatric Otorhinolaryngology
JA - Int J Pediatr Otorhinolaryngol
VL - 75
IS - 2
CY - Ireland

N2 - OBJECTIVES: The prevalence of middle ear disorders in children with Down syndrome is higher than normal children due to the associated craniofacial abnormalities. The goal of this study is to evaluate middle ear function using wideband energy reflectance at ambient pressure in 14 young children with Down syndrome and matched control group (21/2-5 years old; N=19 ears per group) who each have a normal 226Hz tympanogram. METHODS: All children underwent otoscopic examination, hearing screening using play audiometry (500-4000Hz), and middle ear testing using 226Hz tympanometry and wideband energy reflectance. The chirp signals for the wideband energy reflectance were presented to the child's ear at 65dB SPL stimulus level and the recording was done over 220-8000Hz range. The measured energy reflectance represents the ratio of the sound energy reflected from the tympanic membrane to the incident sound energy transmitted to the middle ear at a specific frequency. Paired Samples t-test was computed for the mean, 95th, 75th, 25th, and 5th percentile data of each frequency of the two groups. RESULTS: Despite the presence of normal tympanometric findings in both groups, results revealed abnormal wideband energy reflectance findings in 63% of the children with Down syndrome compared to the normal wideband energy reflectance findings in the control group. The mean energy reflectance ratio of the Down syndrome group was abnormally lower than that of the control at 5700-8000Hz ($p<0.0005$). The 5th and 95th percentile ratios of the Down syndrome group fell outside the 5th and 95th percentile of the control group ($p<0.0005$). CONCLUSIONS: Abnormally low energy reflectance ratios above 4000Hz in the presence of normal tympanograms in the Down syndrome group may suggest associated congenital middle ear anomalies in children with DS. The present findings suggest that wideband energy reflectance has the potential to be of more practical value in children with DS than tympanometry. Further research with a larger number of Down syndrome children will illuminate the potential of wideband energy reflectance in diagnosing middle ear disorders in children with Down syndrome. Copyright A 2010 Elsevier Ireland Ltd. All rights reserved
SN - 1872-8464
AD - Communication Sciences and Disorders Department, Missouri State University, 901 S National Avenue, Springfield, MO 65897, USA. wafaakaf@missouristate.edu
UR - 21129789
ER -

TY - JOUR
ID - 668
T1 - Barriers to and facilitators of healthful eating and physical activity in low-income schools
A1 - Hammerschmidt,P.
A1 - Tackett,W.
A1 - Golzynski,M.
A1 - Golzynski,D.
Y1 - 2011/01//
N1 - Hammerschmidt, Patricia. Tackett, Wendy. Golzynski, Matthew. Golzynski, Diane
Journal of nutrition education and behavior
101132622
IM
Journal Article. Research Support, U.S. Gov't, Non-P.H.S.
English
KW - MEDLINE
KW - Adolescent
KW - Child
KW - Child Nutrition Sciences/ed [Education]
KW - Preschool
KW - Communication Barriers
KW - Consumer Participation
KW - Exercise/ph [Physiology]
KW - Exercise/px [Psychology]
KW - Female
KW - Health Education/mt [Methods]
KW - Health Education/og [Organization & Administration]
KW - Health Promotion/mt [Methods]
KW - Health Promotion/og [Organization & Administration]
KW - Humans

KW - Male
KW - Poverty
KW - Schools
KW - Students/px [Psychology]
KW - Time Factors
RP - NOT IN FILE
SP - 63
EP - 68
JF - Journal of Nutrition Education & Behavior
JA - J Nutr Educ Behav
VL - 43
IS - 1
CY - United States
N2 - OBJECTIVE: To identify barriers to and facilitators of healthful eating and physical activity in low-income schools as perceived by school-affiliated staff. METHODS: Perceptions of barriers and facilitators were measured in low-income schools through two online surveys (Kindergarten-8th grade and high school). Sixty-nine (Kindergarten-8th grade) and 22 (high school) responses were analyzed using descriptive statistics and frequency distributions. RESULTS: Respondents felt students received too little nutrition education, physical activity, and physical education. Time within the school day and lack of funds were barriers. Family programs were highest rated for increasing nutrition education yet reported as implemented the least. CONCLUSIONS AND IMPLICATIONS: Schools are a logical place to promote healthful eating and physical activity; however, time within the school day and lack of funds are major barriers for low-income schools. Health professionals can respect these barriers by holding programs outside of classroom time and finding ways to involve families (eg, during school events). Copyright 2011 Society for Nutrition Education. Published by Elsevier Inc. All rights reserved
SN - 1878-2620
AD - Michigan State University Extension, East Lansing, MI 48824, USA
UR - 20880753
ER -

TY - JOUR
ID - 669
T1 - Ultrafine particle concentrations and exposures in six elementary school classrooms in northern California
A1 - Mullen,N.A.
A1 - Bhangar,S.
A1 - Hering,S.V.
A1 - Kreisberg,N.M.
A1 - Nazaroff,W.W.
Y1 - 2011/02//
N1 - Mullen, N A. Bhangar, S. Hering, S V. Kreisberg, N M. Nazaroff, W W
Indoor air
9423515, da3
IM
Journal Article. Research Support, Non-U.S. Gov't
English
KW - MEDLINE
KW - Air Pollution
KW - Indoor/an [Analysis]
KW - California
KW - Child
KW - Environmental Monitoring
KW - Humans
KW - Particle Size
KW - Schools
KW - Ventilation
RP - NOT IN FILE
SP - 77

EP - 87
JF - Indoor Air
VL - 21
IS - 1
CY - Denmark
N2 - UNLABELLED: Potential health risks may result from environmental exposure to ultrafine particles (UFP), i.e., those smaller than 0.1 mum in diameter. One important exposure setting that has received relatively little attention is school classrooms. We made time-resolved, continuous measurements of particle number (PN) concentrations for 2-4 school days per site (18 days total) inside and outside of six classrooms in northern California during normal occupancy and use. Additional time-resolved information was gathered on ventilation conditions, occupancy, and classroom activity. Across the six classrooms, average indoor PN concentrations when students were present were 5200-16,500/cm(3) (overall average 10,800/cm(3)); corresponding outdoor concentrations were 9000-26,000/cm(3) (overall average 18,100/cm(3)). Average indoor levels were higher when classrooms were occupied than when they were unoccupied because of higher outdoor concentrations and higher ventilation rates during occupancy. In these classrooms, PN exposures appear to be primarily attributable to outdoor sources. Indoor emission sources (candle use, cooking on an electric griddle, use of a heater, use of terpene-containing cleaning products) were seen to affect indoor PN concentrations only in a few instances. The daily-integrated exposure of students in these six classrooms averaged 52,000/cm(3) h/day for the 18 days monitored. PRACTICAL IMPLICATIONS: This study provides data and insight concerning the UFP exposure levels children may encounter within classrooms and the factors that most significantly affect these levels in an urban area in northern California. This information can serve as a basis to guide further study of children's UFP exposure and the potential associated health risks. 2010 John Wiley & Sons A/S
SN - 1600-0668
AD - Department of Civil and Environmental Engineering, University of California, Berkeley, CA, USA
Aerosol Dynamics, Inc., Berkeley, CA, USA
UR - 21029184
ER -

TY - JOUR
ID - 670
T1 - Cerebral reorganization as a function of linguistic recovery in children: An fMRI study
A1 - Elkana,O.
A1 - Frost,R.
A1 - Kramer,U.
A1 - Ben-Bashat,D.
A1 - Hendler,T.
A1 - Schmidt,D.
A1 - Schweiger,A.
Y1 - 2011/02//
N1 - Elkana, Odelia. Frost, Ram. Kramer, Uri. Ben-Bashat, Dafna. Hendler, Talma. Schmidt, David. Schweiger, Avraham
Cortex; a journal devoted to the study of the nervous system and behavior
dsp, 0100725
IM
Journal Article. Research Support, Non-U.S. Gov't
English
KW - MEDLINE
KW - Adolescent
KW - Adult
KW - Age of Onset
KW - Brain Damage
KW - Chronic/pa [Pathology]
KW - Child
KW - Dominance
KW - Cerebral/ph [Physiology]
KW - Female
KW - Functional Laterality/ph [Physiology]

KW - Humans
KW - Image Processing
KW - Computer-Assisted
KW - Intelligence Tests
KW - Language
KW - Language Tests
KW - Linguistics
KW - Longitudinal Studies
KW - Magnetic Resonance Imaging
KW - Male
KW - Middle Cerebral Artery/pa [Pathology]
KW - Neuropsychological Tests
KW - Psychomotor Performance/ph [Physiology]
KW - Reaction Time/ph [Physiology]
KW - Recovery of Function/ph [Physiology]
KW - Young Adult
RP - NOT IN FILE
SP - 202
EP - 216
JF - Cortex
VL - 47
IS - 2
CY - Italy
N2 - Characterizing and mapping the relationship between neuronal reorganization and functional recovery are essential to the understanding of cerebral plasticity and the dynamic processes which occur following brain damage. The neuronal mechanisms underlying linguistic recovery following left hemisphere (LH) lesions are still unknown. Using functional magnetic resonance imaging (fMRI), we investigated whether the extent of brain lateralization of linguistic functioning in specific regions of interest (ROIs) is correlated with the level of linguistic performance following recovery from acquired childhood aphasia. The study focused on a rare group of children in whom lesions occurred after normal language acquisition, but prior to complete maturation of the brain. During fMRI scanning, rhyming, comprehension and verb generation activation tasks were monitored. The imaging data were evaluated with reference to linguistic performance measured behaviorally during imaging, as well as outside the scanner. Compared with normal controls, we found greater right hemisphere (RH) lateralization in patients. However, correlations with linguistic performance showed that increased proficiency in linguistic tasks was associated with greater lateralization to the LH. These results were replicated in a longitudinal case study of a patient scanned twice, 3 years apart. Additional improvement in linguistic performance of the patient was accompanied by increasing lateralization to the LH in the anterior language region. This, however, was the result of a decreased involvement of the RH. These findings suggest that recovery is a dynamic, ongoing process, which may last for years after onset. The role of each hemisphere in the recovery process may continuously change within the chronic stage. Copyright 2009 Elsevier Srl. All rights reserved
SN - 1973-8102
AD - Psychology Department, Hebrew University, Jerusalem, Israel. odelia.elkana@gmail.com
UR - 20138262
ER -

TY - JOUR
ID - 671
T1 - N170 asymmetry as an index of inferior occipital dysfunction in patients with symptomatic occipital lobe epilepsy
A1 - Lopes,R.
A1 - Cabral,P.
A1 - Canas,N.
A1 - Breia,P.
A1 - Foreid,J.P.
A1 - Calado,E.
A1 - Silva,R.

A1 - Leal,A.
Y1 - 2011/01//
N1 - Lopes, Ricardo. Cabral, Pedro. Canas, Nuno. Breia, Paula. Foreid, John P. Calado, Eulalia. Silva, Rita. Leal, Alberto
Clinical neurophysiology : official journal of the International Federation of Clinical Neurophysiology
db4, 100883319
IM
Journal Article. Research Support, Non-U.S. Gov't
English
KW - MEDLINE
KW - Adult
KW - Brain Waves/ph [Physiology]
KW - Child
KW - Preschool
KW - Diagnosis
KW - Differential
KW - Electroencephalography/mt [Methods]
KW - Epilepsies
KW - Partial/di [Diagnosis]
KW - Partial/pp [Physiopathology]
KW - Evoked Potentials/ph [Physiology]
KW - Female
KW - Humans
KW - Male
KW - Middle Aged
KW - Occipital Lobe/pp [Physiopathology]
KW - Predictive Value of Tests
KW - Reaction Time/ph [Physiology]
KW - Temporal Lobe/pp [Physiopathology]
KW - Young Adult
RP - NOT IN FILE
SP - 9
EP - 15
JF - Clinical Neurophysiology
JA - Clin Neurophysiol
VL - 122
IS - 1
CY - Netherlands
N2 - OBJECTIVE: Localizing epileptic foci in posterior brain epilepsy remains a difficult exercise in surgery for epilepsy evaluation. Neither clinical manifestations, neurological, EEG nor neuropsychological evaluations provide strong information about the area of onset, and fast spread of paroxysms often produces mixed features of occipital, temporal and parietal symptoms. We investigated the usefulness of the N170 event-related potential to map epileptic activity in these patients. METHODS: A group of seven patients with symptomatic posterior cortex epilepsy were submitted to a high-resolution EEG (78 electrodes), with recordings of interictal spikes and face-evoked N170. Generators of spikes and N170 were localized by source analysis. Range of normal N170 asymmetry was determined in 30 healthy volunteers. RESULTS: In 3 out of 7 patients the N170 inter-hemispheric asymmetry was outside control values. Those were the patients whose spike sources were nearest (within 3cm) to the fusiform gyrus, while foci further away did not affect the N170 ratio. CONCLUSIONS: N170 event-related potential provides useful information about focal cortical dysfunction produced by epileptic foci located in the close neighborhood of the fusiform gyrus, but are unaffected by foci further away. SIGNIFICANCE: The N170 evoked by faces can improve the epileptic foci localization in posterior brain epilepsy. Copyright 2010 International Federation of Clinical Neurophysiology. Published by Elsevier Ireland Ltd. All rights reserved
SN - 1872-8952
AD - Department of Pediatric Neurology, Hospital Center of Central Lisbon, Rua Jacinta Marto, 1169-045 Lisbon, Portugal. r.lopes@clix.pt
UR - 20566302

ER -

TY - JOUR
ID - 672
T1 - The 'dark' side of sedation: 12 years of office-based pediatric deep sedation for electroretinography in the dark
A1 - Lalwani,K.
A1 - Tompkins,B.D.
A1 - Burnes,K.
A1 - Krahmer,M.R.
A1 - Pennesi,M.E.
A1 - Weleber,R.G.
Y1 - 2011/01//
N1 - Lalwani, Kirk. Tompkins, Brian D. Burnes, Kevin. Krahmer, Melissa R. Pennesi, Mark E. Weleber, Richard G
Paediatric anaesthesia
cg8, 9206575
IM
Journal Article. Multicenter Study
English
KW - MEDLINE
KW - Airway Management
KW - Anesthesia Recovery Period
KW - Child
KW - Preschool
KW - Darkness
KW - Deep Sedation/ae [Adverse Effects]
KW - Deep Sedation
KW - Electroretinography/mt [Methods]
KW - Female
KW - Humans
KW - Hypnotics and Sedatives/ad [Administration & Dosage]
KW - Hypnotics and Sedatives/ae [Adverse Effects]
KW - Infant
KW - Infusions
KW - Intravenous
KW - Male
KW - Patient Discharge
KW - Propofol/ad [Administration & Dosage]
KW - Propofol/ae [Adverse Effects]
KW - Prospective Studies
KW - Retinal Rod Photoreceptor Cells/ph [Physiology]
KW - Vision Disorders/di [Diagnosis]
RP - NOT IN FILE
SP - 65
EP - 71
JF - Paediatric Anaesthesia
JA - Paediatr Anaesth
VL - 21
IS - 1
CY - France
N2 - OBJECTIVES: Analyze pediatric ERG data for adverse events, interventions, and outcomes of propofol sedations performed in near-complete darkness. AIM: To demonstrate that deep sedation with propofol for ERG can be performed efficiently and safely in children in near-total darkness. BACKGROUND: Full-field electroretinography (ERG) is a valuable tool for the diagnosis of vision loss in children. The ERG measures the electrical activity of the retina. In children, ERG quality significantly improves with deep sedation by allowing easier eye electrode placement and decreasing motion artifacts. As this procedure must be performed in

darkness, administering sedation imposes unique challenges. METHODS AND MATERIALS: ERGs are performed outside of the operating room in our hospital's electrophysiology suite. IVs are placed, and patients are allowed to adapt to complete darkness. An anesthesiologist then administers propofol sedation in the dark with the aid of a red-filter light source and monitor light shields. Data were collected on 379 patients (411 ERGs) performed from 1996 to 2008. These records were reviewed and analyzed for demographic, medical, and anesthetic data. RESULTS: Propofol sedation resulted in an ERG completion rate of 99.5%. During sedation, 8.5% (35) of patients experienced minor respiratory complications such as airway obstruction that resulted in an oxygen saturation <90%. A total of 9.7% (40) of patients required minor airway interventions such as a chin lift. CONCLUSIONS: We demonstrated that pediatric sedation is a safe, efficient, and a cost-effective method for measuring ERGs in a challenging environment. The incidence of minor complications is low and appears similar to other studies of propofol sedation. 2010 Blackwell Publishing Ltd
SN - 1460-9592
AD - Department of Anesthesiology and Perioperative Medicine, Oregon Health & Science University, Doernbecher Children's Hospital, Portland, OR 97239, USA. lalwanik@ohsu.edu
UR - 21155929
ER -

TY - JOUR
ID - 673
T1 - Exertional heat-related injuries treated in emergency departments in the U.S., 1997-2006
A1 - Nelson,N.G.
A1 - Collins,C.L.
A1 - Comstock,R.D.
A1 - McKenzie,L.B.
Y1 - 2011/01//
N1 - Nelson, Nicolas G. Collins, Christy L. Comstock, R Dawn. McKenzie, Lara B
American journal of preventive medicine
8704773, apl
IM
Journal Article
English
KW - MEDLINE
KW - Adolescent
KW - Adult
KW - Age Factors
KW - Aged
KW - 80 and over
KW - Athletic Injuries/ep [Epidemiology]
KW - Athletic Injuries/pp [Physiopathology]
KW - Child
KW - Preschool
KW - Emergency Service
KW - Hospital/sn [Statistics & Numerical Data]
KW - Exercise
KW - Female
KW - Heat Stress Disorders/ep [Epidemiology]
KW - Heat Stress Disorders/et [Etiology]
KW - Heat Stress Disorders/pp [Physiopathology]
KW - Humans
KW - Infant
KW - Linear Models
KW - Male
KW - Middle Aged
KW - Retrospective Studies
KW - Risk
KW - United States/ep [Epidemiology]
KW - Young Adult

RP - NOT IN FILE
SP - 54
EP - 60
JF - American Journal of Preventive Medicine
JA - Am J Prev Med
VL - 40
IS - 1
CY - Netherlands
N2 - BACKGROUND: Exertional heat-related injuries are a risk to all physically active individuals in warm or hot environments. Unlike classic heat-related injury, exertional heat-related injuries do not require extreme ambient temperatures to cause injury. Still, exertional heat-related injuries, including heat cramps, heat syncope, heat exhaustion, heat stress, and heat stroke, can result in injuries causing a range of outcomes from minimal discomfort to death. PURPOSE: The purpose of this paper was to describe the epidemiology of exertional heat-related injuries treated in U.S. emergency departments. METHODS: A retrospective analysis was conducted using data from the National Electronic Injury Surveillance System of the U.S. Consumer Product Safety Commission for all ages from 1997 through 2006. Data provided by the National Electronic Injury Surveillance System were used to calculate national estimates of exertional heat-related injuries. Trends of exertional heat-related injuries over time were analyzed using linear regression. RESULTS: Nationally, an estimated 54,983 (95% CI=39995, 69970) patients were treated in U.S. emergency departments for exertional heat-related injuries from 1997 to 2006. The number of exertional heat-related injuries increased significantly from 3192 in 1997 to 7452 in 2006 (p=0.002), representing a 133.5% increase. The overall exertional heat-related injury rate per 100,000 U.S. population more than doubled from 1.2 in 1997 to 2.5 in 2006 (p=0.005). Patients aged <19 years accounted for the largest proportion of exertional heat-related injuries (47.6%). The majority of exertional heat-related injuries were associated with performing a sport or exercising (75.5%) and yard work (11.0%). The majority of patients (90.4%) were treated and released from the emergency department. Patients aged <19 years sustained a larger proportion of sports and recreation exertional heat-related injuries, whereas patients aged 40-59 years and >60 years sustained a larger proportion of exertional heat-related injuries from yard work. CONCLUSIONS: This study confirms that although there is a risk of exertional heat-related injury among all physically active individuals, sports pose a specific risk for people of all ages especially among children and adolescents playing football. Many "everyday" activities such as yard work and home maintenance also pose risks of exertional heat-related injury, particularly to those aged >40 years. Further research on risk factors of exertional heat-related injuries during home maintenance and yard work as well as appropriate prevention practices is needed. Copyright 2011 American Journal of Preventive Medicine. Published by Elsevier Inc. All rights reserved
SN - 1873-2607
AD - Center for Injury Research and Policy, The Research Institute at Nationwide Children's Hospital, Columbus, Ohio 43205, USA
UR - 21146768
ER -

TY - JOUR
ID - 674
T1 - [Applicability of Children's Environment and Health Action Plan in Serbia]. [Serbian]
A1 - Ilic,M.K.
Y1 - 2010/11//
N1 - Ilic, Miroslava Kristoforovic
Medicinski pregled
0134461, 2985249r, m8u
IM
English Abstract. Journal Article
Serbian
KW - MEDLINE
KW - Child
KW - Child Welfare
KW - Environmental Health
KW - Humans
KW - Public Health

KW - Serbia
KW - World Health Organization
RP - NOT IN FILE
SP - 789
EP - 792
JF - Medicinski Pregled
JA - Med Pregl.
VL - 63
IS - 11-12
CY - Serbia
N2 - The Children's Environment and Health Action Plan for Europe was adopted at the 4th Ministry Conference on Environment (the World Health Organization, 2004). It is focused on children health care against hazards originating from the human environment. In its conclusion, the need is expressed for the development of national plans in the field of Environmental and Children Health for European region by 2007. Mutual activities would be obligatory for each country and their realization should be the responsibility of Ministers of Health Care and Environmental Health. In our country, a draft version of this document was recently adopted, where the following priority regional goals are proposed: safe drinking water and adequate sanitation, injury prevention and adequate physical activity, clean indoor and outdoor air, the human environment without chemicals. Every segment has been explained in details through activities, expected results, indicators, sources of verification and the main participants in the project implementation. The end of the action plan period is proposed to be the year 2019. It is also followed by a defined set of indicators: exposure, activities and health status. The analyses of particular activities or data to be used have pointed to some drawbacks of this draft version, which can be overcome by respecting expert opinions
SN - 0025-8105
AD - Medicinski fakultet Novi Sad. kristof@eunet.rs
UR - 21553456
ER -

TY - JOUR
ID - 675
T1 - Continual infusion of intrathecal baclofen (ITB): long-term effect on spasticity
A1 - Al,Khudhairi D.
A1 - Aldin,A.S.
A1 - Hamdan,Y.
A1 - Rababah,A.
A1 - Matthana,M.H.
A1 - Pazdirek,J.
A1 - Abdulsalam,J.
Y1 - 2010/10//
N1 - Al Khudhairi, Dhafir. Aldin, Abdulrahman Shug'a. Hamdan, Yousef. Rababah, Abdullah. Matthana, Mona Hafez. Pazdirek, Jiri. Abdulsalam, Jalal
Middle East journal of anesthesiology
8604187
IM
Journal Article
English
KW - MEDLINE
KW - Adolescent
KW - Adult
KW - Baclofen/ad [Administration & Dosage]
KW - Child
KW - Female
KW - Humans
KW - Infusion Pumps
KW - Implantable
KW - Injections
KW - Spinal

KW - Male
KW - Middle Aged
KW - Muscle Relaxants
KW - Central/ad [Administration & Dosage]
KW - Muscle Spasticity/dt [Drug Therapy]
KW - Time
KW - Treatment Outcome
KW - Young Adult
RP - NOT IN FILE
SP - 851
EP - 855
JF - Middle East Journal of Anesthesiology
JA - Middle East J Anesthesiol
VL - 20
IS - 6
CY - Lebanon
N2 - BACKGROUND: Spasticity is a disorder of muscular function causing muscular tightness or spasm which occurs when there is damage to the central nervous system whether it is of spinal cord or brain origin. This insult could be either pathological or traumatic. METHOD: Thirty-three patients had intrathecal Baclofen pumps implanted for severe spasticity. These patients either did not respond to or tolerate oral medications. RESULTS: All patients showed significant improvement in their spasms following the procedure. Improvement was noted not only in spasticity, but also in pain, management of sleep disturbance, activities of daily living, indoor and outdoor mobility and behavior. CONCLUSION: The number of complications was acceptable, and generally were not life-threatening. Infection was the significant complication in two patients and this led to explantation of their pumps. Patient satisfaction was very high and was related to improvement in the quality of life for the patients
SN - 0544-0440
AD - Anaesthesia Department, Prince Sultan Cardiac Centre, Consultant Chronic Pain Management, Riyadh Military Hospital and Sultan Bin Abdulaziz Humanitarian City, Riyadh, Kingdom of Saudi Arabia. psccanaes@yahoo.com
UR - 21526672
ER -

TY - JOUR
ID - 676
T1 - Acute skin sun damage in children and its consequences in adults. [Review]
A1 - Pustisek,N.
A1 - Sikanic-Dugic,N.
A1 - Hirsl-Hecej,V.
A1 - Domljan,M.L.
Y1 - 2010/04//
N1 - Pustisek, Nives. Sikanic-Dugic, Nives. Hirsl-Hecej, Vlasta. Domljan, Mislav Luka
Collegium antropologicum
8003354
IM
Journal Article. Review
English
KW - MEDLINE
KW - Acute Disease
KW - Adult
KW - Child
KW - Chronic Disease
KW - Humans
KW - Melanoma/ep [Epidemiology]
KW - Melanoma/pc [Prevention & Control]
KW - Risk Factors
KW - Skin Aging/pa [Pathology]

KW - Skin Neoplasms/ep [Epidemiology]
KW - Skin Neoplasms/pc [Prevention & Control]
KW - Sunscreening Agents/tu [Therapeutic Use]
RP - NOT IN FILE
SP - 233
EP - 237
JF - Collegium Antropologicum
JA - Coll Antropol
VL - 34 Suppl 2
CY - Croatia
N2 - Children spend more time outdoors than adults and there is compelling evidence that childhood is a particularly vulnerable time for the photocarcinogenic effects of the sun. The negative effects of solar radiation are accumulated during the entire lifetime; however 80% of total lifetime sun exposure is taking place before the age of 18 years. Child skin is more sensitive than adult skin because natural defense mechanisms are not fully developed. A short exposure to midday sun will result in sunburns. Epidemiologic studies show a higher incidence of malignant melanoma in persons with a history of sunburns during childhood and adolescence. Sun exposure among infants and pre-school children is largely dependent on the discretion of adult care providers. Sun protective habits of mothers may predict the level of sun exposure in children. It is very important to transfer the knowledge and positive habits of proper sun protection to children. The purpose of sun-safety behavior is not to avoid outdoor activities, but rather to protect the skin from detrimental sun effects. Proper sun protection of children includes protection from excessive sun exposure, sunburns and other forms of skin damage caused by sun, which may lead to the future development of skin cancers. This paper reviews acute skin reactivity to sun in childhood and adolescence that causes damage in skin structure and function and produces undesirable chronic changes in adults
SN - 0350-6134
AD - Department of Reproductive Health, Children's Hospital Zagreb, Medical School, University of Zagreb, Zagreb Croatia. nives.pustisek@kdb.hr
UR - 21302727
ER -

TY - JOUR
ID - 677
T1 - Sport education and extracurricular sport participation: an examination using the trans-contextual model of motivation
A1 - Wallhead,T.L.
A1 - Hagger,M.
A1 - Smith,D.T.
Y1 - 2010/12//
N1 - Wallhead, Tristan L. Hagger, Martin. Smith, Derek T
Research quarterly for exercise and sport
r6y, 8006373
IM
Journal Article. Research Support, N.I.H., Extramural. Research Support, Non-U.S. Gov't
English
KW - MEDLINE
KW - Adolescent
KW - Chi-Square Distribution
KW - Child
KW - Female
KW - Humans
KW - Male
KW - Models
KW - Theoretical
KW - Motivation
KW - Peer Group
KW - Personal Autonomy
KW - Prospective Studies

KW - Questionnaires
KW - Social Support
KW - Sports/ph [Physiology]
KW - Students
RP - NOT IN FILE
SP - 442
EP - 455
JF - Research Quarterly for Exercise & Sport
JA - Res Q Exerc Sport
VL - 81
IS - 4
CY - United States
N2 - In this study, we used the trans-contextual model of motivation (TCM) to examine the effect of Sport Education (SE) on students' participation in a voluntary lunch recess sport club. A total of 192 participants (ages 9-14 years) completed measures of the TCM constructs before and after a 12-week SE intervention period. Participants had the opportunity to participate in weekly, voluntary lunch recess sport club sessions during the intervention period. SE elicited a moderate increase in autonomous motives in physical education. The TCM accounted for a significant proportion of the explained variance in lunch recess sport club intention and participation. Autonomy-supportive curricular models, such as SE, may have the potential to facilitate transfer of motivation and participation in physical activity from a physical education to an extracurricular context
SN - 0270-1367
AD - Division of Kinesiology and Health, University of Wyoming, Laramie 82071, USA. wallhead@uwyo.edu
UR - 21268468
ER -

TY - JOUR
ID - 678
T1 - Overweight and obesity among schoolchildren in Manipur, India
A1 - Bishwalata,R.
A1 - Singh,A.B.
A1 - Singh,A.J.
A1 - Devi,L.U.
A1 - Singh,R.K.
Y1 - 2010/09//
N1 - Bishwalata, Rajkumari. Singh, Akoijam Brogen. Singh, Akoijam Joy. Devi, Longjam Usharani. Singh, R K Bikramjit
The National medical journal of India
bnt, 8809315
IM
Journal Article
English
KW - MEDLINE
KW - Adolescent
KW - Body Mass Index
KW - Child
KW - Cross-Sectional Studies
KW - Female
KW - Humans
KW - India/ep [Epidemiology]
KW - Life Style
KW - Male
KW - Obesity/ep [Epidemiology]
KW - Overweight/ep [Epidemiology]
KW - Prevalence
KW - Risk Factors
KW - Young Adult
RP - NOT IN FILE

SP - 263
EP - 266
JF - National Medical Journal of India
JA - Natl Med J India
VL - 23
IS - 5
CY - India
N2 - BACKGROUND: The prevalence of overweight and obesity and their associated factors among schoolchildren in Manipur has not been studied. METHODS: We did a cross-sectional study among 3356 schoolchildren of classes VIII to XII in Imphal West district, Manipur between September 2005 and August 2006. The characteristics of the respondents and related variables such as parental build, watching television, eating habits, playing of video/computer games and outdoor games, dietary pattern and knowledge of obesity were assessed using a questionnaire. Height, weight, waist circumference, hip circumference, fat percentage, fat mass and fat-free mass were measured. Body mass index (BMI) and waist-hip ratio for each student were calculated. RESULTS: The BMI of the sampled students was lower than the corresponding WHO and International Obesity Task Force standards. Using the WHO standard, the prevalence of overweight was 4.2% and of obesity 0.8%. Using multivariate logistic regression, mother being reported to be obese (OR 1.9, 95% CI 1.4-2.6), watching television for > 2 hours a day (OR 2.052, 95% CI 1.191-3.536), higher family income (OR 5.844, 95% CI 2.135-15.99), not eating other type of vegetables in the past 1 week (OR 2.338, 95% CI 1.04-5.24) and waist-hip ratio (OR 7.737, 95% CI 4.429-13.51) were found to be independent predictors of a higher BMI. Mother's literacy below class X (OR 0.6, 95% CI 0.378-0.997) and eating between major meals (OR 0.447, 95% CI 0.293-0.681) were significant predictors of a lower BMI. CONCLUSION: The prevalence of overweight and obesity among schoolchildren in the Imphal West district of Manipur is low. The possible reasons for this include a more traditional low-fat diet, less exposure to sedentary past-times and a greater time spent playing outdoors
SN - 0970-258X
AD - Manipur Health Services, Government Polytechnic, Takyelpat, Imphal, Manipur, India. dr.bishwalata@rediffmail.com
UR - 21250579
ER -

TY - JOUR
ID - 679
T1 - Energy expenditure and perceived effort during brisk walking and running in 8- to 10-year-old children
A1 - Ford,P.
A1 - Bailey,R.
A1 - Coleman,D.
A1 - Stretch,D.
A1 - Winter,E.
A1 - Woolf-May,K.
A1 - Swaine,I.
Y1 - 2010/11//
N1 - Ford, Paul. Bailey, Richard. Coleman, Damian. Stretch, Daniel. Winter, Edward. Woolf-May, Kate. Swaine, Ian
Pediatric exercise science
8909729
IM
Comparative Study. Journal Article
English
KW - MEDLINE
KW - Anthropometry
KW - Child
KW - Energy Metabolism
KW - Female
KW - Humans
KW - Male
KW - Running/ph [Physiology]

KW - Statistics
KW - Nonparametric
KW - Walking/ph [Physiology]
RP - NOT IN FILE
SP - 569
EP - 580
JF - Pediatric Exercise Science
JA - Pediatr Exerc Sci
VL - 22
IS - 4
CY - United States
N2 - There are no previous reports of energy expenditure and perceived effort during brisk-walking and running at speeds self-selected by young children. Fifty four participants (age 8-11 years old) performed 1500 m of brisk-walking and running in a marked school playground, and were given simple instructions to either 'walk quickly' or to 'jog'. During the running the children achieved higher mean speeds and a greater total energy expenditure ($p < .001$). However, there was no difference in the perceived effort between the two activities ($p > .05$). These findings suggest that under certain conditions children find it just as easy to run as they do to walk briskly, even though the speed and energy expenditure is significantly higher
SN - 0899-8493
AD - School of Health and Bioscience, University of East London, Stratford Campus, London, England, UK
UR - 21242606
ER -

TY - JOUR
ID - 680
T1 - Characterization of personal exposure concentration of fine particles for adults and children exposed to high ambient concentrations in Beijing, China
A1 - Du,X.
A1 - Kong,Q.
A1 - Ge,W.
A1 - Zhang,S.
A1 - Fu,L.
Y1 - 2010///
N1 - Du, Xuan. Kong, Qian. Ge, Weihua. Zhang, Shaojun. Fu, Lixin
Journal of environmental sciences (China)
100967627, dvr
IM
Journal Article. Research Support, Non-U.S. Gov't
English
KW - MEDLINE
KW - Adult
KW - Air Pollutants/an [Analysis]
KW - Air Pollution
KW - Indoor/an [Analysis]
KW - Child
KW - China
KW - Environmental Exposure/an [Analysis]
KW - Environmental Monitoring
KW - Female
KW - Humans
KW - Male
KW - Particle Size
RP - NOT IN FILE
SP - 1757
EP - 1764
JF - Journal of Environmental Sciences (China)
JA - J Environ Sci (China)

VL - 22
IS - 11
CY - Netherlands
N2 - In China, the health risk from overexposure to particles is becoming an important public health concern. To investigate daily exposure characteristics to PM2.5 with high ambient concentration in urban area, a personal exposure study was conducted for school children, and office workers in Beijing, China. For all participants (N = 114), the mean personal 24-hr exposure concentration was 102.5, 14.7, 0.093, 0.528, 0.934, 0.174 and 0.703 microg/m3 for PM2.5, black carbon, Mn, Al, Ca, Pb, and Fe. Children's exposure concentrations of PM2.5 were 4-5 times higher than those in related studies. The ambient concentration of PM2.5 (128.5 microg/m3) was significantly higher than the personal exposure concentration (P < 0.05), and exceed the reference concentration (25 microg/m3) of WHO air quality guideline. Good correlation relationships and significant differences were identified between ambient concentration and personal exposure concentration. The relationships indicate that the ambient concentration is the main factor influencing personal exposure concentration, but is not a good indicator of personal exposure concentration. Outdoor activities (commute mode, exposure to heating, workday or weekend travel) influenced personal exposure concentrations significantly, but the magnitude of the influence from indoor activities (exposure to cooking) was masked by the high ambient concentrations
SN - 1001-0742
AD - Department of Environmental Science and Engineering, Tsinghua University, Beijing 100084, China. duxuan@mails.thu.edu.cn
UR - 21235164
ER -

TY - JOUR
ID - 681
T1 - "Sun Safe Kids," implementing a low cost, school-based public policy to protect Hawaii's children from skin cancer risks
A1 - Cassel,K.D.
Y1 - 2010/11//
N1 - Cassel, Kevin D
Hawaii medical journal
g1d, 2984209r
IM
Journal Article
English
KW - MEDLINE
KW - Child
KW - Hawaii
KW - Health Education/og [Organization & Administration]
KW - Humans
KW - Public Policy
KW - School Health Services/og [Organization & Administration]
KW - Skin Neoplasms/et [Etiology]
KW - Skin Neoplasms/pc [Prevention & Control]
KW - Sunlight/ae [Adverse Effects]
RP - NOT IN FILE
SP - 274
EP - 277
JF - Hawaii Medical Journal
JA - Hawaii Med J
VL - 69
IS - 11
CY - United States
N2 - The rates of melanomas and skin cancers are increasing in the United States. Children attending elementary schools are in the most danger of acquiring these diseases later in life, and elementary school children in Hawaii have the greatest risk of all children in the United States. The parents and educators of Hawaii's elementary school age children are unaware of the potential risks for cancer that young children experience every day at school. Effective sun protection policies have been implemented in other jurisdictions,

including Australia, that have similar risks for over-exposure to solar ultraviolet radiation in children. These proven policy models can inform sun protection practices in Hawaii. A simple policy whereby public elementary schools require that children wear ordinary long sleeves shirts and hats during the school's outdoor activities will protect Hawaii's children from overexposure to sun's ultraviolet radiation. Establishment of a state law codifying the implementation of this simple, yet scientifically proven strategy into the policies of Hawaii's public elementary schools can significantly reduce the incidence and deaths from melanoma and skin cancer in the state. Hawaii Medical Journal Copyright 2010
SN - 0017-8594
AD - John A. Burns School of Medicine, University of Hawaii, Honolulu, Hawaii 96813, USA
UR - 21218379
ER -

TY - JOUR
ID - 682
T1 - [Association between socioeconomic status and physical activities in Chinese children]. [Chinese]
A1 - Wu,S.S.
A1 - Wang,H.J.
A1 - Li,B.H.
A1 - Li,S.S.
A1 - Ma,J.
Y1 - 2010/05//
N1 - Wu, Shuang-sheng. Wang, Hai-jun. Li, Bai-hui. Li, Shan-shan. Ma, Jun
Zhonghua liu xing bing xue za zhi = Zhonghua liuxingbingxue zazhi
cqg, 8208604
IM
English Abstract. Journal Article. Research Support, Non-U.S. Gov't
Chinese
KW - MEDLINE
KW - Child
KW - China
KW - Exercise
KW - Family
KW - Female
KW - Humans
KW - Male
KW - Questionnaires
KW - Social Class
KW - Sports
KW - Students/px [Psychology]
RP - NOT IN FILE
SP - 513
EP - 516
JF - Chung-Hua Liu Hsing Ping Hsueh Tsa Chih Chinese Journal of Epidemiology
JA - Chung Hua Liu Hsing Ping Hsueh Tsa Chih
VL - 31
IS - 5
CY - China
N2 - OBJECTIVE: To study the daily physical activity patterns of pupils in Beijing, and to identify the association between the socioeconomic status (SES) and physical activities. METHODS: 1502 pupils were recruited from 2 - 5 grades of eight primary schools in Beijing. Information on physical activity, sedentary activity, and transportation styles inside and outside schools were collected using a "7-day physical activity questionnaire". RESULTS: Gymnastics, running, playing games, walking in queues etc. were the most common activities at school, with the proportion as 94.13%, 85.55%, 77.26%, and 71.32% respectively. Physical labor, playing games, running and walking were the common activities outside schools, with the proportion as 72.14%, 69.70%, 65.05%, and 64.64% respectively. However, pupils seldom participated in the ball games. Significant differences between SES groups were observed on the patterns of both physical and sedentary activities. Compared to low-SES pupils, high-SES pupils more frequently participated in dancing, walking

upstairs or downstairs, swimming, skating, but less frequently (P < 0.05) in walking, rope skipping, kicking shuttlecock or rubber band skipping. CONCLUSION: Data from our study showed that both the quantity and the distribution of different activities did not meet the need of the Chinese children. Physical activity in association with SES was also demonstrated
SN - 0254-6450
AD - School of Public Health/Insitute of Child and Adolescent, Peking University, Beijing 100191, China
UR - 21163027
ER -

TY - JOUR
ID - 683
T1 - Seizures temporally associated with nitrous oxide administration for pediatric procedural sedation
A1 - Zier,J.L.
A1 - Doescher,J.S.
Y1 - 2010/12//
N1 - Zier, Judith L. Doescher, Jason S
Journal of child neurology
ia2, 8606714
IM
Case Reports. Journal Article
English
KW - MEDLINE
KW - Anesthetics
KW - Inhalation/ae [Adverse Effects]
KW - Child
KW - Preschool
KW - Conscious Sedation/ae [Adverse Effects]
KW - Conscious Sedation/mt [Methods]
KW - Conscious Sedation
KW - Female
KW - Humans
KW - Infant
KW - Male
KW - Nitrous Oxide/ae [Adverse Effects]
KW - Pediatrics/mt [Methods]
KW - Seizures/ci [Chemically Induced]
KW - Seizures/di [Diagnosis]
KW - Seizures/ep [Epidemiology]
KW - Time Factors
RP - NOT IN FILE
SP - 1517
EP - 1520
JF - Journal of Child Neurology
JA - J Child Neurol
VL - 25
IS - 12
CY - United States
N2 - Nitrous oxide is an inhaled agent commonly used by dental staff to provide anxiolysis and analgesia for dental procedures and by anesthesia personnel as an adjunct to more potent general anesthetic gases. More recently, nitrous oxide has been used to provide sedation/analgesia for a variety of medical procedures in children outside of the operating room, including lumbar puncture, laceration repair, fracture reduction, and urologic imaging. We report 3 cases of clinical seizure activity associated with nitrous oxide administration for pediatric procedural sedation. Although temporally related, no causality is established. Review of the medical and dental literature confirm the rarity of these events
SN - 1708-8283
AD - Department of Pediatric Critical Care, Children's Hospitals and Clinics of Minnesota, St Paul, Minnesota, USA. judy.zier@childrensmn.org

UR - 20519672
ER -

TY - JOUR
ID - 684
T1 - Identifying what pediatric residents are taught about children and youth with special health care needs and the medical home
A1 - Nazarian,B.L.
A1 - Glader,L.
A1 - Choueiri,R.
A1 - Shipman,D.L.
A1 - Sadof,M.
Y1 - 2010/12//
N1 - Nazarian, Beverly L. Glader, Laurie. Choueiri, Roula. Shipman, Deborah L. Sadof, Matthew
Pediatrics
oxv, 0376422
AIM, IM
Journal Article. Research Support, U.S. Gov't, P.H.S.
English
KW - MEDLINE
KW - Adolescent
KW - Child
KW - Disabled Children
KW - Humans
KW - Internship and Residency
KW - Patient-Centered Care
KW - Pediatrics/ed [Education]
RP - NOT IN FILE
SP - S183
EP - S189
JF - Pediatrics
VL - 126 Suppl 3
CY - United States
N2 - OBJECTIVE: To describe what and how pediatric residents in Massachusetts are taught about children and youth with special health care needs (CYSHCN) and the medical home. PARTICIPANTS AND METHODS: Faculty members and residents at Massachusetts' 5 pediatric residency programs were interviewed to identify current curricula and teaching methods related to care of CYSHCN. In addition, residents were surveyed to quantify these concepts. RESULTS: Thirty-one faculty members and 25 residents were interviewed. Most exposure to CYSHCN was reported to occur in inpatient settings. However, most formal teaching about CYSHCN was described as occurring in the ambulatory setting. Promising educational strategies included home and community visits, inclusion of CYSHCN in resident continuity panels, and simulation and role-playing. Overall, the programs had little training emphasis on the lives and needs of CYSHCN and their families outside the hospital setting. Twenty (80%) of the residents interviewed completed the written survey instrument. They noted a high degree of comfort in caring for CYSHCN in various settings and involving families in decision-making about their child's care but expressed less comfort in identifying community resources and collaborating with community agencies and schools. CONCLUSIONS: Programs offer a variety of successful educational and clinical experiences related to the medical home and CYSHCN. The results of our study indicate that residents and faculty members believe that residents would benefit from more formal training opportunities to learn directly from families and community representatives about caring for CYSHCN
SN - 1098-4275
AD - UMass Memorial Children's Medical Center, 55 Lake Ave North, Worcester, MA 01655, USA. beverly.nazarian@umassmemorial.org
UR - 21123484
ER -

TY - JOUR
ID - 685

T1 - Tularemia outbreak associated with outdoor exposure along the western side of Utah Lake, Utah, 2007
A1 - Calanan,R.M.
A1 - Rolfs,R.T.
A1 - Summers,J.
A1 - Coombs,J.
A1 - Amadio,J.
A1 - Holbrook,J.
A1 - Mead,P.S.
Y1 - 2010/11//
N1 - Calanan, Renee M. Rolfs, Robert T. Summers, JoDee. Coombs, Jana. Amadio, John. Holbrook, Joy. Mead, Paul S
Public health reports (Washington, D.C. : 1974)
9716844, qja
AIM, IM
Journal Article
English
KW - MEDLINE
KW - Adolescent
KW - Adult
KW - Aged
KW - Animals
KW - Camping
KW - Child
KW - Preschool
KW - Cohort Studies
KW - Diptera
KW - Disease Outbreaks
KW - Female
KW - Humans
KW - Infant
KW - Insect Bites and Stings/ep [Epidemiology]
KW - Male
KW - Middle Aged
KW - Retrospective Studies
KW - Risk Factors
KW - Tularemia/ep [Epidemiology]
KW - Utah/ep [Epidemiology]
KW - Young Adult
RP - NOT IN FILE
SP - 870
EP - 876
JF - Public Health Reports
JA - Public Health Rep
VL - 125
IS - 6
CY - United States
N2 - OBJECTIVES: In 2007, a localized outbreak of tularemia occurred among visitors to a lodge on the western side of Utah Lake, Utah. We assessed risk factors for disease and attempted to identify undiagnosed clinically compatible illnesses. METHODS: We conducted a retrospective cohort study by recruiting all people who had visited the lodge on the western side of Utah Lake from June 3 to July 28, 2007. A self-administered questionnaire was distributed to a sub-cohort of people who were part of an organized group that had at least one tularemia patient. Questions assessed risk and protective factors and disease symptoms. RESULTS: During the outbreak period, 14 cases of tularemia were reported from five of Utah's 12 health districts. The weekly attack rate ranged from 0 to 2.1/100 lodge visitors from June 3 to July 28. Illness onset dates ranged from June 15 to July 8. The median delay between onset of symptoms and laboratory test for tularemia was 14 days (range: 7-34 days). Cohort study respondents who reported deer-fly bites while at the lodge (adjusted risk ratio [ARR] = 7.2, 95% confidence interval [CI] 2.4, 22.0) and who reported having worn a hat (ARR = 5.6, 95% CI 1.3, 24.6)

were more likely to become ill. CONCLUSIONS: This was Utah's second documented deer-fly-associated human tularemia outbreak. People participating in outdoor activities in endemic areas should be aware of disease risks and take precautions. Educational campaigns can aid in earlier disease recognition, reporting, and, consequently, outbreak detection
SN - 0033-3549
AD - Epidemic Intelligence Service, Office of Workforce and Career Development, Centers for Disease Control and Prevention, Atlanta, GA, USA. rcalanan@cdc.gov
UR - 21121232
ER -

TY - JOUR
ID - 686
T1 - The bone and mineral disorder of children undergoing chronic peritoneal dialysis
A1 - Borzych,D.
A1 - Rees,L.
A1 - Ha,I.S.
A1 - Chua,A.
A1 - Valles,P.G.
A1 - Lipka,M.
A1 - Zambrano,P.
A1 - Ahlenstiel,T.
A1 - Bakkaloglu,S.A.
A1 - Spizzirri,A.P.
A1 - Lopez,L.
A1 - Ozaltin,F.
A1 - Printza,N.
A1 - Hari,P.
A1 - Klaus,G.
A1 - Bak,M.
A1 - Vogel,A.
A1 - Ariceta,G.
A1 - Yap,H.K.
A1 - Warady,B.A.
A1 - Schaefer,F.
A1 - International Pediatric PD Network (IPPN)
Y1 - 2010/12//
N1 - Borzych, Dagmara. Rees, Lesley. Ha, Il Soo. Chua, Annabelle. Valles, Patricia G. Lipka, Maria. Zambrano, Pedro. Ahlenstiel, Thurid. Bakkaloglu, Sevcan A. Spizzirri, Ana P. Lopez, Laura. Ozaltin, Fatih. Printza, Nikoleta. Hari, Pankaj. Klaus, Gunter. Bak, Mustafa. Vogel, Andrea. Ariceta, Gema. Yap, Hui Kim. Warady, Bradley A. Schaefer, Franz. International Pediatric PD Network (IPPN)
Kidney international
kvb, 0323470
IM
Journal Article. Research Support, Non-U.S. Gov't
English
KW - MEDLINE
KW - Adolescent
KW - Calcium/bl [Blood]
KW - Child
KW - Preschool
KW - Europe/ep [Epidemiology]
KW - Female
KW - Follow-Up Studies
KW - Humans
KW - Hyperphosphatemia/dt [Drug Therapy]
KW - Hyperphosphatemia/ep [Epidemiology]
KW - Hyperphosphatemia/et [Etiology]

KW - Hypocalcemia/dt [Drug Therapy]
KW - Hypocalcemia/ep [Epidemiology]
KW - Hypocalcemia/et [Etiology]
KW - Infant
KW - Kidney Failure
KW - Chronic/bl [Blood]
KW - Chronic/co [Complications]
KW - Latin America/ep [Epidemiology]
KW - Male
KW - Naphthalenes/tu [Therapeutic Use]
KW - North America/ep [Epidemiology]
KW - Parathyroid Hormone/bl [Blood]
KW - Peritoneal Dialysis
KW - Phosphate-Binding Proteins/tu [Therapeutic Use]
KW - Phosphorus/bl [Blood]
KW - Prospective Studies
KW - Registries
KW - Vitamin D/tu [Therapeutic Use]
KW - Young Adult
RP - NOT IN FILE
SP - 1295
EP - 1304
JF - Kidney International
JA - Kidney Int
VL - 78
IS - 12
CY - United States
N2 - The mineral and bone disorder of chronic kidney disease remains a challenging complication in pediatric end-stage renal disease. Here, we assessed symptoms, risk factors and management of this disorder in 890 children and adolescents from 24 countries reported to the International Pediatric Peritoneal Dialysis Network Registry. Signs of this disease were most common in North American patients. The prevalence of hyperphosphatemia increased with age from 6% in young infants to 81% in adolescents. Serum parathyroid hormone (PTH) was outside the guideline targets in the majority of patients and associated with low calcium, high phosphorus, acidosis, dialysis vintage and female gender. Serum calcium was associated with dialytic calcium exposure, serum phosphorus with low residual renal function and pubertal status. PTH levels were highest in Latin America and lowest in Europe. Vitamin D and its active analogs were most frequently administered in Europe; calcium-free phosphate binders and cinacalcet in North America. Clinical and radiological symptoms markedly increased when PTH exceeded 300 pg/ml, the risk of hypercalcemia increased with levels below 100 pg/ml, and time-averaged PTH concentrations above 500 pg/ml were associated with impaired longitudinal growth. Hence, the symptoms and management of the mineral and bone disorder of chronic kidney disease in children on peritoneal dialysis showed substantial regional variation. Our findings support a PTH target range of 100-300 pg/ml in the pediatric age group
SN - 1523-1755
AD - Division of Pediatric Nephrology, Center for Pediatrics and Adolescent Medicine, Heidelberg, Germany
UR - 20811335
ER -

TY - JOUR
ID - 687
T1 - Mediators and moderators of a psychosocial intervention for children affected by political violence
A1 - Tol,W.A.
A1 - Komproe,I.H.
A1 - Jordans,M.J.
A1 - Gross,A.L.
A1 - Susanty,D.
A1 - Macy,R.D.
A1 - de Jong,J.T.

Y1 - 2010/12//
N1 - Tol, Wietse A. Komproe, Ivan H. Jordans, Mark J D. Gross, Alden L. Susanty, Dessy. Macy, Robert D. de Jong, Joop T V M
Journal of consulting and clinical psychology
0136553, hw3
IM
Journal Article. Randomized Controlled Trial. Research Support, Non-U.S. Gov't
English
KW - MEDLINE
KW - Adaptation
KW - Psychological
KW - Adolescent
KW - Age Factors
KW - Child
KW - Family
KW - Female
KW - Humans
KW - Indonesia
KW - Male
KW - Politics
KW - Psychotherapy
KW - Questionnaires
KW - Social Support
KW - Stress Disorders
KW - Post-Traumatic/px [Psychology]
KW - Post-Traumatic/th [Therapy]
KW - Treatment Outcome
KW - Violence/px [Psychology]
RP - NOT IN FILE
SP - 818
EP - 828
JF - Journal of Consulting & Clinical Psychology
JA - J Consult Clin Psychol
VL - 78
IS - 6
CY - United States
N2 - OBJECTIVE: The authors examined moderators and mediators of a school-based psychosocial intervention for children affected by political violence, according to an ecological resilience theoretical framework. METHOD: The authors examined data from a cluster randomized trial, involving children aged 8-13 in Central Sulawesi, Indonesia (treatment condition n = 182, waitlist control condition n = 221). Mediators (hope, coping, peer/emotional/play social support) and moderators (gender, age, family connectedness, household size, other forms of social support, exposure to political violence, and displacement) of treatment outcome on posttraumatic stress symptoms and function impairment were examined in parallel process latent growth curve models. RESULTS: Compared with the waitlist group, those receiving treatment showed maintained hope, increased positive coping, maintained peer social support, and increased play social support. Of these putative mediators, only play social support was found to mediate treatment effects, such that increases in play social support were associated with smaller reductions in posttraumatic stress disorder (PTSD) symptoms. Furthermore, the authors identified a number of moderators: Girls showed larger treatment benefits on PTSD symptoms; girls, children in smaller households, and children receiving social support from adults outside the household showed larger treatment benefits on function impairment. CONCLUSIONS: Findings provide limited evidence for an ecological resilience theoretical framework. On the basis of these findings, the authors recommend a stronger separation between universal prevention (e.g., resilience promotion through play) and selective/indicated prevention (e.g., interventions aimed at decreasing posttraumatic stress symptoms). Play-based interventions should be careful to exclude children with psychological distress. In addition, treatment effects may be augmented by selecting girls and socially vulnerable children. (c) 2010 APA, all rights reserved
SN - 1939-2117

AD - HealthNet TPO, Amsterdam, the Netherlands and Global Health Initiative, Yale University, USA. wietse.tol@yale.edu
UR - 21114342
ER -

TY - JOUR
ID - 688
T1 - [Trihalomethanes in swimming pool water in four areas of Spain participating in the INMA project]. [Spanish]
A1 - Font-Ribera,L.
A1 - Esplugues,A.
A1 - Ballester,F.
A1 - Martinez-Arguelles,B.
A1 - Tardon,A.
A1 - Freire,C.
A1 - Fernandez,M.F.
A1 - Carrasco,G.
A1 - Cases,A.
A1 - Sunyer,J.
A1 - Villanueva,C.M.
Y1 - 2010/11//
N1 - Font-Ribera, Laia. Esplugues, Ana. Ballester, Ferran. Martinez-Arguelles, Begona. Tardon, Adonina. Freire, Carmen. Fernandez, Marina F. Carrasco, Gloria. Cases, Amparo. Sunyer, Jordi. Villanueva, Cristina M
Gaceta sanitaria / S.E.S.P.A.S
0370727, 8901623, gsz, 8901623
IM
English Abstract. Journal Article. Research Support, Non-U.S. Gov't
Spanish
KW - MEDLINE
KW - Child
KW - Child Welfare
KW - Environmental Monitoring
KW - Humans
KW - Spain
KW - Swimming Pools
KW - Trihalomethanes/an [Analysis]
KW - Water Pollutants
KW - Chemical/an [Analysis]
RP - NOT IN FILE
SP - 483
EP - 486
JF - Gaceta Sanitaria
JA - Gac.Sanit.
VL - 24
IS - 6
CY - Spain
N2 - OBJECTIVE: Swimming is one of the most widely practiced sports in Spain among people of all ages and physical conditions. This activity is also a source of exposure to disinfection by-products (DBP), which are potentially toxic. The DBP concentration in swimming pool water is not regulated and is poorly known. The aim of this study was to describe trihalomethane concentrations in swimming pool water in the municipalities of four cohorts of the INMA project. METHODS: In July 2009, trihalomethanes were analyzed in water from 27 swimming pools in Asturias, Granada, Valencia and Sabadell. RESULTS: The mean total trihalomethane concentration was 42.7 mug/L (standard deviation [SD]=19.1) in indoor pools and 151.2 mug/L (SD=80.7) in outdoor pools. In all pools, the most abundant trihalomethane was always chloroform. The lowest levels were found in Granada. CONCLUSION: Trihalomethane concentrations in swimming pool water were highly variable. Outdoor swimming pools showed the highest levels, which were usually above the legal limit for drinking water. Copyright 2010 SESPAS. Published by Elsevier Espana. All rights reserved

SN - 1578-1283
AD - Centre de Recerca en Epidemiologia Ambiental (CREAL), Barcelona, Espana
UR - 21074293
ER -

TY - JOUR
ID - 689
T1 - Increased risk of Blount disease in obese children and adolescents with vitamin D deficiency
A1 - Montgomery,C.O.
A1 - Young,K.L.
A1 - Austen,M.
A1 - Jo,C.H.
A1 - Blasier,R.D.
A1 - Ilyas,M.
Y1 - 2010/12//
N1 - Montgomery, Corey O. Young, Karen L. Austen, Mark. Jo, Chan-Hee. Blasier, Robert Dale. Ilyas, Mohammad
Journal of pediatric orthopedics
hsw, 8109053
IM
Journal Article
English
KW - MEDLINE
KW - Adolescent
KW - Bone Diseases
KW - Developmental/et [Etiology]
KW - Child
KW - Epiphyses
KW - Slipped/et [Etiology]
KW - Female
KW - Femur Head
KW - Fractures
KW - Bone/et [Etiology]
KW - Humans
KW - Male
KW - Obesity/co [Complications]
KW - Osteochondrosis/cn [Congenital]
KW - Osteochondrosis/et [Etiology]
KW - Retrospective Studies
KW - Risk Factors
KW - Vitamin D Deficiency/co [Complications]
RP - NOT IN FILE
SP - 879
EP - 882
JF - Journal of Pediatric Orthopedics
JA - J Pediatr Orthop
VL - 30
IS - 8
CY - United States
N2 - BACKGROUND: Poor dietary habits and decreased outdoor activity has led to an epidemic of obese children and vitamin D deficiency. The lack of vitamin D alters bone development and mineralization by diminishing physiological levels of calcium and phosphorus. Given vitamin D's role in bone and growth plate mineralization and regulation, we hypothesized that vitamin D deficiency would lead to higher rates of fractures, slipped capital femoral epiphysis (SCFE), and Blount disease in obese youth. METHODS: A retrospective review was performed at the obesity clinic using the obesity database (890 patients). Data obtained included body mass index (BMI), vitamin D levels (25-vitamin D), history of fractures, Blount disease, and/or SCFE. The chart review identified 2 populations of obese patients, those with vitamin D deficiency, <16 ng/mL

(198 patients) and those not vitamin D deficient >16 ng/mL (692 patients). Fisher exact, chi2, and 2-sample t tests along with logistic regression were used for statistical analysis. A P value <0.05 was considered statistically significant. RESULTS: Blount disease was found to have a statistically significant (P<0.05) positive association with patient's sex, BMI, and vitamin D level. Specifically, males were 8.16 times more likely than females to be observed with Blount disease (P=0.01). Patients with very low vitamin D levels were 7.33 times more likely to have Blount disease than patients with higher levels (P=0.002). Each whole number increase in BMI increases the likelihood of Blount disease by 3% (P=0.01). There was no association between increased number of fractures or SCFE with vitamin D deficiency in these obese patients. CONCLUSION: As our findings indicate, BMI and vitamin D levels have a strong association with Blount disease, which may be especially important among males. Ours is the first study to show a relationship between vitamin D deficiency and Blount disease, but further prospective studies are needed with larger numbers to confirm this independent association of vitamin D deficiency with Blount disease. LEVEL OF EVIDENCE: Level III retrospective study
SN - 1539-2570
AD - Department of Pediatrics, University of Arkansas for Medical Sciences-Arkansas Children's Hospital, Little Rock, AR 72202, USA
UR - 21102216
ER -

TY - JOUR
ID - 690
T1 - Treatment of children with chronic hepatitis B virus infection in the United States: patient selection and therapeutic options
A1 - Jonas,M.M.
A1 - Block,J.M.
A1 - Haber,B.A.
A1 - Karpen,S.J.
A1 - London,W.T.
A1 - Murray,K.F.
A1 - Narkewicz,M.R.
A1 - Rosenthal,P.
A1 - Schwarz,K.B.
A1 - McMahon,B.J.
A1 - Hepatitis,B.Foundation
Y1 - 2010/12//
N1 - Jonas, Maureen M. Block, Joan M. Haber, Barbara A. Karpen, Saul J. London, W Thomas. Murray, Karen F. Narkewicz, Michael R. Rosenthal, Philip. Schwarz, Kathleen B. McMahon, Brian J. Hepatitis B Foundation Hepatology (Baltimore, Md.)
gbz, 8302946
IM
Consensus Development Conference. Journal Article. Practice Guideline. Research Support, Non-U.S. Gov't
English
KW - MEDLINE
KW - Adenine/aa [Analogs & Derivatives]
KW - Adenine/tu [Therapeutic Use]
KW - Adolescent
KW - Adult
KW - Alanine Transaminase/bl [Blood]
KW - Antiviral Agents/tu [Therapeutic Use]
KW - Child
KW - Preschool
KW - DNA
KW - Viral/an [Analysis]
KW - Drug Resistance
KW - Viral
KW - Hepatitis B Surface Antigens/bl [Blood]
KW - Hepatitis B virus/ge [Genetics]
KW - Hepatitis B

KW - Chronic/dt [Drug Therapy]
KW - Chronic/im [Immunology]
KW - Humans
KW - Infant
KW - Interferon-alpha/tu [Therapeutic Use]
KW - Lamivudine/tu [Therapeutic Use]
KW - Organophosphonates/tu [Therapeutic Use]
KW - Patient Selection
KW - Recombinant Proteins
RP - NOT IN FILE
SP - 2192
EP - 2205
JF - Hepatology
VL - 52
IS - 6
CY - United States
N2 - Chronic hepatitis B virus (HBV) infection in children presents a therapeutic challenge for the practitioner. Decisions regarding selection of patients who may benefit from treatment, appropriate timing of treatment, and the choice of antiviral therapy are complex and are compounded by the limited number of drugs that have been studied in children. An expert panel of nationally recognized pediatric liver specialists was convened by the Hepatitis B Foundation on August 11, 2009, to consider clinical practice relative to the therapeutic options available for children. A detailed account of these discussions is provided, and the opinions expressed are based on consensus of the experts, as well as on published evidence when available. The panel concludes that, at this time, there is no established benefit of treatment of children in the immune tolerant phase, and there is a very high risk of development of drug resistance. In addition, there is no indication for treatment of children in the inactive carrier state. For children in the immune active or reactivation phases, liver histology can help guide treatment decisions, and family history of liver disease, especially hepatocellular carcinoma, may argue for early treatment in some cases. Outside of clinical trials, interferon is the agent of choice in most cases. Nucleos(t)ide analogues are secondary therapies, and children who receive these agents require careful monitoring for development of resistance. There are a few situations when treatment is indicated regardless of HBV DNA or alanine aminotransferase levels. There is still much to be elucidated about the appropriate use of HBV therapy in children. Until more clinical data and therapeutic options are available, a conservative approach is warranted. Copyright 2010 American Association for the Study of Liver Diseases
SN - 1527-3350
AD - Division of Gastroenterology, Children's Hospital Boston, Boston, MA 02115, USA. maureen.jonas@childrens.harvard.edu
UR - 20890947
ER -

TY - JOUR
ID - 691
T1 - Rationale, design and methods for a randomised and controlled trial to evaluate "Animal Fun" - a program designed to enhance physical and mental health in young children
A1 - Piek,J.P.
A1 - Straker,L.M.
A1 - Jensen,L.
A1 - Dender,A.
A1 - Barrett,N.C.
A1 - McLaren,S.
A1 - Roberts,C.
A1 - Reid,C.
A1 - Rooney,R.
A1 - Packer,T.
A1 - Bradbury,G.
A1 - Elsley,S.
Y1 - 2010///

N1 - Piek, Jan P. Straker, Leon M. Jensen, Lynn. Dender, Alma. Barrett, Nicholas C. McLaren, Sue. Roberts, Clare. Reid, Carly. Rooney, Rosie. Packer, Tanya. Bradbury, Greer. Elsley, Sharon
BMC pediatrics
100967804
IM
Journal Article. Randomized Controlled Trial. Research Support, Non-U.S. Gov't
English
KW - MEDLINE
KW - Child Development
KW - Child
KW - Preschool
KW - Female
KW - Humans
KW - Male
KW - Mental Health
KW - Motor Activity
KW - Physical Education and Training/mt [Methods]
KW - Physical Fitness
KW - Research Design
KW - Social Environment
RP - NOT IN FILE
SP - 78
JF - BMC Pediatrics
JA - BMC Pediatr
VL - 10
CY - England
N2 - BACKGROUND: Children with poor motor ability have been found to engage less in physical activities than other children, and a lack of physical activity has been linked to problems such as obesity, lowered bone mineral density and cardiovascular risk factors. Furthermore, if children are confident with their fine and gross motor skills, they are more likely to engage in physical activities such as sports, crafts, dancing and other physical activity programs outside of the school curriculum which are important activities for psychosocial development. The primary objective of this project is to comprehensively evaluate a whole of class physical activity program called Animal Fun designed for Pre-Primary children. This program was designed to improve the child's movement skills, both fine and gross, and their perceptions of their movement ability, promote appropriate social skills and improve social-emotional development. METHODS: The proposed randomized and controlled trial uses a multivariate nested cohort design to examine the physical (motor coordination) and psychosocial (self perceptions, anxiety, social competence) outcomes of the program. The Animal Fun program is a teacher delivered universal program incorporating animal actions to facilitate motor skill and social skill acquisition and practice. Pre-intervention scores on motor and psychosocial variables for six control schools and six intervention schools will be compared with post-intervention scores (end of Pre-Primary year) and scores taken 12 months later after the children's transition to primary school Year 1. 520 children aged 4.5 to 6 years will be recruited and it is anticipated that 360 children will be retained to the 1 year follow-up. There will be equal numbers of boys and girls. DISCUSSION: If this program is found to improve the child's motor and psychosocial skills, this will assist in the child's transition into the first year of school. As a result of these changes, it is anticipated that children will have greater enjoyment participating in physical activities which will further promote long term physical and mental health. TRIAL REGISTRATION: This trial is registered in the Australian and New Zealand Clinical trials Registry (ACTRN12609000869279)
SN - 1471-2431
AD - School of Psychology and Speech Pathology, Curtin University, Perth, Australia. j.piek@curtin.edu.au
UR - 21050483
ER -

TY - JOUR
ID - 692
T1 - Active living for rural children: community perspectives using PhotoVOICE
A1 - Hennessy,E.
A1 - Kraak,V.I.

A1 - Hyatt,R.R.
A1 - Bloom,J.
A1 - Fenton,M.
A1 - Wagoner,C.
A1 - Economos,C.D.
Y1 - 2010/12//
N1 - Hennessy, Erin. Kraak, Vivica I. Hyatt, Raymond R. Bloom, Julia. Fenton, Mark. Wagoner, Colby. Economos, Christina D
American journal of preventive medicine
8704773, apl
IM
Journal Article. Research Support, N.I.H., Extramural. Research Support, Non-U.S. Gov't
English
KW - MEDLINE
KW - Child
KW - Female
KW - Humans
KW - Male
KW - Motor Activity
KW - Organizational Policy
KW - Photography
KW - Poverty/sn [Statistics & Numerical Data]
KW - Residence Characteristics/sn [Statistics & Numerical Data]
KW - Rural Population
KW - Schools/sn [Statistics & Numerical Data]
KW - Sedentary Lifestyle
KW - United States
RP - NOT IN FILE
SP - 537
EP - 545
JF - American Journal of Preventive Medicine
JA - Am J Prev Med
VL - 39
IS - 6
CY - Netherlands
N2 - BACKGROUND: Active living integrates physical activity into one's daily routine. Current understanding of active living among children and their families living in rural communities is limited. A community perspective is critical to understand the contextual factors that influence children's physical activity in rural areas. PURPOSE: The purpose of this study was to identify the perceived environmental factors that support or hinder physical activity among rural children to develop testable hypotheses to inform future interventions for reducing unhealthy weight gain and preventing chronic diseases associated with physical inactivity. METHODS: PhotoVOICE was used to explore active living opportunities and barriers for children living in four low-income, rural U.S. communities. In 2007, parents (n=99) and elementary school staff (n=17) received disposable cameras to document their perspective. Using their photographs and narratives, participants developed emergent themes during a facilitated group discussion. In 2008, study authors used the Analysis Grid for Environments Linked to Obesity (ANGELO) framework to categorize the themes. RESULTS: Microenvironment themes include physical (e.g., natural features, topography); sociocultural (e.g., isolation); policy (e.g., time for school recess); and economic (e.g., funding for physical activity programs). Macroenvironmental themes related to the built and natural environments and transportation infrastructure. CONCLUSIONS: This study identified rural environment elements that community members perceived as influencing children's physical activity patterns. Certain aspects were unique to rural areas, whereas other urban and suburban factors may be generalizable to rural settings. PhotoVOICE was a useful participatory research method to gain insight into perceived factors affecting rural children's physical activity behaviors. Copyright 2010 American Journal of Preventive Medicine. Published by Elsevier Inc. All rights reserved
SN - 1873-2607
AD - National Cancer Institute, National Institutes of Health, Bethesda, Maryland, USA. erin.hennessy@nih.gov

UR - 21084074
ER -

TY - JOUR
ID - 693
T1 - Effects of socioeconomic factors and human activities on children's PM(10) exposure in inner-city households in Korea
A1 - Byun,H.
A1 - Bae,H.
A1 - Kim,D.
A1 - Shin,H.
A1 - Yoon,C.
Y1 - 2010/12//
N1 - Byun, Hyaejeong. Bae, Hyunjoo. Kim, Dongjin. Shin, Hosung. Yoon, Chungsik
International archives of occupational and environmental health
gpn, 7512134
IM
Comparative Study. Journal Article. Research Support, Non-U.S. Gov't
English
KW - MEDLINE
KW - Air Pollutants/an [Analysis]
KW - Air Pollution
KW - Indoor/an [Analysis]
KW - Child
KW - Cities
KW - Cooking
KW - Educational Status
KW - Environmental Monitoring/mt [Methods]
KW - Family Characteristics
KW - Female
KW - Humans
KW - Income
KW - Korea
KW - Linear Models
KW - Male
KW - Particle Size
KW - Particulate Matter/an [Analysis]
KW - Questionnaires
KW - Smoking/px [Psychology]
KW - Socioeconomic Factors
RP - NOT IN FILE
SP - 867
EP - 878
JF - International Archives of Occupational & Environmental Health
JA - Int Arch Occup Environ Health
VL - 83
IS - 8
CY - Germany
N2 - PURPOSE: In this study, we investigated how socioeconomic factors contributed to airborne PM(10) concentrations in living rooms and children's bedrooms in 50 homes in Korea from July to September 2008. METHODS: PM(10) was measured with the personal environmental monitor, and both a questionnaire and time activity diary were used to acquire data on socioeconomic factors and various human activities (i.e., cooking, cleaning, and smoking). Analysis of variance and general linear model were used to identify the effects of socioeconomic and behavioral factors on PM(10) concentrations. RESULTS: Mean PM(10) concentrations in living rooms and children's rooms were 45.3 + 33.3 mug/m(3) and 45.9 + 21.0 mug/m(3), respectively, whereas outdoor PM(10) concentrations were 50.0 + 19.8 mug/m(3). Significant relationships were found between concentrations in children's rooms and living rooms, and also between indoor and outdoor concentrations.

PM(10) concentrations in children's rooms varied significantly by region, parental education, floor of residence, and average monthly household expenses. Concentrations in living rooms varied significantly by the number of children. This implies that lower socioeconomic status can contribute to higher indoor PM(10) concentrations. Indoor PM(10) concentrations in households with cleaning, cooking, and smoking were higher than in homes without these activities. General linear model showed that the effects of socioeconomic factors on PM(10) concentrations were significant in the following order: region (the increment in estimate beta = 24.16), parental education (beta = -18.84), type of housing (beta = -16.97; p < 0.01), and number of children (beta = 19.12; p < 0.05). CONCLUSIONS: We found that indoor PM(10) concentrations were affected by socioeconomic factors rather than human behavioral activities. In determining the environmental policy for indoor air quality, it is important to consider various socioeconomic factors of subjects
SN - 1432-1246
AD - Department of Environmental Health, The Graduate School of Public Health, Seoul National University, 28 Yeonkun-dong Jongno-gu, Seoul, Republic of Korea
UR - 20339864
ER -

TY - JOUR
ID - 694
T1 - Child nutrition in Mozambique in 2003: the role of mother's schooling and nutrition knowledge
A1 - Burchi,F.
Y1 - 2010/12//
N1 - Burchi, Francesco
Economics and human biology
101166135
IM
Journal Article
English
KW - MEDLINE
KW - Age Factors
KW - Anthropometry
KW - Body Mass Index
KW - Child Nutrition Disorders/ep [Epidemiology]
KW - Child Welfare/sn [Statistics & Numerical Data]
KW - Child
KW - Preschool
KW - Educational Status
KW - Female
KW - Health Knowledge
KW - Attitudes
KW - Practice
KW - Humans
KW - Male
KW - Mozambique
KW - Multivariate Analysis
KW - Nutrition Surveys
KW - Nutritional Status
KW - Regression Analysis
KW - Socioeconomic Factors
RP - NOT IN FILE
SP - 331
EP - 345
JF - Economics & Human Biology
JA - Econ Hum Biol
VL - 8
IS - 3
CY - Netherlands

N2 - This paper is a study of the determinants of the anthropometric status of preschool children in Mozambique. Using the 2003 Demographic and Health Survey, we provide insights into two main explanatory factors: the mother's schooling and the mother's nutrition knowledge. Rather than treating the mother's schooling as a black box, we analyze its interaction with the mother's nutrition knowledge and household wealth in order to elucidate the mechanisms underlying their ultimate effect on child height. The estimates obtained through instrumental variable regression show that the direct effect of the mother's schooling is large but that the rate at which it increases declines as her educational level rises. Primary education seems to be a key to enhance the mothers' general knowledge, which then improves the allocation of resources in regard to children's well-being and the care for the child. A higher educational level attained by the mother is likely to play only a minimal and indirect role in her child's nutrition, by expanding her economic opportunities. This is because more educated mothers have also more qualified and time-consuming jobs, which reduces the time spent for childcare. Mothers with higher levels of nutrition knowledge, acquired primarily outside of school, are able to choose a more diversified diet for their children and, broadly speaking, to utilize food more effectively. Based on a second technique, the instrumental variable quantile regression, we are able to draw a double conclusion: that mothers' nutrition knowledge contributes to height increases among extremely deprived children, and that mothers' formal education and household wealth are slightly more important for relatively well-off children.
Copyright 2010 Elsevier B.V. All rights reserved
SN - 1873-6130
AD - Roma Tre University, Department of Economics, via Silvio D'Amico 77, 00145 Rome, Italy.
fra_burchi@hotmail.com
UR - 20646971
ER -

TY - JOUR
ID - 695
T1 - Pattern of fractures across pediatric age groups: analysis of individual and lifestyle factors
A1 - Valerio,G.
A1 - Galle,F.
A1 - Mancusi,C.
A1 - Di,Onofrio,V
A1 - Colapietro,M.
A1 - Guida,P.
A1 - Liguori,G.
Y1 - 2010///
N1 - Valerio, Giuliana. Galle, Francesca. Mancusi, Caterina. Di Onofrio, Valeria. Colapietro, Marianna. Guida, Pasquale. Liguori, Giorgio
BMC public health
100968562
IM
Journal Article
English
KW - MEDLINE
KW - Adolescent
KW - Ambulatory Care Facilities
KW - Child
KW - Preschool
KW - Cross-Sectional Studies
KW - Female
KW - Fractures
KW - Bone/ep [Epidemiology]
KW - Humans
KW - Individuality
KW - Italy/ep [Epidemiology]
KW - Life Style
KW - Male
RP - NOT IN FILE
SP - 656

JF - BMC Public Health
VL - 10
CY - England
N2 - BACKGROUND: Knowledge of the epidemiology of children's fractures is essential to develop preventive strategies. The aim of this study was to analyze the individual/lifestyle determinants of fractures across pediatric age groups. METHODS: A cross-sectional study was performed in the first six months of 2008 through questionnaire on a sample of children from an outpatient clinic for pediatric fractures. Differences in gender, anatomic site, circumstances and location of fracture occurrence, behavioural lifestyle, and calcium intake were investigated among three different age classes (pre-school children, school children, and adolescents). RESULTS: The sample consisted of 382 subjects (2-14 years of age) sustaining a fracture after low or moderate trauma. Males were at a higher risk of fractures than females; greater than two-thirds of injuries occurred after low-energy trauma and the upper limb was more frequently involved. With increasing age, the male/female ratio and time spent in sports participation increased ($p < 0.001$), while calcium intake and time spent in sedentary behaviors decreased ($p < 0.001$ and < 0.003, respectively). Gender discordance existed in pre-school children with respect to the anatomic location, and in school children and adolescents with respect to the dynamics. In the adolescent group, males were more physically active and also more sedentary than females. Fractures most frequently occurred in homes (41.6%), followed by playgrounds and footpaths (26.2%), sports facilities (18.3%), and educational facilities (13.9%), with gender differences existing only in adolescence. Twenty-three percent of the subjects sustained one or more fractures in the past. The percentage of recurrent fractures increased with age ($p = 0.001$), with a similar trend in both genders. CONCLUSIONS: Gender differences were shown in the prevalence of injuries, characteristics, and circumstances across ages. These differences may be explained by the related changes in behaviors, together with attending different places. Individual and lifestyle factors can in part explain the variability in the occurrence of fractures and can also address targeted preventive strategies
SN - 1471-2458
AD - School of Movement Sciences (DiSiST), Parthenope University, via Medina 40, 80133, Naples, Italy
UR - 21034509
ER -

TY - JOUR
ID - 696
T1 - Outdoor recreational fires: a review of 329 adult and pediatric patients
A1 - Neaman,K.C.
A1 - Do,V.H.
A1 - Olenzek,E.K.
A1 - Baca,M.
A1 - Ford,R.D.
A1 - Wilcox,R.M.
Y1 - 2010/11//
N1 - Neaman, Keith C. Do, Viet H. Olenzek, Emily K. Baca, Marissa. Ford, Ronald D. Wilcox, Richard M
Journal of burn care & research : official publication of the American Burn Association
101262774
IM
Journal Article
English
KW - MEDLINE
KW - Adolescent
KW - Adult
KW - Aged
KW - 80 and over
KW - Alcoholic Intoxication/co [Complications]
KW - Burn Units
KW - Burns/co [Complications]
KW - Burns/ep [Epidemiology]
KW - Burns/et [Etiology]
KW - Burns/pc [Prevention & Control]
KW - Child

KW - Preschool
KW - Fires
KW - Humans
KW - Infant
KW - Injury Severity Score
KW - Length of Stay/sn [Statistics & Numerical Data]
KW - Michigan/ep [Epidemiology]
KW - Middle Aged
KW - Recreation
KW - Retrospective Studies
KW - Risk Factors
KW - Seasons
RP - NOT IN FILE
SP - 926
EP - 930
JF - Journal of Burn Care & Research
JA - J Burn Care Res
VL - 31
IS - 6
CY - United States
N2 - Outdoor recreational fires are a frequent occurrence during the summer months and can be associated with burns resulting in significant morbidity. Both pediatric and adult populations can be affected, and their mechanism of injury is often different. Understanding these mechanisms is important when designing prevention programs. It is the goal of this study to review our experience with outdoor recreational fires. All patients who presented to Spectrum Health Blodgett Regional Burn Unit for burns secondary to an outdoor recreational fire over an 8-year period were reviewed. Demographic data, mechanism of injury, body area involved, TBSA burned, treatments undertaken, and subsequent complications were recorded. Pediatric patients (aged 16 years and younger) were analyzed independently, and risk factors were determined. A total of 329 patients suffered burns secondary to outdoor recreational fires over the length of the study. More than 35% required inpatient treatment, with an average length of stay of 4.8 days. Hands were the most frequently affected body part, with the mean TBSA involved being 3.5%. Ninety-four patients (28.6%) required split-thickness skin grafting. The most common mechanism of injury in both adult and pediatric populations was falling into an ongoing fire. Wound infection was the most common complication. Alcohol intoxication was associated with a higher burn severity and complication rate. Pediatric patients represented 39.8% of the sample. Burns secondary to outdoor recreational fires are associated with significant morbidity. Adult prevention programs should target awareness with respect to alcohol consumption and campfires secondary to the morbidity associated with these injuries. Pediatric patients are particularly susceptible, and parents should remain diligent about campfire safety and be educated about the inherent dangers of both active and extinguished fires
SN - 1559-0488
AD - Grand Rapids Medical Education and Research Center, Michigan State University General Surgery Residency, Grand Rapids, Michigan, USA
UR - 20859212
ER -

TY - JOUR
ID - 697
T1 - Physical education and school contextual factors relating to students' achievement and cross-grade differences in aerobic fitness and obesity
A1 - Zhu,W.
A1 - Boiarskaia,E.A.
A1 - Welk,G.J.
A1 - Meredith,M.D.
Y1 - 2010/09//
N1 - Zhu, Weimo. Boiarskaia, Elena A. Welk, Gregory J. Meredith, Marilu D
Research quarterly for exercise and sport
r6y, 8006373
IM

Journal Article
English
KW - MEDLINE
KW - Achievement
KW - Adolescent
KW - Body Mass Index
KW - Child
KW - Exercise Test
KW - Female
KW - Humans
KW - Least-Squares Analysis
KW - Linear Models
KW - Male
KW - Organizational Policy
KW - Physical Education and Training/og [Organization & Administration]
KW - Physical Education and Training/sn [Statistics & Numerical Data]
KW - Physical Fitness
KW - Schools/og [Organization & Administration]
KW - Schools/sn [Statistics & Numerical Data]
KW - Sex Factors
KW - Texas
RP - NOT IN FILE
SP - S53
EP - S64
JF - Research Quarterly for Exercise & Sport
JA - Res Q Exerc Sport
VL - 81
IS - 3 Suppl
CY - United States
N2 - Using two major data sets from the Texas Youth Fitness Study, ordinary least squares regression, and hierarchical linear modeling, we examined the impact of hey correlates in school physical education programs and policies on students'fitness status and cross-grade differences. While a number of factors, such as teachers' training/updates, recess time, available physical activity space, a school wellness policy, and fitness testing before administration, were confirmed, these correlates can explain only limited variance. Other aspects, such as socioeconomic status and community confounding factors, were recognized and illustrated. Future studies should include more factors such as these in data collection and analysis
SN - 0270-1367
AD - Department of Kinesiology and Community Health, University of Illinois at Urbana-Champaign, Urbana 61801, USA. weimozhu@illinois.edu
UR - 21049838
ER -

TY - JOUR
ID - 698
T1 - [Descriptive study of fall among children under the age of 15 in the municipality of Londrina (Parana, Brazil)]. [Portuguese]
A1 - Martins,C.B.
A1 - Andrade,S.M.
Y1 - 2010/10//
N1 - Martins, Christine Baccarat de Godoy. Andrade, Selma Maffei de
Ciencia & saude coletiva
9713483
IM
English Abstract. Journal Article
Portuguese
KW - MEDLINE
KW - Accidental Falls/sn [Statistics & Numerical Data]

KW - Adolescent
KW - Brazil/ep [Epidemiology]
KW - Child
KW - Preschool
KW - Cross-Sectional Studies
KW - Female
KW - Humans
KW - Infant
KW - Male
RP - NOT IN FILE
SP - 3167
EP - 3173
JF - Ciencia & Saude Coletiva
JA - Cienc.
VL - 15 Suppl 2
CY - Brazil
N2 - This study aims at analyzing, from the epidemiological point of view, occurrences of falls among children under the age of 15 who live in Londrina, Parana, and were treated at emergency departments, hospitalized or passed away due to such event in 2001. Data were obtained from registries of general hospitals and from the Mortality Information Nucleus. 2,991 children victims of falls were analyzed, representing an incidence rate of 25.3 per 1000 children. Non-fatal hospitalization rate was 3.9%; one death occurred in an emergency department. The risk of suffering falls was higher among boys, except with children aged less than one year. Most frequently injured by falls were the groups of one year old (coefficient of 46.0 per 1000 children) and of two years old (coefficient of 38.4 per 1000 children). The most affected body region was head/neck (55.2%) and superficial traumatism was the most frequent kind of injury (46.4%). Falls occurred mainly from bed/cradle, furniture/couch, tricycle/skates, staircase/degrees, playground equipment, wall/gate/roof/balcony, hammock, tree and stroller. Findings show a high falls incidence among children and adolescents and contribute to increasing epidemiological knowledge of such events and so grounding the planning of preventive and control measures
SN - 1678-4561
AD - Faculdade de Enfermagem, Universidade Federal de Mato Grosso, Cuiaba, MT.
leocris2001@terra.com.br
UR - 21049157
ER -

TY - JOUR
ID - 699
T1 - The crucial role of recess in schools
A1 - Ramstetter,C.L.
A1 - Murray,R.
A1 - Garner,A.S.
Y1 - 2010/11//
N1 - Ramstetter, Catherine L. Murray, Robert. Garner, Andrew S
The Journal of school health
k13, 0376370
IM, N
Journal Article
English
KW - MEDLINE
KW - Adolescent
KW - Body Mass Index
KW - Child
KW - Child Welfare/sn [Statistics & Numerical Data]
KW - Exercise
KW - Female
KW - Health Promotion/og [Organization & Administration]
KW - Humans

KW - Male
KW - Obesity/ep [Epidemiology]
KW - Obesity/pc [Prevention & Control]
KW - Physical Education and Training/sn [Statistics & Numerical Data]
KW - Play and Playthings
KW - Public Policy
KW - School Health Services/og [Organization & Administration]
KW - Schools/og [Organization & Administration]
KW - United States/ep [Epidemiology]
RP - NOT IN FILE
SP - 517
EP - 526
JF - Journal of School Health
JA - J Sch Health
VL - 80
IS - 11
CY - United States
N2 - BACKGROUND: Recess is at the heart of a vigorous debate over the role of schools in promoting optimal child development and well-being. Reallocating time to accentuate academic concerns is a growing trend and has put recess at risk. Conversely, pressure to increase activity in school has come from efforts to combat childhood obesity. The purpose of this review was to examine the value of recess as an integral component of the school day. METHODS: A comprehensive review of recess-specific literature was conducted, beginning with a Google Scholar search, to cull definitions, position statements, and policy recommendations from national/international associations and organizations. A multi-database search followed. Additional articles were selected from reference lists. RESULTS: The search yielded a range of articles, from those focused on specific aspects of recess to those that examined multiple factors, including how to structure and conduct recess. Several themes emerged supporting recess as beneficial for children's cognitive, social, emotional, and physical functioning. Optimal recess was well-supervised and safe. Crucial components were well-maintained playground equipment and well-trained supervisors. CONCLUSION: Recess serves a critical role in school as a necessary break from the rigors of academic challenges. Recess is a complement to, not a replacement for, physical education. Both promote activity and a healthy lifestyle; however, recess--particularly unstructured recess and free play--provides a unique contribution to a child's creative, social, and emotional development. From the perspective of children's health and well-being, recess time should be considered a child's personal time and should not be withheld for academic or punitive reasons. 2010, American School Health Association
SN - 1746-1561
AD - Center for the Enhancement of Teaching and Learning, Health Promotion and Education, University of Cincinnati, Langsam 480, Mail Location 0033, PO Box 210033, Cincinnati, OH 45221-0033, USA. ramstecl@ucmail.uc.edu
UR - 21039550
ER -

TY - JOUR
ID - 700
T1 - The global pediatric antiretroviral market: analyses of product availability and utilization reveal challenges for development of pediatric formulations and HIV/AIDS treatment in children
A1 - Waning,B.
A1 - Diedrichsen,E.
A1 - Jambert,E.
A1 - Barnighausen,T.
A1 - Li,Y.
A1 - Pouw,M.
A1 - Moon,S.
Y1 - 2010///
N1 - Waning, Brenda. Diedrichsen, Ellen. Jambert, Elodie. Barnighausen, Till. Li, Yun. Pouw, Mieke. Moon, Suerie
BMC pediatrics
100967804

IM
Comparative Study. Journal Article. Research Support, N.I.H., Extramural
English
KW - MEDLINE
KW - Acquired Immunodeficiency Syndrome/dt [Drug Therapy]
KW - Acquired Immunodeficiency Syndrome/ec [Economics]
KW - Anti-Retroviral Agents/ec [Economics]
KW - Anti-Retroviral Agents/tu [Therapeutic Use]
KW - Child
KW - Drug Costs/td [Trends]
KW - Drugs
KW - Generic/ec [Economics]
KW - Generic/tu [Therapeutic Use]
KW - HIV Infections/dt [Drug Therapy]
KW - HIV Infections/ec [Economics]
KW - Health Services Accessibility/ec [Economics]
KW - Humans
KW - Marketing/ec [Economics]
KW - Public Policy
KW - United States
KW - United States Food and Drug Administration/ec [Economics]
KW - World Health Organization/ec [Economics]
RP - NOT IN FILE
SP - 74
JF - BMC Pediatrics
JA - BMC Pediatr
VL - 10
CY - England
N2 - BACKGROUND: Important advances in the development and production of quality-certified pediatric antiretroviral (ARV) formulations have recently been made despite significant market disincentives for manufacturers. This progress resulted from lobbying and innovative interventions from HIV/AIDS activists, civil society organizations, and international organizations. Research on uptake and dispersion of these improved products across countries and international organizations has not been conducted but is needed to inform next steps towards improving child health. METHODS: We used information from the World Health Organization Prequalification Programme and the United States Food and Drug Administration to describe trends in quality-certification of pediatric formulations and used 7,989 donor-funded, pediatric ARV purchase transactions from 2002-2009 to measure uptake and dispersion of new pediatric ARV formulations across countries and programs. Prices for new pediatric ARV formulations were compared to alternative dosage forms. RESULTS: Fewer ARV options exist for HIV/AIDS treatment in children than adults. Before 2005, most pediatric ARVs were produced by innovator companies in single-component solid and liquid forms. Five 2-in1 and four 3-in-1 generic pediatric fixed-dose combinations (FDCs) in solid and dispersible forms have been quality-certified since 2005. Most (67%) of these were produced by one quality-certified manufacturer. Uptake of new pediatric FDCs outside of UNITAID is low. UNITAID accounted for 97-100% of 2008-2009 market volume. In total, 33 and 34 countries reported solid or dispersible FDC purchases in 2008 and 2009, respectively, but most purchases were made through UNITAID. Only three Global Fund country recipients reported purchase of these FDCs in 2008. Prices for pediatric FDCs were considerably lower than liquids but typically higher than half of an adult FDC. CONCLUSION: Pediatric ARV markets are more fragile than adult markets. Ensuring a long-term supply of quality, well-adapted ARVs for children requires ongoing monitoring and improved understanding of global pediatric markets, including country-based research to explain and address low uptake of new, improved formulations. Continued innovation in pediatric ARV development may be threatened by outdated procurement practices failing to connect clinicians making prescribing decisions, supply chain staff dealing with logistics, donors, international organizations, and pharmaceutical manufacturers. Perceptions of global demand must be better informed by accurate estimates of actual country-level demand
SN - 1471-2431
AD - Department of Family Medicine, Boston University School of Medicine, One Boston Medical Center Place, Dowling 5 South, Boston, MA 02118, USA. bwaning@bu.edu
UR - 20950492

ER -

TY - JOUR
ID - 701
T1 - Craniofacial growth disturbance is related to temporomandibular joint abnormality in patients with juvenile idiopathic arthritis, but normal facial profile was also found at the 27-year follow-up
A1 - Arvidsson,L.Z.
A1 - Fjeld,M.G.
A1 - Smith,H.J.
A1 - Flato,B.
A1 - Ogaard,B.
A1 - Larheim,T.A.
Y1 - 2010///
N1 - Arvidsson, L Z. Fjeld, M G. Smith, H-J. Flato, B. Ogaard, B. Larheim, T A
Scandinavian journal of rheumatology
ud1, 0321213
IM
Journal Article. Research Support, Non-U.S. Gov't
English
KW - MEDLINE
KW - Adolescent
KW - Adult
KW - Arthritis
KW - Juvenile/co [Complications]
KW - Juvenile/pa [Pathology]
KW - Case-Control Studies
KW - Child
KW - Preschool
KW - Craniofacial Abnormalities/di [Diagnosis]
KW - Craniofacial Abnormalities/ep [Epidemiology]
KW - Craniofacial Abnormalities/et [Etiology]
KW - Facial Bones/ab [Abnormalities]
KW - Facial Bones/gd [Growth & Development]
KW - Facial Bones/ra [Radiography]
KW - Female
KW - Follow-Up Studies
KW - Humans
KW - Magnetic Resonance Imaging
KW - Male
KW - Micrognathism/ep [Epidemiology]
KW - Prevalence
KW - Prognosis
KW - Severity of Illness Index
KW - Skull/ab [Abnormalities]
KW - Skull/gd [Growth & Development]
KW - Skull/ra [Radiography]
KW - Temporomandibular Joint/ab [Abnormalities]
KW - Temporomandibular Joint/gd [Growth & Development]
KW - Temporomandibular Joint/ra [Radiography]
KW - Tomography
KW - X-Ray Computed
RP - NOT IN FILE
SP - 373
EP - 379
JF - Scandinavian Journal of Rheumatology
JA - Scand J Rheumatol
VL - 39

IS - 5
CY - England
N2 - OBJECTIVES: To assess the long-term outcome of craniofacial morphology related to disease variables and temporomandibular joint (TMJ) involvement as demonstrated with computed tomography (CT) and magnetic resonance imaging (MRI) in adult patients with juvenile idiopathic arthritis (JIA). METHODS: Sixty of 103 patients participated in a re-examination on average 27 years after baseline. Craniofacial morphology, with emphasis on size and position of the mandible, was assessed in lateral cephalographic images and related to disease variables and TMJ involvement by CT and MRI. Definitions of craniofacial growth disturbances were based on measurements outside 2 SD from the mean of healthy adult controls. RESULTS: Sagittal craniofacial growth disturbances were found in 57% and micrognathia in 27% of the 60 patients. Of those with JIA TMJ involvement, 70% had some form of growth disturbance. Micrognathia occurred only in patients with bilateral TMJ involvement. The bilateral TMJ group had significantly different craniofacial morphology than healthy controls and patients without TMJ involvement. Growth disturbances and TMJ involvement were present in all subtypes of JIA, except for one subtype comprising one patient. Patients with growth disturbances had more severe disease than patients with normal craniofacial growth, regarding both present and previous disease activity. Unexpectedly, half of the patients without craniofacial growth disturbances also had TMJ involvement, many from before the age of 12. CONCLUSIONS: Craniofacial growth disturbances were found to be frequent in adult JIA patients, especially in those with bilateral TMJ involvement. However, growth disturbances did not always follow TMJ involvement, not even when affected early
SN - 1502-7732
AD - Department of Maxillofacial Radiology, University of Oslo, Oslo, Norway. l.z.arvidsson@odont.uio.no
UR - 20615158
ER -

TY - JOUR
ID - 702
T1 - Laparoscopic versus open surgery for suspected appendicitis. [Review][Update of Cochrane Database Syst Rev. 2004;(4):CD001546; PMID: 15495014]
A1 - Sauerland,S.
A1 - Jaschinski,T.
A1 - Neugebauer,E.A.
Y1 - 2010///
N1 - Sauerland, Stefan. Jaschinski, Thomas. Neugebauer, Edmund Am
The Cochrane database of systematic reviews
100909747
IM
Journal Article. Meta-Analysis. Review
English
KW - MEDLINE
KW - Acute Disease
KW - Adult
KW - Appendectomy/ae [Adverse Effects]
KW - Appendectomy/mt [Methods]
KW - Appendicitis/di [Diagnosis]
KW - Appendicitis/su [Surgery]
KW - Child
KW - Female
KW - Humans
KW - Laparoscopy/ae [Adverse Effects]
KW - Laparoscopy
KW - Male
KW - Randomized Controlled Trials as Topic
RP - NOT IN FILE
SP - CD001546
JF - Cochrane Database of Systematic Reviews
JA - Cochrane Database Syst Rev
IS - 10

CY - England
N2 - BACKGROUND: Laparoscopic surgery for acute appendicitis has been proposed to have advantages over conventional surgery. OBJECTIVES: To compare the diagnostic and therapeutic effects of laparoscopic and conventional 'open' surgery. SEARCH STRATEGY: We searched the Cochrane Library, MEDLINE, EMBASE, LILACS, CNKI, SciSearch, study registries, and the congress proceedings of endoscopic surgical societies. SELECTION CRITERIA: We included randomized clinical trials comparing laparoscopic (LA) versus open appendectomy (OA) in adults or children. Studies comparing immediate OA versus diagnostic laparoscopy (followed by LA or OA if necessary) were separately identified. DATA COLLECTION AND ANALYSIS: Two reviewers independently assessed trial quality. Missing information or data was requested from the authors. We used odds ratios (OR), relative risks (RR), and 95% confidence intervals (CI) for analysis. MAIN RESULTS: We included 67 studies, of which 56 compared LA (with or without diagnostic laparoscopy) vs. OA in adults. Wound infections were less likely after LA than after OA (OR 0.43; CI 0.34 to 0.54), but the incidence of intraabdominal abscesses was increased (OR 1.87; CI 1.19 to 2.93). The duration of surgery was 10 minutes (CI 6 to 15) longer for LA. Pain on day 1 after surgery was reduced after LA by 8 mm (CI 5 to 11 mm) on a 100 mm visual analogue scale. Hospital stay was shortened by 1.1 day (CI 0.7 to 1.5). Return to normal activity, work, and sport occurred earlier after LA than after OA. While the operation costs of LA were significantly higher, the costs outside hospital were reduced. Seven studies on children were included, but the results do not seem to be much different when compared to adults. Diagnostic laparoscopy reduced the risk of a negative appendectomy, but this effect was stronger in fertile women (RR 0.20; CI 0.11 to 0.34) as compared to unselected adults (RR 0.37; CI 0.13 to 1.01). AUTHORS' CONCLUSIONS: In those clinical settings where surgical expertise and equipment are available and affordable, diagnostic laparoscopy and LA (either in combination or separately) seem to have various advantages over OA. Some of the clinical effects of LA, however, are small and of limited clinical relevance. In spite of the mediocre quality of the available research data, we would generally recommend to use laparoscopy and LA in patients with suspected appendicitis unless laparoscopy itself is contraindicated or not feasible. Especially young female, obese, and employed patients seem to benefit from LA
SN - 1469-493X
AD - Department of Non-Drug Interventions, Institute for Quality and Efficiency in Health Care, Dillenburger Str. 27, Cologne, Germany, 51105
UR - 20927725
ER -

TY - JOUR
ID - 703
T1 - The Stamp-in-Safety programme, an intervention to promote better supervision of children on childcare centre playgrounds: an evaluation in an urban setting
A1 - Chelvakumar,G.
A1 - Sheehan,K.
A1 - Hill,A.L.
A1 - Lowe,D.
A1 - Mandich,N.
A1 - Schwebel,D.C.
Y1 - 2010/10//
N1 - Chelvakumar, Gayathri. Sheehan, Karen. Hill, Amy L. Lowe, Danita. Mandich, Nicole. Schwebel, David C
Injury prevention : journal of the International Society for Child and Adolescent Injury Prevention
crz, 9510056
IM
Journal Article. Research Support, Non-U.S. Gov't
English
KW - MEDLINE
KW - Accident Prevention
KW - Accidental Falls/pc [Prevention & Control]
KW - Accidental Falls/sn [Statistics & Numerical Data]
KW - Behavior Control
KW - Child Day Care Centers
KW - Child

KW - Preschool
KW - Equipment Design
KW - Female
KW - Humans
KW - Male
KW - Play and Playthings/in [Injuries]
KW - Social Environment
KW - United States/ep [Epidemiology]
RP - NOT IN FILE
SP - 352
EP - 354
JF - Injury Prevention
JA - Inj Prev
VL - 16
IS - 5
CY - England
N2 - Using a non-equivalent control group design, this report evaluated a previously studied behavioural intervention, the Stamp-in-Safety programme, which is designed to reduce the injury risk for young children on playgrounds at childcare centres by increasing the quality of adult supervision and rewarding children for safe play. In an urban, commercial childcare centre, 71 children aged 3-5 years and 15 teachers participated. Primary outcome measures were teacher verbalisations (warnings, explanations, redirects), teacher location (core, outskirt, or fringe of playground), child risk-taking behaviours (using equipment appropriately) and the number of injuries on the playground. Analyses revealed that the intervention had a modest positive effect in promoting safer teacher and child playground behaviours. This study reaffirms previous results that the Stamp-in-Safety programme is an effective method to decrease the risk of playground injuries at childcare centres
SN - 1475-5785
AD - Medical College of Wisconsin, Milwaukee, Wisconsin, USA
UR - 20587809
ER -

TY - JOUR
ID - 704
T1 - [Recommendations of prophylaxis of vitamin D deficiency in Poland (2009)]. [Polish]
A1 - Charzewska,J.
A1 - Chlebna-Sokol,D.
A1 - Chybicka,A.
A1 - Czech-Kowalska,J.
A1 - Dobrzanska,A.
A1 - Helwich,E.
A1 - Imiela,J.R.
A1 - Karczmarewicz,E.
A1 - Ksiazyk,J.B.
A1 - Lewinski,A.
A1 - Lorenc,R.S.
A1 - Lukas,W.
A1 - Lukaszkiewicz,J.
A1 - Marcinowska-Suchowierska,E.
A1 - Milanowski,A.
A1 - Milewicz,A.
A1 - Pludowski,P.
A1 - Pronicka,E.
A1 - Radowicki,S.
A1 - Ryzko,J.
A1 - Socha,J.
A1 - Szczapa,J.
A1 - Weker,H.
Y1 - 2010/04//

N1 - Charzewska, Jadwiga. Chlebna-Sokol, Danuta. Chybicka, Alicja. Czech-Kowalska, Justyna. Dobrzanska, Anna. Helwich, Ewa. Imiela, Jacek R. Karczmarewicz, Elzbieta. Ksiazyk, Janusz B. Lewinski, Andrzej. Lorenc, Roman S. Lukas, Witold. Lukaszkiewicz, Jacek. Marcinowska-Suchowierska, Ewa. Milanowski, Andrzej. Milewicz, Andrzej. Pludowski, Pawel. Pronicka, Ewa. Radowicki, Stanislaw. Ryzko, Jozef. Socha, Jerzy. Szczapa, Jerzy. Weker, Halina
Medycyna wieku rozwojowego
100928610, dpo
IM
English Abstract. Journal Article
Polish
KW - MEDLINE
KW - Adolescent
KW - Adult
KW - Child
KW - Preschool
KW - Dietary Supplements
KW - Female
KW - Humans
KW - Infant
KW - Infant Food
KW - Newborn
KW - Lactation/ph [Physiology]
KW - Male
KW - Middle Aged
KW - Poland/ep [Epidemiology]
KW - Practice Guidelines as Topic
KW - Pregnancy
KW - Pregnancy Complications/ep [Epidemiology]
KW - Pregnancy Complications/pc [Prevention & Control]
KW - Prevalence
KW - Skin/me [Metabolism]
KW - Sunlight
KW - Vitamin D/ad [Administration & Dosage]
KW - Vitamin D/bi [Biosynthesis]
KW - Vitamin D Deficiency/ep [Epidemiology]
KW - Vitamin D Deficiency/pc [Prevention & Control]
KW - Young Adult
RP - NOT IN FILE
SP - 218
EP - 223
JF - Medycyna Wieku Rozwojowego
JA - Med Wieku Rozwoj.
VL - 14
IS - 2
CY - Poland
N2 - Adequate vitamin D intake and its status as well outdoor physical activity are important not only for normal bone development and Ca-P metabolism, but for optimal function of many organs and tissues throughout the body. Due to documented changes in dietary habits and physical activity level, both observed in growing children and adults, the prevalence of vitamin D insufficiency is continuously increasing. National Consultants and experts in this field established the Polish recommendations for prophylactic vitamin D supplementation in infants, toddlers, children and adolescents as well as in adults, including pregnant and lactating women based on current literature review. Taking into consideration pleyotropic vitamin D action and safety aspects serum 25-hydroxyvitamin D (25-OHD) level of 20-60 ng/ml (50-750 nmol/l) in children and 30-80 ng/ml (75-200 nmol/I) in adults is considered as optimal. Sunlight exposure inducing vitamin D production in the skin is main endogen source of vitamin D in the body but sunscreens may reduce skin synthesis by 90%. In Poland, skin synthesis is effective only from April to September so other sources of vitamin D such as diet and supplements play an important role. All newborns should be supplemented with 400 IU/d of vitamin D beginning from the first few

days of life and continue during infancy. In formula fed infants vitamin D intake from the diet should be taken into account. In preterm infants higher total vitamin D intake (400-800 IU/day) is recommended till 40 weeks post conception. Total vitamin D intake in children and adolescents required from all sources (diet and/or supplements) should be 400 IU/d between October and March and throughout the whole year in case of inadequate vitamin D skin synthesis during the summer months. In overweight/obese children supplementation with higher dosage of vitamin D up to 800-1000 IU/d should be considered. Adults require 800-1000 IU/d of vitamin D. In pregnant and lactating women such supplementation is recommended in case of inadequate intake from diet and/or skin synthesis supplementation. Monitoring of serum 25-OHD level to define optimal dosage should be considered
SN - 1428-345X
AD - Zakladu Epidemiologii i Norm Zywienia Instytutu zywnosci i Zywienia
UR - 20919473
ER -

TY - JOUR
ID - 705
T1 - [BINet programme as a standard of modern health policy]. [Polish]
A1 - Hryniewicz,W.
A1 - Jackowska,T.
A1 - Skoczynska,A.
Y1 - 2010/04//
N1 - Hryniewicz, Waleria. Jackowska, Teresa. Skoczynska, Anna
Medycyna wieku rozwojowego
100928610, dpo
IM
English Abstract. Journal Article
Polish
KW - MEDLINE
KW - Bacterial Infections/di [Diagnosis]
KW - Bacterial Infections/ep [Epidemiology]
KW - Bacterial Infections/pc [Prevention & Control]
KW - Child
KW - Preschool
KW - Community Networks/og [Organization & Administration]
KW - Community-Acquired Infections/di [Diagnosis]
KW - Community-Acquired Infections/ep [Epidemiology]
KW - Community-Acquired Infections/pc [Prevention & Control]
KW - Health Policy
KW - Humans
KW - Infant
KW - Newborn
KW - Poland/ep [Epidemiology]
KW - Policy Making
KW - Program Development/mt [Methods]
RP - NOT IN FILE
SP - 211
EP - 217
JF - Medycyna Wieku Rozwojowego
JA - Med Wieku Rozwoj.
VL - 14
IS - 2
CY - Poland
N2 - Infectious diseases--despite significant progress, both in their management and prevention--are becoming an increasingly serious problem, and their extent and character frequently constitute a threat for public health. In addition, a significant part of medical decisions regarding treatment and prevention of invasive bacterial infections is not made on the basis of the etiological agent of infection, which increases the chance of therapeutic failures, and makes the right decisions regarding prophylaxis difficult. In June 2008 the National

Reference Centre for Bacterial Meningitis (Krajowy Osrodek Referencyjny ds. Diagnostyki Bakteryjnych Zakazen Osrodkowego Ukladu Nerwowego, KOROUN) started the BINet programme, which is a network monitoring invasive bacterial infections acquired outside the hospital. The programme aims to improve the diagnostic and thus therapeutic standards, in community acquired invasive bacterial infections in Poland. The BINet programme is based on the cooperation of clinicians, epidemiologists, microbiologists, and is headed by a team of the National Reference Centre for Bacterial Meningitis. The BINet programme enables quick and appropriate microbiological diagnosis of infections which is the basis of rational therapy and plays a key role in dealing with the infected patient. In addition, accurate epidemiological data allow to take quick and effective preventive measures at local and national level
SN - 1428-345X
AD - Zaklad Epidemiologii i Mikrobiologii Klinicznej, Narodowy Instytut Lekow
UR - 20919472
ER -

TY - JOUR
ID - 706
T1 - Participation in leisure activities among boys with attention deficit hyperactivity disorder
A1 - Shimoni,M.
A1 - Engel-Yeger,B.
A1 - Tirosh,E.
Y1 - 2010/11//
N1 - Shimoni, Ma'ayan. Engel-Yeger, Batya. Tirosh, Emanuel
Research in developmental disabilities
8709782, rid
IM
Journal Article
English
KW - MEDLINE
KW - Attention Deficit Disorder with Hyperactivity/px [Psychology]
KW - Attention Deficit Disorder with Hyperactivity/th [Therapy]
KW - Child
KW - Child Psychology
KW - Goals
KW - Happiness
KW - Humans
KW - Leisure Activities/px [Psychology]
KW - Male
KW - Patient Participation/px [Psychology]
KW - Questionnaires
RP - NOT IN FILE
SP - 1234
EP - 1239
JF - Research in Developmental Disabilities
JA - Res Dev Disabil
VL - 31
IS - 6
CY - United States
N2 - ADHD is a neural developmental disorder expressed in various life settings. Yet, previous studies have focused mainly on children's function in school and academic achievement. The purpose of the present study was, therefore, to examine participation patterns in outside formal school activities among boys with ADHD compared to typical boys. Participants included 25 boys aged 8-11 years with ADHD and 25 age-matched typical boys. All participants completed the Children's Assessment of Participation and Enjoyment (CAPE). Several aspects of participation were examined: diversity, intensity, enjoyment, place, and partners in 49 extra curricular activities. The findings indicate that boys with ADHD reported significant lower intensity rates of participation in most activity domains. Furthermore, boys with ADHD also reported higher diversity scores and lower enjoyment in 'formal' activities. Yet, no significant differences were found with regard to activity place and partners. These findings enhance the importance of providing therapy that refers to after school activities.

Accordingly, CAPE can be useful for assessing boys with ADHD and planning appropriate intervention programs. Copyright 2010 Elsevier Ltd. All rights reserved
SN - 1873-3379
AD - Occupational Therapy Department, Faculty of Social Welfare & Health Sciences, University of Haifa, Mount Carmel, Haifa 31905, Israel
UR - 20705424
ER -

TY - JOUR
ID - 707
T1 - [The use of acetylcholinesterase inhibitors pesticides in eleven local health institutions, Colombia, 2002-2005]. [Spanish]
A1 - Cardenas,O.
A1 - Silva,E.
A1 - Ortiz,J.E.
Y1 - 2010/01//
N1 - Cardenas, Omayda. Silva, Elizabeth. Ortiz, Jaime Eduardo
Biomedica : revista del Instituto Nacional de Salud
8205605
IM
English Abstract. Journal Article. Research Support, Non-U.S. Gov't
Spanish
KW - MEDLINE
KW - Adolescent
KW - Adult
KW - Aged
KW - 80 and over
KW - Child
KW - Preschool
KW - Cholinesterase Inhibitors/an [Analysis]
KW - Colombia
KW - Environmental Exposure/an [Analysis]
KW - Female
KW - Health Facilities
KW - Humans
KW - Infant
KW - Male
KW - Middle Aged
KW - Occupational Exposure/an [Analysis]
KW - Pesticides/an [Analysis]
KW - Young Adult
RP - NOT IN FILE
SP - 95
EP - 106
JF - Biomedica
JA - Biomedica (Bogota)
VL - 30
IS - 1
CY - Colombia
N2 - INTRODUCTION: Due to the importance of acetylcholinesterase inhibiting chemicals as pesticides in developing countries, the Instituto Nacional de Salud in Colombia designed the organophosphate and carbamate epidemiological surveillance program for the period 2002-2005. OBJECTIVE: The acetylcholinesterase activity was determined in study participants with a history of organophosphate and carbamate exposure and the most commonly used pesticides were identified in each study area. MATERIALS AND METHODS: The information was compiled from reports sent to the Instituto Nacional de Salud organophosphate and carbamate epidemiological surveillance program from each of 11 provinces in Colombia. The analytical determination of the biomarker was performed by acetylcholinestare activity determined with the Lovibond field equipment.

RESULTS: A total of 28,303 people were designated as having risk of exposure to pesticides. Most were men (81.4%). Abnormal determinations averaged 9.3% (9.9% in men and 7.0% in women). The 18-25 year old age group showed the highest prevalence of abnormal results (12.3%), followed by the group of 0-5 year olds (10.7%). The highest prevalence of abnormal acetylcholinesterase activity was in farm workers (27.0%), followed by general outdoor activities (26.1%). In the province of Meta, 80% of participants showed abnormal values of enzyme activity. The most commonly used pesticides were organophosphates (39.7%) and carbamates (16.6%). CONCLUSION: The increase in the prevalence of abnormal values of acetylcholinesterase activity and the risk of exposure to pesticides in children necessitates a lowering of use and commercialization of high risk pesticides, and a need for developing safer methods for pest management
SN - 0120-4157
AD - Grupo Salud Ambiental, Subdireccion Red Nacional de Laboratorios, Instituto Nacional de Salud, Bogota, DC, Colombia
UR - 20890554
ER -

TY - JOUR
ID - 708
T1 - Temporal and spatial patterns of ambient endotoxin concentrations in Fresno, California
A1 - Tager,I.B.
A1 - Lurmann,F.W.
A1 - Haight,T.
A1 - Alcorn,S.
A1 - Penfold,B.
A1 - Hammond,S.K.
Y1 - 2010/10//
N1 - Tager, Ira B. Lurmann, Frederick W. Haight, Thaddeus. Alcorn, Siana. Penfold, Bryan. Hammond, S Katharine
Environmental health perspectives
ei0, 0330411
IM
Journal Article. Research Support, N.I.H., Extramural. Research Support, Non-U.S. Gov't
English
KW - MEDLINE
KW - Air Pollutants/an [Analysis]
KW - Air Pollutants/to [Toxicity]
KW - Asthma/pp [Physiopathology]
KW - California
KW - Child
KW - Cohort Studies
KW - Endotoxins/an [Analysis]
KW - Endotoxins/to [Toxicity]
KW - Humans
RP - NOT IN FILE
SP - 1490
EP - 1496
JF - Environmental Health Perspectives
JA - Environ Health Perspect
VL - 118
IS - 10
CY - United States
N2 - BACKGROUND: Endotoxins are found in indoor dust generated by human activity and pets, in soil, and adsorbed onto the surfaces of ambient combustion particles. Endotoxin concentrations have been associated with respiratory symptoms and the risk of atopy and asthma in children. OBJECTIVE: We characterized the temporal and spatial variability of ambient endotoxin in Fresno/Clovis, California, located in California's Central Valley, to identify correlates and potential predictors of ambient endotoxin concentrations in a cohort of children with asthma [Fresno Asthmatic Children's Environment Study (FACES)]. METHODS: Between May 2001 and October 2004, daily ambient endotoxin and air pollutants were collected at the central ambient

monitoring site of the California Air Resources Board in Fresno and, for shorter time periods, at 10 schools and indoors and outdoors at 84 residences in the community. Analyses were restricted to May-October, the dry months during which endotoxin concentrations are highest. RESULTS: Daily endotoxin concentration patterns were determined mainly by meteorologic factors, particularly the degree of air stagnation. Overall concentrations were lowest in areas distant from agricultural activities. Highest concentrations were found in areas immediately downwind from agricultural/pasture land. Among three other measured air pollutants [fine particulate matter, elemental carbon (a marker of traffic in Fresno), and coarse particulate matter (PMc)], PMc was the only pollutant correlated with endotoxin. Endotoxin, however, was the most spatially variable. CONCLUSIONS: Our data support the need to evaluate the spatial/temporal variability of endotoxin concentrations, rather than relying on a few measurements made at one location, in studies of exposure and and respiratory health effects, particularly in children with asthma and other chronic respiratory diseases
SN - 1552-9924
AD - Division of Epidemiology, School of Public Health, University of California, Berkeley, California 94720, USA. ibt@berkeley.edu
UR - 20494854
ER -

TY - JOUR
ID - 709
T1 - Population density and refractive error among Chinese children
A1 - Zhang,M.
A1 - Li,L.
A1 - Chen,L.
A1 - Lee,J.
A1 - Wu,J.
A1 - Yang,A.
A1 - Chen,C.
A1 - Xu,D.
A1 - Lam,D.S.
A1 - Sharma,A.
A1 - Griffiths,S.
A1 - Gao,Y.
A1 - Congdon,N.
Y1 - 2010/10//
N1 - Zhang, Mingzhi. Li, Liping. Chen, Lizhen. Lee, Jack. Wu, Jiasi. Yang, Amy. Chen, Connie. Xu, Daocheng. Lam, Dennis S C. Sharma, Abhishek. Griffiths, Sian. Gao, Yang. Congdon, Nathan
Investigative ophthalmology & visual science
gwi, 7703701
IM
Journal Article. Research Support, Non-U.S. Gov't
English
KW - MEDLINE
KW - Adolescent
KW - Asian Continental Ancestry Group/eh [Ethnology]
KW - Child
KW - China/ep [Epidemiology]
KW - Female
KW - Humans
KW - Male
KW - Myopia/ep [Epidemiology]
KW - Population Density
KW - Prevalence
KW - Questionnaires
KW - Refraction
KW - Ocular/ph [Physiology]
KW - Risk Factors
KW - Rural Population/sn [Statistics & Numerical Data]

KW - Urban Population/sn [Statistics & Numerical Data]
KW - Visual Acuity/ph [Physiology]
RP - NOT IN FILE
SP - 4969
EP - 4976
JF - Investigative Ophthalmology & Visual Science
JA - Invest Ophthalmol Vis Sci
VL - 51
IS - 10
CY - United States
N2 - PURPOSE: China is urbanizing rapidly, and the prevalence of myopia is high. This study was conducted to identify the reasons for observed differences in the prevalence of myopia among urban versus rural Chinese children. METHODS: All children with uncorrected acuity of 6/12 or worse and a 50% random sample of children with vision better than 6/12 at all secondary schools in mixed rural-urban Liangying Township, Guangdong, underwent cycloplegic refraction, and provided data on age, gender, parental education, weekly near work and time outdoors, and urban development level of respondents' neighborhoods (12-item questionnaire). Population density of 32 villages and urban zones in Liangying was calculated from census figures (mean population density, 217 persons/km(2); range, 94-957; mean for Guangdong, 486). RESULTS: Among 5844 eligible children, 4612 (78.9%) had parental consent and completed examinations; 2957 were refracted per protocol, and 2480 (83.9%) of these had questionnaire data. Those with completed examinations were more likely to be girls (P < 0.001), and questionnaire respondents were more myopic (P = 0.02), but otherwise did not differ significantly from nonrespondents. In multivariate models, older age (P < 0.001), more near work (P = 0.02), and higher population density (P = 0.003), but not development index, parental education, or time outdoors were significantly associated with more myopic refractive error. CONCLUSIONS: Higher population density appears to be associated with myopia risk, independent of academic activity, time spent outdoors, familial educational level, or economic development, factors that have been thought to explain higher myopia prevalence among urban children. Mechanisms for this apparent association should be sought
SN - 1552-5783
AD - Joint Shantou International Eye Center, Shantou, Peoples Republic of China
UR - 20445117
ER -

TY - JOUR
ID - 710
T1 - Effects of outdoor activities on myopia among rural school children in Taiwan
A1 - Wu,P.C.
A1 - Tsai,C.L.
A1 - Hu,C.H.
A1 - Yang,Y.H.
Y1 - 2010/10//
N1 - Wu, Pei-Chang. Tsai, Chia-Ling. Hu, Chia-Huo. Yang, Yi-Hsin
Ophthalmic epidemiology
cg6, 9435674
IM
Journal Article. Research Support, Non-U.S. Gov't
English
KW - MEDLINE
KW - Child
KW - Child Behavior
KW - Cross-Sectional Studies
KW - Female
KW - Humans
KW - Leisure Activities
KW - Male
KW - Myopia/ep [Epidemiology]
KW - Prevalence
KW - Questionnaires

KW - Risk Factors
KW - Rural Population/sn [Statistics & Numerical Data]
KW - Schools
KW - Sedentary Lifestyle
KW - Taiwan/ep [Epidemiology]
KW - Television
KW - Visual Acuity
RP - NOT IN FILE
SP - 338
EP - 342
JF - Ophthalmic Epidemiology
JA - Ophthalmic Epidemiol
VL - 17
IS - 5
CY - England
N2 - PURPOSE: The aim was to identify the prevalence and risk factors of myopia among elementary school students in a rural area of Taiwan. METHODS: A cross-sectional study was conducted. Elementary school students aged 7-12 years were recruited from the two schools located on Chimei Island. Data were obtained by means of a parent questionnaire and ocular evaluations that included axial length and cycloplegic autorefraction. RESULTS: One hundred and forty five students were recruited for this study. Myopia prevalence was 31%. In univariate analysis, myopia was significantly associated with school year, myopic parent, and watching television (TV) (P < .0001, =0.007 and =0.029, respectively). Multiple logistic regression analysis revealed that myopia was significantly associated with school year and myopic parent. However, the effect of watching TV was not statistically significant (P=0.059). Outdoor activity showed significance and was inversely associated with myopia (Odds Ratio [OR]=0.3, 95% Confidence Interval [CI]=0.1-0.9, P=0.025). CONCLUSION: This study suggests that outdoor activities might be an important protecting factor for myopia in rural school children
SN - 1744-5086
AD - Department of Opthalmology, Chang Gung Memorial Hospital-Kaohsiung Medical Center and Chang Gung University College of Medicine, Kaohsiung, Taiwan
UR - 20868261
ER -

TY - JOUR
ID - 711
T1 - A cluster randomised trial to evaluate a physical activity intervention among 3-5 year old children attending long day care services: study protocol
A1 - Finch,M.
A1 - Wolfenden,L.
A1 - Morgan,P.J.
A1 - Freund,M.
A1 - Wyse,R.
A1 - Wiggers,J.
Y1 - 2010///
N1 - Finch, Meghan. Wolfenden, Luke. Morgan, Philip J. Freund, Megan. Wyse, Rebecca. Wiggers, John
BMC public health
100968562
IM
Journal Article. Randomized Controlled Trial. Research Support, Non-U.S. Gov't
English
KW - MEDLINE
KW - Child Day Care Centers
KW - Child
KW - Preschool
KW - Cluster Analysis
KW - Exercise
KW - Health Promotion/og [Organization & Administration]
KW - Humans

KW - Interviews as Topic
KW - New South Wales
KW - Program Evaluation
RP - NOT IN FILE
SP - 534
JF - BMC Public Health
VL - 10
CY - England
N2 - BACKGROUND: Young children are not participating in recommended levels of physical activity and exhibit high levels of sedentary behaviour. Childcare services provide access to large numbers of young children for prolonged periods, yet there is limited experimental evidence regarding the effectiveness of physical activity interventions implemented in this setting. The aim of this study is to assess the effectiveness and acceptability of a multi-component physical activity intervention, delivered by childcare service staff, in increasing the physical activity levels of children attending long day care services. METHODS/DESIGN: The study will employ a cluster randomised controlled trial design. Three hundred children aged between 3-5 years from twenty randomly selected long day care services in the Hunter Region of New South Wales, Australia will be invited to participate in the trial. Ten of the 20 long day care services will be randomly allocated to deliver the intervention with the remaining ten services allocated to a wait list control group. The physical activity intervention will consist of a number of strategies including: delivering structured fundamental movement skill activities, increasing physical activity opportunities, increasing staff role modelling, providing children with a physical activity promoting indoor and outdoor environment and limiting children's small screen recreation and sedentary behaviours. Intervention effectiveness will be measured via child physical activity levels during attendance at long day care. The study also seeks to determine the acceptability and extent of implementation of the intervention by services and their staff participating in the study. DISCUSSION: The trial will address current gaps in the research evidence base and contribute to the design and delivery of future interventions promoting physical activity for young children in long day care settings. TRIAL REGISTRATION: Australian New Zealand Clinical Trials Registry ACTRN12610000087055
SN - 1471-2458
AD - Hunter New England Population Health, Newcastle, NSW, Australia.
Meghan.finch@hnehealth.nsw.gov.au
UR - 20822543
ER -

TY - JOUR
ID - 712
T1 - Case-control study of male germ cell tumors nested in a cohort of car-manufacturing workers: Findings from the occupational history
A1 - Langner,I.
A1 - Schmeisser,N.
A1 - Mester,B.
A1 - Behrens,T.
A1 - Gottlieb,A.
A1 - Ahrens,W.
Y1 - 2010/10//
N1 - Langner, Ingo. Schmeisser, Nils. Mester, Birte. Behrens, Thomas. Gottlieb, Andrea. Ahrens, Wolfgang
American journal of industrial medicine
3in, 8101110
IM
Journal Article. Research Support, Non-U.S. Gov't
English
KW - MEDLINE
KW - Adolescent
KW - Adult
KW - Aged
KW - Automobiles
KW - Case-Control Studies
KW - Child

KW - Confidence Intervals
KW - Germany/ep [Epidemiology]
KW - Humans
KW - Incidence
KW - Industry/sn [Statistics & Numerical Data]
KW - Male
KW - Middle Aged
KW - Multivariate Analysis
KW - Neoplasms
KW - Germ Cell and Embryonal/ep [Epidemiology]
KW - Germ Cell and Embryonal/et [Etiology]
KW - Occupational Exposure/sn [Statistics & Numerical Data]
KW - Occupations/sn [Statistics & Numerical Data]
KW - Odds Ratio
KW - Risk
KW - Young Adult
RP - NOT IN FILE
SP - 1006
EP - 1018
JF - American Journal of Industrial Medicine
JA - Am J Ind Med
VL - 53
IS - 10
CY - United States
N2 - BACKGROUND: To examine whether the previously observed excess risk of male germ cell cancer in a cohort of car-manufacturing workers can be attributed to occupational activities inside and/or outside the car industry. METHODS: A nested case-control study among workers in six plants included 205 cases of germ cell cancer and 1,105 controls, individually matched by year of birth (+2 years). Job periods of the individual occupational histories were coded based on the International Standard Classification of Occupations (ISCO) and the industrial classification of economic activities (NACE). Odds ratios (ORs) and corresponding 95%-confidence intervals (CI) for ever-never and cumulative employment were calculated by conditional multivariate logistic regression adjusted for cryptorchidism. RESULTS: Significantly increased risks were observed for machinery fitters and assemblers (A) (OR=1.8, 95% CI 1.25-2.53) and "workers not elsewhere classified" (OR=2.10, 95% CI 1.27-3.54), but no trend was observed for employment duration in either occupational group. Stratification of job group A by metal-cutting and non-cutting jobs yielded ORs of 1.87 (95% CI 1.31-2.67) and of 1.24 (95% CI 0.68-2.28), respectively. Among "plumbers, welders, sheet & structural metal workers" (adjusted OR 1.4, 95% CI 0.99-1.95) only "structural metal preparers and erectors" showed a substantially increased risk (OR=2.30; 95% CI 1.27-4.27). CONCLUSIONS: Our results do not fully explain the increased incidence of germ cell cancer in the cohort, but support previous findings showing increased risks among metal workers. These risks were most strongly pronounced in metal-cutting activities. 2010 Wiley-Liss, Inc
SN - 1097-0274
AD - Bremen Institute for Prevention Research and Social Medicine, Bremen University, Germany. ingo.langner@bips.uni-bremen.de
UR - 20860055
ER -

TY - JOUR
ID - 713
T1 - Prevalence of overweight and obesity in Indian adolescent school going children: its relationship with socioeconomic status and associated lifestyle factors
A1 - Goyal,R.K.
A1 - Shah,V.N.
A1 - Saboo,B.D.
A1 - Phatak,S.R.
A1 - Shah,N.N.
A1 - Gohel,M.C.

A1 - Raval,P.B.
A1 - Patel,S.S.
Y1 - 2010/03//
N1 - Goyal, Ramesh K. Shah, Vitthaldas N. Saboo, Banshi D. Phatak, Sanjiv R. Shah, Navneet N. Gohel, Mukesh C. Raval, Prashad B. Patel, Snehal S
The Journal of the Association of Physicians of India
hg7, 7505585
IM
Journal Article. Research Support, Non-U.S. Gov't
English
KW - MEDLINE
KW - Adolescent
KW - Age Distribution
KW - Body Mass Index
KW - Child
KW - Diet
KW - Exercise
KW - Female
KW - Humans
KW - India/ep [Epidemiology]
KW - Life Style
KW - Male
KW - Obesity/ec [Economics]
KW - Obesity/ep [Epidemiology]
KW - Overweight/ec [Economics]
KW - Overweight/ep [Epidemiology]
KW - Population Surveillance
KW - Prevalence
KW - Risk Factors
KW - Schools
KW - Sex Distribution
KW - Socioeconomic Factors
RP - NOT IN FILE
SP - 151
EP - 158
JF - Journal of the Association of Physicians of India
JA - J Assoc Physicians India
VL - 58
CY - India
N2 - AIMS AND OBJECTIVES: Obesity and overweight have become a worldwide epidemic, and there is an urgent need to examine childhood obesity and overweight across countries using a standardized international standard. In the present study we have investigated the prevalence of obesity and overweight and their association with socioeconomic status (SES) and the risk factors like diet, physical activity like exercise, sports, sleeping habit in afternoon, eating habits like junk food, chocolate, eating outside at weekend, family history of diabetes and obesity. MATERIAL AND METHODS: The study was carried out in 5664 school children of 12-18 years of age and having different SES. The obesity and overweight were considered using an updated body mass index reference. SES and life style factors were determined using pre-tested questionnaire. RESULTS: Age-adjusted prevalence of overweight was found to be 14.3% among boys and 9.2% among girls where as the prevalence of obesity was 2.9% in boys and 1.5% in girls. The prevalence of overweight among children was higher in middle SES as compared to high SES group in both boys and girls whereas the prevalence of obesity was higher in high SES group as compared to middle SES group. The prevalence of obesity as well as overweight in low SES group was the lowest as compared to other group. Eating habit like junk food, chocolate, eating outside at weekend and physical activity like exercise, sports, sleeping habit in afternoon having remarkable effect on prevalence on overweight and obesity among middle to high SES group. Family history of diabetes and obesity were also found to be positively associated. CONCLUSION: Our data suggest that the prevalence of overweight and obesity varies remarkably with different socioeconomic development levels
SN - 0004-5772

AD - Sterling Hospital and Department of Pharmacology, L.M.College of Pharmacy, Ahmedabad 380 009, India
UR - 20848812
ER -

TY - JOUR
ID - 714
T1 - Leisure-time physical activity in elementary schools: analysis of contextual conditions
A1 - McKenzie,T.L.
A1 - Crespo,N.C.
A1 - Baquero,B.
A1 - Elder,J.P.
Y1 - 2010/10//
N1 - McKenzie, Thomas L. Crespo, Noe C. Baquero, Barbara. Elder, John P
The Journal of school health
k13, 0376370
IM, N
Journal Article
English
KW - MEDLINE
KW - Child
KW - Child Welfare
KW - Confidence Intervals
KW - Curriculum
KW - Female
KW - Health Policy
KW - Humans
KW - Leisure Activities
KW - Logistic Models
KW - Male
KW - Motor Activity
KW - Odds Ratio
KW - Physical Fitness
KW - Poverty
KW - School Health Services/og [Organization & Administration]
KW - Schools/og [Organization & Administration]
KW - Sedentary Lifestyle
KW - Social Environment
KW - Socioeconomic Factors
KW - Sports
KW - Time Factors
RP - NOT IN FILE
SP - 470
EP - 477
JF - Journal of School Health
JA - J Sch Health
VL - 80
IS - 10
CY - United States
N2 - BACKGROUND: Little is known about children's leisure-time physical activity (PA) at school and how it is associated with contextual variables. The purpose of this study was to objectively assess children's voluntary PA during 3 daily periods and examine modifiable contextual factors. METHODS: We conducted SOPLAY (System for Observing Play and Leisure Activity in Youth) observations before school, during recess, and at lunchtime in 137 targeted activity areas in 13 elementary schools over 18 months. During observations, each child was coded as Sedentary, Walking, or Vigorous, and simultaneous entries were made for area characteristics (accessibility, usability, presence of supervision, loose equipment, and organized activities). Logistic regression analysis was used to test associations between PA and area characteristics. RESULTS:

Assessors made 2349 area visits and observed 36,995 children. Boys had more moderate-to-vigorous physical activity (MVPA; 66.2 vs 60.0%, p < .001) and more vigorous PA (29.8 vs 24.6%; p < .001) than girls. Areas were typically accessible and usable, but provided organized activities infrequently (16.5%). Odds of engaging in MVPA were greater during lunch and recess than before school and in areas with play equipment (p < .05). CONCLUSIONS: Children accrued a substantial amount of voluntary PA during leisure time at school. Their PA would likely be increased if school playground equipment was more readily available and if supervisors were taught to provide active games and promote PA rather than suppress it. 2010, American School Health Association
SN - 1746-1561
AD - School of Exercise and Nutritional Sciences, San Diego State University, San Diego, CA 92182-7251, USA. tmckenzie@sdsu.edu
UR - 20840656
ER -

TY - JOUR
ID - 715
T1 - Where can they play? Outdoor spaces and physical activity among adolescents in U.S. urbanized areas
A1 - Boone-Heinonen,J.
A1 - Casanova,K.
A1 - Richardson,A.S.
A1 - Gordon-Larsen,P.
Y1 - 2010/09//
N1 - Boone-Heinonen, Janne. Casanova, Kathleen. Richardson, Andrea S. Gordon-Larsen, Penny
Preventive medicine
pm4, 0322116
IM
Journal Article. Research Support, N.I.H., Extramural. Research Support, U.S. Gov't, P.H.S.
English
KW - MEDLINE
KW - Adolescent
KW - Child
KW - Cross-Sectional Studies
KW - Female
KW - Humans
KW - Logistic Models
KW - Male
KW - Motor Activity
KW - Play and Playthings
KW - Recreation
KW - Residence Characteristics
KW - Sex Factors
KW - Socioeconomic Factors
KW - Sports/sn [Statistics & Numerical Data]
KW - United States/ep [Epidemiology]
KW - Urban Population/sn [Statistics & Numerical Data]
KW - Young Adult
RP - NOT IN FILE
SP - 295
EP - 298
JF - Preventive Medicine
JA - Prev Med
VL - 51
IS - 3-4
CY - United States
N2 - OBJECTIVE: To estimate behavior-specific effects of several objectively measured outdoor spaces on different types of moderate to vigorous physical activity (MVPA) in a large, diverse sample of U.S. adolescents. METHODS: Using data from Wave I (1994-1995) of the National Longitudinal Study of Adolescent Health

(U.S., n=10,359) and a linked geographic information system, we calculated percent greenspace coverage and distance to the nearest neighborhood and major parks. Using sex-stratified multivariable logistic regression, we modeled reported participation in wheel-based activities, active sports, exercise, and > 5 MVPA bouts/week as a function of each outdoor space variable, controlling for individual- and neighborhood-level sociodemographics. RESULTS: Availability of major or neighborhood parks was associated with higher participation in active sports and, in females, wheel-based activity and reporting > 5 MVPA bouts/week [OR (95% CI): up to 1.71 (1.29, 2.27)]. Greater greenspace coverage was associated with reporting > 5 MVPA bouts/week in males and females [OR (95% CI): up to 1.62 (1.10, 2.39) for 10.1 to 20% versus < 10% greenspace] and exercise participation in females [OR (95% CI): up to 1.73 (1.21, 2.49)]. CONCLUSIONS: Provision of outdoor spaces may promote different types of physical activities, with potentially greater benefits in female adolescents, who have particularly low physical activity levels. Copyright 2010 Elsevier Inc. All rights reserved
SN - 1096-0260
AD - Department of Nutrition, Gillings School of Global Public Health, University of North Carolina at Chapel Hill, Chapel Hill, NC 27516-3997, USA. boonej@unc.edu
UR - 20655948
ER -

TY - JOUR
ID - 716
T1 - Cognitive levels of performance account for hemispheric lateralisation effects in dyslexic and normally reading children
A1 - Heim,S.
A1 - Grande,M.
A1 - Meffert,E.
A1 - Eickhoff,S.B.
A1 - Schreiber,H.
A1 - Kukolja,J.
A1 - Shah,N.J.
A1 - Huber,W.
A1 - Amunts,K.
Y1 - 2010/12//
N1 - Heim, Stefan. Grande, Marion. Meffert, Elisabeth. Eickhoff, Simon B. Schreiber, Helen. Kukolja, Juraj. Shah, Nadim Jon. Huber, Walter. Amunts, Katrin
NeuroImage
cpp, 9215515
IM
Journal Article. Research Support, N.I.H., Extramural. Research Support, Non-U.S. Gov't
English
KW - MEDLINE
KW - Brain/ph [Physiology]
KW - Brain Mapping
KW - Child
KW - Cognition/ph [Physiology]
KW - Dyslexia/pp [Physiopathology]
KW - Female
KW - Functional Laterality/ph [Physiology]
KW - Humans
KW - Magnetic Resonance Imaging
KW - Male
KW - Reading
RP - NOT IN FILE
SP - 1346
EP - 1358
JF - Neuroimage
VL - 53
IS - 4
CY - United States

N2 - Recent theories of developmental dyslexia explain reading deficits in terms of deficient phonological awareness, attention, visual and auditory processing, or automaticity. Since dyslexia has a neurobiological basis, the question arises how the reader's proficiency in these cognitive variables affects the brain regions involved in visual word recognition. This question was addressed in two fMRI experiments with 19 normally reading children (Experiment 1) and 19 children with dyslexia (Experiment 2). First, reading-specific brain activation was assessed by contrasting the BOLD signal for reading aloud words vs. overtly naming pictures of real objects. Next, ANCOVAs with brain activation during reading the individuals' scores for all five cognitive variables assessed outside the scanner as covariates were performed. Whereas the normal readers' brain activation during reading showed co-variation effects predominantly in the right hemisphere, the reverse pattern was observed for the dyslexics. In particular, middle frontal gyrus, inferior parietal cortex, and precuneus showed contralateral effects for controls as compared to dyslexics. In line with earlier findings in the literature, these data hint at a global change in hemispheric asymmetry during cognitive processing in dyslexic readers, which, in turn, might affect reading proficiency. Copyright 2010 Elsevier Inc. All rights reserved
SN - 1095-9572
AD - Department of Psychiatry and Psychotherapy, University Hospital Aachen, RWTH Aachen University, Aachen, Germany. sheim@ukaachen.de
UR - 20633659
ER -

TY - JOUR
ID - 717
T1 - Traumatic brain injury in children between 7-12 years of age
A1 - Elgmark,Andersson E.
A1 - Lund,J.
A1 - Mansson,J.
Y1 - 2010///
N1 - Elgmark Andersson, Elisabeth. Lund, Josefinne. Mansson, Josefinne
Developmental neurorehabilitation
101304394
IM
Journal Article. Research Support, Non-U.S. Gov't
English
KW - MEDLINE
KW - Accidents/sn [Statistics & Numerical Data]
KW - Accidents
KW - Home/sn [Statistics & Numerical Data]
KW - Age Factors
KW - Athletic Injuries
KW - Brain Injuries/ep [Epidemiology]
KW - Brain Injuries/et [Etiology]
KW - Child
KW - Female
KW - Humans
KW - Male
KW - Patient Admission/sn [Statistics & Numerical Data]
KW - Play and Playthings/in [Injuries]
KW - Retrospective Studies
KW - Risk Factors
KW - Schools/sn [Statistics & Numerical Data]
KW - Seasons
KW - Sweden/ep [Epidemiology]
KW - Time Factors
RP - NOT IN FILE
SP - 346
EP - 350
JF - Developmental neurorehabilitation
JA - Dev Neurorehabil

VL - 13
IS - 5
CY - England
N2 - OBJECTIVE: The purpose of this study was to describe the amount of traumatic brain injuries among children in the western part of Sweden. METHODS: Based on a randomized controlled study, a retrospective analysis was conducted of data for children of 257 children 7-12 years of age. The journals were collected from 1997-2000. RESULTS: The results showed that the most common causes were play and sports and that the accidents occurred in the school environment in the afternoon, whilst the least amount of accidents took place in the home. Nearly half of all the children were admitted to the hospital. Most accidents occurred in March and the least number of accidents occurred during the summer months. CONCLUSION: Prevention strategies are needed, especially for play and sport activities in recreation centres and playgrounds. These places are a high risk in causing a TBI for children after a day at school
SN - 1751-8431
AD - Jonkoping University, Department of Rehabilitation Medicine, School of Health Sciences, Jonkoping, Sweden
UR - 20828331
ER -

TY - JOUR
ID - 718
T1 - Are overweight and obese youth at increased risk for physical activity injuries?
A1 - Warsh,J.
A1 - Pickett,W.
A1 - Janssen,I.
Y1 - 2010/08//
N1 - Warsh, Joel. Pickett, William. Janssen, Ian
Obesity facts
101469429
IM
Journal Article. Research Support, Non-U.S. Gov't
English
KW - MEDLINE
KW - Adolescent
KW - Athletic Injuries/ep [Epidemiology]
KW - Body Mass Index
KW - Child
KW - Cross-Sectional Studies
KW - Female
KW - Health Surveys
KW - Humans
KW - Male
KW - Motor Activity
KW - Obesity/ep [Epidemiology]
KW - Overweight/ep [Epidemiology]
KW - Risk Factors
KW - Risk-Taking
KW - Schools/sn [Statistics & Numerical Data]
RP - NOT IN FILE
SP - 225
EP - 230
JF - Obesity Facts
JA - Obes Facts
VL - 3
IS - 4
CY - Switzerland
N2 - OBJECTIVE: To determine whether relationships between physical activity and physical activity injuries are modified by BMI status in youth. METHOD: Data were obtained from the 2006 Canadian Health Behaviour

in School-Aged Children survey; a representative study of 7,714 grade 6-10 youth. A sub-sample of 1,814 were re-administered the survey in 2007. Analyses considered relationships among the major variables in theory-driven cross-sectional and longitudinal analyses. RESULT: Among normal weight youth, cross-sectional analyses indicated that those who reported high levels of physical activity outside of school experienced 2.28 (95% confidence interval 1.95-2.68) the relative odds for physical activity injury in comparison to those with low levels of physical activity outside of school. Analogous odds ratios for overweight and obese youth were 1.89 (1.31-2.72) and 3.72 (1.89-7.33), respectively. BMI status was not an effect modifier of the relationship between physical activity and physical activity injury. Similar observations were made in the confirmatory longitudinal analyses. CONCLUSION: Concerns surrounding the design of physical activity programmes include side-effects such as injury risk. This study provides some re-assurance that physical activity participation relates to injury in a consistent manner across BMI groups. Copyright 2010 S. Karger AG, Basel
SN - 1662-4025
AD - Department of Community Health and Epidemiology, Queen's University, Kingston, ON, Canada
UR - 20823685
ER -

TY - JOUR
ID - 719
T1 - Exposures of preschool children to chlorpyrifos, diazinon, pentachlorophenol, and 2,4-dichlorophenoxyacetic acid over 3 years from 2003 to 2005: A longitudinal model
A1 - Wilson,N.K.
A1 - Strauss,W.J.
A1 - Iroz-Elardo,N.
A1 - Chuang,J.C.
Y1 - 2010/09//
N1 - Wilson, Nancy K. Strauss, Warren J. Iroz-Elardo, Nicole. Chuang, Jane C
Journal of exposure science & environmental epidemiology
101262796
IM
Journal Article. Research Support, U.S. Gov't, Non-P.H.S.
English
KW - MEDLINE
KW - 2
KW - 4-Dichlorophenoxyacetic Acid/an [Analysis]
KW - Child
KW - Preschool
KW - Chlorpyrifos/an [Analysis]
KW - Diazinon/an [Analysis]
KW - Eating
KW - Environmental Exposure/an [Analysis]
KW - Environmental Monitoring/mt [Methods]
KW - Female
KW - Food Contamination/an [Analysis]
KW - Gas Chromatography-Mass Spectrometry
KW - Herbicides/an [Analysis]
KW - Humans
KW - Infant
KW - Inhalation Exposure/an [Analysis]
KW - Insecticides/an [Analysis]
KW - Linear Models
KW - Longitudinal Studies
KW - Male
KW - North Carolina
KW - Pentachlorophenol/an [Analysis]
KW - Pyridones/an [Analysis]
KW - Skin Absorption
RP - NOT IN FILE

SP - 546
EP - 558
JF - Journal of Exposure Science & Environmental Epidemiology
JA - J Expo Sci Environ Epidemiol
VL - 20
IS - 6
CY - United States
N2 - The impact of the US EPA-required phase-outs starting in 2000-2001 of residential uses of the organophosphate (OP) pesticides chlorpyrifos (CPF) and diazinon (DZN) on preschool children's pesticide exposures was investigated over 2003-2005, in the Raleigh-Durham-Chapel Hill area of North Carolina. Data were collected from 50 homes, each with a child initially of age 3 years (OCh) and a younger child (YCh). Environmental samples (indoor and outdoor air, dust, soil) and child-specific samples (hand surface residue, urine, diet) were collected annually over 24-h periods at each home. Child time-activity diaries and household pesticide use information were also collected. Analytes included CPF and DZN; pentachlorophenol (PCP); 2,4-dichlorophenoxyacetic acid (2,4-D); the CPF metabolite 3,5,6-trichloro-2-pyridinol (TCP); and the DZN metabolite 2-isopropyl-6-methyl-4-pyrimidinol (IMP). Exposures (ng/day) through the inhalation, dietary ingestion, and indirect ingestion were calculated. Aggregate potential doses in ng/kg body weight per day (ng/kg/day) were obtained by summing the potential doses through the three routes of exposure. Geometric mean aggregate potential doses decreased from 2003 to 2005 for both OCh and YCh, with the exception of 2,4-D. Child-specific longitudinal modeling indicated significant declines across time of the potential doses of CPF, DZN, and PCP for both children; declines of IMP for both children, significant only for OCh; a decline of TCP for OCh but an increase of TCP for YCh; and no significant change of 2,4-D for either child. Age-adjusted modeling indicated significant effects of the child's age for all except CPF, and of time for all except PCP and 2,4-D. Within-home variability was small compared with that between homes; variability was smallest for 2,4-D, both within and between homes. The aggregate potential doses of CPF and DZN were well below published reference dose values. These findings show the success of the US EPA restrictions in reducing young children's pesticide exposures
SN - 1559-064X
AD - Battelle Centers for Public Health Research and Evaluation, Durham, North Carolina 27713-4411, USA. wilsonk@battelle.org
UR - 19724304
ER -

TY - JOUR
ID - 720
T1 - High prevalence of vitamin D insufficiency in athletes and dancers
A1 - Constantini,N.W.
A1 - Arieli,R.
A1 - Chodick,G.
A1 - Dubnov-Raz,G.
Y1 - 2010/09//
N1 - Constantini, Naama W. Arieli, Rakefet. Chodick, Gabriel. Dubnov-Raz, Gal
Clinical journal of sport medicine : official journal of the Canadian Academy of Sport Medicine
b9t, 9103300
IM
Journal Article
English
KW - MEDLINE
KW - Adolescent
KW - Adult
KW - Age Factors
KW - Calcifediol/bl [Blood]
KW - Child
KW - Cross-Sectional Studies
KW - Dancing/ph [Physiology]
KW - Female
KW - Humans

KW - Israel/ep [Epidemiology]
KW - Linear Models
KW - Male
KW - Multivariate Analysis
KW - Prevalence
KW - Risk Factors
KW - Sex Factors
KW - Sports/ph [Physiology]
KW - Statistics as Topic
KW - Vitamin D Deficiency/ep [Epidemiology]
KW - Young Adult
RP - NOT IN FILE
SP - 368
EP - 371
JF - Clinical Journal of Sport Medicine
JA - Clin J Sport Med
VL - 20
IS - 5
CY - United States
N2 - OBJECTIVE: Vitamin D insufficiency is prevalent in various populations worldwide but with scarce data on physically active individuals. Vitamin D is important to athletes, affecting bone mass, immunity, and physical performance. This study evaluated the prevalence of vitamin D insufficiency and deficiency among young athletes and dancers. DESIGN: Cross-sectional study. SETTING: : Sport medicine clinic. PATIENTS: Data on 98 athletes and dancers (age, 14.7 +/- 3.0 years; range, 10-30 years; 53% men), who had undergone screening medical evaluations, were extracted from medical records. INDEPENDENT VARIABLE: Serum 25(OH)D concentrations. MAIN OUTCOME MEASURES: Serum 25(OH)D concentrations, age, sex, sport discipline, month of blood test, and serum ferritin. Vitamin D insufficiency was defined as serum 25(OH)D concentration <30 ng/mL. RESULTS: Mean serum 25(OH)D concentration was 25.3 +/- 8.3 ng/mL. Seventy-three percent of participants were vitamin D insufficient. Prevalence of vitamin D insufficiency was higher among dancers (94%), basketball players (94%), and Tae Kwon Do fighters (67%) and among athletes from indoor versus outdoor sports (80% vs 48%; P = 0.002). 25(OH)D levels adjusted for age and sex correlated with serum ferritin and season. CONCLUSIONS: In this study, conducted among young athletes and dancers from various disciplines in a sunny country, a high prevalence of vitamin D insufficiency was identified. A higher rate of vitamin D insufficiency was found among participants who practice indoors, during the winter months, and in the presence of iron depletion. Given the importance of vitamin D to athletes for several reasons, we suggest that athletes and dancers be screened for vitamin D insufficiency and treated as needed
SN - 1536-3724
AD - Department of Orthopedic Surgery, Hadassah-Hebrew University Medical Center, Jerusalem, Israel. naamacons@gmail.com
UR - 20818195
ER -

TY - JOUR
ID - 721
T1 - Grass pollen, aeroallergens, and clinical symptoms in Ciudad Real, Spain
A1 - Feo,Brito F.
A1 - Mur,Gimeno P.
A1 - Carnes,J.
A1 - Fernandez-Caldas,E.
A1 - Lara,P.
A1 - Alonso,A.M.
A1 - Garcia,R.
A1 - Guerra,F.
Y1 - 2010///
N1 - Feo Brito, F. Mur Gimeno, P. Carnes, J. Fernandez-Caldas, E. Lara, P. Alonso, A M. Garcia, R. Guerra, F Journal of investigational allergology & clinical immunology
9107858, byj, 9107858

IM
Journal Article. Research Support, Non-U.S. Gov't
English
KW - MEDLINE
KW - Adolescent
KW - Adult
KW - Air/an [Analysis]
KW - Antigens
KW - Plant/ae [Adverse Effects]
KW - Plant/im [Immunology]
KW - Plant/ip [Isolation & Purification]
KW - Asthma/di [Diagnosis]
KW - Asthma/ep [Epidemiology]
KW - Asthma/im [Immunology]
KW - Asthma/pp [Physiopathology]
KW - Child
KW - Environmental Exposure/ae [Adverse Effects]
KW - Female
KW - Humans
KW - Male
KW - Middle Aged
KW - Particulate Matter/an [Analysis]
KW - Particulate Matter/me [Metabolism]
KW - Pollen/ae [Adverse Effects]
KW - Pollen/ch [Chemistry]
KW - Pollen/me [Metabolism]
KW - Rhinitis
KW - Allergic
KW - Seasonal/di [Diagnosis]
KW - Seasonal/ep [Epidemiology]
KW - Seasonal/im [Immunology]
KW - Seasonal/pp [Physiopathology]
KW - Seasons
KW - Spain
RP - NOT IN FILE
SP - 295
EP - 302
JF - Journal of Investigational Allergology & Clinical Immunology
JA - J Investig.Allergol Clin Immunol
VL - 20
IS - 4
CY - Spain
N2 - BACKGROUND: In allergic individuals, onset of symptoms is related to atmospheric pollen grain counts and aeroallergen concentrations. However, this relationship is not always clear. OBJECTIVES: To analyze the correlation between grass pollen grain and aeroallergen concentrations in Ciudad Real, Spain, during the year 2004 and establish their association with symptoms in patients with allergic asthma, rhinitis, or both. METHODS: Two different samplers were used to assess allergen exposure: a Burkard spore trap to collect pollen grains and a high-volume air sampler to collect airborne particles. Individual filters were extracted daily in phosphate-buffered serum and analyzed by enzyme-linked immunosorbent assay based on serum containing high titers of specific immunoglobulin (Ig) E to grasses. The study population comprised 27 grass-allergic patients whose symptoms and medication were recorded daily. RESULTS: Grass pollens were detected between April 28 and July 18. There was a positive correlation between pollen grain counts and symptoms (r = 0.62; P > .001). Grass aeroallergens were detected not only during the grass pollination period, but also before and after this period. There was also a very significant correlation between aeroallergen levels and symptoms (r = 0.76; P < .0001). The threshold level for grass pollen was 35 grains/m3. CONCLUSIONS: Grass-related allergenic activity is present throughout the year, demonstrating the existence of aeroallergens outside the pollen season.

Symptoms in allergic patients may be related to airborne particle concentrations. This fact should be taken into account in the clinical follow-up and management of allergic patients
SN - 1018-9068
AD - Allergy Section, General Hospital, Ciudad Real, Spain. feobrito@hotmail.com
UR - 20815307
ER -

TY - JOUR
ID - 722
T1 - Describing patterns of physical activity in adolescents using global positioning systems and accelerometry
A1 - Maddison,R.
A1 - Jiang,Y.
A1 - Vander,Hoorn S.
A1 - Exeter,D.
A1 - Mhurchu,C.N.
A1 - Dorey,E.
Y1 - 2010/08//
N1 - Maddison, Ralph. Jiang, Yannan. Vander Hoorn, Stephen. Exeter, Daniel. Mhurchu, Cliona Ni. Dorey, Enid
Pediatric exercise science
8909729
IM
Journal Article. Multicenter Study. Research Support, Non-U.S. Gov't
English
KW - MEDLINE
KW - Adolescent
KW - Child
KW - Ergometry/is [Instrumentation]
KW - Ergometry/mt [Methods]
KW - Exercise/ph [Physiology]
KW - Female
KW - Geographic Information Systems
KW - Humans
KW - Male
KW - Monitoring
KW - Ambulatory/is [Instrumentation]
KW - Ambulatory/mt [Methods]
KW - New Zealand
RP - NOT IN FILE
SP - 392
EP - 407
JF - Pediatric Exercise Science
JA - Pediatr Exerc Sci
VL - 22
IS - 3
CY - United States
N2 - This study aimed to describe the location and intensity of free-living physical activity in New Zealand adolescents during weekdays and weekend days using Global Positioning Systems (GPS), accelerometry, and Geographical Information Systems (GIS). Participants (n = 79) aged 12-17 years (M = 14.5, SD 1.6) recruited from two large metropolitan high schools each wore a GPS watch and an accelerometer for four consecutive days. GPS and accelerometer data were integrated with GIS software to map the main locations of each participant's episodes of moderate-vigorous physical activity. On average participants performed 74 (SD 36) minutes of moderate and 7.5 (SD 8) minutes of vigorous activity per day, which on weekdays was most likely to occur within a 1 km radius of their school or 150 meters of their home environment. On weekends physical activity patterns were more disparate and took place outside of the home environment. Example maps were generated to display the location of moderate to vigorous activity for weekdays and weekends
SN - 0899-8493

AD - Clinical Trials Research Unit, University of Auckland, Auckland, New Zealand
UR - 20814035
ER -

TY - JOUR
ID - 723
T1 - [Agility in treatment of children with type 1 diabetes--pilot study]. [Polish]
A1 - Niewiadomska,M.
A1 - Radziejewska,M.
A1 - Horodnicka-Jozwa,A.
A1 - Petriczko,E.
Y1 - 2010///
N1 - Niewiadomska, Monika. Radziejewska, Marija. Horodnicka-Jozwa, Anita. Petriczko, Elzbieta
Pediatric endocrinology, diabetes, and metabolism
101518750
IM
English Abstract. Journal Article
Polish
KW - MEDLINE
KW - Animal Assisted Therapy
KW - Animals
KW - Child
KW - Diabetes Mellitus
KW - Type 1/dt [Drug Therapy]
KW - Type 1/pp [Physiopathology]
KW - Type 1/rh [Rehabilitation]
KW - Dogs
KW - Exercise/ph [Physiology]
KW - Exercise Therapy
KW - Female
KW - Humans
KW - Insulin/ad [Administration & Dosage]
KW - Pilot Projects
RP - NOT IN FILE
SP - 89
EP - 93
JF - Pediatric endocrinology, diabetes, & metabolism
JA - Pediatr Endocrinol Diabetes Metab
VL - 16
IS - 2
CY - Poland
N2 - INTRODUCTION: Physical activity is a very important element in treatment of children with type 1 diabetes. However, it is difficult to find suitable exercises for the children due to their specific needs and psychophysiological condition. The aim of this study was to examine the effects of agility as a physical activity used to improve parameters of metabolic control in children with type 1 diabetes. Additionally, we hope that this form of recreation could induce a more health-oriented behavior in children. MATERIAL AND METHODS: The experimental group consisted of seven girls aged 8-10 years, the patients of the Clinic of Pediatrics, Endocrinology, Diabetology, Metabolic Disorders and Cardiology of the Developmental Age in Szczecin. The children were qualified for the study after the prior assessment of their metabolic control under the conditions of the one-day hospital stay program. The physical condition of the patients was controlled with a 6-minute walk test and the test of perceived exertion (Borg scale). All the patients were treated using a personal insulin pump and the basal-bolus therapy. The applied research method used the scheme of physical exercise performed 3 times a week, 45 min each, reaching the intensity of 75% of the maximum heart rate under effort performed by a given patient. Before the exercises and directly after their completion, sugar level was measured in the blood of the patients (Accu-chek Active). During the exercises, the heart rate was measured with a pulsometer Bauer PM 80. The exercises included outdoor games and agility sessions. Especially the latter received a positive response and high involvement. This form includes a dog going through an obstacle course,

with a child as a guide. RESULTS AND CONCLUSIONS: After three months of the exercise scheme, the analysis of the collected results showed a statistically significant (p <0.05) decrease in the insulin doses (bolus) during morning hours (7-8 am), and in the evening at 9 pm and 10.30 pm, with an unchanged basal. No such decrease was observed in the control group. This study showed that an interesting physical activity resulted in a more eager and systematic effort among examined diabetic children. Its proper organization in time may help in the metabolic control in children with type 1 diabetes
SN - 2081-237X
AD - Zaklad Rehabilitacji i Traumatologii Sportowej, Instytut Kultury Fizycnej Wydzialu Nauk Przyrodniczych Uniwersytetu Szczecinski. monikaniewiadomska@wp.pl
UR - 20813085
ER -

TY - JOUR
ID - 724
T1 - [Late effect of treatment of nephroblastoma in patients treated in 1980-2001 in a single centre]. [Czech]
A1 - Radvansky,J.
A1 - Slaby,K.
A1 - Radvanska,J.
A1 - Malis,J.
A1 - Eckschalger,T.
A1 - Sulc,J.
A1 - Safarova,M.
A1 - Kolsky,A.
A1 - Prochazka,M.
A1 - Gilik,J.
Y1 - 2010///
N1 - Radvansky, J. Slaby, K. Radvanska, J. Malis, J. Eckschalger, T. Sulc, J. Safarova, M. Kolsky, A. Prochazka, M. Gilik, J
Klinicka onkologie : casopis Ceske a Slovenske onkologicke spolecnosti
9425213
IM
English Abstract. Journal Article. Research Support, Non-U.S. Gov't
Czech
KW - MEDLINE
KW - Adolescent
KW - Adult
KW - Antineoplastic Agents/ae [Adverse Effects]
KW - Blood Pressure
KW - Child
KW - Preschool
KW - Echocardiography
KW - Female
KW - Follow-Up Studies
KW - Humans
KW - Infant
KW - Kidney/ph [Physiology]
KW - Kidney Neoplasms/th [Therapy]
KW - Lipids/bl [Blood]
KW - Male
KW - Physical Fitness
KW - Quality of Life
KW - Remission Induction
KW - Wilms Tumor/th [Therapy]
KW - Young Adult
RP - NOT IN FILE
SP - 245
EP - 255

JF - Klinicka Onkologie
JA - KLIN
VL - 23
IS - 4
CY - Czech Republic
N2 - BACKGROUNDS: The aim of the study was to describe the late effects in a cohort of patients in long-term remission (> 5 years from end of treatment) diagnosed and treated for nephroblastoma at the single paediatric centre during 1980- 2001. PATIENTS AND METHODS: 151 patients were examined for renal function, lipid profile, sonography, echocardiography, exercise capacity and postural status. Age at diagnosis was 3.7 +/- 2.7 years, median 3.1, range 0.01-17.2 years. The cohort included 55% of women. Age in the study 19.4 +/- 5.8, median 19.6 years, range 7.8-36 years. Anthracyclines (ATC) were administered to 25.9% of the cohort. Abdominal radiotherapy (RTA) was required in 34.2%. RESULTS: Creatinine clearance was on average 1.56 +/- 0.56 ml/s/1.73m2, (median 1.49 ml/s/1.73m2). Proteinuria was 0.18 +/- 0.30 g/24 h/m2, median 0.13 mg/24 h/m2. Three patients had proteinuria above 1 g/24 h with a normal glomerular filtration rate and s-albumin. Hypertension was treated in 8.6% of patients (6% by pharmacotherapy). Increased diastolic blood pressure (DBP) and systolic blood pressure (SBP) was found in adolescents: DBP > 90 Torr was found in 10.2% of patients and SBP > 135 Torr in 8.3%, all of them above the age of 15 years. ECHO did not reveal any pathology even in patients treated by ATC. Plasma cholesterol, LDL, HDL, triglycerides were outside the recommended range in two or more parameters in 28.9% of the cohort. Lipoprotein Lp(a) > 500 mg.l(-1) (more than 200% higher than the upper limit) was found in 15.2%. Low peak oxygen consumption (VO2peak) was found in 40% of the cohort. Scoliosis (Sc) was anamnestically found in 46%. Prevalence of Sc according to the age of treatment has a similar trend as the reduced use of the RTA. Pulmonary function test showed a moderate increase in the functional residual capacity (the average Z-score of 1.35). VO2 peak correlates positively with the inspiratory capacity (p < 0.05). CONCLUSION: Based on the found pathologies and known risks, a schedule of late effect prevention and monitoring of patients in long-term NFB remission was established. It should include clinical examination, kidney function and blood pressure monitoring, sonographic and echocardiography examination, long-term physiotherapeutic care and prevention of cardiovascular diseases
SN - 0862-495X
AD - Klinika rehabilitace a telovychovneho lekarstvi 2. LF UK a FN Motol, Praha.
jiri.radvansky@lfmotol.cuni.cz
UR - 20806823
ER -

TY - JOUR
ID - 725
T1 - [Retrospective analysis of 44 childhood drowning accidents]. [German]
A1 - Bruning,C.
A1 - Siekmeyer,W.
A1 - Siekmeyer,M.
A1 - Merkenschlager,A.
A1 - Kiess,W.
Y1 - 2010/07//
N1 - Bruning, Caroline. Siekmeyer, Werner. Siekmeyer, Manuela. Merkenschlager, Andreas. Kiess, Wieland
Wiener klinische Wochenschrift
xop, 21620870r
IM
English Abstract. Journal Article
German
KW - MEDLINE
KW - Accidents/mo [Mortality]
KW - Adolescent
KW - Brain Damage
KW - Chronic/mo [Mortality]
KW - Cardiopulmonary Resuscitation
KW - Cause of Death
KW - Child
KW - Preschool

KW - Cross-Sectional Studies
KW - Drowning/mo [Mortality]
KW - Female
KW - Germany
KW - Glasgow Coma Scale
KW - Hospitals
KW - Pediatric/sn [Statistics & Numerical Data]
KW - University/sn [Statistics & Numerical Data]
KW - Humans
KW - Incidence
KW - Infant
KW - Male
KW - Neurologic Examination
KW - Retrospective Studies
RP - NOT IN FILE
SP - 405
EP - 412
JF - Wiener Klinische Wochenschrift
JA - Wien Klin Wochenschr.
VL - 122
IS - 13-14
CY - Austria
N2 - BACKGROUND: Worldwide, drowning is the second leading cause of unintentional death and the leading cause of cardiovascular failure for children [1-3]. The number of near-drownings, where the incident is survived for at least 24 hours, is assumed to be four times as high [5]. In the years 1994 until 2008 there were 44 cases of drowning treated at the children's department of the University of Leipzig. This number shows that even in a medical centre drowning incidents are only occasional incidents. Therefore it is important to know the sequelae and handlings to be able to react in case of an emergency. PATIENTS: A total of 44 children suffering a drowning accident within the last 48 hours who were treated during the period of 01.01.1994 through 30.06.2008 at the Children's Centre at the University of Leipzig. METHODS: A retrospective analysis using a structured questionnaire was done. Social demographic data, accident progress, clinical results and progress as well as outcome of the cases were investigated. RESULTS: During the analysed period in the median three children were treated each year after drowning incidents. Clustering in the summer and winter months and on the weekends was recognizable. The median age was 3.33 years and the group of high risk were children aged 1-3 years, especially boys. Sixty percent of the children came from stable social backgrounds. Half of the children suffered from drowning in created swimming pools or ponds, the rest in natural waters, public pools and sources of water in the household. The median submersion lasted 2 minutes. Correlation of submersions below 1 minute with a good, and submersions above 10 minutes with a negative outcome was shown. A Glasgow Coma Scale (GCS) of 3 points (n = 15) and pupils without light reaction (n = 14) were associated with a lethal outcome or residual neurological deficits. Looking at the laboratory values, correlation between severe acidotic pH-values with a very low base excess, high blood sugar as well as high lactate values and a poor outcome is revealed. Six patients died within the first 24 hours, 6 more over the course suffering organ failure or brain death. Five children retained neurological damages. Twenty-seven children could be released from the clinic healthily. CONCLUSION: The risk of suffering a drowning incident is highest for boys aged 1-3 years, playing in the yard by themselves. Prognosis is depending on multiple factors and especially the circumstances of the accident. High influence is seen in the time of submersion and the need for cardiopulmonary resuscitation. Clinics and laboratory values at the time of hospital admittance may hint to the outcome. Basic life support at the scene of the accident has the highest impact on the outcome. Training of parents and supervisors in prevention and first aid after drowning incidents can avoid accidents
SN - 1613-7671
AD - Department fur Frauen- und Kindermedizin, Universitatsklinikum Leipzig, Leipzig, Germany
UR - 20602279
ER -

TY - JOUR
ID - 726
T1 - The effect of caregiving on women in families with Duchenne/Becker muscular dystrophy

A1 - Kenneson,A.
A1 - Bobo,J.K.
Y1 - 2010/09//
N1 - Kenneson, Aileen. Bobo, Janet Kay
Health & social care in the community
9306359
IM
Journal Article. Research Support, U.S. Gov't, P.H.S.
English
KW - MEDLINE
KW - Adaptation
KW - Psychological
KW - Adolescent
KW - Adult
KW - Caregivers/px [Psychology]
KW - Child
KW - Preschool
KW - Cost of Illness
KW - Family Relations
KW - Female
KW - Humans
KW - Infant
KW - Middle Aged
KW - Muscular Dystrophy
KW - Duchenne/nu [Nursing]
KW - Duchenne/px [Psychology]
KW - Personal Satisfaction
KW - Quality of Life/px [Psychology]
KW - Questionnaires
KW - Social Support
KW - Stress
KW - Psychological/px [Psychology]
KW - United States/ep [Epidemiology]
KW - Women's Health
KW - Young Adult
RP - NOT IN FILE
SP - 520
EP - 528
JF - Health & Social Care in the Community
JA - Health Soc Care Community
VL - 18
IS - 5
CY - England
N2 - Duchenne/Becker muscular dystrophy (DBMD) is a disorder of progressive muscle weakness that causes an increasing need for assistance with activities of daily living. Our objective was to assess the psychosocial health and contributing factors among female caregivers in families with DBMD. We conducted a survey of adult women among families with DBMD in the United States (US) from June 2006 through January 2007, collecting data related to the care recipient, perception of caregiving demands, personal factors, and socio-ecologic factors. Life satisfaction, stress, and distress were assessed as outcomes. Existing validated instruments were used when available. We received responses from 1238 women who were caring for someone with DBMD, 24.2% of whom were caring for two or more people with DBMD. Caregivers were more likely to be married/cohabitating than women in the general US population, and a high level of resiliency was reported by 89.3% of caregivers. However, the rate of serious psychological distress was significantly higher among caregivers than among the general population. Likewise, 46.4% reported a high level of stress, and only 61.7% reported that they were satisfied with their life. A high level of caregiving demands based on the Zarit Burden Interview (ZBI) was reported by 50.4% of caregivers. The post-ambulatory phase of DBMD was associated with decreased social support and increased ZBI scores. In multivariate logistic regression modelling, life

satisfaction was dependent on high social support, high resiliency, high income, and form of DBMD. Distress and high stress were predicted by low resiliency, low social support, and low income. Employment outside of the home was also a predictor of high stress. Interventions focused on resiliency and social support are likely to improve the quality of life of DBMD caregivers, and perhaps caregivers of children with other disabilities or special health care needs as well
SN - 1365-2524
AD - McKing Consulting Corporation, Atlanta, GA, USA
UR - 20561071
ER -

TY - JOUR
ID - 727
T1 - Level of purposeful hand function as a marker of clinical severity in Rett syndrome
A1 - Downs,J.
A1 - Bebbington,A.
A1 - Jacoby,P.
A1 - Williams,A.M.
A1 - Ghosh,S.
A1 - Kaufmann,W.E.
A1 - Leonard,H.
Y1 - 2010/09//
N1 - Downs, Jenny. Bebbington, Ami. Jacoby, Peter. Williams, Anne-Marie. Ghosh, Soumya. Kaufmann, Walter E. Leonard, Helen
Developmental medicine and child neurology
0006761, e83
IM
Journal Article. Research Support, N.I.H., Extramural. Research Support, Non-U.S. Gov't
English
KW - MEDLINE
KW - Adolescent
KW - Adult
KW - Age Factors
KW - Child
KW - Preschool
KW - Cross-Sectional Studies
KW - Databases as Topic
KW - Female
KW - Genotype
KW - Hand/ph [Physiology]
KW - Hand
KW - Humans
KW - Methyl-CpG-Binding Protein 2/ge [Genetics]
KW - Motor Activity/ph [Physiology]
KW - Motor Activity
KW - Mutation
KW - Phenotype
KW - Registries
KW - Rett Syndrome/di [Diagnosis]
KW - Rett Syndrome/ge [Genetics]
KW - Rett Syndrome/pp [Physiopathology]
KW - Severity of Illness Index
KW - Video Recording
KW - Walking
KW - Young Adult
RP - NOT IN FILE
SP - 817
EP - 823

JF - Developmental Medicine & Child Neurology
JA - Dev Med Child Neurol
VL - 52
IS - 9
CY - England
N2 - AIM: We investigated relationships between hand function and genotype and aspects of phenotype in Rett syndrome. METHOD: Video assessment in naturalistic settings was supplemented by parent-reported data in a cross-sectional study of 144 females with a mean age of 14 years 10 months (SD 7 y 10 mo; range 2 y-31 y 10 mo), 110 of whom had a mutation of the methyl CpG binding protein 2 (MECP2) gene. Ordinal logistic regression was used to assess relationships between hand function and MECP2 mutation, age, a modified Kerr score, Functional Independence Measure for Children (WeeFIM), ambulation level, and frequency of hand stereotypies. RESULTS: Approximately two-thirds of participants demonstrated purposeful hand function, ranging from simple grasping skills to picking up and manipulating small objects. In participants with a confirmed MECP2 mutation, those with the p.R168X mutation had the poorest hand function on multivariate analysis with C-terminal deletion as the baseline (odds ratio [OR] 0.19; 95% confidence interval [CI] 0.04-0.95), whereas those with the p.R133C or p.R294X mutation had better hand function. Participants aged 19 years or older had lower hand function than those aged less than 8 years (OR 0.36; 95% CI 0.14-0.92). Factors that were associated with better hand function were lower Kerr scores for a 1-point increase in score (OR 0.77; 95% CI 0.69-0.86), higher WeeFIM scores for a 1-point increase in score (OR 1.08; 95% CI 1.04-1.12), and greater ambulation than those completely dependent on carers for mobility (OR 22.64; 95% CI 7.02-73.08). The results for participants with a confirmed pathogenic mutation were similar to results obtained when participants without a mutation were also included. INTERPRETATION: Our novel assessment of hand function in Rett syndrome correlated well with known profiles of common MECP2 mutations and overall clinical severity. This promising assessment could measure clinical responses to therapy
SN - 1469-8749
AD - Centre for Child Health Research, Telethon Institute for Child Health Research, University of Western Australia, Perth, Australia
UR - 20345957
ER -

TY - JOUR
ID - 728
T1 - Determination of exposure to benzene, toluene and xylenes in Turkish primary school children by analysis of breath and by environmental passive sampling
A1 - Scheepers,P.T.
A1 - Konings,J.
A1 - Demirel,G.
A1 - Gaga,E.O.
A1 - Anzion,R.
A1 - Peer,P.G.
A1 - Dogeroglu,T.
A1 - Ornektekin,S.
A1 - Van,Doorn W.
Y1 - 2010/09/15/
N1 - Scheepers, Paul T J. Konings, Joke. Demirel, Gulcin. Gaga, Eftade O. Anzion, Rob. Peer, Petronella G M. Dogeroglu, Tuncay. Ornektekin, Sermin. van Doorn, Wim
The Science of the total environment
uj0, 0330500
IM
Journal Article. Research Support, Non-U.S. Gov't
English
KW - MEDLINE
KW - Air Pollutants/an [Analysis]
KW - Air Pollutants/me [Metabolism]
KW - Benzene/an [Analysis]
KW - Benzene/me [Metabolism]
KW - Breath Tests

KW - Child
KW - Environmental Exposure/an [Analysis]
KW - Environmental Monitoring
KW - Female
KW - Humans
KW - Male
KW - Toluene/an [Analysis]
KW - Toluene/me [Metabolism]
KW - Volatile Organic Compounds/an [Analysis]
KW - Volatile Organic Compounds/me [Metabolism]
KW - Xylenes/an [Analysis]
KW - Xylenes/me [Metabolism]
RP - NOT IN FILE
SP - 4863
EP - 4870
JF - Science of the Total Environment
JA - Sci Total Environ
VL - 408
IS - 20
CY - Netherlands
N2 - Benzene, toluene, m/p-xylene and o-xylene (BTX) are toxic volatile organic compounds and ubiquitous air pollutants. Smoking and consumer products are indoor sources of BTX, whereas traffic and industrial activities are primary sources contributing to outdoor levels of BTX. The aim of this study was to characterize exposure of children to BTX by personal air sampling using diffusive samplers and by analysis of end-exhaled air. For this study, 101 children of 10-11 years of age were recruited from four primary schools in Southern Turkey during the warm season (May 2008). Two schools were situated in a residential area near primary and secondary iron and steel works (Payas) and two schools were located in a non-industrialized city (Iskenderun). The children and their parents were visited at home for an interview and to identify possible sources of BTX in the residence. Median concentrations of benzene determined by diffusive samplers were higher in Payas (4.1 microg/m(3)) than in Iskenderun (2.7 microg/m(3), p<0.001). For toluene, no differences were observed, whereas for xylene isomers air concentrations tended to be lower for children living in Payas. The median end-exhaled air concentrations were 8.2, 29, 3.8, and 5.7 pmol/L for benzene, toluene, m/p-xylene and o-xylene, respectively (Payas), and 6.9, 25, 4.9, and 6.0 pmol/L, respectively (Iskenderun). Concentrations of toluene in end-exhaled air were 50% higher in children living with household members who smoked indoors (p<0.05) and benzene in end-exhaled air was more than 3-fold higher for those children who were exposed to tobacco smoke inside a vehicle (p<0.001). End-exhaled concentrations of benzene were also higher in children living in a residence with an attached garage (p<0.05). These exposure modifying factors were not identified when using the results obtained with diffusive samplers. Copyright 2010 Elsevier B.V. All rights reserved
SN - 1879-1026
AD - Department of Epidemiology, Biostatistics and Health Technology Assessment, Radboud University Nijmegen Medical Centre, P.O. Box 9101, Nijmegen, The Netherlands. p.scheepers@ebh.umcn.nl
UR - 20619876
ER -

TY - JOUR
ID - 729
T1 - Exposure assessment and risk characterization from trace elements following soil ingestion by children exposed to playgrounds, parks and picnic areas
A1 - Guney,M.
A1 - Zagury,G.J.
A1 - Dogan,N.
A1 - Onay,T.T.
Y1 - 2010/10/15/
N1 - Guney, Mert. Zagury, Gerald J. Dogan, Nurten. Onay, Turgut T
Journal of hazardous materials
dde, 9422688
IM

Journal Article. Research Support, Non-U.S. Gov't
English
KW - MEDLINE
KW - Child
KW - Environmental Exposure
KW - Humans
KW - Quality Control
KW - Recreation
KW - Risk Assessment
KW - Soil Pollutants/to [Toxicity]
RP - NOT IN FILE
SP - 656
EP - 664
JF - Journal of Hazardous Materials
JA - J Hazard.Mater.
VL - 182
IS - 1-3
CY - Netherlands
N2 - Soil ingestion is an important pathway for exposure to metals for children. The objectives of this study were to: (1) Assess urban soil contamination by selected metals (As, Cr, Cu, Ni, Pb, and Zn) in 24 sites (127 soil samples) in Istanbul, Turkey, (2) Investigate relationships between soil contamination and site properties (type of site, equipment type, soil properties), (3) Characterize the risk for critically contaminated sites by taking oral metal bioaccessibility and two soil ingestion scenarios into account. Average metal concentrations were similar in the 17 playgrounds, 4 parks and 3 picnic areas sampled. Five out of 24 sites (all equipped with treated wood structures) had systematically higher contamination than background for As, Cu, Cr or Zn, and measured concentrations generally exceeded Turkish regulatory values. High Cu concentrations in these sites were attributed to the leaching from wood treated with Cu-containing preservatives other than chromated copper arsenate (CCA). Risk characterization for these sites showed that hazard index was below one in both involuntary soil ingestion and soil pica behaviour scenarios for all metals. However, probabilistic carcinogenic risk for As uptake exceeded 1x10(-6) in both scenarios. A sensitivity analysis showed that soil ingestion rate was the most important parameter affecting risk estimation. Risk from As uptake for children from soils of parks, playgrounds and picnic areas may be serious, especially if soil pica behaviour is present. 2010 Elsevier B.V. All rights reserved
SN - 1873-3336
AD - Department of Civil, Geological and Mining Engineering, Ecole Polytechnique de Montreal, Montreal, Quebec, H3C 3A7 Canada
UR - 20663608
ER -

TY - JOUR
ID - 730
T1 - Participatory action research (PAR) in middle school: opportunities, constraints, and key processes
A1 - Ozer,E.J.
A1 - Ritterman,M.L.
A1 - Wanis,M.G.
Y1 - 2010/09//
N1 - Ozer, Emily J. Ritterman, Miranda L. Wanis, Maggie G
American journal of community psychology
3fv, 0364535
IM
Journal Article. Research Support, Non-U.S. Gov't
English
KW - MEDLINE
KW - Adolescent
KW - Child
KW - Community-Based Participatory Research/mt [Methods]
KW - Community-Based Participatory Research/og [Organization & Administration]

KW - Faculty
KW - Humans
KW - Program Evaluation
KW - Research Design
KW - San Francisco
KW - Schools/og [Organization & Administration]
KW - Schools
KW - Social Environment
KW - Students/px [Psychology]
RP - NOT IN FILE
SP - 152
EP - 166
JF - American Journal of Community Psychology
JA - Am J Community Psychol
VL - 46
IS - 1-2
CY - United States
N2 - Late childhood and early adolescence represent a critical transition in the developmental and academic trajectory of youth, a time in which there is an upsurge in academic disengagement and psychopathology. PAR projects that can promote youth's sense of meaningful engagement in school and a sense of efficacy and mattering can be particularly powerful given the challenges of this developmental stage. In the present study, we draw on data from our own collaborative implementation of PAR projects in secondary schools to consider two central questions: (1) How do features of middle school settings and the developmental characteristics of the youth promote or inhibit the processes, outcomes, and sustainability of the PAR endeavor? and (2) How can the broad principles and concepts of PAR be effectively translated into specific intervention activities in schools, both within and outside of the classroom? In particular, we discuss a participatory research project conducted with 6th and 7th graders at an urban middle school as a means of highlighting the opportunities, constraints, and lessons learned in our efforts to contribute to the high-quality implementation and evaluation of PAR in diverse urban public schools
SN - 1573-2770
AD - Division of Community Health and Human Development, University of California at Berkeley School of Public Health, Berkeley, CA 94720-7360, USA. eozer@berkeley.edu
UR - 20676754
ER -

TY - JOUR
ID - 731
T1 - A recess evaluation with the players: taking steps toward participatory action research
A1 - Ren,J.Y.
A1 - Langhout,R.D.
Y1 - 2010/09//
N1 - Ren, Julie Yunyi. Langhout, Regina Day
American journal of community psychology
3fv, 0364535
IM
Journal Article
English
KW - MEDLINE
KW - Child
KW - Child Psychology/mt [Methods]
KW - Community-Based Participatory Research/mt [Methods]
KW - Evaluation Studies as Topic
KW - Focus Groups
KW - Humans
KW - Play and Playthings/px [Psychology]
KW - Psychology
KW - Social/mt [Methods]

KW - Schools/og [Organization & Administration]
KW - Schools
KW - Students/px [Psychology]
KW - Teaching
RP - NOT IN FILE
SP - 124
EP - 138
JF - American Journal of Community Psychology
JA - Am J Community Psychol
VL - 46
IS - 1-2
CY - United States
N2 - This playground study conceptualizes recess as a time and space that belongs to students; their inclusion in this evaluation is a notable difference from other recess/playground research. The goal was to help elementary school students make the changes they felt were needed on their playground. After conducting structured observations and student and recess aide focus groups, a report was presented to all stakeholders, and recess changes were made. We seek to show how the process of being inclusive during the evaluation was not only valuable for determining problem definition and potential interventions, but was also necessary to determine the best methods for solutions, move toward second-order change, and to create a space to facilitate children's participation and empowerment
SN - 1573-2770
AD - Wesleyan University, Middletown, CT, USA
UR - 20544270
ER -

TY - JOUR
ID - 732
T1 - Outdoor particulate matter and childhood asthma admissions in Athens, Greece: a time-series study
A1 - Nastos,P.T.
A1 - Paliatsos,A.G.
A1 - Anthracopoulos,M.B.
A1 - Roma,E.S.
A1 - Priftis,K.N.
Y1 - 2010///
N1 - Nastos, Panagiotis T. Paliatsos, Athanasios G. Anthracopoulos, Michael B. Roma, Eleftheria S. Priftis, Kostas N
Environmental health : a global access science source
101147645
IM
Journal Article
English
KW - MEDLINE
KW - Adolescent
KW - Air Pollutants/ae [Adverse Effects]
KW - Asthma/ci [Chemically Induced]
KW - Asthma/ep [Epidemiology]
KW - Child
KW - Preschool
KW - Environmental Exposure
KW - Female
KW - Greece/ep [Epidemiology]
KW - Hospitalization/sn [Statistics & Numerical Data]
KW - Humans
KW - Infant
KW - Newborn
KW - Linear Models
KW - Logistic Models

KW - Male
KW - Particulate Matter
KW - Time Factors
RP - NOT IN FILE
SP - 45
JF - Environmental Health: A Global Access Science Source
JA - Environ Health
VL - 9
CY - England
N2 - BACKGROUND: Particulate matter with diameter less than 10 micrometers (PM10) that originates from anthropogenic activities and natural sources may settle in the bronchi and cause adverse effects possibly via oxidative stress in susceptible individuals, such as asthmatic children. This study aimed to investigate the effect of outdoor PM10 concentrations on childhood asthma admissions (CAA) in Athens, Greece. METHODS: Daily counts of CAA from the three Children's Hospitals within the greater Athens' area were obtained from the hospital records during a four-year period (2001-2004, n = 3602 children). Mean daily PM10 concentrations recorded by the air pollution-monitoring network of the greater Athens area were also collected. The relationship between CAA and PM10 concentrations was investigated using the Generalized Linear Models with Poisson distribution and logistic analysis. RESULTS: There was a statistically significant (95% CL) relationship between CAA and mean daily PM10 concentrations on the day of exposure (+3.8% for 10 microg/m3 increase in PM10 concentrations), while a 1-day lag (+3.4% for 10 microg/m3 increase in PM10 concentrations) and a 4-day lag (+4.3% for 10 microg/m3 increase in PM10 concentrations) were observed for older asthmatic children (5-14 year-old). High mean daily PM10 concentration (the highest 10%; >65.69 microg/m3) doubled the risk of asthma exacerbations even in younger asthmatic children (0-4 year-old). CONCLUSIONS: Our results provide evidence of the adverse effect of PM10 on the rates of paediatric asthma exacerbations and hospital admissions. A four-day lag effect between PM10 peak exposure and asthma admissions was also observed in the older age group
SN - 1476-069X
AD - Laboratory of Climatology and Atmospheric Environment, Department of Geology and Geoenvironment, University of Athens, Panepistimioupolis, 157 84 Athens, Greece. nastos@geol.uoa.gr
UR - 20667130
ER -

TY - JOUR
ID - 733
T1 - Growing up in rural community--children's experiences of social capital from perspectives of wellbeing
A1 - Eriksson,U.
A1 - Asplund,K.
A1 - Sellstrom,E.
Y1 - 2010///
N1 - Eriksson, Ulrika. Asplund, Kenneth. Sellstrom, Eva
Rural and remote health
101174860
IM
Journal Article
English
KW - MEDLINE
KW - Attitude to Health
KW - Child
KW - Child Behavior/px [Psychology]
KW - Child Welfare/px [Psychology]
KW - Female
KW - Humans
KW - Interpersonal Relations
KW - Life Style
KW - Male
KW - Residence Characteristics
KW - Rural Health

KW - Rural Population
KW - Social Environment
KW - Social Identification
KW - Socioeconomic Factors
KW - United States
RP - NOT IN FILE
SP - 1322
JF - Rural & Remote Health
JA - RURAL REMOTE HEALTH
VL - 10
IS - 3
CY - Australia
N2 - INTRODUCTION: People are influenced by the neighborhood in which they live. The neighborhood may be particularly important for children's wellbeing because of the constraints it imposes on their patterns of daily activities. Furthermore, the neighborhood is a central context for social development, being a place where children form networks and learn social skills and values. The aim of this study was to describe how social capital in the neighborhood is perceived by children living in rural areas, and to reveal what this adds to their sense of wellbeing. METHODS: The study had a descriptive research design with a qualitative approach. Seven single-sex focus group interviews were conducted with children the in 6th grade (aged 11-12 years). Data were analyzed using deductive content analysis. RESULTS: The children perceived a lack of social capital due to environmental and social constraints in their everyday lives. However, their wellbeing was enhanced by strong cohesion in the neighborhood. In addition, settings such as the school, the natural environment, and sporting associations were highly valued and emerged as crucial factors for enhancing the children's wellbeing. The spatial isolation that characterizes rural areas created a special context of social network structures, cohesion and trust, but was also a breeding ground for exclusion and social control. The stories revealed paradoxical feelings of living in a good and safe area that simultaneously felt isolated and restricted. CONCLUSIONS: From a rural perspective, this study reveals the complexity of the children's perceptions of their social environment, and the ways in which these perceptions have both positive and negative effects on wellbeing. The results highlight how important it is for health professionals in rural areas to consider the complex influence of bonding social capital on children's wellbeing, and to be aware that it can promote exclusion as well as cohesion
SN - 1445-6354
AD - Department of Health Sciences, Mid Sweden University, Ostersund, Sweden. ulrika.eriksson@miun.se
UR - 20701413
ER -

TY - JOUR
ID - 734
T1 - Power mobility and socialization in preschool: a case study of a child with cerebral palsy
A1 - Ragonesi,C.B.
A1 - Chen,X.
A1 - Agrawal,S.
A1 - Galloway,J.C.
Y1 - 2010///
N1 - Ragonesi, Christina B. Chen, Xi. Agrawal, Sunil. Galloway, James Cole
Pediatric physical therapy : the official publication of the Section on Pediatrics of the American Physical Therapy Association
8912748
IM
Case Reports. Journal Article. Research Support, N.I.H., Extramural. Research Support, U.S. Gov't, Non-P.H.S.
English
KW - MEDLINE
KW - Cerebral Palsy/pp [Physiopathology]
KW - Cerebral Palsy/px [Psychology]
KW - Cerebral Palsy/rh [Rehabilitation]
KW - Child
KW - Preschool
KW - Humans

KW - Male
KW - Motor Activity/ph [Physiology]
KW - Motor Skills/ph [Physiology]
KW - Physical Therapy Modalities/is [Instrumentation]
KW - Socialization
RP - NOT IN FILE
SP - 322
EP - 329
JF - Pediatric Physical Therapy
JA - PEDIATR
VL - 22
IS - 3
CY - United States
N2 - PURPOSE: Power mobility training for young children and infants appears feasible under controlled conditions. Dynamic, natural environments provide the ultimate test of training. The purpose of this case study was to determine whether it was feasible for Will, a 3-year-old boy with cerebral palsy, to use a power mobility device (UD2) in his preschool classroom and to quantify his classroom mobility and socialization. METHODS: Will, 2 peers (typically developing), and 2 teachers were filmed daily in class during a baseline phase without UD2, followed by a mobility phase with UD2. We coded socialization and mobility measures from video recordings. RESULTS: Will was more mobile and interactive when driving UD2 than during the baseline phase; however, he remained notably less mobile and interactive than his peers. CONCLUSIONS: The use and assessment of power mobility in a preschool classroom appear feasible. Issues important to maximizing children's use of power mobility for classroom participation are discussed
SN - 1538-005X
AD - Infant Motor Behavior Laboratory, Department of Physical Therapy, University of Delaware, Newark, USA
UR - 20699785
ER -

TY - JOUR
ID - 735
T1 - Differences in children's recess physical activity: recess activity of the week intervention
A1 - Stellino,M.B.
A1 - Sinclair,C.D.
A1 - Partridge,J.A.
A1 - King,K.M.
Y1 - 2010/09//
N1 - Stellino, Megan Babkes. Sinclair, Christina D. Partridge, Julie A. King, Kristi McClary
The Journal of school health
k13, 0376370
IM, N
Journal Article
English
KW - MEDLINE
KW - Age Factors
KW - Body Mass Index
KW - Child
KW - Exercise
KW - Female
KW - Humans
KW - Male
KW - Schools/og [Organization & Administration]
KW - Sex Factors
KW - Socioeconomic Factors
KW - Sports
RP - NOT IN FILE
SP - 436

EP - 444
JF - Journal of School Health
JA - J Sch Health
VL - 80
IS - 9
CY - United States
N2 - BACKGROUND: The increased prevalence in recent childhood obesity rates raises concern about youth health and the role that lack of physical activity plays in this trend. A focus on how children today choose to spend their discretionary time is one approach that may yield ideas for how to reduce childhood obesity. The purpose of the present study was to examine whether 3 separate recess activities of the week (RAWs) would make a difference in children's discretionary time physical activity levels. METHODS: Children (N = 65: 30 boys, 35 girls; 32 first and second graders; 33 third and fourth graders; 45 healthy body mass index [BMI], 20 overweight BMI) at 1 Midwest elementary school wore pedometers for each 15-minute morning recess period for 4 weeks. Following 1 no RAW (#1), a new RAW was introduced each subsequent week: #2, circuit course; #3, obstacle course; and #4, Frisbees. RESULTS: Repeated measures factorial analysis of variance results revealed that children were significantly more active during the no RAW and circuit course week than the Frisbee week. Males were significantly more physically active than females during the obstacle course week. Older children were significantly more active during the Frisbee week than younger children. Healthy BMI children were significantly more physically active during the circuit course week than children in the overweight/obese BMI category. CONCLUSIONS: Results imply that it is important for schools to consider demographic factors in the creation of recess opportunities to increase physical activity
SN - 1746-1561
AD - School of Sport and Exercise Science, University of Northern Colorado, Gunter Hall, Greeley, CO 80639, USA. megan.stellino@unco.edu
UR - 20690975
ER -

TY - JOUR
ID - 736
T1 - Correlates of physical activity in young American Indian children: lessons learned from the Wisconsin Nutrition and Growth Study
A1 - Adams,A.
A1 - Prince,R.
Y1 - 2010/09//
N1 - Adams, Alexandra. Prince, Ronald
Journal of public health management and practice : JPHMP
c0l, 9505213
T
Journal Article. Research Support, N.I.H., Extramural. Research Support, U.S. Gov't, P.H.S.
English
KW - MEDLINE
KW - Body Weight/eh [Ethnology]
KW - Body Weight/ph [Physiology]
KW - Child
KW - Preschool
KW - Cross-Sectional Studies
KW - Exercise
KW - Female
KW - Humans
KW - Indians
KW - North American/sn [Statistics & Numerical Data]
KW - North American
KW - Male
KW - Nutrition Surveys
KW - Obesity/eh [Ethnology]
KW - Overweight/eh [Ethnology]
KW - Prevalence

KW - Questionnaires
KW - Sex Distribution
KW - Television/ut [Utilization]
KW - Wisconsin/ep [Epidemiology]
RP - NOT IN FILE
SP - 394
EP - 400
JF - Journal of Public Health Management & Practice
JA - J Public Health Manag Pract
VL - 16
IS - 5
CY - United States
N2 - BACKGROUND: Obesity is a serious and growing health problem in American Indian (AI) children. Our study, the Wisconsin Nutrition and Growth Study, aimed to understand the prevalence and contributing factors to pediatric obesity in Wisconsin tribes and provide the foundation for intervention design. OBJECTIVE: This article focuses on associations among age, gender, and 3 measures of weight status with proxy-reported physical activity and TV/screen time in 3 to 8 year-old AI children. DESIGN/METHODS: In a cross-sectional design, 581 AI children (49.1% female, aged 3-8 years) participated in health screenings that included height, weight, waist circumference, percent body fat, and a caregiver survey on demographics and health, with questions on physical activity and TV/screen time. RESULTS: Forty-five percent of children were overweight or obese. Boys were significantly more obese and had higher levels of body fat than girls. There were no differences in weight category across age groups. Boys participated in significantly more weekly sports than girls and sports participation was somewhat higher in younger children. Body mass index and waist circumference were not significantly correlated with TV/screen time or with the 3 activity measures (sports participation, outdoor play time, or physical education classes). Hours of outdoor play significantly predicted child body fat percentage controlling for maternal body mass index and child age and gender. CONCLUSIONS: Young AI children in Wisconsin have high rates of overweight/obesity starting at a very early age, and outdoor play may play a significant role in mediating body fat. There is a need to develop obesity-prevention interventions at early ages
SN - 1550-5022
AD - Department of Family Medicine, Collaborative Center for Health Equity, University of Wisconsin School of Medicine and Public Health, Madison, USA. alex.adams@fammed.wisc.edu
UR - 20689387
ER -

TY - JOUR
ID - 737
T1 - A prescription for better health: go alfresco. Spending time outside might have some health benefits--and the 'greening' of exercise might have some more
Y1 - 2010/07//
N1 - Harvard health letter / from Harvard Medical School
9425764, c2y
K
Journal Article
English
KW - MEDLINE
KW - Aged
KW - Child
KW - Exercise/ph [Physiology]
KW - Exercise/px [Psychology]
KW - Humans
KW - Sunlight
KW - Vitamin D/me [Metabolism]
KW - Young Adult
RP - NOT IN FILE
SP - 1
EP - 2
JF - Harvard Health Letter

JA - Harv Health Lett
VL - 35
IS - 9
CY - United States
SN - 1052-1577
UR - 20821853
ER -

TY - JOUR
ID - 738
T1 - Permanent play facilities in school playgrounds as a determinant of children's activity
A1 - Nielsen,G.
A1 - Taylor,R.
A1 - Williams,S.
A1 - Mann,J.
Y1 - 2010/07//
N1 - Nielsen, Glen. Taylor, Rachael. Williams, Sheila. Mann, Jim
Journal of physical activity & health
101189457
IM
Journal Article
English
KW - MEDLINE
KW - Child
KW - Preschool
KW - Environment Design
KW - Exercise
KW - Humans
KW - Monitoring
KW - Ambulatory
KW - Play and Playthings
KW - Schools
RP - NOT IN FILE
SP - 490
EP - 496
JF - Journal of Physical Activity & Health
JA - J Phys Act Health
VL - 7
IS - 4
CY - United States
N2 - BACKGROUND: To investigate whether the number of permanent playground facilities in schools influences objectively measured physical activity. METHODS: Physical activity was measured using Actical accelerometers over 2 to 5 days in 417 children (5-12 years) from 7 schools. The number of permanent play facilities likely to encourage physical activity in individuals or groups of children (eg, adventure playgrounds, swings, trees, playground markings, courts, sandpits) were counted on 2 occasions in each school. The surface area of each playground (m(2)) was also measured. RESULTS: The number of permanent play facilities in schools ranged from 14 to 35 and was positively associated with all measures of activity. For each additional play facility, average accelerometry counts were 3.8% (P < .001) higher at school and 2.7% (P < .001) higher overall. Each additional play facility was also associated with 2.3% (P = .001) or 4 minutes more moderate/vigorous activity during school hours and 3.4% (P < .001) more (9 minutes) over the course of the day. School playground area did not affect activity independent of the number of permanent play facilities. Findings were consistent across age and sex groups. CONCLUSION: Increasing the number of permanent play facilities at schools may offer a cost-effective and sustainable option for increasing physical activity in young children
SN - 1543-3080
AD - Dept of Exercise and Sport Sciences, University of Copenhagen, Denmark
UR - 20683091

ER -

TY - JOUR
ID - 739
T1 - Associations between neighborhood safety, availability of recreational facilities, and adolescent physical activity among Canadian youth
A1 - Nichol,M.
A1 - Janssen,I.
A1 - Pickett,W.
Y1 - 2010/07//
N1 - Nichol, Marianne. Janssen, Ian. Pickett, William
Journal of physical activity & health
101189457
IM
Journal Article
English
KW - MEDLINE
KW - Adolescent
KW - Canada
KW - Child
KW - Exercise
KW - Female
KW - Health Behavior
KW - Humans
KW - Male
KW - Play and Playthings
KW - Residence Characteristics
KW - Safety
RP - NOT IN FILE
SP - 442
EP - 450
JF - Journal of Physical Activity & Health
JA - J Phys Act Health
VL - 7
IS - 4
CY - United States
N2 - BACKGROUND: The safety of neighborhoods and availability of parks and facilities may influence adolescent physical activity independently or interactively. METHODS: 9114 Canadians in grades 6 to 10 completed the 2006 Health Behavior in School-Aged Children Survey. The outcome of interest was students' self-reported participation in moderate-to-vigorous physical activity outside of school. A composite scale based on questions measuring student perceptions of safety was used to capture individual perceptions of safety. In addition, schools were grouped into quintiles based on the mean of the perceived safety scale, used as a proxy for peer perceptions. The number of parks and recreational facilities within 5 km of schools was abstracted from a geographical information system. RESULTS: Moderate gradients in physical activity were observed according to individual and group perceptions of safety. Boys and girls with the highest perceptions of safety were 1.31 (95% CI: 1.17-1.45) and 1.45 (1.26-1.65) times more likely to be physically active, respectively, than those with the lowest perceptions. Compared with those who perceived the neighborhood as least safe, elementary students in higher quintiles were 1.31, 1.39, 1.37, and 1.56 times more likely to be physically active (P(trend) = 0.012). Increased numbers of recreational features were not related to physical activity irrespective of neighborhood safety. CONCLUSIONS: Individual and group perceptions of neighborhood safety were modestly associated with adolescents' physical activity
SN - 1543-3080
AD - Dept of Community Health and Epidemiology, Queen's University, Kingston, Ontario, Canada
UR - 20683085
ER -

TY - JOUR

ID - 740
T1 - A study on Aspergillus species in houses of asthmatic patients from Sari City, Iran and a brief review of the health effects of exposure to indoor Aspergillus
A1 - Hedayati,M.T.
A1 - Mayahi,S.
A1 - Denning,D.W.
Y1 - 2010/09//
N1 - Hedayati, Mohammad T. Mayahi, Sabah. Denning, David W
Environmental monitoring and assessment
dvk, 8508350
IM
Journal Article. Research Support, Non-U.S. Gov't
English
KW - MEDLINE
KW - Adolescent
KW - Adult
KW - Aged
KW - Air Microbiology
KW - Air Pollution
KW - Indoor/an [Analysis]
KW - Indoor/sn [Statistics & Numerical Data]
KW - Aspergillus/ip [Isolation & Purification]
KW - Asthma/ep [Epidemiology]
KW - Child
KW - Cities
KW - Environmental Monitoring
KW - Epidemiological Monitoring
KW - Female
KW - Housing/sn [Statistics & Numerical Data]
KW - Humans
KW - Inhalation Exposure/an [Analysis]
KW - Inhalation Exposure/sn [Statistics & Numerical Data]
KW - Iran
KW - Male
KW - Middle Aged
KW - Young Adult
RP - NOT IN FILE
SP - 481
EP - 487
JF - Environmental Monitoring & Assessment
JA - Environ Monit Assess
VL - 168
IS - 1-4
CY - Netherlands
N2 - To study the distribution of Aspergillus spp. in outdoor and indoor air of asthmatic patients' houses, as well as a review on the health effects of exposure to indoor Aspergillus. Open plates containing malt extract agar media were used to isolate fungi from the indoor (n = 360) and outdoor (n = 180) air of 90 asthmatic patients' houses living in Sari City, Iran. Plates were incubated at room temperature for 7-14 days. Cultured Aspergillus spp. were identified by standard mycological techniques. All culture plates grew fungi, a testament to the ubiquitous nature of fungal exposure. Cladosporium spp. (29.2%), Aspergillus spp. (19.0%), and Penicillium spp. (18.3%) were most common inside the houses while Cladosporium spp. (44.5%), Aspergillus spp. (12.4%), and Alternaria spp. (11.1%) were most common outside the houses. Aspergillus flavus (30.1%) and A. fumigatus (23.1%) are the most commonly isolated species in indoor air. Aspergillus flavus (44.5%) and A. fumigatus (42.6%) were the most prevalent Aspergillus spp. outside. The most colony numbers of Aspergillus were isolated from kitchens (30.4%) and the least from bedrooms (21.1%). Aspergillus flavus was the most prevalent species in all sampled rooms except in the kitchen where A. fumigatus was the most common. Aspergillus flavus is the most prevalent species among the Aspergillus spp. in the indoor and outdoor

of a warm climate area. In these areas, A. flavus can be a major source of allergen in the air. Therefore, minimizing indoor fungal exposure could play an important role in reducing allergic symptoms in susceptible persons
SN - 1573-2959
AD - Medical Mycology and Parasitology Department, School of Medicine, Mazandaran University of Medical Sciences, Sari, Iran. hedayaty2001@yahoo.co.uk
UR - 19697147
ER -

TY - JOUR
ID - 741
T1 - Coping with parental cancer: web-based peer support in children
A1 - Giesbers,J.
A1 - Verdonck-de,Leeuw,I
A1 - van Zuuren,F.J.
A1 - Kleverlaan,N.
A1 - van der Linden,M.H.
Y1 - 2010/08//
N1 - Giesbers, J. Verdonck-de Leeuw, I M. van Zuuren, F J. Kleverlaan, N. van der Linden, M H
Psycho-oncology
cps, 9214524
IM
Journal Article. Research Support, Non-U.S. Gov't
English
KW - MEDLINE
KW - Adaptation
KW - Psychological
KW - Adolescent
KW - Adult
KW - Child
KW - Child of Impaired Parents/px [Psychology]
KW - Female
KW - Humans
KW - Internet
KW - Male
KW - Middle Aged
KW - Neoplasms/px [Psychology]
KW - Netherlands
KW - Peer Group
KW - Social Support
KW - Software
KW - Young Adult
RP - NOT IN FILE
SP - 887
EP - 892
JF - Psycho-Oncology
JA - Psychooncology.
VL - 19
IS - 8
CY - England
N2 - OBJECTIVE: To investigate the use and content of web-based peer support in children coping with parental cancer. METHODS: In children aged above 12 years, 158 forum discussions on the Dutch website www.kankerspoken.nl in a time-period of 3 months were investigated. Age, gender, number of discussions, participation and main activities on the website were quantitatively assessed. Contents of the discussions were qualitatively analyzed. RESULTS: In total, 129 children and 8 adults participated on the website with an average age of 15.3 years. The majority was female (80%). On average, a child was on-line for 3 days and participated in 4 to 5 forum discussions. Main activities on the website were sharing personal experience,

providing encouragement/support, providing and seeking information or advice and seeking contact outside of the website. Qualitative content analysis revealed that children regularly faced emotional problems and experienced a lack of understanding and communication in their direct environment. CONCLUSIONS: In dealing with parental cancer, children experience a variety of difficulties. This study offers a window into the use of web-based peer support by children dealing with parental cancer. (c) 2009 John Wiley & Sons, Ltd
SN - 1099-1611
AD - Department of Clinical Psychology, VU University, Amsterdam, The Netherlands
UR - 19998331
ER -

TY - JOUR
ID - 742
T1 - Automated vocal analysis of naturalistic recordings from children with autism, language delay, and typical development
A1 - Oller,D.K.
A1 - Niyogi,P.
A1 - Gray,S.
A1 - Richards,J.A.
A1 - Gilkerson,J.
A1 - Xu,D.
A1 - Yapanel,U.
A1 - Warren,S.F.
Y1 - 2010/07/27/
N1 - Oller, D K. Niyogi, P. Gray, S. Richards, J A. Gilkerson, J. Xu, D. Yapanel, U. Warren, S F
Proceedings of the National Academy of Sciences of the United States of America
pv3, 7505876
IM
Journal Article. Research Support, Non-U.S. Gov't
English
KW - MEDLINE
KW - Autistic Disorder/di [Diagnosis]
KW - Autistic Disorder/pp [Physiopathology]
KW - Child
KW - Preschool
KW - Female
KW - Humans
KW - Infant
KW - Language Development
KW - Language Development Disorders/di [Diagnosis]
KW - Language Development Disorders/pp [Physiopathology]
KW - Linear Models
KW - Male
KW - Multivariate Analysis
KW - Speech Disorders/di [Diagnosis]
KW - Speech Disorders/pp [Physiopathology]
KW - Speech Production Measurement/is [Instrumentation]
KW - Speech Production Measurement/mt [Methods]
RP - NOT IN FILE
SP - 13354
EP - 13359
JF - Proceedings of the National Academy of Sciences of the United States of America
JA - Proc Natl Acad Sci U S A
VL - 107
IS - 30
CY - United States
N2 - For generations the study of vocal development and its role in language has been conducted laboriously, with human transcribers and analysts coding and taking measurements from small recorded samples. Our

research illustrates a method to obtain measures of early speech development through automated analysis of massive quantities of day-long audio recordings collected naturalistically in children's homes. A primary goal is to provide insights into the development of infant control over infrastructural characteristics of speech through large-scale statistical analysis of strategically selected acoustic parameters. In pursuit of this goal we have discovered that the first automated approach we implemented is not only able to track children's development on acoustic parameters known to play key roles in speech, but also is able to differentiate vocalizations from typically developing children and children with autism or language delay. The method is totally automated, with no human intervention, allowing efficient sampling and analysis at unprecedented scales. The work shows the potential to fundamentally enhance research in vocal development and to add a fully objective measure to the battery used to detect speech-related disorders in early childhood. Thus, automated analysis should soon be able to contribute to screening and diagnosis procedures for early disorders, and more generally, the findings suggest fundamental methods for the study of language in natural environments
SN - 1091-6490
AD - School of Audiology and Speech-Language Pathology, University of Memphis, Memphis, TN 38105, USA. koller@memphis.edu
UR - 20643944
ER -

TY - JOUR
ID - 743
T1 - Individual differences in auditory sentence comprehension in children: An exploratory event-related functional magnetic resonance imaging investigation
A1 - Yeatman,J.D.
A1 - Ben-Shachar,M.
A1 - Glover,G.H.
A1 - Feldman,H.M.
Y1 - 2010/08//
N1 - Yeatman, Jason D. Ben-Shachar, Michal. Glover, Gary H. Feldman, Heidi M
Brain and language
7506220, b5h
IM
Journal Article. Research Support, N.I.H., Extramural
English
KW - MEDLINE
KW - Adolescent
KW - Auditory Pathways/ph [Physiology]
KW - Cerebral Cortex/ph [Physiology]
KW - Child
KW - Evoked Potentials
KW - Auditory/ph [Physiology]
KW - Female
KW - Humans
KW - Individuality
KW - Language Development
KW - Magnetic Resonance Imaging
KW - Male
KW - Speech Perception/ph [Physiology]
RP - NOT IN FILE
SP - 72
EP - 79
JF - Brain & Language
JA - Brain Lang
VL - 114
IS - 2
CY - United States
N2 - The purpose of this study was to explore changes in activation of the cortical network that serves auditory sentence comprehension in children in response to increasing demands of complex sentences. A further goal is

to study how individual differences in children's receptive language abilities are associated with such changes in cortical responses. Fourteen children, 10-16 years old, participated in an event-related functional magnetic resonance imaging experiment using a cross modal sentence-picture verification paradigm. We manipulated sentence difficulty and length in a 2x2 factorial design. Task-related activation covered large regions of the left and right superior temporal cortex, inferior parietal lobe, precuneous, cingulate, middle frontal gyrus and precentral gyrus. Sentence difficulty, independent of length, led to increased activation in the left temporal-parietal junction and right superior temporal gyrus. Changes in activation in frontal regions positively correlated with age-standardized receptive vocabulary scores and negatively correlated with reaction time on a receptive grammar test outside the scanner. Thus, individual differences in language skills were associated with changes in the network in response to changing task demands. These preliminary findings in a small sample of typically developing children suggest that the investigation of individual differences may prove useful in elucidating the underlying neural mechanisms of language disorders in children. 2009 Elsevier Inc. All rights reserved
SN - 1090-2155
AD - Department of Pediatrics, Stanford University, CA, United States. jyeatman@stanford.edu
UR - 20053431
ER -

TY - JOUR
ID - 744
T1 - Dietary patterns associated with fat and bone mass in young children
A1 - Wosje,K.S.
A1 - Khoury,P.R.
A1 - Claytor,R.P.
A1 - Copeland,K.A.
A1 - Hornung,R.W.
A1 - Daniels,S.R.
A1 - Kalkwarf,H.J.
Y1 - 2010/08//
N1 - Wosje, Karen S. Khoury, Philip R. Claytor, Randal P. Copeland, Kristen A. Hornung, Richard W. Daniels, Stephen R. Kalkwarf, Heidi J
The American journal of clinical nutrition
3ey, 0376027
AIM, IM
Journal Article. Research Support, N.I.H., Extramural
English
KW - MEDLINE
KW - Adipose Tissue
KW - Bone Density
KW - Child
KW - Preschool
KW - Cooking
KW - Diet
KW - Dietary Fats
KW - Female
KW - Food Handling
KW - Humans
KW - Longitudinal Studies
KW - Male
KW - Meat
KW - Obesity/pc [Prevention & Control]
KW - Osteoporosis/pc [Prevention & Control]
KW - Regression Analysis
KW - Vegetables
RP - NOT IN FILE
SP - 294
EP - 303
JF - American Journal of Clinical Nutrition

JA - Am J Clin Nutr
VL - 92
IS - 2
CY - United States
N2 - BACKGROUND: Obesity and osteoporosis have origins in childhood, and both are affected by dietary intake and physical activity. However, there is little information on what constitutes a diet that simultaneously promotes low fat mass and high bone mass accrual early in life. OBJECTIVE: Our objective was to identify dietary patterns related to fat and bone mass in children during the age period of 3.8-7.8 y. DESIGN: A total of 325 children contributed data from 13 visits over 4 separate study years (age ranges: 3.8-4.8, >4.8-5.8, >5.8-6.8, and >6.8-7.8 y). We performed reduced-rank regression to identify dietary patterns related to fat mass and bone mass measured by dual-energy X-ray absorptiometry for each study year. Covariables included race, sex, height, weight, energy intake, calcium intake, physical activity measured by accelerometry, and time spent viewing television and playing outdoors. RESULTS: A dietary pattern characterized by a high intake of dark-green and deep-yellow vegetables was related to low fat mass and high bone mass; high processed-meat intake was related to high bone mass; and high fried-food intake was related to high fat mass. Dietary pattern scores remained related to fat mass and bone mass after all covariables were controlled for (P < 0.001-0.03). CONCLUSION: Beginning at preschool age, diets rich in dark-green and deep-yellow vegetables and low in fried foods may lead to healthy fat and bone mass accrual in young children
SN - 1938-3207
AD - Division of General and Community Pediatrics, Cincinnati Children's Hospital Medical Center, Cincinnati, OH 45229, USA. wosjek@yahoo.com
UR - 20519562
ER -

TY - JOUR
ID - 745
T1 - Greenspace and children's physical activity: a GPS/GIS analysis of the PEACH project
A1 - Wheeler,B.W.
A1 - Cooper,A.R.
A1 - Page,A.S.
A1 - Jago,R.
Y1 - 2010/08//
N1 - Wheeler, Benedict W. Cooper, Ashley R. Page, Angie S. Jago, Russell
Preventive medicine
pm4, 0322116
IM
Journal Article. Research Support, Non-U.S. Gov't
English
KW - MEDLINE
KW - Body Mass Index
KW - Child
KW - Environment Design
KW - Female
KW - Geographic Information Systems
KW - Great Britain
KW - Humans
KW - Male
KW - Monitoring
KW - Physiologic/is [Instrumentation]
KW - Physiologic/mt [Methods]
KW - Motor Activity
KW - Regression Analysis
RP - NOT IN FILE
SP - 148
EP - 152
JF - Preventive Medicine
JA - Prev Med

VL - 51
IS - 2
CY - United States
N2 - OBJECTIVE: To quantify the volume and intensity of children's physical activity after school in greenspace and elsewhere. METHODS: Data were collected between 2006 and 2008 from 1,307 children aged 10-11 in Bristol, UK. Accelerometers and Global Positioning System receivers measured activity and location every 10 s (epoch) after school for four days. Data were mapped in a Geographic Information System with a greenspace dataset. Activity volume (accelerometer counts per minute), time in moderate-vigorous physical activity (MVPA), and the odds of an epoch being MVPA (using logistic regression) were compared for greenspace, non-greenspace and indoors. RESULTS: 13% of monitored time was spent outdoors (2% in greenspace), during which time 30% of activity volume and 35% of MVPA was accumulated. 7% of boys' activity volume and 9% of MVPA were in greenspace with girls slightly lower (5% and 6% respectively). The odds of an epoch being MVPA in greenspace relative to outdoor non-greenspace was 1.37 (95% CI 1.22-1.53) for boys and 1.08 (95% CI 0.95-1.22) for girls. CONCLUSION: Most activity occurring outdoors is not in greenspace and non-green urban environments are therefore very important for children's activity. However, when boys are in greenspace, activity is more likely to be of higher intensity. Copyright 2010 Elsevier Inc. All rights reserved
SN - 1096-0260
AD - DECIPHer Centre, Department of Social Medicine, University of Bristol, Bristol, UK. benedict.wheeler@pcmd.ac.uk
UR - 20542493
ER -

TY - JOUR
ID - 746
T1 - Indoor-outdoor distribution and risk assessment of volatile organic compounds in the atmosphere of industrial and urban areas
A1 - Massolo,L.
A1 - Rehwagen,M.
A1 - Porta,A.
A1 - Ronco,A.
A1 - Herbarth,O.
A1 - Mueller,A.
Y1 - 2010/08//
N1 - Massolo, Laura. Rehwagen, Martina. Porta, Andres. Ronco, Alicia. Herbarth, Olf. Mueller, Andrea
Environmental toxicology
dvq, 100885357
IM
Journal Article. Research Support, Non-U.S. Gov't
English
KW - MEDLINE
KW - Air/an [Analysis]
KW - Air Pollutants/to [Toxicity]
KW - Air Pollution
KW - Indoor
KW - Alkanes/an [Analysis]
KW - Benzene/an [Analysis]
KW - Child
KW - Cities
KW - Environmental Monitoring
KW - Humans
KW - Industry
KW - Risk Assessment
KW - Schools
KW - Urban Population
KW - Volatile Organic Compounds/to [Toxicity]
KW - Xylenes/an [Analysis]

RP - NOT IN FILE
SP - 339
EP - 349
JF - Environmental Toxicology
JA - Environ Toxicol
VL - 25
IS - 4
CY - United States
N2 - Volatile organic compounds (VOCs), which play an important part indoors and outdoors, comprise differing compound groups such as n-alkanes, cycloalkanes, aromatic and chlorinated hydrocarbons and terpenes. In the current study, samples were analyzed from indoor (schools and houses, n = 92) and outdoor (n = 33) air in urban, industrial, semirural and residential areas from the region of La Plata (Argentine) to consider VOC exposure in different types of environments. VOCs were sampled for 1 month during winter for 3 years, with passive 3M monitors. Samples were extracted with CS(2) and analyzed by GC/MS detectors. The results show significant differences in concentration and distribution between indoor and outdoor samples, depending on the study area. Most VOCs predominantly originated indoors in urban, semirural and residential areas, whereas an important outdoor influence in the industrial area was observed. In all areas alkanes and aromatic compounds dominated, even though a different chemical distribution was seen. Traffic burden was determined as the major source of outdoor VOC with a benzene/toluene ratio close to 0.5. Indoors, C9-C11 alkanes, toluene and xylenes dominated, caused by human activities. In contrast, in the industrial area higher concentrations of hexane, heptane and benzene occurred outdoors and affected the indoor air significantly. The lifetime cancer risk (LCR) associated to the benzene exposure was calculated for children from the different study areas. For all groups the study showed a LCR value greater than 1 x 10(-6) related to the benzene exposure indoors as well outdoors. A value two magnitudes higher was detected indoors in the industrial area, what demonstrates the high risk for children living in this area of La Plata
SN - 1522-7278
AD - Facultad de Ciencias Exactas, Centro de Investigaciones del Medio Ambiente-CIMA, Universidad Nacional de La Plata (CICPBA-CONICET), 47 y 115; (1900)-La Plata, Argentina
UR - 19449388
ER -

TY - JOUR
ID - 747
T1 - (99m)Technetium-macroaggregated albumin perfusion lung scan versus contrast enhanced echocardiography in the diagnosis of the hepatopulmonary syndrome in children with chronic liver disease
A1 - El-Shabrawi,M.H.
A1 - Omran,S.
A1 - Wageeh,S.
A1 - Isa,M.
A1 - Okasha,S.
A1 - Mohsen,N.A.
A1 - Zekry,O.
A1 - Bartan,G.
A1 - El-Karaksy,H.M.
Y1 - 2010/08//
N1 - El-Shabrawi, Mortada Hassan. Omran, Salwa. Wageeh, Shereine. Isa, Mona. Okasha, Sawsan. Mohsen, Nabil A. Zekry, Ola. E-Bartan, Gamal. El-Karaksy, Hanaa M
European journal of gastroenterology & hepatology
9000874, B9X
IM
Comparative Study. Journal Article
English
KW - MEDLINE
KW - Anoxia/bl [Blood]
KW - Anoxia/ri [Radionuclide Imaging]
KW - Child
KW - Preschool

KW - Chronic Disease
KW - Echocardiography/mt [Methods]
KW - Female
KW - Hepatitis B/ri [Radionuclide Imaging]
KW - Hepatitis C/ri [Radionuclide Imaging]
KW - Hepatopulmonary Syndrome/ri [Radionuclide Imaging]
KW - Hepatopulmonary Syndrome/us [Ultrasonography]
KW - Humans
KW - Liver Cirrhosis/ri [Radionuclide Imaging]
KW - Male
KW - Metabolism
KW - Inborn Errors/ri [Radionuclide Imaging]
KW - Perfusion Imaging/mt [Methods]
KW - Pulmonary Circulation
KW - Radiopharmaceuticals/du [Diagnostic Use]
KW - Schistosomiasis/ri [Radionuclide Imaging]
KW - Technetium Tc 99m Aggregated Albumin/du [Diagnostic Use]
RP - NOT IN FILE
SP - 1006
EP - 1012
JF - European Journal of Gastroenterology & Hepatology
JA - Eur J Gastroenterol Hepatol
VL - 22
IS - 8
CY - England
N2 - BACKGROUND AND AIMS: The hepatopulmonary syndrome (HPS) is a triad of advanced chronic liver disease (CLD), arterial hypoxemia and intrapulmonary arteriovenous shunting in the absence of a primary cardiopulmonary disease. HPS has been more frequently reported in adults than in children with no data on its prevalence in children with CLD. The aim of this study was to detect the prevalence of the HPS in a cohort of children with CLD because of chronic hepatitis B and/or C virus infection, schistosomiasis as well as inborn metabolic errors. We also aimed to evaluate the role of Technetium labeled macroaggregated albumin (Tc--MAA) perfusion lung scan versus contrast enhanced echocardiography (CEE) with intravenous injection of agitated saline in the diagnosis and quantification of intrapulmonary shunts and their relationship to important clinical and laboratory findings. METHODS: Forty Egyptian children (22 males) were investigated. Their ages ranged from 5 to 12 years (with a mean of 9.5 years). Twenty individuals proved to have cirrhosis. RESULTS: Blood gas determination revealed more significant arterial hypoxemia in cirrhotics than noncirrhotics both under room air and after breathing 100% oxygen for 15 mins. CEE showed comparable cardiac measurements in cirrhotic and noncirrhotic patients, and diagnosed intrapulmonary shunts in three hypoxemic cirrhotic patients; whereas Tc--MAAperfusion lung scan diagnosed shunts in seven patients (five of them cirrhotic). The presence of shunts was significantly correlated with the duration of CLD, clinical findings, presence of cirrhosis and porto-systemic collaterals. We calculated for each patient a shunt index (SI) by the formula: (activity outside thorax/activity outside plus inside thorax) 100; and an SI value of 0.278 was found to be a cutoff value for shunt detection. All patients with SI above this value had shunting associated with hypoxemia and all patients with SI below this value had no hypoxemia (specificity 100%). CONCLUSION: Arterial hypoxemia and intrapulmonary shunts were diagnosed in 17.5% of this cohort of children with cirrhotic or noncirrhotic CLD representing the classic HPS. Tc--MAA perfusion lung scan was more sensitive than CEE in detection of intrapulmonary shunts. SI cutoff value of 0.278 was found to be highly specific for shunt detection and we recommend its validation in further studies
SN - 1473-5687
AD - Department of Pediatrics and Nuclear Medicine, Cairo University, Cairo, Egypt. melshabrawi@medicine.cu.edu.eg
UR - 20101183
ER -

TY - JOUR
ID - 748
T1 - Associations between physical activity and health parameters in adolescent pupils in Egypt

A1 - El,Ansari W.
A1 - El,Ashker S.
A1 - Moseley,L.
Y1 - 2010/04//
N1 - El Ansari, Walid. El Ashker, Said. Moseley, Laurence
International journal of environmental research and public health
101238455
IM
Journal Article
English
KW - MEDLINE
KW - Adolescent
KW - Body Mass Index
KW - Child
KW - Egypt
KW - Exercise
KW - Female
KW - Health Status
KW - Humans
KW - Male
RP - NOT IN FILE
SP - 1649
EP - 1669
JF - International Journal of Environmental Research & Public Health [Electronic Resource]
JA - Int J Environ Res Public Health
VL - 7
IS - 4
CY - Switzerland
N2 - Physical activity (PA) could be protective against hypertension, atherosclerosis, coronary heart disease and cardiovascular disease. This quantitative study assessed the association between a PA intervention and three anthropometric parameters (weight, body mass index, body fat) and four physiological parameters (cholesterol level, systolic blood pressure, diastolic blood pressure, heart rate) among secondary school pupils (N = 160) in Egypt through the school term. The pupils were randomised to an intervention group (80 pupils) and controls (80 pupils). Measurements were obtained for all pupils twice: at baseline; and then again after three months. The PA intervention programme comprised an 'afterschool' one hour of moderate exercise three times a week for three months. Both the controls and the intervention pupils attended the 'normal' exercise schedule provided by the school; in addition, the intervention group attended afterschool PA programme from about 2-3 o'clock in the afternoon. At baseline, employing pupil's BMI, 27.5% and 28.8% of the intervention and control pupils respectively were classified as overweight. After three months, the percentage of overweight decreased to 12.5% in the intervention pupils, while it increased to 37.3% in the controls. At the end of the three months period, there were significant improvements across most anthropometric and physiological parameters of the intervention pupils when compared with the control children. The correlation coefficient of the improvements for the boys and the girls was 0.97, indicating clearly that the intervention was having nearly the same beneficial effect for boys and girls. A moderate PA programme for a modest period of 3 months could be effective in maintaining or enhancing pupil's anthropometric and physiological parameters in comparison to the controls where there was deterioration in both parameters. Policy makers and secondary schools in Egypt might need to pay more attention to PA programmes conducted on school days, in order to motivate pupils to attend such programmes. There is also an urgent need to look at current PA systems within schools in Egypt in order to assess PA outside school times
SN - 1660-4601
AD - Faculty of Sport, Health & Social Care, University of Gloucestershire, Gloucester, GL2 9HW, UK. walidansari@glos.ac.uk
UR - 20617051
ER -

TY - JOUR
ID - 749

T1 - Effects of socioeconomic status on the physical activity in adolescents: a systematic review of the evidence. [Review]
A1 - Stalsberg,R.
A1 - Pedersen,A.V.
Y1 - 2010/06//
N1 - Stalsberg, R. Pedersen, A V
Scandinavian journal of medicine & science in sports
9111504, cac
IM
Journal Article. Review
English
KW - MEDLINE
KW - Adolescent
KW - Child
KW - Exercise
KW - Female
KW - Humans
KW - Male
KW - Social Class
KW - Young Adult
RP - NOT IN FILE
SP - 368
EP - 383
JF - Scandinavian Journal of Medicine & Science in Sports
JA - Scand J Med Sci Sports
VL - 20
IS - 3
CY - Denmark
N2 - The relationship between physical activity and socioeconomic status (SES) is evident in the adult population, but is much discussed with regard to adolescents. The main objective of this review was, therefore, to clarify whether there is a relationship between physical activity and SES in adolescents. Computerized searches were conducted in the databases PubMed, ISI Web of knowledge and SPORT-Discus to identify all relevant articles up to July 2009. Other review articles, descriptive or theoretical articles and articles where the adolescents in the samples were outside the age group of 13-18 years were excluded. Sixty-two articles were included in the end. The main results support the hypothesis that there is an association between SES and physical activity among adolescents, and that adolescents with higher SES are more physically active than those with lower SES. The findings are, however, far from uniform. Forty-two percent of the included studies report no or an opposite relation. There is also an inconsistent use of measures for both variables that complicates explanations and interpretations of the findings. This fortifies the claim that there is no single explanation for a possible difference in physical activity between different socioeconomic groups
SN - 1600-0838
AD - Radiography Programme, Sor-Trondelag University College, Trondheim, Norway
UR - 20136763
ER -

TY - JOUR
ID - 750
T1 - Adverse encounters with alligators in the United States: an update. [Review]
A1 - Langley,R.L.
Y1 - 2010/06//
N1 - Langley, Ricky L
Wilderness & environmental medicine
dbe, 9505185
IM
Journal Article. Review
English
KW - MEDLINE

KW - Adolescent
KW - Adult
KW - Aged
KW - 80 and over
KW - Alligators and Crocodiles/ah [Anatomy & Histology]
KW - Alligators and Crocodiles
KW - Amputation
KW - Traumatic/pa [Pathology]
KW - Animals
KW - Arm Injuries/pa [Pathology]
KW - Behavior
KW - Animal
KW - Bites and Stings/co [Complications]
KW - Bites and Stings/ep [Epidemiology]
KW - Child
KW - Preschool
KW - Female
KW - Humans
KW - Leg Injuries/pa [Pathology]
KW - Male
KW - Middle Aged
KW - Population Density
KW - United States/ep [Epidemiology]
KW - Wound Infection/ep [Epidemiology]
KW - Wound Infection/et [Etiology]
KW - Young Adult
RP - NOT IN FILE
SP - 156
EP - 163
JF - Wilderness & Environmental Medicine
JA - Wilderness Environ Med
VL - 21
IS - 2
CY - United States
N2 - OBJECTIVE: Severe injuries and fatalities can occur from an alligator attack. Encounters with alligators appear to be increasing in the United States. This review provides information from alligator attacks reported in the United States as well as infections that may occur after an alligator bite. METHODS: Telephone interviews were conducted with state wildlife offices in all Southern states in order to collect information on the number of alligator bites, nuisance calls, and the estimated alligator population of each state. Detailed information from alligator attacks in Florida is presented, including basic demographic information on the victims and description of the types of injuries and the activity of the victim at the time of injury. Additional information regarding the size and behavior of the alligator involved in the attack is also provided in many cases. RESULTS: There have been 567 reports of adverse encounters with alligators with 24 deaths reported in the United States from 1928 to January 1, 2009. In addition, thousands of nuisance calls are made yearly and the number of nuisance calls as well as the alligator population is increasing in many states. CONCLUSIONS: Injuries from encounters with alligators may range from minor scratches and punctures to amputations and death. The larger the alligator, the more likely that serious injury will occur. As the human population encroaches on the habitat of the alligator, attacks and nuisance complaints will continue to occur. A uniform reporting system among states should be developed to obtain more complete information on alligator encounters. Guidelines have been developed by many state wildlife officials to reduce adverse encounters with alligators. Copyright (c) 2010 Wilderness Medical Society. Published by Elsevier Inc. All rights reserved
SN - 1545-1534
AD - North Carolina Department of Health and Human Services, Raleigh, NC 27699-1912, USA. Rick.langley@dhhs.nc.gov
UR - 20591380
ER -

TY - JOUR
ID - 751
T1 - Vital status of pre-ART and ART patients defaulting from care in rural Malawi
A1 - McGuire,M.
A1 - Munyenyembe,T.
A1 - Szumilin,E.
A1 - Heinzelmann,A.
A1 - Le,Paih M.
A1 - Bouithy,N.
A1 - Pujades-Rodriguez,M.
Y1 - 2010/06//
N1 - McGuire, Megan. Munyenyembe, Tamika. Szumilin, Elisabeth. Heinzelmann, Annette. Le Paih, Mickael. Bouithy, Nenette. Pujades-Rodriguez, Mar
Tropical medicine & international health : TM & IH
cf5, 9610576
IM
Journal Article. Research Support, Non-U.S. Gov't
English
KW - MEDLINE
KW - Adult
KW - Antiretroviral Therapy
KW - Highly Active/px [Psychology]
KW - Highly Active/sn [Statistics & Numerical Data]
KW - Attitude to Health
KW - CD4 Lymphocyte Count
KW - Child
KW - Cross-Sectional Studies
KW - Female
KW - HIV Infections/dt [Drug Therapy]
KW - HIV Infections/im [Immunology]
KW - HIV Infections/mo [Mortality]
KW - Health Services Accessibility/sn [Statistics & Numerical Data]
KW - Humans
KW - Malawi/ep [Epidemiology]
KW - Male
KW - Medication Adherence/sn [Statistics & Numerical Data]
KW - Patient Dropouts/px [Psychology]
KW - Patient Dropouts/sn [Statistics & Numerical Data]
KW - Rural Health/sn [Statistics & Numerical Data]
KW - Socioeconomic Factors
KW - Treatment Outcome
KW - Young Adult
RP - NOT IN FILE
SP - 55
EP - 62
JF - Tropical Medicine & International Health
JA - Trop Med Int Health
VL - 15 Suppl 1
CY - England
N2 - OBJECTIVES: To ascertain the outcome of pre-Antiretroviral therapy (ART) and ART patients defaulting from care and investigate reasons for defaulting. METHODS: Patients defaulting from HIV care in Chiradzulu between July 2004 and September 2007 were traced at last known home address. Deaths and moves were recorded, and patients found alive were interviewed. Defaulting was defined as missed last appointment by more than 1 month among patients of unknown vital status. RESULTS: A total of 1637 individuals were traced (54%-88% of eligible), 981 pre-ART and 656 ART patients. Of 694 pre-ART patients found, 49% had died (51% of adults and 38% of children), a median of 47 days after defaulting, and 14% had moved away. Of 451 ART patients found, 54% had died (54% of adults and 50% of children), a median of 52 days after defaulting,

and 20% had moved away. Overall, 221 patients were interviewed (90% of those found alive), 42% had worked outside the district in the previous year; 49% of pre-ART and 19% of ART patients had not disclosed their HIV status to other household members. Main reasons for defaulting were stigma (43%), care dissatisfaction (34%), improved health (28%) and for ART discontinuation, poor understanding of disease or treatment (56%) and drug side effects (42%). CONCLUSION: This study in a rural African HIV programme reveals the dynamics related to health service access and use, and it provides information to correct programme mortality estimates for adults and children
SN - 1365-3156
AD - Medecins Sans Frontieres, Chiradzulu, Malawi
UR - 20586961
ER -

TY - JOUR
ID - 752
T1 - Care of children at a large outdoor music festival in the United Kingdom
A1 - McQueen,C.P.
Y1 - 2010/05//
N1 - McQueen, Carl P
Prehospital and disaster medicine
bdf, 8918173
T
Journal Article
English
KW - MEDLINE
KW - Child
KW - Emergency Medical Services/og [Organization & Administration]
KW - England/ep [Epidemiology]
KW - Holidays
KW - Humans
KW - Leisure Activities
KW - Music
KW - Retrospective Studies
KW - Wounds and Injuries/ep [Epidemiology]
RP - NOT IN FILE
SP - 223
EP - 226
JF - Prehospital & Disaster Medicine
JA - Prehospital Disaster Med
VL - 25
IS - 3
CY - United States
N2 - INTRODUCTION: Limited data exist on the standard of care provided for children at mass gatherings and special events (MGSE). Some studies provide valuable insight into the proportion of pediatric patients that can be expected at various types of MGSEs, but an accurate breakdown of the range of pediatric conditions treated at major events has yet to be produced. Such data are essential for the preparation of MGSEs so that the health and safety of children at such events can be adequately safeguarded. The aim of this study is to examine the care requirements for children at a large, outdoor music festival in the United Kingdom. METHODS: A retrospective review of all patient report forms (PRFs) from a large, outdoor music festival held in Leeds (UK) in 2003. Data were extracted from the PRFs using a standardized proforma and analyzed using an Excel computer program. RESULTS: Pediatric cases contributed approximately 15% to the overall workload at the event. Children presented with a range of conditions that varied from those seen in the adult population. Children were more likely than adults to present for medical attention following crush injuries (OR = 2.536; 95% CI = 1.537-4.187); after a collapse/syncopal episode (OR = 2.687; 95% CI = 1.442-5.007); or complaining of nausea (OR = 3.484; 95% CI = 2.089-5.813). Alcohol/drugs were less likely to be involved in the precipitating cause for medical attention in children compared to adults (OR = 0.477; 95% CI = 0.250-0.912). No critical care incidents involving children were encountered during the event. CONCLUSIONS: Mass gatherings and special events in the UK, such as outdoor music festivals, can involve a large number of children who access medical care for a

different range of conditions compared to adults. The care of children at large, outdoor music events should not be overlooked. Event planning in the UK should include measures to ensure that appropriately trained and equipped medical teams are used at music festivals to safeguard the welfare of children who may attend. Further research into this exciting area is required
SN - 1049-023X
AD - Specialty Training Registar East Midlands Workforce Deanery, UK. carl_mcqueen@hotmail.com
UR - 20586015
ER -

TY - JOUR
ID - 753
T1 - Affects of ionizing radiation on T-cell population lymphocyte: a risk factor of irritable bowel syndrome
A1 - Sheikh Sajjadieh,M.R.
A1 - Kuznetsova,L.V.
A1 - Bojenko,V.B.
Y1 - 2010/07//
N1 - Sheikh Sajjadieh, M R. Kuznetsova, L V. Bojenko, V B
Toxicology and industrial health
vws, 8602702
IM
Journal Article
English
KW - MEDLINE
KW - Adolescent
KW - Antigens
KW - CD3/im [Immunology]
KW - CD4-CD8 Ratio
KW - CD4-Positive T-Lymphocytes/re [Radiation Effects]
KW - CD8-Positive T-Lymphocytes/re [Radiation Effects]
KW - Case-Control Studies
KW - Cesium Radioisotopes/ae [Adverse Effects]
KW - Chernobyl Nuclear Accident
KW - Child
KW - Preschool
KW - Dose-Response Relationship
KW - Radiation
KW - Humans
KW - Immunity
KW - Cellular/re [Radiation Effects]
KW - Irritable Bowel Syndrome/et [Etiology]
KW - Lymphocyte Count
KW - Risk Factors
KW - T-Lymphocytes/re [Radiation Effects]
RP - NOT IN FILE
SP - 323
EP - 330
JF - Toxicology & Industrial Health
JA - Toxicol Ind Health
VL - 26
IS - 6
CY - England
N2 - Irritable bowel syndrome is observed mostly in Ukrainian children and may be related to adverse health effects as a result of the Chernobyl disaster. The aim of this study was to determine status of T-cell population lymphocytes in children with clinical symptom of irritable bowel syndrome. The test population consisted of 95 participants: 75 rural patients aged 4 to 18 who lived in a contaminated area exposed to natural environmental radiation with clinical symptom of irritable bowel syndrome (categorized in three groups) and 20 healthy urban participants from Kiev aged 5 to 15 as control group. Internal radiation activity has been measured by gamma-

ray spectrometry. Peripheral blood leukocytes were analyzed for T-lymphocytes subset such as T-lymphocytes (CD3(+)), T-helper (CD4(+)) and T-cytotoxic (CD8(+)) and then CD4/CD8 ratio was calculated. Percentage of CD3(+) and CD4(+) in all study groups decreased significantly in comparison to control group ($p < 0.001$). Percentage of CD8(+) increased significantly in all study groups in comparison to control group ($p < 0.05$). The CD4/CD8 ratio decreased significantly all study groups in comparison to control group ($p < 0.001$). Cellular immune status of study groups that are residing in a contaminated area has changed. Our data have demonstrated that this change may be related to a higher incidence of children with irritable bowel syndrome
SN - 1477-0393
AD - Department of Clinical Immunology & Allergology, National Medical Academy for Post Graduate Education, Kiev, Ukraine. mohammad_esfahan@yahoo.com
UR - 20348276
ER -

TY - JOUR
ID - 754
T1 - Reliability and validity of a school recess physical activity recall in Spanish youth
A1 - Martinez-Gomez,D.
A1 - Calabro,M.A.
A1 - Welk,G.J.
A1 - Marcos,A.
A1 - Veiga,O.L.
Y1 - 2010/05//
N1 - Martinez-Gomez, David. Calabro, M Andres. Welk, Gregory J. Marcos, Ascension. Veiga, Oscar L
Pediatric exercise science
8909729
IM
Journal Article. Research Support, Non-U.S. Gov't. Validation Studies
English
KW - MEDLINE
KW - Adolescent
KW - Child
KW - Female
KW - Humans
KW - Male
KW - Mental Recall
KW - Monitoring
KW - Ambulatory/is [Instrumentation]
KW - Motor Activity
KW - Questionnaires
KW - Reproducibility of Results
KW - Spain
KW - Students
RP - NOT IN FILE
SP - 218
EP - 230
JF - Pediatric Exercise Science
JA - Pediatr Exerc Sci
VL - 22
IS - 2
CY - United States
N2 - Recess is a frequent target in school-based physical activity (PA) promotion research but there are challenges in assessing PA during this time period. The purpose of this study was to evaluate the reliability and validity of a recess PA recall (RPAR) instrument designed to assess total PA and time spent in moderate to vigorous PA (MVPA) during recess. One hundred twenty-five 7th and 8th-grade students (59 females), age 12-14 years, participated in the study. Activity levels were objectively monitored on Mondays using different activity monitors (Yamax Digiwalker, Biotrainer and ActiGraph). On Tuesdays, 2 RPAR self-reports were administered within 1-hr. Test-retest reliability showed ICC = 0.87 and 0.88 for total PA and time spent in

MVPA, respectively. The RPAR was correlated against Yamax (r = .35), Biotrainer (r = .40 and 0.54) and ActiGraph (r = .42) to assess total PA during recess. The RPAR was also correlated against ActiGraph (r = .54) to assess time spent in MVPA during recess. Mean difference between the RPAR and ActiGraph to assess time spent in MVPA during recess was no significant (2.15 +/- 3.67 min, p = .313). The RPAR showed an adequate reliability and a reasonable validity for assessing PA during the school recess in youth
SN - 0899-8493
AD - Immunonutrition Research Group, Dept. of Metabolism and Nutrition, Institute of Food Science, Technology and Nutrition (ICTAN), Spanish National Research Council (CSIC), Madrid, Spain
UR - 20567043
ER -

TY - JOUR
ID - 755
T1 - Efavirenz plasma concentrations at 1, 3, and 6 months post-antiretroviral therapy initiation in HIV type 1-infected South African children
A1 - Viljoen,M.
A1 - Gous,H.
A1 - Kruger,H.S.
A1 - Riddick,A.
A1 - Meyers,T.M.
A1 - Rheeders,M.
Y1 - 2010/06//
N1 - Viljoen, Michelle. Gous, Hermien. Kruger, Herculina S. Riddick, Alison. Meyers, Tammy M. Rheeders, Malie
AIDS research and human retroviruses
art, 8709376
IM, X
Journal Article. Research Support, Non-U.S. Gov't
English
KW - MEDLINE
KW - Adolescent
KW - Ambulatory Care
KW - Anti-HIV Agents/pk [Pharmacokinetics]
KW - Anti-HIV Agents/tu [Therapeutic Use]
KW - Antiretroviral Therapy
KW - Highly Active
KW - Benzoxazines/pk [Pharmacokinetics]
KW - Benzoxazines/tu [Therapeutic Use]
KW - Child
KW - Preschool
KW - Chromatography
KW - Liquid
KW - Female
KW - HIV Infections/dt [Drug Therapy]
KW - HIV Infections/vi [Virology]
KW - HIV-1/ip [Isolation & Purification]
KW - Humans
KW - Male
KW - Plasma/ch [Chemistry]
KW - South Africa
KW - Tandem Mass Spectrometry
KW - Time Factors
RP - NOT IN FILE
SP - 613
EP - 619
JF - AIDS Research & Human Retroviruses
JA - AIDS Res Hum Retroviruses

VL - 26
IS - 6
CY - United States
N2 - The aim of this study was to quantify the plasma efavirenz concentrations over 6 months in black HIV-1-infected South African children (3-14 years), from resource-limited households, attending an outpatient clinic. The children were antiretroviral treatment (ART) naive and received efavirenz in combination with two nucleoside reverse transcriptase inhibitors according to South African national guidelines. Two blood samples were taken between 12 and 20 h after the last efavirenz dose at 1 (n = 58), 3 (n = 54), and 6 (n = 54) months post-ART initiation. A total of 328 efavirenz mid-dose plasma samples from 58 patients was determined with a validated liquid chromatography tandem mass spectrometry method. Viral suppression (<25 copies/ml) was achieved in 95% of the children after 6 months on ART. The median (range) plasma concentration at time points 1 and 2 were 2.06 (0.10-11.14) and 1.80 (0.14-10.70) microg/ml with respective mean (+/-SD) blood sampling times of 15.24 (2.03) and 16.91 (2.03) h post-evening dose. Efavirenz plasma samples within the therapeutic range of 1-4 microg/ml accounted for 58%; 17% were <1 microg/ml and 25% were >4 microg/ml over the 6 months. Efavirenz levels persistently >4 microg/ml were recorded for 13 (23%) children and 3 (5%) children had persistent efavirenz levels <1 microg/ml. Possible reasons for efavirenz plasma levels outside the accepted therapeutic range include genetic variation in drug metabolism, incorrect dosing, drug-drug interactions, and nonadherence. However, these need to be further explored and the importance of sequential plasma levels has been highlighted in this study
SN - 1931-8405
AD - Department of Pharmacology, North-West University, Potchefstroom, South Africa. Michelle.Viljoen@nwu.ac.za
UR - 20507205
ER -

TY - JOUR
ID - 756
T1 - Spatial and genetic epidemiology of hookworm in a rural community in Uganda
A1 - Pullan,R.L.
A1 - Kabatereine,N.B.
A1 - Quinnell,R.J.
A1 - Brooker,S.
Y1 - 2010///
N1 - Pullan, Rachel L. Kabatereine, Narcis B. Quinnell, Rupert J. Brooker, Simon
PLoS neglected tropical diseases
101291488
IM
Journal Article. Research Support, Non-U.S. Gov't
English
KW - MEDLINE
KW - Adolescent
KW - Analysis of Variance
KW - Ancylostomatoidea
KW - Animals
KW - Bayes Theorem
KW - Child
KW - Preschool
KW - Cluster Analysis
KW - Female
KW - Genetic Predisposition to Disease
KW - Geography
KW - Hookworm Infections/ep [Epidemiology]
KW - Hookworm Infections/ge [Genetics]
KW - Humans
KW - Infant
KW - Male
KW - Questionnaires

KW - Regression Analysis
KW - Risk Factors
KW - Rural Population/sn [Statistics & Numerical Data]
KW - Sex Distribution
KW - Uganda/ep [Epidemiology]
RP - NOT IN FILE
SP - e713
JF - PLoS Neglected Tropical Diseases [electronic resource]
JA - PLoS Negl Trop Dis
VL - 4
IS - 6
CY - United States
N2 - There are remarkably few contemporary, population-based studies of intestinal nematode infection for sub-Saharan Africa. This paper presents a comprehensive epidemiological analysis of hookworm infection intensity in a rural Ugandan community. Demographic, kinship, socioeconomic and environmental data were collected for 1,803 individuals aged six months to 85 years in 341 households in a cross-sectional community survey. Hookworm infection was assessed by faecal egg count. Spatial variation in the intensity of infection was assessed using a Bayesian negative binomial spatial regression model and the proportion of variation explained by host additive genetics (heritability) and common domestic environment was estimated using genetic variance component analysis. Overall, the prevalence of hookworm was 39.3%, with the majority of infections (87.7%) of light intensity (<or=1000 eggs per gram faeces). Intensity was higher among older individuals and was associated with treatment history with anthelmintics, walking barefoot outside the home, living in a household with a mud floor and education level of the household head. Infection intensity also exhibited significant household and spatial clustering: the range of spatial correlation was estimated to be 82 m and was reduced by a half over a distance of 19 m. Heritability of hookworm egg count was 11.2%, whilst the percentage of variance explained by unidentified domestic effects was 17.8%. In conclusion, we suggest that host genetic relatedness is not a major determinant of infection intensity in this community, with exposure-related factors playing a greater role
SN - 1935-2735
AD - Department of Infectious and Tropical Diseases, London School of Hygiene and Tropical Medicine, London, United Kingdom. rachel.pullan@lshtm.ac.uk
UR - 20559556
ER -

TY - JOUR
ID - 757
T1 - Can intraoperative TEE correctly measure residual shunt after surgical repair of ventricular septal defects?
A1 - Kurokawa,S.
A1 - Honma,T.
A1 - Taneoka,M.
A1 - Imai,H.
A1 - Baba,H.
A1 - Nomura,M.
Y1 - 2010/06//
N1 - Kurokawa, Satoshi. Honma, Takayuki. Taneoka, Miki. Imai, Hidekazu. Baba, Hiroshi. Nomura, Minoru
Journal of anesthesia
8905667
IM
Journal Article
English
KW - MEDLINE
KW - Adolescent
KW - Algorithms
KW - Arteriovenous Shunt
KW - Surgical
KW - Blood Gas Analysis
KW - Cardiac Catheterization

KW - Child
KW - Preschool
KW - Echocardiography
KW - Transesophageal
KW - Electrocardiography
KW - Female
KW - Follow-Up Studies
KW - Heart Septal Defects
KW - Ventricular/su [Surgery]
KW - Ventricular/us [Ultrasonography]
KW - Humans
KW - Image Processing
KW - Computer-Assisted
KW - Infant
KW - Male
KW - Pulmonary Circulation/ph [Physiology]
KW - Reoperation
KW - Young Adult
RP - NOT IN FILE
SP - 343
EP - 350
JF - Journal of Anesthesia
VL - 24
IS - 3
CY - Japan
N2 - PURPOSE: No groups have yet succeeded in identifying the need for re-repair of residual shunt after surgical repair of ventricular septal defect (VSD) based on quantitative evaluation of the ratio of the pulmonary blood flow to the systemic blood flow (Qp/Qs) by transesophageal echocardiography (TEE). Hence, we studied the accuracy of Qp/Qs as estimated by intraoperative TEE. METHODS: Twenty-six patients undergoing VSD closure were studied. After separation from the cardiopulmonary bypass, the presence and severity of residual leakage was evaluated by color Doppler image, and the Qp/Qs (TEE-derived Qp/Qs) was calculated by measuring the vessel diameter and the velocity-time integral of the flow profiles in the main pulmonary artery and left ventricular outflow tract. Transthoracic echocardiography (TTE) was performed at pre-discharge and at 6-12 months after the correction to confirm the presence and severity of residual leakage. RESULTS: TEE detected only minor leakage, with no indication for re-repair, in 8 of the 26 patients. Nevertheless, TEE-derived Qp/Qs varied from 0.57 to 2.07 and were incorrect in 17 patients (65.4%). This meant that when TEE-derived Qp/Qs was outside the acceptable range, the patient was judged not to be in need of re-repair. TTE at pre-discharge confirmed trivial leakage in 3 patients in whom TEE had also identified similar leakages. These leakages were not observed at the follow-up TTE. CONCLUSION: TEE-derived Qp/Qs lacks the accuracy required to play a crucial role in quantitatively measuring the severity of residual shunt, while two-dimensional TEE can reliably detect residual leakage after VSD closure and lead to optimal judgment on the need for re-repair
SN - 1438-8359
AD - Department of Anesthesiology, Faculty of Medicine, Tokyo Women's Medical University, 8-1 Kawadacho, Shinjuku-ku, Tokyo 162-8666, Japan. satokuro@sea.plala.or.jp
UR - 20229003
ER -

TY - JOUR
ID - 758
T1 - Developing and improving after-school programs to enhance youth's personal growth and adjustment: a special issue of AJCP
A1 - Durlak,J.A.
A1 - Mahoney,J.L.
A1 - Bohnert,A.M.
A1 - Parente,M.E.
Y1 - 2010/06//

N1 - Durlak, Joseph A. Mahoney, Joseph L. Bohnert, Amy M. Parente, Maria E
American journal of community psychology
3fv, 0364535
IM
Journal Article
English
KW - MEDLINE
KW - Child
KW - Child Development
KW - Humans
KW - Program Development
KW - Program Evaluation
KW - Schools/og [Organization & Administration]
KW - Schools
KW - Social Adjustment
RP - NOT IN FILE
SP - 285
EP - 293
JF - American Journal of Community Psychology
JA - Am J Community Psychol
VL - 45
IS - 3-4
CY - United States
N2 - Participating in after-school programs (ASPs) has become a common experience for children. This special issue provides a perspective on the current status of research on ASPs. This introductory article overviews the historical and current context of ASPs and then describes a developmental ecological model to guide research in this area. The model offers a framework from which to organize and synthesize the research presented in this issue. Key principles include a holistic view of development that recognizes interrelations between multiple domains of youth adjustment, attention to multiple, relevant factors within and outside of youth that affect development, examining the dynamic interplay between persons, program features, and other contexts over time, and understanding the active role of youth in affecting their own development. These principles are examined in relation to five main areas: youth characteristics, social ecologies, program features, participation, and short- and long-term outcomes. Recommendations for future research are discussed
SN - 1573-2770
AD - Department of Psychology, Loyola University Chicago, 6525 N. Sheridan Road, Chicago, IL 60626, USA. jdurlak@luc.edu
UR - 20358278
ER -

TY - JOUR
ID - 759
T1 - Assessment of elementary school students' sun protection behaviors
A1 - Hunter,S.
A1 - Wells,K.J.
A1 - Jacobsen,P.B.
A1 - Lee,J.H.
A1 - Boulware,D.
A1 - Love-Jackson,K.
A1 - Abdulla,R.
A1 - Roetzheim,R.G.
Y1 - 2010/03//
N1 - Hunter, Seft. Wells, Kristen J. Jacobsen, Paul B. Lee, Ji-Hyun. Boulware, David. Love-Jackson, Kymia. Abdulla, Rania. Roetzheim, Richard G
Pediatric dermatology
ped, 8406799
IM

Journal Article. Randomized Controlled Trial. Research Support, N.I.H., Extramural
English
KW - MEDLINE
KW - Child
KW - Female
KW - Florida
KW - Health Knowledge
KW - Attitudes
KW - Practice
KW - Humans
KW - Male
KW - Protective Clothing/ut [Utilization]
KW - Skin Neoplasms/pc [Prevention & Control]
KW - Sunburn/pc [Prevention & Control]
KW - Sunscreening Agents/tu [Therapeutic Use]
RP - NOT IN FILE
SP - 182
EP - 188
JF - Pediatric Dermatology
JA - Pediatr Dermatol
VL - 27
IS - 2
CY - United States
N2 - BACKGROUND/OBJECTIVES: Studies suggest that excessive sun exposure in childhood contributes to the development of skin cancer later in life. METHODS: This study explores 4th grade student assessment of their sun protection behaviors. This study used baseline data collected in the Fall of 2006 for the Sun Protection for Florida's Children (SPF) project. In brief, the SPF project is a group randomized trial to test the effectiveness of a school based intervention promoting sun protection in general, and hat use in particular, in Hillsborough County Schools, Florida. The data reported in this study were collected at baseline before any intervention activities was initiated. RESULTS: The self-reported use of various methods of sun protection was low. Only a small percentage of students wore long sleeves or a hat with a brim before leaving for school. In addition, few students wore a hat with a wide brim when outside but not at school. Students spent an average of 59.1 minutes per week outdoors while attending school and 35.5 minutes during peak sun exposure. CONCLUSION: Sun exposure at school poses a significant risk to student health and more needs to be carried out to promote the use of a wide-brimmed hat and limiting student sun exposure
SN - 1525-1470
AD - Department of Family Medicine, University of South Florida, Tampa, Florida 33612, USA
UR - 19686304
ER -

TY - JOUR
ID - 760
T1 - Giardiasis surveillance - United States, 2006-2008
A1 - Yoder,J.S.
A1 - Harral,C.
A1 - Beach,M.J.
A1 - Centers for Disease Control and Prevention (CDC)
Y1 - 2010/06/11/
N1 - Yoder, Jonathan S. Harral, Courtney. Beach, Michael J. Centers for Disease Control and Prevention (CDC)
Morbidity and mortality weekly report. Surveillance summaries (Washington, D.C. : 2002)
nnb, 101142015, 8400155
IM
Journal Article
English
KW - MEDLINE
KW - Adolescent

KW - Adult
KW - Age Factors
KW - Aged
KW - 80 and over
KW - Child
KW - Preschool
KW - Female
KW - Giardia/ip [Isolation & Purification]
KW - Giardiasis/di [Diagnosis]
KW - Giardiasis/ep [Epidemiology]
KW - Giardiasis/tm [Transmission]
KW - Humans
KW - Incidence
KW - Infant
KW - Male
KW - Middle Aged
KW - Population Surveillance
KW - Seasons
KW - United States/ep [Epidemiology]
RP - NOT IN FILE
SP - 15
EP - 25
JF - Morbidity & Mortality Weekly Report
JA - Morb Mortal Wkly Rep Surveill.Summ.
VL - Surveillance Summaries. 59
IS - 6
CY - United States
N2 - PROBLEM/CONDITION: Giardiasis is a nationally notifiable gastrointestinal illness caused by the protozoan parasite Giardia intestinalis. REPORTING PERIOD: 2006-2008. SYSTEM DESCRIPTION: State, commonwealth, territorial, and two metropolitan health departments voluntarily report cases of giardiasis through CDC's National Notifiable Disease Surveillance System. RESULTS: During 2006--2008, the total number of reported cases of giardiasis increased slightly from 19,239 for 2006 to 19,794 for 2007 and decreased slightly to 19,140 for 2008. During this period, 49 jurisdictions reported giardiasis cases; giardiasis is a reportable condition in 45 states (not reportable in Indiana, Kentucky, Mississippi, North Carolina, and Texas). A greater number of case reports were received for children aged 1-9 years and for adults aged 35-44 years compared with other age groups. Incidence of giardiasis was highest in northern states. Peak onset of illness occurred annually during early summer through early fall. Interpretation: Transmission of giardiasis occurs throughout the United States, with more frequent diagnosis or reporting occurring in northern states. However, state incidence figures should be compared with caution because individual state surveillance systems have varying capabilities to detect cases. The seasonal peak in age-specific case reports coincides with the summer recreational water season and likely reflects increased outdoor activities and exposures such as camping and use of communal swimming venues (e.g., lakes, rivers, swimming pools, and water parks) by young children. PUBLIC HEALTH ACTION: Local and state health departments can use giardiasis surveillance data to better understand the epidemiologic characteristics and the disease burden of giardiasis in the United States, design efforts to prevent the spread of disease, and establish research priorities
SN - 1545-8636
AD - Division of Foodborne, Waterborne, and Environmental Diseases, National Center for Emerging and Zoonotic Infectious Diseases, CDC, Atlanta, GA 30341, USA. jey9@cdc.gov
UR - 20535095
ER -

TY - JOUR
ID - 761
T1 - Geographic differences in childhood overweight, physical activity, nutrition and neighbourhood facilities: implications for prevention
A1 - Simen-Kapeu,A.
A1 - Kuhle,S.

A1 - Veugelers,P.J.
Y1 - 2010/03//
N1 - Simen-Kapeu, Aline. Kuhle, Stefan. Veugelers, Paul J
Canadian journal of public health = Revue canadienne de sante publique
ck6, 0372714
IM
Journal Article. Research Support, Non-U.S. Gov't
English
KW - MEDLINE
KW - Alberta/ep [Epidemiology]
KW - Child
KW - Diet
KW - Female
KW - Geography
KW - Humans
KW - Male
KW - Motor Activity
KW - Obesity/ep [Epidemiology]
KW - Overweight/ep [Epidemiology]
KW - Prevalence
KW - Residence Characteristics
KW - Socioeconomic Factors
RP - NOT IN FILE
SP - 128
EP - 132
JF - Canadian Journal of Public Health
JA - Can J Public Health
VL - Revue Canadienne de Sante Publique. 101
IS - 2
CY - Canada
N2 - BACKGROUND: Childhood overweight is a major public health concern. Whereas various studies have documented higher prevalence rates in rural areas compared to urban areas, little is known about what is causing these differences. We sought to identify the factors underlying the overweight differentials by examining physical activity and nutrition behaviours as well as neighbourhood characteristics of urban areas, towns and rural areas across Alberta. METHODS: In 2008, we surveyed 3,421 grade five students and their parents from 148 randomly selected schools. Students completed the Harvard Food Frequency Questionnaire, questions on physical activities, and had their height and weight measured. Parents completed questions on socioeconomic background, child's lifestyle, and neighbourhood perception. We applied multilevel regression methods to quantify the geographic differentials in physical activity, nutrition and neighbourhood facilities. RESULTS: The prevalence of overweight was 28.5% among Albertan grade five students, with 6.7% being obese. Among students attending schools in towns and rural areas, the prevalence of overweight (obesity) was 29.8% (7.9%) and 30.6% (8.0%), respectively. Compared with students attending urban schools, those attending schools in towns and rural areas reported more physical activity despite perceiving less access to playgrounds/parks and recreational programs ($p < 0.01$). These latter students further reported poorer diets and purchasing more energy-dense foods and snacks at their schools ($p < 0.01$). CONCLUSION: Our findings confirmed the existence of geographic differentials in overweight and its underlying causes. The study urges more promotion of healthy eating and active living, particularly in towns and rural areas to reduce geographic inequalities in health
SN - 0008-4263
AD - Population Health Intervention Research Unit, School of Public Health, University of Alberta, Edmonton, AB
UR - 20524377
ER -

TY - JOUR
ID - 762

T1 - Parents' perception of neighbourhood environment as a determinant of screen time, physical activity and active transport
A1 - Carson,V.
A1 - Kuhle,S.
A1 - Spence,J.C.
A1 - Veugelers,P.J.
Y1 - 2010/03//
N1 - Carson, Valerie. Kuhle, Stefan. Spence, John C. Veugelers, Paul J
Canadian journal of public health = Revue canadienne de sante publique
ck6, 0372714
IM
Journal Article. Research Support, Non-U.S. Gov't
English
KW - MEDLINE
KW - Alberta
KW - Child
KW - Environment Design
KW - Female
KW - Humans
KW - Male
KW - Motor Activity
KW - Parents/px [Psychology]
KW - Perception
KW - Residence Characteristics
KW - Safety
KW - Walking
RP - NOT IN FILE
SP - 124
EP - 127
JF - Canadian Journal of Public Health
JA - Can J Public Health
VL - Revue Canadienne de Sante Publique. 101
IS - 2
CY - Canada
N2 - OBJECTIVE: To study the importance of parents' perception of neighbourhood environment for health behaviours such as screen time, physical activity, and active transport in a Canadian context. METHODS: As part of the REAL Kids Alberta project, 3,421 grade five students from 148 randomly selected schools in Alberta and their parents were surveyed in the spring of 2008. Physical activity was assessed by self-report using an adapted version of the Physical Activity Questionnaire for Older Children (PAQ-C). Screen time and active transport (walking and biking) was assessed by parent proxy reports. Parents were also surveyed on their perception of their neighbourhoods. These responses were reduced to three components (satisfaction/services, safety, sidewalks/parks) through principal component analysis. Subsequent multilevel logistic regression analyses were conducted to quantify the associations of these principal neighbourhood components with screen time, physical activity, and active transport. RESULTS: Children residing in neighbourhoods with good satisfaction/services and sidewalks/parks were significantly more likely to engage in 2 hours or less of screen time and to be physically active. Children in neighbourhoods with good sidewalks/parks were also more likely to engage in active transport to and from school. However, perceived neighbourhood safety had little impact on activity. CONCLUSION: The findings suggest physical activity and active transport may be increased and sedentary behaviours reduced through 1) increasing access to parks, playgrounds, and play spaces, 2) increasing access to sports and recreation programs, and 3) provision of sidewalks such that children and youth can walk or bike to school
SN - 0008-4263
AD - Faculty of Physical Education and Recreation, University of Alberta, Edmonton, AB
UR - 20524376
ER -

TY - JOUR

ID - 763
T1 - Children's refractions and visual activities in the school year and summer
A1 - Deng,L.
A1 - Gwiazda,J.
A1 - Thorn,F.
Y1 - 2010/06//
N1 - Deng, Li. Gwiazda, Jane. Thorn, Frank
Optometry and vision science : official publication of the American Academy of Optometry
oiz, 8904931
IM
Journal Article. Research Support, N.I.H., Extramural
English
KW - MEDLINE
KW - Adolescent
KW - Adult
KW - Child
KW - Computers
KW - Environment
KW - Female
KW - Holidays
KW - Humans
KW - Leisure Activities
KW - Male
KW - Myopia/ep [Epidemiology]
KW - Questionnaires
KW - Reading
KW - Refraction
KW - Ocular/ph [Physiology]
KW - Risk Factors
KW - Schools
KW - Seasons
KW - Sports/ph [Physiology]
KW - Television
KW - Video Games
KW - Vision
KW - Visual Fields/ph [Physiology]
RP - NOT IN FILE
SP - 406
EP - 413
JF - Optometry & Vision Science
JA - Optom.Vis Sci
VL - 87
IS - 6
CY - United States
N2 - PURPOSE: To investigate the association of children's refractive errors with their visual activities assessed by questionnaire in the school year and summer break (June, July, and August). METHODS: The parents of 147 children aged 6 to 18 years participating in a longitudinal study of refraction and visual function filled out a questionnaire in 1999 listing the number of weekly hours outside of school that the children read for pleasure, studied, watched TV, used the computer/played video games, and engaged in sports/outdoor activities. They also provided hours for these activities during the summer break. Refractions were measured annually by non-cycloplegic distance retinoscopy. Myopes refer to subjects who were myopic (spherical equivalent < -0.5 diopter) at the time of the survey and non-myopes (spherical equivalent refraction > or = -0.5 diopter) were emmetropic or in a few cases hyperopic at survey time. RESULTS: During the school year, myopes spent significantly fewer hours (8.25 +/- 6.24 h/week) than non-myopes (10.95 +/- 5.95 h/week) in sports/outdoor activity (p < 0.05). In addition, myopes (12.78 +/- 9.28 h/week) watched more television than non-myopes (8.91 +/- 5.95 h/week) (p = 0.02). No significant refractive group differences were found for other activities. During the summer break, no significant differences were found between refractive groups in any visual activity times.

No significant correlations between sports/outdoor activity and TV time were found. Overall, the biggest differences between summer and school activity times were found in outdoor activity (21.76 +/- 13.80 vs.10.34 +/- 6.10 h/week; p < 0.001) and studying (1.69 +/- 3.71 vs. 9.51 +/- 6.96 h/week; p < 0.001). CONCLUSIONS: In agreement with other studies, the non-myopes had more hours of sports/outdoor activity during the school year, which may protect against myopia development. A new finding is the high number of sports/outdoor activity hours for both myopes and non-myopes during the summer break, which may contribute to slowed eye growth in all children during these 3 months
SN - 1538-9235
AD - Department of Vision Science, New England College of Optometry, Boston, Massachusetts 02115, USA. DengL@neco.edu
UR - 20375747
ER -

TY - JOUR
ID - 764
T1 - Are serum levels of trace elements in children with auditory neuropathy within normal limits? - A pilot study
A1 - Muluk,N.B.
A1 - Yalcinkaya,F.
A1 - Arikan,O.K.
A1 - Cirpar,O.
A1 - Kaygusuz,S.
A1 - Ercan,V.
Y1 - 2010///
N1 - Muluk, Nuray Bayar. Yalcinkaya, Fulya. Arikan, Osman Kursat. Cirpar, Ozden. Kaygusuz, Sedat. Ercan, Varol
Clinical and investigative medicine. Medecine clinique et experimentale
dfg, 7804071
IM
Journal Article
English
KW - MEDLINE
KW - Adolescent
KW - Adult
KW - Auditory Diseases
KW - Central/bl [Blood]
KW - Auditory Threshold
KW - Child
KW - Preschool
KW - Female
KW - Humans
KW - Male
KW - Trace Elements/bl [Blood]
KW - Young Adult
RP - NOT IN FILE
SP - E155
EP - E160
JF - Clinical & Investigative Medicine - Medecine Clinique et Experimentale
JA - Clin Invest Med
VL - 33
IS - 3
CY - Canada
N2 - OBJECTIVES: To determine whether trace elements that are essential for neural function play a role in the pathophysiology and etiology of auditory neuropathy (AN). MATERIALS AND METHODS: Patients diagnosed with auditory neuropathy consisted of eight children (two male, six female). The blood tests including the measurement of sodium, potassium, chloride, calcium, phosphorus, iron, copper and magnesium were done in children with AN during their routine care. RESULTS: Of the eight children with AN, many had

serum levels outside the normal range: one had low sodium, two had low potassium, one had low chloride, two had high zinc and three had low zinc, two had low calcium and two had higher than normal phosphorus. CONCLUSION: Although some serum trace element levels in our patients were higher or lower than normal values, the mean values were within normal limits. Thus, we were unable to detect a relationship between serum trace element levels and AN-. In the future, larger studies should be conducted to confirm these findings
SN - 1488-2353
AD - Kirikkale University, Faculty of Medicine, ENT Department, Kirikkale, Turkey. nbayarmuluk@yahoo.com
UR - 20519093
ER -

TY - JOUR
ID - 765
T1 - Heterogeneous neuronal firing patterns during interictal epileptiform discharges in the human cortex
A1 - Keller,C.J.
A1 - Truccolo,W.
A1 - Gale,J.T.
A1 - Eskandar,E.
A1 - Thesen,T.
A1 - Carlson,C.
A1 - Devinsky,O.
A1 - Kuzniecky,R.
A1 - Doyle,W.K.
A1 - Madsen,J.R.
A1 - Schomer,D.L.
A1 - Mehta,A.D.
A1 - Brown,E.N.
A1 - Hochberg,L.R.
A1 - Ulbert,I.
A1 - Halgren,E.
A1 - Cash,S.S.
Y1 - 2010/06//
N1 - Keller, Corey J. Truccolo, Wilson. Gale, John T. Eskandar, Emad. Thesen, Thomas. Carlson, Chad. Devinsky, Orrin. Kuzniecky, Ruben. Doyle, Werner K. Madsen, Joseph R. Schomer, Donald L. Mehta, Ashesh D. Brown, Emery N. Hochberg, Leigh R. Ulbert, Istvan. Halgren, Eric. Cash, Sydney S
Brain : a journal of neurology
0372537
AIM, IM
Journal Article. Research Support, N.I.H., Extramural. Research Support, Non-U.S. Gov't. Research Support, U.S. Gov't, Non-P.H.S.
English
KW - MEDLINE
KW - Action Potentials
KW - Adolescent
KW - Adult
KW - Cerebral Cortex/pp [Physiopathology]
KW - Child
KW - Electrodes
KW - Implanted
KW - Electroencephalography/mt [Methods]
KW - Epilepsy/di [Diagnosis]
KW - Epilepsy/pp [Physiopathology]
KW - Female
KW - Hippocampus/pp [Physiopathology]
KW - Humans
KW - Male
KW - Microelectrodes

KW - Middle Aged
KW - Neurons/ph [Physiology]
KW - Time Factors
KW - Young Adult
RP - NOT IN FILE
SP - 1668
EP - 1681
JF - Brain
VL - 133
IS - Pt 6
CY - England
N2 - Epileptic cortex is characterized by paroxysmal electrical discharges. Analysis of these interictal discharges typically manifests as spike-wave complexes on electroencephalography, and plays a critical role in diagnosing and treating epilepsy. Despite their fundamental importance, little is known about the neurophysiological mechanisms generating these events in human focal epilepsy. Using three different systems of microelectrodes, we recorded local field potentials and single-unit action potentials during interictal discharges in patients with medically intractable focal epilepsy undergoing diagnostic workup for localization of seizure foci. We studied 336 single units in 20 patients. Ten different cortical areas and the hippocampus, including regions both inside and outside the seizure focus, were sampled. In three of these patients, high density microelectrode arrays simultaneously recorded between 43 and 166 single units from a small (4 mm x 4 mm) patch of cortex. We examined how the firing rates of individual neurons changed during interictal discharges by determining whether the firing rate during the event was the same, above or below a median baseline firing rate estimated from interictal discharge-free periods (Kruskal-Wallis one-way analysis, P<0.05). Only 48% of the recorded units showed such a modulation in firing rate within 500 ms of the discharge. Units modulated during the discharge exhibited significantly higher baseline firing and bursting rates than unmodulated units. As expected, many units (27% of the modulated population) showed an increase in firing rate during the fast segment of the discharge (+ or - 35 ms from the peak of the discharge), while 50% showed a decrease during the slow wave. Notably, in direct contrast to predictions based on models of a pure paroxysmal depolarizing shift, 7.7% of modulated units recorded in or near the seizure focus showed a decrease in activity well ahead (0-300 ms) of the discharge onset, while 12.2% of units increased in activity in this period. No such pre-discharge changes were seen in regions well outside the seizure focus. In many recordings there was also a decrease in broadband field potential activity during this same pre-discharge period. The different patterns of interictal discharge-modulated firing were classified into more than 15 different categories. This heterogeneity in single unit activity was present within small cortical regions as well as inside and outside the seizure onset zone, suggesting that interictal epileptiform activity in patients with epilepsy is not a simple paroxysm of hypersynchronous excitatory activity, but rather represents an interplay of multiple distinct neuronal types within complex neuronal networks
SN - 1460-2156
AD - Department of Neurology, 30 Massachusetts General Hospital, 55 Fruit Street, Boston, MA 02114, USA
UR - 20511283
ER -

TY - JOUR
ID - 766
T1 - Utilizing Ecological Momentary Assessment in pediatric obesity to quantify behavior, emotion, and sleep
A1 - Rofey,D.L.
A1 - Hull,E.E.
A1 - Phillips,J.
A1 - Vogt,K.
A1 - Silk,J.S.
A1 - Dahl,R.E.
Y1 - 2010/06//
N1 - Rofey, Dana L. Hull, Ethan E. Phillips, Jennifer. Vogt, Kristen. Silk, Jennifer S. Dahl, Ronald E
Obesity (Silver Spring, Md.)
101264860
IM

Clinical Trial. Journal Article. Research Support, N.I.H., Extramural. Research Support, Non-U.S. Gov't
English
KW - MEDLINE
KW - Adolescent
KW - Adolescent Behavior/ph [Physiology]
KW - Adolescent Psychology
KW - Child
KW - Cognitive Therapy
KW - Diagnostic Techniques
KW - Endocrine/is [Instrumentation]
KW - Emotions/ph [Physiology]
KW - Emotions
KW - Feasibility Studies
KW - Female
KW - Humans
KW - Mental Disorders/co [Complications]
KW - Mental Disorders/di [Diagnosis]
KW - Obesity/co [Complications]
KW - Obesity/di [Diagnosis]
KW - Obesity/px [Psychology]
KW - Obesity/th [Therapy]
KW - Pediatrics/is [Instrumentation]
KW - Pediatrics/mt [Methods]
KW - Psychological Techniques/is [Instrumentation]
KW - Psychological Techniques
KW - Sleep/ph [Physiology]
KW - Sleep Disorders/co [Complications]
KW - Sleep Disorders/di [Diagnosis]
KW - Weight Loss/ph [Physiology]
KW - Young Adult
RP - NOT IN FILE
SP - 1270
EP - 1272
JF - Obesity
JA - Obesity (Silver Spring)
VL - 18
IS - 6
CY - United States
N2 - This study examined the feasibility of using Ecological Momentary Assessment (EMA) to examine important domains relevant to interregulatory health processes in overweight adolescent females in their natural environments. Participants were 20 overweight adolescent females engaged in a cognitive-behavioral and motivational interviewing intervention aimed at weight loss and improving mood (11-19 years old, 80% white, 15% African American, mean BMI = 39). During this EMA protocol, participants were asked to report their physical activity (PA), nutrition, mood, and sleep during 14 cellular phone calls over three extended weekends (Thursday to Monday). Simultaneously, participants wore an actigraph (armband and watch communicator) that provided instantaneous PA feedback (steps taken and kilocalories) and sleep parameters (duration and efficiency). EMA compliance rates for the armband and phone calls were 74.7 +/- 0.3% and 64.2 +/- 0.3%, respectively. Data from the armband and phone calls are presented to illustrate the depth of information acquired by utilizing this innovative methodology
SN - 1930-739X
AD - Children's Hospital of Pittsburgh, Weight Management and Wellness Center, University of Pittsburgh Medical Center, Pittsburgh, Pennsylvania, USA. dana.rofey@chp.edu
UR - 20019675
ER -

TY - JOUR
ID - 767

T1 - Age groups and spread of influenza: implications for vaccination strategy
A1 - Hsieh,Y.H.
Y1 - 2010///
N1 - Hsieh, Ying-Hen
BMC infectious diseases
100968551
IM
Journal Article. Research Support, Non-U.S. Gov't. Research Support, U.S. Gov't, P.H.S.
English
KW - MEDLINE
KW - Adolescent
KW - Adult
KW - Age Factors
KW - Aged
KW - 80 and over
KW - Basic Reproduction Number
KW - Child
KW - Preschool
KW - Computer Simulation
KW - Female
KW - Humans
KW - Incidence
KW - Infant
KW - Newborn
KW - Influenza A Virus
KW - H1N1 Subtype/py [Pathogenicity]
KW - Influenza Vaccines/ad [Administration & Dosage]
KW - Influenza
KW - Human/ep [Epidemiology]
KW - Human/mo [Mortality]
KW - Human/tm [Transmission]
KW - Male
KW - Middle Aged
KW - Models
KW - Statistical
KW - Taiwan/ep [Epidemiology]
KW - Vaccination/mt [Methods]
KW - Young Adult
RP - NOT IN FILE
SP - 106
JF - BMC Infectious Diseases
JA - BMC Infect Dis
VL - 10
CY - England
N2 - BACKGROUND: The unpredictable nature of the potentially devastating impact of 2009 pH1N1 influenza pandemic highlights the need for pandemic preparedness planning, where modeling studies could be most useful for simulations of possible future scenarios. METHODS: A compartmental model with pre-symptomatic and asymptomatic influenza infections is proposed which incorporates age groups as well as intervention measures such as age-specific vaccination, in order to study spread of influenza in a community. RESULTS: We derive the basic reproduction number and other effective reproduction numbers under various intervention measures. For illustration, we make use of the Pneumonia and Influenza (P&I) mortality data and vaccination data of the very young (age 0-2) and the very old (age > 64) during 2004-2005 Taiwan winter influenza season to fit our model and to compute the relevant reproduction numbers. The reproduction number for this winter flu season is estimated to be slightly above one (approximately 1.0001). CONCLUSIONS: Comparatively large errors in fitting the P&I mortality data of the elderly (> 64) were observed shortly after winter school closings in January, which may indicate the impact of younger, more active age groups transmitting influenza to other age groups outside of the school settings; in particular, to the elderly in the

households. Pre-symptomatic infections seemed to have little effect on the model fit, while asymptomatic infection by asymptomatic infectives has a more pronounced impact on the model fit for the elderly mortality, perhaps indicating a larger role in disease transmission by asymptomatic infection. Simulations indicate that the impact of vaccination on the disease incidence might not be fully revealed in the change (or the lack thereof) in the effective reproduction number with interventions, but could still be substantial. The estimated per contact transmission probability for susceptible elderly is significantly higher than that of any other age group, perhaps highlighting the vulnerability of the elderly due to close contacts with their caretakers from other age groups. The relative impact of targeting the very young and the very old for vaccination was weakened by their relative inactivity, thus giving evidence of the lack of impact of vaccinating these two groups on the overall transmissibility of the disease in the community. This further underscores the need for morbidity-based strategy to prevent elderly mortality
SN - 1471-2334
AD - Department of Public Health and Center for Infectious Disease Epidemiology, China Medical University, Taichung, Taiwan. hsieh@mail.cmu.edu.tw
UR - 20429954
ER -

TY - JOUR
ID - 768
T1 - Aetiology of childhood leukaemia. [Review] [141 refs]
A1 - Eden,T.
Y1 - 2010/06//
N1 - Eden, Tim
Cancer treatment reviews
cnn, 7502030
IM
Journal Article. Research Support, Non-U.S. Gov't. Review
English
KW - MEDLINE
KW - Alcohol Drinking/ae [Adverse Effects]
KW - Background Radiation/ae [Adverse Effects]
KW - Child
KW - Electromagnetic Fields
KW - Folic Acid/me [Metabolism]
KW - Genetic Predisposition to Disease
KW - Humans
KW - Incidence
KW - Leukemia/ep [Epidemiology]
KW - Leukemia/et [Etiology]
KW - Leukemia/ge [Genetics]
KW - Seasons
KW - Smoking/ae [Adverse Effects]
RP - NOT IN FILE
SP - 286
EP - 297
JF - Cancer Treatment Reviews
JA - Cancer Treat Rev
VL - 36
IS - 4
CY - Netherlands
N2 - The acute leukaemias account for about 30% of all malignancy seen in childhood across the Western world. A peak incidence of precursor B cell ALL has emerged as socio-economic conditions have improved in countries worldwide. From twin studies and the use of neonatal blood spots it has been possible to back track the first initiating genetic events within critical haemopoietic cells to foetal development in utero for most precursor B cell ALL and some cases of AML. These events may occur as part of normal foetal development. Whether other factors (environmental or constitutional) are involved to increase the chance of these first genetic changes happening is unclear. For some leukaemias (e.g. infant MLL positive ALL) the first event appears

adequate to create a malignant clone but for the majority of ALL and AML further 'genetic' changes are required, probably postnatal. Many environmental factors have been proposed as causative for leukaemia but only ionising irradiation and certain chemicals, e.g. benzene and cytotoxics (alkylators and topoisomerase II inhibitors) have been confirmed and then principally for acute myeloid leukaemia. It appears increasingly likely that delayed, dysregulated responses to 'common' infectious agents play a major part in the conversion of pre-leukaemic clones into overt precursor B cell ALL, the most common form of childhood leukaemia. Constitutional polymorphic alleleic variants in immune response genes (especially the HLA Class II proteins) and cytokines may play a role in determining the type of immune response. High penetrance germ-line mutations are involved in only about 5% of childhood leukaemias (more in AML than ALL). There is little evidence to support any role of viral transformation in causation, unlike in animals. Other environmental factors for which some evidence exists include non-ionising electromagnetic radiation and electric fields, although their mode of action in leukaemogenesis remains unclear. There is no single cause for childhood leukaemia and for most individuals a combination of factors appears to be necessary; all involving gene-environment interactions. To date few clear preventative measures have emerged, except the complete avoidance of first trimester X-rays in pregnancy; a healthy diet with adequate oral folic acid intake both preconception and early in pregnancy; and the early exposure of children to other children outside the home to facilitate stimulation and maturation of the natural immune system. Here then are clear echoes of the "hygiene hypothesis" regarding the initiation of allergies, autoimmune disease and type I diabetes mellitus in children and young people. 2010. Published by Elsevier Ltd. [References: 141]
SN - 1532-1967
AD - University of Manchester, UK. Tim.eden@manchester.ac.uk
UR - 20223594
ER -

TY - JOUR
ID - 769
T1 - Soluble serum HLA-G in children with allergic rhinitis and asthma
A1 - Ciprandi,G.
A1 - De,Amici M.
A1 - Caimmi,S.
A1 - Marseglia,A.
A1 - Marchi,A.
A1 - Castellazzi,A.M.
A1 - Marseglia,G.
Y1 - 2010/04//
N1 - Ciprandi, Giorgio. De Amici, M. Caimmi, S. Marseglia, A. Marchi, A. Castellazzi, A M. Marseglia, G
Journal of biological regulators and homeostatic agents
8809253
IM
Letter
English
KW - MEDLINE
KW - Asthma/bl [Blood]
KW - Asthma/im [Immunology]
KW - Child
KW - Female
KW - HLA Antigens/bl [Blood]
KW - HLA-G Antigens
KW - Histocompatibility Antigens Class I/bl [Blood]
KW - Humans
KW - Immunoenzyme Techniques
KW - Male
KW - Patient Selection
KW - Reference Values
KW - Rhinitis
KW - Allergic
KW - Seasonal/bl [Blood]

KW - Seasonal/im [Immunology]
RP - NOT IN FILE
SP - 221
EP - 224
JF - Journal of Biological Regulators & Homeostatic Agents
JA - J Biol Regul Homeost.Agents
VL - 24
IS - 2
CY - Italy
N2 - Allergic rhinitis (AR) and asthma are characterized by Th2 polarized immune response. Soluble HLA (sHLA) molecules play an immunomodulatory activity. It has been reported that both molecules are increased in sera of patients with pollen-induced allergic rhinitis studied outside the pollen season. To date, however, no study has investigated them in AR children. The aim of this preliminary study is to evaluate serum sHLA-G levels in children with AR or asthma patients and in a group of healthy controls. Forty-seven symptomatic allergic patients were enrolled. A group of 50 healthy subjects was considered as control. Serum sHLA-G levels were determined by the immunoenzymatic method. Children with AR had significantly higher levels of sHLA-G molecules than normal controls or children with allergic asthma. However, there is no difference between children with AR and asthmatics. In conclusion, the present study provides the preliminary evidence that serum sHLA-G molecules are significantly increased in children with AR
SN - 0393-974X
UR - 20487636
ER -

TY - JOUR
ID - 770
T1 - Experience of pediatric rapid response team in a tertiary care hospital in Pakistan
A1 - Anwar,ul Haque
A1 - Saleem,A.F.
A1 - Zaidi,S.
A1 - Haider,S.R.
Y1 - 2010/03//
N1 - Anwar-ul-Haque. Saleem, Ali Faisal. Zaidi, Samreen. Haider, Syed Rizwan
Indian journal of pediatrics
gkt, 0417442
IM
Journal Article
English
KW - MEDLINE
KW - Child
KW - Preschool
KW - Clinical Audit
KW - Hospital Mortality
KW - Hospital Rapid Response Team/sn [Statistics & Numerical Data]
KW - Humans
KW - Infant
KW - Intensive Care Units
KW - Pediatric/ut [Utilization]
KW - Intubation
KW - Intratracheal/ut [Utilization]
KW - Outcome Assessment (Health Care)
KW - Pakistan
KW - Respiration
KW - Artificial/ut [Utilization]
KW - Retrospective Studies
RP - NOT IN FILE
SP - 273
EP - 276

JF - Indian Journal of Pediatrics
JA - Indian J Pediatr
VL - 77
IS - 3
CY - India
N2 - OBJECTIVE: To report our experience before and after implementation of pediatric rapid response team (RRT) in pediatric wards of a tertiary care hospital in Pakistan. METHODS: An audit of RRT activity from December 2007 to August 2008 was conducted and reviewed patient diagnoses at the time of call placement, interventions done and post-intervention clinical outcomes. Clinical Outcomes in the nine months before RRT implementation were compared with those in the first operational nine months after RRT. RESULTS: Eighty-three calls were generated during the post-intervention study period of 9-month (21 calls/1000 admissions). The median age of patients was 27 months; 37% calls were for infants. The majority of patients were under care of medical services (93% vs 7% under care of surgical services). Greater numbers of calls were made during 0800-1600 hours (45%). Respiratory issues were the most common reason for activation of RRT. Because of early interventions, majority (61%) of patients avoided unnecessary PICU stay and expenditure; only 17% required mechanical ventilation in PICU. The code rate per 1000 admissions decreased from 5.2 (pre-RRT) to 2.7 (post-RRT) (p=0.08; OR 1.88 (95%Cl 0.9-3.93). The mortality rate of patients admitted in PICU from wards decreased from 50% to 15% (p=0.25; OR 1.64 (95%Cl 0.63-4.29). CONCLUSION: Our experience with implementation of RRT was associated with reduction in cardiorespiratory arrest, mortality and saved a lot of PICU resource utilization. It is an excellent patient-safety initiative especially in resource-constrained countries by bringing PICU reflexes outside the PICU
SN - 0973-7693
AD - Department of Pediatrics and Child Health, Aga Khan University Hospital, Karachi, Pakistan. anwar.haq@aku.edu
UR - 20177830
ER -

TY - JOUR
ID - 771
T1 - Twelve-month effects of a playground intervention on children's morning and lunchtime recess physical activity levels
A1 - Ridgers,N.D.
A1 - Fairclough,S.J.
A1 - Stratton,G.
Y1 - 2010/03//
N1 - Ridgers, Nicola D. Fairclough, Stuart J. Stratton, Gareth
Journal of physical activity & health
101189457
IM
Journal Article. Research Support, Non-U.S. Gov't
English
KW - MEDLINE
KW - Acceleration
KW - Actigraphy
KW - Case-Control Studies
KW - Child
KW - Child Welfare
KW - Confidence Intervals
KW - Female
KW - Great Britain
KW - Health Behavior
KW - Heart Rate
KW - Humans
KW - Male
KW - Motor Activity
KW - Multivariate Analysis
KW - Play and Playthings

KW - Recreation
KW - Schools/sn [Statistics & Numerical Data]
KW - Students
KW - Time Factors
RP - NOT IN FILE
SP - 167
EP - 175
JF - Journal of Physical Activity & Health
JA - J Phys Act Health
VL - 7
IS - 2
CY - United States
N2 - BACKGROUND: Recess is an opportunity for children to engage in daily physical activity. The aim of this study was to investigate the 12-month effects of a playground intervention on children's moderate-to-vigorous (MVPA) and vigorous physical activity (VPA) during morning and lunchtime recess. METHODS: Four hundred and seventy children (232 boys, 238 girls) from 26 elementary schools participated in the study. Fifteen schools redesigned the playground environment using playground markings and physical structures. Eleven schools served as socioeconomic matched controls. Physical activity levels were quantified using heart rate and accelerometry at baseline, 6 months, and 12 months post-intervention. A 3-level (time, pupil, and school) multilevel analysis was used to determine the effects of the intervention across time on MVPA and VPA. RESULTS: Positive yet nonsignificant intervention effects were found for MVPA and VPA during morning and lunchtime recess. Intervention children were more active during recess than control children. Interactions revealed that the intervention effect was stronger at 6 months than 12 months post-intervention. CONCLUSIONS: A playground markings and physical structures intervention had a positive effect on intervention children's morning and lunchtime MVPA and VPA when assessed using heart rate and accelerometry, but this effect is strongest 6-months post-intervention and decreased between 6 months and 12 months
SN - 1543-3080
AD - Research Institute for Sport and Exercise Sciences, Liverpool John Moores University, Liverpool, United Kingdom
UR - 20484755
ER -

TY - JOUR
ID - 772
T1 - Epidemiological assessment of predictors of caries increment in 7-10- year-olds: a 2-year cohort study
A1 - Kassawara,A.B.
A1 - Tagliaferro,E.P.
A1 - Cortelazzi,K.L.
A1 - Ambrosano,G.M.
A1 - Assaf,A.V.
A1 - Meneghim,Mde C.
A1 - Pereira,A.C.
Y1 - 2010/03//
N1 - Kassawara, Ariana Bellotto Correa. Tagliaferro, Elaine Pereira da Silva. Cortelazzi, Karine Laura. Ambrosano, Glaucia Maria Bovi. Assaf, Andrea Videira. Meneghim, Marcelo de Castro. Pereira, Antonio Carlos
Journal of applied oral science : revista FOB
101189774
D, IM
Journal Article. Research Support, Non-U.S. Gov't
English
KW - MEDLINE
KW - Analysis of Variance
KW - Brazil/ep [Epidemiology]
KW - Chi-Square Distribution
KW - Child

KW - Cohort Studies
KW - DMF Index
KW - Dental Caries/ep [Epidemiology]
KW - Dentition
KW - Permanent
KW - Female
KW - Forecasting
KW - Humans
KW - Male
KW - Observer Variation
KW - Odds Ratio
KW - Prospective Studies
KW - Risk Factors
RP - NOT IN FILE
SP - 116
EP - 120
JF - Journal of Applied Oral Science
VL - 18
IS - 2
CY - Brazil
N2 - OBJECTIVE: The aim of this 2-year cohort study (2003 to 2005) was to investigate how caries experience, at initial lesions (early or non-cavited lesions) and cavited stages, predicts caries increment in permanent teeth in 7-10- year-olds. MATERIAL AND METHODS: The random sample of 765 children attending public schools in the city of Piracicaba, SP, Brazil, was divided into two groups: 423 children aged 7-8 years and 342 children aged 9-10 years. All subjects were examined by a calibrated examiner, using dental mirror and ball-ended probes, after tooth brushing and air-drying in an outdoor setting, based on the World Health Organization criteria. Active caries with intact surfaces were also recorded as initial lesion (IL). Univariate analysis was used for statistical analysis (Odds Ratios and Chi-square). RESULTS: The association between the DMFT (decayed, missing and filled teeth) increment and the presence of IL was significant only for 9-10-year-old children. The children with DMFT>0 at baseline were more prone to have DMFT increment, with the highest risk for caries increment occurring in children aged 7-8 years. CONCLUSIONS: The predictors of caries increment were the presence (at baseline) of caries experience in permanent teeth for both age groups (7-8; 9-10-year-olds) and the presence of the IL (at baseline) for 9-10-year-olds
SN - 1678-7765
AD - Department of Community Dentistry, Piracicaba Dental School, State University of Campinas, Piracicaba, SP, Brazil
UR - 20485921
ER -

TY - JOUR
ID - 773
T1 - Successful treatment of influenza A virus by oseltamivir in bone marrow transplant recipients
A1 - Mellouli,F.
A1 - Ouederni,M.
A1 - Dhouib,N.
A1 - Hajkacem,M.A.
A1 - Slim,A.
A1 - Bejaoui,M.
Y1 - 2010/03//
N1 - Mellouli, F. Ouederni, M. Dhouib, N. Hajkacem, M A. Slim, A. Bejaoui, M
Pediatric transplantation
c8x, 9802574
IM
Case Reports. Journal Article
English
KW - MEDLINE
KW - Antiviral Agents/tu [Therapeutic Use]

KW - Bone Marrow Transplantation/ae [Adverse Effects]
KW - Child
KW - Preschool
KW - Female
KW - Humans
KW - Infant
KW - Influenza A Virus
KW - Influenza
KW - Human/dt [Drug Therapy]
KW - Human/vi [Virology]
KW - Male
KW - Oseltamivir/tu [Therapeutic Use]
RP - NOT IN FILE
SP - 178
EP - 181
JF - Pediatric Transplantation
JA - PEDIATR TRANSPLANT
VL - 14
IS - 2
CY - Denmark
N2 - INFVA is an important cause of pulmonary infections in patients receiving BMT, and is associated with considerable morbidity and mortality for a readily preventable and treatable infection. Few studies have addressed the impact of the new neuraminidase inhibitors in the prognosis of influenza after BMT. The aim of this study is to assess the impact of oseltamivir on the control of INFVA infection in BMT recipients. INFVA was screened in NPA and/or bronchoalveolar lavage using IF in all BMT recipients having respiratory symptoms. Three URTI and one associated upper and LRTI were diagnosed in three BMT recipients out of six patients admitted to the BMT unit, during eight-wk period (March and April 2008). All patients having INFVA respiratory infection were treated by oral oseltamivir 60 mg/day, begun more than 48 h after symptom onset. Respiratory symptoms disappeared within a mean of 60 h (48-96 h) of treatment. However, viral tests had remained positive for 8-39 days. Outside the initial associated URTI and LRTI, no further viral pneumonia occurred. No patient died of INFVA. Oseltamivir was well tolerated outside vomiting during the first three days of treatment in one patient. Oseltamivir appears to play an important role in the outcome of INFVA infection as well in URTI as in severe LRTI in patients receiving BMT
SN - 1399-3046
AD - Service d'Immuno-Hematologie Pediatrique, Centre National de Greffe de Moelle Osseuse, Tunis, Tunisia. melloulif@yahoo.fr
UR - 19170930
ER -

TY - JOUR
ID - 774
T1 - [Plasma antiretroviral levels in children with human immunodeficiency virus infection. Influence of sex and age]. [Spanish]
A1 - Nso-Roca,A.P.
A1 - Larru,B.
A1 - Bellon,J.M.
A1 - Mellado,M.J.
A1 - Ramos,J.T.
A1 - Gonzalez,M.I.
A1 - Navarro,M.L.
A1 - Munoz-Fernandez,M.A.
A1 - de Jose,M.I.
Y1 - 2010/05//
N1 - Nso-Roca, Ana Pilar. Larru, Beatriz. Bellon, Jose Maria. Mellado, Maria Jose. Ramos, Jose Tomas. Gonzalez, Maria Isabel. Navarro, Maria Luisa. Munoz-Fernandez, Maria Angeles. de Jose, Maria Isabel
Enfermedades infecciosas y microbiologia clinica
a10, 9104081

IM
English Abstract. Journal Article. Multicenter Study. Research Support, Non-U.S. Gov't
Spanish
KW - MEDLINE
KW - Age Factors
KW - Anti-Retroviral Agents/bl [Blood]
KW - Anti-Retroviral Agents/tu [Therapeutic Use]
KW - Child
KW - Female
KW - HIV Infections/bl [Blood]
KW - HIV Infections/dt [Drug Therapy]
KW - Humans
KW - Male
KW - Prospective Studies
KW - Sex Factors
RP - NOT IN FILE
SP - 278
EP - 283
JF - Enfermedades Infecciosas y Microbiologia Clinica
JA - Enferm Infecc.Microbiol Clin
VL - 28
IS - 5
CY - Spain
N2 - INTRODUCTION: Pharmacologic studies have shown a relationship between plasma antiretroviral levels and toxicity/viral activity. Nevertheless, pharmacokinetic and pharmacodynamic data are inconsistent and limited in HIV-infected children. An analysis was performed of plasma antiretroviral concentrations in clinical practice and their influence on therapy efficacy in HIV-infected children. METHODS: Observational, prospective, multicenter study, including HIV-infected children followed up at 5 reference hospitals between March 2006 and June 2008. Pre-dose plasma antiretroviral levels were determined and the relationships with various clinical and analytical variables were investigated. RESULTS: A total of 129 patients were included, and 41.3% had antiretroviral plasma levels outside the established range. No differences were found between sexes. Children younger than 1 year had a higher rate of suboptimal levels and higher viral load than the remaining children. CONCLUSION: Antiretroviral plasma concentrations are more frequently suboptimal in children younger than 1 year. This finding is related with greater viral failure and implies a considerable challenge in this population, which requires very long-term treatment. (c) 2009 Elsevier Espana, S.L. All rights reserved
SN - 1578-1852
AD - Servicio de Pediatria-Infecciosas, Hospital Universitario La Paz, Madrid, Espana. ananso@yahoo.es
UR - 20097453
ER -

TY - JOUR
ID - 775
T1 - Playground arsenic dose relatively low
A1 - Pelley,J.
Y1 - 2010/05/15/
N1 - Pelley, Janet
Environmental science & technology
dvo, 0213155
IM
News
English
KW - MEDLINE
KW - Arsenic/an [Analysis]
KW - Child
KW - Environmental Exposure
KW - Humans

KW - Play and Playthings
KW - Soil Pollutants/an [Analysis]
RP - NOT IN FILE
SP - 3650
JF - Environmental Science & Technology
JA - Environ Sci Technol
VL - 44
IS - 10
CY - United States
SN - 0013-936X
UR - 20420447
ER -

TY - JOUR
ID - 776
T1 - Biomonitoring of arsenic in urine and saliva of children playing on playgrounds constructed from chromated copper arsenate-treated wood
A1 - Lew,K.
A1 - Acker,J.P.
A1 - Gabos,S.
A1 - Le,X.C.
Y1 - 2010/05/15/
N1 - Lew, Kristi. Acker, Jason P. Gabos, Stephan. Le, X Chris
Environmental science & technology
dvo, 0213155
IM
Journal Article. Research Support, Non-U.S. Gov't
English
KW - MEDLINE
KW - Arsenic/an [Analysis]
KW - Arsenic/ur [Urine]
KW - Child
KW - Chromates/ch [Chemistry]
KW - Humans
KW - Play and Playthings
KW - Saliva/ch [Chemistry]
KW - Wood
RP - NOT IN FILE
SP - 3986
EP - 3991
JF - Environmental Science & Technology
JA - Environ Sci Technol
VL - 44
IS - 10
CY - United States
N2 - Children may be exposed to arsenic during contact with structures treated with chromated copper arsenate (CCA). A high frequency of hand-to-mouth activity may increase their risk of ingesting arsenic. Previous work showed that arsenic concentrations in the hand-wash samples of children playing on CCA playgrounds were four times higher than those playing on non-CCA playgrounds. It is not clear whether playing on CCA playgrounds results in elevated overall exposure to arsenic. The objective of this study was to perform arsenic biomonitoring in children to determine whether playing on CCA-treated playgrounds substantially contributes to their overall exposure to arsenic. One hundred and twenty five saliva samples from 61 children and 101 urine samples from 45 children were collected after children played on 8 CCA and 8 non-CCA playgrounds. Arsenic speciation analysis was conducted using high performance liquid chromatography combined with inductively coupled plasma mass spectrometry. The arsenic species detected in the urine and saliva samples from children playing on CCA and non-CCA playgrounds were similar. Dimethylarsinic acid and arsenobetaine were the main arsenic species found in urine samples. The sum of inorganic trivalent and pentavalent arsenic,

monomethylarsonic acid, and dimethylarsinic acid in urine was 15 +/- 28 microg/L in the CCA group and 12 +/- 23 microg/L in the non-CCA group (p = 0.60). The sum of these species in saliva was 1.1 +/- 2.1 microg/L in the CCA group and 1.4 +/- 1.1 microg/L in the non-CCA group (p = 0.32). These results show that there is no significant difference in the concentration or speciation of arsenic between the samples from children playing on CCA and non-CCA playgrounds. Contact with CCA playgrounds is not likely to significantly contribute to the overall arsenic exposure in children; other sources such as dietary arsenic may be a main contributor to their overall exposure
SN - 0013-936X
AD - Department of Laboratory Medicine and Pathology, Analytical and Environmental Toxicology Division, Faculty of Medicine and Dentistry, University of Alberta, Edmonton, AB, Canada T6G 2G3
UR - 20377243
ER -

TY - JOUR
ID - 777
T1 - Built environment, parents' perception, and children's vigorous outdoor play
A1 - Bringolf-Isler,B.
A1 - Grize,L.
A1 - Mader,U.
A1 - Ruch,N.
A1 - Sennhauser,F.H.
A1 - Braun-Fahrlander,C.
A1 - SCARPOL team.
Y1 - 2010/05//
N1 - Bringolf-Isler, Bettina. Grize, Leticia. Mader, Urs. Ruch, Nicole. Sennhauser, Felix H. Braun-Fahrlander, Charlotte. SCARPOL team
Preventive medicine
pm4, 0322116
IM
Journal Article. Research Support, Non-U.S. Gov't
English
KW - MEDLINE
KW - Accidents
KW - Traffic
KW - Adolescent
KW - Age Factors
KW - Attitude to Health/eh [Ethnology]
KW - Attitude to Health
KW - Child
KW - Child Welfare/eh [Ethnology]
KW - Child Welfare/sn [Statistics & Numerical Data]
KW - Child Welfare
KW - Cross-Sectional Studies
KW - Environment Design
KW - Family Characteristics
KW - Female
KW - Geographic Information Systems
KW - Humans
KW - Male
KW - Multivariate Analysis
KW - Parents/ed [Education]
KW - Parents/px [Psychology]
KW - Play and Playthings
KW - Population Density
KW - Questionnaires
KW - Regression Analysis
KW - Residence Characteristics

KW - Safety
KW - Sex Factors
KW - Social Environment
KW - Statistics
KW - Nonparametric
KW - Switzerland
KW - Time Factors
KW - Urbanization
RP - NOT IN FILE
SP - 251
EP - 256
JF - Preventive Medicine
JA - Prev Med
VL - 50
IS - 5-6
CY - United States
N2 - OBJECTIVES: To evaluate the combined effects and relative importance of socio-cultural factors as well as parents' subjectively perceived and objectively assessed environment on time children spent vigorously playing outdoors. METHODS: Cross-sectional study conducted in Berne, Biel-Bienne, and Payerne (Switzerland) during the school year 2004/2005. Included 1345 parental questionnaires from children out of three age groups (6/7, 9/10, and 13/14 years). A total of 1081 (80%) provided a home address, which could be linked to environmental data using a geographic information system (GIS). RESULTS: GIS-derived main street density in a buffer of 100 m around the home was inversely associated with time playing outdoors in adolescents and younger children, but only in more urbanized areas. In addition and independently of GIS-based main street density, parental concern about traffic safety was associated with less time playing outdoors in primary school children. Girls, adolescents, and children from the French speaking part of the country spent less time playing outdoors. A non-Swiss nationality and having younger siblings increased time playing vigorously outdoors in adolescents. CONCLUSION: In addition to socio-cultural factors, parents' perceptions and objectively measured environmental factors were significantly associated with the time spent vigorously playing outdoors. These associations differed by age group. Copyright (c) 2010 The Institute For Cancer Prevention. Published by Elsevier Inc. All rights reserved
SN - 1096-0260
AD - Swiss Tropical and Public Health Institute, Basel, Switzerland. Bettina.Bringolf@unibas.ch
UR - 20346370
ER -

TY - JOUR
ID - 778
T1 - Using accelerometers and GPS units to identify the proportion of daily physical activity located in parks with playgrounds in New Zealand children
A1 - Quigg,R.
A1 - Gray,A.
A1 - Reeder,A.I.
A1 - Holt,A.
A1 - Waters,D.L.
Y1 - 2010/05//
N1 - Quigg, Robin. Gray, Andrew. Reeder, Anthony I. Holt, Alec. Waters, Debra L
Preventive medicine
pm4, 0322116
IM
Journal Article. Research Support, Non-U.S. Gov't
English
KW - MEDLINE
KW - Actigraphy/ut [Utilization]
KW - Anthropometry
KW - Child
KW - Child Nutrition Disorders/di [Diagnosis]

KW - Child Nutrition Disorders/ep [Epidemiology]
KW - Child Nutrition Disorders/pc [Prevention & Control]
KW - Child Welfare/sn [Statistics & Numerical Data]
KW - Preschool
KW - Cross-Sectional Studies
KW - Exercise/ph [Physiology]
KW - Feasibility Studies
KW - Female
KW - Geographic Information Systems/ut [Utilization]
KW - Health Surveys
KW - Humans
KW - Linear Models
KW - Male
KW - Medical Record Linkage
KW - Monitoring
KW - Ambulatory/is [Instrumentation]
KW - Ambulatory/mt [Methods]
KW - New Zealand/ep [Epidemiology]
KW - Obesity/di [Diagnosis]
KW - Obesity/ep [Epidemiology]
KW - Obesity/pc [Prevention & Control]
KW - Play and Playthings
KW - Poverty Areas
KW - Public Facilities
KW - Time Factors
KW - Urban Health/sn [Statistics & Numerical Data]
RP - NOT IN FILE
SP - 235
EP - 240
JF - Preventive Medicine
JA - Prev Med
VL - 50
IS - 5-6
CY - United States
N2 - OBJECTIVE: To identify the proportion of children's physical activity occurring in public parks with playgrounds. METHODS: Children (n=184) aged 5 to 10 years were recruited from schools located in two low socio-economic status communities in Dunedin, New Zealand. Accelerometers (Actigraph GT1M) and global positioning system units (Globalsat DG-100) were used to quantify and identify where physical activity had occurred over a 7-day period. Cross-sectional data were collected October to December 2007 (southern hemisphere spring) and the child's height and weight were measured at school. RESULTS: At least 84% of participants had accelerometer and global positioning system data available for five or more hourly periods per day, for at least 4 days. Overall, 1.9% of recorded activity took place at city parks (95% confidence interval: 1.4, 2.4), although this was 2.7% (95% confidence intervals: 0.7, 4.6) among obese children. CONCLUSIONS: Accelerometers and GPS data was able to be successfully recorded and matched among this age group. The proportion of children's activity taking place in parks with playgrounds was low, although this may still be important for some subgroups. Copyright (c) 2010 Elsevier Inc. All rights reserved
SN - 1096-0260
AD - Cancer Society Social & Behavioural Research Unit, Department of Preventive and Social Medicine, University of Otago, New Zealand. robin.quigg@otago.ac.nz
UR - 20153361
ER -

TY - JOUR
ID - 779
T1 - Predictors of time spent outdoors among children: 5-year longitudinal findings
A1 - Cleland,V.
A1 - Timperio,A.

A1 - Salmon,J.
A1 - Hume,C.
A1 - Baur,L.A.
A1 - Crawford,D.
Y1 - 2010/05//
N1 - Cleland, V. Timperio, A. Salmon, J. Hume, C. Baur, L A. Crawford, D
Journal of epidemiology and community health
i1p, 7909766
IM
Journal Article. Research Support, Non-U.S. Gov't
English
KW - MEDLINE
KW - Adolescent
KW - Adult
KW - Australia
KW - Child
KW - Preschool
KW - Exercise
KW - Female
KW - Humans
KW - Leisure Activities
KW - Longitudinal Studies
KW - Male
KW - Parent-Child Relations
KW - Parents/px [Psychology]
KW - Play and Playthings
KW - Questionnaires
KW - Sex Distribution
KW - Social Environment
KW - Time Factors
KW - Urban Population
RP - NOT IN FILE
SP - 400
EP - 406
JF - Journal of Epidemiology & Community Health
JA - J Epidemiol Community Health
VL - 64
IS - 5
CY - England
N2 - BACKGROUND: Given the importance of physical activity for health and age-related declines in physical activity, understanding influences on related behaviours, such as time outdoors, is crucial. This study aimed to understand individual, social and physical environmental influences on longitudinal changes in urban children's time outdoors. METHODS: The time children spent outdoors in 2001, 2004 and 2006 (aged 5-6 and 10-12 years at baseline) was reported by their parents (n=421). In 2001, individual, social and physical environmental factors were self-reported by parents. Generalized estimating equations examined longitudinal relationships between baseline predictors and average change in time outdoors over 5 years. RESULTS: Children's time outdoors significantly declined over time. "Indoor tendencies" inversely predicted time outdoors among younger and older boys, and younger girls. Social opportunities positively predicted time outdoors among younger boys, while "outdoor tendencies" positively predicted time outdoors among older boys. Parental encouragement for activity positively predicted time outdoors among younger and older girls, while lack of adult supervision for active play outdoors after school inversely predicted time outdoors among older girls and older boys. CONCLUSION: Individual (indoor and outdoor tendencies) and social factors (social opportunities, parental encouragement and parental supervision) predicted children's time outdoors over 5 years. Interventions targeting reduced indoor tendencies, increased outdoor play with others, and increased parental encouragement and supervision are warranted
SN - 1470-2738

AD - Center for Physical Activity and Nutrition Research, Deakin University, 221 Burwood Highway, Burwood 3125, Australia. verity.cleland@deakin.edu.au
UR - 19778909
ER -

TY - JOUR
ID - 780
T1 - Time-location patterns of a population living in an air pollution hotspot
A1 - Wu,X.M.
A1 - Fan,Z.T.
A1 - Ohman-Strickland,P.
Y1 - 2010///
N1 - Wu, Xiangmei May. Fan, Zhihua Tina. Ohman-Strickland, Pamela
Journal of environmental and public health
101516361
IM
Journal Article. Research Support, N.I.H., Extramural. Research Support, Non-U.S. Gov't
English
KW - MEDLINE
KW - Adolescent
KW - Adult
KW - Aged
KW - 80 and over
KW - Air Pollutants
KW - Air Pollution
KW - Child
KW - Cohort Studies
KW - Employment
KW - Environmental Exposure
KW - Female
KW - Humans
KW - Male
KW - Middle Aged
KW - New Jersey
KW - Questionnaires
KW - Risk
KW - Seasons
KW - Socioeconomic Factors
KW - Time Factors
KW - Young Adult
RP - NOT IN FILE
SP - 625461
JF - Journal Of Environmental & Public Health
JA - J Environ Public Health
VL - 2010
CY - Egypt
N2 - This study characterized the time-location pattern of 107 residents living in air pollution hotspots, the Waterfront South and Copewood/Davis Streets communities in Camden, NJ. Most residents in the two communities are minority and impoverished individuals. Results showed that employment status played the fundamental role in determining time-location patterns of this study population, and the variations of time-location pattern by season and by day-type were partially attributed to employment status. Compared to the National Human Activity Pattern Survey, the Camden cohort spent significantly more time outdoors (3.8 hours versus 1.8 hours) and less time indoors (19.4 hours versus 20.9 hours) than the general US population, indicating a higher risk of exposure to ambient air pollution for the Camden cohort. The findings of the study are important for understanding exposure routes and sources for the socioeconomically disadvantaged subgroup and ultimately help develop effective strategies to reduce community exposure to ambient air pollution in "hotspots"

SN - 1687-9813
AD - Environmental and Occupational Health Sciences Institute (EOHSI), University of Medicine and Dentistry of New Jersey (UMDNJ) and Rutgers University, Piscataway, NJ 08854, USA
UR - 20445735
ER -

TY - JOUR
ID - 781
T1 - Are children and adolescents less active if parents restrict their physical activity and active transport due to perceived risk?
A1 - Carver,A.
A1 - Timperio,A.
A1 - Hesketh,K.
A1 - Crawford,D.
Y1 - 2010/06//
N1 - Carver, Alison. Timperio, Anna. Hesketh, Kylie. Crawford, David
Social science & medicine (1982)
ut9, 8303205
IM
Journal Article. Research Support, Non-U.S. Gov't
English
KW - MEDLINE
KW - Adolescent
KW - Age Factors
KW - Bicycling/sn [Statistics & Numerical Data]
KW - Child
KW - Cross-Sectional Studies
KW - Exercise
KW - Female
KW - Humans
KW - Linear Models
KW - Male
KW - Parent-Child Relations
KW - Parents/px [Psychology]
KW - Residence Characteristics
KW - Risk
KW - Safety
KW - Sex Factors
KW - Time Factors
KW - Victoria
KW - Walking/sn [Statistics & Numerical Data]
RP - NOT IN FILE
SP - 1799
EP - 1805
JF - Social Science & Medicine
JA - Soc Sci Med
VL - 70
IS - 11
CY - England
N2 - The study examined parental restriction of children's active transport and physical activity outside school hours due to safety concerns, and how this restriction was associated with perceived risk and with youth physical activity levels. We used a cross-sectional design with data from children aged 10-11 years (n=170) and adolescents aged 15-17 years (n=270) who participated in the five-year follow-up of the Children Living in Active Neighbourhoods longitudinal study in Melbourne, Australia. Walking/cycling to local destinations was survey-reported. Moderate-to-vigorous physical activity (MVPA) was recorded during non-school hours using accelerometers. Using surveys, parents reported perceived risk of their children being harmed in their neighbourhood, and restrictions they placed on their children's physical activity. Linear regression analyses

examined how perceived risk, constrained behaviour, and physical activity were related. We found that perceived risk did not vary by age-group and was positively associated with constrained behaviour among adolescents but not children. Children were subject to higher levels of constrained behaviour than adolescents. Constrained behaviour was negatively associated with active transport among younger boys and among girls in both age-groups. Furthermore, it was negatively associated with younger boys' MVPA on weekends and with adolescent girls' MVPA during evenings. There were no associations between constrained behaviour and active transport or MVPA among adolescent boys. The findings demonstrate that constrained behaviour exhibited by parents may result in lower levels of active transport and of MVPA outside school hours. In particular, this was true for children and adolescent girls. Social interventions to improve perceived safety and physical interventions involving redesign of the built environment to improve actual safety may help to ease parental restriction of their children's active transport and physical activity in their neighbourhood. Copyright 2010 Elsevier Ltd. All rights reserved
SN - 1873-5347
AD - Deakin University, Burwood, VIC 3125, Australia. alison.carver@deakin.edu.au
UR - 20347200
ER -

TY - JOUR
ID - 782
T1 - A new method to sample stuttering in preschool children
A1 - O'Brian,S.
A1 - Jones,M.
A1 - Pilowsky,R.
A1 - Onslow,M.
A1 - Packman,A.
A1 - Menzies,R.
Y1 - 2010/06//
N1 - O'Brian, Sue. Jones, Mark. Pilowsky, Rachel. Onslow, Mark. Packman, Ann. Menzies, Ross
International journal of speech-language pathology
101320232
IM
Journal Article. Research Support, Non-U.S. Gov't
English
KW - MEDLINE
KW - Acoustics/is [Instrumentation]
KW - Analysis of Variance
KW - Child Language
KW - Child
KW - Preschool
KW - Humans
KW - Parents
KW - Play and Playthings
KW - Reproducibility of Results
KW - Speech
KW - Speech Production Measurement/is [Instrumentation]
KW - Speech Production Measurement/mt [Methods]
KW - Stuttering/di [Diagnosis]
KW - Telephone
RP - NOT IN FILE
SP - 173
EP - 177
JF - International Journal of Speechlanguage Pathology
JA - Int J Speech Lang Pathol
VL - 12
IS - 3
CY - England

N2 - This study reports a new method for sampling the speech of preschool stuttering children outside the clinic environment. Twenty parents engaged their stuttering children in an everyday play activity in the home with a telephone handset nearby. A remotely located researcher telephoned the parent and recorded the play session with a phone-recording jack attached to a digital audio recorder at the remote location. The parent placed an audio recorder near the child for comparison purposes. Children as young as 2 years complied with the remote method of speech sampling. The quality of the remote recordings was superior to that of the in-home recordings. There was no difference in means or reliability of stutter-count measures made from the remote recordings compared with those made in-home. Advantages of the new method include: (1) cost efficiency of real-time measurement of percent syllables stuttered in naturalistic situations, (2) reduction of bias associated with parent-selected timing of home recordings, (3) standardization of speech sampling procedures, (4) improved parent compliance with sampling procedures, (5) clinician or researcher on-line control of the acoustic and linguistic quality of recordings, and (6) elimination of the need to lend equipment to parents for speech sampling
SN - 1754-9515
AD - Australian Stuttering Research Centre, The University of Sydney, Australia
UR - 20433336
ER -

TY - JOUR
ID - 783
T1 - Does parent report measure performance? A study of the construct validity of the Functional Mobility Scale
A1 - Harvey,A.
A1 - Baker,R.
A1 - Morris,M.E.
A1 - Hough,J.
A1 - Hughes,M.
A1 - Graham,H.K.
Y1 - 2010/02//
N1 - Harvey, Adrienne. Baker, Richard. Morris, Meg E. Hough, Janet. Hughes, Marty. Graham, H Kerr
Developmental medicine and child neurology
0006761, e83
IM
Journal Article. Research Support, Non-U.S. Gov't
English
KW - MEDLINE
KW - Activities of Daily Living
KW - Adolescent
KW - Cerebral Palsy/cl [Classification]
KW - Cerebral Palsy/pp [Physiopathology]
KW - Child
KW - Child Development/ph [Physiology]
KW - Disability Evaluation
KW - Female
KW - Humans
KW - Male
KW - Mobility Limitation
KW - Motor Activity/ph [Physiology]
KW - Observer Variation
KW - Parents/px [Psychology]
KW - Questionnaires
KW - Reproducibility of Results
KW - Time Factors
RP - NOT IN FILE
SP - 181
EP - 185
JF - Developmental Medicine & Child Neurology
JA - Dev Med Child Neurol

VL - 52
IS - 2
CY - England
N2 - AIM: Parental report is often relied on to measure performance of activities in children with cerebral palsy (CP). This study examined whether the Functional Mobility Scale (FMS) accurately reflects performance of mobility in children with CP. METHOD: Eighteen children with spastic CP (11 males, seven females; mean age 12 y 8 mo, SD 2 y 8 mo, range 8-17 y) were recruited from a special development school. Children were in Gross Motor Function Classification System (GMFCS) levels II (n=5), III (n=4), or IV (n=9), and had quadriplegia (n=9), diplegia (n=7), or hemiplegia (n=2). The children's mobility was observed directly around and outside the home and at school and their mobility methods were recorded. The parent's FMS rating was obtained on the telephone by a physiotherapist. Agreement between direct observation and the FMS rating was examined using quadratic weighted kappa (kappa) statistics. RESULTS: Agreement between direct observation and the FMS was as follows: FMS 5m kappa=0.71, 45%; FMS 50m kappa=0.76, 94%; FMS 500m kappa=0.74, 95%. Differences in the range and number of mobility methods were observed by GMFCS level across environmental settings. INTERPRETATION: Substantial agreement was found between FMS ratings and direct observation, particularly over longer distances, providing evidence of the validity of the FMS as a measure of performance in children with CP
SN - 1469-8749
AD - McMaster Child Health Research Institute, Hamilton, Ontario, Canada. harvey.adrienne@gmail.com
UR - 19549200
ER -

TY - JOUR
ID - 784
T1 - The ScanBrit randomised, controlled, single-blind study of a gluten- and casein-free dietary intervention for children with autism spectrum disorders
A1 - Whiteley,P.
A1 - Haracopos,D.
A1 - Knivsberg,A.M.
A1 - Reichelt,K.L.
A1 - Parlar,S.
A1 - Jacobsen,J.
A1 - Seim,A.
A1 - Pedersen,L.
A1 - Schondel,M.
A1 - Shattock,P.
Y1 - 2010/04//
N1 - Whiteley, Paul. Haracopos, Demetrious. Knivsberg, Ann-Mari. Reichelt, Karl Ludvig. Parlar, Sarah. Jacobsen, Judith. Seim, Anders. Pedersen, Lennart. Schondel, Maja. Shattock, Paul
Nutritional neuroscience
100892202
IM
Clinical Trial. Journal Article. Randomized Controlled Trial. Research Support, Non-U.S. Gov't
English
KW - MEDLINE
KW - Aging
KW - Attention
KW - Caseins/ad [Administration & Dosage]
KW - Caseins/ae [Adverse Effects]
KW - Child
KW - Child Development
KW - Child Development Disorders
KW - Pervasive/di [Diagnosis]
KW - Pervasive/dh [Diet Therapy]
KW - Pervasive/ur [Urine]
KW - Preschool
KW - Diet

KW - Gluten-Free
KW - Protein-Restricted
KW - Female
KW - Glycine/aa [Analogs & Derivatives]
KW - Glycine/ur [Urine]
KW - Humans
KW - Hyperkinesis
KW - Male
KW - Reproducibility of Results
KW - Severity of Illness Index
KW - Single-Blind Method
KW - Statistics as Topic
KW - Time Factors
RP - NOT IN FILE
SP - 87
EP - 100
JF - Nutritional Neuroscience
JA - Nutr Neurosci
VL - 13
IS - 2
CY - England
N2 - There is increasing interest in the use of gluten- and casein-free diets for children with autism spectrum disorders (ASDs). We report results from a two-stage, 24-month, randomised, controlled trial incorporating an adaptive 'catch-up' design and interim analysis. Stage 1 of the trial saw 72 Danish children (aged 4 years to 10 years 11 months) assigned to diet (A) or non-diet (B) groups by stratified randomisation. Autism Diagnostic Observation Schedule (ADOS) and the Gilliam Autism Rating Scale (GARS) were used to assess core autism behaviours, Vineland Adaptive Behaviour Scales (VABS) to ascertain developmental level, and Attention-Deficit Hyperactivity Disorder - IV scale (ADHD-IV) to determine inattention and hyperactivity. Participants were tested at baseline, 8, and 12 months. Based on per protocol repeated measures analysis, data for 26 diet children and 29 controls were available at 12 months. At this point, there was a significant improvement to mean diet group scores (time*treatment interaction) on sub-domains of ADOS, GARS and ADHD-IV measures. Surpassing of predefined statistical thresholds as evidence of improvement in group A at 12 months sanctioned the re-assignment of group B participants to active dietary treatment. Stage 2 data for 18 group A and 17 group B participants were available at 24 months. Multiple scenario analysis based on inter- and intra-group comparisons showed some evidence of sustained clinical group improvements although possibly indicative of a plateau effect for intervention. Our results suggest that dietary intervention may positively affect developmental outcome for some children diagnosed with ASD. In the absence of a placebo condition to the current investigation, we are, however, unable to disqualify potential effects derived from intervention outside of dietary changes. Further studies are required to ascertain potential best- and non-responders to intervention. The study was registered with ClincialTrials.gov, number NCT00614198
SN - 1476-8305
AD - Dept of Pharmacy, Health & Well-being, Faculty of Applied Sciences, University of Sunderland, UK. paul.whiteley@espa-research.org.uk
UR - 20406576
ER -

TY - JOUR
ID - 785
T1 - Comparison of the clinical presentation and visual outcome in open globe injuries in adults and children over 30 months
A1 - Gupta,A.
A1 - Srinivasan,R.
A1 - Babu,K.R.
A1 - Setia,S.
Y1 - 2010/05//
N1 - Gupta, Arvind. Srinivasan, Renuka. Babu, K Ramesh. Setia, Sajita
European journal of ophthalmology

bjg, 9110772
IM
Comparative Study. Journal Article
English
KW - MEDLINE
KW - Adolescent
KW - Adult
KW - Child
KW - Preschool
KW - Eye Injuries
KW - Penetrating/di [Diagnosis]
KW - Penetrating/ep [Epidemiology]
KW - Penetrating/pp [Physiopathology]
KW - Female
KW - Humans
KW - Male
KW - Prospective Studies
KW - Risk Factors
KW - Visual Acuity/ph [Physiology]
KW - Young Adult
RP - NOT IN FILE
SP - 590
EP - 595
JF - European Journal of Ophthalmology
JA - Eur J Ophthalmol
VL - 20
IS - 3
CY - Italy
N2 - PURPOSE: To compare the clinical presentation and final visual outcome of open globe injuries in children and adults in a referral hospital over a 30-month period. METHODS: This is an institutional-based prospective study of open globe injuries cases presenting in the emergency department between July 2003 and December 2005. Patients were divided in 2 groups: group 1, children (2-15 years), and group 2, adults (>15 years). All the patients were admitted and emergency surgical interventions were undertaken. The clinical features at presentation and the final visual acuity are compared. Chi-square and Fisher exact tests were used for statistical analysis. RESULTS: Ninety and 84 patients were included in group 1 and group 2, respectively. The most common places of injuries were home or while playing outdoor games in group 1 (67%) and workplace in group 2 (53.5%). The presenting features were significantly more grave in group 2. These included poor presenting visual acuity (p=0.012), vitreous prolapse (p=0.002), presence of relative afferent pupillary defect (p=0.001), and incidence of endophthalmitis (p=0.004). Time interval between injury and surgical intervention (p=0.018) was better in group 2. Other features, such as presence of hyphema, uveal tissue prolapse, cataract, intraocular foreign body, and length or location of laceration were similar in both groups. The final visual outcome was similar in the groups (p = 0.21), with approximately half of the patients achieving vision of 20/60 or better in each group. CONCLUSIONS: The majority of injuries in children and adults occurred in their homes or workplaces, respectively. Although the clinical presentations of open globe injuries were significantly more grave in adults than in children, the final visual outcomes were similar
SN - 1120-6721
AD - Department of Ophthalmology, JIPMER (Jawaharlal Institute of Postgraduate Medical Education and Research), Pondicherry - India. arvind_ophtahl@yahoo.co.in
UR - 20037911
ER -

TY - JOUR
ID - 786
T1 - Evaluation of efficacy and human health risk of aerial ultra-low volume applications of pyrethrins and piperonyl butoxide for adult mosquito management in response to West Nile virus activity in Sacramento County, California
A1 - Macedo,P.A.

A1 - Schleier,J.J.,III
A1 - Reed,M.
A1 - Kelley,K.
A1 - Goodman,G.W.
A1 - Brown,D.A.
A1 - Peterson,R.K.
Y1 - 2010/03//
N1 - Macedo, Paula A. Schleier, Jerome J 3rd. Reed, Marcia. Kelley, Kara. Goodman, Gary W. Brown, David A. Peterson, Robert K D
Journal of the American Mosquito Control Association
jao, 8511299
IM
Evaluation Studies. Journal Article. Research Support, Non-U.S. Gov't
English
KW - MEDLINE
KW - Adolescent
KW - Adult
KW - Aged
KW - Animals
KW - California
KW - Child
KW - Preschool
KW - Culicidae
KW - Female
KW - Humans
KW - Infant
KW - Insect Vectors
KW - Insecticides/ad [Administration & Dosage]
KW - Insecticides/to [Toxicity]
KW - Male
KW - Middle Aged
KW - Mosquito Control
KW - Pesticide Synergists/ad [Administration & Dosage]
KW - Pesticide Synergists/to [Toxicity]
KW - Piperonyl Butoxide/ad [Administration & Dosage]
KW - Piperonyl Butoxide/to [Toxicity]
KW - Pyrethrins/ad [Administration & Dosage]
KW - Pyrethrins/to [Toxicity]
KW - Risk Assessment
KW - West Nile Fever/tm [Transmission]
KW - Young Adult
RP - NOT IN FILE
SP - 57
EP - 66
JF - Journal of the American Mosquito Control Association
JA - J Am Mosq.Control Assoc
VL - 26
IS - 1
CY - United States
N2 - The Sacramento and Yolo Mosquito and Vector Control District (SYMVCD, also referred to as "the District") conducts surveillance and management of mosquitoes in Sacramento and Yolo counties in California. Following an increase in numbers and West Nile virus (WNV) infection rates of Culex tarsalis and Culex pipiens, the District decided on July 26, 2007, to conduct aerial applications of Evergreen EC 60-6 (60% pyrethrins: 6% piperonyl butoxide) over approximately 215 km2 in the north area of Sacramento County on the nights of July 30, July 31, and August 1, 2007. At the same time, the District received notification of the first human WNV case in the area. To evaluate the efficacy of the applications in decreasing mosquito abundance and infection rates, we conducted pre- and post-trapping inside and outside the spray zone and assessed human

health risks from exposure to the insecticide applications. Results showed a significant decrease in abundance of both Cx. tarsalis and Cx. pipiens, and in the minimum infection rate of Cx. tarsalis. Human-health risks from exposure to the insecticide were below thresholds set by the US Environmental Protection Agency
SN - 8756-971X
AD - Sacramento-Yolo Mosquito and Vector Control District, 8631 Bond Road, Elk Grove, CA 95757, USA
UR - 20402352
ER -

TY - JOUR
ID - 787
T1 - [Update on photoprotection in children]. [Review] [38 refs] [Spanish]
A1 - Valdivielso-Ramos,M.
A1 - Herranz,J.M.
Y1 - 2010/04//
N1 - Valdivielso-Ramos, M. Herranz, J M
Anales de pediatria (Barcelona, Spain : 2003)
101162596
IM
English Abstract. Journal Article. Review
Spanish
KW - MEDLINE
KW - Child
KW - Health Education
KW - Humans
KW - Radiation Protection/mt [Methods]
KW - Skin Neoplasms/pc [Prevention & Control]
KW - Sunscreening Agents
RP - NOT IN FILE
SP - 282
EP - 289
JF - Anales de Pediatria
JA - An Pediatr (Barc)
VL - 72
IS - 4
CY - Spain
N2 - Children are one of the population groups in which the photo-protection must be maximised. Firstly, because they take part in outdoor activities more often than adults. Secondly, because the principal risk factor for all types of skin cancer is ultraviolet radiation, and in particular, cumulative exposure during childhood. Hence, decreasing exposure to ultraviolet radiation in childhood has the potential to significantly lower the incidence of most forms of skin cancer. Photoprotection includes behavioural measures to protect the skin from sun exposure, e.g. sun protective clothes, hats, sunglasses, and sunscreens. It is necessary to provide information on aphotoprotection to parents, and, above all, to children, using educational campaigns to increase knowledge of photoprotection to help change attitudes towards sun exposure. Dermatologists and Paediatricians play a essential role in this educational work. In this article we review the latest information regarding paediatric sun protection, the new sunscreens, and the recent sun protection educational programs. 2009 Asociacion Espanola de Pediatria. Published by Elsevier Espana. All rights reserved. [References: 38]
SN - 1695-9531
AD - Hospital Infanta Leonor, Madrid, Espana. mvaldira@yahoo.es
UR - 20053592
ER -

TY - JOUR
ID - 788
T1 - The development of a unique physical activity self-report for young children: challenges and lessons learned
A1 - Parrish,A.M.
A1 - Iverson,D.

A1 - Russell,K.
A1 - Yeatman,H.
Y1 - 2010/01//
N1 - Parrish, Anne-Maree. Iverson, Don. Russell, Ken. Yeatman, Heather
Research in sports medicine (Print)
101167637
IM
Journal Article. Validation Studies
English
KW - MEDLINE
KW - Australia
KW - Child
KW - Preschool
KW - Exercise
KW - Female
KW - Humans
KW - Male
KW - Observation
KW - Questionnaires/st [Standards]
KW - Reproducibility of Results
KW - Schools
RP - NOT IN FILE
SP - 71
EP - 83
JF - Research in Sports Medicine
JA - RES SPORTS MED
VL - 18
IS - 1
CY - United States
N2 - The number of overweight and obese children in the Western world is alarming. Efforts to impact this problem at an individual level have had little effect. Interventions that focus on the whole population may prove more successful. This study reports the development of a unique self-report picture questionnaire designed to assess playground physical activity preferences in young children (4-9 years, N = 1,881) at 14 regional Australian primary schools. Children's picture preferences were compared with observational data using the Children's Activity Scanning tool (CAST2). Results indicate that 62% to 89% of children preferred activities that were moderate or highly active; however, CAST2 data indicated that children spent only 50% to 70% of play time being active. The findings confirm that young children are able to indicate their preferences for physical activity. Important considerations in future attempts to develop a measure of physical activity in young children are discussed
SN - 1543-8635
AD - Faculty of Health and Behavioural Sciences, University of Wollongong, Northfields Ave., New South Wales 2522, Australia. amp17@uow.edu.au
UR - 20391248
ER -

TY - JOUR
ID - 789
T1 - Using nature and outdoor activity to improve children's health. [Review] [141 refs][Erratum appears in Curr Probl Pediatr Adolesc Health Care. 2010 Jul;40(6):152]
A1 - McCurdy,L.E.
A1 - Winterbottom,K.E.
A1 - Mehta,S.S.
A1 - Roberts,J.R.
Y1 - 2010/05//
N1 - McCurdy, Leyla E. Winterbottom, Kate E. Mehta, Suril S. Roberts, James R
Current problems in pediatric and adolescent health care
101134613

IM
Journal Article. Review
English
KW - MEDLINE
KW - Asthma/et [Etiology]
KW - Asthma/pc [Prevention & Control]
KW - Cardiovascular Diseases/et [Etiology]
KW - Cardiovascular Diseases/pc [Prevention & Control]
KW - Child
KW - Child Nutrition Disorders/co [Complications]
KW - Child Nutrition Disorders/ep [Epidemiology]
KW - Child Nutrition Disorders/pc [Prevention & Control]
KW - Child Welfare
KW - Diabetes Mellitus
KW - Type 2/et [Etiology]
KW - Type 2/pc [Prevention & Control]
KW - Fatty Liver/et [Etiology]
KW - Fatty Liver/pc [Prevention & Control]
KW - Health Promotion/mt [Methods]
KW - Humans
KW - Mental Health
KW - Metabolic Syndrome X/et [Etiology]
KW - Metabolic Syndrome X/pc [Prevention & Control]
KW - Obesity/co [Complications]
KW - Obesity/ep [Epidemiology]
KW - Obesity/pc [Prevention & Control]
KW - Pediatrics/mt [Methods]
KW - Play and Playthings/px [Psychology]
KW - Play and Playthings
KW - Sedentary Lifestyle
KW - Sleep Apnea
KW - Obstructive/et [Etiology]
KW - Obstructive/pc [Prevention & Control]
KW - United States/ep [Epidemiology]
KW - Vitamin D Deficiency/pc [Prevention & Control]
RP - NOT IN FILE
SP - 102
EP - 117
JF - Current Problems in Pediatric & Adolescent Health Care
JA - Curr Probl Pediatr Adolesc Health Care
VL - 40
IS - 5
CY - United States
N2 - Childhood obesity affects 17% or 12.5 million of America's children, contributing to the rise in children's health disparities. Type 2 diabetes, asthma, vitamin D deficiency, and attention-deficit/hyperactivity disorder have also increased over the past few decades. A shift toward a sedentary lifestyle is a major contributor to the decline in children's health. Children spend more time indoors using electronic media and less time engaged in outdoor unstructured play. This article reviews the current evidence of the mental and physical health benefits associated with unstructured, outdoor activities and time spent in a natural environment such as a park or other recreational area. Pediatric health care providers should recommend outdoor activities for children and refer families to safe and easily accessible outdoor areas. Pediatric health care providers can incorporate this simple, lifestyle-based intervention into anticipatory guidance. Copyright 2010 Mosby, Inc. All rights reserved. [References: 141]
SN - 1538-3199
AD - National Environmental Education Foundation, Washington, DC, USA
UR - 20381783
ER -

TY - JOUR
ID - 790
T1 - Peripheral TH-17 cells in children with allergic rhinitis: preliminary report
A1 - Ciprandi,G.
A1 - Castellazzi,A.M.
A1 - Fenoglio,D.
A1 - Battaglia,F.
A1 - Marseglia,G.
Y1 - 2010/01//
N1 - Ciprandi, G. Castellazzi, A M. Fenoglio, D. Battaglia, F. Marseglia, G
International journal of immunopathology and pharmacology
8911335
IM
Journal Article
English
KW - MEDLINE
KW - Child
KW - Female
KW - Humans
KW - Interferon-gamma/bl [Blood]
KW - Interleukin-17/bl [Blood]
KW - Male
KW - Rhinitis
KW - Allergic
KW - Perennial/im [Immunology]
KW - Seasonal/im [Immunology]
KW - T-Lymphocytes
KW - Helper-Inducer/im [Immunology]
RP - NOT IN FILE
SP - 379
EP - 382
JF - International Journal of Immunopathology & Pharmacology
VL - 23
IS - 1
CY - Italy
N2 - Th17 is a subset of T helper lymphocytes and exerts pro-inflammatory activities. Recently, it has been reported that serum IL-17 levels are high in the most severe patients with birch allergy studied both outside and during the pollen season. This study aims to compare the frequency of peripheral IL-17-producing T cells in children with allergic rhinitis and in healthy controls. Ten children with allergic rhinitis and 5 healthy non-allergic subjects were evaluated. Th17 were evaluated by intracellular staining in ex-vivo T cell compartment. Ex- vivo PBMNC evaluation showed that allergic patients had higher frequencies of IL-17 producing T cells, both concerning CD4+ and CD8+ cells. In particular, there is a subset co-expressing IL-17 and IFN-gamma both for CD4+ and CD8+ cells. In conclusion, this preliminary study suggests a possible role of Th-17 cells in the response to allergens in children
SN - 0394-6320
AD - Department of Internal Medicine, Azienda Ospedaliera Universitaria San Martino and University of Genoa, Genoa, Italy. gio.cip@libero.it
UR - 20378028
ER -

TY - JOUR
ID - 791
T1 - Maternal perception of neighborhood safety as a predictor of child weight status: The moderating effect of gender and assessment of potential mediators
A1 - Bacha,J.M.
A1 - Appugliese,D.

A1 - Coleman,S.
A1 - Kaciroti,N.
A1 - Bradley,R.H.
A1 - Corwyn,R.F.
A1 - Lumeng,J.C.
Y1 - 2010///
N1 - Bacha, Jason M. Appugliese, Danielle. Coleman, Sharon. Kaciroti, Niko. Bradley, Robert H. Corwyn, Robert F. Lumeng, Julie C
International journal of pediatric obesity : IJPO : an official journal of the International Association for the Study of Obesity
101256330
IM
Journal Article. Multicenter Study
English
KW - MEDLINE
KW - Body Mass Index
KW - Body Weight
KW - Chi-Square Distribution
KW - Child
KW - Child Behavior
KW - Exercise
KW - Female
KW - Humans
KW - Life Style
KW - Longitudinal Studies
KW - Male
KW - Maternal Behavior
KW - Mothers/px [Psychology]
KW - Obesity/et [Etiology]
KW - Obesity/pp [Physiopathology]
KW - Obesity/px [Psychology]
KW - Odds Ratio
KW - Perception
KW - Puberty/px [Psychology]
KW - Questionnaires
KW - Residence Characteristics
KW - Risk Assessment
KW - Risk Factors
KW - Safety
KW - Sedentary Lifestyle
KW - Sex Factors
KW - Television
KW - United States
RP - NOT IN FILE
SP - 72
EP - 79
JF - International Journal of Pediatric Obesity
JA - Int J Pediatr Obes
VL - 5
IS - 1
CY - England
N2 - OBJECTIVE: To determine if there is a relationship between maternal perception of neighborhood safety in 3(rd) grade and weight status in 5(th) grade children, to test if gender moderates this relationship, and to identify potential mediators. METHOD: Data from 868 children and their mothers involved in the National Institute of Child Health and Human Development Study of Early Child Care and Youth Development (NICHD-SECCYD) were used to examine the relationship between maternal perception of neighborhood safety in the 3(rd) grade and child body mass index (BMI) z-score in the 5(th) grade. Multiple regression models tested

this relationship, the effect of gender, and potential mediating variables (time outdoors in neighborhood, television viewing, child behavior problems and puberty status). RESULTS: Neighborhood safety ratings in the least safe tertile, compared with the safest tertile, were associated with an increased risk of obesity independent of gender, race and income-to-needs ratio (OR=1.59; 95% confidence interval [CI]: 1.03, 2.46), and higher child BMI z-scores among girls, but not boys, compared with the safest tertile (beta=0.33; 95% CI: 0.09, 0.57). Neither amount of time spent outdoors in the neighborhood, television viewing, child behavior problems (internalizing or externalizing), nor puberty status altered the relationship. CONCLUSIONS: Maternal perception of the neighborhood as unsafe in 3(rd) grade independently predicted a higher risk of obesity, and a higher BMI z-score among girls, but not boys, in the 5(th) grade. The relationship was not explained by several potential mediators. Further investigation is needed to explore these gender differences and potential mediators
SN - 1747-7174
AD - Department of Pediatrics and Communicable Diseases, University of Michigan, Ann Arbor, MI, USA
UR - 19606373
ER -

TY - JOUR
ID - 792
T1 - Stationary cycling and children with cerebral palsy: case reports for two participants
A1 - Siebert,K.L.
A1 - DeMuth,S.K.
A1 - Knutson,L.M.
A1 - Fowler,E.G.
Y1 - 2010/05//
N1 - Siebert, Kara L. DeMuth, Sharon K. Knutson, Loretta M. Fowler, Eileen G
Physical & occupational therapy in pediatrics
8109120, d0u
IM
Case Reports. Journal Article. Research Support, Non-U.S. Gov't
English
KW - MEDLINE
KW - Bicycling
KW - Cerebral Palsy/pp [Physiopathology]
KW - Cerebral Palsy/rh [Rehabilitation]
KW - Child
KW - Exercise Therapy
KW - Female
KW - Humans
KW - Locomotion
KW - Lower Extremity/pp [Physiopathology]
KW - Male
KW - Muscle Spasticity/co [Complications]
RP - NOT IN FILE
SP - 125
EP - 138
JF - Physical & Occupational Therapy in Pediatrics
JA - Phys Occup Ther Pediatr
VL - 30
IS - 2
CY - England
N2 - These case reports describe a stationary cycling intervention and outcomes for two child participants (P1 and P2) with spastic diplegic cerebral palsy. Each child completed a 12-week, 30-session cycling intervention consisting of strengthening and cardiorespiratory fitness phases. P1 exhibited higher training intensities, particularly during the cardiorespiratory phase. Average training heart rates were 59% and 35% of maximum heart rate for P1 and P2, respectively. Lower extremity peak knee flexor and extensor moments, gross motor function (Gross Motor Function Measure (GMFM-66)), preferred walking speed (thirty-second walk test), and walking endurance (600-yard walk-run test) were measured pre- and postintervention. Changes in outcome measurements corresponded with differences in exercise intensity. Greater gains in peak knee extensor

moments, GMFM-66 scores (+4.2 versus +0.9), 600-yard walk-run test (-29% versus 0%) occurred for P1 versus P2, respectively. Preferred walking speeds did not increase substantially for P1 and decreased for P2
SN - 1541-3144
AD - South Bay Medical Therapy Unit, California Children's Services of Los Angeles County, USA
UR - 20367517
ER -

TY - JOUR
ID - 793
T1 - Catheter-related polymicrobial bloodstream infections among pediatric bone marrow transplant outpatients--Atlanta, Georgia, 2007
A1 - Wiersma,P.
A1 - Schillie,S.
A1 - Keyserling,H.
A1 - Watson,J.R.
A1 - De,A.
A1 - Banerjee,S.N.
A1 - Drenzek,C.L.
A1 - Arnold,K.E.
A1 - Shivers,C.
A1 - Kendrick,L.
A1 - Ryan,L.G.
A1 - Jensen,B.
A1 - Noble-Wang,J.
A1 - Srinivasan,A.
Y1 - 2010/05//
N1 - Wiersma, Petra. Schillie, Sarah. Keyserling, Harry. Watson, J Renee. De, Anindya. Banerjee, Shailendra N. Drenzek, Cherie L. Arnold, Kathryn E. Shivers, Christina. Kendrick, Lea. Ryan, Lydia Gonzalez. Jensen, Bette. Noble-Wang, Judith. Srinivasan, Arjun
Infection control and hospital epidemiology : the official journal of the Society of Hospital Epidemiologists of America
ich, 8804099
IM, N
Journal Article
English
KW - MEDLINE
KW - Adolescent
KW - Bone Marrow Transplantation/ae [Adverse Effects]
KW - Catheter-Related Infections/ep [Epidemiology]
KW - Catheter-Related Infections/mi [Microbiology]
KW - Catheter-Related Infections/pc [Prevention & Control]
KW - Catheterization
KW - Central Venous/ae [Adverse Effects]
KW - Catheters
KW - Indwelling/ae [Adverse Effects]
KW - Indwelling/mi [Microbiology]
KW - Child
KW - Preschool
KW - Cohort Studies
KW - Cross Infection/ep [Epidemiology]
KW - Cross Infection/mi [Microbiology]
KW - Cross Infection/pc [Prevention & Control]
KW - Female
KW - Georgia
KW - Gram-Negative Bacterial Infections/ep [Epidemiology]
KW - Gram-Negative Bacterial Infections/mi [Microbiology]
KW - Gram-Negative Bacterial Infections/pc [Prevention & Control]

KW - Hospitals
KW - Pediatric/sn [Statistics & Numerical Data]
KW - Humans
KW - Infant
KW - Infection Control/mt [Methods]
KW - Male
KW - Outpatient Clinics
KW - Hospital
KW - Outpatients/sn [Statistics & Numerical Data]
KW - Risk Factors
RP - NOT IN FILE
SP - 522
EP - 527
JF - Infection Control & Hospital Epidemiology
JA - Infect Control Hosp Epidemiol
VL - 31
IS - 5
CY - United States
N2 - OBJECTIVE: To identify risk factors for polymicrobial bloodstream infections (BSIs) in pediatric bone marrow transplant (BMT) outpatients attending a newly constructed clinic affiliated with a children's hospital. METHODS: All 30 outpatients treated at a new BMT clinic during September 10-21, 2007, were enrolled in a cohort study. The investigation included interviews, medical records review, observations, and bacterial culture and molecular typing of patient and environmental isolates. Data were analyzed using exact conditional logistic regression. RESULTS: Thirteen patients experienced BSIs caused by 16 different, predominantly gram-negative organisms. Presence of a tunneled catheter (odds ratio [OR], 19.9 [95% confidence interval {CI}, 2.4-infinity), catheter access (OR, 13.7 [95% CI, 1.8-infinity]), and flushing of a catheter with predrawn saline (OR, 12.9 [95% CI, 1.0-766.0]) were independently associated with BSI. The odds of experiencing a BSI increased by a factor of 16.8 with each additional injection of predrawn saline (95% CI, 1.8-827.0). Although no environmental source of pathogens was identified, interviews revealed breaches in recommended infection prevention practice and medication handling. Saline flush solutions were predrawn, and multiple doses were obtained from single-dose preservative-free vials to avoid delays in patient care. CONCLUSION: We speculate that infection prevention challenges in the new clinic, combined with successive needle punctures of vials, facilitated extrinsic contamination and transmission of healthcare-associated pathogens. We recommend that preservative-free single-use vials not be punctured more than once. Use of single-use prefilled saline syringes might prevent multiuse of single-use saline vials. Storage of saline outside a medication supply system might be advisable. Before opening new clinic facilities, hospitals should consider conducting a mock patient flow exercise to identify infection control challenges
SN - 1559-6834
AD - Epidemic Intelligence Service, Centers for Disease Control and Prevention, Atlanta, Georgia, USA. petra.wiersma@gmail.com
UR - 20350149
ER -

TY - JOUR
ID - 794
T1 - Outdoor air pollution and children's health
A1 - Suwanwaiphatthana,W.
A1 - Ruangdej,K.
A1 - Turner-Henson,A.
Y1 - 2010/01//
N1 - Suwanwaiphatthana, Wiparat. Ruangdej, Kannika. Turner-Henson, Anne
Pediatric nursing
oun, 7505804
N
Journal Article
English
KW - MEDLINE

KW - Air Pollutants/ae [Adverse Effects]
KW - Air Pollution/ae [Adverse Effects]
KW - Air Pollution/pc [Prevention & Control]
KW - Air Pollution/sn [Statistics & Numerical Data]
KW - Carbon Monoxide/ae [Adverse Effects]
KW - Child
KW - Child Welfare/sn [Statistics & Numerical Data]
KW - Child Welfare
KW - Federal Government
KW - Government Regulation
KW - Humans
KW - Inhalation Exposure/ae [Adverse Effects]
KW - Inhalation Exposure/pc [Prevention & Control]
KW - Inhalation Exposure/sn [Statistics & Numerical Data]
KW - Lead/ae [Adverse Effects]
KW - Nitrogen Dioxide/ae [Adverse Effects]
KW - Nurse's Role
KW - Ozone/ae [Adverse Effects]
KW - Pediatric Nursing
KW - Respiratory Rate
KW - Risk Factors
KW - Sulfur Dioxide/ae [Adverse Effects]
KW - United States
KW - United States Environmental Protection Agency
RP - NOT IN FILE
SP - 25
EP - 32
JF - Pediatric Nursing
JA - Pediatr Nurs
VL - 36
IS - 1
CY - United States
N2 - Children spend almost 90% of their time indoors, though outside air can be a significant source of potential and actual exposure to outdoor air pollutants. Children are vulnerable to pollutants and toxins because of their size and developing organ systems. Young children have increased respiratory rates and inhale more toxins, and young children often ignore respiratory symptoms and continue play. Outdoor play and recreational activities expose children to outdoor air pollution from sources such as automobiles, power plants, industry, and other combustion sources, which can impact children. Outdoor air pollution has been linked to respiratory illness exacerbations, infant mortality, the development of asthma, and atopy and reduction in lung development in children. This article will examine outdoor air pollution and its impact on children's health, as well as implications for pediatric nursing clinical practice
SN - 0097-9805
AD - Boromarajonani College of Nursing, Nakhonratchasima Praboromarajchanok Institute of Health Workforce Development, Ministry of Public Health, Thailand
UR - 20361442
ER -

TY - JOUR
ID - 795
T1 - Camp jump start: effects of a residential summer weight-loss camp for older children and adolescents
A1 - Huelsing,J.
A1 - Kanafani,N.
A1 - Mao,J.
A1 - White,N.H.
Y1 - 2010/04//
N1 - Huelsing, Jean. Kanafani, Nadim. Mao, Jingnan. White, Neil H
Pediatrics

oxv, 0376422
AIM, IM
Comparative Study. Journal Article
English
KW - MEDLINE
KW - Adolescent
KW - Age Factors
KW - Blood Pressure/ph [Physiology]
KW - Camping/px [Psychology]
KW - Camping
KW - Child
KW - Cohort Studies
KW - Female
KW - Humans
KW - Male
KW - Obesity/px [Psychology]
KW - Obesity/th [Therapy]
KW - Overweight/px [Psychology]
KW - Overweight/th [Therapy]
KW - Physical Fitness/ph [Physiology]
KW - Physical Fitness/px [Psychology]
KW - Seasons
KW - Time Factors
KW - Weight Loss/ph [Physiology]
KW - Weight Loss
RP - NOT IN FILE
SP - e884
EP - e890
JF - Pediatrics
VL - 125
IS - 4
CY - United States
N2 - OBJECTIVE: Residential weight-loss camps offer an opportunity for overweight and obese children to lose weight in a medically safe, supervised, supportive environment. The purpose of this report is to describe short-term outcomes in 76 children participating in a 4- or 8-week residential weight-loss camp for children and adolescents. PATIENTS AND METHODS: The camp program enrolled obese 10- to 18-year-old adolescents. The program consisted of structured and nonstructured physical activities and group educational sessions covering nutrition, physical fitness, and self-esteem. A diet plan of 3 balanced meals and 2 snacks per day was prepared under the supervision of a registered dietitian. Participants had height, weight, and blood pressure measured and performed a 1-mile run at maximum effort on an outdoor track. RESULTS: For all campers, statistically significant ($P < .0001$) reductions were observed for BMI, BMI z score, systolic blood pressure, body weight, and 1-mile run times. Compared with campers in the 4-week session, campers in the 8-week session had greater reductions in BMI, BMI z score, body weight, and systolic blood pressure. Multivariate analysis revealed that gender was a significant predictor for reduction in body weight, BMI, and BMI z score, all of which decreased more in boys than in girls. CONCLUSIONS: This report adds to the evidence that residential weight-loss camps are highly effective in improving measures of health and fitness among overweight and obese children and adolescents. Additional study is needed on the long-term effects of such camps in terms of weight maintenance, behavior change, and metabolic and health outcomes
SN - 1098-4275
AD - Camp Jump Start, Living Well Foundation, Imperial, Missouri, USA
UR - 20194273
ER -

TY - JOUR
ID - 796
T1 - "Pedogeriatrics": a pediatric nephrologist's outlook on common challenges facing pediatric and geriatric nephrologists

A1 - Grunberg,J.
Y1 - 2010/03//
N1 - Grunberg, Jose
International urology and nephrology
guq, 0262521
IM
Editorial
English
KW - MEDLINE
KW - Aged
KW - Child
KW - Geriatrics
KW - Humans
KW - Nephrology
KW - Pediatrics
RP - NOT IN FILE
SP - 253
EP - 257
JF - International Urology & Nephrology
JA - Int Urol Nephrol
VL - 42
IS - 1
CY - Netherlands
N2 - Dramatic demographic changes longevity and medical progress helped create a new population made up of the survivors of previously fatal diseases. These trends pose new major social and economic challenges that should be accounted for in health policy making. This paper discusses the similarities between the specialties of pediatrics and geriatrics, especially in the realm of patient care. Children and the elderly share a limited autonomy and dependence on the human environment (i.e., willing and able caregiving persons) due to age or disease. The long-term care of dependent patients (DP) requires caregiving persons who share with dependent persons the risk of losing autonomy, facing burnout, family disruption, and interference with work and educational activities. Families with DPs may face potential losses of income because both patients and caregivers are partially or completely unable to work, the former for medical reasons and the latter due to the new demands on their time and energy. Additionally, new expenses have to be met because while direct medical expenses might be covered by insurance or the State, other expenses have to be financed by the family, such as co-payments for medicines, new water or electricity home installations, and transport and eventual hotel costs if they have to stay overnight near a hospital outside of their town. The main objectives of long-term care should be to maximize patients' independence and prevent their physical and psychological deterioration while minimizing the social, economic and personal costs to caregivers. To achieve these goals, one needs a holistic approach, a multidisciplinary professional team (doctors, nurses, social workers, nutritionists and psychologists) and auxiliary staff (secretaries, electricians, administrators, messengers, cleaning staff, doormen, nursing aids and coordinators of medical appointments and medical procedures). Optimal management of DPs on chronic treatments such as chronic dialysis requires adequate communication skills, respectful attitudes toward patients and caregivers and effective use of communication and information technologies. Auxiliary personnel require specific training to contribute effectively to the DP attention processes. This paper postulates that pediatric and geriatric teams and their patients would benefit from closer training and sharing of experiences and systems
SN - 1573-2584
UR - 19517264
ER -

TY - JOUR
ID - 797
T1 - Immunohistochemical study of the expression of matrix metalloproteinase-9 in skin lesions of mycosis fungoides
A1 - Rasheed,H.
A1 - Tolba Fawzi,M.M.
A1 - bdel-Halim,M.R.
A1 - Eissa,A.M.

A1 - Mohammed,Salem N.
A1 - Mahfouz,S.
Y1 - 2010/04//
N1 - Rasheed, Hoda. Tolba Fawzi, Marwa Mohsen. Abdel-Halim, Mona R E. Eissa, Amal M. Mohammed Salem, Nahla. Mahfouz, Soheir
The American Journal of dermatopathology
35v, 7911005
IM
Journal Article
English
KW - MEDLINE
KW - Adolescent
KW - Adult
KW - Aged
KW - Case-Control Studies
KW - Child
KW - Preschool
KW - Disease Progression
KW - Female
KW - Humans
KW - Male
KW - Matrix Metalloproteinase 9/me [Metabolism]
KW - Middle Aged
KW - Mycosis Fungoides/me [Metabolism]
KW - Mycosis Fungoides/pa [Pathology]
KW - Severity of Illness Index
KW - Skin/me [Metabolism]
KW - Skin/pa [Pathology]
KW - Skin Neoplasms/me [Metabolism]
KW - Skin Neoplasms/pa [Pathology]
KW - Young Adult
RP - NOT IN FILE
SP - 162
EP - 169
JF - American Journal of Dermatopathology
JA - Am J Dermatopathol
VL - 32
IS - 2
CY - United States
N2 - Matrix metalloproteinase-9 (MMP-9) has been correlated with poor clinical outcome in various malignancies and is associated with enhanced tumor growth and dissemination through its role in angiogenesis. This study was carried out to review the immunohistological staining of MMP-9 in skin lesions of different stages of mycosis fungoides (MF). The study was carried on 22 patients with MF and 10 healthy controls. Immunohistochemical staining using MMP-9 monoclonal anti-human antibodies was performed to determine the intensity of expression and distribution pattern of MMP-9 in MF lesions and in normal control skin. The general intensity of expression of MMP-9 was found to be significantly higher in cases with MF than in controls, and it increased in direct proportion to the increase in disease severity, being greatest in the tumor stages. A significantly greater number of blood vessels were found in cases with MF when compared with controls, and the MMP-9 expression by endothelial cells was significantly higher in endothelial cells within tumor cell aggregates than in endothelial cells outside the tumor cell aggregates. This study raises the possibility that MMP-9 may play an important role in the development of MF lesions, and its significantly higher expression in tumor stages may point to a possible role in disease progression. Further studies are needed to validate these findings and to assess the possible therapeutic role of drugs targeting MMP-9 in the treatment of MF
SN - 1533-0311
AD - Department of Dermatology, Cairo University, Egypt
UR - 20051814

ER -

TY - JOUR
ID - 798
T1 - Travel and implications for the elimination of trachoma in ethiopia
A1 - Shah,N.A.
A1 - House,J.
A1 - Lakew,T.
A1 - Alemayehu,W.
A1 - Halfpenny,C.
A1 - Hong,K.C.
A1 - Keenan,J.D.
A1 - Porco,T.C.
A1 - Whitcher,J.P.
A1 - Lietman,T.M.
A1 - Gaynor,B.D.
Y1 - 2010/03//
N1 - Shah, Neelima A. House, Jenafir. Lakew, Takele. Alemayehu, Wondu. Halfpenny, Colleen. Hong, Kevin C. Keenan, Jeremy D. Porco, Travis C. Whitcher, John P. Lietman, Thomas M. Gaynor, Bruce D
Ophthalmic epidemiology
cg6, 9435674
IM
Journal Article. Research Support, N.I.H., Extramural
English
KW - MEDLINE
KW - Adolescent
KW - Adult
KW - Aged
KW - 80 and over
KW - Anti-Bacterial Agents/tu [Therapeutic Use]
KW - Azithromycin/tu [Therapeutic Use]
KW - Child
KW - Preschool
KW - Chlamydia trachomatis/ph [Physiology]
KW - Ethiopia/ep [Epidemiology]
KW - Female
KW - Humans
KW - Infant
KW - Male
KW - Middle Aged
KW - Questionnaires
KW - Rural Population/sn [Statistics & Numerical Data]
KW - Trachoma/dt [Drug Therapy]
KW - Trachoma/pc [Prevention & Control]
KW - Trachoma/tm [Transmission]
KW - Travel/sn [Statistics & Numerical Data]
KW - Young Adult
RP - NOT IN FILE
SP - 113
EP - 117
JF - Ophthalmic Epidemiology
JA - Ophthalmic Epidemiol
VL - 17
IS - 2
CY - England
N2 - PURPOSE: Trachoma is the leading infectious cause of blindness. The World Health Organization has set a goal of reducing the trachoma disease burden to a level where it is no longer a public health concern by the

year 2020. Some investigators feel that local elimination of ocular chlamydia infection is possible, but little has been done to study the likelihood of reintroduction of infection from neighboring areas. Mass administration of azithromycin has been shown to dramatically reduce the prevalence of infection in many villages in central Ethiopia. However, after treatment is discontinued, infection returns. Reintroduction of infection could occur from the few remaining infected cases in a treated community or from outside the community. People traveling between villages might be responsible thus complicating the elimination of trachoma. Methods: We conducted a survey to assess the travel pattern of the Gurage zone residents in Ethiopia. Seven hundred and seventeen households with at least one child aged 1-5 years in 48 villages were surveyed to collect the details of travel in 1 month prior to the survey. RESULTS: Seventy-eight percent of the surveyed households had at least one traveler, with the majority being women. Pre-school children, the main reservoir of clinically active infection, rarely traveled. Most travel was to the market or to school, and most for less than 1 day. CONCLUSIONS: Travel routinely takes place in these villages. Trachoma control programs in this area might consider treating areas with the same markets and schools in the same period to increase the efficacy of mass treatment
SN - 1744-5086
AD - Department of Public Health Sciences, University of California, Davis, California, USA.
bruce.gaynor@ucsf.edu
UR - 20302432
ER -

TY - JOUR
ID - 799
T1 - Chloroform anesthesia and the Saville Kent murder in 1860
A1 - Hutcheon,D.E.
Y1 - 2010/03//
N1 - Hutcheon, Duncan E
American journal of therapeutics
db7, 9441347
IM
Historical Article. Journal Article
English
KW - MEDLINE
KW - Anesthesia
KW - Inhalation/ae [Adverse Effects]
KW - Inhalation/hi [History]
KW - Child
KW - Preschool
KW - Chloroform/ae [Adverse Effects]
KW - Chloroform/hi [History]
KW - England
KW - History
KW - 19th Century
KW - Homicide/hi [History]
KW - Humans
KW - Male
KW - Substance-Related Disorders/ep [Epidemiology]
RP - NOT IN FILE
SP - 226
EP - 231
JF - American Journal of Therapeutics
JA - Am J Ther
VL - 17
IS - 2
CY - United States
N2 - The discovery of the anesthetic properties of chloroform by Sir James Simpson is one of the therapeutic triumphs of the 19th century. Queen Victoria requested chloroform anesthesia for the birth of her second son, Prince Leopold, and from then until the end of century chloroform was the most popular general anesthetic for obstetrics and surgery. Chloroform was so pleasant to inhale that it became a drug of abuse and was involved in

all sorts of criminal activity. Despite its wide usage, chloroform was not thought to be part of the brutal murder of 3-year-old Saville Kent on the night of June 30, 1860, outside his manor house in southwest England. The events surrounding and after the murder have recently been documented by Kate Summerscale in a book entitled, "The Suspicions of Mr. Whicher," the Detective Inspector assigned to the case by Scotland Yard. According to Ms Summerscale's records, Mr Whicher ignored the possibility that chloroform was involved in Saville's death. However, evidence supports the view that chloroform played a critical role in the crime and indicates that the guilty plea by Samuel Kent's daughter, Constance, for which she spent 20 years to the day in prison, was as inaccurate as it was incomplete
SN - 1536-3686
AD - Peabody Foundation, Chicago, IL
UR - 20305402
ER -

TY - JOUR
ID - 800
T1 - Tick-borne encephalitis in the age of general mobility.[Erratum appears in Wien Med Wochenschr. 2010 Jun;160(11-12):310 Note: Randolph, Sarah [removed]; Steffen, Robert [removed]]
A1 - Suss,J.
A1 - Kahl,O.
A1 - Aspock,H.
A1 - Hartelt,K.
A1 - Vaheri,A.
A1 - Oehme,R.
A1 - Hasle,G.
A1 - Dautel,H.
A1 - Kunz,C.
A1 - Kupreviciene,N.
A1 - Zimmermann,H.P.
A1 - Atkinson,B.
A1 - Dobler,G.
A1 - Kutsar,K.
A1 - Heinz,F.X.
Y1 - 2010/02//
N1 - Suss, Jochen. Kahl, Olaf. Aspock, Horst. Hartelt, Kathrin. Vaheri, Antii. Oehme, Rainer. Hasle, Gunnar. Dautel, Hans. Kunz, Christian. Kupreviciene, Nerija. Zimmermann, Hans-Peter. Atkinson, Barry. Dobler, Gerhard. Kutsar, Kuulo. Heinz, Franz X
Wiener medizinische Wochenschrift (1946)
xou, 8708475
IM
Comparative Study. Congresses
English
KW - MEDLINE
KW - Aged
KW - Child
KW - Cross-Cultural Comparison
KW - Cross-Sectional Studies
KW - Encephalitis Viruses
KW - Tick-Borne
KW - Encephalitis
KW - Tick-Borne/di [Diagnosis]
KW - Tick-Borne/ep [Epidemiology]
KW - Tick-Borne/pc [Prevention & Control]
KW - Tick-Borne/tm [Transmission]
KW - Endemic Diseases
KW - Europe
KW - Global Warming
KW - Humans

KW - Leisure Activities
KW - Population Dynamics
KW - Risk Factors
KW - Travel
KW - Viral Vaccines/ad [Administration & Dosage]
RP - NOT IN FILE
SP - 94
EP - 100
JF - Wiener Medizinische Wochenschrift
JA - Wien Med Wochenschr.
VL - 160
IS - 3-4
CY - Austria
N2 - The 11th meeting of the International Scientific Working Group on Tick-borne Encephalitis (ISW-TBE) was conducted under the title of, "From childhood to golden age: increased mobility - increased risk of contracting TBE?" Participants from 26 countries, including the United States of America and China, presented reports on the latest developments and trends in local TBE cases, vaccination coverage and risk factors. In particular, the situation of children and the elderly (the "golden agers") was discussed. As the current evidence suggests, the location and extension of endemic areas for TBE have changed over the last few years, along with global warming and the shift of infected ticks to higher altitudes. The increased mobility of the human population adds to the heightened exposure; outdoor activities and international travel are on the rise also, and especially, amongst the 50+ generation, who are already per se at higher risk of disease manifestation, complications and case fatality. Most Europeans travel within Europe, often without sufficient awareness of endemic areas. Only high immunization rates can ensure low disease rates in the long run. To achieve this goal, public education is the sole effective approach for raising the level of awareness. Overall, the risk of any given person to contract TBE should not be regarded as a fixed entity, but rather it must be estimated individually, on the basis of knowledge of the TBE virus endemic areas and risk factors
SN - 1563-258X
AD - National Reference Laboratory for Tick-borne diseases, Friedrich-Loeffler-Institute Jena, Jena, Germany. jochen.suess@fli.bund.de
UR - 20300927
ER -

TY - JOUR
ID - 801
T1 - Participation in activities outside of school hours in relation to problem behavior and social skills in middle childhood
A1 - Howie,L.D.
A1 - Lukacs,S.L.
A1 - Pastor,P.N.
A1 - Reuben,C.A.
A1 - Mendola,P.
Y1 - 2010/03//
N1 - Howie, Lajeana D. Lukacs, Susan L. Pastor, Patricia N. Reuben, Cynthia A. Mendola, Pauline
The Journal of school health
k13, 0376370
IM, N
Journal Article
English
KW - MEDLINE
KW - Child
KW - Child Behavior
KW - Data Collection
KW - Female
KW - Humans
KW - Leisure Activities
KW - Male

KW - Social Behavior
KW - Social Problems
KW - Sports
KW - United States
RP - NOT IN FILE
SP - 119
EP - 125
JF - Journal of School Health
JA - J Sch Health
VL - 80
IS - 3
CY - United States
N2 - BACKGROUND: Research has shown that participating in activities outside of school hours is associated with lower dropout rates, enhanced school performance, improved social skills, and reduced problem behaviors. However, most prior studies have been limited to small populations of older children (>12 years). This analysis focuses on children aged 6 to 11 to assess the potential association between participation in activities outside of school hours and behavior in middle childhood in a nationally representative survey. METHODS: Estimates were based on 25,797 children from the 2003-2004 National Survey of Children's Health. Outside of school activity was defined as participating in sports teams/lessons, clubs/organizations, or both at least once in the past year. Analysis of variance was used to evaluate the differences in behavior problems and social skills adjusting for sociodemographic factors, among children classified by participation in outside of school activities. RESULTS: Seventy-five percent of children participated in outside of school activities: 23% in sports, 16% in clubs, and 36% in both clubs and sports. Activity participation differed by gender, race/ethnicity, type of school, poverty status, family structure, household education, and school and community safety. Children participating in both sports and clubs had higher social skills index scores, but no significant difference in problem behavior scores compared with children who did not participate in any outside of school activity. CONCLUSION: Children participating in both sports and clubs had greater social competence during middle childhood compared with children who did not participate in any outside of school activities
SN - 1746-1561
AD - Centers for Disease Control and Prevention, National Center for Health Statistics, 3311 Toledo Road, RM 6110, Hyattsville, MD 20782, USA. LHOWIE@CDC.GOV
UR - 20236413
ER -

TY - JOUR
ID - 802
T1 - Role of individual and school factors in physical activity patterns of secondary-level Spanish students
A1 - Juan,F.R.
A1 - Bengoechea,E.G.
A1 - Montes,M.E.
A1 - Bush,P.L.
Y1 - 2010/02//
N1 - Juan, Francisco Ruiz. Bengoechea, Enrique Garcia. Montes, Maria Elena Garcia. Bush, Paula Louise
The Journal of school health
k13, 0376370
IM, N
Journal Article
English
KW - MEDLINE
KW - Adolescent
KW - Adolescent Behavior
KW - Body Mass Index
KW - Body Weight
KW - Child
KW - Confidence Intervals
KW - Exercise
KW - Female

KW - Health Surveys
KW - Humans
KW - Logistic Models
KW - Male
KW - Odds Ratio
KW - Physical Education and Training
KW - Questionnaires
KW - Schools/sn [Statistics & Numerical Data]
KW - Schools
KW - Self Concept
KW - Spain
KW - Students/px [Psychology]
KW - Students
RP - NOT IN FILE
SP - 88
EP - 95
JF - Journal of School Health
JA - J Sch Health
VL - 80
IS - 2
CY - United States
N2 - BACKGROUND: While the importance of individual and school factors as correlates of overall youth physical activity has been demonstrated by previous research, less is known about the relationship of these factors with specific patterns of physical activity during adolescence. Thus, the purpose of this study was to examine the association of selected individual and school factors with patterns of physical activity based on a sum index of physical activity in a population-based sample of Spanish adolescents. METHODS: One thousand and eighty-four students aged 12 to 17 years completed a self-report survey once during school hours. In addition to participation in physical activity outside of school hours, the following variables were included in the analysis: gender, age, weight status, physical self-perceptions, evaluation of the school physical education experience, and type of school (public vs private). Multinomial logistic regression was used to model the associations among the variables and to calculate odd ratios (ORs) and 95% confidence intervals (CIs) for each pattern of physical activity. RESULTS: The physical self-perceptions variable was the most consistent individual correlate of physical activity across participation patterns (ORs ranging from 4.29 to 1.88, CIs ranging from 2.16-8.54 to 1.10-3.21). Regarding the school variables included in this study, both were linked with participation in physical activity, but evaluation of the physical education experience showed the most consistent associations across activity patterns (ORs 2.49-2.17, CIs 1.49-4.15 to 1.25-3.74). CONCLUSION: Physical education programs may benefit adolescents with different physical activity participation preferences regardless of important individual characteristics and broader school factors
SN - 1746-1561
AD - Faculty of Sport Sciences, University of Murcia, C/Argentina s/n, 30720 San Javier (Murcia), Spain. fruizj@um.es
UR - 20236407
ER -

TY - JOUR
ID - 803
T1 - Plantar shear stress distribution in athletic individuals with frictional foot blisters
A1 - Yavuz,M.
A1 - Davis,B.L.
Y1 - 2010/03//
N1 - Yavuz, Metin. Davis, Brian L
Journal of the American Podiatric Medical Association
8501423, jpa
IM
Comparative Study. Journal Article. Research Support, N.I.H., Extramural
English
KW - MEDLINE

KW - Adolescent
KW - Adult
KW - Age Distribution
KW - Aged
KW - 80 and over
KW - Biomechanical Phenomena
KW - Blister/ep [Epidemiology]
KW - Blister/pp [Physiopathology]
KW - Case-Control Studies
KW - Child
KW - Female
KW - Foot Diseases/ep [Epidemiology]
KW - Foot Diseases/pp [Physiopathology]
KW - Friction
KW - Humans
KW - Incidence
KW - Male
KW - Middle Aged
KW - Probability
KW - Reference Values
KW - Risk Assessment
KW - Sports
KW - Stress
KW - Mechanical
KW - Weight-Bearing
KW - Young Adult
RP - NOT IN FILE
SP - 116
EP - 120
JF - Journal of the American Podiatric Medical Association
JA - J Am Podiatr.Med Assoc
VL - 100
IS - 2
CY - United States
N2 - BACKGROUND: Foot blisters are common and painful nuisances in competitive sports and in military service. The pathogenesis of the problem is related to excessive frictional forces experienced on or under the foot. The incidence of foot blisters in marathon runners can reach 39%. Similarly, up to 42% of cadets in Reserve Officers' Training Corps camps might be prone to foot blisters. Although the problem usually disappears within 5 days, a single blister might be a major problem in competitive sports or in a wilderness setting. Military training and combat effectiveness might also be compromised by foot blisters. This study sought to reveal the distribution of plantar shear forces in athletic individuals and its relevance to foot blisters. METHODS: Three groups of 11 participants each were studied: blister, adult control, and pediatric control. A custom-built shear and pressure platform was used to collect plantar pressure and shear data while the participants walked over the device. Data were analyzed with repeated-measures analysis of variance. RESULTS: The blister group had significantly increased pressure and shear stress magnitudes compared with the other groups, although no significant group-site interaction was found. The shear-time integral values were increased approximately 50% at specific sites of the athletic feet, suggesting that contact time may play a role in blister formation. CONCLUSIONS: The biomechanical interaction on the plantar surface of a blister-prone person is different from that of individuals who are less prone to the problem
SN - 1930-8264
AD - Department of Biomedical Engineering and Orthopedic Research Center (ND20), Lerner Research Institute, Cleveland Clinic, Cleveland, OH 44195, USA
UR - 20237363
ER -

TY - JOUR
ID - 804

T1 - The characteristics of the outdoor school environment associated with physical activity
A1 - Haug,E.
A1 - Torsheim,T.
A1 - Sallis,J.F.
A1 - Samdal,O.
Y1 - 2010/04//
N1 - Haug, Ellen. Torsheim, Torbjorn. Sallis, James F. Samdal, Oddrun
Health education research
bqp, 8608459
T
Journal Article. Research Support, Non-U.S. Gov't
English
KW - MEDLINE
KW - Adolescent
KW - Child
KW - Environment Design
KW - Exercise
KW - Female
KW - Humans
KW - Male
KW - Norway
KW - Play and Playthings
KW - Questionnaires
KW - Schools
RP - NOT IN FILE
SP - 248
EP - 256
JF - Health Education Research
JA - Health Educ Res
VL - 25
IS - 2
CY - England
N2 - The school is an important setting for physical activity. The purpose of the present study was to examine the association between physical environmental characteristics and participation in daily physical activity during school breaks. Data from 130 schools and 16 471 students (Grades 4-10) in Norway were obtained in 2004 through self-administered questionnaires to principals and students. Multilevel logistic regression models revealed that boys at secondary level with a larger number of outdoor facilities at school had 2.69 times [95% confidence interval (CI) = 1.21-5.98] and girls 2.90 times (95% CI = 1.32-6.37) higher odds of being physically active compared with students in schools with fewer facilities. Boys at secondary level with areas for hopscotch/skipping rope had 2.53 times (95% CI = 1.55-4.13), with a soccer field 1.68 times (95% CI = 1.15-2.45), with playground equipment 1.66 times (95% CI = 1.16-2.37) and with a sledding hill 1.70 times (95% CI = 1.23-2.35) higher odds to be physically active compared with students in schools without these facilities. A sledding hill was also associated with girls' physical activity participation in secondary school (odds ratio = 1.58, 95% CI = 1.11-2.24). Outdoor facilities in secondary schools are associated with students' daily physical activity participation during school breaks. Therefore, improving the outdoor environment should be considered in physical activity promotion school programs in secondary schools
SN - 1465-3648
AD - Faculty of Psychology, Research Centre for Health Promotion, University of Bergen, Christiesgate 13, N-5020 Bergen, Norway. ellen.haug@psyhp.uib.no
UR - 18936270
ER -

TY - JOUR
ID - 805
T1 - Reliability and validity of the System for Observing Children's Activity and Relationships during Play (SOCARP)
A1 - Ridgers,N.D.

A1 - Stratton,G.
A1 - McKenzie,T.L.
Y1 - 2010/01//
N1 - Ridgers, Nicola D. Stratton, Gareth. McKenzie, Thomas L
Journal of physical activity & health
101189457
IM
Journal Article. Research Support, Non-U.S. Gov't. Validation Studies
English
KW - MEDLINE
KW - Acceleration
KW - Analysis of Variance
KW - Child
KW - Child Welfare
KW - Energy Metabolism
KW - Female
KW - Group Structure
KW - Humans
KW - Interpersonal Relations
KW - Male
KW - Motor Activity
KW - Movement
KW - Play and Playthings
KW - Reproducibility of Results
KW - Statistics as Topic
KW - Videotape Recording
RP - NOT IN FILE
SP - 17
EP - 25
JF - Journal of Physical Activity & Health
JA - J Phys Act Health
VL - 7
IS - 1
CY - United States
N2 - BACKGROUND: Children frequently engage in diverse activities that are broadly defined as play, but little research has documented children's activity levels during play and how they are influenced by social contexts. Assessing potentially modifiable conditions that influence play behavior is needed to design optimal physical activity interventions. METHODS: System for Observing Children's Activity and Relationships during Play (SOCARP) was developed to simultaneously assess children's physical activity, social group sizes, activity type, and social behavior during play. One hundred and fourteen children (48 boys, 66 girls; 42% overweight) from 8 elementary schools were observed during recess over 24 days, with 12 days videotaped for reliability purposes. Ninety-nine children wore a uni-axial accelerometer during their observation period. RESULTS: Estimated energy expenditure rates from SOCARP observations and mean accelerometer counts were significantly correlated ($r = .67$; $P < .01$), and interobserver reliabilities (ie, percentage agreement) for activity level (89%), group size (88%), activity type (90%) and interactions (88%) met acceptable criteria. Both physical activity and social interactions were influenced by group size, activity type, and child gender and body weight status. CONCLUSIONS: SOCARP is a valid and reliable observation system for assessing physical activity and play behavior in a recess context
SN - 1543-3080
AD - Research Institute for Sport and Exercise Sciences, Liverpool John Moores University, Liverpool, UK
UR - 20231751
ER -

TY - JOUR
ID - 806
T1 - Physical activity levels of children living in different built environments. [Review] [33 refs]
A1 - Sandercock,G.

A1 - Angus,C.
A1 - Barton,J.
Y1 - 2010/04//
N1 - Sandercock, Gavin. Angus, Caroline. Barton, Joanna
Preventive medicine
pm4, 0322116
IM
Journal Article. Review
English
KW - MEDLINE
KW - Adolescent
KW - Child
KW - Preschool
KW - Developed Countries
KW - Environment Design
KW - Exercise
KW - Health Behavior
KW - Health Promotion
KW - Health Status
KW - Humans
KW - Motor Activity
KW - Rural Population
KW - Urban Population
RP - NOT IN FILE
SP - 193
EP - 198
JF - Preventive Medicine
JA - Prev Med
VL - 50
IS - 4
CY - United States
N2 - OBJECTIVE: To review the available literature assessing differences in physical activity levels of children living in different built environments classified according to land use within developed countries. METHODS: A systematic review of published literature up to March 2009. Online searches of five databases yielded 18 studies which met inclusion criteria. Studies provided data on n=129446, 5-18 years old (n=117544 from the United States). RESULTS: From 13 assessments of differences in physical activity between rural and urban children one showed that rural children were significantly more active than urban children. In studies where the built environment was sub-divided further, suburban and small town children showed the highest levels of physical activity, followed by rural, then urban children. Differences in types of physical activity undertaken were evident, showing that rural children spent more time outdoors, involved in unstructured play compared with urban children. These findings were mainly restricted to children <13 years old. CONCLUSIONS: The literature does not show major differences in the physical activity levels between children from rural or urban areas. Where studied, the suburban built environment appears most conducive to promoting physical activity. Further research should use at least a trilateral division of the built environment and should also account for socioeconomic status, racial factors and seasonal effects. Copyright 2010 Elsevier Inc. All rights reserved. [References: 33]
SN - 1096-0260
AD - Centre for Sports and Exercise Sciences, Department of Biological Sciences, University of Essex, Wivenhoe Park, Colchester, UK, CO43SQ. gavins@essex.ac.uk
UR - 20083131
ER -

TY - JOUR
ID - 807
T1 - Japanese encephalitis vaccines: recommendations of the Advisory Committee on Immunization Practices (ACIP)
A1 - Fischer,M.

A1 - Lindsey,N.
A1 - Staples,J.E.
A1 - Hills,S.
A1 - Centers for Disease Control and Prevention (CDC)
Y1 - 2010/03/12/
N1 - Fischer, Marc. Lindsey, Nicole. Staples, J Erin. Hills, Susan. Centers for Disease Control and Prevention (CDC)
MMWR. Recommendations and reports : Morbidity and mortality weekly report. Recommendations and reports / Centers for Disease Control
101124922
IM
Guideline. Journal Article
English
KW - MEDLINE
KW - Adolescent
KW - Adult
KW - Asia/ep [Epidemiology]
KW - Child
KW - Preschool
KW - Encephalitis Virus
KW - Japanese/cl [Classification]
KW - Japanese/ge [Genetics]
KW - Encephalitis
KW - Japanese/di [Diagnosis]
KW - Japanese/ep [Epidemiology]
KW - Japanese/pc [Prevention & Control]
KW - Japanese/tm [Transmission]
KW - Humans
KW - Infant
KW - Japanese Encephalitis Vaccines/ad [Administration & Dosage]
KW - Japanese Encephalitis Vaccines/ae [Adverse Effects]
KW - Japanese Encephalitis Vaccines/im [Immunology]
KW - Japanese Encephalitis Vaccines/sd [Supply & Distribution]
KW - Medical Laboratory Personnel
KW - Risk
KW - Seasons
KW - Travel
RP - NOT IN FILE
SP - 1
EP - 27
JF - Morbidity & Mortality Weekly Report
JA - Morb Mortal Wkly Rep Surveill.Summm.
VL - Recommendations & Reports. 59
IS - RR-1
CY - United States
N2 - This report updates the 1993 recommendations by CDC's Advisory Committee on Immunization Practices (ACIP) regarding the prevention of Japanese encephalitis (JE) among travelers (CDC. Inactivated Japanese encephalitis virus vaccine: recommendations of the Advisory Committee on Immunization Practices [ACIP]. MMWR 1993;42[No. RR-1]). This report summarizes the epidemiology of JE, describes the two JE vaccines that are licensed in the United States, and provides recommendations for their use among travelers and laboratory workers. JE virus (JEV), a mosquito-borne flavivirus, is the most common vaccine-preventable cause of encephalitis in Asia. JE occurs throughout most of Asia and parts of the western Pacific. Among an estimated 35,000-50,000 annual cases, 20%-30% of patients die, and 30%-50% of survivors have neurologic or psychiatric sequelae. No treatment exists. For most travelers to Asia, the risk for JE is very low but varies on the basis of destination, duration, season, and activities. JE vaccine is recommended for travelers who plan to spend a month or longer in endemic areas during the JEV transmission season and for laboratory workers with a potential for exposure to infectious JEV. JE vaccine should be considered for 1) short-term (<1 month) travelers

to endemic areas during the JEV transmission season if they plan to travel outside of an urban area and will have an increased risk for JEV exposure; 2) travelers to an area with an ongoing JE outbreak; and 3) travelers to endemic areas who are uncertain of specific destinations, activities, or duration of travel. JE vaccine is not recommended for short-term travelers whose visit will be restricted to urban areas or times outside of a well-defined JEV transmission season. Two JE vaccines are licensed in the United States. An inactivated mouse brain--derived JE vaccine (JE-VAX [JE-MB]) has been licensed since 1992 to prevent JE in persons aged >or=1 year traveling to JE-endemic countries. Supplies of this vaccine are limited because production has ceased. In March 2009, an inactivated Vero cell culture-derived vaccine (IXIARO [JE-VC]) was licensed for use in persons aged >or=17 years. JE-MB is the only JE vaccine available for use in children aged 1-16 years, and remaining supplies will be reserved for use in this group
SN - 1545-8601
AD - Division of Vector-Borne Infectious Diseases, National Center for Emerging and Zoonotic Infectious Diseases, CDC, Fort Collins, CO 80521, USA. mfischer@cdc.gov
UR - 20224546
ER -

TY - JOUR
ID - 808
T1 - Efficacy and tolerability of EPs 7630 in children and adolescents with acute bronchitis - a randomized, double-blind, placebo-controlled multicenter trial with a herbal drug preparation from Pelargonium sidoides roots
A1 - Kamin,W.
A1 - Maydannik,V.
A1 - Malek,F.A.
A1 - Kieser,M.
Y1 - 2010/03//
N1 - Kamin, W. Maydannik, V. Malek, F A. Kieser, M
International journal of clinical pharmacology and therapeutics
b0d, 9423309
IM
Journal Article. Multicenter Study. Randomized Controlled Trial. Research Support, Non-U.S. Gov't
English
KW - MEDLINE
KW - Acute Disease
KW - Adolescent
KW - Bronchitis/dt [Drug Therapy]
KW - Child
KW - Preschool
KW - Double-Blind Method
KW - Female
KW - Humans
KW - Infant
KW - Male
KW - Patient Satisfaction
KW - Pelargonium/ch [Chemistry]
KW - Plant Extracts/ae [Adverse Effects]
KW - Plant Extracts/tu [Therapeutic Use]
KW - Plant Roots
KW - Treatment Outcome
RP - NOT IN FILE
SP - 184
EP - 191
JF - International Journal of Clinical Pharmacology & Therapeutics
JA - Int J Clin Pharmacol Ther
VL - 48
IS - 3
CY - Germany

N2 - OBJECTIVE: The study aim was to demonstrate the efficacy and to investigate the tolerability of EPs 7630, a herbal drug preparation from Pelargonium sidoides roots, in the treatment of patients (1 - 18 years) with acute bronchitis outside the strict indication for antibiotics. MATERIALS AND METHODS: A total of 200 patients were randomized to receive either active drug containing EPs 7630 (1 - 6 years: 3 x 10 drops/d; > 6 - 12 years: 3 x 20 drops/d; > 12 - 18 years: 3 x 30 drops/d) or placebo for 7 consecutive days. Primary outcome measure: change in the total score of bronchitis-specific symptoms (BSS) from Day 0 to Day 7. Main secondary outcome measures: treatment outcome, patients' satisfaction with treatment, onset of effect, bed rest. RESULTS: From baseline to Day 7, the mean BSS score improved significantly more for EPs 7630 compared with placebo (3.4 +/- 1.8 vs. 1.2 +/- 1.8 points, p < 0.0001). On Day 7, treatment outcome was significantly better (p < 0.0001), satisfaction with treatment more pronounced (77.6% vs. 25.8%, p < 0.0001), onset of effect faster, and time of bed rest shorter as compared with placebo. Tolerability was similarly good in both groups. All adverse events were assessed as non-serious. CONCLUSION: EPs 7630 was shown to be efficacious and safe in the treatment of acute bronchitis in children and adolescents outside the strict indication for antibiotics with patients treated with EPs 7630 perceiving a more favorable course of the disease and a good tolerability as compared with placebo
SN - 0946-1965
AD - University Hospital Pediatrics, Mainz, Germany. wkamin@evkhamm.de
UR - 20197012
ER -

TY - JOUR
ID - 809
T1 - Activation-induced cytidine deaminase expression in diffuse large B-cell lymphoma with a paracortical growth pattern: a lymphoma of possible interfollicular large B-cell origin
A1 - Aguilera,N.S.
A1 - Auerbach,A.
A1 - Barekman,C.L.
A1 - Lichy,J.
A1 - Abbondanzo,S.L.
Y1 - 2010/03//
N1 - Aguilera, Nadine S. Auerbach, Aaron. Barekman, Carol L. Lichy, Jack. Abbondanzo, Susan L
Archives of pathology & laboratory medicine
79z, 7607091
AIM, IM
Journal Article
English
KW - MEDLINE
KW - Adolescent
KW - Adult
KW - Antigens
KW - CD20/me [Metabolism]
KW - Child
KW - Clone Cells
KW - Cytidine Deaminase/bi [Biosynthesis]
KW - Cytidine Deaminase/ge [Genetics]
KW - Enzyme Activation
KW - Female
KW - Gene Expression Regulation
KW - Enzymologic
KW - Humans
KW - Lymph Nodes/pa [Pathology]
KW - Lymphoma
KW - Follicular/en [Enzymology]
KW - Follicular/ge [Genetics]
KW - Follicular/pa [Pathology]
KW - Large B-Cell
KW - Diffuse/en [Enzymology]

KW - Diffuse/ge [Genetics]
KW - Diffuse/pa [Pathology]
KW - Male
KW - Middle Aged
KW - Tumor Markers
KW - Biological/me [Metabolism]
KW - Young Adult
RP - NOT IN FILE
SP - 449
EP - 456
JF - Archives of Pathology & Laboratory Medicine
JA - Arch Pathol Lab Med
VL - 134
IS - 3
CY - United States
N2 - CONTEXT: Activation-induced cytidine deaminase, necessary for immunoglobulin somatic hypermutation and class switch recombination, is usually expressed within the follicular dendritic network but is also expressed in a population of interfollicular large B cells outside the germinal center. OBJECTIVE: To report 7 cases of diffuse large B-cell lymphoma with a distinct paracortical distribution. Expression of activation-induced cytidine deaminase, previously described in interfollicular large B cells, was evaluated. DESIGN: A panel of immunohistochemical markers, including double staining for activation-induced cytidine deaminase and CD20, was used to illustrate the cases. Molecular studies were performed by polymerase chain reaction in the paraffin-embedded tissue for t(14;18) chromosomal translocation and immunoglobulin heavy chain and T-cell receptor rearrangements. RESULTS: Patients included 3 males and 4 females ranging in age from 11 to 59 years (mean, 39 years). All specimens were lymph nodes (4 from the groin, 2 from the neck, and 1 from the axilla). Malignant lymphocytes were positive for CD20 and negative for CD5 and CD10. Staining for CD30, CD43, and BCL-2 was variable. The malignant cells showed at least focal staining with activation-induced cytidine deaminase. All cases were found to be monoclonal by immunoglobulin heavy-chain gene rearrangement or showed light-chain restriction. None of the tested cases showed t(14;18). CONCLUSIONS: Diffuse large B-cell lymphoma with a paracortical distribution is unusual and may be a distinct morphologic variant. More study is necessary to determine the stage of B-cell development and the cell of origin of these tumors. However, activation-induced cytidine deaminase expression suggests they may arise from a putative interfollicular large B cell
SN - 1543-2165
AD - Department of Hematopathology, Armed Forces Institute of Pathology, Washington, DC 20306-6000, USA. aguilera@afip.osd.mil
UR - 20196672
ER -

TY - JOUR
ID - 810
T1 - Increasing school playground physical activity: a mixed methods study combining environmental measures and children's perspectives
A1 - Willenberg,L.J.
A1 - Ashbolt,R.
A1 - Holland,D.
A1 - Gibbs,L.
A1 - MacDougall,C.
A1 - Garrard,J.
A1 - Green,J.B.
A1 - Waters,E.
Y1 - 2010/03//
N1 - Willenberg, Lisa J. Ashbolt, Rosie. Holland, Dionne. Gibbs, Lisa. MacDougall, Colin. Garrard, Jan. Green, Julie B. Waters, Elizabeth
Journal of science and medicine in sport / Sports Medicine Australia
c54, 9812598
IM

Journal Article. Research Support, Non-U.S. Gov't
English
KW - MEDLINE
KW - Child
KW - Environment Design
KW - Exercise
KW - Female
KW - Focus Groups
KW - Humans
KW - Male
KW - Play and Playthings
KW - Research Design
KW - Schools
KW - Victoria
RP - NOT IN FILE
SP - 210
EP - 216
JF - Journal of Science & Medicine in Sport
JA - J Sci Med Sport
VL - 13
IS - 2
CY - Australia
N2 - Schools are integral settings in which children can participate in moderate and vigorous physical activity. This study reports on the relationship between playground characteristics and child activity levels using a combination of quantitative and qualitative methods. The aim of the study is to increase understanding of how children respond to their school playground environments. Twenty-three primary schools located in a predominantly low socio-economic area of Melbourne, Australia were involved. SOPLAY (System for Observing Play and Leisure Activity in Youth) was administered in all 23 schools to measure the levels of student physical activity and playground environmental factors such as equipment provision. In 12 of the 23 schools, a sample of 9-11-year-old students participated in focus group discussions exploring perceptions of their school playgrounds using a photo ordering technique. A larger proportion of students participated in VPA when loose equipment (60% vs. 52%; p<0.001) and teacher supervision (62% vs. 52%; p<0.001) were present in activity settings, compared to when they were unavailable. Fixed play equipment and bitumen with court/play-line markings were seen by children as settings inviting active play but only had the greatest impact on moderate activity (fixed play equipment: 35% vs. 20%, p<0.001; court markings/goals: 62% vs. 52%, p<0.001; play-line markings: 25% vs. 20%, p=0.04). The mixed method design provided a greater understanding of the potential influence of environmental characteristics on children's lunch time activity levels and their perceptions of play areas. The findings indicate that relatively simple changes such as the provision of loose equipment, painting of court and play-line markings, and increased teacher presence on the playground, are likely to provide opportunities for increased physical activity. Copyright 2009 Sports Medicine Australia. Published by Elsevier Ltd. All rights reserved
SN - 1878-1861
AD - The McCaughey Centre, VicHealth Centre for the Promotion of Mental Health and Community Wellbeing, Melbourne School of Population Health, University of Melbourne, Australia. lwillenberg@vichealth.vic.gov.au
UR - 19553158
ER -

TY - JOUR
ID - 811
T1 - Geographic distribution of autism in California: a retrospective birth cohort analysis
A1 - Van Meter,K.C.
A1 - Christiansen,L.E.
A1 - Delwiche,L.D.
A1 - Azari,R.
A1 - Carpenter,T.E.
A1 - Hertz-Picciotto,I.

Y1 - 2010/02//
N1 - Van Meter, Karla C. Christiansen, Lasse E. Delwiche, Lora D. Azari, Rahman. Carpenter, Tim E. Hertz-Picciotto, Irva
Autism research : official journal of the International Society for Autism Research
101461858
IM
Journal Article. Research Support, N.I.H., Extramural. Research Support, Non-U.S. Gov't. Research Support, U.S. Gov't, Non-P.H.S.. Twin Study
English
KW - MEDLINE
KW - Adolescent
KW - California
KW - Child
KW - Child Development Disorders
KW - Pervasive/ep [Epidemiology]
KW - Pervasive/et [Etiology]
KW - Pervasive/ge [Genetics]
KW - Preschool
KW - Cluster Analysis
KW - Cohort Studies
KW - Cross-Sectional Studies
KW - Demography
KW - Diseases in Twins/ep [Epidemiology]
KW - Diseases in Twins/et [Etiology]
KW - Diseases in Twins/ge [Genetics]
KW - Environmental Exposure/ae [Adverse Effects]
KW - Environmental Exposure/sn [Statistics & Numerical Data]
KW - Female
KW - Humans
KW - Infant
KW - Male
KW - Maternal Age
KW - Paternal Age
KW - Risk Factors
KW - Statistics as Topic
RP - NOT IN FILE
SP - 19
EP - 29
JF - Autism research : Official Journal of the International Society for Autism Research
JA - Autism Res
VL - 3
IS - 1
CY - United States
N2 - Prenatal environmental exposures are among the risk factors being explored for associations with autism. We applied a new procedure combining multiple scan cluster detection tests to identify geographically defined areas of increased autism incidence. This procedure can serve as a first hypothesis-generating step aimed at localized environmental exposures, but would not be useful for assessing widely distributed exposures, such as household products, nor for exposures from nonpoint sources, such as traffic. Geocoded mothers' residences on 2,453,717 California birth records, 1996-2000, were analyzed including 9,900 autism cases recorded in the California Department of Developmental Services (DDS) database through February 2006 which were matched to their corresponding birth records. We analyzed each of the 21 DDS Regional Center (RC) catchment areas separately because of the wide variation in diagnostic practices. Ten clusters of increased autism risk were identified in eight RC regions, and one Potential Cluster in each of two other RC regions.After determination of clusters, multiple mixed Poisson regression models were fit to assess differences in known demographic autism risk factors between the births within and outside areas of elevated autism incidence, independent of case status.Adjusted for other covariates, the majority of areas of autism clustering were characterized by high parental education, e.g. relative risks >4 for college-graduate vs. nonhigh-school graduate parents. This

geographic association possibly occurs because RCs do not actively conduct case finding and parents with lower education are, for various reasons, less likely to successfully seek services
SN - 1939-3806
AD - Department of Public Health Sciences, School of Medicine, and Medical Investigations of Neurodevelopmental Disorders Institute, University of California-Davis, One Shields Avenue, Davis, CA 95616, USA. vanmeter@pobox.com
UR - 20049980
ER -

TY - JOUR
ID - 812
T1 - Drug utilization and off-label drug use among Spanish emergency room paediatric patients
A1 - Morales-Carpi,C.
A1 - Estan,L.
A1 - Rubio,E.
A1 - Lurbe,E.
A1 - Morales-Olivas,F.J.
Y1 - 2010/03//
N1 - Morales-Carpi, Cristina. Estan, Luis. Rubio, Elena. Lurbe, Empar. Morales-Olivas, Francisco J
European journal of clinical pharmacology
en4, 1256165
IM
Journal Article
English
KW - MEDLINE
KW - Adolescent
KW - Age Factors
KW - Chi-Square Distribution
KW - Child
KW - Preschool
KW - Drug Utilization/sn [Statistics & Numerical Data]
KW - Drug Utilization Review
KW - Emergency Service
KW - Hospital/sn [Statistics & Numerical Data]
KW - Female
KW - Guideline Adherence
KW - Humans
KW - Infant
KW - Male
KW - Off-Label Use/sn [Statistics & Numerical Data]
KW - Pediatrics/sn [Statistics & Numerical Data]
KW - Physician's Practice Patterns/sn [Statistics & Numerical Data]
KW - Practice Guidelines as Topic
KW - Prospective Studies
KW - Spain
RP - NOT IN FILE
SP - 315
EP - 320
JF - European Journal of Clinical Pharmacology
JA - Eur J Clin Pharmacol
VL - 66
IS - 3
CY - Germany
N2 - OBJECTIVE: To describe the use of medicines and to determine the frequency of off-label use in emergency room paediatric patients. PATIENTS AND METHODS: A prospective, observational and descriptive study was carried out in the setting of the paediatric emergency room of a Spanish general hospital. Medicines used by children <14 years prior to their emergency room visit were analysed based on information

collected from parents/guardians and relatives for each drug prescription. Off-label use was defined as the utilization of a drug at an indication, dosage, frequency or route of administration that differed from the specifications in the Summary of Product Characteristics or by children outside the authorized age group. RESULTS: The patient cohort comprised 462 children, among whom 336 children had been prescribed 667 prescriptions. Of the medicines prescribed, 90% fell into only five 5 Anatomical Therapeutic Chemical Classification System groups. The most frequent active principles were ibuprofen and paracetamol. Of a total of 152 different formulations recorded, no paediatric information was provided for 40 formulations, and one formulation was contraindicated in children. Based on the established criteria, 338 prescriptions were off-label: no paediatric information or contraindication in children were available (82 prescriptions); the drug was used for an indication different from the authorized one (111 prescriptions); drug use was inconsistent with age recommendations (16 prescriptions); drug use was inconsistent with dose/frequency (129 prescriptions). Of the 152 formulations, 107 were occasionally used in an off-label manner. CONCLUSIONS: Although the mean number of drugs used in children is small, off-label use is frequent. Research efforts should target paediatric studies that allow a rational drug use in children
SN - 1432-1041
AD - CS Picassent, Agencia Valenciana de Salud, Valencia, Spain
UR - 19936729
ER -

TY - JOUR
ID - 813
T1 - Childhood injuries in rural north India
A1 - Mohan,D.
A1 - Kumar,A.
A1 - Varghese,M.
Y1 - 2010/03//
N1 - Mohan, Dinesh. Kumar, Adarsh. Varghese, Mathew
International journal of injury control and safety promotion
101247254
IM
Journal Article
English
KW - MEDLINE
KW - Adolescent
KW - Adult
KW - Aged
KW - Child
KW - Preschool
KW - Female
KW - Health Surveys
KW - Humans
KW - India/ep [Epidemiology]
KW - Infant
KW - Newborn
KW - Male
KW - Middle Aged
KW - Rural Population
KW - Wounds and Injuries/cl [Classification]
KW - Wounds and Injuries/ep [Epidemiology]
KW - Young Adult
RP - NOT IN FILE
SP - 45
EP - 52
JF - International Journal of Injury Control & Safety Promotion
JA - Int J Inj Contr Saf Promot
VL - 17
IS - 1

CY - England
N2 - This article reports the results of 100% household injury surveillance project conducted over a 1-year period in nine contiguous villages with a total population of 22,883 persons in north India. Fourteen trained field workers did the health and injury survey by visiting 16-20 households every day. In this article, we document the epidemiology of injuries among children in rural households. A person was considered injured if the injury prevented the victim from continuing a normal daily routine as understood by the family and the victim. A total of 2029 injury cases were recorded. Children in the age group 0-14 years accounted for 611 (30%) of all injury cases of which 42% were injured at home (28% for >14 years), 35% on roads (30% for >14 years), 8% on farms (31% for >14 years) and 6% on playgrounds. The maximum number of injuries was due to falls (35%). Eighty per cent of the injuries were minor (Abbreviated Injury Scale (AIS) 1), 18% were moderate or serious (AIS 2-3); none were severe (AIS 4) and one child had a critical injury (AIS 5). The injury rates per 100,000 children in different age groups were 5354, 6962 and 8060 for 0-4, 5-9 and 10-14 years per year
SN - 1745-7319
AD - Transportation Research and Injury Prevention Programme, Indian Institute of Technology Delhi, Hauz Khas, New Delhi, India. dmohan@cbme.iitd.ac.in
UR - 20182938
ER -

TY - JOUR
ID - 814
T1 - Composite playground safety measure to correlate the rate of supracondylar humerus fractures with safety: an ecologic study
A1 - Park,M.J.
A1 - Baldwin,K.
A1 - Weiss-Laxer,N.
A1 - Christian,J.B.
A1 - Mello,M.J.
A1 - Eberson,C.
A1 - Spiegel,D.A.
Y1 - 2010/03//
N1 - Park, Min Jung. Baldwin, Keith. Weiss-Laxer, Nomi. Christian, Jennifer B. Mello, Michael J. Eberson, Craig. Spiegel, David A
Journal of pediatric orthopedics
hsw, 8109053
IM
Journal Article
English
KW - MEDLINE
KW - Accidental Falls
KW - Child
KW - Preschool
KW - Consumer Product Safety
KW - Female
KW - Humans
KW - Humeral Fractures/ep [Epidemiology]
KW - Humeral Fractures/et [Etiology]
KW - Incidence
KW - Male
KW - Play and Playthings/in [Injuries]
KW - Residence Characteristics
KW - Rhode Island/ep [Epidemiology]
KW - Risk
KW - United States/ep [Epidemiology]
RP - NOT IN FILE
SP - 101
EP - 105
JF - Journal of Pediatric Orthopedics

JA - J Pediatr Orthop
VL - 30
IS - 2
CY - United States
N2 - BACKGROUND: More than 200,000 children are injured at playgrounds in the United States each year. Our goal was to introduce a composite measure of playground safety and use this instrument to correlate the incidence of supracondylar humerus fractures with playground safety in an ecologic study design. METHODS: We used a novel "overall-safety rating," defined as a composite of 3 previously validated instruments (National Program for Playground Safety School score, surface depth compliance, and the use zone compliance) to measure the overall safety of all playgrounds within a region. The regions were rated from most to least safe based on average playground safety as measured by this new method. The incidence of supracondylar fractures was calculated using Hasbro Children's Hospital Emergency Department data and state of Rhode Island Census data from 1998 to 2006. The incidence was then correlated with playground safety as defined by our composite measure. RESULTS: Compared with the neighborhood deemed the safest, the least safe district had 4.7 times greater odds of supracondylar humerus fracture. Overall composite safety score of the district was linearly correlated with the injury rate observed in the population at risk (R=0.98; P=0.04). CONCLUSIONS: Using our novel composite playground safety score, we found that the incidence of supracondylar humerus fractures was increased in districts with playgrounds with lower scores, suggesting that improvements in playground infrastructure may potentially reduce the incidence of supracondylar humerus fractures, and other injuries, in children. LEVEL OF EVIDENCE: Level IV
SN - 1539-2570
AD - Department of Orthopaedic Surgery, Hospital of the University of Pennsylvania, Philadelphia, PA 19104, USA. parkmj82@gmail.com
UR - 20179553
ER -

TY - JOUR
ID - 815
T1 - The elephant in the playground: confronting lead-contaminated soils as an important source of lead burdens to urban populations
A1 - Filippelli,G.M.
A1 - Laidlaw,M.A.
Y1 - 2010///
N1 - Filippelli, Gabriel M. Laidlaw, Mark A
Perspectives in biology and medicine
ozo, 0401132
IM
Journal Article
English
KW - MEDLINE
KW - Child
KW - Preschool
KW - Environmental Exposure/ae [Adverse Effects]
KW - Environmental Exposure/an [Analysis]
KW - Geography
KW - Health Policy
KW - Humans
KW - Lead/an [Analysis]
KW - Lead/bl [Blood]
KW - Lead Poisoning/bl [Blood]
KW - Lead Poisoning/et [Etiology]
KW - Play and Playthings
KW - Poverty
KW - Soil Pollutants/ae [Adverse Effects]
KW - Soil Pollutants/an [Analysis]
KW - United States
KW - Urban Population

RP - NOT IN FILE
SP - 31
EP - 45
JF - Perspectives in Biology & Medicine
JA - Perspect Biol Med
VL - 53
IS - 1
CY - United States
N2 - Although significant headway has been made over the past 50 years in understanding and reducing the sources and health risks of lead, the incidence of lead poisoning remains shockingly high in urban regions of the United States. At particular risk are poor people who inhabit the polluted centers of our older cities without the benefits of adequate nutrition, education, and access to health care. To provide a future with fewer environmental and health burdens related to lead, we need to consider the multiple pathways of lead exposure in children, including their continued contact with dust derived from inner-city soils. Recent research into the causes of seasonal variations in blood-lead levels among children has confirmed the importance of soil in lead exposure. "Capping" lead-contaminated soil with lead-free soil or soil amendment appears to be a simple and cost-effective way to reduce the lead load for urban youth
SN - 1529-8795
AD - Department of Earth Sciences, Indiana University-Purdue University Indianapolis (IUPUI), Indianapolis, IN 46205, USA. gfilippe@iupui.edu
UR - 20173294
ER -

TY - JOUR
ID - 816
T1 - Physical activity in young children: the role of child care
A1 - Ward,D.S.
Y1 - 2010/03//
N1 - Ward, Dianne S
Medicine and science in sports and exercise
8005433, mg8
IM, S
Introductory Journal Article
English
KW - MEDLINE
KW - Child
KW - Child Day Care Centers
KW - Child Development
KW - Exercise
KW - Female
KW - Guidelines as Topic
KW - Health Promotion
KW - Humans
KW - Male
RP - NOT IN FILE
SP - 499
EP - 501
JF - Medicine & Science in Sports & Exercise
JA - Med Sci Sports Exerc
VL - 42
IS - 3
CY - United States
N2 - Young children's regular participation in physical activity is important not only to prevent excess weight gain and avoid chronic health problems but also to promote optimal physical, social, and psychological development. Given the large number of children enrolled in some form of out-of-home child care, this setting holds great potential to make important contributions to the welfare and health of young children, including the promotion of physical activity. The five articles included in this series were originally presented as a symposium

at the 2008 American College of Sports Medicine Annual Meeting. Papers review the current evidence on levels of physical activity at child care, techniques for measuring physical activity in this setting, use of outdoor space for physical activity, the influence of child care environment and policies on children's activity, and child care-based physical activity intervention strategies
SN - 1530-0315
AD - Department of Nutrition and Center for Health Promotion and Disease Prevention, University of North Carolina at Chapel Hill, NC 27599-7426, USA. dsward@email.unc.edu
UR - 20068500
ER -

TY - JOUR
ID - 817
T1 - Behavior mapping: a method for linking preschool physical activity and outdoor design
A1 - Cosco,N.G.
A1 - Moore,R.C.
A1 - Islam,M.Z.
Y1 - 2010/03//
N1 - Cosco, Nilda G. Moore, Robin C. Islam, Mohammed Z
Medicine and science in sports and exercise
8005433, mg8
IM, S
Journal Article. Research Support, N.I.H., Extramural
English
KW - MEDLINE
KW - Child Behavior
KW - Child Day Care Centers
KW - Child
KW - Preschool
KW - Data Collection/st [Standards]
KW - Environment Design
KW - Exercise
KW - Humans
KW - North Carolina
KW - Play and Playthings
RP - NOT IN FILE
SP - 513
EP - 519
JF - Medicine & Science in Sports & Exercise
JA - Med Sci Sports Exerc
VL - 42
IS - 3
CY - United States
N2 - UNLABELLED: The preschool that children attend has been shown to be a significant but variable predictor of physical activity of 3- to 5-yr-olds, whereas the time outdoors has been found to be a strong correlate of physical activity. Researchers speculate that variations in preschool physical activity may be attributed to variations in preschool policies and practices, including the form and content of outdoor physical environments. However, assessment methods linking them to physical activity are limited. Improved understanding of links between environment and activity could be used to influence childcare policy, which is highly regulated, usually at state level, to create outdoor environments more conducive to children's informal play and physical activity. The purpose of this article was to introduce behavior mapping as a direct observation method on the basis of the theories of behavior setting and affordance and to demonstrate its sensitivity to gathering physical activity and associated environmental data at a sufficiently detailed level to affect built environment design policy. METHODS: Behavior mapping data, including outdoor environmental characteristics and children's physical activity levels, were obtained in two preschool centers at the Research Triangle region, NC. RESULTS: Physical activity levels at the two centers varied across different types of behavior settings, including pathways, play structures, and open areas. The same type of setting with different attributes, such as circular versus straight pathways, and open areas with different ground surfaces, such as

asphalt, compacted soil, woodchips, and sand, attracted different levels of physical activity. CONCLUSIONS: Behavior mapping provides a promising method for objectively measuring relationships between physical behavior settings and directly associated activity levels
SN - 1530-0315
AD - College of Design, NC State University, Raleigh, NC 27695, USA. nilda_cosco@ncsu.edu
UR - 20068497
ER -

TY - JOUR
ID - 818
T1 - Effects of child care policy and environment on physical activity. [Review] [14 refs]
A1 - Trost,S.G.
A1 - Ward,D.S.
A1 - Senso,M.
Y1 - 2010/03//
N1 - Trost, Stewart G. Ward, Dianne S. Senso, Meghan
Medicine and science in sports and exercise
8005433, mg8
IM, S
Journal Article. Review
English
KW - MEDLINE
KW - Child Day Care Centers
KW - Child
KW - Preschool
KW - Databases
KW - Factual
KW - Environment Design
KW - Exercise
KW - Female
KW - Humans
KW - Male
KW - Policy Making
KW - Research
RP - NOT IN FILE
SP - 520
EP - 525
JF - Medicine & Science in Sports & Exercise
JA - Med Sci Sports Exerc
VL - 42
IS - 3
CY - United States
N2 - UNLABELLED: Child care centers differ systematically with respect to the quality and quantity of physical activity they provide, suggesting that center-level policies and practices, as well as the center's physical environment, are important influences on children's physical activity behavior. PURPOSE: To summarize and critically evaluate the extant peer-reviewed literature on the influence of child care policy and environment on physical activity in preschool-aged children. METHODS: A computer database search identified seven relevant studies that were categorized into three broad areas: cross-sectional studies investigating the impact of selected center-level policies and practices on moderate-to-vigorous physical activity (MVPA), studies correlating specific attributes of the outdoor play environment with the level and intensity of MVPA, and studies in which a specific center-level policy or environmental attribute was experimentally manipulated and evaluated for changes in MVPA. RESULTS: Staff education and training, as well as staff behavior on the playground, seem to be salient influences on MVPA in preschoolers. Lower playground density (less children per square meter) and the presence of vegetation and open play areas also seem to be positive influences on MVPA. However, not all studies found these attributes to be significant. The availability and quality of portable play equipment, not the amount or type of fixed play equipment, significantly influenced MVPA levels. CONCLUSIONS: Emerging evidence suggests that several policy and environmental factors contribute to the marked between-center

variability in physical activity and sedentary behavior. Intervention studies targeting these factors are thus warranted. [References: 14]
SN - 1530-0315
AD - Department of Nutrition and Exercise Sciences, Oregon State University, Corvallis, OR 97333, USA. stewart.trost@oregonstate.edu
UR - 20068496
ER -

TY - JOUR
ID - 819
T1 - Socioeconomic and personal behavioral factors affecting children's exposure to VOCs in urban areas in Korea
A1 - Byun,H.
A1 - Ryu,K.
A1 - Jang,K.
A1 - Bae,H.
A1 - Kim,D.
A1 - Shin,H.
A1 - Chu,J.
A1 - Yoon,C.
Y1 - 2010/02//
N1 - Byun, Hyaejeong. Ryu, Kyongnam. Jang, Kyungjo. Bae, Hyunjoo. Kim, Dongjin. Shin, Hosung. Chu, Jangmin. Yoon, Chungsik
Journal of environmental monitoring : JEM
d0a, 100968688
IM
Journal Article. Research Support, Non-U.S. Gov't
English
KW - MEDLINE
KW - Air Pollution
KW - Indoor/an [Analysis]
KW - Child
KW - Environmental Exposure/an [Analysis]
KW - Female
KW - Humans
KW - Korea
KW - Male
KW - Principal Component Analysis
KW - Socioeconomic Factors
KW - Urban Population
KW - Volatile Organic Compounds/an [Analysis]
RP - NOT IN FILE
SP - 524
EP - 535
JF - Journal of Environmental Monitoring
JA - J Environ Monit
VL - 12
IS - 2
CY - England
N2 - Volatile organic compounds (VOCs) are known to cause adverse health effects. We investigated the relationships between children's VOC exposure and socioeconomic and human activity factors with passive personal samplers, questionnaires, and time-activity diaries (TAD). Statistical analyses were conducted using SAS 9.1, and the results were organized using SigmaPlot 8.0 software. Chemicals such as benzene, toluene, 2-butanone, ethylbenzene, xylene, chloroform, n-hexane, heptane, and some kinds of decanes, which are known to adversely affect public health, were identified in measured samples. These were mainly emitted from outdoor sources (e.g., vehicular traffic) or indoor sources (e.g., household activities such as cooking and cleaning) or both. We concluded that region was the most important socioeconomic factor affecting children's VOC

exposure, and the significant compounds were n-hexane (p = 0.006), 1,1,1-trichloroethane (p = 0.001), benzene (p = 0.003), toluene (p = 0.002), ethylbenzene (p = 0.020), m-, p-xylene (p = 0.014), dodecane (p = 0.003), and hexadecane (p = 0.001). Parental education, year of home construction and type of housing were also slightly correlated with personal VOC exposure. Only the concentration of o-xylene (p = 0.027) was significantly affected by the parental education, and the concentrations of benzene (p = 0.030) and 2-butanone (p = 0.049) by the type of housing. Also, tridecane (p = 0.049) and n-hexane (p = 0.033) were significantly associated with the year of home construction. When household activities such as cooking were performed indoors, children's VOC concentrations tended to be higher, especially for n-hexane, chloroform, heptane, toluene (p < 0.05), 1,1,1-trichloroethane, benzene, dodecane, and hexadecane (p < 0.01). However, smoking had a significant effect for only dodecane, and cleaning had no impact on any VOC concentrations. Considering both socioeconomic and personal behavioral factors simultaneously, socioeconomic factors such as region had a greater effect on children's VOC exposures than indoor activities. From this study, we can suggest that socioeconomic factors as well as environmental factors should be considered when formulating environmental policy to protect children's health
SN - 1464-0333
AD - Department of Environmental Health, Graduate School of Public Health, Seoul National University, 28 Yeonkun-dong Jongno-gu, Seoul, Republic of Korea
UR - 20145896
ER -

TY - JOUR
ID - 820
T1 - Injuries in youth soccer
A1 - Koutures,C.G.
A1 - Gregory,A.J.
A1 - American Academy of Pediatrics.Council on Sports Medicine and Fitness.
Y1 - 2010/02//
N1 - Koutures, Chris G. Gregory, Andrew J M. American Academy of Pediatrics. Council on Sports Medicine and Fitness
Pediatrics
oxv, 0376422
AIM, IM
Journal Article
English
KW - MEDLINE
KW - Adolescent
KW - Anterior Cruciate Ligament/in [Injuries]
KW - Athletic Injuries/ep [Epidemiology]
KW - Athletic Injuries/pc [Prevention & Control]
KW - Child
KW - Female
KW - Humans
KW - Knee Injuries/ep [Epidemiology]
KW - Male
KW - Risk Factors
KW - Sex Factors
KW - Soccer/in [Injuries]
RP - NOT IN FILE
SP - 410
EP - 414
JF - Pediatrics
VL - 125
IS - 2
CY - United States
N2 - Injury rates in youth soccer, known as football outside the United States, are higher than in many other contact/collision sports and have greater relative numbers in younger, preadolescent players. With regard to musculoskeletal injuries, young females tend to suffer more knee injuries, and young males suffer more ankle

injuries. Concussions are fairly prevalent in soccer as a result of contact/collision rather than purposeful attempts at heading the ball. Appropriate rule enforcement and emphasis on safe play can reduce the risk of soccer-related injuries. This report serves as a basis for encouraging safe participation in soccer for children and adolescents
SN - 1098-4275
UR - 20100755
ER -

TY - JOUR
ID - 821
T1 - Green laser pointers for visual astronomy: how much power is enough?
A1 - Bara,S.
A1 - Robles,M.
A1 - Tejelo,I.
A1 - Marzoa,R.I.
A1 - Gonzalez,H.
Y1 - 2010/02//
N1 - Bara, Salvador. Robles, Marisol. Tejelo, Isabel. Marzoa, Ramon I. Gonzalez, Hector
Optometry and vision science : official publication of the American Academy of Optometry
oiz, 8904931
IM
Journal Article. Research Support, Non-U.S. Gov't
English
KW - MEDLINE
KW - Adolescent
KW - Adult
KW - Astronomy/is [Instrumentation]
KW - Child
KW - Color
KW - Dose-Response Relationship
KW - Radiation
KW - Humans
KW - Lasers
KW - Middle Aged
KW - Safety
KW - Young Adult
RP - NOT IN FILE
SP - 140
EP - 144
JF - Optometry & Vision Science
JA - Optom.Vis Sci
VL - 87
IS - 2
CY - United States
N2 - PURPOSE: Green laser pointers with output powers in the tens to hundreds of milliwatt (mW) range, clearly exceeding the limiting 5 mW of American National Standards Institute class 3a (International Electrotechnical Commission class 3R), are now easily available in the global market. They are increasingly being used in public sky observations and other nighttime outreach activities by educators and science communicators in countries where their use is not well regulated, despite the fact that such high power levels may represent a potential threat to visual health. The purpose of this study was to determine the output power reasonably required to perform satisfactorily this kind of activities. METHODS: Twenty-three observers were asked to vary continuously the output power of a green laser source (wavelength 532 nm) until clearly seeing the laser beam propagating skyward through the atmosphere in a heavily light-polluted urban setting. Measurements were conducted with observers of a wide range of ages (9 to 56 years), refractions (spherical equivalents -8.50 to +1.50 diopters), and previous expertise in using lasers as pointing devices outdoors (from no experience to professional astronomers). Two measurement runs were made in different nights under different meteorological conditions. RESULTS: The output power chosen by observers in the first run (11

observers) averaged to 1.84 mW (+/-0.68 mW, 1 SD). The second run (17 observers) averaged to 2.91 mW (+/-1.54 mW). The global average was 2.38 mW (+/-1.30 mW). Only one observer scored 5.6 mW, just above the class 3a limit. The power chosen by the remaining 22 observers ranged from 1.37 to 3.53 mW. CONCLUSIONS: Green laser pointers with output powers below 5 mW (laser classes American National Standards Institute 3a or International Electrotechnical Commission 3R) appear to be sufficient for use in educational nighttime outdoors activities, providing enough bright beams at reasonable safety levels
SN - 1538-9235
AD - Area de Optica, Dept. Fisica Aplicada, Optics and Optometry School, Universidade de Santiago de Compostela, Galiza, Spain. salva.bara@usc.es
UR - 20035242
ER -

TY - JOUR
ID - 822
T1 - Neighborhood characteristics favorable to outdoor physical activity: disparities by socioeconomic and racial/ethnic composition
A1 - Franzini,L.
A1 - Taylor,W.
A1 - Elliott,M.N.
A1 - Cuccaro,P.
A1 - Tortolero,S.R.
A1 - Janice,Gilliland M.
A1 - Grunbaum,J.
A1 - Schuster,M.A.
Y1 - 2010/03//
N1 - Franzini, Luisa. Taylor, Wendell. Elliott, Marc N. Cuccaro, Paula. Tortolero, Susan R. Janice Gilliland, M. Grunbaum, Joanne. Schuster, Mark A
Health & place
din, 9510067
T
Journal Article. Research Support, U.S. Gov't, P.H.S.
English
KW - MEDLINE
KW - Child
KW - Environment
KW - Ethnic Groups
KW - Humans
KW - Motor Activity
KW - Parents/px [Psychology]
KW - Perception
KW - Poverty Areas
KW - Residence Characteristics
KW - Social Environment
RP - NOT IN FILE
SP - 267
EP - 274
JF - Health & Place
JA - Health Place
VL - 16
IS - 2
CY - England
N2 - This paper uses a socioecological framework to investigate socioeconomic and racial/ethnic disparities in neighborhood characteristics that are associated with outdoor physical activity. We surveyed 632 parents of 5th graders about perceptions of their neighborhood social processes and collected systematic observations of the physical environment on their block-face of residence. Higher poverty neighborhoods and non-White neighborhoods have better accessibility; however, they are less safe, less comfortable, and less pleasurable for outdoor physical activity, and have less favorable social processes. Interventions to reduce disparities in

physical activity should address not only the physical environment, but also social processes favorable to physical activity. Copyright 2009 Elsevier Ltd. All rights reserved
SN - 1873-2054
AD - University of Texas School of Public Health, Houston, TX 77030, USA. Luisa.Franzini@uth.tmc.edu
UR - 19896408
ER -

TY - JOUR
ID - 823
T1 - Effect of early life exposure to air pollution on development of childhood asthma
A1 - Clark,N.A.
A1 - Demers,P.A.
A1 - Karr,C.J.
A1 - Koehoorn,M.
A1 - Lencar,C.
A1 - Tamburic,L.
A1 - Brauer,M.
Y1 - 2010/02//
N1 - Clark, Nina Annika. Demers, Paul A. Karr, Catherine J. Koehoorn, Mieke. Lencar, Cornel. Tamburic, Lillian. Brauer, Michael
Environmental health perspectives
ei0, 0330411
IM
Journal Article. Research Support, Non-U.S. Gov't
English
KW - MEDLINE
KW - Air Pollution/ae [Adverse Effects]
KW - Asthma/ci [Chemically Induced]
KW - British Columbia
KW - Carbon Monoxide/ae [Adverse Effects]
KW - Case-Control Studies
KW - Child
KW - Preschool
KW - Environmental Exposure/ae [Adverse Effects]
KW - Female
KW - Humans
KW - Logistic Models
KW - Male
KW - Nitrogen Dioxide/ae [Adverse Effects]
KW - Nitrogen Oxides/ae [Adverse Effects]
KW - Particulate Matter/ae [Adverse Effects]
KW - Pregnancy
KW - Prenatal Exposure Delayed Effects/ci [Chemically Induced]
KW - Time Factors
KW - United States
RP - NOT IN FILE
SP - 284
EP - 290
JF - Environmental Health Perspectives
JA - Environ Health Perspect
VL - 118
IS - 2
CY - United States
N2 - BACKGROUND: There is increasing recognition of the importance of early environmental exposures in the development of childhood asthma. Outdoor air pollution is a recognized asthma trigger, but it is unclear whether exposure influences incident disease. We investigated the effect of exposure to ambient air pollution in utero and during the first year of life on risk of subsequent asthma diagnosis in a population-based nested case-

control study. METHODS: We assessed all children born in southwestern British Columbia in 1999 and 2000 (n = 37,401) for incidence of asthma diagnosis up to 34 years of age using outpatient and hospitalization records. Asthma cases were age- and sex-matched to five randomly chosen controls from the eligible cohort. We estimated each individual's exposure to ambient air pollution for the gestational period and first year of life using high-resolution pollution surfaces derived from regulatory monitoring data as well as land use regression models adjusted for temporal variation. We used logistic regression analyses to estimate effects of carbon monoxide, nitric oxide, nitrogen dioxide, particulate matter <or= 10 microm and <or= 2.5 microm in aerodynamic diameter (PM10 and PM2.5), ozone, sulfur dioxide, black carbon, woodsmoke, and proximity to roads and point sources on asthma diagnosis. RESULTS: A total of 3,482 children (9%) were classified as asthma cases. We observed a statistically significantly increased risk of asthma diagnosis with increased early life exposure to CO, NO, NO2, PM10, SO2, and black carbon and proximity to point sources. Traffic-related pollutants were associated with the highest risks: adjusted odds ratio = 1.08 (95% confidence interval, 1.041.12) for a 10-microg/m3 increase of NO, 1.12 (1.071.17) for a 10-microg/m3 increase in NO2, and 1.10 (1.061.13) for a 100-microg/m3 increase in CO. These data support the hypothesis that early childhood exposure to air pollutants plays a role in development of asthma
SN - 1552-9924
AD - School of Population and Public Health, University of British Columbia, British Columbia, Canada
UR - 20123607
ER -

TY - JOUR
ID - 824
T1 - Household responses to pandemic (H1N1) 2009-related school closures, Perth, Western Australia
A1 - Effler,P.V.
A1 - Carcione,D.
A1 - Giele,C.
A1 - Dowse,G.K.
A1 - Goggin,L.
A1 - Mak,D.B.
Y1 - 2010/02//
N1 - Effler, Paul V. Carcione, Dale. Giele, Carolien. Dowse, Gary K. Goggin, Leigh. Mak, Donna B
Emerging infectious diseases
cod, 9508155
IM
Journal Article
English
KW - MEDLINE
KW - Adolescent
KW - Child
KW - Preschool
KW - Contact Tracing
KW - Disease Outbreaks/pc [Prevention & Control]
KW - Health Behavior
KW - Health Knowledge
KW - Attitudes
KW - Practice
KW - Humans
KW - Influenza A Virus
KW - H1N1 Subtype
KW - Influenza
KW - Human/ep [Epidemiology]
KW - Human/pc [Prevention & Control]
KW - Leisure Activities
KW - Parents
KW - Quarantine
KW - Schools
KW - Western Australia/ep [Epidemiology]

RP - NOT IN FILE
SP - 205
EP - 211
JF - Emerging Infectious Diseases
JA - Emerg Infect Dis
VL - 16
IS - 2
CY - United States
N2 - School closure is often purported to reduce influenza transmission, but little is known about its effect on families. We surveyed families affected by pandemic (H1N1) 2009-related school closures in Perth, Western Australia, Australia. Surveys were returned for 233 (58%) of 402 students. School closure was deemed appropriate by 110 parents (47%); however, 91 (45%) parents of 202 asymptomatic students reported taking >or=1 day off work to care for their child, and 71 (35%) had to make childcare arrangements because of the class closures. During the week, 172 (74%) students participated in activities outside the home on >or=1 occasion, resulting in an average of 3.7 out-of-home activities for each student. In our survey, activities outside the home were commonly reported by students affected by school closure, the effect on families was substantial, and parental opinion regarding school closures as a means to mitigate the outbreak of pandemic (H1N1) 2009 was divided
SN - 1080-6059
AD - Emerging Infectious Diseases Journal, Centers for Disease Control and Prevention, Atlanta, Georgia, USA. paul.effl er@health.wa.gov.au
UR - 20113548
ER -

TY - JOUR
ID - 825
T1 - Mapping the walk to school using accelerometry combined with a global positioning system
A1 - Cooper,A.R.
A1 - Page,A.S.
A1 - Wheeler,B.W.
A1 - Griew,P.
A1 - Davis,L.
A1 - Hillsdon,M.
A1 - Jago,R.
Y1 - 2010/02//
N1 - Cooper, Ashley R. Page, Angie S. Wheeler, Benedict W. Griew, Pippa. Davis, Laura. Hillsdon, Melvyn. Jago, Russell
American journal of preventive medicine
8704773, apl
IM
Journal Article. Research Support, Non-U.S. Gov't
English
KW - MEDLINE
KW - Acceleration
KW - Child
KW - Female
KW - Geographic Information Systems
KW - Humans
KW - London
KW - Male
KW - Monitoring
KW - Physiologic/is [Instrumentation]
KW - Schools
KW - Walking
RP - NOT IN FILE
SP - 178
EP - 183

JF - American Journal of Preventive Medicine
JA - Am J Prev Med
VL - 38
IS - 2
CY - Netherlands
N2 - BACKGROUND: Walking to school is associated with higher levels of physical activity, but the contribution of the journey itself to physical activity before school is unknown. PURPOSE: This study combined accelerometer and GPS data to investigate the level and location of physical activity in children walking to school. METHODS: Participants were 137 children (aged 11.3 + or - 0.3 years) from London, England, measured in June-July 2006. Physical activity was measured by accelerometry, and location was determined with a GPS receiver. Travel mode was self-reported. Accelerometer and GPS data were time-matched to provide activity level and location for each 10-second epoch where both were available. Journeys were mapped in a GIS. RESULTS: Mean accelerometer counts per minute before school (8:00 am to 9:00 am) were 43% higher in those who walked to school than those traveling by car (878.8 + or - 387.6 vs 608.7 + or - 264.1 counts per minute [cpm], p<0.001). Eleven percent (4.5 minutes) of daily moderate to vigorous physical activity (MVPA) occurred in this hour, with walkers recording 2.1 minutes more than car travelers (p = 0.004). Children followed direct routes between home and the school playground. Total activity during the walk to school was twice that in the playground (2131.3 + or - 1170.7 vs 1089.7 + or - 938.6 cpm, p<0.001), with the journey contributing three times as much MVPA as time in the playground. CONCLUSIONS: Our results provide evidence that the journey to school is purposeful and contributes to higher total physical activity and MVPA in children. Combining accelerometer and GPS data may aid our understanding of the environmental context of physical activity. Copyright 2010 American Journal of Preventive Medicine. Published by Elsevier Inc. All rights reserved
SN - 1873-2607
AD - Department of Exercise, Nutrition, and Health Sciences, University of Bristol, Bristol, United Kingdom. ashley.cooper@bris.ac.uk
UR - 20117574
ER -

TY - JOUR
ID - 826
T1 - Facility provision in elementary schools: correlates with physical education, recess, and obesity
A1 - Fernandes,M.
A1 - Sturm,R.
Y1 - 2010/01//
N1 - Fernandes, Meenakshi. Sturm, Roland
Preventive medicine
pm4, 0322116
IM
Journal Article. Research Support, N.I.H., Extramural. Research Support, Non-U.S. Gov't
English
KW - MEDLINE
KW - Child
KW - Climate
KW - Facility Design and Construction
KW - Humans
KW - Longitudinal Studies
KW - Multivariate Analysis
KW - Obesity/ep [Epidemiology]
KW - Obesity/pc [Prevention & Control]
KW - Physical Education and Training
KW - Public Facilities
KW - Recreation
KW - Residence Characteristics
KW - Schools
KW - Socioeconomic Factors
KW - United States/ep [Epidemiology]

RP - NOT IN FILE
SP - S30
EP - S35
JF - Preventive Medicine
JA - Prev Med
VL - 50 Suppl 1
CY - United States
N2 - OBJECTIVES: The objectives were to: (1) document correlations among facility provision (availability and adequacy) in elementary schools, child sociodemographic factors, and school characteristics nationwide; and (2) investigate whether facility provision is associated with physical education (PE) time, recess time, and obesity trajectory. METHODS: The analytic sample included 8935 fifth graders from the Early Childhood Longitudinal Survey Kindergarten Cohort. School teachers and administrators were surveyed about facility provision, PE, and recess time in April 2004. Multivariate linear and logistic regressions that accounted for the nesting of children within schools were used. RESULTS: Children from disadvantaged backgrounds were more likely to attend a school with worse gymnasium and playground provision. Gymnasium availability was associated with an additional 8.3 min overall and at least an additional 25 min of PE per week for schools in humid climate zones. These figures represent 10.8 and 32.5%, respectively, of the average time spent in PE. No significant findings were obtained for gymnasium and playground adequacy in relation to PE and recess time, and facility provision in relation to obesity trajectory. CONCLUSIONS: Poor facility provision is a potential barrier for school physical activity programs and facility provision is lower in schools that most need them: urban, high minority, and high enrollment schools
SN - 1096-0260
AD - Pardee RAND Graduate School, Santa Monica, CA 90407, USA. meena@rand.org
UR - 19850074
ER -

TY - JOUR
ID - 827
T1 - Relationships of putative endocrine disruptors to human sexual maturation and thyroid activity in youth. [Review] [88 refs]
A1 - Schell,L.M.
A1 - Gallo,M.V.
Y1 - 2010/02/09/
N1 - Schell, Lawrence M. Gallo, Mia V
Physiology & behavior
p72, 0151504
IM
Journal Article. Research Support, N.I.H., Extramural. Review
English
KW - MEDLINE
KW - Adolescent
KW - Adolescent Development/ph [Physiology]
KW - Child
KW - Dichlorodiphenyl Dichloroethylene/bl [Blood]
KW - Endocrine Disruptors/an [Analysis]
KW - Endocrine Disruptors/bl [Blood]
KW - Endocrine Disruptors/pd [Pharmacology]
KW - Female
KW - Hexachlorobenzene/bl [Blood]
KW - Humans
KW - Indians
KW - North American
KW - Iodide Peroxidase/im [Immunology]
KW - Male
KW - Menarche/de [Drug Effects]
KW - Menarche/ph [Physiology]
KW - Metals

KW - Heavy/bl [Blood]
KW - Mirex/bl [Blood]
KW - Polychlorinated Biphenyls/bl [Blood]
KW - Predictive Value of Tests
KW - Retrospective Studies
KW - Sexual Maturation/de [Drug Effects]
KW - Sexual Maturation/ph [Physiology]
KW - Thyroid Gland/de [Drug Effects]
KW - Thyroid Gland/me [Metabolism]
RP - NOT IN FILE
SP - 246
EP - 253
JF - Physiology & Behavior
JA - Physiol Behav
VL - 99
IS - 2
CY - United States
N2 - Endocrine disruption has become a significant human health concern, but is difficult to study outside of the laboratory for several reasons including the multiplicity of exposures, the difficulty in assessing each exposure, and the variety of possible outcomes among human populations. This review summarizes our studies of the relationships of measured persistent organic pollutants (PCBs, p,p'-DDE, HCB and mirex), and heavy metals (lead and mercury), to outcomes directly related to thyroid function and sexual maturation. These studies were conducted in a sample of Native American youth from the Akwesasne Mohawk community. The participants were first studied during puberty (10-16.9 years of age) and then at approximately 18 years of age. Results from these studies show that PCB levels are positively related to TSH and negatively to free T4. Further, these effects are conditioned by breastfeeding history. Anti-thyroid peroxidase antibody levels also are related to PCB levels suggesting elevated risk of autoimmune disease among the exposed. Earlier age at menarche is associated with higher PCB levels while risk of delay is associated with higher lead levels. Some evidence that the timing of exposure produces different effects is presented, and the level of exposure in the participants suggests that effects observed may be relevant to a considerable proportion of the US population. Further investigations are warranted to determine effect thresholds and mechanisms. 2009 Elsevier Inc. All rights reserved. [References: 88]
SN - 1873-507X
AD - University at Albany, Department of Anthropology, Albany, NY 12222, USA. l.schell@albany.edu
UR - 19800354
ER -

TY - JOUR
ID - 828
T1 - WASP plays a novel role in regulating platelet responses dependent on alphaIIbbeta3 integrin outside-in signalling
A1 - Shcherbina,A.
A1 - Cooley,J.
A1 - Lutskiy,M.I.
A1 - Benarafa,C.
A1 - Gilbert,G.E.
A1 - Remold-O'Donnell,E.
Y1 - 2010/02//
N1 - Shcherbina, Anna. Cooley, Jessica. Lutskiy, Maxim I. Benarafa, Charaf. Gilbert, Gary E. Remold-O'Donnell, Eileen
British journal of haematology
axc, 0372544
IM
Journal Article. Research Support, N.I.H., Extramural. Research Support, Non-U.S. Gov't
English
KW - MEDLINE
KW - Adolescent

KW - Adult
KW - Animals
KW - Blood Platelets/me [Metabolism]
KW - Blood Platelets/ph [Physiology]
KW - Child
KW - Preschool
KW - Female
KW - Fibrin/ph [Physiology]
KW - Fibrinogen/me [Metabolism]
KW - Hemostasis/ph [Physiology]
KW - Humans
KW - Infant
KW - Male
KW - Mice
KW - Knockout
KW - Middle Aged
KW - Platelet Aggregation/ph [Physiology]
KW - Platelet Count
KW - Platelet Glycoprotein GPIIb-IIIa Complex/me [Metabolism]
KW - Platelet Glycoprotein GPIIb-IIIa Complex/ph [Physiology]
KW - Signal Transduction/ph [Physiology]
KW - Wiskott-Aldrich Syndrome/bl [Blood]
KW - Wiskott-Aldrich Syndrome/ge [Genetics]
KW - Wiskott-Aldrich Syndrome Protein/df [Deficiency]
KW - Wiskott-Aldrich Syndrome Protein/ge [Genetics]
KW - Wiskott-Aldrich Syndrome Protein/ph [Physiology]
KW - Young Adult
RP - NOT IN FILE
SP - 416
EP - 427
JF - British Journal of Haematology
JA - Br J Haematol
VL - 148
IS - 3
CY - England
N2 - The most consistent feature of Wiskott Aldrich syndrome (WAS) is profound thrombocytopenia with small platelets. The responsible gene encodes WAS protein (WASP), which functions in leucocytes as an actin filament nucleating agent -yet- actin filament nucleation proceeds normally in patient platelets regarding shape change, filopodia and lamellipodia generation. Because WASP localizes in the platelet membrane skeleton and is mobilized by alphaIIbbeta3 integrin outside-in signalling, we questioned whether its function might be linked to integrin. Agonist-induced alphaIIbbeta3 activation (PAC-1 binding) was normal for patient platelets, indicating normal integrin inside-out signalling. Inside-out signalling (fibrinogen, JON/A binding) was also normal for wasp-deficient murine platelets. However, adherence/spreading on immobilized fibrinogen was decreased for patient platelets and wasp-deficient murine platelets, indicating decreased integrin outside-in responses. Another integrin outside-in dependent response, fibrin clot retraction, involving contraction of the post-aggregation actin cytoskeleton, was also decreased for patient platelets and wasp-deficient murine platelets. Rebleeding from tail cuts was more frequent for wasp-deficient mice, suggesting decreased stabilisation of the primary platelet plug. In contrast, phosphatidylserine exposure, a pro-coagulant response, was enhanced for WASP-deficient patient and murine platelets. The collective results reveal a novel function for WASP in regulating pro-aggregatory and pro-coagulant responses downstream of integrin outside-in signalling
SN - 1365-2141
AD - Immune Disease Institute, Harvard Medical School, Boston, MA, USA
UR - 19863535
ER -

TY - JOUR
ID - 829

T1 - Hyperlipidaemia in paediatric patients: the role of lipid-lowering therapy in clinical practice. [Review] [94 refs]
A1 - Wierzbicki,A.S.
A1 - Viljoen,A.
Y1 - 2010/02/01/
N1 - Wierzbicki, Anthony S. Viljoen, Adie
Drug safety : an international journal of medical toxicology and drug experience
ahq, 9002928
IM
Journal Article. Review
English
KW - MEDLINE
KW - Adolescent
KW - Child
KW - Humans
KW - Hydroxymethylglutaryl-CoA Reductase Inhibitors/tu [Therapeutic Use]
KW - Hyperlipidemias/dt [Drug Therapy]
KW - Pediatrics
KW - Risk Factors
RP - NOT IN FILE
SP - 115
EP - 125
JF - Drug Safety
JA - Drug Saf
VL - 33
IS - 2
CY - New Zealand
N2 - Atherosclerosis begins in childhood with the formation of fatty streaks. Early plaques can be found in adolescence and early coronary disease can be found in young adults. It has been suggested that early treatment may lead to great benefits in later life. This article is a narrative review of the role of lipid-lowering drug therapy in paediatric practice. Increased rates of atherosclerosis are known to occur in children with familial hypercholesterolaemia (FH), especially in homozygotes. There is evidence for the efficacy and safety of lipid-lowering therapies in children, particularly with respect to the effects of HMG-CoA reductase inhibitors (statins) on lipids and, to a limited extent, on other surrogate measures of atherosclerosis in patients with FH. Diagnosis of FH and its early treatment are recommended in all guidelines. Lipid-lowering drug therapy is recommended for the treatment of homozygous FH at all ages and from as young as 10 years of age for the treatment of heterozygous FH when there is a family history of very premature coronary heart disease (occurring at age <40 years). Controversy exists about other possible indications. Increased rates of atherosclerosis are seen in autoimmune disorders, including type 1 diabetes mellitus, systemic lupus erythematosus and Kawasaki's disease, and in transplant recipients. All evidence in these areas is derived by extrapolation from studies in adults. These disorders can be divided into those for which percutaneous coronary intervention is performed early and/or for which drugs used to treat the primary disorder increase the rate of atherosclerosis, and those for which this is not the case. In both cardiac transplantation and Kawasaki's disease, increased atherosclerosis can occur as a result of (i) disease-related vasculopathy; or (ii) increased restenosis secondary to interventions. Statins have a good evidence base for reducing rates of re-occlusion following coronary artery procedures, and this justifies their use in these settings. In renal transplantation, statins may have a role to play in patients with persistent dyslipidaemia and additional cardiovascular risk factors. In other disorders, such as type 1 diabetes, the disease process is atherogenic and thus statins may be justified in patients with a long history of disease (>10 years), poor control, and evidence of vascular or endothelial damage or additional cardiovascular risk factors. There is a role for lipid-lowering therapies in children at high risk of atherosclerosis, but the evidence base outside of FH is weak. Lipid-lowering therapy should be prescribed to all children with homozygous or severe heterozygous FH. Based on adult evidence, statin therapy should be considered in patients who have undergone coronary artery procedures or received cardiac transplants, in whom their primary role is to prevent vascular re-occlusion. In diseases associated with a chronic increased atherogenic risk, such as type 1 diabetes, statins should be considered in high-risk cases where additional cardiovascular risk factors are present. At present, the most important need is for trials to be performed in children using accepted surrogate endpoints to define whether lipid-lowering drug therapy is beneficial in this group. [References: 94]

SN - 0114-5916
AD - Guy's and St Thomas' Hospitals, London SE1 7EH, UK. Anthony.Wierzbicki@kcl.ac.uk
UR - 20082538
ER -

TY - JOUR
ID - 830
T1 - Weight status, energy-balance behaviours and intentions in 9-12-year-old inner-city children
A1 - Jansen,W.
A1 - Mackenbach,J.P.
A1 - Joosten-van,Zwanenburg E.
A1 - Brug,J.
Y1 - 2010/02//
N1 - Jansen, W. Mackenbach, J P. Joosten-van Zwanenburg, E. Brug, J
Journal of human nutrition and dietetics : the official journal of the British Dietetic Association
d1s, 8904840
IM
Journal Article. Research Support, Non-U.S. Gov't
English
KW - MEDLINE
KW - Body Weight
KW - Child
KW - Child Behavior
KW - Computers
KW - Diet
KW - Energy Metabolism
KW - Exercise
KW - Female
KW - Health Behavior
KW - Humans
KW - Intention
KW - Logistic Models
KW - Male
KW - Netherlands
KW - Obesity/ep [Epidemiology]
KW - Obesity/px [Psychology]
KW - Overweight/ep [Epidemiology]
KW - Overweight/px [Psychology]
KW - Prevalence
KW - Recreation
KW - Sex Factors
KW - Sports
KW - Transportation
KW - Urban Health
RP - NOT IN FILE
SP - 85
EP - 96
JF - Journal of Human Nutrition & Dietetics
JA - J Hum Nutr Diet
VL - 23
IS - 1
CY - England
N2 - BACKGROUND: Dutch youth health care promotes four so-called energy-balance behaviours for the prevention of obesity: increasing physical activity, reducing sedentary behaviour and sugar-containing drinks, and eating breakfast. However, data on the prevalence of these behaviours and intentions to engage in them among primary schoolchildren is limited, especially for multi-ethnic, inner-city populations. The present study aimed to provide these data and explore differences according to socio-demographic characteristics and weight

status. METHODS: Data on behaviours and accompanying intentions were collected using classroom questionnaires. Stature and body weight were measured by trained staff. Twenty primary schools in Rotterdam participated. Data on 1095 9-12 year olds (81.7% response rate) were available for analysis. Multiple logistic regression analyses were conducted to determine associations between behaviours (favourable or unfavourable), intentions (positive or not), gender, age, ethnicity, neighbourhood income level and weight status. RESULTS: The prevalence of being overweight was 30.4%, including 9.0% obesity. Engagement in energy-balance behaviours varied from 58.6% for outdoor play (>1 h previous day) to 85.9% for active transportation to school (day of survey). The highest positive intentions were reported for taking part in sports (83.9%), and lowest for reducing computer time (41.3%). Small differences in behaviours and intentions according to socio-demographic characteristics were found, most notably a lower engagement in physical activity by girls. Skipping breakfast and total number of energy-balance behaviours were associated with being overweight. CONCLUSIONS: The prevalence of being overweight among Dutch inner-city schoolchildren is high. A general rather than a differentiated approach is needed to improve engagement in energy-balance behaviours among inner-city schoolchildren
SN - 1365-277X
AD - Department of Public Health, Erasmus MC, Rotterdam, The Netherlands. jansenw@ggd.rotterdam.nl
UR - 20078731
ER -

TY - JOUR
ID - 831
T1 - Ecological momentary interventions: incorporating mobile technology into psychosocial and health behaviour treatments. [Review] [74 refs]
A1 - Heron,K.E.
A1 - Smyth,J.M.
Y1 - 2010/02//
N1 - Heron, Kristin E. Smyth, Joshua M
British journal of health psychology
9605409
IM
Journal Article. Review
English
KW - MEDLINE
KW - Adolescent
KW - Adult
KW - Alcoholism/th [Therapy]
KW - Anxiety Disorders/th [Therapy]
KW - Cellular Phone
KW - Child
KW - Computers
KW - Handheld
KW - Diabetes Mellitus/th [Therapy]
KW - Eating Disorders/th [Therapy]
KW - Feeding Behavior
KW - Female
KW - Health Behavior
KW - Health Promotion/mt [Methods]
KW - Humans
KW - Male
KW - Mental Disorders/th [Therapy]
KW - Middle Aged
KW - Motor Activity
KW - Obesity/th [Therapy]
KW - Patient Satisfaction
KW - Psychotherapy/mt [Methods]
KW - Self Care/mt [Methods]
KW - Smoking Cessation/mt [Methods]

KW - Treatment Outcome
KW - Weight Loss
KW - Young Adult
RP - NOT IN FILE
SP - 1
EP - 39
JF - British Journal of Health Psychology
JA - Br J Health Psychol
VL - 15
IS - Pt 1
CY - England
N2 - PURPOSE: Psychosocial and health behaviour treatments and therapies can be extended beyond traditional research or clinical settings by using mobile technology to deliver interventions to individuals as they go about their daily lives. These ecological momentary interventions (EMIs) are treatments that are provided to people during their everyday lives (i.e. in real time) and in natural settings (i.e. real world). The goal of the present review is to synthesize and critique mobile technology-based EMI aimed at improving health behaviours and psychological and physical symptoms. METHODS: Twenty-seven interventions using palmtop computers or mobile phones to deliver ambulatory treatment for smoking cessation, weight loss, anxiety, diabetes management, eating disorders, alcohol use, and healthy eating and physical activity were identified. RESULTS: There is evidence that EMI can be successfully delivered, are accepted by patients, and are efficacious for treating a variety of health behaviours and physical and psychological symptoms. Limitations of the existing literature were identified and recommendations and considerations for research design, sample characteristics, measurement, statistical analyses, and clinical implementation are discussed. CONCLUSIONS: Mobile technology-based EMI can be effectively implemented as interventions for a variety of health behaviours and psychological and physical symptoms. Future research should integrate the assessment and intervention capabilities of mobile technology to create dynamically and individually tailored EMI that are ecologically sensitive. [References: 74]
SN - 1359-107X
AD - Syracuse University, New York, USA
UR - 19646331
ER -

TY - JOUR
ID - 832
T1 - Incidental findings at MRI-enterography in patients with suspected or known Crohn's disease
A1 - Jensen,M.D.
A1 - Nathan,T.
A1 - Kjeldsen,J.
A1 - Rafaelsen,S.R.
Y1 - 2010/01/07/
N1 - Jensen, Michael Dam. Nathan, Torben. Kjeldsen, Jens. Rafaelsen, Soren Rafael
World journal of gastroenterology : WJG
100883448
IM
Journal Article
English
KW - MEDLINE
KW - Adolescent
KW - Adult
KW - Aged
KW - 80 and over
KW - Child
KW - Crohn Disease/di [Diagnosis]
KW - Female
KW - Humans
KW - Incidental Findings
KW - Intestines/pa [Pathology]

KW - Magnetic Resonance Imaging
KW - Male
KW - Middle Aged
KW - Predictive Value of Tests
KW - Retrospective Studies
KW - Unnecessary Procedures
KW - Young Adult
RP - NOT IN FILE
SP - 76
EP - 82
JF - World Journal of Gastroenterology
JA - World J Gastroenterol
VL - 16
IS - 1
CY - China
N2 - AIM: To determine the frequency and clinical impact of incidental findings detected with magnetic resonance imaging (MRI)-enterography in patients with suspected or known Crohn's disease (CD). METHODS: Incidental findings were defined as unexpected lesions outside the small intestine, not previously known or suspected at the time of referral, and not related to inflammatory bowel disease. Through a systematic review of medical charts we analyzed the clinical impact of incidental findings, and compared the MRI findings with subsequent diagnostic procedures. RESULTS: A total of 283 patients were included in the analysis, and MRI detected active CD in 31%, fistula in 1.4% and abscess in 0.7%. Extra-intestinal findings not related to CD were recorded in 72 patients (25%), of which 58 patients (20%) had 74 previously unknown lesions. Important or incompletely characterized findings were detected in 17 patients (6.0%). Incidental findings led to 12 further interventions in 9 patients (3.2%) revealing previously unknown pathological conditions in 5 (1.8%). One patient (0.4%) underwent surgery and one patient was diagnosed with a malignant disease. MRI detected incidental colonic lesions in 16 patients of which additional work-up in 4 revealed normal anatomy. Two patients (0.7%) benefitted from the additional examinations, whereas incidental findings led to unnecessary examinations in 9 (3.2%). CONCLUSION: In a minority of patients with suspected or known CD, important incidental findings are diagnosed at MRI-enterography. However, a substantial number of patients experience unnecessary morbidity because of additional examinations of benign or normal conditions
SN - 1007-9327
AD - Department of Internal Medicine, Section of Gastroenterology, Vejle Hospital part of Lillebaelt Hospital, Kabbeltoft 25, DK-7100 Vejle, Denmark. michael.dam.jensen@slb.regionsyddanmark.dk
UR - 20039452
ER -

TY - JOUR
ID - 833
T1 - Correlates of objectively assessed physical activity and sedentary time in children: a cross-sectional study (The European Youth Heart Study)
A1 - Nilsson,A.
A1 - Andersen,L.B.
A1 - Ommundsen,Y.
A1 - Froberg,K.
A1 - Sardinha,L.B.
A1 - Piehl-Aulin,K.
A1 - Ekelund,U.
Y1 - 2009///
N1 - Nilsson, Andreas. Andersen, Lars Bo. Ommundsen, Yngvar. Froberg, Karsten. Sardinha, Luis B. Piehl-Aulin, Karin. Ekelund, Ulf
BMC public health
100968562
IM
Journal Article. Research Support, Non-U.S. Gov't
English
KW - MEDLINE

KW - Adolescent
KW - Child
KW - Cross-Sectional Studies
KW - Europe
KW - Exercise
KW - Female
KW - Humans
KW - Life Style
KW - Male
RP - NOT IN FILE
SP - 322
JF - BMC Public Health
VL - 9
CY - England
N2 - BACKGROUND: Identifying leisure time activities performed before and after school that influence time in physical activity (PA) and/or time spent sedentary can provide useful information when designing interventions aimed to promote an active lifestyle in young people. The purpose of this study was to examine associations between mode of transportation to school, outdoor play after school, participation in exercise in clubs, and TV viewing with objectively assessed PA and sedentary behaviour in children. METHODS: A total of 1327 nine- and 15-year-old children from three European countries (Norway, Estonia, Portugal) participated as part of the European Youth Heart Study. PA was measured during two weekdays and two weekend days using the MTI accelerometer, and average percent of time in moderate-to-vigorous PA (MVPA) and time spent sedentary were derived. Potential correlates were assessed by self-report. Independent associations between self-reported correlates with percent time in MVPA and percent time sedentary were analysed by general linear models, adjusted by age, gender, country, measurement period, monitored days and parental socio-economic status. RESULTS: In 9-year-olds, playing outdoors after school was associated with higher percent time in MVPA ($P < 0.01$), while participation in sport clubs was associated with higher percent time in MVPA ($P < 0.01$) in 15-year-olds. No associations with percent time sedentary were observed in either age group. CONCLUSION: Frequency of outdoor play after school is a significant correlate for daily time in MVPA in 9-year-olds, while this correlate is attenuated in favour of participation in sport and exercise in clubs in 15-year-olds. Targeting walking to school or reduced TV viewing time in order to increase time in daily MVPA in children is unlikely to be sufficient. Correlates related to time spent sedentary need further examination
SN - 1471-2458
AD - School of Health and Medical Sciences, Orebro University, Orebro, Sweden. andreas.nilsson@oru.se
UR - 19735565
ER -

TY - JOUR
ID - 834
T1 - Morbidity, mortality, and response to treatment by children in the United Kingdom and Ireland with perinatally acquired HIV infection during 1996-2006: planning for teenage and adult care
A1 - Judd,A.
A1 - Doerholt,K.
A1 - Tookey,P.A.
A1 - Sharland,M.
A1 - Riordan,A.
A1 - Menson,E.
A1 - Novelli,V.
A1 - Lyall,E.G.
A1 - Masters,J.
A1 - Tudor-Williams,G.
A1 - Duong,T.
A1 - Gibb,D.M.
A1 - Collaborative HIV Paediatric Study (CHIPS)
A1 - National Study of HIV in Pregnancy and Childhood (NSHPC)
Y1 - 2007/10/01/

N1 - Judd, Ali. Doerholt, Katja. Tookey, Pat A. Sharland, Mike. Riordan, Andrew. Menson, Esse. Novelli, Vas. Lyall, E G Hermione. Masters, Janet. Tudor-Williams, Gareth. Duong, Trinh. Gibb, Di M. Collaborative HIV Paediatric Study (CHIPS). National Study of HIV in Pregnancy and Childhood (NSHPC)
Clinical infectious diseases : an official publication of the Infectious Diseases Society of America
a4j, 9203213
IM
Journal Article. Research Support, Non-U.S. Gov't
English
KW - MEDLINE
KW - Adolescent
KW - Anti-HIV Agents/tu [Therapeutic Use]
KW - Antiretroviral Therapy
KW - Highly Active/sn [Statistics & Numerical Data]
KW - CD4 Lymphocyte Count/sn [Statistics & Numerical Data]
KW - Child
KW - Preschool
KW - Cohort Studies
KW - Female
KW - Great Britain/ep [Epidemiology]
KW - HIV Infections/dt [Drug Therapy]
KW - HIV Infections/mo [Mortality]
KW - HIV Infections/tm [Transmission]
KW - Hospitalization/sn [Statistics & Numerical Data]
KW - Humans
KW - Infant
KW - Newborn
KW - Infectious Disease Transmission
KW - Vertical/sn [Statistics & Numerical Data]
KW - Ireland/ep [Epidemiology]
KW - Male
KW - Needs Assessment
KW - Pregnancy
KW - Prospective Studies
KW - Registries
KW - Survival Analysis
KW - Viral Load/sn [Statistics & Numerical Data]
RP - NOT IN FILE
SP - 918
EP - 924
JF - Clinical Infectious Diseases
JA - Clin Infect Dis
VL - 45
IS - 7
CY - United States
N2 - BACKGROUND: Recent evidence suggests that decreases in morbidity and mortality in cohorts of adults infected with human immunodeficiency virus (HIV) are showing signs of reversal. We describe changes over time in these characteristics and in the response to treatment among children in the United Kingdom and Ireland with perinatally acquired HIV infection, many of whom are now adolescents. METHODS: We analyzed prospective cohort data reported to the National Study of HIV in Pregnancy and Childhood (NSHPC) and the Collaborative HIV Paediatric Study. RESULTS: By mid 2006, 1441 HIV-infected children were reported to NSHPC; 40% were > or = 10 years old at their most recent follow-up visit, and 34% were receiving care outside London. The proportion of children born abroad increased from 24% during 1994-1996 to 64% during 2003-2006. The percentage of total child time during which children received highly active antiretroviral therapy (HAART) increased from 36% during 1997-1999 to 61% during 2000-2002 and 63% during 2003-2006. Of children who were naive to antiretroviral therapy at the start of HAART, the percentage with an HIV-1 RNA load of < 400 copies/mL after 12 months increased from 52% during 1997-1999 to 79% during 2003-2006. In multivariate analysis, only calendar time predicted virological response, whereas both younger age and lower

CD4 cell percentage at HAART initiation predicted increases of > 10% in the CD4 cell percentage. A total of 31% of children aged 5-14 years and 38% aged > or = 15 years at their most recent follow-up visit had been exposed to drugs from each of the 3 main HAART classes. The rate of AIDS and mortality combined decreased from 13.3 cases per 100 person-years before 1997 to 3.1 and 2.5 cases per 100 person-years, respectively, during 2000-2002 and 2003-2006; rates of hospital admission also declined during this interval. Of 18 children known to have died since 2003, 9 died within 1 month after presentation. CONCLUSIONS: Morbidity and mortality rates among HIV-infected children continue to decrease over time. Because these children are increasingly dispersed outside London, specialist care is now provided in national clinical networks. Transition pathways to adolescent and adult services and long-term observation to monitor the effects of prolonged exposure to both HIV and HAART are required
SN - 1537-6591
AD - MRC Clinical Trials Unit, London, United Kingdom. a.judd@ctu.mrc.ac.uk
UR - 17806062
ER -

TY - JOUR
ID - 835
T1 - Nowhere to play? The relationship between the location of outdoor play areas and deprivation in Glasgow
A1 - Ellaway,A.
A1 - Kirk,A.
A1 - Macintyre,S.
A1 - Mutrie,N.
Y1 - 2007/06//
N1 - Ellaway, Anne. Kirk, Alison. Macintyre, Sally. Mutrie, Nanette
Health & place
din, 9510067
T
Journal Article
English
KW - MEDLINE
KW - Adolescent
KW - Child
KW - Preschool
KW - Humans
KW - Obesity/pc [Prevention & Control]
KW - Play and Playthings
KW - Poverty
KW - Public Facilities
KW - Scotland
RP - NOT IN FILE
SP - 557
EP - 561
JF - Health & Place
JA - Health Place
VL - 13
IS - 2
CY - England
N2 - Childhood obesity is rising and this rise has been linked to a decrease in physical activity. Access to appropriate facilities for physical activity is a key determinant of participation. This paper investigates the provision of outdoor play areas for children in relation to area deprivation in Glasgow, Scotland. Analysis of the distribution of outdoor play areas showed significantly higher mean number of play areas per 1000 child population in more deprived areas. However, despite the apparent advantage of deprived areas in terms of actual number of play areas, the quality of play areas in different types of areas may warrant further exploration
SN - 1353-8292
AD - Medical Research Council, MRC Social and Public Health Sciences Unit, 4 Lilybank Gardens, Glasgow, Scotland, G12 8RZ, UK. Anne@msoc.mrc.gla.ac.uk
UR - 16777464

ER -

TY - JOUR
ID - 836
T1 - Efavirenz liquid formulation in human immunodeficiency virus-infected children
A1 - Starr,S.E.
A1 - Fletcher,C.V.
A1 - Spector,S.A.
A1 - Brundage,R.C.
A1 - Yong,F.H.
A1 - Douglas,S.D.
A1 - Flynn,P.M.
A1 - Kline,M.W.
A1 - Study Team.Pediatric AIDS Clinical Trials Group.
Y1 - 2002/07//
N1 - Starr, Stuart E. Fletcher, Courtney V. Spector, Stephen A. Brundage, Richard C. Yong, Florence H. Douglas, Steven D. Flynn, Patricia M. Kline, Mark W. PACTG 382 Study Team. Pediatric AIDS Clinical Trials Group
The Pediatric infectious disease journal
oxj, 8701858
IM
Clinical Trial. Clinical Trial, Phase I. Clinical Trial, Phase II. Comparative Study. Journal Article. Multicenter Study. Research Support, Non-U.S. Gov't. Research Support, U.S. Gov't, P.H.S.
English
KW - MEDLINE
KW - Administration
KW - Oral
KW - Benzoxazines
KW - Child
KW - Preschool
KW - Dose-Response Relationship
KW - Drug
KW - Drug Administration Schedule
KW - Drug Therapy
KW - Combination
KW - Female
KW - Follow-Up Studies
KW - HIV Infections/di [Diagnosis]
KW - HIV Infections/dt [Drug Therapy]
KW - HIV Infections/mo [Mortality]
KW - HIV Protease Inhibitors/ad [Administration & Dosage]
KW - HIV-1/ip [Isolation & Purification]
KW - Humans
KW - Male
KW - Nelfinavir/ad [Administration & Dosage]
KW - Oxazines/ad [Administration & Dosage]
KW - RNA
KW - Viral/an [Analysis]
KW - Sensitivity and Specificity
KW - Severity of Illness Index
KW - Single-Blind Method
KW - Survival Analysis
KW - Treatment Outcome
KW - Viral Load
RP - NOT IN FILE
SP - 659
EP - 663

JF - Pediatric Infectious Disease Journal
JA - Pediatr Infect Dis J
VL - 21
IS - 7
CY - United States
N2 - BACKGROUND: This study determined the safety, pharmacokinetics, antiviral activity and immunologic effects of efavirenz liquid formulation, nelfinavir and nucleoside reverse transcriptase inhibitors (NRTIs) in HIV-infected children, 3 to 9 years of age. METHODS: Plasma HIV-1 RNA and lymphocyte subsets were measured at various intervals after initiation of therapy. Pharmacokinetic studies were performed at Week 2, and doses of efavirenz and nelfinavir were adjusted if area under the curve values fell outside specified target ranges. RESULTS: This combination of antiretrovirals was well-tolerated. Pharmacokinetic values were similar to those observed in a previous study of older children who received efavirenz capsules in combination with nelfinavir and NRTIs. After 48 weeks of therapy 63% of subjects had plasma HIV RNA levels of <400 copies/ml, and 58% had <50 copies/ml in an intent-to-treat analysis. CD4 cell count and percentage rose significantly over this time, whereas the number of activated CD8 cells declined. CONCLUSIONS: Combination therapy with efavirenz liquid formulation, nelfinavir and NRTIs is an attractive treatment option for HIV-infected children >3 years of age who are unable to take efavirenz capsules
SN - 0891-3668
AD - Division of Infectious and Immunologic Diseases, Abramson Research Center, The Children's Hospital of Philadelphia, PA 19104, USA. starr@email.chop.edu
UR - 12237599
ER -

TY - JOUR
ID - 837
T1 - Lead-based paint on playground equipment in public children's parks in Johannesburg, Tshwane and Ekurhuleni
A1 - Mathee,A.
A1 - Singh,E.
A1 - Mogotsi,M.
A1 - Timothy,G.
A1 - Maduka,B.
A1 - Olivier,J.
A1 - Ing,D.
Y1 - 2009/11//
N1 - Mathee, Angela. Singh, Elvira. Mogotsi, Mirriam. Timothy, Geraldine. Maduka, Bryne. Olivier, Jan. Ing, Dennis
South African medical journal = Suid-Afrikaanse tydskrif vir geneeskunde
u4r, 0404520
IM
Journal Article. Research Support, Non-U.S. Gov't
English
KW - MEDLINE
KW - Child
KW - Environmental Pollutants/an [Analysis]
KW - Humans
KW - Lead/an [Analysis]
KW - Paint/an [Analysis]
KW - Play and Playthings
KW - South Africa
KW - Spectrometry
KW - X-Ray Emission
RP - NOT IN FILE
SP - 819
EP - 821
JF - South African Medical Journal
JA - SAMJ, S

VL - 99
IS - 11
CY - South Africa
N2 - OBJECTIVE: To determine the use of lead-based paint in public playgrounds in the municipalities of Johannesburg, Tshwane and Ekurhuleni. METHODS: Forty-nine public parks were selected from the municipalities of Johannesburg, Tshwane and Ekurhuleni. Lead levels in paint on playground equipment were measured in situ using a hand-held Thermo Scientific NITON XLP 700 Series X-ray fluorescence (XRF) analyser. RESULTS: Playground lead levels ranged from 'too low to detect' to 10.4 mg/cm2. The mean and median lead concentrations were 1.9 mg/cm2 and 0.9 mg/cm2 respectively. Forty-eight per cent of lead paint measurements exceeded the internationally accepted reference level of 1 mg/cm2. CONCLUSION: The study shows that lead-based paint is widely used in public playgrounds in the three study municipalities, and most likely throughout South Africa. We suggest key actions to ensure that children's playgrounds in South Africa are lead-free zones, and that childhood lead exposure in these settings is prevented
SN - 0038-2469
AD - South African Medical Research Council, University of the Witwatersrand. Angie.Mathee@mrc.ac.za
UR - 20218484
ER -

TY - JOUR
ID - 838
T1 - Cognitive and behavioural effects of a school breakfast
A1 - Richter,L.M.
A1 - Rose,C.
A1 - Griesel,R.D.
Y1 - 1997/01//
N1 - Richter, L M. Rose, C. Griesel, R D
South African medical journal = Suid-Afrikaanse tydskrif vir geneeskunde
u4r, 0404520
IM
Journal Article
English
KW - MEDLINE
KW - Adolescent
KW - Child
KW - Child Behavior
KW - Child Nutritional Physiological Phenomena
KW - Cognition
KW - Female
KW - Food Services
KW - Humans
KW - Male
KW - Psychometrics
KW - Schools
KW - Socioeconomic Factors
KW - South Africa
RP - NOT IN FILE
SP - 93
EP - 100
JF - South African Medical Journal
JA - SAMJ, S
VL - 87
IS - 1 Suppl
CY - SOUTH AFRICA
N2 - The cognitive and behavioural effects of a school breakfast were explored in a study of 55 children in Grade II and Standard 1 at a farm school outside Johannesburg. A previous study had confirmed widespread undernutrition and micronutrient deficiencies among the children. For comparative purposes, 55 children at an inner-city school, among whom no signs of undernutrition were found, were assessed in the same way. Three

different types of measures of attention, distractibility, short-term memory and activity level were used: psychometric testing of the children; teacher ratings of children's classroom behaviour, and coded video-recorded classroom behaviour. A pre- and post-test design was employed to assess the effects of a school breakfast, continually in place in the experimental school for a period of 6 weeks. The results indicated significant change from pre- to post-test assessment among the experimental children in respect of the psychometric measures, teacher-rated hyperactivity and video-recorded classroom behaviour. With regard to the latter measure, the children showed a decline in both the occurrence and duration of off-task and out-of-seat behaviour, and an increase in active participation in class and positive peer interaction. While the children in the comparison group also showed some changes from pre- to post-test, probably attributable to the effects of observation, familiarity with the test materials and developmental change, the changes were not generalised or consistent. The findings support the conclusion that a school breakfast programme had a beneficial effect on the cognitive and behavioural performance of socially disadvantaged, undernourished children in their first 2 years of school
SN - 0038-2469
AD - Department of Psychology, University of Natal, Pietermaritzburg
UR - 9180808
ER -

TY - JOUR
ID - 839
T1 - EEG spectral changes underlying BOLD responses contralateral to spikes in patients with focal epilepsy
A1 - Yu,J.M.
A1 - Tyvaert,L.
A1 - LeVan,P.
A1 - Zelmann,R.
A1 - Dubeau,F.
A1 - Gotman,J.
A1 - Kobayashi,E.
Y1 - 2009/07//
N1 - Yu, Juming M. Tyvaert, Louise. Levan, Pierre. Zelmann, Rina. Dubeau, Francois. Gotman, Jean. Kobayashi, Eliane
Epilepsia
eix, 2983306r
IM
Comparative Study. Journal Article. Research Support, Non-U.S. Gov't
English
KW - MEDLINE
KW - Adolescent
KW - Adult
KW - Brain Mapping/mt [Methods]
KW - Cerebral Cortex/pp [Physiopathology]
KW - Child
KW - Electroencephalography/mt [Methods]
KW - Electroencephalography/sn [Statistics & Numerical Data]
KW - Epilepsies
KW - Partial/di [Diagnosis]
KW - Partial/pp [Physiopathology]
KW - Female
KW - Functional Laterality/ph [Physiology]
KW - Humans
KW - Image Processing
KW - Computer-Assisted
KW - Magnetic Resonance Imaging/sn [Statistics & Numerical Data]
KW - Male
KW - Middle Aged
KW - Oxygen/bl [Blood]
KW - Spectrum Analysis/sn [Statistics & Numerical Data]

RP - NOT IN FILE
SP - 1804
EP - 1809
JF - Epilepsia
VL - 50
IS - 7
CY - United States
N2 - PURPOSE: Simultaneous electroencephalogram and functional magnetic resonance imaging (EEG-fMRI) in patients with focal epilepsy and unilateral spikes often shows positive blood oxygenation level-dependent (BOLD) responses (activations), not only ipsilateral but also contralateral to the spikes. We aimed to investigate whether minimal EEG changes could underlie these contralateral BOLD responses by using EEG spectral analysis. METHODS: We studied 19 patients with focal epilepsy and unilateral spikes. According to the pattern of BOLD activation, patients were divided into Group 1 (ipsi- and contralateral to the spikes) or Group 2 (only ipsilateral). EEG from outside the scanner was used to mark spikes similar to those recorded in the scanner. Epochs of 640 ms before and after the peak of the spikes were chosen as baseline and spike epochs. Spectral analysis was performed in referential montage (FCz reference), and differences between baselines and spikes were analyzed by paired t-test. RESULTS: Significant EEG changes in electrodes contralateral to the spikes were seen in 9 of 10 patients in Group 1 and in only 2 of 10 patients in Group 2 (one patient had two types of spikes that were analyzed separately). Spectral changes were seen in delta and/or theta bands in all patients except one (in Group 1) who had changes in all bands. DISCUSSION: Significant contralateral EEG changes occurred in 90% of contralateral BOLD activations and in only 20% of patients without contralateral BOLD responses. The reason why these changes predominate in lower frequencies rather than in higher frequencies is unclear. These spectral changes in areas corresponding to contralateral activations might reflect poorly synchronized but possibly intense neuronal activity
SN - 1528-1167
AD - Montreal Neurological Institute and Department of Neurology and Neurosurgery, McGill University, Montreal, Canada
UR - 19389143
ER -

TY - JOUR
ID - 840
T1 - Promoting physical activity during school break times: piloting a simple, low cost intervention
A1 - Loucaides,C.A.
A1 - Jago,R.
A1 - Charalambous,I.
Y1 - 2009/04//
N1 - Loucaides, Constantinos A. Jago, Russell. Charalambous, Ioannis
Preventive medicine
pm4, 0322116
IM
Journal Article. Research Support, Non-U.S. Gov't
English
KW - MEDLINE
KW - Child
KW - Costs and Cost Analysis
KW - Cyprus
KW - Exercise
KW - Health Promotion/ec [Economics]
KW - Health Promotion/mt [Methods]
KW - Humans
KW - Intervention Studies
KW - Leisure Activities
KW - Male
KW - Monitoring
KW - Physiologic/is [Instrumentation]
KW - Pilot Projects

KW - Play and Playthings
KW - Schools
KW - Time Factors
RP - NOT IN FILE
SP - 332
EP - 334
JF - Preventive Medicine
JA - Prev Med
VL - 48
IS - 4
CY - United States
N2 - OBJECTIVE: The purpose of this pilot study was to examine the effectiveness of two interventions on children's physical activity during the 20-min school break. METHODS: Children from three schools participated in this study. In the first intervention school (n=89) the school's courts were allocated to different children on alternate days of the week, playground markings were painted in the school's yard and jump ropes were provided. In the second intervention school (n=89) the school's courts were just allocated to different children on alternate days of the week. The third school served as the control group (n=69). Physical activity was measured with a pedometer during the 20-min break (09:05-09:25) and during the after school period (13:05 till bed time). Data were collected in Cyprus, between January/April of 2007. RESULTS: A significant time by group interaction indicated a change in break-time activity. Four weeks after the intervention, mean steps in the first (1427+/-499) and second (1331+/-651) intervention schools were significantly higher than steps in the control school (1053+/-447). Non-significant increases were observed in the after-school activity of children in the intervention schools. CONCLUSIONS: Implementing simple, low-cost interventions during break periods could help increase children's activity
SN - 1096-0260
AD - Centre for Educational Research and Evaluation, Ministry of Education and Culture, Nicosia, Cyprus. conlou@avacom.net
UR - 19463481
ER -

TY - JOUR
ID - 841
T1 - Double burden of underweight and overweight among children (10-19 years of age) of employees working in Indian industrial units
A1 - Jeemon,P.
A1 - Prabhakaran,D.
A1 - Mohan,V.
A1 - Thankappan,K.R.
A1 - Joshi,P.P.
A1 - Ahmed,F.
A1 - Chaturvedi,V.
A1 - Reddy,K.S.
A1 - Investigators,S.S.I.P.
Y1 - 2009/07//
N1 - Jeemon, P. Prabhakaran, D. Mohan, V. Thankappan, K R. Joshi, P P. Ahmed, F. Chaturvedi, V. Reddy, K S. SSIP Investigators
The National medical journal of India
bnt, 8809315
IM
Journal Article. Research Support, Non-U.S. Gov't
English
KW - MEDLINE
KW - Adolescent
KW - Child
KW - Cross-Sectional Studies
KW - Female
KW - Humans

KW - India/ep [Epidemiology]
KW - Male
KW - Nutritional Status
KW - Overweight/ep [Epidemiology]
KW - Prevalence
KW - Thinness/ep [Epidemiology]
KW - Urban Population/sn [Statistics & Numerical Data]
KW - Young Adult
RP - NOT IN FILE
SP - 172
EP - 176
JF - National Medical Journal of India
JA - Natl Med J India
VL - 22
IS - 4
CY - India
N2 - BACKGROUND: Along with the existing problem of underweight, overweight in children is increasing in the developing world. However, there is little information on its magnitude and pattern in the Indian context. We aimed to study the pattern and correlates of overweight in Indian children and adolescents. METHODS: A total of 3750 children in the age group of 10-19 years, who were family members of randomly selected employees from 10 different industrial sites in India, were surveyed using an interviewer-administered questionnaire. RESULTS: The prevalence of underweight was highest in peri-urban areas (30.2% and 53.2% according to Indian and international criteria, respectively). In urban and highly urban areas, the prevalence of underweight was 14.1% and 9.8%, respectively, according to the Indian criteria, and 27.1% and 19.2%, respectively, according to international criteria. The proportion of overweight children was highest in the highly urban category (19.1% and 13.4% according to Indian and international criteria, respectively). The level of urbanization (OR 3.1 and 4.7 for overweight in urban and highly urban areas, respectively, compared with peri-urban areas, p < 0.001), physical activity (OR 0.4, p < 0.001, in children with physical activity score > or = 75th percentile compared with a score < or = 75th percentile) and frequency of meals outside the home (OR 12, p < 0.001, if > 25% weekly meals taken outside the home compared with < 25% of weekly meals outside home) were significant predictors of overweight. CONCLUSION: There is a double burden of underweight and overweight among Indian children and adolescents
SN - 0970-258X
AD - Initiative for Cardiovascular Health Research in the Developing Countries, C1/52, Second Floor, Safdarjang Development Area, New Delhi 110016, India
UR - 20131480
ER -

TY - JOUR
ID - 842
T1 - National plan for physical activity: education sector
A1 - Siedentop,D.L.
Y1 - 2009/11//
N1 - Siedentop, Daryl L
Journal of physical activity & health
101189457
IM
Journal Article. Research Support, Non-U.S. Gov't
English
KW - MEDLINE
KW - Adolescent
KW - Child
KW - Preschool
KW - Health Policy/ec [Economics]
KW - Health Policy
KW - Health Promotion/mt [Methods]
KW - Humans

KW - Meta-Analysis as Topic
KW - Motor Activity
KW - Overweight/pc [Prevention & Control]
KW - Physical Education and Training/ec [Economics]
KW - Physical Education and Training/mt [Methods]
KW - Physical Fitness
KW - School Health Services
KW - Schools
KW - United States
RP - NOT IN FILE
SP - S168
EP - S180
JF - Journal of Physical Activity & Health
JA - J Phys Act Health
VL - 6 Suppl 2
CY - United States
N2 - BACKGROUND: Schools must play a central role in combating the prevalence of overweight and obesity among children and youths. This cannot be achieved without more robust policy and funding programs at both federal and state levels. METHODS: Reviews of meta-analyses were used to assess the efficacy of improving PA/MVPA through interventions in school physical education programs. Individual research studies were reviewed to assess the efficacy of improving PA/MVPA in preschool settings, recess, and classroom activity breaks. Legislation at the federal and state levels was reviewed along with surveillance and accountability mechanisms at the state level. RESULTS: Physical education interventions produce improvements in PA/MVPA if protocols relating to use of time are followed. PA/MVPA in recess can be increased through careful planning for attractive activity opportunities on carefully designed playgrounds. Classroom activity breaks provide important PA/MVPA daily and improve student on-task behavior. CONCLUSIONS: Federal legislation is needed to provide guidelines and financial support for states to improve the quantity and quality of PA in school programs. States need to develop clear expectations for quantity and quality of PA programs in schools, surveillance systems to monitor district compliance in meeting those expectations, and an accountability system aimed at ensuring that state expectations are being met with assistance for districts that do not meet expectations
SN - 1543-3080
AD - The Ohio State University, Columbus, OH, USA
UR - 20120126
ER -

TY - JOUR
ID - 843
T1 - Feasibility and efficacy of a church-based intervention to promote physical activity in children
A1 - Trost,S.G.
A1 - Tang,R.
A1 - Loprinzi,P.D.
Y1 - 2009/11//
N1 - Trost, Stewart G. Tang, Rebecca. Loprinzi, Paul D
Journal of physical activity & health
101189457
IM
Journal Article
English
KW - MEDLINE
KW - Analysis of Variance
KW - Child
KW - Feasibility Studies
KW - Female
KW - Health Behavior
KW - Health Promotion/mt [Methods]
KW - Humans

KW - Male
KW - Motor Activity
KW - Religion
KW - Urban Health
RP - NOT IN FILE
SP - 741
EP - 749
JF - Journal of Physical Activity & Health
JA - J Phys Act Health
VL - 6
IS - 6
CY - United States
N2 - BACKGROUND: This study evaluated the feasibility and preliminary efficacy of a church-based intervention to promote physical activity (PA) in children. METHODS: The study was conducted in 4 churches located in 2 large metropolitan areas and 2 regional towns in Kansas. Churches in the intervention condition implemented the "Shining Like Stars" physical activity curriculum module during their regularly scheduled Sunday school classes. Churches in the control condition delivered the same content without integrating physical activity into the lessons. In addition to the curriculum, the intervention churches completed a series of weekly family devotional activities designed to promote parental support for PA and increase PA outside of Sunday school. RESULTS: Children completing the Shining Like Stars curriculum exhibited significantly greater amounts of MVPA than those in the control condition (20 steps/min vs. 7 steps/min). No intervention effects were observed for PA levels outside of Sunday school or parental support for PA; however, relative to controls, children in the intervention churches did exhibit a significant reduction in screen time. CONCLUSION: The findings confirm that the integration of physical activity into Sunday school is feasible and a potentially effective strategy for promoting PA in young children
SN - 1543-3080
AD - Dept of Nutrition and Exercise Sciences, Oregon State University, Corvallis, OR, USA
UR - 20101917
ER -

TY - JOUR
ID - 844
T1 - Adaptation of the System for Observing Physical Activity and Recreation in Communities (SOPARC) to assess age groupings of children
A1 - Bocarro,J.N.
A1 - Floyd,M.
A1 - Moore,R.
A1 - Baran,P.
A1 - Danninger,T.
A1 - Smith,W.
A1 - Cosco,N.
Y1 - 2009/11//
N1 - Bocarro, Jason N. Floyd, Myron. Moore, Robin. Baran, Perver. Danninger, Tom. Smith, William. Cosco, Nilda
Journal of physical activity & health
101189457
IM
Journal Article. Research Support, Non-U.S. Gov't
English
KW - MEDLINE
KW - Adolescent
KW - Adolescent Development
KW - Age Factors
KW - Child
KW - Child Development
KW - Preschool
KW - Female

KW - Humans
KW - Infant
KW - Newborn
KW - Male
KW - Motor Activity
KW - Observation
KW - Play and Playthings
KW - Public Facilities
KW - Recreation
RP - NOT IN FILE
SP - 699
EP - 707
JF - Journal of Physical Activity & Health
JA - J Phys Act Health
VL - 6
IS - 6
CY - United States
N2 - BACKGROUND: To better measure physical activity (PA) in outdoor environments, McKenzie and colleagues developed the System for Observing Play and Recreation in Communities (SOPARC). However, previous SOPARC research has focused on adults, seniors, teens and children. One avenue for extending this work is to expand the child age group code to capture important nuances that can influence children's PA and their environments. This study reports on the reliability of a measure designed to account for PA in parks among children in different childhood age groups. METHODS: Three groups were developed: 0 to 5-years-old (Young Children); 6 to 12 (Middle Childhood) and 13 to 18 (Older Children) based on Erikson's stages of child development. Data were obtained by direct observation in 3 neighborhood parks in Raleigh, NC and 20 neighborhood parks in Durham, NC. RESULTS: Kappa coefficients showed high agreement for all age group, gender, and PA codes. For the 3 assessments, the results show that the 3 age group category exhibit acceptable reliability for measuring PA in parks among children. CONCLUSIONS: The reliability of measuring PA among children by segmenting children by 3 age groups was established. This approach is recommended for future studies of PA among children in parks and other outdoor environments
SN - 1543-3080
AD - Dept of Parks, Recreation, and Tourism Management, North Carolina State University, Raleigh, NC, USA
UR - 20101912
ER -

TY - JOUR
ID - 845
T1 - Basketball injuries in children
A1 - Gaca,A.M.
Y1 - 2009/12//
N1 - Gaca, Ana Maria
Pediatric radiology
owi, 0365332
IM
Journal Article
English
KW - MEDLINE
KW - Adolescent
KW - Basketball/in [Injuries]
KW - Child
KW - Preschool
KW - Female
KW - Humans
KW - Magnetic Resonance Imaging/mt [Methods]
KW - Male
KW - Tomography

KW - X-Ray Computed/mt [Methods]
KW - Wounds
KW - Nonpenetrating/di [Diagnosis]
RP - NOT IN FILE
SP - 1275
EP - 1285
JF - Pediatric Radiology
JA - Pediatr Radiol
VL - 39
IS - 12
CY - Germany
N2 - Basketball is a popular, worldwide sport played outdoors and indoors year-round. Patterns of injury are related to abrupt changes in the athlete's direction, jumping, contact between athletes, the hard playing surface and paucity of protective equipment. Intensity of play and training in the quest of scholarships and professional careers is believed to contribute to an increasing occurrence of injury. Radiologists' appreciation of the breadth of injury and its relation to imaging and clinical findings should enhance the care of these children. Some of the patterns of injury are well known to radiologists but vary due to age- and size-related changes; the growing skeleton is affected by differing susceptibilities from biomechanical stresses at different sizes. Beyond screening radiographs, the accuracy of MRI and CT has improved diagnosis and treatment plans in this realm. Investigations to detect symptoms and signs in an attempt to prevent the tragedy of sudden cardiac death in basketball players may lead to MRI and CTA studies that compel radiologists to evaluate cardiac function along with myocardial and coronary artery anatomy. Worthy of mention also is the female athlete triad of disordered eating, amenorrhea, and osteoporosis that is observed in some young women participating in this and other sports
SN - 1432-1998
AD - Division of Pediatric Radiology, Department of Radiology, Duke University Health Systems, Durham, NC 27710, USA. ana.gaca@duke.edu
UR - 19774373
ER -

TY - JOUR
ID - 846
T1 - [The surgical treatment of chronic extension deficits of the knee]. [German]
A1 - Freiling,D.
A1 - Lobenhoffer,P.
Y1 - 2009/12//
N1 - Freiling, Denise. Lobenhoffer, Philipp
Operative Orthopadie und Traumatologie
9604937
IM
Clinical Trial. English Abstract. Journal Article
German
KW - MEDLINE
KW - Ankylosis/su [Surgery]
KW - Arthroplasty/is [Instrumentation]
KW - Arthroplasty/mt [Methods]
KW - Arthroscopy/mt [Methods]
KW - Child
KW - Chronic Disease
KW - Combined Modality Therapy
KW - Female
KW - Humans
KW - Infant
KW - Joint Instability/su [Surgery]
KW - Knee Joint/su [Surgery]
KW - Male
KW - Range of Motion

KW - Articular
KW - Reconstructive Surgical Procedures/mt [Methods]
KW - Treatment Outcome
RP - NOT IN FILE
SP - 545
EP - 556
JF - Operative Orthopadie und Traumatologie
JA - Oper.
VL - 21
IS - 6
CY - Germany
N2 - OBJECTIVE: Restoration of full knee extension in patients with chronic extension deficits, especially in posttraumatic and postoperative cases. INDICATIONS: Chronic knee extension deficits of more than 10 degrees . CONTRAINDICATIONS: Local intraarticular problems caused by cyclops syndrome, graft hypertrophy or graft impingement after anterior cruciate ligament reconstruction (notch impingement). These patients should be treated with arthroscopic procedures. Spastic flexion contracture. Noncompliant patients. Acute or chronic infections. Poor soft-tissue conditions on site of surgery. SURGICAL TECHNIQUE: If necessary, arthroscopy before arthrolysis to assure that the extension deficit is not caused by a local problem (cyclops, osteophytes, graft hypertrophy or graft impingement after anterior cruciate ligament reconstruction). Anterior skin incision at the medial border of the patellar ligament. Resection of Hoffa's fat pad, which is extremely fibrotic in almost all cases. Second skin incision at the posteromedial side of the knee joint. Incision of the medial retinaculum between the posterior border of the medial collateral ligament and the posterior oblique ligament. Posteromedial arthrotomy between the distal part of the tendon of the adductor magnus muscle and the posterior horn of the medial meniscus. Release of all adhesions in the posterior recess of the knee joint. Complete release of the posterior joint capsule from the femoral shaft. POSTOPERATIVE MANAGEMENT: Immobilization for 48 h after surgery in full extension (no knee motion allowed in the first 48 h). For 48 h after surgery only short walks to the bathroom are allowed. Special dynamic extension brace (Dynasplint((R)), CDS((R)) Forte, Albrecht company, Stephanskirchen, Germany) for 4-6 weeks after surgery 6-8 h per day. Painkillers following WHO (World Health Organization) protocol. Manual lymph drainage and electric muscle stimulation help to decrease pain and swelling. Physiotherapy twice daily starting at the 2nd postoperative day. No flexion exercises for the first 7 days after surgery. 15 kg partial weight bearing for 4-6 weeks. Daily physiotherapy is recommended after discharge. RESULTS: 121 patients underwent anterior and posterior arthrolysis between 1990 and 2000. 86 of these patients could be included in this study. The average follow-up was 4.6 years (1-10 years). The extension deficit before surgery averaged 20 degrees compared with the opposite side. At follow-up, the average extension had increased by 17 degrees , no patient had more than 5 degrees of flexion contracture. The Lysholm Score was 84 postoperatively. The Tegner Activity Scale increased from 1.9 to 4.0 after arthrolysis. In the AOSSM Subjective Outcome Score, 35 patients showed excellent and 60 good results. 14 patients were satisfied after surgery and nine were not. Three patients required revision surgery (two synovial fistulas, one hematoma)
SN - 1439-0981
AD - Klinik fur Unfall- und Wiederherstellungschirurgie, Diakoniekrankenhaus Henriettenstiftung Hannover, Hannover, Germany. d.freiling@gmx.de
UR - 20087716
ER -

TY - JOUR
ID - 847
T1 - [Students' physical activity: an analysis according to Pender's health promotion model]. [Portuguese]
A1 - Guedes,N.G.
A1 - Moreira,R.P.
A1 - Cavalcante,T.F.
A1 - de Araujo,T.L.
A1 - Ximenes,L.B.
Y1 - 2009/12//
N1 - Guedes, Nirla Gomes. Moreira, Rafaella Pessoa. Cavalcante, Tahissa Frota. de Araujo, Thelma Leite. Ximenes, Lorena Barbosa
Revista da Escola de Enfermagem da U S P

rss, 0242726
N
English Abstract. Journal Article
Portuguese
KW - MEDLINE
KW - Adolescent
KW - Child
KW - Cross-Sectional Studies
KW - Female
KW - Health Promotion
KW - Humans
KW - Male
KW - Models
KW - Theoretical
KW - Motor Activity
KW - Students
RP - NOT IN FILE
SP - 774
EP - 780
JF - Revista Da Escola de Enfermagem Da Usp
JA - Rev Esc.Enferm USP
VL - 43
IS - 4
CY - Brazil
N2 - The objective of this study was to describe the everyday physical activity habits of students and analyze the practice of physical activity and its determinants, based on the first component of Pender's health promotion model. This cross-sectional study was performed from 2004 to 2005 with 79 students in a public school in Fortaleza, Ceara, Brazil. Data collection was performed by interviews and physical examinations. The data were analyzed according to the referred theoretical model. Most students (n=60) were physically active. Proportionally, adolescents were the most active (80.4%). Those with a sedentary lifestyle had higher rates for overweight and obesity (21.1%). Many students practiced outdoor physical activities, which did not require any physical structure and good financial conditions. The results show that it is possible to associate the first component of Pender's health promotion model with the everyday lives of students in terms of the physical activity practice
SN - 0080-6234
AD - Programa de Pos-Graduacao em Enfermagem da Universidade Federal do Ceara, Fortaleza, CE, Brasil. nirlagomes@hotmail.com
UR - 20085145
ER -

TY - JOUR
ID - 848
T1 - Economic and other barriers to adopting recommendations to prevent childhood obesity: results of a focus group study with parents
A1 - Sonneville,K.R.
A1 - La,Pelle N.
A1 - Taveras,E.M.
A1 - Gillman,M.W.
A1 - Prosser,L.A.
Y1 - 2009///
N1 - Sonneville, Kendrin R. La Pelle, Nancy. Taveras, Elsie M. Gillman, Matthew W. Prosser, Lisa A
BMC pediatrics
100967804
IM
Comparative Study. Journal Article
English
KW - MEDLINE

KW - Adolescent
KW - Child
KW - Preschool
KW - Communication Barriers
KW - Feeding Behavior
KW - Female
KW - Follow-Up Studies
KW - Food Preferences
KW - Humans
KW - Male
KW - Obesity/ec [Economics]
KW - Obesity/pc [Prevention & Control]
KW - Obesity/px [Psychology]
KW - Parent-Child Relations
KW - Parenting
KW - Retrospective Studies
KW - Social Environment
RP - NOT IN FILE
SP - 81
JF - BMC Pediatrics
JA - BMC Pediatr
VL - 9
CY - England
N2 - BACKGROUND: Parents are integral to the implementation of obesity prevention and management recommendations for children. Exploration of barriers to and facilitators of parental decisions to adopt obesity prevention recommendations will inform future efforts to reduce childhood obesity. METHODS: We conducted 4 focus groups (2 English, 2 Spanish) among a total of 19 parents of overweight (BMI >or= 85th percentile) children aged 5-17 years. The main discussion focused on 7 common obesity prevention recommendations: reducing television (TV) watching, removing TV from child's bedroom, increasing physically active games, participating in community or school-based athletics, walking to school, walking more in general, and eating less fast food. Parents were asked to discuss what factors would make each recommendation more difficult (barriers) or easier (facilitators) to follow. Participants were also asked about the relative importance of economic (time and dollar costs/savings) barriers and facilitators if these were not brought into the discussion unprompted. RESULTS: Parents identified many barriers but few facilitators to adopting obesity prevention recommendations for their children. Members of all groups identified economic barriers (time and dollar costs) among a variety of pertinent barriers, although the discussion of dollar costs often required prompting. Parents cited other barriers including child preference, difficulty with changing habits, lack of information, lack of transportation, difficulty with monitoring child behavior, need for assistance from family members, parity with other family members, and neighborhood walking safety. Facilitators identified included access to physical activity programs, availability of alternatives to fast food and TV which are acceptable to the child, enlisting outside support, dietary information, involving the child, setting limits, making behavior changes gradually, and parental change in shopping behaviors and own eating behaviors. CONCLUSIONS: Parents identify numerous barriers to adopting obesity prevention recommendations, most notably child and family preferences and resistance to change, but also economic barriers. Intervention programs should consider the context of family priorities and how to overcome barriers and make use of relevant facilitators during program development
SN - 1471-2431
AD - Department of Nutrition, Harvard School of Public Health, Boston, MA, USA. ksonnevi@hsph.harvard.edu
UR - 20025769
ER -

TY - JOUR
ID - 849
T1 - A cross-curricular physical activity intervention to combat cardiovascular disease risk factors in 11-14 year olds: 'activity knowledge circuit'
A1 - Knox,G.
A1 - Baker,J.S.

A1 - Davies,B.
A1 - Faulkner,S.
A1 - Rance,J.
A1 - Rees,A.
A1 - Morgan,K.
A1 - Thomas,N.
Y1 - 2009///
N1 - Knox, Gareth. Baker, Julien S. Davies, Bruce. Faulkner, Susan. Rance, Jaynie. Rees, Anwen. Morgan, Kelly. Thomas, Non
BMC public health
100968562
IM
Journal Article. Randomized Controlled Trial. Research Support, Non-U.S. Gov't
English
KW - MEDLINE
KW - Adolescent
KW - Cardiovascular Diseases/pc [Prevention & Control]
KW - Child
KW - Curriculum
KW - Exercise
KW - Female
KW - Health Promotion/mt [Methods]
KW - Humans
KW - Interdisciplinary Communication
KW - Intervention Studies
KW - Male
KW - Problem-Based Learning
KW - Risk Factors
KW - Social Class
KW - Wales
RP - NOT IN FILE
SP - 466
JF - BMC Public Health
VL - 9
CY - England
N2 - BACKGROUND: Cardiovascular disease is the leading cause of mortality worldwide. Risk factors associated with cardiovascular disease have been shown to track from childhood through to adulthood. Previous school-based physical activity interventions have demonstrated modest improvements to cardiovascular disease risk factors by implementing extra-curricular activities or improving current physical education curriculum. Few have attempted to increase physical activity in class-room taught curriculum subjects. This study will outline a school-based cross-curricular physical activity intervention to combat cardiovascular disease risk factors in 11-14 year old children. METHOD/DESIGN: A South Wales Valley school of low socio-economic status has been selected to take part. Participants from year eight (12-13 years) are to be assigned to an intervention group, with maturation-matched participants from years seven (11-12 years) and nine (13-14 years) assigned to a control group. A cross-curricular physical activity intervention will be implemented to increase activity by two hours a week for 18 weeks. Participants will briskly walk 3200 m twice weekly during curriculum lessons (60 minutes duration). With the exception of physical education, all curriculum subjects will participate, with each subject delivering four intervention lessons. The intervention will be performed outdoors and on school premises. An indoor course of equal distance will be used during adverse weather conditions. Cardiovascular disease risk factors will be measured pre- and post-intervention for intervention and control groups. These will take place during physical education lessons and will include measures of stature, mass, waist, hip, and neck circumferences, together with skinfold measure's taken at four sites. Blood pressure will be measured, and fitness status assessed via the 20 m multi-stage fitness test. Questionnaires will be used to determine activity behaviour (physical activity questionnaire for adolescence), diet (seven day food diary) and maturation status. Fasting blood variables will include total cholesterol, low-density lipoprotein cholesterol, high density lipoprotein cholesterol, triglycerides, insulin, glucose, high-sensitivity C-reactive protein, interleukin-6, adiponectin, and fibrinogen. Motivational variables and psychological well-being will be assessed by

questionnaire. DISCUSSION: Our study may prove to be a cost effective strategy to increase school time physical activity to combat cardiovascular disease risk factors in children. TRIAL REGISTRATION: [NCT00998478]
SN - 1471-2458
AD - Cardiff School of Sport, University of Wales Institute Cardiff, Cardiff, UK. gaknox@uwic.ac.uk
UR - 20003492
ER -

TY - JOUR
ID - 850
T1 - [Analysis of non-confraternity sports accidents in the surgical department of a former district hospital during the period of 2 years]. [German]
A1 - Raschka,Ch
A1 - Raschka,S.
A1 - Peikert,T.
Y1 - 2009/12/01/
N1 - Raschka, Ch. Raschka, S. Peikert, T
Versicherungsmedizin / herausgegeben von Verband der Lebensversicherungs-Unternehmen e.V. und Verband der Privaten Krankenversicherung e.V
xac, 8803623
IM
English Abstract. Journal Article
German
KW - MEDLINE
KW - Adolescent
KW - Adult
KW - Age Factors
KW - Aged
KW - Athletic Injuries/ep [Epidemiology]
KW - Child
KW - Preschool
KW - Cross-Sectional Studies
KW - Emergency Service
KW - Hospital/sn [Statistics & Numerical Data]
KW - Female
KW - Germany
KW - Hospitals
KW - General/sn [Statistics & Numerical Data]
KW - Humans
KW - Incidence
KW - Leg Injuries/ep [Epidemiology]
KW - Leisure Activities
KW - Male
KW - Middle Aged
KW - Sex Factors
KW - Soccer/in [Injuries]
KW - Sports/sn [Statistics & Numerical Data]
KW - Young Adult
RP - NOT IN FILE
SP - 173
EP - 176
JF - Versicherungsmedizin
VL - 61
IS - 4
CY - Germany
N2 - This epidemiological study analyses all n = 1,659 outpatient and inpatient non-confraternity sports accidents treated during a 2-year period in a former district hospital. The largest share with 40.6% is soccer,

followed by cycling (15%), general fitness sports (7.6%), outdoor sports (6.5%), winter sports (5.5%), and riding (5.2%). Soccer injuries rise steadily until the age of 30. Of 86 horse riding accidents a total of 68 involved women, but only 18 men (ratio 8 : 2). 53% of the horse riding accidents among women concern the age group between 10 and 20 years. 70.6% (79%) of the athletes under (over) 20 years were male, 29.4% (21%) female ($p < 0.05$). Topographically the lower extremities represent the most affected body region in all sports (runners 84.4%, soccer players 60.2%). Most accidents occur on a Sunday. The most common diagnosis is contusion, most commonly in martial arts (60.8%), followed by horse riding (51%). There is an astonishing dominance of soccer accidents given the fact that this study records all athletes, not just club athletes, unlike insurance studies. Important preventive measures would be a comprehensive biological training prophylaxis and the provision of communication of age-specific accident prevention proposals for the mainly affected sports
SN - 0933-4548
AD - Chirurgischen Abteilung der Helios St.Elisabeth-Klinik, Hunfeld
UR - 20052829
ER -

TY - JOUR
ID - 851
T1 - Injury and illness encountered in Shenandoah National Park
A1 - Forrester,J.D.
A1 - Holstege,C.P.
Y1 - 2009///
N1 - Forrester, Joseph D. Holstege, Christopher P
Wilderness & environmental medicine
dbe, 9505185
IM
Journal Article
English
KW - MEDLINE
KW - Adolescent
KW - Adult
KW - Age Distribution
KW - Athletic Injuries/ep [Epidemiology]
KW - Child
KW - Preschool
KW - Conservation of Natural Resources
KW - Female
KW - Humans
KW - Male
KW - Middle Aged
KW - Recreation
KW - Retrospective Studies
KW - Virginia/ep [Epidemiology]
KW - Young Adult
RP - NOT IN FILE
SP - 318
EP - 326
JF - Wilderness & Environmental Medicine
JA - Wilderness Environ Med
VL - 20
IS - 4
CY - United States
N2 - OBJECTIVE: There have been no studies to date exploring the nature of injuries and illness experienced by individuals in a National Park in the southeastern United States. The purpose of this study was to determine the incidence of such illnesses and injuries to visitors in Shenandoah National Park. METHODS: This study was a retrospective review of the case incident reports from Shenandoah National Park from 2003 to 2007. Data obtained included age, sex, time and date report was received, medical symptoms, trauma type, location of injury, mechanism of injury, level of care, time to patient, time to disposition, disposition type, location, and

activity at time of event. RESULTS: There were 159 total cases, corresponding to a reported incident rate of 2.7 persons reported injured or ill per 100 000 visitors to Shenandoah National Park. A total of 23.3% of all reported injuries occurred in persons less than 18 years of age. The most common reported adult injury was soft tissue injury, with the most common anatomical location being the distal lower extremity. The most common activity in which adults were involved at the time of the injury was hiking. Of the pediatric trauma cases, the most common mechanism of injury was a fall. Of the adult medical illnesses, the most common complaint was chest pain. CONCLUSIONS: The pattern of adult and pediatric trauma is consistent among several geographically different National Parks in the United States and represents an injury pattern that all wilderness/outdoor care providers need to be competent to treat. Among adult visitors, the most common medical complaint was chest pain, a complaint more prevalent at Shenandoah National Park compared to other parks. Knowing that trauma injury patterns are relatively similar to those of other parks but that medical illness is more locale specific can help health care providers tailor their resource allotment and health management protocols
SN - 1545-1534
AD - Division of Medical Toxicology, Department of Emergency Medicine, University of Virginia, Charlottesville, VA, USA
UR - 20030438
ER -

TY - JOUR
ID - 852
T1 - School playground surfacing and arm fractures in children: a cluster randomized trial comparing sand to wood chip surfaces
A1 - Howard,A.W.
A1 - Macarthur,C.
A1 - Rothman,L.
A1 - Willan,A.
A1 - Macpherson,A.K.
Y1 - 2009/12//
N1 - Howard, Andrew W. Macarthur, Colin. Rothman, Linda. Willan, Andrew. Macpherson, Alison K
PLoS medicine
101231360
IM
Journal Article. Randomized Controlled Trial. Research Support, Non-U.S. Gov't
English
KW - MEDLINE
KW - Arm Injuries/ep [Epidemiology]
KW - Canada/ep [Epidemiology]
KW - Child
KW - Cluster Analysis
KW - Craniocerebral Trauma/ep [Epidemiology]
KW - Fractures
KW - Bone/ep [Epidemiology]
KW - Humans
KW - Risk Assessment
KW - Schools
KW - Silicon Dioxide
KW - Surface Properties
KW - Wood
RP - NOT IN FILE
SP - e1000195
JF - PLoS Medicine / Public Library of Science
JA - PLoS Med
VL - 6
IS - 12
CY - United States

N2 - BACKGROUND: The risk of playground injuries, especially fractures, is prevalent in children, and can result in emergency room treatment and hospital admissions. Fall height and surface area are major determinants of playground fall injury risk. The primary objective was to determine if there was a difference in playground upper extremity fracture rates in school playgrounds with wood fibre surfacing versus granite sand surfacing. Secondary objectives were to determine if there were differences in overall playground injury rates or in head injury rates in school playgrounds with wood fibre surfacing compared to school playgrounds with granite sand surfacing. METHODS AND FINDINGS: The cluster randomized trial comprised 37 elementary schools in the Toronto District School Board in Toronto, Canada with a total of 15,074 students. Each school received qualified funding for installation of new playground equipment and surfacing. The risk of arm fracture from playground falls onto granitic sand versus onto engineered wood fibre surfaces was compared, with an outcome measure of estimated arm fracture rate per 100,000 student-months. Schools were randomly assigned by computer generated list to receive either a granitic sand or an engineered wood fibre playground surface (Fibar), and were not blinded. Schools were visited to ascertain details of the playground and surface actually installed and to observe the exposure to play and to periodically monitor the depth of the surfacing material. Injury data, including details of circumstance and diagnosis, were collected at each school by a prospective surveillance system with confirmation of injury details through a validated telephone interview with parents and also through collection (with consent) of medical reports regarding treated injuries. All schools were recruited together at the beginning of the trial, which is now closed after 2.5 years of injury data collection. Compliant schools included 12 schools randomized to Fibar that installed Fibar and seven schools randomized to sand that installed sand. Noncompliant schools were added to the analysis to complete a cohort type analysis by treatment received (two schools that were randomized to Fibar but installed sand and seven schools that were randomized to sand but installed Fibar). Among compliant schools, an arm fracture rate of 1.9 (95% confidence interval [CI] 0.04-6.9) per 100,000 student-months was observed for falls into sand, compared with an arm fracture rate of 9.4 (95% CI 3.7-21.4) for falls onto Fibar surfaces (p< or =0.04905). Among all schools, the arm fracture rate was 4.5 (95% CI 0.26-15.9) per 100,000 student-months for falls into sand compared with 12.9 (95% CI 5.1-30.1) for falls onto Fibar surfaces. No serious head injuries and no fatalities were observed in either group. CONCLUSIONS: Granitic sand playground surfaces reduce the risk of arm fractures from playground falls when compared with engineered wood fibre surfaces. Upgrading playground surfacing standards to reflect this information will prevent arm fractures. TRIAL REGISTRATION: Current Controlled Trials ISRCTN02647424
SN - 1549-1676
AD - Division of Orthopaedic Surgery, Hospital for Sick Children, Toronto, Ontario, Canada.
andrew.howard@sickkids.ca
UR - 20016688
ER -

TY - JOUR
ID - 853
T1 - [Mumps transmission control status and inapparent infection rate among middle and high school students during the 2007-2008 mumps outbreak in Daegu]. [Korean]
A1 - Kim,K.H.
A1 - Kim,C.H.
A1 - Choi,B.Y.
A1 - Go,U.Y.
A1 - Lee,D.H.
A1 - Ki,M.
Y1 - 2009/11//
N1 - Kim, Kyo Hyun. Kim, Chang Hwi. Choi, Bo Youl. Go, Un Yeong. Lee, Dong Han. Ki, Moran
Journal of preventive medicine and public health = Yebang Uihakhoe chi
101242972
IM
English Abstract. Journal Article
Korean
KW - MEDLINE
KW - Adolescent
KW - Child
KW - Communicable Disease Control/mt [Methods]
KW - Disease Outbreaks/sn [Statistics & Numerical Data]

KW - Female
KW - Humans
KW - Immunoglobulin G/bl [Blood]
KW - Immunoglobulin M/bl [Blood]
KW - Male
KW - Mumps/ep [Epidemiology]
KW - Mumps/pc [Prevention & Control]
KW - Mumps/tm [Transmission]
KW - Patient Isolation
KW - Questionnaires
KW - Republic of Korea/ep [Epidemiology]
KW - Schools/sn [Statistics & Numerical Data]
KW - Students
RP - NOT IN FILE
SP - 408
EP - 415
JF - Journal of Preventive Medicine & Public Health / Yebang Uihakhoe Chi
JA - J Prev Med Pub Health
VL - 42
IS - 6
CY - Korea (South)
N2 - OBJECTIVES: This study was performed to investigate the mumps transmission control status and inapparent infection rate among middle and high school students in Daegu City during a mumps outbreak. METHODS: Nine schools (two middle schools and seven high schools), which reported a number of mumps cases between 2007 and 2008 were selected for investigation. During March-May 2008, a standard questionnaire was distributed to gather information about case identification, instructed isolation measure, isolation status of mumps cases and related factors, and outdoor activities of non-isolated mumps case. Inapparent infection rate was estimated by serum mumps IgM and IgG antibodies status and self-reported mumps symptoms in three of the nine schools. RESULTS: Among 2,560 respondents, more than half of students answered that they did not receive instructions in mumps transmission control measures during the outbreak. Among the 327 mumps cases identified by the questionnaire, 131 cases (40.1%) were considered as isolated and the isolation rates were significantly different among schools, grades, and gender. Of the non-isolated cases, 88.3% continued attending school. Inapparent mumps infection rates were between 56.3% and 70.2%. CONCLUSIONS: Mumps transmission control was inadequate to control the mumps outbreak. Although high inapparent infection rate would mitigate the transmission control effect of case isolation, this measure is fundamental for infection control. The reasons of this inadequate status need to be explored to develop an effective intervention strategy
SN - 1975-8375
AD - Department of Preventive Medicine, Eulji University School of Medicine, Korea
UR - 20009488
ER -

TY - JOUR
ID - 854
T1 - Ultrafine particles in indoor air of a school: possible role of secondary organic aerosols
A1 - Morawska,L.
A1 - He,C.
A1 - Johnson,G.
A1 - Guo,H.
A1 - Uhde,E.
A1 - Ayoko,G.
Y1 - 2009/12/15/
N1 - Morawska, Lidia. He, Congrong. Johnson, Graham. Guo, Hai. Uhde, Erik. Ayoko, Godwin
Environmental science & technology
dvo, 0213155
IM

Journal Article
English
KW - MEDLINE
KW - Aerosols/ch [Chemistry]
KW - Air Pollution
KW - Indoor/an [Analysis]
KW - Child
KW - Humans
KW - Oxidants
KW - Photochemical/an [Analysis]
KW - Ozone/an [Analysis]
KW - Particle Size
KW - Particulate Matter/an [Analysis]
KW - Schools
KW - Volatile Organic Compounds/an [Analysis]
RP - NOT IN FILE
SP - 9103
EP - 9109
JF - Environmental Science & Technology
JA - Environ Sci Technol
VL - 43
IS - 24
CY - United States
N2 - The aim of this work was to investigate ultrafine particles (<0.1 microm) in primary school classrooms, in relation to the classroom activities. The investigations were conducted in three classrooms during two measuring campaigns, which together encompassed a period of 60 days. Initial investigations showed that under the normal operating conditions of the school there were many occasions in all three classrooms where indoor particle concentrations increased significantly compared to outdoor levels. By far the highest increases in the classroom resulted from art activities (painting, gluing, and drawing), at times reaching over 1.4 x 10(5) particle cm(-3). The indoor particle concentrations exceeded outdoor concentrations by approximately 1 order of magnitude, with a count median diameter ranging from 20 to 50 nm. Significant increases also occurred during cleaning activities, when detergents were used. GC-MS analysis conducted on 4 samples randomly selected from about 30 different paints and glues, as well as the detergent used in the school, showed that d-limonene was one of the main organic compounds of the detergent, however, it was not detected in the samples of the paints and the glue. Controlled experiments showed that this monoterpene, emitted from the detergent, reacted with O(3) (at outdoor ambient concentrations ranging from 0.06 to 0.08 ppm) and formed secondary organic aerosols. Further investigations to identify other liquids that may be potential sources of the precursors of secondary organic aerosols were outside the scope of this project, however, it is expected that the problem identified by this study could be more widely spread, since most primary schools use liquid materials for art classes, and all schools use detergents for cleaning. Further studies are therefore recommended to better understand this phenomenon and also to minimize exposure of school children to ultrafine particles from these indoor sources
SN - 0013-936X
AD - International Laboratory for Air Quality and Health, Queensland University of Technology, Brisbane, Australia. l.morawska@qut.edu.au
UR - 20000499
ER -

TY - JOUR
ID - 855
T1 - Blood lead levels and potental environmental exposures among children under five years in Kibera slums, Nairobi
A1 - Olewe,T.M.
A1 - Mwanthi,M.A.
A1 - Wang'ombe,J.K.
A1 - Griffiths,J.K.
Y1 - 2009/04//

N1 - Olewe, Tom M. Mwanthi, Mutuku A. Wang'ombe, Joseph K. Griffiths, Jeffrey K
East African journal of public health
101302040
IM
Journal Article. Research Support, N.I.H., Extramural
English
KW - MEDLINE
KW - Analysis of Variance
KW - Child
KW - Preschool
KW - Cross-Sectional Studies
KW - Environmental Exposure/sn [Statistics & Numerical Data]
KW - Female
KW - Humans
KW - Infant
KW - Newborn
KW - Kenya/ep [Epidemiology]
KW - Lead/bl [Blood]
KW - Lead Poisoning/ep [Epidemiology]
KW - Lead Poisoning/et [Etiology]
KW - Male
KW - Prevalence
KW - Risk Factors
KW - Socioeconomic Factors
KW - Soil
KW - Water Supply
RP - NOT IN FILE
SP - 6
EP - 10
JF - East African Journal of Public Health
JA - East Afr J Public Health
VL - 6
IS - 1
CY - Tanzania
N2 - OBJECTIVE: Lead exposure has been associated with intellectual impairment in children in a number of international studies. Prevalence of elevated blood lead levels (eBLL > or = 10ug/dL) of between 5 - 15% has been reported among in Nairobi (UNEP, 2006). However, little is known about potential environmental exposure for eBLLs among children in Kibera, Nairobi. METHODS: A descriptive, cross-sectional study of children drawn from Kibera slums who presented at Yes to kids (Y2K) programme of VIPS Health Services at Woodley, Nairobi between June and August 2007 was carried out. The study assessed potential correlates of eBLLs in 387 children aged 6 to 59 months and had lived in Kibera slums since birth. Sampling was purposive. The factors examined were age, sex, breastfeeding history, respondent's education and occupation, type of house walls, sources of drinking water and kales, and awareness of lead poisoning among respondents. Potential risk factors such exposure to paint, contaminated playgrounds, glazed pottery, cosmetics and para-occupational as well as living near lead industry and pica behavior were also examined. Potential environmental sources of lead such as drinking water, soil and kales were analyzed for lead levels. RESULTS: Seven percent (n = 27, N = 387) had BLLs above 10ug/dl. BLL > or = 10ug/dl was associated with non-permanent housing (p = 0.812), playing on potentially lead contaminated grounds (p = 0.627) and pica behavior (p = 0.439). Low risk parental occupation (p = 0.001) and Kales sourced from the market/kiosks (p = 0.001) were significantly associated with BLL > or = 10ug/dl. Soil lead levels (Soil Pb) ranged from 3,000 to 90,000ug/kg, which was very high compared to WHO acceptable range of 100 - 200ug/kg. There was weak linear association (r2 = 0.0160) between Soil Pb and mean BLLs for a given village. There were no detectable levels of lead in kales and tap water. CONCLUSIONS: The study found about 7% (N = 387) of the children tested had eBLL > or = 10ug/dl in an area with very high soil lead levels (range in Kibera slums: 3,365 - 89,570ug/kg; WHO allowable range: 100 - 120ug/kg), raising a health flag that must be addressed using the multi-sectoral approach and further studies. It's important to note that the study design and its inherent limitations could have masked true picture of childhood lead poisoning in Kibera slums, Nairobi

SN - 0856-8960
AD - Vision Integrity & Passion to Serve (VIPS) Health Services at Woodley, Nairobi, Kenya.
tolewe@vipskenya.or.ke
UR - 20000055
ER -

TY - JOUR
ID - 856
T1 - Exposure to environmental tobacco smoke among adolescents in Kampala-Uganda, 2002
A1 - Rudatsikira,E.
A1 - Muula,A.S.
A1 - Siziya,S.
Y1 - 2009/08//
N1 - Rudatsikira, Emmanuel. Muula, Adamson S. Siziya, Seter
East African journal of public health
101302040
IM
Journal Article
English
KW - MEDLINE
KW - Adolescent
KW - Child
KW - Environmental Exposure/ae [Adverse Effects]
KW - Environmental Exposure/sn [Statistics & Numerical Data]
KW - Environmental Exposure
KW - Female
KW - Health Knowledge
KW - Attitudes
KW - Practice
KW - Humans
KW - Logistic Models
KW - Male
KW - Prevalence
KW - Questionnaires
KW - Risk Factors
KW - Sex Factors
KW - Smoking/ep [Epidemiology]
KW - Tobacco Smoke Pollution/ae [Adverse Effects]
KW - Tobacco Smoke Pollution/sn [Statistics & Numerical Data]
KW - Tobacco Smoke Pollution
KW - Uganda/ep [Epidemiology]
RP - NOT IN FILE
SP - 197
EP - 199
JF - East African Journal of Public Health
JA - East Afr J Public Health
VL - 6
IS - 2
CY - Tanzania
N2 - OBJECTIVE: To estimate the prevalence of environmental tobacco smoke (ETS) inside or outside the home among school-going adolescents in Kampala, Uganda. METHODS: Data from the Kampala Global Youth Tobacco Survey (GYTS) of 2002 was used. We estimated frequencies and proportions of self reported exposure to ETS by the study participants. With logistic analysis, we assessed the association between ETS (outcome) in the home or outside the home and the following variables: sex; parental smoking status; and whether best friend was a smoker or not. RESULTS: Of the 2427 non-smoker teenagers who participated in this study, 52.8% were females, 17.9% were exposed to ETS at home while 48.7% were exposed to ETS outside of the home. The majority of the participants (65.8%) were in favour of banning smoking in public places such as in hotels, taxi,

in schools, on playgrounds, in discos, markets, and shops. Compared to female participants, males were more likely to be exposed to ETS outside of the home (OR=1.21; 95% CI (1.02, 1.441). Having parents and close friends who smoked cigarettes was positively associated with exposure to ETS at home or outside of the home. Responders whose parents smoked cigarettes were more than four times likely to be exposed to ETS at home than those whose parents were non-smokers (OR=4.88; 95% CI [3.76, 6.33]). CONCLUSION: Cultural factors may expose boys to ETS than girls. We also found that having parents who were smokers exposed adolescents to ETS outside the home and having best friends who smoked exposed adolescents to ETS. This may suggest that adolescents who are exposed to ETS in one way may also be at risk of exposure through other means
SN - 0856-8960
AD - Division of Epidemiology and Biostatistics, San Diego State University, San Diego, California, USA
UR - 20000029
ER -

TY - JOUR
ID - 857
T1 - Observing children's playground activity levels at 13 Illawarra primary schools using CAST2
A1 - Parrish,A.M.
A1 - Iverson,D.
A1 - Russell,K.
A1 - Yeatman,H.
Y1 - 2009///
N1 - Parrish, Anne-Maree. Iverson, Don. Russell, Ken. Yeatman, Heather
Journal of physical activity & health
101189457
IM
Journal Article. Multicenter Study
English
KW - MEDLINE
KW - Australia
KW - Child
KW - Preschool
KW - Education/og [Organization & Administration]
KW - Education
KW - Environment
KW - Exercise
KW - Female
KW - Humans
KW - Logistic Models
KW - Male
KW - Observation
KW - Odds Ratio
KW - Organizational Policy
KW - Play and Playthings
KW - Sex Distribution
KW - Socioeconomic Factors
KW - Students
RP - NOT IN FILE
SP - S89
EP - S96
JF - Journal of Physical Activity & Health
JA - J Phys Act Health
VL - 6 Suppl 1
CY - United States
N2 - BACKGROUND: Declining levels of children's physical activity may contribute to Australia's increasing childhood obesity epidemic. School recess is an underutilized opportunity to increase children's physical activity. METHODS: Thirteen regional Australian public primary schools participated in the study (2946 children). The Children's Activity Scanning Tool 2 (CAST2) collected observational playground physical

activity data. The research also addressed: length of break, socioeconomic status (SES), gender, number of scanning days, and instrument calibration. RESULTS: The proportions of Moderate or Vigorous Physically Activity (MVPA) children at the observed schools ranged from 0.4 to 0.7. The odds ratio of boys being MVPA relative to girls ranged from 0.8581 to 2.137. There were significant differences between the mean proportions of 3 days of activity (range P = .001 to P = .015) and no association between SES school groupings (deviance ratio: 0.48; P = .503). Interrater reliability for instrument calibration using Spearman correlations coefficients ranged from r = .71 to r = .99. CONCLUSIONS: There were significant differences between proportions of MVPA children at the 13 schools and between male and female populations. There was no association between playground physical activity and SES. The monitoring period for CAST2 should be at least 3 days. Interrater reliability indicates that correlations between observers were consistently high
SN - 1543-3080
AD - Faculty of Health and Behavioural Sciences, University of Wollongong, Australia
UR - 19998854
ER -

TY - JOUR
ID - 858
T1 - A national survey of obesity prevention practices in Head Start
A1 - Whitaker,R.C.
A1 - Gooze,R.A.
A1 - Hughes,C.C.
A1 - Finkelstein,D.M.
Y1 - 2009/12//
N1 - Whitaker, Robert C. Gooze, Rachel A. Hughes, Cayce C. Finkelstein, Daniel M
Archives of pediatrics & adolescent medicine
9422751, bwf
AIM, IM
Journal Article. Research Support, Non-U.S. Gov't
English
KW - MEDLINE
KW - Analysis of Variance
KW - Child
KW - Preschool
KW - Early Intervention (Education)
KW - Female
KW - Health Promotion/mt [Methods]
KW - Humans
KW - Linear Models
KW - Male
KW - Motor Activity
KW - Obesity/ep [Epidemiology]
KW - Obesity/pc [Prevention & Control]
KW - Questionnaires
KW - United States/ep [Epidemiology]
RP - NOT IN FILE
SP - 1144
EP - 1150
JF - Archives of Pediatrics & Adolescent Medicine
JA - Arch Pediatr Adolesc Med
VL - 163
IS - 12
CY - United States
N2 - OBJECTIVE: To describe obesity prevention practices and environments in Head Start, the largest federally funded early childhood education program in the United States. DESIGN: Self-administered survey as part of the Study of Healthy Activity and Eating Practices and Environments in Head Start (SHAPES). SETTING: Head Start, 2008. PARTICIPANTS: Directors of all 1810 Head Start programs, excluding those in US territories. OUTCOME MEASURES: Descriptive measures of reported practices and environments related

to healthy eating and gross motor activity. RESULTS: The 1583 (87%) programs responding to the survey enrolled 828 707 preschool children. Of these programs, 70% reported serving only nonfat or 1% fat milk. Ninety-four percent of programs reported that each day they served children some fruit other than 100% fruit juice; 97% reported serving some vegetable other than fried potatoes; and 91% reported both of these daily practices. Sixty-six percent of programs said they celebrated special events with healthy foods or nonfood treats, and 54% did not allow vending machines for staff. Having an on-site outdoor play area at every center was reported by 89% of programs. Seventy-four percent of programs reported that children were given structured (adult-led or -guided) gross motor activity for at least 30 minutes each day; 73% reported that children were given unstructured gross motor activity for at least 30 minutes each day, and 56% reported both of these daily practices. CONCLUSION: Most Head Start programs report doing more to support healthy eating and gross motor activity than required by federal performance standards in these areas
SN - 1538-3628
AD - Department of Public Health, Temple University, Center for Obesity Research and Education, Philadelphia, PA 19140, USA. rwhitaker@temple.edu
UR - 19996052
ER -

TY - JOUR
ID - 859
T1 - Sex differences in the structure and stability of children's playground social networks and their overlap with friendship relations
A1 - Baines,E.
A1 - Blatchford,P.
Y1 - 2009/09//
N1 - Baines, Ed. Blatchford, Peter
The British journal of developmental psychology
8308022
IM
Journal Article
English
KW - MEDLINE
KW - Child
KW - Female
KW - Friends/px [Psychology]
KW - Gender Identity
KW - Humans
KW - Longitudinal Studies
KW - Male
KW - Models
KW - Psychological
KW - Peer Group
KW - Personality Assessment
KW - Play and Playthings
KW - Social Behavior
KW - Social Identification
KW - Social Support
KW - Sociometric Techniques
RP - NOT IN FILE
SP - 743
EP - 760
JF - British Journal of Developmental Psychology
JA - BR J DEV PSYCHOL
VL - 27
IS - Pt 3
CY - England
N2 - Gender segregated peer networks during middle childhood have been highlighted as important for explaining later sex differences in behaviour, yet few studies have examined the structural composition of these

networks and their implications. This short-term longitudinal study of 119 children (7-8 years) examined the size and internal structure of boys' and girls' social networks, their overlap with friendship relations, and their stability over time. Data collection at the start and end of the year involved systematic playground observations of pupils' play networks during team and non-team activities and measures of friendship from peer nomination interviews. Social networks were identified by aggregating play network data at each time point. Findings showed that the size of boy's play networks on the playground, but not their social networks, varied according to activity type. Social network cores consisted mainly of friends. Girl's social networks were more likely to be composed of friends and boys' networks contained friends and non-friends. Girls had more friends outside of the social network than boys. Stability of social network membership and internal network relations were higher for boys than girls. These patterns have implications for the nature of social experiences within these network contexts
SN - 0261-510X
AD - Institute of Education, University of London, London, UK. e.baines@ioe.ac.uk
UR - 19994579
ER -

TY - JOUR
ID - 860
T1 - Social and community participation of children and youth with cerebral palsy is associated with age and gross motor function classification
A1 - Palisano,R.J.
A1 - Kang,L.J.
A1 - Chiarello,L.A.
A1 - Orlin,M.
A1 - Oeffinger,D.
A1 - Maggs,J.
Y1 - 2009/12//
N1 - Palisano, Robert J. Kang, Lin-Ju. Chiarello, Lisa A. Orlin, Margo. Oeffinger, Donna. Maggs, Jill
Physical therapy
0022623, p6w
AIM, IM
Journal Article. Multicenter Study. Research Support, Non-U.S. Gov't
English
KW - MEDLINE
KW - Adolescent
KW - Age Factors
KW - Cerebral Palsy/pp [Physiopathology]
KW - Child
KW - Cross-Sectional Studies
KW - Disabled Children/cl [Classification]
KW - Female
KW - Humans
KW - Interpersonal Relations
KW - Leisure Activities
KW - Male
KW - Motor Skills/cl [Classification]
KW - Prospective Studies
KW - Sampling Studies
KW - Young Adult
RP - NOT IN FILE
SP - 1304
EP - 1314
JF - Physical Therapy
JA - Phys Ther
VL - 89
IS - 12
CY - United States

N2 - BACKGROUND: Through social and community participation, children and youth with cerebral palsy (CP) form friendships, gain knowledge, learn skills, express creativity, and determine meaning and purpose in life. OBJECTIVE: The purposes of this study were: (1) to determine whether social and community participation of children and youth with CP differ based on age, sex, and gross motor function, and (2) to identify the types of activities in which social and community participation are highest. DESIGN AND METHODS: A prospective cross-sectional analytic design was used. The participants were a sample of convenience of 291 children (6-12 years of age) and 209 youth (13-21 years of age) with CP (55.4% males, 44.6% females) receiving services from 7 children's hospitals. Participants completed the Children's Assessment of Participation and Enjoyment (CAPE) by structured interview. Gross Motor Function Classification System (GMFCS) level was determined by the researchers. RESULTS: Youth did a higher percentage of activities with friends and others and outside the home than children. Children and youth in level I did a higher percentage of activities with friends and others compared with children and youth in levels II and III and in levels IV and V. Children and youth in level I and in levels IV and V did a higher percentage of activities outside the home than children and youth in levels II and III. Differences were not found between females and males. The percentage of activities done with friends and others and outside the home was highest for physical and skill-based activities. LIMITATIONS: Findings cannot be attributed only to GMFCS level. CONCLUSIONS: The ability to walk without restrictions is desirable for social and community participation. For children and youth with CP who have limitations in mobility, physical therapists have roles as consultants for accessibility, activity accommodations, and assistive technology and as advocates for inclusive environments
SN - 1538-6724
AD - Department of Physical Therapy and Rehabilitation Sciences, Drexel University, Philadelphia, PA 19102-1192, USA. robert.j.palisano@drexel.edu
UR - 19815646
ER -

TY - JOUR
ID - 861
T1 - Secondhand smoke and respiratory symptoms among adolescent current smokers
A1 - Lai,H.K.
A1 - Ho,S.Y.
A1 - Wang,M.P.
A1 - Lam,T.H.
Y1 - 2009/11//
N1 - Lai, Hak-Kan. Ho, Sai-Yin. Wang, Man-Ping. Lam, Tai-Hing
Pediatrics
oxv, 0376422
AIM, IM
Journal Article. Research Support, Non-U.S. Gov't
English
KW - MEDLINE
KW - Adolescent
KW - Child
KW - Cough/et [Etiology]
KW - Female
KW - Humans
KW - Male
KW - Respiration Disorders/et [Etiology]
KW - Risk Factors
KW - Smoking/ae [Adverse Effects]
KW - Sputum/se [Secretion]
KW - Tobacco Smoke Pollution/ae [Adverse Effects]
KW - Young Adult
RP - NOT IN FILE
SP - 1306
EP - 1310
JF - Pediatrics
VL - 124

IS - 5
CY - United States
N2 - OBJECTIVE: No study has ever reported the association between persistent respiratory symptoms and exposure to secondhand smoke (SHS) in adolescent smokers. The impact of SHS exposure on child health could be largely underestimated by not taking into account such effects. We investigated the association between exposure to SHS and respiratory symptoms among adolescent current smokers. METHODS: A total of 32506 students aged 11 to 20 years from 85 randomly selected secondary schools in Hong Kong completed a self-administered questionnaire that included persistent respiratory symptoms (for 3 consecutive months in the past 12 months), number of days of SHS exposure per week at home and outside home, smoking status, amount of active smoking, and other basic demographic characteristics and socioeconomic status. RESULTS: Adolescent current smokers who were exposed to SHS at home 1 to 4 and 5 to 7 days/wk were 50% (95% confidence interval [CI]: 3%-121%) and 77% (95% CI: 5%-199%) more likely, respectively, to report respiratory symptoms compared with those who were unexposed (P = .01 for trend). The corresponding figures for exposure outside home were 41% (95% CI: 3%-94%) and 85% (95% CI: 31%-161%; P = .004 for trend). Such associations were also observed among never-smokers, but they were weaker than those among current smokers (P < .01 for interaction). CONCLUSIONS: This is the first evidence that SHS exposure is associated with increased risks for persistent respiratory symptoms among adolescent current smokers. Health promotion programs should aim at SHS reduction as well as smoking cessation among adolescent smokers
SN - 1098-4275
AD - School of Public Health, University of Hong Kong, Hong Kong
UR - 19841127
ER -

TY - JOUR
ID - 862
T1 - From the American Academy of Pediatrics: Policy statement--Tobacco use: a pediatric disease.[Erratum appears in Pediatrics. 2010 Apr;125(4):861]
A1 - Committee on Environmental HealthHealth
A1 - Committee on Substance Abuse
A1 - Committee on Adolescence
A1 - Committee on Native American Child.
Y1 - 2009/11//
N1 - Committee on Environmental HealthHealth. Committee on Substance Abuse. Committee on Adolescence. Committee on Native American Child
Pediatrics
oxv, 0376422
AIM, IM
Journal Article
English
KW - MEDLINE
KW - Cetirizine
KW - Child
KW - Child Welfare
KW - Counseling
KW - Health Policy
KW - Humans
KW - Physician's Role
KW - Smoking/pc [Prevention & Control]
KW - Smoking Cessation
KW - Tobacco Smoke Pollution/pc [Prevention & Control]
RP - NOT IN FILE
SP - 1474
EP - 1487
JF - Pediatrics
VL - 124
IS - 5
CY - United States

N2 - Tobacco use and secondhand tobacco-smoke (SHS) exposure are major national and international health concerns. Pediatricians and other clinicians who care for children are uniquely positioned to assist patients and families with tobacco-use prevention and treatment. Understanding the nature and extent of tobacco use and SHS exposure is an essential first step toward the goal of eliminating tobacco use and its consequences in the pediatric population. The next steps include counseling patients and family members to avoid SHS exposures or cease tobacco use; advocacy for policies that protect children from SHS exposure; and elimination of tobacco use in the media, public places, and homes. Three overarching principles of this policy can be identified: (1) there is no safe way to use tobacco; (2) there is no safe level or duration of exposure to SHS; and (3) the financial and political power of individuals, organizations, and government should be used to support tobacco control. Pediatricians are advised not to smoke or use tobacco; to make their homes, cars, and workplaces tobacco free; to consider tobacco control when making personal and professional decisions; to support and advocate for comprehensive tobacco control; and to advise parents and patients not to start using tobacco or to quit if they are already using tobacco. Prohibiting both tobacco advertising and the use of tobacco products in the media is recommended. Recommendations for eliminating SHS exposure and reducing tobacco use include attaining universal (1) smoke-free home, car, school, work, and play environments, both inside and outside, (2) treatment of tobacco use and dependence through employer, insurance, state, and federal supports, (3) implementation and enforcement of evidence-based tobacco-control measures in local, state, national, and international jurisdictions, and (4) financial and systems support for training in and research of effective ways to prevent and treat tobacco use and SHS exposure. Pediatricians, their staff and colleagues, and the American Academy of Pediatrics have key responsibilities in tobacco control to promote the health of children, adolescents, and young adults
SN - 1098-4275
UR - 19841108
ER -

TY - JOUR
ID - 863
T1 - Short nighttime sleep-duration and hyperactivity trajectories in early childhood
A1 - Touchette,E.
A1 - Cote,S.M.
A1 - Petit,D.
A1 - Liu,X.
A1 - Boivin,M.
A1 - Falissard,B.
A1 - Tremblay,R.E.
A1 - Montplaisir,J.Y.
Y1 - 2009/11//
N1 - Touchette, Evelyne. Cote, Sylvana M. Petit, Dominique. Liu, Xuecheng. Boivin, Michel. Falissard, Bruno. Tremblay, Richard E. Montplaisir, Jacques Y
Pediatrics
oxv, 0376422
AIM, IM
Journal Article. Research Support, Non-U.S. Gov't
English
KW - MEDLINE
KW - Attention Deficit Disorder with Hyperactivity/et [Etiology]
KW - Child
KW - Preschool
KW - Humans
KW - Infant
KW - Male
KW - Maternal Behavior
KW - Motor Activity
KW - Parents
KW - Questionnaires
KW - Risk Factors
KW - Sleep

RP - NOT IN FILE
SP - e985
EP - e993
JF - Pediatrics
VL - 124
IS - 5
CY - United States
N2 - OBJECTIVES: Our objectives were to investigate the developmental trajectories of nighttime sleep duration and hyperactivity over the preschool years and to identify the risk factors associated with short nighttime sleep duration and high hyperactivity scores. DESIGN, SETTING, AND PARTICIPANTS: Nighttime sleep duration and hyperactivity were measured yearly by questionnaires administered to mothers of 2057 children from age 1.5 to 5 years. Developmental trajectories of nighttime sleep duration and hyperactivity throughout early childhood were analyzed to determine interassociations. A multinomial logistic regression was performed to determine which factors among selected child, maternal, and family characteristics and parental practices surrounding sleep periods in early childhood were associated with short nighttime sleep duration and high hyperactivity scores. RESULTS: The trajectories of nighttime sleep duration and hyperactivity were significantly associated. The odds ratio (OR) of reporting short nighttime sleep duration was 5.1 for highly hyperactive children (confidence interval [CI]: 3.2-7.9), whereas the OR of reporting high hyperactivity scores was 4.2 for persistently short sleepers (CI: 2.7-6.6). The risk factors for reporting short nighttime sleep duration and high hyperactivity scores were (1) being a boy, (2) having insufficient household income, (3) having a mother with a low education, and (4) being comforted outside the bed after a nocturnal awakening at 1.5 years of age. CONCLUSIONS: The risk of short nighttime sleep duration in highly hyperactive children is greater than the risk of high hyperactivity scores in short sleepers. Preventive interventions that target boys living in adverse familial conditions could be used to address these concomitant behavioral problems
SN - 1098-4275
AD - International Laboratory for Child and Adolescent Mental Health Development, INSERM U669, France
UR - 19841107
ER -

TY - JOUR
ID - 864
T1 - Evaluation of a pre-existing, 3-year household water treatment and handwashing intervention in rural Guatemala
A1 - Arnold,B.
A1 - Arana,B.
A1 - Mausezahl,D.
A1 - Hubbard,A.
A1 - Colford,J.M.,Jr.
Y1 - 2009/12//
N1 - Arnold, Benjamin. Arana, Byron. Mausezahl, Daniel. Hubbard, Alan. Colford, John M Jr
International journal of epidemiology
gr6, 7802871
IM
Journal Article. Research Support, Non-U.S. Gov't
English
KW - MEDLINE
KW - Adolescent
KW - Case-Control Studies
KW - Child
KW - Preschool
KW - Cohort Studies
KW - Cross-Sectional Studies
KW - Diarrhea/ep [Epidemiology]
KW - Diarrhea/pc [Prevention & Control]
KW - Female
KW - Follow-Up Studies
KW - Fresh Water

KW - Growth/ph [Physiology]
KW - Guatemala/ep [Epidemiology]
KW - Hand Disinfection
KW - Health Behavior
KW - Health Knowledge
KW - Attitudes
KW - Practice
KW - Humans
KW - Hygiene/ed [Education]
KW - Hygiene/st [Standards]
KW - Intervention Studies
KW - Likelihood Functions
KW - Male
KW - Respiratory Tract Infections/ep [Epidemiology]
KW - Respiratory Tract Infections/pc [Prevention & Control]
KW - Rural Health
KW - Soaps
RP - NOT IN FILE
SP - 1651
EP - 1661
JF - International Journal of Epidemiology
JA - Int J Epidemiol
VL - 38
IS - 6
CY - England
N2 - BACKGROUND: The promotion of household water treatment and handwashing with soap has led to large reductions in child diarrhoea in randomized efficacy trials. Currently, we know little about the health effectiveness of behaviour-based water and hygiene interventions after the conclusion of intervention activities. METHODS: We present an extension of previously published design (propensity score matching) and analysis (targeted maximum likelihood estimation) methods to evaluate the behavioural and health impacts of a pre-existing but non-randomized intervention (a 3-year, combined household water treatment and handwashing campaign in rural Guatemala). Six months after the intervention, we conducted a cross-sectional cohort study in 30 villages (15 intervention and 15 control) that included 600 households, and 929 children <5 years of age. RESULTS: The study design created a sample of intervention and control villages that were comparable across more than 30 potentially confounding characteristics. The intervention led to modest gains in confirmed water treatment behaviour [risk difference = 0.05, 95% confidence interval (CI) 0.02-0.09]. We found, however, no difference between the intervention and control villages in self-reported handwashing behaviour, spot-check hygiene conditions, or the prevalence of child diarrhoea, clinical acute lower respiratory infections or child growth. CONCLUSIONS: To our knowledge this is the first post-intervention follow-up study of a combined household water treatment and handwashing behaviour change intervention, and the first post-intervention follow-up of either intervention type to include child health measurement. The lack of child health impacts is consistent with unsustained behaviour adoption. Our findings highlight the difficulty of implementing behaviour-based household water treatment and handwashing outside of intensive efficacy trials
SN - 1464-3685
AD - Division of Epidemiology, University of California, Berkeley, CA 94720-7360, USA. benarnold@berkeley.edu
UR - 19574492
ER -

TY - JOUR
ID - 865
T1 - Intrinsic and extrinsic rewards in a nonformal environmental education program
A1 - Price,E.A.
A1 - Vining,J.
A1 - Saunders,C.D.
Y1 - 2009/09//

N1 - Price, Emily A. Vining, Joanne. Saunders, Carol D
Zoo biology
8807837
IM
Journal Article
English
KW - MEDLINE
KW - Adult
KW - Animals
KW - Zoo
KW - Child
KW - Conservation of Natural Resources
KW - Education/mt [Methods]
KW - Education
KW - Female
KW - Humans
KW - Interpersonal Relations
KW - Male
KW - Motivation
KW - Reward
RP - NOT IN FILE
SP - 361
EP - 376
JF - Zoo Biology
JA - Zoo biol
VL - 28
IS - 5
CY - United States
N2 - Humans are surrounded by threats to the environment, many of their own making. The severity of environmental problems will not decrease unless action is taken to develop and encourage greater environmentally responsible behavior (ERB) in the general populace. Environmental education (EE) is one method for strengthening precursors to ERB such as knowledge and attitudes, but research on the connection is currently unclear. In this paper we present the results of a study investigating the role played by rewards in encouraging ERB precursors for adults and children involved in a zoo-based Nature Swap program. We used semistructured interviews to question 91 participants, including 38 children, 38 adult guardians, and 15 staff members regarding the importance of rewards in the program. We content analyzed the interviews to identify and describe major themes and then coded them. We found that adult guardians and Play Partners perceived intrinsic and extrinsic rewards as aiding in maintaining motivation and interest in the nonformal Nature Swap program. In addition, both children and adult companion participants in the program mentioned strengthened precursors to ERB. Overall we found that adult companions perceived that children who participated in the program spent more quality time outdoors and had a heightened awareness of their surroundings as a result of program-based rewards. Implications for other EE and conservation education programs are discussed
SN - 1098-2361
AD - Utah State University, Logan, Utah 84322, USA. Emily.Price@usu.edu
UR - 19821499
ER -

TY - JOUR
ID - 866
T1 - Supporting adolescent emotional health in schools: a mixed methods study of student and staff views in England
A1 - Kidger,J.
A1 - Donovan,J.L.
A1 - Biddle,L.
A1 - Campbell,R.
A1 - Gunnell,D.
Y1 - 2009///

N1 - Kidger, Judi. Donovan, Jenny L. Biddle, Lucy. Campbell, Rona. Gunnell, David
BMC public health
100968562
IM
Comparative Study. Journal Article. Research Support, Non-U.S. Gov't
English
KW - MEDLINE
KW - Adolescent
KW - Adolescent Health Services/st [Standards]
KW - Child
KW - England
KW - Faculty
KW - Female
KW - Focus Groups
KW - Humans
KW - Interviews as Topic
KW - Male
KW - Mental Health
KW - Mental Health Services/st [Standards]
KW - Questionnaires
KW - School Health Services/st [Standards]
KW - Students/px [Psychology]
KW - Students/sn [Statistics & Numerical Data]
RP - NOT IN FILE
SP - 403
JF - BMC Public Health
VL - 9
CY - England
N2 - BACKGROUND: Schools have been identified as an important place in which to support adolescent emotional health, although evidence as to which interventions are effective remains limited. Relatively little is known about student and staff views regarding current school-based emotional health provision and what they would like to see in the future, and this is what this study explored. METHODS: A random sample of 296 English secondary schools were surveyed to quantify current level of emotional health provision. Qualitative student focus groups (27 groups, 154 students aged 12-14) and staff interviews (12 interviews, 15 individuals) were conducted in eight schools, purposively sampled from the survey respondents to ensure a range of emotional health activity, free school meal eligibility and location. Data were analysed thematically, following a constant comparison approach. RESULTS: Emergent themes were grouped into three areas in which participants felt schools did or could intervene: emotional health in the curriculum, support for those in distress, and the physical and psychosocial environment. Little time was spent teaching about emotional health in the curriculum, and most staff and students wanted more. Opportunities to explore emotions in other curriculum subjects were valued. All schools provided some support for students experiencing emotional distress, but the type and quality varied a great deal. Students wanted an increase in school-based help sources that were confidential, available to all and sympathetic, and were concerned that accessing support should not lead to stigma. Finally, staff and students emphasised the need to consider the whole school environment in order to address sources of distress such as bullying and teacher-student relationships, but also to increase activities that enhanced emotional health. CONCLUSION: Staff and students identified several ways in which schools can improve their support of adolescent emotional health, both within and outside the curriculum. However, such changes should be introduced as part of a wider consideration of how the whole school environment can be more supportive of students' emotional health. Clearer guidance at policy level, more rigorous evaluation of current interventions, and greater dissemination of good practice is necessary to ensure adolescents' emotional health needs are addressed effectively within schools
SN - 1471-2458
AD - Department of Social Medicine, University of Bristol, Canynge Hall, 39, Whatley Road, Bristol BS8 2PS, UK. judi.kidger@bristol.ac.uk
UR - 19878601
ER -

TY - JOUR
ID - 867
T1 - Characteristics influencing participation of Australian children with cerebral palsy
A1 - Imms,C.
A1 - Reilly,S.
A1 - Carlin,J.
A1 - Dodd,K.J.
Y1 - 2009///
N1 - Imms, Christine. Reilly, Sheena. Carlin, John. Dodd, Karen J
Disability and rehabilitation
9207179, a8i
IM
Journal Article. Research Support, Non-U.S. Gov't
English
KW - MEDLINE
KW - Cerebral Palsy/px [Psychology]
KW - Cerebral Palsy/rh [Rehabilitation]
KW - Child
KW - Disabled Children/px [Psychology]
KW - Disabled Children/rh [Rehabilitation]
KW - Family
KW - Female
KW - Humans
KW - Interpersonal Relations
KW - Leisure Activities/px [Psychology]
KW - Leisure Activities
KW - Linear Models
KW - Male
KW - Social Environment
RP - NOT IN FILE
SP - 2204
EP - 2215
JF - Disability & Rehabilitation
JA - Disabil Rehabil
VL - 31
IS - 26
CY - England
N2 - PURPOSE: To investigate the extent to which selected individual, family and environmental variables were associated with participation of children who have cerebral palsy in activities outside school. METHODS: Data were gathered through a population-based survey of 114 children born in 1994 or 1995 in Victoria, Australia. Participation was measured using the Children's Assessment of Participation and Enjoyment. Selected independent variables were classified as related to the child, family or environment. Linear regression analysis was used to identify variables associated with participation in informal (activities that require little planning) and formal (those with structure and leaders) activities. RESULTS: Participation in informal activities tended to be greater in children who preferred informal activities and who had higher manual ability (adjusted R(2) = 36.3%). Girls and those with better gross motor function also tended to participate in more activities. The explanatory power of the regression model for participation in formal activities was limited (adjusted R(2) = 4.2%). CONCLUSION: Knowing a child's activity preferences is critical to intervention planning. Being exposed to a range of activities within supportive environments may provide the opportunity to develop preferences, especially in activities where children with cerebral palsy have reduced participation, such as in physical activities
SN - 0963-8288
AD - La Trobe University, School of Occupational Therapy, La Trobe University, Melbourne, Australia. c.imms@latrobe.edu.au
UR - 19903130
ER -

TY - JOUR
ID - 868
T1 - Do neighborhood and home contexts help explain why low-income children miss opportunities to participate in activities outside of school?
A1 - Dearing,E.
A1 - Wimer,C.
A1 - Simpkins,S.D.
A1 - Lund,T.
A1 - Bouffard,S.M.
A1 - Caronongan,P.
A1 - Kreider,H.
A1 - Weiss,H.
Y1 - 2009/11//
N1 - Dearing, Eric. Wimer, Christopher. Simpkins, Sandra D. Lund, Terese. Bouffard, Suzanne M. Caronongan, Pia. Kreider, Holly. Weiss, Heather
Developmental psychology
0260564, cn8
IM
Journal Article. Research Support, Non-U.S. Gov't
English
KW - MEDLINE
KW - Child
KW - Child Development
KW - Cognition
KW - Family/px [Psychology]
KW - Female
KW - Humans
KW - Male
KW - Odds Ratio
KW - Poverty
KW - Residence Characteristics
KW - Schools
KW - Self Efficacy
KW - Social Environment
KW - Social Support
KW - Socioeconomic Factors
RP - NOT IN FILE
SP - 1545
EP - 1562
JF - Developmental Psychology
JA - Dev Psychol
VL - 45
IS - 6
CY - United States
N2 - In this study, children's participation (N = 1,420) in activities outside of elementary school was examined as a function of disparities in family income using data from the Panel Study of Income Dynamics, Child Development Supplement. Children's neighborhood and home environments were investigated as mechanisms linking income disparities and participation rates. Family income was positively associated with children's participation in activities, with the largest effect sizes evident for children at the lowest end of the income distribution. Affluence in the neighborhood and cognitive stimulation in the home were both important mediators of the association between income and participation, explaining from approximately one tenth to one half of the estimated associations between income and participation
SN - 1939-0599
AD - Counseling, Developmental, and Educational Psychology, Lynch School of Education, Boston College, Chestnut Hill, MA 02467, USA. dearinge@bc.edu
UR - 19899913
ER -

TY - JOUR
ID - 869
T1 - Towards translation of environmental determinants of physical activity in children into multi-sector policy measures: study design of a Dutch project
A1 - Aarts,M.J.
A1 - van,de Goor,I
A1 - van Oers,H.A.
A1 - Schuit,A.J.
Y1 - 2009///
N1 - Aarts, Marie-Jeanne. van de Goor, Ien A M. van Oers, Hans A M. Schuit, Albertine J
BMC public health
100968562
IM
Journal Article. Research Support, Non-U.S. Gov't
English
KW - MEDLINE
KW - Adolescent
KW - Child
KW - Preschool
KW - Cities
KW - Exercise
KW - Feasibility Studies
KW - Female
KW - Health Policy
KW - Health Promotion/mt [Methods]
KW - Humans
KW - Leisure Activities
KW - Male
KW - Netherlands
KW - Questionnaires
KW - Residence Characteristics
KW - Schools
KW - Social Environment
KW - Social Planning
RP - NOT IN FILE
SP - 396
JF - BMC Public Health
VL - 9
CY - England
N2 - BACKGROUND: Physical inactivity in children is a major health problem in The Netherlands as well as in many other Western countries. In addition to health promotion among parents and children, creating "active" neighbourhoods can contribute to the solution of this health problem. However, changing environmental characteristics is often the responsibility of policy sectors outside the Public Health domain. Therefore this project identifies and evaluates the possibilities of multi-sector policy measures to stimulate physical activity in children. METHODS AND DESIGN: The project consists of quantitative as well as qualitative research methods and is conducted in four medium sized Dutch cities. To identify perceived environmental determinants of physical activity in children, a large scale health survey was conducted at 42 primary schools. Written questionnaires including topics on the children's physical activity behaviour (i.e. sports participation, outdoor play, active commuting, television watching and computer usage) and physical and social environmental characteristics were completed by 6,601 parents of children aged 3-13 years old and 3449 children aged 9-13 years old. In addition, 33 neighbourhood audits (systematic observations) were conducted to assess objective neighbourhood characteristics. Furthermore, a policy analysis was conducted in the four participating municipalities to provide an overview of the current local policy measures directed at stimulation of physical activity in children. Policy plans of six different policy sectors (Public Health, Sports, Education & Youth, Spatial Planning, Traffic & Transport, and Safety) were screened for their content on physical activity in children. In addition, semi-structured interviews were conducted with policy makers of each of these sectors to

identify critical success factors in the development and realization of multi-sector policy plans aimed at stimulating physical activity in children. The results of all these research activities will be discussed with local policy makers during interactive workshop sessions in order to identify clear cut multi-sector policy measures that stimulate physical activity in children. DISCUSSION: This paper describes the study design of a project that focuses on multi-sector policy measures that stimulate physical activity in children. Next to extensive research into the environmental determinants of physical activity in children, much emphasis is placed on the translation of the research outcomes into concrete and feasible policy plans
SN - 1471-2458
AD - Tilburg University, Faculty of Social and Behavioural Sciences, Department Tranzo, PO Box 90153, 5000 LE Tilburg, The Netherlands. m.j.aarts@uvt.nl
UR - 19860876
ER -

TY - JOUR
ID - 870
T1 - Physical activity levels of Hungarian children during school recess
A1 - Ridgers,N.D.
A1 - Toth,M.
A1 - Uvacsek,M.
Y1 - 2009/11//
N1 - Ridgers, Nicola D. Toth, Miklos. Uvacsek, Martina
Preventive medicine
pm4, 0322116
IM
Comparative Study. Journal Article
English
KW - MEDLINE
KW - Child
KW - Female
KW - Health Behavior
KW - Humans
KW - Hungary
KW - Leisure Activities
KW - Male
KW - Motor Activity/ph [Physiology]
KW - Physical Fitness
KW - Probability
KW - Risk Factors
KW - School Health Services
KW - Sex Factors
RP - NOT IN FILE
SP - 410
EP - 412
JF - Preventive Medicine
JA - Prev Med
VL - 49
IS - 5
CY - United States
N2 - OBJECTIVE: The purpose of this study was to examine physical activity levels and patterns of physical activity across daily school recess periods, and the contribution of recess to daily physical activity. METHOD: Ninety-eight children (61% boys) from three schools in Hungary had their physical activity quantified using uni-axial accelerometry every 5 s for three consecutive school days (Wednesday to Friday). The proportion of time spent in sedentary, light, moderate-to-vigorous, and vigorous physical activity during 5 daily school recess periods was determined using existing age-appropriate cut-points. The relative contribution of recess to daily moderate-to-vigorous physical activity was also determined. Data were collected between May and October 2008. RESULTS: Boys engaged in significantly more light (30.6+/-5.2%; 27.7+/-5.1%), moderate-to-vigorous (24.9+/-8.9%; 17.5+/-5.2%) and vigorous physical activity (7.6+/-4.7%; 4.3+/-2.9%) than girls during recess.

Girls (54.8+/-8.1%) engaged in more sedentary activity than boys (44.5+/-10.2%). Physical activity levels were generally similar across multiple recess periods. Recess contributed more moderate-to-vigorous physical activity towards weekday physical activity for boys (13.1%) than girls (10.8%). CONCLUSIONS: Since sedentary activity accounted for the largest proportion of recess, interventions may be needed across all recess periods to promote physical activity during the school day
SN - 1096-0260
AD - Research Institute for Sport and Exercise Sciences, Liverpool John Moores University, Henry Cotton Campus, 15-21 Webster Street, Liverpool, L3 2ET, UK. n.ridgers@ljmu.ac.uk
UR - 19716378
ER -

TY - JOUR
ID - 871
T1 - Improving GP diabetes management - A PDSA audit cycle in Western Australia
A1 - Porter,C.
A1 - Greenfield,C.
A1 - Larson,A.
A1 - Gilles,M.
Y1 - 2009/11//
N1 - Porter, Cynthia. Greenfield, Charlie. Larson, Ann. Gilles, Marisa
Australian family physician
9ec, 0326701
IM
Comparative Study. Journal Article. Multicenter Study
English
KW - MEDLINE
KW - Adolescent
KW - Adult
KW - Aged
KW - 80 and over
KW - Child
KW - Commission on Professional and Hospital Activities/st [Standards]
KW - Commission on Professional and Hospital Activities/td [Trends]
KW - Cross-Sectional Studies
KW - Diabetes Mellitus/ep [Epidemiology]
KW - Diabetes Mellitus/th [Therapy]
KW - Disease Management
KW - Family Practice/mt [Methods]
KW - Female
KW - Humans
KW - Male
KW - Middle Aged
KW - Morbidity/td [Trends]
KW - Quality Assurance
KW - Health Care
KW - Retrospective Studies
KW - Western Australia/ep [Epidemiology]
KW - Young Adult
RP - NOT IN FILE
SP - 939
EP - 944
JF - Australian Family Physician
JA - Aust Fam Physician
VL - 38
IS - 11
CY - Australia

N2 - BACKGROUND: Tight glucose, blood pressure and lipid control in patients with diabetes can reduce morbidity and mortality from macro- and micro-vascular complications. However, treatment targets are not being met in a large proportion of patients. Clinical audit involves cycles of evaluation of current activity against standards. It allows problems to be identified and action to be taken to address them. METHODS: Annual retrospective audits over 3 years of random samples of up to 20 patient medical records from 13 general practitioners in the midwest region of Western Australia (n=807). Statistical tests compared the second and third audits with the first in regard to completeness of screening, health indicators, and the proportion of patients within The Royal Australian College of General Practitioners and Diabetes Australia guidelines targets. RESULTS: While there was a significant improvement in lipid monitoring over the study period (p<0.001), monitoring of HbA1c and blood pressure (BP) remained unchanged. Between the first and third audits, a reduction in mean HbA1c (p<0.001), mean total cholesterol (p=0.017), mean LDL cholesterol (p=0.014) and mean systolic BP (p=0.002) was seen. There was an improvement in the proportion of patients achieving cholesterol goals (measured by LDL and reaching a target of HbA1c <7%) between the first and third audits; however the proportion with BP within target declined. In the third audit, 11% of patients on diet alone, 36% on an oral hypoglycaemic agent, 90% on three oral hypoglycaemic agents and 84% of those on insulin were outside the target HbA1c. In the same audit, of those outside target BP, 53% were on no treatment and 65% were only on one type of medication. Eighty-seven percent of patients outside target cholesterol levels had not been prescribed a statin. DISCUSSION: Many of the audited GPs in our study undertreated BP, HbA1c and cholesterol. Improvement in some areas was seen over the study period, which may have been due to the quality assurance activities undertaken. These results reveal a therapeutic opportunity for reducing cardiovascular events in patients with diabetes. More aggressive management of BP and lipids by GPs may see rewards in terms of reducing cardiovascular events in patients with diabetes
SN - 0300-8495
AD - Combined Universities Centre for Rural Health, Geraldton, Western Australia. cindyp@cucrh.uwa.edu.au
UR - 19893848
ER -

TY - JOUR
ID - 872
T1 - Developing a coordinated school health approach to child obesity prevention in rural Appalachia: results of focus groups with teachers, parents, and students
A1 - Schetzina,K.E.
A1 - Dalton,W.T.,III
A1 - Lowe,E.F.
A1 - Azzazy,N.
A1 - Vonwerssowetz,K.M.
A1 - Givens,C.
A1 - Stern,H.P.
Y1 - 2009///
N1 - Schetzina, Karen E. Dalton, William T 3rd. Lowe, Elizabeth F. Azzazy, Nora. Vonwerssowetz, Katrina M. Givens, Connie. Stern, Harold P
Rural and remote health
101174860
IM
Journal Article. Research Support, N.I.H., Extramural
English
KW - MEDLINE
KW - Appalachian Region
KW - Body Mass Index
KW - Child
KW - Diet
KW - Exercise
KW - Faculty
KW - Female
KW - Focus Groups
KW - Humans
KW - Male

KW - Obesity/pc [Prevention & Control]
KW - Parents
KW - Rural Population
KW - School Health Services/og [Organization & Administration]
KW - Students
RP - NOT IN FILE
SP - 1157
JF - Rural & Remote Health
JA - RURAL REMOTE HEALTH
VL - 9
IS - 4
CY - Australia
N2 - INTRODUCTION: High prevalence rates of obesity, particularly among those residing in US rural areas, and associated physical and psychosocial health consequences, direct attention to the need for effective prevention programs. The current study describes an initial step in developing a school-based obesity prevention program in rural Appalachia, USA. The program, modeled on the Centers for Disease Control and Prevention Coordinated School Health (CSH) Program, includes a community-based participatory research approach to addressing the health needs specific to this region. METHODS: Focus groups with teachers, parents, and 4th grade students were used to understand perceptions and school policy related to nutrition, physical activity, and the role of the school in obesity prevention. RESULTS: Results revealed that these community stakeholders were concerned about the problem of child obesity and supported the idea of their school doing more to improve the diet and physical activity of its students. Specifically, all groups thought that foods and drinks consumed by students at school should be healthier and that they should have more opportunities for physical activity. However, they cited limitations of the school environment, academic pressures, and lack of parental support as potential barriers to making such changes. Parents were most concerned that their children were not getting enough to eat and they and the teachers were not in favor of BMI screening at the school. Parents were in favor of increasing physical activity during school and thought that parent volunteers should help students select foods in the cafeteria. Students cited examples of how diet and physical activity affect their health and school performance, and thought that they should have more physical education time and recess. CONCLUSIONS: The data collected in the current study contributed to the limited knowledge base regarding rural populations as well as identified strengths and potential barriers to assist with the development of a pilot program based on the CSH model, Winning with Wellness
SN - 1445-6354
AD - Department of Pediatrics, James H. Quillen College of Medicine, East Tennessee State University, Johnson City, Tennessee, USA. schetzin@etsu.edu
UR - 19877760
ER -

TY - JOUR
ID - 873
T1 - Enzyme replacement therapy for Fabry disease: a systematic review of available evidence. [Review] [65 refs]
A1 - Schaefer,R.M.
A1 - Tylki-Szymanska,A.
A1 - Hilz,M.J.
Y1 - 2009/11/12/
N1 - Schaefer, Roland M. Tylki-Szymanska, Anna. Hilz, Max J
Drugs
ec2, 7600076
IM
Journal Article. Review
English
KW - MEDLINE
KW - Child
KW - Clinical Trials as Topic
KW - Enzyme Replacement Therapy/ae [Adverse Effects]
KW - Enzyme Replacement Therapy/mt [Methods]

KW - Fabry Disease/dt [Drug Therapy]
KW - Fabry Disease/en [Enzymology]
KW - Fabry Disease/pp [Physiopathology]
KW - Female
KW - Humans
KW - Isoenzymes/ae [Adverse Effects]
KW - Isoenzymes/tu [Therapeutic Use]
KW - Male
KW - Trihexosylceramides/me [Metabolism]
KW - alpha-Galactosidase/ae [Adverse Effects]
KW - alpha-Galactosidase/tu [Therapeutic Use]
RP - NOT IN FILE
SP - 2179
EP - 2205
JF - Drugs
VL - 69
IS - 16
CY - New Zealand
N2 - Fabry disease is a progressive and life-threatening glycolipid storage disorder affecting both males and females. The primary driver of the disease is the accumulation of glycolipids (globotriaosylceramide [GL-3]) in a variety of cell types, including vascular endothelial cells, a range of renal cell types, cardiomyocytes and neurons, which is caused by deficient activity of the lysosomal enzyme, alpha-galactosidase. The disease typically presents during childhood or adolescence. First manifestations reflect involvement of small nerve fibres of the peripheral and autonomic nervous systems. With age, severe complications involving the kidneys, heart and brain cause considerable morbidity and premature death. Outside the US, enzyme replacement therapy (ERT) with agalsidase alfa 0.2 mg/kg every other week (EOW) and agalsidase beta 1.0 mg/kg EOW is available for the treatment of patients with Fabry disease, while agalsidase beta 1.0 mg/kg EOW is the only approved drug in the US. To analyse the evidence for ERT, a systematic review of the literature was performed to identify prospectively designed randomized, controlled trials (RCTs) and open-label studies on the efficacy of agalsidase alfa and agalsidase beta. MEDLINE and EMBASE databases were searched; inclusion criteria for the systematic review were prospectively designed clinical studies evaluating ERT with quantifiable endpoints: double-blind and open-label studies were eligible. Exclusion criteria were review articles, case reports, case studies, letters to the editor and articles based on registry data (Fabry Outcome Survey or Fabry Registry). In addition, any studies with a retrospective design or data based on post hoc analyses were excluded. The evidence was reviewed with respect to the clinical benefits of ERT at the level of the end organ. A total of 9 RCTs and 23 open-label studies were identified for inclusion. The efficacy of ERT in Fabry disease has been measured against a variety of endpoints, the majority of which were subclinical parameters rather than clinical outcomes. Plasma levels of GL-3 together with accumulation in the kidney, heart and skin were the most commonly studied endpoints, followed by renal endpoints of proteinuria and glomerular filtration rate, whereas cardiac and neurological endpoints were not commonly studied. To date, only one RCT with ERT defined hard clinical outcomes in the form of cardiac, renal or cerebrovascular events, or death as its primary endpoint. The currently available data from prospective RCTs and open-label studies in patients with Fabry disease are more robust for ERT at a dose of 1 mg/kg EOW than a dose of 0.2 mg/kg EOW, although the beneficial effects of ERT with either dose or preparation are variable. [References: 65]
SN - 0012-6667
AD - Department of Medicine, University of Muenster, Muenster, Germany. schaefe@uni-muenster.de
UR - 19852524
ER -

TY - JOUR
ID - 874
T1 - Association between school closure and subsequent absenteeism during a seasonal influenza epidemic
A1 - Rodriguez,C.V.
A1 - Rietberg,K.
A1 - Baer,A.
A1 - Kwan-Gett,T.
A1 - Duchin,J.

Y1 - 2009/11//
N1 - Rodriguez, Carla V. Rietberg, Krista. Baer, Atar. Kwan-Gett, Tao. Duchin, Jeffrey
Epidemiology (Cambridge, Mass.)
a2t, 9009644
IM
Comparative Study. Journal Article
English
KW - MEDLINE
KW - Absenteeism
KW - Adolescent
KW - Child
KW - Disease Outbreaks
KW - Humans
KW - Influenza
KW - Human/ep [Epidemiology]
KW - Models
KW - Statistical
KW - Organizational Case Studies
KW - Organizational Policy
KW - Population Surveillance/mt [Methods]
KW - Prospective Studies
KW - Retrospective Studies
KW - Schools
KW - Seasons
KW - Sick Role
KW - Washington/ep [Epidemiology]
RP - NOT IN FILE
SP - 787
EP - 792
JF - Epidemiology
VL - 20
IS - 6
CY - United States
N2 - BACKGROUND: Mathematical models suggest that social distancing measures, such as school closures, may mitigate community transmission during an influenza pandemic. Because closures are disruptive to schools and families, they are rarely employed during seasonal influenza outbreaks. A rare circumstance enabled us to examine the association between school closure and absenteeism during a seasonal influenza outbreak when half of King County, Washington public schools closed for a winter recess 19-23 February 2007, while half remained open for all or part of the week. METHODS: Using absenteeism as a proxy for influenza activity, we tested the hypothesis that schools on break would experience lower rates of post-break absenteeism than schools remaining open. We conducted daily retrospective and prospective surveillance from 5 February-9 March 2007 in schools on break (n = 256) and in session (n = 205). We use generalized estimating equations with Poisson distribution to evaluate whether mean absenteeism after the break differed between schools on break and those in session, adjusting for baseline absenteeism and repeated measurements by schools over time. RESULTS: Results indicate no difference in post-break absenteeism in schools on break compared with schools that remained in session (relative risk = 1.07 [95% confidence interval = 0.96-1.20]). This result held in elementary schools (1.00 [0.91-1.10]), where absenteeism patterns are thought to be most representative of community influenza activity. CONCLUSION: We did not find that school closure during a seasonal influenza outbreak reduced subsequent absenteeism. However, limitations in this "natural experiment" hampered our ability to detect a benefit if one truly was present
SN - 1531-5487
AD - Department of Epidemiology, School of Public Health and Community Medicine, University of Washington, Seattle, WA, USA. carlarod@u.washington.edu
UR - 19770773
ER -

TY - JOUR

ID - 875
T1 - An alternative design for small-scale school health experiments: does daily walking produce benefits in physical performance of school children?
A1 - Monness,E.
A1 - Sjolie,A.N.
Y1 - 2009/11//
N1 - Monness, E. Sjolie, A N
Child: care, health and development
7602632, c9x
IM
Comparative Study. Evaluation Studies. Journal Article
English
KW - MEDLINE
KW - Adolescent
KW - Case-Control Studies
KW - Child
KW - Exercise/ph [Physiology]
KW - Female
KW - Health Promotion/mt [Methods]
KW - Humans
KW - Male
KW - Norway
KW - Physical Fitness/ph [Physiology]
KW - Program Evaluation
KW - Rural Health
KW - Schools
KW - Walking/ph [Physiology]
RP - NOT IN FILE
SP - 858
EP - 867
JF - Child: Care, Health & Development
JA - Child Care Health Dev
VL - 35
IS - 6
CY - England
N2 - BACKGROUND: The mainstream randomized clinical trial is not always feasible in a school setting. There might be practical and ethical issues that make dividing school classes into an intervention and a control group impossible or undesirable, and there is a need to explore the validity of alternative designs and analyses. METHODS: An alternative to a randomized clinical trial in a physical performance experiment at a school is introduced and evaluated. The before-intervention data are utilized as control data for the intervention data in addition to adjust for pre-intervention differences. The strict class year structure of school data makes this possible. In a rural school in inland Norway, all school children joined the project of walking in a rugged terrain outside school for 20 min every school day during one school year. Measurements of low back static endurance, hamstrings flexibility, standing balance and cardiovascular fitness were made before and after the intervention. As intervention and 'aging' were confounded, the special use of the pre-intervention data, 'age-adjusted', is proposed to solve this issue. A comparison with having an independent control group is performed. RESULTS: The alternative analysing method is judged to yield valid results without having an independent control group. The age-adjusted analyses showed 11% increase in low back static endurance, 8% increase in hamstrings flexibility, 69% increase in balance and 6-13% increase in cardiovascular fitness. The effects were largest among those children who had the lowest performances before the intervention. CONCLUSION: The introduced statistical methods display that, in a school population, evaluations from an experiment can be made without an independent control group. A 20-min walk during school time for 1 year seemed to improve physical performance
SN - 1365-2214
AD - Faculty of Business Administration, Social Sciences and Computer Science (OSIR), Hedmark University College, Rena, Norway. erik.monness@hihm.no
UR - 19832847

ER -

TY - JOUR
ID - 876
T1 - [Assessment of exposure to polycyclic aromatic hydrocarbons in Japanese children]. [Japanese]
A1 - Mori,T.
A1 - Yoshinaga,J.
A1 - Kawahara,J.
A1 - Mizoi,M.
A1 - Adachi,S.
Y1 - 2009/09//
N1 - Mori, Takuya. Yoshinaga, Jun. Kawahara, Junko. Mizoi, Miho. Adachi, Shuichi
Nihon eiseigaku zasshi. Japanese journal of hygiene
kkn, 0417457
IM
English Abstract. Journal Article
Japanese
KW - MEDLINE
KW - Biological Markers/ur [Urine]
KW - Child
KW - Preschool
KW - Environmental Exposure/an [Analysis]
KW - Female
KW - Humans
KW - Japan
KW - Polycyclic Hydrocarbons
KW - Aromatic/an [Analysis]
KW - Pyrenes/an [Analysis]
RP - NOT IN FILE
SP - 817
EP - 823
JF - Nippon Eiseigaku Zasshi - Japanese Journal of Hygiene
JA - Nippon Eiseigaku Zasshi
VL - 64
IS - 4
CY - Japan
N2 - OBJECTIVES: To estimate the level of exposure to polycyclic aromatic hydrocarbons (PAHs) in Japanese children by urinary metabolite analysis and the possible contribution of soil ingestion and environmental tobacco smoke (ETS) to PAHs exposure. METHODS: Spot urine samples and questionnaire data were collected from 107 kindergarten children (3-6 yrs) and their mother. The urinary concentration of 1-hydroxypyrene (1-OHP), a biomarker of PAHs exposure, was measured using a high performance liquid chromatography-fluorescence detector. RESULTS: The geometric mean (GM) of urinary 1-OHP concentrations in children was 0.065 mumol/mol-cre (geometric standard deviation=1.88). Parental smoking and time of playing outside (surrogate of soil exposure level) did not increase urinary 1-OHP level. Maternal urinary 1-OHP concentration correlated with, whereas GM (0.038 mumol/mol-cre) was significantly lower than, the urinary 1-OHP concentration in children. The latter might be attributable to greater amount of food intake per body weight for children than for adult. CONCLUSIONS: The contribution of ETS and soil ingestion to PAHs exposure seemed to be small and thus they cannot be the major source of PAHs in Japanese children
SN - 0021-5082
AD - Department of Environmental Studies, University of Tokyo, Kashiwa, Chiba, Japan
UR - 19797851
ER -

TY - JOUR
ID - 877
T1 - Directional effects on infants and young children in real life: implications for amplification
A1 - Ching,T.Y.

A1 - O'Brien,A.
A1 - Dillon,H.
A1 - Chalupper,J.
A1 - Hartley,L.
A1 - Hartley,D.
A1 - Raicevich,G.
A1 - Hain,J.
Y1 - 2009/10//
N1 - Ching, Teresa Y C. O'Brien, Anna. Dillon, Harvey. Chalupper, Josef. Hartley, Lisa. Hartley, David. Raicevich, George. Hain, Jens
Journal of speech, language, and hearing research : JSLHR
ct1, 9705610
IM
Journal Article
English
KW - MEDLINE
KW - Adaptation
KW - Psychological
KW - Auditory Threshold
KW - Child
KW - Child Behavior
KW - Preschool
KW - Female
KW - Head Movements
KW - Hearing
KW - Hearing Aids
KW - Hearing Loss/px [Psychology]
KW - Hearing Loss/rh [Rehabilitation]
KW - Humans
KW - Infant
KW - Male
KW - Social Behavior
KW - Sound Localization
KW - Videotape Recording
RP - NOT IN FILE
SP - 1241
EP - 1254
JF - Journal of Speech Language & Hearing Research
JA - J Speech Lang Hear Res
VL - 52
IS - 5
CY - United States
N2 - PURPOSE: This study examined the head orientation of young children in naturalistic settings and the acoustics of their everyday environments for quantifying the potential effects of directionality. METHOD: Twenty-seven children (11 with normal hearing, 16 with impaired hearing) between 11 and 78 months of age were video recorded in naturalistic settings for analyses of head orientation. Reports on daily activities were obtained from caregivers. The effect of directionality in different environments was quantified by measuring the Speech Transmission Index (STI; H. J. M. Steeneken & T. Houtgast, 1980). RESULTS: Averaged across 4 scenarios, children looked in the direction of a talker for 40% of the time when speech was present. Head orientation was not affected by age or hearing status. The STI measurements revealed a directional advantage of 3 dB when a child looked at a talker but a deficit of 2.8 dB when the talker was sideways or behind the child. The overall directional effect in real life was between -0.4 and 0.2 dB. CONCLUSIONS: The findings suggest that directional microphones in personal hearing devices for young children are not detrimental and have much potential for benefits in real life. The benefits may be enhanced by fitting directionality early and by counseling caregivers on ways to maximize benefits in everyday situations
SN - 1092-4388
AD - National Acoustic Laboratories, Chatswood, New South Wales, Australia. teresa.ching@nal.gov.au

UR - 19564437
ER -

TY - JOUR
ID - 878
T1 - The U.S. military wartime pediatric trauma mission: how surgeons and pediatricians are adapting the system to address the need
A1 - Fuenfer,M.M.
A1 - Spinella,P.C.
A1 - Naclerio,A.L.
A1 - Creamer,K.M.
Y1 - 2009/09//
N1 - Fuenfer, Michael M. Spinella, Philip C. Naclerio, Anne L. Creamer, Kevin M
Military medicine
2984771r, n1a
IM
Journal Article
English
KW - MEDLINE
KW - Adolescent
KW - Afghan Campaign 2001-
KW - Child
KW - Preschool
KW - General Surgery/ma [Manpower]
KW - General Surgery/og [Organization & Administration]
KW - Health Services Needs and Demand
KW - Hospitals
KW - Military/ma [Manpower]
KW - Military/og [Organization & Administration]
KW - Humans
KW - Infant
KW - Newborn
KW - Iraq War
KW - 2003-2011
KW - Military Medicine/ma [Manpower]
KW - Military Medicine/og [Organization & Administration]
KW - Pediatrics/ma [Manpower]
KW - Pediatrics/og [Organization & Administration]
KW - United States
KW - Wounds and Injuries/th [Therapy]
RP - NOT IN FILE
SP - 887
EP - 891
JF - Military Medicine
JA - Mil.Med
VL - 174
IS - 9
CY - United States
N2 - PURPOSE: Over 3,500 infants and children, many critically ill and injured, have been admitted to military combat support hospitals (CSH) in Afghanistan and Iraq, which are not doctrinally staffed or equipped to provide their care. This report details how the military medical system is adapting to create a data driven and comprehensive response to optimize the medical and surgical pediatric care being provided. METHODS: Information from multiple sources was used over time to craft the military medical response to the pediatric wartime mission. Pediatric data from both the Joint Theater Trauma Registry (JTTR) and the Patient Administration Systems and Biostatistics Activity (PASBA) database were utilized extensively. The resulting educational, supply, and personnel adaptations implemented by the U.S. military will be highlighted, and innovations currently under development will also be described on the basis of this demonstrated need.

RESULTS: This information helped drive pediatric-specific, just-in-time education for CSH personnel, modified CSH equipment and supply lists, inspired the 24/7 pediatric critical care teleconsultation service, and resulted in new initiatives in the predeployment training for CSH personnel. CONCLUSION: Military physicians are routinely asked to perform outside their traditional scopes of practice while deployed. Given this reality, military pediatric specialists in medicine and surgery have initiated several successful multidisciplinary programs designed to improve in-theater care of injured children. These innovative efforts include drafting a pediatric addendum to the Army's "Emergency War Surgery" manual, development of instructional compact discs, augmenting and refining the pediatric portion of the Joint Forces Combat Trauma Management course, formation of a pediatric augmentation team to the CSH, and a comprehensive hyperlinked Web-based pediatric critical care and trauma educational platform
SN - 0026-4075
AD - Department of Surgery, Walter Reed Army Medical Center, Washington, DC, USA
UR - 19780362
ER -

TY - JOUR
ID - 879
T1 - Extension of organised cervical cancer screening programmes in Italy and their process indicators: 2007 activity
A1 - Ronco,G.
A1 - Giubilato,P.
A1 - Naldoni,C.
A1 - Zorzi,M.
A1 - Anghinoni,E.
A1 - Scalisi,A.
A1 - Dalla,Palma P.
A1 - Zanier,L.
A1 - Barca,A.
A1 - Gaimo,M.D.
A1 - Maglietta,R.
A1 - Mancini,E.
A1 - Pizzuti,R.
A1 - Iossa,A.
A1 - Segnan,N.
A1 - Zappa,M.
Y1 - 2009/05//
N1 - Ronco, Guglielmo. Giubilato, Pamela. Naldoni, Carlo. Zorzi, Manuel. Anghinoni, Emanuela. Scalisi, Aurora. Dalla Palma, Paolo. Zanier, Loris. Barca, Alessandra. Gaimo, Maria Donata. Maglietta, Rocco. Mancini, Ettore. Pizzuti, Renato. Iossa, Anna. Segnan, Nereo. Zappa, Marco
Epidemiologia e prevenzione
8902507, epp
IM
Comparative Study. Journal Article
English
KW - MEDLINE
KW - Adult
KW - Age Factors
KW - Cervical Intraepithelial Neoplasia/di [Diagnosis]
KW - Cervical Intraepithelial Neoplasia/ep [Epidemiology]
KW - Child
KW - Colposcopy/td [Trends]
KW - Female
KW - Humans
KW - Italy/ep [Epidemiology]
KW - Mass Screening/td [Trends]
KW - Middle Aged
KW - Patient Compliance

KW - Practice Guidelines as Topic
KW - Time Factors
KW - Uterine Cervical Neoplasms/di [Diagnosis]
KW - Uterine Cervical Neoplasms/ep [Epidemiology]
KW - Vaginal Smears/td [Trends]
RP - NOT IN FILE
SP - 41
EP - 56
JF - Epidemiologia e Prevenzione
JA - Epidemiol Prev
VL - 33
IS - 3 Suppl 2
CY - Italy
N2 - Italian national guidelines recommend to Regions the implementation of organised screening programmes for cervical cancer. As in previous years since 1998, we collected from Italian organised cervical screening programmes aggregated tables of data in order to centrally compute process indicators. Data on women invited during 2007 and screened up to April 2008 were considered. In 2007, the target population of Italian organised screening programmes included 11,872,810 women, corresponding to 71.8% of Italian women aged 25-64 years. Uptake of invitation was 39.8%, with a clear North-South decreasing trend. It should, however, be considered that many women are screened outside the organised programmes. Of the women screened, 5.0% were referred for repeat cytology and 60.4 % of them complied; 2.4% of screened women were referred to colposcopy. Compliance with colposcopy referral was 82.3% among women referred because of ASCUS or more severe cytology and 89.5% among those referred because of HSIL or more severe cytology. The positive predictive value (PPV) of referral because of ASCUS or more severe cytology for CIN2 or more severe histology was 16.0%. The unadjusted detection rate of CIN2 or more severe histology was 2.9 per 1,000 screened women (3.1 standardised on the Italian population, truncated 25-64)
SN - 1120-9763
AD - CPO Piemonte, Torino
UR - 19776486
ER -

TY - JOUR
ID - 880
T1 - Certification of sun protection practices in a German child day-care centre improves children's sun protection--the 'SunPass' pilot study
A1 - Aulbert,W.
A1 - Parpart,C.
A1 - Schulz-Hornbostel,R.
A1 - Hinrichs,B.
A1 - Kruger-Corcoran,D.
A1 - Stockfleth,E.
Y1 - 2009/11//
N1 - Aulbert, W. Parpart, C. Schulz-Hornbostel, R. Hinrichs, B. Kruger-Corcoran, D. Stockfleth, E
The British journal of dermatology
aw0, 0004041
IM
Evaluation Studies. Journal Article. Research Support, Non-U.S. Gov't
English
KW - MEDLINE
KW - Certification
KW - Child
KW - Child Day Care Centers
KW - Preschool
KW - Female
KW - Health Knowledge
KW - Attitudes
KW - Practice

KW - Humans
KW - Infant
KW - Newborn
KW - Male
KW - Neoplasms
KW - Radiation-Induced/pc [Prevention & Control]
KW - Parenting
KW - Pilot Projects
KW - Preventive Health Services
KW - Protective Clothing/ut [Utilization]
KW - Questionnaires
KW - Skin Neoplasms/pc [Prevention & Control]
KW - Sunburn/pc [Prevention & Control]
KW - Sunscreening Agents/ad [Administration & Dosage]
RP - NOT IN FILE
SP - 5
EP - 12
JF - British Journal of Dermatology
JA - Br J Dermatol
VL - 161 Suppl 3
CY - England
N2 - BACKGROUND: Nonmelanoma skin cancer (NMSC) and malignant melanoma (CMM) are among the most common malignancies in the white population. The major risk factor for those malignancies is ultraviolet radiation (UV) causing directly DNA damage and promoting the development of skin cancer. It is suggested that the exposure to UV during childhood elevates an individual's lifetime risk of developing skin cancer more than exposure in adulthood. Since an increasing number of children spend the time of the most intense UV in a day-care centre, it seems an excellent place for establishing primary skin cancer prevention. Important targets are staff members and parents of the day-care centre, since sun protection of children depends directly on their knowledge and their attitude towards sun protection practices. OBJECTIVES: To establish a feasible certification program for sun protection in a German child day-care centre, for a better sun protection of the children and the reduction of skin cancer incidence in the long term. METHODS: Initially sun protection practices of the centre at baseline were assessed. A written sun protection policy was developed in consultation with all members of the day-care centre as basis for certification. It was followed by training sessions for staff members (n=12) and parents (n=46). After a fixed period of time the final assessment of the child day-care centre was conducted and the centre then was certified for improved sun protection practices and better protection of the children. The primary assessed outcomes were the gain in knowledge of staff members and parents after the training sessions, the number of children wearing a hat when playing outside, the use of sunscreen and the percentage of shaded areas on the playground. RESULTS: Sun protection was an issue more discussed during the time of intervention than before. Staff members (n=12) and parents (n=27) had a significant gain in knowledge (staff members: P=0.002; parents: P=0.001) concerning sun related issues. The number of children wearing a hat increased from 13.2% to 73%. The sunscreen use increased, 58.8% of staff members reported a more regular application of sunscreen to the children. There was a higher percentage of shaded area on the playground (70-80% before intervention, 90% after intervention). The intervention failed in keeping the children inside during the most intense UV and in educating the staff members to be a convincing example of sun protection by wearing appropriate clothes. CONCLUSIONS: The intervention showed that the introduction of a simple certification program including a written sun protection policy and training sessions for staff members and parents helps to improve children's sun protection. We suggest that a certificate for adequate sun protection acts as a motivating factor. It seems important to refresh sun protection practices each year by repeating training sessions and reviewing the sun protection policy
SN - 1365-2133
AD - Department of Dermatology, Charite, Skin Cancer Centre Charite, University Hospital of Berlin, Berlin, Germany
UR - 19775351
ER -

TY - JOUR
ID - 881

T1 - Dysfunctional neural network of spatial working memory contributes to developmental dyscalculia
A1 - Rotzer,S.
A1 - Loenneker,T.
A1 - Kucian,K.
A1 - Martin,E.
A1 - Klaver,P.
A1 - von,Aster M.
Y1 - 2009/11//
N1 - Rotzer, S. Loenneker, T. Kucian, K. Martin, E. Klaver, P. von Aster, M
Neuropsychologia
0020713, nzn
IM
Journal Article. Research Support, Non-U.S. Gov't
English
KW - MEDLINE
KW - Brain/pp [Physiopathology]
KW - Case-Control Studies
KW - Child
KW - Female
KW - Humans
KW - Intelligence Tests
KW - Learning Disorders/pp [Physiopathology]
KW - Magnetic Resonance Imaging
KW - Male
KW - Mathematical Concepts
KW - Memory
KW - Short-Term/ph [Physiology]
KW - Neural Pathways/pp [Physiopathology]
KW - Psychomotor Performance/ph [Physiology]
RP - NOT IN FILE
SP - 2859
EP - 2865
JF - Neuropsychologia
VL - 47
IS - 13
CY - England
N2 - The underlying neural mechanisms of developmental dyscalculia (DD) are still far from being clearly understood. Even the behavioral processes that generate or influence this heterogeneous disorder are a matter of controversy. To date, the few studies examining functional brain activation in children with DD mainly focus on number and counting related tasks, whereas studies on more general cognitive domains that are involved in arithmetical development, such as working memory are virtually absent. There are several studies showing a close relationship between DD and spatial working memory [Camos, V. (2008). Low working memory capacity impedes both efficiency and learning of number transcoding in children. Journal of Experimental Child Psychology, 99(1), 37-57; McLean, J. F., & Hitch, G. J. (1999). Working memory impairments in children with specific arithmetic learning difficulties. Journal of Experimental Child Psychology, 74(3), 240-260; Rosselli, M., Matute, E., Pinto, N., & Ardila, A. (2006). Memory abilities in children with subtypes of dyscalculia. Developmental Neuropsychology, 30(3), 801-818; Siegel, L. S., & Ryan, E. B. (1989). The development of working memory in normally achieving and subtypes of learning disabled children. Child Development, 60(4), 973-980]. The relationship between these two mechanisms is still matter of debate, but this study follows the assumption that poor spatial working memory capacity may hinder the acquisition of spatial number representations in children with DD [Geary, D. C. (1993). Mathematical disabilities: Cognitive, neuropsychological, and genetic components. Psychological Bulletin, 114(2), 345-362; von Aster, M., & Shalev, R. S. (2007). Number development and developmental dyscalculia. Developmental Medicine and Child Neurology, 49(11), 868-873]. Using functional MRI the current study compares brain activity associated with spatial working memory processes in 8-10-year-old children with DD and normally achieving controls. Both groups showed significant spatial working memory related activity in a network including occipital and parietal regions. Children with DD showed weaker neural activation compared to the control group during a spatial

working memory task in the right intraparietal sulcus (IPS), the right insula and the right inferior frontal lobe. Performance tests outside the scanner showed impaired working memory proficiency in children with DD. Bringing behavioral performance and neural activity together we found significant correlations of right IPS activity with performance on the verbal digit span forward and the spatial Corsi Block Tapping test. Our findings demonstrate for the first time an involvement of spatial working memory processes in the neural underpinnings of DD. These poor spatial working memory processes may inhibit the formation of spatial number representations (mental numberline) as well as the storage and retrieval of arithmetical facts
SN - 1873-3514
AD - MR-Center, University Children's Hospital, Steinwiesstrasse 75, CH-8032 Zurich, Switzerland. stephanie.rotzer@kispi.uzh.ch
UR - 19540861
ER -

TY - JOUR
ID - 882
T1 - Time allocation shifts and pollutant exposure due to traffic congestion: an analysis using the national human activity pattern survey
A1 - Zhang,K.
A1 - Batterman,S.A.
Y1 - 2009/10/15/
N1 - Zhang, Kai. Batterman, Stuart A
The Science of the total environment
uj0, 0330500
IM
Journal Article. Research Support, U.S. Gov't, Non-P.H.S.
English
KW - MEDLINE
KW - Adolescent
KW - Adult
KW - Age Factors
KW - Aged
KW - 80 and over
KW - Automobile Driving
KW - Child
KW - Preschool
KW - Environmental Monitoring
KW - Female
KW - Humans
KW - Infant
KW - Newborn
KW - Male
KW - Middle Aged
KW - Models
KW - Theoretical
KW - Particle Size
KW - Particulate Matter/an [Analysis]
KW - Time Factors
KW - Vehicle Emissions/an [Analysis]
RP - NOT IN FILE
SP - 5493
EP - 5500
JF - Science of the Total Environment
JA - Sci Total Environ
VL - 407
IS - 21
CY - Netherlands

N2 - Traffic congestion increases air pollutant exposures of commuters and urban populations due to the increased time spent in traffic and the increased vehicular emissions that occur in congestion, especially "stop-and-go" traffic. Increased time in traffic also decreases time in other microenvironments, a trade-off that has not been considered in previous time activity pattern (TAP) analyses conducted for exposure assessment purposes. This research investigates changes in time allocations and exposures that result from traffic congestion. Time shifts were derived using data from the National Human Activity Pattern Survey (NHAPS), which was aggregated to nine microenvironments (six indoor locations, two outdoor locations and one transport location). After imputing missing values, handling outliers, and conducting other quality checks, these data were stratified by respondent age, employment status and period (weekday/weekend). Trade-offs or time-shift coefficients between time spent in vehicles and the eight other microenvironments were then estimated using robust regression. For children and retirees, congestion primarily reduced the time spent at home; for older children and working adults, congestion shifted the time spent at home as well as time in schools, public buildings, and other indoor environments. Changes in benzene and PM(2.5) exposure were estimated for the current average travel delay in the U.S. (9 min day(-1)) and other scenarios using the estimated time shifts coefficients, concentrations in key microenvironments derived from the literature, and a probabilistic analysis. Changes in exposures depended on the duration of the congestion and the pollutant. For example, a 30 min day(-1) travel delay was determined to account for 21+/-12% of current exposure to benzene and 14+/-8% of PM(2.5) exposure. The time allocation shifts and the dynamic approach to TAPs improve estimates of exposure impacts from congestion and other recurring events
SN - 1879-1026
AD - Environmental Health Sciences, University of Michigan, Ann Arbor, MI 48109-2029, USA
UR - 19683333
ER -

TY - JOUR
ID - 883
T1 - Vitamin D deficiency in healthy children in a sunny country: associated factors
A1 - Bener,A.
A1 - Al-Ali,M.
A1 - Hoffmann,G.F.
Y1 - 2009///
N1 - Bener, Abdulbari. Al-Ali, Mariam. Hoffmann, Georg F
International journal of food sciences and nutrition
ccg, 9432922
IM
Journal Article. Research Support, Non-U.S. Gov't
English
KW - MEDLINE
KW - 25-Hydroxyvitamin D 2/bl [Blood]
KW - Adolescent
KW - Aging
KW - Calcifediol/bl [Blood]
KW - Child
KW - Preschool
KW - Cross-Sectional Studies
KW - Diet
KW - Environmental Exposure
KW - Family Health
KW - Female
KW - Health Status
KW - Humans
KW - Infant
KW - Male
KW - Motor Activity
KW - Nutritional Status/re [Radiation Effects]
KW - Nutritional Status
KW - Prevalence

KW - Qatar/ep [Epidemiology]
KW - Socioeconomic Factors
KW - Sunlight
KW - Time Factors
KW - Vitamin D/ad [Administration & Dosage]
KW - Vitamin D Deficiency/bl [Blood]
KW - Vitamin D Deficiency/ep [Epidemiology]
KW - Vitamin D Deficiency/pp [Physiopathology]
RP - NOT IN FILE
SP - 60
EP - 70
JF - International Journal of Food Sciences & Nutrition
JA - Int J Food Sci Nutr
VL - 60 Suppl 5
CY - England
N2 - OBJECTIVE: The objective of the present study was to determine the factors associated with low concentrations of 25-hydroxy vitamin D (vitamin D deficiency) in healthy children in Qatar. DESIGN: The survey was a cross-sectional study conducted at the Primary Health Care Clinics over the period from August 2007 to March 2008. Subjects The study was carried out among healthy Qatari nationals, male and female, aged below 16 years. A random sample of 650 healthy subjects who visited the Primary Health Care Centers for any reason other than acute or chronic disease were approached and 458 subjects gave consent; a response rate of 70.5%. METHODS: Face-to-face interviews were based on a questionnaire that included variables such as socio-demographic information, assessment of non-dietary covariates, assessment of dietary intake, vitamin D intake, type of feeding, clinical manifestations and laboratory investigations. The subjects' health status was assessed by medical conditions, family history, body mass index, past or present clinical manifestations, 25-hydroxy vitamin D, calcium, alkaline phosphates, phosphorus, HbA1C, Parathyroid Hormone (PTH), magnesium and creatinine analysis. RESULTS: The study revealed that vitamin D deficiency was highly prevalent in Qatari adolescents (11-16 years old; 61.6%), followed by the 5-10 year olds (28.9%) and those below 5 years old (9.5%). Vitamin D deficiency increased with age and there was a significant difference between vitamin D-deficient and normal children in their age groups (P =0.013). The body mass index was significantly lower in vitamin D-deficient children (19.6+/-3.6; P =0.019). A family history of vitamin D deficiency was more frequent in children with vitamin D deficiency (33.7%) than in normal children (24.5%). Most of the vitamin D-deficient children had no physical activity (60.6%) and no exposure to sunlight (57.5%). There was a significant difference between both groups in terms of family history of vitamin D deficiency, physical activity, exposure to sunlight and duration of time spent outside under the sun (P <0.05). The mean values of vitamin D serum concentration, calcium, alkaline phosphates, and phosphorus were very low in vitamin D-deficient children. Vitamin D-deficient children had a very poor diet for vitamin D (cod liver oil, 56.5%; milk fortified with vitamin D, 27.3%; fortified food, 24.1%; and seafood, 5.7%) compared with normal children. Fractures (P =0.006), delayed milestones (P =0.013), rickets (P =0.017) and gastroenteritis (P =0.020) were significantly higher in vitamin D-deficient children. CONCLUSION: The study findings revealed that Qatari children are at high risk for vitamin D deficiency. Lack of exposure to sunlight, outdoor activities under the sun, and physical activity and vitamin D intake are the main associated factors for vitamin D deficiency in the young population of Qatar. Breast-fed infants need to take vitamin D supplements for a longer period
SN - 1465-3478
AD - Department of Medical Statistics & Epidemiology, Hamad General Hospital, Hamad Medical Corporation, Doha, State of Qatar. abener@hmc.org.qa
UR - 18946796
ER -

TY - JOUR
ID - 884
T1 - Late reposition of a lateral luxated maxillary incisor with an immature apex
A1 - Pelka,M.
A1 - Berthold,C.
A1 - van,Waes H.
Y1 - 2009/10//

N1 - Pelka, Matthias. Berthold, Christine. van Waes, Hubertus
Dental traumatology : official publication of International Association for Dental Traumatology
101091305, d2p
D
Journal Article
English
KW - MEDLINE
KW - Child
KW - Preschool
KW - Female
KW - Follow-Up Studies
KW - Humans
KW - Incisor/in [Injuries]
KW - Maxilla
KW - Time Factors
KW - Tooth Apex/gd [Growth & Development]
KW - Tooth Avulsion/pa [Pathology]
KW - Tooth Avulsion/su [Surgery]
KW - Tooth Replantation/mt [Methods]
RP - NOT IN FILE
SP - 550
EP - 554
JF - Dental Traumatology
JA - Dent Traumatol
VL - 25
IS - 5
CY - Denmark
N2 - Here we describe an unusual trauma case. A recently erupted permanent upper-right incisor sustained a lateral luxation when a 5-year-old girl on a playground climbing net dropped off, catching the right upper incisor in the net. The tooth was laterally luxated in vestibular direction, and no other signs of injury occurred. A dental practitioner could not reposition the bony locked tooth. Four days later, the girl came to our clinic, and we performed an incomplete repositioning of the tooth and made a flexible splint. Controls were made at 1, 6, and 12 weeks and at 6, 12, 18, and 24 months later. The 24-month follow-up clinical examination revealed the patient to be asymptomatic and the tooth to be completely functional, and the recall radiograph showed further apical root growth. The implications of a late incomplete reposition of laterally luxated permanent teeth with immature apices are discussed
SN - 1600-9657
AD - Dental Clinic 1, Operative Dentistry and Periodontology, University of Erlangen-Nuremberg, Erlangen, Germany. pelka@dent.uni-erlangen.de
UR - 19754701
ER -

TY - JOUR
ID - 885
T1 - Epidemiology of enterovirus 71 in the Netherlands, 1963 to 2008
A1 - van der,Sanden S.
A1 - Koopmans,M.
A1 - Uslu,G.
A1 - van der,Avoort H.
A1 - Dutch Working Group for Clinical Virology.
Y1 - 2009/09//
N1 - van der Sanden, Sabine. Koopmans, Marion. Uslu, Gokhan. van der Avoort, Harrie. Dutch Working Group for Clinical Virology
Journal of clinical microbiology
hsh, 7505564
IM

Journal Article
English
KW - MEDLINE
KW - Animals
KW - Capsid Proteins/ge [Genetics]
KW - Child
KW - Preschool
KW - Cluster Analysis
KW - Enterovirus A
KW - Human/ge [Genetics]
KW - Human/ip [Isolation & Purification]
KW - Enterovirus Infections/ep [Epidemiology]
KW - Enterovirus Infections/vi [Virology]
KW - Genotype
KW - Humans
KW - Incidence
KW - Molecular Sequence Data
KW - Netherlands/ep [Epidemiology]
KW - Phylogeny
KW - Sequence Analysis
KW - DNA
KW - Sequence Homology
RP - NOT IN FILE
SP - 2826
EP - 2833
JF - Journal of Clinical Microbiology
JA - J Clin Microbiol
VL - 47
IS - 9
CY - United States
N2 - The incidence of enterovirus 71 (EV71) infection has greatly increased in the Asian Pacific region since 1997. Several large outbreaks, caused by different subgenogroups of EV71, occurred with high rates of morbidity and a substantial number of deaths. In 2007, 58 cases of EV71 infection requiring hospitalization were reported in The Netherlands after a period of low endemicity of 21 years. These events triggered a study on the epidemiology of EV71 in The Netherlands. Genetic analysis of the VP1 capsid region of 199 EV71 isolates collected from 1963 to 2008 as part of enterovirus surveillance activities revealed a change in the prevailing subgenogroups over time. From 1963 to 1986 infections were caused by three different and successive lineages belonging to subgenogroup B (the novel lineage designated B0, as well as B1 and B2). In 1987, following a major epidemic the previous year, the B genogroup was replaced by genogroup C strains of lineages C1 and, later, C2. Analyses of the clinical data suggested that there were differences between infection with genogroup B and with genogroup C strains in terms of the age groups affected and the severity of illness. From comparative analysis with genomic data available in the public domain, we concluded that EV71 strain evolution shows a global pattern, which leads to the question of whether the recently emerged C4 lineage strains will also spread outside of Asia
SN - 1098-660X
AD - National Institute for Public Health and the Environment, RIVM, P.O. Box 1, 3720 BA Bilthoven, The Netherlands. sabine.van.der.sanden@rivm.nl
UR - 19625480
ER -

TY - JOUR
ID - 886
T1 - Leisure activity patterns and their associations with overweight: a prospective study among adolescents
A1 - Lajunen,H.R.
A1 - Keski-Rahkonen,A.
A1 - Pulkkinen,L.
A1 - Rose,R.J.

A1 - Rissanen,A.
A1 - Kaprio,J.
Y1 - 2009/10//
N1 - Lajunen, Hanna-Reetta. Keski-Rahkonen, Anna. Pulkkinen, Lea. Rose, Richard J. Rissanen, Aila. Kaprio, Jaakko
Journal of adolescence
hwt, 7808986
IM
Journal Article. Research Support, N.I.H., Extramural. Research Support, Non-U.S. Gov't
English
KW - MEDLINE
KW - Adolescent
KW - Body Mass Index
KW - Child
KW - Cohort Studies
KW - Female
KW - Finland/ep [Epidemiology]
KW - Humans
KW - Leisure Activities
KW - Logistic Models
KW - Male
KW - Overweight/ep [Epidemiology]
KW - Prospective Studies
KW - Questionnaires
KW - Young Adult
RP - NOT IN FILE
SP - 1089
EP - 1103
JF - Journal of Adolescence
JA - J Adolesc
VL - 32
IS - 5
CY - England
N2 - We examined longitudinal associations between individual leisure activities (television viewing, video viewing, computer games, listening to music, board games, musical instrument playing, reading, arts, crafts, socializing, clubs or scouts, sports, outdoor activities) and being overweight using logistic regression and latent class analysis in a cohort of Finnish twins responding to self-report questionnaires at 11-12 (N=5184), 14, and 17 years. We also studied activity patterns ("Active and sociable", "Active but less sociable", "Passive but sociable", "Passive and solitary") thought to represent different lifestyles. Among boys, activity patterns did not predict becoming overweight, but sports and playing an instrument reduced the risk and arts and listening to music increased it. Among girls, few individual leisure activities predicted becoming overweight. However, girls in the "Passive and solitary" cluster carried the greatest risk of becoming overweight in late adolescence. Studying leisure activities related to overweight may help focus specific interventions on high risk groups
SN - 1095-9254
AD - Department of Public Health, University of Helsinki, Helsinki, Finland. hanna-reetta.lajunen@helsinki.fi
UR - 19345989
ER -

TY - JOUR
ID - 887
T1 - Outdoor environmental assessment of attention promoting settings for preschool children
A1 - Martensson,F.
A1 - Boldemann,C.
A1 - Soderstrom,M.
A1 - Blennow,M.
A1 - Englund,J.E.
A1 - Grahn,P.

Y1 - 2009/12//
N1 - Martensson, F. Boldemann, C. Soderstrom, M. Blennow, M. Englund, J-E. Grahn, P
Health & place
din, 9510067
T
Journal Article. Research Support, Non-U.S. Gov't
English
KW - MEDLINE
KW - Attention
KW - Child
KW - Child Psychology
KW - Preschool
KW - Environment Design
KW - Female
KW - Humans
KW - Male
KW - Play and Playthings/px [Psychology]
KW - Schools
KW - Nursery
RP - NOT IN FILE
SP - 1149
EP - 1157
JF - Health & Place
JA - Health Place
VL - 15
IS - 4
CY - England
N2 - The restorative potential of green outdoor environments for children in preschool settings was investigated by measuring the attention of children playing in settings with different environmental features. Eleven preschools with outdoor environments typical for the Stockholm area were assessed using the outdoor play environment categories (OPEC) and the fraction of visible sky from play structures (sky view factor), and 198 children, aged 4.5-6.5 years, were rated by the staff for inattentive, hyperactive and impulsive behaviors with the ECADDES tool. Children playing in large and integrated outdoor areas containing large areas of trees, shrubbery and a hilly terrain showed less often behaviors of inattention (p<.05). The choice of tool for assessment of attention is discussed in relation to outdoor stay and play characteristics in Swedish preschool settings. The results indicate that the restorative potential of green outdoor environments applies also to preschool children and that environmental assessment tools as OPEC can be useful when to locate and develop health-promoting land adjacent to preschools
SN - 1353-8292
AD - Department of Work Science, Business Economics and Environmental Psychology, Swedish University of Agricultural Sciences, Alnarp, Sweden
UR - 19643655
ER -

TY - JOUR
ID - 888
T1 - Magnetoencephalography in Landau-Kleffner syndrome
A1 - Paetau,R.
Y1 - 2009/08//
N1 - Paetau, Ritva
Epilepsia
eix, 2983306r
IM
Comparative Study. Journal Article. Research Support, Non-U.S. Gov't
English
KW - MEDLINE
KW - Acoustic Stimulation

KW - Auditory Cortex/pp [Physiopathology]
KW - Auditory Perception/ph [Physiology]
KW - Brain Mapping
KW - Cerebral Cortex/pp [Physiopathology]
KW - Cerebral Cortex/su [Surgery]
KW - Child
KW - Preschool
KW - Electroencephalography
KW - Evoked Potentials
KW - Auditory/ph [Physiology]
KW - Frontal Lobe/pp [Physiopathology]
KW - Functional Laterality/ph [Physiology]
KW - Humans
KW - Landau-Kleffner Syndrome/di [Diagnosis]
KW - Landau-Kleffner Syndrome/pp [Physiopathology]
KW - Landau-Kleffner Syndrome/su [Surgery]
KW - Magnetoencephalography
KW - Parietal Lobe/pp [Physiopathology]
KW - Preoperative Care
KW - Pyramidal Cells/pp [Physiopathology]
KW - Temporal Lobe/pp [Physiopathology]
KW - Treatment Outcome
RP - NOT IN FILE
SP - 51
EP - 54
JF - Epilepsia
VL - 50 Suppl 7
CY - United States
N2 - Magnetoencephalography (MEG) detects weak magnetic fields outside the head. Spikes generated on the perisylvian convexity are detected only by electroencephalography (EEG), whereas spikes with intrasylvian generators are selectively seen by MEG. Generators of MEG and EEG spikes are determined using equivalent current dipoles (ECDs) that represent local cortical activity in parallel pyramidal neurons. MEG localizes cortical spike generators with a 1-2-cm spatial accuracy, and with a millisecond time resolution. This allows tracking of neural activity over successive synaptic connections in the cortical network. Both EEG and MEG are necessary for comprehensive spatial and temporal description of perisylvian epileptic networks in the Landau-Kleffner syndrome (LKS). MEG studies suggest that in more than 80% of LKS patients, the bilateral epileptic discharges are generated in the auditory- and language-related perisylvian cortex. Approximately 20% of children with LKS children have a unilateral perisylvian pacemaker that triggers secondary bilateral synchrony of spikes. This 20% may regain considerable language skills after multiple subpial transections (MSTs) of the pacemaker area. Outcome data of a few surgery patients suggest an indispensable role of MEG when planning the most efficient therapy for patients with LKS
SN - 1528-1167
AD - BioMag Laboratory, HUSLAB, Hospital District of Helsinki and Uusimaa, Helsinki University Central Hospital, HUS, Finland. ritva.paetau@hus.fi
UR - 19682052
ER -

TY - JOUR
ID - 889
T1 - The validity and reliability of an instrument to assess children's outdoor play in various locations
A1 - Veitch,J.
A1 - Salmon,J.
A1 - Ball,K.
Y1 - 2009/09//
N1 - Veitch, Jenny. Salmon, Jo. Ball, Kylie
Journal of science and medicine in sport / Sports Medicine Australia
c54, 9812598

IM
Journal Article. Research Support, Non-U.S. Gov't. Validation Studies
English
KW - MEDLINE
KW - Child
KW - Exercise
KW - Female
KW - Health Surveys
KW - Humans
KW - Male
KW - Parents
KW - Play and Playthings
KW - Reproducibility of Results
RP - NOT IN FILE
SP - 579
EP - 582
JF - Journal of Science & Medicine in Sport
JA - J Sci Med Sport
VL - 12
IS - 5
CY - Australia
N2 - The aim of this study was to develop and test the reliability and validity of survey items that examine the frequency with which primary school-aged children play in particular outdoor locations. Parents reported the number of days their child spent playing in specified outdoor locations (i.e., yard at home, own street/court/footpath, and park/playground) out-of-school hours on weekdays and weekend days during a typical week. To test the reliability of these items, the survey was administered on two occasions, 2 weeks apart, to a sample of 53 parents of children attending primary schools located in metropolitan Melbourne. The validity study involved the completion of a log book by 46 parents of primary school children over a 1-week period. Two weeks later, the same sample of parents completed the survey items. The test-retest reliability of individual items was determined using intra-class correlation coefficients (ICC). The kappa statistic and percent agreement between responses were used to assess validity by comparing the information provided in the log book with that provided in the survey. Results from the two studies suggest that the survey was generally a reliable and valid instrument for assessing the frequency with which children play in particular locations especially at home or in the street. Evidence of the reliability and validity of items assessing where children play is novel and important considering the need to promote children's physical activity in a variety of settings
SN - 1440-2440
AD - Centre for Physical Activity and Nutrition Research, Deakin University, Australia. jveitch@deakin.edu.au
UR - 19027361
ER -

TY - JOUR
ID - 890
T1 - Specific IgE against Alternaria alternata in atopic dermatitis and asthma patients
A1 - Hedayati,M.T.
A1 - Arabzadehmoghadam,A.
A1 - Hajheydari,Z.
Y1 - 2009/05//
N1 - Hedayati, M T. Arabzadehmoghadam, A. Hajheydari, Z
European review for medical and pharmacological sciences
cyu, 9717360
IM
Journal Article. Research Support, Non-U.S. Gov't
English
KW - MEDLINE
KW - Adolescent
KW - Adult
KW - Air Microbiology

KW - Air Pollutants/im [Immunology]
KW - Allergens
KW - Alternaria/im [Immunology]
KW - Antibodies
KW - Fungal/bl [Blood]
KW - Antigens
KW - Fungal/im [Immunology]
KW - Asthma/im [Immunology]
KW - Asthma/mi [Microbiology]
KW - Child
KW - Preschool
KW - Dermatitis
KW - Atopic/im [Immunology]
KW - Atopic/mi [Microbiology]
KW - Female
KW - Humans
KW - Immunoglobulin E/bl [Blood]
KW - Infant
KW - Male
KW - Middle Aged
KW - Young Adult
RP - NOT IN FILE
SP - 187
EP - 191
JF - European Review for Medical & Pharmacological Sciences
JA - Eur Rev Med Pharmacol Sci
VL - 13
IS - 3
CY - Italy
N2 - Alternaria alternata (A. alternata) is one of the most common airborne fungi in outdoor and indoor environment. A. alternata has also considered as an aeroallergen. So, it could be responsible for an allergen release and may be responsible for allergic reactions in sensitive patients. The aim of this study was the analyzing of specific IgE against A. alternata in atopic dermatitis (AD) and asthma patients. A total of 50 AD patients (male 17 and female 33) and 50 asthma patients (male 20 and female 30) were entered in study. The range age vary from 4 months to 60 years. To analyzing of specific IgE, A. alternata was cultured in Sabouraud's dextrose agar. The grown fungi were harvested and ruptured by liquid nitrogen and glass beads. Samples were centrifuged at 3000rpm in 15 minutes and then at 15,500 rpm (4 degrees C) in 2 hours and then supernatant were collected as crude extract. The crude extract was separated by Sodium Dodecyl Sulfate-Polyacryl Amide Gel Electrophoresis (SDS-PAGE). The separated proteins transferred to nitrocellulose filter and then socked with atopic dermatitis and asthma patient's sera. The responsive bands to IgE were revealed by antihuman IgE antibodies conjugated with enzyme in chromogenic substrate. 16 (32%) and 19 (38%) of AD and asthma patients had specific IgE against A. alternata, respectively. Among the AD and asthma patients who were positive for specific IgE to A. alternata, 14 (87.5%) and 9 (47.4%) were women, respectively. Of the 16 AD patients for specific IgE positive, 9 (56.3%) were >12 years old. Of the 19 asthma patients for specific IgE positive, 10 (52.6%) were 20-39 years old. This study suggests that A. alternata is a major aeroallergen. Our previous studies as well as different studies from other countries have shown that A. alternata is one of the most common indoor and outdoor airborne fungi, so it could permanently present some allergens to susceptible individuals. Therefore, control of A. alternata growth in indoor areas and avoidance with A. alternata propagules could play an important role in reducing allergic reaction in susceptible individuals
SN - 1128-3602
AD - Department of Medical Mycology and Parasitology, School of Medicine, Mazandaran University of Medical Sciences, Sari, Iran. hedayaty2001@yahoo.co.uk
UR - 19673169
ER -

TY - JOUR
ID - 891

T1 - Associations between physical activity of primary school first-graders during leisure time and family socioeconomic status
A1 - Dregval,L.
A1 - Petrauskiene,A.
Y1 - 2009///
N1 - Dregval, Liudmila. Petrauskiene, Ausra
Medicina (Kaunas, Lithuania)
9425208
IM
Comparative Study. Evaluation Studies. Journal Article
English
KW - MEDLINE
KW - Adolescent
KW - Adolescent Behavior
KW - Age Factors
KW - Chi-Square Distribution
KW - Child
KW - Child Behavior
KW - Computers
KW - Dancing
KW - Data Collection
KW - Data Interpretation
KW - Statistical
KW - Education
KW - Exercise
KW - Health Behavior
KW - Humans
KW - Income
KW - Leisure Activities
KW - Obesity/ep [Epidemiology]
KW - Obesity/pc [Prevention & Control]
KW - Parents
KW - Questionnaires
KW - Risk Factors
KW - Socioeconomic Factors
KW - Sports
KW - Television
KW - Time Factors
KW - Video Games
RP - NOT IN FILE
SP - 549
EP - 556
JF - Medicina (Kaunas, Lithuania)
JA - Medicina (Kaunas)
VL - 45
IS - 7
CY - Lithuania
N2 - In 2008, an international survey on obesity among first-graders and its risk factors was performed in Lithuania. The objective of this study was to assess physical activity of first-graders during leisure time according to family socioeconomic status. The study was performed in Siauliai region schools selected randomly in 2008. The anonymous questionnaires were distributed among 630 first-graders and filled out by 515 parents (response rate was 81.8%). It was showed that physical activity of first-graders during leisure time is insufficient. More than half of them (60.4%) did not attend sports or dancing clubs; children spent much time passively watching TV or playing on a computer. Mostly children watched TV for 2 hours on workdays (45.1%) and for 3 hours or more on weekends (41.4%). Mostly children spent about an hour per day playing on a computer: one-third of first-graders spent it on workdays; during weekends, the percentage of children spending about an hour per day playing on a computer was lower (28.5%). One-third of first-graders (36.9%) spent their

leisure time outside for 3 or more hours on workdays and 87.1% on weekends independently of parents' educational level, income, and place of residence. The associations between family socioeconomic status and physical activity of children were observed. The lowest percentage of children attending sports or dancing clubs and playing computer games was seen in low-income families and families where parents had low educational level. They spent more time outside (on workdays) compared with those children whose parents had university education and high income. Fewer first-graders from families living in villages than those living in cities attended sports or dancing clubs and played on a computer, but more of them spent leisure time outside
SN - 1648-9144
AD - Institute for Biomedical Research, Kaunas University of Medicine, Eiveniu 4, 50009 Kaunas, Lithuania. dregval@vector.kmu.lt
UR - 19667750
ER -

TY - JOUR
ID - 892
T1 - [Trachoma in the health district of Douentza, 10 years later the investigation in 1997]. [French]
A1 - Bamani,S.
A1 - Diawara,A.
A1 - Dembele,A.
A1 - Dembele,M.
A1 - Telly,A.
A1 - Simaga,S.Y.
Y1 - 2009///
N1 - Bamani, S. Diawara, A. Dembele, A. Dembele, M. Telly, A. Simaga, S Y
Le Mali medical
18420390r
IM
English Abstract. Journal Article
French
KW - MEDLINE
KW - Adolescent
KW - Adult
KW - Child
KW - Preschool
KW - Cross-Sectional Studies
KW - Female
KW - Humans
KW - Infant
KW - Male
KW - Mali
KW - Middle Aged
KW - Prevalence
KW - Risk Factors
KW - Time Factors
KW - Trachoma/co [Complications]
KW - Trachoma/ep [Epidemiology]
KW - Trichiasis/co [Complications]
KW - Trichiasis/ep [Epidemiology]
KW - Young Adult
RP - NOT IN FILE
SP - 42
EP - 45
JF - Mali Medical
JA - Mali med
VL - 24
IS - 2
CY - Mali

N2 - In Mali one of the options of the fight against blindness is to implement the "CHANCE" strategy. The antibiotherapy is one of the strategy which has always a question who should be treated by which process (mass treatment focused treatment) and when we should treat for both mass treatment or targeted treatment, that is why this study has been initiated to evaluate the level of active trachoma (TI/TI) and the trichiasis in the district of Douentza and undertake the appropriate actions to fight for its reduction. It was a cross sectional survey done in may 2005 following a methodology based on the random size described by WHO (world health Organization) in the evaluation of the health coverage. The study targets 1450 ten years old children, less than 1564 of fifteen years old children and respectively for the estimation of the trachoma prevalence and trichiasis. The families chiefs have been asked or interviewed for the risk factor and the happening of trachoma, mothers were interviewed about their attitude and practices in hygiene. The active trachoma prevalence rate among children is estimated at 13.2 + 2.7% in the district. Kids from 0 to 4 are more affected than those from 5 to 10 years old with $p < 0.001$. The trichiasis rate is 0.6% The pumps (30.3%), the communicative wells (23.8%) the traditional wells not maintained (19.4%), and the maintained wells (15.7%), faucets (9.7%) are the essential sources of water which is not sufficient. The presence of animals in 40.40 to 76.1% families, the low percentage of families with latrines (toilets) 41.40%, the presence of waste water and garbage in 25.3% families, 42.9% outside the families or 31.8% anywhere were observed during the study. Based on all these results, the antibiotic mass treatment to reduce the active trachoma is a necessity; this mass treatment combined with an efficient IEC to improve body and environmental hygiene will have an impact on the trachoma in the district of Douentza
SN - 1993-0836
AD - Sante Publique a la FMPOS de Bamako
UR - 19666368
ER -

TY - JOUR
ID - 893
T1 - Peridomestic Lyme disease prevention: results of a population-based case-control study
A1 - Connally,N.P.
A1 - Durante,A.J.
A1 - Yousey-Hindes,K.M.
A1 - Meek,J.I.
A1 - Nelson,R.S.
A1 - Heimer,R.
Y1 - 2009/09//
N1 - Connally, Neeta P. Durante, Amanda J. Yousey-Hindes, Kimberly M. Meek, James I. Nelson, Randall S. Heimer, Robert
American journal of preventive medicine
8704773, apl
IM
Journal Article. Research Support, Non-U.S. Gov't. Research Support, U.S. Gov't, P.H.S.
English
KW - MEDLINE
KW - Adolescent
KW - Adult
KW - Aged
KW - 80 and over
KW - Animals
KW - Case-Control Studies
KW - Child
KW - Preschool
KW - Connecticut/ep [Epidemiology]
KW - Environment Design
KW - Environmental Exposure/ae [Adverse Effects]
KW - Environmental Exposure/sn [Statistics & Numerical Data]
KW - Female
KW - Humans
KW - Hygiene
KW - Infant

KW - Logistic Models
KW - Lyme Disease/ep [Epidemiology]
KW - Lyme Disease/et [Etiology]
KW - Lyme Disease/pc [Prevention & Control]
KW - Male
KW - Matched-Pair Analysis
KW - Middle Aged
KW - Population Surveillance
KW - Prospective Studies
KW - Questionnaires
KW - Residence Characteristics
KW - Risk Factors
KW - Ticks
KW - Young Adult
RP - NOT IN FILE
SP - 201
EP - 206
JF - American Journal of Preventive Medicine
JA - Am J Prev Med
VL - 37
IS - 3
CY - Netherlands
N2 - BACKGROUND: Peridomestic Lyme disease-prevention initiatives promote personal protection, landscape modification, and chemical control. PURPOSE: A 32-month prospective age- and neighborhood-matched case-control study was conducted in Connecticut to evaluate the effects of peridomestic prevention measures on risk of Lyme disease. METHODS: The study was conducted in 24 disease-endemic Connecticut communities from 2005 to 2007. Subjects were interviewed by telephone using a questionnaire designed to elicit disease-prevention measures during the month prior to the case onset of erythema migrans. Data were analyzed in 2008 by conditional logistic regression. Potential confounders, such as occupational/recreational exposures, were examined. RESULTS: Between April 2005 and November 2007, interviews were conducted with 364 participants with Lyme disease, and 349 (96%) were matched with a suitable control. Checking for ticks within 36 hours of spending time in the yard at home was protective against Lyme disease (OR=0.55; 95% CI=0.32, 0.94). Bathing within 2 hours after spending time in the yard was also protective (OR=0.42; 95% CI=0.23, 0.78). Fencing of any type or height in the yard, whether it was contiguous or not, was protective (OR=0.54; 95% CI=0.33, 0.90). No other landscape modifications or features were significantly protective against Lyme disease. CONCLUSIONS: The results of this study suggest that practical activities such as checking for ticks and bathing after spending time in the yard may reduce the risk of Lyme disease in regions where peridomestic risk is high. Fencing did appear to protect against infection, but the mechanism of its protection is unclear
SN - 1873-2607
AD - Connecticut Emerging Infections Program, Yale School of Public Health, New Haven, Connecticut, USA. neeta.connally@yale.edu
UR - 19595558
ER -

TY - JOUR
ID - 894
T1 - Dietary patterns in relation to socio-economic and lifestyle characteristics among Greek adolescents: a multivariate analysis
A1 - Kourlaba,G.
A1 - Panagiotakos,D.B.
A1 - Mihas,K.
A1 - Alevizos,A.
A1 - Marayiannis,K.
A1 - Mariolis,A.
A1 - Tountas,Y.
Y1 - 2009/09//

N1 - Kourlaba, Georgia. Panagiotakos, Demosthenes B. Mihas, Kostas. Alevizos, Alevizos. Marayiannis, Kostas. Mariolis, Anargiros. Tountas, Yannis
Public health nutrition
9808463, dgy
IM
Journal Article
English
KW - MEDLINE
KW - Adolescent
KW - Adolescent Nutritional Physiological Phenomena/ph [Physiology]
KW - Child
KW - Diet/td [Trends]
KW - Female
KW - Greece
KW - Humans
KW - Life Style
KW - Male
KW - Multivariate Analysis
KW - Principal Component Analysis/mt [Methods]
KW - Questionnaires
KW - Social Class
KW - Socioeconomic Factors
RP - NOT IN FILE
SP - 1366
EP - 1372
JF - Public Health Nutrition
JA - Public Health Nutr
VL - 12
IS - 9
CY - England
N2 - BACKGROUND: We sought to evaluate the relationship between selected lifestyle and socio-economic characteristics and dietary habits of Greek adolescents. METHODS: During 2004, 2118 school adolescents were selected from twelve schools in Vyronas region, Athens. Dietary intake was assessed through a semi-quantitative FFQ. Principal components analysis was applied to extract dietary patterns. RESULTS: Seven components, explaining the 50% of the total variation in intake, were extracted. Component 1 ('junk food' pattern) was heavily loaded by the consumption of 'sweet' and 'salty' snacks, soft drinks and other 'fast foods'. Component 2 was characterized as 'red meat' consumption pattern. Component 3 was characterized by the consumption of vegetables, fruits and juices. Component 4 was characterized by the intake of dairy products, pasta and wholegrain bread. Component 5 can be described as rice, fish, potatoes and poultry intake. Component 6 was characterized by the consumption of 'traditional Greek cooked foods' and legumes, and component 7 was characterized by the consumption of eggs and white bread. Time spent watching television was positively associated with the 'junk food' pattern and inversely associated with the 'vegetarian/healthy' pattern. Moreover, the 'junk food' pattern was positively related to smoking status and the 'vegetarian/healthy' pattern was positively correlated with sports activities outside school. CONCLUSION: An unhealthy dietary behaviour is associated with an overall unhealthy lifestyle. Taking into account the fact that unhealthy eating habits and sedentary lifestyle have been associated with increased obesity prevalence, school- or community-based programmes should be conducted promoting healthy dietary and lifestyle behaviours
SN - 1475-2727
AD - Department of Nutrition Science-Dietetics, Harokopio University, 70 El. Venizelou Street, 17671 Athens, Greece
UR - 19063765
ER -

TY - JOUR
ID - 895
T1 - Expected values for pedometer-determined physical activity in youth. [Review] [63 refs]
A1 - Tudor-Locke,C.

A1 - McClain,J.J.
A1 - Hart,T.L.
A1 - Sisson,S.B.
A1 - Washington,T.L.
Y1 - 2009/06//
N1 - Tudor-Locke, Catrine. McClain, James J. Hart, Teresa L. Sisson, Susan B. Washington, Tracy L
Research quarterly for exercise and sport
r6y, 8006373
IM
Journal Article. Review
English
KW - MEDLINE
KW - Adolescent
KW - Child
KW - Preschool
KW - Female
KW - Humans
KW - Male
KW - Monitoring
KW - Ambulatory/is [Instrumentation]
KW - Motor Activity/ph [Physiology]
RP - NOT IN FILE
SP - 164
EP - 174
JF - Research Quarterly for Exercise & Sport
JA - Res Q Exerc Sport
VL - 80
IS - 2
CY - United States
N2 - This review assembles pedometry literature focused on youth, with particular attention to expected values for habitual, school day, physical education class, recess, lunch break, out-of-school, weekend, and vacation activity. From 31 studies published since 1999, we constructed a youth habitual activity step-curve that indicates: (a) from ages 6 to 18 years, boys typically take more steps per day than girls; (b) for both sexes the youngest age groups appear to take fewer steps per day than those immediately older; and (c) from a young age, boys decline more in steps per day to become more consistent with girls at older ages. Additional studies revealed that boys take approximately 42-49% of daily steps during the school day; girls take 41-47%. Steps taken during physical education class contribute to total steps per day by 8.7-23.7% in boys and 11.4-17.2% in girls. Recess represents 8-11% and lunch break represents 15-16% of total steps per day. After-school activity contributes approximately 47-56% of total steps per day for boys and 47-59% for girls. Weekdays range from approximately 12,000 to 16,000 steps per day in boys and 10,000 to 14,000 steps perday in girls. The corresponding values for weekend days are 12,000-13,000 steps per day in boys and 10,000-12,000 steps per day in girls. [References: 63]
SN - 0270-1367
AD - Pennington Biomedical Research Center, Baton Rouge, LA 70808, USA. Tudor-Locke@pbrc.edu
UR - 19650381
ER -

TY - JOUR
ID - 896
T1 - An evaluation of four automatic methods of segmenting the subcortical structures in the brain.[Erratum appears in Neuroimage. 2010 Jan 1;49(1):1152]
A1 - Babalola,K.O.
A1 - Patenaude,B.
A1 - Aljabar,P.
A1 - Schnabel,J.
A1 - Kennedy,D.
A1 - Crum,W.

A1 - Smith,S.
A1 - Cootes,T.
A1 - Jenkinson,M.
A1 - Rueckert,D.
Y1 - 2009/10/01/
N1 - Babalola, Kolawole Oluwole. Patenaude, Brian. Aljabar, Paul. Schnabel, Julia. Kennedy, David. Crum, William. Smith, Stephen. Cootes, Tim. Jenkinson, Mark. Rueckert, Daniel
NeuroImage
cpp, 9215515
IM
Evaluation Studies. Journal Article. Research Support, Non-U.S. Gov't
English
KW - MEDLINE
KW - Adolescent
KW - Adult
KW - Aged
KW - 80 and over
KW - Algorithms
KW - Artificial Intelligence
KW - Brain/pa [Pathology]
KW - Brain Diseases/pa [Pathology]
KW - Child
KW - Female
KW - Humans
KW - Image Enhancement/mt [Methods]
KW - Image Interpretation
KW - Computer-Assisted/mt [Methods]
KW - Imaging
KW - Three-Dimensional/mt [Methods]
KW - Magnetic Resonance Imaging/mt [Methods]
KW - Male
KW - Middle Aged
KW - Pattern Recognition
KW - Automated/mt [Methods]
KW - Reproducibility of Results
KW - Sensitivity and Specificity
KW - Young Adult
RP - NOT IN FILE
SP - 1435
EP - 1447
JF - Neuroimage
VL - 47
IS - 4
CY - United States
N2 - The automation of segmentation of subcortical structures in the brain is an active research area. We have comprehensively evaluated four novel methods of fully automated segmentation of subcortical structures using volumetric, spatial overlap and distance-based measures. Two methods are atlas-based - classifier fusion and labelling (CFL) and expectation-maximisation segmentation using a brain atlas (EMS), and two incorporate statistical models of shape and appearance - profile active appearance models (PAM) and Bayesian appearance models (BAM). Each method was applied to the segmentation of 18 subcortical structures in 270 subjects from a diverse pool varying in age, disease, sex and image acquisition parameters. Our results showed that all four methods perform on par with recently published methods. CFL performed better than the others according to all three classes of metrics. In summary over all structures, the ranking by the Dice coefficient was CFL, BAM, joint EMS and PAM. The Hausdorff distance ranked the methods as CFL, joint PAM and BAM, EMS, whilst percentage absolute volumetric difference ranked them as joint CFL and PAM, joint BAM and EMS. Furthermore, as we had four methods of performing segmentation, we investigated whether the results obtained by each method were more similar to each other than to the manual segmentations using Williams' Index.

Reassuringly, the Williams' Index was close to 1 for most subjects (mean=1.02, sd=0.05), indicating better agreement of each method with the gold standard than with the other methods. However, 2% of cases (mainly amygdala and nucleus accumbens) had values outside 3 standard deviations of the mean
SN - 1095-9572
AD - University of Manchester, Imaging Science and Biomedical Engineering, Stopford Building, Oxford Road, Manchester M13 9PT, UK. kola.babalola@manchester.ac.uk
UR - 19463960
ER -

TY - JOUR
ID - 897
T1 - Peer responses to stuttering in the preschool setting
A1 - Langevin,M.
A1 - Packman,A.
A1 - Onslow,M.
Y1 - 2009/08//
N1 - Langevin, Marilyn. Packman, Ann. Onslow, Mark
American journal of speech-language pathology / American Speech-Language-Hearing Association
9114726
IM
Journal Article. Research Support, Non-U.S. Gov't
English
KW - MEDLINE
KW - Child
KW - Preschool
KW - Faculty
KW - Female
KW - Humans
KW - Male
KW - Peer Group
KW - Play and Playthings
KW - Psycholinguistics
KW - Schools
KW - Social Behavior
KW - Speech Production Measurement
KW - Stuttering
KW - Time Factors
RP - NOT IN FILE
SP - 264
EP - 276
JF - American Journal of Speech-Language Pathology
JA - Am J Speech Lang Pathol
VL - 18
IS - 3
CY - United States
N2 - PURPOSE: This study investigated peer responses to preschoolers' stuttering in preschool and sought to determine whether specific characteristics of participants' stuttering patterns elicited negative peer responses. METHOD: Four outdoor free-play sessions of 4 preschoolers age 3-4 years who stutter were videotaped. Stutters were identified on transcripts of the play sessions. Peer responses to stuttered utterances were judged to be negative or neutral/positive. Thereafter, participants' stuttering behaviors, durations of stutters, and judgments of the meaningfulness of peer-directed stuttered utterances were analyzed. RESULTS: Between 71.4% and 100% of peer responses were judged to be neutral/positive. In the negative responses across 3 participants, peers were observed to react with confusion or to interrupt, mock, walk away from, or ignore the stuttered utterances. Utterances that elicited negative responses were typically meaningless and contained stutters that were behaviorally complex and/or of longer duration. Other social interaction difficulties also were observed-for example, difficulty leading peers in play, participating in pretend play, and resolving conflicts. CONCLUSIONS: Results indicate that the majority of peer responses to stuttered utterances were

neutral/positive; however, results also indicate that stuttering has the potential to elicit negative peer responses and affect other social interactions in preschool
SN - 1058-0360
AD - Institute for Stuttering Treatment & Research, Faculty of Rehabilitation Medicine, University of Alberta, 1500, 8215-112 Street, Edmonton, Alberta T6G 2C8, Canada. marilyn.langevin@ualberta.ca
UR - 19332523
ER -

TY - JOUR
ID - 898
T1 - The pattern of ankle fractures sustained by outdoor activities at the Snowdonia National Park, North Wales, United Kingdom
A1 - Kumar,A.J.
A1 - Gill,D.S.
A1 - Fairweather,C.
A1 - Dykes,L.
Y1 - 2009///
N1 - Kumar, A J Shyam. Gill, D S. Fairweather, C. Dykes, L
Foot and ankle surgery : official journal of the European Society of Foot and Ankle Surgeons
9308427, bqr, 9609647
IM
Journal Article
English
KW - MEDLINE
KW - Adolescent
KW - Adult
KW - Aircraft
KW - Ankle Injuries/ep [Epidemiology]
KW - Ankle Injuries/su [Surgery]
KW - Child
KW - Emergency Service
KW - Hospital/sn [Statistics & Numerical Data]
KW - Female
KW - Fractures
KW - Bone/ep [Epidemiology]
KW - Bone/su [Surgery]
KW - Open/ep [Epidemiology]
KW - Open/su [Surgery]
KW - Humans
KW - Joint Instability/ep [Epidemiology]
KW - Joint Instability/su [Surgery]
KW - Male
KW - Middle Aged
KW - Mountaineering
KW - Wales
KW - Young Adult
RP - NOT IN FILE
SP - 144
EP - 145
JF - Journal of Foot & Ankle Surgery
JA - J Foot Ankle Surg
VL - 15
IS - 3
CY - France
N2 - OBJECTIVE: The aim of this study was to review the pattern of ankle fractures sustained by patients brought to the Emergency Department at Ysbyty Gwynedd from The Snowdonia National Park. MATERIALS AND METHODS: The study group included all patients with ankle fractures on the mountain medicine

database between March 2004 and December 2006. The presence of talar shift and comminution of the medial malleolus was noted. The pattern of fractures were analysed and compared with the literature. Radiographs were obtained for 20 casualties. RESULTS: 70% of these were injured whilst hill walking. Open fractures represented 12% of injuries. 75% of fractures required operative fixation. Weber B injuries were the commonest followed by Weber C and A. Talar shift was seen in 80% of the cases and 45% showed comminution of the medial malleolus. CONCLUSIONS: In our case series we observed a high proportion of open and unstable ankle fractures, with the majority treated by operative fixation. The high rate of comminution of the medial malleolus has previously not been reported in the literature and has the potential of making operative fixation technically difficult. Encouraging the use of walking poles particularly at the time of descending may help to reduce the incidence of ankle fractures in hill walkers
SN - 1460-9584
AD - Trauma &Orthopaedics-All Wales, Ysbyty Gwynedd, Bangor, North Wales, UK.
ajshyamkumar@hotmail.co.uk
UR - 19635422
ER -

TY - JOUR
ID - 899
T1 - Outdoor activity and myopia in Singapore teenage children
A1 - Dirani,M.
A1 - Tong,L.
A1 - Gazzard,G.
A1 - Zhang,X.
A1 - Chia,A.
A1 - Young,T.L.
A1 - Rose,K.A.
A1 - Mitchell,P.
A1 - Saw,S.M.
Y1 - 2009/08//
N1 - Dirani, M. Tong, L. Gazzard, G. Zhang, X. Chia, A. Young, T L. Rose, K A. Mitchell, P. Saw, S-M
The British journal of ophthalmology
azk, 0421041
IM
Journal Article. Research Support, Non-U.S. Gov't
English
KW - MEDLINE
KW - Adolescent
KW - Age Factors
KW - Child
KW - Female
KW - Humans
KW - Leisure Activities
KW - Male
KW - Myopia/ep [Epidemiology]
KW - Myopia/pc [Prevention & Control]
KW - Sex Factors
KW - Singapore/ep [Epidemiology]
KW - Sports/sn [Statistics & Numerical Data]
KW - Time Factors
KW - Young Adult
RP - NOT IN FILE
SP - 997
EP - 1000
JF - British Journal of Ophthalmology
JA - Br J Ophthalmol
VL - 93
IS - 8

CY - England
N2 - AIM: To investigate the relationship of outdoor activities and myopia in Singapore teenage children. METHODS: Teenage children (1249 participants), examined in the Singapore Cohort study Of Risk factors for Myopia (SCORM), during 2006 were included in analyses. Participants completed questionnaires that quantified total outdoor activity, and underwent an eye examination. RESULTS: The mean total time spent on outdoor activity was 3.24 h/day. The total outdoor activity (h/day) was significantly associated with myopia, odds ratio 0.90 (95% CI 0.84 to 0.96) (p = 0.004), after adjusting for age, gender, ethnicity, school type, books read per week, height, parental myopia, parental education and intelligence quotient. In addition, the total time spent outdoors was associated with significantly less myopic refraction (regression coefficient = 0.17; CI 0.10 to 0.25, p<0.001) and shorter axial length (regression coefficient -0.06 (CI -0.1 to -0.03, p<0.001). Total sports was also significantly negatively associated with myopia (p = 0.008) but not indoor sports (p = 0.16). CONCLUSIONS: Participants who spent more time outdoors were less likely to be myopic. Thus, outdoor activity may protect against development of myopia in children, supporting recent Australian data. As near work did not predict outdoor activity, this can be viewed as an independent factor and not merely the reciprocal of near work
SN - 1468-2079
AD - Singapore Eye Research Institute, and Department of Community, Occupational and Family Medicine, Yong Loo Lin School of Medicine, National University of Singapore, MD3, 16 Medical Drive, Singapore 117597. dirani@unimelb.edu.au
UR - 19211608
ER -

TY - JOUR
ID - 900
T1 - Preventing unintentional injury in children and adolescents--the importance of local injury data collection
A1 - O'Carroll,C.
A1 - Egleston,C.
A1 - Nicholson,A.J.
Y1 - 2009/05//
N1 - O'Carroll, C. Egleston, C. Nicholson, A J
Irish medical journal
gxd, 0430275
IM
Journal Article
English
KW - MEDLINE
KW - Accident Prevention/mt [Methods]
KW - Accident Prevention/sn [Statistics & Numerical Data]
KW - Accidental Falls/pc [Prevention & Control]
KW - Accidental Falls/sn [Statistics & Numerical Data]
KW - Accidents
KW - Traffic/pc [Prevention & Control]
KW - Traffic/sn [Statistics & Numerical Data]
KW - Adolescent
KW - Age Factors
KW - Child
KW - Data Collection
KW - Female
KW - Humans
KW - Ireland
KW - Male
KW - Population Surveillance
KW - Prospective Studies
KW - Risk Factors
RP - NOT IN FILE
SP - 152
EP - 154

JF - Irish Medical Journal
JA - Ir Med J
VL - 102
IS - 5
CY - Ireland
N2 - We sought to prospectively study all injuries in children and adolescents up to 16 years of age presenting to a regional Emergency Department (ED), to ascertain detailed injury patterns and to use this data to recommend injury prevention priorities. Electronic injury surveillance was prospectively collected over a 10 year period (1997-2007) in a hospital with a paediatric catchment population of 75,000 in a region with pockets of high social deprivation. All fatalities were obtained from data provided by the Central Statistics Office (CSO). Over a 10 year period, there were 31 fatalities, 5,408 admissions and 40,817 new attendances due to injury. Males outnumbered females in a 3:2 ratio. Of all injuries 24,317 (60%) occurred at home. Peak injury presentation time was in the evening between 18:00 and 20:00. Minor injuries (bruises, minor head injuries, lacerations and sprains) accounted for 32,456 (80%) of total. Fractures resulting from high falls (n=1,194) tended to result from bunk beds, staircases, horses, walls and playground equipment. Burns (n=630) involved hot liquids (tea, coffee), hot bath water, hot cooking oil and hot cooking plates. Pedestrian injuries (n=251) were predominantly 'dart outs' in urban areas. Car passenger injuries (n=869) showed low rates of documented car restraint use. Poisonings (n= 1,153) were predominantly medicinal products. Cyclist injuries (n=477) indicated low documented use of appropriate helmet wear. Prevention priorities should focus on home injuries, hot liquid burn and scald injuries and high falls from walls, beds and playground equipment. To prevent road-related injuries and deaths, further legislation, urban planning and greater police enforcement is required
SN - 0332-3102
AD - Department of Paediatrics and Emergency Medicine, Our Lady of Lourdes Hospital, Drogheda, Co Louth
UR - 19623812
ER -

TY - JOUR
ID - 901
T1 - Quality of life assessment and disease experience of patient members of a web-based hydroa vacciniforme support group
A1 - Huggins,R.H.
A1 - Leithauser,L.A.
A1 - Eide,M.J.
A1 - Hexsel,C.L.
A1 - Jacobsen,G.
A1 - Lim,H.W.
Y1 - 2009/08//
N1 - Huggins, Richard H. Leithauser, Laurel A. Eide, Melody J. Hexsel, Camile L. Jacobsen, Gordon. Lim, Henry W
Photodermatology, photoimmunology & photomedicine
awp, 9013641
IM
Comparative Study. Journal Article
English
KW - MEDLINE
KW - Adolescent
KW - Adult
KW - Age Factors
KW - Child
KW - Cohort Studies
KW - Female
KW - Humans
KW - Hydroa Vacciniforme/px [Psychology]
KW - Internet
KW - Male
KW - Middle Aged
KW - Quality of Life/px [Psychology]

KW - Questionnaires
RP - NOT IN FILE
SP - 209
EP - 215
JF - Photodermatology, Photoimmunology & Photomedicine
JA - Photodermatol.Photoimmunol.Photomed.
VL - 25
IS - 4
CY - Denmark
N2 - BACKGROUND/PURPOSE: Hydroa vacciniforme (HV) is a rare photodermatosis that primarily affects children. It is characterized by photodistributed vesicles that heal with scarring. The purposes of this study are to perform the initial investigation into the effect of HV on quality of life (QoL) and gain insight into disease diagnosis and management. METHODS: Using the listserv from a web-based, international HV support group, either the Dermatology Life Quality Index (DLQI) or the Children's DLQI (CDLQI), and an HV-specific questionnaire were administered. RESULTS: Fifteen HV patients participated, nine (60%) males and six (40%) females. Median age at onset was 7 years, and 11/15 (73%) were younger than 18 years. The majority of patients were Caucasian (73%). Children cited life quality as being negatively impacted by an inability to play outdoors while adults noted QoL influences due to limitations on clothing choices. The mean CLDLQI and DLQI scores, 12.1 and 8.5, respectively, suggest a higher negative QoL impact than previously reported indices for generalized eczema, atopic dermatitis, and psoriasis. CONCLUSION: When compared with other dermatoses, HV appears to have an equal or greater impact on patients' QoL. Dermatologists should be aware of the psychosociologic impact of this disease, especially on young HV patients
SN - 1600-0781
AD - Department of Dermatology, Henry Ford Hospital, Detroit, MI 48202, USA
UR - 19614900
ER -

TY - JOUR
ID - 902
T1 - Physical activity levels of children with intellectual disabilities during school
A1 - Pitetti,K.H.
A1 - Beets,M.W.
A1 - Combs,C.
Y1 - 2009/08//
N1 - Pitetti, Kenneth H. Beets, Michael W. Combs, Cindy
Medicine and science in sports and exercise
8005433, mg8
IM, S
Journal Article
English
KW - MEDLINE
KW - Child
KW - Preschool
KW - Developmental Disabilities
KW - Exercise/ph [Physiology]
KW - Female
KW - Humans
KW - Intellectual Disability
KW - Male
KW - Physical Exertion/ph [Physiology]
KW - Schools
KW - Telemetry
RP - NOT IN FILE
SP - 1580
EP - 1586
JF - Medicine & Science in Sports & Exercise
JA - Med Sci Sports Exerc

VL - 41
IS - 8
CY - United States
N2 - INTRODUCTION: Current guidelines recommend school-age children accumulate at least 60 min of moderate-to-vigorous physical activity (MVPA) on most days of the week. Little is known about the activity level of school-age children with intellectual disabilities (ID). PURPOSE: To evaluate physical activity patterns of children with ID during three school settings: adapted physical education (APE, 55 min), classroom (CR, 55 min), and recess (RE, 25 min). METHODS: HR was measured by telemetry for 15 youth (6 boys, 9 girls, 8.8 +/- 2.2 yr) with ID during APE, CR, and RE on three nonconsecutive school days. Resting HR (RHR) was measured on three nonconsecutive days between 8:00 and 8:45 a.m. The mean of the five lowest RHR was used to create MVPA cutoffs. Total MVPA was determined by the mean time spent (min) above 1.25% RHR (PAHR-25 Index) in each of the three school settings. Three (one for each environment) random effects two-level models (measures nested within child) were used to describe the proportion of time students spend in MVPA across the three environments and to examine the within-child variation across measures within locations. RESULTS: The total daily MVPA across the three environments was 83.5 min (72.9-94 min). Intraclass correlations ranged from 0.06 to 0.53, suggesting considerable within-child variation across the three observations across settings. CONCLUSIONS: Although variation existed across observations for each child, on average, children met and exceeded recommendations for 60 min of MVPA during the day. Theses findings suggest that through allocation of time for APE and RE, coupled with programming designed to maximize activity opportunities, students with ID can achieve recommended levels of MVPA
SN - 1530-0315
AD - Department of Physical Therapy, College of Health Professions, Wichita State University, Wichita, KS 67260-0043, USA. ken.pitetti@wichita.edu
UR - 19568202
ER -

TY - JOUR
ID - 903
T1 - Validity of a traffic air pollutant dispersion model to assess exposure to fine particles
A1 - Kostrzewa,A.
A1 - Reungoat,P.
A1 - Raherison,C.
Y1 - 2009/08//
N1 - Kostrzewa, Aude. Reungoat, Patrice. Raherison, Chantal
Environmental research
ei2, 0147621
IM
Journal Article. Research Support, Non-U.S. Gov't
English
KW - MEDLINE
KW - Air Pollutants/an [Analysis]
KW - Child
KW - Data Interpretation
KW - Statistical
KW - Environmental Monitoring/mt [Methods]
KW - Environmental Monitoring/sn [Statistics & Numerical Data]
KW - France
KW - Humans
KW - Inhalation Exposure/an [Analysis]
KW - Models
KW - Theoretical
KW - Particle Size
KW - Particulate Matter/an [Analysis]
KW - Reproducibility of Results
KW - Research Design
KW - Schools/st [Standards]
RP - NOT IN FILE

SP - 651
EP - 656
JF - Environmental Research
JA - Environ Res
VL - 109
IS - 6
CY - United States
N2 - INTRODUCTION: Fine particles (PM(2.5)) are an important component of air pollution. Epidemiological studies have shown health effects due to ambient air particles, particularly allergies in children. Since the main difficulty is to determine exposure to such pollution, traffic air pollutant (TAP) dispersions models have been developed to improve the estimation of individual exposure levels. One such model, the ExTra index, has been validated for nitrogen oxide concentrations but not for other pollutants. The purpose of this study was to assess the validity of the ExTra index to assess PM(2.5) exposure. METHODS: We compared PM(2.5) concentrations calculated by the ExTra index to reference measures (passive samplers situated under the covered part of the playground), in 15 schools in Bordeaux, in 2000. First, we collected the input data required by the ExTra index: background and local pollution depending on traffic, meteorology and topography. Second, the ExTra index was calculated for each school. Statistical analysis consisted of a graphic description; then, we calculated an intraclass correlation coefficient. RESULTS: Concentrations calculated with the ExTra index and the reference method were similar. The ExTra index underestimated exposure by 2.2 microg m(-3) on average compared to the reference method. The intraclass correlation coefficient was 0.85 and its 95% confidence interval was [0.62; 0.95]. CONCLUSIONS: The results suggest that the ExTra index provides an assessment of PM(2.5) exposure similar to that of the reference method. Although caution is required in interpreting these results owing to the small number of sites, the ExTra index could be a useful epidemiological tool for reconstructing individual exposure, an important challenge in epidemiology
SN - 1096-0953
AD - Laboratoire Sante Travail Environnement (LSTE), EA 3672, ISPED-Universite Victor Segalen Bordeaux 2, Case 11, 146 Rue Leo Saignat, 33076 Bordeaux Cedex, France. Aude.Kostrzewa@isped.u-bordeaux2.fr
UR - 19545865
ER -

TY - JOUR
ID - 904
T1 - Differentiation between attention-deficit/hyperactivity disorder and pervasive developmental disorders with hyperactivity on objective activity levels using actigraphs
A1 - Tsujii,N.
A1 - Okada,A.
A1 - Kaku,R.
A1 - Kuriki,N.
A1 - Hanada,K.
A1 - Shirakawa,O.
Y1 - 2009/06//
N1 - Tsujii, Noa. Okada, Akira. Kaku, Reigetsu. Kuriki, Noriko. Hanada, Kazushi. Shirakawa, Osamu
Psychiatry and clinical neurosciences
cfs, 9513551
IM
Comparative Study. Journal Article
English
KW - MEDLINE
KW - Attention Deficit Disorder with Hyperactivity/di [Diagnosis]
KW - Case-Control Studies
KW - Child
KW - Child Development Disorders
KW - Pervasive/co [Complications]
KW - Pervasive/di [Diagnosis]
KW - Diagnosis
KW - Differential
KW - Humans

KW - Hyperkinesis/co [Complications]
KW - Hyperkinesis/di [Diagnosis]
KW - Male
KW - Monitoring
KW - Ambulatory/mt [Methods]
KW - Motor Activity
RP - NOT IN FILE
SP - 336
EP - 343
JF - Psychiatry & Clinical Neurosciences
JA - Psychiatry Clin Neurosci
VL - 63
IS - 3
CY - Australia
N2 - AIMS: To clarify differences in objective activity levels between children with attention-deficit/hyperactivity disorder (ADHD) and those with pervasive developmental disorders (PDD) and hyperactivity. METHOD: Eighteen boys with combined type ADHD, 10 boys with PDD with hyperactivity, and 18 control boys wore actigraphs for 1 week while attending elementary school. In addition to the average activity level, the standard deviation in the activity levels were compared for two continuous situations: (i) in-seat classes, in which the participants were expected to sit in their own seats and learn quietly; and (ii) free recess periods following the in-seat classes. RESULTS: All the groups were affected by the situational shift, the average activity level of each the groups was higher and the standard deviation was smaller than those during the in-seat classes. The boys with ADHD exhibited a still smaller standard deviation than the controls and the boys with PDD and hyperactivity during the free recess period; no difference between the controls and the boys with PDD was seen. The boys with PDD exhibited a significantly lower average activity level than the other groups. No differences among the groups in the average activity levels and standard deviation were seen during the in-seat classes. CONCLUSIONS: The observed objective activity levels in each group reflect the degree to which the boys are able to tolerate changes in situations. Objective measurement of activity levels may be useful to differentiate hyperactivity in children with ADHD from that in children without ADHD
SN - 1440-1819
AD - Department of Neuropsychiatry, Nara Hospital, Kinki University School of Medicine, Nara, Japan. tsujii-noa@nara.med.kindai.ac.jp
UR - 19566765
ER -

TY - JOUR
ID - 905
T1 - Relative cost-effectiveness of treatments for adolescent depression: 36-week results from the TADS randomized trial
A1 - Domino,M.E.
A1 - Foster,E.M.
A1 - Vitiello,B.
A1 - Kratochvil,C.J.
A1 - Burns,B.J.
A1 - Silva,S.G.
A1 - Reinecke,M.A.
A1 - March,J.S.
Y1 - 2009/07//
N1 - Domino, Marisa Elena. Foster, E Michael. Vitiello, Benedetto. Kratochvil, Christopher J. Burns, Barbara J. Silva, Susan G. Reinecke, Mark A. March, John S
Journal of the American Academy of Child and Adolescent Psychiatry
hg5, 8704565
IM
Comparative Study. Journal Article. Multicenter Study. Randomized Controlled Trial. Research Support, N.I.H., Extramural
English
KW - MEDLINE

KW - Adolescent
KW - Antidepressive Agents
KW - Second-Generation/ec [Economics]
KW - Second-Generation/tu [Therapeutic Use]
KW - Child
KW - Cognitive Therapy/ec [Economics]
KW - Combined Modality Therapy/ec [Economics]
KW - Cost-Benefit Analysis/sn [Statistics & Numerical Data]
KW - Depressive Disorder
KW - Major/ec [Economics]
KW - Major/th [Therapy]
KW - Double-Blind Method
KW - Drug Costs/sn [Statistics & Numerical Data]
KW - Female
KW - Fluoxetine/ec [Economics]
KW - Fluoxetine/tu [Therapeutic Use]
KW - Follow-Up Studies
KW - Hospitalization/ec [Economics]
KW - Humans
KW - Male
KW - Personality Assessment/sn [Statistics & Numerical Data]
KW - Psychometrics
RP - NOT IN FILE
SP - 711
EP - 720
JF - Journal of the American Academy of Child & Adolescent Psychiatry
JA - J Am Acad Child Adolesc Psychiatry
VL - 48
IS - 7
CY - United States
N2 - OBJECTIVE: The cost-effectiveness of three active interventions for major depression in adolescents was compared after 36 weeks of treatment in the Treatment of Adolescents with Depression Study. METHOD: Outpatients aged 12 to 18 years with a primary diagnosis of major depression participated in a randomized controlled trial conducted at 13 U.S. academic and community clinics from 2000 to 2004. Three hundred twenty-seven participants randomized to 1 of 3 active treatment arms, fluoxetine alone (n = 109), cognitive-behavioral therapy (n = 111) alone, or their combination (n = 107), were evaluated for a 3-month acute treatment and a 6-month continuation/maintenance treatment period. Costs of services received for the 36 weeks were estimated and examined in relation to the number of depression-free days and quality-adjusted life-years. Cost-effectiveness acceptability curves were also generated. Sensitivity analyses were conducted to assess treatment differences on the quality-adjusted life-years and cost-effectiveness measures. RESULTS: Cognitive-behavioral therapy was the most costly treatment component (mean $1,787 [in monotherapy] and $1,833 [in combination therapy], median $1,923 [for both]). Reflecting higher direct and indirect costs associated with psychiatric hospital use, the costs of services received outside Treatment of Adolescents with Depression Study in fluoxetine-treated patients (mean $5,382, median $2,341) were significantly higher than those in participants treated with cognitive-behavioral therapy (mean $3,102, median $1,373) or combination (mean $2,705, median $927). Accordingly, cost-effectiveness acceptability curves indicate that combination treatment is highly likely (>90%) to be more cost-effective than fluoxetine alone at 36 weeks. Cognitive-behavioral therapy is not likely to be more cost-effective than fluoxetine. CONCLUSIONS: These findings support the use of combination treatment in adolescents with depression over monotherapy
SN - 1527-5418
AD - Department of Health Policy and Administration, School of Public Health, University of North Carolina at Chapel Hill, Chapel Hill, NC 27599-7411, USA. domino@unc.edu
UR - 19465880
ER -

TY - JOUR
ID - 906

T1 - Global childhood unintentional injury surveillance in four cities in developing countries: a pilot study
A1 - Hyder,A.A.
A1 - Sugerman,D.E.
A1 - Puvanachandra,P.
A1 - Razzak,J.
A1 - El-Sayed,H.
A1 - Isaza,A.
A1 - Rahman,F.
A1 - Peden,M.
Y1 - 2009/05//
N1 - Hyder, Adnan A. Sugerman, David E. Puvanachandra, Prasanthi. Razzak, Junaid. El-Sayed, Hesham. Isaza, Andres. Rahman, Fazlur. Peden, Margie
Bulletin of the World Health Organization
7507052, c80
IM
Journal Article. Research Support, Non-U.S. Gov't
English
KW - MEDLINE
KW - Accidents/sn [Statistics & Numerical Data]
KW - Child
KW - Preschool
KW - Developing Countries
KW - Female
KW - Humans
KW - Infant
KW - Male
KW - Pilot Projects
KW - Urban Health
KW - World Health
KW - Wounds and Injuries/ep [Epidemiology]
RP - NOT IN FILE
SP - 345
EP - 352
JF - Bulletin of the World Health Organization
JA - Bull World Health Organ
VL - 87
IS - 5
CY - Switzerland
N2 - OBJECTIVE: To determine the frequency and nature of childhood injuries and to explore the risk factors for such injuries in low-income countries by using emergency department (ED) surveillance data. METHODS: This pilot study represents the initial phase of a multi-country global childhood unintentional injury surveillance (GCUIS) project and was based on a sequential sample of children < 11 years of age of either gender who presented to selected EDs in Bangladesh, Colombia, Egypt and Pakistan over a 3-4 month period, which varied for each site, in 2007. FINDINGS: Of 1559 injured children across all sites, 1010 (65%) were male; 941 (60%) were aged >or= 5 years, 32 (2%) were < 1 year old. Injuries were especially frequent (34%) during the morning hours. They occurred in and around the home in 56% of the cases, outside while children played in 63% and during trips in 11%. Of all the injuries observed, 913 (56%) involved falls; 350 (22%), road traffic injuries; 210 (13%), burns; 66 (4%), poisoning; and 20 (1%), near drowning or drowning. Falls occurred most often from stairs or ladders; road traffic injuries most often involved pedestrians; the majority of burns were from hot liquids; poisonings typically involved medicines, and most drowning occurred in the home. The mean injury severity score was highest for near drowning or drowning (11), followed closely by road traffic injuries (10). There were 6 deaths, of which 2 resulted from drowning, 2 from falls and 2 from road traffic injuries. CONCLUSION: Hospitals in low-income countries bear a substantial burden of childhood injuries, and systematic surveillance is required to identify the epidemiological distribution of such injuries and understand their risk factors. Methodological standardization for surveillance across countries makes it possible to draw international comparisons and identify common issues
SN - 1564-0604

AD - Department of International Health, Johns Hopkins University Bloomberg School of Public Health, Baltimore, MD, USA. ahyder@jhsph.edu
UR - 19551252
ER -

TY - JOUR
ID - 907
T1 - Physical activity pattern of prepubescent Filipino school children during school days
A1 - Gonzalez-Suarez,C.B.
A1 - Grimmer-Somers,K.
Y1 - 2009/07//
N1 - Gonzalez-Suarez, Consuelo B. Grimmer-Somers, Karen
The Journal of school health
k13, 0376370
IM, N
Journal Article. Research Support, N.I.H., Extramural. Research Support, Non-U.S. Gov't
English
KW - MEDLINE
KW - Age Factors
KW - Child
KW - Exercise
KW - Female
KW - Humans
KW - Male
KW - Philippines
KW - Schools/sn [Statistics & Numerical Data]
KW - Sex Factors
KW - Sports
KW - Television
RP - NOT IN FILE
SP - 304
EP - 311
JF - Journal of School Health
JA - J Sch Health
VL - 79
IS - 7
CY - United States
N2 - BACKGROUND: Little is known about pre-pubescent Filipino children's involvement in moderate-to-vigorous physical activity (MVPA). There are international guidelines regarding required levels of MVPA for healthy children. METHODS: This study describes participation of 11- to 12-year-olds in randomly selected public and private schools in San Juan, Metromanila, in MVPA and sports during a school day. The Filipino-modified Physical Activity Questionnaire for Older Children (F_PAQ_C) was administered in English and Filipino. Additional data was collected on sex, age, type of school, and amount of time spent using television and computers. Children's self-assessment of physical activities (1 question in the F_PAQ_C) was correlated with their cumulative F_PAQ_C score. RESULTS: Three hundred eighty subjects (167 boys, 213 girls) participated. Participation in MVPA varied between sex and age groups, from 56.1% to 65.0%. Fewer than 10% of participants were very active. The children were more active during physical education classes than at recess or lunch, after class, or in the evening. Walking for exercise, jumping, jogging and running, free play, and dance were most common. Boys, younger children, and private school students most commonly engaged in MVPA. Self-assessed physical activity had modest correlation (r(2)= 0.21) with cumulative F_PAQ_C score, after adjusting for sex, age, and school type. CONCLUSION: Most children were not physically active during the school day, except in physical education classes. To reduce the gap between recommended and current activity levels, more opportunities should be provided for preteen Filipino children to engage in MVPA during and after school
SN - 1746-1561
AD - Center for Research on Movement Science, Tomas Aquinas Research Complex, University of Santo Tomas, Espana, Manila 1008, Philippines. cgsuarez@mnl.ust.edu.ph

UR - 19527412
ER -

TY - JOUR
ID - 908
T1 - [Computed tomographic analysis of frontal recess region]. [Chinese]
A1 - Wang,P.
A1 - Xu,X.
A1 - Zhao,P.
A1 - Zhen,H.
A1 - Gao,Q.
Y1 - 2009/03/20/
N1 - Wang, Peng. Xu, Xunhua. Zhao, Ping. Zhen, Hongtao. Gao, Qixue
Lin chuang er bi yan hou tou jing wai ke za zhi = Journal of clinical otorhinolaryngology, head, and neck surgery
101303164
IM
English Abstract. Journal Article
Chinese
KW - MEDLINE
KW - Adolescent
KW - Adult
KW - Aged
KW - Child
KW - Female
KW - Frontal Lobe/ra [Radiography]
KW - Frontal Sinus/ra [Radiography]
KW - Humans
KW - Image Processing
KW - Computer-Assisted
KW - Male
KW - Middle Aged
KW - Nasal Cavity/ra [Radiography]
KW - Tomography
KW - X-Ray Computed
KW - Turbinates/ra [Radiography]
KW - Young Adult
RP - NOT IN FILE
SP - 214
EP - 215
JF - Lin Chuang Er Bi Yan Hou Tou Jing Wai Ke Za Zhi = Journal Of Clinical Otorhinolaryngology, Head, & Neck Surgery
JA - Lin Chung Er Bi Yan Hou Tou Jing Wai Ke Za Zhi
VL - 23
IS - 5
CY - China
N2 - OBJECTIVE: The purpose of the study was to observe the CT imaging features of the frontal recess regional. METHOD: Eighty-two patients were undergone spiral computed tomography (CT). Then multiplanar reconstruction images were made using standard triplanar reconstruction protocol on a computer workstation. RESULT: The prevalence of agger nasi cell was 87.8% (144/164). One hundred and forty-five (89%, 145/164) uncinate processes had one superior attachment for each uncinate process. The others had two superior attachments for each uncinate process. The uncinate process' single superior attachment of into the surrounding structures was identified to have the following distribution: 90/164 (54.9%): to the lamina papyracea, 50/164 (30.5%) to the middle turbinate, and 5/164 (3.0%) to the skull base. Of all the frontal cells were identified in 144 (87.8%) sides of frontal recesses, the prevalence of type I II, type III IV, were 30.5% and 9.8% respectively. CONCLUSION: T Agger nasi cell and uncinate process play an important role in endoscopic frontal sinus surgery

SN - 1001-1781
AD - Department of Otolaryngology, WISCO General Hospital, Wuhan, 430080, China.
wangpengdragon@yahoo.com.cn
UR - 19522189
ER -

TY - JOUR
ID - 909
T1 - [Hygienic requirements for work organization of full-day schools]. [Review] [7 refs] [Russian]
A1 - Stepanova,M.I.
A1 - Sazaniuk,Z.I.
A1 - Voronova,B.Z.
A1 - Aleksandrova,I.E.
A1 - Berezina,N.O.
A1 - Laponova,E.D.
A1 - Lashneva,I.P.
A1 - Polenova,M.A.
A1 - Sedova,A.S.
A1 - Shumkova,T.V.
Y1 - 2009/03//
N1 - Stepanova, M I. Sazaniuk, Z I. Voronova, B Z. Aleksandrova, I E. Berezina, N O. Laponova, E D. Lashneva, I P. Polenova, M A. Sedova, A S. Shumkova, T V
Gigiena i sanitariia
fpz, 0412700
IM
English Abstract. Journal Article. Review
Russian
KW - MEDLINE
KW - Adolescent
KW - Child
KW - Child Welfare
KW - Humans
KW - Hygiene/st [Standards]
KW - Program Evaluation
KW - Russia
KW - School Health Services/og [Organization & Administration]
KW - Schools/og [Organization & Administration]
RP - NOT IN FILE
SP - 47
EP - 52
JF - Gigiena i Sanitariia
JA - Gig Sanit.
IS - 2
CY - Russia (Federation)
N2 - Physiological and hygienic studies under the conditions of a natural hygienic experiment were conducted to examine different variants of the organization of work of new types of general educational establishments--full-day schools. Over 580 pupils from 5 full-day schools were followed up. Organization of teaching children at full-day schools was found to mainly correspond to the age-related capabilities of pupils from primary and secondary classes. Of vital importance for maintenance of mental performance, good emotional and psychosomatic states are the organization of the intraschool environment, including a school plot, as well as the conditions for realization of additional education, motor activity of children, and recess. Health keeping in pupils from full-day schools is favored by the reduction in the duration of lessons to 35 minutes and day sleep for first-form children, the decrease in the number of pupils in a class, outdoor physical exercises in the middle of a school day (a primary school) and strolls after lessons, three meals a day, balanced additional education, medicopsychological accompaniment, optimization of studies and rest in children during a school year. [References: 7]
SN - 0016-9900

UR - 19514287
ER -

TY - JOUR
ID - 910
T1 - [Current problems of school education and ways of its hygienic optimization]. [Review] [10 refs] [Russian]
A1 - Stepanova,M.I.
A1 - Sazaniuk,Z.I.
A1 - Voronova,B.Z.
A1 - Polenova,M.A.
Y1 - 2009///
N1 - Stepanova, M I. Sazaniuk, Z I. Voronova, B Z. Polenova, M A
Vestnik Rossiiskoi akademii meditsinskikh nauk / Rossiiskaia akademiia meditsinskikh nauk
bl9, 9215641
IM
English Abstract. Journal Article. Review
Russian
KW - MEDLINE
KW - Adolescent
KW - Adolescent Health Services/td [Trends]
KW - Child
KW - Child Health Services/td [Trends]
KW - Education/og [Organization & Administration]
KW - Humans
KW - Hygiene/st [Standards]
KW - Russia
KW - Schools/st [Standards]
RP - NOT IN FILE
SP - 30
EP - 33
JF - Vestnik Rossiiskoi Akademii Meditsinskikh Nauk
JA - Vestn.Ross.Akad.Med Nauk
IS - 5
CY - Russia (Federation)
N2 - The aim of the study was to analyse effects of various innovative forms of school education on the health status and functional abilities of children and adolescents. Enhanced academic loads are shown to be the most unfavourable factor of the school environment. The main consequences of excess teaching load are shortened motor and outdoor activities of the children, smaller duration of night sleep. Optimization of academic routine (alternation of studies and holidays), modular structure of school calendar might help to reduce fatigue during school hours. Hygienic estimates of different variants of specialized education are obtained. Scientifically sound hygienic requirements are proposed to be applied to the organization of academic activities in a new type of educational institutions, full-day schools. [References: 10]
SN - 0869-6047
UR - 19507350
ER -

TY - JOUR
ID - 911
T1 - Do mechanical gait parameters explain the higher metabolic cost of walking in obese adolescents?
A1 - Peyrot,N.
A1 - Thivel,D.
A1 - Isacco,L.
A1 - Morin,J.B.
A1 - Duche,P.
A1 - Belli,A.
Y1 - 2009/06//

N1 - Peyrot, Nicolas. Thivel, David. Isacco, Laurie. Morin, Jean-Benoit. Duche, Pascale. Belli, Alain
Journal of applied physiology (Bethesda, Md. : 1985)
heg, 8502536
IM
Journal Article. Research Support, Non-U.S. Gov't
English
KW - MEDLINE
KW - Adolescent
KW - Body Composition
KW - Body Mass Index
KW - Body Weight
KW - Child
KW - Energy Metabolism/ph [Physiology]
KW - Exercise/ph [Physiology]
KW - Exercise Test
KW - Female
KW - Gait/ph [Physiology]
KW - Humans
KW - Male
KW - Obesity/me [Metabolism]
KW - Obesity/pp [Physiopathology]
KW - Walking/ph [Physiology]
RP - NOT IN FILE
SP - 1763
EP - 1770
JF - Journal of Applied Physiology
JA - J Appl Physiol
VL - 106
IS - 6
CY - United States
N2 - Net metabolic cost of walking normalized by body mass (C(W.BM(-1)); in J.kg(-1).m(-1)) is greater in obese than in normal-weight individuals, and biomechanical differences could be responsible for this greater net metabolic cost. We hypothesized that, in obese individuals, greater mediolateral body center of mass (COM) displacement and lower recovery of mechanical energy could induce an increase in the external mechanical work required to lift and accelerate the COM and thus in net C(W.BM(-1)). Body composition and standing metabolic rate were measured in 23 obese and 10 normal-weight adolescents. Metabolic and mechanical energy costs were assessed while walking along an outdoor track at four speeds (0.75-1.50 m/s). Three-dimensional COM accelerations were measured by means of a tri-axial accelerometer and gyroscope and integrated twice to obtain COM velocities, displacements, and fluctuations in potential and kinetic energies. Last, external mechanical work (J.kg(-1).m(-1)), mediolateral COM displacement, and the mechanical energy recovery of the inverted pendulum were calculated. Net C(W.BM(-1)) was 25% higher in obese than in normal-weight subjects on average across speeds, and net C(W.BM(-67)) (J.kg(-0.67).m(-1)) was significantly related to percent body fat (r(2) = 0.46). However, recovery of mechanical energy and the external work performed (J.kg(-1).m(-1)) were similar in the two groups. The mediolateral displacement was greater in obese subjects and significantly related to percent body fat (r(2) = 0.64). The mediolateral COM displacement, likely due to greater step width, was significantly related to net C(W.BM(-67)) (r(2) = 0.49). In conclusion, we speculate that the greater net C(W.BM(-67)) in obese subjects may be partially explained by the greater step-to-step transition costs associated with wide gait during walking
SN - 8750-7587
AD - Laboratoire de Physiologie de l'Exercice, Medecine du Sport-Myologie, CHU Bellevue, 42055 Saint-Etienne Cedex 02, France. nicolas.peyrot@univ-st-etienne.fr
UR - 19246657
ER -

TY - JOUR
ID - 912
T1 - A tale of two countries: HIV among core groups in Togo

A1 - Sobela,F.
A1 - Pepin,J.
A1 - Gbeleou,S.
A1 - Banla,A.K.
A1 - Pitche,V.P.
A1 - Adom,W.
A1 - Sodji,D.
A1 - Frost,E.
A1 - Deslandes,S.
A1 - Labbe,A.C.
Y1 - 2009/06/01/
N1 - Sobela, Francois. Pepin, Jacques. Gbeleou, Sesso. Banla, Abiba Kere. Pitche, Vincent Palokinam. Adom, Wiyoou. Sodji, Dometo. Frost, Eric. Deslandes, Sylvie. Labbe, Annie-Claude
Journal of acquired immune deficiency syndromes (1999)
100892005
IM, X
Journal Article. Research Support, Non-U.S. Gov't
English
KW - MEDLINE
KW - Adolescent
KW - Adult
KW - Child
KW - Cross-Sectional Studies
KW - Disease Transmission
KW - Infectious
KW - Female
KW - HIV Infections/ep [Epidemiology]
KW - HIV Infections/tm [Transmission]
KW - Humans
KW - Male
KW - Middle Aged
KW - Prevalence
KW - Prostitution
KW - Risk Factors
KW - Togo/ep [Epidemiology]
KW - Young Adult
RP - NOT IN FILE
SP - 216
EP - 223
JF - Journal of Acquired Immune Deficiency Syndromes: JAIDS
JA - J Acquir.Immune Defic.Syndr
VL - 51
IS - 2
CY - United States
N2 - OBJECTIVE: To describe the epidemiology of HIV among core groups in Togo. METHODS: We enumerated sex workers (SWs) and conducted cross-sectional surveys of SWs and their clients in 2003 in Lome and in 2005 in the whole country. RESULTS: Sex work was concentrated in Lome, which comprised 15% of the population, but 52% of the 5397 SWs enumerated in Togo in 2005 and 68% of the estimated 101,376 men who had bought sex in the year before the 2005 survey. HIV prevalence among SWs was highest in Lome (45.4% in 2005) and progressively decreased from south to north. A similar geographical pattern was seen for clients (8.3% were HIV infected in Lome in 2005) and had already been reported for pregnant women. In Lome, the population attributable fraction of prevalent cases of HIV acquired during transactional sex was estimated at 32%; in the rest of the country, this was only 2%. CONCLUSIONS: This is the first study quantifying sex work at a national level in Africa. Variations in HIV prevalence within Togo, with a north-south gradient among SWs, their clients, and pregnant women, may to a large extent reflect the concentration of the sex trade within Lome. Prostitution played only a modest a role in HIV dynamics outside Lome
SN - 1525-4135

AD - Centre de Sante Internationale de l'Universite de Sherbrooke, Quebec, Canada
UR - 19339896
ER -

TY - JOUR
ID - 913
T1 - After-school program impact on physical activity and fitness: a meta-analysis. [Review] [50 refs]
A1 - Beets,M.W.
A1 - Beighle,A.
A1 - Erwin,H.E.
A1 - Huberty,J.L.
Y1 - 2009/06//
N1 - Beets, Michael W. Beighle, Aaron. Erwin, Heather E. Huberty, Jennifer L
American journal of preventive medicine
8704773, apl
IM
Journal Article. Meta-Analysis. Review
English
KW - MEDLINE
KW - Adolescent
KW - Body Composition
KW - Child
KW - Exercise/ph [Physiology]
KW - Exercise
KW - Female
KW - Health Behavior
KW - Health Promotion
KW - Humans
KW - Lipids/bl [Blood]
KW - Male
KW - Physical Fitness
KW - Schools
KW - United States
RP - NOT IN FILE
SP - 527
EP - 537
JF - American Journal of Preventive Medicine
JA - Am J Prev Med
VL - 36
IS - 6
CY - Netherlands
N2 - CONTEXT: The majority of children do not participate in sufficient amounts of daily, health-enhancing physical activity. One strategy to increase activity is to promote it within the after-school setting. Although promising, the effectiveness of this strategy is unclear. A systematic review was performed summarizing the research conducted to date regarding the effectiveness of after-school programs in increasing physical activity. EVIDENCE ACQUISITION: Databases, journals, and review articles were searched for articles published between 1980 and February 2008. Meta-analysis was conducted during July of 2008. Included articles had the following characteristics: findings specific to an after-school intervention in the school setting; subjects aged <or=18 years; an intervention component designed to promote physical activity; outcome measures of physical activity, related constructs, and/or physical fitness. Study outcomes were distilled into six domains: physical activity, physical fitness, body composition, blood lipids, psychosocial constructs, and sedentary activities. Effect sizes (Hedge's g) were calculated within and across studies for each domain, separately. EVIDENCE SYNTHESIS: Of the 797 articles found, 13 unique articles describing findings from 11 after-school interventions were reviewed. Although physical activity was a primary component of all the tested interventions, only eight studies measured physical activity. From the six domains, positive effect sizes were demonstrated for physical activity (0.44 [95% CI=0.28-0.60]); physical fitness (0.16 [95% CI=0.01-0.30]); body composition (0.07 [95% CI=0.03-0.12]); and blood lipids (0.20 [95% CI=0.06-0.33]). CONCLUSIONS: The

limited evidence suggests that after-school programs can improve physical activity levels and other health-related aspects. Additional studies are required that provide greater attention to theoretical rationale, levels of implementation, and measures of physical activity within and outside the intervention. [References: 50]
SN - 1873-2607
AD - Department of Exercise Science, Arnold School of Public Health, University of South Carolina, Columbia, South Carolina 29208, USA. beets@mailbox.sc.edu
UR - 19362799
ER -

TY - JOUR
ID - 914
T1 - [A pilot study to develop a screening system and maltreatment index for elementary schoolchildren focusing on daily habits and dental caries]. [Japanese]
A1 - Morita,I.
A1 - Shibata,T.
A1 - Hane,M.
A1 - Nakai,T.
A1 - Ishigaki,H.
A1 - Mine,M.
A1 - Nakagaki,H.
Y1 - 2009/03//
N1 - Morita, Ichizo. Shibata, Tomiko. Hane, Morito. Nakai, Takayoshi. Ishigaki, Hiroki. Mine, Masahiro. Nakagaki, Haruo
[Nihon koshu eisei zasshi] Japanese journal of public health
19130150r, a9j
IM
English Abstract. Journal Article
Japanese
KW - MEDLINE
KW - Child
KW - Child Abuse/di [Diagnosis]
KW - Female
KW - Humans
KW - Japan
KW - Life Style
KW - Male
KW - Oral Hygiene
KW - Pilot Projects
KW - Toothbrushing
RP - NOT IN FILE
SP - 145
EP - 154
JF - Nippon Koshu Eisei Zasshi - Japanese Journal of Public Health
JA - Nippon Koshu Eisei Zasshi
VL - 56
IS - 3
CY - Japan
N2 - OBJECTIVE: In recent years, the number of consultations for maltreatment cases has been increasing in Japan. The characteristics of maltreated children are important factors for their identification. In an earlier paper, we analysed the daily habits related to maltreated children. In the present study, the objectives were to assess the relative weights of each factor and score the daily habits and treatment percentages for dental caries, and also to develop a screening system to find maltreated children. METHODS: The subjects of this study were 57 elementary schoolchildren who were given temporary protection by the child social welfare authorities. The control group comprised 575 elementary schoolchildren. Multiple logistics regression analysis was used to calculate scores for each item derived from partial correlation coefficients. RESULTS: Two versions of a maltreatment index for elementary schoolchildren (MIES) were developed, a daily habits version (H) and daily habits and treatment percentage of dental caries version (HD) for the 1st to 3rd grades (G1-3) and the 4th to 6th

grades (G4-6). The MIES for G1-3H was: Do you brush your teeth before bedtime (3 points); Do you know that the first molars are important for chewing (2); Have you used a fluoride gel or mouth rinse (2); Do you have any hobbies except video games (2); Do you know the differences between permanent teeth and milk teeth (1). The MIES for G1-3HD were: Have you used a fluoride gel or mouth rinse (4); Do you brush your teeth before bedtime (3); Do you wash your hands after you play outside (2); Is the number of untreated deciduous teeth larger than the number of treated deciduous teeth (1). The MIES for G4-6H were: Do you wash your hands after you play outside (4); Do you brush your teeth after breakfast (3); Do you brush your teeth before bedtime (3). The G4-6HD were: Do you brush your teeth before bedtime (3); Do you wash your hands after you play outside (2); Is the number of untreated permanent teeth greater than the number of treated permanent teeth (1). The availability (sensitivity + specificity) of MIES HD was higher than MIES H when the original values were substituted into MIES. CONCLUSIONS: A screening system for finding maltreated children for MIES was designed. The precision of MIES could be improved by adding the treatment percentage of dental caries to daily habits
SN - 0546-1766
AD - Department of Preventive Dentistry and Dental Public Health, School of Dentistry, Aichi-Gakuin University, Nagoya, Japan
UR - 19455977
ER -

TY - JOUR
ID - 915
T1 - Understanding screen-related sedentary behavior and its contributing factors among school-aged children: a social-ecologic exploration
A1 - He,M.
A1 - Harris,S.
A1 - Piche,L.
A1 - Beynon,C.
Y1 - 2009/05//
N1 - He, Meizi. Harris, Stewart. Piche, Leonard. Beynon, Charlene
American journal of health promotion : AJHP
amj, 8701680
T
Journal Article. Research Support, Non-U.S. Gov't
English
KW - MEDLINE
KW - Attitude to Health
KW - Child
KW - Cross-Sectional Studies
KW - Educational Status
KW - Female
KW - Health Behavior
KW - Humans
KW - Interpersonal Relations
KW - Linear Models
KW - Male
KW - Motor Activity
KW - Obesity/pc [Prevention & Control]
KW - Ontario
KW - Parent-Child Relations
KW - Parents/ed [Education]
KW - Parents/px [Psychology]
KW - Pilot Projects
KW - Questionnaires
KW - Schools
KW - Socioeconomic Factors
KW - Students/px [Psychology]
KW - Students

RP - NOT IN FILE
SP - 299
EP - 308
JF - American Journal of Health Promotion
JA - Am J Health Promot
VL - 23
IS - 5
CY - United States
N2 - PURPOSE: To explore the factors that contribute to children's screen-related sedentary (S-RS) behaviors. SETTINGS: Elementary schools. SUBJECTS: A random sample of children in grades five and six and their parents. MEASURES: The outcome measure was children's S-RS activity level measured by a self-administered questionnaire. A full spectrum of potential contributing factors for children's S-RS behaviors was obtained through surveys. Multilevel linear regression methods were used to determine the associations between these factors and children's screen time (hours per day) and results were expressed as regression coefficients (g). RESULTS: Of 955 child-parent pairs in 14 participating schools, 508 pairs (53%) completed the surveys. At an intrapersonal level, protective factors included being a girl (g = -.71); belonging to a sports team inside (g = -.56) or outside (g = -.49) of school; having a negative attitude toward S-RS activities (g = -.13); and having a positive attitude toward physical activity (g = .48). At the interpersonal and social levels, parental leisure S-RS behaviors (g = .32) were positively associated, whereas strict parental rules on computer use (g = -.27) and family income (g = -.32) were inversely correlated with S-RS behavior. At the environmental level, the presence of TVs in children's bedrooms (g = .44) and owning videogame devices (g = .58) increased the risk of S-RS behaviors, whereas after school programs (g = - .86) and schools' participation in the Turn Off the Screen Week campaign (g = -.91) decreased the risk. CONCLUSIONS: Public health interventions should target multilevel factors, including increasing children's awareness, promoting parental involvement in healthy lifestyle pursuits, and creating less screenogenic environments
SN - 0890-1171
AD - Public Health Research, Education and Development (PHRED) Program, Middlesex-London Health Unit, London, Ontario, Canada. mhe@uwo.ca
UR - 19445431
ER -

TY - JOUR
ID - 916
T1 - Epstein-Barr virus WZhet DNA can induce lytic replication in epithelial cells in vitro, although WZhet is not detectable in many human tissues in vivo
A1 - Ryan,J.L.
A1 - Jones,R.J.
A1 - Elmore,S.H.
A1 - Kenney,S.C.
A1 - Miller,G.
A1 - Schroeder,J.C.
A1 - Gulley,M.L.
Y1 - 2009///
N1 - Ryan, Julie L. Jones, Richard J. Elmore, Sandra H. Kenney, Shannon C. Miller, George. Schroeder, Jane C. Gulley, Margaret L
Intervirology
gw7, 0364265
IM
Journal Article. Research Support, N.I.H., Extramural
English
KW - MEDLINE
KW - Adenocarcinoma/bl [Blood]
KW - Adenocarcinoma/vi [Virology]
KW - Adolescent
KW - Adult
KW - Cell Line
KW - Tumor

KW - Child
KW - Preschool
KW - DNA
KW - Viral/ph [Physiology]
KW - Epithelial Cells/vi [Virology]
KW - Gastrointestinal Neoplasms/bl [Blood]
KW - Gastrointestinal Neoplasms/vi [Virology]
KW - Herpesvirus 4
KW - Human/ge [Genetics]
KW - Humans
KW - Infant
KW - Infectious Mononucleosis/bl [Blood]
KW - Infectious Mononucleosis/vi [Virology]
KW - Lymphoma/bl [Blood]
KW - Lymphoma/vi [Virology]
KW - Sensitivity and Specificity
KW - Virus Replication
RP - NOT IN FILE
SP - 8
EP - 16
JF - Intervirology
VL - 52
IS - 1
CY - Switzerland
N2 - OBJECTIVE: WZhet is a rearranged and partially deleted form of the Epstein-Barr virus (EBV) genome in which the BamH1W region becomes juxtaposed with and activates BZLF1, resulting in constitutive viral replication. We tested whether WZhet induces viral replication in epithelial cells, and we studied its prevalence in a wide range of lesional tissues arising in vivo. METHODS: A quantitative real-time PCR assay targeting EBV WZhet DNA was developed to measure this recombinant form of the EBV genome. RESULTS: WZhet DNA was undetectable in any of 324 plasma or paraffin-embedded tissue samples from patients with EBV-associated and EBV-negative disorders. These included specimens from patients with Hodgkin or non-Hodgkin lymphoma, post-transplant lymphoproliferation, nasopharyngeal or gastric adenocarcinoma, and infectious mononucleosis. However, WZhet DNA was detected in vitro in EBV-infected AGS gastric cancer cells. Additionally, transient transfection of infected AGS gastric cancer cells showed that viral replication could be induced by a WZhet plasmid. CONCLUSION: This is the first evidence that WZhet induces the EBV lytic cycle in an epithelial cell line. Our negative findings in natural settings suggest that WZhet is a defective viral product that thrives in the absence of a host immune system but is rarely present in vivo. Copyright 2009 S. Karger AG, Basel
SN - 1423-0100
AD - Department of Dermatology, University of Rochester Medical Center, Rochester, N.Y., USA
UR - 19349713
ER -

TY - JOUR
ID - 917
T1 - Occupation of motherhood: challenges for mothers with scleroderma
A1 - Poole,J.L.
A1 - Willer,K.
A1 - Mendelson,C.
Y1 - 2009/03//
N1 - Poole, Janet L. Willer, Kelly. Mendelson, Cindy
The American journal of occupational therapy : official publication of the American Occupational Therapy Association
7705978, 3o4
IM
Journal Article. Research Support, Non-U.S. Gov't
English

KW - MEDLINE
KW - Adolescent
KW - Adult
KW - Child
KW - Preschool
KW - Disability Evaluation
KW - Female
KW - Humans
KW - Infant
KW - Middle Aged
KW - Mother-Child Relations
KW - Parenting
KW - Scleroderma
KW - Systemic/co [Complications]
RP - NOT IN FILE
SP - 214
EP - 219
JF - American Journal of Occupational Therapy
JA - Am J Occup Ther
VL - 63
IS - 2
CY - United States
N2 - OBJECTIVE: We sought to identify which aspects of motherhood are difficult for mothers who have scleroderma along with variables associated with mothering. METHOD: Participants were 75 mothers with scleroderma with a child between birth and 18 years old living with them. They completed the Parent Disability Index (PDI); a demographic questionnaire; and measures of pain, fatigue, and activity limitations. RESULTS: Mothers with children ages 5 or younger reported that getting up and down from the floor to play with their children was the most difficult task, and mothers with children between 6 and 18 years old reported that playing with their children outdoors, doing household chores, and shopping were the most difficult tasks. Significant correlations were found between PDI and pain, fatigue, and activity limitations. CONCLUSIONS: Mothers with scleroderma have difficulty with aspects of motherhood. The findings that pain, fatigue, and activity limitations were related to mothering suggest areas for occupational therapy intervention
SN - 0272-9490
AD - Occupational Therapy Graduate Program, School of Medicine, University of New Mexico, Albuquerque, NM 87131-0001, USA. jpoole@salud.unm.edu
UR - 19432060
ER -

TY - JOUR
ID - 918
T1 - Participant and parent experiences in the oral insulin study of the Diabetes Prevention Trial for Type 1 Diabetes
A1 - Johnson,S.B.
A1 - Baughcum,A.E.
A1 - Rafkin-Mervis,L.E.
A1 - Schatz,D.A.
A1 - Study Group.
Y1 - 2009/05//
N1 - Johnson, Suzanne Bennett. Baughcum, Amy E. Rafkin-Mervis, Lisa E. Schatz, Desmond A. DPT-1 Study Group
Pediatric diabetes
100939345
IM
Journal Article. Randomized Controlled Trial. Research Support, N.I.H., Extramural. Research Support, Non-U.S. Gov't
English
KW - MEDLINE

KW - Administration
KW - Oral
KW - Adult
KW - Attitude
KW - Child
KW - Culture
KW - Decision Making
KW - Diabetes Mellitus
KW - Type 1/dt [Drug Therapy]
KW - Type 1/pc [Prevention & Control]
KW - Type 1/px [Psychology]
KW - Female
KW - Health Surveys
KW - Humans
KW - Insulin/ad [Administration & Dosage]
KW - Insulin/tu [Therapeutic Use]
KW - Male
KW - Parent-Child Relations
KW - Parents/px [Psychology]
KW - Patient Compliance
KW - Patient Participation
KW - Placebos
KW - Risk
KW - Stress
KW - Psychological/ep [Epidemiology]
RP - NOT IN FILE
SP - 177
EP - 183
JF - Pediatric Diabetes
JA - Pediatr Diabetes
VL - 10
IS - 3
CY - Denmark
N2 - OBJECTIVE: To assess the experiences of participants and parents of children in the oral insulin study of the Diabetes Prevention Trial - Type 1. METHOD: Before trial results were publicized, surveys were completed by 124 participants and 219 parents of children in the oral trial. RESULTS: Although most of those surveyed were positive about the trial, survey results suggest that participant perspective (adult, child, parent, and gender), study procedures, and beliefs about placebo vs. active drug assignment have important implications for planning future clinical trials. Parents and children reported greater distress, worry, and difficulty making the decision to join the trial compared with adult participants. Mothers and female participants were particularly interested in additional psychosocial support during the study. Random assignment was viewed negatively by both parents and children, and close observation for diabetes onset was viewed as the most favorable aspect of the study. Adherence to study procedures declined over time and behaviors outside the study protocol to prevent/delay diabetes onset were common, particularly among those who believed the participant was taking a placebo. Children and respondents who believed that the participant was taking the active drug expressed confidence in oral insulin's ability to delay or prevent type 1 diabetes. CONCLUSIONS: Although most participants were positive about the trial and many expressed optimism about the intervention's potential for success, future trials need to address negative reactions to random assignment, the unique concerns of children and their parents, declining adherence, and behaviors - external to the trial - designed to delay or prevent diabetes
SN - 1399-5448
AD - Department of Medical Humanities and Social Sciences, Florida State University College of Medicine, Tallahassee, FL 2306-4300, USA. suzanne.johnson@med.fsu.edu
UR - 18823410
ER -

TY - JOUR
ID - 919

T1 - Physical activity patterns among preadolescent children in Cyprus: the CYKIDS study
A1 - Lazarou,C.
A1 - Soteriades,E.S.
Y1 - 2009/03//
N1 - Lazarou, Chrystalleni. Soteriades, Elpidoforos S
Journal of physical activity & health
101189457
IM
Journal Article. Research Support, Non-U.S. Gov't
English
KW - MEDLINE
KW - Adolescent
KW - Age Factors
KW - Child
KW - Child Welfare
KW - Cross-Sectional Studies
KW - Cyprus
KW - Factor Analysis
KW - Statistical
KW - Female
KW - Health Behavior
KW - Health Surveys
KW - Humans
KW - Life Style
KW - Male
KW - Motor Activity
KW - Questionnaires
KW - Statistics as Topic
RP - NOT IN FILE
SP - 185
EP - 194
JF - Journal of Physical Activity & Health
JA - J Phys Act Health
VL - 6
IS - 2
CY - United States
N2 - PURPOSE: The purpose of this study was to summarize patterns of correlations between variables and reduce a large number of variables to a smaller number of factors. METHODS: During 2005 we conducted a national, cross-sectional, multistage study among 1140 children (mean age=10.70+/-0.98). Principal component analysis was employed to extract the main factors out of 21 variables assessing children's duration of physical and sedentary activities. RESULTS: Eight factors emerged as important, explaining 63% of the total variance in children's PA patterns. The combined explained variance of 3 factors for exercise was 24.1% (PA and sports after school [11.6%]; home and outside home chores, aerobics, gymnastics, sports [6.8%]; Sports for All, after-school activities [except sports] [5.7%]) and of 5 factors for sedentary activities was 38.9% (video, electronic games, and computers [10.2%]; watching TV, video, and DVD [9.5%]; homework and private lessons [7.3%]; theater cinema, use of mobile phone [6.2%]; afternoon sleep, less private lessons [5.8%]). Further analyses of the emerged factors by gender showed that boys, on a daily basis, are engaged in higher duration in physical activity and sports after school (factor 1) and in electronic games and computers (factor 2), whereas they are engaged in lower durations in factor 4 type behavior (homework and private lessons) and factor 6 type behavior (theater cinema, use of mobile phone). CONCLUSION: Use of the above factors will inform further research and enable researchers to identify potential differences in physical and sedentary activities patterns by various environmental and sociodemographic variables. Furthermore, associations of factors by gender may inform public health programs
SN - 1543-3080
AD - Department of Nutrition and Dietetics, Harokopio University, Athens, Greece
UR - 19420396
ER -

TY - JOUR
ID - 920
T1 - Does the twenty meter shuttle-run test elicit maximal effort in 11- to 16-year-olds?
A1 - Voss,C.
A1 - Sandercock,G.
Y1 - 2009/02//
N1 - Voss, Christine. Sandercock, Gavin
Pediatric exercise science
8909729
IM
Journal Article. Validation Studies
English
KW - MEDLINE
KW - Adolescent
KW - Body Mass Index
KW - Child
KW - Cohort Studies
KW - Exercise Test/mt [Methods]
KW - Female
KW - Heart Rate
KW - Humans
KW - Male
KW - Obesity
KW - Physical Endurance
KW - Running/ph [Physiology]
KW - Statistics
KW - Nonparametric
KW - Thinness
RP - NOT IN FILE
SP - 55
EP - 62
JF - Pediatric Exercise Science
JA - Pediatr Exerc Sci
VL - 21
IS - 1
CY - United States
N2 - The aim of this study was to determine if maximal effort, evidenced by peak HR was attained during the 20m shuttle-run test in a naturalistic setting. Shuttle-run test performance and peak HR were measured in 208 volunteers (11-16 years). Peak HR was 196 (95% confidence interval (C.I.) 194-198 bpm). The relationship between test performance and peak HR was assessed by regression. There was a weak, but statistically significant relationship between test performance and peak HR (R2 = .029, p = .029) but with such a low coefficient of determination (less than 5% criterion), poor performances were not associated with low peak HR values or underestimation of maximal performance. Peak HR values (196 bpm) were higher than cited criterion values (185 bpm) for maximal effort in laboratory studies. In a naturalistic setting, the 20m shuttle-run test elicits a maximal effort in most children
SN - 0899-8493
AD - Centre for Sports and Exercise Science, School of Biological Sciences, University of Essex, UK
UR - 19411711
ER -

TY - JOUR
ID - 921
T1 - Identification and characterization of novel collagen VI non-canonical splicing mutations causing Ullrich congenital muscular dystrophy
A1 - Martoni,E.
A1 - Urciuolo,A.

A1 - Sabatelli,P.
A1 - Fabris,M.
A1 - Bovolenta,M.
A1 - Neri,M.
A1 - Grumati,P.
A1 - D'Amico,A.
A1 - Pane,M.
A1 - Mercuri,E.
A1 - Bertini,E.
A1 - Merlini,L.
A1 - Bonaldo,P.
A1 - Ferlini,A.
A1 - Gualandi,F.
Y1 - 2009/05//
N1 - Martoni, Elena. Urciuolo, Anna. Sabatelli, Patrizia. Fabris, Marina. Bovolenta, Matteo. Neri, Marcella. Grumati, Paolo. D'Amico, Adele. Pane, Marika. Mercuri, Eugenio. Bertini, Enrico. Merlini, Luciano. Bonaldo, Paolo. Ferlini, Alessandra. Gualandi, Francesca
Human mutation
brd, 9215429
IM
Journal Article. Research Support, Non-U.S. Gov't
English
KW - MEDLINE
KW - Adolescent
KW - Blotting
KW - Western
KW - Case-Control Studies
KW - Cells
KW - Cultured
KW - Child
KW - Preschool
KW - Codon
KW - Nonsense/ge [Genetics]
KW - Collagen Type VI/ge [Genetics]
KW - Collagen Type VI/me [Metabolism]
KW - Exons/ge [Genetics]
KW - Female
KW - Fibroblasts/me [Metabolism]
KW - Fibroblasts/pa [Pathology]
KW - Fluorescent Antibody Technique
KW - Heterozygote
KW - Humans
KW - Inheritance Patterns/ge [Genetics]
KW - Introns/ge [Genetics]
KW - Male
KW - Muscular Dystrophies/cn [Congenital]
KW - Muscular Dystrophies/ge [Genetics]
KW - Muscular Dystrophies/pa [Pathology]
KW - Mutation/ge [Genetics]
KW - RNA Splicing/ge [Genetics]
KW - RNA
KW - Messenger/ge [Genetics]
KW - Messenger/me [Metabolism]
KW - Sequence Deletion
RP - NOT IN FILE
SP - E662
EP - E672

JF - Human Mutation
JA - Hum Mutat
VL - 30
IS - 5
CY - United States
N2 - Splicing mutations occurring outside the invariant GT and AG dinucleotides are frequent in disease genes and the definition of their pathogenic potential is often challenging. We have identified four patients affected by Ullrich congenital muscular dystrophy and carrying unusual mutations of COL6 genes affecting RNA splicing. In three cases the mutations occurred in the COL6A2 gene and consisted of nucleotide substitutions within the degenerated sequences flanking the canonical dinucleotides. In the fourth case, a genomic deletion occurred which removed the exon8-intron8 junction of the COL6A1 gene. These mutations induced variable splicing phenotypes, consisting of exon skipping, intron retention and cryptic splice site activation/usage. A quantitative RNA assay revealed a reduced level of transcription of the mutated in-frame mRNA originating from a COL6A2 point mutation at intronic position +3. At variance, the transcription level of the mutated in-frame mRNA originating from a genomic deletion which removed the splicing sequences of COL6A1 exon 8 was normal. These findings suggest a different transcriptional efficiency of a regulatory splicing mutation compared to a genomic deletion causing a splicing defect. Copyright 2009 Wiley-Liss, Inc
SN - 1098-1004
AD - Department of Experimental and Diagnostic Medicine, Section of Medical Genetics, University of Ferrara, Italy
UR - 19309692
ER -

TY - JOUR
ID - 922
T1 - Seasonal variation of retinal detachment in Lebanon
A1 - Mansour,A.M.
A1 - Hamam,R.N.
A1 - Sibai,T.A.
A1 - Farah,T.I.
A1 - Mehio-Sibai,A.
A1 - Kanaan,M.
Y1 - 2009///
N1 - Mansour, Ahmad M. Hamam, Rola N. Sibai, Tarek A. Farah, Toufic I. Mehio-Sibai, Abla. Kanaan, Mona
Ophthalmic research
oie, 0267442
IM
Journal Article
English
KW - MEDLINE
KW - Adolescent
KW - Adult
KW - Age Distribution
KW - Aged
KW - 80 and over
KW - Child
KW - Female
KW - Humans
KW - Lebanon/ep [Epidemiology]
KW - Male
KW - Middle Aged
KW - Retinal Detachment/ep [Epidemiology]
KW - Retinal Detachment/su [Surgery]
KW - Retrospective Studies
KW - Seasons
KW - Sex Distribution
KW - Young Adult

RP - NOT IN FILE
SP - 170
EP - 174
JF - Ophthalmic Research
JA - Ophthalmic Res
VL - 41
IS - 3
CY - Switzerland
N2 - BACKGROUND: The literature remains controversial regarding seasonal variation of rhegmatogenous retinal detachment (RRD). METHODS: In this retrospective chart review, seasonal variation of RRD was examined based on the records of 211 consecutive patients operated for idiopathic RRD in one referral medical center over a 13-year period. RESULTS: The number of eyes with RRD recorded per season was 46 in autumn, 46 in winter, 62 in spring and 57 in summer. There was a significant seasonal variation ($p < 0.05$) with an increase in RRD in the warm seasons (spring and summer) compared to the cold seasons (winter and autumn; 56 vs. 44%). Right eyes were more likely to be affected than left eyes (54 vs. 46%), and cases presented with a significantly younger age at onset in the warm than in the cold seasons (47 vs. 54 years, $p = 0.007$). A literature review of 8,533 cases including the present case series established further the seasonal variation of RRD ($p < 0.001$). CONCLUSION: There is convincing evidence for a seasonal variation of RRD in Lebanon and in the literature. This is likely to be related to sun exposure and outdoor activities in the warm seasons. Copyright 2009 S. Karger AG, Basel
SN - 1423-0259
AD - Department of Ophthalmology, American University of Beirut, Beirut, Lebanon.
dr.ahmad@cyberia.net.lb
UR - 19339812
ER -

TY - JOUR
ID - 923
T1 - Soft landings: encouraging compliance with safety standards in Local Government Authority playgrounds
A1 - Sherker,S.
A1 - Ritchie,J.
A1 - Eager,D.
A1 - Dennis,R.
Y1 - 2009/04//
N1 - Sherker, Shauna. Ritchie, Jan. Eager, David. Dennis, Rebecca
Health promotion journal of Australia : official journal of Australian Association of Health Promotion Professionals
9710936
IM
Journal Article. Research Support, Non-U.S. Gov't
English
KW - MEDLINE
KW - Child
KW - Preschool
KW - Data Collection
KW - Databases as Topic
KW - Humans
KW - Local Government
KW - Maintenance
KW - New South Wales
KW - Play and Playthings/in [Injuries]
KW - Policy Making
KW - Safety/st [Standards]
KW - Wounds and Injuries/pc [Prevention & Control]
RP - NOT IN FILE
SP - 31
EP - 36

JF - Health Promotion Journal of Australia
JA - HEALTH PROMOT J AUST
VL - 20
IS - 1
CY - Australia
N2 - ISSUES ADDRESSED: Consistent with health promotion principles of good practice, addressing playground injury necessitates the creation of a supportive environment for the enhancement of wellbeing and the prevention of injuries. This study aims to survey local governments to: determine compliance with playground safety standards; establish frequency of playground inspections and maintenance; and identify motivators and barriers to compliance with safety standards. METHODS: A survey of key informants for playground safety in all 152 local government councils in New South Wales (NSW) was undertaken. RESULTS: Of 152 local councils in NSW (43 metropolitan and 109 non-metropolitan), 71.7% (n=109) completed the survey, 12.5% (n=19) refused to participate and no response was received by 15.8% (n=24). Self-reported compliance with key aspects of the standard was generally high. However, only 55% of councils complied with surface impact attenuation <200 gmax and <1,000 HIC. Further, only 14.7% of councils reported impact testing the playground surface during inspections. The main motivators to compliance included: reducing risk of litigation or liability; enhancing community and child safety, and minimising the risk of injury. The main barriers included a lack of: time; personnel, and a lack of adequate and appropriate funding. CONCLUSIONS: Local Government Authorities have a duty of care to ensure the safety of playgrounds in their jurisdiction. They require time, personnel and adequate and appropriate funding in order to achieve this aim
SN - 1036-1073
AD - NSW Injury Risk Management Research Centre, University of New South Wales, Australia. Shauna.Sherker@unsw.edu.au
UR - 19402813
ER -

TY - JOUR
ID - 924
T1 - Tuberculosis and the city
A1 - Carter,A.
A1 - Zwerling,A.
A1 - Olson,S.
A1 - Tannenbaum,T.N.
A1 - Schwartzman,K.
Y1 - 2009/09//
N1 - Carter, Andrew. Zwerling, Alice. Olson, Sherry. Tannenbaum, Terry-Nan. Schwartzman, Kevin
Health & place
din, 9510067
T
Journal Article
English
KW - MEDLINE
KW - Adolescent
KW - Adult
KW - Child
KW - Preschool
KW - Cities/ep [Epidemiology]
KW - Databases as Topic
KW - Female
KW - Humans
KW - Infant
KW - Newborn
KW - Male
KW - Middle Aged
KW - Quebec/ep [Epidemiology]
KW - Tuberculosis/ep [Epidemiology]
KW - Young Adult

RP - NOT IN FILE
SP - 777
EP - 783
JF - Health & Place
JA - Health Place
VL - 15
IS - 3
CY - England
N2 - Unrecognized tuberculosis transmission outside the household has led to "micro-epidemics". We sought to evaluate how frequently locations outside the household were addressed in tuberculosis contact investigations, and to identify associated patient factors. We reviewed all tuberculosis patients reported in Montreal, Canada, during 1996-2004. Among this largely foreign-born patient population, investigation of locations outside the household was limited: there was documented attendance at 1 non-household location for 40% of the most contagious patients. Given complex, dispersed patterns of work, educational attendance, social activity, and transportation, contact investigation strategies may warrant reevaluation in large cities such as Montreal
SN - 1353-8292
AD - Montreal Chest Institute, Montreal, Quebec, Canada H2X 2P4
UR - 19233710
ER -

TY - JOUR
ID - 925
T1 - Everybody needs a break! Responses to a playgarden survey
A1 - Turner,J.
A1 - Fralic,J.
A1 - Newman-Bennett,K.
A1 - Skinner,L.
Y1 - 2009/01//
N1 - Turner, Joan. Fralic, Jessica. Newman-Bennett, Krista. Skinner, Linda
Pediatric nursing
oun, 7505804
N
Journal Article. Research Support, Non-U.S. Gov't
English
KW - MEDLINE
KW - Child
KW - Data Collection
KW - Female
KW - Health Facility Environment
KW - Humans
KW - Life Style
KW - Play and Playthings
KW - Visitors to Patients
RP - NOT IN FILE
SP - 27
EP - 34
JF - Pediatric Nursing
JA - Pediatr Nurs
VL - 35
IS - 1
CY - United States
N2 - The role of outdoor activity in health and development warrants attention as an important feature of quality health care environments. This study of visitor perceptions of a playgarden located in a pediatric and women's health care facility resulted in 52 completed surveys from hospital staff and parents. Most respondents indicated high levels of agreement to questions related to their general experience. The main reason stated for visiting the playgarden revolved around the need for "a break". Time spent outdoors was appreciated relative to specific features, such as the natural environment, play equipment, furniture, and the atmosphere of the

outdoors. Access to outdoor spaces and play are important features of the principle of normalization and also serve as an avenue for the promotion of healthy lifestyles
SN - 0097-9805
AD - Department of Child and Youth Study, Mount Saint Vincent University, Halifax, Nova Scotia, Canada
UR - 19378571
ER -

TY - JOUR
ID - 926
T1 - Physical activity during the school day in public primary schools in Mexico City
A1 - Jennings-Aburto,N.
A1 - Nava,F.
A1 - Bonvecchio,A.
A1 - Safdie,M.
A1 - Gonzalez-Casanova,I.
A1 - Gust,T.
A1 - Rivera,J.
Y1 - 2009/03//
N1 - Jennings-Aburto, Nancy. Nava, Fernanda. Bonvecchio, Anabelle. Safdie, Margarita. Gonzalez-Casanova, Ines. Gust, Tiffany. Rivera, Juan
Salud publica de Mexico
0404371, uy1
IM
Journal Article. Research Support, Non-U.S. Gov't. Research Support, U.S. Gov't, P.H.S.
English
KW - MEDLINE
KW - Child
KW - Child Welfare
KW - Female
KW - Guidelines as Topic
KW - Health Promotion
KW - Humans
KW - Male
KW - Mexico
KW - Motor Activity
KW - Physical Education and Training/sn [Statistics & Numerical Data]
KW - Play and Playthings
KW - Recreation
KW - Schools
KW - Students/sn [Statistics & Numerical Data]
KW - Urban Population
RP - NOT IN FILE
SP - 141
EP - 147
JF - Salud Publica de Mexico
JA - Salud Publica Mex
VL - 51
IS - 2
CY - Mexico
N2 - OBJECTIVE: To quantify the physical activity (PA) of students and describe the school environment surrounding PA. MATERIAL AND METHODS: Between November 2005 and March 2006, in Mexico City, we conducted quantitative and qualitative observations to describe the PA and the school context. RESULTS: Recess and physical education class (PE) were the only opportunities to participate in PA. PE occurred one time per week with a duration of 39.8+/-10.6 minutes which is less than national and international recommendations. Students participated in moderate-to-vigorous PA 29.2+/-17.8% of PE. The dynamics of PE did not promote the inclusion of all students or PA. During recess there was overcrowding of the school patio and no equipment for

PA or organization of PA. DISCUSSION: The PA of students in public schools in Mexico City can be improved by increasing the quantity and quality of PE and increasing opportunities for activity during recess
SN - 1606-7916
AD - Instituto Nacional de Salud Publica, Centro de Investigacion en Nutricion y Salud, Cuernavaca, Mexico
UR - 19377741
ER -

TY - JOUR
ID - 927
T1 - Examining unsupervised time with peers and the role of association with delinquent peers on adolescent smoking
A1 - Greene,K.
A1 - Banerjee,S.C.
Y1 - 2009/04//
N1 - Greene, Kathryn. Banerjee, Smita C
Nicotine & tobacco research : official journal of the Society for Research on Nicotine and Tobacco
drz, 9815751
IM
Journal Article
English
KW - MEDLINE
KW - Adolescent
KW - Adolescent Behavior
KW - Child
KW - Cross-Sectional Studies
KW - Female
KW - Humans
KW - Interpersonal Relations
KW - Juvenile Delinquency
KW - Male
KW - New England
KW - Peer Group
KW - Personal Autonomy
KW - Smoking
RP - NOT IN FILE
SP - 371
EP - 380
JF - Nicotine & Tobacco Research
JA - Nicotine Tob.Res
VL - 11
IS - 4
CY - England
N2 - INTRODUCTION: This study explored the association between unsupervised time with peers and adolescent smoking behavior both directly and indirectly through interaction with delinquent peers, social expectancies about cigarette smoking, and cigarette offers from peers. METHODS: A cross-sectional survey was used for the study and included 248 male and female middle school students. RESULTS: Results of structural equation modeling revealed that unsupervised time with peers is associated indirectly with adolescent smoking behavior through the mediation of association with delinquent peers, social expectancies about cigarette smoking, and cigarette offers from peers. DISCUSSION: Interventions designed to motivate adolescents without adult supervision to associate more with friends who engage in prosocial activities may eventually reduce adolescent smoking. Further implications for structured supervised time for students outside of school time are discussed
SN - 1469-994X
AD - Department of Communication, Rutgers University, 4 Huntington Street, New Brunswick, NJ 08901, USA. kgreene@scils.rutgers.edu
UR - 19307446
ER -

TY - JOUR
ID - 928
T1 - Promoting physical activity at the pre-school playground: the effects of providing markings and play equipment
A1 - Cardon,G.
A1 - Labarque,V.
A1 - Smits,D.
A1 - De,Bourdeaudhuij,I
Y1 - 2009/04//
N1 - Cardon, Greet. Labarque, Valery. Smits, Dirk. De Bourdeaudhuij, Ilse
Preventive medicine
pm4, 0322116
IM
Journal Article. Randomized Controlled Trial
English
KW - MEDLINE
KW - Belgium
KW - Child
KW - Preschool
KW - Color
KW - Environment Design
KW - Exercise
KW - Female
KW - Humans
KW - Male
KW - Monitoring
KW - Physiologic/is [Instrumentation]
KW - Motivation
KW - Play and Playthings
KW - Schools
RP - NOT IN FILE
SP - 335
EP - 340
JF - Preventive Medicine
JA - Prev Med
VL - 48
IS - 4
CY - United States
N2 - OBJECTIVES: We aimed to investigate the effects of providing play equipment and markings at the pre-school playground on physical activity engagement levels. METHODS: We performed a cluster randomised control trial. In November and December 2007, a convenience sample of 40 public pre-schools in Flanders, Belgium, was randomly assigned to one of the following conditions: 1) in 10 pre-schools play equipment was provided, 2) in 10 pre-schools markings were painted on the playground, 3) in 10 schools play equipment was provided and markings were painted, 4) 10 schools served as a control condition. Accelerometer-based physical activity levels during recess were evaluated at baseline and 4 to 6 weeks after the implementation of the intervention in 583 children (52% boys; mean age 5.3 years, SD 0.4). RESULTS: At baseline pre-schoolers spent only 11.2% (average: 4.7 min) of recess time in moderate to vigorous activity, while 61.3% (average: 25.7 min) was spent in sedentary activity. The interventions were not effective in increasing the average activity levels or the percentages of engagement in moderate or vigorous activity, or in decreasing sedentary time. CONCLUSION: Providing playground markings or play equipment is not sufficient to increase activity levels and decrease levels of sedentary activity during pre-school recess. More activating supervision and the inclusion of more structured physical activity seem needed
SN - 1096-0260
AD - Department of Movement and Sports Sciences, Faculty of Medicine and Health Sciences, Ghent University, Watersportlaan 2, Ghent, Belgium. greet.cardon@UGent.be
UR - 19236894

ER -

TY - JOUR
ID - 929
T1 - Implementation of a school-based state policy to increase physical activity*
A1 - Evenson,K.R.
A1 - Ballard,K.
A1 - Lee,G.
A1 - Ammerman,A.
Y1 - 2009/06//
N1 - Evenson, Kelly R. Ballard, Kymm. Lee, Ginny. Ammerman, Alice
The Journal of school health
k13, 0376370
IM, N
Journal Article. Research Support, Non-U.S. Gov't. Research Support, U.S. Gov't, P.H.S.
English
KW - MEDLINE
KW - Adolescent
KW - Child
KW - Data Collection
KW - Education
KW - Continuing
KW - Exercise
KW - Humans
KW - Internet
KW - North Carolina
KW - Policy Making
KW - Schools
KW - State Government
RP - NOT IN FILE
SP - 231
EP - 238
JF - Journal of School Health
JA - J Sch Health
VL - 79
IS - 5
CY - United States
N2 - BACKGROUND: In 2005, the North Carolina State Board of Education updated the Healthy Active Children Policy to include a requirement that all kindergarten through eighth-grade children receive at least 30 minutes of moderate-to-vigorous physical activity each school day through physical education, recess, and other creative approaches. This study describes how districts are meeting the physical activity-related portion of this policy and explores the policy implementation successes and challenges. METHODS: An online survey was conducted in summer 2007 in all school districts in the state (response rate 106/111; 95.5%). Respondents were asked to describe the implementation successes and challenges associated with including 30 minutes of physical activity in the school day. Answers were double coded, checked, and grouped into themes. RESULTS: The physical activity requirement was most often met through recess, physical education, classroom Energizers, and intramural sports. School districts reported numerous positive effects of the policy in elementary and middle schools. Benefits included increased student focus on studies, physical activity participation, awareness of healthy habits, alertness and enjoyment, and higher staff involvement. Implementation challenges to the policy included lack of time in the school day, teacher participation, and concerns about academics. CONCLUSIONS: School districts reported that implementation of the policy produced many positive results for students and staff. Addressing several implementation challenges common across school districts would strengthen the ongoing success of the policy
SN - 1746-1561
AD - Department of Epidemiology, Gillings School of Global Public Health, University of North Carolina-Chapel Hill, Bank of America Center, Chapel Hill, NC 27514, USA. kelly_evenson@unc.edu
UR - 19341442

ER -

TY - JOUR
ID - 930
T1 - Groundwater contamination by microbiological and chemical substances released from hospital wastewater: health risk assessment for drinking water consumers
A1 - Emmanuel,E.
A1 - Pierre,M.G.
A1 - Perrodin,Y.
Y1 - 2009/05//
N1 - Emmanuel, Evens. Pierre, Marie Gisele. Perrodin, Yves
Environment international
du1, 7807270
IM
Journal Article
English
KW - MEDLINE
KW - Adult
KW - Animals
KW - Child
KW - Preschool
KW - Colony Count
KW - Microbial
KW - Drinking
KW - Enterobacteriaceae/ip [Isolation & Purification]
KW - Enterococcus/ip [Isolation & Purification]
KW - Environmental Exposure
KW - Eukaryota/ip [Isolation & Purification]
KW - Hospitals
KW - Humans
KW - Infant
KW - Newborn
KW - Risk Assessment
KW - Viruses/ip [Isolation & Purification]
KW - Water Microbiology
KW - Water Pollutants
KW - Chemical/an [Analysis]
KW - Water Pollution
KW - Young Adult
RP - NOT IN FILE
SP - 718
EP - 726
JF - Environment International
JA - Environ Int
VL - 35
IS - 4
CY - Netherlands
N2 - Contamination of natural aquatic ecosystems by hospital wastewater is a major environmental and human health issue. Disinfectants, pharmaceuticals, radionuclides and solvents are widely used in hospitals for medical purposes and research. After application, some of these substances combine with hospital effluents and, in industrialised countries, reach the municipal sewer network. In certain developing countries, hospitals usually discharge their wastewater into septic tanks equipped with diffusion wells. The discharge of chemical compounds from hospital activities into the natural environment can lead to the pollution of water resources and risks for human health. The aim of this article is to present: (i) the steps of a procedure intended to evaluate risks to human health linked to hospital effluents discharged into a septic tank equipped with a diffusion well; and (ii) the results of its application on the effluents of a hospital in Port-au-Prince. The procedure is based on a scenario that describes the discharge of hospital effluents, via septic tanks, into a karstic formation where water resources

are used for human consumption. COD, Chloroform, dichlomethane, dibromochloromethane, dichlorobromomethane and bromoform contents were measured. Furthermore, the presence of heavy metals (chrome, nickel and lead) and faecal coliforms were studied. Maximum concentrations were 700 NPP/100 ml for faecal coliforms and 112 mg/L for COD. A risk of infection of 10(-5) infection per year was calculated. Major chemical risks, particularly for children, relating to Pb(II), Cr(III), Cr(VI) and Ni(II) contained in the ground water were also characterised. Certain aspects of the scenario studied require improvement, especially those relating to the characterisation of drugs in groundwater and the detection of other microbiological indicators such as protozoa, enterococcus and viruses
SN - 1873-6750
AD - Laboratoire de Qualite de l'Eau et de l'Environnement, Universite Quisqueya, BP 796 Port-au-Prince, Haiti. evemm1@yahoo.fr
UR - 19269687
ER -

TY - JOUR
ID - 931
T1 - Severe neurologic side effects in patients being treated for hemophagocytic lymphohistiocytosis
A1 - Thompson,P.A.
A1 - Allen,C.E.
A1 - Horton,T.
A1 - Jones,J.Y.
A1 - Vinks,A.A.
A1 - McClain,K.L.
Y1 - 2009/05//
N1 - Thompson, Patrick A. Allen, Carl E. Horton, Terzah. Jones, Jeremy Y. Vinks, Alexander A. McClain, Kenneth L
Pediatric blood & cancer
101186624
IM
Journal Article
English
KW - MEDLINE
KW - Adolescent
KW - Central Nervous System Diseases/ci [Chemically Induced]
KW - Central Nervous System Diseases/pa [Pathology]
KW - Child
KW - Preschool
KW - Cyclosporine/ae [Adverse Effects]
KW - Cyclosporine/tu [Therapeutic Use]
KW - Drug-Induced Liver Injury
KW - Female
KW - Humans
KW - Infant
KW - Liver Diseases/pp [Physiopathology]
KW - Lymphohistiocytosis
KW - Hemophagocytic/dt [Drug Therapy]
KW - Magnetic Resonance Imaging
KW - Male
KW - Models
KW - Biological
RP - NOT IN FILE
SP - 621
EP - 625
JF - Pediatric Blood & Cancer
JA - Pediatr Blood Cancer
VL - 52
IS - 5

CY - United States
N2 - BACKGROUND: Hemophagocytic Lymphohistiocytosis (HLH) is characterized by uncontrolled inflammation that is generally fatal without immune modulating chemotherapy. At Texas Children's Hospital, we have observed significant central nervous system (CNS) toxicity in several patients treated for HLH according to the Histiocyte Society protocol HLH-2004 in which cyclosporine is given early in the treatment regimen. METHODS: Patients diagnosed with HLH at Texas Children's Hospital between April 2004 and October 2007 were identified and charts were reviewed. A reference group of patients treated between August 2001 and March 2004, prior to the introduction of HLH-2004, was also evaluated. RESULTS: Five of 17 patients in the study group developed severe neurotoxicity. Four had new onset seizures associated with significant MRI abnormalities, while the fifth died of intracerebral hemorrhage. Timing of the development of neurologic side effects ranged from day 5 to week 6 of therapy. Cyclosporine levels were outside the therapeutic range (200-300 ng/ml) prior to the onset of symptoms in two of the five patients. Systolic blood pressures for all five patients were greater than the 95th percentile for age on at least one measurement within 24 hr of the onset of neurologic symptoms. MRI scans obtained within 24 hr of seizure activity in four patients were consistent with posterior reversible encephalopathy syndrome (PRES). By comparison only one patient in the reference group (n = 15) had neurotoxicity (PRES). CONCLUSIONS: Patients being treated for HLH appear to be at risk for neurotoxicity, particularly PRES. Elevated blood pressure, worsening renal and liver function, increased cyclosporine levels, and CNS involvement of HLH may be triggers for the neurotoxic side effects of treatment. Patients being treated on HLH-2004 require close monitoring of their neurologic status and modifiable risk factors such as hypertension should managed aggressively. If larger studies validate our observations, it will be important to determine if up-front cyclosporine in HLH protocols confers a survival benefit that outweighs the potential risk of increased neurotoxicity. (c) 2009 Wiley-Liss, Inc
SN - 1545-5017
AD - Texas Children's Cancer Center, Baylor College of Medicine, Houston, Texas 77030-2399, USA
UR - 19137570
ER -

TY - JOUR
ID - 932
T1 - [Posttraumatic pseudomeningocele--a case report]. [Japanese]
A1 - Nagaishi,M.
A1 - Suzuki,R.
A1 - Iwatate,K.
A1 - Kowata,K.
A1 - Tanaka,Y.
A1 - Hoya,K.
Y1 - 2009/03//
N1 - Nagaishi, Masaya. Suzuki, Ryotaro. Iwatate, Kensho. Kowata, Kazuma. Tanaka, Yoshihiro. Hoya, Katsumi
Brain and nerve = Shinkei kenkyu no shinpo
101299709
IM
Case Reports. English Abstract. Journal Article
Japanese
KW - MEDLINE
KW - Bandages
KW - Child
KW - Diagnostic Imaging
KW - Drainage/mt [Methods]
KW - Head Injuries
KW - Closed/co [Complications]
KW - Humans
KW - Male
KW - Meningocele/di [Diagnosis]
KW - Meningocele/et [Etiology]
KW - Meningocele/th [Therapy]
KW - Subdural Effusion/et [Etiology]

KW - Subdural Effusion/th [Therapy]
RP - NOT IN FILE
SP - 324
EP - 327
JF - Brain & Nerve / Shinkei Kenkyu no Shinpo
JA - Brain Nerve
VL - 61
IS - 3
CY - Japan
N2 - Pseudomeningocele is a rare complication of head trauma. An 7-year-old male child who fell from playground equipment was admitted to hospital with lambdoid sutural diastatic fracture. As epidural and subgaleal fluid collections gradually developed from the subsequent day of admission, a subgaleal drain was placed for cerebrospinal fluid (CSF) diversion. In addition, the scalp just over the fractured segment was compressed by band after drainage whereby the CSF leak was stopped. On the basis of our experience with this case, we suggest that appropriate management enables repair of posttraumatic pseudomeningocele in early stages of injury
SN - 1881-6096
AD - Department of Neurosurgery, Koshigaya Hospital, Dokkyo University School of Medicine, 2-1-50 Minami-Koshigaya, Koshigaya-shi, Saitama 343-8555, Japan
UR - 19301605
ER -

TY - JOUR
ID - 933
T1 - Schools and obesity prevention: creating school environments and policies to promote healthy eating and physical activity
A1 - Story,M.
A1 - Nanney,M.S.
A1 - Schwartz,M.B.
Y1 - 2009/03//
N1 - Story, Mary. Nanney, Marilyn S. Schwartz, Marlene B
The Milbank quarterly
8607003, m9q
IM
Journal Article
English
KW - MEDLINE
KW - Adolescent
KW - Adolescent Behavior
KW - Body Mass Index
KW - Child
KW - Child Welfare
KW - Curriculum
KW - Female
KW - Health Knowledge
KW - Attitudes
KW - Practice
KW - Health Promotion
KW - Humans
KW - Male
KW - Motor Activity
KW - Nutrition Policy/ec [Economics]
KW - Nutritional Status
KW - Nutritive Value
KW - Obesity/ep [Epidemiology]
KW - Obesity/pc [Prevention & Control]
KW - School Health Services

KW - Schools
KW - Social Environment
KW - Social Marketing
KW - United States/ep [Epidemiology]
RP - NOT IN FILE
SP - 71
EP - 100
JF - Milbank Quarterly
JA - Milbank Q
VL - 87
IS - 1
CY - United States
N2 - CONTEXT: Research consistently shows that the majority of American children do not consume diets that meet the recommendations of the Dietary Guidelines for Americans, nor do they achieve adequate levels of daily physical activity. As a result, more children are overweight today than at any other time in U.S. history. Schools offer many opportunities to develop strategies to prevent obesity by creating environments in which children eat healthfully and engage regularly in physical activity. METHODS: This article discusses the role of schools in obesity prevention efforts. Current issues in schools' food and physical activity environments are examined, as well as federal, state, and local policies related to food and physical activity standards in schools. The article is organized around four key areas: (1) school food environments and policies, (2) school physical activity environments and policies, (3) school body mass index measurements, and (4) school wellness policies. Recommendations for accelerating change also are addressed. FINDINGS: The article found that (1) competitive foods (foods sold outside of federally reimbursed school meals) are widely available in schools, especially secondary schools. Studies have related the availability of snacks and drinks sold in schools to students' high intake of total calories, soft drinks, total fat and saturated fat, and lower intake of fruits and vegetables; (2) physical activity can be added to the school curriculum without academic consequences and also can offer physical, emotional, and social benefits. Policy leadership has come predominantly from the districts, then the states, and, to a much lesser extent, the federal government; (3) few studies have examined the effectiveness or impact of school-based BMI measurement programs; and (4) early comparative analyses of local school wellness policies suggest that the strongest policies are found in larger school districts and districts with a greater number of students eligible for a free or reduced-price lunch. CONCLUSIONS: Studies show that schools have been making some progress in improving the school food and physical activity environments but that much more work is needed. Stronger policies are needed to provide healthier meals to students at schools; limit their access to low-nutrient, energy-dense foods during the school day; and increase the frequency, intensity, and duration of physical activity at school
SN - 1468-0009
AD - Division of Epidemiology and Community Health, 1300 S. 2nd Street, University of Minnesota, Minneapolis, MN 55454, USA. story@umn.edu
UR - 19298416
ER -

TY - JOUR
ID - 934
T1 - Self-reported physical activity levels during a segmented school day in a large multiethnic sample of high school students
A1 - Hohepa,M.
A1 - Scragg,R.
A1 - Schofield,G.
A1 - Kolt,G.S.
A1 - Schaaf,D.
Y1 - 2009/03//
N1 - Hohepa, Maea. Scragg, Robert. Schofield, Grant. Kolt, Gregory S. Schaaf, David
Journal of science and medicine in sport / Sports Medicine Australia
c54, 9812598
IM
Journal Article. Research Support, Non-U.S. Gov't
English

KW - MEDLINE
KW - Adolescent
KW - Adolescent Behavior
KW - Child
KW - Data Collection
KW - Female
KW - Humans
KW - Leisure Activities
KW - Male
KW - Motor Activity
KW - Students
RP - NOT IN FILE
SP - 284
EP - 292
JF - Journal of Science & Medicine in Sport
JA - J Sci Med Sport
VL - 12
IS - 2
CY - Australia
N2 - The school day provides youth several opportunistic time periods to be active. How active youth are during these time periods or whether they engage in physical activity across multiple time periods is not well established. A cross-sectional sample of 12-18-year-old students (n=3471) were recruited from low socioeconomic status high schools within South Auckland, New Zealand. Participants reported their physical activity during lunchtime, morning recess, and after school, as well as their level of active transportation to and from school. For each physical activity opportunity participants were dichotomised as being either "more active" or "less active". Height, weight, and waist circumference measurements were also collected for each participant. Data were analysed using binary logistic regression to understand demographic associations and with kappa coefficient calculations to examine level of association in participation levels between different time periods. The percentage of students considered 'more active' depended on the physical activity variable of interest (after-school, 56.3%; active transportation, 58.1%; morning recess, 26.4%; lunchtime recess, 32.4%). Only 11.1% of participants were classified as 'more active' across all four physical activity opportunities. Substantial agreement (kappa=.68) was only observed in participation levels at lunchtime and morning recess. At-risk groups for lower physical activity were female students, senior students, and students of Asian/Other descent. During a school day multiple opportunistic time periods exist for youth to be active, yet a large proportion of students are not as active as they could be with few youth engaging in physical activity across various segments of the school day
SN - 1440-2440
AD - Centre for Physical Activity and Nutrition Research, Faculty of Health and Environmental Sciences, Auckland University of Technology, New Zealand. maea.hohepa@aut.ac.nz
UR - 18328781
ER -

TY - JOUR
ID - 935
T1 - Substance use and delinquency among fifth graders who have jobs
A1 - Ramchand,R.
A1 - Elliott,M.N.
A1 - Mrug,S.
A1 - Grunbaum,J.A.
A1 - Windle,M.
A1 - Chandra,A.
A1 - Peskin,M.F.
A1 - Cooper,S.P.
A1 - Schuster,M.A.
Y1 - 2009/04//
N1 - Ramchand, Rajeev. Elliott, Marc N. Mrug, Sylvie. Grunbaum, Jo Anne. Windle, Michael. Chandra, Anita. Peskin, Melissa F. Cooper, Sharon P. Schuster, Mark A
American journal of preventive medicine

8704773, apl
IM
Journal Article
English
KW - MEDLINE
KW - Absenteeism
KW - Alcoholism/ep [Epidemiology]
KW - Child
KW - Comorbidity
KW - Cross-Sectional Studies
KW - Employment/sn [Statistics & Numerical Data]
KW - Female
KW - Humans
KW - Juvenile Delinquency/sn [Statistics & Numerical Data]
KW - Logistic Models
KW - Male
KW - Multivariate Analysis
KW - Population Surveillance
KW - Prevalence
KW - Smoking/ep [Epidemiology]
KW - Substance-Related Disorders/ep [Epidemiology]
KW - United States/ep [Epidemiology]
RP - NOT IN FILE
SP - 297
EP - 303
JF - American Journal of Preventive Medicine
JA - Am J Prev Med
VL - 36
IS - 4
CY - Netherlands
N2 - BACKGROUND: Working for pay is associated with substance use and delinquency among older adolescents, although information is scant about younger youth who work. This study investigates associations between self-reports of having a job and substance use and delinquent behaviors in a sample of U.S. 5th graders. METHODS: A total of 5147 5th graders and their parents from three large metropolitan areas were assessed in a cross-sectional survey between Fall 2004 and Summer 2006. Multivariate regression was used to estimate associations between having a job and substance use and delinquency. Analyses were conducted in Fall 2007. RESULTS: Twenty-one percent of 5th graders reported having a job, with most working <5 hours per week. Typical jobs included yard work, babysitting, and cleaning. In multivariate models that controlled for demographic characteristics, household composition, and household income, having a job was significantly associated with past-30-day use of tobacco (OR=2.2), alcohol (OR=1.7), and marijuana (OR=3.1). Having a job was also significantly associated with ever being in a fight (OR=1.5) and with running away from home (OR=1.8). Further analyses indicated that the associations between holding a job and delinquency outcomes were driven largely by young workers who worked >2 hours per week. CONCLUSIONS: Among 5th graders, having a job was associated with substance-using behaviors and delinquency. Clinicians should consider asking young patients whether they work, and stress to parents the importance of monitoring the work activities, workplaces, and associates of their children
SN - 1873-2607
AD - RAND Corporation, Arlington, Virginia, USA. Rajeev_Ramchand@rand.org
UR - 19285195
ER -

TY - JOUR
ID - 936
T1 - Low physical fitness among fifth- and seventh-grade students, Georgia, 2006
A1 - Powell,K.E.
A1 - Roberts,A.M.
A1 - Ross,J.G.

A1 - Phillips,M.A.
A1 - Ujamaa,D.A.
A1 - Zhou,M.
Y1 - 2009/04//
N1 - Powell, Kenneth E. Roberts, Alice M. Ross, James G. Phillips, Mary Ann C. Ujamaa, Dawud A. Zhou, Mei
American journal of preventive medicine
8704773, apl
IM
Journal Article
English
KW - MEDLINE
KW - Adolescent
KW - Body Composition
KW - Child
KW - Female
KW - Georgia
KW - Humans
KW - Male
KW - Muscle Strength
KW - Physical Endurance
KW - Physical Fitness
KW - Population Surveillance
KW - Range of Motion
KW - Articular
KW - Schools/sn [Statistics & Numerical Data]
KW - Students/sn [Statistics & Numerical Data]
RP - NOT IN FILE
SP - 304
EP - 310
JF - American Journal of Preventive Medicine
JA - Am J Prev Med
VL - 36
IS - 4
CY - Netherlands
N2 - BACKGROUND: The nationwide epidemic of obesity may be due, in part, to declining levels of physical activity, raising the possibility that other components of health-related physical fitness may also be in decline. Few data are available to describe and monitor the physical fitness of children and youth. The Georgia Youth Fitness Assessment was conducted to assess health-related fitness in Georgia's fifth- and seventh-grade students, provide a baseline against which future progress could be measured, and guide public and private leaders and decision makers. METHODS: A statewide probability sample of fifth- and seventh-grade students designed to enable grade-specific comparisons by gender, race/ethnicity, and urban/rural status was drawn. Measurements included aerobic capacity; body composition; and muscular strength, endurance, and flexibility. Physical activity during the most recent 3 days was assessed. The survey was conducted in 2006; the data were analyzed in 2007-2008. RESULTS: Ninety-three schools (86% response rate) and 5248 students (77% response rate) participated. Fifty-two percent of students did not meet the standard for healthy aerobic fitness; 23% did not meet the standard for muscular strength, endurance, and flexibility; 30% were outside the recommended range for BMI. Twenty-two percent did not achieve the recommended 60 minutes of daily moderate-to-vigorous physical activity. All subgroups (e.g., boys/girls, urban/rural) scored poorly. CONCLUSIONS: Substantial numbers of Georgia's fifth- and seventh-grade students exhibit unhealthy levels of physical fitness. These data are consistent with the suggestion that physical inactivity has led to deficient levels of health-related fitness in more areas than just body composition. Monitoring all components of health-related fitness would provide helpful information about the health of children and youth
SN - 1873-2607
AD - nekllewop@hotmail.com
UR - 19201145
ER -

TY - JOUR
ID - 937
T1 - Pediatric tree house-related injuries treated in emergency departments in the United States: 1990-2006
A1 - Randazzo,C.
A1 - Stolz,U.
A1 - Hodges,N.L.
A1 - McKenzie,L.B.
Y1 - 2009/03//
N1 - Randazzo, Charles. Stolz, Uwe. Hodges, Nichole L. McKenzie, Lara B
Academic emergency medicine : official journal of the Society for Academic Emergency Medicine
ce1, 9418450
IM
Journal Article
English
KW - MEDLINE
KW - Accidental Falls/sn [Statistics & Numerical Data]
KW - Accidents
KW - Home/sn [Statistics & Numerical Data]
KW - Adolescent
KW - Child
KW - Preschool
KW - Emergency Service
KW - Hospital
KW - Female
KW - Humans
KW - Infant
KW - Male
KW - Play and Playthings
KW - Regression Analysis
KW - Retrospective Studies
KW - Trees
KW - United States/ep [Epidemiology]
KW - Wounds and Injuries/ep [Epidemiology]
KW - Wounds and Injuries/et [Etiology]
RP - NOT IN FILE
SP - 235
EP - 242
JF - Academic Emergency Medicine
JA - Acad Emerg Med
VL - 16
IS - 3
CY - United States
N2 - OBJECTIVES: The objective was to describe the epidemiology of tree house-related injuries in the United States among children and adolescents. METHODS: The authors conducted a retrospective analysis using data from the National Electronic Injury Surveillance System for patients < or =19 years who were treated in an emergency department (ED) for a tree house-related injury from 1990 through 2006. RESULTS: An estimated 47,351 patients < or =19 years of age were treated in EDs for tree house-related injuries over the 17-year study period. Fractures were the most common diagnosis (36.6%), and the upper extremities were the most commonly injured body part (38.8%). The odds of sustaining a head injury were increased for children aged <5 years. Falls were the most common injury mechanism (78.6%) and increased the odds of sustaining a fracture. Falls or jumps from a height > or =10 feet occurred in 29.3% of cases for which height of the fall/jump was recorded. Boys had significantly higher odds of falling or jumping from a height of > or =10 ft than girls, and children 10 to 19 years old also had significantly higher odds of falling or jumping from a height of > or =10 feet, compared to those 9 years old and younger. The odds of hospitalization were tripled if the patient fell or jumped from > or =10 feet and nearly tripled if the patient sustained a fracture. CONCLUSIONS: This study examined tree house-related injuries on a national level. Tree house safety deserves special attention because of the potential for

serious injury or death due to falls from great heights, as well as the absence of national or regional safety standards. The authors provide safety and prevention recommendations based on the successful standards developed for playground equipment
SN - 1553-2712
AD - College of Medicine, The Ohio State University, Columbus, OH, USA
UR - 19281495
ER -

TY - JOUR
ID - 938
T1 - In-home particle concentrations and childhood asthma morbidity
A1 - McCormack,M.C.
A1 - Breysse,P.N.
A1 - Matsui,E.C.
A1 - Hansel,N.N.
A1 - Williams,D.
A1 - Curtin-Brosnan,J.
A1 - Eggleston,P.
A1 - Diette,G.B.
A1 - Center for Childhood Asthma in the Urban Environment.
Y1 - 2009/02//
N1 - McCormack, Meredith C. Breysse, Patrick N. Matsui, Elizabeth C. Hansel, Nadia N. Williams, D'Ann. Curtin-Brosnan, Jean. Eggleston, Peyton. Diette, Gregory B. Center for Childhood Asthma in the Urban Environment
Environmental health perspectives
ei0, 0330411
IM
Journal Article. Research Support, N.I.H., Extramural. Research Support, U.S. Gov't, Non-P.H.S.
English
KW - MEDLINE
KW - Air Pollution
KW - Indoor/ae [Adverse Effects]
KW - Asthma/ep [Epidemiology]
KW - Asthma/pa [Pathology]
KW - Baltimore
KW - Child
KW - Preschool
KW - Environmental Monitoring
KW - Epidemiological Monitoring
KW - Female
KW - Humans
KW - Male
KW - Maryland
KW - Particulate Matter/ae [Adverse Effects]
KW - Questionnaires
RP - NOT IN FILE
SP - 294
EP - 298
JF - Environmental Health Perspectives
JA - Environ Health Perspect
VL - 117
IS - 2
CY - United States
N2 - BACKGROUND: Although outdoor particulate matter (PM) has been linked to mortality and asthma morbidity, the impact of indoor PM on asthma has not been well established. OBJECTIVE: This study was designed to investigate the effect of in-home PM on asthma morbidity. METHODS: For a cohort of 150 asthmatic children (2-6 years of age) from Baltimore, Maryland, a technician deployed environmental

monitoring equipment in the children's bedrooms for 3-day intervals at baseline and at 3 and 6 months. Caregivers completed questionnaires and daily diaries during air sampling. Longitudinal data analyses included regression models with generalized estimating equations. RESULTS: Children were primarily African Americans (91%) from lower socioeconomic backgrounds and spent most of their time in the home. Mean (+/- SD) indoor PM(2.5-10) (PM with aerodynamic diameter 2.5-10 microm) and PM(2.5) (aerodynamic diameter < 2.5 microm) concentrations were 17.4 +/- 21.0 and 40.3 +/- 35.4 microg/m(3). In adjusted models, 10-microg/m(3) increases in indoor PM(2.5-10) and PM(2.5) were associated with increased incidences of asthma symptoms: 6% [95% confidence interval (CI), 1 to 12%] and 3% (95% CI, -1 to 7%), respectively; symptoms causing children to slow down: 8% (95% CI, 2 to 14%) and 4% (95% CI, 0 to 9%), respectively; nocturnal symptoms: 8% (95% CI, 1 to 14%) and 6% (95% CI, 1 to 10%), respectively; wheezing that limited speech: 11% (95% CI, 3 to 19%) and 7% (95% CI, 0 to 14%), respectively; and use of rescue medication: 6% (95% CI, 1 to 10%) and 4% (95% CI, 1 to 8%), respectively. Increases of 10 microg/m(3) in indoor and ambient PM(2.5) were associated with 7% (95% CI, 2 to 11%) and 26% (95% CI, 1 to 52%) increases in exercise-related symptoms, respectively. CONCLUSIONS: Among preschool asthmatic children in Baltimore, increases in in-home PM(2.5-10) and PM(2.5) were associated with respiratory symptoms and rescue medication use. Increases in in-home and ambient PM(2.5) were associated with exercise-related symptoms. Although reducing PM outdoors may decrease asthma morbidity, reducing PM indoors, especially in homes of inner-city children, may lead to improved asthma health
SN - 0091-6765
AD - Department of Medicine, Johns Hopkins University School of Medicine, Baltimore, Maryland 21205 , USA
UR - 19270802
ER -

TY - JOUR
ID - 939
T1 - Environment and obesity in the National Children's Study. [Review] [106 refs][Reprint in Cien Saude Colet. 2010 Jan;15(1):195-210; PMID: 20169246]
A1 - Trasande,L.
A1 - Cronk,C.
A1 - Durkin,M.
A1 - Weiss,M.
A1 - Schoeller,D.A.
A1 - Gall,E.A.
A1 - Hewitt,J.B.
A1 - Carrel,A.L.
A1 - Landrigan,P.J.
A1 - Gillman,M.W.
Y1 - 2009/02//
N1 - Trasande, Leonardo. Cronk, Chris. Durkin, Maureen. Weiss, Marianne. Schoeller, Dale A. Gall, Elizabeth A. Hewitt, Jeanne B. Carrel, Aaron L. Landrigan, Philip J. Gillman, Matthew W
Environmental health perspectives
ei0, 0330411
IM
Journal Article. Research Support, N.I.H., Extramural. Review
English
KW - MEDLINE
KW - Adolescent
KW - Body Mass Index
KW - Child
KW - Preschool
KW - Energy Intake
KW - Energy Metabolism
KW - Environmental Exposure/ae [Adverse Effects]
KW - Humans
KW - Infant
KW - Obesity/ep [Epidemiology]

KW - Prospective Studies
KW - Risk Factors
KW - Young Adult
RP - NOT IN FILE
SP - 159
EP - 166
JF - Environmental Health Perspectives
JA - Environ Health Perspect
VL - 117
IS - 2
CY - United States
N2 - OBJECTIVE: In this review we describe the approach taken by the National Children's Study (NCS), a 21-year prospective study of 100,000 American children, to understanding the role of environmental factors in the development of obesity. DATA SOURCES AND EXTRACTION: We review the literature with regard to the two core hypotheses in the NCS that relate to environmental origins of obesity and describe strategies that will be used to test each hypothesis. DATA SYNTHESIS: Although it is clear that obesity in an individual results from an imbalance between energy intake and expenditure, control of the obesity epidemic will require understanding of factors in the modern built environment and chemical exposures that may have the capacity to disrupt the link between energy intake and expenditure. The NCS is the largest prospective birth cohort study ever undertaken in the United States that is explicitly designed to seek information on the environmental causes of pediatric disease. CONCLUSIONS: Through its embrace of the life-course approach to epidemiology, the NCS will be able to study the origins of obesity from preconception through late adolescence, including factors ranging from genetic inheritance to individual behaviors to the social, built, and natural environment and chemical exposures. It will have sufficient statistical power to examine interactions among these multiple influences, including gene-environment and gene-obesity interactions. A major secondary benefit will derive from the banking of specimens for future analysis. [References: 106]
SN - 0091-6765
AD - Department of Community and Preventive Medicine, Mount Sinai School of Medicine, One Gustave L. Levy Place, Box 1043, New York, NY 10029 USA. leo.trasande@mssm.edu
UR - 19270782
ER -

TY - JOUR
ID - 940
T1 - Socioeconomic, family, and pediatric practice factors that affect level of asthma control
A1 - Bloomberg,G.R.
A1 - Banister,C.
A1 - Sterkel,R.
A1 - Epstein,J.
A1 - Bruns,J.
A1 - Swerczek,L.
A1 - Wells,S.
A1 - Yan,Y.
A1 - Garbutt,J.M.
Y1 - 2009/03//
N1 - Bloomberg, Gordon R. Banister, Christina. Sterkel, Randall. Epstein, Jay. Bruns, Julie. Swerczek, Lisa. Wells, Suzanne. Yan, Yan. Garbutt, Jane M
Pediatrics
oxv, 0376422
AIM, IM
Journal Article. Research Support, N.I.H., Extramural
English
KW - MEDLINE
KW - Administration
KW - Inhalation
KW - Adrenal Cortex Hormones/tu [Therapeutic Use]
KW - Albuterol/tu [Therapeutic Use]

KW - Anti-Asthmatic Agents/tu [Therapeutic Use]
KW - Asthma/dt [Drug Therapy]
KW - Asthma/ep [Epidemiology]
KW - Asthma/ge [Genetics]
KW - Asthma/px [Psychology]
KW - Child
KW - Preschool
KW - Emergency Service
KW - Hospital/ut [Utilization]
KW - Family Characteristics
KW - Female
KW - Guideline Adherence/sn [Statistics & Numerical Data]
KW - Hospitalization/sn [Statistics & Numerical Data]
KW - Humans
KW - Male
KW - Medicaid
KW - Multivariate Analysis
KW - Patient Compliance/sn [Statistics & Numerical Data]
KW - Pediatrics/sn [Statistics & Numerical Data]
KW - Quality of Life/px [Psychology]
KW - Risk Factors
KW - Socioeconomic Factors
KW - United States
KW - Utilization Review/sn [Statistics & Numerical Data]
KW - Women
KW - Working
RP - NOT IN FILE
SP - 829
EP - 835
JF - Pediatrics
VL - 123
IS - 3
CY - United States
N2 - BACKGROUND: Multiple issues play a role in the effective control of childhood asthma. OBJECTIVE: To identify factors related to the level of asthma control in children receiving asthma care from community pediatricians. PATIENTS AND METHODS: Data for 362 children participating in an intervention study to reduce asthma morbidity were collected by a telephone-administered questionnaire. Level of asthma control (well controlled, partially controlled, or poorly controlled) was derived from measures of recent impairment (symptoms, activity limitations, albuterol use) and the number of exacerbations in a 12-month period. Data also included demographic characteristics, asthma-related quality of life, pediatric management practices, and medication usage. Univariable and multivariable analyses were used to identify factors associated with poor asthma control and to explore the relationship between control and use of daily controller medications. RESULTS: Asthma was well controlled for 24% of children, partially controlled for 20%, and poorly controlled for 56%. Medicaid insurance, the presence of another family member with asthma, and maternal employment outside the home were significant univariable factors associated with poor asthma control. Medicaid insurance had an independent association with poor control. Seventy-six percent of children were reported by parents as receiving a daily controller medication. Comparison of guideline recommended controller medication with current level of asthma control indicated that a higher step level of medication would have been appropriate for 74% of these children. Significantly lower overall quality-of-life scores were observed in both parents and children with poor control. CONCLUSIONS: Despite substantial use of daily controller medication, children with asthma continue to experience poorly controlled asthma and reduced quality of life. Although Medicaid insurance and aspects of family structure are significant factors associated with poorly controlled asthma, attention to medication use and quality-of-life indicators may further reduce morbidity
SN - 1098-4275
AD - St Louis Children's Hospital, Washington University School of Medicine, Department of Pediatrics, Division of Allergy and Pulmonary Medicine, One Children's Place, St Louis, MO 63110, USA. bloomberg@kids.wustl.edu

UR - 19255010
ER -

TY - JOUR
ID - 941
T1 - Changes in the limb kinematics and walking-distance estimation after shank elongation: evidence for a locomotor body schema?.[Erratum appears in J Neurophysiol. 2009 May;101(5):2734]
A1 - Dominici,N.
A1 - Daprati,E.
A1 - Nico,D.
A1 - Cappellini,G.
A1 - Ivanenko,Y.P.
A1 - Lacquaniti,F.
Y1 - 2009/03//
N1 - Dominici, Nadia. Daprati, Elena. Nico, Daniele. Cappellini, Germana. Ivanenko, Yuri P. Lacquaniti, Francesco
Journal of neurophysiology
jc7, 0375404
IM
Journal Article. Research Support, Non-U.S. Gov't
English
KW - MEDLINE
KW - Achondroplasia/pp [Physiopathology]
KW - Biomechanical Phenomena
KW - Child
KW - Foot/ph [Physiology]
KW - Humans
KW - Male
KW - Motor Activity/ph [Physiology]
KW - Posture/ph [Physiology]
KW - Psychomotor Performance/ph [Physiology]
KW - Walking/ph [Physiology]
RP - NOT IN FILE
SP - 1419
EP - 1429
JF - Journal of Neurophysiology
JA - J Neurophysiol
VL - 101
IS - 3
CY - United States
N2 - When walking, step length provides critical information on traveled distance along the ongoing path [corrected] Little is known on the role that knowledge about body dimensions plays within this process. Here we directly addressed this question by evaluating whether changes in body proportions interfere with computation of traveled distance for targets located outside the reaching space. We studied locomotion and distance estimation in an achondroplastic child (ACH, 11 yr) before and after surgical elongation of the shank segments of both lower limbs and in healthy adults walking on stilts, designed to mimic shank-segment elongation. Kinematic analysis of gait revealed that dynamic coupling of the thigh, shank, and foot segments changed substantially as a result of elongation. Step length remained unvaried, in spite of the significant increase in total limb length (approximately 1.5-fold). These relatively shorter strides resulted from smaller oscillations of the shank segment, as would be predicted by proportional increments in limb size and not by asymmetrical segmental increment as in the present case (length of thighs was not modified). Distance estimation was measured by walking with eyes closed toward a memorized target. Before surgery, the behavior of ACH was comparable to that of typically developing participants. In contrast, following shank elongation, the ACH walked significantly shorter distances when aiming at the same targets. Comparable changes in limb kinematics, stride length, and estimation of traveled distance were found in adults wearing on stilts, suggesting that path integration errors in both cases were related to alterations in the intersegmental coordination of the walking

limbs. The results are consistent with a dynamic locomotor body schema used for controlling step length and path estimation, based on inherent relationships between gait parameters and body proportions
SN - 0022-3077
AD - Department of Neuromotor Physiology, IRCCS Fondazione Santa Lucia, Rome, Italy. n.dominici@hsantalucia.it
UR - 19091916
ER -

TY - JOUR
ID - 942
T1 - Childhood trauma profile at a tertiary care hospital in India
A1 - Verma,S.
A1 - Lal,N.
A1 - Lodha,R.
A1 - Murmu,L.
Y1 - 2009/02//
N1 - Verma, Sumit. Lal, Neena. Lodha, Rakesh. Murmu, Lakhiram
Indian pediatrics
gm2, 2985062r
IM
Journal Article
English
KW - MEDLINE
KW - Accidents
KW - Home/sn [Statistics & Numerical Data]
KW - Child
KW - Preschool
KW - Craniocerebral Trauma/ep [Epidemiology]
KW - Female
KW - Fractures
KW - Bone/ep [Epidemiology]
KW - Humans
KW - India/ep [Epidemiology]
KW - Infant
KW - Lacerations/ep [Epidemiology]
KW - Male
KW - Neck Injuries/ep [Epidemiology]
KW - Play and Playthings/in [Injuries]
KW - Prospective Studies
KW - Wounds and Injuries/ep [Epidemiology]
KW - Wounds and Injuries/th [Therapy]
RP - NOT IN FILE
SP - 168
EP - 171
JF - Indian Pediatrics
JA - Indian Pediatr
VL - 46
IS - 2
CY - India
N2 - Childhood injuries are the leading cause of morbidity and mortality worldwide. We evaluated the type of pediatric injuries encountered in the emergency room amongst 225 children (boys 151, girls 64; age range, 2 mo-12 yr). Data were collected using a structured injury proforma over a period of 12 months. Injuries occurred at home (n=137, 60.8%), street (n=38, 16.8%), and playground (n=37, 16.4%). Most frequent injuries were falls (n=144, 64%) and road traffic injuries (RTI) (n=37, 16.4%). Injuries mostly consisted of fractures (n=72, 32%), bruises (n=39, 17.3%), and lacerations (n=35, 15.5%). Child abuse was recognized in 7 (3.5%) children. There was an average delay of 2 hour 50 minutes to reach the medical facility
SN - 0019-6061

AD - Departments of Pediatrics and Emergency Medicine, All India Institute of Medical Sciences, Ansari Nagar, New Delhi, India. drsumitverma@yahoo.com
UR - 19242037
ER -

TY - JOUR
ID - 943
T1 - In vivo age dependency of unfractionated heparin in infants and children
A1 - Newall,F.
A1 - Ignjatovic,V.
A1 - Summerhayes,R.
A1 - Gan,A.
A1 - Butt,W.
A1 - Johnston,L.
A1 - Monagle,P.
Y1 - 2009/03//
N1 - Newall, Fiona. Ignjatovic, Vera. Summerhayes, Robyn. Gan, Andrew. Butt, Warwick. Johnston, Linda. Monagle, Paul
Thrombosis research
vrn, 0326377
IM
Journal Article. Research Support, Non-U.S. Gov't
English
KW - MEDLINE
KW - Adolescent
KW - Age Factors
KW - Anticoagulants/pd [Pharmacology]
KW - Antithrombin III/an [Analysis]
KW - Antithrombins/an [Analysis]
KW - Antithrombins/tu [Therapeutic Use]
KW - Child
KW - Preschool
KW - Female
KW - Heparin/me [Metabolism]
KW - Heparin/pd [Pharmacology]
KW - Humans
KW - Infant
KW - Male
KW - Partial Thromboplastin Time
KW - Prospective Studies
KW - Prothrombin/ai [Antagonists & Inhibitors]
KW - Thrombin/an [Analysis]
RP - NOT IN FILE
SP - 710
EP - 714
JF - Thrombosis Research
JA - Thromb.Res
VL - 123
IS - 5
CY - United States
N2 - INTRODUCTION: Unfractionated Heparin (UFH) is used widely in paediatrics. Paediatric specific recommendations for UFH therapy are few, with the majority of recommendations being extrapolated from adult practice. In vitro studies have shown that this practice may be suboptimal. This study aimed to improve the understanding of the impact of age upon UFH response in vivo. MATERIALS AND METHODS: This prospective, observational study, conducted in the Paediatric Intensive Care Unit (PICU), included: patients 16 years or younger; treated with UFH of at least 10 U/Kg/hr. Laboratory analysis included: Antithrombin, APTT, Anti-Xa, Anti-IIa and thrombin generation expressed as the Endogenous Thrombin Potential. Results were

grouped according to patient age (i.e. <1, 1-5, 6-10 and 11-16 years). RESULTS: 85 patients received an equivalent mean UFH dose with a median duration of 3 days. Antithrombin levels were decreased compared to age-related norms in children up to 11 years of age. APTT results were comparable across the age-groups. The Anti-Xa results using two different assays showed a trend for lower values in younger children. All children less than one year old recorded Anti-Xa values outside the therapeutic range for heparin therapy, for both assays. There was a trend for decreased Anti-IIa activity in younger children. Endogenous Thrombin Potential showed a significant trend for increased inhibition in older children. In vitro Antithrombin supplementation did not change the Anti-Xa or thrombin generation. CONCLUSIONS: This study confirms that, in vivo, for the same dose of UFH, the anti Xa and anti IIa effect, as well as the inhibition of endogenous thrombin potential is age dependent and that these differences are not purely AT dependent. The implication is that the anticoagulant and antithrombotic effect of a given dose of UFH differs with age. Clinical outcome studies to determine the optimal dosing for each age group are warranted
SN - 0049-3848
AD - Department of Pathology, The University of Melbourne, Parkville, VIC 3010. fiona.newall@rch.org.au
UR - 18829072
ER -

TY - JOUR
ID - 944
T1 - Social and environmental factors associated with preschoolers' nonsedentary physical activity
A1 - Brown,W.H.
A1 - Pfeiffer,K.A.
A1 - McIver,K.L.
A1 - Dowda,M.
A1 - Addy,C.L.
A1 - Pate,R.R.
Y1 - 2009/01//
N1 - Brown, William H. Pfeiffer, Karin A. McIver, Kerry L. Dowda, Marsha. Addy, Cheryl L. Pate, Russell R
Child development
0372725, d28
IM
Journal Article. Research Support, N.I.H., Extramural
English
KW - MEDLINE
KW - Child Day Care Centers
KW - Child
KW - Preschool
KW - Early Intervention (Education)
KW - Female
KW - Humans
KW - Male
KW - Motor Activity
KW - Peer Group
KW - Play and Playthings
KW - Schools
KW - Nursery
KW - Social Environment
KW - Social Identification
RP - NOT IN FILE
SP - 45
EP - 58
JF - Child Development
JA - Child Dev
VL - 80
IS - 1
CY - United States

N2 - The twofold purposes of the investigation were (a) to describe with direct observation data the physical activity behaviors and the accompanying social and environmental events of those behaviors for children in preschools and (b) to determine which contextual conditions were predictors of moderate to vigorous physical activity (MVPA) and nonsedentary physical activity (i.e., light activity + MVPA) for 3-, 4-, and 5-year-old children during their outdoor play. The results indicate that preschoolers' physical activity is characterized as sedentary in nature throughout their preschool day (i.e., 89% sedentary, 8% light activity, and 3% MVPA). During outdoor play periods, when children are most likely to be physically active, some contextual and social circumstances better predict their physical activity. Implications for policy makers, practitioners, and researchers are discussed
SN - 1467-8624
AD - Department of Educational Studies, University of South Carolina, Columbia, SC 29208, USA. bbrown@sc.edu
UR - 19236392
ER -

TY - JOUR
ID - 945
T1 - Joint drumming: social context facilitates synchronization in preschool children
A1 - Kirschner,S.
A1 - Tomasello,M.
Y1 - 2009/03//
N1 - Kirschner, Sebastian. Tomasello, Michael
Journal of experimental child psychology
2985128r, i2n
IM
Journal Article. Research Support, Non-U.S. Gov't
English
KW - MEDLINE
KW - Acoustics
KW - Arousal
KW - Child
KW - Preschool
KW - Cooperative Behavior
KW - Female
KW - Humans
KW - Male
KW - Movement
KW - Music
KW - Psychomotor Performance/ph [Physiology]
KW - Social Environment
RP - NOT IN FILE
SP - 299
EP - 314
JF - Journal of Experimental Child Psychology
JA - J Exp Child Psychol
VL - 102
IS - 3
CY - United States
N2 - The human capacity to synchronize body movements to an external acoustic beat enables uniquely human behaviors such as music making and dancing. By hypothesis, these first evolved in human cultures as fundamentally social activities. We therefore hypothesized that children would spontaneously synchronize their body movements to an external beat at earlier ages and with higher accuracy if the stimulus was presented in a social context. A total of 36 children in three age groups (2.5, 3.5, and 4.5 years) were invited to drum along with either a human partner, a drumming machine, or a drum sound coming from a speaker. When drumming with a social partner, children as young as 2.5 years adjusted their drumming tempo to a beat outside the range of their spontaneous motor tempo. Moreover, children of all ages synchronized their drumming with higher accuracy in the social condition. We argue that drumming together with a social partner creates a shared

representation of the joint action task and/or elicits a specific human motivation to synchronize movements during joint rhythmic activity
SN - 1096-0457
AD - Department of Developmental and Comparative Psychology, Max Planck Institute for Evolutionary Anthropology, Leipzig, Germany. kirschner@eva.mpg.de
UR - 18789454
ER -

TY - JOUR
ID - 946
T1 - Outdoor air pollution, genetic susceptibility, and asthma management: opportunities for intervention to reduce the burden of asthma
A1 - Gilliland,F.D.
Y1 - 2009/03//
N1 - Gilliland, Frank D
Pediatrics
oxv, 0376422
AIM, IM
Journal Article. Research Support, N.I.H., Extramural. Research Support, Non-U.S. Gov't. Research Support, U.S. Gov't, Non-P.H.S.
English
KW - MEDLINE
KW - Absenteeism
KW - Air Pollution/ae [Adverse Effects]
KW - Air Pollution/an [Analysis]
KW - Asthma/ep [Epidemiology]
KW - Asthma/et [Etiology]
KW - Asthma/pc [Prevention & Control]
KW - Bronchitis/ep [Epidemiology]
KW - Bronchitis/et [Etiology]
KW - California/ep [Epidemiology]
KW - Child
KW - Emergency Service
KW - Hospital/ut [Utilization]
KW - Environmental Exposure/ae [Adverse Effects]
KW - Environmental Exposure/pc [Prevention & Control]
KW - Genetic Predisposition to Disease
KW - Hospitalization
KW - Humans
KW - Nitrogen Dioxide/to [Toxicity]
KW - Ozone/to [Toxicity]
KW - Particle Size
KW - Public Health
KW - Respiratory Sounds
KW - Urban Health
KW - Vehicle Emissions
RP - NOT IN FILE
SP - S168
EP - S173
JF - Pediatrics
VL - 123 Suppl 3
CY - United States
N2 - Outdoor air pollution at levels occurring in many urban areas around the world has substantial adverse effects on health. Children in general, and children with asthma in particular, are sensitive to the adverse effects of outdoor air pollutants, including ozone, nitrogen oxides, and respirable particulate matter. A growing number of studies also show that children living in environments near traffic have increased risks of new-onset asthma, asthma symptoms, exacerbations, school absences, and asthma-related hospitalizations. The large population of

children exposed to high levels of outdoor air pollutants and the substantial risks for adverse health effects present unexploited opportunities to reduce the burden of asthma. Because the evidence indicates significant adverse effects of air pollution at current levels, there is clearly a need to reduce levels of regulated pollutants such as ozone, as well as unregulated pollutants in tailpipe emissions from motor vehicles. Achieving this long-term goal requires the active involvement of physicians and medical providers to ensure that the health of children is at the top of the list of competing priorities for regulatory policy decision-making. Clinical approaches include treatment to control asthma and patient education to reduce adverse effects of the disease. Reduction in exposures also can be approached at a policy level through changes in schools and school bus operations. Beyond clinical and public health approaches to reduce exposure, another strategy to be used before clean air goals are met is to decrease the susceptibility of children to air pollution. Emerging research indicates that dietary supplementation for individuals with low antioxidant levels is one promising approach to reducing susceptibility to air pollution. A second approach involves induction of enzymatic antioxidant defenses, especially for individuals with at-risk genetic variants of key antioxidant enzymes
SN - 1098-4275
AD - University of Southern California, Keck School of Medicine, Department of Preventive Medicine, 1540 Alcazar St, CHP 236, Los Angeles, CA 90033, USA. gillilan@hsc.usc.edu
UR - 19221160
ER -

TY - JOUR
ID - 947
T1 - Utilization and physical activity levels at renovated and unrenovated school playgrounds
A1 - Colabianchi,N.
A1 - Kinsella,A.E.
A1 - Coulton,C.J.
A1 - Moore,S.M.
Y1 - 2009/02//
N1 - Colabianchi, Natalie. Kinsella, Audrey E. Coulton, Claudia J. Moore, Shirley M
Preventive medicine
pm4, 0322116
IM
Comparative Study. Journal Article. Research Support, Non-U.S. Gov't
English
KW - MEDLINE
KW - Child
KW - Exercise
KW - Facility Design and Construction/sn [Statistics & Numerical Data]
KW - Female
KW - Humans
KW - Male
KW - Motor Activity
KW - Ohio
KW - Play and Playthings
KW - Regression Analysis
KW - Residence Characteristics
KW - Schools/sn [Statistics & Numerical Data]
KW - Sex Distribution
KW - Urban Population
RP - NOT IN FILE
SP - 140
EP - 143
JF - Preventive Medicine
JA - Prev Med
VL - 48
IS - 2
CY - United States

N2 - OBJECTIVE: This study examined utilization and physical activity levels at renovated compared to unrenovated school playgrounds. METHODS: Ten unrenovated and ten renovated school playgrounds (renovated at least a year prior) in Cleveland, OH were matched on school and neighborhood characteristics. Using direct observation (SOPLAY), the number of persons attending each playground and their physical activity levels were recorded using separate counts for girls, boys, men and women. Each school was observed ten times for 90 min each time outside of school hours in 2005. Paired t-tests, Wilcoxon Signed Ranks tests, and regression analyses were completed to examine differences across school pairs. RESULTS: More persons overall including adults and children utilized the renovated playgrounds compared to the unrenovated playgrounds. The proportion moderately-to-vigorously active was not different between renovated and unrenovated playgrounds although the proportion of children, in particular boys, who were vigorously active was greater at the renovated playgrounds. Although utilization was higher at the renovated playgrounds, absolute utilization was low across all playgrounds. CONCLUSIONS: This study suggests that playgrounds renovations may have the potential to increase the number of children utilizing the playground outside of school hours and may increase the proportion of children, especially boys, who are vigorously active
SN - 1096-0260
AD - University of South Carolina, Department of Epidemiology and Biostatistics, Columbia, SC 29208, USA. colabianchi@sc.edu
UR - 19063915
ER -

TY - JOUR
ID - 948
T1 - Unchanged thymidine triphosphate pools and thymidine metabolism in two lines of thymidine kinase 2-mutated fibroblasts
A1 - Frangini,M.
A1 - Rampazzo,C.
A1 - Franzolin,E.
A1 - Lara,M.C.
A1 - Vila,M.R.
A1 - Marti,R.
A1 - Bianchi,V.
Y1 - 2009/02//
N1 - Frangini, Miriam. Rampazzo, Chiara. Franzolin, Elisa. Lara, Mari-Carmen. Vila, Maya R. Marti, Ramon. Bianchi, Vera
The FEBS journal
101229646
IM
Journal Article. Research Support, Non-U.S. Gov't
English
KW - MEDLINE
KW - Adolescent
KW - Cells
KW - Cultured
KW - Child
KW - Cytosol/en [Enzymology]
KW - Deoxycytidine Kinase/me [Metabolism]
KW - Fibroblasts/me [Metabolism]
KW - Humans
KW - Mitochondria/en [Enzymology]
KW - Mitochondrial Proteins/ge [Genetics]
KW - Mitochondrial Proteins/me [Metabolism]
KW - Mutation
KW - Nucleotidases/me [Metabolism]
KW - Phosphorylation
KW - Protein Subunits/me [Metabolism]
KW - Ribonucleotide Reductases/me [Metabolism]
KW - Thymidine/me [Metabolism]

KW - Thymidine Kinase/ge [Genetics]
KW - Thymidine Kinase/me [Metabolism]
KW - Thymidine Phosphorylase/me [Metabolism]
KW - Thymine Nucleotides/me [Metabolism]
RP - NOT IN FILE
SP - 1104
EP - 1113
JF - FEBS Journal
JA - FEBS J
VL - 276
IS - 4
CY - England
N2 - Mitochondrial thymidine kinase (TK2) catalyzes the phosphorylation of thymidine in mitochondria. Its function becomes essential for dTTP synthesis in noncycling cells, where cytosolic dTTP synthesis via R1/R2 ribonucleotide reductase and thymidine kinase 1 is turned down. Mutations in the nuclear gene for TK2 cause a fatal mtDNA depletion syndrome. Only selected cell types are affected, suggesting that the other cells compensate for the TK2 deficiency by adapting the enzyme network that regulates dTTP synthesis outside S-phase. Here we looked for such metabolic adaptation in quiescent cultures of fibroblasts from two TK2-deficient patients with a slow-progressing syndrome. In cell extracts, we measured the activities of TK2, deoxycytidine kinase, thymidine phosphorylase, deoxynucleotidases and the amounts of the three ribonucleotide reductase subunits. Patient cells contained 40% or 5% TK2 activity and unchanged activities of the other enzymes. However, their mitochondrial and cytosolic dTTP pools were unchanged, and also the overall composition of the dNTP pools was normal. TK2-dependent phosphorylation of [(3)H]thymidine in intact cells and the turnover of the dTTP pool showed that even the fibroblasts with 5% residual TK2 activity synthesized dTTP at an almost normal rate. Normal fibroblasts apparently contain more TK2 than needed to maintain dTTP during quiescence, which would explain why TK2-mutated fibroblasts do not manifest mtDNA depletion despite their reduced TK2 activity
SN - 1742-4658
AD - Department of Biology, University of Padova, Italy
UR - 19154348
ER -

TY - JOUR
ID - 949
T1 - The analysis of count data: a gentle introduction to poisson regression and its alternatives
A1 - Coxe,S.
A1 - West,S.G.
A1 - Aiken,L.S.
Y1 - 2009/03//
N1 - Coxe, Stefany. West, Stephen G. Aiken, Leona S
Journal of personality assessment
jmz, 1260201
IM
Journal Article
English
KW - MEDLINE
KW - Aggression/px [Psychology]
KW - Aggression
KW - Child
KW - Child Behavior/px [Psychology]
KW - Child Behavior
KW - Data Interpretation
KW - Statistical
KW - Humans
KW - Models
KW - Poisson Distribution
KW - Psychometrics

KW - Regression Analysis
RP - NOT IN FILE
SP - 121
EP - 136
JF - Journal of Personality Assessment
JA - J Pers Assess
VL - 91
IS - 2
CY - United States
N2 - Count data reflect the number of occurrences of a behavior in a fixed period of time (e.g., number of aggressive acts by children during a playground period). In cases in which the outcome variable is a count with a low arithmetic mean (typically < 10), standard ordinary least squares regression may produce biased results. We provide an introduction to regression models that provide appropriate analyses for count data. We introduce standard Poisson regression with an example and discuss its interpretation. Two variants of Poisson regression, overdispersed Poisson regression and negative binomial regression, are introduced that may provide more optimal results when a key assumption of standard Poisson regression is violated. We also discuss the problems of excess zeros in which a subgroup of respondents who would never display the behavior are included in the sample and truncated zeros in which respondents who have a zero count are excluded by the sampling plan. We provide computer syntax for our illustrations in SAS and SPSS. The Poisson family of regression models provides improved and now easy to implement analyses of count data. [Supplementary materials are available for this article. Go to the publisher's online edition of Journal of Personality Assessment for the following free supplemental resources: the data set used to illustrate Poisson regression in this article, which is available in three formats-a text file, an SPSS database, or a SAS database.]
SN - 1532-7752
AD - Department of Psychology, Arizona State University, USA
UR - 19205933
ER -

TY - JOUR
ID - 950
T1 - Urban residence is associated with bronchial hyperresponsiveness in Italian general population samples
A1 - Maio,S.
A1 - Baldacci,S.
A1 - Carrozzi,L.
A1 - Polverino,E.
A1 - Angino,A.
A1 - Pistelli,F.
A1 - Di,Pede F.
A1 - Simoni,M.
A1 - Sherrill,D.
A1 - Viegi,G.
Y1 - 2009/02//
N1 - Maio, Sara. Baldacci, Sandra. Carrozzi, Laura. Polverino, Eva. Angino, Anna. Pistelli, Francesco. Di Pede, Francesco. Simoni, Marzia. Sherrill, Duane. Viegi, Giovanni
Chest
0231335, d1c
AIM, IM
Comparative Study. Journal Article. Research Support, Non-U.S. Gov't
English
KW - MEDLINE
KW - Adolescent
KW - Adult
KW - Age Distribution
KW - Aged
KW - Analysis of Variance
KW - Bronchial Hyperreactivity/di [Diagnosis]
KW - Bronchial Hyperreactivity/ep [Epidemiology]

KW - Bronchial Hyperreactivity/et [Etiology]
KW - Bronchial Provocation Tests
KW - Child
KW - Cross-Sectional Studies
KW - Environmental Pollution/ae [Adverse Effects]
KW - Female
KW - Humans
KW - Hypersensitivity/co [Complications]
KW - Incidence
KW - Italy/ep [Epidemiology]
KW - Logistic Models
KW - Male
KW - Methacholine Chloride/du [Diagnostic Use]
KW - Middle Aged
KW - Odds Ratio
KW - Probability
KW - Respiratory Function Tests
KW - Risk Factors
KW - Sampling Studies
KW - Severity of Illness Index
KW - Sex Distribution
KW - Skin Tests
KW - Smoking/ae [Adverse Effects]
KW - Urban Population/sn [Statistics & Numerical Data]
KW - Young Adult
RP - NOT IN FILE
SP - 434
EP - 441
JF - Chest
VL - 135
IS - 2
CY - United States
N2 - BACKGROUND: The role of different risk factors for bronchial hyperresponsiveness (BHR), such as gender, atopy, IgE, and environmental factors (smoking, occupational exposure, infections), has been described. Indoor and outdoor pollution play an important role too, but few studies have analyzed the association with BHR. The aim of this study was to assess the effect of urban residence on BHR. METHODS: We studied two general population samples enrolled in two cross-sectional epidemiological studies performed in Northern Italy (Po Delta, rural area) and Central Italy (Pisa, urban area). We analyzed 2,760 subjects (age range, 8 to 74 years). We performed analysis of variance and logistic regression analysis using ln slope of the dose-response curve of the methacholine challenge test as dependent variable, and sex, age, smoking habits, respiratory symptoms, skin-prick test results, IgE value, residence, and airway caliber as independent variables. RESULTS: The mean value of ln slope of the dose-response curve adjusted for initial airways caliber (by baseline FEV(1) percentage of predicted value) was significantly higher in female subjects, in smokers, in subjects with respiratory symptoms, in younger and older ages, in subjects with high values of IgE, and in subjects with positive skin-prick test results. After controlling for the independent effects of all these variables, living in urban area was an independent risk factor for having BHR (odds ratio, 1.41; 95% confidence interval, 1.13 to 1.76). CONCLUSION: Living in urban area is a risk factor for increased bronchial responsiveness
SN - 1931-3543
AD - Pulmonary Environmental Epidemiology Unit, Institute of Clinical Physiology, National Research Council, Pisa, Italy
UR - 18719062
ER -

TY - JOUR
ID - 951
T1 - Differences in patterns of participation between youths with cerebral palsy and typically developing peers
A1 - Engel-Yeger,B.

A1 - Jarus,T.
A1 - Anaby,D.
A1 - Law,M.
Y1 - 2009/01//
N1 - Engel-Yeger, Batya. Jarus, Tal. Anaby, Dana. Law, Mary
The American journal of occupational therapy : official publication of the American Occupational Therapy Association
7705978, 3o4
IM
Journal Article
English
KW - MEDLINE
KW - Activities of Daily Living
KW - Adolescent
KW - Case-Control Studies
KW - Cerebral Palsy/px [Psychology]
KW - Child
KW - Female
KW - Humans
KW - Interpersonal Relations
KW - Leisure Activities
KW - Male
KW - Sex Factors
RP - NOT IN FILE
SP - 96
EP - 104
JF - American Journal of Occupational Therapy
JA - Am J Occup Ther
VL - 63
IS - 1
CY - United States
N2 - OBJECTIVE: This study investigated the effects of cerebral palsy (CP) and gender on youth participation in activities outside of formal school. METHOD: Twenty-two participants with CP and 30 typically developing peers, ages 12-16 years, completed the Children's Assessment of Participation and Enjoyment (CAPE; King et al., 2004). RESULTS: Typically developing youths engaged in a broader range of activities and did so more frequently than did youths with CP. Similar levels of enjoyment in activity were found in both groups. In some scales of the CAPE, youths with CP participated in proportionally more activities alone and at home. Gender differences and Group x Gender interaction were found in some scales with respect to participation in and enjoyment of activities. CONCLUSIONS: Physical limitations associated with CP may affect the frequency of a child's participation in activity outside of school. However, youths with CP may express levels of enjoyment similar to those of typically developing peers while participating in activity
SN - 0272-9490
AD - Department of Occupational Therapy, Faculty of Social Welfare and Health Sciences, University of Haifa, Mount Carmel, Haifa, Israel 31950. batya@research.haifa.ac.il
UR - 19192732
ER -

TY - JOUR
ID - 952
T1 - Early impact of the federally mandated Local Wellness Policy on physical activity in rural, low-income elementary schools in Colorado
A1 - Belansky,E.S.
A1 - Cutforth,N.
A1 - Delong,E.
A1 - Ross,C.
A1 - Scarbro,S.
A1 - Gilbert,L.

A1 - Beatty,B.
A1 - Marshall,J.A.
Y1 - 2009///
N1 - Belansky, Elaine S. Cutforth, Nick. Delong, Erin. Ross, Courtney. Scarbro, Sharon. Gilbert, Lynn. Beatty, Bridget. Marshall, Julie A
Journal of public health policy
8006508, hs5
IM
Journal Article. Research Support, Non-U.S. Gov't. Research Support, U.S. Gov't, P.H.S.
English
KW - MEDLINE
KW - Child
KW - Colorado
KW - Federal Government
KW - Female
KW - Health Policy/lj [Legislation & Jurisprudence]
KW - Health Policy/td [Trends]
KW - Health Promotion/lj [Legislation & Jurisprudence]
KW - Health Promotion/sn [Statistics & Numerical Data]
KW - Humans
KW - Male
KW - Motor Activity
KW - Obesity/pc [Prevention & Control]
KW - Poverty/sn [Statistics & Numerical Data]
KW - Program Development
KW - Program Evaluation
KW - Rural Population/sn [Statistics & Numerical Data]
KW - Schools/sn [Statistics & Numerical Data]
KW - Social Marketing
KW - Socioeconomic Factors
KW - Time Factors
RP - NOT IN FILE
SP - S141
EP - S160
JF - Journal of Public Health Policy
JA - J Public Health Policy
VL - 30 Suppl 1
CY - England
N2 - The What's Working project described the initial impact of the United States' federally mandated Local Wellness Policy in rural, low-income elementary schools located in Colorado. Before and after the Local Wellness Policy mandate went into effect, a survey about school features related to nutrition and physical activity was sent to a random sample of 45 rural elementary schools (i.e., schools located outside of urban areas), in which at least 40% of students qualified for free or reduced-cost lunch. Overall, opportunities for physical activity did not change after the policy went into effect: although time in physical education increased by 14 min per week (P=0.10), time for recess decreased by roughly 19 min per week (P=0.10). Policies supporting student participation in physical education and recess (an unstructured time during school hours when students are allowed to play outside) did not change. The researchers coded Local Wellness Policies and found them to have weak wording that produced minimal impact. Content analysis of key informant interviews suggested several barriers to the impact of the Local Wellness Policies: (1) competing pressures facing school districts, (2) lack of resources devoted to the Local Wellness Policy, (3) principals' lack of knowledge about the policy, and (4) lack of accountability mechanisms to ensure policy implementation. Financial resources and more effective communication about Local Wellness Policies among school districts and principals are needed to elevate the importance of and increase opportunities for physical activity in rural, low-income Colorado elementary schools
SN - 0197-5897
AD - Rocky Moutain Prevention Research Center, Colorado School of Public Health, University of Colorado Denver, Anschutz Medical Campus, Denver, CO, USA. elaine.belansky@ucdenver.edu

UR - 19190570
ER -

TY - JOUR
ID - 953
T1 - Once bitten, twice shy? Medically-attended injuries can sensitise parents to children's risk of injuries on playgrounds
A1 - Morrongiello,B.A.
A1 - Howard,A.W.
A1 - Rothman,L.
A1 - Sandomierski,M.
Y1 - 2009/02//
N1 - Morrongiello, B A. Howard, A W. Rothman, L. Sandomierski, M
Injury prevention : journal of the International Society for Child and Adolescent Injury Prevention
crz, 9510056
IM
Journal Article
English
KW - MEDLINE
KW - Adaptation
KW - Psychological
KW - Analysis of Variance
KW - Attitude of Health Personnel
KW - Case-Control Studies
KW - Child
KW - Female
KW - Humans
KW - Male
KW - Parents/px [Psychology]
KW - Perception
KW - Play and Playthings
KW - Risk Assessment
KW - Risk Factors
KW - Wounds and Injuries/px [Psychology]
RP - NOT IN FILE
SP - 50
EP - 54
JF - Injury Prevention
JA - Inj Prev
VL - 15
IS - 1
CY - England
N2 - OBJECTIVE: To compare risk perceptions of parents whose child sustained a medically attended playground injury (cases) with those of parents whose child had not (controls) to address two questions. Does having a child experience a medically attended injury: (1) sensitise parents to children's injury vulnerability and severity; (2) influence parents' appraisal of the injury mechanism (child's behaviour), attributions for injuries or beliefs about strategies for prevention? METHOD: Each case-control parent dyad was assigned to one of two conditions: (1) being presented with 10 common injury-risk playground behaviours specific to the equipment on which their child had been hurt, and asked to appraise injury vulnerability and severity; or (2) being presented with scenarios about playground injuries that varied in severity but were all based on the same child behaviour, and asked questions about this behaviour, attributions for injury and strategies for prevention. RESULTS: The results support the occurrence of a sensitisation process. Compared with control parents, case parents showed higher ratings of injury severity and children's vulnerability to injury, made fewer attributions for injuries to bad luck, and endorsed a greater diversity of prevention strategies, including parent (closer supervision), child (teaching rules about safe play on playgrounds) and environmental (modifications to playgrounds). CONCLUSIONS: A child's medically attended injury can create a "teachable moment" for the parent. Linking injury-prevention programming to this teachable moment may increase the likelihood of evoking changes in

parental supervisory behaviour and their setting of rules limiting their child's risk behaviours to reduce the occurrence of childhood injury
SN - 1475-5785
AD - Psychology Department, University of Guelph, Guelph, Canada. bmorrong@uoguelph.ca
UR - 19190277
ER -

TY - JOUR
ID - 954
T1 - The contribution of childhood emotional abuse to teen dating violence among child protective services-involved youth
A1 - Wekerle,C.
A1 - Leung,E.
A1 - Wall,A.M.
A1 - MacMillan,H.
A1 - Boyle,M.
A1 - Trocme,N.
A1 - Waechter,R.
Y1 - 2009/01//
N1 - Wekerle, Christine. Leung, Eman. Wall, Anne-Marie. MacMillan, Harriet. Boyle, Michael. Trocme, Nico. Waechter, Randall
Child abuse & neglect
can, 7801702
IM
Journal Article. Research Support, Non-U.S. Gov't
English
KW - MEDLINE
KW - Adolescent
KW - Adolescent Behavior/px [Psychology]
KW - Child
KW - Child Abuse/px [Psychology]
KW - Child Welfare
KW - Courtship
KW - Female
KW - Humans
KW - Longitudinal Studies
KW - Male
KW - Stress Disorders
KW - Post-Traumatic/pp [Physiopathology]
KW - Violence
RP - NOT IN FILE
SP - 45
EP - 58
JF - Child Abuse & Neglect
JA - Child Abuse Negl
VL - 33
IS - 1
CY - England
N2 - OBJECTIVE: For child protective services (CPS) youth who may have experienced more than one form of maltreatment, the unique contribution of emotional abuse may be over-looked when other forms are more salient and more clearly outside of accepted social norms for parenting. This study considers the unique predictive value of childhood emotional abuse for understanding adolescent post-traumatic stress disorder (PTSD) symptomatology and dating violence. Further, PTSD symptomatology is assessed as an explanatory bridge in the emotional abuse-teen dating violence link. METHODS: A random sample of 402 youth from the active caseload of a large urban CPS catchment area participated as part of a larger longitudinal study on adolescent health behaviors. Mid-adolescent youth across types of CPS status were targeted. CPS youth reported on lifetime maltreatment experiences, PTSD symptomatology, and past year dating experiences, using published

scales. RESULTS: Over 85% of CPS youth had begun dating. For dating youth, some level of dating violence was common: over half of females (63-67%) and nearly half of males (44-49%). Taking into account other forms of maltreatment, emotional abuse emerged as a significant predictor of both PTSD symptomatology and dating violence among males and females. PTSD symptomatology was a significant mediator of the male emotional abuse-perpetration and the female emotional/physical abuse-victimization links, indicating a gendered patterning to findings. CONCLUSIONS: These results indicate that: (1) CPS youth are a high priority group for dating violence and PTSD-linked intervention; and (2) CPS youth continue to experience the unique negative impact of childhood emotional abuse in their adolescent adjustment. All CPS children should be evaluated for emotional abuse incurred, and appropriate intervention attention be given as to how it specifically impacts on the child's approach to relating to themselves and to others. PRACTICE IMPLICATIONS: The present study directs practice implications in regards to: (1) the problem of teen dating violence, (2) the salience of childhood emotional abuse; and (3) the importance of targeting PTSD symptomatolgy among CPS youth. A substantial number of CPS youth report early engagement in violent romantic relationships and require support towards attaining the non-coercive relationship experiences of their non-CPS-involved age mates. The topic of dating, healthy dating relationships, and dating violence may need to be part of the regular casework, with a view towards supporting youths' conceptualization of and skill set for healthy, close relationships. Further, this knowledge needs to be translated to foster parents and group home staff. With regard to the impact of childhood emotional abuse, CPS workers need to be sensitive to its potential for long-term, unique impact impairing relationship development. Emotional abuse is (a) unique among genders (i.e., for females, it clusters with physical abuse) and (b) uniquely predictive of PTSD symptoms and dating violence. Finally, as is consistent with theory and biopsychosocial evidence, PTSD symptomatology is a key causal candidate for understanding maltreatment-related impairment. Attention to targeting PTSD symptoms may be preventative for dating violence; attention to targeting emotional abuse experiences may be preventative for PTSD symptoms. CPS youth are an important population to involve in research, as their inclusion adds to the evidence-base to achieve evidence-informed practice and policy within child welfare
SN - 1873-7757
AD - Faculty of Education, Department of Psychology, Psychatry Program, University of Western Ontario, Ont., Canada
UR - 19167066
ER -

TY - JOUR
ID - 955
T1 - High prevalence of vitamin D deficiency in young children in a highly sunny humid country: a global health problem
A1 - Bener,A.
A1 - Al-Ali,M.
A1 - Hoffmann,G.F.
Y1 - 2009/02//
N1 - Bener, A. Al-Ali, M. Hoffmann, G F
Minerva pediatrica
nam, 0400740
IM
Journal Article. Research Support, Non-U.S. Gov't
English
KW - MEDLINE
KW - Adolescent
KW - Child
KW - Preschool
KW - Climate
KW - Cross-Sectional Studies
KW - Female
KW - Humans
KW - Humidity
KW - Male
KW - Prevalence
KW - Qatar/ep [Epidemiology]

KW - Sunlight
KW - Vitamin D Deficiency/ep [Epidemiology]
KW - World Health
RP - NOT IN FILE
SP - 15
EP - 22
JF - Minerva Pediatrica
JA - Minerva Pediatr
VL - 61
IS - 1
CY - Italy
N2 - AIM: Several studies indicate a high prevalence of vitamin D deficiency among young populations. Information about the vitamin D status in young adult populations from the Middle East is scarce. The vitamin D status can be expected to be influenced by highly different factors between various countries in Europe, the Middle East and Asia. The aim of this study was to determine the prevalence of vitamin D deficiency in young Qatari children below 16 years of age. METHODS: A cross-sectional study carried out among children below 16 years of age who visited the Primary Health Care Centers (PHCs). The survey was conducted over a period from August 2007 to March 2008. Qatari nationals, male and female, aged below 16 years. A random sample of 650 children who visited the PHC Centers was approached and parents of 458 children expressed their consent to participate in this study, corresponding to a response rate of 70.5%. Face-to-face interviews were based on a questionnaire that included variables such as socio-demographic information, life style, family history and feeding patterns during infancy, and clinical information as well as laboratory investigations for biochemical assessment of vitamin D status. RESULTS: Of the total number of 458 children surveyed, 228 (49.8%) were males and 230 (50.2%) females. The prevalence of vitamin D deficiency among the studied Qatari children was (68.8%), mostly in the age group (11-16) years (61.6%). There was a significant difference between vitamin D deficient and normal children as compared to their age (P=0.013). Vitamin D deficiency was more common among girls (51.4%) than boys (48.6%). Exposure to sunlight was limited in both groups; but even lower in vitamin D deficient children (57.5%) than in normal children (70.6%). The duration of time spent outside was again low in both groups but significantly lower in vitamin D deficient children (23.5 minutes) compared to normal children (28.4 minutes). Low duration of time spent outdoors, breast feeding less than 6 months, a family history of diabetes mellitus and physical activity were significant predictors for vitamin D deficiency in Qatari children. Rickets, fractures, gastroenteritis and delayed milestones were all significantly higher in vitamin D deficient children. CONCLUSIONS: The present study revealed that the prevalence of vitamin D deficiency is high in Qatari children and more common in Qatari girls. In the young population in Qatar, vitamin D deficiency appears to result from a combination of limitations in sunlight exposure and a low oral intake of vitamin D
SN - 0026-4946
AD - Department of Medical Statistics and Epidemiology, Hamad General Hospital Hamad Medical Corporation, Doha, Qatar. abener@hmc.org.qa
UR - 19179999
ER -

TY - JOUR
ID - 956
T1 - School recess and group classroom behavior
A1 - Barros,R.M.
A1 - Silver,E.J.
A1 - Stein,R.E.
Y1 - 2009/02//
N1 - Barros, Romina M. Silver, Ellen J. Stein, Ruth E K
Pediatrics
oxv, 0376422
AIM, IM
Journal Article
English
KW - MEDLINE
KW - Child

KW - Child Behavior
KW - Female
KW - Group Processes
KW - Humans
KW - Male
KW - Play and Playthings
KW - Time Factors
RP - NOT IN FILE
SP - 431
EP - 436
JF - Pediatrics
VL - 123
IS - 2
CY - United States
N2 - OBJECTIVES: This study examines the amount of recess that children 8 to 9 years of age receive in the United States and compares the group classroom behavior of children receiving daily recess with that of children not receiving daily recess. METHODS: This is a secondary analysis of a public-use data set, the Early Childhood Longitudinal Study, Kindergarten Class of 1998-1999, third-grade data set. Children were categorized into 2 levels of recess exposure, that is, none/minimal break (<1 break of 15 minutes/day) or some recess. Some recess was further categorized into 5 levels on the basis of frequency and duration of recess. Child, parent, school, and classroom characteristics of those with and without recess were compared. The group classroom behavior was assessed by using the teacher's rating of class behavior. RESULTS: Complete data were available for 10301 to 11624 children 8 to 9 years of age. There were equal numbers of boys and girls (boys: 50.3%). Children exposed to none/minimal break (30%) were much more likely to be black, to be from families with lower incomes and lower levels of education, to live in large cities, to be from the Northeast or South, and to attend public school, compared with those with recess. Teacher's rating of classroom behavior scores were better for children with some recess than for those with none/minimal break. This finding was maintained in multivariate regression analysis. However, among children receiving daily recess, the teacher's rating of class behavior scores did not differ significantly according to the level of exposure. CONCLUSIONS: These results indicated that, among 8- to 9-year-old children, having > or =1 daily recess period of >15 minutes in length was associated with better teacher's rating of class behavior scores. This study suggests that schoolchildren in this age group should be provided with daily recess
SN - 1098-4275
AD - Department of Pediatrics, Albert Einstein College of Medicine, Children's Hospital at Montefiore and Rose F. Kennedy Center, Bronx, New York, USA. romina_barros@hotmail.com
UR - 19171606
ER -

TY - JOUR
ID - 957
T1 - Policies and characteristics of the preschool environment and physical activity of young children
A1 - Dowda,M.
A1 - Brown,W.H.
A1 - McIver,K.L.
A1 - Pfeiffer,K.A.
A1 - O'Neill,J.R.
A1 - Addy,C.L.
A1 - Pate,R.R.
Y1 - 2009/02//
N1 - Dowda, Marsha. Brown, William H. McIver, Kerry L. Pfeiffer, Karin A. O'Neill, Jennifer R. Addy, Cheryl L. Pate, Russell R
Pediatrics
oxv, 0376422
AIM, IM
Journal Article. Research Support, N.I.H., Extramural
English
KW - MEDLINE

KW - Child
KW - Preschool
KW - Exercise
KW - Female
KW - Humans
KW - Male
KW - Motor Activity
KW - Organizational Policy
KW - Schools/st [Standards]
KW - Schools
RP - NOT IN FILE
SP - e261
EP - e266
JF - Pediatrics
VL - 123
IS - 2
CY - United States
N2 - OBJECTIVE: The purpose of this study was to examine policies and characteristics of preschools and the extent to which they influence the physical activity of 3- to 5-year-old children during the preschool day. METHODS: A total of 299 children from 20 preschools wore accelerometers for an average of 8.1 hours/day (SD: 1.5 hours/day), for 5.5 days (SD: 2.1 days). A researcher completed the Early Childhood Environment Rating Scale-Revised for each preschool to assess quality. Classrooms and playgrounds were measured, and the preschool director was interviewed about physical activity policies. For each policy or characteristic, preschools were divided into 2 groups on the basis of whether the characteristic/policy was presumed to promote or not promote physical activity. RESULTS: Children spent fewer minutes per hour in sedentary activity and more minutes per hour in moderate/vigorous physical activity in preschools that had higher quality scores, less fixed playground equipment, more portable playground equipment, lower use of electronic media, and larger playgrounds. Five preschools had all 5 of these characteristics, and children in those preschools had significantly more moderate/vigorous physical activity minutes per hour and fewer sedentary minutes per hour compared with children in the other preschools. CONCLUSION: Children in the top 5 physical activity-promoting preschools accumulated >60 minutes of moderate/vigorous physical activity per day, compared with the children in the other preschools, who accumulated <60 minutes of moderate/vigorous physical activity per day
SN - 1098-4275
AD - Departments of Exercise Science, University of South Carolina, Columbia, SC 29208, USA. mdowda@gwm.sc.edu
UR - 19171578
ER -

TY - JOUR
ID - 958
T1 - Association between school food environment and practices and body mass index of US public school children
A1 - Fox,M.K.
A1 - Dodd,A.H.
A1 - Wilson,A.
A1 - Gleason,P.M.
Y1 - 2009/02//
N1 - Fox, Mary Kay. Dodd, Allison Hedley. Wilson, Ander. Gleason, Philip M
Journal of the American Dietetic Association
h6f, 7503061
AIM, IM
Journal Article. Research Support, Non-U.S. Gov't. Research Support, U.S. Gov't, Non-P.H.S.
English
KW - MEDLINE
KW - Adolescent
KW - Beverages/ae [Adverse Effects]

KW - Body Mass Index
KW - Child
KW - Child Nutritional Physiological Phenomena/ph [Physiology]
KW - Preschool
KW - Cross-Sectional Studies
KW - Diet/st [Standards]
KW - Dietary Fats/ad [Administration & Dosage]
KW - Dietary Fats/ae [Adverse Effects]
KW - Energy Intake/ph [Physiology]
KW - Environment
KW - Female
KW - Food Dispensers
KW - Automatic/ut [Utilization]
KW - Food Services/st [Standards]
KW - Humans
KW - Male
KW - Nutritive Value
KW - Obesity/ep [Epidemiology]
KW - Obesity/pc [Prevention & Control]
KW - Schools
KW - Socioeconomic Factors
KW - United States/ep [Epidemiology]
RP - NOT IN FILE
SP - S108
EP - S117
JF - Journal of the American Dietetic Association
JA - J Am Diet Assoc
VL - 109
IS - 2 Suppl
CY - United States
N2 - BACKGROUND: With the ongoing interest in implementing school policies to address the problem of childhood obesity, there is a need for information about the relationships between school food environments and practices and children's weight status. OBJECTIVE: To examine the association between school food environments and practices and children's body mass index (BMI; calculated as kg/m(2)). DESIGN: The study used data from the third School Nutrition Dietary Assessment Study, a cross-sectional study that included a national sample of public school districts, schools, and children in the 2004-2005 school year. Data on school food environments and practices were collected through on-site observations and interviews with school principals, and children were weighed and measured by trained data collectors. SUBJECTS/SETTING: The study included 287 schools and 2,228 children in grades 1 through 12. STATISTICAL ANALYSES PERFORMED: Ordinary least squares regression was used to estimate the associations between school food environments and practices and BMI z scores and logistic regression was used to estimate associations between school food environments and practices and the likelihood of obesity (defined as BMI-for-age >or=95th percentile). Models included controls for sociodemographic characteristics of schools and children, to control for potential endogeneity of school environments and practices, as well as controls for children's dietary and physical activity behaviors outside of school. RESULTS: Among elementary school children, offering french fries and similar potato products in subsidized school meals more than once per week and offering dessert more than once per week were each associated with a significantly higher likelihood of obesity. Among middle school children, the availability of low-nutrient, energy-dense foods in vending machines in or near the foodservice area was associated with a higher BMI z score, and the availability of such foods for a la carte purchase in the cafeteria was associated with a lower BMI z score. CONCLUSIONS: Findings from this analysis suggest that limiting children's access to low-nutrient, energy-dense foods at school may hold promise as a tactic for reducing children's total calorie intake and controlling children's BMI
SN - 1878-3570
AD - Mathematica Policy Research, Inc, PO Box 2393, Princeton, NJ 08543-2393, USA. mfox@mathematica-mpr.com
UR - 19166665
ER -

TY - JOUR
ID - 959
T1 - Oscillating devices for airway clearance in people with cystic fibrosis. [Review] [83 refs]
A1 - Morrison,L.
A1 - Agnew,J.
Y1 - 2009///
N1 - Morrison, Lisa. Agnew, Jennifer
The Cochrane database of systematic reviews
100909747
IM
Journal Article. Review
English
KW - MEDLINE
KW - Adolescent
KW - Adult
KW - Breathing Exercises
KW - Child
KW - Cystic Fibrosis/co [Complications]
KW - Cystic Fibrosis/pp [Physiopathology]
KW - Forced Expiratory Volume
KW - Humans
KW - Lung Diseases
KW - Obstructive/et [Etiology]
KW - Obstructive/th [Therapy]
KW - Mucus/se [Secretion]
KW - Physical Therapy Modalities/is [Instrumentation]
KW - Randomized Controlled Trials as Topic
RP - NOT IN FILE
SP - CD006842
JF - Cochrane Database of Systematic Reviews
JA - Cochrane Database Syst Rev
IS - 1
CY - England
N2 - BACKGROUND: Chest physiotherapy is widely prescribed to assist the clearance of airway secretions in people with cystic fibrosis (CF). Oscillating devices generate intra- or extra-thoracic oscillations orally or external to the chest wall. Internally they create variable resistances within the airways, generating controlled oscillating positive pressure which mobilises mucus. Extra-thoracic oscillations are generated by forces outside the respiratory system, e.g. high frequency chest wall oscillation. OBJECTIVES: To determine the effectiveness and acceptability of oscillating devices compared to other forms of physiotherapy to improve respiratory function, mucus clearance and other outcomes in people with CF. SEARCH STRATEGY: We searched the Cochrane Cystic Fibrosis and Genetic Disorders Group Trials Register comprising references identified from comprehensive electronic database searches and hand searches of relevant journals and abstract books of conference proceedings. Most recent search of the Cystic Fibrosis Trials Register: November 2008. SELECTION CRITERIA: Randomised controlled studies and controlled clinical studies of oscillating devices compared with any other form of physiotherapy in people with CF. DATA COLLECTION AND ANALYSIS: Two authors independently applied the inclusion criteria to publications and assessed the quality of the included studies. MAIN RESULTS: Two hundred and sixty-five studies were identified; thirty studies (total of 708 participants) met the inclusion criteria. Single treatment interventions (therapy technique used only once in the comparison) were excluded. Studies varied in duration from up to one week to one year in duration. Nineteen of the studies were cross-over in design. Data were not published in sufficient detail in most of these studies to perform meta-analysis.Forced expiratory volume in one second (FEV(1)) was the most frequently measured outcome. Results did not show significant difference in effect between oscillating devices and other methods of airway clearance on FEV(1) or other lung function parameters. Where there has been a small but significant change in secondary outcome variables such as sputum volume or weight this has not been wholly in favour of oscillating devices. Participant satisfaction was reported in eleven studies, but this was not specifically in favour of an oscillating device as some participants preferred breathing techniques or techniques used prior to the study

interventions. The results for the remaining outcome measures were not examined or reported in sufficient detail to provide any high level evidence. AUTHORS' CONCLUSIONS: There was no clear evidence that oscillation was a more or less effective intervention overall than other forms of physiotherapy. More adequately-powered long-term randomised controlled trials are needed. [References: 83]
SN - 1469-493X
AD - West of Scotland Adult CF Unit, Gartnavel General Hospital, 1053 Great Western Road, Glasgow, UK, G12 0YN. Lisa.Morrison@ggc.scot.nhs.uk
UR - 19160305
ER -

TY - JOUR
ID - 960
T1 - Is there a relationship between physical fitness and academic achievement? Positive results from public school children in the northeastern United States
A1 - Chomitz,V.R.
A1 - Slining,M.M.
A1 - McGowan,R.J.
A1 - Mitchell,S.E.
A1 - Dawson,G.F.
A1 - Hacker,K.A.
Y1 - 2009/01//
N1 - Chomitz, Virginia R. Slining, Meghan M. McGowan, Robert J. Mitchell, Suzanne E. Dawson, Glen F. Hacker, Karen A
The Journal of school health
k13, 0376370
IM, N
Journal Article. Research Support, U.S. Gov't, Non-P.H.S.
English
KW - MEDLINE
KW - Adolescent
KW - Analysis of Variance
KW - Body Mass Index
KW - Child
KW - Educational Status
KW - Female
KW - Humans
KW - Logistic Models
KW - Male
KW - Massachusetts
KW - Physical Education and Training
KW - Physical Fitness
KW - Socioeconomic Factors
KW - Urban Health
RP - NOT IN FILE
SP - 30
EP - 37
JF - Journal of School Health
JA - J Sch Health
VL - 79
IS - 1
CY - United States
N2 - OBJECTIVES: To determine relationships between physical fitness and academic achievement in diverse, urban public school children. METHODS: This cross-sectional study used public school data from 2004 to 2005. Academic achievement was assessed as a passing score on Massachusetts Comprehensive Assessment System (MCAS) achievement tests in Mathematics (fourth, sixth, and eighth grade, n = 1103) and in English (fourth and seventh grade, n = 744). Fitness achievement was assessed as the number of physical fitness tests passed during physical education (PE). Multivariate logistic regression analyses were conducted to assess the

probability of passing the MCAS tests, controlling for students' weight status (BMI z score), ethnicity, gender, grade, and socioeconomic status (school lunch enrollment). RESULTS: The odds of passing both the MCAS Mathematics test and the MCAS English test increased as the number of fitness tests passed increased (p < .0001 and p < .05, respectively). CONCLUSIONS: Results show statistically significant relationships between fitness and academic achievement, though the direction of causation is not known. While more research is required, promoting fitness by increasing opportunities for physical activity during PE, recess, and out of school time may support academic achievement
SN - 1746-1561
AD - Institute for Community Health, Lecturer on Medicine, Harvard Medical School,Boston, MA 02141, USA. vchomitz@challiance.org
UR - 19149783
ER -

TY - JOUR
ID - 961
T1 - Overweight is more prevalent than stunting and is associated with socioeconomic status, maternal obesity, and a snacking dietary pattern in school children from Bogota, Colombia
A1 - McDonald,C.M.
A1 - Baylin,A.
A1 - Arsenault,J.E.
A1 - Mora-Plazas,M.
A1 - Villamor,E.
Y1 - 2009/02//
N1 - McDonald, Christine M. Baylin, Ana. Arsenault, Joanne E. Mora-Plazas, Mercedes. Villamor, Eduardo
The Journal of nutrition
jev, 0404243
IM
Journal Article. Research Support, N.I.H., Extramural. Research Support, Non-U.S. Gov't
English
KW - MEDLINE
KW - Body Height
KW - Child
KW - Preschool
KW - Colombia/ep [Epidemiology]
KW - Eating
KW - Female
KW - Humans
KW - Male
KW - Obesity/pp [Physiopathology]
KW - Overweight/ep [Epidemiology]
KW - Prevalence
KW - Questionnaires
KW - Social Class
RP - NOT IN FILE
SP - 370
EP - 376
JF - Journal of Nutrition
JA - J Nutr
VL - 139
IS - 2
CY - United States
N2 - The objectives of this study were to estimate the prevalence of overweight in school-aged children from Bogota, Colombia and to examine its associations with sociodemographic characteristics, dietary patterns, and indicators of physical activity. We measured height and weight in 3075 children between 5 and 12 y of age who attended public primary schools in 2006 and we obtained information on maternal sociodemographic and anthropometric characteristics. The survey was representative of children from low and middle socioeconomic backgrounds. The prevalences of child overweight (including obesity) and obesity according to the International

Obesity Task Force criteria were 11.1 and 1.8%, respectively. The prevalence of stunting was 9.8%. In multivariate analysis, child overweight was positively associated with indicators of higher socioeconomic status (SES), including low maternal parity and ownership of household assets. The prevalence of overweight was 3.6 times greater in children whose mothers were obese compared with children whose mothers had an adequate BMI (adjusted prevalence ratio = 3.61; 95% CI = 2.64, 4.93). Child overweight was positively associated with adherence to a "snacking" dietary pattern (P-trend = 0.06) and to frequent intake of hamburgers or hot dogs (adjusted prevalence ratio for at least once per week vs. never = 1.93; 95% CI = 1.03, 3.62), independent of total energy intake and other potential confounders. Time spent viewing television or playing outside the household were not significantly related to the prevalence of child overweight. In conclusion, child overweight in Bogota is more common than stunting and is associated with higher SES, maternal obesity, and a snacking dietary pattern
SN - 1541-6100
AD - Department of Nutrition and 4Department of Epidemiology, Harvard School of Public Health, Boston, MA 02115, USA
UR - 19106320
ER -

TY - JOUR
ID - 962
T1 - The impact of a new pediatric epilepsy diagnosis on parents: parenting stress and activity patterns
A1 - Modi,A.C.
Y1 - 2009/01//
N1 - Modi, Avani C
Epilepsy & behavior : E&B
100892858
IM
Journal Article
English
KW - MEDLINE
KW - Adaptation
KW - Psychological
KW - Adult
KW - Child
KW - Preschool
KW - Cues
KW - Epilepsy/px [Psychology]
KW - Family/px [Psychology]
KW - Humans
KW - Parenting/px [Psychology]
KW - Parents/px [Psychology]
KW - Recreation
KW - Socioeconomic Factors
KW - Stress
KW - Psychological/ep [Epidemiology]
KW - Psychological/px [Psychology]
RP - NOT IN FILE
SP - 237
EP - 242
JF - Epilepsy & Behavior
JA - Epilepsy Behav
VL - 14
IS - 1
CY - United States
N2 - The purpose of this study was to compare parenting stress and activity patterns in parents of children with new-onset epilepsy (NOE, n=30) and parents of children without epilepsy (controls, n=29). Participants completed parenting stress measures and Daily Phone Diaries, a cued-recall procedure to track parents through their activities over a 24-h period. No significant differences were found in parenting stress, with the exception of a higher proportion of parents of children with NOE experiencing life stress compared with parents of

controls (P<0.05). Results indicated parents of children with NOE spent more time in medical care (F=4.4, P<0.05) and less time in recreation outside of the home (F=5.6, P<0.05) compared with parents of controls. These findings have important implications for family adaptation to a new epilepsy diagnosis
SN - 1525-5069
AD - Division of Behavioral Medicine and Clinical Psychology, Center for the Promotion of Adherence and Self Management, MLC-7039, Cincinnati Children's Hospital Medical Center, Cincinnati, OH 45229, USA
UR - 18977459
ER -

TY - JOUR
ID - 963
T1 - Altered striatal activation predicting real-world positive affect in adolescent major depressive disorder
A1 - Forbes,E.E.
A1 - Hariri,A.R.
A1 - Martin,S.L.
A1 - Silk,J.S.
A1 - Moyles,D.L.
A1 - Fisher,P.M.
A1 - Brown,S.M.
A1 - Ryan,N.D.
A1 - Birmaher,B.
A1 - Axelson,D.A.
A1 - Dahl,R.E.
Y1 - 2009/01//
N1 - Forbes, Erika E. Hariri, Ahmad R. Martin, Samantha L. Silk, Jennifer S. Moyles, Donna L. Fisher, Patrick M. Brown, Sarah M. Ryan, Neal D. Birmaher, Boris. Axelson, David A. Dahl, Ronald E
The American journal of psychiatry
0370512, 3vg
AIM, IM
Journal Article. Research Support, N.I.H., Extramural. Research Support, Non-U.S. Gov't
English
KW - MEDLINE
KW - Adolescent
KW - Affect/ph [Physiology]
KW - Age Factors
KW - Caudate Nucleus/pp [Physiopathology]
KW - Child
KW - Computers
KW - Handheld
KW - Corpus Striatum/pp [Physiopathology]
KW - Depressive Disorder
KW - Major/di [Diagnosis]
KW - Major/pp [Physiopathology]
KW - Major/px [Psychology]
KW - Dominance
KW - Cerebral/ph [Physiology]
KW - Female
KW - Humans
KW - Image Processing
KW - Computer-Assisted
KW - Magnetic Resonance Imaging
KW - Male
KW - Motivation
KW - Neurons/ph [Physiology]
KW - Oxygen/bl [Blood]
KW - Prefrontal Cortex/pp [Physiopathology]
KW - Prognosis

KW - Reward
KW - Social Environment
RP - NOT IN FILE
SP - 64
EP - 73
JF - American Journal of Psychiatry
JA - Am J Psychiatry
VL - 166
IS - 1
CY - United States
N2 - OBJECTIVE: Alterations in reward-related brain function and phenomenological aspects of positive affect are increasingly examined in the development of major depressive disorder. The authors tested differences in reward-related brain function in healthy and depressed adolescents, and the authors examined direct links between reward-related brain function and positive mood that occurred in real-world contexts. METHOD: Fifteen adolescents with major depressive disorder and 28 adolescents with no history of psychiatric disorder, ages 8-17 years, completed a functional magnetic resonance imaging guessing task involving monetary reward. Participants also reported their subjective positive affect in natural environments during a 4-day cell-phone-based ecological momentary assessment. RESULTS: Adolescents with major depressive disorder exhibited less striatal response than healthy comparison adolescents during reward anticipation and reward outcome, but more response in dorsolateral and medial prefrontal cortex. Diminished activation in a caudate region associated with this depression group difference was correlated with lower subjective positive affect in natural environments, particularly within the depressed group. CONCLUSIONS: Results support models of altered reward processing and related positive affect in young people with major depressive disorder and indicate that depressed adolescents' brain response to monetary reward is related to their affective experience in natural environments. Additionally, these results suggest that reward-processing paradigms capture brain function relevant to real-world positive affect
SN - 1535-7228
AD - University of Pittsburgh School of Medicine, Western Psychiatric Institute and Clinic, 3811 O'Hara St., Loeffler 319, Pittsburgh, PA 15213, USA. forbese@upmc.edu
UR - 19047324
ER -

TY - JOUR
ID - 964
T1 - Analysis of 347 kindergarten-related injuries
A1 - Eberl,R.
A1 - Schalamon,J.
A1 - Singer,G.
A1 - Ainoedhofer,H.
A1 - Petnehazy,T.
A1 - Hoellwarth,M.E.
Y1 - 2009/02//
N1 - Eberl, Robert. Schalamon, Johannes. Singer, Georg. Ainoedhofer, Herwig. Petnehazy, Thomas. Hoellwarth, Michael E
European journal of pediatrics
end, 7603873
IM
Journal Article. Research Support, Non-U.S. Gov't
English
KW - MEDLINE
KW - Accidents/sn [Statistics & Numerical Data]
KW - Austria
KW - Causality
KW - Child Day Care Centers/sn [Statistics & Numerical Data]
KW - Child
KW - Preschool
KW - Circadian Rhythm

KW - Female
KW - Health Surveys
KW - Humans
KW - Male
KW - Play and Playthings
KW - Prospective Studies
KW - Risk Factors
KW - Seasons
KW - Sex Factors
KW - Wounds and Injuries/ep [Epidemiology]
KW - Wounds and Injuries/pc [Prevention & Control]
RP - NOT IN FILE
SP - 163
EP - 166
JF - European Journal of Pediatrics
JA - Eur J Pediatr
VL - 168
IS - 2
CY - Germany
N2 - While it has been shown that non-parental child care is associated with a reduced risk for unintentional injuries, a considerable number of accidents in kindergartens do result in severe injuries. We have collected data on the behavioral and environmental aspects of accidents that occurred in kindergartens in Austria with the aim of determining possible prevention measures. Included in the study were all kindergarten-related injuries (347) from among 21,582 pediatric trauma cases treated in a 22-month period in Graz, Austria. Kindergarten-related injuries that were treated during the same period at six participating hospitals located throughout Austria were used for comparison. A questionnaire was completed at first attendance, and additional information was achieved by interviews with the parents and kindergarten teachers. Only the 347 kindergarten accidents that occurred in Graz were analyzed in detail. Half of the injuries occurred in an outdoor environment (outside), even though most of the time was spent indoors. Boys were more frequently involved in accidents than girls (male:female=3:2). We identified seasonal and circadian differences, with most children being injured during the first 2 months of attendance (September and October), during the first 3 days of the week (Monday to Wednesday) and in the hour before and after lunch, respectively. Of the 347 accidents analyzed, 24% resulted in serious injury, and injuries occurring outdoors were more severe than those occurring indoors. Most parents felt that the accidents were unpreventable (47%), while 18% stated that improved supervision may have prevented the accident. Kindergarten accidents in Austria still result in a significant number of severe injuries. Kindergarten injuries were related to gender, season, time of day and location. Our results indicate the necessity of a continuous child safety training program that involves the participation of all teachers in day-care functions
SN - 1432-1076
AD - Department of Pediatric Surgery, Medical University of Graz, Auenbruggerplatz 34, 8036 Graz, Austria. robert.eberl@meduni-graz.at
UR - 18461361
ER -

TY - JOUR
ID - 965
T1 - A Bayesian approach to probabilistic risk assessment in municipal playgrounds
A1 - Iribarren,I.
A1 - Chacon,E.
A1 - De,Miguel E.
Y1 - 2009/01//
N1 - Iribarren, I. Chacon, E. De Miguel, E
Archives of environmental contamination and toxicology
6yd, 0357245
IM
Journal Article
English
KW - MEDLINE

KW - Arsenicals/ae [Adverse Effects]
KW - Bayes Theorem
KW - Child
KW - Preschool
KW - Environmental Exposure/ae [Adverse Effects]
KW - Environmental Exposure/sn [Statistics & Numerical Data]
KW - Environmental Monitoring/mt [Methods]
KW - Environmental Monitoring/sn [Statistics & Numerical Data]
KW - Environmental Pollutants/ae [Adverse Effects]
KW - Humans
KW - Mercury Compounds/ae [Adverse Effects]
KW - Play and Playthings
KW - Recreation
KW - Risk Assessment/mt [Methods]
KW - Risk Assessment/sn [Statistics & Numerical Data]
KW - Spain
RP - NOT IN FILE
SP - 165
EP - 172
JF - Archives of Environmental Contamination & Toxicology
JA - Arch Environ Contam Toxicol
VL - 56
IS - 1
CY - United States
N2 - A Bayesian risk assessment of the exposure of children up to 6 years of age to trace elements in the sandy substrate of municipal playgrounds in Madrid, Spain was carried out. As a first step, exposure variables and parameters were borrowed from US Environmental Protection Agency (EPA) databases. However, the use of these non-site-specific exposure data introduced a high arbitrariness in the assumed distributions, which, in the Bayes approach, translates into a high uncertainty for those distributions that is also reflected in the risk outcome. In order to reduce this uncertainty, site-specific values for children's body weight and exposure frequency were determined from three surveys of 75, 56, and 34 individuals, respectively, carried out in randomly selected playgrounds in Madrid. This information was used in a Bayesian approach to modify the prior distributions of exposure frequency and body weight adapted from the literature. As a result, the predictive distributions of risk values for arsenic and mercury presented a reduced arbitrariness and a significantly lower uncertainty than those obtained either from the prior distributions borrowed from the US EPA or from the limited data gathered in Madrid separately
SN - 1432-0703
AD - Grupo de Geoquimica Ambiental, E.T.S.I. Minas Madrid, Alenza 4, 28003, Madrid, Spain
UR - 18427709
ER -

TY - JOUR
ID - 966
T1 - Child care as an untapped setting for obesity prevention: state child care licensing regulations related to nutrition, physical activity, and media use for preschool-aged children in the United States
A1 - Kaphingst,K.M.
A1 - Story,M.
Y1 - 2009/01//
N1 - Kaphingst, Karen M. Story, Mary
Preventing chronic disease
101205018
IM
Journal Article
English
KW - MEDLINE
KW - Child Day Care Centers/lj [Legislation & Jurisprudence]
KW - Child Nutritional Physiological Phenomena

KW - Child
KW - Preschool
KW - Exercise
KW - Government Regulation
KW - Humans
KW - Licensure
KW - Mandatory Programs/lj [Legislation & Jurisprudence]
KW - Mandatory Programs/st [Standards]
KW - Mass Media
KW - Nutrition Policy/lj [Legislation & Jurisprudence]
KW - Obesity/pc [Prevention & Control]
KW - State Government
KW - United States
RP - NOT IN FILE
SP - A11
JF - Preventing Chronic Disease
JA - Prev Chronic Dis
VL - 6
IS - 1
CY - United States
N2 - INTRODUCTION: Child care is a potential setting for obesity prevention; 8.6 million preschool-aged children participated in child care in 2001. Each US state creates and enforces its own child care licensing regulations. We analyzed obesity-related child care licensing regulations of US states. METHODS: We downloaded state licensing regulations for children in child care centers (CCCs), small family child care homes (SFHs), and large family or group child care homes (LFGHs) in each state and the District of Columbia (collectively referred to as "states") in 2006 from national and state Web sites. We conducted a quantitative content analysis to identify 13 coding dimensions related to nutrition, physical activity, and media use. RESULTS: We found variability among and within states. CCCs were the most heavily regulated and had the most specific regulations, followed by LFGHs. SFHs had the fewest and most general regulations. Just 2 states, Michigan and West Virginia, specified that CCC menus should be consistent with the Dietary Guidelines for Americans. Only 12 states had regulations that limited foods of low nutritional value in CCCs. Thirty-six states required that children have daily outdoor activity time in CCCs; only 9 states set specific minimum lengths of time that children should be outdoors each day. Eight states set quantified time limits on screen time per day or per week in SFHs. CONCLUSION: Opportunities exist for strengthening state licensing regulations to prevent childhood obesity. The increasing prevalence of childhood obesity underscores the urgency for state policy efforts to create child care environments that foster healthful eating and participation in physical activity
SN - 1545-1151
AD - Division of Epidemiology and Community Health, School of Public Health, University of Minnesota, 1300 S 2nd St, Ste 300, Minneapolis, MN 55454, USA. kaph0004@umn.edu
UR - 19080017
ER -

TY - JOUR
ID - 967
T1 - Indoor air quality investigation according to age of the school buildings in Korea.[Erratum appears in J Environ Manage. 2009 Apr;90(5):1962]
A1 - Sohn,J.
A1 - Yang,W.
A1 - Kim,J.
A1 - Son,B.
A1 - Park,J.
Y1 - 2009/01//
N1 - Sohn, Jongryeul. Yang, Wonho. Kim, Jihwan. Son, Busoon. Park, Jinchul
Journal of environmental management
0401664, du5
IM

Journal Article. Research Support, Non-U.S. Gov't
English
KW - MEDLINE
KW - Adolescent
KW - Air/an [Analysis]
KW - Air Microbiology
KW - Air Pollution
KW - Indoor/an [Analysis]
KW - Bacteria/ip [Isolation & Purification]
KW - Child
KW - Preschool
KW - Computers
KW - Facility Design and Construction/st [Standards]
KW - Humans
KW - Humidity
KW - Korea
KW - Schools
KW - Seasons
KW - Temperature
RP - NOT IN FILE
SP - 348
EP - 354
JF - Journal of Environmental Management
JA - J environ manage
VL - 90
IS - 1
CY - England
N2 - Since the majority of schools are housed in buildings dating from the 1960s and 1970s, a comprehensive construction and renovation program of school buildings has been carried out to improve the educational conditions in Korea. However, classrooms and computer rooms, with pressed wood desks, chairs and furnishings, as well as construction materials, might have negative effects on the indoor air quality. Furthermore, most schools have naturally ventilated classrooms. The purpose of this study was to characterize the concentrations of different indoor air pollutants within Korean schools and to compare their indoor levels within schools according to the age of school buildings. Indoor and outdoor air samples of carbon monoxide (CO), carbon dioxide (CO(2)), particulate matter (PM(10)), total microbial count (TBC), total volatile organic compounds (TVOCs) and formaldehyde (HCHO) were obtained during summer, autumn and winter from three sites; a classroom, a laboratory and a computer classroom at 55 different schools. The selection of the schools was based on the number of years since the schools had been constructed. The problems causing indoor air pollution at the schools were chemicals emitted by building materials or furnishings, and insufficient ventilation rates. The I/O ratio for HCHO was 6.32 during the autumn, and the indoor HCHO concentrations (mean = 0.16 ppm) in schools constructed within 1 year were significantly higher than the Korean Indoor Air Standard, indicating that schools have indoor sources of HCHO. Therefore, increasing the ventilation rate by means of a mechanical system and the use of low-emission furnishings can play key roles in improving the indoor air quality within schools
SN - 0301-4797
AD - Department of Occupational Health, Catholic University of Daegu, Hayang-eup, Gyeongsan-si, Kyongbuk 712-702, South Korea. whyang@cu.ac.kr
UR - 18079045
ER -

TY - JOUR
ID - 968
T1 - Assessment of the current status of playground safety in the midwestern region of Turkey: an effort to provide a safe environment for children
A1 - Uskun,E.
A1 - Kisioglu,A.N.
A1 - Altay,T.

A1 - Cikinlar,R.
A1 - Kocakaya,A.
Y1 - 2008/11//
N1 - Uskun, Ersin. Kisioglu, Ahmet Nesimi. Altay, Tulin. Cikinlar, Rengul. Kocakaya, Asuman
The Turkish journal of pediatrics
wkv, 0417505
IM
Journal Article
English
KW - MEDLINE
KW - Accident Prevention
KW - Child
KW - Preschool
KW - Equipment Design
KW - Equipment Safety
KW - Guideline Adherence
KW - Humans
KW - Play and Playthings
KW - Safety
KW - Turkey
KW - Wounds and Injuries/pc [Prevention & Control]
RP - NOT IN FILE
SP - 559
EP - 565
JF - Turkish Journal of Pediatrics
JA - Turk J Pediatr
VL - 50
IS - 6
CY - Turkey
N2 - This study aimed to identify and evaluate the degree of conformity to the playground standards and the level of compliance with current safety specifications of the playgrounds in the midwestern region of Turkey. An observational technique was used at a total of 57 public playgrounds. A playground safety control form was prepared based on the United States National Program for Playground Safety and the Consumer Product Safety Commission security standards, since there is no national law covering playground equipment and safety in Turkey. The study evaluated the surroundings of the playground, arrangement of equipment in the playground, and characteristics of the equipment. The percentage of playgrounds surveyed with inadequate or hard surfacing was 80.7%. Fifty-two percent of the equipment was found to be inappropriate. Equipment was higher than the recommended heights. The results of our study unfortunately point out that playgrounds for children do not meet many of the safety criteria
SN - 0041-4301
AD - Department of Public Health, Faculty of Medicine, Suleyman Demirel University, Isparta, Turkey
UR - 19227420
ER -

TY - JOUR
ID - 969
T1 - [Influence of smoking cigarettes on the development of the child and its health condition]. [Polish]
A1 - Pirogowicz,I.
A1 - Joniec,L.
A1 - Guzikowski,W.
A1 - Gwiazda,E.
Y1 - 2008///
N1 - Pirogowicz, Iwona. Joniec, Lucja. Guzikowski, Wojciech. Gwiazda, Elzbieta
Przeglad lekarski
q96, 19840720r
IM

English Abstract. Journal Article
Polish
KW - MEDLINE
KW - Adult
KW - Anti-Bacterial Agents/tu [Therapeutic Use]
KW - Causality
KW - Child
KW - Preschool
KW - Comorbidity
KW - Environmental Exposure/sn [Statistics & Numerical Data]
KW - Female
KW - Humans
KW - Parent-Child Relations
KW - Poland/ep [Epidemiology]
KW - Questionnaires
KW - Respiratory Tract Diseases/dt [Drug Therapy]
KW - Respiratory Tract Diseases/ep [Epidemiology]
KW - Smoking/ep [Epidemiology]
KW - Tobacco Smoke Pollution/sn [Statistics & Numerical Data]
RP - NOT IN FILE
SP - 427
EP - 431
JF - Przeglad Lekarski
JA - Przegl Lek
VL - 65
IS - 10
CY - Poland
N2 - 35.5% of Polish society is active smokers and as many as 20% of pregnant women. Influence of passive smoking on fetuses and children is bigger than active smoking on adults. The aim of this scrutiny was to develop relationship between preschool children passive smoking and their health condition. Research was made with the use of 100 anonymous questionnaires among parents of children from nursery schools in Opole. We received the following results: active smokers lived in almost half of examined families (44%), the most numerous group of smokers was parents (80.5%). The most often (43.3%) children were exposed on 20 cigarettes per day. 40.9% parents smoked outside, on the balcony, however the rest of them inside, in various rooms of the house. Most of parents thought that the health condition of their children was good, but more smokers than non-smokers estimated it as bad (11.4% vs. 1.8%). To the question how often are their children sick the most parents in both groups answered that 1-3 times per year. Most frequent illnesses concerned children of smokers and it was mainly respiratory tract diseases. Antibiotics intake was more often among children of smokers than non-smokers. Relationship between their children health condition and cigarette smoking confirmed 90% of parents. Denied it 10% and all of them were smokers. It indicates the need of better education of both, parents and children, in the field of harmful effects of passive smoking on health
SN - 0033-2240
AD - Katedra i Zaklad Medycyny Rodzinnej, Akademia Medyczna we Wroclawiu. iwapir@wp.pl
UR - 19189516
ER -

TY - JOUR
ID - 970
T1 - Association between early childhood caries and behavior as measured by the Child Behavior Checklist
A1 - Williamson,R.
A1 - Oueis,H.
A1 - Casamassimo,P.S.
A1 - Thikkurissy,S.
Y1 - 2008/11//
N1 - Williamson, Robert. Oueis, Hassan. Casamassimo, Paul S. Thikkurissy, Sarat
Pediatric dentistry
pan, 7909102

D, IM
Journal Article. Multicenter Study
English
KW - MEDLINE
KW - Age Factors
KW - Child Behavior/px [Psychology]
KW - Child Behavior Disorders/co [Complications]
KW - Child Behavior Disorders/di [Diagnosis]
KW - Child Behavior Disorders/px [Psychology]
KW - Child
KW - Preschool
KW - Dental Caries/co [Complications]
KW - Dental Caries/px [Psychology]
KW - Female
KW - Humans
KW - Male
KW - Personality Assessment
KW - Psychological Tests
RP - NOT IN FILE
SP - 505
EP - 509
JF - Pediatric Dentistry
JA - Pediatr Dent
VL - 30
IS - 6
CY - United States
N2 - PURPOSE: This study's purpose was to use a standardized behavioral assessment instrument to compare the observed behavior of caries-free (CF) with caries-active (CA) children requiring restoration under general anesthesia (GA). METHODS: Healthy 30- to 60-month-olds, either CF or in need of dental restoration under GA, were selected in 4 equal-size cohorts of 30 subjects from both private practice settings and a clinic. Trained assistants administered the child behavior checklist, a standardized, age-appropriate instrument to assess child behavior problems to parents at the time of appointment. RESULTS: The 60 CF and 60 CA subjects did not differ significantly (P<.05) between clinic and private practice for age, gender, and race and were collapsed into groups of 60 CF and 60 CA children (mean ages=41.2 and 45.2 months, respectively). Compared to CF children, CA children had significantly higher (more problems) scores for categories of: anxious/depressed; sleep problems; aggressive behavior; externalizing and total problems; and attention deficit/hyperactivity problems. CA children scores for withdrawn, sleep problems, and attention deficit/hyperactivity problems were also higher than normal, but neither group had scores outside normative ranges. CONCLUSIONS: No significant age, gender, behavior, or race differences were noted for site of care, but caries-active children had significantly more behavior problems than caries-free children
SN - 0164-1263
AD - Department of Pediatric Dentistry, University of Detroit Mercy Dental School, Detroit, Mich, USA
UR - 19186777
ER -

TY - JOUR
ID - 971
T1 - European perspectives on pediatric formulations
A1 - Breitkreutz,J.
Y1 - 2008/11//
N1 - Breitkreutz, Jorg
Clinical therapeutics
cpe, 7706726
IM
Journal Article. Research Support, Non-U.S. Gov't
English
KW - MEDLINE

KW - Chemistry
KW - Pharmaceutical/lj [Legislation & Jurisprudence]
KW - Pharmaceutical/mt [Methods]
KW - Pharmaceutical/td [Trends]
KW - Child
KW - European Union
KW - Guidelines as Topic
KW - Humans
KW - Pediatrics/mt [Methods]
KW - Pediatrics/st [Standards]
KW - Pediatrics/td [Trends]
RP - NOT IN FILE
SP - 2146
EP - 2154
JF - Clinical Therapeutics
JA - Clin Ther
VL - 30
IS - 11
CY - United States
N2 - BACKGROUND: The 2007 European Union (EU) regulation on medicinal products for pediatric use may change the present unsatisfying situation in the EU by stimulating research and development of medicines for use in children through rewards and incentives. OBJECTIVES: This commentary reflects on the new EU regulations and guidelines, with special attention paid to the impact on pediatric formulation science. The focus of this article is on the EU perspective for pediatric formulations and highlights the differences compared with the pediatric drug formulation situation in the United States. METHODS: Materials for this article were gathered during a literature search of MEDLINE and Chemical Abstracts (1970-October 2008) using the following terms: paediatric/pediatric drug formulations, age-appropriate dosage forms, child-appropriate medicines, and paediatric/pediatric regulation. RESULTS: Since the EU legislation on medicines for children came into force in 2007, a great emphasis has been placed on creating new organizations, scientific networks, and programs dealing with pediatric medicines and child-appropriate drug formulations. Although the US legislation was an appropriate model, the EU introduced some novel measures to improve the current situation, such as the Paediatric Investigation Plan and the Paediatric Use Marketing Authorisation. For globally operating pharmaceutical companies, the peculiarities of the European market have a strong impact on their product development strategies. Because the European approach demands early investigations into drug formulations for children, various issues must be resolved, including the following: choosing formulations for each age group, determining which excipients may be used in the formulation and which delivery device is appropriate, and predicting the taste sensation of an oral formulation. Numerous initiatives and networks are evolving in Europe. An important future task will be the coordination of these activities and the linking to other groups working on pediatric formulations outside the EU. CONCLUSION: Similar to the research in pediatric drug formulations that was stimulated by the US legislation and incentives of the last decade, the 2007 EU legislation promises improvements in the availability of child-appropriate drugs in Europe
SN - 0149-2918
AD - Institute of Pharmaceutics and Biopharmaceutics, Heinrich Heine University, Dusseldorf Germany. joerg.breitkreutz@uni-dusseldorf.de
UR - 19108802
ER -

TY - JOUR
ID - 972
T1 - [A national epidemiological survey on obesity of children under 7 years of age in nine cities of China, 2006]. [Chinese]
A1 - Coordinating Group of Nine Cities Study on the Physical Growth and Development of Children,Capital Institute of Pediatrics
A1 - Li,H.
Y1 - 2008/03//
N1 - Coordinating Group of Nine Cities Study on the Physical Growth and Development of Children, Capital Institute of Pediatrics. Li, Hui

Zhonghua er ke za zhi. Chinese journal of pediatrics
0417427
IM
English Abstract. Journal Article. Research Support, Non-U.S. Gov't
Chinese
KW - MEDLINE
KW - Child
KW - Preschool
KW - China/ep [Epidemiology]
KW - Female
KW - Humans
KW - Infant
KW - Newborn
KW - Male
KW - Obesity/ep [Epidemiology]
KW - Prevalence
KW - Risk Factors
KW - Sampling Studies
KW - Urban Population
RP - NOT IN FILE
SP - 174
EP - 178
JF - Zhonghua Erke Zazhi
JA - Zhonghua Er Ke Za Zhi
VL - 46
IS - 3
CY - China
N2 - OBJECTIVE: To examine the epidemiological features of prevalence and influencing factors on obesity of Chinese children from 1 month to 7 years of age in nine cities. METHODS: Stratified-clustered-random sampling was used, and the sampling size was 112 945 persons. Overweight and obesity were defined by the weight for height of NCSH/WHO reference, being over 10% as overweight and over 20% as obesity. The normal children were matched with obese children by region, gender, age and height in the case-control designs,and the data were analyzed by multiple conditional Logistic regression model. RESULTS: (1) The overall prevalence of overweight was 6.25%, 6.59% for boys and 5.88% for girls. The overall prevalence of obesity was 3.19%, 3.82% for boys and 2.48% for girls. The prevalence of obesity in different age groups was significantly different, 1.86% for 1 month to 1 year old, lower for 1 to 3 years old, higher again after 3 years of age and reached 7.02% for 6 - 7 years of age. There was a significant regional difference in prevalence, which in the central (3.97%) and northern (3.21%) regions was higher than that in the south (2.47%). Most of the obese children belonged to mild obesity. Severe obesity occurred mainly after 3 years of age, and the prevalence of severe obesity for boys was significantly higher than the girls, in the northern part it was higher than those in the other two parts. (2) As compared with the survey data in 1986 and 1996, the prevalence of obesity increased by 2.78 times in the past 20 years, and the annual incremental rate was 6.9%. The prevalence of obesity increased rapidly after 4 years of age and significant gender difference was seen. The higher prevalence shifted from the north to the central part of China. (3) Results of the Logistic regression indicated that appetite, eating speed, high maternal and paternal BMI, child caretaker, mode of delivery, main style of the outdoor activities, hours of watching TV, birth weight and duration of night time sleep were significantly related to obesity. CONCLUSION: (1) During the past 20 years the prevalence of child obesity showed a fast increasing trend in China. Degrees of the increasing trend differed across gender and age groups and regions. Effective strategies for intervention on population should be stipulated. (2) Good appetite, fast eating, high BMI of parents, caesarean birth, high birth-weight and excess hours of watching TV were possibly associated with increased risk of obesity in childhood, and those children looked after by their parents, medium intensity outdoor exercise and sufficient night sleep were associated with decreased risk of obesity
SN - 0578-1310
AD - Department of Growth and Development, Capital Institute of Pediatrics, Beijing 100020, China. Email: huiligrowth@163.com
UR - 19099703
ER -

TY - JOUR
ID - 973
T1 - Neighborhood characteristics in relation to diet, physical activity and overweight of Canadian children
A1 - Veugelers,P.
A1 - Sithole,F.
A1 - Zhang,S.
A1 - Muhajarine,N.
Y1 - 2008///
N1 - Veugelers, Paul. Sithole, Fortune. Zhang, Sharon. Muhajarine, Nazeem
International journal of pediatric obesity : IJPO : an official journal of the International Association for the Study of Obesity
101256330
IM
Journal Article. Research Support, Non-U.S. Gov't
English
KW - MEDLINE
KW - Child
KW - Crime
KW - Cross-Sectional Studies
KW - Diet
KW - Exercise
KW - Health Behavior
KW - Health Surveys
KW - Humans
KW - Life Style
KW - Nova Scotia
KW - Odds Ratio
KW - Overweight/et [Etiology]
KW - Overweight/pp [Physiopathology]
KW - Overweight/pc [Prevention & Control]
KW - Play and Playthings
KW - Public Facilities
KW - Questionnaires
KW - Residence Characteristics
KW - Risk Assessment
KW - Risk Factors
KW - Socioeconomic Factors
KW - Sports
KW - Time Factors
RP - NOT IN FILE
SP - 152
EP - 159
JF - International Journal of Pediatric Obesity
JA - Int J Pediatr Obes
VL - 3
IS - 3
CY - England
N2 - BACKGROUND: Neighborhood infrastructure may provide an important opportunity to prevent overweight among children. In the present study we investigated whether access to shops for modestly priced fresh produce, access to parks and playgrounds, access to recreational facilities and neighborhood safety are related to children's diet, physical and sedentary activities, and body weights. METHODS: Data were obtained from the Children's Lifestyle and School-performance Study, a survey including 5,471 grade five students and their parents in the province of Nova Scotia, Canada. Students completed the Harvard Food Frequency Questionnaire and had their height and weight measured. Parents completed questions on socio-economic background and how they perceived their neighborhood. We applied multilevel regression methods to relate these neighborhood characteristics with children's fruit and vegetable consumption, dietary fat intake, diet

quality, frequency of engaging in sports with and without a coach, screen time, overweight and obesity. RESULTS: Children in neighborhoods with greater perceived access to shops had healthier diets and were less likely to be overweight or obese. Children in neighborhoods with good access to playgrounds, parks and recreational facilities were reportedly more active and were less likely to be overweight or obese, whereas children in safe neighborhoods engaged more in unsupervised sports. CONCLUSIONS: The study demonstrated associations between neighborhood characteristics, health behaviors and childhood overweight. This contributes to the knowledge base that is still too narrow to justify informed preventative public health policy. We advocate the evaluation of natural experiments created by new policy that affect neighborhood infrastructures as the optimal opportunity to enlarge this knowledge base
SN - 1747-7166
AD - School of Public Health, University of Alberta, Alberta, Canada. paul.veugelers@ualberta.ca
UR - 19086297
ER -

TY - JOUR
ID - 974
T1 - [Survey on sports practice and physical activity of primary school children living in the area of Bologna Local Health Unit in relation with some individual and environmental variables]. [Italian]
A1 - Leoni,E.
A1 - Beltrami,P.
A1 - Poletti,G.
A1 - Baldi,E.
A1 - Sacchetti,R.
A1 - Garulli,A.
A1 - Masotti,A.
A1 - Bianco,L.
A1 - Ventura,F.A.
A1 - Pandolfi,P.
A1 - Guberti,E.
Y1 - 2008/09//
N1 - Leoni, E. Beltrami, P. Poletti, G. Baldi, E. Sacchetti, R. Garulli, A. Masotti, A. Bianco, L. Ventura, F A M. Pandolfi, P. Guberti, E
Annali di igiene : medicina preventiva e di comunita
9002865, and
IM
Comparative Study. English Abstract. Journal Article
Italian
KW - MEDLINE
KW - Age Factors
KW - Body Mass Index
KW - Child
KW - Data Interpretation
KW - Statistical
KW - Exercise/ph [Physiology]
KW - Female
KW - Humans
KW - Italy
KW - Life Style
KW - Male
KW - Obesity/ep [Epidemiology]
KW - Overweight/ep [Epidemiology]
KW - Parents
KW - Prevalence
KW - Questionnaires
KW - Sampling Studies
KW - Sex Factors
KW - Sports/sn [Statistics & Numerical Data]

RP - NOT IN FILE
SP - 441
EP - 453
JF - Annali di Igiene
JA - Ann Ig
VL - 20
IS - 5
CY - Italy
N2 - A randomized stratified sample of 522 children attending the third class of primary schools within the area of Bologna Local Health Unit was analysed for physical activity and sports practice. Information about the children's habits and availability of facilities for physical and sports activities were collected by means of structured questionnaires completed by children (507 respondents), parents (491), reference teachers for physical education (26) and class teachers (46) during the school year 2006-07. At the same time, the children's heights and weights were measured in order to calculate BMI values. Regular sports activity is practised by 80.1% of children (males: 82.4%, females: 77.6%), with significant diferences between genders only in children with at least one non-Italian parent (M>F, p < 0.05); the practice of sports is influenced by the area of residence (metropolitan > plain and hills, p < 0.05) and nationality (Italians > non-Italians, p < 0.01). Children with at least one actively sports practising parent are involved more frequently in sports activities (p < 0.001). In free time, sedentary activities are prevalent for both sports-practising children and not. However children not involved in regular sports activities tend to practise outdoor physical activities with a frequency significantly higher than children involved in sports (17.3% vs 10.4% of respondents). The percentage of completely sedentary children, who stated that they practise neither sports nor physical activity in their free time, is 7.3% (metropolitan area: 4.5%, hills: 8.7%, plain: 10.6%). The prevalence of overweight is 24.4%, of obesity 9.7%, with a better distribution of BMI values in the metropolitan area where there is the highest occurrence of positive conditions and behaviours: availability of sports facilities, the highest prevalence of sports practice, and the lowest prevalence of completely sedentary children
SN - 1120-9135
AD - Universita degli Studi di Bologna, Dipartimento di Medicina e Sanita Pubblica, Sezione di Igiene. erica.leoni@unibo.it
UR - 19069250
ER -

TY - JOUR
ID - 975
T1 - Risk factors for obesity development in school children from south-eastern Poland
A1 - Mazur,A.
A1 - Klimek,K.
A1 - Telega,G.
A1 - Hejda,G.
A1 - Wdowiak,L.
A1 - Malecka-Tendera,E.
Y1 - 2008/12//
N1 - Mazur, Artur. Klimek, Katarzyna. Telega, Grzegorz. Hejda, Grazyna. Wdowiak, Leszek. Malecka-Tendera, Ewa
Annals of agricultural and environmental medicine : AAEM
9500166
IM
Journal Article
English
KW - MEDLINE
KW - Adolescent
KW - Birth Weight/ph [Physiology]
KW - Body Mass Index
KW - Child
KW - Child Nutritional Physiological Phenomena
KW - Cross-Sectional Studies
KW - Energy Intake/ph [Physiology]

KW - Exercise/ph [Physiology]
KW - Family Characteristics
KW - Female
KW - Health Surveys
KW - Humans
KW - Male
KW - Obesity/ep [Epidemiology]
KW - Odds Ratio
KW - Parent-Child Relations
KW - Poland/ep [Epidemiology]
KW - Prevalence
KW - Risk Factors
KW - Schools
KW - Sex Factors
KW - Social Class
KW - Socioeconomic Factors
RP - NOT IN FILE
SP - 281
EP - 285
JF - Annals of Agricultural & Environmental Medicine
JA - Ann Agric Environ Med
VL - 15
IS - 2
CY - Poland
N2 - The aim of the study was to determine socio-economic and parental factors affecting odds for development of obesity in school-age children from south-eastern Poland (SEP). 2,182 boys and 2,066 girls from randomly selected elementary schools in SEP were involved in this study. The mean age of the girls was 10.4 years (SD 2.4, range 6.7-14.9). The mean age of the boys was 10.5 years (SD 2.3, range 6.9-14.9). 167 boys (7.7 % , 95 % CI : 6.6-8.8 %) and 208 girls (10.1 % , 95 % CI : 8.8-11.4 %) were obese. The difference in prevalence of obesity between genders was statistically significant. Socioeconomic risk factors (RF) were different from those in Western Europe or the United States. A small number of siblings was RF for obesity. Intact family had a protective effect. No correlation was found between child's obesity and parental education, income per capita or mother working outside the home. Parental obesity was RF for the obesity in children. High BMI at birth was an RF for obesity. A distinct pattern of socio-economic RF underlines the importance of population specific epidemiological studies. Defining RF in a specific region provides information to design specific preventive strategies
SN - 1232-1966
AD - Regional Hospital No. 2, Lwowska 60, Rzeszow. drmazur@poczta.onet.pl
UR - 19061264
ER -

TY - JOUR
ID - 976
T1 - [A personalised health care programme (PANDELAS) operating in Buenos Aires , Argentina , during 2006]. [Spanish]
A1 - Marin,G.
A1 - Silberman,M.
A1 - Etchegoyen,G.
Y1 - 2008/03//
N1 - Marin, Gustavo. Silberman, Martin. Etchegoyen, Graciela
Revista de salud publica (Bogota, Colombia)
100936348
IM
Comparative Study. English Abstract. Journal Article
Spanish
KW - MEDLINE
KW - Adolescent

KW - Adult
KW - Aged
KW - Argentina
KW - Child
KW - Preschool
KW - Consumer Participation
KW - Female
KW - Health Planning
KW - Health Services Accessibility
KW - Humans
KW - Male
KW - Middle Aged
KW - Personal Satisfaction
KW - Pregnancy
KW - Socioeconomic Factors
KW - Software
RP - NOT IN FILE
SP - 203
EP - 214
JF - Revista de Salud Publica
JA - Rev Salud Publica (Bogota)
VL - 10
IS - 2
CY - Colombia
N2 - OBJECTIVE: Identifying families having health risks using GIS technology to plan health care action which would include the whole community and not just that part of the population demanding attention. METHODS: 48,800 inhabitants from Ensenada county near Buenos Aires, Argentina were registered and questioned regarding socio-economic-sanitary aspects and their data was georeferenced to cadastral maps (using Arc-GIS-ESRI-2002 software). Health teams (HT) from each local health centre (LHC) were instructed in how to identify the population at risk and plan and carry out preventative health action using the software. Variables such as the quality of attention received, knowledge of the area and its inhabitants, the effectiveness of LHC action, the amount of extramural activities engaged in, community participation and the degree of satisfaction were evaluated pre- and post-PANDELAS. RESULTS: PANDELAS led to focusing attention on homes having structural disadvantages (i.e. the absence of drinking water or drains), vulnerable inhabitants having special characteristics or sick people suffering from a chronic pathology. Children aged <1 year, patients affected by chronic disease, the aged, pregnant women and people lacking routine controls (such as Papanicolau, etc) were georeferenced. Controls were increased by 300 % based on this information. LHC tripled their knowledge of the area for which they were responsible and the needs of their target population; outdoor community activities increased 6-fold and community participation increased from 0 ,1 % to 3,9 %. LHC satisfaction and that of the community with the work done was reflected in 89 % and 85 % favourable answers, respectively. CONCLUSIONS: PANDELAS brought greater LHC commitment towards their community, planning their activities on the basis of preventative action and increasing accessibility to the heath care system
SN - 0124-0064
AD - Ministerio de Salud de la Provincia de Buenos Aires, Argentina. gmarin@netverk.com.ar
UR - 19039417
ER -

TY - JOUR
ID - 977
T1 - [Value of a clinical test for assessing physical activity in children]. [Spanish]
A1 - Godard,M.C.
A1 - Rodriguez,N.Mdel
A1 - Diaz,N.
A1 - Lera,M.L.
A1 - Salazar,R.G.
A1 - Burrows,A.R.
Y1 - 2008/09//

N1 - Godard M, Claude. Rodriguez N, Maria del Pilar. Diaz, Nora. Lera M, Lydia. Salazar R, Gabriela. Burrows A, Raquel
Revista medica de Chile
shd, 0404312
IM
Comparative Study. English Abstract. Evaluation Studies. Journal Article. Research Support, Non-U.S. Gov't
Spanish
KW - MEDLINE
KW - Acceleration
KW - Adolescent
KW - Anthropometry
KW - Child
KW - Female
KW - Humans
KW - Leisure Activities
KW - Life Style
KW - Male
KW - Motor Activity/ph [Physiology]
KW - Obesity/pp [Physiopathology]
KW - Physical Fitness
KW - Questionnaires/st [Standards]
KW - Reproducibility of Results
KW - Sensitivity and Specificity
RP - NOT IN FILE
SP - 1155
EP - 1162
JF - Revista Medica de Chile
JA - Rev Med Chil.
VL - 136
IS - 9
CY - Chile
N2 - BACKGROUND: An appropriate measurement of physical activity (PA) in children is useful, since inactivity is associated to obesity, cardiovascular and metabolic risk. AIM: To assess the reliability of the INTA questionnaire of PA, to compare the derived PA score with accelerometry and to assess its ability to identify excessively inactive children. MATERIAL AND METHODS: One hundred eighty children aged 8 to 13 years answered an interviewer-administered questionnaire about their usual PA, consisting in 5 items (recumbent, seated, walking, playing outdoor, sports). The answers were converted to a PA score with a 0-10 points scale. Reliability was tested in 87 children by test/retest conducted 3-5 days apart. The PA score was compared with 3-day accelerometry in 77 of 93 children (35 obese and 42 non obese). Receiver operating characteristic (ROC) curves were used to determine the optimal cut-point for identify an excessively sedentary child. RESULTS: The test/retest reliability of the questionnaire was 0.69 to 0.93 (Lin coefficient). Accelerometry was significantly associated with PA score (RHO: 0.60, p =0.008), outdoor plays (RHO: 0.37, p =0.0009) and practicing of sports (RHO: 0.33, p =0.003). Obese children were less active than non obese children, according both to PA score and to accelerometry. The optimal cut-point for classifying a child as too sedentary was a score of 5 (sensitivity =0.89). CONCLUSIONS: The INTA-test is a valuable instrument for measuring usual PA in clinical practice and is easy to administer
SN - 0034-9887
AD - Instituto de Nutricion y Tecnologia de los Alimentos, Universidad de Chile, Santiago, Chile. cgodard@inta.cl
UR - 19030660
ER -

TY - JOUR
ID - 978
T1 - Where are youth active? Roles of proximity, active transport, and built environment
A1 - Grow,H.M.
A1 - Saelens,B.E.

A1 - Kerr,J.
A1 - Durant,N.H.
A1 - Norman,G.J.
A1 - Sallis,J.F.
Y1 - 2008/12//
N1 - Grow, Helene Mollie. Saelens, Brian E. Kerr, Jacqueline. Durant, Nefertiti H. Norman, Gregory J. Sallis, James F
Medicine and science in sports and exercise
8005433, mg8
IM, S
Journal Article. Research Support, Non-U.S. Gov't
English
KW - MEDLINE
KW - Adolescent
KW - Bicycling
KW - Child
KW - Preschool
KW - Exercise
KW - Humans
KW - Parents
KW - Recreation
KW - United States
KW - Walking
RP - NOT IN FILE
SP - 2071
EP - 2079
JF - Medicine & Science in Sports & Exercise
JA - Med Sci Sports Exerc
VL - 40
IS - 12
CY - United States
N2 - PURPOSE: This study examined factors related to two sources of physical activity for youth: active use of recreation sites and active transport to recreation sites. METHODS: Parents of children (n = 87) and matched pairs of parents and adolescents (n = 124 pairs) in three US cities reported on youths' active use of, proximity to, and walking/biking to 12 recreation sites and on neighborhood walkability and safety. Multivariate regression models evaluated factors associated with youths' frequent site use and active transport to sites. RESULTS: Proximity to the site was associated with frequent use of large parks and public open space. Walking/biking to the site was associated with frequent use of most sites (indoor recreation sites, small and large parks, basketball courts, walking/running tracks, school recreation sites, playgrounds, and public open space). After controlling for proximity and demographic factors, active transport to sites remained significantly associated (P < 0.05) with frequent use of four sites for children (indoor recreation, walking/running tracks, school recreation facilities, and public open space) and all but three sites for adolescents (indoor recreation, playfields/courts, and beach/lake/rivers). Adolescents' active transport to more sites was most positively related to higher perceived traffic safety and to better pedestrian infrastructure and was negatively related to crime threat. Adolescents with driver's licenses walked/biked to recreation sites less often. CONCLUSIONS: Active transport was strongly associated with the use of multiple recreation sites by children and adolescents, even when accounting for proximity and demographic factors. Adolescents living in neighborhoods with better traffic safety walked/biked to more recreation sites for physical activity. Findings support the need for built environments and transportation policies that facilitate safe, active transport to recreation sites for youth physical activity
SN - 1530-0315
AD - Department of Pediatrics, University of Washington, Seattle, WA, 98195-4920, USA. greves@u.washington.edu
UR - 18981942
ER -

TY - JOUR
ID - 979

T1 - Expressive communication of children with autism: the use of challenging behaviour
A1 - Chiang,H.M.
Y1 - 2008/11//
N1 - Chiang, Hsu-Min
Journal of intellectual disability research : JIDR
9206090, bga
IM
Journal Article
English
KW - MEDLINE
KW - Adolescent
KW - Analysis of Variance
KW - Australia
KW - Autistic Disorder/co [Complications]
KW - Autistic Disorder/px [Psychology]
KW - Child
KW - Child Behavior/px [Psychology]
KW - Child Behavior
KW - Preschool
KW - Cross-Cultural Comparison
KW - Female
KW - Humans
KW - Interpersonal Relations
KW - Language Development Disorders/et [Etiology]
KW - Language Development Disorders/px [Psychology]
KW - Male
KW - Nonverbal Communication/px [Psychology]
KW - Nonverbal Communication
KW - Observer Variation
KW - Peer Group
KW - Rejection (Psychology)
KW - Schools
KW - Taiwan
KW - Videotape Recording
RP - NOT IN FILE
SP - 966
EP - 972
JF - Journal of Intellectual Disability Research
JA - J Intellect Disabil Res
VL - 52
IS - 11
CY - England
N2 - BACKGROUND: There is a lack of empirical research investigating challenging behaviour in children with autism with severe speech impairments in naturalistic settings. The aim of the present study was to investigate challenging behaviour among Australian and Taiwanese children with autism who are non-verbal or have limited speech (i.e. less than five functional words) in school settings and to address the following questions: (1) What are the communicative characteristics (e.g. communicative functions, partners, activities and partner's responses) of challenging behaviour? (2) Do sample characteristics (e.g. age, severity of autism, adaptive behaviour level, the use of graphic symbols, the use of non-symbolic acts, the use of speech) associate with the amount of challenging behaviour? (3) Do cultural differences exist in communicative variables of challenging behaviour? METHOD: Naturalistic observations were used to investigate challenging behaviour in children with autism. A total of 32 (17 Australian and 15 Taiwanese) children with autism who were non-verbal or had limited speech participated in the current study and each participant was videotaped for 2 h during his/her everyday school routines. RESULTS: Sixteen out of 32 children had challenging behaviour. Challenging behaviour was used for requesting and rejecting communicative functions. A paired-samples t-test revealed that no significant differences existed in the frequency of occurrence between request and reject. Challenging behaviour was directed to adults and peers. A paired-samples t-test revealed that challenging behaviour was

directed to adults significantly more frequently than to peers. Communicative partners showed different responses (e.g. deny, no response, delivered, removed, acknowledge) to challenging behaviour. A repeated-measures analysis of variance (ANOVA) revealed a main effect for partner response. Deny was the most common response. Challenging behaviour occurred across a variety of classroom activities (e.g. academic activities, morning circle, morning tea, lunch, free time). A repeated-measures ANOVA revealed a main effect for activity. Challenging behaviour occurred most often during academic activities. A set of correlation analyses indicated a significant correlation existed between the use of graphic symbols and the amount of challenging behaviour. A series of Mann-Whitney U-tests revealed significant differences between Australian and Taiwanese children on the amount of challenging behaviour, communicative functions and partners. A Chi-squared test revealed that there were significantly more Australian children with autism who displayed challenging behaviour than Taiwanese children. CONCLUSIONS: The current study shows that a high proportion of children with autism with severe speech impairments used challenging behaviour as a form of expressive communication in their school environments. The findings from the present study may have implications for practitioners who work with children with autism
SN - 1365-2788
AD - Australian Centre for Educational Studies, Macquarie University, Sydney, Australia. hsu-min.chiang@students.mq.edu.au
UR - 18205752
ER -

TY - JOUR
ID - 980
T1 - Advances in the prevention of children's injuries: an examination of four common outdoor activities. [Review] [60 refs]
A1 - Parkin,P.C.
A1 - Howard,A.W.
Y1 - 2008/12//
N1 - Parkin, Patricia C. Howard, Andrew W
Current opinion in pediatrics
but, 9000850
IM
Journal Article. Review
English
KW - MEDLINE
KW - Adolescent
KW - Athletic Injuries/pc [Prevention & Control]
KW - Bicycling/in [Injuries]
KW - Child
KW - Preschool
KW - Head Protective Devices
KW - Humans
KW - Play and Playthings/in [Injuries]
KW - Skiing/in [Injuries]
KW - Walking/in [Injuries]
RP - NOT IN FILE
SP - 719
EP - 723
JF - Current Opinion in Pediatrics
JA - Curr Opin Pediatr
VL - 20
IS - 6
CY - United States
N2 - PURPOSE OF REVIEW: Injuries remain the leading cause of death for children. Experts in paediatrics and child health have a current interest in promoting children's healthy active living. This review highlights findings from recent literature regarding the prevention of injuries from four common outdoor activities: bicycling, snowboarding and skiing, walking and playground activity. RECENT FINDINGS: There is sound evidence for the effectiveness of bicycle helmets, the promotion of bicycle helmets at a community level and

through physician counselling, and legislation; for the effectiveness of helmets for skiing and snowboarding; for the effectiveness of implementing playground safety standards; and for the effectiveness of modifications to the pedestrian physical environment. SUMMARY: The science of injury prevention has advanced considerably. The highest level of evidence, including systematic reviews, is now available regarding the effectiveness of protective measures, engineering approaches to the environment and legislation. Healthcare providers caring for children play a leading role in injury prevention through child and family counselling, advocacy and research. [References: 60]
SN - 1531-698X
AD - Division of Pediatric Medicine and the Pediatric Outcomes Research Team and the Child Health Evaluative Sciences, Hospital for Sick Children Research Institute, University of Toronto Faculty of Medicine, Toronto, Canada. patricia.parkin@sickkids.ca
UR - 19023919
ER -

TY - JOUR
ID - 981
T1 - Investigating tuberculosis trends in England
A1 - Crofts,J.P.
A1 - Gelb,D.
A1 - Andrews,N.
A1 - Delpech,V.
A1 - Watson,J.M.
A1 - Abubakar,I.
Y1 - 2008/12//
N1 - Crofts, J P. Gelb, D. Andrews, N. Delpech, V. Watson, J M. Abubakar, I
Public health
qi7, 0376507
IM
Journal Article
English
KW - MEDLINE
KW - Adolescent
KW - Adult
KW - Aged
KW - Child
KW - Preschool
KW - Confidence Intervals
KW - England/ep [Epidemiology]
KW - Female
KW - Humans
KW - Incidence
KW - Infant
KW - Newborn
KW - Male
KW - Middle Aged
KW - Multivariate Analysis
KW - Population Surveillance
KW - Prospective Studies
KW - Public Health/td [Trends]
KW - Risk Factors
KW - Tuberculosis
KW - Pulmonary/ep [Epidemiology]
KW - Pulmonary/pp [Physiopathology]
KW - Pulmonary/tm [Transmission]
KW - Young Adult
RP - NOT IN FILE
SP - 1302

EP - 1310
JF - Public Health
VL - 122
IS - 12
CY - England
N2 - OBJECTIVE: To investigate the factors associated with the increasing incidence of tuberculosis in England. DESIGN: Prospective national surveillance study. METHODS: Measurement of relative trends in demographic, clinical and microbiological characteristics of tuberculosis cases reported in England between 1999 and 2003. RESULTS: Between 1999 and 2003, the number of tuberculosis cases reported in England increased by 19% from 5539 to 6608. Significant increases in tuberculosis cases were seen in the non-UK-born population who were recent entrants to the UK (arrival less than 5 years prior to diagnosis) relative to cases in the UK-born population, both in London [1.08; 95% confidence interval (CI) 1.02-1.14] and outside London (1.22; 95% CI 1.16-1.28). Cases of tuberculosis co-infected with human immunodeficiency virus (HIV) increased significantly both in London (1.19; 95% CI 1.12-1.27) and outside London (1.22; 95% CI 1.12-1.33) relative to cases not known to be co-infected with HIV. Small increases in the number of cases living in less-deprived areas were observed relative to those in the most-deprived areas (1.11; 95% CI 1.02-1.21) in London. The number of cases of isoniazid-resistant (1.09; 95% CI 1.02-1.16) and multi-drug-resistant tuberculosis (1.22; 95% CI 1.02-1.45) increased relative to drug-susceptible cases in London. CONCLUSIONS: Tuberculosis remains strongly associated with deprivation, but no real change in the distribution of tuberculosis cases by deprivation was observed over the study period. The proportion of tuberculosis cases co-infected with HIV has increased, but migration explains most of the recent trends in tuberculosis in England. Measures that target latent tuberculosis infection could have an increased role to play alongside measures against active disease in reducing the incidence of tuberculosis in the UK
SN - 0033-3506
AD - Respiratory Diseases Department, Centre for Infections, Health Protection Agency, 61 Colindale Avenue, London NW9 5EQ, UK
UR - 18672258
ER -

TY - JOUR
ID - 982
T1 - The Classroom Observation Schedule to Measure Intentional Communication (COSMIC): an observational measure of the intentional communication of children with autism in an unstructured classroom setting
A1 - Pasco,G.
A1 - Gordon,R.K.
A1 - Howlin,P.
A1 - Charman,T.
Y1 - 2008/11//
N1 - Pasco, Greg. Gordon, Rosanna K. Howlin, Patricia. Charman, Tony
Journal of autism and developmental disorders
7904301, hgw
IM
Comparative Study. Journal Article. Research Support, Non-U.S. Gov't
English
KW - MEDLINE
KW - Autistic Disorder/di [Diagnosis]
KW - Autistic Disorder/px [Psychology]
KW - Autistic Disorder/th [Therapy]
KW - Child
KW - Child Behavior/px [Psychology]
KW - Preschool
KW - Communication
KW - Communication Disorders
KW - Female
KW - Humans
KW - Intention

KW - Language Development Disorders/di [Diagnosis]
KW - Language Development Disorders/th [Therapy]
KW - Language Tests/sn [Statistics & Numerical Data]
KW - Male
KW - Nonverbal Communication
KW - Observation
KW - Play and Playthings
KW - Social Behavior
KW - Video Recording
KW - Vocabulary
RP - NOT IN FILE
SP - 1807
EP - 1818
JF - Journal of Autism & Developmental Disorders
JA - J Autism Dev Disord
VL - 38
IS - 10
CY - United States
N2 - The Classroom Observation Schedule to Measure Intentional Communication (COSMIC) was devised to provide ecologically valid outcome measures for a communication-focused intervention trial. Ninety-one children with autism spectrum disorder aged 6 years 10 months (SD 16 months) were videoed during their everyday snack, teaching and free play activities. Inter-rater reliability was high and relevant items showed significant associations with comparable items from concurrent Autism Diagnostic Observation Schedule-Generic (Lord et al. 2000, J Autism Dev Disord 30(3):205-223) assessments. In a subsample of 28 children initial differences in rates of initiations, initiated speech/vocalisation and commenting were predictive of language and communication competence 15 months later. Results suggest that the use of observational measures of intentional communication in natural settings is a valuable assessment strategy for research and clinical practice
SN - 0162-3257
AD - Department of Community Health Science, St George's University of London, London, UK. gp310@medschl.cam.ac.uk
UR - 18401692
ER -

TY - JOUR
ID - 983
T1 - A prospective examination of children's time spent outdoors, objectively measured physical activity and overweight
A1 - Cleland,V.
A1 - Crawford,D.
A1 - Baur,L.A.
A1 - Hume,C.
A1 - Timperio,A.
A1 - Salmon,J.
Y1 - 2008/11//
N1 - Cleland, V. Crawford, D. Baur, L A. Hume, C. Timperio, A. Salmon, J
International journal of obesity (2005)
101256108
IM
Journal Article. Multicenter Study. Research Support, Non-U.S. Gov't
English
KW - MEDLINE
KW - Australia/ep [Epidemiology]
KW - Body Mass Index
KW - Body Weights and Measures
KW - Child
KW - Preschool

KW - Cross-Sectional Studies
KW - Female
KW - Health Behavior
KW - Humans
KW - Leisure Activities/px [Psychology]
KW - Life Style
KW - Male
KW - Motor Activity/ph [Physiology]
KW - Obesity/ep [Epidemiology]
KW - Obesity/pc [Prevention & Control]
KW - Prospective Studies
KW - Schools
KW - Seasons
KW - Weight Loss
RP - NOT IN FILE
SP - 1685
EP - 1693
JF - International Journal of Obesity
JA - Int J Obes (Lond)
VL - 32
IS - 11
CY - England
N2 - OBJECTIVE: This study aimed to determine whether time spent outdoors was associated with objectively measured physical activity, body mass index (BMI) z-score and overweight in elementary-school aged children, cross-sectionally and prospectively over 3 years. METHODS: Three-year cohort study with data collected during 2001 and 2004. Nineteen randomly selected state elementary schools across Melbourne, Australia. One hundred and eighty eight 5-6-year-old and 360 10-12-year-old children. Baseline parent reports of children's time spent outdoors during warmer and cooler months, on weekdays and weekends. At baseline and follow-up, children's moderate and vigorous physical activity (MVPA) was objectively assessed by accelerometry, and BMI z-score and overweight was calculated from measured height and weight. RESULTS: Cross-sectionally, each additional hour outdoors on weekdays and weekend days during the cooler months was associated with an extra 27 min week(-1) MVPA among older girls, and with an extra 20 min week(-1) MVPA among older boys. Longitudinally, more time outdoors on weekends predicted higher MVPA on weekends among older girls and boys (5 min week(-1)). The prevalence of overweight among older children at follow-up was 27-41% lower among those spending more time outdoors at baseline. CONCLUSION: Encouraging 10-12-year-old children to spend more time outdoors may be an effective strategy for increasing physical activity and preventing increases in overweight and obesity. Intervention research investigating the effect of increasing time outdoors on children's physical activity and overweight is warranted
SN - 1476-5497
AD - Centre for Physical Activity and Nutrition Research, Deakin University, 221 Burwood Hwy, Burwood, Victoria, Australia. verity.cleland@deakin.edu.au
UR - 18852701
ER -

TY - JOUR
ID - 984
T1 - Contribution of violence to health inequalities in England: demographics and trends in emergency hospital admissions for assault
A1 - Bellis,M.A.
A1 - Hughes,K.
A1 - Anderson,Z.
A1 - Tocque,K.
A1 - Hughes,S.
Y1 - 2008/12//
N1 - Bellis, M A. Hughes, K. Anderson, Z. Tocque, K. Hughes, S
Journal of epidemiology and community health
i1p, 7909766

IM
Journal Article
English
KW - MEDLINE
KW - Adolescent
KW - Adult
KW - Child
KW - Preschool
KW - Cross-Sectional Studies
KW - Demography
KW - Emergency Service
KW - Hospital/td [Trends]
KW - Hospital/ut [Utilization]
KW - England/ep [Epidemiology]
KW - Female
KW - Health Status Disparities
KW - Humans
KW - Infant
KW - Newborn
KW - Male
KW - Middle Aged
KW - Patient Admission/sn [Statistics & Numerical Data]
KW - Patient Admission/td [Trends]
KW - Violence/sn [Statistics & Numerical Data]
KW - Violence/td [Trends]
KW - Young Adult
RP - NOT IN FILE
SP - 1064
EP - 1071
JF - Journal of Epidemiology & Community Health
JA - J Epidemiol Community Health
VL - 62
IS - 12
CY - England
N2 - INTRODUCTION: Violence is increasingly recognised as a major public health issue yet health data are underutilised for describing the problem or developing responses. We use English emergency hospital admissions for assault over four years to examine assault demography and contribution to health inequalities. METHODS: Geodemographic cross-sectional analyses utilising records of all individuals in England (n = 120 643) admitted between 1 April 2002 and 31 March 2006. RESULTS: Over 4 years, rates of admission increased by 29.56% across England. Admissions peaked on Saturdays (22.34%) and Sundays (20.38%). Higher rates were associated with deprivation across all ages, including those <15 years, with a sixfold increase in admission rates between the poorest and wealthiest quintiles of residence. Logistic regression analyses indicate males are 5.59 times more likely to be admitted to hospital for assault and such admissions peak in those aged 15-29 years. Modelling based on national assault admissions and limited Accident and Emergency (A&E) data suggest that while more serious assaults requiring hospital admission have increased, assault attendances at A&Es have fallen. DISCUSSION: Hospital admission and A&E data identify a direct contribution made by violence to health inequalities. Levels of violence inhibit other interventions to improve people's health through, for instance, outdoor exercise or delivery of health-related services in affected areas. With disproportionate exposure to violence in poorer areas even in those under 15, early life primary prevention initiatives are required in disadvantaged communities to reduce childhood harm and the development of adult perpetrators and victims of violence
SN - 1470-2738
AD - Centre for Public Health, Liverpool John Moores University, Castle House, North Street, Liverpool L3 2AY, UK. m.a.bellis@ljmu.ac.uk
UR - 18628267
ER -

TY - JOUR
ID - 985
T1 - Physical activity and healthy eating in the after-school environment
A1 - Coleman,K.J.
A1 - Geller,K.S.
A1 - Rosenkranz,R.R.
A1 - Dzewaltowski,D.A.
Y1 - 2008/12//
N1 - Coleman, Karen J. Geller, Karly S. Rosenkranz, Richard R. Dzewaltowski, David A
The Journal of school health
k13, 0376370
IM, N
Journal Article. Research Support, U.S. Gov't, Non-P.H.S.
English
KW - MEDLINE
KW - Body Mass Index
KW - Child
KW - Diet
KW - Exercise
KW - Female
KW - Health Promotion/og [Organization & Administration]
KW - Humans
KW - Male
KW - Obesity/pc [Prevention & Control]
KW - Schools/og [Organization & Administration]
KW - Socioeconomic Factors
RP - NOT IN FILE
SP - 633
EP - 640
JF - Journal of School Health
JA - J Sch Health
VL - 78
IS - 12
CY - United States
N2 - BACKGROUND: No research to date has extensively described moderate and vigorous physical activity (MVPA) and healthful eating (HE) opportunities in the after-school environment. The current study described the quality of the after-school environment for its impact on children's MVPA and HE. METHODS: An alliance of 7 elementary schools and Boys and Girls Clubs who worked with the Cooperative Extension Service in Lawrence, KS, was selected to participate in a larger intervention study. After-school settings were observed for information regarding session type, session context, leader behavior, physical activity, and snack quality using validated instruments such as the System for Observing Fitness Instruction Time. Data presented are baseline measures for all sites. RESULTS: Participating children (n = 144) were primarily non-Hispanic white (60%) and in fourth grade (69%). After-school sites offered 4 different sessions per day (active recreation, academic time, nonactive recreation, and enrichment activities). Children were provided with a daily snack. On 36% of the days observed, this snack included fruit, fruit juice, or vegetables. There was significantly more time spent in MVPA during free play sessions (69%) compared to organized adult-led sessions (51%). There was also significantly more discouragement of physical activity during organized adult-led sessions (29%) as compared to the free play sessions (6%). CONCLUSIONS: The quality of after-school programs can be improved by providing fruits and vegetables as snacks; offering more free play activities; training the after-school staff in simple, structured games for use in a variety of indoor and outdoor settings; and training after-school staff to promote and model MVPA and HE in and out of the after-school setting
SN - 1746-1561
AD - Southern California Permanente Medical Group, 100 S Los Robles Ave, 2nd Floor, Pasadena, CA 91101, USA. karen.j.coleman@kp.org
UR - 19000239
ER -

TY - JOUR
ID - 986
T1 - Neighborhood greenness and 2-year changes in body mass index of children and youth
A1 - Bell,J.F.
A1 - Wilson,J.S.
A1 - Liu,G.C.
Y1 - 2008/12//
N1 - Bell, Janice F. Wilson, Jeffrey S. Liu, Gilbert C
American journal of preventive medicine
8704773, apl
IM
Journal Article. Research Support, N.I.H., Extramural. Research Support, U.S. Gov't, P.H.S.
English
KW - MEDLINE
KW - Adolescent
KW - Body Mass Index
KW - Body Weight
KW - Child
KW - Preschool
KW - Cohort Studies
KW - Confounding Factors (Epidemiology)
KW - Environment Design
KW - Exercise
KW - Female
KW - Humans
KW - Indiana/ep [Epidemiology]
KW - Logistic Models
KW - Male
KW - Multivariate Analysis
KW - Obesity/ep [Epidemiology]
KW - Plants
KW - Residence Characteristics
KW - Retrospective Studies
KW - Risk Factors
KW - Socioeconomic Factors
RP - NOT IN FILE
SP - 547
EP - 553
JF - American Journal of Preventive Medicine
JA - Am J Prev Med
VL - 35
IS - 6
CY - Netherlands
N2 - BACKGROUND: Available studies of the built environment and the BMI of children and youth suggest a contemporaneous association with neighborhood greenness in neighborhoods with high population density. The current study tests whether greenness and residential density are independently associated with 2-year changes in the BMI of children and youth. METHODS: The sample included children and youth aged 3-16 years who lived at the same address for 24 consecutive months and received well-child care from a Marion County IN clinic network within the years 1996-2002 (n=3831). Multiple linear regression was used to examine associations among age- and gender-specific BMI z-scores in Year 2, residential density, and a satellite-derived measure of greenness, controlling for baseline BMI z-scores and other covariates. Logistic regression was used to model associations between an indicator of BMI z-score increase from baseline to Time 2 and the above-mentioned predictors. RESULTS: Higher greenness was significantly associated with lower BMI z-scores at Time 2 regardless of residential density characteristics. Higher residential density was not associated with Time 2 BMI z-scores in models regardless of greenness. Higher greenness was also associated with lower odds of children's and youth's increasing their BMI z-scores over 2 years (OR=0.87; 95% CI=0.79, 0.97). CONCLUSIONS: Greenness may present a target for environmental approaches to preventing child obesity.

Children and youth living in greener neighborhoods had lower BMI z-scores at Time 2, presumably due to increased physical activity or time spent outdoors. Conceptualizations of walkability from adult studies, based solely on residential density, may not be relevant to children and youth in urban environments
SN - 1873-2607
AD - Department of Health Services, School of Public Health and Community Medicine, University of Washington, Seattle, Washington 98195-7230, USA. jfbell@u.washington.edu
UR - 19000844
ER -

TY - JOUR
ID - 987
T1 - Features of public open spaces and physical activity among children: findings from the CLAN study
A1 - Timperio,A.
A1 - Giles-Corti,B.
A1 - Crawford,D.
A1 - Andrianopoulos,N.
A1 - Ball,K.
A1 - Salmon,J.
A1 - Hume,C.
Y1 - 2008/11//
N1 - Timperio, Anna. Giles-Corti, Billie. Crawford, David. Andrianopoulos, Nick. Ball, Kylie. Salmon, Jo. Hume, Clare
Preventive medicine
pm4, 0322116
IM
Journal Article. Research Support, Non-U.S. Gov't
English
KW - MEDLINE
KW - Adolescent
KW - Child
KW - Cross-Sectional Studies
KW - Environment Design
KW - Exercise
KW - Female
KW - Geographic Information Systems
KW - Humans
KW - Male
KW - Monitoring
KW - Physiologic/is [Instrumentation]
KW - Public Facilities
KW - Victoria
RP - NOT IN FILE
SP - 514
EP - 518
JF - Preventive Medicine
JA - Prev Med
VL - 47
IS - 5
CY - United States
N2 - OBJECTIVE: To examine associations between features of public open spaces, and children's physical activity. PARTICIPANTS: 163 children aged 8-9 years and 334 adolescents aged 13-15 years from Melbourne, Australia participated in 2004. METHODS: A Geographic Information System was used to identify all public open spaces (POS) within 800 m of participants' homes and their closest POS. The features of all POS identified were audited in 2004/5. Accelerometers measured moderate-to-vigorous physical activity (MVPA) after school and on weekends. Linear regression analyses examined associations between features of the closest POS and participants' MVPA. RESULTS: Most participants had a POS within 800 m of their home. The presence of playgrounds was positively associated with younger boys' weekend MVPA (B=24.9 min/day; p< or =0.05), and

lighting along paths was inversely associated with weekend MVPA (B=-54.9 min/day; p< or =0.05). The number of recreational facilities was inversely associated with younger girls' MVPA after school (B=-2.6 min/day; p< or =0.05) and on the weekend (B=-8.7 min/day; p< or =0.05). The presence of trees providing shade (5.8 min/day, p< or =0.01) and signage regarding dogs (B=6.8 min/day, p< or =0.05) were positively associated with adolescent girls' MVPA after school. CONCLUSION: Certain features of POS were associated with participants' MVPA, although mixed associations were evident. Further research is required to clarify these complex relationships
SN - 1096-0260
AD - Centre for Physical Activity and Nutrition Research, Deakin University, Australia
UR - 18718847
ER -

TY - JOUR
ID - 988
T1 - Search and rescue in Alaska's national parks
A1 - Heggie,T.W.
Y1 - 2008/11//
N1 - Heggie, Travis W
Travel medicine and infectious disease
101230758
IM
Journal Article
English
KW - MEDLINE
KW - Adolescent
KW - Adult
KW - Aged
KW - Alaska
KW - Child
KW - Emergency Medical Services/ec [Economics]
KW - Emergency Medical Services/sn [Statistics & Numerical Data]
KW - Female
KW - Humans
KW - Male
KW - Middle Aged
KW - Recreation
KW - Rescue Work/ec [Economics]
KW - Rescue Work/sn [Statistics & Numerical Data]
KW - Retrospective Studies
KW - Wounds and Injuries/ep [Epidemiology]
KW - Young Adult
RP - NOT IN FILE
SP - 355
EP - 361
JF - Travel Medicine & Infectious Disease
JA - Travel Med Infect Dis
VL - 6
IS - 6
CY - Netherlands
N2 - BACKGROUND: Recreational travel to wilderness destinations such as national parks is increasing. The inherent risks present in such destinations can result in injury and illness, have a significant impact on local medical resources, and end with expensive search and rescue operations. In order to increase our understanding of the activities and situations which lead to wilderness search and rescue incidents, this study examines search and rescue operations from National Park Service units in Alaska. METHODS: A retrospective review of all search and rescue incident reports filed by National Park Service units in Alaska during 2002. RESULTS: During 2002 there were 25 reported search and rescue incidents involving 38 individuals. The majority of incidents (19 of 25) occurred at Denali National Park and Preserve. Thirteen fatalities were reported in six

incidents, nine incidents involved traumatic injuries, eight involved illnesses, and two involved both injuries and an illness. Mountain climbing (20) and hiking (8) were the most common subject activities at the time search and rescue assistance was required. Climbing solo (4), uneven and wet terrain (4), falls into crevasses (3), and a lack of experience or ability (3) were the factors most commonly contributing to search and rescue incidents. Nineteen helicopters were utilized in 15 operations and fixed-wing aircraft were utilized in seven operations. Males accounted for 33 of the 38 individuals involved in all search and rescue incidents and United States citizens accounted for 74% of the individuals involved. The mountain environment higher than 4500m was the most common search and rescue environment (11). The average cost was USD $6253. CONCLUSIONS: Search and rescue operations in Alaska can be expensive and end with severe health consequences. Preventive education efforts at park visitor centers and at the lower and upper base camps on Mt. McKinley should be continued. In addition, pre-departure travel education efforts via the internet should be expanded for all park units and match the detail provided on the Denali National Park and Preserve website
SN - 1477-8939
AD - University of North Dakota, Recreation and Tourism Studies Program, 225 Centennial Drive, Mail Stop 7116, Grand Forks, ND 58202, USA. travis.heggie@und.edu
UR - 18984480
ER -

TY - JOUR
ID - 989
T1 - "Practice what you preach": induced hypocrisy as an intervention strategy to reduce children's intentions to risk take on playgrounds
A1 - Morrongiello,B.A.
A1 - Mark,L.
Y1 - 2008/11//
N1 - Morrongiello, Barbara A. Mark, Landa
Journal of pediatric psychology
jl8, 7801773
IM
Journal Article. Multicenter Study. Randomized Controlled Trial. Research Support, Non-U.S. Gov't
English
KW - MEDLINE
KW - Accidental Falls/pc [Prevention & Control]
KW - Adolescent
KW - Age Factors
KW - Behavior Therapy/mt [Methods]
KW - Character
KW - Child
KW - Cognitive Dissonance
KW - Exploratory Behavior
KW - Fear
KW - Female
KW - Generalization (Psychology)
KW - Health Education
KW - Health Knowledge
KW - Attitudes
KW - Practice
KW - Humans
KW - Intention
KW - Judgment
KW - Male
KW - Motivation
KW - Ontario
KW - Play and Playthings
KW - Risk-Taking
KW - Safety
KW - Self Concept

KW - Wounds and Injuries/pc [Prevention & Control]
KW - Wounds and Injuries/px [Psychology]
RP - NOT IN FILE
SP - 1117
EP - 1128
JF - Journal of Pediatric Psychology
JA - J Pediatr Psychol
VL - 33
IS - 10
CY - United States
N2 - OBJECTIVE: An intervention based on induced hypocrisy was conducted to reduce children's intentions to show fall-risk behaviors on playground equipment. METHODS: To induce hypocrisy participants (7-12 years) advocated for safe-play behaviors while being made mindful about past failures to play safely on playgrounds. To measure the impact of the intervention, children created posters indicating which playground behaviors they Would Do and Would Not Do; children believed they would have to demonstrate the behaviors endorsed on their posters. RESULTS: The intervention resulted in significant reductions in the risk behaviors children endorsed both for risk behaviors targeted (specific effects) and nontargeted risk behaviors (generalized effects). Assessing the mechanism by which the intervention produced changes in behavioral intentions revealed it yielded changes in cognitions that predicted subsequent reductions in risk-taking intentions. CONCLUSIONS: The present intervention was successful in reducing children's intentions to engage in risky playground behaviors and it did so by changing cognitions
SN - 1465-735X
AD - Department of Psychology, University of Guelph, MacKinnon Building, 5th Floor, Guelph, ONT, N1G 2W1, Canada. bmorrong@uoguelph.ca
UR - 18296456
ER -

TY - JOUR
ID - 990
T1 - Improving nutrition and physical activity in child care: what parents recommend
A1 - Benjamin,S.E.
A1 - Haines,J.
A1 - Ball,S.C.
A1 - Ward,D.S.
Y1 - 2008/11//
N1 - Benjamin, Sara E. Haines, Jess. Ball, Sarah C. Ward, Dianne S
Journal of the American Dietetic Association
h6f, 7503061
AIM, IM
Journal Article. Research Support, U.S. Gov't, P.H.S.
English
KW - MEDLINE
KW - Adolescent
KW - Adult
KW - Child Day Care Centers/st [Standards]
KW - Child Nutritional Physiological Phenomena/ph [Physiology]
KW - Child
KW - Preschool
KW - Exercise/ph [Physiology]
KW - Female
KW - Food Services/st [Standards]
KW - Fruit
KW - Humans
KW - Male
KW - North Carolina
KW - Obesity/ep [Epidemiology]
KW - Obesity/pc [Prevention & Control]

KW - Parents/px [Psychology]
KW - Questionnaires
KW - Vegetables
KW - Young Adult
RP - NOT IN FILE
SP - 1907
EP - 1911
JF - Journal of the American Dietetic Association
JA - J Am Diet Assoc
VL - 108
IS - 11
CY - United States
N2 - A large percentage of children in the United States spend part of their day in out-of-home child care. As rates of obesity continue to rise, especially among young children, child care has become a focus for nutrition and physical activity intervention. Parental involvement is an important component of these efforts. During summer 2006, parents of children in child care were surveyed to better understand their perceived quality of meals, snacks, and physical activity at the child-care center, and their recommendations for improvement. Parents of children who attended 94 licensed child-care centers in North Carolina were invited to complete a brief survey of perceived quality of meals, snacks, and physical activity at their centers using close-ended questions. Open-ended questions were used to identify suggestions for improvement. Five hundred eight parents from 91 child-care centers completed the questionnaire. The majority of parents reported quality of meals and snacks at the center as either excellent (30% meals, 27% snacks) or good (42% meals, 46% snacks). The main recommendations for improving meals and snacks were to increase fruits and vegetables and provide a variety of healthful foods. The majority of parents categorized the quality of physical activity at the center as excellent (36%) or good (46%), and suggested more structured, outdoor activities for children. Findings from this study provide insight into key areas of concern for parents regarding the nutrition and activity environment of child-care centers. This information may be used to create or modify interventions or policies and to help motivate parents to become advocates for change in child care
SN - 0002-8223
AD - Department of Ambulatory Care and Prevention, Harvard Medical School and Harvard Pilgrim Health Care, 133 Brookline Ave, 6th FL, Boston, MA 02215, USA. sara_benjamin@harvardpilgrim.org
UR - 18954582
ER -

TY - JOUR
ID - 991
T1 - The fraction of total hand surface area involved in young children's outdoor hand-to-object contacts
A1 - AuYeung,W.
A1 - Canales,R.A.
A1 - Leckie,J.O.
Y1 - 2008/11//
N1 - AuYeung, Willa. Canales, Robert A. Leckie, James O
Environmental research
ei2, 0147621
IM
Comparative Study. Journal Article. Research Support, Non-U.S. Gov't. Research Support, U.S. Gov't, Non-P.H.S.
English
KW - MEDLINE
KW - Body Weights and Measures
KW - Child Behavior/ph [Physiology]
KW - Child
KW - Preschool
KW - Environmental Exposure
KW - Equipment Contamination/sn [Statistics & Numerical Data]
KW - Female
KW - Hand/ah [Anatomy & Histology]

KW - Humans
KW - Infant
KW - Male
KW - Video Recording
RP - NOT IN FILE
SP - 294
EP - 299
JF - Environmental Research
JA - Environ Res
VL - 108
IS - 3
CY - United States
N2 - Information on the fraction of total hand surface area touching a contaminated object is necessary in accurately estimating contaminant (e.g., pesticides, pathogens) loadings onto the hands during hand-to-object contacts. While several existing physical-stochastic human exposure models require such surface area data to estimate dermal and non-dietary ingestion exposure, there are very limited data sets. This paper provides statistical distributions of fractional surface areas (FSAs) for children's outdoor hand contacts. These distributions were constructed by combining information collected from two distinct studies exploring children's activity patterns and quantifying hand contact surface area. Results show that for outdoor contacts with "All Objects", a range of 0.13-0.27 captured median FSAs, while a range of 0.12-0.24 captured time-weighted FSAs. Overall, an FSA of 0.31 captured 80-100% of FSAs involved in each child's outdoor hand contacts, depending upon the object of interest. These values are much lower than the often conservative assumptions of up to 1 (i.e., the entire hand) that researchers currently make regarding FSAs involved in indoor and outdoor contacts [USEPA, 1997. Standard operating procedures (SOPs) for residential exposure assessments. Contract no. 68-W6-0030. http://www.epa.gov/pesticides/trac/science/trac6a05.pdf]
SN - 1096-0953
AD - Department of Civil and Environmental Engineering, Stanford University, Environment and Energy Building, Stanford, CA 94305, USA
UR - 18760778
ER -

TY - JOUR
ID - 992
T1 - School time physical activity of students with and without autism spectrum disorders during PE and recess
A1 - Pan,C.Y.
Y1 - 2008/10//
N1 - Pan, Chien-Yu
Adapted physical activity quarterly : APAQ
8701671
IM
Journal Article
English
KW - MEDLINE
KW - Autistic Disorder/pp [Physiopathology]
KW - Autistic Disorder
KW - Child
KW - Female
KW - Humans
KW - Male
KW - Monitoring
KW - Ambulatory/is [Instrumentation]
KW - Motor Activity
KW - Play and Playthings
KW - Schools
RP - NOT IN FILE
SP - 308
EP - 321

JF - Adapted Physical Activity Quarterly
JA - Adapt Phys Act Q
VL - 25
IS - 4
CY - United States
N2 - This study compared moderate-to-vigorous physical activity (MVPA) of students with autism spectrum disorders (ASD) and students without disabilities during inclusive physical education and recess. Students (7-12 years) wore a uniaxial accelerometer in school for 5 consecutive school days. Results indicated a significant difference between settings, F(1,46) = 15.94, p < .01, partial eta2 = 0.26, observed power = 0.97. Students with and without ASD spent a higher proportion of time in MVPA during physical education than during recess, relative to the amount of time spent in those settings. In addition, structured physical education offers opportunities to increase students' MVPA engagement
SN - 0736-5829
AD - Department of Physical Education, National Kaohsiung Normal University, Taiwan. chpan@nknucc.edu.tw
UR - 18955747
ER -

TY - JOUR
ID - 993
T1 - Soil pollution in day-care centers and playgrounds in Norway: national action plan for mapping and remediation
A1 - Ottesen,R.T.
A1 - Alexander,J.
A1 - Langedal,M.
A1 - Haugland,T.
A1 - Hoygaard,E.
Y1 - 2008/12//
N1 - Ottesen, Rolf Tore. Alexander, Jan. Langedal, Marianne. Haugland, Toril. Hoygaard, Erik
Environmental geochemistry and health
8903118
IM
Journal Article
English
KW - MEDLINE
KW - Child Day Care Centers
KW - Child
KW - Preschool
KW - Cities
KW - Environmental Monitoring
KW - Environmental Remediation
KW - Geography
KW - Humans
KW - Infant
KW - Norway
KW - Risk Assessment
KW - Soil Pollutants/an [Analysis]
KW - Urban Health
RP - NOT IN FILE
SP - 623
EP - 637
JF - Environmental Geochemistry & Health
JA - Environ Geochem.Health
VL - 30
IS - 6
CY - Netherlands

N2 - Systematic geochemical mapping based on sampling and analysis of surface soils (0-2 cm) has been carried out in several Norwegian cities. The soils in the oldest parts of the cities are contaminated with metals (especially Pb) and polycyclic aromatic hydrocarbons (PAH). Due to the fact that children are often in contact with surface soil, it was realized that special focus had to be directed towards soils in day-care centers and playgrounds. The first mapping and remedy program was initiated in Trondheim in 1996. Here, the importance of copper-chromium-arsenic (CCA)-pressure-impregnated wood in playing equipment as a pollution source for soils was documented, and a process was started with the aim to ban this product. Soils from day-care centers in the inner city of Bergen were polluted to a degree that required remediation in 45% out of 87 centers, mainly due to high concentrations of Pb and benzo(a)pyrene. In Oslo, 38% of 700 day-care centers needed remediation due to soil pollution by Pb, BaP, Cd, Hg, Ni and PCB. Removal of CCA-impregnated wood was necessary in more than half of the day-care centers The Norwegian parliament has decided to investigate all outdoor playing areas in day-care centers, playgrounds and schoolyards in Norway, starting in 2007 with day-care centers in the ten largest cities and five most important industrial areas. The Norwegian Institute of Public Health has developed quality criteria for soils in day-care centers and playgrounds for As, Cd, Cr(6+), Hg, Ni, Pb, zinc, PAH(sum16), benzo(a)pyrene, and PCB(sum7). The Geological Survey of Norway has developed guidance for mapping of soil pollution (sampling, chemical analysis and reporting) in day-care centers. Especially the sampling strategy has been developed in the period 1996-2007, and the preferred sampling strategy is to collect at least 10 samples of surface soil (0-2 cm) from (1) "original soil" on the site, (2) artificial man-made "hills", and (3) soils used for growing vegetables. A total number of 2,000 day-care-centres are to be investigated, and necessary remediation should be completed before the end of 2010
SN - 0269-4042
AD - Geological Survey of Norway, Norwegian University of Science and Technology (NTNU), Leiv Eirikssonsvei 39, 7491 Trondheim, Norway. rolf.ottesen@ngu.no
UR - 18563591
ER -

TY - JOUR
ID - 994
T1 - Accuracy of spring-levered and piezo-electric pedometers in primary school Japanese children
A1 - Nakae,S.
A1 - Oshima,Y.
A1 - Ishii,K.
Y1 - 2008/09//
N1 - Nakae, Satoshi. Oshima, Yoshitake. Ishii, Kojiro
Journal of physiological anthropology
101269653
IM
Journal Article. Research Support, Non-U.S. Gov't
English
KW - MEDLINE
KW - Asian Continental Ancestry Group
KW - Child
KW - Female
KW - Humans
KW - Male
KW - Monitoring
KW - Physiologic/is [Instrumentation]
KW - Motor Activity/ph [Physiology]
KW - Sports Equipment/st [Standards]
KW - Walking
RP - NOT IN FILE
SP - 233
EP - 239
JF - Journal of Physiological Anthropology
JA - J Physiol Anthropol
VL - 27
IS - 5

CY - Japan
N2 - Pedometers are used in many studies because physical activities can be simply assessed using them. In addition to the spring-levered type, piezo-electric pedometers have recently been used. However, their accuracy in children has not been investigated in previous studies. In this study, we investigated the accuracy of spring-levered pedometer and piezo-electric pedometer step counts in comparison with hand-tallied step counts with children during self-paced walking. First to 6th-grade primary school children (394 subjects; 201 boys and 193 girls) walked with pedometers on an outdoor 50-m course, and the measured values were compared with actual steps at 3 speeds: normal, slow, and fast paces. The counts obtained by the spring-levered pedometer were significantly lower than the actual steps in most grades at all walking paces. In the normal- and fast-pace walking, the counts by spring-levered pedometer were significantly lower than the actual steps in all grades excluding the 5th grade. Moreover, the spring-levered pedometer underestimated by 25% or more in all grades at slow pace. In contrast, the percent error of the counts by the piezo-electric pedometers at normal pace were mostly within +/-3%, confirming their high accuracy. Based on these findings, spring-levered pedometers are not appropriate for children, whereas piezo-electric pedometers are useful for investigation of the physical activity of children
SN - 1880-6805
AD - Health & Human Performance Research Center, Doshisha University. snakae@mail.doshisha.ac.jp
UR - 18838838
ER -

TY - JOUR
ID - 995
T1 - Playful interaction: occupational therapy for all children on the school playground
A1 - Bundy,A.C.
A1 - Luckett,T.
A1 - Naughton,G.A.
A1 - Tranter,P.J.
A1 - Wyver,S.R.
A1 - Ragen,J.
A1 - Singleton,E.
A1 - Spies,G.
Y1 - 2008/09//
N1 - Bundy, Anita C. Luckett, Tim. Naughton, Geraldine A. Tranter, Paul J. Wyver, Shirley R. Ragen, Jo. Singleton, Emma. Spies, Greta
The American journal of occupational therapy : official publication of the American Occupational Therapy Association
7705978, 3o4
IM
Journal Article
English
KW - MEDLINE
KW - Child
KW - Preschool
KW - Cohort Studies
KW - Female
KW - Humans
KW - Male
KW - Occupational Therapy/mt [Methods]
KW - Play and Playthings
KW - Schools
RP - NOT IN FILE
SP - 522
EP - 527
JF - American Journal of Occupational Therapy
JA - Am J Occup Ther
VL - 62
IS - 5

CY - United States
N2 - We examined the impact of an intervention on the playfulness of 5- to 7-year-old children who are developing typically. Materials that had no defined purpose were placed on a school playground for 11 weeks. The Test of Playfulness (ToP) was used to compare videotaped play segments pre- and postintervention. Teachers who did playground duty were interviewed regarding changes in play. ToP data were analyzed using a Wilcoxon signed-ranks test. Interview data were analyzed for themes. ToP scores were significantly higher after intervention (Z= -1.94; p = .025, one-tailed; Cohen's d = 0.55). Teachers reported that children were more social, creative, and resilient when the materials were on the playground. Children who were creative, rather than very physically capable, became leaders in activity. Our results revealed a potential role for occupational therapists with typically developing children in schools. This finding has clear implications for children with disability
SN - 0272-9490
AD - Faculty of Health Sciences, University of Sydney, Lidcombe New South Wales, Australia. a.bundy@usyd.edu.au
UR - 18826012
ER -

TY - JOUR
ID - 996
T1 - School design and physical activity among middle school girls
A1 - Cohen,D.
A1 - Scott,M.
A1 - Wang,F.Z.
A1 - McKenzie,T.L.
A1 - Porter,D.
Y1 - 2008/09//
N1 - Cohen, Deborah. Scott, Molly. Wang, Frank Zhen. McKenzie, Thomas L. Porter, Dwayne
Journal of physical activity & health
101189457
IM
Journal Article. Multicenter Study. Randomized Controlled Trial. Research Support, N.I.H., Extramural
English
KW - MEDLINE
KW - Child
KW - Environment Design
KW - Exercise
KW - Facility Design and Construction
KW - Female
KW - Humans
KW - Questionnaires
KW - Schools
KW - United States
RP - NOT IN FILE
SP - 719
EP - 731
JF - Journal of Physical Activity & Health
JA - J Phys Act Health
VL - 5
IS - 5
CY - United States
N2 - Building design and grounds might contribute to physical activity, and youth spend much of their daylight hours at school. We examined the associations among school building footprints, the size of school grounds, and in-school physical activity of 1566 sixth-grade girls from medium to large middle schools enrolled in the Trial of Activity for Adolescent Girls (TAAG). The school building footprint and the number of active outdoor amenities were associated with physical activity among adolescent girls. On average, the school footprint size accounted for 4% of all light physical activity and 16% of all MET-weight moderate-to-vigorous physical activity (MW-MVPA) during school hours. Active outdoor amenities accounted for 29% of all MW-MVPA

during school. School design appears to be associated with physical activity, but it is likely that programming (eg, physical education, intramurals, club sports), social factors, and school siting are more important determinants of total physical activity
SN - 1543-3080
AD - RAND Corporation, Santa Monica, CA 90405, USA
UR - 18820346
ER -

TY - JOUR
ID - 997
T1 - The 2007 annual report of the Regional Infant and Child Mortality Review Committee
A1 - Randall,B.
A1 - Wilson,A.L.
A1 - Regional Infant and Child Mortality Review Committee.
Y1 - 2007///
N1 - Randall, Brad. Wilson, Ann L. Regional Infant and Child Mortality Review Committee
South Dakota medicine : the journal of the South Dakota State Medical Association
101265265
IM
Journal Article
English
KW - MEDLINE
KW - Adolescent
KW - Cause of Death
KW - Child
KW - Child Mortality
KW - Preschool
KW - Humans
KW - Infant
KW - Infant Mortality
KW - Newborn
KW - South Dakota
KW - Sudden Infant Death/ep [Epidemiology]
KW - Sudden Infant Death/pc [Prevention & Control]
RP - NOT IN FILE
SP - 287
EP - 289
JF - South Dakota Medicine: The Journal of the South Dakota State Medical Association
JA - S D Med
VL - 61
IS - 8
CY - United States
N2 - The mission of the Regional Infant and Child Mortality Review Committee (RICMRC) is to review infant and child deaths so that information can be transformed into action to protect young lives. The 2007 review area includes South Dakota's Minnehaha, Turner, Lincoln, Moody, Lake, McCook, Union, Hansen, Miner and Brookings counties. Although there were no deaths in 2007 that met the criteria of the Sudden Infant Death Syndrome (SIDS) in our region, there were three infant deaths associated with unsafe sleeping environments (including adult co-sleeping) that either caused or potentially may have caused these infants' deaths. We need to continue to promote the "Back to Sleep" campaign message of not only placing infants to sleep on their backs, but also making sure infants are put down to sleep on safe, firm sleeping surfaces and that they are appropriately dressed for the ambient temperature. Parents need to be aware of the potential hazards of co-sleeping with their infants. Compared to nine such deaths in 2006, only four deaths in 2007 involved motor-vehicle crashes, none of which were alcohol related. Two drowning deaths illustrated the rapidity in which even momentary caregiver distractions can lead to deaths in children in and around water. Since 1997 the Regional Infant and Child Mortality Review Committee (RICMRC) has sought to achieve its mission to "review infant and child deaths so that information can be transformed into action to protect young lives." For 2007, the committee reviewed 25 deaths from Minnehaha, Turner, Lincoln, Moody, Lake, McCook, Union, Hansen, Miner and Brookings

counties that met the following criteria: Children under the age of 18 dying subsequent to hospital discharge following delivery. Children who either died in these counties from causes sustained in them, or residents who died elsewhere from causes sustained in the 10-county region. The report that follows reviews the committee's activities for 2007. No deaths meeting the criteria for Sudden Infant Death Syndrome (SIDS) occurred in the review region. The committee has observed a stable decline in the number of deaths due to the SIDS as compared to the eight SIDS or possible SIDS deaths occurring in 1999. Unfortunately, however, there were three infant deaths in SIDS-like settings where asphyxia could not be excluded. Although the committee strives to be consistent year to year in its investigation protocols, we have noticed that the number of deaths classified as "undetermined" has been increasing over the last few years (starting in 1997: 1, 2, 1, 0, 2, 1, 2, 0, 5, 4, 5). Although it is possible that deaths certified as SIDS in earlier years may now be classified as undetermined due to asphyxial risks, we may also be seeing an increase in infants sleeping in unsafe environments. Our data may be mirroring a national trend amongst death investigators to increasingly recognize asphyxial risks in infant death scenes. Infants co-sleeping with adults (two deaths in 2007) may represent a risk factor not as frequently seen as in prior years. We will watch this trend closely in the future. The Committee strenuously advocates that the Back to Sleep message include placing infants on their back to sleep, avoiding soft bedding for infants, making parents aware of the potential hazards of co-sleeping with young infants, and eliminating both pre- and post-delivery fetal/infant cigarette smoke exposure. a decrease in youth motor-vehicle crash deaths to four versus the nine seen in 2006.2 Three of those dying in motor-vehicle crashes in 2007 were not wearing seat belts. The committee continues to feel that some of these deaths could have been prevented had these children been wearing a seat belt. Compared to four alcohol-related motor-vehicle crash fatalities in 2006, no deaths in 2007 were alcohol related. The committee, however, is painfully aware that even one crash involving multiple occupants can rapidly skew the apparent improvements in 2007 in the wrong direction. In 2007, two youths, both with disabilities, drown. One drown in a bath tub and the other in an outdoor pool. It is unfortunate that these deaths bring home the message that even the smallest, momentary lapses in supervision of these children can have fatal consequences. Fortunately, the child and infant homicide rate in our region is very low. Unfortunately, however, one infant did die as a result of being assaulted and shaken by one of its parents. There remains a need for the community at large, and the medical community specifically, to address issues of parental anger management, particularly when caregiver frustration is directed at children
SN - 0038-3317
UR - 18819316
ER -

TY - JOUR
ID - 998
T1 - Parental experience of family resources in single-parent families having a child with cancer
A1 - Huang,I.C.
A1 - Mu,P.F.
A1 - Chiou,T.J.
Y1 - 2008/10//
N1 - Huang, I-Chen. Mu, Pei-Fan. Chiou, Tzeon-Jye
Journal of clinical nursing
bzz, 9207302
N
Journal Article
English
KW - MEDLINE
KW - Adaptation
KW - Psychological
KW - Child
KW - Humans
KW - Neoplasms/px [Psychology]
KW - Parents/px [Psychology]
KW - Single-Parent Family
RP - NOT IN FILE
SP - 2741
EP - 2749
JF - Journal of Clinical Nursing

JA - J Clin Nurs
VL - 17
IS - 20
CY - England
N2 - AIM: The purpose of this study was to explore the essence of family experiences in terms of family resources and how these assist a single-parent caring for a child with cancer. BACKGROUND: When families face stresses caused by cancer, they need to readjust their roles, interactive patterns and relationships, both inside and outside the family. During the adaptation process, family resources may assist recovery from stress and a return to equilibrium. Most research has emphasised the support resources available to two-parent families during the treatment process. There is a lack of information on the experiences of single-parent families and their available resources together with the functions and roles played by family resources during the adjustment process. DESIGN: Qualitative. RESULTS: Five major themes were identified: (i) facing the disease with courage; (ii) hope kindled by professionals; (iii) constructing parental role ability; (iv) assisting the children to live with the illness; and (v) family flexibility. CONCLUSION: The results of the current study demonstrate that single-parent families with a child suffering from cancer employ family resources to assist family adjustment and to maintain family function/equilibrium. These results explain the dynamic interactions between the multiple levels of resources available to the family. RELEVANCE TO CLINICAL PRACTICE: The study results provide evidence-based information that identifies the nature of family resources in single-parent families and describes how these resources can be applied to assist the families
SN - 1365-2702
AD - Institute of Clinical Nursing, National Yang-Ming University, Taipei, Taiwan
UR - 18808643
ER -

TY - JOUR
ID - 999
T1 - [Ecchymotic angioedema revealing childhood systemic lupus erythematosus with anti-C1q antibodies]. [French]
A1 - Frot,A.S.
A1 - Barbarot,S.
A1 - Poignant,S.
A1 - Guyot,C.
A1 - Stalder,J.F.
Y1 - 2008/08//
N1 - Frot, A-S. Barbarot, S. Poignant, S. Guyot, C. Stalder, J-F
Annales de dermatologie et de venereologie
5rc, 7702013
IM
Case Reports. English Abstract. Journal Article
French
KW - MEDLINE
KW - Adrenal Cortex Hormones/ad [Administration & Dosage]
KW - Adrenal Cortex Hormones/tu [Therapeutic Use]
KW - Angioedema/et [Etiology]
KW - Antibodies
KW - Antinuclear/an [Analysis]
KW - Biopsy
KW - Child
KW - Complement C1q/im [Immunology]
KW - Disease Progression
KW - Ecchymosis/et [Etiology]
KW - Female
KW - Fluorescent Antibody Technique
KW - Direct
KW - Follow-Up Studies
KW - Humans
KW - Immunoglobulin M/an [Analysis]

KW - Kidney/pa [Pathology]
KW - Lupus Erythematosus
KW - Systemic/co [Complications]
KW - Systemic/di [Diagnosis]
KW - Systemic/dt [Drug Therapy]
KW - Systemic/im [Immunology]
KW - Lupus Nephritis/di [Diagnosis]
KW - Lupus Nephritis/pa [Pathology]
KW - Time Factors
KW - Treatment Outcome
KW - Urticaria/et [Etiology]
KW - Vasculitis/et [Etiology]
RP - NOT IN FILE
SP - 584
EP - 586
JF - Annales de Dermatologie et de Venereologie
JA - Ann Dermatol Venereol
VL - 135
IS - 8-9
CY - France
N2 - BACKGROUND: Hypocomplement urticarial vasculitis syndrome may be the presenting sign of systemic lupus erythematosus. Hypocomplement urticarial vasculitis presents as atypical urticaria associated in 50% of cases with angioedema. On laboratory investigation, hypocomplementaemia is the characteristic feature, with reduced C3, C4 and C1q. This disease is very rare in children. PATIENTS AND METHODS: An eight-year-old girl was hospitalised for relapsing urticaria with ecchymotic angioedema present for one year, in a setting of impaired general health and fever. Screening for native anti-DNA and antinuclear antibodies was positive. Analysis of complement revealed activation of the classical pathway with reduced CH50, C4 and C3. These anomalies persisted outside active episodes. The C1q fraction was completely depressed and screening for anti-C1q was positive. There was no quantitative or qualitative deficit in C1-esterase inhibitor. Direct immunofluorescence of skin lesions demonstrated deposits of immunoglobulin and complement. These episodes of angioedema persisted despite long-term systemic corticosteroid therapy (1mg/kg per day). DISCUSSION: This is the first reported case of hypocomplement urticarial vasculitis syndrome arising from systemic lupus erythematosus in a child exhibiting anti-C1q antibodies. Furthermore, this case is original because of the highly ecchymotic nature of the lesions. In the presence of angioedema with ecchymotic progression associated with atypical chronic urticaria, a diagnosis of hypocomplement urticarial vasculitis syndrome should be considered
SN - 0151-9638
AD - Service de dermatologie, Hotel-Dieu, place Alexis-Ricordeau, 44093 Nantes cedex 1, France
UR - 18789294
ER -

TY - JOUR
ID - 1000
T1 - Lead poisoning associated with malaria in children of urban areas of Nigeria
A1 - Nriagu,J.
A1 - Afeiche,M.
A1 - Linder,A.
A1 - Arowolo,T.
A1 - Ana,G.
A1 - Sridhar,M.K.
A1 - Oloruntoba,E.O.
A1 - Obi,E.
A1 - Ebenebe,J.C.
A1 - Orisakwe,O.E.
A1 - Adesina,A.
Y1 - 2008/10//
N1 - Nriagu, Jerome. Afeiche, Myriam. Linder, Aaron. Arowolo, Toyin. Ana, Godson. Sridhar, Mynepalli K C. Oloruntoba, E O. Obi, Ejeatulu. Ebenebe, Joy C. Orisakwe, Orish E. Adesina, Adesuwa

International journal of hygiene and environmental health
do6, 100898843
IM
Journal Article
English
KW - MEDLINE
KW - Animals
KW - Child
KW - Preschool
KW - Cross-Sectional Studies
KW - Environmental Exposure/ae [Adverse Effects]
KW - Environmental Monitoring
KW - Epidemiological Monitoring
KW - Female
KW - Helminthiasis/co [Complications]
KW - Helminthiasis/ep [Epidemiology]
KW - Humans
KW - Lead/bl [Blood]
KW - Lead Poisoning/bl [Blood]
KW - Lead Poisoning/co [Complications]
KW - Lead Poisoning/ep [Epidemiology]
KW - Malaria/bl [Blood]
KW - Malaria/co [Complications]
KW - Malaria/ep [Epidemiology]
KW - Male
KW - Nigeria/ep [Epidemiology]
KW - Prevalence
KW - Risk Factors
KW - Urban Health
RP - NOT IN FILE
SP - 591
EP - 605
JF - International Journal of Hygiene & Environmental Health
JA - Int J Hyg Environ Health
VL - 211
IS - 5-6
CY - Germany
N2 - The principal objectives of this study are to (a) investigate the prevalence of elevated blood lead levels (EBLLs) in children of three major cities of Nigeria with different levels of industrial pollution; (b) identify the environmental, social and behavioral risk factors for the EBLLs in the children; and (c) explore the association between malaria (endemic in the study areas) and EBLLs in the pediatric population. The study involved 653 children aged 2-9 years (average, 3.7 years). The mean blood lead level (BLL) for the children was 8.9+/-4.8microg/dL, the median value was 7.8microg/dL, and the range was 1-52microg/dL. About 25% of the children had BLL greater than 10microg/dL. There were important differences in BLLs across the three cities, with the average value in Ibadan (9.9+/-5.2microg/dL) and Nnewi (8.3+/-3.5microg/dL) being higher than that in Port Harcourt (4.7+/-2.2micro/dL). Significant positive associations were found between BLL and a child's town of residence (p<0.001), age of the child (p=0.004), length of time the child played outside (p<0.001), presence of pets in a child's home (p=0.023), but negatively with educational level of caregiver (p<0.001). This study is one of the first to find a significant negative association between BLL and malaria in a pediatric population, and this association remained significant after controlling for confounding diseases and symptoms. The shared environmental and socio-demographic risks factors for lead exposure and Plasmodium (most common malaria parasites) infection in urban areas of Nigeria are discussed along with possible ways that lead exposure may influence the host response to infection with malarial parasites
SN - 1438-4639
AD - Department of Environmental Health Sciences, School of Public Health, University of Michigan, Ann Arbor, MI 48109, USA. jnriagu@umich.edu
UR - 18599348

ER -

TY - JOUR
ID - 1001
T1 - Myopia and the urban environment: findings in a sample of 12-year-old Australian school children
A1 - Ip,J.M.
A1 - Rose,K.A.
A1 - Morgan,I.G.
A1 - Burlutsky,G.
A1 - Mitchell,P.
Y1 - 2008/09//
N1 - Ip, Jenny M. Rose, Kathryn A. Morgan, Ian G. Burlutsky, George. Mitchell, Paul
Investigative ophthalmology & visual science
gwi, 7703701
IM
Journal Article. Research Support, Non-U.S. Gov't
English
KW - MEDLINE
KW - Adolescent
KW - Australia/ep [Epidemiology]
KW - Child
KW - Environment
KW - Female
KW - Humans
KW - Male
KW - Myopia/ep [Epidemiology]
KW - Prevalence
KW - Questionnaires
KW - Socioeconomic Factors
KW - Suburban Population/sn [Statistics & Numerical Data]
KW - Urban Population/sn [Statistics & Numerical Data]
RP - NOT IN FILE
SP - 3858
EP - 3863
JF - Investigative Ophthalmology & Visual Science
JA - Invest Ophthalmol Vis Sci
VL - 49
IS - 9
CY - United States
N2 - PURPOSE: To examine associations between myopia and measures of urbanization in a population-based sample of 12-year-old Australian children. METHODS: Questionnaire data on sociodemographic and environmental factors including ethnicity, parental education, and time spent in near work and outdoor activities were collected from 2367 children (75.0% response) and their parents. Population density data for the Sydney area were used to construct five urban regions. Myopia was defined as spherical equivalent refraction </= -0.50 D. RESULTS: Myopia prevalence was lowest in the outer suburban region (6.9%) and highest in the inner city region (17.8%), with mean refraction tending toward greater myopia by region (outer suburban to inner city), after adjustment for age, sex, ethnicity, near work, outdoor activity, and parental myopia. Multivariate-adjusted analyses confirmed greater odds for myopia in regions of higher population density (P(trend) = 0.0001). Myopia was significantly more prevalent among children living in apartment residences than other housing types (chi(2) < 0.0001), after adjustment for ethnicity, near work, and outdoor activity. Housing density (measured as the number of houses visible from a front door) was not significantly associated with myopia (chi(2) = 0.1). For both European Caucasian and East Asian children, myopia was most prevalent in the inner city region (8.1% and 55.1%, for European Caucasian and East Asian, respectively). CONCLUSIONS: The higher myopia prevalence in inner city-urban areas compared with outer suburban areas for this large childhood sample suggest that even moderate environmental differences within a predominantly urban setting may be associated with increased odds of myopia. These findings are consistent with previous reports of rural-urban differences in childhood myopia

SN - 1552-5783
AD - Centre for Vision Research, Department of Ophthalmology and the Westmead Millennium Institute, University of Sydney, Sydney, Australia
UR - 18469186
ER -

TY - JOUR
ID - 1002
T1 - Correlates of overweight & obesity among school going children of Wardha city, Central India
A1 - Bharati,D.R.
A1 - Deshmukh,P.R.
A1 - Garg,B.S.
Y1 - 2008/06//
N1 - Bharati, D R. Deshmukh, P R. Garg, B S
The Indian journal of medical research
gjf, 0374701
IM
Journal Article
English
KW - MEDLINE
KW - Adolescent
KW - Child
KW - Cross-Sectional Studies
KW - Female
KW - Humans
KW - India/ep [Epidemiology]
KW - Male
KW - Obesity/ep [Epidemiology]
KW - Obesity/pc [Prevention & Control]
KW - Overweight/ep [Epidemiology]
KW - Overweight/pc [Prevention & Control]
KW - Risk Factors
RP - NOT IN FILE
SP - 539
EP - 543
JF - Indian Journal of Medical Research
JA - Indian J Med Res
VL - 127
IS - 6
CY - India
N2 - BACKGROUND & OBJECTIVE: Overweight and obesity are important determinants of health leading to adverse metabolic changes and increase the risk of non communicable diseases. Following the increase in adult obesity, the proportion of overweight and obese children and adolescents has also been increasing. Hence, the present study was undertaken to study the magnitude of overweight/obesity and its correlates among school going children of Wardha city in central India and suggest interventions. METHODS: The cross-sectional study was carried out in all the 31 middle-schools (5th to 7th standard) and high-schools (8th to 10th standard) of Wardha city. Probability proportionate to size of population technique (PPS) was used to decide the number of children to be studied from each school, each class and then each section. Systematic random sampling technique was used to select the children from each section. Pre-designed and pre-tested questionnaire was used to elicit the information on family characteristics and individual characteristics. Height and weight was measured and BMI was calculated. Overweight and obesity was assessed by BMI for age. Student who had BMI for age >or=85th and < 95th percentile of reference population were classified as overweight and BMI for age >or=95th percentile of reference population were classified as obese. RESULTS: Overweight and obesity was found to be 3.1 per cent (95% CI: 2.5-3.8%) and 1.2 per cent (95% CI: 0.8-1.8%) respectively; together constitute 4.3 per cent (95% CI: 3.6-5.2%) for overweight/ obesity. Final model of the multivariate logistic regression showed that the important correlates of overweight/obesity were urban residence, father and/or mother involved in service/business, English medium school and child playing outdoor games for less than 30

min. INTERPRETATION & CONCLUSION: The magnitude of overweight/obesity among school going children of Wardha city was found to be 4.3 per cent. Family characteristics play important role in predisposing the children to overweight/obesity and hence the interventions need to be directed towards the families
SN - 0971-5916
AD - Dr Sushila Nayar School of Public Health, Incorporating Department of Community Medicine, Mahatma Gandhi Institute of Medical Sciences, Sewagram, India
UR - 18765871
ER -

TY - JOUR
ID - 1003
T1 - Activity levels during physical education and recess in two special schools for children with mild intellectual disabilities
A1 - Sit,C.H.
A1 - McKenzie,T.L.
A1 - Lian,J.M.
A1 - McManus,A.
Y1 - 2008/07//
N1 - Sit, Cindy H P. McKenzie, Thomas L. Lian, John M G. McManus, Alison
Adapted physical activity quarterly : APAQ
8701671
IM
Comparative Study. Journal Article. Research Support, Non-U.S. Gov't
English
KW - MEDLINE
KW - Child
KW - Disabled Children
KW - Education
KW - Special/mt [Methods]
KW - Exercise
KW - Female
KW - Hong Kong
KW - Humans
KW - Leisure Activities
KW - Male
KW - Mental Disorders
KW - Motor Activity
KW - Physical Education and Training/mt [Methods]
KW - Sports
RP - NOT IN FILE
SP - 247
EP - 259
JF - Adapted Physical Activity Quarterly
JA - Adapt Phys Act Q
VL - 25
IS - 3
CY - United States
N2 - This study compared physical education (PE) and recess in two markedly different special schools for children with mild intellectual disabilities; one school had a reputation for focusing on sports (High Sport Focus-HSF) and the other did not (Low Sport Focus-LSF). Data were collected in 24 PE classes and 48 recess periods using a validated observation system. During both PE and recess, HSF students engaged in physical activity (PA) at greater intensity levels, but LSF students accrued more total activity min. Differences in PA during PE between the schools were associated with both lesson context and teacher behavior. The results suggest written (e.g., scheduling) and unwritten policies within schools affect children's activity levels
SN - 0736-5829
AD - University of Hong Kong, Institute of Human Performance, Pokfulam, Hong Kong. sithp@hku.hk
UR - 18765864

ER -

TY - JOUR
ID - 1004
T1 - Blood lead and cadmium levels and relevant factors among children from an e-waste recycling town in China
A1 - Zheng,L.
A1 - Wu,K.
A1 - Li,Y.
A1 - Qi,Z.
A1 - Han,D.
A1 - Zhang,B.
A1 - Gu,C.
A1 - Chen,G.
A1 - Liu,J.
A1 - Chen,S.
A1 - Xu,X.
A1 - Huo,X.
Y1 - 2008/09//
N1 - Zheng, Liangkai. Wu, Kusheng. Li, Yan. Qi, Zongli. Han, Dai. Zhang, Bao. Gu, Chengwu. Chen, Gangjian. Liu, Junxiao. Chen, Songjian. Xu, Xijin. Huo, Xia
Environmental research
ei2, 0147621
IM
Journal Article. Research Support, Non-U.S. Gov't
English
KW - MEDLINE
KW - Cadmium/bl [Blood]
KW - Child
KW - Preschool
KW - Conservation of Natural Resources
KW - Electronics
KW - Humans
KW - Infant
KW - Lead/bl [Blood]
KW - Questionnaires
KW - Spectrophotometry
KW - Atomic
RP - NOT IN FILE
SP - 15
EP - 20
JF - Environmental Research
JA - Environ Res
VL - 108
IS - 1
CY - United States
N2 - BACKGROUND: Primitive electronic waste (e-waste) recycling is ongoing in Guiyu, and thus toxic heavy metals may keep on threatening to the health of local children. Some related factors may contribute to the elevation of blood lead levels (BLLs) or blood cadmium levels (BCLs). OBJECTIVE: To investigate the children's BLLs and BCLs in Guiyu and Chendian as compare to discuss the effects of primitive e-waste recycling activities on children's health. METHODS: Two hundred and seventy-eight children less than 8 years who lived in Guiyu and Chendian were observed, and their BLLs and BCLs were determined by graphite atomizer absorption spectrophotometer. Questionnaire survey for risk factors was also performed and data were analyzed using spearman correlation analyses and logistic regression analyses. RESULTS: Children living in Guiyu had significantly higher BLLs and BCLs as compared with those living in Chendian (p<0.01). In Guiyu, 70.8% of children (109/154) had BLLs>10 microg/dL, and 20.1% of children (31/154) had BCLs>2 microg/L, compared with 38.7% of children (48/124) had BLLs>10 microg/dL and 7.3% of children (9/124) had BCLs>2

microg/L in Chendian (p<0.01, respectively). We also observed a significant increasing trend in BLLs with increasing age in Guiyu (p<0.01). Mean height of children in Guiyu was significantly lower than that in Chendian (p<0.01). The risk factors related to children's BLLs and BCLs mainly included father's engagement in the work related to e-waste, children's residence in Guiyu and the amount of time that children played outside near the road everyday. CONCLUSIONS: There are close relationships between the BLLs, BCLs in children and the primitive e-waste recycling activities in Guiyu. Environmental pollution, especially lead pollution, has threatened the health of children living around e-waste recycling site
SN - 1096-0953
AD - Central Laboratory and Key Immunopathology Laboratory of Guangdong Province, Shantou University Medical College, Shantou, PR China
UR - 18514186
ER -

TY - JOUR
ID - 1005
T1 - Using social cognitive theory to predict physical activity in inner-city African American school children.[Erratum appears in J Sport Exerc Psychol. 2009 Feb;31(1):128-9]
A1 - Martin,J.J.
A1 - McCaughtry,N.
Y1 - 2008/08//
N1 - Martin, Jeffrey J. McCaughtry, Nate
Journal of sport & exercise psychology
8809258
IM
Journal Article
English
KW - MEDLINE
KW - Adolescent
KW - African Americans
KW - Child
KW - Cognition
KW - Exercise/px [Psychology]
KW - Exercise
KW - Female
KW - Forecasting
KW - Humans
KW - Male
KW - Midwestern United States
KW - Models
KW - Theoretical
KW - Psychological Theory
KW - Urban Population
RP - NOT IN FILE
SP - 378
EP - 391
JF - Journal of Sport & Exercise Psychology
JA - J Sport Exerc Psychol
VL - 30
IS - 4
CY - United States
N2 - Researchers using social cognitive theory and employing built environment constructs to predict physical activity (PA) in inner-city African American children is quite limited. Thus, the purpose of our investigation was to evaluate the ability of important social cognitive variables (e.g., self-efficacy) and built environment constructs (e.g., neighborhood hazards) to predict African American children's PA. Children (N = 331, ages 10-14) completed questionnaires assessing social cognitive theory constructs and PA. Using multiple regression analyses we were able to account for 19% of the variance in PA. Based on standardized beta weights, the best predictors of PA were time spent outside and social support derived from friends. These findings illuminate the

valuable role of PA support from peers, as well as the simple act of going outside for inner-city African American children
SN - 0895-2779
AD - Division of Kinesiology, Health, and Sport Studies, Wayne State University, Detroit, MI, USA
UR - 18723898
ER -

TY - JOUR
ID - 1006
T1 - Inflatable bouncer-related fractures in children
A1 - Avoian,T.
A1 - Choi,P.D.
A1 - Manjra,N.
A1 - Weiss,J.
Y1 - 2008/09//
N1 - Avoian, Tigran. Choi, Paul D. Manjra, Nahid. Weiss, Jennifer
Journal of pediatric orthopedics
hsw, 8109053
IM
Journal Article
English
KW - MEDLINE
KW - Accidents
KW - Adolescent
KW - Adult
KW - Age Factors
KW - Child
KW - Preschool
KW - Databases
KW - Factual
KW - Female
KW - Fractures
KW - Bone/ep [Epidemiology]
KW - Bone/et [Etiology]
KW - Bone/pc [Prevention & Control]
KW - Humans
KW - Infant
KW - Male
KW - Parenting
KW - Play and Playthings
KW - Retrospective Studies
KW - Risk Factors
KW - Sex Factors
KW - Trauma Centers/sn [Statistics & Numerical Data]
KW - Upper Extremity/in [Injuries]
RP - NOT IN FILE
SP - 656
EP - 659
JF - Journal of Pediatric Orthopedics
JA - J Pediatr Orthop
VL - 28
IS - 6
CY - United States
N2 - BACKGROUND: Inflatable bouncers or moonbouncers are very popular in private and public settings and are usually perceived as very safe attractions, but are associated with frequent fractures in children. To date, there are no publications in the medical literature about these types of injuries. The purpose of this study was to show skeletal injuries related to inflatable bouncer use, describe their characteristics, and determine possible risk

factors and preventive measures. METHODS: Demographic data and injury characteristics were analyzed for all patients who were treated for inflatable bouncer-associated injuries in the pediatric fracture clinic of a level I trauma center from October 2002 to March 2007. RESULTS: Forty-nine patients were treated for inflatable bouncer-related fractures. Children ranged in age from 1.5 to 15 years old (mean age, 7.8 years) with a male-female ratio of approximately 3:1. The most commonly injured region was the upper extremity (65.5%, n = 32). The most commonly injured area was the elbow (31%, n = 15), and the most common single diagnosis was supracondylar humerus fracture (22%, n = 11). Diaphyseal long bone fractures were found in 18% (n = 9) of the patients and nondiaphyseal in 71% (n = 35). One patient (2%) had an open fracture. Mechanisms of injury included collision of 1 person with another (67%), falling out of a bouncer onto a hard object outside the device (19%), and twisting motion to the leg (14%). There was no adult supervision in many of the incidents (43%), and the presence of different-aged children inside the jumper took place in 52% cases. CONCLUSIONS: Inflatable bouncers can cause serious orthopaedic injuries. Children playing in the bouncer should be placed in small groups according to their size and should be closely supervised at all times. STUDY DESIGN: Case series. Level IV evidence
SN - 1539-2570
AD - University of Southern California, Los Angeles, CA, USA. avoian@usc.edu
UR - 18724203
ER -

TY - JOUR
ID - 1007
T1 - Dental trauma among 5th and 6th grade Arab schoolchildren in Eastern Jerusalem
A1 - Sgan-Cohen,H.D.
A1 - Yassin,H.
A1 - Livny,A.
Y1 - 2008/08//
N1 - Sgan-Cohen, Harold D. Yassin, Hillal. Livny, Alon
Dental traumatology : official publication of International Association for Dental Traumatology
101091305, d2p
D
Journal Article
English
KW - MEDLINE
KW - Accidental Falls
KW - Arabs
KW - Athletic Injuries/ep [Epidemiology]
KW - Child
KW - Female
KW - Humans
KW - Injury Severity Score
KW - Israel/ep [Epidemiology]
KW - Jews
KW - Lip/pp [Physiopathology]
KW - Logistic Models
KW - Male
KW - Malocclusion/co [Complications]
KW - Prevalence
KW - Tooth Injuries/ep [Epidemiology]
KW - Tooth Injuries/et [Etiology]
RP - NOT IN FILE
SP - 458
EP - 461
JF - Dental Traumatology
JA - Dent Traumatol
VL - 24
IS - 4
CY - Denmark

N2 - Dental trauma is an irreversible pathology which after occurrence is characterized by life long debilitating effects. The objective of the study was to measure the prevalence of trauma in anterior permanent teeth among children and associations with independent variables. A total of 453 5th and 6th grade Arab Eastern Jerusalem schoolchildren participated. Trauma, incisal overjet and lip competence were measured by one examiner in the schools. Reasons and location of trauma were assessed by telephone interviews with the parents. The total prevalence of dental trauma was 33.8%. Severe trauma prevalence (involving dentine) was 12.6%. Severe trauma was more prevalent among boys (OR = 2.03, P = 0.026); children with lip incompetence (OR = 2.71, P = 0.001); and those with an overjet of 4mm or more (OR = 3.73, P < 0.001). The main reported causes for dental trauma were falling (29.1%), sports (16.4%), violence (20%) and playing (20%). According to the parents' reports, more fall-related injuries were reported at home, more sport injuries at school, more 'playing' injuries 'outside' (P < 0.001). Combination of results with a previous study among Jewish schoolchildren demonstrated: severe dental trauma was related to overjet (OR = 1.73, P = 0.002), lip incompetence (OR = 2.19, P < 0.001), gender (OR for males = 1.54, P = 0.005), but not to ethnicity (Arab or Jewish). The reasons for trauma observed in this study were potentially preventable. A community effort should therefore be pursued, with the aim of educating sports teachers, teachers, health personnel and schoolchildren regarding awareness of dental trauma prevention, with emphasis on utilizing mouthguards and early orthodontic intervention, when financially possible
SN - 1600-9657
AD - Department of Community Dentistry, Hebrew University-Hadassah School of Dental Medicine, Jerusalem, Israel. harolds@cc.huji.ac.il
UR - 18721348
ER -

TY - JOUR
ID - 1008
T1 - Bullying among school children in postwar bosnia and herzegovina: cross-sectional study
A1 - Cerni,Obrdalj E.
A1 - Rumboldt,M.
Y1 - 2008/08//
N1 - Cerni Obrdalj, Edita. Rumboldt, Mirjana
Croatian medical journal
c0s, 9424324
IM
Journal Article
English
KW - MEDLINE
KW - Adolescent
KW - Bosnia-Herzegovina
KW - Child
KW - Cross-Sectional Studies
KW - Female
KW - Humans
KW - Male
KW - Psychological Tests
KW - Psychometrics
KW - Questionnaires
KW - Schools
KW - Stress
KW - Psychological/co [Complications]
KW - Violence/px [Psychology]
KW - War
RP - NOT IN FILE
SP - 528
EP - 535
JF - Croatian Medical Journal
JA - Croat.Med J
VL - 49

IS - 4
CY - Croatia
N2 - AIM: To compare the prevalence and characteristics of bullying between two towns in Bosnia and Herzegovina-Stolac, which was exposed to firearm conflict during the 1992-1995 war in Bosnia and Herzegovina, and Posusje, which was outside of the active combat zone. METHODS: In this cross-sectional study, we included 484 primary school pupils attending 4th-8th grade of elementary school, 217 (44.8%) of them from Stolac and 267 (55.2%) from Posusje. The pupils were interviewed using a standardized questionnaire on the experience of bullying. RESULTS: Every sixth pupil (16.4%) experienced at least one form of bullying almost every day, while 34 (7.0%) pupils constantly bullied other children. Sixth-eighth graders were more often bullies than 4th-5th graders (P=0.044). Girls were most often victims was of bullying, while boys were most often bullies (P=0.036). The expected difference in bullying between the two towns was not observed, except for older pupils in Posusje, who were more violent than their peers in Stolac (P=0.044). Among the analyzed variables of sex, age, town, and school achievement, only male sex was significant predictor of bullying (P=0.010), increasing the relative risk by 3.005 times. CONCLUSION: Bullying among primary school pupils did not differ between areas that experienced war activities in 1992-1995. Our results could be useful in the introduction of specific prevention measures against bullying in postwar situation
SN - 1332-8166
AD - Department of Family Medicine, Mostar University School of Medicine, Bijeli Brijeg bb, 88000 Mostar, Bosnia and Herzegovina. edita1@net.hr
UR - 18717000
ER -

TY - JOUR
ID - 1009
T1 - A scripted activity study of the impact of protective advice on personal exposure to ultra-fine and fine particulate matter and volatile organic compounds
A1 - Stieb,D.M.
A1 - Evans,G.J.
A1 - Sabaliauskas,K.
A1 - Chen,L.I.
A1 - Campbell,M.E.
A1 - Wheeler,A.J.
A1 - Brook,J.R.
A1 - Guay,M.
Y1 - 2008/09//
N1 - Stieb, David M. Evans, Gregory J. Sabaliauskas, Kelly. Chen, L I. Campbell, Monica E. Wheeler, Amanda J. Brook, Jeffrey R. Guay, Mireille
Journal of exposure science & environmental epidemiology
101262796
IM
Journal Article
English
KW - MEDLINE
KW - Adult
KW - Air Pollution/an [Analysis]
KW - Air Pollution
KW - Indoor/an [Analysis]
KW - Child
KW - Preschool
KW - Environmental Exposure/an [Analysis]
KW - Environmental Exposure/pc [Prevention & Control]
KW - Environmental Monitoring
KW - Health Behavior
KW - Health Promotion/mt [Methods]
KW - Humans
KW - Models
KW - Statistical

KW - Ontario
KW - Organic Chemicals/an [Analysis]
KW - Organic Chemicals/ch [Chemistry]
KW - Ozone/an [Analysis]
KW - Particulate Matter/an [Analysis]
KW - Transportation
KW - Volatilization
RP - NOT IN FILE
SP - 495
EP - 502
JF - Journal of Exposure Science & Environmental Epidemiology
JA - J Expo Sci Environ Epidemiol
VL - 18
IS - 5
CY - United States
N2 - We evaluated the impact on personal exposure to air pollutants of following advice which typically accompanies air quality advisories and indices. Scripts prescribed the time, location, duration and nature of activities intended to simulate daily activity patterns for adults and children. Scripts were paired such that one individual would proceed with usual activities (base scenario), whereas the other (intervention scenario) would alter activities as if following advice. Other than commuting, where the intervention group walked or used public transportation rather than riding in personal vehicles, this group generally spent less time outdoors. Ultra-fine particles (UFPs), particulate matter of median aerodynamic diameter less than 2.5 mum (PM(2.5)) and total volatile organic compounds (VOCs) were measured using samplers carried by individuals during the course of daily activities. During daytime activities (e.g., work, daycare) constituting the largest share of sampling time (approximately 6 h per day), the intervention group experienced a 14% reduction in exposure to UFPs (P=0.01), a 21% reduction in exposure to PM(2.5) (P=0.08), and an 86% increase in exposure to VOCs (P=0.02). Other findings included an 89% increase in exposure to UFPs (P=0.02) and a threefold increase in exposure to VOCs (P=0.08) in the intervention group during evening cooking. Following smog advisory advice results in reduced exposures to some pollutants, while at the same time increasing exposure to others. Advice needs to be refined giving consideration to overall personal exposure
SN - 1559-064X
AD - ealthy Environments and Consumer Safety Branch, Health Canada, Ottawa, Ontario, Canada. dave_stieb@hc-sc.gc.ca
UR - 18059426
ER -

TY - JOUR
ID - 1010
T1 - A developmental examination of amygdala response to facial expressions
A1 - Guyer,A.E.
A1 - Monk,C.S.
A1 - Clure-Tone,E.B.
A1 - Nelson,E.E.
A1 - Roberson-Nay,R.
A1 - Adler,A.D.
A1 - Fromm,S.J.
A1 - Leibenluft,E.
A1 - Pine,D.S.
A1 - Ernst,M.
Y1 - 2008/09//
N1 - Guyer, Amanda E. Monk, Christopher S. McClure-Tone, Erin B. Nelson, Eric E. Roberson-Nay, Roxann. Adler, Abby D. Fromm, Stephen J. Leibenluft, Ellen. Pine, Daniel S. Ernst, Monique
Journal of cognitive neuroscience
c1n, 8910747
IM
Journal Article. Research Support, N.I.H., Intramural
English

KW - MEDLINE
KW - Adolescent
KW - Adult
KW - Aging
KW - Amygdala/bs [Blood Supply]
KW - Amygdala/ph [Physiology]
KW - Brain Mapping
KW - Child
KW - Emotions
KW - Eye Movements/ph [Physiology]
KW - Facial Expression
KW - Female
KW - Functional Laterality/ph [Physiology]
KW - Humans
KW - Image Processing
KW - Computer-Assisted/mt [Methods]
KW - Magnetic Resonance Imaging/mt [Methods]
KW - Male
KW - Oxygen/bl [Blood]
KW - Pattern Recognition
KW - Visual/ph [Physiology]
KW - Photic Stimulation/mt [Methods]
RP - NOT IN FILE
SP - 1565
EP - 1582
JF - Journal of Cognitive Neuroscience
JA - J Cogn Neurosci
VL - 20
IS - 9
CY - United States
N2 - Several lines of evidence implicate the amygdala in face-emotion processing, particularly for fearful facial expressions. Related findings suggest that face-emotion processing engages the amygdala within an interconnected circuitry that can be studied using a functional-connectivity approach. Past work also underscores important functional changes in the amygdala during development. Taken together, prior research on amygdala function and development reveals a need for more work examining developmental changes in the amygdala's response to fearful faces and in amygdala functional connectivity during face processing. The present study used event-related functional magnetic resonance imaging to compare 31 adolescents (9-17 years old) and 30 adults (21-40 years old) on activation to fearful faces in the amygdala and other regions implicated in face processing. Moreover, these data were used to compare patterns of amygdala functional connectivity in adolescents and adults. During passive viewing, adolescents demonstrated greater amygdala and fusiform activation to fearful faces than did adults. Functional connectivity analysis revealed stronger connectivity between the amygdala and the hippocampus in adults than in adolescents. Within each group, variability in age did not correlate with amygdala response, and sex-related developmental differences in amygdala response were not found. Eye movement data collected outside of the magnetic resonance imaging scanner using the same task suggested that developmental differences in amygdala activation were not attributable to differences in eye-gaze patterns. Amygdala hyperactivation in response to fearful faces may explain increased vulnerability to affective disorders in adolescence; stronger amygdala-hippocampus connectivity in adults than adolescents may reflect maturation in learning or habituation to facial expressions
SN - 0898-929X
AD - National Institutes of Health (NIH), Bethesda, MD 20892-2670, USA. amandaguyer@mail.nih.gov
UR - 18345988
ER -

TY - JOUR
ID - 1011
T1 - A school-based intervention to reduce overweight and inactivity in children aged 6-12 years: study design of a randomized controlled trial

A1 - Jansen,W.
A1 - Raat,H.
A1 - Zwanenburg,E.J.
A1 - Reuvers,I.
A1 - van,Walsem R.
A1 - Brug,J.
Y1 - 2008///
N1 - Jansen, Wilma. Raat, Hein. Zwanenburg, Evelien Joosten-van. Reuvers, Ivo. van Walsem, Ron. Brug, Johannes
BMC public health
100968562
IM
Journal Article. Randomized Controlled Trial
English
KW - MEDLINE
KW - Child
KW - Exercise
KW - Female
KW - Health Promotion/mt [Methods]
KW - Humans
KW - Male
KW - Overweight/pc [Prevention & Control]
KW - Physical Education and Training/ma [Manpower]
KW - Physical Education and Training
KW - Physical Fitness
KW - Regression Analysis
KW - School Health Services
KW - Sports
RP - NOT IN FILE
SP - 257
JF - BMC Public Health
VL - 8
CY - England
N2 - BACKGROUND: Effective interventions to prevent overweight and obesity in children are urgently needed especially in inner-city neighbourhoods where prevalence of overweight and inactivity among primary school children is high. A school based intervention was developed aiming at the reduction of overweight and inactivity in these children by addressing both behavioural and environmental determinants. METHODS/DESIGN: The main components of the intervention (Lekker Fit!) are the re-establishment of a professional physical education teacher; three (instead of two) PE classes per week; additional sport and play activities outside school hours; fitness testing; classroom education on healthy nutrition, active living and healthy lifestyle choices; and the involvement of parents. The effectiveness of the intervention is evaluated through a cluster randomized controlled trial in 20 primary schools among grades 3 through 8 (6-12 year olds). Primary outcome measures are BMI, waist circumference and fitness. Secondary outcome measures are assessed in a subgroup of grade 6-8 pupils (9-12 year olds) through classroom questionnaires and constitute of nutrition and physical activity behaviours and behavioural determinants. Multilevel regression analyses are used to study differences in outcomes between children in the intervention schools and in control schools, taking clustering of children within schools into account. DISCUSSION: Hypotheses are that the intervention results in a lower prevalence of children being overweight and an improved mean fitness score, in comparison with a control group where the intervention is not implemented. The results of our study will contribute to the discussion on the role of physical education and physical activity in the school curriculum. TRIAL REGISTRATION: [ISRCTN84383524]
SN - 1471-2458
AD - Department of Public Health, Erasmus MC, Rotterdam, The Netherlands. jansenw@ggd.rotterdam.nl
UR - 18657280
ER -

TY - JOUR

ID - 1012
T1 - Recess is time-in: using peers to improve social skills of children with autism
A1 - Harper,C.B.
A1 - Symon,J.B.
A1 - Frea,W.D.
Y1 - 2008/05//
N1 - Harper, Christena Blauvelt. Symon, Jennifer B G. Frea, William D
Journal of autism and developmental disorders
7904301, hgw
IM
Journal Article
English
KW - MEDLINE
KW - Autistic Disorder/px [Psychology]
KW - Child
KW - Communication
KW - Humans
KW - Interpersonal Relations
KW - Male
KW - Peer Group
KW - Social Behavior
KW - Social Perception
KW - Teaching/mt [Methods]
KW - Time Factors
RP - NOT IN FILE
SP - 815
EP - 826
JF - Journal of Autism & Developmental Disorders
JA - J Autism Dev Disord
VL - 38
IS - 5
CY - United States
N2 - Children with autism face enormous struggles when attempting to interact with their typically developing peers. More children are educated in integrated settings; however, play skills usually need to be explicitly taught, and play environments must be carefully prepared to support effective social interactions. This study incorporated the motivational techniques of Pivotal Response Training through peer-mediated practice to improve social interactions for children with autism during recess activities. A multiple baseline design across subjects was used to assess social skills gains in two elementary school children. The results demonstrated an increase in important social skills, namely social initiations and turn taking, during recess
SN - 0162-3257
AD - Garden Grove Unified School District in Orange County, California State University, Los Angeles, CA, USA
UR - 17874290
ER -

TY - JOUR
ID - 1013
T1 - Sun safety: what are the health messages?
A1 - Hedges,T.
A1 - Scriven,A.
Y1 - 2008/07//
N1 - Hedges, Trudy. Scriven, Angela
The journal of the Royal Society for the Promotion of Health
IM
Journal Article
English
KW - MEDLINE

KW - Aged
KW - Child
KW - Great Britain
KW - Health Education
KW - Humans
KW - Infant
KW - Skin Neoplasms/pc [Prevention & Control]
KW - Sunlight/ae [Adverse Effects]
KW - Sunlight
KW - Vitamin D Deficiency/pc [Prevention & Control]
RP - NOT IN FILE
SP - 164
EP - 169
JF - Journal of the Royal Society for the Promotion of Health
JA - J R SOC PROMOT HEALTH
VL - 128
IS - 4
CY - England
N2 - Solar ultraviolet radiation (UVR) is a human carcinogen. The UK and the worldwide incidence of cutaneous (skin) melanoma are rising. It is now the second most common cancer in 15-34 year-olds in the UK, increasing by 49% in 1991-2000. The UK government prioritized skin cancer in 1992 in their public health strategy The Health of the Nation, and set the target for reducing the yearly rise in skin cancer incidence by 2005. In 1998 in the Saving Lives: Our Healthier Nation strategy, targets were set to reduce the death rate from cancer, including skin cancer, by 20% by 2010. As a consequence of these targets, since the mid-1990s public health campaigns to reduce sun exposure and skin cancer incidence in the UK have used specific interventions for at-risk groups and the mass media to reach the general population. Evidence indicates a shift in knowledge, with further work required to increase change in behaviour and attitudes. An associate health problem is vitamin D deficiency, which is prevalent across certain UK population groups. The main childhood health problem associated with vitamin D deficiency is the development of rickets, with evidence indicating those at high risk are exclusively breastfed dark-skinned infants, the elderly, those with dark skin pigmentation, and certain cultural groups that cover their bodies in clothing. Adolescents are the group with the highest incidence of low vitamin D status in the UK, with children of Asian descent at higher risk than Caucasian children. Increasing the outdoor activity levels of children in winter and during the cooler hours in summer is one way of increasing safe sun exposure. It is imperative to educate the general public on the risk of vitamin D deficiency, and offer advice on safe sun exposure, dietary sources of vitamin D, and dietary supplements. More research is required on recommendations for daily vitamin D supplements to determine optimal vitamin D levels in different population groups. There also needs to be reliable and accessible evidence on the links between vitamin D deficiency and lack of sun exposure, with sun safe messages adjusted accordingly
SN - 1466-4240
AD - HCML Health & Case Management Limited, Melrose House, 42 Dingwall Road, Croydon, Surrey CR0 2NE. trudy.hedges@hcml.co.uk
UR - 18678111
ER -

TY - JOUR
ID - 1014
T1 - Outdoor activity reduces the prevalence of myopia in children
A1 - Rose,K.A.
A1 - Morgan,I.G.
A1 - Ip,J.
A1 - Kifley,A.
A1 - Huynh,S.
A1 - Smith,W.
A1 - Mitchell,P.
Y1 - 2008/08//
N1 - Rose, Kathryn A. Morgan, Ian G. Ip, Jenny. Kifley, Annette. Huynh, Son. Smith, Wayne. Mitchell, Paul Ophthalmology

7802443
IM
Journal Article. Research Support, Non-U.S. Gov't
English
KW - MEDLINE
KW - Child
KW - Cross-Sectional Studies
KW - Female
KW - Humans
KW - Hyperopia/ep [Epidemiology]
KW - Leisure Activities
KW - Male
KW - Myopia/ep [Epidemiology]
KW - Myopia/eh [Ethnology]
KW - New South Wales/ep [Epidemiology]
KW - Odds Ratio
KW - Prevalence
KW - Questionnaires
KW - Refraction
KW - Ocular/ph [Physiology]
KW - Sports
KW - Work
RP - NOT IN FILE
SP - 1279
EP - 1285
JF - Ophthalmology
VL - 115
IS - 8
CY - United States
N2 - OBJECTIVE: To assess the relationship of near, midworking distance, and outdoor activities with prevalence of myopia in school-aged children. DESIGN: Cross-sectional study of 2 age samples from 51 Sydney schools, selected using a random cluster design. PARTICIPANTS: One thousand seven hundred sixty-five 6-year-olds (year 1) and 2367 12-year-olds (year 7) participated in the Sydney Myopia Study from 2003 to 2005. METHODS: Children had a comprehensive eye examination, including cycloplegic refraction. Parents and children completed detailed questionnaires on activity. MAIN OUTCOME MEASURES: Myopia prevalence and mean spherical equivalent (SE) in relation to patterns of near, midworking distance, and outdoor activities. Myopia was defined as SE refraction < or = -0.5 diopters (D). RESULTS: Higher levels of outdoor activity (sport and leisure activities) were associated with more hyperopic refractions and lower myopia prevalence in the 12-year-old students. Students who combined high levels of near work with low levels of outdoor activity had the least hyperopic mean refraction (+0.27 D; 95% confidence interval [CI], 0.02-0.52), whereas students who combined low levels of near work with high levels of outdoor activity had the most hyperopic mean refraction (+0.56 D; 95% CI, 0.38-0.75). Significant protective associations with increased outdoor activity were seen for the lowest (P = 0.04) and middle (P = 0.02) tertiles of near-work activity. The lowest odds ratios for myopia, after adjusting for confounders, were found in groups reporting the highest levels of outdoor activity. There were no associations between indoor sport and myopia. No consistent associations between refraction and measures of activity were seen in the 6-year-old sample. CONCLUSIONS: Higher levels of total time spent outdoors, rather than sport per se, were associated with less myopia and a more hyperopic mean refraction, after adjusting for near work, parental myopia, and ethnicity
SN - 1549-4713
AD - School of Applied Vision Sciences, Faculty of Health Sciences, University of Sydney, Sydney, Australia. k.rose@usyd.edu.au
UR - 18294691
ER -

TY - JOUR
ID - 1015
T1 - Correlates of childhood obesity in Athens, Greece

A1 - Lagiou,A.
A1 - Parava,M.
Y1 - 2008/09//
N1 - Lagiou, Areti. Parava, Maria
Public health nutrition
9808463, dgy
IM
Journal Article
English
KW - MEDLINE
KW - Child
KW - Confidence Intervals
KW - Cross-Sectional Studies
KW - Eating/ph [Physiology]
KW - Energy Intake/ph [Physiology]
KW - Exercise/ph [Physiology]
KW - Female
KW - Greece/ep [Epidemiology]
KW - Humans
KW - Logistic Models
KW - Male
KW - Multivariate Analysis
KW - Obesity/ep [Epidemiology]
KW - Obesity/et [Etiology]
KW - Odds Ratio
KW - Predictive Value of Tests
KW - Prevalence
KW - Public Health
KW - Sex Factors
KW - Television
KW - Time Factors
RP - NOT IN FILE
SP - 940
EP - 945
JF - Public Health Nutrition
JA - Public Health Nutr
VL - 11
IS - 9
CY - England
N2 - OBJECTIVE: Childhood obesity is a growing public health problem. We have examined the association between sociodemographic profile and eating and physical activity patterns with overweight among primary-school students in Athens, Greece. DESIGN: Cross-sectional study. SETTING: Eleven primary schools in the greater Athens area, Greece. SUBJECTS: A total of 633 children aged 10-12 years (50 % boys, 50 % girls) were interviewed in person during spring 2003. Multivariate logistic regression was used to investigate the association between eating and physical activity patterns and overweight (> or =85th sex- and age-specific BMI centile). Results are presented as odds ratios and 95 % confidence intervals. RESULTS: Overweight was more common among girls than among boys (OR=1.73; 95% CI 1.11, 2.69) and substantially less common among children born outside Greece (OR=0.46; CI 0.22, 0.95). Reported physical activity (per 1.5 h per day) was unrelated to overweight (OR=0.97; CI 0.85, 1.12) but patent physical inactivity, operationalised as time spent watching television or working/playing with the computer (per 1.5 h per day) was a highly significant predictor of overweight (OR=1.20; CI 1.05, 1.36). Composition of diet was unrelated to overweight but the daily number of eating occasions, controlling for total energy intake, was significantly inversely associated with overweight (OR=0.61; CI 0.48, 0.76). CONCLUSIONS: The principal factor underlying overweight among children in Athens appears to be the extended inactivity imposed by modern childhood lifestyles. An intriguing finding is that spreading a given energy intake over several eating occasions was inversely associated with the likelihood of childhood obesity
SN - 1368-9800

AD - Faculty of Health Professions, Athens Technological Institute (TEI), 274 Thivon Ave, Athens, GR-122 41, Greece. arlagiou@med.uoa.gr
UR - 18489810
ER -

TY - JOUR
ID - 1016
T1 - Fidelity of gamma-glutamyl transferase (GGT) in differentiating skeletal muscle from liver damage
A1 - Rosales,X.Q.
A1 - Chu,M.L.
A1 - Shilling,C.
A1 - Wall,C.
A1 - Pastores,G.M.
A1 - Mendell,J.R.
Y1 - 2008/07//
N1 - Rosales, Xiomara Q. Chu, Mary-Lynn. Shilling, Christopher. Wall, Cheryl. Pastores, Gregory M. Mendell, Jerry R
Journal of child neurology
ia2, 8606714
IM
Comparative Study. Evaluation Studies. Journal Article
English
KW - MEDLINE
KW - Adolescent
KW - Adrenal Cortex Hormones/tu [Therapeutic Use]
KW - Adult
KW - Biological Markers/bl [Blood]
KW - Case-Control Studies
KW - Child
KW - Cohort Studies
KW - Creatine Kinase/bl [Blood]
KW - Diagnosis
KW - Differential
KW - Humans
KW - Liver/en [Enzymology]
KW - Liver Diseases/bl [Blood]
KW - Liver Diseases/di [Diagnosis]
KW - Liver Diseases/en [Enzymology]
KW - Male
KW - Muscle
KW - Skeletal/en [Enzymology]
KW - Muscular Dystrophy
KW - Duchenne/bl [Blood]
KW - Duchenne/dt [Drug Therapy]
KW - Duchenne/en [Enzymology]
KW - Reference Values
KW - gamma-Glutamyltransferase/bl [Blood]
KW - gamma-Glutamyltransferase/du [Diagnostic Use]
RP - NOT IN FILE
SP - 748
EP - 751
JF - Journal of Child Neurology
JA - J Child Neurol
VL - 23
IS - 7
CY - United States

N2 - This study tested the hypothesis that gamma-glutamyl transferase (GGT) can be used as a reliable biomarker to distinguish skeletal muscle from liver damage. Twenty-eight Duchenne muscular dystrophy subjects with proven dystrophin gene mutations were enrolled. Included were 14 ambulatory and 14 nonambulatory patients with approximately half of each cohort taking corticosteroids. Twenty normal males served as controls. Initial blood samples for serum GGT and creatine kinase were taken between 8AM and 9AM and redrawn 8 hours later to test for variability. Between blood draws, subjects resumed normal activities in a play environment or could leave the clinic. Not a single duchenne muscular dystrophy patient showed a GGT outside the control range at any time point, while creatine kinase levels were 14 to 200 times normal. Validation of this finding is essential for management of patients with muscle disorders exposed to potentially hepatotoxic drugs for clinical management or monitoring subjects participating in clinical trials
SN - 1708-8283
AD - Department of Pediatrics, The Ohio State University, Columbus, Ohio, USA
UR - 18354148
ER -

TY - JOUR
ID - 1017
T1 - Objectively measured physical activity between children with autism spectrum disorders and children without disabilities during inclusive recess settings in Taiwan
A1 - Pan,C.Y.
Y1 - 2008/08//
N1 - Pan, Chien-Yu
Journal of autism and developmental disorders
7904301, hgw
IM
Journal Article. Research Support, Non-U.S. Gov't
English
KW - MEDLINE
KW - Asperger Syndrome/di [Diagnosis]
KW - Asperger Syndrome/px [Psychology]
KW - Autistic Disorder/di [Diagnosis]
KW - Autistic Disorder/px [Psychology]
KW - Child
KW - Female
KW - Humans
KW - Male
KW - Motor Activity
KW - Play and Playthings
KW - Reference Values
KW - Social Environment
KW - Taiwan
RP - NOT IN FILE
SP - 1292
EP - 1301
JF - Journal of Autism & Developmental Disorders
JA - J Autism Dev Disord
VL - 38
IS - 7
CY - United States
N2 - The purpose of this study was to compare the percent time children with and without autism spectrum disorders (ASD) spent in moderate-to-vigorous physical activity (MVPA) during inclusive recess settings. Forty-eight children (ASD, 23 boys and 1 girl; Non-ASD, 23 boys and 1 girl) aged 7-12 years from 14 schools had their physical activity during recess quantified using a uniaxial accelerometer over a 5-day in school period. Children with ASD were less active during overall recess, lunchtime, first and second morning recess compared to those without disabilities ($p < .01$). All children in this study did not achieve 40% of recess time in physical activity, suggesting that interventions for increasing physical activity of children during inclusive recess settings are warranted

SN - 0162-3257
AD - Department of Physical Education, National Kaohsiung Normal University, No. 116, He-Ping First Road, Kaohsiung 802, Taiwan. chpan@nknucc.nknu.edu.tw
UR - 18157623
ER -

TY - JOUR
ID - 1018
T1 - The vitamin D receptor Fok1 polymorphism and bone mineral density in Chinese children
A1 - Zhang,C.
A1 - Wang,C.
A1 - Liang,J.
A1 - Zhou,X.
A1 - Zheng,F.
A1 - Fan,Y.
A1 - Shi,Q.
Y1 - 2008/09//
N1 - Zhang, Chunlian. Wang, Chunhong. Liang, Jiancheng. Zhou, Xin. Zheng, Fang. Fan, Yong. Shi, Qun
Clinica chimica acta; international journal of clinical chemistry
dcc, 1302422
IM
Journal Article. Research Support, Non-U.S. Gov't
English
KW - MEDLINE
KW - Bone Density/ge [Genetics]
KW - Child
KW - China
KW - Female
KW - Genotype
KW - Humans
KW - Male
KW - Polymorphism
KW - Genetic/ge [Genetics]
KW - Receptors
KW - Calcitriol/ge [Genetics]
KW - Reference Values
KW - Sex Distribution
RP - NOT IN FILE
SP - 111
EP - 114
JF - Clinica Chimica Acta
JA - Clin Chim Acta
VL - 395
IS - 1-2
CY - Netherlands
N2 - BACKGROUND: To evaluate the relationship between vitamin D receptor (VDR) gene polymorphism and bone mineral density (BMD) in 213 healthy children aged 6-10 year in China. METHODS: A questionnaire survey of dietary pattern, outdoor activity was conducted among 213 children (boys 126, girls 86) randomly selected in Xishui county of Hubei province. The BMD was determined by dual energy X-ray absorptiometry at the distal forearm, calcium, phosphorus, and alkaline phosphatase in serum were immediately analyzed. The FokI polymorphism was detected by using PCR-RFLP. RESULTS: BMD was significantly higher in boys than in girls in 8/9 year group. (2) the frequencies of FF, Ff, and ff genotype were 25.8%, 62.0% and 12.2%, respectively; no difference was found between boys and girls. (3) BMD of children carrying FF genotype was higher (0.256+/-0.03) than those of carrying Ff genotype (0.241+/-0.03), P<0.01; the Ff genotype was associated with lowest forearm BMD in both boys and girls. Outdoor activity also positively affected peak bone mass. CONCLUSION: The Fok1 polymorphism of the VDR receptor seems to directly affect bone mineral mass in Chinese children

SN - 0009-8981
AD - Department of Toxicology, School of Public Health, Wuhan University, Wuhan 430071, People's Republic of China
UR - 18570891
ER -

TY - JOUR
ID - 1019
T1 - Environmental correlates of physical activity in Mexican American children at home
A1 - McKenzie,T.L.
A1 - Baquero,B.
A1 - Crespo,N.C.
A1 - Arredondo,E.M.
A1 - Campbell,N.R.
A1 - Elder,J.P.
Y1 - 2008/07//
N1 - McKenzie, Thomas L. Baquero, Barbara. Crespo, Noe C. Arredondo, Elva M. Campbell, Nadia R. Elder, John P
Journal of physical activity & health
101189457
IM
Journal Article. Research Support, N.I.H., Extramural
English
KW - MEDLINE
KW - Body Mass Index
KW - Child
KW - Child Behavior/eh [Ethnology]
KW - Environment
KW - Family Characteristics/eh [Ethnology]
KW - Female
KW - Food Habits
KW - Humans
KW - Male
KW - Mexican Americans/px [Psychology]
KW - Mexican Americans/sn [Statistics & Numerical Data]
KW - Motor Activity
KW - Overweight/ep [Epidemiology]
KW - Parent-Child Relations
KW - Risk Factors
KW - Sex Factors
KW - Social Environment
KW - Socioeconomic Factors
RP - NOT IN FILE
SP - 579
EP - 591
JF - Journal of Physical Activity & Health
JA - J Phys Act Health
VL - 5
IS - 4
CY - United States
N2 - BACKGROUND: Understanding home environments might shed light on factors contributing to reduced physical activity (PA) in children, particularly minorities. Few studies have used microlevel observations to simultaneously assess children's PA and associated conditions in homes. METHODS: Trained observers assessed PA and associated physical and social environmental variables in the homes of 139 Mexican American children (69 boys, 70 girls; mean age = 6 years) after school. RESULTS: Children spent most time indoors (77%) and being sedentary (74%). Reduced PA was associated with viewing media, being indoors, and parents being present. Increased PA was associated with prompts for PA and other children being present. PA prompts

differed by child gender and location and prompter age status. CONCLUSIONS: Children are frequently sedentary at home. Microlevel observations showed PA is associated with potentially modifiable social and physical factors, including spending time outdoors. Studies to determine whether interventions on these correlates can improve children's PA are needed
SN - 1543-3080
AD - School of Exercise and Nutritional Sciences, San Dieto State University, San Diego, CA 92182, USA
UR - 18648122
ER -

TY - JOUR
ID - 1020
T1 - Young people's use of sports facilities: a Norwegian study on physical activity
A1 - Limstrand,T.
A1 - Rehrer,N.J.
Y1 - 2008/07//
N1 - Limstrand, Torgeir. Rehrer, Nancy J
Scandinavian journal of public health
dew, 100883503
IM
Comparative Study. Journal Article. Research Support, Non-U.S. Gov't
English
KW - MEDLINE
KW - Adolescent
KW - Adolescent Behavior/ph [Physiology]
KW - Child
KW - Child Behavior/ph [Physiology]
KW - Exercise/ph [Physiology]
KW - Exercise/px [Psychology]
KW - Exercise
KW - Female
KW - Health Promotion/ec [Economics]
KW - Health Promotion/sn [Statistics & Numerical Data]
KW - Health Promotion
KW - Humans
KW - Male
KW - Norway
KW - Public Health/ec [Economics]
KW - Public Health/sn [Statistics & Numerical Data]
KW - Public Health
KW - Questionnaires
KW - Sports/px [Psychology]
KW - Sports/sn [Statistics & Numerical Data]
KW - Sports
RP - NOT IN FILE
SP - 452
EP - 459
JF - Scandinavian Journal of Public Health
JA - Scand J Public Health
VL - 36
IS - 5
CY - Sweden
N2 - UNLABELLED: In recent years, sports facilities have formed part of Norwegian public health policies to increase physical activity among children and adolescents. Despite large sums of public money being spent on such facilities, information on usage is limited. AIMS: Our aim was to study the effects of gender, age and relative activity level on young people's use of sports facilities. METHODS: We explored 662 young people's (age 6-16 years) usage of 19 different kinds of sports facilities. A questionnaire was administered to students and teachers, and situation plots of students at recess were made. RESULTS: The findings indicate that sports

facilities in general were less used by girls, adolescents (14-16 years) and the least active (physically active < or = 1 times/week outside school) than by boys, children (6-13 years) and the most active (physically active > or = 4 times/ week outside school). More general, multifunctional facilities were used to a greater extent than specialized facilities, particularly by the least active. Distance to facility was important for the use of common facilities. CONCLUSIONS: These results raise the question of whether sports facilities significantly increase physical activity among "all" young people, which is the government's stated goal. More research on sports facilities use and physical activity levels among males and females of all ages is warranted
SN - 1403-4948
AD - Bodo University College, School of Professional Studies, Bodo, Norway
UR - 18635728
ER -

TY - JOUR
ID - 1021
T1 - Positron emission tomography/computed tomography (PET/CT) in children. [Review] [12 refs]
A1 - Shore,R.M.
Y1 - 2008/06//
N1 - Shore, Richard M
Pediatric annals
oub, 0356657
IM
Journal Article. Review
English
KW - MEDLINE
KW - Child
KW - Child Welfare
KW - Epilepsy/di [Diagnosis]
KW - Epilepsy/pa [Pathology]
KW - Female
KW - Humans
KW - Male
KW - Neoplasms/di [Diagnosis]
KW - Neoplasms/pa [Pathology]
KW - Positron-Emission Tomography/is [Instrumentation]
KW - Positron-Emission Tomography/mt [Methods]
KW - Tomography
KW - Emission-Computed/is [Instrumentation]
KW - Emission-Computed/mt [Methods]
RP - NOT IN FILE
SP - 404
EP - 412
JF - Pediatric Annals
JA - Pediatr Ann
VL - 37
IS - 6
CY - United States
N2 - Although PET imaging has been available for more than two decades, its use has greatly increased lately due to the advent of PET/CT, readily available sources of commercially supplied FDG, and mobile scanners. These features have enabled PET scanning to expand beyond select major university medical centers, with on-site cyclotron facilities to smaller institutions including free- standing children's hospitals. In these settings, imaging is generally limited to FDG, which suffices for most applications, with the majority of studies performed for tumor imaging. FDGI is being used for evaluation of many tumors in children, with its use in lymphoma being the most established. In lymphoma, it has proven quite useful in determining whether active tumor is present in residual masses following treatment, which may otherwise contain only residual fibrous tissue. For brain tumors, FDGI has some relation to tumor grade, although its more important role is distinguishing recurrent or residual tumor from the effects of treatment, particularly radiation necrosis. For neurological evaluation, interictal FDGI is helpful in localizing potential seizure foci for subsequent subdural

EEG monitoring. Because of the relatively long uptake time of FDG, true ictal studies cannot be performed with FDG, and these remain within the domain of SPECT imaging of tracers such as Tc-99m HMPAO. Examinations utilizing radiopharmaceuticals other than FDG are significantly more limited in their availability and are limited to PET centers with on-site cyclotrons. However, these additional agents open the door to many additional studies, including more specific tumor-imaging agents of certain tumors such as neuroblastoma. Another neurotransmitter, F-18-fluoro-L-dopa, is of benefit outside of the central nervous system for evaluating infantile hyperinsulism. The use of PET/CT in children is increasing quickly, particularly FDGI imaging of tumors. It is expected that over the next few years, its role will become increasingly better defined. [References: 12]
SN - 0090-4481
AD - Children's Memorial Hospital, Chicago, USA. rshore@northwestern.edu
UR - 18616194
ER -

TY - JOUR
ID - 1022
T1 - Cultural and gender effects on Israeli children's preferences for activities
A1 - Engel-Yeger,B.
A1 - Jarus,T.
Y1 - 2008/06//
N1 - Engel-Yeger, Batya. Jarus, Tal
Canadian journal of occupational therapy. Revue canadienne d'ergotherapie
0406021, cj0
IM
Journal Article
English
KW - MEDLINE
KW - Arabs/px [Psychology]
KW - Child
KW - Cross-Sectional Studies
KW - Cultural Characteristics
KW - Female
KW - Humans
KW - Israel
KW - Jews/px [Psychology]
KW - Leisure Activities/px [Psychology]
KW - Male
KW - Sex Factors
KW - Socioeconomic Factors
RP - NOT IN FILE
SP - 139
EP - 148
JF - Canadian Journal of Occupational Therapy - Revue Canadienne d Ergotherapie
JA - Can J Occup Ther
VL - 75
IS - 3
CY - Canada
N2 - BACKGROUND: Knowledge about factors that affect participation, as preference of activities, has major intervention implications. PURPOSE: To evaluate culture and gender effects on Israeli Jewish and Druze children's preference of activities performed outside mandated school. This study used the "Preference Assessment of Children" (PAC) (King et al., 2004). FINDINGS: On most scales, the Jewish children showed significantly less interest in activities than the Druze children. Among the Jews, girls showed higher preference in most PAC scales than boys while among the Druze girls showed higher preference than the boys only in social skills. IMPLICATIONS: Culture and gender may influence children's preference of activity. More studies should elaborate the knowledge about individuals' preferred activities; understanding the factors that affect these preferences may enhance occupational therapy evaluation and intervention processes. Canadian occupational therapists, as health care professionals in a multicultural society, must develop cultural competency and explore people's experience as cultural beings

SN - 0008-4174
AD - Occupational Therapy Department, Faculty of Social Welfare & Health Sciences, University of Haifa-Mount Carmel, Haifa, Israel. batya@research.haifa.ac.il
UR - 18615925
ER -

TY - JOUR
ID - 1023
T1 - Lack of adequate sun protection for children with oculocutaneous albinism in South Africa
A1 - Lund,P.M.
A1 - Taylor,J.S.
Y1 - 2008///
N1 - Lund, Patricia M. Taylor, Julie S
BMC public health
100968562
IM
Journal Article. Research Support, Non-U.S. Gov't
English
KW - MEDLINE
KW - Albinism
KW - Child
KW - Female
KW - Humans
KW - Interviews as Topic
KW - Male
KW - Protective Clothing/ut [Utilization]
KW - Rural Population
KW - Sex Factors
KW - South Africa
KW - Sunburn/pc [Prevention & Control]
KW - Sunscreening Agents/ad [Administration & Dosage]
KW - Visually Impaired Persons
RP - NOT IN FILE
SP - 225
JF - BMC Public Health
VL - 8
CY - England
N2 - BACKGROUND: Childhood is a high risk time for ultraviolet induced skin damage as this age group has more time and opportunity to be outdoors in the sun. Children in Africa with the inherited condition oculocutaneous albinism (OCA) are especially vulnerable due to their lack of protective melanin. They are highly susceptible to developing skin lesions that have both cosmetic and health complications, with a high risk of developing skin cancers. The study aimed to explore the adequacy of sun protection strategies of children with albinism in order to inform future provision. METHODS: Community based participatory research methods were employed to investigate sun protection strategies in 90 pupils with OCA (40 female and 50 male) boarding at a special school educating pupils with visual impairment in a rural area of northern South Africa. Hats worn and sunscreen preparations used were examined during semi-structured face to face interviews conducted in small peer groups. The resident nurse interpreted if necessary and provided additional information on monitoring and treatment of skin lesions. RESULTS: Participants with albinism in this study were exposed to high levels of ultraviolet radiation throughout the year and showed skin damage despite wearing protective head gear. All except one pupil possessed at least one hat, with a mean brim width of 5.4 cm. Gender differences in sun avoidance behaviour were documented, with females seeking shade during recreational periods and males playing soccer outside. Although 38% of pupils were using a sunscreen with a sun protection factor (SPF) rating, only 12% had government sponsored tubes of SPF15 cream. Government sponsored sunscreen preparations were only provided if actively sought, involving time consuming trips to regional hospitals, with inadequate availability and insufficient supply. CONCLUSION: Children with albinism living away from home in rural areas appear to have inadequate sun protection strategies. Changes in health policy could address these deficiencies. We recommend providing more detailed health care information, giving advice

on appropriate styles of hat to wear and how to assess commercial SPF products. Health promotional material should also be evaluated to determine its effectiveness among user groups
SN - 1471-2458
AD - Department of Biomolecular and Sport Science, Faculty of Health and Life Sciences, Priory Street, Coventry, UK. p.lund@coventry.ac.uk
UR - 18590551
ER -

TY - JOUR
ID - 1024
T1 - Gender and injury in Finnish comprehensive schools
A1 - Salminen,S.
A1 - Lounamaa,A.
A1 - Kurenniemi,M.
Y1 - 2008/07//
N1 - Salminen, Simo. Lounamaa, Anne. Kurenniemi, Marja
Accident; analysis and prevention
acs, 1254476
IM
Journal Article
English
KW - MEDLINE
KW - Adolescent
KW - Athletic Injuries/ep [Epidemiology]
KW - Child
KW - Female
KW - Finland/ep [Epidemiology]
KW - Humans
KW - Male
KW - Play and Playthings/in [Injuries]
KW - Retrospective Studies
KW - Risk Factors
KW - School Health Services/sn [Statistics & Numerical Data]
KW - Schools/sn [Statistics & Numerical Data]
KW - Sex Distribution
KW - Trauma Severity Indices
RP - NOT IN FILE
SP - 1267
EP - 1272
JF - Accident Analysis & Prevention
JA - Accid Anal Prev
VL - 40
IS - 4
CY - England
N2 - The aim of this study is to analyze the gender differences in injuries at Finnish comprehensive schools. Nine schools reported a total of 1135 injuries to the injury register over two school years. Boys (56%) were injured more often than girls, their injuries happened more often during breaks at school yard, whereas girls hurt themselves during sport lectures in the gymnastic halls. The proportion of boys' injuries also increased with age. More often, boys' injuries were caused by intentional actions by other pupils. Girls injured their lower extremities more often, whereas boys injured their faces and head. Boys also suffered concussion more often than girls, and were also referred for further care to health care centres or hospitals more often than girls. The higher injury frequency among boys at school corresponds to the higher injury rate of males in general, which is true for work, traffic, and leisure-time injuries. Increased adult supervisor control especially during breaks can prevent most of school injuries
SN - 0001-4575
AD - Finnish Institute of Occupational Health, Occupational Safety, Topeliuksenkatu 41a A, Helsinki, Finland. simo.salminen@ttl.fi

UR - 18606255
ER -

TY - JOUR
ID - 1025
T1 - Neighborhood road environments and physical activity among youth: the CLAN study
A1 - Carver,A.
A1 - Timperio,A.F.
A1 - Crawford,D.A.
Y1 - 2008/07//
N1 - Carver, Alison. Timperio, Anna F. Crawford, David A
Journal of urban health : bulletin of the New York Academy of Medicine
c5l, 9809909
IM
Journal Article. Research Support, Non-U.S. Gov't
English
KW - MEDLINE
KW - Adolescent
KW - Australia
KW - Bicycling
KW - Child
KW - Cross-Sectional Studies
KW - Female
KW - Humans
KW - Logistic Models
KW - Longitudinal Studies
KW - Male
KW - Motor Activity
KW - Residence Characteristics
KW - Urban Health
KW - Urban Population
KW - Walking
RP - NOT IN FILE
SP - 532
EP - 544
JF - Journal of Urban Health
JA - J Urban Health
VL - 85
IS - 4
CY - United States
N2 - We examined associations between objective measures of the local road environment and physical activity (including active transport) among youth. There is little empirical evidence of the impact of the road environment on physical activity among children/adolescents in their neighborhoods. Most recent studies have examined perceptions rather than objective measures of the road environment. This was a cross-sectional study of children aged 8-9 years (n = 188) and adolescents aged 13-15 years (n = 346) who were participants in the 3-year follow-up of the Children Living in Active Neighborhoods (CLAN) longitudinal study in Melbourne, Australia. At baseline (2001), they were recruited from 19 state primary schools in areas of varying socioeconomic status across Melbourne. Habitual walking/cycling to local destinations was parent-reported for children and self-reported for adolescents, while moderate-to-vigorous physical activity (MVPA) outside school hours was recorded using accelerometers. Road environment features in each participant's neighborhood (area of radius 800 m around the home) were measured objectively using a geographical information system. Regression analyses found no associations between road environment variables and children's likelihood of making at least seven walking/cycling trips per week to neighborhood destinations. Adolescent girls residing in neighborhoods with two to three traffic/pedestrian lights were more likely to make seven or more walking/cycling trips per week as those whose neighborhoods had fewer traffic lights (OR: 2.7; 95% CI: 1.2-6.2). For adolescent boys, residing on a cul-de-sac, compared with a through road, was associated with increases in MVPA of 9 min after school, 5 min in the evenings, and 22 min on weekend days. Speed humps were positively associated with

adolescent boys' MVPA during evenings. The road environment influences physical activity among youth in different ways, according to age group, sex and type of physical activity
SN - 1099-3460
AD - Deakin University, Burwood, VIC, Australia. alison.carver@deakin.edu.au
UR - 18437579
ER -

TY - JOUR
ID - 1026
T1 - Quantification of the probability of reaching mobility independence at discharge from a rehabilitation hospital in nonwalking early ischemic stroke patients: a multivariate study
A1 - Paolucci,S.
A1 - Bragoni,M.
A1 - Coiro,P.
A1 - De,Angelis D.
A1 - Fusco,F.R.
A1 - Morelli,D.
A1 - Venturiero,V.
A1 - Pratesi,L.
Y1 - 2008///
N1 - Paolucci, Stefano. Bragoni, Maura. Coiro, Paola. De Angelis, Domenico. Fusco, Francesca Romana. Morelli, Daniela. Venturiero, Vincenzo. Pratesi, Luca
Cerebrovascular diseases (Basel, Switzerland)
c2g, 9100851
IM
Journal Article
English
KW - MEDLINE
KW - Activities of Daily Living
KW - Adolescent
KW - Adult
KW - Age Factors
KW - Aged
KW - 80 and over
KW - Aphasia/co [Complications]
KW - Brain Ischemia/co [Complications]
KW - Brain Ischemia/mo [Mortality]
KW - Brain Ischemia/pp [Physiopathology]
KW - Brain Ischemia/rh [Rehabilitation]
KW - Child
KW - Dependent Ambulation
KW - Female
KW - Hospital Units
KW - Humans
KW - Logistic Models
KW - Male
KW - Middle Aged
KW - Mobility Limitation
KW - Odds Ratio
KW - Patient Discharge
KW - Prognosis
KW - Recovery of Function
KW - Rehabilitation Centers
KW - Rehabilitation
KW - Vocational
KW - Risk Assessment
KW - Risk Factors

KW - Severity of Illness Index
KW - Sex Factors
KW - Stroke/et [Etiology]
KW - Stroke/mo [Mortality]
KW - Stroke/pp [Physiopathology]
KW - Stroke/rh [Rehabilitation]
KW - Walking
RP - NOT IN FILE
SP - 16
EP - 22
JF - Cerebrovascular Diseases
JA - Cerebrovasc.Dis
VL - 26
IS - 1
CY - Switzerland
N2 - BACKGROUND: This study was designed to quantify the probability of recovery of mobility in admission nonwalking stroke survivors. METHODS: We evaluated 437 of 500 consecutive patients admitted for sequelae of first ischemic stroke within the first month. We performed several logistic regressions using mobility status at discharge (independence in stair climbing; walking outside and inside, without aid or supervision; walking with cane or other aid, or need for wheelchair) as dependent variable, and several independent variables, including stratification of patients according to their Barthel Index (BI) score into 6 classes (< or =10; 11-20; 21-30; 31-40; 41-50; 51-60). RESULTS: At discharge, 4.58% of patients were independent in stair climbing, 8.70% were able to walk outside, 14.41% to walk inside, and 27.46% to walk with cane or other aid, while 44.85% remained in wheelchair. Very low BI scores at admission were associated with a high risk of need for wheelchair, whereas patients with BI score 51-60 showed a high probability to reach independence in stair climbing (OR = 5.60). Age, severity of neurological impairment, global aphasia, unilateral spatial neglect, male gender and vocational status also played a prognostic role. CONCLUSIONS: The probability of potential mobility recovery can be quantified at admission with better accuracy for independence in stair climbing and walking outside without any aid (percentages correctly predicted 95.4 and 91.8%, respectively). Stratification of BI score may be useful to better quantify the risk for each patient. (c) 2008 S. Karger AG, Basel
SN - 1421-9786
AD - Fondazione IRCCS Santa Lucia, Rome, Italy. s.paolucci@hsantalucia.it
UR - 18511867
ER -

TY - JOUR
ID - 1027
T1 - Validation of the RT3 accelerometer for measuring physical activity of children in simulated free-living conditions
A1 - Sun,D.X.
A1 - Schmidt,G.
A1 - Teo-Koh,S.M.
Y1 - 2008/05//
N1 - Sun, David Xiaoqian. Schmidt, Gordon. Teo-Koh, Sock Miang
Pediatric exercise science
8909729
IM
Journal Article. Validation Studies
English
KW - MEDLINE
KW - Acceleration
KW - Adolescent
KW - Body Mass Index
KW - Calorimetry
KW - Child
KW - Energy Metabolism/ph [Physiology]

KW - Exercise Test
KW - Female
KW - Heart Rate
KW - Humans
KW - Life Style
KW - Male
KW - Motor Activity/ph [Physiology]
KW - Oxygen Consumption
KW - Patient Simulation
KW - Singapore
RP - NOT IN FILE
SP - 181
EP - 197
JF - Pediatric Exercise Science
JA - Pediatr Exerc Sci
VL - 20
IS - 2
CY - United States
N2 - This is a validation study of the RT3 accelerometer for measuring physical activities of children in simulated free-living conditions. Twenty-five children age 12-14 years completed indoor testing, and 18 of them completed outdoor testing. Activity counts from the RT3 accelerometer estimated activity energy expenditure (AEE) and the Cosmed K4b2 analyzer measured oxygen uptake. Correlations were found between activity counts and metabolic cost (r = .95, p < .001), metabolic cost and RT3 estimated AEE (r = .96, p < .001) in the indoor test, activity counts and RT3 estimated AEE (r = .97, p < .001) in the outdoor test, and activity counts and metabolic cost when all activities were combined (r = .77, p < .001). Results indicate that the RT3 accelerometer might be used to provide acceptable estimates of free-living physical activity in children
SN - 0899-8493
AD - Dept. of Physical Education and Sports Sciences, National Institute of Education, Nanyang Technological University, Republic of Singapore
UR - 18579899
ER -

TY - JOUR
ID - 1028
T1 - Exercise aids, neighborhood safety, and physical activity in adolescents and parents
A1 - Kerr,J.
A1 - Norman,G.J.
A1 - Sallis,J.F.
A1 - Patrick,K.
Y1 - 2008/07//
N1 - Kerr, Jacqueline. Norman, Gregory J. Sallis, James F. Patrick, Kevin
Medicine and science in sports and exercise
8005433, mg8
IM, S
Journal Article. Research Support, N.I.H., Extramural
English
KW - MEDLINE
KW - Adolescent
KW - Adolescent Behavior
KW - Adult
KW - Child
KW - Confidence Intervals
KW - Environment Design
KW - Equipment and Supplies
KW - Exercise
KW - Female
KW - Health Behavior

KW - Humans
KW - Logistic Models
KW - Male
KW - Motor Activity
KW - Odds Ratio
KW - Parents
KW - Play and Playthings
KW - Questionnaires
KW - Residence Characteristics
KW - Safety
KW - Sports
RP - NOT IN FILE
SP - 1244
EP - 1248
JF - Medicine & Science in Sports & Exercise
JA - Med Sci Sports Exerc
VL - 40
IS - 7
CY - United States
N2 - PURPOSE: To investigate the relationships among exercise aids available at home, physical activity, and perceived neighborhood safety. METHODS: Physical activity was assessed using the 7-d recall interview for adolescents (n = 878) and the International Physical Activity Questionnaire for parents (n = 853). Parents reported exercise aids such as fitness equipment, running shoes, and dogs in their household using a 16-item checklist and perceptions of neighborhood safety using the Neighborhood Environment Walkability Scale. Physical activity scores were dichotomized to represent meeting weekly guidelines for children (300 min) and adults (150 min). Logistic regression analyses investigated the interaction between exercise equipment and neighborhood safety in relation to the two physical activity outcomes, controlling for participant demographics. RESULTS: The number of home-use (OR = 1.27) and outdoor-use (OR = 1.24) exercise aids was significantly related to physical activity in adolescent girls but not boys. An interaction effect indicated that the relationship between home-use exercise equipment and physical activity levels was specific for girls in neighborhoods perceived as less-safe (OR = 4.40), rather than those perceived as safe (OR = 1.07, P < 0.01). In the parent sample, home-use (OR = 1.24) and outdoor use (OR = 1.16) exercise aids were significantly related to physical activity levels. An interaction between outdoor exercise aids and safety indicated that the effect was specific to parents who lived in neighborhoods perceived as safe (OR = 2.43) compared to those perceived as less-safe (OR = 0.91, P < 0.01). CONCLUSION: Girls living in neighborhoods their parents perceive to be less-safe may benefit from having exercise equipment they can use in the home. Parents living in neighborhoods perceived to be safe may benefit from having exercise aids that they can use outside
SN - 0195-9131
AD - 1Department of Psychology, San Diego State University, University of California, San Diego, CA 92103, USA. jkerr@projects.sdsu.edu
UR - 18580403
ER -

TY - JOUR
ID - 1029
T1 - Perceptions of neighborhood safety and physical activity among youth: the CLAN study
A1 - Carver,A.
A1 - Timperio,A.
A1 - Crawford,D.
Y1 - 2008/05//
N1 - Carver, Alison. Timperio, Anna. Crawford, David
Journal of physical activity & health
101189457
IM
Journal Article. Research Support, Non-U.S. Gov't
English
KW - MEDLINE

KW - Adolescent
KW - Adult
KW - Age Factors
KW - Attitude to Health
KW - Australia
KW - Child
KW - Data Collection
KW - Environment Design
KW - Female
KW - Health Behavior
KW - Humans
KW - Male
KW - Motor Activity
KW - Questionnaires
KW - Residence Characteristics/sn [Statistics & Numerical Data]
KW - Safety
KW - Sex Factors
KW - Social Perception
KW - Socioeconomic Factors
RP - NOT IN FILE
SP - 430
EP - 444
JF - Journal of Physical Activity & Health
JA - J Phys Act Health
VL - 5
IS - 3
CY - United States
N2 - BACKGROUND: The purpose of this study was to examine associations between perceptions of neighborhood safety and physical activity among youth. METHODS: We completed a cross-sectional study of children age 8 to 9 years (n = 188) and adolescents age 13 to 15 years (n = 346) in areas of varying socioeconomic status in Melbourne, Australia. Parents and adolescents completed questionnaires on perceptions of neighborhood safety. Scores were computed for perceptions of road safety, incivilities, and personal safety of the child or adolescent. Moderate-to-vigorous physical activity (MVPA) before or after school, on evenings, and on weekends was recorded using accelerometers. RESULTS: There were no associations between parental perceptions of neighborhood safety and children's MVPA outside school hours. Parental perception of personal safety was positively associated with adolescent boys' MVPA after school. Adolescent girls' concern about road safety was negatively associated with their MVPA during evenings and outside school hours. CONCLUSION: Perceptions of neighborhood safety might influence physical activity among youth in different ways according to age group and sex
SN - 1543-3080
AD - Centre for Physical Activity and Nutrition Research, School of Exercise and Nutrition Sciences, Deakin University, Burwood, VIC, Australia
UR - 18579920
ER -

TY - JOUR
ID - 1030
T1 - Pediatric Heelys injuries
A1 - Aarons,C.
A1 - Iobst,C.
A1 - Lopez,M.
Y1 - 2008/07//
N1 - Aarons, Chad. Iobst, Christopher. Lopez, Miguel
Journal of pediatric orthopedics
hsw, 8109053
IM

Journal Article
English
KW - MEDLINE
KW - Accidental Falls
KW - Child
KW - Preschool
KW - Female
KW - Fracture Fixation/mt [Methods]
KW - Fractures
KW - Bone/ep [Epidemiology]
KW - Bone/et [Etiology]
KW - Humans
KW - Male
KW - Protective Devices/ut [Utilization]
KW - Retrospective Studies
KW - Shoes/ae [Adverse Effects]
KW - Skating/in [Injuries]
KW - Treatment Outcome
RP - NOT IN FILE
SP - 502
EP - 505
JF - Journal of Pediatric Orthopedics
JA - J Pediatr Orthop
VL - 28
IS - 5
CY - United States
N2 - BACKGROUND: To determine the incidence and severity of injuries caused by Heelys. METHODS: A retrospective review of all fractures presenting to an orthopaedic emergency room at a metropolitan children's hospital during a 90-day period. The type of fracture, mechanism of injury, and management were recorded for each patient. For those injuries related to the use of Heelys, further data were collected including total number of visits, cast changes, and cost. Each Heelys patient/family was contacted and answered a questionnaire detailing their use of Heelys and the events surrounding the injury. RESULTS: A total of 953 patients with fractures were evaluated for 90 days. Sixteen patients with 17 fractures (1.68%) were identified as being related to the use of Heelys. This compares to the incidence of fractures in our sample from basketball (6.19%), bicycle (4.41%), football (4.09%), monkeybars (3.78%), skateboarding (3.25%), soccer (2.62%), baseball (2.52%), and trampoline (2.31%). The average age of each Heelys patient was 8.9 years, and 13 patients were girls. There were 16 upper extremity and 1 lower extremity fracture. No patient needed operative treatment or admission. Average number of follow-up visits was 1.6, with an average of 1.4 casts per patient. Average cost per patient was $1368. Ninety-two percent of the Heelys injuries occurred outdoors. Fifty-four percent of children were being supervised when they fell, but only 31% were wearing any safety equipment. Sixty-two percent of parents were not aware that safety equipment was recommended. All 13 parents indicated that they would not purchase another pair of Heelys, and only 23% of the children wanted to keep using Heelys after the injury. CONCLUSIONS: The incidence of Heelys injuries (1.68%) was relatively low compared with other common childhood play activities. The fractures were mostly in the upper extremity, and no fracture required surgical intervention or admission to the hospital. Sixty-two percent of the parents were not aware that safety equipment was recommended, and only 31% of the children were wearing safety equipment
SN - 0271-6798
AD - Department of Orthopaedics, Leonard M. Miller School of Medicine, University of Miami, Miami, FL 33101, USA. caarons2@med.miami.edu
UR - 18580362
ER -

TY - JOUR
ID - 1031
T1 - Role of near work in myopia: findings in a sample of Australian school children
A1 - Ip,J.M.
A1 - Saw,S.M.

A1 - Rose,K.A.
A1 - Morgan,I.G.
A1 - Kifley,A.
A1 - Wang,J.J.
A1 - Mitchell,P.
Y1 - 2008/07//
N1 - Ip, Jenny M. Saw, Seang-Mei. Rose, Kathryn A. Morgan, Ian G. Kifley, Annette. Wang, Jie Jin. Mitchell, Paul
Investigative ophthalmology & visual science
gwi, 7703701
IM
Journal Article. Research Support, Non-U.S. Gov't
English
KW - MEDLINE
KW - Adolescent
KW - Asian Continental Ancestry Group/sn [Statistics & Numerical Data]
KW - Biometry
KW - Child
KW - European Continental Ancestry Group/sn [Statistics & Numerical Data]
KW - Eye/pa [Pathology]
KW - Family Health
KW - Female
KW - Humans
KW - Male
KW - Multivariate Analysis
KW - Myopia/eh [Ethnology]
KW - Myopia/et [Etiology]
KW - Myopia/pa [Pathology]
KW - Myopia/pp [Physiopathology]
KW - Odds Ratio
KW - Parents
KW - Questionnaires
KW - Reading
KW - Refraction
KW - Ocular
KW - Schools
KW - Sex Factors
KW - Students
KW - Time Factors
RP - NOT IN FILE
SP - 2903
EP - 2910
JF - Investigative Ophthalmology & Visual Science
JA - Invest Ophthalmol Vis Sci
VL - 49
IS - 7
CY - United States
N2 - PURPOSE: To examine the association of time spent in near work and reading with spherical equivalent refraction (SER) in a population-based sample of 12-year-old Australian schoolchildren. METHODS: Data on the time spent in near-work or outdoor activities per week and estimates for the duration of continuous reading and reading distances, were collected in questionnaires (2353 participants, 75.3% response) in the Sydney Myopia Study between 2004 and 2005; 2339 children underwent a comprehensive eye examination, including cycloplegia. RESULTS: Longer time spent on reading for pleasure and reports of close reading distance (< 30 cm) were associated with a more myopic refraction after adjustment for age, sex, ethnicity, and school type (P(trend) = 0.02 and P = 0.0003, respectively). Time spent in individual near-work activities, however, correlated poorly with SER (all r < or = 0.2) and was not significant in multivariate analyses for myopia (SER < or = -0.50 D), with adjustment for age, sex, ethnicity, parental myopia, school type, and outdoor activity.

Children of European Caucasian ethnicity reported spending marginally less time in near work than children of East Asian ethnicity (26.0 h/wk vs. 32.5 h/wk, P < 0.0001). East Asian ethnicity, however, was associated with substantially greater odds of having myopia (odds ratio [OR], 11.0; 95% confidence interval [CI], 7.0-17.4). Near work such as close reading distance (< 30 cm) and continuous reading (> 30 minutes) independently increased the odds of having myopia in this sample of children. CONCLUSIONS: Although myopia was not significantly associated with time spent in near work after adjustment for other factors, there were significant independent associations with close reading distance and continuous reading. These associations may indicate that the intensity rather than the total duration of near work is an important factor
SN - 1552-5783
AD - Centre for Vision Research, Department of Ophthalmology and the Westmead Millennium Institute, University of Sydney, Sydney, Australia
UR - 18579757
ER -

TY - JOUR
ID - 1032
T1 - Physical activity as a possible mechanism behind the relationship between green space and health: a multilevel analysis
A1 - Maas,J.
A1 - Verheij,R.A.
A1 - Spreeuwenberg,P.
A1 - Groenewegen,P.P.
Y1 - 2008///
N1 - Maas, Jolanda. Verheij, Robert A. Spreeuwenberg, Peter. Groenewegen, Peter P
BMC public health
100968562
IM
Journal Article. Research Support, Non-U.S. Gov't
English
KW - MEDLINE
KW - Adolescent
KW - Adult
KW - Age Factors
KW - Aged
KW - Child
KW - Environment
KW - Exercise
KW - Female
KW - Health Status Indicators
KW - Health Surveys
KW - Humans
KW - Logistic Models
KW - Male
KW - Middle Aged
KW - Multivariate Analysis
KW - Netherlands
KW - Plants
KW - Residence Characteristics/sn [Statistics & Numerical Data]
KW - Residence Characteristics
KW - Rural Population
KW - Socioeconomic Factors
KW - Urban Population
RP - NOT IN FILE
SP - 206
JF - BMC Public Health
VL - 8
CY - England

N2 - BACKGROUND: The aim of this study was to investigate whether physical activity (in general, and more specifically, walking and cycling during leisure time and for commuting purposes, sports and gardening) is an underlying mechanism in the relationship between the amount of green space in people's direct living environment and self-perceived health. To study this, we first investigated whether the amount of green space in the living environment is related to the level of physical activity. When an association between green space and physical activity was found, we analysed whether this could explain the relationship between green space and health. METHODS: The study includes 4.899 Dutch people who were interviewed about physical activity, self-perceived health and demographic and socioeconomic background. The amount of green space within a one-kilometre and a three-kilometre radius around the postal code coordinates was calculated for each individual. Multivariate multilevel analyses and multilevel logistic regression analyses were performed at two levels and with controls for socio-demographic characteristics and urbanicity. RESULTS: No relationship was found between the amount of green space in the living environment and whether or not people meet the Dutch public health recommendations for physical activity, sports and walking for commuting purposes. People with more green space in their living environment walked and cycled less often and fewer minutes during leisure time; people with more green space garden more often and spend more time on gardening. Furthermore, if people cycle for commuting purposes they spend more time on this if they live in a greener living environment. Whether or not people garden, the time spent on gardening and time spent on cycling for commuting purposes did not explain the relationship between green space and health. CONCLUSION: Our study indicates that the amount of green space in the living environment is scarcely related to the level of physical activity. Furthermore, the amount of physical activity undertaken in greener living environments does not explain the relationship between green space and health
SN - 1471-2458
AD - NIVEL (Netherlands Institute for Health Services Research), PO Box 1568, 3500 BN, Utrecht, The Netherlands. j.maas@nivel.nl
UR - 18544169
ER -

TY - JOUR
ID - 1033
T1 - Concentrations and bioaccessibility of polycyclic aromatic hydrocarbons in wastewater-irrigated soil using in vitro gastrointestinal test
A1 - Khan,S.
A1 - Cao,Q.
A1 - Lin,A.J.
A1 - Zhu,Y.G.
Y1 - 2008/06//
N1 - Khan, Sardar. Cao, Qing. Lin, Ai-Jun. Zhu, Yong-Guan
Environmental science and pollution research international
9441769, dvn
IM
Journal Article. Research Support, Non-U.S. Gov't
English
KW - MEDLINE
KW - Biological Availability
KW - Child
KW - China
KW - Cities
KW - Environmental Monitoring
KW - Gastrointestinal Tract/de [Drug Effects]
KW - Gastrointestinal Tract/me [Metabolism]
KW - Gastrointestinal Tract/pa [Pathology]
KW - Humans
KW - Polycyclic Hydrocarbons
KW - Aromatic/an [Analysis]
KW - Aromatic/pk [Pharmacokinetics]
KW - Aromatic/to [Toxicity]
KW - Aromatic

KW - Risk Assessment
KW - Rivers
KW - Soil Pollutants/an [Analysis]
KW - Soil Pollutants/pk [Pharmacokinetics]
KW - Soil Pollutants/to [Toxicity]
KW - Soil Pollutants
KW - Therapeutic Irrigation
KW - Urban Health
KW - Water Pollutants
KW - Chemical/an [Analysis]
KW - Chemical/pk [Pharmacokinetics]
KW - Chemical/to [Toxicity]
KW - Chemical
RP - NOT IN FILE
SP - 344
EP - 353
JF - Environmental Science & Pollution Research
JA - Environ Sci Pollut.Res Int
VL - 15
IS - 4
CY - Germany
N2 - BACKGROUND, AIM, AND SCOPE: Polycyclic aromatic hydrocarbons (PAHs) are widespread environmental contaminants and contribute to the pollution of soil environment. Soil ingestion is of increasing concern for assessing health risk from PAH-contaminated soils because soil ingestion is one of the potentially important pathways of exposure to environmental pollutants, particularly relevant for children playing at contaminated sites due to their hand-to-mouth activities. In vitro gastro-intestinal tests imitate the human digestive tract, based on the physiology of humans, generally more simple, less time-consuming, and especially more reproducible than animal tests. This study was conducted to investigate the level of PAH contamination and oral bioaccessibility in surface soils, using physiologically based in vitro gastro-intestinal tests regarding both gastric and small intestinal conditions. MATERIALS AND METHODS: Wastewater-irrigated soils were sampled from the metropolitan areas of Beijing and Tianjin, China, which were highly contaminated with PAHs. Reference soil samples were also collected for comparisons. At each site, four soils were sampled in the upper horizon at the depth of 0-20 cm randomly and were bulked together to form one composite sample. PAH concentrations and origin were investigated and a physiologically based in vitro test was conducted using all analytical grade reagents. Linear regression model was used to assess the relationship between total PAH concentrations in soils and soil organic carbon (SOC). RESULTS: A wide range of total PAH concentrations ranging from 1,304 to 3,369 mug kg(-1) in soils collected from different wastewater-irrigated sites in Tianjin, while ranging from 2,687 to 4,916 mug kg(-1) in soils collected from different wastewater-irrigated sites in Beijing, was detected. In general, total PAH concentrations in soils from Beijing sites were significantly higher than those from Tianjin sites, indicating a dominant contribution from both pyrogenic and petrogenic sources. Results indicated that the oral bioaccessibility of PAHs in small intestinal was significantly higher (from P < 0.05 to P < 0.001) than gastric condition. Similarly, the oral bioaccessibility of PAHs in contaminated sites was significantly higher (from P < or = 0.05 to P < 0.001) than in reference sites. Individual PAH ratios (three to six rings), a more accurate and reliable estimation about the emission sources, were used to distinguish the natural and anthropogenic PAH inputs in the soils. Results indicated that PAHs were both pyrogenic and petrogenic in nature. DISCUSSION: The identification of PAH sources and importance of in vitro test for PAH bioaccessibility were emphasized in this study. The oral bioaccessibility of individual PAHs in soils generally decreased with increasing ring numbers of PAHs in both the gastric and small intestinal conditions. However, the ratio of bioaccessibility of individual PAHs in gastric conditions to that in the small intestinal condition generally increased with increasing ring numbers, indicating the relatively pronounced effect of bile extract on improving the bioaccessibility of PAHs with relatively high ring numbers characterized by their high K (ow) values. Similarly, total PAH concentrations in soils were strongly correlated with SOC, indicating that SOC was the key factor determining the retention of PAHs in soils. CONCLUSIONS: Soils were contaminated with PAHs due to long-term wastewater irrigation. PAHs with two to six rings showed high concentrations with a significant increase over reference soils. Based on the molecular indices, it was suggested that PAHs in soils had both pyrogenic and petrogenic sources. It was also concluded that the oral bioaccessibility of total PAHs in the small intestinal condition was significantly higher than that in the gastric condition. Furthermore, the

bioaccessibility of individual PAHs in soils generally decreased with the increasing ring numbers in both the gastric and small intestinal conditions. RECOMMENDATIONS AND PERSPECTIVES: It is suggested that more care should be given while establishing reliable soil criteria for PAHs, especially concerning the health of children who may ingest a considerable amount of PAH-contaminated soil via outdoor hand-to-mouth activities
SN - 0944-1344
AD - Department of Soil Environmental Science, Research Center for Eco-Environmental Sciences, Chinese Academy of Sciences, Beijing 100085, China. sardar.khan2008@yahoo.com
UR - 18465157
ER -

TY - JOUR
ID - 1034
T1 - An investigation of school playground safety practices as reported by school nurses
A1 - Hudson,S.D.
A1 - Olsen,H.M.
A1 - Thompson,D.
Y1 - 2008/06//
N1 - Hudson, Susan D. Olsen, Heather M. Thompson, Donna
The Journal of school nursing : the official publication of the National Association of School Nurses
bgs, 9206498
N
Evaluation Studies. Journal Article
English
KW - MEDLINE
KW - Attitude of Health Personnel
KW - Child
KW - Communication
KW - Documentation
KW - Equipment Safety
KW - Health Knowledge
KW - Attitudes
KW - Practice
KW - Health Services Needs and Demand
KW - Human Engineering
KW - Humans
KW - Nurse's Role/px [Psychology]
KW - Nurse's Role
KW - Nursing Evaluation Research
KW - Nursing Methodology Research
KW - Nursing Staff/ed [Education]
KW - Nursing Staff/og [Organization & Administration]
KW - Nursing Staff/px [Psychology]
KW - Play and Playthings
KW - Questionnaires
KW - Safety Management/og [Organization & Administration]
KW - School Nursing/ed [Education]
KW - School Nursing/og [Organization & Administration]
KW - United States
RP - NOT IN FILE
SP - 138
EP - 144
JF - Journal of School Nursing
JA - J Sch Nurs
VL - 24
IS - 3
CY - United States

N2 - The purpose of this study was to investigate school playground safety practices. The study used a purposeful sample of school nurses who attended a playground safety workshop at the 2006 National Association of School Nurses annual conference. Seventy-five questionnaires were distributed, and 64 useable questionnaires were returned. The responses indicated that little attention is being given to providing safe playground environments in schools as measured by best practices of supervision, age-appropriate design, fall surfacing, and equipment maintenance. Participants pointed to the need for better supervision and supervision training, careful selection of age-appropriate equipment, maintaining adequate fall surfaces under the equipment, and ensuring that equipment is properly maintained and repaired. The study also revealed that school nurses believe they could play a role in playground injury prevention through the collection and analysis of injury data, communication to administrators about the need for comprehensive planning of the play environment, and becoming active members of playground safety committees
SN - 1059-8405
AD - National Program for Playground Safety, University of Northern Iowa, School of Health, Physical Education, and Leisure Services, Cedar Falls, Iowa, USA
UR - 18557672
ER -

TY - JOUR
ID - 1035
T1 - Increasing preschoolers' physical activity intensities: an activity-friendly preschool playground intervention
A1 - Hannon,J.C.
A1 - Brown,B.B.
Y1 - 2008/06//
N1 - Hannon, James C. Brown, Barbara B
Preventive medicine
pm4, 0322116
IM
Journal Article. Research Support, Non-U.S. Gov't
English
KW - MEDLINE
KW - Acceleration
KW - Child Welfare
KW - Child
KW - Preschool
KW - Female
KW - Health Behavior
KW - Humans
KW - Male
KW - Motor Activity
KW - Pilot Projects
KW - Play and Playthings
RP - NOT IN FILE
SP - 532
EP - 536
JF - Preventive Medicine
JA - Prev Med
VL - 46
IS - 6
CY - United States
N2 - OBJECTIVE: The purpose of this study was to see if portable play equipment added to a preschool playground resulted in higher intensities of physical activity among 3-5-year-old children. METHODS: Activity-friendly equipment was added to an outdoor preschool playground. Accelerometry-measured intensities of 15-s epochs of physical activity were tracked for 5 pre-intervention and 5 post-intervention days during outdoor play. Data were collected during fall 2005 in Salt Lake City for 64 preschoolers aged 3, 4, and 5 years. RESULTS: After the intervention, both male and female 3- to 5-year-olds significantly decreased sedentary behavior and significantly increased light, moderate, and vigorous physical activity as measured by

accelerometry. CONCLUSIONS: Results suggest simple interventions, requiring little teacher training, can yield increases in healthy physical activity
SN - 0091-7435
AD - University of Utah, College of Health, Department of Exercise and Sport Science, 250 South, 1850 East, Room 206, Salt Lake City, UT 84112, USA. james.hannon@hsc.utah.edu
UR - 18313741
ER -

TY - JOUR
ID - 1036
T1 - Exploration and validation of clusters of physically abused children
A1 - Sabourin,Ward C.
A1 - Haskett,M.E.
Y1 - 2008/05//
N1 - Sabourin Ward, Caryn. Haskett, Mary E
Child abuse & neglect
can, 7801702
IM
Journal Article. Research Support, N.I.H., Extramural
English
KW - MEDLINE
KW - Child Abuse/px [Psychology]
KW - Child Abuse/rh [Rehabilitation]
KW - Child Abuse/sn [Statistics & Numerical Data]
KW - Child
KW - Preschool
KW - Cluster Analysis
KW - Female
KW - Humans
KW - Male
KW - Object Attachment
KW - Parenting/px [Psychology]
KW - Peer Group
KW - Personal Construct Theory
KW - Personality Assessment/sn [Statistics & Numerical Data]
KW - Problem Solving
KW - Psychometrics
KW - Punishment
KW - Reproducibility of Results
KW - Social Adjustment
KW - Social Behavior
RP - NOT IN FILE
SP - 577
EP - 588
JF - Child Abuse & Neglect
JA - Child Abuse Negl
VL - 32
IS - 5
CY - England
N2 - OBJECTIVE: Cluster analysis was used to enhance understanding of heterogeneity in social adjustment of physically abused children. METHOD: Ninety-eight physically abused children (ages 5-10) were clustered on the basis of social adjustment, as measured by observed behavior with peers on the school playground and by teacher reports of social behavior. Seventy-seven matched nonabused children served as a comparison sample. Clusters were validated on the basis of observed parental sensitivity, parents' self-reported disciplinary tactics, and children's social information processing operations (i.e., generation of solutions to peer relationship problems and attributions of peer intentions in social situations). RESULTS: Three subgroups of physically abused children emerged from the cluster analysis; clusters were labeled Socially Well Adjusted, Hanging in

There, and Social Difficulties. Examination of cluster differences on risk and protective factors provided substantial evidence in support of the external validity of the three-cluster solution. Specifically, clusters differed significantly in attributions of peer intent and in parenting (i.e., sensitivity and harshness of parenting). Clusters also differed in the ways in which they were similar to, or different from, the comparison group of nonabused children. CONCLUSIONS: Results supported the contention that there were clinically relevant subgroups of physically abused children with potentially unique treatment needs. Findings also pointed to the relevance of social information processing operations and parenting context in understanding diversity among physically abused children. PRACTICE IMPLICATIONS: Pending replication, findings provide support for the importance of considering unique treatment of needs among physically abused children. A singular approach to intervention is unlikely to be effective for these children. For example, some physically abused children might need a more intensive focus on development of prosocial skills in relationships with peers while the prosocial skills of other abused children will be developmentally appropriate. In contrast, most physically abused children might benefit from training in social problem-solving skills. Findings also point to the importance of promoting positive parenting practices in addition to reducing harsh discipline of physically abusive parents
SN - 0145-2134
AD - Department of Psychology, NC State University, Raleigh, NC, USA
UR - 18511115
ER -

TY - JOUR
ID - 1037
T1 - Source apportionment of personal exposure of fine particulates among school communities in India
A1 - Gadkari,N.
A1 - Pervez,S.
Y1 - 2008/07//
N1 - Gadkari, Nilima. Pervez, Shamsh
Environmental monitoring and assessment
dvk, 8508350
IM
Journal Article
English
KW - MEDLINE
KW - Air Pollution
KW - Indoor/an [Analysis]
KW - Child
KW - Humans
KW - India
KW - Industry
KW - Particle Size
KW - Particulate Matter/ch [Chemistry]
KW - Schools
KW - Soil
KW - Vehicle Emissions
RP - NOT IN FILE
SP - 227
EP - 241
JF - Environmental Monitoring & Assessment
JA - Environ Monit Assess
VL - 142
IS - 1-3
CY - Netherlands
N2 - Source contribution estimates (SCE) of school community personal Respirable Particulate Matter (RPM) have been investigated. Reported relationships of personal RPM with Ambient-outdoors and indoor RPM levels have given the concept of defining the sources of personal exposure. Ambient-outdoors, indoors, soils and local road- traffic dusts were identified as main routes and principal sources of fine particulates at personal exposure levels. Fifteen subjects (05 from each of three schools) were selected from previous conducted study of interrelationships among classified atmospheric receptors in theses schools located in Bhilai-Durg, District

Durg, India. Samples of RPM collected from identified receptors and sources were analyzed for selected chemical constituents and the chemical data has been utilized in preparation of source-receptor profiles. Chemical mass balance (CMB8) model has been used for source apportionment study. Major dominating source is ambient-outdoors in case of school located near to steel plant downwind. Indoors and road-traffic dusts have also played dominating role in case of school located near to National Highways. Indoor ventilation properties have played an important role in source contribution estimates
SN - 0167-6369
AD - School of Studies in Chemistry, Pt. Ravishankar Shukla University, Raipur, C.G., India 492 010
UR - 17972151
ER -

TY - JOUR
ID - 1038
T1 - Polymorphous light eruption. [Review] [54 refs]
A1 - Honigsmann,H.
Y1 - 2008/06//
N1 - Honigsmann, Herbert
Photodermatology, photoimmunology & photomedicine
awp, 9013641
IM
Journal Article. Review
English
KW - MEDLINE
KW - Child
KW - Dermatologic Agents/tu [Therapeutic Use]
KW - Female
KW - Humans
KW - Male
KW - Photosensitivity Disorders/di [Diagnosis]
KW - Photosensitivity Disorders/et [Etiology]
KW - Photosensitivity Disorders/im [Immunology]
KW - Photosensitivity Disorders/th [Therapy]
KW - Photosensitivity Disorders
KW - Phototherapy
KW - Risk Factors
KW - Skin/pa [Pathology]
KW - Skin Tests
KW - Ultraviolet Rays/ae [Adverse Effects]
RP - NOT IN FILE
SP - 155
EP - 161
JF - Photodermatology, Photoimmunology & Photomedicine
JA - Photodermatol.Photoimmunol.Photomed.
VL - 24
IS - 3
CY - Denmark
N2 - Polymorphous light eruption is the most common photodermatosis, with a prevalence of as high as 10-20% in Western Europe and in the USA. It starts during the second and third decades of life. Although not life-threatening it can severely impair the quality of life, in particular during leisure activities and in outdoors workers. Polymorphous light eruption belongs to the group of so-called idiopathic photodermatoses. This term denotes dermatoses that occur in otherwise healthy individuals from exposure to sunlight or artificial light without the intervention of an exogenous photosensitizing agent. These diseases have two factors in common: they are precipitated by ultraviolet or visible radiation; and their exact pathomechanism remains obscure but is presumably immunologic in nature. [References: 54]
SN - 1600-0781
AD - Department of Dermatology, Medical University of Vienna, Vienna, Austria.
herbert.hoenigsmann@meduniwien.ac.at

UR - 18477138
ER -

TY - JOUR
ID - 1039
T1 - How free of tobacco smoke are 'smoke-free' homes?
A1 - Rumchev,K.
A1 - Jamrozik,K.
A1 - Stick,S.
A1 - Spickett,J.
Y1 - 2008/06//
N1 - Rumchev, K. Jamrozik, K. Stick, S. Spickett, J
Indoor air
9423515, da3
IM
Journal Article. Research Support, Non-U.S. Gov't
English
KW - MEDLINE
KW - Air Pollution
KW - Indoor/an [Analysis]
KW - Child
KW - Preschool
KW - Family Characteristics
KW - Female
KW - Humans
KW - Male
KW - Nicotine/an [Analysis]
KW - Poverty Areas
KW - Questionnaires
KW - Smoking/ep [Epidemiology]
KW - Tobacco Smoke Pollution/an [Analysis]
KW - Western Australia
RP - NOT IN FILE
SP - 202
EP - 208
JF - Indoor Air
VL - 18
IS - 3
CY - Denmark
N2 - UNLABELLED: The risks of exposure to environmental tobacco smoke (ETS) are well established and 'harm reduction' strategies such as smoking outside to protect infants and children from exposure to ETS have been advocated for some time. The aim of this study was to assess the validity of self-reported smoking levels in residential settings. The participants were families (n = 92) randomly selected from lower socioeconomic areas of Perth, Western Australia. Each household was monitored for vapor phase nicotine and particulates with an aerodynamic diameter of < or = 10 microm (PM(10)). Of the 42% (39) households who reported that someone smoked cigarettes at home, only four (4%) said that smoking occurred inside the house. There was a 'moderate' agreement between parental-reported tobacco smoking and levels of nicotine (kappa = 0.55, P < 0.01). There were significant differences in the median levels of air nicotine (P < 0.01) and PM(10) (P < 0.05) between households in which smoking was reported as only occurring outside, and the smoke-free households. PRACTICAL IMPLICATIONS: The study outcome suggests that a strategy based on the separation of children and smoking activity is inadequate to protect the former from ETS at home, and that health professionals should give parents unambiguous advice to give up smoking in order to make their homes a completely smoke-free environment
SN - 1600-0668
AD - School of Public Health, Curtin University of Technology, Perth, Western Australia, Australia. k.rumchev@curtin.edu.au
UR - 18336533

ER -

TY - JOUR
ID - 1040
T1 - The quality of children's home environment and attachment security in Indonesia
A1 - Zevalkink,J.
A1 - Riksen-Walraven,J.M.
A1 - Bradley,R.H.
Y1 - 2008/03//
N1 - Zevalkink, Jolien. Riksen-Walraven, J Marianne. Bradley, Robert H
The Journal of genetic psychology
i9z, 2985112r
IM
Journal Article
English
KW - MEDLINE
KW - Age Distribution
KW - Age Factors
KW - Asian Continental Ancestry Group/px [Psychology]
KW - Caregivers/px [Psychology]
KW - Child
KW - Child Psychology
KW - Preschool
KW - Female
KW - Humans
KW - Indonesia
KW - Infant
KW - Male
KW - Models
KW - Psychological
KW - Mother-Child Relations
KW - Object Attachment
KW - Parenting/px [Psychology]
KW - Personality Inventory
KW - Play and Playthings/px [Psychology]
KW - Population Density
KW - Quality of Life/px [Psychology]
KW - Questionnaires
KW - Residence Characteristics/sn [Statistics & Numerical Data]
KW - Social Environment
KW - Urban Population
RP - NOT IN FILE
SP - 72
EP - 91
JF - Journal of Genetic Psychology
JA - J Genet Psychol
VL - 169
IS - 1
CY - United States
N2 - The authors examined the relation of the Home Observation for Measurement of the Environment (HOME) Inventory (B. M. Caldwell & R. H. Bradley, 1984) for 0- to 6-year-old Sundanese Indonesian children with the quality of the mother-child attachment relationship (n=44) and attachment-related behaviors during play interactions (n=37) and with characteristics of the Indonesian caregiving context (N=77). Results showed that infants and toddlers with secure attachment relationships lived in higher quality home environments than did children with insecure attachment relationships. In particular, children with insecure-resistant attachment relationships lived in more unsafe and less organized homes with less play material available. For preschoolers, a lower quality home environment predicted more negativity and noncompliance toward their mothers in a play

setting outside the home. With regard to the caregiving context, the socioeconomic status of the family was strongly related to the quality of preschoolers' home environment. Scores on the HOME Inventory for Infants/Toddlers and the HOME Inventory for Early Childhood were related to other culture-specific contextual characteristics for 0- to 6-year old Indonesian children as well. As a whole, the HOME was a good indicator of the general quality of the Sundanese Indonesian home environment
SN - 0022-1325
AD - Department of Research and Quality Assurance, Netherlands Psychoanalytic Institute, Amsterdam, The Netherlands. zevalkink@npsai.nl
UR - 18476478
ER -

TY - JOUR
ID - 1041
T1 - Epidemiologic evidence of relationships between reproductive and child health outcomes and environmental chemical contaminants. [Review] [851 refs]
A1 - Wigle,D.T.
A1 - Arbuckle,T.E.
A1 - Turner,M.C.
A1 - Berube,A.
A1 - Yang,Q.
A1 - Liu,S.
A1 - Krewski,D.
Y1 - 2008/05//
N1 - Wigle, Donald T. Arbuckle, Tye E. Turner, Michelle C. Berube, Annie. Yang, Qiuying. Liu, Shiliang. Krewski, Daniel
Journal of toxicology and environmental health. Part B, Critical reviews
c08, 9802627
IM
Journal Article. Research Support, Non-U.S. Gov't. Review
English
KW - MEDLINE
KW - Adult
KW - Child
KW - Child Development/de [Drug Effects]
KW - Environmental Exposure/ae [Adverse Effects]
KW - Environmental Pollutants/to [Toxicity]
KW - Female
KW - Fetal Development/de [Drug Effects]
KW - Hazardous Substances/to [Toxicity]
KW - Humans
KW - Pregnancy
KW - Pregnancy Outcome
KW - Prenatal Exposure Delayed Effects
RP - NOT IN FILE
SP - 373
EP - 517
JF - Journal of Toxicology & Environmental Health Part B: Critical Reviews
JA - J Toxicol Environ Health B Crit Rev
VL - 11
IS - 5-6
CY - United States
N2 - This review summarizes the level of epidemiologic evidence for relationships between prenatal and/or early life exposure to environmental chemical contaminants and fetal, child, and adult health. Discussion focuses on fetal loss, intrauterine growth restriction, preterm birth, birth defects, respiratory and other childhood diseases, neuropsychological deficits, premature or delayed sexual maturation, and certain adult cancers linked to fetal or childhood exposures. Environmental exposures considered here include chemical toxicants in air, water, soil/house dust and foods (including human breast milk), and consumer products. Reports reviewed here

included original epidemiologic studies (with at least basic descriptions of methods and results), literature reviews, expert group reports, meta-analyses, and pooled analyses. Levels of evidence for causal relationships were categorized as sufficient, limited, or inadequate according to predefined criteria. There was sufficient epidemiological evidence for causal relationships between several adverse pregnancy or child health outcomes and prenatal or childhood exposure to environmental chemical contaminants. These included prenatal high-level methylmercury (CH(3)Hg) exposure (delayed developmental milestones and cognitive, motor, auditory, and visual deficits), high-level prenatal exposure to polychlorinated biphenyls (PCBs), polychlorinated dibenzofurans (PCDFs), and related toxicants (neonatal tooth abnormalities, cognitive and motor deficits), maternal active smoking (delayed conception, preterm birth, fetal growth deficit [FGD] and sudden infant death syndrome [SIDS]) and prenatal environmental tobacco smoke (ETS) exposure (preterm birth), low-level childhood lead exposure (cognitive deficits and renal tubular damage), high-level childhood CH(3)Hg exposure (visual deficits), high-level childhood exposure to 2,3,7,8-tetrachlorodibenzo-p-dioxin (TCDD) (chloracne), childhood ETS exposure (SIDS, new-onset asthma, increased asthma severity, lung and middle ear infections, and adult breast and lung cancer), childhood exposure to biomass smoke (lung infections), and childhood exposure to outdoor air pollutants (increased asthma severity). Evidence for some proven relationships came from investigation of relatively small numbers of children with high-dose prenatal or early childhood exposures, e.g., CH(3)Hg poisoning episodes in Japan and Iraq. In contrast, consensus on a causal relationship between incident asthma and ETS exposure came only recently after many studies and prolonged debate. There were many relationships supported by limited epidemiologic evidence, ranging from several studies with fairly consistent findings and evidence of dose-response relationships to those where 20 or more studies provided inconsistent or otherwise less than convincing evidence of an association. The latter included childhood cancer and parental or childhood exposures to pesticides. In most cases, relationships supported by inadequate epidemiologic evidence reflect scarcity of evidence as opposed to strong evidence of no effect. This summary points to three main needs: (1) Where relationships between child health and environmental exposures are supported by sufficient evidence of causal relationships, there is a need for (a) policies and programs to minimize population exposures and (b) population-based biomonitoring to track exposure levels, i.e., through ongoing or periodic surveys with measurements of contaminant levels in blood, urine and other samples. (2) For relationships supported by limited evidence, there is a need for targeted research and policy options ranging from ongoing evaluation of evidence to proactive actions. (3) There is a great need for population-based, multidisciplinary and collaborative research on the many relationships supported by inadequate evidence, as these represent major knowledge gaps. Expert groups faced with evaluating epidemiologic evidence of potential causal relationships repeatedly encounter problems in summarizing the available data. A major driver for undertaking such summaries is the need to compensate for the limited sample sizes of individual epidemiologic studies. Sample size limitations are major obstacles to exploration of prenatal, paternal, and childhood exposures during specific time windows, exposure intensity, exposure-exposure or exposure-gene interactions, and relatively rare health outcomes such as childhood cancer. Such research needs call for investments in research infrastructure, including human resources and methods development (standardized protocols, biomarker research, validated exposure metrics, reference analytic laboratories). These are needed to generate research findings that can be compared and subjected to pooled analyses aimed at knowledge synthesis. [References: 851]
SN - 1521-6950
AD - McLaughlin Centre for Population Health Risk Assessment, University of Ottawa, Ottawa, Ontario, Canada. don.wigle@sympatico.ca
UR - 18470797
ER -

TY - JOUR
ID - 1042
T1 - [On the impact of social stress on concentration performances in kindergarteners: results of naturalistic experiments in kindergarten-settings]. [German]
A1 - Krampen,G.
Y1 - 2008///
N1 - Krampen, Gunter
Praxis der Kinderpsychologie und Kinderpsychiatrie
pj6, 0404246
IM

Comparative Study. English Abstract. Journal Article. Randomized Controlled Trial
German
KW - MEDLINE
KW - Attention
KW - Child
KW - Preschool
KW - Discrimination Learning
KW - Fatigue/px [Psychology]
KW - Female
KW - Group Processes
KW - Humans
KW - Male
KW - Noise/ae [Adverse Effects]
KW - Pattern Recognition
KW - Visual
KW - Social Environment
KW - Stress
KW - Psychological/co [Complications]
RP - NOT IN FILE
SP - 167
EP - 178
JF - Praxis der Kinderpsychologie und Kinderpsychiatrie
JA - Prax Kinderpsychol.Kinderpsychiatr.
VL - 57
IS - 3
CY - Germany
N2 - Results of three experimental studies (implemented in naturalistic kindergarten-settings) on short-term effects of social stress on concentration performance in young children are presented. In all 250 kindergarten-children were tested repeatedly with the Kaseler-Konzentrations-Aufgabe (KKA; Kasel-Concentration-Task for Children Aged 3-8 Years; test-duration: 6-7 minutes). In addition, after each KKA-test the children were asked about their subjective feelings of tiredness and of distractions during the test. In Study I 90 kindergarten-children were randomized to a standard individual test-setting, a triadic test-setting with another child observing the test child, and an individual test-setting within the noisy kindergarten group. Study II refers to 40 kindergarten-children tested individually and repeatedly in a silent standard setting, in a noisy setting with other children playing loudly outside, and in a noisy setting with other children singing very loudly in their group-rooms outside the testing-room. 120 kindergarten-children took part in Study III, in which test time and social setting (shortly after arrival in kindergarten, after joint breakfast, before and after joint lunch) were varied. In Study I results point at large negative effects of social stress on concentration performance. No effects of typical kindergarten noises on concentration were observed in Study II and Study III, with the exception of stress reactions on concentration in the after-joint-lunch kindergarten setting. Results point at good habituations to common social stress in kindergarten-children as well as specific strong stress reactions on concentration in social settings, including observers and noisy group-settings. Subjective ratings of distractions during testing and of tiredness after testing are not correlated with cognitive stress reactions, putting the competence of kindergarten-children to report verbally solid about their stress reactions in question
SN - 0032-7034
AD - Universitat Trier, Fachbereich I Psychologie, Trier. krampen@uni-trier.de
UR - 18461846
ER -

TY - JOUR
ID - 1043
T1 - Production and perception of speech intonation in pediatric cochlear implant recipients and individuals with normal hearing
A1 - Peng,S.C.
A1 - Tomblin,J.B.
A1 - Turner,C.W.
Y1 - 2008/06//

N1 - Peng, Shu-Chen. Tomblin, J Bruce. Turner, Christopher W
Ear and hearing
e9g, 8005585
IM
Journal Article. Research Support, N.I.H., Extramural. Research Support, Non-U.S. Gov't
English
KW - MEDLINE
KW - Adolescent
KW - Adult
KW - Auditory Threshold
KW - Child
KW - Cochlear Implants
KW - Deafness/di [Diagnosis]
KW - Deafness/rh [Rehabilitation]
KW - Female
KW - Humans
KW - Male
KW - Phonetics
KW - Sound Spectrography
KW - Speech Acoustics
KW - Speech Intelligibility
KW - Speech Perception
KW - Speech Production Measurement
RP - NOT IN FILE
SP - 336
EP - 351
JF - Ear & Hearing
JA - Ear Hear
VL - 29
IS - 3
CY - United States
N2 - OBJECTIVES: Current cochlear implant (CI) devices are limited in providing voice pitch information that is critical for listeners' recognition of prosodic contrasts of speech (e.g., intonation and lexical tones). As a result, mastery of the production and perception of such speech contrasts can be very challenging for prelingually deafened individuals who received a CI in their childhood (i.e., pediatric CI recipients). The purpose of this study was to investigate (a) pediatric CI recipients' mastery of the production and perception of speech intonation contrasts, in comparison with their age-matched peers with normal hearing (NH), and (b) the relationships between intonation production and perception in CI and NH individuals. DESIGN: Twenty-six pediatric CI recipients aged from 7.44 to 20.74 yrs and 17 age-matched individuals with NH participated. All CI users were prelingually deafened, and each of them received a CI between 1.48 and 6.34 yrs of age. Each participant performed an intonation production task and an intonation perception task. In the production task, 10 questions and 10 statements that were syntactically matched (e.g., "The girl is on the playground." versus "The girl is on the playground?") were elicited from each participant using interactive discourse involving pictures. These utterances were judged by a panel of eight adult listeners with NH in terms of utterance type accuracy (question versus statement) and contour appropriateness (on a five-point scale). In the perception task, each participant identified the speech intonation contrasts of natural utterances in a two-alternative forced-choice task. RESULTS: The results from the production task indicated that CI participants' scores for both utterance type accuracy and contour appropriateness were significantly lower than the scores of NH participants (both $p < 0.001$). The results from the perception task indicated that CI participants' identification accuracy was significantly lower than that of their NH peers (CI, 70.13% versus NH, 97.11%, $p < 0.001$). The Pearson correlation coefficients (r) between CI participants' performance levels in the production and perception tasks were approximately 0.65 ($p = 0.001$). CONCLUSION: As a group, pediatric CI recipients do not show mastery of speech intonation in their production or perception to the same extent as their NH peers. Pediatric CI recipients' performance levels in the production and perception of speech intonation contrasts are moderately correlated. Intersubject variability exists in pediatric CI recipients' mastery levels in the production and perception of speech intonation contrasts. These findings suggest the importance of addressing both aspects

(production and perception) of speech intonation in the aural rehabilitation and speech intervention programs for prelingually deafened children and young adults who use a CI
SN - 0196-0202
AD - Department of Speech Pathology and Audiology, University of Iowa, Iowa City, IA, USA. speng@hesp.umd.edu
UR - 18344873
ER -

TY - JOUR
ID - 1044
T1 - Exercise-induced changes in respiratory impedance in young wheezy children and nonatopic controls
A1 - Malmberg,L.P.
A1 - Makela,M.J.
A1 - Mattila,P.S.
A1 - Hammaren-Malmi,S.
A1 - Pelkonen,A.S.
Y1 - 2008/06//
N1 - Malmberg, L Pekka. Makela, Mika J. Mattila, Petri S. Hammaren-Malmi, Sari. Pelkonen, Anna S
Pediatric pulmonology
owh, 8510590
IM
Journal Article. Research Support, Non-U.S. Gov't
English
KW - MEDLINE
KW - Airway Resistance
KW - Asthma
KW - Exercise-Induced/di [Diagnosis]
KW - Child
KW - Preschool
KW - Exercise Test
KW - Feasibility Studies
KW - Female
KW - Humans
KW - Male
KW - Oscillometry/mt [Methods]
KW - ROC Curve
RP - NOT IN FILE
SP - 538
EP - 544
JF - Pediatric Pulmonology
JA - Pediatr Pulmonol
VL - 43
IS - 6
CY - United States
N2 - Exercise-induced bronchoconstriction (EIB) is a specific sign of active asthma, but its assessment in young children may be difficult with lung function techniques requiring active cooperation. The aim of the study was to assess the normal pattern of exercise-induced responses of respiratory impedance by using impulse oscillometry (IOS), and to investigate how these responses discriminate wheezy children from control subjects. IOS measurements were performed in a consecutive sample of wheezy children aged 3-7 years (n = 130) and in an aged matched control group of nonatopic children without respiratory symptoms (n = 79) before and after a free running test. After exercise, wheezy children showed significantly larger responses in respiratory resistance (Rrs5), reactance (Xrs5), and the resonance frequency (Fr) than the control subjects. In the control group, the upper 95% confidence limit of the maximal change was 32.5% for Rrs5, 85.7% for Xrs5, and 53.1% for Fr. By using analysis of receiver operating characteristics, the change in Rrs5 distinguished the wheezy children from the control subjects more effectively than change in Xrs5 or Fr. In wheezy children, the response was significantly effected by the outdoor temperature and exercise intensity in terms of maximum heart rate. In conclusion, an increase of 35% in Rrs5 after a free running test can be regarded as an abnormal response.

Wheezy children show an enhanced airway response, which is clearly distinguishable from the control subjects. IOS is a feasible method to detect EIB in young children
SN - 1099-0496
AD - Department of Allergy, Helsinki University Central Hospital, Helsinki, Finland. pekka.malmberg@hus.fi
UR - 18433041
ER -

TY - JOUR
ID - 1045
T1 - Mania profile in a community sample of prepubertal children in Turkey
A1 - Diler,R.S.
A1 - Uguz,S.
A1 - Seydaoglu,G.
A1 - Avci,A.
Y1 - 2008/06//
N1 - Diler, Rasim Somer. Uguz, Sukru. Seydaoglu, Gulsah. Avci, Ayse
Bipolar disorders
dz6, 100883596
IM
Journal Article
English
KW - MEDLINE
KW - Bipolar Disorder/ep [Epidemiology]
KW - Child
KW - Female
KW - Humans
KW - Male
KW - Psychiatric Status Rating Scales
KW - Residence Characteristics
KW - Turkey/ep [Epidemiology]
RP - NOT IN FILE
SP - 546
EP - 553
JF - Bipolar Disorders
JA - Bipolar Disord
VL - 10
IS - 4
CY - Denmark
N2 - BACKGROUND: Mania in youth is increasingly recognized and accompanied by substantial psychiatric and psychosocial morbidity. There are no data on prepubertals in the general population and we aimed to search for mania symptoms and its clinical correlations in a community sample of prepubertal Turkish children. METHODS: Among all children (n = 56,335) aged 7-11 in Adana, Turkey, 2,468 children (48% girls) were randomly included. Parents completed Child Behavior Checklist (CBCL) 4-18 and Parent-Young Mania Rating Scale (P-YMRS). Cut-off scores of 17 and 27 on total P-YMRS were defined as efficient (probable-mania group) and specific (mania group), respectively, for bipolar profile. We searched for clinical correlations and used logistic regression to show how well each CBCL subscale predicted the presence of mania and probable-mania, after adjusting for any demographic differences. RESULTS: Parent-Young Mania Rating Scale scores were > or =17 but <27 (probable-mania) in 155 (6.3%) children and > or =27 (mania) in 32 (1.3%) children. Elevated mood, increased activity levels, and poor insight were the most frequent manic symptoms in our sample. Children with probable-mania and mania had higher scores on all CBCL subscales and the CBCL-Pediatric Bipolar Disorder (CBCL-PBD) profile (sum of attention, aggression, and anxiety/depression subscales). Logistic regression analysis revealed only thought problems on CBCL that predicted probable-mania and mania. CONCLUSION: Our study shows that mania profile is common in the community sample of Turkish prepubertal children and does not support the thought that mania is rare outside the US. We need further population-based studies that will use diagnostic interviews and multiple informants
SN - 1399-5618

AD - Department of Psychiatry, Western Psychiatric Institute and Clinic, University of Pittsburgh, Pittsburgh, PA 15213, USA. dilerrs@yahoo.com
UR - 18452451
ER -

TY - JOUR
ID - 1046
T1 - Self-reported home environmental risk factors for childhood asthma: a cross-sectional study of children in Buffalo, New York
A1 - Lin,S.
A1 - Gomez,M.I.
A1 - Hwang,S.A.
A1 - Munsie,J.P.
A1 - Fitzgerald,E.F.
Y1 - 2008/05//
N1 - Lin, Shao. Gomez, Marta I. Hwang, Syni-An. Munsie, Jean Pierre. Fitzgerald, Edward F
The Journal of asthma : official journal of the Association for the Care of Asthma
hf7, 8106454
IM
Journal Article
English
KW - MEDLINE
KW - Adolescent
KW - Age Distribution
KW - Asthma/ep [Epidemiology]
KW - Asthma/et [Etiology]
KW - Bias (Epidemiology)
KW - Case-Control Studies
KW - Child
KW - Preschool
KW - Cross-Sectional Studies
KW - Environmental Exposure/ae [Adverse Effects]
KW - Environmental Pollutants/ae [Adverse Effects]
KW - Environmental Pollutants/an [Analysis]
KW - Female
KW - Humans
KW - Infant
KW - Logistic Models
KW - Male
KW - New York/ep [Epidemiology]
KW - Prevalence
KW - Questionnaires
KW - Risk Factors
KW - Sex Distribution
KW - Socioeconomic Factors
RP - NOT IN FILE
SP - 325
EP - 332
JF - Journal of Asthma
JA - J Asthma
VL - 45
IS - 4
CY - United States
N2 - We present prevalence estimates of indoor and outdoor environmental risk factors for asthma from a cross-sectional study of children 1 to 17 years of age living in Buffalo, New York. A child's primary caretaker completed a questionnaire about the household's demographics, lifestyle habits, housing, indoor and outdoor environment, and the child's activity patterns, family history of asthma, asthma symptoms and treatment, and

medical care access. Significant environmental risk factors were presence of smokers in the household, humidifier or vaporizer use, chemical odors indoors, frequent truck traffic, and chemical odors outdoors. Most of these risk factors can be mitigated or controlled
SN - 1532-4303
AD - New York State Department of Health, Bureau of Environmental and Occupational Epidemiology, Troy, New York 12180-2216, USA
UR - 18446598
ER -

TY - JOUR
ID - 1047
T1 - Providing sporting experiences for children in Out of School Hours Care (OSHC) environments: sport and physical activity participation and intentions
A1 - Spittle,M.
A1 - O'Meara,J.
A1 - Garnham,J.
A1 - Kerr,M.
Y1 - 2008/06//
N1 - Spittle, Michael. O'Meara, James. Garnham, Jennie. Kerr, Megan
Journal of science and medicine in sport / Sports Medicine Australia
c54, 9812598
IM
Journal Article. Research Support, Non-U.S. Gov't
English
KW - MEDLINE
KW - Child
KW - Child Day Care Centers
KW - Female
KW - Humans
KW - Intention
KW - Leisure Activities
KW - Male
KW - Motor Activity
KW - Parent-Child Relations
KW - Sports
KW - Victoria
RP - NOT IN FILE
SP - 316
EP - 322
JF - Journal of Science & Medicine in Sport
JA - J Sci Med Sport
VL - 11
IS - 3
CY - Australia
N2 - The Out of School Hours Sports Program (OSHSP) aimed to provide structured sporting experiences and community links to local clubs for children in Out of School Hours Care (OSHC). The OSHSP involved 17 State Sporting Associations (SSAs), 71 OSHC Services and local club representatives. This study explored children's participation in sport in and outside the OSHSP and parental intention for participation in sport in and outside the OSHSP. Surveys were received from 211 children (76 girls and 125 boys; mean age=7.9 years, S.D.=1.7) and their parents/guardians (37.9% response rate). OSHC is characterised by freedom of choice of participation in activities by children. The OSHSP was used to provide an opportunity to choose to participate in a sport while attending OSHC. At the OSHC Services surveyed, between 7.1 and 100% of the children attending OSHC chose to participate in the OSHSP. Of those children who chose to participate, 85% were participating in a sport, usually a different sport to the one offered in the OSHSP. This participation was largely club-based (49.8%), most often once a week for training and competition (55.2%). Parental intentions for children's participation in the OSHSP sports varied with respect to the number of years attending the OSHSP, where children played and trained in their main sport, and how many times a week a child played and trained in their

main sport. Older children tended to play and train for sport more times per week and had been attending the OSHC for more years than younger children
SN - 1440-2440
AD - School of Human Movement and Sport Sciences, University of Ballarat, Australia.
m.spittle@ballarat.edu.au
UR - 17569584
ER -

TY - JOUR
ID - 1048
T1 - Lead contamination in Uruguay: the "La Teja" neighborhood case. [Review] [43 refs]
A1 - Manay,N.
A1 - Cousillas,A.Z.
A1 - Alvarez,C.
A1 - Heller,T.
Y1 - 2008///
N1 - Manay, Nelly. Cousillas, Adriana Z. Alvarez, Cristina. Heller, Teresa
Reviews of environmental contamination and toxicology
rec, 8703602
IM
Journal Article. Review
English
KW - MEDLINE
KW - Animals
KW - Child
KW - Preschool
KW - Environmental Exposure/ae [Adverse Effects]
KW - Female
KW - Humans
KW - Lead/an [Analysis]
KW - Lead Poisoning/bl [Blood]
KW - Lead Poisoning/ep [Epidemiology]
KW - Lead Poisoning/et [Etiology]
KW - Male
KW - Mining
KW - Occupational Exposure/ae [Adverse Effects]
KW - Rural Population
KW - Soil Pollutants/an [Analysis]
KW - Urban Population
KW - Uruguay/ep [Epidemiology]
RP - NOT IN FILE
SP - 93
EP - 115
JF - Reviews of Environmental Contamination & Toxicology
JA - Rev Environ Contam Toxicol
VL - 195
CY - United States
N2 - Lead, ubiquitous in the environment as a result of mining and industrialization, is found as a contaminant in humans although it has no known physiological function there. Lead-exposed children are known to be the population with the highest potential health risks. The recommended biomarker to assess environmental lead exposure in animals is lead level in blood. Before 2001, the Department of Toxicology and Environmental Hygiene was the only team to produce human monitoring data on Uruguayan populations (Manay 2001a,b; Manay et al. 1999). Lead pollution in Uruguay first received official attention during the 2001 La Teja poisoning episode. It was in the La Teja neighbourhood of Montevideo that high BLL were found in children (as high as 20 microg/dL), prompting corrective responses from Health and Environmental authorities. Growing awareness of environmental lead pollution and consequential human health effects from that event, resulted in public debate and demands for solutions from Health and Environmental authorities. Citizens demanded public

disclosure of information concerning lead pollution and wanted action to address contaminated Uruguayan sites. In response, the Ministry of Health assembled an interinstitutional multidisciplinary committee, with delegates from health, environmental, labor, educational, and social security authorities, as well as community nongovernmental organizations (NGOs), among others. The University of the Republic was designated to serve as the main responsible entity for technical advice and support. After 2001, new research on lead pollution was undertaken and included multidisciplinary studies with communities in response to health risk alerts. The main emphasis was placed on children exposed to environmental lead. Major sources of Uruguayan lead contamination, similar to those in other developing countries, result from metallurgical industries, lead-acid battery processing, lead wire and pipe factories, metal foundries, metal recyclers, leaded gasoline (before December 2003), lead water pipes in old houses, and scrap and smelter solid wastes, among others. Nonoccupational lead exposure usually results from living in or near current or former manufacturing areas or improper handling of lead-containing materials or solid wastes (a particularly important health risk for children). In this chapter, we reviewed available studies published or reported after the pollution events first announced in 2001. These studies include data on exposure, health, and actions taken to mitigate or prevent lead exposure from pollution events in Uruguay. Uruguay adopted CDC's 10 microg/dL as the reference BLL for children (CDC 1991) and a BLL of 30 microg/dL for workers (from the ACGIH standard). Environmental authorities adopted the Canadian reference concentrations for soil: residential and playgrounds (> 140 mg/kg) or industrial areas (> 600 mg/kg) (CCME 2006). Most studies reviewed addressed soil pollution as the main source of lead exposure. Results of thousands of analyses indicated that most children had BLL above reference intervention limits. A significant decrease in BLL was also found over time in the study results, demonstrating the importance of medical intervention, nutrition, and environmental education. The severity of lead pollution discovered required official governmental actions, both to reduce sources of lead contamination and to address the health implications for children who had been exposed to environmental or industrial lead pollution. Dogs were discovered to be useful sentinels for environmental lead pollution; they had higher BLL than children when exposed to the same polluted environment and developed symptoms of lead intoxication earlier and at lower BLL than did children. This same pattern was also observed in families with children and pet dogs living in the La Teja neighborhood. This discovery renders dogs prospectively useful in lead pollution monitoring and diagnosis, particularly in developing countries. BLL results from similar human lead exposure studies conducted 10 yr apart showed significant BLL reductions, after 10 yr, for nonoccupationally exposed Uruguayans. The phase-out of leaded gasoline is thought to have contributed to this improvement. New laws to address occupational and environmental exposures were passed to prevent new cases of lead contamination, and new research studies are underway to monitor lead pollution. Moreover, a systematic surveillance screening program for lead workers and children is planned, although it is not yet underway. The sensitization of the public to the lead pollution problem has been a key driver of governmental action to mitigate and prevent further lead pollution in Uruguay. The changes made since 2001 appear to have yielded positive results. BLL from different populations studied more recently show decreased lead levels, suggesting a lower contribution of environmental lead to exposure of children and nonoccupationally exposed adults. The diverse analytical data collected on lead pollution in Uruguay between 2001 and 2004 were the main ingredient that allowed effective identification of lead pollution in Uruguay and paved the way for official intervention to prevent new pollution events. Nevertheless, full research studies must still be done, including both spot analysis of environmental soil, air, and water samples, and extensive screening of BLL. Future health and environmental actions are needed, not only to remediate known areas of lead pollution, but also to investigate other sources of potential health risks. [References: 43]
SN - 0179-5953
AD - Department of Toxicology and Environmental Hygiene, Faculty of Chemistry, University of the Republic of Uruguay, Gral. Flores 2124, 11800 Montevideo, Uruguay. nmanay@fq.edu.uy
UR - 18418955
ER -

TY - JOUR
ID - 1049
T1 - Diversity of participation in children with cerebral palsy
A1 - Imms,C.
A1 - Reilly,S.
A1 - Carlin,J.
A1 - Dodd,K.
Y1 - 2008/05//

N1 - Imms, Christine. Reilly, Sheena. Carlin, John. Dodd, Karen
Developmental medicine and child neurology
0006761, e83
IM
Comparative Study. Journal Article. Research Support, Non-U.S. Gov't
English
KW - MEDLINE
KW - Activities of Daily Living
KW - Adolescent
KW - Australia/ep [Epidemiology]
KW - Cerebral Palsy/cl [Classification]
KW - Cerebral Palsy/ep [Epidemiology]
KW - Cerebral Palsy/pp [Physiopathology]
KW - Cerebral Palsy/px [Psychology]
KW - Child
KW - Community Health Planning
KW - Developmental Disabilities/pp [Physiopathology]
KW - Female
KW - Humans
KW - Male
KW - Motor Skills/ph [Physiology]
KW - Questionnaires
KW - Retrospective Studies
KW - Severity of Illness Index
RP - NOT IN FILE
SP - 363
EP - 369
JF - Developmental Medicine & Child Neurology
JA - Dev Med Child Neurol
VL - 50
IS - 5
CY - England
N2 - The aim of this study was to investigate the participation of children with cerebral palsy (CP) in activities outside school and to compare their participation with a large representative sample of children. A population-based survey was conducted of children with CP born in Victoria, Australia in 1994 and 1995. Of 219 living children identified, 114 (52.1%) returned completed surveys. The children (65 males, 49 females) were aged between 10 years 9 months and 12 years 9 months (mean age 11y 9mo, SD 6mo). Thirty-eight per cent had hemiplegia, 23% diplegia, 4% triplegia, 34% quadriplegia, and 1% was of unknown topography. Distribution according to the Gross Motor Function Classification System (GMFCS) was 22.8% Level I, 36% Level II, 10.5% Level III, 8.8% Level IV, and 21.9% Level V. Distribution according to the Manual Ability Classification System (MACS) was: 19.3% Level I, 38.6% Level II, 14.0% Level III, 8.8% Level IV, and 19.3% Level V. Participation was measured using the Children's Assessment of Participation and Enjoyment. Participation in selected sport, cultural, and quiet leisure activities was compared with population-based data for 11-year-olds from the Australian Bureau of Statistics. Children with CP undertook a median of 26.5 activities (interquartile range 10) in 4 months which were commonly informal rather than formal. Intensity of participation was low. Diversity and intensity of participation was similar for children in each level of the MACS and the GMFCS, except for participants in Level V. More children with CP participated in organized sports ($p<0.001$) compared with other Australian children, although with lower frequency ($p<0.001$). Participation diversity and level of intensity of Australian children with CP were similar to those reported in a Canadian study
SN - 0012-1622
AD - School of Occupational Therapy, La Trobe University, Melbourne, Victoria, Australia. c.imms@latrobe.edu.au
UR - 18355337
ER -

TY - JOUR

ID - 1050
T1 - Myopia, lifestyle, and schooling in students of Chinese ethnicity in Singapore and Sydney
A1 - Rose,K.A.
A1 - Morgan,I.G.
A1 - Smith,W.
A1 - Burlutsky,G.
A1 - Mitchell,P.
A1 - Saw,S.M.
Y1 - 2008/04//
N1 - Rose, Kathryn A. Morgan, Ian G. Smith, Wayne. Burlutsky, George. Mitchell, Paul. Saw, Seang-Mei
Archives of ophthalmology
7706534, 830
AIM, IM
Comparative Study. Journal Article. Research Support, Non-U.S. Gov't
English
KW - MEDLINE
KW - Asian Continental Ancestry Group/eh [Ethnology]
KW - Child
KW - Cross-Sectional Studies
KW - Education
KW - Female
KW - Humans
KW - Life Style/eh [Ethnology]
KW - Male
KW - Myopia/eh [Ethnology]
KW - New South Wales/ep [Epidemiology]
KW - Prevalence
KW - Questionnaires
KW - Refraction
KW - Ocular
KW - Risk Factors
KW - Singapore/ep [Epidemiology]
KW - Visual Acuity
RP - NOT IN FILE
SP - 527
EP - 530
JF - Archives of Ophthalmology
JA - Arch Ophthalmol
VL - 126
IS - 4
CY - United States
N2 - OBJECTIVE: To compare the prevalence and risk factors for myopia in 6- and 7-year-old children of Chinese ethnicity in Sydney and Singapore. METHODS: Two cross-sectional samples of age- and ethnicity-matched primary school children participated: 124 from the Sydney Myopia Study and 628 from the Singapore Cohort Study on the Risk Factors for Myopia. Cycloplegic autorefraction was used to determine myopia prevalence (spherical equivalent < or = -0.5 diopter). Lifestyle activities were ascertained by questionnaire. RESULTS: The prevalence of myopia in 6- and 7-year-old children of Chinese ethnicity was significantly lower in Sydney (3.3%) than in Singapore (29.1%) (P < .001). The prevalence of myopia in 1 or more parents was 68% in Sydney and 71% in Singapore. Children in Sydney read more books per week (P < .001) and did more total near-work activity (P = .002). Children in Sydney spent more time on outdoor activities (13.75 vs 3.05 hours per week; P < .001), which was the most significant factor associated with the differences in the prevalence of myopia between the 2 sites. CONCLUSIONS: The lower prevalence of myopia in Sydney was associated with increased hours of outdoor activities. We hypothesize that another factor contributing to the differences in the prevalence of myopia may be the early educational pressures found in Singapore but not in Sydney
SN - 1538-3601

AD - Discipline of Applied Vision Sciences, Faculty of Health Sciences, University of Sydney, Lidcombe, Australia. k.rose@usyd.edu.au
UR - 18413523
ER -

TY - JOUR
ID - 1051
T1 - The demographics of playground equipment injuries in children
A1 - Loder,R.T.
Y1 - 2008/04//
N1 - Loder, Randall T
Journal of pediatric surgery
jmj, 0052631
IM
Journal Article. Research Support, Non-U.S. Gov't
English
KW - MEDLINE
KW - Accidental Falls/sn [Statistics & Numerical Data]
KW - Age Distribution
KW - Athletic Injuries/cl [Classification]
KW - Athletic Injuries/ep [Epidemiology]
KW - Child
KW - Female
KW - Humans
KW - Indiana/ep [Epidemiology]
KW - Logistic Models
KW - Male
KW - Play and Playthings/in [Injuries]
KW - Schools/sn [Statistics & Numerical Data]
KW - Seasons
KW - Sex Distribution
RP - NOT IN FILE
SP - 691
EP - 699
JF - Journal of Pediatric Surgery
JA - J Pediatr Surg
VL - 43
IS - 4
CY - United States
N2 - BACKGROUND/PURPOSE: There have been many different studies of injuries owing to playground equipment but none that have looked in detail using large nationwide databases. It was the purpose of this study to investigate injuries owing to playground equipment using the National Electronic Injury Surveillance System (NEISS) database and further understand their demographics. METHODS: Detailed NEISS injury data from 2002 through 2004 for slides, monkey bars, and swings were analyzed. Appropriate statistical analyses were performed; because of the many analyses on this large data set, P < .01 was considered statistically significant. RESULTS: There were 22728 emergency department visits owing to playground equipment injuries recorded by NEISS between 2002 and 2004; 83.9% were owing to monkey bars, swings, and slides, and the 5 most common diagnoses were fractures (39.3%), contusions/abrasions (20.6%), lacerations (16.6%), strains/sprains (9.9%), and traumatic brain injuries (TBI) (8.5%). There were 9487 boys (54.3%) and 7995 girls (45.7%). The average age was 6.5 +/- 3.0 years. The injuries occurred at school in 38.9%; at a recreation/sporting facility, in 35.5%; and at home, in 25.6%. Most were treated and released (94.4%). Amerindian children were 2 times more likely than blacks to be admitted; compared to contusions, fractures were 9.8 times, and TBIs, 4.7 times more likely to be admitted. Injuries on monkey bars were 1.2 times more likely to be admitted than those on swings or slides. Fractures were 1.9 times more likely to occur on a monkey bar compared with swings or slides. Traumatic brain injuries were 1.4 times more likely to occur on a swing compared to slides or monkey bars. CONCLUSION: Swings at school are the most common mechanism of injury for TBIs, and the seasonal data would suggest that increased supervision of children using swings during school hours might reduce the

occurrence of TBIs. Monkey bars are the most common cause of fracture, and fracture is the most common cause of admission. Prevention strategies to reduce the number of fractures should be directed at monkey bar equipment and landing surfaces. The trend in playground equipment injury also indicates that monkey bars are problematic because the number of injuries per year per capita owing to monkey bars is stable, whereas those from swings and slides is decreasing
SN - 1531-5037
AD - Department of Orthopaedic Surgery, Indiana School of Medicine, Indiana University, IN 46202, USA. rloder@iupui.edu
UR - 18405717
ER -

TY - JOUR
ID - 1052
T1 - Preschool children and physical activity: a review of correlates. [Review] [43 refs]
A1 - Hinkley,T.
A1 - Crawford,D.
A1 - Salmon,J.
A1 - Okely,A.D.
A1 - Hesketh,K.
Y1 - 2008/05//
N1 - Hinkley, Trina. Crawford, David. Salmon, Jo. Okely, Anthony D. Hesketh, Kylie
American journal of preventive medicine
8704773, apl
IM
Journal Article. Research Support, Non-U.S. Gov't. Review
English
KW - MEDLINE
KW - Child Welfare
KW - Child
KW - Preschool
KW - Exercise
KW - Female
KW - Health Behavior
KW - Humans
KW - Male
KW - Motivation
RP - NOT IN FILE
SP - 435
EP - 441
JF - American Journal of Preventive Medicine
JA - Am J Prev Med
VL - 34
IS - 5
CY - Netherlands
N2 - BACKGROUND: Several reviews have summarized the research on correlates of older children's and adolescents' physical activity behaviors, but none have been published on preschool children. Over the past 27 years, a number of studies have investigated the correlates of preschool children's physical activity behaviors. It is timely and necessary to review the extant literature in this area. This paper reviews articles investigating correlates of preschool children's physical activity behaviors published in peer-reviewed journals between 1980 and March 2007. METHODS: A literature search was conducted to identify studies that investigated correlates of preschool children's physical activity. Data were collected and analyzed in 2007. RESULTS: Twenty-four articles were identified that met the inclusion criteria. From those articles, 39 variables were identified across five domains. Results showed that boys were more active than girls, that children with active parents tended to be more active, and that children who spent more time outdoors were more active than children who spent less time outdoors. Age and BMI were consistently shown to have no association with preschool children's physical activity. Other variables produced largely inconclusive results. CONCLUSIONS: The influences on the physical

activity behaviors of preschool children are multidimensional. Further research is required to enhance an understanding of these influences. [References: 43]
SN - 0749-3797
AD - Centre for Physical Activity and Nutrition Research, Deakin University, Burwood, VIC, Australia. thin@deakin.edu.au
UR - 18407012
ER -

TY - JOUR
ID - 1053
T1 - Where do the children play? The influence of playground equipment on physical activity of children in free play
A1 - Farley,T.A.
A1 - Meriwether,R.A.
A1 - Baker,E.T.
A1 - Rice,J.C.
A1 - Webber,L.S.
Y1 - 2008/03//
N1 - Farley, Thomas A. Meriwether, Rebecca A. Baker, Erin T. Rice, Janet C. Webber, Larry S
Journal of physical activity & health
101189457
IM
Journal Article. Research Support, N.I.H., Extramural
English
KW - MEDLINE
KW - Child
KW - Equipment Design
KW - Exercise
KW - Female
KW - Humans
KW - Louisiana
KW - Male
KW - Observation
KW - Play and Playthings
KW - Public Facilities
KW - Urban Population
RP - NOT IN FILE
SP - 319
EP - 331
JF - Journal of Physical Activity & Health
JA - J Phys Act Health
VL - 5
IS - 2
CY - United States
N2 - BACKGROUND: Promotion of physical activity in children depends on an understanding of how children use play equipment. METHODS: We conducted observations over 2 years of children in 2nd through 8th grades in a schoolyard with 5 distinct play areas with different amounts of play equipment. RESULTS: Children were more likely to play in areas with more installed play equipment, with densities of children in equipped areas 3.3 to 12.6 times higher than in an open grassy field. There were no significant differences by play area in the percent of children who were physically active at all, but children were more likely to be very active in areas with basketball goals and an installed play structure than in an open field. CONCLUSIONS: Playground equipment appeared to have a strong influence on where children played and a moderate influence on levels of activity. To maximize physical activity in children, playgrounds should be designed with ample and diverse play equipment
SN - 1543-3080
AD - School of Public Health and Tropical Medicine, Tulane University, New Orleans, LA 70112, USA
UR - 18382040

ER -

TY - JOUR
ID - 1054
T1 - Neighborhood disorder, perceived safety, and readiness to encourage use of local playgrounds
A1 - Miles,R.
Y1 - 2008/04//
N1 - Miles, Rebecca
American journal of preventive medicine
8704773, apl
IM
Journal Article
English
KW - MEDLINE
KW - Adult
KW - Child
KW - Cross-Sectional Studies
KW - Environment Design
KW - Europe
KW - Female
KW - Humans
KW - Male
KW - Middle Aged
KW - Motor Activity
KW - Motor Vehicles
KW - Parents/px [Psychology]
KW - Perception
KW - Play and Playthings
KW - Public Facilities
KW - Residence Characteristics
KW - Safety
KW - Walking
RP - NOT IN FILE
SP - 275
EP - 281
JF - American Journal of Preventive Medicine
JA - Am J Prev Med
VL - 34
IS - 4
CY - Netherlands
N2 - BACKGROUND: Knowledge of the association between the neighborhood physical environment and adults' readiness to encourage children's use of local playgrounds, and the extent to which perceived safety acts as a mediator, can inform efforts to increase children's physical activity. METHODS: Data were obtained from seven European cities based on a cross-sectional household survey conducted between 2001 and 2002. The sample included 2123 household informants (from a total of 2782 households) with a median age of 48 years; 65% were women, 66% were married, and 33% had achieved a secondary education. Indicators of local neighborhood physical disorder (litter, graffiti, lack of greenery), traffic volume, and land use were directly observed by trained surveyors. Perceived safety, encouragement of playground use, and physical activity levels were assessed with self-reported measures. Analyses were conducted in 2007. RESULTS: Respondents in neighborhoods showing signs of low or moderate physical disorder compared to high physical disorder had slightly over twice the odds of encouraging children to use local playgrounds ($p<0.01$). The percentage of the effect of neighborhood physical disorder accounted for by perceived safety was between 15% and 20%. Neighborhood physical disorder was associated only with adults' occasional involvement in sports or exercise and only among women ($p<0.05$); perceived safety was not significantly associated with physical activity for either men or women. CONCLUSIONS: Neighborhood physical environments and perceived safety influence adults' readiness to encourage children's physical activity and women's occasional involvement in sports or

exercise. Health promotion strategies designed to upgrade the environments near where children live and to address parental safety concerns merit further exploration
SN - 0749-3797
AD - Department of Urban and Regional Planning, Florida State University, Tallahassee, Florida 32306-2280, USA. rebecca.miles@fsu.edu
UR - 18374240
ER -

TY - JOUR
ID - 1055
T1 - Cerebral traumatism with a playground rocking toy mimicking shaken baby syndrome
A1 - Sauvageau,A.
A1 - Bourgault,A.
A1 - Racette,S.
Y1 - 2008/03//
N1 - Sauvageau, Anny. Bourgault, Andre. Racette, Stephanie
Journal of forensic sciences
i5z, 0375370
IM
Case Reports. Journal Article
English
KW - MEDLINE
KW - Axons/pa [Pathology]
KW - Brain Edema/pa [Pathology]
KW - Brain Injuries/di [Diagnosis]
KW - Brain Injuries/et [Etiology]
KW - Child
KW - Preschool
KW - Diagnosis
KW - Differential
KW - Forensic Pathology
KW - Hematoma
KW - Subdural/pa [Pathology]
KW - Humans
KW - Male
KW - Meninges/pa [Pathology]
KW - Play and Playthings/in [Injuries]
KW - Retinal Hemorrhage/pa [Pathology]
KW - Shaken Baby Syndrome/di [Diagnosis]
KW - Subarachnoid Hemorrhage/pa [Pathology]
RP - NOT IN FILE
SP - 479
EP - 482
JF - Journal of Forensic Sciences
JA - J Forensic Sci
VL - 53
IS - 2
CY - United States
N2 - Shaken baby syndrome (SBS), one of the most deadly and devastating forms of child abuse, is caused by violent shaking. The combination of subdural hematoma, retinal hemorrhage, brain swelling, and diffuse axonal injury is highly typical of this syndrome and faced with these autopsy findings, induced traumatic lesions are strongly considered. However, it is known that motor-vehicle accidents and falls from great height can also produce this pattern of injury. Nevertheless, stories of arms fall, couch fall, or bumped head while the baby is being carried are generally considered incompatible with SBS. We here report a case of a 2-year-old boy presenting with all the classic autopsy findings of SBS from a playground rocking toy shaken by an older child
SN - 0022-1198

AD - Laboratoire de Sciences Judiciaires et de Medecine Legale, Edifice Wilfrid-Derome, Montreal, QC, Canada. a.sauvageau@msp.gouv.qc.ca
UR - 18366585
ER -

TY - JOUR
ID - 1056
T1 - Management of status epilepticus in infants and children prior to pediatric ICU admission: deviations from the current guidelines
A1 - Tobias,J.D.
A1 - Berkenbosch,J.W.
Y1 - 2008/03//
N1 - Tobias, Joseph D. Berkenbosch, John W
Southern medical journal
uvh, 0404522
AIM, IM
Journal Article. Multicenter Study
English
KW - MEDLINE
KW - Adolescent
KW - Anti-Anxiety Agents/ad [Administration & Dosage]
KW - Anti-Anxiety Agents/ae [Adverse Effects]
KW - Anti-Anxiety Agents
KW - Anticonvulsants/tu [Therapeutic Use]
KW - Benzodiazepines/ad [Administration & Dosage]
KW - Benzodiazepines/ae [Adverse Effects]
KW - Benzodiazepines
KW - Child
KW - Preschool
KW - Cohort Studies
KW - Dose-Response Relationship
KW - Drug
KW - Female
KW - Guideline Adherence/sn [Statistics & Numerical Data]
KW - Humans
KW - Infant
KW - Intensive Care Units
KW - Pediatric
KW - Male
KW - Physician's Practice Patterns
KW - Practice Guidelines as Topic
KW - Prospective Studies
KW - Status Epilepticus/dt [Drug Therapy]
RP - NOT IN FILE
SP - 268
EP - 272
JF - Southern Medical Journal
JA - South Med J
VL - 101
IS - 3
CY - United States
N2 - OBJECTIVES: Despite the availability of guidelines for the treatment of status epilepticus (SE), there may be variability in the initial stabilization, evaluation, and pharmacologic treatment of infants and children with SE. To improve the treatment of such patients, documentation of these problems is needed. The current study is a prospective observational evaluation of the care received by pediatric patients with SE before Pediatric Intensive Care Unit (PICU) admission. METHODS: Following admission to the PICU, the care received was reviewed from the available records and/or direct conversations with the healthcare providers. RESULTS: The

study cohort included 100 consecutive patients with SE. Potential issues in care included lack of laboratory evaluation. The following laboratory parameters were not obtained during the initial evaluation: serum sodium (9% of patients), serum or ionized calcium (22%), and serum glucose (16%). When serum glucose was measured, results were not available for 20 minutes or more in 37% of the patients. Ninety-two patients were treated with anticonvulsant medication as they were having active seizure activity at the initial presentation. Primary drug therapy for SE did not include a benzodiazepine in 11% of the treated patients. When a benzodiazepine was administered, dosing was outside of suggested guidelines in 22.5% of patients. Respiratory compromise was more common in patients who received a benzodiazepine dose greater than suggested guidelines. CONCLUSIONS: Education regarding the initial evaluation and treatment of SE should focus on the appropriate laboratory evaluation required, suggested drug therapy, and dosing guidelines
SN - 1541-8243
AD - Department of Anesthesiology, University of Missouri, Columbia, MO 65212, USA. Tobiasj@health.missouri.edu
UR - 18364657
ER -

TY - JOUR
ID - 1057
T1 - Differences in school-day patterns of daily physical activity in girls according to level of physical activity
A1 - Mota,J.
A1 - Silva,P.
A1 - Aires,L.
A1 - Santos,M.P.
A1 - Oliveira,J.
A1 - Ribeiro,J.C.
Y1 - 2008///
N1 - Mota, Jorge. Silva, Pedro. Aires, Luisa. Santos, Maria Paula. Oliveira, Jose. Ribeiro, Jose C
Journal of physical activity & health
101189457
IM
Journal Article. Research Support, Non-U.S. Gov't
English
KW - MEDLINE
KW - Adolescent
KW - Age Factors
KW - Body Height
KW - Body Mass Index
KW - Body Weight
KW - Child
KW - Cross-Sectional Studies
KW - Exercise
KW - Female
KW - Humans
KW - Physical Exertion
KW - Portugal
KW - Schools
KW - Time Factors
RP - NOT IN FILE
SP - S90
EP - S97
JF - Journal of Physical Activity & Health
JA - J Phys Act Health
VL - 5 Suppl 1
CY - United States
N2 - BACKGROUND: The purpose of this study was to examine whether there are differences in physical activity (PA) during specific periods of the day among active and less-active girls. METHODS: The sample comprised 54 girls age 10 to 15 years. PA was assessed by accelerometry. Girls were grouped as less active,

active, and highly active. RESULTS: Total minutes of moderate-to-vigorous physical activity (MVPA) was significantly higher in more-active girls than in their less-active peers (113 and 72 min/d, respectively). The most-active groups were significantly more engaged in MVPA during the outside-of-school period than were less-active girls. Highly active girls spent a significantly higher amount of their MVPA time outside of school than did the less-active group, which spent a significantly higher proportion of MVPA time during late afternoon. CONCLUSION: Outside-of-school PA is a key point for MVPA engagement. Particularly for the less-active girls, however, schools might provide additional PA
SN - 1543-3080
AD - Research Center of Physical Activity, Health and Leisure, Faculty of Sport Sciences, University of Porto, Portugal
UR - 18364527
ER -

TY - JOUR
ID - 1058
T1 - Comparative research on epidemiological aspects of tsutsugamushi disease (scrub typhus) between Korea and Japan
A1 - Bang,H.A.
A1 - Lee,M.J.
A1 - Lee,W.C.
Y1 - 2008/03//
N1 - Bang, Hyeong-Ae. Lee, Myeong-Jin. Lee, Won-Chang
Japanese journal of infectious diseases
dii, 100893704
IM
Comparative Study. Journal Article
English
KW - MEDLINE
KW - Adolescent
KW - Adult
KW - Age Distribution
KW - Aged
KW - Analysis of Variance
KW - Child
KW - Disease Outbreaks
KW - Female
KW - Humans
KW - Japan/ep [Epidemiology]
KW - Korea/ep [Epidemiology]
KW - Male
KW - Middle Aged
KW - Orientia tsutsugamushi
KW - Prevalence
KW - Risk Factors
KW - Scrub Typhus/ep [Epidemiology]
KW - Scrub Typhus/pc [Prevention & Control]
KW - Scrub Typhus/tm [Transmission]
KW - Seasons
KW - Sex Distribution
RP - NOT IN FILE
SP - 148
EP - 150
JF - Japanese Journal of Infectious Diseases
JA - Jpn J Infect Dis
VL - 61
IS - 2
CY - Japan

N2 - In order to compare the epidemiological aspects of tsutsugamushi disease (scrub typhus) between Korea and Japan, we analyzed the current state of tsutsugamushi disease outbreaks and related risk factors. The average prevalence rate of tsutsugamushi disease from 2001 to 2005 in Korea was higher than that in Japan (7.2 and 0.3 per 100,000 population, respectively) (P<0.01). The seasonal distribution in Korea showed that the incidence of tsutsugamushi disease in autumn (94.5% of total cases) was higher than in Japan (38.2%) (P<0.01), while the incidences of outbreaks in other seasons were much higher in Japan (P<0.01). In Korea, more females (64.8%) were infected than males (35.2%) (P<0.01), while there was no significant difference in Japan. The remarkable difference between the gender distribution in Korea and Japan is believed to reflect cultural differences between the two countries in terms of work, dress and ornamentation. In both countries, elderly people (over 60 years old), especially in rural areas, showed a very high prevalence rate (50%), which is possibly due to increased outdoor activities and a decreased number of young people in those areas. These differences in tsutsugamushi disease risk factors reflect the different influences of vectors/hosts, climate, and geographical and cultural characteristics between the two countries
SN - 1344-6304
AD - Faculty of Public Health, The Korean Public Health Association, Seoul, Korea
UR - 18362409
ER -

TY - JOUR
ID - 1059
T1 - Not enough time? Individual and environmental implications for workplace physical activity programming among women with and without young children
A1 - Tavares,L.S.
A1 - Plotnikoff,R.C.
Y1 - 2008/03//
N1 - Tavares, Leonor S. Plotnikoff, Ronald C
Health care for women international
hcw, 8411543
N
Journal Article
English
KW - MEDLINE
KW - Adult
KW - Alberta
KW - Attitude to Health
KW - Child
KW - Exercise
KW - Female
KW - Focus Groups
KW - Health Promotion/mt [Methods]
KW - Humans
KW - Life Style
KW - Middle Aged
KW - Mother-Child Relations
KW - Questionnaires
KW - Social Environment
KW - Time Management/mt [Methods]
KW - Time Management
KW - Women's Health
KW - Women
KW - Working/cl [Classification]
KW - Workplace/px [Psychology]
RP - NOT IN FILE
SP - 244
EP - 281
JF - Health Care for Women International
JA - Health Care Women Int

VL - 29
IS - 3
CY - United States
N2 - This study sets out to determine the main issues employed women with and without young children voice as influencing their physical activity behaviors, and to identify the environmental dimensions (e.g., physical, social, cultural, organizational, policy) within and outside of the workplace surrounding physical activity promotion that are most pertinent to employed women in Canada. Thirty employed women participated in focus groups, and four senior personnel were interviewed. Worksite observations were carried out and a Workplace Physical Activity Audit Tool was administered. Results indicate that time constraints and demands of personal and work life are barriers to achieving more activity. The issues surrounding family obligations and pressures for women with young children illustrate the main differences between groups. Key environmental factors are addressed and considered for future workplace physical activity programming goals
SN - 1096-4665
AD - Research Assistant, Physical Activity and Population Health Research Lab, Centre of Health Promotion Studies, University of Alberta, Edmonton, Alberta, Canada
UR - 18350427
ER -

TY - JOUR
ID - 1060
T1 - Cultural attitudes toward weight, diet, and physical activity among overweight African American girls
A1 - Boyington,J.E.
A1 - Carter-Edwards,L.
A1 - Piehl,M.
A1 - Hutson,J.
A1 - Langdon,D.
A1 - McManus,S.
Y1 - 2008/04//
N1 - Boyington, Josephine E A. Carter-Edwards, Lori. Piehl, Mark. Hutson, Jeanne. Langdon, Debbe. McManus, Shilpa
Preventing chronic disease
101205018
IM
Journal Article. Research Support, N.I.H., Extramural. Research Support, Non-U.S. Gov't. Research Support, U.S. Gov't, P.H.S.
English
KW - MEDLINE
KW - Adolescent
KW - African Americans/eh [Ethnology]
KW - Child
KW - Culture
KW - Diet/eh [Ethnology]
KW - Female
KW - Health Knowledge
KW - Attitudes
KW - Practice
KW - Humans
KW - Motor Activity
KW - Overweight
RP - NOT IN FILE
SP - A36
JF - Preventing Chronic Disease
JA - Prev Chronic Dis
VL - 5
IS - 2
CY - United States

N2 - INTRODUCTION: The growing epidemic of childhood obesity has led to an increasing focus on strategies for prevention. However, little is known about attitudes and perceptions toward weight, diet, and physical activity among American youth, and particularly among young African American females. This pilot study sought to qualitatively explore cultural attitudes and perceptions toward body image, food, and physical activity among a sample of overweight African American girls. METHODS: We recruited 12 overweight girls, aged 12 to 18 years, from a hospital-based pediatric diabetes screening and prevention program. Five semistructured group interviews were conducted to explore attitudes on weight, diet, and physical activity. Sessions averaged 1 hour and were conducted by trained interviewers. Data were transcribed and evaluated for content and relevant themes. RESULTS: The following themes emerged: weight and body size preferences were primarily determined by the individual and her immediate social circle and were less influenced by opinions of those outside of the social circle; food choices depended on texture, taste, appearance, and context more than on nutritional value; engagement in recreational physical activity was influenced by time constraints from school and extracurricular activities and by neighborhood safety; participation in structured exercise was limited because of the cost and time related to maintenance of personal aesthetics (hair and nails); and celebrities were not perceived as role models for diet and physical activity habits. CONCLUSION: In this sample of girls, the findings imply that perceptions of weight and healthy lifestyle behaviors are largely determined by environmental and personal influences. These factors should be considered in the development of healthy-weight interventions for African American girls
SN - 1545-1151
AD - Institute for Health, Social and Community Research, Shaw University, 900 S Wilmington St, Suite 220, Raleigh, NC 27601, USA. jboyington@shawu.edu
UR - 18341772
ER -

TY - JOUR
ID - 1061
T1 - Understanding school-age obesity: through participatory action research
A1 - DiNapoli,P.P.
A1 - Lewis,J.B.
Y1 - 2008/03//
N1 - DiNapoli, Pamela P. Lewis, James B
MCN. The American journal of maternal child nursing
ma3, 7605941
IM, N
Journal Article. Multicenter Study
English
KW - MEDLINE
KW - Adolescent
KW - Body Mass Index
KW - Child
KW - Community Health Planning
KW - Female
KW - Health Promotion
KW - Health Services Research
KW - Healthy People Programs
KW - Humans
KW - Male
KW - New Hampshire/ep [Epidemiology]
KW - Obesity/ep [Epidemiology]
KW - Obesity/pc [Prevention & Control]
KW - Physical Fitness
KW - Prevalence
RP - NOT IN FILE
SP - 104
EP - 110
JF - MCN, American Journal of Maternal Child Nursing
JA - MCN Am J Matern Child Nurs

VL - 33
IS - 2
CY - United States
N2 - PURPOSE: This study aimed to assess current levels of overweight (obesity) and fitness among school students using objective data. METHODS: School-based action research teams were recruited statewide by the New Hampshire Healthy Schools Coalition, the state team of the National Action for Healthy Kids Coalition. Action teams consisted of a physical education teacher, a school nurse, and a school administrator. Data were collected from 6,511 student participants aged 6 to 14 years, which was a representative cross-section from New Hampshire school districts. Key variables of interest in the study were body mass index, and ability to pass five fitness tests using FITNESSGRAM. Pearson's correlation was used to assess the relationships among body mass index, age, gender, and the percent of students that passed FITNESSGRAM tests. RESULTS: The ability of participants to pass the FITNESSGRAM tests declined markedly with age and differed between boys and girls, although the healthy fitness zones for any particular test was lower for girls. Body mass index was significantly negatively correlated with performance on all tests. Age was also statistically negatively correlated with performance on all tests; the relationship between gender and performance on the tests was less striking. CLINICAL IMPLICATIONS: Results reflected an increase in the prevalence of overweight school children, even in New Hampshire, which is purported to be one of the healthiest states in the nation. Results offered evidence that body mass index is a valid proxy measure for fitness levels and that fitness programs are necessary to effectively combat the obesity epidemic. Evidence-based changes need to be implemented to address obesity-related factors in schools, because children spend many of their waking hours in that setting. Physical activity during recess and physical education classes could help to increase energy expenditure and develop sound minds and bodies. Schools should consider the development of school-based wellness teams to advise and advocate improved school-based wellness policies. School nurses can take an active part in these initiatives
SN - 0361-929X
AD - Department of Nursing, University of New Hampshire, Durham, USA. ppdn@cisunix.unh.edu
UR - 18327109
ER -

TY - JOUR
ID - 1062
T1 - [Overweight and obesity in preschool children (0-4 years): behaviour and views of parents]. [Dutch]
A1 - Boere-Boonekamp,M.M.
A1 - L'Hoir,M.P.
A1 - Beltman,M.
A1 - Bruil,J.
A1 - Dijkstra,N.
A1 - Engelberts,A.C.
Y1 - 2008/02/09/
N1 - Boere-Boonekamp, M M. L'Hoir, M P. Beltman, M. Bruil, J. Dijkstra, N. Engelberts, A C
Nederlands tijdschrift voor geneeskunde
nuk, 0400770
IM
English Abstract. Journal Article
Dutch
KW - MEDLINE
KW - Beverages
KW - Child Nutritional Physiological Phenomena
KW - Child
KW - Preschool
KW - Cross-Sectional Studies
KW - Female
KW - Health Behavior
KW - Humans
KW - Infant
KW - Leisure Activities
KW - Life Style
KW - Male

KW - Obesity/ep [Epidemiology]
KW - Obesity/et [Etiology]
KW - Obesity/pc [Prevention & Control]
KW - Overweight/ep [Epidemiology]
KW - Overweight/et [Etiology]
KW - Overweight/pc [Prevention & Control]
KW - Parents/ed [Education]
KW - Parents/px [Psychology]
KW - Parents
KW - Risk-Taking
KW - Television
RP - NOT IN FILE
SP - 324
EP - 330
JF - Nederlands Tijdschrift voor Geneeskunde
JA - Ned Tijdschr Geneeskd
VL - 152
IS - 6
CY - Netherlands
N2 - OBJECTIVE: Description of unhealthy behaviour and views regarding nutrition, physical exercise and education in families with young children, in relation to specific groups with a high risk of overweight. DESIGN: Cross-sectional study. METHOD: In wellbaby clinics for infants and toddlers in various sites in The Netherlands 534 parents were requested to fill in questionnaires on nutrition, physical exercise and education. Enquiry into background characteristics took place and several hypotheses were tested. Using a bivariate and multivariate analysis each hypothesis was examined with regard to linkage of the answers to specific high-risk groups. RESULTS: 73% (390 parents) responded. Overweight was found in 15% of the 2-4-year-olds, this included 3.7% with obesity. Relatively often, parents held views and showed behaviour characteristic of an unhealthy lifestyle for young children. For example, approximately 1 out of 7 families was not used to having breakfast. 43% of the respondents considered sweetened milk products approprite substitutes for milk and 39% of the children always had soft drinks at their disposal. One fifth of the parents indicated that they did not have enough time to go out with their child. Approximately 1 out of 10 toddlers aged 2-4 years had a TV in their own room; 22% were allowed to determine whether they wanted to watch TV or not and 9% were allowed to decide how long they watched. Such specific high-risk behaviour was seen in particular in families with non-western mothers, mothers with a low level of education or mothers living on social security. Regular use of child care or a nursery seemed to have some protective effect. CONCLUSION: In families with children aged 0-4 years, behaviour patterns related to overweight at a later age were frequently observed. In view of the increased incidence of overweight and obesity in young children, initiation of research aimed at optimisation of education is warranted, which should include special attention for the behaviour of parents with regard to 5 spearheads: breast-feeding, more outside play and more physical exercise, a regular wholesome breakfast, less consumption of sweetened drinks and less TV and computer use. Implementation of interventions in young age groups is able to prevent the development of unhealthy lifestyle and childrearing styles
SN - 0028-2162
AD - Universiteit Twente, faculteit Bestuur en Management, Postbus 217, 7500 AE Enschede. m.m.boere-boonekamp@utwente.nl
UR - 18326414
ER -

TY - JOUR
ID - 1063
T1 - Vitamin D status of apparently healthy schoolgirls from two different socioeconomic strata in Delhi: relation to nutrition and lifestyle
A1 - Puri,S.
A1 - Marwaha,R.K.
A1 - Agarwal,N.
A1 - Tandon,N.
A1 - Agarwal,R.
A1 - Grewal,K.

A1 - Reddy,D.H.
A1 - Singh,S.
Y1 - 2008/04//
N1 - Puri, Seema. Marwaha, Raman K. Agarwal, Neha. Tandon, Nikhil. Agarwal, Rashmi. Grewal, Khushi. Reddy, D H K. Singh, Satveer
The British journal of nutrition
az4, 0372547
IM
Comparative Study. Journal Article. Research Support, Non-U.S. Gov't
English
KW - MEDLINE
KW - Adolescent
KW - Body Mass Index
KW - Calcifediol/bl [Blood]
KW - Chi-Square Distribution
KW - Child
KW - Diet
KW - Female
KW - Humans
KW - India/ep [Epidemiology]
KW - Life Style
KW - Nutritional Status
KW - Prevalence
KW - Social Class
KW - Sunlight
KW - Vitamin D Deficiency/di [Diagnosis]
KW - Vitamin D Deficiency/ep [Epidemiology]
KW - Vitamin D Deficiency/px [Psychology]
KW - Vitamins/bl [Blood]
RP - NOT IN FILE
SP - 876
EP - 882
JF - British Journal of Nutrition
JA - Br J Nutr
VL - 99
IS - 4
CY - England
N2 - Forty to fifty per cent of skeletal mass, accumulated during childhood and adolescence, is influenced by sunlight exposure, physical activity, lifestyle, endocrine status, nutrition and gender. In view of scarce data on association of nutrition and lifestyle with hypovitaminosis D in Indian children and adolescents, an in-depth study on 3,127 apparently healthy Delhi schoolgirls (6-18 years) from the lower (LSES, n 1,477) and upper socioeconomic strata (USES, n 1650) was carried out. These girls were subjected to anthropometry and clinical examination for hypovitaminosis D. Girls randomly selected from the two strata (LSES, n 193; USES, n 211) underwent detailed lifestyle, dietary, biochemical and hormonal assessment. Clinical vitamin D deficiency was noted in 11.5 % girls (12.4 % LSES, 10.7 % USES). USES girls had significantly higher BMI than LSES counterparts. Prevalence of biochemical hypovitaminosis D (serum 25-hydroxyvitamin D < 50 nmol/l) was seen in 90.8 % of girls (89.6 % LSES, 91.9 % USES, NS). Mean intake of energy, protein, fat, Ca, vitamin D and milk/milk products was significantly higher in USES than LSES girls. Conversely, carbohydrate, fibre, phytate and cereal intakes were higher in LSES than USES girls. Physical activity and time spent outdoors was significantly higher in LSES girls (92.8 v. 64 %, P = 0.000). Significant correlation between serum 25-hydroxyvitamin D and estimated sun exposure (r 0.185, P = 0.001) and percentage body surface area exposed (r 0.146, P = 0.004) suggests that these lifestyle-related factors may contribute significantly to the vitamin D status of the apparently healthy schoolgirls. Hence, in the absence of vitamin D fortification of foods, diet alone appears to have an insignificant role
SN - 0007-1145
AD - Department of Foods and Nutrition, Institute of Home Economics, University of Delhi, New Delhi, India
UR - 17903343

ER -

TY - JOUR
ID - 1064
T1 - Mothers of preschoolers report using less pressure in child feeding situations following a newsletter intervention
A1 - Essery,E.V.
A1 - DiMarco,N.M.
A1 - Rich,S.S.
A1 - Nichols,D.L.
Y1 - 2008/03//
N1 - Essery, Eve V. DiMarco, Nancy M. Rich, Shannon S. Nichols, David L
Journal of nutrition education and behavior
101132622
IM
Journal Article. Research Support, Non-U.S. Gov't
English
KW - MEDLINE
KW - Adult
KW - Child Nutrition Sciences/ed [Education]
KW - Child Nutritional Physiological Phenomena
KW - Child
KW - Preschool
KW - Exercise/ph [Physiology]
KW - Exercise/px [Psychology]
KW - Female
KW - Health Promotion
KW - Humans
KW - Male
KW - Mothers/ed [Education]
KW - Mothers/px [Psychology]
KW - Questionnaires
RP - NOT IN FILE
SP - 110
EP - 115
JF - Journal of Nutrition Education & Behavior
JA - J Nutr Educ Behav
VL - 40
IS - 2
CY - Canada
N2 - OBJECTIVE: To determine the impact of written intervention materials on child feeding practices of mothers and on physical activity behaviors of preschoolers. DESIGN: Mothers were divided into 3 groups: newsletter (n = 30), booklet (n = 31), and control (n = 31). Questionnaires were completed before and after a 12-week intervention. SETTING: Mail correspondence. PARTICIPANTS: White (90%), full-time mothers (76%) in their thirties (68%). INTERVENTIONS: Nutrition and activity information was provided as twelve 4-page weekly newsletters or one 52-page booklet. MAIN OUTCOME MEASURES: Child feeding factors, physical activity practices. ANALYSIS: Child feeding factors were analyzed using Kruskal-Wallis and Wilcoxon. Physical activity practices were analyzed using 2-way chi-square and sign tests. The significance level was set at .0125. RESULTS: There was a significant decrease in the "pressure to eat" score for participants in the newsletter group (2.5 +/- 1.0 to 2.1 +/- 1.0, P < .01). No other differences in feeding practices were observed. At baseline, 43% of the mothers said their child spent at least 1 hour per day in active play, and 63% said their child watched television less than 2 hours per day. A significant increase in outdoor play was reported in the newsletter (P < .01) and control (P < .01) groups. CONCLUSIONS AND IMPLICATIONS: This study is one of the first to indicate that child feeding practices may be modifiable
SN - 1499-4046
AD - Institute for Women's Health, Texas Woman's University, Denton, Texas 76204, USA. EEssery@mail.twu.edu

UR - 18314087
ER -

TY - JOUR
ID - 1065
T1 - Small bowel gastric metaplasia: a report of three cases in children. [Review] [6 refs]
A1 - Drut,R.
A1 - Altamirano,E.
Y1 - 2008/03//
N1 - Drut, R. Altamirano, E
Journal of clinical pathology
ht3, 0376601
AIM, IM
Case Reports. Journal Article. Review
English
KW - MEDLINE
KW - Adolescent
KW - Anastomosis
KW - Surgical
KW - Child
KW - Female
KW - Humans
KW - Ileostomy
KW - Inflammation
KW - Intestinal Mucosa/im [Immunology]
KW - Intestinal Mucosa/pa [Pathology]
KW - Intestinal Pseudo-Obstruction/im [Immunology]
KW - Intestinal Pseudo-Obstruction/pa [Pathology]
KW - Intestine
KW - Small
KW - Metaplasia
KW - Precancerous Conditions/im [Immunology]
KW - Precancerous Conditions/pa [Pathology]
KW - Pyloric Antrum/im [Immunology]
KW - Pyloric Antrum/pa [Pathology]
KW - Staining and Labeling
KW - Time Factors
RP - NOT IN FILE
SP - 382
EP - 384
JF - Journal of Clinical Pathology
JA - J Clin Pathol
VL - 61
IS - 3
CY - England
N2 - AIMS: To report three children displaying gastric metaplasia antral pyloric type of the small bowel mucosa. METHODS: Analysis of clinical, histopathological and immunohistochemical data. RESULTS: The first patient was a 14-year-old girl with history of chronic intestinal pseudo-obstruction and chronic jejunitis; the second patient was a 6-year-old girl with a long-lasting jejunostomy; and the third patient was a 5-year-old girl with ileal-rectal anastomosis. The foci of gastric metaplasia were obvious with H&E-stained sections. The cells at the gastric metaplasia mucosa proved to be MUC-1 and sialyl-Tn positive by immunohistochemistry and they were in a pattern that was different from that of the adjacent mucosa; the cells were autofluorescent in H&E-stained sections. CONCLUSIONS: Gastric metaplasia of the small bowel mucosa in these cases seems to have resulted from chronic inflammation and persistent regenerative activity. This has rarely been reported outside Crohn disease, and if ever in children. [References: 6]
SN - 1472-4146

AD - Department of Pathology, Superiora Sor Maria Ludovica Children's Hospital, La Plata, Argentina. patologi@netverk.com.ar
UR - 17693575
ER -

TY - JOUR
ID - 1066
T1 - Changes in lung function and airway inflammation among asthmatic children residing in a woodsmoke-impacted urban area
A1 - Allen,R.W.
A1 - Mar,T.
A1 - Koenig,J.
A1 - Liu,L.J.
A1 - Gould,T.
A1 - Simpson,C.
A1 - Larson,T.
Y1 - 2008/02//
N1 - Allen, Ryan W. Mar, Therese. Koenig, Jane. Liu, L-J Sally. Gould, Timothy. Simpson, Christopher. Larson, Timothy
Inhalation toxicology
8910739, db8
IM
Journal Article. Research Support, U.S. Gov't, Non-P.H.S.
English
KW - MEDLINE
KW - Adolescent
KW - Air Pollution/ae [Adverse Effects]
KW - Asthma/me [Metabolism]
KW - Asthma/pp [Physiopathology]
KW - Child
KW - Cities
KW - Environmental Monitoring
KW - Glucose/aa [Analogs & Derivatives]
KW - Glucose/an [Analysis]
KW - Humans
KW - Inflammation
KW - Inhalation Exposure
KW - Lung/de [Drug Effects]
KW - Lung/me [Metabolism]
KW - Lung/pp [Physiopathology]
KW - Nitric Oxide/me [Metabolism]
KW - Particulate Matter/ae [Adverse Effects]
KW - Particulate Matter/an [Analysis]
KW - Respiratory Function Tests
KW - Smoke
KW - Urban Population
KW - Wood
RP - NOT IN FILE
SP - 423
EP - 433
JF - Inhalation Toxicology
JA - Inhal.Toxicol
VL - 20
IS - 4
CY - United States
N2 - Fine particulate matter (PM(2.5)) is associated with respiratory effects, and asthmatic children are especially sensitive. Preliminary evidence suggests that combustion-derived particles play an important role.

Our objective was to evaluate effect estimates from different PM(2.5) exposure metrics in relation to airway inflammation and lung function among children residing in woodsmoke-impacted areas of Seattle. Nineteen children (ages 6-13 yr) with asthma were monitored during the heating season. We measured 24-h outdoor and personal concentrations of PM(2.5) and light-absorbing carbon (LAC). Levoglucosan (LG), a marker of woodsmoke, was also measured outdoors. We partitioned PM(2.5) exposure into its ambient-generated (E(ag)) and nonambient (E(na)) components. These exposure metrics were evaluated in relation to daily changes in exhaled nitric oxide (FE(NO)), a marker of airway inflammation, and four lung function measures: midexpiratory flow (MEF), peak expiratory flow (PEF), forced expiratory volume in the first second (FEV(1)), and forced vital capacity (FVC). E(ag), but not E(na), was correlated with combustion markers. Significant associations with respiratory health were seen only among participants not using inhaled corticosteroids. Increases in FE(NO) were associated with personal PM(2.5), personal LAC, and E(ag) but not with ambient PM(2.5) or its combustion markers. In contrast, MEF and PEF decrements were associated with ambient PM(2.5), its combustion markers, and E(ag), but not with personal PM(2.5) or personal LAC. FEV(1) was associated only with ambient LG. Our results suggest that lung function may be especially sensitive to the combustion-generated component of ambient PM(2.5), whereas airway inflammation may be more closely related to some other constituent of the ambient PM(2.5) mixture
SN - 1091-7691
AD - Faculty of Health Sciences, Simon Fraser University, Burnaby, British Columbia, Canada. allenr@sfu.ca
UR - 18302050
ER -

TY - JOUR
ID - 1067
T1 - Measuring aerobic cycling power as an assessment of childhood fitness
A1 - Carrel,A.L.
A1 - Sledge,J.S.
A1 - Ventura,S.J.
A1 - Clark,R.R.
A1 - Peterson,S.E.
A1 - Eickhoff,J.C.
A1 - Allen,D.B.
Y1 - 2008/01//
N1 - Carrel, Aaron L. Sledge, Jeffrey S. Ventura, Steve J. Clark, R Randall. Peterson, Susan E. Eickhoff, Jens C. Allen, David B
Journal of strength and conditioning research / National Strength & Conditioning Association
9415084
IM
Journal Article. Research Support, Non-U.S. Gov't
English
KW - MEDLINE
KW - Bicycling/ph [Physiology]
KW - Body Mass Index
KW - Child
KW - Cohort Studies
KW - Diabetes Mellitus
KW - Type 2/pc [Prevention & Control]
KW - Exercise Test/mt [Methods]
KW - Female
KW - Heart Rate/ph [Physiology]
KW - Humans
KW - Insulin Resistance
KW - Male
KW - Obesity/pc [Prevention & Control]
KW - Oxygen Consumption/ph [Physiology]
KW - Physical Endurance/ph [Physiology]
KW - Physical Fitness/ph [Physiology]
KW - School Health Services

KW - Sensitivity and Specificity
KW - Spirometry
RP - NOT IN FILE
SP - 192
EP - 195
JF - Journal of Strength & Conditioning Research
JA - J Strength Cond Res
VL - 22
IS - 1
CY - United States
N2 - The emergence of obesity, insulin resistance, and type 2 diabetes in children requires a rational, effective public health response. Physical activity remains an important component of prevention and treatment for obesity, type 2 diabetes, and insulin resistance. Studies in adults show cardiovascular fitness to be more important than obesity in predicting insulin resistance. We recently demonstrated that a school-based fitness intervention in children who are overweight could improve cardiovascular fitness, body composition, and insulin sensitivity, but it remains unclear whether accurate assessment of fitness could be performed at the school or outside of an exercise laboratory. To determine whether new methodology using measurement of cycling power could estimate cardiovascular aerobic fitness (as defined by VO2max) in middle school children who were overweight. Thirty-five middle school children (mean age 12 +/- 0.4 years) who were overweight underwent testing on a power sensor-equipped Cycle Ops indoor cycle (Saris Cycling Group, Fitchburg, WI) as well as body composition by dual x-ray absorptiometry and VO2max by treadmill determination. Insulin sensitivity was also estimated by fasting glucose and insulin. Maximal heart rate (MHR) was determined during VO2max testing, and power produced at 80%MHR was recorded. Spearman's rank correlation was performed to evaluate associations. Mean power determined on the indoor cycle at 80% of MHR was 129 +/- 77 watts, and average power at 80% MHR divided by total body weight was 1.5 +/- 0.5. A significant correlation between watts and total body weight was seen for VO2max (P = 0.03), and significant negative correlation was seen between watts/total body weight and fasting insulin (P < 0.05). Among middle school children who were overweight, there was a significant relationship between the power component of fitness and cardiovascular aerobic fitness (measured by VO2max). This more accessible and less intimidating field-based measure of power may prove useful in predicting changes in cardiovascular fitness. Thus, accurate assessment of childhood aerobic fitness may be achievable by measurement of power, possibly within the school environment, at substantially less cost and effort than laboratory-based measurements
SN - 1533-4287
AD - Department of Pediatrics, University of Wisconsin Children's Hospital, Madison, Wisconsin, USA. alcarrel@wisc.edu
UR - 18296974
ER -

TY - JOUR
ID - 1068
T1 - Orthodontists' views on indications for and timing of orthodontic treatment in Finnish public oral health care
A1 - Pietila,I.
A1 - Pietila,T.
A1 - Pirttiniemi,P.
A1 - Varrela,J.
A1 - Alanen,P.
Y1 - 2008/02//
N1 - Pietila, Ilpo. Pietila, Terttu. Pirttiniemi, Pertti. Varrela, Juha. Alanen, Pentti
European journal of orthodontics
eos, 7909010
D, IM
Journal Article. Research Support, Non-U.S. Gov't
English
KW - MEDLINE
KW - Activator Appliances
KW - Adolescent

KW - Age Factors
KW - Attitude of Health Personnel
KW - Child
KW - Preschool
KW - Delivery of Health Care
KW - Dentist's Practice Patterns
KW - Dentition
KW - Mixed
KW - Extraoral Traction Appliances
KW - Female
KW - Finland
KW - Humans
KW - Male
KW - Malocclusion/th [Therapy]
KW - Malocclusion
KW - Angle Class II/th [Therapy]
KW - Needs Assessment
KW - Orthodontic Appliances/cl [Classification]
KW - Orthodontics
KW - Corrective/mt [Methods]
KW - Corrective
KW - Public Health Dentistry
KW - Tooth
KW - Deciduous/pa [Pathology]
RP - NOT IN FILE
SP - 46
EP - 51
JF - European Journal of Orthodontics
JA - Eur J Orthod
VL - 30
IS - 1
CY - England
N2 - The aim of this study was to analyse the variation in the views of Finnish orthodontists on the indications for orthodontic treatment, timing of orthodontic assessment, and treatment methods used. The views were elicited by a questionnaire that was sent to all 146 specialist orthodontists under 65 years of age living in Finland in 2001. The response rate was 57 per cent. The association between an orthodontist's experience and timing of treatment was tested by Fisher's exact test. Stepwise logistic regression analysis was used to estimate the association between the demographic characteristics of orthodontists and the tendency to start Class II division I treatment early. Most orthodontists recommended that the first assessment of occlusion should be carried out before 7 years of age. A crossbite was mentioned as the most frequent indication for treatment in the primary and early mixed dentition, and a severe Class II division I malocclusion with an increased overjet as the most frequent indication in the late mixed dentition. Most respondents preferred early treatment, but there was a wide variation in the choice of appliances and in the timing of treatment of malocclusions other than crossbite and Class II malocclusions. A quadhelix, headgear, and the eruption guidance appliance were the most frequently used appliances in early treatment, with fixed appliances being most frequently used during the late mixed and permanent dentition phase. Orthodontists working full time in municipal health centres tended to prefer early treatment more often than those working part-time or outside health centres. There was no statistically significant association between an orthodontist's experience and timing of Class II division I and Class III treatment ($P = 0.142$ and $P = 0.296$, respectively). The preference for an early start in Class II division I treatment might be related to differing professional decisions, but no explaining factors could be found in the regression analysis
SN - 0141-5387
AD - Health Centre of Pori and Institutes of Dentistry, Pori, Finland. ilpo.pietila@pori.fi
UR - 17962314
ER -

TY - JOUR

ID - 1069
T1 - Voluntary drinking and hydration in trained, heat-acclimatized girls exercising in a hot and humid climate.[Erratum appears in Eur J Appl Physiol. 2008 Aug;103(6):739]
A1 - Rivera-Brown,A.M.
A1 - Ramirez-Marrero,F.A.
A1 - Wilk,B.
A1 - Bar-Or,O.
Y1 - 2008/05//
N1 - Rivera-Brown, Anita M. Ramirez-Marrero, Farah A. Wilk, Boguslaw. Bar-Or, Oded
European journal of applied physiology
100954790, dos
IM
Journal Article. Research Support, Non-U.S. Gov't
English
KW - MEDLINE
KW - Acclimatization/ph [Physiology]
KW - Body Temperature/ph [Physiology]
KW - Carbohydrate Metabolism/ph [Physiology]
KW - Child
KW - Dehydration/pp [Physiopathology]
KW - Dehydration/pc [Prevention & Control]
KW - Drinking Behavior/ph [Physiology]
KW - Exercise/ph [Physiology]
KW - Female
KW - Hot Temperature
KW - Humans
KW - Oxygen Consumption/ph [Physiology]
KW - Sodium Chloride/me [Metabolism]
KW - Sweating/ph [Physiology]
KW - Tropical Climate
KW - Water-Electrolyte Balance/ph [Physiology]
RP - NOT IN FILE
SP - 109
EP - 116
JF - European Journal of Applied Physiology
JA - Eur J Appl Physiol
VL - 103
IS - 1
CY - Germany
N2 - This study examined the effects of beverage composition on the voluntary drinking pattern, body fluid balance and body temperature responses of heat-acclimatized trained girls exercising intermittently in outdoor conditions (WBGT = 30.9 +/- 0.2 degrees C). Twelve trained, heat-acclimatized girls (age = 10.6 +/- 0.2 years) performed three 3-h sessions, each consisting of four 20-min cycling bouts at 60% VO2max, alternating with 25-min rest. One of three beverages was assigned: unflavored water (W), flavored water (FW) or flavored water plus 6% carbohydrate and 18 mmol/l NaCl (CNa). Drinking was ad libitum. Total intake was similar among conditions (W = 953.3 +/- 107.8 ; FW = 1026.5 +/- 138.1; CNa = 906.4 +/- 107.5 g). A mild hypohydration occurred during the three conditions (W = -1.12%; FW = -0.95%; CNa = -0.74% BW, P > 0.05). Sweat loss, higher than previously reported for sedentary girls, was not different among conditions (W = 1,051.5 +/- 90.8; FW = 979.9 +/- 72.8; CNa = 1,052.7 +/- 52.6 g). The average amount of urine produced (W = 269.8 +/- 85.9; FW = 320.8 +/- 87.2; CNa = 85.6 +/- 9.3 g) was 73 and 68% lower [corrected] during CNa compared to [corrected] FW and W, respectively, [corrected] (CNa vs. FW, P < 0.05), CNa vs W, P = 0.06) [corrected] The increase in rectal temperature, heart rate and all perceptual variables did not differ among conditions. In conclusion, flavoring of the water and addition of 6% carbohydrate plus 18 mmol/l NaCl do not prevent mild hypohydration in trained, heat-acclimatized girls with high sweating rates. However, there is a tendency towards a greater fluid retention with the CNa beverage
SN - 1439-6319

AD - Center for Sports Health and Exercise Sciences at the Albergue Olimpico, Department of Physical Medicine, Rehabilitation and Sports Medicine, University of Puerto Rico School of Medicine, PO Box 2004, Salinas, Puerto Rico 00751. aniriver@coqui.net
UR - 18247043
ER -

TY - JOUR
ID - 1070
T1 - Perinatal and maternal risk factors for autism spectrum disorders in New South Wales, Australia
A1 - Williams,K.
A1 - Helmer,M.
A1 - Duncan,G.W.
A1 - Peat,J.K.
A1 - Mellis,C.M.
Y1 - 2008/03//
N1 - Williams, K. Helmer, M. Duncan, G W. Peat, J K. Mellis, C M
Child: care, health and development
7602632, c9x
IM
Journal Article. Research Support, Non-U.S. Gov't
English
KW - MEDLINE
KW - Autistic Disorder/ep [Epidemiology]
KW - Birth Weight
KW - Child
KW - Confidence Intervals
KW - Female
KW - Gestational Age
KW - Humans
KW - Male
KW - Maternal Age
KW - New South Wales/ep [Epidemiology]
KW - Odds Ratio
KW - Pregnancy
KW - Risk Factors
KW - Sex Factors
RP - NOT IN FILE
SP - 249
EP - 256
JF - Child: Care, Health & Development
JA - Child Care Health Dev
VL - 34
IS - 2
CY - England
N2 - BACKGROUND: This study was commenced in 1999 with the aim of examining risk factors for autism using established population-based data for comparison. METHODS: Cases were ascertained using active surveillance and compared with birth data. Results: Four risk factors were found to be significantly associated with autism using binary logistic regression analysis; being male [adjusted odds ratio (OR) 4.7, 95% confidence interval (CI) 3.2-7.0], being born prematurely (adjusted OR 2.2, 95% CI 1.5-3.5), having maternal age >/=35 years (adjusted OR 1.7, 95% CI 1.2-2.4) and having a mother born outside Australia (adjusted OR 1.4, 95% CI 1.0-1.9). For analysis completed for pregnancies, rather than live births, multiple birth was also a significant risk factor for one or more children of the pregnancy to be affected by autism (adjusted OR 2.5, 95% CI 1.1-5.5). There was a statistically significant trend towards increasing risk with increasing risk factor 'dose' for gestational age (P = 0.019), multiple birth (P = 0.016) and maternal age (P < 0.001). For mother's country of birth the group with the highest risk were children of mother's born in south-east or north-east Asia. There was a non-significant trend towards a higher proportion of children with developmental disability having risk factors. CONCLUSION: Replication of risk factors from previous studies and a significant risk factor 'dose' effect add to growing

evidence that maternal and perinatal factors are low magnitude risk factors for autism. The association between developmental disability and autism risk factors warrants further examination
SN - 1365-2214
AD - Clinical Epidemiology Unit, Children's Hospital at Westmead, Westmead, Australia. katrina.williams@sesiahs.health.nsw.gov.au
UR - 18257794
ER -

TY - JOUR
ID - 1071
T1 - Pharmacological interventions for recurrent abdominal pain (RAP) and irritable bowel syndrome (IBS) in childhood. [Review] [90 refs][Update of Cochrane Database Syst Rev. 2002;(1):CD003017; PMID: 11869650]
A1 - Huertas-Ceballos,A.
A1 - Logan,S.
A1 - Bennett,C.
A1 - Macarthur,C.
Y1 - 2008///
N1 - Huertas-Ceballos, A. Logan, S. Bennett, C. Macarthur, C
The Cochrane database of systematic reviews
100909747
IM
Journal Article. Meta-Analysis. Review
English
KW - MEDLINE
KW - Abdominal Pain/dt [Drug Therapy]
KW - Adolescent
KW - Analgesics
KW - Non-Narcotic/tu [Therapeutic Use]
KW - Child
KW - Preschool
KW - Famotidine/tu [Therapeutic Use]
KW - Humans
KW - Irritable Bowel Syndrome/dt [Drug Therapy]
KW - Pizotyline/tu [Therapeutic Use]
KW - Plant Oils/tu [Therapeutic Use]
KW - Recurrence
RP - NOT IN FILE
SP - CD003017
JF - Cochrane Database of Systematic Reviews
JA - Cochrane Database Syst Rev
IS - 1
CY - England
N2 - BACKGROUND: Between 4% and 25% of school-age children complain of recurrent abdominal pain (RAP) of sufficient severity to interfere with daily activities. For the majority no organic cause for their pain can be found on physical examination or investigation and although most children are likely managed by reassurance and simple measures, a large range of interventions have been recommended. OBJECTIVES: To determine the effectiveness of medication for recurrent abdominal pain in school-age children. SEARCH STRATEGY: The Cochrane Library (CENTRAL) 2006 (Issue 4), MEDLINE (1966 to Dec 2006), EMBASE (1980 to Dec 2006), CINAHL (1982 to Dec 2007), ERIC (1966 to Dec 2006), PsycINFO (1872 to Dec 2006), LILACS (1982 to Dec 2006), SIGLE (1980 to March 2005), and JICST (1985 to 06/2000) were searched with appropriate filters SELECTION CRITERIA: Studies on school age children with RAP (Apley or the Rome II criteria for gastrointestinal diseases) allocated by random or quasi-random methods to a drug treatment vs. placebo/ no treatment were included. DATA COLLECTION AND ANALYSIS: References identified by the searches were screened against the inclusion criteria by two independent reviewers. Data was extracted and analysed using RevMan 4.2.10. MAIN RESULTS: Three trials met the inclusion criteria. Symon et al report a cross-over trial comparing pizotifen and placebo in 16 children with "abdominal migraine". Data before cross-over was not available. Results for 14 children showed Mean fewer days in pain of 8.21 (95% CI 2.93, 13.48)

while taking the active drug. Kline et al compared peppermint oil capsules with placebo in a randomised trial in 50 children with RAP and IBS. 42 children completed the study. OR for improvement was 3.33 (95% CI 0.93-12.1)See et al compared famotidine with placebo in a randomised cross-over trial in 25 children with RAP and dyspepsia. OR for improvement before cross-over was 11 (95%CI 1.6, 75.5). AUTHORS' CONCLUSIONS: This review provides weak evidence of benefit on medication in children with RAP. The lack of clear evidence of effectiveness for any of the recommended drugs suggests that there is little reason for their use outside of clinical trials. Clinicians may choose to prescribe drugs in children with severe symptoms that have not responded to simple management. However, if using drugs as a "therapeutic trial", clinicians should be aware that, RAP is a fluctuating condition and any "response" may reflect the natural history of the condition or a placebo effect rather than drug efficacy. [References: 90]
SN - 1469-493X
AD - EGA Hospital, Neonatal Unit, UCLH, Huntley Street, London, UK WC1E 6DH. Angela.Huertas-Ceballos@uclh.nhs.uk
UR - 18254013
ER -

TY - JOUR
ID - 1072
T1 - Association of father involvement and neighborhood quality with kindergartners' physical activity: a multilevel structural equation model
A1 - Beets,M.W.
A1 - Foley,J.T.
Y1 - 2008/01//
N1 - Beets, Michael W. Foley, John T
American journal of health promotion : AJHP
amj, 8701680
T
Journal Article
English
KW - MEDLINE
KW - Child
KW - Child Welfare/sn [Statistics & Numerical Data]
KW - Preschool
KW - Cross-Sectional Studies
KW - Exercise
KW - Father-Child Relations
KW - Female
KW - Humans
KW - Longitudinal Studies
KW - Male
KW - Models
KW - Statistical
KW - Parents/px [Psychology]
KW - Residence Characteristics/cl [Classification]
KW - Residence Characteristics/sn [Statistics & Numerical Data]
KW - Role
KW - Safety
KW - Sports
RP - NOT IN FILE
SP - 195
EP - 203
JF - American Journal of Health Promotion
JA - Am J Health Promot
VL - 22
IS - 3
CY - United States

N2 - PURPOSE: Examine the effects of father-child involvement and neighborhood characteristics with young children's physical activity (PA) within a multilevel framework. DESIGN: Cross-sectional analysis of the Early Childhood Longitudinal Study-Kindergarten Cohort 1998. SETTING: Nationally representative sample. SUBJECTS: Data were available for 10,694 kindergartners (5-6 years; 5240 girls) living in 1053 neighborhoods. MEASURES: Parental report of child's PA level, father characteristics (e.g., time spent with child, age, education, socioeconomic status, hours worked), family time spent doing sports/ activities together, and neighborhood quality (e.g., safety, presence of crime violence, garbage). Child weight status, motor skills, ethnicity, and television viewing were used as covariates. ANALYSIS: Multilevel structural equation modeling with children nested within neighborhoods. RESULTS: At the child level father-child time and family time doing sports together were positively associated with children's PA. At the neighborhood level parental perception of a neighborhood's safety for children to play outside fully mediated the effect of neighborhood quality on children's PA. Overall 19.1% and 7.6% of the variance in PA was explained at the child and neighborhood levels, respectively. CONCLUSIONS: Family-based interventions for PA should consider father-child time, with this contributing to a child's overall PA level. Further, neighborhood quality is an important predictor of PA only to the extent by which parents perceive it to be unsafe for their child to play outdoors
SN - 0890-1171
AD - Department of Public Health, Oregon State University, Corvallis, Oregon 97331-6406, USA. beetsm@onid.orst.edu
UR - 18251121
ER -

TY - JOUR
ID - 1073
T1 - Common household activities are associated with elevated particulate matter concentrations in bedrooms of inner-city Baltimore pre-school children
A1 - McCormack,M.C.
A1 - Breysse,P.N.
A1 - Hansel,N.N.
A1 - Matsui,E.C.
A1 - Tonorezos,E.S.
A1 - Curtin-Brosnan,J.
A1 - Williams,D.L.
A1 - Buckley,T.J.
A1 - Eggleston,P.A.
A1 - Diette,G.B.
Y1 - 2008/02//
N1 - McCormack, Meredith C. Breysse, Patrick N. Hansel, Nadia N. Matsui, Elizabeth C. Tonorezos, Emily S. Curtin-Brosnan, Jean. Williams, D'Ann L. Buckley, Timothy J. Eggleston, Peyton A. Diette, Gregory B
Environmental research
ei2, 0147621
IM
Journal Article. Research Support, N.I.H., Extramural
English
KW - MEDLINE
KW - Air Pollutants/ae [Adverse Effects]
KW - Air Pollutants/an [Analysis]
KW - Air Pollution
KW - Indoor/ae [Adverse Effects]
KW - Indoor/an [Analysis]
KW - Asthma/ep [Epidemiology]
KW - Asthma/et [Etiology]
KW - Asthma/pc [Prevention & Control]
KW - Baltimore/ep [Epidemiology]
KW - Child
KW - Child Welfare
KW - Preschool
KW - Environmental Monitoring

KW - Epidemiological Monitoring
KW - Female
KW - Housing
KW - Humans
KW - Male
KW - Particulate Matter/ae [Adverse Effects]
KW - Particulate Matter/an [Analysis]
KW - Poverty
KW - Tobacco Smoke Pollution/ae [Adverse Effects]
KW - Tobacco Smoke Pollution/an [Analysis]
KW - Urban Population
RP - NOT IN FILE
SP - 148
EP - 155
JF - Environmental Research
JA - Environ Res
VL - 106
IS - 2
CY - United States
N2 - Asthma disproportionately affects inner-city, minority children in the U.S. Outdoor pollutant concentrations, including particulate matter (PM), are higher in inner-cities and contribute to childhood asthma morbidity. Although children spend the majority of time indoors, indoor PM exposures have been less extensively characterized. There is a public health imperative to characterize indoor sources of PM within this vulnerable population to enable effective intervention strategies. In the present study, we sought to identify determinants of indoor PM in homes of Baltimore inner-city pre-school children. Children ages 2-6 (n=300) who were predominantly African-American (90%) and from lower socioeconomic backgrounds were enrolled. Integrated PM(2.5) and PM(10) air sampling was conducted over a 3-day period in the children's bedrooms and at a central monitoring site while caregivers completed daily activity diaries. Homes of pre-school children in inner-city Baltimore had indoor PM concentrations that were twice as high as simultaneous outdoor concentrations. The mean indoor PM(2.5) and PM(10) concentrations were 39.5+/-34.5 and 56.2+/-44.8 microg/m(3), compared to the simultaneously measured ambient PM(2.5) and PM(10) (15.6+/-6.9 and 21.8+/-9.53 microg/m(3), respectively). Common modifiable household activities, especially smoking and sweeping, contributed significantly to higher indoor PM, as did ambient PM concentrations. Open windows were associated with significantly lower indoor PM. Further investigation of the health effects of indoor PM exposure is warranted, as are studies to evaluate the efficacy of PM reduction strategies on asthma health of inner-city children
SN - 0013-9351
AD - Department of Medicine, Johns Hopkins University, Pulmonary and Critical Care Medicine, 1830 East Monument Street, 5th Floor, Baltimore, MD 21231, USA
UR - 17927974
ER -

TY - JOUR
ID - 1074
T1 - The epidemiology and cost of falls requiring hospitalisation in children in Western Australia: a study using linked administrative data
A1 - Moorin,R.E.
A1 - Hendrie,D.
Y1 - 2008/01//
N1 - Moorin, Rachael E. Hendrie, Delia
Accident; analysis and prevention
acs, 1254476
IM
Journal Article. Research Support, Non-U.S. Gov't
English
KW - MEDLINE
KW - Accidental Falls/ec [Economics]

KW - Accidental Falls/sn [Statistics & Numerical Data]
KW - Adolescent
KW - Child
KW - Preschool
KW - Cost of Illness
KW - Female
KW - Health Care Costs
KW - Health Priorities
KW - Hospitalization/ec [Economics]
KW - Hospitalization/sn [Statistics & Numerical Data]
KW - Humans
KW - Infant
KW - Male
KW - Risk Factors
KW - Western Australia/ep [Epidemiology]
KW - Wounds and Injuries/ec [Economics]
KW - Wounds and Injuries/ep [Epidemiology]
KW - Wounds and Injuries/th [Therapy]
RP - NOT IN FILE
SP - 216
EP - 222
JF - Accident Analysis & Prevention
JA - Accid Anal Prev
VL - 40
IS - 1
CY - England
N2 - OBJECTIVE: The aim of this study was to examine the epidemiology and health system cost of children's falls resulting in hospitalisation in 2003 in Western Australia. METHODS: The Injury Cost Database was used to identify children who were admitted to hospital with a falls related injury. Adjusted incidence rate ratios (IRR) of hospitalisation were modelled using Poisson regression. In-patient hospital costs were assigned using the published DRG costs for Western Australia. These costs were then extrapolated to health system costs based on previously published relative proportions. RESULTS: When adjusted for other covariates in the model males had a 53% higher incidence of falls requiring hospitalisation compared with females. Aboriginal children had a 36% higher incidence compared with their non-Aboriginal counterparts, and the incidence of falls reduced with increasing age. The total cost of in-patient hospitalisation associated with children's falls in Western Australia was A$4,554,000 with an average cost of A$1876 per case. In children aged 0-4 years and 10-14 years the highest cost resulted from falls on the flat (slips and trips). However, in children aged 5-9 years injuries resulting from falls from playground equipment resulted in both the highest cost group (A$539,000) and the highest cost per case (A$1917). The total cost to the health system of children's falls in Western Australia in 2003 were estimated to be A$21.5 million, with the total cost to the community estimated at A$108.5 million. CONCLUSION: Children's falls impose a considerable burden and cost to both the health care system and the community. This study has provided information on where the burden of risk and the majority of costs lie, namely males, Aboriginal children and for children aged 5-9 years, unlike their younger and older peers, playground equipment
SN - 0001-4575
AD - Australian Centre for Economic Research on Health, School of Population Health, The University of Western Australia, 35 Stirling Highway, Crawley, WA 6009, Australia. rachael.moorin@uwa.edu.au
UR - 18215551
ER -

TY - JOUR
ID - 1075
T1 - Blood levels of polycyclic aromatic hydrocarbons in children of Lucknow, India
A1 - Singh,V.K.
A1 - Patel,D.K.
A1 - Ram,S.
A1 - Mathur,N.

A1 - Siddiqui,M.K.
A1 - Behari,J.R.
Y1 - 2008/02//
N1 - Singh, Vipul K. Patel, Devendra K. Ram, S. Mathur, N. Siddiqui, M K J. Behari, Jai Raj
Archives of environmental contamination and toxicology
6yd, 0357245
IM
Journal Article. Research Support, Non-U.S. Gov't
English
KW - MEDLINE
KW - Child
KW - Preschool
KW - Environmental Monitoring
KW - Environmental Pollutants/bl [Blood]
KW - Environmental Pollutants/ch [Chemistry]
KW - Female
KW - Humans
KW - India
KW - Male
KW - Molecular Weight
KW - Polycyclic Hydrocarbons
KW - Aromatic/bl [Blood]
KW - Aromatic/ch [Chemistry]
RP - NOT IN FILE
SP - 348
EP - 354
JF - Archives of Environmental Contamination & Toxicology
JA - Arch Environ Contam Toxicol
VL - 54
IS - 2
CY - United States
N2 - Polycyclic aromatic hydrocarbons (PAHs) are compounds with two or more fused benzene rings produced by incomplete combustion of organic substances involved in natural and anthropogenic processes. Children are exposed to these compounds through inhalation, dietary ingestion, and, also, soil at the playground. It has been well established that PAHs have carcinogenic, mutagenic, and teratogenic effects. Considering possible health risks due to PAHs exposure among children, the present study was carried out in collaboration with the Pediatrics Department, King George's Medical University (KGMU), Lucknow, to determine its exposure in children by estimating blood PAHs levels. Due to the variable composition of PAHs mixtures emitted from different environmental sources, any single compound or metabolite may not be representative of all exposure conditions. For these reasons, the measurement of blood PAHs levels as a possible biomarker, especially of the EPA (Environmental Protection Agency, USA) priority list, has been proposed. Acenaphthylene, anthracene, phenanthrene, fluoranthene, naphthalene, pyrene, benzo(b)fluoranthene, benzo(k)fluoranthene, and benzo(a)pyrene were determined by HPLC-FD/UV. On the basis of the individual compound, the median (50th percentile) of naphthalene (19 ppb) was highest, however, benzo(a)pyrene (4.0 ppb) level was found to be lowest among all detected PAHs. The median level of total noncarcinogenic PAHs (113.55 ppb) was higher than the total carcinogenic PAHs (32.35 ppb) in blood samples of children. A significant correlation was found between period of time spent in the surrounding breathing zone of the cooking place and total noncarcinogenic PAHs ($p < 0.05$), while the blood carcinogenic PAHs level in children was found to be associated with lower status of their families ($p < 0.05$). It is speculated that there may be chances of health hazards through exposure to PAHs, those not yet declared hazardous and present at higher concentrations in the Indian environment. Further study with a larger sample size and accompanying environmental data is desired to validate the findings of this pilot study and strengthen the database of PAHs exposure in India
SN - 0090-4341
AD - Analytical Toxicology Section, Industrial Toxicology Research Centre, MG Marg, Lucknow, India
UR - 17763887
ER -

TY - JOUR
ID - 1076
T1 - [Helminthes in sandboxes of day care centers of a city in Southeastern Brazil]. [Portuguese]
A1 - Araujo,Nda S.
A1 - Rodrigues,C.T.
A1 - Cury,M.C.
Y1 - 2008/02//
N1 - Araujo, Nubia da Silva. Rodrigues, Cristiane Teixeira. Cury, Marcia Cristina
Revista de saude publica
0135043, t5x
IM
English Abstract. Journal Article
Portuguese
KW - MEDLINE
KW - Animals
KW - Brazil
KW - Child
KW - Child Day Care Centers
KW - Helminthiasis/ps [Parasitology]
KW - Helminthiasis/pc [Prevention & Control]
KW - Helminths/ip [Isolation & Purification]
KW - Humans
KW - Parasite Egg Count
KW - Play and Playthings
KW - Seasons
KW - Silicon Dioxide
KW - Soil/ps [Parasitology]
RP - NOT IN FILE
SP - 150
EP - 153
JF - Revista de Saude Publica
JA - Rev Saude Publica
VL - 42
IS - 1
CY - Brazil
N2 - The objective of the study was to test for helminthes in sandboxes in day care centers in the city of Uberlandia, Southeastern Brazil in 2005. Fourteen private and 14 public day care centers with playgrounds were selected. One collection was carried out during the dry season (June and August), and the second one was in the rainy season (September and November). Samples were tested using Willis and Baermann methods. In the first collection, 17 (61%) day care centers were positive for helminthes larvae and 14 (50%) for eggs. In the second collection, 18 (64%) day care centers were positive for larvae and 10 (36%) for eggs. No influence of either season of the year or day care finance condition was seen
SN - 0034-8910
AD - Laboratorio de Parasitologia, Instituto de Ciencias Biomedicas, Universidade Federal de Uberlandia, Uberlandia, MG, Brasil
UR - 18200354
ER -

TY - JOUR
ID - 1077
T1 - Promotion of physical activity in children. [Review] [50 refs]
A1 - Floriani,V.
A1 - Kennedy,C.
Y1 - 2008/02//
N1 - Floriani, Victoria. Kennedy, Christine
Current opinion in pediatrics
but, 9000850

IM
Journal Article. Review
English
KW - MEDLINE
KW - Child
KW - Exercise/ph [Physiology]
KW - Exercise/px [Psychology]
KW - Health Behavior
KW - Health Promotion
KW - Humans
KW - Life Style
KW - Motor Activity/ph [Physiology]
RP - NOT IN FILE
SP - 90
EP - 95
JF - Current Opinion in Pediatrics
JA - Curr Opin Pediatr
VL - 20
IS - 1
CY - United States
N2 - PURPOSE OF REVIEW: Promotion of physical activity continues to be recommended as an essential component of obesity treatment and prevention interventions. This review explores recent updates in the area of physical activity promotion and its impact on the physical and mental health consequences of childhood obesity. RECENT FINDINGS: Despite the availability of opportunities for physical activity in the school environment, namely recess and physical education classes, students do not appear to be meeting activity recommendations at school alone. Access to neighborhood parks may increase levels of physical activity and reduce time spent in sedentary behaviors at home. Less time spent watching television and in other sedentary behaviors such as playing videogames may contribute to higher rates of physical activity. Frequency of physical activity also appears to be related to improved mental health status, although the direction of this relationship warrants further exploration. SUMMARY: Physical activity is an evidence-based intervention that offers benefits to both physical and mental health. Pediatric health care providers are encouraged to engage in discussions with patients and families on the topic of physical activity and to assist them in finding ways to incorporate activity into daily life. [References: 50]
SN - 1040-8703
AD - University of California, San Francisco, Department of Family Healthcare Nursing, San Francisco, California 94143-0606, USA
UR - 18197046
ER -

TY - JOUR
ID - 1078
T1 - Return to activity after knee arthroscopy
A1 - Lubowitz,J.H.
A1 - Ayala,M.
A1 - Appleby,D.
Y1 - 2008/01//
N1 - Lubowitz, James H. Ayala, Myna. Appleby, David
Arthroscopy : the journal of arthroscopic & related surgery : official publication of the Arthroscopy Association of North America and the International Arthroscopy Association
abt, 8506498
IM
Journal Article
English
KW - MEDLINE
KW - Adolescent
KW - Adult
KW - Aged

KW - Arthroscopy
KW - Child
KW - Female
KW - Humans
KW - Knee Joint/su [Surgery]
KW - Male
KW - Middle Aged
KW - Motor Activity
KW - Recovery of Function
KW - Time Factors
RP - NOT IN FILE
SP - 58
EP - 61
JF - Arthroscopy
VL - 24
IS - 1
CY - United States
N2 - PURPOSE: Although knee arthroscopy is described as minimally invasive, return to activity has been poorly quantitated. Our purpose is to test the hypothesis that most patients return to unrestricted activity within 4 weeks after knee arthroscopy. METHODS: After prospective power analysis, 72 consecutive patients who underwent arthroscopic knee partial medial meniscectomy, partial lateral meniscectomy, chondroplasty, loose body removal, or synovectomy (or some combination thereof) by a single surgeon were included. Patients with Workers' Compensation claims were excluded. Postoperative instructions were standardized. Patients completed a diary preoperatively and at 1, 2, 3, 4, 8, 12, 16, 20, and 24 weeks postoperatively indicating their highest International Knee Documentation Committee (subjective) level of activity, as well as whether activity was restricted for knee-related reasons. RESULTS: Preoperatively, 88% of patients described knee-related activity restriction. By 2 weeks postoperatively, only 74% described knee-related activity restriction, a significant difference (P = .039); this improved to 38% at 4 weeks and was only 4% at 20 weeks. In addition, 82% returned to light activity such as walking, housework, or yard work after 1 week, with 94% after 2 weeks and 100% after 4 weeks. CONCLUSIONS: Our results support the hypothesis: Most patients had no knee-related activity restriction 4 weeks after arthroscopy. LEVEL OF EVIDENCE: Level IV, therapeutic case series
SN - 1526-3231
AD - Taos Orthopaedic Institute Research Foundation, Taos, New Mexico, USA. jlubowitz@kitcarson.net
UR - 18182203
ER -

TY - JOUR
ID - 1079
T1 - Patterns in the received facial UV exposure of school children measured at a subtropical latitude
A1 - Downs,N.
A1 - Parisi,A.
Y1 - 2008/01//
N1 - Downs, Nathan. Parisi, Alfio
Photochemistry and photobiology
0376425, p69
IM
Journal Article
English
KW - MEDLINE
KW - Adolescent
KW - Basketball
KW - Calibration
KW - Child
KW - Face/re [Radiation Effects]
KW - Humans
KW - Seasons
KW - Soccer

KW - Time Factors
KW - Tropical Climate
RP - NOT IN FILE
SP - 90
EP - 100
JF - Photochemistry & Photobiology
JA - Photochem Photobiol
VL - 84
IS - 1
CY - United States
N2 - Polysulfone (PS) dosimeters have been employed to measure the erythemally effective UV exposure to the vertex, nose, cheek, chin and side facial sites of 45 volunteer high school students from Hervey Bay, Australia (25.3 degrees S 152.9 degrees E). The results of a series of 1 h outdoor sport trials (basketball and soccer) found the mean student facial exposure, determined as the arithmetic average of facial site exposures of unprotected students (no hat) to protected students (hat), varied from 140 +/- 82 J m(-2) (1sigma) to 99 +/- 33 J m(-2) (1sigma), respectively. All hourly student facial exposures recorded over the study period were found to exceed the National Health and Medical Research Council's adopted safe daily limit of 30 J m(-2). Facial exposure relative to the received ambient UV increased to the nose at higher (winter) solar zenith angles (SZAs) compared with lower (summer) SZA ranges for both protected and unprotected students. The protection offered by the broad-brimmed hats was reduced significantly to the lower chin facial site at the higher SZA range, indicating that the style of hat used offers best protection in summer to the upper facial regions at most risk of receiving a high exposure when no hat protection is used. Variations to specific student facial exposure sites were measured between both basketball and soccer players. Variation in student facial exposure was further examined with respect to cloud cover and comparisons to manikin headform measurements were also made. The study results indicate that hats alone are not adequate forms of sun protection in a school environment. Schools aiming to achieve acceptable safe limits of facial exposure may need to further consider the effectiveness of hat protection with increasing SZA, cloud cover and head position relative to the sun that is specific to the scheduled outdoor activity
SN - 0031-8655
AD - Faculty of Sciences, University of Southern Queensland, Toowoomba, Queensland, Australia.
downsn@usq.edu.au
UR - 18173708
ER -

TY - JOUR
ID - 1080
T1 - Children's response to air pollutants. [Review] [83 refs]
A1 - Bateson,T.F.
A1 - Schwartz,J.
Y1 - 2008///
N1 - Bateson, Thomas F. Schwartz, Joel
Journal of toxicology and environmental health. Part A
ds5, 100960995
IM
Journal Article. Review
English
KW - MEDLINE
KW - Air Pollutants/ae [Adverse Effects]
KW - Asthma/et [Etiology]
KW - Child
KW - Preschool
KW - Humans
KW - Infant
KW - Infant Mortality
KW - Inhalation Exposure/ae [Adverse Effects]
KW - Lung/de [Drug Effects]
KW - Lung/gd [Growth & Development]

KW - Lung/ph [Physiology]
KW - Lung
RP - NOT IN FILE
SP - 238
EP - 243
JF - Journal of Toxicology & Environmental Health Part A
JA - J Toxicol Environ Health A
VL - 71
IS - 3
CY - England
N2 - It is important to focus on children with respect to air pollution because (1) their lungs are not completely developed, (2) they can have greater exposures than adults, and (3) those exposures can deliver higher doses of different composition that may remain in the lung for greater duration. The undeveloped lung is more vulnerable to assault and less able to fully repair itself when injury disrupts morphogenesis. Children spend more time outside, where concentrations of combustion-generated air pollution are generally higher. Children have higher baseline ventilation rates and are more physically active than adults, thus exposing their lungs to more air pollution. Nasal breathing in adults reduces some pollution concentrations, but children are more typically mouth-breathers--suggesting that the composition of the exposure mixture at the alveolar level may be different. Finally, higher ventilation rates and mouth-breathing may pull air pollutants deeper into children's lungs, thereby making clearance slower and more difficult. Children also have immature immune systems, which plays a significant role in asthma. The observed consequences of early life exposure to adverse levels of air pollutants include diminished lung function and increased susceptibility to acute respiratory illness and asthma. Exposure to diesel exhaust, in particular, is an area of concern for multiple endpoints, and deserves further research. [References: 83]
SN - 1528-7394
AD - National Center for Environmental Assessment, Office of Research and Development, U.S. Environmental Protection Agency, Washington, DC 20460, USA. bateson.thomas@epa.gov
UR - 18097949
ER -

TY - JOUR
ID - 1081
T1 - Multimedia measurements and activity patterns in an observational pilot study of nine young children
A1 - Tulve,N.S.
A1 - Egeghy,P.P.
A1 - Fortmann,R.C.
A1 - Whitaker,D.A.
A1 - Nishioka,M.G.
A1 - Naeher,L.P.
A1 - Hilliard,A.
Y1 - 2008/01//
N1 - Tulve, Nicolle S. Egeghy, Peter P. Fortmann, Roy C. Whitaker, Donald A. Nishioka, Marcia G. Naeher, Luke P. Hilliard, Aaron
Journal of exposure science & environmental epidemiology
101262796
IM
Journal Article. Research Support, U.S. Gov't, Non-P.H.S.
English
KW - MEDLINE
KW - Child
KW - Preschool
KW - Environmental Exposure
KW - Environmental Pollutants/to [Toxicity]
KW - Environmental Pollutants/ur [Urine]
KW - Florida
KW - Food Contamination
KW - Geography

KW - Humans
KW - Permethrin/to [Toxicity]
KW - Permethrin/ur [Urine]
KW - Pesticides/to [Toxicity]
KW - Pesticides/ur [Urine]
KW - Pilot Projects
KW - Pyrethrins/to [Toxicity]
KW - Pyrethrins/ur [Urine]
KW - Risk Assessment
KW - Time Factors
RP - NOT IN FILE
SP - 31
EP - 44
JF - Journal of Exposure Science & Environmental Epidemiology
JA - J Expo Sci Environ Epidemiol
VL - 18
IS - 1
CY - United States
N2 - A pilot observational exposure study was performed to evaluate methods for collecting multimedia measurements (air, dust, food, urine) and activity patterns to assess potential exposures of young children to pesticides in their homes. Nine children (mean age=5 years) and their caregivers participated in this study, performed in the Duval County, Florida, in collaboration with the Centers for Disease Control and Prevention and the Duval County Health Department. For all nine children, the total time reported for sleeping and napping ranged from 9.5 to 14 h per day, indoor quiet time from 0 to 5.5 h per day, indoor active time from 0.75 to 5.5 h per day, outdoor quiet time from 0 to 1.5 h per day, and outdoor active time from 0.5 to 6.5 h per day. Each home had one to three pesticide products present, with aerosols being most common. Pesticide inventories, however, were not useful for predicting pesticide levels in the home. Synthetic pyrethroids were the most frequently identified active ingredients in the products present in each home. Fifteen pesticide active ingredients were measured in the application area wipes (not detected (ND) to 580 ng/cm(2)), 13 in the play area wipes (ND-117 ng/cm(2)), and 14 in the indoor air samples (ND-378 ng/m(3)) and the socks (ND-1000 ng/cm(2)). Cis-permethrin, trans-permethrin, and cypermethrin were measured in all nine homes. Chlorpyrifos was measured in all nine homes even though it was not reported used by the participants. All urine samples contained measurable concentrations of 3-phenoxybenzoic acid (3-PBA). The median 3-PBA urinary concentration for the nine children was 2.2 mug/l. A wide variety of pesticide active ingredients were measured in these nine homes at median concentrations that were often higher than reported previously in similar studies. These data highlight the need for additional observational studies in regions where pesticides are used in order to understand the factors that affect young children's exposures and the education/mitigation strategies that can be used to reduce children's exposures
SN - 1559-064X
AD - National Exposure Research Laboratory, US EPA, MD-E205-04, Research Triangle Park, NC 27711, USA. tulve.nicolle@epa.gov
UR - 17851450
ER -

TY - JOUR
ID - 1082
T1 - Playing it safe: the influence of neighbourhood safety on children's physical activity. A review. [Review] [72 refs]
A1 - Carver,A.
A1 - Timperio,A.
A1 - Crawford,D.
Y1 - 2008/06//
N1 - Carver, Alison. Timperio, Anna. Crawford, David
Health & place
din, 9510067
T

Journal Article. Review
English
KW - MEDLINE
KW - Child
KW - Preschool
KW - Exercise
KW - Humans
KW - Play and Playthings
KW - Residence Characteristics
KW - Safety
KW - Victoria
RP - NOT IN FILE
SP - 217
EP - 227
JF - Health & Place
JA - Health Place
VL - 14
IS - 2
CY - England
N2 - Compared with previous generations, children spend less time playing outdoors and have lower participation rates in active transport. Many studies have identified lack of neighbourhood safety as a potential barrier to children's physical activity. This review describes concerns regarding 'stranger danger' and road safety, and discusses empirical studies that examine associations between neighbourhood safety and physical activity among youth. Variability of perceptions of safety between parents and youth are examined; 'social traps' are identified; and physical/social environmental interventions aimed at improving neighbourhood safety are discussed. A research agenda is suggested for further study of perceived and objective measures of neighbourhood safety and their associations with children's physical activity. [References: 72]
SN - 1353-8292
AD - Centre for Physical Activity and Nutrition Research, School of Exercise and Nutrition Sciences, Deakin University, VIC, Australia. alison.carver@deakin.edu.au
UR - 17662638
ER -

TY - JOUR
ID - 1083
T1 - Physical activity across the curriculum (PAAC): rationale and design
A1 - DuBose,K.D.
A1 - Mayo,M.S.
A1 - Gibson,C.A.
A1 - Green,J.L.
A1 - Hill,J.O.
A1 - Jacobsen,D.J.
A1 - Smith,B.K.
A1 - Sullivan,D.K.
A1 - Washburn,R.A.
A1 - Donnelly,J.E.
Y1 - 2008/01//
N1 - DuBose, Katrina D. Mayo, Matthew S. Gibson, Cheryl A. Green, J Leon. Hill, James O. Jacobsen, Dennis J. Smith, Bryan K. Sullivan, Debra K. Washburn, Richard A. Donnelly, Joseph E
Contemporary clinical trials
101242342
IM
Journal Article. Randomized Controlled Trial. Research Support, N.I.H., Extramural
English
KW - MEDLINE
KW - Body Mass Index
KW - Child

KW - Curriculum
KW - Diet
KW - Exercise
KW - Female
KW - Humans
KW - Male
KW - Physical Fitness
KW - Schools/og [Organization & Administration]
RP - NOT IN FILE
SP - 83
EP - 93
JF - Contemporary Clinical Trials
JA - Contemp Clin Trials
VL - 29
IS - 1
CY - United States
N2 - BACKGROUND: Over the years schools have reduced physical education and recess time in favor of more academic instruction. Due to the drastic rise in obesity levels among children, some states have begun to mandate minimum amounts of physical activity (PA) that school children receive, causing schools to find alternative methods for increasing PA levels. Physical Activity Across the Curriculum (PAAC) is a 3-year randomized clinical trial incorporating moderate-intensity PA in elementary schools to reduce childhood obesity. This paper describes the rational, design, and methods of the PAAC intervention study. METHODS: Twenty-two elementary schools were randomized to either a control or intervention condition. In schools randomized to the intervention condition (PAAC), regular classroom teachers were taught how to incorporate PA into standard academic lessons. Teachers were asked to accumulate 90-100 min/week of PAAC each week through out the 3-year study period. Schools randomized to the control group did not alter their teaching methods. Direct observation of PA levels in the classroom was collected weekly. Height and weight was measured twice a year to calculate BMI. RESULTS: Two years of the intervention have been completed and only one school has left the study. The remaining 21 schools are participating in the final intervention year. CONCLUSIONS: The results from the PAAC intervention may provide schools with an alternative method to increase PA levels in children and reduce childhood obesity
SN - 1551-7144
AD - East Carolina University, USA. dubosek@ecu.edu
UR - 17611168
ER -

TY - JOUR
ID - 1084
T1 - Indoor and outdoor PM mass and number concentrations at schools in the Athens area
A1 - Diapouli,E.
A1 - Chaloulakou,A.
A1 - Mihalopoulos,N.
A1 - Spyrellis,N.
Y1 - 2008/01//
N1 - Diapouli, E. Chaloulakou, A. Mihalopoulos, N. Spyrellis, N
Environmental monitoring and assessment
dvk, 8508350
IM
Journal Article. Research Support, Non-U.S. Gov't
English
KW - MEDLINE
KW - Air Pollutants/an [Analysis]
KW - Air Pollution
KW - Indoor/an [Analysis]
KW - Child
KW - Cities
KW - Environmental Exposure/an [Analysis]

KW - Environmental Monitoring
KW - Greece
KW - Humans
KW - Particulate Matter/an [Analysis]
KW - Schools
RP - NOT IN FILE
SP - 13
EP - 20
JF - Environmental Monitoring & Assessment
JA - Environ Monit Assess
VL - 136
IS - 1-3
CY - Netherlands
N2 - Simultaneous indoor and outdoor PM10 and PM2.5 concentration measurements were conducted in seven primary schools in the Athens area. Both gravimetric samplers and continuous monitors were used. Filters were subsequently analyzed for anion species. Moreover ultrafine particles number concentration was monitored continuously indoors and outdoors. Mean 8-hr PM10 concentration was measured equal to 229 +/- 182 microg/m3 indoors and 166 +/- 133 microg/m3 outdoors. The respective PM2.5 concentrations were 82 +/- 56 microg/m3 indoors and 56 +/- 26 microg/m3 outdoors. Ultrafine particles 8-h mean number concentration was measured equal to 24,000 +/- 17,900 particles/cm3 indoors and 32,000 +/- 14,200 particles/cm3 outdoors. PM10 outdoor concentrations exhibited a greater spatial variability than the corresponding PM2.5 ones. I/O ratios were close or above 1.00 for PM10 and PM2.5 and smaller than 1.00 for ultrafine particles. Very high I/O ratios were observed when intense activities took place. The initial results of the chemical analysis showed that SO4(-2) accounts for the 6.6 +/- 3.5% of the PM10 and NO3(1) for the 3.1 +/- 1.4%.The corresponding results for PM2.5 are 12.0 +/- 7.7% for SO4(-2) and 3.1 +/- 1.9% for NO3-. PM2.5 SO4(-2) indoor concentrations were highly correlated with outdoor ones and the regression line had the largest slope and a very low intercept, indicative of no indoor sources of fine particulate SO4(-2). The results of the statistical analysis of indoor and outdoor concentration data support the use of SO4(-2) as a proper surrogate for indoor PM of outdoor origin
SN - 1573-2959
AD - School of Chemical Engineering, NTUA-Zographos Campus, 9 Heroon Polytechniou Street, Athens 15773, Greece
UR - 17458512
ER -

TY - JOUR
ID - 1085
T1 - Epidemiological aspects and risk factors of toxocariasis in a pediatric population in Sri Lanka
A1 - Fernando,S.D.
A1 - Wickramasinghe,V.P.
A1 - Kapilananda,G.M.
A1 - Devasurendra,R.L.
A1 - Amarasooriya,J.D.
A1 - Dayaratne,H.G.
Y1 - 2007/11//
N1 - Fernando, S D. Wickramasinghe, V P. Kapilananda, G M G. Devasurendra, R L. Amarasooriya, J D M S. Dayaratne, H G A K
The Southeast Asian journal of tropical medicine and public health
uvn, 0266303
IM
Journal Article. Research Support, Non-U.S. Gov't
English
KW - MEDLINE
KW - Animals
KW - Child
KW - Preschool
KW - Cross-Sectional Studies
KW - Female

KW - Humans
KW - Male
KW - Risk Factors
KW - Sri Lanka/ep [Epidemiology]
KW - Toxocariasis/ep [Epidemiology]
KW - Toxocariasis/et [Etiology]
RP - NOT IN FILE
SP - 983
EP - 990
JF - Southeast Asian Journal of Tropical Medicine & Public Health
JA - Southeast Asian J Trop Med Public Health
VL - 38
IS - 6
CY - Thailand
N2 - This cross-sectional study, carried out over a period of 11 months, investigated the relationship between Toxocara seropositivity, socio-demographic and environmental variables in a pediatric population. Risk factors for Toxocara infection were assessed by direct interview of parent or guardian using a structured pre-tested questionnaire. Eosinophilia and presence of helminth eggs or protozoan cysts in a fecal smear were recorded. Diagnosis of Toxocara seropositivity in children was based on IgG Toxocara Microwell Serum Elisa Kits. The ELISA test was regarded as positive if the optical density was 0.3 units or above. Unadjusted and adjusted odds ratios were calculated to determine risk factors for disease. The proportion of children who were positive for Toxocara antibodies in the study population was 20%. Children being exposed to a puppy of less than 3 months at home, visiting a playground frequently, living in a poorly constructed house and dogs having access to playgrounds were significant risk factors on univariate analysis. Of these four variables, only the first three variables (OR 19, OR 4 and OR 3, respectively) remained significant risk factors on the multivariate model. Presence of eosinophilia in seropositive children was significantly higher than the seronegative group (77% vs 40%; $p < 0.001$). This study indicates that dogs contribute significantly to children being seropositive for toxocariasis in Sri Lanka. Implementation of public health programs specifically focused on anti-parasitic treatment of dogs is recommended
SN - 0125-1562
AD - Department of Parasitology, University of Colombo, Sri Lanka. deepfern@slt.lk
UR - 18613538
ER -

TY - JOUR
ID - 1086
T1 - Peer teaching at recess and improvement of disc-throwing skills by children in kindergarten through grade 3
A1 - Murase,T.
A1 - Gallahue,D.L.
A1 - Tosi,S.H.
Y1 - 2007/12//
N1 - Murase, Tomohiko. Gallahue, David L. Tosi, Silvia H
Perceptual and motor skills
ozb, 0401131
IM
Comparative Study. Journal Article
English
KW - MEDLINE
KW - Athletic Performance/ph [Physiology]
KW - Biomechanical Phenomena
KW - Child
KW - Child Development/ph [Physiology]
KW - Preschool
KW - Female
KW - Humans
KW - Male

KW - Motor Skills/ph [Physiology]
KW - Movement/ph [Physiology]
KW - Peer Group
KW - Psychomotor Performance/ph [Physiology]
KW - Teaching
RP - NOT IN FILE
SP - 768
EP - 776
JF - Perceptual & Motor Skills
JA - Percept.Mot.Skills
VL - 105
IS - 3 Pt 1
CY - United States
N2 - This study examined the effect of peer teaching at recess on improving disc-throwing skills for kindergartners to children in Grade 3. 167 boys and girls were instructed in basic disc-throwing skills, backhand and forehand throwing techniques, and peer teaching. The practice group (n = 73) was encouraged to practice with a disc using peer teaching for at least 10 min. during recess for 10 days. The control group (n = 94) took part in other activities without a disc. Two-way analysis of variance with repeated measures gave significant interaction of groups and tests for girls in Grade 3 as well as significant main effects between tests in kindergarten boys and Grade 2 boys and girls. This study suggests that peer teaching for disc-throwing skills is useful for girls in Grade 3 and may be more effective for girls than boys
SN - 0031-5125
AD - Research Center of Physical Education, Aichi University, 370 Kurozasa, Miyoshi, Nishikamo, Aichi 470-0296, Japan. murase@vega.aichi-u.ac.jp
UR - 18229532
ER -

TY - JOUR
ID - 1087
T1 - P-wave body surface isointegral maps in children and in young adults
A1 - Kozlikova,K.
Y1 - 2007///
N1 - Kozlikova, K
Physiological research / Academia Scientiarum Bohemoslovaca
az7, 9112413
IM
Journal Article
English
KW - MEDLINE
KW - Action Potentials
KW - Adolescent
KW - Atrial Function
KW - Body Surface Potential Mapping
KW - Child
KW - Female
KW - Humans
KW - Male
KW - Reference Values
KW - Reproducibility of Results
KW - Signal Processing
KW - Computer-Assisted
KW - Time Factors
RP - NOT IN FILE
SP - S123
EP - S128
JF - Physiological Research
JA - Physiol Res

VL - 56 Suppl 1
CY - Czech Republic
N2 - Only limited data are available on body surface potential distribution during atrial activation. The aim of this study was to establish the distributions and to analyze chosen quantitative parameters of atrial isointegral maps recorded using a limited 24-lead system in a young healthy population. A total of 166 subjects underwent a procedure of body surface potential mapping. Isointegral maps during the P wave were constructed and qualitatively and quantitatively evaluated. Three types of atrial activation in individual maps were found according to the different shape of the zero isointegral line and to mutual positions of extrema. The most frequently occurring type resembled the group mean maps and was in good agreement with published data obtained from full lead systems. The highest extrema were found in the young men group, while, surprisingly, the lowest values in the young women group. All minima and the majority of maxima were recorded outside the ranges of standard chest leads. The usefulness of the limited lead system to record isointegral P wave maps was shown and new data were presented that can be useful in noninvasive evaluation of atrial pathologies
SN - 0862-8408
AD - Institute of Medical Physics and Biophysics, Faculty of Medicine Comenius University, Bratislava, Slovakia. katarina.kozlikova@fmed.uniba.sk
UR - 17552886
ER -

TY - JOUR
ID - 1088
T1 - Physical activity patterns of kindergarten children in comparison to teenagers and young adults
A1 - Sigmund,E.
A1 - De Ste,Croix M.
A1 - Miklankova,L.
A1 - Fromel,K.
Y1 - 2007/12//
N1 - Sigmund, Erik. De Ste Croix, Mark. Miklankova, Ludmila. Fromel, Karel
European journal of public health
d0w, 9204966
IM
Comparative Study. Journal Article. Research Support, Non-U.S. Gov't
English
KW - MEDLINE
KW - Adolescent
KW - Adult
KW - Child
KW - Preschool
KW - Czechoslovakia
KW - Exercise
KW - Female
KW - Humans
KW - Leisure Activities
KW - Male
KW - Monitoring
KW - Ambulatory/is [Instrumentation]
KW - Obesity/pc [Prevention & Control]
RP - NOT IN FILE
SP - 646
EP - 651
JF - European Journal of Public Health
JA - Eur J Public Health
VL - 17
IS - 6
CY - England
N2 - BACKGROUND: Due to the increasing occurrence of obesity and low physical activity (PA) reported in school children there is a need to investigate PA patterns of preschool children. Our aim was to identify the PA

of preschool children in comparison to the PA of teenagers and young adults. METHODS: PA of 104 Czech preschool children (aged 5-7 years), 1174 teenagers (aged 12-17 years) and 787 young adults (aged 18-24 years) was determined using Caltrac accelerometery. PA was evaluated on the basis of activity energy expenditure (AEE--kcal kg(-1) day(-1)). Subjects were monitored over 7 days to include a weekend. RESULTS: The AEE in preschool children was significantly higher in comparison to all age groups of teenagers and young adults during both weekdays and at the weekend (P<0.0001). On weekdays during leisure time, preschoolers were considerably more physically active than all categories of teenagers and young adults (P<0.001). Preschoolers show similarities in PA level between weekdays and weekends, unlike the older age groups. CONCLUSION: It seems to appear that organized daily PA (2x20 min of exercising on carpet, 50-70 min of walking outdoors) in preschool children along with enough space for spontaneous PA establishes prerequisites for their further healthy development
SN - 1101-1262
AD - Center for Kinanthropology Research, Faculty of Physical Culture, Palacky University Olomouc, Czech Republic. erik.sigmund@upol.cz
UR - 17434876
ER -

TY - JOUR
ID - 1089
T1 - Lifestyle-related factors and environmental agents causing cancer: an overview. [Review] [313 refs]
A1 - Irigaray,P.
A1 - Newby,J.A.
A1 - Clapp,R.
A1 - Hardell,L.
A1 - Howard,V.
A1 - Montagnier,L.
A1 - Epstein,S.
A1 - Belpomme,D.
Y1 - 2007/12//
N1 - Irigaray, P. Newby, J A. Clapp, R. Hardell, L. Howard, V. Montagnier, L. Epstein, S. Belpomme, D
Biomedicine & pharmacotherapy = Biomedecine & pharmacotherapie
a59, 8213295
IM
Journal Article. Review
English
KW - MEDLINE
KW - Aging/ph [Physiology]
KW - Air Pollutants/to [Toxicity]
KW - Alcohol Drinking/ae [Adverse Effects]
KW - Child
KW - Diet
KW - Drug-Related Side Effects and Adverse Reactions
KW - Environmental Pollutants/to [Toxicity]
KW - Exercise/ph [Physiology]
KW - Food Contamination
KW - Humans
KW - Leukemia/ep [Epidemiology]
KW - Life Expectancy
KW - Life Style
KW - Neoplasms/ci [Chemically Induced]
KW - Neoplasms/ep [Epidemiology]
KW - Neoplasms/ge [Genetics]
KW - Obesity/co [Complications]
KW - Occupational Diseases/ep [Epidemiology]
KW - Oncogenic Viruses
KW - Overweight/co [Complications]
KW - Smoking/ae [Adverse Effects]

RP - NOT IN FILE
SP - 640
EP - 658
JF - Biomedicine & Pharmacotherapy
JA - Biomed Pharmacother
VL - 61
IS - 10
CY - France
N2 - The increasing incidence of a variety of cancers after the Second World War confronts scientists with the question of their origin. In Western countries, expansion and ageing of the population as well as progress in cancer detection using new diagnostic and screening tests cannot fully account for the observed growing incidence of cancer. Our hypothesis is that environmental factors play a more important role in cancer genesis than it is usually agreed. (1) Over the last 2-3 decades, alcohol consumption and tobacco smoking in men have significantly decreased in Western Europe and North America. (2) Obesity is increasing in many countries, but the growing incidence of cancer also concerns cancers not related to obesity nor to other known lifestyle-related factors. (3) There is evidence that the environment has changed over the time period preceding the recent rise in cancer incidence, and that this change, still continuing, included the accumulation of many new carcinogenic factors in the environment. (4) Genetic susceptibility to cancer due to genetic polymorphism cannot have changed over one generation and actually favours the role of exogenous factors through gene-environment interactions. (5) Age is not the unique factor to be considered since the rising incidence of cancers is seen across all age categories, including children, and adolescents. (6) The fetus is specifically vulnerable to exogenous factors. A fetal exposure during a critical time window may explain why current epidemiological studies may still be negative in adults. We therefore propose that the involuntary exposure to many carcinogens in the environment, including microorganisms (viruses, bacteria and parasites), radiations (radioactivity, UV and pulsed electromagnetic fields) and many xenochemicals, may account for the recent growing incidence of cancer and therefore that the risk attributable to environmental carcinogen may be far higher than it is usually agreed. Of major concern are: outdoor air pollution by carbon particles associated with polycyclic aromatic hydrocarbons; indoor air pollution by environmental tobacco smoke, formaldehyde and volatile organic compounds such as benzene and 1,3 butadiene, which may particularly affect children and food contamination by food additives and by carcinogenic contaminants such as nitrates, pesticides, dioxins and other organochlorines. In addition, carcinogenic metals and metalloids, pharmaceutical medicines and some ingredients and contaminants in cosmetics may be involved. Although the risk fraction attributable to environmental factors is still unknown, this long list of carcinogenic and especially mutagenic factors supports our working hypothesis according to which numerous cancers may in fact be caused by the recent modification of our environment. [References: 313]
SN - 0753-3322
AD - Cancer Research Center, Association for Research and Treatments Against Cancer (ARTAC), 57-59 Rue de la Convention, 75015 Paris, France. philippei.artac@gmail.com
UR - 18055160
ER -

TY - JOUR
ID - 1090
T1 - [Sociocultural factors and physical activity level in early adolescence]. [Portuguese]
A1 - Goncalves,H.
A1 - Hallal,P.C.
A1 - Amorim,T.C.
A1 - Araujo,C.L.
A1 - Menezes,A.M.
Y1 - 2007/10//
N1 - Goncalves, Helen. Hallal, Pedro C. Amorim, Tales C. Araujo, Cora L P. Menezes, Ana M B
Revista panamericana de salud publica = Pan American journal of public health
csl, 9705400
IM
English Abstract. Journal Article
Portuguese
KW - MEDLINE

KW - Adolescent
KW - Child
KW - Culture
KW - Demography
KW - Ethnic Groups/eh [Ethnology]
KW - Female
KW - Humans
KW - Life Style
KW - Male
KW - Motor Activity
KW - Prevalence
RP - NOT IN FILE
SP - 246
EP - 253
JF - Pan American Journal of Public Health
JA - Rev Panam.Salud Publica
VL - 22
IS - 4
CY - United States
N2 - OBJECTIVE: To describe the level of physical activity in adolescents born in 1993 in the city of Pelotas, Brazil, and to evaluate the effect of sociocultural variables on those levels. METHODS: A combined ethno-epidemiological methodology was employed. In the epidemiological study, 4 452 adolescents born in 1993 were interviewed. Physical activity level was evaluated by means of a questionnaire. Adolescents with fewer than 300 minutes of physical activity per week were classified as sedentary. The ethnographic study included 69 adolescents randomly selected from among all cohort participants. On average, three in-depth interviews were carried out (about one every 6 months), with mothers and adolescents being interviewed separately. RESULTS: The prevalence of a sedentary lifestyle was 48.7% (95%CI: 46.5 to 50.8) in boys and 67.5% (95%CI: 65.6 to 69.5) in girls (P < 0.001). The independent variable presenting the strongest association with physical activity level was the weekly frequency with which the adolescent met friends outside of school. The ethnographic study showed that boys have more social and family support to engage in physical activities in adolescence, but that many parents associate poor school performance with the amount of time spent outside the home. CONCLUSIONS: This study suggests that physical activity is often viewed as competing with family values in adolescence. This perspective should be explored in future studies and discussed with families so that physical activity may be adopted as a cultural norm
SN - 1020-4989
AD - Universidade Federal de Pelotas, Programa de Pos-Graduacao em Epidemiologia, Pelotas, RS, Brazil. hdgs@uol.com.br
UR - 18078586
ER -

TY - JOUR
ID - 1091
T1 - Outbreak of cutaneous larva migrans at a children's camp--Miami, Florida, 2006
A1 - Centers for Disease Control and Prevention (CDC)
Y1 - 2007/12/14/
N1 - Centers for Disease Control and Prevention (CDC)
MMWR. Morbidity and mortality weekly report
ne8, 7802429
IM
Journal Article
English
KW - MEDLINE
KW - Adolescent
KW - Animals
KW - Cats
KW - Child
KW - Preschool

KW - Disease Outbreaks
KW - Feces
KW - Florida/ep [Epidemiology]
KW - Humans
KW - Larva Migrans/ep [Epidemiology]
KW - Larva Migrans/tm [Transmission]
KW - Recreation
KW - Silicon Dioxide
RP - NOT IN FILE
SP - 1285
EP - 1287
JF - MMWR - Morbidity & Mortality Weekly Report
JA - MMWR Morb Mortal Wkly Rep
VL - 56
IS - 49
CY - United States
N2 - On July 19, 2006, the director of a children's aquatic sports day camp notified the Miami-Dade County Health Department (MDCHD) of three campers who had received a diagnosis of cutaneous larva migrans (CLM), or "creeping eruption," a skin condition typically caused by dog or cat hookworm larvae of the genus Ancylostoma. MDCHD conducted an investigation to determine the source and magnitude of the outbreak and prevent additional illness. This report summarizes the results of that investigation, which identified exposure to cat feces in a playground sandbox as the likely source of infection. Although CLM outbreaks are reported rarely to the Florida Department of Health, evidence indicates that CLM is a potential health hazard in Florida. This disease cluster highlights the importance of appropriate environmental hygiene practices and education in preventing CLM
SN - 1545-861X
UR - 18075486
ER -

TY - JOUR
ID - 1092
T1 - Physical activity levels of children in special schools
A1 - Sit,C.H.
A1 - McManus,A.
A1 - McKenzie,T.L.
A1 - Lian,J.
Y1 - 2007/12//
N1 - Sit, Cindy H P. McManus, Alison. McKenzie, Thomas L. Lian, John
Preventive medicine
pm4, 0322116
IM
Journal Article. Research Support, Non-U.S. Gov't
English
KW - MEDLINE
KW - Child
KW - Cross-Sectional Studies
KW - Disabled Children/sn [Statistics & Numerical Data]
KW - Education
KW - Special/sn [Statistics & Numerical Data]
KW - Exercise
KW - Female
KW - Health Services Needs and Demand
KW - Health Surveys
KW - Hong Kong
KW - Humans
KW - Male
KW - Physical Education and Training/sn [Statistics & Numerical Data]

RP - NOT IN FILE
SP - 424
EP - 431
JF - Preventive Medicine
JA - Prev Med
VL - 45
IS - 6
CY - United States
N2 - OBJECTIVE: Children's physical activity (PA) has been studied extensively, but little information is available on those with disabilities. We sought to examine the PA of children with disabilities during physical education (PE) and recess while simultaneously documenting environmental conditions. METHOD: Five schools designed for students with four types of special needs (physical disability, mild intellectual disability, hearing impairment, and visual impairment) participated. We used the System for Observing Fitness Instruction Time (SOFIT) to code the PA of children in grades 4 to 6 during both PE and recess and to document teacher behavior and lesson context in PE. Observations were conducted during 2 school days over a 2-week period. RESULTS: Children accrued little moderate-to-vigorous physical activity (MVPA) during PE (7.8 min) and recess (8.9 min). Activity levels varied across disability types, with differences attributed to lesson context and teacher behavior. Children with physical disabilities were the least active during both PE and recess. CONCLUSIONS: Children with disabilities accrue little PA at school. Increased PE frequency and lesson intensity, more PA opportunities during non-structured school time, and collaborations with home and community agencies are needed to reach PA recommendations
SN - 0091-7435
AD - The University of Hong Kong, Institute of Human Performance, Pokfulam, Hong Kong. sithp@hku.hk
UR - 17337044
ER -

TY - JOUR
ID - 1093
T1 - Sports participation outside school in total physical activity of children
A1 - Saar,M.
A1 - Jurimae,T.
Y1 - 2007/10//
N1 - Saar, Meeli. Jurimae, Toivo
Perceptual and motor skills
ozb, 0401131
IM
Journal Article
English
KW - MEDLINE
KW - Adolescent
KW - Age Factors
KW - Aptitude
KW - Body Composition
KW - Child
KW - Competitive Behavior
KW - Estonia
KW - Exercise/px [Psychology]
KW - Female
KW - Humans
KW - Leisure Activities
KW - Male
KW - Personal Satisfaction
KW - Physical Fitness/px [Psychology]
KW - Self Concept
KW - Sports/px [Psychology]
KW - Statistics as Topic
RP - NOT IN FILE

SP - 559
EP - 562
JF - Perceptual & Motor Skills
JA - Percept.Mot.Skills
VL - 105
IS - 2
CY - United States
N2 - This study of associations of sports participation with perceived and actual physical fitness, and total physical activity for 525 10- to 17-yr.-old boys and girls in groups of 10-11-yr. (56 boys and 64 girls), 12-13-yr. (68 boys and 68 girls), 14-15-yr. (70 boys and 71 girls), and 16-17-yr. (68 boys and 60 girls) was based on the Physical Activity Index derived from a questionnaire by Telama, Leskinen, and Young, and self-perceived endurance, strength, flexibility, and body composition. Questions about satisfaction with physical activity, participation in organized physical activity and competitions, or watching competitions were asked. Two EUROFIT tests were used, the 20-m endurance shuttle-run and sit-and-reach, plus the sum of 9 skinfold thicknesses. Children who participated in organized physical activity and in competitions had a higher Physical Activity Index. Passive watching of competitions was not related to children's physical activity or their perceived or measured motor abilities
SN - 0031-5125
AD - Estonian Centre of Behavioural and Health Sciences, University of Tartu
UR - 18065079
ER -

TY - JOUR
ID - 1094
T1 - Relationships of Indoor, Outdoor, and Personal Air (RIOPA): part II. Analyses of concentrations of particulate matter species
A1 - Turpin,B.J.
A1 - Weisel,C.P.
A1 - Morandi,M.
A1 - Colome,S.
A1 - Stock,T.
A1 - Eisenreich,S.
A1 - Buckley,B.
Y1 - 2007/08//
N1 - Turpin, Barbara J. Weisel, Clifford P. Morandi, Maria. Colome, Steven. Stock, Thomas. Eisenreich, Steven. Buckley, Brian
Research report (Health Effects Institute)
8812230, ah8, 8812230
IM
Journal Article. Research Support, U.S. Gov't, Non-P.H.S.
English
KW - MEDLINE
KW - Adolescent
KW - Adult
KW - Aged
KW - 80 and over
KW - Air Pollutants/an [Analysis]
KW - Air Pollution
KW - Indoor/an [Analysis]
KW - Child
KW - Environmental Monitoring
KW - Female
KW - Humans
KW - Inhalation Exposure/an [Analysis]
KW - Los Angeles
KW - Male
KW - Middle Aged

KW - New Jersey
KW - Organic Chemicals/an [Analysis]
KW - Particle Size
KW - Particulate Matter/an [Analysis]
KW - Questionnaires
KW - Spectroscopy
KW - Fourier Transform Infrared
KW - Texas
KW - Urban Health
RP - NOT IN FILE
SP - 1
EP - 77
JF - Research Report - Health Effects Institute
JA - Res Rep Health Eff.Inst
IS - 130 Pt 2
CY - United States
N2 - During the study Relationships of Indoor, Outdoor, and Personal Air (RIOPA*), 48-hour integrated indoor, outdoor, and personal air samples were collected between summer 1999 and spring 2001 in three different areas of the United States: Elizabeth NJ, Houston TX, and Los Angeles County CA. Air samples suitable for analyzing particulate matter 2.5 microm or smaller in aerodynamic diameter (PM2.5) were collected in 219 homes (twice in 169 homes). Indoor and outdoor air samples suitable for gas-phase and particle-phase organic analyses were collected in 152 homes (twice in 132 homes). Samples or subsets of samples were analyzed for PM2.5 mass, organic functional groups, elements, organic carbon (OC), elemental carbon (EC), gas-phase and particle-phase polycyclic aromatic hydrocarbons (PAHs), and chlordanes. Air exchange rate (AER), temperature, and relative humidity were measured for each residence; questionnaire data and time-activity information were collected from the participants. Median indoor, outdoor, and personal PM2.5 mass concentrations were 14.4, 15.5, and 31.4 microg/m3, respectively. Personal PM2.5 concentrations were significantly higher and more variable than indoor and outdoor concentrations. Several approaches were applied to quantify indoor PM2.5 of ambient (outdoor) and nonambient (indoor) origin, some using PM2.5 mass concentrations and others using PM2.5 species concentrations. PM of outdoor origin was estimated in three ways using increasingly accurate assumptions. Comparing estimates from the three approaches enabled us to quantify several types of errors that may be introduced when central-site PM concentrations are used as surrogate estimates for PM exposure. Estimates made using individual measurements produced broader distributions and higher means than those made using a single infiltration factor for all homes and days. The best estimate (produced by the robust regression approach) of the mean contribution of outdoor PM2.5 to the indoor mass concentration was 73% and to personal exposure was 26%. Possible implications of exposure error for epidemiologic assessments of PM are discussed below. Organic particulate matter was the major constituent of PM2.5 generated indoors. After correcting for artifacts, it constituted 48%, 55%, and 61% of PM2.5 mass inside study homes in Los Angeles, Elizabeth, and Houston, respectively. At least 40% but probably closer to 75% of this organic matter, on average, was emitted or formed indoors. Functional group analysis provided some insights into the composition and properties of the indoor-generated organic PM2.5. Chlordane, a very minor but mutagenic semivolatile organic mixture previously used as a termiticide, was found to be mostly of indoor origin. High emission rates were most frequently found in homes built from 1945 to 1959. Analysis of the change in gas-particle partitioning during transport of outdoor PAHs to indoor environments illustrated that chemical thermodynamics can alter the concentration and composition of outdoor PM as it is transported indoors. (This has been previously noted for nitrate [Lunden et al 2003].) In epidemiologic studies that rely on central-site monitoring data, such transformations may result in measurement error, and this possibility warrants further investigation
SN - 1041-5505
AD - Department of Environmental Sciences, Rutgers University, New Brunswick, NJ 08901, USA. turpin@envsci.rutgers.edu
UR - 18064946
ER -

TY - JOUR
ID - 1095
T1 - School accidents in Austria

A1 - Schalamon,J.
A1 - Eberl,R.
A1 - Ainoedhofer,H.
A1 - Singer,G.
A1 - Spitzer,P.
A1 - Mayr,J.
A1 - Schober,P.H.
A1 - Hoellwarth,M.E.
Y1 - 2007/09//
N1 - Schalamon, Johannes. Eberl, Robert. Ainoedhofer, Herwig. Singer, Georg. Spitzer, Peter. Mayr, Johannes. Schober, Peter H. Hoellwarth, Michael E
Pediatric surgery international
cq0, 8609169
IM
Journal Article. Research Support, Non-U.S. Gov't
English
KW - MEDLINE
KW - Accidents/sn [Statistics & Numerical Data]
KW - Adolescent
KW - Age Distribution
KW - Athletic Injuries/ep [Epidemiology]
KW - Austria/ep [Epidemiology]
KW - Child
KW - Preschool
KW - Female
KW - Humans
KW - Incidence
KW - Infant
KW - Newborn
KW - Male
KW - Prospective Studies
KW - Questionnaires
KW - Schools/sn [Statistics & Numerical Data]
KW - Severity of Illness Index
KW - Sex Distribution
KW - Violence/sn [Statistics & Numerical Data]
RP - NOT IN FILE
SP - 861
EP - 865
JF - Pediatric Surgery International
JA - Pediatr Surg Int
VL - 23
IS - 9
CY - Germany
N2 - The aim of this study was to obtain information about the mechanisms and types of injuries in school in Austria. Children between 0 and 18 years of age presenting with injuries at the trauma outpatient in the Department of Pediatric Surgery in Graz and six participating hospitals in Austria were evaluated over a 2-year prospective survey. A total of 28,983 pediatric trauma cases were registered. Personal data, site of the accident, circumstances and mechanisms of accident and the related diagnosis were evaluated. At the Department of Pediatric Surgery in Graz 21,582 questionnaires were completed, out of which 2,148 children had school accidents (10%). The remaining 7,401 questionnaires from peripheral hospitals included 890 school accidents (12%). The male/female ratio was 3:2. In general, sport injuries were a predominant cause of severe trauma (42% severe injuries), compared with other activities in and outside of the school building (26% severe injuries). Injuries during ball-sports contributed to 44% of severe injuries. The upper extremity was most frequently injured (34%), followed by lower extremity (32%), head and neck area (26%) and injuries to thorax and abdomen (8%). Half of all school related injuries occur in children between 10 and 13 years of age. There are typical gender related mechanisms of accident: Boys get frequently injured during soccer, violence, and

collisions in and outside of the school building and during craft work. Girls have the highest risk of injuries at ball sports other than soccer
SN - 0179-0358
AD - Department of Pediatric Surgery, Medical University of Graz, Auenbruggerplatz 34, 8036 Graz, Austria. Johannes.schalamon@meduni-graz.at
UR - 17611761
ER -

TY - JOUR
ID - 1096
T1 - Preschool children's sickness absenteeism from Norwegian regular and outdoor day care centres: a comparative study
A1 - Moen,K.H.
A1 - Bakke,H.K.
A1 - Bakke,O.
A1 - Fors,E.A.
Y1 - 2007///
N1 - Moen, Kiari H. Bakke, Hjordis K. Bakke, Oyvind. Fors, Egil A
Scandinavian journal of public health
dew, 100883503
IM
Journal Article
English
KW - MEDLINE
KW - Air Pollution
KW - Indoor/ae [Adverse Effects]
KW - Child Day Care Centers
KW - Child
KW - Preschool
KW - Environmental Exposure/ae [Adverse Effects]
KW - Female
KW - Health Status
KW - Humans
KW - Male
KW - Morbidity
KW - Norway/ep [Epidemiology]
KW - Play and Playthings
KW - Questionnaires
RP - NOT IN FILE
SP - 490
EP - 496
JF - Scandinavian Journal of Public Health
JA - Scand J Public Health
VL - 35
IS - 5
CY - Sweden
N2 - AIMS: To examine whether there are any differences in sickness absenteeism between children in outdoor day care centres and regular day care centres and also to investigate whether other variables predict sickness absenteeism. METHODS: Data on sickness absence during a 4-week period together with several explanatory variables of 531 children in 32 regular and 37 outdoor day care centres were collected and included in the analysis. The data were analysed by generalized linear modelling. RESULTS: The overall frequency of sickness absence was 5.1%. There was no general significant difference between sickness absenteeism in regular and outdoor day care centres. Of the other possible explanatory variables only two were found to contribute significantly: age, with a negative relationship, and the interaction effect of a child with a chronic disease or disability going to an outdoor day care centre, with a positive relationship. CONCLUSIONS: The present study indicates that sickness absenteeism of a child without a chronic disease or disability is not affected by whether the child attends a regular or an outdoor day care centre. There seem to be no health benefits for children with

chronic diseases or disabilities to attend outdoor day care centres--there is in fact evidence that sickness absence for those children is higher in outdoor centres
SN - 1403-4948
AD - Queen Maud's College of Early Childhood Education, Trondheim, Norway. khm@dmmh.no
UR - 17852981
ER -

TY - JOUR
ID - 1097
T1 - The effects of increasing outdoor play time on physical activity in Latino preschool children
A1 - Alhassan,S.
A1 - Sirard,J.R.
A1 - Robinson,T.N.
Y1 - 2007///
N1 - Alhassan, Sofiya. Sirard, John R. Robinson, Thomas N
International journal of pediatric obesity : IJPO : an official journal of the International Association for the Study of Obesity
101256330
IM
Journal Article. Randomized Controlled Trial. Research Support, N.I.H., Extramural. Research Support, Non-U.S. Gov't
English
KW - MEDLINE
KW - Body Mass Index
KW - Body Weight
KW - California
KW - Child
KW - Preschool
KW - Exercise/ph [Physiology]
KW - Family
KW - Female
KW - Hispanic Americans
KW - Humans
KW - Male
KW - Motor Activity
KW - Play and Playthings
KW - Poverty
KW - Sports
KW - Time Factors
RP - NOT IN FILE
SP - 153
EP - 158
JF - International Journal of Pediatric Obesity
JA - Int J Pediatr Obes
VL - 2
IS - 3
CY - England
N2 - OBJECTIVE: A randomized controlled pilot study to test the hypothesis that increasing preschool children's outdoor free play time increases their daily physical activity levels. METHODS: Physical activity was assessed by accelerometers for four consecutive school days in thirty-two Latino children (3.6+/-0.5 years) attending a preschool for low-income families. After two days of baseline physical activity assessment, participants were randomly assigned to an intervention (RECESS; n =17) or control (CON; n =15) group. The RECESS group received two additional 30-minute periods of outdoor free play time per day for two days. The CON group followed their normal classroom schedule. Between group differences in physical activity variables were tested with a Wilcoxon rank-sum test. RESULTS: There were no statistically significant differences between groups in changes from baseline in average total daily (CON, 48.2+/-114.5; RECESS, 58.2+/-74.6) and during school day (CON, 64.6+/-181.9; RECESS, 59.7+/-79.1) counts per minute, or total daily (CON, 0.4+/-

1.3; RECESS, 0.3+/-0.8) and during school day (CON, 0.6+/-2.1; RECESS, 0.5+/-0.8) percent of time spent in moderate to vigorous physical activity. CONCLUSIONS: Substantially increasing preschoolers' outdoor free play time did not increase their physical activity levels
SN - 1747-7166
AD - Stanford Prevention Research Center, Department of Medicine and Division of General Pediatrics, University School of Medicine, Stanford, California 94305-5705, USA. sofiya.alhassan@stanford.edu
UR - 17852547
ER -

TY - JOUR
ID - 1098
T1 - Effect of a rapid response team on hospital-wide mortality and code rates outside the ICU in a Children's Hospital
A1 - Sharek,P.J.
A1 - Parast,L.M.
A1 - Leong,K.
A1 - Coombs,J.
A1 - Earnest,K.
A1 - Sullivan,J.
A1 - Frankel,L.R.
A1 - Roth,S.J.
Y1 - 2007/11/21/
N1 - Sharek, Paul J. Parast, Layla M. Leong, Kit. Coombs, Jodi. Earnest, Karla. Sullivan, Jill. Frankel, Lorry R. Roth, Stephen J
JAMA : the journal of the American Medical Association
7501160
AIM, IM
Journal Article
English
KW - MEDLINE
KW - Adolescent
KW - Cardiopulmonary Resuscitation
KW - Child
KW - Preschool
KW - Cohort Studies
KW - Female
KW - Heart Arrest/mo [Mortality]
KW - Heart Arrest/th [Therapy]
KW - Hospital Mortality
KW - Hospitals
KW - Pediatric/og [Organization & Administration]
KW - Pediatric/sn [Statistics & Numerical Data]
KW - Humans
KW - Infant
KW - Intensive Care/og [Organization & Administration]
KW - Male
KW - Patient Care Team
RP - NOT IN FILE
SP - 2267
EP - 2274
JF - JAMA
VL - 298
IS - 19
CY - United States
N2 - CONTEXT: Introduction of a rapid response team (RRT) has been shown to decrease mortality and cardiopulmonary arrests outside of the intensive care unit (ICU) in adult inpatients. No published studies to date show significant reductions in mortality or cardiopulmonary arrests in pediatric inpatients. OBJECTIVE: To

determine the effect on hospital-wide mortality rates and code rates outside of the ICU setting after RRT implementation at an academic children's hospital. DESIGN, SETTING, AND PARTICIPANTS: A cohort study design with historical controls at a 264-bed, free-standing, quaternary care academic children's hospital. Pediatric inpatients who spent at least 1 day on a medical or surgical ward between January 1, 2001, and March 31, 2007, were included. A total of 22,037 patient admissions and 102,537 patient-days were evaluated preintervention (before September 1, 2005), and 7257 patient admissions and 34,420 patient-days were evaluated postintervention (on or after September 1, 2005). INTERVENTION: The RRT included a pediatric ICU-trained fellow or attending physician, ICU nurse, ICU respiratory therapist, and nursing supervisor. This team was activated using standard criteria and was available at all times to assess, treat, and triage decompensating pediatric inpatients. MAIN OUTCOME MEASURES: Hospital-wide mortality rates and code (respiratory and cardiopulmonary arrests) rates outside of the ICU setting. All outcomes were adjusted for case mix index values. RESULTS: After RRT implementation, the mean monthly mortality rate decreased by 18% (1.01 to 0.83 deaths per 100 discharges; 95% confidence interval [CI], 5%-30%; P = .007), the mean monthly code rate per 1000 admissions decreased by 71.7% (2.45 to 0.69 codes per 1000 admissions), and the mean monthly code rate per 1000 patient-days decreased by 71.2% (0.52 to 0.15 codes per 1000 patient-days). The estimated code rate per 1000 admissions for the postintervention group was 0.29 times that for the preintervention group (95% likelihood ratio CI, 0.10-0.65; P = .008), and the estimated code rate per 1000 patient-days for the postintervention group was 0.28 times that for the preintervention group (95% likelihood ratio CI, 0.10-0.64; P = .007). CONCLUSION: Implementation of an RRT was associated with a statistically significant reduction in hospital-wide mortality rate and code rate outside of the pediatric ICU setting
SN - 1538-3598
AD - Department of Pediatrics, Stanford University School of Medicine, Palo Alto, CA 94304, USA. psharek@lpch.org
UR - 18029830
ER -

TY - JOUR
ID - 1099
T1 - [Physical activity in school for 11 - 12 year-olds]. [Norwegian]
A1 - Gronningsaeter,I.
A1 - Hallas,O.
A1 - Kristiansen,T.
A1 - Naevdal,F.
Y1 - 2007/11/15/
N1 - Gronningsaeter, Ivar. Hallas, Oddrun. Kristiansen, Torbjorn. Naevdal, Folkvard
Tidsskrift for den Norske laegeforening : tidsskrift for praktisk medicin, ny raekke
0413423, 101086543, vrv
IM
Comparative Study. English Abstract. Journal Article
Norwegian
KW - MEDLINE
KW - Child
KW - Child Welfare
KW - Exercise/ph [Physiology]
KW - Exercise
KW - Heart Rate/ph [Physiology]
KW - Humans
KW - Physical Education and Training
KW - Physical Fitness/ph [Physiology]
KW - Schools
RP - NOT IN FILE
SP - 2927
EP - 2929
JF - Tidsskrift for Den Norske Laegeforening
JA - Tidsskr.Nor Laegeforen
VL - 127
IS - 22

CY - Norway
N2 - BACKGROUND: "Outdoor school" is a method, which moves the teaching out into the open. There has been little research into the relationship between outdoor school, physical activity and improved fitness. MATERIAL AND METHODS: We have compared the level of activity (measured as heart rate) in a class of sixth-grade pupils during an ordinary school day and a day outdoors. The pupils' heart rates were measured during the entire school day. RESULTS: The average heart rate for the pupils was 104 beats/minute during an ordinary school day and 136 beats/minute during a day outdoors. The average percentage of time with a heart rate >or= 160 beats/minute was 5 % for an ordinary school day and 18 % for a day outdoors (p < 0.001). INTERPRETATION: Outdoor school can result in a significant increase in physical activity compared to the level on ordinary school days. Thus, the organisation of outdoor school days may have a positive effect on pupils' aerobic capacity, which is an important factor in physical fitness
SN - 0807-7096
AD - igro@hib.no
UR - 18026238
ER -

TY - JOUR
ID - 1100
T1 - Does short sleep duration favor abdominal adiposity in children?
A1 - Chaput,J.P.
A1 - Tremblay,A.
Y1 - 2007///
N1 - Chaput, Jean-Philippe. Tremblay, Angelo
International journal of pediatric obesity : IJPO : an official journal of the International Association for the Study of Obesity
101256330
IM
Journal Article. Research Support, Non-U.S. Gov't
English
KW - MEDLINE
KW - Abdomen/ah [Anatomy & Histology]
KW - Adipose Tissue/ah [Anatomy & Histology]
KW - Adult
KW - Body Mass Index
KW - Child
KW - Female
KW - Humans
KW - Life Style
KW - Male
KW - Obesity
KW - Overweight
KW - Parents
KW - Questionnaires
KW - Regression Analysis
KW - Sleep/ph [Physiology]
KW - Thinness
KW - Time Factors
RP - NOT IN FILE
SP - 188
EP - 191
JF - International Journal of Pediatric Obesity
JA - Int J Pediatr Obes
VL - 2
IS - 3
CY - England
N2 - The main aim of this cross-sectional study was to determine whether the increased body mass index (BMI) characterizing short-duration sleeping children is related to an increased predisposition to abdominal adiposity.

A total of 422 children (211 boys and 211 girls) involved in the "Quebec en Forme" Project were tested for body weight, height, waist circumference, and sleep duration. As there was no gender interaction with the other factors, a partial regression of waist circumference on hours of sleep was performed for both genders combined, adjusting for age, sex, BMI, parental obesity, parental education, total annual family income, frequency of taking breakfast, watching television, playing videogames, computer use, and frequency of practicing sports activities outside of school. Sleep duration had an independent effect on waist circumference, with the correlation between these variables remaining significant after adjustment for BMI and the several other covariates (r=- 0.17, p<0.001). In conclusion, these results suggest that short sleep duration favors abdominal adiposity in children. This finding is of particular concern since abdominal obesity is an important feature of the metabolic syndrome
SN - 1747-7166
AD - Division of Kinesiology, Department of Social and Preventive Medicine, Faculty of Medicine, Laval University, Quebec City, Quebec, Canada
UR - 17999284
ER -

TY - JOUR
ID - 1101
T1 - The Light Time-Use Diary and preschool activity patterns: exploratory study
A1 - Tey,C.
A1 - Wake,M.
A1 - Campbell,M.
A1 - Hampton,A.
A1 - Williams,J.
Y1 - 2007///
N1 - Tey, Corinne. Wake, Melissa. Campbell, Michele. Hampton, Anne. Williams, Joanne
International journal of pediatric obesity : IJPO : an official journal of the International Association for the Study of Obesity
101256330
IM
Journal Article. Research Support, Non-U.S. Gov't
English
KW - MEDLINE
KW - Body Height
KW - Body Mass Index
KW - Body Weight
KW - Child
KW - Preschool
KW - Cohort Studies
KW - Female
KW - Humans
KW - Life Style
KW - Male
KW - Medical Records
KW - Parent-Child Relations
KW - Time Factors
KW - Victoria
RP - NOT IN FILE
SP - 167
EP - 173
JF - International Journal of Pediatric Obesity
JA - Int J Pediatr Obes
VL - 2
IS - 3
CY - England
N2 - AIM: To conduct an exploratory study of time-use patterns in Australian 5-year-old children, and to pilot the novel Children's Light Time-Use Diary as a potential tool for investigating relationships between children's

time-use and weight status. METHODS: Subjects for the present cross-sectional study were drawn from an established longitudinal cohort and included eighty-four 5-year-old Australian children (36 males) originally recruited as infants in three local government areas of Melbourne. Children were weighed and measured, and body mass index (BMI; kg/m(2)) calculated. Over three to four complete 24-hour periods, parents completed the Children's Light Time-Use Diary to record their child's activities in 15-minute blocks and details about the context in which the activities took place, selecting from a list of predetermined options. RESULTS: The children studied were largely sedentary, with television viewing the most time-consuming activity outside sleep. Only 49% of children spent any time walking for transport or pleasure. Children spent a median of 71% of their time in activities that were likely to be physically active when outdoors, compared with 3% when indoors, but averaged only 110 minutes/day outdoors (excluding passive transport). The 11 overweight/obese children watched significantly more television than non-overweight children. CONCLUSION: The Children's Light Time-Use Diary appears to be a practical and informative tool, which may complement accelerometry as a tool relevant to future studies of the determinants of child overweight/obesity. Further validation studies and larger research trials seem warranted
SN - 1747-7166
AD - Centre for Community Child Health, University of Melbourne & Murdoch Childrens Research Institute, Royal Children's Hospital, Parkville, VIC, Australia
UR - 17999282
ER -

TY - JOUR
ID - 1102
T1 - Risk factors associated with St. Louis encephalitis seroprevalence in two populations from Cordoba, Argentina
A1 - Spinsanti,L.
A1 - Farias,A.
A1 - Aguilar,J.
A1 - Diaz,M.P.
A1 - Ghisiglieri,S.
A1 - Bustos,M.A.
A1 - Vilches,N.
A1 - Gonzalez,B.
A1 - Contigiani,M.
Y1 - 2007/12//
N1 - Spinsanti, L. Farias, A. Aguilar, J. Diaz, M P. Ghisiglieri, S. Bustos, M A. Vilches, N. Gonzalez, B. Contigiani, M
Transactions of the Royal Society of Tropical Medicine and Hygiene
wbu, 7506129
IM
Journal Article. Multicenter Study. Research Support, Non-U.S. Gov't
English
KW - MEDLINE
KW - Adolescent
KW - Adult
KW - Aged
KW - Animals
KW - Antibodies
KW - Viral/bl [Blood]
KW - Argentina/ep [Epidemiology]
KW - Child
KW - Encephalitis Virus
KW - St.Louis/ip [Isolation & Purification]
KW - Encephalitis
KW - St.Louis/ep [Epidemiology]
KW - Female
KW - Humans
KW - Logistic Models

KW - Male
KW - Middle Aged
KW - Prevalence
KW - Risk Factors
KW - Seroepidemiologic Studies
RP - NOT IN FILE
SP - 1248
EP - 1252
JF - Transactions of the Royal Society of Tropical Medicine & Hygiene
JA - Trans R Soc Trop Med Hyg
VL - 101
IS - 12
CY - England
N2 - St. Louis encephalitis virus (SLEV) is endemic in Argentina. The first outbreak was recorded in Cordoba during the late summer of 2005. This study analyzes the association between social and demographic factors and other variables related to exposure to mosquitoes with SLEV infection in 264 individuals who attended two health centers in the city of Cordoba during the period December 2004-January 2005. Multiple logistic regression models were used to estimate the risk coefficients. The seroprevalence was 12.9%, similar to that in previous reports. Risks of infection were associated with the presence of garbage dumps near dwellings, the practice of outdoor activities at night and place of residence. Risk for older people (60-80 years old) was moderate. The identification of risk factors related to SLEV infection would be useful to improve programs for vector control and community health
SN - 0035-9203
AD - Instituto de Virologia Dr. J.M. Vanella, Facultad de Ciencias Medicas, Universidad Nacional de Cordoba, calle Enfermera Gordillo Gomez s/n, 5016 Cordoba, Argentina
UR - 17915268
ER -

TY - JOUR
ID - 1103
T1 - The use of acacia gum in end stage renal failure. [Review] [20 refs]
A1 - Al Mosawi,A.J.
Y1 - 2007/10//
N1 - Al Mosawi, Aamir Jalal
Journal of tropical pediatrics
kaw, 8010948
IM
Case Reports. Journal Article. Review
English
KW - MEDLINE
KW - Child
KW - Child Development
KW - Developing Countries
KW - Diet
KW - Protein-Restricted
KW - Dietary Supplements
KW - Female
KW - Gum Arabic/tu [Therapeutic Use]
KW - Humans
KW - Kidney Failure
KW - Chronic/dt [Drug Therapy]
KW - Chronic/th [Therapy]
KW - Renal Dialysis
KW - Treatment Outcome
RP - NOT IN FILE
SP - 362
EP - 365

JF - Journal of Tropical Pediatrics
JA - J Trop Pediatr
VL - 53
IS - 5
CY - England
N2 - OBJECTIVE: To describe a new model for the management of end-stage renal failure (ESRF) associated with longest period of dialysis freedom (4-year) achieved with this novel form of renal replacement therapy (RRT).The research was done in accordance with Helsinki declaration and approved the scientific committee in the hospital. Conservative management of chronic renal failure (CRF) can only be successful in nonterminal CRF, and patients with ESRF cannot sustain life in the absence of renal replacement therapy. A new regimen combining the traditional conservative management of CRF (dietary and pharmacologic) with addition of Acacia gum (AG) 1 g kg(-1) per day has been reported to provide patients with ESRF dialysis freedom. An 11-year-old girl with ESRF initially required four sessions of intermittent peritoneal dialysis to control uremic symptoms despite conservative measures. The parents refused further treatment by dialysis. Thereafter, she was managed with a new regimen combining the traditional conservative management of CRF with addition of AG. Four year dialysis and improved well-being was achieved. The chronicity of her illness was confirmed by the presence of small contracted kidneys, a finding that has not changed during the subsequent follow-up. During these 4 years she continued experiencing improved well-being and good participation in outdoor activities, had never been acidotic or experienced significant uremic symptoms. CONCLUSION: This is the longest period of dialysis freedom reported in children with ESRF. [References: 20]
SN - 0142-6338
AD - Head of the Department of Pediatrics, University Hospital in Al kadhimiyia, Po Box 70025 Baghdad, Iraq. almosawiAJ@yahoo.com
UR - 17517814
ER -

TY - JOUR
ID - 1104
T1 - Nonspecific verbal cues alleviate forgetting by young children
A1 - Morgan,K.
A1 - Hayne,H.
Y1 - 2007/11//
N1 - Morgan, Kirstie. Hayne, Harlene
Developmental science
9814574
IM
Journal Article. Research Support, Non-U.S. Gov't
English
KW - MEDLINE
KW - Age Factors
KW - Child
KW - Preschool
KW - Cues
KW - Humans
KW - Memory
KW - Mental Recall
KW - Time Factors
RP - NOT IN FILE
SP - 727
EP - 733
JF - Developmental Science
JA - Dev
VL - 10
IS - 6
CY - England
N2 - Verbal reminders play a pervasive role in memory retrieval by human adults. In fact, relatively nonspecific verbal information (e.g. 'Remember the last time we ate at that restaurant?') will often cue vivid recollections of

a past event even when presented outside the original encoding context. Although research has shown that memory retrieval by young children can be initiated by physical cues and by highly specific verbal cues, the effect of less specific verbal cues is not known. Using a Visual Recognition Memory (VRM) procedure, we examined the effect of nonspecific verbal cues on memory retrieval by 4-year-old children. Our findings showed that nonspecific verbal cues were as effective as highly specific nonverbal cues in facilitating memory retrieval after a 2-week delay. We conclude that, at least by 4 years of age, children are able to use nonspecific verbal reminders to cue memory retrieval, and that the VRM paradigm may be particularly valuable in examining the age at which this initially occurs
SN - 1363-755X
AD - Department of Psychology, University of Otago, Dunedin, New Zealand. kmorgan@psy.otago.ac.nz
UR - 17973788
ER -

TY - JOUR
ID - 1105
T1 - Trends in physical activity behaviours and attitudes among South Australian youth between 1985 and 2004
A1 - Lewis,N.
A1 - Dollman,J.
A1 - Dale,M.
Y1 - 2007/12//
N1 - Lewis, Nicole. Dollman, James. Dale, Michael
Journal of science and medicine in sport / Sports Medicine Australia
c54, 9812598
IM
Comparative Study. Journal Article
English
KW - MEDLINE
KW - Adolescent
KW - Attitude
KW - Bicycling/px [Psychology]
KW - Child
KW - Exercise/px [Psychology]
KW - Female
KW - Health Behavior
KW - Humans
KW - Leisure Activities/px [Psychology]
KW - Male
KW - Motor Activity
KW - Physical Education and Training/td [Trends]
KW - Questionnaires
KW - South Australia
KW - Sports/td [Trends]
KW - Walking/px [Psychology]
RP - NOT IN FILE
SP - 418
EP - 427
JF - Journal of Science & Medicine in Sport
JA - J Sci Med Sport
VL - 10
IS - 6
CY - Australia
N2 - Rising youth obesity is a serious public health concern. There is a widespread view that declining physical activity is contributing to this trend. A total of 929 young South Australians (age=9-15 years) were surveyed in 1985 and 2004 on usual physical activity in several contexts, including attitudes to physical activity. Eight of 10 South Australian schools participating in the 1985 Schools Health and Fitness Survey were revisited in 2004. Comparisons were made on: organised sport, active transport, physical education (PE), playground activity, vigorous physical activity (VPA), total leisure-time physical activity (LTPA), and attitudes to PE and school

sport. The questionnaire and method of administration were identical in both surveys. There were no differences between surveys in club and school sport participation, walking to school, and reported enjoyment of PE and school sport. In 2004 fewer children rode to school, but PE classes were more frequent. The percentage of children who 'sit and talk' during school breaks had increased, with a decreased percentage of older girls who 'run around' during school breaks. There was a significantly higher LTPA in MET.min in 2004 for boys, which was particularly evident at higher percentiles. There were no changes in mean or distribution of LTPA for the whole sample or girls. The percentage of respondents reporting >/=3 bouts of VPA in the previous week rose from 51% (1985) to 76% (2004). There is no consistent evidence of declining physical activity among South Australian youth. It is apparent that physical activity in some contexts has declined, while in other contexts levels are the same or higher than in 1985. This underscores the complex nature of physical activity and the influences on this behaviour
SN - 1440-2440
AD - School of Health Sciences, University of South Australia, Australia
UR - 17336154
ER -

TY - JOUR
ID - 1106
T1 - The occurrence and seasonal variation of accelerant-related burn injuries in central Florida
A1 - Rainey,S.
A1 - Cruse,C.W.
A1 - Smith,J.S.
A1 - Smith,K.R.
A1 - Jones,D.
A1 - Cobb,S.
Y1 - 2007/09//
N1 - Rainey, Susan. Cruse, C Wayne. Smith, Jackie S. Smith, Kirk R. Jones, Dawn. Cobb, Sarah
Journal of burn care & research : official publication of the American Burn Association
101262774
IM
Journal Article
English
KW - MEDLINE
KW - Adolescent
KW - Adult
KW - Aged
KW - 80 and over
KW - Burn Units
KW - Burns/ep [Epidemiology]
KW - Burns/et [Etiology]
KW - Child
KW - Preschool
KW - Female
KW - Fires
KW - Florida/ep [Epidemiology]
KW - Humans
KW - Male
KW - Middle Aged
KW - Registries
KW - Retrospective Studies
KW - Risk Assessment
KW - Risk Factors
KW - Risk-Taking
KW - Seasons
KW - Time Factors
RP - NOT IN FILE
SP - 675

EP - 680
JF - Journal of Burn Care & Research
JA - J Burn Care Res
VL - 28
IS - 5
CY - United States
N2 - Accidental burn injuries result in significant economic and public health burdens. The inappropriate use of gasoline and other accelerants has been identified in many studies as dangerous, yet it remains an all-too-common practice resulting in a significant number of injuries annually. Florida's unique climate permits outdoor recreational and maintenance activities, such as burning yard debris and other trash, throughout the year. Additionally, the hurricane season, lasting from June 1 though November 30, produces large amounts of waste in its wake. The purpose of this study was to examine the seasonal pattern of occurrence and develop an understanding of factors related to accelerant-related burn injuries with the goal of prevention. This nonexperimental research involved a retrospective quantitative observational study of data stored in the National Trauma Registry database. All burn patients admitted to the Tampa General Regional Burn Center as inpatients between January 1, 2001, and December 31, 2005, were included. As with previous studies on the occurrence of accelerant related injuries, young men were much more likely to suffer this type of injury. The hurricane season correlates with an increased number of accelerant related burn injuries, which differs somewhat from the seasonal variations in other regions. The size and severity of accelerant-related injuries varies significantly, as does the length of hospital stay. Accelerant use is frequently associated with trash/brush-related accidents. Hurricane seasons can produce an inordinately large amount of debris and therefore are related with an increased incident in this type of burn injury. The results of this study support the development of a community-based educational program directed at burn injury prevention, with special attention to the implications of the hurricane season
SN - 1559-047X
AD - Tampa General Regional Burn Center, Tampa, Florida, USA
UR - 17667487
ER -

TY - JOUR
ID - 1107
T1 - Levels of ultrafine particles in different microenvironments--implications to children exposure
A1 - Diapouli,E.
A1 - Chaloulakou,A.
A1 - Spyrellis,N.
Y1 - 2007/12/15/
N1 - Diapouli, E. Chaloulakou, A. Spyrellis, N
The Science of the total environment
uj0, 0330500
IM
Journal Article
English
KW - MEDLINE
KW - Air Pollutants/an [Analysis]
KW - Air Pollution
KW - Indoor/an [Analysis]
KW - Child
KW - Environmental Exposure/an [Analysis]
KW - Environmental Monitoring
KW - Greece
KW - Housing
KW - Humans
KW - Motor Vehicles
KW - Particle Size
KW - Particulate Matter/an [Analysis]
KW - Schools
KW - Vehicle Emissions

RP - NOT IN FILE
SP - 128
EP - 136
JF - Science of the Total Environment
JA - Sci Total Environ
VL - 388
IS - 1-3
CY - Netherlands
N2 - Indoor and outdoor ultrafine particles (UFPs) (0.01 to greater than 1 microm) concentration levels were examined in the area of Athens during cold period of 2003 and 2004. Seven primary schools, located in areas with different characteristics of urbanization and traffic density, as well as a typical suburban residence, were monitored. Moreover, in-vehicle concentration levels, while driving along major avenues and in the heavy-trafficked centre of Athens, were measured (mean route duration: 45 min). UFPs number concentration was monitored by condensation particle counter (model CPC 3007), with a logging time interval of 1 min. The highest mean indoor concentrations were observed in a small carpet-covered library and a teachers' office (8-hour mean equal to 52.6x10(3) particles/cm(3) and 50.2x10(3) particles/cm(3), respectively), at the same school unit. The highest outdoor concentrations (8-hour mean equal to 36.9x10(3) particles/cm(3) and 38.8x10(3) particles/cm(3)) were measured at two schools, both affected by heavy traffic. Finally, the highest in-vehicle concentrations (148.0x10(3)-173.0x10(3) particles/cm(3)) were measured in central commercial areas of Athens during, on average, 55 min drives. Indoor-to-outdoor concentration (I/O) ratios were below 1.00 at all sites. The largest ratio (0.88) was observed in the residence, during a day when there was cleaning activity in the room monitored. Outdoor concentrations diurnal cycles, both outside the schools and the residence, were closely related to traffic. Indoor concentrations inside schools were relatively stable in classrooms. Nevertheless, number concentrations exhibited variability when there were significant changes in room occupancy. Diurnal variation of indoor concentrations at the residence followed the respective outdoor one with a delay of 1 h or less, in the absence of strong indoor sources, indicating the major contribution of outdoor UFPs to the indoor concentration levels. The present work is the first effort to examine UFPs indoor and outdoor concentration levels in the area of Athens. The obtained concentration data give an insight on the concentration levels to which children may be exposed. They may be also very useful in epidemiological studies, in order to estimate children total personal exposure though the calculation of exposures received in different microenvironments. This kind of studies may contribute to the design of effective policies and mitigation measures for the protection of public health
SN - 0048-9697
AD - National Technical University of Athens, School of Chemical Engineering, 9 Heroon Polytechniou Street, Zographos Campus 15789, Athens, Greece. diapouli@mail.ntua.gr
UR - 17888492
ER -

TY - JOUR
ID - 1108
T1 - Use of the ICECI and ICD-10 E-Coding structures to evaluate causes of head injury and concussion from sport and recreation participation in a school population
A1 - Kozlowski,K.F.
A1 - Leddy,J.J.
A1 - Tomita,M.
A1 - Bergen,A.
A1 - Willer,B.S.
Y1 - 2007///
N1 - Kozlowski, Karl F. Leddy, John J. Tomita, Machiko. Bergen, Anne. Willer, Barry S
NeuroRehabilitation
9113791
IM
Journal Article
English
KW - MEDLINE
KW - Accidental Falls/sn [Statistics & Numerical Data]
KW - Adolescent

KW - Athletic Injuries/cl [Classification]
KW - Athletic Injuries/ep [Epidemiology]
KW - Brain Concussion/et [Etiology]
KW - Child
KW - Humans
KW - Insurance Claim Reporting/sn [Statistics & Numerical Data]
KW - International Classification of Diseases
KW - Ontario/ep [Epidemiology]
KW - Play and Playthings/in [Injuries]
KW - Risk Factors
RP - NOT IN FILE
SP - 191
EP - 198
JF - Neurorehabilitation
VL - 22
IS - 3
CY - Netherlands
N2 - The purpose of this study was to identify the most common causes of head injury and associated symptoms of concussion in a population of school children (n=1,372,979). Using standardized injury report forms, we identified the mechanisms of head injury associated with various sports/recreation activities and assessed each injury for the presence of concussion symptoms. Head injury reports (n=7,765) were coded using the WHO's ICECI and ICD-10 E-codes. 1,338 Cases reported symptoms of concussion. The majority of head injuries occurred during School Free-Play/Recess (59.8%). Combative Sports and Wheeled Non-Motored Sports were the activities most often associated with concussion symptoms (rate of concussion (RC): 48.3% and 44.4%, respectively, p<0.001). School Free-Play/Recess and Physical Education Classes were significantly less likely to have head injuries associated with concussion (RC: 16.0%, p<0.001, and 12.4%, p=0.034, respectively). The most common causes of head injury were (1) Struck by an Object (24.9%) and (2) Falling on the Same Level (22.8%). Falling from a Transport was the only etiological code significantly associated with concussion symptoms after head injury (RC: 28.7%, p<.001). Results were similar when using the two coding structures in combination. Prevention efforts should focus on activities where children are moving at high speeds since these are more likely to cause a concussion when a head injury occurs
SN - 1053-8135
AD - Department of Exercise and Nutrition Sciences - Program in Athletic Training, State University of New York at Buffalo, Buffalo, NY, USA. kfk@buffalo.edu
UR - 17917169
ER -

TY - JOUR
ID - 1109
T1 - Healthy and safe school environment, Part I: Results from the School Health Policies and Programs Study 2006
A1 - Jones,S.E.
A1 - Fisher,C.J.
A1 - Greene,B.Z.
A1 - Hertz,M.F.
A1 - Pritzl,J.
Y1 - 2007/10//
N1 - Jones, Sherry Everett. Fisher, Carolyn J. Greene, Brenda Z. Hertz, Marci F. Pritzl, Jane
The Journal of school health
k13, 0376370
IM, N
Journal Article
English
KW - MEDLINE
KW - Adolescent
KW - Centers for Disease Control and Prevention (U.S.)
KW - Child

KW - Environment Design
KW - Health Policy
KW - Humans
KW - Organizational Policy
KW - Program Evaluation
KW - Questionnaires
KW - Safety
KW - Schools
KW - Substance-Related Disorders/pc [Prevention & Control]
KW - United States
KW - Wounds and Injuries/pc [Prevention & Control]
RP - NOT IN FILE
SP - 522
EP - 543
JF - Journal of School Health
JA - J Sch Health
VL - 77
IS - 8
CY - United States
N2 - BACKGROUND: Policies set at the state, district, and school levels can support and enhance a healthy and safe school environment. METHODS: The Centers for Disease Control and Prevention conducts the School Health Policies and Programs Study every 6 years. In 2006, computer-assisted telephone interviews or self-administered mail questionnaires were completed by state education agency personnel in all 50 states plus the District of Columbia and among a nationally representative sample of school districts (n=461). Computer-assisted personal interviews were conducted with personnel in a nationally representative sample of elementary, middle, and high schools (n=1025). RESULTS: Most districts had adopted a policy on the inspection and maintenance of school facilities and equipment, and most schools had inspected and provided appropriate maintenance for each type of school facility and equipment during the 12 months preceding the study. Nearly all districts and schools had a comprehensive crisis preparedness, response, and recovery plan. Nearly all districts and schools prohibited tobacco, alcohol, and illegal drug use; fighting; weapons use; and weapon possession; but when students broke rules related to those behaviors, punitive measures were taken more often than provision of supportive services. Most schools did not reschedule outdoor activities to avoid times when the sun was at peak intensity, nor did they encourage the use of sunscreen before going outside. CONCLUSIONS: To provide students with a truly healthy and safe school environment in which learning can take place, more schools need to promote a positive school climate and reduce violence, injuries, and the use of tobacco, alcohol, and other substances. States and districts need to continue to provide policy and technical assistance in support of school efforts
SN - 0022-4391
AD - Division of Adolescent and School Health, Centers for Disease Control and Prevention, 4770 Buford Highway, NE, MS K33, Atlanta, GA 30341, USA. sce2@cdc.gov
UR - 17908106
ER -

TY - JOUR
ID - 1110
T1 - Physical education and physical activity: results from the School Health Policies and Programs Study 2006
A1 - Lee,S.M.
A1 - Burgeson,C.R.
A1 - Fulton,J.E.
A1 - Spain,C.G.
Y1 - 2007/10//
N1 - Lee, Sarah M. Burgeson, Charlene R. Fulton, Janet E. Spain, Christine G
The Journal of school health
k13, 0376370
IM, N
Journal Article
English

KW - MEDLINE
KW - Adolescent
KW - Centers for Disease Control and Prevention (U.S.)
KW - Child
KW - Exercise
KW - Health Education
KW - Health Policy
KW - Health Promotion
KW - Humans
KW - Organizational Policy
KW - Physical Education and Training/og [Organization & Administration]
KW - Program Evaluation
KW - Questionnaires
KW - School Health Services
KW - Schools
KW - Staff Development
KW - United States
RP - NOT IN FILE
SP - 435
EP - 463
JF - Journal of School Health
JA - J Sch Health
VL - 77
IS - 8
CY - United States
N2 - BACKGROUND: Comprehensive school-based physical activity programs consist of physical education and other physical activity opportunities including recess and other physical activity breaks, intramurals, interscholastic sports, and walk and bike to school initiatives. This article describes the characteristics of school physical education and physical activity policies and programs in the United States at the state, district, school, and classroom levels. METHODS: The Centers for Disease Control and Prevention conducts the School Health Policies and Programs Study every 6 years. In 2006, computer-assisted telephone interviews or self-administered mail questionnaires were completed by state education agency personnel in all 50 states plus the District of Columbia and among a nationally representative sample of districts (n=453). Computer-assisted personal interviews were conducted with personnel in a nationally representative sample of elementary, middle, and high schools (n=988) and with a nationally representative sample of teachers of required physical education classes and courses (n=1194). RESULTS: Most states and districts had adopted a policy stating that schools will teach physical education; however, few schools provided daily physical education. Additionally, many states, districts, and schools allowed students to be exempt from participating in physical education. Most schools provided some opportunities for students to be physically active outside physical education. Staff development for physical education was offered by states and districts, but physical education teachers generally did not receive staff development on a variety of important topics. CONCLUSIONS: To enhance physical education and physical activity in schools, a comprehensive approach at the state, district, school, and classroom levels is necessary. Policies, practices, and comprehensive staff development at the state and district levels might enable schools to improve opportunities for students to become physically active adults
SN - 0022-4391
AD - Division of Adolescent and School Health, Centers for Disease Control and Prevention, 4770 Buford Highway, NE (MS K-12), Atlanta, GA 30341, USA. skeuplee@cdc.gov
UR - 17908102
ER -

TY - JOUR
ID - 1111
T1 - Pediatric trampoline injuries
A1 - Hurson,C.
A1 - Browne,K.
A1 - Callender,O.
A1 - O'Donnell,T.

A1 - O'Neill,A.
A1 - Moore,D.P.
A1 - Fogarty,E.E.
A1 - Dowling,F.E.
Y1 - 2007/10//
N1 - Hurson, Conor. Browne, Katherine. Callender, Orla. O'Donnell, Turlough. O'Neill, Anthony. Moore, David P. Fogarty, Esmond E. Dowling, Francis E
Journal of pediatric orthopedics
hsw, 8109053
IM
Journal Article
English
KW - MEDLINE
KW - Adolescent
KW - Child
KW - Preschool
KW - Female
KW - Humans
KW - Infant
KW - Ireland/ep [Epidemiology]
KW - Male
KW - Prospective Studies
KW - Sports Equipment/ae [Adverse Effects]
KW - Sports Equipment
KW - Trauma Severity Indices
KW - Wounds and Injuries/ep [Epidemiology]
RP - NOT IN FILE
SP - 729
EP - 732
JF - Journal of Pediatric Orthopedics
JA - J Pediatr Orthop
VL - 27
IS - 7
CY - United States
N2 - INTRODUCTION: The recreational use of trampolines has increased dramatically during the last 10 years. There has been a striking increase in the number of children presenting to fracture clinics with injuries associated with trampoline use. This increase in trampoline injuries has been reported in North America, but there has been a paucity of research in this area in Europe. METHODS: We prospectively recorded details of patients presenting to our institution, Our Lady's Children's Hospital, Crumlin (Dublin, Ireland), during the busy summer months of June, July, and August 2005. Details recorded included type and mechanism of injury, the mode of referral, treatment, inpatient days, outpatient visits, specific details relating to trampoline safety, and an analysis of the cost of medical care. RESULTS: There were 101 patients treated for trampoline-related injuries in 3 months from June to August 2005. This represented 1.5% of the total attendances to the emergency department. The average age was 8.5 years (range, 1.4-17.4 years). There were 55 fractures, 38 soft tissue injuries, 5 head injuries, and 5 neck injuries, with an average Pediatric Trauma Score of 11.4. Fifty seven percent (58/101) of patients were on the trampoline with at least 1 other person. Twenty patients (19.8%) were admitted to hospital requiring 71 inpatient days. Twelve patients were treated in theatre. There were 163 fracture clinic visits, 212 x-rays, and 2 magnetic resonance imaging scans. CONCLUSIONS: Trampolines are a high-risk activity with the potential for significant orthopaedic injury. In Ireland, we have recently seen a dramatic increase in pediatric trampoline-related injuries mirroring the trend in the United States during the last 10 to 15 years. We found that more than 1 individual on a trampoline is a major risk factor for injury, where the lightest person is 14 times more likely to be injured than the heavier. The lighter person also has a greater chance of being injured with smaller numbers on the trampoline. We reiterate the American Academy of Pediatrics policy statement advice that trampolines be used only in supervised training programs--never at home, in outdoor playgrounds, or in schools. The public should be made aware of the potential dangers of trampolines through public health campaigns, radio, and television
SN - 0271-6798

AD - Our Lady's Children's Hospital, Crumlin, Dublin, Ireland. conorhurson@hotmail.com
UR - 17878774
ER -

TY - JOUR
ID - 1112
T1 - Young lungs at play: preventing children's exposure to secondhand smoke in outdoor play areas in a steps to a healthier NY County
A1 - Jacobson,M.
A1 - Beers,R.A.
A1 - Kessler,D.
A1 - Diffley,U.
A1 - Chanler,S.
A1 - Reid,H.
A1 - Facelle,J.
Y1 - 2007/10//
N1 - Jacobson, Melissa. Beers, R Allan. Kessler, Devon. Diffley, Una. Chanler, Shelley. Reid, Hailey. Facelle, Joan
Preventing chronic disease
101205018
IM
Journal Article. Research Support, Non-U.S. Gov't. Research Support, U.S. Gov't, P.H.S.
English
KW - MEDLINE
KW - Child
KW - Consumer Participation
KW - Environmental Exposure/pc [Prevention & Control]
KW - Health Promotion/og [Organization & Administration]
KW - Humans
KW - New York
KW - Play and Playthings
KW - Program Development
KW - Recreation
KW - Tobacco Smoke Pollution/pc [Prevention & Control]
RP - NOT IN FILE
SP - A110
JF - Preventing Chronic Disease
JA - Prev Chronic Dis
VL - 4
IS - 4
CY - United States
SN - 1545-1151
AD - Rockland County Department of Health, 50 Sanatorium Rd, Pomona, NY 10970, USA. jacobsom@co.rockland.ny.us
UR - 17875254
ER -

TY - JOUR
ID - 1113
T1 - Contribution of youth sport to total daily physical activity among 6- to 12-yr-old boys
A1 - Wickel,E.E.
A1 - Eisenmann,J.C.
Y1 - 2007/09//
N1 - Wickel, Eric E. Eisenmann, Joey C
Medicine and science in sports and exercise
8005433, mg8
IM, S

Journal Article. Research Support, Non-U.S. Gov't
English
KW - MEDLINE
KW - Acceleration
KW - Anthropometry
KW - Child
KW - Cross-Sectional Studies
KW - Humans
KW - Leisure Activities
KW - Male
KW - Monitoring
KW - Ambulatory/mt [Methods]
KW - Motor Activity/ph [Physiology]
KW - Physical Exertion/ph [Physiology]
KW - Schools
KW - Sports/ph [Physiology]
RP - NOT IN FILE
SP - 1493
EP - 1500
JF - Medicine & Science in Sports & Exercise
JA - Med Sci Sports Exerc
VL - 39
IS - 9
CY - United States
N2 - PURPOSE: The purpose of this study was twofold: 1) to determine the contribution of organized youth sport to total daily physical activity (PA), and 2) to examine the contribution of daily recess and physical education (PE) to total daily PA. METHODS: Using a cross-sectional design, 119 children wore an accelerometer during a school day in which they participated in organized youth sport. A subsample (N=36) wore the accelerometer on a nonsport day to examine day-to-day differences in PA. Total daily PA and PA during youth sport, recess, and PE were estimated. The contributions of youth sport, recess, and PE were determined by dividing the amount of PA from each activity by the total daily amount of PA. RESULTS: Approximately 110 min of moderate to vigorous physical activity (MVPA) were achieved on the monitoring day. Youth sport contributed approximately 23% of the total MVPA, whereas PE and recess contributed almost 11 and 16%, respectively. Nearly half of the accumulated minutes of MVPA were attributed to unstructured activities (approximately 56 min). For the entire sample, approximately 52% of youth sport time was spent in either sedentary or light-intensity activities, whereas moderate and vigorous physical activity accounted for approximately 27 and 22% of the time, respectively. During a nonsport day, participants engaged in significantly more sedentary activity (P=0.02) and significantly less moderate (P=0.02) and vigorous activity (P<0.001) compared with the sport day. CONCLUSION: Participants in this study averaged 110 min of MVPA during a day in which they participated in youth sport. The additional amount of MVPA accumulated on the sport day (approximately 30 min) was not maintained on a nonsport day
SN - 0195-9131
AD - Exercise and Sport Science, University of Tulsa, Tulsa, OK 74104, USA. eric-wickel@utulsa.edu
UR - 17805079
ER -

TY - JOUR
ID - 1114
T1 - Giardiasis surveillance--United States, 2003-2005.[Erratum appears in MMWR Morb Mortal Wkly Rep. 2007 Nov 2;56(43):1141]
A1 - Yoder,J.S.
A1 - Beach,M.J.
A1 - Centers for Disease Control and Prevention (CDC)
Y1 - 2007/09/07/
N1 - Yoder, Jonathan S. Beach, Michael J. Centers for Disease Control and Prevention (CDC)
Morbidity and mortality weekly report. Surveillance summaries (Washington, D.C. : 2002)
nnb, 101142015, 8400155

IM
Journal Article
English
KW - MEDLINE
KW - Adolescent
KW - Adult
KW - Animals
KW - Child
KW - Preschool
KW - Female
KW - Giardia lamblia/ip [Isolation & Purification]
KW - Giardiasis/ep [Epidemiology]
KW - Giardiasis/pc [Prevention & Control]
KW - Giardiasis/tm [Transmission]
KW - Humans
KW - Incidence
KW - Infant
KW - Male
KW - Middle Aged
KW - Population Surveillance
KW - Swimming
KW - United States/ep [Epidemiology]
KW - Water/ps [Parasitology]
RP - NOT IN FILE
SP - 11
EP - 18
JF - Morbidity & Mortality Weekly Report
JA - Morb Mortal Wkly Rep Surveill.Summ.
VL - Surveillance Summaries. 56
IS - 7
CY - United States
N2 - PROBLEM/CONDITION: Giardiasis, a gastrointestinal illness, is caused by the protozoan parasite Giardia intestinalis. REPORTING PERIOD: 2003-2005. DESCRIPTION OF SYSTEM: State, commonwealth, territorial, and two metropolitan health departments voluntarily reported cases of giardiasis through CDC's National Notifiable Disease Surveillance System. RESULTS: During 2003-2005, the total number of reported cases of giardiasis remained relatively stable. Reporting increased from 20,084 for 2003 to 20,962 for 2004 and then decreased to 20,075 for 2005. A total of 49 jurisdictions reported giardiasis cases; the number of areas reporting >15 cases per 100,000 population increased from four areas in 2003 to seven in 2005. Compared with other age groups, a greater number of case reports were received for children aged 1-9 years and for adults aged 30-39 years. Incidence of giardiasis was highest in northern states. Peak onset of illness occurred annually during early summer through early fall. INTERPRETATION: Transmission of giardiasis occurs throughout the United States, with increased diagnosis or reporting occurring in northern states. State incidence figures should be compared with caution because individual state surveillance systems have varying capabilities to detect cases. The seasonal peak in age-specific case reports coincides with the summer recreational water season and might reflect increased outdoor activity and exposures such as use of communal swimming venues (e.g., lakes, rivers, swimming pools, and water parks) by young children. PUBLIC HEALTH ACTION: Giardiasis surveillance provides data to educate public health practitioners and health-care providers about the epidemiologic characteristics and the disease burden of giardiasis in the United States. These data are used to improve reporting of cases, plan prevention efforts, and establish research priorities
SN - 1545-8636
AD - Division of Parasitic Diseases, National Center for Zoonotic, Vector-Borne, and Enteric Diseases, CDC, Atlanta, GA 30333, USA. jey9@cdc.gov
UR - 17805224
ER -

TY - JOUR
ID - 1115

T1 - Solar UVR exposure, concurrent activities and sun-protective practices among primary schoolchildren
A1 - Wright,C.Y.
A1 - Reeder,A.I.
A1 - Bodeker,G.E.
A1 - Gray,A.
A1 - Cox,B.
Y1 - 2007/05//
N1 - Wright, Caradee Y. Reeder, Anthony I. Bodeker, Gregory E. Gray, Andrew. Cox, Brian
Photochemistry and photobiology
0376425, p69
IM
Journal Article. Research Support, Non-U.S. Gov't
English
KW - MEDLINE
KW - Child
KW - Clothing
KW - Environmental Exposure/an [Analysis]
KW - Humans
KW - New Zealand
KW - Radiation Dosage
KW - Radiation Protection/mt [Methods]
KW - Schools
KW - Sunlight
KW - Sunscreening Agents
KW - Ultraviolet Rays
RP - NOT IN FILE
SP - 749
EP - 758
JF - Photochemistry & Photobiology
JA - Photochem Photobiol
VL - 83
IS - 3
CY - United States
N2 - Comprehensive measures of ultraviolet radiation (UVR) exposure, concurrent activities and sun-protective practices are needed to develop and evaluate skin cancer prevention and sun protection interventions. The UVR exposures of 345 primary schoolchildren at 23 schools around New Zealand were measured using electronic UVR monitors for 1-week periods over 12 weeks in 2004 and 2005. In addition, ambient UVR levels on a horizontal surface were measured on-site at each school. Children completed activity diaries during the period UVR measurements were made and provided information on their indoor and outdoor status and clothing and sun protection worn. Mean total daily UVR exposure (7:00-20:00 h NZST + 1) at the body location where the UVR monitors were worn was 0.9 SED (standard erythemal dose, 1 SED = 100 J m(-2)). This was 4.9% of the ambient UVR on a horizontal surface. Mean time spent outdoors was 2.3 h day(-1). Differences in children's UVR exposure could be explained in part by activity, where outdoor passive pursuits were associated with higher UVR exposure rates than outdoor active and outdoor travel pursuits. Compared with older children, the activities of younger children, although labeled the same, resulted in different UVR exposures, either as a result of reporting differences or a real difference in UVR exposure patterns. UVR exposure rates were generally higher on weekdays compared with the weekend, confirming the important role of school sun protection and skin cancer prevention programs. High UVR exposure activities included physical education, athletics and lunch break
SN - 0031-8655
AD - Social & Behavioural Research in Cancer Group, Department of Preventive and Social Medicine, University of Otago, Dunedin, New Zealand
UR - 17576384
ER -

TY - JOUR
ID - 1116

T1 - Evaluation of playground injuries based on ICD, E codes, international classification of external cause of injury codes (ICECI), and abbreviated injury scale coding systems
A1 - Tan,N.C.
A1 - Ang,A.
A1 - Heng,D.
A1 - Chen,J.
A1 - Wong,H.B.
Y1 - 2007///
N1 - Tan, N C. Ang, A. Heng, D. Chen, J. Wong, H B
Asia-Pacific journal of public health / Asia-Pacific Academic Consortium for Public Health
asj, 8708538
IM
Evaluation Studies. Journal Article. Research Support, Non-U.S. Gov't
English
KW - MEDLINE
KW - Child
KW - Female
KW - Forms and Records Control/st [Standards]
KW - Humans
KW - International Classification of Diseases
KW - International Cooperation
KW - Male
KW - Play and Playthings
KW - Singapore
KW - Wounds and Injuries/cl [Classification]
KW - Wounds and Injuries/et [Etiology]
RP - NOT IN FILE
SP - 18
EP - 27
JF - Asia-Pacific Journal of Public Health
JA - Asia Pac J Public Health
VL - 19
IS - 1
CY - China
N2 - The survey is aimed to describe the epidemiology of playground related injuries in Singapore based on the ICD-9, AIS/ ISS and PTS scoring systems, and mechanisms and causes of such injuries according to E codes and ICECI codes. A cross-sectional questionnaire survey examined children (< 16 years old), who sought treatment for or died of unintentional injuries in the ED of three hospitals, two primary care centers and the sole Forensic Medicine Department of Singapore. A data dictionary was compiled using guidelines from CDC/WHO. The ISS, AIS and PTS, ICD-9, ICECI v1 and E codes were used to describe the details of the injuries. 19,094 childhood injuries were recorded in the database, of which 1617 were playground injuries (8.5%). The injured children (mean age=6.8 years, SD 2.9 years) were predo-minantly male (M:F ratio = 1.71:1). Falls were the most frequent in-juries (70.7%) using ICECI. 25.0% of injuries involved radial and ulnar fractures (ICD-9 code). 99.4% of these injuries were minor, with PTS scores of 9-12. Children aged 6-10 years, were prone to upper limb injuries (71.1%) based on AIS. The use of international coding systems in injury surveillance facilitated standardisation of description and comparison of playground injuries
SN - 1010-5395
AD - SingHealth Polyclinics, Singapore. Tan.Ngiap.Chuan@singhealth.com.sg
UR - 17784655
ER -

TY - JOUR
ID - 1117
T1 - Safe play spaces to promote physical activity in inner-city children: results from a pilot study of an environmental intervention
A1 - Farley,T.A.
A1 - Meriwether,R.A.

A1 - Baker,E.T.
A1 - Watkins,L.T.
A1 - Johnson,C.C.
A1 - Webber,L.S.
Y1 - 2007/09//
N1 - Farley, Thomas A. Meriwether, Rebecca A. Baker, Erin T. Watkins, Liza T. Johnson, Carolyn C. Webber, Larry S
American journal of public health
1254074, 3xw
AIM, IM
Journal Article. Research Support, N.I.H., Extramural
English
KW - MEDLINE
KW - Body Mass Index
KW - Child
KW - Child Behavior/eh [Ethnology]
KW - Child Behavior
KW - Child Welfare/eh [Ethnology]
KW - Child Welfare
KW - Environment Design/st [Standards]
KW - Humans
KW - Intervention Studies
KW - Louisiana
KW - Motor Activity/ph [Physiology]
KW - Pilot Projects
KW - Poverty Areas
KW - Residence Characteristics/cl [Classification]
KW - Safety
KW - Schools
RP - NOT IN FILE
SP - 1625
EP - 1631
JF - American Journal of Public Health
JA - Am J Public Health
VL - 97
IS - 9
CY - United States
N2 - OBJECTIVES: We evaluated the effect of providing a safe play space on the physical activity level of inner-city schoolchildren. METHODS: In 1 of 2 matched neighborhoods, we opened a schoolyard and provided attendants to ensure children's safety. Over the next 2 years we directly observed the number of children and their physical activity levels in the school-yard, as well as in the surrounding intervention and comparison neighborhoods. We also surveyed children in the schools in the intervention and comparison neighborhoods regarding sedentary activities. RESULTS: After the schoolyard was opened, a mean of 71.4 children used it on weekdays and 25.8 used it on weekends during the school year. When observed, 66% of these children were physically active. The number of children who were outdoors and physically active was 84% higher in the intervention neighborhood than the comparison neighborhood. Survey results showed that children in the intervention school reported declines relative to the children in the comparison school in watching television, watching movies and DVDs, and playing video games on weekdays. CONCLUSION: When children were provided with a safe play space, we observed a relative increase in their physical activity. Provision of safe play spaces holds promise as a simple replicable intervention
SN - 1541-0048
AD - School of Public Health and Tropical Medicine, Tulane University, New Orleans, La70112, USA. tfarley@tulane.edu
UR - 17666701
ER -

TY - JOUR

ID - 1118
T1 - Spatial filters and automated spike detection based on brain topographies improve sensitivity of EEG-fMRI studies in focal epilepsy
A1 - Siniatchkin,M.
A1 - Moeller,F.
A1 - Jacobs,J.
A1 - Stephani,U.
A1 - Boor,R.
A1 - Wolff,S.
A1 - Jansen,O.
A1 - Siebner,H.
A1 - Scherg,M.
Y1 - 2007/09/01/
N1 - Siniatchkin, Michael. Moeller, Friederike. Jacobs, Julia. Stephani, Ulrich. Boor, Rainer. Wolff, Stephan. Jansen, Olav. Siebner, Hartwig. Scherg, Michael
NeuroImage
cpp, 9215515
IM
Journal Article
English
KW - MEDLINE
KW - Action Potentials
KW - Algorithms
KW - Artificial Intelligence
KW - Brain Mapping/mt [Methods]
KW - Child
KW - Diagnosis
KW - Computer-Assisted/mt [Methods]
KW - Electroencephalography/mt [Methods]
KW - Epilepsies
KW - Partial/di [Diagnosis]
KW - Partial/ep [Epidemiology]
KW - Female
KW - Humans
KW - Magnetic Resonance Imaging/mt [Methods]
KW - Male
KW - Pattern Recognition
KW - Automated/mt [Methods]
KW - Reproducibility of Results
KW - Sensitivity and Specificity
RP - NOT IN FILE
SP - 834
EP - 843
JF - Neuroimage
VL - 37
IS - 3
CY - United States
N2 - The ballistocardiogram (BCG) represents one of the most prominent sources of artifacts that contaminate the electroencephalogram (EEG) during functional MRI. The BCG artifacts may affect the detection of interictal epileptiform discharges (IED) in patients with epilepsy, reducing the sensitivity of the combined EEG-fMRI method. In this study we improved the BCG artifact correction using a multiple source correction (MSC) approach. On the one hand, a source analysis of the IEDs was applied to the EEG data obtained outside the MRI scanner to prevent the distortion of EEG signals of interest during the correction of BCG artifacts. On the other hand, the topographies of the BCG artifacts were defined based on the EEG recorded inside the scanner. The topographies of the BCG artifacts were then added to the surrogate model of IED sources and a combined source model was applied to the data obtained inside the scanner. The artifact signal was then subtracted without considerable distortion of the IED topography. The MSC approach was compared with the traditional averaged

artifact subtraction (AAS) method. Both methods reduced the spectral power of BCG-related harmonics and enabled better detection of IEDs. Compared with the conventional AAS method, the MSC approach increased the sensitivity of IED detection because the IED signal was less attenuated when subtracting the BCG artifacts. The proposed MSC method is particularly useful in situations in which the BCG artifact is spatially correlated and time-locked with the EEG signal produced by the focal brain activity of interest
SN - 1053-8119
AD - Christian-Albrechts-University, University Hospital of Pediatric Neurology, Schwanenweg 20, D-24105 Kiel, Germany. m.siniatchkin@pedneuro.uni-kiel.de
UR - 17627849
ER -

TY - JOUR
ID - 1119
T1 - Variability in mobility of children with cerebral palsy
A1 - Tieman,B.
A1 - Palisano,R.J.
A1 - Gracely,E.J.
A1 - Rosenbaum,P.L.
Y1 - 2007///
N1 - Tieman, Beth. Palisano, Robert J. Gracely, Edward J. Rosenbaum, Peter L
Pediatric physical therapy : the official publication of the Section on Pediatrics of the American Physical Therapy Association
8912748
IM
Journal Article. Research Support, N.I.H., Extramural. Research Support, Non-U.S. Gov't. Research Support, U.S. Gov't, Non-P.H.S.
English
KW - MEDLINE
KW - Activities of Daily Living
KW - Cerebral Palsy/cl [Classification]
KW - Cerebral Palsy/pp [Physiopathology]
KW - Cerebral Palsy/rh [Rehabilitation]
KW - Chi-Square Distribution
KW - Child
KW - Disability Evaluation
KW - Female
KW - Humans
KW - Male
KW - Motor Activity/ph [Physiology]
KW - Motor Skills/ph [Physiology]
KW - Movement Disorders/cl [Classification]
KW - Movement Disorders/pp [Physiopathology]
KW - Questionnaires
KW - Severity of Illness Index
KW - Social Environment
KW - Statistics
KW - Nonparametric
RP - NOT IN FILE
SP - 180
EP - 187
JF - Pediatric Physical Therapy
JA - PEDIATR
VL - 19
IS - 3
CY - United States
N2 - PURPOSE: To examine the variability in mobility of children with cerebral palsy (CP) within classification levels. METHODS: The subjects were 183 children with CP, ages six to 12 years, at Gross Motor

Function Classification System (GMFCS) levels II to IV. Parents reported their child's mobility methods in the home, school, and outdoors/community. Mobility methods were ranked using empirical data and reflect trunk and lower extremity motor control requirements. These ranks were used for Friedman analysis of variance and post-hoc analyses for each GMFCS level. RESULTS: Children within the same GMFCS level exhibited varying degrees of independence in mobility methods, which differed across setting. Children usually performed higher-ranked mobility methods (requiring the most gross motor control) at home, lower-ranked mobility methods at school, and the lowest ranked mobility methods in the outdoors/community. CONCLUSIONS: Contextual, environmental, and personal factors may explain these findings. Therapists are encouraged to examine contextual features in everyday settings when planning interventions to improve functional mobility
SN - 0898-5669
AD - Department of Physical Therapy, Georgia State University, Atlanta, Georgia 30302-4019, USA. btieman@gsu.edu
UR - 17700346
ER -

TY - JOUR
ID - 1120
T1 - Effects of circumscribed interests on the social behaviors of children with autism spectrum disorders
A1 - Boyd,B.A.
A1 - Conroy,M.A.
A1 - Mancil,G.R.
A1 - Nakao,T.
A1 - Alter,P.J.
Y1 - 2007/09//
N1 - Boyd, Brian A. Conroy, Maureen A. Mancil, G Richmond. Nakao, Taketo. Alter, Peter J
Journal of autism and developmental disorders
7904301, hgw
IM
Case Reports. Journal Article. Research Support, U.S. Gov't, Non-P.H.S.
English
KW - MEDLINE
KW - Attention
KW - Autistic Disorder/di [Diagnosis]
KW - Autistic Disorder/px [Psychology]
KW - Autistic Disorder/th [Therapy]
KW - Behavior Therapy
KW - Child
KW - Preschool
KW - Choice Behavior
KW - Communication
KW - Humans
KW - Intelligence
KW - Language Development Disorders/di [Diagnosis]
KW - Language Development Disorders/px [Psychology]
KW - Male
KW - Peer Group
KW - Reaction Time
KW - Social Behavior
KW - Stereotyped Behavior
RP - NOT IN FILE
SP - 1550
EP - 1561
JF - Journal of Autism & Developmental Disorders
JA - J Autism Dev Disord
VL - 37
IS - 8
CY - United States

N2 - This study compared the effects of circumscribed interests (CI) to less preferred (LP) tangible stimuli on the social behaviors of three children with autism spectrum disorders (ASD). Based on single subject design methodology, the CI experimental sessions resulted in longer durations of target-child initiated social interactions in comparison to LP sessions. In addition, latency of participant's initial social bids to peers was decreased when CI were present. The results suggest that embedding CI into dyadic play situations with typical peers can be used to increase the social behavior children with ASD direct toward typical peers. Future research should examine the specific environmental conditions that must be present in naturalistic settings to facilitate generalization of social behavior
SN - 0162-3257
AD - University of Florida, Gainesville, Florida, USA. brian_boyd@med.unc.edu
UR - 17146704
ER -

TY - JOUR
ID - 1122
T1 - Resale of recalled children's products online: an examination of the world's largest yard sale
A1 - Brown,Kirschman K.
A1 - Smith,G.A.
Y1 - 2007/08//
N1 - Brown Kirschman, Keri. Smith, Gary A
Injury prevention : journal of the International Society for Child and Adolescent Injury Prevention
crz, 9510056
IM
Journal Article
English
KW - MEDLINE
KW - Child
KW - Preschool
KW - Commerce/st [Standards]
KW - Consumer Product Safety
KW - Female
KW - Humans
KW - Infant
KW - Internet
KW - Male
KW - Play and Playthings/in [Injuries]
KW - Risk Management
RP - NOT IN FILE
SP - 228
EP - 231
JF - Injury Prevention
JA - Inj Prev
VL - 13
IS - 4
CY - England
N2 - OBJECTIVE: To examine an online auction site for the presence and sale of children's products and toys previously recalled because of safety concerns. METHODS: Targeted items were randomly selected from US Consumer Product Safety Commission (CPSC) press releases of recalled children's products dated 1992-2004. Auction listings from eBay were searched for the 150 targeted recalled items for 30 days. Item, seller, and buyer information were recorded from the auction listings. RESULTS: 190 auctions contained or were suspected to contain a recalled children's item from the target list. Most of the recalled items were listed for sale from addresses within the United States, with sellers from Canada, Australia, Great Britain, and Ireland also represented. On average, six bids were placed on each recalled item, with 70% of auctions eventuating in a sale. CONCLUSIONS: Recalled children's products were found to be available for sale online and were sold most of the time, presenting a risk of injury to children. Although the CPSC is charged with notifying the public of recalled items, these results suggest that potentially hazardous products are recirculating online. A multi-front initiative to decrease the presence of hazards in online auctions is needed. This initiative should include

increased manufacturer efforts to improve recall return rates, a requirement by online auction sites that sellers verify non-recall status before item posting, and parental checks of government recall websites before item purchase. Investigation of parental understanding and awareness of recalls and the potential risks associated with recall announcements is needed
SN - 1353-8047
AD - Department of Psychology, University of Dayton, Dayton, OH 45409, USA. kirschKe@notes.udayton.edu
UR - 17686931
ER -

TY - JOUR
ID - 1123
T1 - Boys with developmental coordination disorder: loneliness and team sports participation
A1 - Poulsen,A.A.
A1 - Ziviani,J.M.
A1 - Cuskelly,M.
A1 - Smith,R.
Y1 - 2007/07//
N1 - Poulsen, Anne A. Ziviani, Jenny M. Cuskelly, Monica. Smith, Rachel
The American journal of occupational therapy : official publication of the American Occupational Therapy Association
7705978, 3o4
IM
Journal Article
English
KW - MEDLINE
KW - Adolescent
KW - Child
KW - Humans
KW - Leisure Activities
KW - Loneliness
KW - Male
KW - Motor Skills Disorders/px [Psychology]
KW - Psychosocial Deprivation
KW - Queensland
KW - Sports
RP - NOT IN FILE
SP - 451
EP - 462
JF - American Journal of Occupational Therapy
JA - Am J Occup Ther
VL - 61
IS - 4
CY - United States
N2 - OBJECTIVE: This study investigated the mediational role of team sports and other leisure occupations for boys ages 10 to 13 years in the relationship between physical coordination ability and perceptions of loneliness. METHOD: Sixty boys with developmental coordination disorder (DCD) and 113 comparison boys without DCD completed a self-report measure of loneliness. Parents recorded information on leisure involvement over 7 days. RESULTS: Boys with DCD recorded significantly higher loneliness and lower participation rates in all group physical activities, whether structured (e.g., team sports) or unstructured (e.g., informal outdoor play) than boys without DCD. An inverse relationship between physical coordination ability and loneliness was mediated by participation in team sports. No other leisure pursuits were found to be significant mediators. Childhood physical coordination difficulties were significantly associated with loneliness. CONCLUSION: Participation in team sports acted as one potential mechanism mediating the inverse relationship between physical coordination ability and loneliness in boys. Occupational therapists can act as advocates to support boys with DCD who choose to participate in team sports. Further investigations are recommended to determine aspects of team sports environments that promote an optimal fit among child, activity, and environment

SN - 0272-9490
AD - Division of Occupational Therapy, School of Health and Rehabilitation Sciences, The University of Queensland, Brisbane, 4072, Queensland, Australia. a.poulsen@uq.edu.au
UR - 17685178
ER -

TY - JOUR
ID - 1124
T1 - Impact of culture on children's community participation in Israel
A1 - Engel-Yeger,B.
A1 - Jarus,T.
A1 - Law,M.
Y1 - 2007/07//
N1 - Engel-Yeger, Batya. Jarus, Tal. Law, Mary
The American journal of occupational therapy : official publication of the American Occupational Therapy Association
7705978, 3o4
IM
Comparative Study. Journal Article
English
KW - MEDLINE
KW - Arabs
KW - Child
KW - Consumer Participation/sn [Statistics & Numerical Data]
KW - Cultural Diversity
KW - Female
KW - Humans
KW - Israel
KW - Jews
KW - Male
KW - Pediatrics
KW - Sex Factors
RP - NOT IN FILE
SP - 421
EP - 428
JF - American Journal of Occupational Therapy
JA - Am J Occup Ther
VL - 61
IS - 4
CY - United States
N2 - OBJECTIVE: This study compared the participation in activities outside of formal school among Jewish and Druze Israeli children. It also examined the impact of gender and the interaction of culture and gender on participation. METHODS: Participants included 30 Jewish and 30 Druze children ages 8-10 years, who completed the Children's Assessment of Participation and Enjoyment (CAPE). RESULTS: On most of the scales measured, the Jewish children showed a significantly higher participation level than the Druze children, and the Druze children performed more activities alone. The Jewish children demonstrated a higher enjoyment level than the Druze children on half of the scales. Some scales revealed higher participation by girls than by boys as well as a significant cultural group by gender interaction. CONCLUSION: Culture may have an impact on children's level of participation in the community. The CAPE was found to be a sensitive tool in elucidating differences between the Druze and Jewish children in community participation
SN - 0272-9490
AD - Department of Occupational Therapy, Faculty of Welfare and Health Studies, University of Haifa, Mount Carmel, Haifa 31905 Israel. batya@research.haifa.ac.il
UR - 17685175
ER -

TY - JOUR

ID - 1125
T1 - Safety of specific sublingual immunotherapy with SQ standardized grass allergen tablets in children
A1 - Ibanez,M.D.
A1 - Kaiser,F.
A1 - Knecht,R.
A1 - Armentia,A.
A1 - Schopfer,H.
A1 - Tholstrup,B.
A1 - Bufe,A.
Y1 - 2007/09//
N1 - Ibanez, Maria Dolores. Kaiser, Friedrich. Knecht, Roland. Armentia, Alicia. Schopfer, Helmut. Tholstrup, Bente. Bufe, Albrecht
Pediatric allergy and immunology : official publication of the European Society of Pediatric Allergy and Immunology
bu6, 9106718
IM
Journal Article. Multicenter Study. Randomized Controlled Trial
English
KW - MEDLINE
KW - Administration
KW - Sublingual
KW - Allergens/ad [Administration & Dosage]
KW - Allergens/ae [Adverse Effects]
KW - Child
KW - Preschool
KW - Conjunctivitis
KW - Allergic/im [Immunology]
KW - Allergic/th [Therapy]
KW - Desensitization
KW - Immunologic/ae [Adverse Effects]
KW - Immunologic/mt [Methods]
KW - Double-Blind Method
KW - Female
KW - Germany
KW - Humans
KW - Male
KW - Poaceae/im [Immunology]
KW - Rhinitis
KW - Allergic
KW - Seasonal/im [Immunology]
KW - Seasonal/th [Therapy]
KW - Spain
KW - Tablets
KW - Treatment Outcome
RP - NOT IN FILE
SP - 516
EP - 522
JF - Pediatric Allergy & Immunology
JA - Pediatr Allergy Immunol
VL - 18
IS - 6
CY - England
N2 - The aim of the study was to confirm the safety of an orodispersible grass allergen tablet 75,000 SQ-T (Grazax, ALK-Abello A/S, Horsholm, Denmark) in children aged 5-12 yr. The study was randomized, double-blinded and placebo-controlled. Sixty children aged 5-12 yr suffering from grass pollen-induced rhinoconjunctivitis (with or without asthma) from five centres in two countries (three in Germany and two in Spain) participated in the study. They were randomized at the ratio of 3:1 as receiving either Grazax or placebo

tablet given sublingually once daily for 28 days outside the grass pollen season. A total of 810 treatment-related adverse events were reported in the Grazax group. The majority of these were local reactions in the mouth or throat and were mostly mild (71%) to moderate (27%) in severity and resolved within days. Thirty-five (78%) subjects treated with Grazax and five (33%) treated with placebo reported at least one treatment-related adverse event. Oral pruritus, throat irritation, mouth oedema and ear pruritus appeared as the most frequently reported treatment-related adverse events. 62% (28 of 45) of the actively treated subjects reported oral pruritus, 36% (16 of 45) throat irritation, 31% (14 of 45) mouth oedema and 22% (10 of 45) ear pruritus. Two actively treated subjects withdrew from the study: one subject due to four adverse events (moderate eye pruritus, moderate pharyngolaryngeal pain, moderate non-cardiac chest pain and moderate dysphagia) and one subject due to a serious adverse event (asthmatic attack). The subjects recovered completely from the events. In conclusion, in the present study, Grazax was in general tolerated in a paediatric population and considered suitable for further clinical investigations in children
SN - 0905-6157
AD - Servicio de Alergia, Hospital Universitario Nino Jesus, Madrid, Spain. dibanezs@medynet.com
UR - 17680910
ER -

TY - JOUR
ID - 1126
T1 - Falls from playground equipment: will the new Australian playground safety standard make a difference and how will we tell?
A1 - Mitchell,R.
A1 - Sherker,S.
A1 - Cavanagh,M.
A1 - Eager,D.
Y1 - 2007/08//
N1 - Mitchell, Rebecca. Sherker, Shauna. Cavanagh, Margaret. Eager, David
Health promotion journal of Australia : official journal of Australian Association of Health Promotion Professionals
9710936
IM
Comparative Study. Journal Article. Research Support, Non-U.S. Gov't
English
KW - MEDLINE
KW - Accidental Falls/pc [Prevention & Control]
KW - Accidental Falls/sn [Statistics & Numerical Data]
KW - Adolescent
KW - Age Distribution
KW - Child
KW - Preschool
KW - Craniocerebral Trauma/ep [Epidemiology]
KW - Craniocerebral Trauma/et [Etiology]
KW - Female
KW - Hospitalization/sn [Statistics & Numerical Data]
KW - Humans
KW - Incidence
KW - Infant
KW - Newborn
KW - Length of Stay
KW - Male
KW - New South Wales/ep [Epidemiology]
KW - Play and Playthings/in [Injuries]
KW - Risk Assessment
KW - Safety/st [Standards]
RP - NOT IN FILE
SP - 98
EP - 104

JF - Health Promotion Journal of Australia
JA - HEALTH PROMOT J AUST
VL - 18
IS - 2
CY - Australia
N2 - ISSUE ADDRESSED: This study describes the trend in incidence of hospitalised falls from playground equipment of children aged 14 years or less in New South Wales (NSW) and considers the potential effectiveness of playground safety standards in reducing the impact of playground-related injuries. METHOD: Hospitalisations of children aged 14 years or less following a fall from playground equipment were identified from the NSW hospitalisation data for the financial years 1992/93 to 2003/04 and described. RESULTS: During 1992/93 to 2003/04 there were 16,828 hospitalisations of children aged 0-14 years as a result of a fall from playground equipment, at a rate of 106.6 per 100,000 children. The incidence of hospitalisation increased from 83.3 to 130.3 per 100,000 children between 1992/93 to 2003/04. Males aged 5-9 years had the highest rate of hospitalisation at 198.4 per 100,000 children. Injury type varied by age group, but injuries to the elbow and forearm were common for all age groups. The rate of upper limb fractures that resulted in hospitalisation increased, while the rate of serious head injuries decreased. CONCLUSIONS: While severe head injuries have declined between 1992/93 to 2003/04, the increasing trend of upper limb fractures is of concern. Many factors need to be taken into account to assess the effectiveness of playground safety standards. The collection of exposure data is also crucial to be able to calculate the true risk associated with childhood falls from playground equipment
SN - 1036-1073
AD - NSW Injury Risk Management Research Centre, University of New South Wales.
r.mitchell@unsw.edu.au
UR - 17663656
ER -

TY - JOUR
ID - 1127
T1 - Indoor residential chemical emissions as risk factors for respiratory and allergic effects in children: a review. [Review] [103 refs]
A1 - Mendell,M.J.
Y1 - 2007/08//
N1 - Mendell, M J
Indoor air
9423515, da3
IM
Journal Article. Research Support, U.S. Gov't, Non-P.H.S.. Review
English
KW - MEDLINE
KW - Air Pollution
KW - Indoor/ae [Adverse Effects]
KW - Biocompatible Materials/to [Toxicity]
KW - Child
KW - Preschool
KW - Environmental Exposure
KW - Formaldehyde/to [Toxicity]
KW - Housing
KW - Humans
KW - Infant
KW - Newborn
KW - Paint/to [Toxicity]
KW - Phthalic Acids/to [Toxicity]
KW - Plasticizers/to [Toxicity]
KW - Respiratory Hypersensitivity/et [Etiology]
KW - Respiratory Hypersensitivity/im [Immunology]
KW - Respiratory Hypersensitivity/pa [Pathology]
KW - Respiratory Tract Infections/et [Etiology]

KW - Respiratory Tract Infections/im [Immunology]
KW - Respiratory Tract Infections/pa [Pathology]
KW - Risk Assessment
KW - Risk Factors
RP - NOT IN FILE
SP - 259
EP - 277
JF - Indoor Air
VL - 17
IS - 4
CY - Denmark
N2 - UNLABELLED: Most research into effects of residential exposures on respiratory health has focused on allergens, moisture/mold, endotoxin, or combustion products. A growing body of research from outside the US; however, has associated chemical emissions from common indoor materials with risk of asthma, allergies, and pulmonary infections. This review summarizes 21 studies in the epidemiologic literature on associations between indoor residential chemical emissions, or emission-related materials or activities, and respiratory health or allergy in infants or children. Associations, some strong, were reported between many risk factors and respiratory or allergic effects. Risk factors identified most frequently included formaldehyde or particleboard, phthalates or plastic materials, and recent painting. Findings for other risk factors, such as aromatic and aliphatic chemical compounds, were limited but suggestive. Elevated risks were also reported for renovation and cleaning activities, new furniture, and carpets or textile wallpaper. Reviewed studies were entirely observational, limited in size, and variable in quality, and specific risk factors identified may only be indicators for correlated, truly causal exposures. Nevertheless, overall evidence suggests a new class of residential risk factors for adverse respiratory effects, ubiquitous in modern residences, and distinct from those currently recognized. It is important to confirm and quantify any risks, to motivate and guide necessary preventive actions. PRACTICAL IMPLICATIONS: Composite wood materials that emit formaldehyde, flexible plastics that emit plasticizers, and new paint have all been associated with increased risks of respiratory and allergic health effects in children. Although causal links have not been documented, and other correlated indoor-related exposures may ultimately be implicated, these findings nevertheless point to a new class of little recognized indoor risk factors for allergic and respiratory disease, distinct from the current set of indoor risk factors. The available evidence thus raises initial questions about many common residential practices: for instance, using pressed wood furnishings in children's bedrooms, repainting infant nurseries, and encasing mattresses and pillows with vinyl for asthmatic children. The findings summarized here suggest a need for substantially increased research to replicate these findings, identify causal factors, and validate preventive strategies. [References: 103]
SN - 0905-6947
AD - Lawrence Berkeley National Laboratory, Indoor Environment Department, Berkeley, CA 94720, USA. mjmendell@lbl.gov
UR - 17661923
ER -

TY - JOUR
ID - 1128
T1 - Assessing children with autism, mental retardation, and typical development using the Playground Observation Checklist
A1 - Ingram,D.H.
A1 - Mayes,S.D.
A1 - Troxell,L.B.
A1 - Calhoun,S.L.
Y1 - 2007/07//
N1 - Ingram, Daniel H. Mayes, Susan Dickerson. Troxell, Lucinda B. Calhoun, Susan L
Autism : the international journal of research and practice
9713494
IM
Evaluation Studies. Journal Article. Research Support, Non-U.S. Gov't
English
KW - MEDLINE
KW - Age Factors

KW - Autistic Disorder/di [Diagnosis]
KW - Autistic Disorder/px [Psychology]
KW - Child
KW - Child Behavior/px [Psychology]
KW - Child Development
KW - Preschool
KW - Diagnosis
KW - Differential
KW - Employment
KW - Female
KW - Humans
KW - Intellectual Disability/di [Diagnosis]
KW - Intellectual Disability/px [Psychology]
KW - Intelligence
KW - Male
KW - Parents/px [Psychology]
KW - Pilot Projects
KW - Play and Playthings/px [Psychology]
KW - Questionnaires/st [Standards]
KW - Reproducibility of Results
KW - Retrospective Studies
KW - Sex Factors
KW - Social Behavior
RP - NOT IN FILE
SP - 311
EP - 319
JF - Autism
VL - 11
IS - 4
CY - England
N2 - Elementary school children with normal intelligence and autism (n = 20), mental retardation and no autism (n = 24), and typical development (n = 37) were observed for 15 minutes during recess at school. Ten behaviors were scored as present or absent using the Playground Observation Checklist. Children with autism were distinguished from children with mental retardation and typical development by their social problems, whereas children with typical development and mental retardation did not differ significantly in social competency. The four social behaviors on the checklist correctly identified 94 percent of the children as having or not having autism. All of the children with autism and all of the typical children were correctly classified. Our pilot findings suggest that the Playground Observation Checklist has potential as a simple and clinically useful component of a comprehensive evaluation for possible autism
SN - 1362-3613
AD - Lincoln Intermediate Unit, New Oxford, USA
UR - 17656396
ER -

TY - JOUR
ID - 1129
T1 - Bioaccessibility of metals in urban playground soils
A1 - Ljung,K.
A1 - Oomen,A.
A1 - Duits,M.
A1 - Selinus,O.
A1 - Berglund,M.
Y1 - 2007/07/15/
N1 - Ljung, Karin. Oomen, Agnes. Duits, Menno. Selinus, Olle. Berglund, Marika
Journal of environmental science and health. Part A, Toxic/hazardous substances & environmental engineering
dvj, 9812551
IM

Journal Article. Research Support, Non-U.S. Gov't
English
KW - MEDLINE
KW - Biological Availability
KW - Child
KW - Data Interpretation
KW - Statistical
KW - Environmental Monitoring/mt [Methods]
KW - Humans
KW - Metals
KW - Heavy/an [Analysis]
KW - Heavy/pk [Pharmacokinetics]
KW - Models
KW - Biological
KW - Play and Playthings
KW - Soil Pollutants/an [Analysis]
KW - Soil Pollutants/pk [Pharmacokinetics]
KW - Sweden
KW - Urban Health
RP - NOT IN FILE
SP - 1241
EP - 1250
JF - Journal of Environmental Science & Health Part A-Toxic/Hazardous Substances & Environmental Engineering
JA - J Environ Sci Health Part A Tox.Hazard.Subst Environ Eng
VL - 42
IS - 9
CY - United States
N2 - Children ingest soil. The amount ingested varies with the child's behaviour, and daily ingestion rates have been calculated to be between 39 and 270 mg day(-1). During play, children ingest soil both involuntarily and deliberately, and it can be assumed that the latter may result in ingestion of a larger soil particle size fraction and a larger soil mass than the former. Measurements of soil metal contents commonly display the total metal content, where soil sieved to <2 mm has been digested with strong acids. This procedure does not consider differences in metal contents between size fractions or ingested soil masses. Moreover, it does not consider the difference between bioaccessible and total metal content, possibly resulting in an incorrect evaluation of the potential health risks from soil intake. Intervention and guideline values are commonly calculated via tolerable daily intake values, in turn derived from toxicological studies where the contaminant is administered to a test animal in feed or water. It is then assumed that the bioavailability of a contaminant in soil equals the bioavailability in the matrix used in the toxicology study. However, the complexity and heterogeneity of soil often results in a lower bioavailability than from food or water. The current study investigated the bioaccessibility of soil As, Cd, Cr, Ni and Pb from two different particle size fractions representing deliberate (<4 mm) and involuntary (<50 microm) ingestion, as well as from two different soil masses representing deliberate soil intake; 2 g for a child with pica behaviour and 0.6 g for a non-pica child. The bioaccessibility was investigated using an in vitro digestion model and urban playground soils collected away from any point pollution sources. The bioaccessibility (%) of the different metals increased in the order Ni=Cr=Pb<<As<Cd for the involuntary ingestion (<50 microm, 0.6 g) and pica behaviour (<4 mm, 2 g) scenarios, while the deliberate non-pica scenario (<4 mm, 0.6 g) displayed increasing bioaccessibility in the order Ni=Cr<Pb<As=Cd. The difference in bioaccessibility between elements was concluded to be due to differences in origin, sorption behaviour and pH dependence. The study also found that the bioaccessible amount of metal in ingested soil is not always related to particle size or to soil mass in soils with low contaminant levels. Factors such as pH dependence of the metal and the soil's clay content are also significant in determining bioaccessibility
SN - 1093-4529
AD - Department of Soil Science, Swedish University of Agricultural Sciences, Sweden. Karin.Ljung@mv.slu.se
UR - 17654144
ER -

TY - JOUR
ID - 1130
T1 - Parental history of myopia, sports and outdoor activities, and future myopia
A1 - Jones,L.A.
A1 - Sinnott,L.T.
A1 - Mutti,D.O.
A1 - Mitchell,G.L.
A1 - Moeschberger,M.L.
A1 - Zadnik,K.
Y1 - 2007/08//
N1 - Jones, Lisa A. Sinnott, Loraine T. Mutti, Donald O. Mitchell, Gladys L. Moeschberger, Melvin L. Zadnik, Karla
Investigative ophthalmology & visual science
gwi, 7703701
IM
Journal Article. Research Support, N.I.H., Extramural. Research Support, Non-U.S. Gov't
English
KW - MEDLINE
KW - Adult
KW - Child
KW - Family Health
KW - Humans
KW - Leisure Activities
KW - Logistic Models
KW - Longitudinal Studies
KW - Motor Activity
KW - Myopia/ep [Epidemiology]
KW - Myopia/ge [Genetics]
KW - Parents
KW - Predictive Value of Tests
KW - ROC Curve
KW - Sports/sn [Statistics & Numerical Data]
RP - NOT IN FILE
SP - 3524
EP - 3532
JF - Investigative Ophthalmology & Visual Science
JA - Invest Ophthalmol Vis Sci
VL - 48
IS - 8
CY - United States
N2 - PURPOSE: To identify whether parental history of myopia and/or parent-reported children's visual activity levels can predict juvenile-onset myopia. METHODS: Survey-based data from Orinda Longitudinal Study of Myopia subjects from 1989 to 2001 were used to predict future myopia. Univariate and multiple logistic regression analyses were performed, and receiver operator characteristic (ROC) curves were generated. Differences among the areas under the ROC curves were compared using the method of multiple comparison with the best. RESULTS: Of the 514 children eligible for this analysis, 111 (21.6%) became myopic. Differences in the third grade between eventual myopes and nonmyopes were seen for the number of myopic parents ($P < 0.001$) and for the number of sports and outdoor activity hours per week (11.65 +/- 6.97 hours for nonmyopes vs. 7.98 +/- 6.54 hours for future myopes, $P < 0.001$). Analysis of the areas under the ROC curves showed three variables with a predictive value better than chance: the number of myopic parents, the number of sports and outdoor activity hours per week, and the number of reading hours per week. After controlling for sports and outdoor hours per week and parental myopia history, reading hours per week was no longer a statistically significant factor. The area under the curve for the parental myopia history and sports and outdoor activities model was 0.73. A significant interaction in the logistic model showed a differential effect of sport and outdoor activity hours per week based on a child's number of myopic parents. CONCLUSIONS: Parental history of myopia was an important predictor in univariate and multivariate models, with a differential effect of sports and outdoor activity hours per week based on the number of myopic parents. Lower amounts of sports

and outdoor activity increased the odds of becoming myopic in those children with two myopic parents more than in those children with either zero or one myopic parent. The chance of becoming myopic for children with no myopic parents appears lowest in the children with the highest amount of sports and outdoor activity, compared with those with two myopic parents
SN - 0146-0404
AD - College of Optometry, The Ohio State University, Columbus, Ohio 43210, USA. ljones@optometry.osu.edu
UR - 17652719
ER -

TY - JOUR
ID - 1131
T1 - Assessment and intervention for dysphagia in infants and children: beyond the neonatal intensive care unit. [Review] [42 refs]
A1 - Bell,H.R.
A1 - Alper,B.S.
Y1 - 2007/08//
N1 - Bell, Hannah R. Alper, Beth Sheckman
Seminars in speech and language
8405117, cfb, 8405117
IM
Journal Article. Review
English
KW - MEDLINE
KW - Child
KW - Preschool
KW - Deglutition Disorders/di [Diagnosis]
KW - Deglutition Disorders/th [Therapy]
KW - Home Care Services
KW - Hospital-Based
KW - Humans
KW - Infant
KW - Intensive Care Units
KW - Neonatal
KW - Severity of Illness Index
RP - NOT IN FILE
SP - 213
EP - 222
JF - Seminars in Speech & Language
JA - Semin Speech Lang
VL - 28
IS - 3
CY - United States
N2 - Over the last 10 years, the assessment and intervention for feeding and swallowing problems in infants and children have attracted increased attention on a national and international level. Increases in the population of children with dysphagia are due, in large part, to advances in medical and surgical management of at-risk term infants, improved medical support for viability of younger and smaller preterm infants, and increases in the number of children on the autism spectrum. Because of legislative initiatives, settings in which children are seen for assessment and intervention have shifted, with services provided more often in the natural environments of homes, daycares, preschools, and schools, as well as in hospitals and outpatient clinics in the United States. Assessment of infants and children with dysphagia continues to include clinical and instrumental evaluations with clinical assessment including a specific focus on the feeding environment. Speech-language pathologists are increasingly assuming consultative roles to support the needs of children in all settings. Areas for further research in the era of evidence-based practice include efficacy of oral exercises and other intervention strategies. [References: 42]
SN - 0734-0478
AD - KenCrest Children and Family Services, Philadelphia, PA 19119, USA. hbell@kencrest.org

UR - 17647133
ER -

TY - JOUR
ID - 1132
T1 - Physical activity patterns and sedentary behaviors of children from urban and rural areas of Cyprus
A1 - Bathrellou,E.
A1 - Lazarou,C.
A1 - Panagiotakos,D.B.
A1 - Sidossis,L.S.
Y1 - 2007/06//
N1 - Bathrellou, Eirini. Lazarou, Chrystalleni. Panagiotakos, Demosthenes B. Sidossis, Labros S
Central European journal of public health
bo6, 9417324
IM
Comparative Study. Journal Article. Research Support, Non-U.S. Gov't
English
KW - MEDLINE
KW - Child
KW - Cyprus
KW - Exercise
KW - Female
KW - Health Behavior
KW - Humans
KW - Male
KW - Questionnaires
KW - Rural Population
KW - Urban Population
RP - NOT IN FILE
SP - 66
EP - 70
JF - Central European Journal of Public Health
JA - Cent Eur J Public Health
VL - 15
IS - 2
CY - Czech Republic
N2 - BACKGROUND: A sedentary lifestyle among children is becoming increasingly common and has been linked to future risk of degenerative diseases. Urban residence has been suggested to be a contributing factor to a less active lifestyle; however, not all available studies support this link. In the present study we examined the physical activity patterns and sedentary behaviours of children living in urban and rural areas of Cyprus, where major demographic shifts have occurred the last decades. METHODS: We studied 1140 children (531 boys; 609 girls), aged 10-12 years, registered in 24 selected elementary public schools from five urban and rural districts of Cyprus. Children completed a semi-quantitative physical activity questionnaire regarding frequency and duration of everyday physical and sedentary activities. Weight and height of the children, as well as demographic and socioeconomic information was collected from children and their guardians. RESULTS: Rural children reported being slightly more active after school and occupied weekly with outdoors chores compared to urban children, who on the other hand reported engaging in sports on a weekly basis more than their rural peers (all p < 0.10). However, the average weekly time spent by urban and rural children on vigorous (8.6 +/- 4.7 and 9.1 +/- 4.8 h/w, respectively; p = 0.193) or moderate-to-vigorous (14.9 +/- 7.6 and 15.2 +/- 7.6 h/w, respectively; p = 0.612) activities, as well as total screen time, were not different. The distribution of children with regards to most other physical activity and inactivity pursuits was similar between urban and rural areas. CONCLUSION: We found no substantial differences in the physical activity habits and sedentary behaviours among children living in urban and rural areas of Cyprus. Hence public health awareness directed to enhance physical activity and decrease sedentary lifestyle among youngsters should focus equally to urban and rural children
SN - 1210-7778
AD - Department of Nutrition - Dietetics, Harokopio University, Athens, Greece

UR - 17645220
ER -

TY - JOUR
ID - 1133
T1 - Combined EEG and MEG analysis of early somatosensory evoked activity in children and adolescents with focal epilepsies
A1 - Bast,T.
A1 - Wright,T.
A1 - Boor,R.
A1 - Harting,I.
A1 - Feneberg,R.
A1 - Rupp,A.
A1 - Hoechstetter,K.
A1 - Rating,D.
A1 - Baumgartner,U.
Y1 - 2007/08//
N1 - Bast, T. Wright, T. Boor, R. Harting, I. Feneberg, R. Rupp, A. Hoechstetter, K. Rating, D. Baumgartner, U
Clinical neurophysiology : official journal of the International Federation of Clinical Neurophysiology
db4, 100883319
IM
Evaluation Studies. Journal Article. Research Support, Non-U.S. Gov't
English
KW - MEDLINE
KW - Adolescent
KW - Adult
KW - Cerebral Cortex/ab [Abnormalities]
KW - Cerebral Cortex/pa [Pathology]
KW - Cerebral Cortex/pp [Physiopathology]
KW - Child
KW - Preschool
KW - Electroencephalography/st [Standards]
KW - Electroencephalography
KW - Epilepsies
KW - Partial/di [Diagnosis]
KW - Partial/pp [Physiopathology]
KW - Evoked Potentials
KW - Somatosensory
KW - Female
KW - Humans
KW - Magnetic Resonance Imaging
KW - Magnetoencephalography/st [Standards]
KW - Magnetoencephalography
KW - Male
KW - Physical Stimulation
KW - Touch
RP - NOT IN FILE
SP - 1721
EP - 1735
JF - Clinical Neurophysiology
JA - Clin Neurophysiol
VL - 118
IS - 8
CY - Netherlands
N2 - OBJECTIVE: The study aimed to evaluate differences between EEG and MEG analysis of early somatosensory evoked activity in patients with focal epilepsies in localizing eloquent areas of the somatosensory cortex. METHODS: Twenty-five patients (12 male, 13 female; age 4-25 years, mean 11.7 years)

were included. Syndromes were classified as symptomatic in 17, idiopathic in 2 and cryptogenic in 6 cases. 10 patients presented with malformations of cortical development (MCD). 122 channel MEG and simultaneous 33-channel EEG were recorded during tactile stimulation of the thumb (sampling rate 769 Hz, band-pass 0.3-260 Hz). Forty-four hemispheres were analyzed. Hemispheres were classified as type I: normal (15), II: central structural lesion (16), III: no lesion, but central epileptic discharges (ED, 8), IV: lesion or ED outside the central region (5). Analysis of both sides including one normal and one type II or III hemisphere was possible in 15 patients. Recordings were repeated in 18 hemispheres overall. Averaged data segments were filtered (10-250 Hz) and analyzed off-line with BESA. Latencies and amplitudes of N20 and P30 were analyzed. A regional source was fitted for localizing S1 by MRI co-registration. Orientation of EEG N20 was calculated from a single dipole model. RESULTS: EEG and MEG lead to comparable good results in all normal hemispheres. Only EEG detected N20/P30 in 3 hemispheres of types II/III while MEG showed no signal. N20 dipoles had a more radial orientation in these cases. MEG added information in one hemisphere, when EEG source analysis of a clear N20 was not possible because of a low signal-to-noise ratio. Overall N20 dipoles had a more radial orientation in type II when compared to type I hemispheres (p=0.01). Further N20/P30 parameters (amplitudes, latencies, localization related to central sulcus) showed no significant differences between affected and normal hemispheres. Early somatosensory evoked activity was preserved within the visible lesion in 5 of the 10 patients with MCD. CONCLUSIONS: MEG should be combined with EEG when analyzing tactile evoked activities in hemispheres with a central structural lesion or ED focus. SIGNIFICANCE: At time, MEG analysis is frequently applied without simultaneous EEG. Our results clearly show that EEG may be superior under specific circumstances and combination is necessary when analyzing activity from anatomically altered cortex
SN - 1388-2457
AD - Department of Pediatric Neurology, University Children's Hospital, INF 150, 69120 Heidelberg, Germany. Thomas_Bast@med.uni-heidelberg.de
UR - 17572142
ER -

TY - JOUR
ID - 1134
T1 - Pesticides and their metabolites in the homes and urine of farmworker children living in the Salinas Valley, CA.[Erratum appears in J Expo Sci Environ Epidemiol. 2009 Nov;19(7):694-5]
A1 - Bradman,A.
A1 - Whitaker,D.
A1 - Quiros,L.
A1 - Castorina,R.
A1 - Claus,Henn B.
A1 - Nishioka,M.
A1 - Morgan,J.
A1 - Barr,D.B.
A1 - Harnly,M.
A1 - Brisbin,J.A.
A1 - Sheldon,L.S.
A1 - McKone,T.E.
A1 - Eskenazi,B.
Y1 - 2007/07//
N1 - Bradman, Asa. Whitaker, Donald. Quiros, Lesliam. Castorina, Rosemary. Claus Henn, Birgit. Nishioka, Marcia. Morgan, Jeffrey. Barr, Dana B. Harnly, Martha. Brisbin, Judith A. Sheldon, Linda S. McKone, Thomas E. Eskenazi, Brenda
Journal of exposure science & environmental epidemiology
101262796
IM
Journal Article. Research Support, N.I.H., Extramural. Research Support, U.S. Gov't, Non-P.H.S.
English
KW - MEDLINE
KW - Agriculture
KW - Air Pollution/an [Analysis]
KW - Air Pollution
KW - Indoor/an [Analysis]

KW - California
KW - Child
KW - Preschool
KW - Clothing
KW - Dust/an [Analysis]
KW - Environmental Exposure/ae [Adverse Effects]
KW - Environmental Exposure/an [Analysis]
KW - Environmental Monitoring
KW - Environmental Pollutants/an [Analysis]
KW - Environmental Pollutants/po [Poisoning]
KW - Family
KW - Female
KW - Food Contamination/an [Analysis]
KW - Housing
KW - Humans
KW - Infant
KW - Male
KW - Organophosphate Poisoning
KW - Organophosphorus Compounds/an [Analysis]
KW - Organophosphorus Compounds/ur [Urine]
KW - Pesticide Residues/an [Analysis]
KW - Pesticides/an [Analysis]
KW - Pesticides/po [Poisoning]
KW - Pesticides/ur [Urine]
KW - Pyrethrins/an [Analysis]
KW - Pyrethrins/po [Poisoning]
KW - Pyrethrins/ur [Urine]
KW - Risk Assessment/mt [Methods]
RP - NOT IN FILE
SP - 331
EP - 349
JF - Journal of Exposure Science & Environmental Epidemiology
JA - J Expo Sci Environ Epidemiol
VL - 17
IS - 4
CY - United States
N2 - In support of planning efforts for the National Children's Study, we conducted a study to test field methods for characterizing pesticide exposures to 20 farmworker children aged 5-27 months old living in the Salinas Valley of Monterey County, California. We tested methods for collecting house dust, indoor and outdoor air, dislodgeable residues from surfaces and toys, residues on clothing (sock and union suits), food, as well as spot and overnight diaper urine samples. We measured 29 common agricultural and home use pesticides in multiple exposure media samples. A subset of organophosphorus (OP), organochlorine (OC) and pyrethroid pesticides were measured in food. We also analyzed urine samples for OP pesticide metabolites. Finally, we administered four field-based exposure assessment instruments: a questionnaire; food diary; home inspection; and a self-administered child activity timeline. Pesticides were detected more frequently in house dust, surface wipes, and clothing than other media, with chlorpyrifos, diazinon, chlorthal-dimethyl, and cis- and trans-permethrin detected in 90% to 100% of samples. Levels of four of these five pesticides were positively correlated among the house dust, sock, and union suit samples (Spearman's rho=0.18-0.76). Pesticide loading on socks and union suits was higher for the group of 10 toddlers compared to the 10 younger crawling children. Several OP pesticides, as well as 4,4'-DDE, atrazine, and dieldrin were detected in the food samples. The child activity timeline, a novel, low-literacy instrument based on pictures, was successfully used by our participants. Future uses of these data include the development of pesticide exposure models and risk assessment
SN - 1559-0631
AD - Center for Children's Environmental Health Research, School of Public Health, University of California, Berkeley, CA 94720-7380, USA. abradman@socrates.berkeley.edu
UR - 16736054
ER -

TY - JOUR
ID - 1135
T1 - Measuring time costs in interventions designed to reduce behavior problems among children and youth
A1 - Foster,E.M.
A1 - Johnson-Shelton,D.
A1 - Taylor,T.K.
Y1 - 2007/09//
N1 - Foster, E Michael. Johnson-Shelton, Deborah. Taylor, Ted K
American journal of community psychology
3fv, 0364535
IM
Journal Article. Research Support, N.I.H., Extramural
English
KW - MEDLINE
KW - Child
KW - Child Behavior Disorders/pc [Prevention & Control]
KW - Costs and Cost Analysis
KW - Education/ec [Economics]
KW - Education/og [Organization & Administration]
KW - Humans
KW - Time Factors
KW - United States
RP - NOT IN FILE
SP - 64
EP - 81
JF - American Journal of Community Psychology
JA - Am J Community Psychol
VL - 40
IS - 1-2
CY - United States
N2 - The economic evaluation of psychosocial interventions is a growing area of research. Though time costs are central to the economist's understanding of social costs, these costs generally have been ignored by prevention scientists. This article highlights the need to measure such costs and then reviews the principles economists use in valuing time. It then considers the specific time costs that often arise in interventions designed to reduce behavior problems among children and youth. These include classroom time devoted to program activities, the time of parents or other caregivers, the time of teachers (outside of the classroom), and the time of volunteers. We consider the economic principles that govern how economists value these inputs and then apply these principles to data from an evaluation of a prominent intervention in the field, the Incredible Years Program. We find that the time costs are potentially rather large and consider the implications for public policy of ignoring them
SN - 0091-0562
AD - School of Public Health, University of North Carolina Chapel Hill, Rosenau Hall, Campus Box #7445, Chapel Hill, NC 27599-7445, USA. emfoster@unc.edu
UR - 17592769
ER -

TY - JOUR
ID - 1136
T1 - Addressing the issue of falls off playground equipment: an empirically-based intervention to reduce fall-risk behaviors on playgrounds
A1 - Morrongiello,B.A.
A1 - Matheis,S.
Y1 - 2007/08//
N1 - Morrongiello, Barbara A. Matheis, Shawn
Journal of pediatric psychology
jl8, 7801773

IM
Journal Article. Research Support, Non-U.S. Gov't
English
KW - MEDLINE
KW - Accidental Falls/pc [Prevention & Control]
KW - Accidental Falls/sn [Statistics & Numerical Data]
KW - Child
KW - Child Behavior
KW - Empirical Research
KW - Female
KW - Humans
KW - Leisure Activities
KW - Male
KW - Play and Playthings
KW - Questionnaires
KW - Risk-Taking
KW - Safety
KW - Technology
KW - Wounds and Injuries/ep [Epidemiology]
KW - Wounds and Injuries/pc [Prevention & Control]
RP - NOT IN FILE
SP - 819
EP - 830
JF - Journal of Pediatric Psychology
JA - J Pediatr Psychol
VL - 32
IS - 7
CY - United States
N2 - OBJECTIVE: The present study evaluated the impact of an intervention to reduce fall-risk behaviors on playgrounds among children 6-11 years of age. METHODS: Children completed posters indicating risky playground behaviors they would and would not do. In the intervention group, video and audio presentations were used to expose children to injury occurrences so that injury vulnerability was communicated in a fear-evoking way. In the control group, children only completed the pre- and post-intervention measures. RESULTS: Significant decreases in intentions to risk-take were obtained in the intervention, but not the control group. Effectiveness did not vary with children's age or sex, but was greater for those scoring high in sensation-seeking. CONCLUSIONS: A fear-appeals approach proved successful to reduce intended fall-risk behaviors, particularly for children high in sensation-seeking whose risk-taking is motivated by affect arousal
SN - 0146-8693
AD - Psychology Department, University of Guelph, Guelph, Ontario, N1G 2W1, Canada.
bmorrong@uoguelph.ca
UR - 17522112
ER -

TY - JOUR
ID - 1137
T1 - Brief report: designing a playground for children with autistic spectrum disorders--effects on playful peer interactions
A1 - Yuill,N.
A1 - Strieth,S.
A1 - Roake,C.
A1 - Aspden,R.
A1 - Todd,B.
Y1 - 2007/07//
N1 - Yuill, Nicola. Strieth, Sara. Roake, Caroline. Aspden, Ruth. Todd, Brenda
Journal of autism and developmental disorders
7904301, hgw
IM

Journal Article
English
KW - MEDLINE
KW - Autistic Disorder/di [Diagnosis]
KW - Autistic Disorder/px [Psychology]
KW - Autistic Disorder/rh [Rehabilitation]
KW - Child
KW - Preschool
KW - Education
KW - Special
KW - Environment Design
KW - Female
KW - Humans
KW - Imagination
KW - Male
KW - Motor Activity
KW - Motor Skills
KW - Peer Group
KW - Play and Playthings
KW - Social Behavior
KW - Social Identification
KW - Socialization
RP - NOT IN FILE
SP - 1192
EP - 1196
JF - Journal of Autism & Developmental Disorders
JA - J Autism Dev Disord
VL - 37
IS - 6
CY - United States
N2 - This study investigated possible changes in social play and initiations in eight boys (5 to 7-years-old) with autistic spectrum disorders (ASD) who were moving from an old to a new school playground that was designed specifically to enhance playful peer interaction. Each boy was observed for half an hour over three occasions in the old, then the new setting. The playgrounds differed in design, spatial density and identity of potential play partners. As hypothesised, frequency of group play and overall social initiations increased significantly in the new setting. We discuss how playgrounds with appropriate levels of physical challenge and support for both structured, imaginative play and solitary observation may support peer interactions in children with ASD
SN - 0162-3257
AD - Department of Psychology, Centre for Research in Cognitive Science, University of Sussex, Brighton, BN1 9QH, UK. nicolay@sussex.ac.uk
UR - 17063401
ER -

TY - JOUR
ID - 1138
T1 - The perceptions of statutory service providers of a local Sure Start programme: a shared agenda?
A1 - Edgley,A.
A1 - Avis,M.
Y1 - 2007/07//
N1 - Edgley, Alison. Avis, Mark
Health & social care in the community
9306359
IM
Journal Article
English
KW - MEDLINE
KW - Attitude of Health Personnel

KW - Child
KW - Child Health Services/og [Organization & Administration]
KW - Child Welfare
KW - Preschool
KW - Eligibility Determination
KW - Great Britain
KW - Health Personnel
KW - Humans
KW - Infant
KW - Interprofessional Relations
KW - Interviews as Topic
KW - Referral and Consultation
KW - Resource Allocation
KW - Social Work/og [Organization & Administration]
RP - NOT IN FILE
SP - 379
EP - 386
JF - Health & Social Care in the Community
JA - Health Soc Care Community
VL - 15
IS - 4
CY - England
N2 - Sure Start is the UK government's flagship scheme for providing services to children and their families in deprived areas. This study aimed to examine the perceptions of statutory service providers about their experience of working with Sure Start professionals in one Nottinghamshire (UK) Sure Start programme, and to explore the extent to which there was a shared agenda and successful collaboration. A purposive sample of 18 statutory providers working within existing mainstream health, education and social care services were interviewed, between January and March 2005. Data were analysed through thematic content analysis. We found that although interviewees welcomed the additional input provided by Sure Start for the most vulnerable families, a number of tensions arose over key divergences between the philosophical positions of statutory providers and Sure Start. The most important tension was over Sure Start's philosophy of targeting resources on an entire geographical area. This was seen as antithetical to statutory providers' case-by-case approach, and raised questions about access and equity for families living outside Sure Start's boundaries. Sure Start's concentration on young children, and the time-limited nature of their services and activities, frustrated statutory providers who had a broader family focus, and a longer term perspective. The perceived under-resourcing of statutory services in comparison to Sure Start, and statutory providers' responsibility for 'selling' Sure Start services, strained a sense of equality between agencies and professionals, and undermined a sense of a shared agenda. We conclude that successful collaboration and a shared agenda depends upon a mutual understanding of the basic philosophy of service provision. The government's commitment to developing interprofessional collaboration will be furthered significantly by placing Sure Start services under the umbrella of statutory provision, and ensuring that the differing service philosophies are reconciled
SN - 0966-0410
AD - School of Nursing, University of Nottingham, Nottingham, UK. alison.edgley@nottingham.ac.uk
UR - 17578399
ER -

TY - JOUR
ID - 1139
T1 - Physical activity levels of normal-weight and overweight girls and boys during primary school recess
A1 - Stratton,G.
A1 - Ridgers,N.D.
A1 - Fairclough,S.J.
A1 - Richardson,D.J.
Y1 - 2007/06//
N1 - Stratton, Gareth. Ridgers, Nicola D. Fairclough, Stuart J. Richardson, David J
Obesity (Silver Spring, Md.)
101264860

IM
Journal Article. Multicenter Study. Research Support, Non-U.S. Gov't
English
KW - MEDLINE
KW - Body Weight
KW - Child
KW - Female
KW - Heart Rate
KW - Humans
KW - Leisure Activities
KW - Male
KW - Motor Activity/ph [Physiology]
KW - Overweight/ph [Physiology]
KW - Schools
RP - NOT IN FILE
SP - 1513
EP - 1519
JF - Obesity
JA - Obesity (Silver Spring)
VL - 15
IS - 6
CY - United States
N2 - OBJECTIVE: This study aimed to compare moderate-to-vigorous physical activity (MVPA) and vigorous physical activity (VPA) in normal-weight and overweight boys and girls during school recess. RESEARCH METHODS AND PROCEDURES: Four hundred twenty children, age 6 to 10 years, were randomly selected from 25 schools in England. Three hundred seventy-seven children completed the study. BMI was calculated from height and weight measurements, and heart rate reserve thresholds of 50% and 75% reflected children's engagement in MVPA and VPA, respectively. RESULTS: There was a significant main effect for sex and a significant interaction between BMI category and sex for the percent of recess time spent in MVPA and VPA. Normal-weight girls were the least active group, compared with overweight boys and girls who were equally active. Fifty-one boys and 24 girls of normal weight achieved the 40% threshold; of these, 30 boys and 10 girls exceeded 50% of recess time in MVPA. Eighteen overweight boys and 22 overweight girls exceeded the 40% threshold, whereas 8 boys and 8 girls exceeded the 50% threshold. DISCUSSION: Overweight boys were significantly less active than their normal-weight male counterparts; this difference did not hold true for girls. Even though nearly double the number of normal-weight children achieved the 40% of MVPA during recess compared with overweight children, physical activity promotion in school playgrounds needs to be targeted not only at overweight but at other health parameters, as 40 overweight children met the 40% MVPA target proposed for recess
SN - 1930-7381
AD - Research Institute for Sports and Exercise Sciences, 15-21 Webster Street, Liverpool John Moores University, Liverpool, UK. G.Stratton@ljmu.ac.uk
UR - 17557988
ER -

TY - JOUR
ID - 1140
T1 - Factors affecting prevalence of overweight among 12- to 17-year-old urban adolescents in Hyderabad, India
A1 - Laxmaiah,A.
A1 - Nagalla,B.
A1 - Vijayaraghavan,K.
A1 - Nair,M.
Y1 - 2007/06//
N1 - Laxmaiah, Avula. Nagalla, Balakrishna. Vijayaraghavan, Kamasamudram. Nair, Mohanan
Obesity (Silver Spring, Md.)
101264860
IM

Journal Article
English
KW - MEDLINE
KW - Adolescent
KW - Child
KW - Cross-Sectional Studies
KW - Humans
KW - India/ep [Epidemiology]
KW - Motor Activity/ph [Physiology]
KW - Obesity/ep [Epidemiology]
KW - Obesity/et [Etiology]
KW - Overweight
KW - Risk Factors
KW - Socioeconomic Factors
RP - NOT IN FILE
SP - 1384
EP - 1390
JF - Obesity
JA - Obesity (Silver Spring)
VL - 15
IS - 6
CY - United States
N2 - OBJECTIVE: The problem of overweight and obesity is not confined only to developed countries but is also widely prevalent in developing countries. The objective of this study was to assess the prevalence of overweight and obesity as defined by the International Obesity Task Force (IOTF) among school-age children in Hyderabad, India, and identify its associated factors. RESEARCH METHODS AND PROCEDURES: A cross-sectional and institutional study, adopting a multistage stratified cluster sampling procedure, was carried out during 2003 on adolescents 12 to 17 years of age of both sexes from Hyderabad, India. RESULTS: The overall prevalence of overweight was 6.1% [95% confidence interval (CI): 4.2, 8.0] among boys and 8.2% among girls (CI: 6.0, 10.4); 1.6% and 1.0% were obese, respectively. The prevalence was significantly higher (p < 0.05) among adolescents who watched television > or =3 h/d (10.4%) or belonged to a high socioeconomic background (14.9%, p < 0.001), whereas it was significantly lower among those participating regularly in outdoor games > or =6 h/wk (3.1%, p < 0.004) and household activities > or =3 h/d (4.7%, p < 0.001). The logistic regression analysis revealed that the prevalence of overweight was 4 times higher among the adolescents of high socioeconomic status [odds ratio (OR): 4.1; CI: 2.25, 7.52], 3 times higher in those not participating in outdoor games (OR: 2.75; CI: 1.56, 4.72), and 1.92 times higher in those watching television > or =3 h/d (OR: 1.92; CI: 1.16, 3.18). DISCUSSION: This study confirmed the findings of earlier studies carried out in Western countries and emphasizes that regular physical exercise, doing household activities, regulated television viewing, and healthy eating behaviors could contribute to controlling overweight and obesity
SN - 1930-7381
AD - Division of Community Studies, National Institute of Nutrition, Indian Council of Medical Research, Jamai-Osmania (Post), Hyderabad 500007, India. avulalaxman@yahoo.com
UR - 17557974
ER -

TY - JOUR
ID - 1141
T1 - Deltamethrin-impregnated bednets reduce human landing rates of sandfly vector Lutzomyia longipalpis in Amazon households
A1 - Courtenay,O.
A1 - Gillingwater,K.
A1 - Gomes,P.A.
A1 - Garcez,L.M.
A1 - Davies,C.R.
Y1 - 2007/06//
N1 - Courtenay, O. Gillingwater, K. Gomes, P A F. Garcez, L M. Davies, C R
Medical and veterinary entomology

a9o, 8708682
IM
Evaluation Studies. Journal Article
English
KW - MEDLINE
KW - Adolescent
KW - Animals
KW - Bedding and Linens
KW - Child
KW - Preschool
KW - Cross-Over Studies
KW - Female
KW - Humans
KW - Infant
KW - Newborn
KW - Insect Control/mt [Methods]
KW - Insect Vectors/de [Drug Effects]
KW - Insect Vectors/ph [Physiology]
KW - Insect Vectors
KW - Insecticides
KW - Leishmaniasis/pc [Prevention & Control]
KW - Nitriles/ad [Administration & Dosage]
KW - Psychodidae/de [Drug Effects]
KW - Psychodidae/ph [Physiology]
KW - Psychodidae
KW - Pyrethrins/ad [Administration & Dosage]
KW - Questionnaires
KW - Time Factors
RP - NOT IN FILE
SP - 168
EP - 176
JF - Medical & Veterinary Entomology
JA - Med Vet Entomol.
VL - 21
IS - 2
CY - England
N2 - The entomological efficacy of using 25% deltamethrin EC insecticide-treated bednets (ITNs) was evaluated against the sandfly Lutzomyia longipalpis Lutz and Neiva (Diptera: Psychodidae), the principal vector of zoonotic visceral leishmaniasis (ZVL) in Latin America. A crossover field study in Amazon Brazil (Marajo Island, Para State) demonstrated that, compared with untreated nets, the insecticide increased the barrier effect of nets by 39% (95% confidence interval [CI] 34-44%), reduced human landing rates by 80% (95% CI 62-90%) and increased the 24-h mortality rate from 0% to 98% (95% CI 93-99%) inside ITNs. The presence of an ITN also reduced the human landing rate on unprotected persons outside the net in the same room by 56% (95% CI 52-59%), and increased 24-h mortality to 68% (95% CI 62-73%) compared to 0.4% (0.1-2.0%) in untreated houses. The reduction in human landing rates in ITN rooms was associated with a doubling in the proportion of sandflies alighting on walls compared with that in untreated rooms, which was attributed to insecticide-induced excito-repellency. There was no evidence that sandflies were diverted onto unprotected hosts. Human landing catches inside houses peaked between 19.00 hours and 23.00 hours and declined steadily to zero at 02.00 hours and thereafter. House-to-house questionnaires established that only 34% of households owned at least one net (median two, range 1-8), only 20% of the population slept under a net (33% of 0-5-year-old children), and the majority (73%) of the population slept in hammocks. Combined data pertaining to sleeping times for children and sandfly activity period indicate that > 50% of sandfly bites inside houses, and substantially more outside houses, were taken before a third of children were potentially protected by a net. This study demonstrates the clear entomological efficacy of ITNs against Lu. longipalpis in this endemic region. The effectiveness of ITNs at preventing ZVL infection and disease has still to be evaluated
SN - 0269-283X

AD - Ecology and Epidemiology Group, Department of Biological Sciences, University of Warwick, Coventry, UK. orin.courtenay@warwick.ac.uk
UR - 17550436
ER -

TY - JOUR
ID - 1142
T1 - Seasonal and altitudinal variations in fly density and their association with the occurrence of trachoma, in the Gurage zone of central Ethiopia
A1 - Taye,A.
A1 - Alemayehu,W.
A1 - Melese,M.
A1 - Geyid,A.
A1 - Mekonnen,Y.
A1 - Tilahun,D.
A1 - Asfaw,T.
Y1 - 2007/07//
N1 - Taye, A. Alemayehu, W. Melese, M. Geyid, A. Mekonnen, Y. Tilahun, D. Asfaw, T
Annals of tropical medicine and parasitology
68e, 2985178r
IM
Journal Article. Research Support, Non-U.S. Gov't
English
KW - MEDLINE
KW - Altitude
KW - Animals
KW - Child
KW - Preschool
KW - Ethiopia/ep [Epidemiology]
KW - Eye/mi [Microbiology]
KW - Houseflies
KW - Humans
KW - Insect Vectors
KW - Muscidae
KW - Prevalence
KW - Rural Health
KW - Seasons
KW - Trachoma/ep [Epidemiology]
RP - NOT IN FILE
SP - 441
EP - 448
JF - Annals of Tropical Medicine & Parasitology
JA - Ann Trop Med Parasitol
VL - 101
IS - 5
CY - England
N2 - In the Gurage zone of central Ethiopia, the association between fly density and the occurrence of trachoma has been investigated across varying altitudes. The seasonal pattern of fly density in the area was also explored. When, over short sampling periods (10 min/child indoors and 10 min/child outdoors), hand nets were used to collect flies from the eyes of children aged 2-8 years, only Musca sorbens and M. domestica were caught. Almost all of the 13,147 'eye-seeking' flies collected came from villages at low (<2000 m; 40.7%) or mid altitudes (2200-2500 m; 58.6%) with only 0.7% of them caught in the high-altitude villages investigated (at >3000 m). Musca sorbens predominated outdoors and M. domestica indoors. Almost all (99.3%) of the eye-seeking M. sorbens collected were caught outdoors whereas most (76.7%) of the M. domestica were caught indoors (P<0.0001 for each). The median numbers of flies caught, per child, per 10-min collection, in the low-, mid- and high-altitude villages were 9.5, six and zero, respectively, for M. sorbens, and eight, three and zero, respectively, for M. domestica. The altitudinal trends in these numbers of 'eye-seeking' flies matched those in

the prevalences of active trachoma among children aged 1-10 years, which were high in the villages at low (81.6%) and mid altitude (78.7%) but much lower (1.7%) in the high-altitude villages. In conclusion, trachoma is a common disease of public-health importance only in the low- and mid-altitude villages in the Gurage zone, where there are large numbers of eye-seeking flies, and not in the villages that lie >3000 m above sea level, where there is a dearth of such flies
SN - 0003-4983
AD - Department of Infectious and Non-infectious Diseases Research, Ethiopian Health and Nutrition Research Institute, PO Box 1242, Addis Ababa, Ethiopia. asegedt@yahoo.com
UR - 17550650
ER -

TY - JOUR
ID - 1143
T1 - Heelys and street gliders injuries: a new type of pediatric injury
A1 - Vioreanu,M.
A1 - Sheehan,E.
A1 - Glynn,A.
A1 - Casidy,N.
A1 - Stephens,M.
A1 - McCormack,D.
Y1 - 2007/06//
N1 - Vioreanu, Mihai. Sheehan, Eoin. Glynn, Aaron. Casidy, Noelle. Stephens, Michael. McCormack, Damian
Pediatrics
oxv, 0376422
AIM, IM
Journal Article
English
KW - MEDLINE
KW - Accidental Falls/pc [Prevention & Control]
KW - Adolescent
KW - Athletic Injuries/ep [Epidemiology]
KW - Athletic Injuries/et [Etiology]
KW - Athletic Injuries/pc [Prevention & Control]
KW - Child
KW - Female
KW - Follow-Up Studies
KW - Heel
KW - Humans
KW - Male
KW - Play and Playthings/in [Injuries]
KW - Prospective Studies
KW - Protective Devices/td [Trends]
KW - Protective Devices/ut [Utilization]
KW - Skating/td [Trends]
KW - Sports Equipment/ae [Adverse Effects]
RP - NOT IN FILE
SP - e1294
EP - e1298
JF - Pediatrics
VL - 119
IS - 6
CY - United States
N2 - OBJECTIVES: Our goals were to highlight an increasing trend in orthopedic injuries in children as a result of "heeling" or "street gliding," to describe injuries sustained by children using Heelys (HSL, Carrollton, TX) and Street Gliders (Glowgadgets Ltd, Bristol, United Kingdom), and to increase public awareness and prevent such injuries. PATIENTS AND METHODS: We prospectively recorded the data of all roller shoes injuries referred to our department during the summer school holiday. Using a data-collection sheet, we

recorded demographic data, type of injury, mechanism and place of injury, heeling or street-gliding experience, use of safety equipment, methods of treatment, and intention to continue heeling or street gliding after recovery from injury. RESULTS: Over a 10-week period, 67 children suffered orthopedic injuries while using Heelys or Street Gliders. There were 56 girls and 11 boys with a mean age of 9.6 years. Upper limbs were the most common location of injury. Distal radius fractures were the most prevalent, followed by supracondylar fractures, elbow dislocations, and hand fractures. The majority of children suffered the injury while heeling or street gliding outdoors. Interestingly, 20% of the injuries happened while trying Heelys or Street Gliders for the first time, and 36% of the injuries occurred while learning (using 1-5 times) how to use them. None of the children used any sort of protective gear at the time of the injury. The majority of the injured children expressed their intention to continue heeling or street gliding after complete recovery from their injury. CONCLUSIONS: Our study shows that the majority of children with injuries from heeling or street gliding are girls. We recommend close supervision of children using Heelys or Street Gliders during the steep learning curve and usage of protective gear at all times. These new types of injuries have a serious impact on child health and constitute a burden for the pediatric orthopedic service
SN - 1098-4275
AD - Trauma and Orthopaedic Surgery Department, Temple Street Children's University Hospital, Dublin, Ireland. mihaiv@eircom.net
UR - 17545360
ER -

TY - JOUR
ID - 1145
T1 - Ethnic differences in the impact of parental myopia: findings from a population-based study of 12-year-old Australian children
A1 - Ip,J.M.
A1 - Huynh,S.C.
A1 - Robaei,D.
A1 - Rose,K.A.
A1 - Morgan,I.G.
A1 - Smith,W.
A1 - Kifley,A.
A1 - Mitchell,P.
Y1 - 2007/06//
N1 - Ip, Jenny M. Huynh, Son C. Robaei, Dana. Rose, Kathryn A. Morgan, Ian G. Smith, Wayne. Kifley, Annette. Mitchell, Paul
Investigative ophthalmology & visual science
gwi, 7703701
IM
Journal Article. Research Support, Non-U.S. Gov't
English
KW - MEDLINE
KW - Adolescent
KW - Asian Continental Ancestry Group/eh [Ethnology]
KW - Child
KW - Child of Impaired Parents
KW - European Continental Ancestry Group/eh [Ethnology]
KW - Eyeglasses
KW - Female
KW - Humans
KW - Male
KW - Myopia/eh [Ethnology]
KW - Myopia/th [Therapy]
KW - New South Wales/ep [Epidemiology]
KW - Parents
KW - Prevalence
KW - Questionnaires
KW - Refraction

KW - Ocular/ph [Physiology]
RP - NOT IN FILE
SP - 2520
EP - 2528
JF - Investigative Ophthalmology & Visual Science
JA - Invest Ophthalmol Vis Sci
VL - 48
IS - 6
CY - United States
N2 - PURPOSE: To examine the influences of ethnicity, parental myopia, and near work on spherical equivalent refraction (SER) and axial length (AL) in a population-based sample of 12-year-old Australian children. METHODS: Year-7 children in the Sydney Myopia Study (n = 2353, 75.3% response) underwent an ophthalmic examination including cycloplegic autorefraction (1% cyclopentolate) and ocular biometry (IOLMaster; Carl Zeiss Meditec GmbH, Jena, Germany). Data for parental myopia, ethnicity, near work, and outdoor activities were derived from questionnaires and were available for 1781 children. Optical prescriptions of parents were sought if the spectacles were used. RESULTS: The prevalence of myopia in the children increased with the number of myopic parents (7.6%, 14.9%, and 43.6% for no, one, or two myopic parents). In parallel, the mean SER (+/-SE of the mean) was more negative (0.70 +/- 0.08, 0.34 +/- 0.09, and -0.55 +/- 0.34 D), and the mean AL was longer (23.32 +/- 0.05, 23.44 +/- 0.06, and 23.62 +/- 0.16 mm) after adjustment for demographic and environmental factors. In multivariate analyses, odds of childhood myopia did not change with higher levels of near work (odds ratio [OR] = 1.01; 95% confidence interval [CI] = 0.99-1.03). Interactions between parental myopia and ethnicity were significant for SER and AL (both P < 0.0001), reflecting greater decreases in SER and greater increases in AL with the number of myopic parents in the children of East Asian ethnicity than in the children of European Caucasian ethnicity. In the nonmyopic children, there was no association between parental myopia and AL. CONCLUSIONS: In this sample, parental myopia was associated with more myopic SER and longer AL, with significant ethnic interactions
SN - 0146-0404
AD - Centre for Vision Research (Department of Ophthalmology and Westmead Millennium Institute), University of Sydney, and the Vision Co-operative Research Centre, University of New South Wales, Sydney Australia
UR - 17525179
ER -

TY - JOUR
ID - 1146
T1 - The SHEDS-Wood model: incorporation of observational data to estimate exposure to arsenic for children playing on CCA-treated wood structures
A1 - Barraj,L.M.
A1 - Tsuji,J.S.
A1 - Scrafford,C.G.
Y1 - 2007/05//
N1 - Barraj, Leila M. Tsuji, Joyce S. Scrafford, Carolyn G
Environmental health perspectives
ei0, 0330411
IM
Journal Article. Research Support, Non-U.S. Gov't
English
KW - MEDLINE
KW - Arsenates/to [Toxicity]
KW - Child
KW - Environmental Exposure
KW - Hand
KW - Humans
KW - Models
KW - Theoretical
KW - United States
KW - United States Environmental Protection Agency

KW - Wood/ch [Chemistry]
KW - Wood/to [Toxicity]
RP - NOT IN FILE
SP - 781
EP - 786
JF - Environmental Health Perspectives
JA - Environ Health Perspect
VL - 115
IS - 5
CY - United States
N2 - BACKGROUND: Lumber treated with chromated copper arsenate (CCA) compounds has been used in residential outdoor wood structures and public playgrounds. The U.S. Environmental Protection Agency (EPA) has conducted a probabilistic assessment of children's exposure to arsenic using the Stochastic Human Exposure and Dose Simulation model for the wood preservative scenario (SHEDS-Wood). The assessment relied on data derived from an experimental study conducted using adult volunteers and designed to result in maximum hand and wipe loadings to estimate the residue-skin transfer efficiency. Recent analyses of arsenic hand-loading data generated by studies of children actively involved in playing on CCA-treated structures indicate that the transfer efficiency coefficient and hand-loading estimates derived from the experimental study significantly overestimate the amount that occurs during actual play. OBJECTIVES: Our goal was to assess the feasibility of using child hand-loading data in the SHEDS-Wood model and their impact on exposure estimates. METHODS: We used data generated by the larger of the studies of children in SHEDS-Wood, instead of the distributions used by U.S. EPA. We compared our estimates of the lifetime average daily dose (LADD) and average daily dose (ADD) with those derived by the U.S. EPA. RESULTS: Our analysis indicates that data from observational studies of children can be used in SHEDS-Wood. Our estimates of the mean (and 95th percentile) LADD and ADD were 27% (10%) and 29% (15%) of the estimates derived by U.S. EPA. CONCLUSION: We recommend that the SHEDS-Woods model use data from studies of children actively playing on playsets to more accurately estimate children's actual exposures to CCA
SN - 0091-6765
AD - Center for Chemical Regulation and Food Safety, Exponent, Inc., Washington, DC 20036, USA. lbarraj@exponent.com
UR - 17520068
ER -

TY - JOUR
ID - 1147
T1 - The origin of reference intervals
A1 - Friedberg,R.C.
A1 - Souers,R.
A1 - Wagar,E.A.
A1 - Stankovic,A.K.
A1 - Valenstein,P.N.
A1 - College of American Pathologists.
Y1 - 2007/03//
N1 - Friedberg, Richard C. Souers, Rhona. Wagar, Elizabeth A. Stankovic, Ana K. Valenstein, Paul N. College of American Pathologists
Archives of pathology & laboratory medicine
79z, 7607091
AIM, IM
Journal Article
English
KW - MEDLINE
KW - Adult
KW - Blood Chemical Analysis/st [Standards]
KW - Child
KW - Clinical Laboratory Techniques/st [Standards]
KW - Data Collection
KW - Female

KW - Hematologic Tests/st [Standards]
KW - Humans
KW - Laboratories/st [Standards]
KW - Male
KW - Multivariate Analysis
KW - Reference Values
RP - NOT IN FILE
SP - 348
EP - 357
JF - Archives of Pathology & Laboratory Medicine
JA - Arch Pathol Lab Med
VL - 131
IS - 3
CY - United States
N2 - CONTEXT: Standards have been developed for establishing reference intervals, but little is known about how intervals are determined in practice, interlaboratory variation in intervals, or errors that occur while setting reference intervals. OBJECTIVES: To determine (1) methods used by clinical laboratories to establish reference intervals for 7 common analytes, (2) variation in intervals, and (3) factors that contribute to establishment of "outlier" intervals. DESIGN: One hundred sixty-three clinical laboratories provided information about their reference intervals for potassium, calcium, magnesium, thyroid-stimulating hormone, hemoglobin, platelet count, and activated partial thromboplastin time. RESULTS: Approximately half the laboratories reported conducting an internal study of healthy individuals to validate reference intervals for adults. Most laboratories relied on external sources to establish reference intervals for pediatric patients. There was slight variation in intervals used by the central 80% of study laboratories, but some laboratories outside the central 80% had surprisingly low and high limits for their reference intervals. In some cases the intervals used by 2 laboratories had no overlap. For example, one laboratory considered a hemoglobin of 13.8 g/dL in a woman to be "low" while another considered the same value to be "high." Three percent of reference intervals contained a limit that qualified as an "outlier" using standard statistical tests; we could not identify any practice associated with adoption of outlier intervals. CONCLUSIONS: Many laboratories adopt reference intervals from manufacturers without on-site testing of healthy individuals. Reference intervals used by facilities that forgo on-site testing are not statistically different from intervals validated with on-site studies
SN - 1543-2165
AD - Department of Pathology, Baystate Medical Center, Springfield, Mass, USA
UR - 17516737
ER -

TY - JOUR
ID - 1148
T1 - A meta-analysis of children's hand-to-mouth frequency data for estimating nondietary ingestion exposure
A1 - Xue,J.
A1 - Zartarian,V.
A1 - Moya,J.
A1 - Freeman,N.
A1 - Beamer,P.
A1 - Black,K.
A1 - Tulve,N.
A1 - Shalat,S.
Y1 - 2007/04//
N1 - Xue, Jianping. Zartarian, Valerie. Moya, Jacqueline. Freeman, Natalie. Beamer, Paloma. Black, Kathy. Tulve, Nicolle. Shalat, Stuart
Risk analysis : an official publication of the Society for Risk Analysis
8109978, ria
IM
Journal Article. Meta-Analysis. Research Support, U.S. Gov't, Non-P.H.S.
English
KW - MEDLINE
KW - Age Factors

KW - Child
KW - Preschool
KW - Eating
KW - Environmental Exposure
KW - Environmental Monitoring
KW - Environmental Pollutants/to [Toxicity]
KW - Face
KW - Humans
KW - Infant
KW - Motor Activity
KW - Mouth
KW - Play and Playthings
KW - Risk Assessment
KW - Sex Factors
RP - NOT IN FILE
SP - 411
EP - 420
JF - Risk Analysis
JA - Risk Anal
VL - 27
IS - 2
CY - United States
N2 - Because of their mouthing behaviors, children have a higher potential for exposure to available chemicals through the nondietary ingestion route; thus, frequency of hand-to-mouth activity is an important variable for exposure assessments. Such data are limited and difficult to collect. Few published studies report such information, and the studies that have been conducted used different data collection approaches (e.g., videography versus real-time observation), data analysis and reporting methods, ages of children, locations, and even definitions of "mouthing." For this article, hand-to-mouth frequency data were gathered from 9 available studies representing 429 subjects and more than 2,000 hours of behavior observation. A meta-analysis was conducted to study differences in hand-to-mouth frequency based on study, age group, gender, and location (indoor vs. outdoor), to fit variability and uncertainty distributions that can be used in probabilistic exposure assessments, and to identify any data gaps. Results of this analysis indicate that age and location are important for hand-to-mouth frequency, but study and gender are not. As age increases, both indoor and outdoor hand-to-mouth frequencies decrease. Hand-to-mouth behavior is significantly greater indoors than outdoors. For both indoor and outdoor hand-to-mouth frequencies, interpersonal, and intra-personal variability are approximately 60% and approximately 30%, respectively. The variance difference among different studies is much bigger than its mean, indicating that different studies with different methodologies have similar central values. Weibull distributions best fit the observed data for the different variables considered and are presented in this article by study, age group, and location. Average indoor hand-to-mouth behavior ranged from 6.7 to 28.0 contacts/hour, with the lowest value corresponding to the 6 to <11 year olds and the highest value corresponding to the 3 to <6 month olds. Average outdoor hand-to-mouth frequency ranged from 2.9 to 14.5 contacts/hour, with the lowest value corresponding to the 6 to <11 year olds and the highest value corresponding to the 6 to <12 month olds. The analysis highlights the need for additional hand-to-mouth data for the <3 months, 3 to <6 months, and 3 to <6 year age groups using standardized collection and analysis because of lack of data or high uncertainty in available data. This is the first publication to report Weibull distributions as the best fitting distribution for hand-to-mouth frequency; using the best fitting exposure factor distribution will help improve estimates of exposure. The analyses also represent a first comprehensive effort to fit hand-to-mouth frequency variability and uncertainty distributions by indoor/outdoor location and by age groups, using the new standard set of age groups recommended by the U.S. Environmental Protection Agency for assessing childhood exposures. Thus, the data presented in this article can be used to update the U.S. EPA's Child-Specific Exposure Factors Handbook and to improve estimates of nondietary ingestion in probabilistic exposure modeling
SN - 0272-4332
AD - U.S. Environmental Protection Agency, Boston, MA, USA
UR - 17511707
ER -

TY - JOUR

ID - 1149
T1 - Traumatic dental injuries and attention-deficit/hyperactivity disorder: is there a link?. [Review] [52 refs]
A1 - Sabuncuoglu,O.
Y1 - 2007/06//
N1 - Sabuncuoglu, Osman
Dental traumatology : official publication of International Association for Dental Traumatology
101091305, d2p
D
Journal Article. Review
English
KW - MEDLINE
KW - Accident Proneness
KW - Age Factors
KW - Attention Deficit Disorder with Hyperactivity/co [Complications]
KW - Child
KW - Female
KW - Functional Laterality
KW - Humans
KW - Male
KW - Risk Factors
KW - Sex Factors
KW - Stress
KW - Psychological/co [Complications]
KW - Tooth Injuries/co [Complications]
KW - Violence
RP - NOT IN FILE
SP - 137
EP - 142
JF - Dental Traumatology
JA - Dent Traumatol
VL - 23
IS - 3
CY - Denmark
N2 - Traumatic dental injuries (TDIs) constitute a major cause of morbidity among children worldwide. Both TDIs and attention-deficit/hyperactivity disorder (ADHD) are prevalent conditions with similar male-to-female predominance and similar age of presentation. While the commonest causes of TDIs have been identified as playground or sports accidents, a well-known feature of ADHD is accident proneness. Violence, another significant risk factor for TDIs, is more likely to be observed in conduct disorder, a common comorbidity of ADHD. Also, both TDIs and ADHD have been found to be associated with left-handedness. The presence of multiple dental trauma episodes (MDTE), as well as increased risk for non-dental injuries in children with TDIs indicates an underlying persistent condition, which resembles ADHD. Higher point prevalence of mental distress at age 30, found to be associated with increased lifetime prevalence of TDIs, may also be taken as an evidence of a background developmental disorder persisting into adulthood with various comorbidities. In conclusion, this review combines the results of several studies and explains the significance of ADHD as an underlying and predisposing factor for TDIs. Increased awareness and close collaboration between different disciplines involved are essential. [References: 52]
SN - 1600-4469
AD - Department of Child Psychiatry, School of Medicine, Marmara University, Istanbul, Turkey. sabuncuoglu2004@yahoo.com
UR - 17511834
ER -

TY - JOUR
ID - 1150
T1 - Correlations in refractive errors between siblings in the Singapore Cohort Study of Risk factors for Myopia
A1 - Guggenheim,J.A.
A1 - Pong-Wong,R.

A1 - Haley,C.S.
A1 - Gazzard,G.
A1 - Saw,S.M.
Y1 - 2007/06//
N1 - Guggenheim, Jeremy A. Pong-Wong, Ricardo. Haley, Chris S. Gazzard, Gus. Saw, Seang Mei
The British journal of ophthalmology
azk, 0421041
IM
Journal Article. Research Support, Non-U.S. Gov't
English
KW - MEDLINE
KW - Biometry/mt [Methods]
KW - Child
KW - Cohort Studies
KW - Cornea/pa [Pathology]
KW - Environment
KW - Female
KW - Follow-Up Studies
KW - Humans
KW - Male
KW - Myopia/et [Etiology]
KW - Myopia/ge [Genetics]
KW - Myopia/pa [Pathology]
KW - Quantitative Trait
KW - Heritable
KW - Risk Factors
KW - Siblings
KW - Singapore
KW - Vitreous Body/pa [Pathology]
RP - NOT IN FILE
SP - 781
EP - 784
JF - British Journal of Ophthalmology
JA - Br J Ophthalmol
VL - 91
IS - 6
CY - England
N2 - BACKGROUND: The prevalence of myopia in parts of South East Asia has risen dramatically over the past 1-2 generations, suggesting that environmental factors may be particularly important determinants of refractive development in these populations. AIM: To assess the contribution of familial factors (shared genes and/or shared family environment) to refractive error and ocular component dimensions of school-aged children in Singapore. METHODS: Data were available for 315 children who had one or more siblings also participating in the Singapore Cohort Study of the Risk factors for Myopia (SCORM). Refractive error and ocular biometric parameters were measured under cycloplegia at baseline when children were 7-9 years, and at yearly follow-up sessions for the next 3 years, using consistent clinical procedures. The time children spent performing a variety of nearwork-related tasks was obtained from questionnaires. Familial influences were assessed by calculating between-sibling correlations. RESULTS: After adjusting for age and sex, the between-sibling correlation in refractive error was 0.447 (95% CI 0.314 to 0.564), suggesting that familial factors account for 63-100% of the variation in the cohort. The between-sibling correlation for 1-year change in refractive error was similarly high, at 0.420 (95% CI 0.282 to 0.543). All ocular component dimensions were correlated significantly between siblings, especially for corneal curvature and vitreous chamber depth--the major structural determinants of refraction. The amount of time siblings spent engaged in nearwork tasks (reading, watching TV, playing video games, computing) and in outdoor activities was also highly correlated between siblings (p<0.001). CONCLUSION: Shared genes and/or shared environment are important factors in the refractive development of children in Singapore. Because the time spent in nearwork tasks is highly correlated between siblings, epidemiological studies will benefit from precise, quantitative measures of refractive error in parents and more distant relatives in order to begin to dissociate genetic and environmental sources of variation

SN - 0007-1161
AD - School of Optometry and Vision Sciences, Cardiff University, Cardiff, Wales CF10 3NB, UK. guggenheim@cf.ac.uk
UR - 17135339
ER -

TY - JOUR
ID - 1151
T1 - Outdoor activities and diet in childhood and adolescence relate to MS risk above the Arctic Circle
A1 - Kampman,M.T.
A1 - Wilsgaard,T.
A1 - Mellgren,S.I.
Y1 - 2007/04//
N1 - Kampman, M T. Wilsgaard, T. Mellgren, S I
Journal of neurology
jb7, 0423161
IM
Journal Article
English
KW - MEDLINE
KW - Adolescent
KW - Adult
KW - Arctic Regions/ep [Epidemiology]
KW - Case-Control Studies
KW - Child
KW - Cod Liver Oil/ad [Administration & Dosage]
KW - Confidence Intervals
KW - Dietary Supplements
KW - Environmental Exposure
KW - Female
KW - Humans
KW - Male
KW - Middle Aged
KW - Multiple Sclerosis/ep [Epidemiology]
KW - Multiple Sclerosis/et [Etiology]
KW - Multiple Sclerosis/pc [Prevention & Control]
KW - Multiple Sclerosis
KW - Questionnaires
KW - Retrospective Studies
KW - Risk
KW - Sunlight
RP - NOT IN FILE
SP - 471
EP - 477
JF - Journal of Neurology
JA - J Neurol
VL - 254
IS - 4
CY - Germany
N2 - BACKGROUND: A relationship between the latitude related distribution of multiple sclerosis (MS) and exposure to sunlight has long been considered. Higher sun exposure during early life has been associated with decreased risk of MS. OBJECTIVE: Since Norway is an exception to the latitude gradient of MS prevalence, we tested here whether sunlight exposure or vitamin D-related dietary factors in childhood and adolescence are associated with the risk of MS. METHODS: Retrospective recall questionnaire data from 152 MS patients and 402 population controls born at and living at latitudes 66-71 degrees N were analysed by means of conditional logistic regression analysis accounting for the matching variables age, sex, and place of birth. RESULTS: Increased outdoor activities during summer in early life were associated with a decreased risk of MS, most

pronounced at ages 16-20 years (odds ratio (OR) 0.55, 95% CI 0.39-0.78, p = 0.001, adjusted for intake of fish and cod-liver oil). A protective effect of supplementation with cod-liver oil was suggested in the subgroup that reported low summer outdoor activities (OR 0.57, 95% CI 0.31-1.05, p = 0.072). Consumption of fish three or more times a week was also associated with reduced risk of MS (OR 0.55, 95% CI 0.33-0.93, p = 0.024). CONCLUSION: Summer outdoor activities in childhood and adolescence are associated with a reduced risk of MS even north of the Arctic Circle. Supplemental cod-liver oil may be protective when sun exposure is less, suggesting that both climate and diet may interact to influence MS risk at a population level
SN - 0340-5354
AD - Department of Neurology, University Hospital of North Norway, P.O. Box 33, 9038 Tromso, Norway. margitta.kampman@unn.no
UR - 17377831
ER -

TY - JOUR
ID - 1152
T1 - Implementation of a medical emergency team in a large pediatric teaching hospital prevents respiratory and cardiopulmonary arrests outside the intensive care unit
A1 - Brilli,R.J.
A1 - Gibson,R.
A1 - Luria,J.W.
A1 - Wheeler,T.A.
A1 - Shaw,J.
A1 - Linam,M.
A1 - Kheir,J.
A1 - McLain,P.
A1 - Lingsch,T.
A1 - Hall-Haering,A.
A1 - McBride,M.
Y1 - 2007/05//
N1 - Brilli, Richard J. Gibson, Rosemary. Luria, Joseph W. Wheeler, T Arthur. Shaw, Julie. Linam, Matt. Kheir, John. McLain, Patricia. Lingsch, Tammy. Hall-Haering, Amy. McBride, Mary
Pediatric critical care medicine : a journal of the Society of Critical Care Medicine and the World Federation of Pediatric Intensive and Critical Care Societies
100954653
IM
Journal Article
English
KW - MEDLINE
KW - Child
KW - Emergency Service
KW - Hospital/og [Organization & Administration]
KW - Heart Arrest/pc [Prevention & Control]
KW - Hospitals
KW - Pediatric
KW - Teaching
KW - Humans
KW - Intensive Care Units
KW - Retrospective Studies
RP - NOT IN FILE
SP - 236
EP - 246
JF - Pediatric Critical Care Medicine
JA - Pediatr Crit Care Med
VL - 8
IS - 3
CY - United States

N2 - OBJECTIVE: We implemented a medical emergency team (MET) in our free-standing children's hospital. The specific aim was to reduce the rate of codes (respiratory and cardiopulmonary arrests) outside the intensive care units by 50% for >6 months following MET implementation. DESIGN: Retrospective chart review and program implementation. SETTING: A children's hospital. PATIENTS: None. INTERVENTIONS: The records of patients who required cardiorespiratory resuscitation outside the critical care areas were reviewed before MET implementation to determine activation criteria for the MET. Codes were prospectively defined as respiratory arrests or cardiopulmonary arrests. MET-preventable codes were prospectively defined. The incidence of codes before and after MET implementation was recorded. MEASUREMENTS AND MAIN RESULTS: Twenty-five codes occurred during the pre-MET baseline compared with six following MET implementation. The code rate (respiratory arrests + cardiopulmonary arrests) post-MET was 0.11 per 1,000 patient days compared with baseline of 0.27 (risk ratio, 0.42; 95% confidence interval, 0-0.89; p = .03). The code rate per 1,000 admissions decreased from 1.54 (baseline) to 0.62 (post-MET) (risk ratio, 0.41; 95% confidence interval, 0-0.86; p = .02). For MET-preventable codes, the code rate post-MET was 0.04 per 1,000 patient days compared with a baseline of 0.14 (risk ratio, 0.27; 95% confidence interval, 0-0.94; p = .04). There was no difference in the incidence of cardiopulmonary arrests before and after MET. For codes outside the intensive care unit, the pre-MET mortality rate was 0.12 per 1,000 days compared with 0.06 post-MET (risk ratio, 0.48; 95% confidence interval, 0-1.4, p = .13). The overall mortality rate for outside the intensive care unit codes was 42% (15 of 36 patients). CONCLUSIONS: Implementation of a MET is associated with a reduction in the risk of respiratory and cardiopulmonary arrest outside of critical care areas in a large tertiary children's hospital
SN - 1529-7535
AD - Pediatric Intensive Care Unit, Cincinnati College of Medicine, Cincinnati, OH, USA.
rich.brilli@cchmn.org
UR - 17417113
ER -

TY - JOUR
ID - 1153
T1 - Long-term effects of a playground markings and physical structures on children's recess physical activity levels
A1 - Ridgers,N.D.
A1 - Stratton,G.
A1 - Fairclough,S.J.
A1 - Twisk,J.W.
Y1 - 2007/05//
N1 - Ridgers, Nicola D. Stratton, Gareth. Fairclough, Stuart J. Twisk, Jos W R
Preventive medicine
pm4, 0322116
IM
Journal Article. Research Support, Non-U.S. Gov't
English
KW - MEDLINE
KW - Child
KW - England
KW - Exercise
KW - Facility Design and Construction
KW - Female
KW - Humans
KW - Male
KW - Motivation
KW - Play and Playthings
KW - Schools
RP - NOT IN FILE
SP - 393
EP - 397
JF - Preventive Medicine
JA - Prev Med

VL - 44
IS - 5
CY - United States
N2 - OBJECTIVE: The aim of the study was to investigate the impact of a playground redesign intervention across time on children's recess physical activity levels using combined physical activity measures and to evaluate the potential influence of covariates on the intervention effect. METHOD: Fifteen schools located in areas of high deprivation in one large city in England each received 20,000 pounds through a national 10 million pounds Sporting Playgrounds Initiative to redesign the playground environment based on a multicolored zonal design. Eleven schools served as matched socioeconomic controls. Physical activity levels during recess were quantified using heart rate telemetry and accelerometry at baseline, 6 weeks and 6 months following the playground redesign intervention. Data were collected between July 2003 and January 2005 and analyzed using multilevel modeling. RESULTS: Statistically significant intervention effects were found across time for moderate-to-vigorous and vigorous physical activity assessed using both heart rate and accelerometry. CONCLUSIONS: The results suggest that a playground redesign, which utilizes multicolor playground markings and physical structures, is a suitable stimulus for increasing children's school recess physical activity levels
SN - 0091-7435
AD - Research Institute for Sport and Exercise Sciences, Liverpool John Moores University, Henry Cotton Campus, 15-21 Webster Street, Liverpool L3 2ET, UK. n.ridgers@ljmu.ac.uk
UR - 17335891
ER -

TY - JOUR
ID - 1154
T1 - Digital childhood: electronic media and technology use among infants, toddlers, and preschoolers
A1 - Vandewater,E.A.
A1 - Rideout,V.J.
A1 - Wartella,E.A.
A1 - Huang,X.
A1 - Lee,J.H.
A1 - Shim,M.S.
Y1 - 2007/05//
N1 - Vandewater, Elizabeth A. Rideout, Victoria J. Wartella, Ellen A. Huang, Xuan. Lee, June H. Shim, Mi-suk
Pediatrics
oxv, 0376422
AIM, IM
Journal Article. Research Support, N.I.H., Extramural. Research Support, Non-U.S. Gov't. Research Support, U.S. Gov't, Non-P.H.S.
English
KW - MEDLINE
KW - Age Factors
KW - Child
KW - Preschool
KW - Communications Media/td [Trends]
KW - Data Collection
KW - Female
KW - Humans
KW - Infant
KW - Male
KW - Socioeconomic Factors
KW - Technology/td [Trends]
KW - Technology
KW - Television/td [Trends]
KW - Television
KW - United States
RP - NOT IN FILE

SP - e1006
EP - e1015
JF - Pediatrics
VL - 119
IS - 5
CY - United States
N2 - OBJECTIVES: The objectives of this study were to describe media access and use among US children aged 0 to 6, to assess how many young children fall within the American Academy of Pediatrics media-use guidelines, to identify demographic and family factors predicting American Academy of Pediatrics media-use guideline adherence, and to assess the relation of guideline adherence to reading and playing outdoors. METHODS: Data from a representative sample of parents of children aged 0 to 6 (N = 1051) in 2005 were used. Descriptive analyses, logistic regression, and multivariate analyses of covariance were used as appropriate. RESULTS: On a typical day, 75% of children watched television and 32% watched videos/DVDs, for approximately 1 hour and 20 minutes, on average. New media are also making inroads with young children: 27% of 5- to 6-year-olds used a computer (for 50 minutes on average) on a typical day. Many young children (one fifth of 0- to 2-year-olds and more than one third of 3- to 6-year-olds) also have a television in their bedroom. The most common reason given was that it frees up other televisions in the house so that other family members can watch their own shows (54%). The majority of children aged 3 to 6 fell within the American Academy of Pediatrics guidelines, but 70% of 0- to 2-year-olds did not. CONCLUSIONS: This study is the first to provide comprehensive information regarding the extent of media use among young children in the United States. These children are growing up in a media-saturated environment with almost universal access to television, and a striking number have a television in their bedroom. Media and technology are here to stay and are virtually guaranteed to play an ever-increasing role in daily life, even among the very young. Additional research on their developmental impact is crucial to public health
SN - 1098-4275
AD - Population Research Center, University of Texas, 1 University Station, G1800, Austin, TX 78712-0544, USA. elizvan@prc.utexas.edu
UR - 17473074
ER -

TY - JOUR
ID - 1155
T1 - Assessment and treatment of problem behavior occasioned by interruption of free-operant behavior
A1 - Hagopian,L.P.
A1 - Bruzek,J.L.
A1 - Bowman,L.G.
A1 - Jennett,H.K.
Y1 - 2007///
N1 - Hagopian, Louis P. Bruzek, Jennifer L. Bowman, Lynn G. Jennett, Heather K
Journal of applied behavior analysis
0174763, hdr
IM
Case Reports. Journal Article
English
KW - MEDLINE
KW - Aggression/px [Psychology]
KW - Attention Deficit and Disruptive Behavior Disorders/th [Therapy]
KW - Autistic Disorder/th [Therapy]
KW - Behavior Therapy
KW - Child
KW - Child Behavior Disorders/th [Therapy]
KW - Conditioning
KW - Operant
KW - Cooperative Behavior
KW - Extinction
KW - Psychological
KW - Female

KW - Generalization (Psychology)
KW - Humans
KW - Intellectual Disability/th [Therapy]
KW - Male
KW - Motivation
KW - Reinforcement Schedule
KW - Reversal Learning
KW - Self-Injurious Behavior/pc [Prevention & Control]
RP - NOT IN FILE
SP - 89
EP - 103
JF - Journal of Applied Behavior Analysis
JA - J Appl Behav Anal
VL - 40
IS - 1
CY - United States
N2 - The current study describes the assessment and treatment of the problem behavior of 3 individuals with autism for whom initial functional analysis results were inconclusive. Subsequent analyses revealed that the interruption of free-operant behavior using "do" requests (Study 1) as well as "do" and "don't" requests (Study 2) occasioned problem behavior. Initially, treatment involved differential and noncontingent reinforcement without interruption. To make the intervention more sustainable in the natural environment (where interruptions are unavoidable), a two-component multiple-schedule arrangement was used to progressively increase the period of time in which ongoing activities would be interrupted. During generalization sessions, the intervention was applied across a variety of contexts and therapists
SN - 0021-8855
AD - Department of Behavioral Psychology, Kennedy Krieger Institute and Johns Hopkins University School of Medicine, Baltimore, Maryland 21205, USA. hagopian@kennedykrieger.org
UR - 17471795
ER -

TY - JOUR
ID - 1156
T1 - Predicting functional change from preintervention measures in selective dorsal rhizotomy
A1 - Engsberg,J.R.
A1 - Ross,S.A.
A1 - Collins,D.R.
A1 - Park,T.S.
Y1 - 2007/04//
N1 - Engsberg, Jack R. Ross, Sandy A. Collins, David R. Park, Tae Sung
Journal of neurosurgery
jd3, 0253357
AIM, IM
Clinical Trial. Journal Article. Research Support, N.I.H., Extramural
English
KW - MEDLINE
KW - Cerebral Palsy/pp [Physiopathology]
KW - Cerebral Palsy/su [Surgery]
KW - Child
KW - Preschool
KW - Female
KW - Gait/ph [Physiology]
KW - Humans
KW - Male
KW - Motor Activity/ph [Physiology]
KW - Muscle Strength/ph [Physiology]
KW - Predictive Value of Tests
KW - Range of Motion

KW - Articular/ph [Physiology]
KW - Rhizotomy
KW - Spinal Nerve Roots/su [Surgery]
KW - Treatment Outcome
RP - NOT IN FILE
SP - 282
EP - 287
JF - Journal of Neurosurgery
JA - J Neurosurg Pediatrics
VL - 106
IS - 4 Suppl
CY - United States
N2 - OBJECT: In this investigation the authors attempted to predict change in function following selective dorsal rhizotomy (SDR) and intensive physical therapy in patients with spastic diplegic cerebral palsy (CP) based on multidomain preintervention measures. METHODS: Data pertaining to 22 children with CP were collected before the SDR and again 20 months afterward. Although equations for predicting change in gait speed and function (such as the Gross Motor Function Measure) were derived, the 95% confidence interval (CI) widths were too broad to make accurate predictions that were clinically useful outside the study group. CONCLUSIONS: Future work should be focused on developing additional measures such as lower-extremity motor control and balance in an attempt to reduce the CIs to more clinically relevant values
SN - 0022-3085
AD - Department of Physical Therapy, Saint Louis University, Missouri 63104, USA. jengsber@slu.edu
UR - 17465361
ER -

TY - JOUR
ID - 1157
T1 - Determinants of activity-friendly neighborhoods for children: results from the SPACE study
A1 - de Vries,S.I.
A1 - Bakker,I.
A1 - van,Mechelen W.
A1 - Hopman-Rock,M.
Y1 - 2007/03//
N1 - de Vries, Sanne I. Bakker, Ingrid. van Mechelen, Willem. Hopman-Rock, Marijke
American journal of health promotion : AJHP
amj, 8701680
T
Journal Article. Research Support, Non-U.S. Gov't
English
KW - MEDLINE
KW - Activities of Daily Living/px [Psychology]
KW - Anthropometry
KW - Body Mass Index
KW - Child
KW - Child Behavior
KW - Cross-Sectional Studies
KW - Environment Design
KW - Female
KW - Geography
KW - Health Promotion
KW - Humans
KW - Life Style
KW - Male
KW - Medical Records
KW - Motor Activity
KW - Netherlands
KW - Residence Characteristics/cl [Classification]

RP - NOT IN FILE
SP - 312
EP - 316
JF - American Journal of Health Promotion
JA - Am J Health Promot
VL - 21
IS - 4 Suppl
CY - United States
N2 - PURPOSE: To examine the association between children's physical activity and factors of the built environment. DESIGN: Cross-sectional study. Setting. Ten neighborhoods in six cities in the Netherlands. SUBJECTS: Four hundred twenty-two children (age range, 6-11 years; 49% male). MEASURES: Physical activity diary, neighborhood observations, and anthropometric measures. ANALYSIS: Univariate and multivariate linear regression analyses. RESULTS: According to univariate analyses adjusted for age, sex, body mass index, and highest level of maternal education, physical activity (> or = 3 metabolic equivalents) was significantly (p < .05) associated with the proportion of green space, with the residential density, with the general impression of activity-friendliness of the neighborhood, and with the frequency of certain types of residences (e.g., terraced houses), sports fields, water, dog waste, heavy traffic, and safe walking and cycling conditions (e.g., cycle tracks and 30-km speed zones) in the neighborhood. According to adjusted multivariate analyses, physical activity was best predicted by the frequency of parallel parking spaces in the neighborhood and by the general impression of activity-friendliness of the neighborhood (I2 = 0. 193). CONCLUSIONS: Children's physical activity is associated with certain modifiable factors of the built environment. Longitudinal studies should examine whether there is a causal relationship
SN - 0890-1171
AD - Department of Physical Activity and Health, TNO Quality of Life, Leiden, the Netherlands.
sanne.devries@tno.nl
UR - 17465176
ER -

TY - JOUR
ID - 1158
T1 - Fasting plasma glucose levels in healthy preschool children: effects of weight and lifestyle
A1 - Ludvigsson,J.
A1 - Huus,K.
A1 - Eklov,K.
A1 - Klintstrom,R.
A1 - Lahdenpera,A.
Y1 - 2007/05//
N1 - Ludvigsson, Johnny. Huus, Karina. Eklov, Kristina. Klintstrom, Rebecka. Lahdenpera, Anne
Acta paediatrica (Oslo, Norway : 1992)
bgc, 9205968
IM
Journal Article. Multicenter Study. Research Support, Non-U.S. Gov't
English
KW - MEDLINE
KW - Blood Glucose/an [Analysis]
KW - Body Mass Index
KW - Body Weight
KW - Child
KW - Preschool
KW - Female
KW - Humans
KW - Life Style
KW - Male
KW - Television
KW - Time Factors
RP - NOT IN FILE
SP - 706

EP - 709
JF - Acta Paediatrica
JA - Acta Paediatr
VL - 96
IS - 5
CY - Norway
N2 - AIM: To investigate whether a modern lifestyle, with a high-energy intake and a low level of physical activity, influences fasting plasma glucose concentration in healthy children. METHODS: As a part of the prospective study 'All Babies in Southeast Sweden', 127 children from six preschool units chose to participate. The children, 56% girls and 44% boys, were 5-7 years old. Parents answered a questionnaire about their children's heredity, and physical exercise and eating habits. In the morning, before the children ate breakfast, fasting plasma glucose levels and weight, height and waist circumference were measured. RESULTS: Fasting plasma glucose levels varied between 3.7 and 6.1 mmol/L, with both mean and median values of 4.7 mmol/L. There was no association between fasting plasma glucose level and body mass index (BMI), eating habits or degree of physical exercise. BMI and waist circumference were significantly correlated ($p < 0.01$). Children who play outdoors most frequently had a significantly lower BMI ($p < 0.05$) and waist circumference ($p < 0.01$), whereas children who more often watch TV had a significantly higher BMI ($p < 0.01$). CONCLUSION: A modern lifestyle, with low levels of exercise and high-energy consumption, may explain the increasing weight and even obesity of otherwise healthy, preschool children, but does not influence their fasting plasma glucose levels
SN - 0803-5253
AD - Div of Pediatrics and Diabetes Research Centre, Linkoping University, Sweden
UR - 17376178
ER -

TY - JOUR
ID - 1159
T1 - The pattern and genetics of pediatric extrinsic asthma risk factors in polluted environment
A1 - Bener,A.
A1 - Ehlayel,M.
A1 - Sabbah,A.
Y1 - 2007/02//
N1 - Bener, Abdulbari. Ehlayel, Mohammed. Sabbah, Alfred
European annals of allergy and clinical immunology
101466614
IM
Journal Article. Research Support, Non-U.S. Gov't
English
KW - MEDLINE
KW - Adolescent
KW - Air Pollutants/ae [Adverse Effects]
KW - Asthma/ci [Chemically Induced]
KW - Asthma/et [Etiology]
KW - Asthma/ge [Genetics]
KW - Case-Control Studies
KW - Child
KW - Eczema/ci [Chemically Induced]
KW - Eczema/et [Etiology]
KW - Eczema/ge [Genetics]
KW - Environmental Pollution/ae [Adverse Effects]
KW - Female
KW - Genetic Predisposition to Disease
KW - Humans
KW - Male
KW - Rhinitis/ci [Chemically Induced]
KW - Rhinitis/et [Etiology]
KW - Rhinitis/ge [Genetics]

KW - Risk Factors
RP - NOT IN FILE
SP - 58
EP - 63
JF - European Annals of Allergy & Clinical Immunology
JA - Eur Ann Allergy Clin Immunol
VL - 39
IS - 2
CY - France
N2 - AIM: The aim of the present study is to determine the effect of polluted environment on extrinsic of asthma and allergic diseases among school children. DESIGN: This is case and control study. SETTING: The study was carried out among school children living and attending the school in industrial and residential area during the period of October 2004 and June 2005. SUBJECTS: The study based on age, sex, and ethnicity of 716 cases (with asthma) and 716 controls (without asthma) school children living in both urban and in industrial polluted with oil refinery and chemical pollutant. METHODS: The International study of asthma and allergies in childhood (ISAAC) and some additional questionnaires were used to collect the data of the school children. The questionnaire included information about: socio-demographic characteristics; respiratory symptoms; associated respiratory illness; family history of allergic diseases among first-degree relatives of asthmatic children; behavioural factors which could be additive to asthma. Univariate and multivariate statistical analyses were performed. RESULTS: The proportion of children in the asthmatic group who reported symptoms was significantly higher than in non-asthmatic group (<0.0001). The asthmatic group reported that 47.5% morning time breathlessness, shortness of breath (61.4%), wheeze after exercise (65.4%), phlegm (45.3%) and chronic cough (42.2%). Male asthmatics had a average age at onset of symptoms of 6.9% (+/-4.8%) years compared with female asthmatics who had higher age at onset of symptoms, 7.6 (+/-5.9). Male asthmatics also had longer duration of symptoms (7.5 +/- 4.9 in males and 6.4 +/- 4.6 years in females). Significantly odds ratios were found higher in asthmatic compared to controls for pneumonia, bronchitis, atopy (allergic rhinitis and atopic dermatitis), sinusitis, croup, parental asthmas, parental atopy including parental allergic rhinitis, atopic dermatitis and parental smoking (p < 0.0001). The logistic regression model showed that shortness of breath, bronchitis, pneumonia, sinusitis, parental asthma, allergic rhinitis, atopic dermatitis, croup, pets ownership and parental smoking were significant risk factors for asthma CONCLUSION: The present study provides some evidence that exposure to outdoor air pollutants increases the risk of childhood asthma and allergic diseases in school children. The results are consistent with the hypothesis that long term exposure to NOx and CO levels suggests that emissions from photochemical air pollution and oil refinery contributes to adverse health effects in Qatar
SN - 1764-1489
AD - Department of Medical Statistics and Epidemiology, Hamad Medical Corporation, Qatar
UR - 17441417
ER -

TY - JOUR
ID - 1160
T1 - Neurobehavioral development in children with potential exposure to pesticides
A1 - Handal,A.J.
A1 - Lozoff,B.
A1 - Breilh,J.
A1 - Harlow,S.D.
Y1 - 2007/05//
N1 - Handal, Alexis J. Lozoff, Betsy. Breilh, Jaime. Harlow, Sioban D
Epidemiology (Cambridge, Mass.)
a2t, 9009644
IM
Journal Article. Research Support, N.I.H., Extramural. Research Support, Non-U.S. Gov't
English
KW - MEDLINE
KW - Agriculture
KW - Child Development/de [Drug Effects]
KW - Child

KW - Preschool
KW - Ecuador/ep [Epidemiology]
KW - Environmental Exposure/ae [Adverse Effects]
KW - Female
KW - Flowers
KW - Humans
KW - Male
KW - Motor Skills/de [Drug Effects]
KW - Neuropsychological Tests
KW - Pesticides/ae [Adverse Effects]
KW - Problem Solving/de [Drug Effects]
KW - Residence Characteristics
KW - Risk Factors
KW - Socioeconomic Factors
KW - Water
KW - Water Pollutants
KW - Chemical/ae [Adverse Effects]
RP - NOT IN FILE
SP - 312
EP - 320
JF - Epidemiology
VL - 18
IS - 3
CY - United States
N2 - BACKGROUND: Children may be at higher risk than adults from pesticide exposure, due to their rapidly developing physiology, unique behavioral patterns, and interactions with the physical environment. This preliminary study conducted in Ecuador examines the association between household and environmental risk factors for pesticide exposure and neurobehavioral development. METHODS: We collected data over 6 months in the rural highland region of Cayambe, Ecuador (2003-2004). Children age 24-61 months residing in 3 communities were assessed with the Ages and Stages Questionnaire and the Visual Motor Integration Test. We gathered information on maternal health and work characteristics, the home and community environment, and child characteristics. Growth measurements and a hemoglobin finger-prick blood test were obtained. Multiple linear regression analyses were conducted. RESULTS: Current maternal employment in the flower industry was associated with better developmental scores. Longer hours playing outdoors were associated with lower gross and fine motor and problem solving skills. Children who played with irrigation water scored lower on fine motor skills (8% decrease; 95% confidence interval = -9.31 to -0.53), problem-solving skills (7% decrease; -8.40 to -0.39), and Visual Motor Integration test scores (3% decrease; -12.00 to 1.08). CONCLUSIONS: These results suggest that certain environmental risk factors for exposure to pesticides may affect child development, with contact with irrigation water of particular concern. However, the relationships between these risk factors and social characteristics are complex, as corporate agriculture may increase risk through pesticide exposure and environmental contamination, while indirectly promoting healthy development by providing health care, relatively higher salaries, and daycare options
SN - 1044-3983
AD - Department of Epidemiology, University of Michigan School of Public Health, Ann Arbor, MI, USA. handalal@mail.nih.gov
UR - 17435439
ER -

TY - JOUR
ID - 1161
T1 - Attitudes of mothers towards their child with Down syndrome before and after the introduction of prenatal diagnosis
A1 - Lenhard,W.
A1 - Breitenbach,E.
A1 - Ebert,H.
A1 - Schindelhauer-Deutscher,H.J.
A1 - Zang,K.D.

A1 - Henn,W.
Y1 - 2007/04//
N1 - Lenhard, Wolfgang. Breitenbach, Erwin. Ebert, Harald. Schindelhauer-Deutscher, H Joachim. Zang, Klaus D. Henn, Wolfram
Intellectual and developmental disabilities
101299965
IM
Journal Article. Research Support, Non-U.S. Gov't
English
KW - MEDLINE
KW - Adolescent
KW - Adult
KW - Attitude to Health
KW - Child
KW - Preschool
KW - Down Syndrome/di [Diagnosis]
KW - Female
KW - Humans
KW - Infant
KW - Newborn
KW - Mother-Child Relations
KW - Mothers/px [Psychology]
KW - Pregnancy
KW - Prenatal Diagnosis
KW - Questionnaires
KW - Time Factors
RP - NOT IN FILE
SP - 98
EP - 102
JF - Intellectual & Developmental Disabilities
JA - Intellect Dev Disabil
VL - 45
IS - 2
CY - United States
N2 - In 1970, before the introduction of prenatal diagnosis of chromosome anomalies, an unpublished questionnaire study concerning the social and emotional situation of mothers of children with Down syndrome was conducted in southern Germany. To assess the psychosocial impact of the availability of prenatal diagnosis on parents of genetically handicapped children, we re-evaluated and repeated the 1970 study over 30 years later. Although mothers' feelings of guilt for having a child with disabilities remained on a low level, today's mothers have a stronger feeling of being involuntarily segregated in society. On the other hand, they more often experience support and respect from outside, particularly through self-support groups; moreover, tendencies of active withdrawal from social life have decreased
SN - 1934-9491
AD - Institute of Special Education, Wuerzburg University, Wuerzburg, Germany
UR - 17428145
ER -

TY - JOUR
ID - 1162
T1 - Secular trends in the aerobic fitness test performance and body mass index of Korean children and adolescents (1968-2000)
A1 - Tomkinson,G.R.
A1 - Olds,T.S.
A1 - Kang,S.J.
A1 - Kim,D.Y.
Y1 - 2007/04//

N1 - Tomkinson, G R. Olds, T S. Kang, S J. Kim, D Y
International journal of sports medicine
8008349, grk
IM
Comparative Study. Journal Article
English
KW - MEDLINE
KW - Adolescent
KW - Body Mass Index
KW - Child
KW - Energy Metabolism
KW - Exercise Test
KW - Female
KW - Humans
KW - Korea
KW - Male
KW - Physical Fitness/ph [Physiology]
RP - NOT IN FILE
SP - 314
EP - 320
JF - International Journal of Sports Medicine
JA - Int J Sports Med
VL - 28
IS - 4
CY - Germany
N2 - There is increasing evidence that the aerobic fitness performance of children is declining, at least in developed countries. To see if there was evidence of similar trends in a non-Western country, this study analysed data on 6-18-year-old Koreans tested between 1968 and 2000 using distance runs ranging from 600 to 1200 m. All existing data on the results of children's aerobic fitness tests in Korea were collated. In addition to six individual studies, very large datasets were available from the Korean Ministries of Education, and the Ministry of Culture and Tourism. Data on a total of 22,127,265 6-18-year-old children were available in the form of group means and standard deviations. Data were collated for each age x sex x test group, and performances were expressed as percentages of the fitted values for the year 1992 to standardise across tests, ages and sexes. All age x sex x test groups were then combined, and curves were fitted using weighted regression. A two-linear segment model best described the pattern of change ($r = 0.83$, $p < 0.001$). There was a relatively slow decline (0.26 % per year) in the aerobic performance of Korean children between 1968 and 1984. After 1984, however, there was a steep decline in performance, averaging 0.80 % per year. The rate of decline was greater in boys, younger children and children from outside the capital Seoul. Changes in running performance showed a similar pattern to changes in estimated body mass index. Compared to other countries, there has been a sharp decline in Korean children's performance on tests of aerobic fitness, which has been concurrent with increases in estimated body mass index
SN - 0172-4622
AD - Centre for Applied Anthropometry, School of Health Sciences, University of South Australia, Adelaide, Australia. grant.tomkinson@unisa.edu.au
UR - 17024618
ER -

TY - JOUR
ID - 1163
T1 - Pediatric endurance and limb strengthening for children with cerebral palsy (PEDALS)--a randomized controlled trial protocol for a stationary cycling intervention
A1 - Fowler,E.G.
A1 - Knutson,L.M.
A1 - DeMuth,S.K.
A1 - Sugi,M.
A1 - Siebert,K.
A1 - Simms,V.

A1 - Azen,S.P.
A1 - Winstein,C.J.
Y1 - 2007///
N1 - Fowler, Eileen G. Knutson, Loretta M. DeMuth, Sharon K. Sugi, Mia. Siebert, Kara. Simms, Victoria. Azen, Stanley P. Winstein, Carolee J
BMC pediatrics
100967804
IM
Journal Article. Randomized Controlled Trial. Research Support, Non-U.S. Gov't
English
KW - MEDLINE
KW - Adolescent
KW - Bicycling
KW - Cerebral Palsy/pp [Physiopathology]
KW - Cerebral Palsy/th [Therapy]
KW - Child
KW - Clinical Protocols
KW - Exercise Therapy/is [Instrumentation]
KW - Exercise Therapy/mt [Methods]
KW - Humans
KW - Leg
KW - Muscle
KW - Skeletal/pp [Physiopathology]
KW - Physical Endurance
KW - Quality of Life
KW - Randomized Controlled Trials as Topic/mt [Methods]
KW - Walking
RP - NOT IN FILE
SP - 14
JF - BMC Pediatrics
JA - BMC Pediatr
VL - 7
CY - England
N2 - BACKGROUND: In the past, effortful exercises were considered inappropriate for children with spastic cerebral palsy (CP) due to concern that they would escalate abnormalities including spasticity and abnormal movement patterns. Current scientific evidence indicates that these concerns were unfounded and that therapeutic interventions focused on muscle strengthening can lead to improved functional ability. However, few studies have examined the potential benefits of cardiorespiratory fitness exercises in this patient population. METHODS/DESIGN: The rationale and design of a randomized controlled trial examining the effects of a stationary cycling intervention for children with CP are outlined here. Sixty children with spastic diplegic CP between the ages of 7 and 18 years and Gross Motor Function Classification System (GMFCS) levels of I, II, or III will be recruited for this study. Participants will be randomly assigned to either an intervention (cycling) or a control (no cycling) group. The cycling intervention will be divided into strengthening and cardiorespiratory endurance exercise phases. During the strengthening phase, the resistance to lower extremity cycling will be progressively increased using a uniquely designed limb-loaded mechanism. The cardiorespiratory endurance phase will focus on increasing the intensity and duration of cycling. Children will be encouraged to exercise within a target heart rate (HR) range (70-80% maximum HR). Thirty sessions will take place over a 10-12 week period. All children will be evaluated before (baseline) and after (follow-up) the intervention period. Primary outcome measures are: knee joint extensor and flexor moments, or torque; the Gross Motor Function Measure (GMFM); the 600 Yard Walk-Run test and the Thirty-Second Walk test (30 sec WT). DISCUSSION: This paper presents the rationale, design and protocol for Pediatric Endurance and Limb Strengthening (PEDALS); a Phase I randomized controlled trial evaluating the efficacy of a stationary cycling intervention for children with spastic diplegic cerebral palsy
SN - 1471-2431
AD - Department of Orthopedics, UCLA/Orthopaedic Hospital Center for Cerebral Palsy, Los Angeles, CA, USA. efowler@mednet.ucla.edu
UR - 17374171

ER -

TY - JOUR
ID - 1164
T1 - Carbon monoxide poisoning in Florida during the 2004 hurricane season
A1 - Van,Sickle D.
A1 - Chertow,D.S.
A1 - Schulte,J.M.
A1 - Ferdinands,J.M.
A1 - Patel,P.S.
A1 - Johnson,D.R.
A1 - Harduar-Morano,L.
A1 - Blackmore,C.
A1 - Ourso,A.C.
A1 - Cruse,K.M.
A1 - Dunn,K.H.
A1 - Moolenaar,R.L.
Y1 - 2007/04//
N1 - Van Sickle, David. Chertow, Daniel S. Schulte, Joann M. Ferdinands, Jill M. Patel, Prakash S. Johnson, David R. Harduar-Morano, Laurel. Blackmore, Carina. Ourso, Andre C. Cruse, Kelly M. Dunn, Kevin H. Moolenaar, Ronald L
American journal of preventive medicine
8704773, apl
IM
Journal Article
English
KW - MEDLINE
KW - Adolescent
KW - Adult
KW - Aged
KW - Carbon Monoxide Poisoning/ep [Epidemiology]
KW - Child
KW - Preschool
KW - Disasters
KW - Female
KW - Florida/ep [Epidemiology]
KW - Humans
KW - Infant
KW - Interviews as Topic
KW - Male
KW - Medical Audit
KW - Middle Aged
RP - NOT IN FILE
SP - 340
EP - 346
JF - American Journal of Preventive Medicine
JA - Am J Prev Med
VL - 32
IS - 4
CY - Netherlands
N2 - BACKGROUND: During August-September 2004, four major hurricanes hit Florida, resulting in widespread power outages affecting several million households. Carbon monoxide (CO) poisonings during this period were investigated to identify ways to prevent future poisoning. METHODS: Medical records from ten hospitals (two with hyperbaric oxygen chambers) were reviewed to identify individuals diagnosed with unintentional CO poisoning between August 13 and October 15, 2004. Multiple attempts were made to interview one person from each nonfatal incident. Medical examiner records and reports of investigations conducted by the U.S. Consumer Product Safety Commission of six fatal poisonings from five additional

incidents were also reviewed. RESULTS: A total of 167 people treated for nonfatal CO poisoning were identified, representing 51 incidents. A portable, gasoline-powered generator was implicated in nearly all nonfatal incidents and in all fatal poisonings. Generators were most often located outdoors, followed by inside the garage, and inside the home. Telephone interviews with representatives of 35 (69%) incidents revealed that concerns about theft or exhaust most often influenced the choice of location. Twenty-six (74%) households did not own a generator before the hurricanes, and 86% did not have a CO detector at the time of the poisoning. Twenty-one (67%) households reported reading or hearing CO education messages before the incident. CONCLUSIONS: Although exposure to public education messages may have encouraged more appropriate use of generators, a substantial number of people were poisoned even when the devices were operated outdoors. Additional educational efforts and engineering solutions that reduce CO emission from generators should be the focus of public health activities
SN - 0749-3797
AD - Epidemic Intelligence Service, Centers for Disease Control and Prevention, Atlanta, Georgia, USA. vansickle@wisc.edu
UR - 17383566
ER -

TY - JOUR
ID - 1165
T1 - Extremely-low-frequency magnetic field exposure of children at schools near high voltage transmission lines
A1 - Li,C.Y.
A1 - Sung,F.C.
A1 - Chen,F.L.
A1 - Lee,P.C.
A1 - Silva,M.
A1 - Mezei,G.
Y1 - 2007/04/15/
N1 - Li, Chung-Yi. Sung, Fung-Chang. Chen, Fu-Li. Lee, Pei-Chen. Silva, Michael. Mezei, Gabor
The Science of the total environment
uj0, 0330500
IM
Journal Article. Research Support, Non-U.S. Gov't
English
KW - MEDLINE
KW - Child
KW - Electromagnetic Fields
KW - Environmental Exposure/an [Analysis]
KW - Female
KW - Humans
KW - Male
KW - Schools
KW - Taiwan
RP - NOT IN FILE
SP - 151
EP - 159
JF - Science of the Total Environment
JA - Sci Total Environ
VL - 376
IS - 1-3
CY - Netherlands
N2 - Many epidemiological studies have investigated residential exposure to extremely-low-frequency magnetic field (ELF-MF) for children, but exposure at schools where children may stay up to 8 h every weekday was rarely considered. Between March and June 2004, we carried out a field study in Taipei City and County of northern Taiwan to explore ELF-MF exposure pattern among children at schools with high voltage transmission lines (HVTL) running through the campuses. One hundred and one children attending 14 schools with nearby HVTL (exposed group) and 123 children of 18 schools at least 100 m away from HVTL

(unexposed group) were monitored for 24-hour personal ELF-MF exposure. Selected classrooms and playgrounds within the buffer regions (i.e., within 30 m of HVTL) and those away from the buffer regions were also assessed, using spot measurements, to determine the extent to which HVTL may contribute to the environmental exposure on campuses. Results show that the two groups didn't differ significantly for both mean exposure and proportion of exposure greater than 0.4 micro-Tesla (microT) estimated for 24 h. However, we noted that 17.8% of the exposed children had a personal mean exposure greater than 0.4 microT during school hours, significantly (p=0.011) higher than that (6.5%) estimated for the unexposed children. An even higher percentage (27.3%) was observed for 11 students with their classrooms intersecting the buffer regions. Playgrounds near buffer regions showed a significantly higher ELF-MF than the other areas of the exposed schools (0.70 vs 0.18 microT, p=0.043). The study demonstrates a wide range of exposures to ELF-MF among school children at campuses adjacent to HVTL. Children attending the exposed schools may have a higher chance of experiencing a mean exposure greater than 0.4 microT during school hours
SN - 0048-9697
AD - Department of Public Health, College of Medicine, Fu-Jen Catholic University, 510 Chung-Cheng Road, Hsinchuang, Taipei Hsien 24205, Taiwan. chungyi@mails.fju.edu.tw
UR - 17316772
ER -

TY - JOUR
ID - 1166
T1 - Association between activity level and situational factors in children with attention deficit/hyperactivity disorder in elementary school
A1 - Tsujii,N.
A1 - Okada,A.
A1 - Kaku,R.
A1 - Kuriki,N.
A1 - Hanada,K.
A1 - Matsuo,J.
A1 - Kusube,T.
A1 - Hitomi,K.
Y1 - 2007/04//
N1 - Tsujii, Noa. Okada, Akira. Kaku, Reigetsu. Kuriki, Noriko. Hanada, Kazushi. Matsuo, Junko. Kusube, Takeshi. Hitomi, Kazuhiko
Psychiatry and clinical neurosciences
cfs, 9513551
IM
Journal Article
English
KW - MEDLINE
KW - Attention Deficit Disorder with Hyperactivity/px [Psychology]
KW - Child
KW - Female
KW - Humans
KW - Japan
KW - Male
KW - Motor Activity
KW - Psychiatric Status Rating Scales
KW - Questionnaires
KW - Schools
KW - Social Environment
RP - NOT IN FILE
SP - 181
EP - 185
JF - Psychiatry & Clinical Neurosciences
JA - Psychiatry Clin Neurosci
VL - 61
IS - 2

CY - Australia
N2 - The aim of this study was to determine whether the objective activity level of children with attention deficit/hyperactivity disorder (AD/HD) is associated with situational factors in elementary school. A total of 16 children with combined-type AD/HD (13 boys, 3 girls) and 20 controls matched for age and gender participated in the study. All the participants wore the objective measurement of activity level for 1 week while attending elementary school. The average activity levels were calculated for the morning and afternoon classes. The classes were categorized into four types: (i) in-seat classes, in which the participants were expected to learn quietly while sitting in their own seats; (ii) not-in-seat classes, in which the participants were not expected to sit in their seats; (iii) physical education classes; and (iv) lunch/recess periods. During the afternoon in-seat classes, the children with AD/HD were significantly more active than the controls. However, no significant differences based on the group (children with AD/HD or controls) were observed during the other morning or afternoon classes. These findings indicate that in naturalistic settings where the effects of situational demands involving inhibition and the effect of fatigue overlapped, the children with AD/HD exhibited an activity level that was different from that exhibited by the controls. Differences in the activity levels of children with AD/HD and normal controls were identified using an objective measurement of activity level when two situational factors overlapped
SN - 1323-1316
AD - Department of Neuropsychiatry, Kinki University School of Medicine, Osaka, Japan.
noa@pop21.odn.ne.jp
UR - 17362436
ER -

TY - JOUR
ID - 1167
T1 - Increase of exhaled nitric oxide in children exposed to low levels of ambient ozone.[Erratum appears in J Toxicol Environ Health A. 2009;72(5):358 Note: Sylviane, Carbonnelle [corrected to Carbonnelle, Sylviane]; Xavier, Dumont [corrected to Dumont, Xavier]; Alfred, Bernard [corrected to Bernard, Alfred]; Alain, Derouane [corrected to Derouane, Alain]]
A1 - Nickmilder,M.
A1 - de,Burbure C.
A1 - Carbonnelle,S.
A1 - Dumont,X.
A1 - Bernard,A.
A1 - Derouane,A.
Y1 - 2007/02/01/
N1 - Nickmilder, Marc. de Burbure, Claire. Carbonnelle, Sylviane. Dumont, Xavier. Bernard, Alfred. Derouane, Alain
Journal of toxicology and environmental health. Part A
ds5, 100960995
IM
Journal Article
English
KW - MEDLINE
KW - Adolescent
KW - Air Pollutants/ae [Adverse Effects]
KW - Air Pollution/ae [Adverse Effects]
KW - Belgium
KW - Child
KW - Circadian Rhythm
KW - Environmental Exposure/ae [Adverse Effects]
KW - Female
KW - Humans
KW - Lung Diseases/ci [Chemically Induced]
KW - Lung Diseases/di [Diagnosis]
KW - Male
KW - Nitric Oxide/me [Metabolism]
KW - Ozone/ae [Adverse Effects]

KW - Respiratory Function Tests
KW - Time Factors
RP - NOT IN FILE
SP - 270
EP - 274
JF - Journal of Toxicology & Environmental Health Part A
JA - J Toxicol Environ Health A
VL - 70
IS - 3-4
CY - England
N2 - Ozone (O3) is known to induce lung function impairment and airways inflammation during episodes of photochemical smog. The aim of the present study was to assess the inflammatory effect of ambient O3 in healthy children using nitric oxide in exhaled air (eNO) as a noninvasive test. The study was performed on 6 groups of children (n = 11-15), aged 6.5 to 15 yr, who attended summer camps in rural areas of the south of Belgium in 2002. Ambient O3 concentrations continuously monitored in the camps ranged from 48 to 221 microg/m3 (1-h maximal concentration). Children remained outdoors during the experimental days, doing various recreational activities but no sports. Lung function tests (forced expiratory volume in 1 s [FEV1] and forced vital capacity [FVC]) and eNO were measured twice in each child in the morning and in the evening. While lung function tests did not show any consistent pattern of decrease at these O3 levels, a highly significant increase in eNO was found in all subjects from an ambient 1-h O3 level of 167 microg/m3. A multivariate analysis did not reveal any influence of age, gender, height, weight, and body mass index (BMI) of the children. The threshold for this O3-induced increase in eNO estimated benchmark dose analysis was 135 microg/m3 for 1-h exposure and 110 microg/m3 for 8-h exposure. These observations suggest that ambient ozone produces early inflammatory changes in the airways of children at levels slightly below current air quality standards
SN - 1528-7394
AD - Industrial Toxicology Unit, Faculty of Medicine, Catholic University of Louvain, Brussels, Belgium. marc.nickmilder@toxi.ucl.ac.be
UR - 17365589
ER -

TY - JOUR
ID - 1168
T1 - Relationships of video assessments of touching and mouthing behaviors during outdoor play in urban residential yards to parental perceptions of child behaviors and blood lead levels
A1 - Ko,S.
A1 - Schaefer,P.D.
A1 - Vicario,C.M.
A1 - Binns,H.J.
A1 - Safer,Yards Project
Y1 - 2007/01//
N1 - Ko, Stephen. Schaefer, Peter D. Vicario, Cristina M. Binns, Helen J. Safer Yards Project
Journal of exposure science & environmental epidemiology
101262796
IM
Journal Article. Research Support, Non-U.S. Gov't. Research Support, U.S. Gov't, Non-P.H.S.
English
KW - MEDLINE
KW - Child Behavior
KW - Child
KW - Preschool
KW - Female
KW - Humans
KW - Infant
KW - Lead/bl [Blood]
KW - Male
KW - Mouth
KW - Parents/px [Psychology]

KW - Residence Characteristics
KW - Touch
KW - Urban Health
RP - NOT IN FILE
SP - 47
EP - 57
JF - Journal of Exposure Science & Environmental Epidemiology
JA - J Expo Sci Environ Epidemiol
VL - 17
IS - 1
CY - United States
N2 - Childrens' touching and mouthing behaviors during outdoor play in urban residential yards were measured using video observations. Descriptions were made of childrens' outdoor residential play environments. Behaviors assessed were used to examine (1) validity of parental responses to questions on childrens' oral behaviors and outdoor play and (2) relationships of mouthing behaviors to blood lead levels (BLLs). Thirty-seven children aged 1-5 years were recruited for 2 h of video recording in their yard and blood lead measurement. Video assessments included hourly rates of hand touches to ground/walking-level surfaces (cement/stone/steel, porch floor/steps, grass, and bare soil) and oral behaviors. Parental questionnaires assessed their child's outdoor activities, behaviors, and home environment. The children were: mean 39 months; 51% male; 89% Hispanic; and 78% Medicaid or uninsured. Twenty-two children had a blood lead measured (mean 6 microg/dl). During taping, all children had access to cement, 92% to grass, 73% to bare soil, and 59% to an open porch. Children had frequent touching and mouthing behaviors observed (median touches/h: touches to surfaces 81; hand-to-mouth area (with and without food) 26; hand-in-mouth 7; and object-in-mouth 17). Blood lead was directly correlated with log-transformed rates of hand-in-mouth (Pearson's correlation, r=0.564, n=22, P=0.006) and object-in-mouth (Pearson's correlation, r=0.482, n=22, P=0.023) behaviors. Parental questionnaire responses did not accurately reflect childrens' observed oral behaviors, play habits, or play environment. These data confirm the direct relationship between hand-to-mouth activities and BLLs and fail to validate parental perceptions of their child's mouthing behaviors or outdoor play environment
SN - 1559-0631
AD - Medical College of Georgia, Augusta, GA, USA
UR - 16941017
ER -

TY - JOUR
ID - 1169
T1 - Measures of cumulative exposure from a standardized sun exposure history questionnaire: a comparison with histologic assessment of solar skin damage
A1 - Karagas,M.R.
A1 - Zens,M.S.
A1 - Nelson,H.H.
A1 - Mabuchi,K.
A1 - Perry,A.E.
A1 - Stukel,T.A.
A1 - Mott,L.A.
A1 - Andrew,A.S.
A1 - Applebaum,K.M.
A1 - Linet,M.
Y1 - 2007/03/15/
N1 - Karagas, Margaret R. Zens, Michael S. Nelson, Heather H. Mabuchi, Kiyohiko. Perry, Ann E. Stukel, Therese A. Mott, Leila A. Andrew, Angeline S. Applebaum, Katie M. Linet, Martha
American journal of epidemiology
3h3, 7910653
IM
Comparative Study. Journal Article. Research Support, N.I.H., Extramural. Research Support, N.I.H., Intramural. Validation Studies
English
KW - MEDLINE

KW - Aged
KW - Biopsy
KW - Child
KW - Environmental Exposure/ae [Adverse Effects]
KW - Environmental Exposure/sn [Statistics & Numerical Data]
KW - Environmental Monitoring/mt [Methods]
KW - Environmental Monitoring/st [Standards]
KW - Epidemiological Monitoring
KW - Female
KW - Histological Techniques/st [Standards]
KW - Humans
KW - Keratosis/ep [Epidemiology]
KW - Keratosis/et [Etiology]
KW - Keratosis/pa [Pathology]
KW - Leisure Activities
KW - Male
KW - Medical History Taking/mt [Methods]
KW - Medical History Taking/st [Standards]
KW - Middle Aged
KW - New Hampshire/ep [Epidemiology]
KW - Questionnaires/st [Standards]
KW - Risk Factors
KW - Skin Aging/pa [Pathology]
KW - Skin Neoplasms/ep [Epidemiology]
KW - Skin Neoplasms/et [Etiology]
KW - Skin Neoplasms/pa [Pathology]
KW - Skin Pigmentation
KW - Sunburn/co [Complications]
KW - Sunburn/pa [Pathology]
KW - Sunlight/ae [Adverse Effects]
KW - Time Factors
RP - NOT IN FILE
SP - 719
EP - 726
JF - American Journal of Epidemiology
JA - Am J Epidemiol
VL - 165
IS - 6
CY - United States
N2 - Ultraviolet radiation exposure is the dominant environmental determinant of all major forms of skin cancer; however, the nature of the association is incompletely understood. Existing instruments to capture sun exposure history tend to yield reproducible results, but the validity of these responses is unknown. To address this question, the authors examined the relation between responses to a standardized sun exposure instrument and histologic evidence of actinic damage in a population-based study of keratinocyte cancers from New Hampshire diagnosed from July 1, 1997, through March 31, 2000. A single study dermatopathologist histologically reviewed the adjacent skin of 925 skin cancer biopsies for the presence of solar keratoses and the extent of solar elastosis. The authors compared these measures with responses to a personal interview on history of sunburns, sunbathing, and time spent outdoors. Focusing on site-specific exposure, they found variables that estimated cumulative exposure related to histologic evidence of actinic damage. In contrast, measures of acute/intermittent exposure were generally unrelated to solar damage histologically. Findings suggest that cumulative, but not intermittent, measures of sun exposure derived from a personal interview appear to reflect a person's exposure history based on histologic evidence
SN - 0002-9262
AD - Section of Biostatistics and Epidemiology, Department of Community and Family Medicine, Dartmouth Medical School, One Medical Center Drive, Lebanon, NH 03756, USA. margaret.karagas@dartmouth.edu
UR - 17204514
ER -

TY - JOUR
ID - 1170
T1 - Environmental correlates of physical activity in youth - a review and update. [Review] [150 refs]
A1 - Ferreira,I.
A1 - van der,Horst K.
A1 - Wendel-Vos,W.
A1 - Kremers,S.
A1 - van Lenthe,F.J.
A1 - Brug,J.
Y1 - 2007/03//
N1 - Ferreira, I. van der Horst, K. Wendel-Vos, W. Kremers, S. van Lenthe, F J. Brug, J
Obesity reviews : an official journal of the International Association for the Study of Obesity
100897395
IM
Journal Article. Review
English
KW - MEDLINE
KW - Adolescent
KW - Child
KW - Preschool
KW - Cross-Sectional Studies
KW - Educational Status
KW - Environment
KW - Family Relations
KW - Humans
KW - Motor Activity/ph [Physiology]
KW - Obesity/ep [Epidemiology]
KW - Obesity/pp [Physiopathology]
KW - Prevalence
KW - Socioeconomic Factors
KW - World Health
RP - NOT IN FILE
SP - 129
EP - 154
JF - Obesity Reviews
JA - Obes Rev
VL - 8
IS - 2
CY - England
N2 - Obesogenic environments are thought to underlie the increased obesity prevalence observed in youth during the past decades. Understanding the environmental factors that are associated with physical activity (PA) in youth is needed to better inform the development of effective intervention strategies attempting to halt the obesity epidemic. We conducted a systematic semi-quantitative review of 150 studies on environmental correlates of youth PA published in the past 25 years. The ANalysis Grid for Environments Linked to Obesity (ANGELO) framework was used to classify the environmental correlates studied. Most studies retrieved used cross-sectional designs and subjective measures of environmental factors and PA. Variables of the home and school environments were especially associated with children's PA. Most consistent positive correlates of PA were father's PA, time spent outdoors and school PA-related policies (in children), and support from significant others, mother's education level, family income, and non-vocational school attendance (in adolescents). Low crime incidence (in adolescents) was characteristic of the neighbourhood environment associated with higher PA. Convincing evidence of an important role for many other environmental factors was, however, not found. Further research should aim at longitudinal and intervention studies, and use more objective measures of PA and its potential (environmental) determinants. [References: 150]
SN - 1467-7881
AD - Department of Public Health, Erasmus University Medical Center, Rotterdam, The Netherlands
UR - 17300279

ER -

TY - JOUR
ID - 1171
T1 - Prevalence of respiratory symptoms in migrant children to Italy: the results of SIDRIA-2 study
A1 - Migliore,E.
A1 - Pearce,N.
A1 - Bugiani,M.
A1 - Galletti,G.
A1 - Biggeri,A.
A1 - Bisanti,L.
A1 - Caranci,N.
A1 - Dell'Orco,V.
A1 - De,Sario M.
A1 - Sestini,P.
A1 - Piffer,S.
A1 - Viegi,G.
A1 - Forastiere,F.
A1 - Galassi,C.
A1 - Ciccone,G.
A1 - Collaborative Group.
Y1 - 2007/03//
N1 - Migliore, E. Pearce, N. Bugiani, M. Galletti, G. Biggeri, A. Bisanti, L. Caranci, N. Dell'Orco, V. De Sario, M. Sestini, P. Piffer, S. Viegi, G. Forastiere, F. Galassi, C. Ciccone, G. SIDRIA-2 Collaborative Group
Allergy
39c, 7804028
IM
Comparative Study. Journal Article. Multicenter Study. Research Support, Non-U.S. Gov't
English
KW - MEDLINE
KW - Adolescent
KW - Asthma/ep [Epidemiology]
KW - Child
KW - Cross-Sectional Studies
KW - Emigration and Immigration
KW - Humans
KW - Italy/ep [Epidemiology]
KW - Prevalence
KW - Questionnaires
KW - Respiratory Sounds
KW - Risk Factors
KW - Time Factors
RP - NOT IN FILE
SP - 293
EP - 300
JF - Allergy
VL - 62
IS - 3
CY - Denmark
N2 - BACKGROUND: Epidemiological studies have documented large international variations in the prevalence of asthma, and 'westernization' seems to play an important role in the development of the disease. The aims of this study were to compare the prevalence of respiratory symptoms in migrant and nonmigrant children resident in Italy, and to examine the effect of length of time living in Italy. METHODS: Data were collected in a large cross-sectional study (SIDRIA-2) performed in 12 Italian centres, using standardized parental questionnaires. For the 29 305 subjects included in the analysis (6-7 and 13-14 years old), information about place of birth and parental nationality was available. RESULTS: There were 1012 children (3%) born outside of Italy, mainly in East Europe. Lifetime asthma and current wheeze were generally significantly less

common among children born abroad than among children born in Italy (lifetime asthma: 5.4% and 9.7% respectively, P < 0.001; current wheeze: 5.2% and 6.9%, respectively, P = 0.04). Lower risks for lifetime asthma (prevalence odds ratio, POR = 0.39; 95% CI: 0.23-0.66) and current wheeze (POR = 0.72; 95% CI: 0.47-1.10) were found for children who had lived in Italy <5 years, while migrant children who had lived in Italy for 5 years or more had risks very similar to Italian children. CONCLUSIONS: Migrant children have a lower prevalence of asthma symptoms than children born in Italy. Prevalence increased with the number of years of living in Italy, suggesting that exposure to environmental factors may play an important role in the development of asthma in childhood
SN - 0105-4538
AD - Cancer Epidemiology Unit, San Giovanni Battista Hospital - Center for Cancer Prevention Piemonte (CPO), Turin, Italy
UR - 17298347
ER -

TY - JOUR
ID - 1172
T1 - Nevi in children: a practical approach to evaluation
A1 - Prok,L.D.
A1 - Arbuckle,H.A.
Y1 - 2007/01//
N1 - Prok, Lori D. Arbuckle, H Alan
Pediatric annals
oub, 0356657
IM
Journal Article
English
KW - MEDLINE
KW - Child
KW - Humans
KW - Melanoma/di [Diagnosis]
KW - Melanoma/pc [Prevention & Control]
KW - Nevus/cn [Congenital]
KW - Nevus/di [Diagnosis]
KW - Skin Neoplasms/di [Diagnosis]
RP - NOT IN FILE
SP - 39
EP - 45
JF - Pediatric Annals
JA - Pediatr Ann
VL - 36
IS - 1
CY - United States
N2 - Education is the cornerstone of melanoma prevention. Well-child examinations are an excellent opportunity to educate patients and parents about melanoma risk factors and prevention. The only modifiable risk factor for the development of melanoma and acquisition of nevi is level of sun exposure. Every patient who is evaluated for a melanocytic lesion should be educated about sun avoidance during midday, protective clothing including long-sleeved shirts, long pants, and hats with brims, and playing in shaded areas when outdoors. Proper application and appropriate uses of sunscreen should also be reviewed
SN - 0090-4481
AD - Department of Dermatology, University of Colorado School of Medicine, USA. Lori.Prok@uchsc.edu
UR - 17269282
ER -

TY - JOUR
ID - 1173
T1 - Body mass index, physical activity and dietary behaviours among adolescents in the Kingdom of Tonga
A1 - Smith,B.J.

A1 - Phongsavan,P.
A1 - Havea,D.
A1 - Halavatau,V.
A1 - Chey,T.
Y1 - 2007/02//
N1 - Smith, B J. Phongsavan, P. Havea, D. Halavatau, V. Chey, T
Public health nutrition
9808463, dgy
IM
Journal Article. Research Support, Non-U.S. Gov't
English
KW - MEDLINE
KW - Adolescent
KW - Adolescent Behavior
KW - Adolescent Nutritional Physiological Phenomena
KW - Body Mass Index
KW - Child
KW - Cross-Sectional Studies
KW - Diet
KW - Exercise/ph [Physiology]
KW - Female
KW - Food Habits
KW - Humans
KW - Male
KW - Obesity/di [Diagnosis]
KW - Obesity/ep [Epidemiology]
KW - Obesity/eh [Ethnology]
KW - Prevalence
KW - Reference Values
KW - Sensitivity and Specificity
KW - Tonga
RP - NOT IN FILE
SP - 137
EP - 144
JF - Public Health Nutrition
JA - Public Health Nutr
VL - 10
IS - 2
CY - England
N2 - OBJECTIVES: To compare the prevalence of overweight and obesity among adolescents using international and ethnic-specific cut-off points and to examine patterns of physical activity and dietary behaviours. DESIGN: Cross-sectional analytical study. SETTING: Schools on Tongatapu, Vava'u and Hapa'ai islands in the Kingdom of Tonga. SUBJECTS: A total of 443 school students aged 11-16 years underwent anthropometric measures of height and weight and provided self-reported measures of physical activity and dietary behaviours. Results Mean body mass index (BMI) was higher among girls than boys (23.7 kg m(-2) vs. 21.8 kg m(-2) and tended to increase with age. A total of 36.0% of boys and 53.8% of girls were overweight or obese using the international cut-off points, whereas 25.0% of boys and 37.6% of girls were classified in this way using Polynesian-specific cut-off points. Tinned mutton or beef was the food that most participants (56.9%) reported eating once or more per day. Over half of the young people did not eat taro, fruit or vegetables at least once per day. Regular physical activity outside of school hours was reported by 20.7% of respondents, and 58.2% watched 1 h or more of television per day. Physical activity participation was the only behaviour independently associated with a lower risk of overweight or obesity. Conclusions Using Polynesian-specific cut-off points for overweight and obesity the prevalence of these conditions was still among the highest found in adolescents. The prevalence of physical inactivity and poor dietary habits indicate that risk factors for chronic disease are well established during adolescence in Tonga
SN - 1368-9800

AD - School of Public Health, University of Sydney, New South Wales 2006, Australia.
bens@health.usyd.edu.au
UR - 17261222
ER -

TY - JOUR
ID - 1174
T1 - Time spent outdoors at midday and children's body mass index
A1 - Milne,E.
A1 - Simpson,J.A.
A1 - Johnston,R.
A1 - Giles-Corti,B.
A1 - English,D.R.
Y1 - 2007/02//
N1 - Milne, Elizabeth. Simpson, Julie A. Johnston, Robyn. Giles-Corti, Billie. English, Dallas R
American journal of public health
1254074, 3xw
AIM, IM
Controlled Clinical Trial. Journal Article. Research Support, Non-U.S. Gov't
English
KW - MEDLINE
KW - Body Mass Index
KW - Child
KW - Curriculum
KW - Female
KW - Health Education/mt [Methods]
KW - Humans
KW - Male
KW - Melanoma/et [Etiology]
KW - Melanoma/pc [Prevention & Control]
KW - Motor Activity
KW - Obesity/ep [Epidemiology]
KW - Obesity/pc [Prevention & Control]
KW - Outcome Assessment (Health Care)
KW - Program Evaluation
KW - Recreation
KW - Risk Assessment
KW - Risk Factors
KW - School Health Services
KW - Skin Neoplasms/et [Etiology]
KW - Skin Neoplasms/pc [Prevention & Control]
KW - Sunburn/co [Complications]
KW - Sunburn/ep [Epidemiology]
KW - Sunburn/pc [Prevention & Control]
KW - Time Factors
KW - Western Australia/ep [Epidemiology]
RP - NOT IN FILE
SP - 306
EP - 310
JF - American Journal of Public Health
JA - Am J Public Health
VL - 97
IS - 2
CY - United States
N2 - OBJECTIVES: We investigated whether the Kidskin sun protection intervention increased children's body mass index by reducing the time spent outdoors at midday. METHODS: The Kidskin sun protection intervention involved 1614 Australian school children assigned to 1 of 3 groups: a control group, a moderate-

intervention group, or a high-intervention group. Schools in the control group received the standard health curriculum and schools in the intervention groups received a multicomponent intervention. Outcomes included time spent outdoors and nevus development (a marker of melanoma risk). Height and weight were measured at 3 time points. Body mass index was transformed into age- and gender-specific z scores; z scores at each age were modeled simultaneously. Time spent outdoors at ages 10 and 12 years was analyzed using a linear mixed effects modeling. RESULTS: The proportion of children who were overweight or obese increased with age. The moderate-intervention and control groups had a minimal increase in z score over time, and the z score for the high-intervention group decreased over time. There were no differences among groups with respect to total time outdoors at any age. CONCLUSIONS: It is possible to reduce the time children spend outdoors when ultraviolet radiation is high without producing an unfavorable effect on the children's body mass index
SN - 1541-0048
AD - Telethon Institute for Child Health Research, Centre for Child Health Research, University of Western Australia, West Perth. lizm@ichr.uwa.edu.au
UR - 17194858
ER -

TY - JOUR
ID - 1175
T1 - [Trends in vigorous physical activity versus physical inactivity among 11-15 year olds from 1988 to 2002]. [Danish]
A1 - Holstein,B.E.
A1 - Henriksen,P.E.
A1 - Krolner,R.
A1 - Rasmussen,M.
A1 - Due,P.
Y1 - 2007/01/01/
N1 - Holstein, Bjorn E. Henriksen, Pia Elena. Krolner, Rikke. Rasmussen, Mette. Due, Pernille
Ugeskrift for laeger
0141730, wm8
IM
Comparative Study. English Abstract. Journal Article. Research Support, Non-U.S. Gov't
Danish
KW - MEDLINE
KW - Adolescent
KW - Age Factors
KW - Child
KW - Exercise
KW - Female
KW - Health Behavior
KW - Humans
KW - Male
KW - Motor Activity
KW - Physical Education and Training
KW - Physical Fitness
KW - Questionnaires
KW - Sex Factors
RP - NOT IN FILE
SP - 37
EP - 42
JF - Ugeskrift for Laeger
JA - Ugeskr.Laeger
VL - 169
IS - 1
CY - Denmark
N2 - INTRODUCTION: The increasing prevalence of overweight and obesity among children and adolescents suggests a need to study the development in main predictors of obesity, like eating habits and physical activity. The objective was to describe self-reported vigorous physical activity versus physical inactivity among children

and adolescents over a period of 14 years, 1988-2002. MATERIAL AND METHODS: The study population was the Health Behaviour in School-aged Children (HBSC) study among nationally representative samples of 11, 13 and 15 year old students in 1988, 1991, 1994, 1998, and 2002. The data collection used the standardised HBSC questionnaire which included items about vigorous physical activity (VPA) outside school hours per week. We defined physically inactivity as 0 hours of VPA per week and high activity as at least 7 hours of VPA per week. RESULTS: The prevalence of high activity was higher among boys than girls and increased by age. The prevalence of physically inactive students increased from 1988 to 2002, especially among the 15 year olds. The proportion of students with high activity decreased from 1988 to 2002, especially among the 15 year olds. CONCLUSION: A large proportion of children and adolescents do not comply with the national recommendations that children should be physically active at least 60 minutes per day. The physical activity level decreased from 1988 to 2002. There is a need for extraordinary efforts to increase physical activity among children and adolescents
SN - 1603-6824
AD - Kobenhavns Universitet, Institut for Folkesundhedsvidenskab, Afdeling for Social Medicin, Kobenhavn N. holstein@socmed.ku.dk
UR - 17217885
ER -

TY - JOUR
ID - 1176
T1 - Fractures in New Zealand elementary school settings
A1 - Rubie-Davies,C.M.
A1 - Townsend,M.A.
Y1 - 2007/01//
N1 - Rubie-Davies, Christine M. Townsend, Michael A R
The Journal of school health
k13, 0376370
IM, N
Journal Article
English
KW - MEDLINE
KW - Child
KW - Equipment Design/st [Standards]
KW - Female
KW - Fractures
KW - Bone/ep [Epidemiology]
KW - Bone/et [Etiology]
KW - Humans
KW - Male
KW - New Zealand/ep [Epidemiology]
KW - Play and Playthings/in [Injuries]
KW - Safety/sn [Statistics & Numerical Data]
KW - School Health Services/sn [Statistics & Numerical Data]
KW - Schools/st [Standards]
RP - NOT IN FILE
SP - 36
EP - 40
JF - Journal of School Health
JA - J Sch Health
VL - 77
IS - 1
CY - United States
N2 - BACKGROUND: There is a need for greater international understanding of student safety in schools. This New Zealand study investigated the causes and school location of fractures sustained by students attending elementary school, with special emphasis on the types of fractures sustained following falls from playground equipment of various heights. METHODS: Over a 1-year period, 76 participating schools (with a combined roll of over 25,000 students) completed a questionnaire about the nature and circumstances of student fractures

sustained during regular school hours. RESULTS: Some 118 students sustained a total of 131 fractures. Injuries from playground equipment were no more frequent than those from general activities at school, and most were sustained in falls from heights less than 59 inches. Most fractures were to the upper limbs. Fractures were found to vary by gender and school size. CONCLUSIONS: The results are discussed in terms of the conditions and policies present in local schools, and the tension that exists in maintaining safety while offering appropriate challenges to students. Improvements in school safety may be more likely to result from a greater focus on the way that students interact at school, rather than on modifications to playground equipment
SN - 0022-4391
AD - Faculty of Education, The University of Auckland, Private Bag 92019, Auckland, New Zealand. c.rubie@auckland.ac.nz
UR - 17212758
ER -

TY - JOUR
ID - 1177
T1 - Comparison of self-reported lifetime sun exposure with two methods of cutaneous microtopography
A1 - Weiler,L.
A1 - Knight,J.A.
A1 - Vieth,R.
A1 - Barnett,H.
A1 - Wong,A.
Y1 - 2007/01/15/
N1 - Weiler, Lynn. Knight, Julia A. Vieth, Reinhold. Barnett, Heidi. Wong, Ansely
American journal of epidemiology
3h3, 7910653
IM
Journal Article. Research Support, Non-U.S. Gov't
English
KW - MEDLINE
KW - Adolescent
KW - Adult
KW - Aged
KW - Child
KW - Environmental Exposure
KW - Female
KW - Follow-Up Studies
KW - Humans
KW - Middle Aged
KW - Observer Variation
KW - Ontario
KW - Photomicrography/mt [Methods]
KW - Replica Techniques
KW - Retrospective Studies
KW - Skin/cy [Cytology]
KW - Skin/re [Radiation Effects]
KW - Skin Aging/re [Radiation Effects]
KW - Sunlight
RP - NOT IN FILE
SP - 222
EP - 230
JF - American Journal of Epidemiology
JA - Am J Epidemiol
VL - 165
IS - 2
CY - United States
N2 - There is currently no "gold standard" for measuring lifetime sun exposure. Exploration of alternatives to self-reports is important for examining illnesses related to ultraviolet light exposure. Using skin replicas

obtained from 184 controls in a breast cancer case-control study (Toronto, Ontario, Canada, 2004-2005), the authors compared self-reported indicators of lifetime sun exposure with two measures of cutaneous microtopography, the Beagley-Gibson system and skin line counts. With the Beagley-Gibson system, significantly increased odds ratios were found for age (odds ratio (OR) = 1.10, 95% confidence interval (CI): 1.05, 1.16), spending 7 days outside per week during the summer (OR = 3.33, 95% CI: 1.48, 7.50), and lifetime number of sunlamp sessions. Significantly decreased odds ratios were found for having darker skin, ever giving birth, and ever using sunlamps. With the skin line count approach, significant positive associations were found for age (OR = 2.31, 95% CI: 1.23, 4.35), age squared, duration of working in outdoor jobs (OR = 0.88, 95% CI: 0.79, 0.98), and average number of outdoor activities per week at ages 20-29 years (OR = 1.05, 95% CI: 1.00, 1.10). While the Beagley-Gibson method was associated with more variables than the skin line count method, both methods require further refinement before graded skin replicas can be recommended as a substitute for self-report measures
SN - 0002-9262
AD - Department of Public Health Sciences, Faculty of Medicine, University of Toronto, Toronto, Ontario, Canada
UR - 17101707
ER -

TY - JOUR
ID - 1178
T1 - Acute hepatitis delta virus infection in Italy: incidence and risk factors after the introduction of the universal anti-hepatitis B vaccination campaign
A1 - Mele,A.
A1 - Mariano,A.
A1 - Tosti,M.E.
A1 - Stroffolini,T.
A1 - Pizzuti,R.
A1 - Gallo,G.
A1 - Ragni,P.
A1 - Zotti,C.
A1 - Lopalco,P.
A1 - Curtale,F.
A1 - Balocchini,E.
A1 - Spada,E.
A1 - SEIEVA Collaborating Group.
Y1 - 2007/02/01/
N1 - Mele, Alfonso. Mariano, Andrea. Tosti, Maria Elena. Stroffolini, Tommaso. Pizzuti, Renato. Gallo, Giovanni. Ragni, Pietro. Zotti, Carla. Lopalco, Pierluigi. Curtale, Filippo. Balocchini, Emanuela. Spada, Enea. SEIEVA Collaborating Group
Clinical infectious diseases : an official publication of the Infectious Diseases Society of America
a4j, 9203213
IM
Journal Article. Research Support, Non-U.S. Gov't
English
KW - MEDLINE
KW - Adolescent
KW - Adult
KW - Case-Control Studies
KW - Child
KW - Preschool
KW - Hepatitis B/ep [Epidemiology]
KW - Hepatitis B/pc [Prevention & Control]
KW - Hepatitis B Vaccines/tu [Therapeutic Use]
KW - Hepatitis B virus/im [Immunology]
KW - Hepatitis D/ep [Epidemiology]
KW - Hepatitis D/pc [Prevention & Control]
KW - Hepatitis Delta Virus/ip [Isolation & Purification]

KW - Humans
KW - Incidence
KW - Infant
KW - Newborn
KW - Italy/ep [Epidemiology]
KW - Odds Ratio
KW - Population Surveillance
KW - Risk Factors
RP - NOT IN FILE
SP - e17
EP - e24
JF - Clinical Infectious Diseases
JA - Clin Infect Dis
VL - 44
IS - 3
CY - United States
N2 - BACKGROUND: Updates on the incidence of and risk factors for acute hepatitis delta virus infection in Italy, as well as in other countries, are lacking, and the impact of the mandatory anti-hepatitis B vaccination has not been evaluated. METHODS: We performed a case-control study within a population-based surveillance for acute viral hepatitis. RESULTS: During 1993-2004, 344 cases of acute hepatitis delta virus infection were reported. After a peak in 1993 (2.8 cases per 1 million population), the incidence decreased from 1.7 to 0.5 cases per 1 million population. Coinfections were prevalent. The decrease in incidence particularly affected young adults, and it paralleled the decrease in incidence of acute hepatitis B. In 1993, being an injection drug user (adjusted odds ratio [OR(adj)], 67.9; 95% confidence interval [CI], 18.1-254.5) or being a member of a household with a carrier of hepatitis B surface antigen (OR(adj,) 14.8; 95% CI, 3.0-72.9) were the only independent predictors of infection. During 1994-2004, being an injection drug user (OR(adj), 36.8; 95% CI, 20.7-65.4), cohabitation with an injection drug user (OR(adj), 4.2, 95% CI, 1.7-12.3), hospitalization (OR(adj), 3.5; 95% CI, 1.9-6.6), receipt of dental therapy (OR(adj), 2.3; 95% CI, 1.4-3.6), promiscuous sexual activity (OR(adj), 2.2; 95% CI, 1.4-3.6), and receipt of beauty treatment (OR(adj), 2.0; 95% CI, 1.3-3.2) were independently associated with infection. CONCLUSIONS: Incidence of acute hepatitis delta infection is markedly decreasing in Italy. Undergoing invasive medical procedures, engaging in promiscuous sexual activity, and receiving beauty treatments are emerging, in addition to injection drug use, as important risk factors for infection. Further efforts are needed to increase vaccine coverage in high-risk groups and to implement the safety of invasive procedures performed both inside and outside health care facilities
SN - 1537-6591
AD - National Centre of Epidemiology, Surveillance and Health Promotion, Istituto Superiore di Sanita, Rome, 00162, Italy
UR - 17205431
ER -

TY - JOUR
ID - 1179
T1 - The importance of play in promoting healthy child development and maintaining strong parent-child bonds
A1 - Ginsburg,K.R.
A1 - American Academy of Pediatrics Committee on Communications
A1 - American Academy of Pediatrics Committee on Psychosocial Aspects of Child and Family Health.
Y1 - 2007/01//
N1 - Ginsburg, Kenneth R. American Academy of Pediatrics Committee on Communications. American Academy of Pediatrics Committee on Psychosocial Aspects of Child and Family Health
Pediatrics
oxv, 0376422
AIM, IM
Guideline. Journal Article
English
KW - MEDLINE
KW - Child
KW - Child Development

KW - Child Psychology
KW - Child Rearing
KW - Preschool
KW - Humans
KW - Parent-Child Relations
KW - Parenting
KW - Parents/px [Psychology]
KW - Pediatrics
KW - Physician's Role
KW - Play and Playthings
KW - Stress
KW - Psychological
RP - NOT IN FILE
SP - 182
EP - 191
JF - Pediatrics
VL - 119
IS - 1
CY - United States
N2 - Play is essential to development because it contributes to the cognitive, physical, social, and emotional well-being of children and youth. Play also offers an ideal opportunity for parents to engage fully with their children. Despite the benefits derived from play for both children and parents, time for free play has been markedly reduced for some children. This report addresses a variety of factors that have reduced play, including a hurried lifestyle, changes in family structure, and increased attention to academics and enrichment activities at the expense of recess or free child-centered play. This report offers guidelines on how pediatricians can advocate for children by helping families, school systems, and communities consider how best to ensure that play is protected as they seek the balance in children's lives to create the optimal developmental milieu
SN - 1098-4275
UR - 17200287
ER -

TY - JOUR
ID - 1180
T1 - Television watching and frequency of family meals are predictive of overweight onset and persistence in a national sample of school-aged children
A1 - Gable,S.
A1 - Chang,Y.
A1 - Krull,J.L.
Y1 - 2007/01//
N1 - Gable, Sara. Chang, Yiting. Krull, Jennifer L
Journal of the American Dietetic Association
h6f, 7503061
AIM, IM
Journal Article. Research Support, U.S. Gov't, Non-P.H.S.
English
KW - MEDLINE
KW - Body Mass Index
KW - Child
KW - Child Development
KW - Preschool
KW - Cohort Studies
KW - Confidence Intervals
KW - Eating
KW - Exercise/ph [Physiology]
KW - Female
KW - Humans
KW - Logistic Models

KW - Longitudinal Studies
KW - Male
KW - Multivariate Analysis
KW - Obesity/ep [Epidemiology]
KW - Obesity/et [Etiology]
KW - Odds Ratio
KW - Overweight
KW - Play and Playthings
KW - Predictive Value of Tests
KW - Risk Assessment
KW - Social Class
KW - Television
KW - Time Factors
KW - United States/ep [Epidemiology]
KW - Weight Gain/ph [Physiology]
RP - NOT IN FILE
SP - 53
EP - 61
JF - Journal of the American Dietetic Association
JA - J Am Diet Assoc
VL - 107
IS - 1
CY - United States
N2 - OBJECTIVE: To identify eating and activity factors associated with school-aged children's onset of overweight and persistent overweight. DESIGN: Data were gathered at four time points between kindergarten entry and spring of third grade. Children were directly weighed and measured and categorized as not overweight (<95th percentile of body mass index) or overweight (> or =95th percentile body mass index); parents were interviewed by telephone or in person. SUBJECTS/SETTING: Subjects were participants in the Early Childhood Longitudinal Study-Kindergarten Cohort, a nationally representative sample of children who entered kindergarten during 1998-1999. Children who weighed <2,000 g at birth, received therapeutic services before kindergarten, skipped or repeated a grade, or without complete height and weight data were excluded, resulting in 8,459 participants. Children with intermittent overweight were not examined (n=459); analyses addressed 8,000 children. MAIN OUTCOME MEASURES: Three mutually exclusive groups of children were identified: never overweight, overweight onset, and persistent overweight. STATISTICAL ANALYSES PERFORMED: Multilevel, multivariate logistic regression analyses estimated the effects of eating and activity factors on the odds of overweight onset and persistent overweight above child sex, race, and family socioeconomic status. RESULTS: Children who watched more television (odds ratio [OR] 1.02) and ate fewer family meals (OR 1.08) were more likely to be overweight for the first time at spring semester of third grade. Children who watched more television (OR 1.03), ate fewer family meals (OR 1.08), and lived in neighborhoods perceived by parents as less safe for outdoor play (OR 1.32) were more likely to be persistently overweight. Child aerobic exercise and opportunities for activity were not associated with a greater likelihood of weight problems. CONCLUSIONS: This study supports theories regarding the contributions of television watching, family meals, and neighborhood safety to childhood weight status. When working with families to prevent and treat childhood weight problems, food and nutrition professionals should attend to children's time spent with screen media, the frequency of family mealtimes, and parents' perceptions of neighborhood safety for children's outdoor play
SN - 0002-8223
AD - Department of Psychological Sciences, University of Missouri, Columbia, MO 65211-7700, USA. gables@missouri.edu
UR - 17197271
ER -

TY - JOUR
ID - 1181
T1 - Different effects of urban and rural environments in the respiratory status of Greek schoolchildren
A1 - Priftis,K.N.
A1 - Anthracopoulos,M.B.
A1 - Paliatsos,A.G.

A1 - Tzavelas,G.
A1 - Nikolaou-Papanagiotou,A.
A1 - Douridas,P.
A1 - Nicolaidou,P.
A1 - Mantzouranis,E.
Y1 - 2007/01//
N1 - Priftis, Kostas N. Anthracopoulos, Michael B. Paliatsos, Athanasios G. Tzavelas, George. Nikolaou-Papanagiotou, Alexandra. Douridas, Panayiotis. Nicolaidou, Polyxeni. Mantzouranis, Eva
Respiratory medicine
8908438, rme
IM
Comparative Study. Journal Article
English
KW - MEDLINE
KW - Air Pollution
KW - Analysis of Variance
KW - Asthma/di [Diagnosis]
KW - Asthma/et [Etiology]
KW - Child
KW - Cohort Studies
KW - Confounding Factors (Epidemiology)
KW - Cough/di [Diagnosis]
KW - Cough/et [Etiology]
KW - Greece
KW - Health Status
KW - Humans
KW - Logistic Models
KW - Prevalence
KW - Respiratory Sounds
KW - Respiratory Tract Diseases/di [Diagnosis]
KW - Respiratory Tract Diseases/et [Etiology]
KW - Rural Population
KW - Spirometry
KW - Urban Population
RP - NOT IN FILE
SP - 98
EP - 106
JF - Respiratory Medicine
JA - Respir Med
VL - 101
IS - 1
CY - England
N2 - We hypothesized that asthma symptoms and lung function of schoolchildren living in Athens urban area are adversely affected as compared to others living in a rural environment, over a period of 8 years. We recruited 478 and 342 children aged 8-10 years living within a short radius around the urban and rural area monitoring stations, respectively. Respiratory health was assessed by a parent-completed questionnaire in three phases: 1995-1996 (phase-1), 1999-2000 (phase-2), 2003-2004 (phase-3) and by spirometry in phases-1 and 2. Reported asthma and wheeze did not differ in the two areas, whereas cough was more prevalent in the urban area in phase-1. Children from the rural environment had lower levels of percent-predicted forced vital capacity (FVC%) in phase-1 and higher of percent-predicted-forced expiratory flow at mid-FVC (FEF(50)%) in both phases. Independent associations were detected between FVC% as-well-as FEF(50)% and residential area. High FVC% was associated with outdoor systemic athletic activities; there was lower FVC% growth in the urban versus the rural area. Nitrogen dioxide and sulfur dioxide were higher in the urban area, whereas ozone concentrations differed less between the two areas. These results suggest that long-term exposure to urban environment is associated with sub-clinical airway narrowing and slower rate of FVC growth
SN - 0954-6111

AD - Department of Allergy-Pneumonology, Penteli Children's Hospital, 152 36 P Penteli, Greece.
kpriftis@otenet.gr
UR - 16757161
ER -

TY - JOUR
ID - 1182
T1 - Re-assessment of risk factors for sporadic Salmonella serotype Enteritidis infections: a case-control study in five FoodNet Sites, 2002-2003
A1 - Marcus,R.
A1 - Varma,J.K.
A1 - Medus,C.
A1 - Boothe,E.J.
A1 - Anderson,B.J.
A1 - Crume,T.
A1 - Fullerton,K.E.
A1 - Moore,M.R.
A1 - White,P.L.
A1 - Lyszkowicz,E.
A1 - Voetsch,A.C.
A1 - Angulo,F.J.
A1 - Emerging Infections Program FoodNet Working Group.
Y1 - 2007/01//
N1 - Marcus, R. Varma, J K. Medus, C. Boothe, E J. Anderson, B J. Crume, T. Fullerton, K E. Moore, M R. White, P L. Lyszkowicz, E. Voetsch, A C. Angulo, F J. Emerging Infections Program FoodNet Working Group
Epidemiology and infection
epi, 8703737
IM
Journal Article
English
KW - MEDLINE
KW - Adolescent
KW - Adult
KW - Aged
KW - 80 and over
KW - Animals
KW - Case-Control Studies
KW - Chickens/mi [Microbiology]
KW - Child
KW - Preschool
KW - Eggs/mi [Microbiology]
KW - Food Microbiology
KW - Humans
KW - Incidence
KW - Infant
KW - Middle Aged
KW - Population Surveillance/mt [Methods]
KW - Risk Factors
KW - Salmonella Food Poisoning/ep [Epidemiology]
KW - Salmonella Food Poisoning/mi [Microbiology]
KW - Salmonella enteritidis/cl [Classification]
KW - Salmonella enteritidis/ge [Genetics]
KW - Salmonella enteritidis/ip [Isolation & Purification]
KW - Salmonella enteritidis/py [Pathogenicity]
KW - Salmonella enteritidis
KW - Travel
KW - United States/ep [Epidemiology]

RP - NOT IN FILE
SP - 84
EP - 92
JF - Epidemiology & Infection
JA - Epidemiol Infect
VL - 135
IS - 1
CY - England
N2 - Active surveillance for laboratory-confirmed Salmonella serotype Enteritidis (SE) infection revealed a decline in incidence in the 1990s, followed by an increase starting in 2000. We sought to determine if the fluctuation in SE incidence could be explained by changes in foodborne sources of infection. We conducted a population-based case-control study of sporadic SE infection in five of the Foodborne Diseases Active Surveillance Network (FoodNet) sites during a 12-month period in 2002-2003. A total of 218 cases and 742 controls were enrolled. Sixty-seven (31%) of the 218 case-patients and six (1%) of the 742 controls reported travel outside the United States during the 5 days before the case's illness onset (OR 53, 95% CI 23-125). Eighty-one percent of cases with SE phage type 4 travelled internationally. Among persons who did not travel internationally, eating chicken prepared outside the home and undercooked eggs inside the home were associated with SE infections. Contact with birds and reptiles was also associated with SE infections. This study supports the findings of previous case-control studies and identifies risk factors associated with specific phage types and molecular subtypes
SN - 0950-2688
AD - Connecticut Emerging Infections Program, New Haven, CT 06510, USA. Ruthanne.Marcus@yale.edu
UR - 16756692
ER -

TY - JOUR
ID - 1183
T1 - [Measurement of drinking water contaminants and water use activities during pregnancy in a cohort study in Spain]. [Spanish]
A1 - Villanueva,C.M.
A1 - Grimalt,J.O.
A1 - Ballester,F.
A1 - Ibarluzea,J.
A1 - Sala,M.
A1 - Tardon,A.
A1 - Romero-Aliaga,E.
A1 - Fernandez,M.
A1 - Ribas-Fito,N.
A1 - Kogevinas,M.
Y1 - 2006/12//
N1 - Villanueva, Cristina M. Grimalt, Joan O. Ballester, Ferran. Ibarluzea, Jesus. Sala, Maria. Tardon, Adonina. Romero-Aliaga, Elena. Fernandez, Marieta. Ribas-Fito, Nuria. Kogevinas, Manolis
Gaceta sanitaria / S.E.S.P.A.S
0370727, 8901623, gsz, 8901623
IM
English Abstract. Journal Article. Research Support, Non-U.S. Gov't
Spanish
KW - MEDLINE
KW - Child
KW - Preschool
KW - Cohort Studies
KW - Drinking
KW - Female
KW - Follow-Up Studies
KW - Humans
KW - Infant
KW - Pregnancy

KW - Spain
KW - Trihalomethanes/an [Analysis]
KW - Water Pollution
KW - Chemical/an [Analysis]
RP - NOT IN FILE
SP - 1
EP - 9
JF - Gaceta Sanitaria
JA - Gac.Sanit.
VL - 20 Suppl 3
CY - Spain
N2 - OBJECTIVES: Exposure to chlorination by-products during pregnancy has been associated with reproductive outcomes but the evidence remains inconclusive. Levels of trihalomethanes, the most prevalent chlorination by-products, are high in some areas of Spain. Within the framework of a follow-up study in pregnant women, we describe: 1) the personal habits of water use and consumption, and 2) trihalomethane levels in the drinking water of the study areas. METHODS: We performed a follow-up cohort study in pregnant women in Valencia (n = 800), Sabadell (n = 800), the Basque Country (n = 600), Asturias (n = 500), and Granada (n = 700). Interviews were conducted in the third trimester of pregnancy to obtain individual information on the sources and amounts of drinking water, and on the frequency of showering, bathing, and swimming pool attendance. Water samples were collected to analyze trihalomethane levels. RESULTS: The main source of drinking water was bottled water, both inside and outside the home (82% and 94% of the women, respectively). Drinking water for cooking was mainly municipal (91%). Women preferred to take showers (98.2%) and did so, on average, 6.8 times/week for 11.7 minutes. A total of 58.5% of the women went to swimming pools during pregnancy, on average 9.4 times/month to outdoor swimming pools in the summer and 3.1 times/month the rest of the year for 32.6 and 21.7 minutes, respectively. Average trihalomethane levels ranged from 4 microg/l in the province of Granada to 117 microg/l in Sabadell. CONCLUSION: These preliminary results suggest that the main routes of trihalomethane exposure during pregnancy are dermal absorption and inhalation. Ingestion is a minor exposure route
SN - 0213-9111
AD - Centre de Recerca en Epidemiologia Ambiental (CREAL), Institut Municipal d'Investigacio Medica (IMIM), Barcelona, Espana. cvillanueva@imim.es
UR - 17433195
ER -

TY - JOUR
ID - 1184
T1 - Comparison of effects of socioeconomic and geographic variations on survival for adults and children with glioma
A1 - Tseng,M.Y.
A1 - Tseng,J.H.
A1 - Merchant,E.
Y1 - 2006/10//
N1 - Tseng, Ming-Yuan. Tseng, Jen-Ho. Merchant, Edwin
Journal of neurosurgery
jd3, 0253357
AIM, IM
Comparative Study. Journal Article
English
KW - MEDLINE
KW - Adolescent
KW - Adult
KW - Age Factors
KW - Aged
KW - 80 and over
KW - Brain Neoplasms/mo [Mortality]
KW - Brain Neoplasms/pa [Pathology]
KW - Child

KW - Preschool
KW - Female
KW - Geography
KW - Glioma/mo [Mortality]
KW - Glioma/pa [Pathology]
KW - Great Britain/ep [Epidemiology]
KW - Humans
KW - Kaplan-Meier Estimate
KW - Male
KW - Middle Aged
KW - Registries
KW - Sex Factors
KW - Socioeconomic Factors
RP - NOT IN FILE
SP - 297
EP - 305
JF - Journal of Neurosurgery
JA - J Neurosurg Pediatrics
VL - 105
IS - 4 Suppl
CY - United States
N2 - OBJECT: The authors analyzed the effects of socioeconomic status (SES) and geographic variations on survival rates for adults and children with glioma, studying the data of 30,489 adults and 2940 children from the Cancer Registry in England and Wales. METHODS: The median survival time and crude survival rates for eight variables (age, sex, morphology, World Health Organization grade, tumor site, SES, geographic region, and period of diagnosis) were calculated using the Kaplan-Meier method. Distributions among different variables were compared using the chi-square test. Cox regressions were performed to estimate the hazard ratios (HR) to death. The median survival time and 1-, 5-, and 10-year crude survival rates for adults were 0.42 years and 29.1, 12, and 7.7%, respectively; the values for children were 9.33 years and 72.69, 54.32, and 49.5%, respectively. Similar gradients in SES from the south to the north exist in both populations ($p < 0.001$, chi-square test). Multivariate analyses revealed that all eight variables influenced survival in adults, including independent effects of sex (HR 0.94 for female, $p < 0.001$), SES (HR 1.03/quintile of deprivation, $p < 0.001$), and geographic region (HR 1.10 for outside southern England, $p < 0.001$). In children, only five of the eight variables affected survival; sex, SES, and geographic variation did not have an effect. CONCLUSIONS: Although age and tumor characteristics are well-known prognostic factors for both adults and children with glioma, SES and geographic variation also play significant roles in the survival of adults. The effects of SES and geographic variation may be directly related to the National Health Service in the United Kingdom
SN - 0022-3085
AD - Department of Neurosurgery, Addenbrooke's Hospital, University of Cambridge, United Kingdom. myt22@cam.ac.uk
UR - 17328280
ER -

TY - JOUR
ID - 1185
T1 - The Australian Twin ADHD Project: current status and future directions
A1 - Bennett,K.S.
A1 - Hay,D.A.
A1 - Piek,J.
A1 - Pearsall-Jones,J.
A1 - Levy,F.
A1 - Martin,N.
Y1 - 2006/12//
N1 - Bennett, Kellie S. Hay, David A. Piek, Jan. Pearsall-Jones, Jillian. Levy, Florence. Martin, Neilson
Twin research and human genetics : the official journal of the International Society for Twin Studies
101244624
IM

Journal Article. Research Support, N.I.H., Extramural. Research Support, Non-U.S. Gov't
English
KW - MEDLINE
KW - Attention Deficit Disorder with Hyperactivity/ge [Genetics]
KW - Australia
KW - Child
KW - Cohort Studies
KW - Data Collection/td [Trends]
KW - Databases
KW - Factual
KW - Diseases in Twins/ge [Genetics]
KW - Humans
KW - Infant
KW - Newborn
KW - Siblings
KW - Twin Studies as Topic/td [Trends]
KW - Twin Studies as Topic
RP - NOT IN FILE
SP - 718
EP - 726
JF - Twin Research & Human Genetics: the Official Journal of the International Society for Twin Studies
JA - Twin Res Hum Genet
VL - 9
IS - 6
CY - Australia
N2 - This article describes the Australian Twin Attention-deficit/hyperactivity disorder (ADHD) Project (ATAP), the results of research conducted using this database and plans for future studies. Information has been actively collected from Australian families with twin children since 1991 for the ATAP database. The value of assessing siblings as well as twins is emphasized. Much work has gone into continuing the involvement of families in the study though this does become more difficult when twins reach maturity. The main focus of the project is ADHD in children and adolescents plus comorbid conditions including conduct disorder, oppositional defiant disorder, and generalized anxiety disorder. A major challenge has been how to retain continuity in the assessments, while at the same time covering changes in psychiatric classification, such as the move to Diagnostic and Statistical Manual of Mental Disorders (4th ed.; DSM-IV; American Psychiatric Association, 1994). Changes in the scale can affect the reports of twin similarity. Over the years, these twins have become part of other twin studies and future plans include linking different twin databases to investigate the relationships between childhood behavior and adult conditions. Recruitment, assessment and retention of twin families require a major commitment but create a significant resource for collaboration in areas outside the original aim
SN - 1832-4274
AD - School of Psychology, Curtin University of Technology, Perth, Western Australia 6945, Australia. K.Bennett@curtin.edu.au
UR - 17254397
ER -

TY - JOUR
ID - 1186
T1 - [Pre-adolescents and sport: results of a health survey in some secondary schools of Brianza]. [Italian]
A1 - Schieppati,S.
A1 - Rossetti,E.
A1 - Piatti,A.
A1 - Fonte,L.
A1 - Pesenti,E.
A1 - Zecca,G.
A1 - Lattuada,M.
A1 - Candela,A.
A1 - Valagussa,F.

A1 - Pellai,A.
Y1 - 2006/11//
N1 - Schieppati, S. Rossetti, E. Piatti, A. Fonte, L. Pesenti, E. Zecca, G. Lattuada, M. Candela, A. Valagussa, F. Pellai, A
Annali di igiene : medicina preventiva e di comunita
9002865, and
IM
English Abstract. Journal Article. Multicenter Study
Italian
KW - MEDLINE
KW - Adolescent
KW - Child
KW - Female
KW - Health Behavior
KW - Health Surveys
KW - Humans
KW - Italy
KW - Male
KW - Motivation
KW - Questionnaires
KW - Schools
KW - Sports/sn [Statistics & Numerical Data]
KW - Students/sn [Statistics & Numerical Data]
RP - NOT IN FILE
SP - 535
EP - 542
JF - Annali di Igiene
JA - Ann Ig
VL - 18
IS - 6
CY - Italy
N2 - This study is aimed at assessing time dedicated to, motivation and involvement in physical activity on behalf of pre-adolescents. 802 students (49.3% boys and 50.7% girls; mean age: 12.6) attending 43 classes of 11 Brianza's post-elementary schools. A questionnaire was prepared and administered to the involved sample. Classes took part in this study through randomized selection and data were analyzed using program Epi Info 6. Most of the respondents (60.1%) stay involved in sport because "it makes me feel good" and 32.4% because sport is "enjoyable and entertaining". Pre-adolescents chose the sport in which they become engaged on the basis of personal inclination (40%), in order to share experiences and their free time with friends (15.4%); 13.7% declare to prefer sport to be practiced in team. (13.7%). The most practiced sport are: football (51.3% boys, 4.5% girls), volley (3.9%-36.4%), dance (0.8%-25.2%) e swimming (9.4%-14%). 80.8% in the sample practice physical activity in settings outside schools and, among these, 51.7% dedicate three or more hours weekly to extra-school activities. There are significant differences ($p < 0.05$) between males' and females' engagement in physical activity (mean value: 4.1 hours; males: 4.6 h, females: 3.5 h). Among pre-teens, 18% declare to be physically inactive at all; 15.7% do not take part in sport activities because afraid to be bullied or shamed by peers. Research show that males are significantly more involved in physical activity than females
SN - 1120-9135
AD - Scuola di Specializzazione in Igiene e Medicina Preventiva, Universita degli Studi di Milano
UR - 17228610
ER -

TY - JOUR
ID - 1187
T1 - Development and use of behavior and social interaction software installed on Palm handheld for observation of a child's social interactions with the environment
A1 - Sarkar,A.
A1 - Dutta,A.
A1 - Dhingra,U.

A1 - Dhingra,P.
A1 - Verma,P.
A1 - Juyal,R.
A1 - Black,R.E.
A1 - Menon,V.P.
A1 - Kumar,J.
A1 - Sazawal,S.
Y1 - 2006/08//
N1 - Sarkar, Archana. Dutta, Arup. Dhingra, Usha. Dhingra, Pratibha. Verma, Priti. Juyal, Rakesh. Black, Robert E. Menon, Venugopal P. Kumar, Jitendra. Sazawal, Sunil
Behavior research methods
101244316
IM
Evaluation Studies. Journal Article. Research Support, Non-U.S. Gov't
English
KW - MEDLINE
KW - Activities of Daily Living
KW - Adult
KW - Behavioral Sciences/is [Instrumentation]
KW - Behavioral Sciences/mt [Methods]
KW - Child Behavior
KW - Child
KW - Preschool
KW - Computers
KW - Handheld
KW - Data Collection/is [Instrumentation]
KW - Data Collection/mt [Methods]
KW - Humans
KW - Interpersonal Relations
KW - Psychology
KW - Social/is [Instrumentation]
KW - Social/mt [Methods]
KW - Social Behavior
KW - Social Environment
KW - Software
RP - NOT IN FILE
SP - 407
EP - 415
JF - Behavior Research Methods
JA - Behav Res Methods
VL - 38
IS - 3
CY - United States
N2 - In settings in developing countries, children often socialize with multiple socializing agents (peers, siblings, neighbors) apart from their parents, and thus, a measurement of a child's social interactions should be expanded beyond parental interactions. Since the environment plays a role in shaping a child's development, the measurement of child-socializing agents' interactions is important. We developed and used a computerized observational software Behavior and Social Interaction Software (BASIS) with a preloaded coding scheme installed on a handheld Palm device to record complex observations of interactions between children and socializing agents. Using BASIS, social interaction assessments were conducted on 573 preschool children for 1 h in their natural settings. Multiple screens with a set of choices in each screen were designed that included the child's location, broad activity, state, and interactions with child-socializing agents. Data were downloaded onto a computer and systematically analyzed. BASIS, installed on Palm OS (M-125), enabled the recording of the complex interactions of child-socializing agents that could not be recorded with manual forms. Thus, this tool provides an innovative and relatively accurate method for the systematic recording of social interactions in an unrestricted environment
SN - 1554-351X

AD - Annamalai University, New Delhi, India
UR - 17186750
ER -

TY - JOUR
ID - 1188
T1 - Exercise tolerance in a hot and humid climate in heat-acclimatized girls and women
A1 - Rivera-Brown,A.M.
A1 - Rowland,T.W.
A1 - Ramirez-Marrero,F.A.
A1 - Santacana,G.
A1 - Vann,A.
Y1 - 2006/12//
N1 - Rivera-Brown, A M. Rowland, T W. Ramirez-Marrero, F A. Santacana, G. Vann, A
International journal of sports medicine
8008349, grk
IM
Journal Article. Research Support, Non-U.S. Gov't
English
KW - MEDLINE
KW - Acclimatization
KW - Adult
KW - Body Temperature Regulation/ph [Physiology]
KW - Cardiovascular Physiological Phenomena
KW - Child
KW - Exercise/ph [Physiology]
KW - Exercise Tolerance/ph [Physiology]
KW - Female
KW - Hot Temperature
KW - Humans
KW - Humidity
KW - Sweating
KW - Tropical Climate
KW - Water-Electrolyte Balance/ph [Physiology]
RP - NOT IN FILE
SP - 943
EP - 950
JF - International Journal of Sports Medicine
JA - Int J Sports Med
VL - 27
IS - 12
CY - Germany
N2 - This study compared physiological responses associated with exercise tolerance in girls (G) and women (W) of similar fitness and heat acclimatization level during exercise in a hot and humid outdoor environment (33.4 degrees C and 55.1 % RH; WBGT = 29.9 +/- 0.2 degrees C). Nine pre-menarcheal G (age = 11.3 yr) and nine W (age = 26.8 yr), matched for aerobic capacity and heat acclimatization level, performed a cycling session at 60 % VO2max until fatigue. A sports drink was provided periodically to prevent dehydration. Tolerance time was not different between the groups (G = 56.9 +/- 6.3, W = 76.5 +/- 9.9 min, p > 0.05). During exercise, sweat rate (G = 9.1 +/- 1.1, W = 12.0 +/- 1.1 ml.m(-2).min(-1)), the increase in rectal temperature [T(re)] (G = 0.9 +/- 0.1, W = 1.1 +/- 0.1 degrees C), and heat storage (G = 10.6 +/- 5.3, W = 20.5 +/- 4.5 W.m(-2)) did not differ between the groups. At fatigue, T(re) (G = 38.2 +/- 0.1, W = 38.4 +/- 0.1 degrees C), heart rate (G = 167.3 +/- 7.3, W = 171 +/- 3.3 beats.min(-1)), stroke index (G = 48.3 +/- 1.5, W = 52.4 +/- 1.8 ml.m(-2)), and forearm skin blood flow (G = 9.5 +/- 1.3, W = 11.7 +/- 1.5 ml.100 ml(- 1).min(-1)) did not differ between the groups. Similar to women, the main reasons reported by girls to stop exercising in the heat were localized leg fatigue and gluteus muscle discomfort. We conclude that heat-acclimatized girls exhibit an adequate cardiovascular and thermoregulatory adjustment while exercising in a hot and humid outdoor environment when hypohydration is prevented

SN - 0172-4622
AD - Center for Sports Health and Exercise Sciences, Department of Physical Medicine, Rehabilitation and Sports Medicine, University of Puerto Rico School of Medicine, San Juan, Puerto Rico. aniriver@coqui.net
UR - 16739090
ER -

TY - JOUR
ID - 1189
T1 - Water ingestion during swimming activities in a pool: a pilot study
A1 - Dufour,A.P.
A1 - Evans,O.
A1 - Behymer,T.D.
A1 - Cantu,R.
Y1 - 2006/12//
N1 - Dufour, Alfred P. Evans, Otis. Behymer, Thomas D. Cantu, Ricardo
Journal of water and health
101185420
IM
Journal Article
English
KW - MEDLINE
KW - Adolescent
KW - Adult
KW - Child
KW - Preschool
KW - Chlorine
KW - Chromatography
KW - High Pressure Liquid
KW - Disinfectants
KW - Female
KW - Humans
KW - Male
KW - Pilot Projects
KW - Swimming
KW - Swimming Pools
KW - Temperature
KW - Triazines/to [Toxicity]
KW - Triazines/ur [Urine]
KW - Water Pollutants
KW - Chemical/to [Toxicity]
KW - Chemical/ur [Urine]
RP - NOT IN FILE
SP - 425
EP - 430
JF - Journal of Water & Health
JA - J Water Health
VL - 4
IS - 4
CY - England
N2 - Chloroisocyanurates are commonly added to outdoor swimming pools to stabilize chlorine disinfectants. The chloroisocyanurates decompose slowly to release chlorine and cyanuric acid. Studies conducted to determine if the chloroisocyanurates might be toxic to swimmers showed that they were not and that ingested cyanuric acid passed through the body unmetabolized. This fact was used to determine the amount of water swallowed during swimming activity. Fifty-three recreational swimmers, using a community swimming pool disinfected with cyanuric acid stabilized chlorine, participated in the study. The participants did not swim on the day before or after the test swim. The swimmers were asked to actively swim for at least 45 minutes and to collect their urine for the next 24 hours. Cyanuric acid was measured in pool water using high performance

liquid chromatography and porous graphitic carbon columns with UV detection. The urine sample assay required a clean-up procedure to remove urinary proteins and interfering substances. Results of the study indicate that non-adults ingest about twice as much water as adults during swimming activity. The average amount of water swallowed by non-adults and adults was 37 ml and 16 ml, respectively. The design for this study and the analytical methodology used to assay cyanuric acid in swimming pool water and human urine were effective for measuring the volume of water swallowed during swimming activity
SN - 1477-8920
AD - US Environmental Protection Agency, National Exposure Research Laboratory, 26 W. Martin Luther King Drive, Cincinnati, OH 45268, USA. dufour.alfred@epa.gov
UR - 17176813
ER -

TY - JOUR
ID - 1190
T1 - Active surveillance for candidemia, Australia
A1 - Chen,S.
A1 - Slavin,M.
A1 - Nguyen,Q.
A1 - Marriott,D.
A1 - Playford,E.G.
A1 - Ellis,D.
A1 - Sorrell,T.
A1 - Australian,Candidemia Study
Y1 - 2006/10//
N1 - Chen, Sharon. Slavin, Monica. Nguyen, Quoc. Marriott, Deborah. Playford, E Geoffrey. Ellis, David. Sorrell, Tania. Australian Candidemia Study
Emerging infectious diseases
cod, 9508155
IM
Journal Article. Research Support, Non-U.S. Gov't
English
KW - MEDLINE
KW - Adolescent
KW - Adult
KW - Aged
KW - Australia/ep [Epidemiology]
KW - Candidiasis/ep [Epidemiology]
KW - Candidiasis/mo [Mortality]
KW - Candidiasis/th [Therapy]
KW - Child
KW - Preschool
KW - Cross Infection/ep [Epidemiology]
KW - Cross Infection/mi [Microbiology]
KW - Fungemia/ep [Epidemiology]
KW - Fungemia/mi [Microbiology]
KW - Fungemia/mo [Mortality]
KW - Fungemia/th [Therapy]
KW - Humans
KW - Incidence
KW - Infant
KW - Newborn
KW - Middle Aged
KW - Retrospective Studies
KW - Risk Factors
RP - NOT IN FILE
SP - 1508
EP - 1516

JF - Emerging Infectious Diseases
JA - Emerg Infect Dis
VL - 12
IS - 10
CY - United States
N2 - Population-based surveillance for candidemia in Australia from 2001 to 2004 identified 1,095 cases. Annual overall and hospital-specific incidences were 1.81/100,000 and 0.21/1,000 separations (completed admissions), respectively. Predisposing factors included malignancy (32.1%), indwelling vascular catheters (72.6%), use of antimicrobial agents (77%), and surgery (37.1%). Of 919 episodes, 81.5% were inpatient healthcare associated (IHCA), 11.6% were outpatient healthcare associated (OHCA), and 6.9% were community acquired (CA). Concomitant illnesses and risk factors were similar in IHCA and OHCA candidemia. IHCA candidemia was associated with sepsis at diagnosis (p<0.001), death <30 days after infection (p<0.001), and prolonged hospital admission (p<0.001). Non-Candida albicans species (52.7%) caused 60.5% of cases acquired outside hospitals and 49.9% of IHCA candidemia (p = 0.02). The 30-day death rate was 27.7% in those > or =65 years of age. Adult critical care stay, sepsis syndrome, and corticosteroid therapy were associated with the greatest risk for death. Systematic epidemiologic studies that use standardized definitions for IHCA, OHCA, and CA candidemia are indicated
SN - 1080-6040
AD - Westmead Hospital, Westmead, New South Wales, Australia. sharonc@icpmr.wsahs.nsw.gov.au
UR - 17176564
ER -

TY - JOUR
ID - 1191
T1 - Injury risk from popular childhood physical activities: results from an Australian primary school cohort
A1 - Spinks,A.B.
A1 - Macpherson,A.K.
A1 - Bain,C.
A1 - McClure,R.J.
Y1 - 2006/12//
N1 - Spinks, A B. Macpherson, A K. Bain, C. McClure, R J
Injury prevention : journal of the International Society for Child and Adolescent Injury Prevention
crz, 9510056
IM
Journal Article. Research Support, Non-U.S. Gov't
English
KW - MEDLINE
KW - Athletic Injuries/ep [Epidemiology]
KW - Athletic Injuries/et [Etiology]
KW - Child
KW - Preschool
KW - Epidemiologic Methods
KW - Female
KW - Humans
KW - Male
KW - Queensland/ep [Epidemiology]
KW - Sex Factors
KW - Soccer/in [Injuries]
KW - Tennis/in [Injuries]
RP - NOT IN FILE
SP - 390
EP - 394
JF - Injury Prevention
JA - Inj Prev
VL - 12
IS - 6
CY - England

N2 - BACKGROUND: Children engage in various physical activities that pose different injury risks. However, the lack of adequate data on exposure has meant that these risks have not been quantified or compared in young children aged 5-12 years. OBJECTIVES: To measure exposure to popular activities among Australian primary school children and to quantify the associated injury risks. METHOD: The Childhood Injury Prevention Study prospectively followed up a cohort of randomly selected Australian primary and preschool children aged 5-12 years. Time (min) engaged in various physical activities was measured using a parent-completed 7-day diary. All injuries over 12 months were reported to the study. All data on exposure and injuries were coded using the International classification of external causes of injury. Injury rates per 1000 h of exposure were calculated for the most popular activities. RESULTS: Complete diaries and data on injuries were available for 744 children. Over 12 months, 314 injuries relating to physical activity outside of school were reported. The highest injury risks per exposure time occurred for tackle-style football (2.18/1000 h), wheeled activities (1.72/1000 h) and tennis (1.19/1000 h). Overall, boys were injured more often than girls; however, the differences were non-significant or reversed for some activities including soccer, trampolining and team ball sports. CONCLUSION: Although the overall injury rate was low in this prospective cohort, the safety of some popular childhood activities can be improved so that the benefits may be enjoyed with fewer negative consequences
SN - 1353-8047
AD - School of Population Health, University of Queensland, Brisbane, Queensland, Australia. a.spinks@griffith.edu.au
UR - 17170188
ER -

TY - JOUR
ID - 1192
T1 - Risk factors for unintentional injuries due to falls in children aged 0-6 years: a systematic review. [Review] [28 refs]
A1 - Khambalia,A.
A1 - Joshi,P.
A1 - Brussoni,M.
A1 - Raina,P.
A1 - Morrongiello,B.
A1 - Macarthur,C.
Y1 - 2006/12//
N1 - Khambalia, A. Joshi, P. Brussoni, M. Raina, P. Morrongiello, B. Macarthur, C
Injury prevention : journal of the International Society for Child and Adolescent Injury Prevention
crz, 9510056
IM
Journal Article. Research Support, Non-U.S. Gov't. Review
English
KW - MEDLINE
KW - Accidental Falls/pc [Prevention & Control]
KW - Accidental Falls
KW - Age Factors
KW - Beds
KW - Child
KW - Preschool
KW - Female
KW - Humans
KW - Infant
KW - Infant Equipment
KW - Newborn
KW - Male
KW - Poverty
KW - Research Design
KW - Risk Factors
KW - Sex Factors
KW - Social Class
KW - Wounds and Injuries/et [Etiology]

KW - Wounds and Injuries/pc [Prevention & Control]
RP - NOT IN FILE
SP - 378
EP - 381
JF - Injury Prevention
JA - Inj Prev
VL - 12
IS - 6
CY - England
N2 - OBJECTIVE: To identify risk factors for unintentional injuries due to falls in children aged 0-6 years. DESIGN: A systematic review of the literature. METHODS: Electronic databases from 1966 to March 2005 were comprehensively searched to identify empirical research that evaluated risk factors for unintentional injuries due to falls in children aged 0-6 years and included a comparison group. RESULTS: 14 studies met the inclusion criteria. Studies varied by the type of fall injury that was considered (ie, bunk bed, stairway, playground or infant walker) and with respect to the quality of evidence. In general, major risk factors for the incidence or severity of injuries due to falls in children included age of the child, sex, height of the fall, type of surface, mechanism (dropped, stairway or using a walker), setting (day care v home care) and socioeconomic status. CONCLUSION: Despite a high burden, few controlled studies have examined the risk and protective factors for injuries due to falls in children aged 0-6 years. The only study to examine falls from a population health perspective suggests that age, sex and poverty are independent risk factors for injuries due to falls in children. [References: 28]
SN - 1353-8047
AD - Division of Paediatric Medicine and the Paediatric Outcomes Research Team, The Hospital for Sick Children, Toronto, Ontario, Canada
UR - 17170185
ER -

TY - JOUR
ID - 1193
T1 - Aortic stenosis: the spectrum of practice
A1 - Khalid,O.
A1 - Luxenberg,D.M.
A1 - Sable,C.
A1 - Benavidez,O.
A1 - Geva,T.
A1 - Hanna,B.
A1 - Abdulla,R.
Y1 - 2006/11//
N1 - Khalid, O. Luxenberg, D M. Sable, C. Benavidez, O. Geva, T. Hanna, B. Abdulla, R
Pediatric cardiology
pa3, 8003849
IM
Journal Article. Multicenter Study
English
KW - MEDLINE
KW - Aortic Valve Stenosis/cn [Congenital]
KW - Aortic Valve Stenosis/di [Diagnosis]
KW - Aortic Valve Stenosis/pp [Physiopathology]
KW - Aortic Valve Stenosis/th [Therapy]
KW - Cardiac Catheterization
KW - Catheterization
KW - Child
KW - Exercise Test
KW - Heart Function Tests
KW - Humans
KW - Physician's Practice Patterns
RP - NOT IN FILE

SP - 661
EP - 669
JF - Pediatric Cardiology
JA - Pediatr Cardiol
VL - 27
IS - 6
CY - United States
N2 - There is significant variation in practice patterns in managing congenital aortic valve stenosis. Review of medical literature reveals no significant information regarding the current practice methods in the treatment of a simple lesion such as aortic stenosis (AS). Therefore, this survey-based study was conducted in an attempt to better understand the uniformity or heterogeneity of practice in treating AS. A questionnaire was prepared to evaluate the style of management of AS. This survey was designed to assess the practice of follow-up visitations, type and frequency of investigative studies, pharmacological therapy, and exercise recommendations. Questions about therapeutic intervention included those of timing and type of intervention. Questionnaires were sent to all academic pediatric cardiology programs in the United States (48 program) and selected international programs from Europe, Asia, and Australasia (19 program). The total number of surveys sent out was 67, and the total number of respondents was 25 (37%), 15 (31%) from the United States and 9 (53%) from outside the United States. The definition of moderate AS varied among respondents. The range provided for mild AS was identified as that with a peak-to-peak pressure gradient of < 25-30 mmHg, peak instantaneous Doppler gradient of < 36-50 mmHg, or mean Doppler gradient of < 25-40 mmHg. On the other hand, severe AS was defined as that with a peak-to-peak gradient of > 50-60 mmHg, peak instantaneous Doppler gradient of > 64-80 mmHg, or mean Doppler gradient of > 45-64 mmHg. In assessing follow-up patterns, 84% of respondents recommended seeing patients with mild AS annually, the longest time of follow-up listed in the questionnaire, whereas 20% suggested follow-up every 6 months. There was no consensus among survey centers regarding follow-up of patients with moderate AS. For severe AS, 16% recommend immediate intervention, 16% arrange follow-up every 6 months, and 56 and 28% recommend follow-up in 3 and 1 month(s), respectively. In making the decision to proceed with biventricular versus univentricular repair in patients with AS in the neonatal period, many factors were considered. Ninety-two percent of respondents rely on mitral valve z score, 84% on aortic valve z score, 52% on left ventricle length, 48% on the presence of antegrade ascending aorta flow, and only 32% considered significant endocardial fibroelastosis as a factor. Rhodes score was used by 20% of respondents in decision making regarding the approach to management of this subset of AS. This study shows that there is consensus in the management of mild and severe forms of AS. As expected, disagreement is present in the definition, evaluation, and therapy of moderate aortic valve stenosis. There is a tendency for catheter intervention except in the presence of dysplastic aortic valve or moderate to severe aortic regurgitation. There is also disagreement regarding methods used to determine biventricular versus univentricular repair of a borderline hypoplastic left heart
SN - 0172-0643
AD - The University of Chicago, MC 4051, Chicago, IL 60637-1470, USA. rabdulla@uchicago.edu
UR - 17111288
ER -

TY - JOUR
ID - 1194
T1 - Spectrum of noninfectious health effects from molds
A1 - Committee on Environmental Health,American Academy of Pediatrics
A1 - Kim,J.J.
A1 - Mazur,L.J.
Y1 - 2006/12//
N1 - Committee on Environmental Health, American Academy of Pediatrics. Kim, Janice J. Mazur, Lynnette J
Pediatrics
oxv, 0376422
AIM, IM
Journal Article. Practice Guideline
English
KW - MEDLINE
KW - Child
KW - Fungi

KW - Humans
KW - Respiratory Tract Infections/et [Etiology]
KW - Respiratory Tract Infections/pc [Prevention & Control]
RP - NOT IN FILE
SP - 2582
EP - 2586
JF - Pediatrics
VL - 118
IS - 6
CY - United States
N2 - Molds are eukaryotic (possessing a true nucleus) nonphotosynthetic organisms that flourish both indoors and outdoors. For humans, the link between mold exposure and asthma exacerbations, allergic rhinitis, infections, and toxicities from ingestion of mycotoxin-contaminated foods are well known. However, the cause-and-effect relationship between inhalational exposure to mold and other untoward health effects (eg, acute idiopathic pulmonary hemorrhage in infants and other illnesses and health complaints) requires additional investigation. Pediatricians play an important role in the education of families about mold, its adverse health effects, exposure prevention, and remediation procedures
SN - 1098-4275
UR - 17142549
ER -

TY - JOUR
ID - 1195
T1 - For comparison: experience with a children's obesity camp
A1 - Cooper,C.
A1 - Sarvey,S.
A1 - Collier,D.
A1 - Willson,C.
A1 - Green,I.
A1 - Pories,M.L.
A1 - Rose,M.A.
A1 - Escott-Stump,S.
A1 - Pories,W.
Y1 - 2006/11//
N1 - Cooper, Christopher. Sarvey, Sharon. Collier, David. Willson, Charles. Green, Ira. Pories, Mary Lisa. Rose, Mary Ann. Escott-Stump, Sylvia. Pories, Walter
Surgery for obesity and related diseases : official journal of the American Society for Bariatric Surgery
101233161
IM
Comparative Study. Journal Article
English
KW - MEDLINE
KW - Behavior Therapy
KW - Body Mass Index
KW - Camping
KW - Child
KW - Diet
KW - Reducing
KW - Exercise Therapy
KW - Female
KW - Humans
KW - Male
KW - North Carolina
KW - Obesity/su [Surgery]
KW - Obesity/th [Therapy]
KW - Physical Fitness
KW - Treatment Outcome

KW - Weight Loss
RP - NOT IN FILE
SP - 622
EP - 626
JF - Surgery for Obesity & Related Diseases
VL - 2
IS - 6
CY - United States
N2 - BACKGROUND: The alarming rise in childhood obesity has resulted in a number of bariatric surgical initiatives. To interpret the outcomes, a comparison with a nonoperative approach is prudent. METHODS: In 2003, 2004, and 2005, we measured the outcomes produced by an isolated summer camp in North Carolina for 74, 99, and 89 obese children and adolescents with a mean age of 12.7 +/- 2.3, 13.0 +/- 1.9, and 13.2 +/- 1.8 years and initial body mass index (BMI) of 33.1 +/- 5.7, 33.4 +/- 6.4, and 32.9 +/- 7.0 kg/m2, respectively. The camp featured a 1700/d caloric diet, daily aerobic and resistance weight training exercise, nutrition classes, and weekly sessions with a psychologist. The changes in weight, BMI, body shape measurements, and fitness level were assessed. The average length of stay was 4.3, 4.2, and 4.4 weeks, respectively, in 2003, 2004, and 2005. RESULTS: The BMI, body shape, and weight change measures significantly improved during each of the summers. The campers lost 1.6, 2.0, and 1.8 kg/wk during each of the 3 years. Their waist measurements decreased by 9.1 +/- 5.12 cm, 9.9 +/- 5.6 cm, and 8.1 +/- 5.8 cm. Significant improvements occurred in the timed sprints, .5-mile (0.8-km) run, and vertical jump. Of those campers staying for two summers, 4.4% continued to reduce their BMI despite growth, 56.5% maintained their BMI at less than the initial baseline measurement, and 39.1% increased their BMI to greater than the baseline. The co-morbidities also improved. One camper, aged 15 years, weighing 211 kg, progressed from being able to walk 3 steps to managing the 100-yard (91-m) dash in 42 seconds after losing 35 kg in 8 weeks. CONCLUSION: Diets, exercise, and behavioral modification are useful approaches in severely obese children that can, in some, produce significant albeit expensive long-term results. Recidivism is a problem. Surgical outcomes must compare favorably with these outcomes
SN - 1550-7289
AD - East Carolina University Brody School of Medicine, Greenville, North Carolina 27834, USA
UR - 17020822
ER -

TY - JOUR
ID - 1196
T1 - Validation and calibration of an accelerometer in preschool children
A1 - Pate,R.R.
A1 - Almeida,M.J.
A1 - McIver,K.L.
A1 - Pfeiffer,K.A.
A1 - Dowda,M.
Y1 - 2006/11//
N1 - Pate, Russell R. Almeida, Maria J. McIver, Kerry L. Pfeiffer, Karin A. Dowda, Marsha
Obesity (Silver Spring, Md.)
101264860
IM
Journal Article. Research Support, N.I.H., Extramural. Research Support, Non-U.S. Gov't. Validation Studies
English
KW - MEDLINE
KW - Acceleration
KW - Calibration
KW - Child
KW - Preschool
KW - Cross-Sectional Studies
KW - Exercise Test/is [Instrumentation]
KW - Exercise Test/st [Standards]
KW - Female
KW - Humans

KW - Male
KW - Oxygen Consumption/ph [Physiology]
KW - Walking
RP - NOT IN FILE
SP - 2000
EP - 2006
JF - Obesity
JA - Obesity (Silver Spring)
VL - 14
IS - 11
CY - United States
N2 - OBJECTIVE: Obesity rates in young children are increasing, and decreased physical activity is likely to be a major contributor to this trend. Studies of physical activity in young children are limited by the lack of valid and acceptable measures. The purpose of this study was to calibrate and validate the ActiGraph accelerometer for use with 3- to 5-year-old children. RESEARCH METHODS AND PROCEDURES: Thirty preschool children wore an ActiGraph accelerometer (ActiGraph, Fort Walton Beach, FL) and a Cosmed portable metabolic system (Cosmed, Rome, Italy) during a period of rest and while performing three structured physical activities in a laboratory setting. Expired respiratory gases were collected, and oxygen consumption was measured on a breath-by-breath basis. Accelerometer data were collected at 15-second intervals. For cross-validation, the same children wore the same instruments while participating in unstructured indoor and outdoor activities for 20 minutes each at their preschool. RESULTS: In calibrating the accelerometer, the correlation between Vo(2) (ml/kg per min) and counts was r = 0.82 across all activities. The only significant variable in the prediction equation was accelerometer counts (R(2) = 0.90, standard error of the estimate = 4.70). In the cross-validation, the intraclass correlation coefficient between measured and predicted Vo(2) was R = 0.57 and the Spearman correlation coefficient was R = 0.66 (p < 0.001). Cut-off points for moderate- and vigorous-intensity physical activity were identified at 420 counts/15 s (Vo(2) = 20 mL/kg per min) and 842 counts/15 s (Vo(2) = 30 mL/kg per min), respectively. When these cutpoints were applied to the cross-validation data, percentage agreement, kappa, and modified kappa for moderate activity were 0.69, 0.36, and 0.38, respectively. For vigorous activity, the same measures were 0.81, 0.13, and 0.62. DISCUSSION: Accelerometer counts were highly correlated with Vo(2) in young children. Accelerometers can be appropriately used as a measure of physical activity in this population
SN - 1930-7381
AD - Arnold School of Public Health, University of South Carolina, 730 Devine Street, Columbia, SC 29208, USA. rpate@gwm.sc.edu
UR - 17135617
ER -

TY - JOUR
ID - 1197
T1 - Non-fatal sports and recreational violent injuries among children and teenagers, United States, 2001-2003
A1 - Conn,J.M.
A1 - Annest,J.L.
A1 - Bossarte,R.M.
A1 - Gilchrist,J.
Y1 - 2006/12//
N1 - Conn, Judith M. Annest, Joseph L. Bossarte, Robert M. Gilchrist, Julie
Journal of science and medicine in sport / Sports Medicine Australia
c54, 9812598
IM
Journal Article
English
KW - MEDLINE
KW - Adolescent
KW - Adult
KW - Athletic Injuries/ep [Epidemiology]
KW - Athletic Injuries/mo [Mortality]
KW - Child

KW - Preschool
KW - Female
KW - Humans
KW - Infant
KW - Newborn
KW - Male
KW - Recreation
KW - Sports
KW - Time Factors
KW - United States/ep [Epidemiology]
RP - NOT IN FILE
SP - 479
EP - 489
JF - Journal of Science & Medicine in Sport
JA - J Sci Med Sport
VL - 9
IS - 6
CY - Australia
N2 - BACKGROUND: An estimated 2.7 million non-fatal unintentional sports and recreational injuries are treated in U.S. hospital emergency departments (EDs) annually. However, little is known about the number of sports and recreational injuries resulting from violent behavior. METHODS: Data for 2001-2003 on sports and recreational injuries were obtained from the National Electronic Injury Surveillance System-All Injury Program (NEISS-AIP)-a national sample of 66 U.S. EDs. National estimates and rates of persons treated for violence-related sports and recreational injuries in EDs are compared to those treated for unintentional sports and recreational injuries. Types of injuries and injury circumstances are described. RESULTS: During the study period, an estimated 6,705 (8.3 per 100,000; 95% confidence intervals (CI), 6.3-10.3) children and teenagers with violence-related sports and recreational injuries were treated in U.S. EDs annually, compared to 2,698,634 children and teenagers with unintentional sports and recreational injuries. Thus, violent behavior accounted for 0.25% of sports and recreational injuries. The highest incidence rate (13.6 per 100,000) for violence-related sports and recreational injuries was for children aged 10-14 years. Most patients with violence-related sports and recreational injuries were treated and released from the ED. A majority of those with violence-related sports and recreational injuries were injured to the head/neck region (52.2%), of which 24.1% were treated for traumatic brain injuries. Most violent injuries resulted from being pushed or hit (65.6%); the most common sports and recreational activity varied by age: playground (65.2%) for children < or =9 years; bicycling (26.7%) for 10-14-year-olds; basketball (45.3%) for 15-19-year-olds. CONCLUSIONS: National ED surveillance systems can provide useful information pertaining to prevention programs designed to reduce sports and recreational injuries resulting from violent behavior and unintentional causes
SN - 1440-2440
AD - Office of Statistics and Programming, National Center for Injury Prevention and Control, Centers for Disease Control and Prevention, MS-K59, Atlanta, GA 30341, USA. jconn@cdc.gov
UR - 16621700
ER -

TY - JOUR
ID - 1198
T1 - Children's physical activity during recess and outside of school
A1 - Beighle,A.
A1 - Morgan,C.F.
A1 - Le,Masurier G.
A1 - Pangrazi,R.P.
Y1 - 2006/12//
N1 - Beighle, Aaron. Morgan, Charles F. Le Masurier, Guy. Pangrazi, Robert P
The Journal of school health
k13, 0376370
IM, N
Comparative Study. Journal Article
English

KW - MEDLINE
KW - Analysis of Variance
KW - Child
KW - Female
KW - Humans
KW - Male
KW - Motor Activity
KW - Schools
KW - Sex Factors
RP - NOT IN FILE
SP - 516
EP - 520
JF - Journal of School Health
JA - J Sch Health
VL - 76
IS - 10
CY - United States
N2 - The purpose of this study was to examine children's physical activity during recess and outside of school. Third-, fourth-, and fifth-grade students (N = 270; 121 boys, age = 9.5 +/- 0.9 years; 150 girls, age = 9.6 +/- 0.9 years) wore sealed pedometers during a 15-minute recess period and outside of school for 4 consecutive school days. A factorial analysis of variance (grade by gender) was used to examine differences among grades and between genders for the following variables: recess activity time (RAT), recess step counts (RSC), out-of-school activity time (OAT), and out-of-school step counts (OSC). For all outcome variables, there were no significant interactions between grade and gender and no significant main effect for grade. A significant main effect for gender (F(1,264)= 73.1, p < .001) indicated that boys accumulated more RSC and OSC than girls (1268 +/- 341 vs 914 +/- 261 and 7229 +/- 2877 vs 5808 +/- 2059, respectively) and more RAT and OAT than girls (11.7 +/- 2.4 vs 9.4 +/- 2.2 and 77.3 +/- 28 vs 67.4 +/- 21, respectively). Boys spent 78% and girls spent 63% of their recess time engaged in physical activity. Outside of school, girls spent 20% and boys spent 25% of their time engaged in physical activity. RAT comprised 14% and 16% of total discretionary activity time for girls and boys, respectively. Boys in this study are more active during discretionary time periods compared to girls. Study participants spent the majority of their recess time engaged in physical activity
SN - 0022-4391
AD - Department of Kinesiology and Health Promotion, University of Kentucky, 120 Seaton Bldg, Lexington, KY 40509, USA. beighle@uky.edu
UR - 17096825
ER -

TY - JOUR
ID - 1199
T1 - 'Outlook': A psychological service for children with a different appearance
A1 - Maddern,L.H.
A1 - Cadogan,J.C.
A1 - Emerson,M.P.
Y1 - 2006/07//
N1 - Maddern, Lynn H. Cadogan, Julia C. Emerson, Michael P
Clinical child psychology and psychiatry
9604507
IM
Journal Article
English
KW - MEDLINE
KW - Adolescent
KW - Adult
KW - Child
KW - Preschool
KW - Cleft Lip/px [Psychology]
KW - Cleft Palate/px [Psychology]

KW - Disabled Children/px [Psychology]
KW - Female
KW - Great Britain
KW - Humans
KW - Male
KW - Mental Disorders/px [Psychology]
KW - Mental Disorders/th [Therapy]
KW - Mental Health Services/og [Organization & Administration]
KW - Self Concept
KW - Social Desirability
RP - NOT IN FILE
SP - 431
EP - 443
JF - Clinical Child Psychology & Psychiatry
JA - Clin
VL - 11
IS - 3
CY - England
N2 - 'Outlook' is a unit within North Bristol Trust which has been developing a psychosocial service for children, young people and adults with a different appearance resulting from congenital abnormalities such as cleft lip and palate and also burns and other forms of trauma. This study presents data collected from 29 children aged between 5 and 16 who received individual therapeutic interventions. The baseline assessment included a semistructured interview schedule, visual analogue scales and the CBCL (Achenbach, 1991) to provide the parents' view of their children's behaviour profile. Measures were repeated post intervention and at 6 months follow-up. The intervention consisted of four sessions of cognitive-behavioural therapy (CBT) including social skills and problem-solving components. Statistical analyses revealed significant outcomes on a range of measures. At 6 months follow-up children reported a reduction in the frequency of teasing and in the degree of distress it caused both in the classroom and in the playground. Parents reported reductions in their children's' somatizing behaviour and levels of anxiety. These outcomes suggest that this approach represents an effective therapy for children with appearance-related psychological problems
SN - 1359-1045
AD - Child and Adolescent Mental Health Service, Downend Clinic, Bristol, UK. Lynn.Maddern@nbt.nhs.uk
UR - 17080779
ER -

TY - JOUR
ID - 1200
T1 - Who suffers from indoor air pollution? Evidence from Bangladesh
A1 - Dasgupta,S.
A1 - Huq,M.
A1 - Khaliquzzaman,M.
A1 - Pandey,K.
A1 - Wheeler,D.
Y1 - 2006/11//
N1 - Dasgupta, Susmita. Huq, Mainul. Khaliquzzaman, M. Pandey, Kiran. Wheeler, David
Health policy and planning
8610614, f9q
H
Journal Article. Research Support, Non-U.S. Gov't
English
KW - MEDLINE
KW - Adolescent
KW - Adult
KW - Air Pollution
KW - Indoor
KW - Bangladesh
KW - Child

KW - Preschool
KW - Data Collection
KW - Female
KW - Humans
KW - Inhalation Exposure/an [Analysis]
KW - Male
KW - Middle Aged
RP - NOT IN FILE
SP - 444
EP - 458
JF - Health Policy & Planning
JA - Health Policy Plan.
VL - 21
IS - 6
CY - England
N2 - In this paper, we investigate individuals' exposure to indoor air pollution. Using new survey data from Bangladesh, average hours spent by members of households in the cooking area, living area and outdoors in a typical day are combined with the estimates of pollution concentration in different locations in order to estimate exposure. We analyse exposure at two levels: differences within households attributable to family roles, and differences across households attributable to income and education. Within households, we relate individuals' exposure to pollution in different locations during their daily round of activities. We find high levels of exposure for children and adolescents of both sexes, with particularly serious exposure for children under 5 years. Among prime-age adults, we find that men have half the exposure of women (whose exposure is similar to that of children and adolescents). We also find that elderly men have significantly lower exposure than elderly women. Across households, we draw on results from a previous paper, which relate pollution variation across households to choices of cooking fuel, cooking locations, construction materials and ventilation practices. We find that these choices are significantly affected by family income and adult education levels (particularly for women). Overall, we find that the poorest, least-educated households have twice the pollution levels of relatively high-income households with highly educated adults. Our findings further suggest that young children and poorly educated women in poor households face pollution exposures that are four times those for men in higher income households organized by more highly educated women. Since infants and young children suffer the worst mortality and morbidity from indoor air pollution, in this paper we consider measures for reducing their exposure. Our recommendations for reducing the exposure of infants and young children are based on a few simple, robust findings. Hourly pollution levels in cooking and living areas are quite similar because cooking smoke diffuses rapidly and nearly completely into living areas. However, outdoor pollution is far lower. At present, young children are only outside for an average of 3 hours per day. For children in a typical household, pollution exposure can be halved by adopting two simple measures: increasing their outdoor time from 3 to 5 or 6 hours per day, and concentrating outdoor time during peak cooking periods
SN - 0268-1080
AD - Development Research Group, World Bank, NW, Washington DC 20433, USA.
sdasgupta@worldbank.org
UR - 17030552
ER -

TY - JOUR
ID - 1201
T1 - Children's indoor and outdoor play patterns in Ahwaz City: implications for injury prevention
A1 - Soori,H.
Y1 - 2006/05//
N1 - Soori, H
Eastern Mediterranean health journal = La revue de sante de la Mediterranee orientale = al-Majallah al-sihhiyah li-sharq al-mutawassit
daq, 9608387
IM
Journal Article
English
KW - MEDLINE

KW - Accident Prevention
KW - Age Factors
KW - Bicycling/in [Injuries]
KW - Bicycling/sn [Statistics & Numerical Data]
KW - Child
KW - Cross-Sectional Studies
KW - Female
KW - Humans
KW - Iran/ep [Epidemiology]
KW - Leisure Activities
KW - Male
KW - Needs Assessment/og [Organization & Administration]
KW - Parents/px [Psychology]
KW - Play and Playthings/in [Injuries]
KW - Public Facilities/sn [Statistics & Numerical Data]
KW - Questionnaires
KW - Risk Assessment
KW - Risk Factors
KW - Safety Management/og [Organization & Administration]
KW - Sex Factors
KW - Soccer/in [Injuries]
KW - Soccer/sn [Statistics & Numerical Data]
KW - Socioeconomic Factors
KW - Television/sn [Statistics & Numerical Data]
KW - Urban Population/sn [Statistics & Numerical Data]
KW - Wounds and Injuries/ep [Epidemiology]
KW - Wounds and Injuries/et [Etiology]
KW - Wounds and Injuries/pc [Prevention & Control]
RP - NOT IN FILE
SP - 372
EP - 381
JF - Eastern Mediterranean Health Journal
JA - East Mediterr Health J
VL - 12
IS - 3-4
CY - Egypt
N2 - A cross-sectional study in Ahwaz city, Islamic Republic of Iran, described children's indoor and outdoor play patterns. A total of 952 pupils aged 7 and 9 years and 942 parents completed questionnaires. Children's favourite activities were: playing football (22.3%), cycling (13.2%) and watching television (12.3%). This differed from those perceived by the parents who reported watching television (20.3%), playing football (13.3%) and playing in street (11.4%). Children reported their favourite places to play were: street (27.4%), park/playground (18.3%) and home (17.8%). There were significant differences in play patterns by age and sex. The play patterns of older children and boys exposed them to more hazardous situations than younger children and girls
SN - 1020-3397
AD - Department of Epidemiology and Biostatistics, Faculty of Public Health, Shaheed Beheshti University of Medical Sciences, Tehran, Islamic Republic of Iran. hsoori@yahoo.com
UR - 17037706
ER -

TY - JOUR
ID - 1202
T1 - Preterm delivery and asthma: a systematic review and meta-analysis. [Review] [43 refs]
A1 - Jaakkola,J.J.
A1 - Ahmed,P.
A1 - Ieromnimon,A.
A1 - Goepfert,P.

A1 - Laiou,E.
A1 - Quansah,R.
A1 - Jaakkola,M.S.
Y1 - 2006/10//
N1 - Jaakkola, Jouni J K. Ahmed, Parvez. Ieromnimon, Antonia. Goepfert, Petra. Laiou, Elpiniki. Quansah, Reginald. Jaakkola, Maritta S
The Journal of allergy and clinical immunology
h53, 1275002
AIM, IM
Journal Article. Meta-Analysis. Research Support, Non-U.S. Gov't. Review
English
KW - MEDLINE
KW - Asthma/ep [Epidemiology]
KW - Asthma/et [Etiology]
KW - Child
KW - Preschool
KW - Female
KW - Humans
KW - Infant
KW - Newborn
KW - Male
KW - Pregnancy
KW - Premature Birth/pp [Physiopathology]
KW - Research Design
KW - Risk Factors
RP - NOT IN FILE
SP - 823
EP - 830
JF - Journal of Allergy & Clinical Immunology
JA - J Allergy Clin Immunol
VL - 118
IS - 4
CY - United States
N2 - BACKGROUND: Accumulating evidence suggests that reduced duration of pregnancy predicts increased risk of asthma, but the studies published have been inconsistent. OBJECTIVE: We sought to synthesize the evidence on the relation between preterm delivery and the risk of asthma later in life and to assess differences between the studies as potential sources for heterogeneity of the results. METHODS: We conducted a MEDLINE search (until the end of May 2005). The outcome was asthma. The determinant of interest was preterm delivery defined as a gestational age of less than 37 weeks. RESULTS: We identified 19 articles that provided estimates for the meta-analysis. The summary effect estimates for asthma (fixed-effects odds ratio, 1.074 [95% CI, 1.072-1.075]; heterogeneity P = .000; random-effects odds ratio, 1.366 [95% CI, 1.303-1.432]) showed an increased risk in relation to preterm delivery, with substantial heterogeneity between study-specific estimates. The effect of preterm delivery on asthma was stronger in cross-sectional studies; studies with broad outcome criteria, a small sample size, and a younger study population; and studies conducted in English-speaking populations, outside Europe, and published more recently. In metaregression, adjusting for other determinants, the effect estimate was significantly associated only with the mean age of the study population. CONCLUSIONS: The weight of evidence shows that preterm babies have an increased risk of asthma compared with term babies. CLINICAL IMPLICATIONS: Recognition of prematurity as a determinant of asthma emphasizes the importance of active treatment of physiologic airflow obstruction and a need for special preventive measures against known environmental determinants of asthma in preterm babies. [References: 43]
SN - 0091-6749
AD - Institute of Occupational and Environmental Medicine, University of Birmingham, UK. j.jaakkola@bham.ac.uk
UR - 17030233
ER -

TY - JOUR

ID - 1203
T1 - Children's pedometer-determined physical activity during the segmented school day
A1 - Tudor-Locke,C.
A1 - Lee,S.M.
A1 - Morgan,C.F.
A1 - Beighle,A.
A1 - Pangrazi,R.P.
Y1 - 2006/10//
N1 - Tudor-Locke, Catrine. Lee, Sarah M. Morgan, Charles F. Beighle, Aaron. Pangrazi, Robert P
Medicine and science in sports and exercise
8005433, mg8
IM, S
Journal Article. Research Support, Non-U.S. Gov't
English
KW - MEDLINE
KW - Child
KW - Ergometry
KW - Female
KW - Health Behavior
KW - Humans
KW - Male
KW - Motor Activity/ph [Physiology]
KW - Physical Education and Training
KW - Pilot Projects
KW - School Health Services
KW - Students
KW - Time
KW - Time Factors
KW - Walking/ph [Physiology]
RP - NOT IN FILE
SP - 1732
EP - 1738
JF - Medicine & Science in Sports & Exercise
JA - Med Sci Sports Exerc
VL - 38
IS - 10
CY - United States
N2 - PURPOSE: To describe the sex-specific patterns of school children's daily pedometer-determined physical (PA) during physical education (PE), recess and lunchtime, and before and after school. METHODS: Eighty-one sixth-grade students (28 boys, age = 11.9 +/- 0.4 yr, BMI = 18.8 +/- 4.1 kg x m(-2); 53 girls, 11.8 +/- 0.5 yr, BMI = 20.2 +/- 4.6 kg x m(-2)) wore pedometers for four school days and were prompted to record steps accumulated at arrival and departure from school and during pre- and postrecess, lunchtime, and PE class. RESULTS: Boys took significantly (P < 0.001) more steps per day than girls: 16,421 +/- 5,444 vs 12,332 +/- 3,056 steps per day, and more steps during release time (e.g., before-school Delta = 1289 steps, recess Delta = 479 steps, lunchtime Delta = 608 steps, and after-school Delta = 1872 steps) but the same number of steps during structured PE classes (1429 +/- 567 vs 1410 +/- 445 steps; P = 0.87). Lunchtime PA represented the most important source of daily PA (15-16%) obtained during school hours for both boys and girls, whereas recess accounted for 8-9% and PE class accounted for 8-11% of total steps per day. Regardless, almost half of daily steps taken are attributable to after-school activities. CONCLUSION: These data provide greater understanding of sex-specific PA patterns and the relative contribution of distinct segments of the school day to school children's total PA
SN - 0195-9131
AD - Walking Research Laboratory, Department of Exercise and Wellness, Arizona State University, Mesa, AZ 85212-0180, USA. Tudor-Locke@asu.edu
UR - 17019294
ER -

TY - JOUR
ID - 1204
T1 - Infestation of rural houses by Triatoma infestans (Hemiptera: Reduviidae) in southern area of Gran Chaco in Argentina
A1 - Porcasi,X.
A1 - Catala,S.S.
A1 - Hrellac,H.
A1 - Scavuzzo,M.C.
A1 - Gorla,D.E.
Y1 - 2006/09//
N1 - Porcasi, X. Catala, S S. Hrellac, H. Scavuzzo, M C. Gorla, D E
Journal of medical entomology
j1b, 0375400
IM
Comparative Study. Journal Article. Research Support, Non-U.S. Gov't
English
KW - MEDLINE
KW - Adolescent
KW - Animals
KW - Argentina
KW - Child
KW - Cluster Analysis
KW - Geographic Information Systems
KW - Housing
KW - Humans
KW - Insect Control/mt [Methods]
KW - Insect Control/st [Standards]
KW - Insecticides
KW - Nitriles
KW - Plants
KW - Pyrethrins
KW - Rural Population
KW - Temperature
KW - Time Factors
KW - Triatoma/de [Drug Effects]
KW - Triatoma/ph [Physiology]
RP - NOT IN FILE
SP - 1060
EP - 1067
JF - Journal of Medical Entomology
JA - J Med Entomol.
VL - 43
IS - 5
CY - United States
N2 - The impact of control activities against Triatoma infestans (Klug) (Hemiptera: Reduviidae) in South America has a marked contrast within and outside the Gran Chaco region. Development of a geographic information system, as part of an improvement in control program activities, allowed analysis of the spatial pattern of house infestations by T. infestans before and after house spraying with deltamethrin in the San Martin Department (an arid Chaco region of central Argentina). The overall peridomestic infestation index decreased from 48.2 to 28.2% after insecticide application. House infestation was spatially clustered in regions with low or high infestation levels that were located east and southwest of the department, respectively. This pattern was detected both before and after the insecticide application. Three environmental variables calculated from a temporal series of MODIS imagery (average of night temperature, maximum of day temperature, and temporal variation of vegetation index) were capable of correctly discriminating 96% of the places belonging to either high or low house infestation observed after the insecticide application
SN - 0022-2585

AD - Centro Regional de Investigaciones Cientificas y Transferencia Tecnologica (CRILAR), Entre Rios y Mendoza s/n. (5301) Anillaco, La Rioja, Argentina. xporcasi@crilar-conicet.com.ar
UR - 17017246
ER -

TY - JOUR
ID - 1205
T1 - Multiple needle-stick injuries with risk of human immunodeficiency virus exposure in a primary school
A1 - Thomas,H.L.
A1 - Liebeschuetz,S.
A1 - Shingadia,D.
A1 - Addiman,S.
A1 - Mellanby,A.
Y1 - 2006/10//
N1 - Thomas, H Lucy. Liebeschuetz, Susan. Shingadia, Delane. Addiman, Sarah. Mellanby, Alex
The Pediatric infectious disease journal
oxj, 8701858
IM
Journal Article
English
KW - MEDLINE
KW - Adult
KW - Anti-HIV Agents/tu [Therapeutic Use]
KW - Child
KW - Preschool
KW - Counseling
KW - HIV Infections/pc [Prevention & Control]
KW - Humans
KW - Needlestick Injuries
KW - Patient Compliance
KW - Schools
RP - NOT IN FILE
SP - 933
EP - 936
JF - Pediatric Infectious Disease Journal
JA - Pediatr Infect Dis J
VL - 25
IS - 10
CY - United States
N2 - Twenty children received needle-stick injuries with a risk of exposure to human immunodeficiency virus type 1 during an incident in a primary school playground. All were counseled and offered human immunodeficiency virus postexposure prophylaxis. All 20 children started postexposure prophylaxis, and 19 attended for follow-up testing 3 months later. More than one-half of the children completed the full 4-week course of treatment. None of the 19 children tested seroconverted after the incident
SN - 0891-3668
AD - North East and Central London Health Protection Unit, London, United Kingdom. lucy.thomas@btconnect.com
UR - 17006290
ER -

TY - JOUR
ID - 1206
T1 - A prospective study into the demographics and treatment of paediatric facial lacerations
A1 - Islam,S.
A1 - Ansell,M.
A1 - Mellor,T.K.
A1 - Hoffman,G.R.

Y1 - 2006/10//
N1 - Islam, S. Ansell, M. Mellor, T K. Hoffman, G R
Pediatric surgery international
cq0, 8609169
IM
Journal Article
English
KW - MEDLINE
KW - Age Distribution
KW - Child
KW - Preschool
KW - Facial Injuries/co [Complications]
KW - Facial Injuries/ep [Epidemiology]
KW - Facial Injuries/su [Surgery]
KW - Facial Injuries
KW - Female
KW - Follow-Up Studies
KW - Great Britain/ep [Epidemiology]
KW - Humans
KW - Incidence
KW - Infant
KW - Newborn
KW - Lacerations/ep [Epidemiology]
KW - Lacerations/et [Etiology]
KW - Lacerations/su [Surgery]
KW - Lacerations
KW - Male
KW - Prognosis
KW - Prospective Studies
KW - Questionnaires
KW - Reconstructive Surgical Procedures/mt [Methods]
KW - Sex Distribution
KW - Suture Techniques
KW - Time Factors
KW - Trauma Severity Indices
RP - NOT IN FILE
SP - 797
EP - 802
JF - Pediatric Surgery International
JA - Pediatr Surg Int
VL - 22
IS - 10
CY - Germany
N2 - To evaluate the demographics and treatment of facial lacerations occurring in a paediatric patient cohort. We undertook a prospective study of 106 children who sustained a soft tissue facial injury and who presented to an Accident and Emergency department in a UK district general hospital supporting a population of 750,000. Approximately 31,000 are dependent children between the age of 0-12 years. Our results show that the majority of paediatric patients who sustained a facial laceration were male (62%). The frequency of this injury was greatest amongst males across all age groups. The majority of children above 3 years of age sustained their injury outdoors. The peak time for injury varied for different age groups. The 0-3 year olds sustained the highest incidence of injuries around 17:00 h. A bi-modal time pattern was seen in the 4-6 year age group, initially at 12:00 h with a second peak at 17:00 h. The most frequent aetiology was play. A significant finding was that 8% of the injuries that were managed resulted from a dog bite. Almost 50% of children above 4 years of age, who required primary closure of their laceration, were able to tolerate their treatment being performed under local anaesthesia. The pattern of facial lacerations in our study supports the results of previous studies. Our data has provided further insight into the presentation of these injuries. These studies are valuable in targeted injury

prevention programmes aimed at potentially reducing the nature, incidence and severity of facial soft tissue trauma in children in the UK
SN - 0179-0358
AD - Department of Oral and Maxillofacial Surgery, University Hospitals of Coventry and Warwickshire (UHCW) NHS Trust, Clifford Bridge Road, Coventry CV2 2DX, UK. drshafiqislam@hotmail.co.uk
UR - 16947027
ER -

TY - JOUR
ID - 1207
T1 - The Washington aerial spray drift study: children's exposure to methamidophos in an agricultural community following fixed-wing aircraft applications
A1 - Weppner,S.
A1 - Elgethun,K.
A1 - Lu,C.
A1 - Hebert,V.
A1 - Yost,M.G.
A1 - Fenske,R.A.
Y1 - 2006/09//
N1 - Weppner, Sarah. Elgethun, Kai. Lu, Chensheng. Hebert, Vince. Yost, Michael G. Fenske, Richard A
Journal of exposure science & environmental epidemiology
101262796
IM
Journal Article. Research Support, N.I.H., Extramural. Research Support, U.S. Gov't, Non-P.H.S.. Research Support, U.S. Gov't, P.H.S.
English
KW - MEDLINE
KW - Agriculture
KW - Aircraft
KW - Child
KW - Preschool
KW - Environmental Exposure
KW - Environmental Monitoring
KW - Female
KW - Humans
KW - Insecticides/to [Toxicity]
KW - Male
KW - Organophosphorus Compounds/pd [Pharmacology]
KW - Organothiophosphorus Compounds/to [Toxicity]
KW - Rural Population
KW - Time Factors
KW - Washington
RP - NOT IN FILE
SP - 387
EP - 396
JF - Journal of Exposure Science & Environmental Epidemiology
JA - J Expo Sci Environ Epidemiol
VL - 16
IS - 5
CY - United States
N2 - This study characterized exposures of eight children living in an agricultural community near potato fields that were treated by aerial application with the organophosphorus (OP) insecticide, methamidophos (O,S-dimethyl phosphoramidothioate). Exposure monitoring included air and deposition samples in the outdoor community environment, outdoor and indoor air samples at each residence, wipe samples of playground equipment, toys, indoor surfaces, and children's hands, and periodic urine samples. Monitoring occurred prior to, the day of, and 1 day following applications. Methamidophos deposition in the community was very low compared to deposition inside the boundaries of the treated fields. Community air concentrations increased from

0.05 microg/m3 (prespray) to 0.11 and 0.48 microg/m3 (spray day morning and afternoon, respectively), decreasing to 0.10 microg/m3 on the postspray day. Air concentrations outside residences followed a similar pattern; indoor levels did not exceed 0.03 pg/m3. Methamidophos residues were found on playground equipment following applications, but not on indoor residential surfaces. The median hand wipe levels increased from < 0.02 (prespray) to 0.08 microg/sample (spray day), decreasing to 0.05 microg/sample (postspray day). Median concentrations of the primary methamidophos urinary metabolite were 61 microg/l before 1100 hours on the spray day, 170 microg/l after 1100 hours on the spray day, and 114 microg/l on the postspray day. Spray day metabolite levels were correlated with time outside on the spray day (rs = 0.68), with spray day hand wipe levels (rs = 0.67), and with postspray day metabolite levels (rs = 0.64). Postspray day metabolites levels were also positively associated with postspray day hand wipe levels (rs = 0.66). The documentation of children's exposure in this study does not necessarily mean that risks for these children were significantly altered, since nearly all children in the United States are exposed to some level of OP pesticides through dietary intake and other pathways. The association of metabolite levels with time spent outside, and the absence of methamidophos in homes indicates that children's exposures occurred primarily outdoors
SN - 1559-0631
AD - Department of Environmental and Occupational Health Sciences, University of Washington, Seattle, Washington 98195-7234, USA
UR - 16249796
ER -

TY - JOUR
ID - 1208
T1 - Selective BCG vaccination in a country with low incidence of tuberculosis
A1 - Romanus,V.
Y1 - 2006///
N1 - Romanus, V
Euro surveillance : bulletin Europeen sur les maladies transmissibles = European communicable disease bulletin
100887452
IM
Journal Article
English
KW - MEDLINE
KW - Adolescent
KW - Age Distribution
KW - BCG Vaccine/ae [Adverse Effects]
KW - BCG Vaccine
KW - Child
KW - Preschool
KW - Humans
KW - Immunization Programs
KW - Incidence
KW - Infant
KW - Newborn
KW - Sweden/ep [Epidemiology]
KW - Tuberculosis/ep [Epidemiology]
KW - Tuberculosis/pc [Prevention & Control]
KW - Vaccination
RP - NOT IN FILE
SP - 14
EP - 17
JF - Euro Surveillance: Bulletin Europeen sur les Maladies Transmissibles = European Communicable Disease Bulletin
JA - Euro Surveill.
VL - 11
IS - 3
CY - France

N2 - In 1975 the BCG vaccination policy in Sweden changed from routine vaccination of all newborn infants to selective vaccination of groups at higher risk. This report aims to evaluate the present BCG policy, with focus on the tuberculosis situation in Sweden during the period from 1989 to 2005. The population structure in Sweden has changed, with increasing numbers and proportions of people who were born outside Sweden, especially in countries with high prevalence of tuberculosis. BCG vaccination coverage fell from more than 95% before 1975 to less than 2% in 1976 to 1980, and then again increased to around 16% (corresponding to about 88% of the risk group recommended for vaccination). The increasing proportion of foreign born tuberculosis patients among all tuberculosis cases of illness in Sweden, and the high age-specific incidence of tuberculosis in the childbearing age groups in the foreign-born population, indicate the need to continue selective vaccination of children in families originating from countries with high tuberculosis incidence. The cumulative incidence of tuberculosis in the 30 cohorts born in Sweden after 1974 and observed to the end of 2004 was estimated at 0.5 cases per 100 000 person-years. Sweden still has one of the lowest incidences of tuberculosis in the world, which means a minimal average risk of infection for the majority of children born to Swedish parents. The observed increase of tuberculosis in 2005, partly attributed to an outbreak at a day nursery, is a reminder of the serious consequences of delayed diagnosis. Intensified active case finding is the most important action to prevent childhood tuberculosis, by means of eliminating the sources of infection to prevent transmission to the child population. Early detection and treatment of infected children is necessary to prevent development of serious disseminated tuberculosis
SN - 1560-7917
AD - Swedish Institute for Infectious Disease Control, Solna, Sweden
UR - 16567880
ER -

TY - JOUR
ID - 1209
T1 - Young children's hand contact activities: an observational study via videotaping in primarily outdoor residential settings
A1 - AuYeung,W.
A1 - Canales,R.A.
A1 - Beamer,P.
A1 - Ferguson,A.C.
A1 - Leckie,J.O.
Y1 - 2006/09//
N1 - Auyeung, Willa. Canales, Robert A. Beamer, Paloma. Ferguson, Alesia C. Leckie, James O
Journal of exposure science & environmental epidemiology
101262796
IM
Journal Article. Research Support, Non-U.S. Gov't. Research Support, U.S. Gov't, Non-P.H.S.
English
KW - MEDLINE
KW - Behavior
KW - Child
KW - Preschool
KW - Data Interpretation
KW - Statistical
KW - Environmental Exposure
KW - Environmental Pollutants
KW - Female
KW - Hand
KW - Humans
KW - Infant
KW - Male
KW - Play and Playthings
KW - Skin/me [Metabolism]
KW - Videotape Recording
RP - NOT IN FILE
SP - 434

EP - 446
JF - Journal of Exposure Science & Environmental Epidemiology
JA - J Expo Sci Environ Epidemiol
VL - 16
IS - 5
CY - United States
N2 - Microlevel activity time series (MLATS) data were gathered on hand contact activities of 38 children (1-6 years old) by videotaping in primarily outdoor residential environments. The videotape recordings were then translated into text files using a specialized software called VirtualTimingDevicetrade mark. Contact frequency (contacts/h), duration per contact (s/contact), and hourly contact duration (min/h) were summarized for outdoor hand contacts with 15 distinct object/surface categories ("Animal", "Body", "Clothes/Towels", "Fabric", "Floor", "Food", "Footwear", "Metal", "Non-dietary Water", "Paper/Wrapper", "Plastic", "Rock/Brick", "Toys", "Vegetation/Grass", and "Wood") and two aggregate object/surface categories ("Non-dietary objects/surfaces" and "Total objects/surfaces"). For outdoor both hand contacts with "Total objects/surfaces", contact frequencies ranged from 229.9 to 1517.7 contacts/h, median durations/contact ranged from < 1 to 5 s, and hourly contact durations ranged from 42.6 to 102.2 m/h. The data were analyzed for significant differences in hand contact activities as a function of (1) age, (2) location, (3) gender, and (4) hand. Significant differences (P < or = 0.05) were found for all four factors analyzed. Hourly contact durations with "Non-dietary objects/surfaces" and "Total objects/surfaces" increased with age (P = 0.01, rs = 0.42 and P = 0.005, rs = 0.46, respectively), while contact frequencies and hourly contact durations with "Wood" decreased with age (P = 0.02, rs = -0.38 and P = 0.05, rs = -0.32, respectively). Location was found to affect contact frequencies and hourly contact durations with certain objects/surfaces. For example, contact frequencies and hourly contact durations with "Fabric" were higher indoors (P = 0.02 for both), while contact frequencies and hourly contact durations with "Vegetation/Grass" were higher outdoors (P = 0.02 and P = 0.04, respectively). Girls had longer hourly contact durations with "Footwear" (P = 0.02), "Non-dietary objects/surfaces" (P = 0.03), and "Total objects/surfaces" (P = 0.01) than boys. The right hand had longer hourly contact durations with objects that are often manipulated with the hand (e.g., "Toys" (P = 0.0002)), while the left hand had longer hourly contact durations with passively touched objects/surfaces (e.g., "Clothes/Towels" (P = 0.003) and "Floor" (P = 0.04))
SN - 1559-0631
AD - Department of Civil and Environmental Engineering, Stanford University, Stanford, CA 94309, USA. wauyeung@stanford.edu
UR - 16552427
ER -

TY - JOUR
ID - 1210
T1 - A longitudinal approach to assessing urban and suburban children's exposure to pyrethroid pesticides
A1 - Lu,C.
A1 - Barr,D.B.
A1 - Pearson,M.
A1 - Bartell,S.
A1 - Bravo,R.
Y1 - 2006/09//
N1 - Lu, Chensheng. Barr, Dana B. Pearson, Melanie. Bartell, Scott. Bravo, Roberto
Environmental health perspectives
ei0, 0330411
IM
Journal Article. Research Support, U.S. Gov't, Non-P.H.S.. Research Support, U.S. Gov't, P.H.S.
English
KW - MEDLINE
KW - Age Factors
KW - Child
KW - Preschool
KW - Diet
KW - Environmental Monitoring
KW - Environmental Pollutants/to [Toxicity]
KW - Environmental Pollutants/ur [Urine]

KW - Humans
KW - Infant
KW - Longitudinal Studies
KW - Pesticides/to [Toxicity]
KW - Pesticides/ur [Urine]
KW - Pyrethrins/to [Toxicity]
KW - Pyrethrins/ur [Urine]
KW - Risk Assessment
KW - Risk Factors
KW - Suburban Population
KW - Time Factors
KW - United States
KW - Urban Population
RP - NOT IN FILE
SP - 1419
EP - 1423
JF - Environmental Health Perspectives
JA - Environ Health Perspect
VL - 114
IS - 9
CY - United States
N2 - We conducted a longitudinal study to assess the exposure of 23 elementary school-age children to pyrethroid pesticides, using urinary pyrethroid metabolites as exposure biomarkers. We substituted most of the children's conventional diets with organic food items for 5 consecutive days and collected two daily spot urine samples, first morning and before bedtime voids, throughout the 15-day study period. We analyzed urine samples for five common pyrethroid metabolites. We found an association between the parents' self-reported pyrethroid use in the residential environment and elevated pyrethroid metabolite levels found in their children's urine. Children were also exposed to pyrethroids through their conventional diets, although the magnitude was smaller than for the residential exposure. Children's ages appear to be significantly associated with pyrethroids exposure, which is likely attributed to the use of pyrethroids around the premises or in the facilities where older children engaged in the outdoor activities. We conclude that residential pesticide use represents the most important risk factor for children's exposure to pyrethroid insecticides. Because of the wide use of pyrethroids in the United States, the findings of this study are important for both children's pesticide exposure assessment and environmental public health
SN - 0091-6765
AD - Department of Environmental and Occupational Health, Rollins School of Public Health, Emory University, Atlanta, Georgia, USA. clu2@sph.emory.edu
UR - 16966099
ER -

TY - JOUR
ID - 1211
T1 - Parents' perceptions of neighborhood safety and children's physical activity
A1 - Weir,L.A.
A1 - Etelson,D.
A1 - Brand,D.A.
Y1 - 2006/09//
N1 - Weir, Lori A. Etelson, Debra. Brand, Donald A
Preventive medicine
pm4, 0322116
IM
Journal Article. Research Support, U.S. Gov't, P.H.S.
English
KW - MEDLINE
KW - Child
KW - Preschool
KW - Crime

KW - Cross-Sectional Studies
KW - Exercise
KW - Female
KW - Humans
KW - Male
KW - New York
KW - Parents/px [Psychology]
KW - Poverty
KW - Questionnaires
KW - Residence Characteristics/cl [Classification]
KW - Safety
KW - Suburban Population
KW - Urban Population
RP - NOT IN FILE
SP - 212
EP - 217
JF - Preventive Medicine
JA - Prev Med
VL - 43
IS - 3
CY - United States
N2 - OBJECTIVE: The obesity epidemic disproportionately affects minority and poor children. Negative perceptions of neighborhood safety in poor communities may affect overweight by inhibiting children's physical activity. This study investigates the degree to which parents in a poor inner city vs. a middle-class suburban community limit their children's outdoor activity because of neighborhood safety concerns. METHOD: Parents of children aged 5-10 years from an inner city family practice in a poor community and from a suburban pediatric practice in a middle-class community completed a 20-item questionnaire. Parents estimated the amount of their child's activity in various situations and indicated their level of anxiety concerning gangs, child aggression, crime, traffic, and personal safety in their neighborhood. RESULTS: Inner city children (n = 204) engaged in less physical activity than suburban children (N = 103) (P < 0.001). Inner city parents expressed much greater anxiety about neighborhood safety than suburban parents (P < 0.0001). In the inner city population, children's physical activity levels were negatively correlated with parental anxiety about neighborhood safety (r = -0.18, P < 0.05). CONCLUSIONS: Inner city parents have high levels of anxiety about neighborhood safety. While these concerns may not entirely explain the discrepancy in activity levels between inner city and suburban children, a safe environment is crucial to increasing opportunities for physical activity
SN - 0091-7435
AD - Department of Family Medicine, New York Medical College, Munger Pavilion, Room 306, Valhalla, NY 10595, USA. lori_weir@nymc.edu
UR - 16712912
ER -

TY - JOUR
ID - 1212
T1 - Assessment of soil lead exposure in children in Shenyang, China
A1 - Ren,H.M.
A1 - Wang,J.D.
A1 - Zhang,X.L.
Y1 - 2006/11//
N1 - Ren, H M. Wang, J D. Zhang, X L
Environmental pollution (Barking, Essex : 1987)
dvl, 8804476
IM
Comparative Study. Journal Article. Research Support, Non-U.S. Gov't
English
KW - MEDLINE
KW - Age Factors
KW - Child

KW - Child Behavior
KW - Preschool
KW - China
KW - Environmental Exposure
KW - Environmental Monitoring/mt [Methods]
KW - Environmental Pollution/ae [Adverse Effects]
KW - Humans
KW - Lead/bl [Blood]
KW - Soil Pollutants/bl [Blood]
KW - Urban Health
RP - NOT IN FILE
SP - 327
EP - 335
JF - Environmental Pollution
JA - Environ Pollut.
VL - 144
IS - 1
CY - England
N2 - Soil lead pollution is serious in Shenyang, China. The paper brings together the soil work, the bioaccessibility, and the blood lead data to assess the soil lead exposure in children in Shenyang, China. Approximately 15.25% of the samples were above China Environment Protection Agency guideline concentration for soil Pb to protect human from health risk (350 mgkg(-1)). Pb concentrations varied among use scenarios. The main lead contamination sources are industry emission and automobile exhaust. Bioaccessibility also varied among use scenarios. Children, who ingested soil from industrial area, public parks, kindergarten playground, and commercial area, are more susceptible to soil lead toxicity. The industrial area soil samples presented higher bioaccessibility compared to the other use scenario soil samples contaminated by automobile exhaust. The result also suggested a most significant linear relationship between the level of Pb contamination and the amount of Pb mobilized from soil into ingestion juice. Soil pH seemed to have insignificant influence on bioaccessibility in the present study. Bioaccessibility was mainly controlled by other factors that are not investigated in this study. A linear relationship between children blood lead and soil intestinal bioaccessibility was present in the study. Children who are 4-5 years old are more likely to demonstrate the significant relationship between soil lead bioaccessibility and blood lead as their behaviors place them at greatest risk of soil lead toxicity, and their blood lead levels are more likely to represent recent exposure
SN - 0269-7491
AD - Northeast Institute of Geography and Agricultural Ecology, Chinese Academy of Sciences, No.3195 Weishan Road, Changchun 130012, Jilin Province, PR China
UR - 16824656
ER -

TY - JOUR
ID - 1213
T1 - The distribution of cystathionine beta-synthase (CBS) in the eye: implication of the presence of a trans-sulfuration pathway for oxidative stress defense
A1 - Persa,C.
A1 - Osmotherly,K.
A1 - Chao-Wei,Chen K.
A1 - Moon,S.
A1 - Lou,M.F.
Y1 - 2006/10//
N1 - Persa, Cristina. Osmotherly, Kaila. Chao-Wei Chen, Kate. Moon, Sungchur. Lou, Marjorie F
Experimental eye research
epl, 0370707
IM
Journal Article. Research Support, N.I.H., Extramural
English
KW - MEDLINE
KW - Adolescent

KW - Adult
KW - Aged
KW - 80 and over
KW - Aging/me [Metabolism]
KW - Animals
KW - Child
KW - Preschool
KW - Cystathionine beta-Synthase/ge [Genetics]
KW - Cystathionine beta-Synthase/me [Metabolism]
KW - Eye/en [Enzymology]
KW - Eye/me [Metabolism]
KW - Gene Expression
KW - Humans
KW - Middle Aged
KW - Oxidative Stress/ph [Physiology]
KW - RNA
KW - Messenger/ge [Genetics]
KW - Reverse Transcriptase Polymerase Chain Reaction/mt [Methods]
KW - Signal Transduction/ph [Physiology]
KW - Species Specificity
KW - Swine/me [Metabolism]
KW - Tissue Distribution
RP - NOT IN FILE
SP - 817
EP - 823
JF - Experimental Eye Research
JA - Exp Eye Res
VL - 83
IS - 4
CY - England
N2 - Clinical abnormalities in cystathionine-beta-synthase (CBS) deficiency, a key enzyme in the trans-sulfuration pathway, associate with many eye disorders. However, little is known about this enzyme in the eye. The goal of this study is to examine the distribution of CBS in the various regions of the eye, including conjunctiva, cornea, iris, lens, vitreous, retina and optic nerve using fresh eyes from both pigs (6 months) and humans (4-82 years). We have found that pig eye showed the highest CBS protein presence in cornea, conjunctiva and iris, followed by retina and optic nerve. The whole lens had a relatively lower amount and vitreous body had none. CBS protein distribution in the human eyes showed a similar pattern, with high level in the anterior segments but much lower amount in retina and optic nerve. CBS in anterior segments remained high throughout the lifespan, but retinal CBS showed a trend of age-dependent increase. The presence of CBS in human and pig eye tissues was further confirmed by RT-PCR, CBS activity assay, both showed similar distribution profiles as the Western blot analysis. This is the first evidence of the presence of CBS enzyme in the eye outside of the lens, which indicates that a functional trans-sulfuration pathway may be present in various eye tissues
SN - 0014-4835
AD - Department of Veterinary and Biomedical Sciences, University of Nebraska-Lincoln, 134 VBS, 68583-0905, USA
UR - 16769053
ER -

TY - JOUR
ID - 1214
T1 - Gray matter atrophy associated with duration of temporal lobe epilepsy
A1 - Bonilha,L.
A1 - Rorden,C.
A1 - Appenzeller,S.
A1 - Coan,A.C.
A1 - Cendes,F.

A1 - Li,L.M.
Y1 - 2006/09//
N1 - Bonilha, Leonardo. Rorden, Chris. Appenzeller, Simone. Coan, Ana Carolina. Cendes, Fernando. Li, Li Min
NeuroImage
cpp, 9215515
IM
Journal Article
English
KW - MEDLINE
KW - Adolescent
KW - Adult
KW - Aging/ph [Physiology]
KW - Anticonvulsants/tu [Therapeutic Use]
KW - Atrophy
KW - Brain/pa [Pathology]
KW - Child
KW - Disease Progression
KW - Drug Resistance
KW - Epilepsy
KW - Temporal Lobe/dt [Drug Therapy]
KW - Temporal Lobe/pa [Pathology]
KW - Female
KW - Hippocampus/pa [Pathology]
KW - Humans
KW - Image Processing
KW - Computer-Assisted
KW - Limbic System/pa [Pathology]
KW - Male
KW - Middle Aged
RP - NOT IN FILE
SP - 1070
EP - 1079
JF - Neuroimage
VL - 32
IS - 3
CY - United States
N2 - Hippocampal sclerosis is the most common abnormality associated with medial temporal lobe epilepsy (MTLE). Converging evidence supports that hippocampal sclerosis progresses with time. However, it is unclear whether extrahippocampal atrophy in patients with MTLE, similarly to hippocampal sclerosis, is an unremitting progressive process. In this article, we investigate the relationship between duration of epilepsy and gray matter concentration reduction in patients with MTLE within and outside the hippocampus. We employed a voxel-based morphometry study of MRI of the entire brain of 36 patients with drug refractory MTLE and 49 neurologically healthy age-matched controls. We performed a voxel-based parametric and nonparametric investigation of the association between gray matter concentration, age and duration of epilepsy. We complemented the investigation by extracting the gray matter concentration of regions of interest (ROIs) within the limbic system, and we investigated the association between the gray matter concentration on the ROIs and duration of epilepsy. Patients with MTLE exhibited gray matter concentration reduction that is negatively correlated with the duration of epilepsy within the ipsilateral hippocampus, temporal lobes as well as extratemporal limbic structures that are closely connected with the hippocampus. In conclusion, longer duration of refractory epilepsy was associated with a more intense hippocampal and extrahippocampal atrophy in patients with MTLE. The mechanism of progressive neuronal damage in MTLE may be related to active seizure activity within a limbic network, and early seizure control may prevent further brain atrophy in patients with refractory MTLE
SN - 1053-8119
AD - Department of Neuropsychiatry, University of South Carolina, SC 29425, USA
UR - 16872843

ER -

TY - JOUR
ID - 1215
T1 - Parents with first time major depression: perceptions of social support for themselves and their children
A1 - Skarsater,I.
Y1 - 2006/09//
N1 - Skarsater, Ingela
Scandinavian journal of caring sciences
sjo, 8804206
N
Journal Article
English
KW - MEDLINE
KW - Activities of Daily Living
KW - Adolescent
KW - Adult
KW - Attitude to Health
KW - Child
KW - Child Care/px [Psychology]
KW - Child Welfare
KW - Depressive Disorder
KW - Major/di [Diagnosis]
KW - Major/pc [Prevention & Control]
KW - Major/px [Psychology]
KW - Female
KW - Follow-Up Studies
KW - Health Services Needs and Demand
KW - Humans
KW - Male
KW - Mental Health
KW - Middle Aged
KW - Nursing Assessment
KW - Nursing Methodology Research
KW - Parenting/px [Psychology]
KW - Parents/px [Psychology]
KW - Patient Admission
KW - Psychiatric Status Rating Scales
KW - Questionnaires
KW - Severity of Illness Index
KW - Social Support
KW - Sweden
RP - NOT IN FILE
SP - 308
EP - 314
JF - Scandinavian Journal of Caring Sciences
JA - Scand J Caring Sci
VL - 20
IS - 3
CY - Sweden
N2 - The aim of this study was to ascertain the perceptions of parents, with first time major depression, regarding the social support for themselves and their children. Eighteen parents, with children under the age of 19, were interviewed on admission to an inpatient unit and of these 16 were followed up 1 year later. In addition, the severity of parents' depression and their functional status was measured. The results showed that although the parents did regain their mental health and functional capacity to a certain extent, they nevertheless remained in a vulnerable position, increasing the strain on the whole family. The parents' and their children's social support was low and came mostly from outside the family. The parents were worried about their children,

due to problems related to the depressive episode as well as ordinary teenage rebellion. Important questions concerned practical problems such as whether there was someone to take care of the children if the parent is incapable of doing so. There were also concerns pertaining to how the changed family situation might interfere with the child's natural development or whether depression was a question of heredity. This highlights the need to investigate the importance of social support for the family as a whole when one of them suffers from depression. There is a need for improved treatment of parental depression to reduce symptoms such as decreased functional status that may impair parenting. The study also emphasises the need for healthcare practitioners to assess multiple aspects of social support so that care planning will target all relevant domains
SN - 0283-9318
AD - Institute of Health and Care Sciences, The Sahlgrenska Academy at Goteborg University, Goteborg, Sweden. ingela.skarsater@fhs.gu.se
UR - 16922985
ER -

TY - JOUR
ID - 1216
T1 - Comparison of the experience sampling method and questionnaires to assess visual activities in pre-teen and adolescent children
A1 - Rah,M.J.
A1 - Walline,J.J.
A1 - Lynn,Mitchell G.
A1 - Zadnik,K.
Y1 - 2006/09//
N1 - Rah, Marjorie J. Walline, Jeffrey J. Lynn Mitchell, G. Zadnik, Karla
Ophthalmic & physiological optics : the journal of the British College of Ophthalmic Opticians (Optometrists)
onk, 8208839
IM
Comparative Study. Journal Article. Research Support, N.I.H., Extramural. Research Support, Non-U.S. Gov't
English
KW - MEDLINE
KW - Adolescent
KW - Adolescent Behavior
KW - Child
KW - Child Behavior
KW - Contact Lenses
KW - Eyeglasses
KW - Female
KW - Health Surveys
KW - Humans
KW - Leisure Activities
KW - Male
KW - Microcomputers
KW - Myopia/px [Psychology]
KW - Parents
KW - Questionnaires
KW - Reading
KW - Refractive Errors/px [Psychology]
KW - Self-Assessment
KW - Sports
KW - Television
KW - Vision
KW - Ocular
RP - NOT IN FILE
SP - 483
EP - 489
JF - Ophthalmic & Physiological Optics
JA - Ophthalmic Physiol Opt.

VL - 26
IS - 5
CY - England
N2 - AIM: A study was conducted to assess the feasibility of the experience sampling method (ESM) to quantify the daily visual tasks of children. METHODS: Thirty-one children (9-14 years old, 39% male) were randomly paged after school (four times per day) and on weekends (eight times per day) for seven consecutive days. When paged, the children completed a voicemail survey regarding the nature, duration, working distance and type of visual correction worn during the activity. Responses of the subjects were grouped into 14 categories. Before beginning the paging, each child and one parent also reported, via survey, the number of hours and working distance for several visual activities. RESULTS: The usable (complete voicemail responses) response rate was 87.1%. The most commonly reported activity category was watching television (17.8%). Other common responses included distance activities (12.5%), reading/studying (12.2%) and sports/outdoor activities (12.0%). Of the total number of responses, 25% were near work activities (reading/studying, computer use, near hobbies and playing Game Boy). The mean working distance (+/-S.D.) reported was 40.4 +/- 36.9 cm for reading/studying, 232.9 +/- 127.1 cm for watching television, 354.7 +/- 345.8 cm for distance activities and 438.5 +/- 421.0 cm for sports/outdoor activities. When comparing the ESM to surveys, the only significant difference was for the amount of time estimated to be spent in talking/conversation (p < 0.0001). No differences were found for any of the near work activities. CONCLUSIONS: The ESM provides a feasible way to quantify daily near work tasks in children
SN - 0275-5408
AD - The New England College of Optometry, 1255 Boylston Street, Boston, MA 02215, USA. rahm@ncopost.ne-optometry.edu
UR - 16918773
ER -

TY - JOUR
ID - 1217
T1 - Isolation of mesenchymal stem cells from human vermiform appendix
A1 - De,Coppi P.
A1 - Pozzobon,M.
A1 - Piccoli,M.
A1 - Gazzola,M.V.
A1 - Boldrin,L.
A1 - Slanzi,E.
A1 - Destro,R.
A1 - Zanesco,L.
A1 - Zanon,G.F.
A1 - Gamba,P.
Y1 - 2006/09//
N1 - De Coppi, Paolo. Pozzobon, Michela. Piccoli, Martina. Gazzola, Maria Vittoria. Boldrin, Luisa. Slanzi, Elisa. Destro, Roberta. Zanesco, Luigi. Zanon, Giovanni Franco. Gamba, Piergiorgio
The Journal of surgical research
k7b, 0376340
IM
Journal Article. Research Support, Non-U.S. Gov't
English
KW - MEDLINE
KW - Adolescent
KW - Appendix/cy [Cytology]
KW - Appendix/gd [Growth & Development]
KW - Appendix/su [Surgery]
KW - Cell Differentiation
KW - Cell Division
KW - Cell Lineage
KW - Cell Separation/mt [Methods]
KW - Cells
KW - Cultured

KW - Child
KW - Preschool
KW - Chondrocytes/cy [Cytology]
KW - Endothelial Cells/cy [Cytology]
KW - Flow Cytometry
KW - Humans
KW - Immunophenotyping
KW - Infant
KW - Newborn
KW - Laparotomy
KW - Mesenchymal Stromal Cells/cy [Cytology]
KW - Muscle Cells/cy [Cytology]
KW - Osteoblasts/cy [Cytology]
RP - NOT IN FILE
SP - 85
EP - 91
JF - Journal of Surgical Research
JA - J Surg Res
VL - 135
IS - 1
CY - United States
N2 - BACKGROUND: Recent findings have shown that pluripotent stem cells exist in areas outside the bone marrow (BM). Moreover, it has been demonstrated that the appendix is important for the development of mucosal gut immunity, and hematopoietic progenitors have been isolated from animal and human appendices. MATERIALS AND METHODS: Non-inflamed appendices removed during laparotomy were processed and cultured until the appearance of adherent cells. Differentiations (performed under osteogenic, adipogenic, and myogenic conditions) were confirmed by immunohistochemistry and cytochemistry. Polymerase chain reaction and cytofluorimetric analyses were performed to evidence the presence of genes and protein specific lineages in appendix-derived mesenchymal stem cells (ADMCs). RESULTS: ADMCs were present in non-inflamed appendices. ADMCs under osteogenic conditions differentiated in osteoblasts and showed increased alkaline phosphatase expression; at the gene level, we observed the expression of Core binding factor alpha 1 (Cbfa1) and osteocalcin in osteogenic induced ADMCs. Under adipogenic conditions, lipidic drops in the cytoplasm, expression of lipoprotein lipase (LpL), and peroxisome proliferator-activated receptor gamma were observed; under myogenic conditions, myotubes expressing muscle specific proteins like desmin were formed. Myogenic regulatory factor 4 and MyoD were selectively induced in the ADMCs under myogenic conditions. CONCLUSIONS: This study shows for the first time that mesenchymal stem cells can be isolated from normal appendices obtained from a pediatric and adult age group (0-18 years of age). This finding not only may further knowledge of the maturation of the intestinal immunesystem but also could indicate a new physiological role of the human vermiform appendix
SN - 0022-4804
AD - Laboratorio Trapianto del Midollo Osseo and Banca del Sangue di Cordone Ombelicale, Dipartimento di Oncoematologia Pediatrica, Padova, Italy. pdecoppi@hotmail.com
UR - 16650433
ER -

TY - JOUR
ID - 1218
T1 - Using school staff to establish a preventive network of care to improve elementary school students' control of asthma
A1 - Bruzzese,J.M.
A1 - Evans,D.
A1 - Wiesemann,S.
A1 - Pinkett-Heller,M.
A1 - Levison,M.J.
A1 - Du,Y.
A1 - Fitzpatrick,C.
A1 - Krigsman,G.

A1 - Ramos-Bonoan,C.
A1 - Turner,L.
A1 - Mellins,R.B.
Y1 - 2006/08//
N1 - Bruzzese, Jean-Marie. Evans, David. Wiesemann, Sandra. Pinkett-Heller, Marcia. Levison, Moshe J. Du, Yunling. Fitzpatrick, Cecilia. Krigsman, Gary. Ramos-Bonoan, Carmen. Turner, Levonne. Mellins, Robert B
The Journal of school health
k13, 0376370
IM, N
Journal Article. Randomized Controlled Trial. Research Support, N.I.H., Extramural. Research Support, Non-U.S. Gov't
English
KW - MEDLINE
KW - Absenteeism
KW - Asthma/di [Diagnosis]
KW - Asthma/th [Therapy]
KW - Caregivers/ed [Education]
KW - Child
KW - Preschool
KW - Education
KW - Medical
KW - Continuing/mt [Methods]
KW - Humans
KW - Inservice Training/mt [Methods]
KW - Intervention Studies
KW - Mass Screening/mt [Methods]
KW - New York City
KW - Patient Care Team
KW - Physicians
KW - Family
KW - Program Evaluation
KW - Quality of Life
KW - School Health Services
KW - School Nursing/ed [Education]
KW - School Nursing/mt [Methods]
KW - Schools
KW - Urban Population
RP - NOT IN FILE
SP - 307
EP - 312
JF - Journal of School Health
JA - J Sch Health
VL - 76
IS - 6
CY - United States
N2 - School-based asthma interventions delivered by nonschool staff have been successful but are limited in their reach because of the cost and effort of bringing in outside educators and their inability to establish improved communication about asthma between schools, families, and primary care providers (PCPs). To address these problems, Columbia University and the New York City Department of Education and the New York City Department of Health and Mental Hygiene undertook a randomized controlled trial to test the efficacy of a comprehensive school-based asthma program. In this intervention, school nurses were trained to facilitate the establishment of a preventive network of care for children with asthma by coordinating communications and fostering relationships between families, PCPs, and school personnel. PCPs also received training regarding asthma management. There was limited support for this model. While case detection helped nurses identify additional students with asthma and nurses increased the amount of time spent on asthma-related tasks, PCPs did not change their medical management of asthma. Few improvements in health outcomes were achieved. Relative to controls, 12-months posttest intervention students had a reduction in activity limitations

due to asthma (-35% vs -9%, p < .05) and days with symptoms (26% vs 39%, p = .06). The intervention had no impact on the use of urgent health care services, school attendance, or caregiver's quality of life. There were also no improvements at 24-months postintervention. We faced many challenges related to case detection, training, and implementing preventive care activities, which may have hindered our success. We present these challenges, describe how we coped with them, and discuss the lessons we learned
SN - 0022-4391
AD - New York University Child Study Center, 215 Lexington Avenue, 13th Floor, New York, NY 10016, USA. bruzzj01@med.nyu.edu
UR - 16918861
ER -

TY - JOUR
ID - 1219
T1 - Validity and reliability of adult recall of past sun exposure in a case-control study of multiple sclerosis
A1 - van,der Mei,I
A1 - Blizzard,L.
A1 - Ponsonby,A.L.
A1 - Dwyer,T.
Y1 - 2006/08//
N1 - van der Mei, I A F. Blizzard, L. Ponsonby, A-L. Dwyer, T
Cancer epidemiology, biomarkers & prevention : a publication of the American Association for Cancer Research, cosponsored by the American Society of Preventive Oncology
bnj, 9200608
IM
Comparative Study. Journal Article. Research Support, Non-U.S. Gov't. Validation Studies
English
KW - MEDLINE
KW - Adolescent
KW - Adult
KW - Age Distribution
KW - Case-Control Studies
KW - Child
KW - Female
KW - Humans
KW - Interviews as Topic
KW - Male
KW - Middle Aged
KW - Multiple Sclerosis/pa [Pathology]
KW - Questionnaires
KW - Sex Distribution
KW - Sunlight/ae [Adverse Effects]
KW - Vitamin D/bl [Blood]
RP - NOT IN FILE
SP - 1538
EP - 1544
JF - Cancer Epidemiology, Biomarkers & Prevention
JA - Cancer Epidemiol Biomarkers Prev
VL - 15
IS - 8
CY - United States
N2 - BACKGROUND: Measurement of past sun exposure through recall by adults has the potential for measurement error. We aimed to investigate aspects of validity and reliability of self-reported past sun exposure. METHODS: A population-based case-control study was conducted in Tasmania on 136 cases with multiple sclerosis and 272 age- and sex-matched community controls. Repeat interviews on 52 cases and 52 controls were done on average 11 weeks after the initial interview. Sun exposure was assessed by questionnaire and lifetime calendar. Other measurements included serum 25-hydroxyvitamin D, actinic damage, and skin phenotype. RESULTS: There was an association between recent sun exposure and serum vitamin D (time in the

sun: r = 0.22, P < 0.01; activities outside: r = 0.31, P < 0.01 for controls) and between lifetime sun exposure and actinic damage [correlation between 0.34 (P < 0.01) and 0.17 (P = 0.01) for controls]. The test-retest weighted kappa statistic of self-reported sun exposure ranged from 0.43 to 0.74. Recall of childhood/adolescent sun exposure by standardized questioning was no less reproducible than recall of recent adult sun exposure and no less reliable when made with the calendar method. Comparing the questionnaire and calendar method, the measures of childhood/adolescent sun exposure had a similar predictive validity for multiple sclerosis. CONCLUSIONS: The results of this study provide further evidence that adults are able to recall past sun exposure with shown validity and reliability and present information about the possible reasons for the good reliability of recalled sun exposure measures
SN - 1055-9965
AD - Menzies Research Institute, University of Tasmania, Private Bag 23, Hobart, Tasmania 7001, Australia. Ingrid.vanderMei@utas.edu.au
UR - 16896046
ER -

TY - JOUR
ID - 1220
T1 - Long-term personal exposure to traffic-related air pollution among school children, a validation study
A1 - van,Roosbroeck S.
A1 - Wichmann,J.
A1 - Janssen,N.A.
A1 - Hoek,G.
A1 - van Wijnen,J.H.
A1 - Lebret,E.
A1 - Brunekreef,B.
Y1 - 2006/09/15/
N1 - van Roosbroeck, Sofie. Wichmann, Janine. Janssen, Nicole A H. Hoek, Gerard. van Wijnen, Joop H. Lebret, Erik. Brunekreef, Bert
The Science of the total environment
uj0, 0330500
IM
Evaluation Studies. Journal Article. Research Support, Non-U.S. Gov't
English
KW - MEDLINE
KW - Air Pollutants/an [Analysis]
KW - Air Pollution/an [Analysis]
KW - Carbon/an [Analysis]
KW - Child
KW - Dust/an [Analysis]
KW - Environmental Monitoring/mt [Methods]
KW - Humans
KW - Netherlands
KW - Nitrogen Oxides/an [Analysis]
KW - Vehicle Emissions
RP - NOT IN FILE
SP - 565
EP - 573
JF - Science of the Total Environment
JA - Sci Total Environ
VL - 368
IS - 2-3
CY - Netherlands
N2 - Several recent studies suggest an association between long-term exposure to traffic-related air pollution and health. Most studies use indicators of exposure such as outdoor air pollution or traffic density on the street of residence. Little information is available about the validity of these measurements as an estimate of long-term personal exposure to traffic-related air pollution. In this pilot study, we assessed outdoor and personal exposure to traffic-related air pollution in children living in homes on streets with different degree of traffic intensity. The

personal exposure of 14 children aged 9-12 years to 'soot', NO(x) (NO and NO(2)) was assessed in Amsterdam between March and June 2003. Each child's personal exposure was monitored during four repeated 48-h periods. Concurrently, in- and outdoor NO(x) measurements were carried out at the school and at the home of each participating child. Measurements were supplemented by a questionnaire on time activity patterns and possible indoor sources. Flow-controlled battery operated pumps in a made-to-fit backpack were used to sample personal exposure to 'soot', determined from the reflectance of PM(2.5) filters. Exposure to NO(x) was assessed using Ogawa passive samplers. Children living near busy roads were found to have a 35% higher personal exposure to 'soot' than children living at an urban background location, despite that all children attended the same school that was located away from busy roads. Smaller contrasts in personal exposure were found for NO (14%), NO(2) (15%) and NO(x) (14%). This finding supports the use of 'living near a busy road' as a measure of exposure in epidemiological studies on the effects of traffic-related air pollution in children
SN - 0048-9697
AD - Institute for Risk Assessment Sciences, Utrecht University, P.O. Box 80176, 3508 TD Utrecht, The Netherlands. s.a.h.vanroosbroeck@iras.uu.nl
UR - 16650461
ER -

TY - JOUR
ID - 1221
T1 - Effect of physical education and activity levels on academic achievement in children
A1 - Coe,D.P.
A1 - Pivarnik,J.M.
A1 - Womack,C.J.
A1 - Reeves,M.J.
A1 - Malina,R.M.
Y1 - 2006/08//
N1 - Coe, Dawn Podulka. Pivarnik, James M. Womack, Christopher J. Reeves, Mathew J. Malina, Robert M
Medicine and science in sports and exercise
8005433, mg8
IM, S
Journal Article. Randomized Controlled Trial. Research Support, Non-U.S. Gov't
English
KW - MEDLINE
KW - Chi-Square Distribution
KW - Child
KW - Educational Measurement
KW - Educational Status
KW - Female
KW - Humans
KW - Male
KW - Physical Education and Training/mt [Methods]
KW - Physical Fitness/ph [Physiology]
RP - NOT IN FILE
SP - 1515
EP - 1519
JF - Medicine & Science in Sports & Exercise
JA - Med Sci Sports Exerc
VL - 38
IS - 8
CY - United States
N2 - PURPOSE: This study was conducted to determine the effect of physical education class enrollment and physical activity on academic achievement in middle school children. METHODS: Participants were 214 sixth-grade students randomly assigned to physical education during either first or second semesters. Moderate and vigorous physical activity (MVPA) (number of 30-min time blocks) outside of school was assessed using the 3-d physical activity recall (3DPAR). The 3DPAR time blocks were converted to ordinal data with scores of 1 (no activity), 2 (some activity), or 3 (activity meeting Healthy People 2010 guidelines). Academic achievement was assessed using grades from four core academic classes and standardized test scores (Terra Nova percentiles).

RESULTS: Grades were similar regardless of whether students were enrolled in physical education during first or second semesters. Physical education classes averaged only 19 min of MVPA. Students who either performed some or met Healthy People 2010 guidelines for vigorous activity had significantly higher grades ($P < 0.05$) than students who performed no vigorous activity in both semesters. Moderate physical activity did not affect grades. Standardized test scores were not significantly related to physical education class enrollment or physical activity levels. CONCLUSION: Although academic achievement was not significantly related to physical education enrollment, higher grades were associated with vigorous physical activity, particularly activity meeting recommended Healthy People 2010 levels
SN - 0195-9131
AD - Department of Kinesiology, Michigan State University, East Lansing, MI, USA. coed@gvsu.edu
UR - 16888468
ER -

TY - JOUR
ID - 1222
T1 - Increasing children's physical activity levels during recess periods in elementary schools: the effects of providing game equipment
A1 - Verstraete,S.J.
A1 - Cardon,G.M.
A1 - De Clercq,D.L.
A1 - De,Bourdeaudhuij,I
Y1 - 2006/08//
N1 - Verstraete, Stefanie J M. Cardon, Greet M. De Clercq, Dirk L R. De Bourdeaudhuij, Ilse M M
European journal of public health
d0w, 9204966
IM
Journal Article. Randomized Controlled Trial. Research Support, Non-U.S. Gov't
English
KW - MEDLINE
KW - Analysis of Variance
KW - Belgium
KW - Child
KW - Female
KW - Health Promotion/mt [Methods]
KW - Humans
KW - Leisure Activities
KW - Male
KW - Motor Activity
KW - Play and Playthings
KW - Schools
RP - NOT IN FILE
SP - 415
EP - 419
JF - European Journal of Public Health
JA - Eur J Public Health
VL - 16
IS - 4
CY - England
N2 - BACKGROUND: During recess, children can be active on a daily basis, making it an important school environmental factor for the promotion of health-related physical activity. The aim of the present study was to investigate the effects of providing game equipment on children's physical activity levels during morning recess and lunch break in elementary schools. METHODS: Seven elementary schools were randomly assigned to the intervention group (four schools), including 122 children (75 boys, 47 girls, mean age: 10.8 +/- 0.6 years), and to the control group (three schools), including 113 children (46 boys, 67 girls, mean age: 10.9 +/- 0.7 years). Children's activity levels were measured before and three months after providing game equipment, using MTI accelerometers. RESULTS: During lunch break, children's moderate and vigorous physical activity significantly increased in the intervention group (moderate: from 38 to 50%, vigorous: from 10 to 11%), while it decreased in

the control group (moderate: from 44 to 39%, vigorous: from 11 to 5%). At morning recess, providing game equipment was effective in increasing children's moderate physical activity (from 41 to 45%), while it decreased in the control group (from 41 to 34%). CONCLUSION: Providing game equipment during recess periods was found to be effective in increasing children's physical activity levels. This finding suggests that promoting physical activity through game equipment provision during recess periods can contribute to reach the daily activity levels recommended for good health
SN - 1101-1262
AD - Department of Movement and Sports Sciences, Ghent University and Policy Research Center Sport, Physical Activity and Health, Belgium. Stefanie.Verstraete@UGent.be
UR - 16431866
ER -

TY - JOUR
ID - 1223
T1 - Patterns of injury associated with routine childhood falls
A1 - Pitone,M.L.
A1 - Attia,M.W.
Y1 - 2006/07//
N1 - Pitone, Melanie L. Attia, Magdy W
Pediatric emergency care
pau, 8507560
IM
Journal Article
English
KW - MEDLINE
KW - Accidental Falls/sn [Statistics & Numerical Data]
KW - Child
KW - Preschool
KW - Craniocerebral Trauma/ep [Epidemiology]
KW - Craniocerebral Trauma/et [Etiology]
KW - Female
KW - Fractures
KW - Bone/ep [Epidemiology]
KW - Bone/et [Etiology]
KW - Humans
KW - Infant
KW - Male
KW - Retrospective Studies
KW - Soft Tissue Injuries/ep [Epidemiology]
KW - Soft Tissue Injuries/et [Etiology]
RP - NOT IN FILE
SP - 470
EP - 474
JF - Pediatric Emergency Care
JA - Pediatr Emerg Care
VL - 22
IS - 7
CY - United States
N2 - OBJECTIVE: To identify the pattern of injuries associated with routine childhood falls. METHODS: Retrospective chart review of patients at most 12 years presenting to a children's hospital emergency department with complaint of a fall. Patients were classified into 3 age groups (<2, 2-4, and 5-12 years) and analyzed for the type of fall and diagnosis. RESULTS: Seven hundred eighty-seven patients were enrolled. Mean age was 5.7 years. Fifty-six percent were boys. The types of falls reported were categorized as a fall down steps, from patient's own height, from an object, and other. In all 3 groups, the most common fall was fall from an object (50%, 50%, and 48%, respectively). There were 91 (12%) patients in the younger-than-2-year-old age group and 235 (30%) in the 2- to 4-year-old age group. Both groups commonly fell from a bed/chair (35% and 25%, respectively). In the youngest group, the most frequent diagnosis was head injury (41%; odds ratio [OR], 5.0;

95% confidence interval [CI], 3.0-8.1). Children ages 5 to 12 years numbered 461 (58%) and most commonly fell from playground equipment (26%) sustaining a fracture (65%; OR, 3.1; 95% CI, 2.3-4.3). Of these, 77% were in the upper extremity (arm fracture; OR, 41; 95% CI, 22-79). CONCLUSIONS: In children who presented to a children's hospital emergency department with a fall, fall from an object was the most common type. Those younger than 2 years, most commonly fell from a bed/chair and sustained head injury. Children 5 to 12 years old were likely to fall from playground equipment and fracture their arm. These findings may be helpful to clinicians who evaluate routine childhood falls
SN - 1535-1815
AD - Division of Pediatric Emergency Medicine, AI duPont Hospital for Children, Wilmington, DE 19803, USA. mpitone@nemours.org
UR - 16871104
ER -

TY - JOUR
ID - 1224
T1 - Parsonage-Turner syndrome: MR imaging findings and clinical information of 27 patients
A1 - Gaskin,C.M.
A1 - Helms,C.A.
Y1 - 2006/08//
N1 - Gaskin, Cree M. Helms, Clyde A
Radiology
qsh, 0401260
AIM, IM
Journal Article
English
KW - MEDLINE
KW - Adolescent
KW - Adult
KW - Aged
KW - 80 and over
KW - Brachial Plexus Neuritis/di [Diagnosis]
KW - Child
KW - Electromyography
KW - Female
KW - Humans
KW - Magnetic Resonance Imaging/mt [Methods]
KW - Male
KW - Middle Aged
KW - Retrospective Studies
RP - NOT IN FILE
SP - 501
EP - 507
JF - Radiology
VL - 240
IS - 2
CY - United States
N2 - PURPOSE: To review retrospectively the magnetic resonance (MR) imaging findings and clinical information of patients with Parsonage-Turner syndrome (PTS). MATERIALS AND METHODS: The institutional review board did not require its formal approval or informed patient consent at the time of the study. However, the study was HIPAA compliant. The information in a computerized database of 2875 consecutive shoulder MR examinations was retrospectively reviewed. With use of key terms, the database software identified 81 examinations potentially associated with PTS. Both authors together reviewed the 81 imaging reports and the corresponding patients' medical records. In consensus, they made the diagnosis of PTS in 21 patients (two with bilateral involvement) on the basis of MR findings, electromyographic results, and clinical data. They also examined the data of an additional six patients (one with bilateral involvement) obtained from outside facilities. Ultimately, 30 shoulders of 27 patients (18 male, nine female; age range, 12-81 years; mean age, 41 years) were evaluated. The MR findings and clinical information (ie, regarding atrophy, pain,

weakness, electromyographic results, neck and spine history, trauma, excessive overhead activity, recent surgery, vaccination, and illness) of all patients with PTS were reviewed. MR findings of diffuse high T2 signal intensity abnormality and fatty atrophy of muscles were evaluated to assess the pattern of nerve involvement. Structural causes (eg, ganglion cyst or other mass) of neurogenic high T2 signal intensity abnormality were excluded at MR imaging. RESULTS: Twenty-nine (97%) of 30 shoulders had suprascapular nerve involvement; in 15 (50%) shoulders, the involvement was limited to this nerve. Fifteen (50%) shoulders had axillary nerve involvement; in only one (3%) shoulder, the involvement was limited to this nerve. One shoulder (3%) had subscapular nerve involvement. Nine (30%) shoulders demonstrated focal muscular atrophy. Eleven (41%) of 27 patients also underwent electromyography; all of these patients demonstrated neuropathies that matched the patterns of neurogenic high T2 signal intensity abnormality seen at MR imaging. CONCLUSION: The suprascapular nerve was almost invariably involved (in 97% of shoulders) in patients with PTS. Axillary nerve involvement also was commonly observed (in 50% of shoulders). Subscapular nerve involvement was uncommon (in 3% of shoulders). RSNA, 2006
SN - 0033-8419
AD - Department of Radiology, Duke University Medical Center, Durham, NC, USA. cmg9s@virginia.edu
UR - 16864674
ER -

TY - JOUR
ID - 1225
T1 - The effectiveness of low-cost soil treatments to reduce soil and dust lead hazards: The Boston lead safe yards low cost lead in soil treatment, demonstration and evaluation
A1 - Dixon,S.L.
A1 - McLaine,P.
A1 - Kawecki,C.
A1 - Maxfield,R.
A1 - Duran,S.
A1 - Hynes,P.
A1 - Plant,T.
Y1 - 2006/09//
N1 - Dixon, Sherry L. McLaine, Pat. Kawecki, Carolyn. Maxfield, Robert. Duran, Sandra. Hynes, Pat. Plant, Thomas
Environmental research
ei2, 0147621
IM
Journal Article. Research Support, Non-U.S. Gov't. Research Support, U.S. Gov't, Non-P.H.S.
English
KW - MEDLINE
KW - Boston
KW - Child
KW - Preschool
KW - Dust/an [Analysis]
KW - Environmental Exposure/pc [Prevention & Control]
KW - Floors and Floorcoverings
KW - Housing
KW - Humans
KW - Lead/ch [Chemistry]
KW - Lead/ip [Isolation & Purification]
KW - Lead Poisoning/pc [Prevention & Control]
KW - Questionnaires
KW - Soil Pollutants/ip [Isolation & Purification]
KW - Spectrometry
KW - X-Ray Emission
RP - NOT IN FILE
SP - 113
EP - 124
JF - Environmental Research

JA - Environ Res
VL - 102
IS - 1
CY - United States
N2 - The Boston lead safe yards low cost lead in soil treatment, demonstration, and evaluation was developed to explore the viability and effectiveness of low-cost soil interventions to reduce exposure to soil lead hazards. Buildings that had been abated for lead to Massachusetts's deleading standards in the previous 5 yrs and met other program requirements were recruited for the evaluation. Following individual property assessments, yards were treated with application of ground coverings and ground barriers in 2000-2001 and followed up at 1 yr. The treatment cost ranged from 1095 dollars to 5643 dollars with an average of 2798 dollars. Soil lead levels at the building dripline, measured with a field-portable X-ray fluorescence analyzer (Niton Model 702 Spectrum Analyzer), dropped from 2021 PPM at baseline to 206 PPM at 1-yr follow-up. Most of the barrier treatments continued to block access to the lead-contaminated soil at 1yr. At the follow-up, few properties with grass treatment had areas that were completely bare, but 28% had more than a small amount of treated areas bare. Treatments were effective in reducing entryway dust lead in the rear of the building if the residents reported they had maintained the yard treatments. Each additional yard work activity reported was predicted to lower 1-yr floor dust lead loading at the rear common/main and dwelling unit entries by about 20%. Each additional 100 ft2 of yard treated was predicted to lower 1-yr floor dust loading at the rear dwelling unit entry by 19%. Treatments did not show a dust lead effect at 1 yr in the front entryway of the building, but the investigators believe that this may be due to the effect of resident cleaning overshadowing the treatment effect
SN - 0013-9351
AD - National Center for Healthy Housing, Columbia, MD 21044, USA. sdixon@centerforhealthyhousing.org
UR - 16500641
ER -

TY - JOUR
ID - 1226
T1 - [Indoor and outdoor peer relations in preschool children]. [Japanese]
A1 - Hirose,T.
A1 - Shizawa,Y.
A1 - Hinobayashi,T.
A1 - Minami,T.
Y1 - 2006/04//
N1 - Hirose, Toshiya. Shizawa, Yasuhiro. Hinobayashi, Toshihiko. Minami, Tetsuhiro
Shinrigaku kenkyu : The Japanese journal of psychology
kpj, 0413571
IM
English Abstract. Journal Article
Japanese
KW - MEDLINE
KW - Age Factors
KW - Child Psychology
KW - Child
KW - Preschool
KW - Female
KW - Humans
KW - Male
KW - Peer Group
KW - Play and Playthings/px [Psychology]
RP - NOT IN FILE
SP - 40
EP - 47
JF - Shinrigaku Kenkyu - Japanese Journal of Psychology
JA - Shinrigaku Kenkyu
VL - 77
IS - 1
CY - Japan

N2 - This study examined whether the choice of interactors is influenced by indoor and outdoor situations. Peer relations among twenty three-year-old, eighteen four-year-old, and twenty five-year-old children in an urban preschool in Japan were observed during indoor and outdoor free play situations. We analyzed the partners in the interactions, the number, and the stability of interactors. The Shannon-Wiener diversity index (H) was used to measure the stability of interactors. In outdoor situations, three-year-old and four-year-old children were involved with a diversity of interactors, while four-year-old children's preferred friends were stable. Five-year-old children showed a relation with stable interactors in both indoor and outdoor situations, choosing different interactors in each situation. In addition, the children who had few interactors in indoor situations increased their relations with interactors in outdoor situations. These results suggest that three-year-old and four-year-old children are affected by environmental factors that seem to stimulate the children's physiognomic perception, whereas five-year-old children make use of the environment. Opportunities for children to encounter various situations and meet various peers may facilitate the development of social relations
SN - 0021-5236
AD - Department of Comparative and Developmental Psychology, Graduate School of Human Sciences, Osaka University, Yamada-oka, Suita 565-0871, Japan. hirose@hus.osaka-u.ac.jp
UR - 16862965
ER -

TY - JOUR
ID - 1227
T1 - A pilot study of children's exposure to CCA-treated wood from playground equipment
A1 - Shalat,S.L.
A1 - Solo-Gabriele,H.M.
A1 - Fleming,L.E.
A1 - Buckley,B.T.
A1 - Black,K.
A1 - Jimenez,M.
A1 - Shibata,T.
A1 - Durbin,M.
A1 - Graygo,J.
A1 - Stephan,W.
A1 - Van De,Bogart G.
Y1 - 2006/08/15/
N1 - Shalat, S L. Solo-Gabriele, H M. Fleming, L E. Buckley, B T. Black, K. Jimenez, M. Shibata, T. Durbin, M. Graygo, J. Stephan, W. Van De Bogart, G
The Science of the total environment
uj0, 0330500
IM
Journal Article. Research Support, N.I.H., Extramural. Research Support, Non-U.S. Gov't
English
KW - MEDLINE
KW - Arsenates/an [Analysis]
KW - Arsenates/ur [Urine]
KW - Biological Markers/an [Analysis]
KW - Biological Markers/ur [Urine]
KW - Child
KW - Preschool
KW - Cross-Sectional Studies
KW - Environmental Exposure/an [Analysis]
KW - Environmental Monitoring
KW - Florida
KW - Humans
KW - Infant
KW - Pilot Projects
KW - Play and Playthings
KW - Wood
RP - NOT IN FILE

SP - 80
EP - 88
JF - Science of the Total Environment
JA - Sci Total Environ
VL - 367
IS - 1
CY - Netherlands
N2 - Arsenic from chromated copper arsenate (CCA)-treated wood, widely used in playgrounds and other outdoor equipment, can persist as surface residues on wood. This raises concerns about possible health risks associated with children playing on CCA-treated playgrounds. In a Pilot Study, 11 children (13-71 months) in homes with and without CCA-treated playgrounds were evaluated with post-exposure hand rinses and urine for total arsenic. Samples of wood, soil, and mulch, as well as synthetic wipes, were sampled for total arsenic. In non-CCA-treated playgrounds vs. CCA-treated playgrounds, respectively, wood arsenic was <2.0 mg/kg vs. mean arsenic 2370 mg/kg (range 1440-3270 mg/kg); soil arsenic was <3.0 mg/kg vs. mean arsenic of 19 mg/kg (range 4.0-42 mg/kg); mulch arsenic at one non-CCA-treated playground was 0.4 mg/kg vs. two CCA-treated playgrounds of 0.6 and 69 mg/kg. The arsenic removed using a synthetic wipe at non-CCA-treated playgrounds was <0.5 microg, while mean arsenic from CCA-treated wood was 117 microg (range 1.0-313). The arsenic mass from hand rinses for children who played at non-CCA-treated playgrounds was <0.2 microg, while mean arsenic mass was 0.6 microg (range <0.2-1.9) at CCA-treated playgrounds. Mean urinary total arsenic levels were 13.6 pg/ml (range 7.2-23.1 pg/ml) for all children evaluated, but there was no association between access to CCA-playgrounds and urinary arsenic levels. Arsenic speciation was not performed. This preliminary Pilot Study of CCA-treated wood playgrounds observed dislodgeable arsenic on 11 children's hands after brief periods of play exposure. Future efforts should increase the number of children and the play exposure periods, and incorporate speciation in order to discriminate between various sources of arsenic
SN - 0048-9697
AD - Environmental and Occupational Health Sciences Institute (EOHSI), University of Medicine and Dentistry of New Jersey, Robert Wood Johnson Medical School, and Rutgers, the State University of New Jersey, Piscataway, New Jersey, USA
UR - 16487576
ER -

TY - JOUR
ID - 1228
T1 - Navigating social class roles in community research
A1 - Shpungin,E.
A1 - Lyubansky,M.
Y1 - 2006/06//
N1 - Shpungin, Elaine. Lyubansky, Mikhail
American journal of community psychology
3fv, 0364535
IM
Journal Article
English
KW - MEDLINE
KW - Adolescent
KW - Adult
KW - Child
KW - Preschool
KW - Female
KW - Hierarchy
KW - Social
KW - Homeless Persons
KW - Humans
KW - Infant
KW - Interprofessional Relations
KW - Male
KW - Organizational Culture

KW - Psychology
KW - Research Design
KW - Residential Facilities/og [Organization & Administration]
KW - Social Class
KW - Social Identification
KW - Social Values
KW - Social Work
RP - NOT IN FILE
SP - 227
EP - 235
JF - American Journal of Community Psychology
JA - Am J Community Psychol
VL - 37
IS - 3-4
CY - United States
N2 - This paper describes the first author's attempt to collect data in a homeless shelter without attending to her role in the social class hierarchy of the organization. The author's egalitarian approach towards the homeless clients, and her lack of involvement in "staff-only" activities, transgress the social class norms within the organizational structure. While the author is successful in gaining the trust of the homeless clients, her approach alienates shelter staff, especially those in the higher echelons of the social hierarchy. The concepts of classism, world views, and Social Identity Theory, are utilized to discuss the dilemma faced by researchers who want to challenge, or work outside, a setting's status quo
SN - 0091-0562
AD - Department of Psychology, University of Illinois, Urbana-Champaign, Illinois, USA. shpungin@uiuc.edu
UR - 16645901
ER -

TY - JOUR
ID - 1229
T1 - Contribution of main causes of death to social inequalities in mortality in the whole population of Scania, Sweden
A1 - Rosvall,M.
A1 - Chaix,B.
A1 - Lynch,J.
A1 - Lindstrom,M.
A1 - Merlo,J.
Y1 - 2006///
N1 - Rosvall, Maria. Chaix, Basile. Lynch, John. Lindstrom, Martin. Merlo, Juan
BMC public health
100968562
IM
Journal Article. Research Support, Non-U.S. Gov't
English
KW - MEDLINE
KW - Adolescent
KW - Adult
KW - Aged
KW - 80 and over
KW - Cardiovascular Diseases/mo [Mortality]
KW - Cause of Death/td [Trends]
KW - Child
KW - Preschool
KW - Female
KW - Health Status Indicators
KW - Humans
KW - Infant
KW - Newborn

KW - Longitudinal Studies
KW - Male
KW - Middle Aged
KW - Mortality/td [Trends]
KW - Neoplasms/mo [Mortality]
KW - Occupations/cl [Classification]
KW - Risk Factors
KW - Social Class
KW - Social Justice
KW - Socioeconomic Factors
KW - Stroke/mo [Mortality]
KW - Sweden/ep [Epidemiology]
RP - NOT IN FILE
SP - 79
JF - BMC Public Health
VL - 6
CY - England
N2 - BACKGROUND: To more efficiently reduce social inequalities in mortality, it is important to establish which causes of death contribute the most to socioeconomic mortality differentials. Few studies have investigated which diseases contribute to existing socioeconomic mortality differences in specific age groups and none were in samples of the whole population, where selection bias is minimized. The aim of the present study was to determine which causes of death contribute the most to social inequalities in mortality in each age group in the whole population of Scania, Sweden. METHODS: Data from LOMAS (Longitudinal Multilevel Analysis in Skane) were used to estimate 12-year follow-up mortality rates across levels of socioeconomic position (SEP) and workforce participation in 975,938 men and women aged 0 to 80 years, during 1991-2002. RESULTS: The results generally showed increasing absolute mortality differences between those holding manual and non-manual occupations with increasing age, while there were inverted u-shaped associations when using relative inequality measures. Cardiovascular diseases (CVD) contributed to 52% of the male socioeconomic difference in overall mortality, cancer to 18%, external causes to 4% and psychiatric disorders to 3%. The corresponding contributions in women were 55%, 21%, 2% and 3%. Additionally, those outside the workforce (i.e., students, housewives, disability pensioners, and the unemployed) showed a strongly increased risk of future mortality in all age groups compared to those inside the workforce. Even though coronary heart disease (CHD) played a major contributing role to the mortality differences seen, stroke and other types of cardiovascular diseases also made substantial contributions. Furthermore, while the most common types of cancers made substantial contributions to the socioeconomic mortality differences, in some age groups more than half of the differences in cancer mortality could be attributed to rarer cancers. CONCLUSION: CHD made a major contribution to the socioeconomic differences in overall mortality. However, there were also important contributions from diseases with less well understood mechanistic links with SEP such as stroke and less-common cancers. Thus, an increased understanding of the mechanisms connecting SEP with more rare causes of disease might be important to be able to more successfully intervene on socioeconomic differences in health
SN - 1471-2458
AD - Department of Health Sciences, Lund University, Malmo University Hospital, Malmo, Sweden. Maria.rosvall@med.lu.se
UR - 16569222
ER -

TY - JOUR
ID - 1230
T1 - Metals in soils of children's urban environments in the small northern European city of Uppsala
A1 - Ljung,K.
A1 - Selinus,O.
A1 - Otabbong,E.
Y1 - 2006/08/01/
N1 - Ljung, Karin. Selinus, Olle. Otabbong, Erasmus
The Science of the total environment
uj0, 0330500
IM

Journal Article. Research Support, Non-U.S. Gov't
English
KW - MEDLINE
KW - Arsenic/an [Analysis]
KW - Child
KW - Child Day Care Centers
KW - Cities
KW - Environmental Monitoring
KW - Humans
KW - Metals/an [Analysis]
KW - Play and Playthings
KW - Soil Pollutants/an [Analysis]
KW - Sweden
RP - NOT IN FILE
SP - 749
EP - 759
JF - Science of the Total Environment
JA - Sci Total Environ
VL - 366
IS - 2-3
CY - Netherlands
N2 - Metals occur naturally in soil, but contents are generally increased in the urban environment due to anthropogenic activities. The presence of elevated metals in soils of the urban environment has been recognized as an important source of metal intake in children and is linked to elevated metal levels in children's blood. Several metals have undesirable health effects, especially on children due to their still developing nervous system and small body volumes. Playgrounds are where urban children spend most of their time outdoors and are also where children most frequently come in contact with soil. Elevated contents of metals in playgrounds are therefore of great concern for children's wellbeing. This study investigates the soil metal content of 25 playgrounds located in different land use areas in urban Uppsala, Sweden's fourth largest city. Uppsala covers an area of approximately 100 km2 and has a population of 136,000. The soil samples were analysed for 12 metals (Al, As, Cd, Cr, Cu, Fe, Hg, Mn, Ni, Pb, W, Zn) using aqua regia. Median metal contents were found to be 1.8, 3.4, 0.21, 32, 25, 2.5, 0.14, 494, 19, 26, 0.35 and 84 mg kg(-1) soil for each of the above metals, respectively. The median clay content was around 20% while the organic matter content was measured by loss on ignition at a median of 8%. The land use areas included industrial land, the city center, road verges, natural land and former industrial land. The results showed that land use did not have the expected large influence on the total metal contents of the soils tested. The clay content together with the age of the site proved to be a more important factor. Sites with elevated clay contents had in general elevated metal contents, which were explained by the relatively high adsorption capacity of clay particles. The soils at sites where land use had not been altered since the 1800s had increased metal contents compared to playgrounds constructed in the late 1900s. The immobility of metals once they had entered the soil system was the reason for increased metal content in soils of old playgrounds. It was concluded that in cities with few internal pollution sources, the soil characteristics of the site and the time the soil has been on-site to accumulate metal residues become important factors in determining the soil metal content
SN - 0048-9697
AD - Department of Soil Science, Swedish University of Agricultural Sciences, PO Box 7014, SE-750 07 Uppsala, Sweden. Karin.Ljung@mv.slu.se
UR - 16309734
ER -

TY - JOUR
ID - 1231
T1 - Interaction of lexical and grammatical aspect in toddlers' language
A1 - Johnson,B.W.
A1 - Fey,M.E.
Y1 - 2006/05//
N1 - Johnson, Bonnie W. Fey, Marc E
Journal of child language

0425743, hp1
IM
Journal Article. Research Support, N.I.H., Extramural. Research Support, U.S. Gov't, Non-P.H.S.
English
KW - MEDLINE
KW - Analysis of Variance
KW - Child Language
KW - Child
KW - Preschool
KW - Comprehension
KW - Female
KW - Humans
KW - Language Tests
KW - Linguistics
KW - Male
KW - Verbal Behavior
RP - NOT IN FILE
SP - 419
EP - 435
JF - Journal of Child Language
JA - J Child Lang
VL - 33
IS - 2
CY - England
N2 - This study examined the effect of lexical aspect on children's imitation accuracy of English tense-aspect morphology. Thirty-five typically developing children, ages 2;4 to 3;1, imitated sentence-pairs in which the same regular verb was used once in an activity (skip on the rug) and once in an accomplishment (skip out the door). Children imitated past-imperfective morphology equally well in accomplishments and activities, but they imitated past-perfective morphology with higher accuracy in accomplishments than activities. These findings suggest that children's early morphology development is influenced by lexical aspect conveyed at the sentence level, as predicted by the PROTOTYPE HYPOTHESIS
SN - 0305-0009
AD - Department of Communication Sciences and Disorders, University of Florida, 336 Dauer Hall, PO Box 117420, Gainesville, FL 32611, USA
UR - 16826833
ER -

TY - JOUR
ID - 1232
T1 - Physical performance in relation to age, sex, birth order, social class, and sports activities of preschool children
A1 - Krombholz,H.
Y1 - 2006/04//
N1 - Krombholz, Heinz
Perceptual and motor skills
ozb, 0401131
IM
Comparative Study. Journal Article
English
KW - MEDLINE
KW - Age Factors
KW - Birth Order
KW - Child
KW - Child Development
KW - Preschool
KW - Cognition
KW - Female

KW - Humans
KW - Male
KW - Motor Skills
KW - Only Child
KW - Physical Fitness
KW - Psychomotor Performance
KW - Sex Factors
KW - Social Class
KW - Sports
RP - NOT IN FILE
SP - 477
EP - 484
JF - Perceptual & Motor Skills
JA - Percept.Mot.Skills
VL - 102
IS - 2
CY - United States
N2 - Physical performance of 1,194 preschool children, ages 43 to 84 mo. was related to characteristics of physical growth, cognitive performance, and social variables. Correlations between measures of physical growth and physical performance and between motor and cognitive performance were positive and significant. Physical fitness, body coordination, and manual dexterity improved across age groups. Significant sex differences were found, although boys exceeded on some measures and girls on others. Children with older sisters or brothers performed better than only or first-born children, and children who participated in sports activities outside school outperformed those who did not
SN - 0031-5125
AD - State Institute of Early Childhood Education and Research, Munich. h.krombholz@extern.lrz-muenchen.de
UR - 16826670
ER -

TY - JOUR
ID - 1233
T1 - Auditory characteristics of children with autism
A1 - Tharpe,A.M.
A1 - Bess,F.H.
A1 - Sladen,D.P.
A1 - Schissel,H.
A1 - Couch,S.
A1 - Schery,T.
Y1 - 2006/08//
N1 - Tharpe, Anne Marie. Bess, Fred H. Sladen, Douglas P. Schissel, Holly. Couch, Steve. Schery, Teris
Ear and hearing
e9g, 8005585
IM
Journal Article. Research Support, U.S. Gov't, Non-P.H.S.
English
KW - MEDLINE
KW - Acoustic Impedance Tests
KW - Audiometry
KW - Pure-Tone
KW - Auditory Threshold/ph [Physiology]
KW - Autistic Disorder/pp [Physiopathology]
KW - Case-Control Studies
KW - Child
KW - Preschool
KW - Evoked Potentials
KW - Auditory

KW - Brain Stem/ph [Physiology]
KW - Female
KW - Humans
KW - Male
KW - Otoacoustic Emissions
KW - Spontaneous/ph [Physiology]
KW - Reaction Time/ph [Physiology]
KW - Reproducibility of Results
RP - NOT IN FILE
SP - 430
EP - 441
JF - Ear & Hearing
JA - Ear Hear
VL - 27
IS - 4
CY - United States
N2 - OBJECTIVES: The objectives of this study were (1) to describe the auditory characteristics of children with autism relative to those of typically developing children and (2) to describe the test-retest reliability of behavioral auditory test measures with this population of children with autism. DESIGN: Audiometric data were obtained from 22 children diagnosed with autism and 22 of their typically developing peers. The audiologic test battery consisted of behavioral measures (i.e., visual reinforcement audiometry, tangible reinforcement operant conditioning audiometry, and conditioned play audiometry) and physiological measures (auditory brain stem response audiometry, distortion product otoacoustic emissions, and acoustic reflexes). RESULTS: Children with autism had physiologic test results equivalent to their typically developing counterparts. That is, no differences in auditory brain stem response audiometry, distortion product otoacoustic emissions, or acoustic reflex results were noted between the children with autism and typically developing children. However, behavioral measures revealed that about half of the children diagnosed with autism presented pure-tone averages outside of normal limits (i.e., >20 dB HL), although their response thresholds to speech were within normal limits. All behavioral test results were within normal limits (i.e., </=20 dB HL) for the typically developing children. In addition, test-retest variability was typically 15 dB or greater for children with autism as compared with variability of 10 dB or less for most of the typically developing children. CONCLUSIONS: Children with autism demonstrated essentially equivalent results on a battery of physiological auditory tests as those obtained from typically developing children. However, on average, behavioral responses of children with autism were elevated and less reliable relative to those of typically developing children. Furthermore, approximately half of the children with autism demonstrated behavioral pure-tone averages outside of the normal hearing range (i.e., >20 dB HL) despite having normal to near-normal hearing sensitivity as determined by other audiometric measures
SN - 0196-0202
AD - Vanderbilt Bill Wilkerson Center for Otolaryngology and Communication Sciences, Vanderbilt Medical Center, Nashville, Tennessee, USA. anne.m.tharpe@vanderbilt.edu
UR - 16825892
ER -

TY - JOUR
ID - 1234
T1 - Urinary 1-hydroxypyrene in children living in city and rural residences in Denmark
A1 - Hansen,A.M.
A1 - Raaschou-Nielsen,O.
A1 - Knudsen,L.E.
Y1 - 2006/06/15/
N1 - Hansen, Ase Marie. Raaschou-Nielsen, Ole. Knudsen, Lisbeth Ehlert
The Science of the total environment
uj0, 0330500
IM
Comparative Study. Journal Article. Research Support, Non-U.S. Gov't
English
KW - MEDLINE
KW - Adolescent

KW - Air Pollutants/to [Toxicity]
KW - Air Pollutants/ur [Urine]
KW - Child
KW - Preschool
KW - Creatinine/ur [Urine]
KW - Demography
KW - Denmark
KW - Environmental Exposure
KW - Humans
KW - Multivariate Analysis
KW - Pyrenes/an [Analysis]
KW - Regression Analysis
KW - Rural Health
KW - Urban Health
RP - NOT IN FILE
SP - 70
EP - 77
JF - Science of the Total Environment
JA - Sci Total Environ
VL - 363
IS - 1-3
CY - Netherlands
N2 - AIMS: The present study aims to assess the biological uptake in children of polycyclic aromatic hydrocarbons measured as 1-hydroxypyrene in urine from children living in city and rural residences. METHODS: 103 children living in Copenhagen and 101 children living in rural residences of Denmark collected urine samples Monday to Friday morning. Each day, the family filled in a printed diary that included questions about the time and activity patterns of the child. Multiple regression analyses were used to identify predictors of the excreted 1-hydroxypyrene level. RESULTS: During the week, the children excreted on average 0.07 [95% CI: 0.01-0.41] micromol urinary 1-hydroxypyrene per mol creatinine. Children living in urban residences excreted 0.02 [95% CI: 0.01-0.05] micromol more 1-hydroxypyrene than children living in rural residences. This was confirmed in the multiple regression analysis showing a 29% (95% CI: 2-64%) higher excretion among urban children than rural children. Moreover, the regression analysis showed that for each hour per day spent outside the children excreted 58% (1.58 [1.22-2.03]) more 1-hydroxypyrene in urine. CONCLUSION: The present study indicates that children living in urban residences are more exposed to PAH than children living in rural residences. Time spent outdoors increased the excretion of 1-hydroxypyrene, which was most evident among urban children. Higher concentrations of ambient air pollution in urban areas may explain this finding. No influence of environmental tobacco smoke, cooking habits, and heating facilities was detected
SN - 0048-9697
AD - National Institute of Occupational Health, Copenhagen, Denmark. aamh@ami.dk
UR - 16832893
ER -

TY - JOUR
ID - 1235
T1 - Quantifying the association between physical activity and injury in primary school-aged children
A1 - Spinks,A.B.
A1 - McClure,R.J.
A1 - Bain,C.
A1 - Macpherson,A.K.
Y1 - 2006/07//
N1 - Spinks, Anneliese B. McClure, Roderick J. Bain, Chris. Macpherson, Alison K
Pediatrics
oxv, 0376422
AIM, IM
Journal Article. Research Support, Non-U.S. Gov't
English

KW - MEDLINE
KW - Athletic Injuries/ep [Epidemiology]
KW - Child
KW - Preschool
KW - Exercise
KW - Female
KW - Humans
KW - Male
KW - Motor Activity
KW - Prospective Studies
KW - Queensland/ep [Epidemiology]
KW - Socioeconomic Factors
KW - Students/sn [Statistics & Numerical Data]
KW - Wounds and Injuries/ep [Epidemiology]
RP - NOT IN FILE
SP - e43
EP - e50
JF - Pediatrics
VL - 118
IS - 1
CY - United States
N2 - BACKGROUND: Regular physical activity is strongly advocated in children, with recommendations suggesting up to several hours of daily participation. However, an unintended consequence of physical activity is exposure to the risk of injury. To date, these risks have not been quantified in primary school-aged children despite injury being a leading cause for hospitalization and death in this population. OBJECT: Our goal was to quantify the risk of injury associated with childhood physical activity both in and out of the school setting and calculate injury rates per exposure time for organized and non-organized activity outside of school. METHODS: The Childhood Injury Prevention Study prospectively followed a cohort of randomly selected Australian primary school-and preschool-aged children (4 to 12 years). Over 12 months, each injury that required first aid attention was registered with the study. Exposure to physical activity outside school hours was measured by using a parent-completed 7-day diary. The age and gender distribution of injury rates per 10 000 hours of exposure were calculated for all activity and for organized and non-organized activity occurring outside school hours. In addition, child-based injury rates were calculated for physical activity-related injuries both in and out of the school setting. RESULTS: Complete diary and injury data were available for 744 children. There were 504 injuries recorded over the study period, 396 (88.6%) of which were directly related to physical activity. Thirty-four percent of physical activity-related injuries required professional medical treatment. Analysis of injuries occurring outside of school revealed an overall injury rate of 5.7 injuries per 10000 hours of exposure to physical activity and a medically treated injury rate of 1.7 per 10000 hours. CONCLUSION: Injury rates per hours of exposure to physical activity were low in this cohort of primary school-aged children, with <2 injuries requiring medical treatment occurring for every 10000 hours of activity participation outside of school
SN - 1098-4275
AD - School of Population Health, University of Queensland, Brisbane, Australia. a.spinks@uq.edu.au
UR - 16818536
ER -

TY - JOUR
ID - 1236
T1 - [Air pollution and asthma in children]. [Review] [47 refs] [French]
A1 - Just,J.
A1 - Nisakinovic,L.
A1 - Laoudi,Y.
A1 - Grimfeld,A.
Y1 - 2006/07//
N1 - Just, J. Nisakinovic, L. Laoudi, Y. Grimfeld, A
Archives de pediatrie : organe officiel de la Societe francaise de pediatrie
9421356, bwh
IM

English Abstract. Journal Article. Review
French
KW - MEDLINE
KW - Air Pollutants/ae [Adverse Effects]
KW - Allergens/ae [Adverse Effects]
KW - Asthma/et [Etiology]
KW - Child
KW - Humans
KW - Lung/gd [Growth & Development]
KW - Weather
RP - NOT IN FILE
SP - 1055
EP - 1060
JF - Archives de Pediatrie
JA - Arch Pediatr
VL - 13
IS - 7
CY - France
N2 - The prevalence of asthma and allergic diseases has increased world-wide during the last quarter of the 20th century, particularly among children and adolescents. No change common to all sites where asthma has increased throughout the world has been identified, suggesting that this 'epidemic' phenomenon is likely due to multiple factors. The following have been most discussed: exposure to indoor and outdoor allergens, modification of the patterns of respiratory infections, decreasing trends of physical activity, evolution in the make-up of environmental irritants, including tobacco smoke and urban air toxicants. In this review, we point out the role of exposure to air pollutants, in addition to and in combination with other asthma enhancers or precipitators. Whereas concentrations of the 'classical' air quality indicators (SO2, CO) have more or less steadily decreased, asthma prevalence augmented in developed countries during the same period. However, the nature of the air pollution mix has deeply evolved, and should also be considered. Ambient air concentrations of industrial and house heating combustion sources of pollutants in the city have substantially decreased, but by contrast the concentrations of various ultrafine particles have increased. Now, there is in vitro and in vivo evidence that exposure to urban air particles, and particularly to diesel exhausts, elicits chronic oxidative stress and repeated inflammatory responses, so that they may enhance allergic inflammation and airway hyper-responsiveness. Several epidemiological studies suggested an association between traffic density close to places of children's residence and prevalence of respiratory symptoms, and more specifically of asthma or allergic rhinitis symptoms in them. Chronic exposure during infancy to traffic-related pollutants may accelerate or even provoke, among genetically sensitive subjects, disruption of the normal regulatory and repair processes eventually contributing to the increase of asthma incidence. [References: 47]
SN - 0929-693X
AD - Service de pneumologie et allergologie pediatriques, hopital d'enfants Armand-Trousseau, APHP, 26, avenue du Docteur-Arnold-Netter, 75571 Paris cedex 12 et universite Pierre et Marie-Curie Paris-VI, France
UR - 16697622
ER -

TY - JOUR
ID - 1237
T1 - Characteristics of 1494 pediatric burn patients in Shanghai
A1 - Xin,W.
A1 - Yin,Z.
A1 - Qin,Z.
A1 - Jian,L.
A1 - Tanuseputro,P.
A1 - Gomez,M.
A1 - Beveridge,M.
A1 - Zhenjiang,L.
Y1 - 2006/08//
N1 - Xin, Wang. Yin, Zhang. Qin, Zhang. Jian, Liu. Tanuseputro, Peter. Gomez, Manuel. Beveridge, Massey. Zhenjiang, Liao

Burns : journal of the International Society for Burn Injuries
afc, 8913178
IM
Journal Article
English
KW - MEDLINE
KW - Adolescent
KW - Age Distribution
KW - Burns/ep [Epidemiology]
KW - Burns/et [Etiology]
KW - Burns/th [Therapy]
KW - Child
KW - Preschool
KW - China/ep [Epidemiology]
KW - Emigration and Immigration/sn [Statistics & Numerical Data]
KW - Female
KW - Hospitalization
KW - Humans
KW - Infant
KW - Newborn
KW - Male
KW - Prognosis
KW - Retrospective Studies
KW - Sex Distribution
RP - NOT IN FILE
SP - 613
EP - 618
JF - Burns
VL - 32
IS - 5
CY - England
N2 - To analyze the epidemiological characteristics of pediatric burn patients in Shanghai and to determine the targets for a pediatric burn prevention program, a retrospective review of all medical records of acute pediatric burn patients (age</=14 years old) admitted to the Burn Center of the Ruijin Hospital between January 1980 and December 2002 was performed. Patient demographics, etiology of burn, mechanism of injury, extent and anatomical areas burned, number of operations, and length of hospital stay were recorded. A total of 1494 pediatric burn patients were admitted. Six hundred eighty-seven (46%) patients were from the migrant population (non-registered population of temporary workers from rural areas outside of Shanghai). Scalding was the main cause of pediatric burns in the age groups. Children 0-3-year-old were the most common victims of scalding, chemical burns, and contact burns. Domestic burns resulted in 1293 (86.5%) injuries followed by burns occurring while playing in public. The incidence of domestic burns has increased since the beginning of the study period, while the incidence of burns while playing in public has decreased. The median total body surface area was 4% for mild burns, 10% for moderate burns, and 18% for extensive burns. Predominant areas involved were the head, neck, anterior trunk, and right lower limb. Most children received conservative treatment, and their mean hospital stay was 16.1+/-12.2 days. There were 17 (1.1%) deaths, mostly due to sepsis (82.4%). Migrant children are the majority of burn victims since 1996. The education of burn prevention should focus on the migrant population in an industrializing city
SN - 0305-4179
AD - Burn Center, Ruijin Hospital, Shanghai Second Medical University, 200025 Shanghai, PR China
UR - 16713685
ER -

TY - JOUR
ID - 1238
T1 - Enhancing generalized teaching strategy use in daily routines by parents of children with autism
A1 - Kashinath,S.
A1 - Woods,J.

A1 - Goldstein,H.
Y1 - 2006/06//
N1 - Kashinath, Shubha. Woods, Juliann. Goldstein, Howard
Journal of speech, language, and hearing research : JSLHR
ct1, 9705610
IM
Journal Article. Research Support, Non-U.S. Gov't
English
KW - MEDLINE
KW - Activities of Daily Living
KW - Autistic Disorder/th [Therapy]
KW - Child
KW - Preschool
KW - Female
KW - Humans
KW - Language Therapy/mt [Methods]
KW - Male
KW - Parents
KW - Reproducibility of Results
KW - Speech Production Measurement
KW - Teaching/mt [Methods]
KW - Teaching/st [Standards]
KW - Videotape Recording
RP - NOT IN FILE
SP - 466
EP - 485
JF - Journal of Speech Language & Hearing Research
JA - J Speech Lang Hear Res
VL - 49
IS - 3
CY - United States
N2 - PURPOSE: The purpose of this study was to examine the effects of facilitating generalized use of teaching strategies by parents of children with autism within daily routines. METHOD: Five preschool children with autism participated in intervention with a parent within daily routines in the family's home. Parents learned to include 2 teaching strategies in target routines to address their child's communication objectives. Parent-child interactions in routines were videotaped for data coding and analysis. Proactive programming of generalization occurred by systematic selection of intervention routines and by embedding intervention in multiple routines. Generalization data were collected by measuring strategy use in untrained routines. A multiple baseline design across teaching strategies was used to assess experimental effects. RESULTS: All parents demonstrated proficient use of teaching strategies and generalized their use across routines. The intervention had positive effects on child communication outcomes. All parents perceived the intervention to be beneficial. CONCLUSION: Results from this study add to the limited body of evidence supporting parent-implemented interventions in natural environments with young children with autism spectrum disorder. Additional research that replicates this approach with children of varying ages and disabilities and families with diverse characteristics is needed to support the generality of these findings
SN - 1092-4388
AD - Department of Communication Disorders, Florida State University, Tallahassee, 32306-1200, USA. skashina@fsu.edu
UR - 16787891
ER -

TY - JOUR
ID - 1239
T1 - Evaluation of the performance of photochromic spectacle lenses in children and adolescents aged 10 to 15 years
A1 - Lakkis,C.
A1 - Weidemann,K.

Y1 - 2006/07//
N1 - Lakkis, Carol. Weidemann, Kate
Clinical & experimental optometry : journal of the Australian Optometrical Association
8703442
IM
Journal Article. Randomized Controlled Trial. Research Support, Non-U.S. Gov't
English
KW - MEDLINE
KW - Adaptation
KW - Ocular
KW - Adolescent
KW - Child
KW - Cross-Over Studies
KW - Eye Protective Devices
KW - Female
KW - Follow-Up Studies
KW - Humans
KW - Male
KW - Patient Satisfaction
KW - Prospective Studies
KW - Questionnaires
KW - Radiation Injuries/co [Complications]
KW - Radiation Injuries/pc [Prevention & Control]
KW - Ultraviolet Rays/ae [Adverse Effects]
KW - Vision Disorders/et [Etiology]
KW - Vision Disorders/pc [Prevention & Control]
KW - Visual Acuity
RP - NOT IN FILE
SP - 246
EP - 252
JF - Clinical & Experimental Optometry
JA - Clin Exp Optom.
VL - 89
IS - 4
CY - Australia
N2 - BACKGROUND: To compare the performance of clear and photochromic spectacle lenses in children and adolescents, with respect to visual acuity and satisfaction with day-to-day activities. METHODS: Fifty full-time spectacle wearers, aged 10 to 15 years, were randomly assigned to wear clear and Transitions photochromic spectacle lenses for two weeks each in a crossover fashion. Subjects were evaluated at screening, lens delivery and two weeks post-delivery. At each visit, distance and near visual acuity (VA, logMAR) were assessed and subjective questionnaires, using Likert scales, were administered. Parents/guardians also completed questionnaires at entry and exit from the study. RESULTS: There were no significant differences in VA between clear and photochromic lenses ($p > 0.05$) and no difficulties were encountered with completion of the questionnaires. Subjective evaluation of vision in bright sunlight and when playing sport was significantly better with photochromic compared to clear lenses ($p < 0.05$). There were no significant differences between lens designs in subjective performance indoors, such as in the classroom or when reading ($p > 0.05$). Forty-three subjects (88 per cent) chose to continue wearing photochromic lenses on conclusion of the trial. Thirty subjects (61 per cent) preferred photochromic over clear lenses due to reasons such as darkening in sunlight, better vision and less squinting in sunlight. Forty-three parents (88 per cent) rated the children's overall experience with photochromic lenses to be favourable or very favourable. CONCLUSION: Photochromic lenses can be successfully prescribed for children and adolescents aged 10 to 15 years. Clear and photochromic lenses were considered to be equivalent for indoor activities; however, photochromic lenses were significantly preferred over clear lenses for outdoor activities. Likert grading scales can be used effectively in questionnaires for children and adolescents and further development of questionnaires for use in clinical trials evaluating lens performance in children is warranted
SN - 0816-4622
AD - Clinical Vision Research, Carlton, Australia. clakkis@unimelb.edu.au

UR - 16776732
ER -

TY - JOUR
ID - 1240
T1 - Seroepidemiological study of Rickettsia felis, Rickettsia typhi, and Rickettsia conorii infection among the population of southern Spain
A1 - Bernabeu-Wittel,M.
A1 - del,Toro,M.D.
A1 - Nogueras,M.M.
A1 - Muniain,M.A.
A1 - Cardenosa,N.
A1 - Marquez,F.J.
A1 - Segura,F.
A1 - Pachon,J.
Y1 - 2006/06//
N1 - Bernabeu-Wittel, M. del Toro, M D. Nogueras, M M. Muniain, M A. Cardenosa, N. Marquez, F J. Segura, F. Pachon, J
European journal of clinical microbiology & infectious diseases : official publication of the European Society of Clinical Microbiology
em5, 8804297
IM
Journal Article. Research Support, Non-U.S. Gov't
English
KW - MEDLINE
KW - Adolescent
KW - Adult
KW - Aged
KW - Analysis of Variance
KW - Boutonneuse Fever/ep [Epidemiology]
KW - Child
KW - Preschool
KW - Cross-Sectional Studies
KW - Humans
KW - Infant
KW - Newborn
KW - Middle Aged
KW - Rickettsia Infections/ep [Epidemiology]
KW - Rickettsia conorii
KW - Rickettsia felis
KW - Rickettsia typhi
KW - Risk Factors
KW - Seroepidemiologic Studies
KW - Spain/ep [Epidemiology]
KW - Typhus
KW - Endemic Flea-Borne/ep [Epidemiology]
RP - NOT IN FILE
SP - 375
EP - 381
JF - European Journal of Clinical Microbiology & Infectious Diseases
JA - Eur J Clin Microbiol Infect Dis
VL - 25
IS - 6
CY - Germany
N2 - Rickettsia typhi and Rickettsia conorii, the etiologic agents of, respectively, murine typhus and Mediterranean spotted fever, are recognized as frequent causes of fever of intermediate duration in southern Spain; in addition, in recent years Rickettsia felis has been detected in potential vectors in this area.

Nevertheless, limited data exist regarding the actual prevalence of past infection due to these three pathogens. In the present study, the prevalence of past infection due to R. felis, R. typhi, and R. conorii was determined in a representative population of southern Spain during 2002. In addition, the possible risk factors associated with exposure to these pathogens were investigated. An epidemiological survey was completed by all subjects included in the study. Serum samples were tested by indirect immunofluorescence assay. The prevalence of past infection due to R. felis, R. typhi, and R. conorii among the 504 total subjects was 6.5, 3.8 and 8.7%, respectively. In multivariate analysis, infection due to R. felis was independently associated with a high-risk occupation (one that required working outdoors in nature, close contact with domestic animals, or potential contact with rodents) (OR=5.8; 95%CI 2.1-15.6), while infection due to R. typhi was associated with older age (factor of 1.04 [95%CI 1.008-1.068]) and frequent insect bites (OR=10.3; 95%CI 2.3-45.5). Two factors were associated with infection due to R. conorii: a high-risk occupation (OR=9.3; 95%CI 3.7-23.2), and participation in outdoor activities (OR=7.2; 95%CI 1.4-38.5). The results confirm the widespread prevalence of past infection due to R. felis, R. typhi, and R. conorii in the population of southern Spain
SN - 0934-9723
AD - Department of Infectious Diseases, Hospitales Universitarios Virgen del Rocio, Avenida Manuel Siurot s/n, 41013, Seville, Spain. MAXBW@telefonica.net
UR - 16767485
ER -

TY - JOUR
ID - 1241
T1 - Heart and lung transplantation pathology: the Padua experience
A1 - Valente,M.
A1 - Angelini,A.
A1 - Calabrese,F.
A1 - Thiene,G.
Y1 - 2006/05//
N1 - Valente, M. Angelini, A. Calabrese, F. Thiene, G
Transplantation proceedings
we9, 0243532
IM
Journal Article
English
KW - MEDLINE
KW - Adolescent
KW - Adult
KW - Cardiomyopathies/cl [Classification]
KW - Cardiomyopathies/su [Surgery]
KW - Cause of Death
KW - Child
KW - Female
KW - Heart Diseases/cl [Classification]
KW - Heart Diseases/su [Surgery]
KW - Heart Transplantation/mo [Mortality]
KW - Heart Transplantation/pa [Pathology]
KW - Heart-Lung Transplantation/pa [Pathology]
KW - Humans
KW - Italy
KW - Lung Diseases/su [Surgery]
KW - Lung Transplantation/mo [Mortality]
KW - Lung Transplantation/pa [Pathology]
KW - Male
KW - Treatment Outcome
RP - NOT IN FILE
SP - 1163
EP - 1166
JF - Transplantation Proceedings

JA - Transplant Proc
VL - 38
IS - 4
CY - United States
N2 - Physicians caring for heart and lung transplantation patients utilize routine follow-up biopsies on a prearranged schedule unrelated to the suspicion of a clinical diagnosis of rejection. Of course biopsies are also performed outside the prearranged scheme at any time the clinician is puzzled by clinical suspicion of rejection or infections. Technical considerations are important in handling the biopsies; pathologists who are aware of bias produced by tissue processing are forced to serially section the samples to increase the sensitivity for detection of the pathological processes. During the 20 years since the first Italian cardiac transplantation was performed in our center, 600 patients have been transplanted and monitored with 12,386 endomyocardial biopsies. The overall 5-year mortality was 24.5% and 18.4% in pediatric populations; at 10 years, 34%, and at 19 years, 55%. There was a progressive decrease in mortality from 36.8% in 1985 to 1990 to 12% in the 1996 to 2000. During a decade of experience in lung transplantation from May 1995 to May 2005 (n = 129), all patients underwent surveillance bronchoscopy including transbronchial biopsy (n = 722) and bronchoalveolar lavage (n = 629). The ancillary techniques of immunohistochemistry and molecular analysis have allowed the pathologists to play a pivotal role in the pre- and posttransplant management of patients requiring thoracic organ transplantation
SN - 0041-1345
AD - Institute of Pathological Anatomy, University of Padua, Medical School, Italy. marialuisa.valente@unipd.it
UR - 16757295
ER -

TY - JOUR
ID - 1242
T1 - Epidemiology of community-onset candidemia in Connecticut and Maryland
A1 - Sofair,A.N.
A1 - Lyon,G.M.
A1 - Huie-White,S.
A1 - Reiss,E.
A1 - Harrison,L.H.
A1 - Sanza,L.T.
A1 - rthington-Skaggs,B.A.
A1 - Fridkin,S.K.
Y1 - 2006/07/01/
N1 - Sofair, Andre N. Lyon, G Marshall. Huie-White, Sharon. Reiss, Errol. Harrison, Lee H. Sanza, Laurie Thomson. Arthington-Skaggs, Beth A. Fridkin, Scott K
Clinical infectious diseases : an official publication of the Infectious Diseases Society of America
a4j, 9203213
IM
Journal Article. Research Support, U.S. Gov't, P.H.S.
English
KW - MEDLINE
KW - Adolescent
KW - Adult
KW - Aged
KW - Candida/ip [Isolation & Purification]
KW - Candidiasis/ep [Epidemiology]
KW - Child
KW - Community-Acquired Infections/ep [Epidemiology]
KW - Connecticut/ep [Epidemiology]
KW - Female
KW - Fungemia/ep [Epidemiology]
KW - Humans
KW - Male
KW - Maryland/ep [Epidemiology]

KW - Middle Aged
KW - Population Surveillance
KW - Risk Factors
KW - Time Factors
RP - NOT IN FILE
SP - 32
EP - 39
JF - Clinical Infectious Diseases
JA - Clin Infect Dis
VL - 43
IS - 1
CY - United States
N2 - BACKGROUND: Almost one-third of patients with bloodstream infections with Candida species (candidemia) have onset of disease that occurs outside of the hospital or < or = 2 days after hospital admission (i.e., community-onset candidemia). We compared the characteristics of patients who developed candidemia by the timing of onset of infection. METHODS: Incident episodes of candidemia were identified through active, population-based surveillance in Connecticut and in Baltimore and Baltimore County, Maryland, during 1 October 1998-30 September 2000. The molecular subtypes of a sample of 45 Candida parapsilosis isolates were evaluated using Southern blots hybridized with the complex probe Cp3-13. RESULTS: Overall, 356 (31%) of the 1143 incident episodes of candidemia were classified as community-onset disease (occurring < or = 2 days after hospital admission), and 132 (37%) were caused by Candida albicans, 89 (25%) were caused by Candida glabrata, 57 (16%) were caused by C. parapsilosis, and 53 (15%) were caused by Candida tropicalis. Community-onset disease was less likely to be associated with concurrent immunosuppressive therapy, recent surgery, or use of a central venous catheter, compared with inpatient disease. Among patients with community-onset disease, the median time from blood culture to initiation of antifungal treatment was 2.7 days, the 30-day case-fatality rate was 26%, and 262 patients (75%) had been hospitalized at least once in the previous 3 months. Although there were few differences between patients with very recent hospitalization (in the previous 1 month), less recent hospitalization (previous 1-3 months), and no documented past hospitalization, C. parapsilosis was more frequently associated with community-onset disease as hospitalization became more distant. C. parapsilosis strains tended to be unique to the patient, with little similarity found between strain types, on the basis of epidemiologic classification of patients. CONCLUSION: We report that community-onset candidemia is common and occurs in patients with extensive contact with the health care system. Disease caused by C. parapsilosis tends to involve unique strains
SN - 1537-6591
AD - Yale University School of Medicine, New Haven, Connecticut, USA
UR - 16758415
ER -

TY - JOUR
ID - 1243
T1 - What are the etiology and epidemiology of out-of-hospital pediatric cardiopulmonary arrest in Ontario, Canada?
A1 - Gerein,R.B.
A1 - Osmond,M.H.
A1 - Stiell,I.G.
A1 - Nesbitt,L.P.
A1 - Burns,S.
A1 - OPALS Study Group.
Y1 - 2006/06//
N1 - Gerein, Richard Bradley. Osmond, Martin H. Stiell, Ian G. Nesbitt, Lisa P. Burns, Starla. OPALS Study Group
Academic emergency medicine : official journal of the Society for Academic Emergency Medicine
ce1, 9418450
IM
Journal Article. Research Support, Non-U.S. Gov't
English
KW - MEDLINE

KW - Adolescent
KW - Age Distribution
KW - Cardiopulmonary Resuscitation/sn [Statistics & Numerical Data]
KW - Caregivers/sn [Statistics & Numerical Data]
KW - Causality
KW - Child
KW - Preschool
KW - Comorbidity
KW - Emergency Medical Services/sn [Statistics & Numerical Data]
KW - Female
KW - Heart Arrest/ep [Epidemiology]
KW - Hospitalization/sn [Statistics & Numerical Data]
KW - Humans
KW - Incidence
KW - Infant
KW - Newborn
KW - Male
KW - Ontario/ep [Epidemiology]
KW - Pediatrics/sn [Statistics & Numerical Data]
KW - Remission
KW - Spontaneous
KW - Respiratory Tract Diseases/ep [Epidemiology]
KW - Sex Distribution
KW - Survival Analysis
KW - Wounds and Injuries/ep [Epidemiology]
RP - NOT IN FILE
SP - 653
EP - 658
JF - Academic Emergency Medicine
JA - Acad Emerg Med
VL - 13
IS - 6
CY - United States
N2 - BACKGROUND: Pediatric cardiopulmonary arrest (CPA) outside of the hospital has a very high mortality rate. OBJECTIVES: To evaluate the etiology and initial compromise of pediatric CPA cases in hopes of developing strategies to improve out-of-hospital resuscitation. METHODS: The Ontario Prehospital Advanced Life Support (OPALS) study was a large multicenter initiative to evaluate the impact of emergency medical services (EMS) programs on 17 communities with 40,000 critically ill and injured patients who were older than 11 years. As part of this study, the authors conducted a retrospective observational cohort study that included all children younger than 18 years of age with out-of-hospital CPA, during an 11-year period from 1991-2002. CPA was defined as patient being pulseless, apneic, and requiring chest compressions. Data were collected from ambulance call reports and centralized dispatch data and were reviewed by two independent investigators. RESULTS: There were 503 children with CPA in the sample. Mean age was 5.6 years (range, 0-17 yr); 58.4% of patients were male, and 37.8% were younger than 1 year of age. Cardiopulmonary resuscitation (CPR) first was started by a bystander in 32.4% of cases, whereas 66.0% were unwitnessed arrests. Initial rhythms were asystole 77.2% of the time, pulseless electrical activity 16.4% of the time, and ventricular fibrillation or ventricular tachycardia 4% of the time. Annual incidence was 9.1/100,000 children. CPA was witnessed in 34.0% of cases; 80.7% of these were bystander-witnessed, and 18.1% were EMS-witnessed. Primary pathogenic cause of arrest was medical in 61.2% of cases, trauma in 37.2% of cases, and indeterminate in 1.6% of cases. Initial underlying physiologic compromise of witnessed arrests was judged to be respiratory in 39.8% of cases, sudden collapse (presumed electrical) in 16.4% of cases, progressive shock in 1.2% of cases, and indeterminate in 42.6% of cases. Presumed etiology was trauma, 37.6%; sudden infant death syndrome (SIDS), 20.3%; and respiratory disease, 11.6%, most commonly. Survival to hospital discharge was 2.0%. CONCLUSIONS: This is one of the largest population-based, prospective cohorts of pediatric CPA reported to date, and it reveals that most pediatric arrests are unwitnessed and receive no bystander CPR. Those that are witnessed most often are caused by respiratory arrests or trauma. Trauma, SIDS, and respiratory disease are the most common etiologies overall. These data are vital to planning large resuscitation trials looking at specific

interventions (i.e., increasing bystander CPR) and highlight the need for better strategies for prevention and early recognition
SN - 1553-2712
AD - Department of Pediatrics, University of Ottawa, Ottawa, Ontario, Canada. rgerein@cheo.on.ca
UR - 16670256
ER -

TY - JOUR
ID - 1244
T1 - Aggression, dominance, and affiliation: Their relationships with androgen levels and intelligence in 5-year-old children
A1 - Azurmendi,A.
A1 - Braza,F.
A1 - Garcia,A.
A1 - Braza,P.
A1 - Munoz,J.M.
A1 - Sanchez-Martin,J.R.
Y1 - 2006/06//
N1 - Azurmendi, Aitziber. Braza, Francisco. Garcia, Ainhoa. Braza, Paloma. Munoz, Jose M. Sanchez-Martin, Jose R
Hormones and behavior
gb7, 0217764
IM
Comparative Study. Journal Article. Research Support, Non-U.S. Gov't
English
KW - MEDLINE
KW - Aggression/ph [Physiology]
KW - Analysis of Variance
KW - Androgens/me [Metabolism]
KW - Androstenedione/me [Metabolism]
KW - Child
KW - Preschool
KW - Dehydroepiandrosterone/me [Metabolism]
KW - Female
KW - Humans
KW - Intelligence/ph [Physiology]
KW - Leadership
KW - Male
KW - Principal Component Analysis
KW - Reference Values
KW - Regression Analysis
KW - Saliva/me [Metabolism]
KW - Social Behavior
KW - Social Dominance
KW - Testosterone/me [Metabolism]
RP - NOT IN FILE
SP - 132
EP - 140
JF - Hormones & Behavior
JA - Horm Behav
VL - 50
IS - 1
CY - United States
N2 - This study explores the potential relationship between social behavior (aggression, dominance, and affiliation) and testosterone, androstenedione, and DHEA measurements in 5-year-old children while also analyzing the moderating effect of IQ on the hormone-behavior relationship. 129 healthy normal Iberian children (60 boys and 69 girls) were videotaped in free play interactions in the school playground. Their

behavior was then evaluated with particular emphasis on aggression, government, and affiliation. Testosterone, androstenedione, and DHEA levels were measured using an enzyme immunoassay technique in saliva samples. A test (K-BIT) which provides an IQ measurement for children was also administered to subjects. The correlation analysis revealed a positive relationship between the behavioral factor of Provocation and androstenedione in boys, and a regression analysis indicated that this relationship was moderated in a positive direction by the subject's intelligence. In girls, we observed a positive relationship between testosterone and Affectivity, with this relationship being moderated in a negative direction by intelligence
SN - 0018-506X
AD - Area of Psychobiology, Faculty of Psychology, University of the Basque Country, San Sebastian, Spain
UR - 16564527
ER -

TY - JOUR
ID - 1245
T1 - Differing perspectives of inner-city parents and pediatric clinicians impact management of iron-deficiency anemia
A1 - Pierce,M.B.
A1 - Crowell,R.E.
A1 - Ferris,A.M.
Y1 - 2006/05//
N1 - Pierce, Michelle B. Crowell, Rebecca E. Ferris, Ann M
Journal of nutrition education and behavior
101132622
IM
Journal Article. Research Support, Non-U.S. Gov't
English
KW - MEDLINE
KW - Adult
KW - Anemia
KW - Iron-Deficiency/pc [Prevention & Control]
KW - Iron-Deficiency/px [Psychology]
KW - Child
KW - Child Health Services/st [Standards]
KW - Child Nutrition Sciences/ed [Education]
KW - Connecticut
KW - Culture
KW - Ethnic Groups
KW - Female
KW - Focus Groups
KW - Humans
KW - Interviews as Topic
KW - Male
KW - Parents/ed [Education]
KW - Parents/px [Psychology]
KW - Parents
KW - Patient Care Team
KW - Physicians/px [Psychology]
RP - NOT IN FILE
SP - 169
EP - 176
JF - Journal of Nutrition Education & Behavior
JA - J Nutr Educ Behav
VL - 38
IS - 3
CY - Canada
N2 - OBJECTIVE: To ascertain the beliefs and experiences of inner-city pediatric clinicians and parents regarding anemia in young children. DESIGN: Focus groups and in-depth interviews. SETTING: Pediatric

clinics and community agencies in Hartford, Connecticut. PARTICIPANTS: Convenience sample of 41 pediatric clinicians (93% white, 73% female) and 85 parents (100% minority, 88% female, 47% < 12 years education). PHENOMENON OF INTEREST: Identification, understanding, and management of anemia. ANALYSIS: Researcher pairs coded complete transcriptions. Recurrent themes were identified, which were then contrasted and compared between clinicians and parents. RESULTS: Both clinicians and parents were familiar with, but frustrated by the persistence of anemia. They noted time constraints and poor communication during office visits as contributing to the problem. Parents felt alarmed upon initial diagnosis; linked anemia with heredity, food patterns, and activity; reported culturally linked management strategies; but were uncertain of the seriousness. Health clinicians saw physiological processes as outside the parents' understanding and emphasized prevention through feeding recommendations. CONCLUSIONS AND IMPLICATIONS: In order to address childhood anemia effectively, differing socio-cultural perspectives of clinicians and parents need to be incorporated into a unified health care plan. Nutritionists are well suited to collaborate on the health care team to effectively address this issue
SN - 1499-4046
AD - Department of Nutritional Sciences, University of Connecticut, Storrs, CT 06269-4017, USA. michelle.pierce@uconn.edu
UR - 16731452
ER -

TY - JOUR
ID - 1246
T1 - The effect of ageing on the bioaccessibility and fractionation of cadmium in some typical soils of China
A1 - Tang,X.Y.
A1 - Zhu,Y.G.
A1 - Cui,Y.S.
A1 - Duan,J.
A1 - Tang,L.
Y1 - 2006/07//
N1 - Tang, Xiang-Yu. Zhu, Yong-Guan. Cui, Yan-Shan. Duan, Jing. Tang, Lily
Environment international
du1, 7807270
IM
Journal Article. Research Support, Non-U.S. Gov't
English
KW - MEDLINE
KW - Aging/de [Drug Effects]
KW - Aging/me [Metabolism]
KW - Aging
KW - Biological Availability
KW - Cadmium/ae [Adverse Effects]
KW - Cadmium/an [Analysis]
KW - Cadmium/me [Metabolism]
KW - Child
KW - China
KW - Humans
KW - Hydrogen-Ion Concentration
KW - Soil/an [Analysis]
KW - Soil Pollutants/ae [Adverse Effects]
KW - Soil Pollutants/an [Analysis]
KW - Soil Pollutants/me [Metabolism]
RP - NOT IN FILE
SP - 682
EP - 689
JF - Environment International
JA - Environ Int
VL - 32
IS - 5

CY - United States
N2 - Ingestion of contaminated soil has been recognized as an important exposure pathway of cadmium (Cd) for humans, especially for children through outdoor hand-to-mouth activities. The effect of ageing process following the input of Cd into soil on the bioaccessibility of Cd in five typical soils of China was investigated using physiologically based in vitro test in this study. A sequential extraction procedure was employed with attempt to identify the bioaccessible fraction(s) of Cd in soils. The bioaccessibility of Cd in strongly acidic (approximately pH 4.5) soils reached nearly steady levels (76.5-76.9% and 52.0-52.6% in the gastric and small intestinal phases, respectively) after a sharp decline in the first week of ageing. In contrast, the bioaccessibility of Cd in higher pH (>6.0) soils was found to be much lower (53.3-72.7% and 29.9-43.4% in gastric and small intestinal phases, respectively) and took 2 weeks of ageing to reach steady levels. The freshly spiked Cd was more labile than native Cd. The main proportion of spiked Cd was found in exchangeable Cd which was higher in strongly acidic soils (68.6-71.8%) than in higher pH soils (53.4-61.4%) at day 120 after a sharp decline to the nearly steady state in the first 1 and 2 weeks, respectively. Significant correlations between Cd bioaccessibility and either water soluble and exchangeable Cd individually, or the sum of water soluble and exchangeable Cd throughout the incubation period for all soils, indicate that these forms of Cd are likely to constitute the main proportion of bioaccessible Cd in soils
SN - 0160-4120
AD - Department of Soil Environmental Sciences, Research Center for Eco-Environmental Sciences, the Chinese Academy of Sciences, Beijing 100085, PR China
UR - 16616372
ER -

TY - JOUR
ID - 1247
T1 - Day-to-day and seasonal variability of physical activity during school recess
A1 - Ridgers,N.D.
A1 - Stratton,G.
A1 - Clark,E.
A1 - Fairclough,S.J.
A1 - Richardson,D.J.
Y1 - 2006/05//
N1 - Ridgers, Nicola D. Stratton, Gareth. Clark, Emily. Fairclough, Stuart J. Richardson, David J
Preventive medicine
pm4, 0322116
IM
Clinical Trial. Journal Article. Research Support, Non-U.S. Gov't
English
KW - MEDLINE
KW - Child
KW - Exercise
KW - Female
KW - Heart Rate
KW - Humans
KW - Leisure Activities
KW - Male
KW - Schools
KW - Seasons
RP - NOT IN FILE
SP - 372
EP - 374
JF - Preventive Medicine
JA - Prev Med
VL - 42
IS - 5
CY - United States
N2 - OBJECTIVE: Children's physical activity levels are difficult to establish on a day-to-day or season-to-season basis. Most studies have attempted to measure reliability in habitual settings. This study investigated the

variability in children's physical activity during recess. METHODS: Fifteen boys and 19 girls (aged 6 to 11 years) from 2 schools in North West England wore heart rate monitors for 5 consecutive days in summer and winter terms to assess day-to-day and seasonal variability during school recess. Data were collected in 2004. Repeated measures ANOVA's and intraclass correlations (ICC) analysed the day-to-day and seasonal variability in children's moderate-to-vigorous (MVPA) and vigorous physical activity (VPA) data. RESULTS: There were no significant differences in children's MVPA and VPA across days and seasons. ICCs for MVPA across 2 days ranged from 0.75 to 0.85 in summer, and from 0.53 to 0.81 in winter. Three-day MVPA ICCs were 0.83 in summer and 0.71 in winter. CONCLUSIONS: The results revealed no significant variation in children's recess physical activity levels across days and seasons. Whilst children were free to choose their recess activities in school, the results suggested that children were relatively consistent in their choices, limiting physical activity variability
SN - 0091-7435
AD - Research Institute for Sport and Exercise Sciences, Liverpool John Moores University, Henry Cotton Campus, 15-21 Webster Street, Liverpool L3 2ET, UK. n.ridgers@ljmu.ac.uk
UR - 16510176
ER -

TY - JOUR
ID - 1248
T1 - Vehicular air pollution, playgrounds, and youth athletic fields
A1 - Rundell,K.W.
A1 - Caviston,R.
A1 - Hollenbach,A.M.
A1 - Murphy,K.
Y1 - 2006/07//
N1 - Rundell, Kenneth W. Caviston, Renee. Hollenbach, Amanda M. Murphy, Kerri
Inhalation toxicology
8910739, db8
IM
Journal Article
English
KW - MEDLINE
KW - Adolescent
KW - Air Pollutants/ae [Adverse Effects]
KW - Air Pollutants/an [Analysis]
KW - Cardiovascular Diseases/et [Etiology]
KW - Child
KW - Environmental Health
KW - Environmental Monitoring/mt [Methods]
KW - Humans
KW - Ozone/an [Analysis]
KW - Respiratory Tract Diseases/et [Etiology]
KW - Rural Population
KW - Schools
KW - Sports
KW - Temperature
KW - Vehicle Emissions/an [Analysis]
KW - Wind
RP - NOT IN FILE
SP - 541
EP - 547
JF - Inhalation Toxicology
JA - Inhal.Toxicol
VL - 18
IS - 8
CY - United States

N2 - In spite of epidemiological evidence concerning vehicular air pollution and adverse respiratory/cardiovascular health, many athletic fields and school playgrounds are adjacent to high traffic roadways and could present long-term health risks for exercising children and young adults. Particulate matter (PM(1),0.02-1.0 microm diameter) number counts were taken serially at four elementary school athletic/playground fields and at one university soccer field. Elementary school PM1 measurements were taken over 17 days; measurements at the university soccer field were taken over 62 days. The high-traffic-location elementary school field demonstrated higher 17-day [PM1] than the moderate and 2 low traffic elementary school fields (48,890 +/- 34,260, 16,730 +/- 10,550, 11,960 +/- 6680, 10,030 +/- 6280, respective mean counts; p < .05). The 62-day mean PM1 values at the university soccer field ranged from 115,000 to 134,000 particles cm(-3). Lowest mean values were recorded at measurement sites furthest from the highway (approximately 34,000 particles cm(-3)) and followed a second-order logarithmic decay (R2 = .999) with distance away from the highway. Mean NO2 and SO2 levels were below 100 ppb, mean CO was 0.33 +/- 1.87 ppm, and mean O3 was 106 +/- 47 ppb. Ozone increased with rising temperature and was highest in the warmer afternoon hours (R = .61). Although the consequence of daily recess play and athletic activities by school children and young athletes in high ambient [PM1] conditions has not yet been clearly defined, this study is a critical component to evaluating functional effects of chronic combustion-derived PM exposure on these exercising schoolchildren and young adults. Future studies should examine threshold limits and mechanistic actions of real-world particle exposure
SN - 1091-7691
AD - Human Performance Laboratory, Keith J. O'Neill Center for Healthy Families, Marywood University, Scranton, Pennsylvania 18509, USA. rundell@marywood.edu
UR - 16717025
ER -

TY - JOUR
ID - 1249
T1 - Emergency department visits among pediatric patients for sports-related injury: basic epidemiology and impact of race/ethnicity and insurance status
A1 - Simon,T.D.
A1 - Bublitz,C.
A1 - Hambidge,S.J.
Y1 - 2006/05//
N1 - Simon, Tamara D. Bublitz, Caroline. Hambidge, Simon J
Pediatric emergency care
pau, 8507560
IM
Comparative Study. Journal Article. Research Support, Non-U.S. Gov't. Research Support, U.S. Gov't, P.H.S.
English
KW - MEDLINE
KW - Adolescent
KW - Athletic Injuries/ec [Economics]
KW - Athletic Injuries/ep [Epidemiology]
KW - Causality
KW - Child
KW - Preschool
KW - Continental Population Groups/sn [Statistics & Numerical Data]
KW - Cross-Sectional Studies
KW - Emergency Service
KW - Hospital/sn [Statistics & Numerical Data]
KW - Ethnic Groups/sn [Statistics & Numerical Data]
KW - Female
KW - Health Care Surveys
KW - Hospitalization/sn [Statistics & Numerical Data]
KW - Humans
KW - Infant
KW - Insurance
KW - Health/sn [Statistics & Numerical Data]

KW - Male
KW - Multivariate Analysis
KW - United States/ep [Epidemiology]
RP - NOT IN FILE
SP - 309
EP - 315
JF - Pediatric Emergency Care
JA - Pediatr Emerg Care
VL - 22
IS - 5
CY - United States
N2 - OBJECTIVES: (1) To characterize the demographics and external causes of pediatric sports injury-related visits (SIRVs) to emergency departments (EDs). (2) To analyze the effect of race/ethnicity and insurance on SIRVs to EDs. METHODS: A stratified random-sample cross-sectional survey of EDs in the National Hospital Ambulatory Medical Care Survey was conducted from 1997-2001; for patients younger than 19 years, we used all visits [n = 33,654; injury-related visits (IRVs) = 13,496, SIRVs = 2990]. We examined both the external cause codes and the actual verbatim text of all IRVs. National estimates of pediatric IRVs were obtained using the assigned patient visit weights in the National Hospital Ambulatory Medical Care Survey databases and SUDAAN 9.1 software (SAS Institute, Inc., Cary, NC). RESULTS: Sports injuries resulted in 2.5 million visits annually, or 23% of ED IRVs. Male sex, older age (6-18 years), and white race/ethnicity are associated with higher rates of SIRVs. Cycling, basketball, playground injuries, and football resulted in the largest numbers of ED SIRVs. Leading diagnoses for SIRVs included fractures and dislocations, sprains and strains, open wounds, and contusions. Hispanic race/ethnicity was associated with lower rates of SIRVs across all insurance types. After controlling for demographic factors and insurance, Hispanic children were less likely to have an SIRV than white children (odds ratio, 0.7; 95% confidence interval, 0.6-0.9). CONCLUSIONS: Sports and recreation are the leading causes of pediatric ED IRVs. Hispanic children, regardless of insurance status, had lower rates of SIRVs than white children, which helps explain the lower rate of nonfatal IRVs to EDs among Hispanic youth
SN - 1535-1815
AD - Department of Pediatrics, University of Colorado at Denver, USA. Tamara.Simon@uchsc.edu
UR - 16714957
ER -

TY - JOUR
ID - 1250
T1 - New Orleans soil lead (Pb) cleanup using Mississippi River alluvium: need, feasibility, and cost
A1 - Mielke,H.W.
A1 - Powell,E.T.
A1 - Gonzales,C.R.
A1 - Mielke,P.W.,Jr.
A1 - Ottesen,R.T.
A1 - Langedal,M.
Y1 - 2006/04/15/
N1 - Mielke, Howard W. Powell, Eric T. Gonzales, Christopher R. Mielke, Paul W Jr. Ottesen, Rolf Tore. Langedal, Marianne
Environmental science & technology
dvo, 0213155
IM
Journal Article. Research Support, Non-U.S. Gov't. Research Support, U.S. Gov't, Non-P.H.S.
English
KW - MEDLINE
KW - Child
KW - Costs and Cost Analysis
KW - Dust/an [Analysis]
KW - Housing
KW - Humans
KW - Lead/an [Analysis]
KW - Lead/bl [Blood]

KW - Mississippi
KW - Rivers
KW - Soil Pollutants/an [Analysis]
KW - Soil Pollutants/bl [Blood]
KW - Waste Management/ec [Economics]
KW - Waste Management/mt [Methods]
RP - NOT IN FILE
SP - 2784
EP - 2789
JF - Environmental Science & Technology
JA - Environ Sci Technol
VL - 40
IS - 8
CY - United States
N2 - In New Orleans, LA prior to hurricane Katrina 20-30% of inner-city children had elevated blood Pb levels > or =10 microg/ dL and 10 census tracts had a median surface soil level of Pb >1000 mg/kg (2.5 times the U.S. standard). This project tests the feasibility of transporting and grading contaminated properties (n = 25) with 15 cm (6 in.) of clean Mississippi River alluvium from the Bonnet Carre Spillway (BCS) (median soil Pb content 4.7 mg/kg; range 1.7-22.8). The initial median surface soil Pb was 1051 mg/kg (maximum 19 627). After 680 metric tons (750 tons) of clean soil cover was emplaced on 6424 m2 (69 153 ft2), the median surface soil Pb decreased to 6 mg/kg (range 3-18). Interior entrance wipe samples were collected at 10 homes before and after soil treatment and showed a decreasing trend of Pb (p value = 0.048) from a median of 52 microg/ft2 to a median of 36 microg/ft2 (25th and 75th percentiles are 22 and 142 microg/ft2 and 12 and 61 microg/ft2, respectively). Average direct costs for properties with homes were $3,377 ($1.95 per square foot), with a range of $1,910-7,020, vs $2,622 ($0.61 per square foot), with a range of $2,400-3,040 for vacant lots. Approximately 40% (86,000) of properties in New Orleans are in areas of >400 mg Pb/kg soil and estimated direct costs for treatment are between $225.5 and $290.4 million. Annual costs of Pb poisoning in New Orleans are estimated at approximately $76 million in health, education, and societal harm. Urban accumulation of Pb is an international problem; for example, the new Government of Norway established a policy precedence for an isolated soil cleanup program at daycare centers, school playgrounds, and parks to protect children. New Orleans requires a community-wide soil cleanup program because of the extent and quantity of accumulated soil Pb. The post-Katrina benefits of reducing soil Pb are expected to outweigh the foreseeable costs of Pb poisoning to children returning to New Orleans
SN - 0013-936X
AD - College of Pharmacy, Xavier University, New Orleans, Louisiana, USA. howard.mielke@gmail.com
UR - 16683624
ER -

TY - JOUR
ID - 1251
T1 - Pedometer steps in primary school-aged children: a comparison of school-based and out-of-school activity
A1 - Cox,M.
A1 - Schofield,G.
A1 - Greasley,N.
A1 - Kolt,G.S.
Y1 - 2006/05//
N1 - Cox, M. Schofield, G. Greasley, N. Kolt, G S
Journal of science and medicine in sport / Sports Medicine Australia
c54, 9812598
IM
Comparative Study. Journal Article
English
KW - MEDLINE
KW - Age Factors
KW - Child
KW - Preschool
KW - Exercise/ph [Physiology]

KW - Female
KW - Humans
KW - Male
KW - Motor Activity/ph [Physiology]
KW - New Zealand
KW - Schools
KW - Sex Factors
RP - NOT IN FILE
SP - 91
EP - 97
JF - Journal of Science & Medicine in Sport
JA - J Sci Med Sport
VL - 9
IS - 1-2
CY - Australia
N2 - While studies of the physical activity habits of New Zealand children have been carried out, the findings have been restricted by the use of proxy and self-report measures and limited to total overall daily activity. Objective measurement of children's in-school and out-of school physical activity using pedometry is likely to provide more accurate data on habitual daily activity. To date, no such data are available for New Zealand children. In the present study, children from school years 1-6 (girls, n=46; boys, n=45) at a New Zealand primary school wore a Yamax Digiwalker SW-200 pedometer to record school-based and out-of-school steps over a 3-day period. Mean daily steps for the overall sample were 14 333 (S.D.=4110). Boys (X=15 606; S.D.=4601) were significantly more active than girls (X=13 031; S.D.=3079) (p=.00). Mean steps were also significantly higher in older age groups for both boys (p=.03) and in particular, girls (p=.00). Of note, for the overall sample, steps taken out of school made up 52.4% of total daily steps. Girls (53.6%) and boys (51.3%) took a similar proportion of their overall daily steps outside of the school environment. While a significant difference was found between the most and least active tertiles in steps taken during both during school hours (p=.00) and outside of school hours (p=.00), the most active third of the sample completed significantly more of their daily steps outside of school (55.1%) than did their least active (46.7%) counterparts (p=.00). These results suggest that physical activity outside of the school environment is a key contributor to a child's overall level of physical activity, reinforcing the need for interventions targeting the family and community as well as the school environment
SN - 1440-2440
AD - Centre for Physical Activity and Nutrition Research, Faculty of Health and Environmental Sciences, Auckland University of Technology, Private Bag 92006, Auckland 1020, New Zealand. michele.cox@aut.ac.nz
UR - 16580252
ER -

TY - JOUR
ID - 1252
T1 - Coalition-based approaches for addressing environmental issues in childhood asthma
A1 - Nicholas,E.A.
A1 - Dwyer,M.
A1 - Murphy,A.
A1 - Taylor-Fishwick,J.C.
A1 - Cohn,J.H.
A1 - Kelly,C.S.
A1 - Gould,C.
A1 - Di,Stefano D.
Y1 - 2006/04//
N1 - Nicholas, Elisa A. Dwyer, Maura. Murphy, Amy. Taylor-Fishwick, Judith C. Cohn, Jennifer H. Kelly, Cynthia S. Gould, Christine. Di Stefano, Daeman
Health promotion practice
100890609
IM
Journal Article
English

KW - MEDLINE
KW - Adolescent
KW - Asthma
KW - Child
KW - Community Networks/og [Organization & Administration]
KW - Environmental Exposure/pc [Prevention & Control]
KW - Humans
KW - United States
RP - NOT IN FILE
SP - 108S
EP - 116S
JF - Health Promotion Practice
JA - HEALTH PROMOT PRACT
VL - 7
IS - 2 Suppl
CY - United States
N2 - Outdoor air pollution and the indoor environment have been shown to cause or exacerbate childhood asthma. Allies Against Asthma coalitions implemented a broad range of activities to reduce or remediate indoor exposure to asthma environmental triggers for children with asthma including education and trigger remediation, physician and other health care provider education, and policy efforts to improve air quality in homes and schools. Outdoor environmental triggers were addressed through programs to increase education and awareness and efforts to change policies related to environmental conditions. Implementation of such efforts was complicated by the lack of safe and affordable housing and the amount of time and resources necessary to address policy change. Keys to success included promoting coordination and partnerships among coalition members, implementation of consistent messages, and building a broad and unified voice for policy change
SN - 1524-8399
AD - Long Beach Alliance for Children with Asthma (LBACA), The Children's Clinic, Serving Children and Their Families in Long Beach, CA, USA
UR - 16636161
ER -

TY - JOUR
ID - 1253
T1 - Association of parental and children behaviors with the health status of preschool children
A1 - Watanabe,M.
A1 - Nakamura,K.
A1 - Fukuda,Y.
A1 - Takano,T.
Y1 - 2006/04//
N1 - Watanabe, Miki. Nakamura, Keiko. Fukuda, Yoshiharu. Takano, Takehito
Preventive medicine
pm4, 0322116
IM
Journal Article. Research Support, Non-U.S. Gov't
English
KW - MEDLINE
KW - Child Welfare
KW - Child
KW - Preschool
KW - Cross-Sectional Studies
KW - Female
KW - Health Behavior
KW - Health Promotion
KW - Health Status
KW - Health Surveys
KW - Humans
KW - Interviews as Topic

KW - Japan
KW - Male
KW - Maternal Behavior
KW - Parent-Child Relations
KW - Parents/px [Psychology]
KW - Play and Playthings
KW - Social Behavior
KW - Social Environment
KW - Socioeconomic Factors
RP - NOT IN FILE
SP - 297
EP - 300
JF - Preventive Medicine
JA - Prev Med
VL - 42
IS - 4
CY - United States
N2 - BACKGROUND: Parental and children behaviors can be associated with health promotion and illness prevention in preschool children. METHODS: We interviewed mothers of 804 out of 1000 households with children aged 3 to 5 years randomly sampled in Tokyo, in 1997. Child health status was categorized as: "good", that is, good general health with no sick days; "poor", that is, fair to poor with frequent sick days; or "moderate". RESULTS.: Good health status was associated with playing outside (OR=1.19, 95% CI: 1.03, 1.37) and family income (OR=1.38-1.78, depending on family income). Mothers of children with poor health were less likely to have good health behavior themselves (OR=0.76, 95% CI: 0.64, 0.90) and were more often sick (OR=1.55, 95% CI: 1.24, 1.94). CONCLUSION: Outdoor playing, good health practice of the mother and high income are associated with a better health status of urban preschool children
SN - 0091-7435
AD - Health Promotion/International Health and Medicine, Division of Public Health, Graduate School of Tokyo Medical and Dental University, 1-5-45 Yushima, Bunkyo-ku, 113-8519, Tokyo, Japan
UR - 16490240
ER -

TY - JOUR
ID - 1254
T1 - Impact of preschool environment upon children's physical activity and sun exposure
A1 - Boldemann,C.
A1 - Blennow,M.
A1 - Dal,H.
A1 - Martensson,F.
A1 - Raustorp,A.
A1 - Yuen,K.
A1 - Wester,U.
Y1 - 2006/04//
N1 - Boldemann, Cecilia. Blennow, Margareta. Dal, Henrik. Martensson, Fredrika. Raustorp, Anders. Yuen, Katarina. Wester, Ulf
Preventive medicine
pm4, 0322116
IM
Journal Article. Research Support, Non-U.S. Gov't
English
KW - MEDLINE
KW - Child
KW - Preschool
KW - Environment Design
KW - Environmental Exposure/ae [Adverse Effects]
KW - Environmental Exposure/an [Analysis]
KW - Female

KW - Humans
KW - Male
KW - Motor Activity
KW - Plants
KW - Recreation
KW - Schools
KW - Sunlight/ae [Adverse Effects]
KW - Sweden
KW - Time Factors
KW - Trees
KW - Ultraviolet Rays/ae [Adverse Effects]
RP - NOT IN FILE
SP - 301
EP - 308
JF - Preventive Medicine
JA - Prev Med
VL - 42
IS - 4
CY - United States
N2 - BACKGROUND: The physical qualities of outdoor environments are important to trigger healthy behavior in children. We studied the impact of outdoor environments upon spontaneous physical activity and exposure to ultraviolet (UV) radiation in 4- to 6-year-old children at 11 preschools in Stockholm county. METHODS: In May-June 2004, pedometry and measurement of UV radiation were carried out on 197 children from 11 preschools in Stockholm county. Outdoor environments differed regarding vegetation, topography, space, and education. Ambient global UV radiation data were collected, free sky, and ground surface assessed. Arrival, in- and outdoor stay, and departure were recorded. For analysis, linear mixed model analysis was applied. RESULTS: In environments with trees, shrubbery, and broken ground, the mean step count/min was 21.5, and mean exposure to UV radiation as fraction of available UV during play outdoors 14.6%. In delimited environments with little vegetation, the mean step count/min was 17.7 and mean exposure fraction to UV radiation 24.3% ($P < 0.001$, crude). Step/min range was 8.9-30.0 (girls) and 8.8-37.2 (boys), UV radiation exposure range 4-60% (no difference between genders). CONCLUSION: Spacious preschool environments with trees, shrubbery, and broken ground trigger physical activity and yield sun protection in outdoor play. As many children attend preschool, access to such environments is recommended in community architecture
SN - 0091-7435
AD - Center for Public Health, Stockholm County Council, Stockholm, Sweden; Department of Public Health Sciences, Karolinska Institute, Stockholm, Sweden. cecilia.bildemann@sll.se
UR - 16448688
ER -

TY - JOUR
ID - 1255
T1 - Who died as a result of the tsunami? Risk factors of mortality among internally displaced persons in Sri Lanka: a retrospective cohort analysis
A1 - Nishikiori,N.
A1 - Abe,T.
A1 - Costa,D.G.
A1 - Dharmaratne,S.D.
A1 - Kunii,O.
A1 - Moji,K.
Y1 - 2006///
N1 - Nishikiori, Nobuyuki. Abe, Tomoko. Costa, Dehiwala G M. Dharmaratne, Samath D. Kunii, Osamu. Moji, Kazuhiko
BMC public health
100968562
IM
Journal Article. Research Support, Non-U.S. Gov't
English

KW - MEDLINE
KW - Adolescent
KW - Adult
KW - Age Distribution
KW - Aged
KW - Child
KW - Preschool
KW - Cohort Studies
KW - Disasters/sn [Statistics & Numerical Data]
KW - Female
KW - Geography
KW - Humans
KW - Indian Ocean
KW - Infant
KW - Newborn
KW - Logistic Models
KW - Male
KW - Middle Aged
KW - Mortality
KW - Population Dynamics
KW - Population Surveillance/mt [Methods]
KW - Refugees/sn [Statistics & Numerical Data]
KW - Rescue Work
KW - Retrospective Studies
KW - Risk Factors
KW - Sex Distribution
KW - Sri Lanka/ep [Epidemiology]
RP - NOT IN FILE
SP - 73
JF - BMC Public Health
VL - 6
CY - England
N2 - BACKGROUND: Describing adverse health effects and identifying vulnerable populations during and after a disaster are important aspects of any disaster relief operation. This study aimed to describe the mortality and related risk factors which affected the displaced population over a period of two and a half months after the 2004 Indian Ocean tsunami in an eastern coastal district of Sri Lanka. METHODS: A cross-sectional household survey was conducted in 13 evacuation camps for internally displaced persons (IDP). Information on all pre-tsunami family members was collected from householders, and all deaths which occurred during the recall period (77 to 80 days starting from the day of the tsunami) were recorded. The distribution of mortality and associated risk factors were analysed. Logistic regression modelling using the generalized estimating equations method was applied in multivariate analysis. RESULTS: Overall mortality rate out of 3,533 individuals from 859 households was 12.9% (446 deaths and 11 missing persons). The majority of the deaths occurred during and immediately after the disaster. A higher mortality was observed among females (17.5% vs. 8.2% for males, $p < 0.001$), children and the elderly (31.8%, 23.7% and 15.3% for children aged less than 5 years, children aged 5 to 9 years and adults over 50 years, respectively, compared with 7.4% for adults aged 20 to 29 years, $p < 0.001$). Other risk factors, such as being indoors at the time of the tsunami (13.8% vs. 5.9% outdoors, $p < 0.001$), the house destruction level (4.6%, 5.5% and 14.2% in increasing order of destruction, $p < 0.001$) and fishing as an occupation (15.4% vs. 11.2% for other occupations, $p < 0.001$) were also significantly associated with increased mortality. These correlations remained significant after adjusting for the confounding effects by multivariate analysis. CONCLUSION: A significantly high mortality was observed in women and children among the displaced population in the eastern coastal district of Sri Lanka who were examined by us. Reconstruction activities should take into consideration these changes in population structure
SN - 1471-2458
AD - Research Center for Tropical Infectious Diseases, Institute of Tropical Medicine, Nagasaki University, Nagasaki, Japan. nobu@net.nagasaki-u.ac.jp
UR - 16545145
ER -

TY - JOUR
ID - 1256
T1 - Physical activity levels of children during school playtime. [Review] [59 refs]
A1 - Ridgers,N.D.
A1 - Stratton,G.
A1 - Fairclough,S.J.
Y1 - 2006///
N1 - Ridgers, Nicola D. Stratton, Gareth. Fairclough, Stuart J
Sports medicine (Auckland, N.Z.)
8412297, spo
IM
Journal Article. Review
English
KW - MEDLINE
KW - Child
KW - Energy Metabolism/ph [Physiology]
KW - Female
KW - Humans
KW - Male
KW - Motor Activity/ph [Physiology]
KW - Physical Education and Training
KW - Schools
KW - Sex Factors
KW - Time Factors
RP - NOT IN FILE
SP - 359
EP - 371
JF - Sports Medicine
JA - Sports Med
VL - 36
IS - 4
CY - New Zealand
N2 - School represents a suitable setting for intervention programmes aiming to promote physical activity to benefit health. During the school day, physical education and school playtime offer children regular opportunities to engage in physical activity. However, there is growing concern that, internationally, curricular time allocated to physical education is not meeting statutory guidelines. The effectiveness of the playground environment to promote physical activity has been considered as a complementary setting to physical education. Physical activity guidelines state that children should engage in at least 1 hour of moderate intensity physical activity a day. Currently no empirically tested guidelines exist for physical activity levels during playtime. However, studies cited in this article indicate that playtime can contribute between 5-40% of recommended daily physical activity levels when no interventions have been utilised. The limited school-based investigations that have been reported in the literature suggest that boys engage in more physical activity during playtime than girls. Studies that have implemented intervention strategies in order to promote physical activity levels indicate that playtime can substantially contribute towards daily optimal physical activity guidelines. Energy expenditure and physical activity levels have increased during playtime following the implementation of playtime-based interventions. In order to advance knowledge of children's physical activity during playtime, a number of key issues for consideration in future research are detailed. Research on children's use of playtime to be physically active and the extent of the contribution of playtime to daily physical activity guidelines is warranted. [References: 59]
SN - 0112-1642
AD - Research Institute for Sport and Exercise Sciences, Liverpool John Moores University, Liverpool, UK. n.ridgers@ljmu.ac.uk
UR - 16573359
ER -

TY - JOUR

ID - 1257
T1 - A probabilistic arsenic exposure assessment for children who contact chromated copper arsenate (CCA)-treated playsets and decks, Part 2: Sensitivity and uncertainty analyses
A1 - Xue,J.
A1 - Zartarian,V.G.
A1 - Ozkaynak,H.
A1 - Dang,W.
A1 - Glen,G.
A1 - Smith,L.
A1 - Stallings,C.
Y1 - 2006/04//
N1 - Xue, Jianping. Zartarian, Valerie G. Ozkaynak, Haluk. Dang, Winston. Glen, Graham. Smith, Luther. Stallings, Casson
Risk analysis : an official publication of the Society for Risk Analysis
8109978, ria
IM
Journal Article
English
KW - MEDLINE
KW - Arsenates/ae [Adverse Effects]
KW - Arsenic/ad [Administration & Dosage]
KW - Arsenic/ae [Adverse Effects]
KW - Child
KW - Environmental Exposure
KW - Housing
KW - Humans
KW - Models
KW - Statistical
KW - Monte Carlo Method
KW - Play and Playthings
KW - Risk Assessment/sn [Statistics & Numerical Data]
KW - Risk Assessment
KW - Sensitivity and Specificity
KW - Soil Pollutants/ae [Adverse Effects]
KW - Uncertainty
KW - United States
KW - United States Environmental Protection Agency
KW - Wood
RP - NOT IN FILE
SP - 533
EP - 541
JF - Risk Analysis
JA - Risk Anal
VL - 26
IS - 2
CY - United States
N2 - A probabilistic model (SHEDS-Wood) was developed to examine children's exposure and dose to chromated copper arsenate (CCA)-treated wood, as described in Part 1 of this two-part article. This Part 2 article discusses sensitivity and uncertainty analyses conducted to assess the key model inputs and areas of needed research for children's exposure to CCA-treated playsets and decks. The following types of analyses were conducted: (1) sensitivity analyses using a percentile scaling approach and multiple stepwise regression; and (2) uncertainty analyses using the bootstrap and two-stage Monte Carlo techniques. The five most important variables, based on both sensitivity and uncertainty analyses, were: wood surface residue-to-skin transfer efficiency; wood surface residue levels; fraction of hand surface area mouthed per mouthing event; average fraction of nonresidential outdoor time a child plays on/around CCA-treated public playsets; and frequency of hand washing. In general, there was a factor of 8 for the 5th and 95th percentiles and a factor of 4 for the 50th percentile in the uncertainty of predicted population dose estimates due to parameter uncertainty. Data were

available for most of the key model inputs identified with sensitivity and uncertainty analyses; however, there were few or no data for some key inputs. To evaluate and improve the accuracy of model results, future measurement studies should obtain longitudinal time-activity diary information on children, spatial and temporal measurements of residue and soil concentrations on or near CCA-treated playsets and decks, and key exposure factors. Future studies should also address other sources of uncertainty in addition to parameter uncertainty, such as scenario and model uncertainty
SN - 0272-4332
AD - U.S. Environmental Protection Agency, Office of Research and Development, National Exposure Research Laboratory, Research Triangle Park, NC, USA
UR - 16573638
ER -

TY - JOUR
ID - 1258
T1 - Malaria prevention in The Gambia: patterns of expenditure and determinants of demand at the household level
A1 - Wiseman,V.
A1 - McElroy,B.
A1 - Conteh,L.
A1 - Stevens,W.
Y1 - 2006/04//
N1 - Wiseman, V. McElroy, B. Conteh, L. Stevens, W
Tropical medicine & international health : TM & IH
cf5, 9610576
IM
Journal Article. Research Support, Non-U.S. Gov't
English
KW - MEDLINE
KW - Adult
KW - Aerosols/ec [Economics]
KW - Bedding and Linens/ec [Economics]
KW - Child
KW - Preschool
KW - Environment
KW - Female
KW - Gambia/ep [Epidemiology]
KW - Health Expenditures
KW - Health Services Needs and Demand
KW - Humans
KW - Insecticides/ec [Economics]
KW - Malaria/ec [Economics]
KW - Malaria/ep [Epidemiology]
KW - Malaria/pc [Prevention & Control]
KW - Male
KW - Middle Aged
KW - Seasons
KW - Socioeconomic Factors
RP - NOT IN FILE
SP - 419
EP - 431
JF - Tropical Medicine & International Health
JA - Trop Med Int Health
VL - 11
IS - 4
CY - England
N2 - OBJECTIVE: To provide a better understanding of (1) the amounts households in The Gambia spend on a wide variety of malaria prevention measures, (2) how expenditure fluctuates throughout the year and (3) the

main determinants of expenditure. METHODS: A random sample of 1700 households from the Farafenni region were interviewed about their expenditure on malaria prevention over the past 2 weeks. Interviews were staggered over 12 months. Expenditure was measured for bed nets, treating and repairing bed nets, aerosols, coils, indoor spraying, smoke and other prevention strategies such as drinking herbs and cleaning the outside environment. Results Expenditure on bed nets, including treatment and repair, constituted only 10% of total expenditure on malaria prevention. Every fortnight, households spent an average of 8.40 Dalasis (D) on coils, 4.20 D on indoor sprays, 3.09 D on smoke and 3.06 D on aerosols, together making up 81% of total fortnightly expenditure. Of the 442 households that did not own a bed net, 68% said it was because they could not afford one. Every 2 months, the same households spent an average of US 5 dollars, the equivalent to the cost of an insecticide treated bed net, on other forms of prevention. Total expenditure was 42% higher during the wet season than for the rest of the year. For every month of the year, coils were the dominant form of prevention expenditure. Wealth, age, occupation of household head, location of residence and month of the year were significant determinants of prevention expenditure. CONCLUSIONS: Households in The Gambia spend considerable amounts on a range of malaria prevention products and activities throughout the year. Bed nets represent a relatively small proportion of this expenditure even though they are perceived to be the most efficient and effective method of malaria control. A more concerted effort is needed to develop appropriate targeting strategies to encourage bed net use especially for children <5 years of age. Equal emphasis should be given to addressing barriers to purchasing nets such as their relative high upfront cost
SN - 1360-2276
AD - Gates Malaria Partnership, Gates Malaria Partnership and Health Policy Unit, London School of Hygiene and Tropical Medicine, London, UK. virginia.wiseman@lshtm.ac.uk
UR - 16553925
ER -

TY - JOUR
ID - 1259
T1 - Smoking in film and impact on adolescent smoking: with special reference to European adolescents. [Review] [91 refs]
A1 - Sargent,J.D.
Y1 - 2006/02//
N1 - Sargent, J D
Minerva pediatrica
nam, 0400740
IM
Journal Article. Review
English
KW - MEDLINE
KW - Adolescent
KW - Adolescent Behavior
KW - Adolescent Psychology
KW - Advertising as Topic
KW - Child
KW - Cross-Sectional Studies
KW - Europe
KW - Humans
KW - Motion Pictures as Topic
KW - Parenting
KW - Public Policy
KW - Smoking/px [Psychology]
KW - Social Conformity
KW - Tobacco Industry
KW - United States
RP - NOT IN FILE
SP - 27
EP - 45
JF - Minerva Pediatrica
JA - Minerva Pediatr

VL - 58
IS - 1
CY - Italy
N2 - This review examines the evidence supporting an association between seeing smoking depictions in movies and adolescent smoking. The portrayal of tobacco use is common in movies and often modeled by movie stars who, from a social influences standpoint, should be powerful behavior change agents. The results of studies assessing audience responses to tobacco portrayal in movies are remarkably consistent in showing a moderate to strong association between seeing movie smoking and more positive attitudes toward smoking and adolescent smoking initiation. The population-based data include cross sectional samples from different regions of the United States, all supporting a movie smoking-teen smoking link. The 2 published longitudinal studies show an independent link between exposure to movie smoking at baseline and initiation in the future, with estimates of the effect size being remarkably consistent with their cross-sectional counterparts. Experimental research adds support by showing that scene depictions of smoking enhance positive views of smokers and increase intent to smoke in the future. Taken as a whole, this rich research base provides very strong support for the notion that movie smoking plays a role in smoking initiation among adolescents that warrants action at the individual and societal level. A major gap in our understanding is the impact of Hollywood movies on adolescents outside the United States. There is a real need for studies to be conducted in European and other populations to better understand the global reach of smoking in American film, since over half of box office revenues come from outside the United States. [References: 91]
SN - 0026-4946
AD - Unit of Pediatrics, Dartmouth Medical School, Lebanon, NH 03756, USA.
james.D.sargent@hitchcock.org
UR - 16541005
ER -

TY - JOUR
ID - 1260
T1 - [Severe primary congenital unilateral hydronephrosis. A review of 98 cases]. [Spanish]
A1 - Areses,Trapote R.
A1 - Urbieta Garagorri,M.A.
A1 - Ubetagoyena,Arrieta M.
A1 - Arruebarrena,Lizarraga D.
A1 - zueta Beneite,M.T.
A1 - Eizaguirre,Sexmilo,I
A1 - Rodriguez,Mazorriaga F.
A1 - Esparza,Paz P.
A1 - Emparanza Knorr,J.I.
Y1 - 2006/01//
N1 - Areses Trapote, R. Urbieta Garagorri, M A. Ubetagoyena Arrieta, M. Arruebarrena Lizarraga, D. Alzueta Beneite, M T. Eizaguirre Sexmilo, I. Rodriguez Mazorriaga, F. Esparza Paz, P. Emparanza Knorr, J I
Anales de pediatria (Barcelona, Spain : 2003)
101162596
IM
English Abstract. Journal Article
Spanish
KW - MEDLINE
KW - Child
KW - Preschool
KW - Female
KW - Humans
KW - Hydronephrosis/cn [Congenital]
KW - Hydronephrosis/su [Surgery]
KW - Infant
KW - Newborn
KW - Male
KW - Remission
KW - Spontaneous

KW - Retrospective Studies
KW - Treatment Outcome
RP - NOT IN FILE
SP - 11
EP - 20
JF - Anales de Pediatria
JA - An Pediatr (Barc)
VL - 64
IS - 1
CY - Spain
N2 - INTRODUCTION: Currently, consensus is lacking on the indications for surgery in primary congenital unilateral hydronephrosis. OBJECTIVES: To analyze the clinical characteristics, treatment and outcome of severe asymptomatic primary congenital unilateral hydronephrosis (grades 3 and 4). PATIENTS AND METHODS: We performed a retrospective study of 98 neonates with severe primary congenital unilateral hydronephrosis. Diagnostic techniques consisted of ultrasound, renogram, and DMSA scan. RESULTS: The mean age at diagnosis was 24 days. The mean length of follow-up was 4 years. Forty-eight percent of cases were grade 3 and the remaining cases were grade 4. Fifty-six percent of the patients received conservative treatment. Forty-four percent underwent surgery. Surgical indications consisted of prolonged T1/2, reduced differential renal function and/or very severe hydronephrosis. The mean age at surgery was 5.5 months. In 94 % of the patients who received conservative treatment, hydronephrosis resolved spontaneously. In this group, renal parenchyma and renal function were normal in all patients except two. In 97 % of the patients who received surgical treatment, hydronephrosis was corrected or improved after pyeloplasty. At diagnosis, 19 kidneys had reduced function. In the postsurgical renogram, although T1/2 had markedly improved, 8 kidneys showed reduced function. DMSA performed in 63 patients belonging to both groups, outside the active phase of hydronephrosis, revealed 8 atrophic kidneys, 4 with moderately reduced renal size and function and 4 with scarring and normal renal size and function. During the study period, overall function was maintained in all patients and none developed hypertension. CONCLUSIONS: Severe congenital unilateral hydronephrosis resolves spontaneously in most patients. Consequently, clinicians increasingly adopt a conservative approach. The available diagnostic techniques cannot identify patients who will benefit from pyeloplasty. In general, accepted surgical indications are an increase in hydronephrosis and/or worsening of renal function. In our series, 15 % of the patients with hydronephrosis had an irreversible lesion of variable severity, which in some patients seemed to have developed during embryogenesis. Kidneys at risk of presenting a lesion were those that had grade 4 hydronephrosis
SN - 1695-4033
AD - Seccion de Nefrologia Pediatrica, Instituto Oncologico de Guipuzcoa, Hospital Donostia, San Sebastian, Spain. rareses@chdo.osakidetza.net
UR - 16539911
ER -

TY - JOUR
ID - 1261
T1 - Acute pyomyositis of the pelvis: the spectrum of clinical presentations and MR findings
A1 - Karmazyn,B.
A1 - Kleiman,M.B.
A1 - Buckwalter,K.
A1 - Loder,R.T.
A1 - Siddiqui,A.
A1 - Applegate,K.E.
Y1 - 2006/04//
N1 - Karmazyn, Boaz. Kleiman, Martin B. Buckwalter, Kenneth. Loder, Randall T. Siddiqui, Aslam. Applegate, Kimberly E
Pediatric radiology
owi, 0365332
IM
Clinical Trial. Journal Article
English
KW - MEDLINE

KW - Acute Disease
KW - Adolescent
KW - Child
KW - Preschool
KW - Female
KW - Hip Joint/pa [Pathology]
KW - Humans
KW - Infant
KW - Magnetic Resonance Imaging/mt [Methods]
KW - Male
KW - Myositis/di [Diagnosis]
KW - Pelvic Infection/di [Diagnosis]
KW - Prognosis
KW - Reproducibility of Results
KW - Retrospective Studies
KW - Sensitivity and Specificity
KW - Staphylococcal Infections/di [Diagnosis]
RP - NOT IN FILE
SP - 338
EP - 343
JF - Pediatric Radiology
JA - Pediatr Radiol
VL - 36
IS - 4
CY - Germany
N2 - BACKGROUND: Acute pelvic pyomyositis is uncommon in non-tropical areas. OBJECTIVE: To summarize the clinical and MR findings in children with acute pelvic pyomyositis. MATERIALS AND METHODS: We retrospectively identified 20 children (mean age 9.4 years) who were evaluated by MR and diagnosed with acute pelvis pyomyositis during the time period between January 2002 and June 2005. We reviewed clinical, laboratory, and imaging findings. RESULTS: Fifteen of the 20 children had secondary pyomyositis associated with osteomyelitis (n=13), septic hip (n=4) or sacroiliitis (n=4); all were previously healthy except for one child with leukemia. Seven of the children with secondary pyomyositis underwent bone scintigraphy; three (43%) did not show pelvic abnormalities. Staphylococcus aureus was cultured in 13 of the 15 (87%) children. Five of the 20 children had primary pyomyositis. Three had underlying disease and two others were engaged in vigorous physical activity. Bone scintigraphies (n=2) were negative. Cultures were positive for S. aureus in three of the five (60%) children. CONCLUSION: Septic hip should be the first diagnostic consideration in children with fever and acute hip pain. Pyomyositis should be considered if arthrocentesis is negative or there is clinical suspicion of infection outside the hip joint. MR is the preferred imaging modality for evaluating foci of pyomyositis, muscle abscesses, and additional foci of infection within the pelvis
SN - 0301-0449
AD - Department of Radiology, Indiana University School of Medicine, Indianapolis, IN, USA.
bkarmazy@iupui.edu
UR - 16474970
ER -

TY - JOUR
ID - 1262
T1 - The role of schools in obesity prevention
A1 - Story,M.
A1 - Kaphingst,K.M.
A1 - French,S.
Y1 - 2006///
N1 - Story, Mary. Kaphingst, Karen M. French, Simone
The Future of children / Center for the Future of Children, the David and Lucile Packard Foundation
9306342, b4m
IM

Journal Article
English
KW - MEDLINE
KW - Adolescent
KW - Child
KW - Child Nutritional Physiological Phenomena
KW - Preschool
KW - Food Services/lj [Legislation & Jurisprudence]
KW - Food Services
KW - Health Education
KW - Health Policy
KW - Humans
KW - National Health Programs
KW - Obesity/pc [Prevention & Control]
KW - Physical Education and Training/lj [Legislation & Jurisprudence]
KW - Physical Education and Training
KW - School Health Services
KW - Schools
KW - United States
RP - NOT IN FILE
SP - 109
EP - 142
JF - Future of Children
JA - Future Child
VL - 16
IS - 1
CY - United States
N2 - Mary Story, Karen Kaphingst, and Simone French argue that U.S. schools offer many opportunities for developing obesity-prevention strategies by providing more nutritious food, offering greater opportunities for physical activity, and providing obesity-related health services. Meals at school are available both through the U.S. Department of Agriculture's school breakfast and lunch programs and through "competitive foods" sold a la carte in cafeterias, vending machines, and snack bars. School breakfasts and school lunches must meet federal nutrition standards, but competitive foods are exempt from such requirements. And budget pressures force schools to sell the popular but nutritionally poor foods a la carte. Public discomfort with the school food environment is growing. But can schools provide more healthful food options without losing money? Limited evidence shows that they can. Although federal nutrition regulations are inadequate, they permit state and local authorities to impose additional restrictions. And many are doing so. Some states limit sales of nonnutritious foods, and many large school districts restrict competitive foods. Several interventions have changed school food environments, for example, by reducing fat content of food in vending machines and making more fruits and vegetables available. Interventions are just beginning to target the availability of competitive foods. Other pressures can also compromise schools' efforts to encourage physical activity. As states use standardized tests to hold schools and students academically accountable, physical education and recess have become a lower priority. But some states are now mandating and promoting more physical activity in schools. School health services can also help address obesity by providing screening, health information, and referrals to students, especially low-income students, who are at high risk of obesity, tend to be underinsured, and may not receive health services elsewhere
SN - 1054-8289
AD - Division of Epidemiology and Community Health, School of Public Health, University of Minnesota, USA
UR - 16532661
ER -

TY - JOUR
ID - 1263
T1 - Analysis of RAG expression by peripheral blood CD5+ and CD5- B cells of patients with childhood systemic lupus erythematosus
A1 - Morbach,H.

A1 - Singh,S.K.
A1 - Faber,C.
A1 - Lipsky,P.E.
A1 - Girschick,H.J.
Y1 - 2006/04//
N1 - Morbach, H. Singh, S K. Faber, C. Lipsky, P E. Girschick, H J
Annals of the rheumatic diseases
0372355, 62w
IM
Journal Article
English
KW - MEDLINE
KW - Adolescent
KW - Antigens
KW - CD5/bl [Blood]
KW - Antirheumatic Agents/tu [Therapeutic Use]
KW - B-Lymphocyte Subsets/im [Immunology]
KW - CD40 Ligand/bi [Biosynthesis]
KW - CD40 Ligand/ge [Genetics]
KW - Child
KW - Cyclophosphamide/tu [Therapeutic Use]
KW - DNA
KW - Complementary/ge [Genetics]
KW - DNA-Binding Proteins/bi [Biosynthesis]
KW - DNA-Binding Proteins/ge [Genetics]
KW - Female
KW - Flow Cytometry/mt [Methods]
KW - Gene Expression
KW - Gene Library
KW - Homeodomain Proteins/bi [Biosynthesis]
KW - Homeodomain Proteins/ge [Genetics]
KW - Humans
KW - Immunoglobulin D/bl [Blood]
KW - Immunoglobulin Light Chains
KW - Surrogate
KW - Lupus Erythematosus
KW - Systemic/dt [Drug Therapy]
KW - Systemic/im [Immunology]
KW - Male
KW - Membrane Glycoproteins/bi [Biosynthesis]
KW - Membrane Glycoproteins/ge [Genetics]
KW - RNA
KW - Messenger/ge [Genetics]
KW - Reverse Transcriptase Polymerase Chain Reaction/mt [Methods]
RP - NOT IN FILE
SP - 482
EP - 487
JF - Annals of the Rheumatic Diseases
JA - Ann Rheum Dis
VL - 65
IS - 4
CY - England
N2 - BACKGROUND: The assembly of immunoglobulin genes during B cell development in the bone marrow is dependent on the expression of recombination activating genes (RAG) 1 and 2. Recently, RAG expression in peripheral blood IgD+ B cells outside the bone marrow has been demonstrated and is associated with the development of autoimmune diseases. OBJECTIVE: To investigate RAG expression in the CD5+ or CD5- IgD+ B cell compartment in childhood systemic lupus erythematosus (SLE). METHODS: Using a combination of

flow cytometric cell sorting and reverse transcriptase polymerase chain reaction analysis of cDNA libraries generated from individual cells, the expression of RAG, VpreB, and CD154 mRNA by individual peripheral blood B cells of three paediatric SLE patients was examined in detail. RESULTS: While only one patient had a significantly increased frequency of RAG+ B cells in the CD5- B cell population, all patients showed higher frequencies of RAG+ B cells in the CD5+IgD+ B cell population. The frequency of RAG+ IgD+CD5+/- B cells was reduced during intravenous cyclophosphamide treatment. In healthy age matched children, RAG expressing IgD+ B cells were hardly detectable. Coexpression of RAG and VpreB or CD154 mRNA could only be found in SLE B cells. CONCLUSIONS: RAG expression in peripheral blood B cells of SLE patients is particularly increased in the IgD+CD5+ B cell population. CD5+ and CD5- B cells in SLE have the potential to undergo receptor revision leading to the generation of high affinity pathogenic autoantibodies
SN - 0003-4967
AD - Section of Paediatric Rheumatology, Children's Hospital, University of Wurzburg, Josef-Schneider-Str 2, 97080 Wurzburg, Germany
UR - 16126793
ER -

TY - JOUR
ID - 1264
T1 - Emission of ammonia from indoor concrete wall and assessment of human exposure
A1 - Bai,Z.
A1 - Dong,Y.
A1 - Wang,Z.
A1 - Zhu,T.
Y1 - 2006/04//
N1 - Bai, Z. Dong, Y. Wang, Z. Zhu, T
Environment international
du1, 7807270
IM
Journal Article. Research Support, Non-U.S. Gov't
English
KW - MEDLINE
KW - Adult
KW - Air Pollutants/an [Analysis]
KW - Air Pollution
KW - Indoor/an [Analysis]
KW - Ammonia/an [Analysis]
KW - Child
KW - Construction Materials
KW - Environmental Monitoring
KW - Facility Design and Construction
KW - Humans
KW - Inhalation Exposure/an [Analysis]
KW - Urea
KW - Volatilization
RP - NOT IN FILE
SP - 303
EP - 311
JF - Environment International
JA - Environ Int
VL - 32
IS - 3
CY - United States
N2 - Addition of urea-based antifreeze admixtures during cement mixing can make it possible to produce concrete cement in construction of buildings in cold weather; this, however, has led to increasing indoor air pollution due to continuous transformation and emission from urea to gaseous ammonia in indoor concrete wall. It is believed that ammonia is harmful to human body and exposure to ammonia can cause some serious symptoms such as headaches, burns, and even permanent damage to the eyes and lungs. In order to understand

the emission of ammonia from indoor concrete wall in civil building and assess the health risk of people living in these buildings, the experimental pieces of concrete wall were first prepared by concreting cement and urea-based antifreeze admixtures to simulate the indoor wall in civil building in this work. Then environmental chamber was adopted for studying the effect of temperature, relative humility and air exchange rate on emission of ammonia from experimental pieces of concrete wall. Also the field experiment was made at selected rooms in given civil buildings. Exposure and potential dose of adult and children exposed to indoor/outdoor ammonia in summer and in winter are calculated and evaluated by using Scenario Evaluation Approach. The results indicated that high air exchange rate leads to decreased ammonia concentration, and elevation of temperature causes increasing ammonia concentration and volatilizing rate in chamber. The complete emission of ammonia from the wall containing urea-based antifreeze admixtures needs more than 10 years in general. Ventilating or improving air exchange can play a significant role in reducing ammonia concentration in actual rooms in field experiments. Urea-based antifreeze admixtures in concrete wall can give rise to high exposure and potential dose, especially in summer. Generally, adults have a high potential dose than children, while children have personal average dose rate beyond adults in the same conditions
SN - 0160-4120
AD - State Environmental Protection Key Laboratory of Urban Ambient Air Particulate Matter Pollution Prevention and Control, College of Environmental Sciences and Engineering, Nankai University, Tianjin 300071, PR China
UR - 16061286
ER -

TY - JOUR
ID - 1265
T1 - Chromium on the hands of children after playing in playgrounds built from chromated copper arsenate (CCA)-treated wood
A1 - Hamula,C.
A1 - Wang,Z.
A1 - Zhang,H.
A1 - Kwon,E.
A1 - Li,X.F.
A1 - Gabos,S.
A1 - Le,X.C.
Y1 - 2006/03//
N1 - Hamula, Camille. Wang, Zhongwen. Zhang, Hongquan. Kwon, Elena. Li, Xing-Fang. Gabos, Stephan. Le, X Chris
Environmental health perspectives
ei0, 0330411
IM
Journal Article. Research Support, Non-U.S. Gov't
English
KW - MEDLINE
KW - Arsenates
KW - Arsenic/an [Analysis]
KW - Canada
KW - Child
KW - Preschool
KW - Chromium/an [Analysis]
KW - Copper/an [Analysis]
KW - Environmental Exposure/an [Analysis]
KW - Environmental Monitoring
KW - Environmental Pollutants/an [Analysis]
KW - Hand
KW - Humans
KW - Play and Playthings
KW - Silicon Dioxide/an [Analysis]
KW - Soil/an [Analysis]
KW - Wood

RP - NOT IN FILE
SP - 460
EP - 465
JF - Environmental Health Perspectives
JA - Environ Health Perspect
VL - 114
IS - 3
CY - United States
N2 - Children's exposure to arsenic and chromium from playground equipment constructed with chromated copper arsenate (CCA)-treated wood is a potential concern because of children's hand-to-mouth activity. However, there exists no direct measure of Cr levels on the hands of children after playing in such playgrounds. In this study we measured both soluble and total Cr on the hands of 139 children playing in playgrounds, eight of which were constructed with CCA-treated wood and eight of which were not. Children's age and duration of play were recorded. The hands of each child were washed after play with 150 mL deionized water, which was collected in a bag and subsequently underwent analysis of Cr and 20 other elements, using inductively coupled plasma mass spectrometry. Total average Cr on the hands of 63 children who played in CCA playgrounds was 1,112 +/- 1,089 ng (median, 688; range 78-5,875). Total average Cr on the hands of 64 children who played in non-CCA playgrounds was 652 +/- 586 ng (median, 492; range 61-3,377). The difference between the two groups is statistically significant (p < 0.01). Cr levels were highly correlated to both Cu (r = 0.672) and As (r = 0.736) levels in CCA playgrounds (p < or = 0.01), but not non-CCA playgrounds (r = 0.252 and 0.486 for Cu and As, respectively). Principal-component analysis indicates that Cr, Cu, and As are more closely grouped together in CCA than in non-CCA playgrounds. These results suggest that the elevated levels of Cr and As on children's hands are due to direct contact with CCA wood
SN - 0091-6765
AD - Environmental Health Sciences, Department of Public Health Sciences, University of Alberta, Edmonton, Alberta, Canada
UR - 16507472
ER -

TY - JOUR
ID - 1266
T1 - Current management of blunt splenic trauma in children
A1 - Thompson,S.R.
A1 - Holland,A.J.
Y1 - 2006/01//
N1 - Thompson, Stephen R. Holland, Andrew J A
ANZ journal of surgery
dze, 101086634
IM
Journal Article
English
KW - MEDLINE
KW - Adolescent
KW - Child
KW - Female
KW - Humans
KW - Injury Severity Score
KW - Length of Stay
KW - Male
KW - Retrospective Studies
KW - Spleen/in [Injuries]
KW - Spleen/su [Surgery]
KW - Wounds
KW - Nonpenetrating/su [Surgery]
RP - NOT IN FILE
SP - 48
EP - 52

JF - ANZ Journal of Surgery
JA - ANZ J Surg
VL - 76
IS - 1-2
CY - Australia
N2 - BACKGROUND: Non-operative management of the great majority of blunt splenic injuries in children has become routine. Debate continues on the need for intensive care unit (ICU) admission, follow-up imaging and the duration of physical activity restrictions following injury. The purpose of this study was to review the recent experience of an Australian Paediatric Trauma Centre with splenic trauma to define current practice. METHODS: A retrospective chart review of patients with splenic trauma admitted to the Children's Hospital at Westmead between November 1995 and December 2003. RESULTS: A total of 39 patients with blunt splenic trauma were identified: 20 (51%) were multiply injured. Thirty-three (85%) children were managed non-operatively. The most common initial imaging method was computed tomography (n = 28, 72%). Fourteen patients (36%) were admitted to the ICU with a mean length of stay (LOS) of 4.1 days (range 1-13 days). The overall mean LOS was 10.8 days (range 1-43 days). Nineteen patients (50%) had imaging studies performed after diagnosis but before discharge. Further post-discharge imaging was carried out in 21 cases (54%). There were no deaths, but 10 patients developed complications. The mean documented activity restriction was 7.4 weeks (range 1-16 weeks). CONCLUSION: The majority of children who had suffered blunt splenic trauma were safely managed non-operatively outside an ICU. In stable patients, there appeared to be no benefits associated with repeated imaging following the diagnosis of splenic trauma. Physical activity restriction in excess of 3-4 weeks did not appear to be warranted
SN - 1445-1433
AD - Department of Academic Surgery, The Children's Hospital at Westmead, The University of Sydney, Sydney, NSW 2145, Australia
UR - 16483296
ER -

TY - JOUR
ID - 1267
T1 - The Stamp-in-Safety program: a behavioral intervention to reduce behaviors that can lead to unintentional playground injury in a preschool setting
A1 - Schwebel,D.C.
A1 - Summerlin,A.L.
A1 - Bounds,M.L.
A1 - Morrongiello,B.A.
Y1 - 2006/03//
N1 - Schwebel, David C. Summerlin, Adam L. Bounds, Marjorie L. Morrongiello, Barbara A
Journal of pediatric psychology
jl8, 7801773
IM
Clinical Trial. Journal Article
English
KW - MEDLINE
KW - Adult
KW - Alabama
KW - Analysis of Variance
KW - Behavior Control/mt [Methods]
KW - Child Day Care Centers
KW - Child
KW - Preschool
KW - Female
KW - Humans
KW - Male
KW - Play and Playthings/in [Injuries]
KW - Play and Playthings
KW - Token Economy
KW - Wounds and Injuries/pc [Prevention & Control]

RP - NOT IN FILE
SP - 152
EP - 162
JF - Journal of Pediatric Psychology
JA - J Pediatr Psychol
VL - 31
IS - 2
CY - United States
N2 - OBJECTIVE: To introduce and evaluate the Stamp-in-Safety program, a behavioral intervention designed to increase the quality of supervision by teachers on preschool playgrounds and to reduce the risk of unintentional child playground injury. METHODS: A quasi-experimental time series design was used, with observational techniques, to evaluate behavior on the playground before, during, and after the intervention as well as during a 6-month post-intervention assessment. RESULTS: Both applied behavior analysis techniques and inferential statistics suggest that the Stamp-in-Safety program resulted in behavioral changes likely to reduce the risk of child injury on the playgrounds of childcare centers. CONCLUSION: Results indicate promise for the Stamp-in-Safety program as a low-cost, easily implemented intervention to reduce pediatric playground injury risk at childcare centers. Suggestions for future research are offered
SN - 0146-8693
AD - Department of Psychology, University of Alabama at Birmingham, 1300 University Boulevard, CH 415, Birmingham, Alabama 35294, USA. schwebel@uab.edu
UR - 16467315
ER -

TY - JOUR
ID - 1268
T1 - Mucolipidosis II (I-cell disease) and mucolipidosis IIIA (classical pseudo-hurler polydystrophy) are caused by mutations in the GlcNAc-phosphotransferase alpha / beta -subunits precursor gene
A1 - Kudo,M.
A1 - Brem,M.S.
A1 - Canfield,W.M.
Y1 - 2006/03//
N1 - Kudo, Mariko. Brem, Michael S. Canfield, William M
American journal of human genetics
3im, 0370475
IM
Journal Article
English
KW - MEDLINE
KW - Adolescent
KW - Adult
KW - Alternative Splicing/ge [Genetics]
KW - Amino Acid Sequence
KW - Child
KW - Preschool
KW - Exons/ge [Genetics]
KW - Female
KW - Fibroblasts/en [Enzymology]
KW - Gene Frequency
KW - Humans
KW - Infant
KW - Newborn
KW - Male
KW - Middle Aged
KW - Molecular Sequence Data
KW - Mucolipidoses/en [Enzymology]
KW - Mucolipidoses/ge [Genetics]
KW - Mutation

KW - Pedigree
KW - Transferases (Other Substituted Phosphate Groups)/ge [Genetics]
KW - Transferases (Other Substituted Phosphate Groups)/me [Metabolism]
RP - NOT IN FILE
SP - 451
EP - 463
JF - American Journal of Human Genetics
JA - Am J Hum Genet
VL - 78
IS - 3
CY - United States
N2 - Mucolipidosis II (MLII; I-cell disease) and mucolipidosis IIIA (MLIIIA; classical pseudo-Hurler polydystrophy) are diseases in which the activity of the uridine diphosphate (UDP)-N-acetylglucosamine:lysosomal enzyme N-acetylglucosamine-1-phosphotransferase (GlcNAc-phosphotransferase) is absent or reduced, respectively. In the absence of mannose phosphorylation, trafficking of lysosomal hydrolases to the lysosome is impaired. In these diseases, mistargeted lysosomal hydrolases are secreted into the blood, resulting in lysosomal deficiency of many hydrolases and a storage-disease phenotype. To determine whether these diseases are caused by mutations in the GlcNAc-phosphotransferase alpha / beta -subunits precursor gene (GNPTAB), we sequenced GNPTAB exons and flanking intronic sequences and measured GlcNAc-phosphotransferase activity in patient fibroblasts. We identified 15 different mutations in GNPTAB from 18 pedigrees with MLII or MLIIIA and demonstrated that these two diseases are allelic. Mutations in both alleles were identified in each case, which demonstrated that GNPTAB mutations are the cause of both diseases. Some pedigrees had identical mutations. One frameshift mutation (truncation at amino acid 1171) predominated and was found in both MLII and MLIIIA. This mutation was found in combination with severe mutations (i.e., mutations preventing the generation of active enzyme) in MLII and with mild mutations (i.e., mutations allowing the generation of active enzyme) in MLIIIA. Some cases of MLII and MLIIIA were the result of mutations that cause aberrant splicing. Substitutions were inside the invariant splice-site sequence in MLII and were outside it in MLIIIA. When the mutations were analyzed along with GlcNAc-phosphotransferase activity, it was possible to confidently distinguish these two clinically related but distinct diseases. We propose criteria for distinguishing these two disorders by a combination of mutation detection and GlcNAc-phosphotransferase activity determination
SN - 0002-9297
AD - Genzyme Corporation, Oklahoma City, OK 73104, USA
UR - 16465621
ER -

TY - JOUR
ID - 1269
T1 - Applicability of interrupter resistance measurements for evaluation of exercise-induced bronchoconstriction in children
A1 - Song,D.J.
A1 - Woo,C.H.
A1 - Kang,H.
A1 - Kim,H.J.
A1 - Choung,J.T.
Y1 - 2006/03//
N1 - Song, Dae Jin. Woo, Chul Hee. Kang, Hee. Kim, Hyung Jin. Choung, Ji Tae
Pediatric pulmonology
owh, 8510590
IM
Comparative Study. Journal Article
English
KW - MEDLINE
KW - Airway Resistance/ph [Physiology]
KW - Anthropometry
KW - Asthma
KW - Exercise-Induced/di [Diagnosis]

KW - Exercise-Induced/pp [Physiopathology]
KW - Bronchial Hyperreactivity/di [Diagnosis]
KW - Bronchoconstriction
KW - Child
KW - Preschool
KW - Exercise Test/mt [Methods]
KW - Female
KW - Humans
KW - Korea
KW - Male
KW - Peak Expiratory Flow Rate
KW - Prospective Studies
KW - ROC Curve
KW - Reproducibility of Results
KW - Risk Assessment
KW - Sensitivity and Specificity
KW - Spirometry
RP - NOT IN FILE
SP - 228
EP - 233
JF - Pediatric Pulmonology
JA - Pediatr Pulmonol
VL - 41
IS - 3
CY - United States
N2 - The interrupter technique is a noninvasive method for measuring air-flow resistance during tidal breathing. This method requires minimal cooperation, and is therefore promising for use in uncooperative children. The aim of this study was to evaluate applicability interrupter resistance (Rint) measurements in the assessment of exercise-induced bronchoconstriction (EIB). Fifty children aged 5-12 years with mild to moderate asthma were tested by exercise challenge, consisting of free outdoor running for 6 min at 80-90% of maximal predicted heart rate for age. Rint, forced expiratory volume in 1 sec (FEV1), and peak expiratory flow (PEF) were measured before and 10 min after exercise. EIB was defined as a fall of 10% or more in FEV1 after exercise. The repeatability of Rint was assessed, and its response to exercise challenge was compared with current standardized methods. The mean intermeasurement coefficient of variation was 4.6% (SD, +/- 3.0%), and the repeatability coefficient was 0.056 kPa/l/sec. Eighteen (36%) of the 50 children had EIB after exercise challenge test. The area under the receiver-operating characteristic (ROC) curve was 0.953 (95% confidence interval, 0.853-0.992; P < 0.001), and the optimal Rint cutoff value was 15.2%, producing a sensitivity of 88.9% and a specificity of 96.9%. The positive and negative predictive values were 94.1% and 93.9%, respectively. The kappa value between FEV1 and Rint was 0.83. The repeatability of Rint measurements was good, and the results of exercise challenge tests using Rint measurements have excellent agreement with the current standardized methods to detect EIB. Considering that only minimal comprehension and coordination are needed without forced breathing technique, the Rint measurement can provide a useful alternative for assessment of EIB in children unable to perform reliable spirometry. (c) 2006 Wiley-Liss, Inc
SN - 8755-6863
AD - Department of Pediatrics, Korea University Medical College Hospital, Seoul, Korea
UR - 16429434
ER -

TY - JOUR
ID - 1270
T1 - Grass pollen immunotherapy as an effective therapy for childhood seasonal allergic asthma
A1 - Roberts,G.
A1 - Hurley,C.
A1 - Turcanu,V.
A1 - Lack,G.
Y1 - 2006/02//

N1 - Roberts, Graham. Hurley, Catriona. Turcanu, Victor. Lack, Gideon
The Journal of allergy and clinical immunology
h53, 1275002
AIM, IM
Journal Article. Randomized Controlled Trial. Research Support, Non-U.S. Gov't
English
KW - MEDLINE
KW - Adolescent
KW - Allergens/ae [Adverse Effects]
KW - Allergens/im [Immunology]
KW - Asthma/et [Etiology]
KW - Asthma/im [Immunology]
KW - Asthma/pp [Physiopathology]
KW - Asthma/th [Therapy]
KW - Child
KW - Preschool
KW - Conjunctivitis
KW - Allergic/co [Complications]
KW - Allergic/im [Immunology]
KW - Allergic/th [Therapy]
KW - Desensitization
KW - Immunologic/mt [Methods]
KW - Double-Blind Method
KW - Female
KW - Humans
KW - Male
KW - Phleum/ae [Adverse Effects]
KW - Phleum/im [Immunology]
KW - Pollen/ae [Adverse Effects]
KW - Pollen/im [Immunology]
KW - Rhinitis
KW - Allergic
KW - Seasonal/co [Complications]
KW - Seasonal/im [Immunology]
KW - Seasonal/th [Therapy]
KW - Severity of Illness Index
KW - Treatment Outcome
RP - NOT IN FILE
SP - 263
EP - 268
JF - Journal of Allergy & Clinical Immunology
JA - J Allergy Clin Immunol
VL - 117
IS - 2
CY - United States
N2 - BACKGROUND: Few studies have investigated the use of specific immunotherapy (SIT) for childhood seasonal allergic asthma. OBJECTIVE: We sought to examine the efficacy and safety of SIT with Alutard SQ grass pollen (Phleum pratense Alutard SQ; ALK-Abello, Horsholm, Denmark) in children with seasonal allergic asthma. METHODS: A randomized, double-blind, placebo-controlled study assessing the efficacy of grass pollen SIT over 2 pollen seasons was performed. Children (3-16 years) with a history of seasonal allergic asthma sensitized to grass pollen (P pratense) and requiring at least 200 microg of inhaled beclomethasone equivalent per day were enrolled. Subjects with symptomatic asthma or rhinoconjunctivitis outside the grass pollen season were excluded. The primary outcome measure was a combined asthma symptom-medication score during the second pollen season. Secondary outcome measures included end-point titration skin prick testing and conjunctival and bronchial provocation testing to allergen, sputum eosinophilia, exhaled nitric oxide, and adverse events. RESULTS: Thirty-nine subjects were enrolled. Thirty-five subjects provided data for analysis. The use of SIT was associated with a substantial reduction in asthma symptom-medication score compared with

that after placebo (P = .04). There were also significant reductions in cutaneous (P = .002), conjunctival (P = .02), and bronchial (P = .01) reactivity to allergen after SIT compared with that after placebo. The 2 groups had similar levels of airway inflammation, despite a trend toward less inhaled steroid use in the active group. No serious adverse events were reported, and no subjects withdrew because of adverse events. CONCLUSION: The study has shown that SIT is effective and well tolerated in children with seasonal allergic asthma to grass pollen
SN - 0091-6749
AD - University Child Health, University of Southampton, Hants, UK. g.c.roberts@soton.ac.uk
UR - 16461125
ER -

TY - JOUR
ID - 1271
T1 - Screening for speech and language delay in preschool children: systematic evidence review for the US Preventive Services Task Force. [Review] [97 refs][Erratum appears in Pediatrics. 2006 Jun;117(6):2336-7]
A1 - Nelson,H.D.
A1 - Nygren,P.
A1 - Walker,M.
A1 - Panoscha,R.
Y1 - 2006/02//
N1 - Nelson, Heidi D. Nygren, Peggy. Walker, Miranda. Panoscha, Rita
Pediatrics
oxv, 0376422
AIM, IM
Journal Article. Research Support, U.S. Gov't, P.H.S.. Review
English
KW - MEDLINE
KW - Child
KW - Preschool
KW - Developmental Disabilities/di [Diagnosis]
KW - Developmental Disabilities/th [Therapy]
KW - Humans
KW - Language Development Disorders/di [Diagnosis]
KW - Language Development Disorders/th [Therapy]
KW - Language Therapy
KW - Primary Health Care
KW - Speech Disorders/di [Diagnosis]
KW - Speech Disorders/th [Therapy]
KW - Speech Therapy
RP - NOT IN FILE
SP - e298
EP - e319
JF - Pediatrics
VL - 117
IS - 2
CY - United States
N2 - BACKGROUND: PEDIATRICS (ISSN Numbers: Print, 0031-4005; Online, 1098-4275). Published in the public domain by the American Academy of Pediatrics. Speech and language development is a useful indicator of a child's overall development and cognitive ability and is related to school success. Identification of children at risk for developmental delay or related problems may lead to intervention services and family assistance at a young age, when the chances for improvement are best. However, optimal methods for screening for speech and language delay have not been identified, and screening is practiced inconsistently in primary care. PURPOSE: We sought to evaluate the strengths and limits of evidence about the effectiveness of screening and interventions for speech and language delay in preschool-aged children to determine the balance of benefits and adverse effects of routine screening in primary care for the development of guidelines by the US Preventive Services Task Force. The target population includes all children up to 5 years old without previously known conditions associated with speech and language delay, such as hearing and neurologic impairments. METHODS: Studies were identified from Medline, PsycINFO, and CINAHL databases (1966 to November 19, 2004), systematic

reviews, reference lists, and experts. The evidence review included only English-language, published articles that are available through libraries. Only randomized, controlled trials were considered for examining the effectiveness of interventions. Outcome measures were considered if they were obtained at any time or age after screening and/or intervention as long as the initial assessment occurred while the child was < or =5 years old. Outcomes included speech and language measures and other functional and health outcomes such as social behavior. A total of 745 full-text articles met our eligibility criteria and were reviewed. Data were extracted from each included study, summarized descriptively, and rated for quality by using criteria specific to different study designs developed by the US Preventive Services Task Force. RESULTS: The use of risk factors for selective screening has not been evaluated, and a list of specific risk factors to guide primary care physicians has not been developed or tested. Sixteen studies about potential risk factors for speech and language delay in children enrolled heterogeneous populations, had dissimilar inclusion and exclusion criteria, and measured different risk factors and outcomes. The most consistently reported risk factors included a family history of speech and language delay, male gender, and perinatal factors. Other risk factors reported less consistently included educational levels of the mother and father, childhood illnesses, birth order, and family size. The performance characteristics of evaluation techniques that take < or =10 minutes to administer were described in 24 studies relevant to screening. Studies that were rated good to fair quality reported wide ranges of sensitivity and specificity when compared with reference standards (sensitivity: 17-100%; specificity: 45-100%). Most of the evaluations, however, were not designed for screening purposes, the instruments measured different domains, and the study populations and settings were often outside of primary care. No "gold standard" has been developed and tested for screening, reference standards varied across studies, few studies compared the performance of > or =2 screening techniques in 1 population, and comparisons of a single screening technique across different populations are lacking. Fourteen good- and fair-quality randomized, controlled trials of interventions reported significantly improved speech and language outcomes compared with control groups. Improvement was demonstrated in several domains including articulation, phonology, expressive language, receptive language, lexical acquisition, and syntax among children in all age groups studied and across multiple therapeutic settings. Improvement in other functional outcomes such as socialization skills, self-esteem, and improved play themes were demonstrated in some, but not all, of the 4 studies that measured them. In general, studies of interventions were small and heterogeneous, may be subject to plateau effects, and reported short-term outcomes based on various instruments and measures. As a result, long-term outcomes are not known, interventions could not be compared directly, and generalizability is questionable. CONCLUSIONS: Use of risk factors to guide selective screening is not supported by studies. Several aspects of screening have been inadequately studied to determine optimal methods, including which instrument to use, the age at which to screen, and which interval is most useful. Trials of interventions demonstrate improvement in some outcome measures, but conclusions and generalizability are limited. Data are not available addressing other key issues including the effectiveness of screening in primary care settings, role of enhanced surveillance by primary care physicians before referral for diagnostic evaluation, non-speech and language and long-term benefits of interventions, and adverse effects of screening and interventions. [References: 97]
SN - 1098-4275
AD - Department of Medical Informatics, Oregon Health and Science University, Portland, Oregon 97239, USA. nelsonh@ohsu.edu
UR - 16452337
ER -

TY - JOUR
ID - 1272
T1 - Elemental mercury spills. [Review] [25 refs]
A1 - Baughman,T.A.
Y1 - 2006/02//
N1 - Baughman, Thomas A
Environmental health perspectives
ei0, 0330411
IM
Journal Article. Research Support, U.S. Gov't, Non-P.H.S.. Review
English
KW - MEDLINE
KW - Accidents
KW - Adult

KW - Child
KW - Environmental Exposure
KW - Environmental Pollutants
KW - Environmental Pollution/pc [Prevention & Control]
KW - Equipment Contamination
KW - Humans
KW - Inhalation Exposure
KW - Manufactured Materials
KW - Mercury/pk [Pharmacokinetics]
KW - Mercury/to [Toxicity]
KW - Volatilization
KW - Waste Management
RP - NOT IN FILE
SP - 147
EP - 152
JF - Environmental Health Perspectives
JA - Environ Health Perspect
VL - 114
IS - 2
CY - United States
N2 - Sources of elemental mercury (Hg0) include old natural gas regulators, manometers, sphygmomanometers, thermometers, and thermostats. Causes of Hg0 spills include improper storage, container breakage, children playing with Hg0, the breakage of devices containing Hg0, and ritualistic use of Hg0. Inhalation is the primary exposure route for Hg0. Mercury released into the environment can enter lakes and streams, where bacteria convert it into methylmercury, which bioaccumulates in fish. Chronic exposure to Hg0 vapors can damage the kidneys and neurologic system. Short-term exposure to high levels of Hg0 vapors may cause lung damage, nausea, vomiting, diarrhea, increases in blood pressure or heart rate, skin rashes, and eye irritation, among other effects. Minimizing Hg0 dispersal is important after an Hg0 spill. Tracking by shoes or apparel or vacuuming can spread Hg0, increasing airborne concentrations and cleanup costs. The Illinois Department of Public Health's response to an Hg0 spill depends on the size of the spill. Airborne concentrations after large spills are mapped with a mercury vapor analyzer (MVA). The cleanup begins with the spill site and any hot spots that were identified with the MVA. Hard surfaces can usually be cleaned, but contaminated porous items must be discarded. Leaving marginally contaminated items outdoors for a month or more during warm weather may dissipate the Hg0. After a cleanup, clearance sampling is conducted to determine if further cleanup is needed. The best way to prevent Hg0 spills is reduce its use. Key words: cleanup, elemental mercury, health effects, mercury, prevention, remediation, spill, spill management. [References: 25]
SN - 0091-6765
AD - Environmental Toxicology Section, Illinois Department of Public Health, West Chicago, Illinois 60185, USA. tbaughma@idph.state.il.us
UR - 16451846
ER -

TY - JOUR
ID - 1273
T1 - People under 60 living in aged care facilities in Victoria
A1 - Winkler,D.
A1 - Farnworth,L.
A1 - Sloan,S.
Y1 - 2006/02//
N1 - Winkler, Dianne. Farnworth, Louise. Sloan, Sue
Australian health review : a publication of the Australian Hospital Association
9gc, 8214381
H
Journal Article. Research Support, Non-U.S. Gov't
English
KW - MEDLINE
KW - Adolescent

KW - Adult
KW - Child
KW - Data Collection
KW - Female
KW - Homes for the Aged/ut [Utilization]
KW - Humans
KW - Male
KW - Middle Aged
KW - Victoria
RP - NOT IN FILE
SP - 100
EP - 108
JF - Australian Health Review
JA - Aust Health Rev
VL - 30
IS - 1
CY - Australia
N2 - OBJECTIVE: To describe the characteristics of people under 60 years of age living in residential aged care in Victoria and to examine the occupational participation of younger residents in aged care facilities by measuring their social contact, participation in recreation and community access. METHODS: A survey was sent to the Directors of Nursing at all 803 Commonwealth Department of Health and Aged Care registered aged care facilities in Victoria. RESULTS: The survey had a 78% response rate and information was provided about the characteristics of 330 people under 60 years with high clinical needs residing in aged care facilities. This sample was extremely isolated from peers, with 44% receiving a visit from a friend less often than once per year. Sixteen per cent of residents participated in a recreation activity less than once per month and 21% went outside less than once per month. Of the sample, 34% almost never participated in any community-based activities such as shopping, leisure or visiting friends and family. CONCLUSION: Over one third of younger people in aged care are effectively excluded from life in our community. Most younger residents are socially isolated and have limited opportunities for recreation. Placement of younger people in aged care facilities is inappropriate, and alternative care models and settings are required
SN - 0156-5788
AD - Occupational Therapy, School of Primary Health Care, Monash University, Melbourne, Australia. ypiacresearch@optusnet.com.au
UR - 16448382
ER -

TY - JOUR
ID - 1274
T1 - Association of indoor nitrogen dioxide exposure with respiratory symptoms in children with asthma
A1 - Belanger,K.
A1 - Gent,J.F.
A1 - Triche,E.W.
A1 - Bracken,M.B.
A1 - Leaderer,B.P.
Y1 - 2006/02/01/
N1 - Belanger, Kathleen. Gent, Janneane F. Triche, Elizabeth W. Bracken, Michael B. Leaderer, Brian P
American journal of respiratory and critical care medicine
9421642, bzs
AIM, IM
Journal Article. Research Support, N.I.H., Extramural
English
KW - MEDLINE
KW - Air Pollution
KW - Indoor/ae [Adverse Effects]
KW - Asthma/co [Complications]
KW - Child
KW - Preschool

KW - Cohort Studies
KW - Female
KW - Household Articles
KW - Housing
KW - Humans
KW - Inhalation Exposure/ae [Adverse Effects]
KW - Male
KW - Nitrogen Dioxide/ae [Adverse Effects]
KW - Oxidants
KW - Photochemical/ae [Adverse Effects]
KW - Respiration Disorders/et [Etiology]
RP - NOT IN FILE
SP - 297
EP - 303
JF - American Journal of Respiratory & Critical Care Medicine
JA - Am J Respir Crit Care Med
VL - 173
IS - 3
CY - United States
N2 - RATIONALE: Chronic exposure to indoor nitrogen dioxide (NO2) is a public health concern. Over half of U.S. households have a source of NO2, and experimental data suggest potential for adverse respiratory effects. OBJECTIVE: To examine associations of indoor NO2 exposure with respiratory symptoms among children with asthma. METHODS: NO2 was measured using Palmes tubes, and respiratory symptoms in the month before sampling were collected during home interviews of mothers of 728 children with active asthma. All were younger than 12 yr, lived at the sampled home for at least 2 mo, and had asthma symptoms or used maintenance medication within the previous year. MEASUREMENTS: Respiratory symptoms (wheeze, persistent cough, shortness of breath, chest tightness). RESULTS: Mean (SD) NO2 was 8.6 (9.1) ppb in homes with electric ranges and 25.9 (18.1) ppb in homes with gas stoves. In models stratified by housing type (a factor associated with socioeconomic status), gas stove presence and elevated NO2 were each significantly associated with respiratory symptoms, controlling for age, ethnicity, medication, mold/mildew, water leaks, and season of sampling. Among children in multifamily housing, exposure to gas stoves increased likelihood of wheeze (odds ratio [OR], 2.27; 95% confidence interval [95% CI], 1.15, 4.47), shortness of breath (OR, 2.33; 95% CI, 1.12, 5.06), and chest tightness (OR, 4.34; 95% CI, 1.76, 10.69), whereas each 20-ppb increase in NO2 increased both likelihood of any wheeze (OR, 1.52; 95% CI, 1.04, 2.21) or chest tightness (OR, 1.61; 95% CI, 1.04, 2.49), and days of wheeze (rate ratio (RR), 1.33; 95% CI, 1.05, 1.68) or chest tightness (RR, 1.51; 95% CI, 1.18, 1.91). CONCLUSION: Exposure to indoor NO2 at levels well below the Environmental Protection Agency outdoor standard (53 ppb) is associated with respiratory symptoms among children with asthma in multifamily housing
SN - 1073-449X
AD - Yale Center for Perinatal, Pediatric, and Environmental Epidemiology, New Haven, CT 06510, USA. kathleen.belanger@yale.edu
UR - 16254270
ER -

TY - JOUR
ID - 1275
T1 - Loss of the NPM1 gene in myeloid disorders with chromosome 5 rearrangements
A1 - Berger,R.
A1 - Busson,M.
A1 - Baranger,L.
A1 - Helias,C.
A1 - Lessard,M.
A1 - Dastugue,N.
A1 - Speleman,F.
Y1 - 2006/02//
N1 - Berger, R. Busson, M. Baranger, L. Helias, C. Lessard, M. Dastugue, N. Speleman, F
Leukemia
leu, 8704895

IM
Journal Article
English
KW - MEDLINE
KW - Adult
KW - Aged
KW - Child
KW - Preschool
KW - Chromosome Banding
KW - Chromosomes
KW - Human
KW - Pair 3/ge [Genetics]
KW - Pair 5/ge [Genetics]
KW - Female
KW - Hematopoiesis/ge [Genetics]
KW - Humans
KW - In Situ Hybridization
KW - Fluorescence/mt [Methods]
KW - Leukemia
KW - Myeloid/ge [Genetics]
KW - Male
KW - Myelodysplastic Syndromes/ge [Genetics]
KW - Nuclear Proteins/ge [Genetics]
KW - Oncogene Proteins
KW - Fusion/ge [Genetics]
KW - Reverse Transcriptase Polymerase Chain Reaction
KW - Translocation
KW - Genetic
RP - NOT IN FILE
SP - 319
EP - 321
JF - Leukemia
VL - 20
IS - 2
CY - England
N2 - The assignment with chromosome banding techniques of the breakpoints of the recurrent translocation t(3;5) which leads to NPM1/MLF1 gene fusion in myeloid malignancies has not been unequivocal. In order to assess whether this is due to uncertainty in interpretation of the observed banding pattern or whether it reflects true genomic heterogeneity, we decided to analyze the breakpoint positions using fluorescence in situ (FISH) techniques in eight patients with myeloid malignancies and rearrangements of chromosomes 3 and 5. In three patients, colocalization of the NPM1 and MLF1 spanning BACs was demonstrated and NPM1/MLF1 fusion shown by PCR in one while in the remaining cases breakpoints were located outside the NPM1 and MLF1 loci. Interestingly, loss of a copy of the NPM1 gene was found in three of these latter patients. This findings suggest that haploinsufficiency of NPM1 may play a role in subtypes of myelodysplasias and leukemias
SN - 0887-6924
AD - 1EMI 0210 Hopital Necker-Enfants Malades, Paris, France. berger@necker.fr
UR - 16341035
ER -

TY - JOUR
ID - 1276
T1 - Effects of the Sunny Days, Healthy Ways curriculum on students in grades 6 to 8
A1 - Buller,D.B.
A1 - Reynolds,K.D.
A1 - Yaroch,A.
A1 - Cutter,G.R.
A1 - Hines,J.M.

A1 - Geno,C.R.
A1 - Maloy,J.A.
A1 - Brown,M.
A1 - Woodall,W.G.
A1 - Grandpre,J.
Y1 - 2006/01//
N1 - Buller, David B. Reynolds, Kim D. Yaroch, Amy. Cutter, Gary R. Hines, Joan M. Geno, Cristy R. Maloy, Julie A. Brown, Melissa. Woodall, W Gill. Grandpre, Joseph
American journal of preventive medicine
8704773, apl
IM
Journal Article. Randomized Controlled Trial. Research Support, N.I.H., Extramural
English
KW - MEDLINE
KW - Adolescent
KW - Arizona
KW - Child
KW - Colorado
KW - Curriculum
KW - Female
KW - Health Behavior
KW - Health Education/mt [Methods]
KW - Health Knowledge
KW - Attitudes
KW - Practice
KW - Health Promotion/mt [Methods]
KW - Humans
KW - Linear Models
KW - Male
KW - Matched-Pair Analysis
KW - New Mexico
KW - Program Evaluation
KW - Protective Clothing/ut [Utilization]
KW - Safety
KW - School Health Services/og [Organization & Administration]
KW - Students/px [Psychology]
KW - Sunburn/pc [Prevention & Control]
KW - Sunlight/ae [Adverse Effects]
KW - Sunscreening Agents/tu [Therapeutic Use]
RP - NOT IN FILE
SP - 13
EP - 22
JF - American Journal of Preventive Medicine
JA - Am J Prev Med
VL - 30
IS - 1
CY - Netherlands
N2 - BACKGROUND: There are few effective sun-safety education programs for use in secondary schools. Project aims were to create a sun-safety curriculum for grades 6 to 8, and to test whether exposure to the curriculum would increase children's sun-protection behavior. DESIGN: A pair-matched, group-randomized, pre--post test, controlled trial was performed with middle schools as the unit of randomization. Teachers implemented the six-unit sun-safety curriculum in 2001-2003, and analyses were performed in 2003-2004. SETTING/PARTICIPANTS: A total of 2038 children from 30 middle schools in Colorado, New Mexico, and Arizona. MAIN OUTCOME MEASURES: Self-reported sun-protection behavior using frequency ratings and diary. RESULTS: Compared to control schools, children receiving the curriculum reported more frequent sun protection ($p=0.0035$), and a greater proportion wore long-sleeved shirts during recess ($p<0.0001$) and applied sunscreen ($p<0.0001$). Exposure to the curriculum improved knowledge ($p<0.0001$), decreased perceived

barriers to using sunscreen (p=0.0046), enhanced self-efficacy expectations (p=0.0577) about sun safety, and reduced favorable attitudes toward sun tanning (p=0.0026 to <0.0001). In intent-to-treat analyses, the treatment effect was eliminated only under the most conservative assumptions about dropouts. CONCLUSIONS: Educational approaches to sun safety in middle school may be effective for improving children's sun safety. Potential trial limitations include measuring short-term outcomes, focusing on young adolescents, using active parental consent, and testing in the American Southwest
SN - 0749-3797
AD - Klein Buendel, Inc., Golden, Colorado 80401, USA. dbuller@kleinbuendel.com
UR - 16414419
ER -

TY - JOUR
ID - 1277
T1 - Assessment of the bioaccessibility of polycyclic aromatic hydrocarbons in soils from Beijing using an in vitro test
A1 - Tang,X.Y.
A1 - Tang,L.
A1 - Zhu,Y.G.
A1 - Xing,B.S.
A1 - Duan,J.
A1 - Zheng,M.H.
Y1 - 2006/03//
N1 - Tang, Xiang-Yu. Tang, Lily. Zhu, Yong-Guan. Xing, Bao-Shan. Duan, Jing. Zheng, Ming-Hui
Environmental pollution (Barking, Essex : 1987)
dvl, 8804476
IM
Journal Article. Research Support, Non-U.S. Gov't
English
KW - MEDLINE
KW - Biological Availability
KW - Child
KW - Child Behavior
KW - China
KW - Digestion/ph [Physiology]
KW - Environmental Exposure/ae [Adverse Effects]
KW - Humans
KW - Intestine
KW - Small/ch [Chemistry]
KW - Mouth
KW - Polycyclic Hydrocarbons
KW - Aromatic/an [Analysis]
KW - Recreation
KW - Risk Assessment/mt [Methods]
KW - Soil/an [Analysis]
KW - Soil Pollutants/an [Analysis]
KW - Stomach/ch [Chemistry]
KW - Surface Tension
KW - Urban Health
RP - NOT IN FILE
SP - 279
EP - 285
JF - Environmental Pollution
JA - Environ Pollut.
VL - 140
IS - 2
CY - England

N2 - As an important human exposure pathway of contaminants, soil ingestion is of increasing concern for assessing health risk from polycyclic aromatic hydrocarbons (PAHs) in soils. A wide range of total PAH concentrations ranging from 0.112 microg g(-1) to 27.8 microg g(-1) in soils collected from different public sites, including gas stations, roadsides, bus stops, a kindergarten, primary and middle schools, a university and residential area, was detected. In general, total PAHs concentrations in soils from traffic areas were significantly higher than that from the other sites, indicating a dominant contribution from motor vehicles. Physiologically based in vitro tests were used to evaluate the oral bioaccessibility of PAHs in surface soil under different land uses in Beijing regarding both gastric and small intestinal conditions. It was found that the oral bioaccessibility of total PAHs in small intestinal condition, ranging from 9.2% to 60.5% of total PAHs in soil, was significantly higher than gastric condition, ranging from 3.9% to 54.9%. The bioaccessibility of individual PAHs in soils generally decreased with the increasing ring number of PAHs in both gastric and small intestinal conditions. However, the ratio of bioaccessibility of individual PAHs in gastric condition to that in small intestinal condition, generally increased with increasing ring number, indicating the relatively pronounced effect of bile extract on improving bioaccessibility of PAHs with relatively high ring numbers characterized by their high K(ow) values. The observation that bile extract at a level higher than critical micelle concentration could reduce the surface tension of digestive juice substantially, which may cause PAHs to be available for intestinal absorption, calls for more careful establishment of reliable soil criteria for PAHs, especially concerning the health of children who may ingest a considerable amount of PAH-contaminated soil via outdoor hand-mouth activities
SN - 0269-7491
AD - Department of Soil Environmental Science/State Key Lab of Environmental Chemistry and Ecotoxicology, Research Center for Eco-Environmental Sciences, The Chinese Academy of Sciences, Beijing 100085, PR China
UR - 16157432
ER -

TY - JOUR
ID - 1278
T1 - Validation and calibration of the Actical accelerometer in preschool children
A1 - Pfeiffer,K.A.
A1 - McIver,K.L.
A1 - Dowda,M.
A1 - Almeida,M.J.
A1 - Pate,R.R.
Y1 - 2006/01//
N1 - Pfeiffer, Karin A. McIver, Kerry L. Dowda, Marsha. Almeida, Maria J C A. Pate, Russell R
Medicine and science in sports and exercise
8005433, mg8
IM, S
Journal Article. Research Support, N.I.H., Extramural. Research Support, Non-U.S. Gov't. Validation Studies
English
KW - MEDLINE
KW - Acceleration
KW - Calibration/st [Standards]
KW - Child
KW - Preschool
KW - Cross-Sectional Studies
KW - Exercise Test/is [Instrumentation]
KW - Humans
KW - South Carolina
KW - Walking
RP - NOT IN FILE
SP - 152
EP - 157
JF - Medicine & Science in Sports & Exercise
JA - Med Sci Sports Exerc
VL - 38

IS - 1
CY - United States
N2 - PURPOSE: Decreased physical activity (PA) is likely a contributor to the rising prevalence of obesity in children. Lack of valid and acceptable measures of PA has been an issue in studies involving young children. The Actical accelerometer is a promising tool for measurement of PA in young children. The purpose of this study was to calibrate and validate the Actical accelerometer for use with 3- to 5-yr-old children. METHODS: Eighteen preschool children wore an Actical accelerometer and a Cosmed portable metabolic system during a period of rest, while performing three structured activities in a laboratory setting (used for calibration), and during 20 min each of unstructured indoor and outdoor activities at their preschool (used for cross-validation). Expired respiratory gases were collected, and oxygen consumption was measured on a breath-by-breath basis. Accelerometer data were collected in 15-s intervals. RESULTS: For the accelerometer calibration, the correlation between VO2 and counts was r = 0.89 across all activities. The calibration equation established was VO2 = counts x 15 s(-1) (0.01437) + 9.73 (R2 = 0.96, SEE = 3.02). The cut-point for moderate activity (20 mL x kg(-1) x min(-1)) was 715 counts x 15 s(-1) (sensitivity 97.2%, specificity 91.7%), and the cut-point for vigorous activity (30 mL x kg(-1) x min(-1)) was 1411 counts x 15 s(-1) (sensitivity 98.2%, specificity 61.1%). For the cross-validation, the intraclass correlation coefficient was R = 0.59 and the Spearman correlation coefficient was R = 0.80 (P < 0.001) between measured and predicted VO2. Percentage of agreement, kappa, and modified kappa for moderate activity were 0.73, 0.40, and 0.46, respectively. For vigorous activity, the same measures were 0.85, 0.26, and 0.71, respectively. CONCLUSION: The Actical accelerometer is a valid tool for measuring PA in young children
SN - 0195-9131
AD - Department of Exercise Science, Arnold School of Public Health, University of South Carolina, Columbia, SC 29208, USA. kapfeiffer@sc.edu
UR - 16394968
ER -

TY - JOUR
ID - 1279
T1 - A case study of tire crumb use on playgrounds: risk analysis and communication when major clinical knowledge gaps exist
A1 - Anderson,M.E.
A1 - Kirkland,K.H.
A1 - Guidotti,T.L.
A1 - Rose,C.
Y1 - 2006/01//
N1 - Anderson, Mark E. Kirkland, Katherine H. Guidotti, Tee L. Rose, Cecile
Environmental health perspectives
ei0, 0330411
IM
Journal Article
English
KW - MEDLINE
KW - Child
KW - Communication
KW - Conservation of Natural Resources
KW - Environment
KW - Environmental Exposure
KW - Environmental Health
KW - Humans
KW - Mutagenicity Tests
KW - Play and Playthings
KW - Risk Assessment
KW - Rubber
KW - United States
KW - United States Environmental Protection Agency
RP - NOT IN FILE
SP - 1

EP - 3
JF - Environmental Health Perspectives
JA - Environ Health Perspect
VL - 114
IS - 1
CY - United States
N2 - Physicians and public health professionals working with the U.S. Environmental Protection Agency's Region 8 Pediatric Environmental Health Specialty Unit (PEHSU) received several telephone calls requesting information regarding the safety of recycled tire crumb as a playground surface constituent placed below children's play structures. There were no reported symptoms or adverse health effects in exposed children. The literature available on the safety and risk of exposure to crumb rubber constituents was limited and revealed no information quantifying exposures associated with product use. Callers were informed by the PEHSU that no evidence existed suggesting harm from intended use of the product, but gaps in knowledge about the product were identified and communicated. Here the case of crumb rubber on playgrounds is used as a model to present an approach to similar environmental medicine questions. From defining the question, to surveying traditional and nontraditional resources for information, synthesis of findings, and risk communication, the case provides a model to approach similar questions
SN - 0091-6765
AD - Department of Community Health Services, Denver Health, and Department of Pediatrics, University of Colorado Health Science Center, Denver, Colorado 80204, USA. manderso@dhha.org
UR - 16393649
ER -

TY - JOUR
ID - 1280
T1 - Changes in manganese and lead in the environment and young children associated with the introduction of methylcyclopentadienyl manganese tricarbonyl in gasoline--preliminary results
A1 - Gulson,B.
A1 - Mizon,K.
A1 - Taylor,A.
A1 - Korsch,M.
A1 - Stauber,J.
A1 - Davis,J.M.
A1 - Louie,H.
A1 - Wu,M.
A1 - Swan,H.
Y1 - 2006/01//
N1 - Gulson, Brian. Mizon, Karen. Taylor, Alan. Korsch, Michael. Stauber, Jennifer. Davis, J Michael. Louie, Honway. Wu, Michael. Swan, Hilton
Environmental research
ei2, 0147621
IM
Journal Article
English
KW - MEDLINE
KW - Air Pollution
KW - Indoor/an [Analysis]
KW - Australia/ep [Epidemiology]
KW - Child Day Care Centers
KW - Child
KW - Preschool
KW - Diet
KW - Dust/an [Analysis]
KW - Environmental Monitoring
KW - Environmental Pollutants/an [Analysis]
KW - Environmental Pollutants/bl [Blood]
KW - Epidemiological Monitoring

KW - Female
KW - Gasoline
KW - Housing
KW - Humans
KW - Infant
KW - Lead/an [Analysis]
KW - Lead/bl [Blood]
KW - Male
KW - Manganese/an [Analysis]
KW - Manganese/bl [Blood]
KW - Organometallic Compounds
KW - Risk Assessment
KW - Vehicle Emissions
RP - NOT IN FILE
SP - 100
EP - 114
JF - Environmental Research
JA - Environ Res
VL - 100
IS - 1
CY - United States
N2 - A 4-year longitudinal study is being conducted to evaluate potential changes to the environment and exposure of young children associated with the introduction of methylcyclopentadienyl manganese tricarbonyl (MMT) into Australia in 2001. The cohort consists of 57 females and 56 males, with an age range of 0.29-3.9 years. Samples are collected every 6 months from children in residences located at varying distances from major traffic thoroughfares in Sydney. Environmental samples include air, house, and daycare center dustfall, soil, dust sweepings, and gasoline; samples from the children include blood, urine, handwipes prior to and after playing outdoors, and a 6-day duplicate diet. All samples are analyzed for a suite of 20 elements using inductively coupled plasma methods. Results are presented for the first three 6-month sampling periods for lead (Pb) and manganese (Mn). For dustfall accumulation, expressed as metal concentration/m2/30 days, there was no significant difference between homes and daycare centers for either Pb or Mn, no significant change over the three sampling periods (time) for Pb or Mn, and a positive relationship between "traffic exposure" (traffic volume and proximity to the road) and Pb but not Mn. Lead concentrations in soil was a significant predictor for Pb in the house dustfall. For handwipes, the concentrations of Pb and Mn in wipes taken from children after playing outdoors was usually significantly greater than those for wipes taken prior to playing. There was no significant association between the concentrations of either Pb or Mn in handwipes and traffic exposure, and there was no significant association between Pb concentrations in the handwipes and gender, although the latter showed a marginally significant association for Mn (P = 0.053). Age was related to Pb level in the handwipes, with older subjects having higher Pb levels, and there were significant decreases in Pb and Mn concentrations over time. Dustfall accumulation was a significant predictor for Pb in the handwipes, and dust sweepings were a significant predictor of Mn in handwipes. Blood lead (PbB) concentrations ranged from 0.6 to 19 microg/dL (GM 2.6) (n = 269), and manganese in blood (MnB) ranged from 1.8 to 45 microg/L (GM 11.6) (n = 254). There was no significant difference between females and males for either mean PbB or MnB; over time there was a significant decline in PbB but no significant change in MnB. The only significant predictor for PbB was dustfall accumulation, although dietary intake may also be important, and the only significant predictor for MnB was Mn in handwipes prior to playing. At this early stage of the investigation we have not been able to detect any increases in Mn in these environmental samples or blood samples potentially associated with the use of MMT; in fact the Mn levels in handwipes declined over time
SN - 0013-9351
AD - Graduate School of the Environment, Macquarie University, Sydney NSW 2109, Australia. bgulson@gse.mq.edu.au
UR - 16337847
ER -

TY - JOUR
ID - 1281

T1 - Relationship between radical generation by urban ambient particulate matter and pulmonary function of school children.[Reprint in Ned Tijdschr Geneeskd. 2006 Apr 1;150(13):735-40; PMID: 16623348]
A1 - Hogervorst,J.G.
A1 - de Kok,T.M.
A1 - Briede,J.J.
A1 - Wesseling,G.
A1 - Kleinjans,J.C.
A1 - van Schayck,C.P.
Y1 - 2006/02//
N1 - Hogervorst, Janneke G F. de Kok, Theo M C M. Briede, Jacob J. Wesseling, Geertjan. Kleinjans, Jos C S. van Schayck, Constant P
Journal of toxicology and environmental health. Part A
ds5, 100960995
IM
Journal Article
English
KW - MEDLINE
KW - Adolescent
KW - Air Pollutants/an [Analysis]
KW - Air Pollutants/to [Toxicity]
KW - Child
KW - Cities
KW - Dust/an [Analysis]
KW - Electron Spin Resonance Spectroscopy
KW - Environmental Monitoring
KW - Epidemiological Monitoring
KW - Female
KW - Forced Expiratory Volume/de [Drug Effects]
KW - Free Radicals/an [Analysis]
KW - Humans
KW - Inhalation Exposure
KW - Male
KW - Maximal Midexpiratory Flow Rate/de [Drug Effects]
KW - Netherlands/ep [Epidemiology]
KW - Particle Size
KW - Reactive Oxygen Species/an [Analysis]
KW - Schools
KW - Spirometry
RP - NOT IN FILE
SP - 245
EP - 262
JF - Journal of Toxicology & Environmental Health Part A
JA - J Toxicol Environ Health A
VL - 69
IS - 3-4
CY - England
N2 - The mechanisms by which particulate matter (PM) produces adverse effects on the respiratory system, such as pulmonary dysfunction in children, are largely unknown. However, oxidative stress is thought to play an important role. Various chemical compounds in ambient particulate matter, including transition metals and aromatic organic compounds, may contribute to adverse effects through intrinsic generation of reactive oxygen species (ROS). It was hypothesized that ROS generation by PM, as determined through electron spin resonance (ESR) spectroscopy, may be negatively associated with pulmonary function in school children. PM(2.5), PM(10), and total suspended particulates (TSP) were sampled at the playgrounds of six elementary schools in the city of Maastricht, the Netherlands. All children (8-13 yr) from the six schools were asked to undergo spirometry. Multivariate linear regression models were constructed to evaluate associations between oxygen radical formation by PM and lung function. The radical-generating capacity per microgram PM correlated negatively to forced expiratory volume in 1 s (FEV(1)) and forced expiratory flow at 50% (FEF(50%)) of forced

vital capacity (FVC). The data indicate that chemical features that contribute to intrinsic generation of ROS may be relevant for PM risk assessment
SN - 1528-7394
AD - Research Institute CAPHRI, Maastricht
UR - 16263695
ER -

TY - JOUR
ID - 1282
T1 - Twenty years of HIV surveillance in the Pacific--what do the data tell us and what do we still need to know?
A1 - Sladden,T.
Y1 - 2005/09//
N1 - Sladden, Tim
Pacific health dialog
9434923
IM
Journal Article
English
KW - MEDLINE
KW - Adolescent
KW - Adult
KW - Child
KW - Preschool
KW - Female
KW - HIV Infections/ep [Epidemiology]
KW - Humans
KW - Infant
KW - Newborn
KW - Male
KW - Mandatory Reporting
KW - Middle Aged
KW - Pacific Islands/ep [Epidemiology]
KW - Population Surveillance/mt [Methods]
KW - Population Surveillance
RP - NOT IN FILE
SP - 23
EP - 37
JF - Pacific Health Dialog
JA - Pac Health Dialog
VL - 12
IS - 2
CY - New Zealand
N2 - BACKGROUND: Most Pacific Island countries and territories (PICTs) provide some level of HIV testing for their populations and record demographic data for diagnosed cases of HIV and AIDS. These routine HIV data are usually held within the national Department/Ministry of Health or National AIDS Council. Summary HIV data can illustrate important trends in HIV infection over time and by age, gender and exposure. For some countries no other data are available to illustrate the extent and distribution of HIV infection in the region. METHODS: Annual HIV and AIDS data were requested from all PICT reporting authorities disaggregated by year, age group, sex and exposure. Data were grouped to provide regional and sub-regional totals. Descriptive statistics were reported, including age-adjusted rates and trends. RESULTS: The first cases of HIV in the Pacific were reported in the mid-1980s in all three sub-regions (Melanesia, Micronesia and Polynesia). Many early cases were acquired either by men who have sex with men (MSM) or by recipients of blood products. Some early cases migrated from outside the region. Since then numbers have increased, with 12,169 HIV cases reported across the Pacific to December 2004, including 2,335 AIDS cases and 617 AIDS-related deaths. The majority have been observed in Papua New Guinea (PNG)--11,139 HIV, 1,926 AIDS and 353 AIDS deaths). Other countries with relatively high HIV rates are New Caledonia, French Polynesia, Guam and Kiribati. Fiji,

despite a high case count (182), has a mid-range rate of HIV infection due to its large population. Zero or very few cases have been reported in many other PICTs. Nearly two thirds of HIV cases have been diagnosed in the predominant risk group--young, sexually active adults. The ratio of male to female cases is 1.1 to 1 (2.5 to 1 excluding PNG), much lower than in countries like Australia (14.2) and New Zealand (5.5) where MSM predominates exposure. Rates of observed cases in women and young people are increasing with heterosexual contact being an increasingly important mode of spread. Other exposures include injecting drug use (mostly imported), perinatal transmission (rising more recently with increased heterosexual risk) and blood exposures. DISCUSSION: Apart from Papua New Guinea (PNG), which is currently experiencing an exponential increase in HIV infection, observed rates for most of the Pacific are currently low, and are either static or rising very slowly. Low observed HIV prevalence in pregnant women lends some support for this observation. However these data do not reflect the total HIV disease burden due to variable access to and uptake of testing, and incomplete notification. Several factors make the Pacific vulnerable to rapid spread such as close proximity to countries with high HIV prevalence, increasing migration, both in and outward, and expanding economic links with other regions. High levels of STIs and risk behaviours across the Pacific indicate significant potential for rapid spread where HIV is introduced. Efforts are currently under way to improve routine screening, complement with targeted surveillance surveys of at-risk and vulnerable groups. This expanded monitoring of HIV trends is designed to guide increasing efforts to prevent and control HIV in the region
SN - 1015-7867
AD - HIV/AIDS & STIs Public Health Programme, Secretariat of the Pacific Community, New Caledonia. tims@spc.int
UR - 18181491
ER -

TY - JOUR
ID - 1283
T1 - Off-label dermatologic uses of anti-TNF-a therapies. [Review] [67 refs]
A1 - Alexis,A.F.
A1 - Strober,B.E.
Y1 - 2005/12//
N1 - Alexis, Andrew F. Strober, Bruce E
Journal of cutaneous medicine and surgery
c1t, 9614685
IM
Comparative Study. Journal Article. Review
English
KW - MEDLINE
KW - Adolescent
KW - Adult
KW - Antibodies
KW - Monoclonal/ad [Administration & Dosage]
KW - Monoclonal/tu [Therapeutic Use]
KW - Monoclonal
KW - Humanized
KW - Arthritis
KW - Psoriatic/dt [Drug Therapy]
KW - Chi-Square Distribution
KW - Child
KW - Preschool
KW - Female
KW - Humans
KW - Immunoglobulin G/ad [Administration & Dosage]
KW - Immunoglobulin G/tu [Therapeutic Use]
KW - Infant
KW - Male
KW - Psoriasis/dt [Drug Therapy]
KW - Randomized Controlled Trials as Topic
KW - Receptors

KW - Tumor Necrosis Factor/ad [Administration & Dosage]
KW - Tumor Necrosis Factor/tu [Therapeutic Use]
KW - Skin Diseases/dt [Drug Therapy]
KW - Time Factors
KW - Tumor Necrosis Factor-alpha/ai [Antagonists & Inhibitors]
RP - NOT IN FILE
SP - 296
EP - 302
JF - Journal of Cutaneous Medicine & Surgery
JA - J Cutan Med Surg
VL - 9
IS - 6
CY - United States
N2 - BACKGROUND: Tumor necrosis factor-alpha (TNF-a) is a proinflammatory cytokine that plays an immunomodulatory role in a variety of systemic and dermatologic diseases. Currently, three anti-TNF-a drugs are available in North America- infliximab (approved in the U.S. for the treatment of rheumatoid arthritis, Crohn's disease, ankylosing spondylitis, ulcerative colitis, and psoriatic arthritis), etanercept (approved in the U.S. for the treatment of rheumatoid arthritis, juvenile rheumatoid arthritis, psoriatic arthritis, ankylosing spondylitis, and psoriasis), and adalimumab (approved for the treatment of rheumatoid arthritis and psoriatic arthritis). OBJECTIVE: To review the current literature supporting alternative (and currently off-label) dermatologic uses of TNF-a antagonists. METHODS: A MEDLINE search (1966-March 2005) was conducted using the keywords "infliximab," "etanercept," "adalimumab," "TNF inhibitors," and "off-label" to identify published reports of off-label dermatologic uses of TNF-a inhibitors. RESULTS: Anti-TNF-a therapies have been reported in the following dermatologic diseases: sarcoidosis, hidradenitis suppuritiva, cicatricial pemphigoid, Behcet's disease, pyoderma gangrenosum, multicentric reticulohistiocytosis, apthous stomatitis, Sneddon-Wilkinson disease, SAPHO syndrome, pityriasis rubra pilaris, eosinophilic fasciitis, panniculitis, Crohn's disease, necrobiosis lipoidica diabeticorum, dermatomyositis, and scleroderma. The vast majority of these reports are in the form of individual case reports and small case series. Only two published randomized controlled trials involving the off-label use of a TNF inhibitor were found. CONCLUSIONS: A growing number of published reports suggest that anti-TNF-a therapies may be effective in the treatment of numerous inflammatory skin diseases outside their currently approved indications. [References: 67]
SN - 1203-4754
AD - Department of Dermatology, St. Luke's-Roosevelt Hospital Center, New York, NY, USA
UR - 16699906
ER -

TY - JOUR
ID - 1284
T1 - [Evaluation of community-based rehabilitation programs in Ghana and Benin]. [French]
A1 - Jadin,O.
A1 - Agbogbe,N.
A1 - Barima,O.
Y1 - 2005/11//
N1 - Jadin, O. Agbogbe, N. Barima, O
Medecine tropicale : revue du Corps de sante colonial
mhq, 8710146
IM
Comparative Study. English Abstract. Journal Article
French
KW - MEDLINE
KW - Adolescent
KW - Adult
KW - Benin
KW - Child
KW - Preschool
KW - Community Health Services/st [Standards]
KW - Disabled Persons/rh [Rehabilitation]

KW - Ghana
KW - Humans
KW - Infant
KW - Program Evaluation
KW - Rehabilitation Centers
RP - NOT IN FILE
SP - 592
EP - 601
JF - Medecine Tropicale
JA - Med Trop (Mars.)
VL - 65
IS - 6
CY - France
N2 - This article describes two community-based rehabilitation programs set up for disabled persons in Ghana and Benin in the early 1990s. Both programs were based on the model described by the World Health Organization but differed greatly with regard to implementation. This difference had great impact on the respective effectiveness, efficiency and viability. Analysis of 624 disabled persons ranging in age from 0 to 30 years showed better progress in the Beninese group for all areas considered with a resulting improvement in quality of life. Assessment of the cost-effectiveness indicated that spending was higher and less effective in Ghana for globally less benefit than in Benin. Data also suggested that differences in implementation impacted program viability since the Ghana program collapsed in 1999 while the Beninese program is ongoing. The authors speculate that the differences in the two programs were related to failure to observe the basic principles of any community development strategy in Ghana. For successful community response to difficult local situations, populations require technical assistance from outside sources. National authorities must provide players at both lower levels with the necessary resources including subsidies, training, salaries, planning, monitoring and legislation. These basic rules were largely applied in Benin but almost completely ignored in Ghana
SN - 0025-682X
AD - Ecole Superieure de Kinesitherapie, 01 BP 3089 RP Cotonou, Benin. jadin.siebertz@serv.eit.bj
UR - 16555522
ER -

TY - JOUR
ID - 1285
T1 - Parasitic infections in Malaysia: changing and challenges
A1 - Nissapatorn,V.
A1 - Lim,Y.A.
A1 - Jamaiah,I.
A1 - Agnes,L.S.
A1 - Amyliana,K.
A1 - Wen,C.C.
A1 - Nurul,H.
A1 - Nizam,S.
A1 - Quake,C.T.
A1 - Valartmathi,C.
A1 - Woei,C.Y.
A1 - Anuar,A.K.
Y1 - 2005///
N1 - Nissapatorn, V. Lim, Y A L. Jamaiah, I. Agnes, L S H. Amyliana, K. Wen, C Chian. Nurul, H. Nizam, S. Quake, C T. Valartmathi, C. Woei, C Ying. Anuar, A Khairul
The Southeast Asian journal of tropical medicine and public health
uvn, 0266303
IM
Journal Article
English
KW - MEDLINE
KW - Adolescent

KW - Adult
KW - Age Distribution
KW - Animals
KW - Child
KW - Preschool
KW - Cryptosporidiosis/di [Diagnosis]
KW - Cryptosporidiosis/ep [Epidemiology]
KW - Cryptosporidiosis/ps [Parasitology]
KW - Cryptosporidium parvum/ip [Isolation & Purification]
KW - Female
KW - Giardia lamblia/ip [Isolation & Purification]
KW - Giardiasis/di [Diagnosis]
KW - Giardiasis/ep [Epidemiology]
KW - Humans
KW - Malaria/di [Diagnosis]
KW - Malaria/ep [Epidemiology]
KW - Malaysia/ep [Epidemiology]
KW - Male
KW - Middle Aged
KW - Plasmodium/ip [Isolation & Purification]
KW - Protozoan Infections/di [Diagnosis]
KW - Protozoan Infections/ep [Epidemiology]
KW - Protozoan Infections/ps [Parasitology]
KW - Seroepidemiologic Studies
KW - Sex Distribution
KW - Toxoplasmosis/di [Diagnosis]
KW - Toxoplasmosis/ep [Epidemiology]
RP - NOT IN FILE
SP - 50
EP - 59
JF - Southeast Asian Journal of Tropical Medicine & Public Health
JA - Southeast Asian J Trop Med Public Health
VL - 36 Suppl 4
CY - Thailand
N2 - A total of 1,885 blood and stool samples of four main protozoan parasitic infections were retrospectively reviewed from January, 2000 to April, 2004. Eleven of the 1,350 stool samples were shown positive for Cryptosporidium and Giardia infections; one of the 5 cases was clinically diagnosed as gastrointestinal cryptosporidiosis, while 6 cases were giardiasis. In patients with giardiasis, children were among the high-risk groups, making up 66.7% of these patients. The common presenting signs and symptoms were: diarrhea (83.3%), loss of appetite (83.3%), lethargy (83.3%), fever (66.7%), nausea/vomiting (50.0%), abdominal pain (16.7%), dehydration (16.7%) and rigor and chills (16.7%). Metronidazole was the drug of choice and was given to all symptomatic patients (83.3%). For the blood samples, 28 of the 92 peripheral smears for Plasmodium spp infection were diagnosed as malaria. The age range was from 4 to 57, with a median of 32.5 years. The sex ratio (M:F) was 3.6:1, while the age group of 30-44 years was the most commonly affected in both sexes. The majority of patients were foreigners (60.7%) and non-professional (39%). Plasmodium vivax (71%) infection was the most common pathogen found in these patients, along with a history of traveling to an endemic area of malaria (31%). The predominant presenting signs and symptoms were: fever (27%), rigor and chills (24%), nausea/vomiting (15%) and headache (8%). Chloroquine and primaquine was the most common anti-malarial regimen used (78.6%) in these patients. The seroprevalence of toxoplasmosis in different groups was 258/443 (58%): seropositive for IgG 143 (32.3%); IgM 67 (15%); and IgG + IgM 48 (10.8%). The age range was from 1 to 85, with a mean of 34 (+/- SD 16.6) years. The predominant age group was 21 to 40 years (126; 28.4%). The sex ratio (M:F) was 1.2:1. Subjects were predominantly male (142; 32%) and the Malay (117; 26.4%). Of these, 32 cases were clinically diagnosed with ocular toxoplasmosis. The range of age was from 10 to 56 years with a mean of 30.5 (+/- SD 12.05) years. The sex ratio (M:F) was 1:1.7. The majority were in the age group of 21 to 40 years, female (20; 62.5%), and Malay (17; 53%). They were also single (16; 50%), unemployed (12; 37%), and resided outside Kuala Lumpur (21; 65.6%). The more common clinical presentations were blurring of vision (25; 78%), floaters (10; 31%) and pain in the eye (7; 22%). We found that

funduscopic examination (100%) and seropositivity for anti-Toxoplasma antibodies (93.7%) were the main reasons for investigation. Choroidoretinitis was the most common clinical diagnosis (69%), while clindamycin was the most frequently used antimicrobial in all cases. Among HIV-infected patients, 10 cases were diagnosed as AIDS-related toxoplasmic encephalitis (TE) (9 were active and 1 had relapse TE). In addition, 1 case was confirmed as congenital toxoplasmosis
SN - 0125-1562
AD - Department of Parasitology, University of Malaya Medical Center, 50603 Kuala Lumpur, Malaysia. nissapat@hotmail.com
UR - 16438180
ER -

TY - JOUR
ID - 1286
T1 - Commentary: siblings in their families
A1 - Dunn,J.
Y1 - 2005/12//
N1 - Dunn, Judy
Journal of family psychology : JFP : journal of the Division of Family Psychology of the American Psychological Association (Division 43)
8802265, dlv
IM
Journal Article
English
KW - MEDLINE
KW - Adaptation
KW - Psychological/ph [Physiology]
KW - Adolescent
KW - Adolescent Behavior/px [Psychology]
KW - Child Behavior
KW - Child
KW - Preschool
KW - Family/px [Psychology]
KW - Friends/px [Psychology]
KW - Humans
KW - Parent-Child Relations
KW - Peer Group
KW - Sibling Relations
KW - Social Behavior
RP - NOT IN FILE
SP - 654
EP - 657
JF - Journal of Family Psychology
JA - J Fam Psychol
VL - 19
IS - 4
CY - United States
N2 - Three themes in the contributions to the Special Issue are discussed. The first is new evidence for associations between the quality of sibling relationships and children's adjustment problems and positive development, from research which also takes account of parent-child relationships, and genetic associations. The second is the new methodological approaches to studying the complexity of family patterns in which siblings play a central role (for instance with multilevel modelling) and the study of changes over time. The third is the significance of connections with relationships outside the family especially peer relationships and friendships. Copyright 2006 APA, all rights reserved)
SN - 0893-3200
AD - Institute of Psychiatry, King's College London, England. judy.dunn@iop.kcl.ac.uk
UR - 16402881
ER -

TY - JOUR
ID - 1287
T1 - Paediatric home ventilatory support: the Auckland experience
A1 - Edwards,E.A.
A1 - Hsiao,K.
A1 - Nixon,G.M.
Y1 - 2005/12//
N1 - Edwards, E A. Hsiao, K. Nixon, G M
Journal of paediatrics and child health
arp, 9005421
IM
Journal Article
English
KW - MEDLINE
KW - Adolescent
KW - Child
KW - Preschool
KW - Continuous Positive Airway Pressure/sn [Statistics & Numerical Data]
KW - Female
KW - Follow-Up Studies
KW - Home Care Services
KW - Humans
KW - Infant
KW - Newborn
KW - Male
KW - New Zealand
KW - Respiration Disorders/th [Therapy]
KW - Retrospective Studies
KW - Tracheostomy
KW - Treatment Outcome
RP - NOT IN FILE
SP - 652
EP - 658
JF - Journal of Paediatrics & Child Health
JA - J Paediatr Child Health
VL - 41
IS - 12
CY - Australia
N2 - OBJECTIVES: To examine the trend over time, describe the disease categories treated, intervention success and outcomes of the children treated at home with continuous positive airway pressure (CPAP), non-invasive ventilation (NIV) and ventilation via tracheostomy (invasive ventilatory support, IVS) by the Respiratory Service at the Starship Children's Hospital in Auckland. METHODS: A retrospective review was undertaken of the Respiratory Service records from November 1991 to February 2004. RESULTS: Home CPAP, NIV or IVS was initiated in 160 children (89 boys, median age 6 years) in the study period. Sixty-nine (46 boys) remain on support and are still actively managed by the Starship Respiratory Service, of whom 46% live outside the Greater Auckland Region. Despite 42% of children being less than 5 years of age at initiation of therapy, institution of support failed in only 11%. The majority received treatment by non-invasive mask interface (68% (n = 108) CPAP, 29% (n = 47) NIV), with only 3% (n = 5) supported via tracheostomy. The numbers and complexity of support rose over the 12 years. Respiratory support was discontinued in 57% of cases, after a median of 12.5 months (range 3-52 months); in two-thirds, support was no longer required due to an improvement in the medical condition. The most common indication for support in current patients is respiratory parenchymal or airway disease followed by neuromuscular disease. Obesity is not a common indication. CONCLUSION: This review documents the increasing trend in children receiving respiratory support at home. Future planning and resources are needed to address this growing need
SN - 1034-4810
AD - Department of Respiratory Medicine, Starship Children's Hospital, Auckland, New Zealand

UR - 16398869
ER -

TY - JOUR
ID - 1288
T1 - What's for lunch? When's recess? The fight against obesity makes its way into schools
A1 - Winterfeld,A.
Y1 - 2005/12//
N1 - Winterfeld, Amy
State legislatures
100890177, dek
T
Journal Article
English
KW - MEDLINE
KW - Adolescent
KW - Adult
KW - Child
KW - Child Nutritional Physiological Phenomena
KW - Choice Behavior
KW - Exercise
KW - Food Habits
KW - Food Services/lj [Legislation & Jurisprudence]
KW - Health Education
KW - Humans
KW - Life Style
KW - Obesity/pc [Prevention & Control]
KW - Obesity/th [Therapy]
KW - Physical Education and Training/lj [Legislation & Jurisprudence]
KW - Physical Education and Training
KW - School Health Services/lj [Legislation & Jurisprudence]
KW - State Government
KW - Students
RP - NOT IN FILE
SP - 32
EP - 33
JF - State Legislatures
JA - State Legis.
VL - 31
IS - 10
CY - United States
SN - 0147-6041
UR - 16397979
ER -

TY - JOUR
ID - 1289
T1 - Promoting physical activity among children and adolescents: the strengths and limitations of school-based approaches
A1 - Booth,M.
A1 - Okely,A.
Y1 - 2005/04//
N1 - Booth, Michael. Okely, Anthony
Health promotion journal of Australia : official journal of Australian Association of Health Promotion Professionals
9710936
IM

Journal Article
English
KW - MEDLINE
KW - Adolescent
KW - Child
KW - Curriculum
KW - Faculty
KW - Health Promotion
KW - Humans
KW - Life Style
KW - Motor Activity
KW - Schools/og [Organization & Administration]
RP - NOT IN FILE
SP - 52
EP - 54
JF - Health Promotion Journal of Australia
JA - HEALTH PROMOT J AUST
VL - 16
IS - 1
CY - Australia
N2 - Paediatric overweight and obesity is recognised as one of Australia's most significant health problems and effective approaches to increasing physical activity and reducing energy consumption are being sought urgently. Every potential approach and setting should be subjected to critical review in an attempt to maximise the impact of policy and program initiatives. This paper identifies the strengths and limitations of schools as a setting for promoting physical activity. The strengths are: most children and adolescents attend school; most young people are likely to see teachers as credible sources of information; schools provide access to the facilities, infrastructure and support required for physical activity; and schools are the workplace of skilled educators. Potential limitations are: those students who like school the least are the most likely to engage in health-compromising behaviours and the least likely to be influenced by school-based programs; there are about 20 more hours per week available for physical activity outside schools hours than during school hours; enormous demands are already being made on schools; many primary school teachers have low levels of perceived competence in teaching physical education and fundamental movement skills; and opportunities for being active at school may not be consistent with how and when students prefer to be active
SN - 1036-1073
AD - Centre for Research into Adolescents' Health, NSW Centre for Overweight and Obesity, School of Public Health, University of Sydney, New South Wales. Michael_Booth@health.usyd.edu.au
UR - 16389931
ER -

TY - JOUR
ID - 1290
T1 - The relative effects of classwide peer tutoring and peer coaching on the positive social behaviors of children with ADHD
A1 - Plumer,P.J.
A1 - Stoner,G.
Y1 - 2005/08//
N1 - Plumer, Pamela J. Stoner, Gary
Journal of attention disorders
9615686
IM
Journal Article
English
KW - MEDLINE
KW - Attention Deficit Disorder with Hyperactivity/ep [Epidemiology]
KW - Attention Deficit Disorder with Hyperactivity/px [Psychology]
KW - Attention Deficit Disorder with Hyperactivity/th [Therapy]
KW - Child

KW - Consumer Satisfaction
KW - Faculty
KW - Humans
KW - Observer Variation
KW - Peer Group
KW - School Health Services
KW - Social Behavior
KW - Treatment Outcome
RP - NOT IN FILE
SP - 290
EP - 300
JF - Journal of Attention Disorders
JA - J Atten Disord
VL - 9
IS - 1
CY - Canada
N2 - This study investigates the effects of Classwide Peer Tutoring (CWPT) and peer coaching on the peer social behaviors of children with ADHD. A single-subject, multiple-baseline design is used with three elementary-school students in Grades 3 and 4. Following a baseline period, CWPT is implemented in each student's classroom. During the second intervention phase, CWPT is continued and peer coaching is added. Peer social behaviors are observed in both academic and social settings, with a primary focus on intervention effects on the latter setting. Results suggest that students participating in CWPT are actively and positively engaged with their peers while carrying out the CWPT program in the academic setting. However, when only CWPT is implemented, increases in positive peer social behaviors are not observed in social settings. The addition of peer coaching results in enhanced social behaviors during recess and lunch
SN - 1087-0547
AD - School Psychology Program, University of Massachusetts Amherst, MA 01003, USA.
pplumer@educ.umass.edu
UR - 16371675
ER -

TY - JOUR
ID - 1291
T1 - Youth solar ultraviolet radiation exposure, concurrent activities and sun-protective practices: a review.
[Review] [120 refs]
A1 - Wright,C.Y.
A1 - Reeder,A.I.
Y1 - 2005/11//
N1 - Wright, C Y. Reeder, A I
Photochemistry and photobiology
0376425, p69
IM
Journal Article. Research Support, Non-U.S. Gov't. Review
English
KW - MEDLINE
KW - Adolescent
KW - Age Factors
KW - Child
KW - Film Dosimetry
KW - Health Knowledge
KW - Attitudes
KW - Practice
KW - Humans
KW - Radiation Dosage
KW - Research Design
KW - Skin Neoplasms/et [Etiology]
KW - Skin Neoplasms/pc [Prevention & Control]

KW - Sunlight/ae [Adverse Effects]
KW - Sunlight
KW - Time Factors
KW - Ultraviolet Rays/ae [Adverse Effects]
KW - Ultraviolet Rays
RP - NOT IN FILE
SP - 1331
EP - 1342
JF - Photochemistry & Photobiology
JA - Photochem Photobiol
VL - 81
IS - 6
CY - United States
N2 - To assist standardization of procedures, facilitate comparisons, and help guide research efforts to optimally inform development of appropriately targeted interventions, there is a need to review methods used to quantify child and adolescent solar ultraviolet radiation (UV) exposure, related outdoor activities and sun-protective practices. This holistic approach is essential for comprehensive research that will provide all-inclusive, informative and meaningful messages for preventive measures of harmful UV exposure. Two databases were searched and 29 studies were retrieved, and these studies report measurement or assessment techniques documenting UV exposure patterns and related outdoor activities. Polysulfone film badges were the main measurement instrument used in 10 studies, with questionnaire, survey data, observation, a model, electronic dosimeters, biological dosimeters, colorimeter and UV colouring labels used in the remaining studies. Methods used to record activities included self-report, parental report, a logbook and observation. Measurement duration and unit of UV exposure varied in most studies, but a method common to 15 studies was measured UV exposure as a percentage of ambient UV. The studies reviewed do not provide sufficient information for the development and evaluation of targeted youth sun protection programs. Studies are required which document precise UV exposure, concurrent activities and sun protection usage for children and adolescents. [References: 120]
SN - 0031-8655
AD - Social and Behavioural Research in Cancer Group, Department of Preventive and Social Medicine, University of Otago, Dunedin, New Zealand. wrica032@student.otago.ac.nz
UR - 16354111
ER -

TY - JOUR
ID - 1292
T1 - Sun protection policies in Miami-Dade County public schools: opportunities for skin cancer prevention. [Review] [26 refs]
A1 - Kirsner,R.S.
A1 - Parker,D.F.
A1 - Brathwaite,N.
A1 - Thomas,A.
A1 - Tejada,F.
A1 - Trapido,E.J.
Y1 - 2005/11//
N1 - Kirsner, Robert S. Parker, Dorothy F. Brathwaite, Noel. Thomas, Andrea. Tejada, Francisco. Trapido, Edward J
Pediatric dermatology
ped, 8406799
IM
Journal Article. Research Support, U.S. Gov't, P.H.S.. Review
English
KW - MEDLINE
KW - Adolescent
KW - Child
KW - Preschool
KW - Data Collection

KW - Female
KW - Florida
KW - Health Education/og [Organization & Administration]
KW - Health Promotion/og [Organization & Administration]
KW - Humans
KW - Male
KW - Multicenter Studies as Topic
KW - Policy Making
KW - Program Evaluation
KW - Protective Clothing
KW - Questionnaires
KW - Risk Assessment
KW - School Health Services/og [Organization & Administration]
KW - Schools
KW - Skin Neoplasms/pc [Prevention & Control]
KW - Sunlight
KW - Sunscreening Agents/ad [Administration & Dosage]
KW - Ultraviolet Rays
RP - NOT IN FILE
SP - 513
EP - 519
JF - Pediatric Dermatology
JA - Pediatr Dermatol
VL - 22
IS - 6
CY - United States
N2 - Childhood exposure to ultraviolet radiation from the sun and a history of sunburns are risk factors for skin cancer. Because children spend time outdoors when they are at school, school sun protection policies are an important health issue, particularly in areas of the country with year-round warm and sunny climates, such as Florida. To better understand the sun protection policies and practices in South Florida schools, a sample (n = 51) of elementary and middle schools in Miami-Dade County public schools were surveyed as part of a CDC-funded cancer control program at the University of Miami. Of the principals and teachers surveyed, most (78%) knew about the county school system's guidelines for avoiding excessive heat exposure, which include two sun protection measures. Two-thirds reported that they shared these guidelines with teachers; 21% shared them with parents. Few schools monitor implementation of the guidelines, although 70% schedule outdoor activities to avoid peak sun hours. No schools required sunscreen, hats, or protective clothing. Physical education teachers and students spend an average of 4.5 and 0.6 hours per day outdoors, respectively. Improved school sun protection policies and monitoring of such policies is needed to reduce sun exposure and skin cancer risk for both students and staff. [References: 26]
SN - 0736-8046
AD - Department of Dermatology and Cutaneous Surgery, University of Miami School of Medicine, Miami, Florida 33125, USA. rkirsner@med.miami.edu
UR - 16354252
ER -

TY - JOUR
ID - 1293
T1 - Tanning and skin cancer. [Review] [105 refs]
A1 - Abdulla,F.R.
A1 - Feldman,S.R.
A1 - Williford,P.M.
A1 - Krowchuk,D.
A1 - Kaur,M.
Y1 - 2005/11//
N1 - Abdulla, Farah R. Feldman, Steven R. Williford, Phillip M. Krowchuk, Daniel. Kaur, Mandeep
Pediatric dermatology
ped, 8406799

IM
Journal Article. Review
English
KW - MEDLINE
KW - Adolescent
KW - Age Factors
KW - Carcinoma
KW - Basal Cell/ep [Epidemiology]
KW - Basal Cell/et [Etiology]
KW - Basal Cell/pa [Pathology]
KW - Child
KW - Preschool
KW - Female
KW - Humans
KW - Incidence
KW - Male
KW - Melanoma/ep [Epidemiology]
KW - Melanoma/et [Etiology]
KW - Melanoma/pa [Pathology]
KW - Prognosis
KW - Risk Assessment
KW - Skin Neoplasms/ep [Epidemiology]
KW - Skin Neoplasms/et [Etiology]
KW - Skin Neoplasms/pa [Pathology]
KW - Skin Pigmentation/ph [Physiology]
KW - Sunburn/ep [Epidemiology]
KW - Sunburn/pc [Prevention & Control]
KW - Sunlight/ae [Adverse Effects]
KW - Survival Rate
KW - Ultraviolet Rays/ae [Adverse Effects]
KW - United States/ep [Epidemiology]
RP - NOT IN FILE
SP - 501
EP - 512
JF - Pediatric Dermatology
JA - Pediatr Dermatol
VL - 22
IS - 6
CY - United States
N2 - Skin cancer is a large and growing problem in the United States. Sun and other ultraviolet (UV) light exposures play a key role in the development of skin cancer. Pediatricians can play an important role in counseling patients and are in a position to help educate children and their families about skin cancer. The purpose of this review is to familiarize pediatricians with the magnitude of the skin cancer problem and the evidence that ultraviolet light exposure, particularly indoor tanning, contributes to this problem. We reviewed the literature on ultraviolet light and skin cancer (based on a MEDLINE search of articles using the headings "ultraviolet light" and "skin cancer") and found that skin cancer is the most rapidly growing cause of cancer deaths in the United State. There is strong epidemiologic evidence for the relationship between UV exposure and nonmelanoma skin cancer and growing evidence for the relationship between indoor tanning and melanoma. We recommend that pediatricians counsel children and their parents about UV protection. Measures such as use of sunscreen and hats for outdoor play, both at home and in school, should be encouraged. [References: 105]
SN - 0736-8046
AD - Center for Dermatology Research, Department of Dermatology, Wake Forest University School of Medicine, Winston-Salem, North Carolina 27157-1071, USA
UR - 16354251
ER -

TY - JOUR
ID - 1294
T1 - Indoor physical education measured by heart rate monitor. A case study in Portugal
A1 - Wang,G.Y.
A1 - Pereira,B.
A1 - Mota,J.
Y1 - 2005/06//
N1 - Wang, G Y. Pereira, B. Mota, J
The Journal of sports medicine and physical fitness
0376337, k6v
IM
Journal Article. Research Support, Non-U.S. Gov't
English
KW - MEDLINE
KW - Adolescent
KW - Child
KW - Exercise/ph [Physiology]
KW - Female
KW - Heart Rate/ph [Physiology]
KW - Humans
KW - Male
KW - Monitoring
KW - Physiologic/is [Instrumentation]
KW - Physical Education and Training
KW - Physical Fitness
KW - Portugal
KW - Schools
RP - NOT IN FILE
SP - 171
EP - 177
JF - Journal of Sports Medicine & Physical Fitness
JA - J Sports Med Phys Fitness
VL - 45
IS - 2
CY - Italy
N2 - AIM: Reports indicated that children are spending too much time being physically inactive during physical education classes and many school physical education programs are not meeting the recommendation. The purpose of this study was to investigate the physical activity levels in Portuguese indoor physical education classes. METHODS: The physical activity levels of 28 Portuguese 7th grade children were assessed by heart rate monitor during their indoor physical education classes. RESULTS: The study showed that much physical education class time had been wasted, and less than 70% of physical education class time had actually been used in classes. On average, 14.4 minutes of 45-min physical education classes and 27.9 min of 90-min physical education classes had been spent in MVPA with children's heart rates above 139 bpm; 6.7 min of 45-min physical education classes and 15.7 min of 90-min physical education classes had been spent in VPA with children's heart rates above 159 bpm. Children engaged in more MVPA, VPA, and continuous bouts of physical activity in 90-min physical education classes than those of 45-min physical education classes. There were no significant differences in activity levels between genders in co-educational indoor physical education classes. CONCLUSIONS: This indoor PE class study supports the results that children are less active during school physical education classes. Both the quantity and the quality of school physical education needs to improve to encourage children to engage in a substantial amount of physical activity both inside and outside school
SN - 0022-4707
AD - Institute of Child Study, University of Minho, Braga, Portugal. wang@iec.uminho.pt
UR - 16355077
ER -

TY - JOUR
ID - 1295

T1 - The effect of multicolor playground markings on children's physical activity level during recess
A1 - Stratton,G.
A1 - Mullan,E.
Y1 - 2005/11//
N1 - Stratton, Gareth. Mullan, Elaine
Preventive medicine
pm4, 0322116
IM
Journal Article. Research Support, Non-U.S. Gov't
English
KW - MEDLINE
KW - Child
KW - Preschool
KW - Color
KW - England
KW - Female
KW - Humans
KW - Male
KW - Motivation
KW - Physical Exertion/ph [Physiology]
KW - Play and Playthings
KW - Schools
KW - Wales
RP - NOT IN FILE
SP - 828
EP - 833
JF - Preventive Medicine
JA - Prev Med
VL - 41
IS - 5-6
CY - United States
N2 - BACKGROUND: British children do not meet daily physical activity recommendations, and as yet no sustainable intervention has been reported to address their sedentary lifestyle. The purpose of this investigation was twofold: First, to examine whether painting playgrounds with multicolored markings would increase the percent of recess time spent in moderate to vigorous physical activity (MVPA) and vigorous physical activity (VPA) in girls and boys and second to quantify the contribution recess makes to national recommendations for young people's physical activity. METHODS: MVPA and vigorous physical activity (VPA) were measured using short wave heart rate telemetry. Physical activity levels were compared before and after playgrounds were painted in 4 intervention schools and compared to 4 matched schools with unmarked playgrounds. Schools were situated in areas of deprivation. Ninety-nine children completed the study. RESULTS: A significant interaction (group x time) was evident for MVPA and VPA. Time spent in MVPA and VPA increased significantly in intervention schools as a result of playground painting. Boys were more active than girls, and activity decreased with age, but neither difference reached significance. CONCLUSION: Multicolor playground markings can be a low-cost method of increasing children's daily physical activity levels in the short term. If these increases were sustained, then school playgrounds with multicolor markings would make a valuable contribution to physical activity recommendations for young people
SN - 0091-7435
AD - REACH Group, Research Institute for Sport and Exercise Sciences, Liverpool John Moores University, 15-21 Webster St., Liverpool L3 2ET, UK. g.stratton@livjm.ac.uk
UR - 16137756
ER -

TY - JOUR
ID - 1296
T1 - Measuring chromosome breaks in patients with thalassemia
A1 - Offer,T.
A1 - Bhagat,A.

A1 - Lal,A.
A1 - Atamna,W.
A1 - Singer,S.T.
A1 - Vichinsky,E.P.
A1 - Kuypers,F.A.
A1 - Ames,B.N.
Y1 - 2005///
N1 - Offer, Tal. Bhagat, Amrita. Lal, Ashutosh. Atamna, Wafa. Singer, Sylvia T. Vichinsky, Elliott P. Kuypers, Frans A. Ames, Bruce N
Annals of the New York Academy of Sciences
5nm, 7506858
IM
Journal Article
English
KW - MEDLINE
KW - Cell Separation
KW - Child
KW - Chromosome Breakage
KW - DNA Damage
KW - Dactinomycin/an [Analysis]
KW - Flow Cytometry
KW - Fluorescent Dyes/an [Analysis]
KW - Humans
KW - Immunomagnetic Separation
KW - Micronucleus Tests
KW - Oxidative Stress
KW - Reticulocytes/ch [Chemistry]
KW - Reticulocytes/ul [Ultrastructure]
KW - Splenectomy
KW - Staining and Labeling
KW - Thalassemia/bl [Blood]
KW - Thalassemia/ge [Genetics]
KW - Thalassemia/pa [Pathology]
KW - Thalassemia/su [Surgery]
RP - NOT IN FILE
SP - 439
EP - 444
JF - Annals of the New York Academy of Sciences
JA - Ann N Y Acad Sci
VL - 1054
CY - United States
N2 - Iron-mediated oxidative stress plays an important role in the pathophysiology of thalassemia. Oxidative stress can cause lesions in DNA, including double-strand breaks. DNA damage, which is a cause of cancer (although not the only one), is recognized as deleterious. Unlike cancer, DNA damage can be assayed easily and relatively inexpensively in humans. In this study, a sensitive micronucleus assay was used to measure the frequency of chromosomal breaks in patients with alpha- and beta-thalassemia. The micronucleus test is based on the observation that a secondary nucleus (micronucleus) is formed around a chromosomal fragment, outside the main nucleus of a dividing cell. Micronuclei are readily apparent in red blood cells (RBCs), which otherwise lack DNA. We combined an immunomagnetic separation technique with single-laser flow cytometry to isolate and analyze reticulocytes in peripheral blood for the presence of micronuclei before these cells are removed by the spleen. Blood samples were obtained from patients with thalassemia and healthy volunteers. After immunomagnetic enrichment of CD71-positive reticulocytes, the cells were stained for micronuclei using the DNA dye 7-aminoactinomycin D (7-AAD) and evaluated by flow cytometry. Our findings indicate that higher levels of micronuclei frequencies are present in thalassemic RBCs
SN - 0077-8923
AD - Children's Hospital Oakland Research Institute, Oakland, CA 94609, USA
UR - 16339694

ER -

TY - JOUR
ID - 1297
T1 - Tick prevention in a population living in a highly endemic area
A1 - Stjernberg,L.
A1 - Berglund,J.
Y1 - 2005///
N1 - Stjernberg, Louise. Berglund, Johan
Scandinavian journal of public health
dew, 100883503
IM
Comparative Study. Journal Article. Research Support, Non-U.S. Gov't
English
KW - MEDLINE
KW - Adolescent
KW - Adult
KW - Animals
KW - Bacterial Vaccines/ad [Administration & Dosage]
KW - Bites and Stings/ep [Epidemiology]
KW - Bites and Stings/pc [Prevention & Control]
KW - Child
KW - Preschool
KW - Cohort Studies
KW - Encephalitis
KW - Tick-Borne/pc [Prevention & Control]
KW - Endemic Diseases/pc [Prevention & Control]
KW - Female
KW - Humans
KW - Infant
KW - Ixodes
KW - Lyme Neuroborreliosis/pc [Prevention & Control]
KW - Male
KW - Middle Aged
KW - Questionnaires
KW - Risk Factors
KW - Sweden/ep [Epidemiology]
KW - Tick-Borne Diseases/ep [Epidemiology]
KW - Tick-Borne Diseases/pc [Prevention & Control]
KW - Viral Vaccines/ad [Administration & Dosage]
RP - NOT IN FILE
SP - 432
EP - 438
JF - Scandinavian Journal of Public Health
JA - Scand J Public Health
VL - 33
IS - 6
CY - Sweden
N2 - AIMS: To describe environmental and personal tick-preventive measures and their predictors, taken by a population living in a highly tick-endemic area. METHODS: Owing to the recent confirmation of human tick-borne encephalitis cases, vaccination against tick-borne encephalitis was offered to the population living in the endemic area through the use of leaflets and media campaigns. At the time of the initial dose, information and enrollment to this cohort study was carried out. Participants' characteristics, frequency of tick-bites and preventive measures were included in questionnaires. Logistic analysis was used to determine behavioural differences in activities taken in order to prevent tick bites. CONCLUSION: In total, 70% of the permanent residents had themselves vaccinated before the next tick season. Of the studied participants 356/517 (69%) regularly took preventive measures in their environment and/or personally. Women in particular, and those

previously treated for a tick-borne disease, took significantly more preventive measures. When analysing all variables together, spending less time in a tick-endemic area and being tick-bitten during the latest tick season significantly increased the probability of taking preventive measures. After being tick-bitten, men were more inclined to start taking preventive measures than women. Awareness of the risks caused by living in a high tick-endemic area influenced the participant's daily life through preventive activities. Public health action should be considered, thus encouraging out-of-door activities for the population without anxiety as to the risks of contracting tick-borne disease after being tick-bitten
SN - 1403-4948
AD - Department of Community Medicine, Lund University, Malmo, Sweden. louise.stjernberg@bth.se
UR - 16332608
ER -

TY - JOUR
ID - 1298
T1 - Severity of playground fractures: play equipment versus standing height falls
A1 - Fiissel,D.
A1 - Pattison,G.
A1 - Howard,A.
Y1 - 2005/12//
N1 - Fiissel, D. Pattison, G. Howard, A
Injury prevention : journal of the International Society for Child and Adolescent Injury Prevention
crz, 9510056
IM
Comparative Study. Journal Article
English
KW - MEDLINE
KW - Accidental Falls/pc [Prevention & Control]
KW - Accidental Falls/sn [Statistics & Numerical Data]
KW - Adolescent
KW - Canada/ep [Epidemiology]
KW - Case-Control Studies
KW - Child
KW - Preschool
KW - Consumer Product Safety
KW - Female
KW - Fractures
KW - Bone/ep [Epidemiology]
KW - Bone/pc [Prevention & Control]
KW - Humans
KW - Infant
KW - Injury Severity Score
KW - Male
KW - Odds Ratio
KW - Play and Playthings/in [Injuries]
KW - Prevalence
RP - NOT IN FILE
SP - 337
EP - 339
JF - Injury Prevention
JA - Inj Prev
VL - 11
IS - 6
CY - England
N2 - AIM: To compare the severity of fractures from playground equipment falls to the severity of fractures from standing height falls occurring on the playground. METHODS: This case control study used data on all children presenting to the Hospital for Sick Children (Toronto) from 1995 to 2002 with a fracture due to a playground fall. Cases were children who fell from a height off playground equipment. Controls were children

who fell from standing height on a playground. Fractures were major if they required reduction and minor if they did not. RESULTS: Fractures from equipment falls were 3.91 (95% CI 2.76 to 5.54) times more likely to require reduction than were fractures from standing height falls. CONCLUSIONS: Major fractures were strongly associated with falls from playground equipment, whereas minor fractures came from both play equipment and standing height falls. Efforts to prevent major fractures should target playground equipment and the impact absorbing surface beneath it
SN - 1353-8047
AD - The Hospital for Sick Children, Toronto, Canada
UR - 16326766
ER -

TY - JOUR
ID - 1299
T1 - Percutaneous intramedullary decompression, curettage, and grafting with medical-grade calcium sulfate pellets for unicameral bone cysts in children: a new minimally invasive technique
A1 - Dormans,J.P.
A1 - Sankar,W.N.
A1 - Moroz,L.
A1 - Erol,B.
Y1 - 2005/11//
N1 - Dormans, John P. Sankar, Wudbhav N. Moroz, Leslie. Erol, Bulent
Journal of pediatric orthopedics
hsw, 8109053
IM
Clinical Trial. Journal Article
English
KW - MEDLINE
KW - Administration
KW - Cutaneous
KW - Adolescent
KW - Bone Cysts/th [Therapy]
KW - Calcaneus/pa [Pathology]
KW - Calcaneus/ra [Radiography]
KW - Calcaneus/su [Surgery]
KW - Calcium Sulfate/ad [Administration & Dosage]
KW - Calcium Sulfate/tu [Therapeutic Use]
KW - Child
KW - Preschool
KW - Curettage/mt [Methods]
KW - Decompression
KW - Surgical/mt [Methods]
KW - Drug Implants
KW - Female
KW - Femur/pa [Pathology]
KW - Femur/ra [Radiography]
KW - Femur/su [Surgery]
KW - Fibula/pa [Pathology]
KW - Fibula/ra [Radiography]
KW - Fibula/su [Surgery]
KW - Fluoroscopy
KW - Follow-Up Studies
KW - Humans
KW - Humerus/pa [Pathology]
KW - Humerus/ra [Radiography]
KW - Humerus/su [Surgery]
KW - Male
KW - Surgical Procedures

KW - Minimally Invasive/mt [Methods]
KW - Treatment Outcome
RP - NOT IN FILE
SP - 804
EP - 811
JF - Journal of Pediatric Orthopedics
JA - J Pediatr Orthop
VL - 25
IS - 6
CY - United States
N2 - Several treatment options exist for unicameral bone cysts (UBCs), including observation, steroid injection, bone marrow injection, and curettage and bone grafting. These are all associated with high recurrence rates, persistence, and occasional complications. Newer techniques have been described, most with variable success and only short follow-up reported. Because of these factors, a new minimally invasive percutaneous technique was developed for the treatment of UBCs in children. Twenty-eight children with UBCs who underwent percutaneous intramedullary decompression, curettage, and grafting with medical-grade calcium sulfate (MGCS) pellets by the senior author (J.P.D.) between April 2000 and April 2003 were analyzed as part of a pediatric musculoskeletal tumor registry at a large tertiary children's hospital. Four patients were lost to follow-up, and the remaining 24 patients had an average follow-up of 21.9 months (range 4-48 months). Twelve patients were followed for at least 24 months. Six of the 24 children had received previous treatment of their UBC, most often at an outside institution. Follow-up was performed through clinical evaluation and radiographic review. Postoperative radiographs at most recent follow-up showed complete healing, defined as more than 95% opacification, in 22 of 24 patients (91.7%). One patient (4.2%) demonstrated partial healing, defined as 80% to 95% opacification. One patient had less than 80% radiographic healing (4.2%). All 24 patients returned to full activities and were asymptomatic at most recent follow-up. The only complication noted was a superficial suture abscess that occurred in one patient; this resolved with local treatment measures. The new minimally invasive technique of percutaneous intramedullary decompression, curettage, and grafting with MGCS pellets demonstrates favorable results with low complication and recurrence rates compared with conventional techniques. The role of intramedullary decompression as a part of this percutaneous technique is discussed
SN - 0271-6798
AD - Children's Hospital of Philadelphia, Wood Building, Philadelphia, PA 19104, USA. dormans@email.chop.edu
UR - 16294140
ER -

TY - JOUR
ID - 1300
T1 - A miniaturized active sampler for the assessment of personal exposure to nitrogen dioxide
A1 - Staimer,N.
A1 - Delfino,R.J.
A1 - Bufalino,C.
A1 - Fine,P.M.
A1 - Sioutas,C.
A1 - Kleinman,M.T.
Y1 - 2005/11//
N1 - Staimer, Norbert. Delfino, Ralph J. Bufalino, Charles. Fine, Philip M. Sioutas, Constantinos. Kleinman, Michael T
Analytical and bioanalytical chemistry
101134327
IM
Evaluation Studies. Journal Article. Research Support, N.I.H., Extramural
English
KW - MEDLINE
KW - Air Pollutants/an [Analysis]
KW - Child
KW - Preschool

KW - Environmental Monitoring/is [Instrumentation]
KW - Ethanolamines/ch [Chemistry]
KW - Humans
KW - Los Angeles
KW - Miniaturization
KW - Nitrogen Dioxide/an [Analysis]
RP - NOT IN FILE
SP - 955
EP - 962
JF - Analytical & Bioanalytical Chemistry
JA - Anal Bioanal.Chem
VL - 383
IS - 6
CY - Germany
N2 - A personalized, miniaturized air sampling system was evaluated to estimate the daily exposure of pediatric asthmatics to nitrogen dioxide (NO2). The lightweight device (170 g) uses a sampling pump connected to a solid sorbent tube containing triethanolamine (TEA)-impregnated molecular sieve. The pump is powered by a 9 V battery and samples air over a 24 h period at a collection rate of 0.100 L/min. After exposure, the solid sorbent is removed from the tubes for spectrophotometric analysis (Griess Assay). The lower detection limit of the overall method for NO2 is 11 microg/m3. The linearity, precision and accuracy of the sampler was evaluated. Different NO2 concentrations generated in the laboratory (range: 50 to 340 microg/m3) were simultaneously measured by the TEA tube samplers and colocated continuous chemiluminescent NO(x) analyzers (reference method). The coefficient of determination for the laboratory test derived from ordinary linear regression (OLR) was r2 = 0.99 (y(OLR) = 0.94 x -4.58) and the precision 3.6%. Further, ambient NO2 concentrations in the field (range: 10-120 microg/m3) were verified with continuous chemiluminescent monitors next to the active samplers. Re-weighted least squares analysis (RLS) based on the least median squares procedure (LMS) resulted in a correlation of r2 = 0.68 for a field comparison in Riverside, CA (y(RLS) = 1.01 x -0.94) and r2 = 0.92 in Los Angeles, CA (y(RLS) = 1.31 x -7.12). The precision of the TEA tube devices was 7.4% (at 20-60 microg/m3 NO2) under outdoor conditions. Data show that the performance of this small active sampling system was satisfactory for measuring environmental concentrations of NO2 under laboratory and field conditions. It is useful for personal monitoring of NO2 in environmental epidemiology studies where daily measurements are desired
SN - 1618-2642
AD - Epidemiology Division, Department of Medicine, School of Medicine, University of California at Irvine, Irvine, CA 92617, USA. nstaimer@uci.edu
UR - 16231134
ER -

TY - JOUR
ID - 1301
T1 - Importance of the personal endotoxin cloud in school-age children with asthma
A1 - Rabinovitch,N.
A1 - Liu,A.H.
A1 - Zhang,L.
A1 - Rodes,C.E.
A1 - Foarde,K.
A1 - Dutton,S.J.
A1 - Murphy,J.R.
A1 - Gelfand,E.W.
Y1 - 2005/11//
N1 - Rabinovitch, Nathan. Liu, Andrew H. Zhang, Lening. Rodes, Charles E. Foarde, Karin. Dutton, Steven J. Murphy, James R. Gelfand, Erwin W
The Journal of allergy and clinical immunology
h53, 1275002
AIM, IM
Journal Article. Research Support, N.I.H., Extramural. Research Support, Non-U.S. Gov't. Research Support,

U.S. Gov't, Non-P.H.S.
English
KW - MEDLINE
KW - Air Pollutants
KW - Asthma/pp [Physiopathology]
KW - Child
KW - Endotoxins/an [Analysis]
KW - Environmental Monitoring
KW - Female
KW - Forced Expiratory Volume
KW - Humans
KW - Male
KW - Personal Space
KW - Schools
KW - Severity of Illness Index
RP - NOT IN FILE
SP - 1053
EP - 1057
JF - Journal of Allergy & Clinical Immunology
JA - J Allergy Clin Immunol
VL - 116
IS - 5
CY - United States
N2 - BACKGROUND: A number of studies have observed associations between the amount of endotoxin in urban dust and chronic asthma severity, but a direct relationship between personal exposure to household endotoxin and acute asthma worsening has not yet been defined. OBJECTIVE: We sought to investigate the relationship between day-to-day changes in personal endotoxin exposure and asthma severity. METHODS: In the winter and spring of 1999 through 2000, endotoxin exposures were monitored in asthmatic schoolchildren by using portable, as opposed to stationary, monitors designed to measure inhalable and respirable particulate matter less than or equal to 2.5 and 10 microm in diameter. Children were followed with daily measurements of FEV(1) and asthma symptoms. RESULTS: Over a 24-hour period, median daily personal endotoxin exposures ranged from 0.08 EU/m(3) (measured at a particulate matter size range </=2.5 microm in diameter) to 0.37 EU/m(3) (measured at a particulate matter size range </=10 microm in diameter). Personal exposures were significantly (P < .001) higher than endotoxin measurements from either indoor or outdoor stationary monitors. Moreover, individual exposures did not correlate with stationary measurements, suggesting that exposures derived from sources in close proximity to the children's personal activities might be better correlated with disease severity. Increases in personal endotoxin exposures were associated with decreased FEV(1) values and increased symptoms. CONCLUSIONS: These findings demonstrate the importance of using personal monitoring to both measure and correlate endotoxin exposure with asthma severity
SN - 0091-6749
AD - Department of Pediatrics, National Jewish Medical and Research Center, 1400 Jackson St, Denver, CO 80206, USA. rabinovitchn@njc.org
UR - 16275375
ER -

TY - JOUR
ID - 1302
T1 - Administration of epinephrine for life-threatening allergic reactions in school settings
A1 - McIntyre,C.L.
A1 - Sheetz,A.H.
A1 - Carroll,C.R.
A1 - Young,M.C.
Y1 - 2005/11//
N1 - McIntyre, C Lynne. Sheetz, Anne H. Carroll, Constance R. Young, Michael C
Pediatrics
oxv, 0376422
AIM, IM

Journal Article
English
KW - MEDLINE
KW - Anaphylaxis/di [Diagnosis]
KW - Anaphylaxis/dt [Drug Therapy]
KW - Anaphylaxis/et [Etiology]
KW - Child
KW - Emergencies
KW - Epinephrine/tu [Therapeutic Use]
KW - Food Hypersensitivity/co [Complications]
KW - Humans
KW - Insect Bites and Stings/co [Complications]
KW - School Health Services
KW - School Nursing
KW - Schools
RP - NOT IN FILE
SP - 1134
EP - 1140
JF - Pediatrics
VL - 116
IS - 5
CY - United States
N2 - OBJECTIVE: Although the potential for life-threatening allergic reactions in children is a significant health concern for schools, there is little information about the circumstances surrounding anaphylactic events that occur in schools. The objectives of this study were to determine the incidence of anaphylaxis in schools, describe the circumstances around anaphylactic events, assess practices that are used to manage students with life-threatening allergies, and identify opportunities for improvement. METHODS: A total of 109 school districts in Massachusetts completed an Epinephrine Administration Form whenever epinephrine was administered at school. Data were collected from September 2001 to August 2003. RESULTS: Forty-eight school districts reported a total of 115 administrations of epinephrine during the 2-year reporting period. In 24% of the cases, the individual was not known to have a life-threatening allergy. Almost one third (31%) of the students who received epinephrine had allergies to multiple substances, and one quarter (25%) had an allergy to peanuts or tree nuts only. Twenty-two (19%) cases occurred outside the school building on the playground, traveling to and from school, or on field trips. The administration of epinephrine most often occurred in the health office by a registered nurse. The average time from onset of symptoms to administration of epinephrine was 10 minutes. In 92% of the cases, the student was transported to a medical facility via the emergency medical system. CONCLUSIONS: Anaphylactic reactions in schools, although not frequent, are not uncommon events. A systematic review of anaphylactic events that required epinephrine administration identified opportunities for improvement in the treatment of students with life-threatening allergies
SN - 1098-4275
AD - Division of Applied Statistics, Evaluation, and Technical Services, Massachusetts Department of Public Health, Boston, Massachusetts, USA. lynne.mcintyre@state.ma.us
UR - 16264000
ER -

TY - JOUR
ID - 1303
T1 - Biologic monitoring to characterize organophosphorus pesticide exposure among children and workers: an analysis of recent studies in Washington State
A1 - Fenske,R.A.
A1 - Lu,C.
A1 - Curl,C.L.
A1 - Shirai,J.H.
A1 - Kissel,J.C.
Y1 - 2005/11//
N1 - Fenske, Richard A. Lu, Chensheng. Curl, Cynthia L. Shirai, Jeffry H. Kissel, John C
Environmental health perspectives

ei0, 0330411
IM
Journal Article. Research Support, N.I.H., Extramural. Research Support, U.S. Gov't, Non-P.H.S.. Research Support, U.S. Gov't, P.H.S.
English
KW - MEDLINE
KW - Adult
KW - Agriculture
KW - Child
KW - Preschool
KW - Environmental Exposure
KW - Environmental Monitoring
KW - Humans
KW - Insecticides/me [Metabolism]
KW - Insecticides
KW - Occupational Exposure
KW - Organophosphorus Compounds/me [Metabolism]
KW - Organophosphorus Compounds
KW - Organothiophosphates/ur [Urine]
KW - Phosphates/ur [Urine]
KW - Time Factors
KW - Washington
RP - NOT IN FILE
SP - 1651
EP - 1657
JF - Environmental Health Perspectives
JA - Environ Health Perspect
VL - 113
IS - 11
CY - United States
N2 - We examined findings from five organophosphorus pesticide biomonitoring studies conducted in Washington State between 1994 and 1999. We compared urinary dimethylthiophosphate (DMTP) concentrations for all study groups and composite dimethyl alkylphosphate (DMAP) concentrations for selected groups. Children of pesticide applicators had substantially higher metabolite levels than did Seattle children and farmworker children (median DMTP, 25 microg/L; $p < 0.0001$). Metabolite levels of children living in agricultural communities were elevated during periods of crop spraying. Median DMTP concentrations for Seattle children and farmworker children did not differ significantly (6.1 and 5.8 microg/L DMTP, respectively; $p = 0.73$); however, the DMAP concentrations were higher for Seattle children than for farmworker children (117 and 87 nmol/L DMAP, respectively; $p = 0.007$). DMTP concentrations of U.S. children 6-11 years of age (1999-2000 National Health and Nutrition Examination Survey population) were higher than those of Seattle children and farmworker children at the 75th, 90th, and 95th percentiles. DMTP concentrations for workers actively engaged in apple thinning were 50 times higher than DMTP concentrations for farmworkers sampled outside of peak exposure periods. We conclude that workers who have direct contact with pesticides should continue to be the focus of public health interventions and that elevated child exposures in agricultural communities may occur during active crop-spraying periods and from living with a pesticide applicator. Timing of sample collection is critical for the proper interpretation of pesticide biomarkers excreted relatively soon after exposure. We surmise that differences in dietary exposure can explain the similar exposures observed among farmworker children, children living in the Seattle metropolitan area, and children sampled nationally
SN - 0091-6765
AD - Department of Environmental and Occupational Health Sciences, School of Public Health and Community Medicine, University of Washington, Seattle, Washington 98195-7234, USA. rfenske@u.washington.edu
UR - 16263526
ER -

TY - JOUR
ID - 1304

T1 - Reducing arsenic exposure from pressure-treated wood playground equipment
Y1 - 2005/10//
N1 - Child health alert
9881972
K
Journal Article
English
KW - MEDLINE
KW - Arsenic/an [Analysis]
KW - Arsenic Poisoning/pc [Prevention & Control]
KW - Child
KW - Hand Disinfection
KW - Humans
KW - Play and Playthings
KW - Wood
RP - NOT IN FILE
SP - 5
EP - 6
JF - Child Health Alert
VL - 23
CY - United States
SN - 1064-4849
UR - 16299836
ER -

TY - JOUR
ID - 1305
T1 - Fatal blunt trauma of the larynx in a child
A1 - Durak,D.
A1 - Fedakar,R.
A1 - Eren,B.
A1 - Saka,E.
A1 - Durak,V.A.
Y1 - 2005/09//
N1 - Durak, Dilek. Fedakar, Recep. Eren, Bulent. Saka, Esra. Durak, V Aslihan
Journal of forensic sciences
i5z, 0375370
IM
Case Reports. Journal Article
English
KW - MEDLINE
KW - Accidental Falls
KW - Asphyxia/et [Etiology]
KW - Child
KW - Fatal Outcome
KW - Female
KW - Hematoma/pa [Pathology]
KW - Humans
KW - Laryngeal Diseases/pa [Pathology]
KW - Laryngostenosis/et [Etiology]
KW - Larynx/in [Injuries]
KW - Larynx/pa [Pathology]
KW - Play and Playthings/in [Injuries]
KW - Wounds
KW - Nonpenetrating/pa [Pathology]
RP - NOT IN FILE
SP - 1199

EP - 1200
JF - Journal of Forensic Sciences
JA - J Forensic Sci
VL - 50
IS - 5
CY - United States
N2 - We report an autopsy case of an 11-year-old girl who suffered mechanical asphyxia from falling off the metal bars in the playground. This autopsy case is interesting because of the atypical trauma and lesions. To our knowledge there has been no similiar published case reports in the forensic literature. The young age of the victim, the setting and the pattern of the injuries are rare in a fall at playground by a child
SN - 0022-1198
AD - Uludag University Medical Faculty, Department of Forensic Medicine, Gorukle 16059, Bursa, Turkey
UR - 16225230
ER -

TY - JOUR
ID - 1306
T1 - Functional outcome for children with thumb aplasia undergoing pollicization
A1 - Staines,K.G.
A1 - Majzoub,R.
A1 - Thornby,J.
A1 - Netscher,D.T.
Y1 - 2005/10//
N1 - Staines, Kimberly Goldie. Majzoub, Ramsey. Thornby, John. Netscher, David T
Plastic and reconstructive surgery
1306050
AIM, IM
Journal Article
English
KW - MEDLINE
KW - Child
KW - Preschool
KW - Female
KW - Fingers
KW - Hand Strength
KW - Humans
KW - Questionnaires
KW - Range of Motion
KW - Articular
KW - Recovery of Function
KW - Thumb/ab [Abnormalities]
KW - Thumb/pp [Physiopathology]
KW - Thumb/ra [Radiography]
KW - Thumb/su [Surgery]
KW - Transplantation
KW - Autologous
KW - Treatment Outcome
RP - NOT IN FILE
SP - 1314
EP - 1323
JF - Plastic & Reconstructive Surgery
JA - Plast Reconstr Surg
VL - 116
IS - 5
CY - United States
N2 - BACKGROUND: Hand function is difficult to evaluate in young patients. It is helpful to assess young children after surgery for trauma or congenital anomaly to see how they cope as they progress through their

developmental milestones. METHODS: Functional outcome in 10 children (12 upper extremities) who had previous pollicization for a congenitally absent or severely hypoplastic thumb were evaluated by standard radiographs, thumb total active range of motion, grip and pinch strength, parent questionnaire, modified Jebsen functional testing, and a pegboard Functional Dexterity Test. RESULTS: Grip strength was significantly less (p = 0.008) in the hands that had been operated on (mean, 2 kg) compared with the hands that had not been operated on (mean, 5.6 kg). Pinch strength was also significantly less (p = 0.008) in the hands that had been operated on (mean, 1.0 kg) compared with those that had not been operated on (mean, 2.1 kg). In most, hands that had been operated on and those that had not been operated on tested outside the 2-SD range of age-matched normals for pinch and grip strength and also for the Functional Dexterity Test. In contrast, total Jebsen Hand Function Test time was not significantly different from hands that had not been operated on, except that some subtests were significantly different, such as checker stacking (p = 0.016; mean difference, 7.2 seconds) and page turning (p = 0.031; mean difference, -10 seconds). The total active range of motion in hands that had been operated on and those that had not been operated on was also not significantly different. All children used their reconstructed thumbs in a normal pattern. Parent questionnaires revealed satisfaction with appearance and good social interactions. Functional activities showed greatest difficulty handling small objects, especially when simultaneous pinch strength was required, such as fastening buttons and small snaps. CONCLUSIONS: This study demonstrates the importance of evaluating multiple aspects of functional outcome for congenital hand problems and of using comparative age-appropriate validated norms. Pollicization is a rewarding procedure for children with thumb aplasia
SN - 1529-4242
AD - HealthSouth, Baylor College of Medicine, St. Luke's Episcopal Hospital, Houston, Texas, USA
UR - 16217473
ER -

TY - JOUR
ID - 1307
T1 - Self-regulated running using perceived exertion in children
A1 - Groslambert,A.
A1 - Monnier,Benoit P.
A1 - Grange,C.C.
A1 - Rouillon,J.D.
Y1 - 2005/03//
N1 - Groslambert, A. Monnier Benoit, P. Grange, C C. Rouillon, J D
The Journal of sports medicine and physical fitness
0376337, k6v
IM
Journal Article
English
KW - MEDLINE
KW - Analysis of Variance
KW - Child
KW - Preschool
KW - Female
KW - Heart Rate/ph [Physiology]
KW - Humans
KW - Male
KW - Oxygen Consumption/ph [Physiology]
KW - Physical Exertion/ph [Physiology]
KW - Running/ph [Physiology]
RP - NOT IN FILE
SP - 20
EP - 25
JF - Journal of Sports Medicine & Physical Fitness
JA - J Sports Med Phys Fitness
VL - 45
IS - 1
CY - Italy

N2 - AIM: The aim of this study was to examine the ability of 5- to 7-year-old female (n=16) and male (n=16) children to use perceived exertion in order to self-regulate their running intensity. An estimation-production paradigm was used to determine if 1) self-regulation of exercise using OMNI Scale could be administered in young children, 2) children were able to distinguish 3 different intermittent exercise intensities and 3) gender would differentiate the use of perceived exertion. METHODS: Children underwent 1) 1 estimation trial and 2) 3 production trials. During the estimation trial, perceived exertion was estimated at the end of each stage of an incremental running field test. Then, during the production test, the children were requested to run in random order 300 m bouts on an outdoor track at an exercise intensity based on their interpretation of levels 2, 6 and 10 of the OMNI Scale. Heart rate was continuously recorded during both trials. RESULTS: Heart rate did not differ significantly between estimation and production trials at the level 2 (124.1 SD 6 vs 125.3 SD 4 bpm), 6 (164.9 SD 5 vs 166.2 SD 6 bpm) and 10 (200.9 SD 8 vs 203.1 SD 8) of the OMNI scale. Furthermore, HR responses at OMNI 6 were significantly ($p<0.05$) higher than OMNI 2, and OMNI 10 were significantly ($p<0.05$) higher that OMNI 6. No significant difference was found between female and male children. CONCLUSIONS: The present investigation supports the view that the young female and male children tested in this study were able to use perceived exertion to self-regulate during intermittent running exercises
SN - 0022-4707
AD - Laboratory of Sport Sciences, UFRSTAPS, Besancon, France. alain.groslambert@univ-fcomte.fr
UR - 16208286
ER -

TY - JOUR
ID - 1308
T1 - Perfluorinated sulfonamides in indoor and outdoor air and indoor dust: occurrence, partitioning, and human exposure
A1 - Shoeib,M.
A1 - Harner,T.
A1 - Wilford,B.H.
A1 - Jones,K.C.
A1 - Zhu,J.
Y1 - 2005/09/01/
N1 - Shoeib, Mahiba. Harner, Tom. Wilford, Bryony H. Jones, Kevin C. Zhu, Jiping
Environmental science & technology
dvo, 0213155
IM
Journal Article
English
KW - MEDLINE
KW - Air Pollutants/an [Analysis]
KW - Air Pollution
KW - Indoor/an [Analysis]
KW - Canada
KW - Child
KW - Dust/an [Analysis]
KW - Fluorocarbons/an [Analysis]
KW - Fluorocarbons/ch [Chemistry]
KW - Housing
KW - Humans
KW - Inhalation Exposure
KW - Octanols/an [Analysis]
KW - Octanols/ch [Chemistry]
KW - Risk Assessment
KW - Sulfonamides/an [Analysis]
KW - Sulfonamides/ch [Chemistry]
KW - Time Factors
RP - NOT IN FILE
SP - 6599
EP - 6606

JF - Environmental Science & Technology
JA - Environ Sci Technol
VL - 39
IS - 17
CY - United States
N2 - Perfluorinated alkyl sulfonamides (PFASs) which are used in a variety of consumer products for surface protection were investigated through a comprehensive survey of indoor air, house dust, and outdoor air in the city of Ottawa, Canada. This study revealed new information regarding the occurrence and indoor air source strength of several PFASs including N-methylperfluorooctane sulfonamidoethanol (MeFOSE), N-ethylperfluorooctane sulfonamidoethanol (EtFOSE), N-ethylperfluorooctane sulfonamide (EtFOSA), and N-methylperfluorooctane sulfonamidethylacrylate (MeFOSEA). Passive air samplers consisting of polyurethane foam disks were calibrated and used to conduct the indoor and outdoor survey. Indoor air concentrations for MeFOSE and EtFOSE (1490 and 740 pg m(-3), respectively) were about 10-20 times greater than outdoor concentrations, establishing indoor air as an important source to the outside environment. EtFOSA and MeFOSEA concentrations were lower in indoor air (40 and 29 pg m(-3) respectively) and below detection in outdoor air samples. For indoor dust, highest concentrations were recorded for MeFOSE and EtFOSE with geometric mean concentrations of 110 and 120 ng g(-1), while concentrations for EtFOSA and MeFOSEA were below detection and 7.9 ng g(-1) respectively. MeFOSE and EtFOSE concentrations in house dust followed levels in indoor air. However, resolution of the coupled air and dust data (for the same homes) was not successful using existing KoA-based models for surface-air exchange. The partitioning to house dust was greatly underpredicted. The difficulties with existing models may be due to the high activity coefficient of PFASs in octanol and/or a situation where the dust is greatly oversaturated with respect to the air due to components of the dust being contaminated with PFASs. A human exposure assessment based on median air and dust concentrations revealed that human exposure through inhalation (100% absorption assumed) and dust ingestion were approximately 40 and approximately 20 ng d(-1), respectively. However, for children the dust ingestion pathway was dominant and accounted for approximately 44 ng d(-1)
SN - 0013-936X
AD - Meteorological Service of Canada, Air Quality Research Branch, 4905 Dufferin Street, Toronto, Ontario, Canada. Mahiba.Shoeib@ec.gc.ca
UR - 16190217
ER -

TY - JOUR
ID - 1309
T1 - West Nile virus infection among health-fair participants, Wyoming 2003: assessment of symptoms and risk factors
A1 - Murphy,T.D.
A1 - Grandpre,J.
A1 - Novick,S.L.
A1 - Seys,S.A.
A1 - Harris,R.W.
A1 - Musgrave,K.
Y1 - 2005///
N1 - Murphy, Tracy D. Grandpre, Joe. Novick, Sandra L. Seys, Scott A. Harris, Richard W. Musgrave, Karl
Vector borne and zoonotic diseases (Larchmont, N.Y.)
100965525
IM
Journal Article. Research Support, N.I.H., Extramural. Research Support, Non-U.S. Gov't. Research Support, U.S. Gov't, P.H.S.
English
KW - MEDLINE
KW - Adolescent
KW - Adult
KW - Aged
KW - 80 and over
KW - Antibodies
KW - Viral/bl [Blood]

KW - Child
KW - Confidence Intervals
KW - Exanthema/ep [Epidemiology]
KW - Female
KW - Health Surveys
KW - Humans
KW - Immunoglobulin G/bl [Blood]
KW - Immunoglobulin M/bl [Blood]
KW - Male
KW - Middle Aged
KW - Muscle Weakness/ep [Epidemiology]
KW - Odds Ratio
KW - Risk Factors
KW - Seroepidemiologic Studies
KW - West Nile Fever/di [Diagnosis]
KW - West Nile Fever/ep [Epidemiology]
KW - West Nile virus/im [Immunology]
KW - West Nile virus/ip [Isolation & Purification]
KW - Wyoming/ep [Epidemiology]
RP - NOT IN FILE
SP - 246
EP - 251
JF - Vector Borne & Zoonotic Diseases
JA - Vector borne zoonotic dis
VL - 5
IS - 3
CY - United States
N2 - Wyoming experienced heavy West Nile virus (WNV) activity for the first time in 2003 and the area hardest hit was Goshen County. Little was known about the epidemiology of WNV in this region. This study describes the symptomatology of WNV and the association between certain behaviors and infection in Goshen County. Study participants were recruited from attendees of a health-fair sponsored by a local hospital, held October 1-3, 2003. A blood sample for WNV testing was obtained from each participant, and participants completed a questionnaire seeking information about the presence of specified symptoms consistent with WNV infection and risk factors possibly associated with infection. The samples were tested for anti-WNV IgM and IgG at the Wyoming Public Health Laboratory. Eight-hundred sixty-nine residents of Goshen County participated, and 122 (14.0%) were seropositive for anti-WNV IgM or IgG. Sixty (59.4%) of 101 persons seropositive for anti-WNV IgM experienced at least one symptom in the previous 4 months consistent with WNV infection, compared with 323 (43.2%) of 747 seronegative persons, resulting in an attributable risk of WNV seropositivity of 16.2%. Of the many symptoms queried, muscle aches (OR 2.63, 95% CI 1.69-4.09), skin rash (OR 6.35, 95% CI 3.74-10.80), fever (OR 2.56, 95% Cl 1.50-4.36), and muscle weakness (OR 2.33, 95% CI 1.34-4.02) were significantly associated with seropositivity on univariate analysis. By multivariate analysis, only skin rash remained significant. Risk factor analysis showed those spending > or =3 hours outside per day were more likely to be seropositive than those spending less time outside per day ($p < 0.05$). This study corroborates the belief that a minority of persons infected with WNV develop symptoms attributable to WNV, and also demonstrates that some symptoms are more significantly associated with infection than others
SN - 1530-3667
AD - Epidemiology Program Office, Wyoming Department of Health, Cheyenne, Wyoming 82002, USA. tmurph@state.wy.us
UR - 16187893
ER -

TY - JOUR
ID - 1310
T1 - Drowning deaths of zero- to five-year-old children in Victorian dams, 1989-2001
A1 - Bugeja,L.
A1 - Franklin,R.
Y1 - 2005/10//

N1 - Bugeja, Lyndal. Franklin, Richard
The Australian journal of rural health
9305903, c0i
N
Journal Article. Research Support, Non-U.S. Gov't
English
KW - MEDLINE
KW - Age Distribution
KW - Cause of Death
KW - Child Care/sn [Statistics & Numerical Data]
KW - Child
KW - Preschool
KW - Drowning/ep [Epidemiology]
KW - Drowning/pc [Prevention & Control]
KW - Female
KW - Humans
KW - Infant
KW - Newborn
KW - Male
KW - Risk Factors
KW - Seasons
KW - Sex Distribution
KW - Victoria/ep [Epidemiology]
KW - Water Supply/sn [Statistics & Numerical Data]
RP - NOT IN FILE
SP - 300
EP - 308
JF - Australian Journal of Rural Health
JA - Aust J Rural Health
VL - 13
IS - 5
CY - Australia
N2 - OBJECTIVE: To examine drowning deaths of young children in Victorian dams to identify common contributing factors in order to develop strategies for future prevention. DESIGN: Case records of children aged zero to five years from the State Coroner's Office Victoria were reviewed for the 13-year period 1989-2001. Cases where the child drowned in a dam were extracted for analysis. RESULTS: During the 13-year period there were 27 deaths; 11 occurred on farms, five on hobby farms and 11 on properties where it was not specified whether the property was a farm. Almost three quarters of the children were male and the majority were aged between one year and three years. Half of the incidents occurred on the weekend and nearly half occurred during the summer months. Five major factors were common among incidents: stage of the child's development; absence of carer supervision; child playing outside the house; dam within 300 metres of where the child was playing; and lack of effective barriers between the dam and the child. CONCLUSION: The coronial information examined identified patterns of behaviour by both carers and young children that contributed to these deaths. The results support the implementation of strategies such as the promotion of child safe play areas and targeted public awareness campaigns for rural and regional aquatic environments
SN - 1038-5282
AD - State Coroner's Office Victoria, Southbank, Victoria, Australia
UR - 16171505
ER -

TY - JOUR
ID - 1311
T1 - Levofloxacin treatment of active tuberculosis and the risk of adverse events
A1 - Marra,F.
A1 - Marra,C.A.
A1 - Moadebi,S.
A1 - Shi,P.

A1 - Elwood,R.K.
A1 - Stark,G.
A1 - FitzGerald,J.M.
Y1 - 2005/09//
N1 - Marra, Fawziah. Marra, Carlo A. Moadebi, Susanne. Shi, Peilin. Elwood, R Kevin. Stark, Greg. FitzGerald, J Mark
Chest
0231335, d1c
AIM, IM
Journal Article. Research Support, Non-U.S. Gov't
English
KW - MEDLINE
KW - Adolescent
KW - Adult
KW - Aged
KW - 80 and over
KW - Antibiotics
KW - Antitubercular/ae [Adverse Effects]
KW - Antitubercular Agents/ae [Adverse Effects]
KW - Case-Control Studies
KW - Child
KW - Female
KW - Humans
KW - Levofloxacin
KW - Male
KW - Middle Aged
KW - Ofloxacin/ae [Adverse Effects]
KW - Treatment Outcome
KW - Tuberculosis/dt [Drug Therapy]
RP - NOT IN FILE
SP - 1406
EP - 1413
JF - Chest
VL - 128
IS - 3
CY - United States
N2 - BACKGROUND: Fluoroquinolones are commonly used in the treatment of tuberculosis (TB) for drug-sensitive patients who are intolerant to first-line antituberculous agents or who are infected with drug-resistant organisms. Despite increasing use of these agents, there is little information on their tolerance outside of clinical trial settings. OBJECTIVES: To compare overall rate of major adverse events associated with levofloxacin-containing regimen to standard therapy. METHODS: Cases (levofloxacin-containing regimen) were matched by age and sex to their control subjects (standard first-line TB drugs). Eligible patients were identified from the provincial TB database from 2001 to 2004. Drug safety was assessed by evaluation of the nature of the adverse event, the likelihood of association with the study medications, and severity. Only major side effects, that is, those who had a severe or moderate adverse event that was categorized to be definitely, probably, or possibly related to the TB medications, were considered for the analysis. RESULTS: During the 3-year study period, 102 patients received levofloxacin, and 358 patients received first-line agents for treatment of active TB. There were no significant differences between the two groups except for indication (82% of patients in the levofloxacin group had an antecedent adverse event to first-line TB drugs, whereas 18% received levofloxacin because of resistance) and concurrent use of first-line drugs (majority of patients in the levofloxacin arm were not receiving concurrent isoniazid or rifampin). The rate of any major adverse event was almost half among those using levofloxacin as among those on standard therapies (rate ratio, 0.60; 95% confidence interval [CI], 0.44 to 0.82). After adjustment for the differences in exposure of concomitant medications, the rate of any major adverse event was similar between the levofloxacin and control arms (adjusted rate ratio, 0.83; 95% CI, 0.66 to 1.03). Furthermore, there was no difference between the levofloxacin and control arms with respect to CNS (adjusted rate ratio, 0.94; 95% CI, 0.61 to 1.43), GI tract (adjusted rate ratio, 0.81; 95% CI, 0.58 to 1.13), skin (adjusted rate ratio, 0.65; 95% CI, 0.38 to 1.10), or musculoskeletal (MSK) [adjusted rate ratio, 0.87; 95% CI, 0.48 to

1.60] related adverse events when adjusted for concomitant drugs. The results of the secondary analysis for the rate of major adverse events within the first 100 days were similar to the primary analysis. The time to the first major adverse event was similar between the levofloxacin group and the control group (adjusted hazards ratio, 1.01; 95% CI, 0.76 to 1.34). CONCLUSIONS: Concomitant use of a levofloxacin-containing regimen resulted in a similar rate of adverse events compared with conventional first-line regimens when used for treatment of active TB, despite a history of adverse events
SN - 0012-3692
AD - University of British Columbia, Vancouver, BC, Canada. fawziah.marra@bccdc.ca
UR - 16162736
ER -

TY - JOUR
ID - 1312
T1 - Maturation of medial temporal lobe response and connectivity during memory encoding
A1 - Menon,V.
A1 - Boyett-Anderson,J.M.
A1 - Reiss,A.L.
Y1 - 2005/09//
N1 - Menon, V. Boyett-Anderson, J M. Reiss, A L
Brain research. Cognitive brain research
blw, 9214304
IM
Comparative Study. Journal Article. Research Support, N.I.H., Extramural. Research Support, U.S. Gov't, P.H.S.
English
KW - MEDLINE
KW - Adolescent
KW - Adult
KW - Age Factors
KW - Analysis of Variance
KW - Brain Mapping
KW - Child
KW - Female
KW - Functional Laterality/ph [Physiology]
KW - Humans
KW - Image Processing
KW - Computer-Assisted/mt [Methods]
KW - Magnetic Resonance Imaging/mt [Methods]
KW - Male
KW - Memory/ph [Physiology]
KW - Models
KW - Psychological
KW - Neural Pathways/bs [Blood Supply]
KW - Neural Pathways/ph [Physiology]
KW - Neuropsychological Tests/sn [Statistics & Numerical Data]
KW - Oxygen/bl [Blood]
KW - Photic Stimulation/mt [Methods]
KW - Reaction Time/ph [Physiology]
KW - Temporal Lobe/bs [Blood Supply]
KW - Temporal Lobe/ph [Physiology]
RP - NOT IN FILE
SP - 379
EP - 385
JF - Cognitive Brain Research
JA - Brain Res Cogn Brain Res
VL - 25
IS - 1

CY - Netherlands
N2 - The medial temporal lobe (MTL) plays an important role in memory encoding. The development and maturation of MTL and other brain regions involved in memory encoding are, however, poorly understood. We used functional magnetic resonance imaging to examine activation and effective connectivity of the MTL in children and adolescents during encoding of outdoor visual scenes. Here, we show that MTL response decreases with age whereas its connectivity with the left dorsolateral prefrontal cortex (PFC) increases with age. Our findings provide evidence for dissociable maturation of local and distributed memory encoding processes involving the MTL and furthermore suggest that increased functional interactions between the MTL and the PFC may underlie the development of more effective memory encoding strategies
SN - 0926-6410
AD - Department of Psychiatry and Behavioral Sciences, Stanford University School of Medicine, Stanford, CA 94305-5719, USA. menon@stanford.edu
UR - 16122916
ER -

TY - JOUR
ID - 1313
T1 - Teenage asthma after severe early childhood wheezing: an 11-year prospective follow-up
A1 - Hyvarinen,M.K.
A1 - Kotaniemi-Syrjanen,A.
A1 - Reijonen,T.M.
A1 - Korhonen,K.
A1 - Korppi,M.O.
Y1 - 2005/10//
N1 - Hyvarinen, Mari K. Kotaniemi-Syrjanen, Anne. Reijonen, Tiina M. Korhonen, Kaj. Korppi, Matti O
Pediatric pulmonology
owh, 8510590
IM
Journal Article. Research Support, Non-U.S. Gov't
English
KW - MEDLINE
KW - Adolescent
KW - Asthma/et [Etiology]
KW - Asthma/vi [Virology]
KW - Bronchial Provocation Tests
KW - Child
KW - Exercise Test
KW - Female
KW - Follow-Up Studies
KW - Hospitalization
KW - Humans
KW - Male
KW - Prospective Studies
KW - Respiratory Sounds/et [Etiology]
KW - Respiratory Sounds/pp [Physiopathology]
KW - Skin Tests
RP - NOT IN FILE
SP - 316
EP - 323
JF - Pediatric Pulmonology
JA - Pediatr Pulmonol
VL - 40
IS - 4
CY - United States
N2 - The role of factors related to early wheezing and their associations with subsequent development of asthma are controversial. We reevaluated 81 children who had been prospectively followed up since hospitalization for wheezing at less than 2 years of age. The baseline data on characteristics of the children,

family-related factors, and viral causes of wheezing were collected on entry into the study. At the median age of 12.3 years, current symptoms suggestive of asthma and allergy were recorded. As part of the clinical examination, an outdoor exercise challenge test and skin prick tests to common inhalant allergens were performed. Asthma, as indicated by current inhaled anti-inflammatory medication or repeated wheezing and positive result in the challenge test, was present in 32 (40%) children, and 90% of them were sensitized to at least one allergen. Early asthma-predictive factors were atopic dermatitis (odds ratio (OR), 3.5; 95% confidence interval (CI), 1.2-10.1) and the presence of specific IgE to inhalant allergens (OR, 11.3; 95% CI, 1.9-67.6). Respiratory syncytial virus (RSV) identification during wheezing in infancy was relatively rare (20%) among later asthmatics compared with other or no viral identification (52%) or rhinovirus identification (58%). Since the prevalence of childhood asthma in our area is 4.0-5.0%, we conclude that the increased risk of asthma persists until the teenage years after hospitalization for wheezing in infancy. The risk was about 5-fold after respiratory syncytial virus-induced wheezing, and more than 10-fold after rhinovirus-induced wheezing in the present study. 2005 Wiley-Liss, Inc
SN - 8755-6863
AD - Department of Pediatrics, Kuopio University Hospital, Kuopio, Finland
UR - 16082689
ER -

TY - JOUR
ID - 1314
T1 - A national study of neighborhood safety, outdoor play, television viewing, and obesity in preschool children
A1 - Burdette,H.L.
A1 - Whitaker,R.C.
Y1 - 2005/09//
N1 - Burdette, Hillary L. Whitaker, Robert C
Pediatrics
oxv, 0376422
AIM, IM
Journal Article. Research Support, N.I.H., Extramural. Research Support, U.S. Gov't, P.H.S.
English
KW - MEDLINE
KW - Attitude
KW - Body Mass Index
KW - Child
KW - Preschool
KW - Cross-Sectional Studies
KW - Exercise
KW - Humans
KW - Mothers/px [Psychology]
KW - Obesity/ep [Epidemiology]
KW - Obesity/et [Etiology]
KW - Play and Playthings
KW - Poverty
KW - Residence Characteristics
KW - Safety
KW - Socioeconomic Factors
KW - Television
KW - United States/ep [Epidemiology]
RP - NOT IN FILE
SP - 657
EP - 662
JF - Pediatrics
VL - 116
IS - 3
CY - United States

N2 - OBJECTIVE: To test the hypothesis that preschool children have a higher prevalence of obesity, spend less time playing outdoors, and spend more time watching television (TV) when they live in neighborhoods that their mothers perceive as unsafe. METHODS: In a cross-sectional survey in 20 large US cities, mothers reported the average daily time of outdoor play and TV viewing for their 3-year-old children, and the children's BMI was measured. Maternal perception of neighborhood safety was assessed with the Neighborhood Environment for Children Rating Scales; the scale score was used to divide children into tertiles of neighborhood safety. RESULTS: Of the 3141 children studied, 35% lived in households with incomes below the US poverty threshold. After adjustment for sociodemographic factors (household income and mothers' education, race/ethnicity, age, and marital status), obesity prevalence (BMI > or =95th percentile) did not differ in children from the least safe to the safest neighborhood safety tertile (18% vs 17% vs 20%) or in weekday (160 vs 151 vs 156 minutes/day) or weekend (233 vs 222 vs 222 minutes/day) outdoor play time. Children who lived in neighborhoods that were perceived by their mothers as the least safe watched more TV (201 vs 182 vs 185 minutes/day) and were more likely to watch >2 hours/day (66% vs 60% vs 62%). TV viewing and outdoor play minutes were not significantly correlated to each other or to BMI. CONCLUSIONS: In a national sample of preschool children, mothers' perception of neighborhood safety was related to their children's TV viewing time but not to their outdoor play time or risk for obesity
SN - 1098-4275
AD - Division of Gastroenterology and Nutrition, Children's Hospital of Philadelphia, Department of Pediatrics, University of Pennsylvania School of Medicine, Philadelphia, Pennsylvania, USA. burdette@email.chop.edu
UR - 16140705
ER -

TY - JOUR
ID - 1315
T1 - Occurrence of bacteria and biochemical markers on public surfaces
A1 - Reynolds,K.A.
A1 - Watt,P.M.
A1 - Boone,S.A.
A1 - Gerba,C.P.
Y1 - 2005/06//
N1 - Reynolds, Kelly A. Watt, Pamela M. Boone, Stephanie A. Gerba, Charles P
International journal of environmental health research
9106628, dus
IM
Journal Article. Research Support, Non-U.S. Gov't
English
KW - MEDLINE
KW - Adult
KW - Bacteria/ip [Isolation & Purification]
KW - Biological Markers/an [Analysis]
KW - Child
KW - Child Day Care Centers
KW - Commerce
KW - Data Collection
KW - Environmental Exposure
KW - Environmental Monitoring
KW - Feces/mi [Microbiology]
KW - Humans
KW - Hygiene
KW - Industry
KW - Play and Playthings
KW - Proteins/an [Analysis]
KW - Risk Assessment
KW - United States
RP - NOT IN FILE
SP - 225

EP - 234
JF - International Journal of Environmental Health Research
JA - Int J Environ Health Res
VL - 15
IS - 3
CY - England
N2 - From 1999-2003, the hygiene of 1061 environmental surfaces from shopping, daycare, and office environments, personal items, and miscellaneous activities (i.e., gymnasiums, airports, movie theaters, restaurants, etc.), in four US cities, was monitored. Samples were analyzed for fecal and total coliform bacteria, protein, and biochemical markers. Biochemical markers, i.e., hemoglobin (blood marker), amylase (mucus, saliva, sweat, and urine marker), and urea (urine and sweat marker) were detected on 3% (26/801); 15% (120/801), and 6% (48/801) of the surfaces, respectively. Protein (general hygiene marker) levels > or = 200 microg/10 cm2 were present on 26% (200/801) of the surfaces tested. Surfaces from children's playground equipment and daycare centers were the most frequently contaminated (biochemical markers on 36%; 15/42 and 46%; 25/54, respectively). Surfaces from the shopping, miscellaneous activities, and office environments were positive for biochemical markers with a frequency of 21% (69/333), 21% (66/308), and 11% (12/105), respectively). Sixty samples were analyzed for biochemical markers and bacteria. Total and fecal coliforms were detected on 20% (12/60) and 7% (4/ 60) of the surfaces, respectively. Half and one-third of the sites positive for biochemical markers were also positive for total and fecal coliforms, respectively. Artificial contamination of public surfaces with an invisible fluorescent tracer showed that contamination from outside surfaces was transferred to 86% (30/ 35) of exposed individual's hands and 82% (29/35) tracked the tracer to their home or personal belongings hours later. Results provide information on the relative hygiene of commonly encountered public surfaces and aid in the identification of priority environments where contaminant occurrence and risk of exposure may be greatest. Children's playground equipment is identified as a priority surface for additional research on the occurrence of and potential exposure to infectious disease causing agents
SN - 0960-3123
AD - The University of Arizona, Department of Soil, Water and Environmental Science, Environmental Research Laboratory, 2601 E. Airport Drive, Tucson, AZ 85706, USA. reynolds@u.arizona.edu
UR - 16134485
ER -

TY - JOUR
ID - 1316
T1 - Laparoscopically assisted colorectal resection in Hirschsprung's disease
A1 - Ruckauer,K.D.
A1 - von,Dobschuetz E.
Y1 - 2005/08/17/
N1 - Ruckauer, K-D. von Dobschuetz, E
European journal of medical research
coq, 9517857
IM
Journal Article
English
KW - MEDLINE
KW - Adolescent
KW - Child
KW - Preschool
KW - Colorectal Surgery
KW - Female
KW - Hirschsprung Disease/pa [Pathology]
KW - Hirschsprung Disease/su [Surgery]
KW - Humans
KW - Infant
KW - Laparoscopy
KW - Male
KW - Treatment Outcome
RP - NOT IN FILE

SP - 361
EP - 365
JF - European Journal of Medical Research
JA - Eur J Med Res
VL - 10
IS - 8
CY - Germany
N2 - BACKGROUND: Minimally invasive surgery (MIS) has been adapted to virtually every kind of abdominal operation. Colorectal resections of any extent are possible. The reduction in local and systemic surgical trauma in MIS suggests this method could be valuable for rectal resection for Hirschsprung's disease. - METHOD: Diagnostic work-up is similar to that in open surgery. Three trocars are sufficient, and a fourth may be helpful. Dissection encompasses the entire aganglionic segment and is extended orally to normal bowel.. After complete dissection down to the pelvic floor the bowel is everted transanally, resected in due length and the coloanal anastomosis sutured from outside. Since 1996 four children out of nine (age 11 weeks - 18 years) have been operated laparoscopically at our institution. - RESULTS: There were no intraoperative complications. Recovery time is impressively rapid. Oral intake may be begun on the first postoperative day and physical activity is rapidly restored. There are however problems typical to this method: anastomotic leakage, stricture formation, and the necessity of continuing dilation. - CONCLUSIONS: The feasibility of laparoscopic colorectal resections of any extent is unquestionable. Benefits for the patient seem evident. Prospective randomized studies to provide a higher level of evidence for the benefit of laparoscopy as compared to open technique are difficult to perform due to the small number of patients
SN - 0949-2321
AD - Pediatric Surgery, Department of General and Visceral Surgery, Albert-Ludwigs-University, Freiburg, Germany
UR - 16131479
ER -

TY - JOUR
ID - 1317
T1 - Air pollution and young children's inhalation exposure to organophosphorus pesticide in an agricultural community in Japan
A1 - Kawahara,J.
A1 - Horikoshi,R.
A1 - Yamaguchi,T.
A1 - Kumagai,K.
A1 - Yanagisawa,Y.
Y1 - 2005/10//
N1 - Kawahara, Junko. Horikoshi, Ryoko. Yamaguchi, Takashi. Kumagai, Kazukiyo. Yanagisawa, Yukio
Environment international
du1, 7807270
IM
Journal Article. Research Support, Non-U.S. Gov't
English
KW - MEDLINE
KW - Air Pollution
KW - Indoor/an [Analysis]
KW - Child
KW - Preschool
KW - Environmental Exposure
KW - Environmental Monitoring
KW - Female
KW - Humans
KW - Infant
KW - Inhalation Exposure
KW - Japan
KW - Male
KW - Organophosphorus Compounds/an [Analysis]

KW - Pesticides/an [Analysis]
KW - Ventilation
RP - NOT IN FILE
SP - 1123
EP - 1132
JF - Environment International
JA - Environ Int
VL - 31
IS - 8
CY - United States
N2 - Assessment of airborne organophosphorus pesticides in houses of young children (1-6 years old) and childcare facilities was conducted following pesticide applications in an agricultural community in Japan. Trichlorfon and fenitrothion, applied in two separate periods, were frequently detected from outdoor and indoor air. Dichlorvos, the primary degradation product of trichlorfon, was also detected after the application of trichlorfon. Both the outdoors and indoor concentration of applied pesticide were shown to increase with decreasing distance from the pesticide-applied farm. Indoor concentration of these pesticides significantly correlated with outdoor concentration (p=0.001 for trichlorfon and p=0.001 for fenitrothion), indicating infiltration of applied pesticide inside. Ratio of indoor to outdoor concentration (I/O ratio) of fenitrothion was higher for houses with windows open during the application than those with closed windows (median value: 0.74 vs. 0.16, p=0.003). However, a similar trend was not observed for trichlorfon as well as dichlorvos in the first period. Dichlorvos was found to have a higher I/O ratio than trichlorfon during the period, and clear correlation between indoor concentrations of dichlorvos and those of trichlorfon suggested increased decomposition of trichlorfon in the indoor environment. Daily inhalation exposure estimated by using the fixed measurement data and time-activity questionnaire ranged from 0 to 35 ng/kg/day for trichlorfon, from 0 to 26 ng/kg/day for dichlorvos, and from 0 to 44 ng/kg/day for fenitrothion. Median inhalation exposure from indoor air accounted for 74%, 86.3%, and 45% of the daily inhalation exposure, respectively. For kindergarteners or nursery school children, inhalation exposure at childcare facilities was comparable with or more than that at home, indicating that pollution level at childcare facilities had potential of high impact on children's exposure. Estimated daily inhalation exposures were inversely correlated to the proximity of their activity location to the pesticide-applied farm
SN - 0160-4120
AD - Department of Environmental System Institute of Environmental Studies, Graduate School of Frontier Sciences, The University of Tokyo, Japan. junko.kawahara@yy.t.u-tokyo.ac.jp
UR - 15979719
ER -

TY - JOUR
ID - 1318
T1 - [Environmental, social and demographic characteristics of children and adolescents, resident in different Italian areas]. [Italian]
A1 - Chellini,E.
A1 - Talassi,F.
A1 - Corbo,G.
A1 - Berti,G.
A1 - De,Sario M.
A1 - Rusconi,F.
A1 - Piffer,S.
A1 - Caranci,N.
A1 - Petronio,M.G.
A1 - Sestini,P.
A1 - Dell'Orco,V.
A1 - Bonci,E.
A1 - Armenio,L.
A1 - La,Grutta S.
A1 - Gruppo Collaborativo,S.I.D.R.
Y1 - 2005/03//

N1 - Chellini, Elisabetta. Talassi, Fiorella. Corbo, Giuseppe. Berti, Giovanna. De Sario, Manuela. Rusconi, Franca. Piffer, Silvano. Caranci, Nicola. Petronio, M Grazia. Sestini, Piersante. Dell'Orco, Valerio. Bonci, Enea. Armenio, Lucio. La Grutta, Stefania. Gruppo Collaborativo SIDRIA-2
Epidemiologia e prevenzione
8902507, epp
IM
Comparative Study. English Abstract. Journal Article. Multicenter Study
Italian
KW - MEDLINE
KW - Adolescent
KW - Animals
KW - Domestic
KW - Asthma/ep [Epidemiology]
KW - Asthma/et [Etiology]
KW - Child
KW - Child Day Care Centers
KW - Educational Status
KW - Emigration and Immigration/sn [Statistics & Numerical Data]
KW - Employment
KW - Environmental Exposure/ae [Adverse Effects]
KW - Environmental Exposure
KW - Family Characteristics
KW - Health Surveys
KW - Humans
KW - Hypersensitivity/ep [Epidemiology]
KW - Hypersensitivity/et [Etiology]
KW - Infant
KW - Low Birth Weight
KW - Newborn
KW - Italy/ep [Epidemiology]
KW - Maternal Age
KW - Overweight
KW - Parents
KW - Prevalence
KW - Questionnaires
KW - Risk Factors
KW - Rural Population/sn [Statistics & Numerical Data]
KW - Smoking
KW - Urban Population/sn [Statistics & Numerical Data]
RP - NOT IN FILE
SP - 14
EP - 23
JF - Epidemiologia e Prevenzione
JA - Epidemiol Prev
VL - 29
IS - 2 Suppl
CY - Italy
N2 - The second phase of the SIDRIA study provides important information regarding the family characteristics of Italian children (6-7 years old) and adolescents (13-14 years old), and the frequency of risk factors for asthma and allergies, allowing comparisons between study areas according to differences in latitude (North, Centre, South) and urbanization level (metropolitan areas, with at least 500,000 inhabitants, and other areas). Parental education level was higher in metropolitan and central areas. The frequency of children and adolescents born abroad, and the percentage of mothers and fathers employed were higher outside metropolitan areas and there was an increase from the South to the North of Italy. This trend was paralleled by an increase in maternal age at child's birth and in the frequency of low birth weight and day care attendance. The frequency of breastfeeding was greater in children than in adolescents; the opposite was registered for passive smoking, with a frequency of exposure higher in adolescents than in children, especially in the Northern and Central areas,

even if the proportion of subjects having at least one parent who smokes was still high in both age groups. The prevalence of overweight children was striking, especially in the South where physical activity was less frequent and children had the unhealthy habit of consuming a lot of snacks and carbonated beverages. Frequency of exposure to traffic in the area of residence was particularly high, especially in the metropolitan areas
SN - 1120-9763
AD - CSPO, Firenze
UR - 16128549
ER -

TY - JOUR
ID - 1319
T1 - The impact of development and malaria control activities on its vectors in the Kinabatangan area of Sabah, East Malaysia
A1 - Vythilingam,I.
A1 - Chan,S.T.
A1 - Shanmugratnam,C.
A1 - Tanrang,H.
A1 - Chooi,K.H.
Y1 - 2005/10//
N1 - Vythilingam, I. Chan, S T. Shanmugratnam, C. Tanrang, H. Chooi, K H
Acta tropica
23a, 0370374
IM
Journal Article. Research Support, Non-U.S. Gov't
English
KW - MEDLINE
KW - Adolescent
KW - Animals
KW - Anopheles/cl [Classification]
KW - Anopheles/ps [Parasitology]
KW - Bites and Stings
KW - Child
KW - Disease Vectors/cl [Classification]
KW - Humans
KW - Malaria/ep [Epidemiology]
KW - Malaria/pc [Prevention & Control]
KW - Malaysia/ep [Epidemiology]
KW - Mosquito Control/mt [Methods]
KW - Seasons
KW - Sporozoites/ip [Isolation & Purification]
RP - NOT IN FILE
SP - 24
EP - 30
JF - Acta Tropica
JA - Acta Trop
VL - 96
IS - 1
CY - Netherlands
N2 - A study was carried out from July 2001 until January 2003 in the Kinabatangan area of Sabah, part of Borneo island, where malaria used to be mesoendemic. Vector surveys determined that Plasmodium falciparum was the predominant species and Anopheles balabacensis the primary vector. Malaria cases have dropped drastically over the years but P. falciparum is still predominant. In the present study, Anopheles donaldi was the predominant species and was positive for sporozoites. Although An. balabacensis was present, none were infective. An. donaldi bite more outdoors than indoors and have a peak biting time from 18:00 to 19:00 h when most people are still out of their homes. An integrated malaria control programme along with area development has helped in the control of malaria and its vector
SN - 0001-706X

AD - Infectious Diseases Research Centre, Institute for Medical Research, Jalan Pahang, Kuala Lumpur, Malaysia. indra@imr.gov.my
UR - 16076459
ER -

TY - JOUR
ID - 1320
T1 - [UV-radiation--sources, wavelength, environment]. [German]
A1 - Holzle,E.
A1 - Honigsmann,H.
Y1 - 2005/09//
N1 - Holzle, Erhard. Honigsmann, Herbert
Journal der Deutschen Dermatologischen Gesellschaft = Journal of the German Society of Dermatology : JDDG
101164708
IM
Comparative Study. English Abstract. Journal Article
German
KW - MEDLINE
KW - Air Pollution
KW - Atmosphere
KW - Child
KW - Earth (Planet)
KW - Female
KW - Humans
KW - Infrared Rays
KW - Male
KW - Microwaves
KW - Occupations
KW - Ozone
KW - PUVA Therapy
KW - Phototherapy
KW - Posture
KW - Seasons
KW - Skin/re [Radiation Effects]
KW - Skin Diseases/dt [Drug Therapy]
KW - Skin Diseases/th [Therapy]
KW - Solar Energy
KW - Time Factors
KW - Ultraviolet Rays/ae [Adverse Effects]
KW - Ultraviolet Rays
RP - NOT IN FILE
SP - S3
EP - 10
JF - Journal der Deutschen Dermatologischen Gesellschaft
VL - 3 Suppl 2
CY - Germany
N2 - The UV-radiation in our environment is part of the electromagnetic radiation, which emanates from the sun. It is designated as optical radiation and reaches from 290-4,000 nm on the earth's surface. According to international definitions UV irradiation is divided into short-wave UVC (200-280 nm), medium-wave UVB (280-320 nm), and long-wave UVA (320-400 nm). Solar radiation which reaches the surface of the globe at a defined geographical site and a defined time point is called global radiation. It is modified quantitatively and qualitatively while penetrating the atmosphere. Besides atmospheric conditions, like ozone layer and air pollution, geographic latitude, elevation, time of the season, time of the day, cloudiness and the influence of indirect radiation resulting from stray effects in the atmosphere and reflection from the underground play a role in modifying global radiation, which finally represents the biologically effective radiation. The radiation's distribution on the body surface varies according to sun angle and body posture. The cumulative UV exposure is

mainly influenced by outdoor profession and recreational activities. The use of sun beds and phototherapeutic measures additionally may contribute to the cumulative UV dose
SN - 1610-0379
AD - Klinik fur Dermatologie und Allergologie, Klinikum Oldenburg, Germany. hoelzle.erhard@klinikum-oldenburg.de
UR - 16117741
ER -

TY - JOUR
ID - 1321
T1 - Elemental carbon and respirable particulate matter in the indoor air of apartments and nursery schools and ambient air in Berlin (Germany)
A1 - Fromme,H.
A1 - Lahrz,T.
A1 - Hainsch,A.
A1 - Oddoy,A.
A1 - Piloty,M.
A1 - Ruden,H.
Y1 - 2005/10//
N1 - Fromme, H. Lahrz, T. Hainsch, A. Oddoy, A. Piloty, M. Ruden, H
Indoor air
9423515, da3
IM
Journal Article
English
KW - MEDLINE
KW - Adult
KW - Air Pollutants/ae [Adverse Effects]
KW - Air Pollutants/an [Analysis]
KW - Air Pollution
KW - Indoor/ae [Adverse Effects]
KW - Indoor/an [Analysis]
KW - Carbon/an [Analysis]
KW - Child
KW - Preschool
KW - Germany
KW - Housing
KW - Humans
KW - Schools
KW - Nursery
KW - Smoke/ae [Adverse Effects]
KW - Smoke/an [Analysis]
KW - Solvents
KW - Vehicle Emissions/ae [Adverse Effects]
KW - Vehicle Emissions/an [Analysis]
RP - NOT IN FILE
SP - 335
EP - 341
JF - Indoor Air
VL - 15
IS - 5
CY - Denmark
N2 - UNLABELLED: This study was performed to examine exposure to typical carcinogenic traffic air pollutants in the city center of an urban area. In all, 123 apartments and 74 nursery schools were analyzed with and without tobacco smoke interference and the households in two measuring periods. Simultaneously, the air outside 61 apartment windows as well as the average daily traffic volume were measured. Elemental carbon (EC), the marker for particulate diesel exhaust and respirable particulate matter (RPM) were determined. The

thermographic EC analysis was conducted with and without prior solvent extraction of the soluble carbon fraction. Comparison of these two thermographic EC measurements clearly showed that method-related differences in the results, especially for indoor measurements, when high background loads of organic material were present (e.g. tobacco smoke), existed. Solvent extraction prior to EC determination was therefore appropriate. For the first winter measuring period, the EC concentration levels without solvent extraction in the indoor air were about 50% higher than those measured in the spring/summer period. In the second measuring period (i.e. spring/summer), the median EC concentrations after solvent extraction were 1.9 microg/m3 for smokers' apartments and 2.1 microg/m3 for non-smokers' apartments, with RPM concentrations of 57 and 27 microg/m3, respectively. Nursery schools showed high concentrations with median values of 53 microg/m3 for RPM and 2.9 microg/m3 for EC after solvent extraction. A significant correlation between the fine dust and EC concentrations (after solvent extraction) in the indoor and ambient air was determined. Outdoor EC values were also correlated with the average daily traffic volume. The EC ratios between indoor and ambient concentration showed a median of 0.8 (range: 0.3-4.2) in non-smoker households and 0.9 (range: 0.4-1.5) in smoker apartments. Furthermore, the EC/RPM ratio in indoor and ambient air was 0.01-0.15 (median 0.06) and 0.04-0.37 (median 0.09), respectively. PRACTICAL IMPLICATIONS: In the absence of indoor sources a significant correlation with regard to respirable particulate matter (RPM) and elemental carbon concentrations between the indoor and ambient air of apartments was observed. The high degree of certainty resulting from this correlation underscores the importance of ambient air concentrations for indoor air quality. In nursery schools we found higher concentrations of RPM. An explanation of these results could be the high number of occupants in the room, their activity and the cleaning intensity
SN - 0905-6947
AD - Bayerisches Landesamt fur Gesundheit und Lebensmittelsicherheit, Oberschleissheim, Germany. hermann.fromme@lgl.bayem.de
UR - 16108906
ER -

TY - JOUR
ID - 1322
T1 - Influence of family environment on language outcomes in children with myelomeningocele
A1 - Vachha,B.
A1 - Adams,R.
Y1 - 2005/09//
N1 - Vachha, B. Adams, R
Child: care, health and development
7602632, c9x
IM
Journal Article
English
KW - MEDLINE
KW - Adolescent
KW - Child
KW - Child Language
KW - Culture
KW - Family
KW - Female
KW - Follow-Up Studies
KW - Humans
KW - Intelligence
KW - Language Tests
KW - Male
KW - Meningomyelocele/eh [Ethnology]
KW - Meningomyelocele/px [Psychology]
KW - Orientation
KW - Prognosis
KW - Self-Assessment
KW - Social Environment
KW - Socioeconomic Factors

RP - NOT IN FILE
SP - 589
EP - 596
JF - Child: Care, Health & Development
JA - Child Care Health Dev
VL - 31
IS - 5
CY - England
N2 - BACKGROUND: Previously, our studies demonstrated language differences impacting academic performance among children with myelomeningocele and shunted hydrocephalus (MMSH). This follow-up study considers the environmental facilitators within families (achievement orientation, intellectual-cultural orientation, active recreational orientation, independence) among a cohort of children with MMSH and their relationship to language performance. METHODS: Fifty-eight monolingual, English-speaking children (36 females; mean age: 10.1 years; age range: 7-16 years) with MMSH were evaluated. Exclusionary criteria were prior shunt infection; seizure or shunt malfunction within the previous 3 months; uncorrected visual or auditory impairments; prior diagnoses of mental retardation or attention deficit disorder. The Comprehensive Assessment of Spoken Language (CASL) and the Wechsler Abbreviated Scale of Intelligence (WASI) were administered individually to all participants. The CASL Measures four subsystems: lexical, syntactic, supralinguistic and pragmatic. Parents completed the Family Environment Scale (FES) questionnaire and provided background demographic information. Spearman correlation analyses and partial correlation analyses were performed. RESULTS: Mean intelligence scores for the MMSH group: full scale IQ 92.2 (SD = 11.9). The CASL revealed statistically significant difficulty for supralinguistic and pragmatic (or social) language tasks. FES scores fell within the average range for the group. Spearman correlation and partial correlation analyses revealed statistically significant positive relationships for the FES 'intellectual-cultural orientation' variable and performance within the four language subsystems. Socio-economic status (SES) characteristics were analyzed and did not discriminate language performance when the intellectual-cultural orientation factor was taken into account. CONCLUSION: The role of family facilitators on language skills in children with MMSH has not previously been described. The relationship between language performance and the families' value on intellectual/cultural activities seems both statistically and intuitively sound. Focused interest in the integration of family values and practices should assist developmental specialists in supporting families and children within their most natural environment
SN - 0305-1862
AD - Pediatric Developmental Disabilities, Texas Scottish Rite Hospital for Children, Dallas, 75219, USA. Behroze.Vachha@tsrh.org
UR - 16101655
ER -

TY - JOUR
ID - 1323
T1 - Children's injuries in a Scottish district general hospital
A1 - Graham,C.A.
A1 - Macdonald,A.
A1 - Stevenson,J.
Y1 - 2005/09//
N1 - Graham, Colin A. Macdonald, Andrew. Stevenson, James
Injury
0226040, gon
IM
Journal Article. Research Support, Non-U.S. Gov't
English
KW - MEDLINE
KW - Accidental Falls
KW - Accidents
KW - Traffic
KW - Bicycling/in [Injuries]
KW - Child
KW - Child Care

KW - Preschool
KW - Emergencies/ep [Epidemiology]
KW - Head Protective Devices
KW - Hospitalization
KW - Hospitals
KW - General
KW - Humans
KW - Prospective Studies
KW - Questionnaires
KW - Scotland/ep [Epidemiology]
KW - Wounds and Injuries/ep [Epidemiology]
KW - Wounds and Injuries/et [Etiology]
RP - NOT IN FILE
SP - 1040
EP - 1044
JF - Injury
VL - 36
IS - 9
CY - Netherlands
N2 - BACKGROUND: Injury is a common cause of emergency department (ED) attendance but there are few data published on the spectrum of paediatric injury in a typical district general hospital (DGH). This study aimed to provide a complete picture of injury presentations to such a centre. METHODS: Prospective questionnaire study of consecutive paediatric attendances at a DGH ED in Scotland (annual attendance 53,500 patients) due to injury or poisoning. Paediatric in this context was defined as less than 14 years on the day of presentation. Admission rates were identified from the hospital information system and information on deaths was sought from the local Procurator Fiscal (the Scottish equivalent of the Coroner). RESULTS: One thousand three hundred and seventy-eight questionnaires were completed from a potential 10,697 eligible patients. Safety devices (helmets, belts, etc.) were in use in only 99 cases. Cycle helmets were used in 26% of cycle incidents and seat belts were used in 71% of car incidents. Cycling and pedestrian incidents were more common during the summer months and outside school hours. Adult supervision was present in 49% of incidents. Seventy-three percent of incidents at school were unsupervised. There were 5.6 admissions to hospital per day in the 0-13 years age group for all causes, with little seasonal variation in admission rates. There were three deaths during the year, two from SIDS and one due to choking, all in infants. CONCLUSION: Trauma is a common cause of ED attendance in children. Preventative measures are still underutilised and could make a significant impact on the incidence of children's injuries and possibly ED attendances. Cycle helmets could play a major role in injury prevention in school age children in this area
SN - 0020-1383
AD - Crosshouse Hospital, Kilmarnock KA2 0BE, Scotland, UK. cagraham@cuhk.edu.hk
UR - 16051238
ER -

TY - JOUR
ID - 1324
T1 - Who is 'who' in dissociation?: A plea for psychodynamics in a time of trauma
A1 - Gullestad,S.E.
Y1 - 2005/06//
N1 - Gullestad, Siri Erika
The International journal of psycho-analysis
gsl, 2985179r
IM
Case Reports. Journal Article
English
KW - MEDLINE
KW - Adult
KW - Child
KW - Child Abuse
KW - Sexual/px [Psychology]

KW - Conflict (Psychology)
KW - Correspondence as Topic
KW - Countertransference (Psychology)
KW - Dissociative Disorders/di [Diagnosis]
KW - Dissociative Disorders/px [Psychology]
KW - Dissociative Disorders/th [Therapy]
KW - Female
KW - Humans
KW - Hysteria/di [Diagnosis]
KW - Hysteria/px [Psychology]
KW - Life Change Events
KW - Male
KW - Models
KW - Psychological
KW - Multiple Personality Disorder/di [Diagnosis]
KW - Multiple Personality Disorder/px [Psychology]
KW - Multiple Personality Disorder/th [Therapy]
KW - Parent-Child Relations
KW - Personal Autonomy
KW - Professional-Patient Relations
KW - Psychoanalytic Interpretation
KW - Psychoanalytic Theory
KW - Psychoanalytic Therapy/mt [Methods]
KW - Self Psychology
KW - Unconscious (Psychology)
RP - NOT IN FILE
SP - 639
EP - 656
JF - International Journal of Psycho-Analysis
JA - Int J Psychoanal.
VL - 86
IS - Pt 3
CY - England
N2 - Contemporary theories of dissociation and trauma for the most part have evolved outside of psychoanalysis. Psychoanalytic writings have also been regarded as being in opposition to trauma-based notions of human psychopathology. The specific psychoanalytic contribution--the emphasis on unconscious conflict and meaning--is for the most part excluded from the discourse on dissociation, often resulting in a 'mechanic' conceptualisation of trauma. In this paper, based on clinical material, the author argues in favour of including conflict, unconscious intention and personal meaning in understanding the kind of dissociation we see in cases of multiple personality pathology. Textual analysis of letters written to the analyst illustrates how events of abuse are defensively elaborated. The author demonstrates that patterns of affect regulation and dominant object-relational strategies can be captured through analysis of the discourse structure. She focuses on how an organised character pattern, revealed mainly through narrative style and the analyst's countertransference, serves protective purposes as well as wish-fulfillment. She argues that dissociation in the form of multiple personalities may imply an active, strategic agent
SN - 0020-7578
AD - Institute of Psychology, PO Box 1094 Blindern, N-0317 Oslo, Norway. siri.gullestad@psykologi.uio.no
UR - 16096068
ER -

TY - JOUR
ID - 1325
T1 - Physical activity patterns in nonobese and obese children assessed using minute-by-minute accelerometry
A1 - Page,A.
A1 - Cooper,A.R.
A1 - Stamatakis,E.
A1 - Foster,L.J.

A1 - Crowne,E.C.
A1 - Sabin,M.
A1 - Shield,J.P.
Y1 - 2005/09//
N1 - Page, A. Cooper, A R. Stamatakis, E. Foster, L J. Crowne, E C. Sabin, M. Shield, J P H
International journal of obesity (2005)
101256108
IM
Journal Article. Multicenter Study. Research Support, Non-U.S. Gov't
English
KW - MEDLINE
KW - Body Mass Index
KW - Child
KW - Child Behavior
KW - Choice Behavior
KW - Circadian Rhythm/ph [Physiology]
KW - Cross-Sectional Studies
KW - Female
KW - Humans
KW - Male
KW - Monitoring
KW - Ambulatory/is [Instrumentation]
KW - Ambulatory/mt [Methods]
KW - Obesity/pp [Physiopathology]
KW - Physical Exertion/ph [Physiology]
KW - Sex Factors
RP - NOT IN FILE
SP - 1070
EP - 1076
JF - International Journal of Obesity
JA - Int J Obes (Lond)
VL - 29
IS - 9
CY - England
N2 - OBJECTIVE: To determine the levels and patterns of physical activity in a sample of obese (> or =99th percentile body mass index (BMI)) and nonobese (<99th percentile BMI) children. DESIGN: Cross-sectional study. SETTING: Children were recruited from schools in Bristol and from the childhood obesity clinic, Bristol Royal Hospital for Children. Children were instructed in the use of the accelerometer either while at school or in the clinic, and wore the instrument while carrying out their normal daily activities for 7 days. PARTICIPANTS: A total of 133 children (mean age 10.5+/-0.8 y). In all 11 (16.9%) of the 65 girls and 14 (20.6%) of the 68 boys were classified as obese (above the 99th percentile for BMI and corresponding to projected adult BMI of 30). MAIN OUTCOME MEASURES: Objectively measured physical activity volume, intensity and pattern. RESULTS: Obese children were significantly less physically active overall than their nonobese counterparts (31,844+/-13,200 vs 41,844+/-10,430 counts/h; 95% confidence interval 4407 to 15592; P=0.001). Similarly the obese children spent less time in physical activity of moderate or greater intensity than the nonobese children (9.9+/-3.9 vs 12.9+/-4.2 min/h; 95% confidence interval 1.15 to 4.80; P=0.002). Hourly patterns of activity indicated a tendency in obese children to be less active than nonobese children at times when activity was more likely to be determined by free choice, particularly outside of school time. CONCLUSIONS: Obese children demonstrated patterns of physical activity that may have contributed to and are likely to sustain their obesity. Minute-by-minute accelerometry is a valuable tool to investigate physical activity patterns in obese children. It can identify periods when intervention to increase activity may be most appropriate and provide an evidence base for specific exercise prescription in primary and secondary care
SN - 0307-0565
AD - Department of Exercise and Health Sciences, Centre for Sport, Exercise and Health, University of Bristol, Tyndall Avenue, Bristol BS8 1TP, UK. a.s.page@bristol.ac.uk
UR - 15917845
ER -

TY - JOUR
ID - 1326
T1 - Inflammatory chronic otitis media and the anterior epitympanic recess
A1 - Mansour,S.
A1 - Nicolas,K.
A1 - Naim,A.
A1 - Yamine,F.
A1 - Moukarzel,N.
A1 - Nehme,A.
Y1 - 2005/06//
N1 - Mansour, Salah. Nicolas, Karen. Naim, Albert. Yamine, Fady. Moukarzel, Nabil. Nehme, Antoine
The Journal of otolaryngology
kbc, 7610513
IM
Journal Article
English
KW - MEDLINE
KW - Adolescent
KW - Adult
KW - Child
KW - Chronic Disease
KW - Female
KW - Humans
KW - Male
KW - Middle Aged
KW - Otitis Media/pa [Pathology]
KW - Otitis Media/su [Surgery]
KW - Prospective Studies
KW - Temporal Bone/ra [Radiography]
KW - Tomography
KW - X-Ray Computed
KW - Tympanic Membrane/ah [Anatomy & Histology]
KW - Tympanic Membrane/ra [Radiography]
KW - Tympanic Membrane
RP - NOT IN FILE
SP - 149
EP - 159
JF - Journal of Otolaryngology
JA - J Otolaryngol
VL - 34
IS - 3
CY - Canada
N2 - OBJECTIVE: To demonstrate the important role of the anterior epitympanic recess (AER) in the surgery of noncholesteatomateous chronic inflammatory middle ear disorders. To establish selective criteria as to the indication of surgical intervention on the AER, aiming to create a permanent anterior aeration pathway for the attic. In addition, to point out the mandatory role of preoperative temporal bone computed tomography (CT) demonstrating whether the AER is involved and thus contributing, within the clinical context, to the indication for this surgery and its appropriate approach. STUDY DESIGN: Prospective study on patients with persistent or recurring chronic inflammatory middle ear disease for at least 3 years, after failure of conventional medical or surgical treatments. SETTING: Tertiary referral university centre. PATIENTS: Between November 2002 and July 2003, every patient presenting with clinical findings suggestive of an AER pathology was included in this study. SURGERY: Surgical approach of the AER during a mastoatticotomy and tympanoplasty with excision of the Cog and the tensor tympani fold, preserving the ossicular chain in almost all cases. OUTCOME MEASURES: Absence of postoperative otorrhea, satisfactory otoscopic examination, and improvement in the air-bone gap postoperatively in case the presurgery hearing level was abnormal and not due to an ossicular chain abnormality. RESULTS: Eight patients were included in the study. The preoperative CT scan showed AER

opacities in all patients that were either isolated or associated with a diseased meso- or hypotympanum or the mastoid cavity. The measurement of the relevant transverse diameter of the AER is proposed to evaluate preoperatively the distance between the Cog laterally and the facial nerve canal medially to minimize the risk of a perioperative injury. During the operation, we found granulation tissue and adhesions in the AER in all cases A clinical follow-up 3 months after the intervention showed good local control in all patients, absence of otorrhea, and almost complete closure of the air-bone gap at audiometric evaluation. The last clinical follow-up in August 2004, a mean of 18 months after our intervention, did not reveal any relapse of symptoms in any case. CT control could be obtained in five of eight cases, within 13 to 21 months after the intervention, showing a reaerated tympanic cavity and AER. CONCLUSION: The AER plays a major role in sustaining some noncholesteatomateous chronic or recurrent inflammatory middle ear disorders that do not respond to conventional medical treatment. Definitive control of this pathology will be obtained by approaching the AER through an excision of the Cog and the tensor tympani fold, exenterating the inflammatory tissues, and creating a sufficient and permanent anterior atticomesotympanic communication. The indication for such a surgical approach is highly dependent on clinical findings correlated to temporal bone CT. Familiarity with the AER and its critical role should become part of every resident's training program in otology
SN - 0381-6605
AD - Division of Otolaryngology, Sacre-Coeur Hospital, Lebanese University, Hazmieh, Baabada, Lebanon
UR - 16089217
ER -

TY - JOUR
ID - 1328
T1 - Arsenic on children's hands after playing in playgrounds
A1 - Zagury,G.J.
A1 - Pouschat,P.
Y1 - 2005/08//
N1 - Zagury, Gerald J. Pouschat, Priscilla
Environmental health perspectives
ei0, 0330411
IM
Comment. Letter. Research Support, Non-U.S. Gov't
English
KW - MEDLINE
KW - Arsenates
KW - Arsenic/an [Analysis]
KW - Child
KW - Environmental Exposure
KW - Environmental Pollutants/an [Analysis]
KW - Hand
KW - Humans
KW - Play and Playthings
KW - Wood
RP - NOT IN FILE
SP - A508
EP - A509
JF - Environmental Health Perspectives
JA - Environ Health Perspect
VL - 113
IS - 8
CY - United States
SN - 0091-6765
UR - 16079049
ER -

TY - JOUR
ID - 1329
T1 - Auditory brainstem implant (ABI): new frontiers in adults and children

A1 - Colletti,V.
A1 - Carner,M.
A1 - Miorelli,V.
A1 - Guida,M.
A1 - Colletti,L.
A1 - Fiorino,F.
Y1 - 2005/07//
N1 - Colletti, Vittorio. Carner, Marco. Miorelli, Veronica. Guida, Maurizio. Colletti, Liliana. Fiorino, Francesco
Otolaryngology--head and neck surgery : official journal of American Academy of Otolaryngology-Head and Neck Surgery
7909794, 8508176, on7, on8
IM
Journal Article
English
KW - MEDLINE
KW - Adolescent
KW - Adult
KW - Aged
KW - Auditory Brain Stem Implantation/mt [Methods]
KW - Auditory Brain Stem Implantation/td [Trends]
KW - Auditory Brain Stem Implants
KW - Child
KW - Preschool
KW - Cochlear Diseases/co [Complications]
KW - Cochlear Diseases/su [Surgery]
KW - Craniocerebral Trauma/co [Complications]
KW - Craniocerebral Trauma/su [Surgery]
KW - Evoked Potentials
KW - Auditory
KW - Brain Stem/ph [Physiology]
KW - Female
KW - Hearing Loss
KW - Sensorineural/di [Diagnosis]
KW - Sensorineural/et [Etiology]
KW - Sensorineural/pp [Physiopathology]
KW - Sensorineural/su [Surgery]
KW - Hearing Tests
KW - Humans
KW - Infant
KW - Male
KW - Middle Aged
KW - Neuroma
KW - Acoustic/co [Complications]
KW - Acoustic/su [Surgery]
KW - Retrospective Studies
KW - Treatment Outcome
KW - Vestibulocochlear Nerve Diseases/co [Complications]
KW - Vestibulocochlear Nerve Diseases/su [Surgery]
RP - NOT IN FILE
SP - 126
EP - 138
JF - Otolaryngology - Head & Neck Surgery
JA - Otolaryngol Head Neck Surg
VL - 133
IS - 1
CY - United States

N2 - UNLABELLED: Previous studies have considered only patients with neurofibromatosis type 2 (NF2) older than 12 years as candidates for an auditory brainstem implant (ABI). Our study expands the potential criteria to include both children and adult subjects with other cochlear or cochlear nerve malfunctions who either would not benefit at all from a cochlear implant (eg, cochlear nerve aplasia or avulsion) or whose benefit was or would be severely compromised (eg, cochlear ossification, cochlear fracture). STUDY DESIGN: In our department, over the period from April 1997 to September 2002, 29 patients, 20 adults and 9 children, were fitted with ABIs. Their ages ranged from 14 months to 70 years. Thirteen subjects had tumors, 10 NF2 and 3 solitary vestibular schwannoma, and 16 patients had a variety of nontumor (NT) cochlear or cochlear nerve diseases. A retrosigmoid-transmeatal approach was used in T and a retrosigmoid approach in NT patients. The electrode array was inserted into the lateral recess of the fourth ventricle and correct electrode positioning was monitored with the aid of electrically evoked auditory brainstem responses (EABRs). RESULTS: Correct implantation was achieved in all patients. No complications were observed due to implantation surgery or related to ABI activation or long-term use. Auditory sensations were induced in all patients with various numbers of electrodes (from 5 to 15). Different pitch sensations were identifiable with different electrode stimulation. Closed-set word recognition, open-set sentence recognition, and speech tracking scores achieved by the patients are reported in detail. The auditory performance of the patients showed significantly better outcomes than controls (Multicentric European clinical investigations on ABI with NF2). CONCLUSION: We have shown that the indications for the ABI can be extended to include NT patients with severe cochlear and/or cochlear nerve abnormalities. The degree of auditory benefit varies as a function of the underlying pathological conditions, with NT subjects exhibiting significantly better outcomes than the T patients
SN - 0194-5998
AD - ENT Department, University of Verona, Italy. vittoriocolletti@yahoo.com
UR - 16025066
ER -

TY - JOUR
ID - 1330
T1 - Exposures of preschool children to chlorpyrifos and its degradation product 3,5,6-trichloro-2-pyridinol in their everyday environments
A1 - Morgan,M.K.
A1 - Sheldon,L.S.
A1 - Croghan,C.W.
A1 - Jones,P.A.
A1 - Robertson,G.L.
A1 - Chuang,J.C.
A1 - Wilson,N.K.
A1 - Lyu,C.W.
Y1 - 2005/07//
N1 - Morgan, Marsha K. Sheldon, Linda S. Croghan, Carry W. Jones, Paul A. Robertson, Gary L. Chuang, Jane C. Wilson, Nancy K. Lyu, Christopher W
Journal of exposure analysis and environmental epidemiology
bjn, 9111438
IM
Journal Article. Research Support, U.S. Gov't, Non-P.H.S.
English
KW - MEDLINE
KW - Air/an [Analysis]
KW - Air Pollution
KW - Indoor/an [Analysis]
KW - Biological Markers/ur [Urine]
KW - Child Day Care Centers
KW - Child
KW - Preschool
KW - Chlorpyrifos/an [Analysis]
KW - Dust/an [Analysis]
KW - Environmental Monitoring
KW - Environmental Pollutants/an [Analysis]

KW - Floors and Floorcoverings
KW - Food Analysis
KW - Food Contamination
KW - Housing
KW - Humans
KW - Infant
KW - Insecticides/an [Analysis]
KW - North Carolina
KW - Pesticide Residues/an [Analysis]
KW - Pyridones/an [Analysis]
KW - Pyridones/ur [Urine]
KW - Soil/an [Analysis]
RP - NOT IN FILE
SP - 297
EP - 309
JF - Journal of Exposure Analysis and Environmental Epidemiology
JA - J Expo Anal Environ Epidemiol
VL - 15
IS - 4
CY - England
N2 - As part of the Children's Total Exposure to Persistent Pesticides and Other Persistent Organic Pollutants (CTEPP) study, we investigated the exposures of preschool children to chlorpyrifos and its degradation product 3,5,6-trichloro-2-pyridinol (TCP) in their everyday environments. During this study, the participants were still able to purchase and apply chlorpyrifos at their homes or day care centers. Participants were recruited randomly from 129 homes and 13 day care centers in six North Carolina counties. Monitoring was performed over a 48-h period at the children's homes and/or day care centers. Samples that were collected included duplicate plate, indoor and outdoor air, urine, indoor floor dust, play area soil, transferable residues (PUF roller), and surface wipes (hand, food preparation, and hard floor). The samples were extracted and analyzed by gas chromatography/mass spectrometry. Chlorpyrifos was detected in 100% of the indoor air and indoor floor dust samples from homes and day care centers. TCP was detected at homes and day care centers in 100% of the indoor floor dust and hard floor surface wipe, in >97% of the solid food, and in >95% of the indoor air samples. Generally, median levels of chlorpyrifos were higher than those of TCP in all media, except for solid food samples. For these samples, the median TCP concentrations were 12 and 29 times higher than the chlorpyrifos concentrations at homes and day care centers, respectively. The median urinary TCP concentration for the preschool children was 5.3 ng/ml and the maximum value was 104 ng/ml. The median potential aggregate absorbed dose (ng/kg/day) of chlorpyrifos for these preschool children was estimated to be 3 ng/kg/day. The primary route of exposure to chlorpyrifos was through dietary intake, followed by inhalation. The median potential aggregate absorbed dose of TCP for these children was estimated to be 38 ng/kg/day, and dietary intake was the primary route of exposure. The median excreted amount of urinary TCP for these children was estimated to be 117 ng/kg/day. A full regression model of the relationships among chlorpyrifos and TCP for the children in the home group explained 23% of the variability of the urinary TCP concentrations by the three routes of exposure (inhalation, ingestion, dermal absorption) to chlorpyrifos and TCP. However, a final reduced model via step-wise regression retained only chlorpyrifos through the inhalation route and explained 22% of the variability of TCP in the children's urine. The estimated potential aggregate absorbed doses of chlorpyrifos through the inhalation route were low (median value, 0.8 ng/kg/day) and could not explain most of the excreted amounts of urinary TCP. This suggested that there were other possible sources and pathways of exposure that contributed to the estimated potential aggregate absorbed doses of these children to chlorpyrifos and TCP. One possible pathway of exposure that was not accounted for fully is through the children's potential contacts with contaminated surfaces at homes and day care centers. In addition, other pesticides such as chlorpyrifos-methyl may have also contributed to the levels of TCP in the urine. Future studies should include additional surface measurements in their estimation of potential absorbed doses of preschool children to environmental pollutants. In conclusion, the results showed that the preschool children were exposed to chlorpyrifos and TCP from several sources, through several pathways and routes.
SN - 1053-4245
AD - National Exposure Research Laboratory, USEPA, Research Triangle Park, NC 27711, USA. morgan.marsha@epa.gov
UR - 15367928

ER -

TY - JOUR
ID - 1331
T1 - Impacts of ozone on the activities of asthmatics: revisiting the data
A1 - Eiswerth,M.E.
A1 - Douglass,Shaw W.
A1 - Yen,S.T.
Y1 - 2005/10//
N1 - Eiswerth, Mark E. Douglass Shaw, W. Yen, Steven T
Journal of environmental management
0401664, du5
IM
Journal Article
English
KW - MEDLINE
KW - Air Pollutants/ae [Adverse Effects]
KW - Asthma/et [Etiology]
KW - Child
KW - Humans
KW - Leisure Activities
KW - Models
KW - Biological
KW - Oxidants
KW - Photochemical/ae [Adverse Effects]
KW - Ozone/ae [Adverse Effects]
KW - Severity of Illness Index
RP - NOT IN FILE
SP - 56
EP - 63
JF - Journal of Environmental Management
JA - J environ manage
VL - 77
IS - 1
CY - England
N2 - This manuscript develops a conditional demand system for three activities (indoor chores and leisure, outdoor chores and active leisure, and outdoor inactive leisure), and considers how individuals' activity schedules respond to ozone pollution and asthma symptom severity. The data are for a sample of individuals living near Los Angeles. Our approach differs from earlier ones in that we use a system of equations that allows for correlations across activity types. The empirical results suggest that increased ozone levels have a statistically significant influence on the amount of time that asthmatics spend in one of the activities, even though the examined time period exhibited relatively low ozone levels. Elasticities are estimated to better indicate the sensitivity of humans' activity schedules to pollution levels, and the one ozone effect is shown to be fairly weak. This study adds to the existing literature on the influence of ozone on asthma and asthma sufferers, some of which finds a causal link while other studies do not. The study results are also of interest because the asthmatics in the sample are all adults, whereas some recent research showing a link between ozone and asthma has focused on children
SN - 0301-4797
AD - Department of Economics, University of Wisconsin-Whitewater, Whitewater, WI 53190, USA. eiswertm@uww.edu
UR - 15949890
ER -

TY - JOUR
ID - 1332
T1 - Context enrichment of health risk surveillance data for use and application: conceptual considerations from an Australian perspective

A1 - Zubrick,S.R.
Y1 - 2005///
N1 - Zubrick, Stephen R
Sozial- und Praventivmedizin
yf1, 7502479
IM
Comparative Study. Journal Article. Research Support, Non-U.S. Gov't
English
KW - MEDLINE
KW - Adult
KW - Australia
KW - Causality
KW - Child
KW - Cross-Cultural Comparison
KW - Data Collection
KW - Health Education/td [Trends]
KW - Health Knowledge
KW - Attitudes
KW - Practice
KW - Health Status Indicators
KW - Humans
KW - Morbidity/td [Trends]
KW - Population Surveillance
KW - Social Justice
KW - Socioeconomic Factors
KW - Systems Theory
RP - NOT IN FILE
SP - S16
EP - S24
JF - Sozial- und Praventivmedizin
JA - Soz.Praventivmed.
VL - 50 Suppl 1
CY - Switzerland
N2 - Increasing the relevance of health surveillance data for use and application remains a critical issue among users and stakeholders. However methods for displaying and communicating health risk data could do more to enrich these data for use and application. There is too much focus on developers, users and stakeholders and not enough focus on pathway models of disease and health. A greater focus on pathway models would help detail a wider explanation of the health problems across sectors and encourage better acceptance of responsibility for their occurrence within other sectors. Contextual enrichment of health data would encourage a shared theory among these players that would permit greater use and application. Better use of data and its translation into information and increased policy traction are likely to be achieved through processes that effectively: (1) establish a shared theoretical base for risk behaviour surveillance; (2) deliver data about the health and developmental status of individuals living in contexts over time; (3) maintain a systems approach to monitoring and surveillance that builds capacity for development, ownership, access to and dissemination of the data outside of the health system and (4) achieve sustainable partnerships that produce more intersectoral engagement
SN - 0303-8408
AD - Curtin University of Technology Centre for Developmental Health, Telethon Institute for Child Health Research, West Perth, Australia. steve@ichr.uwa.edu.au
UR - 16003914
ER -

TY - JOUR
ID - 1333
T1 - Predictors of self-reported confidence ratings for adult recall of early life sun exposure
A1 - Relova,A.S.
A1 - Marrett,L.D.

A1 - Klar,N.
A1 - McLaughlin,J.R.
A1 - Ashbury,F.D.
A1 - Nishri,D.
A1 - Theis,B.
Y1 - 2005/07/15/
N1 - Relova, Anne-Sharon. Marrett, Loraine D. Klar, Neil. McLaughlin, John R. Ashbury, Fredrick D. Nishri, Diane. Theis, Beth
American journal of epidemiology
3h3, 7910653
IM
Comparative Study. Journal Article. Research Support, N.I.H., Extramural. Research Support, Non-U.S. Gov't. Research Support, U.S. Gov't, P.H.S.
English
KW - MEDLINE
KW - Adolescent
KW - Adult
KW - Age Distribution
KW - Bias (Epidemiology)
KW - Case-Control Studies
KW - Child
KW - Confidence Intervals
KW - Environmental Exposure/ae [Adverse Effects]
KW - Female
KW - Humans
KW - Male
KW - Melanoma/ep [Epidemiology]
KW - Melanoma/et [Etiology]
KW - Mental Recall
KW - Questionnaires
KW - Research Design
KW - Seasons
KW - Skin Neoplasms/ep [Epidemiology]
KW - Skin Neoplasms/et [Etiology]
KW - Sunlight/ae [Adverse Effects]
KW - Time Factors
RP - NOT IN FILE
SP - 183
EP - 192
JF - American Journal of Epidemiology
JA - Am J Epidemiol
VL - 162
IS - 2
CY - United States
N2 - Use of self-reported confidence ratings may be an efficient method for assessing recall bias. In this exploratory application of the method, the authors examined the relation between case-control status and self-reported confidence ratings. In 2002 and 2003, melanoma cases (n = 141) and controls (n = 143) aged 20-44 years residing in Ontario, Canada, estimated the amounts of time they had spent outdoors in summer activities when they were 6-18 years of age and indicated their confidence in the accuracy of each estimate. The generalized estimating equations extension of logistic regression was used to examine dichotomized confidence ratings (more confident vs. less confident) for activities reported for ages 6-11 years and 12-18 years. Types of activity were associated with more confident reporting for both age strata; as the number of stable outdoor activity periods (total number of similar outdoor periods within each activity) reported by respondents increased, confidence decreased. Cumulative time spent outdoors was also associated with more confidence but reached statistical significance only for the age stratum 12-18 years. There was no statistically significant association between case-control status and self-reported confidence for either age stratum (6-11 years: odds

ratio = 0.91; 12-18 years: odds ratio = 1.32), which suggests an absence of recall bias for reported time spent outdoors
SN - 0002-9262
AD - Division of Preventive Oncology, Cancer Care Ontario, Toronto, Ontario, Canada
UR - 15972935
ER -

TY - JOUR
ID - 1334
T1 - Preschool children's accumulated and sustained physical activity
A1 - ham-Deal,T.
Y1 - 2005/04//
N1 - Benham-Deal, Tami
Perceptual and motor skills
ozb, 0401131
IM
Journal Article
English
KW - MEDLINE
KW - Age Factors
KW - Child Behavior/ph [Physiology]
KW - Child
KW - Preschool
KW - Female
KW - Health Behavior
KW - Health Education/mt [Methods]
KW - Heart Rate/ph [Physiology]
KW - Humans
KW - Male
KW - Monitoring
KW - Ambulatory
KW - Motor Activity/ph [Physiology]
KW - Periodicity
KW - Risk-Taking
KW - Time Factors
RP - NOT IN FILE
SP - 443
EP - 450
JF - Perceptual & Motor Skills
JA - Percept.Mot.Skills
VL - 100
IS - 2
CY - United States
N2 - 39 3- to 5-year-old children wore heart-rate monitors for 12 hr. on three consecutive days to examine physical activity patterns. Parent logs supplied additional information on type and location of activity. The highest number of accumulated minutes of moderate to vigorous physical activity was recorded during the weekday (38.6 min.) and weekend (31.4 min.) afternoons, a time period when more activity occurred outdoors. Sustained activity was mostly short in duration, but many children (71%) accumulated recommended amounts of activity on the weekday, fewer (46%) on the weekend. Spearman rank correlations for weekday (r=.50, p=.004) and weekend (r=.58, p=.004) activity were significant. Considering health risk factors associated with physical inactivity, early educators must look to the essence of young children's physical activity patterns in designing programs. Large motor activity that is intense and that occurs outdoors is a good place to start
SN - 0031-5125
AD - Division of Kinesiology and Health, University of Wyoming, Laramie, WY 82071, USA. benham@uwyo.edu
UR - 15974355
ER -

TY - JOUR
ID - 1335
T1 - Multiple insertional events, restricted by the genetic background, have led to acquisition of pathogenicity island IIJ96-like domains among Escherichia coli strains of different clinical origins
A1 - Bidet,P.
A1 - Bonacorsi,S.
A1 - Clermont,O.
A1 - De,Montille C.
A1 - Brahimi,N.
A1 - Bingen,E.
Y1 - 2005/07//
N1 - Bidet, Philippe. Bonacorsi, Stephane. Clermont, Olivier. De Montille, Caroline. Brahimi, Naima. Bingen, Edouard
Infection and immunity
go7, 0246127
IM
Journal Article
English
KW - MEDLINE
KW - Adhesins
KW - Escherichia coli/ge [Genetics]
KW - Adult
KW - Child
KW - Preschool
KW - Escherichia coli/py [Pathogenicity]
KW - Fimbriae Proteins/ge [Genetics]
KW - Genomic Islands
KW - Humans
KW - Infant
KW - Polymerase Chain Reaction
KW - Ribotyping
KW - Urinary Tract Infections/mi [Microbiology]
RP - NOT IN FILE
SP - 4081
EP - 4087
JF - Infection & Immunity
JA - Infect Immun
VL - 73
IS - 7
CY - United States
N2 - We investigated the dissemination of pathogenicity island (PAI) II(J96)-like elements (hra, hly, cnf1, and pap) among 455 Escherichia coli isolates from children and adults with urinary tract infection (UTI), neonates with meningitis or colonized healthy neonates, and 74 reference strains by means of PCR phylogenetic grouping, ribotyping, and PCR analysis of virulence genes. Colocalization of these genes was documented by pulsed-field gel electrophoresis followed by Southern hybridization and long-range PCR (LRPCR) between the hra and the papG alleles. Site-specific insertion of the PAI was determined by LRPCR between hra and tRNA flanking sequences. hra, hly, and cnf1 were found in 113 isolates and consistently colocalized, constituting the backbone of PAI II(J96)-like domains. The prevalence of PAI II(J96)-like domains was significantly higher among UTI isolates than among neonatal meningitis and commensal isolates. These domains were restricted to a few ribotypes of group B2. In contrast to the consistent colocalization of hra, hly, and cnf1, the pap operon was varied: 12% of strains exhibited an allelic exchange of the papG class III allele (papGIII) for the papG class II allele (papGII) (only UTI isolates), and the pap operon was deleted in 23% of strains. No strains harbored papGIII outside the PAI, which appears to be the only source of this allele. PAI II(J96)-like domains were inserted in the vicinities of three different tRNAs--pheU (54%), leuX (29%), and pheV (15%)--depending on the genetic backgrounds and origins of the isolates. Multiple insertional events restricted by the genetic background

have thus led to PAI II(J96) acquisition. Specific genetic backgrounds and insertion sites may have played a role in additional recombination processes for E. coli adaptation to different ecological niches
SN - 0019-9567
AD - Laboratoire d'etudes de genetique bacterienne dans les infections de l'enfant (EA3105), Universite Denis Diderot--Paris 7, Service de Microbiologie, Hopital Robert Debre (AP-HP), Paris, France
UR - 15972496
ER -

TY - JOUR
ID - 1336
T1 - MRSA at an English children's hospital from 1998 to 2003
A1 - Adedeji,A.
A1 - Gray,J.W.
Y1 - 2005/07//
N1 - Adedeji, A. Gray, J W
Archives of disease in childhood
6xg, 0372434
AIM, IM
Journal Article
English
KW - MEDLINE
KW - Age Distribution
KW - Bacteremia/ep [Epidemiology]
KW - Bacteremia/mi [Microbiology]
KW - Child
KW - Preschool
KW - Cross Infection/ep [Epidemiology]
KW - Cross Infection/mi [Microbiology]
KW - England/ep [Epidemiology]
KW - Female
KW - Hospitalization
KW - Hospitals
KW - Pediatric
KW - Teaching
KW - Humans
KW - Infant
KW - Newborn
KW - Male
KW - Medicine/sn [Statistics & Numerical Data]
KW - Methicillin Resistance
KW - Outpatient Clinics
KW - Hospital
KW - Specialization
KW - Staphylococcal Infections/ep [Epidemiology]
KW - Staphylococcal Infections/mi [Microbiology]
KW - Staphylococcus aureus/de [Drug Effects]
RP - NOT IN FILE
SP - 720
EP - 723
JF - Archives of Disease in Childhood
JA - Arch Dis Child
VL - 90
IS - 7
CY - England
N2 - AIMS: To investigate the epidemiological and clinical aspects of MRSA among inpatients and outpatients presenting to hospital. METHODS: Analysis of demographic, epidemiological, and clinical data collected on 385 children first identified as having MRSA between January 1998 and December 2003 in a 250 bed English

children's hospital. RESULTS: There were 267 inpatients and 118 outpatients. The number of new cases of MRSA declined from 72 in 1998 to 52 in 2003, whereas hospital activity increased. Ninety nine (37.1%) inpatients acquired MRSA outside the hospital; a further 90 occurred among 31 clusters of cases. One hundred and seventy eight (66.7%) inpatients were aged <2 years; cardiac services and paediatric & neonatal surgery accounted for 59.6% of cases. Dermatology and A&E accounted for 51.7% of outpatients; 73.8% of outpatients had recently previously attended the hospital. A total of 13.9% of inpatients with MRSA developed bacteraemia; MRSA accounted for 15% of Staphylococcus aureus bacteraemias. The risk of MRSA bacteraemia in colonised patients, and the proportion of S aureus bacteraemias that were MRSA, varied between specialties. Intravascular devices were the most common source of MRSA bacteraemia (63.4% of cases). The mortality rate was 7.3%. CONCLUSIONS: Enhanced surveillance of MRSA can identify at-risk patient groups, thus facilitating targeting of control measures. The absence of a link between numbers of cases of acquisition of MRSA and bacteraemia suggests that the rise in MRSA bacteraemia may not solely reflect an increase in MRSA prevalence in children in the UK. The need for larger epidemiological studies is emphasised
SN - 1468-2044
AD - Birmingham Children's Hospital, Steelhouse Lane, Birmingham B4 6NH, UK. adekolaa@hotmail.com
UR - 15970616
ER -

TY - JOUR
ID - 1337
T1 - Physical activity and school recess time: differences between the sexes and the relationship between children's playground physical activity and habitual physical activity
A1 - Mota,J.
A1 - Silva,P.
A1 - Santos,M.P.
A1 - Ribeiro,J.C.
A1 - Oliveira,J.
A1 - Duarte,J.A.
Y1 - 2005/03//
N1 - Mota, Jorge. Silva, Pedro. Santos, Maria Paula. Ribeiro, Jose Carlos. Oliveira, Jose. Duarte, Jose A
Journal of sports sciences
8405364, kbt
IM
Comparative Study. Journal Article. Research Support, Non-U.S. Gov't
English
KW - MEDLINE
KW - Body Size
KW - Child
KW - Female
KW - Habits
KW - Health Behavior
KW - Health Surveys
KW - Humans
KW - Life Style
KW - Male
KW - Monitoring
KW - Ambulatory
KW - Motor Activity
KW - Play and Playthings
KW - Schools/sn [Statistics & Numerical Data]
KW - Sex Factors
KW - Time Factors
RP - NOT IN FILE
SP - 269
EP - 275
JF - Journal of Sports Sciences
JA - J Sports Sci

VL - 23
IS - 3
CY - England
N2 - The aims of this study were: (1) to observe participation in moderate-to-vigorous physical activity (MVPA) during school recess periods; (2) to determine the relative importance of physical activity during recesses to overall daily physical activity; and (3) to examine differences in physical activity between the sexes during unstructured recess periods. The participants were 22 school children (10 boys, 12 girls) aged 8 - 10 years (mean = 8.9, s = 0.7) in the third and fourth grades. Daily totals for the physical activity variables were calculated by summing the values for each hour of 14 h of physical activity measurements (08:00 to 22:00 h). Recess times (minutes) were as follows: morning 10:30 to 11:00 h and afternoon 15:30 to 16:00 h. We did not differences between boys and girls in daily total accelerometer counts or the overall time spent in MVPA. However, girls were significantly (P < 0.05) more involved (38%) in MVPA during recess time than boys (31%). Participation in MVPA during recess contributes significantly more (P < 0.05) for girls (19%) than boys (15%) to the total amount of physical activity suggested by international health-related physical activity guidelines, while the percentage of time engaged in MVPA during recess time at school accounts for a small amount of the daily MVPA (6% for boys and 8% for girls). The results of this study suggest that school recess time is an important setting to promote MVPA and contributes to daily physical activity in young children, especially in girls
SN - 0264-0414
AD - Research Centre for Physical Activity, Health and Leisure, Faculty of Sport Sciences and Physical Education, University of Porto, Porto, Portugal. jmota@fcdef.up.pt
UR - 15966345
ER -

TY - JOUR
ID - 1338
T1 - Grandmothers' diaries: a glimpse at daily lives
A1 - Musil,C.M.
A1 - Standing,T.
Y1 - 2005///
N1 - Musil, Carol M. Standing, Theresa
International journal of aging & human development
gqf, 0370033
IM
Journal Article. Research Support, N.I.H., Extramural. Research Support, U.S. Gov't, P.H.S.
English
KW - MEDLINE
KW - Caregivers/px [Psychology]
KW - Child
KW - Child Rearing/px [Psychology]
KW - Cross-Sectional Studies
KW - Data Collection
KW - Employment
KW - Female
KW - Humans
KW - Intergenerational Relations
KW - Ohio
KW - Poverty
KW - Stress
KW - Psychological/et [Etiology]
RP - NOT IN FILE
SP - 317
EP - 329
JF - International Journal of Aging & Human Development
JA - Int J Aging Hum Dev
VL - 60
IS - 4

CY - United States
N2 - Little information exists about the daily lives of women who are grandmothers, and the differences in daily stresses based on caregiving status to grandchildren. This content analysis examines the stresses of 64 grandmothers as grouped by caregiver status (grandmothers raising grandchildren, grandmothers living in multigeneration homes, non-caregivers to grandchildren) as recorded in three-week diaries. The nature of salient issues and stressful interactions differed by caregiver groups. Grandmothers raising grandchildren reported more stresses related to grandchildren's routines, activities, and school progress, more time pressure, and difficult interactions with grandchildren. The diary entries of grandmothers in multigenerational homes reflected their supplemental role in childcare, and sometimes stressful interactions with other family members. Grandmothers with no routine caregiving to grandchildren reported more involvement with those outside the immediate family. Many general concerns about the well-being of the family represent commonalities in grandmothers despite differences in current caregiving roles to grandchildren
SN - 0091-4150
AD - Bolton School of Nursing, Case Western Reserve University, Cleveland, Ohio 44106-4904, USA. cmm4@cwru.edu
UR - 15954682
ER -

TY - JOUR
ID - 1339
T1 - Sun protection initiatives in Cornwall
A1 - Morris,J.M.
A1 - Gould,D.
A1 - Bennett,S.
A1 - Bastin,J.
A1 - Salter,L.
A1 - Watt,A.
Y1 - 2005/07//
N1 - Morris, J M. Gould, D. Bennett, S. Bastin, J. Salter, L. Watt, A
Clinical and experimental dermatology
ddu, 7606847
IM
Journal Article. Multicenter Study
English
KW - MEDLINE
KW - Child
KW - England
KW - Family Practice/st [Standards]
KW - Guidelines as Topic
KW - Health Care Surveys
KW - Health Promotion/og [Organization & Administration]
KW - Health Promotion/st [Standards]
KW - Humans
KW - Melanoma/et [Etiology]
KW - Melanoma/pc [Prevention & Control]
KW - Neoplasms
KW - Radiation-Induced/pc [Prevention & Control]
KW - Protective Clothing/ut [Utilization]
KW - Schools/st [Standards]
KW - Skin Neoplasms/et [Etiology]
KW - Skin Neoplasms/pc [Prevention & Control]
KW - Sunburn/pc [Prevention & Control]
KW - Sunlight/ae [Adverse Effects]
RP - NOT IN FILE
SP - 340
EP - 343
JF - Clinical & Experimental Dermatology

JA - Clin Exp Dermatol
VL - 30
IS - 4
CY - England
N2 - Recent evidence indicates that there are significant numbers of cases of malignant melanoma in the UK. In order to assess the current position with regard to sun awareness in Cornwall, a questionnaire survey of all state primary school heads (n = 123) and a survey of a random sample of GP practices (n = 9) was carried out. The data obtained were supported by visits to libraries and Tourist Information Centres at urban and rural centres--this enabled the identification of sun awareness literature. Key health professionals who worked within the field of health promotion were also contacted. The findings showed that in Cornwall public campaigns organized around the issue of sun protection took place only sporadically, although GP surgeries usually organize a display at the appropriate time of the year. None of the public places (e.g. Tourist Information Centres, libraries) surveyed had sun protection messages on display. It is concluded that insufficient sun awareness initiatives were being undertaken in Cornwall. Although most primary schools included sun awareness education in their curriculum in a form based on the Sun Awareness Guidelines produced by the Department of Health in 1995, few schools considered further measures to protect pupils on hot and sunny days. In particular the provision of shade, the scheduling of outdoor activities and the use of sunscreen and protective clothing were not standard
SN - 0307-6938
AD - Faculty of Health and Social Work, University of Plymouth, Truro, Cornwall, UK.
j.m.morris@plymouth.ac.uk
UR - 15953062
ER -

TY - JOUR
ID - 1340
T1 - Acute effects of subclinical epileptiform EEG discharges on cognitive activation
A1 - Aldenkamp,A.P.
A1 - Beitler,J.
A1 - Arends,J.
A1 - van,der Linden,I
A1 - Diepman,L.
Y1 - 2005/01//
N1 - Aldenkamp, Albert Pierre. Beitler, Jolanda. Arends, Johann. van der Linden, Inge. Diepman, Leonie
Functional neurology
8707746, fun
IM
Journal Article. Research Support, Non-U.S. Gov't
English
KW - MEDLINE
KW - Auditory Perception
KW - Child
KW - Cognition Disorders/di [Diagnosis]
KW - Cognition Disorders/et [Etiology]
KW - Diagnosis
KW - Computer-Assisted
KW - Electroencephalography
KW - Epilepsy/px [Psychology]
KW - Humans
KW - Neuropsychological Tests
KW - Prospective Studies
KW - Reaction Time
KW - Time Factors
KW - Visual Perception
RP - NOT IN FILE
SP - 23
EP - 28
JF - Functional Neurology

JA - Funct Neurol
VL - 20
IS - 1
CY - Italy
N2 - In this prospective, open, clinical comparative study we analyzed impairments of cognitive activation occurring during, immediately before and immediately after epochs with epileptiform EEG discharges of 3 seconds or longer, in an attempt to establish whether cognitive slowing occurs in direct association with an epoch with epileptiform EEG discharges and whether cognitive impairments precede or follow such an epoch. All children were assessed with EEG/video (Brainlab) simultaneously with computerized neuropsychological testing (FePsy): a test for cognitive activation (simple visual and auditory reaction time measurement). Thirty-seven epochs with epileptiform EEG discharges without clinical signs of a seizure (subclinical epileptiform EEG discharges) were evaluated. The results showed a statistically significant and clinically relevant slowing (35% compared to the overall reaction time), occurring during the epoch with epileptiform EEG discharges (repeated measurement analysis of variance p = < .05; df = 3; F-value: 3.293). No statistically significant slowing was found for the periods 'post-discharge' or 'pre-discharge'. Type of discharge was important and effects on cognitive activation were found exclusively for generalized discharges. This effect was, however, also seen in the remaining period, outside the 'peri-discharge' periods and thus seemed to represent a more general effect of the type of epilepsy on cognitive activation. Our results show that the acute effect of short epileptiform EEG discharges (duration 4.14 sec; sd 1.38) may be impressive, causing impairment (slowing) of cognitive activation. This effect was limited to generalized discharges. This effect was not observed for focal discharges, even during longer periods with discharges. However, it is reassuring that this impact on cognitive activation is limited to the actual period in which the discharges occur and does not have 'post-discharge' effects. The risk of accumulating effects that may have longer-lasting repercussions on higher-order cognitive functions therefore seems to be negligible
SN - 0393-5264
AD - Department of Behavioural Research and Clinical Neuropsychology, Epilepsy Centre Kempenhaeghe, P.O. Box: 61, NL-5590 A.B. Heeze, The Netherlands. aldenkampB@kempenhaeghe.nl
UR - 15948564
ER -

TY - JOUR
ID - 1341
T1 - Day care in infancy and risk of childhood acute lymphoblastic leukaemia: findings from UK case-control study
A1 - Gilham,C.
A1 - Peto,J.
A1 - Simpson,J.
A1 - Roman,E.
A1 - Eden,T.O.
A1 - Greaves,M.F.
A1 - Alexander,F.E.
A1 - UKCCS,Investigators
Y1 - 2005/06/04/
N1 - Gilham, C. Peto, J. Simpson, J. Roman, E. Eden, T O B. Greaves, M F. Alexander, F E. UKCCS Investigators
BMJ (Clinical research ed.)
8900488, bmj, 101090866
AIM, IM
Journal Article. Multicenter Study. Research Support, Non-U.S. Gov't
English
KW - MEDLINE
KW - Adolescent
KW - Case-Control Studies
KW - Child
KW - Child Day Care Centers/sn [Statistics & Numerical Data]
KW - Preschool
KW - Communicable Diseases/ep [Epidemiology]

KW - Environmental Exposure/ae [Adverse Effects]
KW - Environmental Exposure/sn [Statistics & Numerical Data]
KW - Great Britain/ep [Epidemiology]
KW - Humans
KW - Infant
KW - Odds Ratio
KW - Precursor Cell Lymphoblastic Leukemia-Lymphoma/ep [Epidemiology]
KW - Precursor Cell Lymphoblastic Leukemia-Lymphoma/et [Etiology]
KW - Regression Analysis
KW - Risk Factors
KW - Social Behavior
RP - NOT IN FILE
SP - 1294
JF - BMJ
VL - 330
IS - 7503
CY - England
N2 - OBJECTIVE: To test the hypothesis that reduced exposure to common infections in the first year of life increases the risk of developing acute lymphoblastic leukaemia. Design and setting The United Kingdom childhood cancer study (UKCCS) is a large population based case-control study of childhood cancer across 10 regions of the UK. PARTICIPANTS: 6305 children (aged 2-14 years) without cancer; 3140 children with cancer (diagnosed 1991-6), of whom 1286 had acute lymphoblastic leukaemia (ALL). MAIN OUTCOME MEASURE: Day care and social activity during the first year of life were used as proxies for potential exposure to infection in infancy. RESULTS: Increasing levels of social activity were associated with consistent reductions in risk of ALL; a dose-response trend was seen. When children whose mothers reported no regular activity outside the family were used as the reference group, odds ratios for increasing levels of activity were 0.73 (95% confidence interval 0.62 to 0.87) for any social activity, 0.62 (0.51 to 0.75) for regular day care outside the home, and 0.48 (0.37 to 0.62) for formal day care (attendance at facility with at least four children at least twice a week) (P value for trend < 0.001). Although not as striking, results for non-ALL malignancies showed a similar pattern (P value for trend < 0.001). When children with non-ALL malignancies were taken as the reference group, a significant protective effect for ALL was seen only for formal day care (odds ratio = 0.69, 0.51 to 0.93; P = 0.02). Similar results were obtained for B cell precursor common ALL and other subgroups, as well as for cases diagnosed above and below age 5 years. CONCLUSION: These results support the hypothesis that reduced exposure to infection in the first few months of life increases the risk of developing acute lymphoblastic leukaemia
SN - 1756-1833
AD - Cancer Research UK Epidemiology and Genetics Unit, Institute of Cancer Research, Sutton SM2 5NG
UR - 15849205
ER -

TY - JOUR
ID - 1342
T1 - UV exposure of elementary school children in five Japanese cities
A1 - Ono,M.
A1 - Munakata,N.
A1 - Watanabe,S.
Y1 - 2005/03//
N1 - Ono, Masaji. Munakata, Nobuo. Watanabe, Shaw
Photochemistry and photobiology
0376425, p69
IM
Comparative Study. Journal Article. Research Support, Non-U.S. Gov't
English
KW - MEDLINE
KW - Child
KW - Climate
KW - Dose-Response Relationship

KW - Radiation
KW - Environmental Exposure
KW - Female
KW - Health Surveys
KW - Humans
KW - Japan
KW - Male
KW - Motor Activity/ph [Physiology]
KW - Radiation Monitoring/mt [Methods]
KW - Seasons
KW - Sunlight
KW - Time Factors
KW - Ultraviolet Rays
RP - NOT IN FILE
SP - 437
EP - 445
JF - Photochemistry & Photobiology
JA - Photochem Photobiol
VL - 81
IS - 2
CY - United States
N2 - A 1 week UV-exposure measurement and outdoor-activity pattern survey was conducted for elementary school children for four seasons at five sites in Japan, i.e. Sapporo (43 degrees 05' N, altitude 40 m), Tsukuba (36 degrees 05' N, 20 m), Tokyo (35 degrees 40' N, 45 m), Miyazaki (31 degrees 60' N, 40 m) and Naha (26 degrees 10' N, 5 m), and UV exposure was measured directly and estimated using outdoor-activity records. The study site with largest UV exposure was Miyazaki, a southern rural area. Comparing the results for boys and girls, UV exposure was larger in boys. UV exposure was large in spring and summer and small in winter. The total amount of UV exposure in spring and summer contributed 57.7-73.4% of total exposure for the year. As a whole, 8.1% and 1.8% of the schoolchildren were exposed to more than 1 minimum erythemal dose (MED) and 2 MED of solar UV in a day, respectively. The estimated yearly UV exposure ranged from 49 207 J/m2 in Miyazaki to 31 520 J/m2 in Tsukuba. The actual UV exposure correlated to potential UV exposure, estimated using outdoor-activity records and ambient UV irradiance, but the ratio differed by season and site. The yearly average of percent UV exposure to ambient UV on a horizontal plane ranged from 9.9% in Tokyo to 4.0% in Naha. In the questionnaire survey on outdoor-activity pattern, a short question "How long did you spend time outdoors between 0900 and 1500 h?" gives the best estimates of UV exposure
SN - 0031-8655
AD - Environmental Health Sciences Division, National Institute for Environmental Studies, Tsukuba, Ibaraki, Japan. onomasaj@nies.go.jp
UR - 15581388
ER -

TY - JOUR
ID - 1343
T1 - Evaluation of a community-based handgun safe-storage campaign
A1 - Sidman,E.A.
A1 - Grossman,D.C.
A1 - Koepsell,T.D.
A1 - D'Ambrosio,L.
A1 - Britt,J.
A1 - Simpson,E.S.
A1 - Rivara,F.P.
A1 - Bergman,A.B.
Y1 - 2005/06//
N1 - Sidman, Elanor A. Grossman, David C. Koepsell, Thomas D. D'Ambrosio, Luann. Britt, John. Simpson, Evan S. Rivara, Frederick P. Bergman, Abraham B
Pediatrics
oxv, 0376422

AIM, IM
Journal Article. Research Support, N.I.H., Extramural. Research Support, Non-U.S. Gov't. Research Support, U.S. Gov't, P.H.S.
English
KW - MEDLINE
KW - Accident Prevention/mt [Methods]
KW - Accidents
KW - Home/pc [Prevention & Control]
KW - Adult
KW - Child
KW - Cooperative Behavior
KW - Cross-Sectional Studies
KW - Data Collection
KW - Family Characteristics
KW - Female
KW - Firearms/sn [Statistics & Numerical Data]
KW - Firearms
KW - Health Education/og [Organization & Administration]
KW - Health Knowledge
KW - Attitudes
KW - Practice
KW - Household Articles/sn [Statistics & Numerical Data]
KW - Household Articles
KW - Humans
KW - Intervention Studies
KW - Male
KW - Mass Media
KW - Protective Devices/sn [Statistics & Numerical Data]
KW - Protective Devices/ut [Utilization]
KW - Residence Characteristics/sn [Statistics & Numerical Data]
KW - Safety
KW - Sampling Studies
KW - Washington
KW - Wounds
KW - Gunshot/pc [Prevention & Control]
RP - NOT IN FILE
SP - e654
EP - e661
JF - Pediatrics
VL - 115
IS - 6
CY - United States
N2 - OBJECTIVE: Safe storage of firearms has been recommended as a means of preventing gun-related pediatric injuries, yet few interventions have led to significant improvements in storage practices. This study examined a multifaceted community education campaign to promote safe handgun storage and the campaign's impact on firearm locking and loading practices in households with children. METHODS: Beginning in 1997, a safe-storage campaign consisting of television and radio announcements, educational materials, billboards, and discount coupons for lock boxes was conducted in King County, Washington. The campaign evaluation used a quasi-experimental design and compared the intervention site with 9 control counties outside Washington State and west of the Mississippi River. Cross-sectional, random-digit-dial telephone surveys of handgun-owning households with children were conducted in all study counties both before the intervention in 1996 (n = 302) and again in 2001 (n = 255). The main analyses assessed whether greater improvements in household firearm-storage practices occurred between 1996 and 2001 in the intervention, compared with the control, counties. Primary outcomes were based on up to 3 handguns per household and included (1) all stored with trigger locks, lock boxes, or gun safes (formal locking devices), (2) all stored in lock boxes or gun safes, (3) any stored loaded, (4) any stored loaded without a formal locking device, and (5) any stored loaded and not in a lock box or gun safe. Data were also collected on up to 1 long gun per household; long-gun outcomes included (1) stored

with a trigger lock or gun safe and (2) stored loaded. RESULTS: Overall, handguns and long guns were generally more likely to be stored locked and less likely to be loaded in 2001 compared with 1996, with these trends seeming to be more consistent in the intervention county. Even so, more than one quarter of households with children and handguns in 2001 failed to store all of their handguns with a formal locking device, and up to 8% continued to possess at least 1 loaded handgun that was not stored with a formal device. The majority of households that stored their handguns with formal devices used lock boxes or gun safes. Storage of handguns in lock boxes or gun safes became more common in both the intervention (adjusted odds ratio [aOR]: 1.71; 95% confidence interval [CI]: 1.03-2.84) and control households (aOR: 1.66; 95% CI: 1.01-2.72) between 1996 and 2001. None of the other measured changes reached statistical significance, such as storing any household handgun loaded (aOR: 0.71; 95% CI: 0.35-1.42 [intervention]; aOR: 1.08; 95% CI: 0.58-2.00 [control]) or keeping any household handgun loaded and not stored in a lock box or gun safe (aOR: 0.59; 95% CI: 0.22-1.55 [intervention]; aOR: 0.67; 95% CI: 0.30-1.49 [control]). Moreover, the intervention county did not experience significantly greater overall improvements in household storage practices for handguns or long guns than did control counties. CONCLUSIONS: In both the intervention and control counties, households were more likely to lock all handguns in 2001 compared with 1996. After accounting for temporal trends, this educational campaign, combined with economic incentives to purchase lock boxes, did not seem to significantly change safe storage practices in households with handguns and children. Even if the campaign did result in small improvements in firearm safe storage, simultaneous national and state-specific gun-safety activities or legislative efforts may have drawn increasing attention to gun-related issues in the control counties, thereby making it more difficult to identify effects of our specific handgun storage intervention
SN - 1098-4275
AD - Harborview Injury Prevention and Research Center, Department of Epidemiology, University of Washington, Seattle, Washington, USA
UR - 15930192
ER -

TY - JOUR
ID - 1344
T1 - The health and well-being of caregivers of children with cerebral palsy
A1 - Raina,P.
A1 - O'Donnell,M.
A1 - Rosenbaum,P.
A1 - Brehaut,J.
A1 - Walter,S.D.
A1 - Russell,D.
A1 - Swinton,M.
A1 - Zhu,B.
A1 - Wood,E.
Y1 - 2005/06//
N1 - Raina, Parminder. O'Donnell, Maureen. Rosenbaum, Peter. Brehaut, Jamie. Walter, Stephen D. Russell, Dianne. Swinton, Marilyn. Zhu, Bin. Wood, Ellen
Pediatrics
oxv, 0376422
AIM, IM
Journal Article. Multicenter Study. Research Support, Non-U.S. Gov't
English
KW - MEDLINE
KW - Adaptation
KW - Psychological
KW - Adult
KW - Caregivers/px [Psychology]
KW - Cerebral Palsy/px [Psychology]
KW - Cerebral Palsy
KW - Child
KW - Child Behavior Disorders/et [Etiology]
KW - Cohort Studies
KW - Disabled Children

KW - Family Health
KW - Female
KW - Foster Home Care
KW - Humans
KW - Male
KW - Middle Aged
KW - Models
KW - Theoretical
KW - Ontario
KW - Parent-Child Relations
KW - Parents
KW - Professional-Patient Relations
KW - Questionnaires
KW - Self Concept
KW - Severity of Illness Index
KW - Social Support
KW - Stress
KW - Psychological/ep [Epidemiology]
KW - Psychological/et [Etiology]
RP - NOT IN FILE
SP - e626
EP - e636
JF - Pediatrics
VL - 115
IS - 6
CY - United States
N2 - OBJECTIVE: Most children enjoy healthy childhoods with little need for specialized health care services. However, some children experience difficulties in early childhood and require access to and utilization of considerable health care resources over time. Although impaired motor function is the hallmark of the cerebral palsy (CP) syndromes, many children with this development disorder also experience sensory, communicative, and intellectual impairments and may have complex limitations in self-care functions. Although caregiving is a normal part of being the parent of a young child, this role takes on an entirely different significance when a child experiences functional limitations and possible long-term dependence. One of the main challenges for parents is to manage their child's chronic health problems effectively and juggle this role with the requirements of everyday living. Consequently, the task of caring for a child with complex disabilities at home might be somewhat daunting for caregivers. The provision of such care may prove detrimental to both the physical health and the psychological well-being of parents of children with chronic disabilities. It is not fully understood why some caregivers cope well and others do not. The approach of estimating the "independent" or "direct" effects of the care recipient's disability on the caregiver's health is of limited value because (1) single-factor changes are rare outside the context of constrained experimental situations; (2) assumptions of additive relationships and perfect measurements rarely hold; and (3) such approaches do not provide a complete perspective, because they fail to examine indirect pathways that occur between predictor variables and health outcomes. A more detailed analytical approach is needed to understand both direct and indirect effects simultaneously. The primary objective of the current study was to examine, within a single theory-based multidimensional model, the determinants of physical and psychological health of adult caregivers of children with CP. METHODS: We developed a stress process model and applied structural equation modeling with data from a large cohort of caregivers of children with CP. This design allowed the examination of the direct and indirect relationships between a child's health, behavior and functional status, caregiver characteristics, social supports, and family functioning and the outcomes of caregivers' physical and psychological health. Families (n = 468) of children with CP were recruited from 19 regional children's rehabilitation centers that provide outpatient disability management and supports in Ontario, Canada. The current study drew on a population available to the investigators from a previous study, the Ontario Motor Growth study, which explored patterns of gross motor development in children with CP. Data on demographic variables and caregivers' physical and psychological health were assessed using standardized, self-completed parent questionnaires as well as a face-to-face home interview. Structural equation modeling was used to test specific hypotheses outlined in our conceptual model. This analytic approach involved a 2-step process. In the first step, observed variables that were hypothesized to measure the underlying constructs were tested using confirmatory factor analysis; this step led to the so-called

measurement model. The second step tested hypotheses about relationships among the variables in the structural model. All of the hypothesized paths in the conceptual model were tested and included in the structural model. However, only paths that were significant were shown in the final results. The direct, indirect, and total effects of theoretical constructs on physical and psychological health were calculated using the structural model. RESULTS: The most important predictors of caregivers' well-being were child behavior, caregiving demands, and family function. A higher level of behavior problems was associated with lower levels of both psychological (beta = -.22) and physical health (beta = -.18) of the caregivers, whereas fewer child behavior problems were associated with higher self-perception (beta = -.37) and a greater ability to manage stress (beta = -.18). Less caregiving demands were associated with better physical (beta = .23) and psychological (beta = .12) well-being of caregivers, respectively. Similarly, higher reported family functioning was associated with better psychological health (beta = .33) and physical health (beta = .33). Self-perception and stress management were significant direct predictors of caregivers' psychological health but did not directly influence their physical well-being. Caregivers' higher self-esteem and sense of mastery over the caregiving situation predicted better psychological health (beta = .23). The use of more stress management strategies was also associated with better psychological health of caregivers (beta = .11). Gross income (beta = .08) and social support (beta = .06) had indirect overall effects only on psychological health outcome, whereas self-perception (beta = .22), stress management (beta = .09), gross income (beta = .07), and social support (beta = .06) had indirect total effects only on physical health outcomes. CONCLUSIONS: The psychological and physical health of caregivers, who in this study were primarily mothers, was strongly influenced by child behavior and caregiving demands. Child behavior problems were an important predictor of caregiver psychological well-being, both directly and indirectly, through their effect on self-perception and family function. Caregiving demands contributed directly to both the psychological and the physical health of the caregivers. The practical day-to-day needs of the child created challenges for parents. The influence of social support provided by extended family, friends, and neighbors on health outcomes was secondary to that of the immediate family working closely together. Family function affected health directly and also mediated the effects of self-perception, social support, and stress management. In families of children with CP, strategies for optimizing caregiver physical and psychological health include supports for behavioral management and daily functional activities as well as stress management and self-efficacy techniques. These data support clinical pathways that require biopsychosocial frameworks that are family centered, not simply technical and short-term rehabilitation interventions that are focused primarily on the child. In terms of prevention, providing parents with cognitive and behavioral strategies to manage their child's behaviors may have the potential to change caregiver health outcomes. This model also needs to be examined with caregivers of children with other disabilities
SN - 1098-4275
AD - Department of Clinical Epidemiology and Biostatistics, McMaster Evidence-Based Practice Centre, Hamilton, ON, Canada. praina@mcmaster.ca
UR - 15930188
ER -

TY - JOUR
ID - 1345
T1 - International Resuscitation Network Registry: design, rationale and preliminary results
A1 - Nichol,G.
A1 - Steen,P.
A1 - Herlitz,J.
A1 - Morrison,L.J.
A1 - Jacobs,I.
A1 - Ornato,J.P.
A1 - O'Connor,R.
A1 - Nadkarni,V.
A1 - International,Resuscitation Network,I
Y1 - 2005/06//
N1 - Nichol, G. Steen, P. Herlitz, J. Morrison, L J. Jacobs, I. Ornato, J P. O'Connor, R. Nadkarni, V. International Resuscitation Network Investigators
Resuscitation
r8q, 0332173
IM

Journal Article. Multicenter Study
English
KW - MEDLINE
KW - Adolescent
KW - Adult
KW - Aged
KW - 80 and over
KW - Australia
KW - Cardiopulmonary Resuscitation
KW - Child
KW - Preschool
KW - Europe
KW - Feasibility Studies
KW - Female
KW - Heart Arrest/th [Therapy]
KW - Humans
KW - Infant
KW - Male
KW - Middle Aged
KW - North America
KW - Outcome and Process Assessment (Health Care)
KW - Prospective Studies
KW - Registries
RP - NOT IN FILE
SP - 265
EP - 277
JF - Resuscitation
VL - 65
IS - 3
CY - Ireland
N2 - There is a lack of high-quality information about the effectiveness of resuscitation interventions and international differences in structure, process and outcome after out-of-hospital cardiac arrest and cardiopulmonary resuscitation because data are not collected uniformly. An internet-based international registry could make such evaluations possible, and enable the conduct of large randomized controlled trials of resuscitation therapies. A prospective international cohort study was performed that included 571 infants, children and adults (a) who experienced cardiac arrest requiring chest compressions or external defibrillation, (b) outside the hospital in the study communities and (c) upon whom resuscitation was attempted by EMS personnel. Cardiac arrest was defined as lack of responsiveness, breathing or movement in individuals for whom the EMS system is activated for whom an arrest record is completed. All data were collated via a secure and confidential web-based method by using automated forms processing software with appropriate variable range checks, logic checks and skip rules. Median number of missing responses for each variable was 0 (interquartile range 0, 0). Twenty-seven percent of the patients had a first recorded rhythm of ventricular fibrillation or ventricular tachycardia, 60% had a witnessed arrest, and 34% received bystander CPR. Mean time from call to arrival on scene was 7.1+/-5.1 min. Six percent of the patients survived to hospital discharge. The resuscitation process was highly variable across centers, and survival and neurological outcome were also significantly and independently different across centers. This study shows that it is possible to collect data prospectively describing the structure, process and outcome associated with cardiac arrest in multiple international sites via the internet. Therefore, it is feasible to conduct adequately powered randomized trials of resuscitation therapies in international settings
SN - 0300-9572
AD - University of Washington, Seattle, WA 98104, USA. nichol@u.washington.edu
UR - 15919562
ER -

TY - JOUR
ID - 1346
T1 - Sun protective behaviors and sunburn experiences in parents of youth ages 11 to 18

A1 - Cardinez,C.J.
A1 - Cokkinides,V.E.
A1 - Weinstock,M.A.
A1 - O'Connell,M.C.
Y1 - 2005/07//
N1 - Cardinez, Cheryll J. Cokkinides, Vilma E. Weinstock, Martin A. O'Connell, Mary C
Preventive medicine
pm4, 0322116
IM
Comparative Study. Journal Article
English
KW - MEDLINE
KW - Adolescent
KW - Adult
KW - Age Factors
KW - Child
KW - Confidence Intervals
KW - Cross-Sectional Studies
KW - Female
KW - Health Behavior
KW - Humans
KW - Male
KW - Middle Aged
KW - Parent-Child Relations
KW - Prevalence
KW - Primary Prevention/mt [Methods]
KW - Probability
KW - Protective Clothing/sn [Statistics & Numerical Data]
KW - Questionnaires
KW - Risk Assessment
KW - Severity of Illness Index
KW - Sex Factors
KW - Sunburn/ep [Epidemiology]
KW - Sunburn/pc [Prevention & Control]
KW - Sunscreening Agents/ad [Administration & Dosage]
KW - United States/ep [Epidemiology]
RP - NOT IN FILE
SP - 108
EP - 117
JF - Preventive Medicine
JA - Prev Med
VL - 41
IS - 1
CY - United States
N2 - BACKGROUND: Recent studies suggest that parental sun protective behaviors and communication influence their adolescents. However, there is limited information on sun protection for parents of adolescents. METHODS: A telephone-based, nationally representative prevalence study of sun exposure among youth, aged 11-18, and their parents living in households was conducted in 1998. Separate, independent responses were collected. Weighted prevalence estimates and 95% confidence intervals were estimated and presented for parents only (n = 1187). RESULTS: Approximately one-third of parents planned activities to avoid the sun and used sunscreen. Among parents who used sunscreen, 70% applied it while at the beach or pool, but not as often during other outdoor activities. Almost one-third of parents were participating in water or non-water recreational activities during their most serious sunburn. Differences in sun protection and sunburn experiences were observed by age, gender, sun sensitivity, race and ethnicity, and educational attainment. CONCLUSIONS: Parents have adopted sun protection habits, but have not surpassed national sun-protection goals. Combined use of sun protection behaviors may reduce sunburn prevalence and number of incident skin cancers. These data

may be useful for developing or enhancing current sun protection programs for effective sun protection that include parents and their adolescents
SN - 0091-7435
AD - Department of Epidemiology and Surveillance Research, American Cancer Society, National Home Office, 1599 Clifton Road NE, Atlanta, GA 30329-4251, USA
UR - 15917001
ER -

TY - JOUR
ID - 1347
T1 - Assessing physical activity during recess using accelerometry
A1 - Ridgers,N.D.
A1 - Stratton,G.
A1 - Fairclough,S.J.
Y1 - 2005/07//
N1 - Ridgers, Nicola D. Stratton, Gareth. Fairclough, Stuart J
Preventive medicine
pm4, 0322116
IM
Journal Article. Multicenter Study. Research Support, Non-U.S. Gov't
English
KW - MEDLINE
KW - Body Mass Index
KW - Child
KW - Cohort Studies
KW - Exercise
KW - Female
KW - Great Britain
KW - Health Behavior
KW - Humans
KW - Leisure Activities
KW - Male
KW - Multivariate Analysis
KW - Physical Fitness/ph [Physiology]
KW - Probability
KW - School Health Services
KW - Sensitivity and Specificity
KW - Sex Factors
KW - Students/sn [Statistics & Numerical Data]
KW - Time Factors
RP - NOT IN FILE
SP - 102
EP - 107
JF - Preventive Medicine
JA - Prev Med
VL - 41
IS - 1
CY - United States
N2 - BACKGROUND: Physical activity guidelines recommend children should engage in 60 min of moderate-to-vigorous physical activity (MVPA) a day. School recess presents an opportunity for children to be physically active during the school day. Limited research has investigated children's activity levels during recess and its contribution to physical activity recommendations. Moreover, no target for physical activity during recess has been set. METHODS: One hundred sixteen boys and 112 girls (aged 5-10 years) from 23 schools had their physical activity during recess quantified using a uniaxial accelerometer during three recess breaks on one school day. The percentage of time spent engaged in moderate, high, and very high intensity activity was calculated using existing thresholds. RESULTS: Boys engaged in more moderate, high, and very high intensity activity than girls. On average, boys and girls spent 32.9% and 23% of recess engaged in physical activity,

respectively. CONCLUSIONS: Boys engaged in higher intensity activities than girls. The results suggest that recess can contribute 28 min for boys and 21.5 min for girls toward the accumulation of recommended daily physical activity. However, the physical activity intensities that children engaged in were low during recess. On average, children in this study did not achieve 50% of recess time in physical activity. Interventions for increasing the physical activity of children in the playground are warranted
SN - 0091-7435
AD - School of Sport and Exercise Sciences, Liverpool John Moores University, Henry Cotton Campus, 15-21 Webster Street, Liverpool, L3 2ET, UK. n.ridgers@livjm.ac.uk
UR - 15917000
ER -

TY - JOUR
ID - 1348
T1 - Malaria epidemiology in the Pakaanova (Wari') Indians, Brazilian Amazon
A1 - Sa,D.R.
A1 - Souza-Santos,R.
A1 - Escobar,A.L.
A1 - Coimbra,C.E.,Jr.
Y1 - 2005/04//
N1 - Sa, D Ribeiro. Souza-Santos, R. Escobar, A L. Coimbra, C E A Jr
Bulletin de la Societe de pathologie exotique (1990)
bk9, 9212564
IM
Journal Article. Research Support, Non-U.S. Gov't
English
KW - MEDLINE
KW - Adolescent
KW - Adult
KW - Brazil/ep [Epidemiology]
KW - Child
KW - Female
KW - Humans
KW - Indians
KW - South American
KW - Longitudinal Studies
KW - Malaria/ep [Epidemiology]
KW - Male
RP - NOT IN FILE
SP - 28
EP - 32
JF - Bulletin de la Societe de Pathologie Exotique
JA - Bull Soc Pathol Exot.
VL - 98
IS - 1
CY - France
N2 - This paper reports the results of a longitudinal study of malaria incidence (1998-2002) among the Pakaanova (Wari') Indians, Brazilian southwest Amazon region, based on data routinely gathered by Brazilian National Health Foundation outposts network in conjunction with the Indian health service. Malaria is present yearlong in the Pakaanova. Statistically significant differences between seasons or months were not noticed. A total of 1933 cases of malaria were diagnosed in the Pakaanova during this period. The P. vivax / P. falciparum ratio was 3.4. P. vivax accounted for 76.5% of the cases. Infections with P. malariae were not recorded. Incidence rates did not differ by sex. Most malaria cases were reported in children < 10 years old (45%). About one fourth of all cases were diagnosed on women 10-40 years old. An entomological survey carried out at two Pakaanova villages yielded a total of 3.232 specimens of anophelines. Anopheles darlingi predominated (94.4%). Most specimens were captured outdoors and peak activity hours were noted at early evening and just before sunrise. It was observed that Pakaanova cultural practices may facilitate outdoor exposure of individuals of both sexes and all age groups during peak hours of mosquito activities (e.g., coming to the river early in the

morning for bathing or to draw water, fishing, engaging in hunting camps, etc). In a context in which anophelines are ubiquitous and predominantly exophilic, and humans of both sexes and all ages are prone to outdoor activities during peak mosquito activity hours, malaria is likely to remain endemic in the Pakaanova, thus requiring the development of alternative control strategies that are culturally and ecologically sensitive
SN - 0037-9085
AD - Centro de Estudos em Saude do Indio de Rondonia, Universidade Federal de Rondonia, Av. Presidente Dutra 2965, Porto Velho, RO 78900-500, Brazil
UR - 15915970
ER -

TY - JOUR
ID - 1349
T1 - Arsenic, cadmium and lead levels in hair and toenail samples in pakistan
A1 - Anwar,M.
Y1 - 2005///
N1 - Anwar, Muhammad
Environmental sciences : an international journal of environmental physiology and toxicology
9209787
IM
Journal Article. Research Support, Non-U.S. Gov't
English
KW - MEDLINE
KW - Adolescent
KW - Adult
KW - Age Factors
KW - Arsenic/an [Analysis]
KW - Arsenic/me [Metabolism]
KW - Cadmium/an [Analysis]
KW - Cadmium/me [Metabolism]
KW - Child
KW - Preschool
KW - Environmental Monitoring
KW - Environmental Pollutants/an [Analysis]
KW - Environmental Pollutants/me [Metabolism]
KW - Epidemiological Monitoring
KW - Fabaceae
KW - Female
KW - Food Contamination
KW - Hair/ch [Chemistry]
KW - Humans
KW - Infant
KW - Lead/an [Analysis]
KW - Lead/me [Metabolism]
KW - Male
KW - Nails/ch [Chemistry]
KW - Pakistan/ep [Epidemiology]
KW - Shellfish
KW - Vehicle Emissions
RP - NOT IN FILE
SP - 71
EP - 86
JF - Environmental Sciences
VL - 12
IS - 2
CY - Japan
N2 - We collected toenail and hair samples from 160 subjects, 83 males and 77 females, residing in Lahore city and its suburban areas in Pakistan, and examined the concentrations of arsenic, cadmium, lead and other metals

using inductively coupled plasma mass spectrometry (ICP-MS). The mean hair and toenail arsenic levels were 0.31 ppm and 0.70 ppm, respectively. The correlation coefficient between log-transformed values of hair and toenail arsenic levels was 0.16 (p=0.051). After excluding a subject with hair and toenail arsenic levels as high as 1.12 ppm and 4.72 ppm, respectively, the correlation coefficient was 0.27 (p=0.001). Arsenic levels observed in the present study were relatively higher than in most developed countries. Hair or toenail arsenic concentration was not affected by sex, area of origin, place of residence and education, but toenail arsenic levels increased with age (p=0.029). Fish consumption was inversely related to arsenic levels, suggesting that consumption of beans , which are the main staple of poor people living in the study area who cannot afford to eat fish frequently, may be the source of arsenic exposure among the residents of Lahore and its suburban areas. The mean hair and toenail cadmium levels in the present study were 0.08 ppm and 0.05 ppm, respectively. The correlation coefficient between log-transformed values of hair and toenail cadmium levels was 0.17 (p=0.034). Cadmium levels were lower compared to those reported from developed countries, including Japan. The mean hair and toenail lead levels were 3.53 ppm and 2.11 ppm, respectively. The correlation coefficient between log-transformed values of hair and toenail lead levels was 0.15, which was not statistically significant (p=0.055). When a female subject with toenail lead of 52.4 ppm was excluded, the correlation was 0.17 (p=0.036). Lead levels observed in the present study were similar to those reported from developed countries. In the present study, relatively high lead toenail values were observed among children. The levels of cadmium and lead in hair but not in toenails were higher among those living in central Lahore than among those living outside Lahore, suggesting that dust containing those heavy metals was attached to hair samples due to a typical urban environment with heavy traffic load, congested population and industrial activities
SN - 0915-955X
AD - Department of Epidemiology and Preventive Medicine, Graduate School of Medical and Dental Sciences, Kagoshima University 8-35-1 Sakuragaoka, Kagoshima 890-8544, Japan. manwar@m3.kufm.kagoshima-u.ac.jp
UR - 15915148
ER -

TY - JOUR
ID - 1350
T1 - Clinical evaluation of subjective and objective changes in motor activity and attention in children with attention-deficit/hyperactivity disorder in a double-blind methylphenidate trial
A1 - Konrad,K.
A1 - Gunther,T.
A1 - Heinzel-Gutenbrunner,M.
A1 - Herpertz-Dahlmann,B.
Y1 - 2005/04//
N1 - Konrad, Kerstin. Gunther, Thomas. Heinzel-Gutenbrunner, Monika. Herpertz-Dahlmann, Beate
Journal of child and adolescent psychopharmacology
co4, 9105358
IM
Clinical Trial. Comparative Study. Journal Article. Randomized Controlled Trial. Research Support, Non-U.S. Gov't
English
KW - MEDLINE
KW - Analysis of Variance
KW - Attention/de [Drug Effects]
KW - Attention/ph [Physiology]
KW - Attention Deficit Disorder with Hyperactivity/dt [Drug Therapy]
KW - Attention Deficit Disorder with Hyperactivity/px [Psychology]
KW - Child
KW - Cross-Over Studies
KW - Dose-Response Relationship
KW - Drug
KW - Double-Blind Method
KW - Female
KW - Humans
KW - Male

KW - Methylphenidate/pd [Pharmacology]
KW - Methylphenidate/tu [Therapeutic Use]
KW - Motor Activity/de [Drug Effects]
KW - Motor Activity/ph [Physiology]
KW - Neuropsychological Tests/sn [Statistics & Numerical Data]
RP - NOT IN FILE
SP - 180
EP - 190
JF - Journal of Child & Adolescent Psychopharmacology
JA - J Child Adolesc Psychopharmacol
VL - 15
IS - 2
CY - United States
N2 - The aim of this study was to determine drug-dependent effects on attention and motor activity in children with attention-deficit/hyperactivity disorder (ADHD) in a double-blind methylphenidate (MPH) trial using both subjective behavior ratings and objective measures of function. In particular, we were interested in the relationship between changes of those subjective and objective measures. A total of 44 ADHD children with the combined subtype completed a randomized, double-blind, placebo-controlled, crossover trial with 2 doses of MPH (0.25; 0.5 mg/kg body weight) and placebo. In each condition, computerized attention tasks were performed, teacher-behavior ratings were obtained, and actigraph data were collected in both clinical and naturalistic settings. Trend tests revealed linear effects of MPH dose on actigraph data in the test session ($p = 0.02$) and at school ($p = 0.001$), as well as on sustained attention ($p < 0.001$), while inhibitory control showed a quadratic dose-response curve ($p < 0.001$). Multivariate regression analyses revealed that changes of both, hyperactive-impulsive symptoms (28%) and inattentive symptoms (23%) could be explained by objective changes of motor activity. Thus, for clinical practice, it should be taken into account that behavior ratings of ADHD symptoms seemed to be predominantly influenced by changes of motor activity
SN - 1044-5463
AD - Department of Child and Adolescent Psychiatry, University Hospital Aachen, Aachen, Germany. kkonrad@ukaachen.de
UR - 15910203
ER -

TY - JOUR
ID - 1351
T1 - The removal of patients who live outside the practice boundary: a study of outside-area removals in Northern Ireland in 2001-2002
A1 - O'Reilly,D.
A1 - Steele,K.
Y1 - 2005/05//
N1 - O'Reilly, Dermot. Steele, Keith
The British journal of general practice : the journal of the Royal College of General Practitioners
9005323, ark
IM
Journal Article. Research Support, Non-U.S. Gov't
English
KW - MEDLINE
KW - Adolescent
KW - Adult
KW - Age Distribution
KW - Aged
KW - 80 and over
KW - Child
KW - Preschool
KW - Family Practice/sn [Statistics & Numerical Data]
KW - Female
KW - Humans
KW - Infant

KW - Newborn
KW - Male
KW - Middle Aged
KW - Northern Ireland
KW - Refusal to Treat/sn [Statistics & Numerical Data]
KW - Residence Characteristics
KW - Sex Distribution
RP - NOT IN FILE
SP - 384
EP - 386
JF - British Journal of General Practice
JA - Br J Gen Pract
VL - 55
IS - 514
CY - England
N2 - It has been suggested that there are significant overlaps between removals due to deregistration and removals arising because patients live outside the practice area. If this is true, it would mean that the current estimates of deregistration would need to be revised upwards. All outside-area removals for the calendar years 2001 and 2002 were reviewed and characterised by age, sex and Jarman score of the enumeration district of the patients' residence and distance from the practice. The average outside-area removal rate was just over one removal per practice per year. Removal rates were highest between the ages of 18 and 44 years; there were no significant differences between the sexes. Rates of removal increased exponentially with distance, although even at marked distances from the practice there were about 10 patients remaining on the list for each one removed. Residents in deprived areas were more likely to be removed, although because areas most distal to the practice tend to be affluent, overall there was a predominance of affluent patients among those who are removed. In Northern Ireland rates of outside-area removal are only slightly higher than those of deregistration. It is evident that GPs are exercising some discretion as to which of the outside-area patients they retain on their list. This has the potential to cause some misunderstanding and resentment among patients, as has been reported previously
SN - 0960-1643
AD - Department of Epidemiology & Public Health, The Queen's University of Belfast, Belfast.
d.oreilly@qub.ac.uk
UR - 15904558
ER -

TY - JOUR
ID - 1352
T1 - [Long-term results following fracture of the femoral neck in children]. [Czech]
A1 - Matejka,J.
A1 - Pavelka,T.
A1 - Kostal,J.
A1 - Cervenkova,H.
Y1 - 2005///
N1 - Matejka, J. Pavelka, T. Kostal, J. Cervenkova, H
Acta chirurgiae orthopaedicae et traumatologiae Cechoslovaca
0407123
IM
English Abstract. Journal Article
Czech
KW - MEDLINE
KW - Adolescent
KW - Child
KW - Preschool
KW - Female
KW - Femoral Neck Fractures/pa [Pathology]
KW - Femoral Neck Fractures/su [Surgery]
KW - Femur Neck/ra [Radiography]

KW - Femur Neck/su [Surgery]
KW - Follow-Up Studies
KW - Fracture Fixation
KW - Internal
KW - Fracture Healing
KW - Humans
KW - Male
KW - Osteonecrosis/et [Etiology]
RP - NOT IN FILE
SP - 98
EP - 104
JF - Acta Chirurgiae Orthopaedicae et Traumatologiae Cechoslovaca
JA - Acta Chir Orthop Traumatol Cech.
VL - 72
IS - 2
CY - Czech Republic
N2 - PURPOSE OF THE STUDY: This retrospective study was designed to evaluate the severity and nature of long-term sequelae of femoral neck fractures in children in relation to the strategy and technique of therapy. MATERIAL: The study included 15 patients with a fracture of the femoral neck who, at the time of injury, had an opened proximal physis. The average age at the time of injury was 11.5 years (range, 4 to 16.3 years). There were eight boys and seven girls. Twelve children suffered injury due to a fall varying in gravity, two were knocked down by a vehicle, and one was injured as a co-driver in a car accident. The group involved no type I fracture, six type II, seven type III and two type IV fractures, as classified by the Delbet and Colonna system. METHODS: All children were operated on within an average of 4.1 days after injury. The delay was caused by a late referral from an outside hospital or was due to associated complications.However, the majority of fractures were treated within 24 hours of injury. Surgery was carried out from the anterolateral approach. Miniarthrotomy was performed in 11 patients to remove hematoma and decompress the intra-articular space. The methods of stabilization included Kirschner's wires in four children, compressive osteosynthesis using lag screws inserted extraphyseally in 10 children and the combination of both methods in one child. No plaster of Paris spica or traction was applied after surgery. RESULTS: The long-term results were evaluated at a minimum of 5 years after injury. The average follow-up was 9 years and 11 months, with the range from 5 years and 1 month to 15 years and 5 months, and 12 patients were involved. Six had a type II and six had a type III fracture. Ten patients were treated by arthrotomy. Stabilization of the fracture was performed with lag screws in nine children and Kirschner's wires in three patients. Both subjective and objective findings were evaluated by the modified Rattlif criteria. Concerning pain, eight (67 %) children had excellent outcomes, three (25 %) reported good outcomes and only one (8 %) had a poor outcome. The activity following injury was subjectively evaluated as excellent by 11 (92 %) patients and as good by one (8 %) patient. None of the patients was noticeably limited in their activity, as compared with the pre-injury state. Objective findings were based on X-ray images and the range of hip motion. No or minimal radiographic changes were found in five (42 %) patients and were assessed as excellent outcomes. A good outcome, i. e., a spherical head with a moderate neck deformity, was achieved in five (42 %) patients. A poor outcome, i. e., avascular necrosis free of revitalization, but with collapse, on X-ray images was recorded in two (16 %) patients. The values for the range of motion and limb-length discrepancy were excellent in eight (67 %), good in two (16.5 %) and poor in two (16.5 %) patients. Poor objective and subjective findings were recorded in the patients who had not undergone miniarthrotomy. DISCUSSION: A comparison with the literature data showed that the occurrence of each fracture type was in agreement with the reports of other authors. The finding of a higher frequency of type I fractures can be explained by a pathological slip due to hormonal changes. The opinions on treatment of these fractures have developed to the view that surgery is necessary in the early post-injury period, preferably with the use of compressive osteosynthesis or Kirschner's wires. However, subsequent immobilization in a plaster cast spica is not necessary. The subjective and objective findings were not exactly correlated due to differences in patients' age and different intervals between injury and treatment. CONCLUSIONS: The authors recommend early surgery and stabilization by compressive osteosynthesis or Kirschner's wires, together with miniarthrotomy in order to decompress the articular space
SN - 0001-5415
AD - Klinika ortopedie a traumatologie pohyboveho ustroji LF UK a FN Plzen. matejka@fnplzen.cz
UR - 15890141
ER -

TY - JOUR
ID - 1353
T1 - Childhood obesity -- what we can learn from existing data on societal trends, part 2
A1 - Sturm,R.
Y1 - 2005/04//
N1 - Sturm, Roland
Preventing chronic disease
101205018
IM
Journal Article
English
KW - MEDLINE
KW - Adolescent
KW - Bicycling/td [Trends]
KW - Carbonated Beverages
KW - Child
KW - Culture
KW - Diet/td [Trends]
KW - Dietary Carbohydrates
KW - Dietary Fats
KW - Fruit
KW - Health Behavior
KW - Humans
KW - Life Style
KW - Obesity/ep [Epidemiology]
KW - Physical Education and Training/td [Trends]
KW - Risk Factors
KW - Students/sn [Statistics & Numerical Data]
KW - Transportation/sn [Statistics & Numerical Data]
KW - Vegetables
KW - Walking/sn [Statistics & Numerical Data]
RP - NOT IN FILE
SP - A20
JF - Preventing Chronic Disease
JA - Prev Chronic Dis
VL - 2
IS - 2
CY - United States
N2 - The number of overweight and obese youth has increased in recent decades, yet few data assess how the lives of children have changed during the "obesity epidemic." Part 1 of this two-part study discussed trends in time use, studying at home, and media use. Part 2 focuses on transportation, physical education, and diet. Walking or biking for transportation can expend a large amount of energy, but active transportation is not a major source of physical activity for youth, averaging eight minutes a day in 2001, with little change over the past few decades. For adolescents, there was no clear trend in physical education during the past decade, but there are no data for after-school and day-care programs, which have become more important as children spend more time away from home. For younger children, time spent in organized sports and outdoor activities increased by 73 minutes per week between 1981 and 1997. Eating as a primary activity declined, suggesting a shift toward snacking or eating as a secondary activity. Statistically significant trends exist for carbohydrate intake, especially for chips/crackers/popcorn/pretzels (intake tripled from the mid-1970s to the mid-1990s) and soft drinks (intake doubled during the same period). Price and income data suggest possible economic reasons for these changes. The percentage of disposable income spent on food has declined continuously, and almost all of the decline has been represented by food consumed at home, yet today's disposable income buys more calories than it has in the past. Relative prices have encouraged shifts across food types. From a baseline of 100 during 1982-84, the price index for fresh fruit and vegetables increased to 258 by 2002 (far exceeding general inflation), whereas the price index for soft drinks increased only to 126 by 2002 (below general inflation)
SN - 1545-1151

AD - RAND, 1700 Main St, Santa Monica, CA 90401, USA. Roland_Sturm@rand.org
UR - 15888231
ER -

TY - JOUR
ID - 1354
T1 - Remediation of soil from lead-contaminated kindergartens reduces the amount of lead adhering to children's hands
A1 - Nielsen,J.B.
A1 - Kristiansen,J.
Y1 - 2005/05//
N1 - Nielsen, Jesper B. Kristiansen, Jesper
Journal of exposure analysis and environmental epidemiology
bjn, 9111438
IM
Evaluation Studies. Journal Article. Research Support, Non-U.S. Gov't
English
KW - MEDLINE
KW - Child
KW - Child Welfare
KW - Preschool
KW - Dust
KW - Environmental Exposure
KW - Environmental Pollution/pc [Prevention & Control]
KW - Female
KW - Hand
KW - Humans
KW - Lead/an [Analysis]
KW - Lead/ip [Isolation & Purification]
KW - Male
KW - Play and Playthings
KW - Risk Assessment
KW - Soil Pollutants/an [Analysis]
KW - Soil Pollutants/ip [Isolation & Purification]
RP - NOT IN FILE
SP - 282
EP - 288
JF - Journal of Exposure Analysis and Environmental Epidemiology
JA - J Expo Anal Environ Epidemiol
VL - 15
IS - 3
CY - England
N2 - Risk related to contaminated soil is based on the oral intake of soil and dust among children. This exposure is a consequence of mouthing behaviour, which exposes children to whatever adheres to their hands or toys. This project compared hand exposure of children to lead following outdoor playground activities before and after an intervention. The intervention consisted of replacement of contaminated top soil from the most intensively used playground areas and coverage of bare soil with wood chips or grass. We included children from three kindergartens: one with very low levels of lead in soil and two kindergartens with an average lead concentrations in soil of 100-200 mg/kg. Measurements of lead in soil 5-7 weeks after interventions in two kindergartens verified that the interventions had effectively reduced the potential exposure to lead from the most intensively used areas of the playgrounds. The average lead concentration in soil after intervention was below 10 mg/kg. We found a good agreement between the average concentration of lead in soil and the amount of lead on the hands of the children. Thus, the exposure marker worked and had the advantage compared to a blood sample, that we could evaluate the effect of the interventions shortly after they were accomplished using a noninvasive method. The amount of lead on the hands measured in one of the two kindergartens after the remediation (0.73 microg) was not significantly different from the control kindergarten (0.58 microg). Children from the second kindergarten still had higher median exposures to lead (1.29 microg), but a large overlap

existed with several children having lower amounts of lead on their hands than some children from the control kindergarten. Large variations in the amount of lead on hands were observed. Variations may reflect true differences in concentrations of lead in soil, but may also reflect different behavior and playing patterns. Our study demonstrated, that it was possible in a cost-effective way to reduce exposure significantly and to verify the effect with a sensitive, noninvasive method shortly after the interventions had been implemented.
SN - 1053-4245
AD - Environmental Medicine, University of Southern Denmark, Odense, Denmark
UR - 15316573
ER -

TY - JOUR
ID - 1355
T1 - Children's mouthing and food-handling behavior in an agricultural community on the US/Mexico border
A1 - Black,K.
A1 - Shalat,S.L.
A1 - Freeman,N.C.
A1 - Jimenez,M.
A1 - Donnelly,K.C.
A1 - Calvin,J.A.
Y1 - 2005/05//
N1 - Black, Kathleen. Shalat, Stuart L. Freeman, Natalie C G. Jimenez, Marta. Donnelly, Kirby C. Calvin, James A
Journal of exposure analysis and environmental epidemiology
bjn, 9111438
IM
Journal Article. Research Support, N.I.H., Extramural. Research Support, U.S. Gov't, Non-P.H.S.. Research Support, U.S. Gov't, P.H.S.
English
KW - MEDLINE
KW - Agriculture
KW - Child Behavior
KW - Child
KW - Preschool
KW - Environmental Exposure
KW - Environmental Pollutants/an [Analysis]
KW - Feeding Behavior
KW - Female
KW - Hand
KW - Humans
KW - Infant
KW - Male
KW - Mouth
KW - Pesticides/an [Analysis]
KW - Play and Playthings
KW - Poaceae
KW - Soil
KW - Texas
KW - Time Factors
KW - Video Recording
RP - NOT IN FILE
SP - 244
EP - 251
JF - Journal of Exposure Analysis and Environmental Epidemiology
JA - J Expo Anal Environ Epidemiol
VL - 15
IS - 3
CY - England

N2 - Children's mouthing and food-handling activities were measured during a study of nondietary ingestion of pesticides in a south Texas community. Mouthing data on 52 children, ranging in age from 7 to 53 months, were collected using questionnaires and videotaping. Data on children's play and hand-washing habits were also collected. Children were grouped into four age categories: infants (7-12 months), 1-year-olds (13-24 months), 2-year-olds (25-36 months) and preschoolers (37-53 months). The frequency and type of events prompting hand washing did not vary by age category except for hand washing after using the bathroom; this increased with increasing age category. Reported contact with grass and dirt also increased with increasing age category. The median hourly hand-to-mouth frequency for the four age groups ranged from 9.9 to 19.4, with 2-year-olds having the lowest frequency and preschoolers having the highest. The median hourly object to mouth frequency ranged from 5.5 to 18.1 across the four age categories; the frequency decreased as age increased (adjusted R(2)=0.179; P=0.003). The median hourly hand-to-food frequency for the four age groups ranged from 10.0 to 16.1, with the highest frequency being observed in the 1-year-olds. Hand-to-mouth frequency was associated with food contact frequency, particularly for children over 12 months of age (adjusted R(2)=0.291; P=0.002). The frequency and duration of hand-to-mouth, object-to-mouth and food-handling behaviors were all greater indoors than outdoors. Infants were more likely to remain indoors than children in other age groups. The time children spent playing on the floor decreased with increasing age (adjusted R(2)=0.096; P=0.031). Parental assessment was correlated with hand-to-mouth activity but not with object-to-mouth activity. The highest combined (hand and object) mouthing rates were observed among infants, suggesting that this age group has the greatest potential for exposure to environmental toxins
SN - 1053-4245
AD - Environmental and Occupational Health Sciences Institute, Robert Wood Johnson Medical School, Piscataway, New Jersey, USA. blakka@umdnj.edu
UR - 15292908
ER -

TY - JOUR
ID - 1356
T1 - Parathyroid hormone and growth in children with chronic renal failure
A1 - Waller,S.C.
A1 - Ridout,D.
A1 - Cantor,T.
A1 - Rees,L.
Y1 - 2005/06//
N1 - Waller, Simon C. Ridout, Deborah. Cantor, Tom. Rees, Lesley
Kidney international
kvb, 0323470
IM
Journal Article. Research Support, Non-U.S. Gov't
English
KW - MEDLINE
KW - Adolescent
KW - Calcium Carbonate/ad [Administration & Dosage]
KW - Child
KW - Preschool
KW - Female
KW - Growth
KW - Humans
KW - Hydroxycholecalciferols/ad [Administration & Dosage]
KW - Hypercalcemia/et [Etiology]
KW - Infant
KW - Kidney Failure
KW - Chronic/bl [Blood]
KW - Chronic/pp [Physiopathology]
KW - Male
KW - Parathyroid Hormone/bl [Blood]
KW - Puberty
KW - Reference Values

RP - NOT IN FILE
SP - 2338
EP - 2345
JF - Kidney International
JA - Kidney Int
VL - 67
IS - 6
CY - United States
N2 - BACKGROUND: In pediatric chronic renal failure (CRF) optimal parathyroid hormone (PTH) concentrations that minimize renal osteodystrophy and maximize growth are unknown. The search for optimum concentrations has been complicated as currently used "intact" PTH (iPTH) assays cross-react with long carboxyl-terminal PTH fragments (C-PTH), which antagonize the biologic actions of 1-84 PTH. The purpose of this study was to investigate the relationship between PTH, the 1-84 PTH:C-PTH ratio and growth rate in children with CRF. METHODS: A total of 162 patients, median (range) age 9.9 years (0.3 to 17.1 years), were recruited: 136 with a glomerular filtration rate (GFR) <60 mL/min/1.73 m(2)[96 managed conservatively (CRF group) and 40 transplanted patients], and 26 dialysis patients. Over a median (range) period of 1.1 years (0.5 to 1.7 years), children attended five (three to 15) clinics at which iPTH, cyclase-activating PTH (CAP-PTH), and height were measured. RESULTS: Mean PTH concentrations were within the normal range for both assays for the CRF group and up to twice the upper limit of normal for the dialysis group; CAP-PTH 24.8 pg/mL and 59.9 pg/mL (normal range 5 to 39 pg/mL), iPTH 37.1 pg/mL, and 102.6 pg/mL, respectively (normal range 14 to 66 pg/mL). The patients grew normally (change in height standard deviation score per year (DeltaHtSDS) =-0.01). There was no relationship between PTH concentrations and DeltaHtSDS in any patient group. The 1-84 PTH:C-PTH ratio was lower in dialyzed patients (P= 0.003), with worsening renal function (P= 0.047) and with PTH concentrations outside the normal range (P= 0.01). There was a weak correlation between the 1-84 PTH:C-PTH ratio and the DeltaHtSDS (r= 0.2, P= 0.01). CONCLUSION: Normal range PTH concentrations are appropriate, allowing normal growth in children with CRF managed conservatively. C-PTH may be of clinical significance
SN - 0085-2538
AD - Nephro-Urology Unit, Insitute of Child Health and Great Ormond Street Hospital for Children NHS Trust, London, United Kingdom. swaller@doctors.org.uk
UR - 15882277
ER -

TY - JOUR
ID - 1357
T1 - Are there universal parenting concepts among culturally diverse families in an inner-city pediatric clinic?
A1 - McEvoy,M.
A1 - Lee,C.
A1 - O'Neill,A.
A1 - Groisman,A.
A1 - Roberts-Butelman,K.
A1 - Dinghra,K.
A1 - Porder,K.
Y1 - 2005/05//
N1 - McEvoy, Mimi. Lee, Connie. O'Neill, Allison. Groisman, Adriana. Roberts-Butelman, Kirsten. Dinghra, Kishwar. Porder, Kathleen
Journal of pediatric health care : official publication of National Association of Pediatric Nurse Associates & Practitioners
jml, 8709735
N
Journal Article. Research Support, Non-U.S. Gov't
English
KW - MEDLINE
KW - Acculturation
KW - Adaptation
KW - Psychological
KW - Adolescent
KW - Adult

KW - Anthropology
KW - Cultural
KW - Attitude to Health/eh [Ethnology]
KW - Child
KW - Child Rearing/eh [Ethnology]
KW - Child Welfare/eh [Ethnology]
KW - Preschool
KW - Conflict (Psychology)
KW - Cultural Characteristics
KW - Cultural Diversity
KW - Family/eh [Ethnology]
KW - Female
KW - Health Knowledge
KW - Attitudes
KW - Practice
KW - Humans
KW - Infant
KW - Newborn
KW - Male
KW - New York City
KW - Nursing Methodology Research
KW - Parenting/eh [Ethnology]
KW - Parents/ed [Education]
KW - Parents/px [Psychology]
KW - Parents
KW - Philosophy
KW - Questionnaires
KW - Religion and Psychology
KW - Social Values/eh [Ethnology]
KW - Urban Population
RP - NOT IN FILE
SP - 142
EP - 150
JF - Journal of Pediatric Health Care
JA - J Pediatr Health Care
VL - 19
IS - 3
CY - United States
N2 - INTRODUCTION: Cultural competence is necessary in providing care to culturally diverse families. Numerous studies have emphasized similarities and differences between predetermined cultural groups, yet few have studied groups across cultures. This project aimed to investigate parenting concepts, which in this context pertains to philosophy of parenting and child care practices across cultures. METHOD: Using a grounded theory approach, ethnographic interviews of 46 families representing 27 countries were taped, transcribed, and analyzed. RESULTS: Similarities in parenting concepts were found among families. Teaching values and respect and the need for strict discipline were important. A sense of community, family, and spirituality/religion was strong. Television was viewed as educational and parents anticipated opportunities for jobs and higher education for their children. Parents were more inclined to use medical treatments than home remedies for acute illnesses, which may have been linked to the finding that their providers had a strong influence. Parents feared children playing alone outdoors; distrusted nonfamily babysitters; and felt conflicted between a desire for cultural preservation versus assimilation. DISCUSSION: Universal concepts in parenting philosophies and practices exist among culturally diverse families. Providers may approach anticipatory guidance by addressing global parental concerns that transcend culture in order to relieve time constraints and the overwhelming task of being knowledgeable about all cultures
SN - 0891-5245
AD - Albert Einstein College of Medicine, Bronx, New York, USA. mcevoy@aecom.yu.edu
UR - 15867829
ER -

TY - JOUR
ID - 1358
T1 - Physical activity and exercise in asthma: relevance to etiology and treatment. [Review] [44 refs][Erratum appears in J Allergy Clin Immunol. 2005 Aug;116(2):298]
A1 - Lucas,S.R.
A1 - Platts-Mills,T.A.
Y1 - 2005/05//
N1 - Lucas, Sean R. Platts-Mills, Thomas A E
The Journal of allergy and clinical immunology
h53, 1275002
AIM, IM
Journal Article. Research Support, N.I.H., Extramural. Research Support, U.S. Gov't, P.H.S.. Review
English
KW - MEDLINE
KW - Adolescent
KW - Asthma/ep [Epidemiology]
KW - Asthma/pp [Physiopathology]
KW - Asthma/th [Therapy]
KW - Child
KW - Clinical Trials as Topic
KW - Exercise
KW - Exercise Therapy
KW - Health Surveys
KW - Humans
KW - Inhalation/ph [Physiology]
KW - Muscle
KW - Smooth/pp [Physiopathology]
KW - Physical Fitness
KW - Respiratory System/pp [Physiopathology]
RP - NOT IN FILE
SP - 928
EP - 934
JF - Journal of Allergy & Clinical Immunology
JA - J Allergy Clin Immunol
VL - 115
IS - 5
CY - United States
N2 - There is little doubt that the cause of the increased prevalence and severity of asthma is multifactorial. Although the factors of allergen exposure and hygiene are almost certainly necessary for its development, there is a growing body of literature that implicates lifestyle change, specifically decreased physical activity, as a contributor to the increase in asthma prevalence and severity. Several literature reviews of exercise conditioning in patients with asthma have been published. These reviews and recent controlled trials emphasize that although many of the studies of exercise conditioning in asthmatic patients involved different methods and outcome measures, the overwhelming majority of studies demonstrated the capacity for asthmatic subjects to exercise safely and significantly improve their cardiovascular fitness and quality of life. There are several proposed pathophysiologic mechanisms responsible for the effects of decreased activity on the lung function of patients with asthma. A prescription for exercise has been endorsed for all asthmatic subjects by the American College of Sports Medicine and the American Thoracic Society. The allergy community has placed emphasis on medical therapy and allergen avoidance; in addition, exercise [correction] has not been formally incorporated into the National Asthma Education and Prevention Program guidelines. It is our belief that an exercise prescription should be part of the treatment for all cases of asthma. The real question is whether prolonged physical activity and, in particular, outdoor play of children plays a role in prophylaxis against persistent wheezing. If so, the decrease in physical activity might have played a major role in recent increases in asthma prevalence and severity. [References: 44]
SN - 0091-6749
AD - University of Virginia Asthma and Allergic Diseases Center, Charlottesville, VA 22908-1355, USA

UR - 15867847
ER -

TY - JOUR
ID - 1359
T1 - Unconventional intronic splice site mutation in SCN5A associates with cardiac sodium channelopathy
A1 - Rossenbacker,T.
A1 - Schollen,E.
A1 - Kuiperi,C.
A1 - de Ravel,T.J.
A1 - Devriendt,K.
A1 - Matthijs,G.
A1 - Collen,D.
A1 - Heidbuchel,H.
A1 - Carmeliet,P.
Y1 - 2005/05//
N1 - Rossenbacker, T. Schollen, E. Kuiperi, C. de Ravel, T J L. Devriendt, K. Matthijs, G. Collen, D. Heidbuchel, H. Carmeliet, P
Journal of medical genetics
j1f, 2985087r
IM
Journal Article. Research Support, Non-U.S. Gov't
English
KW - MEDLINE
KW - Adolescent
KW - Adult
KW - Aged
KW - Alleles
KW - Arrhythmias
KW - Cardiac/ge [Genetics]
KW - Child
KW - DNA Mutational Analysis
KW - Female
KW - Humans
KW - Introns/ge [Genetics]
KW - Male
KW - Middle Aged
KW - Models
KW - Biological
KW - Muscle Proteins/ge [Genetics]
KW - Mutation
KW - NAV1.5 Voltage-Gated Sodium Channel
KW - Pedigree
KW - RNA Splice Sites/ge [Genetics]
KW - RNA Splicing/ph [Physiology]
KW - Sodium Channels/ge [Genetics]
KW - Syndrome
RP - NOT IN FILE
SP - e29
JF - Journal of Medical Genetics
JA - J Med Genet
VL - 42
IS - 5
CY - England
N2 - BACKGROUND: Mutations in the cardiac sodium channel, SCN5A, have been associated with one type of long-QT syndrome, with isolated cardiac conduction defects and Brugada syndrome. The sodium channelopathies exhibit marked variation in clinical phenotypes. The mechanisms underlying the phenotypical

diversity, however, remain unknown. Exonic SCN5A mutations can be detected in 20% of Brugada syndrome patients. RESULTS: An intronic mutation (c.4810+3_4810+6dupGGGT) in the SCN5A gene, located outside the consensus splice site, was detected in this study in a family with a highly variable clinical phenotype of Brugada syndrome and/or conduction disease and in a patient with Brugada syndrome. The mutation was not found in a control panel of 100 (200 alleles) ethnically matched normal control subjects. We provide in vivo and in vitro evidence that the mutation can disrupt the splice donor site, activate a cryptic splice site, and create a novel splice site. Notably, our data show that normal transcripts can be also derived from the mutant allele. CONCLUSIONS: This is the first report of an unconventional intronic splice site mutation in the SCN5A gene leading to cardiac sodium channelopathy. We speculate that its phenotypical diversity might be determined by the ratio of normal/abnormal transcripts derived from the mutant allele
SN - 1468-6244
AD - Centre for Transgene Technology and Gene Therapy, Flanders Interuniversitary Institute for Biotechnology, KULeuven, Campus Gasthuisberg, Herestraat 49, B-3000, Leuven, Belgium
UR - 15863661
ER -

TY - JOUR
ID - 1360
T1 - Cadmium exposure pathways in the Czech urban population
A1 - Puklova,V.
A1 - Batariova,A.
A1 - Cerna,M.
A1 - Kotlik,B.
A1 - Kratzer,K.
A1 - Melichercik,J.
A1 - Ruprich,J.
A1 - Rehurkova,I.
A1 - Spevakova,V.
Y1 - 2005/03//
N1 - Puklova, V. Batariova, A. Cerna, M. Kotlik, B. Kratzer, K. Melichercik, J. Ruprich, J. Rehurkova, I. Spevakova, V
Central European journal of public health
bo6, 9417324
IM
Journal Article
English
KW - MEDLINE
KW - Body Fluids/ch [Chemistry]
KW - Cadmium/an [Analysis]
KW - Cadmium/to [Toxicity]
KW - Child
KW - Preschool
KW - Czech Republic
KW - Environmental Exposure
KW - Environmental Monitoring
KW - Food Analysis
KW - Humans
KW - Inhalation Exposure
KW - Quality Control
KW - Soil Pollutants/an [Analysis]
KW - Spectrophotometry
KW - Atomic
KW - Urban Population
KW - Water Supply/an [Analysis]
RP - NOT IN FILE
SP - 11
EP - 19

JF - Central European Journal of Public Health
JA - Cent Eur J Public Health
VL - 13
IS - 1
CY - Czech Republic
N2 - The article describes the exposure pathways of cadmium in the Czech urban population. The data on Cd concentrations originated from the Environmental Health Monitoring System, which has been realized in 30 cities since 1994. The data on cadmium content in particular exposure pathways - diet, drinking water, ambient air and soil -were processed for the period 1994-2003. The estimate of the daily dietary intake for an average adult population amounted to 11-19 microg/d, i.e. 0.17-0.30 microg/kg bw/d, which represents 17%-30% of the PTWI (provisional tolerable weekly intake). The contribution from drinking water to the oral exposure is low; on average 0.5 microg/d. Potential exposure to airborne Cd was estimated at about 0.02 microg/d. The additional Cd intake from urban soil ingestion probable in small children was found to be insignificant based on Cd concentrations in the soil of kindergarten playgrounds. Biomonitoring outputs characterize the recent and life-long cadmium burden of the Czech population from general environment In 1994-2003, the median blood Cd levels ranged in the interval 0.9-0.4 microg/l blood, in smokers being more than double that in non-smokers. Blood Cd levels detected indicate slightly decreasing trend as well as urine Cd levels (range of median values 0.44-0.28 microg/g creatinine). Since 1996 the levels in children have been found in more than 50% cases below the detection limit of the methods used. The estimated total cadmium intake in the Czech urban population does not signalize any increased risk of health impairment considering non-carcinogenic effects
SN - 1210-7778
AD - National Institute of Public Health, Centre of Environmental Health, Srobarova 48, 100 42, Prague 10, Czech Republic. puklova@szu.cz
UR - 15859174
ER -

TY - JOUR
ID - 1361
T1 - Risk factors for lead poisoning among Cuban refugee children
A1 - Trepka,M.J.
A1 - Pekovic,V.
A1 - Santana,J.C.
A1 - Zhang,G.
Y1 - 2005/03//
N1 - Trepka, Mary Jo. Pekovic, Vukosava. Santana, Juan Carlos. Zhang, Guoyan
Public health reports (Washington, D.C. : 1974)
9716844, qja
AIM, IM
Journal Article. Research Support, U.S. Gov't, P.H.S.
English
KW - MEDLINE
KW - Age Distribution
KW - Child
KW - Preschool
KW - Cross-Sectional Studies
KW - Cuba/eh [Ethnology]
KW - Emigration and Immigration/sn [Statistics & Numerical Data]
KW - Environmental Exposure/ae [Adverse Effects]
KW - Environmental Exposure/an [Analysis]
KW - Environmental Exposure/pc [Prevention & Control]
KW - Female
KW - Housing/sn [Statistics & Numerical Data]
KW - Humans
KW - Incidence
KW - Infant
KW - Lead Poisoning/bl [Blood]
KW - Lead Poisoning/di [Diagnosis]

KW - Lead Poisoning/eh [Ethnology]
KW - Lead Poisoning/pc [Prevention & Control]
KW - Logistic Models
KW - Male
KW - Mass Screening
KW - Multivariate Analysis
KW - Population Surveillance
KW - Prevalence
KW - Questionnaires
KW - Refugees/sn [Statistics & Numerical Data]
KW - Residence Characteristics
KW - Risk Assessment
KW - Risk Factors
KW - Sex Distribution
KW - United States/ep [Epidemiology]
KW - Urban Health/sn [Statistics & Numerical Data]
RP - NOT IN FILE
SP - 179
EP - 185
JF - Public Health Reports
JA - Public Health Rep
VL - 120
IS - 2
CY - United States
N2 - OBJECTIVES: This study was designed to explore whether parental activities such as repairing cars, welding, and rebuilding car batteries are risk factors for lead poisoning among Cuban refugee children in Miami-Dade County. METHODS: The authors performed a cross-sectional study of 479 children aged 12-83 months who had lived in Cuba during the six months prior to immigrating to the U.S. Lead levels were obtained, and parents provided information on demographics, home/neighborhood environment in Cuba prior to immigration, family/occupational factors prior to immigration, and child behavior factors. RESULTS: Of 479 children, 30 (6.3%) had elevated blood lead levels (EBLLs), defined as > or = 10 microg/dL, based on the Centers for Disease Control and Prevention action level. In multivariate analysis, racial/ethnic identification other than white, living in a home built after 1979, car repair in the home or yard, eating paint chips, and male sex were independently associated with EBLL. CONCLUSIONS: Risk factors for lead poisoning among immigrant children may differ from those among U.S.-born children. Screening of immigrant children who may have been exposed in their country of origin and education of immigrant parents about lead exposure hazards associated with activities such as car repair should be considered in the design of lead poisoning prevention and control programs
SN - 0033-3549
AD - Robert R. Stempel School of Public Health, Florida International University, Miami, FL 33199, USA. trepkam@fiu.edu
UR - 15842120
ER -

TY - JOUR
ID - 1362
T1 - Maternal appraisal styles, family risk status and anger biases of children
A1 - Root,C.A.
A1 - Jenkins,J.M.
Y1 - 2005/04//
N1 - Root, Carol A. Jenkins, Jennifer M
Journal of abnormal child psychology
0364547, h33
IM
Journal Article. Research Support, Non-U.S. Gov't
English
KW - MEDLINE

KW - Aggression/px [Psychology]
KW - Anger
KW - Child
KW - Preschool
KW - Family/px [Psychology]
KW - Female
KW - Humans
KW - Male
KW - Mood Disorders/di [Diagnosis]
KW - Mood Disorders/ep [Epidemiology]
KW - Mothers/px [Psychology]
KW - Prevalence
KW - Risk Factors
KW - Severity of Illness Index
KW - Social Behavior
KW - Socioeconomic Factors
RP - NOT IN FILE
SP - 193
EP - 204
JF - Journal of Abnormal Child Psychology
JA - J Abnorm Child Psychol
VL - 33
IS - 2
CY - United States
N2 - The present study investigated the relationship between maternal appraisal styles, family risk status, and anger biases in children. Participants included 90 mothers and their children between 3-6 years of age. Eighty families were followed up 1 year later. Maternal appraisal styles were assessed via a naturalistic story-reading method, and Time 1 and Time 2 emotion biases included teacher ratings of anger and aggression, peer ratings of anger, as well as classroom and playground observations of anger. While discussing ambiguous stories with their children, mothers with higher scores on the family risk index utilized more hostile appraisals and fewer prosocial appraisals in their explanations. A higher proportion of hostile-to-prosocial appraisals was also related to higher-risk family status. Prosocial appraisals by mothers were inversely correlated with children's anger biases at school. When the appraisal balance in mothers' talk favored hostile appraisals, children tended to show higher levels of anger biases evidenced at school. Finally, a hostile balance of maternal appraisals was found to predict change in children's anger biases in the school with different effects in high- and low-risk families
SN - 0091-0627
AD - Centre for Addiction and Mental Health and University of Toronto. Toronto, Ontario, Canada. carol_root@camh.net
UR - 15839497
ER -

TY - JOUR
ID - 1363
T1 - Home and leisure activities and childhood knee injuries
A1 - Moustaki,M.
A1 - Pitsos,N.
A1 - Dalamaga,M.
A1 - Dessypris,N.
A1 - Petridou,E.
Y1 - 2005/05//
N1 - Moustaki, M. Pitsos, N. Dalamaga, M. Dessypris, N. Petridou, E
Injury
0226040, gon
IM
Journal Article
English
KW - MEDLINE

KW - Accidents/sn [Statistics & Numerical Data]
KW - Accidents
KW - Home/sn [Statistics & Numerical Data]
KW - Adolescent
KW - Age Distribution
KW - Child
KW - Preschool
KW - Cluster Analysis
KW - Female
KW - Greece/ep [Epidemiology]
KW - Humans
KW - Infant
KW - Newborn
KW - Knee Injuries/ep [Epidemiology]
KW - Leisure Activities
KW - Male
KW - Prevalence
KW - Sex Distribution
RP - NOT IN FILE
SP - 644
EP - 650
JF - Injury
VL - 36
IS - 5
CY - England
N2 - AIM: To assess the relative occurrence of non motor-vehicle knee injuries and identify important clusters that can be targeted for preventive interventions. METHODS: The study subjects covered 2167 children (0-14 years) who suffered non motor-vehicle knee injuries out of 66870 registered during a three-year period in an established Emergency Department Injury Surveillance System (EDISS). A more serious joint injury was identified in 263 (12%) children, whereas the remaining 1904 children had only soft tissue knee injuries. RESULTS: The incidence of non motor-vehicle knee injuries was estimated at 6.5 per 1000 children-years. Both the incidence of knee injuries and the male-to-female ratio increase with increasing age, reflecting the gender and age pattern of physical activity. Three clusters were identified: The first consisted of more serious knee injuries among older children, frequently resulting after a fall from stairs or a collision in school during winter months; the second cluster consisted of rather minor knee injuries occurring mostly among younger girls at home or in playgrounds, following a fall after stumbling or hit by an object while playing, especially during the summer; the third cluster comprised injuries among older boys, sustained mainly subsequent to overexertion in a sports area. CONCLUSION: Knee injuries tend to be more common among boys but more serious among girls. More and less serious knee injuries tend to fall into distinct clusters that could facilitate prioritization of preventive measures
SN - 0020-1383
AD - Department of Hygiene and Epidemiology, Athens University Medical School, 75 M. Asias str., 11527 Athens, Greece
UR - 15826625
ER -

TY - JOUR
ID - 1364
T1 - The in situ performance of playground surfacing: implications for maintenance and injury prevention
A1 - Sherker,S.
A1 - Short,A.
A1 - Ozanne-Smith,J.
Y1 - 2005/03//
N1 - Sherker, Shauna. Short, Andrew. Ozanne-Smith, Joan
International journal of injury control and safety promotion
101247254
IM

Journal Article
English
KW - MEDLINE
KW - Australia
KW - Child
KW - Craniocerebral Trauma/pc [Prevention & Control]
KW - Deceleration
KW - Humans
KW - Maintenance
KW - Materials Testing
KW - Play and Playthings
KW - Safety/st [Standards]
KW - Surface Properties
KW - Time Factors
KW - Wood
RP - NOT IN FILE
SP - 63
EP - 66
JF - International Journal of Injury Control & Safety Promotion
JA - Int J Inj Contr Saf Promot
VL - 12
IS - 1
CY - Netherlands
SN - 1745-7300
AD - Accident Research Centre, Monash University, Victoria, Australia. Shauna.Sherker@UNSW.edu.au
UR - 15814379
ER -

TY - JOUR
ID - 1365
T1 - Spectrum of injuries from glacial sports
A1 - Schindera,S.T.
A1 - Triller,J.
A1 - Steinbach,L.S.
A1 - Zimmermann,H.
A1 - Takala,J.
A1 - Anderson,S.E.
Y1 - 2005///
N1 - Schindera, Sebastian T. Triller, Juergen. Steinbach, Lynne S. Zimmermann, Heinz. Takala, Juka. Anderson, Suzanne E
Wilderness & environmental medicine
dbe, 9505185
IM
Case Reports. Journal Article
English
KW - MEDLINE
KW - Adolescent
KW - Adult
KW - Athletic Injuries/ep [Epidemiology]
KW - Athletic Injuries/et [Etiology]
KW - Athletic Injuries/pa [Pathology]
KW - Athletic Injuries/pc [Prevention & Control]
KW - Athletic Injuries/ra [Radiography]
KW - Child
KW - Female
KW - Fractures
KW - Bone

KW - Glasgow Coma Scale
KW - Humans
KW - Hypothermia
KW - Ice
KW - Male
KW - Middle Aged
KW - Mountaineering/in [Injuries]
KW - Registries
KW - Switzerland/ep [Epidemiology]
KW - Tomography
KW - X-Ray Computed
RP - NOT IN FILE
SP - 33
EP - 37
JF - Wilderness & Environmental Medicine
JA - Wilderness Environ Med
VL - 16
IS - 1
CY - United States
N2 - OBJECTIVE: Glacial sports continue to be a popular form of wilderness activity, but no published studies have commented on this type of sport or focused on the injury patterns of glacial accidents. The objectives of this study were to investigate the types and frequency of injuries associated with a glacial-crevasse or ice-field fall. METHODS: The trauma registry data and radiology record system of a pediatric and adult level 1 trauma center were evaluated from January 1997 through August 2003. All admissions with injuries caused by a glacial-crevasse or ice-field fall were reviewed. RESULTS: During the 5 1/2-year period we identified 12 patients, of whom 9 (75%) were male. The average age was 30.2 years with an age range of 9 to 57 years. Six patients fell into glacial crevasses, and the other 6 slid down an ice field of a glacier. Leading diagnoses were head injury (44.6%), thorax injury (20.1%), and lower extremity injury (18.4%). Serious injuries were observed in 66.6% of the patients with a Glasgow Coma Scale of 3 to 8. CONCLUSIONS: A wide spectrum of injuries is associated with glacial accidents. They range from common extremity injuries with fracture or joint instability and peripheral frostbite to potentially life-threatening nonorthopedic trauma, which requires intensive clinical and radiological work-up. Injury prevention strategies should focus on wearing helmets to reduce head injuries and adequate clothing to forestall or prevent hypothermia, for these are the most severe and life-threatening injuries
SN - 1080-6032
AD - Department of Radiology, University Hospital of Bern, Inselspital, Bern, Switzerland.
sebastian.schindera@insel.ch
UR - 15813145
ER -

TY - JOUR
ID - 1366
T1 - Out on a limb: risk factors for arm fracture in playground equipment falls
A1 - Sherker,S.
A1 - Ozanne-Smith,J.
A1 - Rechnitzer,G.
A1 - Grzebieta,R.
Y1 - 2005/04//
N1 - Sherker, S. Ozanne-Smith, J. Rechnitzer, G. Grzebieta, R
Injury prevention : journal of the International Society for Child and Adolescent Injury Prevention
crz, 9510056
IM
Journal Article
English
KW - MEDLINE
KW - Accidental Falls/sn [Statistics & Numerical Data]
KW - Accidental Falls

KW - Arm Injuries/ep [Epidemiology]
KW - Arm Injuries/et [Etiology]
KW - Case-Control Studies
KW - Child
KW - Environment Design
KW - Equipment Design
KW - Female
KW - Fractures
KW - Bone/ep [Epidemiology]
KW - Bone/et [Etiology]
KW - Humans
KW - Male
KW - Play and Playthings/in [Injuries]
KW - Risk Factors
KW - Stress
KW - Mechanical
KW - Victoria/ep [Epidemiology]
RP - NOT IN FILE
SP - 120
EP - 124
JF - Injury Prevention
JA - Inj Prev
VL - 11
IS - 2
CY - England
N2 - OBJECTIVES: To investigate and quantify fall height, surface depth, and surface impact attenuation as risk factors for arm fracture in children who fall from playground equipment. DESIGN: Unmatched case control study. SETTING: Five case hospitals and 78 randomly selected control schools. PARTICIPANTS: Children aged less than 13 years in Victoria, Australia who fell from school playground equipment and landed on their arm. Cases sustained an upper limb fracture and controls had minor or no injury. A total of 402 cases and 283 controls were included. INTERVENTIONS: Children were interviewed in the playground as soon as possible after their fall. MAIN OUTCOME MEASURES: Falls were recreated on site using two validated impact test devices: a headform (measuring peak G and HIC) and a novel anthropometric arm load dummy. Equipment and fall heights, as well as surface depth and substrate were measured. RESULTS: Arm fracture risk was greatest for critical equipment heights above 1.5 m (OR 2.39, 95% CI 1.49 to 3.84, p<0.01), and critical fall heights above 1.0 m (OR 2.96, 95% CI 1.71 to 5.15, p<0.01). Peak headform deceleration below 100G was protective (OR 0.67, 95% CI 0.45 to 0.99, p = 0.04). Compliance with 20 cm surface depth recommendation was poor for both cases and controls. CONCLUSIONS: Arm fracture-specific criteria should be considered for future standards. These include surface and height conditions where critical headform deceleration is less than 100G. Consideration should also be given to reducing maximum equipment height to 1.5 m. Improved surface depth compliance and, in particular, guidelines for surface maintenance are required
SN - 1353-8047
AD - NSW Injury Risk Management Research Centre, University of New South Wales, Sydney, NSW 2052, Australia. Shauna.Sherker@unsw.edu.au
UR - 15805443
ER -

TY - JOUR
ID - 1367
T1 - What distinguishes autism spectrum disorders from other developmental disorders before the age of four years?
A1 - Trillingsgaard,A.
A1 - Ulsted,Sorensen E.
A1 - Nemec,G.
A1 - Jorgensen,M.
Y1 - 2005/03//

N1 - Trillingsgaard, Anegen. Ulsted Sorensen, Ester. Nemec, Gerda. Jorgensen, Meta
European child & adolescent psychiatry
9212296
IM
Comparative Study. Journal Article
English
KW - MEDLINE
KW - Age Factors
KW - Autistic Disorder/di [Diagnosis]
KW - Autistic Disorder/px [Psychology]
KW - Child Development
KW - Child
KW - Preschool
KW - Communication
KW - Developmental Disabilities/di [Diagnosis]
KW - Developmental Disabilities/px [Psychology]
KW - Diagnosis
KW - Differential
KW - Female
KW - Humans
KW - Imagination
KW - Infant
KW - Interviews as Topic
KW - Male
KW - Observation
KW - Parents
KW - Psychological Tests
KW - Social Behavior
KW - Statistics
KW - Nonparametric
RP - NOT IN FILE
SP - 65
EP - 72
JF - European Child & Adolescent Psychiatry
JA - Eur Child Adolesc Psychiatry
VL - 14
IS - 2
CY - Germany
N2 - The increasing recognition of the benefits of early intervention for children with autism spectrum disorder (ASD) stresses the importance of early identification of children who might benefit from those programs. However, in the early years of life it may be difficult to distinguish children with ASD from children with other developmental disorders. The aim of the present study was to identify behavioural patterns that could facilitate this differentiation. Prior to diagnostic assessment, 2- and 3-year-old children (n=30), all referred to a clinic for "possible autism", were observed in a semi-structured play interaction, and their parents were interviewed about the children's early development from 0 to 24 months. Following diagnostic assessment, the 17 children fulfilling the ICD-10 criteria for ASD were compared to the 13 children diagnosed with other developmental disorders (outside the autism spectrum). On the basis of parent reports only a few distinguishing signs of ASD were found before 24 months of age. On the basis of professional observations in a semi-structured play interaction several distinguishing signs were found for the 2- and 3-year-olds; smiles in response, responds to name, follows pointing, looks to "read" faces, initiates requesting verbal and nonverbal behaviours, and functional play
SN - 1018-8827
AD - Psychiatric Hospital for Children and Adolescents, University Hospital of Aarhus, Harald Selmersvej 66, 8240 Risskov, Denmark. Atr@buh.aaa.dk
UR - 15793685
ER -

TY - JOUR
ID - 1368
T1 - Pseudoexfoliation in southern India: the Andhra Pradesh Eye Disease Study
A1 - Thomas,R.
A1 - Nirmalan,P.K.
A1 - Krishnaiah,S.
Y1 - 2005/04//
N1 - Thomas, Ravi. Nirmalan, Praveen Kumar. Krishnaiah, Sannapaneni
Investigative ophthalmology & visual science
gwi, 7703701
IM
Journal Article. Research Support, Non-U.S. Gov't
English
KW - MEDLINE
KW - Adolescent
KW - Adult
KW - Age Distribution
KW - Aged
KW - Blindness/ep [Epidemiology]
KW - Cataract/ep [Epidemiology]
KW - Child
KW - Preschool
KW - Comorbidity
KW - Cross-Sectional Studies
KW - Exfoliation Syndrome/di [Diagnosis]
KW - Exfoliation Syndrome/ep [Epidemiology]
KW - Female
KW - Humans
KW - India/ep [Epidemiology]
KW - Infant
KW - Newborn
KW - Male
KW - Middle Aged
KW - Prevalence
KW - Sex Distribution
KW - Visual Fields
RP - NOT IN FILE
SP - 1170
EP - 1176
JF - Investigative Ophthalmology & Visual Science
JA - Invest Ophthalmol Vis Sci
VL - 46
IS - 4
CY - United States
N2 - PURPOSE: To report the prevalence of pseudoexfoliation (PXF) and its associations with ocular diseases in a south Indian population. METHODS: This was a population-based, cross-sectional epidemiologic study in the south Indian state of Andhra Pradesh (AP). A total of 10,293 subjects of all ages from one urban and three rural areas representative of the population of AP were interviewed and underwent a comprehensive ophthalmic evaluation. PXF was diagnosed on slit lamp biomicroscopy by the presence of white dandruff-like material in the pupillary margin, on the trabecular meshwork, and/or on the anterior lens capsule of one or both eyes. RESULTS: The age-gender-area-adjusted overall prevalence of PXF was 0.69% (95% CI: 0.53-0.86). The prevalence of PXF increased with increasing age: 3.01% (95% CI: 2.45-3.80), in those 40 years of age or older, and 6.28% (95% CI: 4.80-7.76), in those 60 years of age or older. The prevalence of PXF was significantly higher among people whose occupation involved outdoor activities (adjusted odds ratio [OR], 2.14; 95% CI: 1.10-4.16). After adjustment for age, the prevalence of PXF was significantly higher in those with nuclear cataract (adjusted OR, 2.00; 95% CI: 1.13-3.54). PXF was significantly associated with blindness (adjusted OR, 2.19; 95% CI: 1.16-4.13). Fifteen (20.5%; 95% CI: 11.20-29.80) of those with PXF were blind, with age-

adjusted relative risk (RR) = 4.25 (95% CI: 4.01-4.51). Unilateral blindness (41.2%; 95% CI: 29.81-52.39) and visual impairment (45.21%; 95% CI: 34.29-57.13) were also more common with PXF. Four subjects (5.5%; 95% CI: 0.27-10.2) of those with PXF had glaucoma. The prevalence of PXF in those with glaucoma was 4.2%; (95% CI: 0.17-8.23). In general linear models, the estimated mean +/- SE of IOP with glaucoma and PXF was 24.14 +/- 1.41 mm Hg and was 18.94 +/- 0.26 mm Hg with glaucoma in the absence of PXF; the difference was statistically significant (P < 0.0001). CONCLUSIONS: The association of PXF with blindness and aging has public health implications for India. This is especially so considering the burden of cataract with aging and the association of PXF with cataract as well as complications of cataract surgery. The diagnosis of PXF may also be important in the management of glaucoma in this population
SN - 0146-0404
AD - Glaucoma Services, L. V. Prasad Eye Institute, Hyderabad, India. ravithomas@lvpei.org
UR - 15790875
ER -

TY - JOUR
ID - 1369
T1 - Comment on "Children's exposure to arsenic from CCA-treated wooden decks and playground structures"
A1 - Boyce,C.P.
A1 - Beck,B.D.
A1 - Dube,E.M.
A1 - Lewandowski,T.A.
Y1 - 2005/02//
N1 - Boyce, Catherine Petito. Beck, Barbara D. Dube, Eric M. Lewandowski, Thomas A
Risk analysis : an official publication of the Society for Risk Analysis
8109978, ria
IM
Comment. Letter
English
KW - MEDLINE
KW - Arsenates/ip [Isolation & Purification]
KW - Arsenates/to [Toxicity]
KW - Arsenic/ip [Isolation & Purification]
KW - Arsenic/pk [Pharmacokinetics]
KW - Arsenic/to [Toxicity]
KW - Biological Availability
KW - Child
KW - Environmental Exposure
KW - Hand
KW - Humans
KW - Mouth
KW - Play and Playthings
KW - Risk Assessment
KW - Soil Pollutants/ip [Isolation & Purification]
KW - Soil Pollutants/to [Toxicity]
KW - Wood
RP - NOT IN FILE
SP - 1
EP - 5
JF - Risk Analysis
JA - Risk Anal
VL - 25
IS - 1
CY - United States
SN - 0272-4332
UR - 15787751
ER -

TY - JOUR
ID - 1370
T1 - The WHO 'Roll Back Malaria Project': planning for adverse event monitoring in Africa
A1 - Simooya,O.
Y1 - 2005///
N1 - Simooya, Oscar
Drug safety : an international journal of medical toxicology and drug experience
ahq, 9002928
IM
Journal Article
English
KW - MEDLINE
KW - Adverse Drug Reaction Reporting Systems
KW - Africa South of the Sahara/ep [Epidemiology]
KW - Antimalarials/ae [Adverse Effects]
KW - Antimalarials/tu [Therapeutic Use]
KW - Child
KW - Preschool
KW - Health Planning/og [Organization & Administration]
KW - Humans
KW - Malaria/dt [Drug Therapy]
KW - Malaria/ep [Epidemiology]
KW - Malaria/pc [Prevention & Control]
KW - Mosquito Control
KW - Product Surveillance
KW - Postmarketing/mt [Methods]
RP - NOT IN FILE
SP - 277
EP - 286
JF - Drug Safety
JA - Drug Saf
VL - 28
IS - 4
CY - New Zealand
N2 - Artemisinin combination therapies (ACTs) have been recommended for the treatment of malaria in countries where there is widespread resistance to commonly used antimalarial drugs. Several sub-Saharan African countries are, therefore, in the process of introducing ACTs in their malaria drug policies. However, there is limited information about the safety of ACTs outside South East Asia, where their use has been well documented. As with all other new medicinal compounds, the monitoring of a drug's safety or 'pharmacovigilance' is important, especially in areas where co-morbid conditions, such as HIV/AIDS, malnutrition and tuberculosis, are common. Because in most malaria endemic countries, particularly Africa, there are no pharmacovigilance programmes in place, it has been suggested that the introduction of ACTs offers an opportunity for these countries to put drug safety monitoring systems in place. Backed by the WHO Roll Back Malaria department and other international cooperating partners, five African countries, which are in the process of introducing ACTs (Burundi, Democratic Republic of the Congo, Mozambique, Zambia and Zanzibar), have drawn up action plans to introduce pharmacovigilance in their health sector. It is planned that once the safety monitoring of antimalarials has been established, these activities can then be extended to cover medicinal compounds used in other public health programmes, such as HIV/AIDS, tuberculosis and the immunisation programmes. This article looks at the rationale for pharmacovigilance, the process of setting up monitoring centres and the challenges of implementing the project in the region
SN - 0114-5916
AD - The Copperbelt University, Kitwe, Zambia, Africa. cbumed@zamnet.zm
UR - 15783238
ER -

TY - JOUR
ID - 1371

T1 - Effect of sunscreen and clothing on the number of melanocytic nevi in 1,812 German children attending day care
A1 - Bauer,J.
A1 - Buttner,P.
A1 - Wiecker,T.S.
A1 - Luther,H.
A1 - Garbe,C.
Y1 - 2005/04/01/
N1 - Bauer, Jurgen. Buttner, Petra. Wiecker, Tine Sander. Luther, Heike. Garbe, Claus
American journal of epidemiology
3h3, 7910653
IM
Journal Article
English
KW - MEDLINE
KW - Chi-Square Distribution
KW - Child
KW - Child Day Care Centers
KW - Preschool
KW - Cross-Sectional Studies
KW - Female
KW - Germany/ep [Epidemiology]
KW - Health Behavior
KW - Humans
KW - Infant
KW - Linear Models
KW - Male
KW - Melanosis/ep [Epidemiology]
KW - Melanosis/pc [Prevention & Control]
KW - Nevus
KW - Pigmented/ep [Epidemiology]
KW - Pigmented/pc [Prevention & Control]
KW - Physical Examination
KW - Protective Clothing
KW - Risk Factors
KW - Skin Neoplasms/ep [Epidemiology]
KW - Skin Neoplasms/pc [Prevention & Control]
KW - Statistics
KW - Nonparametric
KW - Sunscreening Agents
KW - Ultraviolet Rays/ae [Adverse Effects]
RP - NOT IN FILE
SP - 620
EP - 627
JF - American Journal of Epidemiology
JA - Am J Epidemiol
VL - 161
IS - 7
CY - United States
N2 - The number of melanocytic nevi is the most important risk factor for cutaneous melanoma. This 1998 cross-sectional study of 1,812 children aged 2-7 years from 78 day-care centers in Germany analyzed the protective effect of sunscreen and clothing on the number of melanocytic nevi. Total body nevi were counted, and pigmentary features were assessed. Parents underwent a standardized interview concerning their children's sun exposure and sun-protective behavior. Almost 95% of the children had used sunscreen previously. Children who used sunscreen and wore more clothing spent significantly longer periods on holidays in sunny climates ($p < 0.001$ and $p = 0.006$, respectively) and had a higher outdoor activity score at home ($p < 0.001$ and $p = 0.012$, respectively). Multivariate analysis adjusted for confounders showed no significant protective effects of

applying sunscreen. Examination of the potential protective effect of clothing revealed an inverse dose-response correlation between the number of clothes worn at the beach or outdoor swimming pool and the number of melanocytic nevi ($p < 0.001$, adjusted for confounding). Randomized controlled trials are needed to confirm these results. Meanwhile, public education should aim to protect children primarily by sun avoidance and protective clothing, while sunscreen should also be used
SN - 0002-9262
AD - Department of Dermatology, Faculty of Medicine, Eberhard-Karls-University, Tubingen, Germany. mail@j-bauer.de
UR - 15781951
ER -

TY - JOUR
ID - 1372
T1 - Perceptions of health status and play activities in parents of overweight Hispanic toddlers and preschoolers
A1 - Rich,S.S.
A1 - DiMarco,N.M.
A1 - Huettig,C.
A1 - Essery,E.V.
A1 - Andersson,E.
A1 - Sanborn,C.F.
Y1 - 2005/04//
N1 - Rich, Shannon S. DiMarco, Nancy M. Huettig, Carol. Essery, Eve V. Andersson, Evelyn. Sanborn, Charlotte F
Family & community health
ese, 7809641
IM
Journal Article. Research Support, U.S. Gov't, P.H.S.
English
KW - MEDLINE
KW - Child
KW - Preschool
KW - Female
KW - Health Knowledge
KW - Attitudes
KW - Practice
KW - Health Status
KW - Hispanic Americans/px [Psychology]
KW - Humans
KW - Male
KW - Obesity/eh [Ethnology]
KW - Obesity/px [Psychology]
KW - Parents/px [Psychology]
KW - Play and Playthings
KW - Poverty
RP - NOT IN FILE
SP - 130
EP - 141
JF - Family & Community Health
JA - Fam Community Health
VL - 28
IS - 2
CY - United States
N2 - Childhood overweight among lower socioeconomic, Hispanic children has increased. Interviews regarding health status and play patterns were conducted with 76 predominantly Hispanic mothers of overweight toddlers and preschoolers served by Women, Infants, and Children (WIC). Most participants believed their child was healthy and half were unconcerned about their child's weight. Most parents reported having a safe place to play and access to a playground, although gender differences were found. Access to an outside play area was related

to amount of active play activities. Children watched an average of 1.7 hours per day of television. Health professionals must partner with parents to address childhood obesity
SN - 0160-6379
AD - Departments of Psychology and Philosophy, Texas Woman's University, Denton, TX 76204, USA. srich@mail.twu.edu
UR - 15778627
ER -

TY - JOUR
ID - 1373
T1 - Prognostic variables for high titres in a fluorescent antibody test to diagnose tuberculosis
A1 - Doveren,R.F.
A1 - Goudswaard,J.
A1 - Hendriks,J.C.
A1 - Bins,M.C.
A1 - Belzen,Cv
Y1 - 2005/04//
N1 - Doveren, Rob F C. Goudswaard, Jan. Hendriks, Jan C M. Bins, Marijn C. Belzen, Cees van
Respiratory medicine
8908438, rme
IM
Journal Article. Multicenter Study
English
KW - MEDLINE
KW - Adolescent
KW - Adult
KW - Aged
KW - 80 and over
KW - Child
KW - Epidemiologic Methods
KW - Female
KW - Fluorescent Antibody Technique/mt [Methods]
KW - Humans
KW - Macrophages/im [Immunology]
KW - Male
KW - Middle Aged
KW - Prognosis
KW - Tuberculin/du [Diagnostic Use]
KW - Tuberculosis/di [Diagnosis]
RP - NOT IN FILE
SP - 477
EP - 484
JF - Respiratory Medicine
JA - Respir Med
VL - 99
IS - 4
CY - England
N2 - SETTING: The four hospitals and a tuberculosis clinic in the province of Zeeland, The Netherlands. OBJECTIVE: To assess the usefulness of PPD antibody measurement in the diagnosis of tuberculosis in patients admitted to hospital. PATIENTS AND METHODS: Sixty-one patients presenting with active tuberculosis, and 215 control patients were included in the study. Initial serum PPD antibody titres were determined with a macrophage uptake Fluorescent antibody test (MuFat) to construct a discrimination model between Tuberculosis (TB) and non-TB. We also retrospectively collected clinical parameters of the TB patients at presentation. Univariate and multivariate logistic regression are used to identify variables predicting high antibody titres. RESULTS: In TB patients, the presence of clinical symptoms (OR=10.63) and the presence of at least two concurrent non-lymph node disease localizations outside thorax and abdomen (OR=13.94) are necessary and sufficient to predict high titres. The logistic model shows a significant contribution of the 2log

(titre) to the discrimination between TB and non-TB patients. At a cut-off value of 128, a specificity, sensitivity, and positive predictive and negative predictive values of 97%, 39%, 80% and 85%, respectively, are calculated in the study cohort. CONCLUSION: Our data suggest an application of the test at high cut-off values for timely diagnosis of difficult-to-diagnose TB patients. The results of this retrospective study will have to be confirmed in further prospective studies
SN - 0954-6111
AD - Emergency Department, St. Vincentius Hospital, St. Vincentiusstraat 20, B-2018 Antwerp, Belgium. r.doveren@zeelandnet.nl
UR - 15763455
ER -

TY - JOUR
ID - 1374
T1 - Everybody else is: Networks, power laws and peer contagion in the aggressive recess behavior of elementary school boys
A1 - Warren,K.
A1 - Craciun,G.
A1 - nderson-Butcher,D.
Y1 - 2005/04//
N1 - Warren, Keith. Craciun, Gheorghe. Anderson-Butcher, Dawn
Nonlinear dynamics, psychology, and life sciences
9704130
IM
Journal Article
English
KW - MEDLINE
KW - Child
KW - Group Processes
KW - Hierarchy
KW - Social
KW - Humans
KW - Male
KW - Neural Networks (Computer)
KW - Nonlinear Dynamics
KW - Peer Group
KW - Play and Playthings/px [Psychology]
KW - Poisson Distribution
KW - Poverty/px [Psychology]
KW - Power (Psychology)
KW - Schools
KW - Social Control
KW - Informal
KW - Social Facilitation
KW - Social Support
KW - Urban Population
RP - NOT IN FILE
SP - 155
EP - 173
JF - Nonlinear Dynamics, Psychology, & Life Sciences
JA - Nonlinear Dynamics Psychol Life Sci
VL - 9
IS - 2
CY - United States
N2 - This paper develops a simple random network model of peer contagion in aggressive behavior among inner-city elementary school boys during recess periods. The model predicts a distribution of aggressive behaviors per recess period with a power law tail beginning at two aggressive behaviors and having a slope of approximately -1.5. Comparison of these values with values derived from observations of aggressive behaviors

during recess at an inner-city elementary school provides empirical support for the model. These results suggest that fluctuations in aggressive behaviors during recess arise from the interactions between students, rather than from variations in the behavior of individual students. The results therefore support those interventions that aim to change the pattern of interaction between students
SN - 1090-0578
AD - The Ohio State University College of Social Work, Stillman Hall, 325Q, 1947 College Rd., Columbus, OH 43210, USA. warren.193@osu.edu
UR - 15757577
ER -

TY - JOUR
ID - 1375
T1 - Prevention of the epidemic increase in child risk of overweight in low-income schools: the El Paso coordinated approach to child health
A1 - Coleman,K.J.
A1 - Tiller,C.L.
A1 - Sanchez,J.
A1 - Heath,E.M.
A1 - Sy,O.
A1 - Milliken,G.
A1 - Dzewaltowski,D.A.
Y1 - 2005/03//
N1 - Coleman, Karen J. Tiller, Claire Lola. Sanchez, Jesus. Heath, Edward M. Sy, Oumar. Milliken, George. Dzewaltowski, David A
Archives of pediatrics & adolescent medicine
9422751, bwf
AIM, IM
Journal Article. Research Support, Non-U.S. Gov't
English
KW - MEDLINE
KW - Anthropometry
KW - Cardiovascular Diseases/pc [Prevention & Control]
KW - Case-Control Studies
KW - Child
KW - Community-Institutional Relations
KW - Disease Outbreaks
KW - Female
KW - Health Promotion/og [Organization & Administration]
KW - Hispanic Americans
KW - Humans
KW - Intervention Studies
KW - Male
KW - Obesity/ep [Epidemiology]
KW - Obesity/pc [Prevention & Control]
KW - Physical Education and Training
KW - Physical Fitness
KW - School Health Services/og [Organization & Administration]
KW - Sex Factors
KW - Socioeconomic Factors
KW - Texas/ep [Epidemiology]
RP - NOT IN FILE
SP - 217
EP - 224
JF - Archives of Pediatrics & Adolescent Medicine
JA - Arch Pediatr Adolesc Med
VL - 159
IS - 3

CY - United States
N2 - OBJECTIVE: To assess the impact on children's health of translating an evidence-based national intervention trial (Child and Adolescent Trial for Cardiovascular Health [CATCH]) to low-income elementary schools with primarily Hispanic students. DESIGN: An untreated, matched control group design with repeated dependent pretest and posttest samples was used. SETTING: Four El Paso CATCH and 4 control elementary schools in El Paso, Tex, along the US-Mexico border region. All had Title I status (most were low-income students). PARTICIPANTS: Participants were 896 third-grade children (473 control schools [224 girls and 249 boys] and 423 CATCH schools [199 girls and 224 boys]); 93% were Hispanic. INTERVENTION: Community-based implementation of the national CATCH program. MAIN OUTCOME MEASURES: Risk of overweight or overweight, body mass index, waist-to-hip ratio, yards run in 9 minutes, passing rates for Fitnessgram national mile standards, moderate to vigorous physical activity and vigorous physical activity in physical education class, and percentage of fat and sodium in school lunches. RESULTS: Girls in control schools had significant increases in percentage of risk of overweight or overweight from third (26%) to fifth (39%) grades, as did girls in CATCH schools (30%-32%); however, the rate of increase for girls in the CATCH schools was significantly lower (2%) compared with the rate for control girls (13%). A similar pattern was seen for boys, with a rate of increase for boys in CATCH schools of 1% (40%-41%), which was significantly less than the 9% increase (40% to 49%) for control boys. CONCLUSIONS: The translation of the national CATCH program to low-income schools with Hispanic students successfully slowed the epidemic increase in risk of overweight or overweight seen in control school children. An emphasis should be placed on community organizing and evaluation feedback when implementing evidence-based school health programs in low-income Hispanic communities
SN - 1072-4710
AD - Graduate School of Public Health, San Diego State University, San Diego, CA 92116, USA. coleman@mail.sdsu.edu
UR - 15753263
ER -

TY - JOUR
ID - 1376
T1 - The accuracy of pedometer steps and time during walking in children
A1 - Beets,M.W.
A1 - Patton,M.M.
A1 - Edwards,S.
Y1 - 2005/03//
N1 - Beets, Michael W. Patton, Megan M. Edwards, Stacey
Medicine and science in sports and exercise
8005433, mg8
IM, S
Clinical Trial. Comparative Study. Journal Article. Validation Studies
English
KW - MEDLINE
KW - Child
KW - Exercise Test
KW - Female
KW - Humans
KW - Male
KW - Monitoring
KW - Physiologic/is [Instrumentation]
KW - Physiologic/mt [Methods]
KW - Physiologic/st [Standards]
KW - Reference Values
KW - Reproducibility of Results
KW - Time Factors
KW - Walking/ph [Physiology]
RP - NOT IN FILE
SP - 513
EP - 520

JF - Medicine & Science in Sports & Exercise
JA - Med Sci Sports Exerc
VL - 37
IS - 3
CY - United States
N2 - PURPOSE: To determine the accuracy of pedometer step counts and time during self-paced walking (SPW) and treadmill walking in children (5-11 yr). Two pedometers were assessed during SPW: Digiwalker SW-200 (DW200) and Walk4Life 2505 (WL), and four pedometers during treadmill walking: DW200, WL, Digiwalker SW-701 (DW701), and the Sun TrekLINQ (SUN). METHODS: Ten boys (8.3+/-1.5 yr) and 10 girls (8.9+/-1.7 yr) completed three single-lap SPW trials around an outdoor athletic track. Treadmill walking was performed at the following speeds: 40, 54, 67, 80, and 94 m.min-1. During treadmill walking, each model was measured on the right and left sides. Pedometer steps and time and observed steps and time were recorded for all trials. RESULTS: Across SPW trials, there was high agreement on observed steps for both the DW200 and WL (intraclass correlation coefficient (ICC) 0.985-0.997). For treadmill walking, at <or=54 m.min-1, low interunit agreement (ICC<or=0.746) and low agreement between pedometer steps and observed steps (ICC<or=0.720) were detected for all models. For pedometer time, the WL exhibited a high level of agreement with observed during SPW (ICC 0.997-0.998). During treadmill walking, the WL was within 5.3% of actual time across all speeds, whereas time with the SUN did not come within 5% agreement until 80 m.min-1. CONCLUSIONS: Findings are consistent with adult studies with step count underestimation during slow (<or=54 m.min-1) walking. Low interunit agreement at slow speeds suggests variations in step count registry within models. The accuracy of time (WL), independent of step count accuracy and speed, suggests that time might be used in addition to steps to quantify the physical activity behavior of children
SN - 0195-9131
AD - Department of Public Health, Oregon State University, Corvallis, OR 97331-6406, USA. beetsm@onid.orst.edu
UR - 15741852
ER -

TY - JOUR
ID - 1377
T1 - Low levels of 25-hydroxy vitamin D are associated with elevated parathyroid hormone in healthy adolescent females
A1 - Harkness,L.
A1 - Cromer,B.
Y1 - 2005/01//
N1 - Harkness, Laura. Cromer, Barbara
Osteoporosis international : a journal established as result of cooperation between the European Foundation for Osteoporosis and the National Osteoporosis Foundation of the USA
9100105, be0
IM
Journal Article. Research Support, U.S. Gov't, P.H.S.
English
KW - MEDLINE
KW - Adolescent
KW - African Continental Ancestry Group
KW - Child
KW - Female
KW - Humans
KW - Parathyroid Hormone/bl [Blood]
KW - Regression Analysis
KW - Seasons
KW - Vitamin D/aa [Analogs & Derivatives]
KW - Vitamin D/bl [Blood]
RP - NOT IN FILE
SP - 109
EP - 113
JF - Osteoporosis International

JA - Osteoporos.Int
VL - 16
IS - 1
CY - England
N2 - This study aimed to investigate the relationship between 25-hydroxyvitamin D [25(OH)D)] and parathyroid hormone (PTH) levels in adolescent females residing in a northern climate. Concern regarding vitamin D status in this population is due to limited sunlight exposure in northern latitudes, decreased outdoor recreational activities, as well as decreased conversion in black girls from increased skin pigmentation. In this cross-sectional analysis, serum samples were assayed for 25(OH)D using competitive protein binding (CPB) assay and PTH with immuno-radiometric (RIA) procedures. Four hundred postmenarcheal females (12-18 years) residing in northeastern Ohio were recruited. Subjects were excluded if they had a history of bone, kidney, or liver disease, or used medications that affect bone. The primary goal was to determine serum 25(OH)D concentrations in relation to circulating PTH levels in a population of adolescent girls. The Spearman correlation test was used to compare PTH and 25(OH)D. Fit multiple split models were run to determine change in slope of the regression line when 25(OH)D and PTH were plotted. Analysis of variance was determined using modeled means with differences by race and season in the final model. Unadjusted mean serum 25(OH)D and PTH levels were 55.0+/-30.4 nmol/l and 39.4+/-20.6 ng/l, respectively. Blacks had lower 25(OH)D and higher PTH compared with non-blacks (P<0.0001), especially during the winter months. Decreasing 25(OH)D was inversely correlated with PTH (r=-0.314) (P<0.0001), and at concentrations of 25(OH)D < or =90 nmol/l, an increase in PTH was observed. Adolescents are at risk for decreased serum 25(OH)D concentrations, especially black girls. We found that the widely used cutoff for vitamin D deficiency is associated with increasing PTH levels and is below the inflection point for a change in the slope of the regression line. Our results support the need for further research to establish optimal vitamin D status in adolescent girls
SN - 0937-941X
AD - Case Western Reserve University School of Medicine, Cleveland, OH 44106, USA.
Lharkness@metrohealth.org
UR - 15175848
ER -

TY - JOUR
ID - 1378
T1 - Distribution of Buruli ulcer lesions over body surface area in a large case series in Ghana: uncovering clues for mode of transmission
A1 - Hospers,I.C.
A1 - Wiersma,I.C.
A1 - Dijkstra,P.U.
A1 - Stienstra,Y.
A1 - Etuaful,S.
A1 - Ampadu,E.O.
A1 - van der Graaf,W.T.
A1 - van der Werf,T.S.
Y1 - 2005/03//
N1 - Hospers, Ilona C. Wiersma, Irene C. Dijkstra, Pieter U. Stienstra, Ymkje. Etuaful, Samuel. Ampadu, Edwin O. van der Graaf, Winette T A. van der Werf, Tjip S
Transactions of the Royal Society of Tropical Medicine and Hygiene
wbu, 7506129
IM
Journal Article
English
KW - MEDLINE
KW - Adolescent
KW - Adult
KW - Age Distribution
KW - Arm
KW - Body Surface Area
KW - Child
KW - Endemic Diseases

KW - Female
KW - Ghana/ep [Epidemiology]
KW - Humans
KW - Leg
KW - Male
KW - Mycobacterium Infections
KW - Nontuberculous/ep [Epidemiology]
KW - Nontuberculous/pa [Pathology]
KW - Nontuberculous/tm [Transmission]
KW - Mycobacterium ulcerans
KW - Sex Distribution
KW - Skin Diseases
KW - Bacterial/ep [Epidemiology]
KW - Bacterial/pa [Pathology]
KW - Bacterial/tm [Transmission]
KW - Skin Ulcer/ep [Epidemiology]
KW - Skin Ulcer/pa [Pathology]
KW - Travel
RP - NOT IN FILE
SP - 196
EP - 201
JF - Transactions of the Royal Society of Tropical Medicine & Hygiene
JA - Trans R Soc Trop Med Hyg
VL - 99
IS - 3
CY - England
N2 - We studied hospital records of 750 consecutive Buruli ulcer patients in a highly endemic area in Amansie West, Ghana. Although more Buruli ulcer lesions were found on the right side of the body, comparison of lesions on arms and legs showed a bilaterally symmetrical distribution. Upper and lower extremities were affected equally by Buruli ulcers, if correction was made for differences in body surface area. Patients from outside the Amansie West district presented significantly more often with ulcerated lesions, which were more often located on a joint, than patients who lived in Amansie West, suggesting that longer travel distance might have caused delay. Our observations of a bilaterally symmetrical distribution of lesions on extremities and equal upper and lower extremity involvement are compatible with a mode of transmission that involves passive exposure of exposed body parts. An asymmetrical distribution of lesions was found in an earlier study, suggesting transmission by vegetation near the ground, through activities like farming or play. Perhaps, transmission in or near water, e.g. by bites of infected aquatic insects, might favour the pattern of distribution of lesions that we found
SN - 0035-9203
AD - Department of Internal Medicine, Groningen University Medical Centre, P.O. Box 30001, 9700 RB Groningen, The Netherlands
UR - 15653121
ER -

TY - JOUR
ID - 1379
T1 - Evaluation and quality control of personal nephelometers in indoor, outdoor and personal environments
A1 - Wu,C.F.
A1 - Delfino,R.J.
A1 - Floro,J.N.
A1 - Samimi,B.S.
A1 - Quintana,P.J.
A1 - Kleinman,M.T.
A1 - Liu,L.J.
Y1 - 2005/01//
N1 - Wu, Chang-Fu. Delfino, Ralph J. Floro, Joshua N. Samimi, Behzad S. Quintana, Penelope J E. Kleinman, Michael T. Liu, L-J Sally

Journal of exposure analysis and environmental epidemiology
bjn, 9111438
IM
Evaluation Studies. Journal Article. Research Support, N.I.H., Extramural. Research Support, U.S. Gov't, Non-P.H.S.. Research Support, U.S. Gov't, P.H.S.
English
KW - MEDLINE
KW - Air Pollutants/an [Analysis]
KW - Air Pollution
KW - Indoor/an [Analysis]
KW - Asthma
KW - Child
KW - Preschool
KW - Data Collection
KW - Environmental Exposure
KW - Equipment Design
KW - Female
KW - Humans
KW - Male
KW - Nephelometry and Turbidimetry/is [Instrumentation]
KW - Nephelometry and Turbidimetry/st [Standards]
KW - Particle Size
KW - Quality Control
KW - Sensitivity and Specificity
RP - NOT IN FILE
SP - 99
EP - 110
JF - Journal of Exposure Analysis and Environmental Epidemiology
JA - J Expo Anal Environ Epidemiol
VL - 15
IS - 1
CY - England
N2 - Personal nephelometers provide useful real-time measurements of airborne particulate matter (PM). Recent studies have applied this tool to assess personal exposures and related health effects. However, a thorough quality control (QC) procedure for data collected from such a device in a large-scale exposure assessment study is lacking. We have evaluated the performance of a personal nephelometer (personal DataRAM or pDR) in the field. We present here a series of post hoc QC procedures for improving the quality of the pDR data. The correlations and the ratios between the pDRs and the collocated gravimetric measurements were used as indices of the pDR data quality. The pDR was operated in four modes: passive (no pump), active (with personal sampling pumps), active with a heated inlet, and a humidistat. The pDRs were worn by 21 asthmatic children, placed at their residences indoors and outdoors, as well as at a central site. All fixed-site pDRs were collocated with Harvard Impactors for PM2.5 (HI2.5). By examining the differences between the time-weighted average concentrations calculated from the real-time pDRs' readings and recorded internally by the pDRs, we identified 9.1% of the pDRs' measurements suffered from negative drifts. By comparing the pDRs' daily base level with the HI2.5 measurements, we identified 5.7% of the pDRs' measurements suffered from positive drifts. High relative humidity (RH) affected outdoor pDR measurements, even when a heater was used. Results from a series of chamber experiments suggest that the heated air stream cooled significantly after leaving the heater and entering the pDR light-scattering chamber. An RH correction equation was applied to the pDR measurements to remove the RH effect. The final R2 values between the fixed-site pDRs and the collocated HI2.5 measurements ranged between 0.53 and 0.72. We concluded that with a carefully developed QC procedure, personal nephelometers can provide high-quality data for assessing PM exposures on subjects and at fixed locations. We also recommend that outdoor pDRs be operated in the active mode without a heater and that the RH effect be corrected with an RH correction equation
SN - 1053-4245
AD - Department of Environmental and Occupational Health Sciences, University of Washington, Seattle, WA 98195, USA. cfwu@u.washington.edu
UR - 15039794

ER -

TY - JOUR
ID - 1380
T1 - Children's perceptions of their home and neighborhood environments, and their association with objectively measured physical activity: a qualitative and quantitative study
A1 - Hume,C.
A1 - Salmon,J.
A1 - Ball,K.
Y1 - 2005/02//
N1 - Hume, C. Salmon, J. Ball, K
Health education research
bqp, 8608459
T
Journal Article. Research Support, Non-U.S. Gov't
English
KW - MEDLINE
KW - Child
KW - Environment
KW - Exercise
KW - Female
KW - Housing
KW - Humans
KW - Male
KW - Qualitative Research
KW - Residence Characteristics
KW - Victoria
RP - NOT IN FILE
SP - 1
EP - 13
JF - Health Education Research
JA - Health Educ Res
VL - 20
IS - 1
CY - England
N2 - Environmental factors may have an important influence on children's physical activity, yet children's perspectives of their home and neighborhood environments have not been widely assessed. The aim of this study was to investigate children's perceptions of their environments, and to examine associations between these perceptions and objectively measured physical activity. The sample consisted of 147, 10-year-old Australian children, who drew maps of their home and neighborhood environments. A subsample of children photographed places and things in these environments that were important to them. The maps were analyzed for themes, and for the frequency with which particular objects and locations appeared. Physical activity was objectively measured using accelerometers. Six themes emerged from the qualitative analysis of the maps and photographs: the family home; opportunities for physical activity and sedentary pursuits; food items and locations; green space and outside areas; the school and opportunities for social interaction. Of the 11 variables established from these themes, one home and two neighborhood factors were associated with children's physical activity. These findings contribute to a broader understanding of children's perceptions of their environment, and highlight the potential importance of the home and neighborhood environments for promoting physical activity behavior
SN - 0268-1153
AD - Centre for Physical Activity.utrition Research, School of Exercise and Nutrition Sciences, Deakin University, Burwood, Victoria 3125, Australia. clareh@deakin.edu.au
UR - 15253992
ER -

TY - JOUR
ID - 1381

T1 - Resurrecting free play in young children: looking beyond fitness and fatness to attention, affiliation, and affect. [Review] [59 refs]
A1 - Burdette,H.L.
A1 - Whitaker,R.C.
Y1 - 2005/01//
N1 - Burdette, Hillary L. Whitaker, Robert C
Archives of pediatrics & adolescent medicine
9422751, bwf
AIM, IM
Journal Article. Review
English
KW - MEDLINE
KW - Affect
KW - Attention
KW - Child
KW - Child Behavior/px [Psychology]
KW - Exercise
KW - Humans
KW - Interpersonal Relations
KW - Learning
KW - Play and Playthings/px [Psychology]
KW - Problem Solving
RP - NOT IN FILE
SP - 46
EP - 50
JF - Archives of Pediatrics & Adolescent Medicine
JA - Arch Pediatr Adolesc Med
VL - 159
IS - 1
CY - United States
N2 - We have observed that the nature and amount of free play in young children has changed. Our purpose in this article is to demonstrate why play, and particularly active, unstructured, outdoor play, needs to be restored in children's lives. We propose that efforts to increase physical activity in young children might be more successful if physical activity is promoted using different language-encouraging play-and if a different set of outcomes are emphasized-aspects of child well-being other than physical health. Because most physical activity in preschoolers is equivalent to gross motor play, we suggest that the term "play" be used to encourage movement in preschoolers. The benefits of play on children's social, emotional, and cognitive development are explored. [References: 59]
SN - 1072-4710
AD - Division of Gastroenterology and Nutrition, Children's Hospital of Philadelphia, Philadelphia, PA 19104, USA. burdette@email.chop.edu
UR - 15630057
ER -

TY - JOUR
ID - 1382
T1 - Is physical fitness decreased in survivors of childhood leukemia? A systematic review. [Review] [38 refs]
A1 - van,Brussel M.
A1 - Takken,T.
A1 - Lucia,A.
A1 - van der,Net J.
A1 - Helders,P.J.
Y1 - 2005/01//
N1 - van Brussel, M. Takken, T. Lucia, A. van der Net, J. Helders, P J M
Leukemia
leu, 8704895
IM

Journal Article. Research Support, Non-U.S. Gov't. Review
English
KW - MEDLINE
KW - Child
KW - Humans
KW - Outcome Assessment (Health Care)
KW - Oxygen Consumption
KW - Physical Fitness
KW - Precursor Cell Lymphoblastic Leukemia-Lymphoma/pp [Physiopathology]
KW - Survivors
RP - NOT IN FILE
SP - 13
EP - 17
JF - Leukemia
VL - 19
IS - 1
CY - England
N2 - The aim of this review is to determine whether physical fitness, assessed by peak oxygen uptake (VO(2peak)) measurement, is reduced in survivors of acute lymphoblastic leukemia (ALL) compared to healthy children. A systematic literature search (up to June 2004) was performed using Medline, Sportdiscus, Cinahl, Embase, Cochrane and PEDro database and reference tracking. The VO(2peak) (ml kg(-1) min(-1)) reached during a maximal exercise test until volitional exhaustion was used as the main outcome for this review. In all, 17 studies were identified in the literature. Data from three studies (102 ALL survivors, age ranging from 7 to 19 years) were pooled in a meta-analysis. Although there was a significant heterogeneity between the included studies (P=0.0006), the standardized mean difference (SMD) value of -0.61 (P=0.07) indicated that VO(2peak) tended to be reduced in survivors of childhood ALL compared to healthy control subjects, that is, decrease of -5.97 ml kg(-1) min(-1) (95% confidence interval (CI): (-12.35, 0.41); P=0.07) or -13% (95 % CI: (-27, 0.004)). Physical fitness tends to be reduced in survivors of ALL during childhood, which suggests the need for this population group to engage in regular physical activities with the purpose of increasing their functional capacity. Although more research is needed, this functional improvement might ameliorate the quality of life of ALL survivors as physical and outdoors activities are an essential part of daily routine during childhood. [References: 38]
SN - 0887-6924
AD - Departments of Pediatric Physical Therapy & Exercise Physiology, University Hospital for Children and Youth Het Wilhelmina Kinderziekenhuis, University Medical Center Utrecht, 3508 AB Utrecht, The Netherlands
UR - 15526028
ER -

TY - JOUR
ID - 1383
T1 - Physical activity is inversely related to waist circumference in 12-y-old French adolescents
A1 - Klein-Platat,C.
A1 - Oujaa,M.
A1 - Wagner,A.
A1 - Haan,M.C.
A1 - Arveiler,D.
A1 - Schlienger,J.L.
A1 - Simon,C.
Y1 - 2005/01//
N1 - Klein-Platat, C. Oujaa, M. Wagner, A. Haan, M C. Arveiler, D. Schlienger, J L. Simon, C
International journal of obesity (2005)
101256108
IM
Journal Article. Research Support, Non-U.S. Gov't
English
KW - MEDLINE

KW - Abdomen
KW - Anthropometry
KW - Body Size/ph [Physiology]
KW - Cardiovascular Diseases/et [Etiology]
KW - Child
KW - Cross-Sectional Studies
KW - Exercise/ph [Physiology]
KW - Female
KW - Humans
KW - Leisure Activities
KW - Male
KW - Multivariate Analysis
KW - Risk Factors
RP - NOT IN FILE
SP - 9
EP - 14
JF - International Journal of Obesity
JA - Int J Obes (Lond)
VL - 29
IS - 1
CY - England
N2 - BACKGROUND AND OBJECTIVE: Waist circumference (W) has been shown to be a good predictor of cardiovascular risk. The aim of this study was to investigate whether physical activity (PA) is related to W in adolescents as previously shown in adults. DESIGN AND SUBJECTS: Cross-sectional population-based survey of 2,714 12-y-old adolescents from the eastern part of France. MEASUREMENTS: Body mass index (BMI) and W were measured. Structured PA, active commuting to and from school and sedentary activities (SED), for example television viewing, computer/video games and reading and different potential confounders (dietary habits, parental overweight, family annual income tax and educational level) were assessed by a questionnaire. RESULTS: The adolescents had a mean BMI of 19.0+/-3.4 kg/m2, and 20.2% of them were overweight, with no gender difference. Boys had a greater W than girls (67.6+/-9.1 vs 65.7+/-8.9 cm, P<0.0001). In all, 42% of the girls and 25% of the boys did not practice any structured PA outside school and less than 40% of the adolescents commuted actively to school more than 20 min/day. About one-third of the adolescents devoted more than 2 h/day to SED. In univariate analyses, BMI was negatively associated with structured PA but significantly only for girls (P<0.01) and positively associated with SED for both genders (P<0.0001 for girls, P<0.01 for boys). W was negatively associated with structured PA and positively associated with SED both in girls (P<0.0001 and P=0.03, respectively) and boys (P<0.01 and P=0.08, respectively). Multiple general linear models show that SED is associated with BMI, independently of structured PA, in both genders. On the other hand, structured PA was inversely associated with W, independently of SED. The inverse relation between structured PA and W persisted after additional adjustment on BMI, with a greater effect of PA for the adolescents with higher BMI. CONCLUSIONS: In 12-y-old adolescents, structured PA is inversely associated with W, an indicator of total adiposity but also more specifically of abdominal fat. This suggests that PA may have a beneficial effect on youth metabolic and cardiovascular risks, in particular in the presence of overweight.
SN - 0307-0565
AD - Groupe d'Etudes en Nutrition, Faculte de Medecine, 4 rue Kirschleger, Strasbourg Cedex, France
UR - 15278108
ER -

TY - JOUR
ID - 1384
T1 - Can we use fixed ambient air monitors to estimate population long-term exposure to air pollutants? The case of spatial variability in the Genotox ER study
A1 - Nerriere,E.
A1 - Zmirou-Navier,D.
A1 - Blanchard,O.
A1 - Momas,I.
A1 - Ladner,J.
A1 - Le,Moullec Y.

A1 - Personnaz,M.B.
A1 - Lameloise,P.
A1 - Delmas,V.
A1 - Target,A.
A1 - Desqueyroux,H.
Y1 - 2005/01//
N1 - Nerriere, Elena. Zmirou-Navier, Denis. Blanchard, Olivier. Momas, Isabelle. Ladner, Joel. Le Moullec, Yvon. Personnaz, Marie-Blanche. Lameloise, Philippe. Delmas, Veronique. Target, Alain. Desqueyroux, Helene
Environmental research
ei2, 0147621
IM
Comparative Study. Journal Article. Research Support, Non-U.S. Gov't
English
KW - MEDLINE
KW - Adult
KW - Air Pollutants/an [Analysis]
KW - Child
KW - Confounding Factors (Epidemiology)
KW - Environmental Exposure/an [Analysis]
KW - Environmental Monitoring/is [Instrumentation]
KW - Environmental Monitoring/mt [Methods]
KW - Female
KW - France
KW - Humans
KW - Linear Models
KW - Male
KW - Models
KW - Theoretical
KW - Nitric Oxide/an [Analysis]
KW - Particle Size
KW - Seasons
KW - Urban Population
RP - NOT IN FILE
SP - 32
EP - 42
JF - Environmental Research
JA - Environ Res
VL - 97
IS - 1
CY - United States
N2 - Associations between average total personal exposures to PM2.5, PM10, and NO2 and concomitant outdoor concentrations were assessed within the framework of the Genotox ER study. It was carried out in four French metropolitan areas (Grenoble, Paris, Rouen, and Strasbourg) with the participation, in each site, of 60-90 nonsmoking volunteers composed of two groups of equal size (adults and children) who carried the personal Harvard Chempass multipollutant sampler during 48 h along two different seasons ("hot" and "cold"). In each center, volunteers were selected so as to live (home and work/school) in three different urban sectors contrasted in terms of air pollution (one highly exposed to traffic emissions, one influenced by local industrial sources, and a background urban environment). In parallel to personal exposure measurements, a fixed ambient air monitoring station surveyed the same pollutants in each local sector. A linear regression model was accommodated where the dependent pollutant-specific variable was the difference, for each subject, between the average ambient air concentrations over 48 h and the personal exposure over the same period. The explanatory variables were the metropolitan areas, the three urban sectors, season, and age group. While average exposures to particles were underestimated by outdoor monitors, in almost all cities, seasons, and age groups, differences were lower for NO2 and, in general, in the other direction. Relationships between average total personal exposures and ambient air levels varied across metropolitan areas and local urban sectors. These results suggest that using ambient air concentrations to assess average exposure of populations, in epidemiological studies of long-term effects or in a risk assessment setting, calls for some caution. Comparison of personal exposures to

PM or NO2 with ambient air levels is inherently disturbed by indoor sources and activities patterns. Discrepancies between measurement devices and local and regional sources of pollution may also strongly influence how the ambient air concentrations relate to population exposure. Much attention should be given to the selection of the most appropriate monitoring sites according to the study objectives
SN - 0013-9351
AD - INSERM, Faculte de Medecine, School of Medicine, 9 avenue de la Foret de Haye, BP 184-54 505 Vandoeuvre-les-Nancy Cedex, France
UR - 15476731
ER -

TY - JOUR
ID - 1385
T1 - Study of young people attending an adolescent friendly centre
A1 - Karki,C.
Y1 - 2004/10//
N1 - Karki, C
Kathmandu University medical journal (KUMJ)
101215359
IM
Journal Article
English
KW - MEDLINE
KW - Adolescent
KW - Adolescent Health Services/ut [Utilization]
KW - Adult
KW - Child
KW - Female
KW - Humans
KW - Male
KW - Morbidity
KW - Nepal/ep [Epidemiology]
KW - Socioeconomic Factors
RP - NOT IN FILE
SP - 324
EP - 330
JF - Kathmandu University Medical Journal
JA - Kathmandu Univ
VL - 2
IS - 4
CY - Nepal
N2 - Adolescents are the individuals between the ages of 10 and 19 years and the youth are those between the ages of 15 to 24 years. World Health Organisation has therefore defined young people as individuals between the age group of 10 to 24 years. In our country Adolescents comprise more than one fifth (22%) of the total population. Therefore Family Health Division, Ministry of Health, established an adolescent friendly centre at Bir Hospital on 5th January 2002. This paper has tried to study and analyse the young population attending this centre at a fixed period of time. This study was carried out to a. Find out the total number of adolescents. b. Estimate their male: female ratio. c. Find out their ethnicity, address, marital status, education, and occupation d. Learn what Health and Development problems these young people have. This is a cross sectional study done at Bir Hospital adolescent friendly centre for a period of one year. All young people (adolescents and youths) coming to this centre within the specified period are included in this study. OPD register was used to collect the data, which was obtained and analysed manually. Total of 956 young people were provided healthcare and counselling services from this centre. Out of them, only 9.21 % were males. 887 (92.78%) of this population are adolescents, 69 (7.22%) are from 19 to 24 years age group and 880 (92.05%) of them are youth. Majority (28.56%) were Brahmins. 313 (32.74%) of these people were from outside the valley. 9 (10.23%) out of 88 boys and 384 (79.34%) out of 484 girls were married. 1.14% of boys and 14.63% of girls were illiterate. 4.55% of boys and 14.17% of girls had not completed the studies of standard five. 17.05% of boys and 47% of girls had not completed their school. 81.82% of boys and 38.36% of girls had joined the college. It also showed that 521

(54.50%) of these young people are students but 35.94% of girls are housewives. 278 (32.03%) girls presented with menstrual problems and 22.12% (192) came with pregnancy related problems and 15.27 % of these young people presented with the symptoms of various infections Twenty six (3%) girls came with features of anaemia and 22 (2.53%) girls had dropped in for family planning services. 20 (2.09%) of these young people had some psychological problem (mainly anxiety) and 9.62% of them had various types of skin problems. Adolescent friendly centre can play a vital role to support and help a good number of adolescents of the society. Key words: Adolescents, youths, and young people
SN - 1812-2027
AD - Department of Obstetrics and Gynaecology, Kathmandu Medical College, Kathmandu.
chandakarki@hotmail.com
UR - 16388244
ER -

TY - JOUR
ID - 1386
T1 - Preventing injuries on horizontal ladders and track rides
A1 - Nixon,J.W.
A1 - Acton,C.H.
A1 - Wallis,B.A.
A1 - Battistutta,D.
A1 - Perry,C.
A1 - Eager,D.B.
Y1 - 2004/12//
N1 - Nixon, James W. Acton, Caroline H C. Wallis, Belinda A. Battistutta, Diana. Perry, Clare. Eager, David B M
Injury control and safety promotion
100941859, 100941859
IM
Journal Article. Research Support, Non-U.S. Gov't
English
KW - MEDLINE
KW - Accidental Falls/pc [Prevention & Control]
KW - Accidental Falls/sn [Statistics & Numerical Data]
KW - Brain Concussion/ep [Epidemiology]
KW - Child
KW - Equipment Design
KW - Facial Injuries/ep [Epidemiology]
KW - Female
KW - Fractures
KW - Bone/ep [Epidemiology]
KW - Humans
KW - Male
KW - Multiple Trauma/ep [Epidemiology]
KW - Odds Ratio
KW - Play and Playthings/in [Injuries]
KW - Prevalence
KW - Queensland/ep [Epidemiology]
KW - Sex Distribution
KW - Surface Properties
KW - Wounds and Injuries/ep [Epidemiology]
KW - Wounds and Injuries/pc [Prevention & Control]
RP - NOT IN FILE
SP - 219
EP - 224
JF - Injury Control & Safety Promotion
JA - Inj Control Saf Promot
VL - 11

IS - 4
CY - Netherlands
N2 - OBJECTIVES: This study examined the type of injury, fall heights and measures of impact attenuation of surfaces on which children fell from horizontal ladders and track rides. METHOD: All injured children who presented to two children's hospitals and received medical attention following a fall from a horizontal ladder or track ride in a public school or park during 1996--1997 were interviewed and the playground visited. RESULTS: The number of children who fell from horizontal ladders and track rides and presented to hospitals with injury was 118. Of those children, 105 were injured when they hit the ground and data were available on 102 of those playground undersurfaces. Fractures to the arm or wrist were the most common injury. The median height fallen by children was 1930 mm, 73% of injuries were from falls greater than 1800mm. In 41% of sites, the surface was deficient in impact absorbing properties for the height of the equipment. Fractures were no more likely on loose surfaces than other surfaces, such as rubber matting (p = 0.556) but more prevalent on compliant than non-compliant surfaces. Relative to falls occurring on noncompliant surfaces, the odds of a fracture occurring on a compliant surface was 2.67 (95% CI 0.88-8.14). CONCLUSIONS: Modification of the height of horizontal ladders and track rides to 1800mm is preferable to removal of such equipment. The prevalence of fractures on compliant surfaces suggests that the threshold of 200g or 1000 head injury criteria (HIC) needs to be revisited, or additional test criteria added to take account of change in momentum that is not presently accounted for with either g-max on HIC calculations
SN - 1566-0974
AD - Department of Paediatrics and Child Health, Royal Children's Hospital, Herston, Australia.
j.nixon@uq.edu.au
UR - 15903155
ER -

TY - JOUR
ID - 1387
T1 - Rearing behavior and rearing stress of fathers with children of preschool and school age
A1 - Lee,J.H.
A1 - Kim,H.Y.
A1 - Park,Y.A.
Y1 - 2004/12//
N1 - Lee, Ja-Hyung. Kim, Hye-Young. Park, Young-Ae
Taehan Kanho Hakhoe chi
101191388
IM, N
Comparative Study. Journal Article
English
KW - MEDLINE
KW - Adolescent
KW - Adult
KW - Age Factors
KW - Analysis of Variance
KW - Child
KW - Child Rearing/eh [Ethnology]
KW - Child Rearing/px [Psychology]
KW - Preschool
KW - Father-Child Relations/eh [Ethnology]
KW - Fathers/ed [Education]
KW - Fathers/px [Psychology]
KW - Gender Identity
KW - Health Services Needs and Demand
KW - Health Surveys
KW - Humans
KW - Korea/ep [Epidemiology]
KW - Middle Aged
KW - Nursing Methodology Research
KW - Paternal Behavior/eh [Ethnology]

KW - Play and Playthings
KW - Questionnaires
KW - Risk Factors
KW - Severity of Illness Index
KW - Social Support
KW - Stress
KW - Psychological/eh [Ethnology]
KW - Psychological/pc [Prevention & Control]
KW - Psychological/px [Psychology]
RP - NOT IN FILE
SP - 1491
EP - 1498
JF - Daehan Ganho Haghoeji
JA - Taehan Kanho Hakhoe Chi
VL - 34
IS - 8
CY - Korea (South)
N2 - PURPOSE: This study was conducted to compare the paternal rearing behavior and rearing stress level between fathers with a preschooler and fathers with school children so that it can be utilized as a basic source for developing parental rearing education programs. METHODS: A descriptive comparative method was conducted to identify the paternal rearing behavior and paternal rearing stress. Respondents were 361 fathers who had either preschoolers (n=189) or children of elementary age (n=172). RESULTS: Comparing the two group's means, the rearing activity score and rearing stress there were significant differences. In the school children's group's father, 'outdoor activity' and 'guidance on discipline activity' were significantly higher than the other group. In the preschool children's fathers group, 'play interaction activity' was statistically significant higher than the other, and the child-part mean score of paternal rearing stress was significantly higher than the other group. The correlation between paternal rearing behavior and paternal rearing stress, indicates that more paternal rearing behavior means less paternal rearing stress. CONCLUSION: These results of this study will help design more effective rearing programs for fathers that have either preschool children or school children by providing the basic data for paternal rearing behaviors and paternal rearing stress
SN - 1598-2874
AD - College of Nursing Science, Ewha Womans University, Seoul 120-750, Korea. jahlee@ewha.ac.kr
UR - 15687792
ER -

TY - JOUR
ID - 1388
T1 - Sun protection policy in elementary schools in Hawaii
A1 - Eakin,P.
A1 - Maddock,J.
A1 - Techur-Pedro,A.
A1 - Kaliko,R.
A1 - Derauf,D.C.
Y1 - 2004/07//
N1 - Eakin, Paul. Maddock, Jay. Techur-Pedro, Angela. Kaliko, Raphael. Derauf, D Christian
Preventing chronic disease
101205018
IM
Journal Article. Research Support, Non-U.S. Gov't
English
KW - MEDLINE
KW - Child
KW - Hawaii
KW - Humans
KW - Schools
KW - Sunburn/pc [Prevention & Control]
RP - NOT IN FILE

SP - A05
JF - Preventing Chronic Disease
JA - Prev Chronic Dis
VL - 1
IS - 3
CY - United States
N2 - INTRODUCTION: Childhood sun exposure is a major risk factor for skin cancer, the most common form of cancer in the United States. Schools in locations that receive high amounts of ultraviolet radiation have been identified as important sites for reducing excessive sun exposure. METHODS: The objective of this study was to determine the prevalence of sun protection policies, environmental features, and attitudes in public elementary schools in Hawaii. Surveys were sent to all (n = 177) public elementary school principals in Hawaii. Non-respondents were called three weeks after the initial mailing. The survey asked about sun protection policies, environmental features, and attitudes toward sun protection. The survey was designed to measure all seven components of Guidelines for School Programs to Prevent Skin Cancer, issued by the Centers for Disease Control and Prevention. RESULTS: Seventy-eight percent of schools responded to the survey. Only one school had a written school policy. Almost all schools (99.3%) scheduled outdoor activities during peak sun hours. School uniforms rarely included long pants (6.5%), long-sleeved shirts (5.1%), or hats (1.5%). Current policies did not support or restrict sun protection habits. Almost one third of those surveyed were in favor of a statewide policy (28.1%), and most believed excessive sun exposure was an important childhood risk (78.9%), even among non-white students (74.5%). CONCLUSION: Results of this study suggest the following: 1) school personnel in Hawaii are concerned about childhood sun exposure; 2) current school policies fail to address the issue; 3) most schools are receptive to developing sun protection policies and programs; and 4) students appear to be at high risk for sun exposure during school hours
SN - 1545-1151
AD - Department of Pediatrics, John A. Burns School of Medicine University of Hawaii at Manoa, USA
UR - 15670426
ER -

TY - JOUR
ID - 1389
T1 - [A case of Williams syndrome who exhibited fetishism]. [Japanese]
A1 - Noguchi,M.
A1 - Kato,S.
Y1 - 2004///
N1 - Noguchi, Masayuki. Kato, Satoshi
Seishin shinkeigaku zasshi = Psychiatria et neurologia Japonica
qam, 9801787
IM
Case Reports. English Abstract. Journal Article
Japanese
KW - MEDLINE
KW - Adolescent
KW - Adult
KW - Child
KW - Fetishism (Psychiatric)/px [Psychology]
KW - Humans
KW - Male
KW - Williams Syndrome/px [Psychology]
RP - NOT IN FILE
SP - 1232
EP - 1241
JF - Seishin Shinkeigaku Zasshi - Psychiatria et Neurologia Japonica
JA - Seishin Shinkeigaku Zasshi
VL - 106
IS - 10
CY - Japan

N2 - Williams syndrome is a rare congenital disease in which the etiological locus is a micro-deletion in chromosome 7. Here, we describe the case of a 22-year-old male who was diagnosed with Williams syndrome at the age of 3 years. As a child, the patient exhibited patterns of behavior characteristic of this syndrome including hyperactivity, attention deficit, and over-friendliness. He also showed persistent interest in construction vehicles, playgrounds, and gloves. He became interested in gloves after watching a television program in which the heroine fought her enemies while wearing gloves. Watching pornographic movies allowed him to attach strong sexual significance to gloves when he was 19 years old. Since that time, he has assaulted women wearing gloves four times to rob them of the gloves. The current paper discusses both the role of the cognitive profile unique to Williams syndrome and that of environmental factors in the development of fetishism in this case
SN - 0033-2658
AD - Department of Psychiatry, Sano Kosei General Hospital
UR - 15669216
ER -

TY - JOUR
ID - 1390
T1 - Evaluation and determinants of airborne bacterial concentrations in school classrooms
A1 - Bartlett,K.H.
A1 - Kennedy,S.M.
A1 - Brauer,M.
A1 - van,Netten C.
A1 - Dill,B.
Y1 - 2004/10//
N1 - Bartlett, Karen H. Kennedy, Susan M. Brauer, Michael. van Netten, Chris. Dill, Barbara
Journal of occupational and environmental hygiene
101189458
IM
Journal Article. Research Support, Non-U.S. Gov't
English
KW - MEDLINE
KW - Aerosols/an [Analysis]
KW - Air Pollution
KW - Indoor/an [Analysis]
KW - Bacteria/ip [Isolation & Purification]
KW - British Columbia
KW - Child
KW - Environmental Exposure
KW - Environmental Monitoring
KW - Humans
KW - Regression Analysis
KW - Schools
KW - Ventilation
RP - NOT IN FILE
SP - 639
EP - 647
JF - Journal of Occupational & Environmental Hygiene
JA - J Occup Environ Hyg
VL - 1
IS - 10
CY - United States
N2 - A survey of 39 elementary schools was undertaken to determine indoor air concentrations of bioaerosols within a coastal, temperate climatic zone in British Columbia, Canada. This article reports the results for airborne bacteria. Determinants of exposure were grouped into environmental (outdoor temperature, relative humidity, season, weather), ventilation and comfort parameter (indoor relative humidity, temperature, indoor CO2 concentration, indoor fungal concentration), and occupancy (number of occupants, activity levels, occupancy patterns) variables. Regression modeling was used to evaluate the association between these factors

and measured concentrations of indoor mesophilic bacteria. Naturally ventilated rooms (47% of the total rooms surveyed) had higher bacterial counts than mechanically ventilated rooms (geometric mean 325 vs. 166 CFU/m3, respectively, $p < 0.001$). In univariate analyses, bacterial counts were negatively correlated with supply and exhaust flow rates, air exchange rates, and the percentage of the day that occupants spent quietly sitting at their desks. Analysis of bacterial groups indicated various sources of the bacterial aerosol, with micrococci and staphylococci closely associated with occupancy variables, Bacillus with site and occupancy, and coryneform bacteria with site variables only. Approximately 60% of the variance in the outcome measurement of total bacteria was accountedfor by indoor CO2, lower air exchange rate, the age of the building, signs of current or old moisture stains, room volume, indoor relative humidity, and occupant activity level in a multiple linear regression model, with ventilation factors accounting for 40% of the variance
SN - 1545-9624
AD - School of Occupational and Environmental Hygiene, University of British Columbia, Vancouver, British Columbia, Canada. kbartlet@interchange.ubc.ca
UR - 15631055
ER -

TY - JOUR
ID - 1391
T1 - Transcription factor AP-2gamma is a developmentally regulated marker of testicular carcinoma in situ and germ cell tumors
A1 - Hoei-Hansen,C.E.
A1 - Nielsen,J.E.
A1 - Almstrup,K.
A1 - Sonne,S.B.
A1 - Graem,N.
A1 - Skakkebaek,N.E.
A1 - Leffers,H.
A1 - Rajpert-De,Meyts E.
Y1 - 2004/12/15/
N1 - Hoei-Hansen, Christina E. Nielsen, John E. Almstrup, Kristian. Sonne, Si Brask. Graem, Niels. Skakkebaek, Niels E. Leffers, Henrik. Rajpert-De Meyts, Ewa
Clinical cancer research : an official journal of the American Association for Cancer Research
c2h, 9502500
IM
Journal Article. Research Support, Non-U.S. Gov't
English
KW - MEDLINE
KW - Adolescent
KW - Adult
KW - Carcinoma in Situ/ge [Genetics]
KW - Carcinoma in Situ/me [Metabolism]
KW - Cell Differentiation
KW - Child
KW - Preschool
KW - DNA-Binding Proteins/ge [Genetics]
KW - DNA-Binding Proteins/me [Metabolism]
KW - Female
KW - Gene Expression Regulation
KW - Developmental
KW - Neoplastic
KW - Germinoma/et [Etiology]
KW - Germinoma/me [Metabolism]
KW - Gonadal Dysgenesis/co [Complications]
KW - Gonadal Dysgenesis/pa [Pathology]
KW - Humans
KW - Infant
KW - Newborn

KW - Male
KW - Middle Aged
KW - Ovarian Neoplasms/ge [Genetics]
KW - Ovarian Neoplasms/me [Metabolism]
KW - Pregnancy
KW - Steroids/pd [Pharmacology]
KW - Testicular Neoplasms/ge [Genetics]
KW - Testicular Neoplasms/me [Metabolism]
KW - Transcription Factor AP-2
KW - Transcription Factors/ge [Genetics]
KW - Transcription Factors/me [Metabolism]
KW - Tretinoin/pd [Pharmacology]
KW - Tumor Markers
KW - Biological
RP - NOT IN FILE
SP - 8521
EP - 8530
JF - Clinical Cancer Research
JA - Clin Cancer Res
VL - 10
IS - 24
CY - United States
N2 - PURPOSE: Transcription factor activator protein-2gamma (TFAP2C, AP-2gamma) was reported previously in extraembryonic ectoderm and breast carcinomas but not in the testis. In our recent gene expression study we detected AP-2gamma in carcinoma in situ testis (CIS, or intratubular germ cell neoplasia), precursor of testicular germ cell tumors. In this study we aimed to investigate the expression pattern of AP-2gamma and to shed light on this factor in germ cell differentiation and the pathogenesis of germ cell neoplasia. EXPERIMENTAL DESIGN: We analyzed expression pattern of AP-2gamma at the RNA and protein level in normal human tissues and a panel of tumors and tumor-derived cell lines. In the gonads, we established the ontogeny of expression of AP-2gamma in normal and dysgenetic samples. We also investigated the regulation of AP-2gamma by steroids and retinoic acid. RESULTS: We detected abundant AP-2gamma in testicular CIS and in testicular germ cell tumors of young adults and confirmed differential expression of AP-2gamma in somatic tumors. We found that AP-2gamma expression was regulated by retinoic acid in an embryonal carcinoma cell line (NT2). The investigation of ontogeny of AP-2gamma protein expression in fetal gonads revealed that it was confined to oogonia/gonocytes and was down-regulated with germ cell differentiation. In some prepubertal intersex cases, AP-2gamma was detected outside of the normal window of expression, probably marking neoplastic transformation of germ cells. CONCLUSIONS: AP-2gamma is developmentally regulated and associated with the undifferentiated phenotype in germ cells. This transcription factor may be involved in self-renewal and survival of immature germ cells and tissue-specific stem cells. AP-2gamma is a novel marker of testicular CIS and CIS-derived tumors
SN - 1078-0432
AD - University Department of Growth and Reproduction, Rigshospitalet, Denmark. cehh@biobase.dk
UR - 15623634
ER -

TY - JOUR
ID - 1392
T1 - Assessing and managing exposure from arsenic in CCA-treated wood play structures
A1 - Ursitti,F.
A1 - Vanderlinden,L.
A1 - Watson,R.
A1 - Campbell,M.
Y1 - 2004/11//
N1 - Ursitti, Franca. Vanderlinden, Loren. Watson, Robert. Campbell, Monica
Canadian journal of public health = Revue canadienne de sante publique
ck6, 0372714
IM

Journal Article
English
KW - MEDLINE
KW - Arsenic/an [Analysis]
KW - Child
KW - Environmental Exposure
KW - Humans
KW - Ontario
KW - Play and Playthings
KW - Soil Pollutants/an [Analysis]
KW - Urban Health
KW - Wood
RP - NOT IN FILE
SP - 429
EP - 433
JF - Canadian Journal of Public Health
JA - Can J Public Health
VL - Revue Canadienne de Sante Publique. 95
IS - 6
CY - Canada
N2 - BACKGROUND: Chromated copper arsenate (CCA)-treated wood has been widely used for outdoor play structures. There is a growing scientific concern about children's exposure to the arsenic that leaches from these structures. The purpose of this study was to measure arsenic from CCA-treated wood play structures owned by the City of Toronto to guide an appropriate exposure reduction strategy. METHODS: In the fall of 2002, 4 soil and 2 wood surface samples (dislodgeable arsenic) were collected from 217 play structures and analyzed for total arsenic content. Soil arsenic concentrations were compared to the federal soil guideline of 12 microg/g. Dislodgeable arsenic concentrations were compared to a Toronto Public Health-derived interim action level of 100 microg/100 cm2. RESULTS: Soil arsenic levels in samples taken from within one metre of CCA-treated wood were low (mean 2.1; range 0.5-10 microg/g). However, the means of the arsenic level in the composite soil samples taken from beneath an elevated platform were significantly greater (p<0.01) than the background soil sample and soil from within one metre of the CCA-treated wood (mean 20.3; range 12.4-47.5 microg/g). Composite soil samples exceeded the federal soil guideline of 12 microg/g at 32 play structures. Dislodgeable arsenic values varied widely (mean 41.9 microg/100 cm2; non-detectable to 521.5 microg/100 cm2). 32 play structures had dislodgeable arsenic levels that exceeded the interim action level. Mean arsenic concentrations on vertical surfaces were significantly higher than on horizontal surfaces (p<0.01). DISCUSSION: Our soil analysis indicates that arsenic does not migrate laterally but accumulates under elevated platforms at levels that can exceed the soil guideline. Dislodgeable arsenic values varied greatly and were not a useful predictor of soil arsenic (R2 = 0.0015)
SN - 0008-4263
AD - Toronto Public Health - Health Promotion and Environmental Protection Office, Community & Neighbourhood Services, City of Toronto, Toronto, ON. fursitti@toronto.ca
UR - 15622791
ER -

TY - JOUR
ID - 1393
T1 - [Assessment of health-risk groups by the german public health service and realisation of preventive measures]. [German]
A1 - Hennighausen,R.H.
Y1 - 2004/12//
N1 - Hennighausen, R H
Gesundheitswesen (Bundesverband der Arzte des Offentlichen Gesundheitsdienstes (Germany))
bfd, 9204210
IM
English Abstract. Journal Article
German
KW - MEDLINE

KW - Body Burden
KW - Child
KW - Environmental Monitoring
KW - Environmental Pollution/ae [Adverse Effects]
KW - Environmental Pollution/an [Analysis]
KW - Germany
KW - Health Status Indicators
KW - Humans
KW - Metals
KW - Heavy/ae [Adverse Effects]
KW - Heavy/an [Analysis]
KW - Population Surveillance
KW - Threshold Limit Values
RP - NOT IN FILE
SP - 821
EP - 826
JF - Gesundheitswesen
VL - 66
IS - 12
CY - Germany
N2 - Recognizing concealed health risks and pertaining high-risk groups is a cardinal task of the Public Health Services. As far as relevant environmental pollutions concerned protection of high-risk groups on the basis of appreciation of the average load and also of the 90th-percentile is insufficient. Public health strategies must be found which also include protection of the most highly exposed 10 % of the population. More than thousand years of mining and metallurgical engineering in the Harz mountains is the reason why heavy metal contamination in soil and air by arsenic, cadmium and lead is a problem in parts of the Harz region. Small children playing in soil with their "Pica-behaviour", hand to mouth contact, to the most endangered group. Therefore it was the duty of the District Community Physician to declare limit values for heavy metals and arsenic in the soil of child playgrounds. Experiences in Goslar/Harz region are reported
SN - 0941-3790
AD - Gesundheitsamt des Landkreises Goslar. rolf.hennighausen@landkreis-goslar.de
UR - 15609219
ER -

TY - JOUR
ID - 1394
T1 - Energy intake and meal portions: associations with BMI percentile in U.S. children
A1 - Huang,T.T.
A1 - Howarth,N.C.
A1 - Lin,B.H.
A1 - Roberts,S.B.
A1 - McCrory,M.A.
Y1 - 2004/11//
N1 - Huang, Terry T-K. Howarth, Nancy C. Lin, Biing-Hwan. Roberts, Susan B. McCrory, Megan A
Obesity research
cde, 9305691
IM
Journal Article. Research Support, U.S. Gov't, Non-P.H.S.
English
KW - MEDLINE
KW - Adolescent
KW - Adult
KW - Aging
KW - Body Mass Index
KW - Child
KW - Preschool
KW - Diet

KW - Diet Records
KW - Energy Intake
KW - Female
KW - Food
KW - Humans
KW - Male
KW - Mental Recall
KW - Sex Characteristics
RP - NOT IN FILE
SP - 1875
EP - 1885
JF - Obesity Research
JA - Obes Res
VL - 12
IS - 11
CY - United States
N2 - OBJECTIVE: We examined relationships of eating patterns and reported energy intake (rEI) with BMI percentile in U.S. children. RESEARCH METHODS AND PROCEDURES: Two 24-hour dietary recalls from the Continuing Surveys of Food Intakes by Individuals 1994 to 1996 and 1998 (1005 boys, 990 girls) were averaged, and children were categorized into three age groups: 3 to 5 years (n = 1077), 6 to 11 years (n = 537), and 12 to 19 years (n = 381). Physiologically implausible reports due to reporting bias or abnormal intake (rEI outside +/-18% to 23% of predicted energy requirements; pER) were identified. RESULTS: rEI averaged 109 +/- 34% and 100 +/- 10% of pER in the total and plausible samples, respectively. EI was overreported more in younger children and underreported more in overweight older children. Children with plausible rEI (45.3% of sample) averaged 4.7 eating occasions/d, 589 kcal/meal, 223 kcal/snack, and 2038 kcal/d. rEI was not associated with BMI percentile in the total sample. In the plausible sample, rEI, meal portion size, and meal energy were positively associated with BMI percentile in boys 6 to 11 years and in children 12 to 19 years. No relationships were found in children 3 to 5 years and girls 6 to 11 years. Relationships were more consistent and stronger in the plausible compared with the total sample. DISCUSSION: Excluding implausible dietary reports may be necessary for discerning dietary associations with BMI percentile. EI and meal, but not snack, patterns may play a quantitatively greater role in weight regulation as children age
SN - 1071-7323
AD - Jean Mayer U.S. Department of Agriculture Human Nutrition Research Center on Aging, Tufts University, Boston, MA, USA
UR - 15601985
ER -

TY - JOUR
ID - 1395
T1 - Cerebral folate deficiency
A1 - Ramaekers,V.T.
A1 - Blau,N.
Y1 - 2004/12//
N1 - Ramaekers, Vincent Th. Blau, Nenad
Developmental medicine and child neurology
0006761, e83
IM
Journal Article. Research Support, Non-U.S. Gov't
English
KW - MEDLINE
KW - Brain/me [Metabolism]
KW - Brain/pa [Pathology]
KW - Carrier Proteins/me [Metabolism]
KW - Cerebrospinal Fluid Proteins/me [Metabolism]
KW - Child
KW - Chromosomal Proteins
KW - Non-Histone/ge [Genetics]

KW - DNA-Binding Proteins/ge [Genetics]
KW - Diagnosis
KW - Differential
KW - Female
KW - Folate Receptors
KW - GPI-Anchored
KW - Folic Acid Deficiency/di [Diagnosis]
KW - Folic Acid Deficiency/ge [Genetics]
KW - Glycosylation
KW - Humans
KW - Infant
KW - Magnetic Resonance Imaging
KW - Male
KW - Metabolism
KW - Inborn Errors/cf [Cerebrospinal Fluid]
KW - Inborn Errors/di [Diagnosis]
KW - Inborn Errors/ge [Genetics]
KW - Methyl-CpG-Binding Protein 2
KW - Protein Processing
KW - Post-Translational/ph [Physiology]
KW - Receptors
KW - Cell Surface/me [Metabolism]
KW - Repressor Proteins/ge [Genetics]
KW - Terminology as Topic
RP - NOT IN FILE
SP - 843
EP - 851
JF - Developmental Medicine & Child Neurology
JA - Dev Med Child Neurol
VL - 46
IS - 12
CY - England
N2 - Cerebral folate deficiency (CFD) can be defined as any neurological syndrome associated with low cerebrospinal fluid (CSF) 5-methyltetrahydrofolate (5MTHF), the active folate metabolite, in the presence of normal folate metabolism outside the nervous system. CFD could result from either disturbed folate transport or from increased folate turnover within the central nervous system (CNS). We report on a novel neurometabolic syndrome in 20 children, which we term 'idiopathic CFD'. Typical features became manifest from the age of 4 months, starting with marked unrest, irritability, and sleep disturbances followed by psychomotor retardation, cerebellar ataxia, spastic paraplegia, and dyskinesia; epilepsy developed in about one third of the children. Most children showed deceleration ofhead growth from the age of 4 to 6 months. Visual disturbances began to develop around the age of 3 years and progressive sensorineural hearing loss started from the age of 6 years. Neuroimaging showed atrophy of frontotemporal regions and periventricular demyelination in seven children, slowly progressive supra- and infratentorial atrophy in three children, and normal findings in the remainder. Because active folate transport to the CNS occurs through receptor-mediated folate receptor protein 1 (FR1) endocytosis, DNA sequencing of the FR1 gene was performed and found to be normal. However, CSF protein analysis revealed a non-functional FR1 protein, suspected to result from either post-translational defects of FR1 protein N-glycosylation, the presence of folate antagonists with irreversible binding, or autoantibodies blocking the folate binding site of FR1. Oral treatment with 5-formyltetrahydrofolate (folinic acid) should be started in low doses at 0.5-1mg/kg/day, but in some patients higher daily doses of folinic acid at 2-3 mg/kg/day are required to normalize CSF 5MTHF values. This proposed treatment protocol resulted in a favourable clinical response in patients identified before the age of six years while partial recovery with poorer outcome was found beyond the age of 6 years. Careful clinical and EEG monitoring should be performed 1, 3, and 6 months after the beginning of treatment. After four to six months of folinic acid treatment, CSF analysis should be repeated in order to prevent over- or under-dosage of folinic acid. Secondary forms of CFD have been recognized during chronic use of antifolate and anticonvulsant drugs and in various known conditions such as Rett syndrome, Aicardi-Goutieres syndrome, 3-phosphoglycerate dehydrogenase deficiency, dihydropteridine reductase

deficiency, aromatic amino acid decarboxylase deficiency, and Kearns-Sayre syndrome. The pathogenic link between these underlying specific disease entities and the observed secondary CFD has not been resolved
SN - 0012-1622
AD - University Hospital Aachen, Germany. vramaekers@skynet.be
UR - 15581159
ER -

TY - JOUR
ID - 1396
T1 - Pulmonary epithelial integrity in children: relationship to ambient ozone exposure and swimming pool attendance
A1 - Lagerkvist,B.J.
A1 - Bernard,A.
A1 - Blomberg,A.
A1 - Bergstrom,E.
A1 - Forsberg,B.
A1 - Holmstrom,K.
A1 - Karp,K.
A1 - Lundstrom,N.G.
A1 - Segerstedt,B.
A1 - Svensson,M.
A1 - Nordberg,G.
Y1 - 2004/12//
N1 - Lagerkvist, Birgitta Json. Bernard, Alfred. Blomberg, Anders. Bergstrom, Erik. Forsberg, Bertil. Holmstrom, Karin. Karp, Kjell. Lundstrom, Nils-Goran. Segerstedt, Bo. Svensson, Mona. Nordberg, Gunnar
Environmental health perspectives
ei0, 0330411
IM
Journal Article. Research Support, Non-U.S. Gov't
English
KW - MEDLINE
KW - Air Pollutants/po [Poisoning]
KW - Child
KW - Chlorine Compounds/po [Poisoning]
KW - Disinfectants/po [Poisoning]
KW - Environmental Exposure
KW - Epithelial Cells/pa [Pathology]
KW - Female
KW - Humans
KW - Inflammation
KW - Lung/im [Immunology]
KW - Lung/pa [Pathology]
KW - Male
KW - Oxidants
KW - Photochemical/po [Poisoning]
KW - Ozone/po [Poisoning]
KW - Respiratory Function Tests
KW - Swimming
KW - Swimming Pools
KW - Uteroglobin/an [Analysis]
RP - NOT IN FILE
SP - 1768
EP - 1771
JF - Environmental Health Perspectives
JA - Environ Health Perspect
VL - 112
IS - 17

CY - United States
N2 - Airway irritants such as ozone are known to impair lung function and induce airway inflammation. Clara cell protein (CC16) is a small anti-inflammatory protein secreted by the nonciliated bronchiolar Clara cells. CC16 in serum has been proposed as a noninvasive and sensitive marker of lung epithelial injury. In this study, we used lung function and serum CC16 concentration to examine the pulmonary responses to ambient O3 exposure and swimming pool attendance. The measurements were made on 57 children 10-11 years of age before and after outdoor exercise for 2 hr. Individual O3 exposure was estimated as the total exposure dose between 0700 hr until the second blood sample was obtained (mean O3 concentration/m3 times symbol hours). The maximal 1-hr value was 118 microg/m3 (59 ppb), and the individual exposure dose ranged between 352 and 914 microg/m3hr. These O3 levels did not cause any significant changes in mean serum CC16 concentrations before or after outdoor exercise, nor was any decrease in lung function detected. However, children who regularly visited chlorinated indoor swimming pools had significantly lower CC16 levels in serum than did nonswimming children both before and after exercise (respectively, 57 +/- 2.4 and 53 +/- 1.7 microg/L vs. 8.2 +/- 2.8 and 8.0 +/- 2.6 microg/L; p < 0.002). These results indicate that repeated exposure to chlorination by-products in the air of indoor swimming pools has adverse effects on the Clara cell function in children. A possible relation between such damage to Clara cells and pulmonary morbidity (e.g., asthma) should be further investigated
SN - 0091-6765
AD - Environmental and Occupational Medicine, Department of Public Health and Clinical Medicine, Umea University, Umea, Sweden. Birgitta.Lagerkvist@envmed.umu.se
UR - 15579425
ER -

TY - JOUR
ID - 1397
T1 - Reorganization of language-specific cortex in patients with lesions or mesial temporal epilepsy
A1 - Pataraia,E.
A1 - Simos,P.G.
A1 - Castillo,E.M.
A1 - Billingsley-Marshall,R.L.
A1 - McGregor,A.L.
A1 - Breier,J.I.
A1 - Sarkari,S.
A1 - Papanicolaou,A.C.
Y1 - 2004/11/23/
N1 - Pataraia, E. Simos, P G. Castillo, E M. Billingsley-Marshall, R L. McGregor, A L. Breier, J I. Sarkari, S. Papanicolaou, A C
Neurology
0401060, nz0
AIM, IM
Journal Article. Research Support, N.I.H., Extramural. Research Support, Non-U.S. Gov't. Research Support, U.S. Gov't, P.H.S.
English
KW - MEDLINE
KW - Adolescent
KW - Adult
KW - Aged
KW - Anterior Temporal Lobectomy
KW - Atrophy
KW - Brain Mapping/mt [Methods]
KW - Child
KW - Cohort Studies
KW - Dominance
KW - Cerebral/ph [Physiology]
KW - Epilepsy
KW - Temporal Lobe/pp [Physiopathology]
KW - Temporal Lobe/su [Surgery]

KW - Female
KW - Humans
KW - Language
KW - Language Tests
KW - Magnetic Resonance Imaging
KW - Magnetoencephalography
KW - Male
KW - Middle Aged
KW - Neuronal Plasticity
KW - Pattern Recognition
KW - Physiological
KW - Preoperative Care
KW - Retrospective Studies
KW - Sclerosis/pp [Physiopathology]
KW - Temporal Lobe/pa [Pathology]
RP - NOT IN FILE
SP - 1825
EP - 1832
JF - Neurology
VL - 63
IS - 10
CY - United States
N2 - OBJECTIVE: To examine brain activation profiles for receptive language function, using magnetoencephalography (MEG), in patients with left hemisphere space-occupying lesions and patients with left temporal lobe epilepsy due to mesial temporal sclerosis (MTS) and to evaluate whether cross- and intrahemispheric plasticity for language varied as a function of lesion type or location. METHODS: Twenty-one patients with MTS and 23 lesional patients underwent preoperative language mapping while performing a word recognition task. The anatomic location of late activity sources was determined by co-registering MEG coordinates onto structural MRI scans. A language laterality index was calculated based on the number of activity sources in each hemisphere. The location of language-specific activity was examined in relation to its proximity or overlap with Wernicke's area. RESULTS: A higher incidence of atypical language lateralization was noted among patients with MTS than lesional patients (43 vs 13%). The majority of MTS patients with early seizure onset (before age 5) showed atypical language lateralization. In contrast, the precise location of receptive language-specific cortex within the dominant hemisphere was found to be atypical (outside of Wernicke's area) in 30% of lesional patients and only 14% of MTS patients. CONCLUSIONS: There is an increased probability of a partial or total displacement of key components of the brain mechanism responsible for receptive language function to the nondominant hemisphere in mesial temporal sclerosis patients. Early onset of seizures is strongly associated with atypical language lateralization. Lesions in the dominant hemisphere tend to result in an intrahemispheric reorganization of linguistic function
SN - 1526-632X
AD - Department of Neurosurgery, Vivian L. Smith Center for Neurologic Research, University of Texas Medical Center at Houston, 1333 Moursund St., H 114, Houston, TX 77030, USA. epataraia1@uth.tmc.edu
UR - 15557497
ER -

TY - JOUR
ID - 1398
T1 - Social skills assessment of children with autism in free-play situations
A1 - Anderson,A.
A1 - Moore,D.W.
A1 - Godfrey,R.
A1 - Fletcher-Flinn,C.M.
Y1 - 2004/12//
N1 - Anderson, Angelika. Moore, Dennis W. Godfrey, Rebecca. Fletcher-Flinn, Claire M
Autism : the international journal of research and practice
9713494
IM

Journal Article. Research Support, Non-U.S. Gov't
English
KW - MEDLINE
KW - Adaptation
KW - Psychological
KW - Autistic Disorder/px [Psychology]
KW - Child
KW - Preschool
KW - Data Collection
KW - Female
KW - Humans
KW - Interpersonal Relations
KW - Male
KW - Play and Playthings
KW - Social Behavior
RP - NOT IN FILE
SP - 369
EP - 385
JF - Autism
VL - 8
IS - 4
CY - England
N2 - Poor social functioning and limited play are characteristic of children with autism. Increasingly, education for children with autism is provided within mainstream settings, but given their particular difficulties, the adequate provision of educational services in such settings is challenging. This study presents observational data of the play behaviour and social interaction patterns of 10 children with autism in mainstream kindergartens and primary school playgrounds. The target children differed significantly in terms of their play and social interactions from typically developing children in the same settings. The adequacy of the provision of services for children with autism in mainstream provision is discussed
SN - 1362-3613
AD - Research Centre for Interventions in Teaching and Learning, The University of Auckland, Private Bag 92019 Auckland, New Zealand. an.anderson@auckland.ac.nz
UR - 15556957
ER -

TY - JOUR
ID - 1399
T1 - Hazards in our environment: the continuing problem of lead in toys...and pressure-treated playground equipment may not pose a risk
Y1 - 2004/11//
N1 - Child health alert
9881972
K
Journal Article
English
KW - MEDLINE
KW - Arsenic/an [Analysis]
KW - Child
KW - Environmental Exposure/an [Analysis]
KW - Humans
KW - Lead Poisoning/et [Etiology]
KW - Play and Playthings
RP - NOT IN FILE
SP - 4
EP - 5
JF - Child Health Alert
VL - 22

CY - United States
SN - 1064-4849
UR - 15612096
ER -

TY - JOUR
ID - 1400
T1 - Paediatric trauma teams in Australia
A1 - Wong,K.
A1 - Petchell,J.
Y1 - 2004/11//
N1 - Wong, Kenneth. Petchell, Jeffrey
ANZ journal of surgery
dze, 101086634
IM
Journal Article
English
KW - MEDLINE
KW - Australia
KW - Child
KW - Emergency Service
KW - Hospital/og [Organization & Administration]
KW - Hospital
KW - Hospitals
KW - Pediatric
KW - Humans
KW - Patient Care Team
KW - Questionnaires
KW - Referral and Consultation
KW - Trauma Centers
KW - Wounds and Injuries/th [Therapy]
RP - NOT IN FILE
SP - 992
EP - 996
JF - ANZ Journal of Surgery
JA - ANZ J Surg
VL - 74
IS - 11
CY - Australia
N2 - BACKGROUND: Trauma teams have been associated with improved survival probability of paediatric trauma patients. The present study seeks to estimate the use of trauma teams in Australian paediatric tertiary referral centres and describe their medical composition, leadership and criteria for activation. METHODS: Australian paediatric tertiary referral centres were identified. A structured questionnaire assessing the presence, composition and means of activation of a trauma team was mailed to the 'Director, Emergency Department' of all identified hospitals. Three months later, all hospitals were contacted by telephone to complete and verify data collection. RESULTS: Questionnaires were distributed to eight hospitals. Seventy-five per cent had an established trauma team. Hospitals without a trauma team claimed to have insufficient doctors to form a team and insufficient trauma caseload to justify a team. All trauma teams were potentially activated by prehospital paramedic data (field triage) and required a combination of anatomical, physiological and mechanistic criteria for activation. The two methods of mobilizing a trauma team were by dispatching a common call onto individual pagers (66%) or a specific trauma pager (33%) carried by trauma team members. Fifty per cent of hospitals had a two-tier, stratified trauma team response. All teams consisted of emergency, surgical and intensive care unit registrars. Trauma team leaders were emergency medicine specialists/registrars (33%), surgical registrars (33%) and non-defined (33%). Consultant surgeons were not members of any trauma team. Eighty-three per cent of trauma teams consisted of more junior members after hours. Fifty per cent of hospitals did not have a surgical registrar on site outside of business hours. Eighty-eight per cent of hospitals engaged in some form of trauma audit. CONCLUSIONS: Trauma teams are utilized by most Australian paediatric tertiary

referral centres, with fairly uniform medical composition and criteria for activation. Paediatric surgeons presently have limited leadership roles and membership of Australian paediatric trauma teams
SN - 1445-1433
AD - Department of Trauma, Royal Prince Alfred Hospital, Sydney, New South Wales, Australia. kennethwo@yahoo.com
UR - 15550090
ER -

TY - JOUR
ID - 1401
T1 - Intervention centred on adolescents' physical activity and sedentary behaviour (ICAPS): concept and 6-month results
A1 - Simon,C.
A1 - Wagner,A.
A1 - DiVita,C.
A1 - Rauscher,E.
A1 - Klein-Platat,C.
A1 - Arveiler,D.
A1 - Schweitzer,B.
A1 - Triby,E.
Y1 - 2004/11//
N1 - Simon, C. Wagner, A. DiVita, C. Rauscher, E. Klein-Platat, C. Arveiler, D. Schweitzer, B. Triby, E
International journal of obesity and related metabolic disorders : journal of the International Association for the Study of Obesity
btx, 9313169
IM
Clinical Trial. Journal Article. Randomized Controlled Trial. Research Support, Non-U.S. Gov't
English
KW - MEDLINE
KW - Adolescent
KW - Adolescent Behavior
KW - Adolescent Psychology
KW - Anthropometry/mt [Methods]
KW - Behavior Control
KW - Child
KW - Eating
KW - Humans
KW - Obesity/pc [Prevention & Control]
KW - Obesity/px [Psychology]
KW - Physical Exertion
RP - NOT IN FILE
SP - S96
EP - S103
JF - International Journal of Obesity & Related Metabolic Disorders: Journal of the International Association for the Study of Obesity
JA - Int J Obes Relat Metab Disord
VL - 28 Suppl 3
CY - England
N2 - OBJECTIVE: To evaluate the 6-month impact of a physical activity (PA) multilevel intervention on activity patterns and psychological predictors of PA among adolescents. The intervention was directed at changing knowledge and attitudes and at providing social support and environmental conditions that encourage PA of adolescents inside and outside school. SUBJECTS AND DESIGN: Randomised, controlled ongoing field trial (ICAPS) in middle-school's first-level adolescents from eight schools selected in the department of the Bas-Rhin (Eastern France) with a cohort of 954 adolescents (92% of the eligible students) initially aged 11.7+/-0.6 y. The 6-month changes in participation in leisure organised PA (LOPA), high sedentary (SED) behaviour (>3 h/day), self-efficacy (SELF) and intention (INTENT) towards PA were analysed after controlling for baseline measures and different covariables (age, overweight, socioprofessional occupation), taking into account the

cluster randomisation design. RESULTS: The proportion of intervention adolescents not engaged in organised PA was reduced by 50% whereas it was unchanged among control students. After adjustment for baseline covariables, LOPA participation significantly increased among the intervention adolescents (odds ratio (95% confidence interval) (OR)=3.38 (1.42-8.05) in girls; 1.73 (1.12-2.66) in boys), while high SED was reduced (OR=0.54 (0.38-0.77) in girls; 0.52 (0.35-0.76) in boys). The intervention improved SELF in girls, whatever their baseline LOPA (P<10(-4)) and INTENT in girls with no baseline LOPA (P=0.04). SELF tended to improve in boys with no baseline LOPA, without reaching statistical significance. When included in the regression, follow-up LOPA was associated with improvement of SELF in girls (P=0.02) and of INTENT in girls with no baseline PA (P<0.02). The intervention effect was then attenuated. CONCLUSION: After 6 months of intervention, ICAPS was associated with a significant improvement of activity patterns and psychological predictors, indicating a promising approach for modifying the long-term PA level of adolescents
SN - 0307-0565
AD - Groupe d'Etudes en Nutrition, Faculte de Medecine, 67085 Strasbourg Cedex, France. chantal.simon@medicine.u-strasbg.fr
UR - 15543228
ER -

TY - JOUR
ID - 1402
T1 - Assessment of different methods for diagnosing dental caries in epidemiological surveys
A1 - Assaf,A.V.
A1 - Meneghim,Mde C.
A1 - Zanin,L.
A1 - Mialhe,F.L.
A1 - Pereira,A.C.
A1 - Ambrosano,G.M.
Y1 - 2004/12//
N1 - Assaf, Andrea Videira. Meneghim, Marcelo de Castro. Zanin, Luciane. Mialhe, Fabio Luiz. Pereira, Antonio Carlos. Ambrosano, Glaucia Maria Bovi
Community dentistry and oral epidemiology
dnf, 0410263
D, IM
Comparative Study. Journal Article
English
KW - MEDLINE
KW - Analysis of Variance
KW - Child
KW - DMF Index
KW - Dental Caries/di [Diagnosis]
KW - Dental Caries/ep [Epidemiology]
KW - Dental Caries Activity Tests/mt [Methods]
KW - Dental Instruments
KW - Humans
KW - Toothbrushing
RP - NOT IN FILE
SP - 418
EP - 425
JF - Community Dentistry & Oral Epidemiology
JA - Community Dent Oral Epidemiol
VL - 32
IS - 6
CY - Denmark
N2 - AIMS: The aims of the study were: (i) to assess different clinical diagnostic methods of dental caries during epidemiological surveys; (ii) to determine which combinations of methods and diagnostic adjuncts show the best performances in epidemiological surveys when compared with examinations performed in a traditional dental setting (standard); (iii) to evaluate the influence of including noncavitated (NC) lesions in dental caries estimation. METHODS: Forty 12-year-old children were divided into low and moderate caries prevalence

groups. The individuals were submitted to 12 epidemiological examinations (in an outdoor setting), which combined three methods (blade, mirror and mirror + CPI (Community Periodontal Index) dental probe) with or without diagnostic adjuncts (previous dental brushing and dental drying). The last examination was performed in a traditional dental setting (standard examination). The unit of measure was the DMFS (decayed, missing and filled surfaces) index according to WHO criteria. The variance analysis, Dunnet's and Tukey's tests were applied. RESULTS: For the DMFS analysis, the visual/tactile method, with or without diagnostic adjuncts, was the best method for both groups, presenting a performance higher than 90% when compared with the standard examination, except for the examinations without previous dental brushing for the low caries prevalence group. Previous dental brushing was more relevant than dental drying (P = 0.0054). All of the epidemiological examinations underestimated the NC diagnosis even with the association of diagnostic adjuncts when compared with the standard examination. CONCLUSION: The visual-tactile (for both groups) and the visual (mirror) methods plus dental brushing (for the moderate group) are appropriate for diagnosing cavitated lesions, but not NC lesions
SN - 0301-5661
AD - Department of Community Dentistry, FOP/UNICAMP, Departamento de Odontologia Social, Avenida Limeira 901, CEP: 13414-903 Piracicaba, SP, Brazil. thalesam@hotmail.com
UR - 15541157
ER -

TY - JOUR
ID - 1403
T1 - Intervention study of airborne fungal spora in homes with portable HEPA filtration units
A1 - Cheong,C.D.
A1 - Neumeister-Kemp,H.G.
A1 - Dingle,P.W.
A1 - Hardy,G.S.
Y1 - 2004/11//
N1 - Cheong, C D. Neumeister-Kemp, H G. Dingle, P W. Hardy, G St J
Journal of environmental monitoring : JEM
d0a, 100968688
IM
Journal Article
English
KW - MEDLINE
KW - Air Microbiology
KW - Air Pollution
KW - Indoor/an [Analysis]
KW - Australia
KW - Child
KW - Colony Count
KW - Microbial
KW - Environmental Monitoring/mt [Methods]
KW - Filtration/mt [Methods]
KW - Floors and Floorcoverings
KW - Housing
KW - Humans
KW - Inhalation Exposure
KW - Spores
KW - Fungal
RP - NOT IN FILE
SP - 866
EP - 873
JF - Journal of Environmental Monitoring
JA - J Environ Monit
VL - 6
IS - 11
CY - England

N2 - The concentrations and composition of airborne fungal spores in homes fitted with portable HEPA filtration units were examined to provide information to evaluate the importance of varying levels of fungal spores in residential environments in Perth, Australia. A novel method for simulating activity/impaction on carpeted environments was also investigated. Reductions in fungal (35%) and particulate (38%) levels were achieved in the air filter homes. Penicillium, Cladosporium and yeasts were the most common and widespread fungi recovered indoors and outdoors. Fungal range decreased over the study period but this could be due to an overall reduced dissemination of spores (less spores in the air)
SN - 1464-0325
AD - School of Environmental Science, Murdoch University, South Street, WA 6150, Australia. futora@wn.com.au
UR - 15536499
ER -

TY - JOUR
ID - 1404
T1 - [Overweight and obesity in children and adolescents and preventative measures]. [Review] [38 refs] [Dutch]
A1 - Renders,C.M.
A1 - Seidell,J.C.
A1 - van,Mechelen W.
A1 - Hirasing,R.A.
Y1 - 2004/10/16/
N1 - Renders, C M. Seidell, J C. van Mechelen, W. Hirasing, R A
Nederlands tijdschrift voor geneeskunde
nuk, 0400770
IM
English Abstract. Journal Article. Review
Dutch
KW - MEDLINE
KW - Adolescent
KW - Adolescent Nutritional Physiological Phenomena
KW - Breast Feeding
KW - Carbonated Beverages/ae [Adverse Effects]
KW - Child
KW - Child Nutritional Physiological Phenomena
KW - Exercise/ph [Physiology]
KW - Female
KW - Health Promotion
KW - Humans
KW - Male
KW - Obesity/co [Complications]
KW - Obesity/pc [Prevention & Control]
KW - Risk Factors
KW - Television
RP - NOT IN FILE
SP - 2066
EP - 2070
JF - Nederlands Tijdschrift voor Geneeskunde
JA - Ned Tijdschr Geneeskd
VL - 148
IS - 42
CY - Netherlands
N2 - The prevalence of overweight and obesity in children is increasing rapidly. This is alarming because obesity is associated with severe chronic diseases, such as type 2 diabetes mellitus. Obesity at young age is related to obesity at adult age. Consequently, the prevention of overweight from childhood onwards is an important issue. Apart from diabetes mellitus type 2 there is an increased risk of orthopaedic complications, respiratory problems, fertility problems, cardiovascular diseases and psychosocial consequences in the form of a

negative self-image, emotional and behavioural problems and depression. Environmental and behavioural factors are regarded as the most important causes of the rapid increase in the prevalence of overweight and as the most important starting points for prevention. Most prevention programmes are still in the initial stages. Prevention programmes aimed at stimulating breast feeding and daily physical activity (playing outside) and the restriction of sweetened drinks and watching TV are very promising. With such preventive measures the involvement of both the school and the parents is important. [References: 38]
SN - 0028-2162
AD - Vrije Universiteit Medisch Centrum, afd. Sociale Geneeskunde, Instituut voor Extramuraal Geneeskundig Onderzoek, Van der Boechorststraat 7, 1081 BT Amsterdam. cm.renders@vumc.nl
UR - 15532328
ER -

TY - JOUR
ID - 1405
T1 - Parent-child physical activity relationships in 12-year old French students do not depend on family socioeconomic status
A1 - Wagner,A.
A1 - Klein-Platat,C.
A1 - Arveiler,D.
A1 - Haan,M.C.
A1 - Schlienger,J.L.
A1 - Simon,C.
Y1 - 2004/09//
N1 - Wagner, A. Klein-Platat, C. Arveiler, D. Haan, M C. Schlienger, J L. Simon, C
Diabetes & metabolism
cki, 9607599
IM
Journal Article
English
KW - MEDLINE
KW - Adolescent
KW - Adult
KW - Body Mass Index
KW - Child
KW - Cross-Sectional Studies
KW - Female
KW - France
KW - Humans
KW - Leisure Activities
KW - Male
KW - Motor Activity/ph [Physiology]
KW - Obesity/ep [Epidemiology]
KW - Parent-Child Relations
KW - Sex Characteristics
KW - Sex Distribution
KW - Socioeconomic Factors
KW - Sports
KW - Television
RP - NOT IN FILE
SP - 359
EP - 366
JF - Diabetes & Metabolism
JA - Diabetes Metab
VL - 30
IS - 4
CY - France

N2 - OBJECTIVES: Regular physical activity (PA) in young needs to be promoted to prevent obesity and subsequent diabetes. Influences of parental PA on adolescents' activity level have been studied with discordant results and the effect of socioeconomic status (SES) on these relationships is not clarified. METHODS: Cross-sectional population-based survey of about 3000 12-year old French students and their parents. Familial associations were assessed with logistic regression models taking into account SES and children's and parents' corpulence. Sport involvement and sedentary behaviors were assessed by standardized questionnaires filled out separately by children and parents, the latter also reporting their educational data and family income tax. RESULTS: Participation in structured PA outside school was higher for boys (74%) than for girls (58%, p<10-5) and a high sedentary behavior (watching television, playing computer/video games and reading > or =2h/day) was observed in one-third of the sample, both in boys and in girls. Children were more likely to participate in structured PA outside school when both parents practiced sport as compared to neither parent practicing it, with an odd ratio OR (95%CI) of 1.97 (1.4-2.8) for boys and 1.56 (1.2-2.1) for girls. Familial associations of inactivity were significant for boys only. A greater percentage of adolescents had a high level of sedentary behavior when both parents versus no parents watched television > 2h/day (OR 1.95 (1.52.6)). Parent-child physical activity relationships were not modified by the family SES or the children's or parents' weight status. CONCLUSION: Parental involvement in sport is an important correlate of a young adolescent's participation in structured PA outside school, whatever the family SES or corpulence of the family members
SN - 1262-3636
AD - Groupe d'etudes et de recherche en nutrition, Faculte de Medecine, Strasbourg, France
UR - 15525880
ER -

TY - JOUR
ID - 1406
T1 - [Gastric lavage, hemodialysis and specific antidotes in acutely poisoned patients treated at the Specialist Hospital in Biala Podlaska (1993, 2001 and 2002)]. [Polish]
A1 - Chwaluk,P.
A1 - Mikusek,I.
A1 - Nosalska,D.
Y1 - 2004///
N1 - Chwaluk, Pawel. Mikusek, Ireneusz. Nosalska, Dorota
Przeglad lekarski
q96, 19840720r
IM
English Abstract. Journal Article
Polish
KW - MEDLINE
KW - Acute Disease
KW - Adolescent
KW - Antidotes/tu [Therapeutic Use]
KW - Charcoal/tu [Therapeutic Use]
KW - Child
KW - Preschool
KW - Decontamination/mt [Methods]
KW - Decontamination/sn [Statistics & Numerical Data]
KW - Ethanol/tu [Therapeutic Use]
KW - Female
KW - Gastric Lavage/sn [Statistics & Numerical Data]
KW - Humans
KW - Infant
KW - Male
KW - Poisoning/ep [Epidemiology]
KW - Poisoning/th [Therapy]
KW - Poland/ep [Epidemiology]
KW - Renal Dialysis/sn [Statistics & Numerical Data]
KW - Time Factors
RP - NOT IN FILE

SP - 256
EP - 260
JF - Przeglad Lekarski
JA - Przegl Lek
VL - 61
IS - 4
CY - Poland
N2 - Gut decontamination, the methods of extracorporeal elimination of poisons and antidote use are the specific measures in clinical toxicology. Statistically they are not often used in intoxicated patients. It is not known, in how many cases of poisoning treated outside toxicological units are they applied. Approximately 110 poisoned patients were hospitalised each year in the Specialist Hospital in Biala Podlaska in years 1993, 2001 and 2002. Gastric lavage was performed in about 50 percent of those cases, especially often in children up till third year of life and in the cases of poisoning with medical drugs. The Gut Decontamination Position Statement of AACT and EAPCCT, published in 1997, had no significant impact on use of decontamination procedures in our hospital. Haemodialyses were performed in 3-5% of poisoned patients. All those patients were poisoned with ethylene glycol or methanol. Only 4 specific antidotes were used in years 1993, 2001 and 2002. The most commonly used antidote was ethanol, despite its level is not measured in our laboratory. Recommendations for gut decontamination should be widely popularised. The use of activated charcoal may be in many cases more suitable as a gastric lavage. Safe and effective use of ethanol as an antidote in a hospital requires its measuring in own laboratory
SN - 0033-2240
AD - Oddzial Chorob Wewnetrznych, Wojewodzkiego Szpitala Specjalistycznego w Bialej Podlaskiej. pchwaluk@poczta.fm
UR - 15521578
ER -

TY - JOUR
ID - 1407
T1 - The Copenhagen Prospective Study on Asthma in Childhood (COPSAC): design, rationale, and baseline data from a longitudinal birth cohort study
A1 - Bisgaard,H.
Y1 - 2004/10//
N1 - Bisgaard, Hans
Annals of allergy, asthma & immunology : official publication of the American College of Allergy, Asthma, & Immunology
cbm, 9503580
IM
Journal Article. Research Support, Non-U.S. Gov't
English
KW - MEDLINE
KW - Asthma/di [Diagnosis]
KW - Asthma/et [Etiology]
KW - Asthma/ge [Genetics]
KW - Child
KW - Preschool
KW - Cohort Studies
KW - Dermatitis
KW - Atopic/di [Diagnosis]
KW - Environment
KW - Female
KW - Humans
KW - Hypersensitivity/di [Diagnosis]
KW - Infant
KW - Newborn
KW - Life Style
KW - Longitudinal Studies
KW - Male

KW - Pregnancy
KW - Prospective Studies
KW - Research Design
KW - Respiratory Function Tests
KW - Rhinitis/di [Diagnosis]
KW - Sweden
RP - NOT IN FILE
SP - 381
EP - 389
JF - Annals of Allergy, Asthma, & Immunology
JA - Ann Allergy Asthma Immunol
VL - 93
IS - 4
CY - United States
N2 - BACKGROUND: The atopic diseases asthma, atopic dermatitis, and allergic rhinitis are the most common chronic diseases in children, and their prevalence has increased recently in industrialized nations. Little is known about the genetic-environmental interaction factors driving such proliferation. OBJECTIVE: To investigate the relationships among genetic, environmental, and lifestyle factors in the development of atopic diseases in high-risk children with the aim of developing evidence-based prevention strategies. METHODS: The Copenhagen Prospective Study on Asthma in Childhood is a single-center, birth cohort study of children of asthmatic mothers. Objective assessments begin at birth, with scheduled visits every 6 months and when acute symptoms manifest. Clinical outcomes comprise preasthma, asthma, atopic dermatitis, allergic rhinitis, allergy, lung function, and bronchial responsiveness. Exposure assessments comprise respiratory, intestinal, and skin microbiology; the child's diet; indoor and outdoor air quality; allergens; and indicators of lifestyle. Genetic characteristics of probands and parents are evaluated. Quality assurance follows Good Clinical Practice guidelines. RESULTS: Four hundred eleven infants of asthmatic mothers were enrolled at the age of 1 month. The children were born between August 2, 1998, and December 28, 2001. Compared with the Copenhagen population, mothers of the cohort population were less likely to have given natural childbirth. The households were slightly less affluent, with fewer children and fewer pets. Whites may be overrepresented. At age 2 years, 93% of the infants were still actively participating in the cohort. CONCLUSIONS: This longitudinal birth cohort study of high-risk Danish infants consists of objective phenotyping, detailed information on exposure, high data quality, and a high participant retention rate
SN - 1081-1206
AD - Department of Pediatrics, Copenhagen University Hospital, Copenhagen, Denmark.
Bisgaarad@copsac.dk
UR - 15521375
ER -

TY - JOUR
ID - 1408
T1 - Injuries to the head among children enrolled in special education
A1 - Limbos,M.A.
A1 - Ramirez,M.
A1 - Park,L.S.
A1 - Peek-Asa,C.
A1 - Kraus,J.F.
Y1 - 2004/11//
N1 - Limbos, Mary Ann P. Ramirez, Marizen. Park, Laura S. Peek-Asa, Corinne. Kraus, Jess F
Archives of pediatrics & adolescent medicine
9422751, bwf
AIM, IM
Journal Article
English
KW - MEDLINE
KW - Adolescent
KW - Adult
KW - Child

KW - Preschool
KW - Craniocerebral Trauma/et [Etiology]
KW - Disabled Children
KW - Education
KW - Special
KW - Female
KW - Humans
KW - Incidence
KW - Los Angeles/ep [Epidemiology]
KW - Male
KW - Schools
KW - Wounds and Injuries/cl [Classification]
RP - NOT IN FILE
SP - 1057
EP - 1061
JF - Archives of Pediatrics & Adolescent Medicine
JA - Arch Pediatr Adolesc Med
VL - 158
IS - 11
CY - United States
N2 - BACKGROUND: Injuries to the head comprise 20% to 39% of all school-related injuries. Head injuries among special education students have not been adequately described. OBJECTIVES: (1) To examine the incidence and characteristics of head injuries in children enrolled in special education and (2) to determine the factors that increase the risk of sustaining a head injury compared with an injury to another part of the body. METHODS: Pupil Accident Reports for 6769 students enrolled in 17 of 18 special education schools in 1 large urban school district during the academic years 1994-1998 were reviewed, and information on the nature of injury, external cause, and activity was abstracted. Head-injured and nonhead-injured cases were identified and compared by race, sex, age, characteristics of injury, and disability category. RESULTS: Six hundred ninety-seven injury events were reported during the 4-year study period. The overall injury rate was 4.7 injuries per 100 student-years. Two hundred five children (29.4%) sustained injuries to the head, and the rate of head injury was 1.3 injuries per 100 student-years. Falls were the leading cause of injury. Head injuries were most commonly associated with physical education and unstructured play and usually occurred on the playground. Disproportionately more head than nonhead injuries were sustained in the classroom (12% vs 8%) and the bathroom (9% vs 3%). Compared with children with emotional/mental disabilities, children with multiple disabilities had the highest risk of a head injury (incidence density ratio, 2.4 [95% confidence interval, 1.6-3.5]), followed by children with physical disabilities (incidence density ratio, 1.8 [95% confidence interval, 1.1-3.1]). There appeared to be no significant difference in the rate of head injury by sex and age. CONCLUSIONS: Modifications of the classroom, bathroom, and playground environments might reduce the risk of head injuries in children enrolled in special education. Special modifications and increased supervision may, in particular, reduce the risk of head injury for children with physical and multiple disabilities
SN - 1072-4710
AD - Division of General Pediatrics, Childrens Hospital Los Angeles, Los Angeles, Calif, USA. mlimbos@chla.usc.edu
UR - 15520343
ER -

TY - JOUR
ID - 1409
T1 - Smoking at home is strongly associated with symptoms of asthma and rhinitis in children of primary school age in Trinidad and Tobago
A1 - Monteil,M.A.
A1 - Joseph,G.
A1 - Chang,Kit C.
A1 - Wheeler,G.
A1 - Antoine,R.M.
Y1 - 2004/09//

N1 - Monteil, Michele A. Joseph, Gina. Chang Kit, Catherine. Wheeler, Gillian. Antoine, Robin M
Revista panamericana de salud publica = Pan American journal of public health
csl, 9705400
IM
Journal Article. Research Support, Non-U.S. Gov't
English
KW - MEDLINE
KW - Adult
KW - Asthma/ep [Epidemiology]
KW - Asthma/et [Etiology]
KW - Child
KW - Cough
KW - Ethnic Groups
KW - Female
KW - Humans
KW - Male
KW - Prevalence
KW - Questionnaires
KW - Respiratory Sounds
KW - Rhinitis
KW - Allergic
KW - Perennial/ep [Epidemiology]
KW - Perennial/et [Etiology]
KW - Seasonal/ep [Epidemiology]
KW - Seasonal/et [Etiology]
KW - Sampling Studies
KW - Sinusitis/ep [Epidemiology]
KW - Tobacco Smoke Pollution/ae [Adverse Effects]
KW - Trinidad and Tobago/ep [Epidemiology]
RP - NOT IN FILE
SP - 193
EP - 198
JF - Pan American Journal of Public Health
JA - Rev Panam.Salud Publica
VL - 16
IS - 3
CY - United States
N2 - OBJECTIVE: To compare, in the twin-island republic of Trinidad and Tobago, the prevalence of symptoms of asthma and rhinitis among children of primary school age who are exposed to household environmental tobacco smoke with the prevalence of these symptoms in their colleagues without this exposure. METHODS: Between September and December 2002, questionnaires based on the instrument developed for the International Study of Asthma and Allergies in Childhood (ISAAC) were distributed, via the children in their schools, to parents of 6,611 Year 2 pupils (typically 6 years old) or Year 3 pupils (typically 7 years old) in 106 randomly selected schools in Trinidad and Tobago (5,511 pupils on Trinidad and 1,100 pupils on Tobago). We added to the standard ISAAC questionnaires two questions, one on household smoking and one on the ethnicity of the children. RESULTS: A total of 3 170 completed questionnaires were suitable for further analysis (2,618 from Trinidad and 552 from Tobago). On Trinidad 782 of the children (29.9%) lived in homes where one or both parents smoked, and 513 (19.6%) had other relatives in the household who smoked. On Tobago 94 of the pupils (17.0%) had parents who smoked, and 84 (15.4%) came from homes where other residents smoked. Parental smoking was significantly associated with wheezing (odds ratio (OR): 1.43; 95% confidence interval (CI): 1.11-1.83), exercise-induced wheezing (OR: 2.12; 95% CI: 1.59-2.82), nocturnal coughing (OR: 1.64; 95% CI: 1.37-1.97), and symptoms of rhinitis (OR: 1.35; 95% CI: 1.10-1.65) in the last 12 months as well as a history of hay fever/sinus problems (OR: 1.39; 95% CI: 1.11-1.74). Smoking in the home by adult residents other than parents was also significantly associated with all of these symptoms as well as a history of asthma (OR: 1.49; 95% CI: 1.13-1.97). In terms of ethnic differences, parental smoking was most prevalent in the homes of South Asian students, while smoking by other adults in the home occurred most commonly in the households of pupils of mixed race. CONCLUSIONS: Even in Trinidad and Tobago, which is a tropical

environment where more time is spent outdoors and homes have more open ventilation than in temperate climates, environmental tobacco smoke exposure is closely associated with an increased prevalence of symptoms of asthma and rhinitis in primary-school-aged children
SN - 1020-4989
AD - University of the West Indies, Faculty of Medical Sciences-St. Augustine Campus, Champs Fleurs, Trinidad. mmonteil@tstt.net.tt
UR - 15507187
ER -

TY - JOUR
ID - 1410
T1 - [Tuberculosis in the San Jose University Hospital in Popayan, Colombia, 1998-2000]. [Spanish]
A1 - Diaz,M.L.
A1 - Munoz,S.
A1 - Garciad,L.B.
Y1 - 2004/06//
N1 - Diaz, Maria Lilia. Munoz, Sulma. Garciad, Liz Betty
Biomedica : revista del Instituto Nacional de Salud
8205605
IM
English Abstract. Journal Article
Spanish
KW - MEDLINE
KW - Adolescent
KW - Adult
KW - Aged
KW - 80 and over
KW - Child
KW - Preschool
KW - Colombia
KW - Female
KW - Hospitals
KW - University
KW - Humans
KW - Infant
KW - Male
KW - Middle Aged
KW - Retrospective Studies
KW - Tuberculosis/di [Diagnosis]
KW - Tuberculosis/dt [Drug Therapy]
KW - Tuberculosis/ep [Epidemiology]
KW - Tuberculosis
RP - NOT IN FILE
SP - 92
EP - 101
JF - Biomedica
JA - Biomedica (Bogota)
VL - 24 Supp 1
CY - Colombia
N2 - TB is a public health problem in the world. In Colombia the Health 100 Law caused changes in prevention programs. The TB control program was compromised too. The actions in relation with active looking and examination of respiratory symptomatic people has been lowering. This retrospective study describes the clinical, epidemiological and medical care characteristics of tuberculosis patients attended in the Hospital Universitario San Jose de-Popayan. The clinical expedients and laboratory registers were investigated. 120 cases of 187 registered patients were included; finally only 89 were tuberculosis cases: 39 pulmonary (43.8%) and 50 extrapulmonary (56.2%). The extrapulmonary forms were: miliar, 39 patients; pleural, 4: of lymph nodes, 3; osteoarticular, 2, and meningitis, 2 cases. 49% of patients were 15-59 years old, 28% more than 59; males 64%

and 65% coming from country areas. Ziehl Neelsen (ZN) smear was positive in 22% of 59 sputums of pulmonary TB and 20.2% of 94 extrapulmonary TB samples: mycobacteria culture positive in 14.3% of 14 pulmonary TB sputum, and 29.6% of 54 extrapulmonary TB samples. The media days into hospital/yard was 4-26 and the media days to begin tuberculosis treatment/yard was 4-8. In summary there are important request for TB medical care to universitary hospital, however the diagnosis is difficult and takes a long time while mycobacteria can be transmitted to hospital contacts. It is necessary to get a better efficiency of diagnostic tests in the hospital and appropriated survey of the cases that begin treatment
SN - 0120-4157
AD - Facultad de Ciencias de la Salud, Universidad del Cauca, Popayan, Colombia. mdiaz@unicauca.edu.co
UR - 15495577
ER -

TY - JOUR
ID - 1411
T1 - Changing epidemiology of methicillin-resistant Staphylococcus aureus in Finland
A1 - Kerttula,A.M.
A1 - Lyytikainen,O.
A1 - Salmenlinna,S.
A1 - Vuopio-Varkila,J.
Y1 - 2004/10//
N1 - Kerttula, A-M. Lyytikainen, O. Salmenlinna, S. Vuopio-Varkila, J
The Journal of hospital infection
id6, 8007166
IM
Historical Article. Journal Article
English
KW - MEDLINE
KW - Adult
KW - Aged
KW - 80 and over
KW - Child
KW - Preschool
KW - Community-Acquired Infections/ep [Epidemiology]
KW - Community-Acquired Infections/mi [Microbiology]
KW - Community-Acquired Infections/pc [Prevention & Control]
KW - Female
KW - Finland/ep [Epidemiology]
KW - Health Services for the Aged/st [Standards]
KW - History
KW - 15th Century
KW - Homes for the Aged/sn [Statistics & Numerical Data]
KW - Humans
KW - Incidence
KW - Infant
KW - Newborn
KW - Infection Control/mt [Methods]
KW - Laboratories/sn [Statistics & Numerical Data]
KW - Male
KW - Methicillin Resistance
KW - Middle Aged
KW - Nursing Homes/sn [Statistics & Numerical Data]
KW - Outcome Assessment (Health Care)
KW - Questionnaires
KW - Registries
KW - Staphylococcal Infections/ep [Epidemiology]
KW - Staphylococcal Infections/mi [Microbiology]
KW - Staphylococcal Infections/pc [Prevention & Control]

KW - Staphylococcus aureus/cl [Classification]
KW - Staphylococcus aureus/ip [Isolation & Purification]
RP - NOT IN FILE
SP - 109
EP - 114
JF - Journal of Hospital Infection
JA - J Hosp Infect
VL - 58
IS - 2
CY - England
N2 - Data on methicillin-resistant Staphylococcus aureus (MRSA) cases notified to the National Infectious Disease Register (NIDR) and antibiotic resistance profiles of MRSA isolates sent to the national reference laboratory between 1997 and 2002 were analysed. In addition, the diagnostic methods used for MRSA identification in Finnish microbiology laboratories, the number of MRSA screening specimens studied, and the MRSA situation in long-term care facilities in 2001 were reviewed. MRSA cases notified to the NIDR rose from 120 in 1997 to 597 in 2002 (from 2.3 to 11.5 cases per 100,000 population). The increase was greatest in elderly people and outside Helsinki metropolitan area, in the districts where the proportion of non-multiresistant strains was most prominent. The National Committee for Clinical Laboratory Standard's guidelines for the oxacillin disk diffusion test were followed, except for the incubation temperature and time, which may have hindered detection of some MRSA strains. There was a wide geographic variation in the rates of MRSA, but this was not related to screening activity. MRSA isolates from long-term facilities accounted for more than half of the notifications to the NIDR in 2001
SN - 0195-6701
AD - Department of Microbiology, National Public Health Institute, Mannerheimintie 166, 00300 Helsinki, Finland. anne-marie.kerttula@ktl.fi
UR - 15474181
ER -

TY - JOUR
ID - 1412
T1 - Outdoor, indoor, and personal exposure to VOCs in children
A1 - Adgate,J.L.
A1 - Church,T.R.
A1 - Ryan,A.D.
A1 - Ramachandran,G.
A1 - Fredrickson,A.L.
A1 - Stock,T.H.
A1 - Morandi,M.T.
A1 - Sexton,K.
Y1 - 2004/10//
N1 - Adgate, John L. Church, Timothy R. Ryan, Andrew D. Ramachandran, Gurumurthy. Fredrickson, Ann L. Stock, Thomas H. Morandi, Maria T. Sexton, Ken
Environmental health perspectives
ei0, 0330411
IM
Journal Article. Research Support, Non-U.S. Gov't. Research Support, U.S. Gov't, Non-P.H.S.
English
KW - MEDLINE
KW - Air Pollutants/an [Analysis]
KW - Air Pollution
KW - Indoor/an [Analysis]
KW - Child
KW - Environmental Monitoring
KW - Ethnic Groups
KW - Female
KW - Humans
KW - Male

KW - Minnesota
KW - Organic Chemicals/an [Analysis]
KW - Risk Assessment
KW - Urban Population
KW - Volatilization
RP - NOT IN FILE
SP - 1386
EP - 1392
JF - Environmental Health Perspectives
JA - Environ Health Perspect
VL - 112
IS - 14
CY - United States
N2 - We measured volatile organic compound (VOC) exposures in multiple locations for a diverse population of children who attended two inner-city schools in Minneapolis, Minnesota. Fifteen common VOCs were measured at four locations: outdoors (O), indoors at school (S), indoors at home (H), and in personal samples (P). Concentrations of most VOCs followed the general pattern O approximately equal to S < P less than or equal to H across the measured microenvironments. The S and O environments had the smallest and H the largest influence on personal exposure to most compounds. A time-weighted model of P exposure using all measured microenvironments and time-activity data provided little additional explanatory power beyond that provided by using the H measurement alone. Although H and P concentrations of most VOCs measured in this study were similar to or lower than levels measured in recent personal monitoring studies of adults and children in the United States, p-dichlorobenzene was the notable exception to this pattern, with upper-bound exposures more than 100 times greater than those found in other studies of children. Median and upper-bound H and P exposures were well above health benchmarks for several compounds, so outdoor measurements likely underestimate long-term health risks from children's exposure to these compounds
SN - 0091-6765
AD - Division of Environmental Health Sciences, School of Public Health, University of Minnesota, 520 Delaware Street SE, Minneapolis, MN 55455, USA. jadgate@umn.edu
UR - 15471730
ER -

TY - JOUR
ID - 1413
T1 - Arsenic on the hands of children after playing in playgrounds
A1 - Kwon,E.
A1 - Zhang,H.
A1 - Wang,Z.
A1 - Jhangri,G.S.
A1 - Lu,X.
A1 - Fok,N.
A1 - Gabos,S.
A1 - Li,X.F.
A1 - Le,X.C.
Y1 - 2004/10//
N1 - Kwon, Elena. Zhang, Hongquan. Wang, Zhongwen. Jhangri, Gian S. Lu, Xiufen. Fok, Nelson. Gabos, Stephan. Li, Xing-Fang. Le, X Chris
Environmental health perspectives
ei0, 0330411
IM
Journal Article. Research Support, Non-U.S. Gov't
English
KW - MEDLINE
KW - Arsenates/an [Analysis]
KW - Arsenic/an [Analysis]
KW - Case-Control Studies
KW - Child

KW - Preschool
KW - Environmental Exposure
KW - Environmental Pollutants/an [Analysis]
KW - Female
KW - Hand
KW - Humans
KW - Male
KW - Play and Playthings
KW - Solubility
KW - Wood
RP - NOT IN FILE
SP - 1375
EP - 1380
JF - Environmental Health Perspectives
JA - Environ Health Perspect
VL - 112
IS - 14
CY - United States
N2 - Increasing concerns over the use of wood treated with chromated copper arsenate (CCA) in playground structures arise from potential exposure to arsenic of children playing in these playgrounds. Limited data from previous studies analyzing arsenic levels in sand samples collected from CCA playgrounds are inconsistent and cannot be directly translated to the amount of children's exposure to arsenic. The objective of this study was to determine the quantitative amounts of arsenic on the hands of children in contact with CCA-treated wood structures or sand in playgrounds. We compared arsenic levels on the hands of 66 children playing in eight CCA playgrounds with levels of arsenic found on the hands of 64 children playing in another eight playgrounds not constructed with CCA-treated wood. The children's age and duration of playtime were recorded at each playground. After play, children's hands were washed in a bag containing 150 mL of deionized water. Arsenic levels in the hand-washing water were quantified by inductively coupled plasma mass spectrometry. Our results show that the ages of the children sampled and the duration of play in the playgrounds were similar between the groups of CCA and non-CCA playgrounds. The mean amount of water-soluble arsenic on children's hands from CCA playgrounds was 0.50 microg (range, 0.0078-3.5 microg). This was significantly higher (p < 0.001) than the mean amount of water-soluble arsenic on children's hands from non-CCA playgrounds, which was 0.095 microg (range, 0.011-0.41 microg). There was no significant difference in the amount of sand on the children's hands and the concentration of arsenic in the sand between the CCA and non-CCA groups. The higher values of arsenic on the hands of children playing in the CCA playgrounds are probably due to direct contact with CCA-treated wood. Washing hands after play would reduce the levels of potential exposure because most of the arsenic on children's hands was washed off with water. The maximum amount of arsenic on children's hands from the entire group of study participants was < 4 microg, which is lower than the average daily intake of arsenic from water and food
SN - 0091-6765
AD - Department of Public Health Sciences, Faculty of Medicine, University of Alberta, Edmonton, Alberta, Canada T6G 2G3
UR - 15471728
ER -

TY - JOUR
ID - 1414
T1 - Impact of school nurse case management on students with asthma
A1 - Taras,H.
A1 - Wright,S.
A1 - Brennan,J.
A1 - Campana,J.
A1 - Lofgren,R.
Y1 - 2004/08//
N1 - Taras, Howard. Wright, Sandra. Brennan, Jesse. Campana, Jack. Lofgren, RoseMarie
The Journal of school health
k13, 0376370

IM, N
Journal Article
English
KW - MEDLINE
KW - Absenteeism
KW - Asthma/ep [Epidemiology]
KW - Asthma/nu [Nursing]
KW - California/ep [Epidemiology]
KW - Case Management
KW - Child
KW - Humans
KW - Nurses
KW - Nursing Records
KW - Prevalence
KW - School Nursing/ma [Manpower]
RP - NOT IN FILE
SP - 213
EP - 219
JF - Journal of School Health
JA - J Sch Health
VL - 74
IS - 6
CY - United States
N2 - This project determined asthma prevalence in a large school district, absentee rates, and potential effects of school nurse case management for student asthma over three years. Data were derived from an asthma tracking tool used by nurses in one school district for every student reported as having asthma by their parent. School nurses began collecting data in their schools in 1999-2000 when an asthma-management protocol was first developed. Nurses documented perceived asthma severity for each student, presence of medication and peak flow meters in school, and case management activities provided. This data base was cross matched with percentage of days students were absent for any illness. Prevalence of asthma, based on school nurse records of parent report, was between 5.1% to 6.2% during the three years. Between 13.5% and 15% were moderate or severe. Students with asthma were absent between one-half to one and one-quarter days more often than those without asthma. In year three, 39% of students with asthma had medication at school, and 12% had a peak flow meter. Contacting a parent was the nurse case management activity provided for the largest number of students (27% of students with asthma), followed by asthma education (16.5%), contact with physician (6%), and home visits (1%). Students who received at least one school nurse case management intervention were more likely the next year to have an asthma medication at school, to use a peak flow meter at school, and to have a change in asthma severity. School nurse case management activity had no association with student absences. Availability of medication and peak flow meters at school was low, suggesting standards of care for asthma were not followed. School nurse case management, when performed outside a project or intervention, offers a promising strategy to improve asthma management
SN - 0022-4391
AD - Community Pediatrics, University of California, San Diego, 9500 Gilman Drive, #0927, La Jolla, CA 92093-0927, USA. htaras@ucsd.edu
UR - 15468525
ER -

TY - JOUR
ID - 1415
T1 - Restoration of the orbital aesthetic subunit in complex midface defects
A1 - Chepeha,D.B.
A1 - Wang,S.J.
A1 - Marentette,L.J.
A1 - Bradford,C.R.
A1 - Boyd,C.M.
A1 - Prince,M.E.
A1 - Teknos,T.N.

Y1 - 2004/10//
N1 - Chepeha, Douglas B. Wang, Steven J. Marentette, Lawrence J. Bradford, Carol R. Boyd, Charles M. Prince, Mark E. Teknos, Theodoros N
The Laryngoscope
8607378, l1w
IM
Journal Article
English
KW - MEDLINE
KW - Adolescent
KW - Adult
KW - Aged
KW - Algorithms
KW - Child
KW - Female
KW - Humans
KW - Male
KW - Middle Aged
KW - Orbit Evisceration/ae [Adverse Effects]
KW - Orbital Neoplasms/su [Surgery]
KW - Reconstructive Surgical Procedures/mt [Methods]
KW - Retrospective Studies
KW - Surgical Flaps/bs [Blood Supply]
KW - Treatment Outcome
KW - Wounds and Injuries/et [Etiology]
KW - Wounds and Injuries/su [Surgery]
RP - NOT IN FILE
SP - 1706
EP - 1713
JF - Laryngoscope
VL - 114
IS - 10
CY - United States
N2 - OBJECTIVES/HYPOTHESIS: Although various options exist for restoration of the orbital defect in complex craniofacial resections, the aesthetic appearance and functional result of the orbit are optimized when the bony orbital architecture, orbital volume, and facial contour are specifically addressed. The study describes an approach using free tissue transfer for restoration of the native orbital aesthetic subunit. STUDY DESIGN: Retrospective case series. METHODS: Nineteen patients (male-to-female ratio, 14:5; mean age, 52 y [age range, 8-79 y]) in the study period between 1997 and 2001 had orbital defects that could be classified into one of the following categories: 1) orbital exenteration cavities only, 2) orbital exenteration cavities with resection of less than 30% of the bony orbital rim, or 3) radical orbital exenteration cavities with resection of overlying skin and bony malar eminence. Group 1 had reconstructions with fasciocutaneous forearm flaps; group 2, with osseocutaneous forearm flaps; and group 3, with osseocutaneous scapula flaps. RESULTS: Eighteen of 19 patients achieved a closed orbital reconstruction with restoration of the orbital aesthetic subunit. Among 16 patients with more than 4 months of follow-up, 10 patients had minimal or no resulting facial contour deformity and 8 patients engaged in social activities outside the home on a frequent basis. Five of the nine patients who were working before their surgery were able to return to work. CONCLUSION: Patients with complex midface defects involving the orbit can undergo free tissue transfer and have successful restoration of the native orbital aesthetic subunit without an orbital prosthesis
SN - 0023-852X
AD - Department of Otolaryngology, University of Michigan Health System, 1904 Taubman Health Center, 1500 East Medical Center Drive, Ann Arbor, MI 48109, U.S.A. Dchepeha@umich.edu
UR - 15454758
ER -

TY - JOUR
ID - 1416

T1 - [The outdoor environment of day care centers. Its importance to play and development]. [Danish]
A1 - Soderstrom,M.
A1 - Martensson,F.
A1 - Grahn,P.
A1 - Blennow,M.
Y1 - 2004/08/30/
N1 - Soderstrom, Margareta. Martensson, Fredrika. Grahn, Patrik. Blennow, Margareta
Ugeskrift for laeger
0141730, wm8
IM
Journal Article
Danish
KW - MEDLINE
KW - Child Day Care Centers
KW - Child
KW - Preschool
KW - Climate
KW - Environment
KW - Health Status
KW - Humans
KW - Infant
KW - Morbidity
KW - Play and Playthings
KW - Questionnaires
KW - Seasons
KW - Sweden/ep [Epidemiology]
RP - NOT IN FILE
SP - 3089
EP - 3092
JF - Ugeskrift for Laeger
JA - Ugeskr.Laeger
VL - 166
IS - 36
CY - Denmark
SN - 0041-5782
AD - Lunds universitet, Avdelningen for allmanmedicin. m.soderstrom@pubhealth.ku.dk
UR - 15387307
ER -

TY - JOUR
ID - 1417
T1 - Children's asthma experience and the importance of place
A1 - Rudestam,K.
A1 - Brown,P.
A1 - Zarcadoolas,C.
A1 - Mansell,C.
Y1 - 2004/10//
N1 - Rudestam, Kirsten. Brown, Phil. Zarcadoolas, Christina. Mansell, Catherine
Health (London, England : 1997)
9800465
IM
Journal Article. Research Support, Non-U.S. Gov't. Research Support, U.S. Gov't, Non-P.H.S.
English
KW - MEDLINE
KW - Asthma/px [Psychology]
KW - Child
KW - Environment

KW - Female
KW - Humans
KW - Male
KW - Motor Activity
KW - Poverty
KW - Quality of Life/px [Psychology]
KW - Self Concept
KW - Social Isolation/px [Psychology]
KW - Urban Population
RP - NOT IN FILE
SP - 423
EP - 444
JF - Health: an Interdisciplinary Journal for the Social Study of Health, Illness & Medicine
JA - HEALTH (LOND)
VL - 8
IS - 4
CY - England
N2 - Asthma is increasing dramatically among poor, inner-city children. Fourteen children with, and twelve without, asthma were interviewed and observed to examine how children with asthma understand their illness and how the asthma experience shapes their sense of place. Quantitative data showed no difference in objective preferences for particular places, but qualitative analysis showed important differences. We identified eight place-related themes, and on seven there were clear differences between children with and without asthma--offering freedom, being aesthetically pleasing, being comfortable, nature of social contact, being isolating, being familiar and being safe. Children experienced asthma as an interruption in daily life that influences time spent outside, being active and being with friends. Children with asthma are more sensitive to places that are 'dirty' or contain asthma triggers, are less likely to explore new or people-free places and emphasize family interactions, while children without asthma emphasize friendships and are sensitive to neighborhood violence
SN - 1363-4593
AD - Santa Barbara Community Environmental Council, USA
UR - 15358897
ER -

TY - JOUR
ID - 1418
T1 - School-based opportunities for physical activity in West Virginia public schools
A1 - O'Hara,Tompkins N.
A1 - Zizzi,S.
A1 - Zedosky,L.
A1 - Wright,J.
A1 - Vitullo,E.
Y1 - 2004/10//
N1 - O'Hara Tompkins, Nancy. Zizzi, Sam. Zedosky, Lenore. Wright, Jessica. Vitullo, Elizabeth
Preventive medicine
pm4, 0322116
IM
Journal Article
English
KW - MEDLINE
KW - Adolescent
KW - Child
KW - Exercise/ph [Physiology]
KW - Health Promotion/mt [Methods]
KW - Health Promotion/sn [Statistics & Numerical Data]
KW - Humans
KW - Questionnaires
KW - Schools/og [Organization & Administration]
KW - Schools/sn [Statistics & Numerical Data]

KW - West Virginia
RP - NOT IN FILE
SP - 834
EP - 840
JF - Preventive Medicine
JA - Prev Med
VL - 39
IS - 4
CY - United States
N2 - BACKGROUND: Schools have the unique potential to provide numerous opportunities for promoting physical activity. This article describes findings from a statewide survey of opportunities for physical activity in West Virginia (WV) schools. The purpose was to provide baseline data for two of the WV Healthy People 2010 objectives related to schools and youth to identify priorities for action. METHOD: Survey questions were adapted from the 2000 School Health Policies and Programs Study (SHPPS), conducted by CDC. Random stratified sampling across school level and size resulted in a final sample of 296 elementary schools, 146 middle and junior high schools, and 124 high schools (total = 566). The overall response rate was 73%. RESULTS: Eleven percent of elementary, 2% of middle or junior high, and 31% of senior high schools met the SHPP's criterion of providing daily physical education. Ninety-four percent of elementary schools reported offering daily recess. Overall, 42.3% of schools provided student and community access to indoor facilities outside of normal school hours, while 80.7% of schools provided access to outdoor facilities beyond normal school hours. CONCLUSIONS: Survey results are being used to target increased physical education in elementary schools and increased opportunities beyond physical education at all school levels
SN - 0091-7435
AD - Prevention Research Center, West Virginia University, Morgantown 26506, USA. NTompkins@hsc.wvu.edu
UR - 15351553
ER -

TY - JOUR
ID - 1419
T1 - Comparison of two programs to teach firearm injury prevention skills to 6- and 7-year-old children
A1 - Gatheridge,B.J.
A1 - Miltenberger,R.G.
A1 - Huneke,D.F.
A1 - Satterlund,M.J.
A1 - Mattern,A.R.
A1 - Johnson,B.M.
A1 - Flessner,C.A.
Y1 - 2004/09//
N1 - Gatheridge, Brian J. Miltenberger, Raymond G. Huneke, Daniel F. Satterlund, Melisa J. Mattern, Amanda R. Johnson, Brigette M. Flessner, Christopher A
Pediatrics
oxv, 0376422
AIM, IM
Clinical Trial. Comparative Study. Journal Article. Randomized Controlled Trial
English
KW - MEDLINE
KW - Child
KW - Child Behavior
KW - Female
KW - Firearms
KW - Humans
KW - Male
KW - Play and Playthings
KW - Role Playing
KW - Safety
KW - Teaching/mt [Methods]

KW - Wounds
KW - Gunshot/pc [Prevention & Control]
RP - NOT IN FILE
SP - e294
EP - e299
JF - Pediatrics
VL - 114
IS - 3
CY - United States
N2 - BACKGROUND: Each year, hundreds of children unintentionally kill or injure other children while playing with firearms in the United States. Although the numbers of these deaths and injuries are distressing, few prevention programs have been developed to prevent gun play among children. OBJECTIVE: This study compared the efficacy of 2 programs designed to prevent gun play among young children. DESIGN: A posttest-only, control group design with 2 treatment groups was used. Children were randomly assigned to 1 of 2 treatment groups or a control group. For all children who did not exhibit the skills after training, 1 in situ (ie, real-life situation) training session was conducted. SETTING: Participant recruitment, training sessions, and assessments were all conducted in the children's after-school program facility. PARTICIPANTS: Forty-five children, 6 or 7 years of age, were recruited for participation. INTERVENTIONS: The efficacy of the Eddie Eagle GunSafe Program, developed by the National Rifle Association, and a behavioral skills training program that emphasized the use of instruction, modeling, rehearsal, and feedback was evaluated. MAIN OUTCOME MEASURES: The criterion firearm safety behaviors included both motor and verbal responses, which were assessed in a naturalistic setting and then assigned a numerical value based on a scale of 0 to 3. RESULTS: Both programs were effective for teaching children to verbalize the safety skills message (don't touch the gun, get away, and tell an adult). However, children who received behavioral skills training were significantly more likely to demonstrate the desired safety skills in role-playing assessments and in situ assessments than were children who received Eddie Eagle program training. In addition, in situ training was found to be effective for teaching the desired safety skills for both groups of children. CONCLUSIONS: Injury prevention programs using education-based learning materials are less effective for teaching children the desired safety skills, compared with programs incorporating active learning approaches (eg, modeling, rehearsal, and feedback). The efficacy of both types of injury prevention programs for teaching the desired skills could be significantly enhanced with the use of in situ training. This program, when implemented with 6- and 7-year-old children, was effective in teaching the desired safety skills
SN - 1098-4275
AD - Department of Psychology, North Dakota State University, Fargo, ND 58105, USA
UR - 15342889
ER -

TY - JOUR
ID - 1420
T1 - A stair-climbing test for ambulatory assessment of children with cerebral palsy
A1 - Bar-Haim,S.
A1 - Belokopytov,M.
A1 - Harries,N.
A1 - Frank,A.
Y1 - 2004/10//
N1 - Bar-Haim, Simona. Belokopytov, Mark. Harries, Netta. Frank, Alexander
Gait & posture
9416830, dcm
IM
Journal Article
English
KW - MEDLINE
KW - Biomechanical Phenomena
KW - Cerebral Palsy/pp [Physiopathology]
KW - Child
KW - Preschool
KW - Female

KW - Heart Rate
KW - Humans
KW - Locomotion/ph [Physiology]
KW - Male
KW - Motor Activity/ph [Physiology]
KW - Oxygen Consumption/ph [Physiology]
RP - NOT IN FILE
SP - 183
EP - 188
JF - Gait & Posture
JA - Gait Posture
VL - 20
IS - 2
CY - Netherlands
N2 - The purpose of this study was to develop a stair-climbing test to measure energy cost (EC) and mechanical efficiency (ME) in children with cerebral palsy (CP) to evaluate ambulation-related motor function and its changes after intervention or maturation. Five normally developed (ND) and 10 children with CP were tested. The gross ME (MEg) was calculated from the work done (W) and the total energy cost (oxygen consumption) measured while repeatedly ascending and descending four steps for approximately 5 min without subtracting the resting metabolic rate. The MEg was significantly lower in CP than ND (3% versus 20%, P < 0.001). The test was repeated in the 10 children with CP after a 4-month therapy recess. The MEg values correlated with the initial tests, with a small, significant increase of 2%. When calculating net ME (MEn) from W and the energy cost above resting, the correlation of MEn values before and after therapy was inferior to that using MEg values. Similarly, individual ME values obtained by estimating energy cost from the increase in heart rate (HR) during stair-climbing also correlated poorly, with large variability. These results show that MEg may be used to evaluate changes in motor function resulting from age-related development or therapy. MEg is as good or superior to MEn; the extra time required to obtain resting energy cost and heart rate values is not necessary when measurements are desired within the same individual
SN - 0966-6362
AD - Faculty of Health Science, Ben-Gurion University, Beer-Sheba, Tzrifin 70300, Israel. adi-star@barak-online.net
UR - 15336289
ER -

TY - JOUR
ID - 1421
T1 - A potential natural treatment for attention-deficit/hyperactivity disorder: evidence from a national study
A1 - Kuo,F.E.
A1 - Taylor,A.F.
Y1 - 2004/09//
N1 - Kuo, Frances E. Taylor, Andrea Faber
American journal of public health
1254074, 3xw
AIM, IM
Journal Article. Research Support, U.S. Gov't, Non-P.H.S.
English
KW - MEDLINE
KW - Adolescent
KW - Attention Deficit Disorder with Hyperactivity/ep [Epidemiology]
KW - Attention Deficit Disorder with Hyperactivity/th [Therapy]
KW - Child
KW - Child Behavior/px [Psychology]
KW - Child Behavior
KW - Preschool
KW - Environment
KW - Female
KW - Humans

KW - Infant
KW - Male
KW - Parent-Child Relations
KW - Play and Playthings
KW - Questionnaires
KW - Sports
KW - Time Factors
KW - United States/ep [Epidemiology]
RP - NOT IN FILE
SP - 1580
EP - 1586
JF - American Journal of Public Health
JA - Am J Public Health
VL - 94
IS - 9
CY - United States
N2 - OBJECTIVES: We examined the impact of relatively "green" or natural settings on attention-deficit/hyperactivity disorder (ADHD) symptoms across diverse subpopulations of children. METHODS: Parents nationwide rated the aftereffects of 49 common after-school and weekend activities on children's symptoms. Aftereffects were compared for activities conducted in green outdoor settings versus those conducted in both built outdoor and indoor settings. RESULTS: In this national, nonprobability sample, green outdoor activities reduced symptoms significantly more than did activities conducted in other settings, even when activities were matched across settings. Findings were consistent across age, gender, and income groups; community types; geographic regions; and diagnoses. CONCLUSIONS: Green outdoor settings appear to reduce ADHD symptoms in children across a wide range of individual, residential, and case characteristics
SN - 0090-0036
AD - Department of Natural Resources and Environmental Sciences, University of Illinois at Urbana-Champaign, Urbana, IL 61801, USA. fekuo@uiuc.edu
UR - 15333318
ER -

TY - JOUR
ID - 1422
T1 - [Ticks and the pediatrician]. [Review] [0 refs] [Italian]
A1 - Occorsio,P.
A1 - Orso,G.
A1 - di,Martino L.
Y1 - 2004/06//
N1 - Occorsio, P. Orso, G. di Martino, L
Parassitologia
or7, 0413724
IM
English Abstract. Journal Article. Review
Italian
KW - MEDLINE
KW - Animals
KW - Arachnid Vectors/mi [Microbiology]
KW - Bites and Stings/ep [Epidemiology]
KW - Bites and Stings/th [Therapy]
KW - Child
KW - Preschool
KW - Dogs/ps [Parasitology]
KW - Female
KW - Humans
KW - Italy/ep [Epidemiology]
KW - Lyme Disease/di [Diagnosis]
KW - Lyme Disease/ep [Epidemiology]

KW - Lyme Disease/tm [Transmission]
KW - Male
KW - Pediatrics
KW - Tick Control
KW - Tick Infestations/co [Complications]
KW - Tick Infestations/th [Therapy]
KW - Tick-Borne Diseases/di [Diagnosis]
KW - Tick-Borne Diseases/ep [Epidemiology]
KW - Tick-Borne Diseases/pc [Prevention & Control]
KW - Ticks/mi [Microbiology]
KW - Ticks
RP - NOT IN FILE
SP - 115
EP - 118
JF - Parassitologia
VL - 46
IS - 1-2
CY - Italy
N2 - The match between ticks and pediatricians in Italy is usually a seasonal event related to the spring and summer trips and to the increasing of outdoor activity that sun and warm weather allow, both for children and ticks. So cared parents reach emergencies asking for tick removal but more often after the tick has yet been removed by empirical manoeuvres and after the killing and the destruction of the "enemy". We have scheduled, in the years 2002-2003, the 167 children that reached our unit for a tick bite; they where 92 males (mean age four years) and 75 females (mean age five years). Two of them had only a questionable tick bite but one had erythema on the lateral side of the left ankle, fever of obscure origin and weakness with generalised malaise. She was six years old and she lived with a dog on which, in more instances, ticks where found, but she never noticed ticks on her body. The dog had positive levels of anti rickettsial and anti borrelia antibodies but no evidence of conclamate illness. In this girl we found high titers of anti borrelia antibodies that WB testing confirmed to be anti B. burgdoferi. We diagnosed Lyme disease and treated the girl with amoxicillin (50 mg/kg/day) for three weeks achieving the complete relief of symptomatology till today. Three children with conclamate tick bite had positive levels of anti Rickettsia conorii antibodies with increasing levels at a twenty days control; two (aged four and five years) of them had a full symptomatology (tache noire, satellite lymphadenopathy, fever, and maculopapular erythema) for Meditarranean spotted fever; the latter (three years old) had an atypical form with the complete absence of maculopapular erythema. The therapy was clarithromycin (15 mg/kg/day) for three weeks. In Campania, in the years 2002/2003 on 167 children with tick bite that reached our unit, we found only four pathological correlable events; so are our ticks not infected? But we don't know how many children where tick bited and if there were other children with Lyme disease or Rickettsial disease because of the lack of a pediatric reference centre for "tick bite diseases" and the incomplete and late diffusion of knowledge on these diseases. Other Italian regions and such as Trentino Alto Adige, Veneto, and Friuli Venezia Giulia had a remarkable officially notified number of cases of Lyme disease and in Sicilia, Sardegna and Lazio there were numerous officially notified Rickettsial diseases. So we hope that Campania may be an happy island but we also think that a more effective cooperation between pediatricians and veterinarians and the creation of regional reference centres for tick bite diseases may arise a surveillance net to prevent the diffusion of these world wide emerging diseases and so that parents can achieve an univoque and useful information, the only way to defeat the fear of ignorance. [References: 0]
SN - 0048-2951
AD - A.O.R.N. Santobono-Pausilipon III Pediatria, P.O. Santobono Napoli
UR - 15305698
ER -

TY - JOUR
ID - 1423
T1 - Low frequency noise and stress: bronchitis and cortisol in children exposed chronically to traffic noise and exhaust fumes
A1 - Ising,H.
A1 - Lange-Asschenfeldt,H.
A1 - Moriske,H.J.

A1 - Born,J.
A1 - Eilts,M.
Y1 - 2004/04//
N1 - Ising, H. Lange-Asschenfeldt, H. Moriske, H-J. Born, J. Eilts, M
Noise & health
9815620
IM
Journal Article. Research Support, Non-U.S. Gov't
English
KW - MEDLINE
KW - Bronchitis/et [Etiology]
KW - Child
KW - Preschool
KW - Germany
KW - Humans
KW - Hydrocortisone/an [Analysis]
KW - Nitrogen Dioxide/an [Analysis]
KW - Noise/ae [Adverse Effects]
KW - Retrospective Studies
KW - Saliva/me [Metabolism]
KW - Vehicle Emissions/ae [Adverse Effects]
KW - Vehicle Emissions/an [Analysis]
RP - NOT IN FILE
SP - 21
EP - 28
JF - Noise & Health
JA - Noise Health
VL - 6
IS - 23
CY - England
N2 - A correlation of respiratory diseases to traffic related air pollution and noise was observed in an interview study. Since in that study the exposure was subjectively assessed, in the present field study nitrogen dioxide as indicator for vehicle exhausts and the mean night-time noise level were measured outside the children's windows in representative locations. Based on these measurements each child was placed in one of the following categories: low, medium or high traffic immission (ambient emissions). The physician contacts due to bronchitis of 68 children were assessed retrospectively from the files of the participating paediatricians. Saliva samples were collected from all children and the cortisol concentration was estimated. Children under high noise exposure (L(night, 8h) = 54-70dB(A)) had in comparison to all other children significantly increased morning saliva cortisol concentrations, indicating an activation of the hypothalamus-pituitary-adrenal (HPA) axis. Analysing a subgroup of children without high noise exposure showed, that children with frequent physician contacts due to bronchitis did not have increased morning saliva cortisol. However, multiple regression analysis with stepwise exclusion of variables showed that bronchitis was correlated more closely to morning salvia cortisol than to traffic immissions. On the other hand, the rate of physician contacts due to bronchitis increased in a dose dependent manner and significantly with increasing traffic immissions. From these results it can be concluded that high exposure to traffic noise, especially at nighttime, activates the HPA axis and this leads in the long term to an aggravation of bronchitis in children. This seems to be more important than the effect of exhaust fumes on bronchitis symptoms. The results of the present study should be subjected to further investigation using specially designed studies
SN - 1463-1741
AD - Berlin Centre for Public Health, Germany. hmising1@aol.com
UR - 15273021
ER -

TY - JOUR
ID - 1424
T1 - Environmental prevention in atopic eczema dermatitis syndrome (AEDS) and asthma: avoidance of indoor allergens. [Review] [112 refs]

A1 - Capristo,C.
A1 - Romei,I.
A1 - Boner,A.L.
Y1 - 2004/08//
N1 - Capristo, C. Romei, I. Boner, A L
Allergy
39c, 7804028
IM
Journal Article. Review
English
KW - MEDLINE
KW - Air Pollution
KW - Indoor/ae [Adverse Effects]
KW - Indoor
KW - Allergens
KW - Animals
KW - Asthma/di [Diagnosis]
KW - Asthma/et [Etiology]
KW - Asthma/pc [Prevention & Control]
KW - Child
KW - Dermatitis
KW - Atopic/di [Diagnosis]
KW - Atopic/et [Etiology]
KW - Atopic/pc [Prevention & Control]
KW - Humans
KW - Mites
KW - Risk Factors
KW - Skin Tests
RP - NOT IN FILE
SP - 53
EP - 60
JF - Allergy
VL - 59 Suppl 78
CY - Denmark
N2 - Indoor allergens represent an important precipitating factor for both asthma and atopic eczema dermatitis syndromes (AEDS). There is also accumulating evidence that sensitization to those allergens is associated with the onset of atopic disorders. Patients with AEDS present aeroallergen-specific T-cell responses associated with worsening of symptoms when exposed to specific aeroallergens. Furthermore, application of indoor allergens to the skin of patient with AEDS induces a local eczematous response in one-third of these patients. Exposure to high concentrations of mite allergens in early infancy have been demonstrated to be a risk factor for developing atopic dermatitis during the first 3 years of life. Moreover, a clear dose-response relationship has been documented between mite exposure and disease activity. Primary prevention of AEDS by avoiding indoor allergen exposure has been proved to be effective only when allergenic foods have also been avoided. Mite allergen avoidance in infants with AEDS and food allergy may however, prevent mite sensitization and the onset of asthma. Indoor allergen avoidance has been demonstrated to be effective in the majority of studies performed in patients with established AEDS. Negative results may be explained either by individual susceptibility variation, by long duration of disease with the consequent irreversible pathological changes in the target tissue or by exposure to allergens outside the house. Education of the patients and public consciousness of the problems are crucial for the efficacy of indoor allergen avoidance in allergic diseases. Copyright 2004 Blackwell Munksgaard [References: 112]
SN - 0105-4538
AD - Department of Pediatris Second University of Naples, Italy
UR - 15245359
ER -

TY - JOUR
ID - 1425

T1 - Risk of Giardia intestinalis infection in children from an artificially recharged groundwater area in Mexico City
A1 - Cifuentes,E.
A1 - Suarez,L.
A1 - Espinosa,M.
A1 - Juarez-Figueroa,L.
A1 - Martinez-Palomo,A.
Y1 - 2004/07//
N1 - Cifuentes, Enrique. Suarez, Leticia. Espinosa, Martha. Juarez-Figueroa, Luis. Martinez-Palomo, Adolfo
The American journal of tropical medicine and hygiene
3zq, 0370507
AIM, IM
Journal Article. Research Support, Non-U.S. Gov't
English
KW - MEDLINE
KW - Animals
KW - Child
KW - Preschool
KW - Female
KW - Giardia lamblia/ip [Isolation & Purification]
KW - Giardiasis/ep [Epidemiology]
KW - Humans
KW - Male
KW - Mexico/ep [Epidemiology]
KW - Rain
KW - Risk Assessment
KW - Seasons
KW - Soil Pollutants/ae [Adverse Effects]
KW - Urban Population
KW - Water/ps [Parasitology]
KW - Water Supply
RP - NOT IN FILE
SP - 65
EP - 70
JF - American Journal of Tropical Medicine & Hygiene
JA - Am J Trop Med Hyg
VL - 71
IS - 1
CY - United States
N2 - The objective of this study was to assess the risk of infection with Giardia intestinalis in children living in an area with artificial groundwater recharge and potable water reuse in Mexico City. Eligible wells and surrounding homesteads were defined by using a geographic information system. Five wells were tested for G. intestinalis cysts per 400 liters of water. A total of 750 eligible households were visited during two cross-sectional surveys. Stool samples were provided by 986 children in the rainy season study and 928 children during the dry season survey for parasitologic tests. Their guardians provided information on water, sanitation, hygiene, and socioeconomic variables. The prevalence rates of G. intestinalis infection were 9.4% in the rainy season and 4.4% in the dry season. Higher rates of infection were observed in older individuals (9.5% and 10.6%) and girls had a lower risk of infection than boys (odds ratio [OR] =0.55, 95% confidence interval [CI] = 0.34, 0.88 in the rainy season and OR = 0.47, 95% CI = 0.25, 0.90 in the dry season). During the wet season survey, a health risk was detected among those storing water in unprotected receptacles (OR = 4.00, 4.69, and 5.34 for those using uncovered jars, cisterns or tanks, and buckets, respectively), and bathing outside the dwelling, i.e., using a tap (OR = 1.93, 95% CI = 1.10, 3.39). A health risk was also detected among children from households with unsafe food hygiene practices (OR =2.41, 95% CI =1.10, 5.30) and those with no hand-washing habits (OR = 2.27, 95% CI = 1.00, 5.20). Groundwater reserves are at risk of fecal pollution, as indicated by the presence of G. intestinalis cysts. However, the endemic pattern of intestinal infection reflects low standards of personal hygiene and unsafe drinking water storage and food-related practices at household level. Prevention activities must address health education and environmental protection policies

SN - 0002-9637
AD - Instituto Nacional de Salud Publica CENSA/CISP, Av. Universidad 655, Santa Maria Ahuacatitlan, Cuernavaca, CP 62508 Mexico. ecifuent@correo.insp.mx
UR - 15238691
ER -

TY - JOUR
ID - 1426
T1 - An evaluation of radiation exposure from portal films taken during definitive course of pediatric radiotherapy.[Erratum appears in Int J Radiat Oncol Biol Phys. 2004 Sep 1;60(1):350]
A1 - Kudchadker,R.J.
A1 - Chang,E.L.
A1 - Bryan,F.
A1 - Maor,M.H.
A1 - Famiglietti,R.
Y1 - 2004/07/15/
N1 - Kudchadker, Rajat J. Chang, Eric L. Bryan, Fatima. Maor, Moshe H. Famiglietti, Robin
International journal of radiation oncology, biology, physics
g97, 7603616
IM
Journal Article
English
KW - MEDLINE
KW - Adolescent
KW - Child
KW - Preschool
KW - Humans
KW - Infant
KW - Neoplasms/ra [Radiography]
KW - Neoplasms/rt [Radiotherapy]
KW - Neoplasms
KW - Radiation-Induced/pc [Prevention & Control]
KW - Second Primary/pc [Prevention & Control]
KW - Radiation Dosage
KW - Retrospective Studies
RP - NOT IN FILE
SP - 1229
EP - 1235
JF - International Journal of Radiation Oncology, Biology, Physics
JA - Int J Radiat Oncol Biol Phys
VL - 59
IS - 4
CY - United States
N2 - PURPOSE: Recently, considerable attention has been directed toward computed tomography radiation doses (estimated 1 to 4 cGy) received by pediatric patients, because of the potential for increasing a pediatric patient's risk for developing a secondary malignancy. However, minimal attention has been given to the radiation exposure outside the treatment field resulting from the use of portal films to visualize surrounding anatomy. The objective of this study was to quantify the radiation dose from portal imaging delivered within and outside the radiation treatment field during a course of radiation therapy. METHODS AND MATERIALS: A retrospective review was conducted of the port film dose for 56 consecutive pediatric patients who underwent definitive radiation therapy between January 2001 and January 2002. Treatment locations were classified as brain, 27 patients; abdomen, 11 patients; extremities, 9 patients; pelvis, 6 patients; and thorax, 3 patients. Using the dose per monitor unit and total number of port films taken, the total port film dose for each patient was calculated. In addition, port film dose was quantified for 5 pediatric patients undergoing intensity modulated radiation therapy. RESULTS: The mean total port dose varied from a maximum of 46 cGy for brain to a minimum of 17 cGy for thorax. The mean total port dose as a percentage of prescribed dose was less than 1.25% for all locations in this study; however, most of the port dose is a result of the open-field dose from the double-

exposure technique. CONCLUSIONS: Care should be exercised while exposing port films of pediatric patients to minimize both the number of films and corresponding radiation exposure without compromising the quality of treatment delivery. Specifically, the number of monitor units used to image regions outside the treatment field should be kept to a minimum, because such exposure could lead to an increased risk of development of secondary neoplasms
SN - 0360-3016
AD - Department of Radiation Physics, Box 94, The University of Texas M.D. Anderson Cancer Center, 1515 Holcombe Boulevard, Houston, TX 77030, USA. rkudchad@mdanderson.org
UR - 15234060
ER -

TY - JOUR
ID - 1427
T1 - Photorefractive keratectomy for pediatric anisometropia: safety and impact on refractive error, visual acuity, and stereopsis
A1 - Paysse,E.A.
A1 - Hamill,M.B.
A1 - Hussein,M.A.
A1 - Koch,D.D.
Y1 - 2004/07//
N1 - Paysse, Evelyn A. Hamill, M Bowes. Hussein, Mohamed A W. Koch, Douglas D
American journal of ophthalmology
3oq, 0370500
AIM, IM
Journal Article. Research Support, Non-U.S. Gov't
English
KW - MEDLINE
KW - Amblyopia/pp [Physiopathology]
KW - Anisometropia/pp [Physiopathology]
KW - Anisometropia/su [Surgery]
KW - Child
KW - Preschool
KW - Corneal Topography
KW - Depth Perception/ph [Physiology]
KW - Female
KW - Humans
KW - Hyperopia/pp [Physiopathology]
KW - Lasers
KW - Excimer
KW - Male
KW - Myopia/pp [Physiopathology]
KW - Photorefractive Keratectomy
KW - Pilot Projects
KW - Prospective Studies
KW - Safety
KW - Visual Acuity/ph [Physiology]
RP - NOT IN FILE
SP - 70
EP - 78
JF - American Journal of Ophthalmology
JA - Am J Ophthalmol
VL - 138
IS - 1
CY - United States
N2 - PURPOSE: To establish the safety and possible efficacy of excimer laser photorefractive keratectomy (PRK) for treatment of pediatric anisometropia. DESIGN: Interventional case series METHODS: This is a prospective, noncomparative interventional case series at an individual university practice of photorefractive

keratectomy in 11 children aged 2 and 11 years with anisometropic amblyopia who were unable or unwilling to use contact lens, glasses, and occlusion therapy to treat the amblyopia. The eye with the higher refractive error was treated with PRK using a standard adult nomogram. The refractive treatment goal was to decrease the anisometropia to 3 diopters or less. Main outcome measures were cycloplegic refraction, refractive correction, degree of corneal haze, uncorrected and best spectacle-corrected visual acuity, and stereopsis over 12 months. RESULTS: All patients tolerated the procedure well. The mean refractive target reduction was -10.10 +/- 1.39 diopters for myopia and +4.75 +/- 0.50 diopters for hyperopia. The mean achieved refractive error reduction at 12 months for myopia was -10.56 +/- 3.00 diopters and for hyperopia was +4.08 +/- 0.8 diopters. Corneal haze at 12 months was minimal. Uncorrected visual acuity improved by 2 or more lines in 6 (75%) of the eight children able to perform psychophysical acuity tests. Best spectacle-corrected visual acuity improved by 2 lines in 3 (38%) of patients. Stereopsis improved in 3 (33%) of nine patients. CONCLUSIONS: Pediatric PRK can be safely performed for anisometropia. The refractive error response in children appears to be similar to that of adults with comparable refractive errors. Visual acuity and stereopsis improved despite several children being outside the standard age of visual plasticity. Photorefractive keratectomy may play a role in the management of anisometropia in selected pediatric patients
SN - 0002-9394
AD - Cullen Eye Institute, Baylor College of Medicine, Department of Ophthalmology, Texas Children's Hospital, 6621 Fannin Street, CC 640.00, Houston, Texas 77030, USA. epaysse@bcm.tmc.edu
UR - 15234284
ER -

TY - JOUR
ID - 1428
T1 - Hepatitis A in Hispanic children who live along the United States-Mexico border: the role of international travel and food-borne exposures
A1 - Weinberg,M.
A1 - Hopkins,J.
A1 - Farrington,L.
A1 - Gresham,L.
A1 - Ginsberg,M.
A1 - Bell,B.P.
Y1 - 2004/07//
N1 - Weinberg, Michelle. Hopkins, Jackie. Farrington, Leigh. Gresham, Louise. Ginsberg, Michele. Bell, Beth P
Pediatrics
oxv, 0376422
AIM, IM
Journal Article. Research Support, Non-U.S. Gov't
English
KW - MEDLINE
KW - Adolescent
KW - California/ep [Epidemiology]
KW - Case-Control Studies
KW - Child
KW - Preschool
KW - Female
KW - Food Microbiology
KW - Hepatitis A/eh [Ethnology]
KW - Hepatitis A/tm [Transmission]
KW - Hepatitis A Virus
KW - Human
KW - Humans
KW - Infant
KW - Male
KW - Mexican Americans
KW - Multivariate Analysis
KW - Risk Factors

KW - Travel
RP - NOT IN FILE
SP - e68
EP - e73
JF - Pediatrics
VL - 114
IS - 1
CY - United States
N2 - OBJECTIVES: Hispanic children who live along the United States-Mexico border historically have had among the highest hepatitis A rates in the United States, but risk factors have not been well characterized. The objective of this study was to examine risk factors associated with acute hepatitis A virus (HAV) infection in Hispanic children who live along the United States-Mexico border in San Diego County, California. METHODS: In this case-control study, hepatitis A cases among Hispanic children who were younger than 18 years reported from June 1998 through August 2000 were matched by age group and exposure period to Hispanic children who were susceptible to HAV infection. Participants and their families were interviewed about demographic information and potential sources of HAV infection, including attending child care, food and waterborne exposures, cross-border and other international travel, and travel-related activities. RESULTS: Participants included 132 children with hepatitis A and 354 control subjects. The median age of study participants was 7 years (range: 1-17). Sixty-seven percent of case-patients traveled outside the United States during the incubation period, compared with 25% of the children without hepatitis A (odds ratio [OR]: 6.3; 95% confidence interval [CI]: 4.0-9.7); all children, except 1, had traveled to Mexico. In multivariate analysis, hepatitis A was associated with having eaten food from a taco stand or street food vendor (adjusted OR: 17.0; 95% CI: 4.1-71.1) and having eaten salad/lettuce (adjusted OR: 5.2; 95% CI: 1.3-20.1) during travel. CONCLUSIONS: Hepatitis A among Hispanic children who live in an urban area of the United States-Mexico border is associated with cross-border travel to Mexico and food-borne exposures during travel. Travelers to areas where hepatitis A is endemic should receive hepatitis A vaccine before travel
SN - 1098-4275
AD - Division of Global Migration and Quarantine, Centers for Disease Control and Prevention, Atlanta, Georgia 30333, USA. mpw5@cdc.gov
UR - 15231975
ER -

TY - JOUR
ID - 1429
T1 - Portable lead analyzer to locate source of lead
A1 - Kuruvilla,A.
A1 - Pillay,V.V.
A1 - Venkatesh,T.
A1 - Adhikari,P.
A1 - Chakrapani,M.
A1 - Clark,C.S.
A1 - D'Souza,H.
A1 - Menezes,G.
A1 - Nayak,N.
A1 - Clark,R.
A1 - Sinha,S.
Y1 - 2004/06//
N1 - Kuruvilla, A. Pillay, V V. Venkatesh, T. Adhikari, P. Chakrapani, M. Clark, C S. D'Souza, H. Menezes, G. Nayak, N. Clark, R. Sinha, S
Indian journal of pediatrics
gkt, 0417442
IM
Journal Article. Research Support, Non-U.S. Gov't
English
KW - MEDLINE
KW - Child
KW - Environmental Exposure/an [Analysis]

KW - Environmental Monitoring/is [Instrumentation]
KW - Environmental Monitoring/mt [Methods]
KW - Fluorescence
KW - Humans
KW - India
KW - Lead/bl [Blood]
KW - Soil Pollutants/an [Analysis]
RP - NOT IN FILE
SP - 495
EP - 499
JF - Indian Journal of Pediatrics
JA - Indian J Pediatr
VL - 71
IS - 6
CY - India
N2 - OBJECTIVE: To investigate the sources of lead in the environment in children with elevated blood, with the help of a Field Portable X-Ray Fluorescence Analyzer. METHODS: One hundred and seven school children were chosen for this study on a random basis, from Mangalore and Karnataka. Their blood lead was analyzed. Of the cases analyzed, 10 students whose blood lead level was more than 40 microg/dl were investigated using a field portable X-Ray Fluorescence Analyzer. This is the first time such a device has been available for this purpose in India. RESULTS: The 'likely' source of lead exposure could be determined in eight cases which was from the immediate environment of the children like 'lead-based' paint on surfaces in the house, on playground and other exterior equipment; lead storage batteries, contaminated dust and soil and other lead-containing substances. CONCLUSION: The use of an X-Ray Fluorescence Analyser appeared to be useful in determining the source of lead
SN - 0019-5456
AD - Kasturba Medical College, Mangalore, India. ajeenellickal@hotmail.com
UR - 15226557
ER -

TY - JOUR
ID - 1430
T1 - [Patterns of behaviour of juvenile smokers and non-smokers]. [German]
A1 - Pruss,U.
A1 - Brandenburg,A.
A1 - von,Ferber C.
A1 - Lehmkuhl,G.
Y1 - 2004/05//
N1 - Pruss, Ulrike. Brandenburg, Alexander. von Ferber, Christian. Lehmkuhl, Gerd
Praxis der Kinderpsychologie und Kinderpsychiatrie
pj6, 0404246
IM
English Abstract. Journal Article. Multicenter Study. Research Support, Non-U.S. Gov't
German
KW - MEDLINE
KW - Adolescent
KW - Age Factors
KW - Aggression/px [Psychology]
KW - Antisocial Personality Disorder/di [Diagnosis]
KW - Antisocial Personality Disorder/ep [Epidemiology]
KW - Antisocial Personality Disorder/px [Psychology]
KW - Child
KW - Coal Mining
KW - Comorbidity
KW - Female
KW - Germany
KW - Health Education

KW - Health Knowledge
KW - Attitudes
KW - Practice
KW - Humans
KW - Juvenile Delinquency/px [Psychology]
KW - Juvenile Delinquency/sn [Statistics & Numerical Data]
KW - Male
KW - Personality Assessment/sn [Statistics & Numerical Data]
KW - Psychometrics
KW - Risk Factors
KW - Smoking/ep [Epidemiology]
KW - Smoking/pc [Prevention & Control]
KW - Smoking/px [Psychology]
KW - Social Environment
RP - NOT IN FILE
SP - 305
EP - 318
JF - Praxis der Kinderpsychologie und Kinderpsychiatrie
JA - Prax Kinderpsychol.Kinderpsychiatr.
VL - 53
IS - 5
CY - Germany
N2 - Preventing habitual smoking by young people forms an important goal of health promotion in schools. In achieving this target, information is needed about the age at which smoking becomes a temptation for young people as well as knowledge about the attitudes that are associated with smoking. At two elementary schools and at one grammar school in a community of a former coalmining area, 985 German pupils aged 11-18 years were interviewed with the Youth Self Report (YSR) together with a questionnaire concerning their smoking habits. The rate of smoking among the pupils rose from the age group 11-15 years to the age group 16-18 years (in this age access to cigarettes is no longer restricted by law) from 12% to 63%; between the sexes there is no difference in smoking habits. According to the scales of the YSR in both types of school, smokers scored significantly higher on scales of "antisocial" and „aggressive behaviour". On the other hand non-smokers scored significantly higher on the scale "social problems". The significantly higher rate of smokers in both elementary schools compared with the corresponding rate in the grammar school will be interpreted by two interrelated explanations: the influence of lower social class as well as the lack of the deferred gratification pattern as an ingredient of the culture in elementary schools. The difference between smoking and non-smoking juveniles according to the scales of the YSR can be explained by the association of smoking behaviour with the juvenile culture of deviant groups on the one hand and by the attitudes of the non-smokers, who are more reluctant in joining peer groups. The results support a strategy for effectively preventing habitual smoking habits of juveniles in schools by combining youth activities inside and outside the schools
SN - 0032-7034
AD - Forschungsgruppe Primarmedizinische Versorgung, Klinik und Poliklinik fur Psychiatrie und Psychotherapie des Kindes- und Jugendalters, Klinikum der Universitat zu Koln. ulrike.pruess@medizin.uni-koeln.de
UR - 15216962
ER -

TY - JOUR
ID - 1431
T1 - Modeling of occupant-generated CO2 dynamics in naturally ventilated classrooms
A1 - Bartlett,K.H.
A1 - Martinez,M.
A1 - Bert,J.
Y1 - 2004/03//
N1 - Bartlett, Karen H. Martinez, Mark. Bert, Joel
Journal of occupational and environmental hygiene
101189458
IM

Journal Article. Research Support, Non-U.S. Gov't
English
KW - MEDLINE
KW - Carbon Dioxide/an [Analysis]
KW - Child
KW - Environmental Monitoring
KW - Humans
KW - Models
KW - Theoretical
KW - Respiration
KW - Schools
KW - Ventilation
RP - NOT IN FILE
SP - 139
EP - 148
JF - Journal of Occupational & Environmental Hygiene
JA - J Occup Environ Hyg
VL - 1
IS - 3
CY - United States
N2 - A numerical method is presented to estimate the concentration of occupant-generated CO(2) for the (time-varying) occupancy typically found in nonforced ventilated elementary school classrooms. Here, the governing mass balance was solved numerically and compared to experimental measurements in order to estimate the respiration and (time-varying) infiltration rates. For the cases studied, we estimate an average CO(2) generation rate per child as 404 mg/min(-1). This is similar to estimates found in the literature for the age and activity level of elementary students, the classroom occupants. The average estimated infiltration rates were found to be larger than those measured from the decay of the tracer gas SF(6) under closed-door static conditions. The in-use infiltration rates were increased by additional air exchange due to people entering and leaving the room. In addition, we show that the difference (or error) between the instantaneous concentration of CO(2) and the time-averaged value recorded by a data-logging CO(2) monitor varies depending on the infiltration rate and sampling time. Therefore, the time interval selected for averaging may increase the overall error of the apparent CO(2) concentration. We conclude that the methods used to measure air exchange rates in naturally ventilated rooms underestimate the actual ventilation rate of a room under "in-use" conditions. However, even with the addition of uncontrolled outdoor air, the concentration of CO(2) in the classrooms studied was higher than recommended to meet air quality objectives
SN - 1545-9624
AD - School of Occupational and Environmental Hygiene, University of British Columbia, Vancouver, Canada. kbartlet@interchange.ubc.ca
UR - 15204871
ER -

TY - JOUR
ID - 1432
T1 - Auditory brainstem implant in posttraumatic cochlear nerve avulsion
A1 - Colletti,V.
A1 - Carner,M.
A1 - Miorelli,V.
A1 - Colletti,L.
A1 - Guida,M.
A1 - Fiorino,F.
Y1 - 2004/07//
N1 - Colletti, V. Carner, M. Miorelli, V. Colletti, L. Guida, M. Fiorino, F
Audiology & neuro-otology
cy0, 9606930
IM
Case Reports. Journal Article
English

KW - MEDLINE
KW - Adolescent
KW - Adult
KW - Aged
KW - Auditory Brain Stem Implants
KW - Child
KW - Preschool
KW - Cochlea/in [Injuries]
KW - Cochlear Nerve/in [Injuries]
KW - Craniocerebral Trauma/co [Complications]
KW - Evoked Potentials
KW - Auditory
KW - Brain Stem
KW - Female
KW - Hearing Loss
KW - Sensorineural/et [Etiology]
KW - Sensorineural/th [Therapy]
KW - Humans
KW - Infant
KW - Male
KW - Middle Aged
KW - Prosthesis Fitting
KW - Speech Perception
KW - Telemetry
KW - Tomography
KW - X-Ray Computed
KW - Treatment Outcome
RP - NOT IN FILE
SP - 247
EP - 255
JF - Audiology & Neuro-Otology
JA - Audiol Neurootol
VL - 9
IS - 4
CY - Switzerland
N2 - Patients aged over 12 years with neurofibromatosis type 2 are considered candidates for an auditory brainstem implant (ABI). This study extends the indication criteria of ABI to subjects with profound hearing loss due to damaged cochleas and/or cochlear nerves (CNs) following head injuries. In our department, over the period from April 1997 to November 2002, 32 patients, 23 adults and 9 children, were fitted with ABIs. Their ages ranged from 14 months to 70 years. These patients were suffering from a variety of tumor (13 subjects) and nontumor CN or cochlear diseases (19 subjects). Six patients, 5 adults and 1 child, had profound hearing loss following head injury. Their mean age was 25 years (range: 16-48 years). Five were male and 1 female. The retrosigmoid approach was used in all 6 patients. The electrode array was inserted into the lateral recess of the fourth ventricle and correct electrode positioning was monitored with the aid of electrically evoked auditory brainstem responses and neural response telemetry. Correct implantation was achieved in all patients. No complications were observed due to implantation surgery or related to ABI activation and stimulation of the cochlear nuclei. At activation, an average of 9.8 electrodes (range 5-13) were switched on without side effects. One to 6 electrodes were activated in the following sessions after time periods ranging from 2 to 16 months. All patients achieved auditory-alone-mode closed-set word recognition scores ranging from 40 to 100%; 3 had auditory-alone-mode open-set sentence recognition scores of 60-100%; 2 of these even had speech-tracking performance scores of 38 and 43 words, respectively, showing an ability to engage in normal conversation and converse over the phone. The present study demonstrates that the ABI is a useful rehabilitation instrument in subjects with damaged cochleas and/or CN avulsion following head injury who are unamenable or poorly responsive to auditory rehabilitation using cochlear implants. Copyright 2004 S. Karger AG, Basel
SN - 1420-3030
AD - ENT Department, University of Verona, Verona, Italy. vittoriocolletti@yahoo.com
UR - 15205552

ER -

TY - JOUR
ID - 1433
T1 - Relationship between illness-associated absence in day-care children and weather parameters
A1 - Sennerstam,R.B.
A1 - Moberg,K.
Y1 - 2004/07//
N1 - Sennerstam, R B. Moberg, K
Public health
qi7, 0376507
IM
Journal Article
English
KW - MEDLINE
KW - Absenteeism
KW - Child Day Care Centers/sn [Statistics & Numerical Data]
KW - Child
KW - Preschool
KW - Communicable Diseases/ep [Epidemiology]
KW - Communicable Diseases/tm [Transmission]
KW - Humans
KW - Infant
KW - Newborn
KW - Sweden/ep [Epidemiology]
KW - Weather
RP - NOT IN FILE
SP - 349
EP - 353
JF - Public Health
VL - 118
IS - 5
CY - England
N2 - This 5-year study demonstrated a statistically significant correlation between morbidity among children attending day-care centres and various weather parameters. The study was undertaken in south-central Sweden, where sharp seasonal contrasts in climatic and weather conditions occur. Illness-associated absence (IAA) decreased significantly when the weather was bright, sunny and warm, determined using average monthly weather parameters analysed over 60 months. This may be because good weather encourages outdoor activities which, in turn, reduce the risk of spreading respiratory tract infections as outdoor play areas are larger, body contact is less frequent, and any bacteria and viruses present will be readily dispersed. In multiple regression analysis, outdoor temperature was the most prominent parameter linked with IAA. This may reflect the behaviour of the staff with regard to various weather conditions. It is therefore to be recommended that children attending day-care centres should be allowed and encouraged to play outdoors when conditions allow
SN - 0033-3506
AD - Department of Women and Child Health, Astrid Lindgren's Child Hospital, Karolinska Institutet and Karolinska Hospital H2:03, 171 76 Stockholm, Sweden
UR - 15178142
ER -

TY - JOUR
ID - 1434
T1 - Comparative performance of playground surfacing materials including conditions of extreme non-compliance
A1 - Gunatilaka,A.H.
A1 - Sherker,S.
A1 - Ozanne-Smith,J.
Y1 - 2004/06//

N1 - Gunatilaka, A H. Sherker, S. Ozanne-Smith, J
Injury prevention : journal of the International Society for Child and Adolescent Injury Prevention
crz, 9510056
IM
Journal Article. Research Support, Non-U.S. Gov't
English
KW - MEDLINE
KW - Accident Prevention
KW - Child
KW - Construction Materials/st [Standards]
KW - Craniocerebral Trauma/pc [Prevention & Control]
KW - Environment Design/st [Standards]
KW - Guidelines as Topic
KW - Humans
KW - Mechanics
KW - Play and Playthings
KW - Risk Factors
KW - Rubber
RP - NOT IN FILE
SP - 174
EP - 179
JF - Injury Prevention
JA - Inj Prev
VL - 10
IS - 3
CY - England
N2 - OBJECTIVE: A recent case series study found that only 4.7% of 402 playgrounds in which arm fractures occurred in Victorian schools complied with the recommended 20 cm depth of tanbark. Tanbark depths at fall sites varied between 0-27 cm and the mean was 11.1 (5.0) cm. The purposes of the present study were to (1) measure impact attenuation properties of shallow and compacted depths of tanbark; (2) validate laboratory measurements with in situ data; (3) compare impact attenuation properties of compacted tanbark with an Australian manufactured rubber based surface material; and (4) study the impact performance of rubber and tanbark hybrid surfacing. METHODS: A standard test headform was dropped on tanbark and rubber surfaces in a laboratory setting to measure peak impact deceleration and head injury criterion (HIC) values. Variations in surface depth ranged from 2 cm-20 cm (tanbark) and 2 cm-9 cm (rubber). Drop height ranged from 0.5 m-2.5 m. RESULTS: Peak deceleration and HIC increased with increasing drop height and decreasing surface depth. Laboratory measurements at depths less than 8 cm overestimated peak deceleration and HIC values compared with in situ playground measurements. Impact attenuation of a 9 cm thick bilaminate rubber material was comparable to that of an 18 cm depth of compacted tanbark. Rubber-tanbark hybrid surfaces showed improved impact attenuation over individual surfaces. CONCLUSIONS: Compacted tanbark of depth less than 8 cm is ineffective in attenuating playground falls, resulting in excessive impact deceleration and HIC values. Shallow and compacted tanbark found in many Victorian school playgrounds poses a high risk for severe head injury. This calls for stricter enforcement of playground surface depth compliance
SN - 1353-8047
AD - Accident Research Centre, Monash University, Melbourne, Australia. gunatilaka@general.monash.edu.au
UR - 15178675
ER -

TY - JOUR
ID - 1435
T1 - Are current playground safety standards adequate for preventing arm fractures?.[Erratum appears in Med J Aust. 2004 Nov 15;181(10):532]
A1 - Sherker,S.
A1 - Ozanne-Smith,J.
Y1 - 2004/06/07/
N1 - Sherker, Shauna. Ozanne-Smith, Joan
The Medical journal of Australia

0400714, m26
IM
Journal Article. Research Support, Non-U.S. Gov't
English
KW - MEDLINE
KW - Accidental Falls/pc [Prevention & Control]
KW - Arm Injuries/pc [Prevention & Control]
KW - Child
KW - Female
KW - Fractures
KW - Bone/pc [Prevention & Control]
KW - Hospitalization/td [Trends]
KW - Humans
KW - Humeral Fractures/pc [Prevention & Control]
KW - Male
KW - Play and Playthings
KW - Radius Fractures/pc [Prevention & Control]
KW - Safety/st [Standards]
KW - Ulna Fractures/pc [Prevention & Control]
KW - Victoria
RP - NOT IN FILE
SP - 562
EP - 565
JF - Medical Journal of Australia
JA - Med J Aust
VL - 180
IS - 11
CY - Australia
N2 - OBJECTIVE: To assess compliance with current standards of playgrounds where children have sustained a fall-related arm fracture. DESIGN, SETTING AND PARTICIPANTS: Between October 2000 and December 2002, a consecutive prospective series of 402 children aged under 13 years who fell from playground equipment and sustained an arm fracture was identified by emergency department staff in five Victorian hospitals. Trained field testers measured playground equipment height, surface type and depth, and surface impact attenuation factors to determine compliance with safety standards. MAIN OUTCOME MEASURES: Playground compliance with current Australian safety standards. RESULTS: Ninety-eight percent of playgrounds had a recommended type of surface material. The mean surface depth was 11.1 cm (SD, 5.0 cm) and the mean equipment height was 2.04 m (SD, 0.43 m). Although over 85% of playgrounds complied with recommended maximum equipment height and surface impact attenuation characteristics, only 4.7% complied with recommended surface depth. CONCLUSION: Playgrounds where children have sustained an arm fracture generally comply with all important safety recommendations except surface depth. Playground fall-related arm fracture requires specific countermeasures for prevention, distinct from head injury prevention guidelines
SN - 0025-729X
AD - Accident Research Centre, Building 70, Monash University, Melbourne, VIC 3800, Australia. ShaunaSherker@yahoo.com.au
UR - 15174986
ER -

TY - JOUR
ID - 1436
T1 - Association of FEV1 in asthmatic children with personal and microenvironmental exposure to airborne particulate matter
A1 - Delfino,R.J.
A1 - Quintana,P.J.
A1 - Floro,J.
A1 - Gastanaga,V.M.
A1 - Samimi,B.S.
A1 - Kleinman,M.T.

A1 - Liu,L.J.
A1 - Bufalino,C.
A1 - Wu,C.F.
A1 - McLaren,C.E.
Y1 - 2004/06//
N1 - Delfino, Ralph J. Quintana, Penelope J E. Floro, Josh. Gastanaga, Victor M. Samimi, Behzad S. Kleinman, Michael T. Liu, L-J Sally. Bufalino, Charles. Wu, Chang-Fu. McLaren, Christine E
Environmental health perspectives
ei0, 0330411
IM
Journal Article. Research Support, U.S. Gov't, P.H.S.
English
KW - MEDLINE
KW - Adolescent
KW - Air Pollutants/po [Poisoning]
KW - Air Pollution
KW - Indoor/an [Analysis]
KW - Asthma/et [Etiology]
KW - Child
KW - Environment
KW - Environmental Exposure
KW - Female
KW - Forced Expiratory Volume
KW - Humans
KW - Male
KW - Models
KW - Theoretical
KW - Nitrogen Dioxide/po [Poisoning]
KW - Ozone/po [Poisoning]
KW - Particle Size
KW - Prospective Studies
KW - Sex Factors
RP - NOT IN FILE
SP - 932
EP - 941
JF - Environmental Health Perspectives
JA - Environ Health Perspect
VL - 112
IS - 8
CY - United States
N2 - Exposure to particulate matter (PM) air pollution has been shown to exacerbate children's asthma, but the exposure sources and temporal characteristics are still under study. Children's exposure to PM is likely to involve both combustion-related ambient PM and PM related to a child's activity in various indoor and outdoor microenvironments. Among 19 children with asthma, 9-17 years of age, we examined the relationship of temporal changes in percent predicted forced expiratory volume in 1 sec (FEV1) to personal continuous PM exposure and to 24-hr average gravimetric PM mass measured at home and central sites. Subjects were followed for 2 weeks during either the fall of 1999 or the spring of 2000, in a southern California region affected by transported air pollution. FEV(subscript)1(/subscript) was measured by subjects in the morning, afternoon, and evening. Exposure measurements included continuous PM using a passive nephelometer carried by subjects; indoor, outdoor home, and central-site 24-hr gravimetric PM2.5 (PM of aerodynamic diameter < 2.5 microm) and PM10; and central-site hourly PM10, nitrogen dioxide, and ozone. Data were analyzed with linear mixed models controlling for within-subject autocorrelation, FEV1 maneuver time, and exposure period. We found inverse associations of FEV1 with increasing PM exposure during the 24 hr before the FEV1 maneuver and with increasing multiday PM averages. Deficits in percent predicted FEV1 (95% confidence interval) for given PM interquartile ranges measured during the preceding 24-hr were as follows: 128 microg/m3 1-hr maximum personal PM, -6.0% (-10.5 to -1.4); 30 microg/m3 24-hr average personal PM, -5.9% (-10.8 to -1.0); 6.7 microg/m3 indoor home PM2.5, -1.6% (-2.8 to -0.4); 16 microg/m3 indoor home PM10, -2.1% (-3.7 to -0.4);

7.1 microg/m3 outdoor home PM2.5, -1.1% (-2.4 to 0.1); and 7.5 microg/m3 central-site PM2.5, -0.7% (-1.9 to 0.4). Stronger associations were found for multiday moving averages of PM for both personal and stationary-site PM. Stronger associations with personal PM were found in boys allergic to indoor allergens. FEV1 was weakly associated with NO2 but not with O3. Results suggest mixed respiratory effects of PM in asthmatic children from both ambient background exposures and personal exposures in various microenvironments
SN - 0091-6765
AD - Epidemiology Division, Department of Medicine, College of Medicine, University of California at Irvine, 224 Irvine hall, Irvine, CA 92697-7550, USA. rdelfino@uci.edu
UR - 15175185
ER -

TY - JOUR
ID - 1437
T1 - Early childhood computer experience and cognitive and motor development
A1 - Li,X.
A1 - Atkins,M.S.
Y1 - 2004/06//
N1 - Li, Xiaoming. Atkins, Melissa S
Pediatrics
oxv, 0376422
AIM, IM
Journal Article. Research Support, Non-U.S. Gov't
English
KW - MEDLINE
KW - Aptitude Tests
KW - Child Development
KW - Child
KW - Preschool
KW - Cognition
KW - Computer Literacy
KW - Female
KW - Humans
KW - Male
KW - Motor Skills
KW - Multivariate Analysis
KW - Sex Factors
KW - Social Class
KW - Software
RP - NOT IN FILE
SP - 1715
EP - 1722
JF - Pediatrics
VL - 113
IS - 6
CY - United States
N2 - OBJECTIVES: To explore the association between early computer experience (both accessibility and frequency of use) and cognitive and psychomotor development among young children. METHODS: The participants were 122 preschool children enrolled in a rural county Head Start program in the United States during 2001-2002. The following tests were administered to the children: the Bender Visual Motor Gestalt Test; the Boehm Test of Basic Concepts, Third Edition Preschool; the Test of Gross Motor Development, Second Edition; and a short form of the Wechsler Preschool and Primary Scales of Intelligence-Revised. Information pertaining to family characteristics and children's early computer experience was collected from parents. Both bivariate and multivariate analyses were used to assess the association between early computer experience and cognitive and motor development. RESULTS: Of the participating children, 53% had a computer at home. Among families who had a computer, 83% had children's software on the computer. According to parents' reports, 29% of these children played on the home computer on a daily basis, and an additional 44% of the children played on the computer at least weekly. Of those families who did not have a home computer, 49%

reported that their children had access to a computer somewhere outside home. Among these children, 10% had daily access to the computer and 33% had weekly access. The presence of a computer in the home was significantly associated with the family's income and the educational attainment of the parents. There was no gender difference in computer accessibility and frequency use among the participating children. Children who had access to a computer performed better on measures of school readiness and cognitive development, controlling for children's developmental stage and family socioeconomic status. The data in the current study did not suggest a relationship between computer experience and visual motor or gross motor skills among the participating children. CONCLUSION: The findings in the present study suggest that early computer exposure before or during the preschool years is associated with development of preschool concepts and cognition among young children. However, frequency of use did not reveal such a relationship; neither did the ownership of other child electronic or video games in the household
SN - 1098-4275
AD - Wayne State University Pediatric Prevention Research Center, Detroit, Michigan 48201, USA. xiaoming_li@wayne.edu
UR - 15173496
ER -

TY - JOUR
ID - 1438
T1 - Electrophysiological evidence of intuition: Part 2. A system-wide process?
A1 - McCraty,R.
A1 - Atkinson,M.
A1 - Bradley,R.T.
Y1 - 2004/04//
N1 - McCraty, Rollin. Atkinson, Mike. Bradley, Raymond Trevor
Journal of alternative and complementary medicine (New York, N.Y.)
9508124, cy7
IM
Clinical Trial. Journal Article. Randomized Controlled Trial
English
KW - MEDLINE
KW - Adolescent
KW - Adult
KW - Aged
KW - Arousal
KW - Brain/ph [Physiology]
KW - Child
KW - Consciousness
KW - Cross-Over Studies
KW - Electrocardiography
KW - Electroencephalography
KW - Evoked Potentials
KW - Visual/ph [Physiology]
KW - Visual
KW - Female
KW - Galvanic Skin Response
KW - Heart Rate
KW - Humans
KW - Intuition
KW - Male
KW - Middle Aged
KW - Pattern Recognition
KW - Photic Stimulation/mt [Methods]
KW - Reaction Time/ph [Physiology]
KW - Time Factors
RP - NOT IN FILE
SP - 325

EP - 336
JF - Journal of Alternative & Complementary Medicine
JA - J Altern Complement Med
VL - 10
IS - 2
CY - United States
N2 - OBJECTIVES: This study aims to contribute to a scientific understanding of intuition, a process by which information normally outside the range of conscious awareness is perceived by the body's psychophysiological systems. The first objective, presented in two empirical reports (Part 1 and Part 2), was to replicate and extend the results of previous experiments demonstrating that the body can respond to an emotionally arousing stimulus seconds before it is actually experienced. The second objective, to be presented in a forthcoming publication (Part 3), is to develop a theory that explains how the body receives and processes information involved in intuitive perception. DESIGN: The study used a counterbalanced crossover design, in which 30 calm and 15 emotionally arousing pictures were presented to 26 participants under two experimental conditions: a baseline condition of "normal" psychophysiologic function and a condition of physiological coherence. Primary measures included: skin conductance; the electroencephalogram (EEG), from which cortical event-related potentials (ERP) and heartbeatevoked potentials (HBEP) were derived; and the electrocardiogram (ECG), from which cardiac decelerations/ accelerations were derived. These measures were used to investigate where and when in the brain and body intuitive information is processed. RESULTS: The main findings presented here are: (1) surprisingly, both the heart and brain appear to receive and respond to intuitive information; (2) even more surprisingly, there is compelling evidence that the heart appears to receive intuitive information before the brain; (3) there were significant differences in prestimulus ERPs for calm versus emotional stimuli; (4) the frontal cortex, temporal, occipital, and parietal areas appear to be involved in the processing of prestimulus information; (5) there were significant differences in prestimulus calm/emotional HBEPs, primarily in the coherent mode; (6) there were significant gender differences in the processing of prestimulus information. Especially noteworthy is the apparent interaction between the HBEPs and ERPs in the females, which suggests that the heart modulates the ERP and that females are more attuned to intuitive information from the heart. CONCLUSIONS: Overall, our data suggest that the heart and brain, together, are involved in receiving, processing, and decoding intuitive information. On the basis of these results and those of other research, it would thus appear that intuitive perception is a system-wide process in which both the heart and brain (and possibly other bodily systems) play a critical role. To account for the study's results, Part 3 will develop a theory based on holographic principles explaining how intuitive perception accesses a field of energy into which information about "future" events is spectrally enfolded
SN - 1075-5535
AD - HeartMath Research Center, Institute of HeartMath, Boulder Creek, CA 95006, USA.
rollin@heartmath.org
UR - 15165413
ER -

TY - JOUR
ID - 1439
T1 - Exercise, stress, and inflammation in the growing child: from the bench to the playground. [Review] [86 refs]
A1 - Cooper,D.M.
A1 - Nemet,D.
A1 - Galassetti,P.
Y1 - 2004/06//
N1 - Cooper, Dan Michael. Nemet, Dan. Galassetti, Pietro
Current opinion in pediatrics
but, 9000850
IM
Journal Article. Review
English
KW - MEDLINE
KW - Age Factors
KW - Child
KW - Chronic Disease

KW - Exercise/ph [Physiology]
KW - Growth/ph [Physiology]
KW - Growth Hormone/bl [Blood]
KW - Humans
KW - Inflammation/bl [Blood]
KW - Insulin-Like Growth Factor I/me [Metabolism]
KW - Interleukin-6/bl [Blood]
KW - Oxidative Stress/ph [Physiology]
KW - Reactive Oxygen Species/me [Metabolism]
KW - Stress
KW - Physiological/bl [Blood]
KW - Physiological/im [Immunology]
KW - Time Factors
RP - NOT IN FILE
SP - 286
EP - 292
JF - Current Opinion in Pediatrics
JA - Curr Opin Pediatr
VL - 16
IS - 3
CY - United States
N2 - PURPOSE OF REVIEW: It is becoming increasingly clear that physical activity in children plays a critical role in growth and development, therapy for certain chronic diseases and disabilities, and in the pediatric origins of a variety of bone, metabolic, and cardiovascular diseases. New mechanistic insights have created the opportunity for a phase shift in understanding of the links between exercise and health in the context of the growing child. RECENT FINDINGS: Exercise even in healthy children profoundly alters stress, immune, and inflammatory mediators including peripheral blood mononuclear cells and circulating pro- and anti-inflammatory cytokines (like interleukin-6). Moreover, exercise even in healthy adults stimulates the production of reactive oxygen species (ROS) and mediators that attenuate them. Oxidative stress, in turn, alters growth and stress mediators. Both ROS and stress/inflammatory factors interact with powerful growth mediators like growth hormone and insulinlike growth factor-I. These findings suggest specific ways in which the balance between pro- and anti-inflammatory, catabolic, and anabolic factors associated with exercise can influence health and growth in children. SUMMARY: To address the current epidemic of physical inactivity and obesity in children and to optimize the therapeutic effects of exercise in children with disease and disability will require real changes in environments (eg, schools and playgrounds); innovative approaches to rehabilitation of children with chronic disease and disability; and enlightened training of child health professionals. Identifying novel exercise mechanisms involving stress, inflammation, and growth factors will help guide these efforts. [References: 86]
SN - 1040-8703
AD - Department of Pediatrics, Center for the Study of Health Effects of Exercise in Children, College of Medicine, University of California, Irvine, Orange, 92868, USA. dcooper@uci.edu
UR - 15167015
ER -

TY - JOUR
ID - 1440
T1 - Effects of a computer-based intervention program on the communicative functions of children with autism
A1 - Hetzroni,O.E.
A1 - Tannous,J.
Y1 - 2004/04//
N1 - Hetzroni, Orit E. Tannous, Juman
Journal of autism and developmental disorders
7904301, hgw
IM
Clinical Trial. Comparative Study. Journal Article
English
KW - MEDLINE

KW - Activities of Daily Living/px [Psychology]
KW - Arabs/eh [Ethnology]
KW - Autistic Disorder/px [Psychology]
KW - Autistic Disorder/th [Therapy]
KW - Child
KW - Communication
KW - Computers
KW - Echolalia/px [Psychology]
KW - Education
KW - Special/mt [Methods]
KW - Female
KW - Humans
KW - Israel
KW - Male
KW - Software
KW - Speech
KW - Statistics as Topic
KW - Treatment Outcome
RP - NOT IN FILE
SP - 95
EP - 113
JF - Journal of Autism & Developmental Disorders
JA - J Autism Dev Disord
VL - 34
IS - 2
CY - United States
N2 - This study investigated the use of computer-based intervention for enhancing communication functions of children with autism. The software program was developed based on daily life activities in the areas of play, food, and hygiene. The following variables were investigated: delayed echolalia, immediate echolalia, irrelevant speech, relevant speech, and communicative initiations. Multiple-baseline design across settings was used to examine the effects of the exposure of five children with autism to activities in a structured and controlled simulated environment on the communication manifested in their natural environment. Results indicated that after exposure to the simulations, all children produced fewer sentences with delayed and irrelevant speech. Most of the children engaged in fewer sentences involving immediate echolalia and increased the number of communication intentions and the amount of relevant speech they produced. Results indicated that after practicing in a controlled and structured setting that provided the children with opportunities to interact in play, food, and hygiene activities, the children were able to transfer their knowledge to the natural classroom environment. Implications and future research directions are discussed
SN - 0162-3257
AD - Special Education, Faculty of Education, University of Haifa, Mount Carmel, Haifa 31905, Israel. hetzroni@construct.haifa.ac.il
UR - 15162930
ER -

TY - JOUR
ID - 1441
T1 - Assessment of lead exposure and associated risk factors in urban children in Silesia, Poland
A1 - Jarosinska,D.
A1 - Peddada,S.
A1 - Rogan,W.J.
Y1 - 2004/06//
N1 - Jarosinska, Dorota. Peddada, Shyamal. Rogan, Walter J
Environmental research
ei2, 0147621
IM
Comparative Study. Journal Article. Research Support, Non-U.S. Gov't
English

KW - MEDLINE
KW - Air Pollutants/an [Analysis]
KW - Analysis of Variance
KW - Child
KW - Preschool
KW - Environmental Exposure
KW - Environmental Monitoring
KW - Humans
KW - Lead/an [Analysis]
KW - Lead/bl [Blood]
KW - Poland
KW - Questionnaires
KW - Regression Analysis
KW - Risk Factors
KW - Seasons
KW - Urban Health
RP - NOT IN FILE
SP - 133
EP - 142
JF - Environmental Research
JA - Environ Res
VL - 95
IS - 2
CY - United States
N2 - A program of childhood lead poisoning prevention was conducted in six cities of Silesia, the most industrialized region of Poland. We analyzed records of 11,877 children aged 24-84 months, tested between 1993 and 1998, to assess children's exposure to lead and associations of blood lead levels (BLL) with season of sampling, questionnaire data, and environmental levels of lead. Air lead concentrations and lead fallout, as measured in the ambient air monitoring system, were below current Polish air quality standards and gradually decreased. The geometric mean (GM) BLL was 6.3 microg/dL (range from 0.6 to 48 microg/dL), and >13% of children had BLL > or =10 microg/dL. Mean BLL declined from 6.8 microg/dL in 1993-1994 to almost 5.5 microg/dL in 1998. GM BLL in spring and summer ('nonheating' season) months were 10% higher than BLL in children tested in fall and winter ('heating' season), although air lead concentrations were lower in nonheating and higher in the heating season. For both the heating and nonheating seasons, there was a significant increase in the mean BLL with increasing air lead concentrations. Poor housing (odds ratio (OR): 1.82; 95% CI: 1.4, 2.3), two or more siblings in the family (OR: 3.12; 95% CI: 2.6, 3.7), lack of recreational trips outside the region (OR: 1.66; 95% CI: 1.47, 1.89), and the time child spent outdoors were associated with elevated BLL. Variables found to affect BLL in the Silesian children should be used to propose criteria to improve identification of children at risk and to focus prevention activities more effectively
SN - 0013-9351
AD - Institute of Occupational Medicine and Environmental Health, 13 Koscielna, 41-200 Sosnowiec, Poland. d-jarosinska@imp.sosnowiec.pl
UR - 15147918
ER -

TY - JOUR
ID - 1442
T1 - Physical activity as a preventive measure for coronary heart disease risk factors in early childhood
A1 - Saakslahti,A.
A1 - Numminen,P.
A1 - Varstala,V.
A1 - Helenius,H.
A1 - Tammi,A.
A1 - Viikari,J.
A1 - Valimaki,I.
Y1 - 2004/06//

N1 - Saakslahti, Arja. Numminen, Pirkko. Varstala, Vaino. Helenius, Hans. Tammi, Anne. Viikari, Jorma. Valimaki, Ilkka
Scandinavian journal of medicine & science in sports
9111504, cac
IM
Journal Article. Research Support, Non-U.S. Gov't
English
KW - MEDLINE
KW - Blood Pressure
KW - Child
KW - Preschool
KW - Coronary Disease/pc [Prevention & Control]
KW - Exercise
KW - Female
KW - Finland
KW - Growth
KW - Humans
KW - Lipids/bl [Blood]
KW - Male
KW - Risk Factors
RP - NOT IN FILE
SP - 143
EP - 149
JF - Scandinavian Journal of Medicine & Science in Sports
JA - Scand J Med Sci Sports
VL - 14
IS - 3
CY - Denmark
N2 - Physical activity causes acute physiological and long-term adaptive responses in the body. It is a protective factor for coronary heart disease (CHD) in adults. It has been assumed that children younger than 8 years of age may be in general active enough and there would be hardly any relationships between physical activity and CHD risk factors in early childhood. One hundred and fifty-five children (age 4-7 years) participated in this physical activity study during three consecutive years. Physical activity was examined twice a year with a special-purpose physical activity diary. CHD risk factors were measured during annual health care visits in the Specific Turku Coronary Risk-Factor Intervention Project (STRIP). We found that physical activity was related to CHD risk factors in early childhood. Among the girls, low-activity playing was related to a higher BMI. At the mean age of 6 years, high-activity playing was negatively related to serum total cholesterol (r=-0.32*) and positively to the high-density lipoprotein (HDL)/total cholesterol ratio (r=0.37**). The negative relationship between high-activity playing and triglycerides was highest (r=-0.32*) at the mean age of six. Among 4-year-old boys, playing outdoors correlated positively with serum HDL cholesterol concentration (r=0.29*) and the HDL/total ratio (r=0.35**). At the age of 5 years, physically active playing correlated positively with systolic blood pressure (r=0.25*). Playing outdoors and high-activity playing already have important health-maintaining effects in 4-7-year-old children. These positive effects differ between genders. (*P<0.05 **P<0.01)
SN - 0905-7188
AD - Department of Physical Education, University of Jyvaskyla, Jyvaskyla, Finland. saakslahti@norssi.jyu.fi
UR - 15144353
ER -

TY - JOUR
ID - 1443
T1 - Understanding variability in time spent in selected locations for 7-12-year old children
A1 - Xue,J.
A1 - McCurdy,T.
A1 - Spengler,J.
A1 - Ozkaynak,H.
Y1 - 2004/05//

N1 - Xue, Jianping. McCurdy, Thomas. Spengler, John. Ozkaynak, Haluk
Journal of exposure analysis and environmental epidemiology
bjn, 9111438
IM
Journal Article. Research Support, U.S. Gov't, P.H.S.
English
KW - MEDLINE
KW - Activities of Daily Living
KW - Child
KW - Cohort Studies
KW - Environmental Exposure
KW - Environmental Pollutants
KW - Female
KW - Humans
KW - Male
KW - Models
KW - Theoretical
KW - Movement
KW - Reproducibility of Results
RP - NOT IN FILE
SP - 222
EP - 233
JF - Journal of Exposure Analysis and Environmental Epidemiology
JA - J Expo Anal Environ Epidemiol
VL - 14
IS - 3
CY - England
N2 - This paper summarizes a series of analyses of clustered, sequential activity/location data collected by Harvard University for 160 children aged 7-12 years in Southern California (Geyh et al., 2000). The main purpose of the paper is to understand intra- and inter-variability in the time spent by the sample in the outdoor location, the location exhibiting the most variability of the ones evaluated. The data were analyzed using distribution-free hypothesis-testing (K-S tests of the distributions), generalized linear modeling techniques, and random-sampling schemes that produced "cohorts" whose descriptive statistical characteristics were evaluated against the original dataset. Most importantly, our analyses indicate that subdividing the population into appropriate cohorts better replicates parameters of the original data, including the interclass correlation coefficient (ICC), which is a relative measure of the intra- and inter-individual variability inherent in the original data. While the findings of our analyses are consistent with previous assessments of "time budget" and physical activity data, they are constrained by the rather homogeneous sample available to us. Owing to a general lack of longitudinal human activity/location data available for other age/gender cohorts, we are unable to generalize our findings to other population subgroups
SN - 1053-4245
AD - Human Exposure and Atmospheric Sciences Division, National Exposure Research Laboratory, US Environmental Protection Agency, Research Triangle Park, North Carolina, USA
UR - 15141151
ER -

TY - JOUR
ID - 1444
T1 - Paediatric open globe injuries. Visual outcome and risk factors for endophthalmitis
A1 - Narang,S.
A1 - Gupta,V.
A1 - Simalandhi,P.
A1 - Gupta,A.
A1 - Raj,S.
A1 - Dogra,M.R.
Y1 - 2004/03//

N1 - Narang, Subina. Gupta, Vishali. Simalandhi, Prabhat. Gupta, Amod. Raj, Srishti. Dogra, Mangat R
Indian journal of ophthalmology
gk4, 0405376
IM
Comparative Study. Journal Article
English
KW - MEDLINE
KW - Adolescent
KW - Child
KW - Preschool
KW - Endophthalmitis/ep [Epidemiology]
KW - Endophthalmitis/et [Etiology]
KW - Endophthalmitis/su [Surgery]
KW - Eye Injuries
KW - Penetrating/co [Complications]
KW - Penetrating/ep [Epidemiology]
KW - Penetrating/su [Surgery]
KW - Female
KW - Humans
KW - Incidence
KW - India/ep [Epidemiology]
KW - Male
KW - Retrospective Studies
KW - Risk Factors
KW - Treatment Outcome
KW - Visual Acuity
RP - NOT IN FILE
SP - 29
EP - 34
JF - Indian Journal of Ophthalmology
JA - Indian J Ophthalmol
VL - 52
IS - 1
CY - India
N2 - PURPOSE: To study the incidence of open globe injuries and the outcome in children, and to study the risk factors for post-traumatic endophthalmitis. METHODS: Paediatric patient population. Retrospective analysis of 72 consecutive cases of open globe injury over 3 years (January 1998 to December 2000). RESULTS: The cause of trauma was sports related (n = 18), home-made bow and arrow (n = 16), household kitchen injuries (n = 10), cracker injuries (n = 7) and other miscellaneous outdoor activities (n = 16). In 5 children the cause could not be ascertained. Visual acuity of > or = 3/60 in the injured eye at the last follow-up examination was recorded in 37 of 70 patients (52.86%) whose visual acuity could be tested. The final visual acuity was significantly poorer in eyes where primary repair was delayed beyond 24 hours of injury (P < 0.05). Post- traumatic endophthalmitis developed in 39 of 72 (54.16%) eyes. Bow and arrow and household injuries (P < 0.5) and eyes in which primary repair was delayed beyond 24 hours of injury (P < 0.01) had a higher risk of endophthalmitis in univariate analysis. In multivariate analysis delayed repair was the only significant risk factor for the occurrence of endophthalmitis (P = 0.014). CONCLUSION: Delayed repair, bow and arrow injuries and household injuries were associated with significantly higher risk of endophthalmitis. The incidence of endophthalmitis can be reduced by early referral of trauma cases and parental supervision
SN - 0301-4738
AD - Department of Ophthalmology, Post Graduate Institute of Medical Education and Research, Chandigarh, India
UR - 15132376
ER -

TY - JOUR
ID - 1445

T1 - Gross motor capability and performance of mobility in children with cerebral palsy: a comparison across home, school, and outdoors/community settings
A1 - Tieman,B.L.
A1 - Palisano,R.J.
A1 - Gracely,E.J.
A1 - Rosenbaum,P.L.
Y1 - 2004/05//
N1 - Tieman, Beth L. Palisano, Robert J. Gracely, Edward J. Rosenbaum, Peter L
Physical therapy
0022623, p6w
AIM, IM
Journal Article. Research Support, Non-U.S. Gov't. Research Support, U.S. Gov't, P.H.S.
English
KW - MEDLINE
KW - Cerebral Palsy/pp [Physiopathology]
KW - Child
KW - Disability Evaluation
KW - Environment
KW - Female
KW - Humans
KW - Male
KW - Motor Activity/ph [Physiology]
KW - Motor Skills/ph [Physiology]
KW - Questionnaires
RP - NOT IN FILE
SP - 419
EP - 429
JF - Physical Therapy
JA - Phys Ther
VL - 84
IS - 5
CY - United States
N2 - BACKGROUND AND PURPOSE: Mobility of children with cerebral palsy (CP) has generally been examined in terms of capability (what a child can do) in a controlled environment, rather than performance (what a child does do) in everyday settings. The purpose of this study was to compare gross motor capability and performance across environmental settings in children with CP. SUBJECTS: The subjects were 307 children with CP, aged 6 to 12 years, who were randomly selected across Ontario, Canada. METHODS: Children were grouped by capability (the highest of 3 items achieved on the Gross Motor Function Measure). Performance was measured via a parent-completed questionnaire on usual mobility methods in the home, at school, and in the outdoors or community. RESULTS: There were statistically significant differences in performance across settings for children in all capability groups. Children who were capable of crawling performed crawling more at home than at school or in the outdoors or community. Children who were capable of walking with support performed walking with support more at school than in the outdoors or community. Children who were capable of walking alone performed walking alone more at home than at school or in the outdoors or community, and more at school than in the outdoors or community. DISCUSSION AND CONCLUSION: The results provide evidence that children with CP with similar capability demonstrate differences in performance across settings. The results suggest that physical therapists should examine performance in the settings that are important to the child's daily life
SN - 0031-9023
AD - Department of Physical Therapy, Georgia State University, MSC 8L0388, 33 Gilmer St SE, Unit 8, Atlanta, GA 30303-3088, USA. btieman@gsu.edu
UR - 15113275
ER -

TY - JOUR
ID - 1446

T1 - Online pediatric information seeking among mothers of young children: results from a qualitative study using focus groups
A1 - Bernhardt,J.M.
A1 - Felter,E.M.
Y1 - 2004/03/01/
N1 - Bernhardt, Jay M. Felter, Elizabeth M
Journal of medical Internet research
100959882
IM
Evaluation Studies. Journal Article
English
KW - MEDLINE
KW - Adult
KW - Child
KW - Preschool
KW - Female
KW - Focus Groups/mt [Methods]
KW - Humans
KW - Information Services/sn [Statistics & Numerical Data]
KW - Information Services/td [Trends]
KW - Internet/sn [Statistics & Numerical Data]
KW - Internet/td [Trends]
KW - Mothers/sn [Statistics & Numerical Data]
KW - Professional-Family Relations
KW - Research Design
RP - NOT IN FILE
SP - e7
JF - Journal of Medical Internet Research
JA - J Med Internet Res
VL - 6
IS - 1
CY - United States
N2 - BACKGROUND: Pre-natal and post-natal periods are times when many women actively seek health information from multiple sources, including the Internet. However, little is known about how pregnant women and mothers of young children seek and process online pediatric health information. OBJECTIVE: To explore why and where mothers of young children look for online health information and how they determine if the information they receive is trustworthy. METHODS: Focus groups were conducted in a Southeastern US city to provide an in-depth exploration of web-related behaviors and beliefs among mothers who work inside and outside of the home. Data from the focus groups were coded using deductive and inductive coding schemes and content was analyzed for the existence of themes. RESULTS: Twenty mothers of young children participated in four focus groups. Most participants sought information on the Internet during pregnancy and nearly all sought online information after their child was born, primarily to diagnose or treat pediatric conditions and to seek advice on parenting and development. Participants mainly used commercial information websites for health information and many expressed disdain for commercial product websites. Many also expressed concerns about the reliability of health information on the web and described strategies for determining how much they trust each website. CONCLUSIONS: Women appear to be high information seekers during pregnancy and the first few years following delivery, and this period represents an important window of time for providing online health information. Participants suggested that online information sources and motives for providing online information should be clear in order to increase perceptions of trust. Participants expressed preference for online clinical health information that is presented by clinical professionals, and online parenting advice that is presented from other parents
SN - 1438-8871
AD - Department of Behavioral Sciences and Health Education, Rollins School of Public Health, Emory University, 1518 Clifton Road NE, Atlanta, GA 30322, USA. jbernha@sph.emory.edu
UR - 15111273
ER -

TY - JOUR
ID - 1447
T1 - Effect of physical and mental activity on blood pressure
A1 - Thakor,H.G.
A1 - Kumar,P.
A1 - Desai,V.K.
Y1 - 2004/04//
N1 - Thakor, Hitendrasinh G. Kumar, Predeep. Desai, Vikas K
Indian journal of pediatrics
gkt, 0417442
IM
Journal Article. Multicenter Study
English
KW - MEDLINE
KW - Adolescent
KW - Blood Pressure/ph [Physiology]
KW - Body Constitution/ph [Physiology]
KW - Body Mass Index
KW - Child
KW - Cross-Sectional Studies
KW - Exercise/ph [Physiology]
KW - Female
KW - Humans
KW - Male
KW - Mental Health
KW - Random Allocation
KW - Sex Factors
RP - NOT IN FILE
SP - 307
EP - 312
JF - Indian Journal of Pediatrics
JA - Indian J Pediatr
VL - 71
IS - 4
CY - India
N2 - OBJECTIVES: The authors measured and recorded the physical (outdoor playing) and mental (home study) activity behavior and blood pressure amongst school going children and observed their association with the blood pressure levels. METHOD: It was a cross sectional school-based study from 12 primary schools; selected by stratified random sampling. Participants included 2250 children aged 10 years and above. Explanatory variables were age, sex, outdoor playing (hours/ day) and home-study (hours/ day). Outcome variables were mean and standard deviation (SD) of systolic and diastolic blood pressures in various activity groups. Statistical tools used were proportions, correlation co-efficient (r), coefficient of determination (r2) and Regression analysis. RESULTS: 47.8% boys and 2.5 % girls were engaged in outdoor playing for more than two hours. No trend was observed for systolic blood pressure (SBP) in both the sexes in various activity groups of outdoor playing. Both mean diagnostic blood pressure (DBP) and mean BMI showed decreasing trend in boys. Mean BMI had an inverse relationship with outdoor playing in girls. Correlation of age and weight with both SBP and DBP was highly significant in both the sexes and in all the age groups except that of the 15-year age. Both SBP and DBP were not associated with home study in all sex and age groups except the age group of 10 and 12 years. Both SBP and DBP were significantly associated with the outdoor playing taking the whole sample as one group, but not in different sex or age groups; except that the SBP was significantly correlated with outdoor playing in 10 to 13 years age groups in boys. CONCLUSION: Boys spent twice the time in outdoor playing than girls. They were spending more time on outdoor playing than on home study. Conversely girls spent more time in home study. A weak negative correlation was observed between outdoor playing and blood pressures. Less time spent in physical activities by the girls may have contributed to their higher BMI. They need behavior change communication for involvement in physical activities
SN - 0019-5456
AD - Community Medicine Department, Government Medical College, Surat, Gujarat, India

UR - 15107510
ER -

TY - JOUR
ID - 1448
T1 - Associations between specific serum IgE response and 6 variants within the genes IL4, IL13, and IL4RA in German children: the German Multicenter Atopy Study
A1 - Liu,X.
A1 - Beaty,T.H.
A1 - Deindl,P.
A1 - Huang,S.K.
A1 - Lau,S.
A1 - Sommerfeld,C.
A1 - Fallin,M.D.
A1 - Kao,W.H.
A1 - Wahn,U.
A1 - Nickel,R.
Y1 - 2004/03//
N1 - Liu, Xin. Beaty, Terri H. Deindl, Philipp. Huang, Shau-Ku. Lau, Susanne. Sommerfeld, Christine. Fallin, M Daniele. Kao, W H Linda. Wahn, Ulrich. Nickel, Renate
The Journal of allergy and clinical immunology
h53, 1275002
AIM, IM
Journal Article. Multicenter Study. Research Support, Non-U.S. Gov't. Research Support, U.S. Gov't, P.H.S.
English
KW - MEDLINE
KW - Allergens
KW - Animals
KW - Case-Control Studies
KW - Cats
KW - Child
KW - Preschool
KW - Female
KW - Food Hypersensitivity/ge [Genetics]
KW - Food Hypersensitivity/im [Immunology]
KW - Genetic Variation
KW - Germany
KW - Humans
KW - Hypersensitivity
KW - Immediate/ge [Genetics]
KW - Immediate/im [Immunology]
KW - Immunoglobulin E/bl [Blood]
KW - Infant
KW - Interleukin-13/ge [Genetics]
KW - Interleukin-4/ge [Genetics]
KW - Male
KW - Mites/im [Immunology]
KW - Receptors
RP - NOT IN FILE
SP - 489
EP - 495
JF - Journal of Allergy & Clinical Immunology
JA - J Allergy Clin Immunol
VL - 113
IS - 3
CY - United States

N2 - BACKGROUND: Among many published studies of specific IgE response or atopy, only a few showed positive marginal effects for 6 potentially functional single nucleotide polymorphisms (SNPs; C-590T in the IL4 gene, C-1055T and Arg130Gln in the IL13 gene, and Ile50Val, Ser478Pro, and Gln551Arg in the IL4RA gene). SNPs were commonly considered individually, and therefore the true effect could be masked by other genes or environmental factors. OBJECTIVE: We tested the relationship between these 6 SNPs and sensitization to food, mite, cat, and outdoor allergens in unrelated German children drawn from the Multicenter Atopy Study. Gene-gene and gene-environment interactions were also evaluated. METHODS: Multiple logistic regression models were used for the analyses of 4 sensitization outcomes. RESULTS: The variant C-1055T was significantly associated with increased risk of sensitization to food and outdoor allergens, with odds ratios of 3.49 (95% CI, 1.52-8.02) and 2.27 (95% CI, 1.04-4.94), respectively. The effects of the TT genotype on food sensitization appear to depend on variants in the IL4RA gene, in which marginally significant interaction terms were observed. Significant evidence supported an interaction between exposure to maternal smoking and variant Gln551Arg on risk of cat sensitization. In addition, we found that the effect of variant C-590T on sensitization to mite depended on Der p 1 allergen levels in carpet dust samples. CONCLUSIONS: These findings not only suggested that variants in the IL4, IL13, and IL4RA genes play an important role in controlling specific IgE response but also strengthened our understanding of gene-gene and gene-environment interaction on the development of specific sensitization in this study population
SN - 0091-6749
AD - Department of Epidemiology, Johns Hopkins Bloomberg School of Public Health, Baltimore, MD, USA
UR - 15007352
ER -

TY - JOUR
ID - 1449
T1 - Fluoroquinolone-resistant Campylobacter infections: eating poultry outside of the home and foreign travel are risk factors
A1 - Kassenborg,H.D.
A1 - Smith,K.E.
A1 - Vugia,D.J.
A1 - Rabatsky-Ehr,T.
A1 - Bates,M.R.
A1 - Carter,M.A.
A1 - Dumas,N.B.
A1 - Cassidy,M.P.
A1 - Marano,N.
A1 - Tauxe,R.V.
A1 - Angulo,F.J.
A1 - Emerging Infections Program FoodNet Working Group.
Y1 - 2004/04/15/
N1 - Kassenborg, Heidi D. Smith, Kirk E. Vugia, Duc J. Rabatsky-Ehr, Therese. Bates, Martha R. Carter, Michael A. Dumas, Nellie B. Cassidy, Maureen P. Marano, Nina. Tauxe, Robert V. Angulo, Frederick J. Emerging Infections Program FoodNet Working Group
Clinical infectious diseases : an official publication of the Infectious Diseases Society of America
a4j, 9203213
IM
Journal Article. Research Support, U.S. Gov't, Non-P.H.S.. Research Support, U.S. Gov't, P.H.S.
English
KW - MEDLINE
KW - Adolescent
KW - Adult
KW - Aged
KW - 80 and over
KW - Animals
KW - Campylobacter/de [Drug Effects]
KW - Campylobacter Infections/ep [Epidemiology]
KW - Child
KW - Preschool

KW - Drug Resistance
KW - Bacterial
KW - Fluoroquinolones/pd [Pharmacology]
KW - Humans
KW - Infant
KW - Information Services
KW - Microbial Sensitivity Tests
KW - Middle Aged
KW - Population Surveillance
KW - Poultry/mi [Microbiology]
KW - Risk Factors
KW - Travel
KW - United States/ep [Epidemiology]
RP - NOT IN FILE
SP - S279
EP - S284
JF - Clinical Infectious Diseases
JA - Clin Infect Dis
VL - 38 Suppl 3
CY - United States
N2 - A 12-month, population-based, case-control study of Campylobacter infections was conducted at Foodborne Disease Active Surveillance Network surveillance areas during 1998-1999. Of 858 Campylobacter isolates tested for antimicrobial susceptibility to the fluoroquinolone ciprofloxacin, 94 (11%) were resistant. Travel outside of the United States was reported by 27 (42%) of 64 patients with fluoroquinolone-resistant Campylobacter infection and by 51 (9%) of 582 patients with fluoroquinolone-susceptible Campylobacter infection (odds ratio [OR], 7.6; 95% confidence interval [CI], 4.3-13.4). When patients with domestically acquired fluoroquinolone-resistant Campylobacter infection were compared with matched healthy control subjects in a multivariate analysis, those infected were 10 times more likely to have eaten chicken or turkey cooked at a commercial establishment (18 [55%] of 33 case patients vs. 7 [21%] of 33 controls; matched OR, 10.0; 95% CI, 1.3-78). Although travel outside of the United States was associated with fluoroquinolone-resistant Campylobacter infection, most infections among study participants were domestically acquired. This study provides additional evidence that poultry is an important source of domestically acquired fluoroquinolone-resistant Campylobacter infection. Control measures should include efforts to improve food handling in commercial establishments
SN - 1537-6591
AD - Minnesota Department of Health, Minneapolis, MN, USA. heidi.kassenborg@state.mn.us
UR - 15095200
ER -

TY - JOUR
ID - 1450
T1 - Egg consumption is the principal risk factor for sporadic Salmonella serotype Heidelberg infections: a case-control study in FoodNet sites
A1 - Hennessy,T.W.
A1 - Cheng,L.H.
A1 - Kassenborg,H.
A1 - Ahuja,S.D.
A1 - Mohle-Boetani,J.
A1 - Marcus,R.
A1 - Shiferaw,B.
A1 - Angulo,F.J.
A1 - Emerging Infections Program FoodNet Working Group.
Y1 - 2004/04/15/
N1 - Hennessy, Thomas W. Cheng, Lay Har. Kassenborg, Heidi. Ahuja, Shama D. Mohle-Boetani, Janet. Marcus, Ruthanne. Shiferaw, Beletshachew. Angulo, Frederick J. Emerging Infections Program FoodNet Working Group
Clinical infectious diseases : an official publication of the Infectious Diseases Society of America

a4j, 9203213
IM
Journal Article. Research Support, U.S. Gov't, Non-P.H.S.. Research Support, U.S. Gov't, P.H.S.
English
KW - MEDLINE
KW - Adolescent
KW - Adult
KW - Case-Control Studies
KW - Child
KW - Preschool
KW - Disease Outbreaks
KW - Female
KW - Food Microbiology
KW - Humans
KW - Infant
KW - Information Services
KW - Male
KW - Middle Aged
KW - Ovum/mi [Microbiology]
KW - Risk Factors
KW - Salmonella Food Poisoning/ep [Epidemiology]
KW - Salmonella Infections/ep [Epidemiology]
KW - Serotyping
KW - United States/ep [Epidemiology]
RP - NOT IN FILE
SP - S237
EP - S243
JF - Clinical Infectious Diseases
JA - Clin Infect Dis
VL - 38 Suppl 3
CY - United States
N2 - To determine risk factors for sporadic Salmonella serotype Heidelberg diarrheal disease, we conducted a population-based case-control study in 5 Foodborne Diseases Active Surveillance Network (FoodNet) surveillance areas in 1996-1997. Forty-four case patients and 83 control subjects matched by age and telephone exchange were asked about exposures during the 5-day period before onset of illness in the case patient. Risk factors for infection were evaluated using conditional logistic regression analysis. Eating eggs prepared outside the home remained the only significant risk factor for illness (matched odds ratio [MOR], 6.0; 95% confidence interval [CI], 1.2-29.6). The population-attributable fraction of S. Heidelberg infections associated with eating eggs prepared outside the home was 37%. Eliminating the risk associated with out-of-home egg consumption could substantially reduce the incidence of S. Heidelberg infections. Control measures to prevent S. Heidelberg infection should include advising consumers to avoid eating undercooked eggs and educating food handlers about proper egg handling and cooking
SN - 1537-6591
AD - Foodborne and Diarrheal Diseases Branch, Centers for Disease Control and Prevention, Atlanta, Georgia 30333, USA. tbh0@cdc.gov
UR - 15095195
ER -

TY - JOUR
ID - 1451
T1 - Policy issues and risk-benefit trade-offs of 'safer surfacing' for children's playgrounds
A1 - Ball,D.J.
Y1 - 2004/07//
N1 - Ball, David J
Accident; analysis and prevention
acs, 1254476
IM

Journal Article. Research Support, Non-U.S. Gov't
English
KW - MEDLINE
KW - Adolescent
KW - Child
KW - Preschool
KW - Construction Materials
KW - Environment Design
KW - Great Britain/ep [Epidemiology]
KW - Health Policy
KW - Humans
KW - Infant
KW - Models
KW - Theoretical
KW - Play and Playthings
KW - Risk Assessment
KW - Risk Management
KW - Wounds and Injuries/mo [Mortality]
KW - Wounds and Injuries/pc [Prevention & Control]
RP - NOT IN FILE
SP - 661
EP - 670
JF - Accident Analysis & Prevention
JA - Accid Anal Prev
VL - 36
IS - 4
CY - England
N2 - The case for the universal application of 'safer surfacing' in playgrounds is assessed in terms of absolute risk, cost-benefit, and qualitative factors, and found to be open to question on each front. In parallel, it is noted that members of the child welfare and play communities are increasingly of the view that playgrounds are losing their appeal for children, which in turn has its own health, safety and developmental consequences. In part, this loss of attractiveness may have linkages with the recent concern over play safety and the imposition of measures such as 'safer surfacing'. It is proposed that the drive for play safety, which has existed for some 20 years, should be succeeded by a more holistic approach which, rather than considering play safety in isolation, acknowledges the importance of all attributes of play including child safety both on and off playgrounds, potential beneficial aspects of childhood risk exposure, adventure, and play value
SN - 0001-4575
AD - Centre for Decision Analysis and Risk Management, School of Health and Social Sciences, Middlesex University, Queensway, Enfield, Middlesex EN3 4SF, UK. d.ball@mdx.ac.uk
UR - 15094421
ER -

TY - JOUR
ID - 1452
T1 - Measuring parent attributes and supervision behaviors relevant to child injury risk: examining the usefulness of questionnaire measures
A1 - Morrongiello,B.A.
A1 - House,K.
Y1 - 2004/04//
N1 - Morrongiello, B A. House, K
Injury prevention : journal of the International Society for Child and Adolescent Injury Prevention
crz, 9510056
IM
Journal Article. Research Support, Non-U.S. Gov't
English
KW - MEDLINE
KW - Accident Prevention

KW - Attitude to Health
KW - Child Behavior
KW - Child Care/mt [Methods]
KW - Child
KW - Preschool
KW - Female
KW - Humans
KW - Male
KW - Parent-Child Relations
KW - Parenting
KW - Parents/px [Psychology]
KW - Parents
KW - Play and Playthings
KW - Questionnaires
KW - Risk Factors
KW - Safety
KW - Self-Assessment
KW - Wounds and Injuries/et [Etiology]
KW - Wounds and Injuries/pc [Prevention & Control]
RP - NOT IN FILE
SP - 114
EP - 118
JF - Injury Prevention
JA - Inj Prev
VL - 10
IS - 2
CY - England
N2 - OBJECTIVE: This study aimed to identify self report questionnaire measures of parent attributes and behaviors that have relevance for understanding injury risk among children 2-5 years of age, and test a new Parent Supervision Attributes Profile Questionnaire (PSAPQ) that was developed to measure aspects of protectiveness and parent supervision. METHODS: Naturalistic observations were conducted of parents' supervision of children on playgrounds, with questionnaires subsequently completed by the parent to measure parent education, family income, parent personality attributes, attributes relevant to parent supervision, and beliefs about parents' control over the child's health status. These measures were then related to children's risk taking and injury history. RESULTS: Visual supervision, auditory supervision, and physical proximity were highly intercorrelated, indicating that parents employed all types of behaviors in service of supervision, rather than relying predominantly on one type of supervisory behavior. Physical proximity was the only aspect of supervision behavior that served a protective function and related to children's risk taking behaviors: parents who remained close to their children had children who engaged in less risk taking. On questionnaires, parents who reported more conscientiousness, protectiveness, worry about safety, vigilance in supervision, confidence in their ability to keep their child safe, and belief in control over their child's health had children who showed less risk taking and/or experienced fewer injuries. The new PSAPQ measure was associated with specific aspects of supervision as well as children's risk taking and injury history. CONCLUSIONS: This study reveals several parent attributes and behaviors with relevance for child injury risk that can be measured via self report questionnaires, including the new PSAPQ
SN - 1353-8047
AD - Psychology Department, University of Guelph, Guelph, Ontario, Canada. bmorrong@uoguelph.ca
UR - 15066978
ER -

TY - JOUR
ID - 1453
T1 - Parental report of outdoor playtime as a measure of physical activity in preschool-aged children
A1 - Burdette,H.L.
A1 - Whitaker,R.C.
A1 - Daniels,S.R.
Y1 - 2004/04//

N1 - Burdette, Hillary L. Whitaker, Robert C. Daniels, Stephen R
Archives of pediatrics & adolescent medicine
9422751, bwf
AIM, IM
Comparative Study. Journal Article. Research Support, U.S. Gov't, P.H.S.. Validation Studies
English
KW - MEDLINE
KW - Child
KW - Preschool
KW - Female
KW - Humans
KW - Male
KW - Monitoring
KW - Physiologic/is [Instrumentation]
KW - Physiologic/mt [Methods]
KW - Motor Activity/ph [Physiology]
KW - Parents/px [Psychology]
KW - Play and Playthings
KW - Questionnaires
KW - Seasons
KW - Time Factors
RP - NOT IN FILE
SP - 353
EP - 357
JF - Archives of Pediatrics & Adolescent Medicine
JA - Arch Pediatr Adolesc Med
VL - 158
IS - 4
CY - United States
N2 - BACKGROUND: Keeping young children physically active is an important strategy to promote their health and well-being. To our knowledge, survey measures of physical activity in preschool-aged children are unavailable. Time spent playing outdoors is a potential surrogate measure of physical activity in preschoolers, but parental-report measures of outdoor playtime have not been evaluated. OBJECTIVE: To compare a direct measure of physical activity in preschool-aged children with 2 parental-report measures of children's outdoor playtime. MAIN OUTCOME MEASURES: Three days of recording with a 3-dimensional accelerometer were used to directly measure physical activity in 250 preschool-aged children. We calculated each child's average vector magnitude per minute while awake. Parental report of outdoor playtime was measured in 2 ways: (1). the score from a checklist used to record outdoor playtime over 3 days and (2). a recall of the usual minutes of daily outdoor playtime during the prior month. We calculated Spearman rank correlation coefficients among these 3 measures. RESULTS: The mean age of the children was 44 months, 87.7% were white, and 12.3% were black. Parents reported that their children spent a mean (+/-SD) of 146 (+/-113) minutes playing outdoors each day. Physical activity as measured by the accelerometer was significantly correlated to the time spent playing outdoors, as measured by the checklist (r = 0.33, P <.001) and recall (r = 0.20, P =.003). CONCLUSION: Parental-report measures of outdoor playtime were significantly correlated to a direct measure of physical activity in preschool-aged children, and are worthy of future evaluation as a survey measure
SN - 1072-4710
AD - Divisions of General and Community Pediatrics and Cardiology, Cincinnati Children's Hospital Medical Center, Philadelphia, PA 19104, USA. burdette@email.chop.edu
UR - 15066875
ER -

TY - JOUR
ID - 1454
T1 - Outdoor/Indoor/Personal ozone exposures of children in Nashville, Tennessee
A1 - Lee,K.
A1 - Parkhurst,W.J.
A1 - Xue,J.

A1 - Ozkaynak,A.H.
A1 - Neuberg,D.
A1 - Spengler,J.D.
Y1 - 2004/03//
N1 - Lee, Kiyoung. Parkhurst, William J. Xue, Jianping. Ozkaynak, A Haluk. Neuberg, Donna. Spengler, John D
Journal of the Air & Waste Management Association (1995)
9503111, cbi
IM
Evaluation Studies. Journal Article. Research Support, Non-U.S. Gov't. Research Support, U.S. Gov't, Non-P.H.S.
English
KW - MEDLINE
KW - Air Pollutants/an [Analysis]
KW - Air Pollution
KW - Indoor/an [Analysis]
KW - Child
KW - Child Behavior
KW - Environmental Exposure
KW - Environmental Monitoring/mt [Methods]
KW - Female
KW - Humans
KW - Male
KW - Oxidants
KW - Photochemical/an [Analysis]
KW - Ozone/an [Analysis]
KW - Reproducibility of Results
KW - Tennessee
RP - NOT IN FILE
SP - 352
EP - 359
JF - Journal of the Air & Waste Management Association
JA - J Air Waste Manag Assoc
VL - 54
IS - 3
CY - United States
N2 - An ozone (O3) exposure study was conducted in Nashville, TN, using passive O3 samplers to measure six weekly outdoor, indoor, and personal O3 exposure estimates for a group of 10- to 12-yr-old elementary school children. Thirty-six children from two Nashville area communities (Inglewood and Hendersonville) participated in the O3 sampling program, and 99 children provided additional time-activity information by telephone interview. By design, this study coincided with the 1994 Nashville/Middle Tennessee Ozone Study conducted by the Southern Oxidants Study, which provided enhanced continuous ambient O3 monitoring across the Nashville area. Passive sampling estimated weekly average outdoor O3 concentrations from 0.011 to 0.O30 ppm in the urban Inglewood community and from 0.015 to 0.042 ppm in suburban Hendersonville. The maximum 1- and 8-hr ambient concentrations encountered at the Hendersonville continuous monitor exceeded the levels of the 1- and 8-hr metrics for the O3 National Ambient Air Quality Standard. Weekly average personal O3 exposures ranged from 0.0013 to 0.0064 ppm (7-31% of outdoor levels). Personal O3 exposures reflected the proportional amount of time spent in indoor and outdoor environments. Air-conditioned homes displayed very low indoor O3 concentrations, and homes using open windows and fans for ventilation displayed much higher concentrations
SN - 1096-2247
AD - Department of Epidemiology and Preventive Medicine, University of California, Davis, California 95616, USA. lee@ucdavis.edu
UR - 15061616
ER -

TY - JOUR

ID - 1455
T1 - A genomewide screen in a four-generation Dutch family with celiac disease: evidence for linkage to chromosomes 6 and 9
A1 - van Belzen,M.J.
A1 - Vrolijk,M.M.
A1 - Meijer,J.W.
A1 - Crusius,J.B.
A1 - Pearson,P.L.
A1 - Sandkuijl,L.A.
A1 - Houwen,R.H.
A1 - Wijmenga,C.
Y1 - 2004/03//
N1 - van Belzen, Martine J. Vrolijk, Martine M. Meijer, Jos W R. Crusius, J Bart A. Pearson, Peter L. Sandkuijl, Lodewijk A. Houwen, Roderick H J. Wijmenga, Cisca
The American journal of gastroenterology
3he, 0421030
IM
Journal Article. Research Support, Non-U.S. Gov't
English
KW - MEDLINE
KW - Adolescent
KW - Adult
KW - Aged
KW - 80 and over
KW - Celiac Disease/ge [Genetics]
KW - Child
KW - Chromosomes
KW - Human
KW - Pair 6/ge [Genetics]
KW - Pair 9/ge [Genetics]
KW - Female
KW - Genetic Linkage
KW - Genome
KW - Humans
KW - Male
KW - Middle Aged
KW - Netherlands
KW - Pedigree
RP - NOT IN FILE
SP - 466
EP - 471
JF - American Journal of Gastroenterology
JA - Am J Gastroenterol
VL - 99
IS - 3
CY - United States
N2 - OBJECTIVES: Celiac disease is caused by the interaction of multiple genes and environmental factors. Inheritance of the disease shows a complex pattern with a 10% sibling recurrence risk. The HLA-region is a major genetic risk locus in celiac disease, but genes outside this region are expected to contribute to the disease risk as well. The aim of this study was to identify the loci causing celiac disease in one large Dutch family with apparent dominant transmission of the disease. METHODS: The family comprised 17 patients in four generations, with possible transmission of the disease by both grandparents. Microsatellite markers evenly spread over all chromosomes were genotyped and linkage analysis was performed using both dominant and recessive disease models and a model-free analysis. RESULTS: Disease susceptibility in the family was linked to the HLA-region (lod score of 2.33) and all patients were HLA-DQ2. A dominantly inherited non-HLA locus with a maximum lod score of 2.61 was detected at 9p21-13, which was shared by 16 patients. Model-free analysis identified another possible non-HLA locus, at 6q25.3, which was shared by 14 patients (p = 0.01).

Neither of these regions was detected in a genomewide screen in Dutch affected sibpairs, but the 9p21 locus has been implicated in Scandinavian families. CONCLUSIONS: Two potential non-HLA loci for celiac disease were identified in this large Dutch family. Our results provide replication of the Scandinavian 9p21 locus, and suggest that this locus plays a role in celiac disease patients from different Caucasian populations
SN - 0002-9270
AD - Department of Biomedical Genetics, University Medical Center Utrecht, Utrecht, The Netherlands
UR - 15056087
ER -

TY - JOUR
ID - 1456
T1 - Neurodevelopmental outcomes of infant intestinal transplant recipients
A1 - Thevenin,D.M.
A1 - Mittal,N.
A1 - Kato,T.
A1 - Tzakis,A.
Y1 - 2004/03//
N1 - Thevenin, D M. Mittal, N. Kato, T. Tzakis, A
Transplantation proceedings
we9, 0243532
IM
Journal Article
English
KW - MEDLINE
KW - Aging
KW - Child
KW - Preschool
KW - Developmental Disabilities/ep [Epidemiology]
KW - Female
KW - Follow-Up Studies
KW - Humans
KW - Infant
KW - Intestines/tr [Transplantation]
KW - Liver Transplantation
KW - Longitudinal Studies
KW - Male
KW - Motor Activity
KW - Nervous System/gd [Growth & Development]
KW - Retrospective Studies
KW - Treatment Outcome
KW - Viscera/tr [Transplantation]
RP - NOT IN FILE
SP - 319
EP - 320
JF - Transplantation Proceedings
JA - Transplant Proc
VL - 36
IS - 2
CY - United States
N2 - Little is known about the impact of intestinal transplantation on development of the infant brain. In this study we report four neurodevelopmental studies on children receiving either liver or intestinal/multivisceral transplants. Our preliminary investigation examined the pretransplant status of 27 infants, who were either liver or intestinal/multivisceral candidates, using the Bayley Scales of Infant Development. A second study examined 23 infants after liver or intestinal/multivisceral transplant. A third study included pre- and posttransplant evaluations on 5 multivisceral infant transplants. In the fourth study, 10 children were tested several years after intestinal/multivisceral transplantation. Some children are able to achieve a normal development. However, even several years posttransplant most children can still experience significant cognitive delays. Children

receiving a transplant during infancy may also suffer severe motor delays. Infants undergoing intestinal/multivisceral transplantation show significantly more cognitive delays than those undergoing single-organ liver transplantation. In addition, multivisceral transplanted infants are more likely to continue to be severely developmentally delayed at the time of hospital discharge. With improved survival rates for infant transplants, both cognitive and motor development must be evaluated to determine the need for early intervention. In addition, educating families on the importance of compliance with intervention services outside the hospital is essential to maximize long-term neurodevelopmental outcomes for these infants
SN - 0041-1345
AD - Department of Pediatrics, University of Miami School of Medicine, Miami, Florida 33131, USA. dthevenin@med.miami.edu
UR - 15050145
ER -

TY - JOUR
ID - 1457
T1 - Influence of fall height and impact surface on biomechanics of feet-first free falls in children
A1 - Bertocci,G.E.
A1 - Pierce,M.C.
A1 - Deemer,E.
A1 - Aguel,F.
A1 - Janosky,J.E.
A1 - Vogeley,E.
Y1 - 2004/04//
N1 - Bertocci, Gina E. Pierce, Mary Clyde. Deemer, Ernest. Aguel, Fernando. Janosky, Janine E. Vogeley, Eva
Injury
0226040, gon
IM
Journal Article. Research Support, Non-U.S. Gov't. Research Support, U.S. Gov't, Non-P.H.S.
English
KW - MEDLINE
KW - Acceleration
KW - Accidental Falls
KW - Biomechanical Phenomena
KW - Child Abuse/di [Diagnosis]
KW - Child
KW - Preschool
KW - Craniocerebral Trauma/et [Etiology]
KW - Craniocerebral Trauma/pp [Physiopathology]
KW - Foot
KW - Humans
KW - Manikins
KW - Surface Properties
KW - Wounds and Injuries/et [Etiology]
KW - Wounds and Injuries/pp [Physiopathology]
RP - NOT IN FILE
SP - 417
EP - 424
JF - Injury
VL - 35
IS - 4
CY - England
N2 - OBJECTIVE: The objectives of our study were to assess biomechanics associated with feet-first free falls in 3-year-old children and to investigate the influence of impact surface type and fall height on key biomechanical measures associated with injury risk. METHODS: Repeatable feet-first free fall experiments were conducted in a laboratory mock-up environment using an instrumented Hybrid II 3-year-old test dummy. Impact surface type and fall height were varied to examine their influence on biomechanical measures. RESULTS: Feet-first falls from short distances (27 in.) (0.69 m) were found to have a low risk of contact-type

head injury, regardless of impact surface type. When comparing different types of impact surfaces in a 27 in. (0.69 m) fall, head acceleration associated with falls onto playground foam was significantly less than that associated with falls onto wood, linoleum or padded carpet. For falls onto playground foam, femoral compressive loads and bending moments were found to significantly increase as fall height increased. CONCLUSIONS: Impact surface type and fall height were found to influence biomechanics associated with injury risk in feet-first free falls as assessed through experimental mock-ups using an instrumented child test dummy. Feet-first falls from short distances (27 in.) (0.69 m) were associated with a low risk of contact-type head injury as assessed using HIC, irrespective of impact surface type
SN - 0020-1383
AD - Child Advocacy and Injury Research Center, Childrens' Hospital of Pittsburgh, Pittsburgh, PA, USA. ginaber@pitt.edu
UR - 15037378
ER -

TY - JOUR
ID - 1458
T1 - Epidemiology of major injury in the population of Friuli Venezia Giulia-Italy
A1 - Di,Bartolomeo S.
A1 - Sanson,G.
A1 - Michelutto,V.
A1 - Nardi,G.
A1 - Burba,I.
A1 - Francescutti,C.
A1 - Lattuada,L.
A1 - Scian,F.
A1 - Regional Study-Group on Major Injury.
Y1 - 2004/04//
N1 - Di Bartolomeo, Stefano. Sanson, Gianfranco. Michelutto, Vanni. Nardi, Giuseppe. Burba, Ivana. Francescutti, Carlo. Lattuada, Luca. Scian, Franca. Regional Study-Group on Major Injury
Injury
0226040, gon
IM
Journal Article
English
KW - MEDLINE
KW - Adolescent
KW - Adult
KW - Aged
KW - Child
KW - Emergency Medical Services/ut [Utilization]
KW - Female
KW - Hospital Mortality
KW - Humans
KW - Incidence
KW - Injury Severity Score
KW - Italy/ep [Epidemiology]
KW - Male
KW - Middle Aged
KW - Prospective Studies
KW - Survival Analysis
KW - Time Factors
KW - Wounds and Injuries/ep [Epidemiology]
KW - Wounds and Injuries/et [Etiology]
RP - NOT IN FILE
SP - 391
EP - 400
JF - Injury

VL - 35
IS - 4
CY - England
N2 - OBJECTIVE: To provide reliable and comparable information on major injury (MIJ) (Injury Severity Score (ISS) > 15) by establishing a comprehensive and Utstein-style compliant registry of all occurrences in a defined geographical area. METHODS: Prospective, population-based, 12-month study targeting the 1,200,000 inhabitants of the Italian region Friuli Venezia Giulia (FVG). Deliberate self-harm was excluded. RESULTS: The total number of MIJ cases was 627, the resulting incidence 522 per million per year. Trauma was mostly blunt (98.4%). Young (15-44 years) adults (54.8%) and males (78.6%) were most affected. Leading mechanisms of injury were traffic accidents (81%) and falls (9.1%). Most events occurred in rural (80.9%) areas despite one third of the regional population living in major urban centres. Summer and weekends carried the highest frequency. The mean ISS (n = 455) was 30.0, median 25. On-scene vital parameters were often subnormal, e.g. 53.9%, GCS < 14. The Emergency Medical System was nearly always activated (98.4%). The time intervals were within standards although in part susceptible of improvement. The percentage of direct triage to the definitive hospital was 79.8%. Overall mortality was 45.6% or 238 per million per year. Most fatalities were found already dead (171/300) and no trimodal distribution was verified. Only 1.5% of the patients found alive died outside hospital. Mean GOS was 4.4 +/- 1 (S.D.), median 5. CONCLUSION: A considerable amount of information on MIJ in FVG has been gathered, of both local and general interest because it can help to assess the local trauma system and also, given the relative scarcity of prospective, population-based information on MIJ, contribute to scientific research
SN - 0020-1383
AD - Italian Resuscitation Council, Trauma Committee, c/o ICU 2nd Service, Az. Osp. SM della Misericordia, 33100 Udine, Italy. dibartolomeo.stefano@aoud.sanita.fvg.it
UR - 15037374
ER -

TY - JOUR
ID - 1459
T1 - School-based obesity prevention in Chilean primary school children: methodology and evaluation of a controlled study
A1 - Kain,J.
A1 - Uauy,R.
A1 - Albala
A1 - Vio,F.
A1 - Cerda,R.
A1 - Leyton,B.
Y1 - 2004/04//
N1 - Kain, J. Uauy, R. Albala. Vio, F. Cerda, R. Leyton, B
International journal of obesity and related metabolic disorders : journal of the International Association for the Study of Obesity
btx, 9313169
IM
Evaluation Studies. Journal Article. Multicenter Study. Research Support, Non-U.S. Gov't
English
KW - MEDLINE
KW - Anthropometry
KW - Body Mass Index
KW - Child
KW - Child Nutrition Sciences/ed [Education]
KW - Chile
KW - Diet
KW - Female
KW - Follow-Up Studies
KW - Health Education/mt [Methods]
KW - Humans
KW - Male
KW - Obesity/pc [Prevention & Control]

KW - Physical Education and Training
KW - Physical Fitness
KW - Schools
RP - NOT IN FILE
SP - 483
EP - 493
JF - International Journal of Obesity & Related Metabolic Disorders: Journal of the International Association for the Study of Obesity
JA - Int J Obes Relat Metab Disord
VL - 28
IS - 4
CY - England
N2 - OBJECTIVE: To assess the impact of a 6 months nutrition education and physical activity intervention on primary school children through changes in adiposity and physical fitness. DESIGN: Longitudinal school-based controlled evaluation study. SUBJECTS: Children from 1st to 8th grade, 2141 in intervention and 945 in control schools. INTERVENTION: Nutrition education for children and parents, 'healthier' kiosks, 90 min of additional physical activity (PA) weekly, behavioral PA program and active recess. MEASUREMENTS: Adiposity indices (BMI, BMI Z-score, triceps skinfold thickness (TSF), waist circumference and physical fitness (20 m shuttle run test and lower back flexibility). RESULTS: Positive effect on adiposity indices (except TSF) was observed in boys (P<0.001 for BMI Z), while both physical fitness parameters increased significantly in both boys (P<0.001 for each test) and girls (P<0.0001 for each test). A differential effect in BMI Z was observed according to baseline nutritional status. CONCLUSIONS: This intervention showed a robust effect on physical fitness in both genders and decreased adiposity only in boys
SN - 0307-0565
AD - Institute of Nutrition and Food Technology (INTA), University of Chile, Santiago, Casilla 138-11, Santiago, Chile. jkain@inta.cl
UR - 14993915
ER -

TY - JOUR
ID - 1460
T1 - Children's exposure to arsenic from CCA-treated wooden decks and playground structures
A1 - Hemond,H.F.
A1 - Solo-Gabriele,H.M.
Y1 - 2004/02//
N1 - Hemond, Harold F. Solo-Gabriele, Helena M
Risk analysis : an official publication of the Society for Risk Analysis
8109978, ria
IM
Journal Article. Research Support, Non-U.S. Gov't. Research Support, U.S. Gov't, P.H.S.
English
KW - MEDLINE
KW - Arsenates/ip [Isolation & Purification]
KW - Arsenates/to [Toxicity]
KW - Arsenic/ip [Isolation & Purification]
KW - Arsenic/pk [Pharmacokinetics]
KW - Arsenic/to [Toxicity]
KW - Biological Availability
KW - Child
KW - Environmental Exposure
KW - Hand
KW - Humans
KW - Mouth
KW - Play and Playthings
KW - Risk Assessment
KW - Soil Pollutants/ip [Isolation & Purification]
KW - Soil Pollutants/to [Toxicity]

KW - Wood
RP - NOT IN FILE
SP - 51
EP - 64
JF - Risk Analysis
JA - Risk Anal
VL - 24
IS - 1
CY - United States
N2 - CCA-treated wood is widely used in the fabrication of outdoor decks and playground equipment. Because arsenic can be removed from the surface of CCA-treated wood both by physical contact and by leaching, it is important to determine whether children who play on such structures may ingest arsenic in quantities sufficient to be of public health concern. Based on a review of existing studies, it is estimated that arsenic doses in amounts of tens of micrograms per day may be incurred by children having realistic levels of exposure to CCA-treated decks and playground structures. The most important exposure pathway appears to be oral ingestion of arsenic that is first dislodged from the wood by direct hand contact, then transferred to the mouth by children's hand-to-mouth activity. The next most important pathway appears to be dermal absorption of arsenic, while ingestion of soil that has become contaminated by leaching from CCA-treated structures appears to be of lesser importance, except possibly in the case of children with pica. Considerable uncertainty, however, is associated with quantitative estimates of children's arsenic exposure from CCA-treated wood. Priorities for refining estimates of arsenic dose include detailed studies of the hand-to-mouth transfer of arsenic, studies of the dermal and gastrointestinal absorption of dislodgeable arsenic, and studies in which doses of arsenic to children playing in contact with CCA-treated wood are directly determined by measurement of arsenic in their urine, hair, and nails
SN - 0272-4332
AD - RM Parsons Laboratory, Department of Civil and Environmental Engineering, Massachusetts Institute of Technology, Cambridge, MA, USA
UR - 15028000
ER -

TY - JOUR
ID - 1461
T1 - Creating SunSmart schools
A1 - Giles-Corti,B.
A1 - English,D.R.
A1 - Costa,C.
A1 - Milne,E.
A1 - Cross,D.
A1 - Johnston,R.
Y1 - 2004/02//
N1 - Giles-Corti, B. English, D R. Costa, C. Milne, E. Cross, D. Johnston, R
Health education research
bqp, 8608459
T
Comparative Study. Journal Article. Research Support, Non-U.S. Gov't
English
KW - MEDLINE
KW - Child
KW - Environmental Exposure/pc [Prevention & Control]
KW - Female
KW - Humans
KW - Male
KW - Program Development
KW - Protective Clothing
KW - School Health Services/og [Organization & Administration]
KW - Skin Neoplasms/pc [Prevention & Control]
KW - Western Australia

RP - NOT IN FILE
SP - 98
EP - 109
JF - Health Education Research
JA - Health Educ Res
VL - 19
IS - 1
CY - England
N2 - Kidskin was a sun-protection intervention study involving 1776 children attending 33 primary schools in Perth, Western Australia. There were three study groups: a control group, a moderate intervention group and a high intervention group. In addition to receiving a specially designed curricular intervention (1995-1998), the moderate and high intervention groups received an environmental intervention aimed at creating SunSmart schools (1996-1998). The environmental intervention focused on encouraging implementation of 'No hat, no play' policies and reducing sun exposure at lunchtime. In 1995 and 1998, observational methods were used to measure children's lunchtime sun exposure (i.e. polysulfone film badges) and hat wearing (i.e. video-taping of children). The proportion of children wearing broad-brimmed hats or legionnaire caps increased in seven of the eight high intervention schools between 1995 and 1998. In three schools, however, the impact was very positive with almost all children wearing these hats in 1998. There was no improvement in wearing these types of hats in either the moderate intervention group or the control group. In terms of sun exposure, there were only small non-significant differences among the three groups in terms of lunchtime sun exposure. The Kidskin program had a positive effect on hat wearing in the playground, but did not change children's use of shade at lunchtime. In this study, disseminating policy guidelines to schools using a mail-only strategy was ineffective, even when combined with an awards program. More information on 'champions' who bring about change in schools is required
SN - 0268-1153
AD - School of Population Health, University of Western Australia, Crawley, Western Australia 6009. billie@cyllene.uwa.edu.au
UR - 15020549
ER -

TY - JOUR
ID - 1462
T1 - Evening activities as a potential confound in research on the adrenocortical system in children
A1 - Kertes,D.A.
A1 - Gunnar,M.R.
Y1 - 2004/01//
N1 - Kertes, Darlene A. Gunnar, Megan R
Child development
0372725, d28
IM
Journal Article. Research Support, U.S. Gov't, Non-P.H.S.. Research Support, U.S. Gov't, P.H.S.
English
KW - MEDLINE
KW - Arousal/ph [Physiology]
KW - Child
KW - Circadian Rhythm/ph [Physiology]
KW - Female
KW - Humans
KW - Hydrocortisone/me [Metabolism]
KW - Hypothalamo-Hypophyseal System/ph [Physiology]
KW - Leisure Activities
KW - Life Change Events
KW - Male
KW - Personality Assessment
KW - Pituitary-Adrenal System/ph [Physiology]
KW - Reference Values
KW - Saliva/ch [Chemistry]

KW - Sex Factors
KW - Sleep Stages/ph [Physiology]
KW - Social Isolation
KW - Sports/ph [Physiology]
KW - Temperament
RP - NOT IN FILE
SP - 193
EP - 204
JF - Child Development
JA - Child Dev
VL - 75
IS - 1
CY - United States
N2 - The relation among children's evening activities, behavioral characteristics, and activity of the hypothalamic-pituitary-adrenocortical axis was assessed in normally developing children ages 7 to 10 years. Salivary cortisol at bedtime was compared on evenings when children had structured activities outside of the home with unstructured evenings at home in relation to parental reports of children's behavioral characteristics. Participating in evening activities, particularly sport activities, was associated with small increases in bedtime cortisol levels in boys but not in girls. Differences in cortisol on activity versus no-activity nights were negatively related to children's social isolation. These results show that in studies with children, nights on which participants engage in sport activities should be avoided when collecting ambulatory measures of salivary cortisol concentrations
SN - 0009-3920
AD - Institute of Child Development, University of Minnesota. Minneapolis, MN 55455, USA
UR - 15015684
ER -

TY - JOUR
ID - 1463
T1 - Children at risk: risk factors for low back pain in the elementary school environment
A1 - Limon,S.
A1 - Valinsky,L.J.
A1 - Ben-Shalom,Y.
Y1 - 2004/03/15/
N1 - Limon, Susan. Valinsky, Liora J. Ben-Shalom, Yael
Spine
7610646, uxk, 7610649
IM
Journal Article
English
KW - MEDLINE
KW - Body Weight
KW - Child
KW - Female
KW - Health Promotion
KW - Health Surveys
KW - Human Engineering
KW - Humans
KW - Interior Design and Furnishings
KW - Israel
KW - Low Back Pain/ep [Epidemiology]
KW - Male
KW - Motor Activity
KW - Posture
KW - Prevalence
KW - Questionnaires
KW - Risk Factors

KW - Schools/cl [Classification]
KW - Schools/sn [Statistics & Numerical Data]
KW - Students
KW - Weight-Bearing
RP - NOT IN FILE
SP - 697
EP - 702
JF - Spine
VL - 29
IS - 6
CY - United States
N2 - STUDY DESIGN: A prevalence survey of 101 randomly selected elementary schools in the Israel Central District. OBJECTIVES: To identify and describe risk factors for low back pain that exist in the elementary school environment. SUMMARY OF BACKGROUND DATA: Recent surveys report a high prevalence of low back pain in children and adolescents that increases with age, with a correlation between low back pain in adolescence and that experienced in adulthood. Environmental risk factors have been associated with the development of low back pain in children. Because of the significant amount of time children spend in their school environment, risk factors need to be identified in this environment. METHODS: A questionnaire, completed by school nurses, measured risk factors among 10,000 children in elementary schools in Israel. These included backpack and student weight, the availability of storage facilities, the appropriateness of chair and desk height to student height, seating arrangements during frontal lessons, and physical activity at recess. RESULTS: Between 30% and 54% of students carried >15% of their body weight. Nearly 15% of the first graders and 20% of sixth graders had inappropriate chairs. In 74% of the classes, students sat with their side facing the teacher and in 35% students sat with their backs. In 6% of schools, no physical activity is offered at recess. CONCLUSIONS: Shortcomings were found in all areas investigated. There is an urgent need for health promotion programs to increase awareness and reduce risks in the school environment
SN - 1528-1159
AD - Petach Tikva Area Ministry of Health, Petach Tikva, Israel. limonfamily@hotmail.com
UR - 15014281
ER -

TY - JOUR
ID - 1464
T1 - 'Thinking outside the box': complementary and alternative therapies use in paediatric oncology patients
A1 - Molassiotis,A.
A1 - Cubbin,D.
Y1 - 2004/03//
N1 - Molassiotis, Alexander. Cubbin, Denise
European journal of oncology nursing : the official journal of European Oncology Nursing Society
100885136
IM, N
Journal Article
English
KW - MEDLINE
KW - Adolescent
KW - Hospitalized
KW - Adult
KW - Attitude to Health
KW - Child
KW - Preschool
KW - Complementary Therapies/ut [Utilization]
KW - Cross-Sectional Studies
KW - Decision Making
KW - Female
KW - Great Britain
KW - Humans
KW - Male

KW - Middle Aged
KW - Neoplasms/th [Therapy]
KW - Parents
RP - NOT IN FILE
SP - 50
EP - 60
JF - European Journal of Oncology Nursing
JA - EUR J ONCOL NURS
VL - 8
IS - 1
CY - Scotland
N2 - The aim of this study was to determine the prevalence of complementary and alternative medicine (CAM) use among children with cancer who had received or were receiving treatment at a large hospital in the UK, including the identification of the most commonly used therapies and parental motives for doing so. Using a cross-sectional survey design, questionnaires were sent to parents of paediatric patients diagnosed with cancer. Of the 49 respondents, 32.7% reported using some type of CAM. The most commonly used therapies included multivitamins, aromatherapy massage, diets and music as therapy. Most children had used more than one therapy. Many of the factors that motivated parents to use CAM were related to helping or supporting their child's medical treatment. The main benefits identified from using CAM included increased confidence, pain relief and relaxation. The longer the time since diagnosis the more children tended to use CAM. The reasons for parents not using CAM included the child doing well and therefore not seeing the need for CAM use; not being aware of CAM; CAM not being offered and lack of information available. Parents identified a need for more information to be available both at ward level and for information about CAM to be discussed by medical staff, particularly at the start of treatment. The results indicate that CAM is frequently used by children and young people with cancer and that their use plays a substantial role in helping children through their conventional cancer treatment
SN - 1462-3889
AD - School of Nursing, Midwifery & Health Visiting, University of Manchester, UK
UR - 15003744
ER -

TY - JOUR
ID - 1465
T1 - External and internal noise surveys of London primary schools
A1 - Shield,B.
A1 - Dockrell,J.E.
Y1 - 2004/02//
N1 - Shield, Bridget. Dockrell, Julie E
The Journal of the Acoustical Society of America
7503051, h3k
IM
Journal Article. Research Support, Non-U.S. Gov't
English
KW - MEDLINE
KW - Child
KW - Health Surveys
KW - Humans
KW - London
KW - Maximum Allowable Concentration
KW - Noise/lj [Legislation & Jurisprudence]
KW - Noise
KW - Transportation/lj [Legislation & Jurisprudence]
KW - Schools/lj [Legislation & Jurisprudence]
KW - Schools
KW - Social Environment
KW - Sound Spectrography
KW - Urban Health

RP - NOT IN FILE
SP - 730
EP - 738
JF - Journal of the Acoustical Society of America
JA - J Acoust.Soc Am
VL - 115
IS - 2
CY - United States
N2 - Internal and external noise surveys have been carried out around schools in London, UK, to provide information on typical levels and sources to which children are exposed while at school. Noise levels were measured outside 142 schools, in areas away from flight paths into major airports. Here 86% of the schools surveyed were exposed to noise from road traffic, the average external noise level outside a school being 57 dB L(Aeq). Detailed internal noise surveys have been carried out in 140 classrooms in 16 schools, together with classroom observations. It was found that noise levels inside classrooms depend upon the activities in which the children are engaged, with a difference of 20 dB L(Aeq) between the "quietest" and "noisiest" activities. The average background noise level in classrooms exceeds the level recommended in current standards. The number of children in the classroom was found to affect noise levels. External noise influenced internal noise levels only when children were engaged in the quietest classroom activities. The effects of the age of the school buildings and types of window upon internal noise were examined but results were inconclusive
SN - 0001-4966
AD - Faculty of Engineering Science and Technology, London South Bank University, 103 Borough Road, London SE1 0AA, United Kingdom. shieldbm@lsbu.ac.uk
UR - 15000185
ER -

TY - JOUR
ID - 1466
T1 - Environmental changes may be needed for prevention of overweight in minority children. [Review] [31 refs]
A1 - Fitzgibbon,M.L.
A1 - Stolley,M.R.
Y1 - 2004/01//
N1 - Fitzgibbon, Marian L. Stolley, Melinda R
Pediatric annals
oub, 0356657
IM
Journal Article. Review
English
KW - MEDLINE
KW - Child
KW - Diet
KW - Health Behavior
KW - Health Promotion/mt [Methods]
KW - Humans
KW - Life Style
KW - Minority Groups
KW - Motor Activity
KW - Obesity/eh [Ethnology]
KW - Obesity/pc [Prevention & Control]
KW - Schools
KW - Social Environment
KW - United States/ep [Epidemiology]
RP - NOT IN FILE
SP - 45
EP - 49
JF - Pediatric Annals
JA - Pediatr Ann

VL - 33
IS - 1
CY - United States
N2 - In minority populations, those who do not maintain normal weight outnumber those who do; thus, overweight is often the community norm. Compared to many white populations, minority populations are transitioning from poverty, food scarcity, and jobs that require significant amounts of energy expenditure. This may mean food and activity habits of both black and Latino populations may have been developed during a more physically-active era. Caloric restriction and leisure-time physical activity are not routine in minority communities. In these communities, there are often few supermarkets that carry fresh produce, many fast food establishments and small grocery stores that sell high-fat, energy-dense foods, and high neighborhood crime rates that discourage outdoor activities and limit safe places for walking and bicycling, including school routes. Although specific dietary and activity behaviors related to weight control ultimately are undertaken by individuals, our current environment makes such individual choices difficult when it contains substantial barriers to establishing healthy lifestyles. Substantial environmental changes need to be made, especially in minority communities. [References: 31]
SN - 0090-4481
AD - Feinberg School of Medicine, Northwestern University, Chicago, IL, USA
UR - 14981869
ER -

TY - JOUR
ID - 1467
T1 - Monkey bars are for monkeys: a study on playground equipment related extremity fractures in Singapore
A1 - Mahadev,A.
A1 - Soon,M.Y.
A1 - Lam,K.S.
Y1 - 2004/01//
N1 - Mahadev, A. Soon, M Y H. Lam, K S
Singapore medical journal
uri, 0404516
IM
Journal Article
English
KW - MEDLINE
KW - Adolescent
KW - Age Distribution
KW - Child
KW - Preschool
KW - Equipment Safety/sn [Statistics & Numerical Data]
KW - Extremities/in [Injuries]
KW - Female
KW - Fractures
KW - Bone/cl [Classification]
KW - Bone/ep [Epidemiology]
KW - Bone/pc [Prevention & Control]
KW - Humans
KW - Incidence
KW - Male
KW - Play and Playthings/in [Injuries]
KW - Retrospective Studies
KW - Risk Factors
KW - Sex Distribution
KW - Singapore/ep [Epidemiology]
RP - NOT IN FILE
SP - 9
EP - 13
JF - Singapore Medical Journal

JA - Singapore Med J
VL - 45
IS - 1
CY - Singapore
N2 - Studies in Caucasian populations have shown that a significant percentage of childhood extremity fractures occur at the playground. There are no comparable studies in Asian populations. Thus this study sets out to determine the pattern of playground related extremity fractures in Asian populations and to suggest modifications to prevent or reduce these injuries. This study involved a retrospective review of 390 patients with these fractures who visited our Department from May 1997 to December 1998. This accounted for 19.5% of all fractures seen in the same period. The largest age group affected were the five through 12-year-old patients with a male to female ratio of 2:1. Monkey bars or upper body devices were the most common cause (66%). The most common fracture was supracondylar fractures (43%). Further studies to determine the actual dimensions of playground equipment will be carried to ascertain with greater certainty the safety of these equipment in our playgrounds
SN - 0037-5675
AD - Department of Orthopaedic Surgery, KK Women's and Children's Hospital, 100 Bukit Timah Road, Singapore 229899. arjandas@kkh.com.sg
UR - 14976576
ER -

TY - JOUR
ID - 1468
T1 - Reducing the risks of children living near the site of a former lead smeltery
A1 - Louekari,K.
A1 - Mroueh,U.M.
A1 - Maidell-Munster,L.
A1 - Valkonen,S.
A1 - Tuomi,T.
A1 - Savolainen,K.
Y1 - 2004/02/05/
N1 - Louekari, K. Mroueh, U-M. Maidell-Munster, L. Valkonen, S. Tuomi, T. Savolainen, K
The Science of the total environment
uj0, 0330500
IM
Comparative Study. Journal Article
English
KW - MEDLINE
KW - Child
KW - Environmental Exposure
KW - Environmental Monitoring
KW - Environmental Pollutants/an [Analysis]
KW - Finland
KW - Humans
KW - Lead/bl [Blood]
KW - Metallurgy
KW - Soil/an [Analysis]
RP - NOT IN FILE
SP - 65
EP - 75
JF - Science of the Total Environment
JA - Sci Total Environ
VL - 319
IS - 1-3
CY - Netherlands
N2 - The sources of lead exposure, soil, household dust, diet and ambient air near a former lead smeltery were studied. The blood lead level of small children was also determined. The aim of the study was to define, based primarily on blood lead measurements, whether children living in the contaminated area may be at risk. Within

500 m from the site of the smeltery, there were several areas where the Finnish limit value for soil Pb, i.e. 300 mg/kg, was exceeded. In the recently built areas, the surface soil has been replaced and soil remediation has taken place in schoolyards and the playgrounds of children's day-care centres. Lead content in household dust was clearly elevated in the contaminated areas. In approximately 20 years, after the smeltery was closed in 1984, the lead concentrations of the fruits and berries in local gardens have decreased to one-tenth. In some samples, the limit values are still exceeded. The lead concentration in ambient air is now 50 times lower than in the 1970s. The blood lead level of the children living in the area is slightly but statistically significantly higher than that of the children in the control areas. The critical blood lead level, i.e. 10 microg/100 ml, was not exceeded in any of the children examined. The average and maximum lead concentrations of 63 analysed blood samples were 2.2 and 5 microg/100 ml, respectively. In contrast, the average and maximum blood lead levels of school children in 1981 were 6.7 and 13.0 microg/100 ml, respectively. The risk reduction measures undertaken during the past 20 years are described
SN - 0048-9697
AD - Finnish Institute of Occupational Health, Topeliuksenkatu 41 B, FIN-00250 Helsinki, Finland. kimmo.louekari@ttl.fi
UR - 14967502
ER -

TY - JOUR
ID - 1469
T1 - Clinico-epidemiological features of snakebite: a study from Eastern Nepal
A1 - Sharma,S.K.
A1 - Koirala,S.
A1 - Dahal,G.
A1 - Sah,C.
Y1 - 2004/01//
N1 - Sharma, Sanjib Kumar. Koirala, Sekhar. Dahal, Gehraj. Sah, Chandra
Tropical doctor
wgc, 1301706
IM
Journal Article
English
KW - MEDLINE
KW - Adolescent
KW - Adult
KW - Aged
KW - 80 and over
KW - Child
KW - Preschool
KW - Female
KW - Humans
KW - Infant
KW - Newborn
KW - Male
KW - Middle Aged
KW - Nepal/ep [Epidemiology]
KW - Snake Bites/ep [Epidemiology]
KW - Snake Bites/th [Therapy]
RP - NOT IN FILE
SP - 20
EP - 22
JF - Tropical Doctor
JA - Trop Doct.
VL - 34
IS - 1
CY - England

N2 - We studied 604 suspected snakebite cases seen at the peripheral snakebite treatment centre, Damak, Eastern Nepal from January to December 2000 using pre-designed proforma. Seventy-five per cent of the patients were in the age group 11-40 years. The common victims were farmers (44.2%) and housewives (31.7%). The bite was commonly encountered during farming (29.4%), working in the field (16.2%), walking (13.7%) and feeding cattle (9.2%). Sixty-five per cent were bitten during outdoor and agriculture related activities. Bite during sleep was recorded in 7%: 62% had sustained bite in lower limb. Forty-two per cent of the victims arrived at the treatment centre within 2 hours of being bitten. Fourteen patients arrived 7 hours after being bitten and six were dead on arrival. A common local practice (seen in 56.7%) was to eat chillies to confirm envenomation. Application of a tourniquet, often multiple, was the common (90%) first aid measure. Neurotoxicity was present in 11% (n=66) of the victims. Five had local features of envenomation. No case of coagulopathy was recorded. Reaction to anti-snake venom was noticed in 28.16% (n=20). Two patients died during treatment. Mortality among patients with features of neurotoxicity was 3%
SN - 0049-4755
AD - Department of Medicine, B P Koirala Institute of Health Sciences, Dharan, Nepal. drsanjib@yahoo.com
UR - 14959965
ER -

TY - JOUR
ID - 1470
T1 - Identification and characterization of melanocortin-4 receptor gene mutations in morbidly obese finnish children and adults
A1 - Valli-Jaakola,K.
A1 - Lipsanen-Nyman,M.
A1 - Oksanen,L.
A1 - Hollenberg,A.N.
A1 - Kontula,K.
A1 - Bjorbaek,C.
A1 - Schalin-Jantti,C.
Y1 - 2004/02//
N1 - Valli-Jaakola, Kaisa. Lipsanen-Nyman, Marita. Oksanen, Laura. Hollenberg, Anthony N. Kontula, Kimmo. Bjorbaek, Christian. Schalin-Jantti, Camilla
The Journal of clinical endocrinology and metabolism
hrb, 0375362
AIM, IM
Journal Article. Research Support, Non-U.S. Gov't. Research Support, U.S. Gov't, P.H.S.
English
KW - MEDLINE
KW - Adolescent
KW - Adult
KW - Cell Line
KW - Child
KW - Cohort Studies
KW - Female
KW - Finland
KW - Heterozygote
KW - Humans
KW - Male
KW - Middle Aged
KW - Mutation
KW - Obesity
KW - Morbid/ge [Genetics]
KW - Morbid/pp [Physiopathology]
KW - Pedigree
KW - Phenotype
KW - Polymorphism
KW - Genetic
KW - Receptor

KW - Melanocortin
KW - Type 4/ge [Genetics]
KW - Type 4/me [Metabolism]
KW - Signal Transduction
KW - Tissue Distribution
RP - NOT IN FILE
SP - 940
EP - 945
JF - Journal of Clinical Endocrinology & Metabolism
JA - J Clin Endocrinol Metab
VL - 89
IS - 2
CY - United States
N2 - Two Finnish cohorts, comprising 56 children with severe early-onset obesity (relative weight for height greater than or equal to +70% before age 10) and 252 morbidly obese adults (body mass index, > or = 40 kg/m(2)), were screened for melanocortin-4 receptor (MC4R) mutations. We identified a pathogenic mutation (S127L) in one child, causing severe early-onset obesity. We describe the phenotype of this particular mutation for the first time. We also identified a novel (I226T) polymorphism in the coding and two new variations (-439delGC and 1059C>T) outside the coding region of the MC4R gene. Three previously described polymorphisms (V103I, T112M, and I125L) were identified. In vitro functional studies of variants T112M, S127L, and I226T supported a pathogenic role of the S127L mutation, because signaling properties of the receptor in response to the MC4R agonists alpha-MSH, beta-MSH, and gamma(1)-MSH were impaired. The S127L mutation did not affect receptor inhibition by the antagonist agouti-related protein. Localization of the three variant receptors was similar to that of wild type. In conclusion, a pathogenic MC4R mutation was found among subjects with severe early-onset obesity but not among morbidly obese adults. Impaired function of the S127L receptor was due to reduced activation, not a defect of protein transport to the cell membrane
SN - 0021-972X
AD - Department of Medicine and Research Program in Molecular Medicine, University of Helsinki, FIN-00290 Helsinki, Finland
UR - 14764818
ER -

TY - JOUR
ID - 1471
T1 - Acute insulin responses to calcium and tolbutamide do not differentiate focal from diffuse congenital hyperinsulinism
A1 - Giurgea,I.
A1 - Laborde,K.
A1 - Touati,G.
A1 - Bellanne-Chantelot,C.
A1 - Nassogne,M.C.
A1 - Sempoux,C.
A1 - Jaubert,F.
A1 - Khoa,N.
A1 - Chigot,V.
A1 - Rahier,J.
A1 - Brunelle,F.
A1 - Nihoul-Fekete,C.
A1 - Dunne,M.J.
A1 - Stanley,C.
A1 - Saudubray,J.M.
A1 - Robert,J.J.
A1 - de,Lonlay P.
Y1 - 2004/02//
N1 - Giurgea, Irina. Laborde, Kathleen. Touati, Guy. Bellanne-Chantelot, Christine. Nassogne, Marie-Cecile. Sempoux, Christine. Jaubert, Francis. Khoa, Nguyen. Chigot, Valerie. Rahier, Jacques. Brunelle, Francis. Nihoul-Fekete, Claire. Dunne, Mark J. Stanley, Charles. Saudubray, Jean-Marie. Robert, Jean-Jacques. de

Lonlay, Pascale
The Journal of clinical endocrinology and metabolism
hrb, 0375362
AIM, IM
Journal Article. Research Support, Non-U.S. Gov't
English
KW - MEDLINE
KW - Calcium Gluconate/ad [Administration & Dosage]
KW - Calcium Gluconate/du [Diagnostic Use]
KW - Child
KW - Preschool
KW - Diagnosis
KW - Differential
KW - Female
KW - Glucose Tolerance Test
KW - Humans
KW - Hyperinsulinism/cl [Classification]
KW - Hyperinsulinism/cn [Congenital]
KW - Hyperinsulinism/di [Diagnosis]
KW - Hyperinsulinism/me [Metabolism]
KW - Hypoglycemic Agents/du [Diagnostic Use]
KW - Infant
KW - Newborn
KW - Injections
KW - Intravenous
KW - Insulin/se [Secretion]
KW - Male
KW - Tolbutamide/du [Diagnostic Use]
RP - NOT IN FILE
SP - 925
EP - 929
JF - Journal of Clinical Endocrinology & Metabolism
JA - J Clin Endocrinol Metab
VL - 89
IS - 2
CY - United States
N2 - Congenital hyperinsulinism (CHI) is related to two main histological pancreas anomalies: focal adenomatous hyperplasia and diffuse beta-cell hypersecretion. Pharmacological tests to measure acute insulin responses (AIR) to peripheral i.v. injections of glucose, calcium, and tolbutamide have been reported as potential means to distinguish between these histological forms. In patients with defects in ATP-sensitive potassium channels, tolbutamide will fail to induce insulin release in affected portions of the pancreas, whereas calcium gluconate will enhance insulin release through spontaneously active voltage-gated Ca(2+) channels. Consequently, in focal CHI patients, calcium should promote AIRs from the lesion, whereas tolbutamide should act to promote insulin secretion from the healthy region of the pancreas (outside the focal hyperplasia). We therefore studied AIRs to calcium and tolbutamide stimulation tests in 16 children with focal (n = 9) or diffuse (n = 7) CHI before pancreatic surgery. We found hypervariable AIRs to glucose and calcium stimulation in both focal and diffuse CHI patients. AIRs to tolbutamide stimulation were found modest in focal CHI patients, which might account for beta-cell quiescence in the healthy portion of the pancreas of these patients. We conclude that AIRs to calcium and tolbutamide stimulation tests are not sufficient to differentiate the focal from the diffuse CHI patients
SN - 0021-972X
AD - Department of Pediatrics, Hopital Necker Enfants Malades, 75743 Paris, France
UR - 14764815
ER -

TY - JOUR
ID - 1472

T1 - Disability and risk of school related injury
A1 - Ramirez,M.
A1 - Peek-Asa,C.
A1 - Kraus,J.F.
Y1 - 2004/02//
N1 - Ramirez, M. Peek-Asa, C. Kraus, J F
Injury prevention : journal of the International Society for Child and Adolescent Injury Prevention
crz, 9510056
IM
Journal Article. Multicenter Study. Research Support, U.S. Gov't, P.H.S.
English
KW - MEDLINE
KW - Accidental Falls/sn [Statistics & Numerical Data]
KW - Adolescent
KW - Adult
KW - Athletic Injuries/ep [Epidemiology]
KW - Athletic Injuries/et [Etiology]
KW - California/ep [Epidemiology]
KW - Child
KW - Preschool
KW - Disabled Children/sn [Statistics & Numerical Data]
KW - Education
KW - Special
KW - Female
KW - Humans
KW - Male
KW - Odds Ratio
KW - Risk Factors
KW - Schools
KW - Wounds and Injuries/ep [Epidemiology]
KW - Wounds and Injuries/et [Etiology]
RP - NOT IN FILE
SP - 21
EP - 26
JF - Injury Prevention
JA - Inj Prev
VL - 10
IS - 1
CY - England
N2 - OBJECTIVE: Approximately six million children with disabilities attend school in the United States. Cognitive and physical limitations may compromise their ability to handle environmental hazards and hence increase their risk for injury. The objective of this study was to describe the epidemiology of school related injury among children enrolled in 17 special education schools in one large, urban school district. DESIGN: Altogether 6769 schoolchildren with disabilities were followed up from 1994-98. Injury and population data were collected from pupil accident reports and existing school records. Associations were estimated through generalized estimating equations. RESULTS: A total of 697 injuries were reported for a rate of 4.7/100 students per year. Children with multiple disabilities had a 70% increased odds of injury compared with the developmentally disabled (odds ratio (OR) 1.7, 95% confidence interval (CI) 1.3 to 2.3). The physically disabled (OR 1.4, 95% CI 1.0 to 1.9) had a modest increased odds of injury. Cuts, bruises, and abrasions composed almost three fourths of all injuries; almost half of these injuries were to the face. Falls (34%) and insults by other students (31%) were the most common external causes. More than a fourth of injuries were sports related, and 21% occurred on the playground/athletic field. Injury patterns differed across disabilities. CONCLUSIONS: Although limited to one school district, the population studied is the largest cohort thus far of schoolchildren with disabilities. With this large study base, potentially high risk groups were identified and circumstances of injury described. This information is imperative for developing and improving school based injury prevention measures
SN - 1353-8047

AD - Division of Research on Children, Youth and Families, Childrens Hospital Los Angeles and the Southern California Injury Prevention Research Center, UCLA School of Public Health, Los Angeles, California 90027, USA. MarizenRamirez@chla.usc.edu
UR - 14760022
ER -

TY - JOUR
ID - 1473
T1 - Swedish pre-school children's UVR exposure - a comparison between two outdoor environments
A1 - Boldeman,C.
A1 - Dal,H.
A1 - Wester,U.
Y1 - 2004/02//
N1 - Boldeman, C. Dal, H. Wester, U
Photodermatology, photoimmunology & photomedicine
awp, 9013641
IM
Comparative Study. Journal Article. Research Support, Non-U.S. Gov't
English
KW - MEDLINE
KW - Child
KW - Preschool
KW - Environmental Exposure
KW - Female
KW - Humans
KW - Infant
KW - Male
KW - Play and Playthings
KW - Radiation Dosage
KW - Radiometry
KW - Sweden
KW - Ultraviolet Rays
RP - NOT IN FILE
SP - 2
EP - 8
JF - Photodermatology, Photoimmunology & Photomedicine
JA - Photodermatol.Photoimmunol.Photomed.
VL - 20
IS - 1
CY - Denmark
N2 - BACKGROUND: Overexposure to ultraviolet radiation (UVR) in childhood is a major risk factor for skin cancer. Shady environments are recommended as one method of protection. METHODS: Environmental exposure to UVR and environmental protection were assessed by dosimeter measurements on 64 children aged 1-6 years at two geographically close and topographically similar pre-schools outside Stockholm. Outdoor play constructions of site 1 (34 children) were mainly exposed to the sun, and those of site 2 (30 children) were mainly shaded. Dosimetry was carried out during 11 work days in May-June 2002 under clear weather conditions. The reliability of dosimeters was tested with meteorologically modelled data from SMHI, and with stationary dosimeters exposed to free sky, and compared with other UV instruments. The differences between children's outdoor stays were adjusted for. RESULTS: The children's average daily exposures were approximately 200 J(CIE)/m(2) erythemally effective UVR. The average relative UVR exposure (% total available UVR 08:30-18:30) was 6.4% (7.0% at site 1, 5.7% at site 2). Fractions of available UVR during outdoor stay were 14.4% (both sites), 15.3% (site 1), and 13.3% (site 2). In terms of relative differences, 5-6-year-old children at site 2 were exposed to 41% less UVR, and 1-4-year-old children 6% less than those at site 1. CONCLUSION: The difference can be explained by the children's outdoor pre-school environments, and the behaviors linked to these environments. It is recommended to consider the attractiveness of shady environments in the design of children's pre-school playgrounds, particularly if these are extremely exposed to the sun
SN - 0905-4383

AD - Stockholm County Council, Center of Public Health, Stockholm, Sweden. cecilia.boldeman@smd.sll.de
UR - 14738526
ER -

TY - JOUR
ID - 1474
T1 - Developing meaningful cohorts for human exposure models
A1 - Graham,S.E.
A1 - McCurdy,T.
Y1 - 2004/01//
N1 - Graham, Stephen E. McCurdy, Thomas
Journal of exposure analysis and environmental epidemiology
bjn, 9111438
IM
Journal Article. Research Support, U.S. Gov't, Non-P.H.S.
English
KW - MEDLINE
KW - Adolescent
KW - Adult
KW - Age Factors
KW - Aged
KW - 80 and over
KW - Child
KW - Preschool
KW - Cohort Studies
KW - Cross-Sectional Studies
KW - Databases
KW - Factual
KW - Environmental Exposure/an [Analysis]
KW - Environmental Monitoring/mt [Methods]
KW - Environmental Monitoring
KW - Ethnic Groups
KW - Female
KW - Human Activities/sn [Statistics & Numerical Data]
KW - Humans
KW - Infant
KW - Newborn
KW - Male
KW - Middle Aged
KW - Models
KW - Theoretical
KW - Sex Factors
KW - Time Factors
KW - Weather
RP - NOT IN FILE
SP - 23
EP - 43
JF - Journal of Exposure Analysis and Environmental Epidemiology
JA - J Expo Anal Environ Epidemiol
VL - 14
IS - 1
CY - England
N2 - This paper summarizes numerous statistical analyses focused on the US Environmental Protection Agency's Consolidated Human Activity Database (CHAD), used by many exposure modelers as the basis for data on what people do and where they spend their time. In doing so, modelers tend to divide the total population being analyzed into "cohorts", to reduce extraneous interindividual variability by focusing on people with common characteristics. Age and gender are typically used as the primary cohort-defining attributes, but

more complex exposure models also use weather, day-of-the-week, and employment attributes for this purpose. We analyzed all of these attributes and others to determine if statistically significant differences exist among them to warrant their being used to define distinct cohort groups. We focused our attention mostly on the relationship between cohort attributes and the time spent outdoors, indoors, and in motor vehicles. Our results indicate that besides age and gender, other important attributes for defining cohorts are the physical activity level of individuals, weather factors such as daily maximum temperature in combination with months of the year, and combined weekday/weekend with employment status. Less important are precipitation and ethnic data. While statistically significant, the collective set of attributes does not explain a large amount of variance in outdoor, indoor, or in-vehicle locational decisions. Based on other research, parameters such as lifestyle and life stages that are absent from CHAD might have reduced the amount of unexplained variance. At this time, we recommend that exposure modelers use age and gender as "first-order" attributes to define cohorts followed by physical activity level, daily maximum temperature or other suitable weather parameters, and day type possibly beyond a simple weekday/weekend classification
SN - 1053-4245
AD - Exposure Modeling Research Branch, Human Exposure and Atmospheric Sciences Division, National Exposure Research Laboratory, US Environmental Protection Agency, RTP, North Carolina 27711, USA. graham.stephen@epa.gov
UR - 14726943
ER -

TY - JOUR
ID - 1475
T1 - Differential diagnosis of cutaneous infiltrates of B lymphocytes with follicular growth pattern
A1 - Leinweber,B.
A1 - Colli,C.
A1 - Chott,A.
A1 - Kerl,H.
A1 - Cerroni,L.
Y1 - 2004/02//
N1 - Leinweber, Bernd. Colli, Claudia. Chott, Andreas. Kerl, Helmut. Cerroni, Lorenzo
The American Journal of dermatopathology
35v, 7911005
IM
Journal Article
English
KW - MEDLINE
KW - Adolescent
KW - Adult
KW - Aged
KW - 80 and over
KW - B-Lymphocytes/im [Immunology]
KW - B-Lymphocytes/me [Metabolism]
KW - B-Lymphocytes/pa [Pathology]
KW - Child
KW - DNA
KW - Neoplasm/an [Analysis]
KW - Diagnosis
KW - Differential
KW - Female
KW - Gene Rearrangement
KW - B-Lymphocyte
KW - Heavy Chain
KW - Humans
KW - Immunoenzyme Techniques
KW - Lymphoma
KW - B-Cell/ge [Genetics]
KW - B-Cell/me [Metabolism]

KW - B-Cell/pa [Pathology]
KW - Follicular/ge [Genetics]
KW - Follicular/me [Metabolism]
KW - Follicular/pa [Pathology]
KW - Male
KW - Middle Aged
KW - Polymerase Chain Reaction
KW - Pseudolymphoma/ge [Genetics]
KW - Pseudolymphoma/me [Metabolism]
KW - Pseudolymphoma/pa [Pathology]
KW - Skin Neoplasms/ge [Genetics]
KW - Skin Neoplasms/me [Metabolism]
KW - Skin Neoplasms/pa [Pathology]
KW - Tumor Markers
KW - Biological/me [Metabolism]
RP - NOT IN FILE
SP - 4
EP - 13
JF - American Journal of Dermatopathology
JA - Am J Dermatopathol
VL - 26
IS - 1
CY - United States
N2 - The differential diagnosis of cutaneous B-cell infiltrates with follicular pattern of growth is one of the most vexing problems in dermatopathology. In this study we focused on histopathologic, immunophenotypic, and molecular differential diagnostic criteria between Borrelia burgdorferi (Bb)-associated lymphocytoma cutis (LC), primary cutaneous follicle center cell lymphoma (FCCL), and primary cutaneous marginal zone lymphoma (MZL) with reactive germinal centers (GCs). A total of 47 patients were included in the study, including 12 cases of LC (M:F = 2:1; mean age: 38.0; median: 31; range: 9-75), 29 cases of FCCL (M:F = 1.2:1; mean age: 57.5; median: 57; range: 24-97), and 6 cases of MZL (M:F = 1:1; mean age: 63.8; median: 67.5; range: 38-86). In all cases complete phenotypic data were available. In addition, the IgH gene rearrangement and the t(14;18) were analyzed using the polymerase chain reaction technique (PCR) in 41 (FCCL = 27, LC = 10, MZL = 4) and 18 cases (FCCL = 15, LC = 2, MZL = 1), respectively. Histology revealed in all cases of FCCL one or more atypical feature of the follicles including the lack of or a reduced mantle zone, lack of polarization, tendency to confluence, and absence of tingible body macrophages. In most cases of Bb-associated LC, the GCs were devoid of mantle zone, lacked polarization, and revealed tendency to confluence as well, but all cases showed the presence of several tingible body macrophages. In MZL, follicles showed typical features of reactive GCs. Immunohistology revealed a reduced proliferative activity of neoplastic follicles as detected by MIB-1 antibody in 23 of 29 cases of FCCL (79.3%), but only in 1 case of LC (8.3%). Proliferation of the GCs was normal in all cases of MZL. Positivity for CD10 and/or Bcl-6 was found in small clusters outside the follicles in 19 cases of FCCL (65.5%), and in 3 cases of LC (25%), but in no case of MZL. The intensity of CD10 staining on follicular cells on average was stronger in cases of FCCL, but overlapping features could be observed. Finally, staining for Bcl-2 protein was consistently negative on GC cells in cases of LC and MZL, and was positive on a variable proportion of the cells in 8 cases of FCCL (28.6%). Molecular analyses showed no evidence of the t(14;18) in all cases tested. Analysis of the IgH gene rearrangement revealed a monoclonal pattern in 1 of 10 cases of LC (10%), 14 of 27 cases of FCCL (51.9%), and 2 of 4 cases of MZL (50%) tested. In summary, Bb-associated LC and FCCL show sometimes overlapping histopathologic, immunohistochemical, and molecular features, whereas follicles in MZL show clear-cut aspects of reactive GCs. Absence of tingible body macrophages within follicles, reduced proliferation of the follicles as detected by immunohistology, presence of positivity for Bcl-2 protein within follicular cells, and monoclonality by PCR are the main criteria suggestive of malignancy. Diagnosis of cutaneous infiltrates of B lymphocytes with follicular growth pattern should be achieved by integration of clinical data with histopathologic, immunohistochemical, and molecular features of the lesions
SN - 0193-1091
AD - Department of Dermatology, University of Graz, Austria
UR - 14726817
ER -

TY - JOUR
ID - 1476
T1 - Neighborhood playgrounds, fast food restaurants, and crime: relationships to overweight in low-income preschool children
A1 - Burdette,H.L.
A1 - Whitaker,R.C.
Y1 - 2004/01//
N1 - Burdette, Hillary L. Whitaker, Robert C
Preventive medicine
pm4, 0322116
IM
Comparative Study. Journal Article. Research Support, Non-U.S. Gov't. Research Support, U.S. Gov't, Non-P.H.S.
English
KW - MEDLINE
KW - Child
KW - Preschool
KW - Crime
KW - Cross-Sectional Studies
KW - Exercise
KW - Female
KW - Humans
KW - Male
KW - Obesity/ep [Epidemiology]
KW - Ohio/ep [Epidemiology]
KW - Play and Playthings
KW - Poverty
KW - Public Facilities
KW - Public Health
KW - Residence Characteristics
KW - Restaurants/cl [Classification]
RP - NOT IN FILE
SP - 57
EP - 63
JF - Preventive Medicine
JA - Prev Med
VL - 38
IS - 1
CY - United States
N2 - BACKGROUND: We examined the relationship between overweight in preschool children and three environmental factors--the proximity of the children's residences to playgrounds and to fast food restaurants and the safety of the children's neighborhoods. We hypothesized that children who lived farther from playgrounds, closer to fast food restaurants, and in unsafe neighborhoods were more likely to be overweight. METHODS: This was a cross-sectional study of 7,020 low-income children, 36 through 59 months of age living in Cincinnati, OH. Overweight was defined as a measured body mass index > or =95th percentile. The distance between each child's residence and the nearest public playground and fast food restaurant was determined with geographic information systems. Neighborhood safety was defined by the number of police-reported crimes per 1,000 residents per year in each of 46 city neighborhoods. RESULTS: Overall, 9.2% of the children were overweight, 76% black, and 23% white. The mean (+/- SD) distances from a child's home to the nearest playground and fast food restaurant were 0.31 (+/- 0.22) and 0.70 (+/- 0.38) miles, respectively. There was no association between child overweight and proximity to playgrounds, proximity to fast food restaurants, or level of neighborhood crime. The association between child overweight and playground proximity did not differ by neighborhood crime level. CONCLUSIONS: Within a population of urban low-income preschoolers, overweight was not associated with proximity to playgrounds and fast food restaurants or with the level of neighborhood crime
SN - 0091-7435

AD - Division of General and Community Pediatrics, Cincinnati Children's Hospital Medical Center, Cincinnati, OH 45229-3039, USA. burdette@email.chop.edu
UR - 14672642
ER -

TY - JOUR
ID - 1477
T1 - Investigation of the level of safety and appropriateness of playgrounds in Elazig city in Turkey
A1 - AcIk,Y.
A1 - Gulbayrak,C.
A1 - Turaci,Celik G.
Y1 - 2004/02//
N1 - AcIk, Yasemin. Gulbayrak, Canan. Turaci Celik, Gulen
International journal of environmental health research
9106628, dus
IM
Journal Article
English
KW - MEDLINE
KW - Accident Prevention
KW - Child
KW - Preschool
KW - Equipment Design
KW - Guideline Adherence
KW - Humans
KW - Play and Playthings
KW - Safety
KW - Turkey
KW - Wounds and Injuries/pc [Prevention & Control]
RP - NOT IN FILE
SP - 75
EP - 82
JF - International Journal of Environmental Health Research
JA - Int J Environ Health Res
VL - 14
IS - 1
CY - England
N2 - This study was conducted to identify and evaluate the degree of appropriateness of children's playground standards and the level of compliance with the current safety specifications of the playgrounds in Elazig city in Turkey. This was accomplished by on-site observation and measurement in the total of 24 playgrounds present in this city centre. It was determined that 83.3% of the playgrounds were next to a street and in 60% of these playgrounds there were no fences or any barrier to keep children from running into the street. In 87.5% of playgrounds the surfacing was not in proper depth and good order. Separate playgrounds for different ages were provided in only 12.5% of the playgrounds. In 62.5% of the playgrounds children were supervised to make sure they were safe. There was unsafe equipment in 95.8% of the playgrounds. In 83.3% of the playgrounds there were sharp edges, pinch points, or playground debris that might injure a child. The findings of the present study indicate that nearly all of the playgrounds in Elazig city center do not meet the criteria for playground standards and are not in compliance with the current safety specifications and need improvement
SN - 0960-3123
AD - Department of Public Health, Faculty of Medicine, Firat University, Elazig, Turkey. yacik@firat.edu.tr
UR - 14660120
ER -

TY - JOUR
ID - 1478
T1 - Childhood overweight: an expanding problem. [Review] [145 refs]
A1 - Kaur,H.

A1 - Hyder,M.L.
A1 - Poston,W.S.
Y1 - 2003///
N1 - Kaur, Harsohena. Hyder, Melissa L. Poston, W S Carlos
Treatments in endocrinology
101132977
IM
Journal Article. Review
English
KW - MEDLINE
KW - Adolescent
KW - Child
KW - Humans
KW - Obesity/ep [Epidemiology]
KW - Obesity/pc [Prevention & Control]
KW - Obesity/th [Therapy]
KW - Obesity
KW - Prevalence
KW - United States/ep [Epidemiology]
RP - NOT IN FILE
SP - 375
EP - 388
JF - Treatments in Endocrinology
JA - Treat
VL - 2
IS - 6
CY - New Zealand
N2 - Childhood overweight is a significant and growing health problem in the US and other parts of the world. Secular trend data in the US suggest that children have become substantially heavier over the last several decades and that their risk for a number of health problems is increasing as a result. Defining obesity in children has been difficult as assessing body fat is expensive and impractical. Body mass index (BMI), derived from weight and height, is used as a surrogate indicator in adults. In children, the consensus is to use BMI percentiles statistically derived from a reference population. There is evidence that the prenatal, early childhood, and adolescent periods are critical in the development of obesity but the mechanisms involved are yet to be elucidated. The recent rapid increase in childhood overweight and obesity is attributed to the modern obesogenic environment. Changes in dietary constituents including higher derivation of energy from nutritionally poor and energy dense foods, increased sweetened drink consumption, larger portion sizes, and more frequent intake of food outside the home have been associated with poorer diets and higher weights. Further, physical activity has reduced with decreases in school physical education classes and organized sports, fewer opportunities to expend energy for daily living activity due to more mechanization, lower frequency of walking and biking, and greater use of sedentary activities for leisure. Television watching remains the most common activity for children. There are significant health outcomes associated with childhood obesity, including the presence of cardiovascular risk factors, and greater prevalence of various medical problems including insulin resistance, type 2 diabetes mellitus, the metabolic syndrome, orthopedic problems, and pseudotumor cerebri. Of further concern is the increased risk for obesity in adulthood with its attendant co-morbidities. Interventions are imperative but not widely studied. The most effective interventions include comprehensive behavioral management, dietary modification, and exercise. Family-based interventions have been most successful at maintaining long-term weight loss. School-based interventions have the potential to significantly impact childhood overweight as large numbers of children can be reached. However, such programs require long-term follow-up and are expensive. Very low calorie diets, pharmacotherapy, and surgery remain experimental options for children. More recently, metformin has shown promise in promoting weight loss and improving insulin sensitivity among adolescents. Combining multiple approaches for treatment, addressing obesity-promoting sociocultural practices and policies, and focusing on prevention strategies will be necessary to address this epidemic. [References: 145]
SN - 1175-6349
AD - Department of Pediatrics, University of Kansas School of Medicine, Kansas City, Kansas 66160, USA. khaur@kumc.edu

UR - 15981942
ER -

TY - JOUR
ID - 1479
T1 - [Protein losing enteropathy (PLE) detected by Tc99m-labelled human serum albumin abdominal scintigraphy--case report]. [Polish]
A1 - Hubalewska-Hola,A.
A1 - Sowa-Staszczak,A.
A1 - Szczerbinski,T.
A1 - Lis,G.
A1 - Huszno,B.
A1 - Szybinski,Z.
Y1 - 2003///
N1 - Hubalewska-Hola, Alicja. Sowa-Staszczak, Anna. Szczerbinski, Tomasz. Lis, Grzegorz. Huszno, Bohdan. Szybinski, Zbigniew
Przeglad lekarski
q96, 19840720r
IM
Case Reports. English Abstract. Journal Article
Polish
KW - MEDLINE
KW - Anemia
KW - Hypochromic/et [Etiology]
KW - Child
KW - Female
KW - Humans
KW - Hypoalbuminemia/et [Etiology]
KW - Hypoproteinemia/et [Etiology]
KW - Immunoglobulin G/bl [Blood]
KW - Intestinal Secretions/ri [Radionuclide Imaging]
KW - Intestines/ri [Radionuclide Imaging]
KW - Protein-Losing Enteropathies/bl [Blood]
KW - Protein-Losing Enteropathies/ri [Radionuclide Imaging]
KW - Protein-Losing Enteropathies/su [Surgery]
KW - Sensitivity and Specificity
KW - Serum Albumin/du [Diagnostic Use]
KW - T-Lymphocytes/me [Metabolism]
KW - Technetium Tc 99m Aggregated Albumin/du [Diagnostic Use]
KW - Time Factors
RP - NOT IN FILE
SP - 606
EP - 608
JF - Przeglad Lekarski
JA - Przegl Lek
VL - 60
IS - 9
CY - Poland
N2 - Protein losing enteropathy (PLE) is a gastrointestinal disorder that is associated with excessive loss of plasma protein into the gut resulting from abnormal mucosal permeability. The disease is usually caused by inflammation. The loss of protein in PLE is a nonselective process affecting albumin, globulin and transferrin. Abdominal scintigraphy with human serum albumin marked by Tc99m seems to be an easy and sensitive method for diagnosing PLE. An 4-year-old girl was presented to an outside Pediatric Department due to hypoproteinemia and recurrent pneumonia which had caused several prior hospitalizations. The laboratory tests revealed hypoproteinemia, hypoalbuminemia, low level of IgG, sideropenia, and a decreased level of T lymphocytes. The loss of protein into the gut was confirmed by fecal clearance of alfa-1 antitrypsin. Only nonspecific inflammation was detected by biopsy of the small intestine. These clinical and laboratory findings,

quickly decreasing IgG and albumin levels in spite of i.v. supplementation and the lack of proteinuria permitted PLE diagnosis. The abdominal scintigraphy was planned to assess and localise protein losing through GIT and for strategy of possible surgical treatment. Abdominal dynamic scintigraphy was performed immediately after the injection of 300 MBq Tc99m human albumin. 90 images were taken within 180 minutes. Delayed abdominal images were obtained 6 and 24 hours after the tracer injection. Anterior abdominal scintigraphy showed pathological activity of Tc99m-albumin in small bowel in the upper left segment of the abdomen in the 40th minute after injection. Extensive accumulation of albumin was seen in the 160th minute. Delayed images, after 3 and 6 hours, revealed translocation of the tracer into the lower right abdominal segment. The further passage and tracer concentration was detected in ascendant and transverse colon. Based on the laboratory tests and scintigraphic images the girl was suspected to have segmental lymphangiectasia of small intestine and was qualified for laparatomy and, possibly, for surgical resection of the pathologically changed bowel. However, diffuse pathological changes revealed in the guts during the operation, rendered the operation impossible. Tc99m-labelled human serum albumin scintigraphy may be considered the method-of-choice in the diagnosis of protein-losing enteropathy. However, in this test the assessment of the enteropathy dimension is difficult and surgical treatment should be planned with caution
SN - 0033-2240
AD - Katedra i Klinika Endokrynologii, Collegium Medicum, Uniwersytetu Jagiellonskiego w Krakowie. alahuh@endo.cm-uj.krakow.pl
UR - 15065343
ER -

TY - JOUR
ID - 1480
T1 - An analysis of a developmentally delayed young girl. Coordinating analytic and developmental processes
A1 - Olesker,W.
Y1 - 2003///
N1 - Olesker, Wendy
The Psychoanalytic study of the child
qdi, 0376472
IM
Case Reports. Journal Article
English
KW - MEDLINE
KW - Child
KW - Preschool
KW - Developmental Disabilities/px [Psychology]
KW - Female
KW - Gender Identity
KW - Humans
KW - Psychoanalytic Therapy/mt [Methods]
RP - NOT IN FILE
SP - 89
EP - 111
JF - Psychoanalytic Study of the Child
JA - Psychoanal.Study Child
VL - 58
CY - United States
N2 - Clinical material is presented from a multi-year treatment of a five-year-old girl with a variety of developmental interferences, making it necessary to consider whether standard technique would suffice. History includes the fact that she was adopted five days after birth and told as early as possible about her adoption; she was placed in a restrictive brace from four months to twenty months because of congenital hip displasia. Sandy's ability to let in the outside world was limited by her intense denial, not looking, not taking in, and by her detachment. Her passivity--whether a defense (modeled on her experience of physical restraint) or an arrest--was a formidable obstacle to the development of active transference moments. I use this case as an opportunity to look at the role of developmental sequences in the context of the analytic process. While I consciously did not do anything different than I would with any child analytic patient, I intuitively stressed certain kinds of interventions

SN - 0079-7308
AD - New York Psychoanalytic Institute, New York University Postdoctoral Program in Psychoanalysis and Psychotherapy, USA
UR - 14982015
ER -

TY - JOUR
ID - 1481
T1 - [Environment and child health: from health transition to shared risk?]. [Review] [80 refs] [French]
A1 - Revault,P.
A1 - Monjour,L.
Y1 - 2003///
N1 - Revault, P. Monjour, L
Medecine tropicale : revue du Corps de sante colonial
mhq, 8710146
IM
English Abstract. Journal Article. Research Support, Non-U.S. Gov't. Review
French
KW - MEDLINE
KW - Child
KW - Child Welfare
KW - Decision Making
KW - Developing Countries
KW - Environmental Health
KW - Environmental Pollutants/po [Poisoning]
KW - Health Policy
KW - Health Status
KW - Humans
KW - Policy Making
KW - Quality of Life
KW - Risk Assessment
KW - Risk Factors
RP - NOT IN FILE
SP - 422
EP - 434
JF - Medecine Tropicale
JA - Med Trop (Mars.)
VL - 63
IS - 4-5
CY - France
N2 - Children under the age of 18 account for almost half of the world's population, with most living in developing countries. Young people are especially sensitive to acute and chronic environmental conditions and 43% of environmental diseases occur in the 12% of the world's population under age 5. The main environmental threats to the health of children in developing countries are inadequate access to clean water for drinking and hygiene, exposure to air pollution: primarily indoors and secondarily outdoors, risk of accidents and wounds, and poisoning due to toxic products. Recent data suggest that the number and diversity of environmental risk factors affecting child health is increasing as a result of increasing malnutrition, pollution, and violence and consequently that the level of health and quality of life of future generations will decrease. Due to the complexity of the interactions between environmental factors and socio-economic determinants, the epidemiological transition model is poorly suited to analyzing and predicting the concurring risks of infectious disease and chronic disease (diabetes, cancer...). This article presents a number of recommendations for training health professional, developing environmental reference centers, implementing risk assessment, coordinating decentralized activities and policy, and involving parents and children in the decisional process with emphasis on divulgating study findings and developing interfaces between the various stakeholders. [References: 80]
SN - 0025-682X

AD - Groupe de Recherche et de Realisation pour le Developpement en milieu Rural (GRDR), ESTHER (Ensemble pour une Solidarite Therapeutique Hospitaliere En Reseau), 36, rue de Charenton, 75012 Paris, France. pascal.revault@esther.fr
UR - 14763297
ER -

TY - JOUR
ID - 1482
T1 - Allergic reactions to Japanese encephalitis vaccine. [Review] [76 refs][Erratum appears in Immunol Allergy Clin North Am. 2004 May;24(2):335]
A1 - Plesner,A.M.
Y1 - 2003/11//
N1 - Plesner, Anne-Marie
Immunology and allergy clinics of North America
8805635
IM
Journal Article. Review
English
KW - MEDLINE
KW - Adolescent
KW - Adult
KW - Adverse Drug Reaction Reporting Systems
KW - Child
KW - Preschool
KW - Encephalitis
KW - Japanese/pc [Prevention & Control]
KW - Female
KW - Humans
KW - Hypersensitivity/et [Etiology]
KW - Hypersensitivity/im [Immunology]
KW - Japanese Encephalitis Vaccines/ae [Adverse Effects]
KW - Male
KW - Middle Aged
KW - Nervous System Diseases/et [Etiology]
RP - NOT IN FILE
SP - 665
EP - 697
JF - Immunology & Allergy Clinics of North America
JA - Immunol Allergy Clin North Am
VL - 23
IS - 4
CY - United States
N2 - The JEV widely is used in Asian countries each year and is an important vaccine for travelers to the East from other parts of the world. JE virus is a zoonotic disease with natural reservoirs and cannot be eliminated. Although a declining incidence of JE has been observed in Asia because of reduced transmission by agricultural approaches and vaccination, the most important control measure now, and in the future, is vaccination of humans against JE. The inactivated vaccine, produced from infected mouse-brain-derived tissue, is the only commercially available vaccine. There are several concerns with the use of this vaccine. It is expensive, requires two or three doses to achieve protective efficacy, and, in practice, requires further booster doses to maintain immunity. The apparent increase in allergic reactions in the first part of the 1990s has set focus on the safety of the JEV. A cheap, live attenuated SA 14-14-2 vaccine is used almost exclusively in China and parts of Korea, but there have been no trials of SA 14-14-2 vaccine outside JE endemic countries. The vaccine seems to be highly efficient, and few adverse events have been observed; however, PHK cells are used for the production of this vaccine, and these cells are not approved by the WHO. A satisfactory cell substrate is needed. A committee under the WHO has proposed that for the live JEV, there should be validity of the assays for retrovirus when applied to PHK cell substrate and validity of the mouse assays for neurovirulence. Further information should be reviewed on the long-term follow-up of recipients of the vaccine. Several new types of vaccines have reached

the phase of clinical trials; however, studies remain to be completed. Until a new vaccine is available, the priority of surveillance of adverse events and the continuous reporting of such events to the users of the vaccines must be of importance. This fact is highlighted by the possibility of the varying frequency of adverse events with different batches over the years. The WHO offers information and recommendations for vaccines in the EPI and issues a series of updated papers on other vaccines that are of international public health importance (eg, JEV). The development of alternative efficient, safe, and appropriately priced JEVs is recommended, as is intensified surveillance of adverse events. Prospective vaccine studies of safety may be limited because of sample size and because rare adverse events may not be detected. Several new initiatives have been taken to improve surveillance of adverse events to vaccines within the past 10 years. In Japan, there is an increasing awareness of the importance of efforts taken to improve vaccine safety, and surveillance of adverse events and possibilities of compensation for vaccine-related injuries are in place. In Vietnam, a database to detect adverse events after vaccination has been established; the project involves active visits to data collectors at the vaccination sites. Comparative studies of adverse events, such as one recent study from Japan and the United States, are important for the evaluation of the reporting systems. The reporting rate for JEV adverse events from Japan was approximately one order of magnitude lower than that in the United States. Japan had strict predefined reporting criteria and time limits for observations. If time limits for the observation are too strict (eg, defining a possible neurologic reaction to occur within 1 week after vaccination), later reactions will not be included (eg, if ADEM is elicited by a vaccine, the symptoms cannot be expected to occur until weeks after the vaccination). The passive surveillance systems have limitations with an underreporting of adverse events, depending on clinical seriousness, temporal proximity to vaccination, awareness of healthcare workers, and tradition of reporting particular events. In developed countries, surveillance of adverse events is formalized, although not necessarily optimal. An increase in reporting would be expected when the reporting of adverse events is mandatory. Reports have been sent to VAERS, the Vaccine Safety Datalink Project, and the European Union Pharmacovigilance System. A Brighton collaboration has been implemented to enhance comparability of vaccine safety data. Public health authorities in specific countries, such as the CDC in the United States and the National Advisory Committee in Canada, regularly have published information on the JE situation in Asia and the preventive measures to be taken, including information on the vaccines and adverse reactions. The conventional recommendation is that travelers should be vaccinated if they will spend more than 1 month in a JE endemic area or in areas with epidemic transmission with even shorter periods. Although the risk for JE for short-term travelers is considered small (1 case per 1 million travelers per year), sporadic cases, including deaths, have been reported among tourists traveling to endemic areas. Risk for travelers in rural districts in the season of risk is considerably higher (range, 1 case per 5000 travelers to 1 case per 20,000 travelers per week). Doctors who advise travelers should be updated on the latest JE occurrences in Asia. Updates on the JE situation can be found on bulletins at http://www.promedmail.org or are available from the WHO or CDC. The allergic reactions primarily described after vaccination with the inactivated mouse-brain-derived JEV have been observed in several countries during the 1900s. Allergic reactions, including the mucocutaneous and neurologic reactions reported after JE vaccination, may vary in frequency, and these reactions should be evaluated meticulously yearly. This step enables recommendations, including information on possible side effects, to be given in an optimal way. [References: 76]
SN - 0889-8561
AD - Department of Medical Officers of Health, Copenhagen County, Islands Brygge 67 DK-2300 Copenhagen S, Denmark. apl@kbh.eli.dk
UR - 14753386
ER -

TY - JOUR
ID - 1483
T1 - Environmental conditions and variation in levels of sun exposure among children in child care
A1 - Stanton,W.R.
A1 - Saleheen,H.N.
A1 - O'Riordan,D.
A1 - Roy,C.R.
Y1 - 2003///
N1 - Stanton, Warren R. Saleheen, Hassan Nazmus. O'Riordan, David. Roy, Colin R
International journal of behavioral medicine
9421097
IM

Comparative Study. Journal Article
English
KW - MEDLINE
KW - Australia
KW - Child Day Care Centers/ut [Utilization]
KW - Child Day Care Centers
KW - Child
KW - Preschool
KW - Environment Design
KW - Environmental Monitoring/mt [Methods]
KW - Epidemiological Monitoring
KW - Female
KW - Humans
KW - Male
KW - Protective Clothing/ut [Utilization]
KW - Risk Assessment
KW - Skin Neoplasms/et [Etiology]
KW - Skin Neoplasms/pc [Prevention & Control]
KW - Sunburn/ep [Epidemiology]
KW - Sunburn/pc [Prevention & Control]
KW - Sunlight/ae [Adverse Effects]
KW - Sunscreening Agents/tu [Therapeutic Use]
KW - Time Factors
RP - NOT IN FILE
SP - 285
EP - 298
JF - International Journal of Behavioral Medicine
JA - Int J Behav Med
VL - 10
IS - 4
CY - United States
N2 - Sun exposure in childhood is 1 of the risk factors for developing skin cancer, yet little is known about levels of exposure at this age. This is particularly important in countries with high levels of ultraviolet radiation (UVR) such as Australia. Among 49 children 3 to 5 years of age attending child care centers, UVR exposure was studied under 4 conditions in a repeated measures design; sunny days, cloudy days, teacher's instruction to stay in the shade, and a health professionals instruction to apply sunscreen. Three different data collection methods were employed: (a) completion of questionnaire or diary by parents and researcher, (b) polysulphone dosimeter readings, and (c) observational audits (video recording). Results of this study indicated that more than half the children had been sunburnt (pink or red) and more than a third had experienced painful sunburn (sore or tender) in the last summer. Most wore short sleeve shirts, short skirts or shorts and cap, that do not provide optimal levels of skin protection. However, sunscreen was applied to all exposed parts before the children went out to the playground. Over the period of 1 hr (9-10 a.m.) the average amount of time children spent in full sun was 22 min. On sunny days there was more variation across children in the amount of sun exposure received. While the potential amount of UVR exposure for young children during the hour they were outside on a sunny day was 1.45 MED (Minimum Erythemal Dose), they received on average 0.35 MED, which is an insufficient amount to result in an erythemal response on fair skin even without the use of sunscreen
SN - 1070-5503
AD - Centre for Health Promotion and Cancer Prevention Research, School of Population Health, Faculty of Health Science, University of Queensland, Herston, Australia. w.stanton@mailbox.uq.edu.au
UR - 14734259
ER -

TY - JOUR
ID - 1484
T1 - Influence of diagnostic adjuncts to the measurement of caries prevalence at different caries thresholds
A1 - Assaf,A.V.
A1 - Meneghim,Mde C.

A1 - Zanin,L.
A1 - Pardi,V.
A1 - Pereira,A.C.
A1 - Ambrosano,G.M.
Y1 - 2003/12//
N1 - Assaf, Andrea Videira. Meneghim, Marcelo de Castro. Zanin, Luciane. Pardi, Vanessa. Pereira, Antonio Carlos. Ambrosano, Glaucia Maria Bovi
International dental journal
gpt, 0374714
D, IM
Journal Article
English
KW - MEDLINE
KW - Analysis of Variance
KW - Brazil/ep [Epidemiology]
KW - Child
KW - DMF Index
KW - Dental Caries/di [Diagnosis]
KW - Dental Caries/ep [Epidemiology]
KW - Dental Caries Activity Tests/mt [Methods]
KW - Humans
KW - Prevalence
RP - NOT IN FILE
SP - 485
EP - 490
JF - International Dental Journal
JA - Int Dent J
VL - 53
IS - 6
CY - England
N2 - OBJECTIVES: To determine the influence of diagnostic adjuncts (dental brushing and drying) in dental caries diagnosis. To analyse the underestimation levels in epidemiological examinations when compared to traditional dental setting examinations. To evaluate the influence of including noncavitated (NC) carious lesions in the estimation of the disease. PARTICIPANTS: 51, 12-year-old children. INTERVENTIONS/METHODS: Four epidemiological exams performed in an outdoor setting and one performed in a traditional dental setting (visual-tactile method associated or not with diagnostic adjuncts). MAIN OUTCOME MEASURES: DMFS (Decayed, Missing and Filled Surfaces) index, according to the WHO criteria. RESULTS: When compared to exams carried out in a traditional dental setting, the epidemiological examinations showed no significant statistical differences for the DMFS score ($p>0.05$), presenting underestimation levels lower than 10% (except for the low prevalence caries group in the WHO exam). For the DMFS, previous dental brushing was more relevant than previous dental drying, although drying was the most important resource for NC diagnosis ($p<0.05$). CONCLUSION: The visual/tactile method, with or without diagnostic adjuncts, can diagnose cavitated lesions efficiently, but not NC lesions. However, for the low caries prevalence group, the implementation of dental brushing prior to an exam could be suggested
SN - 0020-6539
AD - Departamento de Odontologia Social, Avenida Limeira 901, Piracicaba, SP, Brazil
UR - 14725377
ER -

TY - JOUR
ID - 1485
T1 - Risk factors for norovirus, Sapporo-like virus, and group A rotavirus gastroenteritis
A1 - de Wit,M.A.
A1 - Koopmans,M.P.
A1 - van Duynhoven,Y.T.
Y1 - 2003/12//

N1 - de Wit, Matty A S. Koopmans, Marion P G. van Duynhoven, Yvonne T H P
Emerging infectious diseases
cod, 9508155
IM
Journal Article
English
KW - MEDLINE
KW - Adolescent
KW - Adult
KW - Animals
KW - Caliciviridae Infections/ep [Epidemiology]
KW - Caliciviridae Infections/pc [Prevention & Control]
KW - Caliciviridae Infections/tm [Transmission]
KW - Caliciviridae Infections/vi [Virology]
KW - Case-Control Studies
KW - Child
KW - Preschool
KW - Cohort Studies
KW - Disease Transmission
KW - Infectious
KW - Female
KW - Food Handling/mt [Methods]
KW - Gastroenteritis/ep [Epidemiology]
KW - Gastroenteritis/pc [Prevention & Control]
KW - Gastroenteritis/vi [Virology]
KW - Humans
KW - Infant
KW - Male
KW - Middle Aged
KW - Multivariate Analysis
KW - Netherlands/ep [Epidemiology]
KW - Norovirus/ip [Isolation & Purification]
KW - Prospective Studies
KW - Risk Factors
KW - Rotavirus/ip [Isolation & Purification]
KW - Rotavirus Infections/ep [Epidemiology]
KW - Rotavirus Infections/pc [Prevention & Control]
KW - Rotavirus Infections/tm [Transmission]
KW - Rotavirus Infections/vi [Virology]
KW - Sapovirus/ip [Isolation & Purification]
RP - NOT IN FILE
SP - 1563
EP - 1570
JF - Emerging Infectious Diseases
JA - Emerg Infect Dis
VL - 9
IS - 12
CY - United States
N2 - Viral pathogens are the most common causes of gastroenteritis in the community. To identify modes of transmission and opportunities for prevention, a case-control study was conducted and risk factors for gastroenteritis attributable to norovirus (NV), Sapporo-like virus (SLV), and rotavirus were studied. For NV gastroenteritis, having a household member with gastroenteritis, contact with a person with gastroenteritis outside the household, and poor food-handling hygiene were associated with illness (population attributable risk fractions [PAR] of 17%, 56%, and 47%, respectively). For SLV gastroenteritis, contact with a person with gastroenteritis outside the household was associated with a higher risk (PAR 60%). For rotavirus gastroenteritis, contact with a person with gastroenteritis outside the household and food-handling hygiene were associated with

a higher risk (PAR 86% and 46%, respectively). Transmission of these viral pathogens occurs primarily from person to person. However, for NV gastroenteritis, foodborne transmission seems to play an important role
SN - 1080-6040
AD - National Institute of Public Health and the Environment, Bilthoven, The Netherlands
UR - 14720397
ER -

TY - JOUR
ID - 1486
T1 - Wilderness event medicine
A1 - Burdick,T.E.
A1 - Brozen,R.
Y1 - 2003///
N1 - Burdick, Timothy E. Brozen, Reed
Wilderness & environmental medicine
dbe, 9505185
IM
Journal Article. Research Support, Non-U.S. Gov't
English
KW - MEDLINE
KW - Adolescent
KW - Adult
KW - Aged
KW - Athletic Injuries/ep [Epidemiology]
KW - Athletic Injuries/et [Etiology]
KW - Athletic Injuries/th [Therapy]
KW - Child
KW - Emergency Medical Services/og [Organization & Administration]
KW - Emergency Treatment/sn [Statistics & Numerical Data]
KW - Female
KW - Humans
KW - Male
KW - Middle Aged
KW - Mountaineering/in [Injuries]
KW - New Hampshire
RP - NOT IN FILE
SP - 236
EP - 239
JF - Wilderness & Environmental Medicine
JA - Wilderness Environ Med
VL - 14
IS - 4
CY - United States
N2 - OBJECTIVES: Wilderness Event Medicine (WEM), that is, the care of large groups of people participating in events in remote areas, is a rapidly growing subspecialty of wilderness medicine. Our goals are to report the injury rates from one wilderness event and to suggest ways to advance this emerging field. METHODS: We describe a 1-day wilderness hike and compare the injury rates from this one event with rates from other urban and wilderness events. RESULTS: Of the total 350 hikers, 6 persons presented for medical evaluation (5 dehydration and 1 orthopedic), yielding a rate of 17 evaluations per 1000 person-days of exposure. Only 1 person (or 2.9 per 1000) required medical assistance beyond oral rehydration. These rates are comparable to rates reported for urban events and other wilderness activities. CONCLUSIONS: According to very limited reports, the rates of injuries in wilderness events are similar to those for urban events. We recommend defining a wilderness event as an event with more than 200 participants and where the time from injury to care at a medical facility is likely to be greater than 1 hour. We also suggest the creation of a database of wilderness events, including standardized terms for the descriptions of activities, terrain, injuries, and medical response. Such a database could be used to help event planners anticipate and perhaps prevent injuries and to prepare effectively for those injuries that do occur

SN - 1080-6032
AD - Dartmouth Medical School, Hanover, NH, USA. tim.burdick@alum.dartmouth.org
UR - 14719858
ER -

TY - JOUR
ID - 1487
T1 - Propagation of interictal epileptiform activity can lead to erroneous source localizations: a 128-channel EEG mapping study
A1 - Lantz,G.
A1 - Spinelli,L.
A1 - Seeck,M.
A1 - de Peralta Menendez,R.G.
A1 - Sottas,C.C.
A1 - Michel,C.M.
Y1 - 2003/09//
N1 - Lantz, Goran. Spinelli, Laurent. Seeck, Margitta. de Peralta Menendez, Rolando Grave. Sottas, Cyrille C. Michel, Christoph M
Journal of clinical neurophysiology : official publication of the American Electroencephalographic Society
hsp, 8506708
IM
Comparative Study. Journal Article. Research Support, Non-U.S. Gov't
English
KW - MEDLINE
KW - Adolescent
KW - Adult
KW - Brain Mapping
KW - Child
KW - Preschool
KW - Diagnostic Errors
KW - Electrodes
KW - Electroencephalography/is [Instrumentation]
KW - Epilepsies
KW - Partial/di [Diagnosis]
KW - Partial/pp [Physiopathology]
KW - Partial/su [Surgery]
KW - Female
KW - Humans
KW - Magnetic Resonance Imaging/mt [Methods]
KW - Male
KW - Middle Aged
KW - Models
KW - Neurological
KW - Signal Processing
KW - Computer-Assisted
RP - NOT IN FILE
SP - 311
EP - 319
JF - Journal of Clinical Neurophysiology
JA - J Clin Neurophysiol
VL - 20
IS - 5
CY - United States
N2 - The relationship between interictal epileptiform activity and the epileptogenic zone is complex. Despite the fact that intraspike propagation may occur, the peak of the spike is often used as indicator of the site of ictal onset. In this investigation, spatio-temporal segmentation was used to demonstrate this intraspike propagation and to determine at which time point the voltage pattern corresponded best to the epileptogenic zone. Sixteen

patients with focal epilepsy were recorded with 125-channel EEG. Between one and five different map topographies were identified during the rising phase of the spike. A distributed source model (EPIFOCUS) was used to localize the source of each map, and the distance from the EPIFOCUS maximum to the anatomic lesion was calculated. In only 3 of 16 cases was the entire rising phase of the spike accounted for by one single map. In another five patients, several maps were obtained, although all were located within the epileptogenic lesion. In the remaining eight patients, however, parts of the rising phase had locations outside the epileptogenic lesion. On the average, 80% of the rising time had within lesion locations the most reliable time period being halfway between onset and peak. The results illustrate that intraspike propagation has to be considered in source localizations, and they also illustrate the usefulness of spatio-temporal segmentation for visualizing this propagation
SN - 0736-0258
AD - Functional Brain Mapping Laboratory and dagger Unit of Presurgical Epilepsy Evaluation, Neurology Clinic, University Hospital, Geneva, Switzerland. Goran.Lantz@medecine.unige.ch
UR - 14701992
ER -

TY - JOUR
ID - 1488
T1 - Physical activity interventions in the prevention and treatment of paediatric obesity: systematic review and critical appraisal. [Review] [48 refs]
A1 - Reilly,J.J.
A1 - McDowell,Z.C.
Y1 - 2003/08//
N1 - Reilly, John J. McDowell, Zoe C
The Proceedings of the Nutrition Society
pw6, 7505881
IM
Journal Article. Research Support, Non-U.S. Gov't. Review
English
KW - MEDLINE
KW - Adolescent
KW - Child
KW - Evidence-Based Medicine
KW - Exercise/ph [Physiology]
KW - Female
KW - Humans
KW - Intervention Studies
KW - Male
KW - Meta-Analysis as Topic
KW - Obesity/ep [Epidemiology]
KW - Obesity/et [Etiology]
KW - Obesity/pc [Prevention & Control]
KW - Randomized Controlled Trials as Topic
KW - Risk Reduction Behavior
KW - Scotland/ep [Epidemiology]
RP - NOT IN FILE
SP - 611
EP - 619
JF - Proceedings of the Nutrition Society
JA - Proc Nutr Soc
VL - 62
IS - 3
CY - England
N2 - Interventions for prevention and treatment of childhood obesity typically target increases in physical activity and, more recently, reductions in physical inactivity (sedentary behaviour such as television viewing). However, the evidence base for such strategies is extremely limited. The main aim of the present review was to update the systematic review and critical appraisal of evidence in the light of the recent rapid expansion of

research in this area. Randomised controlled trials (RCT) that targeted activity or inactivity, that followed up children or adolescents for at least 1 year and that included an objective weight-related outcome measure were included. Trials were appraised using previously published criteria (Harbour & Miller, 2001), and literature search strategies described previously (Reilly et al. 2002) were updated to May 2002. A total of four new RCT, two new systematic reviews and one meta-analysis were identified. The evidence base has increased markedly since the completion of earlier reviews, although high-quality evidence is still lacking. The evidence on childhood obesity prevention is not encouraging, although promising targets for prevention are now clear, notably reduction in sedentary behaviour. There is stronger evidence that targeting activity and/or inactivity might be effective in paediatric obesity treatment, but doubts as to the generalisability of existing interventions, and the clinical relevance of the interventions is unclear. Further research in settings outside the USA is urgently needed, and two ongoing RCT in Scotland are summarised. [References: 48]
SN - 0029-6651
AD - University of Glasgow Department of Human Nutrition, Royal Hospital for Sick Children, Yorkhill, Glasgow G3 8SJ, UK. jjr2y@clinmed.gla.ac.uk
UR - 14692597
ER -

TY - JOUR
ID - 1489
T1 - Comparative intervention study among Danish daycare children: the effect on illness of time spent outdoors
A1 - Mygind,O.
A1 - Ronne,T.
A1 - Soe,A.L.
A1 - Henrik,Wachmann C.
A1 - Ricks,P.
Y1 - 2003///
N1 - Mygind, Ole. Ronne, Tove. Soe, Anne-Lise. Henrik Wachmann, Carsten. Ricks, Philip
Scandinavian journal of public health
dew, 100883503
IM
Comparative Study. Journal Article. Research Support, Non-U.S. Gov't
English
KW - MEDLINE
KW - Child
KW - Child Care
KW - Preschool
KW - Communicable Diseases/cl [Classification]
KW - Communicable Diseases/ep [Epidemiology]
KW - Denmark/ep [Epidemiology]
KW - Environmental Exposure
KW - Female
KW - Humans
KW - Male
KW - Play and Playthings
RP - NOT IN FILE
SP - 439
EP - 443
JF - Scandinavian Journal of Public Health
JA - Scand J Public Health
VL - 31
IS - 6
CY - Norway
N2 - AIMS: The purpose of the study was to examine the correlation between illnesses, and in particular contagious illnesses, among daycare children and the amount of time spent outdoors, and whether through the intervention of increasing the amount of time spent outdoors it is possible to lower the incidence of illness. METHODS: The study was conducted among eight daycare centres within the same county. A total of 326

children, 17 homeroom groups, were divided into two study groups. Each of the two study groups had a two-month period in which the percentage of time spent outdoors per day was almost double that of the other study group. RESULTS: The overall frequency of sick days was 4%. There was no significant effect of spending more time outdoors on the number of illness episodes, length of the episode, or cause of illness. CONCLUSION: Our negative findings are in accordance with the theory that illness among daycare children is largely a result of contact among children and the hygiene standards of the institution, and thus these are the factors that may need to be addressed to reduce the level of illness among daycare children
SN - 1403-4948
AD - Medical Office of Health, Nykobing, Denmark
UR - 14675935
ER -

TY - JOUR
ID - 1490
T1 - Insured versus uninsured patients in the emergency room: is there a difference?
A1 - Pisarek,W.
A1 - Auwera,J.C.
A1 - Smet,M.
A1 - Damme,P.V.
A1 - Stroobants,J.
Y1 - 2003/12//
N1 - Pisarek, Wojciech. Auwera, Jean-Claude Van der. Smet, Mike. Damme, Pierre Van. Stroobants, Jan
European journal of emergency medicine : official journal of the European Society for Emergency Medicine
cl2, 9442482
IM
Comparative Study. Journal Article
English
KW - MEDLINE
KW - Adolescent
KW - Adult
KW - After-Hours Care/ut [Utilization]
KW - Age Distribution
KW - Aged
KW - 80 and over
KW - Belgium
KW - Child
KW - Preschool
KW - Emergency Service
KW - Hospital/ec [Economics]
KW - Hospital/ut [Utilization]
KW - Health Care Surveys
KW - Humans
KW - Infant
KW - Newborn
KW - Insurance Coverage/sn [Statistics & Numerical Data]
KW - Insurance
KW - Health/sn [Statistics & Numerical Data]
KW - Logistic Models
KW - Medically Uninsured/sn [Statistics & Numerical Data]
KW - Middle Aged
KW - Patient Admission/sn [Statistics & Numerical Data]
KW - Retrospective Studies
RP - NOT IN FILE
SP - 314
EP - 317
JF - European Journal of Emergency Medicine
JA - Eur J Emerg Med

VL - 10
IS - 4
CY - England
N2 - OBJECTIVE: To define the differences in emergency room usage patterns between patients with and without medical insurance coverage. METHODS: A retrospective analysis of the database of 34 642 consecutive patient visits to an urban hospital emergency room over a period of 509 days. Arrival times and admission rates were compared for insured and uninsured patients. RESULTS: A total of 46.8% of insured patients arrived at night or during the weekend versus 51.7% of the uninsured. Slightly more insured patients were admitted (18.6 versus 15.4%), both after their visit during the daytime (20.6 versus 17.1%) and outside daytime hours (16.3 versus 13.8%). The uninsured population was younger. CONCLUSION: Uninsured patients arrived more frequently during weekends and at night than insured patients. They were on average less likely to be admitted to the hospital. Demographic differences between both groups seemed to play an important role in the admission rate. Despite differences in emergency room usage patterns, it cannot be concluded that either group used the emergency room in excess of the other
SN - 0969-9546
AD - Emergency Department, Middelheim General Hospital, Antwerp, Belgium. wojciech.pisarek@skynet.be
UR - 14676511
ER -

TY - JOUR
ID - 1491
T1 - [Eight days of hand emergencies. Report of the audit carried out at the FESUM centers from June 3 to June 9, 2002]. [French]
A1 - Dubert,T.
A1 - Allieu,Y.
A1 - Bellemere,P.
A1 - Egloff,D.
A1 - Nonnenmacher,J.
A1 - Baudet,J.
A1 - Haloua,J.P.
A1 - Masmejean,E.
A1 - Marin-Braun,F.
A1 - Poirier,P.
A1 - Sassoon,D.
Y1 - 2003/10//
N1 - Dubert, T. Allieu, Y. Bellemere, P. Egloff, D. Nonnenmacher, J. Baudet, J. Haloua, J P. Masmejean, E. Marin-Braun, F. Poirier, P. Sassoon, D
Chirurgie de la main
dlc, 100937750
IM
English Abstract. Journal Article. Multicenter Study
French
KW - MEDLINE
KW - Adolescent
KW - Adult
KW - Aged
KW - 80 and over
KW - Child
KW - Preschool
KW - Emergency Treatment
KW - Europe
KW - Female
KW - Hand Injuries/su [Surgery]
KW - Health Facilities
KW - Humans
KW - Infant
KW - Male

KW - Medical Audit
KW - Middle Aged
KW - Societies
KW - Medical
KW - Time Factors
RP - NOT IN FILE
SP - 225
EP - 232
JF - Chirurgie de la Main
JA - Chir Main
VL - 22
IS - 5
CY - France
N2 - All the FESUM centers in France, Belgium and Switzerland were invited to participate in this prospective audit, during 1 week in June 2002. In these FESUM centers, the patients are operated by senior hand surgeons or trainees graduated with a microsurgical and a hand surgery University degrees. All acute hand disorders, requiring surgery or not, were to be included. For every case, a standardized form was to be filled. This form included 22 fields concerning the specificities of the patient, the circumstances of the accident, the lesions and initial treatment up to exit of the patient out of the Hand Center. Out of the 43 French centers, 38 (90%) participated in this study, but only 30% in the other French speaking countries. A total of 2360 forms were completed and analyzed, representing a mean of 8 forms per day center (6-147). The population was predominantly active men with a mean age of 31. Manual workers represented 41%, scholars 33%. Most of them came to the Hand Center with a non-specilized vehicle (86%). Emergency medical transportation was required in 130 cases (5.8%). A majority of the patients were treated on an outdoor basis. A 1-day admission concerned 29% of the patients, and 4.6% have been admitted on an indoor basis during several days. Work accident represented 28% of all the cases, while the majority was daily living (62%) or sport (15%) accidents. Closed trauma represented 50% of the cases. Amongst open trauma (974 cases), 862 were simple skin lacerations, 156 skin loss, 140 extensor tendon lacerations, 70 flexor tendon lacerations. A preliminary wound exploration had been performed in a non-specialized center in 124 cases (12%). Complete amputation of some part was observed in 33 cases. In 32%, the initial severity of the lesion led to expect some degree of definitive consequences. Some kind of anesthesia was required in 43% of the cases (local in 41%, troncular in 19%, plexical in 28% and general in 9%). A surgical procedure was performed in 45% of the patients. Microsurgery was necessary in 15%, six of which were replantations. The period between presentation to the Hand Center and treatment was less than 1 day in 95% of the cases. Time of treatment was considered to be delayed in 113 cases (5%). Following this audit, it is considered that the FESUM centers make provision for the care of 120,000 cases per year, 54,000 of which needing a surgical procedure. This may be a small part of the total load of emergency hand surgery throughout the country (generally estimated over 1.4 million), but compares quite favorably with other European studies. We believe that improvement relies essentially on a better orientation of the patients whether they need a simple skill or specialist skill treatment. An information leaflet about orientation of hand trauma has been distributed to non-specialized emergency centers. Hand surgery training must be reevaluated inside the universitary system to avoid a dramatic lack of hand surgeons within a few years. A new audit will be presented next year
SN - 1297-3203
AD - Clinique la Francilienne, 16, avenue de l'hotel-de-ville, 77340 Pontault-Combault, France. thierry.dubert@noos.fr
UR - 14653016
ER -

TY - JOUR
ID - 1492
T1 - Measuring mood and complex behavior in natural environments: use of ecological momentary assessment in pediatric affective disorders
A1 - Axelson,D.A.
A1 - Bertocci,M.A.
A1 - Lewin,D.S.
A1 - Trubnick,L.S.
A1 - Birmaher,B.

A1 - Williamson,D.E.
A1 - Ryan,N.D.
A1 - Dahl,R.E.
Y1 - 2003///
N1 - Axelson, David A. Bertocci, Michele A. Lewin, Daniel S. Trubnick, Laura S. Birmaher, Boris. Williamson, Douglas E. Ryan, Neal D. Dahl, Ronald E
Journal of child and adolescent psychopharmacology
co4, 9105358
IM
Case Reports. Clinical Trial. Journal Article. Research Support, U.S. Gov't, P.H.S.
English
KW - MEDLINE
KW - Adolescent
KW - Affect/ph [Physiology]
KW - Bipolar Disorder/px [Psychology]
KW - Cellular Phone
KW - Child
KW - Depressive Disorder
KW - Major/px [Psychology]
KW - Environment
KW - Female
KW - Humans
KW - Male
KW - Mood Disorders/px [Psychology]
KW - Motivation
KW - Motor Activity/ph [Physiology]
KW - Pilot Projects
KW - Psychiatric Status Rating Scales
KW - Social Environment
RP - NOT IN FILE
SP - 253
EP - 266
JF - Journal of Child & Adolescent Psychopharmacology
JA - J Child Adolesc Psychopharmacol
VL - 13
IS - 3
CY - United States
N2 - This article describes the theoretical background and methodology of ecological momentary assessment (EMA) and reports results from a pilot study using EMA techniques in 16 children and adolescents with affective disorders and 5 subjects who were healthy and at low risk to develop future affective disorders. Multiple daily assessments of the subjects' mood, thoughts, and behaviors were performed in their natural environments using brief interviews on cellular phone calls by the study staff and by wrist actigraphy. The pilot results demonstrated that the EMA methodology is feasible in this population, as 17 of 21 subjects were able to complete the entire 8-week protocol. The potential usefulness of the EMA methodology is illustrated by specific case reports. Potential applications of the EMA methodology to the study of neurobehavioral systems and the pathophysiology of pediatric affective disorders are discussed
SN - 1044-5463
AD - Department of Psychiatry, University of Pittsburgh Medical Center, Western Psychiatric Institute and Clinic, Pittsburgh, Pennsylvania 15213, USA
UR - 14642013
ER -

TY - JOUR
ID - 1493
T1 - Body composition and physical activity in New Zealand Maori, Pacific and European children aged 5-14 years
A1 - Rush,E.C.

A1 - Plank,L.D.
A1 - Davies,P.S.
A1 - Watson,P.
A1 - Wall,C.R.
Y1 - 2003/12//
N1 - Rush, Elaine C. Plank, Lindsay D. Davies, Peter S W. Watson, Patsy. Wall, Clare R
The British journal of nutrition
az4, 0372547
IM
Journal Article. Research Support, Non-U.S. Gov't
English
KW - MEDLINE
KW - Adolescent
KW - Body Composition/ph [Physiology]
KW - Calorimetry
KW - Indirect
KW - Child
KW - Preschool
KW - Energy Metabolism/ph [Physiology]
KW - Europe/eh [Ethnology]
KW - European Continental Ancestry Group
KW - Female
KW - Humans
KW - Male
KW - New Zealand
KW - Obesity/eh [Ethnology]
KW - Oceanic Ancestry Group
KW - Pacific Islands/eh [Ethnology]
KW - Physical Exertion/ph [Physiology]
KW - Pilot Projects
RP - NOT IN FILE
SP - 1133
EP - 1139
JF - British Journal of Nutrition
JA - Br J Nutr
VL - 90
IS - 6
CY - England
N2 - Body fatness and the components of energy expenditure in children aged 5-14 years were investigated. In a group of seventy-nine healthy children (thirty-nine female, forty male), mean age 10.0 (sd 2.8) years, comprising twenty-seven Maori, twenty-six Pacific Island and twenty-six European, total energy expenditure (TEE) was determined over 10 d using the doubly-labelled water method. Resting metabolic rate (RMR) was measured by indirect calorimetry and physical activity level (PAL) was calculated as TEE:RMR. Fat-free mass (FFM), and hence fat mass, was derived from the (18)O-dilution space using appropriate values for FFM hydration in children. Qualitative information on physical activity patterns was obtained by questionnaire. Maori and Pacific children had a higher BMI than European children (P<0.003), but % body fat was similar for the three ethnic groups. The % body fat increased with age for girls (r 0.42, P=0.008), but not for boys. Ethnicity was not a significant predictor of RMR adjusted for FFM and fat mass. TEE and PAL, adjusted for body weight and age, were higher in Maori than European children (P<0.02), with Pacific children having intermediate values. PAL was inversely correlated with % body fat in boys (r -0.43, P=0.006), but was not significantly associated in girls. The % body fat was not correlated with reported time spent inactive or outdoors. Ethnic-related differences in total and activity-related energy expenditure that might account for higher obesity rates in Maori and Pacific children were not seen. Low levels of physical activity were associated with increased body fat in boys but not in girls
SN - 0007-1145
AD - Department of Applied Science, Auckland University of Technology, Private Bag, 92006, Auckland 1020, New Zealand. elaine.rush@aut.ac.nz

UR - 14641973
ER -

TY - JOUR
ID - 1494
T1 - Parathyroid hormone and its fragments in children with chronic renal failure
A1 - Waller,S.
A1 - Reynolds,A.
A1 - Ridout,D.
A1 - Cantor,T.
A1 - Gao,P.
A1 - Rees,L.
Y1 - 2003/12//
N1 - Waller, Simon. Reynolds, Anthony. Ridout, Deborah. Cantor, Tom. Gao, Ping. Rees, Lesley
Pediatric nephrology (Berlin, Germany)
avr, 8708728
IM
Journal Article. Research Support, Non-U.S. Gov't
English
KW - MEDLINE
KW - Child
KW - Preschool
KW - Female
KW - Glomerular Filtration Rate
KW - Humans
KW - Kidney Failure
KW - Chronic/bl [Blood]
KW - Chronic/pp [Physiopathology]
KW - Kidney Function Tests
KW - Male
KW - Parathyroid Hormone/bl [Blood]
KW - Peptide Fragments/bl [Blood]
KW - Radioimmunoassay
KW - Reference Values
RP - NOT IN FILE
SP - 1242
EP - 1248
JF - Pediatric Nephrology
JA - Pediatr Nephrol
VL - 18
IS - 12
CY - Germany
N2 - Parathyroid hormone (PTH) immunoradiometric assays (IRMA) exhibit cross-reactivity between 1-84 PTH and long carboxyl-terminal-PTH (C-PTH) molecules. C-PTH antagonizes the biological actions of 1-84 PTH and circulates in excess in chronic renal failure (CRF), partially explaining why supra-physiological PTH levels are recommended to maintain bone turnover. Furthermore, the ratio 1-84 PTH/C-PTH may be related to bone turnover. This study characterizes the 1-84 PTH/C-PTH ratio in children with varying severity of CRF and levels of PTH. Two hundred and forty-one children with CRF, managed with the aim of preventing the development of hyperparathyroidism, had PTH measured by 'intact' IRMA and a new more specific Cyclase-Activating-PTH (CAP) IRMA. C-PTH levels were calculated by subtracting CAP-IRMA from 'intact' IRMA. Fifty-three controls with normal renal function were also recruited. Mean 'intact' IRMA correlated with CAP-IRMA (r=0.98), but was higher (P<0.001). The mean 1-84 PTH/C-PTH ratio was lower than controls in dialysis patients (P=0.022) and those with a glomerular filtration rate <30 ml/min per m(2)(P=0.033). This ratio was comparable to controls when the PTH level was normal, but was lower with PTH levels outside the normal range (P<0.01). These data suggest that CAP-IRMA gives a more accurate assessment of actual PTH levels than 'intact' IRMA in CRF. Maintenance of normal PTH levels throughout the course of CRF permits the

maintenance of a normal 1-84 PTH/C-PTH ratio, the clinical significance of which requires further investigation in children
SN - 0931-041X
AD - Department of Nephro-Urology, Institute of Child Health and Great Ormond Street Hospital for Children NHS Trust, London, UK. s.waller@doctors.org.uk
UR - 14579141
ER -

TY - JOUR
ID - 1495
T1 - Residential arsenic and lead levels in an agricultural community with a history of lead arsenate use
A1 - Wolz,S.
A1 - Fenske,R.A.
A1 - Simcox,N.J.
A1 - Palcisko,G.
A1 - Kissel,J.C.
Y1 - 2003/11//
N1 - Wolz, Sarah. Fenske, Richard A. Simcox, Nancy J. Palcisko, Gary. Kissel, John C
Environmental research
ei2, 0147621
IM
Journal Article. Research Support, Non-U.S. Gov't. Research Support, U.S. Gov't, Non-P.H.S.. Research Support, U.S. Gov't, P.H.S.
English
KW - MEDLINE
KW - Adult
KW - Agriculture
KW - Air Pollutants/an [Analysis]
KW - Arsenates
KW - Arsenic/an [Analysis]
KW - Child
KW - Child Welfare
KW - Dust
KW - Environmental Exposure
KW - Environmental Monitoring
KW - Housing
KW - Humans
KW - Hygiene
KW - Lead/an [Analysis]
KW - Public Health
KW - Risk Assessment
KW - Soil Pollutants/an [Analysis]
KW - United States
KW - United States Environmental Protection Agency
RP - NOT IN FILE
SP - 293
EP - 300
JF - Environmental Research
JA - Environ Res
VL - 93
IS - 3
CY - United States
N2 - Lead arsenate (PbHAsO4) was used as an insecticide in Washington fruit orchards from 1905 to 1947. We examined exposure potential for children living in an agricultural community with historic PbHAsO4 use. Soil and housedust samples were collected from 58 residences. Families were asked about land use history, age of home, and remodeling activities. Median concentrations of arsenic were higher in housedust than in soil (9.0 and 4.2 microg/g, respectively; P=0.05), as were lead concentrations (129 and 46 microg/g, respectively;

P=0.0001). Significant associations were observed between indoor and outdoor levels of each metal, indicating track-in as an important exposure pathway. Homes on or near land use for pear or apple production between 1905 and 1947 had significantly higher soil (P=0.005) and housedust (P=0.004) lead, and soil arsenic (P=0.04) than did the other homes. Homes more than 30 years old had significantly higher soil and housedust lead than did newer homes (P=0.01). Homes remodeled within the past two years had significantly higher soil (P=0.01) and housedust (P=0.04) lead. Child doses extrapolated from these data indicate that 36% of homes had soil or dust arsenic levels above the minimum risk level estimated by the Agency for Toxic Substances and Disease Registry. None of the measured lead levels exceeded current US Environmental Protection Agency guidelines. Public health education programs focused on residential hygiene would be of value in areas of historic PbHAsO4 use
SN - 0013-9351
AD - Department of Environmental Health, School of Public Health and Community Medicine, Health Sciences Building, Room F-233, University of Washington, Box 357234, Seattle, WA 98195-7234, USA
UR - 14615240
ER -

TY - JOUR
ID - 1496
T1 - Assessing lay health advisor activity in an intervention to prevent lead poisoning in Native American children
A1 - Kegler,M.C.
A1 - Stern,R.
A1 - Whitecrow-Ollis,S.
A1 - Malcoe,L.H.
Y1 - 2003/04//
N1 - Kegler, Michelle Crozier. Stern, Rachel. Whitecrow-Ollis, Sally. Malcoe, Lorraine Halinka
Health promotion practice
100890609
IM
Evaluation Studies. Journal Article. Research Support, U.S. Gov't, P.H.S.
English
KW - MEDLINE
KW - Child
KW - Child Welfare
KW - Community Health Workers
KW - Environmental Exposure/ae [Adverse Effects]
KW - Environmental Exposure/pc [Prevention & Control]
KW - Health Education/ma [Manpower]
KW - Health Education/mt [Methods]
KW - Health Educators
KW - Health Services
KW - Indigenous/ma [Manpower]
KW - Indigenous/og [Organization & Administration]
KW - Humans
KW - Indians
KW - North American/ed [Education]
KW - Lead Poisoning/eh [Ethnology]
KW - Lead Poisoning/pc [Prevention & Control]
KW - Mining
KW - Oklahoma
KW - Program Evaluation
KW - Social Support
RP - NOT IN FILE
SP - 189
EP - 196
JF - Health Promotion Practice
JA - HEALTH PROMOT PRACT

VL - 4
IS - 2
CY - United States
N2 - The purpose of this study is to assess patterns of lay health advisor (LHA) activity in an intervention to reduce lead exposure in Native American children exposed to mine waste. A total of 39 LHAs were recruited and trained to become LHAs from eight tribes in northeastern Oklahoma. LHAs completed activity tracking forms over a 2-year intervention period to document contacts made with community groups and individuals in their social networks. They engaged in an average of 5.4 activities per month, reaching an average of 39 persons. Close members of their social networks were reached in 40.4% of the contacts; persons outside of their networks were reached in 24% of the contacts. This study suggests that 1 to 3 contacts per week may be a reasonable expectation for LHA activity. Findings also suggest that LHA interventions are a promising approach for engaging Native American communities in addressing an environmental health problem
SN - 1524-8399
AD - Department of Behavioral Sciences and Health Education, Rollins School of Public Health, Emory University, USA
UR - 14610989
ER -

TY - JOUR
ID - 1497
T1 - Detection of distribution of copper inside and outside of lysosomes in cultured hepatolenticular degeneration fibroblasts by electron probe X-ray microanalysis
A1 - Liu,W.
A1 - Li,J.Y.
A1 - Jin,J.
A1 - Zuo,J.
Y1 - 2003/05//
N1 - Liu, Wen. Li, Jin-Yan. Jin, Ji. Zuo, Ji
Hepatobiliary & pancreatic diseases international : HBPD INT
101151457
IM
Journal Article
English
KW - MEDLINE
KW - Adolescent
KW - Adult
KW - Carrier Proteins/an [Analysis]
KW - Cells
KW - Cultured
KW - Child
KW - Copper/an [Analysis]
KW - Copper/me [Metabolism]
KW - Electron Probe Microanalysis
KW - Female
KW - Fibroblasts/ch [Chemistry]
KW - Fibroblasts/me [Metabolism]
KW - Fibroblasts/pa [Pathology]
KW - Hepatolenticular Degeneration/me [Metabolism]
KW - Hepatolenticular Degeneration/pa [Pathology]
KW - Humans
KW - Lysosomes/ch [Chemistry]
KW - Lysosomes/me [Metabolism]
KW - Male
RP - NOT IN FILE
SP - 270
EP - 273
JF - Hepatobiliary & Pancreatic Diseases International

JA - Hepatobiliary Pancreat.Dis Int
VL - 2
IS - 2
CY - China
N2 - OBJECTIVE: To observe the distribution of copper in the subcellular structure for the understanding of primary pathogenesis of hepatolenticular degeneration (HLD). METHODS: Skin fibroblasts taken from HLD patients were cultured as an in vitro model of HLD, and the control cells taken from healthy volunteers were cultured in the same way. The distribution of copper inside and outside of lysosomes in fibroblasts was detected by quantitative electron probe X-ray microanalysis. The relationship between the subcellular location of copper and the genotype of the patients, and relationship between the distribution of copper and the course of the disease were analyzed. RESULTS: The content of Cu2+inside lysosomes of HLD cells (14.6+/-2.1 mmol/kg) and of heterozygote cells (11.6+/-0.6 mmol/kg) was higher than that of normal cells (4.5+/-1.2 mmol/kg) (P<0.01). The content of Cu2+outside lysosomes of HLD cells (17.5+/-4.2 mmol/kg) and of heterozygote cells (12.0+/-0.9 mmol/kg) was higher than that of normal cells (4.7+/-1.2 mmol/kg) (P<0.01). The distribution of copper in the subcellular structure was correlated with disease courses of HLD patients. With the progression of the disease, more copper was deposited in lysosomes (r=0.85, P<0.01). The content of copper in the diffused cytoplasmic compartment in HLD cells was correlated with that of sulfur (r=0.86, P<0.05), but not in heterozygote and normal cells. CONCLUSIONS: In the early stage of HLD, copper is accumulated outside lysosome, which is paralleled with increase of metallothionein-like proteins (copper and sulfur-binding proteins). With the development of the disease, more copper is deposited inside lysosome than outside lysosome. We conclude that the up-regulation expression of copper and sulfur-binding proteins and copper accumulation in lysosomes may play an important role in lowering the ATP7B gene mutation-induced toxic effects of free copper on the cell
SN - 1499-3872
AD - Department of Medical Genetics, School of Medicine, Fudan University, Shanghai 200032, China
UR - 14599982
ER -

TY - JOUR
ID - 1498
T1 - Urban-rural contrasts in the physical fitness of school children in Oaxaca, Mexico
A1 - Pena Reyes,M.E.
A1 - Tan,S.K.
A1 - Malina,R.M.
Y1 - 2003/11//
N1 - Pena Reyes, Maria Eugenia. Tan, Swee Kheng. Malina, Robert M
American journal of human biology : the official journal of the Human Biology Council
d3k, 8915029
IM
Comparative Study. Journal Article. Research Support, U.S. Gov't, Non-P.H.S.
English
KW - MEDLINE
KW - Adolescent
KW - Age Factors
KW - Body Constitution
KW - Child
KW - Female
KW - Humans
KW - Life Style
KW - Male
KW - Mexico
KW - Physical Education and Training
KW - Physical Fitness/ph [Physiology]
KW - Rural Population
KW - Schools
KW - Urban Population
RP - NOT IN FILE

SP - 800
EP - 813
JF - American Journal of Human Biology
JA - Am J Human Biol
VL - 15
IS - 6
CY - United States
N2 - The physical fitness of school children resident in an urban colonia and in a rural indigenous community in Oaxaca, southern Mexico, was compared. Two measures of performance-related fitness (standing long jump, 35-yard dash [32 m]) and four measures of health-related fitness (grip strength, sit and reach, timed sit-ups, distance run) were taken on 355 rural (175 boys, 184 girls) and 324 urban (163 boys, 161 girls) school children, 6-13 years of age. Urban children were significantly taller and heavier than rural children. Absolute grip strength did not consistently differ between rural and urban children, but when adjusted for age and body size, strength was greater in rural children. Explosive power (standing long jump) and abdominal strength and endurance (timed sit-ups) were better in urban than in rural children without and with adjustment for age and body size. Urban-rural differences in running speed (dash) and flexibility (sit and reach) varied by age group and sex. Younger rural children and older urban girls performed better in the distance run, whereas older rural and urban boys did not differ in endurance. The size advantage of urban children does not necessarily translate into better levels of performance- and health-related physical fitness. The observed differences may be related to activity habits associated with school physical education and lifestyle in the respective communities. Copyright 2003 Wiley-Liss, Inc
SN - 1042-0533
AD - Escuela Nacional de Antropologia e Historia, Mexico, DF
UR - 14595872
ER -

TY - JOUR
ID - 1499
T1 - The prolonged epidemic of anthroponotic cutaneous leishmaniasis in Kabul, Afghanistan: 'bringing down the neighbourhood'
A1 - Reyburn,H.
A1 - Rowland,M.
A1 - Mohsen,M.
A1 - Khan,B.
A1 - Davies,C.
Y1 - 2003/03//
N1 - Reyburn, Hugh. Rowland, Mark. Mohsen, Mohammmed. Khan, Bismulla. Davies, Clive
Transactions of the Royal Society of Tropical Medicine and Hygiene
wbu, 7506129
IM
Journal Article. Research Support, Non-U.S. Gov't
English
KW - MEDLINE
KW - Adolescent
KW - Adult
KW - Afghanistan/ep [Epidemiology]
KW - Age Distribution
KW - Aged
KW - Child
KW - Preschool
KW - Disease Outbreaks
KW - Emigration and Immigration
KW - Female
KW - Humans
KW - Incidence
KW - Infant
KW - Newborn

KW - Leishmaniasis
KW - Cutaneous/ep [Epidemiology]
KW - Male
KW - Middle Aged
KW - Prevalence
KW - Residence Characteristics
KW - Retrospective Studies
KW - Risk Factors
KW - Sex Distribution
KW - War
RP - NOT IN FILE
SP - 170
EP - 176
JF - Transactions of the Royal Society of Tropical Medicine & Hygiene
JA - Trans R Soc Trop Med Hyg
VL - 97
IS - 2
CY - England
N2 - In order to investigate the distribution and causes of the spread of anthroponotic cutaneous leishmaniasis (ACL) in Kabul, Afghanistan, a cross-sectional study was conducted during 1997-98 amongst 75,787 residents in the 13 central districts of the city. Using data on active lesions and scars with their times of onset, migration patterns and age of subjects, 2 independent methods were used to estimate, retrospectively, the annual incidence of ACL in recent years. Results indicated a rapid increase in incidence from 1987, peaking in 1996 when an estimated 12% of the population had active disease. Active prevalence was lowest in infants (aged < 2 years), and while risk was gender-independent in children and adolescents, active prevalence in those aged > 20 years was significantly higher amongst females than males (odds ratio [OR] = 1.51, 95% CI 1.34-1.70). About 44% of lesions were located on the head, 38% on upper limbs, 16% on lower limbs and 2% elsewhere. The relative frequency of head lesions dropped with age (P < 0.001), and amongst adults was lowest amongst males (P < 0.001), possibly due to the protective effect of a beard. Within the study population, 32% reported that they had immigrated from outside Kabul, 34% that they had been born in Kabul but had since migrated to another district of the city, and 34% that they had been born in the district of their present residence. Active prevalence amongst those born in their current district of residence was positively associated with the percentage of immigrants in their district (P = 0.027), indicating that a 1% increase in the percentage of immigrants increased the odds of an active lesion by 12% (OR = 1.12, 95% CI 1.01-1.24), but there was no association with the percentage of migrants from other districts in Kabul (P = 0.65) or with war damage (P = 0.33). As active prevalence was not significantly greater in immigrants than local Kabulis, these results support the hypothesis that the epidemic in Kabul has been maintained by a steady influx of susceptible immigrants. It is important that the new opportunities for social development that now exist in Kabul are not hampered by this unpleasant and stigmatizing disease. As population movement is clearly a contributing factor to its transmission, this threat is very real
SN - 0035-9203
AD - HealthNet International, Kabul, Afghanistan
UR - 14584372
ER -

TY - JOUR
ID - 1500
T1 - Respiratory symptoms and exposure to wood smoke in an isolated northern community
A1 - Guggisberg,M.
A1 - Hessel,P.A.
A1 - Michaelchuk,D.
A1 - Ahmed,I.
Y1 - 2003/09//
N1 - Guggisberg, Michael. Hessel, Patrick A. Michaelchuk, Dennis. Ahmed, Iqbal
Canadian journal of public health = Revue canadienne de sante publique
ck6, 0372714
IM

Comparative Study. Journal Article. Research Support, Non-U.S. Gov't
English
KW - MEDLINE
KW - Adolescent
KW - Adult
KW - Air Pollution
KW - Indoor/ae [Adverse Effects]
KW - Indoor/sn [Statistics & Numerical Data]
KW - Child
KW - Cooking/mt [Methods]
KW - Environmental Exposure/ae [Adverse Effects]
KW - Female
KW - Heating/mt [Methods]
KW - Humans
KW - Inuits/sn [Statistics & Numerical Data]
KW - Male
KW - Middle Aged
KW - Northwest Territories/ep [Epidemiology]
KW - Prevalence
KW - Respiratory Tract Diseases/eh [Ethnology]
KW - Respiratory Tract Diseases/et [Etiology]
KW - Smoke/ae [Adverse Effects]
KW - Tobacco Smoke Pollution/ae [Adverse Effects]
KW - Wood
RP - NOT IN FILE
SP - 372
EP - 376
JF - Canadian Journal of Public Health
JA - Can J Public Health
VL - Revue Canadienne de Sante Publique. 94
IS - 5
CY - Canada
N2 - BACKGROUND: Wood smoke has been associated with respiratory symptoms. This study examined the prevalence of respiratory symptoms and health effects of wood smoke exposures (from home heating, curing meat, and tanning hides) among residents of Deline, Northwest Territories (NWT). METHODS: A survey was conducted of all residents. Relationships between wood smoke and respiratory symptoms were examined. RESULTS: The response rate was 70.2% (n=402). 71% of people at least 18 years old were current smokers. Prevalence of symptoms was higher for women (odds ratios (ORs) 1.3-3.1). Women who smoked were more likely to be exposed to indoor smoke from curing and tanning. ORs for respiratory symptoms were higher for females, increased with age, and were strongly affected by smoking. Among those at least 18 years old, phlegm on winter mornings (6.5 (95% CI: 2.3-18.1)), dyspnoea (5.1 (95% CI: 1.9-13.2)), and watery or itchy eyes (3.6 (95% CI: 1.4-9.0)) were significantly related to self-reported outdoor wood smoke and smoke curing. Home heating was marginally associated with wheeze. No significant associations were found for males. CONCLUSIONS: Women engaged in curing/tanning demonstrated increased prevalence of respiratory symptoms. The cultural importance of these activities precludes abandoning them. Smoking cessation, limiting wood smoke exposure times, and process modifications in curing and tanning could reduce risk of adverse health effects
SN - 0008-4263
AD - Department of Environmental Sciences, Swiss Federal Institute of Technology, Zurich, Switzerland
UR - 14577748
ER -

TY - JOUR
ID - 1501
T1 - [Travelling immigrants]. [Spanish]
A1 - Valerio,L.
A1 - Guerrero,L.

A1 - Martinez,O.
A1 - Sabria,M.
A1 - Garrido,P.
A1 - Fabregat,A.
A1 - Reina,M.D.
Y1 - 2003/10/15/
N1 - Valerio, L. Guerrero, L. Martinez, O. Sabria, M. Garrido, P. Fabregat, A. Reina, M D
Atencion primaria / Sociedad Espanola de Medicina de Familia y Comunitaria
9111075, a5f
IM
English Abstract. Journal Article
Spanish
KW - MEDLINE
KW - Adolescent
KW - Adult
KW - Child
KW - Preschool
KW - Emigration and Immigration/sn [Statistics & Numerical Data]
KW - Female
KW - Humans
KW - Infant
KW - Male
KW - Retrospective Studies
KW - Risk Factors
KW - Spain
KW - Travel/sn [Statistics & Numerical Data]
RP - NOT IN FILE
SP - 330
EP - 336
JF - Atencion Primaria
JA - Aten.Primaria
VL - 32
IS - 6
CY - Spain
N2 - OBJECTIVES: To characterise travelling immigrants and the international movements they undertake. To identify the risk journeys that this population makes and to assess the international preventive activities for them compared with those for autochthonous travellers. DESIGN: Retrospective, observational study. SETTING: Unit of International Health (UIH) Santa Coloma de Gramenet (Barcelona), in the Barcelones Nord i Maresme Health Area, Primary Care Division of the Catalan Institute of Health. PARTICIPANTS: Patients seen between June 1999 and June 2002 (n=1163) at the UIH. They were divided into two groups, depending on their origins: those born inside (autochthonous group; no=1019) or outside (immigrant group; n1=144) the European Union. MAIN MEASUREMENTS: Age, country of origin, length of residence in Spain, interval between consultation and journey, reason for journey, destination, duration of the journey, kind of journey, stay in rural or isolated area, exposure to malaria, vaccinations and anti-malaria chemoprophylaxis prescribed, and overall risk of journey. RESULTS: Immigrants spent more time abroad than autochthonous people (mean 43.5 days; 95% CI, 37.9-49.1; and 15.8 days, 95% CI, 14.9-16.7, respectively; P<.0001). Periods between the journey and the consultation at the UIH were generally similar in the two groups (mean of 26.8 days for immigrants and 30.41 days for autochthonous people; ns), although the immigrants consulted more often in periods of less than 15 and 7 days (P<.005). 31.9% of immigrants stayed in areas of high malaria transmission (95% CI, 24.4%-39.6%) vs 21.3% (95% CI, 18.8%-23.8%) of autochthonous people (P=.04). Overall, immigrants made more risk journeys than autochthonous people (54.8% vs 43.1%; P<.01). No significant differences between the two groups were found in terms of quality in the administration of the international vaccinations prescribed. Compliance with the guidelines for anti-malaria chemoprophylaxis was less in the immigrant group than in the autochthonous one (36% vs 84%; P<.005). CONCLUSIONS: The immigrant group in our environment needs to be thought of as a risk group for catching and importing tropical diseases during their international movements. They form a population for whom international preventive measures should be prioritized
SN - 0212-6567

AD - Unidad de Salud Internacional, Servicio de Atencion Primaria Santa, Coloma de Gramenet, Institut Catala de la Salut, Barcelona, Espana. lvalerio.bnm.ics@gencat.net
UR - 14572395
ER -

TY - JOUR
ID - 1502
T1 - Relating improvisational music therapy with severely and multiply disabled children to communication development
A1 - Rainey Perry,M.M.
Y1 - 2003///
N1 - Rainey Perry, Mary M
Journal of music therapy
izv, 0014162
IM
Journal Article
English
KW - MEDLINE
KW - Adolescent
KW - Cerebral Palsy/co [Complications]
KW - Cerebral Palsy/th [Therapy]
KW - Child
KW - Communication
KW - Developmental Disabilities/th [Therapy]
KW - Disabled Children
KW - Female
KW - Humans
KW - Language Development Disorders/th [Therapy]
KW - Male
KW - Music Therapy/mt [Methods]
KW - Time Factors
KW - Video Recording
RP - NOT IN FILE
SP - 227
EP - 246
JF - Journal of Music Therapy
JA - J Music Ther
VL - 40
IS - 3
CY - United States
N2 - The effect of different levels of preintentional and intentional communication development on musical interaction with children with severe and multiple disabilities has not been explored in the music therapy literature. Aside from stage of communication development, what are the particular influences of disability on musical interaction with children who have preintentional and early intentional communication? A qualitative research project explored these issues. Ten school-aged children with severe and multiple disabilities participated in the project. The most common medical diagnosis was cerebral palsy. Analysis of video recordings and other data confirmed that the children's level of communication development was reflected in individual music therapy. Specifically, children at different levels of communication development varied in their abilities to initiate, anticipate, and sustain participation in turn taking, and to maintain attention to and engagement in the interaction. Both turn taking and playing and singing together were found to be important forms of communication during music therapy. Communication problems related to disability included: difficulties in using objects as a focus of joint attention, difficulties in interpreting the interactive environment, being sufficiently motivated to communicate, severely limited means of interaction, attaining and maintaining an appropriate level of arousal, and lack of interest in interaction and the outside environment. Further study of how music therapy can be related to general issues in communication for individuals with severe and multiple disabilities is recommended
SN - 0022-2917

AD - University of Melbourne, Australia
UR - 14567732
ER -

TY - JOUR
ID - 1503
T1 - Is there progression from irritability/dyscontrol to major depressive and manic symptoms? A retrospective community survey of parents of bipolar children
A1 - Fergus,E.L.
A1 - Miller,R.B.
A1 - Luckenbaugh,D.A.
A1 - Leverich,G.S.
A1 - Findling,R.L.
A1 - Speer,A.M.
A1 - Post,R.M.
Y1 - 2003/10//
N1 - Fergus, Emily L. Miller, Rachel B. Luckenbaugh, David A. Leverich, Gabriele S. Findling, Robert L. Speer, Andrew M. Post, Robert M
Journal of affective disorders
h3v, 7906073
IM
Journal Article. Research Support, Non-U.S. Gov't
English
KW - MEDLINE
KW - Adolescent
KW - Adult
KW - Bipolar Disorder/di [Diagnosis]
KW - Bipolar Disorder/px [Psychology]
KW - Child
KW - Child Psychology
KW - Depressive Disorder
KW - Major/di [Diagnosis]
KW - Major/px [Psychology]
KW - Diagnostic and Statistical Manual of Mental Disorders
KW - Disease Progression
KW - Female
KW - Humans
KW - Irritable Mood
KW - Male
KW - Parents
KW - Questionnaires
KW - Residence Characteristics
KW - Retrospective Studies
KW - Self Efficacy
KW - Time Factors
RP - NOT IN FILE
SP - 71
EP - 78
JF - Journal of Affective Disorders
JA - J Affect.Disord
VL - 77
IS - 1
CY - Netherlands
N2 - BACKGROUND: Although previous studies have discussed age-related changes in the presentation of early onset bipolar illness, the developmental progression of early symptoms remains unclear. The current study sought to trace parents' retrospective report of yearly occurrence of symptoms in a sample of children with and without a diagnosis of bipolar disorder in the community. METHODS: Parents retrospectively rated the

occurrence of 37 activated and withdrawn symptoms causing dysfunction for each year of their child's life (mean age 12.6 +/- 6.9). Children were divided into three groups based on parent report of diagnosis by a community clinician: bipolar (n=78); non-bipolar diagnosis (n=38); and well (no psychiatric diagnosis) (n=82). Principal components analysis was performed to understand the relationship among the symptom variables and their potential differences among the three groups as a function of age. RESULTS: Four symptom components were derived and these began to distinguish children with bipolar disorder from the other groups at different ages. Component II (irritability/dyscontrol), which included temper tantrums, poor frustration tolerance, impulsivity, increased aggression, decreased attention span, hyperactivity and irritability, began to distinguish bipolar children from the others the earliest (i.e., from ages 1 to 6). The other components (I, III, and IV) which included symptoms more typical of adult depression (I), mania (III), and psychosis (IV), distinguished the children with a bipolar diagnosis from the others much later (between ages 7 and 12). LIMITATIONS: The data were derived from retrospective reports by parents of their children's symptoms on a yearly symptom check list instrument which has not been previously utilized. Parents' ratings were not validated by an outside rater. Moreover, the children were diagnosed in the community and a formal diagnostic interview was not given. CONCLUSIONS: By parental report, the cluster of symptoms in the irritability/dyscontrol component may characterize the earliest precursors to an illness eventually associated with more classic manic and depressive components that are diagnosed and treated as bipolar disorder in the community. These retrospective survey data suggesting a longitudinal evolution of symptom clusters in childhood bipolar-like illness identify a number of areas for prospective research and validation
SN - 0165-0327
AD - Biological Psychiatry Branch, National Institute of Mental Health, NIH, Bethesda, MD 20892, USA
UR - 14550937
ER -

TY - JOUR
ID - 1504
T1 - [Childhood injuries in northern Israel--prevalance and risk factors]. [Hebrew]
A1 - Miron,D.
A1 - Shinnawi,F.
A1 - Meenes,R.
A1 - Avishai,I.
A1 - Sarid,Y.
A1 - Rotem,M.
Y1 - 2003/09/20/
N1 - Miron, Dan. Shinnawi, Fatmeh. Meenes, Rahel. Avishai, Idit. Sarid, Yosi. Rotem, Menachem
Harefuah
0034351, fzf
IM
English Abstract. Journal Article
Hebrew
KW - MEDLINE
KW - Accident Prevention
KW - Accidents
KW - Home/sn [Statistics & Numerical Data]
KW - Adolescent
KW - Child
KW - Preschool
KW - Humans
KW - Infant
KW - Israel/ep [Epidemiology]
KW - Morbidity
KW - Prevalence
KW - Risk Factors
KW - Wounds and Injuries/ep [Epidemiology]
RP - NOT IN FILE
SP - 579
EP - 582

JF - Harefuah
VL - 142
IS - 8-9
CY - Israel
N2 - BACKGROUND: Injuries resulting from accidents in the home are among the most common causes of morbidity in children. Preventing these accidents by educating parents has proved to be the most successful way of reducing such accidents and protecting children. AIMS: To study the prevalence, demographic variables and risk factors of childhood injuries in Northern Israel and to determine risk groups for preventive activities. METHODS: The study was prospective and conducted during the year 2000 in 36 urban (not including Arab clinics) and rural community clinics of Clalit Health Services in Northern Israel that provided health care for approximately 62,000 children (about 55% of all children residing in these communities). The survey included all children 0-18 years of age admitted to the clinics with home injuries during the study period. Data regarding clinical, demographic and socioeconomic variables was obtained from parents or guardians by a detailed questionnaire. Statistical analysis of the data was conducted using Chi-Square to compare the different groups and logistic regression for analysis of risk factors for injury. RESULTS: An overall number of 2,086 (prevalence of 3.2%) children were included in the study of whom 61.5% and 38.5% were non Jews and Jews, respectively, (prevalence of 4.2% and 2.2% respectively) and 66.5% of all the children were boys ($p < 0.01$). The most common injuries among both populations were falls, blows, burns, and cuts (43%, 23%, 15%, and 13% respectively). The injuries occurred at home, or close to the home (31%), at school or kindergarten (32%), or elsewhere (37%). Seventy eight percent and 49% of the injuries in the non-Jewish and Jewish populations, respectively, occurred at or around the home ($p < 0.001$). Logistic regression analysis revealed that falling was most prevalent outside the home in non-Jewish males older than 3 years. Burns were more prevalent at home in non-Jewish girls under the age of 3, and blows were prevalent in the Jewish population outside the home in children over the age of 3. CONCLUSIONS: This study provides data that could direct preventive measures in the community in order to decrease the prevalence of injury to children. In order to be effective and have significant impact, these preventive activities should concentrate on specific risk groups
SN - 0017-7768
AD - Pediatric Department A, HaEmek Medical Center, Afula
UR - 14518157
ER -

TY - JOUR
ID - 1505
T1 - Development of a multidisciplinary method to determine risk factors for arm fracture in falls from playground equipment
A1 - Sherker,S.
A1 - Ozanne-Smith,J.
A1 - Rechnitzer,G.
A1 - Grzebieta,R.
Y1 - 2003/09//
N1 - Sherker, S. Ozanne-Smith, J. Rechnitzer, G. Grzebieta, R
Injury prevention : journal of the International Society for Child and Adolescent Injury Prevention
crz, 9510056
IM
Journal Article. Research Support, Non-U.S. Gov't
English
KW - MEDLINE
KW - Accidental Falls
KW - Anthropometry/is [Instrumentation]
KW - Anthropometry/mt [Methods]
KW - Arm/ph [Physiology]
KW - Arm Injuries/et [Etiology]
KW - Arm Injuries/pc [Prevention & Control]
KW - Case-Control Studies
KW - Child
KW - Equipment Design
KW - Fractures

KW - Bone/et [Etiology]
KW - Bone/pc [Prevention & Control]
KW - Humans
KW - Manikins
KW - Patient Care Team
KW - Play and Playthings/in [Injuries]
KW - Reproducibility of Results
KW - Risk Factors
KW - Stress
KW - Mechanical
RP - NOT IN FILE
SP - 279
EP - 283
JF - Injury Prevention
JA - Inj Prev
VL - 9
IS - 3
CY - England
N2 - OBJECTIVES: To present the development of a novel multidisciplinary method to investigate physical risk factors for playground related arm fracture. RATIONALE: Previous playground injury research has been limited in its ability to determine risk factors for arm fractures, despite their common and costly occurrence. Biomechanical studies have focused exclusively on head injury. Few epidemiological studies have quantified surface impact attenuation and none have investigated specific injury outcomes such as arm fracture. DESIGN: An unmatched case-control study design was developed. An instrumented child dummy and rig were designed to simulate real playground falls in situ. Validated output from the dummy was used to quantify arm load. Other field measurements included equipment height, fall height, surface depth, headform deceleration, and head injury criterion. DISCUSSION: Validated methods of biomechanics and epidemiology were combined in a robust design. The principle strength of this method was the use of a multidisciplinary approach to identify and quantify risk and protective factors for arm fracture in falls from playground equipment. Application of this method will enable countermeasures for prevention of playground related arm fracture to be developed
SN - 1353-8047
AD - Accident Research Centre, Monash University, Melbourne, Australia.
shauna.sherker@general.monash.edu.au
UR - 12966022
ER -

TY - JOUR
ID - 1506
T1 - Evaluation of North Carolina child care safety regulations
A1 - Kotch,J.B.
A1 - Hussey,J.M.
A1 - Carter,A.
Y1 - 2003/09//
N1 - Kotch, J B. Hussey, J M. Carter, A
Injury prevention : journal of the International Society for Child and Adolescent Injury Prevention
crz, 9510056
IM
Evaluation Studies. Journal Article. Research Support, Non-U.S. Gov't
English
KW - MEDLINE
KW - Child
KW - Child Care/lj [Legislation & Jurisprudence]
KW - Child Day Care Centers/lj [Legislation & Jurisprudence]
KW - Child Day Care Centers/st [Standards]
KW - Child Day Care Centers/td [Trends]
KW - Environment Design/st [Standards]
KW - Humans

KW - North Carolina
KW - Patient Acceptance of Health Care
KW - Play and Playthings/in [Injuries]
KW - Questionnaires
KW - Risk Factors
KW - Safety/lj [Legislation & Jurisprudence]
RP - NOT IN FILE
SP - 220
EP - 225
JF - Injury Prevention
JA - Inj Prev
VL - 9
IS - 3
CY - England
N2 - INTRODUCTION: The goal of this study was to track any changes in injuries and injury hazards during the first 3.5 years of implementation of the North Carolina Child Care Commission's 1996 playground safety regulations. METHODS: All reports (n=5402) of medically attended injuries in regulated child care settings in North Carolina during the period 1 January 1997 through 30 June 2000 were reviewed and analyzed. A total of 294 playground safety inspections were conducted in November and December 1998 in randomly selected North Carolina child care centers, and the playground safety inspections were repeated in 76 child care centers in August 2000. Finally, in 1999 a 1992 child care center director self assessment of safety features in classrooms was sent to the directors of 291 of the 294 centers. RESULTS: The annual rate of reported, medically attended injuries occurring in regulated child care facilities in North Carolina declined by 22% from 1997 to 1999. The playground safety inspections in the year 2000 revealed that, for nine of 10 playground concerns and 12 of 18 playground safety hazards, average ratings were equal to or better than those of 1998. Finally, the director surveys revealed no dramatic changes in classroom safety hazards since 1992. DISCUSSION: This study represents the first time that the authors are aware of that a significant decline in state-wide child care injury rates has been associated with improved regulation of playground safety in the US
SN - 1353-8047
AD - Department of Maternal and Child Health, School of Public Health, University of North Carolina at Chapel Hill, 27599, USA. jonathan_kotch@unc.edu
UR - 12966009
ER -

TY - JOUR
ID - 1507
T1 - Injury and frequency of use of playground equipment in public schools and parks in Brisbane, Australia
A1 - Nixon,J.W.
A1 - Acton,C.H.
A1 - Wallis,B.
A1 - Ballesteros,M.F.
A1 - Battistutta,D.
Y1 - 2003/09//
N1 - Nixon, J W. Acton, C H C. Wallis, B. Ballesteros, M F. Battistutta, D
Injury prevention : journal of the International Society for Child and Adolescent Injury Prevention
crz, 9510056
IM
Journal Article. Research Support, Non-U.S. Gov't
English
KW - MEDLINE
KW - Australia/ep [Epidemiology]
KW - Child
KW - Emergencies/ep [Epidemiology]
KW - Environmental Exposure
KW - Equipment Design
KW - Humans
KW - Play and Playthings/in [Injuries]

KW - Recreation
KW - Risk Factors
KW - Safety
KW - Schools
KW - Urban Health
RP - NOT IN FILE
SP - 210
EP - 213
JF - Injury Prevention
JA - Inj Prev
VL - 9
IS - 3
CY - England
N2 - OBJECTIVE: The purpose of this study was to determine the frequency of use of play equipment in public schools and parks in Brisbane, Australia, and to estimate an annual rate of injury per use of equipment, overall and for particular types of equipment. METHODS: Injury data on all children injured from playground equipment and seeking medical attention at the emergency department of either of the two children's hospitals in the City of Brisbane were obtained for the years 1996 and 1997. Children were observed at play on five different pieces of play equipment in a random sample of 16 parks and 16 schools in the City of Brisbane. Children injured in the 16 parks and schools were counted, and rates of injury and use were calculated. RESULTS: The ranked order for equipment use in the 16 schools was climbing equipment (3762 uses), horizontal ladders (2309 uses), and slides (856 uses). Each horizontal ladder was used 2.6 times more often than each piece of climbing equipment. Each horizontal ladder was used 7.8 times more than each piece of climbing equipment in the sample of public parks. Slides were used 4.6 times more than climbing equipment in parks and 1.2 times more in public schools. The annual injury rate for the 16 schools and 16 parks under observation was 0.59/100 000 and 0.26/100 000 uses of equipment, respectively. CONCLUSIONS: This study shows that annual number of injuries per standardized number of uses could be used to determine the relative risk of particular pieces of playground equipment. The low overall rate of injuries/100 000 uses of equipment in this study suggests that the benefit of further reduction of injury in this community may be marginal and outweigh the economic costs in addition to reducing challenging play opportunities
SN - 1353-8047
AD - Department of Paediatrics and Child Health, University of Queensland, Brisbane, Australia. j.nixon@uq.edu.au
UR - 12966007
ER -

TY - JOUR
ID - 1508
T1 - Playground injuries
A1 - MacKay,M.
Y1 - 2003/09//
N1 - MacKay, M
Injury prevention : journal of the International Society for Child and Adolescent Injury Prevention
crz, 9510056
IM
Comment. Editorial
English
KW - MEDLINE
KW - Accidental Falls/pc [Prevention & Control]
KW - Child
KW - Environmental Exposure
KW - Equipment Design
KW - Fractures
KW - Bone/et [Etiology]
KW - Bone/pc [Prevention & Control]
KW - Humans
KW - Play and Playthings/in [Injuries]

KW - Public Health
KW - Risk Factors
RP - NOT IN FILE
SP - 194
EP - 196
JF - Injury Prevention
JA - Inj Prev
VL - 9
IS - 3
CY - England
SN - 1353-8047
UR - 12966004
ER -

TY - JOUR
ID - 1509
T1 - Influence of socioeconomic status on the effectiveness of bicycle helmet legislation for children: a prospective observational study
A1 - Parkin,P.C.
A1 - Khambalia,A.
A1 - Kmet,L.
A1 - Macarthur,C.
Y1 - 2003/09//
N1 - Parkin, Patricia C. Khambalia, Amina. Kmet, Leanne. Macarthur, Colin
Pediatrics
oxv, 0376422
AIM, IM
Journal Article. Research Support, Non-U.S. Gov't
English
KW - MEDLINE
KW - Accidents
KW - Traffic/ec [Economics]
KW - Traffic/pc [Prevention & Control]
KW - Adolescent
KW - Athletic Injuries/ec [Economics]
KW - Athletic Injuries/pc [Prevention & Control]
KW - Bicycling/ec [Economics]
KW - Bicycling/in [Injuries]
KW - Bicycling/lj [Legislation & Jurisprudence]
KW - Bicycling/td [Trends]
KW - Canada
KW - Child
KW - Preschool
KW - Female
KW - Head Protective Devices/ec [Economics]
KW - Head Protective Devices/td [Trends]
KW - Humans
KW - Male
KW - Observation/mt [Methods]
KW - Prospective Studies
KW - Sex Factors
KW - Socioeconomic Factors
RP - NOT IN FILE
SP - e192
EP - e196
JF - Pediatrics
VL - 112

IS - 3 Pt 1
CY - United States
N2 - OBJECTIVE: To evaluate the influence of average family income in a geographic area on the effectiveness of helmet legislation on observed helmet use by children (5-14 years). METHODS: The study was conducted in East York, a health district of Metropolitan Toronto, in collaboration with the East York Health Unit. In 1996, the total population was 107 822, 11 340 of which were children 5 to 14 years. Census data were used to group the 21 census tracts in East York into 7 geographically distinct areas. The boundaries of these areas are natural barriers to travel, such as expressways, ravines, railway tracks, and hydroelectric power lines. The areas were also ranked according to average family income (based on Statistics Canada data). For analytical purposes, areas were defined as low-, mid-, and high-income areas. Census data profiles of the areas have been previously described. For each consecutive year from 1990 to 1997 inclusive, direct observations of children riding bicycles in East York during the months of April through October were made. In 1995, observations were completed before the introduction of the law on October 1, 1995. Only children who were between 5 and 14 years of age and riding a 2-wheeled bicycle were included in the study. In total, 111 sites across all 7 areas were selected for observation. Observational sites included school yards of all elementary and middle schools (kindergarten to grade 8) and all parks in East York. In addition, 5 major intersections and 5 residential streets from each area were randomly selected. Observers were trained and used a standardized data collection form. A pilot study showed that the data collected by observers were reliable and valid. Observers remained at each site for 1 hour and collected data on helmet use and sex. Ethical approval for the study was obtained from the Hospital for Sick Children Research Ethics Board, the East York Board of Education, and the Metropolitan Separate School Board. The proportion of children who were wearing a bicycle helmet was estimated by year (1990-1997, inclusive), sex (male, female), location (school, park, major intersection, residential street), and income area (low, mid, high). For estimating the effect of legislation on helmet use, data from the year immediately after legislation (1996) were compared with data from the year preceding legislation (1995). The relative risk (RR) of helmet use (after vs before legislation) was calculated along with a 95% confidence interval (CI). Logistic regression analysis was used to adjust for potential confounding variables (sex and location). RESULTS: During the 8-year study period, 9768 observations were made (range: 914-1879 observations per year). The proportion of child cyclists who wore a bicycle helmet increased steadily during the first 4 years of the study period, from 4% in 1990 (34 of 914), to 16% in 1991 (303 of 1879), to 25% in 1992 (383 of 1563), and to 45% in 1993 (438 of 984). During 1994 (460 of 1083) and 1995 (568 of 1227), helmet use remained relatively stable at approximately 44%. Helmet use rose markedly in 1996 (the first year after helmet legislation was introduced) to 68% (818 of 1202) and remained stable at 66% (609 of 916) in 1997. Throughout the study period, girls were consistently more likely to wear helmets than were boys. In total, 47% (1420 of 3047) of girls wore helmets, compared with 33% (2193 of 6721) of boys (RR: 1.43; 95% CI: 1.36-1.50). In addition, children who were riding to school were more likely to use helmets, compared with children who were riding on residential streets, major intersections, and parks. Overall, 48% (1497 of 3129) of children who were riding to school wore bicycle helmets, compared with 32% (2116 of 6639) of children who were riding at other locations (RR: 1.50; 95% CI: 1.43-1.58). Children in the high-income areas were consistently more likely to wear helmets, compared with children in the mid- and low-income areas. Helmet legislation was associated with a significant increase in helmet use by children in East York. In 1995, 46% (ast York. In 1995, 46% (568 of 1227) of children wore bicycle helmets, compared with 68% (818 of 1202) of children in 1996 (RR: 1.47; 95% CI: 1.37-1.58). The effect of legislation, however, varied by income area. In low-income areas, helmet use increased by 28% after legislation, from 33% (213 of 646) in 1995 to 61% (442 of 721) in 1996 (RR: 1.86; 95% CI: 1.64-2.11). In mid-income areas, helmet use increased by 29% after legislation, from 50% (150 of 300) in 1995 to 79% (185 of 234) in 1996 (RR: 1.58; 95% CI: 1.39-1.80). In high-income areas, helmet use increased by only 4%, from 73% (205 of 281) in 1995 to 77% (191 of 247) in 1996 (RR: 1.06; 95% CI: 0.96-1.17). This finding of a significant increase in helmet use after legislation in low- and mid-income areas but not in high-income areas remained even after logistic regression analysis adjusted for sex and location. CONCLUSIONS: This study showed that bicycle helmet use by children increased significantly after helmet legislation. In this urban area with socioeconomic diversity and in the context of prelegislation promotion and educational activities, the legislative effect was most powerful among children who resided in low-income areas
SN - 1098-4275
AD - Department of Paediatrics, University of Toronto Faculty of Medicine, Ontario, Canada. patricia.parkin@sickkids.ca
UR - 12949311
ER -

TY - JOUR
ID - 1510
T1 - Octachlorodipropyl ether (s-2) mosquito coils are inadequately studied for residential use in Asia and illegal in the United States
A1 - Krieger,R.I.
A1 - Dinoff,T.M.
A1 - Zhang,X.
Y1 - 2003/09//
N1 - Krieger, Robert I. Dinoff, Travis M. Zhang, Xiaofei
Environmental health perspectives
ei0, 0330411
IM
Journal Article. Research Support, Non-U.S. Gov't
English
KW - MEDLINE
KW - Air Pollution
KW - Indoor/an [Analysis]
KW - Asia
KW - Child
KW - Child Welfare
KW - Decision Making
KW - Environmental Exposure
KW - Ethers/an [Analysis]
KW - Ethers/to [Toxicity]
KW - Humans
KW - Incineration
KW - Insecticides/an [Analysis]
KW - Insecticides/to [Toxicity]
KW - Lung Neoplasms/ep [Epidemiology]
KW - Lung Neoplasms/et [Etiology]
KW - Mosquito Control
KW - Product Labeling
KW - Public Policy
KW - Risk Factors
KW - Tropical Climate
KW - United States
KW - Volatilization
RP - NOT IN FILE
SP - 1439
EP - 1442
JF - Environmental Health Perspectives
JA - Environ Health Perspect
VL - 111
IS - 12
CY - United States
N2 - Children and their parents in residences are often protected by insecticides from nuisance and disease-bearing mosquitoes. The annual worldwide consumption of the four major types of residential insecticide products--aerosols, mosquito coils, liquid vaporizers, and vaporizing mats--is in the billions of units. Mosquito coils are burned indoors and outdoors in East Asia and to a limited extent in other parts of the world, including the United States. Coils consist of an insecticide/repellant, organic fillers capable of burning with smoldering, binder, and additives such as synergists, dyes, and fungicide. The number of coil users in China is in the millions. In Indonesia alone, an estimated seven billion coils are purchased annually. Coils containing pyrethroid insecticides, particularly d-allethrin, may contain octachlorodipropyl ether (S-2, S-421) as a synergist or active ingredient. Use of those coils likely exposes children and adults to some level of bis(chloromethyl)ether (BCME). BCME is formed from formaldehyde and hydrogen chloride, combustion products formed from the slow smoldering (about 8 hr/coil) of the mosquito coils. Because BCME is an extremely potent lung carcinogen, the nature and extent of prolonged exposures that recur in homes during the

mosquito season in tropical regions must be evaluated with respect to health. In a small analytical study, coils purchased in Indonesia and in the United States contained highly variable amounts of S-2. Some coils that contained S-2 were not labeled, making it impossible for consumers to make an informed decision about coil contents. Mosquito coils containing S-2 are unregistered, and their use is illegal in the United States. Indoor air monitoring under conditions that represent conditions of use in tropical settings and epidemiology to assess health impacts of coil use are essential to permit responsible regulatory decisions regarding continuing S-2 use
SN - 0091-6765
AD - Department of Entomology, Personal Chemical Exposure Program, Graduate Program in Environmental Toxicology, University of California, Riverside, CA 92521, USA. bob.Krieger@ucr.edu
UR - 12948880
ER -

TY - JOUR
ID - 1511
T1 - The effects of active infant orthopedics on occlusal relationships in unilateral complete cleft lip and palate
A1 - Chan,K.T.
A1 - Hayes,C.
A1 - Shusterman,S.
A1 - Mulliken,J.B.
A1 - Will,L.A.
Y1 - 2003/09//
N1 - Chan, Kathleen T. Hayes, Catherine. Shusterman, Stephen. Mulliken, John B. Will, Leslie A
The Cleft palate-craniofacial journal : official publication of the American Cleft Palate-Craniofacial Association
a0r, 9102566
D, IM
Clinical Trial. Comparative Study. Journal Article. Randomized Controlled Trial
English
KW - MEDLINE
KW - Activator Appliances
KW - Cephalometry
KW - Child
KW - Cleft Lip/co [Complications]
KW - Cleft Lip/rh [Rehabilitation]
KW - Cleft Lip/su [Surgery]
KW - Cleft Palate/co [Complications]
KW - Cleft Palate/rh [Rehabilitation]
KW - Cleft Palate/su [Surgery]
KW - Dental Arch/pa [Pathology]
KW - Dental Models
KW - Dental Occlusion
KW - Female
KW - Follow-Up Studies
KW - Humans
KW - Jaw Relation Record
KW - Male
KW - Malocclusion/et [Etiology]
KW - Malocclusion/pc [Prevention & Control]
KW - Maxilla/pa [Pathology]
KW - Maxilla/su [Surgery]
KW - Maxillofacial Development
KW - Observer Variation
KW - Orthopedic Procedures/mt [Methods]
KW - Palatal Obturators
KW - Regression Analysis
KW - Treatment Outcome
RP - NOT IN FILE
SP - 511

EP - 517
JF - Cleft Palate-Craniofacial Journal
JA - Cleft Palate Craniofac J
VL - 40
IS - 5
CY - United States
N2 - OBJECTIVE: To evaluate the effects of active infant orthopedic treatment on dental arch relationships and determine the effect on maxillary growth in children born with unilateral complete cleft lip and palate (UCCLP). DESIGN: The GOSLON Yardstick was used to assess dental models taken on patients treated with and without active infant orthopedics. PATIENTS: Two groups of nonsyndromic Caucasian children born with UCCLP (total n = 40), all treated by the same surgeon and ranging from 5 to 10 years of age, were evaluated. INTERVENTIONS: One group had a Latham dentomaxillary alignment (DMA) appliance inserted at 5 to 6 weeks of age, after which a lip-nasal adhesion was performed at an average age of 3.5 months. This was followed by more definitive nasolabial repair at the average age of 5.9 months. Those patients treated without preoperative orthopedics underwent a lip-nasal adhesion at average age 1.5 months followed by nasolabial repair at average age 5.1 months. MAIN OUTCOME MEASURES: Randomized assessments using the GOSLON Yardstick were done independently at two separate times by three different examiners. Differences in GOSLON scores between the active orthopedic group and nonorthopedic group were evaluated by both categorical and continuous statistical analyses. RESULTS: The mean GOSLON score was 3.30 for the orthopedic group and 3.21 for the nonorthopedic group. There was no significant group difference in the modal scores of the two groups. CONCLUSIONS: This study showed that active infant orthopedics does not affect the dental arch relationships in preadolescent children with repaired UCCLP, compared with a similar group treated without orthopedic intervention at this center
SN - 1055-6656
AD - Department of Growth and Development, Harvard School of Dental Medicine, Boston, MA, USA
UR - 12943436
ER -

TY - JOUR
ID - 1512
T1 - Physical activity and inactivity in Chinese school-aged youth: the China Health and Nutrition Survey
A1 - Tudor-Locke,C.
A1 - Ainsworth,B.E.
A1 - Adair,L.S.
A1 - Du,S.
A1 - Popkin,B.M.
Y1 - 2003/09//
N1 - Tudor-Locke, C. Ainsworth, B E. Adair, L S. Du, S. Popkin, B M
International journal of obesity and related metabolic disorders : journal of the International Association for the Study of Obesity
btx, 9313169
IM
Journal Article. Research Support, Non-U.S. Gov't. Research Support, U.S. Gov't, P.H.S.
English
KW - MEDLINE
KW - Adolescent
KW - Age Factors
KW - Child
KW - China/ep [Epidemiology]
KW - Cohort Studies
KW - Exercise
KW - Female
KW - Health Surveys
KW - Humans
KW - Male
KW - Nutrition Surveys
KW - Obesity/ep [Epidemiology]

KW - Physical Exertion
KW - Questionnaires
KW - Television
KW - Video Games
KW - Walking
RP - NOT IN FILE
SP - 1093
EP - 1099
JF - International Journal of Obesity & Related Metabolic Disorders: Journal of the International Association for the Study of Obesity
JA - Int J Obes Relat Metab Disord
VL - 27
IS - 9
CY - England
N2 - OBJECTIVE: To describe physical activity (PA) and inactivity levels and patterns in Chinese school children (aged 6-18 y). DESIGN: PA and inactivity were assessed in a youth cohort enrolled in the China Health and Nutrition Survey (CHNS) in 1997. SUBJECTS: A total of 1423 males (11.5+/-3.2 y) and 1252 females (11.5+/-3.3 y). MEASUREMENTS: PA and inactivity were assessed by self-reported usual activity (questionnaire). Children under 10 y were assisted by parents. RESULTS: Approximately 84% of Chinese youth actively commute to school for a median of 100-150 min/week. A total of 72% engage in in-school moderate/vigorous (MOD/VIG) PA for a median of 90-110 min/week. Relatively few children (approximately 8%) participate in any MOD/VIG PA outside of school. A total of 72% engage in study-related activities outside of school for a median of 420 min/week. Only 8% of Chinese school children, regardless of gender, watch television > or =2 h/day; less than 1% watch > or =4 h/day. Chores related to housework are not a part of life for Chinese school children; fewer than 20% performed these tasks. CONCLUSION: Chinese youth are unique compared to those in other developing countries because they do not perform household chores. Instead, they are under pressure to achieve scholastically. Participation in MOD/VIG PA outside of school is almost nonexistent. Current television watching habits are relatively low compared to developed countries and walking/biking is a common form of commuting. The descriptive analysis herein represents the foundation upon which future longitudinal studies of PA in this population will be based
SN - 0307-0565
AD - Department of Exercise and Wellness, Arizona State University East, Mesa, AZ 85212, USA. Tudor-Locke@asu.edu
UR - 12917716
ER -

TY - JOUR
ID - 1513
T1 - Physical activity in middle school-aged children participating in a school-based recreation program
A1 - Kien,C.L.
A1 - Chiodo,A.R.
Y1 - 2003/08//
N1 - Kien, C Lawrence. Chiodo, Andrew R
Archives of pediatrics & adolescent medicine
9422751, bwf
AIM, IM
Journal Article. Research Support, Non-U.S. Gov't
English
KW - MEDLINE
KW - Breath Tests
KW - Child
KW - Energy Metabolism
KW - Female
KW - Humans
KW - Leisure Activities
KW - Male
KW - Motor Activity

KW - Ohio
KW - Physical Education and Training/mt [Methods]
KW - School Health Services
RP - NOT IN FILE
SP - 811
EP - 815
JF - Archives of Pediatrics & Adolescent Medicine
JA - Arch Pediatr Adolesc Med
VL - 157
IS - 8
CY - United States
N2 - BACKGROUND: The increasing incidence of obesity in children may be attributed in part to increased sedentary behavior, such as watching television, which leads to less energy expended in physical activity. We have theorized that by middle school, many children lack the physical skills or self-confidence to participate in competitive physical activities. Thus, we hypothesized that if we provided a summer and after-school program featuring noncompetitive, outdoor activities such as gardening and adventure education, we would observe increased physical activity relative to habitual physical activities at home. METHODS: To test this hypothesis, 2 experiments were conducted. In the first, 4 children aged 12 years were evaluated while they participated in a summer recreation program for 2 hours and again while they watched a videotape. They wore a uniaxial accelerometer to assess physical movement, and we used a bicarbonate labeled with 13C tracer technique to assess energy expenditure. In a second experiment, we evaluated 8 children (aged 10-12 years) twice using uniaxial accelerometry only, once while they attended the after-school program for 2 hours and then during a similar period at home. RESULTS: The first study showed that the estimated energy expenditure (kilocalories. kilograms-1. hours-1) was 60% increased during the program (mean +/- SD) (2.6 +/- 0.5) compared with watching a videotape (1.6 +/- 0.3) (P =.02). Physical movement (accelerations per minute) also was significantly increased (3959 +/- 896 vs 513 +/- 182) (P =.004). In the second experiment, movement was 95% increased during the program (4578 +/- 1004) compared with the behavior at home (2345 +/- 746) (P =.005). CONCLUSION: These results show that an organized, noncompetitive, leisure-time program can increase physical activity in children
SN - 1072-4710
AD - Children's Research Institute and the Department of Pediatrics, Ohio State University College of Medicine, Columbus, USA. clkien@utmb.edu
UR - 12912788
ER -

TY - JOUR
ID - 1514
T1 - Trachoma, flies and environmental factors in Burkina Faso
A1 - Schemann,J.F.
A1 - Guinot,C.
A1 - Ilboudo,L.
A1 - Momo,G.
A1 - Ko,B.
A1 - Sanfo,O.
A1 - Ramde,B.
A1 - Ouedraogo,A.
A1 - Malvy,D.
Y1 - 2003/01//
N1 - Schemann, J F. Guinot, C. Ilboudo, L. Momo, G. Ko, B. Sanfo, O. Ramde, B. Ouedraogo, A. Malvy, D
Transactions of the Royal Society of Tropical Medicine and Hygiene
wbu, 7506129
IM
Journal Article
English
KW - MEDLINE
KW - Animals
KW - Burkina Faso/ep [Epidemiology]

KW - Child
KW - Preschool
KW - Chlamydia trachomatis/ip [Isolation & Purification]
KW - Diptera/mi [Microbiology]
KW - Diptera
KW - Environmental Health
KW - Epidemiologic Methods
KW - Face
KW - Female
KW - Humans
KW - Hygiene
KW - Infant
KW - Newborn
KW - Insect Control
KW - Male
KW - Trachoma/ep [Epidemiology]
KW - Trachoma/ps [Parasitology]
RP - NOT IN FILE
SP - 63
EP - 68
JF - Transactions of the Royal Society of Tropical Medicine & Hygiene
JA - Trans R Soc Trop Med Hyg
VL - 97
IS - 1
CY - England
N2 - The presence of flies is one of the earliest risk factors for trachoma and it has been suggested that flies could act as vectors for transmission of chlamydiae. A national trachoma survey was conducted in 1997 in Burkina Faso to (i) study the relationship between trachoma occurrence, flies, dirty faces and some environmental factors, and (ii) investigate the role of flies in the presence of trachoma. The country was stratified into eight groups of provinces and a random sample of 30 clusters was selected in each group. All children aged < 10 years were examined for the diagnosis of active trachoma (trachomatous inflammation which was follicular and/or intense) and the dirtiness of the face and the presence of flies on the face were recorded. The children's carers were questioned about the number of baths given and daily face-washing. Household heads were asked about ownership of cattle and small ruminants. The presence of latrines, a stable, and garbage collection inside the yard was noted. Among 16,514 children examined, 27.0% had active trachoma and 3.3% intense inflammatory trachoma. Flies were present on 11.2% of children's faces and 82.4% and 19.7% of these children had active and intense inflammatory trachoma, respectively. Among the 30.2% of children with dirty faces, 70.2% had active and 10.2% intense inflammatory trachoma. In multivariate analysis, at least one daily bath showed a protective effect on both active and intense inflammatory trachoma. Face-washing twice daily was found to be significantly protective for active trachoma in some regions. A strong association was demonstrated between the presence of flies and dirty faces (odds ratio = 334, 95% confidence interval 202-546). The presence of flies on children's faces, dirty faces and trachoma appeared to be strongly associated. Although the presence of flies may be a marker of socio-economic status and is probably linked with other trachoma risk factors, our data indicated that interventions targeting fly control should be an important issue in controlling trachoma
SN - 0035-9203
AD - IOTA, Bamako, Mali. jfschemann@wanadoo.fr
UR - 12886807
ER -

TY - JOUR
ID - 1515
T1 - Estimation of malaria transmission from humans to mosquitoes in two neighbouring villages in south Cameroon: evaluation and comparison of several indices
A1 - Bonnet,S.
A1 - Gouagna,L.C.
A1 - Paul,R.E.

A1 - Safeukui,I.
A1 - Meunier,J.Y.
A1 - Boudin,C.
Y1 - 2003/01//
N1 - Bonnet, S. Gouagna, L C. Paul, R E. Safeukui, I. Meunier, J Y. Boudin, C
Transactions of the Royal Society of Tropical Medicine and Hygiene
wbu, 7506129
IM
Journal Article. Research Support, Non-U.S. Gov't
English
KW - MEDLINE
KW - Adolescent
KW - Adult
KW - Aged
KW - Animals
KW - Cameroon/ep [Epidemiology]
KW - Child
KW - Preschool
KW - Culicidae/ps [Parasitology]
KW - Disease Vectors
KW - Female
KW - Humans
KW - Immunity
KW - Infant
KW - Newborn
KW - Insect Vectors
KW - Malaria
KW - Falciparum/ep [Epidemiology]
KW - Falciparum/im [Immunology]
KW - Falciparum/tm [Transmission]
KW - Male
KW - Middle Aged
KW - Plasmodium falciparum/ip [Isolation & Purification]
KW - Prevalence
KW - Rural Health
RP - NOT IN FILE
SP - 53
EP - 59
JF - Transactions of the Royal Society of Tropical Medicine & Hygiene
JA - Trans R Soc Trop Med Hyg
VL - 97
IS - 1
CY - England
N2 - Malaria transmission from humans to mosquitoes was assessed in two neighbouring villages in a rural area near Yaounde, Cameroon during high and low transmission seasons during 1998-2000, using several indices previously evaluated in different areas endemic for malaria but never directly compared. These indices were estimated from human parasitological data and mosquito infection rates and, for each individual, thick blood films were prepared at the same time as experimental infection of laboratory-bred mosquitoes. Among the 685 volunteers examined, the prevalence of Plasmodium falciparum gametocyte carriers was 16%, and 8% of individuals were able to infect mosquitoes. The percentage of mosquitoes that became infected by feeding on the infectious individuals was 21%. Children aged < 10 years contributed to about 75% of the infectious reservoir, although they constituted only 35% of the total population. Differences were found between the transmission seasons and the villages, and varied according to the index examined. Although there were more infectious individuals in one of the two villages, they were less infectious than those in the other village during the high transmission season. Comparative analysis of the transmission indices suggests the existence of functioning transmission-blocking immunity in one of the villages, which until now has been only hypothetically considered to play a role in malaria transmission in a natural setting. The epidemiological value

of all the indices used and their accuracy in estimating the human infectious reservoir and its natural or induced variations are discussed
SN - 0035-9203
AD - OCEAC, Aupelf-Uref LAF 302, Yaounde, Cameroon. sbonnet@pasteur.fr
UR - 12886806
ER -

TY - JOUR
ID - 1516
T1 - Toxicological evaluation for the hazard assessment of tire crumb for use in public playgrounds
A1 - Birkholz,D.A.
A1 - Belton,K.L.
A1 - Guidotti,T.L.
Y1 - 2003/07//
N1 - Birkholz, Detlef A. Belton, Kathy L. Guidotti, Tee L
Journal of the Air & Waste Management Association (1995)
9503111, cbi
IM
Journal Article. Research Support, Non-U.S. Gov't
English
KW - MEDLINE
KW - Animals
KW - Bacteria
KW - Biological Assay
KW - Carcinogens/an [Analysis]
KW - Child
KW - Child Welfare
KW - Chlorophyta
KW - Conservation of Natural Resources
KW - Fishes
KW - Humans
KW - Invertebrates
KW - Models
KW - Theoretical
KW - Motor Vehicles
KW - Recreation
KW - Refuse Disposal
KW - Risk Assessment
KW - Rubber
KW - Toxicity Tests
RP - NOT IN FILE
SP - 903
EP - 907
JF - Journal of the Air & Waste Management Association
JA - J Air Waste Manag Assoc
VL - 53
IS - 7
CY - United States
N2 - Disposal of used tires has been a major problem in solid waste management. New uses will have to be found to consume recycled tire products. One such proposed use is as ground cover in playgrounds. However, concern has been expressed regarding exposure of children to hazardous chemicals and the environmental impact of such chemicals. We designed a comprehensive hazard assessment to evaluate and address potential human health and environmental concerns associated with the use of tire crumb in playgrounds. Human health concerns were addressed using conventional hazard analyses, mutagenicity assays, and aquatic toxicity tests of extracted tire crumb. Hazard to children appears to be minimal. Toxicity to all aquatic organisms (bacteria, invertebrates, fish, and green algae) was observed; however, this activity disappeared with aging of the tire

crumb for three months in place in the playground. We conclude that the use of tire crumb in playgrounds results in minimal hazard to children and the receiving environment
SN - 1096-2247
AD - Enviro-Test Laboratories, Edmonton, Alberta, Canada
UR - 12880077
ER -

TY - JOUR
ID - 1517
T1 - How safe are childcare products, toys and playground equipment? A Swedish analysis of mild brain injuries at home and during leisure time 1998-1999
A1 - Emanuelson,I.
Y1 - 2003/09//
N1 - Emanuelson, I
Injury control and safety promotion
100941859, 100941859
IM
Journal Article. Research Support, Non-U.S. Gov't
English
KW - MEDLINE
KW - Accidental Falls/sn [Statistics & Numerical Data]
KW - Accidental Falls
KW - Accidents
KW - Home/sn [Statistics & Numerical Data]
KW - Home
KW - Brain Concussion/ep [Epidemiology]
KW - Brain Concussion/et [Etiology]
KW - Child
KW - Preschool
KW - Female
KW - Humans
KW - Infant
KW - Infant Equipment
KW - Interior Design and Furnishings
KW - Male
KW - Play and Playthings
KW - Sweden/ep [Epidemiology]
RP - NOT IN FILE
SP - 139
EP - 144
JF - Injury Control & Safety Promotion
JA - Inj Control Saf Promot
VL - 10
IS - 3
CY - Netherlands
N2 - The aim was to highlight the role of childcare products as causes for mild brain injury (concussion) in small children (0-4 years of age) and to determine the most dangerous products. By childcare products this report means the following items: child and baby furniture, nursing tables, baby walkers, toys, baby carriages, sport equipment for children, playground equipment and security equipment for children. The data were derived from the EHLASS (European Home and Leisure Accident Surveillance System) for 1998 and 1999 and covered a restricted population of Sweden (approximately 5 per cent). According to this register 182 mild brain injuries (concussions) were recorded following a fall, an accident or a blow to the head among children (0-4 years of age) during 1998 and 158 for 1999. Of those injuries, childcare products were the cause of the accident in 84 (46 per cent) and 76 (48 per cent) cases respectively for 1998 and 1999. The number of children admitted for hospital care was 68 (57/84) and 74 (56/76) per cent respectively. The home was the most common place of the accident and play and leisure activity were the most common activities. More than 50 per cent of these accidents took place during daytime. The product type that caused most accidents was nursery furniture and, in this

category, the baby walker was the most dangerous. The product type that caused the second most frequent accidents was playground equipment
SN - 1566-0974
AD - Queen Silvia Children's Hospital, Regional Pediatric Rehabilitation Centre, PB 210 62, SE-418 04 Gothenburg, Sweden. ingrid.emanuelson@vgregion.se
UR - 12861912
ER -

TY - JOUR
ID - 1518
T1 - Measuring chronic health condition and disability as distinct concepts in national surveys of school-aged children in Canada: a comprehensive review with recommendations based on the ICD-10 and ICF. [Review] [62 refs]
A1 - McDougall,J.
A1 - Miller,L.T.
Y1 - 2003/08//
N1 - McDougall, Janette. Miller, Linda T
Disability and rehabilitation
9207179, a8i
IM
Journal Article. Review
English
KW - MEDLINE
KW - Adolescent
KW - Canada
KW - Child
KW - Chronic Disease
KW - Disabled Children/cl [Classification]
KW - Disabled Children/sn [Statistics & Numerical Data]
KW - Disabled Children
KW - Environment
KW - Health Surveys
KW - Humans
KW - International Classification of Diseases
RP - NOT IN FILE
SP - 922
EP - 939
JF - Disability & Rehabilitation
JA - Disabil Rehabil
VL - 25
IS - 16
CY - England
N2 - PURPOSE: With the aim of improving the measurement of child health and disability in survey research, this paper reviews the coverage of chronic health conditions and the domains of disability and related environmental factors as they are laid out in the ICD-10 and ICF, respectively, in national surveys of school-aged children conducted in Canada since 1980. Recommendations are made for future survey use and construction. METHODS: Two reviewers independently examined each of the surveys. Coverage of chronic health conditions, the domains of disability, and environmental factors in survey questions was identified by mapping question content onto ICD-10 and ICF codes. The reviewers then compared their findings and came to a final consensus. RESULTS: Surveys vary in the range and depth of coverage of the ICD-10 and ICF chapters. Disability surveys and health surveys for persons aged 12 and over contain the most comprehensive lists of chronic conditions. Coverage of impairments is limited. Coverage of activity limitations and participation restrictions is most limited in the domains of personal care and domestic life. Environmental factors not covered include natural environmental changes, attitudes, and policies. CONCLUSIONS: Development of a comprehensive standard list of chronic health conditions based on the ICD-10 and development of standard survey measures of the domains of disability and environmental factors based on the ICF for use in surveys of

school-aged children would facilitate an understanding of children's health and disability in the context of the current international health framework provided by the World Health Organization. [References: 62]
SN - 0963-8288
AD - Research Program, Thames Valley Children's Centre, London, Ontario, Canada. janettem@tvcc.on.ca
UR - 12857580
ER -

TY - JOUR
ID - 1519
T1 - Dangerous activities by children in grocery carts: is adult supervision important?
A1 - Harrell,W.A.
Y1 - 2003/06//
N1 - Harrell, W Andrew
Psychological reports
qf6, 0376475
IM
Journal Article
English
KW - MEDLINE
KW - Accidental Falls/pc [Prevention & Control]
KW - Adult
KW - Alberta
KW - Child
KW - Preschool
KW - Dangerous Behavior
KW - Data Collection/sn [Statistics & Numerical Data]
KW - Female
KW - Humans
KW - Infant
KW - Male
KW - Observer Variation
KW - Parenting/px [Psychology]
KW - Risk-Taking
KW - Social Environment
KW - Wounds and Injuries/pc [Prevention & Control]
RP - NOT IN FILE
SP - 957
EP - 962
JF - Psychological Reports
JA - Psychol Rep
VL - 92
IS - 3 Pt 1
CY - United States
N2 - Systematic observations of 231 children (infancy to 5 years) and the 231 adults accompanying them were made in 29 supermarkets in Edmonton, Alberta, Canada. The objective of the study was to describe the influence of two indices of adult supervision in supermarkets on children's activities in shopping carts. Adult supervision was measured by whether the adult ever lost sight of the child and whether the adult was 10 feet or more from the child at any time during a shopping trip. 23% of the children stood on the ends or sides of carts; 49% climbed or tried to climb out of carts. Adults lost sight of a child, whether in or outside the cart, a mean of 3.3 times and were 10 feet away 2.7 times during an average shopping trip. Logistic regression showed that a child rather than an adult pushing a shopping cart through the store was the strongest factor related to standing on ends or sides of carts. Pushing by a child was more likely to occur when adult monitoring was low and when children were older. Climbing out was predicted by the number of times an adult was > or = 10 feet away, older children, and a child being in the cart basket as opposed to outside the cart or in the child safety seat
SN - 0033-2941
AD - University of Alberta, Centre for Experimental Sociology, Edmonton, Canada. aharrell@gpu.srv.ualberta.ca

UR - 12841471
ER -

TY - JOUR
ID - 1520
T1 - US adult attitudes and practices regarding smoking restrictions and child exposure to environmental tobacco smoke: changes in the social climate from 2000-2001
A1 - McMillen,R.C.
A1 - Winickoff,J.P.
A1 - Klein,J.D.
A1 - Weitzman,M.
Y1 - 2003/07//
N1 - McMillen, Robert C. Winickoff, Jonathan P. Klein, Jonathan D. Weitzman, Michael
Pediatrics
oxv, 0376422
AIM, IM
Journal Article. Research Support, Non-U.S. Gov't. Research Support, U.S. Gov't, P.H.S.
English
KW - MEDLINE
KW - Adult/px [Psychology]
KW - Attitude to Health
KW - Child
KW - Cross-Sectional Studies
KW - Environmental Exposure
KW - Female
KW - Health Knowledge
KW - Attitudes
KW - Practice
KW - Humans
KW - Male
KW - Middle Aged
KW - Sampling Studies
KW - Smoking/lj [Legislation & Jurisprudence]
KW - Smoking/px [Psychology]
KW - Smoking Cessation
KW - Tobacco Industry
KW - Tobacco Smoke Pollution/pc [Prevention & Control]
KW - United States
RP - NOT IN FILE
SP - e55
EP - e60
JF - Pediatrics
VL - 112
IS - 1 Pt 1
CY - United States
N2 - OBJECTIVE: A substantial proportion of homes and automobiles serve as settings for environmental tobacco smoke (ETS) exposure, and many public settings that children frequent are still not smoke-free. Tobacco control efforts are attempting to increase smoking bans. The objective of this study was to describe the knowledge, attitudes, and practices of smokers and nonsmokers regarding smoking bans and child ETS exposure in multiple public and private settings and to report changes from 2000-2001. METHODS: Cross-sectional data from the annual Social Climate Survey of Tobacco Control were analyzed for changes in knowledge, attitudes, and practices regarding tobacco. These data were collected via automated, random-digit-dialing telephone surveys that were conducted in the summers of 2000 and 2001. The samples were weighted by race and gender to be representative of the US population. RESULTS: Response rates for eligible adults actually contacted were 1501 (75%) of 1876 in 2000 and 3002 (84%) of 3566 in 2001. The majority of adults, both smokers and nonsmokers, support smoking bans in a wide variety of places. The percentage of all respondents reporting the presence of smoking bans in several public and private places increased from 2000-2001: the

household (69%-74%), in the presence of children (84%-88%), convenience stores (68%-74%), fast-food restaurants (52%-58%), and non-fast-food restaurants (25%-28%). Support for smoking bans also increased in shopping malls (71%-75%), fast-food restaurants (77%-80%), and indoor sporting events (78%-80%). There were no significant changes in support for smoking bans in convenience stores, restaurants, or outdoor parks. Adults' knowledge of the harm caused by tobacco was unchanged, with the vast majority of adults recognizing the dangers of exposure to ETS from parental smoking (95%) and exposure to ETS in cars (77%). CONCLUSIONS: Small improvements in adult attitudes and practices regarding children's ETS exposure occurred from 2000-2001. However, a significant number of adults in the United States still report ignorance of the harmful effects of child ETS exposure, and there was no improvement in reported knowledge in this 1-year period. In contrast, a growing majority of smokers and nonsmokers favor restrictions on smoking in public settings, suggesting that states and communities have public support for broad public smoking restriction policies. There are significant roles that pediatricians can play in preventing children's ETS exposure, through both patient and family education and by moving smoking restriction policies forward on their community's agenda
SN - 1098-4275
AD - Social Science Research Center, Mississippi State University, Starkville, Mississippi 39762, USA. robert.mcmillen@ssrc.msstate.edu
UR - 12837906
ER -

TY - JOUR
ID - 1521
T1 - Patterns of daily physical activity during school days in children and adolescents
A1 - Mota,J.
A1 - Santos,P.
A1 - Guerra,S.
A1 - Ribeiro,J.C.
A1 - Duarte,J.A.
Y1 - 2003/07//
N1 - Mota, Jorge. Santos, Paula. Guerra, Sandra. Ribeiro, Jose C. Duarte, Jose A
American journal of human biology : the official journal of the Human Biology Council
d3k, 8915029
IM
Comparative Study. Journal Article. Research Support, Non-U.S. Gov't. Validation Studies
English
KW - MEDLINE
KW - Adolescent
KW - Child
KW - Cross-Sectional Studies
KW - Energy Metabolism/ph [Physiology]
KW - Female
KW - Humans
KW - Male
KW - Motor Activity/ph [Physiology]
KW - Physical Exertion/ph [Physiology]
KW - Portugal
KW - Risk Factors
KW - Schools
KW - Students/sn [Statistics & Numerical Data]
KW - Time Factors
RP - NOT IN FILE
SP - 547
EP - 553
JF - American Journal of Human Biology
JA - Am J Human Biol
VL - 15
IS - 4

CY - United States
N2 - The purpose of this study was to examine the weekday patterns of moderate-to-vigorous physical activity (MVPA) in school children and adolescents and determine if there are periods of the day that are representative of their typical MVPA. The sample comprised 84 subjects (boys, n = 30; girls, n = 54), age 8-15 years old. Daily totals for the physical activity variables were calculated by summing the values from 13 hr of physical activity (PA) measurements (9:00-22:00), with 60-min time blocks comprising each day. The MVPA data values were categorized in four daily periods: morning (9:00-11:59), noon (12:00-14:59), late afternoon (15:00-17.59), and evening (18:00-21.59). Our data show that boys participated significantly more in MVPA than girls. Despite no clear patterns or differences among sex being found, girls showed higher percent of time engaged in MVPA during the morning and early afternoon periods (sum of two periods 51.0%), while boys' percent of time engaged in MVPA is higher at late afternoon and evening periods (sum of two periods 53.8%). The principal components analyses showed four distinct components that accounted for 67% of the variance, as follows: school hours (component 1); lunchtime and outside-school activities (component 2); morning time before school period (component 4); and period before bedtime (component 3) appear as distinct periods of the day. In conclusion, the present study shows that boys engaged more in MVPA than girls. Girls tend to be more active during school periods, while boys are more active after school. Copyright 2003 Wiley-Liss, Inc
SN - 1042-0533
AD - Research Center of Physical Activity, Health and Leisure, Faculty of Sport Sciences, University of Porto, Portugal. jmota@fcdef.up.pt
UR - 12820196
ER -

TY - JOUR
ID - 1522
T1 - Exposure assessment of particulate matter for susceptible populations in Seattle
A1 - Liu,L.J.
A1 - Box,M.
A1 - Kalman,D.
A1 - Kaufman,J.
A1 - Koenig,J.
A1 - Larson,T.
A1 - Lumley,T.
A1 - Sheppard,L.
A1 - Wallace,L.
Y1 - 2003/06//
N1 - Liu, L-J Sally. Box, Michael. Kalman, David. Kaufman, Joel. Koenig, Jane. Larson, Tim. Lumley, Thomas. Sheppard, Lianne. Wallace, Lance
Environmental health perspectives
ei0, 0330411
IM
Journal Article. Research Support, U.S. Gov't, Non-P.H.S.. Research Support, U.S. Gov't, P.H.S.
English
KW - MEDLINE
KW - Age Factors
KW - Aged
KW - Air Pollutants/an [Analysis]
KW - Air Pollution/an [Analysis]
KW - Air Pollution
KW - Indoor/an [Analysis]
KW - Asthma
KW - Child
KW - Coronary Disease
KW - Environmental Exposure/an [Analysis]
KW - Environmental Monitoring/mt [Methods]
KW - Humans
KW - Pulmonary Disease
KW - Chronic Obstructive

KW - Time Factors
KW - Urban Population
KW - Washington
RP - NOT IN FILE
SP - 909
EP - 918
JF - Environmental Health Perspectives
JA - Environ Health Perspect
VL - 111
IS - 7
CY - United States
N2 - In this article we present results from a 2-year comprehensive exposure assessment study that examined the particulate matter (PM) exposures and health effects in 108 individuals with and without chronic obstructive pulmonary disease (COPD), coronary heart disease (CHD), and asthma. The average personal exposures to PM with aerodynamic diameters < 2.5 microm (PM2.5) were similar to the average outdoor PM2.5 concentrations but significantly higher than the average indoor concentrations. Personal PM2.5 exposures in our study groups were lower than those reported in other panel studies of susceptible populations. Indoor and outdoor PM2.5, PM10 (PM with aerodynamic diameters < 10 microm), and the ratio of PM2.5 to PM10 were significantly higher during the heating season. The increase in outdoor PM10 in winter was primarily due to an increase in the PM2.5 fraction. A similar seasonal variation was found for personal PM2.5. The high-risk subjects in our study engaged in an equal amount of dust-generating activities compared with the healthy elderly subjects. The children in the study experienced the highest indoor PM2.5 and PM10 concentrations. Personal PM2.5 exposures varied by study group, with elderly healthy and CHD subjects having the lowest exposures and asthmatic children having the highest exposures. Within study groups, the PM2.5 exposure varied depending on residence because of different particle infiltration efficiencies. Although we found a wide range of longitudinal correlations between central-site and personal PM2.5 measurements, the longitudinal r is closely related to the particle infiltration efficiency. PM2.5 exposures among the COPD and CHD subjects can be predicted with relatively good power with a microenvironmental model composed of three microenvironments. The prediction power is the lowest for the asthmatic children
SN - 0091-6765
AD - Department of Environmental and Occupational Health Sciences, University of Washington, Seattle, Washington 98195, USA. sliu@u.washington.edu
UR - 12782491
ER -

TY - JOUR
ID - 1523
T1 - A comparison of assistive technology and personal care in alleviating disability and unmet need
A1 - Agree,E.M.
A1 - Freedman,V.A.
Y1 - 2003/06//
N1 - Agree, Emily M. Freedman, Vicki A
The Gerontologist
fp5, 0375327
IM
Comparative Study. Evaluation Studies. Journal Article. Research Support, U.S. Gov't, P.H.S.
English
KW - MEDLINE
KW - Activities of Daily Living
KW - Adult
KW - Child
KW - Disabled Persons/rh [Rehabilitation]
KW - Disabled Persons/sn [Statistics & Numerical Data]
KW - Fatigue
KW - Female
KW - Humans
KW - Long-Term Care/mt [Methods]

KW - Male
KW - Needs Assessment
KW - Outcome and Process Assessment (Health Care)
KW - Pain Measurement
KW - Patient Satisfaction
KW - Self Care/is [Instrumentation]
KW - Self Care/mt [Methods]
KW - Self-Help Devices/sn [Statistics & Numerical Data]
KW - Self-Help Devices
KW - Time
KW - United States
RP - NOT IN FILE
SP - 335
EP - 344
JF - Gerontologist
VL - 43
IS - 3
CY - United States
N2 - PURPOSE: The authors examine differences in reports of residual disability and unmet need by type of long-term care arrangement (assistive technology or personal care). DESIGN AND METHODS: This study compares three specific dimensions of residual difficulty (pain, fatigue, and time intensity) and reports of unmet need across care arrangements. Samples from the U. S. 1994-1995 National Health Interview Survey Phase 2 Disability Supplements include adults with limitations in bathing, transferring, walking, and getting outside. RESULTS: Even when differences in underlying disability are accounted for, assistive technology (AT) confers no additional benefit in the three dimensions of residual difficulty analyzed here. AT users equally or more often report that tasks are tiring, time consuming, or painful, even when they use assistance. Though this would appear to indicate unmet needs for care, fewer AT users report a desire for hands-on personal care. IMPLICATIONS: Though disability alleviation by technology is no better on specific dimensions of difficulty, technology users report less unmet need for personal care. Designing appropriate and cost-effective home care for adults with disabilities requires a better understanding of the ways in which technology users may differ from others and the circumstances under which technology can be most effective
SN - 0016-9013
AD - Department of Population and Family Health Sciences, Johns Hopkins Bloomberg School of Public Health, Baltimore, MD 21205, USA. eagree@jhsph.edu
UR - 12810897
ER -

TY - JOUR
ID - 1524
T1 - Intensive positive behavior supports for adolescents with acquired brain injury: long-term outcomes in community settings
A1 - Gardner,R.M.
A1 - Bird,F.L.
A1 - Maguire,H.
A1 - Carreiro,R.
A1 - Abenaim,N.
Y1 - 2003/01//
N1 - Gardner, Rita M. Bird, Frank L. Maguire, Helena. Carreiro, Rui. Abenaim, Nicole
The Journal of head trauma rehabilitation
8702552, c3t
IM
Case Reports. Journal Article
English
KW - MEDLINE
KW - Adolescent
KW - Behavior Therapy
KW - Brain Injuries/co [Complications]

KW - Brain Injuries/px [Psychology]
KW - Brain Injuries/rh [Rehabilitation]
KW - Child
KW - Child Behavior Disorders/et [Etiology]
KW - Child Behavior Disorders/px [Psychology]
KW - Child Behavior Disorders/rh [Rehabilitation]
KW - Humans
KW - Life Style
KW - Male
KW - Outcome Assessment (Health Care)
KW - Residence Characteristics
KW - Social Support
KW - Time Factors
RP - NOT IN FILE
SP - 52
EP - 74
JF - Journal of Head Trauma Rehabilitation
JA - J Head Trauma Rehabil
VL - 18
IS - 1
CY - United States
N2 - BACKGROUND: Based on the technologies of applied behavior analysis and person-centered planning, positive behavior support is a process for designing and implementing proactive behavioral interventions with the goal of positive lifestyle changes. PARTICIPANTS: The two adolescents who received the intensive, longitudinal, multicomponent intervention had experienced escalating behavior challenges over several years after brain injury in early childhood. MAIN OUTCOME MEASURES: Quantitative data included episodes of aggression and property destruction. Qualitative data included intensity of supports, family involvement, peer relationships, medication regime, vocational status, educational status, community access, and self-help skills. RESEARCH DESIGN: A long-term, natural-environment, case-study method was used. RESULTS: The targeted challenging behaviors were reduced to zero. In addition, the participants' domains of activity increased and self-management improved even as supports were systematically withdrawn. CONCLUSIONS: These results illustrate the potential for successfully treating extreme chronic behavior disorders after childhood brain injury
SN - 0885-9701
AD - Melmark New England, Woburn, Mass 01801, USA
UR - 12802237
ER -

TY - JOUR
ID - 1525
T1 - Dog keeping in Taiwan: its contribution to the problem of free-roaming dogs
A1 - Hsu,Y.
A1 - Severinghaus,L.L.
A1 - Serpell,J.A.
Y1 - 2003///
N1 - Hsu, Yuying. Severinghaus, Lucia Liu. Serpell, James A
Journal of applied animal welfare science : JAAWS
9804404
IM
Journal Article. Research Support, Non-U.S. Gov't
English
KW - MEDLINE
KW - Adult
KW - Animal Husbandry/sn [Statistics & Numerical Data]
KW - Animal Welfare
KW - Animals
KW - Anthropology

KW - Cultural
KW - Child
KW - Dogs
KW - Female
KW - Health Education
KW - Humans
KW - Male
KW - Ownership/sn [Statistics & Numerical Data]
KW - Questionnaires
KW - Taiwan/ep [Epidemiology]
RP - NOT IN FILE
SP - 1
EP - 23
JF - Journal of Applied Animal Welfare Science
JA - J Appl Anim Welf.Sci
VL - 6
IS - 1
CY - United States
N2 - This study conducted a quantitative ethnographic analysis of the influence of demographic factors and early experience (childhood exposure to dogs) on Taiwanese dog-keeping practices and behavior. A telephone survey of a randomly selected sample of 2001 Taiwan residents determined their dog ownership histories, current patterns of dog ownership and disposal, and other dog-related activities. The results suggest that low rates of neutering, easy availability of low- or no-cost puppies, a tendency to allow owned dogs free access to the outdoors, unrealistic expectations of dog ownership, canine behavioral problems, and religious and cultural taboos against euthanasia and shelter relinquishment have contributed to the recent increase in the numbers of free-roaming dogs in Taiwan. Logistic regression analyses determined that a relatively small number of demographic and experiential variables predicted dog ownership and disposal patterns. The most important of these was childhood experience of living with household dogs. In light of these findings, future efforts to reduce the stray dog problem should focus on enforcing registration fees, particularly for unsterilized animals; low-cost neutering schemes; and educational programs designed to promote neutering, improve knowledge of canine behavior and behavior problems, and develop more realistic expectations and attitudes toward dog ownership. Marked Taiwanese resistance to canine euthanasia and shelter relinquishment suggests a need for alternative methods of managing the existing free-roaming dog population
SN - 1088-8705
AD - Department of Biology, National Taiwan Normal University, Taipei. serpell@vet.upenn.edu
UR - 12795855
ER -

TY - JOUR
ID - 1526
T1 - Postexposure prophylaxis in children and adolescents for nonoccupational exposure to human immunodeficiency virus. [Review] [122 refs]
A1 - Havens,P.L.
A1 - American Academy of Pediatrics Committee on Pediatric AIDS.
Y1 - 2003/06//
N1 - Havens, Peter L. American Academy of Pediatrics Committee on Pediatric AIDS
Pediatrics
oxv, 0376422
AIM, IM
Journal Article. Review
English
KW - MEDLINE
KW - Adolescent
KW - Animals
KW - Child
KW - Disease Models
KW - Animal

KW - Female
KW - HIV
KW - HIV Infections/pc [Prevention & Control]
KW - HIV Infections/tm [Transmission]
KW - Humans
KW - Needle Sharing/ae [Adverse Effects]
KW - Practice Guidelines as Topic
KW - Sexual Behavior
RP - NOT IN FILE
SP - 1475
EP - 1489
JF - Pediatrics
VL - 111
IS - 6 Pt 1
CY - United States
N2 - Exposure to human immunodeficiency virus (HIV) can occur in a number of situations unique to, or more common among, children and adolescents. Guidelines for postexposure prophylaxis (PEP) for occupational and nonoccupational (eg, sexual, needle-sharing) exposures to HIV have been published by the US Public Health Service, but they do not directly address nonoccupational HIV exposures unique to children (such as accidental exposure to human milk from a woman infected with HIV or a puncture wound from a discarded needle on a playground), and they do not provide antiretroviral drug information relevant to PEP in children. This clinical report reviews issues of potential exposure of children and adolescents to HIV and gives recommendations for PEP in those situations. The risk of HIV transmission from nonoccupational, nonperinatal exposure is generally low. Transmission risk is modified by factors related to the source and extent of exposure. Determination of the HIV infection status of the exposure source may not be possible, and data on transmission risk by exposure type may not exist. Except in the setting of perinatal transmission, no studies have demonstrated the safety and efficacy of postexposure use of antiretroviral drugs for the prevention of HIV transmission in nonoccupational settings. Antiretroviral therapy used for PEP is associated with significant toxicity. The decision to initiate prophylaxis needs to be made in consultation with the patient, the family, and a clinician with experience in treatment of persons with HIV infection. If instituted, therapy should be started as soon as possible after an exposure-no later than 72 hours-and continued for 28 days. Many clinicians would use 3 drugs for PEP regimens, although 2 drugs may be considered in certain circumstances. Instruction for avoiding secondary transmission should be given. Careful follow-up is needed for psychologic support, encouragement of medication adherence, toxicity monitoring, and serial HIV antibody testing. [References: 122]
SN - 1098-4275
UR - 12777574
ER -

TY - JOUR
ID - 1527
T1 - Effects of misclassification of causes of death on the power of a trial to assess the efficacy of a pneumococcal conjugate vaccine in The Gambia
A1 - Jaffar,S.
A1 - Leach,A.
A1 - Smith,P.G.
A1 - Cutts,F.
A1 - Greenwood,B.
Y1 - 2003/06//
N1 - Jaffar, Shabbar. Leach, Amanda. Smith, Peter G. Cutts, Felicity. Greenwood, Brian
International journal of epidemiology
gr6, 7802871
IM
Journal Article. Research Support, Non-U.S. Gov't
English
KW - MEDLINE
KW - Africa/ep [Epidemiology]
KW - Autopsy

KW - Cause of Death
KW - Child
KW - Preschool
KW - Classification
KW - Developing Countries
KW - Humans
KW - Infant
KW - Pneumococcal Infections/mo [Mortality]
KW - Pneumococcal Vaccines/tu [Therapeutic Use]
KW - Randomized Controlled Trials as Topic
KW - Respiratory Tract Infections/mi [Microbiology]
KW - Respiratory Tract Infections/mo [Mortality]
KW - Rural Population
KW - Sample Size
KW - Sensitivity and Specificity
RP - NOT IN FILE
SP - 430
EP - 436
JF - International Journal of Epidemiology
JA - Int J Epidemiol
VL - 32
IS - 3
CY - England
N2 - BACKGROUND: A reduction in cause-specific mortality may be the most important public health measure of the efficacy of a new vaccine. However, in developing countries, assignment of causes of deaths occurring outside hospitals can be assessed often only through the questioning of relatives about the signs and symptoms leading to death ('post-mortem questionnaire'). Causes assigned in this way have poor sensitivity and specificity. We illustrate the effects of this misclassification on the power of a large trial of a pneumococcal polysaccharide/protein conjugate vaccine with a mortality endpoint. METHODS: Required sample sizes to achieve a study with specified power were calculated for all-cause and acute lower respiratory tract infection (ALRI) mortality for different levels of sensitivity and specificity of post-mortem questionnaires. Data from active community-based surveillance and post-mortem questionnaires collected 1989-1993 from the study area were used in the calculations. FINDINGS: The mortality rate among children aged 6-29 months from all causes was 34.2 per 1000 child-years; 19% of deaths were attributable to ALRI. Assuming that pneumococci would be responsible for 50% of ALRI deaths and that the vaccine would cover 70% of disease serotypes and would be 90% effective against these serotypes, the expected efficacy of the vaccine would be 6.0% (19% x 50% x 70% x 90%) against all causes combined and 31.5% (50% x 70% x 90%) against deaths from ALRI. If, as suggested by various reports, the sensitivity and specificity of assigning a death to ALRI by post-mortem questionnaire are about 40% and 90% respectively, then the observed vaccine efficacy against ALRI (as classified using the post-mortem questionnaire) would fall to 20%, and the power to detect this would be reduced by approximately 40%. Furthermore, low sensitivity of diagnosis would lead to a falsely low estimate of the burden of ALRI mortality in the population and the trial might have greater power to detect a reduction in mortality from all causes combined than that estimated at the outset. CONCLUSIONS: Low sensitivity and specificity of diagnosis by post-mortem questionnaire may mean that the power of a trial to detect a reduction in all-cause mortality is similar to that to detect a reduction in ALRI mortality. Since the latter is more susceptible to bias from misclassification of cause of death, all-cause mortality may be the most suitable endpoint. Similar considerations apply to trials of interventions against other diseases for which a cause-specific endpoint is subject to substantial misclassification
SN - 0300-5771
AD - Medical Research Council Tropical Epidemiology Group, London School of Hygiene and Tropical Medicine, Keppel Street, London WC1 E7HT, UK. shabbar.jaffar@lshtm.ac.uk
UR - 12777432
ER -

TY - JOUR
ID - 1528

T1 - Severe meningococcal disease is characterized by early neutrophil but not platelet activation and increased formation and consumption of platelet-neutrophil complexes
A1 - Peters,M.J.
A1 - Heyderman,R.S.
A1 - Faust,S.
A1 - Dixon,G.L.
A1 - Inwald,D.P.
A1 - Klein,N.J.
Y1 - 2003/06//
N1 - Peters, M J. Heyderman, R S. Faust, S. Dixon, G L J. Inwald, D P. Klein, N J
Journal of leukocyte biology
iwy, 8405628
IM
Journal Article. Research Support, Non-U.S. Gov't
English
KW - MEDLINE
KW - Antigens
KW - CD/an [Analysis]
KW - CD/im [Immunology]
KW - Differentiation
KW - Myelomonocytic/an [Analysis]
KW - Myelomonocytic/im [Immunology]
KW - Biopsy
KW - Blood
KW - Blood Platelets/cy [Cytology]
KW - Blood Platelets/ph [Physiology]
KW - Cell Adhesion
KW - Cell Adhesion Molecules/me [Metabolism]
KW - Child
KW - Preschool
KW - Humans
KW - Immunohistochemistry
KW - Infant
KW - Newborn
KW - Integrins/me [Metabolism]
KW - Kinetics
KW - Macromolecular Substances
KW - Meningococcal Infections/di [Diagnosis]
KW - Meningococcal Infections/im [Immunology]
KW - Meningococcal Infections/pp [Physiopathology]
KW - Neutrophil Activation
KW - Neutrophils/cy [Cytology]
KW - Neutrophils/im [Immunology]
KW - Platelet Activation
KW - Platelet Count
KW - Platelet Transfusion
KW - Skin/pa [Pathology]
RP - NOT IN FILE
SP - 722
EP - 730
JF - Journal of Leukocyte Biology
JA - J Leukoc.Biol
VL - 73
IS - 6
CY - United States
N2 - Approximately 25% of polymorphonuclear leukocytes (PMNL) circulate in heterotypic complexes with one or more activated platelets. These platelet-neutrophil complexes (PNC) require platelet CD62P expression

for their formation and represent activated subpopulations of both cell types. In this study, we have investigated the presence, time course, and mechanisms of PNC formation in 32 cases of severe pediatric meningococcal disease (MD) requiring intensive care. There were marked early increases in PMNL CD11b/CD18 expression and activation, and reduced CD62L expression compared with intensive care unit control cases. Minimal platelet expression of the active form of alphaIIbbeta3 (GpIIb/IIIa) was seen. PNC were reduced on presentation and fell to very low levels after 24 h. Immunostaining of skin biopsies demonstrated that PNC appear outside the circulation in MD. In vitro studies of anticoagulated whole blood inoculated with Neisseria meningitidis supported these clinical findings with marked increases in PMNL CD11b/CD18 expression and activation but no detectable changes in platelet-activated alphaIIbbeta3 or CD62P expression. In vitro PMNL activation with N. meningitidis (or other agonists) potentiated the formation of PNC in response to platelet activation with adenine diphosphate. Therefore, in severe MD, PMNL activation is likely to promote PNC formation, and we suggest that the reduced levels of PNC seen in established MD reflect rapid loss of PNC from the circulation rather than reduced formation
SN - 0741-5400
AD - Infection and Microbiology Unit and. Portex Unit Critical Care Group, Institute of Child Health, London, United Kingdom. m.peters@ich.ac.uk
UR - 12773504
ER -

TY - JOUR
ID - 1529
T1 - A review of 2,517 childhood injuries seen in a Singapore emergency department in 1999--mechanisms and injury prevention suggestions
A1 - Ong,M.E.
A1 - Ooi,S.B.
A1 - Manning,P.G.
Y1 - 2003/01//
N1 - Ong, M E H. Ooi, S B S. Manning, P G
Singapore medical journal
uri, 0404516
IM
Journal Article
English
KW - MEDLINE
KW - Chi-Square Distribution
KW - Child
KW - Preschool
KW - Emergency Service
KW - Hospital
KW - Female
KW - Humans
KW - Infant
KW - Newborn
KW - Injury Severity Score
KW - Male
KW - Retrospective Studies
KW - Singapore/ep [Epidemiology]
KW - Statistics
KW - Nonparametric
KW - Wounds and Injuries/ep [Epidemiology]
KW - Wounds and Injuries/et [Etiology]
KW - Wounds and Injuries/pc [Prevention & Control]
RP - NOT IN FILE
SP - 12
EP - 19
JF - Singapore Medical Journal
JA - Singapore Med J

VL - 44
IS - 1
CY - Singapore
N2 - BACKGROUND: Childhood injuries cause significant mortality and morbidity in Singapore. With injury surveillance, patterns of repeated injury can be identified and injury prevention strategies devised. METHODS: We conducted a retrospective study of all children aged 12 and below seen for trauma in an Emergency Department over one year. Data captured in the real-time computer system was studied with regards to patient profile, mechanism of injury and patient disposition. Clinical summaries were extracted with follow-up telephone interviews done. RESULTS: Two thousand five hundred and seventeen children aged 12 and below were seen for accidental trauma in 1999, accounting for 37.1% of the total attendance for that age. Mean age was 7.7 years with males making up 62.7%. Home injuries (56.4%) were the most common, followed by road-related (14.4%), sports (8.2%) and playground injuries (7.4%). 48.5% sustained head and face injuries. Pre-school children (age <5) were more likely to sustain home injuries (p<0.0001), a higher proportion of head injuries (p<0.0001), foreign bodies, burns and poisoning compared to school-going children (age 6-12), who were more likely to sustain injuries in road accidents, sports, at playgrounds or schools, with more limb, trunk and multi-trauma. We highlight drownings, falls from height, rollover falls from beds, slamming door injuries, the low use of child car restraints, bicycle injuries and playground falls as areas of concern. CONCLUSION: Several injury prevention strategies have been suggested and it is hoped these may contribute to addressing preventable childhood injuries in Singapore. We also advocate the establishment of a national childhood injury surveillance database
SN - 0037-5675
AD - Department of Emergency Medicine, Singapore General Hospital, Outram Road, Singapore 169608. gaeoeh@sgh.com.sg
UR - 12762558
ER -

TY - JOUR
ID - 1530
T1 - Aggregate exposures of nine preschool children to persistent organic pollutants at day care and at home
A1 - Wilson,N.K.
A1 - Chuang,J.C.
A1 - Lyu,C.
A1 - Menton,R.
A1 - Morgan,M.K.
Y1 - 2003/05//
N1 - Wilson, Nancy K. Chuang, Jane C. Lyu, Christopher. Menton, Ronald. Morgan, Marsha K
Journal of exposure analysis and environmental epidemiology
bjn, 9111438
IM
Journal Article. Research Support, Non-U.S. Gov't
English
KW - MEDLINE
KW - Biological Markers/ur [Urine]
KW - Child Day Care Centers
KW - Child
KW - Preschool
KW - Environmental Exposure/an [Analysis]
KW - Environmental Monitoring/mt [Methods]
KW - Environmental Pollutants/an [Analysis]
KW - Food Contamination/an [Analysis]
KW - Hand
KW - Housing
KW - Humans
KW - Hydrocarbons
KW - Aromatic
KW - North Carolina
KW - Pesticides/an [Analysis]

KW - Polychlorinated Biphenyls/an [Analysis]
KW - Polycyclic Hydrocarbons
KW - Aromatic/an [Analysis]
RP - NOT IN FILE
SP - 187
EP - 202
JF - Journal of Exposure Analysis and Environmental Epidemiology
JA - J Expo Anal Environ Epidemiol
VL - 13
IS - 3
CY - England
N2 - In the summer of 1997, we measured the aggregate exposures of nine preschool children, aged 2-5 years, to a suite of organic pesticides and other persistent organic pollutants that are commonly found in the home and school environment. The children attended either of two child day care centers in the Raleigh-Durham-Chapel Hill area of North Carolina and were in day care at least 25 h/week. Over a 48-h period, we sampled indoor and outdoor air, play area soil and floor dust, as well as duplicate diets, hand surface wipes, and urine for each child at day care and at home. Our target analytes were several polycyclic aromatic hydrocarbons (PAH), organochlorine pesticides, and polychlorinated biphenyls (PCB); two organophosphate pesticides (chlorpyrifos and diazinon), the lawn herbicide 2,4-dichlorophenoxyacetic acid (2,4-D), three phenols (pentachlorophenol (PCP), nonyl phenols, and bisphenol-A), 3,5,6-trichloro-2-pyridinol (TCP), and two phthalate esters (benzylbutyl and dibutyl phthalate). In urine, our target analytes were hydroxy-PAH, TCP, 2,4-D, and PCP. To allow estimation of each child's aggregate exposures over the 48-h sampling period, we also used time-activity diaries, which were filled out by each child's teacher at day care and the parent or other primary caregiver at home. In addition, we collected detailed household information that related to potential sources of exposure, such as pesticide use or smoking habits, through questionnaires and field observation. We found that the indoor exposures were greater than those outdoors, that exposures at day care and at home were of similar magnitudes, and that diet contributed greatly to the exposures. The children's potential aggregate doses, calculated from our data, were generally well below established reference doses (RfDs) for those compounds for which RfDs are available
SN - 1053-4245
AD - Battelle Memorial Institute, Durham, NC 27713-4411, USA. wilsonk@battelle.org
UR - 12743613
ER -

TY - JOUR
ID - 1531
T1 - Using test dummy experiments to investigate pediatric injury risk in simulated short-distance falls
A1 - Bertocci,G.E.
A1 - Pierce,M.C.
A1 - Deemer,E.
A1 - Aguel,F.
A1 - Janosky,J.E.
A1 - Vogeley,E.
Y1 - 2003/05//
N1 - Bertocci, Gina E. Pierce, Mary Clyde. Deemer, Ernest. Aguel, Fernando. Janosky, Janine E. Vogeley, Ev
Archives of pediatrics & adolescent medicine
9422751, bwf
AIM, IM
Journal Article. Research Support, Non-U.S. Gov't. Research Support, U.S. Gov't, P.H.S.
English
KW - MEDLINE
KW - Accidental Falls
KW - Biomechanical Phenomena
KW - Child Abuse/di [Diagnosis]
KW - Child
KW - Preschool
KW - Craniocerebral Trauma/et [Etiology]

KW - Humans
KW - Leg Injuries/et [Etiology]
KW - Manikins
RP - NOT IN FILE
SP - 480
EP - 486
JF - Archives of Pediatrics & Adolescent Medicine
JA - Arch Pediatr Adolesc Med
VL - 157
IS - 5
CY - United States
N2 - BACKGROUND: Short-distance falls, such as from a bed, are often falsely reported scenarios in child abuse. In attempting to differentiate between abusive and nonabusive injury, knowledge of factors that affect injury risk in falls could prove useful. OBJECTIVES: To assess the biomechanics associated with simulated short-distance falls in children (one fall scenario, without attempting to maximize injury potential) and to investigate the effect of impact surface type on injury risk. METHODS: Repeatable fall experiments from bed height (0.68 m) onto different surfaces were conducted using an instrumented side-lying Hybrid II 3-year-old test dummy. Biomechanical measures assessed in falls included head acceleration, pelvis acceleration, femur loading, and head injury criteria. RESULTS: Fall dynamics resulted in the pelvis or legs making first contact. Biomechanical measures assessed in simulated bed falls were below known head injury criteria and lower extremity injury thresholds. The impact surface type had a significant effect on head injury risk and lower extremity loading. Playground foam proved to have the lowest associated injury risk of all the tested surfaces. CONCLUSIONS: The biomechanics of a child falling from a short distance, such as from a bed, were investigated using an experimental laboratory mock-up and an instrumented test dummy. Despite the impact surface having an effect on injury risk, rolling from a 0.68-m (27-in) horizontal surface from a side-lying posture presented low risk of contact-type head injury and leg injury on all tested impact surfaces
SN - 1072-4710
AD - Child Advocacy Center, Centers for Disease Control and Prevention: Center for Injury Research & Control, Children's Hospital of Pittsburgh, PA, USA. ginaber@pitt.edu
UR - 12742885
ER -

TY - JOUR
ID - 1532
T1 - Soccer-related ocular injuries
A1 - Capao Filipe,J.A.
A1 - Fernandes,V.L.
A1 - Barros,H.
A1 - Falcao-Reis,F.
A1 - Castro-Correia,J.
Y1 - 2003/05//
N1 - Capao Filipe, Joao A. Fernandes, Vitor L. Barros, Henrique. Falcao-Reis, Fernando. Castro-Correia, Jose
Archives of ophthalmology
7706534, 830
AIM, IM
Journal Article
English
KW - MEDLINE
KW - Adolescent
KW - Adult
KW - Child
KW - Eye Injuries/ep [Epidemiology]
KW - Eye Injuries/et [Etiology]
KW - Eye Injuries/pc [Prevention & Control]
KW - Eye Protective Devices
KW - Female
KW - Hospitals

KW - University/sn [Statistics & Numerical Data]
KW - Humans
KW - Male
KW - Middle Aged
KW - Portugal/ep [Epidemiology]
KW - Prospective Studies
KW - Soccer/in [Injuries]
KW - Trauma Severity Indices
KW - Visual Acuity
RP - NOT IN FILE
SP - 687
EP - 694
JF - Archives of Ophthalmology
JA - Arch Ophthalmol
VL - 121
IS - 5
CY - United States
N2 - OBJECTIVE: To outline the severity and long-term sequelae of eye injuries in soccer. DESIGN: Prospective observational study of 163 patients who sustained soccer-related ocular injuries between April 1, 1992, and March 31, 2000 (8 years). METHODS: Patients were observed at a sports ophthalmology unit located in the largest university hospital of the northern region of the country and central to all major soccer fields in town. The data were recorded using the United States Eye Injury Registry report forms for initial and follow-up observation. MAIN OUTCOME MEASURES: (1) Self-reported history surrounding the ocular trauma, initial visual acuity, diagnosis, and operations and (2) final visual acuity, late diagnosis, and additional operations. RESULTS: Injuries occurred predominantly in young men (mean +/- SD age, 23.2 +/- 8.8 years) practicing indoor soccer (50.9%) or outdoor soccer (47.2%), and most resulted from a kicked ball (79.1%) near the goal post (60.1%). Angle recession and peripheral vitreoretinal lesions were more likely to occur in the superotemporal quadrant (54.7%; 95% confidence interval, 44.2%-65.0%; and 57.6%; 95% confidence interval, 48.4%-66.4%; respectively). Vitreoretinal lesions were present in 42.2% (95% confidence interval, 33.1%-51.8%) of patients with "normal" visual acuity (> or =20/40) and in 50.0% (95% confidence interval, 38.1%-61.8%) of patients without hyphema. No significant association was found between severity of injury and age, sex, type of soccer, level of athletic expertise, or player position. CONCLUSIONS: Severe ocular lesions can occur in soccer players without symptoms and at all skill levels. The development of laboratory models will be essential to explain the tendency for lesions to be in the superotemporal quadrant. The data support the need for protective eyewear designed specifically for soccer
SN - 0003-9950
AD - Sports Ophthalmology Unit, Department of Ophthalmology, University of Porto School of Medicine, S Joao Hospital, Porto, Portugal. jacapaofilipe@netcabo.pt
UR - 12742847
ER -

TY - JOUR
ID - 1533
T1 - Is there a link between children's motor abilities and unintentional injuries?
A1 - Schwebel,D.C.
A1 - Binder,S.C.
A1 - Sales,J.M.
A1 - Plumert,J.M.
Y1 - 2003///
N1 - Schwebel, David C. Binder, Stephanie C. Sales, Jessica McDermott. Plumert, Jodie M
Journal of safety research
1264241
IM
Journal Article. Research Support, Non-U.S. Gov't
English
KW - MEDLINE
KW - Accidents

KW - Child
KW - Female
KW - Humans
KW - Male
KW - Motor Skills/ph [Physiology]
KW - United States/ep [Epidemiology]
KW - Wounds and Injuries/ep [Epidemiology]
KW - Wounds and Injuries/et [Etiology]
RP - NOT IN FILE
SP - 135
EP - 141
JF - Journal of Safety Research
JA - J Safety Res
VL - 34
IS - 2
CY - United States
N2 - PROBLEM: The common view is that clumsy children experience unintentional injury more frequently. Empirical evidence supporting this position is mixed. METHOD: One hundred 6- and 8-year-olds completed a battery of nine tasks designed to assess motor ability. Mothers completed a lifetime injury history measure about their children and families completed a 2-week injury diary assessing frequency and severity of daily injuries. RESULTS: Internal reliability for the motor ability battery was good. Correlations between motor ability measures and injury risk were nonsignificant and near zero. DISCUSSION: Motor ability does not appear to be directly related to injury risk. Possible explanations include: (a) coordinated and clumsy children engage in hazardous activities with differing frequency; or (b) other individual difference factors may interact with motor ability to explain children's injury risk. IMPACT ON INDUSTRY: Children's motor abilities do not appear to be directly linked to rate of unintentional injury, but instead may influence risk for injury in conjunction with other factors. Results could have implications to the engineering of children's toys and playground equipment and to the design of appropriate supervision strategies for children engaging in potentially dangerous activities
SN - 0022-4375
AD - Department of Psychology, University of Alabama at Birmingham, 35294, USA. schwebel@uab.edu
UR - 12737952
ER -

TY - JOUR
ID - 1534
T1 - Presumed Pseudomonas folliculitis outbreak in children following an outdoor games event
A1 - Evans,M.R.
A1 - Wilkinson,E.J.
A1 - Jones,R.
A1 - Mathias,K.
A1 - Lenartowicz,P.
Y1 - 2003/04//
N1 - Evans, M R. Wilkinson, E J. Jones, R. Mathias, K. Lenartowicz, P
Communicable disease and public health / PHLS
c1k, 9808711
IM, X
Journal Article
English
KW - MEDLINE
KW - Child
KW - Disease Outbreaks
KW - Folliculitis/ep [Epidemiology]
KW - Folliculitis/mi [Microbiology]
KW - Great Britain/ep [Epidemiology]
KW - Humans
KW - Play and Playthings
KW - Pseudomonas Infections/ep [Epidemiology]

KW - Pseudomonas Infections/mi [Microbiology]
KW - Pseudomonas aeruginosa/ip [Isolation & Purification]
KW - Students
KW - Water Microbiology
RP - NOT IN FILE
SP - 18
EP - 21
JF - Communicable Disease & Public Health
JA - Commun Dis Public Health
VL - 6
IS - 1
CY - England
N2 - In early summer 2001, an outbreak of atypical rash occurred among children from 24 junior schools who attended an outdoor games event. The event comprised a series of five water games including a water slide, and within 24 hours of attending the event 151/593 (26%) children developed a papulopustular rash. The rash had a characteristic distribution, predominantly over the lower trunk and buttocks, with some involvement of the arms and legs. Pseudomonas aeruginosa was isolated from a water butt used to draw water for the games, and from the tank of a fire engine that supplied the water. Similar outbreaks, due to Pseudomonas folliculitis, have been previously described and in immunocompromised people this can be associated with serious illness. We recommend that superchlorinated water be used for water play events and that care be taken to avoid abrasive surfaces
SN - 1462-1843
AD - Communicable Disease Surveillance Centre (Wales), Abton House, Wedal Road, Cardiff CF14 3QX. meirion.evans@phls.wales.nhs.uk
UR - 12736966
ER -

TY - JOUR
ID - 1535
T1 - Improving the measurement of child neglect for survey research: issues and recommendations. [Review] [41 refs]
A1 - Slack,K.S.
A1 - Holl,J.
A1 - Altenbernd,L.
A1 - McDaniel,M.
A1 - Stevens,A.B.
Y1 - 2003/05//
N1 - Slack, Kristen Shook. Holl, Jane. Altenbernd, Lisa. McDaniel, Marla. Stevens, Amy Bush
Child maltreatment
dw4, 9602869
IM
Journal Article. Research Support, U.S. Gov't, Non-P.H.S.. Research Support, U.S. Gov't, P.H.S.. Review
English
KW - MEDLINE
KW - Child
KW - Child Abuse/cl [Classification]
KW - Child Abuse/px [Psychology]
KW - Data Collection/mt [Methods]
KW - Epidemiologic Research Design
KW - Health Planning Guidelines
KW - Humans
KW - Models
KW - Theoretical
KW - United States
RP - NOT IN FILE
SP - 98
EP - 111

JF - Child Maltreatment
JA - Child Maltreat
VL - 8
IS - 2
CY - United States
N2 - There is a great need for developing and validating measures of child neglect that can be applied to survey samples outside of a child welfare context. A prospective assessment of child neglect would afford a better estimation of the etiology of various types of child neglect and would greatly inform the development of primary prevention strategies related to child maltreatment. This article offers guidance on the tasks involved with constructing new measures of neglect for prospective survey research. Methodological issues pertaining to child neglect measurement are discussed, and a framework is offered for developing neglect measures for survey research. A discussion is also offered on how this framework is being applied in an ongoing longitudinal study of low-income families with young children. The intended result of this exercise is to encourage the development of new child neglect measures for survey research with both high-risk and general populations. [References: 41]
SN - 1077-5595
AD - School of Social Work, University of Wisconsin-Madison, 53706, USA
UR - 12735712
ER -

TY - JOUR
ID - 1536
T1 - Optimizing out-of-school time: the role of free-choice learning
A1 - Dierking,L.D.
A1 - Falk,J.H.
Y1 - 2003///
N1 - Dierking, Lynn D. Falk, John H
New directions for youth development
101090644
IM
Journal Article
English
KW - MEDLINE
KW - Adolescent
KW - Child
KW - Choice Behavior
KW - Humans
KW - Learning
KW - Leisure Activities
KW - Schools
KW - Time Factors
RP - NOT IN FILE
SP - 75
EP - 88
JF - New Directions for Youth Development
JA - New Dir.Youth Dev
IS - 97
CY - United States
N2 - Free-choice learning, a new paradigm for the learning that youth and their families engage in outside school, can play an important role in the healthy development of youth, their families, and communities
SN - 1533-8916
AD - Institute for Learning Innovation, Annapolis, Maryland, USA
UR - 12728821
ER -

TY - JOUR
ID - 1537

T1 - Domestic poultry-raising practices in a Peruvian shantytown: implications for control of Campylobacter jejuni-associated diarrhea
A1 - Harvey,S.A.
A1 - Winch,P.J.
A1 - Leontsini,E.
A1 - Torres,Gayoso C.
A1 - Lopez,Romero S.
A1 - Gilman,R.H.
A1 - Oberhelman,R.A.
Y1 - 2003/04//
N1 - Harvey, Steven A. Winch, Peter J. Leontsini, Elli. Torres Gayoso, Cecilia. Lopez Romero, Sonia. Gilman, Robert H. Oberhelman, Richard A
Acta tropica
23a, 0370374
IM
Journal Article. Research Support, Non-U.S. Gov't
English
KW - MEDLINE
KW - Adult
KW - Animal Husbandry/ec [Economics]
KW - Animal Husbandry/ed [Education]
KW - Animal Husbandry/mt [Methods]
KW - Animals
KW - Campylobacter Infections/mi [Microbiology]
KW - Campylobacter Infections/pc [Prevention & Control]
KW - Campylobacter Infections/ve [Veterinary]
KW - Campylobacter jejuni/gd [Growth & Development]
KW - Child
KW - Diarrhea/mi [Microbiology]
KW - Diarrhea/pc [Prevention & Control]
KW - Female
KW - Humans
KW - Interviews as Topic
KW - Male
KW - Peru
KW - Poultry/gd [Growth & Development]
KW - Poultry Diseases/mi [Microbiology]
KW - Poverty
RP - NOT IN FILE
SP - 41
EP - 54
JF - Acta Tropica
JA - Acta Trop
VL - 86
IS - 1
CY - Netherlands
N2 - Raising poultry at home is common in many periurban communities in low-income countries. Studies demonstrate that free-range domestic poultry increase children's risk of infection with diarrhea-causing organisms such as Campylobacter jejuni. Corralling might reduce risk, but research on the socioeconomic acceptability of corralling is lacking. To explore this issue, we studied local knowledge and practices related to poultry-raising in a Peruvian shantytown. Our objectives were to understand: (1). motives for raising domestic poultry; (2). economic and cultural factors that affect the feasibility of corralling; and (3). local perceptions about the relationship between domestic poultry and disease. During 1999-2000, we met with community health volunteers and conducted ethnographic and structured interviews with residents about poultry-raising practices. We then enrolled 12 families in a 2-month trial of corral use during which field workers made biweekly surveillance visits to each family. Most participants reported that they raise birds because home-grown poultry and eggs taste better and are more nutritious and because they enjoy living around animals. Some want to teach

their children about raising animals. To prevent theft, many residents shut their birds in provisional enclosures at night, but most stated that birds are healthier, happier, and produce better meat and eggs when let loose by day. Many view bird feces in the house and yard as dirty, but few see a connection to illness. Residents consider chicks and ducklings more innocuous than adult birds and are more likely to allow them inside the house and permit children to play with them. After extensive orientation and technical assistance, participants were willing to corral birds more often. But due to perceived disadvantages, many kept birds penned only intermittently. Additional food and water costs were a significant obstacle for some. Adequate space, bird care and corral hygiene would also need to be addressed to make this intervention viable. Developing a secure, acceptable and affordable corral remains a challenge in this population
SN - 0001-706X
AD - Department of International Health, Johns Hopkins Bloomberg School of Public Health, Baltimore, MD 21025, USA. sharvey@jhsph.edu
UR - 12711102
ER -

TY - JOUR
ID - 1538
T1 - [Emotion regulatory processes in evoked play narratives]. [German]
A1 - Lemche,E.
A1 - Lennertz,I.
A1 - Orthmann,C.
A1 - Ari,A.
A1 - Grote,K.
A1 - Hafker,J.
A1 - Klann-Delius,G.
Y1 - 2003/03//
N1 - Lemche, Erwin. Lennertz, Ilka. Orthmann, Claudia. Ari, Alkim. Grote, Katja. Hafker, Jessika. Klann-Delius, Gisela
Praxis der Kinderpsychologie und Kinderpsychiatrie
pj6, 0404246
IM
English Abstract. Journal Article. Research Support, Non-U.S. Gov't
German
KW - MEDLINE
KW - Adaptation
KW - Psychological
KW - Child
KW - Preschool
KW - Emotions
KW - Female
KW - Humans
KW - Male
KW - Mother-Child Relations
KW - Narration
KW - Object Attachment
KW - Personality Assessment
KW - Personality Development
KW - Play Therapy
RP - NOT IN FILE
SP - 156
EP - 171
JF - Praxis der Kinderpsychologie und Kinderpsychiatrie
JA - Prax Kinderpsychol.Kinderpsychiatr.
VL - 52
IS - 3
CY - Germany

N2 - Evoked play narratives have been demonstrated to provide a novel window towards internal emotion regulation and mental representations. The present study evaluates covariations between emotion themes and mother-child interaction, as well as child behavior problems. An exploratory study in non-referred children in the 3-6 age span utilizing the MacArthur-method was conducted by taking emotional, conflictive and moral themes as indices of emotion-regulatory processes. Emotion themes were linked to external measures of dyadic Emotional Availability, interparental relationship quality, and behavior problems employing the 4/18 version of the Child Behavior Checklist. Mental representations were aggregated using the Person Representation Coding System. Of a principal components analysis with subsequent varimax-rotation for narrative content codes resulted four emotion theme composites: social conflicting, a prosocial aggregate, an antisocial aggregate, and a composite conflict solving/-understanding. The 4-factor solution displayed meaningful correlation patterns with the mental representations of self and parents, as well as with most of the external measures. Although subsequent studies ought to be methodologically improved both in design and sample size, the results of the present investigation give rise to the assumption that future efforts of validating emotion-regulatory processes with more established outside measures are likely to be successful
SN - 0032-7034
AD - Klinik und Poliklinik fur Psychotherapie und Psychosomatik, Medizinischen Fakultat Carl Gustav Carus, Technische Universitat Dresden. erwin.lemche@mailbox.tu-dresden.de
UR - 12693352
ER -

TY - JOUR
ID - 1539
T1 - Ultraviolet radiation exposure of children and adolescents in Durban, South Africa
A1 - Guy,C.
A1 - Diab,R.
A1 - Martincigh,B.
Y1 - 2003/03//
N1 - Guy, Caradee. Diab, Roseanne. Martincigh, Bice
Photochemistry and photobiology
0376425, p69
IM
Journal Article. Research Support, Non-U.S. Gov't
English
KW - MEDLINE
KW - Adolescent
KW - Child
KW - Environmental Exposure
KW - Female
KW - Humans
KW - Male
KW - Meteorological Concepts
KW - Photobiology
KW - Radiation Dosage
KW - Skin/re [Radiation Effects]
KW - South Africa
KW - Ultraviolet Rays/ae [Adverse Effects]
RP - NOT IN FILE
SP - 265
EP - 270
JF - Photochemistry & Photobiology
JA - Photochem Photobiol
VL - 77
IS - 3
CY - United States
N2 - The solar ultraviolet radiation (UVR) exposure of 30 children and adolescents in three age groups (4-6 years, 7-9 years and 13-14 years) was measured for 1 week in late summer (February-March) in Durban, South Africa, using UVR-sensitive polysulfone film badges (PSFB) attached to the lapel region of the body. The mean

and median values for all ages over the study period were 2.0 and 1.2 standard erythemal dose (SED) units, respectively, where 1 SED = 100 J x m(-2). Individual PSFB doses were analyzed as a function of age, gender and behavior. No significant statistical differences were found between different age groups; however, there was a statistical difference between males and females, with males generally receiving higher PSFB doses. Subjects completed UVR exposure journals documenting their time outdoors, shade versus sun conditions, nature of their activities, clothing worn and their use of sunscreen for each day of the study. Activity patterns were noted as the most important factor influencing individual UVR dose. Ambient erythemal UVR was measured by a Yankee Environmental Systems UVB pyranometer, and a relationship between ambient UVR and individual UVR dose was derived. On average, subjects received a dose of 4.6% of the total daily erythemal UVR. Based on this factor, the potential dose of an individual over a full annual cycle was estimated. Accordingly, there were 139 days during the year when an individual with skin type I (light skin) would be likely to experience minimal erythema and 97 and 32 days for individuals with skin types II and III, respectively
SN - 0031-8655
AD - School of Life and Environmental Sciences, University of Natal, Durban, South Africa
UR - 12685653
ER -

TY - JOUR
ID - 1540
T1 - Comparing unintentional and intentional injuries in a school setting
A1 - Limbos,M.A.
A1 - Peek-Asa,C.
Y1 - 2003/03//
N1 - Limbos, Mary Ann P. Peek-Asa, Corinne
The Journal of school health
k13, 0376370
IM, N
Comparative Study. Journal Article
English
KW - MEDLINE
KW - Accident Prevention
KW - Accidents/sn [Statistics & Numerical Data]
KW - Adolescent
KW - Age Distribution
KW - Child
KW - Female
KW - Humans
KW - Incidence
KW - Los Angeles/ep [Epidemiology]
KW - Male
KW - Play and Playthings
KW - Population Surveillance
KW - Risk Factors
KW - Risk Management
KW - Schools/sn [Statistics & Numerical Data]
KW - Sex Distribution
KW - Students/sn [Statistics & Numerical Data]
KW - Time Factors
KW - Urban Health/sn [Statistics & Numerical Data]
KW - Violence/pc [Prevention & Control]
KW - Violence/sn [Statistics & Numerical Data]
KW - Wounds and Injuries/ep [Epidemiology]
KW - Wounds and Injuries/et [Etiology]
KW - Wounds and Injuries/pc [Prevention & Control]
KW - Wounds and Injuries/px [Psychology]
RP - NOT IN FILE
SP - 101

EP - 106
JF - Journal of School Health
JA - J Sch Health
VL - 73
IS - 3
CY - United States
N2 - This study determined the incidence of violence-related injuries in an urban school district, and compared characteristics of unintentional and intentional school injuries. A sample of student Accident Report Forms completed for a school district in 1997 were reviewed for demographic characteristics of the student and injury characteristics. Injuries were categorized as unintentional, intentional, or of unknown intent. Annual incidence rates of injury per 100 students were calculated by intention, grade, and gender. Comparisons between unintentional and intentional injuries were made using odds ratios and 95% confidence intervals. Of 11,674 annualized injuries, 77.2% were unintentional, 16.8% were intentional, and 6.0% were of unknown intent. The overall annualized injury rate was 1.74 injuries per 100 students/year. The unintentional injury rate was almost five times the intentional injury rate of 0.29 injuries per 100 students/year. High school students had both the highest unintentional and intentional injury rates. Males in all grade levels had the highest rates of injury. Most injuries occurred during school hours. Intentional injuries were almost three times more likely to be associated with unstructured play or after school playground hours; were less likely to be witnessed events; and were more likely to occur on the surrounding school grounds than unintentional injuries. Unintentional injuries represent a greater risk to school children than do intentional injuries. A potential area to focus interventions for intentional injuries are modifications of the school environment and surrounding grounds to improve supervision and monitoring
SN - 0022-4391
AD - USC Keck School of Medicine, Division of General Pediatrics, Childrens Hospital of Los Angeles, 4650 Sunset Blvd., MS# 76, Los Angeles, CA 90027, USA. mlimbos@chla.usc.edu
UR - 12677728
ER -

TY - JOUR
ID - 1541
T1 - A Lyme disease case study and individualized healthcare plan
A1 - Cavendish,R.
Y1 - 2003/04//
N1 - Cavendish, Roberta
The Journal of school nursing : the official publication of the National Association of School Nurses
bgs, 9206498
N
Case Reports. Journal Article
English
KW - MEDLINE
KW - Child
KW - Female
KW - Health Promotion
KW - Humans
KW - Lyme Disease/di [Diagnosis]
KW - Lyme Disease/dt [Drug Therapy]
KW - Lyme Disease/ep [Epidemiology]
KW - Lyme Disease/nu [Nursing]
KW - Nursing Assessment
KW - Nursing Diagnosis
KW - Patient Care Planning
KW - Patient Education as Topic
KW - Referral and Consultation
KW - Risk Assessment
KW - Risk Factors
KW - School Nursing/mt [Methods]
KW - United States/ep [Epidemiology]

RP - NOT IN FILE
SP - 81
EP - 88
JF - Journal of School Nursing
JA - J Sch Nurs
VL - 19
IS - 2
CY - United States
N2 - The Atlantic and Pacific coasts are the boundaries of Lyme disease with the Northeastern and Midwestern regions of the United States continuing to report the majority of cases. New reported cases of Lyme disease doubled from 1991 to 2001 according to statistics published by the Centers for Disease Control and Prevention. Within that population are more children between the ages of 5 and 9. The younger the child, the more difficult it is to diagnose Lyme disease. Children under the age of 19 are at high risk for Lyme disease because of the amount of time spent in outdoor activities during the late spring and summer. These months correlate with the tick breeding cycle and the peak time for outdoor recreational activities. Lyme disease can pose serious health risks in late stage illness. A Lyme disease case study on a school-age child provides comprehensive assessment data, interventions, and educational information for parents. A template of an individualized healthcare plan using standardized language is a guide for school nurses. The value of school nurses as guardians of the public's health is addressed
SN - 1059-8405
AD - The College of Staten Island/City University of New York, Staten Island, NY, USA
UR - 12653619
ER -

TY - JOUR
ID - 1542
T1 - Joint attention training for children with autism using behavior modification procedures
A1 - Whalen,C.
A1 - Schreibman,L.
Y1 - 2003/03//
N1 - Whalen, Christina. Schreibman, Laura
Journal of child psychology and psychiatry, and allied disciplines
hp3, 0375361
IM
Clinical Trial. Controlled Clinical Trial. Journal Article. Research Support, U.S. Gov't, P.H.S.
English
KW - MEDLINE
KW - Attention
KW - Autistic Disorder/th [Therapy]
KW - Behavior Therapy/mt [Methods]
KW - California
KW - Child Development
KW - Child
KW - Preschool
KW - Humans
KW - Play and Playthings
KW - Reproducibility of Results
KW - Social Behavior
RP - NOT IN FILE
SP - 456
EP - 468
JF - Journal of Child Psychology & Psychiatry & Allied Disciplines
JA - J Child Psychol Psychiatry
VL - 44
IS - 3
CY - England

N2 - BACKGROUND: Deficits in joint attention are considered by many researchers to be an early predictor of childhood autism (e.g., Osterling & Dawson, 1994) and are considered to be pivotal to deficits in language, play, and social development in this population (Mundy, 1995). Although many researchers have noted the importance of joint attention deficits in the development of children with autism (e.g., Mundy, Sigman, & Kasari, 1994) and have called for intervention strategies (e.g., Mundy & Crowson, 1997), few studies have attempted to target joint attention. In this study, joint attention behaviors were taught to children with autism using a behavior modification procedure. METHODS: A multiple-baseline design was implemented to evaluate intervention effects. The following target behaviors were included in the intervention: 1) Responding to showing, pointing, and gaze shifting of adult; 2) Coordinated gaze shifting (i.e., coordinated joint attention); and 3) Pointing (with the purpose of sharing, not requesting). Generalization to setting and parent, follow-up sessions, and social validation measures were also analyzed. RESULTS: Joint attention behaviors were effectively trained and targeted behaviors generalized to other settings. In addition, positive changes were noted by naive observers using social validation measures. CONCLUSIONS: Integrating joint attention training into existing interventions may be important for children with autism. In addition, training parents in these techniques may help to maintain joint attention skills outside of the treatment setting
SN - 0021-9630
AD - Autism Spectrum Therapies, Los Angeles, CA 90034, USA. cdallaire@autismtherapies.com
UR - 12635974
ER -

TY - JOUR
ID - 1543
T1 - Health risk for children from lead and cadmium near a non-ferrous smelter in Bulgaria
A1 - Fischer,A.B.
A1 - Georgieva,R.
A1 - Nikolova,V.
A1 - Halkova,J.
A1 - Bainova,A.
A1 - Hristeva,V.
A1 - Penkov,D.
A1 - Alandjiisk,D.
Y1 - 2003/01//
N1 - Fischer, A B. Georgieva, R. Nikolova, V. Halkova, J. Bainova, A. Hristeva, V. Penkov, D. Alandjiisk, D
International journal of hygiene and environmental health
do6, 100898843
IM
Journal Article
English
KW - MEDLINE
KW - Adolescent
KW - Bulgaria
KW - Cadmium/ae [Adverse Effects]
KW - Cadmium/an [Analysis]
KW - Child
KW - Child Welfare
KW - Preschool
KW - Environmental Pollutants/ae [Adverse Effects]
KW - Environmental Pollutants/an [Analysis]
KW - Erythrocytes/ch [Chemistry]
KW - Female
KW - Food Contamination
KW - Hematologic Tests
KW - Humans
KW - Lead/ae [Adverse Effects]
KW - Lead/an [Analysis]
KW - Life Style
KW - Male

KW - Nutritional Status
KW - Protoporphyrins/an [Analysis]
KW - Risk Assessment
RP - NOT IN FILE
SP - 25
EP - 38
JF - International Journal of Hygiene & Environmental Health
JA - Int J Hyg Environ Health
VL - 206
IS - 1
CY - Germany
N2 - In an interdisciplinary approach the exposure to lead and cadmium of the population living in the vicinity of a Bulgarian non-ferrous metallurgical plant was studied to determine the routes of exposure. Two exposed villages situated 4 and 6 km SW and W, respectively,--in the plume of the plant's emissions--and a village for comparison situated 14 km SE of the plant were selected for the study. The blood of children aged 3-13 years from the exposed villages (n = 111) and the village for comparison (n = 18) was analyzed for lead (PbB) and cadmium (CdB) as well as for free erythrocyte protoporphyrin (FEP) and haematological parameters. A personal questionnaire concerning nutrition habits and life-style was filled in by interviewers of the children's parents. The cadmium levels in blood were within the variation range stated for Bulgaria. The exposed children had a mean CdB of 0.38 +/- 0.18 microgram/l (range 0.10-0.90 microgram/l) and the comparison group 0.31 +/- 0.35 microgram/l (0.06-1.42 micrograms/l). The mean PbB value measured in the exposed children was 240 +/- 96 micrograms/l (77-631 micrograms/l), while the mean value of PbB for the reference group was 149 +/- 57 micrograms/l (63-285 micrograms/l). Individual PbB levels > 150 and > 200 micrograms/l were found in 85% and 62%, respectively, of the exposed children and FEP > 80 micrograms/dl erythrocytes was detected in 21%. There was a concentration-response and a concentration-effect relationship (r = 0.51, D > 0.001) between PbB and FEP. According to the questionnaire only 10.3% of the interviewed families in the two exposed villages buy all food from the market, the others produce a significant part of their food themselves. The PbB levels of children who consume certain foods of local production (cabbage, cereals, poultry, and beef) were significantly higher compared to the PbB values of children whose families purchase these foods from the market. No significant relationship between lead in children's blood and the use of tin and ceramic utensils, drinking and irrigation water, hygiene habits, family tobacco smoking habits, playgrounds and traffic intensity were revealed. There was a significantly higher incidence of prematurely born babies in the exposed population. The proven relationship between lead intake via the food chain and existing significant health risks is the basis for risk communication. Measures aimed at healthier nutrition and life-style intend to contribute to decreasing the risk of the population living in the smelter region
SN - 1438-4639
AD - Institute of Hygiene and Environmental Medicine, Justus-Liebig-University Giessen, Friedrichstr. 16, D-35392 Giessen, Germany. Anna.B.Fischer@hygiene.med.uni-giessen.de
UR - 12621900
ER -

TY - JOUR
ID - 1544
T1 - The focal form of persistent hyperinsulinemic hypoglycemia of infancy: morphological and molecular studies show structural and functional differences with insulinoma
A1 - Sempoux,C.
A1 - Guiot,Y.
A1 - Dahan,K.
A1 - Moulin,P.
A1 - Stevens,M.
A1 - Lambot,V.
A1 - de,Lonlay P.
A1 - Fournet,J.C.
A1 - Junien,C.
A1 - Jaubert,F.
A1 - Nihoul-Fekete,C.
A1 - Saudubray,J.M.

A1 - Rahier,J.
Y1 - 2003/03//
N1 - Sempoux, Christine. Guiot, Yves. Dahan, Karin. Moulin, Pierre. Stevens, Martine. Lambot, Virginie. de Lonlay, Pascale. Fournet, Jean-Christophe. Junien, Claudine. Jaubert, Francis. Nihoul-Fekete, Claire. Saudubray, Jean-Marie. Rahier, Jacques
Diabetes
e8x, 0372763
AIM, IM
Comparative Study. Journal Article. Research Support, Non-U.S. Gov't
English
KW - MEDLINE
KW - ATP-Binding Cassette Transporters
KW - Adolescent
KW - Adult
KW - Aged
KW - 80 and over
KW - Alleles
KW - Child
KW - Chromosomes
KW - Human
KW - Pair 11/ge [Genetics]
KW - Cyclin-Dependent Kinase Inhibitor p57
KW - Humans
KW - Hyperinsulinism/co [Complications]
KW - Hyperinsulinism/ge [Genetics]
KW - Hyperinsulinism/pa [Pathology]
KW - Hypoglycemia/et [Etiology]
KW - Hypoglycemia/ge [Genetics]
KW - Hypoglycemia/pa [Pathology]
KW - Immunohistochemistry
KW - In Situ Hybridization
KW - Infant
KW - Insulin/me [Metabolism]
KW - Insulin-Like Growth Factor II/an [Analysis]
KW - Insulin-Like Growth Factor II/ge [Genetics]
KW - Insulinoma/ge [Genetics]
KW - Insulinoma/pa [Pathology]
KW - Insulinoma
KW - Islets of Langerhans/ch [Chemistry]
KW - Islets of Langerhans/me [Metabolism]
KW - Islets of Langerhans/pa [Pathology]
KW - Loss of Heterozygosity
KW - Microsatellite Repeats
KW - Middle Aged
KW - Mutation
KW - Nuclear Proteins/an [Analysis]
KW - Nuclear Proteins/ge [Genetics]
KW - Pancreatic Neoplasms/ge [Genetics]
KW - Pancreatic Neoplasms/pa [Pathology]
KW - Pancreatic Neoplasms
KW - Potassium Channels/an [Analysis]
KW - Potassium Channels/ge [Genetics]
KW - Potassium Channels
KW - Inwardly Rectifying
KW - Proinsulin/bi [Biosynthesis]
KW - Proinsulin/ge [Genetics]
KW - RNA

KW - Messenger/an [Analysis]
KW - Receptors
KW - Drug/an [Analysis]
KW - Drug/ge [Genetics]
KW - Sulfonylurea Receptors
RP - NOT IN FILE
SP - 784
EP - 794
JF - Diabetes
VL - 52
IS - 3
CY - United States
N2 - Paternal mutation of ATP-sensitive K(+) (K(ATP)) channel genes and loss of heterozygosity (LOH) of the 11p15 region including the maternal alleles of ABCC8, IGF2, and CDKN1C characterize the focal form of persistent hyperinsulinemic hypoglycemia of infancy (FoPHHI). We aimed to understand the actual nature of FoPHHI in comparison with insulinoma. In FoPHHI, the lesion consists in clusters of beta-cells surrounded by non-beta-cells. Compared with adjacent islets, proinsulin mRNA is similar and proinsulin production higher (P < or = 0.02), indicating regulation at a translational level, with slightly lower insulin stock and lower ABCC8 peptide labeling (P<0.05). Insulinomas, composed of beta-cell nests or cords, have similar proinsulin mRNA compared with adjacent islets, highly variable proinsulin production, lower insulin stock (P < or = 0.02), and higher ABCC8 peptide labeling (P<0.05). Proinsulin mRNA is lower than in FoPHHI (P<0.001). Islets adjacent to FoPHHI appear to be resting, in contrast to those adjacent to insulinomas, evidencing intrapancreatic regulation of islet beta-cell activity. IGF2 peptide is present inside and outside both lesions, but IGF2 mRNA is restricted to the lesions. The 11p15 LOH and absence of CDKN1C peptide staining are demonstrated in all FoPHHI but also in three of eight insulinomas. Despite some molecular similarities, FoPHHI is thus fundamentally different from insulinoma
SN - 0012-1797
AD - Department of Pathology (ANPS 1712), Cliniques Universitaires St-Luc, University Hospital, U.C.L. Avenue Hippocrate 10, B-1200 Brussels, Belgium. christine.sempoux@anps.ucl.ac.be
UR - 12606521
ER -

TY - JOUR
ID - 1545
T1 - Dental anomalies of the permanent lateral incisors and prevalence of hypodontia outside the cleft area in complete unilateral cleft lip and palate
A1 - Lourenco,Ribeiro L.
A1 - Teixeira Das,Neves L.
A1 - Costa,B.
A1 - Ribeiro,Gomide M.
Y1 - 2003/03//
N1 - Lourenco Ribeiro, Luciana. Teixeira Das Neves, Lucimara. Costa, Beatriz. Ribeiro Gomide, Marcia
The Cleft palate-craniofacial journal : official publication of the American Cleft Palate-Craniofacial Association
a0r, 9102566
D, IM
Journal Article
English
KW - MEDLINE
KW - Anodontia/et [Etiology]
KW - Child
KW - Preschool
KW - Cleft Lip/co [Complications]
KW - Cleft Palate/co [Complications]
KW - Female
KW - Humans
KW - Incisor/ab [Abnormalities]
KW - Male

KW - Tooth
KW - Supernumerary/et [Etiology]
RP - NOT IN FILE
SP - 172
EP - 175
JF - Cleft Palate-Craniofacial Journal
JA - Cleft Palate Craniofac J
VL - 40
IS - 2
CY - United States
N2 - OBJECTIVE: To determine in complete unilateral cleft lip and palate (UCLP) subjects the characteristics (location, shape) of the cleft-side lateral incisor. The presence of a supernumerary tooth at the cleft side and the prevalence of hypodontia outside the cleft area were evaluated. A comparison was made of the shape of the cleft side lateral incisor to its contralateral incisor. SETTING: Hospital for Rehabilitation of Craniofacial Anomalies (HRCA), Sao Paulo, Brazil. PATIENTS: Orthopantomograms of 203 subjects with UCLP and without syndromes were chronologically selected from the HRCA data bank, within an age range of 5 to 10 years. Outcome Measure: Orthopantomograms were analyzed by the same observer according to established criteria. RESULTS: There were no statistically significant differences between sexes for any of the criteria studied. The cleft-side lateral incisor was present in 50.2%, and it was more commonly located at the distal side (76.5%). The congenital absence of the cleft-side lateral incisor was observed in 49.8% of the sample, and its antimere was congenitally missing in 10.9%, this difference being statistically significant. The most commonly missing tooth outside the cleft area was the maxillary second premolar. CONCLUSIONS: The high prevalence of hypodontia of the permanent lateral incisor in the cleft side showed that the cleft could play an important role in this absence. There were different patterns for the presence of the cleft-side lateral incisor
SN - 1055-6656
AD - Department of Dentistry for Children, Craniofacial Anomalies Rehabilitation Hospital, University of Sao Paulo, Brazil
UR - 12605523
ER -

TY - JOUR
ID - 1546
T1 - Proton radiotherapy for paediatric tumours: potential areas for clinical research
A1 - Taylor,R.E.
Y1 - 2003/02//
N1 - Taylor, R E
Clinical oncology (Royal College of Radiologists (Great Britain))
ayi, 9002902
IM
Journal Article
English
KW - MEDLINE
KW - Biomedical Research
KW - Brain Neoplasms/rt [Radiotherapy]
KW - Central Nervous System Neoplasms/rt [Radiotherapy]
KW - Child
KW - Humans
KW - Neoplasms/rt [Radiotherapy]
KW - Protons
KW - Radiation Oncology
KW - Radiotherapy
KW - High-Energy
KW - Skull Base Neoplasms/rt [Radiotherapy]
RP - NOT IN FILE
SP - S32
EP - S36
JF - Clinical Oncology (Royal College of Radiologists)

JA - Clin Oncol (R Coll Radiol)
VL - 15
IS - 1
CY - England
N2 - Radiotherapy plays an important role in the management of children with cancer. The aim is to achieve local tumour control while minimizing long-term effects. In the treatment of tumours of the central nervous system (CNS) the most important long-term effects are neuropsychological. Elsewhere orthopaedic long-term effects may compromise function or be cosmetically harmful. Proton therapy has the potential for homogeneous irradiation of the target volume while reducing the magnitude and/or extent of the low dose area outside the target volume. This may be clinically relevant for long-term effects in children. Proton radiotherapy has an established role in the treatment of children with chordomas and chondrosarcomas of the base of skull. Planning studies have demonstrated the potential for improving the therapeutic ratio for radiotherapy for tumours of the central nervous system by achieving a uniform dose within the target volume while minimizing the severity of neuropsychological sequelae. Clinical experience of proton radiotherapy for children remains limited with potential areas for clinical research
SN - 0936-6555
AD - Cookridge Hospital, Leeds LS16 6QB, UK. roger.taylor@leedsth.nhs.uk
UR - 12602562
ER -

TY - JOUR
ID - 1547
T1 - Fostering generalization and maintenance in school settings
A1 - Yaruss,J.S.
A1 - Reardon,N.A.
Y1 - 2003/02//
N1 - Yaruss, J Scott. Reardon, Nina A
Seminars in speech and language
8405117, cfb, 8405117
IM
Journal Article
English
KW - MEDLINE
KW - Child
KW - Generalization (Psychology)
KW - Humans
KW - School Health Services
KW - Speech Therapy/mt [Methods]
KW - Stuttering/th [Therapy]
RP - NOT IN FILE
SP - 33
EP - 40
JF - Seminars in Speech & Language
JA - Semin Speech Lang
VL - 24
IS - 1
CY - United States
N2 - One of the most challenging aspects of therapy for school-age children who stutter is generalizing the skills learned in the therapy room to other settings such as the classroom, lunchroom, playground, or home. An additional challenge is seen in maintaining gains over the long term. This article reviews common roadblocks to generalization and maintenance, including the goals of therapy, the nature of the treatment strategies that require generalization, the scheduling and implementation of generalization activities within the overall therapy process, and the child's understanding of the treatment goals. Specific strategies for overcoming these roadblocks include: desensitizing children to both stuttering and treatment strategies designed to improve fluency, using hierarchies as a way of structuring treatment and moving children toward success in their daily activities, integrating the child's real world and clinical settings, and using structured practice activities to help children solidify all of the lessons they learn in treatment. This article highlights the importance of taking a broad-based

view of stuttering to help children improve their overall communication across a variety of settings and over time
SN - 0734-0478
AD - University of Pittsburgh, Children's Hospital of Pittsburgh, Stuttering Center of Western Pennsylvania, Pittsburgh, Pennsylvania 15260, USA. jsyaruss@csd.pitt.edu
UR - 12601585
ER -

TY - JOUR
ID - 1548
T1 - [Assessment and intervention of preschool children with attention deficit hyperactivity disorder symptoms and disruptive behavior]. [Review] [65 refs] [Spanish]
A1 - Miranda-Casas,A.
A1 - Uribe,L.H.
A1 - Gil-Llario,M.D.
A1 - Jarque,S.
Y1 - 2003/02//
N1 - Miranda-Casas, A. Uribe, L H. Gil-Llario, M D. Jarque, S
Revista de neurologia
cg9, 7706841
IM
English Abstract. Journal Article. Review
Spanish
KW - MEDLINE
KW - Attention Deficit Disorder with Hyperactivity/di [Diagnosis]
KW - Attention Deficit Disorder with Hyperactivity/th [Therapy]
KW - Attention Deficit and Disruptive Behavior Disorders/di [Diagnosis]
KW - Attention Deficit and Disruptive Behavior Disorders/th [Therapy]
KW - Child
KW - Preschool
KW - Humans
RP - NOT IN FILE
SP - S85
EP - S94
JF - Revista de Neurologia
JA - Rev Neurol
VL - 36 Suppl 1
CY - Spain
N2 - Attention deficit hyperactivity disorder (ADHD) constitutes one of the most common childhood syndromes and its negative outcomes on all the child's functional domains have been consistently reported in the literature. As a result, their early identification is becoming a topic of increasing concern among the researchers from the field. However, given that many of the behaviors of interest are normative behaviors during this period, diagnosis in preschool years is controversial. Specifically, from a developmental perspective, although it is well known that high levels of motor activity, poor self control, and lack of attention are typical during these years, both expression and intensity of these behaviors are markedly higher on the group of ADHD preschool children. Consequently, their negative interference with daily living, produce significant maladjustments in the child's natural settings. All these ideas justify the need of considering and studying the most appropriate assessment techniques to reliably identify the deficits of ADHD in preschool children. On the basis of these statements, this paper offers a theoretical overview of the most recent developments regarding ADHD assessment and intervention techniques directed to the prevention of cognitive deficits as well as the achievement of a better school and social adjustment of ADHD preschool children. [References: 65]
SN - 0210-0010
AD - Departamento de Psicologia Evolutiva y de la Educacion, Universidad de Valencia. Facultad de Psicologia, Burjassot, Espana. ana.miranda@uv.es
UR - 12599108
ER -

TY - JOUR
ID - 1549
T1 - Risks to children from exposure to lead in air during remedial or removal activities at Superfund sites: a case study of the RSR lead smelter Superfund site
A1 - Khoury,G.A.
A1 - Diamond,G.L.
Y1 - 2003/01//
N1 - Khoury, Ghassan A. Diamond, Gary L
Journal of exposure analysis and environmental epidemiology
bjn, 9111438
IM
Journal Article. Research Support, Non-U.S. Gov't
English
KW - MEDLINE
KW - Air Movements
KW - Air Pollutants/an [Analysis]
KW - Child
KW - Child Welfare
KW - Dust
KW - Environmental Exposure
KW - Environmental Monitoring
KW - Facility Design and Construction
KW - Hazardous Waste
KW - Humans
KW - Industry
KW - Inhalation Exposure
KW - Lead/ae [Adverse Effects]
KW - Lead/an [Analysis]
KW - Lead/bl [Blood]
KW - Metallurgy
KW - Models
KW - Theoretical
KW - Risk Assessment
KW - Vehicle Emissions/an [Analysis]
RP - NOT IN FILE
SP - 51
EP - 65
JF - Journal of Exposure Analysis and Environmental Epidemiology
JA - J Expo Anal Environ Epidemiol
VL - 13
IS - 1
CY - England
N2 - Superfund sites that are contaminated with lead and undergoing remedial action generate lead-enriched dust that can be released into the air. Activities that can emit lead-enriched dust include demolition of lead smelter buildings, stacks, and baghouses; on-site traffic of heavy construction vehicles; and excavation of soil. Typically, air monitoring stations are placed around the perimeter of a site of an ongoing remediation to monitor air lead concentrations that might result from site emissions. The National Ambient Air Quality (NAAQ) standard, established in 1978 to be a quarterly average of 1.5 microg/m(3), is often used as a trigger level for corrective action to reduce emissions. This study explored modeling approaches for assessing potential risks to children from air lead emissions from the RSR Superfund site in West Dallas, TX, during demolition and removal of a smelter facility. The EPA Integrated Exposure Uptake Biokinetic (IEUBK) model and the International Commission of Radiologic Protection (ICRP) lead model were used to simulate blood lead concentrations in children, based on monitored air lead concentrations. Although air lead concentrations at monitoring stations located in the downwind community intermittently exceeded the NAAQ standard, both models indicated that exposures to children in the community areas did not pose a significant long-term or acute risk. Long-term risk was defined as greater than 5% probability of a child having a long-term blood lead concentration that exceeded 10 microg/dl, which is the CDC and the EPA blood lead concern level. Short-term

or acute risk was defined as greater than 5% probability of a child having a blood lead concentration on any given day that exceeded 20 microg/dl, which is the CDC trigger level for medical evaluation (this is not intended to imply that 20 microg/dl is a threshold for health effects in children exposed acutely to airborne lead). The estimated potential long-term and short-term exposures at the downwind West Dallas community did not result in more than 5% of children exceeding the target blood lead levels. The models were also used to estimate air lead levels for short-term and long-term exposures that would not exceed specified levels of risk (risk-based concentrations, RBCs). RBCs were derived for various daily exposure durations (3 or 8 h/day) and frequencies (1-7 days/week). RBCs based on the ICRP model ranged from 0.3 (7 days/week, 8 h/day) to 4.4 microg/m(3) (1 day/week, 3 h/day) for long-term exposures and were lower than those based on the IEUBK model. For short-term exposures, the RBCs ranged from 3.5 to 29.0 microg/m(3). Recontamination of remediated residential yards from deposition of air lead emitted during remedial activities at the RSR Superfund site was also examined. The predicted increase in soil concentration due to lead deposition at the monitoring station, which represented the community at large, was 3.0 mg/kg. This potential increase in soil lead concentration was insignificant, less than 1% increase, when compared to the clean-up level of 500 mg/kg developed for residential yards at the site
SN - 1053-4245
AD - U.S. EPA, Region 6 Superfund Branch, Mail Code (6SF-LT), 1445 Ross Avenue, Dallas, Texas 75202, USA. khoury.ghassan@epamail.epa.gov
UR - 12595884
ER -

TY - JOUR
ID - 1550
T1 - Assessing remedial effectiveness through the blood lead:soil/dust lead relationship at the Bunker Hill Superfund Site in the Silver Valley of Idaho
A1 - von,Lindern,I
A1 - Spalinger,S.
A1 - Petroysan,V.
A1 - von,Braun M.
Y1 - 2003/02/15/
N1 - von Lindern, Ian. Spalinger, Susan. Petroysan, Varduhi. von Braun, Margrit
The Science of the total environment
uj0, 0330500
IM
Journal Article. Research Support, Non-U.S. Gov't. Research Support, U.S. Gov't, Non-P.H.S.. Research Support, U.S. Gov't, P.H.S.
English
KW - MEDLINE
KW - Absorption
KW - Biological Availability
KW - Child
KW - Preschool
KW - Dust
KW - Environmental Exposure
KW - Environmental Pollution/pc [Prevention & Control]
KW - Female
KW - Hazardous Waste
KW - Humans
KW - Idaho
KW - Infant
KW - Lead/an [Analysis]
KW - Lead/bl [Blood]
KW - Lead Poisoning/pc [Prevention & Control]
KW - Male
KW - Mass Screening
KW - Program Evaluation
KW - Reference Values

KW - Soil Pollutants/an [Analysis]
RP - NOT IN FILE
SP - 139
EP - 170
JF - Science of the Total Environment
JA - Sci Total Environ
VL - 303
IS - 1-2
CY - Netherlands
N2 - The 21 square mile Bunker Hill Superfund Site in northern Idaho includes several thousand acres of contaminated hillsides and floodplain, a 365-acre abandoned lead/zinc smelter and is home to more than 7000 people in 5 residential communities. Childhood lead poisoning was epidemic in the 1970s with >75% of children exceeding 40 microg/dl blood lead. Health response activities have been ongoing for three decades. In 1991, a blood lead goal of 95% of children with levels less than 10 microg/dl was adopted. The cleanup strategy, based on biokinetic pathways models, was to reduce house dust lead exposure through elimination of soil-borne sources. An interim health intervention program, that included monitoring blood lead and exposures levels, was instituted to reduce exposures through parental education during the cleanup. In 1989 and 2001, 56% and 3% of children, respectively, exceeded the blood lead criteria. More than 4000 paired blood lead/environmental exposure observations were collected during this period. Several analyses of these data were accomplished. Slope factors derived for the relationship between blood lead, soil and dust concentrations are age-dependent and similar to literature reported values. Repeat measures analysis assessing year to year changes found that the remediation effort (without intervention) had approximately a 7.5 microg/dl effect in reducing a 2-year-old child's mean blood lead level over the course of the last ten years. Those receiving intervention had an additional 2-15 microg/dl decrease. Structural equations models indicate that from 40 to 50% of the blood lead absorbed from soils and dusts is through house dust with approximately 30% directly from community-wide soils and 30% from the home yard and immediate neighborhood. Both mean blood lead levels and percent of children to exceed 10 microg/dl have paralleled soil/dust lead intake rates estimated from the pathways model. Application of the IEUBK model for lead indicates that recommended USEPA default parameters overestimate mean blood lead levels, although the magnitude of over-prediction is diminished in recent years. Application of the site-specific model, using the soil and dust partitions suggested in the pathways model and an effective bioavailability of 18%, accurately predicts mean blood lead levels and percent of children to exceed 10 microg/dl throughout the 11-year cleanup period. This reduced response rate application of the IEUBK is consistent with the analysis used to originally develop the cleanup criteria and indicates the blood lead goal will be achieved
SN - 0048-9697
AD - TerraGraphics Environmental Engineering, Inc., 121 S. Jackson, Moscow, ID 83843, USA. vonlindi@tgenviro.com
UR - 12568769
ER -

TY - JOUR
ID - 1551
T1 - Effect of smelter emission reductions on children's blood lead levels
A1 - Hilts,S.R.
Y1 - 2003/02/15/
N1 - Hilts, Steven R
The Science of the total environment
uj0, 0330500
IM
Journal Article. Research Support, Non-U.S. Gov't
English
KW - MEDLINE
KW - Air Pollutants/ae [Adverse Effects]
KW - Air Pollutants/pk [Pharmacokinetics]
KW - Air Pollution/pc [Prevention & Control]
KW - Biological Availability
KW - Child

KW - Child Welfare
KW - Preschool
KW - Environmental Exposure
KW - Female
KW - Humans
KW - Infant
KW - Lead/bl [Blood]
KW - Lead/pk [Pharmacokinetics]
KW - Lead Poisoning/pc [Prevention & Control]
KW - Male
KW - Metallurgy
KW - Seasons
RP - NOT IN FILE
SP - 51
EP - 58
JF - Science of the Total Environment
JA - Sci Total Environ
VL - 303
IS - 1-2
CY - Netherlands
N2 - Trail, British Columbia has been the site of an active lead-zinc smelter for approximately 95 years. Since 1989, the community has been monitoring blood lead levels in children, studying exposure pathways and conducting comprehensive education and case management programs. From 1989 through 1996, mean blood lead levels of pre-school children declined at an average rate of 0.6 microg/dl per year. From 1996 to 1999, mean blood lead levels fell at an average rate of 1.8 microg/dl per year, from 11.5 in 1996 to 5.9 in 1999. The recent rapid decline appears to be mainly attributable to the start-up of a new lead smelter using modern flash-smelting technology in May of 1997. In 1998, the annual arithmetic mean air lead level in Trail was 0.28 microg/m(3), compared with 1.1 microg/m(3) in 1996. Reductions of approximately 50% were observed in lead loadings and concentrations in outdoor dustfall, street dust and indoor dustfall after smelter emissions were reduced. Slight reductions (statistically insignificant) have been observed in carpet dust and soil lead concentrations. During the summer of 2001, the smelting and refining operations at Trail were shut down completely for 3 months. During this period, average air lead levels in Trail dropped to 0.03 microg/m(3). The average blood lead level in Trail pre-school children at the end of the shutdown was 4.7 microg/dl. These results challenge prevailing theories about the relative importance of various environmental lead sources. For example, the US EPA Integrated Biokinetic Uptake Model for Lead (IEUBK), with its emphasis on soil concentrations, would not have predicted the dramatic decline in children's blood lead levels seen in Trail following the reductions in air lead levels. The Trail experience suggests that increased attention should be paid to the importance of active sources of highly bioavailable and mobile lead bearing dusts
SN - 0048-9697
AD - Teck Cominco Metals Ltd., P.O. Box 1000, Trail, BC, Canada V1R 4L8. steven.hilts@teckcominco.com
UR - 12568764
ER -

TY - JOUR
ID - 1552
T1 - Utility of antiepileptic drug monitoring in the pediatric emergency department
A1 - Kozer,E.
A1 - Scolnik,D.
A1 - Agamata,W.M.
A1 - Weiss,S.K.
A1 - Verjee,Z.H.
A1 - Koren,G.
Y1 - 2003/02//
N1 - Kozer, Eran. Scolnik, Dennis. Agamata, Whitty M. Weiss, Shelly K. Verjee, Zulfikaral H. Koren, Gideon
Therapeutic drug monitoring
v8p, 7909660
IM

Journal Article. Research Support, Non-U.S. Gov't
English
KW - MEDLINE
KW - Adolescent
KW - Anticonvulsants/bl [Blood]
KW - Chi-Square Distribution
KW - Child
KW - Preschool
KW - Drug Monitoring/mt [Methods]
KW - Drug Monitoring/sn [Statistics & Numerical Data]
KW - Emergency Service
KW - Hospital/sn [Statistics & Numerical Data]
KW - Hospitals
KW - Pediatric/sn [Statistics & Numerical Data]
KW - Humans
KW - Infant
KW - Retrospective Studies
KW - Statistics
KW - Nonparametric
RP - NOT IN FILE
SP - 17
EP - 21
JF - Therapeutic Drug Monitoring
JA - Ther Drug Monit
VL - 25
IS - 1
CY - United States
N2 - Convulsive disorders are common in the pediatric age group, and measurement of serum concentration of an antiepileptic drug (AED) is frequently ordered for epileptic patients in the emergency department (ED). The objective of this study was to develop a better understanding of the indications for, and consequences of, monitoring AED serum concentrations in the pediatric ED. Charts of 116 patients who visited the ED and were tested for blood levels of AED were retrospectively reviewed. Main outcome measures were number and percentage of levels outside the therapeutic range, discontinuation of an AED or introduction of a new one, dosage modifications, and admission to hospital. Two pediatricians and a pediatric neurologist aware only of patients' age, weight, diagnosis, history, clinical presentation, and drug details reviewed each case and on the basis of predetermined criteria decided whether measurement of AED was indicated. Mean age (+/- SD) of the study population was 7 +/- 5 years (range, 2 months-17 years). Forty-two patients (36%) were on monotherapy, and 74 (64%) were on polytherapy. Sixty-eight patients (59%) presented with increased seizure frequency, 7 (6%) with status epilepticus, and 13 (11%) with suspected AED toxicity. The remainder of the children presented with problems unrelated to epilepsy. No significant difference was found between patients with AED levels within the therapeutic range and those with levels outside it in the proportion of children needing dosage change, change in medication, or hospital admission (P = 0.5, 0.8, and 0.8, respectively). None of the patients presenting with status epilepticus and only 15% of those with increased seizure activity had subtherapeutic levels. Review of the cases suggested that measuring serum AED level was not indicated in 57 (49.1%) patients. In the pediatric ED, abnormal AED levels do not correlate with clinical management. Before ordering tests, physicians should consider whether their results would alter patient treatment
SN - 0163-4356
AD - Division of Emergency Services, The Hospital for Sick Children, Toronto, Ontario, Canada
UR - 12548139
ER -

TY - JOUR
ID - 1553
T1 - Moderate sun exposure and nevus counts in parents are associated with development of melanocytic nevi in childhood: a risk factor study in 1,812 kindergarten children
A1 - Wiecker,T.S.
A1 - Luther,H.

A1 - Buettner,P.
A1 - Bauer,J.
A1 - Garbe,C.
Y1 - 2003/02/01/
N1 - Wiecker, Tine Sander. Luther, Heike. Buettner, Petra. Bauer, Jurgen. Garbe, Claus
Cancer
0374236, clz
AIM, IM
Journal Article. Research Support, Non-U.S. Gov't
English
KW - MEDLINE
KW - Adult
KW - Child
KW - Preschool
KW - Cross-Sectional Studies
KW - Humans
KW - Nevus/ep [Epidemiology]
KW - Nevus/ge [Genetics]
KW - Nevus
KW - Pigmented/ep [Epidemiology]
KW - Pigmented/ge [Genetics]
KW - Regression Analysis
KW - Risk Factors
KW - Skin Neoplasms/ep [Epidemiology]
KW - Skin Neoplasms/ge [Genetics]
KW - Sunlight
RP - NOT IN FILE
SP - 628
EP - 638
JF - Cancer
VL - 97
IS - 3
CY - United States
N2 - BACKGROUND: Melanocytic nevi have been identified as the most important risk factor for cutaneous melanoma. Sun exposure, sunburns, and light pigmentation have been found to be associated with their development in childhood. To the authors' knowledge, nevus proneness of parents and the exact type of ultraviolet (UV) exposure have not yet been investigated in this context. The authors' objective was to determine independent risk factors and their impact for nevus development in childhood. METHODS: The current study was conducted by two university departments of dermatology in 49 public nursery schools in Stuttgart, Germany and in 38 public nursery schools in Bochum, Germany. The cross-sectional study included 1,812 children aged 2-7 years and their parents. Total body nevus counts in children, assessment of pigmentary features, and nevus counts on the arms of parents were performed. Parents underwent a standardized interview concerning national origin and lifestyle features, as well as habits and magnitude of sun exposure of children. Analysis was performed by multivariate linear regression analysis and by multiple logistic regression analysis. RESULTS: The number of nevi was found to steadily increase with age from a median of 3 at age 2 years to 19 at age 7 years ($P < 0.0001$). High numbers of nevi in children were associated with the number of weeks on sunny holidays, outdoor activities at home, skin type, facial freckling, ethnicity of parents, and the number of nevi on the arms of parents. Previously experienced sunburns failed significance ($P = 0.0620$). CONCLUSIONS: The authors found a strong association between nevus development in children and the number of parental moles, which most likely points to an inherited factor. Moderate sun exposure such as outdoor activities during a German summer without sunburns seemed to be sufficient for induction of melanocytic nevi. The authors believe that these findings will have direct impact on concepts for preventive strategies. Copyright 2003 American Cancer Society.DOI 10.1002/cncr.11114
SN - 0008-543X
AD - Department of Dermatology, Eberhard-Karls-University, Tuebingen, Germany
UR - 12548604
ER -

TY - JOUR
ID - 1554
T1 - Death due to electrocution in childhood and early adolescence
A1 - Byard,R.W.
A1 - Hanson,K.A.
A1 - Gilbert,J.D.
A1 - James,R.A.
A1 - Nadeau,J.
A1 - Blackbourne,B.
A1 - Krous,H.
Y1 - 2003/01//
N1 - Byard, R W. Hanson, K A. Gilbert, J D. James, R A. Nadeau, J. Blackbourne, B. Krous, H F
Journal of paediatrics and child health
arp, 9005421
IM
Journal Article
English
KW - MEDLINE
KW - Accidents
KW - Home/sn [Statistics & Numerical Data]
KW - Adolescent
KW - Child
KW - Preschool
KW - Electric Injuries/mo [Mortality]
KW - Electric Injuries/pp [Physiopathology]
KW - Electric Wiring
KW - Female
KW - Humans
KW - Infant
KW - Male
KW - South Australia/ep [Epidemiology]
KW - Suicide
RP - NOT IN FILE
SP - 46
EP - 48
JF - Journal of Paediatrics & Child Health
JA - J Paediatr Child Health
VL - 39
IS - 1
CY - Australia
N2 - OBJECTIVES: To delineate the clinicopathological features of fatal childhood electrocutions and to identify specific risk factors. METHODS: Coronial files in Adelaide (Australia) were searched from 1967 to 2001 and Medical Examiners' files in San Diego (USA) were searched from 1988 to 2001, for cases of deaths of children and adolescents younger than 16 years attributed to electrocution. RESULTS: Sixteen cases were identified aged between 10 months and 15 years (mean 8.0 years) with a male : female ratio of 5 : 3. Deaths were due to accidents occurring while playing with or near faulty electrical equipment at home or at school (n = 8), electrical equipment while in the bath (n = 2), damaged outdoor electrical equipment (n = 1), overhead wires (n = 1), and a high voltage electricity substation (n = 1). In addition, one death was due to suicide involving an electrical appliance placed in a bath, and two other deaths occurred in older children who were moving equipment under overhead wires. No homicides were identified. CONCLUSIONS: Childhood deaths due to electrocution are rare and are more likely to occur when children are playing around electrical wires or equipment, and often result from either faulty apparatus, or a lack of understanding of the potential dangers involved. The majority of deaths (11/16; 69%) occur in the home environment. In contrast to adult electrical deaths, high-voltage electrocutions, suicides and workplace deaths are uncommon. Strategies for eliminating childhood electrocution should concentrate on ensuring safe domestic environments with properly maintained electrical devices

SN - 1034-4810
AD - Forensic Science Centre, Adelaide, South Australia, Australia. byard.roger@saugov.sa.gov.au
UR - 12542812
ER -

TY - JOUR
ID - 1555
T1 - Time-location analysis for exposure assessment studies of children using a novel global positioning system instrument
A1 - Elgethun,K.
A1 - Fenske,R.A.
A1 - Yost,M.G.
A1 - Palcisko,G.J.
Y1 - 2003/01//
N1 - Elgethun, Kai. Fenske, Richard A. Yost, Michael G. Palcisko, Gary J
Environmental health perspectives
ei0, 0330411
IM
Journal Article. Research Support, U.S. Gov't, Non-P.H.S.. Research Support, U.S. Gov't, P.H.S.
English
KW - MEDLINE
KW - Child
KW - Child Welfare
KW - Preschool
KW - Environmental Exposure/an [Analysis]
KW - Environmental Monitoring/is [Instrumentation]
KW - Environmental Monitoring/mt [Methods]
KW - Epidemiological Monitoring
KW - Female
KW - Geographic Information Systems
KW - Humans
KW - Male
KW - Maps as Topic
KW - Pilot Projects
KW - Population Surveillance/mt [Methods]
KW - Risk Assessment
KW - Satellite Communications/is [Instrumentation]
KW - Time
KW - Washington/ep [Epidemiology]
RP - NOT IN FILE
SP - 115
EP - 122
JF - Environmental Health Perspectives
JA - Environ Health Perspect
VL - 111
IS - 1
CY - United States
N2 - Global positioning system (GPS) technology is used widely for business and leisure activities and offers promise for human time-location studies to evaluate potential exposure to environmental contaminants. In this article we describe the development of a novel GPS instrument suitable for tracking the movements of young children. Eleven children in the Seattle area (2-8 years old) wore custom-designed data-logging GPS units integrated into clothing. Location data were transferred into geographic information systems software for map overlay, visualization, and tabular analysis. Data were grouped into five location categories (in vehicle, inside house, inside school, inside business, and outside) to determine time spent and percentage reception in each location. Additional experiments focused on spatial resolution, reception efficiency in typical environments, and sources of signal interference. Significant signal interference occurred only inside concrete/steel-frame buildings and inside a power substation. The GPS instruments provided adequate spatial resolution (typically about 2-3 m

outdoors and 4-5 m indoors) to locate subjects within distinct microenvironments and distinguish a variety of human activities. Reception experiments showed that location could be tracked outside, proximal to buildings, and inside some buildings. Specific location information could identify movement in a single room inside a home, on a playground, or along a fence line. The instrument, worn in a vest or in bib overalls, was accepted by children and parents. Durability of the wiring was improved early in the study to correct breakage problems. The use of GPS technology offers a new level of accuracy for direct quantification of time-location activity patterns in exposure assessment studies
SN - 0091-6765
AD - Department of Environmental Health, School of Public Health and Community Medicine, University of Washington, Seattle, Washington, USA. elgethun@u.washington.edu
UR - 12515689
ER -

TY - JOUR
ID - 1556
T1 - Population-based surveillance and a case-control study of risk factors for endemic lymphocutaneous sporotrichosis in Peru
A1 - Lyon,G.M.
A1 - Zurita,S.
A1 - Casquero,J.
A1 - Holgado,W.
A1 - Guevara,J.
A1 - Brandt,M.E.
A1 - Douglas,S.
A1 - Shutt,K.
A1 - Warnock,D.W.
A1 - Hajjeh,R.A.
A1 - Sporotrichosis in Peru Investigation Team.
Y1 - 2003/01/01/
N1 - Lyon, G M. Zurita, S. Casquero, J. Holgado, W. Guevara, J. Brandt, M E. Douglas, S. Shutt, K. Warnock, D W. Hajjeh, R A. Sporotrichosis in Peru Investigation Team
Clinical infectious diseases : an official publication of the Infectious Diseases Society of America
a4j, 9203213
IM
Journal Article
English
KW - MEDLINE
KW - Adolescent
KW - Case-Control Studies
KW - Child
KW - Endemic Diseases
KW - Female
KW - Humans
KW - Male
KW - Multivariate Analysis
KW - Peru/ep [Epidemiology]
KW - Population Surveillance
KW - Risk Factors
KW - Sporotrichosis/ep [Epidemiology]
RP - NOT IN FILE
SP - 34
EP - 39
JF - Clinical Infectious Diseases
JA - Clin Infect Dis
VL - 36
IS - 1
CY - United States

N2 - Population-based surveillance and a case-control study were conducted in Abancay, Peru, to estimate the burden of disease and to determine risk factors for sporadic lymphocutaneous sporotrichosis (LS). Laboratory records from local hospitals were reviewed for the years of 1997 and 1998, and prospective surveillance was conducted for the period of September 1998 through September 1999. A case-control study was conducted with 2 matched control subjects per case patient. The mean annual incidence was 98 cases per 100,000 persons. Children had an incidence 3 times higher than that for adults and were more likely to have LS lesions on the face and neck. Identified risk factors included owning a cat, playing in crop fields, having a dirt floor in the house, working mainly outdoors, and having a ceiling made of raw wood or conditions associated with a lower socioeconomic status. Decreased environmental exposure, such wearing protective clothing during construction activities for adults or limiting contact with cats and soil for children, and improvements in living spaces may decrease the incidence of LS
SN - 1537-6591
AD - Epidemic Intelligence Service, Epidemiology Program Office, Division of Bacterial and Mycotic Diseases, National Center for Infectious Diseases, Centers for Disease Control and Prevention, Atlanta, Georgia, USA. mlyon@partners.org
UR - 12491199
ER -

TY - JOUR
ID - 1557
T1 - Parental fears of Pseudomonas infection and measures to prevent its acquisition
A1 - Ullrich,G.
A1 - Wiedau-Gors,S.
A1 - Steinkamp,G.
A1 - Bartig,H.J.
A1 - Schulz,W.
A1 - Freihorst,J.
Y1 - 2002/09//
N1 - Ullrich, Gerald. Wiedau-Gors, Susanne. Steinkamp, Gratiana. Bartig, Hans-Jurgen. Schulz, Wolfgang. Freihorst, Joachim
Journal of cystic fibrosis : official journal of the European Cystic Fibrosis Society
101128966
IM
Journal Article. Research Support, Non-U.S. Gov't
English
KW - MEDLINE
KW - Activities of Daily Living/px [Psychology]
KW - Adaptation
KW - Psychological
KW - Child
KW - Preschool
KW - Cross-Sectional Studies
KW - Cystic Fibrosis/co [Complications]
KW - Cystic Fibrosis/px [Psychology]
KW - Cystic Fibrosis/th [Therapy]
KW - Health Knowledge
KW - Attitudes
KW - Practice
KW - Humans
KW - Interviews as Topic
KW - Lung Diseases/mi [Microbiology]
KW - Lung Diseases/pc [Prevention & Control]
KW - Lung Diseases/px [Psychology]
KW - Parents/px [Psychology]
KW - Patient Care/mt [Methods]
KW - Pilot Projects
KW - Pseudomonas Infections/co [Complications]

KW - Pseudomonas Infections/pc [Prevention & Control]
KW - Pseudomonas Infections/px [Psychology]
KW - Quality of Life
KW - Self Care/mt [Methods]
RP - NOT IN FILE
SP - 122
EP - 130
JF - Journal of Cystic Fibrosis
JA - J Cyst.Fibros.
VL - 1
IS - 3
CY - Netherlands
N2 - BACKGROUND AND AIM OF THE STUDY: Chronic infection with Pseudomonas aeruginosa (PA) is associated with accelerated worsening of lung disease in patients with cystic fibrosis (CF). Fears of PA are widespread among parents of CF children, and many parents take precautions at home to prevent acquisition of the bacterium from the environment. The present study was undertaken to describe the type and intensity of these activities. METHODS: Parents of 21 CF children (7 without prior PA infection, 10 with intermittent and 4 with chronic PA infection) were investigated using semistructured interviews. These were analyzed descriptively and with respect to predominant themes. Additionally, a German personality test was used to evaluate the influence of psychological factors. RESULTS: The clinical impression of widespread parental anxieties of PA infection was confirmed. Misunderstandings concerning PA infections were related to a simplistic concept of the underlying biological mechanisms. Some parents which we classified as 'bacterium-focussed' thought that each contact with PA would lead to bacterial infection. These parents used a large variety of measures, which concerned both domiciliary and outdoor surroundings and activities. At the other end of the spectrum were parents which we classified as 'child-focussed' who mostly supported (and relied on) the child's defense mechanism instead of hygienic measures. CONCLUSIONS: Recommendations by physicians on how to prevent PA acquisition from the environment should take into account possible non-intended side effects, since some parents will exaggerate daily precautions to the detriment of the child's (and the parent's) quality of life
SN - 1569-1993
AD - Hannover Medical School, Department of Paediatrics, Division of Pediatric Pulmonology and Neonatology, Hannover, Germany. ullrich.Gerald@mh-hannover.de
UR - 15463818
ER -

TY - JOUR
ID - 1558
T1 - Leisure time activities in teenagers in urban and rural areas
A1 - Borzecki,A.
A1 - Nieradko,B.
A1 - Gnass,B.
A1 - Sieklucka-Dziuba,M.
Y1 - 2002///
N1 - Borzecki, Andrzej. Nieradko, Barbara. Gnass, Bogumila. Sieklucka-Dziuba, Maria
Annales Universitatis Mariae Curie-Sklodowska. Sectio D: Medicina
69m, 0414101
IM
Comparative Study. Journal Article
English
KW - MEDLINE
KW - Adolescent
KW - Child
KW - Female
KW - Humans
KW - Leisure Activities
KW - Male
KW - Poland

KW - Rural Population/sn [Statistics & Numerical Data]
KW - Urban Population/sn [Statistics & Numerical Data]
RP - NOT IN FILE
SP - 375
EP - 380
JF - Annales Universitatis Mariae Curie-Sklodowska - Sectio d - Medicina
JA - Ann Univ Mariae Curie Sklodowska [Med]
VL - 57
IS - 1
CY - Poland
N2 - The work aimed to determine the leisure time activities in teenagers on weekdays, weekends, during winter and summer holidays. Vast majority of teenagers spend their leisure time resting in a passive way, i.e. watching TV or playing computer games irrespectively of the season. As a result of this, the number of kids with posture defects increase. On weekdays the country teenagers spend much more time doing outdoor sports and games than town children. They also more often help their parents and less frequently travel away from their homes than children living in towns
SN - 0066-2240
AD - Department of Hygiene, Medical University of Lublin
UR - 12898947
ER -

TY - JOUR
ID - 1559
T1 - Students consuming sack lunches devote more time to eating than those consuming school lunches
A1 - Buergel,N.S.
A1 - Bergman,E.A.
Y1 - 2002/09//
N1 - Buergel, Nancy S. Bergman, Ethan A
Journal of the American Dietetic Association
h6f, 7503061
AIM, IM
Comparative Study. Journal Article
English
KW - MEDLINE
KW - Child
KW - Child Nutritional Physiological Phenomena
KW - Eating/ph [Physiology]
KW - Eating/px [Psychology]
KW - Eating
KW - Female
KW - Food Habits
KW - Food Services
KW - Humans
KW - Male
KW - Play and Playthings
KW - Schools
KW - Sex Factors
KW - Time Factors
KW - Washington
RP - NOT IN FILE
SP - 1283
EP - 1286
JF - Journal of the American Dietetic Association
JA - J Am Diet Assoc
VL - 102
IS - 9
CY - United States

N2 - Concern has been raised about the amount of time provided for students to consume school lunch. This study analyzed the amount of time used by elementary students to consume school lunch vs sack lunch. Data collectors used stop watches to collect wait time, opportunity time to eat and consumption time for elementary students consuming school lunch, sack lunch, and sack lunch with a separate milk purchase. Students consuming school lunch had a significantly longer wait time than those consuming sack lunch or sack lunch with milk (P<.0001). In contrast, students consuming sack lunch and sack lunch with milk had significantly longer opportunity times to eat (P<.0001) and consumption times (P<.001). There was also a significant decrease in opportunity time to eat and consumption time as grade level increased, particularly in boys (P<.05). Older boys appeared more anxious to finish eating so they could go out to recess
SN - 0002-8223
AD - Department of Family and Consumer Sciences, Central Washington University, 400 E 8th Ave, Ellensburg, WA 98926-7565, USA. buergeln@cwu.edu
UR - 12792627
ER -

TY - JOUR
ID - 1560
T1 - Nasopharyngeal carriage and susceptibility patterns of Streptococcus pneumoniae in Kumasi, Ghana
A1 - Denno,D.M.
A1 - Frimpong,E.
A1 - Gregory,M.
A1 - Steele,R.W.
Y1 - 2002/07//
N1 - Denno, Donna M. Frimpong, Enoch. Gregory, Michael. Steele, Russell W
West African journal of medicine
ayq, 8301891
IM
Journal Article
English
KW - MEDLINE
KW - Anti-Bacterial Agents/ae [Adverse Effects]
KW - Anti-Bacterial Agents/tu [Therapeutic Use]
KW - Carrier State/ep [Epidemiology]
KW - Carrier State/mi [Microbiology]
KW - Carrier State/pc [Prevention & Control]
KW - Child
KW - Child Welfare/sn [Statistics & Numerical Data]
KW - Developing Countries
KW - Drug Prescriptions
KW - Drug Resistance
KW - Multiple
KW - Bacterial
KW - Drug Utilization
KW - Female
KW - Ghana/ep [Epidemiology]
KW - Humans
KW - Male
KW - Nasopharyngitis/ep [Epidemiology]
KW - Nasopharyngitis/mi [Microbiology]
KW - Nasopharyngitis/pc [Prevention & Control]
KW - Penicillin Resistance
KW - Population Surveillance
KW - Prevalence
KW - Streptococcal Infections/ep [Epidemiology]
KW - Streptococcal Infections/mi [Microbiology]
KW - Streptococcal Infections/pc [Prevention & Control]
KW - Streptococcus pneumoniae

KW - Urban Health/sn [Statistics & Numerical Data]
RP - NOT IN FILE
SP - 233
EP - 236
JF - West African Journal of Medicine
JA - West Afr J Med
VL - 21
IS - 3
CY - Nigeria
N2 - UNLABELLED: Penicillin resistant Streptococcus pneunmoniae poses an increasing problem in paediatrics, particularly in less developed countries. Outside of South Africa, little is known about S. pneumoniae susceptibilities in Sub-Saharan Africa. The objective of this study was to determine the prevalence of pneumococcal colonization and antimicrobial susceptibility among children in urban Ghana. METHODS: Nasopharyngeal pneumococcal colonization was examined in 311 children attending a polyclinic for sick children and an immunization clinic in Kumasi, Ghana. Isolates were tested for antibiotic susceptibility to penicillin, tetracycline, erythromycin, chloramphenicol, cefuroxime, cefotaxime, ceftriaxone, and trimethoprim-sulfamethoxazole. RESULTS: Over half (51.4%) of subjects were colonized with S. pneumoniae and 17% of isolates were resistant to penicillin, all demonstrating intermediate resistance. S. pneumoniae strains were also frequently resistant to trimethoprim-sulfamethoxazole and tetracycline, less so to chloramphenicol and cefuroxime and were almost uniformly sensitive to cefotaxime, cefriaxone and erythromycin. CONCLUSIONS: Our study shows a high rate of pneumococcal nasopharyngeal colonization and a concerning level of penicillin resistance although at a less alarming rate than seen in some other countries. Multiple antimicrobial resistance was also noted especially among drugs readily available and commonly used. These data impact treatment choices in pneumococcal disease. Vaccine may play an important role in disease limitation. An effort to curtail the misuse of antibiotics, by prescription and otherwise, may prevent further increases in resistance rates
SN - 0189-160X
AD - Harborview Medical Center, MS 359774 325 Ninth Ave., Seattle, WA 98104-2499, USA
UR - 12744576
ER -

TY - JOUR
ID - 1561
T1 - Unintentional injuries among children aged 1-4 years at home
A1 - Chaveepojnkamjorn,W.
A1 - Pichainarong,N.
A1 - Pooltawee,S.
Y1 - 2002/09//
N1 - Chaveepojnkamjorn, Wisit. Pichainarong, Natchaporn. Pooltawee, Siriwan
The Southeast Asian journal of tropical medicine and public health
uvn, 0266303
IM
Journal Article
English
KW - MEDLINE
KW - Accidental Falls/sn [Statistics & Numerical Data]
KW - Accidents
KW - Home/sn [Statistics & Numerical Data]
KW - Adolescent
KW - Case-Control Studies
KW - Child
KW - Preschool
KW - Female
KW - Humans
KW - Infant
KW - Logistic Models
KW - Male
KW - Multivariate Analysis

KW - Risk Factors
KW - Thailand/ep [Epidemiology]
KW - Wounds and Injuries/ep [Epidemiology]
KW - Wounds and Injuries/et [Etiology]
RP - NOT IN FILE
SP - 642
EP - 646
JF - Southeast Asian Journal of Tropical Medicine & Public Health
JA - Southeast Asian J Trop Med Public Health
VL - 33
IS - 3
CY - Thailand
N2 - A hospital based case-control study was performed to determine causes of unintentional injuries among children aged 1-4 years at home. Data were obtained by interviewing parents and guardians of 242 children at Lerdsin Hospital in Bangkok, from August to October 1999. The results showed that falls were the most common type of injuries (54.6%). The age of 2 years comprised the major group (28.9%). The study subjects were injured within the home (60.7%). Sunday and Saturday were the most frequent days of injuries (18.2% and 16.5% respectively). The proportion of cases involving playing with others was 71.1%. Most of the injuries occurred in the period 16.00-19.00 hours (32.2%). Head and neck were the main injured area (45.5%). The multivariate model showed that children who had previous injuries were 19.22 times more likely to be injured again than those who had not previously been injured. Children with vigorous physical activities also had a higher risk (OR = 19.73, 95% CI 6.11-63.74). Outdoor working mothers were at higher risk than houseworking mothers (OR = 4.14, 95% CI 1.57-10.93) of having children affects. Children who spent most of the daytime at their relatives' houses had a higher risk than those who stayed at their home (OR = 3.48, 95% CI 1.02-11.81), while, 37.9% of children injured at their relatives' houses and 50.8% of all injured children were without supervision
SN - 0125-1562
AD - Department of Epidemiology, Faculty of Public Health, Mahidol University, Bangkok, Thailand
UR - 12693604
ER -

TY - JOUR
ID - 1562
T1 - The use of a laboratory school protocol to evaluate concepts about efficacy and side effects of new formulations of stimulant medications
A1 - Swanson,J.M.
A1 - Lerner,M.
A1 - Wigal,T.
A1 - Steinhoff,K.
A1 - Greenhill,L.
A1 - Posner,K.
A1 - Freid,J.
A1 - Wigal,S.
Y1 - 2002///
N1 - Swanson, J M. Lerner, M. Wigal, T. Steinhoff, K. Greenhill, L. Posner, K. Freid, J. Wigal, S
Journal of attention disorders
9615686
IM
Comparative Study. Evaluation Studies. Journal Article
English
KW - MEDLINE
KW - Amphetamines/ad [Administration & Dosage]
KW - Amphetamines/ae [Adverse Effects]
KW - Attention Deficit Disorder with Hyperactivity/di [Diagnosis]
KW - Attention Deficit Disorder with Hyperactivity/dt [Drug Therapy]
KW - Attention Deficit Disorder with Hyperactivity/px [Psychology]
KW - Central Nervous System Stimulants/ad [Administration & Dosage]

KW - Central Nervous System Stimulants/ae [Adverse Effects]
KW - Child
KW - Delayed-Action Preparations
KW - Dexmethylphenidate
KW - Dose-Response Relationship
KW - Drug
KW - Double-Blind Method
KW - Drug Administration Schedule
KW - Humans
KW - Methylphenidate/ad [Administration & Dosage]
KW - Methylphenidate/ae [Adverse Effects]
KW - Methylphenidate/aa [Analogs & Derivatives]
KW - Randomized Controlled Trials as Topic
KW - Structure-Activity Relationship
KW - Treatment Outcome
RP - NOT IN FILE
SP - S73
EP - S88
JF - Journal of Attention Disorders
JA - J Atten Disord
VL - 6 Suppl 1
CY - Canada
N2 - Recently, new long-acting formulations of racemic methylphenidate (MPH: Ritalin LA, Metadate CD and Concerta) and amphetamine (AMP: Adderall XR) were developed and are now approved by the Food and Drug Administration (FDA). In addition, dexmethylphenidate (Focalin), the pharmacologically active d-threo enantiomer of MPH, also was approved by the FDA. In the initial phases of development, prototypes of these five new formulations were evaluated using the University of California, Irvine (UCI) Laboratory School Protocol (LSP), in which surrogate measures of efficacy are collected in highly controlled settings rather than clinical measures of effectiveness in the less-controlled, natural environments of home or school. The LSP studies were followed by large effectiveness and safety studies required for gaining FDA approval. These initial efficacy and side effect studies in the LSP provided missing information about the basic pharmacokinetic (PK) and pharmacodynamic (PD) properties of MPH and AMP and produced some new discoveries (i.e., acute tolerance) that were used to help design the final products. The final once-a-day formulations used different drug delivery systems to achieve long-acting efficacy (Ritalin LA, Metadate CD, Concerta, Adderall XR). All four drug delivery systems were based on two processes: first, a bolus delivery (BD) process to achieve rapid onset of efficacy (mg), and second, a controlled delivery (CD) process to achieve rates of delivery (mg/hr) or a delayed bolus (mg) to maintain efficacy. A theoretical approach was used to compare and contrast the new once-a day formulations of MPH by selecting total daily doses (mg/d) that would equate drug delivery by the first process (mg of the initial bolus) and the second process (mg/hr over specified time period). In addition to efficacy, applications of the LSP to measure common side effects related to eating and sleeping were described and discussed
SN - 1087-0547
AD - Child Development Center, University of California, Department of Pediatrics, Irvine 92697-4480, USA. jmswanso@uci.edu
UR - 12685522
ER -

TY - JOUR
ID - 1563
T1 - Assessment of physical education time and after-school outdoor time in elementary and middle school students in south Mexico City: the dilemma between physical fitness and the adverse health effects of outdoor pollutant exposure
A1 - Villarreal-Calderon,A.
A1 - Acuna,H.
A1 - Villarreal-Calderon,J.
A1 - Garduno,M.
A1 - Henriquez-Roldan,C.F.

A1 - Calderon-Garciduenas,L.
A1 - Valencia-Salazar,G.
Y1 - 2002/09//
N1 - Villarreal-Calderon, Anna. Acuna, Hilda. Villarreal-Calderon, Jessica. Garduno, Monica. Henriquez-Roldan, Carlos F. Calderon-Garciduenas, Lilian. Valencia-Salazar, Gildardo
Archives of environmental health
6yo, 0212627
AIM, IM
Journal Article
English
KW - MEDLINE
KW - Adolescent
KW - Air Pollutants/ae [Adverse Effects]
KW - Air Pollutants/an [Analysis]
KW - Aldehydes/ae [Adverse Effects]
KW - Aldehydes/an [Analysis]
KW - Body Mass Index
KW - Child
KW - Child Nutrition Disorders/di [Diagnosis]
KW - Child Nutrition Disorders/ep [Epidemiology]
KW - Child Nutrition Disorders/et [Etiology]
KW - Child Nutrition Disorders/pc [Prevention & Control]
KW - Child Welfare/sn [Statistics & Numerical Data]
KW - Environmental Exposure/ae [Adverse Effects]
KW - Environmental Exposure/an [Analysis]
KW - Environmental Monitoring
KW - Epidemiological Monitoring
KW - Female
KW - Health Education
KW - Health Surveys
KW - Humans
KW - Leisure Activities
KW - Life Style
KW - Male
KW - Mexico/ep [Epidemiology]
KW - Obesity/di [Diagnosis]
KW - Obesity/ep [Epidemiology]
KW - Obesity/et [Etiology]
KW - Obesity/pc [Prevention & Control]
KW - Ozone/ae [Adverse Effects]
KW - Ozone/an [Analysis]
KW - Physical Education and Training/sn [Statistics & Numerical Data]
KW - Physical Fitness
KW - Protein-Energy Malnutrition/di [Diagnosis]
KW - Protein-Energy Malnutrition/ep [Epidemiology]
KW - Protein-Energy Malnutrition/et [Etiology]
KW - Protein-Energy Malnutrition/pc [Prevention & Control]
KW - Questionnaires
KW - Regression Analysis
KW - Students/sn [Statistics & Numerical Data]
KW - Time Factors
KW - Tobacco Smoke Pollution/ae [Adverse Effects]
KW - Tobacco Smoke Pollution/sn [Statistics & Numerical Data]
KW - Urban Health/sn [Statistics & Numerical Data]
RP - NOT IN FILE
SP - 450
EP - 460

JF - Archives of Environmental Health
JA - Arch Environ Health
VL - 57
IS - 5
CY - United States
N2 - Strategies to promote lifelong physical activity among children are needed to stem the adverse health consequences of inactivity. However, the health effects in growing children of long-term exposure to a polluted atmosphere are of deep concern. The atmosphere of south Mexico City (SMC) is characterized by a complex mixture of air pollutants, including ozone, particulate matter, and aldehydes. Radiological evidence suggests that small-airway disease could be present in clinically healthy, tobacco unexposed SMC children. The aim of this study was to assess, by means of a self-reported questionnaire, the physical education class times, daily outdoor after-school exposure time, and tobacco exposure in students attending public elementary and middle schools in SMC. Additionally, the time each student spent viewing television was assessed, and the authors measured each student's weight and height to determine body mass index (BMI, weight in kg divided by height in m2). The survey included 1,159 students in grades 7-9. The authors identified 2 critical periods of outdoor exposure in SMC children that coincided with significant concentrations of both ozone and particulate matter with diameters less than 10 micrometers (PM10): during school time after 11:00 A.M. and in the after-school outdoor activity period, usually extending from 1:00 P.M. to 6:00 P.M. Thirty-two percent of elementary and 61% of middle school students have physical education classes after 11:00 A.M. Students in SMC spend an average of 19.6 hr/wk outdoors in the after-school period, during which time they are engaged in light to moderate physical activities. Half of the students are exposed to tobacco smoke at home, and 7% of middle school students smoke. On the basis of BMI, 60% of students were classified as undernourished, overweight, or obese. No correlations were found between BMI and time spent viewing TV, time outdoors (on weekdays and weekends), or exposure to environmental tobacco smoke. Children and adolescents in SMC are participating in physical activities that enhance multiple components of health-related fitness. However, their activities occur outdoors, where they are exposed to high concentrations of air pollutants throughout the year. The authors believe that SMC children and adolescents must be educated, through both the school and health systems, regarding ways to obtain the necessary exercise while protecting themselves from the high concentrations of pollutants. Individuals should instruct and encourage young people to be involved in lifetime fitness activities and to eat balanced diets, if the goal is to control health-care costs, reduce disease incidence, and improve the overall quality of life of the Mexico City population
SN - 0003-9896
AD - Facultad de Medicina, Programa NUCE, Universidad Nacional Autonoma de Mexico, Mexico City, Mexico
UR - 12641189
ER -

TY - JOUR
ID - 1564
T1 - Impact of cattle keeping on human biting rate of anopheline mosquitoes and malaria transmission around Ziway, Ethiopia
A1 - Seyoum,A.
A1 - Balcha,F.
A1 - Balkew,M.
A1 - Ali,A.
A1 - Gebre-Michael,T.
Y1 - 2002/09//
N1 - Seyoum, A. Balcha, F. Balkew, M. Ali, A. Gebre-Michael, T
East African medical journal
edg, 0372766
IM
Journal Article. Research Support, Non-U.S. Gov't
English
KW - MEDLINE
KW - Adult
KW - Animal Husbandry/sn [Statistics & Numerical Data]
KW - Animals

KW - Domestic
KW - Anopheles/ps [Parasitology]
KW - Anopheles/ph [Physiology]
KW - Cattle
KW - Child
KW - Endemic Diseases/sn [Statistics & Numerical Data]
KW - Ethiopia/ep [Epidemiology]
KW - Feeding Behavior
KW - Goats
KW - Housing/sn [Statistics & Numerical Data]
KW - Housing
KW - Animal/sn [Statistics & Numerical Data]
KW - Humans
KW - Insect Bites and Stings/co [Complications]
KW - Insect Bites and Stings/ep [Epidemiology]
KW - Insect Vectors/ps [Parasitology]
KW - Insect Vectors/ph [Physiology]
KW - Malaria
KW - Falciparum/ep [Epidemiology]
KW - Falciparum/ps [Parasitology]
KW - Falciparum/tm [Transmission]
KW - Vivax/ep [Epidemiology]
KW - Vivax/ps [Parasitology]
KW - Vivax/tm [Transmission]
KW - Seasons
KW - Splenomegaly/ep [Epidemiology]
KW - Splenomegaly/ps [Parasitology]
KW - Suburban Health/sn [Statistics & Numerical Data]
RP - NOT IN FILE
SP - 485
EP - 490
JF - East African Medical Journal
JA - East Afr Med J
VL - 79
IS - 9
CY - Kenya
N2 - OBJECTIVE: To assess the impact of livestock keeping on the human biting rate (HBR) of anopheline mosquitoes and malaria transmission around Ziway in the middle course of the Ethiopian Rift Valley. DESIGN: As a passive experiment, man landing captures were done in homesteads with mixed dwelling, separate cattle shed and without livestock; and as an active experiment, captures were in experimental tukuls (huts) of cattle, goats, and without livestock. Parasite and spleen rates of children were compared among those residents under variable living conditions mentioned for passive experiment. SUBJECTS: For entomological study, human-baits were used for man-landing captures of mosquitoes. Study subjects for parasitological and clinical studies were children below 10 years old. MAIN OUTCOME MEASURES: Human-biting rate (HBR) of anopheline mosquitoes; and the parasite and spleen rates of the study subjects in different living conditions. RESULTS: In the passive experiment, the mean HBR of Anopheles arabiensis in mixed dwelling, separate cattle shed and without livestock was 8.45, 4.64 and 5.97, respectively. Similarly, the HBR of An. pharoensis was 2.88, 1.79 and 1.61, respectively. In the active experiment, the mean HBR of An. arabiensis in tukuls with cattle, goats, and without livestock was 3.50, 3.38 and 1.43 respectively; while that of An. pharoensis was 0.37, 0.70 and 0.55 respectively. Parasitologically, mean parasite rates of 26.67%, 15.05% and 23.85% were, respectively, recorded from children living under the above conditions stated for passive experiment. Similarly, the mean spleen rates of 50.0%, 26.9%, and 47.37% were recorded, respectively. CONCLUSION: These observations in the present study indicate that the presence of cattle in homesteads tends to increase the man biting rate of An. arabiensis, although differences in the mean HBR of vector mosquitoes were not statistically significant for all groups. In contrast, cattle keeping in separate cattle sheds outside of the human dwellings tends to reduce the man biting rate of An. arabiensis and malaria transmission in the study area
SN - 0012-835X

AD - Institute of Pathobiology, Faculty of Medicine, Addis Ababa University, P.O. Box 1176, Addis Ababa, Ethiopia
UR - 12625690
ER -

TY - JOUR
ID - 1565
T1 - Executive functions and the natural habitat behaviors of children with autism
A1 - Ruble,L.A.
A1 - Scott,M.M.
Y1 - 2002/12//
N1 - Ruble, Lisa A. Scott, M M
Autism : the international journal of research and practice
9713494
IM
Comparative Study. Journal Article. Research Support, U.S. Gov't, Non-P.H.S.
English
KW - MEDLINE
KW - Autistic Disorder/px [Psychology]
KW - Child
KW - Child Development
KW - Cognition
KW - Cognition Disorders/et [Etiology]
KW - Down Syndrome/px [Psychology]
KW - Female
KW - Goals
KW - Humans
KW - Male
KW - Models
KW - Psychological
RP - NOT IN FILE
SP - 365
EP - 381
JF - Autism
VL - 6
IS - 4
CY - England
N2 - Research suggests that impairments in executive functions play a role in the cognitive deficit in autism. Possible autism-specific impairments include an inability to engage in goal-directed behaviors and adjust behaviors given environmental demands. What has been described as executive functions is based largely on observations of performance in the laboratory rather than in natural settings. An ecological method first described by Barker and Wright and adapted by Scott was used to assess the patterns of goal-directed behaviors of eight children with autism and eight chronological and mental age comparable children with Down syndrome. Quantitative and qualitative features of naturalistic behaviors were collected, and coded using previously described categories of children's behavior. Results indicated that children with autism exhibited shorter and less overlapping goal-directed behaviors. These data suggest a cognitive difference rather than developmental delay, and lend support for impaired executive functions in autism. Practical implications for educators and caregivers are discussed
SN - 1362-3613
AD - Vanderbilt University, USA. larubl01@gwise.louisville.edu
UR - 12540128
ER -

TY - JOUR
ID - 1566
T1 - [Prevalence of exercise-induced asthma in school children]. [French]
A1 - Debrock,C.

A1 - Menetrey,C.
A1 - Bonavent,M.
A1 - Antonini,M.T.
A1 - Preux,P.M.
A1 - Bonnaud,F.
A1 - Vergnenegre,A.
Y1 - 2002/12//
N1 - Debrock, C. Menetrey, C. Bonavent, M. Antonini, M-T. Preux, P-M. Bonnaud, F. Vergnenegre, A
Revue d'epidemiologie et de sante publique
rst, 7608039
IM
Comparative Study. English Abstract. Journal Article. Research Support, Non-U.S. Gov't
French
KW - MEDLINE
KW - Adolescent
KW - Age Factors
KW - Asthma
KW - Exercise-Induced/di [Diagnosis]
KW - Exercise-Induced/ep [Epidemiology]
KW - Child
KW - Cluster Analysis
KW - Cross-Sectional Studies
KW - Data Interpretation
KW - Statistical
KW - Female
KW - France/ep [Epidemiology]
KW - Health Surveys
KW - Humans
KW - Male
KW - Questionnaires
KW - Respiratory Function Tests
KW - Sampling Studies
RP - NOT IN FILE
SP - 519
EP - 529
JF - Revue d Epidemiologie et de Sante Publique
JA - Rev Epidemiol Sante Publique
VL - 50
IS - 6
CY - France
N2 - BACKGROUND: Although asthma is the most common pulmonary condition in pediatrics, the incidence of exercise-induced asthma (EIA) in school children is not well documented and few studies have been devoted to the condition. There are considerable variations in morbidity and mortality between countries. This study was designed to evaluate the prevalence of EIA in children in the 6th grade (11-14 year-olds) in one French department (Haute-Vienne) and to identify undiagnosed cases. METHODS: The representative sample was obtained over a period of 28 days (February 2(nd) to April 10(th) 1998) by cluster sampling method, stratified by size of the schools. The number of subjects planned was 891. The selected children filled in a questionnaire on their asthma history and were subjected to an outdoor exercise test (6 minute run). Respiratory function was measured with a peak flow-meter. RESULTS: Seven hundred and eighty two school children were included in this survey. Participation rate was 87.8%. The prevalence of asthma from the questionnaire was 10.7% (CI 95%: 8.7 - 12.8). After exercise, 68 school children presented exercise-induced bronchospasm: the prevalence of the EIA was 8.7% (CI 95%: 6.9 - 10.5). Among these 68 school children, 27 were known asthmatics and 41 were not. With the 10 treated asthmatics, the overall prevalence of EIA was therefore estimated at 9.9% (CI 95%: 8.2 - 11.7). CONCLUSION: The prevalence of asthma and EIA was close to that found in other studies using a similar methodology. EIA remains under-diagnosed since 41 undeclared asthmatic school children were identified in our population. Nurses'information is necessary to initiate the use of systematically testing

respiratory function (with a peak flow meter) at least once a year. Efficient management of the asthmatic child requires cooperation between the various professionals dealing with school children
SN - 0398-7620
AD - Institut d'Epidemiologie Neurologique et de Neurologie Tropicale, Faculte de Medecine, Limoges
UR - 12515922
ER -

TY - JOUR
ID - 1567
T1 - Cumulative trauma disorder risk for children using computer products: results of a pilot investigation with a student convenience sample
A1 - Burke,A.
A1 - Peper,E.
Y1 - 2002/07//
N1 - Burke, Adam. Peper, Erik
Public health reports (Washington, D.C. : 1974)
9716844, qja
AIM, IM
Journal Article
English
KW - MEDLINE
KW - Adolescent
KW - Adolescent Behavior/px [Psychology]
KW - Aggression/px [Psychology]
KW - Child
KW - Child Behavior/px [Psychology]
KW - Consumer Product Safety
KW - Cumulative Trauma Disorders/ep [Epidemiology]
KW - Cumulative Trauma Disorders/et [Etiology]
KW - Depression/ep [Epidemiology]
KW - Depression/et [Etiology]
KW - Electronic Mail
KW - Female
KW - Human Engineering
KW - Humans
KW - Hyperkinesis/ep [Epidemiology]
KW - Hyperkinesis/et [Etiology]
KW - Logistic Models
KW - Male
KW - Microcomputers/ut [Utilization]
KW - Pilot Projects
KW - Questionnaires
KW - Risk Factors
KW - San Francisco/ep [Epidemiology]
KW - Social Isolation/px [Psychology]
KW - Students/px [Psychology]
KW - Students/sn [Statistics & Numerical Data]
KW - Time Factors
KW - Video Games
RP - NOT IN FILE
SP - 350
EP - 357
JF - Public Health Reports
JA - Public Health Rep
VL - 117
IS - 4
CY - United States

N2 - OBJECTIVES: Cumulative trauma disorder is a major health problem for adults. Despite a growing understanding of adult cumulative trauma disorder, however, little is known about the risks for younger populations. This investigation examined issues related to child/adolescent computer product use and upper body physical discomfort. METHODS: A convenience sample of 212 students, grades 1-12, was interviewed at their homes by a college-age sibling or relative. One of the child's parents was also interviewed. A 22-item questionnaire was used for data-gathering. Questionnaire items included frequency and duration of use, type of computer products/games and input devices used, presence of physical discomfort, and parental concerns related to the child's computer use. RESULTS: Many students experienced physical discomfort attributed to computer use, such as wrist pain (30%) and back pain (15%). Specific computer activities-such as using a joystick or playing noneducational games-were significantly predictive of physical discomfort using logistic multiple regression. Many parents reported difficulty getting their children off the computer (46%) and that their children spent less time outdoors (35%). CONCLUSIONS: Computer product use within this cohort was associated with self-reported physical discomfort. Results suggest a need for more extensive study, including multiyear longitudinal surveys
SN - 0033-3549
AD - Institute for Holistic Healing Studies/Health Education, San Francisco State Univ., San Francisco, CA 94132, USA. aburke@sfsu.edu
UR - 12477916
ER -

TY - JOUR
ID - 1568
T1 - Characteristics and risk factors for accident injury in Canada from 1986 to 1996: an analysis of the Canadian Accident Injury Reporting and Evaluation (CAIRE) database
A1 - Mo,F.
A1 - Choi,B.C.
A1 - Clottey,C.
A1 - LeBrun,B.
A1 - Robbins,G.
Y1 - 2002/06//
N1 - Mo, Frank. Choi, Bernard C K. Clottey, Clarence. LeBrun, Barbara. Robbins, Glenn
Injury control and safety promotion
100941859, 100941859
IM
Journal Article
English
KW - MEDLINE
KW - Accident Prevention
KW - Accidents/cl [Classification]
KW - Accidents/sn [Statistics & Numerical Data]
KW - Adolescent
KW - Adult
KW - Age Distribution
KW - Canada/ep [Epidemiology]
KW - Child
KW - Preschool
KW - Consumer Product Safety
KW - Databases
KW - Factual
KW - Female
KW - Humans
KW - Infant
KW - Newborn
KW - Length of Stay
KW - Male
KW - Middle Aged
KW - Risk Factors

KW - Sex Distribution
KW - Statistics as Topic
KW - Wounds and Injuries/cl [Classification]
KW - Wounds and Injuries/ep [Epidemiology]
KW - Wounds and Injuries/pc [Prevention & Control]
RP - NOT IN FILE
SP - 73
EP - 81
JF - Injury Control & Safety Promotion
JA - Inj Control Saf Promot
VL - 9
IS - 2
CY - Netherlands
N2 - This study analyzed the database of Canadian Accident Injury Reporting and Evaluation (CAIRE) for the injuries reported from January 1986 to March 1996 in seven provinces at children's or general hospitals in Canada. In order to describe the characteristics of injuries, we compared the different categories of injuries by sex and by age groups, identified patterns of injuries, and detected the products causing injury to Canadian people. The results showed that there were 130,489 injury cases in Canada during the 10 years from 1986 to 1996. The 10-19 year age group had 57,582 cases, representing 44.13% of total injuries, and making it the group with the highest occurrence of injuries. The male injury rate (69.75%) was significantly higher than the female rate (30.25%) (P = 0.0001). Six areas were identified as priorities for intervention: 1) injuries occurring on playgrounds among children and youth; 2) sports and playground apparatus injuries and injuries sustained in transit among young people; 3) the top five causes of injuries; 4) diagnosis and treatment of injuries; 5) consumer products and safety; and 6) nature and physical sites of injuries. Further work is needed in: evaluating injury causes, comparing the results with reports from other countries and the necessary approaches and prevention measures to reduce and control injury occurrences to improve the quality of consumer products, and to protect the health of the population in Canada
SN - 1566-0974
AD - Centre for Chronic Disease Prevention and Control, Population and Public Health Branch, Health Canada, AL # 1910C1, Tunney's Pasture, Ottawa, Ont., Canada K1A 0K9. Frank_Mo@hc-sc.gc.ca
UR - 12461833
ER -

TY - JOUR
ID - 1569
T1 - The association of abnormal cerebellar function in children with developmental coordination disorder and reading difficulties
A1 - O'Hare,A.
A1 - Khalid,S.
Y1 - 2002/10//
N1 - O'Hare, Anne. Khalid, Shabana
Dyslexia (Chichester, England)
9511375, dlz
IM
Journal Article. Research Support, Non-U.S. Gov't
English
KW - MEDLINE
KW - Cerebellum/pp [Physiopathology]
KW - Child
KW - Dyslexia/co [Complications]
KW - Dyslexia/di [Diagnosis]
KW - Dyslexia/pp [Physiopathology]
KW - Female
KW - Humans
KW - Mainstreaming (Education)
KW - Male
KW - Memory

KW - Motor Skills Disorders/co [Complications]
KW - Motor Skills Disorders/di [Diagnosis]
KW - Motor Skills Disorders/pp [Physiopathology]
KW - Phonetics
KW - Questionnaires
KW - Visual Perception/ph [Physiology]
RP - NOT IN FILE
SP - 234
EP - 248
JF - Dyslexia: the Journal of the British Dyslexia Association
JA - Dyslexia
VL - 8
IS - 4
CY - England
N2 - Children with developmental coordination disorder/dyspraxia (DCD) are at high risk of reading and writing delay. The difficulties with motor skills are heterogeneous and many children have features of poor cerebellar function, reflected in problems with posture, balance and fast accurate control of movement. This study confirmed a high level of parental reporting of reading and writing delay in a clinical group of 23 children with DCD, defined on the basis of both clinical examination and standardized testing of motor function. Direct measurement of reading delay, identified still further children in the group. Those children with reading delay had associated findings typical of phonological awareness difficulties. The children also underwent a standardized test of neurological function and although they all had difficulties with cerebellar function, no distinctive pattern emerged for those whose presentation was complicated by delayed reading and writing. Both the children with DCD and 136 typically developing children, completed the pilot parental questionnaire on gross motor skills. The three skills of catching a ball, jumping on a moving playground roundabout and handwriting, distinguished the children with DCD. This study therefore confirms that children with DCD should be assessed for difficulties in phonological awareness. Additionally, children aged between 7 and 12 years are on the whole, highly competent in a range of gross motor skills and further study might determine whether a simple parental questionnaire might detect children who would benefit from further assessment. The study also suggests that all the children with DCD have cerebellar dysfunction and further work with a larger group might determine particular patterns associated with reading delay
SN - 1076-9242
AD - Reproductive and Developmental Sciences, Child Life and Health, Community Child Health, Royal Hospital for Sick Children, 10 Chalmers Crescent, Edinburgh EH9 ITS, UK. A.O'Hare@ed.ac.uk
UR - 12455853
ER -

TY - JOUR
ID - 1570
T1 - [Toxocariasis - an underestimated problem in paediatrics]. [Polish]
A1 - Hozyasz,K.
A1 - Milanowski,A.
Y1 - 2002/04//
N1 - Hozyasz, Kamil. Milanowski, Andrzej
Medycyna wieku rozwojowego
100928610, dpo
IM
Case Reports. English Abstract. Journal Article
Polish
KW - MEDLINE
KW - Albendazole/tu [Therapeutic Use]
KW - Animals
KW - Anthelmintics/tu [Therapeutic Use]
KW - Cats
KW - Child
KW - Preschool
KW - Diagnosis

KW - Differential
KW - Dogs
KW - Female
KW - Humans
KW - Poland
KW - Time Factors
KW - Toxocara canis/ip [Isolation & Purification]
KW - Toxocariasis/bl [Blood]
KW - Toxocariasis/ps [Parasitology]
KW - Treatment Outcome
RP - NOT IN FILE
SP - 155
EP - 162
JF - Medycyna Wieku Rozwojowego
JA - Med Wieku Rozwoj.
VL - 6
IS - 2
CY - Poland
N2 - Aspects of Toxocara spp. epidemiology and paratenic infestation of children are described. Human toxocariasis is caused by infestation of zoonotic roundworms of dogs and cats. Soil sampling showes widespread contamination of the environment, particularly parks and playgrounds, with the eggs of Toxocara spp. Toxocara seroprevelance values in children vary between 3.5% in the West Poland and 19% in the South-East Poland. Children more frequently have clinical symptoms o the disease because of the closer contact with contaminated soil in sand-boxes and relatively frequent geophagia. A 3 years old girl with covert toxocariasis is described. As this case illustrates, this disease can be present without its classic visceral, ocular or neurological manifestations. On admission to the hospital the results of blood tests were as follows: white blood cells count, 43x10(3)/ mm3 (norm <13x10(3)) and hyperosinophilia, 81%. The girl was treated with albendazole 15 mg/kg for five days, after one year the treatment was repeated. The patient was followed with blood samples throughout a period of one and half year. During that time results of serological tests for Toxocara canis were strongly positive. The lowest blood count was 20.0x10(3)/mm3 and eosinophilia, 19%. Possible association between high eosinophilia in toxocariasis and development of allergic and cardiovascular diseases is discussed
SN - 1428-345X
AD - Klinika Pediatrii, Instytut Matki i Dziecka, ul. Kasprzaka 17a, Warszawa, Poland. khozyasz@alpha.net.pl
UR - 12426460
ER -

TY - JOUR
ID - 1571
T1 - [Sports in children and adolescents]. [French]
A1 - Edelman,R.
Y1 - 2002/09//
N1 - Edelman, R
Revue medicale de Bruxelles
slk, 8003474
IM
English Abstract. Journal Article
French
KW - MEDLINE
KW - Adolescent/ph [Physiology]
KW - Child
KW - Child Welfare
KW - Humans
KW - Obesity/ep [Epidemiology]
KW - Obesity/pc [Prevention & Control]
KW - Physical Fitness
KW - Sports
RP - NOT IN FILE

SP - A203
EP - A205
JF - Revue Medicale de Bruxelles
JA - Rev Med Brux.
VL - 23
IS - 4
CY - Belgium
N2 - The current conditions in Belgium are paradoxical. Indeed, adequate measures are now in place to allow sports activities in a favorable environment: increase in leisure time, widespread availability of indoor and outdoor facilities. However, there has been a significant increase in obesity among young people as well as a gradual erosion in physical fitness of young Belgians. Therefore, physical activity is mandated, not only as a general personal health issue, but also as a public health issue. It should be encouraged, taking into account psychological and physiological growth parameters of teenagers
SN - 0035-3639
UR - 12422435
ER -

TY - JOUR
ID - 1572
T1 - It's about time: a comparison of Canadian and American time-activity patterns
A1 - Leech,J.A.
A1 - Nelson,W.C.
A1 - Burnett,R.T.
A1 - Aaron,S.
A1 - Raizenne,M.E.
Y1 - 2002/11//
N1 - Leech, Judith A. Nelson, William C. Burnett, Richard T. Aaron, Shawn. Raizenne, Mark E
Journal of exposure analysis and environmental epidemiology
bjn, 9111438
IM
Comparative Study. Journal Article
English
KW - MEDLINE
KW - Activities of Daily Living
KW - Adolescent
KW - Adult
KW - Aged
KW - Canada
KW - Child
KW - Preschool
KW - Environmental Exposure
KW - Epidemiologic Studies
KW - Female
KW - Health Surveys
KW - Humans
KW - Male
KW - Middle Aged
KW - Reproducibility of Results
KW - United States
RP - NOT IN FILE
SP - 427
EP - 432
JF - Journal of Exposure Analysis and Environmental Epidemiology
JA - J Expo Anal Environ Epidemiol
VL - 12
IS - 6
CY - United States

N2 - This study compares two North American time-activity data bases: the National Human Activity Pattern Survey (NHAPS) of 9386 interviewees in 1992-1994 in the continental USA with the Canadian Human Activity Pattern Survey (CHAPS) of 2381 interviewees in 1996-1997 in four major Canadian cities. Identical surveys and methodology were used to collect this data: random sample telephone selection within the identified telephone exchanges, computer-assisted telephone interviews, overselection of children and weekends in the 24-h recall diary and the same interviewers. Very similar response rates were obtained: 63% (NHAPS) and 64.5% (CHAPS). Results of comparisons by age within major activity and location groups suggest activity and location patterns are very similar (most differences being less than 1% or 14 min in a 24-h day) with the exception of seasonal differences. Canadians spend less time outdoors in winter and less time indoors in summer than their U.S. counterparts. When exposure assessments use time of year or outdoor/indoor exposure gradients, these differences may result in significant differences in exposure assessments. Otherwise, the 24-h time activity patterns of North Americans are remarkably similar and use of the combined data set for some exposure assessments may be feasible
SN - 1053-4245
AD - Air Quality Health Effects Division, Bureau of Chemical Hazards, Health Canada, Ottawa, Ontario, Canada. jleech@ottowahospital.on.ca
UR - 12415491
ER -

TY - JOUR
ID - 1573
T1 - Efficacy of a new pattern of delivery of methylphenidate for the treatment of ADHD: effects on activity level in the classroom and on the playground.[Erratum appears in J Am Acad Child Adolesc Psychiatry. 2003 Feb;42(2):260.]
A1 - Swanson,J.M.
A1 - Gupta,S.
A1 - Williams,L.
A1 - Agler,D.
A1 - Lerner,M.
A1 - Wigal,S.
Y1 - 2002/11//
N1 - Swanson, James M. Gupta, Suneel. Williams, Lillie. Agler, Dave. Lerner, Marc. Wigal, Sharon
Journal of the American Academy of Child and Adolescent Psychiatry
hg5, 8704565
IM
Clinical Trial. Randomized Controlled Trial. Research Support, Non-U.S. Gov't
English
KW - MEDLINE
KW - Attention Deficit Disorder with Hyperactivity/di [Diagnosis]
KW - Attention Deficit Disorder with Hyperactivity/dt [Drug Therapy]
KW - Central Nervous System Stimulants/ad [Administration & Dosage]
KW - Central Nervous System Stimulants/tu [Therapeutic Use]
KW - Child
KW - Cross-Over Studies
KW - Double-Blind Method
KW - Drug Administration Schedule
KW - Drug Delivery Systems
KW - Female
KW - Humans
KW - Male
KW - Methylphenidate/tu [Therapeutic Use]
KW - Play and Playthings
KW - Social Environment
KW - Treatment Outcome
RP - NOT IN FILE
SP - 1306
EP - 1314

JF - Journal of the American Academy of Child & Adolescent Psychiatry
JA - J Am Acad Child Adolesc Psychiatry
VL - 41
IS - 11
CY - United States
N2 - OBJECTIVES: To evaluate the pharmacodynamic effects of an experimental (EXP) delivery of methylphenidate (MPH) in children with attention-deficit/hyperactivity disorder and to investigate the situational nature of effects in laboratory classroom and playground settings. METHOD: A "sipping" study methodology was used to deliver a bolus followed by small but increasing MPH doses as the EXP regimen. A randomized, double-blind crossover design was used to compare the EXP regimen to a thrice-daily (TID) regimen and a placebo (PLA) regimen. Measures of efficacy were obtained from a Motionlogger actigraph to quantify activity and from the Swanson, Kotkin, Agler, M-Flynn, and Pelham (SKAMP) rating scale to quantify two domains of behavior (attention and deportment). RESULTS: Compared with PLA, both EXP and TID delivery of MPH produced large, significant reductions in activity and inappropriate behavior in the classroom, but the two MPH regimens did not differ in onset or duration of effects across the day. The MPH effects were situationally dependent and were smaller for the playground than for the classroom settings. CONCLUSIONS: The findings of this "proof of concept" study support the efficacy of a continuous, ascending delivery of MPH. The situational effects of MPH provide support for the theory of selective effects of stimulants, dependent on the demands of the environment
SN - 0890-8567
AD - Child Development Center, University of California, Irvine, CA, USA
UR - 12410072
ER -

TY - JOUR
ID - 1574
T1 - Using an injury diary to describe the ecology of children's daily injuries
A1 - Schwebel,D.C.
A1 - Binder,S.C.
A1 - Plumert,J.M.
Y1 - 2002///
N1 - Schwebel, David C. Binder, Stephanie C. Plumert, Jodie M
Journal of safety research
1264241
IM
Journal Article. Research Support, Non-U.S. Gov't
English
KW - MEDLINE
KW - Accident Prevention
KW - Accidental Falls/pc [Prevention & Control]
KW - Accidental Falls/sn [Statistics & Numerical Data]
KW - Accidents
KW - Home/pc [Prevention & Control]
KW - Home/sn [Statistics & Numerical Data]
KW - Analysis of Variance
KW - Causality
KW - Child
KW - Child Welfare
KW - Data Collection/mt [Methods]
KW - Data Interpretation
KW - Statistical
KW - Female
KW - Human Engineering
KW - Humans
KW - Male
KW - Medical Records/st [Standards]
KW - Parents

KW - Play and Playthings
KW - Safety Management
KW - Wounds and Injuries/et [Etiology]
RP - NOT IN FILE
SP - 301
EP - 319
JF - Journal of Safety Research
JA - J Safety Res
VL - 33
IS - 3
CY - United States
N2 - PROBLEM: Two problems were addressed in this study. First, are daily injury diaries an effective means to gather information about children's unintentional injuries? Second, what are the circumstances of children's injuries as recorded through the diary method? METHOD: Two studies were conducted with a total of 172 children to describe the ecology of children's daily unintentional injuries. Families completed a daily diary for 14 days, describing the circumstances surrounding the injuries children experienced each day. RESULTS: Descriptive data is provided on the locations, causes, and types of injuries children incurred, as well as who was present when they were injured. Daily injury rate was modestly related to the number of major injuries children had incurred in their lifetimes. DISCUSSION: The diary methodology was an effective means to study the ecology of children's daily injuries. Children's injuries occur in a wide range of circumstances that can be quantified through diary techniques. IMPACT ON INDUSTRY: Data obtained from daily injury diaries may be useful in a variety of areas, including study of the etiology of childhood injury, design of interventions to prevent injury, and engineering of toys and playground equipment for children
SN - 0022-4375
AD - Department of Psychology, University of Alabama at Birmingham, 1300 University Boulevard, 415 CH, Birmingham, AL 35294, USA. schwebel@uab.edu
UR - 12404995
ER -

TY - JOUR
ID - 1575
T1 - ADHD outside the laboratory: boys' executive function performance on tasks in videogame play and on a visit to the zoo
A1 - Lawrence,V.
A1 - Houghton,S.
A1 - Tannock,R.
A1 - Douglas,G.
A1 - Durkin,K.
A1 - Whiting,K.
Y1 - 2002/10//
N1 - Lawrence, Vivienne. Houghton, Stephen. Tannock, Rosemary. Douglas, Graham. Durkin, Kevin. Whiting, Ken
Journal of abnormal child psychology
0364547, h33
IM
Journal Article. Research Support, Non-U.S. Gov't
English
KW - MEDLINE
KW - Analysis of Variance
KW - Attention Deficit Disorder with Hyperactivity/px [Psychology]
KW - Child
KW - Cohort Studies
KW - Humans
KW - Inhibition (Psychology)
KW - Internal-External Control
KW - Male
KW - Memory/ph [Physiology]

KW - Neuropsychological Tests/sn [Statistics & Numerical Data]
KW - Psychomotor Performance/ph [Physiology]
KW - Task Performance and Analysis
KW - Video Games/px [Psychology]
RP - NOT IN FILE
SP - 447
EP - 462
JF - Journal of Abnormal Child Psychology
JA - J Abnorm Child Psychol
VL - 30
IS - 5
CY - United States
N2 - One current theory of attention-deficit/hyperactivity disorder (ADHD) proposes that a primary deficit in behavioral inhibition gives rise to secondary deficits in 4 executive functions and motor control. To date, empirical support for this model is based primarily on laboratory-based cognitive methods. This study assessed behavioral inhibition and executive functioning in children with ADHD in 2 real-life contexts: videogames (motor-skill target game, cognitively demanding adventure game) and an outing at the zoo (route tasks). Participants were a community sample of 57 boys diagnosed with ADHD (20 inattentive, 37 combined type) and 57 normally developing control boys, matched individually for age and nonverbal IQ. Operationally defined measures of behavioral inhibition and specific executive functions were derived from these activities and assessed under contrasting conditions of low or high working memory and distractor loads. There were no group differences in basic motor skills on the target game, nor in terms of the ability to inhibit a prepotent or ongoing response in the adventure videogame. However, boys with ADHD exhibited more self-talk, more effortful response preparation, and completed fewer challenges in the latter videogame. Also, they manifested inhibition deficits in terms of interference control during the route task at the zoo and took longer to complete the tasks. Typically, these differences were greatest under conditions of high working memory and distractor loads. Findings from this study suggest that cognitive difficulties in ADHD may be context dependent and that ADHD is associated with deficits in some but not all aspects of behavioral inhibition
SN - 0091-0627
AD - The Graduate School of Education, The University of Western Australia, Perth
UR - 12403149
ER -

TY - JOUR
ID - 1576
T1 - Seasonal variability in exercise test responses in Ghana
A1 - ddo-Yobo,E.O.
A1 - Custovic,A.
A1 - Taggart,S.C.
A1 - safo-Agyei,A.P.
A1 - Woodcock,A.
Y1 - 2002/08//
N1 - Addo-Yobo, Emmanuel O D. Custovic, Adnan. Taggart, Simon C O. Asafo-Agyei, A P. Woodcock, Ashley
Pediatric allergy and immunology : official publication of the European Society of Pediatric Allergy and Immunology
bu6, 9106718
IM
Journal Article
English
KW - MEDLINE
KW - Adolescent
KW - Asthma
KW - Exercise-Induced/ep [Epidemiology]
KW - Exercise-Induced/pp [Physiopathology]
KW - Child
KW - Exercise Test

KW - Female
KW - Ghana/ep [Epidemiology]
KW - Humans
KW - Male
KW - Poverty
KW - Rural Population
KW - Seasons
KW - Urban Population
RP - NOT IN FILE
SP - 303
EP - 306
JF - Pediatric Allergy & Immunology
JA - Pediatr Allergy Immunol
VL - 13
IS - 4
CY - Denmark
N2 - Exercise-induced bronchospasm (EIB) is widely used in epidemiological studies to investigate the prevalence of asthma. We aimed to determine seasonal variations in the prevalence of EIB in Ghanaian school children from urban-rich (UR), urban-poor (UP), and rural (R) schools. We have previously reported the prevalence of EIB in 9-16-year-old children to be 3.1% in the dry season, with UR children having a significantly higher prevalence of both EIB and atopy compared to UP or R children. In the current study, the prevalence of EIB was assessed in the same 1,095 children in the wet season (5 months following the initial study) using the same methodology. Exercise provocation consisted of free running outdoors for 6 min. In the wet season, 17/1,095 children [mean 1.55%, 95% confidence interval (CI): 0.91-2.47] had a positive response to exercise, compared to our previous report of 34 children (mean 3.1%, 95% CI: 2.15-4.32) with EIB in the dry season (dry vs. wet season, difference 1.55, 95% CI: 0.41-2.69). The proportion of children with a positive response to exercise in the UR school fell from 4.2% (25/599) to 1.3% (8/599) (difference 2.9, 95% CI: 1.2-4.5). In the wet season, there was no difference in the prevalence of EIB among the UR, UP, and R children. Only five of 1,095 subjects (mean 0.5%, 95% CI: 0.15-1.07) demonstrated EIB in both seasons. In conclusion, although exercise challenge remains a useful tool for determining asthma prevalence in epidemiological studies, seasonal variations in the pattern of responses may occur and the results should be interpreted with caution
SN - 0905-6157
AD - Department of Child Health, Komfo Anokye Teaching Hospital, Kumasi, Ghana
UR - 12390448
ER -

TY - JOUR
ID - 1577
T1 - Contribution of indoor and outdoor environments to PM2.5 personal exposure of children--VESTA study
A1 - Gauvin,S.
A1 - Reungoat,P.
A1 - Cassadou,S.
A1 - Dechenaux,J.
A1 - Momas,I.
A1 - Just,J.
A1 - Zmirou,D.
Y1 - 2002/10/07/
N1 - Gauvin, S. Reungoat, P. Cassadou, S. Dechenaux, J. Momas, I. Just, J. Zmirou, D
The Science of the total environment
uj0, 0330500
IM
Journal Article. Research Support, Non-U.S. Gov't
English
KW - MEDLINE
KW - Adolescent
KW - Air Pollutants/an [Analysis]
KW - Air Pollution

KW - Indoor/an [Analysis]
KW - Animals
KW - Domestic
KW - Child
KW - Child Welfare
KW - Environmental Exposure
KW - Female
KW - Humans
KW - Male
KW - Particle Size
KW - Regression Analysis
KW - Tobacco Smoke Pollution/an [Analysis]
KW - Urban Population
KW - Vehicle Emissions/an [Analysis]
RP - NOT IN FILE
SP - 175
EP - 181
JF - Science of the Total Environment
JA - Sci Total Environ
VL - 297
IS - 1-3
CY - Netherlands
N2 - Several studies among adult populations showed that an array of outdoor and indoor sources of particles emissions contributed to personal exposures to atmospheric particles, with tobacco smoke playing a prominent role (J. Expo. Anal. Environ. Epidemiol. 6 (1996) 57, Environ. Int. 24 (1998) 405, Arch. Environ. Health 54 (1999) 95). The Vesta study was carried out to assess the role of exposure to traffic emissions in the development of childhood asthma. In this paper, we present data on 68 children aged 8-14 years, living in the metropolitan areas of Paris (n = 30), Grenoble (n = 15) and Toulouse (n = 23), France, who continuously carried, over 48 h, a rucksack that contained an active PM2.5 sampler. Data about home indoor sources were collected by questionnaires. In parallel, daily concentrations of PM10 in ambient air were monitored by local air quality networks. The contribution of indoor and outdoor factors to personal exposures was assessed using multiple linear regression models. Average personal exposure across all children was 23.7 microg/m3 (S.D. = 19.0 microg/m3), with local means ranging from 18.2 to 29.4 microg/m3. The final model explains 36% of the total between-subjects variance, with environmental tobacco smoke contributing for more than a third to this variability; presence of pets at home, proximity of the home to urban traffic emissions, and concomitant PM10 ambient air concentrations were the other main determinants of personal exposure
SN - 0048-9697
AD - Public Health Laboratory, Grenoble University School of Medicine, La Tronch, France.
stephanie.gauvin@cstb.fr
UR - 12389789
ER -

TY - JOUR
ID - 1578
T1 - Risk factors for Burkholderia cepacia complex colonization and infection among patients with cystic fibrosis
A1 - Walsh,N.M.
A1 - Casano,A.A.
A1 - Manangan,L.P.
A1 - Sinkowitz-Cochran,R.L.
A1 - Jarvis,W.R.
Y1 - 2002/10//
N1 - Walsh, Nancy Mychalak. Casano, Annelise A. Manangan, Lilia P. Sinkowitz-Cochran, Ronda L. Jarvis, William R
The Journal of pediatrics
jlz, 0375410
AIM, IM

Comparative Study. Journal Article. Multicenter Study
English
KW - MEDLINE
KW - Administration
KW - Inhalation
KW - Adolescent
KW - Adult
KW - Burkholderia Infections/dt [Drug Therapy]
KW - Burkholderia Infections/ep [Epidemiology]
KW - Burkholderia Infections/et [Etiology]
KW - Burkholderia cepacia
KW - Case-Control Studies
KW - Child
KW - Preschool
KW - Cystic Fibrosis/dt [Drug Therapy]
KW - Cystic Fibrosis/ep [Epidemiology]
KW - Cystic Fibrosis/mi [Microbiology]
KW - Female
KW - Follow-Up Studies
KW - Humans
KW - Infant
KW - Logistic Models
KW - Male
KW - Multivariate Analysis
KW - North America/ep [Epidemiology]
KW - Odds Ratio
KW - Prospective Studies
KW - Risk Factors
KW - Severity of Illness Index
RP - NOT IN FILE
SP - 512
EP - 517
JF - Journal of Pediatrics
JA - J Pediatr
VL - 141
IS - 4
CY - United States
N2 - OBJECTIVES: To determine risk factors for acquiring Burkholderia cepacia complex among patients with cystic fibrosis (CF). STUDY DESIGN: A case-control study was conducted with active surveillance for B cepacia complex colonization/infection among patients at 21 CF centers from April 1986 to March 1989 (study period). A case-patient was defined as any CF patient with B cepacia complex colonization for the first time during the study period. Control patients were patients with CF not B cepacia complex colonized during the study period. For each patient, a questionnaire was completed semiannually. RESULTS: In multivariate analyses, hospitalization for pulmonary exacerbations, living with a B cepacia complex-positive person, attending a CF summer camp, and direct contact with a B cepacia complex-colonized CF person outside of camp and home were associated with B cepacia complex acquisition. Receiving antimicrobial aerosol therapy or cleaning and drying a home-used nebulizer between uses were associated with a decrease in B cepacia complex acquisition. CONCLUSIONS: Numerous factors inside and outside the health care setting are associated with person-to-person transmission of B cepacia complex among patients with CF. Prevention programs should reduce direct or indirect contact between noncolonized and B cepacia complex-colonized/infected patients with CF
SN - 0022-3476
AD - Division of Healthcare Quality Promotion, National Center for Infectious Diseases, Centers for Disease Control and Prevention, United States Department of Health and Human Services, Atlanta, Georgia 30333, USA
UR - 12378190
ER -

TY - JOUR
ID - 1579
T1 - Systematic review of the effect of therapeutic dietary supplements and drugs on cognitive function in subjects with Down syndrome. [Review] [36 refs]
A1 - Salman,M.
Y1 - 2002///
N1 - Salman, Michael
European journal of paediatric neurology : EJPN : official journal of the European Paediatric Neurology Society
9715169, dgs
IM
Clinical Trial. Journal Article. Randomized Controlled Trial. Review
English
KW - MEDLINE
KW - Adolescent
KW - Child
KW - Preschool
KW - Cognition/de [Drug Effects]
KW - Databases as Topic
KW - Dietary Supplements
KW - Down Syndrome/dh [Diet Therapy]
KW - Down Syndrome/dt [Drug Therapy]
KW - Female
KW - Humans
KW - Male
KW - Neuroprotective Agents/pd [Pharmacology]
KW - Neuroprotective Agents/tu [Therapeutic Use]
KW - Piracetam/pd [Pharmacology]
KW - Piracetam/tu [Therapeutic Use]
KW - Psychomotor Performance/de [Drug Effects]
KW - Vitamins/pd [Pharmacology]
KW - Vitamins/tu [Therapeutic Use]
RP - NOT IN FILE
SP - 213
EP - 219
JF - European Journal of Paediatric Neurology
JA - Europ.J Paediatr Neurol
VL - 6
IS - 4
CY - England
N2 - The objective was to evaluate the effects of therapeutic dietary supplements and drugs on cognitive function in subjects with Down syndrome. The study design was a systematic review of randomized controlled trials of dietary supplements and/or drugs reporting any assessment of cognitive function in subjects with Down syndrome. Eleven trials were identified with 373 randomized participants. None of the trials reported cognitive enhancing effect in subjects with Down syndrome. Meta-analysis was not conducted due to the heterogeneous nature of the population, interventions and outcome measures used. Overall, the quality of the trials was poor with few subjects and generally inadequate allocation concealment of the treatments given. This comprehensive systematic review provides no positive evidence that any combination of drugs, vitamins and minerals enhance either cognitive function or psychomotor development in people with Down syndrome. However, because of the small number of subjects involved and the overall unsatisfactory quality of the trials, an effect cannot be excluded at this point. At present there is no justification for the use of such regimes outside the context of large well designed trials. Parents of children with Down syndrome should be actively discouraged from giving these 'miracle drugs' to their children. [References: 36]
SN - 1090-3798
AD - Department of Paediatric Neurosciences, King's College Hospital, London, UK.
Michael.Salman@sickkids.ca
UR - 12374588
ER -

TY - JOUR
ID - 1580
T1 - Dietary and physical activity patterns of 8- to 10-year-old urban schoolchildren in Manila, Philippines
A1 - Florentino,R.F.
A1 - Villavieja,G.M.
A1 - Lana,R.D.
Y1 - 2002/09//
N1 - Florentino, Rodolfo F. Villavieja, Gracia M. Lana, Ruby D
Food and nutrition bulletin
7906418
IM
Comparative Study. Journal Article. Research Support, Non-U.S. Gov't
English
KW - MEDLINE
KW - Child
KW - Child Nutritional Physiological Phenomena
KW - Diet
KW - Diet Surveys
KW - Dietary Fats/ad [Administration & Dosage]
KW - Dietary Proteins/ad [Administration & Dosage]
KW - Energy Intake
KW - Exercise
KW - Female
KW - Health Education
KW - Health Knowledge
KW - Attitudes
KW - Practice
KW - Humans
KW - Male
KW - Philippines
KW - Private Sector
KW - Schools
KW - Urban Population
RP - NOT IN FILE
SP - 267
EP - 273
JF - Food & Nutrition Bulletin
JA - Food Nutr Bull
VL - 23
IS - 3
CY - Japan
N2 - This report is the second part of a study on the nutrition of urban schoolchildren in the city of Manila; the first part was on nutritional status. The primary purpose of the study was to gather data that will assist authorities in planning and implementing nutrition education programs focused on the growing problem of obesity and overweight in children. The study included 1,208 children 8 to 10 years of age, who were randomly selected from all public and private schools in the city of Manila. Data on the children's dietary and physical activity patterns, together with information on nutrition-related knowledge, attitudes, and preferences of the children and their parents, were gathered by interviews and self-administered questionnaires. There were distinct differences in dietary and physical activity patterns between the two groups of children. Children from private schools, who are generally of higher socioeconomic status than those from public schools, tended to consume more total food, more animal foods, fats, and oils, and more beverages, resulting in higher intakes of calories, protein, iron, and vitamin A than public schoolchildren. Moreover, children from private schools were apparently less physically active, were more likely to be driven to school instead of walking, and were more likely to prefer television and computer games over outdoor games. These differences agree with an earlier report on the nutritional status of the children as determined by anthropometry, which showed a higher proportion of overnutrition and a lower proportion of undernutrition among private schoolchildren than among

public schoolchildren. Although the emphasis in public schools should be on prevention and control of undernutrition in children, private schools should begin to look at the emerging problem of overnutrition and the role of physical activity programs in the health of children. The information on the knowledge, attitudes, and preferences of the children and their parents showed that parents or guardians, teachers, and television influence the nutritional practices of schoolchildren. These groups should therefore be the major targets for nutrition education programs meant to improve the nutrition and health of schoolchildren
SN - 0379-5721
AD - Food and Nutrition Research Institute, Department of Science and Technology, Manila, Philippines
UR - 12362590
ER -

TY - JOUR
ID - 1581
T1 - Association of asthma symptoms with peak particulate air pollution and effect modification by anti-inflammatory medication use
A1 - Delfino,R.J.
A1 - Zeiger,R.S.
A1 - Seltzer,J.M.
A1 - Street,D.H.
A1 - McLaren,C.E.
Y1 - 2002/10//
N1 - Delfino, Ralph J. Zeiger, Robert S. Seltzer, James M. Street, Donald H. McLaren, Christine E
Environmental health perspectives
ei0, 0330411
IM
Journal Article. Research Support, U.S. Gov't, P.H.S.
English
KW - MEDLINE
KW - Activities of Daily Living
KW - Adolescent
KW - Adult
KW - Air Pollutants/ae [Adverse Effects]
KW - Allergens
KW - Anti-Inflammatory Agents/pd [Pharmacology]
KW - Anti-Inflammatory Agents/tu [Therapeutic Use]
KW - Asthma/dt [Drug Therapy]
KW - Asthma/et [Etiology]
KW - Asthma/pa [Pathology]
KW - Child
KW - Environmental Exposure
KW - Female
KW - Humans
KW - Inflammation
KW - Male
KW - Particle Size
KW - Rural Population
KW - Severity of Illness Index
KW - Steroids
KW - Time Factors
KW - Urban Population
RP - NOT IN FILE
SP - A607
EP - A617
JF - Environmental Health Perspectives
JA - Environ Health Perspect
VL - 110
IS - 10

CY - United States
N2 - Maxima of hourly data from outdoor monitors may capture adverse effects of outdoor particulate matter (PM) exposures in asthmatic children better than do 24-hr PM averages, which form the basis of current regulations in the United States. Also, asthmatic children on anti-inflammatory medications may be protected against the proinflammatory effects of air pollutants and aeroallergens. We examined strengths of pollutant associations with asthma symptoms between subgroups of asthmatic children who were on versus not on regularly scheduled anti-inflammatory medications, and tested associations for different particle averaging times. This is a daily panel study of 22 asthmatic children (9-19 years of age) followed March through April 1996 (1,248 person-days). They lived in nonsmoking households in a semirural area of Southern California within the air inversion mixing zone (range, 1,200-2,100 feet) with transported air pollution from urban areas of Southern California. The dependent variable derived from diary ordinal scores is episodes of asthma symptoms that interfered with daily activities. Minimum to 90th-percentile levels of exposures at the outdoor monitoring site were 12-63 microg/m(3) for 1-hr PM < 10 microm in aerodynamic diameter (PM(10)); 8-46 microg/m(3) for 8-hr PM(10); 7-32 microg/m(3) for 24-hr PM(10); 45-88 ppb for 1-hr O(3); 6-26 ppb for 8-hr NO(2); 70-4,714 particles/m(3) for 12-hr daytime fungi; and 12-744 particles/m(3) for 24-hr pollen. Data were analyzed with generalized estimating equations controlling for autocorrelation. There was no confounding by weather, day of week, or linear time trend. Associations were notably stronger in 12 asthmatic children who were not taking anti-inflammatory medications versus 10 subjects who were. Odds ratios (95% confidence intervals) for asthma episodes in relation to lag 0 minimum to 90th-percentile pollutant changes were, respectively, 1-hr maximum PM(10), 1.92 (1.22-3.02) versus 0.96 (0.25-3.69); 8-hr maximum PM(10), 1.68 (0.91-3.09) versus 0.75 (0.18-3.04); 24-hr average PM(10), 1.35 (0.82-2.22) versus 0.80 (0.24-2.69); 1-hr maximum O(3), 1.28 (0.75-2.17) versus 0.76 (0.24-2.44); 8-hr maximum NO(2), 1.91 (1.07-3.39) versus 1.08 (0.30-3.93); 12-hr fungi, 1.89 (1.24-2.89) versus 0.90 (0.35-2.30); 24-hr pollen, 1.90 (0.99-3.67) versus 0.85 (0.18-3.91). Pollutant associations were stronger during respiratory infections in subjects not on anti-inflammatory medications. Although lag 0 1-hr maximum PM(10) showed the strongest association, the most robust associations were for lag 0 and 3-day moving averages (lags 0-2) of 8-hr maximum and 24-hr mean PM(10) in sensitivity analyses testing for thresholds. Most pollutant effects were largely driven by concentrations in the upper quintile. The divergence of exposure-response relationships by anti-inflammatory medication use is consistent with experimental data on inflammatory mechanisms of airborne pollutants and allergens
SN - 0091-6765
AD - Epidemiology Division, Department of Medicine, University of California, Irvine, Irvine, California 92697-7550, USA. rdelfino@uci.edu
UR - 12361942
ER -

TY - JOUR
ID - 1582
T1 - [Radiotherapy treatment under anesthesia in pediatrics]. [Spanish]
A1 - Fernandez,Baena M.
A1 - Gualda,Canton L.
A1 - Fernandez Jurado,M.I.
A1 - Castilla,Moreno M.
Y1 - 2002/06//
N1 - Fernandez Baena, M. Gualda Canton, L. Fernandez Jurado, M I. Castilla Moreno, M
Revista espanola de anestesiologia y reanimacion
rsx, 0134516
IM
Case Reports. English Abstract. Journal Article
Spanish
KW - MEDLINE
KW - Anesthesia
KW - General
KW - Intravenous
KW - Anesthetics
KW - Intravenous/ad [Administration & Dosage]
KW - Brain Stem Neoplasms/rt [Radiotherapy]
KW - Capnography

KW - Cerebellar Neoplasms/rt [Radiotherapy]
KW - Child
KW - Preschool
KW - Humans
KW - Immobilization
KW - Male
KW - Midazolam/ad [Administration & Dosage]
KW - Oximetry
KW - Propofol/ad [Administration & Dosage]
KW - Radiotherapy
KW - Conformal
KW - High-Energy
KW - Supine Position
RP - NOT IN FILE
SP - 324
EP - 327
JF - Revista Espanola de Anestesiologia y Reanimacion
JA - Rev Esp Anestesiol.Reanim.
VL - 49
IS - 6
CY - Spain
N2 - A four-year-old boy received 30 sessions of radiotherapy, at a rate of one session daily for five days every week, under anesthesia with midazolam and propofol in spontaneous ventilation. Monitoring was by pulse oximetry and capnography; no adverse events were recorded. The presence of an anesthesiologist during diagnostic and therapeutic procedures outside the operating room is essential, given that lack of cooperation can make the procedure difficult or impossible to perform. Radiotherapy under anesthesia is a special case because it is scheduled daily, and care must be taken to interfere as little as possible with a child's meals or other activities
SN - 0034-9356
AD - Servicio de Anestesiologia, Reanimacion y Terapia del Dolor, Complejo Hospitalario Carlos Haya, Malaga. auroragp@teleline.es
UR - 12353410
ER -

TY - JOUR
ID - 1583
T1 - Environmental exposure to endotoxin and its relation to asthma in school-age children
A1 - Braun-Fahrlander,C.
A1 - Riedler,J.
A1 - Herz,U.
A1 - Eder,W.
A1 - Waser,M.
A1 - Grize,L.
A1 - Maisch,S.
A1 - Carr,D.
A1 - Gerlach,F.
A1 - Bufe,A.
A1 - Lauener,R.P.
A1 - Schierl,R.
A1 - Renz,H.
A1 - Nowak,D.
A1 - von,Mutius E.
A1 - Allergy and Endotoxin Study Team.
Y1 - 2002/09//
N1 - Braun-Fahrlander, Charlotte. Riedler, Josef. Herz, Udo. Eder, Waltraud. Waser, Marco. Grize, Leticia. Maisch, Soyoun. Carr, David. Gerlach, Florian. Bufe, Albrecht. Lauener, Roger P. Schierl, Rudolf. Renz, Harald. Nowak, Dennis. von Mutius, Erika. Allergy and Endotoxin Study Team

The New England journal of medicine
0255562, now
AIM, IM
Journal Article. Research Support, Non-U.S. Gov't
English
KW - MEDLINE
KW - Adolescent
KW - Agriculture
KW - Allergens/an [Analysis]
KW - Asthma/ep [Epidemiology]
KW - Asthma/im [Immunology]
KW - Child
KW - Cross-Sectional Studies
KW - Cytokines/bl [Blood]
KW - Dust/an [Analysis]
KW - Endotoxins/an [Analysis]
KW - Endotoxins/im [Immunology]
KW - Environmental Exposure/an [Analysis]
KW - Environmental Exposure
KW - Female
KW - Humans
KW - Hypersensitivity
KW - Immediate/ep [Epidemiology]
KW - Immediate/im [Immunology]
KW - Immunoglobulin E/bl [Blood]
KW - Leukocytes/im [Immunology]
KW - Male
KW - Prevalence
KW - Questionnaires
KW - Risk Factors
RP - NOT IN FILE
SP - 869
EP - 877
JF - New England Journal of Medicine
JA - N Engl J Med
VL - 347
IS - 12
CY - United States
N2 - BACKGROUND: In early life, the innate immune system can recognize both viable and nonviable parts of microorganisms. Immune activation may direct the immune response, thus conferring tolerance to allergens such as animal dander or tree and grass pollen. METHODS: Parents of children who were 6 to 13 years of age and were living in rural areas of Germany, Austria, or Switzerland where there were both farming and nonfarming households completed a standardized questionnaire on asthma and hay fever. Blood samples were obtained from the children and tested for atopic sensitization; peripheral-blood leukocytes were also harvested from the samples for testing. The levels of endotoxin in the bedding used by these children were examined in relation to clinical findings and to the cytokine-production profiles of peripheral-blood leukocytes that had been stimulated with lipopolysaccharide and staphylococcal enterotoxin B. Complete data were available for 812 children. RESULTS: Endotoxin levels in samples of dust from the child's mattress were inversely related to the occurrence of hay fever, atopic asthma, and atopic sensitization. Nonatopic wheeze was not significantly associated with the endotoxin level. Cytokine production by leukocytes (production of tumor necrosis factor alpha, interferon-gamma, interleukin-10, and interleukin-12) was inversely related to the endotoxin level in the bedding, indicating a marked down-regulation of immune responses in exposed children. CONCLUSIONS: A subject's environmental exposure to endotoxin may have a crucial role in the development of tolerance to ubiquitous allergens found in natural environments. Copyright 2002 Massachusetts Medical Society
SN - 1533-4406
AD - Institute of Social and Preventive Medicine, Basel, Switzerland. c.braun@unibas.ch
UR - 12239255

ER -

TY - JOUR
ID - 1584
T1 - Residential camps as a setting for nutrition education of Australian girls
A1 - Payne,J.
A1 - Capra,S.
A1 - Hickman,I.
Y1 - 2002/08//
N1 - Payne, Jan. Capra, Sandra. Hickman, Ingrid
Australian and New Zealand journal of public health
ck2, 9611095
IM
Journal Article
English
KW - MEDLINE
KW - Adolescent
KW - Camping
KW - Child
KW - Child Nutrition Sciences/ed [Education]
KW - Data Collection
KW - Female
KW - Food Habits
KW - Health Education/mt [Methods]
KW - Health Knowledge
KW - Attitudes
KW - Practice
KW - Health Promotion/og [Organization & Administration]
KW - Humans
KW - Queensland
RP - NOT IN FILE
SP - 383
EP - 388
JF - Australian & New Zealand Journal of Public Health
JA - Aust N Z J Public Health
VL - 26
IS - 4
CY - Australia
N2 - OBJECTIVE: To implement a planned nutrition education program aiming to promote healthy eating and consumption of a variety of foods in a residential camp setting for Australian girls aged 9-15 years. METHODS: 1,600 girls attending a seven-day residential camp in Queensland (2000) participated in a program involving the provision of healthy, tasty foods based on the Australian Dietary Guidelines for Children and Adolescents and a nutrition education package for use at the camp restaurants each evening that included nutrition information together with individual 'passport booklets involving puzzles and questions with incentives for completion. Process and impact evaluations were conducted by surveying a sample of participants using questionnaires and focus groups. RESULTS: Of those surveyed, 77% felt they had leamed something from the health promotion material; 94% said they had changed their eating habits to include more core food groups during the camp, with more than 40% stating they had increased vegetable consumption compared with their usual intake. However, approximately 60% of campers were apparently unaware of the incentives offered and less than 30% demonstrated completion of their passports. CONCLUSIONS: Barriers to the involvement of this target group in the health promotion activities need to be explored further. Involvement and training of key personnel is suggested to ensure consistent implementation and encouragement for all participants. Long-term behaviour changes outside the camp environment should also be assessed in any future programs. Implications: Using residential camp settings to target children is an innovative strategy for nutrition education. Integration of such strategies into longer time frame programs may benefit participants and improve effectiveness of nutrition education programs aimed at this group
SN - 1326-0200

AD - School of Public Health, Queensland University of Technology. j.payne@qut.edu.au
UR - 12233962
ER -

TY - JOUR
ID - 1585
T1 - Exposure to traffic among urban children injured as pedestrians
A1 - Posner,J.C.
A1 - Liao,E.
A1 - Winston,F.K.
A1 - Cnaan,A.
A1 - Shaw,K.N.
A1 - Durbin,D.R.
Y1 - 2002/09//
N1 - Posner, J C. Liao, E. Winston, F K. Cnaan, A. Shaw, K N. Durbin, D R
Injury prevention : journal of the International Society for Child and Adolescent Injury Prevention
crz, 9510056
IM
Journal Article. Research Support, Non-U.S. Gov't
English
KW - MEDLINE
KW - Accidents
KW - Traffic/sn [Statistics & Numerical Data]
KW - Adolescent
KW - Age Factors
KW - Child
KW - Preschool
KW - Cross-Sectional Studies
KW - Female
KW - Humans
KW - Male
KW - Risk Factors
KW - Urban Health/sn [Statistics & Numerical Data]
KW - Walking/sn [Statistics & Numerical Data]
RP - NOT IN FILE
SP - 231
EP - 235
JF - Injury Prevention
JA - Inj Prev
VL - 8
IS - 3
CY - England
N2 - OBJECTIVES: To explore the immediate pre-crash activities and the routine traffic exposure (street crossing and play) in a sample of urban children struck by automobiles. In particular, the traffic exposure of children who were struck while playing was compared with that of those struck while crossing streets. DESIGN: Cross sectional survey. SETTING: Urban pediatric emergency department. PATIENTS: A total of 139 children ages 4-15 years evaluated for acute injuries resulting from pedestrian-motor vehicle collisions during a 14 month period. MAIN OUTCOME MEASURES: Sites of outdoor play, daily time in outdoor play, weekly number of street crossings, pre-crash circumstance (play v walking). RESULTS: Altogether 39% of the children routinely used the street and 64% routinely used the sidewalks as play areas. The median number of street crossings per week per child was 27. There were no differences in exposures for the 29% who were hit while playing compared with the 71% who were hit while walking. Although 84% of the children walked to or from school at least one day per week, only 15% of the children were struck while on the school walking trip. The remainder were injured either while playing outdoors or while walking to other places. CONCLUSIONS: Urban children who are victims of pedestrian crashes have a high level of traffic exposure from a variety of circumstances related to their routine outdoor playing and street crossing activities. The distributions of traffic exposures were similar across the sample, indicating that the sample as a whole had high traffic exposure,

regardless of the children's activity preceding the crash. Future pedestrian injury programs should address the pervasive nature of children's exposure to traffic during their routine outdoor activities
SN - 1353-8047
AD - Department of Pediatrics, Children's Hospital of Philadelphia, University of Pennsylvania School of Medicine, Philadelphia 19104, USA. posner@email.chop.edu
UR - 12226122
ER -

TY - JOUR
ID - 1586
T1 - Effect of mattress encasings on atopic dermatitis outcome measures in a double-blind, placebo-controlled study: the Dutch mite avoidance study
A1 - Oosting,A.J.
A1 - de Bruin-Weller,M.S.
A1 - Terreehorst,I.
A1 - Tempels-Pavlica,Z.
A1 - Aalberse,R.C.
A1 - de Monchy,J.G.
A1 - van Wijk,R.G.
A1 - Bruijnzeel-Koomen,C.A.
Y1 - 2002/09//
N1 - Oosting, Albert-Jan. de Bruin-Weller, Marjolein S. Terreehorst, Ingrid. Tempels-Pavlica, Zana. Aalberse, Rob C. de Monchy, Jan G R. van Wijk, Roy Gerth. Bruijnzeel-Koomen, Carla A F M
The Journal of allergy and clinical immunology
h53, 1275002
AIM, IM
Clinical Trial. Journal Article. Randomized Controlled Trial
English
KW - MEDLINE
KW - Adolescent
KW - Adult
KW - Aged
KW - Air Pollution
KW - Indoor/an [Analysis]
KW - Allergens/an [Analysis]
KW - Animals
KW - Antigens
KW - Dermatophagoides
KW - Bedding and Linens
KW - Beds
KW - Child
KW - Preschool
KW - Dermatitis
KW - Atopic/di [Diagnosis]
KW - Atopic/pc [Prevention & Control]
KW - Double-Blind Method
KW - Dust/an [Analysis]
KW - Floors and Floorcoverings
KW - Glycoproteins/an [Analysis]
KW - Humans
KW - Middle Aged
KW - Mites/im [Immunology]
KW - Netherlands
KW - Treatment Outcome
RP - NOT IN FILE
SP - 500
EP - 506

JF - Journal of Allergy & Clinical Immunology
JA - J Allergy Clin Immunol
VL - 110
IS - 3
CY - United States
N2 - BACKGROUND: House dust mite (HDM) allergen might induce and maintain atopic dermatitis (AD). Reduction of allergen load by applying encasings might improve the clinical symptoms of AD. OBJECTIVE: We sought to investigate, in a randomized, double-blind, placebo-controlled study, whether reducing HDM allergen levels by using mattress, duvet, and pillow encasings for 12 months will result in improvement in AD symptoms. METHODS: Patients with AD (8-50 years old and allergic to HDM), having a Leicester sign score (a dermatitis score) of at least 1% extent and a severity score of 6 points or greater, were randomly allocated to an active (n = 45) or a placebo allergen-avoidance group (n = 41). Avoidance measures consisted of applying HDM-impermeable encasings for mattresses, pillows, and duvets for the active treatment group and cotton encasings for the placebo group. Effect on allergen concentrations (Der p 1 and Der p 1 plus Der f 1), Leicester sign score extent and severity, visual analogue scale scores for itching and sleeplessness, intradermal test results, atopy patch test results, total serum IgE levels, anti-Der p 1-specific IgE levels, and total blood eosinophil counts were studied. RESULTS: The active encasings reduced the Der p 1 allergen concentration in the mattress after 12 months with a factor 2.1 (P =.007) and the Der p 1 plus Der f 1 allergen concentration with a factor of 2.5 (P =.005); no significant change in allergen concentrations in mattresses was seen in the placebo group. Although the decrease in allergen load was significant, no differences in treatment-induced changes were seen between the placebo and active groups. CONCLUSIONS: Use of HDM-impermeable encasings resulted in a significant decrease in Der p 1 and Der p 1 plus Der f 1 allergen concentrations. However, this reduction in allergen load did not result in significant changes in clinical parameters between the groups. Reduction of allergens in other environments (work, school, and outdoors) might be equally important in improving symptoms of AD
SN - 0091-6749
AD - Department of Dermatology and Allergology, University Medical Centre Utrecht, The Netherlands
UR - 12209102
ER -

TY - JOUR
ID - 1587
T1 - [Risk factors: environment, tobacco smoke]. [Review] [62 refs] [French]
A1 - Heraud,M.C.
A1 - Herbelin-Wagner,M.L.
Y1 - 2002/08//
N1 - Heraud, M C. Herbelin-Wagner, M L
Archives de pediatrie : organe officiel de la Societe francaise de pediatrie
9421356, bwh
IM
English Abstract. Journal Article. Review
French
KW - MEDLINE
KW - Asthma/ep [Epidemiology]
KW - Asthma/et [Etiology]
KW - Asthma/im [Immunology]
KW - Child
KW - Preschool
KW - Environment
KW - Geography
KW - Humans
KW - Hypersensitivity
KW - Infant
KW - Newborn
KW - Life Style
KW - Prevalence
KW - Respiratory Syncytial Virus Infections/co [Complications]

KW - Risk Factors
KW - Smoking/ae [Adverse Effects]
KW - Tobacco Smoke Pollution/ae [Adverse Effects]
RP - NOT IN FILE
SP - 377s
EP - 383s
JF - Archives de Pediatrie
JA - Arch Pediatr
VL - 9 Suppl 3
CY - France
N2 - Environmental factors are usually considered as risk factors for increase of asthma prevalence. They may act isolately but are frequently associated. They act either directly by inducing asthma or more likely by increasing allergenic sensitization. Geographic situation is a well known risk factor. Important differences are noted between countries. The western lifestyle is evocated, including type of alimentation, small size of siblings, increased allergen exposure in houses. Intrauterine environment may play a role, particularly tobacco smoke during pregnancy and its respiratory effects on infant. Maternal allergenic exposure during pregnancy is an important factor because of maternofetal immunologic interactions. Outdoor pollution acts by enhancing bronchial responsiveness, allergenic sensitization and worsening respiratory diseases. Its effect is probably less important in infants and small children who are living indoor most of the time. Infections seems to have a complex action. Some virus, including respiratory syncytial virus, act to induce asthma or sensitization. Other type of infections (viral ou microbial) have a protector effect. Exposure to tobacco smoke, particularly maternal smoking, is identified in all studies, as one of the most important factors to be considered in childhood asthma. Exposure to allergen increases the risk of sensitization. Its direct responsibility to induce asthma is not established. Some of recent studies are suggesting the concept of a protective effect of early exposure. As far as preventive intervention is concerned, the recognition of these factors is important to limit the prevalence of childhood asthma. [References: 62]
SN - 0929-693X
AD - Unite de reanimation et des maladies respiratoires de l'enfant, Hotel-Dieu, BP 69, 63003 Clermont-Ferrand, France. mcheraud@chu-clermontferrand.fr
UR - 12205812
ER -

TY - JOUR
ID - 1588
T1 - Therapeutic drug monitoring of indinavir and nelfinavir to assess adherence to therapy in human immunodeficiency virus-infected children
A1 - van Rossum,A.M.
A1 - Bergshoeff,A.S.
A1 - Fraaij,P.L.
A1 - Hugen,P.W.
A1 - Hartwig,N.G.
A1 - Geelen,S.P.
A1 - Wolfs,T.F.
A1 - Weemaes,C.M.
A1 - De,Groot R.
A1 - Burger,D.M.
Y1 - 2002/08//
N1 - van Rossum, Annemarie M. Bergshoeff, Alina S. Fraaij, Pieter L. Hugen, Patricia W. Hartwig, Nico G. Geelen, Sobyl P. Wolfs, Tom F. Weemaes, Corry M. De Groot, Ronald. Burger, David M
The Pediatric infectious disease journal
oxj, 8701858
IM
Clinical Trial. Journal Article
English
KW - MEDLINE
KW - Adolescent
KW - Child

KW - Preschool
KW - Drug Administration Schedule
KW - HIV Infections/dt [Drug Therapy]
KW - HIV Infections/vi [Virology]
KW - HIV Protease Inhibitors/ad [Administration & Dosage]
KW - HIV Protease Inhibitors/bl [Blood]
KW - HIV Protease Inhibitors/tu [Therapeutic Use]
KW - HIV-1/ge [Genetics]
KW - HIV-1/ip [Isolation & Purification]
KW - Humans
KW - Indinavir/ad [Administration & Dosage]
KW - Indinavir/bl [Blood]
KW - Indinavir/tu [Therapeutic Use]
KW - Infant
KW - Nelfinavir/ad [Administration & Dosage]
KW - Nelfinavir/bl [Blood]
KW - Nelfinavir/tu [Therapeutic Use]
KW - Patient Compliance
KW - RNA
KW - Viral/an [Analysis]
KW - Viral Load
RP - NOT IN FILE
SP - 743
EP - 747
JF - Pediatric Infectious Disease Journal
JA - Pediatr Infect Dis J
VL - 21
IS - 8
CY - United States
N2 - INTRODUCTION: Adherence to highly active antiretroviral therapy is required to obtain an optimal long term virologic response rate of HIV-1-infected children. Plasma concentrations of protease inhibitors (PIs) outside the limits of the reference values indicate nonadherence to antiretroviral therapy in adults. We studied during a 2-year follow-up period routinely taken plasma protease inhibitor concentrations to assess adherence to antiretroviral therapy in HIV-1-infected children. METHODS: In 40 children (ages 3 months to 18 years) blood samples were taken at regular outpatient visits every 12 weeks after the start of highly active antiretroviral therapy and analyzed for plasma concentrations of indinavir or nelfinavir by high performance liquid chromatography and for plasma HIV-1 RNA load. The percentage of samples fulfilling the criteria for adherence was assessed for each child by three methods. For each sample a concentration ratio was calculated by dividing the concentration in that sample by the time-adjusted population value. According to Method 1 concentration ratios below or above concentration ratio limits (CORALs) of population data obtained in adults were highly indicative of nonadherence. Because many children have high PI levels, Method 2 evaluated plasma samples of PIs with only the lower CORAL. According to Method 3 only children with plasma samples below the limit of quantification (0.04 mg/l) were considered noncompliant. Differences in adherence rate between virologic responders and virologic nonresponders and between adherence rates and the two protease inhibitors were analyzed. The cumulative incidence of HIV-1 RNA levels >500 copies/ml in children was calculated. RESULTS: Thirty-one children started treatment with indinavir, and nine children started treatment with nelfinavir. The median adherence rates for indinavir as determined by methods 1, 2 and 3 were 54% [interquartile range (IQR), 25 to 69%], 67% (IQR 50 to 92%) and 80% (IQR 63 to 100%), respectively. For nelfinavir median adherence rates of 60% (IQR 39 to 75%), 100% (IQR 67 to 100%) and 100% (IQR 100 to 100%) were observed. Adherence rates calculated with Method 2 were significantly higher in virologic responders (= 0.04). Adherence rates calculated with Methods 2 and 3 were significantly lower in children using indinavir compared with those using nelfinavir (= 0.02 and = 0.02, respectively). CONCLUSION: Calculation of adherence rates using the lower limit of CORALs of indinavir or nelfinavir in children may be a useful measurement for the assessment of nonadherence to antiretroviral therapy in children
SN - 0891-3668
AD - Department of Pediatrics, Sophia Children's Hospital/Erasmus University Medical Center, Rotterdam, The Netherlands

UR - 12192162
ER -

TY - JOUR
ID - 1589
T1 - Environmental NO2 concentration and exposure in daily life along main roads in Tokyo
A1 - Kodama,Y.
A1 - Arashidani,K.
A1 - Tokui,N.
A1 - Kawamoto,T.
A1 - Matsuno,K.
A1 - Kunugita,N.
A1 - Minakawa,N.
Y1 - 2002/07//
N1 - Kodama, Yasushi. Arashidani, Keiichi. Tokui, Noritaka. Kawamoto, Toshihiro. Matsuno, Koji. Kunugita, Naoki. Minakawa, Naoto
Environmental research
ei2, 0147621
IM
Journal Article
English
KW - MEDLINE
KW - Activities of Daily Living
KW - Adolescent
KW - Adult
KW - Air Pollutants/an [Analysis]
KW - Air Pollution
KW - Indoor/an [Analysis]
KW - Child
KW - Environmental Exposure
KW - Family Health
KW - Female
KW - Housing
KW - Humans
KW - Japan
KW - Male
KW - Middle Aged
KW - Nitrogen Dioxide/an [Analysis]
KW - Oxidants
KW - Photochemical/an [Analysis]
KW - Seasons
KW - Students
KW - Urban Population
RP - NOT IN FILE
SP - 236
EP - 244
JF - Environmental Research
JA - Environ Res
VL - 89
IS - 3
CY - United States
N2 - Environmental suspended particulate matter and NO2 are possible factors causing chronic obstructive pulmonary diseases. These and other pollutants are monitored at the National Ambient Air Pollution Monitoring Stations and local monitoring stations. Environmental NO2 concentrations in large cities exceed the Japanese Environmental Quality Standards (JEQS); in particular, more than 30% of the Roadside Air Pollution Monitoring Stations (RAMS) do not achieve JEQS. To evaluate the exposure levels to environmental NO2 and its health effects, the data from the monitoring stations are useful. However, there are few reports on the

relationships between these data and the exposure level in daily life. In this study, environmental NO2 concentrations in homes and its exposure levels were investigated by using passive samplers. Two areas along main roads in the south and north of metropolitan Tokyo were selected and about 150 junior high school students and their family members took part in the study. The investigation was conducted five times seasonally, 3 days each, from February 1998 to January 1999. The residence of each subject was plotted on a map, and the distance from the main road was entered on the same map. Environmental NO2 was measured outside of the house and indoors, that is, living room, kitchen, bedroom, and student's room. The average NO2 concentration of outdoors was within JEQS, and the highest value was 52.9 ppb. There was a tendency for outdoor NO2 concentrations to decrease with distance from the roadside, but the NO2 concentration differences between the roadside and the site far from the roadside were less than 10 ppb. The average outdoor NO2 concentrations and the value obtained at RAMS are slightly correlated, but not significant. The stations' data showed about 10 ppb higher values than the average outdoor concentrations obtained at each house. NO2 concentrations in living rooms based on the distance from the main road and NO2 exposure levels in daily life of students and family members were also investigated. This study clearly showed that in wintertime we are highly exposed to NO2 emitted from many types of heaters such as kerosene heater, and the family members' study suggested that the indoor NO2 levels were similar to the personal exposure levels in all seasons
SN - 0013-9351
AD - University of Occupational and Environmental Health, Japan, Kitakyushu City, Japan
UR - 12176007
ER -

TY - JOUR
ID - 1590
T1 - Enlarged SI and SII somatosensory evoked responses in the CLN5 form of neuronal ceroid lipofuscinosis
A1 - Lauronen,L.
A1 - Huttunen,J.
A1 - Kirveskari,E.
A1 - Wikstrom,H.
A1 - Sainio,K.
A1 - Autti,T.
A1 - Santavuori,P.
Y1 - 2002/09//
N1 - Lauronen, Leena. Huttunen, J. Kirveskari, E. Wikstrom, H. Sainio, K. Autti, T. Santavuori, P
Clinical neurophysiology : official journal of the International Federation of Clinical Neurophysiology
db4, 100883319
IM
Clinical Trial. Controlled Clinical Trial. Journal Article. Research Support, Non-U.S. Gov't
English
KW - MEDLINE
KW - Adolescent
KW - Brain Mapping
KW - Child
KW - Evoked Potentials
KW - Somatosensory
KW - Female
KW - Genotype
KW - Humans
KW - Magnetic Resonance Imaging
KW - Magnetoencephalography
KW - Male
KW - Membrane Proteins/ge [Genetics]
KW - Neuronal Ceroid-Lipofuscinoses/di [Diagnosis]
KW - Neuronal Ceroid-Lipofuscinoses/ge [Genetics]
KW - Neuronal Ceroid-Lipofuscinoses/pp [Physiopathology]
KW - Phenotype
KW - Reaction Time
KW - Reference Values

KW - Somatosensory Cortex/pp [Physiopathology]
RP - NOT IN FILE
SP - 1491
EP - 1500
JF - Clinical Neurophysiology
JA - Clin Neurophysiol
VL - 113
IS - 9
CY - Netherlands
N2 - OBJECTIVES: To examine in detail the activation of the primary (SI) and secondary (SII) somatosensory cortex in CLN5, the Finnish variant of late infantile neuronal ceroid lipofuscinoses (NCL). METHODS: Somatory evoked magnetic fields were recorded with a 122-channel planar gradiometer in response to median nerve stimulation in 5 CLN5 patients (aged 8.8-16.7 years) and in 10 healthy age-matched controls. RESULTS: The first two responses from contralateral SI, N20m and P35m, were 6-20 times stronger in the patients than in the controls. The morphology of the subsequent deflections from SI was abnormal in the patients: a prominent N45m was detected, while the normally present P60m deflection was missing. In 4 patients the contra- and in two patients the ipsilateral SII responses were also enlarged. Furthermore, the SII activation was detected at shorter latency in patients than in controls. CONCLUSIONS: At SI, CLN5 is associated with a selective enhancement of the early cortical responses. We propose that the enlargement of N20m most likely reflects increased synchronous input from thalamus, whereas the altered morphology of the following responses may reflect defective interneuronal inhibition at the cortex. The enlargement of SII responses shows that the imbalance between excitation and inhibition in CLN5 extends outside the primary somatosensory areas
SN - 1388-2457
AD - Pediatric Neurology, Hospital for Children and Adolescents, University of Helsinki, Helsinki, Finland. leena.lauronen@hus.fi
UR - 12169332
ER -

TY - JOUR
ID - 1591
T1 - Injuries in public and private playgrounds: the relative contribution of structural, equipment and human factors
A1 - Petridou,E.
A1 - Sibert,J.
A1 - Dedoukou,X.
A1 - Skalkidis,I.
A1 - Trichopoulos,D.
Y1 - 2002///
N1 - Petridou, E. Sibert, J. Dedoukou, X. Skalkidis, I. Trichopoulos, D
Acta paediatrica (Oslo, Norway : 1992)
bgc, 9205968
IM
Journal Article
English
KW - MEDLINE
KW - Accident Prevention
KW - Accidental Falls/sn [Statistics & Numerical Data]
KW - Adolescent
KW - Age Distribution
KW - Case-Control Studies
KW - Child
KW - Child Welfare
KW - Preschool
KW - Cluster Analysis
KW - Consumer Product Safety
KW - Female
KW - Fractures

KW - Bone/ep [Epidemiology]
KW - Greece/ep [Epidemiology]
KW - Humans
KW - Incidence
KW - Injury Severity Score
KW - Leisure Activities
KW - Logistic Models
KW - Male
KW - Odds Ratio
KW - Play and Playthings
KW - Privatization
KW - Probability
KW - Risk Factors
KW - Sex Distribution
KW - Wounds and Injuries/di [Diagnosis]
KW - Wounds and Injuries/ep [Epidemiology]
RP - NOT IN FILE
SP - 691
EP - 697
JF - Acta Paediatrica
JA - Acta Paediatr
VL - 91
IS - 6
CY - Norway
N2 - UNLABELLED: The aim of this case-control study was to identify and quantify risk factors of injuries in playgrounds, where children spend an increasing amount of time in developed countries. The study took place in Greater Athens during 1999. A continuous Emergency Departments Injury Surveillance System (EDISS) of hospitals that cover about 30% of the children's time at risk in Greater Athens identified 777 injuries in public and private playgrounds out of a total of 17 497 injuries. Public playgrounds differ from private ones, because the former generally have more equipment, usually of greater height, with less resilient surfaces, and supervision relies mainly on parents or guardians. Patterns of type of playground use were assessed in a sample of 294 children from the same study base who served as a control group in a hierarchical case-control design. The annual incidence of playground injuries in Greater Athens was about 7 in 1000 among boys and 4 in 1000 among girls, with a 2.2 times higher risk for an injury in public than in private playgrounds (95% confidence interval 1.61-3.07). Children in public vs private playgrounds had a statistically significant eight times higher odds for concussion and six times higher for open wounds, whereas the odds for long bone fractures were four and for other fractures two; swings, slides and seesaws were the types of equipment most frequently associated with injuries. It was further shown that supervision of children was suboptimal (< 60%) in both public and private playgrounds, and children in private playgrounds sustained an unduly high frequency of sprain/ dislocation injuries (odds ratio 1.75) because they were encouraged to play bare-footed. CONCLUSION: Close to 50% of playground injuries could be prevented by structural and equipment changes, while further reduction could be accomplished through simple measures including closer supervision and encouraging children to wear proper shoes and use protective equipment whenever necessary
SN - 0803-5253
AD - Department of Hygiene and Epidemiology, Athens University Medical School, Greece. epetrid@med.uoa.gr
UR - 12162604
ER -

TY - JOUR
ID - 1592
T1 - Ceramide-dominant barrier repair lipids alleviate childhood atopic dermatitis: changes in barrier function provide a sensitive indicator of disease activity
A1 - Chamlin,S.L.
A1 - Kao,J.
A1 - Frieden,I.J.
A1 - Sheu,M.Y.

A1 - Fowler,A.J.
A1 - Fluhr,J.W.
A1 - Williams,M.L.
A1 - Elias,P.M.
Y1 - 2002/08//
N1 - Chamlin, Sarah L. Kao, Jack. Frieden, Ilona J. Sheu, Mary Y. Fowler, Ashley J. Fluhr, Joachim W. Williams, Mary L. Elias, Peter M
Journal of the American Academy of Dermatology
hvg, 7907132
IM
Journal Article. Research Support, Non-U.S. Gov't. Research Support, U.S. Gov't, Non-P.H.S.. Research Support, U.S. Gov't, P.H.S.
English
KW - MEDLINE
KW - Adolescent
KW - Ceramides/ad [Administration & Dosage]
KW - Ceramides/pd [Pharmacology]
KW - Ceramides/tu [Therapeutic Use]
KW - Child
KW - Preschool
KW - Dermatitis
KW - Atopic/dt [Drug Therapy]
KW - Emollients/tu [Therapeutic Use]
KW - Female
KW - Humans
KW - Infant
KW - Male
KW - Severity of Illness Index
KW - Skin/de [Drug Effects]
KW - Treatment Outcome
KW - Water Loss
KW - Insensible
RP - NOT IN FILE
SP - 198
EP - 208
JF - Journal of the American Academy of Dermatology
JA - J Am Acad Dermatol
VL - 47
IS - 2
CY - United States
N2 - BACKGROUND: It is currently fashionable to consider atopic dermatitis (AD), like other inflammatory dermatoses, as immunologic in pathogenesis ("inside-outside" hypothesis). Accordingly, topical glucocorticoids and other immunosuppressive agents are mainstays of therapy, but the risk of toxicity from these agents is not insignificant, particularly in children. Alternatively, because stratum corneum (SC) permeability barrier function is also abnormal in AD, it has been hypothesized that the barrier abnormality could drive disease activity. Yet commonly used emollients and moisturizers do not correct the SC ceramide deficiency, the putative cause of the barrier abnormality. OBJECTIVES: We assessed the efficacy of a newly developed, ceramide-dominant, physiologic lipid-based emollient, when substituted for currently used moisturizers, in 24 children who were also receiving standard therapy for stubborn-to-recalcitrant AD. METHODS: All subjects continued prior therapy (eg, topical tacrolimus or corticosteroids), only substituting the barrier repair emollient for their prior moisturizer. Follow-up evaluations, which included severity scoring of atopic dermatitis (SCORAD) values and several biophysical measures of SC function, were performed every 3 weeks for 20 to 21 weeks. RESULTS: SCORAD values improved significantly in 22 of 24 patients by 3 weeks, with further progressive improvement in all patients between 6 and 20 or 21 weeks. Transepidermal water loss levels (TEWL), which were elevated over involved and uninvolved areas at entry, decreased in parallel with SCORAD scores and continued to decline even after SCORAD scores plateaued. Both SC integrity (cohesion) and hydration also improved slowly but significantly during therapy. Finally, the ultrastructure of the SC, treated with ceramide-dominant emollient,

revealed extracellular lamellar membranes, which were largely absent in baseline SC samples. CONCLUSION: These studies suggest that (1) a ceramide-dominant, barrier repair emollient represents a safe, useful adjunct to the treatment of childhood AD and (2) TEWL is at least as sensitive an indicator of fluctuations in AD disease activity as are SCORAD values. These studies support the outside-inside hypothesis as a component of pathogenesis in AD and other inflammatory dermatoses that are accompanied by a barrier abnormality
SN - 0190-9622
AD - Department of Dermatology, University of California, San Francisco, USA
UR - 12140465
ER -

TY - JOUR
ID - 1593
T1 - Friendships and early relationships: links to sexual initiation among American adolescents born to young mothers
A1 - Cooksey,E.C.
A1 - Mott,F.L.
A1 - Neubauer,S.A.
Y1 - 2002/05//
N1 - Cooksey, Elizabeth C. Mott, Frank L. Neubauer, Stefanie A
Perspectives on sexual and reproductive health
101140654
IM
Journal Article. Research Support, U.S. Gov't, P.H.S.
English
KW - MEDLINE
KW - Adolescent
KW - Adolescent Behavior/eh [Ethnology]
KW - Adolescent Behavior
KW - Adult
KW - African Americans
KW - Age Factors
KW - Child
KW - European Continental Ancestry Group
KW - Female
KW - Friends/eh [Ethnology]
KW - Friends
KW - Humans
KW - Longitudinal Studies
KW - Male
KW - Maternal Age
KW - Questionnaires
KW - Regression Analysis
KW - Sex Factors
KW - Sexual Behavior/eh [Ethnology]
KW - Sexual Behavior/sn [Statistics & Numerical Data]
KW - United States/ep [Epidemiology]
RP - NOT IN FILE
SP - 118
EP - 126
JF - Perspectives on Sexual & Reproductive Health
JA - Perspect Sex Reprod Health
VL - 34
IS - 3
CY - United States
N2 - CONTEXT: Preadolescent friendships and early teenage dating relationships have implications for adolescent sexual initiation that may differ by race and gender. METHODS: Data on participants in the National Longitudinal Survey of Youth and their children are used to profile friendship and dating patterns among a

sample of youth born to relatively young mothers. Logistic regression analyses examine whether these patterns predict early sexual initiation, and whether there are differences associated with gender and race. RESULTS: As youth moved from late childhood to mid-adolescence, they shifted from having almost exclusively same-sex, same-grade friends to having more relationships with persons who are of the opposite sex and older. By ages 15-16,34% had had sexual intercourse; the proportion was significantly higher among blacks (45%) than among others (31%). Most adolescents reported neither frequent dating nor a steady partner by ages 15-16, although the prevalence of such reports was related to friendship patterns in late childhood. Twelve percent of youth who initiated sex in early adolescence did so outside of a dating relationship. For most subgroups examined, the odds of initiating intercourse during early adolescence were associated with going steady, but not with frequency of dating. CONCLUSIONS: Prior social networking is an important element in predicting early sexual activity. Overall, youth whose mothers gave birth at young ages remain sexually inexperienced into middle adolescence, but certain subgroups are more likely than others to initiate early sexual activity
SN - 1538-6341
AD - The Ohio State University, Columbus, USA. Cooksey.1@osu.edu
UR - 12137125
ER -

TY - JOUR
ID - 1594
T1 - One- and two-year predictors of decline in physical activity among inner-city schoolchildren
A1 - Barnett,T.A.
A1 - O'Loughlin,J.
A1 - Paradis,G.
Y1 - 2002/08//
N1 - Barnett, Tracie A. O'Loughlin, Jennifer. Paradis, Gilles
American journal of preventive medicine
8704773, apl
IM
Journal Article. Research Support, Non-U.S. Gov't
English
KW - MEDLINE
KW - Age Factors
KW - Canada
KW - Child
KW - Cohort Studies
KW - Female
KW - Humans
KW - Male
KW - Physical Fitness
KW - Primary Prevention
KW - Prospective Studies
KW - Questionnaires
KW - Residence Characteristics
KW - Risk Factors
KW - Statistics as Topic
KW - Time Factors
KW - Urban Population
RP - NOT IN FILE
SP - 121
EP - 128
JF - American Journal of Preventive Medicine
JA - Am J Prev Med
VL - 23
IS - 2
CY - Netherlands
N2 - BACKGROUND: Alarming secular declines in physical activity (PA) have been observed among youth over the last decade. A better understanding of the predictors of these declines is crucial to identifying those

children most at risk and to developing interventions that target youth before the onset of decline. This report identifies 1- and 2-year predictors of decline in PA among fourth- and fifth-grade students from inner-city neighborhoods in Montreal, Canada. METHODS: Data for this study were collected in classroom questionnaires each May/June from 1993 to 1997. Analyses for this paper were completed in 2001. The cohort included active (at least one PA per day) children with baseline and 1-year (n =1873) or 2-year (n =509) follow-up data. RESULTS: In boys, 1-year predictors of decline to an inactive status identified in generalized estimating equations analysis included moderate (vs high) baseline PA (odds ratio [OR]=1.66, 95% confidence interval [CI]=0.91-3.05); low PA self-efficacy (OR=1.67, 95% CI=1.03-2.71); born outside Canada (OR=2.13; 95% CI=1.31-3.46); Asian origin (OR=1.81; 95% CI=1.03-3.16) and no participation in school teams (OR=1.81, 95% CI=0.93-3.55). In girls, these 1-year predictors included moderate PA (OR=1.91, 95% CI=1.10-3.32); low PA self-efficacy (OR=1.70, 95% CI=1.15-2.49); watching four or more TV programs per day (OR=1.40, 95% CI=0.97-2.02); mother unemployed (OR=1.54, 95% CI=1.07-2.23); and grade five (vs grade four) (OR=1.35, 95% CI=0.94-1.93). Two-year predictors in boys included moderate baseline PA (OR=2.52, 95% CI=0.84-7.50), and born outside Canada (OR=1.96, 95% CI=0.91-4.20). In girls, these 2-year predictors included moderate baseline PA (OR=2.75, 95% CI=1.01-7.49); no participation in school teams (OR=2.14, 95% CI=0.92-5.00); watching four or more TV programs per day (OR=1.93, 95% CI=0.99-3.74); and born outside Canada (OR=1.85, 95% CI=0.96-3.55). CONCLUSIONS: Reduced TV viewing among girls and increased participation in school sports teams in boys and girls may prevent declines in PA among pre-adolescents from inner-city neighborhoods
SN - 0749-3797
AD - Public Health Directorate, Regie regionale de la sante et des services sociaux de Montreal-Centre (Barnett, O'Loughlin, Paradis), Montreal, Quebec, Canada. tbernett@santepub-mtl.qc.ca
UR - 12121800
ER -

TY - JOUR
ID - 1595
T1 - Social integration of children with epilepsy in rural India
A1 - Pal,D.K.
A1 - Chaudhury,G.
A1 - Sengupta,S.
A1 - Das,T.
Y1 - 2002/06//
N1 - Pal, Deb K. Chaudhury, Gautam. Sengupta, Suryanil. Das, Tulika
Social science & medicine (1982)
ut9, 8303205
IM
Journal Article. Research Support, Non-U.S. Gov't
English
KW - MEDLINE
KW - Activities of Daily Living
KW - Adolescent
KW - Child
KW - Preschool
KW - Cross-Sectional Studies
KW - Disabled Children/px [Psychology]
KW - Disabled Children/rh [Rehabilitation]
KW - Epilepsy/px [Psychology]
KW - Epilepsy/rh [Rehabilitation]
KW - Family/px [Psychology]
KW - Female
KW - Humans
KW - India
KW - Interpersonal Relations
KW - Leisure Activities
KW - Male
KW - Peer Group

KW - Questionnaires
KW - Rural Population
KW - Social Adjustment
RP - NOT IN FILE
SP - 1867
EP - 1874
JF - Social Science & Medicine
JA - Soc Sci Med
VL - 54
IS - 12
CY - England
N2 - Only a small fraction of people with epilepsy in developing countries has access to medical facilities. Even with effective treatment, their psychosocial needs are often overlooked in the absence of obvious disability. In rural areas, community-based rehabilitation programmes assist in the integration of people with disabilities into employment and the community. However, the functional impairment associated with epilepsy is not well recognised in intervention programmes in developing countries. We report, for the first time, the social activities of children with epilepsy and their peers in rural India. We employed a cross-sectional design using a new age and sex-specific social activity questionnaire. Population screening in the context of a community-based rehabilitation programme identified 88 children with epilepsy and 250 randomly selected controls. A trained interviewer administered the questionnaire to mothers in Bengali. Girls' activities were principally domestic, whilst boys' were mostly outdoors and involved peers. All groups of children with epilepsy had significant social deficits, equally for boys and girls in the age range from 2 to 18 years ($p < 0.05$). Boys with epilepsy had limited peer group activities, and parents conferred fewer responsibilities to school age and adolescent children compared to controls. The nature and degree of deficits were beyond the constraints imposed by neurological impairment. Our findings in pre-schoolers were consistent with parental attitudes of overprotection found in previous research. We conclude that social integration needs active and early promotion among children with epilepsy. The assessment of remediable risk and protective factors in the family and community is an important practical area for research in community-based rehabilitation
SN - 0277-9536
AD - Neurosciences Unit, Institute of Child Health, University College, London, UK.
deb@shallot.salad.mssm.edu
UR - 12113441
ER -

TY - JOUR
ID - 1596
T1 - Temperature-related overestimation of energy expenditure, based on heart-rate monitoring in obese boys
A1 - Kriemler,S.
A1 - Hebestreit,H.
A1 - Bar-Or,O.
Y1 - 2002/07//
N1 - Kriemler, Susi. Hebestreit, Helge. Bar-Or, Oded
European journal of applied physiology
100954790, dos
IM
Clinical Trial. Journal Article
English
KW - MEDLINE
KW - Adolescent
KW - Child
KW - Climate
KW - Energy Metabolism
KW - Exercise Test
KW - Heart Rate
KW - Hot Temperature
KW - Humans
KW - Male

KW - Obesity/pp [Physiopathology]
KW - Pulmonary Ventilation
KW - Reproducibility of Results
KW - Seasons
KW - Sensitivity and Specificity
KW - Sleep
KW - Temperature
KW - Wakefulness
RP - NOT IN FILE
SP - 245
EP - 250
JF - European Journal of Applied Physiology
JA - Eur J Appl Physiol
VL - 87
IS - 3
CY - Germany
N2 - An equation has been developed to reduce the error in predicting energy expenditure (EE) from heart rate (HR) monitoring, by correcting for climate-related HR increases. To evaluate the effects of such a correction in a "real life" situation, a group of 9- to 14-year-old obese boys [mean (SD) 36.6 (3.3)% body fat; n=14] was monitored for 6 days during the summertime. An activity interview was taken daily. During times of outdoor activities, measurements of HR were corrected for the influence of ambient temperature (T) using the aforementioned equation. Time spent outdoors was 196.7 (21.1) min/day at a T of 17-34 degrees C. Temperature correction reduced the time spent at HR>140 beats/min and >160 beats/min by 20-25% during outdoor activity. Estimated outdoor EE corrected for the influence of temperature was 2.46 (2.37) MJ; the uncorrected value was 2.68 (2.45) MJ. The respective values for the waking hours and over 24 h were 7.34 (2.77) MJ versus 7.55 (2.81) MJ and 10.83 (2.72) MJ versus 11.05 (2.77) MJ, respectively. The excessive HR measured in a warm summer climate could explain 8.8 (3.5)% of EE during outdoor activities, which resulted in a 2.9 (2.7)% overestimate of EE during daily waking hours and a 1.9 (1.8)% overestimate of the total 24-h EE. Thus, the influence of temperature on HR may, at least in part, explain the previously reported overestimate of EE calculated using the HR/oxygen consumption relationship versus that calculated using the doubly labelled water method. We recommend correcting HR for the influence of climate in studies using HR profiles to determine outdoor physical activity levels and/or EE during summertime. However, when total daily EE or waking hours EE is assessed, even in subjects who are engaging in long intervals of outdoor play, the temperature correction required is minimal
SN - 1439-6319
AD - Children's Exercise and Nutrition Centre, McMaster University, Hamilton, Ontario, Canada. kriemlers@swissonline.ch
UR - 12111285
ER -

TY - JOUR
ID - 1597
T1 - Erythrocyte thiopurine methyl transferase assessment prior to azathioprine use in the UK
A1 - Holme,S.A.
A1 - Duley,J.A.
A1 - Sanderson,J.
A1 - Routledge,P.A.
A1 - Anstey,A.V.
Y1 - 2002/07//
N1 - Holme, S A. Duley, J A. Sanderson, J. Routledge, P A. Anstey, A V
QJM : monthly journal of the Association of Physicians
9438285, B4V
IM
Journal Article. Research Support, Non-U.S. Gov't
English
KW - MEDLINE
KW - Adolescent

KW - Adult
KW - Aged
KW - 80 and over
KW - Azathioprine/ae [Adverse Effects]
KW - Child
KW - Preschool
KW - Erythrocytes/en [Enzymology]
KW - Female
KW - Great Britain
KW - Humans
KW - Immunosuppressive Agents/ae [Adverse Effects]
KW - Infant
KW - Male
KW - Methyltransferases/an [Analysis]
KW - Middle Aged
KW - Retrospective Studies
RP - NOT IN FILE
SP - 439
EP - 444
JF - Qjm
VL - 95
IS - 7
CY - England
N2 - BACKGROUND: Individuals with low activity of a key metabolic enzyme, thiopurine methyl transferase (TPMT), are more susceptible to azathioprine-induced myelosuppression. Aim: To determine the pattern of use of TPMT activity estimation, with respect to azathioprine use, by medical practitioners in the UK. DESIGN: Retrospective analysis of assay use. METHODS: We analysed all test results (n=3291), and patient and practitioner details, from inception of TPMT assay in 1990 to the end of December 2000, held at the Purine Research Laboratory, Guy's Hospital, London. Patient details were anonymized. Repeat analyses and requests from outside the UK were excluded. RESULTS: The male:female ratio was approximately equal and the mean age was 46.6 (range 0.5-97) years. Thirteen different medical specialities requested assays; Dermatology and Gastroenterology were the most frequent users, together accounting for 86% of requests. The numbers of centres requesting the assay varied widely both within and between different specialities. Some 80% of individuals had normal TPMT activity, 9% enzymic activity above normal, and 10% low activity. Fifteen had no detectable enzymic activity: 0.45% (1:220) of the study population. DISCUSSION: This incidence of undetectable enzyme activity is significantly higher than the previously reported level of 1:300 derived from smaller studies, and makes the economics of screening more attractive
SN - 1460-2725
AD - Department of Dermatology, Royal Gwent Hospital, Newport, Purine Research Laboratory and Department of Gastroenterology, Guy's & St Thomas' Hospitals Trust, London, UK. saholme@hotmail.com
UR - 12096148
ER -

TY - JOUR
ID - 1598
T1 - Helmet use: a survey of 4 common childhood leisure activities
A1 - Forjuoh,S.N.
A1 - Fiesinger,T.
A1 - Schuchmann,J.A.
A1 - Mason,S.
Y1 - 2002/07//
N1 - Forjuoh, Samuel N. Fiesinger, Troy. Schuchmann, John A. Mason, Saundra
Archives of pediatrics & adolescent medicine
9422751, bwf
AIM, IM
Journal Article. Research Support, Non-U.S. Gov't
English

KW - MEDLINE
KW - Accident Prevention
KW - Adolescent
KW - Bicycling/px [Psychology]
KW - Child
KW - Child Welfare
KW - Preschool
KW - Craniocerebral Trauma/et [Etiology]
KW - Craniocerebral Trauma/pc [Prevention & Control]
KW - Cross-Sectional Studies
KW - Female
KW - Head Protective Devices/ut [Utilization]
KW - Health Behavior
KW - Health Education
KW - Humans
KW - Male
KW - Observation
KW - Play and Playthings/px [Psychology]
KW - Questionnaires
KW - Risk
KW - Skating/px [Psychology]
KW - Texas
KW - United States
RP - NOT IN FILE
SP - 656
EP - 661
JF - Archives of Pediatrics & Adolescent Medicine
JA - Arch Pediatr Adolesc Med
VL - 156
IS - 7
CY - United States
N2 - OBJECTIVE: To compare the distribution and observation of helmet use patterns among 4 common childhood leisure activities-bicycle riding, in-line skating, skateboarding, and scooter riding-in a setting with no helmet use legislation. DESIGN: A cross-sectional, unobtrusive, direct observation of children engaged in 1 of the 4 activities was conducted during an 8-week period. SETTING: Eight communities of at least 1000 persons each in a 2-county Central Texas area. MAIN OUTCOME MEASURES: Prevalence of activity, helmet use, and correct helmet use. RESULTS: Of 841 children observed, most rode bicycles (74.8%); only 4.2% rode skateboards. They included 127 children estimated to be younger than 6 years (15.1%) and 495 estimated to be aged between 6 and 12 years (58.9%). Most were male (67.3%) and observed in urban communities (61.7%). The locations of highest concentration of the 4 activities were minor streets for bicycle riding, playgrounds for in-line skating, and sidewalks for skateboarding and scooter riding. The rate of overall helmet use was 13.6% and did not vary significantly by activity: in-line skating, 18.2%; skateboarding, 14.3%; bicycle riding, 13.5%; and scooter riding, 11.5%. However, the observed correct helmet use prevalence of 72.6% differed significantly by activity. Most children riding scooters wore their helmets incorrectly. Helmet use was positively associated with helmet use by accompanying children and/or adults. CONCLUSIONS: While bicycle riding was the most popular activity observed, helmet use was most prevalent among in-line skaters and least prevalent among scooter riders, many of whom also wore them incorrectly. Observed helmet use was influenced mostly by adult and peer helmet use, indicating that public education and interventions should target children and their parents as well as scooter riders
SN - 1072-4710
AD - Department of Family Medicine, Scott and White Memorial Hospital, Scott, Sherwood, and Brindley Foundation, Texas A & M University System Health Science Center, College of Medicine, Temple, USA. sforjuoh@swmail.sw.org
UR - 12090831
ER -

TY - JOUR

ID - 1599
T1 - Early elementary school intervention to reduce conduct problems: a randomized trial with Hispanic and non-Hispanic children
A1 - Barrera,M.,Jr.
A1 - Biglan,A.
A1 - Taylor,T.K.
A1 - Gunn,B.K.
A1 - Smolkowski,K.
A1 - Black,C.
A1 - Ary,D.V.
A1 - Fowler,R.C.
Y1 - 2002/06//
N1 - Barrera, Manuel Jr. Biglan, Anthony. Taylor, Ted K. Gunn, Barbara K. Smolkowski, Keith. Black, Carol. Ary, Dennis V. Fowler, Rollen C
Prevention science : the official journal of the Society for Prevention Research
d4q, 100894724
IM
Clinical Trial. Comparative Study. Journal Article. Randomized Controlled Trial. Research Support, U.S. Gov't, P.H.S.
English
KW - MEDLINE
KW - Child
KW - Child Behavior/eh [Ethnology]
KW - Child Behavior/px [Psychology]
KW - Conduct Disorder/eh [Ethnology]
KW - Conduct Disorder/pc [Prevention & Control]
KW - Dyslexia/eh [Ethnology]
KW - Dyslexia/pc [Prevention & Control]
KW - Early Intervention (Education)
KW - European Continental Ancestry Group/px [Psychology]
KW - Female
KW - Hispanic Americans/px [Psychology]
KW - Humans
KW - Juvenile Delinquency/pc [Prevention & Control]
KW - Male
KW - Risk Factors
KW - Socialization
KW - United States
RP - NOT IN FILE
SP - 83
EP - 94
JF - Prevention Science
JA - Prev Sci
VL - 3
IS - 2
CY - United States
N2 - Children's aggressive behavior and reading difficulties during early elementary school years are risk factors for adolescent problem behaviors such as delinquency, academic failure, and substance use. This study determined if a comprehensive intervention that was designed to address both of these risk factors could affect teacher, parent, and observer measures of internalizing and externalizing problems. European American (n = 116) and Hispanic (n = 168) children from 3 communities who were selected for aggressiveness or reading difficulties were randomly assigned to an intervention or no-intervention control condition. Intervention families received parent training, and their children received social behavior interventions and supplementary reading instruction over a 2-year period. At the end of intervention, playground observations showed that treated children displayed less negative social behavior than controls. At the end of a 1-year follow-up, treated children showed less teacher-rated internalizing and less parent-rated coercive and antisocial behavior than controls. The study's limitations and implications for prevention are discussed

SN - 1389-4986
AD - Oregon Research Institute, Eugene, USA. manuel.barrera@asu.edu
UR - 12088139
ER -

TY - JOUR
ID - 1600
T1 - An analysis of factors that influence personal exposure to nitrogen oxides in residents of Richmond, Virginia
A1 - Zipprich,J.L.
A1 - Harris,S.A.
A1 - Fox,J.C.
A1 - Borzelleca,J.F.
Y1 - 2002/07//
N1 - Zipprich, Jennifer L. Harris, Shelley A. Fox, J Clifford. Borzelleca, Joseph F
Journal of exposure analysis and environmental epidemiology
bjn, 9111438
IM
Journal Article. Research Support, Non-U.S. Gov't. Research Support, U.S. Gov't, Non-P.H.S.
English
KW - MEDLINE
KW - Activities of Daily Living
KW - Adult
KW - Air Pollutants/an [Analysis]
KW - Air Pollution
KW - Indoor/an [Analysis]
KW - Child
KW - Environmental Exposure
KW - Female
KW - Humans
KW - Male
KW - Nitrogen Oxides/an [Analysis]
KW - Risk Factors
KW - Virginia
RP - NOT IN FILE
SP - 273
EP - 285
JF - Journal of Exposure Analysis and Environmental Epidemiology
JA - J Expo Anal Environ Epidemiol
VL - 12
IS - 4
CY - United States
N2 - Nitrogen oxides (NO(x)) are ubiquitous pollutants in outdoor and indoor air. However, epidemiologic studies that evaluate health effects associated with NO(x) commonly rely upon outdoor concentrations of NO(x), nitrogen dioxide (NO(2)), or residence characteristics as surrogates for personal exposure. In this study, personal exposures (48 h) and corresponding indoor and outdoor concentrations of nitric oxide (NO), NO(2), and NO(x) were measured (July-September) in 39 adults and 9 children from 23 households in Richmond, Virginia, using Ogawa passive NO(x) monitors. Demographic, time-activity patterns, and household data were collected by questionnaire and used to develop exposure prediction models. Adults had higher NO(2), NO, and NO(x) exposures (means: 16, 63, and 79 ppb, respectively) than children (13, 49, and 62 ppb). Measurements taken in bedrooms (18, 57, and 75 ppb) and living rooms (19, 65, and 84 ppb) surpassed measurements taken outdoors (15, 21, and 36 ppb). In indoor locations, NO(x) concentrations were influenced largely by NO, and consequently, personal exposure prediction models for NO(x) were reflective of models for NO. Statistical models that best predicted personal exposures included indoor measurements; outdoor measurements contributed relatively little to personal exposure. Close to 70% of the variation in personal NO(2) and NO(x) exposure was explained by two variable models (bedroom NO(2) and time spent in other indoor locations; bedroom NO(x) and time spent in kitchen). Given appropriate resources, measurement error in epidemiologic

studies can be reduced significantly with the use of personal exposure measurements or prediction models developed from indoor measurements and survey data
SN - 1053-4245
AD - Center for Environmental Studies, Virginia Commonwealth University, Richmond, Virginia 23248, USA
UR - 12087434
ER -

TY - JOUR
ID - 1601
T1 - Environmental lead contamination in Miami inner-city area
A1 - Gasana,J.
A1 - Chamorro,A.
Y1 - 2002/07//
N1 - Gasana, Janvier. Chamorro, Armando
Journal of exposure analysis and environmental epidemiology
bjn, 9111438
IM
Journal Article. Research Support, Non-U.S. Gov't
English
KW - MEDLINE
KW - Child
KW - Child Welfare
KW - Cities
KW - Construction Materials
KW - Dust
KW - Environmental Exposure
KW - Environmental Monitoring
KW - Florida
KW - Hispanic Americans
KW - Housing
KW - Humans
KW - Lead/an [Analysis]
KW - Lead Poisoning/et [Etiology]
KW - Risk Assessment
KW - Soil Pollutants
KW - Urban Population
RP - NOT IN FILE
SP - 265
EP - 272
JF - Journal of Exposure Analysis and Environmental Epidemiology
JA - J Expo Anal Environ Epidemiol
VL - 12
IS - 4
CY - United States
N2 - OBJECTIVES: The purpose of the study was to evaluate the magnitude of environmental lead contamination in the downtown area of Miami. METHODS: Lead inspections took place at 121 homes in Little Haiti and Liberty City and involved the collection of representative samples from floors, window wells, tap water, soil and air. Community health workers (CHWs) trained in interview and safety techniques went from door to door to enlist participation. On-site investigations were tailored to areas most utilized by children under the age of 6 years. The presence of lead-containing paint was also investigated in situ via X-ray fluorescence (XRF) analysis. RESULTS: Of the sampling areas, the window wells area had the most abundant occurrence of lead. On analysis, 24% of sites returned window well samples with lead levels above Department of Housing and Urban Development (HUD) guidelines. Of the soil samples, the playgrounds around the house had the highest concentration of lead. Soil sampling demonstrated that 27.5% of sites returned samples with lead levels (400 to 1600 ppm) in excess of HUD/Environmental Protection Agency (EPA) standards. Positive XRF readings in one or more components were returned by 18% of sites. CONCLUSIONS: More than half of the houses in these two neighborhoods exhibited unacceptably high levels of lead dust and soil in areas where

children live and play. Limitations of this study did not allow the assessment of how many children in this area are affected. A more comprehensive study including other areas of Miami-Dade County with older housing stock is recommended
SN - 1053-4245
AD - Department of Public Health, Florida International University, 3000 NE 151 St., ACI 394H, North Miami, Florida 33181, USA. gasanaj@fiu.edu
UR - 12087433
ER -

TY - JOUR
ID - 1602
T1 - Differences between Japanese pre-school and school-age pedestrian mortality and morbidity trends
A1 - Nakahara,S.
A1 - Wakai,S.
Y1 - 2002/05//
N1 - Nakahara, S. Wakai, S
Public health
qi7, 0376507
IM
Comparative Study. Journal Article
English
KW - MEDLINE
KW - Accidents
KW - Traffic/td [Trends]
KW - Child
KW - Preschool
KW - Female
KW - Humans
KW - Infant
KW - Infant Mortality/td [Trends]
KW - Japan/ep [Epidemiology]
KW - Male
KW - Morbidity/td [Trends]
KW - Walking
RP - NOT IN FILE
SP - 166
EP - 172
JF - Public Health
VL - 116
IS - 3
CY - England
N2 - The risk of pedestrian injury during childhood in industrialised countries is decreasing in a trend that is related to exposure to traffic. Examining the differences between age groups and their behaviour provides an insight into exposure to risks. Using data for the period 1968-1998, we compared the changes in pedestrian mortality and morbidity in pre-school children (aged 0-6) with those of school-age children (aged 7-12). Pedestrian mortality and morbidity decreased in children of all ages in the early 1970s. However, after the mid-1970s, both mortality and morbidity continued to decrease only in pre-schoolers. In school-age children, mortality continued to decrease but morbidity did not change. These age-related differences in mortality and morbidity indicate that over the past 30 y the environment for child pedestrians did not become safer. Instead, some of the decrease in children's injuries was probably achieved at the expense of their outdoor activities. Efforts to protect children from vehicular traffic should focus on changing the outdoor environment rather than on restricting children's activities
SN - 0033-3506
AD - Department of International Community Health, Graduate School of Medicine, The University of Tokyo, Japan. shinji@m.u-tokyo.ac.jp
UR - 12082599
ER -

TY - JOUR
ID - 1603
T1 - Parental permission for children's independent outdoor activities. Implications for injury prevention
A1 - Soori,H.
A1 - Bhopal,R.S.
Y1 - 2002/06//
N1 - Soori, H. Bhopal, R S
European journal of public health
d0w, 9204966
IM
Journal Article
English
KW - MEDLINE
KW - Accident Prevention
KW - Chi-Square Distribution
KW - Child
KW - Child Welfare
KW - Cross-Sectional Studies
KW - Female
KW - Humans
KW - Leisure Activities
KW - Logistic Models
KW - Male
KW - Parent-Child Relations
KW - Questionnaires
KW - Risk Factors
KW - Risk-Taking
KW - Safety
RP - NOT IN FILE
SP - 104
EP - 109
JF - European Journal of Public Health
JA - Eur J Public Health
VL - 12
IS - 2
CY - England
N2 - BACKGROUND: Parental supervision is an important factor related to childhood injuries. However, little research has been done on; what outdoor activities children believe they are allowed or their parents allow them to do; parents' ideas about the age children can do activities, and the age they would allow their children to do them. This study described children's independent outdoor activities after-school, comparing age groups (school year 5 and 3), boys with girls, and the less socio-economically well off with the better off. METHODS: A cross-sectional study using questionnaires completed by 476 children aged seven and nine years and 471 parents in nine primary schools in Newcastle-upon-Tyne, England. RESULTS: Children reported they were permitted many outdoor activities such as cycling (78.8%), playing (78.6%) and roller skating in the street (62.7%). Parents of older children, boys, and more socio-economically deprived children were more likely than their comparison groups to allow children to do outdoor activities independently e.g. 34% of 9-year-olds were allowed to cross a road without an adult compared with 8% of 7-year-olds. Mostly, parents of boys and socio-economically deprived children, perceived children can do outdoor activities slightly younger than parents of girls and the socio-economically better off. CONCLUSION: Children and parents value and support children's independent outdoor activity. Differences in children's and parents' understanding of what activities are permissible and in their judgements about the appropriate age for children's independent activities, may partly underline variations by sex, age and socio-economic status in injury patterns
SN - 1101-1262
AD - Department of Community Medicine, University of Ahwaz Medical Sciences, Ahwaz, Iran
UR - 12073747
ER -

TY - JOUR
ID - 1604
T1 - Multichannel auditory brainstem implant: update on performance in 61 patients
A1 - Otto,S.R.
A1 - Brackmann,D.E.
A1 - Hitselberger,W.E.
A1 - Shannon,R.V.
A1 - Kuchta,J.
Y1 - 2002/06//
N1 - Otto, Steven R. Brackmann, Derald E. Hitselberger, William E. Shannon, Robert V. Kuchta, Johannes
Journal of neurosurgery
jd3, 0253357
AIM, IM
Journal Article
English
KW - MEDLINE
KW - Adolescent
KW - Adult
KW - Aged
KW - Brain Stem/pp [Physiopathology]
KW - Brain Stem/su [Surgery]
KW - Child
KW - Cochlear Nerve/pp [Physiopathology]
KW - Cochlear Nerve/su [Surgery]
KW - Deafness/et [Etiology]
KW - Deafness/pp [Physiopathology]
KW - Deafness/su [Surgery]
KW - Electric Stimulation
KW - Equipment Design
KW - Female
KW - Follow-Up Studies
KW - Humans
KW - Male
KW - Middle Aged
KW - Neurofibromatosis 2/co [Complications]
KW - Neurofibromatosis 2/pp [Physiopathology]
KW - Neurofibromatosis 2/su [Surgery]
KW - Neuroma
KW - Acoustic/et [Etiology]
KW - Acoustic/pp [Physiopathology]
KW - Acoustic/su [Surgery]
KW - Outcome Assessment (Health Care)
KW - Postoperative Complications
KW - Prostheses and Implants
KW - Recovery of Function/ph [Physiology]
RP - NOT IN FILE
SP - 1063
EP - 1071
JF - Journal of Neurosurgery
JA - J Neurosurg Pediatrics
VL - 96
IS - 6
CY - United States
N2 - OBJECT: Neurofibromatosis Type 2 (NF2) has typically resulted in deafness after surgical removal of bilateral vestibular schwannomas (VSs). Cochlear implants are generally ineffective for this kind of deafness because of the loss of continuity in the auditory nerve after tumor removal. The first auditory brainstem implant

(ABI) in such a patient was performed in 1979 at the House Ear Institute, and this individual continues to benefit from electrical stimulation of the cochlear nucleus complex. In 1992, an advanced multichannel ABI was developed and a series of patients with NF2 received this implant to study the safety and efficacy of the device. METHODS: At the time of first- or second-side VS removal, patients received an eight-electrode array applied to the surface of the cochlear nucleus within the confines of the lateral recess of the fourth ventricle. The device was activated approximately 6 weeks after implantation. and patients were tested every 3 months for the 1st year after the initial stimulation, and annually thereafter. The protocol included a comprehensive battery of psychophysical and speech perception tests. CONCLUSIONS: The multichannel ABI proved to be effective and safe in providing useful auditory sensations in most patients with NF2. The ABI improved patients' ability to communicate compared with the lipreading-only condition, it allowed the detection and recognition of many environmental sounds, and in some cases it provided significant ability to understand speech by using just the sound from the ABI (with no lipreading cues). Its performance in most patients has continued to improve for up to 8 years after implantation
SN - 0022-3085
AD - House Ear Institute, Los Angeles, California 90057, USA. otto@hei.org
UR - 12066908
ER -

TY - JOUR
ID - 1605
T1 - Re: Phelan KJ, et al. Trends and patterns of playground injuries in United States children and adolescents. Ambulatory Pediatrics 2001;1:227-233
A1 - DeFrancesco,S.
Y1 - 2002///
N1 - DeFrancesco, Susan
Ambulatory pediatrics : the official journal of the Ambulatory Pediatric Association
101089367
IM
Comment. Letter
English
KW - MEDLINE
KW - Accidental Falls/sn [Statistics & Numerical Data]
KW - Child
KW - Humans
KW - Play and Playthings
KW - United States/ep [Epidemiology]
KW - Wounds and Injuries/ep [Epidemiology]
RP - NOT IN FILE
SP - 234
EP - author reply 235
JF - Ambulatory Pediatrics
JA - Ambul Pediatr
VL - 2
IS - 3
CY - United States
SN - 1530-1567
UR - 12049060
ER -

TY - JOUR
ID - 1606
T1 - Levels of agreement between parents' and children's reports of near work
A1 - Rah,M.J.
A1 - Mitchell,G.L.
A1 - Mutti,D.O.
A1 - Zadnik,K.
Y1 - 2002/07//

N1 - Rah, Marjorie J. Mitchell, G Lynn. Mutti, Donald O. Zadnik, Karla
Ophthalmic epidemiology
cg6, 9435674
IM
Journal Article. Research Support, Non-U.S. Gov't. Research Support, U.S. Gov't, P.H.S.
English
KW - MEDLINE
KW - Adolescent
KW - California/ep [Epidemiology]
KW - Child
KW - Female
KW - Humans
KW - Male
KW - Myopia/ep [Epidemiology]
KW - Myopia/et [Etiology]
KW - Observer Variation
KW - Parents
KW - Prevalence
KW - Questionnaires
KW - Reading
KW - Reproducibility of Results
KW - Risk Factors
KW - Work/sn [Statistics & Numerical Data]
KW - Work
RP - NOT IN FILE
SP - 191
EP - 203
JF - Ophthalmic Epidemiology
JA - Ophthalmic Epidemiol
VL - 9
IS - 3
CY - Netherlands
N2 - PURPOSE: To assess whether parents and children report the same information regarding children's near vision activities when given similar questionnaires. METHODS: Data from questionnaires administered to 406 children and their parents were analyzed to evaluate the agreement between parent and child reports. The questionnaires were completed during the 1994 testing of the Orinda Longitudinal Study of Myopia. Each child and parent was asked to categorize how often the child reads for pleasure. In addition, each subject was asked to estimate how many hours per week were spent in each of five visual activities. The weighted kappa statistic, paired t-tests, and polytomous logistic regression were used for analyses. RESULTS: A weighted kappa = 0.42 (moderate agreement) was found in the comparison of child-reported vs. parent-reported classification of reading. Agreement between parent- and child-reported number of hours spent doing homework (0.21), reading for pleasure (0.31), watching television (0.31), playing video games (0.31), and engaging in sports/outdoor activities (0.26) was fair for each activity. CONCLUSIONS: Although the results indicate fair to moderate levels of agreement, better methods of reporting near-work activities are needed for future myopia research
SN - 0928-6586
AD - New England College of Optometry, Boston, MA 02215, USA. rahm@ncopost.ne-optometry.edu
UR - 12045886
ER -

TY - JOUR
ID - 1607
T1 - Preschool and school age activities: comparison of urban and suburban populations
A1 - Damore,D.T.
Y1 - 2002/06//
N1 - Damore, Dorothy T
Journal of community health
7600747, hut

IM
Comparative Study. Journal Article. Research Support, Non-U.S. Gov't. Research Support, U.S. Gov't, P.H.S.
English
KW - MEDLINE
KW - Camping/sn [Statistics & Numerical Data]
KW - Child
KW - Preschool
KW - Ethnic Groups
KW - Exercise
KW - Humans
KW - Leisure Activities
KW - Libraries/ut [Utilization]
KW - New York City
KW - Prospective Studies
KW - Questionnaires
KW - Reading
KW - Recreation
KW - Social Environment
KW - Sports/sn [Statistics & Numerical Data]
KW - Suburban Population/sn [Statistics & Numerical Data]
KW - Television/ut [Utilization]
KW - Urban Population/sn [Statistics & Numerical Data]
RP - NOT IN FILE
SP - 203
EP - 211
JF - Journal of Community Health
JA - J Community Health
VL - 27
IS - 3
CY - United States
N2 - The objective of this study was to compare urban and suburban preschool and school age activities. A prospective survey using a convenience sample was conducted at one urban and one suburban primary care pediatric office. Questionnaires were completed for 66 urban preschool children, 70 suburban preschool children, 57 urban school age children and 61 suburban school age children during the school year. Also, questionniaires were completed for 63 suburban school age children during the summer. The suburban preschool children spent more time outdoors, were read to more frequently, visited the library more frequently and more often attended summer camp. The suburban school age children spent more time outdoors, more frequently participated in a community sport league and more often attended summer camp. The urban school age children watched more television or videos. During the summer, suburban school age children spent more time outdoors, while during the school year, suburban school age children used the library more frequently. Important differences exist between the activities of urban and suburban children in two practices in the New York metropolitan area. Pediatricians caring for urban children may have an important opportunity to promote participation in sports and educational activities
SN - 0094-5145
AD - Weill Cornell Medical College, New York, New York, USA. DJT2001@med.cornell.edu
UR - 12027270
ER -

TY - JOUR
ID - 1608
T1 - The Environmental Protection Agency's National SunWise School Program: sun protection education in US schools (1999-2000)
A1 - Geller,A.C.
A1 - Cantor,M.
A1 - Miller,D.R.
A1 - Kenausis,K.
A1 - Rosseel,K.

A1 - Rutsch,L.
A1 - Brooks,D.R.
A1 - Zhang,Z.
A1 - Demierre,M.F.
Y1 - 2002/05//
N1 - Geller, Alan C. Cantor, Maura. Miller, Donald R. Kenausis, Kristin. Rosseel, Kevin. Rutsch, Linda. Brooks, Daniel R. Zhang, Zi. Demierre, Marie France
Journal of the American Academy of Dermatology
hvg, 7907132
IM
Comparative Study. Journal Article. Research Support, U.S. Gov't, Non-P.H.S.
English
KW - MEDLINE
KW - Chi-Square Distribution
KW - Child
KW - Preschool
KW - Confidence Intervals
KW - Female
KW - Government Programs/og [Organization & Administration]
KW - Health Education/og [Organization & Administration]
KW - Health Knowledge
KW - Attitudes
KW - Practice
KW - Humans
KW - Male
KW - Melanoma/pc [Prevention & Control]
KW - Probability
KW - Program Evaluation
KW - School Health Services/og [Organization & Administration]
KW - Skin Neoplasms/pc [Prevention & Control]
KW - Sunburn/pc [Prevention & Control]
KW - Sunscreening Agents
KW - United States
KW - United States Environmental Protection Agency
RP - NOT IN FILE
SP - 683
EP - 689
JF - Journal of the American Academy of Dermatology
JA - J Am Acad Dermatol
VL - 46
IS - 5
CY - United States
N2 - BACKGROUND: Melanoma, the most fatal form of skin cancer, is rising at a rate faster than that of all preventable cancers except lung cancer in the United States. Childhood exposure to ultraviolet (UV) light increases the risk for skin cancer as an adult; thus starting positive sun protection habits early may be key to reducing incidence. METHODS: We evaluated the US Environmental Protection Agency's SunWise School Program, a national, environmental education program for sun safety of children in primary and secondary schools (kindergarten through eighth grade). The program was evaluated with surveys administered to participating students. An identical 18-question, self-administered survey was completed by students (median age, 10 years) in the classroom before and immediately after the SunWise educational program. RESULTS: Surveys were completed by students in 40 schools before (pretests; n = 1894) and after the program was presented (post-tests; n = 1815). Significant improvement was noted for the 3 knowledge variables: appropriate type of sunscreen to be used for outdoor play, highest UV Index number, and need for hats and shirts outside. Intentions to play in the shade increased from 73% to 78% (P <.001), with more modest changes in intentions to use sunscreen. Attitudes regarding healthiness of a tan also decreased significantly. CONCLUSIONS: Brief, standardized sun protection education can be efficiently interwoven into school health education and result in improvements in knowledge and positive intentions for sun protection

SN - 0190-9622
AD - Department of Dermatology, Boston University School of Medicine, Boston, MA 02118, USA
UR - 12004307
ER -

TY - JOUR
ID - 1609
T1 - Health-related quality of life after 5-12 years of continent ileal urostomy (Kock reservoir) in children and adolescents
A1 - bd-el-Gawad,G.
A1 - Abrahamsson,K.
A1 - Hellstrom,A.L.
A1 - Hjalmas,K.
A1 - Hanson,E.
Y1 - 2002/02//
N1 - Abd-el-Gawad, G. Abrahamsson, K. Hellstrom, A L. Hjalmas, K. Hanson, E
Scandinavian journal of urology and nephrology
uda, 0114501
IM
Journal Article. Research Support, Non-U.S. Gov't
English
KW - MEDLINE
KW - Activities of Daily Living
KW - Adolescent
KW - Body Image
KW - Child
KW - Preschool
KW - Female
KW - Humans
KW - Ileum/su [Surgery]
KW - Interpersonal Relations
KW - Male
KW - Quality of Life
KW - Questionnaires
KW - Self Concept
KW - Urinary Reservoirs
KW - Continent/ae [Adverse Effects]
KW - Continent
RP - NOT IN FILE
SP - 40
EP - 45
JF - Scandinavian Journal of Urology & Nephrology
JA - Scand J Urol Nephrol
VL - 36
IS - 1
CY - Sweden
N2 - OBJECTIVE: To assess quality of life after continent ileal reservoir diversion in children and adolescents. MATERIAL AND METHODS: Nine boys and 9 girls (10.8-18 years old at surgery) were operated with a Kock reservoir and followed for 5-12 years. They participated in an interview using a questionnaire based on the Swedish self-esteem inventory, "I think I am". The questionnaire consisted of 76 questions that covered medical, physical, psychological, social, body image and sexual aspects. RESULTS: Six of 18 patients had some difficulties on catheterization. Five complained of pain or bleeding during evacuation. Two patients had slight urinary leakage between evacuations and 6 had mucus secretion. Ten patients had diarrhea with different frequencies. Physical activities were not hampered by the operation in any patient. Instead, activities such as full day outside home, friend at home overnight, using all types of clothes, and participation in camps were increased in 8, 6, 5 and 5 patients, respectively. All patients but 2 had an excellent relation with friends and family members. More than half of patients had excellent positive self-esteem and body image and the rest had

very good self-esteem. However, some patients were concerned about how they looked and felt that life was unfair to them. CONCLUSIONS: The patients were satisfied with their operation. They were physically active and declared a positive perspective regarding self-esteem and body image. The medical problems related to the operation did not have any negative influence on the self-esteem or body image. The choice of the operation can only be determined on the basis of an attentive dialogue between surgeon, child and parents
SN - 0036-5599
AD - Department of Paediatric Surgery, Queen Silvia Children's Hospital, Goteborg, Sweden. gamal.abd-el-gawad@vgregion.se
UR - 12002356
ER -

TY - JOUR
ID - 1610
T1 - Triatomines involved in domestic and wild Trypanosoma cruzi transmission in Concepcion, Corrientes, Argentina
A1 - Bar,M.E.
A1 - Pieri,Damborsky M.
A1 - Oscherov,E.B.
A1 - Milano,A.
A1 - Francisco,M.
A1 - Avalos,G.
A1 - Wisnivesky-Colli,C.
Y1 - 2002/01//
N1 - Bar, Maria Esther. Pieri Damborsky, Miryam. Oscherov, Elena Beatriz. Milano, Alicia. Francisco, Maria. Avalos, Gilberto. Wisnivesky-Colli, Cristina
Memorias do Instituto Oswaldo Cruz
mry, 7502619
IM
Journal Article
English
KW - MEDLINE
KW - Adolescent
KW - Adult
KW - Aged
KW - 80 and over
KW - Animals
KW - Argentina/ep [Epidemiology]
KW - Chagas Disease/bl [Blood]
KW - Chagas Disease/ep [Epidemiology]
KW - Chagas Disease/tm [Transmission]
KW - Child
KW - Preschool
KW - Female
KW - Housing
KW - Humans
KW - Infant
KW - Newborn
KW - Insect Vectors/ps [Parasitology]
KW - Male
KW - Middle Aged
KW - Prevalence
KW - Seasons
KW - Trees
KW - Triatominae/ps [Parasitology]
KW - Trypanosoma cruzi/ip [Isolation & Purification]
RP - NOT IN FILE
SP - 43

EP - 46
JF - Memorias do Instituto Oswaldo Cruz
JA - Mem.Inst Oswaldo Cruz
VL - 97
IS - 1
CY - Brazil
N2 - An entomological and serological survey was performed in three localities of the Department of Concepcion, Province of Corrientes, Argentina in 1998 and 1999, to identify triatomines species involved in domestic and wild transmission of Chagas disease. Triatomines were collected by man/hour capture in 32 houses randomly selected and 44 nearby outdoor ecotopes. Trypanosoma cruzi infection in triatomines was assessed by direct microscopic observation (400x) of feces and polymerase chain reaction. Serological techniques used for people were Indirect Hemagglutination Test and Indirect Fluorescent Test. Triatomines were collected in 28.1% of the houses and 31.8% of the wild biotopes. Triatoma infestans (Klug 1834) was exclusively found indoors and T. cruzi infected 60% of them. Triatoma sordida (Stal 1859) was mainly found in extradomestic ecotopes where trypanosome infection rate reached 12.7%. Serological study of 98 local people showed that 29.6% were seroreactive; most of their houses were closed to wild biotopes colonized by T. sordida. Results indicate that there is an active T. infestans mediated transmission of Chagas disease in this zone that yields important human prevalence and that the populations of T. sordida in wild biotopes not only sustain the wild T. cruzi cycle but also represent an actual risk for people living in the area
SN - 0074-0276
AD - Catedra de Artropodos, Facultad de Ciencias Exactas y Naturales y Agrimensura, Universidad Nacional del Nordeste, Av. Libertad 5470 (3400) Corrientes, Argentina. mebar@exa.unne.edu.ar
UR - 11992145
ER -

TY - JOUR
ID - 1611
T1 - Observations of summer sun protection among children in New Zealand: 1998-2000
A1 - McGee,R.
A1 - Reeder,A.
A1 - Williams,S.
A1 - Bandaranayake,M.
A1 - Tan,A.H.
Y1 - 2002/03/08/
N1 - McGee, Rob. Reeder, Anthony. Williams, Sheila. Bandaranayake, Michele. Tan, Aik Haw
The New Zealand medical journal
obq, 0401067
IM
Journal Article. Research Support, Non-U.S. Gov't
English
KW - MEDLINE
KW - Adult
KW - Child
KW - Preschool
KW - Female
KW - Health Behavior
KW - Humans
KW - Infant
KW - Male
KW - New Zealand
KW - Parenting
KW - Proportional Hazards Models
KW - Protective Clothing
KW - Risk Factors
KW - Sunburn/pc [Prevention & Control]
KW - Sunscreening Agents
RP - NOT IN FILE

SP - 103
EP - 106
JF - New Zealand Medical Journal
JA - N Z Med J
VL - 115
IS - 1149
CY - New Zealand
N2 - AIMS: To examine sun protection among New Zealand children (ten years and under) at beaches and playgrounds. METHODS: In the summers of 1998, 1999 and 2000, observations were made of 753 children at selected beaches and playgrounds in Dunedin and Hawkes Bay to determine the extent of sun protection. Parents/carers were also interviewed about sun protection. RESULTS: Across most body sites, levels of protection were high. Clothing and SPF15+ sunscreen were used most often, while the use of shade was relatively low. About four in every ten parents/caregivers were aware of the UV index or burn-time. Level of sun protection among children at beaches was best predicted by parent/carer's own level of protection, the child's perceived susceptibility to burning, and being a preschooler. CONCLUSIONS: Observed levels of sun protection among the children were high and most likely reflected an increased awareness among parents and carers of the social acceptability of protective behaviours. Nevertheless, opportunities to seek shade were limited, and the provision of shade at beaches and playgrounds represents an important next step in a community wide approach to sun protection
SN - 0028-8446
AD - Department of Preventive & Social Medicine, University of Otago Medical School, Dunedin. rob.mcgee@stonebow.otago.ac.nz
UR - 11999220
ER -

TY - JOUR
ID - 1612
T1 - Measurements of children's exposures to particles and nitrogen dioxide in Santiago, Chile
A1 - Rojas-Bracho,L.
A1 - Suh,H.H.
A1 - Oyola,P.
A1 - Koutrakis,P.
Y1 - 2002/03/27/
N1 - Rojas-Bracho, Leonora. Suh, Helen H. Oyola, Pedro. Koutrakis, Petros
The Science of the total environment
uj0, 0330500
IM
Journal Article. Research Support, Non-U.S. Gov't
English
KW - MEDLINE
KW - Air Pollutants/an [Analysis]
KW - Air Pollution
KW - Indoor/an [Analysis]
KW - Child
KW - Child Welfare
KW - Chile
KW - Cooking
KW - Environmental Exposure
KW - Female
KW - Humans
KW - Inhalation Exposure
KW - Male
KW - Nitrogen Dioxide/an [Analysis]
KW - Oxidants
KW - Photochemical/an [Analysis]
KW - Particle Size
KW - Urban Population

KW - Ventilation
RP - NOT IN FILE
SP - 249
EP - 264
JF - Science of the Total Environment
JA - Sci Total Environ
VL - 287
IS - 3
CY - Netherlands
N2 - An exposure study of children (aged 10-12 years) living in Santiago, Chile, was conducted. Personal, indoor and outdoor fine and inhalable particulate matter (< 2.5 .m in diameter, PM2.5 and < 10 microm in diameter, PM10, respectively), and nitrogen dioxide (NO2) were measured during pilot (N = 8) and main (N = 20) studies, which were conducted during the winters of 1998 and 1999, respectively. For the main study, personal, indoor and outdoor 24-h samples were collected for five consecutive days. Similar mean personal, indoor and outdoor PM2.5 concentrations (69.5, 68.5 and 68.1 microg/m3, respectively) were found. However, for coarse particles (calculated as the difference between measured PM10 and PM2.5, PM2.5-10), indoor and outdoor levels (35.4 and 47.4 microg/m3) were lower than their corresponding personal exposures (76.3 microg/m3). Indoor and outdoor NO2 concentrations were comparable (35.8 and 36.9 ppb) and higher than personal exposures (25.9 ppb). Very low ambient indoor and personal O3 levels were found, which were mostly below the method's limit of detection (LOD). Outdoor particles contributed significantly to indoor concentrations, with effective penetration efficiencies of 0.61 and 0.30 for PM2.5 and PM2.5-10, respectively. Personal exposures were strongly associated with indoor and outdoor concentrations for PM2.5, but weakly associated for PM2.5-10. For NO2, weak associations were obtained for indoor-outdoor and personal-outdoor relationships. This is probably a result of the presence of gas cooking stoves in all the homes. Median I/O, P/I and P/O ratios for PM2.5 were close to unity, and for NO2 they ranged between 0.64 and 0.95. These ratios were probably due to high ambient PM2.5 and NO2 levels in Santiago, which diminished the relative contribution of indoor sources and subjects' activities to indoor and personal PM2.5 and NO2 levels
SN - 0048-9697
AD - Harvard University, School of Public Health, Boston, MA 02115, USA. lrojasbr@mail.ssa.gob.mx
UR - 11993967
ER -

TY - JOUR
ID - 1613
T1 - Welfare reform, fertility, and father involvement. [Review] [71 refs]
A1 - McLanahan,S.S.
A1 - Carlson,M.J.
Y1 - 2002///Winter
N1 - McLanahan, Sara S. Carlson, Marcia J
The Future of children / Center for the Future of Children, the David and Lucile Packard Foundation
9306342, b4m
IM
Journal Article. Review
English
KW - MEDLINE
KW - Adolescent
KW - Child
KW - Child Care/sn [Statistics & Numerical Data]
KW - Child Welfare/ec [Economics]
KW - Child Welfare/lj [Legislation & Jurisprudence]
KW - Family Characteristics
KW - Father-Child Relations
KW - Female
KW - Health Planning/lj [Legislation & Jurisprudence]
KW - Humans
KW - Male
KW - Pregnancy

KW - Pregnancy in Adolescence/sn [Statistics & Numerical Data]
KW - Public Assistance/lj [Legislation & Jurisprudence]
KW - Public Assistance/og [Organization & Administration]
KW - United States
RP - NOT IN FILE
SP - 146
EP - 165
JF - Future of Children
JA - Future Child
VL - 12
IS - 1
CY - United States
N2 - Recognizing that most poor families are single-parent families, the federal welfare reform law of 1996 emphasized the responsibility of both parents to support their children. In addition to strengthening the child support enforcement system, the law included several provisions designed to decrease childbearing outside of marriage and to promote two-parent families. This article focuses on the important role that fathers play in children's lives and how public policies have affected childbearing and father involvement. Key observations are: Compared with children living with both biological parents, children in father-absent families often have fewer economic and socioemotional resources from their parents, and do not fare as well on many outcome measures. Efforts to reduce the rising number of father-absent families by focusing on preventing unwanted pregnancy among unmarried women, especially teen girls, have met with some success; those programs seeking to alter adolescents' life opportunities in addition to providing education or family planning services appear to hold the most promise. Efforts to encourage greater father involvement by focusing almost exclusively on increasing absent parents' child support payments reap only minimal benefits for poor children because their absent parents often have few resources and little incentive to make support payments. To date, efforts to increase the emotional involvement of unmarried fathers with their children have produced disappointing results, but new research suggests that such programs can make a difference when targeting fathers at the time of a child's birth. Many children spend some time living away from their fathers, deprived of the financial and emotional resources they can provide. Because of the importance of fathers to child well-being, the authors conclude that new directions in research and public policies are needed to encourage greater father involvement across the wide diversity of family arrangements in society today. [References: 71]
SN - 1054-8289
AD - Bendheim-Thoman Center for Research on Child Wellbeing, Princeton University, Princeton, NJ, USA
UR - 11980034
ER -

TY - JOUR
ID - 1614
T1 - [Detection of exercise-induced bronchospasm in sixth-grade students: impact of health education]. [French]
A1 - Charton,C.
A1 - Droulers,I.
A1 - Girault,E.
A1 - Laurent,C.
A1 - Housset,B.
A1 - Delacourt,C.
Y1 - 2002/03//
N1 - Charton, C. Droulers, I. Girault, E. Laurent, C. Housset, B. Delacourt, C
Archives de pediatrie : organe officiel de la Societe francaise de pediatrie
9421356, bwh
IM
English Abstract. Journal Article
French
KW - MEDLINE
KW - Adolescent
KW - Bronchial Spasm/di [Diagnosis]
KW - Bronchial Spasm/et [Etiology]

KW - Child
KW - Exercise
KW - Female
KW - Health Education
KW - Humans
KW - Male
KW - Questionnaires
RP - NOT IN FILE
SP - 245
EP - 254
JF - Archives de Pediatrie
JA - Arch Pediatr
VL - 9
IS - 3
CY - France
N2 - UNLABELLED: Underdiagnosis of asthma is frequent in children and may be improved by the development of school-based health programs. MATERIAL AND METHODS: We developed an educational program in 11-year-old schoolchildren who participated in a screening test for exercise-induced asthma (EIA). All children were given an asthma questionnaire before and after two educational sessions. RESULTS: Mean score for asthma knowledge quiz increased from 63 to 85% ($p < 0.001$). Thirty-six children (3.7%) were initially considered as asthmatic and showed better responses than non-asthmatic children. Peak expiratory flow (PEF) was measured before and after an outside running exercise. A fall in PEF of at least 15% was considered an abnormal result. Seventy-height children (8%) had a decrease in PEF, including 65 children initially not recognized as asthmatic. For these latter, a medical evaluation was recommended to parents. Only 28 of these children gave informations on follow-up: 19 had lung function tests, two received a treatment without preliminary function test, and seven had neither lung function test nor treatment. Among the children who realized lung function tests, five had criteria for airway obstruction at baseline, and ten had significant bronchial hyperreactivity. CONCLUSIONS: However, a new screening test revealed that only a small minority of children initially not recognized as asthmatic but having decreased their PEF, were consequently considered as asthmatic by their practitioner, even in case of positive lung function test
SN - 0929-693X
AD - Service de promotion de la sante en faveur des eleves, inspection academique du Val-de-Marne, 68, avenue du General-de-Gaulle, 94010 Creteil, France
UR - 11938535
ER -

TY - JOUR
ID - 1615
T1 - Risk factors associated with asymptomatic infection by Leishmania chagasi in north-east Brazil
A1 - Caldas,A.J.
A1 - Costa,J.M.
A1 - Silva,A.A.
A1 - Vinhas,V.
A1 - Barral,A.
Y1 - 2002/01//
N1 - Caldas, A J M. Costa, J M L. Silva, A A M. Vinhas, V. Barral, A
Transactions of the Royal Society of Tropical Medicine and Hygiene
wbu, 7506129
IM
Journal Article. Research Support, Non-U.S. Gov't. Research Support, U.S. Gov't, P.H.S.
English
KW - MEDLINE
KW - Animals
KW - Brazil/ep [Epidemiology]
KW - Child
KW - Preschool
KW - Enzyme-Linked Immunosorbent Assay

KW - Female
KW - Humans
KW - Hypersensitivity
KW - Delayed/ep [Epidemiology]
KW - Delayed/ps [Parasitology]
KW - Incidence
KW - Infant
KW - Newborn
KW - Leishmania infantum
KW - Leishmaniasis
KW - Visceral/ep [Epidemiology]
KW - Male
KW - Prevalence
KW - Prospective Studies
KW - Risk Factors
RP - NOT IN FILE
SP - 21
EP - 28
JF - Transactions of the Royal Society of Tropical Medicine & Hygiene
JA - Trans R Soc Trop Med Hyg
VL - 96
IS - 1
CY - England
N2 - Various factors have been associated with a predisposition to the development of clinical American visceral leishmaniasis (AVL). However, little information is available about the factors that predispose to asymptomatic infection. To identify the risk factors associated with asymptomatic infection, a study was carried out between July 1997 and June 1998 on children aged 0-5 years in the districts of Vila Nova and Bom Viver in the municipality of Raposa in the island of Sao Luis, State of Maranhao, Brazil. A questionnaire containing socioeconomic, demographic and epidemiological data was used. The delayed-type hypersensitivity (DTH) test was carried out on 639 children in the first phase, and on 572 in the second, 7 months after the first survey, using Leishmania amazonensis antigen. Infection was determined by enzyme-linked immunosorbent assay (ELISA) in 638 children during the first phase, and in 572 during the second. Six outcome measures were used: initial prevalence, final prevalence and incidence, each determined by DTH and ELISA. The incidence of infection was 10.8% when determined by DTH and 28.5% when determined by ELISA. After adjustment for confounding variables using Cox regression, infection by L. chagasi was associated with child's age (> or = 2 years), location of the dwellings (Vila Nova) and reporting of relatives with AVL. Bathing outside the house and playing outdoors between 18:00 and 20:00 were identified as risk factors in some analyses but not in others. Presence of intra- and peridomestic Lutzomyia sandflies and animals such as dogs or chickens in the house or in the neighbourhood appeared as risk factors in some analyses but in others they unexpectedly seemed to protect from infection. Malnutrition was not found to be associated with infection
SN - 0035-9203
AD - Federal University of Maranhao, Departamento de Patologia, Praca Madre Deus, No. 2, Madre Deus, 65.025-560, Sao Luis-Maranhao, Brazil. npatufma@elo.com.br
UR - 11925984
ER -

TY - JOUR
ID - 1616
T1 - Extracorporeal suture technique for suprahepatic caval anastomosis in liver transplantation
A1 - Salizzoni,M.
A1 - Romagnoli,R.
A1 - Zamboni,F.
Y1 - 2002/02//
N1 - Salizzoni, Mauro. Romagnoli, Renato. Zamboni, Fausto
American journal of surgery
3z4, 0370473
AIM, IM

Journal Article
English
KW - MEDLINE
KW - Adult
KW - Anastomosis
KW - Surgical/mt [Methods]
KW - Child
KW - Female
KW - Humans
KW - Liver Transplantation
KW - Male
KW - Suture Techniques
KW - Treatment Outcome
KW - Venae Cavae/su [Surgery]
RP - NOT IN FILE
SP - 156
EP - 159
JF - American Journal of Surgery
JA - Am J Surg
VL - 183
IS - 2
CY - United States
N2 - BACKGROUND: During liver transplantation, an oversized graft or active bleeding in the hepatic area can make performance of the suprahepatic caval anastomosis extremely difficult or even impossible. In other instances, a brief as possible warm ischemia is desired to provide a marginal graft with maximum chances of good functioning. METHODS: In order to manage those conditions, a suture technique was devised that allows the construction of a substantial part of the suprahepatic caval anastomosis keeping the graft outside the abdomen of the recipient. RESULTS: Over a 12-month period, the technique was applied in 7 out of 148 transplants (5%). The 7 cases presented one or more of the following conditions: significant oversize mismatch (n = 6), active bleeding in the hepatic area (n = 1), and marginal graft (n = 4). Warm ischemia time averaged 27 minutes, a value not significantly different from the mean warm ischemia time of 25 minutes recorded in the easier transplants in which the conventional technique was used (P = 0.2467). CONCLUSIONS: This extracorporeal suture technique allows construction of the suprahepatic caval anastomosis in critical situations arising during liver transplantation and avoidance of the prolonged warm ischemia that could be expected in such cases
SN - 0002-9610
AD - Liver Transplantation Center, San Giovanni Battista Hospital, Corso Bramante 88, 10126 Turin, Italy. msalizzoni@molinette.piemonte.it
UR - 11918880
ER -

TY - JOUR
ID - 1617
T1 - Classroom-based assessment: validation for the school AMPS
A1 - Fingerhut,P.
A1 - Madill,H.
A1 - Darrah,J.
A1 - Hodge,M.
A1 - Warren,S.
Y1 - 2002/03//
N1 - Fingerhut, Pat. Madill, Helen. Darrah, Johanna. Hodge, Megan. Warren, Sharon
The American journal of occupational therapy : official publication of the American Occupational Therapy Association
7705978, 3o4
IM
Evaluation Studies. Journal Article. Multicenter Study
English

KW - MEDLINE
KW - Child
KW - Preschool
KW - Developmental Disabilities/di [Diagnosis]
KW - Humans
KW - Logistic Models
KW - Motor Skills
KW - North America
KW - Occupational Therapy/mt [Methods]
KW - Reproducibility of Results
KW - School Health Services
RP - NOT IN FILE
SP - 210
EP - 213
JF - American Journal of Occupational Therapy
JA - Am J Occup Ther
VL - 56
IS - 2
CY - United States
N2 - The role of the occupational therapist working in the school system is to facilitate a student's task performance or ability to do purposeful and meaningful activities so that the student benefits from the educational experience. To fulfill this role, occupational therapists need assessments that address functional performance issues in the classroom and provide information for effective programming and consultation. The School Version of the Assessment of Motor and Process Skills (School AMPS) is an observational evaluation of functional skills in the classroom. Common classroom activities are observed to assess a child's school motor and school process skills. This study examined the validity of the School AMPS by comparing it to the Peabody Developmental Motor Scale-Fine Motor (PDMS-FM), a common assessment used in local area school districts in Edmonton, Alberta. Results show a higher correlation of the PDMS-FM with the motor scale of the School AMPS than with the process scale of the School AMPS, which was expected. The School AMPS appears to be a promising instrument for measuring the constructs of school motor and school process skills within a naturalistic setting
SN - 0272-9490
AD - Pediatric Therapy Center, 8323 Southwest Freeway, Suite 101, Houston, Texas 77074, USA
UR - 11905306
ER -

TY - JOUR
ID - 1618
T1 - Actigraph placement and sleep estimation in children
A1 - Paavonen,E.J.
A1 - Fjallberg,M.
A1 - Steenari,M.R.
A1 - Aronen,E.T.
Y1 - 2002/03/15/
N1 - Paavonen, E Juulia. Fjallberg, Mika. Steenari, Maija-Riikka. Aronen, Eeva T
Sleep
sws, 7809084
IM
Journal Article. Research Support, Non-U.S. Gov't
English
KW - MEDLINE
KW - Adolescent
KW - Child
KW - Female
KW - Humans
KW - Male
KW - Polysomnography/is [Instrumentation]

KW - Sleep Disorders/di [Diagnosis]
KW - Sleep Disorders/ep [Epidemiology]
RP - NOT IN FILE
SP - 235
EP - 237
JF - Sleep
VL - 25
IS - 2
CY - United States
N2 - STUDY OBJECTIVES: To determine whether actigraph placement affects sleep estimation in children. DESIGN: Descriptive study. SETTING: Naturalistic setting. PARTICIPANTS: Twenty children aged 7-12 years from primary schools. INTERVENTIONS: N/A. MEASUREMENTS: Motor activity was measured from the waist and non-dominant wrist with actigraphs for three consecutive days during a school week. RESULTS: The minute-by-minute agreement of sleep-wake states between the two measurement sites was 92.5%. Wrist- and waist-recorded sleep parameters correlated well and the mean values did not differ. CONCLUSIONS: Although the placement of the actigraph slightly affected the measured activity parameters, its influence on 3-night mean sleep estimates in children was not statistically significant
SN - 0161-8105
AD - Department of Child Psychiatry, University of Helsinki, Finland
UR - 11902433
ER -

TY - JOUR
ID - 1619
T1 - Psychological status of Sarajevo children after war: 1999-2000 survey
A1 - Barath,A.
Y1 - 2002/04//
N1 - Barath, Arpad
Croatian medical journal
c0s, 9424324
IM
Comparative Study. Journal Article. Research Support, Non-U.S. Gov't
English
KW - MEDLINE
KW - Adaptation
KW - Psychological
KW - Adolescent
KW - Bosnia-Herzegovina
KW - Child
KW - Child Behavior
KW - Preschool
KW - Female
KW - Humans
KW - Male
KW - Population Surveillance
KW - Probability
KW - Prognosis
KW - Psychology
KW - Regression Analysis
KW - Risk Factors
KW - Sampling Studies
KW - Self Concept
KW - Severity of Illness Index
KW - Stress Disorders
KW - Post-Traumatic/di [Diagnosis]
KW - Post-Traumatic/ep [Epidemiology]
KW - Stress

KW - Urban Population
KW - War
RP - NOT IN FILE
SP - 213
EP - 220
JF - Croatian Medical Journal
JA - Croat.Med J
VL - 43
IS - 2
CY - Croatia
N2 - AIM: To make a survey of children's health and psychosocial needs after the 1992-1995 war in Sarajevo, Bosnia and Herzegovina. METHODS: Representative samples of school-age children (n=310) from 6 public schools in the Sarajevo Canton, their parents (n=280), and teachers (n=156) were surveyed by means of self-administered questionnaires and standardized psychometric scale (Ryan-Wengers Schoolagers Coping Strategies Inventory). The survey was conducted in October-November 1999, approximately four years after the war. RESULTS: At the time of survey, well-being of children in Sarajevo was still heavily impacted by many various unhealthy life conditions and psychosocial stressors. Many school-age children lived in unhealthy and dangerous environment, including overcrowded living conditions (40%), unsafe playgrounds (68%), and no access to sports fields (52%). Most felt unsafe on streets (74%), many (73%) coped with one or more school problems, and even 84% were ill at least once during the past 12 months. General poverty was the prime stressor (common variance explained: 23.5%), followed by school- and health-related risks (common variance explained: 17.0%). At the third place were family-associated risk factors impacting children's health and development, such as overcrowded living conditions and lack of social support within their own family (common variance explained: 10.5%). Parents and teachers also lived and worked in stressful life conditions and were concerned for both their children's and their own well-being. Despite all that, most children tended to use healthy strategies in coping with stressful events in their everyday lives. CONCLUSION: The reinforcement of children with positive (healthy) coping skills and strengthening of their social support networks seems to be the most important intervention strategy to help the war-traumatized children in Bosnia and Herzegovina
SN - 0353-9504
AD - Department of Sociology and Social Policy, Pecs University Faculty of Arts and Humanities, Rokus u. 2, 7632 Pecs, Hungary. arpad@btk.pte.hu
UR - 11885050
ER -

TY - JOUR
ID - 1620
T1 - Use of an ecologically based program for teaching motor skills in a naturalistic setting to individuals with disabilities
A1 - Zhang,J.
A1 - Berkey,D.
Y1 - 2002/02//
N1 - Zhang, Jiabei. Berkey, Debra
Perceptual and motor skills
ozb, 0401131
IM
Journal Article
English
KW - MEDLINE
KW - Adolescent
KW - Adult
KW - Child
KW - Ecology
KW - Female
KW - Health Planning
KW - Humans
KW - Male
KW - Mentally Disabled Persons/px [Psychology]

KW - Motor Skills/ph [Physiology]
KW - Physical Education and Training
KW - Psychomotor Performance
KW - Teaching/mt [Methods]
RP - NOT IN FILE
SP - 235
EP - 240
JF - Perceptual & Motor Skills
JA - Percept.Mot.Skills
VL - 94
IS - 1
CY - United States
N2 - The purpose of this action study was to apply an ecological program for teaching motor skills to individuals with disabilities in naturalistic settings. The program was developed based on an ecological survey. Participants were 22 individuals with disabilities, ages 7 to 26 years in the Fall of 1998 and 18 individuals with disabilities, ages 7 to 24 in the Winter of 1999. In each semester, a participant received one or two 50-min, training session(s) per week for 10 weeks. The number of steps in a motor-activity task performed correctly by a participant was measured in the first and last sessions in each semester. The magnitude of increase in the motor performance by participants and answers to questions in a short survey by participants' parents were used to describe the group performance. Analysis showed group performance of targeted motor skills could be improved using this ecological program in this local learning lab for special physical education
SN - 0031-5125
AD - Department of Health, Physical Education, and Recreation, Western Michigan University, Kalamazoo 49008, USA. zhangj@wmich.edu
UR - 11883568
ER -

TY - JOUR
ID - 1621
T1 - Clinical profile and spectrum of commotio cordis
A1 - Maron,B.J.
A1 - Gohman,T.E.
A1 - Kyle,S.B.
A1 - Estes,N.A.,III
A1 - Link,M.S.
Y1 - 2002/03/06/
N1 - Maron, Barry J. Gohman, Thomas E. Kyle, Susan B. Estes, N A Mark 3rd. Link, Mark S
JAMA : the journal of the American Medical Association
7501160
AIM, IM
Journal Article. Research Support, Non-U.S. Gov't
English
KW - MEDLINE
KW - Adolescent
KW - Adult
KW - Arrhythmias
KW - Cardiac/et [Etiology]
KW - Cardiac/th [Therapy]
KW - Athletic Injuries/mo [Mortality]
KW - Cardiopulmonary Resuscitation
KW - Child
KW - Preschool
KW - Death
KW - Sudden
KW - Cardiac/ep [Epidemiology]
KW - Cardiac/pc [Prevention & Control]
KW - Electric Countershock

KW - Female
KW - Humans
KW - Infant
KW - Male
KW - Protective Clothing
KW - Registries
KW - Thoracic Injuries/co [Complications]
KW - Thoracic Injuries/mo [Mortality]
KW - Ventricular Fibrillation/et [Etiology]
KW - Wounds
KW - Nonpenetrating/co [Complications]
KW - Nonpenetrating/mo [Mortality]
RP - NOT IN FILE
SP - 1142
EP - 1146
JF - JAMA
VL - 287
IS - 9
CY - United States
N2 - CONTEXT: Although blunt, nonpenetrating chest blows causing sudden cardiac death (commotio cordis) are often associated with competitive sports, dangers implicit in such blows can extend into many other life activities. OBJECTIVE: To describe the comprehensive spectrum of commotio cordis events. DESIGN AND SETTING: Analysis of confirmed cases from the general community assembled in the US Commotio Cordis Registry occurring up to September 1, 2001. MAIN OUTCOME MEASURE: Commotio cordis event. RESULTS: Of 128 confirmed cases, 122 (95%) were in males and the mean (SD) age was 13.6 (8.2) years (median, 14 years; range, 3 months to 45 years); only 28 (22%) cases were aged 18 years or older. Commotio cordis events occurred most commonly during organized sporting events (79 [62%]), such as baseball, but 49 (38%) occurred as part of daily routine and recreational activities. Fatal blows were inflicted with a wide range of velocities but often occurred inadvertently and under circumstances not usually associated with risk for sudden death in informal settings near the home or playground. Twenty-two (28%) participants were wearing commercially available chest barriers, including 7 in whom the projectile made direct contact with protective padding (baseball catchers and lacrosse/hockey goalies), and 2 in whom the projectile was a baseball specifically designed to reduce risk. Only 21 (16%) individuals survived their event, with particularly prompt cardiopulmonary resuscitation/defibrillation (most commonly reversing ventricular fibrillation) the only identifiable factor associated with a favorable outcome. CONCLUSIONS: The expanded spectrum of commotio cordis illustrates the potential dangers implicit in striking the chest, regardless of the intent or force of the blow. These findings also suggest that the safety of young athletes will be enhanced by developing more effective preventive strategies (such as chest wall barriers) to achieve protection from ventricular fibrillation following precordial blows
SN - 0098-7484
AD - Minneapolis Heart Institute Foundation, 920 E 28th St, Suite 60, Minneapolis, MN 55407, USA. hcm.maron@mhif.org
UR - 11879111
ER -

TY - JOUR
ID - 1622
T1 - Effects of air pollution on changes in lung function induced by exercise in children with chronic respiratory symptoms
A1 - Timonen,K.L.
A1 - Pekkanen,J.
A1 - Tiittanen,P.
A1 - Salonen,R.O.
Y1 - 2002/02//
N1 - Timonen, K L. Pekkanen, J. Tiittanen, P. Salonen, R O
Occupational and environmental medicine
bwc, 9422759

IM
Journal Article. Research Support, Non-U.S. Gov't. Research Support, U.S. Gov't, Non-P.H.S.
English
KW - MEDLINE
KW - Air Pollution/ae [Adverse Effects]
KW - Asthma/pp [Physiopathology]
KW - Bronchial Hyperreactivity/et [Etiology]
KW - Child
KW - Chronic Disease
KW - Cohort Studies
KW - Environmental Exposure/ae [Adverse Effects]
KW - Exercise/ph [Physiology]
KW - Female
KW - Forced Expiratory Volume/ph [Physiology]
KW - Humans
KW - Male
KW - Respiration Disorders/pp [Physiopathology]
KW - Vital Capacity/ph [Physiology]
RP - NOT IN FILE
SP - 129
EP - 134
JF - Occupational & Environmental Medicine
JA - Occup Environ Med
VL - 59
IS - 2
CY - England
N2 - OBJECTIVE: To investigate how daily variations in ambient air pollution, especially in particles, during the cold of winter affect repeated measurements of baseline lung function and exercise induced bronchial responsiveness among primary school children with chronic respiratory symptoms. METHODS: During alternate school weeks (maximum five) from February to April 1994, 33 children took part in exercise challenge tests (n=141 tests). The exercise challenges were conducted outdoors in a school yard in the centre of Kuopio, Finland. Spirometric lung functions were measured indoors before the exercise, and 3 and 10 minutes after. Daily mean concentrations of PM(10), black smoke (BS), NO(2), CO, SO(2), and particle size and numbers were monitored at a nearby fixed monitoring site. RESULTS: Daily variations in ambient air pollution were not associated with enhanced bronchial responsiveness. However, increased concentrations of BS, PM(10), particle numbers, NO(2), and CO were consistently associated with an impairment of baseline lung functions. The reductions in forced vital capacity (FVC) and forced expiratory volume in 1 second (FEV(1)) were 0.5% and 0.6%, respectively, for each 10 microg/m(3) increase in BS (lag 2). CONCLUSION: Particles derived from combustion affect baseline lung function rather than bronchial responsiveness among children with chronic respiratory symptoms
SN - 1351-0711
AD - Unit of Environmental Epidemiology, National Public Health Institute, Kuopio, Finland. Kirsi.Timonen@ktl.fi
UR - 11850557
ER -

TY - JOUR
ID - 1623
T1 - Asthma in exercising children exposed to ozone: a cohort study.[Erratum appears in Lancet 2002 Mar 9;359(9309):896]
A1 - McConnell,R.
A1 - Berhane,K.
A1 - Gilliland,F.
A1 - London,S.J.
A1 - Islam,T.
A1 - Gauderman,W.J.
A1 - Avol,E.

A1 - Margolis,H.G.
A1 - Peters,J.M.
Y1 - 2002/02/02/
N1 - McConnell, Rob. Berhane, Kiros. Gilliland, Frank. London, Stephanie J. Islam, Talat. Gauderman, W James. Avol, Edward. Margolis, Helene G. Peters, John M
Lancet
2985213r, l0s, 0053266
AIM, IM
Journal Article. Research Support, Non-U.S. Gov't. Research Support, U.S. Gov't, Non-P.H.S.. Research Support, U.S. Gov't, P.H.S.
English
KW - MEDLINE
KW - Adolescent
KW - Air Pollutants/an [Analysis]
KW - Asthma
KW - Exercise-Induced/et [Etiology]
KW - California
KW - Child
KW - Cohort Studies
KW - Female
KW - Humans
KW - Male
KW - Oxidants
KW - Photochemical/ae [Adverse Effects]
KW - Photochemical/an [Analysis]
KW - Ozone/ae [Adverse Effects]
KW - Ozone/an [Analysis]
KW - Social Class
KW - Sports
RP - NOT IN FILE
SP - 386
EP - 391
JF - Lancet
VL - 359
IS - 9304
CY - England
N2 - BACKGROUND: Little is known about the effect of exposure to air pollution during exercise or time spent outdoors on the development of asthma. We investigated the relation between newly-diagnosed asthma and team sports in a cohort of children exposed to different concentrations and mixtures of air pollutants. METHODS: 3535 children with no history of asthma were recruited from schools in 12 communities in southern California and were followed up for up to 5 years. 265 children reported a new diagnosis of asthma during follow-up. We assessed risk of asthma in children playing team sports at study entry in six communities with high daytime ozone concentrations, six with lower concentrations, and in communities with high or low concentrations of nitrogen dioxide, particulate matter, and inorganic-acid vapour. FINDINGS: In communities with high ozone concentrations, the relative risk of developing asthma in children playing three or more sports was 3.3 (95% CI 1.9-5.8), compared with children playing no sports. Sports had no effect in areas of low ozone concentration (0.8, 0.4-1.6). Time spent outside was associated with a higher incidence of asthma in areas of high ozone (1.4, 1.0-2.1), but not in areas of low ozone. Exposure to pollutants other than ozone did not alter the effect of team sports. INTERPRETATION: Incidence of new diagnoses of asthma is associated with heavy exercise in communities with high concentrations of ozone, thus, air pollution and outdoor exercise could contribute to the development of asthma in children
SN - 0140-6736
AD - Department of Preventive Medicine, University of Southern California School of Medicine, Los Angeles, CA 90089-9011, USA. mcconne@hsc.usc.edu
UR - 11844508
ER -

TY - JOUR
ID - 1624
T1 - Powered mobility and preschoolers with complex developmental delays
A1 - Deitz,J.
A1 - Swinth,Y.
A1 - White,O.
Y1 - 2002/01//
N1 - Deitz, Jean. Swinth, Yvonne. White, Owen
The American journal of occupational therapy : official publication of the American Occupational Therapy Association
7705978, 3o4
IM
Case Reports. Journal Article. Research Support, Non-U.S. Gov't
English
KW - MEDLINE
KW - Child Behavior
KW - Child
KW - Preschool
KW - Developmental Disabilities/di [Diagnosis]
KW - Developmental Disabilities/px [Psychology]
KW - Developmental Disabilities/rh [Rehabilitation]
KW - Equipment Design
KW - Female
KW - Humans
KW - Interpersonal Relations
KW - Male
KW - Movement
KW - Muscle Spasticity
KW - Occupational Therapy/mt [Methods]
KW - Play and Playthings
KW - Quadriplegia/di [Diagnosis]
KW - Quadriplegia/px [Psychology]
KW - Quadriplegia/rh [Rehabilitation]
KW - Therapy
KW - Computer-Assisted
RP - NOT IN FILE
SP - 86
EP - 96
JF - American Journal of Occupational Therapy
JA - Am J Occup Ther
VL - 56
IS - 1
CY - United States
N2 - OBJECTIVE: The purpose of this study was to explore the effects of a powered mobility riding toy on the participation behaviors of young children with complex developmental delays. METHOD: A single-subject withdrawal design was used to study the effects of powered mobility on child-initiated movement occurrences, initiation of contact with others, and affect. The participants were two young children with complex developmental delays, including spastic quadriplegia. The intervention consisted of having the children use a powered mobility riding toy in their school settings during gym class and outdoor recess. RESULTS: Primary findings were that use of the powered mobility riding toy (a) increased the number of self-initiated movement occurrences; (b) appeared to have some effect on initiation of contacts with adults and, for one child, negative adult initiations and positive peer initiations; and (c) did not have a clear impact on the amount of positive affect. CONCLUSION: For some young children with severe motor impairments and developmental delay, use of a powered mobility device may increase self-initiated movement occurrences during free play
SN - 0272-9490
AD - Department of Rehabilitation Medicine, Box 356490, University of Washington, Seattle, Washington 98195, USA. deitz@u.washington.edu

UR - 11833405
ER -

TY - JOUR
ID - 1625
T1 - Testing night vision goggles in a dark outside environment
A1 - Spandau,U.H.
A1 - Wechsler,S.
A1 - Blankenagel,A.
Y1 - 2002/01//
N1 - Spandau, Ulrich H M. Wechsler, Stefanie. Blankenagel, Anita
Optometry and vision science : official publication of the American Academy of Optometry
oiz, 8904931
IM
Evaluation Studies. Journal Article
English
KW - MEDLINE
KW - Adolescent
KW - Adult
KW - Aged
KW - Child
KW - Contrast Sensitivity
KW - Environment
KW - Eyeglasses
KW - Female
KW - Humans
KW - Male
KW - Middle Aged
KW - Night Blindness/et [Etiology]
KW - Night Blindness/th [Therapy]
KW - Quality of Life
KW - Retinal Diseases/co [Complications]
KW - Sensory Aids
KW - Visual Acuity
KW - Visual Fields
RP - NOT IN FILE
SP - 39
EP - 45
JF - Optometry & Vision Science
JA - Optom.Vis Sci
VL - 79
IS - 1
CY - United States
N2 - BACKGROUND: People with degenerative retinal diseases such as retinitis pigmentosa, may have adequate day vision but suffer from poor night vision. We have tested newly developed night vision goggles (NVG) to help these patients overcome their night blindness, thereby promoting more opportunities for normal activities at night or in the dark. METHODS: A total of 42 subjects with night blindness due to retinitis pigmentosa, choroideremia, cone rod dystrophy, or Bardet Biedl syndrome were recruited and clinically examined (visual acuity, visual field, and contrast sensitivity). Using an experienced mobility trainer, we tested binocular NVG on the subjects in two locations: a dark room and a 1 to 2 h outside course at night that provided different levels of difficulties (i.e., obstacles, brightness, and contrast). The assessment of which patients benefited from the NVG was predominantly based on the subjective evaluation of the mobility trainer, followed by their graded responses on two questionnaires. RESULTS: Based on the evaluation of the mobility trainer, 23 (61%) of the 42 subjects experienced improved mobility and orientation with the NVG outdoors, and 19 (39%) subjects did not. The ophthalmic data demonstrated that a visual acuity better than 20/100 and a visual field > 5 degrees (Goldmann perimetry III4) is necessary to benefit from NVG usage. In addition, subjective responses on increased mobility and independence were positively correlated with successful NVG testing.

CONCLUSIONS: Night vision goggles have the ability to improve poor night vision in subjects with visual acuity > 20/100 and a visual field > or = 5 degrees (Goldmann: III4). In so doing, NVG can help overcome the obstacles experienced by many people suffering from night blindness. NVG, therefore, have the potential to greatly improve quality of life
SN - 1040-5488
AD - Department of Ophthalmology, University of Heidelberg, Heidelberg, Germany. ulrich.spandau@augen.ma.med.uni-heidelberg.de
UR - 11828897
ER -

TY - JOUR
ID - 1626
T1 - Primary care interventions to reduce television viewing in African-American children
A1 - Ford,B.S.
A1 - McDonald,T.E.
A1 - Owens,A.S.
A1 - Robinson,T.N.
Y1 - 2002/02//
N1 - Ford, B Sophia. McDonald, Tiffany E. Owens, Ayisha S. Robinson, Thomas N
American journal of preventive medicine
8704773, apl
IM
Clinical Trial. Journal Article. Randomized Controlled Trial. Research Support, Non-U.S. Gov't. Research Support, U.S. Gov't, P.H.S.
English
KW - MEDLINE
KW - African Americans/px [Psychology]
KW - California
KW - Child
KW - Child Behavior/eh [Ethnology]
KW - Counseling
KW - Exercise
KW - Feasibility Studies
KW - Female
KW - Humans
KW - Incidence
KW - Leisure Activities
KW - Male
KW - Pilot Projects
KW - Primary Health Care/mt [Methods]
KW - Probability
KW - Risk Assessment
KW - Risk Factors
KW - Television/ut [Utilization]
KW - Time Factors
KW - Urban Population
RP - NOT IN FILE
SP - 106
EP - 109
JF - American Journal of Preventive Medicine
JA - Am J Prev Med
VL - 22
IS - 2
CY - Netherlands
N2 - BACKGROUND: Data are lacking on primary care interventions to reduce children's television viewing. Low-income African-American children watch greater amounts of television than their peers.
DESIGN/METHODS: A randomized controlled pilot and feasibility trial was conducted. Twenty-eight families

with 7- to 12-year-old African-American children receiving primary care at an urban community clinic serving a low-income population were randomized to receive counseling alone or counseling plus a behavioral intervention that included an electronic television time manager. The main outcome was hours of children's television, videotape, and video game use. Parents/guardians and children completed baseline and 4-week follow-up self-report surveys. Additional outcomes included overall household television use, time spent in organized physical activity and playing outside, and meals eaten by the child while watching television. RESULTS: Both intervention groups reported similar decreases in children's television, videotape, and video game use (mean changes of -13.7, SD=26.1 and -14.1, SD=16.8 hours per week). The behavioral intervention group reported significantly greater increases in organized physical activity (changes of +2.5, SD=5.9 and -3.6, SD=4.7 hours per week; p =0.004) and nearly significant greater increases in playing outside (changes of 1.0, SD=5.9 and -4.7, SD=9.4 hours per week; p <0.06). Changes in overall household television use and meals eaten while watching television also appeared to favor the behavioral intervention, with small to medium effect sizes, but differences were not statistically significant. CONCLUSIONS: This small pilot and feasibility study evaluated two promising primary care-based interventions to reduce television, videotape, and video game use among low-income African-American children. The effects on physical activity suggest that the behavioral intervention may be more effective
SN - 0749-3797
AD - School of Medicine, Stanford University, Palo Alto, CA 94304, USA
UR - 11818179
ER -

TY - JOUR
ID - 1627
T1 - [Distribution of sources of Toxocara spp. infection in urban and rural environments in Poland]. [Polish]
A1 - Mizgajska,H.
A1 - Jarosz,W.
A1 - Rejmenciak,A.
Y1 - 2001///
N1 - Mizgajska, H. Jarosz, W. Rejmenciak, A
Wiadomosci parazytologiczne
xof, 0420554
IM
English Abstract. Journal Article
Polish
KW - MEDLINE
KW - Adult
KW - Animals
KW - Cats
KW - Child
KW - Disease Vectors
KW - Dogs
KW - Environmental Monitoring/sn [Statistics & Numerical Data]
KW - Epidemiological Monitoring
KW - Humans
KW - Parasite Egg Count/mt [Methods]
KW - Poland/ep [Epidemiology]
KW - Prevalence
KW - Public Facilities
KW - Rural Population/sn [Statistics & Numerical Data]
KW - Soil/ps [Parasitology]
KW - Species Specificity
KW - Suburban Population/sn [Statistics & Numerical Data]
KW - Toxocara/cl [Classification]
KW - Toxocara/ip [Isolation & Purification]
KW - Toxocara canis/ip [Isolation & Purification]
KW - Toxocariasis/ep [Epidemiology]
KW - Urban Population/sn [Statistics & Numerical Data]

RP - NOT IN FILE
SP - 399
EP - 404
JF - Wiadomosci Parazytologiczne
JA - Wiad.Parazytol.
VL - 47
IS - 3
CY - Poland
N2 - Soil examinations made in 5 regions of Poland in the 1990s comprised 1184 samples taken from urban areas and 590 samples from suburban and rural areas. Toxocara spp. eggs were found more often in urban areas (14% positive samples) than in suburban and rural once (12% positive samples). The average ratio of positive samples was: in the streets and roads--19.3% (0.36 eggs/100g of soil), near houses (backyards, gardens)--18.6% (1.11 eggs/100g), in sandpits--13.0% (0.23/100g), in parks and public gardens--10.5% (0.46 eggs/100g), on playgrounds and playing fields 9,4% (0,06 eggs/100g) and on the beaches--3.4% (0.03 eggs/100g). In children habitual play areas the prevalence of T. cati eggs was higher than T. canis eggs
SN - 0043-5163
AD - Zaklad Biologii i Ochrony Przyrody, Akademia Wychowania fizycznego im. Eugeniusza Piaseckiego w Poznaniu, ul. Krolowej Jadwigi 27/39, 61-871 Poznan
UR - 16894754
ER -

TY - JOUR
ID - 1628
T1 - [Lumbar lateral recess stenosis. Clinical symptoms, diagnostic modalities and surgical management]. [German]
A1 - Worner,B.
A1 - Lange,M.
A1 - Fink,U.
A1 - Oeckler,R.
Y1 - 2001/07//
N1 - Worner, B. Lange, M. Fink, U. Oeckler, R
Fortschritte der Medizin. Originalien
101120496
IM
Comparative Study. English Abstract. Journal Article
German
KW - MEDLINE
KW - Adult
KW - Aged
KW - 80 and over
KW - Child
KW - Decompression
KW - Surgical
KW - Diagnosis
KW - Differential
KW - Female
KW - Follow-Up Studies
KW - Humans
KW - Intervertebral Disc Displacement/di [Diagnosis]
KW - Laminectomy
KW - Lumbar Vertebrae
KW - Magnetic Resonance Imaging
KW - Middle Aged
KW - Patient Satisfaction
KW - Spinal Fusion
KW - Spinal Stenosis/di [Diagnosis]
KW - Spinal Stenosis/ra [Radiography]

KW - Spinal Stenosis/su [Surgery]
KW - Spinal Stenosis
KW - Time Factors
KW - Tomography
KW - X-Ray Computed
KW - Treatment Outcome
RP - NOT IN FILE
SP - Erganzungsband
EP - 4
JF - Fortschritte der Medizin
JA - Fortschr.Med Orig.
VL - Originalien
CY - Germany
N2 - BACKGROUND: In spite of its importance for lumbar radicular syndroms only a few reports about lumbar lateral recess stenosis exist in literature. Most important clinical symptom is radicular pain, which may be exercise-dependent. Confirmation of diagnosis and differentiation from disc herniation requires imaging like CT, MRI or post-myelo-CT. Surgical treatment options include selective decompression techniques like foraminotomy, medial facettectomy and undercutting as well as hemilaminectomy, laminotomy and wide laminectomy or fusion. METHOD: We treated 35 patients with selective decompression techniques. RESULTS: In the follow-up 5 up to 18 months after surgery 25 patients for themselves described the results as good or excellent. Therefore, individually tailored selective microsurgical decompression is an appropriate and successful treatment strategy for lumbar lateral recess stenosis, if conservative treatment fails
AD - Klinik fur Neurochirurgie der Stadt Villingen-Schwenningen
UR - 15704356
ER -

TY - JOUR
ID - 1629
T1 - [The experience of institutionalized children]. [Portuguese]
A1 - Zem-Mascarenhas,S.H.
A1 - Dupas,G.
Y1 - 2001/12//
N1 - Zem-Mascarenhas, S H. Dupas, G
Revista da Escola de Enfermagem da U S P
rss, 0242726
N
English Abstract. Journal Article
Portuguese
KW - MEDLINE
KW - Adolescent
KW - Child
KW - Institutionalized/px [Psychology]
KW - Humans
RP - NOT IN FILE
SP - 413
EP - 419
JF - Revista Da Escola de Enfermagem Da Usp
JA - Rev Esc.Enferm USP
VL - 35
IS - 4
CY - Brazil
N2 - The aim of this study was to know the experience of the institutionalized child and was guided by the theoretical referential of the "Symbolic Interactionism" and by the methodological referential of the "Grounded Theory". The data had been collected in two stages: the first one understood group activities and second stage consisted of personal interviews. The results had shown that the institutionalization occurred for abandonment and reject of the parents or for legal decision as a measure of child protection, having as context the poverty of

its family. The child's experience is conflicting in relation to being institutionalized, because while he/she perceives the necessity of material gain, they miss their family, their home, their friends, and simply life outside
SN - 0080-6234
AD - Escola de Enfermagem de Ribeirao Preto, USP. silviazen@mzo.com.br
UR - 12483987
ER -

TY - JOUR
ID - 1630
T1 - Nutrition and the life cycle: nutrition and the school child. [Review] [30 refs]
A1 - Coutts,A.
Y1 - 2001/01/11/
N1 - Coutts, A
British journal of nursing (Mark Allen Publishing)
big, 9212059
N
Journal Article. Review
English
KW - MEDLINE
KW - Adolescent
KW - Age Factors
KW - Behavior Therapy
KW - Cardiovascular Diseases/et [Etiology]
KW - Cardiovascular Diseases/pc [Prevention & Control]
KW - Child
KW - Child Nutritional Physiological Phenomena
KW - Preschool
KW - Diet
KW - Reducing
KW - Exercise Therapy
KW - Family
KW - Humans
KW - Nurse's Role
KW - Nutritional Requirements
KW - Obesity/et [Etiology]
KW - Obesity/pc [Prevention & Control]
RP - NOT IN FILE
SP - 26
EP - 31
JF - British Journal of Nursing
JA - Br J Nurs
VL - 10
IS - 1
CY - England
N2 - This is the third in this series of articles examining nutrition and the life cycle. The first considered current thinking regarding nutrition in pregnancy (Vol 9(17): 1133-8), and the second discussed nourishing the infant, particularly in relation to weaning (Vol 9(21): 2205-16). This article considers nutrition and the older child. The school years are characterized by increasing independence from parents and exposure to values from outside the home. After being neglected for some years, childhood nutrition is again giving cause for concern. Some children are not eating enough, or not the right nutrients at the right time, while others are becoming overweight, with possible severe long-term effects on their health. Many of these problems are not related to poverty, but rather to changes in lifestyle. Some older children, however, are undernourishing themselves in an attempt to obtain a particular body shape. The last two articles in this series will review nutrition in adulthood and in older years. [References: 30]
SN - 0966-0461
AD - City University, St Bartholomew School of Nursing and Midwifery, London
UR - 12170481

ER -

TY - JOUR
ID - 1631
T1 - Dynamics of malaria transmission under changing ecological scenario in and around Nanak Matta Dam, Uttaranchal, India
A1 - Shukla,R.P.
A1 - Sharma,S.N.
A1 - Kohli,V.K.
A1 - Nanda,N.
A1 - Sharma,V.P.
A1 - Subbarao,S.K.
Y1 - 2001/09//
N1 - Shukla, R P. Sharma, S N. Kohli, V K. Nanda, N. Sharma, V P. Subbarao, S K
Indian journal of malariology
e0c, 17540010r
IM
Journal Article
English
KW - MEDLINE
KW - Adult
KW - Animals
KW - Anopheles/ps [Parasitology]
KW - Antimalarials/tu [Therapeutic Use]
KW - Child
KW - Chloroquine/tu [Therapeutic Use]
KW - Disease Vectors/cl [Classification]
KW - Ecology
KW - Humans
KW - India/ep [Epidemiology]
KW - Malaria
KW - Falciparum/dt [Drug Therapy]
KW - Falciparum/ep [Epidemiology]
KW - Falciparum/tm [Transmission]
KW - Seasons
RP - NOT IN FILE
SP - 91
EP - 98
JF - Indian Journal of Malariology
JA - Indian J Malariol.
VL - 38
IS - 3-4
CY - India
N2 - To understand the transmission dynamics of malaria in three different ecotypes, namely watershed (forest), seepage (Nanak Matta Dam) and plain (non-forest, non-dam) areas of Nainital and Udham Singh Nagar districts of Uttaranchal, entomological and parasitological investigations were carried out from July 1996 to June 1997. In the three ecotypes, average per man hour densities of adult vector species in human dwellings and cattlesheds recorded were high for Anopheles culicifacies from April to September and October to March for An. fluviatilis. Prevalence of both An. culicifacies and An. fluviatilis was higher in the forest area as compared to other two areas. Observations on gonotrophic condition revealed endophilic tendency of both vector species. Higher number of both vector species were found in outdoor than indoor during night human bait collections. Out of 864 specimens of An. fluviatilis dissected, one showed natural infection of sporozoites in salivary glands in the month of November from the forest area only. Sibling species study of An. fluviatilis revealed the presence of species S for the first time in the forest area. Parasitological investigations also depicted high incidence of malaria in the forest area as compared to other two areas. Overall results from the study indicated active malaria transmission in the forest area
SN - 0367-8326

AD - Malaria Research Centre (Field Station), Inderjeet Garden, Bhotia Parao, Haldwani-263 141, India
UR - 12125521
ER -

TY - JOUR
ID - 1632
T1 - [Monitoring of renal function in epileptic children and teenagers treated with valproic acid or carbamazepine in concomitant therapy with tiagabine]. [Polish]
A1 - Hurkacz,M.
A1 - Wiela-Hojenska,A.
A1 - Orzechowska-Juzwenko,K.
A1 - Kozik,A.
A1 - Unolt,J.
Y1 - 2001/12//
N1 - Hurkacz, M. Wiela-Hojenska, A. Orzechowska-Juzwenko, K. Kozik, A. Unolt, J
Polski merkuriusz lekarski : organ Polskiego Towarzystwa Lekarskiego
9705469
IM
English Abstract. Journal Article
Polish
KW - MEDLINE
KW - Acetylglucosaminidase/de [Drug Effects]
KW - Acetylglucosaminidase/ur [Urine]
KW - Adolescent
KW - Adult
KW - Anticonvulsants/ae [Adverse Effects]
KW - Anticonvulsants/tu [Therapeutic Use]
KW - Carbamazepine/ae [Adverse Effects]
KW - Carbamazepine/tu [Therapeutic Use]
KW - Case-Control Studies
KW - Child
KW - Controlled Clinical Trials as Topic
KW - Dose-Response Relationship
KW - Drug
KW - Drug Therapy
KW - Combination
KW - Epilepsy/dt [Drug Therapy]
KW - Epilepsy/me [Metabolism]
KW - Female
KW - Humans
KW - Kidney/de [Drug Effects]
KW - Kidney Function Tests
KW - Kidney Glomerulus/de [Drug Effects]
KW - Kidney Tubules/de [Drug Effects]
KW - Male
KW - Nipecotic Acids/ae [Adverse Effects]
KW - Nipecotic Acids/tu [Therapeutic Use]
KW - Renal Insufficiency/ci [Chemically Induced]
KW - Renal Insufficiency/ur [Urine]
KW - Statistics
KW - Nonparametric
KW - Time Factors
KW - Valproic Acid/ae [Adverse Effects]
KW - Valproic Acid/tu [Therapeutic Use]
KW - beta 2-Microglobulin/de [Drug Effects]
KW - beta 2-Microglobulin/ur [Urine]
RP - NOT IN FILE

SP - 480
EP - 483
JF - Polski Merkuriusz Lekarski
JA - Pol Merkuriusz Lek
VL - 11
IS - 66
CY - Poland
N2 - The aim of the study was the evaluation of the influence of 4-month concomitant tiagabine (TGB) and valproic acid (VPA) or carbamazepine (CBZ) therapy on renal function of epileptic children and teenagers. Initial parameter values, indicated on renal disfunction, were compared with these obtained after VPA and TGB or CBZ and TGB therapy and with values in healthy children and teenagers. Investigation group was composed of 22 children and teenagers with drug-resistant focal epilepsy. We observed that in the time of concomitant VPA and TGB therapy increased the NAG/g creatinine activity index. In spite the fact of statistical significance of these changes, they were not outside the normal range. beta 2-microglobulin concentrations in urine of epileptic children treated with VPA in monotherapy before concomitant therapy with TGB were higher than in control group. That difference was statistically significant. Addition of TGB to the therapy normalized this parameter. During concomitant VPA and TGB or CBZ and TGB therapy we didn't observe statistically significant changes of parameters indicating on glomerular disfunction. In the VPA therapy before concomitant treatment with tiagabine the disfunction of tubules and glomerules was observed. On the other side in the concomitant VPA and TGB therapy the disfunction of tubules and glomerules didn't occurred. We can conclude that concomitant therapy VPA or CBZ with tiagabine don't affect the renal function in clinical significant manner. Therapy with VPA could leads to minimal disfunction of tubules what is represented by increasing of beta 2-microglobulin level in urine
SN - 1426-9686
AD - Katedra i Zaklad Farmakologii Klinicznej Akademii Medycznej we Wroclawiu
UR - 11899842
ER -

TY - JOUR
ID - 1633
T1 - Risk factors for lyme disease in Chester County, Pennsylvania
A1 - Smith,G.
A1 - Wileyto,E.P.
A1 - Hopkins,R.B.
A1 - Cherry,B.R.
A1 - Maher,J.P.
Y1 - 2001///
N1 - Smith, G. Wileyto, E P. Hopkins, R B. Cherry, B R. Maher, J P
Public health reports (Washington, D.C. : 1974)
9716844, qja
AIM, IM
Journal Article. Research Support, U.S. Gov't, P.H.S.
English
KW - MEDLINE
KW - Adolescent
KW - Adult
KW - Age Factors
KW - Aged
KW - Bias (Epidemiology)
KW - Case-Control Studies
KW - Child
KW - Environmental Exposure/an [Analysis]
KW - Environmental Exposure/sn [Statistics & Numerical Data]
KW - Family Characteristics
KW - Female
KW - Health Behavior
KW - Humans

KW - Insect Repellents/ad [Administration & Dosage]
KW - Interviews as Topic
KW - Lyme Disease/ep [Epidemiology]
KW - Lyme Disease/pc [Prevention & Control]
KW - Male
KW - Middle Aged
KW - Pennsylvania/ep [Epidemiology]
KW - Primary Prevention
KW - Protective Clothing/ut [Utilization]
KW - Questionnaires
KW - Risk Assessment
KW - Risk Factors
KW - Rural Health
KW - Telephone
RP - NOT IN FILE
SP - 146
EP - 156
JF - Public Health Reports
JA - Public Health Rep
VL - 116 Suppl 1
CY - United States
N2 - OBJECTIVE: To identify factors associated with increased or decreased risk of infection for Lyme disease in Chester County, Pennyslvania. METHODS: The authors designed an unmatched case-control study involving 294 incident cases reported to the Chester County Health Department in 1998 and 449 controls selected by random digit dialing. All case and control participants were interviewed by telephone. RESULTS: Age is a risk factor for Lyme disease for groups aged 10-19 years old and 50 years or older. Sex was not a risk factor. Incidence of Lyme disease in a rural setting was three times the incidence in an urban setting. Increased risk also was associated with living in single family homes, homes with yards or attached land, woods on the land, signs of tick hosts seen on the land, and homes within 100 feet of woodland. Gardening for more than four hours per week was also a risk factor, but most other outdoor activities were not. Twice as many participants took protective measures against tick bites before outdoor employment than those who merely ventured into the yard or land associated with the home. Only checking for ticks during outdoor activity and the use of repellents prior to outdoor activities outside the yard were unequivocally associated with a reduced risk of Lyme disease. CONCLUSIONS: It is important to increase public awareness about the risk of acquiring Lyme disease from ticks in the immediate environment of the home
SN - 0033-3549
AD - Section of Epidemiology and Public Health, University of Pennsylvania School of Veterinary Medicine, Kennett Square, Pennsylvania, USA. garys@vet.upenn.edu
UR - 11889282
ER -

TY - JOUR
ID - 1634
T1 - Trends and patterns of playground injuries in United States children and adolescents
A1 - Phelan,K.J.
A1 - Khoury,J.
A1 - Kalkwarf,H.J.
A1 - Lanphear,B.P.
Y1 - 2001/07//
N1 - Phelan, K J. Khoury, J. Kalkwarf, H J. Lanphear, B P
Ambulatory pediatrics : the official journal of the Ambulatory Pediatric Association
101089367
IM
Journal Article. Research Support, Non-U.S. Gov't. Research Support, U.S. Gov't, P.H.S.
English
KW - MEDLINE
KW - Accidental Falls/pc [Prevention & Control]

KW - Accidental Falls/sn [Statistics & Numerical Data]
KW - Adolescent
KW - Adult
KW - Child
KW - Preschool
KW - Equipment Design
KW - Female
KW - Humans
KW - Infant
KW - Male
KW - Play and Playthings
KW - Prevalence
KW - Trauma Severity Indices
KW - United States/ep [Epidemiology]
KW - Wounds and Injuries/ep [Epidemiology]
KW - Wounds and Injuries/pc [Prevention & Control]
RP - NOT IN FILE
SP - 227
EP - 233
JF - Ambulatory Pediatrics
JA - Ambul Pediatr
VL - 1
IS - 4
CY - United States
N2 - OBJECTIVE: To determine the prevalence, trends, and severity of injuries attributable to playground falls relative to other common unintentional mechanisms that resulted in an emergency department (ED) visit in the United States. DESIGN AND SETTING: Data from the emergency subset of the National Hospital Ambulatory Medical Care Survey collected from 1992 to 1997 for children <20 years. METHODS: Injury rates and 95% confidence intervals (CIs) were estimated and injury severity scores were computed. RESULTS: There were 920551 (95% CI: 540803 to 1300299) ED visits over the 6-year study period by children and adolescents that were attributable to falls from playground equipment. The annual incidence of visits for playground injuries did not significantly decrease over the course of the study (187000 to 98000, P =.053). Injury visits for playground falls were twice as prevalent as pedestrian mechanisms, but they were less prevalent than visits for motor vehicle-- and bicycle-related injuries. A larger proportion of playground falls resulted in "moderate-to-severe" injury than did bicycle or motor vehicle injuries. Children aged 5 to 9 years had the highest number of playground falls (P =.0014). Playground falls were most likely to occur at school compared to home, public, and other locations (P =.0016). CONCLUSIONS: Playground injury emergency visits have not significantly declined and remain a common unintentional mechanism of injury. Injury visits for playground falls were proportionally more severe than injury visits attributable to other common unintentional mechanisms. Interventions targeting schools and 5- to 9-year-old children may have the greatest impact in reducing emergency visits for playground injuries
SN - 1530-1567
AD - Department of Pediatrics, Children's Hospital Medical Center, Cincinnati, OH 45229, USA.
kj.phelan@chmcc.org
UR - 11888406
ER -

TY - JOUR
ID - 1635
T1 - Different types of smiles and laughter in preschool children
A1 - Sarra,S.
A1 - Otta,E.
Y1 - 2001/12//
N1 - Sarra, S. Otta, E
Psychological reports
qf6, 0376475
IM

Journal Article. Research Support, Non-U.S. Gov't
English
KW - MEDLINE
KW - Brazil
KW - Child
KW - Preschool
KW - Female
KW - Humans
KW - Laughter/px [Psychology]
KW - Male
KW - Personality Assessment
KW - Personality Development
KW - Smiling/px [Psychology]
KW - Social Environment
RP - NOT IN FILE
SP - 547
EP - 558
JF - Psychological Reports
JA - Psychol Rep
VL - 89
IS - 3
CY - United States
N2 - The relationship among morphologically different forms of smiling and laughter was examined. The participants were 19 Brazilian preschool children. Each child was observed a total of 60 min. in three 10-min. sessions on the playground and three 10-min. sessions in the classroom. Analysis suggests that the various forms of smiling do not simply express different intensities of a single emotion. A two-dimensional structure was indicated by factorial analysis. The first dimension, which could be called playfulness-mock aggression, consisted of a broad smile and laughter. The second dimension, which could be called friendliness-appeasement, consisted of a closed and upper smile. The pattern of correlation found between expressive behaviors and both teacher's and peers' evaluations gives further support to the interpretation that smiling is an heterogeneous category
SN - 0033-2941
AD - Department of Experimental Psychology, University of Sao Paulo, Brazil
UR - 11824715
ER -

TY - JOUR
ID - 1636
T1 - [The anxiety-phobic disorder classification in children]. [Russian]
A1 - Savost'ianova,O.L.
Y1 - 2001///
N1 - Savost'ianova, O L
Zhurnal nevrologii i psikhiatrii imeni S.S. Korsakova / Ministerstvo zdravookhraneniia i meditsinskoi promyshlennosti Rossiiskoi Federatsii, Vserossiiskoe obshchestvo nevrologov [i] Vserossiiskoe obshchestvo psikhiatrov
cwz, 9712194
IM
English Abstract. Journal Article
Russian
KW - MEDLINE
KW - Adolescent
KW - Affect
KW - Anxiety Disorders/cl [Classification]
KW - Anxiety Disorders/ep [Epidemiology]
KW - Child
KW - Preschool
KW - Comorbidity

KW - Female
KW - Humans
KW - Impulse Control Disorders/ep [Epidemiology]
KW - Male
KW - Phobic Disorders/cl [Classification]
KW - Phobic Disorders/ep [Epidemiology]
KW - Severity of Illness Index
RP - NOT IN FILE
SP - 25
EP - 28
JF - Zhurnal Nevrologii i Psikhiatrii Imeni S
JA - Zh Nevrol Psikhiatr.Im S S Korsakova
VL - S. Korsakova. 101
IS - 12
CY - Russia
N2 - The study on development regularities and psychopathological structure of anxiety phobic disorders has been conducted in 92 patients, aged 5-15 years. The structure of anxiety-phobic complex is represented. A level of anxiety affect development (primary or secondary, presence and direction of affect cognitive component) and negative effectiveness were emerged in the children as common obligate anxiety-phobic characteristics, which contributed to complex structure stability. Three anxiety-phobic disorder types--situation-dependent, personality-dependent and vital--were distinguished. The first one is characterized by the combination of secondary anxiety with cognitive vector expression, the vector being outside directed, and emotionally unstable negative effectiveness variant (particular sensitivity to negative situations with disability for negative emotion control). The second type is distinguished by secondary anxiety manifestations, the anxiety vector being directed towards the self-ego. In these cases, personality component--rigid variant of negative effectiveness (higher sensitivity to negative events, along with disability for negative emotion modulation) dominates. The third type is defined by the combination of primary anxiety (with vital disturbance, diffusion, psychic activity disorganization in the absence of cognitive component) with rigid variant of negative effectiveness. The types of manifesting anxiety-phobic disorders described correspond to reactive (situation-dependent type), endoreactive (personality-dependent type) and autochthonous (vital type) variants of the disease course
SN - 1997-7298
UR - 11811121
ER -

TY - JOUR
ID - 1637
T1 - [The campaign against malaria in central western Madagascar: comparison of lambda-cyhalothrin and DDT house spraying. II--Parasitological and clinical study]. [French]
A1 - Cot,M.
A1 - Brutus,L.
A1 - Le,Goff G.
A1 - Rajaonarivelo,V.
A1 - Raveloson,A.
Y1 - 2001/12//
N1 - Cot, M. Brutus, L. Le Goff, G. Rajaonarivelo, V. Raveloson, A
Parasite (Paris, France)
bz6, 9437094
IM
Comparative Study. English Abstract. Journal Article
French
KW - MEDLINE
KW - Adolescent
KW - Aerosols
KW - Animals
KW - Anopheles
KW - Child
KW - Preschool

KW - Cross-Sectional Studies
KW - DDT
KW - Female
KW - Housing
KW - Humans
KW - Infant
KW - Insect Vectors/gd [Growth & Development]
KW - Insect Vectors
KW - Insecticides
KW - Longevity
KW - Madagascar/ep [Epidemiology]
KW - Malaria/ep [Epidemiology]
KW - Malaria/pc [Prevention & Control]
KW - Malaria/tm [Transmission]
KW - Male
KW - Mosquito Control/mt [Methods]
KW - Mosquito Control/st [Standards]
KW - Nitriles
KW - Pyrethrins
KW - Risk Factors
KW - Seasons
KW - Time Factors
RP - NOT IN FILE
SP - 309
EP - 316
JF - Parasite
VL - 8
IS - 4
CY - France
N2 - For malaria vector control in Madagascar, 10 WP (lambda-cyhalothrin 10% wettable powder) was compared with DDT 75% WP for house-spraying, from November 1997 to September 1998. This study was implemented at the fringe of the malaria epidemic zone, in villages on western slopes of the central highlands, outside the area covered for the past five years by routine DDT house-spraying (OPID). Four types of treatment were compared in different areas: 1) DDT 2 g ai/m2 and 2) lambda-cyhalothrin 30 mg ai/m2 in previously unsprayed villages, 3) no intervention (control); 4) yearly DDT spraying (OPID fifth cycle). To investigate the malariological impact of spraying, cross-sectional surveys of the village populations were performed in each study area at intervals of two months, before and after spraying. In the newly sprayed areas, from December to June, malaria indices decreased by 62% in the ICON area and 44% in the DDT area, whereas in the unsprayed village malaria increased by 32% during the same season. There was a similar decrease in the number of gametocyte carriers in the newly sprayed areas. Active malaria case detection among febrile individuals was performed fortnightly in each village outside the OPID area. Results showed decreased malaria incidence from February (two months post-spraying) in the sprayed villages, despite the rainy season, whereas in the unsprayed area the decline occurred only after the main transmission season. This study demonstrated that, parasitologically as well as entomologically, house-spraying with residual insecticide (DDT or ICON) was an effective method for controlling malaria on the western fringes of the Madagascar highlands epidemic zone. Both products were effective, but ICON had slightly better impact than DDT, i.e. more reduction of malaria indices and of vector longevity, less irritancy of mosquitoes. For best results in this area of transition between stable and unstable malaria, we recommend earlier annual spraying (as soon as November) and extension of the OPID barrier towards western and northern slopes of the Plateau
SN - 1252-607X
AD - Institut de Recherche pour le Developpement (IRD), Programme Paludisme, BP 434, Antananarivo, Madagascar
UR - 11802267
ER -

TY - JOUR
ID - 1638

T1 - Brief report: improvements in the behavior of children with autism following massage therapy
A1 - Escalona,A.
A1 - Field,T.
A1 - Singer-Strunck,R.
A1 - Cullen,C.
A1 - Hartshorn,K.
Y1 - 2001/10//
N1 - Escalona, A. Field, T. Singer-Strunck, R. Cullen, C. Hartshorn, K
Journal of autism and developmental disorders
7904301, hgw
IM
Clinical Trial. Journal Article. Randomized Controlled Trial. Research Support, Non-U.S. Gov't. Research Support, U.S. Gov't, P.H.S.
English
KW - MEDLINE
KW - Analysis of Variance
KW - Autistic Disorder/px [Psychology]
KW - Autistic Disorder/th [Therapy]
KW - Child
KW - Preschool
KW - Female
KW - Humans
KW - Male
KW - Massage/px [Psychology]
KW - Psychiatric Status Rating Scales
KW - Relaxation Therapy
KW - Treatment Outcome
RP - NOT IN FILE
SP - 513
EP - 516
JF - Journal of Autism & Developmental Disorders
JA - J Autism Dev Disord
VL - 31
IS - 5
CY - United States
N2 - Twenty children with autism, ages 3 to 6 years, were randomly assigned to massage therapy and reading attention control groups. Parents in the massage therapy group were trained by a massage therapist to massage their children for 15 minutes prior to bedtime every night for 1 month and the parents of the attention control group read Dr. Seuss stories to their children on the same time schedule. Conners Teacher and Parent scales, classroom and playground observations, and sleep diaries were used to assess the effects of therapy on various behaviors, including hyperactivity, stereotypical and off-task behavior, and sleep problems. Results suggested that the children in the massage group exhibited less stereotypic behavior and showed more on-task and social relatedness behavior during play observations at school, and they experienced fewer sleep problems at home
SN - 0162-3257
AD - Touch Research Institute, University of Miami School of Medicine, FL 33101, USA
UR - 11794416
ER -

TY - JOUR
ID - 1639
T1 - Quantitative analysis of children's microactivity patterns: The Minnesota Children's Pesticide Exposure Study
A1 - Freeman,N.C.
A1 - Jimenez,M.
A1 - Reed,K.J.
A1 - Gurunathan,S.
A1 - Edwards,R.D.

A1 - Roy,A.
A1 - Adgate,J.L.
A1 - Pellizzari,E.D.
A1 - Quackenboss,J.
A1 - Sexton,K.
A1 - Lioy,P.J.
Y1 - 2001/11//
N1 - Freeman, N C. Jimenez, M. Reed, K J. Gurunathan, S. Edwards, R D. Roy, A. Adgate, J L. Pellizzari, E D. Quackenboss, J. Sexton, K. Lioy, P J
Journal of exposure analysis and environmental epidemiology
bjn, 9111438
IM
Journal Article. Research Support, U.S. Gov't, Non-P.H.S.. Research Support, U.S. Gov't, P.H.S.
English
KW - MEDLINE
KW - Activities of Daily Living
KW - Administration
KW - Cutaneous
KW - Oral
KW - Adolescent
KW - Child
KW - Child Behavior
KW - Child Welfare
KW - Preschool
KW - Environment
KW - Environmental Exposure
KW - Female
KW - Hand
KW - Health Surveys
KW - Housing
KW - Humans
KW - Male
KW - Mouth
KW - Pesticides/an [Analysis]
KW - Sex Factors
KW - Video Recording
RP - NOT IN FILE
SP - 501
EP - 509
JF - Journal of Exposure Analysis and Environmental Epidemiology
JA - J Expo Anal Environ Epidemiol
VL - 11
IS - 6
CY - United States
N2 - The National Human Exposure Assessment Survey (NHEXAS)/Minnesota Children's Pesticide Exposure Study (MNCPES) was a population-based study designed to characterize children's exposure to residential pesticides and to evaluate the contribution of residential and children's activities to children's exposure. Families of 168 children were surveyed for residential use of pesticides and children's activities. From these homes, families of 102 children between the ages of 3 and 13 years participated in a week-long intensive exposure study. Of the 102 children, 19 children were videotaped for four consecutive hours in their normal daily activities. The survey responses indicated that the youngest children were more likely to exhibit behaviors that would foster exposure to environmental contaminants. Comparison of questionnaire responses indicated that the videotaped subsample was representative of the exposure study population. The microactivities of the videotaped children that might contribute to their exposure via ingestion or dermal routes were quantified. Hand-to-mouth and object-to-mouth activities were observed most frequently among the youngest children. The youngest children were also most likely to be barefoot both indoors and outside. Gender differences were found in mouthing behavior and the proportion of observed time spent outdoors

SN - 1053-4245
AD - Robert Wood Johnson Medical School, Piscataway, New Jersey, USA. nfreeman@eohsi.rutgers.edu
UR - 11791166
ER -

TY - JOUR
ID - 1640
T1 - A population exposure model for particulate matter: case study results for PM(2.5) in Philadelphia, PA
A1 - Burke,J.M.
A1 - Zufall,M.J.
A1 - Ozkaynak,H.
Y1 - 2001/11//
N1 - Burke, J M. Zufall, M J. Ozkaynak, H
Journal of exposure analysis and environmental epidemiology
bjn, 9111438
IM
Journal Article. Research Support, U.S. Gov't, Non-P.H.S.
English
KW - MEDLINE
KW - Activities of Daily Living
KW - Adolescent
KW - Adult
KW - Aged
KW - 80 and over
KW - Air Pollutants/an [Analysis]
KW - Child
KW - Environment
KW - Environmental Exposure
KW - Female
KW - Forecasting
KW - Humans
KW - Male
KW - Middle Aged
KW - Models
KW - Theoretical
KW - Particle Size
KW - Philadelphia
KW - Seasons
KW - Time Factors
KW - Tobacco Smoke Pollution/an [Analysis]
KW - Urban Population
RP - NOT IN FILE
SP - 470
EP - 489
JF - Journal of Exposure Analysis and Environmental Epidemiology
JA - J Expo Anal Environ Epidemiol
VL - 11
IS - 6
CY - United States
N2 - A population exposure model for particulate matter (PM), called the Stochastic Human Exposure and Dose Simulation (SHEDS-PM) model, has been developed and applied in a case study of daily PM(2.5) exposures for the population living in Philadelphia, PA. SHEDS-PM is a probabilistic model that estimates the population distribution of total PM exposures by randomly sampling from various input distributions. A mass balance equation is used to calculate indoor PM concentrations for the residential microenvironment from ambient outdoor PM concentrations and physical factor data (e.g., air exchange, penetration, deposition), as well as emission strengths for indoor PM sources (e.g., smoking, cooking). PM concentrations in nonresidential microenvironments are calculated using equations developed from regression analysis of available indoor and

outdoor measurement data for vehicles, offices, schools, stores, and restaurants/bars. Additional model inputs include demographic data for the population being modeled and human activity pattern data from EPA's Consolidated Human Activity Database (CHAD). Model outputs include distributions of daily total PM exposures in various microenvironments (indoors, in vehicles, outdoors), and the contribution from PM of ambient origin to daily total PM exposures in these microenvironments. SHEDS-PM has been applied to the population of Philadelphia using spatially and temporally interpolated ambient PM(2.5) measurements from 1992-1993 and 1990 US Census data for each census tract in Philadelphia. The resulting distributions showed substantial variability in daily total PM(2.5) exposures for the population of Philadelphia (median=20 microg/m(3); 90th percentile=59 microg/m(3)). Variability in human activities, and the presence of indoor-residential sources in particular, contributed to the observed variability in total PM(2.5) exposures. The uncertainty in the estimated population distribution for total PM(2.5) exposures was highest at the upper end of the distribution and revealed the importance of including estimates of input uncertainty in population exposure models. The distributions of daily microenvironmental PM(2.5) exposures (exposures due to time spent in various microenvironments) indicated that indoor-residential PM(2.5) exposures (median=13 microg/m(3)) had the greatest influence on total PM(2.5) exposures compared to the other microenvironments. The distribution of daily exposures to PM(2.5) of ambient origin was less variable across the population than the distribution of daily total PM(2.5) exposures (median=7 microg/m(3); 90th percentile=18 microg/m(3)) and similar to the distribution of ambient outdoor PM(2.5) concentrations. This result suggests that human activity patterns did not have as strong an influence on ambient PM(2.5) exposures as was observed for exposure to other PM(2.5) sources. For most of the simulated population, exposure to PM(2.5) of ambient origin contributed a significant percent of the daily total PM(2.5) exposures (median=37.5%), especially for the segment of the population without exposure to environmental tobacco smoke in the residence (median=46.4%). Development of the SHEDS-PM model using the Philadelphia PM(2.5) case study also provided useful insights into the limitations of currently available data for use in population exposure models. In addition, data needs for improving inputs to the SHEDS-PM model, reducing uncertainty and further refinement of the model structure, were identified
SN - 1053-4245
AD - US EPA, National Exposure Research Laboratory, Research Triangle Park, North Carolina 27711, USA. burke.janet@epa.gov
UR - 11791164
ER -

TY - JOUR
ID - 1641
T1 - Levels of persistent organic pollutants in several child day care centers
A1 - Wilson,N.K.
A1 - Chuang,J.C.
A1 - Lyu,C.
Y1 - 2001/11//
N1 - Wilson, N K. Chuang, J C. Lyu, C
Journal of exposure analysis and environmental epidemiology
bjn, 9111438
IM
Journal Article. Research Support, U.S. Gov't, Non-P.H.S.
English
KW - MEDLINE
KW - Activities of Daily Living
KW - Air Pollution
KW - Indoor/an [Analysis]
KW - Carcinogens/an [Analysis]
KW - Child
KW - Child Day Care Centers
KW - Preschool
KW - Dust
KW - Endocrine System/de [Drug Effects]
KW - Environmental Exposure
KW - Female
KW - Gas Chromatography-Mass Spectrometry

KW - Humans
KW - Inhalation Exposure
KW - Insecticides/an [Analysis]
KW - Male
KW - Organic Chemicals/an [Analysis]
KW - Poverty
KW - Soil Pollutants/an [Analysis]
KW - Xenobiotics/an [Analysis]
RP - NOT IN FILE
SP - 449
EP - 458
JF - Journal of Exposure Analysis and Environmental Epidemiology
JA - J Expo Anal Environ Epidemiol
VL - 11
IS - 6
CY - United States
N2 - The concentrations of a suite of persistent organic chemicals were measured in multiple media in 10 child day care centers located in central North Carolina. Five centers served mainly children from low-income families, as defined by the federal Women, Infants, and Children (WIC) assistance program, and five served mainly children from middle-income families. The targeted chemicals were chosen because of their probable carcinogenicity, acute or chronic toxicity, or hypothesized potential for endocrine system disruption. Targeted compounds included polycyclic aromatic hydrocarbons (PAHs), pentachloro- and nonyl-phenol, bisphenol-A, dibutyl and butylbenzyl phthalate, polychlorinated biphenyls (PCBs), organochlorine pesticides, the organophosphate pesticides diazinon and chlorpyrifos, and the herbicide 2,4-dichlorophenoxyacetic acid (2,4D). Sampled media were indoor and outdoor air, food and beverages, indoor dust, and outdoor play area soil. Concentrations of the targeted compounds were determined using a combination of extraction and analysis methods, depending on the media. Analysis was predominantly by gas chromatography/mass spectrometry (GC/MS) or gas chromatography with electron capture detection (GC/ECD). Concentrations of the targeted pollutants were low and well below the levels generally considered to be of concern as possible health hazards. Potential exposures to the target compounds were estimated from the concentrations in the various media, the children's daily time-activity schedules at day care, and the best currently available estimates of the inhalation rates (8.3 m(3)/day) and soil ingestion rates (100 mg/day) of children ages 3-5. The potential exposures for the target compounds differed depending on the compound class and the sampled media. Potential exposures through dietary ingestion were greater than those through inhalation, which were greater than those through nondietary ingestion, for the total of all PAHs, the phenols, the organophosphate pesticides, and the organochlorine pesticides. Potential exposures through dietary ingestion were greater than those through nondietary ingestion, which were greater than those through inhalation, for those PAHs that are probable human carcinogens (B2 PAH), the phthalate esters, and 2,4D. For the PCBs, exposures through inhalation were greater than those through nondietary ingestion, and exposures through dietary ingestion were smallest. Differences in targeted compound levels between the centers that serve mainly low-income clients and those that serve mainly middle-income clients were small and depended on the compound class and the medium
SN - 1053-4245
AD - Battelle, Durham, North Carolina, USA
UR - 11791162
ER -

TY - JOUR
ID - 1642
T1 - UV doses of American children and adolescents
A1 - Godar,D.E.
Y1 - 2001/12//
N1 - Godar, D E
Photochemistry and photobiology
0376425, p69
IM
Journal Article
English

KW - MEDLINE
KW - Adolescent
KW - Child
KW - Preschool
KW - Female
KW - Humans
KW - Infant
KW - Newborn
KW - Male
KW - Neoplasms
KW - Radiation-Induced/et [Etiology]
KW - Photobiology
KW - Radiation Dosage
KW - Risk Factors
KW - Skin/re [Radiation Effects]
KW - Skin Neoplasms/et [Etiology]
KW - Ultraviolet Rays/ae [Adverse Effects]
KW - United States
RP - NOT IN FILE
SP - 787
EP - 793
JF - Photochemistry & Photobiology
JA - Photochem Photobiol
VL - 74
IS - 6
CY - United States
N2 - The ultraviolet (UV) doses of American young adults were never measured, but are needed for assessing UV-related health risks. These doses were calculated using a novel approach. The National Human Activity Pattern Survey recorded the daily minute-by-minute activities of about 2000 young adults (0-19 years) over the course of 2 years to assess their exposure to environmental pollutants. From that survey, only the outdoor daylight data of northern and southern girls and boys were extracted and stratified by season and age to find the time American children (0-5 and 6-12 years) and adolescents (13-19 years) spend outside. They spend about 10% of the day outdoors, but only get about 30% of the available terrestrial UV radiation (on a horizontal plane). American children have about the same percent personal ambients as adults (3.1%), 2.8% for girls and 3.4% for boys. Adolescents have the lowest personal ambients (2.6%), 2.1% for girls and 3.1% for boys. To get their UV doses, their percent ambients are multiplied by the total available terrestrial UV. Excluding vacation, the erythemally weighted UV doses for American children are 25 kJ/m2/year, 23 for girls and 28 for boys. Adolescents get the lowest UV exposure of any group, 21 kJ/m2/year, 18 for girls and 24 for boys. Young adult northern girls get 18 kJ/m2/year and boys get 21 kJ/m2/year, whereas southern girls get 24 kJ/m2/year and boys get 31 kJ/m2/year. The youngest children (0-5 years) get slightly higher summer doses. Thus, we can now assess the UV-related health risks for American children and adolescents
SN - 0031-8655
AD - US Food and Drug Administration, Center for Devices and Radiological Health, Rockville, MD 20852, USA. deg@cdrh.fda.gov
UR - 11783934
ER -

TY - JOUR
ID - 1643
T1 - Incidence patterns of vivax malaria in civilians residing in a high-risk county of Kyonggi-do (province), Republic of Korea
A1 - Moon,J.J.
A1 - Cho,S.Y.
Y1 - 2001/12//
N1 - Moon, J J. Cho, S Y
The Korean journal of parasitology
b3d, 9435800

IM
Journal Article
English
KW - MEDLINE
KW - Adolescent
KW - Adult
KW - Age Factors
KW - Aged
KW - Animals
KW - Child
KW - Culicidae/ps [Parasitology]
KW - Female
KW - Humans
KW - Incidence
KW - Insect Vectors
KW - Korea/ep [Epidemiology]
KW - Malaria
KW - Vivax/ep [Epidemiology]
KW - Male
KW - Middle Aged
KW - Risk
KW - Seasons
KW - Sex Factors
RP - NOT IN FILE
SP - 293
EP - 299
JF - Korean Journal of Parasitology
JA - Korean J Parasitol
VL - 39
IS - 4
CY - Korea (South)
N2 - The characteristics of vivax malaria epidemics along the demilitarized zone (DMZ) in the Republic of Korea has been established by the early surveillance data. To further characterize the epidemic, data of civilian patients microscopically diagnosed with malaria from 1995 through 2000 were analyzed in Yonchon-gun (county). Malaria incidence was greater in male civilians > 30 years of age ($p < 0.05$). The annual parasite index was significantly higher in those living in the administrative areas (Myeon) traversed by DMZ than those living in Myons not traversed by DMZ ($p < 0.05$). Analysis according to the distance (4 to 14 km) from DMZ showed that people living in villages close to DMZ had higher annual parasite indices than those living in villages remote from DMZ (p for trend < 0.05). Civilians living in Myeons with plains and located in northwestern part of the county had higher annual parasite indices than those living in hilly Myeons located in southeastern part of the county (p for trend < 0.05). These findings suggest that the contraction of vivax malaria is related with night-time outdoor activities, and that the distance from DMZ is a risk factor. In this area, the flying distance of infected vector mosquitos can explain the annually repeating occurrence of civilian cases
SN - 0023-4001
AD - Yonchon County Health Center, Yonchon 486-900, Korea
UR - 11775329
ER -

TY - JOUR
ID - 1644
T1 - Free- time activities in middle childhood: links with adjustment in early adolescence
A1 - McHale,S.M.
A1 - Crouter,A.C.
A1 - Tucker,C.J.
Y1 - 2001/11//
N1 - McHale, S M. Crouter, A C. Tucker, C J
Child development

0372725, d28
IM
Journal Article. Research Support, U.S. Gov't, P.H.S.
English
KW - MEDLINE
KW - Adolescent
KW - Adolescent Behavior/px [Psychology]
KW - Child
KW - Female
KW - Follow-Up Studies
KW - Humans
KW - Leisure Activities
KW - Male
KW - Parents
KW - Social Adjustment
KW - Socioeconomic Factors
RP - NOT IN FILE
SP - 1764
EP - 1778
JF - Child Development
JA - Child Dev
VL - 72
IS - 6
CY - United States
N2 - This study assessed links between free-time activities in middle childhood (hobbies, sports, toys and games, outdoor play, reading, television viewing, and hanging out) and school grades, conduct, and depression symptoms both concurrently and 2 years later, in early adolescence. It also explored two mechanisms that might underlie activity-adjustment links: whether the social contexts of children's activities mediate these links, child effects explain these connections, or both. Participants were 198 children (M = 10.9 years, SD = .54 years) in Year 1, and their parents. In home interviews in Years 1 and 3 of the study, mothers rated children's conduct problems, children reported on their depression symptoms, and information was collected on school grades from report cards. In seven evening phone interviews, children reported on the time they spent in free-time activities during the day of the call and their companions in each activity. Links were found between the nature of children's free-time activities and their adjustment. The social contexts of free-time activities explained activity-adjustment links to a limited degree; with respect to child effects, evidence also suggested that better adjusted children became more involved in adaptive activities over time
SN - 0009-3920
AD - Pennsylvania State University, University Park, 16802, USA. x2u@psu.edu
UR - 11768144
ER -

TY - JOUR
ID - 1645
T1 - Signal transduction for clinicians: why should we care?
A1 - Schor,N.F.
Y1 - 2001/11//
N1 - Schor, N F
Pediatric neurology
aa5, 8508183
IM
Journal Article. Research Support, U.S. Gov't, P.H.S.
English
KW - MEDLINE
KW - Animals
KW - Apoptosis/de [Drug Effects]
KW - Apoptosis/ph [Physiology]
KW - Child

KW - Humans
KW - Nervous System Diseases/dt [Drug Therapy]
KW - Nervous System Diseases/pp [Physiopathology]
KW - Neurons/de [Drug Effects]
KW - Neurons/ph [Physiology]
KW - Signal Transduction/de [Drug Effects]
KW - Signal Transduction/ph [Physiology]
RP - NOT IN FILE
SP - 361
EP - 367
JF - Pediatric Neurology
JA - Pediatr Neurol
VL - 25
IS - 5
CY - United States
N2 - Neurons must respond to a bewildering array of external and internal stimuli and must distinguish among them to generate an appropriate response or change in metabolic or electrical activity. Furthermore, the response of a cell to a given stimulus must depend on what else is happening inside and outside the cell at the time of arrival of that stimulus. The process of signal transduction is what gives the cell and organism the flexibility and "knowledge base" to carry out these functions. Conversely, aberrations of signal transduction underlie an increasing array of developmental, genetic, and acquired diseases and conditions of the nervous system. Pharmacological modulation of signal transduction pathways and their effectors holds great promise for the remediation of these neurologic disorders
SN - 0887-8994
AD - Pediatric Center for Neuroscience and Division of Child Neurology, Children's Hospital of Pittsburgh, and Department of Pediatrics, University of Pittsburgh, Pittsburgh, PA 15213, USA
UR - 11744310
ER -

TY - JOUR
ID - 1646
T1 - Otitis media in Greenland. Studies on historical, epidemiological, microbiological, and immunological aspects. [Review] [162 refs]
A1 - Homoe,P.
Y1 - 2001///
N1 - Homoe, P
International journal of circumpolar health
ctg, 9713056
IM
Historical Article. Journal Article. Research Support, Non-U.S. Gov't. Review
English
KW - MEDLINE
KW - Carrier Proteins/ip [Isolation & Purification]
KW - Child
KW - Preschool
KW - Cholesteatoma
KW - Middle Ear/co [Complications]
KW - Collectins
KW - Epidemiologic Studies
KW - Greenland/ep [Epidemiology]
KW - Herpesvirus 4
KW - Human/ip [Isolation & Purification]
KW - History
KW - 18th Century
KW - 19th Century
KW - 20th Century
KW - Humans

KW - Infant
KW - Mass Screening
KW - Otitis Media/ep [Epidemiology]
KW - Otitis Media/hi [History]
KW - Otitis Media/im [Immunology]
KW - Otitis Media/mi [Microbiology]
KW - Otitis Media
KW - Risk Factors
RP - NOT IN FILE
SP - 1
EP - 54
JF - International Journal of Circumpolar Health
JA - Int J Circumpolar Health
VL - 60 Suppl 2
CY - Finland
N2 - This thesis describes the different aspects of otitis media (OM) in the population of Greenland viewed in a historical and modern clinical perspective. Chapter 1 outlines the addressed problems and aims while chapters 2 and 3 deal with historical studies and an evaluation of the present knowledge based on the literature. Physical anthropological studies, using skeletal samples of adult Eskimo crania from before and after the colonization of Greenland in 1721 and information about modern living Eskimos (Inuit), have shown that OM sequelae of the temporal bones were significantly less common in pre-colonization Eskimos and that the mean area size of the pneumatized cell system in the temporal bone was significantly larger in pre-colonization Eskimos. These findings indicated an increase in OM after the colonization most likely caused by the social, cultural, habitary, and dietary changes due to increased contact with the outside world. Historical reports after the colonization confirm a high prevalence of OM especially in children. Modern epidemiological studies from the 1960's to 1980's in the Arctic region of Alaska, Canada, and Greenland along with reports from visiting consultant otologists in Greenland almost uniformly mention prevalent OM problems in children as well as in adults. The aim was therefore to further describe the epidemiological pattern of the different OM disease entities (acute OM (AOM), chronic OM (COM), COM with suppuration (CSOM), secretory OM (SOM), and cholesteatoma) and investigate the potentially associated risk factors in especially Greenlandic children because these diseases are primarily established and problematical in childhood. Chapter 4 describes the definitions used in the thesis and chapter 5 describes the studies included. Section 5.1 describes a study of cholesteatoma in Greenlanders. The study revealed an almost similar incidence of hospital treated children with cholesteatoma (6.6 per 100,000) as seen in comparable studies from other parts of the world. Furthermore, childhood cholesteatomas were the most aggressive. The frequency of residuals or recurrences after otosurgical treatment was high with a trend for better results when using the extensive canal wall-down procedure. It could be concluded that these patients urgently need close follow-up for at least five years postoperatively, if not lifelong. Section 5.2 describes a hearing screening survey of 167 school children using school registration charts. A high prevalence of hearing loss (HL) was found. A total of 43% of the children had hearing thresholds exceeding 20 dB at one or more frequencies between 250-8000 Hz in one or both ears, and 19% had the same type of HL in the frequencies 500-2000 Hz. HL was significantly associated with episodes of OM. These findings were in accordance with reports from Alaska and Canada. It is therefore concluded that a hearing screening programme of school children is important and that OM seems to have an impact on hearing in school children in Greenland. In section 5.3 an epidemiological survey is described concerning the prevalence of the different OM disease entities. The survey was carried out in Nuuk and Sisimiut and involved 740 children aged 3, 4, 5, and 8 years. A total of 591 children participated and selection bias was not found when controlling for age, sex, and episodes of AOM. The survey revealed that 52% of children in Nuuk and 54% in Sisimiut had some kind of pathological affection of their middle ear. COM and CSOM were found in 9%, but more prevalent among children in Sisimiut (12%) than in Nuuk (7%). Middle ear effusion (MEE) diagnosed by tympanometry was found in 23% in Nuuk and 28% in Sisimiut while simple tubal dysfunction (STD) was found in 13% and 8%, respectively. MEE and STD were associated with young age. Sequelae of OM was apparent in 11% in both towns. When comparing the results with a 10-year-older, almost similar survey of 142 children, it was evident that the OM situation had not changed in the period between the studies. The survey underlines the need for increased focus on the different OM entities in Greenlandic children. Section 5.4 deals with microbiological aspects. The nasopharyngeal microflora and ear discharge microflora of potential pathogens were evaluated in 54 children with AOM and in 201 control children without AOM. Very high carriage rates expressed qualitatively and semiquantitatively of potentially pathogenio bacteria were found in the nasopharynx of children with AOM (98%) but also in that of

the control children (91%) and even in children denoted as being very healthy (94%). However, the same bacterial species were cultured from the nasopharynx and ear discharge as in comparable studies world-wide. Only S. pneumoniae was carried significantly more often in the nasopharynx of AOM children compared with age matched control children. Chlamydiae, M. pneumoniae, adenovirus, respiratory syncytial virus, parainfluenza- type 1, 2, and 3 virus, and influenza- type A and B virus were not major pathogens. In contrast, entero- and rhinoviruses were detected significantly more frequent in nasopharyngeal specimens from AOM children (59%) compared with age matched controls (33%) and also in 29% of the examined ear discharge specimens. It is therefore concluded that the potentially pathogenic bacterial load is early and massive. This alone or in interplay with entero- and rhinovirus infection and occasionally with other viruses may play an important role in the high prevalence of OM among children in Greenland. Section 5.5 deals with an examination of potential risk factors for AOM, recurrent AOM (rAOM), and COM in the same 591 children as studied in section 5.3. Early age at first AOM episode was associated with rAOM episodes (> or = 5 episodes since birth). Thus, the relative risk of developing rAOM was eight times higher if the first episode of AOM occurred before 7 months of age than after 24 months of age. Furthermore, compared with studies elsewhere in the world, a high proportion (40%) of the children in this survey had their first AOM episode during their first year of life and 41% of these children developed rAOM. It was also found that children had an increased risk of AOM, rAOM, or COM when both parents were born in Greenland, when parents also have had OM, when living in very crowded households, and when having experienced a long period of exclusive breast feeding, or when recalling of breast feeding was not possible. Gender, type, and size of housing, insulation standard of housing, daycare, exposure to passive cigarette smoking, and dietary habits were not associated with AOM, rAOM, or COM in the surveyed children. It is concluded that early onset of AOM occurs frequently in Greenlandic children and that a high proportion of these children develop rAOM. The study confirms that AOM is a highly multifactorial disease determined by a number of genetic and environmental factors. Finally, section 5.6 is a hypothesis generating study attempting to explain the high prevalence of early episodes of AOM in community-based children in Nuuk. The hypothesis is based on a possible association between findings of mannose-binding lectin genotypes, early Epstein-Barr virus infections and episodes of AOM, rAOM, or nasopharyngeal colonization with potentially pathogenic bacteria. However, the study does not support any of this hypothesis. In chapter 6, future studies are suggested and chapter 7 presents concluding remarks. [References: 162]
SN - 1239-9736
UR - 11725622
ER -

TY - JOUR
ID - 1647
T1 - Seasonality of birth in patients with childhood Type I diabetes in 19 European regions
A1 - McKinney,P.A.
A1 - EURODIAB Seasonality Of Birth Group.Europe and Diabetes.
Y1 - 2001/10//
N1 - McKinney, P A. EURODIAB Seasonality Of Birth Group. Europe and Diabetes
Diabetologia
e93, 0006777
IM
Journal Article. Multicenter Study. Research Support, Non-U.S. Gov't
English
KW - MEDLINE
KW - Adolescent
KW - Age of Onset
KW - Birth Rate/eh [Ethnology]
KW - Child
KW - Preschool
KW - Diabetes Mellitus
KW - Type 1/ep [Epidemiology]
KW - Europe/ep [Epidemiology]
KW - Female
KW - Great Britain/ep [Epidemiology]
KW - Humans

KW - Incidence
KW - Infant
KW - Male
KW - Seasons
KW - Sex Characteristics
RP - NOT IN FILE
SP - B67
EP - B74
JF - Diabetologia
VL - 44 Suppl 3
CY - Germany
N2 - AIMS/HYPOTHESIS: Differences in seasonality of birth patterns between the general population and the group who develop Type I (insulin-dependent) diabetes mellitus indicate that environmental factors operating around the antenatal and perinatal period could be important. We investigated whether the same unusual patterns in seasonality of birth observed in children with Type I diabetes in Great Britain could also be found in other European populations. METHODS: Population-based incidence cohorts of children diagnosed with Type I diabetes under 15 years of age from 1989 onwards were analysed. Previously reported data sets from Great Britain were also included together with data on children diagnosed over an additional 5 year period (1988-1992). To assess the role of seasonality in diabetes, we used the method of Walter and Elwood to examine monthly birth figures for each country or region. RESULTS: Outside of Great Britain, no seasonality of birth was seen for any single or combination of European countries. Significant sinusoidal patterns were observed in Scotland, Yorkshire and Leicester, although the peak for Leicester appeared around autumn rather than spring. There was little evidence that sex or age at diagnosis played a part in differences in seasonal patterns, either overall or for any individual country. CONCLUSIONS/INTERPRETATION: We found no uniform seasonal pattern of birth in childhood diabetes patients across European populations, either overall or according to sex and age. This study provides no consistent evidence that environmental factors, which vary from season to season, have any influence on the fetal or neonatal life to determine the onset of Type I diabetes. However, a study of seasonality that takes into account possible changes both over time and over geographical areas could provide more insights
SN - 0012-186X
AD - Paediatric Epidemiology Group, Institute of Epidemiology, University of Leeds, UK
UR - 11724420
ER -

TY - JOUR
ID - 1648
T1 - Activation of the coagulation system occurs within rather than outside cutaneous haemangiomas
A1 - Antovic,J.
A1 - Bakic,M.
A1 - Milicevic,R.
A1 - Gojkovic,G.
A1 - Blomback,M.
Y1 - 2001/10//
N1 - Antovic, J. Bakic, M. Milicevic, R. Gojkovic, G. Blomback, M
Acta paediatrica (Oslo, Norway : 1992)
bgc, 9205968
IM
Journal Article
English
KW - MEDLINE
KW - Blood Coagulation
KW - Child
KW - Preschool
KW - Female
KW - Fibrinolysis
KW - Hemangioma/bl [Blood]
KW - Humans

KW - Infant
KW - Male
KW - Skin Neoplasms/bl [Blood]
RP - NOT IN FILE
SP - 1137
EP - 1140
JF - Acta Paediatrica
JA - Acta Paediatr
VL - 90
IS - 10
CY - Norway
N2 - UNLABELLED: Haemangiomas are the commonest tumours of infancy. They can become even more serious if followed by consumption coagulopathy and even life-threatening in cases of Kasabach-Merritt syndrome, with thrombocytopenia and haemorrhage. Data exist concerning systemic coagulation abnormalities in children with haemangiomas but to our knowledge there are no data on local consumption coagulopathy in haemangioma per se. We examined blood coagulation and fibrinolysis parameters in blood withdrawn from haemangioma blood vessels and blood withdrawn from the systemic vein in 14 children with cutaneous haemangiomas (3M, 11F; age range 3 mo to 10y). Compared with controls, significant decreases in fibrinogen levels, FVII activity, antithrombin and plasmin inhibitor levels and increases in international normalized ratio (INR) and D-dimer levels were observed in the blood samples withdrawn directly from haemangioma blood vessels. Fibrinogen and antithrombin levels in samples withdrawn from systemic veins were reduced in relation to control values whilst INR values increased, but within normal ranges. D-dimer levels were increased in peripheral blood. The fibrinogen level was significantly lower and the INR and D-dimer levels were significantly higher in blood samples from haemangiomas compared to systemic blood. Clinical signs of systemic disseminated intravascular coagulation were not observed. CONCLUSIONS: Our results suggest a strong local activation and local consumption coagulopathy in haemangioma, along with less conspicuous but observable systemic changes in coagulation and fibrinolysis parameters, although without signs of consumptive coagulopathy. These systemic changes could be a reflection of intra-lesion coagulation activation although there is no evidence to suggest truly systemic disseminated intravascular coagulation
SN - 0803-5253
AD - Department of Surgical Sciences, Karolinska Institutet, Stockholm, Sweden. Jovan.Antovic@kirurgi.ki.se
UR - 11697424
ER -

TY - JOUR
ID - 1649
T1 - Epidemiology, morbidity, mortality and treatment of lightning injuries in a Turkish burns units
A1 - Aslar,A.K.
A1 - Soran,A.
A1 - Yildiz,Y.
A1 - Isik,Y.
Y1 - 2001/10//
N1 - Aslar, A K. Soran, A. Yildiz, Y. Isik, Y
International journal of clinical practice
cvt, 9712381
IM
Journal Article
English
KW - MEDLINE
KW - Adolescent
KW - Adult
KW - Aged
KW - Burn Units/sn [Statistics & Numerical Data]
KW - Child
KW - Female
KW - Humans
KW - Lightning Injuries/ep [Epidemiology]

KW - Lightning Injuries/mo [Mortality]
KW - Lightning Injuries/th [Therapy]
KW - Male
KW - Middle Aged
KW - Turkey/ep [Epidemiology]
RP - NOT IN FILE
SP - 502
EP - 504
JF - International Journal of Clinical Practice
JA - Int J Clin Pract
VL - 55
IS - 8
CY - England
N2 - Deaths from lightning injuries are infrequent--0.2-0.8 per million per year. The victims are mostly young, active people who are struck during various outdoor activities in the summer months. From November 1975 to October 1998; 22 lightning burns were treated in Ankara Numune Teaching and Research Hospital. The mean age of the patients was 32.9 (12-65) years, the female/male ratio 9/13 and the mean duration of hospital stay 15.4 (1-62) days. The commonest clinical symptoms were confusion, amnesia (5 patients), neurological dysfunction (2 patients), cystitis (4 patients), and cardiac arrhythmias (1 patient). There were no deaths. Sixteen surgical procedures were carried out on 14 patients; this was significantly fewer than from any other cause of burns. The commonest long-term complication was chronic pain. Because complications are frequently seen in lightning injuries, our results revealed that patients should be hospitalised and treated as soon as possible after the accident with fluid resuscitation, cardiac resuscitation, tetanus prophylaxis and antibiotics where necessary
SN - 1368-5031
AD - Department of Surgery, Ankara Numune Teaching and Research Hospital, Ankara, Turkey
UR - 11695067
ER -

TY - JOUR
ID - 1650
T1 - Randomised controlled trial of primary school based intervention to reduce risk factors for obesity
A1 - Sahota,P.
A1 - Rudolf,M.C.
A1 - Dixey,R.
A1 - Hill,A.J.
A1 - Barth,J.H.
A1 - Cade,J.
Y1 - 2001/11/03/
N1 - Sahota, P. Rudolf, M C. Dixey, R. Hill, A J. Barth, J H. Cade, J
BMJ (Clinical research ed.)
8900488, bmj, 101090866
AIM, IM
Clinical Trial. Journal Article. Multicenter Study. Randomized Controlled Trial. Research Support, Non-U.S. Gov't
English
KW - MEDLINE
KW - Body Mass Index
KW - Child
KW - Child Nutrition Sciences/ed [Education]
KW - Cross-Over Studies
KW - Diet
KW - England
KW - Exercise
KW - Female
KW - Focus Groups
KW - Food Habits
KW - Growth

KW - Health Promotion/mt [Methods]
KW - Humans
KW - Male
KW - Obesity/et [Etiology]
KW - Obesity/pc [Prevention & Control]
KW - Obesity/px [Psychology]
KW - Program Evaluation
KW - Risk Factors
KW - School Health Services
KW - Vegetables
RP - NOT IN FILE
SP - 1029
EP - 1032
JF - BMJ
VL - 323
IS - 7320
CY - England
N2 - OBJECTIVE: To assess if a school based intervention was effective in reducing risk factors for obesity. DESIGN: Group randomised controlled trial. SETTING: 10 primary schools in Leeds. PARTICIPANTS: 634 children aged 7-11 years. INTERVENTION: Teacher training, modification of school meals, and the development of school action plans targeting the curriculum, physical education, tuck shops, and playground activities. MAIN OUTCOME MEASURES: Body mass index, diet, physical activity, and psychological state. RESULTS: Vegetable consumption by 24 hour recall was higher in children in the intervention group than the control group (weighted mean difference 0.3 portions/day, 95% confidence interval 0.2 to 0.4), representing a difference equivalent to 50% of baseline consumption. Fruit consumption was lower in obese children in the intervention group (-1.0, -1.8 to -0.2) than those in the control group. The three day diary showed higher consumption of high sugar foods (0.8, 0.1 to 1.6)) among overweight children in the intervention group than the control group. Sedentary behaviour was higher in overweight children in the intervention group (0.3, 0.0 to 0.7). Global self worth was higher in obese children in the intervention group (0.3, 0.3 to 0.6). There was no difference in body mass index, other psychological measures, or dieting behaviour between the groups. Focus groups indicated higher levels of self reported behaviour change, understanding, and knowledge among children who had received the intervention. CONCLUSION: Although it was successful in producing changes at school level, the programme had little effect on children's behaviour other than a modest increase in consumption of vegetables
SN - 0959-8138
AD - School of Health Sciences, Leeds Metropolitan University
UR - 11691759
ER -

TY - JOUR
ID - 1651
T1 - Longitudinal study of elbow and shoulder pain in youth baseball pitchers
A1 - Lyman,S.
A1 - Fleisig,G.S.
A1 - Waterbor,J.W.
A1 - Funkhouser,E.M.
A1 - Pulley,L.
A1 - Andrews,J.R.
A1 - Osinski,E.D.
A1 - Roseman,J.M.
Y1 - 2001/11//
N1 - Lyman, S. Fleisig, G S. Waterbor, J W. Funkhouser, E M. Pulley, L. Andrews, J R. Osinski, E D. Roseman, J M
Medicine and science in sports and exercise
8005433, mg8
IM, S

Journal Article
English
KW - MEDLINE
KW - Alabama/ep [Epidemiology]
KW - Arm Injuries/ep [Epidemiology]
KW - Arthralgia/ep [Epidemiology]
KW - Athletic Injuries/ep [Epidemiology]
KW - Baseball/in [Injuries]
KW - Baseball/ph [Physiology]
KW - Biomechanical Phenomena
KW - Child
KW - Elbow/in [Injuries]
KW - Functional Laterality
KW - Humans
KW - Longitudinal Studies
KW - Physical Education and Training/mt [Methods]
KW - Risk Factors
KW - Shoulder Pain/ep [Epidemiology]
KW - Task Performance and Analysis
KW - Time Factors
RP - NOT IN FILE
SP - 1803
EP - 1810
JF - Medicine & Science in Sports & Exercise
JA - Med Sci Sports Exerc
VL - 33
IS - 11
CY - United States
N2 - PURPOSE: Previous studies among young pitchers have focused on the frequency and description of elbow injuries. The purpose of this study was to evaluate the frequency of elbow and shoulder complaints in young pitchers and to identify the associations between pitch types, pitch volume, and other risk factors for these conditions. METHODS: A prospective cohort study of 298 youth pitchers was conducted over two seasons. Each participant was contacted via telephone after each game pitched to identify arm complaints. Generalized estimating equations were used to assess associations between arm complaints and independent variables. RESULTS: The frequency of elbow pain was 26%; that of shoulder pain, 32%. Risk factors for elbow pain were increased age, increased weight, decreased height, lifting weights during the season, playing baseball outside the league, decreased self-satisfaction, arm fatigue during the game pitched, and throwing fewer than 300 or more than 600 pitches during the season. Risk factors for shoulder pain included decreased satisfaction, arm fatigue during the game pitched, throwing more than 75 pitches in a game, and throwing fewer than 300 pitches during the season. CONCLUSION: Arm complaints are common, with nearly half of the subjects reporting pain. The factors associated with elbow and shoulder pain were different, suggesting differing etiologies. Developmental factors may be important in both. To lower the risk of pain at both locations, young pitchers probably should not throw more than 75 pitches in a game. Other recommendations are to remove pitchers from a game if they demonstrate arm fatigue and limit pitching in nonleague games
SN - 0195-9131
AD - American Sports Medicine Institute, Birmingham, AL, USA. Slyman@iihs.org
UR - 11689728
ER -

TY - JOUR
ID - 1652
T1 - Epidemiology of acute hepatitis in the Stann Creek District of Belize, Central America
A1 - Bryan,J.P.
A1 - Reyes,L.
A1 - Hakre,S.
A1 - Gloria,R.
A1 - Kishore,G.M.

A1 - Tillett,W.
A1 - Engle,R.
A1 - Tsarev,S.
A1 - Cruess,D.
A1 - Purcell,R.H.
Y1 - 2001/10//
N1 - Bryan, J P. Reyes, L. Hakre, S. Gloria, R. Kishore, G M. Tillett, W. Engle, R. Tsarev, S. Cruess, D. Purcell, R H
The American journal of tropical medicine and hygiene
3zq, 0370507
AIM, IM
Journal Article. Research Support, U.S. Gov't, Non-P.H.S.
English
KW - MEDLINE
KW - Acute Disease
KW - Adolescent
KW - Adult
KW - Belize/ep [Epidemiology]
KW - Child
KW - Preschool
KW - Disease Transmission
KW - Infectious/pc [Prevention & Control]
KW - Female
KW - Hepatitis/ep [Epidemiology]
KW - Hepatitis/et [Etiology]
KW - Hepatitis/im [Immunology]
KW - Hepatitis B/ep [Epidemiology]
KW - Hepatitis B/im [Immunology]
KW - Hepatitis B/pc [Prevention & Control]
KW - Hepatitis B Vaccines
KW - Humans
KW - Incidence
KW - Jaundice/di [Diagnosis]
KW - Jaundice/et [Etiology]
KW - Malaria/co [Complications]
KW - Malaria/ep [Epidemiology]
KW - Male
KW - Middle Aged
KW - Population Surveillance
KW - Pregnancy
KW - Questionnaires
KW - Serologic Tests
RP - NOT IN FILE
SP - 318
EP - 324
JF - American Journal of Tropical Medicine & Hygiene
JA - Am J Trop Med Hyg
VL - 65
IS - 4
CY - United States
N2 - Hepatitis is common in the Stann Creek District of southern Belize. To determine the etiologies, incidence, and potential risk factors for acute jaundice, we conducted active surveillance for cases. Cases of jaundice diagnosed by a physician within the previous 6 weeks were enrolled. Evaluation included a questionnaire and laboratory tests for hepatitis A, B, C, D, and E, a blood film for malaria, and a serologic test for syphilis. Etiologies of jaundice among 62 evaluable patients included acute hepatitis A, 6 (9.7%), acute hepatitis B, 49 (79.0%), hepatitis non-A-E, 2 (3.2%), and malaria, 5 (8.1%). There were no cases of acute hepatitis E. One patient each with antibody to hepatitis C and D were detected. The annualized incidence of

hepatitis A was 0.26 per 1,000. All cases of hepatitis A were in children 4-16 years of age. The annualized incidence of hepatitis B, 2.17 per 1,000, was highest in adults aged 15-44 years (4.4 per 1,000) and was higher in men (36 cases; 3.09 per 1,000) than women (13 cases; 1.19 per 1,000). Four (31%) of the women with hepatitis B were pregnant. The annualized incidence was significantly higher in Mestizo (6.18 per 1000) and Maya (6.79 per 1,000) than Garifuna (0.38 per 1,000) or Creole (0.36 per 1,000). Persons with hepatitis B were significantly more likely to be born outside of Belize (82%), had been in Belize < 5 years (73%), and lived and worked in rural areas (96%) than was the general population. Of those > or = 14 years of age with hepatitis B, only 36% were married. Few persons admitted to transfusions, tattoos, IV drug use, multiple sexual partners, visiting prostitutes, or sexually transmitted diseases. Only 1 of 49 had a reactive test for syphilis. Six patients were hospitalized (including 3 with acute hepatitis B and one with hepatitis A), and none to our knowledge died. Acute hepatitis B is the most common cause of viral hepatitis in the Stann Creek District, but the modes of transmission remain obscure. Infants, women attending prenatal clinics, and new workers are potential targets for immunization with hepatitis B vaccine
SN - 0002-9637
AD - Department of Preventive Medicine and Biometrics, Uniformed Services University of the Health Sciences, Bethesda, Maryland 20814-4799, USA
UR - 11693876
ER -

TY - JOUR
ID - 1653
T1 - Practitioner review: The role of direct observation in the assessment of young children. [Review] [41 refs]
A1 - Pellegrini,A.D.
Y1 - 2001/10//
N1 - Pellegrini, A D
Journal of child psychology and psychiatry, and allied disciplines
hp3, 0375361
IM
Journal Article. Research Support, Non-U.S. Gov't. Review
English
KW - MEDLINE
KW - Age Factors
KW - Child
KW - Child Development
KW - Preschool
KW - Education
KW - Special
KW - Educational Measurement/ec [Economics]
KW - Educational Measurement/mt [Methods]
KW - Humans
KW - Observation/mt [Methods]
KW - Play and Playthings/px [Psychology]
KW - Social Behavior
KW - United States
RP - NOT IN FILE
SP - 861
EP - 869
JF - Journal of Child Psychology & Psychiatry & Allied Disciplines
JA - J Child Psychol Psychiatry
VL - 42
IS - 7
CY - England
N2 - In this review I examine the role of play and assessment in the lives of young children. These two seemingly opposing constructs are first defined. Next. I address a particularly important form of assessment, "high stakes" assessment, and argue that if it must be used, we should use a variety of assessment methods. One such method that I discuss in great detail is the use of direct observations of children's play. Examples of reliable and valid inferences about children's social cognitive functioning made with this family of methods are

provided. Two specific observational instruments (one for observing play in the classroom and one for observing play in the playground) are presented. Recommendations for their use by practitioners are made. [References: 41]
SN - 0021-9630
AD - Department of Educational Psychology, University of Minnesota, Minneapolis 55455, USA. pelle013@tc.umn.edu
UR - 11693582
ER -

TY - JOUR
ID - 1654
T1 - Active school playgrounds-myth or reality? Results of the "move it groove it" project
A1 - Zask,A.
A1 - van,Beurden E.
A1 - Barnett,L.
A1 - Brooks,L.O.
A1 - Dietrich,U.C.
Y1 - 2001/11//
N1 - Zask, A. van Beurden, E. Barnett, L. Brooks, L O. Dietrich, U C
Preventive medicine
pm4, 0322116
IM
Comparative Study. Journal Article
English
KW - MEDLINE
KW - Child
KW - Preschool
KW - Exercise
KW - Female
KW - Humans
KW - Male
KW - New South Wales
KW - Play and Playthings
KW - Reproducibility of Results
KW - Rural Population
KW - Schools/sn [Statistics & Numerical Data]
KW - Students/sn [Statistics & Numerical Data]
KW - Teaching
RP - NOT IN FILE
SP - 402
EP - 408
JF - Preventive Medicine
JA - Prev Med
VL - 33
IS - 5
CY - United States
N2 - BACKGROUND: School playgrounds during break times provide potential opportunities for children to be active during the school day. However, there is limited research on how active children really are during these breaks. METHODS: The CAST (Children's Activity Scanning Tool) instrument was developed, validated, and used to assess the percentages of children engaged in moderate to vigorous physical activity (MVPA) and vigorous physical activity (VPA-a subset of MVPA) in 18 primary schools (children ages 5-12) in rural Australia. Related environmental factors were also measured. RESULTS: For a school of median size (200 students), 51.4% of boys and 41.6% of girls were engaged in MVPA while 14.7% of boys and 9.4% of girls were engaged in VPA. Levels of engagement in MVPA and VPA were significantly higher during lunch periods than during recess. MVPA and VPA engagement in smaller schools was significantly higher than in larger schools. CONCLUSIONS: With growing concern about children's physical activity (PA), school playgrounds offer an opportunity to increase children's MVPA engagement especially among girls. Consideration may be

given to the lengthening of recess periods in order to increase PA levels. More research is needed to investigate factors affecting PA levels in larger schools. Copyright 2001 American Health Foundation and Academic Press
SN - 0091-7435
AD - Health Promotion Unit, Lismore, New South Wales, Australia
UR - 11676580
ER -

TY - JOUR
ID - 1655
T1 - [Simulation of homicide to hide child's suicide]. [German]
A1 - Schmidt,P.
A1 - Driever,F.
A1 - Madea,B.
Y1 - 2001/07//
N1 - Schmidt, P. Driever, F. Madea, B
Archiv fur Kriminologie
7r2, 0002256
IM
Case Reports. English Abstract. Journal Article
German
KW - MEDLINE
KW - Autopsy/lj [Legislation & Jurisprudence]
KW - Cause of Death
KW - Child
KW - Child Abuse/lj [Legislation & Jurisprudence]
KW - Deception
KW - Diagnosis
KW - Differential
KW - Expert Testimony/lj [Legislation & Jurisprudence]
KW - Female
KW - Germany
KW - Homicide/lj [Legislation & Jurisprudence]
KW - Humans
KW - Suicide/lj [Legislation & Jurisprudence]
RP - NOT IN FILE
SP - 54
EP - 61
JF - Archiv fur Kriminologie
JA - Arch Kriminol.
VL - 208
IS - 1-2
CY - Germany
N2 - A case of pretending a homicide to conceal a child suicide is reported in which characteristic findings of the postmortem examination and conclusions from the analysis of forensic autopsy series provided substantial support for the police investigation. In the late night hours of a spring day the police authorities were informed that a 10-year-old girl had been found dead on a playground near its parents' house. As injuries of the neck were recognized by the police officers a homicide investigation was initiated. The post mortem examination showed a furrow symmetrically slanting from the front to the nape of the neck, discrete punctate haemorrhages of the skin of the face and numerous 'tram-line' bruises of the back, buttocks and extremities. These findings indicated as cause and manner of death suicidal hanging following corporal punishment. Confronted with these conclusions the mother immediately made a corresponding confession
SN - 0003-9225
AD - Institut fur Rechtsmedizin, Universitat Bonn
UR - 11591060
ER -

TY - JOUR

ID - 1656
T1 - Environmental exposure of small children to polycyclic aromatic hydrocarbons
A1 - Fiala,Z.
A1 - Vyskocil,A.
A1 - Krajak,V.
A1 - Viau,C.
A1 - Ettlerova,E.
A1 - Bukac,J.
A1 - Fialova,D.
A1 - Emminger,S.
Y1 - 2001/08//
N1 - Fiala, Z. Vyskocil, A. Krajak, V. Viau, C. Ettlerova, E. Bukac, J. Fialova, D. Emminger, S
International archives of occupational and environmental health
gpn, 7512134
IM
Journal Article. Research Support, Non-U.S. Gov't
English
KW - MEDLINE
KW - Child
KW - Preschool
KW - Czech Republic
KW - Environmental Exposure/an [Analysis]
KW - Environmental Pollutants/ae [Adverse Effects]
KW - Humans
KW - Polycyclic Compounds/ae [Adverse Effects]
KW - Seasons
RP - NOT IN FILE
SP - 411
EP - 420
JF - International Archives of Occupational & Environmental Health
JA - Int Arch Occup Environ Health
VL - 74
IS - 6
CY - Germany
N2 - OBJECTIVES: The aim of the study was to assess the intake (by various routes of exposure) of polycyclic aromatic hydrocarbons (PAH) by children living in a Czech city, and its effect on excretion of 1-hydroxypyrene (1-OHP) in summer and winter periods. METHODS: Four groups of children (3-6 years old) were chosen: (1) two groups from a kindergarten situated in the city center with a higher traffic density ("polluted" area); (2) two groups from a kindergarten situated in a green zone of the same city ("non-polluted" area). Food consumption was recorded in all children and PAH intake from foodstuffs was estimated. Ambient air samples were collected from the playground and inside the kindergartens. Soil samples were collected too. Morning and evening urine samples were collected during sampling days. RESULTS: In both seasons, the mean outdoor total PAH concentration (sum of 12 individual PAH) in the -polluted" area was approximately three-times higher than that in the "non-polluted" area. Indoor concentration in the "polluted" area was more than six-times higher than that in the "non-polluted" area in summer, and almost three-times higher in winter. The same trend was observed for pyrene and for the sum of carcinogenic PAH. The contribution to the total pyrene absorbed dose from food consumption was much more important than that from inhalation and from ingestion of soil dust. Significantly higher urinary concentrations of 1-OHP (evening samples) were found in children from the "polluted" kindergarten in both seasons. The number of significant relationships between 1-OHP and pyrene absorbed dose was weak. CONCLUSIONS: Food seems to be the main source of total pyrene and total PAH intake in small children, even under relatively higher air PAH exposure in the city. Estimated pyrene ingestion from soil had a negligible contribution to the total pyrene absorbed dose. Urinary 1-OHP seems to be an uncertain (non-sensitive) marker of the environmental inhalation exposure to pyrene (PAH) if the pollution of air by pyrene (PAH) is not excessive and the pyrene (PAH) dose by this route is much less than by ingestion. Usefulness of the urinary 1-OHP as an indicator of overall environmental exposure to PAH needs further investigation
SN - 0340-0131

AD - Department of Hygiene and Preventive Medicine, Charles University in Prague, Medical Faculty in Hradec Kralove, Czech Republic. fiala@lfhk.cuni.cz
UR - 11563604
ER -

TY - JOUR
ID - 1657
T1 - Rates of pediatric and adolescent injuries by year of age
A1 - Agran,P.F.
A1 - Winn,D.
A1 - Anderson,C.
A1 - Trent,R.
A1 - Walton-Haynes,L.
Y1 - 2001/09//
N1 - Agran, P F. Winn, D. Anderson, C. Trent, R. Walton-Haynes, L
Pediatrics
oxv, 0376422
AIM, IM
Journal Article. Research Support, U.S. Gov't, P.H.S.
English
KW - MEDLINE
KW - Accidental Falls/sn [Statistics & Numerical Data]
KW - Accidents
KW - Traffic/sn [Statistics & Numerical Data]
KW - Adolescent
KW - Age Distribution
KW - California
KW - Cause of Death
KW - Child
KW - Preschool
KW - Female
KW - Hospitalization/sn [Statistics & Numerical Data]
KW - Humans
KW - Incidence
KW - Infant
KW - Newborn
KW - Male
KW - Poisoning/ep [Epidemiology]
KW - Risk Assessment
KW - Survival Rate
KW - Wounds and Injuries/cl [Classification]
KW - Wounds and Injuries/ep [Epidemiology]
KW - Wounds
KW - Penetrating/ep [Epidemiology]
RP - NOT IN FILE
SP - E45
JF - Pediatrics
VL - 108
IS - 3
CY - United States
N2 - OBJECTIVE: The purpose of this study was to analyze causes of injury hospitalization/death by individual year of age and by specific causes of injury and to examine how well aggregate age groups represented individual year-of-age rates. METHODS: Hospital discharge data and death certificate data for California residents age 0 to 19 years with a principal external cause of injury code (E-code) of E800 to E869, E880 to E929, or E950 to E999, calendar year 1997, were analyzed. Annual rates of injury hospitalization/death by year of age were calculated using combined hospital discharges and deaths as the numerator for major causes and important subcategories. For comparison, rates of injury hospitalization/death were calculated for

conventional vital statistics age groups: <1 year, 1 to 4 years; 5 to 9 years, 10 to 14 years, and 15 to 19 years. RESULTS: In 1997 in California, 35 277 children and adolescents 0 to 19 years were hospitalized and 1934 died as a result of injury, a ratio of 17 hospitalizations to 1 death. The distribution was bimodal with rates highest among 18-year-olds (732/100 000) and 1-year-olds (495/100 000). Except for children who were 5 to 9 years of age, the group rates for all injuries were not reflective of the individual year-of-age rates. In specific categories of injuries, variation in rates by year of age were masked by age group rates for unintentional poisoning among 1- to 4-year-olds, self-inflicted poisoning for 10- to 19-year-olds, falls from playground equipment among 5- to 9-year-olds, falls from furniture among 1- to 4-year-olds, and motor vehicle occupant injury rates among 10- to 19-year-olds. The peak rate of falls from playground equipment among 6-year-olds (34/100 000) was more than twice the rate for 9-year-olds (15/1000,000). Motor vehicle occupant injury rates doubled between 10 and 14 years of age and quadrupled between 14 and 18 years of age. CONCLUSIONS: Analyses using conventional age groups did not identify the age of highest risk for many causes of childhood injury. Changes in the rates often transected the traditional age groups and were not apparent with conventional age group analysis. These data can inform on the age at which to begin a specific injury intervention and on how to allocate resources. These data allow pediatricians and other health professionals to be anticipatory in providing injury prevention counseling. The greatest impact can be achieved by making the counseling topic most age appropriate in anticipation of the high-risk period
SN - 1098-4275
AD - University of California, Center for Health Policy and Research, Pediatric Injury Prevention Research Group, Irvine 92697-5800, USA. pagran@uci.edu
UR - 11533363
ER -

TY - JOUR
ID - 1658
T1 - Severe anaphylaxis induced by latex as a contaminant of plastic balls in play pits
A1 - Fiocchi,A.
A1 - Restani,P.
A1 - Ballabio,C.
A1 - Bouygue,G.R.
A1 - Serra,A.
A1 - Travaini,M.
A1 - Terracciano,L.
Y1 - 2001/08//
N1 - Fiocchi, A. Restani, P. Ballabio, C. Bouygue, G R. Serra, A. Travaini, M. Terracciano, L
The Journal of allergy and clinical immunology
h53, 1275002
AIM, IM
Case Reports. Journal Article
English
KW - MEDLINE
KW - Anaphylaxis/et [Etiology]
KW - Child
KW - Preschool
KW - Environmental Exposure
KW - Female
KW - Humans
KW - Latex Hypersensitivity/et [Etiology]
KW - Male
KW - Play and Playthings
RP - NOT IN FILE
SP - 298
EP - 300
JF - Journal of Allergy & Clinical Immunology
JA - J Allergy Clin Immunol
VL - 108
IS - 2

CY - United States
N2 - BACKGROUND: Latex causes anaphylaxis in specific contexts among children. We present 2 cases that show that severe reactions may occur in everyday circumstances with latex as a contaminant. OBJECTIVE: Because 2 cases of severe reactions to latex suggested similar circumstances of exposure, we investigated the immediate environment in which episodes occurred. METHODS: A 5-year-old girl presented to our casualty department with anaphylaxis after playing in a ball pit filled with approximately 10-cm diameter plastic balls in an American-style fast-food outlet. Two months later, a 9-year-old boy had severe anaphylaxis followed by an asthma attack with loss of consciousness while playing in the playpen of a different outlet belonging to the same company. Latex sensitization was confirmed in both cases by means of skin prick testing, latex glove skin prick testing, and 1-glove finger testing. Immunoblotting of elutions from a ball, the natural rubber latex foam pit lining, and its polyvinyl chloride sheet were performed. RESULTS: In the girl's immunoblot high levels of IgE specific to Hev b 4, Hev b 7, and Hev b 2 were found. The boy's immunoblot showed positivity to Hev b 7. The polyvinyl chloride ball sample showed a high concentration of specific Hevea species allergen similar to that of the foam layer sample. CONCLUSION: Severe anaphylaxis can result from contact with latex proteins as a contaminant, rather than as a component, of play area ball pits and therefore outside the reported settings. Emergency health care workers should be aware of this kind of risk. A latex-reduced environment might prevent potentially severe reactions in young customers of fast-food outlets
SN - 0091-6749
AD - Department of Child and Maternal Medicine, The Melloni Hospital, Milan, Italy
UR - 11496251
ER -

TY - JOUR
ID - 1659
T1 - Compliance of Dunedin school playground equipment with the New Zealand playground standard
A1 - Chalmers,D.J.
A1 - Parry,M.L.
A1 - Crawford,A.I.
A1 - Wright,C.S.
Y1 - 2001/06//
N1 - Chalmers, D J. Parry, M L. Crawford, A I. Wright, C S
Australian and New Zealand journal of public health
ck2, 9611095
IM
Journal Article. Research Support, Non-U.S. Gov't
English
KW - MEDLINE
KW - Accidental Falls/pc [Prevention & Control]
KW - Child
KW - Equipment Design/st [Standards]
KW - Equipment Safety/st [Standards]
KW - Female
KW - Guideline Adherence
KW - Humans
KW - Male
KW - New Zealand
KW - Play and Playthings
KW - Schools/st [Standards]
KW - Wounds and Injuries/pc [Prevention & Control]
RP - NOT IN FILE
SP - 253
EP - 255
JF - Australian & New Zealand Journal of Public Health
JA - Aust N Z J Public Health
VL - 25
IS - 3
CY - Australia

N2 - OBJECTIVE: Injuries resulting from falls from playground equipment are a public health concern in New Zealand. Like many other countries, New Zealand has a safety standard aimed at reducing the incidence and severity of these injuries by limiting the height from which children can fall from playground equipment and requiring the provision of impact-absorbing surfaces beneath equipment from which falls are possible. The purpose of this study was to examine progress towards achieving compliance with these requirements in Dunedin school playgrounds. METHODS: Sixty-two schools were audited over the summer of 1997/98 and information recorded on equipment type, maximum fall height, surface type, and depth of loose-fill surface materials. Comparisons were made with audits conducted in 1989 and 1981. RESULTS: Substantial increases in the amount of playground equipment and in the provision of impact-absorbing surfaces were observed. A small increase in compliance with the requirement that the maximum fall height of equipment not exceed 2.5 metres was also observed. CONCLUSIONS: Any gains in safety achieved through increased compliance with the height and surface requirements of the New Zealand Standard have been counteracted by the substantial increase in the amount of equipment available in playgrounds. IMPLICATIONS: A more drastic measure is needed to achieve a meaningful reduction in the incidence of injury following falls from playground equipment
SN - 1326-0200
AD - Department of Preventive and Social Medicine, Dunedin School of Medicine, University of Otago, New Zealand. david.chalmers@ipru.otago.ac.nz
UR - 11494995
ER -

TY - JOUR
ID - 1660
T1 - Modeled estimates of chlorpyrifos exposure and dose for the Minnesota and Arizona NHEXAS populations
A1 - Buck,R.J.
A1 - Ozkaynak,H.
A1 - Xue,J.
A1 - Zartarian,V.G.
A1 - Hammerstrom,K.
Y1 - 2001/05//
N1 - Buck, R J. Ozkaynak, H. Xue, J. Zartarian, V G. Hammerstrom, K
Journal of exposure analysis and environmental epidemiology
bjn, 9111438
IM
Journal Article. Research Support, U.S. Gov't, Non-P.H.S.
English
KW - MEDLINE
KW - Age Distribution
KW - Air Pollutants/an [Analysis]
KW - Arizona/ep [Epidemiology]
KW - Child
KW - Preschool
KW - Chlorpyrifos/ad [Administration & Dosage]
KW - Environmental Exposure/sn [Statistics & Numerical Data]
KW - Female
KW - Humans
KW - Insecticides/ad [Administration & Dosage]
KW - Male
KW - Minnesota/ep [Epidemiology]
KW - Models
KW - Statistical
RP - NOT IN FILE
SP - 253
EP - 268
JF - Journal of Exposure Analysis and Environmental Epidemiology
JA - J Expo Anal Environ Epidemiol
VL - 11

IS - 3
CY - United States
N2 - This paper presents a probabilistic, multimedia, multipathway exposure model and assessment for chlorpyrifos developed as part of the National Human Exposure Assessment Survey (NHEXAS). The model was constructed using available information prior to completion of the NHEXAS study. It simulates the distribution of daily aggregate and pathway-specific chlorpyrifos absorbed dose in the general population of the State of Arizona (AZ) and in children aged 3-12 years residing in Minneapolis-St. Paul, Minnesota (MSP). Pathways included were inhalation of indoor and outdoor air, dietary ingestion, non-dietary ingestion of dust and soil, and dermal contact with dust and soil. Probability distributions for model input parameters were derived from the available literature, and input values were chosen to represent chlorpyrifos concentrations and demographics in AZ and MSP to the extent possible. When the NHEXAS AZ and MSP data become available, they can be compared to the distributions derived in this and other prototype modeling assessments to test the adequacy of this pre-NHEXAS model assessment. Although pathway-specific absorbed dose estimates differed between AZ and MSP due to differences in model inputs between simulated adults and children, the aggregate model results and general findings for simulated AZ and MSP populations were similar. The major route of chlorpyrifos intake was food ingestion, followed by indoor air inhalation. Two-stage Monte Carlo simulation was used to derive estimates of both inter-individual variability and uncertainty in the estimated distributions. The variability in the model results reflects the difference in activity patterns, exposure factors, and concentrations contacted by individuals during their daily activities. Based on the coefficient of variation, indoor air inhalation and dust ingestion were most variable relative to the mean, primarily because of variability in concentrations due to use or no-use of pesticides. Uncertainty analyses indicated a factor of 10-30 for uncertainty of model predictions of 10th, 50th, and 90th percentiles. The greatest source of uncertainty in the model stems from the definition of no household pesticide use as no use in the past year. Because chlorpyrifos persists in the residential environment for longer than a year, the modeled estimates are likely to be low. More information on pesticide usage and environmental concentrations measured at different post-application times is needed to refine and evaluate this and other pesticide exposure models
SN - 1053-4245
AD - Department of Mathematics and Statistics, Western Michigan University, Kalamazoo, MI 49008, USA
UR - 11477522
ER -

TY - JOUR
ID - 1661
T1 - Participation of a preschooler with visual impairments on the playground: effects of musical adaptations and staff development
A1 - Kern,P.
A1 - Wolery,PhD M.
Y1 - 2001///
N1 - Kern, P. Wolery PhD, M
Journal of music therapy
izv, 0014162
IM
Clinical Trial. Journal Article
English
KW - MEDLINE
KW - Blindness/cn [Congenital]
KW - Blindness/rh [Rehabilitation]
KW - Child
KW - Preschool
KW - Cues
KW - Facility Design and Construction
KW - Humans
KW - Interpersonal Relations
KW - Male
KW - Microphthalmos/rh [Rehabilitation]
KW - Movement
KW - Music Therapy

KW - Sensory Aids
KW - Stereotyped Behavior
RP - NOT IN FILE
SP - 149
EP - 164
JF - Journal of Music Therapy
JA - J Music Ther
VL - 38
IS - 2
CY - United States
N2 - The purpose of this study was to evaluate the adaptations of a playground, and subsequently staff development, on the participation of a 3-year-old boy with congenital blindness. A single-subject design with three conditions (baseline, adaptations of the playground, and staff development) was used. The playground adaptation involved adding musical stations in strategic locations on the playground and connecting them with a "path" that provided auditory feedback. The staff training involved the music therapist providing individualized instruction to the staff who supervised the child. The child's participation was measured in terms of social interaction with peers or adults, play and engagement with materials, movement on the playground, and stereotypic behaviors. The playground adaptation resulted in no changes in the child's social interactions with peers or adults, increases in engagement, no change in movement on the playground, and a decrease in stereotypic responses. Staff training resulted in increased but variable interactions with adults and peers, in additional increases in engagement, less movement, and similar levels of stereotypic behavior. The findings suggest that musical adaptations of physical environments may he helpful but not sufficient for promoting desired outcomes
SN - 0022-2917
AD - University of North Carolina at Chapel Hill, USA
UR - 11469920
ER -

TY - JOUR
ID - 1662
T1 - New graphical method to measure silent periods evoked by transcranial magnetic stimulation
A1 - Garvey,M.A.
A1 - Ziemann,U.
A1 - Becker,D.A.
A1 - Barker,C.A.
A1 - Bartko,J.J.
Y1 - 2001/08//
N1 - Garvey, M A. Ziemann, U. Becker, D A. Barker, C A. Bartko, J J
Clinical neurophysiology : official journal of the International Federation of Clinical Neurophysiology
db4, 100883319
IM
Journal Article
English
KW - MEDLINE
KW - Adolescent
KW - Cerebral Cortex/ph [Physiology]
KW - Child
KW - Electric Stimulation
KW - Evoked Potentials/ph [Physiology]
KW - Female
KW - Humans
KW - Male
KW - Models
KW - Statistical
KW - Observer Variation
KW - Sensitivity and Specificity
KW - Transcranial Magnetic Stimulation

RP - NOT IN FILE
SP - 1451
EP - 1460
JF - Clinical Neurophysiology
JA - Clin Neurophysiol
VL - 112
IS - 8
CY - Netherlands
N2 - OBJECTIVES: Manual methods of measuring duration of cortical silent periods (CSP) evoked by transcranial magnetic stimulation (TMS) depend upon subjective visual estimation of onset and offset. Because of this, the measurements are susceptible to poor rater reliability. We describe a graphical method to measure silent periods with greater precision. The statistical process underlying this new method is simple and particularly suited to signal detection in serially dependent data. METHODS: TMS-evoked silent periods were recorded in 13 healthy subjects. Two investigators subjectively measured silent period duration on each subject to estimate rater reliability. Using the graphical method, the mean and 99.76% variation limits of pre-stimulus electromyogram (EMG) activity were computed. Each averaged trial was displayed and CSP onset and offset detected when post-stimulus EMG activity moved outside the 99.76% limits. RESULTS: Maximum variation in silent period duration was 21.8 ms between the two investigators' subjective measurements. Silent period duration measured with the graphical method closely approximated measurements obtained using the manual method. It was possible to automate the procedure. CONCLUSIONS: This graphical method allowed precise measurement of CSP duration, independent of subjective estimations of onset or offset points. Further studies are necessary to determine if this method can provide a framework for other physiologic measures
SN - 1388-2457
AD - Pediatric Movement Disorders Unit, Pediatrics and Developmental Neuropsychiatry Branch, NIMH, National Institutes of Health, 10 Center Drive, 4N208, MSC 1255, Bethesda, MD 20892-1255, USA. garveym@intra.nimh.nih.gov
UR - 11459685
ER -

TY - JOUR
ID - 1663
T1 - Effects of the CATCH physical education intervention: teacher type and lesson location
A1 - McKenzie,T.L.
A1 - Stone,E.J.
A1 - Feldman,H.A.
A1 - Epping,J.N.
A1 - Yang,M.
A1 - Strikmiller,P.K.
A1 - Lytle,L.A.
A1 - Parcel,G.S.
Y1 - 2001/08//
N1 - McKenzie, T L. Stone, E J. Feldman, H A. Epping, J N. Yang, M. Strikmiller, P K. Lytle, L A. Parcel, G S
American journal of preventive medicine
8704773, apl
IM
Clinical Trial. Journal Article. Multicenter Study. Randomized Controlled Trial. Research Support, U.S. Gov't, P.H.S.
English
KW - MEDLINE
KW - Analysis of Variance
KW - Child
KW - Exercise
KW - Humans
KW - Physical Education and Training/mt [Methods]
KW - Questionnaires
RP - NOT IN FILE
SP - 101

EP - 109
JF - American Journal of Preventive Medicine
JA - Am J Prev Med
VL - 21
IS - 2
CY - Netherlands
N2 - BACKGROUND: Substantial differences exist in how and where physical education (PE) is conducted in elementary schools throughout the United States. Few effectiveness studies of large-scale interventions to improve PE have been reported. DESIGN: Multicenter randomized trial. SETTING/ PARTICIPANTS: The Child and Adolescent Trial for Cardiovascular Health (CATCH) was implemented in PE classes in 96 schools (56 intervention, 40 control) in four study centers: California, Louisiana, Minnesota, and Texas. INTERVENTION: The 2.5-year PE intervention consisted of professional development sessions, curricula, and follow-up consultations. MAIN OUTCOME MEASURES: Intervention effects on student physical activity and lesson context in PE were examined by teacher type (PE specialists and classroom teachers) and lesson location (indoors and outdoors). RESULTS: Differential effects by teacher type and lesson location were evidenced for both physical activity and lesson context. Observations of 2016 lessons showed that intervention schools provided more moderate-to-vigorous physical activity (p=0.002) and vigorous physical activity (p=0.02) than controls. Classroom teachers improved physical activity relatively more than PE specialists, but PE specialists still provided longer lessons and more physical activity. Classroom teachers increased lesson length (p=0.02) and time for physical fitness (p=0.03). CONCLUSIONS: The intervention improved PE of both specialists' and classroom teachers' lessons. States and districts should ensure that the most qualified staff teaches PE. Interventions need to be tailored to meet local needs and conditions, including teacher type and location of lessons
SN - 0749-3797
AD - Department of Exercise and Nutritional Sciences, San Diego State University, San Diego, California 92182, USA. tmckenzie@sdsu.edu
UR - 11457629
ER -

TY - JOUR
ID - 1664
T1 - Links between social understanding and social behavior in verbally able children with autism
A1 - Travis,L.
A1 - Sigman,M.
A1 - Ruskin,E.
Y1 - 2001/04//
N1 - Travis, L. Sigman, M. Ruskin, E
Journal of autism and developmental disorders
7904301, hgw
IM
Comparative Study. Journal Article. Research Support, U.S. Gov't, P.H.S.
English
KW - MEDLINE
KW - Adolescent
KW - Autistic Disorder/px [Psychology]
KW - Child
KW - Developmental Disabilities/px [Psychology]
KW - Empathy
KW - Female
KW - Humans
KW - Intelligence
KW - Interpersonal Relations
KW - Male
KW - Peer Group
KW - Social Behavior
KW - Social Perception
KW - Verbal Behavior

RP - NOT IN FILE
SP - 119
EP - 130
JF - Journal of Autism & Developmental Disorders
JA - J Autism Dev Disord
VL - 31
IS - 2
CY - United States
N2 - This study investigated the relations between various measures of social understanding and social interaction competence in verbally able children with autism. Measures of social understanding included measures of verbalizable knowledge (false belief understanding, affective perspective taking), as well as measures of more intuitive forms of social responsiveness (empathy, concern to distress, and initiating joint attention). Two measures of social interaction competence were employed: level of engagement with peers on the playground, and prosocial behavior in a structured laboratory task. For children with autism, initiating joint attention and empathy were strongly related to both measures of social interaction competence. No understanding-behavior links were identified for a language-age matched comparison sample of developmentally delayed children. Several accounts of these understanding-behavior links are considered, including the possibility that for children with autism, more impaired forms of understanding are more closely linked to behavior because they serve as limits on competence
SN - 0162-3257
AD - Department of Psychology, University of California, Los Angeles, 90095-1563, USA
UR - 11450811
ER -

TY - JOUR
ID - 1665
T1 - Blood lead levels of primary school children in Dhaka, Bangladesh
A1 - Kaiser,R.
A1 - Henderson,A.K.
A1 - Daley,W.R.
A1 - Naughton,M.
A1 - Khan,M.H.
A1 - Rahman,M.
A1 - Kieszak,S.
A1 - Rubin,C.H.
Y1 - 2001/06//
N1 - Kaiser, R. Henderson, A K. Daley, W R. Naughton, M. Khan, M H. Rahman, M. Kieszak, S. Rubin, C H
Environmental health perspectives
ei0, 0330411
IM
Journal Article
English
KW - MEDLINE
KW - Air Pollution/ae [Adverse Effects]
KW - Bangladesh/ep [Epidemiology]
KW - Child
KW - Preschool
KW - Environmental Exposure
KW - Female
KW - Housing
KW - Humans
KW - Hygiene
KW - Incidence
KW - Lead/ae [Adverse Effects]
KW - Lead/bl [Blood]
KW - Lead Poisoning/ep [Epidemiology]
KW - Lead Poisoning/et [Etiology]

KW - Male
KW - Public Policy
KW - Social Class
KW - Vehicle Emissions
RP - NOT IN FILE
SP - 563
EP - 566
JF - Environmental Health Perspectives
JA - Environ Health Perspect
VL - 109
IS - 6
CY - United States
N2 - Dhaka, Bangladesh, has one of the highest air lead levels in the world. In February 2000, we evaluated children at five primary schools in Dhaka to determine blood lead (BPb) levels, sources of environmental exposure, and potential risk factors for lead poisoning. Selected schools represented a range of geographic and socioeconomic strata. A total of 779 students 4-12 years of age participated. The mean BPb level was 15.0 microg/dL (range 4.2-63.1 microg/dL). Most students (87.4%) had BPb levels above the Centers for Disease Control and Prevention's level of concern (10 microg/dL). Elevated BPb levels correlated with soil eating [odds ratio (OR) = 3.31; 95% confidence interval (CI), 1.30-8.39], low parental education (OR = 2.72; 95% CI, 1.97-3.75), living close to major roads (OR = 2.30; 95% CI, 1.23-4.29), and increasing age (OR = 1.11; 95% CI, 1.06-1.16). BPb levels measured were similar to those in other countries that use leaded gasoline. No other potential sources of lead exposure were consistently identified. Combustion of leaded gasoline is the main source of lead exposure in Dhaka, resulting in ubiquitous contamination of the environment. The increase in BPb levels with age, a finding contrary to observations in the United States and Australia, may be related to increased outdoor activities. The Bangladeshi government recently announced a plan to eliminate leaded gasoline. Baseline BPb surveys are critical to develop and evaluate intervention policies. Strategies to reduce BPb levels need to address variations in socioeconomic status, construction type and location of housing, and levels of hygiene
SN - 0091-6765
AD - Epidemic Intelligence Service, Epidemiology Program Office, National Center for Environmental Health, Centers for Disease Control and Prevention, Atlanta, Georgia, USA. rik9@cdc.gov
UR - 11445508
ER -

TY - JOUR
ID - 1666
T1 - UV doses of Americans
A1 - Godar,D.E.
A1 - Wengraitis,S.P.
A1 - Shreffler,J.
A1 - Sliney,D.H.
Y1 - 2001/06//
N1 - Godar, D E. Wengraitis, S P. Shreffler, J. Sliney, D H
Photochemistry and photobiology
0376425, p69
IM
Journal Article
English
KW - MEDLINE
KW - Adolescent
KW - Adult
KW - Aged
KW - Child
KW - Preschool
KW - Data Collection
KW - Female
KW - Humans

KW - Infant
KW - Newborn
KW - Male
KW - Middle Aged
KW - Radiation Dosage
KW - Risk Assessment
KW - Ultraviolet Rays/ae [Adverse Effects]
KW - United States
RP - NOT IN FILE
SP - 621
EP - 629
JF - Photochemistry & Photobiology
JA - Photochem Photobiol
VL - 73
IS - 6
CY - United States
N2 - The UV doses of Americans were never measured, but are needed for assessing the risks of UV-related health effects. We calculated these doses using a novel approach. The Environmental Protection Agency's (EPA) National Human Activity Pattern Survey (NHAPS) recorded the activity profiles of 9386 Americans over 24 months to assess their exposure to environmental pollutants, one of which is UV radiation. NHAPS used randomized telephone interviews to get their previous day's minute-by-minute activities. From NHAPS we extracted only the outdoor-daylight data of the northern and southern indoor workers (95%), stratifying by season, sex and age (0-21, 22-40, 41-59 and 60+ years) to find the average time Americans spend outdoors. Knowing the total daylight time and that while outdoors Americans are exposed to about 30% of the available solar UV (on a horizontal plane), we calculated their percent ambients. The average American's percent ambients are 2.6 and 2.5% for northern and southern females, respectively, and 3.5 and 3.6% for northern and southern males, respectively. Men over 40 years of age have the highest ambients (4%). From their ambients we calculated their annual doses using seasonal averages of UV measurements taken daily for over 2 years by EPA Brewer spectrophotometers located in four quadrants of the United States: Atlanta, GA; Boston, MA; Bozeman, MT and Riverside, CA. The average erythemal UV doses of Americans are about 25,000 J/m2/year, 22,000 for females and 28,000 for males, or 33,000 J/m2/year including a conservative continental U.S. vacation (7800 J/m2). Thus, we can now assess the risks of UV-related health effects for Americans
SN - 0031-8655
AD - US Food and Drug Administration, Center for Devices and Radiological Health, Rockville, MD 20852, USA. deg@cdrh.fda.gov
UR - 11421067
ER -

TY - JOUR
ID - 1667
T1 - Usage of real-time ultraviolet radiation data to modify the daily erythemal exposure of primary schoolchildren
A1 - Kimlin,M.
A1 - Parisi,A.
Y1 - 2001/06//
N1 - Kimlin, M. Parisi, A
Photodermatology, photoimmunology & photomedicine
awp, 9013641
IM
Journal Article
English
KW - MEDLINE
KW - Child
KW - Diagnosis
KW - Computer-Assisted/mt [Methods]
KW - Environmental Exposure/pc [Prevention & Control]
KW - Erythema/et [Etiology]

KW - Face
KW - Female
KW - Humans
KW - Male
KW - Queensland
KW - Radiation Dosage
KW - Seasons
KW - Shoulder
KW - Skin/re [Radiation Effects]
KW - Skin Neoplasms/pc [Prevention & Control]
KW - Ultraviolet Rays/ae [Adverse Effects]
RP - NOT IN FILE
SP - 130
EP - 135
JF - Photodermatology, Photoimmunology & Photomedicine
JA - Photodermatol.Photoimmunol.Photomed.
VL - 17
IS - 3
CY - Denmark
N2 - BACKGROUND/AIMS: Primary schoolchildren in their everyday school life are exposed to solar ultraviolet radiation. This may be through time spent outdoors whilst having meal breaks, physical education classes and other class orientated outdoor activities. METHODS: This research investigates the UV exposure of primary schoolchildren and the effect real-time UV irradiances data and an associated software package, UVGUIDE, have on UV exposure. This software utilises scientifically collected data, such as facial distribution of UV, as well as accessing real-time on-line UV irradiances data to estimate the UV distribution to the head region. The students can also enter other parameters such as hat usage and hat type to show the effect of using such a UV protective device on their facial UV distribution. CONCLUSIONS: The results from this study found that the average 3-day erythemal UV exposure in late summer and early autumn to the left shoulder was 33% higher for the students not having access to the on-line UV data and software package
SN - 0905-4383
AD - Centre for Astronomy and Atmospheric Research, Faculty of Sciences, University of Southern Queensland, Toowoomba, Australia
UR - 11419541
ER -

TY - JOUR
ID - 1668
T1 - Clinical approach to a patient with cough
A1 - Chugh,K.
Y1 - 2001/04//
N1 - Chugh, K
Indian journal of pediatrics
gkt, 0417442
IM
Journal Article
English
KW - MEDLINE
KW - Asthma/co [Complications]
KW - Asthma/di [Diagnosis]
KW - Bronchoscopy
KW - Child
KW - Cough/di [Diagnosis]
KW - Cough/et [Etiology]
KW - Humans
KW - Magnetic Resonance Imaging
KW - Recurrence
KW - Spirometry

KW - Tomography
KW - X-Ray Computed
KW - Tuberculosis
KW - Pulmonary/co [Complications]
KW - Pulmonary/di [Diagnosis]
RP - NOT IN FILE
SP - S11
EP - S19
JF - Indian Journal of Pediatrics
JA - Indian J Pediatr
VL - 68 Suppl 2
CY - India
N2 - A number of disorders of the respiratory tract and some even outside the respiratory tract can cause cough. A systematic approach towards a patient of chronic cough consisting of detailed history, physical examination of upper as well as lower respiratory tract, complete blood counts, tuberculin test, chest X-ray, and peak flow rate testing will give the diagnosis in majority of children. Pulmonary tuberculosis and asthma are the two commonest conditions diagnosed. If the initial work up is inconclusive, further laboratory testing and imaging studies should be considered. Thus, radiolabelled milk scan, barium swallow and 24-hour pH monitoring would diagnose gastroesophageal reflux. Spirometry, methacholine/exercise challenge test or a therapeutic trial may be required for confirming bronchial asthma. Flexible bronchoscopy is useful for evaluation for suspected aspiration syndromes and any anatomical or dynamic problem of the airway (e.g. tracheomalacia). Spiral and high resolution computed tomography (HRCT) along with magnetic resonance imaging are the modern day imaging techniques used for studying mediastinal masses, airway obstruction and even lung parenchyma (HRCT). Sputum examination for type of cells and bacteria can be useful, especially if pseudomonas or acid-fast bacilli are identified. Pseudomonas suggests cystic fibrosis (an uncommon disease in India) which can be confirmed by sweat chloride test and gene mutation studies
SN - 0019-5456
AD - Department of Pediatrics, Sir Ganga Ram Hospital, New Delhi. chughk@bol.net.in
UR - 11411372
ER -

TY - JOUR
ID - 1669
T1 - Sport and recreation-related head injuries treated in the emergency department
A1 - Kelly,K.D.
A1 - Lissel,H.L.
A1 - Rowe,B.H.
A1 - Vincenten,J.A.
A1 - Voaklander,D.C.
Y1 - 2001/04//
N1 - Kelly, K D. Lissel, H L. Rowe, B H. Vincenten, J A. Voaklander, D C
Clinical journal of sport medicine : official journal of the Canadian Academy of Sport Medicine
b9t, 9103300
IM
Journal Article
English
KW - MEDLINE
KW - Adolescent
KW - Adult
KW - Age Distribution
KW - Alberta/ep [Epidemiology]
KW - Ambulances/sn [Statistics & Numerical Data]
KW - Athletic Injuries/ep [Epidemiology]
KW - Bicycling/in [Injuries]
KW - Brain Concussion/ep [Epidemiology]
KW - Child
KW - Cohort Studies

KW - Craniocerebral Trauma/ep [Epidemiology]
KW - Emergency Service
KW - Hospital/ut [Utilization]
KW - Female
KW - Football/in [Injuries]
KW - Hockey/in [Injuries]
KW - Hospitalization/sn [Statistics & Numerical Data]
KW - Humans
KW - Male
KW - Play and Playthings/in [Injuries]
KW - Recreation
KW - Retrospective Studies
KW - Sex Distribution
KW - Skull Fractures/ep [Epidemiology]
KW - Soccer/in [Injuries]
KW - Sports/sn [Statistics & Numerical Data]
KW - Treatment Outcome
KW - Unconsciousness/ep [Epidemiology]
RP - NOT IN FILE
SP - 77
EP - 81
JF - Clinical Journal of Sport Medicine
JA - Clin J Sport Med
VL - 11
IS - 2
CY - United States
N2 - OBJECTIVE: Head injury (HI) in sport is common and can have serious consequences. This study examines the epidemiology of sport/recreational (SR)-related HI presenting to the emergency department (ED). DESIGN: Retrospective review of medical records. SETTING: Five EDs in the Capital Health Region (Edmonton) located in the province of Alberta, Canada. PATIENTS: All persons in a 1-year period reporting to the EDs with an HI. HI was defined as IC9-CM coded skull fracture, loss of consciousness, or concussion. MAIN OUTCOME MEASURES: Hospitalization, utilization of diagnostic testing, and discharge destination. RESULTS: In total, 10,877 (3%) of 288,948 ED encounters were for sports and recreational injuries; 358 (3%) were for HI. Males (71%) were more frequently injured; patients < 20 years old were involved in 66% of all HI cases. The highest proportion of HI occurred during ice hockey (21%), cycling (13%), and playground-related activities (8%). 9% of HI were hospitalized (versus 4% admission rate for other SR injuries: p < 0.01). CONCLUSIONS: These results demonstrate the utility of an ED-based injury registry and indicate that patients with HI presenting to the ED from SR activities are common. These injuries appear to be more severe than other types of SR injuries treated in the ED
SN - 1050-642X
AD - Department of Rural Health, Faculty of Medicine, Dentistry and Health Sciences, University of Melbourne, Shepparton, Victoria, Australia. karendk@unimelb.edu.au
UR - 11403118
ER -

TY - JOUR
ID - 1670
T1 - Near-work activity and myopia in rural and urban schoolchildren in China
A1 - Saw,S.M.
A1 - Hong,R.Z.
A1 - Zhang,M.Z.
A1 - Fu,Z.F.
A1 - Ye,M.
A1 - Tan,D.
A1 - Chew,S.J.
Y1 - 2001/05//

N1 - Saw, S M. Hong, R Z. Zhang, M Z. Fu, Z F. Ye, M. Tan, D. Chew, S J
Journal of pediatric ophthalmology and strabismus
jmi, 7901143
IM
Comparative Study. Journal Article
English
KW - MEDLINE
KW - Child
KW - China/ep [Epidemiology]
KW - Cohort Studies
KW - Cross-Sectional Studies
KW - Female
KW - Humans
KW - Male
KW - Myopia/ep [Epidemiology]
KW - Prevalence
KW - Questionnaires
KW - Refraction
KW - Ocular
KW - Rural Population/sn [Statistics & Numerical Data]
KW - Schools
KW - Social Class
KW - Urban Population/sn [Statistics & Numerical Data]
KW - Work/sn [Statistics & Numerical Data]
RP - NOT IN FILE
SP - 149
EP - 155
JF - Journal of Pediatric Ophthalmology & Strabismus
JA - J Pediatr Ophthalmol Strabismus
VL - 38
IS - 3
CY - United States
N2 - PURPOSE: To examine the prevalence of myopia in rural and urban schoolchildren in Xiamen, China, and to assess the impact of environmental factors on rates of myopia. METHODS: Second-grade children attending either a city (n=119) or rural (n=91) school in Xiamen, China, were examined using cycloplegic autorefraction and biometry. Detailed questions on socioeconomic status, near-work activity, reading and writing habits, and family histories of myopia were asked in a face-to-face interview using a standard questionnaire. RESULTS: The prevalence of myopia was 19.3% (95% confidence interval [CI], 12.3, 29) in the city and 6.6% (95% CI, 2.4, 14.3) in the countryside. The average hours per day children spent reading and writing outside of school was 2.2 hours in the city compared with 1.6 hours in the countryside (P<.0001). In both schools, the odds ratio for total reading and writing, adjusted for parental history of myopia, was 2.2 (95% CI, 1.1, 4). CONCLUSION: These data suggest the prevalence of myopia is higher in the city than in the countryside. One possible explanation for these different rates could be that schoolchildren in the city spend more time reading and writing outside of school compared with children in the countryside. Myopic children in both the city and the countryside spent more time reading and writing compared with nonmyopic children. This increased near-work activity may contribute to the prevalence of myopia
SN - 0191-3913
AD - Department of Community, Occupational, and Family Medicine, National University of Singapore, Singapore
UR - 11386647
ER -

TY - JOUR
ID - 1671
T1 - Late-onset Stargardt disease is associated with missense mutations that map outside known functional regions of ABCR (ABCA4)
A1 - Yatsenko,A.N.

A1 - Shroyer,N.F.
A1 - Lewis,R.A.
A1 - Lupski,J.R.
Y1 - 2001/04//
N1 - Yatsenko, A N. Shroyer, N F. Lewis, R A. Lupski, J R
Human genetics
ged, 7613873
IM
Journal Article. Research Support, Non-U.S. Gov't. Research Support, U.S. Gov't, P.H.S.
English
KW - MEDLINE
KW - ATP-Binding Cassette Transporters/ge [Genetics]
KW - Adolescent
KW - Adult
KW - Age of Onset
KW - Aged
KW - Child
KW - Chromosome Mapping
KW - Chromosome Segregation
KW - Female
KW - Humans
KW - Male
KW - Middle Aged
KW - Mutation
KW - Missense
KW - Pedigree
KW - Retinal Diseases/ge [Genetics]
RP - NOT IN FILE
SP - 346
EP - 355
JF - Human Genetics
JA - Hum Genet
VL - 108
IS - 4
CY - Germany
N2 - Based on recent studies of the photoreceptor-specific ABC transporter gene ABCR (ABCA4) in Stargardt disease (STGD1) and other retinal dystrophies, we and others have developed a model in which the severity of retinal disease correlates inversely with residual ABCR activity. This model predicts that patients with late-onset STGDI may retain partial ABCR activity attributable to mild missense alleles. To test this hypothesis, we used late-onset STGDI patients (onset: > or =35 years) to provide an in vivo functional analysis of various combinations of mutant alleles. We sequenced directly the entire coding region of ABCR and detected mutations in 33/50 (66%) disease chromosomes, but surprisingly, 11/33 (33%) were truncating alleles. Importantly, all 22 missense mutations were located outside the known functional domains of ABCR (ATP-binding or transmembrane), whereas in our general cohort of STGDI subjects, alterations occurred with equal frequency across the entire protein. We suggest that these missense mutations in regions of unknown function are milder alleles and more susceptible to modifier effects. Thus, we have corroborated a prediction from the model of ABCR pathogenicity that (1) one mutant ABCR allele is always missense in late-onset STGD1 patients, and (2) the age-of-onset is correlated with the amount of ABCR activity of this allele. In addition, we report three new pseudodominant families that now comprise eight of 178 outbred STGD1 families and suggest a carrier frequency of STGD1-associated ABCR mutations of about 4.5% (approximately 1/22)
SN - 0340-6717
AD - Department of Molecular and Human Genetics, Baylor College of Medicine, Houston, Texas 77030, USA
UR - 11379881
ER -

TY - JOUR
ID - 1672

T1 - Assessment of knowledge of melanoma risk factors, prevention, and detection principles in Texas teenagers
A1 - Lucci,A.
A1 - Citro,H.W.
A1 - Wilson,L.
Y1 - 2001/05/15/
N1 - Lucci, A. Citro, H W. Wilson, L
The Journal of surgical research
k7b, 0376340
IM
Journal Article
English
KW - MEDLINE
KW - Adolescent
KW - Adolescent Behavior
KW - Child
KW - Female
KW - Genetic Predisposition to Disease
KW - Humans
KW - Incidence
KW - Male
KW - Melanoma/di [Diagnosis]
KW - Melanoma/ep [Epidemiology]
KW - Melanoma/pc [Prevention & Control]
KW - Needs Assessment
KW - Patient Education as Topic
KW - Risk Factors
KW - Skin Neoplasms/di [Diagnosis]
KW - Skin Neoplasms/ep [Epidemiology]
KW - Skin Neoplasms/pc [Prevention & Control]
KW - Skin Pigmentation/ge [Genetics]
KW - Sunlight/ae [Adverse Effects]
KW - Texas
RP - NOT IN FILE
SP - 179
EP - 183
JF - Journal of Surgical Research
JA - J Surg Res
VL - 97
IS - 2
CY - United States
N2 - INTRODUCTION: The incidence of melanoma has increased in the past 10 years more rapidly than any other cancer. Exposure to intense solar radiation in youth significantly increases the lifetime risk of developing melanoma. We postulate that teenagers have little awareness of melanoma prevention or detection principles. The purpose of this study was to assess the knowledge of teenagers about melanoma and to identify which age groups are most receptive to altering their sun exposure behaviors. METHODS: Two hundred and ten examinations testing general knowledge of sun exposure and melanoma were completed and returned by junior high and high school students ages 12 to 18 in Dallas and Houston, Texas. All students completing and returning the examination were provided with the correct answers to the test and a detailed explanation of each of the test items as part of an educational exercise. A second questionnaire was then administered to determine the effect of the educational exercise on future sun exposure practices. Students were divided into two age groups (12 to 15 and >or=16 years old) for comparison of scores on the knowledge examination and responses to behavioral items. Comparison of response rates between age groups was performed using chi(2) analysis. RESULTS: The return rate was 100%, with 109 students age 12-15 years, and 101 students >or=16 years. Seventy-six percent of all respondents sunbathed outdoors, and 18% had used a tanning bed in the past 6 months. Thirty-three percent of students admitted to at least three blistering sunburns in the past. The average score on the knowledge assessment examination was 65% correct for students >or=16 years old and 54%

correct for those 12-15 years old. Students 12 to 15 years old were significantly more likely to indicate they planned to change future behaviors regarding performance of skin self-examinations and limiting sun exposure as compared to the older students. CONCLUSION: A significant number of teenagers have already enhanced their risk for future melanoma by suffering severe sunburns. Students younger than 16 years of age were significantly more likely to indicate they planned to change future behaviors after receiving information about melanoma. The data from this pilot study support education aimed at younger age groups to most effectively achieve risk reduction and prevent future melanomas. Copyright 2001 Academic Press
SN - 0022-4804
AD - Michael E. Debakey Department of Surgery, Baylor College of Medicine, Houston, Texas 77030, USA. alucci@bcm.tmc.edu
UR - 11341796
ER -

TY - JOUR
ID - 1673
T1 - Fungal exposure of children at homes and schools: a health perspective
A1 - Su,H.J.
A1 - Wu,P.C.
A1 - Lin,C.Y.
Y1 - 2001/03//
N1 - Su, H J. Wu, P C. Lin, C Y
Archives of environmental health
6yo, 0212627
AIM, IM
Journal Article. Research Support, Non-U.S. Gov't
English
KW - MEDLINE
KW - Air Pollution
KW - Indoor
KW - Asthma/ep [Epidemiology]
KW - Asthma/et [Etiology]
KW - Child
KW - Environmental Microbiology
KW - Female
KW - Fungi/ip [Isolation & Purification]
KW - Health Surveys
KW - Housing
KW - Humans
KW - Male
KW - Prevalence
KW - Schools
KW - Seasons
KW - Taiwan/ep [Epidemiology]
RP - NOT IN FILE
SP - 144
EP - 149
JF - Archives of Environmental Health
JA - Arch Environ Health
VL - 56
IS - 2
CY - United States
N2 - The authors investigated airborne fungal exposure of Taiwanese children at homes and schools-locations where they spent the majority of their time. Pairs of asthmatic and healthy children, matched by age, gender, classes (schools), and residence, participated in the study. Airborne fungal concentrations at homes and schools, and time-activity data of the study subjects were collected. In winter, the indoor geometric mean concentration was 9,672.1 colony-forming units per cubic meter and 4,380.9 colony-forming units per cubic meter in summer. The indoor/outdoor concentration ratio was similar in all homes for both seasons. Fungal concentrations in

southern Taiwan were high during both winter and summer. Personal fungal exposure was not statistically different between asthmatic and nonasthmatic children, after adjustment of time-activity patterns. Higher symptom scores were shown for children in homes with higher fungal exposures
SN - 0003-9896
AD - Department of Environmental and Occupational Health, National Cheng Kung University Medical College, Tainan, Taiwan, Republic of China
UR - 11339678
ER -

TY - JOUR
ID - 1674
T1 - A rural ambulance helicopter system in northern Sweden
A1 - Vesterbacka,J.
A1 - Eriksson,A.
Y1 - 2001/05//
N1 - Vesterbacka, J. Eriksson, A
Air medical journal
bs3, 9312325
H
Journal Article
English
KW - MEDLINE
KW - Adolescent
KW - Adult
KW - Aged
KW - Air Ambulances/ut [Utilization]
KW - Catchment Area (Health)/sn [Statistics & Numerical Data]
KW - Child
KW - Emergencies/cl [Classification]
KW - Emergencies/ep [Epidemiology]
KW - Female
KW - Health Services Research
KW - Humans
KW - Male
KW - Middle Aged
KW - Program Evaluation
KW - Rural Health Services/og [Organization & Administration]
KW - Rural Health Services/ut [Utilization]
KW - Sweden/ep [Epidemiology]
KW - Time and Motion Studies
KW - Transportation of Patients
KW - Utilization Review
RP - NOT IN FILE
SP - 28
EP - 31
JF - Air Medical Journal
JA - Air Med J
VL - 20
IS - 3
CY - United States
N2 - INTRODUCTION: In Sweden, several ambulance helicopter programs have been used during the past 3 decades. This article describes one of these programs. SETTING: The catchment area of the helicopter system under study was a typical rural setting with a population density of less than 5 people per square kilometer. METHODS: We describe in detail, with special reference to medical aspects, the activities of an helicopter ambulance program in 1997. RESULTS: A total of 288 missions, including 328 patients (204 men), were analyzed. Trauma cases (29%) and patients with cardiovascular (24%) and neurologic problems (13%) dominated the missions. One-sixth of the cases occurred at night. The median response time was 12 minutes,

and the median on-scene time was 28 minutes. The medical interventions performed included intubations; central venous catheter, arterial needle, and duodenal tube insertions; and medication administered intravenously, orally, and intrabronchially. CONCLUSION: We detected a higher rate of trauma cases than in previously published investigations, probably related to the outdoor activities of the area. We believe that the rapid institution of thrombolytic treatment in suspected acute myocardial infarction and a more rapid and flexible transport of trauma cases in a rural area are important. We estimate that, in most cases, the helicopter service could provide a quicker transport than a ground ambulance; however, for short distance transports, ground ambulance is faster than helicopter ambulance
SN - 1067-991X
AD - Department of Community Medicine and Rehabilitation, Unit of Forensic Medicine, Umea University, Sweden
UR - 11331824
ER -

TY - JOUR
ID - 1675
T1 - Active management in patients with ocular manifestations of myasthenia gravis
A1 - Bentley,C.R.
A1 - Dawson,E.
A1 - Lee,J.P.
Y1 - 2001/02//
N1 - Bentley, C R. Dawson, E. Lee, J P
Eye (London, England)
eye, 8703986
IM
Case Reports. Journal Article
English
KW - MEDLINE
KW - Adolescent
KW - Adult
KW - Botulinum Toxins/ae [Adverse Effects]
KW - Botulinum Toxins/tu [Therapeutic Use]
KW - Child
KW - Diplopia/et [Etiology]
KW - Diplopia/th [Therapy]
KW - Esthetics
KW - Female
KW - Humans
KW - Male
KW - Middle Aged
KW - Myasthenia Gravis/co [Complications]
KW - Ocular Motility Disorders/et [Etiology]
KW - Ocular Motility Disorders/th [Therapy]
KW - Oculomotor Muscles/su [Surgery]
KW - Retrospective Studies
KW - Strabismus/et [Etiology]
KW - Strabismus/th [Therapy]
KW - Treatment Outcome
RP - NOT IN FILE
SP - 18
EP - 22
JF - Eye
VL - 15
IS - Pt 1
CY - England
N2 - PURPOSE: Myasthenia gravis can cause variable strabismus with disabling diplopia and/or poor cosmesis. A retrospective study of a group of patients with myasthenia gravis or 'myasthenia gravis like'

syndromes was made. METHODS: The study group consisted of patients who had undergone botulinum toxin treatment and/or surgery for disabling diplopia, poor cosmesis or both. Surgical treatment was by conventional techniques including recess/resect, posterior fixation, superior oblique tenotomy and adjustable sutures. RESULTS: There were 9 patients in the study group (8 female, 1 male). Age at surgery ranged from 21 to 59 years (mean 46 years). Six were symptom-free following treatment. Two, although symptomatically improved, had occasional diplopia. One patient failed treatment and required an occlusive contact lens. CONCLUSIONS: The ocular manifestations of myasthenia gravis or 'myasthenia gravis like' syndromes may respond to surgery and/or botulinum toxin injection. Active intervention should be considered when deviations become stable. To our knowledge this is the first report of the use of botulinum toxin in such patients
SN - 0950-222X
AD - Moorfields Eye Hospital, London, UK
UR - 11318287
ER -

TY - JOUR
ID - 1676
T1 - Evidence for a cerebellar role in reduced exploration and stereotyped behavior in autism
A1 - Pierce,K.
A1 - Courchesne,E.
Y1 - 2001/04/15/
N1 - Pierce, K. Courchesne, E
Biological psychiatry
a3s, 0213264
IM
Journal Article. Research Support, U.S. Gov't, P.H.S.
English
KW - MEDLINE
KW - Autistic Disorder/pa [Pathology]
KW - Autistic Disorder/pp [Physiopathology]
KW - Autistic Disorder/px [Psychology]
KW - Cerebellum/pa [Pathology]
KW - Cerebellum/pp [Physiopathology]
KW - Child
KW - Preschool
KW - Exploratory Behavior/ph [Physiology]
KW - Female
KW - Frontal Lobe/pa [Pathology]
KW - Frontal Lobe/pp [Physiopathology]
KW - Humans
KW - Magnetic Resonance Imaging
KW - Male
KW - Motor Activity/ph [Physiology]
KW - Stereotyped Behavior/ph [Physiology]
KW - Time Factors
RP - NOT IN FILE
SP - 655
EP - 664
JF - Biological Psychiatry
JA - Biol Psychiatry
VL - 49
IS - 8
CY - United States
N2 - BACKGROUND: Although limited environmental exploration in autism is an obvious behavioral feature and may be a manifestation of "restricted interests" as described in DSM-IV criteria, there have been no behavioral or neurobiological studies of this important aspect of the disorder. Given consistent reports of cerebellar abnormality in autism, combined with animal research showing a relationship between exploration and the cerebellum, this study aimed to test the possible link between cerebellar abnormality and exploration in

autism. METHODS: The relationship between visuospatial exploration, stereotyped motor movements, and magnetic resonance imaging measures of the cerebellar vermis, whole brain volume, and frontal lobes in 14 autistic and 14 normal children was investigated. Children were exposed to a large room with several exploration containers and instructed to play. Exploration behavior was videotaped and scored for percentage of time engaged in exploration, number of containers explored, as well as stereotyped movements. RESULTS: Children with autism spent significantly less time in active exploration and explored fewer containers overall than normal children. Measures of decreased exploration were significantly correlated with the magnitude of cerebellar hypoplasia of vermal lobules VI-VII in the autistic children, but no relationship to vermis size was found with normal control children. Further, measures of rates of stereotyped behavior were significantly negatively correlated with area measures of cerebellar vermis lobules VI-VII and positively correlated with frontal lobe volume in the autism sample. CONCLUSIONS: Reduced environmental exploration and repetitive behavior may have particularly important developmental consequences for children with autism because it may lead them to miss learning opportunities that fall outside their scope of interest. Our findings represent the first documented link between the restricted range of interests and stereotyped behaviors pathognomonic of autism and particular neuroanatomic sites
SN - 0006-3223
AD - Department of Neurosciences, School Of Medicine, University of California, 8110 La Jolla Shores Drive, La Jolla, CA 92037, USA
UR - 11313033
ER -

TY - JOUR
ID - 1677
T1 - Bungee cord-associated ocular trauma. [Review] [7 refs]
A1 - Aldave,A.J.
A1 - Gertner,G.S.
A1 - Davis,G.H.
A1 - Regillo,C.D.
A1 - Jeffers,J.B.
Y1 - 2001/04//
N1 - Aldave, A J. Gertner, G S. Davis, G H. Regillo, C D. Jeffers, J B
Ophthalmology
oi5, 7802443
IM
Journal Article. Review
English
KW - MEDLINE
KW - Adolescent
KW - Adult
KW - Child
KW - Equipment Design
KW - Eye Enucleation
KW - Eye Foreign Bodies/et [Etiology]
KW - Eye Foreign Bodies/pa [Pathology]
KW - Eye Foreign Bodies/pc [Prevention & Control]
KW - Eye Foreign Bodies/su [Surgery]
KW - Eye Injuries
KW - Penetrating/et [Etiology]
KW - Penetrating/pa [Pathology]
KW - Penetrating/pc [Prevention & Control]
KW - Penetrating/su [Surgery]
KW - Female
KW - Humans
KW - Incidence
KW - Leisure Activities
KW - Male
KW - Middle Aged

KW - Ophthalmologic Surgical Procedures
KW - Retrospective Studies
KW - Rubber
KW - Visual Acuity
RP - NOT IN FILE
SP - 788
EP - 792
JF - Ophthalmology
VL - 108
IS - 4
CY - United States
N2 - PURPOSE: To discuss the incidence and to characterize the clinical features of ocular trauma associated with bungee cord injuries. DESIGN: Retrospective case series. PARTICIPANTS: The authors identified 67 subjects (60 males) treated at Wills Eye Hospital for ocular trauma resulting from bungee cords between September 1, 1994, and September 1, 1999. METHODS: The authors reviewed subjects' records from Wills Eye Hospital, records from outside physicians involved in the subjects' care, and conducted telephone interviews when additional information was needed. MAIN OUTCOME MEASURES: Each chart was reviewed for demographic information, mechanism of injury, presenting visual acuity, anterior segment, posterior segment and periocular injuries, medical and surgical intervention, final visual acuity, and length of follow-up. RESULTS: Mean visual acuity on presentation was 20/150. The most common anterior, posterior, and periocular injuries were hyphema (42/67, 63%), commotio retinae (30/55, 55%), and eyelid edema/ecchymosis (40/67, 60%), respectively. Seven subjects (10%) sustained open globe injuries, three (4%) of whom required enucleation. Eighteen subjects (27%) required medical treatment, and 4 subjects (6%) required surgical treatment for increased intraocular pressure. A total of 21 subjects (31%) required some form of surgical intervention. Mean final visual acuity was 20/60, with an average follow-up of 34 weeks. CONCLUSIONS: This series, the largest to date, demonstrates a wide spectrum of serious ocular injuries related to bungee cord use. Medical and surgical treatments are commonly necessary to manage these injuries. We advocate a modification in the design of these cords and appropriate printed warnings directed to the users of bungee cords regarding the potential for severe ocular trauma. [References: 7]
SN - 0161-6420
AD - Retina Service, Wills Eye Hospital, Thomas Jefferson University, 900 Walnut Street, Philadelphia, PA 19107, USA
UR - 11297499
ER -

TY - JOUR
ID - 1678
T1 - Playground injuries in children: a review and Pennsylvania Trauma Center experience. [Review] [23 refs]
A1 - Bernardo,L.M.
A1 - Gardner,M.J.
A1 - Seibel,K.
Y1 - 2001/01//
N1 - Bernardo, L M. Gardner, M J. Seibel, K
Journal of the Society of Pediatric Nurses : JSPN
cky, 9615684
IM, N
Journal Article. Review
English
KW - MEDLINE
KW - Accidental Falls/sn [Statistics & Numerical Data]
KW - Child
KW - Child Welfare
KW - Preschool
KW - Female
KW - Humans
KW - Length of Stay/sn [Statistics & Numerical Data]
KW - Male

KW - Patient Education as Topic
KW - Pediatric Nursing
KW - Pennsylvania/ep [Epidemiology]
KW - Play and Playthings/in [Injuries]
KW - Population Surveillance
KW - Primary Prevention
KW - Registries
KW - Retrospective Studies
KW - Risk Factors
KW - Safety Management
KW - Seasons
KW - Time Factors
KW - Trauma Centers/sn [Statistics & Numerical Data]
KW - Wounds and Injuries/ep [Epidemiology]
KW - Wounds and Injuries/et [Etiology]
KW - Wounds and Injuries/pc [Prevention & Control]
RP - NOT IN FILE
SP - 11
EP - 20
JF - Journal of the Society of Pediatric Nurses
JA - J Soc Pediatr Nurs
VL - 6
IS - 1
CY - United States
N2 - ISSUES AND PURPOSE: To describe patient demographics, injury characteristics, and circumstances of playground injuries in children admitted to Pennsylvania trauma centers and to identify injury prevention strategies. DESIGN AND METHODS: Retrospective, descriptive study of 234 children ages 1 to 18 years sustaining playground-related injuries and whose hospital data were entered into the Pennsylvania Trauma Outcome Study. RESULTS: Most of the injuries occurred between April and September (77%), and noon to 6 P.M. (69%). Falls from playground equipment constituted the highest proportion of incidents (73%). Of 421 injuries (M = 1.8/patient), most were upper extremity (n = 117) and head (n = 110) injuries. PRACTICE IMPLICATIONS: Nurses can advocate for playground safety by teaching children to play safely and recommending age-appropriate equipment and protective surfacing. [References: 23]
SN - 1088-145X
AD - School of Nursing, University of Pittsburgh, PA, USA
UR - 11288499
ER -

TY - JOUR
ID - 1679
T1 - Evaluation of the Latrobe Valley Better Health Injury Prevention Program
A1 - Day,L.M.
A1 - Ozanne-Smith,J.
A1 - Cassell,E.
A1 - Li,L.
Y1 - 2001/03//
N1 - Day, L M. Ozanne-Smith, J. Cassell, E. Li, L
Injury prevention : journal of the International Society for Child and Adolescent Injury Prevention
crz, 9510056
IM
Evaluation Studies. Journal Article. Research Support, Non-U.S. Gov't
English
KW - MEDLINE
KW - Accident Prevention
KW - Adolescent
KW - Adult
KW - Aged

KW - Australia/ep [Epidemiology]
KW - Child
KW - Preschool
KW - Emergency Service
KW - Hospital/ut [Utilization]
KW - Female
KW - Health Education
KW - Health Promotion
KW - Humans
KW - Infant
KW - Male
KW - Middle Aged
KW - Population Surveillance
KW - Risk Factors
KW - Safety
KW - Wounds and Injuries/ep [Epidemiology]
KW - Wounds and Injuries/pc [Prevention & Control]
RP - NOT IN FILE
SP - 66
EP - 69
JF - Injury Prevention
JA - Inj Prev
VL - 7
IS - 1
CY - England
N2 - OBJECTIVE: To evaluate the Latrobe Valley Better Health Injury Prevention Program, a regional community based intervention in south east Victoria, Australia. METHOD: The evaluation design was quasiexperimental including pre-intervention and post-intervention observations in a predominately town dwelling population of approximately 76,000. There was no comparison community. Process measures included key informant interviews. Impact evaluation utilised self reported changes in injury risk and protective factors, gathered by a random household telephone survey. Outcome evaluation was based on five years of emergency department injury surveillance data for the Latrobe Valley. RESULTS: The program built strategic partnerships, increasing the emphasis on local safety. Activities were implemented in the targeted areas of home, sport, and playground injuries. Some 47,000 educational contacts were made with the community and at least 6,000 resource items distributed. There were significant increases in home safety knowledge. Some changes in the areas of playground and sport safety were achieved after partnership development with relevant agencies. Poisson regression models showed significant decreases in the presentation rate for all home injury and for the more severe home injuries. CONCLUSION: This study clearly demonstrates the difficulty of conducting robust evaluation in the absence of readily available and reliable data and adequate budgets. The Latrobe Valley Better Health Program activities contributed to structural, environmental, and organisational changes that have the potential to reduce injury. The extent of this contribution beyond that made by the statewide injury prevention strategy is not able to be determined
SN - 1353-8047
AD - Accident Research Centre, Monash University, Victoria, Australia. lesley.day@general.monash.edu.au
UR - 11289539
ER -

TY - JOUR
ID - 1680
T1 - Surface characteristics, equipment height, and the occurrence and severity of playground injuries
A1 - Laforest,S.
A1 - Robitaille,Y.
A1 - Lesage,D.
A1 - Dorval,D.
Y1 - 2001/03//
N1 - Laforest, S. Robitaille, Y. Lesage, D. Dorval, D
Injury prevention : journal of the International Society for Child and Adolescent Injury Prevention

crz, 9510056
IM
Journal Article. Research Support, Non-U.S. Gov't
English
KW - MEDLINE
KW - Accidental Falls/pc [Prevention & Control]
KW - Accidental Falls/sn [Statistics & Numerical Data]
KW - Adolescent
KW - Canada
KW - Child
KW - Preschool
KW - Consumer Product Safety
KW - Female
KW - Humans
KW - Infant
KW - Male
KW - Play and Playthings
KW - Population Surveillance
KW - Risk
KW - Wounds and Injuries/ep [Epidemiology]
KW - Wounds and Injuries/et [Etiology]
KW - Wounds and Injuries/pc [Prevention & Control]
RP - NOT IN FILE
SP - 35
EP - 40
JF - Injury Prevention
JA - Inj Prev
VL - 7
IS - 1
CY - England
N2 - OBJECTIVES: To evaluate whether surface characteristics (absorption level (g-max), material) and the height of play equipment are related to the occurrence and severity of injuries from falls. SETTING AND METHODS: During the summers of 1991 and 1995, conformity of play equipment to Canadian standards was assessed in a random sample (n = 102) of Montreal public playgrounds. Surface absorption (g-max) was tested using a Max Hic instrument and the height of equipment was measured. Concurrently, all injuries presenting at the emergency department of Montreal's two children's hospitals were recorded and parents were interviewed. Inspected equipment was implicated in 185 injuries. The g-max measurements (1995 only) were available for 110 of these playground accidents. RESULTS: One third of falls (35 %) occurred on a surface exceeding 200 g and the risk of injury was three times greater than for g level lower than 150 (95% confidence interval (CI) 1.45 to 6.35). On surfaces having absorption levels between 150 g and 200 g, injuries were 1.8 times more likely (95% CI 0.91 to 3.57). Injuries were 2.56 times more likely to occur on equipment higher than 2 m compared with equipment lower than 1.5 m. Analysis of risk factors by severity of injury failed to show any positive relationships between the g-max or height and severity, whereas surface material was a good predictor of severity. CONCLUSIONS: This study confirms the relationships between risk of injury, surface resilience, and height of equipment, as well as between type of material and severity of injury. Our data suggest that acceptable limits for surface resilience be set at less than 200 g, and perhaps even less than 150 g, and not exceed 2 m for equipment height. These findings reinforce the importance of installing recommended materials, such as sand, beneath play equipment
SN - 1353-8047
AD - Montreal Public Health Department, Montreal, Quebec, Canada. lucsofi@hotmail.com
UR - 11289532
ER -

TY - JOUR
ID - 1681
T1 - Lead exposure in children living in a smelter community in region Lagunera, Mexico
A1 - Garcia Vargas,G.G.

A1 - Rubio,Andrade M.
A1 - Del Razo,L.M.
A1 - Borja,Aburto,V
A1 - Vera,Aguilar E.
A1 - Cebrian,M.E.
Y1 - 2001/03/23/
N1 - Garcia Vargas, G G. Rubio Andrade, M. Del Razo, L M. Borja Aburto, V. Vera Aguilar, E. Cebrian, M E
Journal of toxicology and environmental health. Part A
ds5, 100960995
IM
Journal Article. Research Support, Non-U.S. Gov't
English
KW - MEDLINE
KW - Air Pollutants
KW - Occupational/an [Analysis]
KW - Body Burden
KW - Child
KW - Dust/an [Analysis]
KW - Environmental Exposure/an [Analysis]
KW - Humans
KW - Lead/an [Analysis]
KW - Lead/bl [Blood]
KW - Lead/ur [Urine]
KW - Metallurgy
KW - Mexico
KW - Soil Pollutants/an [Analysis]
KW - Water Pollutants
KW - Chemical/an [Analysis]
RP - NOT IN FILE
SP - 417
EP - 429
JF - Journal of Toxicology & Environmental Health Part A
JA - J Toxicol Environ Health A
VL - 62
IS - 6
CY - England
N2 - Industrial growth has created the potential for environmental problems in Mexico, since attention to environmental controls and urban planning has lagged behind the pace of industrialization. The aim of this cross-sectional study was to assess lead exposure in children aged 6-9 yr attending 3 primary schools and living in the vicinity of the largest smelter complex in Mexico. One of the schools is located 650 m distant from a smelter complex that includes a lead smelter (close school); the second is located 1750 m away from the complex and at the side of a heavy traffic road (intermediate school) in Torreon, Coahuila. The third school is located in Comez Palacio, Durango, 8100 m away from the smelter complex and distant from heavy vehicular traffic or industrial areas (remote school). Lead was measured in air, soil, dust, and well water. Lead in blood (PbB) was determined in 394 children attending the above mentioned schools. Determinations were performed by atomic absorption spectrometry. Diet, socioeconomic status, hygienic habits, and other variables were assessed by questionnaire. Median (range) PbB values were 7.8 microg/dl (3.54-29.61) in the remote school, 21.8 microg/dl (8.37-52.08) in the intermediate school and 27.6 microg/dl (7.37-58.53) in children attending the close school. The percentage of children with PbB > 15 microg/dl was 6.80%, 84.9%, and 92.1% respectively. In this order, the geometric means (range) of Pb concentrations in air were 2.5 microg/m3 (1.1-7.5), 5.8 microg/m3 (4.3-8.5), and 6.1 microg/m3 (1.6-14.9). The Pb concentrations in dust from playgrounds areas in the intermediate and close school settings ranged from 1,457 to 4,162.5 mg/kg. Pb concentrations in drinking water were less than 5 microg/L. Soil and dust ingestion and inhalation appear to be the main routes of exposure. Our results indicate that environmental contamination has resulted in an increased body burden of Pb, suggesting that children living in the vicinity of the smelter complex are at high risk for adverse effects of lead
SN - 1528-7394

AD - Facultad de Medicina, Universidad Juarez del Estado de Durango, Gomez Palacio, Mexico. ggarcia_vargas@hotmail.com
UR - 11289316
ER -

TY - JOUR
ID - 1682
T1 - [Professionals and managers in face of training]. [Spanish]
A1 - March Cerda,J.C.
A1 - Prieto,M.A.
A1 - Gutierrez,P.
A1 - Carmona,G.
A1 - Equipo de Evaluacion del PSMI.
Y1 - 2001/02/28/
N1 - March Cerda, J C. Prieto, M A. Gutierrez, P. Carmona, G. Equipo de Evaluacion del PSMI
Atencion primaria / Sociedad Espanola de Medicina de Familia y Comunitaria
9111075, a5f
IM
English Abstract. Journal Article. Research Support, Non-U.S. Gov't
Spanish
KW - MEDLINE
KW - Child
KW - Cross-Sectional Studies
KW - Education
KW - Medical
KW - Continuing
KW - Female
KW - Humans
KW - Maternal Health Services/og [Organization & Administration]
KW - Maternal Health Services/st [Standards]
KW - Patient Care Team
KW - Primary Health Care/og [Organization & Administration]
KW - Primary Health Care/st [Standards]
KW - Questionnaires
KW - Spain
KW - Staff Development
RP - NOT IN FILE
SP - 172
EP - 177
JF - Atencion Primaria
JA - Aten.Primaria
VL - 27
IS - 3
CY - Spain
N2 - OBJECTIVES: To analyse ongoing education within primary care teams from managers' perspective. DESIGN: Cross-sectional descriptive study. SETTING: The Mother-Infant Health Programme of Andalusia in primary care. INTERVENTION: The views of health centre professionals were obtained through 8 groups. The type of health district and the number of years in operation were used as criteria for division. The views of the area managers were collected through a self-filled questionnaire from all the managers in the autonomous community. MEASUREMENTS AND MAIN RESULTS: Professionals believed it necessary to keep up to date and train in new technology. They thought that training activities should focus on questions such as evaluation of programmes, focus of risk, counselling and communication, and certain techniques such as IUD insertion. Both managers and professionals coincided in recognising the effect of training on the development of mother-child services. There was consensus among professionals in affirming that training increases the quality of care delivered. Access to training was the main incentive element used by most area managers and one of those most highly valued by professionals. The professionals affirmed that in recent years there had been obstacles to their education by attending scientific events outside their areas. Many professionals stated that the economic

assistance of the pharmaceutical industry for courses caused inequalities between doctors and nurses in access to training. Professionals were very critical of training combined with specialist training. CONCLUSIONS: Professionals think they have sufficient training for mother-child health care. It is generally recognised that family doctors are better trained for working on health programmes than other professionals at the centres. Ongoing training was rated very highly by both professionals and directors, as it was seen as a motivating element and thought to be an activity almost solely carried out at health centres
SN - 0212-6567
AD - Escuela Andaluza de Salud Publica, Granada
UR - 11262322
ER -

TY - JOUR
ID - 1683
T1 - Exposure to Schistosoma mansoni infection in a rural area of Brazil. I: water contact
A1 - Gazzinelli,A.
A1 - Bethony,J.
A1 - Fraga,L.A.
A1 - LoVerde,P.T.
A1 - Correa-Oliveira,R.
A1 - Kloos,H.
Y1 - 2001/02//
N1 - Gazzinelli, A. Bethony, J. Fraga, L A. LoVerde, P T. Correa-Oliveira, R. Kloos, H
Tropical medicine & international health : TM & IH
cf5, 9610576
IM
Comparative Study. Journal Article. Research Support, Non-U.S. Gov't. Research Support, U.S. Gov't, P.H.S.
English
KW - MEDLINE
KW - Adolescent
KW - Adult
KW - Age Factors
KW - Aged
KW - 80 and over
KW - Animals
KW - Baths
KW - Brazil/ep [Epidemiology]
KW - Child
KW - Preschool
KW - Female
KW - Food Handling
KW - Health Behavior
KW - Health Surveys
KW - Humans
KW - Hygiene
KW - Laundering
KW - Male
KW - Middle Aged
KW - Observation/mt [Methods]
KW - Rural Health
KW - Schistosomiasis mansoni/ep [Epidemiology]
KW - Schistosomiasis mansoni/ps [Parasitology]
KW - Schistosomiasis mansoni/tm [Transmission]
KW - Sex Factors
KW - Water/ps [Parasitology]
KW - Water
RP - NOT IN FILE
SP - 126

EP - 135
JF - Tropical Medicine & International Health
JA - Trop Med Int Health
VL - 6
IS - 2
CY - England
N2 - The study of water contact patterns in rural Brazil presents unique challenges due to widely dispersed settlement patterns, the ubiquity of water contact sites, and the privatization of water resources. This study addresses these challenges by comparing the two most widely used methods of assessing water contact behaviour: direct observation and survey. The results of a 7-day direct observation of water contact were compared with water contact surveys administered 1 week after and then 1 year after the direct observation study. The direct observation study recorded a water contact rate higher than reported by other investigators (3.2 contacts per person per day); however, 75% of these contacts were for females and consisted mainly of domestic activities occurring around the household. A comparison of the frequency of water contact activities between the direct observation and the two surveys revealed several important points. First, no significant differences were found between methods for routine water contact activities (e.g. bathing), indicating that participants were able to accurately self-report some types of water contact activities. Second, significant differences were found in the recording of water contact activities that took place outside the observation area, indicating that direct observation may under-report water contact activities in areas where contact sites are dispersed widely. Third, significant differences between the direct observation and the survey method were more common for males than for females, indicating that the combination of widespread water contact sites and gender-specific division of labour may result in under-reporting of male contacts by direct observation methods. In short, despite the limitations in the recording of duration and body exposure, the survey method may more accurately record the frequency of water contact activities than direct observation methods in areas of widely dispersed water contact sites. Hence, surveys may be more suitable for the unique challenges of water contact in rural areas of Brazil
SN - 1360-2276
AD - Universidade Federal de Minas Gerais, Belo Horizonte, Minas Gerais, Brazil
UR - 11251909
ER -

TY - JOUR
ID - 1684
T1 - Wilderness mortalities: a 13-year experience
A1 - Goodman,T.
A1 - Iserson,K.V.
A1 - Strich,H.
Y1 - 2001/03//
N1 - Goodman, T. Iserson, K V. Strich, H
Annals of emergency medicine
4z7, 8002646
AIM, IM
Journal Article
English
KW - MEDLINE
KW - Adolescent
KW - Adult
KW - Aged
KW - 80 and over
KW - Alcoholic Intoxication/mo [Mortality]
KW - Athletic Injuries/mo [Mortality]
KW - Cause of Death
KW - Child
KW - Preschool
KW - Female
KW - Humans
KW - Leisure Activities
KW - Male

KW - Middle Aged
KW - Mortality
KW - Relief Work
KW - Retrospective Studies
KW - Rural Population/sn [Statistics & Numerical Data]
KW - Substance-Related Disorders/mo [Mortality]
RP - NOT IN FILE
SP - 279
EP - 283
JF - Annals of Emergency Medicine
JA - Ann Emerg Med
VL - 37
IS - 3
CY - United States
N2 - STUDY OBJECTIVE: To analyze the epidemiology of wilderness mortalities in a localized area with diverse terrain. METHODS: We conducted a retrospective review of the Pima County (Arizona) Sheriff's Office (PCSO) search and rescue logs and case reports, hospital records, and autopsy reports for all wilderness deaths from 1980 to 1992. The study group comprised all victims of injury or illness in Pima County wilderness who died during a 13-year period in a location remote enough so that standard ground-based emergency medical services units could not extract the body. RESULTS: One hundred fatalities occurred during the 13-year study period. There were 59 unintentional traumas, 18 suicides, 9 homicides, 12 medically related deaths, and 2 deaths of unknown causes. Toxicology tests performed on body fluids yielded positive findings for alcohol in a total of 50 (50%) cases and positive findings for drugs of abuse in 12 (12%) cases. It was estimated that alcohol was "a very probable" or "a probable" causative factor in 23 (40%) of the 59 unintentional trauma deaths, and in 1 (8.3%) of the 12 medically related deaths. Fifty-five (55%) deaths were witnessed events, with 45 (80%) of these victims reported as dying immediately or before arrival of search and rescue personnel. Ten (10%) victims received resuscitation in the field, and according to a review of hospital charts and autopsy reports, only 2 victims had a potentially survivable injury or illness. CONCLUSION: Many wilderness mortalities are related to incidents involving alcohol. Once the accident or injury has occurred, the majority of deaths are immediate, or at least before the arrival of medical personnel. Higher levels of medical care would not have improved the outcomes of those who did survive long enough to receive medical care. Therefore, primary efforts to reduce mortalities in the wilderness should be directed toward prevention, especially diminishing alcohol use in wilderness areas
SN - 0196-0644
AD - Section of Emergency Medicine, Department of Surgery, University of Arizona College of Medicine, Tucson, AZ, USA. tgood808@lava.net
UR - 11223764
ER -

TY - JOUR
ID - 1685
T1 - Epidemiological investigation of school-related injuries in Koprivnica County, Croatia
A1 - Vorko-Jovic,A.
A1 - Rimac,M.
A1 - Jovic,F.
A1 - Strnad,M.
A1 - Solaja,D.
Y1 - 2001/02//
N1 - Vorko-Jovic, A. Rimac, M. Jovic, F. Strnad, M. Solaja, D
Croatian medical journal
c0s, 9424324
IM
Journal Article. Research Support, Non-U.S. Gov't
English
KW - MEDLINE
KW - Adolescent
KW - Age Distribution

KW - Chi-Square Distribution
KW - Child
KW - Preschool
KW - Croatia/ep [Epidemiology]
KW - Female
KW - Humans
KW - Injury Severity Score
KW - Male
KW - Population Surveillance
KW - Prevalence
KW - Probability
KW - Risk Assessment
KW - Risk Factors
KW - Rural Population
KW - School Health Services/sn [Statistics & Numerical Data]
KW - Schools/sn [Statistics & Numerical Data]
KW - Sex Distribution
KW - Wounds and Injuries/di [Diagnosis]
KW - Wounds and Injuries/ep [Epidemiology]
RP - NOT IN FILE
SP - 58
EP - 63
JF - Croatian Medical Journal
JA - Croat.Med J
VL - 42
IS - 1
CY - Croatia
N2 - AIM: To assess the prevalence of injuries in elementary schools and determine specific risk groups of school-age children. METHODS: According to the 1991 census, there were 6,398 children between 7 and 14 years of age in the study area of the former Koprivnica district. During the 1992-1997 period, 354 children were injured in school. The registration of injured children was performed via structured questionnaires filled out at the emergency clinic and outpatient surgical clinic of the General Hospital in Koprivnica. The mechanism of accident and activities preceding it were categorized according to the Nordic Medico-Statistical Committee classification. Chi-square test was used to determine groups of school children at specific risk and a classification tree was made on the basis of minimum entropy values for age, sex, activity, and mechanism of injury. RESULTS: The highest injury rate of was recorded in 12-year-olds (21.7%). Upper extremities were most common site of injury (52.8%), whereas the most common type of injury was contusion (45.2%). The rate of head injuries was 3.2 times higher in younger (aged 7-10) children, whereas the rate of sports injuries was 3.5-fold higher in older (aged 11-14) children (p=0.001). Entropy classification revealed younger school-age children to be at the highest risk of contusion due to a blow from a ball, an object, or contact during sports activities. CONCLUSION: In Koprivnica County, most school-related injuries occurred during sport activities (42%) and play during recess (55%), with specific differences in age and sex
SN - 0353-9504
AD - Department of Epidemiology, Statistics, and Informatics, Andrija Stampar School of Public Health, Zagreb University School of Medicine, Rockeffellerova 4, 10000 Zagreb, Croatia. avorko@andrija.snz.hr
UR - 11172657
ER -

TY - JOUR
ID - 1686
T1 - Effects of reducing children's television and video game use on aggressive behavior: a randomized controlled trial
A1 - Robinson,T.N.
A1 - Wilde,M.L.
A1 - Navracruz,L.C.
A1 - Haydel,K.F.
A1 - Varady,A.

Y1 - 2001/01//
N1 - Robinson, T N. Wilde, M L. Navracruz, L C. Haydel, K F. Varady, A
Archives of pediatrics & adolescent medicine
9422751, bwf
AIM, IM
Clinical Trial. Journal Article. Randomized Controlled Trial. Research Support, Non-U.S. Gov't. Research Support, U.S. Gov't, P.H.S.
English
KW - MEDLINE
KW - Aggression/px [Psychology]
KW - California
KW - Child
KW - Child Psychology
KW - Curriculum
KW - Female
KW - Health Education/og [Organization & Administration]
KW - Humans
KW - Male
KW - Mass Media
KW - Parents/px [Psychology]
KW - Peer Group
KW - Program Evaluation
KW - Questionnaires
KW - School Health Services
KW - Social Perception
KW - Television
KW - Video Games/ae [Adverse Effects]
KW - Video Games/px [Psychology]
RP - NOT IN FILE
SP - 17
EP - 23
JF - Archives of Pediatrics & Adolescent Medicine
JA - Arch Pediatr Adolesc Med
VL - 155
IS - 1
CY - United States
N2 - CONTEXT: The relationship between exposure to aggression in the media and children's aggressive behavior is well documented. However, few potential solutions have been evaluated. OBJECTIVE: To assess the effects of reducing television, videotape, and video game use on aggressive behavior and perceptions of a mean and scary world. DESIGN: Randomized, controlled, school-based trial. SETTING: Two sociodemographically and scholastically matched public elementary schools in San Jose, Calif. PARTICIPANTS: Third- and fourth-grade students (mean age, 8.9 years) and their parents or guardians. INTERVENTION: Children in one elementary school received an 18-lesson, 6-month classroom curriculum to reduce television, videotape, and video game use. MAIN OUTCOME MEASURES: In September (preintervention) and April (postintervention) of a single school year, children rated their peers' aggressive behavior and reported their perceptions of the world as a mean and scary place. A 60% random sample of children were observed for physical and verbal aggression on the playground. Parents were interviewed by telephone and reported aggressive and delinquent behaviors on the child behavior checklist. The primary outcome measure was peer ratings of aggressive behavior. RESULTS: Compared with controls, children in the intervention group had statistically significant decreases in peer ratings of aggression (adjusted mean difference, -2.4%; 95% confidence interval [CI], -4.6 to -0.2; P =.03) and observed verbal aggression (adjusted mean difference, -0.10 act per minute per child; 95% CI, -0.18 to -0.03; P =.01). Differences in observed physical aggression, parent reports of aggressive behavior, and perceptions of a mean and scary world were not statistically significant but favored the intervention group. CONCLUSIONS: An intervention to reduce television, videotape, and video game use decreases aggressive behavior in elementary schoolchildren. These findings support the causal influences of these media on aggression and the potential benefits of reducing children's media use

SN - 1072-4710
AD - Department of Pediatrics and Center for Research in Disease Prevention, Stanford University School of Medicine, 1000 Welch Rd, Palo Alto, CA 94304, USA. tom.robinson@stanford.edu
UR - 11177057
ER -

TY - JOUR
ID - 1687
T1 - Preventable illness and out-of-area travel of children in New York counties
A1 - Basu,J.
A1 - Friedman,B.
Y1 - 2001/01//
N1 - Basu, J. Friedman, B
Health economics
bvq, 9306780
IM
Journal Article
English
KW - MEDLINE
KW - Ambulatory Care
KW - Catchment Area (Health)/sn [Statistics & Numerical Data]
KW - Child
KW - Geography
KW - Health Services Accessibility
KW - Hospitals
KW - Urban/ut [Utilization]
KW - Humans
KW - Insurance
KW - Health
KW - Logistic Models
KW - Medically Underserved Area
KW - Models
KW - Economic
KW - Multivariate Analysis
KW - New York
KW - Odds Ratio
KW - Patient Admission/sn [Statistics & Numerical Data]
KW - Rural Population/sn [Statistics & Numerical Data]
KW - Socioeconomic Factors
KW - Travel
RP - NOT IN FILE
SP - 67
EP - 78
JF - Health Economics
JA - Health Econ
VL - 10
IS - 1
CY - England
N2 - The long-distance travellers could be important to any cost-benefit assessment of policies to increase or improve local resources. This study examines the out-of-area hospital admission pattern for patients with Ambulatory Care Sensitive (ACS) conditions, also known as preventable conditions. The availability of local resources play a significant role for hospitalization for these conditions. Despite a growing literature investigating hospital choice, little is known about the effects of resource availabilities in local areas and patient characteristics prompting people to seek care at a longer distance from home for these conditions. Based on hospital discharge data for New York residents (children) admitted to hospitals in New York, Pennsylvania, New Jersey or Connecticut in 1994, the study uses logistic regression to predict travel out of the local area for ACS admission. The actual distance between residence and hospital is a highly skewed and problematic

measure, but the crossing of county boundaries is a related and very useful dichotomous measure of distant hospitalization. The study finds a strong association of types of insurance and availability of primary care with episodes of hospitalization for children outside the area of residence, after controlling for severity of illness and several other patient and county characteristics. Copyright 2001 John Wiley & Sons, Ltd
SN - 1057-9230
AD - Center for Primary Care Research, Agency for Healthcare Research and Quality, Rockville, MD 20852, USA. jbasu@ahrq.gov
UR - 11180570
ER -

TY - JOUR
ID - 1688
T1 - The work environment impact assessment: a methodologic framework for evaluating health-based interventions
A1 - Rosenberg,B.J.
A1 - Barbeau,E.M.
A1 - Moure-Eraso,R.
A1 - Levenstein,C.
Y1 - 2001/02//
N1 - Rosenberg, B J. Barbeau, E M. Moure-Eraso, R. Levenstein, C
American journal of industrial medicine
3in, 8101110
IM
Journal Article
English
KW - MEDLINE
KW - Adult
KW - Agriculture
KW - Child
KW - Cost-Benefit Analysis
KW - Employment
KW - Environment
KW - Environmental Monitoring/ec [Economics]
KW - Environmental Monitoring/mt [Methods]
KW - Evaluation Studies as Topic
KW - Humans
KW - Occupational Diseases/pc [Prevention & Control]
KW - Plant Growth Regulators
KW - Succinates/ae [Adverse Effects]
KW - United States
KW - Workplace
RP - NOT IN FILE
SP - 218
EP - 226
JF - American Journal of Industrial Medicine
JA - Am J Ind Med
VL - 39
IS - 2
CY - United States
N2 - BACKGROUND: A new conceptual framework is needed to evaluate health-based interventions based on the premise that like the environment, workplaces are complex ecologies. The proposed Work Environment Impact Assessment (WEIA) is analogous to Environmental Impact Assessment (EIA), a concept and method developed 30 years ago in the environmental policy arena to evaluate potential consequences of human activity for the natural environment. WEIA entails identifying and evaluating both intended and unintended consequences, or outcomes, associated with a particular intervention. Because the workplace is a complex ecology, changes in one aspect may lead to changes in other aspects. WEIA calls for a systematic and comprehensive approach to the total work environment. METHODS: To illustrate the utility of WEIA we use

this approach to evaluate an intervention to reduce the public's exposure to the pesticide Alar, which had been used on apples until it was withdrawn from the market in 1989. RESULTS: While this intervention did indeed reduce the public's exposure to Alar, it also led to other unintended consequences, namely new ergonomic hazards for apple pickers, increased stress levels in the orchards for both pickers and growers, as well as new worker, and perhaps public, exposure to potent neurotoxins. CONCLUSIONS: The goal of using WEIA is not to engage in a risk-risk debate that stalls worthwhile interventions. Rather, we propose that by conducting a Work Environment Impact Assessment, all possible positive and negative "ripple" effects stemming from an intervention can be considered, so that the intervention can be designed to achieve maximum benefit. Copyright 2001 Wiley-Liss, Inc
SN - 0271-3586
AD - Department of Family Medicine and Community Health, Tufts University School of Medicine, Boston, Massachusetts 02111, USA
UR - 11170164
ER -

TY - JOUR
ID - 1689
T1 - Pilot study of the use of community volunteers to distribute azithromycin for trachoma control in Ghana
A1 - Solomon,A.W.
A1 - Akudibillah,J.
A1 - Abugri,P.
A1 - Hagan,M.
A1 - Foster,A.
A1 - Bailey,R.L.
A1 - Mabey,D.C.
Y1 - 2001///
N1 - Solomon, A W. Akudibillah, J. Abugri, P. Hagan, M. Foster, A. Bailey, R L. Mabey, D C
Bulletin of the World Health Organization
7507052, c80
IM
Journal Article. Research Support, Non-U.S. Gov't
English
KW - MEDLINE
KW - Adolescent
KW - Adult
KW - Anti-Bacterial Agents/ad [Administration & Dosage]
KW - Azithromycin/ad [Administration & Dosage]
KW - Child
KW - Preschool
KW - Community Health Services/ma [Manpower]
KW - Community Health Services/og [Organization & Administration]
KW - Female
KW - Forms and Records Control
KW - Ghana
KW - Humans
KW - Male
KW - Pilot Projects
KW - Sensitivity and Specificity
KW - Trachoma/di [Diagnosis]
KW - Trachoma/dt [Drug Therapy]
KW - Volunteers/ed [Education]
KW - Volunteers
RP - NOT IN FILE
SP - 8
EP - 14
JF - Bulletin of the World Health Organization
JA - Bull World Health Organ

VL - 79
IS - 1
CY - Switzerland
N2 - OBJECTIVE: To assess the skills of community health volunteers in diagnosing active trachoma and distributing azithromycin in the Northern Region of Ghana. METHODS: Six community health volunteers from Daboya were trained to diagnose trachoma and to treat the disease using azithromycin. They were also informed of the drug's possible side-effects. Under supervision, each volunteer then examined, and if necessary treated, 15 households. The dose of azithromycin was determined by weight; height was also measured. Tablets were given in preference to suspension when possible. RESULTS: The volunteers' diagnostic sensitivity for active trachoma was 63%; their specificity was 96%. At the household level, their "decision to treat" was correct in 83% of households. In 344 treatment episodes, volunteers planned a dose of azithromycin outside the range 15-30 mg/kg on only seven occasions (2.0% of all planned treatments). The volunteers' drug management skills were good, the response of the community was excellent, and adverse reactions were infrequent. Diagnosis of active trachoma, record-keeping skills, and knowledge of side-effects were found to need greater emphasis in any future education programme. Most people aged four years or older were able to swallow tablets. For those taking tablets, the correlation between the data gathered for height and weight shows that calculating azithromycin doses by height is a valid alternative to calculating it by weight. CONCLUSION: Trained community health volunteers have a potential role in identifying active trachoma and distributing azithromycin. To simplify training and logistics, it may be better to base dosage schedules on height rather than weight for those taking tablets, which included most people aged four years or more in the population studied
SN - 0042-9686
AD - Clinical Research Unit, London School of Hygiene and Tropical Medicine, England
UR - 11217675
ER -

TY - JOUR
ID - 1690
T1 - Novel missense mutations outside the allosteric domain of glutamate dehydrogenase are prevalent in European patients with the congenital hyperinsulinism-hyperammonemia syndrome
A1 - Santer,R.
A1 - Kinner,M.
A1 - Passarge,M.
A1 - Superti-Furga,A.
A1 - Mayatepek,E.
A1 - Meissner,T.
A1 - Schneppenheim,R.
A1 - Schaub,J.
Y1 - 2001/01//
N1 - Santer, R. Kinner, M. Passarge, M. Superti-Furga, A. Mayatepek, E. Meissner, T. Schneppenheim, R. Schaub, J
Human genetics
ged, 7613873
IM
Journal Article
English
KW - MEDLINE
KW - Adolescent
KW - Adult
KW - Allosteric Regulation
KW - Child
KW - Preschool
KW - DNA Mutational Analysis
KW - Europe
KW - Female
KW - Glutamate Dehydrogenase/ge [Genetics]
KW - Humans
KW - Hyperammonemia/ge [Genetics]

KW - Hyperinsulinism/ge [Genetics]
KW - Male
KW - Middle Aged
KW - Mutation
KW - Missense
KW - Pedigree
KW - Syndrome
RP - NOT IN FILE
SP - 66
EP - 71
JF - Human Genetics
JA - Hum Genet
VL - 108
IS - 1
CY - Germany
N2 - The hyperinsulinism-hyperammonemia syndrome (HHS) has been shown to result from 'gain-of-function' mutations of the glutamate dehydrogenase (GlDH) gene, GLUD1. In the original report, all mutations were found in a narrow range of 27 base pairs within exons 11 and 12 which predicted an effect on the presumed allosteric domain of the enzyme and all these mutations were associated by a diminished inhibitory effect of guanosine triphosphate (GTP) on GlDH activity. We have investigated 14 patients from seven European families with mild hyperinsulinism. In four families, more than one member was affected. In eight cases hyperammonemia was documented, and eight cases had signs of significant leucine sensitivity. In one of the families, a novel heterozygous missense mutation in exon 6 [c.833C>T (R221C)] was detected, and in all other cases from six unrelated families the novel heterozygous missense mutation c.978G>A (R269H) was found in exon 7. When GIDH activity was measured in lymphocytes isolated from affected patients, both mutations were shown to result in a normal basal activity but a diminished sensitivity to GTP. It is the first time that this effect is reported for mutations located in the presumed catalytic site and outside the GTP allosteric domain of the enzyme. The observation of the high prevalence of the exon 7 mutation both in familial and sporadic cases of HHS suggests a mutation hot spot and justifies a mutation screening for this novel mutation by mismatch PCR-based restriction enzyme digestion in patients with hyperinsulinism
SN - 0340-6717
AD - Department of Pediatrics, University Children's Hospital, Kiel, Germany. santer@pediatrics.uni-kiel.de
UR - 11214910
ER -

TY - JOUR
ID - 1691
T1 - Stroke rehabilitation: application and analysis of the modified Barthel index in an Arab community
A1 - Nazzal,M.
A1 - Sa'adah,M.A.
A1 - Al-Ansari,D.
A1 - Al-Awadi,O.
A1 - Eyadah,A.A.
A1 - Al-Kadiri,M.A.
A1 - Inshasi,J.
A1 - Trebinjac,S.
A1 - Khader,S.
Y1 - 2001/01/15/
N1 - Nazzal, M. Sa'adah, M A. Al-Ansari, D. Al-Awadi, O. Eyadah, A A. Al-Kadiri, M A. Inshasi, J. Trebinjac, S. Khader, S
Disability and rehabilitation
9207179, a8i
IM
Comparative Study. Journal Article
English
KW - MEDLINE
KW - Activities of Daily Living/cl [Classification]

KW - Adolescent
KW - Adult
KW - Aged
KW - 80 and over
KW - Child
KW - Disability Evaluation
KW - Female
KW - Humans
KW - Jordan
KW - Male
KW - Middle Aged
KW - Occupational Therapy/mt [Methods]
KW - Physical Therapy Modalities/mt [Methods]
KW - Probability
KW - Prognosis
KW - Prospective Studies
KW - Recovery of Function
KW - Rehabilitation Centers
KW - Retrospective Studies
KW - Sensitivity and Specificity
KW - Sickness Impact Profile
KW - Stroke/di [Diagnosis]
KW - Stroke/rh [Rehabilitation]
RP - NOT IN FILE
SP - 36
EP - 42
JF - Disability & Rehabilitation
JA - Disabil Rehabil
VL - 23
IS - 1
CY - England
N2 - PURPOSE: The primary aim was to provide experience with a functional evaluation instrument (modified Barthel index MBI) that assures the quality of work and identify its deficiencies, familiarize our staff with the feasibility of its application on our local inpatients and educate our personnel in the field of stroke rehabilitation. The secondary aim was to collect data that are measurable and reproducible, identify specific local factors that adversely affect outcomes and serve as a feedback system to our national organizations. METHODS: In this prospective/retrospective study we evaluated 80 hemiplegic patients with completed stroke, admitted to hospital during the year 1989 1990. They were assessed by a neurologist, physiatrist and physiotherapist on admission and discharge using the MBI. All patients received comprehensive inpatient rehabilitation. The study was interrupted at the beginning of the Gulf crisis. However, the data were revived and retrospectively studied in the year 1994-1995. RESULTS: The MBI proved to be fully acceptable and easily applicable in our community. The rehabilitation staff became rapidly familiarized with its application and the reproduction of its data. The goals of rehabilitation were achieved through the reduction in the number of individuals in the more severe MBI scores and the increase in the number of individuals in the less severe ones. Significant improvements occurred in dressing of the upper and lower body, washing, grooming, care of perineum, transfer chair, toilet and walking on a level of 50 yards (p < or = 0.0005). CONCLUSIONS: We conclude that MBI is simple, convenient, efficient, gives exact and accurate information about daily activities and ambulation and could be used in inpatient follow up sittings, in the Arab and culturally similar Middle East countries
SN - 0963-8288
AD - Physical department, Hamad Hospital, Doha, Qatar
UR - 11213322
ER -

TY - JOUR
ID - 1692
T1 - Fungal peritonitis caused by Curvularia species in a child undergoing peritoneal dialysis
A1 - Canon,H.L.

A1 - Buckingham,S.C.
A1 - Wyatt,R.J.
A1 - Jones,D.P.
Y1 - 2001/01//
N1 - Canon, H L. Buckingham, S C. Wyatt, R J. Jones, D P
Pediatric nephrology (Berlin, Germany)
avr, 8708728
IM
Case Reports. Journal Article
English
KW - MEDLINE
KW - Amphotericin B/tu [Therapeutic Use]
KW - Antifungal Agents/tu [Therapeutic Use]
KW - Catheters
KW - Indwelling/mi [Microbiology]
KW - Child
KW - Device Removal
KW - Equipment Contamination
KW - Female
KW - Humans
KW - Mitosporic Fungi
KW - Mycoses/dt [Drug Therapy]
KW - Mycoses
KW - Peritoneal Dialysis/ae [Adverse Effects]
KW - Peritoneal Dialysis/is [Instrumentation]
KW - Peritonitis/mi [Microbiology]
RP - NOT IN FILE
SP - 35
EP - 37
JF - Pediatric Nephrology
JA - Pediatr Nephrol
VL - 16
IS - 1
CY - Germany
N2 - We report the first case of peritonitis caused by Curvularia species in a child undergoing peritoneal dialysis. He presented with gray-black proteinaceous material obstructing the lumen of his Tenckhoff catheter. Although the peritoneal fluid was cloudy, the patient suffered neither significant abdominal tenderness nor systemic symptoms. Catheter removal and treatment with amphotericin B allowed complete recovery and return to peritoneal dialysis within 7 days. Outdoor play in a wooded environment may have allowed contact of this saprophytic fungus with the child's indwelling catheter transfer set
SN - 0931-041X
AD - Le Bonheur Children's Medical Center, University of Tennessee, Memphis, Department of Pediatrics, Crippled Children's Research Center, Memphis, Tennessee, USA
UR - 11198600
ER -

TY - JOUR
ID - 1693
T1 - Immune biomarkers in relation to exposure to particulate matter: a cross-sectional survey in 17 cities of Central Europe
A1 - Leonardi,G.S.
A1 - Houthuijs,D.
A1 - Steerenberg,P.A.
A1 - Fletcher,T.
A1 - Armstrong,B.
A1 - Antova,T.
A1 - Lochman,I.

A1 - Lochmanova,A.
A1 - Rudnai,P.
A1 - Erdei,E.
A1 - Musial,J.
A1 - Jazwiec-Kanyion,B.
A1 - Niciu,E.M.
A1 - Durbaca,S.
A1 - Fabianova,E.
A1 - Koppova,K.
A1 - Lebret,E.
A1 - Brunekreef,B.
A1 - van,Loveren H.
Y1 - 2000///
N1 - Leonardi, G S. Houthuijs, D. Steerenberg, P A. Fletcher, T. Armstrong, B. Antova, T. Lochman, I. Lochmanova, A. Rudnai, P. Erdei, E. Musial, J. Jazwiec-Kanyion, B. Niciu, E M. Durbaca, S. Fabianova, E. Koppova, K. Lebret, E. Brunekreef, B. van Loveren, H
Inhalation toxicology
8910739, db8
IM
Journal Article. Research Support, Non-U.S. Gov't
English
KW - MEDLINE
KW - Air Pollutants/im [Immunology]
KW - Air Pollution/ae [Adverse Effects]
KW - Biological Markers/bl [Blood]
KW - Child
KW - Cities
KW - Cross-Sectional Studies
KW - Environmental Exposure
KW - Europe/ep [Epidemiology]
KW - Female
KW - Humans
KW - Immunoglobulins/im [Immunology]
KW - Lymphocyte Count
KW - Lymphocytes/cy [Cytology]
KW - Lymphocytes/im [Immunology]
KW - Male
KW - Neutrophils/im [Immunology]
KW - Respiratory Tract Diseases/ep [Epidemiology]
KW - Respiratory Tract Diseases/im [Immunology]
KW - Seroepidemiologic Studies
KW - Urban Population
RP - NOT IN FILE
SP - 1
EP - 14
JF - Inhalation Toxicology
JA - Inhal.Toxicol
VL - 12 Suppl 4
CY - United States
N2 - Human population data on air pollution and its effects on the immune system are scarce. A survey was conducted within the framework of the Central European Study of Air Quality and Respiratory Health (CESAR) to measure a panel of immune biomarkers in children of Bulgaria, Czech Republic, Hungary, Poland, Romania, and Slovakia. Seventeen cities were chosen to represent a wide range of exposure to outdoor air pollution. In each, ambient particulate matter of less than 10 microns diameter and less than 2.5 microns diameter (PM10 and PM2.5) were measured with a Harvard impactor. Blood was collected from 366 school children aged 9 to 11 yr between 11 April and 10 May 1996. The percentage of B, total T, CD4+, CD8+, and natural killer (NK) lymphocytes was determined by flow cytometry (Becton Dickinson); total immunoglobulins of class G, M, A

and E (IgG, IgM, IgA, and IgE) were measured in serum using nephelometry (Behring). Associations between PM and each log-transformed biomarker concentration were studied by linear regression, in a two-stage model. The yearly average concentrations varied from 41 to 96 micrograms/m3 for PM10 across the 17 study areas, from 29 to 67 micrograms/m3 for PM2.5, and from 12 to 38 micrograms/m3 for PM10-2.5 (coarse). Number of B, CD4+, CD8+, and NK lymphocytes increased with increasing concentration of PM, having adjusted for age, gender, parental smoking, laboratory of analysis, and recent respiratory illness. Differences in lymphocyte number were larger and statistically significant for exposure to PM2.5. Similar results were found when we examined the association between PM and lymphocyte number separately for each laboratory. Total IgG was increased with increasing concentration of PM, significantly in the case of PM2.5. When we repeated the analyses with two other statistical approaches the results did not differ from those reported here. The effect of coarse PM on lymphocyte numbers appears small in comparison to PM2.5. One possible interpretation of our findings is that long-term exposure to airborne particulates leads to inflammation of the airways and activation of the cellular and humoral immune system
SN - 0895-8378
AD - Environmental Epidemiology Unit, London School of Hygiene and Tropical Medicine, London WC1E 7HT. giovanni.leonardi@lshtm.ac.uk
UR - 12881884
ER -

TY - JOUR
ID - 1694
T1 - Penetration of nitrogen oxides and particles from outdoor into indoor air and removal of the pollutants through filtration of incoming air
A1 - Partti-Pellinen,K.
A1 - Marttila,O.
A1 - Ahonen,A.
A1 - Suominen,O.
A1 - Haahtela,T.
Y1 - 2000/06//
N1 - Partti-Pellinen, K. Marttila, O. Ahonen, A. Suominen, O. Haahtela, T
Indoor air
9423515, da3
IM
Journal Article. Research Support, Non-U.S. Gov't
English
KW - MEDLINE
KW - Air Pollutants/an [Analysis]
KW - Air Pollution
KW - Indoor/an [Analysis]
KW - Indoor/pc [Prevention & Control]
KW - Child
KW - Child Day Care Centers
KW - Environmental Monitoring
KW - Filtration
KW - Humans
KW - Nitrogen Oxides/an [Analysis]
KW - Particle Size
KW - Ventilation
RP - NOT IN FILE
SP - 126
EP - 132
JF - Indoor Air
VL - 10
IS - 2
CY - Denmark
N2 - We studied the effect of ventilation and air filtration systems on indoor air quality in a children's day-care center in Finland. Ambient air nitrogen oxides (NO, NO2) and particles (TSP, PM10) were simultaneously

measured outdoors and indoors with automatic nitrogen oxide analyzers and dust monitoring. Without filtration nitrogen oxides and particulate matter generated by nearby motor traffic penetrated readily indoors. With chemical filtration 50-70% of nitrogen oxides could be removed. Mechanical ventilation and filtration also reduced indoor particle levels. During holidays and weekends when there was no opening of doors and windows and no particle-generating activity indoors, the indoor particle level was reduced to less than 10% of the outdoor level. At times when outdoor particle concentrations were high during weekdays, the indoor level was about 25% of the outdoor level. Thus, the possible adverse health effects of nitrogen oxides and particles indoors could be countered by efficient filtration. We also showed that inclusion of heat recovery equipment can make new ventilation installations economical
SN - 0905-6947
AD - South Karelia Allergy and Environment Institute, 55330 Tiuruniemi, Finland
UR - 11980102
ER -

TY - JOUR
ID - 1695
T1 - Children's risk perception and parents' views on levels of risk that children attach to outdoor activities
A1 - Soori,H.
Y1 - 2000/05//
N1 - Soori, H
Saudi medical journal
dyw, 7909441
IM
Journal Article
English
KW - MEDLINE
KW - Accidents/sn [Statistics & Numerical Data]
KW - Adult
KW - Age Distribution
KW - Attitude to Health
KW - Child
KW - Child Psychology
KW - Child Welfare
KW - Cross-Sectional Studies
KW - England
KW - Female
KW - Humans
KW - Leisure Activities
KW - Male
KW - Parents/px [Psychology]
KW - Questionnaires
KW - Risk-Taking
KW - Safety
KW - Sex Distribution
KW - Socioeconomic Factors
KW - Wounds and Injuries/ep [Epidemiology]
KW - Wounds and Injuries/et [Etiology]
KW - Wounds and Injuries/pc [Prevention & Control]
RP - NOT IN FILE
SP - 455
EP - 460
JF - Saudi Medical Journal
JA - Saudi Med J
VL - 21
IS - 5
CY - Saudi Arabia

N2 - OBJECTIVE: Higher rates of outdoor injuries are found in older children, boys and children from more deprived areas. Some of the causes of these variations have been studied in some age groups and in different situations. To study children's perception of safety and danger and parents' views on the level of risk that their children attach to outdoor activities comparing age groups, boys and girls, and the less economically well off with the better off in order to understand outdoor accidental injuries. METHODS: A cross-sectional study, obtained information from children aged 7 and 9 (n=471) and their parents (n=416) were living in Newcastle upon Tyne by self-completed questionnaires. Nine schools in different socio-economic areas were selected. Children were asked to paste different stickers on to the pictures in the classrooms. The parents' questionnaires were delivered by children and then returned to school after completion. The deprivation of an individual was accessed by Z-score of the households. RESULTS: Older children compared to younger ones, boys versus girls and more economically deprived children compared to less deprived children had less perception of the dangers of outdoor activities. For example, older children compared to younger ones were more likely to perceive crossing a busy road with their friends (38.0% vs. 26.4%, P=0.007) as being safe. Boys compared to girls were more likely to perceive climbing wall (31.5% vs. 13.2%, P<0.001) as being safe. More economically deprived children were more likely to perceive cycling without a helmet (25.8% vs. 11.2%, P<0.001) as being safe than less economically deprived children. CONCLUSION: These findings may partly explain the higher rate of accidental injuries among older children, boys and more deprived children. The results may inform how education of primary schoolchildren about dealing with activities such as crossing a busy road, climbing walls and playing in street and how parents should be conscious of their children's outdoor activities
SN - 0379-5284
AD - Department of Community Medicine, The Medical School, Ahwaz University of Medical Sciences, Ahwaz, Iran
UR - 11500680
ER -

TY - JOUR
ID - 1696
T1 - Telemedical care reduces hypoglycemias and improves glycemic control in children and adolescents with type 1 diabetes
A1 - Liesenfeld,B.
A1 - Renner,R.
A1 - Neese,M.
A1 - Hepp,K.D.
Y1 - 2000///
N1 - Liesenfeld, B. Renner, R. Neese, M. Hepp, K D
Diabetes technology & therapeutics
100889084, d3x
IM
Clinical Trial. Journal Article. Research Support, Non-U.S. Gov't
English
KW - MEDLINE
KW - Adolescent
KW - Adult
KW - Blood Glucose/an [Analysis]
KW - Blood Glucose/me [Metabolism]
KW - Blood Glucose Self-Monitoring
KW - Caregivers/ed [Education]
KW - Child
KW - Preschool
KW - Counseling
KW - Diabetes Mellitus
KW - Type 1/bl [Blood]
KW - Type 1/dt [Drug Therapy]
KW - Type 1/rh [Rehabilitation]
KW - Family Practice
KW - Female
KW - Germany

KW - Humans
KW - Infant
KW - Insulin/ad [Administration & Dosage]
KW - Insulin/tu [Therapeutic Use]
KW - Internal Medicine
KW - Male
KW - Patient Education as Topic/mt [Methods]
KW - Patient Selection
KW - Rural Population
KW - Self Care
KW - Telemedicine/mt [Methods]
KW - Telephone
KW - Treatment Outcome
RP - NOT IN FILE
SP - 561
EP - 567
JF - Diabetes Technology & Therapeutics
JA - Diabetes Technol Ther
VL - 2
IS - 4
CY - United States
N2 - Education programs for intensive insulin therapy were found to be valuable in improving glycemic control, but, due to low prevalence of type 1 diabetes in children and adolescents, access to those programs varies considerably in rural areas. We report on a telemedical care program that overcomes geographical isolation of patients on intensive insulin therapy. Sixty-one children and adolescents under the age of 26 participated in a telemedical care program. They stored daily information on blood glucose, injected insulin, meals and exercise in a glucosemeter with electronic memory and transferred the data via modem go a remote diabetes center outside of the region. By individual telephone consultations from home, they reviewed the data with a diabetologist at the diabetes center and adjusted their intensive insulin therapy in order to achieve predefined treatment goals. Patients were trained for 19 (6-48) weeks in the program and measured blood glucose 4.9 (1.7-4.9) times daily. Compared to the run-in-period, mean blood glucose had decreased (167 to 158 mg/dL, p < 0.01), standard deviation of blood glucose had decreased (81 to 70 mg/dL, p < 0.001), and frequency of hypoglycemia had decreased (5.2 to 3.3 in 4 weeks, p = 0.01) at the end of the program. The proportion of blood glucose values within the target range (80-180 mg/dL) had increased (47-55%, p < 0.001). HbAlc was reduced by 0.4% (-3.8 to +2.2%, p < 0.05). Telemedical care for intensive insulin therapy is safe, can improve glycemic control, and reduce the number of hypoglycemias in children and adolescents with type 1 diabetes
SN - 1520-9156
AD - Diabetes Center, II Department of Medicine, Bruderkrankenhaus, Trier, Germany. b.liesenfeld@gmx.de
UR - 11469619
ER -

TY - JOUR
ID - 1697
T1 - [The pediatrician facing the problem of polluted air]. [Italian]
A1 - Battistini,A.
Y1 - 2000///
N1 - Battistini, A
Acta bio-medica de L'Ateneo parmense : organo della Societa di medicina e scienze naturali di Parma
8106323, 101295064
IM
English Abstract. Journal Article
Italian
KW - MEDLINE
KW - Air Pollution/an [Analysis]
KW - Air Pollution
KW - Child
KW - Humans

KW - Italy
KW - Pediatrics
KW - Seasons
RP - NOT IN FILE
SP - 27
EP - 36
JF - Acta Bio-Medica de l Ateneo Parmense
JA - Acta Biomed Ateneo Parmense
VL - 71
IS - 3-4
CY - Italy
N2 - The enormous amount of data concerning outdoor pollution has allowed quantifying the damage caused by single pollutants. On the basis of these data, and knowing the concentration of a pollutant in a given area, to date it is possible to foresee the expected risk for a given disease in our community. For instance, in an average size town like Parma, it can be calculated that in the middle of the winter, suspended particulate pollution (PM10), one day/three, will increase by 30% the number of symptomatic asthmatic children. In the long term, in a town like Parma, PM10 and NO2 pollution increases the number of children with more than 4 episodes of bronchitis or persistent cough by 30-50%. With regard to the typical summer pollution by O3, extremely harmful as it expands outside towns, in Parma and surroundings in August one should expect in 1/3 days double the number of symptomatic asthmatic patients, and every other day a 25% reduction of the respiratory function in asthmatic children exercising. With regard to indoor pollution besides the well known and severe problem of passive smoking, and that of volatile organic compounds still under investigation, one must consider NO2 pollution originating from the burning processes for heating and cooking. As this latter is extremely important in Italy, as gas is used in practically every house, the Author has done a specific research in which it is shown that if this kind of pollution was eliminated one could reduce the incidence of asthma in childhood from 7 to 5%, and noticeably the severity of the disease in affected subjects. Thus, paediatricians have two new aims. The first is to identify in pollution one of the possible causes of respiratory problems in each single patient, giving recommendations (ex. advise asthmatic children to avoid physical activity in the open and in the sunny hours during the summer, or avoid having children in the kitchen while cooking, or have boilers, heaters, etc. checked). Second, paediatricians should make aware, using the local actual data on pollution, not only the local administrators, but also and may be more important the parents of children with respiratory problems. These are in fact the ones who can and must become the most active in facing the big problem of pollution
AD - Centro di Fisiopatologia Respiratoria Infantile, Universita di Parma
UR - 11424610
ER -

TY - JOUR
ID - 1698
T1 - Environmental and serological evidence for the presence of toxocariasis in the urban area of Ancona, Italy
A1 - Giacometti,A.
A1 - Cirioni,O.
A1 - Fortuna,M.
A1 - Osimani,P.
A1 - Antonicelli,L.
A1 - Del Prete,M.S.
A1 - Riva,A.
A1 - D'Errico,M.M.
A1 - Petrelli,E.
A1 - Scalise,G.
Y1 - 2000///
N1 - Giacometti, A. Cirioni, O. Fortuna, M. Osimani, P. Antonicelli, L. Del Prete, M S. Riva, A. D'Errico, M M. Petrelli, E. Scalise, G
European journal of epidemiology
ere, 8508062
IM
Journal Article
English

KW - MEDLINE
KW - Adolescent
KW - Adult
KW - Chi-Square Distribution
KW - Child
KW - Preschool
KW - Enzyme-Linked Immunosorbent Assay
KW - Female
KW - Humans
KW - Infant
KW - Newborn
KW - Italy/ep [Epidemiology]
KW - Male
KW - Middle Aged
KW - Seroepidemiologic Studies
KW - Soil/ps [Parasitology]
KW - Toxocariasis/ep [Epidemiology]
KW - Urban Population
RP - NOT IN FILE
SP - 1023
EP - 1026
JF - European Journal of Epidemiology
JA - Eur J Epidemiol
VL - 16
IS - 11
CY - Netherlands
N2 - To evaluate the Toxocara spp. eggs environmental contamination of the soil of the urban or suburban area Ancona (Italy), 22 public playgrounds were selected and several cores of soil were taken from any selected areas. To study the Toxocara seroprevalence in the inhabitants of Ancona, blood samples were collected from selected groups of individuals. One hundred and sixty-three blood samples were tested using an enzyme linked immunosorbent assay (ELISA) technique (Lofarma Lab, Milan, Italy) for the detection of IgG-specific antibodies to T. canis excretory-secretory antigens. Toxocara spp. eggs were found in the soil samples from 14 (63.6%) playgrounds. Toxocara seroprevalence was detected in blood specimens from six (8.1%) out of 74 symptomatic individuals and from one (1.1%) out of 89 asymptomatic controls. Among symptomatic individuals, the association between Toxocara seroprevalence and eosinophilia resulted statistically significant (p = 0.029). The high environmental contamination frequency found make necessary to prompt preventive public health measures, such as control of stray animals, treatment of infected pets and hygiene education of the population
SN - 0393-2990
AD - Department of Infectious Diseases, Institute of Infectious Diseases and Public Health, University of Ancona, Italy
UR - 11421470
ER -

TY - JOUR
ID - 1699
T1 - Aspiration of an avulsed primary incisor. A case report
A1 - Holan,G.
A1 - Ram,D.
Y1 - 2000/06//
N1 - Holan, G. Ram, D
International journal of paediatric dentistry / the British Paedodontic Society [and] the International Association of Dentistry for Children
9107511, az2
D
Case Reports. Journal Article
English

KW - MEDLINE
KW - Airway Obstruction/et [Etiology]
KW - Airway Obstruction/ra [Radiography]
KW - Bronchi
KW - Bronchography
KW - Bronchoscopy
KW - Child
KW - Cough/et [Etiology]
KW - Diagnosis
KW - Differential
KW - Dyspnea/et [Etiology]
KW - Female
KW - Foreign Bodies/et [Etiology]
KW - Foreign Bodies/ra [Radiography]
KW - Humans
KW - Incisor/in [Injuries]
KW - Maxilla
KW - Pulmonary Atelectasis/et [Etiology]
KW - Respiratory Tract Infections/di [Diagnosis]
KW - Tooth Avulsion/co [Complications]
KW - Tooth
KW - Deciduous/in [Injuries]
RP - NOT IN FILE
SP - 150
EP - 152
JF - International Journal of Paediatric Dentistry
JA - Int J Paediatr Dent
VL - 10
IS - 2
CY - England
N2 - A 7-year-old girl had injured her maxillary primary incisors in a playground. One of the already-mobile incisors had been avulsed. The child who had a cough and breathing difficulties during sleep was diagnosed as suffering from upper respiratory infection and was treated with antibiotics. A few days later because her body temperature was elevated a chest radiograph was taken. The radiograph revealed an aspirated tooth in her right bronchus with atelectasis of the lower lobe. The tooth was removed by bronchoscopy. Dentists should suspect any tooth that has been avulsed and not found as possibly aspirated
SN - 0960-7439
AD - Department of Paediatric Dentistry, Hebrew University-Hadassah School of Dental Medicine, Jerusalem, Israel. holan@cc.huji.ac.il
UR - 11310100
ER -

TY - JOUR
ID - 1700
T1 - [Presence of larva migrans in sand boxes of public elementary schools, Aracatuba, Brazil]. [Portuguese]
A1 - Nunes,C.M.
A1 - Pena,F.C.
A1 - Negrelli,G.B.
A1 - Anjo,C.G.
A1 - Nakano,M.M.
A1 - Stobbe,N.S.
Y1 - 2000/12//
N1 - Nunes, C M. Pena, F C. Negrelli, G B. Anjo, C G. Nakano, M M. Stobbe, N S
Revista de saude publica
0135043, t5x
IM

English Abstract. Journal Article. Research Support, Non-U.S. Gov't
Portuguese
KW - MEDLINE
KW - Ancylostoma/ip [Isolation & Purification]
KW - Animals
KW - Brazil
KW - Child
KW - Humans
KW - Larva Migrans/tm [Transmission]
KW - Leisure Activities
KW - Schools
KW - Soil/ps [Parasitology]
KW - Toxocara/ip [Isolation & Purification]
RP - NOT IN FILE
SP - 656
EP - 658
JF - Revista de Saude Publica
JA - Rev Saude Publica
VL - 34
IS - 6
CY - Brazil
N2 - There are sandboxes in public elementary school playground areas in Brazil, which can be harmful to children. They are at risk of cutaneous and visceral larva migrans infection caused by Ancylostoma spp. and Toxocara spp., respectively. The study was designed to investigate contamination by Toxocara spp. and/or their eggs and Ancylostoma spp. larvae in sand samples collected from the schools' sandboxes. Five hundred and thirty-five sand samples from 28 public elementary schools were collected during summer and winter and analyzed by both Baerman's method and centrifugal flotation technique. Ancylostoma spp. larvae were found in 35.7% (10/28) schools in summer time and in 46.4% (13/28) schools in the winter time. Eggs of Toxocara spp. could not be recovered from the samples analyzed and eggs from Ancylostoma spp. were seen in 0.56% (3/535) of the samples
SN - 0034-8910
AD - Departamento de Apoio, Producao e Saude Animal do Curso de Medicina Veterinaria, Universidade Estadual de Sao Paulo--Unesp, Aracatuba, SP, Brasil. caris.fmva.unesp.br
UR - 11175613
ER -

TY - JOUR
ID - 1701
T1 - [A study of food-dependent exercise-induced anaphylaxis by analyzing the Japanese cases reported in the literature]. [Japanese]
A1 - Harada,S.
A1 - Horikawa,T.
A1 - Icihashi,M.
Y1 - 2000/11//
N1 - Harada, S. Horikawa, T. Icihashi, M
Arerugi = [Allergy]
0241212, 8l8
IM
English Abstract. Journal Article
Japanese
KW - MEDLINE
KW - Adolescent
KW - Adult
KW - Aged
KW - Anaphylaxis/ep [Epidemiology]
KW - Anaphylaxis/et [Etiology]
KW - Animals

KW - Anti-Inflammatory Agents
KW - Non-Steroidal/ae [Adverse Effects]
KW - Aspirin/ae [Adverse Effects]
KW - Child
KW - Decapoda (Crustacea)
KW - Exercise
KW - Female
KW - Food Hypersensitivity/co [Complications]
KW - Humans
KW - Male
KW - Middle Aged
KW - Triticum/ae [Adverse Effects]
RP - NOT IN FILE
SP - 1066
EP - 1073
JF - Arerugi - Japanese Journal of Allergology
JA - Arerugi
VL - 49
IS - 11
CY - Japan
N2 - We surveyed and analyzed cases of food-dependent exercise-induced anaphylaxis (FDEIA) in the Japanese literature. We found 167 cases which were reported as FDEIA since 1983. Analyzing these case, following characteristic features were revealed: 1) Recent upward trend in the number of reports of FDEIA was noted. 2) Male cases were more frequent than female cases, while about half of the cases were teenager. 3) More than half of the cases were proved to be induced by wheat followed by shrimp. In those under 20, shrimp was the most popular cause rather than wheat. 4) Skin test and/or IgE RAST showed positive results in most cases, suggesting that the response itself is linked to type I allergic reaction. 5) About 40% of the patients had history of atopic disease, indicating that atopic condition may play some role in FDEIA. 6) All cases who performed provocation test with aspirin responded to food challenge and/or exercise in combination with aspirin. This indicates that aspirin plays a key provoking factor in FDEIA. 7) In 17 cases FDEIA attacks were observed during noon recess or physical education class after lunch at school. We stress here the importance of a nation-wide education to school teachers the potential danger of this disorder
SN - 0021-4884
AD - Department of Dermatology, Sanda City Hospital
UR - 11193458
ER -

TY - JOUR
ID - 1702
T1 - [A review of Norwegian studies--how much do we exercise during our leisure time?]. [Review] [40 refs] [Norwegian]
A1 - Sogaard,A.J.
A1 - Bo,K.
A1 - Klungland,M.
A1 - Jacobsen,B.K.
Y1 - 2000/11/20/
N1 - Sogaard, A J. Bo, K. Klungland, M. Jacobsen, B K
Tidsskrift for den Norske laegeforening : tidsskrift for praktisk medicin, ny raekke
0413423, 101086543, vrv
IM
Comparative Study. English Abstract. Journal Article. Review
Norwegian
KW - MEDLINE
KW - Adolescent
KW - Adult
KW - Age Factors
KW - Aged

KW - Child
KW - Emigration and Immigration
KW - Exercise
KW - Female
KW - Humans
KW - Leisure Activities
KW - Male
KW - Middle Aged
KW - Norway
KW - Sex Factors
KW - Socioeconomic Factors
RP - NOT IN FILE
SP - 3439
EP - 3446
JF - Tidsskrift for Den Norske Laegeforening
JA - Tidsskr.Nor Laegeforen
VL - 120
IS - 28
CY - Norway
N2 - BACKGROUND: Low levels of physical activity is associated with major health problems. However, no overview of the level of physical activity outside work or school in Norway has been made, which is important in order to implement targeted interventions. MATERIAL AND METHODS: Based on published as well as unpublished data, we present data on physical activity in Norway--among children and adolescents, adults and elderly people. RESULTS: Results from different studies differ markedly. However, the main findings are these: The level of physical activity increases in childhood up until about 13 years of age. Approximately 70% of adolescents are active two hours or more a week beyond school-hours. The proportion of physical active youngsters then decreases towards young adulthood. Adults and elderly people get more inactive with increasing age. Data on physical activity from the past 10-15 years suggest a slight increase in the proportion of moderately active Norwegians. INTERPRETATION: Research on physical activity is impeded by the fact that different studies have operationalized physical activity differently and used different age grouping. There is a need for surveys in representative samples with standardised and validated questions, with uniform age classification, covering the full spectre of physical activity. In addition, we need smaller studies with more comprehensive measures of physical activity in Norway. [References: 40]
SN - 0029-2001
AD - Senter for forebyggende medisin Ulleval sykehus 0407 Oslo. a.j.sogaard@samfunnsmed.uio.no
UR - 11187202
ER -

TY - JOUR
ID - 1703
T1 - Stimulant drugs. [Review] [111 refs]
A1 - Santosh,P.J.
A1 - Taylor,E.
Y1 - 2000///
N1 - Santosh, P J. Taylor, E
European child & adolescent psychiatry
9212296
IM
Journal Article. Review
English
KW - MEDLINE
KW - Adolescent
KW - Attention Deficit Disorder with Hyperactivity/dt [Drug Therapy]
KW - Attention Deficit Disorder with Hyperactivity/px [Psychology]
KW - Central Nervous System Stimulants/ae [Adverse Effects]
KW - Central Nervous System Stimulants/pk [Pharmacokinetics]
KW - Central Nervous System Stimulants/tu [Therapeutic Use]

KW - Child
KW - Child Psychiatry
KW - Preschool
KW - Combined Modality Therapy
KW - Cost-Benefit Analysis
KW - Drug Costs
KW - Drug Therapy
KW - Combination
KW - Eating Disorders/dt [Drug Therapy]
KW - Evidence-Based Medicine
KW - Female
KW - Humans
KW - Male
KW - Practice Guidelines as Topic
KW - Psychotherapy
RP - NOT IN FILE
SP - I27
EP - I43
JF - European Child & Adolescent Psychiatry
JA - Eur Child Adolesc Psychiatry
VL - 9 Suppl 1
CY - Germany
N2 - Stimulants are a key element in the treatment of ADHD. Carefully designed trials of stimulants have found substantial improvement in ADHD core behaviours in 65-75 % of subjects with ADHD. Most standard stimulants are rapidly absorbed, with their behavioural effects appearing within 30 minutes, reaching a peak within one to three hours and disappearing within five hours. Doses at school are often necessary, in spite of the risk of peer ridicule and added adult supervision requirements. The mechanism by which stimulants act to reduce hyperactivity is not completely understood, but they improve impulsivity and activity levels. Several controlled evaluations made over periods of time greater than a year show a clear persistence of medication effects over time. A carefully crafted programme of treatment with methylphenidate is more effective in the reduction of hyperactivity symptoms than an intensive programme of behavioural and cognitive intervention. The combination of stimulants with psychosocial interventions in ADHD offers few advantages over medication alone. Unchallengeable guides to practice that would be appropriate everywhere are difficult to propose. It is imperative that clinicians prescribing stimulants should monitor the use of the drug properly, making sure that it is not being abused by the child's family, peers or those dispensing medication at school. Polypharmacy should only be embarked on by a specialist service and the combination of methylphenidate and clonidine should be used cautiously. Apart from ADHD, stimulants are useful in narcolepsy, resistant depression and partial syndromes of attention and hyperactivity. Major gaps in knowledge remain; pharmacokinetics, pharmacodynamics and pharmacogenetics of stimulant effects need further study. Details of stimulant administration regimes seem to have a major effect on the response achieved. Further research is needed, preferably in realistic practice settings, comparing different forms of combination with psychological interventions, investigating the effects in groups of children outside the core of schoolaged children with typical ADHD: preschool children, adults, those with partial syndromes (such as inattentiveness) and those with co-morbid disorders. [References: 111]
SN - 1018-8827
AD - Department of Child & Adolescent Psychiatry, Institute of Psychiatry, De Crespigny Park, Denmark Hill, London, UK
UR - 11140778
ER -

TY - JOUR
ID - 1704
T1 - Adult proxy responses to a survey of children's dermal soil contact activities
A1 - Wong,E.Y.
A1 - Shirai,J.H.
A1 - Garlock,T.J.
A1 - Kissel,J.C.

Y1 - 2000/11//
N1 - Wong, E Y. Shirai, J H. Garlock, T J. Kissel, J C
Journal of exposure analysis and environmental epidemiology
bjn, 9111438
IM
Journal Article. Research Support, U.S. Gov't, Non-P.H.S.
English
KW - MEDLINE
KW - Activities of Daily Living
KW - Adolescent
KW - Adult
KW - Child
KW - Child Welfare
KW - Preschool
KW - Climate
KW - Environmental Exposure/an [Analysis]
KW - Female
KW - Health Surveys
KW - Humans
KW - Infant
KW - Newborn
KW - Male
KW - Proxy
KW - Soil Pollutants/ae [Adverse Effects]
KW - Weather
RP - NOT IN FILE
SP - 509
EP - 517
JF - Journal of Exposure Analysis and Environmental Epidemiology
JA - J Expo Anal Environ Epidemiol
VL - 10
IS - 6 Pt 1
CY - United States
N2 - Contaminated site cleanup decisions may require estimation of dermal exposures to soil. Telephone surveys represent one means of obtaining relevant activity pattern data. The initial Soil Contact Survey (SCS-I), which primarily gathered information on the activities of adults, was conducted in 1996. Data describing adult behaviors have been previously reported. Results from a second Soil Contact Survey (SCS-II), performed in 1998-1999 and focused on children's activity patterns, are reported here. Telephone surveys were used to query a randomly selected sample of U.S. households. A randomly chosen child, under the age of 18 years, was targeted in each responding household having children. Play activities as well as bathing patterns were investigated to quantify total exposure time, defined as activity time plus delay until washing. Of 680 total survey respondents, 500 (73.5%) reported that their child played outdoors on bare dirt or mixed grass and dirt surfaces. Among these "players," the median reported play frequency was 7 days/week in warm weather and 3 days/week in cold weather. Median play duration was 3 h/day in warm weather and 1 h/day in cold weather. Hand washes were reported to occur a median of 4 times per day in both warm and cold weather months. Bath or shower median frequency was seven times per week in both warm and cold weather. Finally, based on clothing choice data gathered in SCS-I, a median of about 37% of total skin surface is estimated to be exposed during young children's warm weather outdoor play
SN - 1053-4245
AD - Department of Environmental Health, University of Washington, Seattle 98195-7234, USA
UR - 11140435
ER -

TY - JOUR
ID - 1705
T1 - Measurement of multi-pollutant and multi-pathway exposures in a probability-based sample of children: practical strategies for effective field studies

A1 - Adgate,J.L.
A1 - Clayton,C.A.
A1 - Quackenboss,J.J.
A1 - Thomas,K.W.
A1 - Whitmore,R.W.
A1 - Pellizzari,E.D.
A1 - Lioy,P.J.
A1 - Shubat,P.
A1 - Stroebel,C.
A1 - Freeman,N.C.
A1 - Sexton,K.
Y1 - 2000/11//
N1 - Adgate, J L. Clayton, C A. Quackenboss, J J. Thomas, K W. Whitmore, R W. Pellizzari, E D. Lioy, P J. Shubat, P. Stroebel, C. Freeman, N C. Sexton, K
Journal of exposure analysis and environmental epidemiology
bjn, 9111438
IM
Journal Article. Research Support, Non-U.S. Gov't. Research Support, U.S. Gov't, Non-P.H.S.
English
KW - MEDLINE
KW - Adolescent
KW - Child
KW - Child Welfare
KW - Preschool
KW - Data Collection/mt [Methods]
KW - Environmental Exposure/an [Analysis]
KW - Environmental Pollutants/ae [Adverse Effects]
KW - Environmental Pollutants/an [Analysis]
KW - Female
KW - Health Surveys
KW - Housing
KW - Humans
KW - Male
KW - Questionnaires
KW - Research Design
KW - Sample Size
KW - Sampling Studies
RP - NOT IN FILE
SP - 650
EP - 661
JF - Journal of Exposure Analysis and Environmental Epidemiology
JA - J Expo Anal Environ Epidemiol
VL - 10
IS - 6 Pt 2
CY - United States
N2 - The purpose of this manuscript is to describe the practical strategies developed for the implementation of the Minnesota Children's Pesticide Exposure Study (MNCPES), which is one of the first probability-based samples of multi-pathway and multi-pesticide exposures in children. The primary objective of MNCPES was to characterize children's exposure to selected pesticides through a combination of questionnaires, personal exposure measurements (i.e., air, duplicate diet, hand rinse), and complementary monitoring of biological samples (i.e., pesticide metabolites in urine), environmental samples (i.e., residential indoor/outdoor air, drinking water, dust on residential surfaces, soil), and children's activity patterns. A cross-sectional design employing a stratified random sample was used to identify homes with age-eligible children and screen residences to facilitate oversampling of households with higher potential exposures. Numerous techniques were employed in the study, including in-person contact by locally based interviewers, brief and highly focused home visits, graduated subject incentives, and training of parents and children to assist in sample collection. It is not feasible to quantify increases in rates of subject recruitment, retention, or compliance that resulted from the

techniques employed in this study. Nevertheless, results indicate that the total package of implemented procedures was instrumental in obtaining a high percentage of valid samples for targeted households and environmental media
SN - 1053-4245
AD - School of Public Health, University of Minnesota, Minneapolis 55455, USA
UR - 11138657
ER -

TY - JOUR
ID - 1706
T1 - Preseasonal local allergoid immunotherapy to grass pollen in children: a double-blind, placebo-controlled, randomized trial
A1 - Caffarelli,C.
A1 - Sensi,L.G.
A1 - Marcucci,F.
A1 - Cavagni,G.
Y1 - 2000/12//
N1 - Caffarelli, C. Sensi, L G. Marcucci, F. Cavagni, G
Allergy
39c, 7804028
IM
Clinical Trial. Journal Article. Randomized Controlled Trial
English
KW - MEDLINE
KW - Administration
KW - Oral
KW - Adolescent
KW - Allergens/ad [Administration & Dosage]
KW - Allergens/im [Immunology]
KW - Allergens/tu [Therapeutic Use]
KW - Antibodies
KW - Monoclonal/im [Immunology]
KW - Asthma/im [Immunology]
KW - Asthma/th [Therapy]
KW - Blood Proteins/me [Metabolism]
KW - Child
KW - Preschool
KW - Conjunctivitis
KW - Allergic/im [Immunology]
KW - Allergic/th [Therapy]
KW - Desensitization
KW - Immunologic
KW - Double-Blind Method
KW - Eosinophil Granule Proteins
KW - Female
KW - Humans
KW - Male
KW - Nasal Mucosa/im [Immunology]
KW - Phytotherapy
KW - Placebos
KW - Poaceae/im [Immunology]
KW - Pollen/im [Immunology]
KW - Pollen/tu [Therapeutic Use]
KW - Rhinitis
KW - Allergic
KW - Seasonal/im [Immunology]
KW - Seasonal/th [Therapy]

KW - Ribonucleases
KW - Seasons
RP - NOT IN FILE
SP - 1142
EP - 1147
JF - Allergy
VL - 55
IS - 12
CY - Denmark
N2 - BACKGROUND: We assessed the efficacy of preseasonal local allergoid immunotherapy in a group of children with asthma and/or rhinitis and/or rhinoconjunctivitis due to grass pollen. METHODS: We randomly assigned 24 children allergic to grass pollen to receive local allergoid immunotherapy for 3 months before the pollen season and 24 such patients to receive identically appearing placebo. The immunotherapy consisted of tablets of monomeric allergoid grass pollen allergens held in the mouth until they dissolved and then swallowed. The study was double-blind. Symptoms and medications were scored on diary cards during the pollen season. Nasal eosinophil cationic protein levels were measured by the monoclonal antibodies EG1 and EG2 outside the pollen season and at low and at high pollen concentration during the pollen season. RESULTS: The active-treatment group had a statistically significant reduction of total symptoms (P<0.05), especially bronchial symptoms (P<0.05), in comparison with the placebo group. Immunotherapy was well tolerated and compliance was good. Nasal levels of EG2 and EG1 increased significantly during the pollen season, but there was no difference between groups. EG2/EG1 increased significantly only in the placebo group during natural allergen exposure (P<0.01). CONCLUSIONS: Our results suggest that this immunotherapy is effective for the treatment of asthma due to grass pollen in children
SN - 0105-4538
AD - Pediatric Department, Parma
UR - 11117271
ER -

TY - JOUR
ID - 1707
T1 - Personal exposure to allergenic pollen and mould spores in inland New South Wales, Australia
A1 - Mitakakis,T.Z.
A1 - Tovey,E.R.
A1 - Xuan,W.
A1 - Marks,G.B.
Y1 - 2000/12//
N1 - Mitakakis, T Z. Tovey, E R. Xuan, W. Marks, G B
Clinical and experimental allergy : journal of the British Society for Allergy and Clinical Immunology
ceb, 8906443
IM
Comparative Study. Journal Article. Research Support, Non-U.S. Gov't
English
KW - MEDLINE
KW - Adult
KW - Allergens/an [Analysis]
KW - Ascomycota
KW - Asthma/ep [Epidemiology]
KW - Asthma/im [Immunology]
KW - Cereals
KW - Child
KW - Environmental Exposure/an [Analysis]
KW - Humans
KW - Immunologic Techniques
KW - New South Wales/ep [Epidemiology]
KW - Poaceae
KW - Pollen/ch [Chemistry]
KW - Risk Factors

KW - Spores
KW - Fungal/ip [Isolation & Purification]
RP - NOT IN FILE
SP - 1733
EP - 1739
JF - Clinical & Experimental Allergy
JA - Clin Exp Allergy
VL - 30
IS - 12
CY - ENGLAND
N2 - BACKGROUND: In inland NSW, Australia, allergic sensitization to the fungi Alternaria and Cladosporium and to pollen is common and an important risk factor for asthma. OBJECTIVE: We report the results of a series of experiments designed to assess the nature of personal exposure to these airborne allergenic particles. We have tested the effect of exposure conditions and level of activity on measurements of the personal exposure. METHOD: Personal Air Samplers (PAS) and Nasal Air Samplers (NAS) were employed. NAS are fitted just inside the nose and collect inhaled particles by impaction, while the PAS use a pump-operated filter with constant air flow (2 L/min). Thirty-three subjects (adults and children) used both NAS and PAS simultaneously for four one hour periods during which they performed activities or rested, both inside and outside their homes. Samples were analysed by light microscopy. Alternaria spores, Cladosporium spores, grass pollen and nongrass pollen were counted. RESULTS: Both samplers detected substantial variation in exposure between subjects. Between members of the same household, the intrahouse correlation coefficient ranged from < 0 - 0.38. Levels of pollen grains and fungal spores inhaled were higher during periods of activity than during rest, and higher while subjects were outdoors than indoors. During the active outdoor period, the number of Alternaria spores inhaled ranged from 4 to 794 (median 11) spores/hr, Cladosporium from 0 to 396 (median 4) spores/hr, grass pollen from 0 to 81 (median 1) grains/hr and nongrass pollen from 0 to 72 (median 5) grains/hr. CONCLUSION: This is the first study to quantify individual inhaled levels of allergenic fungal spores and pollen under normal domestic circumstances. Exposure can be substantial and highly variable between individuals. The amount of particles inhaled relates both to location of the individual and activity being performed, independent of age group
SN - 0954-7894
AD - Institute of Respiratory Medicine, The Royal Prince Alfred Hospital, Sydney, Australia
UR - 11122211
ER -

TY - JOUR
ID - 1708
T1 - Split liver is an effective tool to transplant paediatric patients
A1 - Porta,E.
A1 - Cardillo,M.
A1 - Pizzi,C.
A1 - Poli,F.
A1 - Scalamogna,M.
A1 - Sirchia,G.
Y1 - 2000///
N1 - Porta, E. Cardillo, M. Pizzi, C. Poli, F. Scalamogna, M. Sirchia, G
Transplant international : official journal of the European Society for Organ Transplantation
ady, 8908516
IM
Journal Article
English
KW - MEDLINE
KW - Adolescent
KW - Adult
KW - Age Factors
KW - Cadaver
KW - Child
KW - Follow-Up Studies

KW - Graft Survival
KW - Hepatectomy/mt [Methods]
KW - Humans
KW - Italy
KW - Liver Transplantation/mo [Mortality]
KW - Liver Transplantation/ph [Physiology]
KW - Liver Transplantation/sn [Statistics & Numerical Data]
KW - Middle Aged
KW - Survival Rate
KW - Time Factors
KW - Tissue Donors/sn [Statistics & Numerical Data]
KW - Tissue and Organ Harvesting/mt [Methods]
KW - Tissue and Organ Procurement/og [Organization & Administration]
KW - Treatment Outcome
RP - NOT IN FILE
SP - S144
EP - S146
JF - Transplant International
JA - Transpl Int
VL - 13 Suppl 1
CY - GERMANY
N2 - Transplantation activity is dependent upon organ procurement; although great efforts are made to enlarge the cadaver donors' pool, it still remains far too small to meet the recipients' need. Waiting time is a particular problem for paediatric patients, and mortality on the waiting list for liver transplantation is very high. The number of paediatric donors is far too small to satisfy the request. To enlarge the liver pool, the split-liver procedure was introduced in several Transplant Centers. In November 1997, the North Italy Transplant program (NITp) Working Group for Liver Transplantation decided to start an official Split-liver Program. A protocol was therefore defined and criteria for donor's and recipient's eligibility were established to minimize the risk. The Working Group also standardized the technical procedure and defined collaboration between centers. Out of 410 cadaver liver donors used in the NITp, from 1 November 1997 until 31 May 1999, 49 patients (37 males and 12 females) were chosen for the split-liver procedure. Mean age was 29.9 +/- 17.5 years. Mean ICU stay of the donors was considerably short (2.5 +/- 2.1 days), and the other conditions foreseen for donor eligibility were met. In all cases (except two) an "in situ" technique was performed. Forty-nine adult recipients and 43 children were transplanted by the split-liver technique in our Transplant Centers. One right lobe and five left liver lobes were sent to Transplant Centers outside the NITp. Adult recipient age ranged from 18 to 60 years (mean 46.4 +/- 11.7 years), and the paediatric one from 2 to 144 months (mean 24.8). Mean patient follow-up was 8.3 +/- 5.5 months. In the paediatric group, the graft was successful in 34 cases (79%), five patients (10.2%) died and four (9.3%) were re-transplanted. In the adult group, graft survival was 67.3%, 11 (22%) patients died and 5 (10%) were re-transplanted. On 1 November 1997, 30 paediatric patients were on the liver waiting list. In the preceding 19 months, 52 patients were newly enrolled, and 36 transplants were performed. The mean waiting time of paediatric patients was 259 days (range 1-919 says). From 1 November 1997 to 31 May 1999, 61 paediatric patients were newly enrolled. In this period 70 patients were transplanted. The mean waiting time was 185 days (1-1010 days). At present, the liver waiting list includes eight paediatric patients. Split-liver transplantation is a successful procedure, effective in reducing waiting time for paediatric patients. It should be established if this may be a tool to enlarge the organ pool also for adult liver transplantation
SN - 0934-0874
AD - Centro Transfusionale e di Immunologia dei Trapianti, Istituto di Ricovero e Cura a Carattere Scientifico, Ospedale Maggiore Policlinico, Milano, Italy. nitpmi@polic.cilea.it
UR - 11111983
ER -

TY - JOUR
ID - 1709
T1 - Promoting physical activity for youth
A1 - Pangrazi,R.P.
Y1 - 2000/09//

N1 - Pangrazi, R P
Journal of science and medicine in sport / Sports Medicine Australia
c54, 9812598
IM
Journal Article
English
KW - MEDLINE
KW - Adolescent
KW - Child
KW - Health Promotion
KW - Humans
KW - Physical Fitness
KW - United States
RP - NOT IN FILE
SP - 280
EP - 286
JF - Journal of Science & Medicine in Sport
JA - J Sci Med Sport
VL - 3
IS - 3
CY - AUSTRALIA
N2 - The challenge for helping others enjoy a healthy and active life is to move the focus of instruction from physical fitness toward physical activity. Participation in regular physical activity offers a number of benefits including reduction of the risk of premature mortality. coronary heart disease, diabetes mellitus, hypertension, and colon cancer. The physical fitness of American children has not declined over the years even though teachers and parents often believe it to be true. A significant amount of fitness test performance is explained by heredity. Both the response to training and genetic limitations are limiting factors outside the control of individuals. Not all people can reach a high fitness level, but all can be physically active. The Children's Lifetime Physical Activity Model (C-LPAM) offers guidance in how to prescribe activity for youth. Guidelines suggest youngsters should receive at least 60 minutes or more of physical activity on a daily basis
SN - 1440-2440
AD - Arizona State University, Tempe, USA
UR - 11101267
ER -

TY - JOUR
ID - 1710
T1 - Factors related to the progression of myopia in Singaporean children
A1 - Saw,S.M.
A1 - Nieto,F.J.
A1 - Katz,J.
A1 - Schein,O.D.
A1 - Levy,B.
A1 - Chew,S.J.
Y1 - 2000/10//
N1 - Saw, S M. Nieto, F J. Katz, J. Schein, O D. Levy, B. Chew, S J
Optometry and vision science : official publication of the American Academy of Optometry
oiz, 8904931
IM
Clinical Trial. Comparative Study. Journal Article. Randomized Controlled Trial. Research Support, Non-U.S. Gov't
English
KW - MEDLINE
KW - Child
KW - Cohort Studies
KW - Disease Progression
KW - Female

KW - Humans
KW - Male
KW - Myopia/ep [Epidemiology]
KW - Myopia/et [Etiology]
KW - Myopia/rh [Rehabilitation]
KW - Prevalence
KW - Prognosis
KW - Questionnaires
KW - Refraction
KW - Ocular
KW - Risk Factors
KW - Severity of Illness Index
KW - Singapore/ep [Epidemiology]
KW - Socioeconomic Factors
RP - NOT IN FILE
SP - 549
EP - 554
JF - Optometry & Vision Science
JA - Optom.Vis Sci
VL - 77
IS - 10
CY - UNITED STATES
N2 - PURPOSE: To examine the possible factors related to the progression of myopia in Singapore children. METHODS: One hundred fifty-three Singapore children aged 6 to 12 years were recruited to participate in a concurrent cohort study of the risk factors for the progression of myopia. Socioeconomic status, outdoor activity, and near-work activity were documented in a face-to-face clinic interview. The changes in cycloplegic subjective refraction and autorefraction were ascertained with the use of a Nidek ARK 900 over a 2-year period. RESULTS: The average rate of progression of myopia as measured by subjective refraction was -0.59 D per year (95% confidence interval -0.52, -0.66). Younger children and children who were more myopic at the beginning (refractive error worse than -2.0 D) of the study had higher myopia progression rates. CONCLUSIONS: Myopia progression was faster for younger children and for children who had more severe myopia at baseline. Socioeconomic status and near-work activity were not related to myopia progression
SN - 1040-5488
AD - Department of Community, Occupational, and Family Medicine, National University of Singapore, Faculty of Medicine. cofsawsm@nus.edu.sg
UR - 11100893
ER -

TY - JOUR
ID - 1711
T1 - Age-related changes in preschool children's systematic use of private speech in a natural setting
A1 - Winsler,A.
A1 - Carlton,M.P.
A1 - Barry,M.J.
Y1 - 2000/10//
N1 - Winsler, A. Carlton, M P. Barry, M J
Journal of child language
0425743, hp1
IM
Journal Article. Research Support, Non-U.S. Gov't
English
KW - MEDLINE
KW - Adult
KW - Age Factors
KW - Child Language
KW - Child
KW - Preschool

KW - Female
KW - Humans
KW - Male
KW - Parent-Child Relations
KW - Random Allocation
KW - Speech/ph [Physiology]
KW - Verbal Behavior
RP - NOT IN FILE
SP - 665
EP - 687
JF - Journal of Child Language
JA - J Child Lang
VL - 27
IS - 3
CY - ENGLAND
N2 - This study set out to explore the contexts in which preschool children use private speech, or self-talk, in the naturalistic setting of the preschool classroom, and age-related changes in the contexts in which preschoolers talk to themselves. A total of 2752 naturalistic observations of fourteen three-year-old and fourteen four-year-old children were conducted using a time-sampling procedure in two preschool classrooms over the course of one semester. Results from logistic regression analyses revealed that both age groups were (a) more likely to use private speech during the self-selected activity classroom context as opposed to both large group and outside free play classroom contexts, and (b) most likely to talk to themselves when alone, next likely in the presence of peers, and least likely when in the presence of a teacher. Although the probability of private speech among three-year-old children did not vary as a function of the child's immediate activity, four-year-old children's private speech was more likely to occur during sustained and focused goal-directed activity as opposed to rapidly-changing and non goal-directed activity. The findings suggest that private speech appears systematically in young children and that, in several ways, four-year-old children use private speech more selectively than three-year-olds
SN - 0305-0009
AD - Department of Psychology, George Mason University, Fairfax, VA 22030, USA. awinsler@gmu.edu
UR - 11089343
ER -

TY - JOUR
ID - 1712
T1 - Promoting children's physical activity in primary school: an intervention study using playground markings
A1 - Stratton,G.
Y1 - 2000/10//
N1 - Stratton, G
Ergonomics
0373220, elz
IM, S
Evaluation Studies. Journal Article
English
KW - MEDLINE
KW - Child
KW - Preschool
KW - Exercise
KW - Female
KW - Heart Rate
KW - Humans
KW - Male
KW - Physical Fitness
KW - Play and Playthings
KW - Schools
RP - NOT IN FILE
SP - 1538

EP - 1546
JF - Ergonomics
VL - 43
IS - 10
CY - ENGLAND
N2 - The physical activity levels of 47, 5- to 7-year-old children were assessed before and after a school playground was painted with fluorescent markings. Children's physical activity was measured using heart rate telemetry during three playtimes before and after the markings were laid down. Children in the experimental and control groups spent 27 and 29 min, respectively, in moderate to vigorous physical activity (MVPA) before the intervention, increasing to 45 and 36 min, respectively, during the intervention period. MVPA, vigorous physical activity (VPA) and mean heart rate remained relatively stable in the control group compared to respective increases of 10 and 5% of playtime and 6 beats min(-1) in the experimental group during the intervention period. The ANCOVA analysis revealed significant interactions and main effects for the intervention for MVPA, VPA and mean heart rate. Conversely there were no main effect differences between groups. These results suggest that while playground markings had a significant and positive influence on children's physical activity, factors other than playground markings may also influence children's physically active play
SN - 0014-0139
AD - Centre for Physical Education, Sport and Dance, Liverpool John Moores University, UK
UR - 11083134
ER -

TY - JOUR
ID - 1713
T1 - One- and two-year predictors of excess weight gain among elementary schoolchildren in multiethnic, low-income, inner-city neighborhoods
A1 - O'Loughlin,J.
A1 - Gray-Donald,K.
A1 - Paradis,G.
A1 - Meshefedjian,G.
Y1 - 2000/10/15/
N1 - O'Loughlin, J. Gray-Donald, K. Paradis, G. Meshefedjian, G
American journal of epidemiology
3h3, 7910653
IM
Journal Article. Research Support, Non-U.S. Gov't
English
KW - MEDLINE
KW - Body Mass Index
KW - Child
KW - Ethnic Groups
KW - Exercise
KW - Female
KW - Follow-Up Studies
KW - Health Promotion
KW - Humans
KW - Logistic Models
KW - Male
KW - Obesity/ep [Epidemiology]
KW - Poverty
KW - Predictive Value of Tests
KW - Prospective Studies
KW - Quebec/ep [Epidemiology]
KW - Questionnaires
KW - Sex Factors
KW - Urban Population
KW - Weight Gain

RP - NOT IN FILE
SP - 739
EP - 746
JF - American Journal of Epidemiology
JA - Am J Epidemiol
VL - 152
IS - 8
CY - UNITED STATES
N2 - Longitudinal studies are needed to increase understanding of the causes of childhood obesity. To identify 1- and 2-year predictors of excess weight gain among preadolescents, the authors conducted a prospective cohort study of fourth- and fifth-grade students in 16 elementary schools located in multiethnic, low-income neighborhoods in Montreal, Quebec, Canada, that were participating in the evaluation of a school-based heart health promotion program. Subjects included 2,318 children aged 9-12 years with baseline and 1-year follow-up data and 633 children aged 9-11 years with baseline and 2-year follow-up data. One-year predictors of highest decile of change in body mass index (BMI) identified in logistic regression analyses included baseline BMI of 90th percentile or more (odds ratio (OR) = 2.66, 95% confidence interval: 1.80, 3.94) in boys and baseline BMI of 90th percentile or more (OR = 2.34, 95% confidence interval: 1.46, 3.76), no sports outside school (OR = 1.90, 95% confidence interval: 1.18, 3.06), and playing video games everyday (OR = 2.48, 95% confidence interval: 1.04, 5.92) in girls. Two-year predictors included baseline BMI of 90th percentile or more (OR = 3.26, 95% confidence interval: 1.52, 7.01), no sports outside school (OR = 2.14, 95% confidence interval: 0.96, 4.77), and least active (OR = 2.18, 95% confidence interval: 1.01, 4.71) in boys; only baseline BMI of 90th percentile or more (OR = 2.22, 95% confidence interval: 1.02, 4.81) was significant in girls. Results suggest the need for interventions to promote increased physical activity in children
SN - 0002-9262
AD - Department of Public Health, Regie regionale de la sante et des services sociaux de Montreal-Centre, Quebec, Canada. joloug@po-box.mcgill.ca
UR - 11052551
ER -

TY - JOUR
ID - 1714
T1 - Personal exposure to particles in Banska Bystrica, Slovakia
A1 - Brauer,M.
A1 - Hruba,F.
A1 - Mihalikova,E.
A1 - Fabianova,E.
A1 - Miskovic,P.
A1 - Plzikova,A.
A1 - Lendacka,M.
A1 - Vandenberg,J.
A1 - Cullen,A.
Y1 - 2000/09//
N1 - Brauer, M. Hruba, F. Mihalikova, E. Fabianova, E. Miskovic, P. Plzikova, A. Lendacka, M. Vandenberg, J. Cullen, A
Journal of exposure analysis and environmental epidemiology
bjn, 9111438
IM
Journal Article. Research Support, Non-U.S. Gov't. Research Support, U.S. Gov't, Non-P.H.S.
English
KW - MEDLINE
KW - Adult
KW - Air Pollutants/an [Analysis]
KW - Air Pollution
KW - Indoor
KW - Child
KW - Environmental Exposure
KW - Environmental Monitoring

KW - Female
KW - Humans
KW - Male
KW - Occupations
KW - Seasons
KW - Slovakia
RP - NOT IN FILE
SP - 478
EP - 487
JF - Journal of Exposure Analysis and Environmental Epidemiology
JA - J Expo Anal Environ Epidemiol
VL - 10
IS - 5
CY - UNITED STATES
N2 - Epidemiological studies have associated adverse health impacts with ambient concentrations of particulate matter (PM), though these studies have been limited in their characterization of personal exposure to PM. An exposure study of healthy nonsmoking adults and children was conducted in Banska Bystrica, Slovakia, to characterize the range of personal exposures to air pollutants and to determine the influence of occupation, season, residence location, and outdoor and indoor concentrations on personal exposures. Twenty-four-hour personal, at-home indoor, and ambient measurements of PM10, PM2.5, sulfate (SO4(2-)) and nicotine were obtained for 18 office workers, 16 industrial workers, and 15 high school students in winter and summer. Results showed that outdoor levels of pollutants were modest, with clear seasonal differences: outdoor PM10 summer/winter mean = 35/45 microg/m3; PM2.5 summer/winter mean = 22/32 microg/m3. SO4(2-) levels were low (4-7 microg/m3) and relatively uniform across the different sample types (personal, indoor, outdoor), areas, and occupational groups. This suggests that SO4(2-) may be a useful marker for combustion mode particles of ambient origin, although the relationship between personal exposures and ambient SO4(2-) levels was more complex than observed in North American settings. During winter especially, the central city area showed higher concentrations than the suburban location for outdoor, personal, and indoor measures of PM10, PM2.5, and to a lesser extent for SO4(2-), suggesting the importance of local sources. For PM2.5 and PM10, ratios consistent with expectations were found among exposure indices for all three subject groups (personal>indoor>outdoor), and between work type (industrial>students>office workers). The ratio of PM2.5 personal to indoor exposures ranged from 1.0 to 3.9 and of personal to outdoor exposures from 1.6 to 4.2. The ratio of PM10 personal to indoor exposures ranged from 1.1 to 2.9 and the ratio of personal to outdoor exposures from 2.1 to 4.1. For a combined group of office workers and students, personal PM10/PM2.5 levels were predicted by statistically significant multivariate models incorporating indoor (for PM2.5) or outdoor (for PM10) PM levels, and nicotine exposure (for PM10). Small but significant fractions of the overall variability, 15% for PM2.5 and 17% for PM10, were explained by these models. The results indicate that central site monitors underpredict actual human exposures to PM2.5 and PM10. Personal exposure to SO4(2-) was found to be predicted by outdoor or indoor SO4(2-) levels with 23-71% of the overall variability explained by these predictors. We conclude that personal exposure measurements and additional demographic and daily activity data are crucial for accurate evaluation of exposure to particles in this setting
SN - 1053-4245
AD - School of Occupational and Environmental Hygiene, The University of British Columbia, Vancouver, Canada. brauer@interchange.ubc.ca
UR - 11051537
ER -

TY - JOUR
ID - 1715
T1 - Evaluation of a real-time passive personal particle monitor in fixed site residential indoor and ambient measurements
A1 - Quintana,P.J.
A1 - Samimi,B.S.
A1 - Kleinman,M.T.
A1 - Liu,L.J.
A1 - Soto,K.
A1 - Warner,G.Y.

A1 - Bufalino,C.
A1 - Valencia,J.
A1 - Francis,D.
A1 - Hovell,M.H.
A1 - Delfino,R.J.
Y1 - 2000/09//
N1 - Quintana, P J. Samimi, B S. Kleinman, M T. Liu, L J. Soto, K. Warner, G Y. Bufalino, C. Valencia, J. Francis, D. Hovell, M H. Delfino, R J
Journal of exposure analysis and environmental epidemiology
bjn, 9111438
IM
Evaluation Studies. Journal Article. Research Support, U.S. Gov't, P.H.S.
English
KW - MEDLINE
KW - Adolescent
KW - Air Pollutants/ae [Adverse Effects]
KW - Air Pollutants/an [Analysis]
KW - Air Pollution
KW - Indoor/ae [Adverse Effects]
KW - Indoor/an [Analysis]
KW - Asthma/et [Etiology]
KW - California
KW - Child
KW - Environmental Monitoring/is [Instrumentation]
KW - Equipment Design
KW - Humans
KW - Particle Size
RP - NOT IN FILE
SP - 437
EP - 445
JF - Journal of Exposure Analysis and Environmental Epidemiology
JA - J Expo Anal Environ Epidemiol
VL - 10
IS - 5
CY - UNITED STATES
N2 - Recent experimental findings in animals and humans indicate adverse respiratory effects from short-term exposures to particulate air pollutants, especially in sensitive subpopulations such as asthmatics. The relationship between air pollution and asthma has mainly been determined using particulate matter (PM) measurements from central sites. Validated tools are needed to assess exposures most relevant to health effects. Recently, a personal passive particulate sampler (personal Data-RAM, pDR, MIE Inc., Bedford, MA) has become available for studying personal exposures to PM with time resolution at 1 min. The pDR measures light scatter from PM in the 0.1-10 microM range, the significant range for health effects. In order to assess the ability of the pDR in predicting gravimetric mass, pDRs were collocated with PM2.5 and PM10 Harvard Impactors (HI) inside and outside nine homes of asthmatic children and at an outdoor central Air Pollution Control District site. Results are presented of comparisons between the HI samplers and the pDR in various modes of operation: passive, active, and active with a heated inlet. When used outdoors at fixed sites the pDR readings exhibit interference from high relative humidity (RH) unless operated with a method for drying inlet air such as a heater, or if readings at times of high RH are adjusted. The pDR correlates more highly with the HI PM2.5 than with the HI PM10 (r2 = 0.66 vs. 0.13 for outdoors, r2 = 0.42 vs. 0.20 for indoors). The pDR appears to be a useful tool for an epidemiologic study that aims to examine the relationship between health outcomes and personal exposure to peaks in PM
SN - 1053-4245
AD - Graduate School of Public Health, San Diego State University, California 92182-4162, USA. jquintan@mail.sdsu.edu
UR - 11051534
ER -

TY - JOUR
ID - 1716
T1 - Findings from case studies of state and local immunization programs
A1 - Fairbrother,G.
A1 - Kuttner,H.
A1 - Miller,W.
A1 - Hogan,R.
A1 - McPhillips,H.
A1 - Johnson,K.A.
A1 - Alexander,E.R.
Y1 - 2000/10//
N1 - Fairbrother, G. Kuttner, H. Miller, W. Hogan, R. McPhillips, H. Johnson, K A. Alexander, E R
American journal of preventive medicine
8704773, apl
IM
Journal Article
English
KW - MEDLINE
KW - Adult
KW - Age Factors
KW - Child
KW - Preschool
KW - Communicable Disease Control/ec [Economics]
KW - Communicable Disease Control/og [Organization & Administration]
KW - Communicable Disease Control/sn [Statistics & Numerical Data]
KW - Delivery of Health Care
KW - Financing
KW - Government
KW - Humans
KW - Immunization Programs/ec [Economics]
KW - Immunization Programs/og [Organization & Administration]
KW - Immunization Programs/sn [Statistics & Numerical Data]
KW - Infant
KW - Local Government
KW - Managed Care Programs/og [Organization & Administration]
KW - Medicare/ec [Economics]
KW - Organizational Case Studies
KW - Registries/sn [Statistics & Numerical Data]
KW - Schools
KW - Social Welfare/lj [Legislation & Jurisprudence]
KW - State Government
KW - United States
KW - Vaccines/ec [Economics]
RP - NOT IN FILE
SP - 54
EP - 77
JF - American Journal of Preventive Medicine
JA - Am J Prev Med
VL - 19
IS - 3 Suppl
CY - NETHERLANDS
N2 - BACKGROUND: As part of its examination of federal support for immunization services during the past decade, the Institute of Medicine (IOM) Committee on Immunization Finance Policies and Practices (IFPP) commissioned eight case studies of the states of Alabama, Maine, Michigan, New Jersey, North Carolina, Texas, and Washington; and a two-county study of Los Angeles and San Diego in California. Specifically, the IOM Committee and these studies reviewed the use of Section 317 grants by the states. Section 317 is a discretionary grant program that supports vaccine purchase and other immunization-related program activities.

These studies afforded the Committee an in-depth look at local policy choices, the performance of immunization programs, and federal and state spending for immunization during the past decade. METHODS: The case-study reports were developed through interviews with state and local health department officials, including immunization program directors, Medicaid agency staff, budget analysts, and Centers for Disease Control and Prevention public health advisors to the jurisdiction. Other sources included state and federal administrative records and secondary sources on background factors and state-level trends. The case studies were supplemented by site visits to Detroit, Houston, Los Angeles, Newark, and San Diego. OBSERVATIONS: The nature of immunization "infrastructure" supported by the Section 317 program is shifting from primarily service delivery to a broader set of roles that puts the public effort at the head of a broad immunization partnership among public health, health financing, and other entities in both the public and private sectors. The rate and intensity of transition vary across the case-study areas. In the emerging pattern, service delivery increasingly takes place in the private sector and is related to managed care. "Infrastructure" is moving beyond supporting a core state staff and local health department service delivery to include such activities as immunization registries, quality improvement, and coordination with programs outside public health agencies. At the same time, the recent decline in federal Section 317 support is forcing difficult choices between old and new activities at the state and local levels. CONCLUSIONS: Immunization programs function as an organic component of the local health care financing and delivery systems of which they are a part. Immunization efforts are organized and conducted within distinctive state and local fiscal, economic, and health care contexts. Section 317 Financial Assistance grants, while playing a vital role in supporting immunization "infrastructure," have been too unstable and unpredictable to elicit the strategic planning, programming, and own-source spending that would be optimal for state and local programs. The predominant immunization function of state and local public health agencies is becoming assurance of age-appropriate immunization throughout the lifespan. To be successful in this emerging role, the health agencies must be supported with appropriate staffing, interagency collaboration, and clearly articulated authority
SN - 0749-3797
AD - Department of Epidemiology and Social Medicine, Albert Einstein College of Medicine/Montefiore Medical Center, New York, New York, USA
UR - 11024331
ER -

TY - JOUR
ID - 1717
T1 - Restricting opportunities to be active during school time: do children compensate by increasing physical activity levels after school?
A1 - Dale,D.
A1 - Corbin,C.B.
A1 - Dale,K.S.
Y1 - 2000/09//
N1 - Dale, D. Corbin, C B. Dale, K S
Research quarterly for exercise and sport
r6y, 8006373
IM
Clinical Trial. Journal Article. Randomized Controlled Trial. Research Support, Non-U.S. Gov't
English
KW - MEDLINE
KW - Child
KW - Curriculum
KW - Exercise
KW - Female
KW - Humans
KW - Leisure Activities
KW - Male
KW - Physical Education and Training
KW - Students
RP - NOT IN FILE
SP - 240
EP - 248

JF - Research Quarterly for Exercise & Sport
JA - Res Q Exerc Sport
VL - 71
IS - 3
CY - UNITED STATES
N2 - Opportunities for children to be physically active during school time are sparse and becoming increasingly so. The purpose of this investigation was to determine if children would compensate for school days (9 a.m.-3 p.m.) of restricted physical activity opportunities by increasing activity levels after school (3 p.m.-7:30 p.m.). Third and fourth grade children (N = 76) each wore a CSA accelerometer for 4 nonconsecutive days. Two days were categorized as active--during school, all children participated in outdoor recess and physical education class. Two days were categorized as restricted--all children spent their recess time indoors at a computer terminal, and no physical education class was scheduled. Dependent t tests revealed that children did not compensate for a sedentary school day by increasing their levels of physical activity after school. In fact, average movement counts per minute were higher in the 3 p.m.-7:30 p.m. period following the active day (525 counts.min-1) versus the restricted day (186 counts.min-1). These findings suggest cause for concern if children's opportunities to be active within school time are limited. Several reasons are given as to why children did not compensate or "make up" for the physical activity opportunities missed during the restricted school day
SN - 0270-1367
AD - Department of Health, Kinesiology, and Leisure Studies, Purdue University, USA. ddale@sla.purdue.edu
UR - 10999261
ER -

TY - JOUR
ID - 1718
T1 - [Cross-sectional study of school integration of asthmatic children in a general population sample]. [French]
A1 - Pin,I.
A1 - Guerin-Develay,S.
A1 - Cans,C.
A1 - Vivier,S.
A1 - Pequegnot,C.
A1 - Lerendu,B.
A1 - Pison,C.
A1 - Paramelle,B.
Y1 - 2000/08//
N1 - Pin, I. Guerin-Develay, S. Cans, C. Vivier, S. Pequegnot, C. Lerendu, B. Pison, C. Paramelle, B
Archives de pediatrie : organe officiel de la Societe francaise de pediatrie
9421356, bwh
IM
English Abstract. Journal Article
French
KW - MEDLINE
KW - Activities of Daily Living
KW - Adolescent
KW - Asthma
KW - Child
KW - Child Welfare
KW - Preschool
KW - Female
KW - Humans
KW - Interpersonal Relations
KW - Male
KW - Peer Group
KW - Schools
RP - NOT IN FILE
SP - 817
EP - 824

JF - Archives de Pediatrie
JA - Arch Pediatr
VL - 7
IS - 8
CY - FRANCE
N2 - AIMS: Asthma is one of the most frequently encountered chronic illnesses in children. The aim of the present study was to investigate the problem of integration of asthmatic subjects at school by assessing the reactions of parents and teachers, and also by considering the medical aspects. POPULATION AND METHODS: A cross-sectional random study was carried out during autumn 1994 including 4,251 primary school children aged between five and 14 years, and including teachers from 188 classes and 26 schools in Grenoble and its inner suburbs. The first screening questionnaire was completed by the parents, and data on 3,799 children were obtained; from this, 191 currently asthmatic children were identified. One hundred and forty-six parents of the identified asthmatics subsequently answered a second questionnaire on the severity of the illness and its effects on school attendance and educational activities. The 186 teachers from the schools involved were interviewed about their knowledge of the disorder, and about what they knew of the treatment and management of asthmatic children in school. RESULTS: It was found that the cumulative prevalence of asthma amounted to 7.6%, and that the prevalence over the previous 12-month period was 4.3%. Sixteen percent of the asthmatic children reported an asthma-associated school absence of more than six days during the six preceding months. In 45.2% of cases, physical exercise was responsible for an asthmatic attack, but premedication was used in only 21% of cases. Nine percent of the children had obtained a medical certificate which exempted them from participating in sports activities and physical exercise. Seventy-three percent of the parents had informed the teachers of their child's illness, but the information communicated was incomplete. It was found that the teachers did not have a thorough knowledge of the disorder, but that many of them (92.7%) would appreciate further information on the subject and on its management. Eighty-three percent of the teaching staff were unaware that the ministerial circular No. 93-248 of 22 July 1993 had been issued on the proposed management of chronic disorders at school, advocating a combined effort on the part of the parents, the child's physician, the school doctors and the teachers in setting up a concerted plan of action. CONCLUSIONS: This study has demonstrated that the school integration of asthmatic children remains problematical both as regards treatment and the pursuit of indoor and outdoor school activities. Integration could be improved by using parent-doctor-teacher liaison forms, thereby maintaining closer communication regarding the child's needs. When this is not sufficient, an individualized asthma management plan could be introduced in collaboration with the parents, doctors and teachers, which might result in improved treatment and integration of asthmatic children in school
SN - 0929-693X
AD - Departement de pediatrie, CHU de Grenoble, France
UR - 10985181
ER -

TY - JOUR
ID - 1719
T1 - Sexual consent: the criminal law in Europe and overseas
A1 - Graupner,H.
Y1 - 2000/10//
N1 - Graupner, H
Archives of sexual behavior
8gr, 1273516
IM
Journal Article
English
KW - MEDLINE
KW - Adolescent
KW - Adult
KW - Age Factors
KW - Attitude
KW - Child
KW - Coitus
KW - Criminal Law/lj [Legislation & Jurisprudence]

KW - Europe
KW - Female
KW - Humans
KW - Male
KW - Sexual Behavior/px [Psychology]
RP - NOT IN FILE
SP - 415
EP - 461
JF - Archives of Sexual Behavior
JA - Arch Sex Behav
VL - 29
IS - 5
CY - UNITED STATES
N2 - What role can the criminal law play in the battle against child sexual abuse? Should sexual relations of and with, persons under a certain age be criminalized regardless of the circumstances, even if they are consensual ("age of consent," "minimum age")? Where should such a minimum age-limit be fixed? Should there be a special, higher age-limit for particular conditions (e.g., "seduction," "corruption")? Should sexual contacts with minors within a relationship of authority be criminalized generally, or just if authority is abused? Should criminal proceedings be instituted ex officio or upon complaint only? Should authorities be provided with a power of discretion or should they be obliged to prosecute and sentence in each case? In answering these questions, it is highly beneficial to have a look across the borders to the solutions other countries have reached. This study presents an overview on the criminal law governing the sexual behavior of, and with, children and adolescents in all European jurisdictions and in selected jurisdictions outside of Europe. It shows which categories of offences exist and from which age onward young people can effectively consent to various kinds of sexual behavior and relations in the different countries. All states in Europe and all of the studied jurisdictions overseas have minimum age limits for sexual relations, and punish sexual relations with persons under a certain age. Nowhere is this age set lower than 12 years. In Europe, in one-half of the jurisdictions, consensual sexual relations with 14-year-old adolescents are legal; in three-quarters, with 15-year olds; in a majority, this is also the case when the older partner has started the relation (and also when the initiative contains an offer of remuneration). In nearly all jurisdictions, such relations are legal from age 16 onward. Most states apply a higher age limit for contacts in relationships of authority. If the authority is not misused, the age limit in most jurisdictions is set between 14 and 16; if it is misused, between 16 and 18. Most states make no difference between heterosexual and homosexual relations
SN - 0004-0002
AD - hg@graupner
UR - 10983249
ER -

TY - JOUR
ID - 1720
T1 - Potential interaction between methotrexate and omeprazole
A1 - Beorlegui,B.
A1 - Aldaz,A.
A1 - Ortega,A.
A1 - Aquerreta,I.
A1 - Sierrasesumega,L.
A1 - Giraldez,J.
Y1 - 2000/09//
N1 - Beorlegui, B. Aldaz, A. Ortega, A. Aquerreta, I. Sierrasesumega, L. Giraldez, J
The Annals of pharmacotherapy
bbx, 9203131
IM
Case Reports. Journal Article
English
KW - MEDLINE
KW - Child
KW - Drug Interactions

KW - Enzyme Inhibitors/pd [Pharmacology]
KW - Enzyme Inhibitors/tu [Therapeutic Use]
KW - Folic Acid Antagonists/pk [Pharmacokinetics]
KW - Folic Acid Antagonists/tu [Therapeutic Use]
KW - Half-Life
KW - Humans
KW - Male
KW - Metabolic Detoxication
KW - Drug
KW - Methotrexate/pk [Pharmacokinetics]
KW - Methotrexate/tu [Therapeutic Use]
KW - Omeprazole/pd [Pharmacology]
KW - Omeprazole/tu [Therapeutic Use]
KW - Osteosarcoma/dt [Drug Therapy]
RP - NOT IN FILE
SP - 1024
EP - 1027
JF - Annals of Pharmacotherapy
JA - Ann Pharmacother
VL - 34
IS - 9
CY - UNITED STATES
N2 - OBJECTIVE: To report a case of delayed elimination of high-dose methotrexate (MTX) associated with concomitant omeprazole administration. CASE SUMMARY: Delayed MTX elimination was observed in an 11-year-old white boy who concomitantly received high-dose MTX and omeprazole. The patient's serum creatinine and liver function tests were normal during treatment and follow-up. The only medication we suspected of inhibiting MTX elimination was omeprazole 20 mg every 12 hours. Twenty-four hours after the first high-dose MTX cycle (15 g), omeprazole was discontinued. Thereafter, the patient received one high-dose MTX cycle without omeprazole every month for five months; MTX elimination was normal throughout MTX cycles 2 to 5. DISCUSSION: MTX is actively secreted in the distal tubules. The renal hydrogen/potassium adenosine triphosphatase (H+/K(+)-ATPase) pump makes the urine more acidic, by secreting hydrogen ions into the renal tubule and reabsorbing potassium ions. Active tubular secretion of MTX requires the activity of this pump because MTX is excreted with hydrogen ions. Omeprazole can inhibit renal elimination of the hydrogen ion and block the active tubular secretion of MTX. Therefore, the elimination half-life of MTX increases, which may result in potentially toxic concentrations of MTX. At a pH of approximately 5, as found in the renal tubules, pantoprazole is more slowly activated than omeprazole, reducing the incidence of unwanted reactions with sulfhydryl groups and adverse effects occurring outside of the gastric hydrogen pump. CONCLUSIONS: Based on the Naranjo probability scale, a probable drug interaction was observed. Omeprazole may delay MTX elimination; therefore, when prescribing MTX, an alternative to omeprazole should be considered
SN - 1060-0280
AD - Pharmacy Department, University Hospital of Navarra, Pamplona, Spain
UR - 10981249
ER -

TY - JOUR
ID - 1721
T1 - Subliminal stimulation research and its implications for psychoanalytic theory and treatment
A1 - Slipp,S.
Y1 - 2000///
N1 - Slipp, S
The Journal of the American Academy of Psychoanalysis
hac, 7505570
IM
Journal Article
English
KW - MEDLINE
KW - Adult

KW - Affect/ph [Physiology]
KW - Amygdala/pp [Physiopathology]
KW - Brain Mapping
KW - Child
KW - Female
KW - Freudian Theory
KW - Humans
KW - Mental Recall/ph [Physiology]
KW - Psychoanalytic Theory
KW - Psychoanalytic Therapy
KW - Subliminal Stimulation
KW - Unconscious (Psychology)
RP - NOT IN FILE
SP - 305
EP - 320
JF - Journal of the American Academy of Psychoanalysis
JA - J Am Acad Psychoanal.
VL - 28
IS - 2
CY - UNITED STATES
N2 - As a tribute to Freud's genius, he was able to recognize not only the importance of implicit unconscious learning but also the way it currently impacts on the patient in psychoanalytic therapy. He prevented the patient from viewing the analyst's facial expressions by placing the patient on the couch. In addition, the analyst was instructed not to say too much, to be neutral, nonjudgmental, and calmly reflective. Thus the patient's response to the facial expressions and voice tone of the analyst, even when subliminally perceived, were minimized. This may be even more significant in women, who seem to be more sensitive to nonverbal cues of emotional states. Ferenczi further elaborated on the importance of establishing a connective empathic relationship, using a kindly, soothing voice tone and being emotionally available. Sandler and Sandler (1994) also emphasized the importance of tolerance and acceptance when making interpretations. These clinical instructions diminish external threats to survival and thereby minimize the activation of the amygdala. These procedures in psychoanalytic treatment seem similar to a subliminal MIO message and to the subliminal exposure of a happy face, as mentioned above in the fMRI study by Whalen et al. (1998). The empathic responses of the therapist need to create a condition of safety. In turn, this decreases the patient's vigilance and defensiveness and allows for the emergence of unconscious material that can be worked through verbally and rectified by explicit memory. Another fascinating finding is that Freud (1914) became aware, in his paper on the repetition compulsion, that repressed traumatic and conflictual relationships are acted out behaviorally outside of conscious awareness. He then considered that the focus of analysis should be on analyzing the transference relationship, where this enactment was manifested. Brockman (1998) notes that modern empirical findings confirm Freud's clinical hypotheses regarding the repetition compulsion. Repressed traumatic emotional memories are encoded in the amygdala, and they are unconsciously enacted through behavior, especially in the transference. In summary, childhood and other traumatic memories become encoded in the amygdala and are later enacted and expressed behaviorally, especially in the transference relationship. Working through of the emotional trauma makes these implicit memories explicit and exposes them to adult judgment. When the therapist creates a condition of safety, old memories are reexperienced and detoxified. The memories are experienced as nonthreatening now, thus calming the amygdala and diminishing its activity. Biologically, new neural pathways from the cortex to the amygdala can be established, since the cortex is plastic. This process is slow and may account for the need to repeatedly work through in analysis old conflicted relationships that had threatened security and survival. As imaging techniques improve, we may soon be able to evaluate therapy outcomes scientifically by measuring these actual brain changes. We are on the threshold of establishing a scientific psychoanalysis, as empirical research is providing us with data that integrate the mind and the brain. Subliminal stimulation and brain imaging techniques provide us with important tools for developing an empirical base for psychoanalytic theory and treatment. These techniques were not available to Freud at the turn of the last century, and as we center the new millennium Freud's dream of psychoanalysis having a firm scientific foundation is becoming a reality
SN - 0090-3604
AD - New York University School of Medicine, USA
UR - 10976426

ER -

TY - JOUR
ID - 1722
T1 - The human behavioral and socioeconomic determinants of malaria in Bacan Island, North Maluku, Indonesia.[Erratum appears in J Epidemiol 2000 Sep;10(5):361]
A1 - Roosihermiatie,B.
A1 - Nishiyama,M.
A1 - Nakae,K.
Y1 - 2000/07//
N1 - Roosihermiatie, B. Nishiyama, M. Nakae, K
Journal of epidemiology / Japan Epidemiological Association
cl8, 9607688
IM
Comparative Study. Journal Article
English
KW - MEDLINE
KW - Adolescent
KW - Adult
KW - Behavior
KW - Case-Control Studies
KW - Child
KW - Preschool
KW - Duffy Blood-Group System/bl [Blood]
KW - Female
KW - Health Knowledge
KW - Attitudes
KW - Practice
KW - Humans
KW - Indonesia
KW - Infant
KW - Malaria
KW - Falciparum/bl [Blood]
KW - Falciparum/pc [Prevention & Control]
KW - Falciparum/tm [Transmission]
KW - Vivax/bl [Blood]
KW - Vivax/pc [Prevention & Control]
KW - Vivax/tm [Transmission]
KW - Male
KW - Middle Aged
KW - Risk Factors
KW - Socioeconomic Factors
RP - NOT IN FILE
SP - 280
EP - 289
JF - Journal of Epidemiology
JA - J Epidemiol
VL - 10
IS - 4
CY - JAPAN
N2 - In eastern Indonesia, malaria control activities mainly depend on residual spraying but the situation is almost unchanged since the past decade. Understanding the socioeconomic and human behavior determinants is needed to implement an effective malaria control in accordance with the local condition and development. Hence we conducted an unmatched case control study. Two hundred samples were recruited from all, 11 villages surrounding the centre in Bacan Island, Maluku. For children aged 0 to 15 years old, the association of socioeconomic determinants: crowding and poor type of houses with malaria remained significant in the multivariate analysis. Meanwhile for persons above 15 years old, younger persons and regular going outside at

night remained significant in the multivariate analysis. And for persons above 15 years old, a higher proportion of controls (14%) than cases (4%) slept under mosquito net regularly. The Indonesia Family Program should be promoted. There was a better quality of life in small family. For persons above 15 years old, going outside at night should be discouraged because exposed to mosquito bites. The malaria control strategy use of effective personal, regular use of mosquito net could be used as a completion for the present activities. Considering the low malaria knowledge among samples, inhabitants should be enhanced the malaria knowledge on causation, transmission, prevention and to provide proper knowledge on residual spraying
SN - 0917-5040
AD - The National Institute of Health System and Department of Public Health Science, Dokkyo University. Research and Development Surabaya, Indonesia [corrected]
UR - 10959611
ER -

TY - JOUR
ID - 1723
T1 - Evaluation of differences in ultraviolet exposure during weekend and weekday activities
A1 - Parisi,A.V.
A1 - Meldrum,L.R.
A1 - Kimlin,M.G.
A1 - Wong,J.C.
A1 - Aitken,J.
A1 - Mainstone,J.S.
Y1 - 2000/08//
N1 - Parisi, A V. Meldrum, L R. Kimlin, M G. Wong, J C. Aitken, J. Mainstone, J S
Physics in medicine and biology
p6j, 0401220
IM
Comparative Study. Journal Article. Research Support, Non-U.S. Gov't
English
KW - MEDLINE
KW - Adolescent
KW - Adult
KW - Age Factors
KW - Arm/re [Radiation Effects]
KW - Child
KW - Environmental Exposure
KW - Hand/re [Radiation Effects]
KW - Humans
KW - Middle Aged
KW - Models
KW - Statistical
KW - Neck/re [Radiation Effects]
KW - Occupational Exposure
KW - Protective Clothing
KW - Queensland
KW - Radiation Dosage
KW - Seasons
KW - Skin/re [Radiation Effects]
KW - Time Factors
KW - Ultraviolet Rays/ae [Adverse Effects]
RP - NOT IN FILE
SP - 2253
EP - 2262
JF - Physics in Medicine & Biology
JA - Phys Med Biol
VL - 45
IS - 8

CY - ENGLAND
N2 - The weekday UV exposures to anatomical sites were evaluated for outdoor workers, home workers, adolescents, indoor workers, school staff and students in south-east Queensland, Australia. Additionally, the UV exposures at weekends of school staff, school students, indoor workers and outdoor workers were evaluated. The weekday exposures per day ranged from 1.0 to 11.0 SED for winter to summer respectively. During spring, the ratios of the personal exposures divided by the ambient exposures at the weekend to the personal exposures divided by the ambient exposures on the weekdays to the neck, hand and left arm were at least 3.4, 2.0 and 0.67 for the indoor workers, school staff and students and outdoor workers respectively. The same ratios for the erythemal UV exposures over the year, estimated from the exposures on four days in each of the four seasons, were at least 2.3 for the school staff and at least 1.3 for the 13 to 19 year old school students. These results reinforce the importance of targeting prevention programmes to both weekend and weekday exposures
SN - 0031-9155
AD - Centre for Astronomy and Atmospheric Research, Faculty of Sciences, University of Southern Queensland, Toowoomba, Australia
UR - 10958193
ER -

TY - JOUR
ID - 1724
T1 - Injuries to boys and girls in Swedish schools: different activities, different results?
A1 - Menckel,E.
A1 - Laflamme,L.
Y1 - 2000/06//
N1 - Menckel, E. Laflamme, L
Scandinavian journal of public health
dew, 100883503
IM
Comparative Study. Journal Article. Research Support, Non-U.S. Gov't
English
KW - MEDLINE
KW - Adolescent
KW - Athletic Injuries/ep [Epidemiology]
KW - Child
KW - Female
KW - Humans
KW - Male
KW - Schools/sn [Statistics & Numerical Data]
KW - Sex Factors
KW - Sweden/ep [Epidemiology]
KW - Wounds and Injuries/cl [Classification]
KW - Wounds and Injuries/ep [Epidemiology]
RP - NOT IN FILE
SP - 132
EP - 136
JF - Scandinavian Journal of Public Health
JA - Scand J Public Health
VL - 28
IS - 2
CY - NORWAY
N2 - The study examines gender-related injury frequency by the type of school activity. Data were taken from an injury register created during the school year 1996/97 with the help of 79 Swedish public-sector schools. Injury ratios (IRs) by gender, type of municipality, and type of school were computed for injuries during all types of activities, for sports-related injuries, and for injuries sustained during recess periods. IRs were higher among boys for recess activities, and among girls for sports activities. IRs showed major variation by type of school, and minor variation by type of municipality. Schools catering for pupils aged 7-12 years had the highest IRs for all groups of injuries, both on aggregate and by gender. Gender-related injury-event occurrence at school

clearly varies with kind of activity, but less so with age of pupils. The extent to which this reflects individual differences rather than varying exposure is uncertain
SN - 1403-4948
AD - National Institute for Working Life, Department for Work and Health, Karolinska Institutet, Stockholm, Sweden. ewa.menckel@niwl.se
UR - 10954140
ER -

TY - JOUR
ID - 1725
T1 - Exposure of preschoolers to lead in the Makati area of Metro Manila, the Philippines
A1 - Sharma,K.
A1 - Reutergardh,L.B.
Y1 - 2000/07//
N1 - Sharma, K. Reutergardh, L B
Environmental research
ei2, 0147621
IM
Journal Article
English
KW - MEDLINE
KW - Child
KW - Preschool
KW - Eating
KW - Environmental Exposure/an [Analysis]
KW - Female
KW - Food Contamination
KW - Hair/ch [Chemistry]
KW - Humans
KW - Lead/ae [Adverse Effects]
KW - Lead/an [Analysis]
KW - Male
KW - Philippines
KW - Play and Playthings
KW - Soil Pollutants/ae [Adverse Effects]
KW - Soil Pollutants/an [Analysis]
KW - Urban Population
KW - Vehicle Emissions
RP - NOT IN FILE
SP - 322
EP - 332
JF - Environmental Research
JA - Environ Res
VL - 83
IS - 3
CY - UNITED STATES
N2 - A study was conducted in the Makati area of Metro Manila, the Philippines. The study evaluated the significance of playground soil lead intake to the total daily lead burden in preschool children. The lead concentration was measured in residential playground soils, food, water, and hair samples by atomic absorption spectrometer and the data were used to draw conclusions. All of the playground soils were contaminated with lead levels ranging from a minimum of 34.54 microg/g to a maximum of 283.13 microg/g in comparison to the naturally occurring lead level of 15 microg/g in soils. Further, the study conducted a lead analysis on the hair of the study population to determine the accumulated lead intake. Ingestion of 25 mg/day of soil contributed to 4% of the total lead intake per day by children exposed to the study sites. Food (83%) was found to be the major source of lead compared to other sources. The study also includes economic valuation and cost benefit analysis from reducing lead in gasoline
SN - 0013-9351

AD - Division of Geography, University of Sydney, New South Wales, Australia
UR - 10944076
ER -

TY - JOUR
ID - 1727
T1 - Public health and preventive healthcare in children: current practices of Victorian GPs and barriers to participation
A1 - Waters,E.B.
A1 - Haby,M.M.
A1 - Wake,M.
A1 - Salmon,L.A.
Y1 - 2000/07/17/
N1 - Waters, E B. Haby, M M. Wake, M. Salmon, L A
The Medical journal of Australia
0400714, m26
IM
Journal Article
English
KW - MEDLINE
KW - Adult
KW - Attitude of Health Personnel
KW - Child
KW - Child Health Services
KW - Preschool
KW - Cross-Sectional Studies
KW - Family Practice
KW - Female
KW - Humans
KW - Infant
KW - Newborn
KW - Logistic Models
KW - Male
KW - Mass Screening
KW - Middle Aged
KW - Odds Ratio
KW - Patient Education as Topic
KW - Physician's Practice Patterns
KW - Preventive Health Services
KW - Victoria
RP - NOT IN FILE
SP - 68
EP - 71
JF - Medical Journal of Australia
JA - Med J Aust
VL - 173
IS - 2
CY - AUSTRALIA
N2 - OBJECTIVES: To assess general practitioners' attitudes to and involvement in child public health activities and identify barriers affecting their participation. DESIGN: Cross-sectional written questionnaire survey of a representative sample of actively practising GPs. SETTING: General practices in the State of Victoria. PARTICIPANTS: 840 GPs returned questionnaires (65% response rate), and, of these, 792 (94%) saw children 0-12 years. MAIN OUTCOME MEASURES: Attitudes to and involvement in public health promotion; predictive factors for GPs' involvement. RESULTS: The odds of GPs' involvement in child public health issues were increased by being female (odds ratio [OR], 1.88), receiving basic medical qualifications outside Australia (OR, 1.55), attending continuing education and postgraduate training (OR, 1.60), and having confidence in dealing with newborns and infants (OR, 1.93) and preschool children (OR, 2.94) (both $P < 0.05$). Older GPs,

compared with younger GPs, had significantly lower odds of involvement (P < 0.01). After adjustment, GPs had higher odds of involvement if they agreed it was important for them to take part in health promotion, or in screening and surveillance (OR, 2.76 and 1.94, respectively; P < or = 0.05); and lower odds if they agreed that screening and surveillance should be mainly done by maternal and child health nurses (OR, 0.60). The most common barriers to involvement were insufficient time, inadequate financial reimbursement for long consultations, inappropriateness of raising these issues in children presenting with illness, and lack of community resources. CONCLUSIONS: Increasing GPs' involvement in child public health will require attention to barriers (time, remuneration and perceived appropriateness), continuing education and changes in workforce composition and patterns
SN - 0025-729X
AD - Centre for Community Child Health, University of Melbourne, Royal Children's Hospital, Vic. waterse@cryptic.rch.unimelb.edu.au
UR - 10937029
ER -

TY - JOUR
ID - 1728
T1 - Evidence-based implementation of free phenytoin therapeutic drug monitoring
A1 - Burt,M.
A1 - Anderson,D.C.
A1 - Kloss,J.
A1 - Apple,F.S.
Y1 - 2000/08//
N1 - Burt, M. Anderson, D C. Kloss, J. Apple, F S
Clinical chemistry
dbz, 9421549
IM
Journal Article
English
KW - MEDLINE
KW - Adolescent
KW - Adult
KW - Aged
KW - Anticonvulsants/bl [Blood]
KW - Anticonvulsants/me [Metabolism]
KW - Blood Proteins/me [Metabolism]
KW - Child
KW - Evidence-Based Medicine
KW - Female
KW - Humans
KW - Male
KW - Middle Aged
KW - Monitoring
KW - Physiologic
KW - Phenytoin/bl [Blood]
KW - Phenytoin/me [Metabolism]
KW - Pregnancy
KW - Protein Binding
RP - NOT IN FILE
SP - 1132
EP - 1135
JF - Clinical Chemistry
JA - Clin Chem
VL - 46
IS - 8 Pt 1
CY - UNITED STATES

N2 - BACKGROUND: The majority of laboratories measure total phenytoin concentration for therapeutic drug monitoring. However, there are substantial interindividual variations in free phenytoin concentrations, the pharmacologically active component. METHODS: We describe the process and data used to implement monitoring of free phenytoin only in an urban medical center. Over a 6-week period, total and free phenytoin concentrations were measured, clinical charts reviewed, and indications for alterations in the percentage of free phenytoin fraction were determined. RESULTS: Of the 189 phenytoin requests from 139 patients, 136 data points were analyzed. Free phenytoin concentrations were 6.8-35.3%, with 50% outside the expected range of 8-12%. Clinical indications likely responsible for variations were hypoalbuminemia, drug interactions, uremia, pregnancy, and age. Overall, 30% of patients demonstrated a discrepancy between therapeutic, subtherapeutic, or supratherapeutic concentrations between free and total phenytoin concentrations. The largest discordance (53%) occurred in the patient group with free phenytoin <8% or >12%. CONCLUSIONS: This study supports previous clinical findings that monitoring total phenytoin is not as reliable as free phenytoin as a clinical indicator for therapeutic and nontherapeutic concentrations. Thus, we recommend that therapeutic monitoring should use free phenytoin concentrations only
SN - 0009-9147
AD - Departments of Laboratory Medicine and Pathology and Neurology, Hennepin County Medical Center, University of Minnesota School of Medicine, Minneapolis, MN 55415, USA
UR - 10926893
ER -

TY - JOUR
ID - 1729
T1 - The determinants of sexual intercourse before age 16
A1 - Paul,C.
A1 - Fitzjohn,J.
A1 - Herbison,P.
A1 - Dickson,N.
Y1 - 2000/08//
N1 - Paul, C. Fitzjohn, J. Herbison, P. Dickson, N
The Journal of adolescent health : official publication of the Society for Adolescent Medicine
a0j, 9102136
IM, J
Journal Article. Research Support, Non-U.S. Gov't
English
KW - MEDLINE
KW - Adolescent
KW - Adolescent Behavior
KW - Child
KW - Decision Making
KW - Family Relations
KW - Female
KW - Humans
KW - Intelligence
KW - Longitudinal Studies
KW - Male
KW - Risk Factors
KW - Self Concept
KW - Sex Factors
KW - Sexual Behavior
KW - Social Class
KW - Social Support
RP - NOT IN FILE
SP - 136
EP - 147
JF - Journal of Adolescent Health
JA - J Adolesc Health
VL - 27

IS - 2
CY - UNITED STATES
N2 - PURPOSE: To identify risk and protective factors for initiation of sexual intercourse before age 16 years at the level of the individual, family, and school. METHODS: A longitudinal study based on a cohort of 1020 people born in Dunedin, New Zealand in 1972/73 and followed up to age 21 years. Demographic characteristics of the sample were similar to the New Zealand population of that age, except that a smaller proportion (3%) were Maori or Pacific Island Polynesian. Information on individual, family and school factors was collected by interview with parents at ages 3, 5, 7 and 9 years and then by postal questionnaire two-yearly up to 15 years. Subjects were assessed two-yearly from age 3 years and interviewed about their behaviours and ambitions at ages 11, 13, and 15 years. Questions about age at first intercourse were asked by computer at age 21 years. Multivariate logistic regression was used to model associations with age of first intercourse less than 16 years. RESULTS: Data on age at first intercourse were available for 926/1020 (91%) of surviving members of the cohort assembled at age 3 years. Overall 27.5% of males and 31.7% of females reported sexual intercourse before age 16 years. In multivariate analyses the independent predictors for early sexual initiation for males were: not having outside home interests at age 13 years, no religious activity at age 11 years, not being attached to school at age 15 years, a low reading score, and a diagnosis of conduct disorder in early adolescence. For females, independent predictors were: socioeconomic status in the middle range, mother having her first child before age 20 years, IQ in the middle range, not being attached to school, being in trouble at school, planning to leave school early, cigarette smoking and higher self-esteem score. CONCLUSIONS: Individual and school factors appear to be more important than family composition or socioeconomic status in the decision to have sexual intercourse before age 16 years. The lowering of age at first intercourse may be partly a cohort effect related to high rates of teenage childbearing in the mothers' generation, and to changes in social acceptability of early sexual behaviour
SN - 1054-139X
AD - AIDS Epidemiology Group, Department of Preventive & Social Medicine, University of Otago Medical School, Dunedin, New Zealand
UR - 10899475
ER -

TY - JOUR
ID - 1730
T1 - Pediatric burns induced by psoralens in Saudi Arabia
A1 - Al-Qattan,M.M.
Y1 - 2000/11//
N1 - Al-Qattan, M M
Burns : journal of the International Society for Burn Injuries
afc, 8913178
IM
Journal Article
English
KW - MEDLINE
KW - Burns
KW - Chemical/ep [Epidemiology]
KW - Chemical/et [Etiology]
KW - Child
KW - Female
KW - Humans
KW - Incidence
KW - Male
KW - Photosensitizing Agents/ae [Adverse Effects]
KW - Photosensitizing Agents/tu [Therapeutic Use]
KW - Psoralens/ae [Adverse Effects]
KW - Psoralens/tu [Therapeutic Use]
KW - Retrospective Studies
KW - Risk Factors
KW - Saudi Arabia/ep [Epidemiology]
KW - Sunburn/ep [Epidemiology]

KW - Sunburn/et [Etiology]
KW - Vitiligo/dt [Drug Therapy]
RP - NOT IN FILE
SP - 653
EP - 655
JF - Burns
VL - 26
IS - 7
CY - ENGLAND
N2 - Over a 5-year period, three children with psoralen-induced burns were seen. All children were on treatment for vitiligo with a psoralen oil and burns were sustained from unsupervised sun exposure while playing outdoors. The burn wound was partial thickness and involved 6-40% of the total body surface area. All wounds healed without infection or contracture with topical silver sulfadiazine. However, the previously normal skin in phototoxic areas became significantly hyperpigmented while the areas with vitiligo remained hypopigmented. Differences between psoralen-induced burns and other second degree sunburns unrelated to psoralens are discussed
SN - 0305-4179
AD - King Saud University, PO Box 18097, Riyadh 11415, Saudi Arabia
UR - 10925190
ER -

TY - JOUR
ID - 1731
T1 - Measurement issues in the assessment of physical activity in children. [Review] [96 refs]
A1 - Welk,G.J.
A1 - Corbin,C.B.
A1 - Dale,D.
Y1 - 2000/06//
N1 - Welk, G J. Corbin, C B. Dale, D
Research quarterly for exercise and sport
r6y, 8006373
IM
Journal Article. Review
English
KW - MEDLINE
KW - Child
KW - Child Welfare
KW - Exercise
KW - Heart Rate
KW - Humans
KW - Life Style
KW - Monitoring
KW - Ambulatory
KW - Physical Fitness
KW - Questionnaires/st [Standards]
KW - Reproducibility of Results
KW - Sensitivity and Specificity
RP - NOT IN FILE
SP - S59
EP - S73
JF - Research Quarterly for Exercise & Sport
JA - Res Q Exerc Sport
VL - 71
IS - 2 Suppl
CY - UNITED STATES
N2 - This paper reviewed the nature of children's physical activity patterns and how the unique nature of children can impact the assessment of physical activity. To accurately assess children's activity patterns, an

instrument must be sensitive enough to detect, code, or record sporadic and intermittent activity. Care also must be used to select criterion measures that reflect appropriate physical activity guidelines for children. A number of different measurement approaches have been described for assessing children's activity, but no specific method can be identified as the best option for all studies. Selection of an appropriate instrument depends on the specific research question being addressed as well as the relative importance of accuracy and practicality (Baranowski & Simons-Morton, 1991). For example, accurate measures of energy expenditure using doubly-labeled water, indirect calorimetry, or heart rate calibration equations may be needed for certain clinical studies, but the cost and inconvenience would make them impractical for field-based assessments on larger samples. The "accuracy-practicality" trade-off presents a more challenging predicament with children than for adults. In adults, a number of self-report instruments have been found useful for large epidemiological studies or interventions where less precision is needed. Because of developmental differences, especially in ability to think abstractly and perform detailed recall (Going et al., 1999; Sallis, 1991), children are less likely to make accurate self-report assessment than adults. Though self-report methods are still likely to be a principal source of information for many studies, other approaches (or the use of combined measures) may be needed to better characterize children's activity levels. While objective instruments (e.g., direct observation or activity monitoring) require more time and resources than self-report, there are options available to simplify data collection. One approach may be to focus assessments on key times or places that allow children to be active. The time after school, for example, appears to be a critical period that defines their propensity for physical activity (Hager, 1999). Monitoring of entire groups for discrete periods of time (e.g., recess or physical education) may also be useful to understand variability in activity patterns since children would all be exposed to the same stimulus or opportunity to be active. Proxy measures may also be useful in studying activity in children. For example, several studies (Baranowski, Thompson, DuRant, Baranowski, & Puhl, 1993; Sallis et al., 1993) have demonstrated that time spent outside is strongly predictive of activity in children. Involvement in community sports programs may also be a useful proxy measure as sports programs have been found to account for approximately 55-65% of children's moderate to vigorous activity (Katzmarzyk & Malina, 1999). Another option for improving assessments in children is to employ multiple measures of physical activity. A number of studies (Coleman, Saelens, Wiedrich-Smith, Finn, & Epstein, 1997; McMurray et al., 1998; Sallis et al., 1998; Simons-Morton et al., 1994) have reported differences in levels of activity when activity monitors were compared with self-report data. The method of measurement has also been shown to influence the results of studies on the determinants of physical activity in children (Epstein, Paluch, Coleman, Vito, & Anderson, 1996). While we do not currently know which measure is most accurate, reporting the results with different instruments provides a more complete description of children's activity and permit a triangulation of outcomes. In summary, there remains no single way of obtaining a highly accurate account of physical activity or energy expenditure in children. The nature of children's movement patterns, the various types of activities engaged in, and the inherent limitations of each assessment tool limit the ultima [References: 96]
SN - 0270-1367
AD - Iowa State University, USA
UR - 10925827
ER -

TY - JOUR
ID - 1732
T1 - Prescribing books for immigrant children: a pilot study to promote emergent literacy among the children of Hispanic immigrants
A1 - Sanders,L.M.
A1 - Gershon,T.D.
A1 - Huffman,L.C.
A1 - Mendoza,F.S.
Y1 - 2000/08//
N1 - Sanders, L M. Gershon, T D. Huffman, L C. Mendoza, F S
Archives of pediatrics & adolescent medicine
9422751, bwf
AIM, IM
Journal Article. Research Support, Non-U.S. Gov't
English
KW - MEDLINE
KW - Adult

KW - Books
KW - California
KW - Child
KW - Preschool
KW - Education
KW - Emigration and Immigration
KW - Hispanic Americans
KW - Humans
KW - Infant
KW - Mexican Americans
KW - Parents
KW - Pediatrics
KW - Physician's Role
KW - Socioeconomic Factors
RP - NOT IN FILE
SP - 771
EP - 777
JF - Archives of Pediatrics & Adolescent Medicine
JA - Arch Pediatr Adolesc Med
VL - 154
IS - 8
CY - UNITED STATES
N2 - OBJECTIVES: To assess book-sharing activities within first-generation Hispanic immigrant families, and to assess the effect of pediatricians giving books to their patients. DESIGN: Survey. PARTICIPANTS: Convenience sample of 122 predominantly Hispanic immigrant parents of children aged 2 months to 5 years. Of these parents, 56 had received children's books from the pediatrician, and 66 had not. SETTING: House staff continuity clinic at a university children's hospital. MAIN OUTCOME MEASURE: Frequent Book Sharing (FBS) was defined as a parent's reporting more than 3 days per week of sharing books with the child. Main independent variables included the following: (1) exposure to the Reach Out and Read program, defined as having received a children's book from the pediatrician; (2) socioeconomics, as measured by parents' years of education and Medicaid enrollment; (3) acculturation, as defined by 4 questions relating to parents' proficiency with the English language; (4) parent's country of origin; (5) parent literacy, as measured by a parent's reporting more than 3 days per week of reading alone; (6) parent's age; (7) marital status; (8) household size; (9) child's age; (10) child's sex. RESULTS: Ninety percent of the parents were born outside of the United States (71% in Mexico), 85% spoke Spanish in the home, and 63% had completed less than a high-school education. Seventy-five percent of children's medical insurance was provided by Medi-Cal (Medicaid), and 9% of children were uninsured. Sixty-seven percent spoke exclusively Spanish at home, and 84% of parents want their children to learn to read in both English and Spanish. High FBS was reported among parents whose children had received books from the physician when compared with parents whose children had received no books. The odds ratio (OR) was 3.62 (95% confidence interval [CI], 1.40-9.37; P<.05). Also associated with FBS were parents reading frequently to themselves (OR = 9.52; 95% CI, 2.09-43.27; P<.05) and national origin outside Mexico (OR = 5.54; 95% CI, 1.59-19.27; P<.05). These findings were independent of parent's educational level, parent's employment, parent's age, acculturation, and family size. CONCLUSIONS: Pediatricians can promote literacy development among Hispanic immigrant children through the provision of free books at well-child visits. Our findings also suggest the independent effects of adult literacy and child age. Further research is needed to understand the effect of pediatric literacy programs on Hispanic immigrant children, their bilingual environments, and their readiness for school entry. Arch Pediatr Adolesc Med. 2000;154:771-777
SN - 1072-4710
AD - Department of Pediatrics, Stanford University, Palo Alto, CA, USA
UR - 10922272
ER -

TY - JOUR
ID - 1733
T1 - Tuberculosis preventive therapy: perspective from a multi-ethnic community
A1 - Sebastian,M.S.
A1 - Bothamley,G.H.

Y1 - 2000/07//
N1 - Sebastian, M S. Bothamley, G H
Respiratory medicine
8908438, rme
IM
Journal Article. Research Support, Non-U.S. Gov't
English
KW - MEDLINE
KW - Adolescent
KW - Adult
KW - Antitubercular Agents/tu [Therapeutic Use]
KW - Child
KW - Preschool
KW - Female
KW - Health Knowledge
KW - Attitudes
KW - Practice
KW - Humans
KW - Infant
KW - Isoniazid/tu [Therapeutic Use]
KW - London
KW - Male
KW - Patient Compliance
KW - Questionnaires
KW - Tuberculosis
KW - Pulmonary/eh [Ethnology]
KW - Pulmonary/pc [Prevention & Control]
KW - Pulmonary/px [Psychology]
RP - NOT IN FILE
SP - 648
EP - 653
JF - Respiratory Medicine
JA - Respir Med
VL - 94
IS - 7
CY - ENGLAND
N2 - This study explores the knowledge, attitudes and perception of tuberculosis (TB) and how they affect adherence to preventive treatment. A qualitative study of 24 subjects taking preventive therapy for TB was undertaken. Interviews were conducted in English using a structured questionnaire in a private room adjoining the outpatient clinic before the subject's appointment. The questionnaire included; (i) demographic details; (ii) background information on TB; (iii) knowledge and perception of TB and chemoprophylaxis. Data was analysed descriptively and thematically and subjected to statistical evaluation where appropriate. Outcome was assessed >6 months after the start of preventive treatment. The sample was representative of the ethnic mix of those attending the clinic. Knowledge of TB-was gained outside the family and covered transmission but few symptoms of active TB were recognized. The perceived threat from TB was high, although the estimated risk was low. Knowledge of preventive therapy exceeded the general knowledge of TB, although the latter was associated with better adherence. Most denied knowledge of the risk of hepatitis from isoniazid. Defaulters failed to attend their first appointment, attributed more side effects to isoniazid and perceived a longer waiting time in clinic. Adherence is better with a general knowledge of TB and attendance at the first appointment and could be improved by a single daily tablet, warnings about dizziness and discussion of the difficulties of taking a 6 month course of treatment
SN - 0954-6111
AD - London School of Hygiene and Tropical Medicine, UK
UR - 10926335
ER -

TY - JOUR

ID - 1734
T1 - Spatial distribution of neuronal complexity loss in neocortical lesional epilepsies
A1 - Widman,G.
A1 - Lehnertz,K.
A1 - Urbach,H.
A1 - Elger,C.E.
Y1 - 2000/07//
N1 - Widman, G. Lehnertz, K. Urbach, H. Elger, C E
Epilepsia
eix, 2983306r
IM
Journal Article. Research Support, Non-U.S. Gov't
English
KW - MEDLINE
KW - Adolescent
KW - Adult
KW - Brain Mapping
KW - Child
KW - Electrodes
KW - Implanted
KW - Electroencephalography/sn [Statistics & Numerical Data]
KW - Epilepsy/di [Diagnosis]
KW - Epilepsy/pp [Physiopathology]
KW - Epilepsy/su [Surgery]
KW - Epilepsy
KW - Temporal Lobe/di [Diagnosis]
KW - Temporal Lobe/pp [Physiopathology]
KW - Temporal Lobe/su [Surgery]
KW - Female
KW - Humans
KW - Magnetic Resonance Imaging
KW - Male
KW - Middle Aged
KW - Neocortex/pp [Physiopathology]
KW - Neocortex/su [Surgery]
KW - Nonlinear Dynamics
RP - NOT IN FILE
SP - 811
EP - 817
JF - Epilepsia
VL - 41
IS - 7
CY - UNITED STATES
N2 - PURPOSE: Nonlinear EEG analysis is valuable in characterizing the spatiotemporal dynamics of the epileptogenic process in mesial temporal lobe epilepsy. We examined the ability of the measure neuronal complexity loss (L*) to characterize the primary epileptogenic area of neocortical lesional epilepsies during the interictal state. METHODS: Spatial distribution of L* (L* map) was extracted from electrocorticograms (n = 52) recorded during presurgical assessment via subdural 64-contact grid electrodes covering lesions in either frontal, parietal, or temporal neocortex in 15 patients. The exact location of recording contacts on the brain surface was identified by matching a postimplant lateral x-ray of the skull with a postoperatively obtained sagittal MRI scan. Reprojecting L* maps onto the subject's brain surface allowed us to compare the spatial distribution of L* with the resection range of the extended lesionectomy. RESULTS: In each of the six patients who became seizure-free, maximum values of L* were restricted to recording sites coinciding with the area of resection. In contrast, L* maps of most patients who had no benefit from the resection indicated a more widespread extent or the existence of additional, probably autonomous, foci. The mean of L* values obtained from recording sites outside the area of resection correctly distinguished 13 patients (86.7 %) with respect to seizure outcome. CONCLUSIONS: Relevant information obtained from long-lasting interictal

electrocorticographic recordings can be compressed to a single L* map that contributes to a spatial characterization of the primary epileptogenic area. In neocortical lesional epilepsies, L* allows for identification and characterization of epileptogenic activity and thus provides an additional diagnostic tool for presurgical assessment
SN - 0013-9580
AD - Clinic for Neurology, University Hospital Essen, Germany. Guido.Widman@uni-essen.de
UR - 10897151
ER -

TY - JOUR
ID - 1735
T1 - Current action for skin cancer risk reduction in English schools: a report on a survey carried out for the Department of Health
A1 - Horsley,L.
A1 - Charlton,A.
A1 - Wiggett,C.
Y1 - 2000/06//
N1 - Horsley, L. Charlton, A. Wiggett, C
Health education research
bqp, 8608459
T
Journal Article. Research Support, Non-U.S. Gov't
English
KW - MEDLINE
KW - Adolescent
KW - Child
KW - Curriculum
KW - Data Collection
KW - England
KW - Health Education/mt [Methods]
KW - Humans
KW - School Health Services
KW - Skin Neoplasms/pc [Prevention & Control]
KW - Sunburn/pc [Prevention & Control]
RP - NOT IN FILE
SP - 249
EP - 259
JF - Health Education Research
JA - Health Educ Res
VL - 15
IS - 3
CY - ENGLAND
N2 - The objective of this study was to identify current action for skin cancer risk reduction in English schools and thus set a baseline for future skin cancer risk reduction interventions. A postal questionnaire survey was sent to 1295 primary, 59 middle and 216 secondary school headteachers, a 10% sample of schools in England in 1998. Since the Health Education Authority/Department of Health/British Association of Dermatologists introduced the Sun Awareness Guidelines for Schools in 1995, seven items from the Guidelines, i.e. education, uniform, shade, outdoor activities, sunscreens, staff awareness, and parent and governor alliances were chosen as outcome measures. The results of the survey showed that most schools had taken at least one of the seven actions (mean 2.67, SD 0.88). Of the schools which addressed sun protection, the majority started to do so after the release of the Sun Awareness Guidelines in 1995. Judging from the length of time schools had been covering sun awareness issues, the proportion of schools which were just beginning to implement sun protection in 1995 was greater than those who began in the previous year. Teaching in the curriculum was the most frequent action taken, but the approach used was usually information giving. Brimmed hats and long sleeves were rarely part of summer school wear. Most schools had less than 25% of their outdoor break areas in shade, but action was being taken to increase this. Sports days were usually scheduled for the afternoon. Sunscreen use was allowed in over 80% of schools, but its application presented teachers with a dilemma. Few staff manuals

contained sun awareness issues; few staff had attended in-service courses on the subject; two-thirds of headteachers would support their staff attending them. Few schools had developed parent and governor alliances. We conclude that help is needed for schools in the form of materials, courses, funds and clear Government policy if their action is to play a major role in reducing the incidence of skin cancer
SN - 0268-1153
AD - University of Manchester, UK
UR - 10977373
ER -

TY - JOUR
ID - 1736
T1 - Exposure to air pollution is associated with lung hyperinflation in healthy children and adolescents in Southwest Mexico City: a pilot study
A1 - Calderon-Garciduenas,L.
A1 - Mora-Tiscareno,A.
A1 - Chung,C.J.
A1 - Valencia,G.
A1 - Fordham,L.A.
A1 - Garcia,R.
A1 - Osnaya,N.
A1 - Romero,L.
A1 - Acuna,H.
A1 - Villarreal-Calderon,A.
A1 - Devlin,R.B.
A1 - Koren,H.S.
Y1 - 2000/06//
N1 - Calderon-Garciduenas, L. Mora-Tiscareno, A. Chung, C J. Valencia, G. Fordham, L A. Garcia, R. Osnaya, N. Romero, L. Acuna, H. Villarreal-Calderon, A. Devlin, R B. Koren, H S
Inhalation toxicology
8910739, db8
IM
Comparative Study. Journal Article
English
KW - MEDLINE
KW - Adolescent
KW - Air Pollutants/ae [Adverse Effects]
KW - Child
KW - Preschool
KW - Environmental Monitoring
KW - Female
KW - Humans
KW - Inhalation Exposure
KW - Lung Diseases/ci [Chemically Induced]
KW - Lung Diseases/pp [Physiopathology]
KW - Lung Diseases/ra [Radiography]
KW - Male
KW - Maximum Allowable Concentration
KW - Mexico
KW - Ozone/an [Analysis]
KW - Pilot Projects
KW - Radiography
KW - Thoracic
KW - United States
KW - United States Environmental Protection Agency
KW - Urban Health
RP - NOT IN FILE
SP - 537

EP - 561
JF - Inhalation Toxicology
JA - Inhal.Toxicol
VL - 12
IS - 6
CY - UNITED STATES
N2 - Air pollution produces adverse health effects. The consequences of lifelong daily exposures to atmospheric pollutants upon the respiratory apparatus of healthy children are of considerable clinical importance. We investigated the association between exposure to a highly polluted urban environment with a complex mixture of air pollutants-ozone and particulate matter the predominant ones-and chest x-ray abnormalities in 59 healthy Mexican children who are lifelong residents of Southwest Metropolitan Mexico City (SWMMC), with a negative history of tobacco exposure and respiratory illnesses. Their clinical results and x-ray findings were compared to those of 19 Mexican control children, residents of a low-pollution area, with a similar negative history of tobacco exposure and respiratory illnesses. Ozone concentrations in SWMMC exceeded the U.S. Environmental Protection Agency (U.S. EPA) National Ambient Air Quality Standard (NAAQS) for O(3): 0.08 ppm as 1-h maximal concentration, not to be exceeded more than 4 times a year, on 71% of days in 1986 and 95% in 1997, with values as high as 0.48 ppm. Ozone maximal peaks are usually recorded between 2 and 5 pm coinciding with children's outdoor physical activities. Children in the control group reported no upper or lower respiratory symptomatology. Every SWMMC child complained of upper and/or lower respiratory symptoms, including epistaxis, nasal dryness and crusting, cough, shortness of breath, and chest discomfort. Children aged 7-13 yr had the most symptomatology, while 5- to 6-year olds and adolescents with the lowest number of statistically significant outdoor exposure hours had less respiratory symptoms. Bilateral symmetric mild lung hyperinflation was significantly associated with exposure to the SWMMC atmosphere (p = .0004). Chronic and sustained inhalation of a complex mixture of air pollutants, including ozone and particulate matter (PM), is associated with lung hyperinflation, suggestive of small airway disease, in a population of clinically healthy children and adolescents. Small airways are a target of air pollutants in SWMMC children, with ozone and PM being most likely responsible, based on experimental animal, controlled-chamber, and epidemiological data available. Our main concern is the potential likelihood for the development of chronic lung disease in this highly exposed population
SN - 0895-8378
AD - Curriculum in Toxicology, University of North Carolina, Chapel Hill, USA.
caldeon.lilian@epamail.epa.gov
UR - 10880143
ER -

TY - JOUR
ID - 1737
T1 - Shaping oral feeding in a gastronomy tube-dependent child in natural settings
A1 - Gutentag,S.
A1 - Hammer,D.
Y1 - 2000/07//
N1 - Gutentag, S. Hammer, D
Behavior modification
9l5, 7803043
IM
Case Reports. Journal Article. Research Support, Non-U.S. Gov't
English
KW - MEDLINE
KW - Behavior Therapy/mt [Methods]
KW - Child
KW - Preschool
KW - Developmental Disabilities/co [Complications]
KW - Eating Disorders/et [Etiology]
KW - Eating Disorders/px [Psychology]
KW - Eating Disorders/th [Therapy]
KW - Enteral Nutrition/px [Psychology]
KW - Esophageal Motility Disorders/co [Complications]

KW - Feeding Behavior/px [Psychology]
KW - Female
KW - Humans
KW - Parent-Child Relations
KW - Treatment Outcome
RP - NOT IN FILE
SP - 395
EP - 410
JF - Behavior Modification
JA - Behav Modif
VL - 24
IS - 3
CY - UNITED STATES
N2 - A 3-year-old medically fragile girl who refused to eat after prolonged and frequent hospitalizations was started on a feeding program in the home and school settings. She exhibited food aversions and received all nourishment via a gastronomy tube. Preevaluation observations of her feeding behavior revealed that she refused all presented drinks and foods. Treatment was two-fold. First, food acceptance was followed by social praise and access to preferred toy play, and second, food refusal and disruptive behaviors were ignored. Gagging, vomiting, and crying occurred periodically during initial feedings. In addition, there were medical complications during the course of treatment necessitating continuous modifications of the program. Results of a multiple-phase design showed marked increases in the amount of food consumed at home, which then generalized to the school setting
SN - 0145-4455
AD - Clinical Center for the Study of Development and Learning, University of North Carolina, Chapel Hill 27599-7255, USA. sgutentag@css.unc.edu
UR - 10881384
ER -

TY - JOUR
ID - 1738
T1 - Evaluating 'Safe in the Sun', a curriculum programme for primary schools
A1 - McWhirter,J.M.
A1 - Collins,M.
A1 - Bryant,I.
A1 - Wetton,N.M.
A1 - Newton,Bishop J.
Y1 - 2000/04//
N1 - McWhirter, J M. Collins, M. Bryant, I. Wetton, N M. Newton Bishop, J
Health education research
bqp, 8608459
T
Clinical Trial. Controlled Clinical Trial. Journal Article. Multicenter Study. Research Support, Non-U.S. Gov't
English
KW - MEDLINE
KW - Child
KW - Preschool
KW - Curriculum
KW - England
KW - Health Education/mt [Methods]
KW - Humans
KW - School Health Services
KW - Sunburn/pc [Prevention & Control]
KW - Teaching/mt [Methods]
RP - NOT IN FILE
SP - 203
EP - 217
JF - Health Education Research

JA - Health Educ Res
VL - 15
IS - 2
CY - ENGLAND
N2 - Eleven schools in the south of England took part in a trial of 'Safe in the Sun', a curriculum programme for primary school aged pupils. Case study methodology and the 'draw and write' technique were combined to evaluate changes in pupils' perceptions of the effects of the sun on their skin. Teachers were free to use the materials, consisting of a teacher's handbook and video, as they wished. Pupils in all schools showed higher levels of awareness of sun-safety measures in post-intervention studies compared with the levels recorded 4 months earlier. Chi-square analysis revealed that those pupils whose teachers had used the materials as recommended by the authors had significant increases in awareness and knowledge about keeping safe in the sun, compared with other levels of intervention. More pupils also appeared able to transfer their awareness to the context of the school playground, although the differences were not significant for any level of intervention. The discussion focuses on the value of the 'draw and write' technique as a tool for illuminative evaluation, on the factors which contributed to the pupils increased awareness of sun safety and on the importance of a whole school approach to sun safety
SN - 0268-1153
AD - Health Education Unit, School of Education, University of Southampton, UK
UR - 10751379
ER -

TY - JOUR
ID - 1739
T1 - A modeling framework for estimating children's residential exposure and dose to chlorpyrifos via dermal residue contact and nondietary ingestion
A1 - Zartarian,V.G.
A1 - Ozkaynak,H.
A1 - Burke,J.M.
A1 - Zufall,M.J.
A1 - Rigas,M.L.
A1 - Furtaw,E.J.,Jr.
Y1 - 2000/06//
N1 - Zartarian, V G. Ozkaynak, H. Burke, J M. Zufall, M J. Rigas, M L. Furtaw, E J Jr
Environmental health perspectives
ei0, 0330411
IM
Journal Article
English
KW - MEDLINE
KW - Administration
KW - Cutaneous
KW - Oral
KW - Child
KW - Child Welfare
KW - Preschool
KW - Chlorpyrifos/ad [Administration & Dosage]
KW - Chlorpyrifos/ae [Adverse Effects]
KW - Environmental Exposure/an [Analysis]
KW - Female
KW - Humans
KW - Infant
KW - Newborn
KW - Insecticides/ad [Administration & Dosage]
KW - Insecticides/ae [Adverse Effects]
KW - Male
KW - Models
KW - Statistical

KW - Pesticide Residues
KW - Reference Values
RP - NOT IN FILE
SP - 505
EP - 514
JF - Environmental Health Perspectives
JA - Environ Health Perspect
VL - 108
IS - 6
CY - UNITED STATES
N2 - To help address the Food Quality Protection Act of 1996, a physically based probabilistic model has been developed to quantify and analyze dermal and nondietary ingestion exposure and dose to pesticides. The Residential Stochastic Human Exposure and Dose Simulation Model for Pesticides (Residential-SHEDS) simulates the exposures and doses of children contacting residues on surfaces in treated residences and on turf in treated residential yards. The simulations combine sequential time-location-activity information from children's diaries with microlevel videotaped activity data, probability distributions of measured surface residues and exposure factors, and pharmacokinetic rate constants. Model outputs include individual profiles and population statistics for daily dermal loading, mass in the blood compartment, ingested residue via nondietary objects, and mass of eliminated metabolite, as well as contributions from various routes, pathways, and media. To illustrate the capabilities of the model framework, we applied Residential-SHEDS to estimate children's residential exposure and dose to chlorpyrifos for 12 exposure scenarios: 2 age groups (0-4 and 5-9 years); 2 indoor pesticide application methods (broadcast and crack and crevice); and 3 postindoor application time periods (< 1, 1-7, and 8-30 days). Independent residential turf applications (liquid or granular) were included in each of these scenarios. Despite the current data limitations and model assumptions, the case study predicts exposure and dose estimates that compare well to measurements in the published literature, and provides insights to the relative importance of exposure scenarios and pathways
SN - 0091-6765
AD - National Exposure Research Laboratory, Office of Research and Development, U.S. Environmental Protection Agency, Research Triangle Park, North Carolina 20192, USA. zartarian.valerie@epamail.epa.gov
UR - 10856023
ER -

TY - JOUR
ID - 1740
T1 - [Anesthetic management in pediatric interventional cardiology]. [Japanese]
A1 - Handa,F.
A1 - Tanaka,M.
A1 - Shiga,M.
A1 - Suzuki,T.
A1 - Masuda,Y.
A1 - Enya,S.
A1 - Sakai,K.
Y1 - 2000/05//
N1 - Handa, F. Tanaka, M. Shiga, M. Suzuki, T. Masuda, Y. Enya, S. Sakai, K
Masui. The Japanese journal of anesthesiology
khr, 0413707
IM
English Abstract. Journal Article
Japanese
KW - MEDLINE
KW - Anesthesia
KW - General
KW - Cardiac Catheterization/ae [Adverse Effects]
KW - Child
KW - Preschool
KW - Coronary Angiography/ae [Adverse Effects]
KW - Coronary Angiography

KW - Humans
KW - Infant
KW - Radiography
KW - Interventional/ae [Adverse Effects]
KW - Interventional
KW - Risk
RP - NOT IN FILE
SP - 509
EP - 513
JF - Masui - Japanese Journal of Anesthesiology
JA - Masui
VL - 49
IS - 5
CY - JAPAN
N2 - Interventional cardiology (n = 108) and cardiac angiography (n = 481) procedures in pediatrics performed between January 1996 and December 1998 were reviewed. Means (SD) of duration of anesthesia for interventional cardiology and cardiac angiography were 245 (130) and 152 (48) minutes, respectively (P < 0.001). Incidences of long operations requiring over 6 hours of anesthesia were 12 and 0%, respectively (P < 0.001). Incidences of hemodynamic derangements were 17 and 2.9%, and catheter-related complications were 7.4 and 0.83%, respectively (P < 0.001). Incidences of ICU admission were 7.4 and 0.62% those of emergency surgery were 4.6 and 0%, and those of blood transfusion were 4.6 and 0%, respectively (P < 0.001). Incidences of cardiopulmonary resuscitation, and cardioversion were also higher in interventional cardiology (P < 0.05), and all these emergency cases were rescued successfully. The risk of cardiac angiography is higher compared with general surgery, and the risk of interventional cardiology is higher than cardiac angiography in pediatrics. This study reconfirms that anesthesiologists should play an active role in care of pediatric patients undergoing high-risk procedures outside the operating room
SN - 0021-4892
AD - Department of Anesthesia, Kobe Children's Hospital
UR - 10846382
ER -

TY - JOUR
ID - 1741
T1 - Distance telescopes: a survey of user success
A1 - Lowe,J.B.
A1 - Rubinstein,M.P.
Y1 - 2000/05//
N1 - Lowe, J B. Rubinstein, M P
Optometry and vision science : official publication of the American Academy of Optometry
oiz, 8904931
IM
Journal Article
English
KW - MEDLINE
KW - Adaptation
KW - Physiological
KW - Adolescent
KW - Adult
KW - Aged
KW - 80 and over
KW - Child
KW - Eyeglasses/ut [Utilization]
KW - Female
KW - Health Surveys
KW - Humans
KW - Male
KW - Middle Aged

KW - Prostheses and Implants/ut [Utilization]
KW - Questionnaires
KW - Retrospective Studies
KW - Vision
KW - Low/rh [Rehabilitation]
KW - Visual Acuity
KW - Visually Impaired Persons/sn [Statistics & Numerical Data]
RP - NOT IN FILE
SP - 260
EP - 269
JF - Optometry & Vision Science
JA - Optom.Vis Sci
VL - 77
IS - 5
CY - UNITED STATES
N2 - The distance telescope has a historical reputation for causing difficulties in prescribing and adaptation. Hence, we considered that a retrospective survey of patients at Nottingham Low Vision Clinic might elucidate specific attributes that influence an individual patient's success in using a distance telescope. From 142 patients prescribed distance telescopes since the Clinic's inception, 133 apparently remained users and were mailed a preliminary three-question enquiry about usage of their distance telescopes. The 87 respondents were followed up with questionnaire 2, requesting explicit information about usage, namely frequency, degree of ease or difficulty, and purpose. Older patients required higher magnification ($p < 0.025$). Seventeen of 74 respondents to questionnaire 2 had various adaptational problems, which are discussed; 57 of 74 patients found their distance telescopes easy to use, and 49 of 57 were frequent users. Thus, ease and frequency are linked ($p < 0.05$). People tended to use their distance telescopes outdoors and indoors with similar frequency (p > or = 0.29). Adaptation was found to be unrelated to visual acuity, binocularity/monocularity, ocular pathology, or restricted mobility; magnification seemed to be influential, although not significantly. Aging did not significantly impede adaptation. We infer that the universal criterion for selecting treatable patients seems to be personality type. We conclude that adaptation to a device is dependent upon active recognition of its benefits, paralleled with a tolerance of its constraints, which combine to make usage easy and regular on at least one common task
SN - 1040-5488
AD - Directorate of Ophthalmology, University Hospital, Nottingham, United Kingdom
UR - 10831216
ER -

TY - JOUR
ID - 1742
T1 - Severity of fall injuries on sand or grass in playgrounds
A1 - Laforest,S.
A1 - Robitaille,Y.
A1 - Dorval,D.
A1 - Lesage,D.
A1 - Pless,B.
Y1 - 2000/06//
N1 - Laforest, S. Robitaille, Y. Dorval, D. Lesage, D. Pless, B
Journal of epidemiology and community health
i1p, 7909766
IM
Journal Article. Research Support, Non-U.S. Gov't
English
KW - MEDLINE
KW - Accidental Falls
KW - Adolescent
KW - Child
KW - Preschool
KW - Female
KW - Humans

KW - Infant
KW - Injury Severity Score
KW - Male
KW - Odds Ratio
KW - Poaceae
KW - Quebec/ep [Epidemiology]
KW - Risk Assessment
KW - Schools
KW - Silicon Dioxide
KW - Wounds and Injuries/ep [Epidemiology]
RP - NOT IN FILE
SP - 475
EP - 477
JF - Journal of Epidemiology & Community Health
JA - J Epidemiol Community Health
VL - 54
IS - 6
CY - ENGLAND
SN - 0143-005X
AD - Montreal Public Health Department, 1301 Sherbrooke est, Montreal, Quebec, Canada, H2L 1M3
UR - 10818126
ER -

TY - JOUR
ID - 1743
T1 - Ciprofloxacin for treatment of tularemia in children
A1 - Johansson,A.
A1 - Berglund,L.
A1 - Gothefors,L.
A1 - Sjostedt,A.
A1 - Tarnvik,A.
Y1 - 2000/05//
N1 - Johansson, A. Berglund, L. Gothefors, L. Sjostedt, A. Tarnvik, A
The Pediatric infectious disease journal
oxj, 8701858
IM
Journal Article. Research Support, Non-U.S. Gov't
English
KW - MEDLINE
KW - Administration
KW - Oral
KW - Anti-Infective Agents/ae [Adverse Effects]
KW - Anti-Infective Agents/tu [Therapeutic Use]
KW - Child
KW - Preschool
KW - Ciprofloxacin/ae [Adverse Effects]
KW - Ciprofloxacin/tu [Therapeutic Use]
KW - Female
KW - Francisella tularensis/de [Drug Effects]
KW - Francisella tularensis/ge [Genetics]
KW - Francisella tularensis/ip [Isolation & Purification]
KW - Humans
KW - Infant
KW - Male
KW - Microbial Sensitivity Tests
KW - Polymerase Chain Reaction
KW - Treatment Outcome

KW - Tularemia/dt [Drug Therapy]
KW - Tularemia/ep [Epidemiology]
KW - Tularemia/mi [Microbiology]
RP - NOT IN FILE
SP - 449
EP - 453
JF - Pediatric Infectious Disease Journal
JA - Pediatr Infect Dis J
VL - 19
IS - 5
CY - UNITED STATES
N2 - BACKGROUND: Children with tularemia are, irrespective of severity of disease, usually subjected to parenteral treatment with aminoglycosides. Based on available susceptibility testing, quinolones might be effective oral alternatives of parenteral therapy. These drugs cause arthropathy in immature animals, but this risk is currently regarded to be low in humans. PATIENTS AND METHODS: In 12 patients (median age, 4 years; range, 1 to 10) with ulceroglandular tularemia, a 10- to 14-day course of oral ciprofloxacin, 15 to 20 mg/kg daily in 2 divided doses, was prescribed. Microbiologic investigations included identification of the infectious agent by PCR and culture of wound specimens, as well as determination of antibiotic susceptibility of isolates of Francisella tularensis. RESULTS: Defervescence occurred within 4 days of institution of oral ciprofloxacin in all patients. After a median period of 4.5 days (range, 2 to 24), the patients were capable of outdoor activities. In 2 cases, treatment was withdrawn after 3 and 7 days because of rash. In both cases a second episode of fever occurred. All children recovered without complications. In 7 cases F. tularensis was successfully cultured from ulcer specimens and tested for susceptibility to ciprofloxacin. MIC values for all isolates were 0.03 mg/l. CONCLUSION: In our sample of 12 patients ciprofloxacin was satisfactory for outpatient treatment of tularemia in children
SN - 0891-3668
AD - Department of Clinical Microbiology, Infectious Diseases, Umea University, Sweden
UR - 10819342
ER -

TY - JOUR
ID - 1744
T1 - The epidemiology of malaria in an epidemic area of the Peruvian Amazon
A1 - Roper,M.H.
A1 - Torres,R.S.
A1 - Goicochea,C.G.
A1 - Andersen,E.M.
A1 - Guarda,J.S.
A1 - Calampa,C.
A1 - Hightower,A.W.
A1 - Magill,A.J.
Y1 - 2000/02//
N1 - Roper, M H. Torres, R S. Goicochea, C G. Andersen, E M. Guarda, J S. Calampa, C. Hightower, A W. Magill, A J
The American journal of tropical medicine and hygiene
3zq, 0370507
AIM, IM
Journal Article. Research Support, U.S. Gov't, Non-P.H.S.
English
KW - MEDLINE
KW - Adolescent
KW - Adult
KW - Age Factors
KW - Animals
KW - Anopheles/ph [Physiology]
KW - Antimalarials/tu [Therapeutic Use]
KW - Child

KW - Preschool
KW - Female
KW - Humans
KW - Incidence
KW - Insect Vectors/ph [Physiology]
KW - Life Style
KW - Longitudinal Studies
KW - Malaria
KW - Falciparum/bl [Blood]
KW - Falciparum/ep [Epidemiology]
KW - Falciparum/ps [Parasitology]
KW - Falciparum/tm [Transmission]
KW - Vivax/bl [Blood]
KW - Vivax/ep [Epidemiology]
KW - Vivax/ps [Parasitology]
KW - Vivax/tm [Transmission]
KW - Male
KW - Occupational Exposure
KW - Parasitemia/ep [Epidemiology]
KW - Peru/ep [Epidemiology]
KW - Plasmodium falciparum/gd [Growth & Development]
KW - Plasmodium vivax/gd [Growth & Development]
KW - Prevalence
KW - Questionnaires
KW - Risk Factors
KW - Seasons
RP - NOT IN FILE
SP - 247
EP - 256
JF - American Journal of Tropical Medicine & Hygiene
JA - Am J Trop Med Hyg
VL - 62
IS - 2
CY - UNITED STATES
N2 - A longitudinal study of malariometric indicators and their association with potential risk factors was conducted during August 1997-July 1998 at Padre Cocha, a village of 1,400 residents in the Peruvian Amazon. The incidence of Plasmodium falciparum infections during the study year was 166/1,000 persons; that of P. vivax was 826/1,000 persons. The mean duration of symptoms prior to diagnosis was 2 days; presenting geometric mean parasite densities were 3,976 parasites/microl for P. falciparum infections and 2,282 parasites/microl for P. vivax. There were no malaria-associated deaths. Consistent with the epidemic nature of malaria in the area, the incidence of both parasite species increased with age and there were no age-specific differences in mean parasite densities. No specific occupational risks for malaria were identified. Activities significantly associated with malaria risk reflected local vector behavior and included strolling outdoors after 6:00 PM and arising before 6:00 AM for adults, and attending evening church services for children
SN - 0002-9637
AD - U.S. Naval Medical Research Center Detachment, Lima, Peru
UR - 10813480
ER -

TY - JOUR
ID - 1745
T1 - Skin cancer prevention in outdoor recreation settings: effects of the Hawaii SunSmart Program
A1 - Glanz,K.
A1 - Lew,R.A.
A1 - Song,V.
A1 - Murakami-Akatsuka,L.
Y1 - 2000/03//

N1 - Glanz, K. Lew, R A. Song, V. Murakami-Akatsuka, L
Effective clinical practice : ECP
9815774, c51, 9815774
H
Clinical Trial. Journal Article. Randomized Controlled Trial. Research Support, Non-U.S. Gov't. Research Support, U.S. Gov't, P.H.S.
English
KW - MEDLINE
KW - Adult
KW - Child
KW - Cohort Studies
KW - Female
KW - Hawaii
KW - Health Behavior
KW - Health Knowledge
KW - Attitudes
KW - Practice
KW - Humans
KW - Male
KW - Process Assessment (Health Care)
KW - Recreation
KW - Skin Neoplasms/pc [Prevention & Control]
KW - Sunscreening Agents/ad [Administration & Dosage]
RP - NOT IN FILE
SP - 53
EP - 61
JF - Effective Clinical Practice
JA - Eff.Clin Pract
VL - 3
IS - 2
CY - UNITED STATES
N2 - CONTEXT: Skin cancer is the most common form of cancer in the United States, and it is one of the most preventable. Interventions for young children and their parents can help prevent future cases of skin cancer. OBJECTIVE: To determine whether a skin cancer prevention program implemented at outdoor recreation sites improved children's sun-protection behaviors and site sun-protection policies. DESIGN: Randomized trial of 14 outdoor recreation sites on the island of Oahu, Hawaii. The trial had three arms: control, education only, and education/environment. INTERVENTION: The education arm included staff training, on-site activities, take-home booklets, behavior-monitoring boards, and incentives. The education/environment arm included all education components plus provision of sunscreen and promotion of sun-safe environments. PARTICIPANTS: Children 6 to 8 years of age and their parents. OUTCOME MEASURES: Reports from parents of children's sun-protection behaviors and the sun-protection policies of recreation sites. The cohort for analysis from baseline to 6 weeks after testing had 383 participants; the cohort from 6 weeks after testing to 3 months of follow-up had 285 participants. RESULTS: Program implementation was high in the education only and the education/environment sites. Compared with control sites, children's sun-protection behaviors and, in particular, the use of sunscreen improved significantly at sites where the two interventions were implemented. In addition, sun-protection policies of recreation sites were markedly higher at intervention arm sites. The education/environment intervention was not superior to education alone. Changes were partly maintained at 3 months of follow-up. CONCLUSION: A creative, engaging, multicomponent skin cancer prevention program in outdoor recreation settings can lead to modest improvements in children's sun-protection behaviors
SN - 1099-8128
AD - Cancer Research Center of Hawaii, University of Hawaii, Honolulu, USA. kglanz@hawaii.edu
UR - 10915324
ER -

TY - JOUR
ID - 1746
T1 - A family with extrinsic allergic alveolitis caused by wild city pigeons: A case report

A1 - du Marchie Sarvaas,G.J.
A1 - Merkus,P.J.
A1 - de Jongste,J.C.
Y1 - 2000/05//
N1 - du Marchie Sarvaas, G J. Merkus, P J. de Jongste, J C
Pediatrics
oxv, 0376422
IM
Case Reports. Journal Article
English
KW - MEDLINE
KW - Adolescent
KW - Adult
KW - Animals
KW - Anti-Inflammatory Agents/tu [Therapeutic Use]
KW - Antigens/im [Immunology]
KW - Asthma/et [Etiology]
KW - Bird Fancier's Lung/di [Diagnosis]
KW - Bird Fancier's Lung/dt [Drug Therapy]
KW - Bird Fancier's Lung/pp [Physiopathology]
KW - Blood Proteins/im [Immunology]
KW - Budesonide/tu [Therapeutic Use]
KW - Child
KW - Columbidae/im [Immunology]
KW - Columbidae
KW - Family Health
KW - Female
KW - Follow-Up Studies
KW - Glucocorticoids/tu [Therapeutic Use]
KW - Humans
KW - Lung/pp [Physiopathology]
KW - Lung/ra [Radiography]
KW - Male
KW - Prednisolone/tu [Therapeutic Use]
KW - Treatment Outcome
KW - Urban Health
RP - NOT IN FILE
SP - E62
JF - Pediatrics
VL - 105
IS - 5
CY - UNITED STATES
N2 - We describe a family in which the mother died of unresolved lung disease and whose 5 children, some of whom had previous signs of asthma, were subsequently affected by extrinsic allergic alveolitis caused by contact with wild city pigeon antigens. The children received systemic corticosteroids for 1 month and inhaled steroids for 24 months, while antigen exposure was reduced as much as feasible. This was followed by a quick clinical recovery and a slow normalization of chest radiographs and pulmonary function indices, especially of diffusion capacity, during a follow-up of 24 months. Because pigeon-breeder's lung caused by free-roaming city pigeons has not been previously described, it remains unclear whether this family developed the disease because of high antigen exposure or because of increased susceptibility. None of the supposedly high-risk human leukocyte antigen types were found in the children. Whether human leukocyte antigen B7 in 1 child played a role in the course of the illness remains speculative. It is unknown to what extent pigeon-breeder's lung caused by nondomestic birds remains undetected and misdiagnosed as difficult or steroid-resistant asthma. The question remains whether free-roaming city pigeons are indeed a public health risk. We suggest that atypical outdoor antigens be considered in all patients with nonresolving chest disease or therapy-resistant asthma
SN - 1098-4275

AD - Department of Paediatrics, Subdivision of Respiratory Medicine, Erasmus University and University Hospital/Sophia Children's Hospital, Rotterdam, The Netherlands
UR - 10799626
ER -

TY - JOUR
ID - 1747
T1 - A review of correlates of physical activity of children and adolescents. [Review] [131 refs]
A1 - Sallis,J.F.
A1 - Prochaska,J.J.
A1 - Taylor,W.C.
Y1 - 2000/05//
N1 - Sallis, J F. Prochaska, J J. Taylor, W C
Medicine and science in sports and exercise
8005433, mg8
IM, S
Comparative Study. Journal Article. Review
English
KW - MEDLINE
KW - Adolescent
KW - Child
KW - Child Behavior
KW - Preschool
KW - Exercise/px [Psychology]
KW - Exercise
KW - Female
KW - Humans
KW - Male
RP - NOT IN FILE
SP - 963
EP - 975
JF - Medicine & Science in Sports & Exercise
JA - Med Sci Sports Exerc
VL - 32
IS - 5
CY - UNITED STATES
N2 - BACKGROUND: Understanding the factors that influence physical activity can aid the design of more effective interventions. Previous reviews of correlates of youth physical activity have produced conflicting results. METHODS: A comprehensive review of correlates of physical activity was conducted, and semiquantitative results were summarized separately for children (ages 3-12) and adolescents (ages 13-18). The 108 studies evaluated 40 variables for children and 48 variables for adolescents. RESULTS: About 60% of all reported associations with physical activity were statistically significant. Variables that were consistently associated with children's physical activity were sex (male), parental overweight status, physical activity preferences, intention to be active, perceived barriers (inverse), previous physical activity, healthy diet, program/facility access, and time spent outdoors. Variables that were consistently associated with adolescents' physical activity were sex (male), ethnicity (white), age (inverse), perceived activity competence, intentions, depression (inverse), previous physical activity, community sports, sensation seeking, sedentary after school and on weekends (inverse), parent support, support from others, sibling physical activity, direct help from parents, and opportunities to exercise. CONCLUSION: These consistently related variables should be confirmed in prospective studies, and interventions to improve the modifiable variables should be developed and evaluated. [References: 131]
SN - 0195-9131
AD - Joint Doctoral Program in Clinical Psychology, San Diego State University/University of California, 92120, USA. sallis@mail.sdsu.edu
UR - 10795788
ER -

TY - JOUR
ID - 1748
T1 - Mothers' responses to sons and daughters engaging in injury-risk behaviors on a playground: implications for sex differences in injury rates
A1 - Morrongiello,B.A.
A1 - Dawber,T.
Y1 - 2000/06//
N1 - Morrongiello, B A. Dawber, T
Journal of experimental child psychology
2985128r, i2n
IM
Journal Article. Research Support, Non-U.S. Gov't
English
KW - MEDLINE
KW - Child
KW - Female
KW - Gender Identity
KW - Humans
KW - Male
KW - Mother-Child Relations
KW - Parenting/px [Psychology]
KW - Play and Playthings
KW - Reinforcement
KW - Verbal
KW - Risk-Taking
KW - Socialization
KW - Wounds and Injuries/pc [Prevention & Control]
KW - Wounds and Injuries/px [Psychology]
RP - NOT IN FILE
SP - 89
EP - 103
JF - Journal of Experimental Child Psychology
JA - J Exp Child Psychol
VL - 76
IS - 2
CY - UNITED STATES
N2 - Videotapes of children engaging in injury-risk activities on a playground were shown to mothers, who were asked to intervene by stopping the tape and saying whatever they would to their child in the situation shown. Results revealed that mothers of daughters were more likely to judge behaviors as posing some degree of injury risk, and they intervened more frequently and quickly than mothers of sons. Mothers' speed to intervene positively correlated with both children's injury history and their risk-taking tendencies, indicating that mothers of children who were previously injured and who often engaged in injury-risk behaviors had a higher degree of tolerance for children's risk taking than mothers of children who experienced fewer injuries and less frequently engaged in injury-risk behaviors. Mothers' verbalizations to children's risk taking revealed that daughters received more cautions and statements communicating vulnerability for injury, whereas sons received more statements encouraging risk-taking behavior. Copyright 2000 Academic Press
SN - 0022-0965
AD - University of Guelph, Ontario, Canada
UR - 10788304
ER -

TY - JOUR
ID - 1749
T1 - Design strategy for assessing multi-pathway exposure for children: the Minnesota Children's Pesticide Exposure Study (MNCPES)
A1 - Quackenboss,J.J.
A1 - Pellizzari,E.D.

A1 - Shubat,P.
A1 - Whitmore,R.W.
A1 - Adgate,J.L.
A1 - Thomas,K.W.
A1 - Freeman,N.C.
A1 - Stroebel,C.
A1 - Lioy,P.J.
A1 - Clayton,A.C.
A1 - Sexton,K.
Y1 - 2000/03//
N1 - Quackenboss, J J. Pellizzari, E D. Shubat, P. Whitmore, R W. Adgate, J L. Thomas, K W. Freeman, N C. Stroebel, C. Lioy, P J. Clayton, A C. Sexton, K
Journal of exposure analysis and environmental epidemiology
bjn, 9111438
IM
Journal Article. Research Support, Non-U.S. Gov't. Research Support, U.S. Gov't, Non-P.H.S.
English
KW - MEDLINE
KW - Biological Markers/an [Analysis]
KW - Child
KW - Child Welfare
KW - Preschool
KW - Data Collection
KW - Environmental Exposure/an [Analysis]
KW - Female
KW - Health Surveys
KW - Humans
KW - Male
KW - Minnesota
KW - Pesticides/ae [Adverse Effects]
KW - Pesticides/an [Analysis]
KW - Research Design
KW - Rural Population
KW - Sensitivity and Specificity
KW - Urban Population
RP - NOT IN FILE
SP - 145
EP - 158
JF - Journal of Exposure Analysis and Environmental Epidemiology
JA - J Expo Anal Environ Epidemiol
VL - 10
IS - 2
CY - UNITED STATES
N2 - Although children are exposed to a variety of environmental hazards, including pesticides, there is a scarcity of information available to estimate exposures realistically. This article reports on one of the first attempts to measure multi-pathway pesticide exposures in a population-based sample of urban and non-urban children. A design strategy was developed to assess multi-pathway pesticide exposures in children using personal exposure measurements in combination with complimentary measurements of biological markers of exposure, concentrations in relevant environmental media, and time spent in important microenvironments and participating in exposure-related activities. Sample collection and analysis emphasized measurement of three insecticides (i.e., chlorpyrifos, diazinon, and malathion) and one herbicide (i.e., atrazine). These compounds were selected because of their frequent use, presence in multiple environmental media, expected population exposures, and related hazard/toxicity. The study was conducted during the summer of 1997 in Minnesota and involved a stratified sample of households with children ages 3-12 years. Participants resided in either (a) the cities of Minneapolis and St. Paul (urban households), or (b) Rice and Goodhue Counties just south of the metropolitan area (non-urban households). Results from a residential inventory documenting storage and use of products containing the target pesticides were used to preferentially select households where children were

likely to have higher exposures. The study successfully obtained pesticide exposure data for 102 children, including measurements of personal exposures (air, hand rinse, duplicate diet), environmental concentrations (residential indoor/outdoor air, drinking water, residential surfaces, soil), activity patterns (obtained by questionnaire, diary, videotaping), and internal dose (metabolites in urine)
SN - 1053-4245
AD - U.S. Environmental Protection Agency, National Exposure Research Laboratory, Las Vegas, Nevada 89193-3478, USA. quackenboss.james@epa.gov
UR - 10791596
ER -

TY - JOUR
ID - 1750
T1 - Detecting and describing preventive intervention effects in a universal school-based randomized trial targeting delinquent and violent behavior
A1 - Stoolmiller,M.
A1 - Eddy,J.M.
A1 - Reid,J.B.
Y1 - 2000/04//
N1 - Stoolmiller, M. Eddy, J M. Reid, J B
Journal of consulting and clinical psychology
0136553, hw3
IM
Clinical Trial. Journal Article. Randomized Controlled Trial. Research Support, U.S. Gov't, P.H.S.
English
KW - MEDLINE
KW - Aggression/px [Psychology]
KW - Antisocial Personality Disorder/pc [Prevention & Control]
KW - Antisocial Personality Disorder/px [Psychology]
KW - Behavior Therapy
KW - Child
KW - Female
KW - Follow-Up Studies
KW - Humans
KW - Juvenile Delinquency/pc [Prevention & Control]
KW - Juvenile Delinquency/px [Psychology]
KW - Male
KW - Risk Factors
KW - Social Environment
KW - Violence/pc [Prevention & Control]
KW - Violence/px [Psychology]
RP - NOT IN FILE
SP - 296
EP - 306
JF - Journal of Consulting & Clinical Psychology
JA - J Consult Clin Psychol
VL - 68
IS - 2
CY - UNITED STATES
N2 - This study examined theoretical, methodological, and statistical problems involved in evaluating the outcome of aggression on the playground for a universal preventive intervention for conduct disorder. Moderately aggressive children were hypothesized most likely to benefit. Aggression was measured on the playground using observers blind to the group status of the children. Behavior was micro-coded in real time to minimize potential expectancy biases. The effectiveness of the intervention was strongly related to initial levels of aggressiveness. The most aggressive children improved the most. Models that incorporated corrections for low reliability (the ratio of variance due to true time-stable individual differences to total variance) and censoring (a floor effect in the rate data due to short periods of observation) obtained effect sizes 5 times larger

than models without such corrections with respect to children who were initially 2 SDs above the mean on aggressiveness
SN - 0022-006X
AD - Oregon Social Learning Center, Eugene 97401, USA. mikes@.oslc.org
UR - 10780130
ER -

TY - JOUR
ID - 1751
T1 - Risk factors for severe injuries associated with falls from playground equipment
A1 - Macarthur,C.
A1 - Hu,X.
A1 - Wesson,D.E.
A1 - Parkin,P.C.
Y1 - 2000/05//
N1 - Macarthur, C. Hu, X. Wesson, D E. Parkin, P C
Accident; analysis and prevention
acs, 1254476
IM
Journal Article
English
KW - MEDLINE
KW - Accidental Falls/sn [Statistics & Numerical Data]
KW - Case-Control Studies
KW - Child
KW - Female
KW - Humans
KW - Injury Severity Score
KW - Male
KW - Play and Playthings
KW - Risk Factors
KW - Wounds and Injuries/ep [Epidemiology]
RP - NOT IN FILE
SP - 377
EP - 382
JF - Accident Analysis & Prevention
JA - Accid Anal Prev
VL - 32
IS - 3
CY - ENGLAND
N2 - A case control study design was used to determine the risk factors for severe injuries associated with falls from playground equipment. Children presenting to the Hospital for Sick Children in Toronto because of falls from playground equipment (1995-1996) were identified through daily review of admissions and emergency department visits. Cases were defined as children with a severe injury (AIS > or = 2), whereas, controls were children with a minor injury (AIS < 2). Data on age, sex, socioeconomic status, prior experience on the equipment, previous playground injury, type of equipment, height of fall, undersurface, nature of injury, body part involved, and disposition were collected via telephone interview, field trip measurement, and mailed questionnaire. A total of 126 children were studied--67 cases and 59 controls. There were no differences between the two groups on age, sex, socioeconomic status, prior exposure to the equipment, or previous playground injury. Extremity fractures predominated in the case group, while, facial lacerations predominated in the control group. The median height of fall for cases was 199 cm, compared with 160 cm for controls (P = 0.021). Cases were also more likely to have fallen from a height of > 150 cm (73%), compared with controls (54%), P = 0.027. The majority of cases (82%) and controls (86%) fell onto an impact absorbing undersurface (P = 0.540). The median depth of impact absorbing undersurface, however, for both case and control injuries was 3 cm--well below the recommended safety standards. Height of fall was an important risk factor for severe injury associated with falls from playground equipment. Above 150 cm, the risk of severe injury was increased 2-fold

SN - 0001-4575
AD - Department of Community Health Sciences, Faculty of Medicine, Health Sciences Center, University of Calgary, Alta., Canada. colinmac@ucalgary.ca
UR - 10776854
ER -

TY - JOUR
ID - 1752
T1 - [Cutaneous larva migrans in children in a school of center-western Brazil]. [Portuguese]
A1 - Araujo,F.R.
A1 - Araujo,C.P.
A1 - Werneck,M.R.
A1 - Gorski,A.
Y1 - 2000/02//
N1 - Araujo, F R. Araujo, C P. Werneck, M R. Gorski, A
Revista de saude publica
0135043, t5x
IM
English Abstract. Journal Article
Portuguese
KW - MEDLINE
KW - Ancylostoma/ip [Isolation & Purification]
KW - Animals
KW - Brazil/ep [Epidemiology]
KW - Cats
KW - Child
KW - Preschool
KW - Disease Outbreaks
KW - Dogs
KW - Feces/ps [Parasitology]
KW - Female
KW - Humans
KW - Larva Migrans/ep [Epidemiology]
KW - Larva Migrans/ps [Parasitology]
KW - Male
RP - NOT IN FILE
SP - 84
EP - 85
JF - Revista de Saude Publica
JA - Rev Saude Publica
VL - 34
IS - 1
CY - BRAZIL
N2 - This paper reports an outbreak of cutaneous larva migrans in children of a school located in Campo Grande, MS. Six out of the 16 students (37.5%) acquired this parasitic dermatitis in two playgrounds contaminated by cat feces, in which ancylostomid larvae were found. The serpiginous lesions and/or papules were located in the hands, feet, buttocks, thighs, vulva and scrotum. Control measures of this parasitosis are discussed
SN - 0034-8910
AD - Centro de Ciencias Biologicas, Agrarias e da Saude, Universidade para o Desenvolvimento do Estado e da Regiao do Pantanal, Campo Grande, MS, Brasil
UR - 10769366
ER -

TY - JOUR
ID - 1753
T1 - Asthma and allergy among schoolchildren in a mountainous, dry, non-polluted area in Norway

A1 - Nja,F.
A1 - Roksund,O.D.
A1 - Svidal,B.
A1 - Nystad,W.
A1 - Carlsen,K.H.
Y1 - 2000/02//
N1 - Nja, F. Roksund, O D. Svidal, B. Nystad, W. Carlsen, K H
Pediatric allergy and immunology : official publication of the European Society of Pediatric Allergy and Immunology
bu6, 9106718
IM
Journal Article. Research Support, Non-U.S. Gov't
English
KW - MEDLINE
KW - Adolescent
KW - Air Pollution/ae [Adverse Effects]
KW - Air Pollution
KW - Altitude
KW - Asthma/di [Diagnosis]
KW - Asthma/ep [Epidemiology]
KW - Case-Control Studies
KW - Child
KW - Climate
KW - Eczema/di [Diagnosis]
KW - Eczema/ep [Epidemiology]
KW - Female
KW - Humans
KW - Hypersensitivity/di [Diagnosis]
KW - Hypersensitivity/ep [Epidemiology]
KW - Male
KW - Norway/ep [Epidemiology]
KW - Prevalence
KW - Questionnaires
KW - Reproducibility of Results
KW - Rural Population
KW - Skin Tests
RP - NOT IN FILE
SP - 40
EP - 48
JF - Pediatric Allergy & Immunology
JA - Pediatr Allergy Immunol
VL - 11
IS - 1
CY - DENMARK
N2 - The aim of this study was to assess prevalence of asthma and allergy in the non-polluted mountain area of Upper Hallingdal, Norway. All schoolchildren (7-16 years) who in a previous questionnaire survey (n = 1177) reported 'sometime' asthma were enrolled in group I (n = 80), the 59 who reported asthma-like symptoms in the past 12 months formed group II, and 77 of the healthy controls were randomly selected as group III. All 216 children underwent clinical examination, skin prick test, spirometry, bronchial provocation (PD20 metacholine) and treadmill exercise test. Subsequently they were reclassified as (1) healthy, never had asthma or symptoms, (2) symptoms not confirmed as asthma, (3) previous asthma, now healthy, (4) current asthma. Lifetime asthma prevalence was 10.2%. Based upon clinical examination, the specificity and sensitivity of the questionnaire for asthma diagnosis were 88 and 74%, respectively. Forced vital capacity was significantly higher among the asthmatics (group 4 versus 1), whereas forced expiratory volume in one second (FEV1) and forced expiratory flow at 50% of vital capacity were similar in all groups. More than 10% reduction in FEV1 following treadmill-run was found in 20% of children. Children with current asthma compared to controls had significantly; lower mean values of PD20 (9.1 versus 16.5 micromol), higher eosinophil cationic protein (13.4 versus 7.7 micromol)

and more frequent sensitization to animal dander (56% versus 10%). In conclusion, despite a favorable climate, little mite sensitization and low outdoor pollution, asthma prevalence was surprisingly high in Upper Hallingdal. Sensitization to animal dander was the most important contributing factor for current asthma
SN - 0905-6157
AD - Geilomo Children's Hospital for Asthma and Allergy, Geilo and Sandvika, Norway
UR - 10768734
ER -

TY - JOUR
ID - 1754
T1 - Differences between burns in rural and in urban areas: implications for prevention
A1 - Vidal-Trecan,G.
A1 - Tcherny-Lessenot,S.
A1 - Grossin,C.
A1 - Devaux,S.
A1 - Pages,M.
A1 - Laguerre,J.
A1 - Wassermann,D.
Y1 - 2000/06//
N1 - Vidal-Trecan, G. Tcherny-Lessenot, S. Grossin, C. Devaux, S. Pages, M. Laguerre, J. Wassermann, D
Burns : journal of the International Society for Burn Injuries
afc, 8913178
IM
Comparative Study. Journal Article
English
KW - MEDLINE
KW - Accidents/sn [Statistics & Numerical Data]
KW - Adolescent
KW - Adult
KW - Age Factors
KW - Aged
KW - Body Surface Area
KW - Burns/cl [Classification]
KW - Burns/ep [Epidemiology]
KW - Burns/mo [Mortality]
KW - Burns/pc [Prevention & Control]
KW - Causality
KW - Chi-Square Distribution
KW - Child
KW - Preschool
KW - Cooking/sn [Statistics & Numerical Data]
KW - Cross-Sectional Studies
KW - Databases as Topic
KW - Explosions/sn [Statistics & Numerical Data]
KW - Female
KW - Fires/sn [Statistics & Numerical Data]
KW - France/ep [Epidemiology]
KW - Hand Injuries/ep [Epidemiology]
KW - Humans
KW - Male
KW - Middle Aged
KW - Occupational Diseases/ep [Epidemiology]
KW - Perineum/in [Injuries]
KW - Questionnaires
KW - Rural Health/sn [Statistics & Numerical Data]
KW - Urban Health/sn [Statistics & Numerical Data]
RP - NOT IN FILE

SP - 351
EP - 358
JF - Burns
VL - 26
IS - 4
CY - ENGLAND
N2 - This study aimed to identify typical features of burns in rural areas and to improve their prevention by comparing the characteristics of burnt patients and their burns in rural and urban areas. A cross-sectional survey was conducted in 19 of the 23 French burns units over one year, using a structured questionnaire. We analysed the resulting database. Of the 1422 patients, excluding 188 burnt in unclassified areas, 420 (34%) were burnt in rural areas. Burns were more frequent in rural than in urban areas. Occupational burns were more frequent in rural than in urban areas. The characteristics of patients and burns did not differ between the areas. Patients burnt during everyday activities were older and more frequently had predisposing factors in rural than in urban areas. Burns occurred outdoors more often in rural than in urban areas and were frequently due to flames or explosions or to the use of barbecues or open fires. The hands and perineum were burnt more frequently in rural than in urban areas. Rural burns were more severe than urban burns: they were deeper, involved a larger body surface area and caused more deaths. Preventive measures in rural areas should take into account occupation and everyday activities. They should be specifically adapted to the profiles of burnt patients
SN - 0305-4179
AD - Departement de Sante Publique, CHU Cochin, Port Royal, Universite Rene Descartes, 24-27 Rue du Faubourg Saint Jacques, 75014, Paris, France. gwenaelle.Vidal-Trecan@cochin.univ-paris5.fr
UR - 10751703
ER -

TY - JOUR
ID - 1755
T1 - Ozone. The good, the bad, and the ugly
A1 - Williams,L.K.
A1 - Langley,R.
A1 - Howell,R.J.
Y1 - 2000/03//
N1 - Williams, L K. Langley, R. Howell, R J
North Carolina medical journal
ntx, 2984805r
IM
Journal Article
English
KW - MEDLINE
KW - Adult
KW - Air Pollutants/ae [Adverse Effects]
KW - Air Pollution
KW - Indoor
KW - Child
KW - Environmental Exposure/ae [Adverse Effects]
KW - Environmental Exposure/pc [Prevention & Control]
KW - Environmental Monitoring
KW - Humans
KW - North Carolina
KW - Ozone/ae [Adverse Effects]
KW - Respiration Disorders/et [Etiology]
KW - Respiration Disorders/pc [Prevention & Control]
RP - NOT IN FILE
SP - 84
EP - 89
JF - North Carolina Medical Journal
JA - N C Med J
VL - 61

IS - 2
CY - UNITED STATES
N2 - Sensitive individuals are at risk of adverse health effects from exposure to even modest levels of ozone. Such individuals are likely to develop respiratory symptoms and are more likely to be hospitalized when exposed to ozone at currently accepted levels. A review of scientific documents regarding the health effects of ozone has led the DHHS to agree with the EPA that the primary ambient air standard for ozone should be changed from 0.12 ppm (1-hour average) to 0.08 ppm (8-hour average). Achieving the proposed 0.08 ppm standard should decrease physician visits, hospitalization, and use of respiratory medications. In addition, because very susceptible healthy individuals may experience adverse health effects of the current standard, it is clear that the current standard provides little or no margin of safety, particularly for sensitive individuals. In order to avoid exposure to unhealthy levels of ozone, people should take precautions to minimize exposure from 2:00 to 6:00 pm on days when ozone levels are high. This is especially so for children, adults who are active outdoors, those with respiratory disease such as asthma, and people with unusual susceptibility to ozone
SN - 0029-2559
AD - Occupational and Environmental Epidemiology Branch, NC Department of Health and Human Services
UR - 10737029
ER -

TY - JOUR
ID - 1756
T1 - [Epidemiology of alveolar hydatid disease (AHD) and estimation of infected period of AHD in Rebun Island, Hokkaido]. [Japanese]
A1 - Doi,R.
A1 - Nakao,M.
A1 - Nihei,N.
A1 - Kutsumi,H.
Y1 - 2000/02//
N1 - Doi, R. Nakao, M. Nihei, N. Kutsumi, H
[Nihon koshu eisei zasshi] Japanese journal of public health
19130150r, a9j
IM
English Abstract. Journal Article. Research Support, Non-U.S. Gov't
Japanese
KW - MEDLINE
KW - Adolescent
KW - Adult
KW - Animals
KW - Child
KW - Preschool
KW - Dogs/ps [Parasitology]
KW - Echinococcosis
KW - Hepatic/mo [Mortality]
KW - Hepatic/tm [Transmission]
KW - Female
KW - Foxes/ps [Parasitology]
KW - Humans
KW - Japan/ep [Epidemiology]
KW - Male
KW - Zoonoses
RP - NOT IN FILE
SP - 145
EP - 152
JF - Nippon Koshu Eisei Zasshi - Japanese Journal of Public Health
JA - Nippon Koshu Eisei Zasshi
VL - 47
IS - 2
CY - JAPAN

N2 - An epidemiological study was performed of endemic alveolar hydatid disease (AHD, multilocular echinococcosis), Rebun Island, Hokkaido and the period of AHD infection of patients was estimated. Death certificates of the residents of the island were analyzed, and 74 deaths (43 males and 31 females) by AHD were found out of the 3,126 deaths that occurred during the period from 1948 to 1990. The red fox population of the island was estimated on the basis of past researchs. The deaths due to AHD distributed around a major peak (n = 67) between 1948-1975 and there were 7 sporadic cases between 1976-90. The red fox population on the island had been estimated to be largest in 1935. The mean infection period from initial AHD infection to death was estimated to be 26 +/- 7 years (x +/- SD) on the basis of the period between the year in which the peak red fox population was observed (1935) and the major peak of patient death (1962). The mean symptomatic period was 5 +/- 5 years, and the mean latent period from infection to the onset of AHD was 21 +/- 7 years. Sex ratio (M/F = 28/13 = 2.15) was higher (P < 0.05) at the age groups below 10 and 26-45 years than the other age groups (15/18 = 0.83), and playing outdoors during childhood and working outdoors in the prime of life were assumed to be the causes of infection
SN - 0546-1766
AD - Department of Hygiene, Yokohama City University, School of Medicine
UR - 10734729
ER -

TY - JOUR
ID - 1757
T1 - Predicting elementary school participation in children with disabilities
A1 - Mancini,M.C.
A1 - Coster,W.J.
A1 - Trombly,C.A.
A1 - Heeren,T.C.
Y1 - 2000/03//
N1 - Mancini, M C. Coster, W J. Trombly, C A. Heeren, T C
Archives of physical medicine and rehabilitation
2985158r, 8bk
AIM, IM
Journal Article. Research Support, Non-U.S. Gov't. Research Support, U.S. Gov't, Non-P.H.S.
English
KW - MEDLINE
KW - Child
KW - Disabled Children
KW - Female
KW - Humans
KW - Mainstreaming (Education)
KW - Male
KW - Psychometrics
KW - Students
RP - NOT IN FILE
SP - 339
EP - 347
JF - Archives of Physical Medicine & Rehabilitation
JA - Arch Phys Med Rehabil
VL - 81
IS - 3
CY - UNITED STATES
N2 - OBJECTIVE: To identify predictors of participation in school activities from two sets of functional variables using classification and regression tree analysis. DESIGN: Relational study. PARTICIPANTS: A nationwide sample of 341 children with various disabling conditions, including physical and cognitive/behavioral types of impairment and various severity levels. Children attended public elementary school in 40 states in the United States. MAIN OUTCOME MEASURE: Overall participation in elementary school, combining children's participation in six different environments (transportation, transitions, classroom, cafeteria, bathroom, and playground), as measured by the newly developed School Function Assessment. The children were dichotomized into full (n = 117) and limited (n = 224) participation categories. RESULTS: Two

classification trees were developed identifying a small set of predictors from variables measuring performance of functional tasks and discrete activities. Final predictive models included physical and cognitive-behavioral variables, suggested important interactions among predictors, and identified meaningful cut-off points that classified the sample into the outcome categories with about 85% accuracy. CONCLUSIONS: Limited participation was predicted by information about children's physical capabilities. Full participation was predicted by a combination of physical and cognitive-behavioral variables. Findings underscore the relative utility of functional performance compared with impairment information to predict the outcome, and suggest pathways of influence to consider in future research and intervention efforts
SN - 0003-9993
AD - Department of Occupational Therapy, Universidade Federal de Minas Gerais, Belo Horizonte, Brazil
UR - 10724080
ER -

TY - JOUR
ID - 1758
T1 - The Harvard Southern California Chronic Ozone Exposure Study: assessing ozone exposure of grade-school-age children in two Southern California communities
A1 - Geyh,A.S.
A1 - Xue,J.
A1 - Ozkaynak,H.
A1 - Spengler,J.D.
Y1 - 2000/03//
N1 - Geyh, A S. Xue, J. Ozkaynak, H. Spengler, J D
Environmental health perspectives
ei0, 0330411
IM
Journal Article. Research Support, U.S. Gov't, P.H.S.
English
KW - MEDLINE
KW - Age Distribution
KW - Air Pollutants/an [Analysis]
KW - California
KW - Child
KW - Environmental Exposure/an [Analysis]
KW - Environmental Monitoring/mt [Methods]
KW - Female
KW - Humans
KW - Longitudinal Studies
KW - Male
KW - Oxidants
KW - Photochemical/an [Analysis]
KW - Ozone/an [Analysis]
KW - Residence Characteristics/sn [Statistics & Numerical Data]
KW - Seasons
KW - Sex Distribution
KW - Time Factors
RP - NOT IN FILE
SP - 265
EP - 270
JF - Environmental Health Perspectives
JA - Environ Health Perspect
VL - 108
IS - 3
CY - UNITED STATES
N2 - The Harvard Southern California Chronic Ozone Exposure Study measured personal exposure to, and indoor and outdoor ozone concentrations of, approximately 200 elementary school children 6-12 years of age for 12 months (June 1995-May 1996). We selected two Southern California communities, Upland and several

towns located in the San Bernardino mountains, because certain characteristics of those communities were believed to affect personal exposures. On 6 consecutive days during each study month, participant homes were monitored for indoor and outdoor ozone concentrations, and participating children wore a small passive ozone sampler to measure personal exposure. During each sampling period, the children recorded time-location-activity information in a diary. Ambient ozone concentration data were obtained from air quality monitoring stations in the study areas. We present ozone concentration data for the ozone season (June-September 1995 and May 1996) and the nonozone season (October 1995-April 1996). During the ozone season, outdoor and indoor concentrations and personal exposure averaged 48.2, 11.8, and 18.8 ppb in Upland and 60.1, 21.4, and 25.4 ppb in the mountain towns, respectively. During the nonozone season, outdoor and indoor concentrations and personal exposure averaged 21.1, 3.2, and 6.2 ppb in Upland, and 35.7, 2.8, and 5.7 ppb in the mountain towns, respectively. Personal exposure differed by community and sex, but not by age group
SN - 0091-6765
AD - Health Effects Institute, Cambridge, Massachusetts, USA
UR - 10706534
ER -

TY - JOUR
ID - 1759
T1 - Family responsibilities and domestic activities of US women physicians
A1 - Frank,E.
A1 - Harvey,L.
A1 - Elon,L.
Y1 - 2000/02//
N1 - Frank, E. Harvey, L. Elon, L
Archives of family medicine
bx6, 9300357
IM
Journal Article
English
KW - MEDLINE
KW - Adult
KW - Child Care
KW - Child
KW - Preschool
KW - Conflict (Psychology)
KW - Cooking
KW - Female
KW - Housekeeping
KW - Humans
KW - Income
KW - Job Satisfaction
KW - Marital Status
KW - Medicine
KW - Mental Health
KW - Middle Aged
KW - Physicians
KW - Women/px [Psychology]
KW - Women/sn [Statistics & Numerical Data]
KW - Specialization
KW - Stress
KW - Psychological/et [Etiology]
KW - United States
KW - Work Schedule Tolerance
RP - NOT IN FILE
SP - 134
EP - 140
JF - Archives of Family Medicine

JA - Arch Fam Med
VL - 9
IS - 2
CY - UNITED STATES
N2 - BACKGROUND: Women physicians may have a multiplicity of domestic roles (eg, cook, housekeeper, child care provider) that are of inherent interest and that may affect their professional lives, but are largely unstudied. DESIGN, SETTING, PARTICIPANTS, AND MAIN OUTCOME MEASURES: We report data from respondents (N = 4501) to the Women Physicians' Health Study, a cross-sectional, questionnaire-based study of a stratified random sample of US women MDs. RESULTS: Women physicians with children aged 0 to 17 years spent a median of 24.4 hours per week on child care. Women physicians typically spent half an hour per day cooking, and another half-hour per day on other housework. Little time was spent on gardening: a median of 0.05 hours (3 minutes) per week. Those performing more domestic tasks are likely to work fewer hours outside the home and to be on call less often. Women physicians who are married or widowed, have more children, have lower personal incomes, and have more highly educated and higher-earning spouses perform more domestic activities. We found no significant adverse relationship between time spent on any domestic activity and career satisfaction or mental or physical health. CONCLUSIONS: Women physicians spend little time on domestic activities that can be done for them by others, including cooking, housework, and especially gardening. Women physicians spend somewhat less time on child care and substantially less time on housework than do other US women. Despite abundant editorializing about role conflicts of women physicians, our measures of career satisfaction and mental health were not adversely affected by time spent on domestic obligations
SN - 1063-3987
AD - Department of Family and Preventive Medicine, Emory University School of Medicine, Atlanta, GA 30303-3219, USA. efrank@fpm.eushc.org
UR - 10693730
ER -

TY - JOUR
ID - 1760
T1 - Evidence-based guidelines for resource utilization in children with isolated spleen or liver injury. The APSA Trauma Committee
A1 - Stylianos,S.
Y1 - 2000/02//
N1 - Stylianos, S
Journal of pediatric surgery
jmj, 0052631
IM
Journal Article. Multicenter Study. Research Support, Non-U.S. Gov't
English
KW - MEDLINE
KW - Adolescent
KW - Child
KW - Preschool
KW - Evidence-Based Medicine
KW - Female
KW - Hospitalization
KW - Humans
KW - Infant
KW - Injury Severity Score
KW - Liver/in [Injuries]
KW - Liver/ra [Radiography]
KW - Male
KW - Physician's Practice Patterns
KW - Practice Guidelines as Topic
KW - Spleen/in [Injuries]
KW - Spleen/ra [Radiography]
KW - Sports

KW - Tomography
KW - X-Ray Computed/ut [Utilization]
RP - NOT IN FILE
SP - 164
EP - 167
JF - Journal of Pediatric Surgery
JA - J Pediatr Surg
VL - 35
IS - 2
CY - UNITED STATES
N2 - PURPOSE: This study is intended to resolve the disparity and reach consensus on issues regarding the treatment of children with isolated spleen or liver injuries. To maximize patient safety and assure efficient, cost-effective utilization of resources, it was essential to determine current practice. METHODS: Data from the case records of 856 children with isolated spleen or liver injury treated at 32 pediatric surgical centers from July 1995 to June 1997 were collected. The severity of injury was classified by computed tomography (CT) grade and the data analyzed for intensive care unit (ICU) stay, length of hospital stay, transfusion requirement, need for operation, pre- and postdischarge imaging, and restriction of physical activity. Patients with grade V injuries (2.8%) were excluded leaving 832 patients for detailed review. These data and available literature were analyzed for consensus by the 1998 APSA Trauma Committee. RESULTS: Resource utilization increased with injury severity (see Table 2). Based on the data analysis, literature search, and consensus conference, the authors propose guidelines (see Table 3) for the safe and optimal utilization of resources in routine cases. It is important to emphasize that no recommendation falls outside the 25th percentile of current practice at participating centers. CONCLUSIONS: Diversity of treatment, with attendant variation in resource utilization in children with isolated spleen and liver injury of comparable severity is confirmed. This analysis has stimulated a prospective outcomes study with the objective of validating the evidence-based guidelines proposed. This evidence-based study design can bring order and conformity to patient management resulting in optimal utilization of resources while maximizing patient safety
SN - 0022-3468
AD - Babies and Children's Hospital, New York, NY 10032, USA
UR - 10693659
ER -

TY - JOUR
ID - 1761
T1 - Personal UV dosimetry by Bacillus subtilis spore films
A1 - Moehrle,M.
A1 - Garbe,C.
Y1 - 2000///
N1 - Moehrle, M. Garbe, C
Dermatology (Basel, Switzerland)
bbv, 9203244
IM
Journal Article
English
KW - MEDLINE
KW - Adolescent
KW - Adult
KW - Altitude
KW - Bacillus subtilis/re [Radiation Effects]
KW - Child
KW - Preschool
KW - Dose-Response Relationship
KW - Radiation
KW - Humans
KW - Infant
KW - Mountaineering
KW - Occupational Exposure

KW - Radiation Dosage
KW - Radiation Monitoring/is [Instrumentation]
KW - Radiation Monitoring/mt [Methods]
KW - Rescue Work
KW - Spores
KW - Bacterial/re [Radiation Effects]
KW - Swimming Pools
KW - Ultraviolet Rays
RP - NOT IN FILE
SP - 1
EP - 5
JF - Dermatology
VL - 200
IS - 1
CY - SWITZERLAND
N2 - BACKGROUND: Ultraviolet radiation (UVR) is known to be the most important risk factor for melanoma and non-melanoma skin cancers. Until today it has been impossible to measure reliably UVR in the frame of epidemiological studies. The recent development of a spore film containing spores of Bacillus subtilis resulted in a new method of UV measurement by personal dosimetry. METHODS: The practical application of dosimeters was tested in 18 study persons under different circumstances of UV exposure and in 4 different geographical regions. RESULTS: Eleven children carried dosimeters on their shoulders for 1 day, playing in- and outdoors on a sunny day in summertime. Their whole-day values ranged from 0.1 to 1.5 minimal erythema doses (MED) per day with a mean of 0.71 MED (+/-0.44). Four lifeguards in a public swimming-pool carried dosimeters on their shoulders for 11 days and received UV exposures ranging from 3.6 to 9.5 MED (mean 5.9 +/- 1.88). Three mountain guides with dosimeters attached to the lateral head in different mountain regions at 23 mountaineering activities received daily exposures of 4.44-17.07 MED (mean 11.9 +/- 3.8). CONCLUSION: B. subtilis spore film dosimeters can be applied to different study persons including children and mountain guides under different climatic conditions. A broad range of UV exposures can reliably be measured with this method. Copyright 2000 (R) S.Karger AG, Basel
SN - 1018-8665
AD - Section of Dermatological Oncology, Department of Dermatology, Eberhard Karls University, Tubingen, Germany
UR - 10681605
ER -

TY - JOUR
ID - 1762
T1 - Seasonal influences on childhood lead exposure
A1 - Yiin,L.M.
A1 - Rhoads,G.G.
A1 - Lioy,P.J.
Y1 - 2000/02//
N1 - Yiin, L M. Rhoads, G G. Lioy, P J
Environmental health perspectives
ei0, 0330411
IM
Journal Article. Research Support, Non-U.S. Gov't. Research Support, U.S. Gov't, Non-P.H.S.. Research Support, U.S. Gov't, P.H.S.
English
KW - MEDLINE
KW - Child
KW - Preschool
KW - Dust/an [Analysis]
KW - Female
KW - Humans
KW - Infant
KW - Lead/an [Analysis]

KW - Lead/bl [Blood]
KW - Lead Poisoning
KW - Nervous System
KW - Childhood/bl [Blood]
KW - Male
KW - New Jersey
KW - Regression Analysis
KW - Seasons
KW - Urban Health
RP - NOT IN FILE
SP - 177
EP - 182
JF - Environmental Health Perspectives
JA - Environ Health Perspect
VL - 108
IS - 2
CY - UNITED STATES
N2 - We conducted a study to examine seasonal changes in residential dust lead content and its relationship to blood lead in preschool children. We collected blood and dust samples (floors, windowsills, and carpets) to assess lead exposure. The geometric mean blood lead concentrations are 10.77 and 7.66 microg/dL for the defined hot and cold periods, respectively ($p < 0.05$). Lead loading (milligrams per square meter) is the measure derived from floor and windowsill wipe samples that is most correlated with blood lead concentration, whereas lead concentration (micrograms per gram) is the best variable derived from carpet vacuum samples. The variation of dust lead levels for these three dust variables (floor lead loading, windowsill lead loading, and carpet lead concentration) are consistent with the variation of blood lead levels, showing the highest levels in the hottest months of the year, June, July, and August. The regression analysis, including the three representative dust variables in the equations to predict blood lead concentration, suggests that the seasonality of blood lead levels in children is related to the seasonal distributions of dust lead in the home. In addition, the outdoor activity patterns indicate that children are likely to contact high leaded street dust or soil during longer outdoor play periods in summer. Consequently, our results show that children appear to receive the highest dust lead exposure indoors and outdoors during the summer, when they have the highest blood lead levels. We conclude that at least some of the seasonal variation in blood lead levels in children is probably due to increased exposure to lead in dust and soil
SN - 0091-6765
AD - Environmental and Occupational Health Sciences Institute, Department of Environmental Sciences, Rutgers, The State University of New Jersey, Piscataway, New Jersey, USA
UR - 10656860
ER -

TY - JOUR
ID - 1763
T1 - Canine visceral leishmaniasis in Sicily
A1 - Orndorff,G.R.
A1 - Cooper,B.A.
A1 - Smith,W.
A1 - Ryan,J.R.
Y1 - 2000/01//
N1 - Orndorff, G R. Cooper, B A. Smith, W. Ryan, J R
Military medicine
2984771r, n1a
IM
Journal Article
English
KW - MEDLINE
KW - Animals
KW - Domestic/ps [Parasitology]
KW - Antibodies

KW - Protozoan/bl [Blood]
KW - Child
KW - Dog Diseases/ep [Epidemiology]
KW - Dog Diseases/ps [Parasitology]
KW - Dogs
KW - Environmental Exposure
KW - Enzyme-Linked Immunosorbent Assay
KW - Female
KW - Humans
KW - Immunoglobulin G/bl [Blood]
KW - Immunoglobulin M/bl [Blood]
KW - Insect Vectors/ps [Parasitology]
KW - Leishmania infantum/im [Immunology]
KW - Leishmaniasis
KW - Visceral/ep [Epidemiology]
KW - Visceral/ve [Veterinary]
KW - Male
KW - Military Personnel
KW - Prevalence
KW - Psychodidae/ps [Parasitology]
KW - Risk Factors
KW - Sicily/ep [Epidemiology]
RP - NOT IN FILE
SP - 29
EP - 32
JF - Military Medicine
JA - Mil.Med
VL - 165
IS - 1
CY - UNITED STATES
N2 - The Sicilian province of Catania is an active foci for human visceral leishmaniasis (VL) in the Mediterranean area. Approximately 10 to 15 cases of VL are diagnosed via hospital admissions each year in this community. Recently, an increase in VL case reporting by Sicilian physicians was noted, with 38 and 37 VL cases in 1996 and 1997, respectively. Before 1995, there were no reported VL cases among U.S. military personnel or their family members living in Sicily. However, since 1996, there have been four cases referred to Walter Reed Army Medical Center for diagnosis and treatment, all involving the children of personnel assigned to Naval Air Station Sigonella. Exposure histories for all infected individuals excluded exposure to Leishmania parasites outside of Sicily. All patients lived in areas where vectoring sandflies are present. All had dogs as family pets. To evaluate the level of infection among dogs owned by Navy personnel and their families, U.S. Navy Environmental and Preventive Medicine Unit 7, in a collaborative study with the U.S. Army Veterinary Clinic, Naval Air Station Sigonella, and the Walter Reed Army Institute of Research, performed clinical evaluation and serological testing of 50 dogs residing with U.S. personnel assigned to Naval Air Station Sigonella. The data indicate a high exposure rate to Leishmania (60% of the animals tested had elevated immunoglobulin M antibody levels) in the study population, suggesting that they were infected with Leishmania infantum. Distribution of seropositive dogs by sex was equal. Most of the dogs studied appeared to be in good health. However, inapparent infection of dogs, seen by Italian veterinarians, has been observed throughout all areas of Catania. Sandflies responsible for vectoring L. infantum were trapped in the same locations as the dogs sampled in this study. The level of subclinical infection was 75% among seropositive dogs. The overall level of canine infection observed was higher than expected. This study demonstrates an increased risk to military working dogs and companion dogs of U.S. personnel for infection with L. infantum during a 2- to 3-year tour in Sicily
SN - 0026-4075
AD - Department of Epidemiology, U.S. Navy Environmental and Preventive Medicine Unit 7, Sigonella, Sicily
UR - 10658425
ER -

TY - JOUR
ID - 1764
T1 - Cortical motor and somatosensory representation: effect of cerebral lesions
A1 - Bittar,R.G.
A1 - Olivier,A.
A1 - Sadikot,A.F.
A1 - Andermann,F.
A1 - Reutens,D.C.
Y1 - 2000/02//
N1 - Bittar, R G. Olivier, A. Sadikot, A F. Andermann, F. Reutens, D C
Journal of neurosurgery
jd3, 0253357
AIM, IM
Journal Article. Research Support, Non-U.S. Gov't
English
KW - MEDLINE
KW - Adolescent
KW - Adult
KW - Afferent Pathways/pp [Physiopathology]
KW - Afferent Pathways/su [Surgery]
KW - Aged
KW - Arousal/ph [Physiology]
KW - Brain Mapping
KW - Child
KW - Dominance
KW - Cerebral/ph [Physiology]
KW - Epilepsy/di [Diagnosis]
KW - Epilepsy/pp [Physiopathology]
KW - Epilepsy/su [Surgery]
KW - Evoked Potentials
KW - Motor/ph [Physiology]
KW - Somatosensory/ph [Physiology]
KW - Female
KW - Foot/ir [Innervation]
KW - Hand/ir [Innervation]
KW - Humans
KW - Image Processing
KW - Computer-Assisted
KW - Magnetic Resonance Imaging
KW - Male
KW - Middle Aged
KW - Motor Cortex/pp [Physiopathology]
KW - Motor Cortex/su [Surgery]
KW - Neuronal Plasticity/ph [Physiology]
KW - Psychosurgery
KW - Somatosensory Cortex/pp [Physiopathology]
KW - Somatosensory Cortex/su [Surgery]
KW - Supratentorial Neoplasms/di [Diagnosis]
KW - Supratentorial Neoplasms/pp [Physiopathology]
KW - Supratentorial Neoplasms/su [Surgery]
KW - Tomography
KW - Emission-Computed
RP - NOT IN FILE
SP - 242
EP - 248
JF - Journal of Neurosurgery
JA - J Neurosurg Pediatrics

VL - 92
IS - 2
CY - UNITED STATES
N2 - OBJECT: Changes in cortical representation in patients with cerebral lesions may alter the correlation between cortical anatomy and function. This is of potential clinical significance when the extent of cortical resection is based on surface anatomical landmarks. METHODS: Fifty-one patients with supratentorial lesions were studied. Nineteen harbored noncentral lesions (no involvement of pre- or postcentral gyrus), whereas 32 had central lesions. Control studies consisted of stimulation of the hand contralateral to the unaffected hemisphere. Positron emission tomography activation studies were performed using the [15O]H2O tracer. Somatosensory stimulation of the hand or foot was performed using a mechanical vibrator. Motor activation consisted of hand clenching or foot tapping. The t-statistic volumes were generated from images showing the mean change in regional cerebral blood flow, and coregistered with a T1-weighted magnetic resonance image. At the threshold selected, exclusive contralateral primary sensorimotor cortex activation was elicited in 100% of the control studies. A different pattern of cortical activation was associated with central lesions in 35 (78%) of 45 patients, which occurred significantly more often than with noncentral lesions (eight [31%] of 26 patients). The most common difference in the pattern of activation with central lesions was activation of cortical regions outside the central area (including the supplementary sensorimotor area and the secondary somatosensory cortex). No sensorimotor activation was observed in gyri adjacent to the pre- or postcentral gyrus. CONCLUSIONS: Central lesions are more frequently associated with altered patterns in activation than lesions in noncentral locations. Characteristic patterns include activation of secondary sensorimotor areas. The absence of activation in gyri adjacent to the sensorimotor strip has clinical significance for the planning of resections in the central area
SN - 0022-3085
AD - Montreal Neurological Institute and Hospital, Quebec, Canada
UR - 10659010
ER -

TY - JOUR
ID - 1765
T1 - Evaluation of the interrupter technique in measuring post-exercise bronchodilator responses in children
A1 - Kannisto,S.
A1 - Vanninen,E.
A1 - Korppi,M.
Y1 - 2000/01//
N1 - Kannisto, S. Vanninen, E. Korppi, M
Clinical physiology (Oxford, England)
dkg, 8309768
IM
Clinical Trial. Journal Article
English
KW - MEDLINE
KW - Adolescent
KW - Asthma/di [Diagnosis]
KW - Bronchoconstriction
KW - Child
KW - Preschool
KW - Exercise Test/mt [Methods]
KW - Female
KW - Humans
KW - Male
KW - Physical Exertion
KW - Respiratory Function Tests/mt [Methods]
KW - Sensitivity and Specificity
RP - NOT IN FILE
SP - 62
EP - 68
JF - Clinical Physiology

JA - Clin Physiol
VL - 20
IS - 1
CY - ENGLAND
N2 - A significant response in a bronchodilation test is one of the main diagnostic criteria of asthma. However, it is not known what the significant bronchodilator response (BDR) is by the interrupter technique (IR), measuring respiratory resistance (Rint) during tidal breathing. Fifty children with symptoms suggestive for asthma underwent an 8 min free running test outdoors. Flow-volume spirometry (FVS) and IR measurements were performed before and 10 min after running. Thereafter, all children received a salbutamol inhalation, and pulmonary function measurements were repeated 15 min later. The study population was classified into three groups according to the post-exercise pulmonary function: FEV1 > 100% of predicted (group I, n=15), FEV1 86-99% of predicted (group II, n=20) and FEV1 < 85% of predicted (group III, n=15). There were no differences in BDRs between groups I and II; the mean increases in FEV1 were 4-6% and in MMEF 20-23%, and the mean decreases in Rint were 23-26%. The mean changes in group III were significantly higher: 15% (P=0.004) in FEV1, 55% (P=0.021) in MMEF and 38% (P=0. 014) in Rint. BDR was positive (FEV1 rise >10%) in 15 children; five were in group II and 10 in group III. For MMEF and Rint, the best combination of sensitivity and specificity was achieved by a limit of 35%. By this limit, MMEF identified 14 and Rint 17 positive cases. The IR technique agreed with both FEV1 and MMEF in 73% of the cases. The IR technique provides an alternative to conventional ventilatory function measurements in bronchodilation tests in children. We suggest a decrease of 35% or more in Rint as diagnostic in post-exercise bronchodilation tests
SN - 0144-5979
AD - Department of Paediatrics, Kuopio University Hospital, Finland
UR - 10651794
ER -

TY - JOUR
ID - 1766
T1 - Mutations of the GNAS1 gene, stromal cell dysfunction, and osteomalacic changes in non-McCune-Albright fibrous dysplasia of bone
A1 - Bianco,P.
A1 - Riminucci,M.
A1 - Majolagbe,A.
A1 - Kuznetsov,S.A.
A1 - Collins,M.T.
A1 - Mankani,M.H.
A1 - Corsi,A.
A1 - Bone,H.G.
A1 - Wientroub,S.
A1 - Spiegel,A.M.
A1 - Fisher,L.W.
A1 - Robey,P.G.
Y1 - 2000/01//
N1 - Bianco, P. Riminucci, M. Majolagbe, A. Kuznetsov, S A. Collins, M T. Mankani, M H. Corsi, A. Bone, H G. Wientroub, S. Spiegel, A M. Fisher, L W. Robey, P G
Journal of bone and mineral research : the official journal of the American Society for Bone and Mineral Research
8610640
IM
Journal Article. Research Support, Non-U.S. Gov't
English
KW - MEDLINE
KW - Adolescent
KW - Adult
KW - Amino Acid Sequence
KW - Animals
KW - Base Sequence
KW - Cell Transplantation

KW - Child
KW - DNA
KW - Female
KW - Fibrous Dysplasia of Bone/ge [Genetics]
KW - Fibrous Dysplasia of Bone/pa [Pathology]
KW - GTP-Binding Protein alpha Subunits
KW - Gs/ge [Genetics]
KW - Humans
KW - Male
KW - Mice
KW - Mutation
KW - Osteomalacia/pa [Pathology]
KW - Polymerase Chain Reaction
KW - Stromal Cells/pa [Pathology]
RP - NOT IN FILE
SP - 120
EP - 128
JF - Journal of Bone & Mineral Research
JA - J Bone Miner.Res
VL - 15
IS - 1
CY - UNITED STATES
N2 - Activating missense mutations of the GNAS1 gene, encoding the alpha subunit of the stimulatory G protein (Gs), have been identified in patients with the McCune-Albright syndrome (MAS; characterized by polyostotic fibrous dysplasia, cafe au lait skin pigmentation, and endocrine disorders). Because fibrous dysplasia (FD) of bone also commonly occurs outside of the context of typical MAS, we asked whether the same mutations could be identified routinely in non-MAS FD lesions. We analyzed a series of 8 randomly obtained, consecutive cases of non-MAS FD and identified R201 mutations in the GNAS1 gene in all of them by sequencing cDNA generated by amplification of genomic DNA using a standard primer set and by using a novel, highly sensitive method that uses a protein nucleic acid (PNA) primer to block amplification of the normal allele. Histologic findings were not distinguishable from those observed in MAS-related FD and included subtle changes in cell shape and collagen texture putatively ascribed to excess endogenous cyclic adenosine monophosphate (cAMP). Osteomalacic changes (unmineralized osteoid) were prominent in lesional FD bone. In an in vivo transplantation assay, stromal cells isolated from FD failed to recapitulate a normal ossicle; instead, they generated a miniature replica of fibrous dysplasia. These data provide evidence that occurrence of GNAS1 mutations, previously noted in individual cases of FD, is a common and perhaps constant finding in non-MAS FD. These findings support the view that FD, MAS, and nonskeletal isolated endocrine lesions associated with GNAS1 mutations represent a spectrum of phenotypic expressions (likely reflecting different patterns of somatic mosaicism) of the same basic disorder. We conclude that mechanisms underlying the development of the FD lesions, and hopefully mechanism-targeted therapeutic approaches to be developed, must also be the same in MAS and non-MAS FD
SN - 0884-0431
AD - Dipartimento di Medicina Sperimentale, Universita dell'Aquila, Italy
UR - 10646121
ER -

TY - JOUR
ID - 1767
T1 - Physical activity and stages of change in fifth and sixth graders
A1 - Walton,J.
A1 - Hoerr,S.
A1 - Heine,L.
A1 - Frost,S.
A1 - Roisen,D.
A1 - Berkimer,M.
Y1 - 1999/09//

N1 - Walton, J. Hoerr, S. Heine, L. Frost, S. Roisen, D. Berkimer, M
The Journal of school health
k13, 0376370
IM, N
Comparative Study. Journal Article
English
KW - MEDLINE
KW - Adolescent
KW - Age Factors
KW - Child
KW - Data Collection
KW - Exercise
KW - Female
KW - Health Knowledge
KW - Attitudes
KW - Practice
KW - Humans
KW - Life Style
KW - Male
KW - Michigan
KW - Physical Education and Training/st [Standards]
KW - Physical Education and Training/td [Trends]
KW - Physical Fitness
KW - Rural Population
KW - Sampling Studies
KW - Urban Population
RP - NOT IN FILE
SP - 285
EP - 289
JF - Journal of School Health
JA - J Sch Health
VL - 69
IS - 7
CY - UNITED STATES
N2 - The Stage of Change (SC) paradigm was adapted to assess readiness to be or stay physically active among fifth and sixth graders. Students completed a four-item SC survey, the Past Year Leisure Time Physical Activity Questionnaire, and the Modifiable Physical Activity Questionnaire for Adolescents. Precontemplation, contemplation, and preparation stages were grouped as "pre-action" (PRE), and action and maintenance as "post-action" (AX) stages. Nearly 40% of all students were in PRE, compared to 60% of students in AX stages. Twenty-two percent of all students were in the sedentary precontemplation or contemplation stages. Significantly more boys were in maintenance than girls, and more girls than boys were in contemplation. Students averaged 14-21 hours/week of television, video, or computer work, and 1.6 hours/week of physical activity outside of school. Interventions may be targeted at a specific SC to enable a child to move forward along the SC continuum toward an active lifestyle
SN - 0022-4391
AD - Spectrum Health HeartReach Program, Grand Rapids, MI 49503, USA. julie.walton@spectrum-health.org
UR - 10529967
ER -

TY - JOUR
ID - 1768
T1 - "Troo, the Traumaroo" bicycle and playground safety program: a pilot study of kindergarten through second graders in the southwest
A1 - Knight-Bohnhoff,K.
A1 - Smith,J.
A1 - Deis,J.

A1 - Chavez,Y.
A1 - Horne-Lucero,L.
Y1 - 1999/04//
N1 - Knight-Bohnhoff, K. Smith, J. Deis, J. Chavez, Y. Horne-Lucero, L
Journal of trauma nursing : the official journal of the Society of Trauma Nurses
cfl, 9512997, 9512997
N
Journal Article. Research Support, Non-U.S. Gov't
English
KW - MEDLINE
KW - Bicycling/in [Injuries]
KW - Child
KW - Preschool
KW - Health Education/og [Organization & Administration]
KW - Health Knowledge
KW - Attitudes
KW - Practice
KW - Humans
KW - Pilot Projects
KW - Play and Playthings
KW - Program Evaluation
KW - Safety Management/og [Organization & Administration]
KW - School Health Services/og [Organization & Administration]
KW - Southwestern United States
RP - NOT IN FILE
SP - 28
EP - 36
JF - Journal of Trauma Nursing
JA - J Trauma Nurs
VL - 6
IS - 2
CY - UNITED STATES
N2 - "Troo, the Traumaroo" bicycle and playground safety education program created for kindergarten, first, and second grade students, was provided to a convenience sample of seven elementary schools in a Southwestern city. Favorable principal, classroom teacher, and school nurse evaluations indicated that the "Troo, the Traumaroo" program was successful in providing young children with bicycle and playground safety in a fun and entertaining way. Results of this pilot study indicated that kindergarten children pre-tested for bicycle safety knowledge prior to participating in the program, had significantly higher bicycle safety knowledge scores 30 days after participating in the program ($p < .0001$)
SN - 1078-7496
AD - Troo, the Traumaroo Bicycle and Playground Safety Program, Albuquerque, N.M., USA
UR - 10524047
ER -

TY - JOUR
ID - 1769
T1 - Home and recess physical activity of Hong Kong children
A1 - Johns,D.P.
A1 - Ha,A.S.
Y1 - 1999/09//
N1 - Johns, D P. Ha, A S
Research quarterly for exercise and sport
r6y, 8006373
IM
Journal Article. Research Support, Non-U.S. Gov't
English
KW - MEDLINE

KW - Child
KW - Female
KW - Health Behavior
KW - Hong Kong
KW - Humans
KW - Leisure Activities
KW - Male
KW - Physical Fitness
KW - Urban Population
RP - NOT IN FILE
SP - 319
EP - 323
JF - Research Quarterly for Exercise & Sport
JA - Res Q Exerc Sport
VL - 70
IS - 3
CY - UNITED STATES
SN - 0270-1367
AD - Department of Sport Science and Physical Education, Chinese University of Hong Kong. djohns@cuhk.edu.hk
UR - 10522290
ER -

TY - JOUR
ID - 1770
T1 - The relationships among environmental allergen sensitization, allergen exposure, pulmonary function, and bronchial hyperresponsiveness in the Childhood Asthma Management Program
A1 - Nelson,H.S.
A1 - Szefler,S.J.
A1 - Jacobs,J.
A1 - Huss,K.
A1 - Shapiro,G.
A1 - Sternberg,A.L.
Y1 - 1999/10//
N1 - Nelson, H S. Szefler, S J. Jacobs, J. Huss, K. Shapiro, G. Sternberg, A L
The Journal of allergy and clinical immunology
h53, 1275002
AIM, IM
Journal Article. Multicenter Study. Research Support, Non-U.S. Gov't. Research Support, U.S. Gov't, P.H.S.
English
KW - MEDLINE
KW - Adolescent
KW - Allergens/ae [Adverse Effects]
KW - Alternaria/im [Immunology]
KW - Animals
KW - Asthma/et [Etiology]
KW - Asthma/th [Therapy]
KW - Bronchi/im [Immunology]
KW - Cats
KW - Child
KW - Preschool
KW - Disease Management
KW - Dogs
KW - Dust/ae [Adverse Effects]
KW - Environmental Exposure
KW - Female
KW - Government Programs/sn [Statistics & Numerical Data]

KW - Humans
KW - Immunization
KW - Male
KW - National Institutes of Health (U.S.)
KW - Respiratory Function Tests
KW - Skin Tests
KW - United States
RP - NOT IN FILE
SP - 775
EP - 785
JF - Journal of Allergy & Clinical Immunology
JA - J Allergy Clin Immunol
VL - 104
IS - 4 Pt 1
CY - UNITED STATES
N2 - BACKGROUND: Sensitivity and exposure to indoor allergens constitutes a risk factor for the development and persistence of asthma in children. OBJECTIVE: Our purpose was to evaluate the relationship between sensitivity and exposure to inhalant allergens and lung function and bronchial responsiveness in a group of children (n = 1041) aged 8.9 +/- 2.1 years with mild to moderate asthma enrolled in the Childhood Asthma Management Program (CAMP). METHODS: With use of the extensive CAMP baseline cross-sectional data on spirometry, bronchial responsiveness, allergen sensitivities, and household allergen levels, the relationship of sensitization and exposure to allergens to lung function and methacholine sensitivity was evaluated. Children who enrolled in CAMP stopped all antiasthma medication except rescue use of albuterol and prednisone for exacerbations during the 5- to 16-week screening period. During the last 2 of these weeks they underwent spirometry and methacholine challenge. Indoor allergen exposures were determined from questionnaires completed by the parent. Household levels of indoor allergens (mite, cat, dog, cockroach, mold) were determined on house dust samples. Allergen sensitivity was determined by percutaneous skin testing with a standard battery of allergens plus locally important pollen and fungal spores. Lung function and bronchial hyperresponsiveness were compared for children sensitive and not sensitive to both indoor and outdoor allergens on skin testing and, if sensitive, for exposed and not exposed to the allergens to which they were positive on skin testing. RESULTS: There was a strong direct correlation between increased sensitivity to inhaled methacholine and skin test sensitivity to tree, weed, Alternaria, cat, dog, and indoor molds. When the relationship was examined by stepwise regression, the skin test sensitivities showing the strongest associations with the concentration of methacholine that caused a 20% fall in FEV(1) were dog (P =.003), Alternaria (P =.01), and cat (P =.05). Children sensitive to any one of the aeroallergens tested were compared for the presence or absence of exposure to that allergen at the time that the methacholine challenge was performed. Those who were sensitive and exposed to weed and cat had greater methacholine sensitivity than those similarly sensitive but not exposed (P =.003 and P =.02, respectively). CONCLUSIONS: Sensitivity to dog or cat dander or Alternaria by skin testing was associated with increased bronchial responsiveness but not decreased lung function in children with mild to moderate asthma. These findings support the important role that sensitization to certain allergens plays in modulating bronchial responsiveness
SN - 0091-6749
AD - National Jewish Medical and Research Center, Denver, Colo. 80206, USA
UR - 10518821
ER -

TY - JOUR
ID - 1771
T1 - [Daycare centers in a medium-sized Brazilian city: operations, child-care practices, infrastructure, and safety]. [Portuguese]
A1 - Barros,A.J.
A1 - Goncalves,E.V.
A1 - de Borba,C.R.
A1 - Lorenzatto,C.S.
A1 - Motta,D.B.
A1 - da,Silva,V
A1 - Schiroky,V.M.

Y1 - 1999/07//
N1 - Barros, A J. Goncalves, E V. de Borba, C R. Lorenzatto, C S. Motta, D B. da Silva, V R. Schiroky, V M
Cadernos de saude publica
c4a, 8901573
IM
English Abstract. Journal Article. Research Support, Non-U.S. Gov't
Portuguese
KW - MEDLINE
KW - Administrative Personnel
KW - Brazil
KW - Caregivers/st [Standards]
KW - Child
KW - Child Care/lj [Legislation & Jurisprudence]
KW - Child Care/og [Organization & Administration]
KW - Child Day Care Centers/lj [Legislation & Jurisprudence]
KW - Child Day Care Centers/og [Organization & Administration]
KW - Preschool
KW - Educational Status
KW - Humans
KW - Infant
KW - Newborn
KW - Questionnaires
KW - Urban Population
RP - NOT IN FILE
SP - 597
EP - 604
JF - Cadernos de Saude Publica
JA - Cad Saude Publica
VL - 15
IS - 3
CY - BRAZIL
N2 - Ninety-two daycare centers (23 public, 10 charitable, and 59 private) were identified in the municipality of Pelotas, RS, Southern Brazil. These centers were studied in relation to their organization, services, child-care practices, and infrastructure. All public centers worked full-time, while most private centers operated in the afternoon and received a smaller proportion of children under the age of two. The child-to-attendant ratio was similar in both types of centers, and the nurseries were the classes most frequently above the recommended maximum. Group and center sizes were larger in the public centers. Attendant schooling was higher in the private centers, but task specificity was lower. The infrastructure deficiencies most commonly observed in the public centers were lack of an internal recreational area and playground equipment. Private centers lacked adequate diapering facilities and exclusive toilets for the children. A small proportion of centers had staff trained to handle fire emergencies and administer first aid, revealing insufficient concern over safety issues
SN - 0102-311X
AD - Departamento de Medicina Social, Universidade Federal de Pelotas, C. P. 464, Pelotas, RS 96001-970, Brasil. a.barros@zaz.com.br
UR - 10502156
ER -

TY - JOUR
ID - 1772
T1 - [The Children's Pastoral in Criciuma, Southern Brazil: coverage and socio-demographic characteristics of participating families]. [Portuguese]
A1 - Neumann,N.A.
A1 - Victora,C.G.
A1 - Halpern,R.
A1 - Guimaraes,P.R.
A1 - Cesar,J.A.
Y1 - 1999/07//

N1 - Neumann, N A. Victora, C G. Halpern, R. Guimaraes, P R. Cesar, J A
Cadernos de saude publica
c4a, 8901573
IM
English Abstract. Journal Article
Portuguese
KW - MEDLINE
KW - Adolescent
KW - Adult
KW - Brazil
KW - Child
KW - Child Care/mt [Methods]
KW - Child Care/og [Organization & Administration]
KW - Child Care
KW - Abandoned
KW - Preschool
KW - Cross-Sectional Studies
KW - Female
KW - Humans
KW - Infant
KW - Newborn
KW - Male
KW - Pastoral Care/og [Organization & Administration]
KW - Program Evaluation
KW - Socioeconomic Factors
KW - Urban Health
KW - Urban Population
RP - NOT IN FILE
SP - 543
EP - 552
JF - Cadernos de Saude Publica
JA - Cad Saude Publica
VL - 15
IS - 3
CY - BRAZIL
N2 - This study describes the main social, economic, biological, and demographic characteristics of children and families who participate or have participated in the Children's Pastoral as compared to the overall urban population of Criciuma (Southern Brazil). A population-based cross-sectional study with a probabilistic sample of 2208 children under three years of age was conducted; 16.7% of the mothers confirmed having participated in the Pastoral at any given time, of whom 4.8% were currently participating, while the rest had dropped out. Black children and those over 12 months old or with two or more older siblings participated more frequently in the Pastoral. The main family factors associated with participation were mother's age (over 25), mother not working outside the home, low per capita income, low parental schooling, living in the neighborhood for more than 4 years, and death of sibling before age five. Reasons most often given by mothers for dropping out were migration, lack of time, and interruption of the Pastoral's activities in the neighborhood. The conclusion was that the Pastoral should prioritize the poorest of the poor and adopt measures to reduce drop-out rates
SN - 0102-311X
AD - Pastoral da Crianca, Organismo da Conferencia Nacional dos Bispos do Brasil (CNBB), Rua Pasteur 279, Curitiba, PR 80250-902, Brasil
UR - 10502150
ER -

TY - JOUR
ID - 1773
T1 - Prevalence and correlates of physical activity behaviors among elementary schoolchildren in multiethnic, low income, inner-city neighborhoods in Montreal, Canada
A1 - O'Loughlin,J.

A1 - Paradis,G.
A1 - Kishchuk,N.
A1 - Barnett,T.
A1 - Renaud,L.
Y1 - 1999/10//
N1 - O'Loughlin, J. Paradis, G. Kishchuk, N. Barnett, T. Renaud, L
Annals of epidemiology
9100013, bx8
IM
Comparative Study. Journal Article. Research Support, Non-U.S. Gov't
English
KW - MEDLINE
KW - Adolescent
KW - Age Factors
KW - Cardiovascular Diseases/pc [Prevention & Control]
KW - Child
KW - Ethnic Groups
KW - Female
KW - Health Promotion
KW - Humans
KW - Life Style
KW - Male
KW - Physical Exertion
KW - Prevalence
KW - Quebec
KW - Socioeconomic Factors
KW - Sports
KW - Statistics as Topic
KW - Urban Population
RP - NOT IN FILE
SP - 397
EP - 407
JF - Annals of Epidemiology
JA - Ann Epidemiol
VL - 9
IS - 7
CY - UNITED STATES
N2 - PURPOSE: To describe the prevalence and correlates of physical inactivity and of participation in organized sports at and outside school among elementary schoolchildren in multiethnic, low income, urban neighborhoods in Montreal, Canada. METHODS: As part of the evaluation of a school-based heart health promotion program, baseline data on physical activity behaviors and potential correlates of these behaviors, were collected from 2285 students aged 9-13 in all 130 grade 4 to 6 classes in 24 inner-city elementary schools from May to June 1993. RESULTS: One-fifth of boys (20.5%) and 24.4% of girls were inactive; 40.0% and 33.3% of boys and girls respectively, participated in school sports teams; 82.5% and 74.7% participated in organized sports outside school. Declines in activity levels with age were apparent in both genders. Children who participated in organized sports programs at and outside school, those with higher perceived self-efficacy for physical activity, and those with more parental support for engaging in physical activity were more active. Children of Asian family origin were less active. Socioeconomic status was related to participation in organized sports outside school. CONCLUSIONS: To reach children in socio-economically disadvantaged areas and to prevent age-related declines in activity levels, interventions promoting physical activity should focus on increasing availability and access to community-based organized sports programs at and outside school. Also they should include components to increase parental support and to improve perceived self-efficacy for physical activity
SN - 1047-2797
AD - Department of Public Health, McGill University Hospital Centre, Montreal, Quebec, Canada. jenniol@epid.lan.mcgill.ca
UR - 10501407

ER -

TY - JOUR
ID - 1774
T1 - [Sun exposure behavior of children between 3-15 years of age living in Montpellier]. [French]
A1 - Vergnes,C.
A1 - Daures,J.P.
A1 - Sancho-Garnier,H.
A1 - Bousquet,J.
A1 - Pourin-Bourdonneau,C.
A1 - Grabar,S.
A1 - Picot,E.
A1 - Meynadier,J.
Y1 - 1999/06//
N1 - Vergnes, C. Daures, J P. Sancho-Garnier, H. Bousquet, J. Pourin-Bourdonneau, C. Grabar, S. Picot, E. Meynadier, J
Annales de dermatologie et de venereologie
5rc, 7702013
IM, N
Comparative Study. English Abstract. Journal Article
French
KW - MEDLINE
KW - Adolescent
KW - Age Factors
KW - Child
KW - Preschool
KW - Female
KW - France
KW - Health Behavior
KW - Health Education
KW - Humans
KW - Male
KW - Questionnaires
KW - Random Allocation
KW - Skin Neoplasms/pc [Prevention & Control]
KW - Sunburn/pc [Prevention & Control]
KW - Sunlight/ae [Adverse Effects]
KW - Sunscreening Agents/ad [Administration & Dosage]
RP - NOT IN FILE
SP - 505
EP - 512
JF - Annales de Dermatologie et de Venereologie
JA - Ann Dermatol Venereol
VL - 126
IS - 6-7
CY - FRANCE
N2 - OBJECTIVE: Increasing prevalence of skin cancer over the past few decades and their relationship with childhood sun exposure, show that educational campaigns on sun protection are urgently needed. The aim of this study is to estimate the degree of children's sun exposure in the south of France (43 degrees latitude north) and to study sun protection behaviors according to phenotypic characteristics. POPULATION AND METHODS: The population was a randomly selected sample of 573 schoolchildren between 3 and 15 years of age living in Montpellier. The questionnaire provided information about sociodemographic characteristics, summer sun exposure and sun protection. Phenotypic characteristics, reactivity to sunlight and number of sunburns since birth were also reported. RESULTS: Exposure time was very high: half of children spend over 6 hours per day outside in summer. The youngest children were the most protected either by sunscreen, or by shirt. On the beach, sunscreens were used more than half of the time of exposure by 70 p. 100 of the children. At the swimming pool, sunscreens were used by 45 p. 100 of the children and only by 14 p. 100 of them during

other outdoor activities. A tee-shirt was only wore by 50 p. 100 of the children when playing on the beach, and by 75 p. 100 of them at the swimming pool. In addition, the application of sunscreen varied by body site. The adolescents, although they were exposed less than the youngest children, were very poorly protected. Light skinned children who do not tan easily had highest risk of sunburns, although they were generally more protected. CONCLUSION: The parents are able to identify highly sensitive children according to phenotype, but in practice the use of sunscreens and tee-shirt is very low. Parents and adolescents must be the target population but also insufficiently protected children. Health education at school must be an important measure for prevention
SN - 0151-9638
AD - IURC Unite de Biostatistique Epidemiologie et Recherche Clinique, Montpellier
UR - 10495860
ER -

TY - JOUR
ID - 1775
T1 - Magnetoencephalographic patterns of epileptiform activity in children with regressive autism spectrum disorders
A1 - Lewine,J.D.
A1 - Andrews,R.
A1 - Chez,M.
A1 - Patil,A.A.
A1 - Devinsky,O.
A1 - Smith,M.
A1 - Kanner,A.
A1 - Davis,J.T.
A1 - Funke,M.
A1 - Jones,G.
A1 - Chong,B.
A1 - Provencal,S.
A1 - Weisend,M.
A1 - Lee,R.R.
A1 - Orrison,W.W.,Jr.
Y1 - 1999/09//
N1 - Lewine, J D. Andrews, R. Chez, M. Patil, A A. Devinsky, O. Smith, M. Kanner, A. Davis, J T. Funke, M. Jones, G. Chong, B. Provencal, S. Weisend, M. Lee, R R. Orrison, W W Jr
Pediatrics
oxv, 0376422
AIM, IM
Comparative Study. Journal Article. Research Support, Non-U.S. Gov't
English
KW - MEDLINE
KW - Autistic Disorder/di [Diagnosis]
KW - Autistic Disorder/pp [Physiopathology]
KW - Brain/pp [Physiopathology]
KW - Child
KW - Child Development Disorders
KW - Pervasive/di [Diagnosis]
KW - Pervasive/pp [Physiopathology]
KW - Preschool
KW - Electroencephalography
KW - Epilepsy/di [Diagnosis]
KW - Epilepsy/pp [Physiopathology]
KW - Female
KW - Humans
KW - Landau-Kleffner Syndrome/di [Diagnosis]
KW - Landau-Kleffner Syndrome/pp [Physiopathology]
KW - Magnetoencephalography

KW - Male
KW - Sleep/ph [Physiology]
RP - NOT IN FILE
SP - 405
EP - 418
JF - Pediatrics
VL - 104
IS - 3 Pt 1
CY - UNITED STATES
N2 - BACKGROUND: One-third of children diagnosed with autism spectrum disorders (ASDs) are reported to have had normal early development followed by an autistic regression between the ages of 2 and 3 years. This clinical profile partly parallels that seen in Landau-Kleffner syndrome (LKS), an acquired language disorder (aphasia) believed to be caused by epileptiform activity. Given the additional observation that one-third of autistic children experience one or more seizures by adolescence, epileptiform activity may play a causal role in some cases of autism. OBJECTIVE: To compare and contrast patterns of epileptiform activity in children with autistic regressions versus classic LKS to determine if there is neurobiological overlap between these conditions. It was hypothesized that many children with regressive ASDs would show epileptiform activity in a multifocal pattern that includes the same brain regions implicated in LKS. DESIGN: Magnetoencephalography (MEG), a noninvasive method for identifying zones of abnormal brain electrophysiology, was used to evaluate patterns of epileptiform activity during stage III sleep in 6 children with classic LKS and 50 children with regressive ASDs with onset between 20 and 36 months of age (16 with autism and 34 with pervasive developmental disorder-not otherwise specified). Whereas 5 of the 6 children with LKS had been previously diagnosed with complex-partial seizures, a clinical seizure disorder had been diagnosed for only 15 of the 50 ASD children. However, all the children in this study had been reported to occasionally demonstrate unusual behaviors (eg, rapid blinking, holding of the hands to the ears, unprovoked crying episodes, and/or brief staring spells) which, if exhibited by a normal child, might be interpreted as indicative of a subclinical epileptiform condition. MEG data were compared with simultaneously recorded electroencephalography (EEG) data, and with data from previous 1-hour and/or 24-hour clinical EEG, when available. Multiple-dipole, spatiotemporal modeling was used to identify sites of origin and propagation for epileptiform transients. RESULTS: The MEG of all children with LKS showed primary or secondary epileptiform involvement of the left intra/perisylvian region, with all but 1 child showing additional involvement of the right sylvian region. In all cases of LKS, independent epileptiform activity beyond the sylvian region was absent, although propagation of activity to frontal or parietal regions was seen occasionally. MEG identified epileptiform activity in 41 of the 50 (82%) children with ASDs. In contrast, simultaneous EEG revealed epileptiform activity in only 68%. When epileptiform activity was present in the ASDs, the same intra/perisylvian regions seen to be epileptiform in LKS were active in 85% of the cases. Whereas primary activity outside of the sylvian regions was not seen for any of the children with LKS, 75% of the ASD children with epileptiform activity demonstrated additional nonsylvian zones of independent epileptiform activity. Despite the multifocal nature of the epileptiform activity in the ASDs, neurosurgical intervention aimed at control has lead to a reduction of autistic features and improvement in language skills in 12 of 18 cases. CONCLUSIONS: This study demonstrates that there is a subset of children with ASDs who demonstrate clinically relevant epileptiform activity during slow-wave sleep, and that this activity may be present even in the absence of a clinical seizure disorder. MEG showed significantly greater sensitivity to this epileptiform activity than simultaneous EEG, 1-hour clinical EEG, and 24-hour clinical EEG. The multifocal epileptiform pattern identified by MEG in the ASDs typically includes the same perisylvian brain regions identified as abnormal in LKS. When epileptiform activity is present in the ASDs, therapeutic strategies (antiepileptic drugs, steroids, and even neurosurgery) aimed at its control can lead to a significa
SN - 0031-4005
AD - Department of Radiology, University of Utah, Salt Lake City, Utah 84108, USA. lewine@doug.med.utah.edu
UR - 10469763
ER -

TY - JOUR
ID - 1776
T1 - Peer involvement in bullying: insights and challenges for intervention
A1 - O'Connell,P.
A1 - Pepler,D.

A1 - Craig,W.
Y1 - 1999/08//
N1 - O'Connell, P. Pepler, D. Craig, W
Journal of adolescence
hwt, 7808986
IM
Journal Article. Research Support, Non-U.S. Gov't
English
KW - MEDLINE
KW - Aggression/px [Psychology]
KW - Child
KW - Child Psychology
KW - Preschool
KW - Dominance-Subordination
KW - Female
KW - Humans
KW - Male
KW - Ontario
KW - Peer Group
KW - Psychological Theory
KW - Videotape Recording
RP - NOT IN FILE
SP - 437
EP - 452
JF - Journal of Adolescence
JA - J Adolesc
VL - 22
IS - 4
CY - ENGLAND
N2 - The purpose of this research was to examine the peer processes that occur during bullying episodes on the school playground. These processes were examined from a social learning perspective, allowing us to consider the effects of various types of reinforcement among bullies, victims, and peers. Fifty-three segments of video tape were examined. Each segment contained a peer group (two or more peers) that viewed bullying on the school playground. Peers were coded for actively joining or passively reinforcing the bully, and for actively intervening on behalf of the victim. On average, four peers viewed the schoolyard bullying, with a range from two to 14 peers. Averaged across all episodes, peers spent 54% of their time reinforcing bullies by passively watching, 21% of their time actively modelling bullies, and 25% of their time intervening on behalf of victims. Older boys (grades 4-6) were more likely to actively join with the bully than were younger boys (grades 1-3) and older girls. Both younger and older girls were more likely to intervene on behalf of victims than were older boys. The results were interpreted as confirming peers' central roles in the processes that unfold during playground bullying episodes. We discuss the results in terms of the challenges posed to peer-led interventions. Peers' anti-bullying initiatives must be reinforced by simultaneous whole-school interventions. Copyright 1999 The Association for Professionals in Services for Adolescents
SN - 0140-1971
AD - LaMarsh Research Centre on Violence and Conflict Resolution, York University, Toronto, Ont., Canada
UR - 10469508
ER -

TY - JOUR
ID - 1777
T1 - Promoting generalized social interactions using puppets and script training in an integrated preschool. A single-case study using multiple baseline design
A1 - Gronna,S.S.
A1 - Serna,L.A.
A1 - Kennedy,C.H.
A1 - Prater,M.A.
Y1 - 1999/07//

N1 - Gronna, S S. Serna, L A. Kennedy, C H. Prater, M A
Behavior modification
9l5, 7803043
IM
Case Reports. Journal Article
English
KW - MEDLINE
KW - Behavior Therapy
KW - Child
KW - Preschool
KW - Deinstitutionalization
KW - Esotropia/px [Psychology]
KW - Esotropia/rh [Rehabilitation]
KW - Female
KW - Generalization (Psychology)
KW - Humans
KW - Male
KW - Peer Group
KW - Play and Playthings
KW - Role Playing
KW - Schools
KW - Nursery
KW - Social Behavior
RP - NOT IN FILE
SP - 419
EP - 440
JF - Behavior Modification
JA - Behav Modif
VL - 23
IS - 3
CY - UNITED STATES
N2 - Puppet script training was used to teach the social skills of greeting, responding to conversations, and initiating conversations to a preschool child with visual impairments. Susie and four peers without disabilities were taught social skills utilizing puppets enacting sociodramatic scripts within group training sessions. Training sessions were immediately followed by free-play activities among peers without disabilities to assess skill generalization. A single-case study using a multiple-baseline design demonstrated that the intervention increased performance of social skills during recess with peers. Results demonstrated that Susie learned the target behaviors and generalized their use to free-play activities with her peers
SN - 0145-4455
AD - University of Hawaii, USA
UR - 10467891
ER -

TY - JOUR
ID - 1778
T1 - Sun exposure at school
A1 - Moise,A.F.
A1 - Buttner,P.G.
A1 - Harrison,S.L.
Y1 - 1999/08//
N1 - Moise, A F. Buttner, P G. Harrison, S L
Photochemistry and photobiology
0376425, p69
IM
Journal Article
English
KW - MEDLINE

KW - Adolescent
KW - Child
KW - Female
KW - Humans
KW - Male
KW - Melanoma/et [Etiology]
KW - Queensland
KW - Questionnaires
KW - Risk Factors
KW - Schools
KW - Skin Neoplasms/et [Etiology]
KW - Students
KW - Sunlight/ae [Adverse Effects]
RP - NOT IN FILE
SP - 269
EP - 274
JF - Photochemistry & Photobiology
JA - Photochem Photobiol
VL - 70
IS - 2
CY - UNITED STATES
N2 - There is strong evidence that sun exposure during childhood and adolescence plays an important role in the etiology of skin cancer, in particular cutaneous melanoma. Between the age of 6 and 18, most children and adolescents will spend around 200 days per year at school and may receive a substantial fraction of their daily total solar ultraviolet radiation (UVR) exposure while at school. This study estimated the average daily erythemally effective dose of 70 grade 8 students from a high school in Townsville during 5 school days in July 1998. Through UV measurements of shade locations at the school and a combination of frequency counts and a questionnaire of grade 8 students, it was possible to determine the fraction of solar UVR reaching under the shade structures during lunch breaks and routine outdoor activities. Also, a routinely operating UV-Biometer provided the annual variation of the daily dose that was used to calculate exposure levels for the 70 students. Our results suggest that up to 47% of the daily total dose fell within the time periods where students were outdoors during school hours. For students not seeking shade structures during the breaks (which usually was the case when involved in sport activities such as basketball or soccer), the average daily dose could have been as high as 14 SED (standard erythemal dose). Using results from the questionnaire of 70 grade 8 students, their average annual dose while at school was 414 SED or 2 SED per school day. However, the distribution of average daily erythemal effective dose per grade 8 student over the whole year showed that on 31% of all school days in 1998, this dose was exceeded. Because most previous attempts to change arguably poor sun-protective behavior of young Australian children and adolescents at school showed little success, one way of decreasing the amount of harmful UVR reaching unprotected skin is the more careful design of shade structures at schools
SN - 0031-8655
AD - School of Public Health and Tropical Medicine, James Cook University, Townsville, Queensland, Australia. aurel.moise@jcu.edu.au
UR - 10461467
ER -

TY - JOUR
ID - 1779
T1 - Injury surveillance in an accident and emergency department: a year in the life of CHIRPP
A1 - Morrison,A.
A1 - Stone,D.H.
A1 - Doraiswamy,N.
A1 - Ramsay,L.
Y1 - 1999/06//
N1 - Morrison, A. Stone, D H. Doraiswamy, N. Ramsay, L
Archives of disease in childhood
6xg, 0372434
AIM, IM

Journal Article
English
KW - MEDLINE
KW - Adolescent
KW - Age Distribution
KW - Child
KW - Preschool
KW - Emergency Service
KW - Hospital/og [Organization & Administration]
KW - Hospital/ut [Utilization]
KW - Female
KW - Hospitals
KW - Pediatric/og [Organization & Administration]
KW - Pediatric/ut [Utilization]
KW - Humans
KW - Infant
KW - Newborn
KW - Male
KW - Population Surveillance/mt [Methods]
KW - Scotland/ep [Epidemiology]
KW - Seasons
KW - Sex Distribution
KW - Wounds and Injuries/ep [Epidemiology]
KW - Wounds and Injuries/et [Etiology]
KW - Wounds and Injuries/pa [Pathology]
RP - NOT IN FILE
SP - 533
EP - 536
JF - Archives of Disease in Childhood
JA - Arch Dis Child
VL - 80
IS - 6
CY - ENGLAND
N2 - BACKGROUND: The design of childhood injury prevention programmes is hindered by a dearth of valid and reliable information on injury frequency, cause, and outcome. A number of local injury surveillance systems have been developed to address this issue. One example is CHIRPP (Canadian Hospitals Injury Reporting and Prevention Program), which has been imported into the accident and emergency department at the Royal Hospital for Sick Children, Glasgow. This paper examines a year of CHIRPP data. METHODS: A CHIRPP questionnaire was completed for 7940 children presenting in 1996 to the accident and emergency department with an injury or poisoning. The first part of the questionnaire was completed by the parent or accompanying adult, the second part by the clinician. These data were computerised and analysed using SPSSPC for Windows. RESULTS: Injuries commonly occurred in the child's own home, particularly in children aged 0-4 years. These children commonly presented with bruising, ingestions, and foreign bodies. With increasing age, higher proportions of children presented with injuries occurring outside the home. These were most commonly fractures, sprains, strains, and inflammation/oedema. Seasonal variations were evident, with presentations peaking in the summer. CONCLUSIONS: There are several limitations to the current CHIRPP system in Glasgow: it is not population based, only injuries presented to the accident and emergency department are included, and injury severity is not recorded. Nevertheless, CHIRPP is a valuable source of information on patterns of childhood injury. It offers local professionals a comprehensive dataset that may be used to develop, implement, and evaluate child injury prevention activities
SN - 1468-2044
AD - Paediatric Epidemiology and Community Health (PEACH) Unit, Department of Child Health, University of Glasgow, Royal Hospital for Sick Children, Yorkhill, Glasgow G3 8SJ, UK
UR - 10332002
ER -

TY - JOUR

ID - 1780
T1 - Concurrent and longitudinal relations between children's playground behavior and social preference, victimization, and bullying
A1 - Boulton,M.J.
Y1 - 1999/07//
N1 - Boulton, M J
Child development
0372725, d28
IM
Journal Article
English
KW - MEDLINE
KW - Child
KW - Child Behavior Disorders/di [Diagnosis]
KW - Child Behavior Disorders/px [Psychology]
KW - Child Psychology
KW - Choice Behavior/ph [Physiology]
KW - Crime Victims
KW - Female
KW - Humans
KW - Interpersonal Relations
KW - Longitudinal Studies
KW - Male
KW - Peer Group
KW - Play and Playthings
KW - Sex Factors
KW - Social Desirability
KW - Social Perception
KW - Time Factors
RP - NOT IN FILE
SP - 944
EP - 954
JF - Child Development
JA - Child Dev
VL - 70
IS - 4
CY - UNITED STATES
N2 - Prior research with contrived play groups suggests that how children interact with one another can influence their social relationships, but few studies have been carried out to determine if this is the case in naturalistic settings. This study examined the concurrent and longitudinal links between four observational measures of 8- to 9-year-old children's (N = 89) playground behaviors/peer contacts (Alone, Conversation, Group Size, and Network) and three sociometric measures of their peer relationships (Social Preference, Bully, and Victim). The latter were assessed at 2 points within a school year, the first corresponding with the playground observations and the second 5 months later. Several of the concurrent correlations were found to be significant. For boys, Group Size was positively correlated with Bully Score and Social Preference Score, but negatively correlated with Victim Score; and time Alone was positively correlated with Victim Score. For girls, time Alone was positively correlated with Victim Score; Network was negatively correlated with Bully Score but positively correlated with Social Preference Score; and Group Size was positively correlated with Social Preference Score. The longitudinal analyses indicated that for boys, elevated levels of time Alone and, separately, Conversation, predicted the highest increases in Victim Score. For girls, low levels of time Alone predicted the sharpest increases in Social Preference Score. There was also some marginally significant evidence, $p < .06$, that among girls, high levels of time Alone predicted the greatest increases in Bully Score, and high levels of Conversation predicted the greatest increases in Social Preference Score. The theoretical and practical importance of these links between children's nonaggressive playground behaviors and their emerging peer relationships are discussed
SN - 0009-3920
AD - Department of Psychology, Keele University, UK. psa23@keele.ac.uk

UR - 10446727
ER -

TY - JOUR
ID - 1781
T1 - [Evolution of the weight-height and weight-age relationship in children 3-6 years of age receiving preschool care in Sorocaba (SP), Brazil]. [Portuguese]
A1 - Correa,A.M.
A1 - da Silva e Goncalves NN
A1 - Goncalves,A.
A1 - Leite,G.P.
A1 - Padovani,C.R.
Y1 - 1999/07//
N1 - Correa, A M. da Silva e Goncalves, N N. Goncalves, A. Leite, G P. Padovani, C R
Revista panamericana de salud publica = Pan American journal of public health
csl, 9705400
IM
English Abstract. Journal Article. Research Support, Non-U.S. Gov't
Portuguese
KW - MEDLINE
KW - Age Factors
KW - Body Height
KW - Body Weight
KW - Brazil
KW - Child
KW - Child Development
KW - Child Nutritional Physiological Phenomena
KW - Female
KW - Humans
KW - Infant
KW - Male
KW - Nurseries
KW - Nutrition Assessment
KW - Nutritional Status
RP - NOT IN FILE
SP - 26
EP - 33
JF - Pan American Journal of Public Health
JA - Rev Panam.Salud Publica
VL - 6
IS - 1
CY - UNITED STATES
N2 - The nutritional status of Brazilians has improved over the last three decades. Still, little is known about the role played by nutritional assistance programs that public institutions and philanthropic organizations provide for low-income preschool children, who face greater nutritional risk. Therefore, we carried out a quasi-experimental study to evaluate the nutritional impact of the municipal preschool nutritional assistance program in the city of Sorocaba, Sao Paulo, Brazil. Over the course of a year, we performed quarterly measurements of weight and height on 444 children, whose ages ranged from 3 months to 6 years. In this population, 164 children were assisted by the preschool nutritional program (intervention group), and 280 children were not (nonintervention group). The children in the nonintervention group were identified by a special census for that purpose; they did not attend the preschool but lived nearby. After obtaining informed consent and carrying out a pretest, professionals and university students interviewed the mothers of the participating children, following standard techniques and conditions. Anthropometric measurements were done by teams of two interviewers, one of whom was a permanent member of the team, using equipment calibrated by the appropriate regional technical organization. The correspondence between the first measurement and subsequent measurements was evaluated by stratifying the population into three groups by z scores for weight-for-age and for weight-for-height, using U.S. National Center for Health Statistics reference curves. The z score groupings were established at the

beginning of the study for three different age groups: younger than 24 months, 24-36 months, and older than 36 months. The two study groups (intervention and nonintervention) were similar sociodemographically, with the following exceptions: maternal employment (more of the mothers of children in the intervention group had jobs outside the home); age (children in the intervention group were significantly older); nutritional status (it was worse in the intervention group); and prevalence of hospital admittance (it was higher in the intervention group). After just the first three months of the study, 32% of the children in the intervention group had moved to a higher nutritional stratum, whereas only 13% of children in the nonintervention group had shown improved nutritional status. The difference in favor of the intervention group increased with the length of the intervention and was higher among older children. These results show that the preschool nutritional assistance program helped to improve the assisted children's nutritional status
SN - 1020-4989
AD - UNICAMP, Departamento de Medicina Preventiva e Social, Faculdade de Ciencias Medicas, Campinas, Sao Paulo, Brasil
UR - 10446512
ER -

TY - JOUR
ID - 1782
T1 - "Block the sun, not the fun": evaluation of a skin cancer prevention program for child care centers
A1 - Crane,L.A.
A1 - Schneider,L.S.
A1 - Yohn,J.J.
A1 - Morelli,J.G.
A1 - Plomer,K.D.
Y1 - 1999/07//
N1 - Crane, L A. Schneider, L S. Yohn, J J. Morelli, J G. Plomer, K D
American journal of preventive medicine
8704773, apl
IM
Clinical Trial. Comparative Study. Journal Article. Multicenter Study. Randomized Controlled Trial. Research Support, U.S. Gov't, P.H.S.
English
KW - MEDLINE
KW - Adult
KW - Attitude to Health
KW - Carcinoma/pc [Prevention & Control]
KW - Child Day Care Centers
KW - Child
KW - Preschool
KW - Female
KW - Health Education
KW - Health Knowledge
KW - Attitudes
KW - Practice
KW - Humans
KW - Male
KW - Melanoma/pc [Prevention & Control]
KW - Skin Neoplasms/pc [Prevention & Control]
KW - Sunburn/pc [Prevention & Control]
RP - NOT IN FILE
SP - 31
EP - 37
JF - American Journal of Preventive Medicine
JA - Am J Prev Med
VL - 17
IS - 1
CY - NETHERLANDS

N2 - INTRODUCTION: This paper describes the evaluation of a skin cancer prevention program for preschools and daycare centers. The intervention was targeted primarily at staff of child care centers, with the aim of increasing use of sun protection practices for young children while attending these centers. Secondary target groups included parents and the children themselves. The intervention, which adopted the slogan, "Block the Sun, Not the Fun," included workshops for child care center staff, and information/activity packets for parents. METHODS: Twenty-seven preschools and daycare centers were randomly assigned to an intervention or wait-list control group. The intervention group received the intervention during the spring of 1994; the wait-list control group received the intervention during the spring of 1995. Evaluation consisted of interviews with center directors, observations of practices, and review of written policies before the intervention (in summer, 1993) and after the intervention (in summer, 1994). A survey of 201 parents was conducted during late summer 1994. RESULTS: While the intervention did not appear to change the sun protection attitudes or practices of parents, or use of clothing and shade at child care centers, results suggested significant changes in the sun protection knowledge/attitudes of center directors and the use of sunscreen at child care centers. Additionally, parents with children attending centers in the intervention group were more likely to be satisfied with sun protection practices at their centers. CONCLUSION: This low-intensity intervention appears to be effective at changing sun protection attitudes and sunscreen use at child care centers, and can be easily replicated. However, high staff turnover at child care centers would suggest that "boosters" will be necessary to sustain the impact. More intensive efforts directed at social norms are likely to be necessary to change clothing and outdoor play practices
SN - 0749-3797
AD - Department of Preventive Medicine and Biometrics, University of Colorado Health Sciences Center, Denver 80262, USA. lori.crane@uchsc.edu
UR - 10429750
ER -

TY - JOUR
ID - 1783
T1 - Direct measurement of sun protection in primary schools
A1 - Milne,E.
A1 - English,D.R.
A1 - Corti,B.
A1 - Cross,D.
A1 - Borland,R.
A1 - Gies,P.
A1 - Costa,C.
A1 - Johnston,R.
Y1 - 1999/07//
N1 - Milne, E. English, D R. Corti, B. Cross, D. Borland, R. Gies, P. Costa, C. Johnston, R
Preventive medicine
pm4, 0322116
IM
Journal Article. Research Support, Non-U.S. Gov't
English
KW - MEDLINE
KW - Analysis of Variance
KW - Child
KW - Preschool
KW - Confidence Intervals
KW - Environment Design
KW - Environmental Monitoring
KW - Female
KW - Health Behavior
KW - Health Promotion/mt [Methods]
KW - Health Promotion/st [Standards]
KW - Humans
KW - Intervention Studies
KW - Male

KW - Protective Clothing/ut [Utilization]
KW - School Health Services/st [Standards]
KW - Skin Neoplasms/pc [Prevention & Control]
KW - Sunlight/ae [Adverse Effects]
KW - Western Australia
RP - NOT IN FILE
SP - 45
EP - 52
JF - Preventive Medicine
JA - Prev Med
VL - 29
IS - 1
CY - UNITED STATES
N2 - BACKGROUND: "Kidskin" is an intervention study involving children at 33 primary schools in Perth, Western Australia. This study includes measurement of changes in implementation of schools' sun protection policies. This paper reports on measurement of observable aspects of sun protection. METHODS: Hat use was assessed from videos of children in the playground. Shade use was measured using UVR-sensitive polysulfone badges worn by a random sample of children. Shade provision was measured from aerial photographs of the schools. Principals were surveyed about school policies and practices. RESULTS: Eighty-seven percent of children wore a hat during lunch time at school, although only 14% wore the most protective styles of hats. The mean proportion of ambient UVR exposure received by Year 1 children was 15.5%; children spent less time in the sun on sunnier days. On average, 14.5% of the playground was shaded; this was not associated with children's sun exposure. Correlations between these results and the principals' estimates were poor. CONCLUSIONS: Children should be encouraged to wear more protective styles of hats and to avoid sun exposure, even on less sunny days during spring and summer. Principals' estimates of shade provision and children's sun protection behavior at school are of little value. Copyright 1999 American Health Foundation and Academic Press
SN - 0091-7435
AD - Department of Public Health, The University of Western Australia, Nedlands, 6907, Western Australia
UR - 10419799
ER -

TY - JOUR
ID - 1784
T1 - The environmental influence on childhood asthma
A1 - Bjorksten,B.
Y1 - 1999///
N1 - Bjorksten, B
Allergy
39c, 7804028
IM
Journal Article
English
KW - MEDLINE
KW - Air Pollution
KW - Indoor
KW - Allergens/im [Immunology]
KW - Asthma/ep [Epidemiology]
KW - Asthma/et [Etiology]
KW - Asthma/ge [Genetics]
KW - Child
KW - Preschool
KW - Environmental Exposure
KW - Geography
KW - Humans
KW - Incidence
KW - Risk Factors

KW - Tobacco Smoke Pollution
RP - NOT IN FILE
SP - 17
EP - 23
JF - Allergy
VL - 54 Suppl 49
CY - DENMARK
N2 - The pathogenesis of allergy depends on the interaction between the time and amount of allergen exposure and the presence of nonspecific "adjuvant" factors in genetically susceptible individuals. There seems to be a period in early life during which the individual is particularly susceptible to sensitization, and there are variations in susceptibility over time. Allergens are almost ubiquitous, but the relative importance of the individual allergens varies between regions. In many temperate regions, house-dust mites used to be absent but are now more common. This may be due to modern methods of building houses. Differences in the prevalence of a particular allergy cannot explain variations in the prevalence of allergy in general. Various environmental factors that may enhance sensitization include tobacco smoke, NO2, SO2, ozone, and diesel particles. Passive smoking is by far the best established risk factor, particularly in early childhood. The indoor environment probably plays a larger role than outdoor air pollution in the development of allergic disease. The mother is not only a source of genetic information, but also an "environmental factor", as there is a close immunologic interaction between the mother and her offspring, mediated through the placenta and the breast milk, which may affect the likelihood of allergic disease. The concepts of "lifestyle" and "environment" should be expanded to include, for example, dietary changes, the microbial environment, and extensive traveling, as all the currently suspected risk factors taken together can only explain a small proportion of the geographic differences in and increasing prevalence of allergy. The future search for significant environmental factors should be interdisciplinary and be directed toward areas that have not yet been explored, thus giving "lifestyle" a broader interpretation
SN - 0105-4538
AD - Department of Health and Environment, University Hospital, Linkoping, Sweden
UR - 10422743
ER -

TY - JOUR
ID - 1785
T1 - [Prescription and use of antibiotics in ambulatory care. Drug Agency]. [Review] [14 refs] [French]
Y1 - 1999/04//
N1 - Revue de pneumologie clinique
syr, 8406312
IM
Comparative Study. English Abstract. Journal Article. Review
French
KW - MEDLINE
KW - Adult
KW - Age Factors
KW - Anti-Bacterial Agents/tu [Therapeutic Use]
KW - Child
KW - Data Collection
KW - Drug Prescriptions
KW - France
KW - Germany
KW - Great Britain
KW - Humans
KW - Outpatients
KW - Respiratory Tract Infections/dt [Drug Therapy]
KW - Virus Diseases/dt [Drug Therapy]
RP - NOT IN FILE
SP - 65
EP - 74
JF - Revue de Pneumologie Clinique

JA - Rev Pneumol Clin
VL - 55
IS - 2
CY - FRANCE
N2 - OBJECTIVE: This study was conducted to describe changes in prescription practices outside the hospital, to evaluate the adaptation of such prescriptions to current scientific knowledge, and to compare medical practices in France with those in other European countries. METHODS: Data were collected from several sources: analysis of the literature, surveys conducted in the Loiret department and in the Rhone-Alps region, ten-year healt surveys (INSEE), data from the Sentinel network, sales statements from pharmaceutical firms, the Permanent Survey of Medical Prescription (EPPM) of the Medical Information and Statistics (IMS) firm. Comparisons between France, the United Kingdom and Germany were conducted by the French Medicine Agency's Pharmaco-economic Studies and Information Department using data furnished by the IMS firm and by pharmaceutical firms. RESULTS: In France, antibiotic sales increased by a mean annual rate of 2.1%, expressed in antibiotic units, and 2.6%, expressed in turnover (manufacturer price) between 1991 and 1996. The majority of these antibiotics were prescribed for respiratory and ENT infections with a presumed viral etiology such as rhino-pharyngitis and acute bronchitis. The results of the different surveys were in agreement showing that antibiotic prescriptions are made in approximately 40% of all consultations for rhino-pharyngitis and in 80% of those for acute bronchitis. Antibiotics were prescribed in more than 90% of cases of pharyngitis whatever the age of the patient. The situation was different for acute middle ear infections as the number of consultations has remained relatively unchanged over the last 10 years while antibiotic prescriptions have strongly increased, reaching 80% of the consultations. The number of consultations for pharyngitis and acute rhino-pharyngitis appears to be greater in France than in the United Kingdom and in Germany. Likewise, the proportion of patients using antibiotics after consulting for presumed viral conditions would be higher in France with different antibiotic classes being used. CONCLUSIONS: There is a gap between official guidelines (product description documents, therapeutic information documents, good practice guidelines, consensus conferences) and the state of current practices. Excessive and poorly-adapted antibiotic prescription favors the disturbing phenomenon of resistance which is all the more alarming because the emergence of resistant strains is difficult to predict and concerns bacteria causing the most common infections. To improve medical practices and achieve a persistent reduction in the use of antibiotics for viral infections, validated recommendations should be distributed to physicians. An effort should be made to prescribe the most appropriate active substance at optimal dose and treatment duration to limit the development of bacterial resistance. In addition, patients and the general public should be informed of the absence of any beneficial effect and the individual and collective risks involved in using antibiotics for viral infections in order to help them better understand and comply to their physician's prescription. [References: 14]
SN - 0761-8417
UR - 10418049
ER -

TY - JOUR
ID - 1786
T1 - Solar ultraviolet radiation exposure of infants and small children
A1 - Moise,A.F.
A1 - Harrison,S.L.
A1 - Gies,H.P.
Y1 - 1999/06//
N1 - Moise, A F. Harrison, S L. Gies, H P
Photodermatology, photoimmunology & photomedicine
awp, 9013641
IM
Journal Article. Research Support, Non-U.S. Gov't
English
KW - MEDLINE
KW - Child
KW - Preschool
KW - Cohort Studies
KW - Film Dosimetry
KW - Humans

KW - Infant
KW - Neoplasms
KW - Radiation-Induced/et [Etiology]
KW - Queensland
KW - Radiation Dosage
KW - Skin/re [Radiation Effects]
KW - Skin Neoplasms/et [Etiology]
KW - Sunlight/ae [Adverse Effects]
KW - Ultraviolet Rays
RP - NOT IN FILE
SP - 109
EP - 114
JF - Photodermatology, Photoimmunology & Photomedicine
JA - Photodermatol.Photoimmunol.Photomed.
VL - 15
IS - 3-4
CY - DENMARK
N2 - Townsville, in Queensland, Australia, experiences very high levels of ambient solar ultraviolet radiation (UVR) throughout the year and has a predominantly white population which is prone to developing skin cancer. The UVR exposure of 1-year-old and 2 1/2-year-old children raised in Townsville was measured using UVR-sensitive polysulphone film badges. In two separate exposure studies undertaken for 7 days in October 1995 and 5 days in April 1997, exposure at the chest and shoulder for each subject was determined. The chest exposures for the 1-year-olds were significantly higher at weekends than on weekdays, whereas for the 2 1/2-year-old children the shoulder exposures were significantly higher at weekends than on weekdays. The median daily total exposure for 1-year-old infants was 0.4 SED (standard erythemal dose) for the chest and 0.4 SED for the shoulder. The median daily total exposure for 2 1/2-year-olds was 0.6 SED for the chest and 0.9 SED for the shoulder. Although the median daily total exposures were comparatively low, the maximum values for the chest and shoulder were 6.5 SED and 2.4 SED, respectively, for the 1-year-old infants, and 20.6 SED and 8.4 SED, respectively, for the 2 1/2-year-olds. While the 2 1/2-year-old children spent most of their time outside between 9 am and 4 pm, the 1-year-old infants spent more time outside before 9 am and after 4 pm. Exposure increases with age in early childhood. Increased mobility and a greater tendency to play outdoors is likely to account for the higher exposure levels in 2 1/2-year-old children, compared to 1-year-old infants
SN - 0905-4383
AD - School of Public Health and Tropical Medicine, James Cook University, Townsville, Queensland, Australia
UR - 10404720
ER -

TY - JOUR
ID - 1787
T1 - Impact of a single exercise bout on energy expenditure and spontaneous physical activity of obese boys
A1 - Kriemler,S.
A1 - Hebestreit,H.
A1 - Mikami,S.
A1 - Bar-Or,T.
A1 - Ayub,B.V.
A1 - Bar-Or,O.
Y1 - 1999/07//
N1 - Kriemler, S. Hebestreit, H. Mikami, S. Bar-Or, T. Ayub, B V. Bar-Or, O
Pediatric research
owl, 0100714
IM
Journal Article. Research Support, Non-U.S. Gov't
English
KW - MEDLINE
KW - Adolescent
KW - Analysis of Variance

KW - Basal Metabolism
KW - Child
KW - Energy Metabolism
KW - Exercise/ph [Physiology]
KW - Exercise Test
KW - Heart Rate
KW - Humans
KW - Male
KW - Obesity/pp [Physiopathology]
KW - Oxygen Consumption
KW - Physical Exertion/ph [Physiology]
RP - NOT IN FILE
SP - 40
EP - 44
JF - Pediatric Research
JA - Pediatr Res
VL - 46
IS - 1
CY - UNITED STATES
N2 - The main objective of the present study was to determine whether a structured, laboratory-based exercise task would modify the energy expenditure (EE) and the pattern of spontaneous physical activity (PA) of obese boys on the day of an exercise laboratory visit and on the following day. Fourteen 10- to 15-y-old moderately obese (36.6+/-3.3% fat) boys volunteered. They each had three laboratory visits, I wk apart. In one visit, they performed a strenuous 50-min cycling task; in another, a 30-min medium-intensity cycling task; and in another (which served as placebo), they did not exercise. PA was monitored the day before (d 1), during (d 2), and after (d 3) each laboratory visit by use of a heart rate monitor and a 12-h recall interview. EE was calculated from minute-by-minute heart rate and each child's predetermined relationship between oxygen uptake and heart rate. EE and PA were analyzed from 1300 to 1900 h each day using 15-min intervals. EE tended to decrease ($p < 0.087$) in the afternoon of all d 2 compared with d 1, and it increased on d 3 after the medium-intensity exercise ($p < 0.0005$). EE during d 2 and 3 combined, compared with d 1, decreased after the high-intensity exercise (534.2 versus 564.3 kJ/h, $p < 0.05$). It increased after the medium-intensity exercise (561.8 versus 526.7 kJ/h, $p = 0.052$) and was not affected after the placebo visit (589.4 versus 574.3 kJ/h). Time spent outdoors was consistently reduced on the day of laboratory visit compared with the day before and after the visit, regardless of the contents of intervention. In conclusion, a single laboratory visit is followed by a reduction in EE and PA on the day of intervention. However, its effect on EE the following day may be dose dependent: medium-intensity exercise induces an increase in EE, but high-intensity exercise causes a decrease in EE. One implication is that intervention by physical training should employ medium-intensity exercise to enhance the EE of obese boys
SN - 0031-3998
AD - Children's Exercise and Nutrition Centre, McMaster University, Hamilton, Canada
UR - 10400132
ER -

TY - JOUR
ID - 1788
T1 - Children's fractures: a population based study
A1 - Lyons,R.A.
A1 - Delahunty,A.M.
A1 - Kraus,D.
A1 - Heaven,M.
A1 - McCabe,M.
A1 - Allen,H.
A1 - Nash,P.
Y1 - 1999/06//
N1 - Lyons, R A. Delahunty, A M. Kraus, D. Heaven, M. McCabe, M. Allen, H. Nash, P
Injury prevention : journal of the International Society for Child and Adolescent Injury Prevention
crz, 9510056
IM

Comparative Study. Journal Article
English
KW - MEDLINE
KW - Adolescent
KW - Age Distribution
KW - Bone and Bones/in [Injuries]
KW - Child
KW - Preschool
KW - Female
KW - Fractures
KW - Bone/cl [Classification]
KW - Bone/ep [Epidemiology]
KW - Bone/et [Etiology]
KW - Bone/pc [Prevention & Control]
KW - Great Britain/ep [Epidemiology]
KW - Humans
KW - Incidence
KW - Male
KW - Population Surveillance
KW - Registries
KW - Risk Factors
KW - Sex Distribution
KW - Wales/ep [Epidemiology]
RP - NOT IN FILE
SP - 129
EP - 132
JF - Injury Prevention
JA - Inj Prev
VL - 5
IS - 2
CY - ENGLAND
N2 - OBJECTIVE: To measure the incidence of childhood fractures in a defined population. SETTING: Accident and emergency (A&E) departments covering the Swansea and Neath Port Talbot areas of South Wales in 1996. METHODS: Linkage of data from A&E departments with population data to produce fracture incidence rates by anatomical site and cause in children aged 0-14 years. RESULTS: During 1996, 2463 new fractures occurred in 2399 residents yielding a fracture rate of 36.1/1000 children. Fractures were more common in boys than girls and increased with age in both groups. Sports and leisure activities accounted for 36% of fractures, assaults for 3.5%, and road traffic accidents 1.4%. Fractures of the radius/ulna were most frequent (36%). CONCLUSIONS: The fracture rate in South Wales children is twice the rate reported in previous studies. Further research is required to elucidate the reasons behind this high rate. Many fractures could be prevented by the use of safer surfaces in school playgrounds, and wrist protection in in-line skaters and possibly in soccer players
SN - 1353-8047
AD - Welsh Combined Centres for Public Health, University of Wales College of Medicine. ronan.lyons@morgannwg-ha.wales.nhs.uk
UR - 10385833
ER -

TY - JOUR
ID - 1789
T1 - An intervention to reduce playground equipment hazards
A1 - Roseveare,C.A.
A1 - Brown,J.M.
A1 - Barclay McIntosh,J.M.
A1 - Chalmers,D.J.
Y1 - 1999/06//

N1 - Roseveare, C A. Brown, J M. Barclay McIntosh, J M. Chalmers, D J
Injury prevention : journal of the International Society for Child and Adolescent Injury Prevention
crz, 9510056
IM
Clinical Trial. Comparative Study. Journal Article. Randomized Controlled Trial
English
KW - MEDLINE
KW - Accident Prevention
KW - Child
KW - Preschool
KW - Consumer Product Safety
KW - Data Collection
KW - Equipment Design
KW - Female
KW - Health Promotion/og [Organization & Administration]
KW - Humans
KW - Male
KW - New Zealand
KW - Play and Playthings/in [Injuries]
KW - Primary Prevention/og [Organization & Administration]
KW - Program Evaluation
KW - Public Health/st [Standards]
KW - Random Allocation
KW - School Health Services
KW - Schools/st [Standards]
KW - Wounds and Injuries/pc [Prevention & Control]
RP - NOT IN FILE
SP - 124
EP - 128
JF - Injury Prevention
JA - Inj Prev
VL - 5
IS - 2
CY - ENGLAND
N2 - OBJECTIVES: A community intervention trial was carried out to evaluate the relative effectiveness of two methods of reducing playground hazards in schools. The study hypotheses were: (1) a health promotion programme addressing barriers to implementing the New Zealand Playground Safety Standard will reduce playground hazards and (2) the intervention programme will be more successful than providing information alone. METHODS: Twenty four schools in Wellington, New Zealand were randomly allocated into two groups of 12 and their playgrounds audited for hazards. After the audit, the intervention group received a health promotion programme consisting of information about the hazards, an engineer's report, regular contact and encouragement to act on the report, and assistance in obtaining funding. The control group only received information about hazards in their playground. RESULTS: After 19 months, there was a significant fall in hazards in the intervention schools compared with the control schools (Mann-Whitney U test, p = 0.027). No intervention schools had increased hazards and eight out of 12 had reduced them by at least three. In contrast, only two of the control schools had reduced their hazards by this amount, with three others increasing their hazards in that time. CONCLUSIONS: It is concluded that working intensively with schools to overcome barriers to upgrading playground equipment can lead to a reduction in hazards, and that this form of intensive intervention is more effective than providing information alone
SN - 1353-8047
AD - Public Health Service, Hutt Valley Health, Wellington, New Zealand. Christine.roseveare@hvh.co.nz
UR - 10385832
ER -

TY - JOUR
ID - 1790
T1 - Playground safety--United States, 1998-1999

A1 - Centers for Disease Control and Prevention (CDC)
Y1 - 1999/04/30/
N1 - Centers for Disease Control and Prevention (CDC)
MMWR. Morbidity and mortality weekly report
ne8, 7802429
IM
Journal Article
English
KW - MEDLINE
KW - Child
KW - Preschool
KW - Humans
KW - Infant
KW - Recreation
KW - Safety
KW - United States
RP - NOT IN FILE
SP - 329
EP - 332
JF - MMWR - Morbidity & Mortality Weekly Report
JA - MMWR Morb Mortal Wkly Rep
VL - 48
IS - 16
CY - UNITED STATES
N2 - Each year approximately 211,000 U.S. children receive emergency department care for injuries sustained on playground equipment, making the use of this equipment the leading cause of injuries to children in school and child care environments. In response to the problem, the National Program for Playground Safety (NPPS) at the University of Northern Iowa (UNI) developed a national action plan that focuses on four areas of playground injury prevention: supervision, age-appropriateness of equipment, suitable fall surfaces, and equipment maintenance. During 1998-1999, NPPS surveyed a sample of the nation's child care, elementary school, and park playgrounds. This report summarizes the survey results, which indicate that playground injuries could be reduced by measures such as resilient surfacing below equipment, better equipment maintenance, improved supervision, and use of age-appropriate equipment
SN - 0149-2195
UR - 10366141
ER -

TY - JOUR
ID - 1791
T1 - Epidemiology and prevention of traffic injuries to urban children and adolescents
A1 - Durkin,M.S.
A1 - Laraque,D.
A1 - Lubman,I.
A1 - Barlow,B.
Y1 - 1999/06//
N1 - Durkin, M S. Laraque, D. Lubman, I. Barlow, B
Pediatrics
oxv, 0376422
IM
Journal Article. Research Support, Non-U.S. Gov't. Research Support, U.S. Gov't, Non-P.H.S.. Research Support, U.S. Gov't, P.H.S.
English
KW - MEDLINE
KW - Accident Prevention
KW - Accidents
KW - Traffic/cl [Classification]
KW - Traffic/mo [Mortality]

KW - Traffic/pc [Prevention & Control]
KW - Traffic/sn [Statistics & Numerical Data]
KW - Adolescent
KW - Age Distribution
KW - Bicycling/in [Injuries]
KW - Child
KW - Craniocerebral Trauma/cl [Classification]
KW - Craniocerebral Trauma/ep [Epidemiology]
KW - Female
KW - Health Promotion
KW - Humans
KW - Incidence
KW - Male
KW - New York City/ep [Epidemiology]
KW - Population Surveillance
KW - Program Evaluation
KW - Seasons
KW - Urban Population
RP - NOT IN FILE
SP - e74
JF - Pediatrics
VL - 103
IS - 6
CY - UNITED STATES
N2 - OBJECTIVES: To describe the incidence of severe traffic injuries before and after implementation of a comprehensive, hospital-initiated injury prevention program aimed at the prevention of traffic injuries to school-aged children in an urban community. MATERIALS AND METHODS: Hospital discharge and death certificate data on severe pediatric injuries (ie, injuries resulting in hospital admission and/or death to persons age <17 years) in northern Manhattan over a 13-year period (1983-1995) were linked to census counts to compute incidence. Rate ratios with 95% CIs, both unadjusted and adjusted for annual trends, were calculated to test for a change in injury incidence after implementation of the Harlem Hospital Injury Prevention Program. This program was initiated in the fall of 1988 and continued throughout the study period. It included 1) school and community based traffic safety education implemented in classroom settings in a simulated traffic environment, Safety City, and via theatrical performances in community settings; 2) construction of new playgrounds as well as improvement of existing playgrounds and parks to provide expanded off-street play areas for children; 3) bicycle safety clinics and helmet distribution; and 4) a range of supervised recreational and artistic activities for children in the community. PRIMARY RESULTS: Traffic injuries were a leading cause of severe childhood injury in this population, accounting for nearly 16% of the injuries, second only to falls (24%). During the preintervention period (1983-1988), severe traffic injuries occurred at a rate of 147.2/100 000 children <17 years per year. Slightly <2% of these injuries were fatal. Pedestrian injuries accounted for two thirds of all severe traffic injuries in the population. Among school-aged children, average annual rates (per 100 000) of severe injuries before the intervention were 127.2 for pedestrian, 37.4 for bicyclist, and 25.5 for motor vehicle occupant injuries. Peak incidence of pedestrian and bicyclist injuries occurred during the summer months and afternoon hours, whereas motor vehicle occupant injuries showed little seasonal variation and were more common during evening and night-time hours. Age-specific rates showed peak incidence of pedestrian injuries among 6- to 10-year-old children, of bicyclist injuries among 9- to 15-year-old children, and of motor vehicle occupant injuries among adolescents between the ages of 12 and 16 years. The peak age for all traffic injuries combined was 15 years, an age at which nearly 3 of every 1000 children each year in this population sustained a severe traffic injury. Among children hospitalized for traffic injuries during the preintervention period, 6.3% sustained major head trauma (including concussion with loss of consciousness for >/=1 hour, cerebral laceration and/or cerebral hemorrhage), and 36.9% sustained minor head trauma (skull fracture and/or concussion with no loss of consciousness >/=1 hour and no major head injury). The percentage of injured children with major and minor head trauma was higher among those injured in traffic than among those injured by all other means (43.2% vs 14.2%, respectively; chi2 = 336; degrees of freedom = 1). The percentages of children sustaining head trauma were 45.4% of those who were injured as pedestrians, 40.2% of those who were injured as bicyclists, and 38.9% of those who were injured as motor vehicle occupants. During the intervention period, the average incidence of traffic injuries among school aged children declined by 36% relative to the preintervention

period (rate ratio:.64; 95% CI:.58,.72). After adjusting for annual trends in incidence, pedestrian injuries declined during the intervention period among school aged children by 45% (adjusted rate ratio:.55; 95% CI:.38,.79). No comparable reduction occurred in nontargeted injuries among school-aged children (adjusted rate ratio:.89; 95% CI:.72, 1.09) or in traffic injuries among younger children who
SN - 1098-4275
AD - Gertrude H. Sergievsky Center, Faculty of Medicine, Columbia University,New York, NY 10032, USA. durkinm@sergievsky.cpmc.columbia.edu
UR - 10353971
ER -

TY - JOUR
ID - 1792
T1 - Communicating about youth's sun exposure risk to soccer coaches and parents: a pilot study in Georgia
A1 - Parrott,R.
A1 - Duggan,A.
A1 - Cremo,J.
A1 - Eckles,A.
A1 - Jones,K.
A1 - Steiner,C.
Y1 - 1999/06//
N1 - Parrott, R. Duggan, A. Cremo, J. Eckles, A. Jones, K. Steiner, C
Health education & behavior : the official publication of the Society for Public Health Education
cql, 9704962
IM
Journal Article. Research Support, Non-U.S. Gov't. Research Support, U.S. Gov't, P.H.S.
English
KW - MEDLINE
KW - Adolescent
KW - Adult
KW - Analysis of Variance
KW - Child
KW - Curriculum
KW - Female
KW - Georgia
KW - Health Education/mt [Methods]
KW - Health Knowledge
KW - Attitudes
KW - Practice
KW - Humans
KW - Male
KW - Middle Aged
KW - Parents
KW - Pilot Projects
KW - Self Efficacy
KW - Skin Neoplasms/pc [Prevention & Control]
KW - Soccer
KW - Sunburn/pc [Prevention & Control]
RP - NOT IN FILE
SP - 385
EP - 395
JF - Health Education & Behavior
JA - Health Educ Behav
VL - 26
IS - 3
CY - UNITED STATES
N2 - Efforts to increase the sun-protective behaviors of children were extended to outdoor recreational sports and youth soccer settings in this study. The pretest results of a pilot survey of coaches (n = 12), parents (n = 50),

and youths (n = 61) on eight soccer teams in south Georgia were used to guide the development of a health education program for coaches. In the pilot programs, half the coaches were trained to be involved in soccer-playing youths' sun protection by acting as positive role models and promoting sun protection to youths and their parents. The pilot demonstrated coaches' willingness to participate in sun protection promotion to youth: Youths indicated that coaches and parents were more likely to tell youths to wear sunscreen after the training than before, and coaches perceived getting youths to wear sunscreen to be less difficult than before
SN - 1090-1981
AD - University of Georgia, Athens 30602-1725, USA. rparrott@arches.uga.edu
UR - 10349575
ER -

TY - JOUR
ID - 1793
T1 - How physically active are American children and what can we do about it?. [Review] [20 refs]
A1 - Luepker,R.V.
Y1 - 1999/03//
N1 - Luepker, R V
International journal of obesity and related metabolic disorders : journal of the International Association for the Study of Obesity
btx, 9313169
IM
Journal Article. Review
English
KW - MEDLINE
KW - Adolescent
KW - Age Distribution
KW - Child
KW - Exercise
KW - Humans
KW - Obesity/ep [Epidemiology]
KW - Physical Fitness
KW - Sex Distribution
KW - United States/ep [Epidemiology]
RP - NOT IN FILE
SP - S12
EP - S17
JF - International Journal of Obesity & Related Metabolic Disorders: Journal of the International Association for the Study of Obesity
JA - Int J Obes Relat Metab Disord
VL - 23 Suppl 2
CY - ENGLAND
N2 - Any discussion of obesity in youth must consider physical activity level or energy output. Many suggest that increasing levels of obesity among children is the direct result of declining levels of physical activity. There is accumulating evidence that physical activity among youth has declined over the past several decades. Less time is spent on physical education classes in school and many popular activities among youth such as television and computer games, involve minimal physical exertion. These observations are confirmed by studies of physical fitness and endurance. These measures have also declined among youth. Much has been learned about the factors associated with physical activity among youth. These factors were used to develop several innovative school-based programs which demonstrate increased physical activity and performance. Recommendations are made for increasing physical activity both in the school curriculum and outside the school day. Potential health benefits and likely effects on obesity are substantial. [References: 20]
SN - 0307-0565
AD - Division of Epidemiology, School of Public Health, University of Minnesota, Minneapolis 55454-1015, USA
UR - 10340799
ER -

TY - JOUR
ID - 1794
T1 - The use of observational methods for monitoring sun-protection activities in schools
A1 - Milne,E.
A1 - Corti,B.
A1 - English,D.R.
A1 - Cross,D.
A1 - Costa,C.
A1 - Johnston,R.
Y1 - 1999/04//
N1 - Milne, E. Corti, B. English, D R. Cross, D. Costa, C. Johnston, R
Health education research
bqp, 8608459
T
Journal Article
English
KW - MEDLINE
KW - Child
KW - Data Collection/mt [Methods]
KW - Health Promotion/sn [Statistics & Numerical Data]
KW - Humans
KW - Longitudinal Studies
KW - Pilot Projects
KW - Program Evaluation/sn [Statistics & Numerical Data]
KW - Reproducibility of Results
KW - Research Design
KW - Sunburn/pc [Prevention & Control]
KW - Western Australia
RP - NOT IN FILE
SP - 167
EP - 175
JF - Health Education Research
JA - Health Educ Res
VL - 14
IS - 2
CY - ENGLAND
N2 - Evaluation of health promotion interventions aimed at behavioural or environmental change involves assessing change that occurs as a result of the program. Direct observational methods can be used for this purpose and this paper describes three such methods that we pilot tested for use in a 5-year intervention study aimed at reducing sun exposure in primary school children. (1) Monitoring 'No hat, no play' policies. This method involved video taping children in selected school play areas during lunch time and analysing the content of the videos to assess the proportion of children wearing various types of hats. (2) Assessing shade provision in the playground. This method involved taking aerial photographs of each school and using them to estimate the proportion of shade in play areas available to children at lunchtime. (3) Shade use. This involved children wearing polysulphone film badges to measure the amount of UV-B exposure they received during one lunch period, relative to the total possible dose registered on index badges. Each method was implemented successfully, and we demonstrated that the video and aerial photography methods produced highly reproducible results and that all three methods were feasible. These three methods will be used in our intervention study to assess longitudinal change in schools' sun-protection policy and practice
SN - 0268-1153
AD - Department of Public Health, University of Western Australia, Nedlands, Australia
UR - 10387497
ER -

TY - JOUR
ID - 1795
T1 - Initial field evaluation of the Harvard active ozone sampler for personal ozone monitoring

A1 - Geyh,A.S.
A1 - Roberts,P.T.
A1 - Lurmann,F.W.
A1 - Schoell,B.M.
A1 - Avol,E.L.
Y1 - 1999/03//
N1 - Geyh, A S. Roberts, P T. Lurmann, F W. Schoell, B M. Avol, E L
Journal of exposure analysis and environmental epidemiology
bjn, 9111438
IM
Journal Article. Research Support, Non-U.S. Gov't. Research Support, U.S. Gov't, Non-P.H.S.
English
KW - MEDLINE
KW - Air Pollutants/an [Analysis]
KW - California
KW - Child
KW - Environmental Monitoring/is [Instrumentation]
KW - Environmental Monitoring/mt [Methods]
KW - Equipment Design
KW - Humans
KW - Ozone/an [Analysis]
KW - Play and Playthings
RP - NOT IN FILE
SP - 143
EP - 149
JF - Journal of Exposure Analysis and Environmental Epidemiology
JA - J Expo Anal Environ Epidemiol
VL - 9
IS - 2
CY - UNITED STATES
N2 - Assessing personal exposure to ozone has only been feasible recently with the introduction of passive ozone samplers. These devices are easy to use, but changes in air velocity across their collection surfaces can affect performance. The Harvard active ozone sampler (AS) was developed in response to problems with the passive methods. This active sampler has been tested extensively as a microenvironmental sampler. To test for personal sampling, 40 children attending summer day-camp in Riverside, California wore the active ozone sampler for approximately 2.6 h on July 19 and 21, 1994, when ozone concentrations were about 100 ppb and 140 ppb, respectively. The children spent 94-100% of the sampling period outside, staying within a well-defined area while participating in normal camp activities. Ambient ozone concentrations across this area were monitored by two UV photometric ozone monitors. The active sampler was worn in a small backpack that was also equipped with a passive ozone sampler. Device precision, reported as the percent difference between duplicate pairs of samplers, was +/- 3.7% and +/- 4.2% for the active and passive samplers, respectively. The active sampler measured, on average, 94.5 +/- 8.2% of the ambient ozone while the passive samplers measured, on average, 124.5 +/- 18.8%. The samplers were worn successfully for the entire sampling period by all participating children
SN - 1053-4245
AD - Department of Environmental Health, Harvard University, School of Public Health, Boston, Massachusetts, USA
UR - 10321353
ER -

TY - JOUR
ID - 1796
T1 - Monkeybar injuries: complications of play
A1 - Waltzman,M.L.
A1 - Shannon,M.
A1 - Bowen,A.P.
A1 - Bailey,M.C.

Y1 - 1999/05//
N1 - Waltzman, M L. Shannon, M. Bowen, A P. Bailey, M C
Pediatrics
oxv, 0376422
IM
Journal Article
English
KW - MEDLINE
KW - Age Distribution
KW - Age Factors
KW - Athletic Injuries/ep [Epidemiology]
KW - Child
KW - Preschool
KW - Craniocerebral Trauma/ep [Epidemiology]
KW - Data Collection
KW - Equipment Design
KW - Female
KW - Fractures
KW - Bone/ep [Epidemiology]
KW - Hospitalization/sn [Statistics & Numerical Data]
KW - Humans
KW - Infant
KW - Male
KW - Massachusetts/ep [Epidemiology]
KW - Play and Playthings/in [Injuries]
KW - Retrospective Studies
RP - NOT IN FILE
SP - e58
JF - Pediatrics
VL - 103
IS - 5
CY - UNITED STATES
N2 - BACKGROUND: Playground equipment resulted in >200 000 injuries from 1990 to 1994, according to the Consumer Product Safety Commission; 88% were attributable to climbers (monkeybars/jungle gyms [MB/JGs]), swings, and slides. Equipment-specific injury requiring emergency department (ED) evaluation has not been reported previously. OBJECTIVE: To describe the spectrum of significant MB/JG-related injuries. METHODS: A 2-year retrospective chart review was performed using the computerized charting system at a large urban Children's Hospital/Regional Pediatric Trauma Center with 50 000 ED visits per year. A telephone survey also was conducted after the chart review to obtain additional information concerning the injury location, the surface type below the equipment, and the presence of adult supervision. RESULTS: A total of 204 patients were identified. Mean age was 6.2 years (range, 20 months to 12 years); 114 (56%) were male. A seasonal variation was noted with June to August accounting for 43% of visits. Injuries included fractures in 124 (61%), contusions in 20 (10%), neck and back strains in 17 (8%), lacerations in 16 (8%), closed head injuries in 10 (5%), abdominal trauma in 5 (3%), genitourinary injuries in 5 (3%), and miscellaneous injuries in the remainder. Among fractures, 90% were fractures of the upper extremity; 48 (40%) were supracondylar fractures. One child sustained a C7 compression fracture. Abdominal injuries included 1 child who sustained a splenic laceration. All genitourinary injuries (2 vaginal hematomas, 1 vaginal contusion, 1 penile laceration, and 1 urethral injury) were from straddle-type injuries. Fifty-one (25%) patients were admitted to the hospital. Of these, 47 (92%) required an operative procedure (orthopedic reduction or vaginal examination under anesthesia). Analysis of the telephone data revealed that the surface did not influence the injury type. Of the 79 fractures, 30 occurred on "soft surfaces." Injury type was associated significantly with chronologic age. Younger children (1 to 4 years of age) sustained more long-bone fractures than did older children. The presence of adult (at least 18 years of age) supervision, did not influence the occurrence of fractures. CONCLUSIONS: These data suggest that 1) a significant proportion (25%) of MB/JG-related injuries that are evaluated in the ED require hospitalization; 2) most of the injuries resulting in admission will require operative intervention (92%); 3) the surface below the equipment has no influence on the type or severity of the injury; 4) younger children are more likely to sustain

long-bone fractures than are older children; and 5) adult supervision does not influence the injury pattern. These data identify the need for additional investigation of means of making MB/JGs safer for child use
SN - 1098-4275
AD - Children's Hospital, Boston, Division of Emergency Medicine, Harvard Medical School, Boston MA 02115, USA. waltzmanvm@a1.tch.harvard.edu
UR - 10224202
ER -

TY - JOUR
ID - 1797
T1 - Trampolines at home, school, and recreational centers. American Academy of Pediatrics. Committee on Injury and Poison Prevention and Committee on Sports Medicine and Fitness
Y1 - 1999/05//
N1 - Pediatrics
oxv, 0376422
AIM, IM
Guideline. Journal Article. Practice Guideline
English
KW - MEDLINE
KW - Accidents
KW - Home
KW - Adolescent
KW - Adult
KW - Athletic Injuries/ep [Epidemiology]
KW - Athletic Injuries/pc [Prevention & Control]
KW - Child
KW - Preschool
KW - Consumer Product Safety
KW - Female
KW - Health Policy
KW - Humans
KW - Infant
KW - Male
KW - Middle Aged
KW - Physical Education and Training
KW - Recreation
KW - Schools
KW - Sports Equipment
KW - United States/ep [Epidemiology]
RP - NOT IN FILE
SP - 1053
EP - 1056
JF - Pediatrics
VL - 103
IS - 5 Pt 1
CY - UNITED STATES
N2 - The latest available data indicate that an estimated 83 400 trampoline-related injuries occurred in 1996 in the United States. This represents an annual rate 140% higher than was reported in 1990. Most injuries were sustained on home trampolines. In addition, 30% of trampoline-related injuries treated in an emergency department were fractures often resulting in hospitalization and surgery. These data support the American Academy of Pediatrics' reaffirmation of its recommendation that trampolines should never be used in the home environment, in routine physical education classes, or in outdoor playgrounds. Design and behavioral recommendations are made for the limited use of trampolines in supervised training programs
SN - 0031-4005
UR - 10224191
ER -

TY - JOUR
ID - 1798
T1 - Interrupter technique for evaluation of exercise-induced bronchospasm in children
A1 - Kannisto,S.
A1 - Vanninen,E.
A1 - Remes,K.
A1 - Korppi,M.
Y1 - 1999/03//
N1 - Kannisto, S. Vanninen, E. Remes, K. Korppi, M
Pediatric pulmonology
owh, 8510590
IM
Clinical Trial. Comparative Study. Journal Article. Research Support, Non-U.S. Gov't
English
KW - MEDLINE
KW - Adolescent
KW - Asthma
KW - Exercise-Induced/di [Diagnosis]
KW - Exercise-Induced/pp [Physiopathology]
KW - Bronchial Hyperreactivity/di [Diagnosis]
KW - Child
KW - Preschool
KW - Confidence Intervals
KW - Exercise Test/mt [Methods]
KW - Female
KW - Humans
KW - Male
KW - Reproducibility of Results
KW - Respiratory Function Tests
KW - Sensitivity and Specificity
KW - Software
RP - NOT IN FILE
SP - 203
EP - 207
JF - Pediatric Pulmonology
JA - Pediatr Pulmonol
VL - 27
IS - 3
CY - UNITED STATES
N2 - The free running test is a useful method for evaluation of exercise-induced bronchospasm in children. In young children this test simulates real-life circumstances and can be done more easily than histamine or methacholine challenges. The interrupter technique is a noninvasive method for measuring airflow resistance during tidal breathing. This approach requires minimal cooperation, and is therefore promising for use in young children. Fifty children aged 5-15 years with asthma symptoms were tested by exercise challenge consisting of free outdoor running for 8 min at 85% of maximal predicted heart rate for age. Pulmonary function was measured by using the interrupter technique (IR), with a Wright's peak flow meter (WPEF), and by flow-volume spirometry (FVS). The measurements were done before and 10 min after exercise. In addition, WPEF was measured at 5, 15, and 20 min after exercise. A fall of 15% or more in WPEF associated with wheezing or cough symptoms was considered a positive test. The exercise challenge was positive in 16 (32%) of the 50 children. Measurements at 10 min by WPEF identified 9 positive cases. At the same time point the IR identified 10 positive cases; a rise in resistance of 15% or more was considered positive, giving it 80% sensitivity and 93% specificity. The repeatability coefficient (CoR) for the interrupter technique was 0.06 kPa x L(-1) x s (13%) before and 0.07 kPa x L(-1) x s (14%) after exercise. The IR provides a useful alternative for estimation of airway obstruction in children following exercise challenge. The results were comparable with the current reference methods of forced expiratory volume in 1 s and peak flow measurements
SN - 8755-6863
AD - Department of Pediatrics, Kuopio University Hospital, Finland

UR - 10213260
ER -

TY - JOUR
ID - 1799
T1 - Social implications of childhood Crohn's disease
A1 - Moody,G.
A1 - Eaden,J.A.
A1 - Mayberry,J.F.
Y1 - 1999/04//
N1 - Moody, G. Eaden, J A. Mayberry, J F
Journal of pediatric gastroenterology and nutrition
jl6, 8211545
IM
Journal Article
English
KW - MEDLINE
KW - Child
KW - Crohn Disease/px [Psychology]
KW - Humans
KW - Interpersonal Relations
KW - Quality of Life
KW - Questionnaires
KW - Social Behavior
KW - Social Isolation
RP - NOT IN FILE
SP - S43
EP - S45
JF - Journal of Pediatric Gastroenterology & Nutrition
JA - J Pediatr Gastroenterol Nutr
VL - 28
IS - 4
CY - UNITED STATES
N2 - BACKGROUND: Children with Crohn's disease may have an impaired quality of life. METHODS: A validated questionnaire was mailed to the parents of all child members of Crohn's in Childhood Research Association (CICRA) (age range, 5-17 years). It investigated aspects of the quality of life of children with Crohn's disease. Sixty-four questionnaires were returned together with another 35 from those outside the defined age range. Patients in the latter group were not included in the analysis. RESULTS: Of the children investigated, 60% had significant absences from school, with a mean of 3+/-2.8 months' absence in the previous year. Most (53%) thought that their teachers knew nothing about Crohn's disease, and 50% thought that teachers were unsympathetic. Of the 24 who had taken examinations 80% thought they had underachieved because of ill health. Sixty-seven percent of children were unable to participate in sports on a regular basis, 60% felt unable to leave the house, and 50% were unable to play with their friends. Information about Crohn's disease had predominantly come from doctors (89%), information booklets (83%), and nursing staff (42%). The commonest questions asked by the children were: what is Crohn's disease (50%), why me (64%), will it affect my schooling (52%), and will it affect my chances of getting a job (67%). CONCLUSION: Children with Crohn's disease have a significantly impaired quality of life. They fear everyday childhood activities and fear for their future. These children need sympathetic management, and effort should be concentrated on improving their day-to-day existence to enable them to lead as normal a life as possible
SN - 0277-2116
AD - The Gastrointestinal Research Unit, Leicester General Hospital, United Kingdom
UR - 10204525
ER -

TY - JOUR
ID - 1800
T1 - A comparison of New York City playground hazards in high- and low-income areas

A1 - Suecoff,S.A.
A1 - Avner,J.R.
A1 - Chou,K.J.
A1 - Crain,E.F.
Y1 - 1999/04//
N1 - Suecoff, S A. Avner, J R. Chou, K J. Crain, E F
Archives of pediatrics & adolescent medicine
9422751, bwf
AIM, IM
Comparative Study. Journal Article
English
KW - MEDLINE
KW - Child
KW - Preschool
KW - Environment Design/st [Standards]
KW - Humans
KW - Income
KW - New York City
KW - Play and Playthings/in [Injuries]
RP - NOT IN FILE
SP - 363
EP - 366
JF - Archives of Pediatrics & Adolescent Medicine
JA - Arch Pediatr Adolesc Med
VL - 153
IS - 4
CY - UNITED STATES
N2 - OBJECTIVE: To compare playground hazards in high- and low-income neighborhoods. DESIGN: Forty-five playgrounds were randomly selected from the 9 New York City community districts that met our study criteria and were divided into high-and low-income groups based on comparison to the median of the median incomes ($24452 per year) of the 9 districts. Playgrounds are maintained by the City of New York Parks and Recreation Department and were assessed by one of us (S.A.S.) using a standardized on-site survey based on the US Consumer Product Safety Commission's guidelines for public playground safety. MAIN OUTCOME MEASURES: Total hazards per play area were subdivided into 3 categories: park design hazards, equipment maintenance hazards, and equipment hazards relating to fall injuries. A play area was defined as an individual set of equipment. RESULTS: Twenty-five (56%) of the parks were located in low-median-income districts and contained 98 (53%) of the total play areas. High- and low-income playgrounds did not differ significantly in the amount or type of equipment, mean fall injury hazards per play area, or mean park design hazards per play area. Low-income districts had a significantly higher mean total hazards per play area (6.1 vs. 4.2; P = .02) and mean equipment maintenance hazards per play area (2.1 vs. 1.0; P = .02). CONCLUSION: Significantly more hazards per play area were identified in the low-income group compared with the high-income group
SN - 1072-4710
AD - Department of Pediatrics, Albert Einstein College of Medicine, Bronx, NY, USA. suecoff@inch.com
UR - 10201718
ER -

TY - JOUR
ID - 1801
T1 - Conditions underlying parents' knowledge about children's daily lives in middle childhood: between- and within-family comparisons
A1 - Crouter,A.C.
A1 - Helms-Erikson,H.
A1 - Updegraff,K.
A1 - McHale,S.M.
Y1 - 1999/01//
N1 - Crouter, A C. Helms-Erikson, H. Updegraff, K. McHale, S M
Child development

0372725, d28
IM
Comparative Study. Journal Article. Research Support, U.S. Gov't, P.H.S.
English
KW - MEDLINE
KW - Adult
KW - Child
KW - Preschool
KW - Cognition
KW - Family/px [Psychology]
KW - Female
KW - Humans
KW - Life Change Events
KW - Male
KW - Parents
KW - Temperament
RP - NOT IN FILE
SP - 246
EP - 259
JF - Child Development
JA - Child Dev
VL - 70
IS - 1
CY - UNITED STATES
N2 - This study examined the correlates of mothers' and fathers' knowledge about the daily experiences of their firstborn (M = 10.9 years) and secondborn (M = 8.3 years) children in 198 nondivorced, predominantly dual-earner families. Results revealed between- and within-family differences in knowledge as a function of mothers' work involvement, sibship composition (i.e., sex, birth order), children's personal qualities (e.g., temperament), and parents' personal qualities (e.g., education, gender role attitudes). Mothers' knowledge did not vary as a function of how much they worked outside the home, but fathers knew more about their children's activities, whereabouts, and companions when their wives worked longer hours. Parents knew more about their younger than their older offspring. Both mothers and fathers knew more about offspring of the same sex than about opposite-sex children, leading to greater within-family differences in families with mixed-sex siblings. Perhaps because parental involvement and monitoring are more "scripted" for mothers than fathers, fathers' knowledge was more consistently related to their children's characteristics than was mothers.'
SN - 0009-3920
AD - Department of Human Development and Family Studies, Pennsylvania State University, University Park 16802, USA. AC1@psu.edu
UR - 10191526
ER -

TY - JOUR
ID - 1802
T1 - Child centered literacy orientation: a form of social capital?
A1 - High,P.
A1 - Hopmann,M.
A1 - LaGasse,L.
A1 - Sege,R.
A1 - Moran,J.
A1 - Guiterrez,C.
A1 - Becker,S.
Y1 - 1999/04//
N1 - High, P. Hopmann, M. LaGasse, L. Sege, R. Moran, J. Guiterrez, C. Becker, S
Pediatrics
oxv, 0376422
IM

Journal Article. Research Support, Non-U.S. Gov't
English
KW - MEDLINE
KW - Analysis of Variance
KW - Case-Control Studies
KW - Child Rearing/eh [Ethnology]
KW - Child Rearing
KW - Child
KW - Preschool
KW - Cross-Sectional Studies
KW - Female
KW - Humans
KW - Infant
KW - Logistic Models
KW - Male
KW - Parents
KW - Reading
KW - Socioeconomic Factors
KW - United States
RP - NOT IN FILE
SP - e55
JF - Pediatrics
VL - 103
IS - 4
CY - UNITED STATES
N2 - OBJECTIVE: To describe the home literacy environment and to identify financial, human, and social capital variables associated with the presence or lack of Child Centered Literacy Orientation (CCLO) in families with young children who regularly attend pediatric primary care clinics. DESIGN: Cross-sectional case-control analysis of structured parent interviews conducted in two hospital-based and four community-based pediatric clinics in New England. SUBJECTS: Parents of 199 healthy 1- to 5-year-old children whose mean age was 30 +/- 15 (SD) months were interviewed. Parents were primarily mothers (94%) with a mean age of 28 +/- 7 (SD) years 60% of whom were single. Educational levels of study parents varied: 43% had not graduated from high school, 29% had a high school equivalency, and 28% had at least a year of college or vocational training. This was a multiethnic parent group. Sixty-five percent were bilingual or non-English speaking. Fifty-eight percent were born outside of the continental United States. Parents were primarily of low-income status with 85% receiving Women, Infant, and Children (WIC) food supplements, Aid to Families With Dependent Children, and/or Medicaid. RESULTS: Half of the parents interviewed reported that they rarely read books. Sixty percent of children had fewer than 10 books at home and two-thirds of these households contained fewer than 50 books total. When asked open-ended questions, 28% of parents said that sharing books with their child was one of their three favorite activities together, 14% said that looking at books was one of their child's three favorite things to do, and 19% reported sharing books at bedtime at least six times each week. Thirty-nine percent of families had at least one of these three literacy-related responses present and so were said to have a CCLO. A backwards stepwise multiple logistic regression on CCLO was performed with family financial, human, and social capital variables. Parents married or living together (odds ratio [OR] 2.56, 95% confidence interval [CI] = 1.21-5.42), higher adult-to-child ratios in the home (OR 1.92, 95% CI = 1.20-3.05), households speaking only English (OR 2.67, 95% CI = 1.24-5.76), parents reading books themselves at least a few times a week (OR 2.86, 95% CI = 1.38-5.91), and homes with more than 10 children's books (OR 3.3, 95% CI = 1.6-6.83), were all independently and significantly associated with the presence of CCLO. Older child age and higher parent education remain in the model but were not significant at the P <.05 level. Ethnicity and income status were dropped for lack of additional significance from this model, which described 24% of the variance in CCLO. CONCLUSION: Although two-parent families and higher adult-to-child ratios in the home appear to be social capital variables with protective effects, low-income, single-parent, and minority or immigrant families are at significant risk for lacking both children's books and a CCLO. We suggest that CCLO may itself be another form of social capital reflecting parental goals and expectations for their children. We speculate that interventions which provide children's books and information about reading with children to impoverished families with young children may facilitate more parent-child book sharing. Pediatricians and other primary care

providers serving underserved populations may have a unique opportunity to encourage activities focusing on young children and promoting literacy
SN - 1098-4275
AD - Women & Infants' Hospital, Providence, Rhode Island 02905, USA
UR - 10103347
ER -

TY - JOUR
ID - 1803
T1 - Influence factors on the development of obesity in 3-year-old children based on the Toyama study
A1 - Takahashi,E.
A1 - Yoshida,K.
A1 - Sugimori,H.
A1 - Miyakawa,M.
A1 - Izuno,T.
A1 - Yamagami,T.
A1 - Kagamimori,S.
Y1 - 1999/03//
N1 - Takahashi, E. Yoshida, K. Sugimori, H. Miyakawa, M. Izuno, T. Yamagami, T. Kagamimori, S
Preventive medicine
pm4, 0322116
IM
Journal Article
English
KW - MEDLINE
KW - Body Mass Index
KW - Case-Control Studies
KW - Child Welfare
KW - Child
KW - Preschool
KW - Exercise
KW - Female
KW - Food Habits
KW - Health Knowledge
KW - Attitudes
KW - Practice
KW - Humans
KW - Life Style
KW - Logistic Models
KW - Male
KW - Multivariate Analysis
KW - Obesity/et [Etiology]
KW - Occupations
KW - Parents/ed [Education]
KW - Parents/px [Psychology]
KW - Questionnaires
KW - Risk Factors
KW - Socioeconomic Factors
RP - NOT IN FILE
SP - 293
EP - 296
JF - Preventive Medicine
JA - Prev Med
VL - 28
IS - 3
CY - UNITED STATES

N2 - BACKGROUND: Obesity in childhood increases the risk of obesity in adulthood. Obesity in adulthood is a risk factor for chronic diseases such as hypertension and atherosclerosis. Therefore, it is important to eliminate factors influencing the development of obesity in children from the viewpoint of preventive medicine. The purpose of this study is to elucidate the relationship between obesity and lifestyle in 3-year-old children in a case-control study. METHODS: Subjects were selected from the Toyama study at health checkups for 3-year-old children which are done routinely by local governments in Japan on all children of that age. A special questionnaire consisting of items on lifestyle and environmental factors of the children and past histories of parents was distributed to the Toyama study participants beforehand and was collected at the checkup. Matched-pair comparisons were performed between obese children whose body mass index was 18 or more (N = 427) and control children (N = 854) matched by sex and birth month. Multivariate stepwise conditional logistic regression analysis was also applied to assess the influence of confounding factors. RESULTS: The following six factors significantly influenced the development of obese 3-year-old children using the Mantel-Haenszel method: the mother's job, limited playtime outdoors (1 hour or less), snacking irregularity, an overweight father (body mass index >/= 24), an overweight mother (body mass index >/= 24), and overweight at birth (birth weight >/= 3,500 g). An overweight mother or father, limited playtime outdoors, overweight at birth, and snacking irregularity were significantly related to obesity in 3-year-old children after adjusting for confounding factors by multivariate stepwise conditional logistic regression analysis. CONCLUSIONS: We have suggested several factors influencing the development of obesity in 3-year-old Japanese children: parental overweight and overweight at birth as host factors, physical inactivity and snacking irregularity as behavioral factors, the mother's job as an environmental factor. Copyright 1999 American Health Foundation and Academic Press
SN - 0091-7435
AD - Department of Preventive Medicine, St. Marianna University School of Medicine, Kawasaki, Japan. etaka@anet.ne.jp
UR - 10072748
ER -

TY - JOUR
ID - 1804
T1 - Community-wide outbreak of enteroviral illness caused by echovirus 30: a cross-sectional survey and a case-control study
A1 - Reintjes,R.
A1 - Pohle,M.
A1 - Vieth,U.
A1 - Lyytikainen,O.
A1 - Timm,H.
A1 - Schreier,E.
A1 - Petersen,L.
Y1 - 1999/02//
N1 - Reintjes, R. Pohle, M. Vieth, U. Lyytikainen, O. Timm, H. Schreier, E. Petersen, L
The Pediatric infectious disease journal
oxj, 8701858
IM
Journal Article. Research Support, Non-U.S. Gov't
English
KW - MEDLINE
KW - Adolescent
KW - Case-Control Studies
KW - Child
KW - Preschool
KW - Cross-Sectional Studies
KW - Disease Outbreaks
KW - Echovirus Infections/ep [Epidemiology]
KW - Echovirus Infections/vi [Virology]
KW - Enterovirus B
KW - Human/ip [Isolation & Purification]
KW - Germany/ep [Epidemiology]
KW - Humans

KW - Infant
KW - Newborn
KW - Meningitis
KW - Aseptic/ep [Epidemiology]
KW - Aseptic/vi [Virology]
KW - Risk Factors
RP - NOT IN FILE
SP - 104
EP - 108
JF - Pediatric Infectious Disease Journal
JA - Pediatr Infect Dis J
VL - 18
IS - 2
CY - UNITED STATES
N2 - BACKGROUND: In June, 1997, 21 children from a single community in Germany were hospitalized with aseptic meningitis. An epidemiologic investigation was conducted to determine the extent of the outbreak and risk factors for illness. METHOD: The extent of the outbreak was assessed with a cross-sectional survey of every 10th child listed in the town register among the 2240 town children < 16 years old. A case-control study determined risk factors for illness. Sixty-two cases were identified through the cross-sectional survey from hospitalized persons and from persons seen by local physicians. Controls were 114 asymptomatic persons identified from the cross-sectional survey. RESULTS: The overall attack rate was 16%, with the highest attack rates (24%) among the 6- to 8-year olds. Onsets occurred during a 37-day period. Among the 2240 town children <16 years of age, an estimated 353 met the case definition for enteroviral illness, 168 visited a doctor and 21 were hospitalized. Data from the case-control study indicated that contact with an ill household member [odds ratio (OR) = 6.3; 95% confidence interval (CI) 2.6 to 15.5], day-care attendance (OR = 2.6; 95% CI 1.1 to 6.2) and playground use, either two to three times per week (OR = 3.7; 95% CI 1.3 to 10.2) or daily (OR = 4.3; 95% CI 1.6 to 11.3), were risk factors for illness. CONCLUSION: Echovirus 30 caused substantial morbidity during this community outbreak caused by person-to-person spread. Household contacts, day-care centers and playgrounds were prominent risk factors for transmission
SN - 0891-3668
AD - European Programme for Intervention Epidemiology Training, National Institute for Public Health and the Environment, Bilthoven, The Netherlands. Ralf.Reintjes@rivm.nl
UR - 10048680
ER -

TY - JOUR
ID - 1805
T1 - Hyperfractionated craniospinal radiation therapy for primitive neuroectodermal tumors: results of a Phase II study
A1 - Prados,M.D.
A1 - Edwards,M.S.
A1 - Chang,S.M.
A1 - Russo,C.
A1 - Davis,R.
A1 - Rabbitt,J.
A1 - Page,M.
A1 - Lamborn,K.
A1 - Wara,W.M.
Y1 - 1999/01/15/
N1 - Prados, M D. Edwards, M S. Chang, S M. Russo, C. Davis, R. Rabbitt, J. Page, M. Lamborn, K. Wara, W M
International journal of radiation oncology, biology, physics
g97, 7603616
IM
Clinical Trial. Clinical Trial, Phase II. Journal Article. Research Support, U.S. Gov't, P.H.S.
English
KW - MEDLINE

KW - Adolescent
KW - Adult
KW - Antineoplastic Combined Chemotherapy Protocols/tu [Therapeutic Use]
KW - Brain Neoplasms/dt [Drug Therapy]
KW - Brain Neoplasms/rt [Radiotherapy]
KW - Chemotherapy
KW - Adjuvant
KW - Child
KW - Preschool
KW - Cranial Irradiation/mt [Methods]
KW - Disease Progression
KW - Dose Fractionation
KW - Ependymoma/dt [Drug Therapy]
KW - Ependymoma/rt [Radiotherapy]
KW - Female
KW - Humans
KW - Male
KW - Medulloblastoma/dt [Drug Therapy]
KW - Medulloblastoma/rt [Radiotherapy]
KW - Middle Aged
KW - Neoplasm Staging
KW - Neuroectodermal Tumors
KW - Primitive/dt [Drug Therapy]
KW - Primitive/rt [Radiotherapy]
KW - Pineal Gland
KW - Pinealoma/dt [Drug Therapy]
KW - Pinealoma/rt [Radiotherapy]
KW - Recurrence
RP - NOT IN FILE
SP - 279
EP - 285
JF - International Journal of Radiation Oncology, Biology, Physics
JA - Int J Radiat Oncol Biol Phys
VL - 43
IS - 2
CY - UNITED STATES
N2 - PURPOSE: To report the results of a Phase II study of hyperfractionated craniospinal radiation therapy, with and without adjuvant chemotherapy for primitive neuroectodermal brain tumors (PNETs) and malignant ependymomas. METHODS AND MATERIALS: Newly diagnosed PNET or malignant ependymomas were treated with hyperfractionated craniospinal radiation therapy. The primary tumor site was treated to a dose of 72 Gy, with 30 Gy given to the rest of the craniospinal axis. The fraction size was 1.0 Gy, given twice a day. Patients with poor risk factors also received adjuvant chemotherapy with CCNU, cisplatin, and vincristine. Patients had follow-up for survival, time to tumor progression, and patterns of relapse. RESULTS: A total of 39 patients (21 males/18 females) were treated between March 12, 1990 and October 29, 1992. The median age was 16 years (range 3-59 years). Tumor types included 25 medulloblastomas, 5 pineoblastomas, 5 cerebral PNETs, 1 spinal cord PNET, and 3 malignant ependymomas. Twenty cases were staged as poor-risk and received adjuvant chemotherapy following radiation. Three-year progression-free survival (PFS) was 60% and 63% for poor-risk and good-risk patients, respectively. Overall 3-year survival for these groups was 70% and 79%, respectively. For the 25 patients with medulloblastoma, there were 16 good-risk and 9 poor-risk patients. Three-year PFSs were 63% and 56%, respectively. The 5-year survival for good-risk medulloblastoma was 69% with 43.7% of these patients having failures outside the primary site. CONCLUSIONS: Survival in patients with good-risk medulloblastoma was no better than that seen in previous studies with single-fraction radiation, and the rate of failure outside the primary site is excessive. Those with poor-risk features had comparable survival to that seen in patients with good risk factors, but these patients were treated with chemotherapy, and the role that hyperfractionated radiation played in their outcome is uncertain
SN - 0360-3016
AD - Department of Neurological Surgery, University of California, San Francisco 94117, USA

UR - 10030250
ER -

TY - JOUR
ID - 1806
T1 - Drink composition, voluntary drinking, and fluid balance in exercising, trained, heat-acclimatized boys
A1 - Rivera-Brown,A.M.
A1 - Gutierrez,R.
A1 - Gutierrez,J.C.
A1 - Frontera,W.R.
A1 - Bar-Or,O.
Y1 - 1999/01//
N1 - Rivera-Brown, A M. Gutierrez, R. Gutierrez, J C. Frontera, W R. Bar-Or, O
Journal of applied physiology (Bethesda, Md. : 1985)
heg, 8502536
IM
Clinical Trial. Controlled Clinical Trial. Journal Article. Research Support, Non-U.S. Gov't
English
KW - MEDLINE
KW - Acclimatization/ph [Physiology]
KW - Adolescent
KW - Body Temperature Regulation/ph [Physiology]
KW - Child
KW - Drinking/ph [Physiology]
KW - Exercise/ph [Physiology]
KW - Flavoring Agents
KW - Hot Temperature
KW - Humans
KW - Male
KW - Oxygen Consumption/ph [Physiology]
KW - Physical Fitness/ph [Physiology]
KW - Stress
KW - Physiological/pp [Physiopathology]
KW - Water-Electrolyte Balance/ph [Physiology]
RP - NOT IN FILE
SP - 78
EP - 84
JF - Journal of Applied Physiology
JA - J Appl Physiol
VL - 86
IS - 1
CY - UNITED STATES
N2 - This study examined the effects of beverage composition on the voluntary drinking pattern, body fluid balance, and thermoregulation of heat-acclimatized trained boys exercising intermittently in outdoor conditions (wet bulb globe temperature 30.4 +/- 1.0 degreesC). Twelve boys (age 13.4 +/- 0.4 yr) performed two 3-h sessions, each consisting of four 20-min cycling bouts at 60% maximal aerobic power alternating with 25-min rest. One of two beverages was assigned: unflavored water (W) or flavored water plus 6% carbohydrate and 18 mmol/l Na (CNa). Drinking was ad libitum. Total intake was higher (P < 0.05) during CNa (1,943 +/- 190 g) compared with W (1,470 +/- 143 g). Euhydration was maintained with CNa (+0.18% body wt), but a mild dehydration resulted with W (-0.94% body wt; P < 0.05). Sweat loss, much higher than previously published for children of similar age, was similar between conditions (CNa = 1,644.7 +/- 117.5; W = 1,750.2 +/- 152.7 g). The increase in rectal temperature (CNa = 0.86 +/- 0.3; W = 0.76 +/- 0.1 degreesC), heart rate, and all perceptual variables did not differ between conditions. In conclusion, a flavored carbohydrate-electrolyte drink prevents voluntary dehydration in trained heat-acclimatized boys exercising in a tropical climate despite their large sweat losses. Because hydration changes were minor, the thermoregulatory strain observed was similar between conditions
SN - 8750-7587

AD - Department of Physical Medicine, Rehabilitation and Sports Medicine, Center for Sports Health and Exercise Sciences at the Albergue Olimpico, University of Puerto Rico School of Medicine, Salinas, Puerto Rico 00751, USA.anriver@coqui.net
UR - 9887116
ER -

TY - JOUR
ID - 1807
T1 - Effect of natural grass pollen exposure on exhaled nitric oxide in asthmatic children
A1 - Baraldi,E.
A1 - Carra,S.
A1 - Dario,C.
A1 - Azzolin,N.
A1 - Ongaro,R.
A1 - Marcer,G.
A1 - Zacchello,F.
Y1 - 1999/01//
N1 - Baraldi, E. Carra, S. Dario, C. Azzolin, N. Ongaro, R. Marcer, G. Zacchello, F
American journal of respiratory and critical care medicine
9421642, bzs
AIM, IM
Journal Article
English
KW - MEDLINE
KW - Adolescent
KW - Asthma/pp [Physiopathology]
KW - Child
KW - Environmental Exposure
KW - Female
KW - Humans
KW - Hypersensitivity/pp [Physiopathology]
KW - Longitudinal Studies
KW - Lung/pp [Physiopathology]
KW - Male
KW - Nitric Oxide
KW - Poaceae/im [Immunology]
KW - Poaceae
KW - Pollen/im [Immunology]
KW - Pollen
KW - Reference Values
KW - Respiration
KW - Seasons
KW - Spirometry
RP - NOT IN FILE
SP - 262
EP - 266
JF - American Journal of Respiratory & Critical Care Medicine
JA - Am J Respir Crit Care Med
VL - 159
IS - 1
CY - UNITED STATES
N2 - Exhaled nitiric oxide (NO) is increased in exhaled breath of asthmatic patients. The aim of this study was to investigate the longitudinal changes of exhaled NO outside and during the pollen season in pollen-allergic asthmatic children. Twenty-one children (age 6 to 16 yr), with a seasonal allergic asthma sensitive to grass pollen, underwent measurements of exhaled NO and pulmonary function before (March), during (May), and after (November) the pollen season. Exhaled NO was measured by a tidal breathing method with a chemiluminescence analyzer and NO steady-state levels were recorded. The timing of the measurements during

the pollen season was based on the atmospheric pollen count. Exhaled NO values of asthmatic children were compared with those of 21 sex- and age-matched healthy children. Pulmonary function and symptoms of asthma were also evaluated at each visit. The mean value of exhaled NO before the grass season was 12.7 +/- 5.1 ppb (mean +/- SD), significantly higher when compared with controls (7.8 +/- 2.7 ppb, p < 0.001). In the pollen season there was a significant (p < 0.001) twofold increase in exhaled NO (21.4 +/- 7.6 ppb) that, after the season, returned to values similar (12.8 +/- 5.8 ppb, p = NS) to those found before the season. There were no significant changes in FEV1 before and during the season (98.6% predicted versus 101% predicted, p = NS). We conclude that natural allergen exposure is related to an increase of exhaled NO in asthmatic grass pollen-allergic children even in absence of significant changes in airways function. We speculate that measurement of exhaled NO could be a sensitive noninvasive marker of asthma disease activity
SN - 1073-449X
AD - University Department of Pediatrics, Department of Occupational Health, School of Medicine, Padova, Italy. eugi@child.pedi.unipd.it
UR - 9872848
ER -

TY - JOUR
ID - 1808
T1 - [Effects of environmental lead exposure in kindergartens on children's blood lead level]. [Chinese]
A1 - Gao,W.
A1 - Li,Z.
A1 - Wang,Z.
A1 - Wang,N.
A1 - Zhao,X.
A1 - Chen,Y.
Y1 - 1999/09//
N1 - Gao, W. Li, Z. Wang, Z. Wang, N. Zhao, X. Chen, Y
Zhonghua yu fang yi xue za zhi [Chinese journal of preventive medicine]
d74, 7904962
IM
English Abstract. Journal Article
Chinese
KW - MEDLINE
KW - Child Day Care Centers
KW - Child
KW - Preschool
KW - Environmental Exposure/ae [Adverse Effects]
KW - Environmental Exposure/an [Analysis]
KW - Environmental Pollution/an [Analysis]
KW - Female
KW - Humans
KW - Infant
KW - Lead/an [Analysis]
KW - Lead/bl [Blood]
KW - Male
RP - NOT IN FILE
SP - 272
EP - 274
JF - Chung-Hua Yu Fang i Hsueh Tsa Chih [Chinese Journal of Preventive Medicine]
JA - Chung Hua Yu Fang I Hsueh Tsa Chih
VL - 33
IS - 5
CY - China
N2 - OBJECTIVE: To determine if kindergartens' environmental status can influence children's lead exposure. METHODS: Environmental specimens, such as floor dust, peeled-off paint chip, soil and drinking water, as well as children's hand dust and blood samples, were collected and measured for their lead levels in 19 kindergartens, to analyze the relationship between children's blood lead levels and their environmental lead

exposure. RESULTS: Geometric means of lead concentrations in indoor floor dust, peeled-off paint chip, dust fallout, outdoor floor dust, soil and drinking water were 86.5 microg/m(2), 235.5 microg/g, 445.9 microg/g, 172.4 microg/m(2), 70.1 microg/g and 12.5 microg/L, respectively. Lead level on children's hands averaged 3.4 microg at both hands. Blood lead levels in children correlated positively with the lead concentrations of outdoor floor dust and their hand dust, with correlation coefficients of 0.5186 and 0.2206, respectively. Multiple regression analysis showed that hand dust lead level in children entered the regression model with a largest standardized partial regression coefficient of 0.3842 and a coefficient of determination of 0.673 for the full equation with F = 6.52 and P < 0.01. CONCLUSION: Status of environmental health in kindergartens plays an important role in children's lead exposure. It is necessary to offer health education for children and make them wash their hands often and overcome unhealthy behavior of sucking their fingers
SN - 0253-9624
AD - National Center for Maternal and Infant Health, Beijing Medical University, Beijing, 100083
UR - 11864489
ER -

TY - JOUR
ID - 1809
T1 - Girl domestic workers in Kenya
A1 - Mzungu,M.
Y1 - 1999/03//
N1 - Mzungu, M
Links : a newsletter on gender for Oxfam GB staff and partners
101572830
J
Journal Article
English
KW - MEDLINE
KW - Adolescent
KW - Africa
KW - Africa South of the Sahara
KW - Eastern
KW - Age Factors
KW - Child
KW - Crime
KW - Demography
KW - Developing Countries
KW - Domestic Violence
KW - Economics
KW - Employment
KW - Family Characteristics
KW - Family Relations
KW - Health Manpower
KW - Housekeeping
KW - Kenya
KW - Nuclear Family
KW - Population
KW - Population Characteristics
KW - Poverty
KW - Social Change
KW - Social Class
KW - Social Problems
KW - Socioeconomic Factors
RP - NOT IN FILE
SP - 6
JF - Links : A Newsletter on Gender for Oxfam GB Staff and Partners
JA - Links (Oxford)
CY - ENGLAND

UR - 12295034
ER -

TY - JOUR
ID - 1810
T1 - Dopa-responsive dystonia induced by a recessive GTP cyclohydrolase I mutation
A1 - Hwu,W.L.
A1 - Wang,P.J.
A1 - Hsiao,K.J.
A1 - Wang,T.R.
A1 - Chiou,Y.W.
A1 - Lee,Y.M.
Y1 - 1999/09//
N1 - Hwu, W L. Wang, P J. Hsiao, K J. Wang, T R. Chiou, Y W. Lee, Y M
Human genetics
ged, 7613873
IM
Case Reports. Journal Article. Research Support, Non-U.S. Gov't
English
KW - MEDLINE
KW - Amino Acid Substitution
KW - Cell Line
KW - Child
KW - DNA/ch [Chemistry]
KW - DNA/ge [Genetics]
KW - DNA Mutational Analysis
KW - DNA
KW - Recombinant/ge [Genetics]
KW - Dihydroxyphenylalanine/tu [Therapeutic Use]
KW - Dystonia/dt [Drug Therapy]
KW - Dystonia/en [Enzymology]
KW - Dystonia/ge [Genetics]
KW - Escherichia coli/ge [Genetics]
KW - Female
KW - GTP Cyclohydrolase/df [Deficiency]
KW - GTP Cyclohydrolase/ge [Genetics]
KW - GTP Cyclohydrolase/me [Metabolism]
KW - Gene Expression Regulation
KW - Enzymologic
KW - Genes
KW - Recessive
KW - HeLa Cells
KW - Homozygote
KW - Humans
KW - Mutation
KW - Plasmids
KW - Point Mutation
KW - RNA
KW - Messenger/bl [Blood]
KW - Messenger/ge [Genetics]
KW - Messenger/me [Metabolism]
RP - NOT IN FILE
SP - 226
EP - 230
JF - Human Genetics
JA - Hum Genet
VL - 105

IS - 3
CY - GERMANY
N2 - GTP cyclohydrolase I (GTPCH) catalyzes the rate-limiting step of tetrahydrobiopterin (BH4) biosynthesis. GTPCH has been associated with two clinically distinct human diseases: the recessive hyperphenylalaninemia (HPA) and the dominant dopa-responsive dystonia (DRD). We found a recessive GTPCH mutation (R249S, 747C-->G in a dystonia patient. Her PHA-stimulated mononuclear blood cells had a normal amount of GTPCH mRNA, but low GTPCH activity. Arginine 249 is located at the C-terminus of GTPCH, outside the catalytic site. E. coli expressed recombinant R249S mutant protein possessed normal enzyme activity and kinetics. However, in transfected eukaryotic cells, R249S mutant protein expression level was lower than the wild-type protein. Therefore, this is suspected to be a destabilizing mutation. Our data suggest that DRD could be either dominantly or recessively inherited, and the inheritance might be determined by the mechanism of mutation
SN - 0340-6717
AD - Department of Medical Genetics and Pediatrics, College of Medicine, National Taiwan University, Taipei, Republic of China
UR - 10987649
ER -

TY - JOUR
ID - 1811
T1 - Children in Japan and multimedia
A1 - Shimauchi,Y.
Y1 - 1999///
N1 - Shimauchi, Y
The Turkish journal of pediatrics
wkv, 0417505
IM
Journal Article
English
KW - MEDLINE
KW - Adult
KW - Attitude to Health
KW - Child
KW - Child Development
KW - Child Welfare
KW - Communication
KW - Education/td [Trends]
KW - Forecasting
KW - Health Knowledge
KW - Attitudes
KW - Practice
KW - Humans
KW - Information Systems/td [Trends]
KW - Japan
KW - Life Style
KW - Multimedia/td [Trends]
KW - Multimedia
KW - Parents/px [Psychology]
KW - Questionnaires
KW - Video Games/td [Trends]
KW - Video Games
RP - NOT IN FILE
SP - 7
EP - 12
JF - Turkish Journal of Pediatrics
JA - Turk J Pediatr
VL - 41 Suppl
CY - TURKEY

N2 - Many parents in Japan today are very concerned and have daily problems with their children over the following kind of issues concerning video games, multimedia, and similar devices. 1) Is children's enthusiasm for video games actually harming their growth (physically or mentally)? 2) Is there any way that video games can help children in the future? For example, will playing these games help children learn to use computers or develop their power of thought? 3) Will excessive enthusiasm for TV games undermine our basic lifestyle? Is the time they spend studying at home or playing outside actually decreasing? 4) Will the methods of communication brought about by computers, pagers, and cellular phones weaken human relations among children? 5) Will being immersed in a virtual world created by computers result in a diminished sense of reality? The causes of this uneasiness may be traced to parents' own anxieties and concerns about the exceedingly rapid growth of the "information society" of today, together with the fact that their children are directly caught up in the deluge of this information and multimedia society. The development of media has coincided with the growth of today's adults, but children are immersed in a media-filled environment from the day they are born. In Japan, there are now children who can play video games before even learning to write. No one can predict what kind of adults these children will grow to be in the future, and it is fair to say that the most parents and children in Japan are being tossed about on the waves of this information explosion. At this international symposium, we hope to hear the many views that are held around the world concerning children and multimedia
SN - 0041-4301
AD - Benesse Corporation, Japan
UR - 10770069
ER -

TY - JOUR
ID - 1812
T1 - Home and hospital treatment of acute seizures in children with nasal midazolam
A1 - Jeannet,P.Y.
A1 - Roulet,E.
A1 - Maeder-Ingvar,M.
A1 - Gehri,M.
A1 - Jutzi,A.
A1 - Deonna,T.
Y1 - 1999///
N1 - Jeannet, P Y. Roulet, E. Maeder-Ingvar, M. Gehri, M. Jutzi, A. Deonna, T
European journal of paediatric neurology : EJPN : official journal of the European Paediatric Neurology Society
9715169, dgs
IM
Journal Article
English
KW - MEDLINE
KW - Acute Disease
KW - Administration
KW - Rectal
KW - Adolescent
KW - Anticonvulsants/ad [Administration & Dosage]
KW - Anticonvulsants/ae [Adverse Effects]
KW - Child
KW - Preschool
KW - Diazepam/ad [Administration & Dosage]
KW - Diazepam/ae [Adverse Effects]
KW - Electroencephalography/de [Drug Effects]
KW - Epilepsy/dt [Drug Therapy]
KW - Epilepsy/et [Etiology]
KW - Female
KW - Home Nursing
KW - Hospitalization
KW - Humans
KW - Hypnotics and Sedatives/ad [Administration & Dosage]
KW - Hypnotics and Sedatives/ae [Adverse Effects]

KW - Infant
KW - Newborn
KW - Male
KW - Midazolam/ad [Administration & Dosage]
KW - Midazolam/ae [Adverse Effects]
KW - Treatment Outcome
RP - NOT IN FILE
SP - 73
EP - 77
JF - European Journal of Paediatric Neurology
JA - Europ.J Paediatr Neurol
VL - 3
IS - 2
CY - ENGLAND
N2 - Rectal diazepam is widely used in the treatment of acute seizures in children but has some disadvantages. Nasal/sublingual midazolam administration has been recently investigated for this purpose but never at home or in a general paediatric hospital. The aim of this open study was to determine the efficacy, the tolerance and the applicability of nasal midazolam during acute seizures in children both in hospital and at home. We included known epileptic children for treatment at home and all children with acute seizures in the hospital. In all, 26 children were enrolled, 11 at home and 17 in the hospital (including two treated in both locations); only one had simple febrile seizure. They had a total of 125 seizures; 122 seizures (98%) stopped within 10 minutes (average 3.6 minutes). Two patients in the hospital did not respond and in three, seizures recurred within 3 hours. None had serious adverse effects. Parents had no difficulties administering the drug at home. Most of those who were using rectal diazepam found that nasal midazolam was easier to use and that postictal recovery was faster. Among 15 children who received the drug under electroencephalogram monitoring (six without clinical seizures), the paroxysmal activity disappeared in ten and decreased in three. Nasal midazolam is efficient in the treatment of acute seizures. It appears to be safe and most useful outside the hospital in severe epilepsies, particularly in older children because it is easy for parents to use. These data should be confirmed in a larger sample of children. Its usefulness in febrile convulsions also remains to be evaluated. Copyright 1999 European Paediatric Neurology Society
SN - 1090-3798
AD - Department of Pediatrics, Neuropediatric Unit, CHUV, Lausanne, Switzerland
UR - 10700542
ER -

TY - JOUR
ID - 1813
T1 - Use of pocket-sized turbine spirometer in monitoring exercise-induced bronchospasm and bronchodilator responses in children
A1 - Kannisto,S.
A1 - Vanninen,E.
A1 - Remes,K.
A1 - Korppi,M.
Y1 - 1999/11//
N1 - Kannisto, S. Vanninen, E. Remes, K. Korppi, M
Pediatric allergy and immunology : official publication of the European Society of Pediatric Allergy and Immunology
bu6, 9106718
IM
Comparative Study. Journal Article. Research Support, Non-U.S. Gov't
English
KW - MEDLINE
KW - Adolescent
KW - Albuterol/du [Diagnostic Use]
KW - Bronchial Hyperreactivity/di [Diagnosis]
KW - Bronchial Hyperreactivity/et [Etiology]
KW - Bronchial Hyperreactivity/pp [Physiopathology]

KW - Bronchial Spasm/di [Diagnosis]
KW - Bronchial Spasm/et [Etiology]
KW - Bronchial Spasm/pp [Physiopathology]
KW - Bronchodilator Agents/du [Diagnostic Use]
KW - Bronchospirometry/is [Instrumentation]
KW - Child
KW - Preschool
KW - Exercise
KW - Female
KW - Forced Expiratory Volume
KW - Humans
KW - Male
RP - NOT IN FILE
SP - 266
EP - 271
JF - Pediatric Allergy & Immunology
JA - Pediatr Allergy Immunol
VL - 10
IS - 4
CY - DENMARK
N2 - For field studies of asthma, portable hand-held pulmonary function testing devices are required. Other than for peak flow measurements, little has been done to validate their use in children. Fifty children aged 5-15 years having asthma symptoms were examined using an exercise challenge (8 min free running outdoors) and a bronchodilation test (salbutamol inhalation at a dose of 0.15 mg/kg). Pulmonary function was measured with a turbine spirometer, with a Wright peak flow meter (WPEF) and with a flow-volume spirometer (FVS). A fall of 15% or more in peak expiratory flow associated with wheezing or cough was considered diagnostic for bronchial hyper-responsiveness to exercise (BHRE). A rise of 15% or more from baseline in peak expiratory flow after salbutamol inhalation was considered as a positive bronchodilator response (BDR). BHRE was present in 16 children (32%). Using the limit of a 15% or greater fall in FEV1, turbine spirometry identified 12 as BHRE-positive and no additional cases, giving a sensitivity of 75% and a specificity of 100%. The turbine spirometer showed lower FEV1 values than the FVS, the difference increasing with airway obstruction. BDR was positive in eight children (16%). Using the limit of a 10% or greater rise in FEV1, turbine spirometry was positive in six cases. FEV1 measured by turbine spirometry could not be used interchangeably with conventional FVS. However, the turbine spirometer offers the possibility to measure FEV1 repeatedly in field conditions, such as during exercise challenges outdoors
SN - 0905-6157
AD - Department of Paediatrics, Kuopio University Hospital, Finland
UR - 10678724
ER -

TY - JOUR
ID - 1814
T1 - The role of the school in children's out-of-school time
A1 - Dryfoos,J.G.
Y1 - 1999///
N1 - Dryfoos, J G
The Future of children / Center for the Future of Children, the David and Lucile Packard Foundation
9306342, b4m
IM
Journal Article
English
KW - MEDLINE
KW - Adolescent
KW - Child
KW - Child Advocacy
KW - Community-Institutional Relations/td [Trends]
KW - Community-Institutional Relations

KW - Forecasting
KW - Humans
KW - Leisure Activities
KW - Schools/st [Standards]
KW - United States
RP - NOT IN FILE
SP - 117
EP - 134
JF - Future of Children
JA - Future Child
VL - 9
IS - 2
CY - UNITED STATES
N2 - As the primary community institution in the lives of children, schools have much to contribute to plans for addressing the needs of today's youngsters during the time when classes are not being held. In recent years, demands have escalated for after-school child care, educational enrichment, and safe havens that also foster positive youth development. Many programs that respond to these needs are housed in school buildings. Some are operated by the schools, some by community-based organizations, and others by partnerships between schools and outside groups. New public funding for after-school programs often flows through the school system. This article reports the prevalence of school-based programs and discusses extracurricular activities, child care and extended-day programs, enrichment programs, and ambitious efforts to transform the schools into full-time community hubs offering something for residents of all ages. Implementation challenges accompany program expansion, and this article also examines major issues that arise in school-based programs: governance, space, program quality, funding, and accountability. If these programs fulfill their promise, the school of the future may, indeed, be open extended hours for the enrichment of the children and the sustenance of the family
SN - 1054-8289
UR - 10646263
ER -

TY - JOUR
ID - 1815
T1 - [Alcohol and drug consumption among students in Pachuca, Hidalgo]. [Spanish]
A1 - Rojas-Guiot,E.
A1 - Fleiz-Bautista,C.
A1 - Medina-Mora Icaza,M.E.
A1 - Moron,Mde L.
A1 - Domenech-Rodriguez,M.
Y1 - 1999/07//
N1 - Rojas-Guiot, E. Fleiz-Bautista, C. Medina-Mora Icaza, M E. Moron, M de los A. Domenech-Rodriguez, M
Salud publica de Mexico
0404371, uy1
IM
Comparative Study. English Abstract. Journal Article. Research Support, Non-U.S. Gov't
Spanish
KW - MEDLINE
KW - Adolescent
KW - Alcohol Drinking/ep [Epidemiology]
KW - Alcohol Drinking/px [Psychology]
KW - Child
KW - Female
KW - Humans
KW - Male
KW - Mexico/ep [Epidemiology]
KW - Prevalence
KW - Questionnaires
KW - Sex Distribution

KW - Socioeconomic Factors
KW - Students/px [Psychology]
KW - Students/sn [Statistics & Numerical Data]
KW - Substance-Related Disorders/ep [Epidemiology]
KW - Substance-Related Disorders/px [Psychology]
KW - Urban Population/sn [Statistics & Numerical Data]
RP - NOT IN FILE
SP - 297
EP - 308
JF - Salud Publica de Mexico
JA - Salud Publica Mex
VL - 41
IS - 4
CY - MEXICO
N2 - OBJECTIVE: To determine the prevalence of alcohol and drug consumption and its relationship to sociodemographic variables, leisure activities, antisocial behavior, family norms and conflicts, among others. MATERIAL AND METHODS: Data derive from a representative survey of 1,929 students of junior high and high school, conducted in 1996 in the city of Pachuca, Hidalgo, Mexico. Of these, 44.9% were boys and 52.5% were girls; mean age was 14. A self-applied questionnaire, prepared by the WHO together with some countries, among them Mexico, was completed by the studied subjects, and included indicators of alcohol and drug consumption. RESULTS: Of the total sample, 47.9% had tried alcohol, and 12.6% had drunk large quantities--5 drinks or more per sitting--during the month previous to the survey. Preferred drinks are beer and "coolers", which they buy at shops where no identification is required and drink at home or at friend's homes. With respect to drugs, 5.1% had tried illegal or medical drugs without prescription, in particular inhalants, marihuana and tranquilizers. More boys consumed illegal drugs, and more girls medical drugs without prescription. Boys, who are also older, more frequently consumed alcohol and drugs and were more often employed during the previous year at part-time jobs. High alcohol level and drug consumers were characterized by their frequent report of being bored in their free time, drinking with friends and enrolling in antisocial behavior. With respect to family norms, they follow them less and show less interest in doing so. An elevated percentage informed that their parents fight frequently, that they have sought help for this reason and have intended separation. CONCLUSIONS: Groups who drink more alcohol and use other drugs, in contrast with nonusers, presented more behavioral problems, more outdoors activities that included drinking with friends, more antisocial behavior, had a distant relationship with their families sharing few activities with them, an showed little interest in following family rules and perceiving conflicts within their families
SN - 0036-3634
AD - Division de Investigaciones Epidemiologicas y Sociales, Instituto Mexicano de Psiquiatria (IMP), Mexico, D.F. rojasgm@imp.edu.mx
UR - 10624141
ER -

TY - JOUR
ID - 1816
T1 - Reinforcing value of physical activity as a determinant of child activity level
A1 - Epstein,L.H.
A1 - Kilanowski,C.K.
A1 - Consalvi,A.R.
A1 - Paluch,R.A.
Y1 - 1999/11//
N1 - Epstein, L H. Kilanowski, C K. Consalvi, A R. Paluch, R A
Health psychology : official journal of the Division of Health Psychology, American Psychological Association
ejl, 8211523
IM
Journal Article. Research Support, U.S. Gov't, P.H.S.
English
KW - MEDLINE
KW - Child
KW - Child Welfare

KW - Exercise
KW - Female
KW - Humans
KW - Male
KW - Motor Activity
KW - Obesity/pc [Prevention & Control]
KW - Physical Fitness
KW - Reinforcement (Psychology)
KW - Time Factors
RP - NOT IN FILE
SP - 599
EP - 603
JF - Health Psychology
JA - Health Psychol
VL - 18
IS - 6
CY - UNITED STATES
N2 - This study examined whether the relative reinforcing value of physical activity (RRVPA) measured in the laboratory predicted physical activity measured in the natural environment in 32 6-11-year-old children. RRVPA correlated with physical activity level (r = .42, p < .05), and contributed to the prediction of physical activity level along with child obesity. RRVPA was related to the number of moderate to vigorous physical activity exercise bouts (r = .35, p < .05), not to the duration of those bouts. These results suggest that RRVPA is a determinant of child physical activity level, and that it may be naturally more reinforcing for children to engage in multiple, short exercise bouts than fewer, more extended bouts
SN - 0278-6133
AD - Department of Psychology, State University of New York at Buffalo, 14260, USA. lhenet@acsu.buffalo.edu
UR - 10619533
ER -

TY - JOUR
ID - 1817
T1 - Prevalence of epilepsy in rural Bolivia: a door-to-door survey
A1 - Nicoletti,A.
A1 - Reggio,A.
A1 - Bartoloni,A.
A1 - Failla,G.
A1 - Sofia,V.
A1 - Bartalesi,F.
A1 - Roselli,M.
A1 - Gamboa,H.
A1 - Salazar,E.
A1 - Osinaga,R.
A1 - Paradisi,F.
A1 - Tempera,G.
A1 - Dumas,M.
A1 - Hall,A.J.
Y1 - 1999/12/10/
N1 - Nicoletti, A. Reggio, A. Bartoloni, A. Failla, G. Sofia, V. Bartalesi, F. Roselli, M. Gamboa, H. Salazar, E. Osinaga, R. Paradisi, F. Tempera, G. Dumas, M. Hall, A J
Neurology
0401060, nz0
AIM, IM
Journal Article. Research Support, Non-U.S. Gov't
English
KW - MEDLINE
KW - Adolescent

KW - Adult
KW - Aged
KW - Bolivia/ep [Epidemiology]
KW - Child
KW - Preschool
KW - Cross-Sectional Studies
KW - Developing Countries
KW - Epilepsies
KW - Partial/ep [Epidemiology]
KW - Epilepsy/ep [Epidemiology]
KW - Epilepsy
KW - Generalized/ep [Epidemiology]
KW - Female
KW - Health Surveys
KW - Humans
KW - Incidence
KW - Infant
KW - Male
KW - Middle Aged
KW - Rural Population/sn [Statistics & Numerical Data]
RP - NOT IN FILE
SP - 2064
EP - 2069
JF - Neurology
VL - 53
IS - 9
CY - UNITED STATES
N2 - OBJECTIVE: To carry out a door-to-door survey in rural areas of the Cordillera Province, Santa Cruz Department, Bolivia, to determine the prevalence of neurologic diseases (epilepsy, stroke, parkinsonism, and peripheral neuropathy) in a sample of approximately 10,000 inhabitants. METHODS: A team of nondoctor health workers administered a standard screening instrument for neurologic diseases-a slightly modified version of the World Health Organization protocol. All subjects found positive during the screening underwent a neurologic examination. RESULTS: On screening, the authors found 1,130 positive subjects, of whom 1,027 were then investigated by neurologists. On the basis of the definition proposed by the International League Against Epilepsy, we detected 124 epileptic patients (prevalence, 12.3/1,000), 112 of whom had active epilepsy (prevalence, 11.1/1,000) on the prevalence day (November 1, 1994). Peak age-specific prevalence occurred in the 15 to 24-year age group (20.4/1,000). Sex-specific prevalence was higher in women (13.1/1,000) than men (11.4/1,000). Eighty-nine patients (71.8%) underwent a standard EEG recording. Considering both EEG and clinical data, partial seizures were the most common type (53.2%) based on the classification of the International League Against Epilepsy. The mean age at onset was 20.7 years for partial seizures and 13.6 years for generalized seizures. Only 10.5% of patients had received specific treatment for more than 2 months of their life. CONCLUSION: This report on epilepsy prevalence in Bolivia confirms that epilepsy is a major health problem in rural areas of developing countries
SN - 0028-3878
AD - Institutes of Neurological Science, University of Catania, Italy
UR - 10599782
ER -

TY - JOUR
ID - 1818
T1 - Body proportions during long-term growth hormone treatment in girls with Turner syndrome participating in a randomized dose-response trial
A1 - Sas,T.C.
A1 - Gerver,W.J.
A1 - de,Bruin R.
A1 - Stijnen,T.
A1 - de Muinck Keizer-Schrama SM

A1 - Cole,T.J.
A1 - van,Teunenbroek A.
A1 - Drop,S.L.
Y1 - 1999/12//
N1 - Sas, T C. Gerver, W J. de Bruin, R. Stijnen, T. de Muinck Keizer-Schrama, S M. Cole, T J. van Teunenbroek, A. Drop, S L
The Journal of clinical endocrinology and metabolism
hrb, 0375362
AIM, IM
Clinical Trial. Journal Article. Randomized Controlled Trial. Research Support, Non-U.S. Gov't
English
KW - MEDLINE
KW - Body Constitution
KW - Body Height
KW - Child
KW - Preschool
KW - Dose-Response Relationship
KW - Drug
KW - Female
KW - Foot/ah [Anatomy & Histology]
KW - Hand/ah [Anatomy & Histology]
KW - Human Growth Hormone/ad [Administration & Dosage]
KW - Human Growth Hormone/ae [Adverse Effects]
KW - Human Growth Hormone/tu [Therapeutic Use]
KW - Humans
KW - Turner Syndrome/dt [Drug Therapy]
RP - NOT IN FILE
SP - 4622
EP - 4628
JF - Journal of Clinical Endocrinology & Metabolism
JA - J Clin Endocrinol Metab
VL - 84
IS - 12
CY - UNITED STATES
N2 - To assess body proportions in girls with Turner syndrome (TS) during long term GH treatment, height, sitting height (SH), hand (Hand) and foot (Foot) lengths, and biacromial (Biac) and biiliacal (Biil) diameters were measured in 68 girls with TS participating in a GH dose-response trial. These previously untreated girls with TS, aged 2-11 yr, were randomly assigned to 1 of 3 GH dosage groups: group A, 4 IU/m2 x day; group B, first year 4 and thereafter 6 IU/m2 x day; group C, first year 4, second year 6, and thereafter 8 IU/m2 x day. Seven-year data were evaluated to assess the effect of GH treatment on body proportions during childhood. In addition, data from all girls who had reached adult height were evaluated to determine the effect on the adult body proportions. All results were adjusted for age and sex and expressed as SD scores using reference values of healthy Dutch girls. To describe the proportions of SH, Hand, Foot, Biac, and Biil to height, these values were adjusted for the SD score of height and were expressed as shape values, using the formula, e.g. for SH: shape SH = (SH SD score - height SD score)/square root(2 - 2 x correlation coefficient between SH and height in the reference population). Furthermore, SD scores using references of untreated girls with TS were calculated for height and SH. Values less than -2 or more than +2 were considered outside the normal range. At baseline, the shape values of all measurements were significantly higher than zero, but most mean shape values were still within the normal range. Seven-year data of 64 girls and adult height data of 32 girls showed that an increase in height was accompanied by an even higher increase in Foot, resulting in mean SD scores above zero and shape values of +2 and higher. The increase in the shape value of Foot was significantly higher in groups B and C compared to that in group A after 7 yr of GH treatment, but there were no significant differences between the GH dosage groups in the girls who had reached adult height. The shape values of SH had decreased to values closer to zero after reaching adult height, especially in group A. A similar pattern in the relationship of SH to height was seen using references of girls with TS. No significant changes in the other proportions were found after reaching adult height. In conclusion, on the average, untreated girls with TS have relatively large trunk, hands, and feet, and broad shoulders and pelvis compared to height. The increase in height after long term GH

treatment is accompanied by an even higher increase in Foot and a moderate improvement of the disproportion between height and SH. Recently published reference data from untreated adults with TS and the results of a different patient group receiving a comparable GH dosage suggest that the disproportionate growth of feet has to be considered a part of the natural development in TS, but might be influenced by higher GH dosages. The development of large feet can play a role in the decision of the girl to discontinue GH treatment in the last phase of growth
SN - 0021-972X
AD - Department of Pediatrics, Sophia Children's Hospital, Rotterdam, The Netherlands
UR - 10599729
ER -

TY - JOUR
ID - 1819
T1 - Skewed sex ratios in familial holoprosencephaly and in people with isolated single maxillary central incisor
A1 - Suthers,G.
A1 - Smith,S.
A1 - Springbett,S.
Y1 - 1999/12//
N1 - Suthers, G. Smith, S. Springbett, S
Journal of medical genetics
j1f, 2985087r
IM
Journal Article
English
KW - MEDLINE
KW - Adult
KW - Central Nervous System/ab [Abnormalities]
KW - Child
KW - Preschool
KW - Female
KW - Genetic Markers
KW - Hedgehog Proteins
KW - Holoprosencephaly/ge [Genetics]
KW - Humans
KW - Incisor/ab [Abnormalities]
KW - Male
KW - Maxilla
KW - Proteins/ge [Genetics]
KW - Risk Factors
KW - Sex Distribution
KW - Trans-Activators
RP - NOT IN FILE
SP - 924
EP - 926
JF - Journal of Medical Genetics
JA - J Med Genet
VL - 36
IS - 12
CY - ENGLAND
N2 - Autosomal dominant holoprosencephaly is a rare but well documented entity and it can be the result of mutations in the Sonic Hedgehog gene (SHH). The transmitting parent may be normal or have a single maxillary central incisor. We describe a skewed sex ratio among the transmitting parents with SHH mutations, with more mothers than fathers having the mutation (p=0.002). The mechanism underlying this skewed sex ratio is not clear; the SHH mutations do not involve triplet repeats, imprinting is plausible but untested, and there is no evidence that the risk of holoprosencephaly is greater among males carrying such a mutation (p=0.15). We considered the possibility that males with such a mutation are at greater risk of other malformations outside the

central nervous system, which could reduce their reproductive fitness. To avoid ascertainment bias in identifying children with various malformations in kindreds with familial holoprosencephaly, we reviewed the reports of people with single maxillary central incisor and no other congenital malformations. Of the 16 cases identified, 13 were female (p=0.0085). We suggest that boys with mutations associated with autosomal dominant holoprosencephaly may be at greater risk of major malformations outside the central nervous system than girls
SN - 0022-2593
AD - South Australian Clinical Genetics Service, Centre for Medical Genetics, Women's & Children's Hospital, North Adelaide, SA 5006, Australia
UR - 10594002
ER -

TY - JOUR
ID - 1820
T1 - Levels of physical activity and inactivity in children and adults in the United States: current evidence and research issues. [Review] [51 refs]
A1 - Pratt,M.
A1 - Macera,C.A.
A1 - Blanton,C.
Y1 - 1999/11//
N1 - Pratt, M. Macera, C A. Blanton, C
Medicine and science in sports and exercise
8005433, mg8
IM, S
Journal Article. Review
English
KW - MEDLINE
KW - Adult
KW - Age Factors
KW - Child
KW - Health Behavior
KW - Health Surveys
KW - Humans
KW - Life Style
KW - Physical Exertion
KW - Physical Fitness
KW - Research
KW - Sex Factors
KW - United States
RP - NOT IN FILE
SP - S526
EP - S533
JF - Medicine & Science in Sports & Exercise
JA - Med Sci Sports Exerc
VL - 31
IS - 11 Suppl
CY - UNITED STATES
N2 - PURPOSE: The purpose was to describe current levels of physical activity and inactivity among adults and young people in the United States. METHODS: Estimates of participation in regular physical activity were derived from three national surveys for adults (National Health Interview Survey, National Health and Nutrition Examination Survey, and the Behavioral Risk Factor Surveillance System) and from the Youth Risk Behavior Survey for high school students. RESULTS: Overall, 63.8% of high school students surveyed on the 1997 YRBS reported participating in vigorous physical activity for at least 20 min on 3 or more days per week. Participation in vigorous activity was higher for boys (72.3%) than girls (53.5%), whites (66.8%) compared with blacks (53.9%) and Hispanics (60.4%), and decreased with advancing grade. Among adults, 27.7% meet recommended levels of either moderate or vigorous physical activity, whereas 29.2% report no regular physical activity outside of their work. Gender differences in participation in physical activity are less pronounced than

in youth, and age-related patterns were complex. Whites are more active than blacks and Hispanics, and persons with higher family incomes and more education report being more physically active. There have been only minor changes in reported participation in leisure time physical activity over the past 15 yr. CONCLUSION: National estimates of physical activity appear to be reliable and valid for adults but may be less so for adolescents and are poor measures for children. Research is needed to determine the role that objective monitoring with accelerometers may play in surveillance. Reliable and valid measures of occupational, household, and transportation-related physical activity and sedentary behaviors are needed to better characterize the range of activity that is associated with health. [References: 51]
SN - 0195-9131
AD - Physical Activity and Health Branch, Division of Nutrition and Physical Activity, Centers for Disease Control and Prevention, Atlanta, GA 30341-3717, USA. mxp4@cdc.gov
UR - 10593523
ER -

TY - JOUR
ID - 1821
T1 - The epidemiology of injuries in 4 child care centers
A1 - Alkon,A.
A1 - Genevro,J.L.
A1 - Tschann,J.M.
A1 - Kaiser,P.
A1 - Ragland,D.R.
A1 - Boyce,W.T.
Y1 - 1999/12//
N1 - Alkon, A. Genevro, J L. Tschann, J M. Kaiser, P. Ragland, D R. Boyce, W T
Archives of pediatrics & adolescent medicine
9422751, bwf
AIM, IM
Journal Article. Research Support, U.S. Gov't, P.H.S.
English
KW - MEDLINE
KW - Age Distribution
KW - California/ep [Epidemiology]
KW - Chi-Square Distribution
KW - Child Day Care Centers
KW - Child
KW - Preschool
KW - Cohort Studies
KW - Female
KW - Humans
KW - Injury Severity Score
KW - Male
KW - Risk Factors
KW - Sex Distribution
KW - Wounds and Injuries/ep [Epidemiology]
KW - Wounds and Injuries/et [Etiology]
KW - Wounds and Injuries/pc [Prevention & Control]
RP - NOT IN FILE
SP - 1248
EP - 1254
JF - Archives of Pediatrics & Adolescent Medicine
JA - Arch Pediatr Adolesc Med
VL - 153
IS - 12
CY - UNITED STATES
N2 - OBJECTIVES: (1) To describe the pattern of injury in preschool-aged children in 4 child care centers as compared with the results of other studies; (2) to compare injury rates by sex, age, and child care center; and (3)

to examine environmental and child factors contributing to injury severity. DESIGN: A 2-year cohort study of 362 preschool-aged children attending 4 urban child care centers. Teachers completed standardized injury forms on the type of injury, body location, site of injury, and contributing factors. RESULTS: During the 2 years of the study, 1886 injuries were reported. The mean and median child injury rate was 6 and 4 injuries per 2000 exposure hours (equivalent to 1 full-time child care year), respectively. The majority of injuries (87%) were minor, occurred during free play (81%) and on the playground (74%), and were precipitated by child-related factors (59%), such as being pushed. Boys had significantly higher median injury rates than girls. Age-adjusted injury rates for each child care center were significantly different by center (F3 = 61, P<.001). While moderate to severe injuries were more often precipitated by combinations of child and environmental factors (chi2(4) = 20, P<.001), minor injuries were usually precipitated by child-related factors. CONCLUSIONS: Injury data from child care centers are important for identifying common risk factors for frequent or severe injury events and for designing injury prevention programs. More research is needed to identify factors contributing to injuries, such as children's behavior and the child care centers' physical and socioemotional environments
SN - 1072-4710
AD - Department of Family Health Care Nursing, University of California, San Francisco 94143-0606, USA. abbeyalk@uclink4.berkeley.edu
UR - 10591301
ER -

TY - JOUR
ID - 1822
T1 - Relationship between kinetic properties of mutant enzyme and biochemical and clinical responsiveness to biotin in holocarboxylase synthetase deficiency
A1 - Sakamoto,O.
A1 - Suzuki,Y.
A1 - Li,X.
A1 - Aoki,Y.
A1 - Hiratsuka,M.
A1 - Suormala,T.
A1 - Baumgartner,E.R.
A1 - Gibson,K.M.
A1 - Narisawa,K.
Y1 - 1999/12//
N1 - Sakamoto, O. Suzuki, Y. Li, X. Aoki, Y. Hiratsuka, M. Suormala, T. Baumgartner, E R. Gibson, K M. Narisawa, K
Pediatric research
owl, 0100714
IM
Journal Article. Research Support, Non-U.S. Gov't
English
KW - MEDLINE
KW - Biotin/me [Metabolism]
KW - Carbon-Nitrogen Ligases/df [Deficiency]
KW - Carbon-Nitrogen Ligases/ge [Genetics]
KW - Child
KW - Preschool
KW - Female
KW - Humans
KW - Infant
KW - Newborn
KW - Kinetics
KW - Male
KW - Mutation
KW - Substrate Specificity
RP - NOT IN FILE
SP - 671
EP - 676

JF - Pediatric Research
JA - Pediatr Res
VL - 46
IS - 6
CY - UNITED STATES
N2 - Holocarboxylase synthetase (HCS) deficiency is a metabolic disorder that causes a biotin-responsive multiple carboxylase deficiency. We analyzed the kinetic properties of seven mutant HCS proteins. Two of these enzymes harbored mutations within the putative biotin-binding region of HCS and showed elevated Km values for biotin compared with that of the wild-type form (Km mutant; Gly581Ser: 45 times, delThr610: 3 times). The remaining five mutations (Arg183Pro, Leu216Arg, Leu237Pro, Val333Glu, and Val363Asp) were located outside the biotin-binding region. The enzymes containing these mutations showed normal or low Km values for biotin (non-Km mutant). Symptoms of patients who have the non-Km, mutants, as well as those of patients who have the Km, mutants, responded to biotin therapy. This is probably because the Km value for biotin of normal HCS is higher than the physiologic concentration of biotin in human cells. The Vmax values of all mutant HCS proteins were considerably decreased, but to a variable degree. The responsiveness to biotin supplementation of propionyl-CoA carboxylase activity in cultured cells bearing the mutations correlated well with the degree of reduction in the Vmax of HCS. Patients who have mutant HCS proteins with lower Vmax showed poorer clinical and biochemical responses to biotin therapy. These observations suggest that the reduction of Vmax is an essential factor for pathophysiology and prognosis of HCS deficiency under treatment with large amounts of biotin. The determination of HCS genotype can be valuable for characterizing the clinical phenotype in HCS deficient patients
SN - 0031-3998
AD - Department of Medical Genetics, Tohoku University School of Medicine, Sendai, Japan
UR - 10590022
ER -

TY - JOUR
ID - 1823
T1 - Use of personal passive samplers for measurement of NO(2), NO, and O(3) levels in panel studies
A1 - Liard,R.
A1 - Zureik,M.
A1 - Le,Moullec Y.
A1 - Soussan,D.
A1 - Glorian,M.
A1 - Grimfeld,A.
A1 - Neukirch,F.
Y1 - 1999/11//
N1 - Liard, R. Zureik, M. Le Moullec, Y. Soussan, D. Glorian, M. Grimfeld, A. Neukirch, F
Environmental research
ei2, 0147621
IM
Journal Article. Research Support, Non-U.S. Gov't
English
KW - MEDLINE
KW - Activities of Daily Living
KW - Adolescent
KW - Adult
KW - Air Pollution/an [Analysis]
KW - Asthma
KW - Child
KW - Environmental Exposure/an [Analysis]
KW - Equipment Design
KW - Female
KW - Humans
KW - Male
KW - Middle Aged
KW - Nitric Oxide/an [Analysis]

KW - Nitrogen Dioxide/an [Analysis]
KW - Ozone/an [Analysis]
RP - NOT IN FILE
SP - 339
EP - 348
JF - Environmental Research
JA - Environ Res
VL - 81
IS - 4
CY - UNITED STATES
N2 - We measured personal exposure to nitrogen dioxide (NO(2)), nitrogen monoxide (NO), and ozone (O(3)), using personal passive samplers during three 4-day periods, in a panel study of asthmatics continuing the normal activities of everyday life. Fifty-five adults, mean age 42 years, 53% men, and 39 children, mean age 11 years, 67% boys, wore two Ogawa passive samplers simultaneously: one for O(3), the other for NO(2) and NO. Mean outdoor pollution was measured at a regional monitoring network. Personal exposure levels were scattered; they were (on average) higher than stationary-site levels for NO and lower for NO(2) and O(3). In adults, 41% of the variance of personal exposure to NO(2) was explained by mean stationary-site measurement levels (P<0.0001). Twenty-one percent additional variance was explained by living near a main road, not having an extractor fan over the cooker, older age, and male sex. NO and O(3) personal exposures correlated poorly with stationary-site measurements. In panel studies of the health effects of air pollution, personal exposure to NO(2) and NO can be measured satisfactorily by passive samplers: such measurements are necessary for NO but not for NO(2). For O(3), accurate personal exposure measurement remains a challenge and further technical development is required. Copyright 1999 Academic Press
SN - 0013-9351
AD - Institut National de la Sante et de la Recherche Medicale U408, Paris, France
UR - 10581112
ER -

TY - JOUR
ID - 1824
T1 - Helping children adjust--a Tri-Ministry Study: II. Program effects
A1 - Hundert,J.
A1 - Boyle,M.H.
A1 - Cunningham,C.E.
A1 - Duku,E.
A1 - Heale,J.
A1 - McDonald,J.
A1 - Offord,D.R.
A1 - Racine,Y.
Y1 - 1999/10//
N1 - Hundert, J. Boyle, M H. Cunningham, C E. Duku, E. Heale, J. McDonald, J. Offord, D R. Racine, Y
Journal of child psychology and psychiatry, and allied disciplines
hp3, 0375361
IM
Journal Article. Research Support, Non-U.S. Gov't
English
KW - MEDLINE
KW - Child
KW - Child Behavior Disorders/pc [Prevention & Control]
KW - Child Behavior Disorders/px [Psychology]
KW - Dyslexia/th [Therapy]
KW - Female
KW - Humans
KW - Intervention Studies
KW - Longitudinal Studies
KW - Male
KW - Program Evaluation

KW - School Health Services
KW - Social Behavior
RP - NOT IN FILE
SP - 1061
EP - 1073
JF - Journal of Child Psychology & Psychiatry & Allied Disciplines
JA - J Child Psychol Psychiatry
VL - 40
IS - 7
CY - ENGLAND
N2 - This report describes program effects of the Tri-Ministry Study a school-based, longitudinal trial carried out over a 5-year period to assess the effectiveness of classwide social skills training (SS), partner reading (RE), and a combination of both (SS & RE) to reduce maladjustment among children in the primary division (up to grade 3) of Ontario schools. It also places these effects in the context of other school-based prevention studies and discusses them in view of important methodological and programmatic issues. The incremental effects attributable to the intervention programs were small and sporadic. There were statistically significant increases in prosocial behaviour observed in the playgrounds of intervention schools with no differentiation by program type. Furthermore, there was some evidence--a reduction in teacher and parent-rated externalising problems--that the combination of SS & RE and SS alone may have had modest beneficial effects. A review of nine other school-based studies, which evaluated universally delivered mental health prevention programs in general populations of students, revealed similar mixed results. There are both methodologic and programmatic issues implicated in the weak findings that have been reported to date. These issues need to be addressed to advance knowledge about the potential impact of mental-health prevention initiatives delivered universally through school-based programs. A companion paper gives the specific details on the programs, randomisation of schools, selection of subjects, measurements, and analysis
SN - 0021-9630
AD - McMaster University and Hamilton Health Sciences Corporation, Ontario, Canada
UR - 10576536
ER -

TY - JOUR
ID - 1825
T1 - Description and immediate impacts of a preventive intervention for conduct problems
A1 - Reid,J.B.
A1 - Eddy,J.M.
A1 - Fetrow,R.A.
A1 - Stoolmiller,M.
Y1 - 1999/08//
N1 - Reid, J B. Eddy, J M. Fetrow, R A. Stoolmiller, M
American journal of community psychology
3fv, 0364535
IM
Clinical Trial. Journal Article. Randomized Controlled Trial. Research Support, U.S. Gov't, P.H.S.
English
KW - MEDLINE
KW - Behavior Therapy/mt [Methods]
KW - Child
KW - Conduct Disorder/pc [Prevention & Control]
KW - Conduct Disorder/px [Psychology]
KW - Female
KW - Humans
KW - Intervention Studies
KW - Male
KW - Observer Variation
KW - Parenting
KW - Parents/ed [Education]
KW - Primary Prevention/mt [Methods]

KW - Reward
KW - Social Adjustment
KW - Socioenvironmental Therapy/mt [Methods]
RP - NOT IN FILE
SP - 483
EP - 517
JF - American Journal of Community Psychology
JA - Am J Community Psychol
VL - 27
IS - 4
CY - UNITED STATES
N2 - A population-based randomized intervention trial for the prevention of conduct problems (i.e., oppositional defiant disorder and conduct disorder) is described. The LIFT (Linking the Interests of Families and Teachers) intervention was designed for all first- and fifth-grade elementary school boys and girls and their families living in at-risk neighborhoods characterized by high rates of juvenile delinquency. The 10-week intervention strategy was carefully targeted at proximal and malleable antecedents in three social domains that were identified by a developmental model of conduct problems. From 12 elementary schools, 671 first and fifth graders and their families participated either in the theory-based universal preventive intervention or in a control condition. The intervention consisted of parent training, a classroom-based social skills program, a playground behavioral program, and systematic communication between teachers and parents. A multiple measure assessment strategy was used to evaluate participant satisfaction and participation, fidelity of implementation, and the immediate impacts of the program on targeted antecedents
SN - 0091-0562
AD - Oregon Prevention Research Center, Oregon Social Learning Center, Eugene 97401, USA
UR - 10573832
ER -

TY - JOUR
ID - 1826
T1 - Temporal variability of microenvironmental time budgets in Maryland
A1 - Echols,S.L.
A1 - MacIntosh,D.L.
A1 - Hammerstrom,K.A.
A1 - Ryan,P.B.
Y1 - 1999/09//
N1 - Echols, S L. MacIntosh, D L. Hammerstrom, K A. Ryan, P B
Journal of exposure analysis and environmental epidemiology
bjn, 9111438
IM
Journal Article. Research Support, Non-U.S. Gov't. Research Support, U.S. Gov't, Non-P.H.S.
English
KW - MEDLINE
KW - Adolescent
KW - Adult
KW - Aged
KW - Analysis of Variance
KW - Child
KW - Environmental Exposure/an [Analysis]
KW - Female
KW - Human Activities
KW - Humans
KW - Linear Models
KW - Longitudinal Studies
KW - Male
KW - Maryland
KW - Middle Aged
KW - Periodicity

KW - Risk Assessment/mt [Methods]
KW - Socioeconomic Factors
KW - Statistics
KW - Nonparametric
KW - Time Factors
RP - NOT IN FILE
SP - 502
EP - 512
JF - Journal of Exposure Analysis and Environmental Epidemiology
JA - J Expo Anal Environ Epidemiol
VL - 9
IS - 5
CY - UNITED STATES
N2 - Information on human time-activity patterns is often required to interpret environmental exposure data fully and to implement exposure assessment models. Data on short-term time-activity patterns for individuals, such as 1-day measurements, are relatively abundant. The reliability of such data for use in chronic exposure (e.g., 1 or more years) assessments performed for evaluation of health risks is not well understood. As part of the NHEXAS-Maryland investigation, daily time budget data for seven microenvironments were collected from 80 people during as many as six 1-week Cycles over a 12-month period. The data were summarized and analyzed statistically by sampling Cycle, day of week, and individual to characterize long-term average microenvironmental time budgets and to identify their determinants. Median times spent in transit, indoors at home, outside at home, indoors at work or school, outdoors at work or school, indoors at other locations, and outdoors at other locations were found to vary significantly, although not substantively in many cases, by time of year (i.e., Cycle), by day of week, and by individuals. Time budgets for most of the microenvironments also exhibited significant variability by gender, age group, education level, annual household income, and work status. The results indicate that short-term (e.g., 1-day) measures of microenvironmental time budgets for individuals are unlikely to be representative of their long-term patterns. Thus, health risk or epidemiological assessments performed for a population mean or specific quantile may be relatively insensitive to when time budget data were collected. However, the accuracy of such assessments performed for individuals is likely to be greatly improved by collection of time budget data from numerous points in time
SN - 1053-4245
AD - Department of Environmental Health Science, University of Georgia, Athens, USA
UR - 10554152
ER -

TY - JOUR
ID - 1827
T1 - Spatial distributions of arsenic exposure and mining communities from NHEXAS Arizona. National Human Exposure Assessment Survey
A1 - O'Rourke,M.K.
A1 - Rogan,S.P.
A1 - Jin,S.
A1 - Robertson,G.L.
Y1 - 1999/09//
N1 - O'Rourke, M K. Rogan, S P. Jin, S. Robertson, G L
Journal of exposure analysis and environmental epidemiology
bjn, 9111438
IM
Journal Article. Research Support, U.S. Gov't, Non-P.H.S.
English
KW - MEDLINE
KW - Adult
KW - Air Pollutants/an [Analysis]
KW - Air Pollution
KW - Indoor/an [Analysis]
KW - Animals
KW - Arizona

KW - Arsenic/an [Analysis]
KW - Biological Markers/an [Analysis]
KW - Child
KW - Dust/an [Analysis]
KW - Environmental Exposure/an [Analysis]
KW - Female
KW - Fishes
KW - Food Contamination/an [Analysis]
KW - Humans
KW - Male
KW - Medical Records
KW - Mining
KW - Questionnaires
KW - Residence Characteristics
KW - Soil/an [Analysis]
KW - Statistics
KW - Nonparametric
KW - Water Pollutants/an [Analysis]
RP - NOT IN FILE
SP - 446
EP - 455
JF - Journal of Exposure Analysis and Environmental Epidemiology
JA - J Expo Anal Environ Epidemiol
VL - 9
IS - 5
CY - UNITED STATES
N2 - Within the context of the National Human Exposure Assessment Survey (NHEXAS), metals were evaluated in the air, soil, dust, water, food, beverages, and urine of a single respondent. Potential doses were calculated for five metals including arsenic. In this paper, we seek to validate the potential dose calculations through spatial analysis of the data. Others report elevated arsenic concentrations in biological and environmental samples from residents of mining towns, particularly Ajo, Arizona. These reports led us to expect potential arsenic doses above the 90th percentile of the NHEXAS exposure distribution to be from residents of mining communities. Arsenic dose was calculated using media concentrations, time activity patterns, and published exposure factors. Of the 179 homes evaluated, 54 were in mining communities; 11 of these were considered separately for reasons of population bias. Of the 17 homes with the greatest potential arsenic doses, almost half (47%) were in mining communities. We evaluated the potential doses by media from nonmining and mining areas using the nonparametric Mann-Whitney U test. Statistically significant (p = 0.05) differences were found between mining (n = 43) and nonmining sites (n = 122) for total exposure and for each of the following media: house dust, yard soil, outdoor air, beverage consumed, and water consumed. No differences were found in either food or indoor air of mining and nonmining areas. We eliminated outliers and repeated the test for all media; significance increased. Dietary, organic arsenic from fish consumption contributed to elevated arsenic exposure among people from nonmining communities and acted as an initial confounder. When controlling for fish consumption, we were able to validate our potential dose model using arsenic, particularly in Ajo. Further, we identified three mining communities lacking elevated arsenic exposure. Additional work is needed speciating the arsenic and evaluating health risks. The utilization of Geographic Information System facilitated spatial this project and paves the way for more sophisticated future spatial analyses
SN - 1053-4245
AD - Environmental and Occupational Health, Arizona Prevention Center, The University of Arizona, Tucson 85721-0468, USA. maryk@hrp.arizona.edu
UR - 10554147
ER -

TY - JOUR
ID - 1828
T1 - Effects of two types of activity on the performance of second-, third-, and fourth-grade students on a test of concentration
A1 - Caterino,M.C.

A1 - Polak,E.D.
Y1 - 1999/08//
N1 - Caterino, M C. Polak, E D
Perceptual and motor skills
ozb, 0401131
IM
Clinical Trial. Journal Article. Randomized Controlled Trial
English
KW - MEDLINE
KW - Analysis of Variance
KW - Attention
KW - Child
KW - Child Behavior/px [Psychology]
KW - Discrimination (Psychology)
KW - Exercise
KW - Female
KW - Form Perception
KW - Humans
KW - Male
KW - Psychological Tests/sn [Statistics & Numerical Data]
KW - Students/px [Psychology]
RP - NOT IN FILE
SP - 245
EP - 248
JF - Perceptual & Motor Skills
JA - Percept.Mot.Skills
VL - 89
IS - 1
CY - UNITED STATES
N2 - The appropriateness of recess in the elementary program continues to be questioned although generally it is believed to be useful by elementary principals despite a dearth of supportive data. This study was a developmental study of the effects of physical activity on concentration. Comparison of passive and directed physical education activities on the concentration of second-, third-, and fourth-grade children was made. The Woodcock-Johnson Test of Concentration showed better performance by the fourth grades and within Grade 4 in favor of the physical activity group. A structured physical activity or a classroom activity immediately prior to a concentration task was not detrimental to children in Grades 2 and 3. Fourth-grade children performed significantly better on a test of concentration after engaging in a physical activity
SN - 0031-5125
AD - West Deptford Middle School, NJ 08066, USA
UR - 10544425
ER -

TY - JOUR
ID - 1829
T1 - Epidemiological study of brucellosis in eight Greek villages using a computerised mapping programme
A1 - Hadjichristodoulou,C.
A1 - Papatheodorou,C.
A1 - Soteriades,E.
A1 - Panagakos,G.
A1 - Kastritis,I.
A1 - Goutziana,G.
A1 - Charvalos,E.
A1 - Tselentis,Y.
Y1 - 1999/08//
N1 - Hadjichristodoulou, C. Papatheodorou, C. Soteriades, E. Panagakos, G. Kastritis, I. Goutziana, G. Charvalos, E. Tselentis, Y
European journal of epidemiology

ere, 8508062
IM
Journal Article
English
KW - MEDLINE
KW - Adolescent
KW - Adult
KW - Age Distribution
KW - Brucellosis/di [Diagnosis]
KW - Brucellosis/ep [Epidemiology]
KW - Brucellosis/tm [Transmission]
KW - Child
KW - Cluster Analysis
KW - Computer Graphics
KW - Cross-Sectional Studies
KW - Databases
KW - Factual
KW - Disease Transmission
KW - Infectious
KW - Epidemiologic Methods
KW - Female
KW - Greece/ep [Epidemiology]
KW - Humans
KW - Incidence
KW - Information Systems
KW - Male
KW - Population Surveillance
KW - Prevalence
KW - Risk Factors
KW - Rural Population
KW - Sampling Studies
KW - Sensitivity and Specificity
KW - Sex Distribution
RP - NOT IN FILE
SP - 671
EP - 680
JF - European Journal of Epidemiology
JA - Eur J Epidemiol
VL - 15
IS - 7
CY - NETHERLANDS
N2 - A Computerised Mapping Programme (CMP) was created step by step to cover all the needs of a cross sectional population survey conducted in eight villages of Fokida, a rural area of central Greece. The maps of Greece (boundary) and the topographical maps of the eight villages were created using the CMP. A volunteer sample of 1121 out of 2607 inhabitants of the study area participated in the population survey. The participants were tested for brucellosis using serological tests (ELISA and Rose Bengal) and the intradermal reaction test. A questionnaire was used to obtain information concerning the risk factors for brucellosis. The risk factors found through statistical analysis were occupation (RR: 5.81, $p < 0.00001$), consumption of raw milk (RR: 1.98, $p < 0.001$) and unpasteurised fresh cheese (RR: 2.13, $p < 0.01$). The same factors were indicated by the CMP. The CMP also indicated manure-contaminated playgrounds in residential yards as a potential risk factor for children. The origin and dissemination were delineated using time space association display. The CMP proved to be a useful tool in this epidemiological study
SN - 0393-2990
AD - Laboratory of Clinical Bacteriology, Parasitology, Zoonoses and Geographical Medicine, Faculty of Medicine, University of Crete, Heraklion, Greece. hadjich@ncsi.gr
UR - 10543359
ER -

TY - JOUR
ID - 1830
T1 - [A new organization of school health services: its twilight or renaissance?]. [Croatian]
A1 - Prebeg,Z.
Y1 - 1998/09//
N1 - Prebeg, Z
Lijecnicki vjesnik
l6c, 0074253
IM
English Abstract. Journal Article
Croatian
KW - MEDLINE
KW - Adolescent
KW - Child
KW - Croatia
KW - Humans
KW - School Health Services/og [Organization & Administration]
RP - NOT IN FILE
SP - 257
EP - 261
JF - Lijecnicki Vjesnik
JA - Lijec.Vjesn.
VL - 120
IS - 9
CY - CROATIA
N2 - Starting from the beginning of January 1998, school health service in Croatia became exclusively a preventive activity. This change provoked a lot of controversial discussions among general public and medical profession. This paper gives an outline of the historic course, the aims and content of school health in different countries. From its very beginning in the middle of the 19th century, school health started to develop as a preventive activity directed primarily towards organic etiology dominant in the morbidity of school children at that time (infectious diseases, tuberculosis, malnutrition). The morbidity has changed over time. Nowadays, the problems of psychosocial etiology have emerged into the first plane. Moreover, schooling has been prolonged. It covers a great part of childhood and almost the entire adolescence. The problems of adolescents are different and complex. Health care of school children and adolescents is functionally bound to the school as their natural environment. The most efficient health care is provided in close cooperation between medical personnel and teachers. Besides, the classical model of school health offers, particularly with respect to current problems, large possibilities for health promotion of children and adolescents, and in the long run, of the whole population. Health care of school children is not equally organized in different countries. The main difference depends on whether the school health team is directed only towards preventive and specific activities or towards comprehensive primary health care. In Croatia, traditionally, the integrated health care has developed. Beside preventive health care, the school doctors provided curative care for students in their schools. Within the reorganization of health care a new situation has arisen. Compulsory integration of preventive and curative activities at the school level is completely incompatible with the right to a free choice of primary care physician. However, by the strict interpretation of the principle of indivisibility of preventive and curative care, one school should cooperate with numerous personal doctors for its students. The essential principle of school health would be lost. The school represents a community which is exposed to the same health risks but keeps a great potential for health promotion. So, if this advantage is to be utilized, preventive care must be organized at the school level. This is the very nature of the reorganization in school health service which is performed in Croatia under the auspices of the Ministry of Health. Providing the preventive and specific health care for all school children and adolescents is the responsibility of school doctors who have become the employees of the Institutes of Public Health, while the curative care is provided by the personal physician of a student's or his parents' choice
SN - 0024-3477
AD - Skola narodnog zdravlja Andrija Stampar Medicinskog fakulteta Sveucilista u Zagrebu
UR - 10099665
ER -

TY - JOUR
ID - 1831
T1 - Perisylvian dysgenesis. Clinical, EEG, MRI and glucose metabolism features in 10 patients
A1 - Van,Bogaert P.
A1 - David,P.
A1 - Gillain,C.A.
A1 - Wikler,D.
A1 - Damhaut,P.
A1 - Scalais,E.
A1 - Nuttin,C.
A1 - Wetzburger,C.
A1 - Szliwowski,H.B.
A1 - Metens,T.
A1 - Goldman,S.
Y1 - 1998/12//
N1 - Van Bogaert, P. David, P. Gillain, C A. Wikler, D. Damhaut, P. Scalais, E. Nuttin, C. Wetzburger, C. Szliwowski, H B. Metens, T. Goldman, S
Brain : a journal of neurology
0372537, b5f
AIM, IM
Journal Article. Research Support, Non-U.S. Gov't
English
KW - MEDLINE
KW - Adolescent
KW - Adult
KW - Brain/me [Metabolism]
KW - Cerebral Aqueduct/ab [Abnormalities]
KW - Cerebral Aqueduct/pa [Pathology]
KW - Cerebral Aqueduct/pp [Physiopathology]
KW - Child
KW - Preschool
KW - Congenital Abnormalities/di [Diagnosis]
KW - Electroencephalography
KW - Female
KW - Fluorodeoxyglucose F18/du [Diagnostic Use]
KW - Glucose/me [Metabolism]
KW - Humans
KW - Image Processing
KW - Computer-Assisted
KW - Magnetic Resonance Imaging
KW - Male
KW - Tomography
KW - Emission-Computed
RP - NOT IN FILE
SP - 2229
EP - 2238
JF - Brain
VL - 121
IS - Pt 12
CY - ENGLAND
N2 - We studied 10 patients who had neurological disorders with a MRI-based diagnosis of perisylvian dysgenesis based on the fact that the parasagittal and centrifugal extremity of the sylvian fissure was abnormally mesial. This abnormality was bilateral in seven cases; in the other three patients, the contralateral sylvian fissure appeared either normal (two cases) or enlarged (open operculum). The perisylvian cortex had a polymicrogyric appearance in most patients. Potential aetiopathogenic factors were determined in four patients. In two of them, ischaemia at mid-gestation was ascribed to death of a co-twin in a context of monozygotic twinning. In the other two patients, who were siblings, genetic factors were suspected. Pseudobulbar palsy was found in eight patients

and epilepsy in five patients. We used PET with [18F]fluorodeoxyglucose to test the hypothesis that, despite this clinical and MRI heterogeneity, regional cerebral glucose distribution could have common features in these patients. The analysis of PET data was performed by visual inspection in two cases and by using statistical parametric mapping (SPM) in eight patients compared with a control group. Segmented grey matter MRIs of seven out these patients were also analysed using SPM. We found that the abnormal perisylvian cortex had normal grey matter activity in eight patients and in the other two there was a heterogeneous pattern with areas of preserved metabolism and of decreased metabolism. Metabolic changes were also detected outside the polymicrogyric-like cortex; three patients had hypometabolic areas in cortical regions where the MRI appeared normal and had a normal intensity. When polymicrogyria extended into the white matter, this ectopic dysgenetic cortex was associated with a grey matter pattern within the white matter territory, and was detected by SPM as areas of PET hypermetabolism and MRI hyperintensity. In order to detect possible metabolic changes undetected by the individual analyses, the group of patients was compared with the control group. This comparison revealed bilateral hypometabolism in the frontal opercular cortex. We propose that these PET data be considered in light of the presumed cyto-architectonic pattern of perisylvian dysgenesis, i.e. polymicrogyria. In this malformation, two dense cell layers are separated by a necrotic sparse cell layer. We speculate that the amount of synaptic activity preserved in these dense cell layers depends on the importance and timing of the necrotic process; this hypothesis accounts for the large range of metabolic patterns found, from profoundly decreased glucose metabolism to nearly normal activity
SN - 0006-8950
AD - Department of Pediatric Neurology, Hopital Erasme, Universite Libre de Bruxelles, Brussels, Belgium
UR - 9874476
ER -

TY - JOUR
ID - 1832
T1 - Seroprevalence of human granulocytic ehrlichiosis among permanent residents of northwestern Wisconsin
A1 - Bakken,J.S.
A1 - Goellner,P.
A1 - Van,Etten M.
A1 - Boyle,D.Z.
A1 - Swonger,O.L.
A1 - Mattson,S.
A1 - Krueth,J.
A1 - Tilden,R.L.
A1 - Asanovich,K.
A1 - Walls,J.
A1 - Dumler,J.S.
Y1 - 1998/12//
N1 - Bakken, J S. Goellner, P. Van Etten, M. Boyle, D Z. Swonger, O L. Mattson, S. Krueth, J. Tilden, R L. Asanovich, K. Walls, J. Dumler, J S
Clinical infectious diseases : an official publication of the Infectious Diseases Society of America
a4j, 9203213
IM
Journal Article. Research Support, Non-U.S. Gov't
English
KW - MEDLINE
KW - Adolescent
KW - Adult
KW - Aged
KW - 80 and over
KW - Child
KW - Cohort Studies
KW - Ehrlichiosis/ep [Epidemiology]
KW - Female
KW - Fluorescent Antibody Technique
KW - Indirect
KW - Granulocytes/mi [Microbiology]

KW - Humans
KW - Male
KW - Middle Aged
KW - Risk Factors
KW - Seroepidemiologic Studies
KW - Wisconsin/ep [Epidemiology]
RP - NOT IN FILE
SP - 1491
EP - 1496
JF - Clinical Infectious Diseases
JA - Clin Infect Dis
VL - 27
IS - 6
CY - UNITED STATES
N2 - Four-hundred seventy-five permanent residents of Wisconsin were tested for antibodies to the agent of human granulocytic ehrlichiosis (HGE) by indirect immunofluorescent antibody (IFA) testing with Ehrlichia equi as antigen marker. Each resident completed a standard survey questionnaire about outdoor activities, animal and tick exposure, and any febrile illness during the preceding 12 months. Seventy-one serum samples (14.9%) contained E. equi antibodies. The mean IFA titer for seropositive residents was 250 (range, 80-10,240). Seropositive residents were older than seronegative ones (62 vs. 56 years; P = .019). None of the seropositive residents had a history suggestive of ehrlichiosis. There was no association between the IFA test outcome and specific demographic variables or history of tick bites. HGE appears to be a common subclinical or mild infection among residents in northwestern Wisconsin
SN - 1058-4838
AD - Section of Infectious Diseases, Laboratory Medicine, and Education and Research, Saint Mary's Duluth Clinic Health System, Minnesota, USA
UR - 9868666
ER -

TY - JOUR
ID - 1833
T1 - [Injuries in sledding]. [German]
A1 - Grober,J.
A1 - Maurer,F.
A1 - Eingartner,C.
A1 - Weise,K.
Y1 - 1998/09//
N1 - Grober, J. Maurer, F. Eingartner, C. Weise, K
Sportverletzung Sportschaden : Organ der Gesellschaft fur Orthopadisch-Traumatologische Sportmedizin
vcl, 8904133
IM
English Abstract. Journal Article
German
KW - MEDLINE
KW - Acceleration
KW - Adolescent
KW - Adult
KW - Aged
KW - Athletic Injuries/ep [Epidemiology]
KW - Athletic Injuries/et [Etiology]
KW - Child
KW - Cross-Sectional Studies
KW - Female
KW - Fractures
KW - Bone/ep [Epidemiology]
KW - Bone/et [Etiology]
KW - Germany/ep [Epidemiology]

KW - Humans
KW - Incidence
KW - Male
KW - Middle Aged
KW - Prospective Studies
KW - Risk Factors
KW - Snow
RP - NOT IN FILE
SP - 114
EP - 117
JF - Sportverletzung Sportschaden
JA - Sportverletz.Sportschaden
VL - 12
IS - 3
CY - GERMANY
N2 - Tobogganing is a very popular outdoor winter recreational activity. In order to elucidate the patterns of injuries associated with tobogganing all patients with an injury caused by falls from or collisions while on or being hit by a sled were sampled prospectively between the period of November 1996 and March 1997. 50 patients were included in this study, aged from 7 to 69 years (mean 25.5 years). Of these, 14 (= 28%) patients required admission to hospital lasting from 1 to 31 days (mean 13.5 days), 11 (22%) needed an operation. Over all we could register 55 injuries; the lower extremity was the region most commonly injured (63.6%), followed by upper extremity with 21.8%. The most common injury was the sprain of the knee. The most severe injuries could be found at the lower limb and at the vertebral column, including four fractures of the lower leg and 8 ankle-joint fractures as well as two fractures of the lumbar spine. The most common single procedure was the open reduction and internal fixation of a fibular fracture. In 48.6% of the cases the riders struck an object (tree, wall, post), while 32.4% fell from the toboggan caused by environmental conditions such as a bump or a ditch. The most important risk factor was an unadjusted speed referred to the environmental circumstances. Preventive strategies include tobogganing in adequate environmental conditions with no trees, no post or other stationary objects that could result in a collision. Speed should be adapted to the slope conditions
SN - 0932-0555
AD - Berufsgenossenschaftliche Unfallklinik Tubingen
UR - 9842678
ER -

TY - JOUR
ID - 1834
T1 - Effects of physical activity interventions in youth. Review and synthesis. [Review] [108 refs]
A1 - Stone,E.J.
A1 - McKenzie,T.L.
A1 - Welk,G.J.
A1 - Booth,M.L.
Y1 - 1998/11//
N1 - Stone, E J. McKenzie, T L. Welk, G J. Booth, M L
American journal of preventive medicine
8704773, apl
IM
Comparative Study. Journal Article. Review
English
KW - MEDLINE
KW - Adolescent
KW - Adult
KW - African Americans
KW - Age Factors
KW - Child
KW - Cohort Studies
KW - Curriculum
KW - Exercise

KW - Female
KW - Health Promotion
KW - Hispanic Americans
KW - Humans
KW - Intervention Studies
KW - Leisure Activities
KW - Male
KW - Physical Education and Training
KW - Physical Fitness
KW - Public Health
KW - Random Allocation
KW - Research
KW - Sampling Studies
KW - Schools
KW - United States
RP - NOT IN FILE
SP - 298
EP - 315
JF - American Journal of Preventive Medicine
JA - Am J Prev Med
VL - 15
IS - 4
CY - NETHERLANDS
N2 - INTRODUCTION: Physical inactivity has been identified as an important public health concern for youth. School and community settings can be important infrastructures for promoting physical activity (PA). This paper reviews studies of physical activity in school and community settings among preschool through college-aged persons to determine characteristics and effects of interventions. Studies in progress are included. METHODS: Studies from 1980 to 1997 testing physical activity interventions in schools and community settings were identified by computerized search methods and reference lists of published reviews. Studies needed to have used a quantitative assessment of PA, used a comparison or control group, included participants who were preschool through college age, and be conducted in the United States or foreign school or community settings. Significance of effects was examined overall and for various types of interventions. RESULTS: Twenty-two school-based studies were reviewed, 14 completed and 8 in progress. Three studies were in countries other than the United States. The 8 studies in progress were all in the United States. Only 7 community studies were reviewed, all in the United States. Four studies were in progress. Several community studies involved a high percentage of African-American or Hispanic youth and their families. Studies showing the best results used randomized designs, valid and reliable measurements, and more extensive interventions. Some follow-up results showed PA was sustained after interventions ended. CONCLUSIONS: The collection of school and community studies is limited for several age groups with none below third grade and only three at college age. There are few community studies. The most is known about upper-elementary-age-students, including the first multicenter randomized trial to report significant results for increasing moderate to vigorous physical activity (MVPA) in physical education (PE) and increase vigorous PA outside of school. A number of older study designs were weak and assessments less than optimal, but studies in progress are stronger. Special attention is needed for girls, middle schools, and community settings for all youth. More objective assessments are needed for measuring PA outside of school and in younger children, since they cannot provide reliable self-report. [References: 108]
SN - 0749-3797
AD - Division of Epidemiology and Clinical Applications, National Heart, Lung, and Blood Institute, Bethesda, Maryland 20892-7936, USA
UR - 9838974
ER -

TY - JOUR
ID - 1835
T1 - Epidemiology of giardiasis in Tasmania: a potential risk to residents and visitors
A1 - Kettlewell,J.S.
A1 - Bettiol,S.S.

A1 - Davies,N.
A1 - Milstein,T.
A1 - Goldsmid,J.M.
Y1 - 1998/09//
N1 - Kettlewell, J S. Bettiol, S S. Davies, N. Milstein, T. Goldsmid, J M
Journal of travel medicine
c7w, 9434456
IM
Journal Article. Research Support, Non-U.S. Gov't
English
KW - MEDLINE
KW - Adolescent
KW - Adult
KW - Age Distribution
KW - Aged
KW - 80 and over
KW - Animals
KW - Child
KW - Preschool
KW - Endemic Diseases/sn [Statistics & Numerical Data]
KW - Female
KW - Giardiasis/ep [Epidemiology]
KW - Giardiasis/et [Etiology]
KW - Giardiasis/tm [Transmission]
KW - Humans
KW - Infant
KW - Male
KW - Middle Aged
KW - Population Surveillance
KW - Prevalence
KW - Residence Characteristics
KW - Risk Factors
KW - Seasons
KW - Tasmania/ep [Epidemiology]
KW - Travel
KW - Zoonoses/ep [Epidemiology]
KW - Zoonoses/tm [Transmission]
RP - NOT IN FILE
SP - 127
EP - 130
JF - Journal of Travel Medicine
JA - J Travel Med
VL - 5
IS - 3
CY - CANADA
N2 - BACKGROUND: Giardia infection is an ongoing problem in Tasmania and occurs throughout the state. It has been postulated that part of the problem is the reservoir of infection in native animals which is suggested to contribute to infections in humans. To date, however, no detailed study on the epidemiology of giardiasis in Tasmania has been carried out. METHODS: Information regarding the prevalence of Giardia infection in humans and the risk of intrafamilial transmission was obtained from the Department of Community and Health Services and from the direct examination of human or animal fecal samples. RESULTS: Giardia has been found to be present in a wide range of native and domestic animals and in humans of all ages from all over the state. CONCLUSIONS: Giardiasis is endemic in Tasmania and poses a risk to locals as well as to visitors to the state, especially those who participate in wilderness activities such as bushwalking. These people must be aware of the possible risk of Giardia infection and should take precautionary measures to avoid infection
SN - 1195-1982
AD - Division of Pathology, University of Tasmania, Hobart, Tasmania, Australia

UR - 9772330
ER -

TY - JOUR
ID - 1836
T1 - [Load on the low back of teachers in kindergartens]. [Japanese]
A1 - Kumagai,S.
A1 - Tabuchi,T.
A1 - Tainaka,H.
A1 - Miyajima,K.
A1 - Matsunaga,I.
A1 - Kosaka,H.
A1 - Andoh,K.
A1 - Seo,A.
Y1 - 1998/09//
N1 - Kumagai, S. Tabuchi, T. Tainaka, H. Miyajima, K. Matsunaga, I. Kosaka, H. Andoh, K. Seo, A
Sangyo eiseigaku zasshi = Journal of occupational health
cd5, 9507473
IM
English Abstract. Journal Article
Japanese
KW - MEDLINE
KW - Adult
KW - Child
KW - Preschool
KW - Female
KW - Humans
KW - Japan
KW - Lifting
KW - Lumbosacral Region/ph [Physiology]
KW - Schools
KW - Nursery
KW - Teaching
KW - Video Recording
KW - Workload
RP - NOT IN FILE
SP - 204
EP - 211
JF - Sangyo Eiseigaku Zasshi
VL - 40
IS - 5
CY - JAPAN
N2 - In order to evaluate the load on the low back of teachers in kindergartens, basic activity and working posture were analyzed for four teachers by means of video recording. The trunk inclination angle (TIA) was also measured continuously during full workshifts for 12 kindergarten teachers by means of an inclination monitor. The kindergarten teachers spent 67% of the workshift on activities in contact with children, "indoor group childcare", "indoor free playing", "outdoor childcare", "preparation and clearing away" and "help and care", and did not take a recess during the workshift. They spent 36% of the workshift in three working postures with the load on the low back, "standing bent forward", "squatting" and "kneeling". Cumulative time at a TIA of 20 degrees or more represented 43% of the workshift. The frequency of trunk-lifting from severe bending forward (TIA > 45 degrees) was 95 times/hr on average. A comparison of the kindergarten teachers and nursery teachers in 4-5 year age classes showed that the time distributions of basic activities were generally similar to each other. Although the time distributions of working postures were also similar, time spent "standing bent forward", "squatting" and "kneeling" was longer in the kindergarten teachers than in the nursery teachers. Cumulative time at a TIA of 45 degrees or more was significantly longer in the kindergarten teachers. Although the frequency of trunklifting was not significantly different, the kindergarten teachers tended to lift their trunk more frequently. The present study found that the load on the low back was considerably great in the kindergarten teachers

SN - 1341-0725
AD - Department of Occupational Health, Osaka Prefectural Institute of Public Health, Japan
UR - 9836331
ER -

TY - JOUR
ID - 1837
T1 - Extrathymic T cell differentiation in the human intestine early in life
A1 - Howie,D.
A1 - Spencer,J.
A1 - DeLord,D.
A1 - Pitzalis,C.
A1 - Wathen,N.C.
A1 - Dogan,A.
A1 - Akbar,A.
A1 - MacDonald,T.T.
Y1 - 1998/12/01/
N1 - Howie, D. Spencer, J. DeLord, D. Pitzalis, C. Wathen, N C. Dogan, A. Akbar, A. MacDonald, T T
Journal of immunology (Baltimore, Md. : 1950)
ifb, 2985117r
AIM, IM
Journal Article. Research Support, Non-U.S. Gov't
English
KW - MEDLINE
KW - Aging/ge [Genetics]
KW - Aging/im [Immunology]
KW - Animals
KW - Base Sequence
KW - Cell Differentiation/im [Immunology]
KW - Cell Division/im [Immunology]
KW - Child
KW - Preschool
KW - Embryonic and Fetal Development/ge [Genetics]
KW - Embryonic and Fetal Development/im [Immunology]
KW - Fetal Blood/cy [Cytology]
KW - Fetal Blood/im [Immunology]
KW - Gene Rearrangement
KW - beta-Chain T-Cell Antigen Receptor/im [Immunology]
KW - Genes
KW - T-Cell Receptor beta/im [Immunology]
KW - Humans
KW - Immunophenotyping
KW - Infant
KW - Intestinal Mucosa/cy [Cytology]
KW - Intestinal Mucosa/im [Immunology]
KW - Jejunum/cy [Cytology]
KW - Jejunum/im [Immunology]
KW - Jejunum/tr [Transplantation]
KW - Lymphocyte Activation
KW - Lymphocyte Count
KW - Mice
KW - Inbred BALB C
KW - SCID
KW - Molecular Sequence Data
KW - T-Lymphocyte Subsets/cy [Cytology]
KW - T-Lymphocyte Subsets/im [Immunology]
KW - T-Lymphocyte Subsets/tr [Transplantation]

KW - Thymus Gland/cy [Cytology]
KW - Transcription
KW - Genetic/im [Immunology]
RP - NOT IN FILE
SP - 5862
EP - 5872
JF - Journal of Immunology
JA - J Immunol
VL - 161
IS - 11
CY - UNITED STATES
N2 - It is clear from experimental studies in mice that T cell maturation can occur outside the thymus, especially in the intestine. There is little sound evidence so far that extrathymic T cell maturation occurs to any significant extent in human gut, and, postnatally, there is abundant evidence that the gut mucosa is an immune effector organ. Here, we describe a large population of T lymphocytes in human fetal intestinal mucosa that are proliferating (Ki67+) in the absence of foreign Ag (CD3+, Ki67+ lamina propria lymphocytes (LPL) 22 +/- 1.8% and CD3+, Ki67+ intraepithelial lymphocytes (IEL) 9.1 +/- 1.4%), that express the T cell activation markers CD103, HLA-DR, and L-selectin(low), and that express mRNA transcripts for pre-TCR-alpha. There is also a substantial proportion of CD7+ LPLs that do not express CD3 (CD3-7+, 14 +/- 7% of all LPLs) in the fetal gut that may be differentiating into CD3+ cells. Rearranged TCR-beta transcripts of fetal LPLs, IELs, and paired blood lymphocytes were cloned and sequenced, and virtually no overlap of clonality was observed between blood and intestine, suggesting that gut T cells may not be derived from the blood. In addition, 30 days after engraftment of SCID mice with fetal intestine, CD3-7+ cells, proliferating T cells, and pre-TCR-alpha transcripts were abundant, and there is a threefold increase in CD3+ IELs. These data show that in the human intestine before birth a population of precursor T cells exists that may be differentiating into mature T cells in situ
SN - 0022-1767
AD - Department of Paediatric Gastroenterology, St. Bartholomew's and the Royal London School of Medicine and Dentistry, United Kingdom
UR - 9834065
ER -

TY - JOUR
ID - 1838
T1 - The growth hormone response to hexarelin in patients with different hypothalamic-pituitary abnormalities
A1 - Maghnie,M.
A1 - Spica-Russotto,V.
A1 - Cappa,M.
A1 - Autelli,M.
A1 - Tinelli,C.
A1 - Civolani,P.
A1 - Deghenghi,R.
A1 - Severi,F.
A1 - Loche,S.
Y1 - 1998/11//
N1 - Maghnie, M. Spica-Russotto, V. Cappa, M. Autelli, M. Tinelli, C. Civolani, P. Deghenghi, R. Severi, F. Loche, S
The Journal of clinical endocrinology and metabolism
hrb, 0375362
AIM, IM
Clinical Trial. Comparative Study. Controlled Clinical Trial. Journal Article
English
KW - MEDLINE
KW - Adolescent
KW - Child
KW - Preschool
KW - Female

KW - Growth Substances/tu [Therapeutic Use]
KW - Human Growth Hormone/df [Deficiency]
KW - Human Growth Hormone/se [Secretion]
KW - Humans
KW - Hypothalamo-Hypophyseal System/de [Drug Effects]
KW - Magnetic Resonance Imaging
KW - Male
KW - Oligopeptides/tu [Therapeutic Use]
KW - Pituitary Hormones/df [Deficiency]
KW - Secretory Rate/de [Drug Effects]
RP - NOT IN FILE
SP - 3886
EP - 3889
JF - Journal of Clinical Endocrinology & Metabolism
JA - J Clin Endocrinol Metab
VL - 83
IS - 11
CY - UNITED STATES
N2 - We evaluated the GH-releasing effect of hexarelin (Hex; 2 microg/kg, i.v.) and GHRH (1 microg/kg, i.v.) in 18 patients (11 males and 7 females, aged 2.5-20.4 yr) with GH deficiency (GHD) whose hypothalamic pituitary abnormalities had been previously characterized by dynamic magnetic resonance imaging (MRI). Ten patients had isolated GHD, and 8 had multiple pituitary hormone deficiency. All patients were receiving appropriate hormone replacement therapy. Twenty-four prepubertal short normal children (11 boys and 13 girls, aged 5.9-13 yr, body weight within +/-10% of ideal weight) served as controls. MRI studies revealed an ectopic posterior pituitary at the infundibular recess in all patients. A residual vascular component of the pituitary stalk was visualized in 8 patients with isolated GHD (group 1), whereas MRI showed the absence of the pituitary stalk (vascular and neural components) in the remaining 10 patients (group 2), of whom 8 had multiple pituitary hormone deficiency and 2 had isolated GHD. In the short normal children, the mean peak GH response to GHRH (24.8 +/- 4.4 microg/L) was significantly lower than that observed after Hex treatment (48.1 +/- 4.9 microg/L; P < 0.0001). In the GHD patients of group 2, the mean peak GH responses to GHRH (1.4 +/- 0.3 microg/L) and Hex (0.9 +/- 0.3 microg/L) were similar and markedly low. In the patients of group 1, the GH responses to GHRH (8.7 +/- 1.3 microg/L) and Hex (7.0 +/- 1.3 microg/L) were also similar, but were significantly higher that those observed in group 2 (P < 0.0001). In the whole group of patients, a significant correlation was found between the GH peaks after Hex and those after GHRH (r = 0.746; P < 0.0001). In this study we have confirmed that the integrity of the hypothalamic pituitary connections is essential for Hex to express its full GH-releasing activity and that Hex is able to stimulate GH secretion in patients with GHD but with a residual vascular component of the pituitary stalk
SN - 0021-972X
AD - Clinica Pediatrica, Policlinico S. Matteo, Universita di Pavia, Italy
UR - 9814463
ER -

TY - JOUR
ID - 1839
T1 - Symptoms in pediatric asthmatics and air pollution: differences in effects by symptom severity, anti-inflammatory medication use and particulate averaging time
A1 - Delfino,R.J.
A1 - Zeiger,R.S.
A1 - Seltzer,J.M.
A1 - Street,D.H.
Y1 - 1998/11//
N1 - Delfino, R J. Zeiger, R S. Seltzer, J M. Street, D H
Environmental health perspectives
ei0, 0330411
IM
Comparative Study. Journal Article. Research Support, U.S. Gov't, P.H.S.
English

KW - MEDLINE
KW - Adolescent
KW - Air Pollutants/ae [Adverse Effects]
KW - Anti-Asthmatic Agents/tu [Therapeutic Use]
KW - Asthma/dt [Drug Therapy]
KW - Asthma/ep [Epidemiology]
KW - Asthma/et [Etiology]
KW - California/ep [Epidemiology]
KW - Child
KW - Dust/ae [Adverse Effects]
KW - Female
KW - Humans
KW - Male
KW - Ozone/ae [Adverse Effects]
KW - Severity of Illness Index
RP - NOT IN FILE
SP - 751
EP - 761
JF - Environmental Health Perspectives
JA - Environ Health Perspect
VL - 106
IS - 11
CY - UNITED STATES
N2 - Experimental research in humans and animals points to the importance of adverse respiratory effects from short-term particle exposures and to the importance of proinflammatory effects of air pollutants, particularly O(subscript)3. However, particle averaging time has not been subjected to direct scientific evaluation, and there is a lack of epidemiological research examining both this issue and whether modification of air pollutant effects occurs with differences in asthma severity and anti-inflammatory medication use. The present study examined the relationship of adverse asthma symptoms (bothersome or interfered with daily activities or sleep) to O(3) and particles (less than or equal to)10 micrometer (PM10) in a Southern California community in the air inversion zone (1200-2100 ft) with high O(3) and low PM (R = 0.3). A panel of 25 asthmatics 9-17 years of age were followed daily, August through October 1995 (n = 1,759 person-days excluding one subject without symptoms). Exposures included stationary outdoor hourly PM10 (highest 24-hr mean, 54 microgram/m(3), versus median of 1-hr maximums, 56 microgram/m(3) and O(3) (mean of 1-hr maximums, 90 ppb, 5 days (greater than or equal to)120 ppb). Longitudinal regression analyses utilized the generalized estimating equations (GEE) model controlling for autocorrelation, day of week, outdoor fungi, and weather. Asthma symptoms were significantly associated with both outdoor O(3) and PM(10) in single pollutant- and co-regressions, with 1-hr and 8-hr maximum PM(10) having larger effects than the 24-hr mean. Subgroup analyses showed effects of current day PM(10) maximums were strongest in 10 more frequently symptomatic (MS) children: the odds ratios (ORs) for adverse symptoms from 90th percentile increases were 2.24 [95% confidence interval (CI), 1.46-3.46] for 1-hr PM10 (47 microgram/m(3); 1.82 (CI, 1.18-2.81) for 8-hr PM10 (36 microgram/m(3); and 1.50 (CI, 0.80-2.80) for 24-hr PM10 (25 microgram/m(3). Subgroup analyses also showed the effect of current day O(subscript)3 was strongest in 14 less frequently symptomatic (LS) children: the ORs were 2.15 (CI, 1.04-4.44) for 1-hr O(3) (58 ppb) and 1.92 (CI, 0.97-3.80) for 8-hr O(3) (46 ppb). Effects of 24-hr PM10 were seen in both groups, particularly with 5-day moving averages (ORs were 1.95 for MS and 4. 03 for LS; p(less than or equal to)0.05). The largest effects were in 7 LS children not on anti-inflammatory medications [5-day, 8-hr PM10, 9.66 (CI, 2.80-33.21); current day, 1-hr O(3), 4.14 (CI, 1.71-11.85)]. Results suggest that examination of short-term particle excursions, medication use, and symptom severity in longitudinal studies of asthma yields sensitive measures of adverse respiratory effects of air pollution
SN - 0091-6765
AD - Epidemiology Division, Department of Medicine, University of California, Irvine, CA 92697 USA
UR - 9799192
ER -

TY - JOUR
ID - 1840
T1 - [Particle exposure modeling in the general population and risk of respiratory disease]. [Spanish]

A1 - Santos-Burgoa,C.
A1 - Rojas-Bracho,L.
A1 - Rosas-Perez,I.
A1 - Ramirez-Sanchez,A.
A1 - Sanchez-Rico,G.
A1 - Mejia-Hernandez,S.
Y1 - 1998/07//
N1 - Santos-Burgoa, C. Rojas-Bracho, L. Rosas-Perez, I. Ramirez-Sanchez, A. Sanchez-Rico, G. Mejia-Hernandez, S
Gaceta medica de Mexico
fff, 0010333
IM
English Abstract. Journal Article. Research Support, Non-U.S. Gov't
Spanish
KW - MEDLINE
KW - Adolescent
KW - Adult
KW - Aged
KW - Air Pollution
KW - Bronchitis/et [Etiology]
KW - Child
KW - Chronic Disease
KW - Cross-Sectional Studies
KW - Environmental Exposure
KW - Humans
KW - Mexico
KW - Middle Aged
KW - Models
KW - Theoretical
KW - Risk Factors
KW - Rural Health
RP - NOT IN FILE
SP - 407
EP - 417
JF - Gaceta Medica de Mexico
JA - Gac.Med Mex
VL - 134
IS - 4
CY - MEXICO
N2 - Total exposure assessment identifies the various sources of exposure to air pollution which affect each individual, thus functioning as a useful tool in clinical and environmental exposure management. A cross sectional survey was performed in order to create a total exposure model of inhaled particulates. This survey involved 544 subjects located within the Mexico City Merced monitoring station, an area covering three kilometers in diameter. Two questionnaires were developed one on housing and the other on respiratory symptoms. A diary on daily activity was kept, and an outdoor monitoring station was also installed. A new technology was adapted and incorporated for personal and indoor monitoring. A correlation of up to 0.38 was identified between the environmental concentrations. Exposures at home and at other extramural activities are most predictive of personal exposure. It was determined that 8.8% of the subjects studied had chronic bronchitis, and that this percentage increases with greater levels of exposure and outdoor sports. These findings conclude that the models are useful for risk assessment and management in Mexico
SN - 0016-3813
AD - Escuela de Salud Publica de Mexico, Instituto Nacional de Salud Publica, Mexico, D.F. rtn0523@rtn.net.mx
UR - 9789385
ER -

TY - JOUR

ID - 1841
T1 - First recorded outbreak of yellow fever in Kenya, 1992-1993. I. Epidemiologic investigations
A1 - Sanders,E.J.
A1 - Marfin,A.A.
A1 - Tukei,P.M.
A1 - Kuria,G.
A1 - Ademba,G.
A1 - Agata,N.N.
A1 - Ouma,J.O.
A1 - Cropp,C.B.
A1 - Karabatsos,N.
A1 - Reiter,P.
A1 - Moore,P.S.
A1 - Gubler,D.J.
Y1 - 1998/10//
N1 - Sanders, E J. Marfin, A A. Tukei, P M. Kuria, G. Ademba, G. Agata, N N. Ouma, J O. Cropp, C B. Karabatsos, N. Reiter, P. Moore, P S. Gubler, D J
The American journal of tropical medicine and hygiene
3zq, 0370507
AIM, IM
Journal Article
English
KW - MEDLINE
KW - Adolescent
KW - Adult
KW - Aged
KW - Child
KW - Disease Outbreaks
KW - Female
KW - Humans
KW - Kenya/ep [Epidemiology]
KW - Male
KW - Middle Aged
KW - Time Factors
KW - Vaccination
KW - Yellow Fever/ep [Epidemiology]
KW - Yellow Fever/pc [Prevention & Control]
KW - Yellow Fever/tm [Transmission]
RP - NOT IN FILE
SP - 644
EP - 649
JF - American Journal of Tropical Medicine & Hygiene
JA - Am J Trop Med Hyg
VL - 59
IS - 4
CY - UNITED STATES
N2 - Outbreaks of yellow fever (YF) have never been recorded in Kenya. However, in September 1992, cases of hemorrhagic fever (HF) were reported in the Kerio Valley to the Kenya Ministry of Health. Early in 1993, the disease was confirmed as YF and a mass vaccination campaign was initiated. Cases of suspected YF were identified through medical record review and hospital-based disease surveillance by using a clinical case definition. Case-patients were confirmed serologically and virologically. We documented 55 persons with HF from three districts of the Rift Valley Province in the period of September 10, 1992 through March 11, 1993 (attack rate = 27.4/100,000 population). Twenty-six (47%) of the 55 persons had serologic evidence of recent YF infection, and three of these persons were also confirmed by YF virus isolation. No serum was available from the other 29 HF cases. In addition, YF virus was isolated from a person from the epidemic area who had a nonspecific febrile illness but did not meet the case definition. Five patients with confirmed cases of YF died, a case-fatality rate of 19%. Women with confirmed cases of YF were 10.9 times more likely to die than men (P =

0.010, by Fisher's exact test). Of the 26 patients with serologic or virologic evidence of YF, and for whom definite age was known, 21 (81%) were between 10 and 39 years of age, and 19 (73%) were males. All patients with confirmed YF infection lived in rural areas. There was only one instance of multiple cases within a single family, and this was associated with bush-clearing activity. This was the first documented outbreak of YF in Kenya, a classic example of a sylvatic transmission cycle. Surveillance in rural and urban areas outside the vaccination area should be intensified
SN - 0002-9637
AD - Virus Research Centre, Kenya Medical Research Institute, Nairobi
UR - 9790446
ER -

TY - JOUR
ID - 1842
T1 - Objective method for localization of cortical asymmetries using positron emission tomography to aid surgical resection of epileptic foci
A1 - Muzik,O.
A1 - Chugani,D.C.
A1 - Shen,C.
A1 - da Silva,E.A.
A1 - Shah,J.
A1 - Shah,A.
A1 - Canady,A.
A1 - Watson,C.
A1 - Chugani,H.T.
Y1 - 1998///
N1 - Muzik, O. Chugani, D C. Shen, C. da Silva, E A. Shah, J. Shah, A. Canady, A. Watson, C. Chugani, H T
Computer aided surgery : official journal of the International Society for Computer Aided Surgery
cuk, 9708375
IM
Comparative Study. Journal Article. Research Support, U.S. Gov't, P.H.S.
English
KW - MEDLINE
KW - Adolescent
KW - Adult
KW - Cerebral Cortex/ri [Radionuclide Imaging]
KW - Cerebral Cortex/su [Surgery]
KW - Child
KW - Preschool
KW - Electrodes
KW - Implanted
KW - Electroencephalography/is [Instrumentation]
KW - Epilepsy/ri [Radionuclide Imaging]
KW - Epilepsy/su [Surgery]
KW - False Positive Reactions
KW - Female
KW - Fluorodeoxyglucose F18/du [Diagnostic Use]
KW - Humans
KW - Image Processing
KW - Computer-Assisted/mt [Methods]
KW - Infant
KW - Magnetic Resonance Imaging
KW - Male
KW - Middle Aged
KW - Patient Care Planning
KW - ROC Curve
KW - Radiopharmaceuticals/du [Diagnostic Use]
KW - Sensitivity and Specificity

KW - Therapy
KW - Computer-Assisted
KW - Tomography
KW - Emission-Computed
KW - Treatment Outcome
RP - NOT IN FILE
SP - 74
EP - 82
JF - Computer Aided Surgery
JA - Comput Aided Surg
VL - 3
IS - 2
CY - UNITED STATES
N2 - We designed a semiautomated method for the objective detection of abnormal regions of tracer accumulation in the brain. The purpose of the present study was to examine the diagnostic performance of this method by applying it to patients with clinically intractable epilepsy of unilateral origin; they underwent [F-18] deoxyglucose positron emission tomography (PET) prior to surgical resection of epileptic foci. A semiautomated method for assessment of asymmetries in the brain cortex was developed that compares activity concentrations in homotopic cortical areas. When these differences exceeded a predefined threshold, the areas with lower activity were marked and 3-dimensional surface rendered images were created to guide placement of intracranial electrodes (ECoG) followed by surgical resection. The normal amount of asymmetry between small (0.5-0.7 cm2) homotopic cortical regions was determined as 5.9 +/- 4.0% (mean +/- SD). The false-positive fraction was determined for cutoff thresholds of 1 SD (10%), 1.5 SD (12%), and 2 SD (15%) outside the mean and was found to be 89, 44, and 0%, respectively. The obtained sensitivity-specificity pairs for correct localization of epileptogenic lobes based on the ECoG results were best for the 15% threshold (80/94%, accuracy 0.90). This objective PET method allows the accurate determination of cortical asymmetries, and it proved to be highly efficient in guiding epilepsy surgery
SN - 1092-9088
AD - Department of Radiology, Children's Hospital of Michigan, Detroit 48201, USA. otto@pet.wayne.edu
UR - 9784955
ER -

TY - JOUR
ID - 1843
T1 - Recreational-outdoor burns: the impact and severity--a retrospective review of 107 patients
A1 - Phillips,B.J.
A1 - Kassir,A.
A1 - Anderson,B.
A1 - Schiller,W.R.
Y1 - 1998/09//
N1 - Phillips, B J. Kassir, A. Anderson, B. Schiller, W R
Burns : journal of the International Society for Burn Injuries
afc, 8913178
IM
Comparative Study. Journal Article
English
KW - MEDLINE
KW - Accidents/mo [Mortality]
KW - Accidents/sn [Statistics & Numerical Data]
KW - Accidents
KW - Adolescent
KW - Adult
KW - Aged
KW - 80 and over
KW - Arizona/ep [Epidemiology]
KW - Burns/di [Diagnosis]
KW - Burns/ep [Epidemiology]

KW - Burns/et [Etiology]
KW - Child
KW - Preschool
KW - Female
KW - Follow-Up Studies
KW - Humans
KW - Incidence
KW - Infant
KW - Length of Stay
KW - Male
KW - Middle Aged
KW - Recreation
KW - Retrospective Studies
KW - Survival Rate
KW - Trauma Severity Indices
RP - NOT IN FILE
SP - 559
EP - 561
JF - Burns
VL - 24
IS - 6
CY - ENGLAND
N2 - INTRODUCTION: Campfires, bonfires and barbecues play a prominent role in outdoor activities and serve a variety of decorative and functional purposes. Given all of it's various forms, uses and benefits, the outdoor fire can turn dangerous in a matter of seconds: a stumble or fall (the child running that trips) leads to direct contact with the flames and subsequent burn injury. With it's everyday occurrence, we were surprised to find a paucity of information regarding this type of burn in the literature, including the NBIE analysis. METHODS: A retrospective review of 107 patients admitted to the Maricopa Medical Bum Center, from 1987 to 1996, was conducted. Each chart was analyzed for the patient demographics, mechanism of injury, percent total body surface area burned, operative versus non-operative treatment and overall outcome. RESULTS: An average burn injury involved 5.7% TBSA, with a wide range from 1 to 47%. The majority of our population involved either small children or intoxicated adults and a total of 50 patients required 92 operations. Severe morbidity, including 4 amputations and 2 mortalities, were seen. The average hospital stay was 14.3 days, ranging from 1 to 52 days. CONCLUSIONS: Recreational outdoor burns were extremely common injuries leading to loss of functional employment and prolonged physical therapy requirements. Small children and intoxicated adults comprised the majority of our study population. The impact and severity of such outdoor fires could be greatly affected by appropriate attention to prevention
SN - 0305-4179
AD - Department of Surgery, Maricopa Medical Center, Phoenix, AZ, USA
UR - 9776095
ER -

TY - JOUR
ID - 1844
T1 - [Toxocara canis infection. Environmental parasitologic and epidemiologic studies]. [German]
A1 - Tost,F.
A1 - Hellmann,A.
A1 - Ockert,G.
Y1 - 1998/07//
N1 - Tost, F. Hellmann, A. Ockert, G
Der Ophthalmologe : Zeitschrift der Deutschen Ophthalmologischen Gesellschaft
bgv, 9206148
IM
English Abstract. Journal Article
German
KW - MEDLINE
KW - Adult

KW - Animals
KW - Child
KW - Dogs/ps [Parasitology]
KW - Eye Infections
KW - Parasitic/di [Diagnosis]
KW - Parasitic/ps [Parasitology]
KW - Parasitic/tm [Transmission]
KW - Feces/ps [Parasitology]
KW - Germany
KW - Host-Parasite Interactions
KW - Humans
KW - Parasite Egg Count
KW - Risk Factors
KW - Toxocara canis
KW - Toxocariasis/di [Diagnosis]
KW - Toxocariasis/ps [Parasitology]
KW - Toxocariasis/tm [Transmission]
KW - Uveitis/di [Diagnosis]
KW - Uveitis/ps [Parasitology]
KW - Zoonoses/ps [Parasitology]
KW - Zoonoses/tm [Transmission]
RP - NOT IN FILE
SP - 486
EP - 489
JF - Ophthalmologe
VL - 95
IS - 7
CY - GERMANY
N2 - UNLABELLED: Due to the large number of differential diagnostic possibilities, the etiology of endogenous uveitis is still hard to determine. One reason for uveitis may be the occurrence of parasites. However, too little attention is paid to this underlying disease. METHODS: To identify certain sources of infection, ovoscopic probes of 98 dog feces from the urban area of Halle were taken. The material was collected from children's playgrounds, parks and swimming pools. A retrospective analysis of patient case data from the records of the University Eye Hospital for the years 1986-1995 complemented the environmental and parasitological examination of patients with Toxocara canis and T. cati infections. RESULTS: From the ovoscopic examinations, 16% of the fecal samples contained T. canis eggs. Seventeen percent contained coccicidal sporocysts. Eggs of Trichuris vulpis and not clearly identifiable nematodal larvae were found in 1% of the samples. The reasons for the morphology was, in one instance, suspected Echinococcus eggs. The fact that 12 or 15 parasitologically positive dog excrement samples were taken from the immediate vicinity of children's playgrounds and another three directly from these grounds should be regarded as particularly critical. The investigated patient case data records revealed for the period studied nine uveitic patients with T. conis infection. The diagnosis was based on the larva precipitation test in all cases. After correct diagnosis and appropriate treatment, the prognosis was favorable. The specific treatment had to be repeated in two patients. CONCLUSIONS: The environmental parasitological study presented proves the relatively high prevalence of pathogenic causal agents in dog excrement samples from the area of urban recreation and leisure grounds, in particular, children's playgrounds. Parasite infestation is a mirror of the efficiency of hygienic and social measures. Public health education must be reinforced. Differential diagnosis of chronic endogenous eye diseases must pay more attention to infections by parasites
SN - 0941-293X
AD - Klinik und Poliklinik fur Augenheilkunde, Universitat Halle-Wittenberg
UR - 9738379
ER -

TY - JOUR
ID - 1845
T1 - Outdoor tobacco advertising in six Boston neighborhoods. Evaluating youth exposure
A1 - Pucci,L.G.

A1 - Joseph,H.M.,Jr.
A1 - Siegel,M.
Y1 - 1998/08//
N1 - Pucci, L G. Joseph, H M Jr. Siegel, M
American journal of preventive medicine
8704773, apl
IM
Journal Article. Research Support, Non-U.S. Gov't
English
KW - MEDLINE
KW - Adolescent
KW - Advertising as Topic/mt [Methods]
KW - Advertising as Topic/sn [Statistics & Numerical Data]
KW - Boston
KW - Child
KW - Ethnic Groups/sn [Statistics & Numerical Data]
KW - Humans
KW - Persuasive Communication
KW - Residence Characteristics/cl [Classification]
KW - Residence Characteristics/sn [Statistics & Numerical Data]
KW - Schools/sn [Statistics & Numerical Data]
KW - Tobacco Industry/mt [Methods]
KW - Tobacco Industry/st [Standards]
KW - Urban Health
RP - NOT IN FILE
SP - 155
EP - 159
JF - American Journal of Preventive Medicine
JA - Am J Prev Med
VL - 15
IS - 2
CY - NETHERLANDS
N2 - BACKGROUND: The Food and Drug Administration (FDA), in its 1996 regulations to restrict certain forms of cigarette advertising likely to appeal to adolescents, prohibited outdoor tobacco advertising within 1,000 feet of schools and playgrounds. No published studies have determined the density of outdoor tobacco advertising within the FDA's prescribed 1,000-foot buffer zone around schools. OBJECTIVE: To determine the prevalence, type, and proximity to public schools of all stationary, outdoor tobacco advertising in six Boston neighborhoods. DESIGN: A cross-sectional field survey conducted in six Boston neighborhoods with varying ethnic, cultural, and socioeconomic characteristics. The main outcome measure was advertising density within buffer zones around public schools. RESULTS: Youth in the six neighborhoods are heavily exposed to stationary, outdoor cigarette advertising. This exposure is intense in areas close to public schools, and more intense in neighborhoods with more children, with significant Black and Hispanic/Latino populations, and with low socioeconomic status. Advertising strategies employed by the tobacco industry are in line with accepted professional marketing practice that targets adolescents for other products. CONCLUSIONS: Given the pervasive nature of the outdoor tobacco advertising we observed in the present study, it appears that the only way to protect youth from exposure is by eliminating it from the community
SN - 0749-3797
AD - Social and Behavioral Sciences Department, Boston University School of Public Health, Massachusetts 02118, USA
UR - 9713672
ER -

TY - JOUR
ID - 1846
T1 - The effects of primary division, student-mediated conflict resolution programs on playground aggression
A1 - Cunningham,C.E.
A1 - Cunningham,L.J.

A1 - Martorelli,V.
A1 - Tran,A.
A1 - Young,J.
A1 - Zacharias,R.
Y1 - 1998/07//
N1 - Cunningham, C E. Cunningham, L J. Martorelli, V. Tran, A. Young, J. Zacharias, R
Journal of child psychology and psychiatry, and allied disciplines
hp3, 0375361
IM
Journal Article. Research Support, Non-U.S. Gov't
English
KW - MEDLINE
KW - Aggression/px [Psychology]
KW - Child
KW - Preschool
KW - Conflict (Psychology)
KW - Female
KW - Follow-Up Studies
KW - Humans
KW - Male
KW - Negotiating
KW - Peer Group
KW - Play and Playthings
KW - Social Environment
KW - Students/px [Psychology]
KW - Treatment Outcome
RP - NOT IN FILE
SP - 653
EP - 662
JF - Journal of Child Psychology & Psychiatry & Allied Disciplines
JA - J Child Psychol Psychiatry
VL - 39
IS - 5
CY - ENGLAND
N2 - This study examined the effects of a student-mediated conflict resolution program on primary school (junior kindergarten to grade 5) playground aggression. Mediation teams of grade 5 students (approximately age 10) participated in 15 hours of training according to the model developed by Cunningham, Cunningham, and Martorelli (1997). Following baseline observations, mediation was introduced onto the playgrounds of three schools according to a multiple baseline design. Mediators successfully resolved approximately 90% of the playground conflicts in which they intervened. Direct observations suggest that the student mediation program reduced physically aggressive playground behavior by 51% to 65%. These effects were sustained at 1-year follow-up observations. Teacher and mediator satisfaction questionnaires provided strong support for impact, feasibility, and acceptability of this program
SN - 0021-9630
AD - Hamilton Health Sciences Corporation and McMaster University, Canada.
cunningh@fhs.csu.mcmaster.ca
UR - 9690929
ER -

TY - JOUR
ID - 1847
T1 - Victimization, use of violence, and drug use at school among male adolescents who engage in same-sex sexual behavior
A1 - DuRant,R.H.
A1 - Krowchuk,D.P.
A1 - Sinal,S.H.
Y1 - 1998/07//

N1 - DuRant, R H. Krowchuk, D P. Sinal, S H
The Journal of pediatrics
jlz, 0375410
AIM, IM
Journal Article
English
KW - MEDLINE
KW - Adolescent
KW - Alcohol Drinking/ep [Epidemiology]
KW - Child
KW - Crime Victims/sn [Statistics & Numerical Data]
KW - Data Collection
KW - Fear
KW - Homosexuality
KW - Male/px [Psychology]
KW - Male/sn [Statistics & Numerical Data]
KW - Humans
KW - Male
KW - Regression Analysis
KW - Schools
KW - Sexual Partners
KW - Smoking/ep [Epidemiology]
KW - Vermont/ep [Epidemiology]
KW - Violence/sn [Statistics & Numerical Data]
RP - NOT IN FILE
SP - 113
EP - 118
JF - Journal of Pediatrics
JA - J Pediatr
VL - 133
IS - 1
CY - UNITED STATES
N2 - OBJECTIVE: To examine the relationship between the number of male sexual partners of adolescent males and the frequency of victimization at school, missed school because of fear, used drugs at school, and engagement of fighting and weapon carrying both in and out of school. STUDY DESIGN: Sexually active male adolescents (N = 3886) in 8th through 12th grades were administered the 1995 Vermont Youth Risk Behavior Survey. RESULTS: A total of 8.7% of male adolescents reported one or more male sexual partners. Alcohol, marijuana, and smokeless tobacco use at school, not attending school because of fear, having been threatened or injured with a weapon at school, and weapon carrying at school accounted for 15.8% of the variation in the number of male sexual partners ($p < 0.0001$). Suicide attempts, school absence because of fear, cigarette smoking, alcohol use, and smokeless tobacco use at school, frequency of fighting requiring medical treatment, carrying a weapon,aand carrying a weapon at school accounted for 17.2% out of 100% of the variation in the number of male sexual partners (p < or 0.00001). CONCLUSION: The number of male sexual partners reported by sexually active male adolescents correlated with a higher frequency of victimization, use of violence and drug use at school. Frequency of suicide attempts and fighting outside of school were also correlated with the number of same-sex sexual partners
SN - 0022-3476
AD - Department of Pediatrics, Brenner Children's Hospital, Wake Forest University School of Medicine, Winston-Salem, North Carolina 27157, USA
UR - 9672522
ER -

TY - JOUR
ID - 1848
T1 - A comparative study of Greek children in long-term residential group care and in two-parent families: I. Social, emotional, and behavioural differences
A1 - Vorria,P.

A1 - Rutter,M.
A1 - Pickles,A.
A1 - Wolkind,S.
A1 - Hobsbaum,A.
Y1 - 1998/02//
N1 - Vorria, P. Rutter, M. Pickles, A. Wolkind, S. Hobsbaum, A
Journal of child psychology and psychiatry, and allied disciplines
hp3, 0375361
IM
Comparative Study. Journal Article
English
KW - MEDLINE
KW - Affective Symptoms
KW - Child
KW - Child Behavior Disorders/px [Psychology]
KW - Institutionalized/px [Psychology]
KW - Female
KW - Greece
KW - Humans
KW - Male
KW - Parent-Child Relations
KW - Residential Facilities
KW - Social Behavior
RP - NOT IN FILE
SP - 225
EP - 236
JF - Journal of Child Psychology & Psychiatry & Allied Disciplines
JA - J Child Psychol Psychiatry
VL - 39
IS - 2
CY - ENGLAND
N2 - The social, behavioural, and school adjustment of 41 9-year-old children in long-term residential group care in Greece was compared with that of children of the same sex and age brought up in two-parent families. Observational, interview, and questionnaire measures were employed. Observations in school classrooms showed that compared with their classmates, the group care children were more inattentive, participated less often in the classroom activities, were more likely to be passive, and tended to be involved in alternative and nonproductive activities. In the playground, they rarely interacted with non-institutional children. On both parent and teacher scales the group care children showed significantly more overall disturbance. The boys showed poor task involvement in the classroom and more emotional difficulties, conduct problems, and hyperactivity, whereas the between-group differences for girls were statistically significant only for emotional disturbance and poor task involvement in the classroom. Both boys and girls in long-term residential care showed less harmonious, confiding relationships with peers than those reared in families, and were more affection-seeking with teachers
SN - 0021-9630
AD - University of Ioannina, Faculty of Philosophy, Department of Philosophy-Education and Psychology, Greece
UR - 9669235
ER -

TY - JOUR
ID - 1849
T1 - [When children fall]. [Norwegian]
A1 - Solheim,K.
Y1 - 1998/06/20/
N1 - Solheim, K
Tidsskrift for den Norske laegeforening : tidsskrift for praktisk medicin, ny raekke
0413423, 101086543, vrv

IM
Case Reports. English Abstract. Journal Article
Norwegian
KW - MEDLINE
KW - Accidental Falls/pc [Prevention & Control]
KW - Accidental Falls
KW - Cause of Death
KW - Child
KW - Preschool
KW - Female
KW - Humans
KW - Male
KW - Norway/ep [Epidemiology]
KW - Wounds and Injuries/di [Diagnosis]
KW - Wounds and Injuries/et [Etiology]
KW - Wounds and Injuries/mo [Mortality]
KW - Wounds and Injuries/pc [Prevention & Control]
RP - NOT IN FILE
SP - 2481
EP - 2482
JF - Tidsskrift for Den Norske Laegeforening
JA - Tidsskr.Nor Laegeforen
VL - 118
IS - 16
CY - NORWAY
N2 - Second only to traffic accidents, accidental falling is the most significant cause of death in children. Included in this category is a special group of accidents--falling from a height--where preventative measures would give good results. Cases of child abuse have also been found in this group. From 1989 to 1994 64 children, 19 girls and 45 boys, were treated in our surgical department after accidental falls. There were 99 injuries in all, most of them caused by falls from heights less than 2.5 m. Only two children suffered penetrating injuries. Two of the 64 children died from cerebral injuries after falling from great heights. One child died from heart tamponade, caused by rupture of the right auricle after falling down a steep staircase. One child survived a fall from a great height despite multiple injuries. In such cases, treatment is dependent on a multiple trauma team being available on a 24-hour basis. Measures to prevent falls in the home, as well as in children's playgrounds and in kindergartens are not only very important, but also easy to apply. Suspicion of child abuse must be raised where unusual injuries are observed in children who have reportedly fallen from low heights
SN - 0029-2001
AD - Kirurgisk klinikk Ulleval sykehus, Oslo
UR - 9667124
ER -

TY - JOUR
ID - 1850
T1 - Evaluation of a bicycle skills training program for young children: a randomized controlled trial
A1 - Macarthur,C.
A1 - Parkin,P.C.
A1 - Sidky,M.
A1 - Wallace,W.
Y1 - 1998/06//
N1 - Macarthur, C. Parkin, P C. Sidky, M. Wallace, W
Injury prevention : journal of the International Society for Child and Adolescent Injury Prevention
crz, 9510056
IM
Clinical Trial. Journal Article. Randomized Controlled Trial. Research Support, Non-U.S. Gov't
English
KW - MEDLINE
KW - Accident Prevention

KW - Bicycling/ed [Education]
KW - Child
KW - Preschool
KW - Female
KW - Humans
KW - Male
RP - NOT IN FILE
SP - 116
EP - 121
JF - Injury Prevention
JA - Inj Prev
VL - 4
IS - 2
CY - ENGLAND
N2 - OBJECTIVE: To evaluate the effectiveness of a skills training program in improving safe cycling behavior, knowledge, and attitudes in young children. METHODS: Grade 4 children from six elementary schools in East York (a borough of Metropolitan Toronto) participated. The intervention--playground based instruction on bicycle handling skills by certified instructors--was randomly allocated to three schools. Altogether 141 children participated: 73 in the intervention group and 68 in the control group, with follow up evaluations available on 117 (83%). The primary outcome was safe cycling behavior (straight line riding, coming to a complete stop, and shoulder checking before a left turn). A self report questionnaire collected data on knowledge and attitudes. Baseline assessments were made in June, with follow up evaluations in September, 1995. RESULTS: The prevalence of safe cycling behaviors at follow up in the intervention and control groups respectively, were: straight line riding (90% v 88%; p = 0.782), coming to a complete stop (90% v 76%; p = 0.225), and shoulder checking (0% v 2%; p = 1.000). Over time (from baseline to follow up) children in both groups were more likely to maintain straight line riding, less likely to ride on the sidewalk, and less likely to consider that a car had more right to the road. CONCLUSIONS: This brief skills training program was not effective in improving safe cycling behavior, knowledge, or attitudes among grade 4 children
SN - 1353-8047
AD - Department of Paediatrics, University of Calgary, Alberta, Canada. colinmac@acs.ucalgary.ca
UR - 9666365
ER -

TY - JOUR
ID - 1851
T1 - Road injuries in school age children: relation to environmental factors amenable to interventions
A1 - von,Kries R.
A1 - Kohne,C.
A1 - Bohm,O.
A1 - von,Voss H.
Y1 - 1998/06//
N1 - von Kries, R. Kohne, C. Bohm, O. von Voss, H
Injury prevention : journal of the International Society for Child and Adolescent Injury Prevention
crz, 9510056
IM
Journal Article. Research Support, Non-U.S. Gov't
English
KW - MEDLINE
KW - Accident Prevention
KW - Accidents
KW - Traffic/sn [Statistics & Numerical Data]
KW - Case-Control Studies
KW - Child
KW - Environment
KW - Female
KW - Humans
KW - Male

KW - Risk
KW - Statistics
KW - Nonparametric
RP - NOT IN FILE
SP - 103
EP - 105
JF - Injury Prevention
JA - Inj Prev
VL - 4
IS - 2
CY - ENGLAND
N2 - OBJECTIVES: To assess the impact of potentially modifiable environmental factors on the risk for pedestrian and cyclist injuries among school age children. SETTING: Population of school age children in Dusseldorf (population 570,000) in the west of Germany. All pedestrian and cyclist injuries involving children between 6 and 14 years brought to the attention of the police between January 1993 and March 1995 were eligible. METHODS: A case-control design was used, with controls matched by age and sex. Criteria for inclusion of cases were: residence in Dusseldorf, and injury within 500 meters from home. A random sample of 174 cases was selected. For each an age-sex matched child, resident in Dusseldorf, was randomly selected from a list of all school age children. The environment within a radius of 500 meters around the homes of cases and controls was analysed by blinded on site investigators. These used a standardized questionnaire to assess the number of streets with speed limits of 30 kph, the number of pedestrian crossings with traffic lights per street with speed limits of 50 kph or above, and the number of playgrounds for children. RESULTS: Complete information was available for 170 cases and 168 controls. There were significantly more streets with a speed limit of 30 kph around the homes of controls (p = 0.0003; mean 9.5; 95% confidence interval (CI) 8.8 to 10.1) than cases (mean 7.8; 95% CI 7.3 to 8.3). For every five streets with a speed limit of 30 kph injury risk was reduced by nearly 50% (odds ratio 0.57; 95% CI 0.43 to 0.76). There were also significantly more pedestrian crossings with traffic lights on streets with a speed limit 50 kph or above around the homes of controls (p = 0.0004; mean 2.7; 95% CI 2.4 to 2.9) compared with cases (mean 2.1; 95% CI 1.9 to 2.3). Finally there were significantly more playgrounds around the houses of controls (p = 0.04; mean 1.9; 95% CI 1.7 to 2.2) compared with the houses of cases (mean 1.7; 95% CI 1.4 to 2.0). CONCLUSIONS: Significant associations with injury risk were identified for some prespecified modifiable environmental factors
SN - 1353-8047
AD - Institute for Social Paediatrics and Adolescent Medicine, Ludwig-Maximilians University of Munich, Germany. R.vonKries@lrz.uni-muenchen.de
UR - 9666362
ER -

TY - JOUR
ID - 1852
T1 - Evaluation of quality of life of childhood cancer survivors: a methodological conundrum
A1 - Parsons,S.K.
A1 - Brown,A.P.
Y1 - 1998///
N1 - Parsons, S K. Brown, A P
Medical and pediatric oncology
m6p, 7506654
IM
Journal Article. Research Support, Non-U.S. Gov't
English
KW - MEDLINE
KW - Adolescent
KW - Adult
KW - Child
KW - Disease-Free Survival
KW - Humans
KW - Medical Oncology/mt [Methods]
KW - Neoplasms/co [Complications]

KW - Neoplasms/px [Psychology]
KW - Pediatrics/mt [Methods]
KW - Quality of Life
RP - NOT IN FILE
SP - 46
EP - 53
JF - Medical & Pediatric Oncology
JA - Med Pediatr Oncol
VL - Suppl 1
CY - UNITED STATES
N2 - QOL assessment in pediatric oncology is seriously understudied, especially compared with the adult population. The limited progress is due to the methodological complexity of the task, which should not be viewed as insurmountable. Given a precise study question, the methodological issues can be clarified simply, piece by piece. Researchers must consider very carefully the specific characteristics that define a study population in order to choose an instrument that is domain-appropriate and valid for the assessment paradigm. The first priority should be that a researcher must identify the means of accessing the information of interest. In the pediatric population, information about children's status may be elicited from parents, medical personnel, teachers, or the children themselves. Clearly, the type of instrument to be used for assessment is dependent on the choice of reporter. Researchers must also account for developmental age and disease; in assessing generic and disease-specific functioning, the "functional scale" against which an individual is compared must implicitly reflect the types of activities and/or levels of functioning that are realistic norms for the patient. Equally important is the analysis of independent domains in order to characterize the dynamics/divergence of clinical status and functional status. What are the merits of conducting QOL research for the pediatric cancer-survivor population? The policy implications are profound and pervasive both for the individual survivors (regarding treatment, care, and his/her ultimate ability to reintegrate into society) and for society (regarding resource allocation, cost planning, and productivity). Commensurate with the rapid advancement of oncologic therapy, there is now an expanding cohort of pediatric cancer survivors. Current estimates suggest that, by the turn of the century, 200,000 children will be in this category. The long-term survivorship of this cohort is still poorly defined. However, as the survivors mature, it is likely that their needs will evolve as well-whether for treatment of secondary malignancies, long-term morbidities, and fertility issues or for neuropsychological dysfunction, emotional counseling, or occupational issues. Children, as survivors, are unique, in that their future (the context within which long-term outcome is defined) spans decades. Based on a median age at diagnosis of 6 years, survivors can expect to live an additional 66 years. From a cost or policy perspective, children represent enormous future potential. The implications of children's long-term outcomes must be considered regarding the change in future potential secondary to survivorship. Pediatric QOL research plays a role both inside and outside the health care system. Clearly, in the provision of health care, QOL data may be used to improve or modify patient care by supplementing information about the clinical status of individual patients. Information about an individual's general functioning, particularly as it diverges from disease-specific functioning, complements clinical data to facilitate comprehensive care. Information about the long-term outcomes of pediatric cancer, as a whole, will influence the policies of health care institutions and the allocation of health care resources. By expanding the scope of survivorship (or cure) to include long-term clinical and general "costs" the "cost of cure" is shifted: this shift will ultimately impact estimations of cost effectiveness, with ramifications for the evaluation of hospital-wide protocols, utilization priorities, and cost policies. Outside of the hospital, the implications of QOL research are equally ubiquitous. Pediatric survivors will live an estimated 7 decades after "cure," during which time they will exist almost entirely outside the realm of health care; yet, their condition as a survivor, with or without the long-term clinical toxicities secondary to treatment, will continue to affect some or all of thei
SN - 0098-1532
AD - Division of Hematology/Oncology, Children's Hospital, Boston, Massachusetts, USA
UR - 9659946
ER -

TY - JOUR
ID - 1853
T1 - Post traumatic paraplegics living in Athens: the impact of pressure sores and UTIs on everyday life activities
A1 - Sapountzi-Krepia,D.

A1 - Soumilas,A.
A1 - Papadakis,N.
A1 - Sapkas,G.
A1 - Nomicos,J.
A1 - Theodossopoulou,E.
A1 - Dimitriadou,A.
Y1 - 1998/06//
N1 - Sapountzi-Krepia, D. Soumilas, A. Papadakis, N. Sapkas, G. Nomicos, J. Theodossopoulou, E. Dimitriadou, A
Spinal cord
9609749, ckk
IM
Journal Article
English
KW - MEDLINE
KW - Activities of Daily Living
KW - Adolescent
KW - Adult
KW - Child
KW - Female
KW - Greece
KW - Humans
KW - Interpersonal Relations
KW - Male
KW - Middle Aged
KW - Occupations
KW - Paraplegia/co [Complications]
KW - Paraplegia/px [Psychology]
KW - Patient Education as Topic
KW - Pressure Ulcer/et [Etiology]
KW - Pressure Ulcer/px [Psychology]
KW - Questionnaires
KW - Spinal Cord Injuries/co [Complications]
KW - Spinal Cord Injuries/px [Psychology]
KW - Urinary Tract Infections/et [Etiology]
KW - Urinary Tract Infections/px [Psychology]
RP - NOT IN FILE
SP - 432
EP - 437
JF - Spinal Cord
VL - 36
IS - 6
CY - ENGLAND
N2 - This paper contains the findings of a scientific research which was done on post-traumatic paraplegics who live in the area of Athens. Greece, and measured the impact of pressure sores and UTIs on their everyday life activities. The target population was 127 individuals out of which 98 were observed and interviewed. A semi-structured questionnaire which consisted of two parts used; the first part of the questionnaire included general questions whereas the second contained the Sarno Functional Life Scale (SFLS) which is a tool measuring the level of independence of disabled people. Sixty-two per cent of the population was male. The mean age of the sample population at the time they had the accident which caused the paraplegia was 31.5, while the median was 28.5. The majority of the subjects comes from the lower socioeconomic class. Traffic accidents accounted for the 55% of the occurrences of paraplegia, falls for 37%, surgical complication for 4% and athletic activities for the remaining 4%. Forty-nine percent of the sample had been hospitalised in a rehabilitation centre. However, the multiple regressions used did not reveal any statistically significant relation between the hospitalisation in a rehabilitation centre and the paraplegic's scores in any of the Sarno Functional Life Scale's (SFLS) variables. Instead, the multiple regressions employed yielded paraplegics' scores in the SFLS's everyday life activities that were negatively related to age (P = 0.004) and pressure sores (P = 0.021).

The paraplegics' scores on the SFLS's indoors activities are positively related to the years since injury (P = 0.048) and health education on daily fluid consumption (P = 0.003). The scores of the subjects on the SFLS's outdoor activities are positively related to the years of education (P = 0.008), the years since injury (P = 0.011), while are negatively related to pressure sores (P = 0.034) and UTIs (0.044). The subjects' scores on the SFLS variables regarding social relations had a negative relation to sex, (female) (P = 0.0001), age (P = 0.001) and pressure sores (P = 0.019), while they have a positive relation with the years since injury (P = 0.024)
SN - 1362-4393
AD - Health Visiting Department, Technological Educational Institute (TEI) of Athens, Greece
UR - 9648201
ER -

TY - JOUR
ID - 1854
T1 - "I want my mummy". Changes in the care of children in hospital
A1 - Shields,L.
A1 - Nixon,J.
Y1 - 1998/04//
N1 - Shields, L. Nixon, J
Collegian (Royal College of Nursing, Australia)
9612493
N, Q
Historical Article. Journal Article. Portraits. Research Support, Non-U.S. Gov't
English
KW - MEDLINE
KW - Child
KW - Child Care/hi [History]
KW - Child Care/mt [Methods]
KW - Hospitalized/hi [History]
KW - Great Britain
KW - History
KW - 20th Century
KW - Hospitals
KW - Pediatric/hi [History]
KW - Humans
KW - Pediatric Nursing/ed [Education]
KW - Pediatric Nursing/hi [History]
KW - United States
RP - NOT IN FILE
SP - 16
EP - 23
JF - Collegian: Journal of the Royal College of Nursing, Australia
JA - Collegian
VL - 5
IS - 2
CY - AUSTRALIA
N2 - The Welfare of Sick Children in Hospital' was the formal title of a report published in Britain in 1959 which came to be known as the Platt Report, after Sir Harry Platt, the chairman of the parliamentary committee which investigated the care of children in hospital. The committee found the hospitals were miserable places for children, where they were expected to conform to ward routines, not allowed to play; where to lie quietly was the accepted norm, and where, under no circumstances, were the parents allowed to visit outside the declared visiting hours. Subsequent to the publication of the Platt Report a revolution occurred in children's hospitals in Britain and other western countries, including Australia. This paper explores these changes, and discusses implications for paediatric care in hospitals and paediatric nursing education today
SN - 1322-7696
AD - Department of Paediatrics and Child Health, University of Queensland, Mater Children's Hospital, Brisbane
UR - 9644334

ER -

TY - JOUR
ID - 1855
T1 - Acute effects of ambient ozone on asthmatic, wheezy, and healthy children
A1 - Avol,E.L.
A1 - Navidi,W.C.
A1 - Rappaport,E.B.
A1 - Peters,J.M.
Y1 - 1998/05//
N1 - Avol, E L. Navidi, W C. Rappaport, E B. Peters, J M
Research report (Health Effects Institute)
8812230, ah8, 8812230
IM
Journal Article. Research Support, U.S. Gov't, Non-P.H.S.
English
KW - MEDLINE
KW - Acute Disease
KW - Air Pollutants/ae [Adverse Effects]
KW - Air Pollutants/an [Analysis]
KW - Asthma/di [Diagnosis]
KW - Asthma/et [Etiology]
KW - Case-Control Studies
KW - Child
KW - Environmental Monitoring
KW - Female
KW - Humans
KW - Los Angeles
KW - Male
KW - Ozone/ae [Adverse Effects]
KW - Ozone/an [Analysis]
KW - Respiratory Function Tests
KW - Respiratory Sounds/di [Diagnosis]
KW - Respiratory Sounds/et [Etiology]
RP - NOT IN FILE
SP - iii
EP - 18
JF - Research Report - Health Effects Institute
JA - Res Rep Health Eff.Inst
IS - 82
CY - UNITED STATES
N2 - Southern California children (10 to 12 years old) participated in a two-season study to assess the potential acute respiratory effects of ambient ozone (O3). Asthmatic (n = 49), wheezy (n = 53), and healthy (n = 93) children completed a four-day (Friday through Monday) study protocol, once in spring and again in summer, that included the use of daily activity and symptom diaries, heart rate recording devices, personal O3 samplers, and maximal effort spirometry several times per day. Data from regional monitoring stations were used to establish ambient hourly O3 concentrations. Analyses revealed that the children spent more time outdoors and were more physically active in the spring. Girls spent less time outdoors and were less physically active than boys. Personal O3 samplers correlated poorly with, and generally gave lower readings than, outdoor ambient monitors. Higher personal O3 exposures were associated generally with increased inhaler use, more outdoor time, and more physical activity. Children with asthma spent more time outdoors and were more active in the spring on high-O3 days (measured by personal sampler), and had the most trouble breathing, the most wheezing, and the most inhaler use on these days. Activity pattern data suggested that children with asthma protected themselves by being less physically active outdoors during the summer on high-O3 days. Wheezy children had the most trouble breathing during the summer on low-O3 days (measured by personal sampler). Observed relationships between O3 and pulmonary function were erratic and difficult to reconcile with existing knowledge about the acute respiratory effects of air pollution. We conclude that although asthmatic and wheezy

children behave differently from their healthy peers with regard to symptoms and patterns of activity when challenged by ambient ozone, the nature of these changes remains inconsistent and ill-defined
SN - 1041-5505
AD - University of Southern California, Division of Occupational and Environmental Medicine, School of Medicine, Los Angeles, USA
UR - 9635336
ER -

TY - JOUR
ID - 1856
T1 - Hemiparesis and altered mental status in a child after glyburide ingestion
A1 - Spiller,H.A.
A1 - Schroeder,S.L.
A1 - Ching,D.S.
Y1 - 1998/05//
N1 - Spiller, H A. Schroeder, S L. Ching, D S
The Journal of emergency medicine
ibo, 8412174
IM
Case Reports. Journal Article
English
KW - MEDLINE
KW - Child
KW - Cognition Disorders/ci [Chemically Induced]
KW - Drug-Related Side Effects and Adverse Reactions/di [Diagnosis]
KW - Drug-Related Side Effects and Adverse Reactions/th [Therapy]
KW - Female
KW - Glyburide/po [Poisoning]
KW - Hemiplegia/ci [Chemically Induced]
KW - Humans
KW - Hypoglycemic Agents/po [Poisoning]
RP - NOT IN FILE
SP - 433
EP - 435
JF - Journal of Emergency Medicine
JA - J Emerg Med
VL - 16
IS - 3
CY - UNITED STATES
N2 - A normally healthy 6-year-old woke in an agitated state, limp, and moving only her left extremities. Upon arrival at the emergency department, a blood glucose measurement was 34 mg/dL. The child was lethargic, not responding to questions appropriately, and not moving her right extremities. The right arm was flexed, and the right leg was flexed and abducted. Pupils were equal and reactive, and eyes were deviated to the left. Six loose tablets of the grandmother's glyburide were found at home in the child's outdoor playhouse. Administration of glucose produced no change in the child's clinical condition. Intravenous glucose was begun at 4 mg glucose/kg/min, and the blood glucose level did not fall below 74 mg/dL after that. Over the next 48 h, the hemiparesis and mental status changes resolved without sequelae. The events of the case suggest a hypoglycemia-induced seizure with subsequent Todd's paralysis. Early direct medical evaluation in suspected glyburide ingestions in children is suggested
SN - 0736-4679
AD - Kentucky Regional Poison Center, Louisville 40232-5070, USA
UR - 9610973
ER -

TY - JOUR
ID - 1857
T1 - Validation of cardiovascular fitness field tests in children with mental retardation

A1 - Fernhall,B.
A1 - Pitetti,K.H.
A1 - Vukovich,M.D.
A1 - Stubbs,N.
A1 - Hensen,T.
A1 - Winnick,J.P.
A1 - Short,F.X.
Y1 - 1998/05//
N1 - Fernhall, B. Pitetti, K H. Vukovich, M D. Stubbs, N. Hensen, T. Winnick, J P. Short, F X
American journal of mental retardation : AJMR
ame, 8800463
IM
Journal Article. Research Support, U.S. Gov't, Non-P.H.S.
English
KW - MEDLINE
KW - Adolescent
KW - Analysis of Variance
KW - Child
KW - Evaluation Studies as Topic
KW - Exercise Test/mt [Methods]
KW - Exercise Test/st [Standards]
KW - Female
KW - Humans
KW - Intellectual Disability/pp [Physiopathology]
KW - Linear Models
KW - Male
KW - Physical Fitness/ph [Physiology]
KW - Reproducibility of Results
RP - NOT IN FILE
SP - 602
EP - 612
JF - American Journal of Mental Retardation
JA - Am J Ment Retard.
VL - 102
IS - 6
CY - UNITED STATES
N2 - The validity of the 600-yard walk/run, the 20-m shuttle run, and a modified 16-m shuttle run was determined to measure aerobic capacity (VO2peak) in children with mild and moderate mental retardation. Practice sessions for all tests were conducted. All field tests were very reliable, and VO2peak was significantly related to them all. A stepwise multiple regression showed that field test performance, body mass index (BMI), and gender, but not age, were also significant predictors of VO2peak. All field tests were valid and reliable indicators of aerobic capacity, suggesting that these tests can be used to predict VO2max in children with mild and moderate mental retardation
SN - 0895-8017
AD - George Washington University Medical Center, Washington, DC 20052, USA
UR - 9606469
ER -

TY - JOUR
ID - 1858
T1 - Monitoring of solar-UV exposure among schoolchildren in five Japanese cities using spore dosimeter and UV-coloring labels
A1 - Munakata,N.
A1 - Ono,M.
A1 - Watanabe,S.
Y1 - 1998/03//

N1 - Munakata, N. Ono, M. Watanabe, S
Japanese journal of cancer research : Gann
hba, 8509412
IM
Journal Article. Research Support, Non-U.S. Gov't
English
KW - MEDLINE
KW - Air Pollution
KW - Radioactive
KW - Bacillus subtilis/re [Radiation Effects]
KW - Child
KW - Humans
KW - Japan
KW - Radiation Monitoring/mt [Methods]
KW - Radiometry
KW - Spores/re [Radiation Effects]
KW - Sunlight
KW - Ultraviolet Rays
RP - NOT IN FILE
SP - 235
EP - 245
JF - Japanese Journal of Cancer Research
JA - Jpn J Cancer Res
VL - 89
IS - 3
CY - JAPAN
N2 - To monitor personal exposure to biologically effective solar-UV radiation, Bacillus subtilis spores on a membrane filter and UV-coloring labels were incorporated into a monitoring badge. The samples were covered with one of three types of filter sheet, dependent on the season, to reduce the amounts of exposure to measurable levels. Five fifth- or sixth-grade classes of primary schools, each consisting of 30-40 children, were chosen in northern (Sapporo), central (Tsukuba and Tokyo), and southern (Miyazaki and Naha) cities in Japan. In all four season, each child wore a badge on an upper arm for the entire waking hours, changing it daily, for a week. Upon collection of the badges, the survival of spores and the extent of coloration of the label were determined. The results were used to estimate the amount of daily exposure to biologically effective UV radiation, expressed as the value of spore inactivation dose. Unexpectedly, the average amounts of exposure were not directly correlated with the outdoor UV irradiance: in the two southern cities, despite high outdoor irradiance from spring to autumn, the average amounts of exposure were less than 3.1% of the average irradiance. Highly concentrated exposures occurred in two central cities on three days when extensive outdoor exercise took place. These results contradict the simple notion that children's exposure is in proportion to the outdoor UV irradiance, and support the view that the extent of solar-UV exposure is primarily determined by life-style rather than living location
SN - 0910-5050
AD - Radiobiology Division, National Cancer Center Research Institute, Tokyo
UR - 9600116
ER -

TY - JOUR
ID - 1859
T1 - [Children in a day care center that concentrates on outdoor activities are less frequently sick]. [Swedish]
A1 - Soderstrom,M.
A1 - Blennow,M.
Y1 - 1998/04/08/
N1 - Soderstrom, M. Blennow, M
Lakartidningen
l0n, 0027707
IM

Journal Article
Swedish
KW - MEDLINE
KW - Child Day Care Centers
KW - Child
KW - Preschool
KW - Environmental Exposure
KW - Humans
KW - Infant
KW - Morbidity
KW - Play and Playthings
RP - NOT IN FILE
SP - 1670
EP - 1672
JF - Lakartidningen
VL - 95
IS - 15
CY - SWEDEN
SN - 0023-7205
AD - Avdelningen for allmanmedicin, Vasa sjukhus, Goteborg
UR - 9599472
ER -

TY - JOUR
ID - 1860
T1 - A case-control study of risk factors for playground injuries among children in Kingston and area
A1 - Mowat,D.L.
A1 - Wang,F.
A1 - Pickett,W.
A1 - Brison,R.J.
Y1 - 1998/03//
N1 - Mowat, D L. Wang, F. Pickett, W. Brison, R J
Injury prevention : journal of the International Society for Child and Adolescent Injury Prevention
crz, 9510056
IM
Journal Article. Research Support, Non-U.S. Gov't
English
KW - MEDLINE
KW - Adolescent
KW - Case-Control Studies
KW - Child
KW - Preschool
KW - Female
KW - Humans
KW - Infant
KW - Leisure Activities
KW - Male
KW - Multivariate Analysis
KW - Ontario/ep [Epidemiology]
KW - Risk Factors
KW - Wounds and Injuries/ep [Epidemiology]
RP - NOT IN FILE
SP - 39
EP - 43
JF - Injury Prevention
JA - Inj Prev
VL - 4

IS - 1
CY - ENGLAND
N2 - OBJECTIVE: To determine the risk for injury associated with environmental hazards in public playgrounds. SETTING: One hundred and seventeen playgrounds operated by municipalities or school boards in and around Kingston, Ontario, Canada. METHODS: A regional surveillance database was used to identify children presenting to emergency departments who were injured on public playgrounds; each case was individually matched (by sex, age, and month of occurrence) with two controls--one non-playground injury control, and one child seen for non-injury emergency medical care. Exposure data were obtained from an audit of playgrounds conducted using Canadian and US safety guidelines. Exposure variables included the nature of playground hazards, number of hazards, frequency of play, and total family income. No difference in odds ratios (ORs) were found using the two sets of controls, which were therefore combined for subsequent analysis. RESULTS: Multivariate analysis showed strong associations between injuries and the use of inappropriate surface materials under and around equipment (OR 21.0, 95% confidence interval (CI) 3.4 to 128.1), appropriate materials with insufficient depth (OR 18.2, 95% CI 3.3 to 99.9), and inadequate handrails or guardrails (OR 6.7, 95% CI 2.6 to 17.5). CONCLUSION: This study confirms the validity of guidelines for playground safety relating to the type and depth of surface materials and the provision of handrails and guardrails. Compliance with these guidelines is an important means of preventing injury in childhood
SN - 1353-8047
AD - Kingston, Frontenac and Lennox and Addington Health Unit, Queen's University, Kingston, Ontario, Canada
UR - 9595330
ER -

TY - JOUR
ID - 1861
T1 - Exposure to lead and other metals in children from Katowice district, Poland
A1 - Osman,K.
A1 - Zejda,J.E.
A1 - Schutz,A.
A1 - Mielzynska,D.
A1 - Elinder,C.G.
A1 - Vahter,M.
Y1 - 1998/05//
N1 - Osman, K. Zejda, J E. Schutz, A. Mielzynska, D. Elinder, C G. Vahter, M
International archives of occupational and environmental health
gpn, 7512134
IM
Comparative Study. Journal Article. Research Support, Non-U.S. Gov't
English
KW - MEDLINE
KW - Child
KW - Environmental Exposure
KW - Environmental Pollutants
KW - Female
KW - Humans
KW - Industry
KW - Lead/bl [Blood]
KW - Male
KW - Metals
KW - Heavy/bl [Blood]
KW - Poland
KW - Social Class
KW - Sweden
RP - NOT IN FILE
SP - 180
EP - 186
JF - International Archives of Occupational & Environmental Health

JA - Int Arch Occup Environ Health
VL - 71
IS - 3
CY - GERMANY
N2 - OBJECTIVES: To assess the exposure to toxic metals and to evaluate its possible association with essential elements and socioeconomic status in children from the town of Bytom in the Katowice area; this area is one of the most polluted industrialized regions in Poland. METHODS: Concentrations of lead, cadmium, mercury, selenium, magnesium, copper, and zinc were determined in whole blood of 211 children aged 9 years. The samples were analyzed using inductively coupled plasma mass spectrometry (ICP-MS). Information on socioeconomic factors was collected using questionnaires. Concentrations of trace elements in the blood of 24 Swedish children aged 9-10 years were used for comparisons. RESULTS: The concentrations of lead detected in the blood of the Polish children ranged from 0.09 to 1.9 mumol/l, with the median value being 0.27 mumol/l. Statistically significant associations were found between lead and such socioeconomic factors as the number of siblings, trips outside the region, maternal smoking, playing outdoors, and apartment standard. The average blood lead level was about 3 times higher in the Polish children than in the Swedish group. The median blood concentration of cadmium found in the Polish children was 3.4 nmol/l (range 1.1-41 nmol/l; almost 3 times higher than that detected in the Swedish children), and that of mercury was 3 nmol/l (range 0.5-11 nmol/l). The median blood levels and ranges of the essential elements were 1.1 (0.7-2.0) mumol/l for selenium, 1.5 (1.2-1.9) mmol/l for magnesium, 17 (13-22) mumol/l for copper, and 78 (54-104) mumol/l for zinc, respectively. The concentrations of selenium and magnesium were significantly lower in the Polish group as compared with the Swedish children. CONCLUSIONS: In all, 7% of the Polish children had blood lead levels exceeding 0.5 mumol/l, the concentration above which negative effects on mental development have been reported. However, the findings indicate a decrease in lead exposure during recent years among the Bytom children
SN - 0340-0131
AD - Institute of Environmental Medicine, Karolinska Institutet, Stockholm, Sweden.
Katarina.Osman@imm.ki.se
UR - 9591159
ER -

TY - JOUR
ID - 1862
T1 - Eosinophil cationic protein (ECP) and tryptase in the nasal lavage fluid (NLF) of children with grass pollen rhinitis: levocabastine effect
A1 - Bernardini,R.
A1 - Novembre,E.
A1 - Mugnaini,L.
A1 - Rossi,M.E.
A1 - Vierucci,A.
Y1 - 1998/03//
N1 - Bernardini, R. Novembre, E. Mugnaini, L. Rossi, M E. Vierucci, A
Allergy and asthma proceedings : the official journal of regional and state allergy societies
ci9, 9603640
IM
Comparative Study. Journal Article
English
KW - MEDLINE
KW - Blood Proteins/an [Analysis]
KW - Child
KW - Chymases
KW - Eosinophil Granule Proteins
KW - Female
KW - Histamine H1 Antagonists/tu [Therapeutic Use]
KW - Humans
KW - Male
KW - Nasal Lavage Fluid/ch [Chemistry]
KW - Piperidines/tu [Therapeutic Use]
KW - Poaceae

KW - Rhinitis
KW - Allergic
KW - Seasonal/dt [Drug Therapy]
KW - Seasonal/me [Metabolism]
KW - Ribonucleases
KW - Serine Endopeptidases/an [Analysis]
KW - Tryptases
RP - NOT IN FILE
SP - 75
EP - 80
JF - Allergy & Asthma Proceedings
JA - Allergy Asthma Proc
VL - 19
IS - 2
CY - UNITED STATES
N2 - This study was carried out to determine the concentrations of eosinophil cationic protein (ECP) and tryptase in the nasal lavage fluid (NLF) of 24 children (C) with grass pollen rhinitis as well as rhinitis symptoms before and after nasal provocation tests with or without a levocabastine (anti-H1 topical antihistamine) pretreatment. All C were monosensitized to grasses only. Twelve patients (Active Group = AG) were tested with a nasal provocation test with grass pollen (NPT) carried out by the insufflation of increasingly higher doses of an allergenic extract powder, while the other 12 patients (Placebo Group = PG) underwent just a nasal provocation test with lactose (placebo) (NPTp). The prechallenge NLF, obtained both before (C) and after (AG) levocabastine pretreatment, was compared to that obtained after periods of 2 and 24 hours postchallenge. In the AG, before and after levocabastine pretreatment, the tryptase concentrations had not significantly increased, whereas the ECP concentrations were found to be significantly higher ($p < 0.05$) in just the 24-hour postchallenge samples. In the PG the rhinitis symptoms were not induced by the NPTp and there was no significant change in either ECP or tryptase concentrations. In the AG a levocabastine pretreatment induced a significant increase ($p < 0.05$) in the cumulative allergen doses administered by the NPT. There was a reduction of the nasal symptoms in 7 patients, while in 3 subjects there was only a slight improvement, but in 2 subjects no effect was encountered. In conclusion this study shows that a levocabastine pretreatment before an NPT in patients with grass pollen rhinitis, outside the grass pollen season, induces a significant increase in the cumulative allergen doses (administered by the NPT) which provoked rhinitis symptoms but is not able to demonstrate any significant reduction in the ECP concentrations of the NLF
SN - 1088-5412
AD - Department of Pediatrics, A. Meyer Hospital, University of Florence, Italy
UR - 9578915
ER -

TY - JOUR
ID - 1863
T1 - Tobacco advertisements: one of the strongest risk factors for smoking in Hong Kong students
A1 - Lam,T.H.
A1 - Chung,S.F.
A1 - Betson,C.L.
A1 - Wong,C.M.
A1 - Hedley,A.J.
Y1 - 1998/04//
N1 - Lam, T H. Chung, S F. Betson, C L. Wong, C M. Hedley, A J
American journal of preventive medicine
8704773, apl
IM
Journal Article. Research Support, Non-U.S. Gov't
English
KW - MEDLINE
KW - Adolescent
KW - Advertising as Topic
KW - Child

KW - Cross-Sectional Studies
KW - Female
KW - Health Knowledge
KW - Attitudes
KW - Practice
KW - Health Status
KW - Hong Kong
KW - Humans
KW - Logistic Models
KW - Male
KW - Odds Ratio
KW - Plants
KW - Toxic
KW - Questionnaires
KW - Risk Factors
KW - Smoking/ep [Epidemiology]
KW - Smoking/pc [Prevention & Control]
KW - Smoking/px [Psychology]
KW - Students/px [Psychology]
KW - Tobacco
RP - NOT IN FILE
SP - 217
EP - 223
JF - American Journal of Preventive Medicine
JA - Am J Prev Med
VL - 14
IS - 3
CY - NETHERLANDS
N2 - STUDY OBJECTIVE: To describe the prevalence of smoking in junior secondary school students in Hong Kong and to analyse the relationship between a range of risk factors and ever-smoking experience, including tobacco advertisements. METHODS: Cross-sectional survey using an anonymous standardised self-administered questionnaire. SETTING: 61 randomly selected secondary schools in Hong Kong. PARTICIPANTS: 6,304 form 1 to 3 Chinese students from 172 classes (51% girls and 49% boys; 90% were aged 12 to 15 years). RESULTS: The smoking status (95% confidence interval) was: (1) never smoker, 71.1% (70.0%-72.2%), (2) tried only, 15.5% (14.6%-16.4%), (3) used to smoke but not now, 4.2% (3.7%-4.7%), (4) smoked < 1 cigarette per week, 2.9% (2.5%-3.4%), (5) smoked 1-6 per week, 1.7% (1.4%-2.0%), and (6) smoked > 6 per week, 4.5% (4.0%-5.0%). In a backward stepwise logistic regression model, ever-smoking (including categories 2 to 6 above) was independently associated with thirteen factors, including gender (boys), increasing age, place of birth (outside Hong Kong, mainly China), poor knowledge of the hazards of smoking, positive attitudes to smoking, smoking in family members, participation in tobacco promotional activities, and perception of cigarette advertisements as attractive. Among the strongest associations observed was the youth's perception of cigarette advertisements as attractive, with the adjusted odds ratio (95% confidence interval) of 2.68 (2.33-3.07). CONCLUSIONS: Smoking among young people is an important public health problem. Although the causes are multifactorial, in Hong Kong environmental tobacco advertising is an important risk factor that can be removed by banning all forms of tobacco promotion to young people
SN - 0749-3797
AD - Department of Community Medicine, The University of Hong Kong-Hong Kong
UR - 9569223
ER -

TY - JOUR
ID - 1864
T1 - Diagnosis and treatment of dental trauma in a children's hospital
A1 - Lombardi,S.
A1 - Sheller,B.
A1 - Williams,B.J.
Y1 - 1998/03//

N1 - Lombardi, S. Sheller, B. Williams, B J
Pediatric dentistry
pan, 7909102
D, IM
Journal Article. Research Support, Non-U.S. Gov't
English
KW - MEDLINE
KW - Accidental Falls/sn [Statistics & Numerical Data]
KW - Accidents
KW - Home/sn [Statistics & Numerical Data]
KW - Adolescent
KW - Adult
KW - Age Factors
KW - Athletic Injuries/ep [Epidemiology]
KW - Bandages/sn [Statistics & Numerical Data]
KW - Child
KW - Preschool
KW - Dental Care for Children/sn [Statistics & Numerical Data]
KW - Emergencies
KW - Female
KW - Hospitals
KW - Pediatric/sn [Statistics & Numerical Data]
KW - Humans
KW - Incisor/in [Injuries]
KW - Infant
KW - Jaw Fractures/di [Diagnosis]
KW - Jaw Fractures/ep [Epidemiology]
KW - Jaw Fractures/th [Therapy]
KW - Male
KW - Maxilla
KW - Restraint
KW - Physical
KW - Retrospective Studies
KW - Splints/sn [Statistics & Numerical Data]
KW - Tooth Extraction/sn [Statistics & Numerical Data]
KW - Tooth Injuries/di [Diagnosis]
KW - Tooth Injuries/ep [Epidemiology]
KW - Tooth Injuries/th [Therapy]
KW - Washington/ep [Epidemiology]
RP - NOT IN FILE
SP - 112
EP - 120
JF - Pediatric Dentistry
JA - Pediatr Dent
VL - 20
IS - 2
CY - UNITED STATES
N2 - A comprehensive review of 487 emergency dental trauma visits seen at a children's hospital during a 3-year period was performed to investigate aspects of trauma care that have not been previously reported. Patient characteristics, diagnoses, and injury treatment were analyzed. General patient characteristics and diagnoses were consistent with other studies. Young children were often injured in falls within the home whereas older children were often injured in sports and activities outside the home. The emergency visit was the first contact with the dentist for 80% of children 3.5 years old and younger. Maxillary incisors accounted for 84% of primary tooth injuries: examination and extraction were common treatments. Maxillary incisors accounted for 87% of permanent tooth injuries: bandage restorations and splint placement were frequent treatments. An assistive restraint device was used most often for children 4 years and younger who required extractions. The restraint

was used more frequently during clinic hours with support staff present than after hours with a sole provider. Hospital facilities were not essential for treatment of the patients in this study
SN - 0164-1263
AD - Department of Dental Medicine, Children's Hospital and Medical Center, University of Washington, Seattle, USA
UR - 9566015
ER -

TY - JOUR
ID - 1865
T1 - Refugee children from the Middle East
A1 - Montgomery,E.
Y1 - 1998///
N1 - Montgomery, E
Scandinavian journal of social medicine. Supplementum
udt, 0412776
IM
Journal Article
English
KW - MEDLINE
KW - Adolescent
KW - Anxiety
KW - Child
KW - Child Psychology
KW - Preschool
KW - Denmark
KW - Humans
KW - Interviews as Topic
KW - Middle East/eh [Ethnology]
KW - Parents
KW - Refugees
KW - Stress Disorders
KW - Post-Traumatic
KW - Stress
KW - Psychological
KW - Torture
KW - Violence
KW - War
RP - NOT IN FILE
SP - 1
EP - 152
JF - Scandinavian Journal of Social Medicine
JA - Scand J Soc Med Suppl
VL - Supplementum. 54
CY - SWEDEN
N2 - OBJECTIVE: To map the frequency (prevalence) of torture victims among parents in asylum seeking Middle Eastern refugee families, to map the occurrence (prevalence) of experiences of war and other forms of organised violence among the children in these families, to map the occurrence (prevalence) of emotional symptoms and behavioural problems among the children, and to identify risk indicators and modifying factors for anxiety symptoms among the children. DESIGN: Interview with parents using a structured interview questionnaire developed for this study. Validated through a blinded semi-structured interview conducted with approximately 1/3 of the families. AUSPICES: The study has been carried out by the Rehabilitation and Research Centre for Torture Victims (RCT) in cooperation with the Danish Red Cross. MATERIAL: Structured interviews with parents regarding 311 children aged 3-15 from 149 families, all registered as asylum seekers from the Middle East between February 1, 1992 and April 30, 1993. The response was 90.4%. PRINCIPAL VARIABLES: Background (past-past)--social and demographic data; trauma complex (past)--war-related life circumstances (conditions) and experiences of war and other forms of organized violence such as loss,

separation, direct exposure to violence and witnessing acts of violence (specific events and changes of life conditions); present life context (past-present)--family circumstances upon arrival in Denmark; effect (present)--the child's current psychological state. RESULTS: 28% of the parents (44% of the fathers and 13% of the mothers) had been tortured, to the effect that 51% of the children were part of a family including a survivor of torture. The most frequent specific types of violence-related events or circumstances were 'lived in a refugee camp outside the home country' (92%), 'lived under conditions of war' (89%) and 'been on the run with parents' (89%). Twenty percent of the children had lost one parent, and another 60% had been separated from one parent for more than a month. The highest prevalence of emotional symptoms were found within the anxiety dimension, as 67% of the children were assessed as being clinically anxious. The most important risk indicators for anxiety were 'lived in a refugee camp outside the home country', 'part of a torture surviving family', 'lack of opportunities for play with other children', 'beaten/kicked by an official', and 'loss of father'. Current parental behaviour was also an important risk indicator for anxiety, if the mother or father hit or punished the child more than was the case prior to arrival in Denmark. The most important anxiety-modifying factor was arrival in Denmark in the company of both parents. CONCLUSIONS: Asylum seeking refugee children from the Middle East have had many experiences of war and other forms of organised violence. The children frequently reacted with anxiety and with other symptoms of emotional instability. Prevalent anxiety symptoms correlated both with previous living conditions and present family situation. Living under prolonged conditions influenced by war and other forms of organised violence (prevalence) were found to a higher degree to be risk indicators for anxiety than were specific events or changes of life conditions (incidence)
SN - 0301-7311
AD - Rehabilitation and Research Centre for Torture Victims, Copenhagen
UR - 9526769
ER -

TY - JOUR
ID - 1866
T1 - Epidemiology of tuberculosis in Montreal
A1 - Rivest,P.
A1 - Tannenbaum,T.
A1 - Bedard,L.
Y1 - 1998/03/10/
N1 - Rivest, P. Tannenbaum, T. Bedard, L
CMAJ : Canadian Medical Association journal = journal de l'Association medicale canadienne
9711805
AIM, IM
Journal Article
English
KW - MEDLINE
KW - Adolescent
KW - Adult
KW - Aged
KW - Antitubercular Agents/tu [Therapeutic Use]
KW - Child
KW - Preschool
KW - Drug Resistance
KW - Multiple
KW - Emigration and Immigration/sn [Statistics & Numerical Data]
KW - Humans
KW - Incidence
KW - Infant
KW - Middle Aged
KW - Mycobacterium tuberculosis/ip [Isolation & Purification]
KW - Population Surveillance
KW - Quebec/ep [Epidemiology]
KW - Time Factors
KW - Tuberculosis/dt [Drug Therapy]
KW - Tuberculosis/ep [Epidemiology]

KW - Tuberculosis/eh [Ethnology]
RP - NOT IN FILE
SP - 605
EP - 609
JF - CMAJ Canadian Medical Association Journal
JA - CMAJ
VL - 158
IS - 5
CY - CANADA
N2 - OBJECTIVE: To identify the epidemiologic characteristics of tuberculosis (TB) in Montreal and the patterns of resistance to antituberculous drugs in order to improve TB control in the region. DESIGN: Descriptive analysis of surveillance data for TB cases reported in Montreal by physicians and laboratories between 1992 and 1995. SETTING: Region of Montreal, population 1,775,889. PARTICIPANTS: All cases of active TB among Montreal residents reported to the Department of Public Health between Jan. 1, 1992, and Dec. 31, 1995. OUTCOME MEASURES: Epidemiologic characteristics, proportion of cases resistant to antituberculous drugs and types of resistance. RESULTS: A total of 798 cases of TB (mean annual incidence 11.2 per 100,000) were reported in Montreal during the study period. Of these patients, 617 (77.3%) were born outside Canada. The annual incidence of TB in the foreign-born population (37.5 per 100,000) was 10 times the rate in the Canadian-born population, and the highest rate among foreign-born residents (62.8 per 100,000) occurred in those 15-29 years of age. In general, annual incidence in Montreal's foreign-born population reflected the reported incidence of TB in their regions of birth. In 8.7% of all cases, the disease was resistant to isoniazid, and the proportion of cases resistant to this drug was greater than 4% in almost all age groups, among both foreign-born and Canadian-born patients. CONCLUSIONS: TB remains a major problem in Montreal, as in other large cities. Surveillance data give opportunities to public health agencies to adapt their prevention and control strategies to local situations and can also help clinicians in their clinical decision-making
SN - 0820-3946
AD - Direction de la sante publique, Regie regionale de la sante et des services sociaux de Montreal-centre, Montreal, Que. privest@santepub-mtl.qc.ca
UR - 9526474
ER -

TY - JOUR
ID - 1867
T1 - Skin cancer prevention for children, parents, and caregivers: a field test of Hawaii's SunSmart program
A1 - Glanz,K.
A1 - Chang,L.
A1 - Song,V.
A1 - Silverio,R.
A1 - Muneoka,L.
Y1 - 1998/03//
N1 - Glanz, K. Chang, L. Song, V. Silverio, R. Muneoka, L
Journal of the American Academy of Dermatology
hvg, 7907132
IM
Journal Article. Research Support, Non-U.S. Gov't. Research Support, U.S. Gov't, P.H.S.
English
KW - MEDLINE
KW - Caregivers
KW - Child
KW - Hawaii
KW - Health Education/mt [Methods]
KW - Humans
KW - Outcome and Process Assessment (Health Care)
KW - Parents
KW - Skin Neoplasms/pc [Prevention & Control]
KW - Sunlight
RP - NOT IN FILE

SP - 413
EP - 417
JF - Journal of the American Academy of Dermatology
JA - J Am Acad Dermatol
VL - 38
IS - 3
CY - UNITED STATES
N2 - BACKGROUND: Children are at particular risk for sun exposure, a major cause of skin cancer. Parents and caregivers can help protect children by educating them about practicing safe sun habits, serving as role models, and providing supportive environments. OBJECTIVE: We evaluated SunSmart, a cancer prevention program for 6- to 8-year-old children, their parents, and outdoor recreation staff. METHODS: The intervention included staff training, on-site activities for children, interactive take-home booklets, behavior-monitoring scoreboards, incentives, providing sunscreen, and encouraging sun safe environments and policies. The program was evaluated by baseline and follow-up surveys of parents and recreation staff, monitoring forms, and on-site observations. RESULTS: There were positive changes in all major outcomes, including knowledge; sun protection habits of parents, children, and staff; readiness to change; sun protection policies; and sun protection norms. The changes ranged from improvements of 3% to more than 20%, and several changes were statistically significant. CONCLUSION: The results demonstrated the feasibility and short-term impact of Hawaii's SunSmart program and suggested areas for refinement and expansion
SN - 0190-9622
AD - Cancer Research Center of Hawaii, University of Hawaii at Manoa, Honolulu 96813, USA
UR - 9520022
ER -

TY - JOUR
ID - 1868
T1 - A wilderness medicine course for pediatric residents
A1 - Newman,R.D.
A1 - Shubkin,C.D.
A1 - Chapman,S.H.
A1 - Diekema,D.S.
Y1 - 1998/02//
N1 - Newman, R D. Shubkin, C D. Chapman, S H. Diekema, D S
Pediatric emergency care
pau, 8507560
IM
Journal Article
English
KW - MEDLINE
KW - Child
KW - Curriculum
KW - Emergency Medicine/ed [Education]
KW - Environment
KW - Humans
KW - Internship and Residency
KW - Pediatrics/ed [Education]
KW - Recreation
KW - Washington
RP - NOT IN FILE
SP - 58
EP - 61
JF - Pediatric Emergency Care
JA - Pediatr Emerg Care
VL - 14
IS - 1
CY - UNITED STATES

N2 - OBJECTIVE: To design a structured curriculum to teach pediatric residents about wilderness medicine. BACKGROUND: An increasing number of children are involved in more rigorous and potentially risky outdoor activities. Despite the breadth of exposure characteristic of most pediatric residences, we are aware of no formalized syllabus that prepares residents to both treat injuries sustained in outdoor pursuits, and help parents and children to prepare safely for such activities. METHODS: The first half of the course was designed to teach a broad range of topics in wilderness medicine through a series of readings, lectures, and field trips. The second half of the course involved a six-day course in wilderness skills. RESULTS: Over a three-week period, the major topics of wilderness medicine were thoroughly covered. The three residents involved in the planning and execution of the course felt that the course succeeded in filling an important gap in their pediatric residency training. CONCLUSIONS: The addition of a structured wilderness medicine elective to pediatric residencies, with or without a field component, may provide a valuable opportunity for pediatric residents to broaden their skills and knowledge base to include these increasingly important topics
SN - 0749-5161
AD - Department of Pediatrics, University of Washington, Seattle 98195-6320, USA
UR - 9516634
ER -

TY - JOUR
ID - 1869
T1 - The effect of lead in tap water on blood lead in children in a smelter town
A1 - Meyer,I.
A1 - Heinrich,J.
A1 - Trepka,M.J.
A1 - Krause,C.
A1 - Schulz,C.
A1 - Meyer,E.
A1 - Lippold,U.
Y1 - 1998/01//
N1 - Meyer, I. Heinrich, J. Trepka, M J. Krause, C. Schulz, C. Meyer, E. Lippold, U
The Science of the total environment
uj0, 0330500
IM
Journal Article. Research Support, Non-U.S. Gov't
English
KW - MEDLINE
KW - Adolescent
KW - Child
KW - Preschool
KW - Drinking
KW - Environmental Exposure
KW - Female
KW - Germany
KW - Humans
KW - Lead/an [Analysis]
KW - Lead/bl [Blood]
KW - Lead Poisoning/et [Etiology]
KW - Male
KW - Mining
KW - Water Pollutants
KW - Chemical/an [Analysis]
KW - Water Supply/an [Analysis]
RP - NOT IN FILE
SP - 255
EP - 271
JF - Science of the Total Environment
JA - Sci Total Environ
VL - 209

IS - 2-3
CY - NETHERLANDS
N2 - Hettstedt, a city in eastern Germany with a long history of mining and smelting of non-ferrous ores, has multiple lead waste deposits and the remains of a former lead smelter and a copper-silver smelter. As part of a cross-sectional study, an analysis of lead concentrations in drinking water and in blood was undertaken to determine the impact of lead in drinking water on the internal burden of lead in children. The geometric mean of blood lead levels among children 5-14 years old was 35.0 micrograms/l with a 95% confidence interval (C.I.) of 33.4-36.7. The geometric mean of lead in the random tap water samples was 0.5 microgram/l (95% C.I., 0.5-0.6) and 0.7 microgram/l (95% C.I., 0.6-0.8) in the stagnant tap water samples. Blood lead levels were somewhat correlated with the random water measures but not the stagnant water measures (random sample: r = 0.12, P = 0.012; stagnant sample: r = 0.04, P = 0.396). After adjustment for relevant confounders, lead in drinking water (random sample) was not significantly associated with blood lead levels. Factors that were significantly associated with blood lead included gender, the city area of residence, lead in house dust, regular contact with dogs and dirtiness of the child after playing outdoors. Based on this study, lead in domestic tap water contributed little to the lead exposure of children in the lead contaminated region of Hettstedt
SN - 0048-9697
AD - GSF Forschungszentrum fur Umwelt und Gesundheit, Institut fur Epidemiologie, Neuherberg, Germany
UR - 9514044
ER -

TY - JOUR
ID - 1870
T1 - Personal sampling of airborne particles: method performance and data quality
A1 - Janssen,N.A.
A1 - Hoek,G.
A1 - Harssema,H.
A1 - Brunekreef,B.
Y1 - 1998/01//
N1 - Janssen, N A. Hoek, G. Harssema, H. Brunekreef, B
Journal of exposure analysis and environmental epidemiology
bjn, 9111438
IM
Journal Article
English
KW - MEDLINE
KW - Activities of Daily Living
KW - Aged
KW - Air Pollution/an [Analysis]
KW - Child
KW - Environmental Exposure
KW - Environmental Monitoring/is [Instrumentation]
KW - Female
KW - Humans
KW - Male
KW - Middle Aged
KW - Particle Size
KW - Sampling Studies
KW - Sensitivity and Specificity
RP - NOT IN FILE
SP - 37
EP - 49
JF - Journal of Exposure Analysis and Environmental Epidemiology
JA - J Expo Anal Environ Epidemiol
VL - 8
IS - 1
CY - UNITED STATES

N2 - A study of personal exposure to respirable particles (PM10) and fine particles (FP) was conducted in groups of 50-70 year-old adults and primary school children in the Netherlands. Four to eight personal measurements per subject were conducted, on weekdays only. Averaging time was 24 hours. Method performance was evaluated regarding compliance, flow, weighing procedure, field blanks and co-located operation of the personal samplers with stationary methods. Furthermore, the possibility that subjects change their behavior due to the wearing of personal sampling equipment was studied by comparing time activity on days of personal sampling with time activity other weekdays. Compliance was high; 95% of the subjects who agreed to continue participating after the first measurement, successfully completed the study, and, expect for the first two days of FP sampling, over 90% of all personal measurements were successful. All pre and post sampling flow readings were within 10% of the required flow rate of 4 L/min. For PM10 precision of the gravimetric analyses was 2.8 microgram/m3 and 0.7 micrograms/m3 for filters weighted on an analytical and a micro-balance respectively. The detection limit was 10.8 micrograms/m3 and 8.6 micrograms/m3 respectively. For FP, weighing precision was 0.4 micrograms/m3 and the detection limit was 5.3 micrograms/m3. All measurements were above the detection limit. Co-located operation of the personal sampler with stationary samplers gave highly correlated concentration (R > 0.90). Outdoor PM10 concentrations measured with the personal sampler were on average 4% higher compared to a Sierra Anderson (SA) inlet and 9% higher compared to a PM10 Harvard Impactor (HI). With the FP cyclone 6% higher classroom concentrations were measured compared to a PM2.5 HI. Adults spent significantly less time outdoor (0.5 hour) and more time at home (0.9 hour) on days of personal sampling compared to other weekdays. For children no significant differences in time activity were found
SN - 1053-4245
AD - Department of Environmental Sciences, Environmental and Occupational Health University of Wageningen, The Netherlands NICOLE.JANSSEN@STAFF.EOH.WAU.NL
UR - 9470103
ER -

TY - JOUR
ID - 1871
T1 - [A study on fall accident]. [Korean]
A1 - Lee,H.S.
A1 - Kim,M.J.
Y1 - 1997/11//
N1 - Lee, H S. Kim, M J
Taehan kanho. The Korean nurse
kxa, 1264143
N, X
English Abstract. Journal Article
Korean
KW - MEDLINE
KW - Accidental Falls/pc [Prevention & Control]
KW - Accidental Falls/sn [Statistics & Numerical Data]
KW - Adolescent
KW - Adult
KW - Age Distribution
KW - Aged
KW - Child
KW - Preschool
KW - Female
KW - Health Education
KW - Hospitals
KW - General
KW - Humans
KW - Infant
KW - Korea
KW - Male
KW - Middle Aged
KW - Retrospective Studies

KW - Risk Factors
KW - Sex Distribution
KW - Time Factors
RP - NOT IN FILE
SP - 45
EP - 62
JF - Taehan Kanho - Korean Nurse
JA - Taehan Kanho
VL - 36
IS - 5
CY - KOREA (SOUTH)
N2 - The study was conducted from November 1995 to May 1996 at the one general hospital in Seoul. The total subjects of this study were 412 patients who have the experience of fall accident, among them 31 was who have fallen during hospitalization and 381 was who visited emergency room and out patient clinic. The purposes of this study were to determine the characteristics, risk factors and results of fall accident and to suggest the nursing strategies for prevention of fall. Data were collected by reviewing the medical records and interviewing with the fallers and their family members. For data analysis spss/pc+ program was utilized for descriptive statistics, adjusted standardized X2-test. The results of this study were as follows: 1) Total subjects were 412 fallers, of which 245 (59.5%) were men and 167 (40.5%) were women. Age were 0-14 years 79 (19.2%), 15-44 years 125 (30.4%), 45-64 years 104 (25.2%), over 65 years 104 (25.2%). 2) There was significant association between age and the sexes (X2 = 39.17, P = 0.00). 3) There was significant association between age and history of falls (X2 = 44.41, P = .00). And history of falls in the elderly was significantly associated with falls. 4) There was significant association with age and medical diagnosis (X2 = 140.66, P = .00), chief medical diagnosis were hypertension (34), diabetes mellitus (22), arthritis (11), stroke (8), fracture (7), pulmonary tuberculosis (6), dementia (5) and cataract (5). 5) There was significant association between age and intrinsic factors: cognitive impairment, mobility impairment, insomnia, emotional problems, urinary difficulty, visual impairments, hearing impairments, use of drugs (sedatives, antihypertensive drugs, diuretics, antidepressants) (P < 0.05). But there was no significant association between age and dizziness (X2 = 2.87, P = .41). 6) 15.3% of total fallers were drunken state when they were fallen. 7) Environmental factors of fall accident were unusual posture (50.9%), slips (35.2%), trips (9.5%) and collision (4.4%). 8) Most of falls occurred during the day time, peak frequencies of falls occurred from 1 pm to 6 pm and 7 am to 12 am. 9) The places of fall accident were roads (22.6%), house-stairs (16.7%), rooms, floors, kitchen (11.2%), the roof-top, veranda, windows (10.9%), hospital (7.5%), ice or snowy ways (5.8%), bathroom (4.9%), playground, park (4.9%), subway-stairs (4.4%) and public-bathrooms (2.2%). 10) Activities at the time of fall accident were walking (37.6%), turning around or reaching for something (20.9%), going up or down stairs (19.2%), exercise, working (17.4%), up or down from a bed (2.7%), using wheelchair or walking aids, standing up or down from a chair (2.2%) and standing still (2.2%). 11) Anatomical locations of injuries by falls were head, face, neck (31.3%), lower extremities (29.9%), upper extremities (20.6%), spine, thorax, abdomen or pelvic contents (11.4%) and unspecified (2.9%). 12) Types of injures were fracture (47.6%), bruises (13.8%), laceration (13.3%), sprains (9.0%), headache (6.6%), abrasions (2.9%), intracranial hemorhage (2.4%) and burns (0.5%). 13) 41.5% of the fallers were hospitalized and average of hospitalization was 22.3 days. 14) The six fallers (1.46%) died from fall injuries. The two fallers died from intracranial hemorhage and the four fallers died of secondary infection; pneumonia (2), sepsis (1) and cellulitis (1). It is suggested that 1) Further study is needed with larger sample size to identify the fall risk factors. 2) After the fall accident, comprehensive nursing care and regular physical exercise should be emphasized for the elderly person. 3) Safety education and safety facilities of the public place and home is necessary for fall prevention
SN - 0047-3618
UR - 10437605
ER -

TY - JOUR
ID - 1872
T1 - Patterns of change in vector-borne diseases. [Review] [59 refs]
A1 - Molyneux,D.H.
Y1 - 1997/10//
N1 - Molyneux, D H
Annals of tropical medicine and parasitology

68e, 2985178r
IM
Journal Article. Review
English
KW - MEDLINE
KW - Animals
KW - Chagas Disease/pc [Prevention & Control]
KW - Chagas Disease/tm [Transmission]
KW - Child
KW - Preschool
KW - Disease Vectors
KW - Endemic Diseases
KW - Humans
KW - Immunity
KW - Insect Control
KW - Malaria/pc [Prevention & Control]
KW - Onchocerciasis/pc [Prevention & Control]
KW - Onchocerciasis/tm [Transmission]
KW - Parasitic Diseases/ep [Epidemiology]
KW - Parasitic Diseases/pc [Prevention & Control]
KW - Parasitic Diseases/tm [Transmission]
KW - Population Dynamics
KW - Refugees
KW - Trypanosomiasis
KW - African/pc [Prevention & Control]
KW - African/tm [Transmission]
KW - Water Supply
KW - Weather
KW - World Health
RP - NOT IN FILE
SP - 827
EP - 839
JF - Annals of Tropical Medicine & Parasitology
JA - Ann Trop Med Parasitol
VL - 91
IS - 7
CY - ENGLAND
N2 - The statuses of vector-borne diseases have changed over recent years. How a few such diseases have changed and the primary causes of change (urbanization, increased conflict, changes in water-resource management, ecological and environmental change, and reduced health service resourcing) are the subjects of the present review. The key impacts which these primary causes have on selected vectors and the infections they transmit are tabulated. The success of vector-control programmes against onchocerciasis and Chagas disease is discussed, and the methods used to evaluate the epidemiological impact of such controls are described. Bednet programmes for control of malaria are recognized as a potential future means of reducing morbidity and mortality in children. In contrast to the success achieved in limiting Simulium and Triatoma populations through vertical programmes, control of tsetse, whilst successful in Uganda, has not been utilized to stem recent epidemics of sleeping sickness in resource-stressed settings in Central Africa. Vector-borne diseases will continue to be a problem because of the adaptability of vectors, the potential problems of managing effective vector controls within decentralized health systems, and the influence of activities outside the health sector itself. Changes beyond the health sector can increase the problem posed by a vector and increase the frequency of transmission. [References: 59]
SN - 0003-4983
AD - Liverpool School of Tropical Medicine, U.K. fahy@liv.ac.uk
UR - 9625939
ER -

TY - JOUR

ID - 1873
T1 - Serological markers for treponemal infection in children in rural Kilimanjaro, Tanzania: evidence of syphilis or non-venereal treponematoses?
A1 - Klouman,E.
A1 - Masenga,E.J.
A1 - Sam,N.E.
Y1 - 1997/12//
N1 - Klouman, E. Masenga, E J. Sam, N E
Genitourinary medicine
gem, 8503853
IM, X
Journal Article. Research Support, Non-U.S. Gov't
English
KW - MEDLINE
KW - Adolescent
KW - Adult
KW - Child
KW - Preschool
KW - Cohort Studies
KW - Cross-Sectional Studies
KW - Female
KW - Humans
KW - Infant
KW - Newborn
KW - Male
KW - Prevalence
KW - Risk Factors
KW - Rural Health
KW - Sex Factors
KW - Sexual Behavior
KW - Syphilis/ep [Epidemiology]
KW - Syphilis/mi [Microbiology]
KW - Tanzania/ep [Epidemiology]
KW - Treponemal Infections/ep [Epidemiology]
KW - Treponemal Infections/mi [Microbiology]
KW - Treponemal Infections/tm [Transmission]
RP - NOT IN FILE
SP - 522
EP - 527
JF - Genitourinary Medicine
JA - Genitourin.Med
VL - 73
IS - 6
CY - ENGLAND
N2 - OBJECTIVE: To determine the seroprevalence of treponemal infection and possible risk factors among children aged 0-14 in the general population of a rural Tanzanian village. METHODS: The survey was conducted as a part of a cross section study of a total village population on HIV and sexually transmitted disease. Among 1708 registered children aged 0-14, the 553 first attending were tested for treponemal infection with both rapid plasma reagin test (RPR) and Treponema pallidum Haemagglutination test (TPHA). These children belonged to a household cohort--also including their parents, siblings, and other household members--with 1339 members; 1224 (91.4% participated in the survey and 82.1% of these were tested for treponemal infection. RESULTS: The overall prevalence for the TPHA test was 6.4% among girls and 1.1% among boys (odds ratio, OR = 6.5; 95% confidence interval, CI: 1.9-22.3). The sex difference was most pronounced in the age group 10-14; 11.1% among girls versus 1.0% among boys (OR = 12.8; CI: 1.6-101.9). Among the 20 children who were TPHA positive, we found two cases of active, congenital syphilis. There was a lack of association between positive serology in children and positive serology in their parents. CONCLUSION: The highly significant predominance of girls testing positive for TPHA, and the concomitant lack of association

between parents' and children's serostatus might point to sexual transmission as being the most common route of transmission of treponemal infection in girls during childhood in this village. The sources of infection for the seropositive girls are possibly found outside the family
SN - 0266-4348
AD - Institute of General Practice and Community Medicine, University of Oslo, Norway
UR - 9582474
ER -

TY - JOUR
ID - 1874
T1 - [Favorable results with intravenous antimicrobial therapy outside the hospital]. [Dutch]
A1 - van den Broek,P.J.
A1 - Haerkens,H.M.
A1 - van Weert,N.J.
A1 - Vermeij,P.
Y1 - 1997/11/22/
N1 - van den Broek, P J. Haerkens, H M. van Weert, N J. Vermeij, P
Nederlands tijdschrift voor geneeskunde
nuk, 0400770
IM
Clinical Trial. English Abstract. Journal Article. Research Support, Non-U.S. Gov't
Dutch
KW - MEDLINE
KW - Adolescent
KW - Adult
KW - Aged
KW - 80 and over
KW - Anti-Bacterial Agents/ad [Administration & Dosage]
KW - Antiviral Agents/ad [Administration & Dosage]
KW - Child
KW - Preschool
KW - Evaluation Studies as Topic
KW - Female
KW - Home Care Services
KW - Humans
KW - Infection/dt [Drug Therapy]
KW - Infusion Pumps
KW - Infusions
KW - Intravenous/is [Instrumentation]
KW - Male
KW - Middle Aged
KW - Patient Satisfaction
KW - Self Administration
RP - NOT IN FILE
SP - 2297
EP - 2301
JF - Nederlands Tijdschrift voor Geneeskunde
JA - Ned Tijdschr Geneeskd
VL - 141
IS - 47
CY - NETHERLANDS
N2 - OBJECTIVE: To determine whether intravenous home treatment with antimicrobial drugs of patients with an infection is feasible. DESIGN: Descriptive. SETTING: Academic Hospital, Leiden, the Netherlands. METHODS: In October 1992 a programme for intravenous home treatment of patients with infectious diseases was started. The programme was based on self-administration of the intravenous antibiotic therapy by the patient. The hospital pharmacy provided the antibiotics and infusion devices, an infectious diseases specialist supervised the home treatment and the nursing staff of the First Aid Department was available to change

infusion cannulas. After assessment of their medical and psychosocial fitness in which the general practitioner was involved, thorough instruction on use of the infusion device and care of the infusion cannula, patients were sent home. RESULTS: In the period October 1992-September 1996, 162 patients (median age: 45.0 years (range: 3-82)) were treated at home for a median period of 15 days (1-221). Osteomyelitis, arthritis, Lyme disease, septicaemia with secondary foci, and herpes virus infections were the most frequent indications for home treatment. Penicillins and cephalosporins were given in 70% of the cases, teicoplanin and antiviral drugs in 12% and 14%, respectively. For intermittent administration bolus injection, a mechanical or elastomeric pump were used, for continuous infusion a syringe or cassette pump. The intention of the programme that the patient performed the home treatment as much as possible without extra professional help, was realised in 82% of the patients. In 60% of the cases the patients were fully self supporting, in 22% family or friends played an important role. CONCLUSION: Home treatment with antimicrobial drugs intravenously is quite feasible. If this were fully implemented, about 1400 patients in the Netherlands could be treated in this manner
SN - 0028-2162
AD - Afd. Infectieziekten, Academisch Ziekenhuis, Leiden
UR - 9550815
ER -

TY - JOUR
ID - 1875
T1 - Variations in health behaviours among inner city 12-year-olds from four ethnic groups
A1 - Rogers,A.
A1 - Adamson,J.E.
A1 - McCarthy,M.
Y1 - 1997/11//
N1 - Rogers, A. Adamson, J E. McCarthy, M
Ethnicity & health
cy1, 9608374
IM
Comparative Study. Journal Article. Research Support, Non-U.S. Gov't
English
KW - MEDLINE
KW - African Americans/sn [Statistics & Numerical Data]
KW - African Continental Ancestry Group
KW - Alcohol Drinking/eh [Ethnology]
KW - Attitude to Health
KW - Bangladesh/eh [Ethnology]
KW - Child
KW - Ethnic Groups/sn [Statistics & Numerical Data]
KW - European Continental Ancestry Group/sn [Statistics & Numerical Data]
KW - Exercise
KW - Female
KW - Food Habits
KW - Health Behavior
KW - Humans
KW - Life Style
KW - London
KW - Male
KW - Nutrition Surveys
KW - Smoking/eh [Ethnology]
KW - Urban Population
RP - NOT IN FILE
SP - 309
EP - 316
JF - Ethnicity & Health
JA - Ethn Health
VL - 2
IS - 4

CY - ENGLAND
N2 - OBJECTIVES: To describe factors that contribute to variations in health-related behaviours and attitudes among inner city 12-year-olds. To see if there was an identifiable patterning by ethnic group. DESIGN: Semi-structured interviews with a stratified sample of 12-year-old students and their parents from four ethnic groups, attending state secondary schools in two inner London boroughs. RESULTS: Bangladeshi young people were significantly more likely to receive school meals. There was no variation in reported snacking between the groups. Girls and Bangladeshi students were less likely to report exercising outside school (33% of Bangladeshi boys reported not exercising outside school compared to 5% of boys from all other groups). Bangladeshi boys and their parents were more likely to report that bullying or worries about racial violence prevented them from going out after school. White young people were more likely to report experimenting with and the regular use of cigarettes and alcohol. Use of alcohol and cigarettes was also associated with gender, religion and strength of religious observance. White parents were the least likely to report restricting their child's social activities as a way of influencing behaviour and expressed more concerns about their child's potential for health-damaging behaviour than parents in all other groups. CONCLUSION: This study shows that ethnicity alone is insufficient and inadequate in explaining variations in health behaviours among inner city teenagers. A complex mix of personal, cultural and social factors including ethnicity shape the behaviours and attitudes of these young people
SN - 1355-7858
AD - Department of Epidemiology & Public Health, University College London, UK
UR - 9526693
ER -

TY - JOUR
ID - 1876
T1 - Childhood exposure to PM10: relation between personal, classroom, and outdoor concentrations
A1 - Janssen,N.A.
A1 - Hoek,G.
A1 - Harssema,H.
A1 - Brunekreef,B.
Y1 - 1997/12//
N1 - Janssen, N A. Hoek, G. Harssema, H. Brunekreef, B
Occupational and environmental medicine
bwc, 9422759
IM
Journal Article
English
KW - MEDLINE
KW - Air Pollutants/an [Analysis]
KW - Air Pollution
KW - Indoor
KW - Child
KW - Environmental Exposure
KW - Female
KW - Humans
KW - Male
KW - Netherlands
KW - Particle Size
KW - Regression Analysis
KW - Schools
KW - Tobacco Smoke Pollution
RP - NOT IN FILE
SP - 888
EP - 894
JF - Occupational & Environmental Medicine
JA - Occup Environ Med
VL - 54
IS - 12
CY - ENGLAND

N2 - OBJECTIVES: To investigate the validity of outdoor concentrations of particulate matter < 10 microns diameter (PM10) as a measure of exposure in time series studies, and to study the extent to which differences between personal and outdoor PM10 concentrations can be explained. METHODS: Four to eight repeated measurements of personal and outdoor PM10 concentrations were conducted for 45 children, aged 10-12 years, from four schools in Wageningen and Amsterdam, The Netherlands. Repeated PM10 measurements in the classrooms were conducted in three of the schools. Averaging time was 24 hours for the personal and outdoor measurements, and eight hours (daytime) and 24 hours for the classroom measurements. For each child separately, personal exposures were related to outdoor concentrations in a regression analysis. The distribution of the individual correlation and regression coefficients was investigated. Information about factors that might influence personal exposures was obtained by questionnaire. RESULTS: Median Pearson's correlations between personal and outdoor concentrations were 0.63 for children with parents who did not smoke and 0.59 for children with parents who smoked. For children with parents who did not smoke, excluding days with exposure to environmental tobacco smoke (ETS) improved the correlation to a median R of 0.73. The mean personal PM10 concentration was 105 micrograms/m3; on average 67 micrograms/m3 higher than the corresponding outdoor concentrations. The main part of this difference could be attributed to exposure to ETS, to high PM10 concentrations in the classrooms, and to (indoor) physical activity. CONCLUSIONS: The results show a reasonably high correlation between repeated personal and outdoor PM10 measurements within children, providing support for the use of fixed site measurements as a measure of exposure to PM10 in epidemiological time series studies. The large differences between personal and outdoor PM10 concentrations probably result from a child's proximity to particle generating sources and particles resuspended by personal activities
SN - 1351-0711
AD - Department of Environmental Sciences, University of Wageningen, The Netherlands
UR - 9470897
ER -

TY - JOUR
ID - 1877
T1 - Ambient air levels and the exposure of children to benzene, toluene, and xylenes in Denmark
A1 - Raaschou-Nielsen,O.
A1 - Lohse,C.
A1 - Thomsen,B.L.
A1 - Skov,H.
A1 - Olsen,J.H.
Y1 - 1997/11//
N1 - Raaschou-Nielsen, O. Lohse, C. Thomsen, B L. Skov, H. Olsen, J H
Environmental research
ei2, 0147621
IM
Journal Article. Research Support, Non-U.S. Gov't
English
KW - MEDLINE
KW - Air Pollutants/an [Analysis]
KW - Air Pollutants/to [Toxicity]
KW - Air Pollution
KW - Indoor/ae [Adverse Effects]
KW - Indoor/an [Analysis]
KW - Benzene/an [Analysis]
KW - Benzene/to [Toxicity]
KW - Child
KW - Denmark
KW - Environmental Exposure
KW - Environmental Health
KW - Female
KW - Humans
KW - Male
KW - Nitrogen Dioxide/an [Analysis]
KW - Nitrogen Dioxide/to [Toxicity]

KW - Regression Analysis
KW - Reproducibility of Results
KW - Rural Health
KW - Toluene/an [Analysis]
KW - Toluene/to [Toxicity]
KW - Urban Health
KW - Xylenes/an [Analysis]
KW - Xylenes/to [Toxicity]
RP - NOT IN FILE
SP - 149
EP - 159
JF - Environmental Research
JA - Environ Res
VL - 75
IS - 2
CY - UNITED STATES
N2 - The aims of the study were to evaluate if the front-door concentrations of benzene, toluene, and xylenes can be used to classify the personal exposures of Danish children and to identify factors that affect their personal exposure. Average concentrations were measured over 1 week with diffusive samplers, and the personal exposures of 98 children and the concentrations outside the front doors of their homes were measured simultaneously. Time and activity patterns were noted in diaries. The front-door concentrations were significantly higher in Copenhagen than in rural areas (all $P < 0.0001$), but the personal exposures were only slightly higher. Even though the personal exposures were highly significantly associated with front-door concentrations in urban areas (all $P < 0.004$), use of the residential front-door concentration as an exposure surrogate would imply misclassification, as it cannot be used for rural children. Multiple regression analyses brought to light several factors that affect the exposure of children independently, including front-door concentration, riding in cars, and activities involving potential exposure to gasoline vapors like motocross, moped driving, and refueling of cars
SN - 0013-9351
AD - Division for Cancer Epidemiology, Danish Cancer Society, Copenhagen, Denmark. ole@cancer.dk
UR - 9417846
ER -

TY - JOUR
ID - 1878
T1 - Prevalence, epidemiology and geographical distribution of Sporothrix schenckii infections in Gauteng, South Africa
A1 - Vismer,H.F.
A1 - Hull,P.R.
Y1 - 1997///
N1 - Vismer, H F. Hull, P R
Mycopathologia
no4, 7505689
IM
Journal Article
English
KW - MEDLINE
KW - Adolescent
KW - Adult
KW - Aged
KW - 80 and over
KW - Child
KW - Preschool
KW - Epidemiologic Factors
KW - Female
KW - Humans
KW - Infant

KW - Male
KW - Middle Aged
KW - Occupational Diseases/ep [Epidemiology]
KW - Recreation
KW - Risk Factors
KW - Seasons
KW - Soil Microbiology
KW - South Africa/ep [Epidemiology]
KW - Sporothrix/ip [Isolation & Purification]
KW - Sporotrichosis/ep [Epidemiology]
RP - NOT IN FILE
SP - 137
EP - 143
JF - Mycopathologia
VL - 137
IS - 3
CY - NETHERLANDS
N2 - Sporotrichosis is a subcutaneous fungal infection caused by the traumatic implantation of the dimorphic, pathogenic fungus, Sporothrix schenkii. It constitutes the most common subcutaneous fungal infection in the general population in South Africa. Sporotrichosis in South Africa dates back to 1914, when the disease was first diagnosed in the gold mines. Occupational and recreational circumstances of infection are well established, and the environmental requirements for contracting the disease are better understood. Sporotrichosis cases were recorded from 42 suburbs in the greater Pretoria area as well as from 23 towns outside the Pretoria municipal boundary. It occurred in 154 patients with ages ranging from less than 1 year to 90 years old, with males predominating. Females in the area seemed to be at lesser risk, mainly becoming infected through gardening injuries, insect bites or other minor injuries due to outdoor activities. Exposure to possible sources of the fungus, either from recreational or occupational activities in males, was the main determining factor in acquiring the disease. The lymphocutaneous and localized forms of the disease were most often recorded. Our study indicates that, while there is no pronounced seasonal variation, the onset of the disease seemed to be mainly in the cooler and dryer months of the year
SN - 0301-486X
AD - Programme on Mycotoxins and Experimental Carcinogenesis (PROMEC), Medical Research Council, Tygerberg, South Africa. HVISMER@Eagle.MRC.ac.za
UR - 9368407
ER -

TY - JOUR
ID - 1879
T1 - Old paint removal and blood lead levels in children
A1 - Bates,M.N.
A1 - Wyatt,R.
A1 - Garrett,N.
Y1 - 1997/10/10/
N1 - Bates, M N. Wyatt, R. Garrett, N
The New Zealand medical journal
obq, 0401067
IM
Journal Article. Research Support, Non-U.S. Gov't
English
KW - MEDLINE
KW - Child
KW - Preschool
KW - Cross-Sectional Studies
KW - Dust/an [Analysis]
KW - Environmental Exposure
KW - Female
KW - Hobbies

KW - Housing
KW - Humans
KW - Infant
KW - Lead/an [Analysis]
KW - Lead/bl [Blood]
KW - Lead Poisoning/ep [Epidemiology]
KW - Lead Poisoning/et [Etiology]
KW - Lead Poisoning/pc [Prevention & Control]
KW - Male
KW - Paint/ae [Adverse Effects]
KW - Paint
KW - Risk Factors
RP - NOT IN FILE
SP - 373
EP - 377
JF - New Zealand Medical Journal
JA - N Z Med J
VL - 110
IS - 1053
CY - NEW ZEALAND
N2 - AIM: To identify risk factors, particularly paint removal and clean up practices, for elevated blood lead levels in children, 12 to 24 months old, living in Wellington city. METHODS: Children living in residences more than 50 years old, where residential paint removal had taken place in the last two years, were recruited. Care givers were interviewed, a blood sample was taken from the child's arm and a dust wipe sample was collected from the kitchen floor. Blood and dust samples were analysed for lead content. RESULTS: Data were collected for 141 children (75% of those eligible). The mean blood lead level was 0.24 mumol/L (5.06 micrograms/dL). Higher blood levels were associated with lower income of the main family earner, playing outside, eating dirt and parental hobbies involving lead use. Children receiving regular medical treatment were at lower risk of lead absorption. With the exception of paint removal involving blow torches, none of the paint removal, clean up, or disposal practices was more strongly associated than the other methods with elevated blood lead levels in children living in the household. Dust wipes from the kitchen floor had little predictive value for blood lead level. CONCLUSIONS: High temperature methods of paint removal and parental hobbies involving lead are associated with higher lead levels in children. Given the availability of alternative means of paint removal, continuing use of high temperature methods is unnecessary. Further work is needed to assess lead absorption risks associated with hobbies involving lead
SN - 0028-8446
AD - Epidemiology Group, Institute of Environmental Science and Research Ltd, Porirua
UR - 9364184
ER -

TY - JOUR
ID - 1880
T1 - Tuberculin reactivity among California Hispanic migrant farm workers
A1 - McCurdy,S.A.
A1 - Arretz,D.S.
A1 - Bates,R.O.
Y1 - 1997/12//
N1 - McCurdy, S A. Arretz, D S. Bates, R O
American journal of industrial medicine
3in, 8101110
IM
Journal Article. Research Support, Non-U.S. Gov't. Research Support, U.S. Gov't, P.H.S.
English
KW - MEDLINE
KW - Adolescent
KW - Adult
KW - Agriculture

KW - California
KW - Child
KW - Preschool
KW - Cross-Sectional Studies
KW - Female
KW - Hispanic Americans
KW - Humans
KW - Infant
KW - Logistic Models
KW - Male
KW - Occupational Diseases/ep [Epidemiology]
KW - Prevalence
KW - Transients and Migrants
KW - Tuberculosis/ep [Epidemiology]
RP - NOT IN FILE
SP - 600
EP - 605
JF - American Journal of Industrial Medicine
JA - Am J Ind Med
VL - 32
IS - 6
CY - UNITED STATES
N2 - We conducted a cross-sectional study of tuberculin reactivity among residents of two northern California migrant-farm-worker housing centers. Participants completed a brief health questionnaire and were offered tuberculin skin testing with radiologic and medical follow-up. Four hundred and sixty-nine persons (estimated participation rate: 70%) completed questionnaires. All but one were Hispanic. Two hundred and ninety-six (63%) participants completed tuberculin skin testing and 49 (16.6%) showed reactivity (> or = 10 mm induration at 48-72 hours). Increased prevalence was seen for the 15-39-year age group (vs. persons younger than 15: OR 2.59; 95% CI 0.79-8.47), former smokers (vs. never smokers: OR 3.11; 95% CI 1.20-8.09), and persons born outside the U.S. (OR 2.09; 95% CI 0.66-6.61). Prophylaxis with isoniazid was recommended for 23 persons; nine (39%) completed therapy. No cases of active tuberculosis were found. Prevalence of tuberculin reactivity in this population is lower than reported among Hispanic farm workers in the eastern and midwestern U.S. Higher prevalence may obtain among California farm workers not included in the study population, including homeless, single, and highly mobile persons. Public-health efforts in this population should focus on ever-smokers, young adults, and persons born outside the U.S
SN - 0271-3586
AD - University of California Agricultural Health and Safety Center, Department of Epidemiology and Preventive Medicine, University of California, Davis School of Medicine 95616-8638, USA. samccurdy@ucdavis.edu
UR - 9358916
ER -

TY - JOUR
ID - 1881
T1 - Excessive daytime sleepiness associated with idiopathic alterations of consciousness
A1 - Oseas-McNamara,M.E.
A1 - Rosner,B.
A1 - Regestein,Q.R.
Y1 - 1997/10//
N1 - Oseas-McNamara, M E. Rosner, B. Regestein, Q R
Clinical EEG (electroencephalography)
dcg, 0236454
IM
Journal Article
English
KW - MEDLINE
KW - Adolescent

KW - Adult
KW - Aged
KW - Child
KW - Consciousness Disorders/co [Complications]
KW - Consciousness Disorders/pp [Physiopathology]
KW - Electroencephalography
KW - Female
KW - Humans
KW - Male
KW - Middle Aged
KW - Polysomnography
KW - Sleep Disorders/co [Complications]
KW - Sleep Disorders/pp [Physiopathology]
RP - NOT IN FILE
SP - 207
EP - 213
JF - Clinical Electroencephalography
JA - Clin Electroencephalogr.
VL - 28
IS - 4
CY - UNITED STATES
N2 - Sleep/wake patterns were recorded by continuous 24-hour ambulatory polysomnography in 339 patients, who had episodes of altered consciousness. Patients were recorded while they were outside the hospital. From a seven-channel montage of electrodes affixed below the hairline, sleep polygraphic EEG was easily read from T3-T4, EOG from F2-F8 and EMG from T3-T6. Sleep was staged by analysis of aural signals on 60 times real time playback, augmented by continuous visual display and selected frozen frames. Patient major sleep period patterns reflected those reported for general populations. Unexpectedly, 47% of the patients took daytime naps and 44% of the nappers took more than one nap. Naps had a mean duration of 71 minutes. Those who took no naps slept significantly longer at night by 23 min. Napping reduced night sleep much more in patients who did not take CNS-acting medications. We conclude that excessive sleepiness may in part explain complaints of episodically altered consciousness
SN - 0009-9155
AD - Community Integration, Inc., Erie, PA, USA
UR - 9343713
ER -

TY - JOUR
ID - 1882
T1 - Developing assistive technology strategies for infants and toddlers with communication difficulties
A1 - Reinhartsen,D.B.
A1 - Edmondson,R.
A1 - Crais,E.R.
Y1 - 1997///
N1 - Reinhartsen, D B. Edmondson, R. Crais, E R
Seminars in speech and language
8405117, cfb, 8405117
IM
Case Reports. Journal Article
English
KW - MEDLINE
KW - Child Development
KW - Child Language
KW - Child
KW - Preschool
KW - Communication Aids for Disabled
KW - Communication Disorders/rh [Rehabilitation]
KW - Female

KW - Humans
KW - Infant
KW - Language Development
KW - Male
KW - Play and Playthings
KW - Speech Therapy
KW - Verbal Behavior
RP - NOT IN FILE
SP - 283
EP - 301
JF - Seminars in Speech & Language
JA - Semin Speech Lang
VL - 18
IS - 3
CY - UNITED STATES
N2 - Communication is a process that begins at birth and continues throughout life. For young children with developmental delay or disability, the acquisition of expected communication skills may be compromised. Compromised communication skills, in turn, may lead to additional challenges when participating in daily activities and routines, forming social relationships, and developing independence. Assistive technology (AT) is one means by which a child's current communication skills can be expanded and enhanced within the child's natural environment. Increased awareness by both parents and professionals of the availability of AT has led to the inclusion of AT strategies within routine day-to-day early intervention practices. This article briefly highlights early aspects of communication development in typically developing infants and toddlers, then provides a framework through which AT strategies can be identified and utilized by parents and professionals to improve a child's existing communication skills
SN - 0734-0478
AD - Clinical Center for the Study of Development and Learning, University of North Carolina at Chapel Hill 27599, USA
UR - 9306520
ER -

TY - JOUR
ID - 1883
T1 - Time-activity-patterns of some selected small groups as a basis for exposure estimation: a methodological study
A1 - Dorre,W.H.
Y1 - 1997/10//
N1 - Dorre, W H
Journal of exposure analysis and environmental epidemiology
bjn, 9111438
IM
Journal Article
English
KW - MEDLINE
KW - Activities of Daily Living
KW - Adult
KW - Air Pollutants/ae [Adverse Effects]
KW - Air Pollutants/an [Analysis]
KW - Child
KW - Preschool
KW - Data Collection/mt [Methods]
KW - Environmental Exposure/an [Analysis]
KW - Female
KW - Humans
KW - Locomotion
KW - Male
KW - Mathematical Computing

KW - Time Factors
RP - NOT IN FILE
SP - 471
EP - 491
JF - Journal of Exposure Analysis and Environmental Epidemiology
JA - J Expo Anal Environ Epidemiol
VL - 7
IS - 4
CY - UNITED STATES
N2 - In preparation for large-scale investigations, we have conducted some methodological studies with small groups in order to determine patterns of stay and time activity patterns in various microenvironments at different times of the day. A diary technique was used. This paper reports some results of this methodological study: In one study we investigated a group of toddlers (n = 52, aged 2-3 years) attending a nursery school, and in a second study we compared two groups of medical students (n = 79 and 54, respectively). The reported data was analyzed by descriptive statistical methods. The toddlers spent an average of 87.6% of their time indoors (66.2% at home, 20.4% at nursery schools, 1% at other indoor areas), 11.2% of their time outdoors, and 1.2% of their time using various means of enclosed transportation. In the student study, time budgets of the two student groups differ only slightly. The students spent about 87% of their time indoors (about 66% at home, 14% in rooms connected with their study, 7% in other indoor areas), about 6% of their time using various means of enclosed transportation, and about 7% of their time outdoors. In order to describe the physical activity, the students were asked to evaluate their physical activity according to a low, medium or high physical strain. These evaluations were used to calculate a "mean activity coefficient" for each microenvironment and each hour of day. Not every individual in a group spent time in every given microenvironment during the period of investigation. Therefore, the mean over all the individuals of the group (the "group mean") and the mean over the individuals who in fact did spend time in any given microenvironment (the "actual doers mean") may be very different. There are two kinds of frequency distributions of time spent in various microenvironments: (1) The distribution of the individual's mean time spent in the microenvironment across the entire investigation period (distribution of the individual means) and (2) the distribution of the duration of time spent in the microenvironment for all participants at all days (distribution of the person-days). Both types of frequency distributions should be determined for the purpose of exposure and risk estimation for pollutants
SN - 1053-4245
AD - Universitatsklinikum Charite, Institut fur Mikrobiologie und Hygiene, Medizinische Fakultat, Humboldt-Universitat zu Berlin, Germany. goebel@rz.charite.hu-berlin.de
UR - 9306232
ER -

TY - JOUR
ID - 1884
T1 - Skin sensitization in asthmatic children less than 36 months of age
A1 - Song,C.H.
Y1 - 1997/09//
N1 - Song, C H
Annals of allergy, asthma & immunology : official publication of the American College of Allergy, Asthma, & Immunology
cbm, 9503580
IM
Journal Article
English
KW - MEDLINE
KW - Age of Onset
KW - Aging/im [Immunology]
KW - Asthma/di [Diagnosis]
KW - Asthma/ep [Epidemiology]
KW - Child
KW - Preschool
KW - Female
KW - Humans

KW - Immunization
KW - Infant
KW - Male
KW - Respiratory Sounds/pp [Physiopathology]
KW - Skin/im [Immunology]
KW - Skin Tests
RP - NOT IN FILE
SP - 273
EP - 276
JF - Annals of Allergy, Asthma, & Immunology
JA - Ann Allergy Asthma Immunol
VL - 79
IS - 3
CY - UNITED STATES
N2 - BACKGROUND: Atopy is reported to play an insignificant role in wheezing during infancy in contrast to later childhood. For this reason skin testing may not be included in a workup of wheezing infants. OBJECTIVE/METHOD: In order to evaluate the degree and evolution of skin sensitization to allergens in infants with asthma, we have retrospectively analyzed the skin test results from 40 referred asthmatic children less than 36 months of age, who had had more than three wheezing episodes and whose symptoms improved on treatment with beta-agonist and anti-inflammatory agents. RESULTS: Skin sensitization (epicutaneous) to common indoor and outdoor aeroallergens and foods were demonstrated in 23 (58%) of these patients. Asthmatic children with the onset before 12 months of age and duration of less than 12 months were sensitive predominantly to foods. Those with later onset asthmas and longer duration developed sensitivity first to indoor and then to outdoor allergens. The most common food, indoor, and outdoor allergens were egg, dust mite, and pollens, respectively. Six of these patients had repeat skin tests later. Two demonstrated a decrease in skin sensitivities to foods, one gained a new sensitivity to grass, while three remained unsensitized. CONCLUSION: Skin sensitization to allergens was common in selected asthmatic infants and evolves in the order of exposures: foods, indoor allergens, and outdoor allergens. Repeat skin tests showed changing skin test patterns in some patients
SN - 1081-1206
AD - Department of Pediatrics, Harbor-UCLA Medical Center, Torrance, USA
UR - 9305237
ER -

TY - JOUR
ID - 1885
T1 - Front-door concentrations and personal exposures of Danish children to nitrogen dioxide
A1 - Raaschou-Nielsen,O.
A1 - Skov,H.
A1 - Lohse,C.
A1 - Thomsen,B.L.
A1 - Olsen,J.H.
Y1 - 1997/09//
N1 - Raaschou-Nielsen, O. Skov, H. Lohse, C. Thomsen, B L. Olsen, J H
Environmental health perspectives
ei0, 0330411
IM
Journal Article
English
KW - MEDLINE
KW - Air Pollutants/an [Analysis]
KW - Child
KW - Child Welfare
KW - Preschool
KW - Denmark
KW - Environmental Exposure/an [Analysis]
KW - Environmental Monitoring

KW - Epidemiologic Measurements
KW - Female
KW - Humans
KW - Male
KW - Mathematical Computing
KW - Nitrogen Dioxide/an [Analysis]
KW - Risk Assessment
KW - Vehicle Emissions/an [Analysis]
RP - NOT IN FILE
SP - 964
EP - 970
JF - Environmental Health Perspectives
JA - Environ Health Perspect
VL - 105
IS - 9
CY - UNITED STATES
N2 - The aims of the study were to evaluate the front-door concentration of traffic exhaust fumes as a surrogate for the personal exposure of children and to study factors in the behavior and the environment of children that affect their personal exposure to nitrogen dioxide (NO(2)). The exposure to NO(2) of 103 children living in Copenhagen and 101 children living in rural areas of Denmark was studied by measuring average concentrations over 1 week with diffusive badge samplers placed outside the front door of the home, inside the child's bedroom, and on each child. Detailed information about the activities of the children involving potential exposure to NO(2) was noted in diaries. The results indicated that the front-door concentration of traffic pollution might be used to classify the personal exposure of urban children, although misclassification would be introduced. Multiple regression analysis showed several factors that affected the personal NO(2) exposure of the children independently, including the front-door concentration, the bedroom concentration, time spent outdoors, gas appliances used at home, passive smoking, and burning candles
SN - 0091-6765
AD - Division for Cancer Epidemiology, Danish Cancer Society, Copenhagen, Denmark
UR - 9300931
ER -

TY - JOUR
ID - 1886
T1 - Physical activity levels and prompts in young children at recess: a two-year study of a bi-ethnic sample
A1 - McKenzie,T.L.
A1 - Sallis,J.F.
A1 - Elder,J.P.
A1 - Berry,C.C.
A1 - Hoy,P.L.
A1 - Nader,P.R.
A1 - Zive,M.M.
A1 - Broyles,S.L.
Y1 - 1997/09//
N1 - McKenzie, T L. Sallis, J F. Elder, J P. Berry, C C. Hoy, P L. Nader, P R. Zive, M M. Broyles, S L
Research quarterly for exercise and sport
r6y, 8006373
IM
Comparative Study. Journal Article. Research Support, U.S. Gov't, P.H.S.
English
KW - MEDLINE
KW - Age Factors
KW - Child
KW - Child Behavior/eh [Ethnology]
KW - Preschool
KW - European Continental Ancestry Group
KW - Exercise

KW - Female
KW - Humans
KW - Longitudinal Studies
KW - Male
KW - Mexican Americans
KW - Motivation
KW - Motor Activity
KW - Observer Variation
KW - Peer Group
KW - Recreation
KW - Sex Factors
KW - Time Factors
RP - NOT IN FILE
SP - 195
EP - 202
JF - Research Quarterly for Exercise & Sport
JA - Res Q Exerc Sport
VL - 68
IS - 3
CY - UNITED STATES
N2 - We investigated physical activity and encouragement for activity in a bi-ethnic cohort during recess. Activity and associated interactions of 287 children were recorded at preschool and again 2.2 years later. Children expended nearly twice as much energy at preschool recess than at elementary recess. Activity levels declined as recess time elapsed. At preschool, European-American children engaged in more moderate to vigorous activity than Mexican-Americans. As participants moved to elementary school, teachers' prompts to be active decreased and prompts from peers increased. Boys and girls received similar amounts of activity prompts at preschool, but prompts to boys increased over time. The findings suggest that school environments could be altered to promote healthful physical activity among young children
SN - 0270-1367
AD - Department of Exercise and Nutritional Sciences, San Diego State University, USA. tmckenzie@sdsu.edu
UR - 9294873
ER -

TY - JOUR
ID - 1887
T1 - [Auto-transplantation of tooth germs. Discussion and presentation of 3 treated cases]. [Italian]
A1 - Massei,G.
A1 - Cardesi,E.
Y1 - 1997/07//
N1 - Massei, G. Cardesi, E
Minerva stomatologica
nb2, 0421071
D, IM
Case Reports. English Abstract. Journal Article
Italian
KW - MEDLINE
KW - Adolescent
KW - Adult
KW - Child
KW - Female
KW - Follow-Up Studies
KW - Humans
KW - Male
KW - Radiography
KW - Panoramic
KW - Tooth Germ/tr [Transplantation]
KW - Tooth Loss/ra [Radiography]

KW - Tooth Loss/su [Surgery]
KW - Tooth
KW - Supernumerary/ra [Radiography]
KW - Supernumerary/su [Surgery]
KW - Unerupted/su [Surgery]
KW - Transplantation
KW - Autologous/mt [Methods]
KW - Treatment Outcome
RP - NOT IN FILE
SP - 407
EP - 414
JF - Minerva Stomatologica
JA - Minerva Stomatol
VL - 46
IS - 7-8
CY - ITALY
N2 - The authors examine the theoretical possibilities of human dental transplants: autologous, homologous and heterologous. They, then discuss-with reference to autologous transplants-an autotransplant as an alternative to prosthodontic treatment. This would apply both to traditional prosthodontic treatment and on implants or orthodontic treatment aiming at filling dental gaps. They show both general and local counterindications against this operational method the knowledge of which is necessary for an adequate selection of patients. They stress the determining factors for a successful autotransplant: 1) particular care with the choice of the germ to be transplanted taking into account its morphology and the stage of root development; 2) adequate surgical preparation of the receiving site in relation to the size of the germ to be transplanted; 3) suitable surgical technique entailing a particular care in the manipulation of soft and hard tissues and of the germ and appropriate conditions of sterilization; 4) use of appropriate retention means to ensure stability of the transplanted germ so as to favour cellular proliferation and reduce osteoclastic activity; 5) reduction of occlusal pressure on the transplanted germ. The authors describe for example's sake 3 out of 32 cases treated with the documentation of the achieved long-term success. They also analyse the possible causes of failure of such operational method (careless manipulation of the germ, incorrect surgical technique, removal of the germ in a too early stage of its development, too long exposure of the germ outside the oral cavity, poor oral hygiene, caries, periodontal disease, occlusal trauma
SN - 0026-4970
AD - Servizio di Anatomia Patologica, Ospedale Martini, Torino
UR - 9297076
ER -

TY - JOUR
ID - 1888
T1 - Children's perceptions concerning school injuries. [Review] [28 refs]
A1 - Coppens,N.M.
A1 - Koziara,D.M.
Y1 - 1997/08//
N1 - Coppens, N M. Koziara, D M
The Journal of school nursing : the official publication of the National Association of School Nurses
bgs, 9206498
N
Journal Article. Review
English
KW - MEDLINE
KW - Adolescent
KW - Attitude to Health
KW - Child
KW - Child Psychology
KW - Preschool
KW - Female
KW - Humans

KW - Male
KW - Prospective Studies
KW - Questionnaires
KW - Risk Factors
KW - School Nursing
KW - Schools
KW - Wounds and Injuries/et [Etiology]
KW - Wounds and Injuries/pc [Prevention & Control]
RP - NOT IN FILE
SP - 14
EP - 19
JF - Journal of School Nursing
JA - J Sch Nurs
VL - 13
IS - 3
CY - UNITED STATES
N2 - This prospective study examined characteristics of school-related injuries in grades K-6 and children's perceptions regarding prevention and environmental influences. Injuries were most likely to occur on the playground, were most frequent for first and second graders, and for boys. Only 30% of the sample perceived that they could have prevented their injury. This perception was more frequent for children in higher grades. Fifty percent perceived an environmental influence. The Health Promotion Model was used as the theoretical framework to guide this investigation and provide implications for practice. School nurses are in an ideal position to counsel children on taking responsibility for their safety through preventive thinking. [References: 28]
SN - 1059-8405
AD - Department of Nursing, College of Health Professions, University of Massachusetts, Lowell, USA
UR - 9283378
ER -

TY - JOUR
ID - 1889
T1 - The effects of a 2-year physical education program (SPARK) on physical activity and fitness in elementary school students. Sports, Play and Active Recreation for Kids
A1 - Sallis,J.F.
A1 - McKenzie,T.L.
A1 - Alcaraz,J.E.
A1 - Kolody,B.
A1 - Faucette,N.
A1 - Hovell,M.F.
Y1 - 1997/08//
N1 - Sallis, J F. McKenzie, T L. Alcaraz, J E. Kolody, B. Faucette, N. Hovell, M F
American journal of public health
1254074, 3xw
AIM, IM
Clinical Trial. Comparative Study. Controlled Clinical Trial. Journal Article. Research Support, U.S. Gov't, P.H.S.
English
KW - MEDLINE
KW - California
KW - Child
KW - Cohort Studies
KW - Female
KW - Humans
KW - Male
KW - Motor Activity
KW - Physical Education and Training/mt [Methods]
KW - Physical Education and Training/og [Organization & Administration]

KW - Physical Education and Training/sn [Statistics & Numerical Data]
KW - Physical Fitness
KW - Program Evaluation/mt [Methods]
KW - Program Evaluation/sn [Statistics & Numerical Data]
KW - Random Allocation
KW - Sex Characteristics
KW - Time Factors
RP - NOT IN FILE
SP - 1328
EP - 1334
JF - American Journal of Public Health
JA - Am J Public Health
VL - 87
IS - 8
CY - UNITED STATES
N2 - OBJECTIVES: This study evaluated a health-related physical education program for fourth- and fifth-grade students designed to increase physical activity during physical education classes and outside of school. METHODS: Seven schools were assigned to three conditions in a quasi-experimental design. Health-related physical education was taught by physical education specialists or trained classroom teachers. Students from these classes were compared with those in control classes. Analyses were conducted on 955 students with complete data. RESULTS: Students spent more minutes per week being physically active in specialist-led (40 min) and teacher-led (33 min) physical education classes than in control classes (18 min; P < .001). After 2 years, girls in the specialist-led condition were superior to girls in the control condition on abdominal strength and endurance (P < .001) and cardiorespiratory endurance (P < .001). There were no effects on physical activity outside of school. CONCLUSIONS: A health-related physical education curriculum can provide students with substantially more physical activity during physical education classes. Improved physical education classes can potentially benefit 97% of elementary school students
SN - 0090-0036
AD - Department of Psychology, Graduate School of Public Health, San Diego State University, California 92120, USA
UR - 9279269
ER -

TY - JOUR
ID - 1890
T1 - Assessment of respiratory effect of air pollution: study design on general population samples
A1 - Baldacci,S.
A1 - Carrozzi,L.
A1 - Viegi,G.
A1 - Giuntini,C.
Y1 - 1997///
N1 - Baldacci, S. Carrozzi, L. Viegi, G. Giuntini, C
Journal of environmental pathology, toxicology and oncology : official organ of the International Society for Environmental Toxicology and Cancer
jou, 8501420
IM
Journal Article. Research Support, Non-U.S. Gov't
English
KW - MEDLINE
KW - Adolescent
KW - Adult
KW - Aged
KW - 80 and over
KW - Air Pollutants/to [Toxicity]
KW - Child
KW - Preschool
KW - Humans

KW - Italy/ep [Epidemiology]
KW - Middle Aged
KW - Prospective Studies
KW - Respiration/de [Drug Effects]
KW - Respiratory Function Tests
KW - Respiratory Tract Diseases/ci [Chemically Induced]
KW - Respiratory Tract Diseases/ep [Epidemiology]
KW - Respiratory Tract Diseases/pp [Physiopathology]
KW - Rural Population
KW - Urban Population
RP - NOT IN FILE
SP - 77
EP - 83
JF - Journal of Environmental Pathology, Toxicology & Oncology
JA - J Environ Pathol Toxicol Oncol
VL - 16
IS - 2-3
CY - UNITED STATES
N2 - The aim of this paper is to describe an epidemiological model to investigate the relationship between respiratory diseases and environmental air pollution. In the Po Delta prospective study, subjects were investigated before and after a large thermoelectric power plant began operating, in 1980 to 1982 and in 1988 to 1991, respectively. The Pisa prospective study was performed in 1986 to 1988 and in 1991 to 1993, before and after the construction of a new expressway that encircles the city from the North to the Southeast. In each survey, subjects completed the interviewer-administered standardized CNR questionnaire on respiratory symptoms/diseases and risk factors, and performed lung function tests. In the second survey of each study, skin prick tests, total serum IgE determination, methacholine challenge test and biomarkers (such as sister chromatide exchanges, micronuclei, chromosomal abnormalities, DNA and hemoglobin adducts) were also performed. Concentrations of total suspended particulate and SO2 in both surveys were higher in urban than in rural areas, as well as symptom/disease prevalences and bronchial reactivity. Subgroups of subjects from the two samples were enrolled to perform a specific study on the acute respiratory effects of indoor pollution; the daily presence of symptoms and measurements of peak expiratory flow (PEF), daily activity pattern, and assessment of the indoor air quality (particulates < 2.5 mu and NO2) were evaluated. Higher symptom prevalences and PEF variability level were observed in subjects with the highest levels of NO2 or particulates, especially asthmatics. In conclusion, these studies represent a basis for further analyses to better define the relationship between respiratory health and indoor/outdoor pollutant levels
SN - 0731-8898
AD - CNR Institute of Clinical Physiology, Pisa, Italy
UR - 9275987
ER -

TY - JOUR
ID - 1891
T1 - [Prevention of melanoma by sun protective measures in childhood. Temporal changes in awareness of parents]. [German]
A1 - Kolmel,K.F.
A1 - Pfahlberg,A.
A1 - Gefeller,O.
Y1 - 1997/06//
N1 - Kolmel, K F. Pfahlberg, A. Gefeller, O
Der Hautarzt; Zeitschrift fur Dermatologie, Venerologie, und verwandte Gebiete
g13, 0372755
IM
English Abstract. Journal Article. Research Support, Non-U.S. Gov't
German
KW - MEDLINE
KW - Child
KW - Preschool

KW - Clothing
KW - Cross-Sectional Studies
KW - Female
KW - Follow-Up Studies
KW - Germany
KW - Health Education
KW - Health Knowledge
KW - Attitudes
KW - Practice
KW - Humans
KW - Male
KW - Melanoma/et [Etiology]
KW - Melanoma/pc [Prevention & Control]
KW - Neoplasms
KW - Radiation-Induced/et [Etiology]
KW - Radiation-Induced/pc [Prevention & Control]
KW - Parents/ed [Education]
KW - Skin Neoplasms/et [Etiology]
KW - Skin Neoplasms/pc [Prevention & Control]
KW - Sunburn/et [Etiology]
KW - Sunburn/pc [Prevention & Control]
KW - Sunlight/ae [Adverse Effects]
KW - Sunscreening Agents/ad [Administration & Dosage]
RP - NOT IN FILE
SP - 391
EP - 396
JF - Hautarzt
VL - 48
IS - 6
CY - GERMANY
N2 - Numerous epidemiological studies on risk factors of malignant melanoma confirm the etiologic role of excessive UV-exposure especially in childhood. Preventive educational campaigns directed to parents of pre-school children have been inaugurated in several countries. In Germany the information was distributed by the "Working group for Preventive Measures in Dermatology" in cooperation with different public health institutions and the media starting in 1993. To evaluate the influence of these efforts on the knowledge and behaviour of the parents, two successive cross-sectional studies at all 56 nursery schools using the same standardised questionnaire were performed. The first interview took place in spring 1993 (before the campaign) with 1341 evaluable questionnaires', the second in fall 1994 (after the campaign) with 1150 evaluable questionnaire. The knowledge of the parents on melanoma risk factors was significantly improved in the second interview. Also the parental behavior regarding sun-protective measures when their children were outdoor at the beach or in the garden definitely changed. In 1993 the best textile sun protection was used by 21% of the parents at the beach and 36% in the garden. These numbers rose to 34% (beach) and 57% (garden) by the second interview. The percentage of children with no sunburn recorded during the preceding summer rose from 39% to 51%. According to the child's gender the parental behavior was different between the sexes; boys were always better protected than girls. The design of this study with two cross-sectional surveys in the same populations does not provide a methodologically sound basis for attributing the observed positive changes to the campaign. Without any doubt it can be stated that the parental knowledge and their attention to sun protection in their children showed substantial improvement in the second survey after the campaign. Thus, these results provide some evidence for the success of the preventive activities and confirm the necessity to continue with such activities
SN - 0017-8470
AD - Universitats-Hautklinik, Gottingen
UR - 9333614
ER -

TY - JOUR
ID - 1892

T1 - School accidents to children: time to act
A1 - Maitra,A.
Y1 - 1997/07//
N1 - Maitra, A
Journal of accident & emergency medicine
b0u, 9433751
IM
Journal Article
English
KW - MEDLINE
KW - Accident Prevention
KW - Accidents/sn [Statistics & Numerical Data]
KW - Adolescent
KW - Age Distribution
KW - Athletic Injuries/ep [Epidemiology]
KW - Athletic Injuries/pc [Prevention & Control]
KW - Child
KW - Preschool
KW - Emergency Service
KW - Hospital
KW - England/ep [Epidemiology]
KW - Female
KW - Humans
KW - Male
KW - Prospective Studies
KW - Schools
KW - Sex Distribution
KW - Urban Population
KW - Wounds and Injuries/ep [Epidemiology]
KW - Wounds and Injuries/et [Etiology]
KW - Wounds and Injuries/pc [Prevention & Control]
RP - NOT IN FILE
SP - 240
EP - 242
JF - Journal of Accident & Emergency Medicine
JA - J Accid Emerg Med
VL - 14
IS - 4
CY - ENGLAND
N2 - OBJECTIVE: To describe the profile of injuries sustained by children in school accidents and suggest preventive measures. DESIGN: A five month prospective study of children attending an urban accident and emergency (A&E) department. SUBJECTS: 500 children who sustained injuries in school due to a variety of activities. RESULTS: 10 and 12 year old pupils suffered most injuries in school grounds/playgrounds, on concrete, or on grass/soil surfaces due to random activities resulting in striking or being struck by objects/persons, tripping or slipping, and sports (mainly football); 65.5% of these activities were not supervised and 67.4% occurred "out of lessons"; 22% sustained fractures or dislocations, 28.2% needed follow up treatment, and 1.4% were admitted. CONCLUSIONS: Injuries to children in school are a cause for concern. Effective preventive measures should concentrate on (a) specific target areas using schemes based on individual school, and (b) establishing a credible system of monitoring of their effectiveness
SN - 1351-0622
AD - Accident and Emergency Department, Royal Victoria Infirmary, Newcastle Upon Tyne
UR - 9248914
ER -

TY - JOUR
ID - 1893

T1 - Relationship between allergic manifestations and Toxocara seropositivity: a cross-sectional study among elementary school children
A1 - Buijs,J.
A1 - Borsboom,G.
A1 - Renting,M.
A1 - Hilgersom,W.J.
A1 - van Wieringen,J.C.
A1 - Jansen,G.
A1 - Neijens,J.
Y1 - 1997/07//
N1 - Buijs, J. Borsboom, G. Renting, M. Hilgersom, W J. van Wieringen, J C. Jansen, G. Neijens, J
The European respiratory journal
8803460, ery
IM
Comparative Study. Journal Article
English
KW - MEDLINE
KW - Animals
KW - Antibodies
KW - Helminth/bl [Blood]
KW - Child
KW - Preschool
KW - Cross-Sectional Studies
KW - Eosinophilia/et [Etiology]
KW - Female
KW - Humans
KW - Immunoglobulin E/bl [Blood]
KW - Larva Migrans
KW - Visceral/ep [Epidemiology]
KW - Visceral/im [Immunology]
KW - Male
KW - Netherlands/ep [Epidemiology]
KW - Prevalence
KW - Respiratory Hypersensitivity/ep [Epidemiology]
KW - Respiratory Hypersensitivity/im [Immunology]
KW - Respiratory Hypersensitivity/ps [Parasitology]
KW - Risk Factors
KW - Rural Health
KW - Seroepidemiologic Studies
KW - Toxocara/im [Immunology]
KW - Urban Health
RP - NOT IN FILE
SP - 1467
EP - 1475
JF - European Respiratory Journal
JA - Eur Respir J
VL - 10
IS - 7
CY - DENMARK
N2 - Toxocara (the cause of visceral larva migrans in humans) and allergy have in common both elevated immunoglobulin E (IgE) levels and eosinophilia. In the present study, we investigated: 1) associations between Toxocara seropositivity and allergic manifestations; 2) risk factors for Toxocara infection; and 3) differences in Toxocara seroprevalence, allergic manifestations and the associations between these two, in children from urban and rural environments. Blood samples from 1,379 Dutch urban and rural elementary schoolchildren, were examined for Toxocara antibodies, eosinophil numbers, total IgE concentrations, and the occurrence of inhaled allergen-specific IgE. Questionnaires investigating respiratory health and putative risk factors for infection were completed. It was found that 8% of the children had Toxocara antibodies, occurring significantly less often in

females than in males. The means of total serum IgE levels and blood eosinophils were significantly higher in the Toxocara-seropositive than in the seronegative group. Allergic asthma/recurrent bronchitis was found in 7% of the children, allergic reaction on animal contact in 4%, and IgE to at least one inhaled allergen in 16%. These variables were associated with Toxocara seroprevalence. Inhaled allergen-specific IgE and asthma/recurrent bronchitis occurred significantly less often in rural than in urban areas, and significantly less often among girls than among boys. Furthermore, occurrence of allergen-specific IgE increased significantly with age. No association existed between Toxocara seroprevalence and assumed risks, i.e. contact with pet animals and public playgrounds. In conclusion, our results indicate that allergic manifestations occur more often in Toxocara-seropositive children. A relationship with an already existing allergic condition is plausible
SN - 0903-1936
AD - Laboratory for Parasitology and Mycology, National Institute of Public Health and Environment, Bilthoven, The Netherlands
UR - 9230232
ER -

TY - JOUR
ID - 1894
T1 - Physical performance in relation to age, sex, social class and sports activities in kindergarten and elementary school
A1 - Krombholz,H.
Y1 - 1997/06//
N1 - Krombholz, H
Perceptual and motor skills
ozb, 0401131
IM
Journal Article
English
KW - MEDLINE
KW - Age Factors
KW - Child
KW - Child Development/ph [Physiology]
KW - Preschool
KW - Cognition
KW - Female
KW - Growth/ph [Physiology]
KW - Humans
KW - Male
KW - Motor Skills/ph [Physiology]
KW - Physical Fitness
KW - Sex Factors
KW - Social Class
KW - Sports/ph [Physiology]
RP - NOT IN FILE
SP - 1168
EP - 1170
JF - Perceptual & Motor Skills
JA - Percept.Mot.Skills
VL - 84
IS - 3 Pt 2
CY - UNITED STATES
N2 - Physical performance of preschool children and elementary school pupils (N = 2309, age: 61 to 108 mo.) was related to characteristics of physical growth and cognitive performance and to ecological variables. Correlations between measures of physical growth and physical performance and between physical and cognitive performance were positive and significant. Measurements of physical fitness and body coordination increased across ages. Significant differences were found between boys and girls; however, boys exceeded on some items, girls on others. Children of higher socioeconomic status performed better than children of lower status and children who participated in sports outside school outperformed those who did not

SN - 0031-5125
AD - State Institute of Early Childhood Education and Research, Munich, Federal Republic of Germany.
KL21101@sunmail.lrz-muenchen.de
UR - 9229430
ER -

TY - JOUR
ID - 1895
T1 - Safety of surfaces and equipment for children in playgrounds
A1 - Mott,A.
A1 - Rolfe,K.
A1 - James,R.
A1 - Evans,R.
A1 - Kemp,A.
A1 - Dunstan,F.
A1 - Kemp,K.
A1 - Sibert,J.
Y1 - 1997/06/28/
N1 - Mott, A. Rolfe, K. James, R. Evans, R. Kemp, A. Dunstan, F. Kemp, K. Sibert, J
Lancet
2985213r, l0s, 0053266
AIM, IM
Journal Article. Research Support, Non-U.S. Gov't
English
KW - MEDLINE
KW - Accidental Falls/sn [Statistics & Numerical Data]
KW - Adolescent
KW - Arm Injuries/ep [Epidemiology]
KW - Arm Injuries/et [Etiology]
KW - Child
KW - Child Welfare
KW - Preschool
KW - Construction Materials
KW - Fractures
KW - Bone/ep [Epidemiology]
KW - Bone/et [Etiology]
KW - Humans
KW - Infant
KW - Newborn
KW - Play and Playthings
KW - Rubber
KW - Safety
KW - Surface Properties
KW - Wales/ep [Epidemiology]
KW - Wounds and Injuries/ep [Epidemiology]
KW - Wounds and Injuries/et [Etiology]
KW - Wounds and Injuries/pc [Prevention & Control]
RP - NOT IN FILE
SP - 1874
EP - 1876
JF - Lancet
VL - 349
IS - 9069
CY - ENGLAND
N2 - BACKGROUND: The safety of playgrounds is important to protect children from injury, but studies are mostly done mainly under laboratory conditions without epidemiological data. We investigated the safety of different playground surfaces, and types and heights of equipment in public playgrounds in the City of Cardiff,

UK. METHODS: We did a correlational study of 330 children aged between 0 and 14 years. All children were hurt when playing in playgrounds in Cardiff and presented to the Accident and Emergency Department in Cardiff Royal Infirmary during summer (April to September) 1992 and 1993, and the whole of 1994. We studied the children's hospital records to establish the type of injury and interviewed their parents to find out the playground and type of equipment involved. The main outcome measures were the number of children injured whilst playing, and injury rates per observed number of children on different surfaces, types, and heights of equipment. FINDINGS: Children sustained significantly more injuries in playgrounds with concrete surfaces than in those with bark or rubberised surfaces ($p < 0.001$). Playgrounds with rubber surfaces had the lowest rate of injury, with a risk half that of bark and a fifth of that of concrete. Bark surfaces were not significantly more protective against arm fractures than concrete. Most injuries were equipment related. Injury risk due to falls from monkey bars (suspended parallel bars or rings between which children swing) was twice that for climbing-frames and seven times that for swings or slides. The height of the equipment correlated significantly with the number of fractures ($p = 0.005$) from falls. INTERPRETATION: Rubber or bark surfacing is associated with a low rate of injuries and we support their use in all public playgrounds. Bark alone is insufficient, however, to prevent all injuries, particularly arm fractures. Rubberised impact-absorbing surfaces are safer than bark. We believe that playing on monkeys bars increases the risk of injury in playgrounds and that they should generally not be installed. Safety standards should be based on physical and epidemiological data. Our data suggest that the proposed raising of the maximum fall height from 2.5 m to 3.0 m in Europe is worrying
SN - 0140-6736
AD - Department of Child Health, University of Wales College of Medicine Academic Centre, Llandough Hospital, Penarth, Vale of Glamorgan, UK
UR - 9217759
ER -

TY - JOUR
ID - 1896
T1 - Primary monosymptomatic nocturnal enuresis in Singapore--parental perspectives in an Asian community
A1 - Chao,S.M.
A1 - Yap,H.K.
A1 - Tan,A.
A1 - Ong,E.K.
A1 - Murugasu,B.
A1 - Low,E.H.
A1 - Tan,S.P.
Y1 - 1997/03//
N1 - Chao, S M. Yap, H K. Tan, A. Ong, E K. Murugasu, B. Low, E H. Tan, S P
Annals of the Academy of Medicine, Singapore
53f, 7503289
IM
Clinical Trial. Journal Article. Multicenter Study
English
KW - MEDLINE
KW - Adolescent
KW - Asia/eh [Ethnology]
KW - Attitude to Health
KW - Child
KW - Chronic Disease
KW - Deamino Arginine Vasopressin/tu [Therapeutic Use]
KW - Enuresis/dt [Drug Therapy]
KW - Enuresis/eh [Ethnology]
KW - Enuresis/et [Etiology]
KW - Enuresis/px [Psychology]
KW - Female
KW - Humans
KW - Male
KW - Parents/px [Psychology]
KW - Questionnaires

KW - Renal Agents/tu [Therapeutic Use]
KW - Singapore
RP - NOT IN FILE
SP - 179
EP - 183
JF - Annals of the Academy of Medicine, Singapore
JA - Ann Acad Med Singapore
VL - 26
IS - 2
CY - SINGAPORE
N2 - Primary monosymptomatic nocturnal enuresis (PMNE) is often not openly discussed in Asian societies. We report the parental view of PMNE in Singapore, its impact on patients and their families and the traditional beliefs and its influence on subsequent management. A screening questionnaire was used in evaluating 30 children enrolled in a clinical trial on the use of oral Desmopressin for the treatment of PMNE. Primary monosymptomatic nocturnal enuresis was familial in 56.7% of patients. Fifty per cent of them were previously unevaluated. Earlier remedial attempts included bedtime fluid restriction and voiding (100%), incentive measures (43.3%), traditional practices (26.7%), punishment (20%), drugs (16.7%), psychotherapy (100%) and bladder training (3.3%). Perceived causes of PMNE were maturational delay (50%), deep sleep (50%), familial (43.3%), behavioural problems (43.3%) and excessive fluid intake (26.7%). Reasons for seeking treatment included restricted outdoor activities (90%), parental fatigue (86.7%), disrupted sleep for the household (46.7%) and fear of underlying pathology (26.7%). Perceived adverse effects on patients included social stigma (83.3%), disrupted sleep (33.3%) and impaired school performance (13.3%). Primary monosymptomatic nocturnal enuresis can thus be a chronic distressing problem in Asian communities
SN - 0304-4602
AD - Department of Paediatrics, Singapore General Hospital, Singapore
UR - 9208070
ER -

TY - JOUR
ID - 1897
T1 - Preschool injuries in child care centers: nursing strategies for prevention. [Review] [24 refs]
A1 - Ulione,M.S.
A1 - Dooling,M.
Y1 - 1997/05//
N1 - Ulione, M S. Dooling, M
Journal of pediatric health care : official publication of National Association of Pediatric Nurse Associates & Practitioners
jml, 8709735
N
Journal Article. Review
English
KW - MEDLINE
KW - Child
KW - Child Day Care Centers
KW - Preschool
KW - Humans
KW - Nursing Assessment
KW - Primary Prevention/mt [Methods]
KW - Risk Factors
KW - Safety
KW - Wounds and Injuries/nu [Nursing]
KW - Wounds and Injuries/pc [Prevention & Control]
RP - NOT IN FILE
SP - 111
EP - 116
JF - Journal of Pediatric Health Care
JA - J Pediatr Health Care

VL - 11
IS - 3
CY - UNITED STATES
N2 - Injuries to children 0 to 12 years of age pose a national health problem. Injuries are a particular problem in child care settings. Both research and anecdotal reports confirm that most injuries in the child care setting are cuts, scratches, and abrasions caused by falls indoors and in playgrounds. Other injuries are caused by human bites and motor vehicle pedestrian injuries. Child development centers are an obvious focal point to direct injury prevention services by nurses. The nurse's role in injury prevention is to educate the child care providers about injuries and then teach them the skills to assess and monitor injury prevention strategies. This article discusses the problem of injuries in child care centers in general and discusses injury prevention strategies the nurse can share with the child care provider. Educational resources are included to help the child care providers assess and monitor their own center's injury risk. J Pediatr Health Care. [References: 24]
SN - 0891-5245
AD - Barnes College of Nursing, University of Missouri-St. Louis, St. Louis 63121, USA
UR - 9197625
ER -

TY - JOUR
ID - 1898
T1 - Extent and provision of orthodontic services for children and adolescents in Finland
A1 - Pietila,T.
A1 - Pietila,I.
A1 - Widstrom,E.
A1 - Varrela,J.
A1 - Alanen,P.
Y1 - 1997/04//
N1 - Pietila, T. Pietila, I. Widstrom, E. Varrela, J. Alanen, P
Community dentistry and oral epidemiology
dnf, 0410263
D, IM
Journal Article. Research Support, Non-U.S. Gov't
English
KW - MEDLINE
KW - Adolescent
KW - Age Factors
KW - Chi-Square Distribution
KW - Child
KW - Dental Health Services/sd [Supply & Distribution]
KW - Finland/ep [Epidemiology]
KW - Humans
KW - Logistic Models
KW - Malocclusion/ep [Epidemiology]
KW - Odds Ratio
KW - Orthodontic Appliances/ut [Utilization]
KW - Orthodontics/ma [Manpower]
KW - Orthodontics/og [Organization & Administration]
KW - Orthodontics/sn [Statistics & Numerical Data]
KW - Orthodontics
KW - Corrective/sn [Statistics & Numerical Data]
KW - Corrective/ut [Utilization]
KW - Questionnaires
RP - NOT IN FILE
SP - 150
EP - 155
JF - Community Dentistry & Oral Epidemiology
JA - Community Dent Oral Epidemiol
VL - 25

IS - 2
CY - DENMARK
N2 - Orthodontic services for Finnish children and adolescents up to the age of 18 in 1992 were monitored by a questionnaire sent to all municipal health centres responsible for children's dental care in Finland, and 96% responded. All health centres provided some orthodontic treatment, but the percentage of 0 18-year-old children receiving treatment ranged from 1% to 19%. One-quarter of all dental visits of the 0 18-year-olds were in connection with orthodontics. The timing of treatment was early, the average age for starting was 9.5 years. In statistical analyses, the number of children receiving treatment was associated with the timing of treatment. At the age of 7, the most frequently used appliances were quad helix, removable appliance and orthopaedic headgear and, at the age of 13, fixed appliance and activator. Most treatments were provided by nonspecialists. The specialist expertise needed was usually purchased from an outside orthodontist by means of consultation contracts. Every fifth health centre, usually the largest, had employed one or more specialist orthodontists. The regional distribution of orthodontists was uneven, emphasizing regional variation in the delivery of orthodontic services
SN - 0301-5661
AD - Varkaus Health Centre, Finland
UR - 9181289
ER -

TY - JOUR
ID - 1899
T1 - Examining the gender gap in nutrition: an example from rural Mexico
A1 - Backstrand,J.R.
A1 - Allen,L.H.
A1 - Pelto,G.H.
A1 - Chavez,A.
Y1 - 1997/06//
N1 - Backstrand, J R. Allen, L H. Pelto, G H. Chavez, A
Social science & medicine (1982)
ut9, 8303205
IM
Comparative Study. Journal Article. Research Support, U.S. Gov't, Non-P.H.S.
English
KW - MEDLINE
KW - Basal Metabolism
KW - Body Composition
KW - Child
KW - Child Nutritional Physiological Phenomena
KW - Preschool
KW - Energy Intake
KW - Exercise
KW - Female
KW - Food Habits/eh [Ethnology]
KW - Gender Identity
KW - Humans
KW - Infant
KW - Male
KW - Mexico
KW - Nutrition Surveys
KW - Prejudice
KW - Rural Health
KW - Sex
KW - Socioeconomic Factors
RP - NOT IN FILE
SP - 1751
EP - 1759
JF - Social Science & Medicine

JA - Soc Sci Med
VL - 44
IS - 11
CY - ENGLAND
N2 - Gender differences in nutrient and food intake were examined in Mexican Nutrition CRSP (Collaborative Research Support Program) infants (N = 75), preschoolers (N = 80), and school children (N = 91). No significant gender differences in dietary quality or quantity were seen for infants and preschoolers. For school children, the contribution of various foods to total energy intake (dietary quality) was also quite similar for girls and boys. Equity in dietary quality remained even under conditions of economic and demographic stress. Nevertheless, school girls consumed significantly less energy per day than boys (-300 kcal/d or 1.3 mJ/d), and less of all micronutrients examined. Gender differences in estimated basal metabolic rates of school children were slight (-20 kcal/d), and body composition and size were similar. When energy intakes were expressed as a percent of estimated requirement (calculated from age, sex and weight using WHO/FAO/UNU equations), intakes were adequate and not significantly different between girls (mean = 111%) and boys (mean = 113%). Playground observations showed girls to be less active than boys, which may reflect both cultural and biological influences. Apparently due to this lower activity, school girls consumed less energy, and may have been at much higher risk than boys of micronutrient deficiency. The lower food intakes of girls did not appear to be due to purposeful dietary discrimination, but rather to culturally patterned sex roles involving lower activity
SN - 0277-9536
AD - Department of Nutrition and Food Studies, New York University, NY 10012-1172, USA
UR - 9178417
ER -

TY - JOUR
ID - 1900
T1 - Effectiveness of a violence prevention curriculum among children in elementary school. A randomized controlled trial
A1 - Grossman,D.C.
A1 - Neckerman,H.J.
A1 - Koepsell,T.D.
A1 - Liu,P.Y.
A1 - Asher,K.N.
A1 - Beland,K.
A1 - Frey,K.
A1 - Rivara,F.P.
Y1 - 1997/05/28/
N1 - Grossman, D C. Neckerman, H J. Koepsell, T D. Liu, P Y. Asher, K N. Beland, K. Frey, K. Rivara, F P
JAMA : the journal of the American Medical Association
7501160
AIM, IM
Clinical Trial. Journal Article. Randomized Controlled Trial. Research Support, U.S. Gov't, P.H.S.
English
KW - MEDLINE
KW - Aggression
KW - Child
KW - Child Behavior
KW - Curriculum
KW - Female
KW - Humans
KW - Interpersonal Relations
KW - Male
KW - Schools
KW - Socioeconomic Factors
KW - Violence/pc [Prevention & Control]
RP - NOT IN FILE
SP - 1605
EP - 1611

JF - JAMA
VL - 277
IS - 20
CY - UNITED STATES
N2 - OBJECTIVE: To determine if a commonly used violence prevention curriculum, Second Step: A Violence Prevention Curriculum, leads to a reduction in aggressive behavior and an increase in prosocial behavior among elementary school students. DESIGN: Randomized controlled trial. SETTING: Urban and suburban elementary schools in the state of Washington. PARTICIPANTS: Six matched pairs of schools with 790 second-grade and third-grade students. The students were 53% male and 79% white. INTERVENTION: The curriculum uses 30 specific lessons to teach social skills related to anger management, impulse control, and empathy. MAIN OUTCOME MEASURES: Aggressive and prosocial behavior changes were measured 2 weeks and 6 months after participation in the curriculum by parent and teacher reports (Achenbach Child Behavior Checklist and Teacher Report Form, the School Social Behavior Scale, and the Parent-Child Rating Scale) and by observation of a random subsample of 588 students in the classroom and playground/cafeteria settings. RESULTS: After adjusting for sex, age, socioeconomic status, race, academic performance, household size, and class size, change scores did not differ significantly between the intervention and control schools for any of the parent-reported or teacher-reported behavior scales. However, the behavior observations did reveal an overall decrease 2 weeks after the curriculum in physical aggression (P=.03) and an increase in neutral/prosocial behavior (P=.04) in the intervention group compared with the control group. Most effects persisted 6 months later. CONCLUSIONS: The Second Step violence prevention curriculum appears to lead to a moderate observed decrease in physically aggressive behavior and an increase in neutral and prosocial behavior in school
SN - 0098-7484
AD - Department of Pediatrics, University of Washington, Seattle, USA
UR - 9168290
ER -

TY - JOUR
ID - 1901
T1 - Heat-related illness and death, Wisconsin, 1995
A1 - Knobeloch,L.
A1 - Anderson,H.
A1 - Morgan,J.
A1 - Nashold,R.
Y1 - 1997/05//
N1 - Knobeloch, L. Anderson, H. Morgan, J. Nashold, R
Wisconsin medical journal
xpj, 0110663
IM
Journal Article
English
KW - MEDLINE
KW - Adolescent
KW - Adult
KW - Aged
KW - 80 and over
KW - Cause of Death
KW - Child
KW - Preschool
KW - Emergency Medical Services/ut [Utilization]
KW - Female
KW - Heat Exhaustion/co [Complications]
KW - Heat Exhaustion/mo [Mortality]
KW - Humans
KW - Infant
KW - Male
KW - Middle Aged
KW - Risk Factors

KW - Wisconsin/ep [Epidemiology]
RP - NOT IN FILE
SP - 33
EP - 38
JF - Wisconsin Medical Journal
JA - Wis.Med J
VL - 96
IS - 5
CY - UNITED STATES
N2 - This article examines heat-related illnesses and deaths that occurred in Wisconsin during the summer of 1995, which was among the warmest seasons on record. Death certificates that listed heat exposure as a contributing or underlying cause of 154 deaths and ambulance run reports for 454 heat-related emergency calls were analyzed. The time and place of these events and patient information were used to evaluate risk factors such as age, medical condition, physical activity, and environmental temperature. Three-fourths of the deaths and nearly half of the heat-related illnesses occurred during or shortly after two days of intense heat. Major risk factors for these heat-related illnesses and deaths included age, underlying chronic illness, physical exertion, and lack of air-conditioning. To reduce the number of illnesses and deaths that occur during future heat waves, all residents should be advised to install air-conditioning systems in their homes or apartments and encouraged to use them when indoor temperatures are uncomfortably warm. Summer weather forecasts should include heat index information. During periods of extreme heat, public health advisories should be issued encouraging residents to seek shelter in air-conditioned areas and to avoid strenuous outdoor activities and prolonged sun exposure. Region-specific criteria and procedures for issuing public health advisories should be developed jointly by the National Weather Service and the US Centers for Disease Control and Prevention
SN - 0043-6542
AD - Wisconsin Department of Health and Social Services, Madison 53703-3044, USA
UR - 9167436
ER -

TY - JOUR
ID - 1902
T1 - Children's exposure to violence in an urban setting
A1 - Sheehan,K.
A1 - DiCara,J.A.
A1 - LeBailly,S.
A1 - Christoffel,K.K.
Y1 - 1997/05//
N1 - Sheehan, K. DiCara, J A. LeBailly, S. Christoffel, K K
Archives of pediatrics & adolescent medicine
9422751, bwf
AIM, IM
Journal Article. Research Support, U.S. Gov't, P.H.S.
English
KW - MEDLINE
KW - Adolescent
KW - Case-Control Studies
KW - Chi-Square Distribution
KW - Child
KW - Data Collection
KW - Female
KW - Humans
KW - Male
KW - Poverty Areas
KW - Urban Population
KW - Violence/pc [Prevention & Control]
KW - Violence/sn [Statistics & Numerical Data]
RP - NOT IN FILE
SP - 502

EP - 504
JF - Archives of Pediatrics & Adolescent Medicine
JA - Arch Pediatr Adolesc Med
VL - 151
IS - 5
CY - UNITED STATES
N2 - OBJECTIVE: To assess the exposure to violence of a representative sample of children living in an inner-city public housing development. DESIGN: Self-report survey. SETTING: Chicago public housing development that covers 4 census tracts; population, 95% African American, 75% below the poverty level. PARTICIPANTS: One hundred forty-six African American youth, aged 7 through 13 years, completed the survey; 53% were male, mean and median ages, 11 years. Seventy-two children (case subjects) are involved in a community-based health and recreation program. They completed the survey prior to participating in a peer-mentoring violence prevention curriculum. The other 74 children (control subjects) were recruited by a community member going door to door. Control subjects were matched to case subjects for age, sex, and census tract. RESULTS: The case and control subjects were similar in their exposure to violence and so were grouped for analysis. Of the 146 children, 42% had seen someone shot and 37% had seen someone stabbed; 21% lived with someone who had been shot and 16% lived with someone who had been stabbed. Forty-seven percent of the girls and 55% of the boys had witnessed violence (P > .25). Almost all subjects (90%) felt safe at home. Two thirds (65%) of the children were not afraid to play outside, but almost half (43%) worried about getting hurt at school. CONCLUSIONS: These data, which describe a representative sample of children from an inner-city housing project, confirm the results from older clinic- and school-based convenience samples. In this low-income community, children are frequently exposed to deadly violence. In contrast with other reports, girls here are not spared
SN - 1072-4710
AD - Division of General Academic Pediatrics, Children's Memorial Hospital, Northwestern Unviersity Medical School, Chicago, Ill, USA
UR - 9158444
ER -

TY - JOUR
ID - 1903
T1 - Lead poisoning of children in Africa, III. Kaduna, Nigeria
A1 - Nriagu,J.
A1 - Oleru,N.T.
A1 - Cudjoe,C.
A1 - Chine,A.
Y1 - 1997/04/30/
N1 - Nriagu, J. Oleru, N T. Cudjoe, C. Chine, A
The Science of the total environment
uj0, 0330500
IM
Journal Article
English
KW - MEDLINE
KW - Child
KW - Preschool
KW - Demography
KW - Environmental Exposure
KW - Female
KW - Humans
KW - Lead/bl [Blood]
KW - Lead Poisoning/bl [Blood]
KW - Lead Poisoning/ep [Epidemiology]
KW - Male
KW - Nigeria/ep [Epidemiology]
KW - Questionnaires
KW - Social Class

KW - Socioeconomic Factors
KW - Urban Population
RP - NOT IN FILE
SP - 13
EP - 19
JF - Science of the Total Environment
JA - Sci Total Environ
VL - 197
IS - 1-3
CY - NETHERLANDS
N2 - This study investigated the prevalence of elevated blood lead (PbB) levels in children 1-6 years old in Kaduna, a medium size city in northern Nigeria. Mean PbB was found to be 10.6 micrograms/dl, and 2% of the children had PbB levels greater than 30 micrograms/dl. Highest average PbB levels were found in children 5 years old and was attributed to the tendency for this age group to play longer in contaminated outdoor environments. The strongest associations were found between PbB and whether the family owned a car or lived in a house on a tarred road. Potential sources of lead in the city as well as household and behavior risk factors likely to result in exposure of children to lead are discussed. This study provides additional data pointing to childhood lead poisoning as being a major public health problem in urban areas of Africa
SN - 0048-9697
AD - Department of Environmental and Industrial Health, School of Public Health, University of Michigan, Ann Arbor 48109, USA
UR - 9151435
ER -

TY - JOUR
ID - 1904
T1 - Summer sun exposure: knowledge, attitudes, and behaviors of Midwest adolescents
A1 - Robinson,J.K.
A1 - Rademaker,A.W.
A1 - Sylvester,J.A.
A1 - Cook,B.
Y1 - 1997/05//
N1 - Robinson, J K. Rademaker, A W. Sylvester, J A. Cook, B
Preventive medicine
pm4, 0322116
IM
Journal Article. Research Support, Non-U.S. Gov't
English
KW - MEDLINE
KW - Adolescent
KW - Adolescent Behavior
KW - Adult
KW - Analysis of Variance
KW - Chi-Square Distribution
KW - Child
KW - Cross-Sectional Studies
KW - Environmental Exposure/ae [Adverse Effects]
KW - Environmental Exposure/pc [Prevention & Control]
KW - Environmental Exposure/sn [Statistics & Numerical Data]
KW - Female
KW - Health Education/sn [Statistics & Numerical Data]
KW - Health Knowledge
KW - Attitudes
KW - Practice
KW - Health Surveys
KW - Heliotherapy/ae [Adverse Effects]
KW - Heliotherapy/px [Psychology]

KW - Heliotherapy/sn [Statistics & Numerical Data]
KW - Humans
KW - Illinois/ep [Epidemiology]
KW - Male
KW - Protective Clothing/sn [Statistics & Numerical Data]
KW - Sampling Studies
KW - Sex Distribution
KW - Skin Care/px [Psychology]
KW - Skin Care/sn [Statistics & Numerical Data]
KW - Skin Neoplasms/pc [Prevention & Control]
KW - Skin Pigmentation
KW - Sunburn/ep [Epidemiology]
KW - Sunburn/pc [Prevention & Control]
KW - Sunlight/ae [Adverse Effects]
KW - Sunscreening Agents
RP - NOT IN FILE
SP - 364
EP - 372
JF - Preventive Medicine
JA - Prev Med
VL - 26
IS - 3
CY - UNITED STATES
N2 - BACKGROUND: Extensive print, radio, and television coverage about the dangers of sun exposure and benefits of sun protection occurred over the past decade. Illinois teen knowledge and attitudes about sun exposure/protection, sun-exposure/protection behavior, and information sources were determined by a summer telephone survey. METHODS: Telephone interviews with 658 teenagers between ages 11 and 19 included African-American, Asian, Hispanic, Native American, and white teenagers. RESULTS: Teens knew that too much sun was harmful as it caused skin cancer and sunburn. Sunburn was mentioned more often by those with skin types that burned easily and tanned poorly (I,II) ($P < 0.001$), was better known to girls than to boys ($P < 0.001$), and was recognized more by those with higher socioeconomic status ($P < 0.001$) but was not associated with age. Widely held sun exposure attitudes were socializing with friends and feeling better when outdoors. On weekdays, boys averaged 5.3 hr (SD, 1.65 hr) outside compared with 3.9 hr (SD, 0.75 hr) for girls ($P < 0.001$). Teenage boys were more likely to obtain occupational sun exposure, and girls sunbathed. Subjects with skin types I and II reported an average of 3.3 sunburns in the past year. During unprotected sun exposure, extensive numbers of teens with moderate-risk skin type experienced at least 1 sunburn per year. Indoor tanning use was more prevalent among older girls and those with skin types I and II. Sunscreen use was associated with water recreational activities (swimming, water sports, and going to the beach) by girls slightly more than by boys ($P < 0.001$). Hat-wearing was more common among boys than among girls. CONCLUSIONS: Teen knowledge that excessive sun exposure causes skin cancer and sunburns and that wearing sunscreens and hats were sun-protective methods did not enable sun protection that prevented burning. This is particularly troublesome because severe sunburns in youth are associated with an increased risk of melanoma. Existing teen sunscreen use could be broadened by educating teens to use adequate quantities of sunscreen prior to daily sun exposure to prevent painful burns. Messages to teens that emphasize the short-term consequence of painful sunburns because of inadequate protection during outdoor occupational and non-water-related recreational exposure would increase the relevance of the message and may enable behavioral change. Parents and physicians need to be included in messages that are directed to teens and to become part of their education. Parents could ensure an adequate sunscreen supply for daily use by the family, encourage teens not to deliberately tan, and serve as role models for the use of protective clothing
SN - 0091-7435
AD - American Cancer Society, Illinois Division, Inc., Chicago 60603, USA
UR - 9144761
ER -

TY - JOUR
ID - 1905

T1 - Children with ADHD and tic disorder and their classmates: behavioral normalization with methylphenidate
A1 - Nolan,E.E.
A1 - Gadow,K.D.
Y1 - 1997/05//
N1 - Nolan, E E. Gadow, K D
Journal of the American Academy of Child and Adolescent Psychiatry
hg5, 8704565
IM
Clinical Trial. Journal Article. Randomized Controlled Trial. Research Support, Non-U.S. Gov't. Research Support, U.S. Gov't, P.H.S.
English
KW - MEDLINE
KW - Analysis of Variance
KW - Attention Deficit Disorder with Hyperactivity/co [Complications]
KW - Attention Deficit Disorder with Hyperactivity/dt [Drug Therapy]
KW - Attention Deficit Disorder with Hyperactivity/px [Psychology]
KW - Central Nervous System Stimulants/tu [Therapeutic Use]
KW - Child
KW - Dose-Response Relationship
KW - Drug
KW - Double-Blind Method
KW - Female
KW - Humans
KW - Male
KW - Methylphenidate/tu [Therapeutic Use]
KW - Peer Group
KW - Social Behavior
KW - Tic Disorders/co [Complications]
KW - Tic Disorders/dt [Drug Therapy]
KW - Tic Disorders/px [Psychology]
RP - NOT IN FILE
SP - 597
EP - 604
JF - Journal of the American Academy of Child & Adolescent Psychiatry
JA - J Am Acad Child Adolesc Psychiatry
VL - 36
IS - 5
CY - UNITED STATES
N2 - OBJECTIVES: To examine behavioral differences between children with attention-deficit hyperactivity disorder (ADHD) and tics and their peers and the extent to which methylphenidate (0.1, 0.3, and 0.5 mg/kg) normalized the behavior of probands and indirectly influenced the behavior of peers (treatment spillover). METHOD: Thirty-four prepubertal children with ADHD and chronic tic disorder (who were participating in a double-blind, placebo-controlled methylphenidate evaluation) and their peers were observed for approximately 20 hours in the school setting (classroom seatwork activities, lunchroom, and playground). RESULTS: Children with ADHD and tics were more inattentive and more disruptive in the classroom and more aggressive in all school settings than their peers. Although treatment with methylphenidate made probands less easily distinguished from their peers (normalization), many children still scored in the deviant range for at least one ADHD behavior when receiving the 0.5-mg/kg dose. There was little evidence that peer behavior improved as a function of the proband's dose of medication. CONCLUSIONS: Although conventional doses of methylphenidate produced dramatic clinical improvement in ADHD-related behavior, complete behavioral normalization is often not attained
SN - 0890-8567
AD - Department of Psychiatry and Behavioral Science, SUNY at Stony Brook 11794-8790, USA
UR - 9136493
ER -

TY - JOUR
ID - 1906
T1 - Work and workload of nursing personnel in a nursery school and two institutions for handicapped children
A1 - Okuno,M.
A1 - Uketa,S.
A1 - Nakaseko,M.
A1 - Tokunaga,R.
Y1 - 1997/04//
N1 - Okuno, M. Uketa, S. Nakaseko, M. Tokunaga, R
Industrial health
g9l, 2985065r
IM
Journal Article. Research Support, Non-U.S. Gov't
English
KW - MEDLINE
KW - Adult
KW - Biomechanical Phenomena
KW - Child Day Care Centers
KW - Child
KW - Preschool
KW - Electromyography
KW - Female
KW - Humans
KW - Japan
KW - Low Back Pain/et [Etiology]
KW - Nursing Homes
KW - Nursing Staff
KW - Occupational Diseases/et [Etiology]
KW - Posture
KW - Risk Factors
KW - Schools
KW - Nursery
KW - Task Performance and Analysis
KW - Workload
RP - NOT IN FILE
SP - 202
EP - 211
JF - Industrial Health
JA - Ind Health
VL - 35
IS - 2
CY - JAPAN
N2 - Work-related low back pain and cervicobrachial disorders have become increasingly common among nursing personnel with the increase in the number of social welfare institutions and workers. The aim of this study is to assess the burden on the low backs of personnel caring for children. We measured work content, working time, posture and muscular activity in 16 nursery workers in three different types of institutions for healthy and handicapped children. The working time and posture of 7 nursery teachers in a public nursery school for healthy children (A school), 7 nursing workers (4 nursery teachers and 3 nursery instructors) in a public daycare center for mentally and physically handicapped children (B center), and 2 nursery instructors in a private home for severely handicapped children (C home) were analyzed. The muscular activity of the erectores spinae muscle of three subjects was measured using a newly developed EMG monitor (portable EMG monitor, PEMM). The following results were obtained. The average length of a lesson or activity in A school was greater than that in B center and C home. The time required by care for meals was longest in C home. The arrangement and rearrangement times were longest in the classes of children aged 3-5 in A school. The time occupied for clerical work and other work was relatively longer, while recess including lunch time was approximately one hour. The most commonly observed posture was sitting on the floor in all kinds of classes, with this occurring most often in C home. Hazardous postures such as bending forward, squatting, and kneeling appeared in 11-

18% (48-81 min) of a day's working time. The average muscular activity of the erectores spinae muscle of three subjects throughout a day was approximately 10% of the maximum isometric contraction (% maximum work load, %MWL), when muscular activities were measured by PEMM. The relatively high %MWL values (19-27%MWL) were obtained at a slightly bending posture and a kneeling posture, while lower (1-9%MWL) values were observed at a sitting posture. The %MWL values at a sitting posture varied according to such conditions as duration of sitting, back rest, weight on the arms, twisting of the trunk, and hip movement. The risky nature of the sitting posture are also discussed
SN - 0019-8366
AD - Department of Hygiene, Kansai Medical University, Moriguchi, Japan
UR - 9127552
ER -

TY - JOUR
ID - 1907
T1 - Playground injuries in children
A1 - Lillis,K.A.
A1 - Jaffe,D.M.
Y1 - 1997/04//
N1 - Lillis, K A. Jaffe, D M
Pediatric emergency care
pau, 8507560
IM
Comparative Study. Journal Article
English
KW - MEDLINE
KW - Adolescent
KW - Age Distribution
KW - Age Factors
KW - Canada/ep [Epidemiology]
KW - Child
KW - Preschool
KW - Craniocerebral Trauma/ep [Epidemiology]
KW - Equipment and Supplies
KW - Fractures
KW - Bone/ep [Epidemiology]
KW - Bone/et [Etiology]
KW - Hospitalization/sn [Statistics & Numerical Data]
KW - Humans
KW - Infant
KW - Play and Playthings
KW - Prospective Studies
KW - United States/ep [Epidemiology]
KW - Wounds and Injuries/ep [Epidemiology]
KW - Wounds and Injuries/et [Etiology]
KW - Wounds and Injuries/pc [Prevention & Control]
RP - NOT IN FILE
SP - 149
EP - 153
JF - Pediatric Emergency Care
JA - Pediatr Emerg Care
VL - 13
IS - 2
CY - UNITED STATES
N2 - Childhood injuries are a major source of morbidity and mortality in industrialized countries, and many injuries occur on playgrounds. Our purpose was to examine childhood playground injuries in a metropolitan center in Canada. All children injured on playground equipment who were seen in the emergency department (ED) at The Hospital for Sick Children between March 1990 and July 1991 and were entered in the Children's

Hospital Injury Research and Prevention Project (CHIRPP) database were included. The type, body part, and mechanism of injury were determined as well as the type of equipment, location, and surface. Among the 289 children injured on playground equipment, the mean age was 5.9 years with 39% < 5 years (range: 1 to 18 years). The most common injuries included fractures (28%), lacerations (24%), and hematomas (14%). The head and neck were injured 43% of the time, the upper extremity 41%, lower extremity 10%, and the trunk 6%. Climbing apparatus injuries occurred in 29% of children < 5 years compared with 47% of those injured who were > or = 5 years (P = 0.002). Injuries related to slides occurred in 40% of children < 5 years compared to 26% of children > or = 5 years (P = 0.033). Of children < 5 years, 58% had head and neck injuries compared to 32% of children > or = 5 years (P = 0.0006). Of children < 5 years, 28% had upper extremity injuries compared to 49% of children > or = 5 years (P = 0.0005). There were no fatalities and the overall hospitalization rate was 18%. Of those children hospitalized, 77% had fractures, compared to 16% of those not hospitalized (P = 0.00001). Of all children hospitalized, 62% were injured on climbing apparatus, compared to 37% of those not hospitalized (P = 0.0004). There were no significant differences between nonprotective and natural protective surfaces with respect to hospitalization. We conclude that: 1) upper extremity injuries, especially fractures, accounted for the majority of hospitalizations resulting from injuries on playground equipment; 2) climbing apparatus-related injuries accounted for nearly two thirds of hospitalizations; 3) older children sustained more injuries on climbing apparatus, where younger children sustained more injuries on slides; and 4) younger children sustained more head injuries on playground equipment than older children, but most of these were minor
SN - 0749-5161
AD - Department of Pediatrics and Emergency Medicine, Children's Hospital of Buffalo, State University of New York at Buffalo School of Medicine and Biomedical Sciences, USA
UR - 9127428
ER -

TY - JOUR
ID - 1908
T1 - Factors parents use in selecting play spaces for young children
A1 - Sallis,J.F.
A1 - McKenzie,T.L.
A1 - Elder,J.P.
A1 - Broyles,S.L.
A1 - Nader,P.R.
Y1 - 1997/04//
N1 - Sallis, J F. McKenzie, T L. Elder, J P. Broyles, S L. Nader, P R
Archives of pediatrics & adolescent medicine
9422751, bwf
AIM, IM
Comparative Study. Journal Article. Research Support, U.S. Gov't, P.H.S.
English
KW - MEDLINE
KW - Adult
KW - California
KW - Child
KW - Preschool
KW - Data Collection
KW - Decision Making
KW - European Continental Ancestry Group/px [Psychology]
KW - Female
KW - Humans
KW - Male
KW - Mexican Americans/px [Psychology]
KW - Parents/px [Psychology]
KW - Play and Playthings
KW - Safety
KW - Socioeconomic Factors
RP - NOT IN FILE

SP - 414
EP - 417
JF - Archives of Pediatrics & Adolescent Medicine
JA - Arch Pediatr Adolesc Med
VL - 151
IS - 4
CY - UNITED STATES
N2 - BACKGROUND: The amount of time children spend in play spaces (ie, physical locations that are appropriate for children's physical activity) near their homes is correlated with their level of physical activity. OBJECTIVE: To examine factors used in parents' decisions about the selection of play spaces for their children. SUBJECTS: Parents (primarily mothers) of 178 Mexican American and 122 white children who were a mean age of 4.9 years old at the first measurement. MEASURES: In individual interviews, parents rated 24 factors on their importance in selecting for their children a play space that is away from their home or yard. Decision factors were rated from 1 (ie, not important at all) to 5 (ie, very important). RESULTS: The most important factors, with ratings ranging from 4.8 to 4.2, were safety and availability of toilets, drinking water, lighting, and shade. Mexican American parents rated 8 of 24 items significantly higher than did white parents, including lighted at night, organized activities, play supplies, and drinking water. White parents rated 5 of 24 items significantly higher than did Mexican American parents, including distance from home, cost of admission, and child's friends go there. The rated importance of 7 of 24 items increased during 1 year, including play supplies, drinking water, distance from home, and parents' friends or relatives go there. CONCLUSIONS: These results indicate that parents can identify factors they use in selecting places for their young children to play, and selection factors differ somewhat by ethnicity or socioeconomic status. Further studies are needed to determine whether improvements on the most important selection factors might be effective in increasing the use of play spaces by children and their parents. Clinicians may be able to use the most highly rated decision factors to help parents assess the acceptability of play spaces in their areas
SN - 1072-4710
AD - Department of Psychology, San Diego State University, USA
UR - 9111442
ER -

TY - JOUR
ID - 1909
T1 - Ozone exposure assessment in a southern California community
A1 - Liu,L.J.
A1 - Delfino,R.
A1 - Koutrakis,P.
Y1 - 1997/01//
N1 - Liu, L J. Delfino, R. Koutrakis, P
Environmental health perspectives
ei0, 0330411
IM
Comparative Study. Journal Article. Research Support, U.S. Gov't, P.H.S.
English
KW - MEDLINE
KW - Adolescent
KW - Adult
KW - California
KW - Child
KW - Environmental Monitoring
KW - Female
KW - Humans
KW - Male
KW - Middle Aged
KW - Models
KW - Statistical
KW - Oxidants
KW - Photochemical/an [Analysis]

KW - Ozone/an [Analysis]
RP - NOT IN FILE
SP - 58
EP - 65
JF - Environmental Health Perspectives
JA - Environ Health Perspect
VL - 105
IS - 1
CY - UNITED STATES
N2 - An ozone exposure assessment study was conducted in a Southern California community. The Harvard ozone passive sampler was used to monitor cohorts of 22 and 18 subjects for 8 weeks during the spring and fall of 1994, respectively. Ozone exposure variables included 12-hr personal O3 measurements, stationary outdoor O3 measurements from a continuous UV photometer and from 12-hr Harvard active monitors, and time-activity information. Results showed that personal O3 exposure levels averaged one-fourth of outdoor stationary O3 levels, attributable to high percentages of time spent indoors. Personal O3 levels were not predicted well by outdoor measurements. A random-effect general linear model analysis indicated that variance in personal exposure measurements was largely accounted for by random error (59-82%), followed by inter-subject (9-18%) and between-day (9-23%) random effects. The microenvironmental model performs differently by season, with the regression model for spring cohorts exhibiting two times the R2 of the fall cohorts (R2 = 0.21 vs. 0.09). When distance from the stationary monitoring site, elevation, and traffic are taken into account in the microenvironmental models, the adjusted R2 increased almost twofold for the fall personal exposure data. The low predictive power is due primarily to the apparent spatial variation of outdoor O3 and errors in O3 measurements and in time-activity records (particularly in recording the use of air conditioning). This study highlights the magnitude of O3 exposure misclassification in epidemiological settings and proposes an approach to reduce exposure uncertainties in assessing air pollution health effects
SN - 0091-6765
AD - Department of Environmental Health Sciences, University of South Carolina, Columbia 29208, USA
UR - 9074882
ER -

TY - JOUR
ID - 1910
T1 - Medical limitations to wilderness travel. [Review] [73 refs]
A1 - Backer,H.
Y1 - 1997/02//
N1 - Backer, H
Emergency medicine clinics of North America
egd, 8219565
IM
Journal Article. Review
English
KW - MEDLINE
KW - Adult
KW - Aged
KW - Child
KW - Disability Evaluation
KW - Environmental Exposure/ae [Adverse Effects]
KW - Humans
KW - Infant
KW - Newborn
KW - Mass Screening
KW - Medical History Taking
KW - Patient Education as Topic
KW - Patient Selection
KW - Risk Factors
KW - Travel
RP - NOT IN FILE

SP - 17
EP - 41
JF - Emergency Medicine Clinics of North America
JA - Emerg Med Clin North Am
VL - 15
IS - 1
CY - UNITED STATES
N2 - Medical conditions may be exacerbated by environmental stresses or may limit tolerance to them. To advise patients who are planning foreign or wilderness travel of potential health risks, medical providers must first understand the interactions between physiologic demands from environmental factors and from exercise, and the patient's medical problems. This article considers medical limitations owing to specific environmental stresses and limitations imposed by specific medical problems. The challenge is to match the prospective participant with the proposed activity to minimize the risk of failure caused by medical problems. [References: 73]
SN - 0733-8627
AD - Department of Emergency and Sports Medicine, Kaiser Permanente Medical Center, Hayward, California, USA
UR - 9056569
ER -

TY - JOUR
ID - 1911
T1 - Total hip arthroplasty with cement for juvenile rheumatoid arthritis. Results at a minimum of ten years in patients less than thirty years old
A1 - Chmell,M.J.
A1 - Scott,R.D.
A1 - Thomas,W.H.
A1 - Sledge,C.B.
Y1 - 1997/01//
N1 - Chmell, M J. Scott, R D. Thomas, W H. Sledge, C B
The Journal of bone and joint surgery. American volume
hjr, 0014030
AIM, IM
Journal Article
English
KW - MEDLINE
KW - Adolescent
KW - Adult
KW - Arthritis
KW - Juvenile/su [Surgery]
KW - Child
KW - Female
KW - Follow-Up Studies
KW - Hip Prosthesis
KW - Humans
KW - Life Tables
KW - Male
KW - Prosthesis Failure
KW - Reoperation
KW - Retrospective Studies
KW - Treatment Outcome
RP - NOT IN FILE
SP - 44
EP - 52
JF - Journal of Bone & Joint Surgery - American Volume
JA - J Bone Joint Surg Am
VL - 79

IS - 1
CY - UNITED STATES
N2 - We retrospectively reviewed the clinical and radiographic results of total hip arthroplasty with cement in patients with juvenile rheumatoid arthritis who were less than thirty years old at the time of the index procedure. Thirty-nine patients (sixty-six hips) were managed with this procedure at our institution between 1971 and 1983. Six patients (eleven hips) died before a minimum of ten years of follow-up; the remaining thirty-three patients (fifty-five hips) were followed for at least eleven years. Twenty-eight patients (forty-six hips) had at least one original component in situ after an average duration of clinical follow-up of 15.1 years, and twenty-three of these patients (thirty-eight hips) were followed radiographically for an average of 14.7 years. At the time of the latest follow-up examination, all twenty-eight patients were able to walk outside the home; twenty of these patients (thirty-five hips; 76 per cent) had no pain with activity, and eight patients (eleven hips; 24 per cent) had mild-to-moderate pain with activity. Over-all, twelve (18 per cent) of the sixty-six femoral components and twenty-three (35 per cent) of the sixty-six acetabular components were revised after an average of 12.8 and 11.8 years, respectively. The fifteen-year survival rate for the femoral components was 85 per cent with revision or radiographic loosening as the end point. The fifteen-year survival rate for the acetabular components was 70 per cent with revision as the end point and 61 per cent with revision or radiographic loosening as the end point. The benefits of total hip arthroplasty were maintained over the long term in most of our patients who had juvenile rheumatoid arthritis. However, the durability of the components in these young patients remains a concern
SN - 0021-9355
AD - Rockford Orthopedic Associates, Illinois 61108, USA
UR - 9010185
ER -

TY - JOUR
ID - 1912
T1 - Evaluation of a systematic approach for identifying injury scenarios. Kids'n' Cars Teams
A1 - Christoffel,K.K.
A1 - Schofer,J.L.
Y1 - 1996/09//
N1 - Christoffel, K K. Schofer, J L
Injury prevention : journal of the International Society for Child and Adolescent Injury Prevention
crz, 9510056
IM
Journal Article
English
KW - MEDLINE
KW - Accidents
KW - Traffic
KW - Causality
KW - Chicago/ep [Epidemiology]
KW - Child
KW - Preschool
KW - Female
KW - Humans
KW - Male
KW - Reproducibility of Results
KW - Risk Factors
KW - Wounds and Injuries/ep [Epidemiology]
KW - Wounds and Injuries/et [Etiology]
RP - NOT IN FILE
SP - 221
EP - 227
JF - Injury Prevention
JA - Inj Prev
VL - 2
IS - 3
CY - ENGLAND

N2 - OBJECTIVE: To assess the effectiveness of a new multidisciplinary method for reconstructing the causal sequences that lead to child pedestrian injuries. SETTING: Subjects were 5-12 year old residents of Chicago, Illinois, USA, presenting for care due to pedestrian injury at one pediatric trauma center. METHODS: The interactions of medical, child, psychosocial, and traffic factors contributing to the injury were analysed. For 142 cases, information about the victim, his/her family, the injury site, and the activities just before the injury, was used in a structured manner by a multidisciplinary team to produce injury scenarios. Each scenario comprised a list of contributing factors, an estimate of the importance of each, and a narrative description of the causal sequence leading to the injury event. Face validity was assessed by two outside teams that performed a structured review of a subsample of cases (n = 11). Reliability was evaluated by comparison of the results of parallel teams assessing the same cases (n = 14). Process consistency and bias were assessed by analysis of the correlations of factor-importance rating patterns between members and over time. RESULTS: The outside team's agreement scores were based on a 1-5 Likert scale; these showed a mean of 3.6 and median of 4.0. Parallel teams consistently showed agreement greater than 85% on global attributes of cases. Intraclass correlation coefficient scores showed fair or better agreement for all classes of contributors, and excellent agreement for more than one third. Rating pattern analyses showed strong agreement by team members. Agreement did not increase over the period of the study. CONCLUSIONS: This causal sequence reconstruction method has acceptable face validity, reliability, and internal consistency. Although labor intensive and thus costly, it can produce unique, rich information for understanding injury causation and for guiding the search for promising interventions
SN - 1353-8047
AD - Children's Memorial Hospital, Chicago, Illinois, USA
UR - 9346095
ER -

TY - JOUR
ID - 1913
T1 - A statewide survey of hazards in child care centers
A1 - Browning,K.S.
A1 - Runyan,C.W.
A1 - Kotch,J.B.
Y1 - 1996/09//
N1 - Browning, K S. Runyan, C W. Kotch, J B
Injury prevention : journal of the International Society for Child and Adolescent Injury Prevention
crz, 9510056
IM
Journal Article. Research Support, Non-U.S. Gov't
English
KW - MEDLINE
KW - Child
KW - Child Day Care Centers/lj [Legislation & Jurisprudence]
KW - Child Day Care Centers/st [Standards]
KW - Preschool
KW - Confounding Factors (Epidemiology)
KW - Female
KW - Humans
KW - Infant
KW - Male
KW - North Carolina
KW - Questionnaires
KW - Safety Management
RP - NOT IN FILE
SP - 202
EP - 207
JF - Injury Prevention
JA - Inj Prev
VL - 2
IS - 3

CY - ENGLAND
N2 - OBJECTIVES: The purpose of this study was to determine adherence to selected recommended safety standards in North Carolina child care centers. METHODS: A self administered questionnaire eliciting information about safety practices in child care was mailed to a randomly selected sample of 409 North Carolina child care centers. RESULTS: One hundred and ninety five usable questionnaires were returned from child care centers in 75 counties. Results indicated that all of the standards included in the state's child regulations were being adhered to by at least 80% of the centers. However, adherence to recommended standards not included in the state's regulations was quite variable, with one standard implemented by less than 5% of the centers. The lowest rates of adherence were found for standards specifying that resilient surface material be used under playground equipment (4%) and that certain foods that may present a choking hazard to small children not be served (27%). CONCLUSIONS: Many hazards not addressed in North Carolina child care regulations are present in child care centers. Some safety standards are not adhered to due to lack of knowledge or limited resources. Inclusion of national standards in state child care regulations appears to reduce, but not eliminate, the likelihood of hazards being reported. Further research should include on-site inspections and attention to safety in family child care
SN - 1353-8047
AD - Department of Health Behavior and Health Education, University of North Carolina School of Public Health, Chapel Hill 27599-7505, USA
UR - 9346091
ER -

TY - JOUR
ID - 1914
T1 - Injuries and their relation to potential hazards in child day care
A1 - Cummings,P.
A1 - Rivara,F.P.
A1 - Boase,J.
A1 - MacDonald,J.K.
Y1 - 1996/06//
N1 - Cummings, P. Rivara, F P. Boase, J. MacDonald, J K
Injury prevention : journal of the International Society for Child and Adolescent Injury Prevention
crz, 9510056
IM
Journal Article. Research Support, U.S. Gov't, P.H.S.
English
KW - MEDLINE
KW - Accident Prevention
KW - Child Day Care Centers/sn [Statistics & Numerical Data]
KW - Child
KW - Preschool
KW - Cohort Studies
KW - Cross-Sectional Studies
KW - Female
KW - Humans
KW - Incidence
KW - Infant
KW - Male
KW - Prospective Studies
KW - Risk Assessment
KW - Washington/ep [Epidemiology]
KW - Wounds and Injuries/ep [Epidemiology]
KW - Wounds and Injuries/et [Etiology]
KW - Wounds and Injuries/pc [Prevention & Control]
RP - NOT IN FILE
SP - 105
EP - 108
JF - Injury Prevention

JA - Inj Prev
VL - 2
IS - 2
CY - ENGLAND
N2 - OBJECTIVES: To prospectively determine the incidence rate of injuries that required medical attention among children in day care and to identify possible hazards related to these injuries. SETTING: King County, Washington. METHODS: Prospective cohort study of children in a sample of licensed day care facilities. RESULTS: From 1 July 1992 to 30 June 1993, 53 medically attended injuries were reported by 133 day care sites; incidence rate 1.9 per 100,000 hours of day care attendance. The rate of injury in 91 small family day care homes was essentially the same as that in 42 larger day care centers; relative rate 1.0 (95% confidence interval 0.6 to 1.9). Injuries that required sutures accounted for 39% of the cases, while 17% required a cast, splint, or sling. No child was hospitalized. Sixty nine sites were inspected and all had potentially correctable physical hazards, with a median of 15 hazards per site (range 7 to 26). These potential hazards had little relationship to the risk of injury and a case-by-case review identified only two injuries that might have been prevented by a more energy absorbent playground surface. CONCLUSIONS: The incidence of medically attended injuries found in this study is consistent with other studies from the United States. Most injuries were minor and had little relation to physical hazards at day care locations
SN - 1353-8047
AD - Department of Epidemiology, School of Public Health and Community Medicine, University of Washington, Seattle, USA
UR - 9346070
ER -

TY - JOUR
ID - 1915
T1 - Height and surfacing as risk factors for injury in falls from playground equipment: a case-control study
A1 - Chalmers,D.J.
A1 - Marshall,S.W.
A1 - Langley,J.D.
A1 - Evans,M.J.
A1 - Brunton,C.R.
A1 - Kelly,A.M.
A1 - Pickering,A.F.
Y1 - 1996/06//
N1 - Chalmers, D J. Marshall, S W. Langley, J D. Evans, M J. Brunton, C R. Kelly, A M. Pickering, A F
Injury prevention : journal of the International Society for Child and Adolescent Injury Prevention
crz, 9510056
IM
Journal Article. Research Support, Non-U.S. Gov't
English
KW - MEDLINE
KW - Accidental Falls/pc [Prevention & Control]
KW - Accidental Falls/sn [Statistics & Numerical Data]
KW - Adolescent
KW - Case-Control Studies
KW - Child
KW - Preschool
KW - Consumer Product Safety
KW - Data Interpretation
KW - Statistical
KW - Emergency Service
KW - Hospital/ut [Utilization]
KW - Equipment Design
KW - Female
KW - Humans
KW - Infant
KW - Male

KW - New Zealand/ep [Epidemiology]
KW - Play and Playthings
KW - Risk Factors
KW - Surface Properties
KW - Wounds and Injuries/ep [Epidemiology]
KW - Wounds and Injuries/et [Etiology]
KW - Wounds and Injuries/pc [Prevention & Control]
RP - NOT IN FILE
SP - 98
EP - 104
JF - Injury Prevention
JA - Inj Prev
VL - 2
IS - 2
CY - ENGLAND
N2 - OBJECTIVES: Despite the widespread promotion of safety standards no epidemiological studies have adequately evaluated their effectiveness in preventing injury in falls from playground equipment. This study evaluated the effectiveness of the height and surfacing requirements of the New Zealand standard for playgrounds and playground equipment. SETTING: Early childhood education centres and schools in two major cities in the South Island of New Zealand. METHODS: Data were collected on 300 children aged 14 years or less who had fallen from playground equipment. Of these, 110 (cases) had sustained injury and received medical attention, while 190 (controls) had not sustained injury requiring medical attention. RESULTS: Logistic regression models fitted to the data indicated that the risk of injury being sustained in a fall was increased if the equipment failed to comply with the maximum fall height (odds ratio (OR) = 3.0; 95% confidence interval (CI) 0.7 to 13.1), surfacing (OR = 2.3; 95% CI 1.0 to 5.0), or safe fall height (OR = 2.1; 95% CI 1.1 to 4.0) requirements. Falls from heights in excess of 1.5 metres increased the risk of injury 4.1 times that of falls from 1.5 metres or less and it was estimated that a 45% reduction in children attending emergency departments could be achieved if the maximum fall height was lowered to 1.5 metres. CONCLUSIONS: Although the height and surfacing requirements of the New Zealand standard are effective in preventing injury in falls from playground equipment, consideration should be given to lowering the maximum permissible fall height to 1.5 metres
SN - 1353-8047
AD - Department of Preventive and Social Medicine, University of Otago Medical School, Dunedin, New Zealand
UR - 9346069
ER -

TY - JOUR
ID - 1916
T1 - Cross cultural child injury prevention awareness
A1 - Ritzel,D.O.
A1 - Klein,K.
A1 - Gast,J.
A1 - Sarvela,P.D.
Y1 - 1996/03//
N1 - Ritzel, D O. Klein, K. Gast, J. Sarvela, P D
Injury prevention : journal of the International Society for Child and Adolescent Injury Prevention
crz, 9510056
IM
Comparative Study. Journal Article
English
KW - MEDLINE
KW - Accident Prevention
KW - Awareness
KW - Belgium
KW - Child
KW - Preschool
KW - Cross-Cultural Comparison

KW - Female
KW - Germany
KW - Health Education
KW - Health Knowledge
KW - Attitudes
KW - Practice
KW - Humans
KW - Male
KW - United States
KW - Wounds and Injuries/pc [Prevention & Control]
RP - NOT IN FILE
SP - 44
EP - 46
JF - Injury Prevention
JA - Inj Prev
VL - 2
IS - 1
CY - ENGLAND
N2 - PURPOSE: The purpose of this study was to determine how injury prevention awareness of children ages 3, 4, and 5, based on recognition of hazards in pictures differs in the United States, Belgium, East Germany and West Germany. METHODS: Children from these four countries were presented with 10 different pictures. Each picture represented a common injury producing situation to which children are exposed in traffic, home, and recreation. RESULTS: Results indicate that for pictures relating to home hazards, less than 22% of children from Belgium (21.5%), West Germany (4.7%), and the United States (20.3%) clearly recognized the essential hazards in the pictures, whereas over 40% of the East German children clearly recognized these dangers. A higher proportion of the children from all countries recognized the traffic hazards. Only 23.9% of all children had a clear recognition of the playground situations. The child's age had a bearing on ability to recognize hazards overall. CONCLUSIONS: Children need to be provided with better injury prevention education at an early age, especially those from West Germany
SN - 1353-8047
AD - Center for Injury Control and Worksite Health Promotion, Southern Illinois University, Carbondale 62901, USA
UR - 9346054
ER -

TY - JOUR
ID - 1917
T1 - Recent findings concerning liver transplantation in the United States
A1 - Belle,S.H.
A1 - Beringer,K.C.
A1 - Detre,K.M.
Y1 - 1996///
N1 - Belle, S H. Beringer, K C. Detre, K M
Clinical transplants
ayj, 8812419
IM
Journal Article
English
KW - MEDLINE
KW - Adolescent
KW - Adult
KW - Age Factors
KW - Child
KW - Preschool
KW - Continental Population Groups
KW - Disease-Free Survival
KW - Female

KW - Humans
KW - Infant
KW - Liver Diseases/cl [Classification]
KW - Liver Diseases/su [Surgery]
KW - Liver Transplantation/mo [Mortality]
KW - Liver Transplantation/sn [Statistics & Numerical Data]
KW - Liver Transplantation/td [Trends]
KW - Male
KW - Middle Aged
KW - Probability
KW - Registries
KW - Reoperation
KW - Survival Rate
KW - Time Factors
KW - United States
RP - NOT IN FILE
SP - 15
EP - 29
JF - Clinical Transplants
JA - Clin Transpl
CY - UNITED STATES
N2 - UNLABELLED: CENTERS: The growth in liver transplantation activity recorded by the Pitt-UNOS Liver Transplant Registry since October 1987 continued. However, for the first time since the establishment of the LTR, there was no net gain in the number of centers in 1995. The large differences in volume per center also diminished. PATIENTS: The age of pediatric recipients increased significantly in 1995, due to a decrease in the proportion of recipients under age one. For the first time, in 1995 fewer than half of transplantations in children were for biliary atresia. The addition of bone marrow transplantations, for which collection began in 1994, accounted for half of the multi-organ transplantations in 1995. Many of the characteristics examined for adult recipients changed between 1994 and 1995. The proportion of Hispanic recipients increased. The mean age of adult recipients continued to increase, but there was not a significant change in the prevalence of positive CMV serology. Reversing a trend, the proportion of adult recipients awaiting transplantation outside of the hospital decreased between 1994 and 1995. As with children, the proportion of adult multi-organ transplantations which included bone marrow increased. Hepatitis non-A, non-B, or C and alcoholic liver disease (ALD) were the most common reasons for LTX in 1995. While the proportion of recipients with ALD alone decreased slightly, the proportion with ALD and hepatitis C increased from 1994 to 1995. OUTCOME: The cumulative probability of surviving (without retransplantation) for 8 years after initial transplantation was .71 (.60) for pediatric recipients. The one-year survival for pediatric recipients changed significantly over time with the increase from 1994 to 1995 being similar to the increase between 1994 and prior years. Independent risk factors for survival among children included age, race, location awaiting transplantation, primary liver disease, and serum creatinine. Year of transplantation and bilirubin were independently associated with retransplantation-free survival, whereas multi-organ transplantation was associated with poorer patient survival. The cumulative probability of adults surviving (without retransplantation) for 7 years following LTX was .58 (.50). Independent risk factors were year of transplantation, age, location awaiting transplantation, primary liver disease, albumin, creatinine, and ABO match. Black recipients had poorer patient survival rates than other recipients whereas increased prothrombin time and CMV-positive donors were risk factors for retransplantation or death
SN - 0890-9016
AD - Department of Epidemiology, University of Pittsburgh, Pennsylvania, USA
UR - 9286556
ER -

TY - JOUR
ID - 1918
T1 - [Electrical burns in children. 3 years of case histories]. [Portuguese]
A1 - Caneira,E.
A1 - Serafim,Z.
A1 - Duarte,R.
A1 - Leal,M.J.

Y1 - 1996/10//
N1 - Caneira, E. Serafim, Z. Duarte, R. Leal, M J
Acta medica portuguesa
7906803
IM
English Abstract. Journal Article
Portuguese
KW - MEDLINE
KW - Adolescent
KW - Age Distribution
KW - Burns
KW - Electric/ep [Epidemiology]
KW - Electric/et [Etiology]
KW - Electric/th [Therapy]
KW - Child
KW - Preschool
KW - Female
KW - Humans
KW - Infant
KW - Length of Stay
KW - Male
KW - Portugal/ep [Epidemiology]
KW - Seasons
KW - Sex Distribution
RP - NOT IN FILE
SP - 325
EP - 330
JF - Acta Medica Portuguesa
JA - Acta Med Port
VL - 9
IS - 10-12
CY - PORTUGAL
N2 - The Burn Unit of Dona Estefania Hospital admitted a total of 454 patients from January 1992 to January 1995, 24 of these patients suffered from electric shock. Of these 24 patients 3 suffered burns in the mouth, 15 in one or both hands and 6 multiple burns. In 19 patients the burns were up to 1%. A description is made of 5 cases, male children between the ages of 9 and 13 years, which were deemed severe. The incidents occurred outdoors with different voltages and in activities considered of ludic or experimental nature: two on the roof of a house, two with railway cables and one with an electrical cable in a port zone. The burnt areas vary between 4% and 70%, all of them 2nd and 3rd degree, with hospitalization lasting from 36 to 116 days. In addition to early and coordinated medical and rehabilitative treatment, according to individual needs, a description is also made of the cutaneous sequelae (deforming cicatrices, bridles), neurologic and psychologic sequelae, with emphasis on a patient who underwent amputation of the lower left leg and 4th and 5th ranges of the right foot. It was concluded that measures should be taken in education and legislation to prevent these accidents. Relevance is given to the need for a multidisciplinary team and specialized center for the treatment of these patients
SN - 0870-399X
AD - Servico de Cirurgia Pediatrica, Hospital de Dona Estefania, Lisboa
UR - 9254529
ER -

TY - JOUR
ID - 1919
T1 - Fractures in children in south eastern Nigeria
A1 - Archibong,A.E.
A1 - Onuba,O.
Y1 - 1996/12//
N1 - Archibong, A E. Onuba, O
The Central African journal of medicine

cqo, 0372566
IM
Journal Article
English
KW - MEDLINE
KW - Accidents/sn [Statistics & Numerical Data]
KW - Adolescent
KW - Child
KW - Preschool
KW - Female
KW - Fractures
KW - Bone/et [Etiology]
KW - Bone/pc [Prevention & Control]
KW - Hospitals
KW - University
KW - Humans
KW - Infant
KW - Male
KW - Nigeria
KW - Retrospective Studies
KW - Risk Factors
RP - NOT IN FILE
SP - 340
EP - 343
JF - Central African Journal of Medicine
JA - Cent Afr J Med
VL - 42
IS - 12
CY - ZIMBABWE
N2 - OBJECTIVES: To evaluate the pattern of fractures in children in the south eastern region of Nigeria, seeking to highlight the planning and designs of buildings, roads and playgrounds to prevent paediatric fractures. DESIGN: Retrospective review of paediatric patients with fractures who presented at the only tertiary referral centre in the region. SETTING: A university teaching hospital situated in the south eastern corner of Nigeria. SUBJECTS: 102 paediatric patients treated for fractures between January 1993 and December 1995. MAIN OUTCOME MEASURES: Causes of fractures and their anatomical distribution. RESULTS: 71 boys and 31 girls were treated for fractures. The commonest cause of fractures was road traffic accidents accounting for 50% of the patients, followed by falls and collapsing mud walls. Most of the fractures seen (58 patients) were of the green stick type. The femur was the commonly fractured bone (25 patients) followed by the radius (22 patients) and humerus (20 patients). Road traffic accident victims presented earlier at the hospital than victims of other causes. Also lower limb fracture patients presented earlier than patients with upper limb fractures. CONCLUSION: Paediatric fractures are amenable to conventional conservative therapy and open reduction became necessary in long neglected cases with associated malunion. Since the causes of fractures are largely preventable, public enlightenment campaigns, provision of pedestrian pathways, care on the part of school authorities and proper structural construction of the traditional houses may help reduce the number of cases of fracture in our environment
SN - 0008-9176
AD - Paediatric Surgery Unit College of Medical Sciences, University of Calabar, Nigeria
UR - 9164014
ER -

TY - JOUR
ID - 1920
T1 - The Canadian Human Activity Pattern Survey: report of methods and population surveyed
A1 - Leech,J.A.
A1 - Wilby,K.
A1 - McMullen,E.
A1 - Laporte,K.

Y1 - 1996///
N1 - Leech, J A. Wilby, K. McMullen, E. Laporte, K
Chronic diseases in Canada
8506870, cuc
IM
Journal Article. Research Support, Non-U.S. Gov't
English
KW - MEDLINE
KW - Adolescent
KW - Adult
KW - Aged
KW - Canada
KW - Child
KW - Preschool
KW - Environmental Exposure
KW - Female
KW - Health Surveys
KW - Humans
KW - Infant
KW - Life Style
KW - Male
KW - Middle Aged
RP - NOT IN FILE
SP - 118
EP - 123
JF - Chronic Diseases in Canada
JA - Chronic Dis Can
VL - 17
IS - 3-4
CY - CANADA
N2 - The assessment of health risk due to environmental contaminants depends upon accurate estimates of the distribution of population exposures. Exposure assessment, in turn, requires information on the time people spend in micro-environments and their activities during periods of exposure. This paper describes preliminary results including study methodology and population sampled in a large Canadian survey of time-activity patterns. A 24-hour diary recall survey was performed in 2381 households (representing a 65% response rate) to describe in detail the timing, location and activity pattern of one household member (the adult or child with the next birthday). Four cities (Toronto, Vancouver, Edmonton and Saint John, NB) and their suburbs were sampled by random-digit dialling over a nine-month period in 1994/1995. Supplemental questionnaires inquiring about sociodemographic information, house and household characteristics and potential exposure to toxins in the air and water were also administered. In general, the results show that respondents spend the majority of their time indoors (88.6%) with smaller proportions of time outdoors (6.1%) and in vehicles (5.3%). Children under the age of 12 spend more time both indoors and outdoors and less time in transit than do adults. The data from this study will be used to define more accurately the exposure of Canadians to a variety of toxins in exposure assessment models and to improve upon the accuracy of risk assessment for a variety of acute and chronic health effects known or suspected to be related to environmental exposures
SN - 0228-8699
AD - Division of Environmental and Occupational Toxicology, Health Canada
UR - 9097012
ER -

TY - JOUR
ID - 1921
T1 - Short-term air pollution exposures and responses in Los Angeles area schoolchildren
A1 - Linn,W.S.
A1 - Shamoo,D.A.
A1 - Anderson,K.R.
A1 - Peng,R.C.

A1 - Avol,E.L.
A1 - Hackney,J.D.
A1 - Gong,H.,Jr.
Y1 - 1996/10//
N1 - Linn, W S. Shamoo, D A. Anderson, K R. Peng, R C. Avol, E L. Hackney, J D. Gong, H Jr
Journal of exposure analysis and environmental epidemiology
bjn, 9111438
IM
Journal Article. Research Support, Non-U.S. Gov't
English
KW - MEDLINE
KW - Air Pollution/ae [Adverse Effects]
KW - Analysis of Variance
KW - California/ep [Epidemiology]
KW - Child
KW - Cross-Sectional Studies
KW - Environmental Exposure/ae [Adverse Effects]
KW - Environmental Health
KW - Environmental Monitoring/sn [Statistics & Numerical Data]
KW - Epidemiological Monitoring
KW - Female
KW - Health Surveys
KW - Humans
KW - Likelihood Functions
KW - Longitudinal Studies
KW - Male
KW - Nitrogen Dioxide/ae [Adverse Effects]
KW - Ozone/ae [Adverse Effects]
KW - Particle Size
KW - Regression Analysis
KW - Respiration Disorders/ci [Chemically Induced]
KW - Respiration Disorders/ep [Epidemiology]
KW - Respiratory Function Tests/sn [Statistics & Numerical Data]
KW - Seasons
KW - Sex Distribution
KW - Time Factors
KW - Weather
RP - NOT IN FILE
SP - 449
EP - 472
JF - Journal of Exposure Analysis and Environmental Epidemiology
JA - J Expo Anal Environ Epidemiol
VL - 6
IS - 4
CY - UNITED STATES
N2 - We studied 269 school children from three Southern California communities of contrasting air quality in two successive school years, to investigate short-term effects of ambient ozone (O3), nitrogen dioxide (NO2), or particulate matter (PM) on respiratory health. We measured lung function and symptoms twice daily for one week each in fall, winter and spring; and concurrently assessed time-activity patterns and personal exposures. Average daily personal exposures correlated with pollutant concentrations at central sites (r = 0.61 for O3, 0.63 for NO2, 0.48 for PM). Questionnaire-reported outdoor activity increased slightly in communities/seasons with higher pollution. Lung function differences between communities were explainable by age differences. Morning forced vital capacity (FVC) decreased significantly with increase in PM or NO2 measured over the preceding 24 hours. Morning-to-afternoon change of forced expired volume in one second (FEV1) became significantly more negative with increase in PM, NO2, or O3 on the same day. Predicted FVC or FEV1 loss on highest- vs lowest-pollution days was < 2%. Daily symptoms showed no association with current or prior 24-hour pollution, but increased with decreasing temperature. Parents' questionnaire responses suggested excess asthma and allergy in

children from one polluted community while children in the other polluted community reported more symptoms, relative to the cleaner community. We conclude that Los Angeles area children may experience slight lung function changes in association with day-to-day air quality changes, reasonably similar to responses seen by others in less polluted areas. Although short-term pollution effects appear small, they should be assessed in longitudinal lung function studies when possible, to allow maximally accurate measurement of longer-term function changes
SN - 1053-4245
AD - Environmental Health Service, Rancho Los Amigos, Medical Center, Downey, California 90242, USA
UR - 9087865
ER -

TY - JOUR
ID - 1922
T1 - A descriptive study of childhood injuries in Kingston, Ontario, using data from a computerized injury surveillance system
A1 - Bienefeld,M.
A1 - Pickett,W.
A1 - Carr,P.A.
Y1 - 1996///
N1 - Bienefeld, M. Pickett, W. Carr, P A
Chronic diseases in Canada
8506870, cuc
IM
Journal Article. Research Support, Non-U.S. Gov't
English
KW - MEDLINE
KW - Accidents/sn [Statistics & Numerical Data]
KW - Adolescent
KW - Athletic Injuries/ep [Epidemiology]
KW - Bicycling/in [Injuries]
KW - Child
KW - Preschool
KW - Female
KW - Humans
KW - Infant
KW - Male
KW - Ontario/ep [Epidemiology]
KW - Population Surveillance
KW - Prevalence
KW - Wounds and Injuries/ep [Epidemiology]
KW - Wounds and Injuries/et [Etiology]
RP - NOT IN FILE
SP - 21
EP - 27
JF - Chronic Diseases in Canada
JA - Chronic Dis Can
VL - 17
IS - 1
CY - CANADA
N2 - This report uses data from the Kingston and Region Injury Surveillance Program (KRISP), a subset of the Canadian Hospitals Injury Reporting and Prevention Program (CHIRPP), to describe rates and identify patterns of injury among children aged 0-19 years in Kingston and area. During 1994, there were 7572 reported injury events, resulting in an overall rate of 173.6 injuries per 1000 children per year (males: 202 per 1000; females: 143.7 per 1000). Four major patterns of injury were identified as priorities for intervention: 1) household injuries among children aged 0-4 years; 2) injuries occurring on playgrounds to children aged 5-14 years; 3) sports injuries among 10-19-year-old children and youth; and 4) bicycle-related injuries among children 5-14

years of age. Discussion focuses on the use of the surveillance system in prioritizing interventions and evaluating injury prevention programs for this population
SN - 0228-8699
AD - Department of Community Health and Epidemiology, Queen's University, Kingston, Ontario
UR - 9079349
ER -

TY - JOUR
ID - 1923
T1 - CD4 and CD8 lymphocytes in diagnosis and disease progression of pediatric HIV infection
A1 - Aldhous,M.C.
A1 - Raab,G.M.
A1 - Mok,J.Y.
A1 - Doherty,K.V.
A1 - Bird,A.G.
A1 - Froebel,K.S.
Y1 - 1996/02//
N1 - Aldhous, M C. Raab, G M. Mok, J Y. Doherty, K V. Bird, A G. Froebel, K S
Pediatric AIDS and HIV infection
9107942
X
Journal Article. Research Support, Non-U.S. Gov't
English
KW - MEDLINE
KW - Biological Markers
KW - CD4 Lymphocyte Count
KW - CD8-Positive T-Lymphocytes
KW - Case-Control Studies
KW - Child
KW - Preschool
KW - Disease Progression
KW - HIV Infections/cl [Classification]
KW - HIV Infections/di [Diagnosis]
KW - HIV Infections/im [Immunology]
KW - HIV Infections/tm [Transmission]
KW - Humans
KW - Immunophenotyping
KW - Infant
KW - Newborn
KW - Infectious Disease Transmission
KW - Vertical
KW - Prospective Studies
KW - Severity of Illness Index
KW - T-Lymphocyte Subsets
RP - NOT IN FILE
SP - 20
EP - 30
JF - Pediatric AIDS & HIV Infection
JA - Pediatr AIDS HIV Infect
VL - 7
IS - 1
CY - UNITED STATES
N2 - Vertical infection with human immunodeficiency virus-1 (HIV-1) causes profound changes in the proportions of subpopulations of lymphocytes in the peripheral circulation. In this study the percentages in whole blood of CD4 and CD8 cells, and of immunologically important subpopulations, were measured in 19 HIV-infected children over periods of up to 4 years and compared to our recently published ranges for normal children of various ages. The rate of CD4 decline and of CD8 increase differed between clinically fast and slow

progressors. On CD8 cells, cytotoxic, memory (CD11abright and CD45R0), and activation (HLA-DR) markers were raised soon after birth to levels outside the normal range, and compared favorably with HIV culture as a method for early diagnosis of HIV infection. Mean levels of naive (CD45RA) and memory (CD45R0, CD29) markers on CD4 cells became significantly altered after 48 months of age, suggesting that these are markers of more advanced disease. Despite different ages of enrollment into the study, in the cohort as a whole, the levels of the lymphocyte subpopulations studied changed consistently. Thus, their measurement could be useful both in the diagnosis and prognosis of HIV infection in individual children. This is the first report showing that lymphocyte subpopulation analysis can play a major role in the diagnosis of pediatric HIV infection
SN - 1045-5418
AD - Centre for HIV Research, University of Edinburgh, Scotland
UR - 11361469
ER -

TY - JOUR
ID - 1924
T1 - Passive smoking and risk of pulmonary tuberculosis in children immediately following infection. A case-control study
A1 - Altet,M.N.
A1 - Alcaide,J.
A1 - Plans,P.
A1 - Taberner,J.L.
A1 - Salto,E.
A1 - Folguera,L.I.
A1 - Salleras,L.
Y1 - 1996/12//
N1 - Altet, M N. Alcaide, J. Plans, P. Taberner, J L. Salto, E. Folguera, L I. Salleras, L
Tubercle and lung disease : the official journal of the International Union against Tuberculosis and Lung Disease
a8c, 9212467
IM
Journal Article
English
KW - MEDLINE
KW - Adolescent
KW - Case-Control Studies
KW - Child
KW - Preschool
KW - Cotinine/ur [Urine]
KW - Family Health
KW - Female
KW - Humans
KW - Infant
KW - Newborn
KW - Logistic Models
KW - Male
KW - Socioeconomic Factors
KW - Tobacco Smoke Pollution/ae [Adverse Effects]
KW - Tuberculosis
KW - Pulmonary/et [Etiology]
KW - Pulmonary/ra [Radiography]
RP - NOT IN FILE
SP - 537
EP - 544
JF - Tubercle & Lung Disease
JA - Tuber.Lung Dis
VL - 77
IS - 6

CY - SCOTLAND
N2 - SETTING: Passive smoking-related respiratory disorders in children. OBJECTIVES: To assess the effect of passive smoking on the development of active pulmonary tuberculosis (PTB) in children immediately following infection by Mycobacterium tuberculosis within the family. DESIGN: An unmatched case-control study in which 93 contacts who became cases (active PTB diagnosed) and 95 contacts who did not became cases (tuberculin-positive children without evidence of active disease) were included. All were household contacts of a new case of pulmonary bacillary tuberculosis. Smoking habits were investigated by a questionnaire. Urinary cotinine was analysed. Odds Ratio (OR) was adjusted for age and socio-economic status using multiple logistic regression analysis. RESULTS: Passive smoking was a risk factor for PTB (OR: 5.29; 95% confidence interval (CI): 2.33-12.82; P < 0.00005). The adjusted OR was 5.39 (95% CI: 2.44-11.91; P < 0.00001). The risk increased when contacts were passive smokers both at home and outside the home within the family (OR: 6.35; 95% CI: 3.20, 12.72; P < 0.00001). Contacts 0-4 and 5-9 years old showed a significantly higher risk than those aged > or = 10. There was a dose-response relationship between the risk of developing active PTB immediately following infection and the number of cigarettes smoked daily by the household adults (P < 0.001). Mean (SD) urinary continine detectable concentrations (ng/ml) were different between disease contacts (119.46 [68.61]) and non diseased contacts (91.87 [73.10]). The difference was statistically significant (P < 0.001). CONCLUSIONS: Passive exposure to tobacco smoke in children was associated with an increased risk of developing pulmonary tuberculosis immediately following infection. This is an association of great concern requiring health education programmes and antitobacco medical advice
SN - 0962-8479
AD - Centre de Prevencio i Control de la Tuberculosi Dr I.I. Saye, Barcelona, Spain
UR - 9039447
ER -

TY - JOUR
ID - 1925
T1 - [Response of testosterone to chorionic gonadotropin stimulus in prepubertal cryptorchidism and retractile testes. Age-related changes in gonadal steroidogenesis. Authors'experience]. [Italian]
A1 - Rossodivita,A.
A1 - Becerra,Merchan L.
A1 - Giordano,P.
A1 - Latella,C.
A1 - Chiaretti,A.
A1 - Colabucci,F.
Y1 - 1996/10//
N1 - Rossodivita, A. Becerra Merchan, L. Giordano, P. Latella, C. Chiaretti, A. Colabucci, F
Minerva pediatrica
nam, 0400740
IM
English Abstract. Journal Article
Italian
KW - MEDLINE
KW - Age Factors
KW - Child
KW - Preschool
KW - Chorionic Gonadotropin/tu [Therapeutic Use]
KW - Cryptorchidism/bl [Blood]
KW - Cryptorchidism/dt [Drug Therapy]
KW - Gonadal Dysgenesis/bl [Blood]
KW - Gonadal Dysgenesis/dt [Drug Therapy]
KW - Humans
KW - Infant
KW - Male
KW - Testicular Hormones/bi [Biosynthesis]
KW - Testicular Hormones/bl [Blood]
KW - Testosterone/bi [Biosynthesis]
KW - Testosterone/bl [Blood]

RP - NOT IN FILE
SP - 439
EP - 443
JF - Minerva Pediatrica
JA - Minerva Pediatr
VL - 48
IS - 10
CY - ITALY
N2 - The aim of this analysis is to evaluate the gonadal function in children with true undescended testes and in those with retractile testes, in order to verify a possible impairment of the testicular steroidogenesis due to the permanent or transitory anomalous position of the gonad outside the scrotum. The authors carried out a prospective study on 29 prepubertal children affected by true undescended testes (monolateral in 20 cases and bilateral in 9), as well as on 25 prepubertal children with retractile testes (monolateral in 10 cases and bilateral in 15), assaying the testosterone (T) levels, basal and 72 hours after stimulus with human chorionic gonadotrophin (HCG) administered in a single dose of 100 U/kg i.m. Further-more, to verify the hypothesis of a possible progressive reduction of the Leydig cells function, particularly in the gonads bilaterally affected, the authors also evaluated the testosterone response to gonadotrophic stimulus compared to age (> 0 < 4 years). This study in agreement with data already published, confirms the normality of gonadal function both in children with mono or bilateral true undescended testes and in those with retractile testes. The lower the age of the subject the higher is the peak of testosterone after stimulus, confirming the active steroidogenesis of the gonads in infants and small children and sustaining the "non quiescence" of this organ during infancy, even in cases of true undescended testes
SN - 0026-4946
AD - Istituto di Clinica Pediatrica, Universita Cattolica del Sacro Cuore di Roma
UR - 9053722
ER -

TY - JOUR
ID - 1926
T1 - The epidemiology of atopic diseases in Germany: an east-west comparison. [Review] [49 refs]
A1 - Trepka,M.J.
A1 - Heinrich,J.
A1 - Wichmann,H.E.
Y1 - 1996/07//
N1 - Trepka, M J. Heinrich, J. Wichmann, H E
Reviews on environmental health
rsr, 0425754
IM
Comparative Study. Journal Article. Review
English
KW - MEDLINE
KW - Adolescent
KW - Adult
KW - Age Distribution
KW - Asthma/ep [Epidemiology]
KW - Asthma/et [Etiology]
KW - Child
KW - Preschool
KW - Dermatitis
KW - Atopic/ep [Epidemiology]
KW - Atopic/et [Etiology]
KW - Female
KW - Germany
KW - East/ep [Epidemiology]
KW - West/ep [Epidemiology]
KW - Humans
KW - Male

KW - Population Surveillance
KW - Residence Characteristics
KW - Rhinitis
KW - Allergic
KW - Seasonal/ep [Epidemiology]
KW - Seasonal/et [Etiology]
RP - NOT IN FILE
SP - 119
EP - 131
JF - Reviews on Environmental Health
JA - Rev Environ Health
VL - 11
IS - 3
CY - ISRAEL
N2 - This review summarizes studies comparing the epidemiology of asthma, allergic rhinitis, and atopic dermatitis in eastern versus western German populations. These studies clearly revealed that the prevalence of physician-diagnosed allergic rhinitis was higher in western Germany than in eastern Germany. The rate for asthma also tended to be higher in western than in eastern populations, whereas a tendency for less atopic dermatitis was found in western German children. When sensitization rates, as measured by RAST and skin-prick tests, were compared, a clear increase was found in western versus eastern young-adult German populations. This phenomenon may be related to exposure to different allergens, such as those associated with nutritional patterns or pet ownership, as well as to different environmental factors, such as outdoor and indoor pollutants. If lifestyle or environmental factors play a role in developing allergic sensitization, then one would expect the rates of sensitization in eastern and western Germany to converge as the two societies become more similar. [References: 49]
SN - 0048-7554
AD - GSF, Forschungszentrum fur Umwelt and Gesundheit, Institut fur Epidemiologie, Neuherberg, Germany
UR - 9000303
ER -

TY - JOUR
ID - 1927
T1 - Posterior fossa lateral ependymoma in childhood
A1 - Nagib,M.G.
A1 - O'Fallon,M.T.
Y1 - 1996/06//
N1 - Nagib, M G. O'Fallon, M T
Pediatric neurosurgery
axe, 9114967
IM
Journal Article
English
KW - MEDLINE
KW - Cerebellar Neoplasms/dt [Drug Therapy]
KW - Cerebellar Neoplasms/rt [Radiotherapy]
KW - Cerebellar Neoplasms/su [Surgery]
KW - Cerebral Ventricle Neoplasms/dt [Drug Therapy]
KW - Cerebral Ventricle Neoplasms/rt [Radiotherapy]
KW - Cerebral Ventricle Neoplasms/su [Surgery]
KW - Chemotherapy
KW - Adjuvant
KW - Child
KW - Preschool
KW - Combined Modality Therapy
KW - Cranial Fossa
KW - Posterior
KW - Cranial Irradiation

KW - Ependymoma/dt [Drug Therapy]
KW - Ependymoma/rt [Radiotherapy]
KW - Ependymoma/su [Surgery]
KW - Facial Nerve/pp [Physiopathology]
KW - Facial Nerve Injuries
KW - Female
KW - Glossopharyngeal Nerve/pp [Physiopathology]
KW - Glossopharyngeal Nerve Injuries
KW - Humans
KW - Infant
KW - Male
KW - Monitoring
KW - Intraoperative
KW - Neurologic Examination
KW - Radiotherapy
KW - Treatment Outcome
RP - NOT IN FILE
SP - 299
EP - 305
JF - Pediatric Neurosurgery
JA - Pediatr Neurosurg
VL - 24
IS - 6
CY - SWITZERLAND
N2 - Over a period extending from 1984 to 1993, 16 children ranging in ages from 2 months to 12 years with posterior fossa ependymoma were treated. Four of these tumors were classified as lateral ependymomas because of their configuration and suspected site of origin. These 4 patients' ages ranged from 2 months to 5 years. Signs and symptoms of increased intracranial pressure and cranial nerve dysfunction were the hallmark of their presentation. Their imaging evaluation included a preoperative and postoperative CT san and/or MRI. A gross total surgical resection' was completed in the 4 children. At least a 1-year follow-up was available for all the patients. Intraoperative brain stem evoked potentials and facial and glossopharyngeal nerve monitoring were used in all the surgeries. Three children required a ventriculoperitoneal shunt postoperatively. Chemotherapy was used postoperatively in 3 children. The 4th child did not receive chemotherapy due to parental refusal and succumbed to a recurrence 1 years postoperatively. Radiation therapy was given to 2 children. Tumor recurrences developed in all children at 12 months, 18 months, 3 years and 4 years postoperatively in proximity to the original tumor site. Histologic sections in 3 patients, including those at the time of recurrence, were consistent with a low mitotic index. Only 1 child's histologic diagnosis was consistent with a high-grade ependymoma. The lower cranial nerve dysfunction transiently worsened in all the patients and was permanent in 2. The child's age, tumor histology and extent of resection play an important role in the prognosis of posterior fossa ependymoma. The lateral posterior fossa ependymoma has a particularly poor prognosis due to its location and its significant postoperative morbidity. The authors propose a combined midline and lateral suboccipital approach in order to expose the fourth ventricular floor, upper cervical spine, lateral recess, cerebellomedullary fissure, cerebellopontine and the cerebellomedullary cistern in an attempt at a "total' resection with a focus on the site of origin. The usage of neurophysiological monitoring appears to be useful in limiting and predicting the extent of postoperative complications. However, prolonged morbidity is likely and is commonly related to lower cranial nerve deficits. The author proposes a proactive approach in order to limit the sequelae of these complications
SN - 1016-2291
AD - Neurosurgical Associates, Ltd., Minneapolis, MN, USA
UR - 8988495
ER -

TY - JOUR
ID - 1928
T1 - A nursing intervention to ensure a safe playground environment
A1 - McEvoy,M.
A1 - Montana,B.

A1 - Panettieri,M.
Y1 - 1996/09//
N1 - McEvoy, M. Montana, B. Panettieri, M
Journal of pediatric health care : official publication of National Association of Pediatric Nurse Associates & Practitioners
jml, 8709735
N
Journal Article
English
KW - MEDLINE
KW - Child
KW - Child Behavior
KW - Humans
KW - Nurse Practitioners/og [Organization & Administration]
KW - Nursing Assessment
KW - Pediatric Nursing/og [Organization & Administration]
KW - Pilot Projects
KW - Play and Playthings
KW - Safety
KW - School Nursing/og [Organization & Administration]
KW - Wounds and Injuries/pc [Prevention & Control]
RP - NOT IN FILE
SP - 209
EP - 216
JF - Journal of Pediatric Health Care
JA - J Pediatr Health Care
VL - 10
IS - 5
CY - UNITED STATES
N2 - Unintentional injuries sustained on school playgrounds are a continual problem not only for children but also for the school nurses who are concerned with their safety. A group of school nurses organized by the local health department's pediatric nurse practitioner responded proactively to this problem by enlisting both school officials and students to accept joint responsibility for a safe play environment. The school nurses assisted the staff in providing a safe play environment and encouraged the students to behave in a safe and acceptable manner on the playground. The nurses developed (a) a Playground Safety Assessment Guide for the school staff to establish and maintain a safe play environment, (b) an in-service session for playground monitors to learn how to supervise children at recess, and (c) a student-oriented program for the children to improve behavior on the playground based on a program conceived by Utah State University (Daug & Fukui, 1988). School nurses and pediatric nurse practitioners can use this program as a model for other schools to improve the physical play environment and behavior of the children at recess
SN - 0891-5245
UR - 8920353
ER -

TY - JOUR
ID - 1929
T1 - [Limit values for polycyclic aromatic hydrocarbons in soil of children's playgrounds--basic criteria and recommendations]. [German]
A1 - Roscher,E.
A1 - Liebl,B.
A1 - Schwegler,U.
A1 - Schmied,R.
A1 - Kerscher,G.
Y1 - 1996/08//
N1 - Roscher, E. Liebl, B. Schwegler, U. Schmied, R. Kerscher, G
Gesundheitswesen (Bundesverband der Arzte des Offentlichen Gesundheitsdienstes (Germany))
bfd, 9204210

IM
English Abstract. Journal Article
German
KW - MEDLINE
KW - Benzo(a)pyrene/ae [Adverse Effects]
KW - Benzo(a)pyrene/an [Analysis]
KW - Child
KW - Germany
KW - Humans
KW - Maximum Allowable Concentration
KW - Play and Playthings
KW - Polycyclic Hydrocarbons
KW - Aromatic/ae [Adverse Effects]
KW - Aromatic/an [Analysis]
KW - Risk Factors
KW - Social Environment
KW - Soil Pollutants/ae [Adverse Effects]
KW - Soil Pollutants/an [Analysis]
RP - NOT IN FILE
SP - 470
EP - 476
JF - Gesundheitswesen
VL - 58
IS - 8-9
CY - GERMANY
N2 - Elevated concentrations of polycyclic aromatic hydrocarbons (PAK) are often found in the soil of former waste disposal sites, industrial areas, etc. It is desirable and useful to determine orientation values to facilitate and unify the evaluation of contaminations under the aspects of present or planned uses of an area, health protection and decision-making on remedial measures. In the present paper we wish to draw attention to, and discuss problems resulting from, particular characteristics of PAK, e.g. the toxicological property "complete carcinogens" or the necessity of taking into account oral, inhalative and dermal exposure of children on a playground. Based on the discussion, orientation values for benzo[a]pyrene and PAK ("normal" pattern) of 0.5 mg/kg soil and 5 mg/kg soil, respectively, are recommended for top soil of vegetation-free playgrounds. In comparison, deductions carried out by other working groups are presented
SN - 0941-3790
AD - Landesuntersuchungsamt fur das Gesundheitswesen Sudbayem, Oberschleissheim
UR - 9011264
ER -

TY - JOUR
ID - 1930
T1 - Childhood asthma and exposure to indoor allergens: low mite levels are associated with sensitivity
A1 - Warner,A.M.
A1 - Bjorksten,B.
A1 - Munir,A.K.
A1 - Moller,C.
A1 - Schou,C.
A1 - Kjellman,N.I.
Y1 - 1996/05//
N1 - Warner, A M. Bjorksten, B. Munir, A K. Moller, C. Schou, C. Kjellman, N I
Pediatric allergy and immunology : official publication of the European Society of Pediatric Allergy and Immunology
bu6, 9106718
IM
Journal Article. Research Support, Non-U.S. Gov't
English
KW - MEDLINE

KW - Adolescent
KW - Air Pollution
KW - Indoor
KW - Allergens/an [Analysis]
KW - Allergens/im [Immunology]
KW - Animals
KW - Antigens
KW - Plant
KW - Asthma/et [Etiology]
KW - Cats
KW - Child
KW - Preschool
KW - Climate
KW - Dogs
KW - Dust
KW - Environmental Exposure
KW - Female
KW - Glycoproteins/an [Analysis]
KW - Humans
KW - Immunoglobulin E/bl [Blood]
KW - Male
KW - Mites/im [Immunology]
KW - Sweden
RP - NOT IN FILE
SP - 61
EP - 67
JF - Pediatric Allergy & Immunology
JA - Pediatr Allergy Immunol
VL - 7
IS - 2
CY - DENMARK
N2 - The prevalence and level of sensitivity to indoor allergens were studied in relation to current exposure at home in 124 children with perennial asthma living in three climatic zones of Sweden. The house dust mite (HDM) allergen levels were higher in the South than in the North ($p < 0.001$), while cat and dog allergen levels tended to be higher in the North than the South (n.s.). Thirty-four percent of the children were sensitive to the HDM Dermatophagoides pteronyssinus, as determined by IgE antibodies in vitro, 27% were sensitive to D. farinae, 57% to cat and 55% to dog. Sensitivity to HDM was significantly more prevalent in Southern, than in Central and Northern Sweden ($p = 0.001$) where the children were more often sensitive to pets (cat $p = 0.005$, dog $p = 0.002$). A significant association between the concentration of Der p I and Der fI in the house dust and both the prevalence of sensitivity to HDM and the IgE antibody levels against mites was found even at concentrations well below the commonly suggested risk level for sensitisation of 2 micrograms/g dust. No relationship was found between pet allergen concentration in the home dust and sensitivity to pets, possibly because of exposure outside home, e.g. in schools and meeting places for leisure activities. Similarly, there was no consistent association between the level of mite or pet allergen exposure at home and asthma severity as judged by symptom and medication score. The study indicates that there is no threshold value for sensitisation to mite allergens in asthmatic children, and therefore, dust allergen levels at home should be kept as low as possible in homes of children at risk for asthma
SN - 0905-6157
AD - Department of Paediatrics, University Hospital Linkoping, Sweden
UR - 8902855
ER -

TY - JOUR
ID - 1931
T1 - Antimicrobial therapy of pneumonia in infants and children
A1 - Harris,J.A.
Y1 - 1996/09//

N1 - Harris, J A
Seminars in respiratory infections
sei, 8700961
IM
Journal Article
English
KW - MEDLINE
KW - Age Factors
KW - Anti-Bacterial Agents/ad [Administration & Dosage]
KW - Anti-Bacterial Agents/pd [Pharmacology]
KW - Anti-Bacterial Agents/tu [Therapeutic Use]
KW - Child
KW - Preschool
KW - Community-Acquired Infections/dt [Drug Therapy]
KW - Community-Acquired Infections/et [Etiology]
KW - Humans
KW - Infant
KW - Newborn
KW - Microbial Sensitivity Tests
KW - Pneumonia/dt [Drug Therapy]
KW - Pneumonia/et [Etiology]
KW - Prognosis
RP - NOT IN FILE
SP - 139
EP - 147
JF - Seminars in Respiratory Infections
JA - Semin Respir Infect
VL - 11
IS - 3
CY - UNITED STATES
N2 - To provide optimal management for the child with community-acquired pneumonia, the clinician must take multiple factors into consideration. The etiology of pneumonia is difficult to determine and initial choice of therapy is based on the frequency of pathogens in various age groups, local antibiotic resistance patterns of the organisms, clinical presentation, and epidemiological data. Streptococcus pneumoniae and Haemophilus influenzae remain the most common bacterial pathogens outside the newborn period. Increasing numbers of multidrug-resistant strains of S pneumoniae in the United States and Europe, the decline in H influenzae type b because of current vaccination strategies, and increasing recognition of nontypeable H influenzae as etiologic agents of pneumonia have prompted reconsideration of the drug of choice. Amoxicillin and its derivatives or oral cephalosporins are the drugs of choice for initial therapy for mild to moderate disease. For severe disease or if beta-lactamase producing organisms are a concern, extended spectrum cephalosporins are indicated. Pneumococcal pneumonia unresponsive to penicillin therapy may warrant the use of extended spectrum cephalosporins or vancomycin. For older children in whom mycoplasma is a significant cause of pneumonia, the new macrolides have provided additional options for the clinician. Azithromycin and clarithromycin are efficacious, well tolerated, and require less frequent dosing intervals. The introduction of ceftriaxone, a third-generation cephalosporin with a broad spectrum of activity and prolonged half-life, allows once-a-day intramuscular therapy that can be administered on an outpatient basis. With the availability of parenteral outpatient therapy, hospital admission is no longer required for the treatment of most cases of serious community-acquired pneumonia
SN - 0882-0546
AD - Maxwell Finland Laboratory for Infectious Disease, Boston City Hospital, MA 02118, USA
UR - 8883171
ER -

TY - JOUR
ID - 1932
T1 - A controlled trial with ORG 2766, an ACTH-(4-9) analog, in 50 relatively able children with autism
A1 - Buitelaar,J.K.

A1 - Dekker,M.E.
A1 - van Ree,J.M.
A1 - van,Engeland H.
Y1 - 1996/03//
N1 - Buitelaar, J K. Dekker, M E. van Ree, J M. van Engeland, H
European neuropsychopharmacology : the journal of the European College of Neuropsychopharmacology
bjh, 9111390
IM
Clinical Trial. Controlled Clinical Trial. Journal Article
English
KW - MEDLINE
KW - Adolescent
KW - Adrenocorticotropic Hormone/aa [Analogs & Derivatives]
KW - Adrenocorticotropic Hormone/tu [Therapeutic Use]
KW - Anticonvulsants/tu [Therapeutic Use]
KW - Autistic Disorder/dt [Drug Therapy]
KW - Autistic Disorder/pp [Physiopathology]
KW - Autistic Disorder/px [Psychology]
KW - Child
KW - Double-Blind Method
KW - Humans
KW - Peptide Fragments/tu [Therapeutic Use]
KW - Prognosis
KW - Psychiatric Status Rating Scales
KW - Time Factors
RP - NOT IN FILE
SP - 13
EP - 19
JF - European Neuropsychopharmacology
JA - Eur Neuropsychopharmacol.
VL - 6
IS - 1
CY - NETHERLANDS
N2 - The aim of the present study was to replicate earlier findings of beneficial effects of ORG 2766, an ACTH-(4-9) analog, in autistic children. Fifty children with autism, 7-15 years old and with a Performance IQ of more than 60, participated in a double-blind placebo controlled parallel trial. Active treatment was 40 mg ORG 2766 for 6 weeks. The outcome was assessed on the basis of the Aberrant Behavior Checklist completed by parents and teachers, and by means of a detailed behavioral observation (30 subjects). ORG 2766 failed to improve social and communicative behavior at a group level. The rate of individual response, defined as a reliable change in social withdrawal at home and at school, to ORG 2766 (10 out of 30) and placebo (4 out of 20) was not significant either. The children who responded to ORG 2766, but not those who responded to placebo, manifested significant improvements outside the changes in the defining variables, including a decrease in hyperactivity at school. The responders to ORG 2766 were characterized mainly by a relatively lower PIQ; further by more initial hyperactivity, stereotypies and abnormal speech, and less initial eye contact. The responders to placebo could not be differentiated from the non-responders to placebo. Future studies should examine whether ORG 2766 differentially affects various subtypes of autism
SN - 0924-977X
AD - Department of Child and Adolescent Psychiatry, University of Utrecht, Netherlands
UR - 8866933
ER -

TY - JOUR
ID - 1933
T1 - Fixation patterns and reading rates in eyes with central scotomas from advanced atrophic age-related macular degeneration and Stargardt disease
A1 - Sunness,J.S.
A1 - Applegate,C.A.

A1 - Haselwood,D.
A1 - Rubin,G.S.
Y1 - 1996/09//
N1 - Sunness, J S. Applegate, C A. Haselwood, D. Rubin, G S
Ophthalmology
oi5, 7802443
IM
Comparative Study. Journal Article. Research Support, Non-U.S. Gov't. Research Support, U.S. Gov't, P.H.S.
English
KW - MEDLINE
KW - Adolescent
KW - Adult
KW - Aged
KW - 80 and over
KW - Atrophy
KW - Child
KW - Fixation
KW - Ocular/ph [Physiology]
KW - Fluorescein Angiography
KW - Fovea Centralis/pa [Pathology]
KW - Fundus Oculi
KW - Humans
KW - Macular Degeneration/co [Complications]
KW - Ophthalmoscopy
KW - Reading
KW - Scotoma/et [Etiology]
KW - Scotoma/pp [Physiopathology]
KW - Visual Acuity
KW - Visual Fields
RP - NOT IN FILE
SP - 1458
EP - 1466
JF - Ophthalmology
VL - 103
IS - 9
CY - UNITED STATES
N2 - PURPOSE: To study fixation patterns and reading rates in eyes with central scotomas from geographic atrophy (GA) of age-related macular degeneration and to compare fixation patterns with those of patients with Stargardt disease. METHODS: Scanning laser ophthalmoscope analysis of fixation patterns in eyes with 20/80 to 20/200 visual acuity. Included were 41 eyes of 35 patients with GA and 10 eyes of 5 patients with Stargardt disease. The patients with GA also were tested for maximum reading rate, and the size of the areas of atrophy were measured by fundus photograph analysis. RESULTS: Sixty-three percent of GA eyes fixating outside the atrophy placed the scotoma to the right of fixation in visual field space, 22% placed the scotoma above fixation, and 15% placed it to the left, regardless of the laterality of the GA eye. Fixation was stable in subsequent years of testing for scotoma placement to the right of or above fixation. All GA eyes fixated immediately adjacent to the atrophy. In contrast, seven of ten eyes with Stargardt disease fixated at a considerable distance from the scotoma border, with the dense scotoma far above the fixation site in visual field space. For the patients with GA, the maximum reading rate was highly correlated with size of the atrophic area, but not with age or visual acuity within the limited visual acuity range tested. There was a trend to more rapid reading with the scotoma above fixation and slower reading with the scotoma to the left. CONCLUSION: There is a preference for fixation with the scotoma to the right in eyes with GA. Patients with Stargardt disease use different strategies for fixation, perhaps due to subclinical pathology adjacent to the atrophic regions. The size of the atrophic area in GA plays the predominant role in reading rate for eyes that have already lost foveal vision
SN - 0161-6420
AD - Lions Vision Center, Wilmer Ophthalmological Institute, Johns Hopkins University School of Medicine, Baltimore, USA
UR - 8841306

ER -

TY - JOUR
ID - 1934
T1 - Environmental and life-style risk factors for Lyme disease in children
A1 - Klein,J.D.
A1 - Eppes,S.C.
A1 - Hunt,P.
Y1 - 1996/07//
N1 - Klein, J D. Eppes, S C. Hunt, P
Clinical pediatrics
dhe, 0372606, 8407647
AIM, IM
Journal Article
English
KW - MEDLINE
KW - Adolescent
KW - Analysis of Variance
KW - Animals
KW - Borrelia burgdorferi Group/im [Immunology]
KW - Case-Control Studies
KW - Child
KW - Preschool
KW - Confidence Intervals
KW - Ecology
KW - Environment
KW - Female
KW - Humans
KW - Ixodes
KW - Life Style
KW - Lyme Disease/bl [Blood]
KW - Lyme Disease/ep [Epidemiology]
KW - Lyme Disease/pc [Prevention & Control]
KW - Male
KW - Mid-Atlantic Region/ep [Epidemiology]
KW - Odds Ratio
KW - Residence Characteristics
KW - Risk Factors
KW - Tick Infestations
RP - NOT IN FILE
SP - 359
EP - 363
JF - Clinical Pediatrics
JA - Clin Pediatr (Phila)
VL - 35
IS - 7
CY - UNITED STATES
N2 - Risk factors for Lyme disease and/or infection with Borrelia burgdorferi in children are not defined. A case-control study was performed using Lyme disease patients from our Lyme Clinic population. Age- and sex-matched controls from the same neighborhood were identified by the Lyme patients' families. A site visit was made to each case/control neighborhood. Twenty-four environmental variables, especially the ecologic characteristics of the home environment were examined. Forty-five items were surveyed by parent questionnaire, including certain "high-risk" activities and behaviors, whether ticks or certain animals were present on the property, and whether antitick measures were used. Control subjects had serologic assays for antibodies to B. burgdorferi. Forty-four Lyme disease patients and 44 well-matched control subjects participated; 2 controls were seropositive. Significant associations with Lyme disease were found for deer ticks in the home environment, ground cover containing moist humus, and leaf litter in the yard. Among the 45 items

related to life-style, there was no correlation with Lyme disease for the use of any antitick measures or for any childhood activities; in fact, an inverse correlation was observed for camping and fishing. We conclude that conditions in the immediate home environment, including certain ecologic factors that favor the presence of Ixodes ticks, are associated with an increased risk for Lyme disease in children
SN - 0009-9228
AD - Department of Pediatrics, Jefferson Medical College, Wilmington, Delaware, USA
UR - 8829006
ER -

TY - JOUR
ID - 1935
T1 - Trends in the nutritional status of Salvadorian children: the post-war experience
A1 - Grummer-Strawn,L.M.
A1 - Caceres,J.M.
A1 - Herrera de Jaimes,B.P.
Y1 - 1996///
N1 - Grummer-Strawn, L M. Caceres, J M. Herrera de Jaimes, B P
Bulletin of the World Health Organization
7507052, c80
IM, J
Journal Article
English
KW - MEDLINE
KW - Adult
KW - Anthropometry
KW - Child
KW - Child Nutrition Disorders/ep [Epidemiology]
KW - Child Nutritional Physiological Phenomena
KW - Preschool
KW - Demography
KW - El Salvador/ep [Epidemiology]
KW - Female
KW - Humans
KW - Infant
KW - Male
KW - Nutrition Surveys
KW - Nutritional Status
KW - Socioeconomic Factors
KW - War
RP - NOT IN FILE
SP - 369
EP - 374
JF - Bulletin of the World Health Organization
JA - Bull World Health Organ
VL - 74
IS - 4
CY - SWITZERLAND
N2 - This article examines trends in the nutritional status of children in EI Salvador between 1988 and 1993 (before and after the signing of a peace accord that ended the civil war.) The data derive from two national surveys, each of which included measurements of the height and weight of children aged 3-59 months. The prevalence of low weight-for-age (< -2 SD) dropped from 15% in 1988 to 10.5% in 1993. The prevalence of low weight-for-height (< -2 SD) was minimal in both surveys: falling from 3.9% to 2.9%. The prevalence of low height-for-age (< -2 SD) fell from 28.1% to 22%. These declines in malnutrition indicators resulted from an upward shift in the distributions of weight and height of children, not from thinner lower tails of the distributions. The quality of anthropometric data appears to be high for both surveys: < 1% of surveyed children had heights or weights outside the expected range. This analysis demonstrates the value of repeated surveys of nutritional status

SN - 0042-9686
AD - Division of Nutrition, Centers for Disease Control and Prevention, Atlanta, GA 30341-3724 USA
UR - 8823958
ER -

TY - JOUR
ID - 1936
T1 - The role of the family physician in the day care setting. [Review] [32 refs]
A1 - Olsen,C.G.
A1 - Wong,C.P.
A1 - Gordon,R.E.
A1 - Harper,D.J.
A1 - Whitecar,P.S.
Y1 - 1996/08/09/
N1 - Olsen, C G. Wong, C P. Gordon, R E. Harper, D J. Whitecar, P S
American family physician
3bt, 1272646
AIM, IM, X
Journal Article. Review
English
KW - MEDLINE
KW - Child
KW - Child Day Care Centers
KW - Preschool
KW - Humans
KW - Infant
KW - Infection
KW - Physician's Role
KW - Physicians
KW - Family
KW - Wounds and Injuries
RP - NOT IN FILE
SP - 1257
EP - 1265
JF - American Family Physician
JA - Am Fam Physician
VL - 54
IS - 4
CY - UNITED STATES
N2 - Injuries and infectious respiratory, gastrointestinal and dermatologic diseases are common in day care settings. Most day care injuries are contusions, abrasions and cuts involving the head and extremities. Impact-absorbing surfaces under playground equipment, safely-proofing of all play areas, increased staff supervision, and staff and parental education might reduce injuries by as much as 75 percent. Respiratory illnesses are the most common day care infections. Chemoprophylaxis with rifampin is required for all close contacts of children infected with Haemophilus influenzae type B and Neisseria meningitidis. Diarrheal illness may be caused by viral pathogens, bacterial agents such as Shigella, Campylobacter or Salmonella, or parasitic infections caused by Giardia lamblia and Cryptosporidium. Strict hand-washing procedures, especially before food preparation and after toileting, may reduce diarrheal illness by 50 percent. Head lice (Pediculosis capitis) and scabies are common dermatologic infections spread by direct contact and through clothing, bedding and hair brushes. Screening and treating affected children with permethrin preparations and thoroughly washing bedding and clothing are necessary to stop outbreaks. Use of universal precautions for the handling of stool is essential to prevent the spread of both ordinary diarrheal illnesses and serious infections such as hepatitis A and B, human immunodeficiency virus and cytomegalovirus. [References: 32]
SN - 0002-838X
AD - Wright State University School of Medicine, Dept. of Family Medicine, Dayton, OH 45408, USA
UR - 8816571
ER -

TY - JOUR
ID - 1937
T1 - School physical education: effect of the Child and Adolescent Trial for Cardiovascular Health
A1 - McKenzie,T.L.
A1 - Nader,P.R.
A1 - Strikmiller,P.K.
A1 - Yang,M.
A1 - Stone,E.J.
A1 - Perry,C.L.
A1 - Taylor,W.C.
A1 - Epping,J.N.
A1 - Feldman,H.A.
A1 - Luepker,R.V.
A1 - Kelder,S.H.
Y1 - 1996/07//
N1 - McKenzie, T L. Nader, P R. Strikmiller, P K. Yang, M. Stone, E J. Perry, C L. Taylor, W C. Epping, J N. Feldman, H A. Luepker, R V. Kelder, S H
Preventive medicine
pm4, 0322116
IM
Clinical Trial. Journal Article. Multicenter Study. Randomized Controlled Trial. Research Support, U.S. Gov't, P.H.S.
English
KW - MEDLINE
KW - Adolescent
KW - Cardiovascular Diseases/pc [Prevention & Control]
KW - Child
KW - Curriculum
KW - Energy Metabolism
KW - Female
KW - Health Promotion/og [Organization & Administration]
KW - Humans
KW - Intervention Studies
KW - Male
KW - Physical Education and Training/og [Organization & Administration]
KW - Program Development
KW - Program Evaluation
KW - School Health Services/og [Organization & Administration]
KW - Time Factors
KW - United States
RP - NOT IN FILE
SP - 423
EP - 431
JF - Preventive Medicine
JA - Prev Med
VL - 25
IS - 4
CY - UNITED STATES
N2 - BACKGROUND: Physical inactivity is a risk behavior for cardiovascular and other diseases. Schools can promote public health objectives by increasing physical activity among youth. METHODS: The Child and Adolescent Trial for Cardiovascular Health (CATCH) was a multicenter, randomized trial to test the effectiveness of a cardiovascular health promotion program in 96 public schools in four states. A major component of CATCH was an innovative, health-related physical education (P+) program. For 2.5 years, randomly assigned schools received a standardized PE intervention, including curriculum, staff development, and follow-up. RESULTS: Systematic analysis of 2,096 PE lessons indicated students engaged in more moderate-to-vigorous physical activity (MVPA) in intervention than in control schools ($P = 0.002$). MVPA

during lessons in intervention schools increased from 37.4% at baseline to 51.9%, thereby meeting the established Year 2000 objective of 50%. Intervention children reported 12 more min of daily vigorous physical activity (P = 0.003) and ran 18.6 yards more than control children on a 9-min run test of fitness (P = 0.21). CONCLUSIONS: The implementation of a standardized curriculum and staff development program increased children's MVPA in existing school PE classes in four geographic and ethnically diverse communities. CATCH PE provides a tested model for improving physical education in American schools
SN - 0091-7435
AD - San Diego State University, San Diego, California, 92120, USA
UR - 8818066
ER -

TY - JOUR
ID - 1938
T1 - Physical activity levels of 5-11-year-old children in England: cumulative evidence from three direct observation studies
A1 - Sleap,M.
A1 - Warburton,P.
Y1 - 1996/05//
N1 - Sleap, M. Warburton, P
International journal of sports medicine
8008349, grk
IM
Journal Article
English
KW - MEDLINE
KW - Child
KW - Preschool
KW - England
KW - Female
KW - Humans
KW - Life Style
KW - Male
KW - Physical Education and Training
KW - Recreation
KW - Time
RP - NOT IN FILE
SP - 248
EP - 253
JF - International Journal of Sports Medicine
JA - Int J Sports Med
VL - 17
IS - 4
CY - GERMANY
N2 - The aim of this study was to determine the physical activity levels of a sample of young children. Data were collected using direct, continuous observation by trained observers. The observational method utilised a points system to differentiate intensity of physical activity. Observations were carried out during school break times, lunch times and physical education lessons and during free time outside of school. The sample consisted of 93 girls and 86 boys aged between 5 and 11 years. Only 38 children (21%) engaged in a sustained 20-min period of moderate to vigorous physical activity (MVPA), but nearly all children (95%) took part in a 5-min period of MVPA. The highest recordings of MVPA occurred during school break times but MVPA was less prominent during free time outside of school. One hundred and seventy eight children were observed during school physical education lessons. Sustained MVPA was particularly low during physical education lessons with only 13 children (8%) participating in at least one sustained 10-min period. There appeared to be no difference in activity levels between boys and girls or between children of differing ages. During all observation periods the main activity of the child was recorded. Soccer, brisk walking, general play and chasing games were the most common activities. The results are disturbing since preadolescent children appear to be engaging in very little sustained, playful physical activity during their free time outside of school. If childhood is considered

to be the most active stage of life there must be concern for the future. There is a need for health professionals to promote active lifestyles from birth
SN - 0172-4622
AD - Research Centre for PE, Exercise and Health, School of Education, University of Hull, England
UR - 8814504
ER -

TY - JOUR
ID - 1939
T1 - Papular urticaria in children. [Review] [15 refs]
A1 - Howard,R.
A1 - Frieden,I.J.
Y1 - 1996/05//
N1 - Howard, R. Frieden, I J
Pediatric dermatology
ped, 8406799
IM
Journal Article. Review
English
KW - MEDLINE
KW - Child
KW - Diagnosis
KW - Differential
KW - Humans
KW - Insect Bites and Stings/co [Complications]
KW - Urticaria/di [Diagnosis]
KW - Urticaria/et [Etiology]
KW - Urticaria/th [Therapy]
RP - NOT IN FILE
SP - 246
EP - 249
JF - Pediatric Dermatology
JA - Pediatr Dermatol
VL - 13
IS - 3
CY - UNITED STATES
N2 - Papular urticaria is a common pruritic hypersensitivity reaction to the bites of insects of many different types, including fleas, mosquitoes, chiggers, and, more rarely, avian or rodent mites. Which particular insect is the cause varies with geographic location. In San Francisco, children with papular urticaria due to flea bites are part of dermatologic practice year round. With its relatively warm winters and wet, foggy summers, San Francisco is an environment where fleas thrive, and their bites afflict children at day care centers and park playgrounds, as well as in homes with pets. We review the clinical approach to papular urticaria in general, and focus on flea bite prevention and management. [References: 15]
SN - 0736-8046
AD - Department of Dermatology, University of California, San Francisco 94143-0316, USA
UR - 8806128
ER -

TY - JOUR
ID - 1940
T1 - Prevalence of abnormal eating attitudes and behaviors in hospital-based primary and tertiary care clinics: a window of opportunity?
A1 - Rome,E.S.
A1 - Imrie,R.K.
A1 - Rybicki,L.A.
A1 - Gidwani,G.
Y1 - 1996/08//

N1 - Rome, E S. Imrie, R K. Rybicki, L A. Gidwani, G
Journal of pediatric and adolescent gynecology
cmf, 9610774
IM
Journal Article
English
KW - MEDLINE
KW - Adolescent
KW - Adolescent Behavior
KW - Adult
KW - Attitude
KW - Child
KW - Eating/px [Psychology]
KW - Female
KW - Humans
KW - Menstruation Disturbances
KW - Outpatients
KW - Pregnancy
RP - NOT IN FILE
SP - 133
EP - 138
JF - Journal of Pediatric & Adolescent Gynecology
JA - J Pediatr Adolesc Gynecol
VL - 9
IS - 3
CY - UNITED STATES
N2 - OBJECTIVE: The "athletic triad" of amenorrhea, osteopenia, and eating disorders (EDs) has received increasing attention in the past decade. Adolescents may seek care for amenorrhea or other menstrual irregularity before disclosure of an eating disorder to a primary care clinician. The purpose of this study was to determine the prevalence of abnormal eating attitudes and behaviors in adolescent girls going to a clinic for reproductive endocrinology (RE) visits versus the prevalence in girls going for health maintenance (HM) visits. DESIGN: All patients aged 8-22 years going to RE or HM clinics were given a 26-item modified eating attitudes test (EAT) and two pages of questions on other eating issues and demographics. Informed consent was obtained from all participants, and also from the parents of those less than 18 years of age. SETTING: All subjects were seen at a hospital-based ambulatory (HM) or subspecialty (RE) clinic, with both clinics located on the same floor. PARTICIPANTS: The study included 53 girls from the RE clinic and 108 girls from the HM clinic. All demographics were similar except that the RE patients were slightly older and had significantly more parents with masters or doctorate degrees. MAIN OUTCOME MEASURES: Study variables were compared between RE and HM groups using either the Student's t test or the Chi-square test, with statistical significance defined as $p < 0.05$. A score of 20 or higher on the modified EAT was correlated with a high risk of EDs and was used as a measure of prevalence of abnormal eating attitudes and behaviors. RESULTS: Modified EAT scores were 11.8 +/- 8.9 in RE patients vs 7.0 +/- 7.2 in HM patients ($p < 0.001$), with 10 (18.9%) RE patients and 7 (6.5%) HM patients having scores of at least 20 ($p = 0.016$). No differences in daily exercise, dairy consumption, or use of vomiting or laxatives to control weight were found. As expected, RE patients were significantly more likely to experience menstrual irregularities than the HM patients ($p < 0.001$). CONCLUSIONS: Adolescents attending a reproductive endocrinology clinic showed a higher prevalence of abnormal eating attitudes and behaviors than did those going to a clinic for HM visits. Early detection of eating disorders may occur both within and outside of the primary care setting through use of a modified EAT
SN - 1083-3188
AD - Section of Adolescent Medicine, Cleveland Clinic Foundation, Ohio 44195, USA
UR - 8795789
ER -

TY - JOUR
ID - 1941
T1 - Food waste is reduced when elementary-school children have recess before lunch
A1 - Getlinger,M.J.

A1 - Laughlin,V.T.
A1 - Bell,E.
A1 - Akre,C.
A1 - Arjmandi,B.H.
Y1 - 1996/09//
N1 - Getlinger, M J. Laughlin, V T. Bell, E. Akre, C. Arjmandi, B H
Journal of the American Dietetic Association
h6f, 7503061
AIM, IM
Clinical Trial. Controlled Clinical Trial. Journal Article
English
KW - MEDLINE
KW - Child
KW - Preschool
KW - Exercise/ph [Physiology]
KW - Feeding Behavior/ph [Physiology]
KW - Female
KW - Food Services/ec [Economics]
KW - Food Services/og [Organization & Administration]
KW - Food Services/st [Standards]
KW - Humans
KW - Male
KW - Time Factors
KW - United States
KW - United States Department of Agriculture
RP - NOT IN FILE
SP - 906
EP - 908
JF - Journal of the American Dietetic Association
JA - J Am Diet Assoc
VL - 96
IS - 9
CY - UNITED STATES
SN - 0002-8223
AD - Child Nutrition Programs, Washington Magnet and Gifted School in Rockford, Ill., USA
UR - 8784336
ER -

TY - JOUR
ID - 1942
T1 - Two years of school injuries in a Scottish education sub-division
A1 - Stark,C.
A1 - Wright,J.
A1 - Lee,J.
A1 - Watt,L.
Y1 - 1996/07//
N1 - Stark, C. Wright, J. Lee, J. Watt, L
Public health
qi7, 0376507
IM
Journal Article
English
KW - MEDLINE
KW - Adolescent
KW - Child
KW - Female
KW - Hospitalization/sn [Statistics & Numerical Data]

KW - Humans
KW - Incidence
KW - Male
KW - Population Surveillance
KW - Risk Factors
KW - Risk Management
KW - Schools
KW - Scotland/ep [Epidemiology]
KW - Wounds and Injuries/ep [Epidemiology]
KW - Wounds and Injuries/et [Etiology]
RP - NOT IN FILE
SP - 229
EP - 235
JF - Public Health
VL - 110
IS - 4
CY - ENGLAND
N2 - Injuries sustained in schools account for 20-30% of all accident and emergency attendances in school age children. Little information has been available on the epidemiology of school accidents in the United Kingdom. Two years of routine school incident reports were analysed from Renfrew Sub-Region, an area with a school roll of 55,521 children attending 135 schools. Schools returned 1,660 report forms in the two year period, of which 1,440 referred to injuries to school children. The peak incidence of injuries was in the 10-12 year age group. The male:female ratio was 1.37:1. Cuts/ laceration and fractures were the commonest diagnoses reported for both Primary and Secondary Schools. Injuries to face and features were commonest in Primary, and upper limb injuries in Secondary Schools. Uncontrolled areas, e.g. playgrounds, stairways and corridors were the most frequent places of occurrence in Primary Schools (Relative Risk 5.24, 95% C.I. 3.28-8.35). Report accuracy was assessed by comparing one year of school reports in a Local Government District to records in the local District General Hospital. This identified 156 children who had attended hospital as the result of a school accident. Schools overestimated the number of fractures by 27%, but where schools had not provided a diagnosis, 15.4% were identified as fractures in hospital records. Each child seen at the hospital received an average of 2.1 X-rays. Nine children underwent manipulations under general anaesthetic. Seventeen children were admitted to hospital, and the group required 103 outpatient follow-up appointments
SN - 0033-3506
AD - Ayrshire and Arran Health Board, Ayr, Scotland
UR - 8757704
ER -

TY - JOUR
ID - 1943
T1 - Environmental factors and respiratory hypersensitivity: the Americas. [Review] [28 refs]
A1 - Bascom,R.
Y1 - 1996/08//
N1 - Bascom, R
Toxicology letters
vxn, 7709027
IM
Journal Article. Review
English
KW - MEDLINE
KW - Adolescent
KW - Adult
KW - Air Pollution
KW - Indoor/ae [Adverse Effects]
KW - Allergens/ae [Adverse Effects]
KW - Central America/ep [Epidemiology]
KW - Child
KW - Environmental Pollution/ae [Adverse Effects]

KW - Humans
KW - North America/ep [Epidemiology]
KW - Odds Ratio
KW - Prevalence
KW - Respiratory Hypersensitivity/ep [Epidemiology]
KW - Respiratory Hypersensitivity/et [Etiology]
KW - Skin Tests
KW - South America/ep [Epidemiology]
RP - NOT IN FILE
SP - 115
EP - 130
JF - Toxicology Letters
JA - Toxicol Lett
VL - 86
IS - 2-3
CY - NETHERLANDS
N2 - Diverse environmental exposure profiles exist in the Americas because of widely different climates, ambient pollutants, and bioaerosols in these continents. This paper reviews selected studies from the Americas that support the broad hypothesis that environmental factors contribute to respiratory hypersensitivity. Processes influenced by environmental factors include primary immunologic sensitization, the development and exacerbation of specific immunologic diseases and the activation of nonspecific mechanisms with tissue inflammation, injury and remodeling. Endpoints resulting from these processes include respiratory symptoms, diseases such as asthma, with measures of disease severity including medication use and hospitalization rates, and death due to cardiorespiratory disease. Studies associate sensitization rates to specific allergens with environmental factors such as humidity and indices of allergen exposure. Regional variation occurs with exposure to outdoor source pollutants such as ozone, but varies by household to bioaerosols such as dust mite, cat or cockroach allergen. Indoor allergens are associated with asthma while outdoor allergens are associated with allergic rhinitis. In a national survey, the atopic sensitization rate in the USA increased with urban residence (defined as towns of population > 2500) and varied by region. Controlled human challenge studies show that ozone increases the response of allergic subjects to allergen. Increased ambient photochemical pollution concentrations, of which ozone is an important component, are associated with increased emergency room visits for asthma in cities such as Toronto, New York, Atlanta, and Mexico City. In Sao Paolo, Brazil, mortality due to childhood respiratory disease was influenced by the ambient levels of NO2. Epidemiologic studies including the recent meta-analysis of a large, longitudinal study population associate ambient concentrations of particulate matter < 10 microns and respiratory symptoms, disease severity and increased cardiorespiratory deaths. Toxicology studies show that individual variation in responsiveness is important in nonspecific inflammatory responses to irritant pollutants such as ozone and environmental tobacco smoke. These studies indicate that environmental factors influence primary allergen sensitization, secondary allergic responses, the activation of nonspecific inflammatory responses, and the severity of respiratory diseases, including asthma. [References: 28]
SN - 0378-4274
AD - Environmental and Airway Disease Research Facility, University of Maryland School of Medicine, Baltimore 21201, USA
UR - 8711764
ER -

TY - JOUR
ID - 1944
T1 - The effects of maternal education on child nutritional status depend on socio-environmental conditions
A1 - Reed,B.A.
A1 - Habicht,J.P.
A1 - Niameogo,C.
Y1 - 1996/06//
N1 - Reed, B A. Habicht, J P. Niameogo, C
International journal of epidemiology
gr6, 7802871
IM, J

Journal Article. Research Support, Non-U.S. Gov't. Research Support, U.S. Gov't, P.H.S.
English
KW - MEDLINE
KW - Adult
KW - Body Weight
KW - Child Nutritional Physiological Phenomena
KW - Child
KW - Preschool
KW - Educational Status
KW - Female
KW - Humans
KW - Infant
KW - Linear Models
KW - Mothers
KW - Nutritional Status
KW - Rural Population
KW - Rwanda
KW - Socioeconomic Factors
RP - NOT IN FILE
SP - 585
EP - 592
JF - International Journal of Epidemiology
JA - Int J Epidemiol
VL - 25
IS - 3
CY - ENGLAND
N2 - BACKGROUND: Previous studies have shown an inconsistency in the association between maternal education and child nutritional status across socioeconomic levels. This may be because the beneficial effects of education are only significant when resources are sufficient but not abundant. METHODS: Associations were examined for differences across socioeconomic levels using data collected from 41 rural communities of Benin for 435 children aged 13-36 months. Village level indicators of household wealth were used together with child z-scores to partition the sample into three levels of socio-environment relative to conditions more or less conducive to child growth. RESULTS: Using an interactive linear regression model it was shown that for the population of children of women who had no more than 4 years of formal schooling, the association of maternal education and child weight differed significantly across the socio-environment. The relationship was flat and non-significant in the lowest socio-environment, positive and significant ($P < 0.05$) in intermediate conditions, and weakly positive under the best socio-environment conditions. Among children of mothers attaining higher levels of education, an unexpected negative association was found. It could be that maternal education had enabled women to participate in activities outside the home without simultaneously ensuring adequate child care
SN - 0300-5771
AD - Division of Nutritional Sciences, Cornell University, Ithaca, New York 14853, USA
UR - 8671560
ER -

TY - JOUR
ID - 1945
T1 - Hypoendemic malaria in Rondonia (Brazil, western Amazon region): seasonal variation and risk groups in an urban locality
A1 - Camargo,L.M.
A1 - dal Colletto,G.M.
A1 - Ferreira,M.U.
A1 - Gurgel,Sde M.
A1 - Escobar,A.L.
A1 - Marques,A.
A1 - Krieger,H.
A1 - Camargo,E.P.
A1 - da Silva,L.H.

Y1 - 1996/07//
N1 - Camargo, L M. dal Colletto, G M. Ferreira, M U. Gurgel, S de M. Escobar, A L. Marques, A. Krieger, H. Camargo, E P. da Silva, L H
The American journal of tropical medicine and hygiene
3zq, 0370507
AIM, IM
Journal Article. Research Support, Non-U.S. Gov't
English
KW - MEDLINE
KW - Adolescent
KW - Adult
KW - Aged
KW - Brazil/ep [Epidemiology]
KW - Child
KW - Preschool
KW - Cross-Sectional Studies
KW - Female
KW - Humans
KW - Infant
KW - Newborn
KW - Longitudinal Studies
KW - Malaria
KW - Falciparum/ep [Epidemiology]
KW - Falciparum/ps [Parasitology]
KW - Vivax/ep [Epidemiology]
KW - Vivax/ps [Parasitology]
KW - Male
KW - Middle Aged
KW - Prevalence
KW - Risk Factors
KW - Seasons
KW - Urban Population
RP - NOT IN FILE
SP - 32
EP - 38
JF - American Journal of Tropical Medicine & Hygiene
JA - Am J Trop Med Hyg
VL - 55
IS - 1
CY - UNITED STATES
N2 - A longitudinal epidemiologic survey (1989-1991) plus a cross-sectional parasitologic, clinical, and sociodemographic survey (July-October 1990) were conducted in Candeias do Jamary, a village with approximately 7,000 inhabitants in Rondonia, Brazil. Analysis of the results revealed hypoendemic malaria with a complex epidemiology. Plasmodium vivax predominated over P. falciparum infections while infections with P. malariae were absent. Malaria is present throughout the year but was clearly seasonal with epidemic outbreaks in the dry season from June to August. Malaria prevalence was lower in children less than 10 years of age and significantly higher in young adult males, which represent the high-risk group. The incidence of locally acquired infections (autochthonous cases) was significantly lower in the rainy season as compared with the dry season. This is not true with respect to heterotochthonous (imported) malaria cases, that is, malaria acquired elsewhere by Candeias residents, most of whom are male adults working outside the town. In both cases, however, the age and sex distribution of prevalence and its relationship with occupational activities indicate a predominance of outdoor transmission. The results of the cross-sectional survey are in agreement with those of the longitudinal passive survey and, in addition, disclose the absence of asymptomatic infection
SN - 0002-9637
AD - Departamento de Parasitologia, Universidade de Sao Paulo, Brazil
UR - 8702019
ER -

TY - JOUR
ID - 1946
T1 - Nutrition and physical activity program to attenuate obesity and promote physical and metabolic fitness in elementary school children
A1 - Donnelly,J.E.
A1 - Jacobsen,D.J.
A1 - Whatley,J.E.
A1 - Hill,J.O.
A1 - Swift,L.L.
A1 - Cherrington,A.
A1 - Polk,B.
A1 - Tran,Z.V.
A1 - Reed,G.
Y1 - 1996/05//
N1 - Donnelly, J E. Jacobsen, D J. Whatley, J E. Hill, J O. Swift, L L. Cherrington, A. Polk, B. Tran, Z V. Reed, G
Obesity research
cde, 9305691
IM
Journal Article. Research Support, Non-U.S. Gov't
English
KW - MEDLINE
KW - Blood Glucose/an [Analysis]
KW - Blood Pressure/ph [Physiology]
KW - Body Composition/ph [Physiology]
KW - Body Weight/ph [Physiology]
KW - Child
KW - Cholesterol/bl [Blood]
KW - Cohort Studies
KW - Dietary Carbohydrates/ad [Administration & Dosage]
KW - Dietary Fats/ad [Administration & Dosage]
KW - Dietary Fiber/ad [Administration & Dosage]
KW - Energy Intake/ph [Physiology]
KW - Food Services/st [Standards]
KW - Humans
KW - Insulin/bl [Blood]
KW - Nebraska/ep [Epidemiology]
KW - Nutritional Sciences/ed [Education]
KW - Obesity/ep [Epidemiology]
KW - Obesity/pp [Physiopathology]
KW - Obesity/pc [Prevention & Control]
KW - Physical Education and Training/og [Organization & Administration]
KW - Physical Education and Training/st [Standards]
KW - Physical Fitness
KW - Risk Factors
KW - School Health Services/og [Organization & Administration]
KW - School Health Services/st [Standards]
KW - Sodium
KW - Dietary/ad [Administration & Dosage]
KW - Weight Gain/ph [Physiology]
RP - NOT IN FILE
SP - 229
EP - 243
JF - Obesity Research
JA - Obes Res
VL - 4

IS - 3
CY - UNITED STATES
N2 - Obesity and low levels of physical and metabolic fitness are risk factors for cardiovascular disease and diabetes. The purpose of this investigation was to attenuate obesity and improve physical and metabolic fitness in elementary school children. Schools have the opportunity, mechanisms, and personnel in place to deliver nutrition education, fitness activities, and a school food service that is nutritious and healthy. Cohorts from grades 3 to 5 in two school districts in rural Nebraska (Intervention/Control) participated in a 2-year study of physical activity and modified school lunch program. Data collection for aerobic capacity, body composition, blood chemistry, nutrition knowledge, energy intake, and physical activity was at the beginning and end of each year. Int received enhanced physical activity, grade specific nutrition education, and a lower fat and sodium school lunch program. Con continued with a regular school lunch and team sports activity program. At year 2, Int lunches had significantly less energy (9%), fat (25%), sodium (21%), and more fiber (17%). However, measures of 24-hour energy intake for Int and Con showed significant differences for sodium only. Physical activity in the classroom was 6% greater for Int compared to Con ($p < 0.05$) but physical activity outside of school was approximately 16% less for Int compared to Con ($p < 0.05$). Body weight and body fat were not different between schools for normal weight or obese children. No differences were found for cholesterol, insulin, and glucose; however, HDL cholesterol was significantly greater and cholesterol/HDL was significantly less for Int compared to Con ($p < 0.05$). It appears that compensation in both energy intake and physical activity outside of school may be responsible for the lack of differences between Int and Con
SN - 1071-7323
AD - Human Performance Laboratory, University of Nebraska-Kearney 68849, USA
UR - 8732957
ER -

TY - JOUR
ID - 1947
T1 - Analysis of risk factors in a cluster of childhood acute lymphoblastic leukemia
A1 - Cocco,P.
A1 - Rapallo,M.
A1 - Targhetta,R.
A1 - Biddau,P.F.
A1 - Fadda,D.
Y1 - 1996/05//
N1 - Cocco, P. Rapallo, M. Targhetta, R. Biddau, P F. Fadda, D
Archives of environmental health
6yo, 0212627
AIM, IM
Journal Article
English
KW - MEDLINE
KW - Antiemetics/ad [Administration & Dosage]
KW - Case-Control Studies
KW - Child
KW - Cluster Analysis
KW - Female
KW - Humans
KW - Italy/ep [Epidemiology]
KW - Precursor Cell Lymphoblastic Leukemia-Lymphoma/ep [Epidemiology]
KW - Pregnancy
KW - Prenatal Exposure Delayed Effects
KW - Risk Factors
KW - Water Supply
RP - NOT IN FILE
SP - 242
EP - 244
JF - Archives of Environmental Health
JA - Arch Environ Health

VL - 51
IS - 3
CY - UNITED STATES
N2 - A case-control investigation of childhood acute lymphoblastic leukemia was conducted in the town of Carbonia (Sardinia, Italy). Parents of 9 cases diagnosed between 1980 and 1989 and 36 controls were interviewed at their respective residences. None of the risk factors analyzed was associated significantly with childhood acute lymphoblastic leukemia. The following were associated with an increased risk for childhood acute lymphoblastic leukemia: parents born outside of Carbonia, family history of cancer, alcohol consumption by fathers that exceeded 60 g/d, exposure of fathers to solvents at their workplaces, maternal smoking, use of antinausea medications during pregnancy, and presence of a well in the backyard. Chance and recall bias likely played a role in generating positive associations. The increases in childhood leukemia risk associated with the presence of a well and with use of antinausea medications during pregnancy are consistent with previous reports and require further investigation
SN - 0003-9896
AD - Instituto di Medicina del Lavoro, Universita di Cagliari, Italy
UR - 8687246
ER -

TY - JOUR
ID - 1948
T1 - Personal exposure to airborne particles and metals: results from the Particle TEAM study in Riverside, California
A1 - Ozkaynak,H.
A1 - Xue,J.
A1 - Spengler,J.
A1 - Wallace,L.
A1 - Pellizzari,E.
A1 - Jenkins,P.
Y1 - 1996/01//
N1 - Ozkaynak, H. Xue, J. Spengler, J. Wallace, L. Pellizzari, E. Jenkins, P
Journal of exposure analysis and environmental epidemiology
bjn, 9111438
IM
Comparative Study. Journal Article
English
KW - MEDLINE
KW - Adolescent
KW - Adult
KW - Aged
KW - Air Pollution/an [Analysis]
KW - Air Pollution
KW - Indoor/an [Analysis]
KW - California
KW - Child
KW - Dust/an [Analysis]
KW - Environmental Monitoring
KW - Humans
KW - Linear Models
KW - Metals/an [Analysis]
KW - Middle Aged
KW - Particle Size
KW - Tobacco Smoke Pollution/an [Analysis]
RP - NOT IN FILE
SP - 57
EP - 78
JF - Journal of Exposure Analysis and Environmental Epidemiology
JA - J Expo Anal Environ Epidemiol

VL - 6
IS - 1
CY - UNITED STATES
N2 - The PTEAM Study was the first large-scale probability-based study of personal exposure to particles. Sponsored by the U.S. Environmental Protection Agency (EPA) and the Air Resources Board of California, it was carried out by the Research Triangle Institute (RTI) and the Harvard University School of Public Health (HSPH). HSPH designed and constructed a 4-lpm, battery-operated personal monitor for inhalable particles (PM10) that could be worn comfortably for up to 14 hours by persons from 10 to 70 years old. The monitor was worn for two consecutive 12-hour periods (day and night) during the fall of 1990 by 178 participants representing 139,000 nonsmoking residents of Riverside, California. Nearly identical monitors were employed to collect concurrent indoor and outdoor samples. The monitors were equipped with a different sampling nozzle to collect fine particles (PM2.5). Population-weighted daytime personal PM10 exposures averaged 150 +/- 9 (SE) micrograms/m3, compared to concurrent indoor and outdoor concentrations of 95 +/- 6 micrograms/m3. This suggested the existence of excess mass near the person, a "personal cloud" that appeared related to personal activities. Fourteen of 15 prevalent elements also were evaluated in the personal samples. The two major indoor sources of indoor particles were smoking and cooking; even in these homes, however, more than half of the indoor particles came from outdoors, and a substantial portion of the indoor particles were of undetermined indoor origin. Outdoor concentrations near the homes were well correlated with outdoor concentrations at the central site, supporting the idea of using the central site as an indicator of of ambient concentrations over a wider area. Indoor concentrations were only weakly correlated with outdoor concentrations, however, and personal exposures were even more poorly correlated with outdoor concentrations. Elemental profiles were obtained for environmental tobacco smoke (ETS) (major contributions from potassium and chlorine) and cooking emissions (aluminum, iron, calcium, and chlorine). These profiles can be used in future source apportionment studies
SN - 1053-4245
AD - Department of Environmental Health, Environmental Science and Engineering Program, Harvard University School of Public Health, Boston, MA 02115, USA
UR - 8777374
ER -

TY - JOUR
ID - 1949
T1 - Recurrent limb pain in schoolchildren
A1 - bu-Arafeh,I.
A1 - Russell,G.
Y1 - 1996/04//
N1 - Abu-Arafeh, I. Russell, G
Archives of disease in childhood
6xg, 0372434
AIM, IM
Journal Article
English
KW - MEDLINE
KW - Adolescent
KW - Child
KW - Preschool
KW - Extremities/in [Injuries]
KW - Extremities
KW - Female
KW - Humans
KW - Male
KW - Migraine Disorders/co [Complications]
KW - Pain/co [Complications]
KW - Pain/ep [Epidemiology]
KW - Pain/et [Etiology]
KW - Prevalence
KW - Questionnaires

KW - Recurrence
KW - Scotland/ep [Epidemiology]
RP - NOT IN FILE
SP - 336
EP - 339
JF - Archives of Disease in Childhood
JA - Arch Dis Child
VL - 74
IS - 4
CY - ENGLAND
N2 - OBJECTIVES: To determine the prevalence, causes and clinical features of short lasting recurrent limb pain (recurrent limb pain) in children. DESIGN: Population-based study in two stages, with an initial screening questionnaire followed by clinical interviews and physical examination of symptomatic children. SETTING: 67 primary and secondary schools in the city of Aberdeen. SUBJECTS: 2165 children representing a random 10% sample of all schoolchildren aged between 5-15 years. MAIN OUTCOME MEASURES: (a) The causes of limb pain in children, (b) the prevalence of recurrent limb pain in schoolchildren, (c) the relationship of recurrent limb pain to childhood migraine. RESULTS: Sports and playground injuries were the most common cause of limb pain, affecting 9% of all children. The prevalence rate of recurrent limb pain was 2.6% (95% confidence interval 1.9 to 3.4). Episodes of recurrent limb pain had similar trigger factors, associated symptoms, and relieving factors to episodes of headache in children with migraine. CONCLUSIONS: Recurrent limb pain is a common cause of limb pain, with a prevalence rate of 2.6%. The close clinical and epidemiological similarities between recurrent limb pain and childhood migraine suggest a common pathogenesis
SN - 1468-2044
AD - Department of Medical Paediatrics, Royal Aberdeen Children's Hospital, United Kingdom
UR - 8669936
ER -

TY - JOUR
ID - 1950
T1 - The multicentre south European study 'Helios'. II: Different sun exposure patterns in the aetiology of basal cell and squamous cell carcinomas of the skin
A1 - Rosso,S.
A1 - Zanetti,R.
A1 - Martinez,C.
A1 - Tormo,M.J.
A1 - Schraub,S.
A1 - Sancho-Garnier,H.
A1 - Franceschi,S.
A1 - Gafa,L.
A1 - Perea,E.
A1 - Navarro,C.
A1 - Laurent,R.
A1 - Schrameck,C.
A1 - Talamini,R.
A1 - Tumino,R.
A1 - Wechsler,J.
Y1 - 1996/06//
N1 - Rosso, S. Zanetti, R. Martinez, C. Tormo, M J. Schraub, S. Sancho-Garnier, H. Franceschi, S. Gafa, L. Perea, E. Navarro, C. Laurent, R. Schrameck, C. Talamini, R. Tumino, R. Wechsler, J
British journal of cancer
av4, 0370635
IM
Comparative Study. Journal Article. Multicenter Study. Research Support, Non-U.S. Gov't
English
KW - MEDLINE
KW - Carcinoma
KW - Basal Cell/ep [Epidemiology]

KW - Basal Cell/et [Etiology]
KW - Squamous Cell/ep [Epidemiology]
KW - Squamous Cell/et [Etiology]
KW - Case-Control Studies
KW - Child
KW - Environmental Exposure
KW - Europe/ep [Epidemiology]
KW - Holidays
KW - Humans
KW - Interviews as Topic
KW - Occupational Exposure
KW - Odds Ratio
KW - Skin Neoplasms/ep [Epidemiology]
KW - Skin Neoplasms/et [Etiology]
KW - Sports
KW - Sunlight
KW - Time Factors
RP - NOT IN FILE
SP - 1447
EP - 1454
JF - British Journal of Cancer
JA - Br J Cancer
VL - 73
IS - 11
CY - SCOTLAND
N2 - The role of sun exposure in development of basal cell and squamous cell carcinomas among different populations from south Europe was investigated. Between 1989 and 1993 we interviewed incident cases and a random population sample of controls from five centres where a cancer registry was operating, whereas we selected a sample of hospital-based cases and controls from the other three centres. We gathered information on life-long exposure to sunlight during different activities. Results are analysed for 1549 basal cell carcinoma (BCC) cases and 228 squamous cell carcinoma (SCC) cases compared with 1795 controls. We observed a statistically significant increase of risk of SCC with increasing sun exposure beyond a threshold of 70,000 cumulated hours of exposure in a lifetime. Sun exposures during work and holidays were, however, inversely correlated. Odds ratios (ORs) of SCC were up to eight or nine times the reference for the highest exposures (200,000 cumulated hours or more). BCC exhibited a 2-fold increase of risk for lower exposure (8000-10,000 cumulated hours in a lifetime) with a plateau and a slight decrease of risk for the highest exposures (100,000 cumulated hours or more). Outdoor work showed a significantly increased risk of SCC (OR 1.6 for more than 54,000 cumulated hours of exposure in a lifetime), whereas recreational activities such as sun exposure during holidays at the beach (OR 1.6 for more than 2600 cumulated hours of exposure in a lifetime) or during water sports (OR 1.6 for more than 2600 cumulated hours of exposure in a lifetime) were associated with an increased risk of BCC. Risk patterns were different in poor or good tanners with a significant risk trend for good tanners, whereas poor tanners were on a plateau of increased risk at any level of exposure. Solar radiation is associated with a risk of BCC even for relatively short periods of exposure such as during holidays and sports, whereas SCC develops later if exposure continues. The skin's ability to tan modulates the risk of BCC; subjects who tan poorly have a steady risk increase, whereas people who tan easily develop cancer only after prolonged exposures
SN - 0007-0920
AD - Registro de Cancer de Granada, Escuela Andaluza de Salud Publica, Spain
UR - 8645596
ER -

TY - JOUR
ID - 1951
T1 - School-associated violent deaths in the United States, 1992 to 1994
A1 - Kachur,S.P.
A1 - Stennies,G.M.
A1 - Powell,K.E.

A1 - Modzeleski,W.
A1 - Stephens,R.
A1 - Murphy,R.
A1 - Kresnow,M.
A1 - Sleet,D.
A1 - Lowry,R.
Y1 - 1996/06/12/
N1 - Kachur, S P. Stennies, G M. Powell, K E. Modzeleski, W. Stephens, R. Murphy, R. Kresnow, M. Sleet, D. Lowry, R
JAMA : the journal of the American Medical Association
7501160
AIM, IM
Journal Article
English
KW - MEDLINE
KW - Adolescent
KW - Age Distribution
KW - Child
KW - Female
KW - Homicide/sn [Statistics & Numerical Data]
KW - Humans
KW - Male
KW - Mortality
KW - Risk
KW - Schools/sn [Statistics & Numerical Data]
KW - Sex Distribution
KW - Socioeconomic Factors
KW - Suicide/sn [Statistics & Numerical Data]
KW - United States/ep [Epidemiology]
KW - Urban Population
KW - Violence/sn [Statistics & Numerical Data]
RP - NOT IN FILE
SP - 1729
EP - 1733
JF - JAMA
VL - 275
IS - 22
CY - UNITED STATES
N2 - OBJECTIVES: To conduct the first nationwide investigation of violent deaths associated with schools in the United States, to quantify the risk of school-associated violent death, and to identify epidemiologic features of these deaths. DESIGN: Descriptive case series. SETTING: United States, July 1, 1992, through June 30, 1994. METHODS: School-associated violent deaths were identified by study collaborators and through 2 online news databases. Police reports, medical examiners' records, and interviews with police and school officials provided detailed information about each case. RESULTS: In a 2-year period, 105 school-associated violent deaths were identified. The estimated incidence of school-associated violent death was 0.09 per 100 000 student-years. Students in secondary schools, students of minority racial and ethnic backgrounds, and students in urban school districts had higher levels of risk. The deaths occurred in communities of all sizes in 25 different states. Homicide was the predominant cause of death (n = 85 [80.9%]), and firearms were responsible for a majority (n = 81 [77.1%]) of the deaths. Most victims were students (n = 76 [72.4%]). Both victims and offenders tended to be young (median ages, 16 and 17 years, respectively) and male (82.9% and 95.6%, respectively). Approximately equal numbers of deaths occurred inside school buildings (n = 31 [29.5%]), outdoors but on school property (n = 37 [35.2%]), and at off-campus locations while the victim was in transit to or from school (n = 37 [35.2%]). Equal numbers of deaths occurred during classes or other school activities (n = 46 [43.8%]) and before or after official school activities (n = 46 [43.8%]). CONCLUSIONS: School-associated violent deaths were more common than previously estimated. The epidemiologic features of these deaths were similar to those of homicides and suicides that occur elsewhere. A comprehensive approach that addresses violent injury and death among young people at school and elsewhere in the community is suggested

SN - 0098-7484
AD - National Center for Injury Prevention and Control, Centers for Disease Control and Prevention, Atlanta, GA 30341, USA
UR - 8637169
ER -

TY - JOUR
ID - 1953
T1 - Distribution and determinants of personal exposure to nitrogen dioxide in school children
A1 - Linaker,C.H.
A1 - Chauhan,A.J.
A1 - Inskip,H.
A1 - Frew,A.J.
A1 - Sillence,A.
A1 - Coggon,D.
A1 - Holgate,S.T.
Y1 - 1996/03//
N1 - Linaker, C H. Chauhan, A J. Inskip, H. Frew, A J. Sillence, A. Coggon, D. Holgate, S T
Occupational and environmental medicine
bwc, 9422759
IM
Journal Article
English
KW - MEDLINE
KW - Child
KW - England
KW - Environmental Exposure/an [Analysis]
KW - Environmental Monitoring/mt [Methods]
KW - Humans
KW - Nitrogen Dioxide/an [Analysis]
RP - NOT IN FILE
SP - 200
EP - 203
JF - Occupational & Environmental Medicine
JA - Occup Environ Med
VL - 53
IS - 3
CY - ENGLAND
N2 - OBJECTIVES: To assess the distribution of personal exposures to nitrogen dioxide (NO2) in school children, and to investigate factors that might influence personal exposure. METHODS: NO2 exposures were assessed by use of passive diffusion tubes for 46 children aged 9-11 years, selected from two Southampton schools. The tubes were worn for seven days, and parallel measurements were made with static samplers in the child's kitchen, living room, classroom, and playground. Information about potential exposures was collected by questionnaire. RESULTS: Personal exposures to NO2, averaged over seven days, ranged from 11 to 257 micrograms/m3 (6 to 137 ppb) with a geometric mean of 36 micrograms/m3 (19 ppb). Exposures correlated with concentrations of NO2 recorded in the home, but the relation was far from exact. Factors associated with increased personal exposure included the use of gas appliances in the home, living with one or more smokers, and travel to school by means other than a car. However, together these variables only explained a small part of the variation in personal exposures. CONCLUSIONS: These findings reinforce the need for personal monitoring of exposure in studies investigating potential health effects of NO2 in children
SN - 1351-0711
AD - University Medicine, Southampton General Hospital
UR - 8704862
ER -

TY - JOUR
ID - 1954

T1 - Pediatric resident training in a school environment. A prescription for learning
A1 - Zenni,E.A.
A1 - Sectish,T.C.
A1 - Martin,B.N.
A1 - Prober,C.G.
Y1 - 1996/06//
N1 - Zenni, E A. Sectish, T C. Martin, B N. Prober, C G
Archives of pediatrics & adolescent medicine
9422751, bwf
AIM, IM
Journal Article
English
KW - MEDLINE
KW - Adult
KW - Child
KW - Child Development
KW - Health Education
KW - Health Knowledge
KW - Attitudes
KW - Practice
KW - Humans
KW - Internship and Residency
KW - Learning
KW - Pediatrics/ed [Education]
KW - Questionnaires
KW - School Health Services
KW - Schools
RP - NOT IN FILE
SP - 632
EP - 637
JF - Archives of Pediatrics & Adolescent Medicine
JA - Arch Pediatr Adolesc Med
VL - 150
IS - 6
CY - UNITED STATES
N2 - OBJECTIVE: To describe our experience with developing, implementing, and evaluating the educational effect of a school health experience for pediatric residents. DESIGN: Descriptive. SETTING: University-based pediatric residency program and five public elementary and middle schools in surrounding communities. PARTICIPANTS: Eleven pediatric residents. INTERVENTION: A school health experience for pediatric residents was developed in response to the report of the American Academy of Pediatrics Task Force on Pediatric Education and the new training recommendations of the Residency Review Committee of the American Council for Graduate Medical Education. Residents spent 3 weeks in the schools engaged in teaching and observational activities. MAIN OUTCOME MEASURES: Questionnaires of residents' attitudes and knowledge, structured resident interviews, and teacher questionnaires. RESULTS: Positive effect on resident's knowledge of school structure, child development, communication with children, school-related problems, and special education. Positive effects on resident's attitudes about teamwork between teachers and pediatricians and roles of pediatricians in schools. Teacher feedback showed acceptance by the school community. CONCLUSIONS: Pediatric residents benefit from exposure to children in school settings. Schools provide an opportunity to observe normal childhood development and behavior in a more natural setting than that provided in the hospital
SN - 1072-4710
AD - Department of Pediatrics, Stanford (Calif) University School of Medicine, USA
UR - 8646315
ER -

TY - JOUR
ID - 1955

T1 - Nodular fasciitis and related pseudosarcomatous lesions of soft tissues
A1 - Samaratunga,H.
A1 - Searle,J.
A1 - O'Loughlin,B.
Y1 - 1996/01//
N1 - Samaratunga, H. Searle, J. O'Loughlin, B
The Australian and New Zealand journal of surgery
0373115, 9ic
IM
Journal Article
English
KW - MEDLINE
KW - Adolescent
KW - Adult
KW - Aged
KW - Child
KW - Preschool
KW - Fasciitis/di [Diagnosis]
KW - Fasciitis/pa [Pathology]
KW - Fasciitis/su [Surgery]
KW - Female
KW - Humans
KW - Infant
KW - Male
KW - Middle Aged
KW - Retrospective Studies
KW - Sarcoma/di [Diagnosis]
RP - NOT IN FILE
SP - 22
EP - 25
JF - Australian & New Zealand Journal of Surgery
JA - Aust N Z J Surg
VL - 66
IS - 1
CY - AUSTRALIA
N2 - BACKGROUND: Nodular fasciitis is characterized by a proliferation of spindle cells which may be misdiagnosed as sarcomas due to the rich cellularity, mitotic activity and variant morphologic patterns. METHODS: This report includes 42 cases of nodular fasciitis and related conditions of skin and soft tissue from the pathology files of the Royal Brisbane Hospital, Queensland, Australia. There were 33 cases of nodular fasciitis, three cases of ossifying fasciitis, three cases of proliferative fasciitis, two cases of proliferative myositis and one case of intravascular fasciitis. Two-thirds of cases were referred from outside Royal Brisbane Hospital. RESULTS: Nodular fasciitis and ossifying occurred most commonly in young adults with 16 patients (44%) between the ages of 20 and 29. However, the other variants of nodular fasciitis including proliferative myositis and intravascular fasciitis occurred in older people (six patients being older than 49 years) and more commonly occurring in men (n=5). A painless rapidly growing mass was most common. The lesions of nodular fasciitis were most often located in the upper extremity with the forearm most commonly affected. CONCLUSIONS: Nodular fasciitis and related conditions are benign. All patients are alive and well 3 months to 13 years (mean 7.2 years) after simple local resection
SN - 0004-8682
AD - Department of Anatomical Pathology, Royal Brisbane Hospital, Brisbane, Queensland, Australia
UR - 8629974
ER -

TY - JOUR
ID - 1956
T1 - Comparative outdoor study of the efficacy, onset and duration of action, and safety of cetirizine, loratadine, and placebo for seasonal allergic rhinitis

A1 - Meltzer,E.O.
A1 - Weiler,J.M.
A1 - Widlitz,M.D.
Y1 - 1996/02//
N1 - Meltzer, E O. Weiler, J M. Widlitz, M D
The Journal of allergy and clinical immunology
h53, 1275002
AIM, IM
Clinical Trial. Comparative Study. Journal Article. Randomized Controlled Trial. Research Support, Non-U.S. Gov't
English
KW - MEDLINE
KW - Adolescent
KW - Adult
KW - Cetirizine/ae [Adverse Effects]
KW - Cetirizine/tu [Therapeutic Use]
KW - Child
KW - Double-Blind Method
KW - Female
KW - Humans
KW - Loratadine/ae [Adverse Effects]
KW - Loratadine/tu [Therapeutic Use]
KW - Male
KW - Middle Aged
KW - Placebos
KW - Pollen
KW - Rhinitis
KW - Allergic
KW - Seasonal/dt [Drug Therapy]
KW - Seasonal/pp [Physiopathology]
KW - Severity of Illness Index
KW - Time Factors
RP - NOT IN FILE
SP - 617
EP - 626
JF - Journal of Allergy & Clinical Immunology
JA - J Allergy Clin Immunol
VL - 97
IS - 2
CY - UNITED STATES
N2 - BACKGROUND: Cetirizine, a new once-daily highly specific H1-antagonist, has been shown in conventional studies to be efficacious in the treatment of seasonal and perennial allergic rhinitis and chronic idiopathic urticaria. OBJECTIVE: The efficacy, duration and onset of action, and safety of cetirizine, 10 mg once daily, was compared with that of loratadine, 10 mg once daily, and placebo in a field study of patients with seasonal allergic rhinitis. METHODS: This was a randomized, double-blind, parallel, double-dummy study conducted over 2 days in spring allergy season at outdoor parks in San Diego and Iowa City. Study medication was administered at 10:00 AM on both days. After screening, eligible patients completed rhinitis symptom diaries in the park hourly from 7:30 to 9:30 AM (baseline); at 10:30 AM and hourly from 11:00 AM to 4:00 PM (period I); at 6:00, 8:00, and 10:00 PM at home (period II); and the next day in the park hourly from 8:00 to 10:00 AM (period III), and from 11:00 AM to 4:00 PM (period IV). Major and total symptom complex scores, global efficacy and overall satisfaction, and adverse events were assessed. RESULTS: Of the 279 patients (140 men and 139 women; mean age, 29 years) randomized to treatment, 278 were included in the efficacy analysis. Cetirizine produced significantly greater mean reductions than loratadine or placebo in major symptom complex severity scores at all periods ($p <$ or $= 0.05$), except period I for placebo. Cetirizine also produced mean reductions in total symptom complex severity scores that were superior to loratadine at every evaluation period ($p < 0.05$) and were statistically different from placebo at period II ($p < 0.01$). A rapid onset of action was observed with cetirizine, as was a better response pattern in the patient global assessment of efficacy compared

with loratadine. Study medications were well tolerated; no patient stopped treatment because of side effects. The incidence of somnolence with cetirizine was 13% versus 2% with placebo (p < 0.05); headache occurred more frequently with loratadine (23%) than with cetirizine (11%, p = 0.03). CONCLUSIONS: Cetirizine relieved rhinitis symptoms more effectively and quickly than loratadine and placebo in this field study of seasonal allergic rhinitis. Both active agents were generally well tolerated
SN - 0091-6749
AD - Allergy and Asthma Medical Group and Research Center, San Diego, CA 92123, USA
UR - 8621847
ER -

TY - JOUR
ID - 1957
T1 - Utility of a risk assessment questionnaire in identifying children with lead exposure
A1 - Dalton,M.A.
A1 - Sargent,J.D.
A1 - Stukel,T.A.
Y1 - 1996/02//
N1 - Dalton, M A. Sargent, J D. Stukel, T A
Archives of pediatrics & adolescent medicine
9422751, bwf
AIM, IM
Journal Article. Research Support, Non-U.S. Gov't. Research Support, U.S. Gov't, P.H.S.
English
KW - MEDLINE
KW - Centers for Disease Control and Prevention (U.S.)
KW - Child
KW - Child Behavior
KW - Preschool
KW - Environmental Exposure
KW - Humans
KW - Lead/bl [Blood]
KW - Lead Poisoning/bl [Blood]
KW - Lead Poisoning/et [Etiology]
KW - Lead Poisoning/pc [Prevention & Control]
KW - Mass Screening/mt [Methods]
KW - Massachusetts
KW - Prevalence
KW - Questionnaires
KW - Risk Assessment
KW - Risk Factors
KW - United States
KW - Urban Health
RP - NOT IN FILE
SP - 197
EP - 202
JF - Archives of Pediatrics & Adolescent Medicine
JA - Arch Pediatr Adolesc Med
VL - 150
IS - 2
CY - UNITED STATES
N2 - OBJECTIVE: To evaluate the utility of the Centers for Disease Control and Prevention (CDC) Risk Questionnaire and a behavioral risk factor questionnaire in identifying children with blood lead concentrations of 0.48 mumol/L (10 micrograms/dL) or more. DESIGN: Cross-sectional study of 463 urban Massachusetts children (6 to 72 months of age) screened for lead with venous blood. RESULTS: Twenty-two percent of the children had elevated blood lead concentrations. Of the five CDC questions, only one was significantly associated with an increased adjusted odds ratio for elevated blood lead: having a sibling, housemate, or playmate who was followed up or treated for lead poisoning (odds ratio, 2.7; 95% confidence interval, 1.7 to

4.2; P < .001). Children who had at least one positive or equivocal response to any of the five CDC questions (n = 318 [68.7%]) were not at higher risk than were children who displayed a negative response to all five questions (odds ratio, 1.1; 95% confidence interval, 0.7 to 1.8; P = .69). Of nine behaviors surveyed, two were associated with an increased adjusted odds for elevated blood lead: use of a pacifier (odds ratio, 2.4; 95% confidence interval, 1.3 to 4.4; P = .01) and playing near the outside of the home (odds ratio, 3.4; 95% confidence interval, 2.0 to 5.8; P < .001). CONCLUSIONS: In this population of children, the CDC risk questionnaire did not identify a group at higher risk for lead exposure. We suggest that practitioners in urban communities screen all children according to the same schedule. We conclude that risk factors differ by community and no risk questionnaire developed at the national level should be applied across communities to target screening
SN - 1072-4710
AD - Department of Pediatrics, Dartmouth-Hitchcock Medical Center, Lebanon, NH, USA
UR - 8556126
ER -

TY - JOUR
ID - 1958
T1 - Effective doses due to external irradiation from the Chernobyl accident for different population groups of Ukraine
A1 - Likhtariov,I.
A1 - Kovgan,L.
A1 - Novak,D.
A1 - Vavilov,S.
A1 - Jacob,P.
A1 - Paretzke,H.G.
Y1 - 1996/01//
N1 - Likhtariov, I. Kovgan, L. Novak, D. Vavilov, S. Jacob, P. Paretzke, H G
Health physics
g2h, 2985093r
IM
Journal Article
English
KW - MEDLINE
KW - Adult
KW - Child
KW - Preschool
KW - Gamma Rays
KW - Humans
KW - Models
KW - Biological
KW - Power Plants
KW - Radiation Dosage
KW - Radioactive Hazard Release
KW - Ukraine
RP - NOT IN FILE
SP - 87
EP - 98
JF - Health Physics
JA - Health Phys
VL - 70
IS - 1
CY - UNITED STATES
N2 - A model for the external exposure of the Ukrainian population after the Chernobyl accident was developed. It is based on extensive measurements of external gamma-exposure rates (EGER) in air and of external effective doses of members of five population groups. Questionnaires were used to determine the occupancy times of members of the population groups at three types of locations; inside houses, outdoors, and outside of the home settlement. Behavior factors are defined as the ratio of individual external doses to a

reference dose for a phantom standing permanently over an open field with the same average 137Cs activity per unit area as in the settlement. The behavior factors were derived for five population groups (children younger than seven years, the age group from eight to seventeen years, employees, agricultural workers, and pensioners) by two methods: first from direct measurements of individual doses by thermoluminescent dosimetry and an experimental determination of the average 137Cs activity per unit area in the settlement of interest; and second from external gamma-exposure rates in air at various types of locations and from the questionnaire data. The methods were found to be consistent and the results were used to calculate external exposures of the five population groups in the years 1987 through 1991
SN - 0017-9078
AD - Ukrainian Scientific Center for Radiation Medicine, Department of Dosimetry and Radiation Hygiene, Ukraine
UR - 7499158
ER -

TY - JOUR
ID - 1959
T1 - Estimating human exposure to selected motor vehicle pollutants using the NEM series of models: lessons to be learned
A1 - McCurdy,T.
Y1 - 1995/10//
N1 - McCurdy, T
Journal of exposure analysis and environmental epidemiology
bjn, 9111438
IM
Journal Article
English
KW - MEDLINE
KW - Adult
KW - Algorithms
KW - Carbon Monoxide/an [Analysis]
KW - Carbon Monoxide/pd [Pharmacology]
KW - Child
KW - Environmental Monitoring/mt [Methods]
KW - Exercise/ph [Physiology]
KW - Female
KW - Humans
KW - Lead/an [Analysis]
KW - Lead/pd [Pharmacology]
KW - Male
KW - Models
KW - Biological
KW - Nitrogen Dioxide/an [Analysis]
KW - Nitrogen Dioxide/pd [Pharmacology]
KW - Ozone/an [Analysis]
KW - Ozone/pd [Pharmacology]
KW - Pulmonary Ventilation/ph [Physiology]
KW - Regression Analysis
KW - Stochastic Processes
KW - Time Factors
KW - Vehicle Emissions/an [Analysis]
RP - NOT IN FILE
SP - 533
EP - 550
JF - Journal of Exposure Analysis and Environmental Epidemiology
JA - J Expo Anal Environ Epidemiol
VL - 5
IS - 4

CY - UNITED STATES
N2 - This paper reviews the use of exposure modeling by the Ambient Standards Branch (ASB) of EPA's Office of Air Quality Planning and Standards. The Branch uses exposure assessments to evaluate health risks associated with attainment of alternative National Ambient Air Quality Standards (NAAQS). This paper examines the history of the NAAQS Exposure Model (NEM) and probabilistic NEM (pNEM) models and the role that they have played in NAAQS reviews of lead, carbon monoxide, nitrogen dioxide, and oxygen. Trends in how the following substantive issues were addressed in the NEM series of models are reviewed: (1) exposure and dose metrics; (2) microenvironmental (mu e) concentration estimation; and (3) human activity and breathing rate simulation. In response to an outside peer review of its recent exposure assessments, ASB is deemphasizing modeling the entire population in favor of limited modeling of narrowly defined "sensitive groups." In addition, ASB increasingly is focusing its exposure assessments on those human activities that lead to high intake dose, or high intake dose rate. Examples are provided that highlight these changes in emphasis
SN - 1053-4245
AD - U.S. Environmental Protection Agency, Research Triangle Park, North Carolina 27711, USA
UR - 8938248
ER -

TY - JOUR
ID - 1960
T1 - Validity of a mother's history of measles in children: a review of Indian literature
A1 - Singh,J.
A1 - Jain,D.C.
A1 - Datta,K.K.
Y1 - 1995/12//
N1 - Singh, J. Jain, D C. Datta, K K
The Journal of communicable diseases
ibn, 0261652
IM
Journal Article
English
KW - MEDLINE
KW - Child
KW - Preschool
KW - Female
KW - Humans
KW - Immunity
KW - Active
KW - Immunization
KW - India
KW - Infant
KW - Male
KW - Measles/im [Immunology]
RP - NOT IN FILE
SP - 239
EP - 241
JF - Journal of Communicable Diseases
JA - J Commun Dis
VL - 27
IS - 4
CY - INDIA
N2 - A review of the available information on the validity of a mother's history of measles in children and its importance in Indian context is presented. A history of measles was found to have a median sensitivity (SN) of about 60% (n = 6; range 33-75) and a median specificity (SP) of about 95% (n = 5; range 79-100). A subclinical infection seems to be the most plausible explanation for the low SN. Two mechanisms for the development of subclinical infection have been suggested: (i) In infants, exposure to the virus in the households when maternal antibodies are just sufficient to prevent the disease but not the infection. (ii) In older children, exposure to a mild dose of virus outside the households. However, due to the misclassification of non measles cases as measles, the

SN increases and SP decreases as the child grows older. The implications of low SN, high SP and varying predictive values of the mother's history of measles are discussed
SN - 0019-5138
AD - National Institute of Communicable Diseases, Delhi
UR - 8866988
ER -

TY - JOUR
ID - 1961
T1 - [Head injuries in children and adolescents: causes and natures]. [Chinese]
A1 - Chen,C.L.
A1 - Wong,M.K.
A1 - See,L.C.
A1 - Chong,C.K.
Y1 - 1995/12//
N1 - Chen, C L. Wong, M K. See, L C. Chong, C K
Changgeng yi xue za zhi / Changgeng ji nian yi yuan = Chang Gung medical journal / Chang Gung Memorial Hospital
chg, 9809559
IM
English Abstract. Journal Article. Research Support, Non-U.S. Gov't
Chinese
KW - MEDLINE
KW - Accidental Falls
KW - Accidents
KW - Traffic
KW - Adolescent
KW - Child
KW - Preschool
KW - Craniocerebral Trauma/ep [Epidemiology]
KW - Craniocerebral Trauma/et [Etiology]
KW - Female
KW - Humans
KW - Male
KW - Retrospective Studies
KW - Taiwan/ep [Epidemiology]
RP - NOT IN FILE
SP - 353
EP - 360
JF - Changgeng Yi Xue Za Zhi
VL - 18
IS - 4
CY - TAIWAN
N2 - To investigate the nature of exposure factors, and effectiveness of countermeasures in head injuries in children and adolescent of Taiwan area, we conducted a retrospective study on 820 children and adolescents with head injuries consecutively admitted to Chang Gung Memorial Hospital from January 1985 to December 1989. We analyzed the data by chart review and divided the cases into four age-groups: group A (0-4 years old), group B (5-9 years old), group C (10-14 year old) and group D (15-17 years old). The boy to girl ratio was 2.43. The largest proportions of head injuries were the adolescents in group D. The most common cause of head injury was traffic accidents (74%); while the falls were contributed the main cause in other studies. In all group A, B and D, the majority of traffic accidents were pedestrian injury; while in group D, the motorcyclists or car drivers were responsible for the accidents. Fall from height was the major cause of fall in all four age-groups, but it was fall from bicycle or during play or sports in group C. Injuries were most common in summer and clustered at afternoon in all patients. Majority of head injuries occurred outdoors. Most children and adolescents suffered from mild head injury, but there were still 203 cases with severe head injury (24%). Eighty-one cases (9.9%) were either discharged in critical condition or died at the hospital. There were no significant differences in mortality among four age-groups

AD - Department of Rehabilitation, Chang Gung Memorial Hospital, Taipei, Taiwan
UR - 8851985
ER -

TY - JOUR
ID - 1962
T1 - The effects of air pollution on children. [Review] [49 refs]
A1 - Bates,D.V.
Y1 - 1995/09//
N1 - Bates, D V
Environmental health perspectives
ei0, 0330411
IM
Journal Article. Review
English
KW - MEDLINE
KW - Adult
KW - Air Pollutants/ae [Adverse Effects]
KW - Asthma/et [Etiology]
KW - Child
KW - Humans
KW - Infant
KW - Newborn
KW - Lung/pp [Physiopathology]
KW - Respiratory Tract Diseases/et [Etiology]
RP - NOT IN FILE
SP - 49
EP - 53
JF - Environmental Health Perspectives
JA - Environ Health Perspect
VL - 103 Suppl 6
CY - UNITED STATES
N2 - Air pollutants have been documented to be associated with a wide variety of adverse health impacts in children. These include increases in mortality in very severe episodes; an increased risk of perineonatal mortality in regions of higher pollution, and an increased general rate of mortality in children; increased acute respiratory disease morbidity; aggravation of asthma, as shown by increased hospital emergency visits or admissions as well as in longitudinal panel studies; increased prevalence of respiratory symptoms in children, and infectious episodes of longer duration; lowered lung function in children when pollutants increase; lowered lung function in more polluted regions; increased sickness rates as indicated by kindergarten and school absences; the adverse effects of inhaled lead from automobile exhaust. These impacts are especially severe when high levels of outdoor pollution (usually from uncontrolled coal burning) are combined with high levels of indoor pollution. In developed countries, where indoor pollution levels are lower, increasing traffic density and elevated NO2 levels with secondary photochemical and fine particulate pollution appear to be the main contemporary problem. By virtue of physical activity out of doors when pollution levels may be high, children may experience higher exposures than adults. Air pollution is likely to have a greater impact on asthmatic children if they are without access to routine medical care. [References: 49]
SN - 0091-6765
AD - Department of Health Care and Epidemiology, University of British Columbia, Vancouver, Canada
UR - 8549489
ER -

TY - JOUR
ID - 1963
T1 - Colorado pediatricians' involvement in community activities
A1 - Brown,J.
A1 - Westrick,M.C.
A1 - Rushton,F.E.

A1 - Siegel,C.
A1 - LaMont,R.
Y1 - 1995/11//
N1 - Brown, J. Westrick, M C. Rushton, F E. Siegel, C. LaMont, R
The Western journal of medicine
0410504, xn5
AIM, IM
Journal Article. Research Support, Non-U.S. Gov't
English
KW - MEDLINE
KW - Adult
KW - Child
KW - Colorado
KW - Community Networks
KW - Female
KW - Humans
KW - Male
KW - Middle Aged
KW - Pediatrics/sn [Statistics & Numerical Data]
KW - Physician's Practice Patterns/sn [Statistics & Numerical Data]
KW - Physician's Role
KW - Volunteers/sn [Statistics & Numerical Data]
RP - NOT IN FILE
SP - 451
EP - 453
JF - Western Journal of Medicine
JA - West J Med
VL - 163
IS - 5
CY - UNITED STATES
N2 - To determine Colorado American Academy of Pediatrics (AAP) pediatricians' involvement in community-based activities and awareness of and interest in the AAP Community Access to Child Health (CATCH) program, a 22-item survey was mailed to all general pediatrician AAP fellows and candidate fellows practicing in Colorado (n = 434). The return rate was 65%. Of the respondents, 73% provide direct patient care as their primary professional activity, 58% reported either current or past involvement in community-based programs outside of their practices, 91% of this community-based work was voluntary, and 80% of the respondents described this work as moderate to very rewarding. Half of the respondents (51%) were aware of the AAP CATCH program, and 68% were interested in attending a statewide CATCH meeting. We conclude that Colorado AAP pediatrician survey respondents participate heavily in community programs outside of their clinical practices and that among this group there is substantial interest in the AAP CATCH program
SN - 0093-0415
AD - Department of Community Health Services, Denver Health and Hospitals, USA
UR - 8533408
ER -

TY - JOUR
ID - 1964
T1 - Potties, pits and pipes: explaining hygiene behaviour in Burkina Faso
A1 - Curtis,V.
A1 - Kanki,B.
A1 - Mertens,T.
A1 - Traore,E.
A1 - Diallo,I.
A1 - Tall,F.
A1 - Cousens,S.
Y1 - 1995/08//

N1 - Curtis, V. Kanki, B. Mertens, T. Traore, E. Diallo, I. Tall, F. Cousens, S
Social science & medicine (1982)
ut9, 8303205
IM
Journal Article. Research Support, Non-U.S. Gov't
English
KW - MEDLINE
KW - Burkina Faso
KW - Child
KW - Preschool
KW - Developing Countries
KW - Diarrhea
KW - Infantile/et [Etiology]
KW - Infantile/pc [Prevention & Control]
KW - Female
KW - Health Education
KW - Health Knowledge
KW - Attitudes
KW - Practice
KW - Humans
KW - Infant
KW - Life Style
KW - Male
KW - Sanitation
KW - Socioeconomic Factors
KW - Toilet Facilities
KW - Water Supply
RP - NOT IN FILE
SP - 383
EP - 393
JF - Social Science & Medicine
JA - Soc Sci Med
VL - 41
IS - 3
CY - ENGLAND
N2 - Stool disposal practices have been shown to be associated with childhood diarrhoea. However, efforts to promote improved hygiene behaviour are hampered by a lack of understanding of what determines those behaviours. Data from 2793 household interviews with mothers of children from the town of Bobo-Dioulasso in Burkina Faso were analyzed to examine what differentiated mothers who reported using safer stool disposal practices from those who did not. Three 'outcomes' were considered: where the child was reported to defaecate; where the mother reported disposing of the child stools; and whether excreta were observed in the compound. Regression models were developed to identify those factors with the strongest independent associations with the outcomes. There was a consistent association between the source of water and the outcomes. Mothers with access to a tap in the yard reported using safe hygiene practices three times more often than mothers using wells outside the compound and twice as often as mothers who used public standpipes or wells within the yard. The source of water showed a similar pattern of association with observations of faecal matter in the environment. Improved sources of water may contribute to safer stool hygiene by reducing the time spent on water collection or by encouraging mothers to conform to higher standards of hygiene. Other factors which played a role in predicting the hygiene behaviour of mothers were the husbands' occupation, the number of health education sessions that she had attended, her zone of residence and family ownership of certain valuable objects. These factors are likely to be related and to be, to some extent, proxies for the real determinants of her behaviour. A model of the cultural, psycho-social and infrastructural proximate determinants of hygiene behaviour is proposed. Data from focus group discussions suggested that the main purpose of hygienic behaviour is to conform to existing norms of social etiquette. Trials of interventions based on changing such norms are needed to test whether this is an effective means of promoting of safer hygiene practices
SN - 0277-9536

AD - Maternal and Child Epidemiology Unit, London School of Hygiene and Tropical Medicine/Centre Muraz, Bobo-Dioulasso, Burkina Faso
UR - 7481932
ER -

TY - JOUR
ID - 1965
T1 - Acute effects of ozone on the pulmonary function of exercising schoolchildren from Mexico City
A1 - Castillejos,M.
A1 - Gold,D.R.
A1 - Damokosh,A.I.
A1 - Serrano,P.
A1 - Allen,G.
A1 - McDonnell,W.F.
A1 - Dockery,D.
A1 - Ruiz,Velasco S.
A1 - Hernandez,M.
A1 - Hayes,C.
Y1 - 1995/11//
N1 - Castillejos, M. Gold, D R. Damokosh, A I. Serrano, P. Allen, G. McDonnell, W F. Dockery, D. Ruiz Velasco, S. Hernandez, M. Hayes, C
American journal of respiratory and critical care medicine
9421642, bzs
AIM, IM
Comparative Study. Journal Article. Research Support, Non-U.S. Gov't. Research Support, U.S. Gov't, Non-P.H.S.
English
KW - MEDLINE
KW - Air Pollutants/ae [Adverse Effects]
KW - Air Pollutants/an [Analysis]
KW - Child
KW - Chronic Disease
KW - Common Cold/pp [Physiopathology]
KW - Cough/pp [Physiopathology]
KW - Exercise/ph [Physiology]
KW - Exercise Test/mt [Methods]
KW - Exercise Test/sn [Statistics & Numerical Data]
KW - Female
KW - Forced Expiratory Volume/de [Drug Effects]
KW - Humans
KW - Logistic Models
KW - Lung/de [Drug Effects]
KW - Lung/ph [Physiology]
KW - Male
KW - Mexico
KW - Ozone/ae [Adverse Effects]
KW - Ozone/an [Analysis]
KW - Respiratory Sounds/pp [Physiopathology]
KW - Urban Population
KW - Vital Capacity/de [Drug Effects]
RP - NOT IN FILE
SP - 1501
EP - 1507
JF - American Journal of Respiratory & Critical Care Medicine
JA - Am J Respir Crit Care Med
VL - 152
IS - 5 Pt 1

CY - UNITED STATES
N2 - The acute effects of ozone (O3) on the change in lung function before and after exercise was assessed in 22 boys and 18 girls from 7 1/2 to 11 yr of age tested up to eight times over a 1 1/2-yr period outdoors (under a tarpaulin) at a school in Mexico City. Ozone and particulates were monitored at an adjacent government station, in the school yard, and under the tarp. Subjects were selected to oversample children with chronic respiratory symptoms, although children with active asthma under regular medication or FEV1 < 80% predicted were excluded. Of the participants, 21 had chronic cough, chronic phlegm, or ever wheeze with colds or apart from colds. Children performed two cycles of treadmill exercise (15 min) and rest (15 min) for a total of 1 h of intermittent exercise. Most subjects attained the target minute ventilation of 35 L/min/m2. Subjects exercised alternately during low ozone hours (8:00-10:00 A.M.) and during peak O3 hours (12:00-2:00 P.M.), to assure a range of exposures. On 85% of exercise days, the maximum daily 1-h average for ambient O3 exceeded the Mexican guideline of 110 parts per billion (ppb). O3 exposure during the hour of exercise was divided into quintiles, and the response was adjusted for repeated measures, subject having a cold, and prior outdoor exercise. Ambient O3 in the fifth quintile (mean = 229 ppb) was associated with a percentage change in FVC (-1.43% +/- 0.70), FEV1 (-2.85% +/- 0.79), FEF25-75% (-6.32 +/- 1.87) and FEV1 (-1.41% +/- 0.46).(ABSTRACT TRUNCATED AT 250 WORDS)
SN - 1073-449X
AD - Universidad Autonoma Metropolitana-Xochimilco (UAM-X), Mexico City, Mexico
UR - 7582284
ER -

TY - JOUR
ID - 1966
T1 - Case-control study of risk factors for incident Lyme disease in California
A1 - Ley,C.
A1 - Olshen,E.M.
A1 - Reingold,A.L.
Y1 - 1995/11/01/
N1 - Ley, C. Olshen, E M. Reingold, A L
American journal of epidemiology
3h3, 7910653
IM
Journal Article. Research Support, U.S. Gov't, P.H.S.
English
KW - MEDLINE
KW - Adolescent
KW - Adult
KW - California/ep [Epidemiology]
KW - Case-Control Studies
KW - Child
KW - Preschool
KW - Female
KW - Humans
KW - Incidence
KW - Infant
KW - Lyme Disease/ep [Epidemiology]
KW - Lyme Disease/et [Etiology]
KW - Male
KW - Matched-Pair Analysis
KW - Middle Aged
KW - Odds Ratio
KW - Risk Factors
RP - NOT IN FILE
SP - S39
EP - S47
JF - American Journal of Epidemiology
JA - Am J Epidemiol

VL - 142
IS - 9 Suppl
CY - UNITED STATES
N2 - To identify risk factors for incident Lyme disease in California during the period June 1, 1991 to December 31, 1992, the authors compared the activities of 101 cases of physician-diagnosed erythema migrans ascertained via both active and passive surveillance with those of 107 controls matched on sex, age, and neighborhood. Questions asked by telephone pertained to location of home, presence of wildlife around the house, hours of outdoor work and outdoor leisure activities, pet ownership, precautionary measures to avoid tick bites, tick removal methods, and knowledge about Lyme disease. For cases, activities pertained to the month prior to the onset of erythema migrans; controls were interviewed about the same activities during the same one-month period. The observation of deer and lizards around the home and a history of exposure to ticks were associated with Lyme disease (deer, odds ratio (OR) = 2.53, 95% confidence interval (CI) 1.25-5.15; lizards, OR = 2.14, 95% CI 1.14-4.04). However, the only activity associated with Lyme disease was the use for more than 5 hours per week of wide maintained trails (OR = 11.33, 95% CI 1.33-123.5); this association occurred only in persons with other outdoor leisure activities. No other behaviors or activities were identified as risk factors for acquisition of Lyme disease in California
SN - 0002-9262
AD - Epidemiology Program, School of Public Health, University of California, Berkeley 94720, USA
UR - 7572986
ER -

TY - JOUR
ID - 1967
T1 - Variability and tracking of physical activity over 2 yr in young children
A1 - Sallis,J.F.
A1 - Berry,C.C.
A1 - Broyles,S.L.
A1 - McKenzie,T.L.
A1 - Nader,P.R.
Y1 - 1995/07//
N1 - Sallis, J F. Berry, C C. Broyles, S L. McKenzie, T L. Nader, P R
Medicine and science in sports and exercise
8005433, mg8
IM, S
Journal Article. Research Support, U.S. Gov't, P.H.S.
English
KW - MEDLINE
KW - Child Behavior
KW - Child
KW - Preschool
KW - Data Interpretation
KW - Statistical
KW - Exercise/ph [Physiology]
KW - Female
KW - Humans
KW - Male
KW - Play and Playthings
RP - NOT IN FILE
SP - 1042
EP - 1049
JF - Medicine & Science in Sports & Exercise
JA - Med Sci Sports Exerc
VL - 27
IS - 7
CY - UNITED STATES
N2 - The purpose of this study was to examine tracking of physical activity at home and recess in young children. Three hundred fifty-one Mexican-American and Anglo-American children entered the study (mean

age = 4.4 yr), and 83% completed 2 yr of measurement. Physical activity was directly observed on 10 d over 2 yr. Measurement waves occurred every 6 months, and each wave consisted of 2 d of observation within 1 wk. Children were observed for 60 min at home on a weekday evening and up to 30 min during recess at preschool or school. Maximum likelihood procedures using a linear mixed-effects model indicated that most of the variance in home and recess physical activity was accounted for by short-term/weekly factors. Tracking of physical activity accounted for 15% of the total variance at home and 8% at recess. Pearson correlations for physical activity over time were higher at home than at recess. Tracking of home physical activity was r = 0.15 when single days were correlated and r = 0.36 when the means of 4 d were correlated. There was a small, but detectable, stable component of physical activity in young children, at least at home
SN - 0195-9131
AD - Department of Pediatrics, University of California, San Diego 92093-0927, USA
UR - 7564971
ER -

TY - JOUR
ID - 1968
T1 - The validity of the Tritrac-R3D Activity Monitor for the assessment of physical activity in children
A1 - Welk,G.J.
A1 - Corbin,C.B.
Y1 - 1995/09//
N1 - Welk, G J. Corbin, C B
Research quarterly for exercise and sport
r6y, 8006373
IM
Journal Article. Research Support, Non-U.S. Gov't
English
KW - MEDLINE
KW - Child
KW - Energy Metabolism
KW - Exercise/ph [Physiology]
KW - Exercise
KW - Heart Rate
KW - Humans
KW - Male
KW - Monitoring
KW - Physiologic/is [Instrumentation]
KW - Reproducibility of Results
RP - NOT IN FILE
SP - 202
EP - 209
JF - Research Quarterly for Exercise & Sport
JA - Res Q Exerc Sport
VL - 66
IS - 3
CY - UNITED STATES
N2 - The purpose of this study was to evaluate the validity of the Tritrac-R3D Activity Monitor, a new instrument designed to improve assessments of physical activity. Comparisons were made with a heart rate monitor and with a Caltrac Activity Monitor. Thirty-five children (ages 9-11 years) were monitored on 3 different school days with all 3 instruments. The Tritrac was moderately correlated with the heart rate monitor (r = .58) and highly correlated with the Caltrac monitor (r = .88). By taking advantage of the minute-by-minute timing capability of the Tritrac and the heart rate monitors, it was discovered that the correlations between these instruments were highest during free play situations (lunch/recess, recess, after school) and were lower when activity was more limited (class time) or structured (physical education). The ability of the Tritrac to assess activity on a minute-by-minute basis may greatly enhance its overall utility
SN - 0270-1367
AD - Department of Health, Physical Education, Recreation and Dance, Eastern Michigan University, Ypsilanti, USA

UR - 7481081
ER -

TY - JOUR
ID - 1969
T1 - Children's descriptions of their feelings and what they found helpful during bereavement
A1 - Lehna,C.R.
Y1 - 1995/09//
N1 - Lehna, C R
The American journal of hospice & palliative care
9008229, atd
N
Journal Article. Research Support, Non-U.S. Gov't
English
KW - MEDLINE
KW - Anger
KW - Art Therapy
KW - Bereavement
KW - Child
KW - Child Psychology
KW - Preschool
KW - Female
KW - Humans
KW - Male
KW - Nursing Methodology Research
KW - Self-Help Groups/og [Organization & Administration]
RP - NOT IN FILE
SP - 24
EP - 30
JF - American Journal of Hospice & Palliative Medicine
JA - Am J Hosp Palliat Care
VL - 12
IS - 5
CY - UNITED STATES
N2 - Although clinicians have described the value of support groups in assisting children mourn the death of someone close, little is known about helping children with bereavement. By understanding what helps children mourn, nurses can better facilitate a healthy bereavement process. The purpose of this mini-ethnographic study, which used observation and participation procedures, was to identify what 16 children found helpful during their bereavement. Initially, the children's expressions of feelings were demonstrated both verbally and through art work. The patterns of the children's emotions were labeled as affective distress, somatic complaints, and for the boys, disruptive behavior. The children then described their mourning processes as being facilitated by out-of-door activity, yelling, and talking to others (e.g. God, mom, grandmother or grandfather)
SN - 1049-9091
UR - 7546977
ER -

TY - JOUR
ID - 1970
T1 - Injury prevention in an urban setting: challenges and successes
A1 - Laraque,D.
A1 - Barlow,B.
A1 - Durkin,M.
A1 - Heagarty,M.
Y1 - 1995///
N1 - Laraque, D. Barlow, B. Durkin, M. Heagarty, M
Bulletin of the New York Academy of Medicine
bqo, 7505398

IM
Journal Article. Research Support, Non-U.S. Gov't. Research Support, U.S. Gov't, P.H.S.
English
KW - MEDLINE
KW - Accident Prevention
KW - Adolescent
KW - Adult
KW - Burns/pc [Prevention & Control]
KW - Child
KW - Community-Institutional Relations
KW - Consumer Participation
KW - Environment
KW - Government Agencies
KW - Health Care Coalitions
KW - Health Education
KW - Humans
KW - New York City
KW - Organizational Objectives
KW - Outcome Assessment (Health Care)
KW - Parents
KW - Personnel
KW - Hospital
KW - Play and Playthings
KW - Program Development
KW - Program Evaluation
KW - Safety
KW - Schools
KW - Stress
KW - Physiological/pc [Prevention & Control]
KW - Urban Health Services
KW - Violence
KW - Wounds and Injuries/pc [Prevention & Control]
RP - NOT IN FILE
SP - 16
EP - 30
JF - Bulletin of the New York Academy of Medicine
JA - Bull N Y Acad Med
VL - 72
IS - 1
CY - UNITED STATES
N2 - The Harlem Hospital Injury Prevention Program (HHIPP) was established in 1988 with the goal of reducing injuries to children in central Harlem by providing safe play areas, supervised activities, and injury prevention education. To achieve this goal, a broad-based coalition was formed with state and local governmental agencies interested in injury prevention and with community groups, schools, parents, and hospital staff. An evaluation of the program in terms of both process and outcome formed a critical element of this effort. Since 1988 the HHIPP, as the lead agency for the Healthy Neighborhoods/Safe Kids Coalition, developed or participated in two types of programs: injury-prevention education programs and programs that provide safe activities and/or environments for children. The educational programs included Window Guards campaign; Safety City Program; Kids, Injuries and Street Smarts Program (KISS); Burn Prevention Curriculum and Smoke Detector Distribution; Harlem Alternative to Violence Program; Adolescent Outreach Program; and Critical Incident Stress Management Teams. The safe activities and environmental programs included the Bicycle Safety Program/Urban Youth Bike Corps; Playground Injury Prevention Program; the Greening of Harlem Program; the Harlem Horizon Art Studio; Harlem Hospital Dance Clinic; Unity through Murals project; baseball at the Harlem Little League; winter baseball clinic; and the soccer league. Each program was conceived using injury data, coupled with parental concern and activism, which acted as catalysts to create a community coalition to respond to a specific problem. Data systems developed over time, which monitored the prevalence and incidence of childhood injuries in northern Manhattan, including central Harlem, became essential not only

to identify specific types of childhood injuries in this community but also to evaluate these programs for the prevention of injuries in children
SN - 0028-7091
AD - Harlem Hospital Center, Columbia University College of Physicians and Surgeons, USA
UR - 7581311
ER -

TY - JOUR
ID - 1971
T1 - Developmental, behavioural, and environmental risk factors for diarrhoea among rural Bangladeshi children of less than two years
A1 - Zeitlin,M.F.
A1 - Ahmed,N.U.
A1 - Beiser,A.S.
A1 - Zeitlin,J.A.
A1 - Super,C.M.
A1 - Guldan,G.S.
Y1 - 1995/06//
N1 - Zeitlin, M F. Ahmed, N U. Beiser, A S. Zeitlin, J A. Super, C M. Guldan, G S
Journal of diarrhoeal diseases research
iba, 8402695
IM
Journal Article
English
KW - MEDLINE
KW - Bangladesh/ep [Epidemiology]
KW - Child
KW - Preschool
KW - Diarrhea
KW - Infantile/ep [Epidemiology]
KW - Female
KW - Humans
KW - Incidence
KW - Infant
KW - Newborn
KW - Longitudinal Studies
KW - Male
KW - Risk Factors
KW - Rural Population
KW - Socioeconomic Factors
RP - NOT IN FILE
SP - 99
EP - 105
JF - Journal of Diarrhoeal Diseases Research
JA - J Diarrhoeal Dis Res
VL - 13
IS - 2
CY - BANGLADESH
N2 - One hundred and eighty-five rural Bangladeshi children (80 boys and 105 girls) aged 4-27 months were observed to investigate developmental, behavioural, and environmental risk factors for diarrhoea during a 6-month period. Incidence of diarrhoea was found to be the highest among children aged 10-12 months. Children of this age group had the greatest exposure to environmental contaminants in the neighbourhood. Incidence of diarrhoea was the highest in hot, dry months. Risk factors for diarrhoea included: faecal contamination and garbage disposal in infant's outdoor play compound, crawling, contact of hand and mouth with contaminated materials, greater distance of household from water source, inadequate cleaning after defecation; dirt of child's face, presence of flies, feeding rotten food; insufficient washing of infant's and caretaker's hands before feeding rice meals or soft, wet foods; and lack of mothers' willingness to visit a modern (allopathic) health practitioner

SN - 0253-8768
AD - Tufts University School of Nutrition, Medford, MA 02155, USA
UR - 7594318
ER -

TY - JOUR
ID - 1972
T1 - A summer camp for childhood obesity in Thailand
A1 - Jirapinyo,P.
A1 - Limsathayourat,N.
A1 - Wongarn,R.
A1 - Limsathayourat,N.
A1 - Bunnag,A.
A1 - Chockvivatvanit,S.
Y1 - 1995/05//
N1 - Jirapinyo, P. Limsathayourat, N. Wongarn, R. Limsathayourat, N. Bunnag, A. Chockvivatvanit, S
Journal of the Medical Association of Thailand = Chotmaihet thangphaet
izr, 7507216
IM
Journal Article
English
KW - MEDLINE
KW - Adolescent
KW - Camping
KW - Child
KW - Female
KW - Humans
KW - Male
KW - Obesity/px [Psychology]
KW - Obesity/th [Therapy]
KW - Thailand
KW - Weight Loss
RP - NOT IN FILE
SP - 238
EP - 246
JF - Journal of the Medical Association of Thailand
JA - J Med Assoc Thai
VL - 78
IS - 5
CY - THAILAND
N2 - The results of a 4-week summer camp for childhood obesity were reported. Twenty-one children with moderate to severe obesity, aged between 8-13 years, joined the program. Dietary restriction during the official hours and dietary self-control at home were implemented throughout the program. Exercise, swimming and group therapy were also implemented throughout the program. Weekly sight-seeing outside the camp was very interesting for the participants. After the program, all participants had lost weight which was about 5 per cent of their initial weight. Most of the weight loss was due to loss of body fat but not lean body mass. No complications occurred during the program. The 4-week summer camp is then practical for initiating weight loss for obese children. Long-term follow-up which is underway will be reported later
SN - 0125-2208
AD - Clinical Research Center, Department of Pediatrics, Faculty of Medicine, Siriraj Hospital, Mahidol University, Bangkok, Thailand
UR - 7561546
ER -

TY - JOUR
ID - 1973

T1 - The effect of remediation of chromium waste sites on chromium levels in urine of children living in the surrounding neighborhood
A1 - Freeman,N.C.
A1 - Wainman,T.
A1 - Lioy,P.J.
A1 - Stern,A.H.
A1 - Shupack,S.I.
Y1 - 1995/08//
N1 - Freeman, N C. Wainman, T. Lioy, P J. Stern, A H. Shupack, S I
Journal of the Air & Waste Management Association (1995)
9503111, cbi
IM
Journal Article. Research Support, Non-U.S. Gov't
English
KW - MEDLINE
KW - Child
KW - Chromium/ur [Urine]
KW - Hazardous Waste/ae [Adverse Effects]
KW - Hazardous Waste/an [Analysis]
KW - Humans
KW - New Jersey
KW - Refuse Disposal
RP - NOT IN FILE
SP - 604
EP - 614
JF - Journal of the Air & Waste Management Association
JA - J Air Waste Manag Assoc
VL - 45
IS - 8
CY - UNITED STATES
N2 - Exposure to chromium was assessed for 40 children living near chromium waste sites. Sampling was conducted in one Jersey City, New Jersey neighborhood during the summer and fall of 1991. Household dust samples from residences and urine samples from children living near chromium waste sites were collected and analyzed for chromium. During the summer and fall visits when the samples were collected, the children were also interviewed about lifestyle/activity patterns. Comparisons were made with similar samples collected from children and homes in other areas of New Jersey outside of Hudson County with no known chromium waste sites. Household dust masses and chromium loadings and concentrations in the dust showed a significant decline in this Hudson County neighborhood since the area was first sampled in 1990. Interim remediation of neighborhood sites and an active community education program in the interval between the first and second year of sampling may have contributed to the reduction in dust masses and chromium levels in dust. Children's urine chromium concentrations were consistent across the two sampling periods despite reported changes in activity patterns. Chromium concentrations in urine were found to be age-dependent and related to home location. In this sample of Jersey City children less than six years old, time spent playing outdoors was a weak secondary contributor to urine chromium levels
SN - 1096-2247
AD - Department of Environmental and Community Medicine, Robert Wood Johnson Medical School, Piscataway, New Jersey, USA
UR - 7664166
ER -

TY - JOUR
ID - 1974
T1 - Sports and recreation injuries in US children and adolescents
A1 - Bijur,P.E.
A1 - Trumble,A.
A1 - Harel,Y.
A1 - Overpeck,M.D.

A1 - Jones,D.
A1 - Scheidt,P.C.
Y1 - 1995/09//
N1 - Bijur, P E. Trumble, A. Harel, Y. Overpeck, M D. Jones, D. Scheidt, P C
Archives of pediatrics & adolescent medicine
9422751, bwf
AIM, IM
Journal Article. Research Support, U.S. Gov't, P.H.S.
English
KW - MEDLINE
KW - Adolescent
KW - Adult
KW - Age Distribution
KW - Athletic Injuries/ep [Epidemiology]
KW - Child
KW - Cumulative Trauma Disorders/ep [Epidemiology]
KW - Data Collection
KW - Female
KW - Humans
KW - Male
KW - Recreation
KW - Sex Distribution
KW - United States
RP - NOT IN FILE
SP - 1009
EP - 1016
JF - Archives of Pediatrics & Adolescent Medicine
JA - Arch Pediatr Adolesc Med
VL - 149
IS - 9
CY - UNITED STATES
N2 - OBJECTIVES: To estimate and describe morbidity from sports and recreation injuries in children and adolescents. DESIGN: Survey conducted by the National Center for Health Statistics--the Child Health Supplement to the 1988 National Health Interview Survey. SETTING: The general community. PARTICIPANTS: Representative sample of the noninstitutionalized civilian US population. Five percent of the eligible households did not participate. The subject of this report is 11,840 children and adolescents aged 5 to 17 years. MAIN OUTCOME MEASURES: Medically attended nonfatal injuries resulting from sports and recreation, and serious sports injuries, defined as injuries resulting in hospitalization, surgical treatment, missed school, or half a day or more in bed. Sports and recreation injuries were defined as those occurring in a place of recreation or sports, or receiving any of the following International Classification of Diseases, Ninth Revision (ICD-9) E-codes: struck in sports, fall in sports, bicycle-related injury, riding an animal, water sports, overexertion, fall from playground equipment or other vehicles, primarily skates and skateboards. RESULTS: The estimated annual number of all injuries from sports and recreation in US children and adolescents is 4,379,000 (95% confidence interval = 3,147,000 to 5,611,000); from serious sport injuries, 1,363,000 (95% confidence interval = 632,000 to 2,095,000). Sports account for 36% of injuries from all causes. Cause and nature of injury are strongly related to age. Sports do not account for a disproportionate number of serious or repeated injuries compared with other causes of injuries. CONCLUSION: Sports activities account for a large number and substantial proportion of all injuries to children and youth
SN - 1072-4710
AD - Department of Pediatrics, Albert Einstein College of Medicine, Bronx, NY, USA
UR - 7655585
ER -

TY - JOUR
ID - 1975
T1 - Skull fractures in children: altered conscious level is the main indication for urgent CT scanning
A1 - Read,H.S.

A1 - Johnstone,A.J.
A1 - Scobie,W.G.
Y1 - 1995/06//
N1 - Read, H S. Johnstone, A J. Scobie, W G
Injury
0226040, gon
IM
Journal Article
English
KW - MEDLINE
KW - Child
KW - Preschool
KW - Female
KW - Glasgow Coma Scale
KW - Humans
KW - Infant
KW - Male
KW - Patient Selection
KW - Skull Fractures/co [Complications]
KW - Skull Fractures/px [Psychology]
KW - Skull Fractures/ra [Radiography]
KW - Tomography
KW - X-Ray Computed
KW - Unconsciousness/et [Etiology]
RP - NOT IN FILE
SP - 333
EP - 334
JF - Injury
VL - 26
IS - 5
CY - ENGLAND
N2 - It has been suggested that all children with skull fractures require urgent CT scanning to exclude intracranial injury. Adhering to such a policy could both tax limited scanning facilities and result in unnecessary exposure to radiation. The aim of this study is to assess the level of consciousness in determining the need for urgent CT scanning, and the possible role of the mechanism of injury as a secondary risk factor. We identified 140 children admitted during a 7-year period with a skull fracture after falling outside the home. Thirteen children had a diminished level of consciousness. Scans were performed in nine, and seven of the scans revealed significant intracranial injuries. By comparison, all 127 children with a normal level of consciousness recovered fully. Scanning was performed in only eight of them; seven scans were normal and one revealed a small subarachnoid heamorrhage which did not require active treatment. In addition, all 13 children with a diminished level of consciousness had sustained their injuries by falling from greater than their own standing height. By comparison, none of the children who had sustained their skull fractures by tripping up had a diminished level of consciousness or a significant intracranial injury. We conclude that all children with a diminished level of consciousness need to undergo an urgent CT scan. Children with skull fractures and a normal conscious level may be managed initially by neuroobservations and the clinician may be further reassured if the child's injury resulted from tripping up
SN - 0020-1383
AD - Department of Paediatric Surgery, Royal Hospital for Sick Children, Edinburg, Scotland, UK
UR - 7649651
ER -

TY - JOUR
ID - 1976
T1 - Nasal inflammatory responses in children exposed to a polluted urban atmosphere
A1 - Calderon-Garciduenas,L.
A1 - Rodriguez-Alcaraz,A.
A1 - Garcia,R.

A1 - Ramirez,L.
A1 - Barragan,G.
Y1 - 1995/08//
N1 - Calderon-Garciduenas, L. Rodriguez-Alcaraz, A. Garcia, R. Ramirez, L. Barragan, G
Journal of toxicology and environmental health
kaa, 7513622
IM
Journal Article
English
KW - MEDLINE
KW - Air Pollutants/ae [Adverse Effects]
KW - Child
KW - Environmental Exposure/ae [Adverse Effects]
KW - Environmental Exposure/an [Analysis]
KW - Female
KW - Humans
KW - Inflammation/ci [Chemically Induced]
KW - Inflammation/ep [Epidemiology]
KW - Inflammation/pa [Pathology]
KW - Leukocytes
KW - Mononuclear/pa [Pathology]
KW - Male
KW - Mexico/ep [Epidemiology]
KW - Nasal Lavage Fluid/cy [Cytology]
KW - Nasal Mucosa/pa [Pathology]
KW - Ozone/ae [Adverse Effects]
KW - Urban Health
RP - NOT IN FILE
SP - 427
EP - 437
JF - Journal of Toxicology & Environmental Health
JA - J Toxicol Environ Health
VL - 45
IS - 4
CY - UNITED STATES
N2 - Southwest Metropolitan Mexico City (SWMMC) preadolescent children have been exposed to a highly polluted urban atmosphere most of their lives. The main objective of this study was to determine by nasal lavage (NAL) the acute inflammatory nasal influx elicited in these children upon exposure to three different polluted days. Ozone, the main criteria pollutant for SWMMC, varied both in the number of hours above the National Ambient Air Quality Standard (NAAQS), which is 0.12 ppm as a 1-h maximum concentration not to be exceeded more than once per year, and in the maximal concentrations in the preceding three NAL sampling dates. Nasal neutrophilic influx, the surface expression of the B2 integrin CD11b on the nasal polymorphonuclear leukocytes (PMNs), rhinoscopic findings, respiratory symptoms, and nasal cytologies were evaluated in the 38 exposed children and in the 28 control children living in a nonpolluted Pacific coast port. SWMMC children had an average daily outdoor exposure of 7.7 h and complained of nasal mucus secretion, epistaxis, intermittent nasal obstruction, diurnal cough episodes, and chest discomfort. Nasal mucosal atrophy by rhinoscopy was present in 37/38, and all children had an abnormal nasal cytology. Exposed children had significantly higher nasal PMNs and nasal PMN-CD11b expression than controls. PMN median values in exposed children were higher than controls on all sampling dates (November 12, $p < .001$; November 17, $p < .001$; and November 24, $p < .00001$). Interestingly, a lower nasal neutrophilic response ($p < .0004$) was recorded in the SWMMC children 18 h after exposure to the highest O3 concentrations (up to 0.307 ppm) and the largest number of hours with O3 > 0.12 ppm (7 h). The question of a competing inflammatory response at the bronchioalveolar level with structural damage is raised. These NAL findings underscore the need to restrict outdoor activity in SWMMC children during the months of greater potential exposure to ozone
SN - 0098-4108
AD - Instituto Nacional de Pediatria, Experimental Pathology Section, Mexico City, Mexico
UR - 7643430

ER -

TY - JOUR
ID - 1977
T1 - Injuries from falls on playgrounds. Effects of day care center regulation and enforcement
A1 - Briss,P.A.
A1 - Sacks,J.J.
A1 - Addiss,D.G.
A1 - Kresnow,M.J.
A1 - O'neil,J.
Y1 - 1995/08//
N1 - Briss, P A. Sacks, J J. Addiss, D G. Kresnow, M J. O'Neil, J
Archives of pediatrics & adolescent medicine
9422751, bwf
AIM, IM
Comparative Study. Journal Article
English
KW - MEDLINE
KW - Accidental Falls/sn [Statistics & Numerical Data]
KW - Child
KW - Child Day Care Centers
KW - Child Welfare
KW - Preschool
KW - Health Surveys
KW - Humans
KW - Wounds and Injuries/pc [Prevention & Control]
RP - NOT IN FILE
SP - 906
EP - 911
JF - Archives of Pediatrics & Adolescent Medicine
JA - Arch Pediatr Adolesc Med
VL - 149
IS - 8
CY - UNITED STATES
N2 - OBJECTIVES: To measure the incidence of playground fall injuries among children attending licensed US day care centers and to evaluate how injury incidence varies with center characteristics and with the regulatory and enforcement climate in which centers operate. DESIGN: Telephone surveys of directors of day care centers and enforcement agencies and review of written day care regulations. SETTING: Probability sample of licensed day care centers in 50 states and the District of Columbia. PARTICIPANTS: Children attending day care centers with playgrounds. MAIN OUTCOME MEASURES: Medically attended playground fall injuries. RESULTS: Among the 1740 day care centers studied, a weighted total of 89.2 injuries occurred during the 2-month study period (0.25/100,000 child-hours in day care). The most important risk factor for injury was height of the tallest piece of climbing equipment on the playground in both bivariate (P = .01) and multivariate (P = .02) analyses. Neither regulations addressing playground safety or playground surfaces nor enforcement patterns were associated with lower injury rates. CONCLUSIONS: Additional effort is needed to develop and evaluate regulations and enforcement that reduce injury risks for children while minimizing burden on day care centers. In the meantime, limiting climbing equipment heights may reduce playground injury rates
SN - 1072-4710
AD - National Center for Injury Prevention and Control, Preventive Medicine Residency Program, Atlanta, Ga., USA
UR - 7633546
ER -

TY - JOUR
ID - 1978
T1 - Pregnancy among Hispanic teenagers: is good parental communication a deterrent?
A1 - Adolph,C.

A1 - Ramos,D.E.
A1 - Linton,K.L.
A1 - Grimes,D.A.
Y1 - 1995/05//
N1 - Adolph, C. Ramos, D E. Linton, K L. Grimes, D A
Contraception
dqn, 0234361
IM, J
Journal Article
English
KW - MEDLINE
KW - Adolescent
KW - Attitude
KW - Child
KW - Communication
KW - Female
KW - Hispanic Americans
KW - Humans
KW - Los Angeles
KW - Parent-Child Relations
KW - Pregnancy
KW - Pregnancy in Adolescence
KW - Questionnaires
KW - Regression Analysis
KW - Risk Factors
KW - Sexual Behavior
RP - NOT IN FILE
SP - 303
EP - 306
JF - Contraception
VL - 51
IS - 5
CY - UNITED STATES
N2 - Effective communication between Hispanic parents and teens about sexual issues may deter adolescent pregnancy, yet little is known about the prevalence or impact of such communication. The study examined this potential relationship in a cohort of urban Hispanic adolescents. A questionnaire was administered to a non-random sample of pregnant and non-pregnant Hispanic women aged 12-18 years attending inner city schools in Los Angeles to obtain demographic, sexual activity and communication information. Logistic regression analysis was used to evaluate the independent contribution of risk factors to teenage pregnancy. Good communication with one's mother was inversely related to pregnancy; the adjusted odds ratio of pregnancy if the mother told the daughter about sex was 0.3 (95% CI 0.2-0.6). Friends' love was also inversely related to pregnancy (odds ratio 0.7; 95% CI 0.6-0.8). In order of increasing strength, alcohol and drug use, favorable attitude toward premarital sex, receipt of welfare, older age at menarche, and older age were all significantly related to pregnancy. Pregnant Hispanic teenagers have poorer communication with their parents than do other Hispanic teens. Efforts to reduce the incidence of adolescent pregnancy among Hispanics may need to address not only family communication but also issues outside the home such as alcohol and recreational drugs
SN - 0010-7824
AD - Department of Obstetrics and Gynecology, University of Southern California School of Medicine, Los Angeles 90033, USA
UR - 7628205
ER -

TY - JOUR
ID - 1979
T1 - Household and community determinants of exposure to involuntary smoking: a study of urinary cotinine in children and adolescents
A1 - Dell'Orco,V.

A1 - Forastiere,F.
A1 - Agabiti,N.
A1 - Corbo,G.M.
A1 - Pistelli,R.
A1 - Pacifici,R.
A1 - Zuccaro,P.
A1 - Pizzabiocca,A.
A1 - Rosa,M.
A1 - Altieri,I.
Y1 - 1995/08/15/
N1 - Dell'Orco, V. Forastiere, F. Agabiti, N. Corbo, G M. Pistelli, R. Pacifici, R. Zuccaro, P. Pizzabiocca, A. Rosa, M. Altieri, I. et al
American journal of epidemiology
3h3, 7910653
IM
Journal Article. Research Support, Non-U.S. Gov't
English
KW - MEDLINE
KW - Adolescent
KW - Child
KW - Cotinine/ur [Urine]
KW - Cross-Sectional Studies
KW - Environmental Monitoring
KW - Family Characteristics
KW - Female
KW - Humans
KW - Linear Models
KW - Male
KW - Socioeconomic Factors
KW - Tobacco Smoke Pollution/sn [Statistics & Numerical Data]
KW - Tobacco Smoke Pollution
RP - NOT IN FILE
SP - 419
EP - 427
JF - American Journal of Epidemiology
JA - Am J Epidemiol
VL - 142
IS - 4
CY - UNITED STATES
N2 - The study examines the role of several potential predictors of urinary cotinine levels in a cross-sectional sample of 1,072 nonsmoking children and adolescents in Latium, Italy, during 1990-1991. As expected, there was a strong relation between passive exposure to smoking and the amount of maternal and paternal self-reported smoking. The urinary cotinine level increased with a decreasing level of paternal education and with an increasing index of household crowding; self-report of recent exposure to smoking outside the home was a strong predictor of the biologic marker. The analysis was then restricted to 346 subjects whose parents claimed that they were nonsmokers and that there were no smokers at home. In this group, however, 57 children reported some active smoking at home by their parents. Those with parents suspected to be "deceivers" had higher level of urinary cotinine than did those truly not exposed. In addition, urinary cotinine in this group was clearly associated with duration of exposure to smoking outside home. The study indicates that both factors related to family circumstances and exposure outside the household setting are strong determinants of urinary cotinine levels. The finding may be considered a direct confirmation that passive smoking among children should be viewed as a specific community responsibility
SN - 0002-9262
AD - Respiratory Physiology, Catholic University, Rome, Italy
UR - 7625407
ER -

TY - JOUR
ID - 1980
T1 - Use of ethnography in the evaluation and targeting of HIV/AIDS education among Latino farm workers
A1 - Bletzer,K.V.
Y1 - 1995/04//
N1 - Bletzer, K V
AIDS education and prevention : official publication of the International Society for AIDS Education
auy, 9002873
IM, X
Journal Article
English
KW - MEDLINE
KW - Acquired Immunodeficiency Syndrome/eh [Ethnology]
KW - Acquired Immunodeficiency Syndrome/pc [Prevention & Control]
KW - Acquired Immunodeficiency Syndrome/px [Psychology]
KW - Acquired Immunodeficiency Syndrome/tm [Transmission]
KW - Adolescent
KW - Adult
KW - Agricultural Workers' Diseases/et [Etiology]
KW - Agricultural Workers' Diseases/pc [Prevention & Control]
KW - Agricultural Workers' Diseases/px [Psychology]
KW - Anthropology
KW - Cultural
KW - Child
KW - Defense Mechanisms
KW - Female
KW - HIV Infections/eh [Ethnology]
KW - HIV Infections/pc [Prevention & Control]
KW - HIV Infections/px [Psychology]
KW - HIV Infections/tm [Transmission]
KW - Health Education
KW - Health Knowledge
KW - Attitudes
KW - Practice
KW - Hispanic Americans/ed [Education]
KW - Hispanic Americans/px [Psychology]
KW - Humans
KW - Language
KW - Male
KW - Michigan
KW - Pregnancy
KW - Risk Factors
KW - Sexual Behavior
KW - Transients and Migrants/ed [Education]
KW - Transients and Migrants/px [Psychology]
RP - NOT IN FILE
SP - 178
EP - 191
JF - AIDS Education & Prevention
JA - AIDS Educ Prev
VL - 7
IS - 2
CY - UNITED STATES
N2 - Ethnography can be utilized to assess the impact of HIV/AIDS education simultaneous with the implementation of program activities. An ethnographic analysis based on field methods adapted in a Michigan program that targets migrant farmworkers highlights responses to showings of a bilingual AIDS education video; the things to which migrants attend while they are interacting with the educator and each other in HIV

education presentations; the tactics they employ to direct discussion when talking about HIV/AIDS, and the manner in which they use language to "distance" themselves from the topic of HIV infection and AIDS. Migrants in Michigan experience the same risks to health as farmworkers in other states; their pay is low, their hours are long, and the time they spend in the state is seasonal (summer). They engage in risk behavior while in the state (primarily consensual/contracted sex). Some migrants through use of drugs may come closer to exposure to the HIV virus outside the state than when they are working as migrants in Michigan
SN - 0899-9546
AD - Comprehensive Drug Research Center, University of Miami School of Medicine, FL 33136, USA
UR - 7619646
ER -

TY - JOUR
ID - 1981
T1 - Ehrlichiosis in a golf-oriented retirement community
A1 - Standaert,S.M.
A1 - Dawson,J.E.
A1 - Schaffner,W.
A1 - Childs,J.E.
A1 - Biggie,K.L.
A1 - Singleton,J.,Jr.
A1 - Gerhardt,R.R.
A1 - Knight,M.L.
A1 - Hutcheson,R.H.
Y1 - 1995/08/17/
N1 - Standaert, S M. Dawson, J E. Schaffner, W. Childs, J E. Biggie, K L. Singleton, J Jr. Gerhardt, R R. Knight, M L. Hutcheson, R H
The New England journal of medicine
0255562, now
AIM, IM
Journal Article
English
KW - MEDLINE
KW - Adolescent
KW - Adult
KW - Aged
KW - Animals
KW - Antibodies
KW - Bacterial/bl [Blood]
KW - Arachnid Vectors
KW - Base Sequence
KW - Case-Control Studies
KW - Child
KW - Disease Outbreaks
KW - Ehrlichia chaffeensis/im [Immunology]
KW - Ehrlichia chaffeensis
KW - Ehrlichiosis/ep [Epidemiology]
KW - Female
KW - Golf
KW - Housing for the Elderly
KW - Humans
KW - Male
KW - Middle Aged
KW - Molecular Sequence Data
KW - Retirement
KW - Seroepidemiologic Studies
KW - Tennessee/ep [Epidemiology]
KW - Ticks

RP - NOT IN FILE
SP - 420
EP - 425
JF - New England Journal of Medicine
JA - N Engl J Med
VL - 333
IS - 7
CY - UNITED STATES
N2 - BACKGROUND: Ehrlichiosis due to Ehrlichia chaffeensis usually occurs sporadically or in small clusters, with an annual incidence estimated at 3 to 5 cases per 100,000 population in areas of endemic disease. The putative principal vector is the Lone Star tick (Amblyomma americanum). We investigated an outbreak of ehrlichiosis that occurred in June 1993 among members of a golf-oriented retirement community (community A) in Tennessee. The community is densely wooded and borders a wildlife-management area where deer are numerous. METHODS: We conducted a case-control study, using medical-history reviews, serologic testing, and testing with the polymerase chain reaction for E. chaffeensis infection. We also surveyed a sample of 10 percent of the households in community A and in another golf-oriented community (community B) more than 20 miles (32 km) from the wildlife-management area. Survey participants completed a questionnaire and provided specimens for serologic testing. In both communities, searches for ticks were undertaken. RESULTS: Eleven cases of symptomatic ehrlichiosis were identified in the case-control study, 10 of which were in community A (attack rate, 330 per 100,000). Of 311 surveyed residents of community A, 12.5 percent had serologic evidence of past E. chaffeensis infection, as compared with 3.3 percent of 92 in community B (relative risk in community A as compared with community B, 3.9; 95 percent confidence interval, 1.2 to 12.2). The risk of infection was associated with tick bites, exposure to wildlife, golfing, and among golfers, retrieving lost golf balls from the rough. Persons who never used insect repellent were more likely to have had infection than persons who did. In community A, thousands of Lone Star ticks were found; in community B, only three ticks were found. CONCLUSIONS: The high rate of E. chaffeensis infection in community A resulted from its proximity to a wildlife reserve. When outdoor recreational activities are common and concentrations of ticks are high, outbreaks of arthropod-borne zoonoses can be anticipated
SN - 0028-4793
AD - Epidemic Intelligence Service, Centers for Disease Control and Prevention, Atlanta, USA
UR - 7616991
ER -

TY - JOUR
ID - 1982
T1 - Blunt impact to the chest leading to sudden death from cardiac arrest during sports activities
A1 - Maron,B.J.
A1 - Poliac,L.C.
A1 - Kaplan,J.A.
A1 - Mueller,F.O.
Y1 - 1995/08/10/
N1 - Maron, B J. Poliac, L C. Kaplan, J A. Mueller, F O
The New England journal of medicine
0255562, now
AIM, IM
Journal Article
English
KW - MEDLINE
KW - Adolescent
KW - Adult
KW - Arrhythmias
KW - Cardiac/et [Etiology]
KW - Athletic Injuries/co [Complications]
KW - Autopsy
KW - Baseball/in [Injuries]
KW - Cardiopulmonary Resuscitation
KW - Child

KW - Preschool
KW - Death
KW - Sudden
KW - Female
KW - Football/in [Injuries]
KW - Hockey/in [Injuries]
KW - Humans
KW - Male
KW - Martial Arts/in [Injuries]
KW - Protective Devices
KW - Thoracic Injuries/co [Complications]
KW - Wounds
KW - Nonpenetrating/co [Complications]
RP - NOT IN FILE
SP - 337
EP - 342
JF - New England Journal of Medicine
JA - N Engl J Med
VL - 333
IS - 6
CY - UNITED STATES
N2 - BACKGROUND: Sudden death from cardiac arrest in a young person may occur during sports play after a blunt blow to the chest in the absence of structural cardiovascular disease or traumatic injury (cardiac concussion or commotio cordis). We studied the clinical features of this apparently uncommon but important phenomenon. METHODS: We identified cases from the registries of relevant agencies and organizations, as well as newsmedia accounts, and developed a clinical profile of 25 children and young adults, 3 to 19 years of age. RESULTS: Each victim collapsed with cardiac arrest immediately after an unexpected blow to the chest, which was usually inflicted by a projectile (such as a baseball or hockey puck). Incidents took place during organized competitive sports in 16 cases and in recreational settings at home, at school, or on the playground in 9. In each instance, the impact to the chest was not judged to be extraordinary for the sport involved and did not appear to have sufficient force to cause death. Twelve victims collapsed virtually instantaneously on impact, whereas 13 remained conscious and physically active for a brief time before cardiac arrest. Cardiopulmonary resuscitation was administered within about three minutes to 19 victims, but normal cardiac rhythm could be restored in only 2 (both incurred irreversible brain damage and died shortly thereafter). Seven victims (28 percent) were wearing some form of protective chest padding. CONCLUSIONS: We speculate that most sudden deaths related to impact to the chest (not associated with traumatic injury) are due to ventricular dysrhythmia induced by an abrupt, blunt precordial blow, presumably delivered at an electrically vulnerable phase of ventricular excitability. This profile of blunt chest impact leading to cardiac arrest adds to our understanding of the range of causes of sudden death on the athletic field and may help in the development of preventive measures
SN - 0028-4793
AD - Cardiovascular Research Division, Minneapolis Heart Institute Foundation, MN 55407-3984, USA
UR - 7609749
ER -

TY - JOUR
ID - 1983
T1 - Renal effects in children living in the vicinity of a lead smelter
A1 - Bernard,A.M.
A1 - Vyskocil,A.
A1 - Roels,H.
A1 - Kriz,J.
A1 - Kodl,M.
A1 - Lauwerys,R.
Y1 - 1995/02//
N1 - Bernard, A M. Vyskocil, A. Roels, H. Kriz, J. Kodl, M. Lauwerys, R
Environmental research

ei2, 0147621
IM
Journal Article. Research Support, Non-U.S. Gov't
English
KW - MEDLINE
KW - Adolescent
KW - Age Distribution
KW - Analysis of Variance
KW - Chemical Industry
KW - Child
KW - Cross-Sectional Studies
KW - Environmental Exposure/ae [Adverse Effects]
KW - Female
KW - Humans
KW - Kidney/de [Drug Effects]
KW - Kidney/me [Metabolism]
KW - Lead/ae [Adverse Effects]
KW - Lead/bl [Blood]
KW - Lead/ur [Urine]
KW - Male
KW - Multivariate Analysis
KW - Sex Distribution
RP - NOT IN FILE
SP - 91
EP - 95
JF - Environmental Research
JA - Environ Res
VL - 68
IS - 2
CY - UNITED STATES
N2 - A cross-sectional study was carried out to determine whether environmental exposure of children to lead may cause renal effects. The study involved a total of 195 children aged 12 to 15 years. One hundred forty-four children (63 boys and 81 girls) were recruited from two schools in the vicinity of a lead smelter and 51 (25 boys and 26 girls) from a school in a rural area. Compared to their referents, boys and girls from the two schools in the polluted area had significantly higher levels of lead in blood (PbB) but similar levels of cadmium (CdB) and zinc protoporphyrins (ZPP). The functional integrity of the kidney was assessed by measuring the urinary excretion of beta 2-microglobulin, Clara cell protein, retinol-binding protein (RBP), albumin and beta-N-acetyl-D-glucosaminidase. The most significant and consistent finding of the study was that children from the two schools in the polluted area showed a significant elevation of the urinary excretion of RBP that paralleled the level of lead in blood or in the dust collected on the school playgrounds. A similar pattern was observed for the prevalence of elevated values of urinary RBP which increased from 3.9% in the control area up to 17% in the most polluted school. Urinary RBP was found to be associated with PbB (partial r2 = 0.046, P = 0.005) in a stepwise regression analysis testing also the influence of age, sex, CdB, and ZPP. In conclusion, the present study suggests that lead contaminating the environment may cause slight effects on the proximal tubule function in children at exposure levels close to those associated with CNS deficit
SN - 0013-9351
AD - Industrial Toxicology and Occupational Medicine Unit, University of Louvain, Brussels, Belgium
UR - 7601077
ER -

TY - JOUR
ID - 1984
T1 - Parietal lobe epilepsy. Clinical manifestations and outcome in 82 patients treated surgically between 1929 and 1988. [Review] [63 refs]
A1 - Salanova,V.
A1 - Andermann,F.
A1 - Rasmussen,T.

A1 - Olivier,A.
A1 - Quesney,L.F.
Y1 - 1995/06//
N1 - Salanova, V. Andermann, F. Rasmussen, T. Olivier, A. Quesney, L F
Brain : a journal of neurology
0372537, b5f
AIM, IM
Journal Article. Review
English
KW - MEDLINE
KW - Adolescent
KW - Adult
KW - Brain Damage
KW - Chronic/et [Etiology]
KW - Child
KW - Preschool
KW - Electric Stimulation
KW - Electroencephalography
KW - Epilepsies
KW - Partial/co [Complications]
KW - Partial/di [Diagnosis]
KW - Partial/ep [Epidemiology]
KW - Partial/su [Surgery]
KW - Hallucinations/et [Etiology]
KW - Humans
KW - Infant
KW - Kindling
KW - Neurologic
KW - Pain/et [Etiology]
KW - Paresthesia/et [Etiology]
KW - Parietal Lobe/pp [Physiopathology]
KW - Parietal Lobe/su [Surgery]
KW - Parietal Lobe
KW - Quebec/ep [Epidemiology]
KW - Retrospective Studies
KW - Treatment Outcome
RP - NOT IN FILE
SP - 607
EP - 627
JF - Brain
VL - 118
IS - Pt 3
CY - ENGLAND
N2 - We report the clinical manifestations and outcome of 82 patients with nontumoural parietal lobe epilepsy treated surgically at the Montreal Neurological Institute between 1929 and 1988. Patients with extensive resections extending outside the parietal lobe were excluded. Ninety-four percent exhibited aurae: the most common were somatosensory, described by 52 patients; 13 of these also described pain. Other aurae included disturbances of body image, visual illusions, vertiginous sensations and aphasia or dysphasia. A few patients exhibited complex visual or auditory hallucinations and elementary visual hallucinations. Intraoperative cortical stimulation reproduced the habitual aurae in 44 patients. Often the clinical manifestations indicated ictal spread to the frontal, supplementary motor area, or temporo-limbic areas: 28% of patients exhibited tonic posturing of the extremities, 57% unilateral clonic activity, 17% oral or gestural automatisms and 4% complex automatisms. Sixty-one percent of patients with tonic posturing had epileptogenic zones which included the superior parietal lobe, and in 79% of patients with automatisms the epileptogenic zones extended to the inferior parietal lobe, suggesting different spread patterns. Forty-three patients underwent right, and 39 left parietal corticectomies. Postoperative sensory deficits were seen only when the corticectomy extended into the post-central gyrus. Early in the series extensive nondominant inferior parietal resections led to disturbances of body image in a few

patients. Follow-up ranging from 2 to 50 years was available for 79 patients. Sixty-five percent had a complete or nearly complete cessation of seizures. Those patients with no post-resection electrocorticographic epileptiform discharges had a more favourable outcome. [References: 63]
SN - 0006-8950
AD - Department of Neurology and Neurosurgery, McGill University, Montreal, Canada
UR - 7600082
ER -

TY - JOUR
ID - 1985
T1 - Dietary calcium, saturated fat, fiber and vitamin C as predictors of forearm cortical and trabecular bone mineral density in healthy children and adolescents
A1 - Gunnes,M.
A1 - Lehmann,E.H.
Y1 - 1995/04//
N1 - Gunnes, M. Lehmann, E H
Acta paediatrica (Oslo, Norway : 1992)
bgc, 9205968
IM
Journal Article. Research Support, Non-U.S. Gov't
English
KW - MEDLINE
KW - Absorptiometry
KW - Photon
KW - Adolescent
KW - Ascorbic Acid/ad [Administration & Dosage]
KW - Bone Density
KW - Calcium
KW - Dietary/ad [Administration & Dosage]
KW - Child
KW - Dietary Fats/ad [Administration & Dosage]
KW - Dietary Fiber/ad [Administration & Dosage]
KW - Female
KW - Forearm
KW - Humans
KW - Male
KW - Questionnaires
RP - NOT IN FILE
SP - 388
EP - 392
JF - Acta Paediatrica
JA - Acta Paediatr
VL - 84
IS - 4
CY - NORWAY
N2 - Diet, weight-bearing physical activity (WPA) and daylight hours spent outdoors (DE) were related to bone mineral density in the distal (BMDd) and ultradistal (BMDud) forearm of 495 healthy children (8-11 years) and adolescents (11-17 years). BMD was assessed by single photon absorptiometry. Using multiple regression, BMDd was predicted by saturated fat and BMDud by fiber in children. In adolescents, BMDd was predicted by saturated fat and vitamin C, and BMDud by calcium and vitamin C. WPA and DE were not related to BMD. In conclusion, our results showed that current intake of calcium, saturated fat, fiber and vitamin C were positively associated with forearm BMD in children and adolescents. These associations depended on age and bone type. If our findings are verified, dietary recommendations, especially when aimed at young people, may have to be reconsidered
SN - 0803-5253
AD - Cecor AS, Haugesund, Norway
UR - 7795347

ER -

TY - JOUR
ID - 1986
T1 - [Relationship between daily high temperature and mortality in Kyushu, Japan]. [Japanese]
A1 - Honda,Y.
A1 - Ono,M.
A1 - Sasaki,A.
A1 - Uchiyama,I.
Y1 - 1995/04//
N1 - Honda, Y. Ono, M. Sasaki, A. Uchiyama, I
[Nihon koshu eisei zasshi] Japanese journal of public health
19130150r, a9j
IM
English Abstract. Journal Article
Japanese
KW - MEDLINE
KW - Adolescent
KW - Adult
KW - Aged
KW - Cause of Death
KW - Child
KW - Preschool
KW - Female
KW - Humans
KW - Infant
KW - Newborn
KW - Japan
KW - Male
KW - Middle Aged
KW - Mortality
KW - Temperature
RP - NOT IN FILE
SP - 260
EP - 268
JF - Nippon Koshu Eisei Zasshi - Japanese Journal of Public Health
JA - Nippon Koshu Eisei Zasshi
VL - 42
IS - 4
CY - JAPAN
N2 - We analyzed the relationship between daily high temperature and mortality in Kyushu, Japan, 1972-1990. Mortality rate was obtained by dividing the number of deaths in a certain daily high temperature category by person-days in the same category. The results were as follows: (1) With daily high temperature on the x-axis and mortality rate on the y-axis, the relationship between the two variables appeared to be V-shaped. The minimum mortality rate was observed at the 28 degrees C-33 degrees C level. (2) Among all deaths at 33+ degrees C level, the proportion of deaths from "excessive heat" never exceeded 0.4%. This suggested that heat stroke is not a major contributor to the V-shape formation. (3) The V-shape was observed even in 1990, suggesting that the rapid spread of air conditioners did not alter the relationship between daily high temperature and mortality rate. (4) For the 0-4 years age group, mortality was not temperature dependent. However, the 5-14 years age group showed high mortality rates at 33+ degrees C. This may be attributed to outdoor activities during summer. After 30 years of age, the overall mortality rates rose with age, with greater increases at lower ambient temperature levels. As a result, the 65+ years age group had the greatest temperature dependence. (5) Deaths from respiratory diseases, circulatory diseases, and senility were highly temperature dependent, whereas death from cancer was not temperature dependent. Deaths from digestive diseases, infectious diseases, and accidents showed moderate temperature dependence
SN - 0546-1766
UR - 7780169

ER -

TY - JOUR
ID - 1987
T1 - Mercury exposure in a residential community--Florida, 1994
A1 - Centers for Disease Control and Prevention (CDC)
Y1 - 1994/09/16/
N1 - Centers for Disease Control and Prevention (CDC)
MMWR. Morbidity and mortality weekly report
ne8, 7802429
IM
Journal Article
English
KW - MEDLINE
KW - Adult
KW - Child
KW - Environmental Exposure
KW - Environmental Monitoring
KW - Epidemiological Monitoring
KW - Florida/ep [Epidemiology]
KW - Housing
KW - Humans
KW - Mercury
KW - Mercury Poisoning/di [Diagnosis]
KW - Mercury Poisoning/ep [Epidemiology]
KW - Schools
RP - NOT IN FILE
SP - 436
EP - 437
JF - MMWR - Morbidity & Mortality Weekly Report
JA - MMWR Morb Mortal Wkly Rep
VL - 44
IS - 23
CY - UNITED STATES
N2 - Residential exposure to elemental mercury typically involves small amounts (e.g., the approximately 0.3 mL in a thermometer). During August 1994, five children residing in a neighborhood in Palm Beach County, Florida, found 5 pints of elemental mercury in an abandoned van. During the ensuing 25 days, the children shared and played with the mercury outdoors, inside homes, and at local schools. On August 25, 1994, a parent notified local police and fire authorities that her children had brought mercury into the home. On the same day, 50 homes were immediately vacated and an assessment of environmental and health impacts was initiated by the State of Florida Department of Environmental Protection, the Health and Rehabilitative Services of the Palm Beach County Public Health Unit, and the U.S. Environmental Protection Agency. This report summarizes the investigation of this incident.(ABSTRACT TRUNCATED AT 250 WORDS)
SN - 0149-2195
UR - 7770028
ER -

TY - JOUR
ID - 1988
T1 - Hypothesis: the risk of childhood leukemia is related to combinations of power-frequency and static magnetic fields
A1 - Bowman,J.D.
A1 - Thomas,D.C.
A1 - London,S.J.
A1 - Peters,J.M.
Y1 - 1995///

N1 - Bowman, J D. Thomas, D C. London, S J. Peters, J M
Bioelectromagnetics
9z7, 8008281
IM
Comparative Study. Journal Article. Research Support, Non-U.S. Gov't. Research Support, U.S. Gov't, P.H.S.
English
KW - MEDLINE
KW - Calcium/me [Metabolism]
KW - Calcium/re [Radiation Effects]
KW - Case-Control Studies
KW - Cell Movement/re [Radiation Effects]
KW - Child
KW - Preschool
KW - Diatoms/re [Radiation Effects]
KW - Electromagnetic Fields/ae [Adverse Effects]
KW - Environmental Exposure
KW - Housing
KW - Humans
KW - Infant
KW - Newborn
KW - Leukemia/ep [Epidemiology]
KW - Leukemia
KW - Radiation-Induced/ep [Epidemiology]
KW - Los Angeles/ep [Epidemiology]
KW - Magnetics/ae [Adverse Effects]
KW - Models
KW - Theoretical
KW - Registries
KW - Risk Factors
RP - NOT IN FILE
SP - 48
EP - 59
JF - Bioelectromagnetics
VL - 16
IS - 1
CY - UNITED STATES
N2 - We present a hypothesis that the risk of childhood leukemia is related to exposure to specific combinations of static and extremely-low-frequency (ELF) magnetic fields. Laboratory data from calcium efflux and diatom mobility experiments were used with the gyromagnetic equation to predict combinations of 60 Hz and static magnetic fields hypothesized to enhance leukemia risk. The laboratory data predicted 19 bands of the static field magnitude with a bandwidth of 9.1 microT that, together with 60 Hz magnetic fields, are expected to have biological activity. We then assessed the association between this exposure metric and childhood leukemia using data from a case-control study in Los Angeles County. ELF and static magnetic fields were measured in the bedrooms of 124 cases determined from a tumor registry and 99 controls drawn from friends and random digit dialing. Among these subjects, 26 cases and 20 controls were exposed to static magnetic fields lying in the predicted bands of biological activity centered at 38.0 microT and 50.6 microT. Although no association was found for childhood leukemia in relation to measured ELF or static magnetic fields alone, an increasing trend of leukemia risk with measured ELF fields was found for subjects within these static field bands (P for trend = 0.041). The odds ratio (OR) was 3.3 [95% confidence interval (CI) = 0.4-30.5] for subjects exposed to static fields within the derived bands and to ELF magnetic field above 0.30 microT (compared to subjects exposed to static fields outside the bands and ELF magnetic fields below 0.07 microT). When the 60 Hz magnetic fields were assessed according to the Wertheimer-Leeper code for wiring configurations, leukemia risks were again greater with the hypothesized exposure conditions (OR = 9.2 for very high current configurations within the static field bands; 95% CI = 1.3-64.6). Although the risk estimates are based on limited magnetic field measurements for a small number of subjects, these findings suggest that the risk of childhood leukemia may be related to the combined effects of the static and ELF magnetic fields. Further tests of the hypothesis are proposed

SN - 0197-8462
AD - Department of Preventive Medicine, University of Southern California, Los Angeles, USA
UR - 7748203
ER -

TY - JOUR
ID - 1989
T1 - Child fostering and children's nutritional outcomes in rural Mali: the role of female status in directing child transfers
A1 - Castle,S.E.
Y1 - 1995/03//
N1 - Castle, S E
Social science & medicine (1982)
ut9, 8303205
IM
Comparative Study. Journal Article. Research Support, Non-U.S. Gov't
English
KW - MEDLINE
KW - Adolescent
KW - Adult
KW - Child
KW - Child Welfare
KW - Preschool
KW - Female
KW - Foster Home Care
KW - Humans
KW - Infant
KW - Newborn
KW - Male
KW - Mali
KW - Middle Aged
KW - Nutritional Status
KW - Rural Population
KW - Socioeconomic Factors
KW - Women
KW - Women's Health
RP - NOT IN FILE
SP - 679
EP - 693
JF - Social Science & Medicine
JA - Soc Sci Med
VL - 40
IS - 5
CY - ENGLAND
N2 - Research in West Africa has begun to document the phenomenon of child fostering although little attention has focussed on other types of non-maternal child care arrangements and their impact on child health. Evidence from a sample of 77 weaned children under five in rural Mali found that over one third (35%) of children were not the prime responsibility of both their biological parents. Nineteen per cent (N = 15) of the sample were formally fostered children, known as sukaabe bambaabe, who lived neither with their biological mothers nor with their biological fathers. Others lived under flexible or semi-permanent non-maternal care arrangements both within and outside the agnatic family. Factors precipitating fostering are outlined and are divided into: (i) those under which the child is fostered away from its biological family through force of circumstance; and (ii) those under which the child is actively requested by its foster mother. Rather than fostering providing a universal option for over-burdened mothers with too many or too closely-spaced children, the field evidence shows that the movement of children within and between households is rigorously controlled by the female social hierarchy. Children are transferred in a uni-directional fashion from the care of their low status biological mothers to high status foster mothers. Their transfer serves to visibly reinforce socio-political

power differentials between these women who are at different stages of the female life-cycle and in different sets of household circumstances. Economic factors are not associated with the decision to keep the child or to foster it away, but do appear to determine whether the children living under non-maternal care are fostered out under a structured or unstructured fostering arrangement. Contrary to findings in other West African settings, fostering per se has little impact on children's nutritional outcomes as measured by their weight-for-age Z-Scores, probably because the use of surrogate or sibling caretakers is common in this environment even for children who are the full responsibility of their biological mothers. The context of the fostering, however, does influence fostered children's nutritional status. Those children who were requested by their foster mothers had better nutritional outcomes than those who were fostered in by force of circumstances. It is concluded that the concept of 'maternal and child health' and the 'maternal-child' dyad around which both health interventions and demographic evaluations are centred, may not always be appropriate in areas of high fostering prevalence
SN - 0277-9536
AD - Population Studies and Training Center, Brown University, Providence, RI 02912, USA
UR - 7747203
ER -

TY - JOUR
ID - 1990
T1 - Assessment of ozone exposures in the greater metropolitan Toronto area
A1 - Liu,L.J.
A1 - Koutrakis,P.
A1 - Leech,J.
A1 - Broder,I.
Y1 - 1995/04//
N1 - Liu, L J. Koutrakis, P. Leech, J. Broder, I
Journal of the Air & Waste Management Association (1995)
9503111, cbi
IM
Journal Article. Research Support, Non-U.S. Gov't
English
KW - MEDLINE
KW - Adolescent
KW - Adult
KW - Air Pollutants
KW - Occupational/an [Analysis]
KW - Child
KW - Preschool
KW - Environmental Exposure
KW - Environmental Monitoring
KW - Humans
KW - Middle Aged
KW - Ontario
KW - Ozone/an [Analysis]
RP - NOT IN FILE
SP - 223
EP - 234
JF - Journal of the Air & Waste Management Association
JA - J Air Waste Manag Assoc
VL - 45
IS - 4
CY - UNITED STATES
N2 - An ozone (O3) exposure assessment study was conducted in Toronto, Ontario, Canada during the winter and summer of 1992. A new passive O3 sampler developed by Harvard was used to measure indoor, outdoor, and personal O3 concentrations. Measurements were taken weekly and daily during the winter and summer, respectively. Indoor samples were collected at a total of 50 homes and workplaces of study participants. Outdoor O3 concentrations were measured both at home sites using the passive sampler and at 20 ambient monitoring sites with continuous monitors. Personal O3 measurements were collected from 123 participants,

who also completed detailed time-activity diaries. A total of 2,274 O3 samples were collected. In addition, weekly air exchange rates of homes were measured
SN - 1096-2247
AD - Harvard University, School of Public Health, Boston, Massachusetts, USA
UR - 7743405
ER -

TY - JOUR
ID - 1991
T1 - Unintentional perineal injury in prepubescent girls: a multicenter, prospective report of 56 girls
A1 - Bond,G.R.
A1 - Dowd,M.D.
A1 - Landsman,I.
A1 - Rimsza,M.
Y1 - 1995/05//
N1 - Bond, G R. Dowd, M D. Landsman, I. Rimsza, M
Pediatrics
oxv, 0376422
AIM, IM
Journal Article. Multicenter Study
English
KW - MEDLINE
KW - Accidents
KW - Bicycling/in [Injuries]
KW - Child
KW - Child Abuse
KW - Sexual/di [Diagnosis]
KW - Preschool
KW - Female
KW - Humans
KW - Hymen/in [Injuries]
KW - Infant
KW - Perineum/in [Injuries]
KW - Play and Playthings
KW - Prospective Studies
KW - Vulva/in [Injuries]
KW - Wounds and Injuries/et [Etiology]
RP - NOT IN FILE
SP - 628
EP - 631
JF - Pediatrics
VL - 95
IS - 5
CY - UNITED STATES
N2 - OBJECTIVE: To determine the pattern of perineal injuries and frequency of hymenal involvement resulting from unintentional trauma in prepubescent girls. DESIGN: Prospective, multicenter. Observation by skilled observers. Determination of the circumstance and physical pattern of injury, with specific attention to the hymen. SETTING: Children's emergency department or acute care clinic. PATIENTS: Tanner stage 1 girls presenting with acute perineal injury. Criteria for unintentional injury: observation of the event or knowledge of the girl's engagement in a risky activity (eg, biking or climbing monkey bars) immediately before the injury. RESULTS: Fifty-six girls were evaluated. Age range: 1 to 12 years (median, 6 years; mean, 6.2 years). Associations: bicycle, 39%; other outdoor injuries, 25% (climbing apparatus, straddling an object, and falls); indoor injuries, 36% (straddling furniture and falls). Most injuries were minor. In each group the labia minora was the most frequent structure involved. The majority of injuries were anterior or lateral to the hymen. However, in 34% some or all of the injuries were posterior to the hymen. Thigh injuries were observed only in older children engaged in bicycle riding or outdoor play. In only one patient was the hymen involved. That patient was a 2 year old who fell outdoors, at a park, abducting her legs in a splits-type mechanism. She had a

pinpoint abraded area on the hymenal surface at three o'clock. Otherwise, no unique pattern of injury was associated with age or circumstance of injury. CONCLUSIONS: Hymenal injuries are rarely the result of unintentional injury. The presence of a hymenal injury should suggest sexual abuse. Involvement of other perineal structures was commonly associated with unintended injury. Given the limited resources for prevention, the relative infrequency of perineal injuries and the minor nature of most of these injuries, significant preventive efforts are not justified
SN - 0031-4005
AD - Department of Pediatrics, University of Virginia, Charlottesville 22908, USA
UR - 7724296
ER -

TY - JOUR
ID - 1992
T1 - Childhood burns in Ghana: epidemiological characteristics and home-based treatment
A1 - Forjuoh,S.N.
A1 - Guyer,B.
A1 - Smith,G.S.
Y1 - 1995/02//
N1 - Forjuoh, S N. Guyer, B. Smith, G S
Burns : journal of the International Society for Burn Injuries
afc, 8913178
IM, J
Journal Article. Research Support, Non-U.S. Gov't
English
KW - MEDLINE
KW - Age Distribution
KW - Burns/ep [Epidemiology]
KW - Burns/et [Etiology]
KW - Burns/pp [Physiopathology]
KW - Burns/th [Therapy]
KW - Child
KW - Preschool
KW - Data Collection
KW - Female
KW - Ghana/ep [Epidemiology]
KW - Health Services/ut [Utilization]
KW - Home Nursing/sn [Statistics & Numerical Data]
KW - Humans
KW - Incidence
KW - Infant
KW - Newborn
KW - Male
KW - Sex Distribution
RP - NOT IN FILE
SP - 24
EP - 28
JF - Burns
VL - 21
IS - 1
CY - ENGLAND
N2 - The objectives of this research were to study the epidemiological characteristics and home-based treatment of childhood burns in the Ashanti Region of Ghana. Children aged 0-5 years with a burn history were identified through a community-based, multisite survey. A standard questionnaire was administered to mothers of 630 of these children to elicit information on their sociodemographic characteristics and the circumstances of the burn event. Ninety-two per cent of the burns occurred in the home, particularly in the kitchen (51 per cent) and the house yard (36 per cent), with most of them happening in the late morning and around the evening meal. The main causes of the burns were scalds (45 per cent), contact with a hot object (34 per cent) and flame (20 per

cent). 'Cool' water was applied to the burned area in 30 per cent of cases. Otherwise, treatment with a traditional preparation was the most popular first-aid choice. Since a considerable proportion of burns happened between meals when children 'play with fire' in the house yard, the provision of alternative play activities and community play areas may reduce the incidence of burns to these children. Secondly, we recommend that education on first-aid management of burns be intensified, with special emphasis on alternatives to the use of traditional preparations
SN - 0305-4179
AD - Department of Maternal and Child Health, John Hopkins School of Hygiene and Public Health, Baltimore, Maryland, USA
UR - 7718113
ER -

TY - JOUR
ID - 1993
T1 - Community infection ratio as an indicator for tuberculosis control
A1 - Madico,G.
A1 - Gilman,R.H.
A1 - Checkley,W.
A1 - Cabrera,L.
A1 - Kohlstadt,I.
A1 - Kacena,K.
A1 - Diaz,J.F.
A1 - Black,R.
Y1 - 1995/02/18/
N1 - Madico, G. Gilman, R H. Checkley, W. Cabrera, L. Kohlstadt, I. Kacena, K. Diaz, J F. Black, R
Lancet
2985213r, l0s, 0053266
AIM, IM
Journal Article. Research Support, Non-U.S. Gov't
English
KW - MEDLINE
KW - Adolescent
KW - Age Factors
KW - Case-Control Studies
KW - Child
KW - Preschool
KW - Communicable Disease Control/ec [Economics]
KW - Community-Acquired Infections/ep [Epidemiology]
KW - Contact Tracing
KW - Developing Countries
KW - Female
KW - Humans
KW - Infant
KW - Male
KW - Odds Ratio
KW - Peru/ep [Epidemiology]
KW - Poverty Areas
KW - Prevalence
KW - Tuberculosis
KW - Pulmonary/ep [Epidemiology]
KW - Pulmonary/pc [Prevention & Control]
KW - Pulmonary/tm [Transmission]
KW - Urban Health
RP - NOT IN FILE
SP - 416
EP - 419
JF - Lancet

VL - 345
IS - 8947
CY - ENGLAND
N2 - The epidemiology of tuberculosis remains poorly understood. We investigated the relative importance of within-household and community transmission of infection among children aged 6 months to 14 years living in a Peruvian shanty-town. The prevalence of Mycobacterium tuberculosis exposure among 175 contact children (sharing a household with a person who had confirmed pulmonary tuberculosis) and 382 control children (living in nearby households free of active tuberculosis) was defined as the proportion of children with a positive purified protein derivative (PPD) skin-test. 97 (55%) contact children and 129 (34%) controls were PPD positive. Living in a contact household (odds ratio 1.74, 95% CI 1.11-2.73) and age (1.11, 1.06-1.18) were significant risk factors for PPD positivity. We calculated the community infection ratio (CIR) as the odds ratio of PPD-positive controls to PPD-positive contacts: CIR = [formula: see text] A low CIR therefore suggests mainly household spread of infection, whereas a high value suggests frequent transmission outside the household. The adjusted odds ratio (for age, sex, within-household correlation, and household size) was 0.40 (95% CI 0.26-0.64), compared with values of 0.18-0.37 in studies elsewhere. Currently recommended tuberculosis control strategies are suitable for areas with low CIRs. Different strategies may be needed for areas, such as that we studied, with high values
SN - 0140-6736
AD - AB Prisma, Lima, Peru
UR - 7853950
ER -

TY - JOUR
ID - 1994
T1 - Bioenergetics of skeletal muscle in mitochondrial myopathy
A1 - Taylor,D.J.
A1 - Kemp,G.J.
A1 - Radda,G.K.
Y1 - 1994/12/20/
N1 - Taylor, D J. Kemp, G J. Radda, G K
Journal of the neurological sciences
jbj, 0375403
IM
Clinical Trial. Journal Article
English
KW - MEDLINE
KW - Adenosine Diphosphate/me [Metabolism]
KW - Adenosine Triphosphate/me [Metabolism]
KW - Adolescent
KW - Adult
KW - Aged
KW - 80 and over
KW - Child
KW - DNA
KW - Mitochondrial/me [Metabolism]
KW - Energy Metabolism/ph [Physiology]
KW - Exercise/ph [Physiology]
KW - Female
KW - Humans
KW - Hydrogen-Ion Concentration
KW - Magnetic Resonance Imaging
KW - Male
KW - Middle Aged
KW - Mitochondrial Myopathies/me [Metabolism]
KW - Muscle
KW - Skeletal/me [Metabolism]
KW - Ophthalmoplegia

KW - Chronic Progressive External/me [Metabolism]
KW - Phosphates/me [Metabolism]
KW - Phosphocreatine/me [Metabolism]
RP - NOT IN FILE
SP - 198
EP - 206
JF - Journal of the Neurological Sciences
JA - J Neurol Sci
VL - 127
IS - 2
CY - NETHERLANDS
N2 - 31Phosphorus nuclear magnetic resonance spectroscopy was used to examine skeletal muscle in 29 patients with mitochondrial myopathy, 9 male and 20 female. Gastrocnemius was investigated in 15 patients and 30 normal subjects and finger flexor muscle (flexor digitorum superficialis, fds) in 24 patients and 35 normal controls. Both muscles were studied in 10 of the patients. Results were abnormal (outside the full range of normal values) in all but 2 patients. In 86% of patients (25/29) abnormalities were detected in resting muscle. In most cases there was a low phosphocreatine/ATP ratio, high calculated free [ADP] and low phosphorylation potential. At rest, abnormality was detected with equal ease in fds and gastrocnemius. Exercise and recovery increased the sensitivity of MRS in detecting abnormal metabolism. Finger flexion was better tolerated by patients than plantar flexion and gave bigger changes in metabolite concentrations and intracellular pH. Thus, results from fds were more easily differentiated from normal. Exercise duration was significantly shorter than in controls while phosphocreatine depletion was more rapid than normal, consistent with a shortfall in mitochondrial ATP synthesis. Nearly all patients (25/27, 93%) showed abnormalities during recovery from exercise. [ADP] was high during exercise and its recovery was delayed, providing increased drive for oxidative phosphorylation. Phosphocreatine resynthesis during recovery (which reflects oxidative ATP synthesis) was slow both in absolute terms and in relation to [ADP]. Recovery of intracellular pH after exercise was significantly more rapid than normal, consistent with an upregulation of proton efflux.(ABSTRACT TRUNCATED AT 250 WORDS)
SN - 0022-510X
AD - MRC Biochemical and Clinical Magnetic Resonance Unit, John Radcliffe Hospital, Oxford, UK
UR - 7707079
ER -

TY - JOUR
ID - 1995
T1 - A move from hospital to community-based homes for people with learning disabilities: activities outside the home
A1 - Dagnan,D.
A1 - Howard,B.
A1 - Drewett,R.F.
Y1 - 1994/12//
N1 - Dagnan, D. Howard, B. Drewett, R F
Journal of intellectual disability research : JIDR
9206090, bga
IM
Clinical Trial. Controlled Clinical Trial. Journal Article. Research Support, Non-U.S. Gov't
English
KW - MEDLINE
KW - Activities of Daily Living/px [Psychology]
KW - Adult
KW - Child
KW - Deinstitutionalization
KW - Female
KW - Group Homes
KW - Humans
KW - Intellectual Disability/px [Psychology]
KW - Intellectual Disability/rh [Rehabilitation]

KW - Interpersonal Relations
KW - Middle Aged
KW - Social Behavior
KW - Social Environment
RP - NOT IN FILE
SP - 567
EP - 576
JF - Journal of Intellectual Disability Research
JA - J Intellect Disabil Res
VL - 38
IS - Pt 6
CY - ENGLAND
N2 - Eighteen people with learning disabilities who moved from hospital to community-based homes were compared with a matched group of 18 who did not. A week-long time budget diary was used to record each occasion that the person left their home, the people accompanying them on each trip, their mode of transport and their destination. The diary was completed before they left the hospital and was repeated at least 1 year later. There was a strong correlation between the number of trips made from hospital wards at baseline and the number made at follow-up in both groups. Moving from the hospital had no significant effect on the number of trips made, but it did change their nature. Movers made more trips to unsegregated facilities; they made fewer trips alone and more in groups which included both staff and residents
SN - 0964-2633
AD - Learning Difficulties Research Group, School of Psychology, University of Birmingham
UR - 7881228
ER -

TY - JOUR
ID - 1996
T1 - The absence of gamma-interferon production of S. mansoni antigens in patients with schistosomiasis
A1 - Araujo,M.I.
A1 - Bacellar,O.
A1 - Ribeiro-de-Jesus,A.
A1 - Carvalho,E.M.
Y1 - 1994/07//
N1 - Araujo, M I. Bacellar, O. Ribeiro-de-Jesus, A. Carvalho, E M
Brazilian journal of medical and biological research = Revista brasileira de pesquisas medicas e biologicas / Sociedade Brasileira de Biofisica ... [et al.]
bof, 8112917
IM
Journal Article. Research Support, Non-U.S. Gov't. Research Support, U.S. Gov't, P.H.S.
English
KW - MEDLINE
KW - Adolescent
KW - Adult
KW - Animals
KW - Antigens
KW - Helminth/im [Immunology]
KW - Cells
KW - Cultured
KW - Child
KW - Female
KW - Humans
KW - Interferon-gamma/bi [Biosynthesis]
KW - Interferon-gamma/ph [Physiology]
KW - Lymphocyte Activation
KW - Male
KW - Middle Aged
KW - Schistosoma mansoni/im [Immunology]

KW - Schistosomiasis mansoni/im [Immunology]
KW - T-Lymphocytes/ph [Physiology]
RP - NOT IN FILE
SP - 1619
EP - 1625
JF - Brazilian Journal of Medical & Biological Research
JA - Braz J Med Biol Res
VL - 27
IS - 7
CY - BRAZIL
N2 - No gamma-interferon production was observed in peripheral blood mononuclear cells (PBMC) cultures from 45 patients living in an endemic area of schistosomiasis in Brazil following in vitro stimulation with schistosomula or adult worm antigens from Schistosoma mansoni (4.9 +/- 24 and 1.0 +/- 3.4 pg/ml, respectively). This immunological abnormality was observed in patients both with a high degree of infection (> or = 400 eggs/g feces) and with a low degree of infection (< 400 eggs/g feces), and was independent of the degree of natural exposure to infection. This absence of gamma-interferon production was antigen specific since high levels of this cytokine were detected in the same patients when their cells were stimulated with PPD (247 +/- 179 pg/ml) or PHA (408 +/- 328 pg/ml). In two of four subjects cured of a previous S. mansoni infection and currently living outside the endemic area, gamma-IFN was produced when their PBMC were stimulated with adult worm antigen (75 +/- 2.5 pg/ml)
SN - 0100-879X
AD - Servico de Immunologia, Hospital Universitario Prof. Edgard Santos, Universidade Federal da Bahia, Salvador, Brasil
UR - 7874028
ER -

TY - JOUR
ID - 1997
T1 - Pressor reactivity, ethnicity, and 24-hour ambulatory monitoring in children from hypertensive families
A1 - Treiber,F.A.
A1 - Murphy,J.K.
A1 - Davis,H.
A1 - Raunikar,R.A.
A1 - Pflieger,K.
A1 - Strong,W.B.
Y1 - 1994///
N1 - Treiber, F A. Murphy, J K. Davis, H. Raunikar, R A. Pflieger, K. Strong, W B
Behavioral medicine (Washington, D.C.)
beh, 8804264
IM
Comparative Study. Journal Article. Research Support, U.S. Gov't, P.H.S.
English
KW - MEDLINE
KW - Adolescent
KW - African Americans
KW - African Continental Ancestry Group
KW - Analysis of Variance
KW - Blood Pressure
KW - Blood Pressure Monitoring
KW - Ambulatory
KW - Child
KW - Diastole
KW - European Continental Ancestry Group
KW - Exercise Test
KW - Family Health
KW - Follow-Up Studies
KW - Heart Function Tests

KW - Humans
KW - Hypertension/ep [Epidemiology]
KW - Hypertension/pc [Prevention & Control]
KW - Play and Playthings
KW - Reference Values
KW - Systole
KW - Time Factors
RP - NOT IN FILE
SP - 133
EP - 142
JF - Behavioral Medicine
JA - Behav Med
VL - 20
IS - 3
CY - UNITED STATES
N2 - We assessed blood pressure responses of a multiethnic (Black and White) sample of 120 children of hypertensive families to orthostasis, video game, forehead cold, and dynamic exercise, and monitored the children's ambulatory pressure 24 hours later. Thirteen children were studied twice (1-year stability). The Black children exhibited higher 24-hour ambulatory systolic and diastolic pressures than the White children. Regardless of ethnicity, peak and mean systolic pressures during each task were generally positively correlated with mean systolic pressure while the children were awake and asleep. Associations between diastolic pressor responses and ambulatory measurements were somewhat dependent upon ethnicity and task. Relatively few reactivity-ambulatory correlations were significant, using pressor reactivity change scores. The children who participated twice exhibited significant 1-year stability for most ambulatory and pressor measurements. Children's pressor responses to laboratory tasks may generalize to the natural environment
SN - 0896-4289
AD - Department of Pediatrics, Medical College of Georgia in Augusta
UR - 7865933
ER -

TY - JOUR
ID - 1998
T1 - Neuroblastomas in Denmark 1943-80. Epidemiological and clinical studies. [Review] [204 refs]
A1 - Carlsen,N.L.
Y1 - 1994/10//
N1 - Carlsen, N L
Acta paediatrica (Oslo, Norway : 1992). Supplement
bd5, 9315043, 14540280r
IM
Journal Article. Research Support, Non-U.S. Gov't. Review
English
KW - MEDLINE
KW - Adolescent
KW - Age Distribution
KW - Child
KW - Preschool
KW - Denmark/ep [Epidemiology]
KW - Female
KW - Humans
KW - Incidence
KW - Infant
KW - Male
KW - Neoplasm Staging
KW - Neuroblastoma/ep [Epidemiology]
KW - Neuroblastoma/pa [Pathology]
KW - Neuroblastoma/th [Therapy]
KW - Prognosis

KW - Retrospective Studies
KW - Socioeconomic Factors
RP - NOT IN FILE
SP - 1
EP - 27
JF - Acta Paediatrica Supplement
JA - Acta Paediatr Suppl
VL - 403
CY - NORWAY
N2 - Two-hundred-and-fifty patients were registered in a population-based study of neuroblastomas in Denmark in the period 1943-80. The major epidemiological findings were an increased incidence with an unchanged mortality rate during the study period. The increase in incidence related solely to children 0-4 years of age and was most pronounced in infants under 1 year of age. Several reasons for the observed epidemiological rates include (i) changes in the composition of the population, (ii) improved diagnostic procedures, (iii) a shift in the diagnostic criteria, and (iv) an increase in environmental carcinogens of importance in the induction of neuroblastoma. Associated with an increased risk were lower socio-economic levels and young or advanced parental age, suggesting the importance of environmental as well as genetic factors for the induction of neuroblastoma. The epidemiological findings of an increased incidence with an unchanged mortality rate, which suggests the inclusion of borderline lesions in recent years, are of major importance in interpreting the results of mass screening for the disease. The clinical findings in 253 patients treated in Denmark from 1943 to 1980 (including 5 patients resident outside Denmark and excluding 2 patients without available hospital records) were analysed. The major finding was a zero-time shift, that is, earlier diagnosis of the disease during the study period, with increasing survival rates from decade to decade mostly due to a better age and stage distribution, a zero-time shift which was also apparent in the changes of the symptomatology. Independent prognostic variables were age, stage, and treatment with chemotherapy for children over 1 year of age with stage II disease, and for infants with stages III-IV disease. Analysis of data from a subset of the 253 children suggested that high proliferative activity detected by flow cytometry may be an important prognostic variable. The study also suggests that the pattern of metastatic spread might have changed as a consequence of prolonged survival obtained by improved treatment modalities, stressing the importance of a high frequency of autopsy among cancer patients. Hypotheses generated by the study included (i) that most neuroblastomas might be congenital, (ii) that the age influence on prognosis might be explained in terms of growth rate, cell cycle transit time, and duration of the disease, and (iii) that some neuroblastomas might be borderline lesions. [References: 204]
SN - 0803-5326
UR - 7841631
ER -

TY - JOUR
ID - 1999
T1 - Traumatically ruptured globes in children
A1 - Rudd,J.C.
A1 - Jaeger,E.A.
A1 - Freitag,S.K.
A1 - Jeffers,J.B.
Y1 - 1994/09//
N1 - Rudd, J C. Jaeger, E A. Freitag, S K. Jeffers, J B
Journal of pediatric ophthalmology and strabismus
jmi, 7901143
IM
Journal Article
English
KW - MEDLINE
KW - Adolescent
KW - Child
KW - Preschool
KW - Eye Injuries/co [Complications]
KW - Eye Injuries/su [Surgery]

KW - Eye Injuries
KW - Penetrating/co [Complications]
KW - Penetrating/su [Surgery]
KW - Female
KW - Humans
KW - Infant
KW - Male
KW - Prognosis
KW - Retrospective Studies
KW - Risk Factors
KW - Rupture
KW - Visual Acuity
RP - NOT IN FILE
SP - 307
EP - 311
JF - Journal of Pediatric Ophthalmology & Strabismus
JA - J Pediatr Ophthalmol Strabismus
VL - 31
IS - 5
CY - UNITED STATES
N2 - This retrospective study was designed to document the etiology of traumatically ruptured globes in children and to determine the prognostic value of several clinical parameters with respect to visual outcome. Forty-six children 16 years of age and under seen in the emergency room over a 2-year period were found to have full thickness penetration of the globe. Fifty-nine percent of injuries occurred during recreational activities, and 59% occurred outside of the home. Boys outnumbered girls by a 6:1 ratio. For children, initial visual acuity proved to be less valuable as a prognostic indicator with regard to final vision than has been reported in adults. Smaller corneal wounds offered better visual outcomes. Four eyes were enucleated. Ten ruptures (22%) were related to activity involving guns. Four of six BB gun injuries were the result of a ricocheted BB. Visual outcomes in gun-related injuries were particularly poor
SN - 0191-3913
AD - Department of Ophthalmology, Jefferson Medical College, Philadelphia, Pa
UR - 7837018
ER -

TY - JOUR
ID - 2000
T1 - A history of psychiatric consultation in America
A1 - Friedman,R.S.
A1 - Molay,F.
Y1 - 1994/09//
N1 - Friedman, R S. Molay, F
The Psychiatric clinics of North America
pbn, 7708110
IM, Q
Historical Article. Journal Article. Portraits
English
KW - MEDLINE
KW - Adult
KW - Child
KW - History
KW - 19th Century
KW - 20th Century
KW - Hospitals
KW - Psychiatric/hi [History]
KW - Humans
KW - Mental Disorders/hi [History]
KW - Patient Care Team/hi [History]

KW - Psychiatry/hi [History]
KW - Referral and Consultation/hi [History]
KW - United States
RP - NOT IN FILE
SP - 667
EP - 681
JF - Psychiatric Clinics of North America
JA - Psychiatr Clin North Am
VL - 17
IS - 3
CY - UNITED STATES
N2 - Consultation-liaison psychiatry emerged in a particular socio-historical context, following a major shift of medicine into a more scientific, hospital-based system. Psychiatrists realized that they needed to move outside of the asylum and reintegrate themselves with the new structures of medicine, especially the general hospital and the medical school. Psychosomatic theory and psychoanalytic influence shaped the emergence of consultation-liaison activities in the 1930s through 1950s as they shaped most psychiatric services in the United States at that time, but they were an epiphenomenon to this powerful underlying dynamic that dictated psychiatry's move into the medical hospital. The experience of other countries tends to confirm this hypothesis, though a comparative history is limited by the strong postwar US influence. Pediatric consultation-liaison had a somewhat different evolution, reflecting the later emergence of pediatrics itself as a field and its strong psychosocial and preventive medicine emphasis
SN - 0193-953X
AD - Department of Psychiatry, Beth Israel Hospital, Boston, Massachusetts
UR - 7824389
ER -

TY - JOUR
ID - 2001
T1 - Atopic sensitization and respiratory symptoms among Polish and Swedish school children
A1 - Braback,L.
A1 - Breborowicz,A.
A1 - Dreborg,S.
A1 - Knutsson,A.
A1 - Pieklik,H.
A1 - Bjorksten,B.
Y1 - 1994/09//
N1 - Braback, L. Breborowicz, A. Dreborg, S. Knutsson, A. Pieklik, H. Bjorksten, B
Clinical and experimental allergy : journal of the British Society for Allergy and Clinical Immunology
ceb, 8906443
IM
Comparative Study. Journal Article. Research Support, Non-U.S. Gov't
English
KW - MEDLINE
KW - Air Pollutants/ae [Adverse Effects]
KW - Child
KW - Female
KW - Humans
KW - Hypersensitivity
KW - Immediate/ep [Epidemiology]
KW - Industry
KW - Male
KW - Peak Expiratory Flow Rate
KW - Poland/ep [Epidemiology]
KW - Prevalence
KW - Respiratory Hypersensitivity/ep [Epidemiology]
KW - School Health Services
KW - Skin Tests

KW - Social Conditions
KW - Sweden/ep [Epidemiology]
RP - NOT IN FILE
SP - 826
EP - 835
JF - Clinical & Experimental Allergy
JA - Clin Exp Allergy
VL - 24
IS - 9
CY - ENGLAND
N2 - Allergic sensitization and symptoms from the airways in relation to air pollution were compared in 10-12-year-old school children (n = 1113) from urban Konin in central Poland and both urban and rural parts of Sundsvall in northern Sweden. The measurements included parental questionnaires, skin-prick tests and serial peak flow measurements during 2 weeks with simultaneous monitoring of outdoor air pollutants. The skin-prick test technique was validated by IgE antibody determinations. The levels of common industrial pollutants, SO2 and smoke particles were much higher in Konin than in urban Sundsvall and the levels of NO2 were similar. Various respiratory symptoms were more often reported among school children in Konin (except for wheezing and diagnosed asthma). Multiple logistic regression analyses yielded the following increased odds ratios for children in Konin as compared with the reference group (rural Sundsvall): chest tightness and breathlessness 3.48 (95% confidence interval 2.08-5.82), exercise-induced coughing attacks 3.69 (95% confidence interval 1.68-8.10), recurrent episodes of common cold 2.79 (95% confidence interval 1.53-5.09) and prolonged cough 4.89 (95% confidence interval 2.59-9.23). In contrast, as compared with rural Sundsvall, the adjusted odds ratio for a positive skin-prick test was decreased in Konin, but increased in urban Sundsvall, 0.58 (95% confidence interval 0.37-0.91) and 1.67 (95% confidence interval 1.15-2.42) respectively. The study confirms that living in urban, as compared with rural areas, is associated with an increased prevalence of respiratory symptoms and sensitization to allergens. These differences could be explained by air pollution. Respiratory symptoms were more common in a similar urban group of Polish children who were exposed to even higher levels of air pollution. These children, however, had a much lower prevalence of sensitization to allergens, as compared with the Swedish children. This indicates that differences in lifestyle and standard of living between western Europe and a former socialist country influences the prevalence of atopy
SN - 0954-7894
AD - Department of Paediatrics, Sundsvall Hospital, Sweden
UR - 7812884
ER -

TY - JOUR
ID - 2002
T1 - Play behavior and communication between deaf and hard of hearing children and their hearing peers in an integrated preschool
A1 - Minnett,A.
A1 - Clark,K.
A1 - Wilson,G.
Y1 - 1994/10//
N1 - Minnett, A. Clark, K. Wilson, G
American annals of the deaf
0414670, 2ba, 3ba
IM
Journal Article
English
KW - MEDLINE
KW - Child
KW - Preschool
KW - Communication
KW - Communication Methods
KW - Total
KW - Deafness
KW - Hearing

KW - Hearing Disorders
KW - Humans
KW - Interpersonal Relations
KW - Mainstreaming (Education)
KW - Peer Group
KW - Play and Playthings
RP - NOT IN FILE
SP - 420
EP - 429
JF - American Annals of the Deaf
JA - Am Ann Deaf
VL - 139
IS - 4
CY - UNITED STATES
N2 - Sixty preschoolers (30 deaf and hard of hearing, 30 hearing) were observed in their integrated school during "centers" and outdoor play. Half the children experienced auditory communication and half total communication modes of communication. All children had known their classmates for six months to three years. It was found that all children preferred to play and communicate with same-hearing status children, however 63% of all children communicated with children of other-hearing status. Amounts of social play and communication differed somewhat between the two communication environments, and context of interaction was related to the behavior and communication of children who were deaf and hard of hearing. Implications are discussed for the education of young children who are deaf and hard of hearing--specifically that educational and social benefits accrue to those who are integrated throughout the school day (across context) and have access to hearing classmates and classmates who are deaf and hard of hearing
SN - 0002-726X
AD - Salesmanship Club Youth and Family Centers, Dallas, Texas
UR - 7810460
ER -

TY - JOUR
ID - 2003
T1 - [Influence of a CNS pathology on the electrocochleography response]. [Italian]
A1 - Arslan,E.
A1 - Lupi,G.
A1 - Rosignoli,M.
Y1 - 1994/05//
N1 - Arslan, E. Lupi, G. Rosignoli, M
Acta otorhinolaryngologica Italica : organo ufficiale della Societa italiana di otorinolaringologia e chirurgia cervico-facciale
1hx, 8213019
IM
Comparative Study. English Abstract. Journal Article
Italian
KW - MEDLINE
KW - Audiometry
KW - Evoked Response
KW - Brain/pp [Physiopathology]
KW - Brain Diseases/di [Diagnosis]
KW - Brain Diseases/pp [Physiopathology]
KW - Child
KW - Preschool
KW - Cochlea/ph [Physiology]
KW - Electroencephalography
KW - Humans
KW - Infant
KW - Newborn
KW - Magnetic Resonance Imaging

KW - Retrospective Studies
KW - Tomography
KW - X-Ray Computed
RP - NOT IN FILE
SP - 315
EP - 328
JF - Acta Otorhinolaryngologica Italica
JA - Acta Otorhinolaryngol Ital
VL - 14
IS - 3
CY - ITALY
N2 - This study analyzed 73 electrocochleographic recordings made in children with a normal hearing threshold, selected retrospectively from 1563 recordings made between 1973 and 1990. The aim of the study was to check the original findings for any correlation between the various response parameters which might be indicative of a pathological condition. Compound action potential (AP) latency and amplitude, presynaptic summation potential (SP) and cochlear microphonic (CM) amplitudes and AP rapid adaptation behavior were calculated and recordings were associated with clinical information on aetiologic diagnosis, otoscopic examination, impedance measurement data and the finding of any central nervous system (CNS) pathology. The trend of the amplitudes as a function of the intensity of all three potentials (input-output functions), CM and SP in particular, demonstrated unexpected scattered values especially towards the high intensities. This was found correlated to the presence of CNS pathology. The comparison between the two groups (with vs without CNS pathology) with the aid of the Student's t-test proved statically significant, especially for CM and SP amplitudes while rather less so for AP amplitude. In particular, all CM and SP amplitude values outside the confidence intervals (calculated as 95% of normal cases) revealed CNS pathology. It has been suggested that the influence of the CNS on cochlear function is due to a disturbed function of the olicocochlear bundle, which is known to have an inhibitory effect on cochlear dynamics; furthermore, there is also proof that it can be activated regardless of any ipso-and/or contra-lateral acoustic stimulation. The effects observed on the electrocochleography in cases with CNS disorders would thus be explained by an interruption of the olivocochlear bundle at the CNS level or a disruption of the CNS mechanism capable of controlling its activation
SN - 0392-100X
AD - Servizio di Foniatria, Universita di Padova
UR - 7810323
ER -

TY - JOUR
ID - 2004
T1 - A dose-ranging study of the efficacy and safety of azelastine nasal spray in the treatment of seasonal allergic rhinitis with an acute model
A1 - Weiler,J.M.
A1 - Meltzer,E.O.
A1 - Benson,P.M.
A1 - Weiler,K.
A1 - Widlitz,M.D.
A1 - Freitag,J.
Y1 - 1994/12//
N1 - Weiler, J M. Meltzer, E O. Benson, P M. Weiler, K. Widlitz, M D. Freitag, J
The Journal of allergy and clinical immunology
h53, 1275002
AIM, IM
Clinical Trial. Journal Article. Multicenter Study. Randomized Controlled Trial. Research Support, Non-U.S. Gov't
English
KW - MEDLINE
KW - Administration
KW - Intranasal
KW - Adolescent

KW - Adult
KW - Aged
KW - Child
KW - Chlorpheniramine/ae [Adverse Effects]
KW - Chlorpheniramine/tu [Therapeutic Use]
KW - Dose-Response Relationship
KW - Drug
KW - Double-Blind Method
KW - Female
KW - Histamine H1 Antagonists/ad [Administration & Dosage]
KW - Humans
KW - Male
KW - Middle Aged
KW - Phthalazines/ad [Administration & Dosage]
KW - Phthalazines/ae [Adverse Effects]
KW - Rhinitis
KW - Allergic
KW - Seasonal/dt [Drug Therapy]
RP - NOT IN FILE
SP - 972
EP - 980
JF - Journal of Allergy & Clinical Immunology
JA - J Allergy Clin Immunol
VL - 94
IS - 6 Pt 1
CY - UNITED STATES
N2 - BACKGROUND: Oral azelastine, a nonsteroidal antiinflammatory respiratory investigational drug has demonstrated activity in the treatment of allergic rhinitis and asthma with a good safety profile. METHODS: Azelastine nasal spray was compared with sustained-release oral chlorpheniramine maleate and placebo for efficacy and safety in the treatment of seasonal allergic rhinitis in a double-dummy, two-center, 2-day, double-blind, randomized, dose-ranging, parallel-groups, onset and duration of action study. Two hundred sixty-four subjects reported to an outdoor park on Saturday morning during the height of the fall pollen season and remained there for 8 hours that day and the next to ensure maximal exposure to seasonal aeroallergens. Symptom diary cards were collected hourly Saturday from 8:00 AM to 10:00 AM (baseline period). Subjects who had sufficient symptoms were randomized into five groups and received medication at 10:00 AM and 10:00 PM on Saturday and at 10:00 AM on Sunday: azelastine 0.1% (1 spray [0.12 mg] per nostril every 12 hours, 2 sprays per nostril every every 12 hours, or 2 sprays per nostril once daily), Chlor-Trimeton Repetabs (12 mg twice daily), or placebo (twice daily). Diary cards were completed hourly (11:00 AM to 4:00 PM) and at 6:00, 8:00, and 10:00 PM on Saturday and again hourly on Sunday (from 8:00 AM to 4:00 PM) to evaluate rhinitis symptoms and adverse events. RESULTS: Two hundred fifty-nine subjects completed the study. The groups that received 2 sprays of azelastine per nostril once and twice daily and the chlorpheniramine group had statistically significantly more improvement in total rhinitis symptoms than the placebo group without serious adverse events. CONCLUSIONS: This study supports a once to twice daily dosing regimen for 2 sprays of 0.1% azelastine in the acute treatment of allergic rhinitis with onset of action within 2 to 3 hours
SN - 0091-6749
AD - College of Medicine, University of Iowa, Iowa City
UR - 7798545
ER -

TY - JOUR
ID - 2005
T1 - [Bacteremia at a medium-sized Norwegian hospital]. [Norwegian]
A1 - Hallan,S.
A1 - Naustdal,T.
Y1 - 1994/08/10/
N1 - Hallan, S. Naustdal, T
Tidsskrift for den Norske laegeforening : tidsskrift for praktisk medicin, ny raekke

0413423, 101086543, vrv
IM
Comparative Study. English Abstract. Journal Article
Norwegian
KW - MEDLINE
KW - Adult
KW - Anti-Bacterial Agents/ad [Administration & Dosage]
KW - Bacteremia/dt [Drug Therapy]
KW - Bacteremia/ep [Epidemiology]
KW - Bacteremia/mi [Microbiology]
KW - Child
KW - Cross Infection/dt [Drug Therapy]
KW - Cross Infection/ep [Epidemiology]
KW - Cross Infection/mi [Microbiology]
KW - Drug Resistance
KW - Microbial
KW - Hospitals
KW - District/sn [Statistics & Numerical Data]
KW - Humans
KW - Infant
KW - Norway/ep [Epidemiology]
RP - NOT IN FILE
SP - 2102
EP - 2106
JF - Tidsskrift for Den Norske Laegeforening
JA - Tidsskr.Nor Laegeforen
VL - 114
IS - 18
CY - NORWAY
N2 - This report, which is based on laboratory reports and patient records, covers the epidemiology, etiology and antibiotic susceptibility of all bacteremic episodes at a community hospital during 1988-90. The overall incidence was eight episodes per 1000 patient admissions, though this figure varied considerably between departments and between age groups. Most organisms were community acquired, and we had few Klebsiella spp., Pseudomonas spp., anaerobic bacteria, fungi and polymicrobial episodes. There were fewer antibiotic resistant strains than reported from outside Scandinavia. The combination of benzylpenicillin and aminoglycoside (plus metronidazole in surgical wards) has a broad enough spectrum of activity for 95% of our patients with sepsis of unknown origin. Thus, our rather "old-fashioned" prescribing policy, with the new and broadspectrum cephalosporins and betalactam drugs constituting only a minor part, is still good practice. The study also shows that cumulative reports issued by the local microbiological laboratory bring awareness to these issues and affect the prescribing pattern in a positive way
SN - 0029-2001
AD - Medisinsk avdeling, Innherred sykehus, Levanger
UR - 7992265
ER -

TY - JOUR
ID - 2006
T1 - Patterns of injuries to children on public playgrounds
A1 - Mott,A.
A1 - Evans,R.
A1 - Rolfe,K.
A1 - Potter,D.
A1 - Kemp,K.W.
A1 - Sibert,J.R.
Y1 - 1994/10//
N1 - Mott, A. Evans, R. Rolfe, K. Potter, D. Kemp, K W. Sibert, J R
Archives of disease in childhood

6xg, 0372434
AIM, IM
Journal Article
English
KW - MEDLINE
KW - Accidents/sn [Statistics & Numerical Data]
KW - Adolescent
KW - Child
KW - Preschool
KW - Environment
KW - Fractures
KW - Bone/ep [Epidemiology]
KW - Humans
KW - Incidence
KW - Infant
KW - Newborn
KW - Play and Playthings
KW - Wales/ep [Epidemiology]
KW - Wounds and Injuries/cl [Classification]
KW - Wounds and Injuries/ep [Epidemiology]
RP - NOT IN FILE
SP - 328
EP - 330
JF - Archives of Disease in Childhood
JA - Arch Dis Child
VL - 71
IS - 4
CY - ENGLAND
N2 - The incidence and pattern of injuries to children in public playgrounds presenting to the accident and emergency department were reviewed over two six month summer periods in Cardiff. A total of 178 children (mean age 7.5 years) attended with a playground injury representing 1.1% of all the children attending. One hundred and five children fell from equipment, of which the commonest was the climbing frame; 125 children had playground surface related injuries, 86 on bark and 30 on concrete. The pattern of injuries on the playground surfaces was different: fractures and sprains were more common on bark surfaces and lacerations and abrasions on concrete surfaces. Comparison of total injury rates showed there were fewer injuries on modernised playgrounds than expected but these differences were not significant. Modernised playgrounds are more popular, have new exciting equipment, and offer good play opportunities for children. However, the high fracture rate on modernised bark playgrounds is concerning and requires further research
SN - 1468-2044
AD - Department of Child Health, University of Wales College of Medicine, Lansdowne Hospital, Cardiff
UR - 7979526
ER -

TY - JOUR
ID - 2007
T1 - Toxocara seroprevalence in 5-year-old elementary schoolchildren: relation with allergic asthma
A1 - Buijs,J.
A1 - Borsboom,G.
A1 - van Gemund,J.J.
A1 - Hazebroek,A.
A1 - van Dongen,P.A.
A1 - van,Knapen F.
A1 - Neijens,H.J.
Y1 - 1994/11/01/
N1 - Buijs, J. Borsboom, G. van Gemund, J J. Hazebroek, A. van Dongen, P A. van Knapen, F. Neijens, H J
American journal of epidemiology
3h3, 7910653

IM
Journal Article
English
KW - MEDLINE
KW - Animals
KW - Antibodies
KW - Helminth/an [Analysis]
KW - Asthma/ep [Epidemiology]
KW - Asthma/et [Etiology]
KW - Child
KW - Preschool
KW - Female
KW - Humans
KW - Immunoglobulin E/an [Analysis]
KW - Male
KW - Netherlands/ep [Epidemiology]
KW - Prevalence
KW - Seroepidemiologic Studies
KW - Toxocara/im [Immunology]
KW - Toxocariasis/ep [Epidemiology]
RP - NOT IN FILE
SP - 839
EP - 847
JF - American Journal of Epidemiology
JA - Am J Epidemiol
VL - 140
IS - 9
CY - UNITED STATES
N2 - Toxocara seroprevalence and the relation between Toxocara seroprevalence and allergic asthma were investigated in Dutch schoolchildren aged 4-6 years. Data on Toxocara antibodies, allergen-specific immunoglobulin E, allergic manifestations, and risk factors (pets and playgrounds) were obtained from 235 children from The Hague and 477 from Rotterdam, the Netherlands. The surveys were carried out from September 1987 to January 1988 in The Hague and in March and April 1989 in Rotterdam. Logistic regression was used to evaluate putative relations. Toxocara seroprevalence was higher in The Hague (11%) than in Rotterdam (6%), but this difference was not quite significant. Seroprevalences varied widely among schools. No differences between socioeconomic categories or between the sexes were found. Occurrences of asthma/recurrent bronchitis and hospitalization due to asthma/recurrent bronchitis were significantly associated with seroprevalence. Furthermore, a marginally significant relation with eczema was found. Immunoglobulin E specific for inhaled allergens occurred significantly more often in the Toxocara-seropositive group. The risk factors investigated were not related to seroprevalence. It is suggested that Toxocara, among other environmental factors, may stimulate polyclonally immunoglobulin E production, including allergen-specific immunoglobulin E, and thus may contribute to the manifestation of allergic asthma and possibly of eczema in children predisposed to allergy
SN - 0002-9262
AD - National Institute of Public Health and Environmental Protection, Laboratory for Parasitology and Mycology, Bilthoven, The Netherlands
UR - 7977294
ER -

TY - JOUR
ID - 2008
T1 - An international perspective on child day-care health
A1 - Olin,P.
A1 - Tandon,B.N.
A1 - Meme,J.S.
A1 - Ford-Jones,E.L.
A1 - Belsey,M.

A1 - Chang,A.
Y1 - 1994/12//
N1 - Olin, P. Tandon, B N. Meme, J S. Ford-Jones, E L. Belsey, M. Chang, A
Pediatrics
oxv, 0376422
AIM, IM
Journal Article
English
KW - MEDLINE
KW - Canada
KW - Child
KW - Child Day Care Centers
KW - Preschool
KW - Female
KW - Health Promotion
KW - Health Status
KW - Humans
KW - India
KW - Infant
KW - Kenya
KW - Sweden
KW - Women
KW - Working
KW - World Health
RP - NOT IN FILE
SP - 1085
EP - 1087
JF - Pediatrics
VL - 94
IS - 6 Pt 2
CY - UNITED STATES
N2 - If we are committed to the health and development of children, we need to recognize that the vast majority of the world's women are working women. In Africa, 80% of the women are actively engaged in economic activities outside the home. The "economic miracle" in Southeast Asia was made possible by the nimble fingers of thousands of women working in textile and electronics factories. There is need for pre-day-care advocacy for infants, through promotion of breast feeding and maternity leave. When the mother returns to work, the standard of the International Labor Organization should be applied, namely" ...the care of children while the parents are working cannot be ignored because it forms a focal point on which three main concerns of development policy--work, health, and education--converge." Several principles emerged from the presentations in the international panel: 1. Child-care programs must be community based, using the resources of the families and the community organizations themselves. 2. Programs require the active involvement of the communities, women's groups, and other partners. 3. Programs are modified by innovations created by community organizations, universities, and other groups. 4. Programs require the mobilization of trained young men and women into the field of early childhood education and development. This international panel provided an overall uniting theme, that throughout the world the hope for the survival and better life for children unites parents of every country and every creed. This is one of the most powerful and strongest motivational resources in the world.(ABSTRACT TRUNCATED AT 250 WORDS)
SN - 0031-4005
AD - Department of Pediatrics, Stockholm, Sweden
UR - 7971069
ER -

TY - JOUR
ID - 2009
T1 - American Society of Clinical Oncology. Recommendations for the use of hematopoietic colony-stimulating factors: evidence-based, clinical practice guidelines. [Review] [256 refs]
Y1 - 1994/11//

N1 - Journal of clinical oncology : official journal of the American Society of Clinical Oncology
jco, 8309333
IM
Comparative Study. Guideline. Journal Article. Practice Guideline. Review
English
KW - MEDLINE
KW - Adult
KW - Child
KW - Clinical Trials as Topic
KW - Colony-Stimulating Factors/ae [Adverse Effects]
KW - Colony-Stimulating Factors/ec [Economics]
KW - Colony-Stimulating Factors/tu [Therapeutic Use]
KW - Drug-Related Side Effects and Adverse Reactions
KW - Humans
KW - Medical Oncology
KW - Neoplasms/th [Therapy]
KW - Societies
KW - Medical
KW - United States
RP - NOT IN FILE
SP - 2471
EP - 2508
JF - Journal of Clinical Oncology
JA - J Clin Oncol
VL - 12
IS - 11
CY - UNITED STATES
N2 - PURPOSE: Standard practice in protecting against chemotherapy-associated infection has been chemotherapy dose modification or dose delay, administration of progenitor-cell support, or selective use of prophylactic antibiotics. Therapy of chemotherapy-associated neutropenic fever or infection has customarily involved treatment with intravenous antibiotics, usually accompanied by hospitalization. The hematopoietic colony-stimulating factors (CSFs) have been introduced into clinical practice as additional supportive measures that can reduce the likelihood of neutropenic complications due to chemotherapy. Clinical benefit has been shown, but the high cost of CSFs has led to concern about their appropriate use. The American Society of Clinical Oncology (ASCO) wishes to establish evidence-based, clinical practice guidelines for the use of CSFs in patients who are not enrolled on clinical trials. METHODS: An expert multidisciplinary panel reviewed the clinical data documenting the activity of CSFs. For each common clinical situation, the Panel formulated a guideline to encourage reasonable use of CSFs to preserve effectiveness but discourage excess use when little marginal benefit is anticipated. Consensus was reached after critically appraising the available evidence. Guidelines were validated by comparing them with recommendations for CSF use developed in other countries and by several academic institutions. Outcomes considered in evaluating CSF benefit included duration of neutropenia, incidence of febrile neutropenia, incidence and duration of antibiotic use, frequency and duration of hospitalization, infectious mortality, chemotherapy dose-intensity, chemotherapy efficacy, quality of life, CSF toxicity, and economic impact. To the extent that these data were available, the Panel placed greatest value on survival benefit, reduction in rates of febrile neutropenia, decreased hospitalization, and reduced costs. Lesser value was placed on alterations in absolute neutrophil counts (ANC). CONCLUSIONS: CSFs are recommended in some situations, eg, to reduce the likelihood of febrile neutropenia when the expected incidence is > or = 40%; after documented febrile neutropenia in a prior chemotherapy cycle to avoid infectious complications and maintain dose-intensity in subsequent treatment cycles when chemotherapy dose-reduction is not appropriate; and after high-dose chemotherapy with autologous progenitor-cell transplantation. CSFs are also effective in the mobilization of peripheral-blood progenitor cells. Therapeutic initiation of CSFs in addition to antibiotics at the onset of febrile neutropenia should be reserved for patients at high risk for septic complications. CSF use in patients with myelodysplastic syndromes may be reasonable if they are experiencing neutropenic infections. Administration of CSFs after initial chemotherapy for acute myeloid leukemia does not appear to be detrimental, but clinical benefit has been variable and caution is advised. Available data support use of CSFs in pediatric cancer patients similar to that recommended for adult patients. Outside of clinical trials, CSFs should not be used concurrently with chemotherapy and radiation, or to support increasing chemotherapy

dose-intensity. Further research is warranted as a means to improve the cost-effective administration of the CSFs and identify clinical predictors of infectious complications that may direct their use. [References: 256]
SN - 0732-183X
UR - 7964965
ER -

TY - JOUR
ID - 2010
T1 - Initial sociometric impressions of attention-deficit hyperactivity disorder and comparison boys: predictions from social behaviors and from nonbehavioral variables
A1 - Erhardt,D.
A1 - Hinshaw,S.P.
Y1 - 1994/08//
N1 - Erhardt, D. Hinshaw, S P
Journal of consulting and clinical psychology
0136553, hw3
IM
Comparative Study. Journal Article. Research Support, Non-U.S. Gov't. Research Support, U.S. Gov't, P.H.S.
English
KW - MEDLINE
KW - Attention Deficit Disorder with Hyperactivity/di [Diagnosis]
KW - Attention Deficit Disorder with Hyperactivity/px [Psychology]
KW - Child
KW - Cohort Studies
KW - Humans
KW - Intelligence
KW - Intelligence Tests
KW - Male
KW - Motor Skills
KW - Peer Group
KW - Social Behavior
RP - NOT IN FILE
SP - 833
EP - 842
JF - Journal of Consulting & Clinical Psychology
JA - J Consult Clin Psychol
VL - 62
IS - 4
CY - UNITED STATES
N2 - This study systematically compared the influence of naturalistic social behaviors and nonbehavioral variables on the development of peer status in 49 previously unfamiliar boys, aged 6-12 years, who attended a summer research program. Twenty-five boys with attention-deficit hyperactivity disorder (ADHD) and 24 comparison boys participated. Physical attractiveness, motor competence, intelligence, and academic achievement constituted the nonbehavioral variables; social behaviors included noncompliance, aggression, prosocial actions, and isolation, measured by live observations of classroom and playground interactions. As early as the first day of interaction, ADHD and comparison boys displayed clear differences in social behaviors, and the ADHD youngsters were overwhelmingly rejected. Whereas prosocial behavior independently predicted friendship ratings during the first week, the magnitude of prediction was small. In contrast, the boys' aggression (or noncompliance) strongly predicted negative nominations, even with nonbehavioral factors, group status (ADHD versus comparison), and other social behaviors controlled statistically. Implications for understanding and remediating negative peer reputations are discussed
SN - 0022-006X
AD - Department of Psychology, University of California, Los Angeles, Berkeley 94720
UR - 7962888
ER -

TY - JOUR

ID - 2011
T1 - Brachytherapy for recurrent malignant astrocytoma
A1 - Bernstein,M.
A1 - Laperriere,N.
A1 - Glen,J.
A1 - Leung,P.
A1 - Thomason,C.
A1 - Landon,A.E.
Y1 - 1994/12/01/
N1 - Bernstein, M. Laperriere, N. Glen, J. Leung, P. Thomason, C. Landon, A E
International journal of radiation oncology, biology, physics
g97, 7603616
IM
Clinical Trial. Journal Article
English
KW - MEDLINE
KW - Adolescent
KW - Adult
KW - Aged
KW - Astrocytoma/mo [Mortality]
KW - Astrocytoma/rt [Radiotherapy]
KW - Brachytherapy/mt [Methods]
KW - Brain Neoplasms/mo [Mortality]
KW - Brain Neoplasms/rt [Radiotherapy]
KW - Child
KW - Preschool
KW - Humans
KW - Iodine Radioisotopes/ad [Administration & Dosage]
KW - Iodine Radioisotopes/tu [Therapeutic Use]
KW - Middle Aged
KW - Neoplasm Recurrence
KW - Local
KW - Stereotaxic Techniques
KW - Survival Rate
KW - Time Factors
RP - NOT IN FILE
SP - 1213
EP - 1217
JF - International Journal of Radiation Oncology, Biology, Physics
JA - Int J Radiat Oncol Biol Phys
VL - 30
IS - 5
CY - UNITED STATES
N2 - PURPOSE: To assess the efficacy of interstitial brachytherapy in the treatment of patients with recurrent malignant astrocytoma. METHODS AND MATERIALS: Forty-six patients with recurrent malignant astrocytoma were treated with stereotactic high-activity temporary iodine-125 implants between September, 1986 and October, 1992. All patients had been initially treated for malignant astrocytoma (44) or low-grade astrocytoma (2) with surgery and external fractionated radiation. The median time between initial diagnosis and recurrence treated with brachytherapy was 12.5 months. Twenty-five patients received chemotherapy prior to brachytherapy. RESULTS: All but four patients have died; median survival time following brachytherapy is 46 weeks. Twelve patients underwent reoperation for radiation necrosis at a median interval of 6.5 months after treatment (26%). Five patients incurred complications directly due to brachytherapy (11%). Forty-four patients are evaluable regarding pattern of failure following brachytherapy. Six of these 44 patients (13.6%) recurred at a distance from the treatment volume (4 in brain and 2 in spinal subarachnoid space). CONCLUSION: Brachytherapy confers modest but meaningful prolongation of survival in selected patients with recurrent malignant astrocytoma, but complications are significant, reoperation frequently required, and recurrence outside the treatment volume common

SN - 0360-3016
AD - Division of Neurosurgery, Toronto Hospital, Ontario, Canada
UR - 7961031
ER -

TY - JOUR
ID - 2012
T1 - The relationship among television watching, physical activity, and body composition of young children
A1 - DuRant,R.H.
A1 - Baranowski,T.
A1 - Johnson,M.
A1 - Thompson,W.O.
Y1 - 1994/10//
N1 - DuRant, R H. Baranowski, T. Johnson, M. Thompson, W O
Pediatrics
oxv, 0376422
AIM, IM
Journal Article. Multicenter Study
English
KW - MEDLINE
KW - African Americans
KW - Body Composition
KW - Child
KW - Preschool
KW - Energy Metabolism
KW - European Continental Ancestry Group
KW - Exercise
KW - Female
KW - Humans
KW - Longitudinal Studies
KW - Male
KW - Mexican Americans
KW - Obesity/ep [Epidemiology]
KW - Obesity/et [Etiology]
KW - Seasons
KW - Television
KW - Texas/ep [Epidemiology]
KW - Time Factors
RP - NOT IN FILE
SP - 449
EP - 455
JF - Pediatrics
VL - 94
IS - 4 Pt 1
CY - UNITED STATES
N2 - PURPOSE: Television watching has been reported to be associated with obesity, resting energy expenditure, and lower daily physical activity among both children and adolescents. However, most of these studies were based on self report or data collected in laboratory settings. This study examined the relationship among observed time of television watching, observed physical activity level and body composition among 3- or 4-year-old children. METHODS: African-American (41.4%), Mexican-American (23%), and Anglo-American (35.6%) children (N = 191, males = 90) from the Texas site of the Studies of Child Activity and Nutrition program were observed from 6 to 12 hours per day up to 4 days over 1 year. Activity level each minute of the day was measured with the Children's Activity Rating Scale (interobserver reliability = .84 +/- .001). The interobserver reliability of time of television watching was .96 +/- .08. RESULTS: The median of the longest number of consecutive minutes of television watching was 15 (range = 1 to 79). The median percent of minutes of television watching of total observed minutes was 14.8% (0% to 58%) and the median percent of minutes of inside minutes was 17.9% (0% to 80.9%). There were no gender or ethnic differences in time

watching television or physical activity during television watching. Physical activity during television watching was lowest during the longest bout of television watching (mean = 1.48 +/- .28) compared to outside minutes (mean = 2.38 +/- .21), inside non-television minutes (mean = 1.96 +/- .13) and inside television minutes (mean = 1.65 +/- .18). The level of physical activity during television-watching times was highest (P < .0031) during October and November and lowest during March, April, June, and July. Longest bout of television watching and percent of minutes watching television to total observed minutes were inversely associated with mean physical activity, percent of minutes of physical activity levels 3, 4, or 5, and percent of physical activity levels 4 or 5. Percent of television watching to inside minutes was negatively correlated with physical activity levels 4 or 5. Television-watching behavior was not associated with body composition. CONCLUSIONS: Television watching was weakly negatively correlated with physical activity levels, and physical activity was lower during television-watching than non-television-watching time in this sample of children. Television viewing behavior was not associated with body composition
SN - 0031-4005
AD - Division of Adolescent/Young Adult Medicine, Children's Hospital, Harvard Medical School, Boston, MA 02115
UR - 7936851
ER -

TY - JOUR
ID - 2013
T1 - [Respirable mineral fibers in atmospheric air of Wroclaw]. [Polish]
A1 - Wozniak,H.
A1 - Wiecek,E.
A1 - Pelc,W.
A1 - Dobrucka,D.
A1 - Krol,M.
A1 - Opalska,B.
Y1 - 1994///
N1 - Wozniak, H. Wiecek, E. Pelc, W. Dobrucka, D. Krol, M. Opalska, B
Medycyna pracy
moj, 0376642
IM
English Abstract. Journal Article
Polish
KW - MEDLINE
KW - Air Pollutants/an [Analysis]
KW - Asbestos
KW - Serpentine/an [Analysis]
KW - Child
KW - Dust/an [Analysis]
KW - Environmental Monitoring
KW - Humans
KW - Microscopy
KW - Electron
KW - Mining
KW - Particle Size
KW - Poland
KW - Spectrophotometry
KW - Infrared
KW - X-Ray Diffraction
RP - NOT IN FILE
SP - 239
EP - 247
JF - Medycyna Pracy
JA - Med Pr
VL - 45
IS - 3

CY - POLAND
N2 - Serpentine asbestos from the Naslawice mine, which contains mineral impurities of the serpentinite group--chrysotile and antigortie, has been used to built school sports grounds as well as roads and playgrounds within residential areas. The study was aimed to measure concentrations of respirable mineral fibres in the atmospheric air at the time children were playing on one of the playgrounds as well as at four other sites of Wroclaw. Air samples were collected using individual dosimeters and distributions of length and concentration of fibres were measured by means of a laser fiber monitor FM7400. X-ray diffraction and infrared spectrometry were used to determine the mineral composition of raw material collected from the playground. Morphology of particles of dust from the atmospheric air was analysed by means of electronic microscopy. It was found that the concentration of mineral fibres in the air in question depended on the number of children playing, namely when 7 children were playing the concentration value was equal to 165 fibres/litre and 549 fibres/litre with the number of 20 children. The concentration of fibres in a flat in the vicinity of the playground covered with serpentinite was about 11 times higher than at a street crossing with heavy traffic of motor vehicles. Antigorite and chrysotile were found in samples of raw material used to cover the playground. Numerous particles of fibrous structure were observed in the electronic microscopy image of air dust samples collected from the playground
SN - 0465-5893
AD - Zakladu Aerozoli, Instytutu Medycyny Pracy im. prof. dra med. Jerzego Nofera, Lodzi
UR - 8084263
ER -

TY - JOUR
ID - 2014
T1 - Asthma ski day: cold air sports safe with peak flow monitoring
A1 - Silvers,W.
A1 - Morrison,M.
A1 - Wiener,M.
Y1 - 1994/08//
N1 - Silvers, W. Morrison, M. Wiener, M
Annals of allergy
4xc, 0372346
IM
Journal Article
English
KW - MEDLINE
KW - Adolescent
KW - Asthma/dt [Drug Therapy]
KW - Asthma/pp [Physiopathology]
KW - Asthma/pc [Prevention & Control]
KW - Bronchodilator Agents/tu [Therapeutic Use]
KW - Child
KW - Preschool
KW - Cold Temperature
KW - Colorado
KW - Humans
KW - Monitoring
KW - Physiologic
KW - Peak Expiratory Flow Rate
KW - Seasons
KW - Skiing
KW - Temperature
RP - NOT IN FILE
SP - 105
EP - 108
JF - Annals of Allergy
JA - Ann Allergy
VL - 73

IS - 2
CY - UNITED STATES
N2 - The Colorado Asthma Ski Day, an annual cross-country and alpine skiing event, encourages children with asthma to participate fully in outdoor winter sports. Since cold air and exercise can trigger bronchospasm, we examined the peak expiratory flow rates of 80 children who attended Asthma Ski Day 1992 or Asthma Ski Day 1993 to establish a safety profile for this event. Peak expiratory flow rates were measured prior to skiing, at lunchtime, and at the end of the day's activities. We asked the children to pretreat with their regular medications, as prescribed by their physicians, to use their bronchodilator inhalers p.r.n., and to report to our medical station if an episode of acute asthma occurred. The average age of the participants was 9.5 years, and the average baseline daytime peak flow rate was 100.03% of predicted. The average percent change in peak flow rates during the day was an increase of 5.00%. Our results demonstrate that with medical supervision, peak expiratory flow rate monitoring, and properly administered medications, peak flow rates can be stabilized and even improve during cold-weather exercise to an extent that safety concerns need not restrict children with asthma from engaging in exercise or cold-weather sports. The Colorado Asthma Ski Day can serve as a model event for other organizations that want to promote outdoor activities for children with asthma
SN - 0003-4738
AD - Allergy, Asthma and Immunology Clinic of Colorado, PC, Greenwood Medical Center, Englewood 80111-1726
UR - 8067591
ER -

TY - JOUR
ID - 2015
T1 - Patterns of power mower injuries in children compared with adults and the elderly
A1 - Letton,R.W.
A1 - Chwals,W.J.
Y1 - 1994/08//
N1 - Letton, R W. Chwals, W J
The Journal of trauma
kaf, 0376373
AIM, IM
Case Reports. Journal Article
English
KW - MEDLINE
KW - Accidents
KW - Home
KW - Adolescent
KW - Adult
KW - Age Factors
KW - Aged
KW - 80 and over
KW - Amputation
KW - Traumatic/su [Surgery]
KW - Artificial Limbs
KW - Child
KW - Preschool
KW - Debridement
KW - Female
KW - Humans
KW - Leg/su [Surgery]
KW - Male
KW - Middle Aged
RP - NOT IN FILE
SP - 182
EP - 186
JF - Journal of Trauma-Injury Infection & Critical Care
JA - J Trauma

VL - 37
IS - 2
CY - UNITED STATES
N2 - Power mower trauma remains an alarmingly frequent cause of serious injury in young children. The patterns of mower-related injuries in children < 15 years old (n = 13) were compared with those of adults aged 15-64 (n = 16) and elderly victims > or = 65 years old (n = 6), who were similarly injured over the past 5 years. Children were more likely to be injured in accidents involving high-energy riding mowers. Of those children injured, 69% (9 of 13) were playing in the yard while 31% (4 of 13) were riding on the mower with a guardian when the injury occurred. Amputations in children were more frequent and more extensive than in the adults and included one forearm, two Symes, and three below-knee amputations. The need for transfusion was also significantly increased in children (62% vs. 6% adults, p < 0.005), who were also more likely to require prolonged hospitalization (11.8 days vs. 5 days in adults, p < 0.005). Aggressive efforts to increase public awareness regarding the cause and nature of power mower injuries are warranted to decrease the incidence of this debilitating but preventable trauma in young children
SN - 0022-5282
AD - Department of Surgery, Bowman Gray School of Medicine, Winston Salem, NC 27157-1095
UR - 8064912
ER -

TY - JOUR
ID - 2016
T1 - Application of pyrolysis mass spectroscopy and SDS-PAGE in the study of the epidemiology of Pseudomonas cepacia in cystic fibrosis
A1 - Corkill,J.E.
A1 - Sisson,P.R.
A1 - Smyth,A.
A1 - Deveney,J.
A1 - Freeman,R.
A1 - Shears,P.
A1 - Heaf,D.
A1 - Hart,C.A.
Y1 - 1994/08//
N1 - Corkill, J E. Sisson, P R. Smyth, A. Deveney, J. Freeman, R. Shears, P. Heaf, D. Hart, C A
Journal of medical microbiology
j2n, 0224131
IM
Journal Article
English
KW - MEDLINE
KW - Adolescent
KW - Adult
KW - Bacterial Proteins/an [Analysis]
KW - Burkholderia cepacia/ch [Chemistry]
KW - Burkholderia cepacia/cl [Classification]
KW - Case-Control Studies
KW - Child
KW - Cross Infection/ep [Epidemiology]
KW - Cross Infection/mi [Microbiology]
KW - Cystic Fibrosis/co [Complications]
KW - Electrophoresis
KW - Polyacrylamide Gel
KW - Female
KW - Humans
KW - Male
KW - Mass Spectrometry
KW - Pseudomonas Infections/ep [Epidemiology]
KW - Pseudomonas Infections/mi [Microbiology]

KW - Pseudomonas Infections/tm [Transmission]
RP - NOT IN FILE
SP - 106
EP - 111
JF - Journal of Medical Microbiology
JA - J Med Microbiol
VL - 41
IS - 2
CY - SCOTLAND
N2 - Representative isolates of Pseudomonas cepacia from 15 cystic fibrosis (CF) patients attending the Respiratory Unit of Alder Hey Childrens' Hospital were investigated by SDS-PAGE of whole-cell polypeptides and by pyrolysis mass spectroscopy (PMS). SDS-PAGE was less discriminatory than PMS. Eleven isolates were indistinguishable by PMS and considered to represent re-isolates of an endemic strain; four isolates were distinct from this group, and from one another. P. cepacia was first isolated on the unit in July 1989 from a patient who had attended a UK selection meeting for a Canadian CF camp. A ward and outpatient segregation policy was introduced, but colonisation of further patients occurred. In August 1991, the Adult CF Association recommended that all social activities involving colonised patients should cease. This, and an increased awareness amongst older CF patients of the risks of person-to-person transmission, was associated with a marked decline in new cases. Social activity and hospital admissions were compared for colonised patients during the year before colonisation with P. cepacia, and matched patients who did not acquire the endemic strain. This showed a significantly higher attendance at CF social events for colonised patients, but no significant association between colonisation and hospital admission. These results are strong indirect evidence that transmission of P. cepacia occurs through social contact outside the hospital environment
SN - 0022-2615
AD - Royal Liverpool University Hospital, Department of Medical Microbiology
UR - 7519269
ER -

TY - JOUR
ID - 2017
T1 - Inter-rater agreement of two functional independence scales: the Functional Independence Measure (FIM) and a subjective uniform continuous scale
A1 - Chau,N.
A1 - Daler,S.
A1 - Andre,J.M.
A1 - Patris,A.
Y1 - 1994/04//
N1 - Chau, N. Daler, S. Andre, J M. Patris, A
Disability and rehabilitation
9207179, a8i
IM
Comparative Study. Journal Article
English
KW - MEDLINE
KW - Activities of Daily Living
KW - Adolescent
KW - Adult
KW - Child
KW - Preschool
KW - Disability Evaluation
KW - Disabled Persons
KW - Education
KW - Special
KW - Female
KW - France
KW - Humans
KW - Infant

KW - Male
KW - Observer Variation
KW - Occupational Therapy
KW - Physical Therapy Modalities
KW - Rehabilitation Centers
RP - NOT IN FILE
SP - 63
EP - 71
JF - Disability & Rehabilitation
JA - Disabil Rehabil
VL - 16
IS - 2
CY - ENGLAND
N2 - The needs and clinical decisions of care centres are related to patients' ability to carry out daily living activities. Most of the functional scales are not easy to use. This study examined the inter-rater agreement of the Functional Independence Measure (FIM) and a subjective uniform continuous scale (UCS) (the rating varied between 0 = complete dependence and 9 = complete independence) between 'educators', physiotherapists and occupational therapists. Two hundred and fifty-four patients aged below 20 in a rehabilitation centre were rated by professionals who were most familiar with them. For the two scales, inter-rater agreement was very good for all activities except for locomotion outside the centre for the UCS. The rating differences were slightly smaller for the FIM than for the UCS. Physiotherapists rated similarly to occupational therapists. The educators rated slightly though significantly lower than the other raters. The differences could be explained by their professional activities
SN - 0963-8288
AD - INSERM U115, Faculte de Medecine, Vandoeuvre-les-Nancy, France
UR - 8043886
ER -

TY - JOUR
ID - 2018
T1 - Acute respiratory infections (ARI) in rural Bangladesh: perceptions and practices
A1 - Stewart,M.K.
A1 - Parker,B.
A1 - Chakraborty,J.
A1 - Begum,H.
Y1 - 1994/05//
N1 - Stewart, M K. Parker, B. Chakraborty, J. Begum, H
Medical anthropology
7707343, mb1
IM, J
Journal Article. Research Support, Non-U.S. Gov't
English
KW - MEDLINE
KW - Acute Disease
KW - Adult
KW - Anthropology
KW - Cultural
KW - Bangladesh/ep [Epidemiology]
KW - Child
KW - Preschool
KW - Community Health Services
KW - Female
KW - Health Knowledge
KW - Attitudes
KW - Practice
KW - Humans
KW - Infant

KW - Male
KW - Medicine
KW - Traditional
KW - Mothers/px [Psychology]
KW - Respiratory Tract Infections/mo [Mortality]
KW - Respiratory Tract Infections/px [Psychology]
KW - Respiratory Tract Infections/th [Therapy]
KW - Rural Health
RP - NOT IN FILE
SP - 377
EP - 394
JF - Medical Anthropology
JA - Med Anthropol
VL - 15
IS - 4
CY - UNITED STATES
N2 - Acute Respiratory Infections (ARI) are a major cause of death in children under five in rural Bangladesh. A popular strategy for lowering ARI mortality in such settings includes detecting and managing pneumonia in children at the community level. The success of programs using this approach requires a well-trained community-based cadre of health workers and the appropriate utilization of services provided. Determinants of health care seeking behavior are clearly of interest in this regard. A qualitative study was conducted in Matlab, Bangladesh to describe community perceptions of signs and symptoms of ARI, case management behavior, and constraints to service utilization. Mothers recognized pneumonia and thought it to be caused by "exposure to cold." They were able to identify labored breathing, chest retractions, lethargy, and inability to feed as signs of severe disease needing treatment outside the home. Nevertheless, similar illnesses were sometimes believed to be due to attack by evil influences. In these cases, spiritual healers were sought and allopathic treatment was avoided or delayed. The mothers' observance of purdah and "proper" behavior were reported to play a role in prevention of child death from disease. Implications of this belief and its impact on service utilization are discussed. Suggestions for program managers are made in addition to recommendations for further research
SN - 0145-9740
AD - Johns Hopkins University, School of Hygiene and Public Health, Department of International Health, Baltimore, MD 21205
UR - 8041236
ER -

TY - JOUR
ID - 2019
T1 - Deaf children's spoken language output with a hearing peer and with a hearing mother
A1 - Niver,J.M.
A1 - Schery,T.K.
Y1 - 1994/03//
N1 - Niver, J M. Schery, T K
American annals of the deaf
0414670, 2ba, 3ba
IM
Comparative Study. Journal Article
English
KW - MEDLINE
KW - Child
KW - Child Language
KW - Preschool
KW - Communication
KW - Communication Methods
KW - Total
KW - Deafness/rh [Rehabilitation]
KW - Hearing Aids
KW - Humans

KW - Language Tests
KW - Mother-Child Relations
KW - Peer Group
KW - Speech
KW - Speech Intelligibility
RP - NOT IN FILE
SP - 96
EP - 103
JF - American Annals of the Deaf
JA - Am Ann Deaf
VL - 139
IS - 2
CY - UNITED STATES
N2 - Knowledge of how deaf children use speech outside the classroom or therapy room many enhance our ability to improve their speech in a variety of situations and with a variety of communication partners. Past research has shown that deaf children's interactions in informal settings with their mothers and hearing peers create situations that can foster improved linguistic skills. In the current study, the amount and intelligibility of spoken language output in 15 deaf children ages 4-5 to 9-8 years during 15 minutes of free play with their mothers was compared with output and intelligibility during a similar session with a self-selected hearing peer. The results, which indicated that significantly more speech was produced during the children's interaction with their mothers, are discussed in terms of future research
SN - 0002-726X
AD - Department of Human Resources, Peabody College of Vanderbilt University, Nashville, Tennessee
UR - 8037083
ER -

TY - JOUR
ID - 2020
T1 - Low-income children's after-school care: are there beneficial effects of after-school programs?
A1 - Posner,J.K.
A1 - Vandell,D.L.
Y1 - 1994/04//
N1 - Posner, J K. Vandell, D L
Child development
0372725, d28
IM
Comparative Study. Journal Article. Research Support, Non-U.S. Gov't
English
KW - MEDLINE
KW - Child
KW - Child Day Care Centers
KW - Educational Status
KW - Female
KW - Humans
KW - Male
KW - Parenting/px [Psychology]
KW - Personality Assessment
KW - Poverty/px [Psychology]
KW - Reading
KW - Self Care/px [Psychology]
KW - Social Environment
KW - Socialization
RP - NOT IN FILE
SP - 440
EP - 456
JF - Child Development
JA - Child Dev

VL - 65
IS - 2 Spec No
CY - UNITED STATES
N2 - 4 types of after-school care (formal after-school programs, mother care, informal adult supervision, and self-care) were examined for 216 low-income children (M age = 9.1 years). After-school care was associated with maternal education, race, and family income but not with child gender, family marital status, neighborhood safety, or parenting style. When maternal education, race, and family income were controlled, attending a formal after-school program was associated with better academic achievement an social adjustment in comparison to other types of after-school care. Children's activities and experiences also varied in different after-school settings. Children in formal programs spent more time in academic activities and enrichment lessons and less time watching TV and playing outside unsupervised than other children. They also spent more time doing activities with peers and adults and less time with siblings than did other children. The time that children spent in these activities was correlated with their academic and conduct grades, peer relations, and emotional adjustment
SN - 0009-3920
AD - University of Wisconsin-Madison
UR - 8013233
ER -

TY - JOUR
ID - 2021
T1 - Impact of a temporary food shortage on children and their mothers
A1 - McDonald,M.A.
A1 - Sigman,M.
A1 - Espinosa,M.P.
A1 - Neumann,C.G.
Y1 - 1994/04//
N1 - McDonald, M A. Sigman, M. Espinosa, M P. Neumann, C G
Child development
0372725, d28
IM
Journal Article. Research Support, Non-U.S. Gov't. Research Support, U.S. Gov't, Non-P.H.S.
English
KW - MEDLINE
KW - Child
KW - Child Behavior
KW - Preschool
KW - Developing Countries
KW - Female
KW - Food Deprivation
KW - Humans
KW - Infant
KW - Intelligence
KW - Kenya
KW - Male
KW - Mother-Child Relations
KW - Mothers/px [Psychology]
KW - Play and Playthings
KW - Poverty/px [Psychology]
KW - Social Behavior
RP - NOT IN FILE
SP - 404
EP - 415
JF - Child Development
JA - Child Dev
VL - 65
IS - 2 Spec No

CY - UNITED STATES
N2 - Kenya experienced a severe drought and temporary food shortage during a study on mild malnutrition. Effects of the temporary food shortage on energy intake, weight, and behaviors were evaluated in schoolchildren and in toddlers and their mothers. Schoolchildren were seriously affected, showing significant declines in their energy intake, age-corrected weight, activity on the playground, and classroom attention. Toddlers appear to have been somewhat protected since their energy intake, weight, and play and language behaviors were stable. Maternal caregiving of toddlers declined for the group as a whole, but individually those mothers who maintained family food levels delegated responsibility for toddlers to other caregivers. While the food shortage affected poorer families more than those of higher SES, declines in the behaviors of schoolchildren occurred regardless of SES and previous level of nutrition, suggesting that food shortages can have behavioral consequences for schoolchildren in all communities
SN - 0009-3920
AD - University of California, Los Angeles
UR - 8013230
ER -

TY - JOUR
ID - 2022
T1 - Acute effects of ambient ozone on respiratory function of Swiss schoolchildren after a 10-minute heavy exercise
A1 - Braun-Fahrlander,C.
A1 - Kunzli,N.
A1 - Domenighetti,G.
A1 - Carell,C.F.
A1 - ckermann-Liebrich,U.
Y1 - 1994/03//
N1 - Braun-Fahrlander, C. Kunzli, N. Domenighetti, G. Carell, C F. Ackermann-Liebrich, U
Pediatric pulmonology
owh, 8510590
IM
Comparative Study. Journal Article. Research Support, Non-U.S. Gov't
English
KW - MEDLINE
KW - Atmosphere
KW - Child
KW - Confidence Intervals
KW - Environmental Exposure
KW - Exercise
KW - Exercise Test
KW - Female
KW - Forced Expiratory Volume/de [Drug Effects]
KW - Humans
KW - Linear Models
KW - Lung/de [Drug Effects]
KW - Lung/ph [Physiology]
KW - Male
KW - Ozone/an [Analysis]
KW - Ozone/pd [Pharmacology]
KW - Peak Expiratory Flow Rate/de [Drug Effects]
KW - Regression Analysis
KW - Respiratory Function Tests
KW - Spirometry
KW - Switzerland
KW - Vital Capacity/de [Drug Effects]
RP - NOT IN FILE
SP - 169
EP - 177

JF - Pediatric Pulmonology
JA - Pediatr Pulmonol
VL - 17
IS - 3
CY - UNITED STATES
N2 - This study was conducted in Switzerland between May and October 1989 to assess possible decrements in lung function occurring as a result of 10 minute exposure to ambient air containing different ozone concentrations. Once a month, 128 children in two different areas of Southern Switzerland (Chiasso and Aurigeno) had a pulmonary function test before and after a standardized 10 minute exercise (pulse rate, 170/min) on a cycle ergometer, outdoors. Ozone concentrations were similar in both areas, ranging from 40 to 157 micrograms/m3 (1/2h means) during the exercise tests. The two communities differed with respect to long-term average pollution levels. The mean NO2 concentration over the six months study period was 70 micrograms/m3 in Chiasso and 18 micrograms/m3 in rural Aurigeno. Of the eligible children 85% participated and attended 4-6 tests. Parents completed a standardized questionnaire on family background, home characteristics and the child's early and present illness history. A total of 500 acceptable pairs of spirograms and corresponding ozone concentrations (average 3.8 per child) were available for analysis. Regressions of each individual's pre-post differences of FVC, FEV1 and peak flow on ozone concentrations measured during the outdoor exercise indicated that elevated ozone levels significantly reduced peak flow values. Adjustments for temperature and relative humidity increased the magnitude of the peak flow slopes. The average adjusted regression coefficient for delta-peak flow on ozone was -2.28 mL/s/micrograms/m3 (95% CI, -0.57 -3.99). It is noteworthy that the observed relationships occurred at ozone concentrations below 160 micrograms/m3 and after an exercise duration of only 10 minutes
SN - 8755-6863
AD - Institute of Social and Preventive Medicine, University of Basel, Switzerland
UR - 8196997
ER -

TY - JOUR
ID - 2023
T1 - Mild haemophilia A in Iceland: clinical genetic studies of three families with the same mutation
A1 - Jensson,O.
A1 - Stenbjerg,Bernvil S.
A1 - Jonsdottir,S.
A1 - Ingerslev,J.
Y1 - 1994/05//
N1 - Jensson, O. Stenbjerg Bernvil, S. Jonsdottir, S. Ingerslev, J
Journal of internal medicine
i2g, 8904841
IM
Journal Article. Research Support, Non-U.S. Gov't
English
KW - MEDLINE
KW - Adolescent
KW - Adult
KW - Aged
KW - Child
KW - Preschool
KW - Female
KW - Hemophilia A/co [Complications]
KW - Hemophilia A/ge [Genetics]
KW - Hemorrhage/ge [Genetics]
KW - Humans
KW - Iceland
KW - Male
KW - Middle Aged
KW - Mutation
KW - Pedigree

KW - Phenotype
RP - NOT IN FILE
SP - 443
EP - 450
JF - Journal of Internal Medicine
JA - J Intern Med
VL - 235
IS - 5
CY - ENGLAND
N2 - OBJECTIVES: The aim of the study was to define the phenotype in three families with mild haemophilia A and to determine restriction fragment length polymorphisms (RFLP), which could support a hypothesis of a common progenitor of the families. DESIGN: Family survey. SETTING: Index cases were identified in and outside hospital and a family survey for symptoms and signs of bleeding in family members and sampling for coagulation and RFLP studies were mostly carried out in the field. SUBJECT: Family members with and without symptoms of bleeding were selected for investigation and normal spouses and unrelated individuals were investigated for control. INTERVENTIONS: Medical advice regarding affected family members were given to the families and their physicians. MAIN OUTCOME MEASURES: Bleeding time, factor VIII activity, quantification of factor VIII:Ag, von Willebrand factor (vWF) Ag and vWF ristocetin assay. Typing of RFLP polymorphisms for genetic homogeneity. RESULTS: Bleeding manifestations are present in both sexes in the three families although more frequent and more severe in the males. The level of factor VIII activity is between 10 and 20% in most affected males whereas 35-60% is found approximately in 2/3 of female carriers and in 1/3 of them factor VIII activity is within the normal range. It is suggested that screening for this mild haemophilia A gene by a molecular genetic method would be of clinical value now, its mutation having been detected. CONCLUSIONS: Transmission of mild haemophilia A through six to seven generations is demonstrated by the study. The mild haemophilia A type described is the most prevalent of haemophilia A types in Iceland (population 260,000, 1992). The founder effect was confirmed by studies of RFLP polymorphisms
SN - 0954-6820
AD - Department of Medical Genetics, Blood Bank, National University Hospital, Reykjavik, Iceland
UR - 8182400
ER -

TY - JOUR
ID - 2024
T1 - Childhood lightning injuries on the playing field. [Review] [19 refs]
A1 - Cherington,M.
A1 - Martorano,F.J.
A1 - Siebuhr,L.V.
A1 - Stieg,R.L.
A1 - Yarnell,P.R.
Y1 - 1994/01//
N1 - Cherington, M. Martorano, F J. Siebuhr, L V. Stieg, R L. Yarnell, P R
The Journal of emergency medicine
ibo, 8412174
IM
Case Reports. Journal Article. Review
English
KW - MEDLINE
KW - Adolescent
KW - Camping
KW - Child
KW - Coma/pp [Physiopathology]
KW - Fatal Outcome
KW - Female
KW - Humans
KW - Lightning Injuries/pp [Physiopathology]
KW - Lightning Injuries/pc [Prevention & Control]
KW - Male

KW - Sports
RP - NOT IN FILE
SP - 39
EP - 41
JF - Journal of Emergency Medicine
JA - J Emerg Med
VL - 12
IS - 1
CY - UNITED STATES
N2 - Two young boys were struck by lightning during summer outdoor activities. One of them died. A literature review reveals that the most commonly reported locations of childhood lightning injuries are on the playing field, at the swimming pool, and in tents. Knowledge of measures to lower the risks of lightning strikes as well as the use of new lightning detection technology should help to reduce the numbers of these tragic events in the future. [References: 19]
SN - 0736-4679
AD - Lightning Data Center, St. Anthony Hospital, Denver, Colorado 80204
UR - 8163803
ER -

TY - JOUR
ID - 2025
T1 - Head injuries incurred by children and young adults during informal recreation
A1 - Baker,S.P.
A1 - Fowler,C.
A1 - Li,G.
A1 - Warner,M.
A1 - Dannenberg,A.L.
Y1 - 1994/04//
N1 - Baker, S P. Fowler, C. Li, G. Warner, M. Dannenberg, A L
American journal of public health
1254074, 3xw
AIM, IM
Journal Article. Research Support, Non-U.S. Gov't. Research Support, U.S. Gov't, P.H.S.
English
KW - MEDLINE
KW - Adolescent
KW - Adult
KW - Bicycling/in [Injuries]
KW - Child
KW - Preschool
KW - Craniocerebral Trauma/ep [Epidemiology]
KW - Craniocerebral Trauma/et [Etiology]
KW - Craniocerebral Trauma/pc [Prevention & Control]
KW - Female
KW - Head Protective Devices
KW - Humans
KW - Infant
KW - Male
KW - Maryland/ep [Epidemiology]
KW - Play and Playthings
KW - Recreation
KW - Sex Ratio
RP - NOT IN FILE
SP - 649
EP - 652
JF - American Journal of Public Health
JA - Am J Public Health

VL - 84
IS - 4
CY - UNITED STATES
N2 - To examine injuries related to playground equipment, children's vehicles, roller skates, and skateboards, 1991 data on emergency room patients younger than 25 years in the Consumer Product Safety Commission's 91 surveillance hospitals were used. Head injury was the primary diagnosis for an estimated 58,480 patients, exceeding the total number of head injuries to bicyclists younger than 25 years. The head injury rate decreased with age. The large number and high rate of head injuries in children involved in a variety of recreational activities suggests the value of multipurpose helmets
SN - 0090-0036
AD - Injury Prevention Center, Johns Hopkins School of Public Health, Baltimore, MD
UR - 8154572
ER -

TY - JOUR
ID - 2026
T1 - [Digoxin content in blood lipids of children, athletes, sports ground attendants and residents after content with dioxin-containing surface slag (Kieselrot)]. [German]
A1 - Ewers,U.
A1 - Wittsiepe,J.
A1 - Schrey,P.
A1 - Exner,M.
A1 - Selenka,F.
A1 - Hofbauer,M.
A1 - Schmeer,D.
A1 - Holwitt,L.
A1 - Eck,R.
Y1 - 1994/01//
N1 - Ewers, U. Wittsiepe, J. Schrey, P. Exner, M. Selenka, F. Hofbauer, M. Schmeer, D. Holwitt, L. Eck, R Gesundheitswesen (Bundesverband der Arzte des Offentlichen Gesundheitsdienstes (Germany))
bfd, 9204210
IM
English Abstract. Journal Article
German
KW - MEDLINE
KW - Adolescent
KW - Adult
KW - Benzofurans/pk [Pharmacokinetics]
KW - Child
KW - Dioxins/pk [Pharmacokinetics]
KW - Environmental Exposure/ae [Adverse Effects]
KW - Environmental Monitoring
KW - Female
KW - Hazardous Waste/an [Analysis]
KW - Humans
KW - Lipids/bl [Blood]
KW - Male
KW - Middle Aged
KW - Play and Playthings
KW - Polymers/pk [Pharmacokinetics]
KW - Reference Values
KW - Soil Pollutants/an [Analysis]
KW - Sports/ph [Physiology]
KW - Tetrachlorodibenzodioxin/aa [Analogs & Derivatives]
KW - Tetrachlorodibenzodioxin/pk [Pharmacokinetics]
RP - NOT IN FILE
SP - 14

EP - 20
JF - Gesundheitswesen
VL - 56
IS - 1
CY - GERMANY
N2 - In 1991 it was discovered, that a large number of sporting grounds and playgrounds in Germany were covered with a waste slag material from a former copper smelter located at Marsberg, Germany. This material was found to contain high levels of PCDD/F ranging up to 100,000 TE/kg. The objective of the present study was to assess whether subjects sporting on such grounds had elevated levels of PCDD/F in blood. PCDD/F in blood fat was used as an indicator of the PCDD/F body burden. Additionally, six children and seven residents of a contaminated sporting and playground were examined. Generally, the levels of PCDD/F in blood fat were in the range of background levels in all subjects. Taking into account the effect of age, slightly elevated blood levels of PCDD/F were detected in children. The results show that the bioavailability of PCDD/F in the slag material is very low. However, from the preventive point of view children who might ingest slag material by hand-to-mouth-activities, should not play on such contaminated playgrounds
SN - 0941-3790
AD - Hygiene-Institut des Ruhrgebiets, Institut fur Umwelthygiene und Umweltmedizin, Gelsenkirchen
UR - 8148582
ER -

TY - JOUR
ID - 2027
T1 - An exercise challenge protocol for epidemiological studies of asthma in children: comparison with histamine challenge
A1 - Haby,M.M.
A1 - Anderson,S.D.
A1 - Peat,J.K.
A1 - Mellis,C.M.
A1 - Toelle,B.G.
A1 - Woolcock,A.J.
Y1 - 1994/01//
N1 - Haby, M M. Anderson, S D. Peat, J K. Mellis, C M. Toelle, B G. Woolcock, A J
The European respiratory journal
8803460, ery
IM
Comparative Study. Journal Article. Research Support, Non-U.S. Gov't
English
KW - MEDLINE
KW - Asthma/pp [Physiopathology]
KW - Asthma
KW - Exercise-Induced/pp [Physiopathology]
KW - Bronchial Hyperreactivity/pp [Physiopathology]
KW - Child
KW - Epidemiologic Methods
KW - Histamine/du [Diagnostic Use]
KW - Humans
KW - Questionnaires
KW - Skin Tests
RP - NOT IN FILE
SP - 43
EP - 49
JF - European Respiratory Journal
JA - Eur Respir J
VL - 7
IS - 1
CY - DENMARK

N2 - We investigated whether an exercise challenge protocol is suitable for measuring bronchial responsiveness in epidemiological studies of asthma in children, and determined its comparability with histamine challenge. The exercise challenge was 6 minutes of outdoor, free-range running at 85-90% of maximum heart rate, measured by heart rate monitor. Nose clips were worn. Distance run was measured to estimate oxygen consumption. Water content of the inspired air was < 10 mg H2O.l-1. Histamine challenge was by the rapid method. We used questionnaires to measure respiratory symptoms and skin prick tests to measure atopy. A total of 96 children aged 8-11 years were studied. Bronchial hyperresponsiveness (BHR) to exercise challenge was defined as a fall in forced expiratory volume in one second (FEV1) of 13% of greater. Eleven children had a positive response to exercise challenge and 11 to histamine challenge but 12 responded to one challenge and not to the other. The correlation coefficient between the two tests was 0.65 (p = 0.0001). Exercise challenge thus proved to be a practical epidemiological tool for objective measurements of bronchial responsiveness in children. In this sample, some children responded to one challenge and not to the other which suggests that the two challenges identify different abnormalities of the airways
SN - 0903-1936
AD - Department of Medicine, University of Sydney, Australia
UR - 8143831
ER -

TY - JOUR
ID - 2028
T1 - Heparin therapy in pediatric patients: a prospective cohort study
A1 - Andrew,M.
A1 - Marzinotto,V.
A1 - Massicotte,P.
A1 - Blanchette,V.
A1 - Ginsberg,J.
A1 - Brill-Edwards,P.
A1 - Burrows,P.
A1 - Benson,L.
A1 - Williams,W.
A1 - David,M.
Y1 - 1994/01//
N1 - Andrew, M. Marzinotto, V. Massicotte, P. Blanchette, V. Ginsberg, J. Brill-Edwards, P. Burrows, P. Benson, L. Williams, W. David, M. et al
Pediatric research
owl, 0100714
IM
Clinical Trial. In Vitro. Journal Article. Research Support, Non-U.S. Gov't
English
KW - MEDLINE
KW - Adolescent
KW - Child
KW - Preschool
KW - Clinical Protocols
KW - Cohort Studies
KW - Female
KW - Heart Defects
KW - Congenital/bl [Blood]
KW - Congenital/dt [Drug Therapy]
KW - Heparin/ad [Administration & Dosage]
KW - Heparin/tu [Therapeutic Use]
KW - Humans
KW - Infant
KW - Male
KW - Partial Thromboplastin Time
KW - Prospective Studies
KW - Pulmonary Embolism/bl [Blood]

KW - Pulmonary Embolism/dt [Drug Therapy]
KW - Thrombophlebitis/bl [Blood]
KW - Thrombophlebitis/dt [Drug Therapy]
KW - Thrombosis/bl [Blood]
KW - Thrombosis/dt [Drug Therapy]
RP - NOT IN FILE
SP - 78
EP - 83
JF - Pediatric Research
JA - Pediatr Res
VL - 35
IS - 1
CY - UNITED STATES
N2 - Current guidelines for heparin therapy in pediatric patients have been extrapolated from trials in adult patients without rigorous evaluation of efficacy and safety. We prospectively monitored consecutive pediatric patients receiving systemic doses of heparin over 10 mo at one institution using a predetermined nomogram to monitor maintenance therapy. Sixty-five consecutive children; 38 males and 27 females, received systemic doses of heparin. Thirty children had deep venous thrombosis and/or pulmonary embolism; 11 had arterial thrombi, most frequently after diagnostic angiography; and the remaining 24 received heparin prophylactically, for congenital heart disease. Twenty-nine (45%) of the 65 patients were less than 1 y of age and 22 (34%) were 10 y or older. Congenital heart disease was the predominant diagnosis under 1 y and deep venous thrombosis in older children. After a bolus dose of 50 U/kg, 39% of children (n = 30) achieved a minimal level activated partial thromboplastin time (APTT). Sixty-eight percent of children achieved a minimal level APTT by 24 h and 81% by 48 h. For all 65 children, APTT values were within the therapeutic range 43% of the time. APTT values outside the therapeutic range were twice as likely to be low as high. The average amount of heparin required to maintain therapeutic APTT values for children was 22 U/kg/h: 28 U/kg/h for infants < 1 y and 20 U/kg/h for the rest. Bleeding was rare (2%) and mild. Documented recurrent thrombotic disease was more common (7%) with associated morbidity.(ABSTRACT TRUNCATED AT 250 WORDS)
SN - 0031-3998
AD - Department of Pediatrics, McMaster University, Hamilton, Ontario
UR - 8134203
ER -

TY - JOUR
ID - 2029
T1 - Measuring exposure to injury risk in schoolchildren aged 11-14
A1 - Towner,E.M.
A1 - Jarvis,S.N.
A1 - Walsh,S.S.
A1 - ynsley-Green,A.
Y1 - 1994/02/12/
N1 - Towner, E M. Jarvis, S N. Walsh, S S. Aynsley-Green, A
BMJ (Clinical research ed.)
8900488, bmj, 101090866
AIM, IM
Clinical Trial. Journal Article. Randomized Controlled Trial. Research Support, Non-U.S. Gov't
English
KW - MEDLINE
KW - Accident Prevention
KW - Accidents
KW - Traffic
KW - Adolescent
KW - Age Factors
KW - Bicycling/in [Injuries]
KW - Child
KW - England/ep [Epidemiology]
KW - Female

KW - Humans
KW - Male
KW - Risk Factors
KW - Sex Factors
KW - Socioeconomic Factors
KW - Wounds and Injuries/ep [Epidemiology]
KW - Wounds and Injuries/pc [Prevention & Control]
RP - NOT IN FILE
SP - 449
EP - 452
JF - BMJ
VL - 308
IS - 6926
CY - ENGLAND
N2 - OBJECTIVE: To apply a measure of exposure to injury risk for schoolchildren aged 11-14 across a population and to examine how risk factors vary with sex, age, and affluence. DESIGN: Self completion questionnaire survey administered in schools in May 1990. SETTING: 24 schools in Newcastle upon Tyne. SUBJECTS: 5334 pupils aged 11-14, of whom 4637 (87%) completed the questionnaire. RESULTS: Boys were exposed to greater risk than girls in journeys to places to play outdoors; they took longer trips and were more likely to ride bicycles (relative risk 5.30 (95% confidence interval 4.23 to 6.64)) and less likely to travel by public transport or car. Younger pupils (aged 11-12) were less exposed to traffic during journeys to and from school: their journeys were shorter, they were less likely to walk (trip to school, relative risk 0.88 (0.83 to 0.94)), and they were more likely to travel by car (trip to school, relative risk 1.33 (1.13 to 1.56)) or school bus (1.33 (1.10 to 1.62)). Poorer children were exposed to greater risk than affluent children (from families that owned a car and a telephone): they were less likely to travel to school by car (relative risk 0.26 (0.20 to 0.33)) or to be accompanied by an adult (0.39 (0.32 to 0.48)). CONCLUSION: Injury risk data can provide useful information on child injury prevention and can be used to identify priorities and target resources for injury prevention on a citywide scale or for an individual school
SN - 0959-8138
AD - Department of Child Health, University of Newcastle Medical School, Newcastle Upon Tyne
UR - 8124176
ER -

TY - JOUR
ID - 2030
T1 - Personal exposure to nitrogen dioxide in the Los Angeles Basin
A1 - Spengler,J.
A1 - Schwab,M.
A1 - Ryan,P.B.
A1 - Colome,S.
A1 - Wilson,A.L.
A1 - Billick,I.
A1 - Becker,E.
Y1 - 1994/01//
N1 - Spengler, J. Schwab, M. Ryan, P B. Colome, S. Wilson, A L. Billick, I. Becker, E
Air & waste : journal of the Air & Waste Management Association
b2l, 9433394
IM
Journal Article. Research Support, Non-U.S. Gov't
English
KW - MEDLINE
KW - Adolescent
KW - Adult
KW - Aged
KW - Air Pollution/sn [Statistics & Numerical Data]
KW - Air Pollution
KW - Indoor/sn [Statistics & Numerical Data]

KW - Child
KW - Environmental Exposure
KW - Humans
KW - Los Angeles
KW - Middle Aged
KW - Nitric Oxide/an [Analysis]
RP - NOT IN FILE
SP - 39
EP - 47
JF - Air & Waste
JA - Air Waste
VL - 44
IS - 1
CY - UNITED STATES
N2 - A large scale study of human exposure to nitrogen dioxide (NO2) was conducted in the Los Angeles Basin, the only metropolitan area in the United States that exceeds the NO2 NAAQS. Data are available for a population representative sample of 482 households and 682 individuals. Personal exposures, as well as indoor and outdoor home levels, were monitored using passive time-integrating filter badges. Monitoring extended over a one-year period (May 1987 to May 1988), with each individual providing two consecutive days of data. Information was also collected on activity patterns, household and personal characteristics, and spatial and temporal variables. This paper describes the study design, summarizes the sample characteristics and representativeness, and presents the distribution of personal, indoor, and outdoor NO2 concentrations recorded by the monitors. Over the entire sample, median personal and outdoor levels were 35 ppb; median indoor levels were 24 ppb. Personal exposures for those in homes with gas ranges with pilot lights average 10 ppb greater than those with electric ranges, and 4 ppb greater than those with gas ranges without pilot lights. Forty percent of the variation in indoor concentrations is explained by outdoor levels; 59 percent of the variation in personal exposures is explained by indoor levels; and 48 percent of the variation in personal exposures is explained by outdoor levels
SN - 1073-161X
AD - Harvard University, School of Public Health Boston, Massachusetts
UR - 8117430
ER -

TY - JOUR
ID - 2031
T1 - A nationwide study of the risk of injury associated with day care center attendance. [Review] [24 refs]
A1 - Briss,P.A.
A1 - Sacks,J.J.
A1 - Addiss,D.G.
A1 - Kresnow,M.
A1 - O'neil,J.
Y1 - 1994/03//
N1 - Briss, P A. Sacks, J J. Addiss, D G. Kresnow, M. O'Neil, J
Pediatrics
oxv, 0376422
AIM, IM
Journal Article. Review
English
KW - MEDLINE
KW - Child Day Care Centers/sn [Statistics & Numerical Data]
KW - Child
KW - Preschool
KW - Humans
KW - Risk Factors
KW - United States/ep [Epidemiology]
KW - Wounds and Injuries/ep [Epidemiology]
RP - NOT IN FILE

SP - 364
EP - 368
JF - Pediatrics
VL - 93
IS - 3
CY - UNITED STATES
N2 - OBJECTIVE: Because an increasing proportion of US children spends time in day care center environments, a national estimate of injury risks in day care centers is needed. METHODS: We interviewed directors of 1797 day care centers from every state and the District of Columbia from October to December 1990 and analyzed medically attended injuries and center characteristics reported by the directors. RESULTS: The centers were attended by 138,404 children. In the 2 months before the center directors were interviewed, 556 children sustained injuries requiring medical attention while attending the centers. The injury rate was 1.5 injuries per 100,000 child hours in day care. The most common injuries were cuts or lacerations (31%), bumps or bruises (15%), fractures (10%), and dental injuries (8%). Most injuries (51%) occurred on the playground. Many injuries (18%), and more than half of fractures and concussions (53%) were due to falls from climbing equipment. CONCLUSIONS: Day care center injury rates estimated by this study were relatively low. Many injuries that occur in this setting are probably minor. However, lowering the height of playground equipment and providing more resilient playground surfaces could further reduce injury risks in day care centers. [References: 24]
SN - 0031-4005
AD - National Center for Injury Prevention and Control, Centers for Disease Control and Prevention, Atlanta, GA
UR - 8115192
ER -

TY - JOUR
ID - 2032
T1 - Evaluation of methods for monitoring the potential exposure of small children to pesticides in the residential environment
A1 - Lewis,R.G.
A1 - Fortmann,R.C.
A1 - Camann,D.E.
Y1 - 1994/01//
N1 - Lewis, R G. Fortmann, R C. Camann, D E
Archives of environmental contamination and toxicology
6yd, 0357245
IM
Journal Article
English
KW - MEDLINE
KW - Child
KW - Preschool
KW - Environmental Exposure
KW - Environmental Monitoring/mt [Methods]
KW - Humans
KW - Infant
KW - Pesticides/an [Analysis]
KW - Pilot Projects
RP - NOT IN FILE
SP - 37
EP - 46
JF - Archives of Environmental Contamination & Toxicology
JA - Arch Environ Contam Toxicol
VL - 26
IS - 1
CY - UNITED STATES

N2 - A nine-home pilot study was conducted to evaluate monitoring methods in the field that may be used to assess the potential exposures of children aged 6 months to 5 years to pesticides found in the home environment. Several methods, some of which were newly developed in this study, were tested for measuring pesticide residues in indoor air, carpet dust, outdoor soil, and on the children's hands. Information was also collected on household characteristics, pesticides used and stored at the residence, and children's activities. Pesticides were detected at all nine study homes. With the exception of one home, at least one pesticide was detected in all matrices sampled at each house. Of the 30 target pesticides, 23 were detected during the study. The most frequently detected pesticides were chlordane, chlorpyrifos, dieldrin, hepatachlor, and pentachlorophenol. The greatest number of pesticides and highest concentrations were found in carpet dust. The results of these investigations will be discussed in terms of performance of the methods and the distribution of pesticides across the various media sampled
SN - 0090-4341
AD - U.S. Environmental Protection Agency, Research Triangle Park, North Carolina 27711-2055
UR - 8110022
ER -

TY - JOUR
ID - 2033
T1 - Detection of varicella-zoster virus DNA in air samples from hospital rooms
A1 - Sawyer,M.H.
A1 - Chamberlin,C.J.
A1 - Wu,Y.N.
A1 - Aintablian,N.
A1 - Wallace,M.R.
Y1 - 1994/01//
N1 - Sawyer, M H. Chamberlin, C J. Wu, Y N. Aintablian, N. Wallace, M R
The Journal of infectious diseases
ih3, 0413675
AIM, IM
Journal Article
English
KW - MEDLINE
KW - Adolescent
KW - Adult
KW - Air Microbiology
KW - Air Pollution
KW - Indoor
KW - Chickenpox/tm [Transmission]
KW - Child
KW - Preschool
KW - Cross Infection/tm [Transmission]
KW - DNA
KW - Viral/ip [Isolation & Purification]
KW - Electrophoresis
KW - Agar Gel
KW - Herpes Zoster/tm [Transmission]
KW - Herpesvirus 3
KW - Human/ge [Genetics]
KW - Hospitals
KW - Humans
KW - Infant
KW - Male
KW - Polymerase Chain Reaction
RP - NOT IN FILE
SP - 91
EP - 94
JF - Journal of Infectious Diseases

JA - J Infect Dis
VL - 169
IS - 1
CY - UNITED STATES
N2 - Varicella-zoster virus (VZV) is a highly contagious infectious agent that causes outbreaks in institutional settings. Transmission of VZV is felt to occur following direct contact with an infected individual and by aerosol spread. To document the aerosolization of VZV, a polymerase chain reaction (PCR) assay was used to detect VZV DNA in air samples obtained from hospital rooms of patients with active VZV infection. VZV DNA was detected in 64 (82%) of 78 air samples from rooms housing patients with active varicella and 9(70%) of 13 samples from rooms of patients with herpes zoster. VZV was detected 1.2-5.5 m from patients' beds and for 1-6 days following onset of rash. On some occasions, VZV DNA could be detected outside the hospital isolation rooms housing patients. This PCR-based method allows the detection and semiquantitation of VZV aerosolization and can be a useful tool for monitoring efforts to control VZV aerosols in the environment
SN - 0022-1899
AD - Department of Pediatrics, University of California, San Diego, La Jolla 92093-0672
UR - 8277202
ER -

TY - JOUR
ID - 2034
T1 - Risk factors for exposure to arsenic at a hazardous waste site
A1 - Reif,J.S.
A1 - Tsongas,T.A.
A1 - Mitchell,J.
A1 - Keefe,T.J.
A1 - Tessari,J.D.
A1 - Metzger,L.
A1 - Amler,R.
Y1 - 1993///
N1 - Reif, J S. Tsongas, T A. Mitchell, J. Keefe, T J. Tessari, J D. Metzger, L. Amler, R
Journal of exposure analysis and environmental epidemiology
bjn, 9111438
IM
Journal Article. Research Support, Non-U.S. Gov't
English
KW - MEDLINE
KW - Adolescent
KW - Adult
KW - Aged
KW - Arsenic/ur [Urine]
KW - Child
KW - Preschool
KW - Colorado
KW - Cross-Sectional Studies
KW - Environmental Monitoring/mt [Methods]
KW - Environmental Monitoring
KW - Female
KW - Hazardous Substances/ur [Urine]
KW - Hazardous Waste/an [Analysis]
KW - Humans
KW - Infant
KW - Logistic Models
KW - Male
KW - Middle Aged
KW - Multivariate Analysis
KW - Poisons/ur [Urine]
KW - Population Surveillance

KW - Questionnaires
KW - Residence Characteristics
KW - Risk Assessment
KW - Risk Factors
KW - Sampling Studies
RP - NOT IN FILE
SP - 73
EP - 86
JF - Journal of Exposure Analysis and Environmental Epidemiology
JA - J Expo Anal Environ Epidemiol
VL - 3 Suppl 1
CY - UNITED STATES
N2 - Communities surrounding the Rocky Mountain Arsenal (RMA), a Superfund site in Colorado, were studied in order to determine whether exposures to arsenic were greater among persons who resided there than among residents of a comparison area. A census was conducted in areas adjacent to the RMA and in a comparison area 12-15 miles distant. From a stratified random sample, 469 persons were interviewed and urine samples obtained. Arsenic was detected in urine from 43 (9.2%) of the 469 persons sampled at a detection limit of 10 ppb. Trace levels of arsenic (detectable, but non-quantifiable) were found in 184 (39.2%) of those persons sampled. Neither the frequency of detection, the arithmetic mean nor the geometric mean values for urine arsenic was found to be statistically different when persons living near the site were compared to persons from the more distant comparison area. Therefore, the data were pooled across the study areas to evaluate risk factors for exposure to arsenic in this population-based sample. Multivariate logistic regression analyses were conducted to evaluate the risk of arsenic exposure associated with variables included in the interviews while controlling for confounding. Pathways for exposure to arsenic were evaluated through analysis of residence history, occupation, hobbies, dietary habits, water supply, housing and activity patterns. Children of Hispanic origin or non-white race, children who drank less than three glasses of water daily, and children who spent more time outdoors had an increased risk of having > or = 10 ppb of arsenic in their urine. Among adults, younger persons, especially those less than 40 years of age, persons of Hispanic origin or nonwhite race, and those employed in occupations where arsenic is likely to be found had an increased risk of having > or = 10 ppb of urine arsenic. Consumption of red wine or fish during the week prior to sampling was associated with trace levels of arsenic in urine
SN - 1053-4245
AD - Department of Environmental Health, Colorado State University, Fort Collins 80523, USA
UR - 9857295
ER -

TY - JOUR
ID - 2035
T1 - Obstruction of the St. Jude Medical valve--diagnostic and therapeutic values of cineradiography
A1 - Aoyagi,S.
A1 - Higa,Y.
A1 - Matsuzoe,S.
A1 - Nishi,Y.
A1 - Tanaka,K.
A1 - Kawara,T.
A1 - Oryoji,A.
A1 - Kosuga,K.
A1 - Oishi,K.
Y1 - 1993/12//
N1 - Aoyagi, S. Higa, Y. Matsuzoe, S. Nishi, Y. Tanaka, K. Kawara, T. Oryoji, A. Kosuga, K. Oishi, K
The Thoracic and cardiovascular surgeon
vr0, 7903387
IM
Journal Article
English
KW - MEDLINE
KW - Adult

KW - Child
KW - Preschool
KW - Cineradiography
KW - Female
KW - Heart Valve Diseases/dt [Drug Therapy]
KW - Heart Valve Diseases/ra [Radiography]
KW - Heart Valve Prosthesis
KW - Humans
KW - Male
KW - Middle Aged
KW - Postoperative Complications/dt [Drug Therapy]
KW - Postoperative Complications/ra [Radiography]
KW - Thrombolytic Therapy
KW - Urokinase-Type Plasminogen Activator/tu [Therapeutic Use]
RP - NOT IN FILE
SP - 357
EP - 363
JF - Thoracic & Cardiovascular Surgeon
JA - Thorac Cardiovasc Surg
VL - 41
IS - 6
CY - GERMANY
N2 - Between 1984, and 1992, we observed 8 patients with an obstructed St. Jude Medical (SJM) valve. Of these, 1 had an obstructed SJM valve in the aortic position, 3 in the mitral position, and 4 in the tricuspid position. Diagnosis of obstruction of the SJM valve was made by cineradiography combined with echocardiography in all 8 patients. Restriction or absence of movement of the leaflet of the SJM valve was observed by cineradiography in all of the 8 patients. Normal range of leaflet mobility of the SJM valve was measured by conventional cineradiography in 70 patients with a normal SJM valve, and the results were compared with the leaflet mobility obtained from the 8 patients with an obstructed SJM valve. In this study, leaflet function in the obstructed SJM valve was strikingly abnormal, with both opening and closing angles, and leaflet motion clearly outside the normal range. Of the 8 patients, 4 underwent urgent prosthetic valve replacements after cineradiography and echocardiography were carried out. Thrombolysis using urokinase was performed in 4 patients, and this treatment was successful in 1 patient. Efficacy of thrombolytic therapy was evaluated by repeat cineradiography. Three of the 4 patients who received thrombolysis showed no significant improvement of leaflet mobility after at least 72 hours of thrombolytic therapy, and finally required surgical correction for the obstructed SJM valve. We believe that cineradiography combined with echocardiography is the optimal method for the diagnosis of obstruction of the SJM valve, and to follow the effect of thrombolytic therapy on prosthetic valve function.(ABSTRACT TRUNCATED AT 250 WORDS)
SN - 0171-6425
AD - Second Department of Surgery, Kurume University School of Medicine, Japan
UR - 8128465
ER -

TY - JOUR
ID - 2036
T1 - [Epidemiology and diagnosis of allergic diseases]. [Review] [5 refs] [German]
A1 - Aberer,W.
A1 - Geusau,A.
A1 - Pirkhammer,D.
A1 - Schreiber,S.
Y1 - 1993///
N1 - Aberer, W. Geusau, A. Pirkhammer, D. Schreiber, S
Wiener klinische Wochenschrift
xop, 21620870r
IM
English Abstract. Journal Article. Review
German

KW - MEDLINE
KW - Adolescent
KW - Adult
KW - Allergens/du [Diagnostic Use]
KW - Austria/ep [Epidemiology]
KW - Child
KW - Cross-Sectional Studies
KW - Female
KW - Humans
KW - Hypersensitivity/di [Diagnosis]
KW - Hypersensitivity/ep [Epidemiology]
KW - Hypersensitivity/et [Etiology]
KW - Incidence
KW - Intradermal Tests
KW - Male
KW - Patch Tests
RP - NOT IN FILE
SP - 624
EP - 627
JF - Wiener Klinische Wochenschrift
JA - Wien Klin Wochenschr.
VL - 105
IS - 22
CY - AUSTRIA
N2 - Epidemiological studies indicate a world-wide and significant increase in atopic diseases over the past decades, which has adopted alarming dimensions within the industrialized world. However, allergic asthma and pollinosis, in particular, are on the increase in Third world countries, in parallel to the industrialization and westernization of their life-style. Since both antigen exposure and the presence of additional realization factors are required for the manifestation of atopic diseases, this increase in prevalence is not surprising. In addition to increased indoor and outdoor pollution, changes in the way of living--causing increased allergen exposure--certainly play an important role as cofactor in the increased incidence of allergies. Accurate diagnostic procedures permit a better understanding of the realization factors for allergic diseases in epidemiological studies and identification of the causative agent in the individual so that effective therapeutic and prophylactic steps can be taken. Despite improved in vivo and in vitro test methods, the case history is still of paramount importance in reaching a diagnosis; the potential hazards of skin and provocation testing, as well as the high costs and limitations of in vitro methods prohibit "blind allergy screening". [References: 5]
SN - 0043-5325
AD - Abteilung fur Immundermatologie und infektiose Hautkrankheiten, Universitatsklinik fur Dermatologie, Wien
UR - 8291256
ER -

TY - JOUR
ID - 2037
T1 - [Malaria indices, larval ecology and trophic activity of Anopheles mosquitoes in Djohong (Adamaoua, Cameroon) in the rainy season]. [French]
A1 - Raccurt,C.P.
A1 - Bourianne,C.
A1 - Lambert,M.T.
A1 - Tribouley,J.
A1 - Mandji,O.
A1 - Amadou,A.
A1 - Bouloumie,J.
A1 - Ripert,C.
Y1 - 1993/07//
N1 - Raccurt, C P. Bourianne, C. Lambert, M T. Tribouley, J. Mandji, O. Amadou, A. Bouloumie, J. Ripert, C Medecine tropicale : revue du Corps de sante colonial

mhq, 8710146
IM
English Abstract. Journal Article
French
KW - MEDLINE
KW - Adolescent
KW - Adult
KW - Age Factors
KW - Aged
KW - 80 and over
KW - Animals
KW - Anopheles/cl [Classification]
KW - Anopheles/gd [Growth & Development]
KW - Anopheles
KW - Cameroon/ep [Epidemiology]
KW - Child
KW - Preschool
KW - Ecology
KW - Feeding Behavior
KW - Female
KW - Humans
KW - Infant
KW - Newborn
KW - Larva/gd [Growth & Development]
KW - Malaria/ep [Epidemiology]
KW - Malaria/ps [Parasitology]
KW - Malaria/pc [Prevention & Control]
KW - Malaria
KW - Falciparum/ep [Epidemiology]
KW - Falciparum/ps [Parasitology]
KW - Falciparum/pc [Prevention & Control]
KW - Male
KW - Middle Aged
KW - Plasmodium malariae
KW - Population Surveillance
KW - Prevalence
KW - Rain
KW - Seasons
KW - Socioeconomic Factors
RP - NOT IN FILE
SP - 355
EP - 362
JF - Medecine Tropicale
JA - Med Trop (Mars.)
VL - 53
IS - 3
CY - FRANCE
N2 - In Djohong in the wet season the prevalence of malaria is 17.5% for Plasmodium falciparum and 1.1% for Plasmodium malariae. In children 2 to 9 years of age the plasmodic index is 38.6% (mesoendemicity) for the children of the peasants and 9.4% for those belonging to other socioeconomical groups. In infants less than 12 months old, the plasmodic index is 9.3%, this relatively high rate corresponding to the high transmission period of the rainy season. Anopheles gambiae is the mosquito species most often found in the area (2/3 of the mosquitoes caught in the houses). The breeding sites in he surrounding of the houses are rainwater holes linked to human activity. At a larger distance from the houses, in the valley, the breeding sites are water holes borrowed for the retting of cassava tubercle or natural rock pools found in the basaltic shores of the Mbere river. The trophic activity of Anopheles gambiae and Anopheles funestus is high between 0 and 4 hours a.m. In

October the mean number of anopheline mosquitoes bites per night per inhabitant is 33 inside the houses and 7 outside
SN - 0025-682X
AD - Laboratoire de Parasitologie, Universite de Bordeaux II, France
UR - 8289630
ER -

TY - JOUR
ID - 2038
T1 - Prevalence of Toxocara eggs in sandpits in Tokushima city and its outskirts
A1 - Shimizu,T.
Y1 - 1993/10//
N1 - Shimizu, T
The Journal of veterinary medical science / the Japanese Society of Veterinary Science
a27, 9105360
IM
Journal Article
English
KW - MEDLINE
KW - Animals
KW - Child
KW - Preschool
KW - Dog Diseases/ep [Epidemiology]
KW - Dogs/ps [Parasitology]
KW - Female
KW - Humans
KW - Incidence
KW - Japan
KW - Microscopy
KW - Electron
KW - Scanning
KW - Ovum/cy [Cytology]
KW - Ovum/ul [Ultrastructure]
KW - Parasite Egg Count
KW - Seasons
KW - Soil
KW - Suburban Population
KW - Toxocara/ip [Isolation & Purification]
KW - Toxocara canis/ip [Isolation & Purification]
KW - Toxocariasis/ep [Epidemiology]
KW - Toxocariasis/tm [Transmission]
KW - Urban Health
KW - Urban Population
RP - NOT IN FILE
SP - 807
EP - 811
JF - Journal of Veterinary Medical Science
JA - J Vet Med Sci
VL - 55
IS - 5
CY - JAPAN
N2 - To demonstrate the prevalence of Toxocara eggs in sandpits in Tokushima Prefecture, Japan, sand samples were examined. Of 46 sandpits surveyed, 29 sandpits (63.3%) had Toxocara eggs. Sandpits in public parks and playgrounds in residential areas were more contaminated (87.5%) with the eggs than those in kindergartens, schools, and children's centers (36.4%). To examine the seasonal change in the contamination, sand samples obtained from five sandpits were examined monthly from May 1990 to April 1991. Toxocara eggs were found in the majority of sandpits (3/5-5/5) in the spring, early summer, and autumn. In the summer and winter, the

rates of sandpits contaminated were lower (2/5-3/5) than in other seasons. Egg counts became smaller in the summer and winter, especially in summer. The ratio of T. canis to T. cati eggs recovered was 2:3. Fecal examination of 144 puppies revealed that 98 (68.0%) were positive for T. canis eggs. These findings show that Toxocara eggs, a possible causative agent of visceral larva migrans, commonly present in sandpits of the urban area. To prevent the contamination of sandpits with these eggs, control measures are needed
SN - 0916-7250
AD - Tokushima Health Center, Japan
UR - 8286535
ER -

TY - JOUR
ID - 2039
T1 - Use of personal measurements for ozone exposure assessment: a pilot study
A1 - Liu,L.J.
A1 - Koutrakis,P.
A1 - Suh,H.H.
A1 - Mulik,J.D.
A1 - Burton,R.M.
Y1 - 1993/09//
N1 - Liu, L J. Koutrakis, P. Suh, H H. Mulik, J D. Burton, R M
Environmental health perspectives
ei0, 0330411
IM
Journal Article. Research Support, Non-U.S. Gov't. Research Support, U.S. Gov't, Non-P.H.S.
English
KW - MEDLINE
KW - Air Pollution
KW - Indoor/an [Analysis]
KW - Child
KW - Environmental Monitoring/mt [Methods]
KW - Humans
KW - Models
KW - Biological
KW - Ozone/ae [Adverse Effects]
KW - Pilot Projects
KW - Specimen Handling/mt [Methods]
RP - NOT IN FILE
SP - 318
EP - 324
JF - Environmental Health Perspectives
JA - Environ Health Perspect
VL - 101
IS - 4
CY - UNITED STATES
N2 - During summer 1991, we collected indoor, outdoor, and personal ozone concentration data as well as time-activity data in State College, Pennsylvania. These concentrations were measured for 23 children and their homes using passive ozone samplers. Outdoor concentrations were also measured at a stationary ambient monitoring site. Results from this pilot study demonstrate that fixed-site ambient measurements may not adequately represent individual exposures. Outdoor ozone concentrations showed substantial spatial variation between rural and residential regions. Ignoring this spatial variation by using fixed-site measurements to estimate personal exposures can result in an error as high as 127%. In addition, evidence from our pilot study indicates that ozone concentrations of a single indoor microenvironment may not represent those of other indoor microenvironments. Personal exposures were significantly correlated with both indoor (r = 0.55) and outdoor (r = 0.41) concentrations measured at home sites. Multiple regression analyses identified indoor ozone concentrations as the most important predictors of personal exposures. However, models based on time-weighted indoor and outdoor concentrations explained only 40% of the variability in personal exposures. When the model included observations for only those participants who spent the majority of their day in or near their

homes, an R2 of 0.76 resulted when estimates were regressed on measured personal exposures. It is evident that contributions from diverse indoor and outdoor microenvironments must be considered to estimate personal ozone exposures accurately
SN - 0091-6765
AD - Department of Environmental Health, Harvard School of Public Health, Boston, MA 02115
UR - 8275989
ER -

TY - JOUR
ID - 2040
T1 - [Handling of red silica gravel on sports, play and resort surfaces with special reference to new toxicologic results]. [German]
A1 - Heudorf,U.
Y1 - 1993/10//
N1 - Heudorf, U
Gesundheitswesen (Bundesverband der Arzte des Offentlichen Gesundheitsdienstes (Germany))
bfd, 9204210
IM
English Abstract. Journal Article
German
KW - MEDLINE
KW - Adolescent
KW - Adult
KW - Aged
KW - 80 and over
KW - Benzofurans/an [Analysis]
KW - Benzofurans/to [Toxicity]
KW - Body Burden
KW - Child
KW - Preschool
KW - Environmental Pollutants/an [Analysis]
KW - Female
KW - Humans
KW - Infant
KW - Leisure Activities
KW - Male
KW - Maximum Allowable Concentration
KW - Middle Aged
KW - Play and Playthings
KW - Polymers/an [Analysis]
KW - Polymers/to [Toxicity]
KW - Soil Pollutants/an [Analysis]
KW - Sports
KW - Tetrachlorodibenzodioxin/aa [Analogs & Derivatives]
KW - Tetrachlorodibenzodioxin/an [Analysis]
KW - Tetrachlorodibenzodioxin/to [Toxicity]
RP - NOT IN FILE
SP - 521
EP - 526
JF - Gesundheitswesen
VL - 55
IS - 10
CY - GERMANY
N2 - In 1991 "Kieselrot", a material with extremely high PCDD/PCDF-contamination up to about 200,000 ng TEQ/kg was detected on many leisure centres and sports fields in Germany. The contaminated grounds were closed, and covered up. The following examinations to prepare the planned decontamination are described here. The need for decontamination of those "Kieselrot"-areas is discussed with special reference to its relevance on

behalf of environmental toxicology and preventive medicine. Risk estimates, based on mathematical models had shown a considerable health and cancer risk for the users of such play- and sports grounds. However, recent studies conducted with sportsmen, groundsmen, and residents after many years of contact with "Kieselrot"-covered grounds did not reveal any additional PCDD/PCDF blood fat burden. But raised TEQ-levels and a typical congeneric pattern pointing to a "Kieselrot"-load could be seen in some of the examined children. Following these results it was concluded that children play grounds contaminated with "Kieselrot" should remain closed and should be decontaminated, whereas sports fields could be used again. Another possibility could be the adaption of the decontamination concepts and methods to the proven low bioavailability of PCDD/PCDF from "Kieselrot"
SN - 0941-3790
AD - Gesundheitsamt der Stadt Frankfurt am Main
UR - 8268705
ER -

TY - JOUR
ID - 2041
T1 - Cerebral palsy
A1 - Chandra,R.
A1 - Srivastava,A.K.
A1 - Mishra,P.C.
A1 - Rahman,H.
A1 - Sharan,R.
Y1 - 1993/09//
N1 - Chandra, R. Srivastava, A K. Mishra, P C. Rahman, H. Sharan, R
Journal of the Indian Medical Association
ifr, 7505608
IM
Journal Article
English
KW - MEDLINE
KW - Cerebral Palsy/ep [Epidemiology]
KW - Cerebral Palsy
KW - Child
KW - Preschool
KW - Female
KW - Humans
KW - India/ep [Epidemiology]
KW - Infant
KW - Male
KW - Prospective Studies
RP - NOT IN FILE
SP - 236
EP - 237
JF - Journal of the Indian Medical Association
JA - J Indian Med Assoc
VL - 91
IS - 9
CY - INDIA
N2 - In a prospective study it was found that cerebral palsy constituted 0.29% of total paediatric outdoor cases during the study period of 1988-1990. Seventy-five children suffering from cerebral palsy were studied at SN Children Hospital, Allahabad. Male to female ratio was 1:2. There were 42.8% cases who belonged to the age between one and 2 years. Hypertonic cases were predominant over hypotonic cases (3:1). Hemiplegia was commonest (68%) while triplegia was least common (2.7%). There were 24.5% cases who were mentally retarded. Hypertonic cases improved more frequently than hypotonic cases. An early management by paediatric physiotherapy may play an important role in the treatment of cerebral palsy
SN - 0019-5847
AD - Department of Paediatrics, SN Children Hospital, MLN Medical College, Allahabad

UR - 8263349
ER -

TY - JOUR
ID - 2042
T1 - Effect of continuous slow-speed running for 12 weeks on 10-14-year-old Indian boys
A1 - Chatterjee,S.
A1 - Bandyopadhyay,A.
Y1 - 1993/09//
N1 - Chatterjee, S. Bandyopadhyay, A
British journal of sports medicine
0432520, b2w
IM
Journal Article
English
KW - MEDLINE
KW - Adolescent
KW - Child
KW - Heart Rate
KW - Humans
KW - India
KW - Male
KW - Physical Education and Training
KW - Physical Endurance/ph [Physiology]
KW - Physical Fitness/ph [Physiology]
KW - Running/ph [Physiology]
RP - NOT IN FILE
SP - 179
EP - 185
JF - British Journal of Sports Medicine
JA - BJSM online
VL - 27
IS - 3
CY - ENGLAND
N2 - Endurance training was conducted on a group of 41 East Indian boys aged 10-14 years and was compared with 25 untrained boys of the same age. A continuous slow-running method was adopted for 12 weeks. The intensity of the training was 80-85% of maximum heart rate and frequency was 3 days per week. The boys were trained for a 1500-m event and therefore they covered three to five times their racing distance. For psychological reasons the training was carried out in a playground. The investigations included different physical and motor fitness tests: measurement of flexibility, agility, speed, leg muscle strength etc. Their performance times were also recorded before and after training. From statistical analysis we concluded that this particular type of training programme did not produce any detrimental effects on 10-14-year-old boys. On the other hand, this type of training did have some influence on improving physiological parameters in this age group of boys when compared with untrained boys of the same age
SN - 0306-3674
AD - Department of Physiology, University College of Science, Calcutta, India
UR - 8242276
ER -

TY - JOUR
ID - 2043
T1 - The distribution of lifetime cumulative exposures to radon for California residents
A1 - Liu,K.S.
A1 - Chang,Y.L.
A1 - Hayward,S.B.
A1 - Gadgil,A.J.
A1 - Nero,A.V.

Y1 - 1993/04//
N1 - Liu, K S. Chang, Y L. Hayward, S B. Gadgil, A J. Nero, A V
Journal of exposure analysis and environmental epidemiology
bjn, 9111438
IM
Journal Article. Research Support, U.S. Gov't, Non-P.H.S.
English
KW - MEDLINE
KW - Adolescent
KW - Adult
KW - Aged
KW - California
KW - Child
KW - Preschool
KW - Computer Simulation
KW - Environmental Exposure
KW - Female
KW - Humans
KW - Infant
KW - Male
KW - Middle Aged
KW - Monte Carlo Method
KW - Population Dynamics
KW - Radiation Monitoring
KW - Radon/an [Analysis]
RP - NOT IN FILE
SP - 165
EP - 179
JF - Journal of Exposure Analysis and Environmental Epidemiology
JA - J Expo Anal Environ Epidemiol
VL - 3
IS - 2
CY - UNITED STATES
N2 - The distribution of individual lifetime exposures to radon was estimated using data from studies on radon concentrations, mobility, and time-activity patterns in the state of California. The distributions of radon concentrations in various geographic regions were obtained from the results of year-long radon measurements of 310 residences. The mobility patterns were acquired through a survey of the moving histories of the members of 507 households. The indoor and outdoor time-activity data were collected for 1,780 individuals in 1,596 households. Based on these data, a computer simulation technique was used to estimate the distribution of radon exposures with a parametric (lognormal model) and a nonparametric approach (bootstrap method). The estimated average lifetime exposure for radon was 2,448 Bq.m-3.yr for the lognormal model and 2,487 Bq.m-3.yr for the bootstrap method. The standard deviation was 1,130 and 1,145 Bq.m-3.yr respectively. Assuming no move over the lifetime, the estimated average lifetime exposure to radon was 2,052 Bq.m-3.yr for the lognormal model and 2,078 Bq.m-3.yr for the bootstrap method, while the standard deviation increased to 1,378 Bq.m-3.yr for the lognormal model and 1,514 Bq.m-3.yr for the bootstrap method
SN - 1053-4245
AD - Air & Industrial Hygiene Laboratory, California Department of Health Services, Berkeley 94704
UR - 8241780
ER -

TY - JOUR
ID - 2044
T1 - Nitrogen dioxide and respiratory illness in children. Part II: Assessment of exposure to nitrogen dioxide
A1 - Lambert,W.E.
A1 - Samet,J.M.
A1 - Hunt,W.C.
A1 - Skipper,B.J.

A1 - Schwab,M.
A1 - Spengler,J.D.
Y1 - 1993/06//
N1 - Lambert, W E. Samet, J M. Hunt, W C. Skipper, B J. Schwab, M. Spengler, J D
Research report (Health Effects Institute)
8812230, ah8, 8812230
IM
Journal Article. Research Support, Non-U.S. Gov't. Research Support, U.S. Gov't, Non-P.H.S.
English
KW - MEDLINE
KW - Air Pollution
KW - Indoor/ae [Adverse Effects]
KW - Indoor/an [Analysis]
KW - Child
KW - Preschool
KW - Cooking/mt [Methods]
KW - Environmental Monitoring/mt [Methods]
KW - Environmental Monitoring/st [Standards]
KW - Epidemiological Monitoring
KW - Female
KW - Humans
KW - Infant
KW - Male
KW - Nitrogen Dioxide/ae [Adverse Effects]
KW - Nitrogen Dioxide/an [Analysis]
KW - Prospective Studies
KW - Quality Control
KW - Residence Characteristics
KW - Respiratory Tract Diseases/ci [Chemically Induced]
KW - Respiratory Tract Diseases/ep [Epidemiology]
KW - Time Factors
RP - NOT IN FILE
SP - 33
EP - 50
JF - Research Report - Health Effects Institute
JA - Res Rep Health Eff.Inst
IS - 58
CY - UNITED STATES
N2 - Repeated measurements of nitrogen dioxide were obtained from 1988 to 1991 in the homes of 1,205 infants living in Albuquerque, NM. Passive diffusion samplers were used to obtain a series of two-week integrated measurements from the home of each infant for use in a cohort study of the relation of residential exposure to nitrogen dioxide and respiratory illnesses. Information on stove use and time spent inside the residence was collected at two-week and two-month intervals, respectively. During the winter, in the bedrooms of homes with gas cooking stoves, mean nitrogen dioxide concentrations were 21 parts per billion (ppb); mean concentrations in the living room and kitchen were 29 ppb and 34 ppb, respectively. In homes with electric cooking stoves, the mean bedroom concentration was 7 ppb during the winter. Lower indoor concentrations were observed during the summer in homes with both gas and electric stoves. On average, infants spent approximately 12.3 hours per day in their bedrooms, 7.3 hours in the living rooms, 35 minutes in the kitchens, and 3.8 hours out of their homes. (As a condition of participation, none of the infants spent more than 20 hours per week in day care outside of their homes). The mean time infants spent in the kitchen during cooking was approximately nine minutes per day. We tested whether exposures of infants living in homes with gas stoves could be reasonably estimated by measurements in the bedroom in comparison with time-weighted average concentrations based on time-activity data and simultaneous nitrogen dioxide measurements in the kitchen, living room, and bedroom. In 1,937 two-week intervals from 587 infants, 90% of time-weighted exposure (on the three-level classification used in this study) estimates were in agreement with estimates based on bedroom concentrations alone. The agreement of the time-weighted nitrogen dioxide exposure estimates with the bedroom concentrations is attributed to limited amounts of cooking stove use (the mean was 29 minutes per

day), small room-to-room differences in nitrogen dioxide concentrations (the mean kitchen-bedroom difference was 12 ppb), and the relatively large proportion of time that infants spent in their bedrooms
SN - 1041-5505
AD - Department of Medicine, University of New Mexico Medical Center, Albuquerque
UR - 8240759
ER -

TY - JOUR
ID - 2045
T1 - [Post-traumatic stress syndrome in children]. [Review] [23 refs] [French]
A1 - Mouren-Simeoni,M.C.
Y1 - 1993/10//
N1 - Mouren-Simeoni, M C
Annales de pediatrie
5uc, 2984696r
IM
Case Reports. English Abstract. Journal Article. Review
French
KW - MEDLINE
KW - Adolescent
KW - Adult
KW - Child
KW - Child Behavior Disorders/pp [Physiopathology]
KW - Child Behavior Disorders/px [Psychology]
KW - Child Behavior Disorders/th [Therapy]
KW - Preschool
KW - Humans
KW - Male
KW - Risk Factors
KW - Stress Disorders
KW - Post-Traumatic/pp [Physiopathology]
KW - Post-Traumatic/px [Psychology]
KW - Post-Traumatic/th [Therapy]
RP - NOT IN FILE
SP - 489
EP - 495
JF - Annales de Pediatrie
JA - Ann Pediatr (Paris)
VL - 40
IS - 8
CY - FRANCE
N2 - Posttraumatic stress disorder is a form of anxiety disorder which is poorly known in children and manifests as a series of symptoms occurring after an extraordinary event outside the range of usual human experiences and responsible for a feeling of terror. Virtually pathogmonic symptoms include re-experiencing the event through play and ceaselessly repeated behaviors (re-enactments), cognitive distortions when relating the facts (chronological errors, belief in omens), changes in attitudes towards others and life in general, and neurovegetative hyperactivity (hypervigilance, startle responses, difficulties controlling impulses). Although adequate follow-up data are lacking, the disorder can probably become chronic in children, as in adults. This nosographic entity raises the theoretical issue of the roles of life events and individual vulnerability. A number of factors may either "predispose" or "protect" the child: degree and duration of exposure to the trauma, nature of the event, preexistence of psychiatric disorders, level of cognitive development, sex, degree of social support, and containing or noncontaining attitude of the family. Treatments advocated in adults (pharmacotherapy, cognitive and behavioral therapies, group therapy) remain to be used and evaluated in children. [References: 23]
SN - 0066-2097
AD - Service de Psychiatrie de l'Enfant et de l'Adolescent, Hopital Necker-Enfants Malades, Paris
UR - 8239402
ER -

TY - JOUR
ID - 2046
T1 - The role of cognitive appraisal and friendship provisions in adolescents' affect and motivation toward activity in physical education
A1 - Duncan,S.C.
Y1 - 1993/09//
N1 - Duncan, S C
Research quarterly for exercise and sport
r6y, 8006373
IM
Journal Article
English
KW - MEDLINE
KW - Adolescent
KW - Adolescent Psychology
KW - Affect
KW - Child
KW - Female
KW - Humans
KW - Internal-External Control
KW - Interpersonal Relations
KW - Male
KW - Models
KW - Theoretical
KW - Motivation
KW - Physical Education and Training
KW - Self Concept
RP - NOT IN FILE
SP - 314
EP - 323
JF - Research Quarterly for Exercise & Sport
JA - Res Q Exerc Sport
VL - 64
IS - 3
CY - UNITED STATES
N2 - This study investigated the mediational role of affect in the influence of perceived success, causal attributions, and friendship provisions on choice and future expectancies for success and enjoyment, using a multi-sample structural equation modeling approach. Subjects were 422 seventh- and eighth-grade students participating in physical education classes. Higher levels of perceived success, personally controllable attributions, companionship, and esteem support were found to positively influence levels of affect experienced, which in turn enhanced future expectancies for success and enjoyment in physical education and choices to participate in physical activity outside of school. Furthermore, participation experience outside of school influenced adolescents' perceptions within the physical education setting and their consequent affect and motivation
SN - 0270-1367
AD - Oregon Research Institute, Eugene 97403
UR - 8235053
ER -

TY - JOUR
ID - 2047
T1 - Correlates of physical activity at home in Mexican-American and Anglo-American preschool children
A1 - Sallis,J.F.
A1 - Nader,P.R.
A1 - Broyles,S.L.
A1 - Berry,C.C.

A1 - Elder,J.P.
A1 - McKenzie,T.L.
A1 - Nelson,J.A.
Y1 - 1993/09//
N1 - Sallis, J F. Nader, P R. Broyles, S L. Berry, C C. Elder, J P. McKenzie, T L. Nelson, J A
Health psychology : official journal of the Division of Health Psychology, American Psychological Association
ejl, 8211523
IM
Comparative Study. Journal Article. Research Support, U.S. Gov't, P.H.S.
English
KW - MEDLINE
KW - Child Behavior
KW - Child
KW - Preschool
KW - Family
KW - Feeding Behavior
KW - Female
KW - Humans
KW - Leisure Activities
KW - Male
KW - Mexican Americans
KW - Mothers
KW - Parenting
KW - Questionnaires
RP - NOT IN FILE
SP - 390
EP - 398
JF - Health Psychology
JA - Health Psychol
VL - 12
IS - 5
CY - UNITED STATES
N2 - Twenty-two potential correlates of children's physical activity were examined. Two hundred and one Mexican-American and 146 Anglo-American families with 4-year-old children were studied. Children's physical activity was directly observed in the evening at home on 4 visits for 1 hr each time. Anglo-American children and male children were found to be more active. Demographic variables explained 11% of the variance in children's physical activity. After adjusting for demographics, 3 children's variables and 6 social-family variables did not account for significantly more variance. Five environmental variables accounted for 11% additional variance. Variables observed concurrently with physical activity, such as time spent outdoors and prompts to be active, were highly associated with children's physical activity
SN - 0278-6133
AD - Department of Psychology, San Diego State University, California
UR - 8223363
ER -

TY - JOUR
ID - 2048
T1 - Childhood fitness: what is happening? What needs to be done?. [Review] [58 refs]
A1 - Kuntzleman,C.T.
Y1 - 1993/07//
N1 - Kuntzleman, C T
Preventive medicine
pm4, 0322116
IM
Journal Article. Research Support, Non-U.S. Gov't. Review
English
KW - MEDLINE

KW - Adolescent
KW - Adult
KW - Body Composition
KW - Child
KW - Exercise
KW - Female
KW - Health Education
KW - Humans
KW - Life Style
KW - Male
KW - Physical Education and Training
KW - Physical Endurance
KW - Physical Fitness
RP - NOT IN FILE
SP - 520
EP - 532
JF - Preventive Medicine
JA - Prev Med
VL - 22
IS - 4
CY - UNITED STATES
N2 - BACKGROUND: There is some controversy as to the fitness levels of U.S. school-age youth. Some experts claim that U.S. youth are fit. Others feel that there has been a decline, despite a recent adult fitness boom. METHODS: The purpose of this article is to examine the research conducted on the cardiovascular endurance and body composition levels of U.S. youth. Research studies noting the activity patterns of youth outside and during physical education are presented. The frequency and duration of physical education requirements are also reviewed. The effects of these patterns and requirements on cardiovascular endurance and body composition are presented. RESULTS: U.S. youth do not engage in physical activity, within our outside physical education, sufficient to develop cardiovascular endurance. Our youth are, therefore, at risk of developing a myriad of diseases associated with sedentary lifestyles. CONCLUSIONS: Exercise is known to have a prophylactic effect on disease, death, and disability. Young people must be instructed and encouraged to be involved in lifetime fitness activities if we are going to control health care costs, reduce disease incidence, and improve the overall quality of life of our citizens. [References: 58]
SN - 0091-7435
AD - University of Michigan, Fitness for Youth, Ann Arbor 48109
UR - 8415504
ER -

TY - JOUR
ID - 2049
T1 - Patterns of hand fractures and dislocations in a district general hospital
A1 - Packer,G.J.
A1 - Shaheen,M.A.
Y1 - 1993/08//
N1 - Packer, G J. Shaheen, M A
Journal of hand surgery (Edinburgh, Scotland)
ire, 8403839
IM
Journal Article
English
KW - MEDLINE
KW - Adolescent
KW - Adult
KW - Aged
KW - 80 and over
KW - Child
KW - Preschool

KW - Dislocations/ep [Epidemiology]
KW - England/ep [Epidemiology]
KW - Female
KW - Finger Injuries/ep [Epidemiology]
KW - Fractures
KW - Bone/ep [Epidemiology]
KW - Hand Injuries/ep [Epidemiology]
KW - Hospitals
KW - District
KW - General
KW - Humans
KW - Infant
KW - Male
KW - Middle Aged
RP - NOT IN FILE
SP - 511
EP - 514
JF - Journal of Hand Surgery - British Volume
JA - J Hand Surg [Br]
VL - 18
IS - 4
CY - SCOTLAND
N2 - In a retrospective survey of patients with fractures and dislocations attending the Accident department of a District General hospital, comparison was made between patients with fractures and dislocations in the hand and fractures and dislocations of other sites. Patients with hand injuries accounted for 28% of the total patients seen. They were more likely than other fracture patients to be male and between the ages of 10 and 40 years. Almost a quarter of hand injuries attended on Monday. Leisure activities outside the home formed the commonest aetiological factor. Only 66% of patients with hand injuries attended the Accident department within 24 hours of injury. The little finger was the commonest site of injury. This survey emphasizes the differences between hand injuries and injuries of other sites, identifies the risk factors for hand injuries and has implications for the management of hand injuries in a District General Hospital
SN - 0266-7681
AD - Department of Orthopaedic Surgery, Hartlepool General Hospital, Cleveland, UK
UR - 8409670
ER -

TY - JOUR
ID - 2050
T1 - Decreased physical activity in Pima Indian compared with Caucasian children
A1 - Fontvieille,A.M.
A1 - Kriska,A.
A1 - Ravussin,E.
Y1 - 1993/08//
N1 - Fontvieille, A M. Kriska, A. Ravussin, E
International journal of obesity and related metabolic disorders : journal of the International Association for the Study of Obesity
btx, 9313169
IM
Comparative Study. Journal Article
English
KW - MEDLINE
KW - Adipose Tissue/ah [Anatomy & Histology]
KW - Anthropometry
KW - Arizona
KW - Body Composition
KW - Body Constitution
KW - Body Mass Index

KW - Child
KW - Cross-Sectional Studies
KW - European Continental Ancestry Group
KW - Exercise
KW - Female
KW - Humans
KW - Indians
KW - North American
KW - Male
KW - Obesity/eh [Ethnology]
KW - Obesity/et [Etiology]
KW - Regression Analysis
KW - Sex Factors
RP - NOT IN FILE
SP - 445
EP - 452
JF - International Journal of Obesity & Related Metabolic Disorders: Journal of the International Association for the Study of Obesity
JA - Int J Obes Relat Metab Disord
VL - 17
IS - 8
CY - ENGLAND
N2 - Since reduced physical activity might be a risk factor for body weight gain, we studied the relationship between physical activity and body composition in 43 Pima Indian children (22 male/21 female, mean +/- s.d.: 9.9 +/- 1.1 years) and 42 Caucasian children (21 male/21 female, 9.7 +/- 1.2 years). A list of usual sport leisure activities was established (e.g. bicycling, swimming, basketball) and the subjects were asked how much time they had devoted to each activity over the past week and the last year. Data on time spent playing outside (excluding sport leisure activities for the estimation of physical activity) and watching television/videos were also collected. Pima Indians were taller (143 +/- 9 vs. 137 +/- 8 cm, P < 0.001), heavier (48.6 +/- 15.8 vs. 32.9 +/- 7.8 kg, P < 0.0001) and fatter (39 +/- 16 vs. 24 +/- 7% fat, P < 0.001) than Caucasians. Pima Indian girls showed significantly lower past year and past week sport leisure activity than Caucasian girls (P < 0.01) and spent significantly more time watching television/videos (P < 0.05). Pima boys also showed significantly lower past week sport leisure activity than Caucasian boys (P < 0.05). In Pima Indian boys, past year sport leisure activity correlated negatively (P < 0.05) with body mass index (r = -0.49) and percentage body fat (r = -0.56). However, such correlations were not found in Pima Indian girls, possibly due their very low levels of activity.(ABSTRACT TRUNCATED AT 250 WORDS)
SN - 0307-0565
AD - Clinical Diabetes and Nutrition Section, National Institute of Diabetes and Digestive and Kidney Diseases, National Institutes of Health, Phoenix, AZ 85016
UR - 8401746
ER -

TY - JOUR
ID - 2051
T1 - Hypoglycaemia in the diabetic child. [Review] [45 refs]
A1 - Soltesz,G.
Y1 - 1993/07//
N1 - Soltesz, G
Bailliere's clinical endocrinology and metabolism
9jl, 8704785
IM
Journal Article. Review
English
KW - MEDLINE
KW - Child
KW - Diabetes Mellitus
KW - Type 1/co [Complications]

KW - Female
KW - Humans
KW - Hypoglycemia/co [Complications]
KW - Hypoglycemia/et [Etiology]
KW - Hypoglycemia/th [Therapy]
KW - Male
KW - Risk Factors
RP - NOT IN FILE
SP - 741
EP - 755
JF - Baillieres Clinical Endocrinology & Metabolism
JA - Baillieres Clin Endocrinol Metab
VL - 7
IS - 3
CY - ENGLAND
N2 - Hypoglycaemia is the most frequent acute complication of insulin-dependent diabetes mellitus. The clinical symptoms of insulin-induced hypoglycaemia can be grouped into those attributable to the sympathetic and adrenergic responses, e.g. tremor, pallor, palpitation, sweating, mydriasis ('hypoglycaemia awareness'), and those attributable to brain dysfunction, ranging from headache to convulsions and coma. Hypoglycaemia in diabetic children can occur at any time of the day, but nocturnal hypoglycaemia is a particular fear and worry. The frequency of mild hypoglycaemia is almost impossible to ascertain and the incidence of severe hypoglycaemia varies between 0.07 and 3.6 episodes per patient-year, though most authors report a range of 0.1-0.2 episode per patient-year. The most frequent causes of hypoglycaemia in diabetic children are deviations from treatment routine such as strenuous exercise, omission of snacks or skipped meals, and gross deviations from the prescribed times of insulin injections and recommended doses of insulin. Other predisposing factors include intensified insulin treatment, improved glycaemic control, young age, longer duration of diabetes and defective counterregulation. The available paediatric studies do not seem to support the suggestion that human insulin impairs the perception of hypoglycaemic symptoms ('hypoglycaemia unawareness') and increases the frequency of hypoglycaemic episodes, but further conclusive studies are needed. Prolonged and recurrent severe hypoglycaemia, particularly in younger children, can cause permanent neuropsychological dysfunction (e.g. learning disabilities) and permanent electroencephalographic abnormalities. Mild hypoglycaemia has also been documented to affect cognitive function, and the performance of neuropsychological tasks can remain decreased for some time (up to several hours) after full clinical recovery from hypoglycaemia. An impending hypoglycaemic attack can usually be averted by the ingestion of 20 g of rapidly absorbed carbohydrate. A severe episode can be effectively treated outside hospital with subcutaneous or intramuscular glucagon (0.5-1.0 mg) or in the hospital by an intravenous bolus of 0.2-0.5 g/kg glucose followed by a continuous glucose infusion. Patient and parent education and vigilant application of diabetes self-care principles are perhaps the most effective means of prevention, but in very young children a less strict metabolic control (higher glycosylated haemoglobin levels) may be necessary. [References: 45]
SN - 0950-351X
AD - Department of Paediatrics, University Medical School, Pecs, Hungary
UR - 8379914
ER -

TY - JOUR
ID - 2052
T1 - Housing factors and perceived health status among Japanese women living in aggregated dwelling units
A1 - Saito,K.
A1 - Iwata,N.
A1 - Hosokawa,T.
A1 - Ohi,G.
Y1 - 1993///
N1 - Saito, K. Iwata, N. Hosokawa, T. Ohi, G
International journal of health services : planning, administration, evaluation
1305035, grj
IM

Journal Article. Research Support, Non-U.S. Gov't
English
KW - MEDLINE
KW - Adolescent
KW - Adult
KW - Aged
KW - Attitude to Health
KW - Caregivers/px [Psychology]
KW - Child
KW - Crowding/px [Psychology]
KW - Female
KW - Health Status
KW - Housing
KW - Humans
KW - Japan/ep [Epidemiology]
KW - Leisure Activities
KW - Life Change Events
KW - Middle Aged
KW - Personality Inventory/sn [Statistics & Numerical Data]
KW - Psychometrics
KW - Social Environment
KW - Somatoform Disorders/ep [Epidemiology]
KW - Somatoform Disorders/px [Psychology]
KW - Urban Population/sn [Statistics & Numerical Data]
KW - Urban Population
RP - NOT IN FILE
SP - 541
EP - 554
JF - International Journal of Health Services
JA - Int J Health Serv
VL - 23
IS - 3
CY - UNITED STATES
N2 - An epidemiologic survey was conducted of the 818 households in a community consisting of aggregated dwelling units in Tokyo, Japan, in order to assess the relationship between housing environment and psychological or physical health status of the female residents. Psychological health status was assessed by the 28-item General Health Questionnaire in Japanese translation. Main housing factors such as internal density (person-to-room ratio) and floor level (vertical location) of the dwelling unit did not have significant effects on the psychological health status of the sample of Japanese women, but the internal density generally did not exceed 1.5 persons per room. However, physical health status might be associated with floor level: women living at the highest levels complained of more symptoms than did those at lower floor levels, when the type of dwelling unit (i.e., high-rise, low or medium height building) was not taken into account. Poor psychological health status was found among women who were dissatisfied with the plan of their house or with the room arrangement, were anxious about earthquake or other accidents, perceived the house as inadequate for the children, were annoyed by indoor noise, and complained of outdoor noise
SN - 0020-7314
AD - Department of Ergonomics, University of Occupational and Environmental Health, Kitakyushu, Japan
UR - 8375954
ER -

TY - JOUR
ID - 2053
T1 - [No increased urinary arsenic levels in children living on naturally arsenic-rich soil]. [Dutch]
A1 - van den Hazel,P.J.
A1 - ten,Hove M.
A1 - Peerenboom,P.B.
Y1 - 1993/08/14/

N1 - van den Hazel, P J. ten Hove, M. Peerenboom, P B
Nederlands tijdschrift voor geneeskunde
nuk, 0400770
IM
English Abstract. Journal Article
Dutch
KW - MEDLINE
KW - Arsenic/ur [Urine]
KW - Arsenic Poisoning
KW - Child
KW - Preschool
KW - Creatinine/ur [Urine]
KW - Environmental Exposure
KW - Female
KW - Humans
KW - Male
KW - Netherlands
KW - Soil Pollutants/po [Poisoning]
RP - NOT IN FILE
SP - 1667
EP - 1669
JF - Nederlands Tijdschrift voor Geneeskunde
JA - Ned Tijdschr Geneeskd
VL - 137
IS - 33
CY - NETHERLANDS
N2 - In order to assess the health effect of environmental exposure to arsenic, the urinary arsenic concentrations were determined in 25 children aged 2-5 yr in the De Huet quarter in Doetinchem, an area with an increased amount of naturally occurring arsenic in the upper soil, and in 25 children from the other quarters of Doetinchem. With a questionnaire information was obtained regarding playing outdoors and eating fish (also a source of arsenic intake). There were no statistically significant differences in urinary arsenic concentrations between the subgroups of children
SN - 0028-2162
AD - Gemeente Arnhem, dienst Welzijn en Volksgezondheid
UR - 8371797
ER -

TY - JOUR
ID - 2054
T1 - Care-seeking for fatal illnesses in young children in Indramayu, west Java, Indonesia
A1 - Sutrisna,B.
A1 - Reingold,A.
A1 - Kresno,S.
A1 - Harrison,G.
A1 - Utomo,B.
Y1 - 1993/09/25/
N1 - Sutrisna, B. Reingold, A. Kresno, S. Harrison, G. Utomo, B
Lancet
2985213r, l0s, 0053266
AIM, IM, J
Journal Article. Research Support, Non-U.S. Gov't
English
KW - MEDLINE
KW - Child Health Services
KW - Child
KW - Preschool
KW - Cohort Studies

KW - Culture
KW - Female
KW - Health Knowledge
KW - Attitudes
KW - Practice
KW - Humans
KW - Indonesia/ep [Epidemiology]
KW - Infant
KW - Infant Mortality
KW - Newborn
KW - Interviews as Topic
KW - Male
KW - Maternal-Child Health Centers
KW - Medicine
KW - Traditional
KW - Mortality
KW - Mothers/px [Psychology]
KW - Patient Acceptance of Health Care
KW - Pneumonia/mo [Mortality]
KW - Pneumonia/th [Therapy]
RP - NOT IN FILE
SP - 787
EP - 789
JF - Lancet
VL - 342
IS - 8874
CY - ENGLAND
N2 - To examine patterns of seeking care for gravely ill infants and children, we studied all deaths in children under 5 in 10,000 households in Indramayu, West Java, Indonesia, between July 1, 1991, and Dec 31, 1992. 141 deaths were identified (mortality rate 80.7 per 1000), of which 139 were due to causes other than trauma. No treatment of any kind was sought outside the home for 30 (22%) of the children who died from natural causes, and for 59 (42%) others only a traditional healer or other source of non-western medical advice was consulted. Whether or not a mother sought western medical care was strongly associated with the age of the child, the duration of the terminal illness, the previous attendance of the mother at a community-based maternal-and-child-health facility, and the mother's response to a prospectively asked question about what care should be sought for a hypothetical 1-month-old baby with signs of severe pneumonia. Household income, maternal age, and education, and distance between home and government health post were not associated with whether or not western medical care was sought
SN - 0140-6736
AD - Centre for Child Survival, University of Indonesia
UR - 8103880
ER -

TY - JOUR
ID - 2055
T1 - Mosaic tetrasomy 8p: molecular cytogenetic confirmation and measurement of glutathione reductase and tissue plasminogen activator levels. [Review] [27 refs]
A1 - Fisher,A.M.
A1 - Barber,J.C.
A1 - Crolla,J.A.
A1 - James,R.S.
A1 - Lestas,A.N.
A1 - Jennings,I.
A1 - Dennis,N.R.
Y1 - 1993/08/01/
N1 - Fisher, A M. Barber, J C. Crolla, J A. James, R S. Lestas, A N. Jennings, I. Dennis, N R
American journal of medical genetics

3l4, 7708900
IM
Case Reports. Journal Article. Research Support, Non-U.S. Gov't. Review
English
KW - MEDLINE
KW - Abnormalities
KW - Multiple/ge [Genetics]
KW - Centromere
KW - Child
KW - Preschool
KW - Chromosome Aberrations
KW - Chromosome Banding
KW - Chromosome Disorders
KW - Chromosomes
KW - Human
KW - Pair 8
KW - DNA
KW - Satellite/an [Analysis]
KW - Female
KW - Glutathione Reductase/bl [Blood]
KW - Glutathione Reductase/ge [Genetics]
KW - Humans
KW - Intellectual Disability/ge [Genetics]
KW - Karyotyping
KW - Mosaicism
KW - Mothers
KW - Nondisjunction
KW - Genetic
KW - Polymerase Chain Reaction
KW - Tissue Plasminogen Activator/bl [Blood]
RP - NOT IN FILE
SP - 100
EP - 105
JF - American Journal of Medical Genetics
JA - Am J Med Genet
VL - 47
IS - 1
CY - UNITED STATES
N2 - We report the case of a 5-year-old girl with severe developmental disabilities, skeletal anomalies, hypotonia, rectal atresia, malrotation of the intestine, horseshoe kidney, vesicoureteric reflux, and minor facial anomalies. Conventional cytogenetic techniques suggested that she had a mosaic 46,XX/47,XX,+i(8p) constitution, and the identity of the isochromosome was confirmed by in situ hybridization and chromosome painting. Polymorphic DNA markers are consistent with the i(8p) having arisen as the result of a segregation error and centromere misdivision at the second maternal meiotic division. The i(8p) was seen in 17/25 (68%) lymphocytes at the age of one month but had declined to 31/100 (31%) cells by the age of 5 years. At this time the i(8p) was seen in 30/68 (44%) cultured skin fibroblasts. The proposita had an approximately twofold increase in red cell glutathione reductase activity but a normal level of tissue-plasminogen activator. These enzyme results are consistent with the known localisation of the glutathione reductase gene on the short arm of chromosome 8 but suggest that the tissue-plasminogen activator gene may map outside this region. [References: 27]
SN - 0148-7299
AD - Wessex Regional Genetics Laboratory, Salisbury District Hospital, Odstock, Wiltshire, England
UR - 8368238
ER -

TY - JOUR
ID - 2056

T1 - Bicycle accidents often cause disability--an analysis of medical and social consequences of nonfatal bicycle accidents
A1 - Olkkonen,S.
A1 - Lahdenranta,U.
A1 - Slatis,P.
A1 - Honkanen,R.
Y1 - 1993/06//
N1 - Olkkonen, S. Lahdenranta, U. Slatis, P. Honkanen, R
Scandinavian journal of social medicine
ud5, 0365610
IM
Journal Article. Research Support, Non-U.S. Gov't
English
KW - MEDLINE
KW - Accidents
KW - Traffic/ec [Economics]
KW - Traffic/sn [Statistics & Numerical Data]
KW - Activities of Daily Living
KW - Adolescent
KW - Adult
KW - Aged
KW - Ambulatory Care/ec [Economics]
KW - Athletic Injuries/ec [Economics]
KW - Athletic Injuries/ep [Epidemiology]
KW - Athletic Injuries/rh [Rehabilitation]
KW - Bicycling/in [Injuries]
KW - Child
KW - Preschool
KW - Cost of Illness
KW - Costs and Cost Analysis
KW - Disabled Persons/sn [Statistics & Numerical Data]
KW - Female
KW - Finland/ep [Epidemiology]
KW - Follow-Up Studies
KW - Humans
KW - Infant
KW - Male
KW - Middle Aged
KW - Patient Admission/ec [Economics]
RP - NOT IN FILE
SP - 98
EP - 106
JF - Scandinavian Journal of Social Medicine
JA - Scand J Soc Med Suppl
VL - 21
IS - 2
CY - SWEDEN
N2 - Social and medical consequences of 278 children and 264 adults injured in bicycle accidents and seen in two hospitals in Helsinki in 1985-86 were analyzed. Information was collected from patient records, by means of a special questionnaire and by telephone interview. A child outpatient required 1.7 and a child inpatient 3.0 physician visits on an average, while adults required 2.2 and 4.9 visits, respectively. The average duration of hospital stay was 8 days for hospitalized adults and 6 days for children. Rehabilitative care outside the hospital was received by 6% of the adult outpatients and 25% of the inpatients, but none of the injured children. The mean duration of work disability was 82 days among inpatients, 11 days among outpatients, 127 days among the inpatients injured in motor vehicle collisions and 65 days among inpatients injured in other bicycle accidents. Of inpatients 32% and of outpatients 5% reported persistent (> 6 months) disability. Persistent disability was recorded in 11% of children, in 47% of adults and in 67% of elderly inpatients. Most serious consequences were

due to intracranial injuries in motor vehicle-bicycle collisions. Of the hospitalized bicyclists 4% suffered from severe cognitive and behavioural changes or sense impairment and of adult inpatients 3% suffered from permanent work disability. The average costs of health and social services were about FIM 1000 per adult outpatient and FIM 13000 per adult inpatient. In prevention high priority should be given to motor vehicle collisions, head injuries and injuries among the elderly bicyclists
SN - 0300-8037
AD - University of Kuopio Research Institute of Public Health, Injury Research Project, Finland
UR - 8367689
ER -

TY - JOUR
ID - 2057
T1 - [The risk factors for home accidents in children. A case-control study]. [Spanish]
A1 - Hijar-Medina,M.C.
A1 - Tapia-Yanez,J.R.
A1 - Lopez-Lopez,M.V.
A1 - Solorzano-Flores,L.I.
A1 - Lozano-Ascencio,R.
Y1 - 1993/07//
N1 - Hijar-Medina, M C. Tapia-Yanez, J R. Lopez-Lopez, M V. Solorzano-Flores, L I. Lozano-Ascencio, R
Boletin medico del Hospital Infantil de Mexico
ag0, 0414106
IM
English Abstract. Journal Article
Spanish
KW - MEDLINE
KW - Accidents
KW - Home/sn [Statistics & Numerical Data]
KW - Home
KW - Age Factors
KW - Case-Control Studies
KW - Child
KW - Preschool
KW - Confidence Intervals
KW - Family Characteristics
KW - Female
KW - Humans
KW - Infant
KW - Logistic Models
KW - Male
KW - Mexico/ep [Epidemiology]
KW - Residence Characteristics
KW - Risk Factors
KW - Sex Factors
RP - NOT IN FILE
SP - 463
EP - 474
JF - Boletin Medico del Hospital Infantil de Mexico
JA - Bol.Med Hosp Infant Mex
VL - 50
IS - 7
CY - MEXICO
N2 - INTRODUCTION: The main objective was to determine the risk factors involved in unintentional injuries in children under 10 years old occurring at home. MATERIAL AND METHODS: A Case Control study was conducted in the emergency room of three pediatrics hospitals in Mexico City. The sample size was 160 cases and 320 controls. RESULTS: Boys were more affected than girls (OR) 1.7, C.I. (95%) 1.2-2.5. The age group more affected was two-three years old (OR) 1.6, C.I.1.1-2.2. When children play outside home (roof, stairs,

patio) the risk was OR 2.48, C.I. 1.63-3.76. If mother is not encharged of children care (OR) 2.9, C.I. 1.8-4.8. The age > 40 years old of the person encharged of children care (OR) 2.2, C.I. 1.4-3.5. CONCLUSIONS: The results showed us how necessary is to develop injury prevention programs especially to implement passive measures, with target populations people encharged of children care
SN - 0539-6115
AD - Escuela de Salud Publica de Mexico, Instituto Nacional de Salud Publica, Cuernavaca, Morelos
UR - 8363745
ER -

TY - JOUR
ID - 2058
T1 - Proximate causes of aggressive fighting in middle school children
A1 - Boulton,M.J.
Y1 - 1993/06//
N1 - Boulton, M J
The British journal of educational psychology
awn, 0370636
IM
Comparative Study. Journal Article
English
KW - MEDLINE
KW - Age Factors
KW - Aggression/px [Psychology]
KW - Child
KW - Child Behavior Disorders/et [Etiology]
KW - Child Behavior Disorders/px [Psychology]
KW - Preschool
KW - Female
KW - Humans
KW - Infant
KW - Interpersonal Relations
KW - Male
KW - Questionnaires
KW - Schools
KW - Sex Factors
KW - Students/px [Psychology]
RP - NOT IN FILE
SP - 231
EP - 244
JF - British Journal of Educational Psychology
JA - Br J Educ Psychol
VL - 63
IS - Pt 2
CY - ENGLAND
N2 - In study 1, 8- and 11-year-old girls and boys (N = 110) were interviewed individually and asked about why children in general, and themselves in particular, fight with fellow pupils in the playground. A substantial proportion of children (51 per cent) reported having had at least one aggressive fight during the previous year. The most common reasons for children resorting to fighting were retaliations to teasing, retaliations to unprovoked assaults, disagreements over aspects of the game that was being played, because another child was disliked, and to settle dominance disputes. In study 2, a direct observational methodology was employed to examine how often children of these ages engage in aggressive fighting, how long fights last, and their proximate causes. Among the younger pupils, a large proportion of fights (43.3 per cent) had no obvious immediate cause to an adult observer, but others were caused when a playful assault received an aggressive retaliation (15.4 per cent), and by disputes over space in the playground (13.5 per cent). Among the 11-year-old pupils, a smaller percentage of fights (14.8 per cent) had no obvious immediate cause. The two most common causes of fighting at this age were aggressive retaliations to teasing (25.9 per cent) and aggressive retaliations to accidental injury/hurt (18.5 per cent). In study 3, in situ interviews were combined with direct playground

observations in order to reveal participants' views (or onlookers' views if participants were not available to be interviewed) about the causes of fights. Again, the most common causes of fighting were disputes over some aspect of the game being played (33.3 per cent), aggressive retaliations to teasing (25.0 per cent), disagreements over possession of toys/equipment (14.6 per cent), and aggressive retaliations to accidental injury/hurt (14.6 per cent). Age and sex differences are reported in some measures across all three studies. The results are discussed in terms of their implications for interventions aimed at improving children's experiences of life on the school playground
SN - 0007-0998
AD - Psychology Section, School of Health and Community Studies, Sheffield Hallam University
UR - 8353057
ER -

TY - JOUR
ID - 2059
T1 - Randomised controlled trial of single-dose azithromycin in treatment of trachoma
A1 - Bailey,R.L.
A1 - Arullendran,P.
A1 - Whittle,H.C.
A1 - Mabey,D.C.
Y1 - 1993/08/21/
N1 - Bailey, R L. Arullendran, P. Whittle, H C. Mabey, D C
Lancet
2985213r, l0s, 0053266
AIM, IM, J
Clinical Trial. Comparative Study. Journal Article. Randomized Controlled Trial. Research Support, Non-U.S. Gov't
English
KW - MEDLINE
KW - Administration
KW - Oral
KW - Adolescent
KW - Adult
KW - Antigens
KW - Bacterial/an [Analysis]
KW - Azithromycin
KW - Child
KW - Preschool
KW - Chlamydia trachomatis/im [Immunology]
KW - Erythromycin/ad [Administration & Dosage]
KW - Erythromycin/ae [Adverse Effects]
KW - Erythromycin/aa [Analogs & Derivatives]
KW - Erythromycin/tu [Therapeutic Use]
KW - Female
KW - Follow-Up Studies
KW - Humans
KW - Infant
KW - Male
KW - Middle Aged
KW - Ointments
KW - Patient Compliance
KW - Recurrence
KW - Single-Blind Method
KW - Tetracycline/ad [Administration & Dosage]
KW - Tetracycline/ae [Adverse Effects]
KW - Tetracycline/tu [Therapeutic Use]
KW - Trachoma/dt [Drug Therapy]
KW - Trachoma/mi [Microbiology]

RP - NOT IN FILE
SP - 453
EP - 456
JF - Lancet
VL - 342
IS - 8869
CY - ENGLAND
N2 - Blindness due to trachoma is a serious public health issue world wide. The currently recommended treatment of active trachoma with repeated doses of tetracycline eye ointment has many disadvantages. The new azalide antibiotic azithromycin is effective as a single oral dose in the chemotherapy of genital Chlamydia trachomatis infections, and we have assessed its efficacy for trachoma treatment. We carried out a randomised single-blind comparison of azithromycin (a single oral dose of 20 mg/kg) with conventional treatment (6 weeks of topical tetracycline plus erythromycin for severe cases) in two villages with endemic trachoma in The Gambia. The patients were followed up for 26 weeks from the start of treatment by an observer unaware of treatment allocation. By 6 months' follow-up, trachoma had resolved in 76 (78%) of 97 subjects who received azithromycin compared with 70 (72%) of 97 who were treated conventionally (95% CI for difference -6% to 18%). Compliance with both treatments was good, but that for conventional treatment could probably not be achieved outside the research setting. There were no significant differences in treatment effect, baseline characteristics, or re-emergent disease between the treatment groups. Azithromycin was well tolerated. As a systemic treatment effective in a single dose it has important potential for trachoma control
SN - 0140-6736
AD - Department of Clinical Sciences, London School of Hygiene and Tropical Medicine, UK
UR - 8102427
ER -

TY - JOUR
ID - 2060
T1 - Myopia and myopic progression among schoolchildren: a three-year follow-up study
A1 - Parssinen,O.
A1 - Lyyra,A.L.
Y1 - 1993/08//
N1 - Parssinen, O. Lyyra, A L
Investigative ophthalmology & visual science
gwi, 7703701
IM
Clinical Trial. Journal Article. Randomized Controlled Trial. Research Support, Non-U.S. Gov't
English
KW - MEDLINE
KW - Accommodation
KW - Ocular
KW - Age Factors
KW - Child
KW - Convergence
KW - Female
KW - Follow-Up Studies
KW - Humans
KW - Life Style
KW - Longitudinal Studies
KW - Male
KW - Myopia/pp [Physiopathology]
KW - Myopia/th [Therapy]
KW - Reading
KW - Regression Analysis
KW - Sex Factors
RP - NOT IN FILE
SP - 2794
EP - 2802

JF - Investigative Ophthalmology & Visual Science
JA - Invest Ophthalmol Vis Sci
VL - 34
IS - 9
CY - UNITED STATES
N2 - PURPOSE: To discuss the factors that might explain the rate of myopic progression and the degree of myopia after a 3-year follow-up among schoolchildren with myopia. METHODS: Myopic progression among 238 schoolchildren was followed up in a randomized clinical trial of myopia treatment. The associations between the explanatory factors and myopic progression and the final value of the spherical equivalent after the follow-up were studied by analysis of variance and regression analysis. RESULTS: Myopia progressed faster among girls than boys. According to the regression models for the boys, 25% of the variation of myopic progression and 57% of the final spherical equivalent could be explained by initial spherical equivalent, age at receiving first spectacles, time spent on sports and outdoor activities, and on reading and close work. Among the girls, 30% of myopic progression could be explained by age at receiving first spectacles, time spent on reading and close work, and reading distance. Similarly, 49% of the final spherical equivalent could be explained by age at receiving first spectacles, initial spherical equivalent, time spent on reading and close work, and reading distance. The rest of the variations could not be explained by the variables measured in this study. CONCLUSIONS: The factors with the most significant relationships to myopic progression were sex, age of onset, and degree of myopia at the beginning of the follow-up. Myopic progression and final myopia were related to time spent on reading and close work and to reading distance but not, however, to accommodation stimulus
SN - 0146-0404
AD - Department of Ophthalmology, Central Hospital of Central Finland, Jyvaskyla
UR - 8344801
ER -

TY - JOUR
ID - 2061
T1 - Observations on physical activity in physical locations: age, gender, ethnicity, and month effects
A1 - Baranowski,T.
A1 - Thompson,W.O.
A1 - DuRant,R.H.
A1 - Baranowski,J.
A1 - Puhl,J.
Y1 - 1993/06//
N1 - Baranowski, T. Thompson, W O. DuRant, R H. Baranowski, J. Puhl, J
Research quarterly for exercise and sport
r6y, 8006373
IM
Comparative Study. Journal Article. Research Support, U.S. Gov't, P.H.S.
English
KW - MEDLINE
KW - Activity Cycles
KW - African Americans
KW - Age Factors
KW - Child
KW - Preschool
KW - Culture
KW - Exercise
KW - Female
KW - Hispanic Americans
KW - Humans
KW - Male
KW - Seasons
KW - Sex Factors
RP - NOT IN FILE
SP - 127

EP - 133
JF - Research Quarterly for Exercise & Sport
JA - Res Q Exerc Sport
VL - 64
IS - 2
CY - UNITED STATES
N2 - The level of physical activity of 3- and 4-year-old children was assessed in alternative physical locations by month and time of day and by age, gender, and ethnicity. Physical activity was assessed by observation with the Children's Activity Rating Scale (CARS) for up to 12 hours from 7:00 am to 7:00 pm. A sample of 191 three- and four-year-old children was observed for up to four times in the course of a year. The sample was tri-ethnic. Boys were significantly more active than girls. Activity was consistently higher outside than inside. There were significant differences in the amount of time children in this age group spent inside versus outside by time of year; the activity levels of boys and girls differed by time of year, particularly when outside. A model including gender, month, and location terms accounted for 75% of the variance in physical activity. These data further documented gender differences in physical activity among very young children using measures not subject to self-report biases but did not explain or clarify the gender differences. The substantial differences by physical location and time of year deserve future attention, but more refined methods will be needed to balance data by location and important seasonal times. An inference from these results is that activity levels among young children may be increased by encouraging them to spend more time outdoors
SN - 0270-1367
AD - Division of Behavioral Sciences and Health Education, Emory University's School of Public Health, Atlanta, GA
UR - 8341835
ER -

TY - JOUR
ID - 2062
T1 - Follow-up in patients with aspartylglucosaminuria. Part II. Adaptive skills
A1 - Arvio,M.
Y1 - 1993/06//
N1 - Arvio, M
Acta paediatrica (Oslo, Norway : 1992)
bgc, 9205968
IM
Journal Article. Research Support, Non-U.S. Gov't
English
KW - MEDLINE
KW - Activities of Daily Living
KW - Adaptation
KW - Psychological
KW - Adolescent
KW - Adult
KW - Aspartylglucosylaminase/ur [Urine]
KW - Child
KW - Female
KW - Finland
KW - Follow-Up Studies
KW - Humans
KW - Language
KW - Lysosomal Storage Diseases/pp [Physiopathology]
KW - Lysosomal Storage Diseases/px [Psychology]
KW - Male
KW - Middle Aged
KW - Motor Skills
KW - Socialization
RP - NOT IN FILE
SP - 590

EP - 594
JF - Acta Paediatrica
JA - Acta Paediatr
VL - 82
IS - 6-7
CY - NORWAY
N2 - Aspartylglucosaminuria is a lysosomal storage disorder inherited as an autosomal recessive trait. Progressive mental retardation is the main symptom, and the lifespan of the patient is abnormally short. The adaptive skills and maladaptive behaviour of 110 patients aged from 7 to 56 years were analysed using the Portage scale and the AAMD Adaptive Behaviour Scale Part two. The sample was divided into four groups; school-aged, young adults, adults and middle-aged. All patients needed help in the household and transactions outside the home. The school-aged turned out to be superior and middle-aged inferior to other groups concerning adaptive skills. The school-aged and young adults were quite independent in dressing, toileting, bathing and walking alone near the home. Out-of-home replacement became necessary in the majority in adulthood. Twenty-eight patients (25%) were regarded as having behavioural disturbances and the amount of personal disturbances increased significantly with age
SN - 0803-5253
AD - Paajarvi Rehabilitation Centre, Lammi, Finland
UR - 8338997
ER -

TY - JOUR
ID - 2063
T1 - Child day care and home injuries involving playground equipment
A1 - Kotch,J.B.
A1 - Chalmers,D.J.
A1 - Langley,J.D.
A1 - Marshall,S.W.
Y1 - 1993/06//
N1 - Kotch, J B. Chalmers, D J. Langley, J D. Marshall, S W
Journal of paediatrics and child health
arp, 9005421
IM
Comparative Study. Journal Article. Research Support, Non-U.S. Gov't
English
KW - MEDLINE
KW - Accidents/sn [Statistics & Numerical Data]
KW - Accidents
KW - Home/sn [Statistics & Numerical Data]
KW - Child Day Care Centers
KW - Child
KW - Preschool
KW - Craniocerebral Trauma/et [Etiology]
KW - Female
KW - Humans
KW - Infant
KW - Injury Severity Score
KW - Male
KW - New Zealand/ep [Epidemiology]
KW - Play and Playthings
KW - Sex Factors
KW - Wounds and Injuries/ep [Epidemiology]
KW - Wounds and Injuries/et [Etiology]
RP - NOT IN FILE
SP - 222
EP - 227
JF - Journal of Paediatrics & Child Health

JA - J Paediatr Child Health
VL - 29
IS - 3
CY - AUSTRALIA
N2 - The increasing number of children attending child day care has led to a corresponding concern for their safety in the absence of parental care. Previous studies have documented that the majority of injuries occurring in child day care involve falls, and that the most common consumer product associated with such falls is playground equipment. This study describes New Zealand children less than 5 years of age admitted to hospital between 1979 and 1988 for injuries associated with playground equipment located at home or a child care facility. There were 528 hospitalized home injuries involving playground equipment, and 145 such day care injuries. Fractures were the most common injury, and the head was the most commonly involved body region. Lower limb injuries were the most severe. Among the differences between home and day care injuries were the type of equipment involved. Swings were disproportionately associated with head injuries
SN - 1034-4810
AD - Department of Maternal and Child Health, University of North Carolina, School of Public Health, Chapel Hill 27599-7400
UR - 8518007
ER -

TY - JOUR
ID - 2064
T1 - [Polycyclic aromatic hydrocarbons on play grounds--presentation of current status, toxicologic viewpoints and attempt at risk assessment for benzo(a)pyrene]. [Review] [104 refs] [German]
A1 - Fromme,H.
A1 - Geggel,K.
A1 - Gudzuhn,C.
Y1 - 1993/04//
N1 - Fromme, H. Geggel, K. Gudzuhn, C
Gesundheitswesen (Bundesverband der Arzte des Offentlichen Gesundheitsdienstes (Germany))
bfd, 9204210
IM
English Abstract. Journal Article. Review
German
KW - MEDLINE
KW - Benzo(a)pyrene/an [Analysis]
KW - Carcinogens
KW - Environmental/an [Analysis]
KW - Child
KW - Environmental Monitoring
KW - Humans
KW - Play and Playthings
KW - Polycyclic Compounds/an [Analysis]
KW - Risk Factors
KW - Soil Pollutants/an [Analysis]
RP - NOT IN FILE
SP - 195
EP - 205
JF - Gesundheitswesen
VL - 55
IS - 4
CY - GERMANY
N2 - Polycyclic aromatic hydrocarbons (PAH) have become an ubiquitous upper soil component in consequence of industrialisation involving a multitude of combustion processes. Meaningful contaminations can be found on child playgrounds, for example. These may enter into the organism of small children playing there, as children are capable of unintentionally swallowing minute quantities of foreign matter - be that as it may, we cannot exclude such a possibility. Suitable rules are mandatory in particular to prevent such occurrences in an area that is susceptible to them, as preventive health care should always be the order of the day. In the following

article we report on the present scientific-toxicological state of the art, on update data on environmental pollution and reasons for a quantitative risk assessment for different paths of pollution. [References: 104]
SN - 0941-3790
AD - Senatsverwaltung fur Gesundheit Berlin
UR - 8507997
ER -

TY - JOUR
ID - 2065
T1 - Ciliary beating frequency in chronic sinusitis
A1 - Nuutinen,J.
A1 - Rauch-Toskala,E.
A1 - Saano,V.
A1 - Joki,S.
Y1 - 1993/06//
N1 - Nuutinen, J. Rauch-Toskala, E. Saano, V. Joki, S
Archives of otolaryngology--head & neck surgery
8603209, alq
AIM, IM
Comparative Study. Journal Article. Research Support, Non-U.S. Gov't
English
KW - MEDLINE
KW - Adolescent
KW - Adult
KW - Aged
KW - 80 and over
KW - Analysis of Variance
KW - Child
KW - Preschool
KW - Chronic Disease
KW - Ethmoid Sinusitis/ep [Epidemiology]
KW - Ethmoid Sinusitis/pp [Physiopathology]
KW - Ethmoid Sinusitis/su [Surgery]
KW - Female
KW - Humans
KW - Male
KW - Maxillary Sinusitis/ep [Epidemiology]
KW - Maxillary Sinusitis/pp [Physiopathology]
KW - Maxillary Sinusitis/su [Surgery]
KW - Middle Aged
KW - Mucociliary Clearance/ph [Physiology]
KW - Nasal Mucosa/pp [Physiopathology]
KW - Recurrence
KW - Regression Analysis
RP - NOT IN FILE
SP - 645
EP - 647
JF - Archives of Otolaryngology -- Head & Neck Surgery
JA - Arch Otolaryngol Head Neck Surg
VL - 119
IS - 6
CY - UNITED STATES
N2 - Ciliary beating frequency, one component of mucociliary function, was measured outside of the sinus in vitro with a photoelectric method in 150 patients with chronic sinusitis and in 26 control subjects. In the mucosal samples of 35 patients (23%), no ciliary activity was seen. Ciliary beating frequency in maxillary sinus mucosa of the rest of the 115 patients (77%) was, as an average, 16.6 +/- 3.0 Hz (range, 10.9 to 23.3 Hz) and it was 15.9 +/- 2.6 Hz (range, 13.2 to 22.8 Hz) for the control patients. No differences in ciliary beating frequency

were found according to quality of sinus secretion, prevalent respiratory allergy, or mucosal thickness. However, in sinuses with purulent secretion, ciliary beating frequency was slightly better (P < .05) than in "empty" sinuses. The study suggests that in many cases of chronic sinusitis, the sinus mucosa still has a capacity to recure
SN - 0886-4470
AD - Department of Otorhinolaryngology, University of Kuopio, Finland
UR - 8499095
ER -

TY - JOUR
ID - 2066
T1 - The risk of childhood injury on Boston's playground equipment and surfaces
A1 - Bond,M.T.
A1 - Peck,M.G.
Y1 - 1993/05//
N1 - Bond, M T. Peck, M G
American journal of public health
1254074, 3xw
AIM, IM
Journal Article
English
KW - MEDLINE
KW - Boston
KW - Child
KW - Preschool
KW - Equipment Safety
KW - Humans
KW - Infant
KW - Play and Playthings
KW - Risk
KW - Sampling Studies
KW - Wounds and Injuries/ep [Epidemiology]
KW - Wounds and Injuries/pc [Prevention & Control]
RP - NOT IN FILE
SP - 731
EP - 733
JF - American Journal of Public Health
JA - Am J Public Health
VL - 83
IS - 5
CY - UNITED STATES
N2 - The Childhood Injury Prevention Program of the Boston Department of Health and Hospitals conducted a survey of injury risk in Boston's playgrounds. A standardized checklist was used to assess a 25% sample of public playgrounds for hazards. Climbers accounted for 34% of the hazards observed and had the greatest proportion of significant hazards. The surfacing material in 100% of the playgrounds observed was unsafe. Urban children risk exposure to hazardous playground equipment frequently situated above unsafe surfacing material
SN - 0090-0036
AD - Boston Department of Health and Hospitals, Mass
UR - 8484458
ER -

TY - JOUR
ID - 2067
T1 - Injuries in primary care practices
A1 - Gofin,R.
A1 - Lison,M.

A1 - Morag,C.
Y1 - 1993/02//
N1 - Gofin, R. Lison, M. Morag, C
Archives of disease in childhood
6xg, 0372434
AIM, IM
Journal Article. Research Support, Non-U.S. Gov't
English
KW - MEDLINE
KW - Adolescent
KW - Child
KW - Preschool
KW - Female
KW - Humans
KW - Incidence
KW - Infant
KW - Newborn
KW - Israel/ep [Epidemiology]
KW - Male
KW - Primary Health Care
KW - Referral and Consultation
KW - Social Class
KW - Wounds and Injuries/ep [Epidemiology]
KW - Wounds and Injuries/et [Etiology]
KW - Wounds and Injuries/th [Therapy]
RP - NOT IN FILE
SP - 223
EP - 226
JF - Archives of Disease in Childhood
JA - Arch Dis Child
VL - 68
IS - 2
CY - ENGLAND
N2 - A study of injuries among 0-15 year old children attending primary care clinics in a low social class and a middle class neighbourhood was carried out during a period of one year. Data were collected in a structured form by the physicians while the patient was in the clinic. The rate of injuries was 121.3/1000 children per year in the clinic from the lower social class neighbourhood and 70.7/1000 children per year in the clinic from the middle class neighbourhood. Injuries in playgrounds, and on pavements and streets occurred in higher proportions in the low social class than in the middle class neighbourhood clinics. The most frequent causes of injuries were falls and being struck and injured by cutting/piercing instruments; these caused mostly contusions and lacerations. The clinic was the first place of treatment in 65% of the cases in the middle class area and in 45% in the lower social class area; 28% and 10% respectively were referred to the hospital for additional treatment. Data from primary care clinics should be considered when estimating the incidence of injuries in the community, in planning intervention programmes, and future research
SN - 1468-2044
AD - Department of Social Medicine, Hadassah Medical Organization, Braun School of Public Health and Community Medicine of Hadassah, Jerusalem 91010, Israel
UR - 8481045
ER -

TY - JOUR
ID - 2068
T1 - Intercellular adhesion molecule-1 (ICAM-1) staining of reactive and neoplastic follicles. ICAM-1 expression of neoplastic follicle differs from that of reactive germinal center and is independent of follicular dendritic cells
A1 - Ree,H.J.
A1 - Khan,A.A.

A1 - Elsakr,M.
A1 - Liau,S.
A1 - Teplitz,C.
Y1 - 1993/05/01/
N1 - Ree, H J. Khan, A A. Elsakr, M. Liau, S. Teplitz, C
Cancer
0374236, clz
AIM, IM
Journal Article. Research Support, Non-U.S. Gov't
English
KW - MEDLINE
KW - Adolescent
KW - Adult
KW - Aged
KW - 80 and over
KW - Cell Adhesion Molecules/an [Analysis]
KW - Child
KW - Female
KW - Humans
KW - Intercellular Adhesion Molecule-1
KW - Lymph Nodes/ch [Chemistry]
KW - Lymphoma
KW - Follicular/pa [Pathology]
KW - Follicular
KW - Male
KW - Middle Aged
KW - Staining and Labeling/mt [Methods]
RP - NOT IN FILE
SP - 2817
EP - 2822
JF - Cancer
VL - 71
IS - 9
CY - UNITED STATES
N2 - BACKGROUND: Intercellular adhesion molecule-1 (ICAM-1) is expressed on follicular dendritic cells (FDC), and the ICAM-1/LFA-1 pathway is essential for affinity selection of activated B-cells in germinal centers (GC). The expression of ICAM-1 has been studied by immunostaining methods in GC, but not in neoplastic follicles (NF). METHODS: The authors studied the expression of ICAM-1 by the avidin-biotin-peroxidase complex (ABC) method with frozen sections in GC of reactive nodes (n = 22) and NF of follicular lymphomas (n = 19), in comparison with FDC staining. RESULTS: GC stained uniformly for ICAM-1, with staining intensity varying little from node to node, and follicular borders were well demarcated from the surroundings. Endothelial cells within GC did not stain whereas those outside stained strongly. In contrast, NF stained variably from tumor to tumor, and in any given tumor. Characteristically, follicular borders were not demarcated, and stained areas were difficult to define due to the lack of demarcation, with the exception of large cell type with diffuse areas (n = 2). Endothelium of intrafollicular vessels stained prominently (12 of 19), and appeared to contribute to the staining intensity of the follicles. FDC staining revealed a meshwork pattern in GC which was similar to that of ICAM-1. The FDC mesh-work pattern was present in 15 tumors. The pattern was remarkably uniform with sharply defined borders, which contrasted starkly to the variable staining and lack of follicular borders in the ICAM-1 stain of the same tumors. CONCLUSIONS: The staining pattern of follicles for ICAM-1 was similar to that for FDC in reactive nodes, but distinct from the latter in follicular lymphomas. An appropriate expression of ICAM-1 appears to be essential for normal GC, as the alteration of the expression coincides with the malignant transformation of GC. The altered expression of ICAM-1 may be useful in distinguishing NF from reactive GC
SN - 0008-543X
AD - Department of Pathology, Beth Israel Medical Center, New York, NY 10003
UR - 7682151
ER -

TY - JOUR
ID - 2069
T1 - Relations between children's playground and classroom behaviour
A1 - Pellegrini,A.D.
A1 - Davis,P.D.
Y1 - 1993/02//
N1 - Pellegrini, A D. Davis, P D
The British journal of educational psychology
awn, 0370636
IM
Journal Article. Research Support, Non-U.S. Gov't. Research Support, U.S. Gov't, P.H.S.
English
KW - MEDLINE
KW - Attention
KW - Child
KW - Child Behavior
KW - Female
KW - Gender Identity
KW - Humans
KW - Male
KW - Motor Activity
KW - Play and Playthings
KW - Social Behavior
KW - Social Environment
RP - NOT IN FILE
SP - 88
EP - 95
JF - British Journal of Educational Psychology
JA - Br J Educ Psychol
VL - 63
IS - Pt 1
CY - ENGLAND
N2 - In this within-subjects design field experiment children's behaviour in the playground was charted as a function of gender and time in the classroom immediately preceding playtime. Playground behaviour was also related to post-recess classroom behaviour. Twenty-three 9-year-old children were observed for 14 weeks. Classroom behaviour (i.e., task relevant behaviour on standardised seat work immediately before and after playtime) and playground behaviour (i.e., social and non-social exercise and sedentary behaviour) were observed. Time in classroom before playtime was manipulated so that there was a shorter and a longer confinement period. Results indicated that children were less attentive to seat work as a function of time and that longer confinement resulted in more exercise for boys and more social sedentary behaviour for girls. Social behaviour at playtime and post-playtime attention to seat work were significantly related. Results are discussed in terms of Novelty Theory
SN - 0007-0998
AD - University of Sheffield
UR - 8466835
ER -

TY - JOUR
ID - 2070
T1 - Assessment of blood lead levels in children living in a historic mining and smelting community
A1 - Cook,M.
A1 - Chappell,W.R.
A1 - Hoffman,R.E.
A1 - Mangione,E.J.
Y1 - 1993/02/15/

N1 - Cook, M. Chappell, W R. Hoffman, R E. Mangione, E J
American journal of epidemiology
3h3, 7910653
IM
Journal Article. Research Support, Non-U.S. Gov't. Research Support, U.S. Gov't, Non-P.H.S.
English
KW - MEDLINE
KW - Child
KW - Child Behavior
KW - Preschool
KW - Colorado
KW - Environmental Exposure/ae [Adverse Effects]
KW - Environmental Exposure/an [Analysis]
KW - Environmental Pollutants/ae [Adverse Effects]
KW - Environmental Pollutants/an [Analysis]
KW - Humans
KW - Infant
KW - Lead/an [Analysis]
KW - Lead/bl [Blood]
KW - Lead Poisoning/bl [Blood]
KW - Lead Poisoning/ep [Epidemiology]
KW - Lead Poisoning/et [Etiology]
KW - Metallurgy
KW - Mining
KW - Regression Analysis
KW - Risk Factors
KW - Soil Pollutants/an [Analysis]
RP - NOT IN FILE
SP - 447
EP - 455
JF - American Journal of Epidemiology
JA - Am J Epidemiol
VL - 137
IS - 4
CY - UNITED STATES
N2 - Lead poisoning in childhood is an important public health problem, and thus, it is important to determine how children are exposed to lead. In 1987, the authors conducted an exposure assessment and blood lead screening for children aged 6-71 months living in Leadville, Colorado. High levels of lead had been found in the soil as a result of both past mining and smelting activities and natural mineralization. Blood was collected from each child for lead analysis, and behavioral characteristics were identified through an interview with a parent or guardian. Three sources of exposure to lead were associated with blood lead levels: lead in a core sample taken from the backyard of the family's home, lead brought home on the clothes of a miner, and lead from soldering in the home. Two pathways of exposure were associated with blood lead levels: the child swallowing things other than food, and taking food or a bottle outside to play. Multivariate regression using these variables found effect modification by age. For children aged 6-36 months, only sources of exposure were independent predictors of blood lead levels, while in children aged 37-71 months, a pathway of exposure in addition to sources of exposure independently predicted blood lead levels
SN - 0002-9262
AD - Division of Disease Control and Environmental Epidemiology, Colorado Department of Health, Denver 80222-1530
UR - 8460625
ER -

TY - JOUR
ID - 2071
T1 - Effect of ambient ozone on peak expiratory flow of exercising children in The Netherlands
A1 - Hoek,G.

A1 - Brunekreef,B.
A1 - Kosterink,P.
A1 - Van den,Berg R.
A1 - Hofschreuder,P.
Y1 - 1993/01//
N1 - Hoek, G. Brunekreef, B. Kosterink, P. Van den Berg, R. Hofschreuder, P
Archives of environmental health
6yo, 0212627
AIM, IM
Journal Article
English
KW - MEDLINE
KW - Air Pollutants/an [Analysis]
KW - Air Pollutants/pd [Pharmacology]
KW - Child
KW - Environmental Exposure/an [Analysis]
KW - Exercise
KW - Female
KW - Humans
KW - Male
KW - Netherlands
KW - Ozone/an [Analysis]
KW - Ozone/pd [Pharmacology]
KW - Peak Expiratory Flow Rate/de [Drug Effects]
KW - Physical Education and Training
KW - Regression Analysis
KW - Temperature
RP - NOT IN FILE
SP - 27
EP - 32
JF - Archives of Environmental Health
JA - Arch Environ Health
VL - 48
IS - 1
CY - UNITED STATES
N2 - The potential effects of elevated ozone concentrations in The Netherlands were evaluated by the measurement of peak expiratory flow (PEF) of exercising children. Peak expiratory flow was measured with mini-Wright peak flow meters, both before and after out-door sports training. The relationship between PEF and ozone was investigated with individual regression analysis. The difference of PEF after and before training (delta PEF) and the PEF after training were used as dependent variables. The ozone concentration during the training and the 1-h maximum ozone concentration of the same and the previous day were used as independent variables. The highest observed 1-h maximum ozone concentration was 236 micrograms/m3. delta PEF was unrelated to the ambient ozone concentration during training. Peak flow measured after the training was positively correlated with ambient temperature. The high correlation between ozone and temperature prevented the evaluation of effects of the maximum ozone concentration of the same day on PEF after training. A small negative association of borderline statistical significance between PEF after training and previous-day maximum ozone was observed
SN - 0003-9896
AD - Department of Epidemiology and Public Health, University of Wageningen, The Netherlands
UR - 8452396
ER -

TY - JOUR
ID - 2072
T1 - Lymph and blood supply of the human intervertebral disc. Cadaver study of correlations to discitis
A1 - Rudert,M.
A1 - Tillmann,B.

Y1 - 1993/02//
N1 - Rudert, M. Tillmann, B
Acta orthopaedica Scandinavica
0370352, 1go
IM
Journal Article. Research Support, Non-U.S. Gov't
English
KW - MEDLINE
KW - Adolescent
KW - Adult
KW - Aged
KW - 80 and over
KW - Aging/ph [Physiology]
KW - Basement Membrane/ch [Chemistry]
KW - Child
KW - Preschool
KW - Female
KW - Fluorescent Antibody Technique
KW - Humans
KW - Immunohistochemistry
KW - Infant
KW - Intervertebral Disc/bs [Blood Supply]
KW - Intervertebral Disc/gd [Growth & Development]
KW - Laminin/an [Analysis]
KW - Lumbar Vertebrae
KW - Lymphatic System/ah [Anatomy & Histology]
KW - Lymphatic System/gd [Growth & Development]
KW - Male
KW - Middle Aged
KW - Pregnancy
KW - Reference Values
RP - NOT IN FILE
SP - 37
EP - 40
JF - Acta Orthopaedica Scandinavica
JA - Acta Orthop Scand
VL - 64
IS - 1
CY - DENMARK
N2 - Immunohistochemical (antibodies against laminin) and histochemical methods (Ulex europaeus lectin, 5'-nucleotidase activity) were used to describe the vascular pattern of human intervertebral discs and the surrounding tissue at different ages. Blood and lymph vessels were found in the connective tissue outside the annulus in all age groups. In the annulus blood vessels and lymphatics were detected up to 20 years of age, in the cartilage end-plate blood vessels appeared up to 7 years of age (cartilage canals). In the nucleus pulposus neither blood nor lymph vessels could be seen at any age. The occurrence of blood and lymph vessels in growing intervertebral discs help us to understand childhood discitis without simultaneous affection of the vertebral body
SN - 0001-6470
AD - Institute of Anatomy, Christian-Albrechts-University, Kiel, Germany
UR - 8451943
ER -

TY - JOUR
ID - 2073
T1 - Impact of migraine in the United States: data from the National Health Interview Survey
A1 - Stang,P.E.
A1 - Osterhaus,J.T.

Y1 - 1993/01//
N1 - Stang, P E. Osterhaus, J T
Headache
2985091r, g1n
IM
Journal Article
English
KW - MEDLINE
KW - Activities of Daily Living
KW - Adolescent
KW - Adult
KW - Aged
KW - Child
KW - Preschool
KW - Demography
KW - Female
KW - Health Services/ut [Utilization]
KW - Health Surveys
KW - Humans
KW - Infant
KW - Male
KW - Middle Aged
KW - Migraine Disorders/ep [Epidemiology]
KW - Migraine Disorders/pp [Physiopathology]
KW - Prevalence
KW - United States/ep [Epidemiology]
RP - NOT IN FILE
SP - 29
EP - 35
JF - Headache
VL - 33
IS - 1
CY - UNITED STATES
N2 - Data from the 1989 National Health Interview Survey concerning migraine occurrence and impairment were analyzed to assess the impact of migraine on the US population. About four of every one hundred persons in the United States were found to have migraine, accounting for nearly 10 million individuals. Migraine was most prevalent in those aged 25 to 44 years and was about 2.5 times more frequent in females than males. Migraine was most common in whites (85%) and those with low household income. In women, migraine prevalence increased with the level of education. About 10% of migrainous children missed at least one day of school over a two-week period due to migraine; nearly 1% missed four days. Migraineurs were bedridden for about three million days per month and had an estimated 74.2 million days per year of restricted activity due to migraine. The potential cost of lost productivity was estimated at $1.4 billion per year for the estimated 6,196,378 migraineurs who worked outside the home. It is difficult to derive similar estimates for costs of lost productivity in housewives; however, housewives experienced an estimated 38 million days per year of restricted activity. Eighty-five percent of females and 77% of males reported a physician visit at some point for their migraine. Migraine is a relatively common disease whose social and financial impact has been poorly understood
SN - 0017-8748
AD - Pharmacoeconomic Research, Glaxo Research Institute, Research Triangle Park, NC 27709
UR - 8436495
ER -

TY - JOUR
ID - 2074
T1 - Black spots on the scalps of schoolchildren. A recurrent condition in the windy west
A1 - Cobb,N.
A1 - Etzel,R.A.

A1 - Hudson,R.
Y1 - 1993/02//
N1 - Cobb, N. Etzel, R A. Hudson, R
The Western journal of medicine
0410504, xn5
AIM, IM
Journal Article
English
KW - MEDLINE
KW - Air Pollutants/ae [Adverse Effects]
KW - Air Pollutants/an [Analysis]
KW - Child
KW - Preschool
KW - Construction Materials/ae [Adverse Effects]
KW - Construction Materials/an [Analysis]
KW - Diagnosis
KW - Differential
KW - Disease Outbreaks
KW - Female
KW - Humans
KW - Lice Infestations/pa [Pathology]
KW - Male
KW - Nevus
KW - Pigmented/pa [Pathology]
KW - Pigmentation Disorders/ep [Epidemiology]
KW - Pigmentation Disorders/et [Etiology]
KW - Pigmentation Disorders/pa [Pathology]
KW - Recurrence
KW - Scalp Dermatoses/ep [Epidemiology]
KW - Scalp Dermatoses/et [Etiology]
KW - Scalp Dermatoses/pa [Pathology]
KW - Tars/ae [Adverse Effects]
KW - Tars/an [Analysis]
KW - Tinea Versicolor/pa [Pathology]
KW - Wind
KW - Wyoming/ep [Epidemiology]
RP - NOT IN FILE
SP - 139
EP - 141
JF - Western Journal of Medicine
JA - West J Med
VL - 158
IS - 2
CY - UNITED STATES
N2 - During the past 10 years, epidemics of black spots on the scalps of schoolchildren have caused considerable concern in at least 4 communities in the Rocky Mountain states. We describe the clinical presentation of "black spots" in a group of Wyoming elementary school students and the epidemiologic investigation that revealed the cause. Our study included a questionnaire survey of students' parents, examination of students at the affected school and at two other schools, observation of playground activity patterns, and laboratory analysis of specimens taken from affected children and from the school environment. The black material in the scalp spots was chemically identical to flakes of black material found on the playground and tar from the school roof. We concluded that the spots were caused by flakes of windblown tar from the school roof. Previous outbreaks of black spots may have had a similar cause
SN - 0093-0415
AD - Division of Environmental Hazards and Health Effects, Centers for Disease Control and Prevention, Atlanta, Georgia
UR - 8434463

ER -

TY - JOUR
ID - 2075
T1 - Family correlates of a 10-year pulmonary health trend in cystic fibrosis
A1 - Patterson,J.M.
A1 - Budd,J.
A1 - Goetz,D.
A1 - Warwick,W.J.
Y1 - 1993/02//
N1 - Patterson, J M. Budd, J. Goetz, D. Warwick, W J
Pediatrics
oxv, 0376422
AIM, IM
Journal Article. Research Support, U.S. Gov't, Non-P.H.S.. Research Support, U.S. Gov't, P.H.S.
English
KW - MEDLINE
KW - Adaptation
KW - Psychological
KW - Adolescent
KW - Adult
KW - Child
KW - Preschool
KW - Cystic Fibrosis/pp [Physiopathology]
KW - Cystic Fibrosis/ra [Radiography]
KW - Cystic Fibrosis/th [Therapy]
KW - Employment/sn [Statistics & Numerical Data]
KW - Family/px [Psychology]
KW - Female
KW - Follow-Up Studies
KW - Forced Expiratory Volume
KW - Health Services Needs and Demand/sn [Statistics & Numerical Data]
KW - Health Status
KW - Home Care Services/st [Standards]
KW - Humans
KW - Male
KW - Minnesota
KW - Patient Compliance
KW - Regression Analysis
KW - Social Support
KW - Wisconsin
RP - NOT IN FILE
SP - 383
EP - 389
JF - Pediatrics
VL - 91
IS - 2
CY - UNITED STATES
N2 - In this study, 33% of the variance in the 10-year trend of forced expiratory volume in 1 second (FEV1), used as an index of pulmonary health, was explained by differences in family characteristics at the start of the study. Balanced family coping, a family emphasis on personal growth, and compliance with treatment for 91 children with cystic fibrosis were assessed at the start of the study and the FEV1 was observed at every clinic visit over the next 10 years. When both parents' coping emphasized family integration, support for self, and medical consultation, the FEV1 trend was better. Compliance with daily chest physical therapy and with quarterly clinic visits was associated with a better FEV1 trend. Poorer FEV1 trend was associated with active social involvement of family members. Older patients and patients whose parents worked more hours outside

the home had lower compliance. These findings support the importance of encouraging families to balance their resources between the child's health needs and family needs
SN - 0031-4005
AD - School of Public Health, University of Minnesota, Minneapolis 55455
UR - 8424015
ER -

TY - JOUR
ID - 2076
T1 - Child care center's new playground proves that safety isn't child's play
A1 - Minnich,J.S.
Y1 - 1993/01//
N1 - Minnich, J S
Health facilities management
8906517, hfm
H
Journal Article
English
KW - MEDLINE
KW - Child Day Care Centers
KW - Child
KW - Preschool
KW - Facility Design and Construction
KW - Hospital Bed Capacity
KW - 500 and over
KW - Humans
KW - Infant
KW - Play and Playthings
KW - Safety
KW - Virginia
RP - NOT IN FILE
SP - 14
EP - 15
JF - Health Facilities Management
JA - Health Facil.Manage
VL - 6
IS - 1
CY - UNITED STATES
SN - 0899-6210
UR - 10123278
ER -

TY - JOUR
ID - 2077
T1 - Improving the quality of survival for infants of birthweight < 1000 g born in non-level-III centres in Victoria. The Victorian Infant Collaborative Study Group
Y1 - 1993/01/04/
N1 - The Medical journal of Australia
0400714, m26
IM
Comparative Study. Journal Article. Research Support, Non-U.S. Gov't
English
KW - MEDLINE
KW - Blood Gas Monitoring
KW - Transcutaneous
KW - Child
KW - Preschool

KW - Developmental Disabilities/ep [Epidemiology]
KW - Developmental Disabilities/et [Etiology]
KW - Disabled Persons
KW - Humans
KW - Infant Care/st [Standards]
KW - Infant Care/sn [Statistics & Numerical Data]
KW - Infant Mortality
KW - Infant
KW - Low Birth Weight
KW - Newborn
KW - Intensive Care Units
KW - Neonatal/ut [Utilization]
KW - Nurseries
KW - Hospital/st [Standards]
KW - Hospital/sn [Statistics & Numerical Data]
KW - Patient Transfer
KW - Referral and Consultation/td [Trends]
KW - Time Factors
KW - Transportation of Patients
KW - Treatment Outcome
KW - Victoria
RP - NOT IN FILE
SP - 24
EP - 27
JF - Medical Journal of Australia
JA - Med J Aust
VL - 158
IS - 1
CY - AUSTRALIA
N2 - OBJECTIVE: To compare between eras the early care of extremely low birthweight (birthweight < 1000 g) infants born in non-level-III centres, that is, centres without a neonatal intensive care nursery, in Victoria, to identify changes associated with the improved quality of survival over time. DESIGN AND SETTING: The early care and outcome for infants of birthweight 500 to 999 g born in a non-level-III centre in Victoria were compared between two distinct eras, 1979-1980 and 1985-1987. PATIENTS AND OUTCOMES: Mortality data to two years of age were available for all infants liveborn in non-level-III centres in the two eras (1979-1980, n = 106; 1985-1987, n = 129). In 1979-1980, 47 of 52 infants transferred to a level-III centre were transported by the Newborn Emergency Transport Service (NETS); in 1985-1987, all 49 infants transferred were transported by NETS. Data concerning the immediate care after birth and during transport to a level-III centre were available for all infants transferred by NETS. All survivors were assessed for sensorineural impairments and disabilities at two years of age, corrected for prematurity. RESULTS: In both eras, 18 children born outside and transferred to a level-III centre survived to two years of age. Survivors in both eras had almost identical mean birthweights and gestational ages. There were trends for more survivors to be referred by paediatricians--1979-1980, 61%; 1985-1987, 83%; odds ratio (OR), 2.94; 95% confidence interval (CI), 0.7-12.4--and for quicker referral times to NETS in 1985-1987 (1979-1980, median 34.5 minutes after birth; 1985-1987, median 21.5 minutes after birth; z = 1.91, P = 0.056). It was possible only during 1985-1987 to monitor transcutaneous PO2 during transport. Durations of transport were similar in both eras. However, only in 1985-1987 was it possible in survivors to reduce significantly the inspired oxygen concentration during transport (median reductions in inspired oxygen, 1979-1980 3.5%; 1985-1987 20%; P = 0.028). Neurological impairment rates were substantially lower in survivors transported in the latter era (1979-1980 72% impaired; 1985-1987 22% impaired; OR 0.14, 95% CI 0.04-0.52). Of the neurological impairments, fewer had severe developmental delay alone (1979-1980 22%; 1985-1987 0%; OR 0.09, 95% CI 0.018-0.46), and the rate of blindness was lower, but the latter difference was not quite statistically significant (1979-1980 28%; 1985-1987 6%; OR 0.21, 95% CI 0.037-1.19). Over all, neurological disabilities were significantly reduced in the latter era (z = 2.93, P < 0.005). CONCLUSIONS: More active management of extremely low birthweight infants before transfer to a level-III centre, including prompt referral and transcutaneous PO2 monitoring during transport, may be important in improving the sensorineural outcome of survivors
SN - 0025-729X

UR - 7678050
ER -

TY - JOUR
ID - 2078
T1 - How young Europeans sleep
A1 - Tynjala,J.
A1 - Kannas,L.
A1 - Valimaa,R.
Y1 - 1993/03//
N1 - Tynjala, J. Kannas, L. Valimaa, R
Health education research
bqp, 8608459
T
Comparative Study. Journal Article. Research Support, Non-U.S. Gov't
English
KW - MEDLINE
KW - Adolescent
KW - Child
KW - Cross-Cultural Comparison
KW - Europe
KW - Female
KW - Health Behavior
KW - Health Status
KW - Humans
KW - Male
KW - Questionnaires
KW - Sleep/ph [Physiology]
RP - NOT IN FILE
SP - 69
EP - 80
JF - Health Education Research
JA - Health Educ Res
VL - 8
IS - 1
CY - ENGLAND
N2 - This study investigated sleeping habits, difficulties in being able to fall asleep and their connections to self-reported health conditions, as well as other selected health behaviours and use of leisure time, among 11-16 year old Europeans from 11 countries. The study was part of a larger, comparative, WHO coordinated project on the health and life-style of school children (Health Behaviour of School Age Children--A WHO Cross-National Survey, The HBSC Study). In most of the countries, research data were collected from samples representative of the whole country. Using a standardized survey questionnaire, the data were collected anonymously in schools. Altogether 40,202 students responded to the survey. Sleeping habits and an inability to fall asleep varied significantly between countries as well as between age groups but only slightly between the sexes. Finnish school children experienced the most difficulty in being able to fall asleep. After the Israeli youth, the Finnish school children had the shortest night's sleep. At least a fifth of Finnish and Norwegian school children also reported that they felt tired almost every morning, the corresponding figure being smaller in other countries. A frequent use of psychoactive substances (alcohol and tobacco), lack of physical activity, excessive watching of TV/videos together with numerous evenings spent outside the home were all connected with going to bed late as well as with frequent difficulty in not being able to fall asleep. The results of the study offer an important challenge to health promotion and health education. Much more attention must be paid to this essential and exciting health habit!
SN - 0268-1153
AD - University of Jyvaskyla, Department of Health Sciences, Finland
UR - 11067187
ER -

TY - JOUR
ID - 2079
T1 - So-called accidental firearm fatalities in children and teenagers in Tennessee, 1961-1988
A1 - Harruff,R.C.
Y1 - 1992/12//
N1 - Harruff, R C
The American journal of forensic medicine and pathology
3hc, 8108948
IM
Journal Article
English
KW - MEDLINE
KW - Accidents/sn [Statistics & Numerical Data]
KW - Adolescent
KW - Child
KW - Preschool
KW - Female
KW - Firearms
KW - Humans
KW - Infant
KW - Male
KW - Odds Ratio
KW - Rural Health
KW - Seasons
KW - Survival Rate
KW - Tennessee/ep [Epidemiology]
KW - Urban Health
KW - Wounds
KW - Gunshot/mo [Mortality]
RP - NOT IN FILE
SP - 290
EP - 298
JF - American Journal of Forensic Medicine & Pathology
JA - Am J Forensic Med Pathol
VL - 13
IS - 4
CY - UNITED STATES
N2 - Tennessee State medical examiner reports of firearm fatalities classified as accidents involving victims aged 19 year or less from 1961 through 1988, were reviewed to evaluate circumstantial and contributing factors. A total of 225 were analyzed. The peak age for victims was 17 years, and there were more than five times more male than female victims. In Shelby County (Memphis), the racial distribution of fatalities was approximately that of the general population. Playing with a gun was the most frequent circumstance. The person responsible for pulling the trigger was equally likely to be a friend, a family member, or the victim. Head or neck were injured in most cases. The urban mortality rate was nearly twice that of the rural rate. More than half of the deaths in urban counties occurred indoors and involved handguns, whereas in rural counties only a third were due to handguns and the location was more often outdoors. Deaths in rural counties showed a seasonal variation that corresponded with the hunting season; by contrast, a peak in early summer was noted in the urban deaths. Defective guns or guns unsafe in design caused several deaths. (Semiautomatic pistols, which can be fired after unloading the ammunition magazine, are conspicuous examples of guns unsafe in design.) An important observation of this study is that medical examiners vary considerably in their classification of accidental manner, particularly when children are involved. Furthermore, medical examiner reports need to include much more information than is currently recorded if they are to be useful in guiding public policy to reduce firearm injuries
SN - 0195-7910
AD - Department of Pathology, Indiana University Medical Center, Indianapolis
UR - 1288257
ER -

TY - JOUR
ID - 2080
T1 - [Health care. From village to concrete suburb]. [Danish]
A1 - Mandrup,G.H.
Y1 - 1992/07/29/
N1 - Mandrup, G H
Sygeplejersken
vf9, 0421366
J, N
English Abstract. Journal Article
Danish
KW - MEDLINE
KW - Acculturation
KW - Child
KW - Child Health Services
KW - Community Health Nursing
KW - Denmark
KW - Emigration and Immigration
KW - Female
KW - Health Education
KW - Humans
KW - Infant
KW - Maternal Health Services
KW - Peer Group
KW - Pregnancy
KW - Turkey/eh [Ethnology]
RP - NOT IN FILE
SP - 8
EP - 11
JF - Sygeplejersken
VL - 92
IS - 31
CY - DENMARK
SN - 0106-8350
UR - 1492312
ER -

TY - JOUR
ID - 2081
T1 - [Requirements and risk profile of soccer-playing children. Orthopedic aspects]. [German]
A1 - Betz,M.
A1 - Klimt,F.
Y1 - 1992/12//
N1 - Betz, M. Klimt, F
Schweizerische Zeitschrift fur Sportmedizin
ufl, 2984794r
IM
English Abstract. Journal Article
German
KW - MEDLINE
KW - Adolescent
KW - Age Factors
KW - Anthropometry
KW - Athletic Injuries/pc [Prevention & Control]
KW - Child
KW - Preschool

KW - Environment
KW - Humans
KW - Male
KW - Physical Exertion
KW - Soccer/in [Injuries]
RP - NOT IN FILE
SP - 169
EP - 173
JF - Schweizerische Zeitschrift fur Sportmedizin
JA - Schweiz.Z Sportmed
VL - 40
IS - 4
CY - SWITZERLAND
N2 - Although the rate of injuries in soccer is high, this sport puts children only at moderate risk of getting injured. In younger children this risk is very low but the number and severity of injuries increases with puberty. The most common cause for injuries is a collision with another player, the most common injury is distortion of the talocrural joint. Playing soccer indoors is more dangerous than playing outdoors. The danger of accidents and injuries can be reduced by preventive measures, such as weight categories in children leagues
SN - 0036-7885
AD - Philipps-Universitat, D-Marburg
UR - 1475676
ER -

TY - JOUR
ID - 2082
T1 - Medical illness in school-age Kenyans in relation to nutrition, cognition, and playground behaviors
A1 - Neumann,C.
A1 - McDonald,M.A.
A1 - Sigman,M.
A1 - Bwibo,N.
Y1 - 1992/12//
N1 - Neumann, C. McDonald, M A. Sigman, M. Bwibo, N
Journal of developmental and behavioral pediatrics : JDBP
htf, 8006933
IM
Journal Article. Research Support, U.S. Gov't, Non-P.H.S.
English
KW - MEDLINE
KW - Affective Symptoms/px [Psychology]
KW - Child
KW - Cognition Disorders/px [Psychology]
KW - Developing Countries
KW - Educational Status
KW - Female
KW - Gender Identity
KW - Humans
KW - Intelligence Tests
KW - Kenya
KW - Male
KW - Nutritional Status
KW - Play and Playthings
KW - Protein-Energy Malnutrition/px [Psychology]
KW - Sick Role
KW - Social Environment
KW - Socioeconomic Factors
RP - NOT IN FILE
SP - 392

EP - 398
JF - Journal of Developmental & Behavioral Pediatrics
JA - J Dev Behav Pediatr
VL - 13
IS - 6
CY - UNITED STATES
N2 - Because medical illness is associated with malnutrition, it might contribute to the poor outcomes seen in malnourished children. This study explored relations between frequency of mild illness and development in 133 mild-to-moderately malnourished school-age Kenyan children. Morbidity information was collected for one year. Concurrent assessments of cognitive status and playground behaviors were obtained. Nutritional and environmental variables were also assessed. Girls with more reported days of mild illness performed less well on developmental measures than did their healthier female peers. They were less cognitively advanced, and, on the playground, girls with more mild illness were less active, happy, and social. Furthermore, relations between frequency of mild illness and playground behaviors (but not cognitive score) in the girls remained statistically significant when other variables, such as socioeconomic status, parental literacy, food intake, and anthropometry, were considered. For the boys, frequency of mild illness was not related to cognition or play behaviors
SN - 0196-206X
AD - Department of Public Health, University of California, Los Angeles 90024-1772
UR - 1469106
ER -

TY - JOUR
ID - 2083
T1 - The magnitude of mortality from acute respiratory infections in children under 5 years in developing countries
A1 - Garenne,M.
A1 - Ronsmans,C.
A1 - Campbell,H.
Y1 - 1992///
N1 - Garenne, M. Ronsmans, C. Campbell, H
World health statistics quarterly. Rapport trimestriel de statistiques sanitaires mondiales
xp0, 7900237
IM, J
Comparative Study. Journal Article
English
KW - MEDLINE
KW - Acute Disease
KW - Age Factors
KW - Child
KW - Preschool
KW - Developing Countries
KW - Europe/ep [Epidemiology]
KW - Humans
KW - Infant
KW - Newborn
KW - Measles/mo [Mortality]
KW - Pneumonia/mo [Mortality]
KW - Respiratory Tract Infections/di [Diagnosis]
KW - Respiratory Tract Infections/mo [Mortality]
KW - Whooping Cough/mo [Mortality]
RP - NOT IN FILE
SP - 180
EP - 191
JF - World Health Statistics Quarterly - Rapport Trimestriel de Statistiques Sanitaires Mondiales
JA - World Health Stat Q
VL - 45

IS - 2-3
CY - SWITZERLAND
N2 - This article reviews the available evidence of mortality from acute respiratory infections (ARI) among children aged under 5 years in contemporary developing countries and compares the findings with European populations before 1965. In European populations before 1965, the level of mortality was found to be a determinant of the proportion of deaths due to ARI. There were marked differences according to regional patterns of mortality. Deaths from ARI played a smaller role after 1950, when the use of antibiotics became generalized. In developing countries, the role of ARI mortality seems to be similar to the European experience. The age pattern is very marked. In absolute values, ARI mortality is highest in the neonatal period and decreases with age. In relative values, ARI mortality is highest in the postneonatal period. ARI, mainly pneumonia, accounts for about 18% of underlying causes of death in developing countries. Pneumonia and other ARI are frequent complications of measles and pertussis; ARI is also commonly found after other infections and in association with severe malnutrition. Virtually no data are available in developing countries to provide final estimates of the role of ARI in mortality of children aged under 5 years. However, the WHO figure of 1 out of 3 deaths due to--or associated with--ARI may be close to the real range of the ARI-proportional mortality in children of developing countries. Results are discussed in light of the definitions of ARI used in various studies, the difficulties in ascertaining and coding multiple causes of death and the quality of data from some sources
SN - 0379-8070
AD - Harvard University, Center for Population and Development Studies, Cambridge, MA
UR - 1462653
ER -

TY - JOUR
ID - 2084
T1 - Prevalence of hepatitis B markers in the population of Catalonia (Spain). Rationale for universal vaccination of adolescents
A1 - Salleras,L.
A1 - Bruguera,M.
A1 - Vidal,J.
A1 - Taberner,J.L.
A1 - Plans,P.
A1 - Bayas,J.M.
A1 - Pumarola,T.
A1 - Jimenez de Anta,M.T.
A1 - Rodes,J.
Y1 - 1992/09//
N1 - Salleras, L. Bruguera, M. Vidal, J. Taberner, J L. Plans, P. Bayas, J M. Pumarola, T. Jimenez de Anta, M T. Rodes, J
European journal of epidemiology
ere, 8508062
IM
Journal Article
English
KW - MEDLINE
KW - Adolescent
KW - Adult
KW - Age Factors
KW - Aged
KW - Child
KW - Educational Status
KW - Female
KW - Hepatitis B/ep [Epidemiology]
KW - Hepatitis B/pc [Prevention & Control]
KW - Hepatitis B Antigens/an [Analysis]
KW - Hepatitis B Vaccines
KW - Humans
KW - Male

KW - Middle Aged
KW - Prevalence
KW - Rural Population
KW - Sex Factors
KW - Social Class
KW - Spain/ep [Epidemiology]
KW - Urban Population
KW - Vaccination
RP - NOT IN FILE
SP - 640
EP - 644
JF - European Journal of Epidemiology
JA - Eur J Epidemiol
VL - 8
IS - 5
CY - ITALY
N2 - The prevalence of hepatitis B markers was determined in a representative sample of the general population of Catalonia (Spain). HBsAg was found in 0.5% of children (less than 15 years of age) and in 1.7% of adults (more than 15 years of age), and anti HBs in 1.6% and 18%, respectively. Age-specific prevalence for both markers showed a low risk for hepatitis B before puberty, and a progressive rise since adolescence, suggesting that perinatal transmission and horizontal transmission in children are relatively uncommon in Spain. Prevalence of hepatitis B markers was significantly higher among subjects with low education level, residing in an urban area and born outside Catalonia, but in the stratified analysis, a statistical significant difference was only maintained in the prevalence of HBV markers between those who live in urban and rural areas, and between those who were born outside Catalonia and in Catalonia. These data may be used as a basis for a strategy of hepatitis B prevention in Spain which include universal vaccination of adolescents, passive-active immunization of newborns to HBsAg positive mothers and vaccination of susceptible adults subjects from high-risk groups
SN - 0393-2990
AD - Preventive Medicine Unit, Hospital Clinic i Provincial de Barcelona, Spain
UR - 1426162
ER -

TY - JOUR
ID - 2085
T1 - Activity patterns in elementary and high school students exposed to oxidant pollution
A1 - Spier,C.E.
A1 - Little,D.E.
A1 - Trim,S.C.
A1 - Johnson,T.R.
A1 - Linn,W.S.
A1 - Hackney,J.D.
Y1 - 1992/07//
N1 - Spier, C E. Little, D E. Trim, S C. Johnson, T R. Linn, W S. Hackney, J D
Journal of exposure analysis and environmental epidemiology
bjn, 9111438
IM
Comparative Study. Journal Article. Research Support, Non-U.S. Gov't
English
KW - MEDLINE
KW - Activities of Daily Living
KW - Adolescent
KW - Air Pollutants/ae [Adverse Effects]
KW - Child
KW - Environmental Exposure/ae [Adverse Effects]
KW - Exercise/ph [Physiology]
KW - Female
KW - Heart Rate/de [Drug Effects]

KW - Heart Rate/ph [Physiology]
KW - Humans
KW - Los Angeles
KW - Male
KW - Ozone/ae [Adverse Effects]
KW - Respiration/de [Drug Effects]
KW - Respiration/ph [Physiology]
RP - NOT IN FILE
SP - 277
EP - 293
JF - Journal of Exposure Analysis and Environmental Epidemiology
JA - J Expo Anal Environ Epidemiol
VL - 2
IS - 3
CY - UNITED STATES
N2 - We investigated activity patterns of 17 elementary school students aged 10-12, and 19 high school students aged 13-17, in suburban Los Angeles during the oxidant pollution season. Individuals' relationships between ventilation rate (VR) and heart rate (HR) were "calibrated" in supervised outdoor walking/jogging. Log VR was consistently proportional to HR; although "calibrations" were limited by a restricted range of exercise, and possibly by artifact due to mouthpiece breathing, which may cause overestimation of VR at rest. Each subject then recorded activities in diaries, and recorded HR once per minute by wearing Heart Watches, over 3 days (Saturday-Monday). For each activity the subject estimated a breathing rate--slow (like slow walking), medium (like fast walking), or fast (like running). VR ranges for each breathing rate and activity type were estimated from HR recordings. High-school students' diaries showed their aggregate distribution of waking hours as 68% slow inside, 8% slow outside, 10% medium inside, 9% medium outside, 1.5% fast inside, 1.5% fast outside. Elementary students' distribution was 47% slow inside, 15% slow outside, 20% medium inside, 12% medium outside, 2.5% fast inside, 3.5% fast outside. Sleep occupied 38% of high-school students' and 40% of elementary students' time; HR were generally lower in sleep than in slow waking activity. High school students' mean VR estimates were 13 L/min for slow breathing, 18 for medium, and 23 for fast; elementary students' were 14 slow, 18 medium, and 19 fast. VR distributions were approximately lognormal. Maximum estimated VR were approximately 70 L/min in elementary and approximately 100 L/min in high school students. Compared to adults studied similarly, students reported more medium or fast breathing, and had equal or higher VR estimates during slow and medium breathing despite their smaller size. These results suggest that, relative to body size, young people inhale larger doses of outdoor air pollutants than adults
SN - 1053-4245
AD - Environmental Health Service, Rancho Los Amigos Medical Center, Downey, California
UR - 1422159
ER -

TY - JOUR
ID - 2086
T1 - Rehabilitation after severe head injury
A1 - Frowein,R.A.
A1 - Terhaag,D.
A1 - auf der,Haar K.
A1 - Richard,K.E.
A1 - Firsching,R.
Y1 - 1992///
N1 - Frowein, R A. Terhaag, D. auf der Haar, K. Richard, K E. Firsching, R
Acta neurochirurgica. Supplementum
0140560
IM
Journal Article
English
KW - MEDLINE
KW - Activities of Daily Living
KW - Adolescent

KW - Adult
KW - Brain Injuries/rh [Rehabilitation]
KW - Child
KW - Coma/rh [Rehabilitation]
KW - Female
KW - Follow-Up Studies
KW - Glasgow Coma Scale
KW - Head Injuries
KW - Closed/rh [Rehabilitation]
KW - Humans
KW - Male
KW - Neurologic Examination
KW - Neuropsychological Tests
RP - NOT IN FILE
SP - 72
EP - 74
JF - Acta Neurochirurgica - Supplementum
JA - Acta Neurochir Suppl (Wien)
VL - 55
CY - AUSTRIA
N2 - 123 survivors of severely head injured patients presenting with coma grade III show a decreasing mean duration of coma with increasing age. The numbers and frequency of good recovery decrease, whereas poor recovery increases with age. Increase of the duration of coma grade III produces an increase of the mean latency and time of recovery and of the frequency of poor recovery, regardless of the age of the patients. Increasing age does not increase the mean latency and time of recovery systematically. The important conclusion of this analysis is, that the clinical feature of coma grade III, corresponding to GCS score of 4 and RLS of 6 and 7, indicates a different kind of brain damage at various age groups. It represents a lesser degree of brain damage for younger patients under 20, than for those over 20. In our opinion our observations do not demonstrate a better capacity of recovery of the young patients: but the young patients show a more severe clinical picture than the older patients do, if only the clinical syndrome of coma grade III with extensor rigidity, is considered as a yardstick for comparison
SN - 0065-1419
AD - Neurosurgical Department, University of Cologne, Koln, Federal Republic of Germany
UR - 1414550
ER -

TY - JOUR
ID - 2087
T1 - [Is outdoor running exercise suitable for detecting bronchial hyperreactivity in children with mild episodic bronchial asthma?]. [German]
A1 - Riedler,J.
A1 - Golser,A.
A1 - Huttegger,I.
Y1 - 1992/09//
N1 - Riedler, J. Golser, A. Huttegger, I
Klinische Padiatrie
kwe, 0326144
IM
English Abstract. Journal Article
German
KW - MEDLINE
KW - Airway Resistance/ph [Physiology]
KW - Asthma/di [Diagnosis]
KW - Asthma/pp [Physiopathology]
KW - Asthma
KW - Exercise-Induced/di [Diagnosis]
KW - Exercise-Induced/pp [Physiopathology]

KW - Bronchial Hyperreactivity/di [Diagnosis]
KW - Bronchial Hyperreactivity/pp [Physiopathology]
KW - Bronchial Provocation Tests
KW - Child
KW - Exercise Test
KW - Female
KW - Humans
KW - Male
KW - Muscle
KW - Smooth/pp [Physiopathology]
KW - Reference Values
RP - NOT IN FILE
SP - 373
EP - 377
JF - Klinische Padiatrie
JA - Klin Padiatr
VL - 204
IS - 5
CY - GERMANY
N2 - Bronchial hyperreactivity (BHR) can be proved by various methods. 21 children, 14 asthmatics and 7 healthy subjects were submitted to inhalative methacholine challenge as well as to 'free running' as a form of exercise challenge in a randomized sequence. For the methacholine inhalation a standardized procedure was followed and the provocative concentration defined (PC20) at which a decrease of more than 20% in FEV1 was found. There is no real standardisation for 'free running' (concerning temperature and humidity of the inspired air; individual level of exercise) but subjects had to run for 6 minutes while the heart rate should have been between 170 and 180 beats/minute. This increase in pulse rate relates to a submaximal work at which 60-85% of maximal O2 uptake are obtained. A decrease of 15% from basic value of FEV1 was defined as a positive result. By the use of methacholine inhalations we found 16 children (14 diseased, 2 controls) to be hyperreactive, whereas only 3 of them showed a positive result after 'free running'. We conclude, that firstly, methacholine provocations and exercise challenges assess different kinds of bronchial reactivity, secondly, 'free running' as a form of exercise is very difficult to standardize and therefore prone to errors and thirdly, 'free running' is not sensitive enough to assess BHR in children with mild asthma bronchiale if used as the only form of challenge. Problems concerning measurement of BHR are discussed
SN - 0300-8630
AD - Kinderspital der Landeskrankenanstalten, Salzburg
UR - 1405426
ER -

TY - JOUR
ID - 2088
T1 - [Morbidity of children attending day care centers in Copenhagen illustrated by social, physical, environmental and hygienic factors]. [Danish]
A1 - Rindel,A.K.
A1 - Kronborg,D.
A1 - Jensen,S.T.
Y1 - 1992/07/27/
N1 - Rindel, A K. Kronborg, D. Jensen, S T
Ugeskrift for laeger
0141730, wm8
IM
English Abstract. Journal Article
Danish
KW - MEDLINE
KW - Air Pollution
KW - Indoor/ae [Adverse Effects]
KW - Child Day Care Centers
KW - Child

KW - Preschool
KW - Denmark/ep [Epidemiology]
KW - Environment
KW - Facility Design and Construction
KW - Humans
KW - Hygiene
KW - Infant
KW - Models
KW - Statistical
KW - Morbidity
KW - Social Environment
RP - NOT IN FILE
SP - 2140
EP - 2146
JF - Ugeskrift for Laeger
JA - Ugeskr.Laeger
VL - 154
IS - 31
CY - DENMARK
N2 - Sick leave among the children in 24 day nurseries in Copenhagen was registered during a period of one year. The physical, environmental and hygienic conditions of the institutions were assessed by means of interviews and inspection, supplemented by information from the local authorities. Sick leave constitutes 7.5% of the days during which the institutions were open and the average number of sick days per child varied from 14.6 to 24.4. Comparisons with similar investigations are inclusive on account of variations in the methods of registration and age distributions. It was characteristic of the institutions investigated that these included a number of old institutions in flats, many in streets with considerable traffic and where the conditions of ventilation and space do not conform with present-day standards. In some of the institutions, the hygiene is unsatisfactory, particularly where ventilation, outdoor activities and hand-washing are concerned. A statistically significant connection was found between amount of traffic at the institution, the hygiene and playroom area per child, on one hand, and absence due to sickness, on the other, as assessed by a statistical model for sick leave for child in a given institution. In order to prevent sickness, it is concluded that efforts to improve hygiene and space are desirable and, similarly, further research concerning the effects of atmospheric pollution is relevant
SN - 0041-5782
AD - Stadslaegen, Kobenhavns Kommune
UR - 1509594
ER -

TY - JOUR
ID - 2089
T1 - Maternal and paternal disciplinary styles: relations with preschoolers' playground behavioral orientations and peer status
A1 - Hart,C.H.
A1 - DeWolf,D.M.
A1 - Wozniak,P.
A1 - Burts,D.C.
Y1 - 1992/08//
N1 - Hart, C H. DeWolf, D M. Wozniak, P. Burts, D C
Child development
0372725, d28
IM
Journal Article. Research Support, Non-U.S. Gov't
English
KW - MEDLINE
KW - Child
KW - Preschool
KW - Father-Child Relations
KW - Female

KW - Gender Identity
KW - Humans
KW - Male
KW - Mother-Child Relations
KW - Peer Group
KW - Personality Development
KW - Play and Playthings
KW - Social Adjustment
KW - Social Environment
KW - Socialization
KW - Sociometric Techniques
RP - NOT IN FILE
SP - 879
EP - 892
JF - Child Development
JA - Child Dev
VL - 63
IS - 4
CY - UNITED STATES
N2 - To explore relations among parents' self-reported disciplinary styles, preschoolers' playground behavioral orientations, and peer status, 106 mothers and fathers of preschool-age children (age range = 40-71 months) participated in home disciplinary style interviews. Observations of their children's playground behavior in preschool settings and measures of sociometric status were also obtained. Results indicated that children of more inductive mothers and fathers (i.e., less power assertive) exhibited fewer disruptive playground behaviors. In addition, daughters and older preschoolers of inductive mothers exhibited more prosocial behavior. Children of inductive mothers were also more preferred by peers. Few significant relations were found between paternal discipline and child behavior/peer status. Age-related patterns of behavior also indicated that older preschoolers who engaged in more prosocial and less antisocial and disruptive playground behavior were more preferred by peers. In addition, child behaviors were found to mediate maternal discipline and peer status
SN - 0009-3920
AD - Family, Child, and Consumer Sciences, School of Human Ecology, Louisiana State University, Baton Rouge 70803
UR - 1505246
ER -

TY - JOUR
ID - 2090
T1 - [Prevention of post-exertion airway obstruction in children and adolescents with bronchial asthma]. [Czech]
A1 - Zapletal,A.
A1 - Zbojan,J.
A1 - Pohanka,V.
Y1 - 1992/05//
N1 - Zapletal, A. Zbojan, J. Pohanka, V
Ceskoslovenska pediatrie
cw3, 0403576
IM
English Abstract. Journal Article
Czech
KW - MEDLINE
KW - Administration
KW - Inhalation
KW - Adolescent
KW - Asthma
KW - Exercise-Induced/pp [Physiopathology]
KW - Exercise-Induced/pc [Prevention & Control]
KW - Bronchodilator Agents/ad [Administration & Dosage]

KW - Child
KW - Humans
KW - Physical Exertion
KW - Respiratory Mechanics
RP - NOT IN FILE
SP - 272
EP - 279
JF - Ceskoslovenska Pediatrie
JA - Cesk Pediatr
VL - 47
IS - 5
CY - CZECHOSLOVAKIA
N2 - In 86 asthmatics (age 8-16 years), divided into several groups, the protective effects of pharmacological and non-pharmacological agents on the exercise-induced airway obstruction (e.i.a.o.) by 5 minutes free running were studied. There were studied the preventive inhalations of bronchodilators, i.e. fenoterol (0.2 mg), ipratropium-bromide (0.06 mg) and both combined of 0.15 mg of fenoterol and 0.06 mg of ipratropium-bromide on the exercise-induced airway obstruction. The protective effects of breathing through nose as well as the effect of the temperature in the surrounding environment on the e.i.a.o. were studied too. The exercise-induced airway obstruction was assessed on the basis of parameters measured from maximum expiratory flow-volume curves. Maximum expiratory flow at 25% of vital capacity was most sensitive in the assessment of e.i.a.o. The studied bronchodilators protected all patients against e.i.a.o. The textile face mask (surgical mask) put on the nose and mouth during 5 min. free running outdoors and in a closed room protected some asthmatics completely against e.i.a.o. or reduced substantially e.i.a.o. in others. The nose breathing had a similar effect on e.i.a.o. as the textile face mask. The surrounding temperature of air was not decisive in the induction of post-exercise airway obstruction. Non-pharmacological agents ought to be thus more included among the therapeutical means in pediatric and adolescent asthmatics
SN - 0069-2328
AD - Oddeleni kardiopulmonalni funkcni diagnostiky, Kardiocentrum, Fakultni nemocnice Motol, Praha
UR - 1638650
ER -

TY - JOUR
ID - 2091
T1 - Training supervisors in a collaborative team approach to promote peer interaction of children with disabilities in integrated preschools
A1 - Hundert,J.
A1 - Hopkins,B.
Y1 - 1992///
N1 - Hundert, J. Hopkins, B
Journal of applied behavior analysis
0174763, hdr
IM
Journal Article. Research Support, Non-U.S. Gov't
English
KW - MEDLINE
KW - Child Behavior Disorders/px [Psychology]
KW - Child Behavior Disorders/th [Therapy]
KW - Child
KW - Preschool
KW - Disabled Persons/ed [Education]
KW - Disabled Persons/px [Psychology]
KW - Education of Intellectually Disabled/mt [Methods]
KW - Education
KW - Special
KW - Female
KW - Generalization (Psychology)
KW - Humans

KW - Language Development Disorders/px [Psychology]
KW - Language Development Disorders/th [Therapy]
KW - Mainstreaming (Education)
KW - Male
KW - Patient Care Team
KW - Peer Group
KW - Social Adjustment
KW - Social Behavior
KW - Socialization
RP - NOT IN FILE
SP - 385
EP - 400
JF - Journal of Applied Behavior Analysis
JA - J Appl Behav Anal
VL - 25
IS - 2
CY - UNITED STATES
N2 - Three supervisors of integrated preschools were trained in a collaborative team approach to encourage resource and classroom teachers to develop strategies that promote peer interaction of all children, including children with disabilities. The focus of classroom teachers' behaviors and the interactive play of children with disabilities were measured daily in both a training (indoor play period) and a generalization (outdoor play period) setting. In a multiple baseline design, supervisors were individually trained in a collaborative team approach using a manual, modeling, and role playing; then they implemented the approach with classroom and resource teachers. We found that after supervisor training, classroom teachers increased their behaviors directed towards children with disabilities and decreased their behaviors directed towards nondisabled children. Moreover, we found a doubling of the interactive play of children with disabilities and, for two of the three classes, an increase in the interactive play of comparison children, randomly selected by the classroom teachers. Changes in both teachers' and children's behaviors were also found in the generalization setting. The implications of the results for interventions in community settings are discussed
SN - 0021-8855
AD - McMaster University, Hamilton, Ontario, Canada
UR - 1386070
ER -

TY - JOUR
ID - 2092
T1 - The epidemiology of school-related injuries: new perspectives
A1 - Lenaway,D.D.
A1 - Ambler,A.G.
A1 - Beaudoin,D.E.
Y1 - 1992/05//
N1 - Lenaway, D D. Ambler, A G. Beaudoin, D E
American journal of preventive medicine
8704773, apl
IM
Comparative Study. Journal Article
English
KW - MEDLINE
KW - Athletic Injuries/ep [Epidemiology]
KW - Athletic Injuries/pc [Prevention & Control]
KW - Child
KW - Colorado/ep [Epidemiology]
KW - Female
KW - Humans
KW - Incidence
KW - Male
KW - Odds Ratio

KW - Prospective Studies
KW - Risk Factors
KW - Schools
KW - Students
KW - Wounds and Injuries/cl [Classification]
KW - Wounds and Injuries/ep [Epidemiology]
KW - Wounds and Injuries/pc [Prevention & Control]
RP - NOT IN FILE
SP - 193
EP - 198
JF - American Journal of Preventive Medicine
JA - Am J Prev Med
VL - 8
IS - 3
CY - UNITED STATES
N2 - Among school-aged children, unintentional injuries are the leading cause of morbidity and mortality. We began this prospective study in a sample of nine schools within the Boulder Valley School District (Colorado) during the 1988-1989 school year in an effort to explore the etiology of school-related injuries and to provide information relevant to their prevention. During the study period, 509 injuries were reported among a population of 5,518 students, yielding an incidence of 9.22 per 100 students. Boys were nearly one and one-half times more likely to have sustained a school-related injury than girls (risk ratio (RR) = 1.41; 95% confidence intervals [CIs] 1.18, 1.68). We found a significant difference among injury rates by school level (P less than .001). Middle/junior high students had the highest rate, followed closely by elementary students and distantly by high school students. Sports activities accounted for the largest percentage of school injuries (53%), and the percentage of sports-related injuries increased with increasing grade level. Analysis of injury rates by school location revealed that high school students were most frequently injured in the gym (1.52 per 100), middle/junior high students on the athletic field (4.26 per 100), and elementary students on the playground (6.12 per 100). Using injury location as the focal point, we examined relationships among the variables sport/activity, body site, and nature of injury. We found that a large percentage of injuries sustained on the athletic field or in the gym were similar and affected similar body sites, whereas playground injuries differed in their nature and in body site affected.(ABSTRACT TRUNCATED AT 250 WORDS)
SN - 0749-3797
AD - Boulder County Health Department, Colorado 80304
UR - 1633008
ER -

TY - JOUR
ID - 2093
T1 - Lead levels in settled dusts of Tripoli, Libya
A1 - el Hinshery,A.K.
A1 - Kumar,N.S.
Y1 - 1992/06/01/
N1 - el Hinshery, A K. Kumar, N S
The Science of the total environment
uj0, 0330500
IM
Comparative Study. Journal Article
English
KW - MEDLINE
KW - Child
KW - Dust/an [Analysis]
KW - Environmental Pollution/an [Analysis]
KW - Humans
KW - Lead/an [Analysis]
KW - Libya
KW - Spectrophotometry
KW - Atomic/mt [Methods]

KW - Urban Health
RP - NOT IN FILE
SP - 51
EP - 56
JF - Science of the Total Environment
JA - Sci Total Environ
VL - 119
CY - NETHERLANDS
N2 - The levels of lead in settled dust samples collected near small streets, playgrounds, gas stations and main streets in the Tripoli area have been determined by atomic absorption spectrophotometry. The overall mean values observed were 417, 533, 553 and 797 micrograms g-1 dry wt for the above locations respectively. The overall mean concentration for main streets was significantly higher (P less than 0.05) than for other sites. The data were also compared with those reported in the literature for other cities
SN - 0048-9697
AD - Petroleum Research Centre, Tripoli, Libya
UR - 1631533
ER -

TY - JOUR
ID - 2094
T1 - Periodic activity in cerebral arousal mechanisms--the relationship to sleep and brain damage
A1 - Evans,B.M.
Y1 - 1992/08//
N1 - Evans, B M
Electroencephalography and clinical neurophysiology
eeh, 0375035
IM
Journal Article
English
KW - MEDLINE
KW - Adolescent
KW - Adult
KW - Aged
KW - Arousal/ph [Physiology]
KW - Brain Damage
KW - Chronic/pp [Physiopathology]
KW - Cerebral Cortex/ph [Physiology]
KW - Child
KW - Electroencephalography
KW - Female
KW - Humans
KW - Middle Aged
KW - Periodicity
KW - Sleep Stages/ph [Physiology]
RP - NOT IN FILE
SP - 130
EP - 137
JF - Electroencephalography & Clinical Neurophysiology
JA - Electroencephalogr.Clin Neurophysiol
VL - 83
IS - 2
CY - IRELAND
N2 - Periodic activity during light sleep is well recognised in many physiological systems, particularly respiration. In damaged brains this activity can become exaggerated. It involves the autonomic nervous system, the muscles, the cerebrospinal fluid (CSF) pressure, the cerebral blood flow and the electroencephalogram (EEG). It is related to the level of arousal. The EEGs of 52 subjects were studied. In stage 0-1 sleep, periods of alpha activity alternated with periods of theta activity related to the level of arousal. The intervals between the

alpha onsets were measured and the data pooled. There was a dominant interval of about 16 sec. It is suggested that this is a physiological cerebral rhythm involving the cortex and the brain-stem activating mechanisms, responsive to outside stimuli but essentially endogenous. It is related to the controls of the autonomic, motor, and some cerebral auto-regulatory mechanisms. It may be severely disturbed in brain damage
SN - 0013-4694
AD - Regional Dept. of Clinical Neurophysiology, Brook General Hospital, London, U.K
UR - 1378378
ER -

TY - JOUR
ID - 2095
T1 - Anglo- and Mexican-American preschoolers at home and at recess: activity patterns and environmental influences
A1 - McKenzie,T.L.
A1 - Sallis,J.F.
A1 - Nader,P.R.
A1 - Broyles,S.L.
A1 - Nelson,J.A.
Y1 - 1992/06//
N1 - McKenzie, T L. Sallis, J F. Nader, P R. Broyles, S L. Nelson, J A
Journal of developmental and behavioral pediatrics : JDBP
htf, 8006933
IM
Journal Article. Research Support, U.S. Gov't, P.H.S.
English
KW - MEDLINE
KW - California
KW - Cardiovascular Diseases/pc [Prevention & Control]
KW - Child Development
KW - Child
KW - Preschool
KW - Female
KW - Gender Identity
KW - Hispanic Americans/px [Psychology]
KW - Humans
KW - Male
KW - Mexico/eh [Ethnology]
KW - Motor Activity
KW - Physical Fitness/px [Psychology]
KW - Social Environment
RP - NOT IN FILE
SP - 173
EP - 180
JF - Journal of Developmental & Behavioral Pediatrics
JA - J Dev Behav Pediatr
VL - 13
IS - 3
CY - UNITED STATES
N2 - Habitual physical activity in children is related to physical fitness and appears to mediate cardiovascular disease (CVD) risk factors. We studied the physical activity patterns and associated variables of a large bi-ethnic cohort of 4-year-old children from low to middle socioeconomic families. Trained observers coded the behavior of 351 children (150 Anglo-American, 201 Mexican-American; 182 boys, 169 girls) during two 60-minute home visits and two unstructured recesses lasting up to 30 minutes each at 63 different preschools. Findings indicated that although children were much less active at home, there were low but significant correlations between their activity patterns at home and during recess (r = .13). Children who had activity-promoting toys at home also tended to have them available during preschool recess (r = .20). Ethnic differences were evident for both activity and environmental variables. Mexican-American children were less active than

Anglo children at home (p less than .002) and during recess (p less than .03), thus adding to the adult literature that has found Mexican-Americans to be less active than Anglos, and supporting to the notion that physical activity life-style habits may be established in early childhood. In both settings, Mexican-American children spent more time in presence of adults (home, p less than .04; recess, p less than .03) and had access to fewer active toys (home, p less than .001; recess, p less than .05). Gender differences were also evident for both activity and environmental variables.(ABSTRACT TRUNCATED AT 250 WORDS)
SN - 0196-206X
AD - San Diego State University, California
UR - 1613112
ER -

TY - JOUR
ID - 2096
T1 - Wilderness injuries and illnesses
A1 - Gentile,D.A.
A1 - Morris,J.A.
A1 - Schimelpfenig,T.
A1 - Bass,S.M.
A1 - Auerbach,P.S.
Y1 - 1992/07//
N1 - Gentile, D A. Morris, J A. Schimelpfenig, T. Bass, S M. Auerbach, P S
Annals of emergency medicine
4z7, 8002646
AIM, IM
Journal Article
English
KW - MEDLINE
KW - Adolescent
KW - Adult
KW - Americas/ep [Epidemiology]
KW - Child
KW - Female
KW - First Aid
KW - Health Education
KW - Humans
KW - Hygiene
KW - Leisure Activities
KW - Male
KW - Middle Aged
KW - Morbidity
KW - Mountaineering
KW - Primary Prevention
KW - Skiing
KW - Wounds and Injuries/ep [Epidemiology]
KW - Wounds and Injuries/mo [Mortality]
RP - NOT IN FILE
SP - 853
EP - 861
JF - Annals of Emergency Medicine
JA - Ann Emerg Med
VL - 21
IS - 7
CY - UNITED STATES
N2 - STUDY OBJECTIVE: To determine injury and illness patterns and occurrence rates during wilderness recreation. DESIGN: Prospective injury and illness surveillance study. SETTING: Wilderness areas throughout the Western hemisphere. TYPE OF PARTICIPANTS: All students and instructors on National Outdoor Leadership School courses over a five-year period. MAIN RESULTS: A single fatality occurred, resulting in a

death rate of 0.28 per 100,000 person-days of exposure. Injuries occurred at a rate of 2.3 per 1,000 person-days of exposure. Sprains and strains and soft tissue injuries accounted for 80% of the injuries. The illness rate was 1.5 per 1,000 person-days of exposure. Sixty percent of illnesses were due to nonspecific viral illnesses or diarrhea; hygiene appeared to have a significant impact on the incidence of these illnesses. Thirty-nine percent of the injuries and illnesses required evacuation (1.5 per 1,000 person-days of exposure). CONCLUSION: The injury and illness patterns indicate that wilderness medical efforts should concentrate on wilderness hygiene and management of musculoskeletal injuries and soft tissue wounds. The data also indicate that wilderness activities can be conducted relatively safely, but the decision to participate should be individualized, with an understanding of risks versus benefits
SN - 0196-0644
AD - Department of Surgery, Stanford University School of Medicine, Stanford, California
UR - 1376973
ER -

TY - JOUR
ID - 2097
T1 - [Orofacial injuries in skateboard accidents]. [German]
A1 - Frohberg,U.
A1 - Bonsmann,M.
Y1 - 1992/04//
N1 - Frohberg, U. Bonsmann, M
Aktuelle Traumatologie
2m5, 0421405
IM
English Abstract. Journal Article
German
KW - MEDLINE
KW - Adolescent
KW - Child
KW - Preschool
KW - Cross-Sectional Studies
KW - Female
KW - Germany/ep [Epidemiology]
KW - Humans
KW - Incidence
KW - Male
KW - Maxillofacial Injuries/ep [Epidemiology]
KW - Maxillofacial Injuries/et [Etiology]
KW - Skating/in [Injuries]
RP - NOT IN FILE
SP - 84
EP - 86
JF - Aktuelle Traumatologie
JA - Aktuelle Traumatol
VL - 22
IS - 2
CY - GERMANY
N2 - In a clinical study, 25 accidents involving injuries by a fall with a skateboard were investigated and classified in respect of epidemiology, accident mechanism and injury patterns in the facial region. Accident victims are predominantly boys between 7 and 9 years of age. A multiple trauma involving the teeth and the dental system in general and the soft parts of the face is defined as a characteristic orofacial injury pattern in skateboard accidents. The high proportion of damage to the front teeth poses problems of functional and aesthetic rehabilitation necessitating long-term treatment courses in children and adolescents. Effective prevention of facial injuries may be possible by evolving better facial protection systems and by creating areas of playgrounds where skateboarders can practise safely
SN - 0044-6173

AD - Klinik fur Mund-, Kiefer- und Gesichtschirurgie, Plastische Operationen, Ev. Krankenhauses Bethesda Monchengladbach
UR - 1351349
ER -

TY - JOUR
ID - 2098
T1 - Children and chess expertise: the role of calibration
A1 - Horgan,D.D.
Y1 - 1992///
N1 - Horgan, D D
Psychological research
qf9, 0435062
IM
Journal Article. Research Support, Non-U.S. Gov't
English
KW - MEDLINE
KW - Achievement
KW - Adolescent
KW - Attention
KW - Child
KW - Female
KW - Game Theory
KW - Humans
KW - Judgment
KW - Male
KW - Probability Learning
KW - Problem Solving
RP - NOT IN FILE
SP - 44
EP - 50
JF - Psychological Research
JA - Psychol Res
VL - 54
IS - 1
CY - GERMANY
N2 - Three studies of calibration are reported. Calibration refers to the accuracy with which one can predict one's own performance. In the first study child chess players, non-chess playing parents, and statistics students were asked to predict chances of winning chess games against hypothetical opponents. These subjective probabilities were compared to the actual probabilities, based on the Elo rating system. Better players' predictions were better calibrated. Confidence and ratings are negatively correlated, indicating that with lower ratings, players re overconfident. Skilled child players' predictions were better calibrated than any of the adults'. In the second study subjects were asked to estimate chances of winning in conjunctive situations, e.g., winning all the rounds in a tournament. Again, better child players were more accurate in their predictions and more accurate than adults. In the third study, child players were asked to predict their chances of winning in a non-chess domain after hearing a hypothetical win/loss history. Higher-rated players' predictions were again better calibrated, even though the domain was outside their expertise. The motivational and cognitive implications of calibration are discussed
SN - 0340-0727
AD - Foundation of Education Department, Memphis State University, TN 38152
UR - 1603888
ER -

TY - JOUR
ID - 2099
T1 - An examination of spontaneous pointing in 20- to 50-month-old children
A1 - Hannan,T.E.

Y1 - 1992/04//
N1 - Hannan, T E
Perceptual and motor skills
ozb, 0401131
IM
Journal Article
English
KW - MEDLINE
KW - Attention
KW - Child Development
KW - Child
KW - Preschool
KW - Female
KW - Humans
KW - Individuality
KW - Infant
KW - Male
KW - Orientation
KW - Psychomotor Performance
KW - Reference Values
KW - Social Environment
RP - NOT IN FILE
SP - 651
EP - 658
JF - Perceptual & Motor Skills
JA - Percept.Mot.Skills
VL - 74
IS - 2
CY - UNITED STATES
N2 - Occurrences of spontaneous pointing in 20-, 30-, 40-, and 50-mo.-old children were observed during the children's indoor or outdoor free-play periods. Observers recorded the sex of the pointer, the behavioral pattern observed, the reason for the point, the response of the adult, and the distance of the child from the adult at the time of a point. Analysis indicated a significant dip in the rate of pointing among the 30-mo.-olds while the 20- and 50-mo.-olds were observed to point most often. With the exception of the 30-mo.-olds, pointing was significantly more likely to be observed in boys. For all age groups, the point was used primarily to name. After 20 months adults were significantly less likely to respond to the point, and when they did respond they were more likely to name or demonstrate irrespective of the apparent reason for the child's point. Finally, the probability of adults' response appeared to be unrelated to the distance from the child to the adult, and further, the likelihood of an adult's response appeared to exert little influence on the rate of pointing. The results suggest that pointing may be an expressive act that can be used as a communicative gesture, but there was little evidence that any of these age groups were using the point as a ritualized gesture, that is, as an action conventionalized within the context of social interaction
SN - 0031-5125
AD - Department of Psychology, Duke University, Durham, NC 27706
UR - 1594427
ER -

TY - JOUR
ID - 2100
T1 - Cognitive-behavioral pain management in children with juvenile rheumatoid arthritis
A1 - Walco,G.A.
A1 - Varni,J.W.
A1 - Ilowite,N.T.
Y1 - 1992/06//
N1 - Walco, G A. Varni, J W. Ilowite, N T
Pediatrics
oxv, 0376422

AIM, IM
Journal Article. Research Support, Non-U.S. Gov't
English
KW - MEDLINE
KW - Adolescent
KW - Arthritis
KW - Juvenile/co [Complications]
KW - Child
KW - Preschool
KW - Cognitive Therapy/mt [Methods]
KW - Female
KW - Follow-Up Studies
KW - Humans
KW - Male
KW - Pain/et [Etiology]
KW - Pain Management
KW - Pain Measurement
KW - Questionnaires
RP - NOT IN FILE
SP - 1075
EP - 1079
JF - Pediatrics
VL - 89
IS - 6 Pt 1
CY - UNITED STATES
N2 - Decreasing chronic joint pain is a major goal in the management of juvenile rheumatoid arthritis. Cognitive-behavioral self-regulatory techniques were taught to children with juvenile rheumatoid arthritis to reduce musculoskeletal pain intensity and to facilitate better adaptive functioning. Subjects were 13 children between the ages of 4.5 and 16.9 years who had pauciarticular or systemic onset juvenile rheumatoid arthritis. Baseline data included an initial comprehensive assessment of pain, disease activity, and level of functional disability, as well as pain intensity ratings gathered over a 4-week period. Subjects were seen for eight individual sessions in which self-regulatory techniques (progressive muscle relaxation, guided imagery, meditative breathing) were taught, and parents were seen for two sessions in which key aspects of behavioral pain management techniques were reviewed. Results indicated that these techniques led to substantial reduction of pain intensity, which generalized to outside the clinic setting. Six- and 12-month follow-up data showed consistent decreases in pain as well as improved adaptive functioning. The data suggest that cognitive-behavioral interventions for pain are an effective adjunct to standard pharmacologic interventions for pain in patients with juvenile rheumatoid arthritis
SN - 0031-4005
AD - Schneider Children's Hospital of Long Island Jewish Medical Center, Albert Einstein College of Medicine, New Hyde Park, NY
UR - 1594351
ER -

TY - JOUR
ID - 2101
T1 - Methylphenidate in hyperactive boys with comorbid tic disorder: II. Short-term behavioral effects in school settings
A1 - Gadow,K.D.
A1 - Nolan,E.E.
A1 - Sverd,J.
Y1 - 1992/05//
N1 - Gadow, K D. Nolan, E E. Sverd, J
Journal of the American Academy of Child and Adolescent Psychiatry
hg5, 8704565
IM
Clinical Trial. Journal Article. Randomized Controlled Trial. Research Support, Non-U.S. Gov't. Research

Support, U.S. Gov't, P.H.S.
English
KW - MEDLINE
KW - Attention Deficit Disorder with Hyperactivity/dt [Drug Therapy]
KW - Attention Deficit Disorder with Hyperactivity/ep [Epidemiology]
KW - Attention Deficit Disorder with Hyperactivity/px [Psychology]
KW - Child
KW - Child Behavior Disorders/dt [Drug Therapy]
KW - Child Behavior Disorders/ep [Epidemiology]
KW - Child Behavior Disorders/px [Psychology]
KW - Comorbidity
KW - Dose-Response Relationship
KW - Drug
KW - Double-Blind Method
KW - Humans
KW - Male
KW - Methylphenidate/ad [Administration & Dosage]
KW - Neurologic Examination/de [Drug Effects]
KW - Personality Assessment
KW - Social Environment
KW - Tic Disorders/dt [Drug Therapy]
KW - Tic Disorders/ep [Epidemiology]
KW - Tic Disorders/px [Psychology]
KW - Tourette Syndrome/dt [Drug Therapy]
KW - Tourette Syndrome/ep [Epidemiology]
KW - Tourette Syndrome/px [Psychology]
RP - NOT IN FILE
SP - 462
EP - 471
JF - Journal of the American Academy of Child & Adolescent Psychiatry
JA - J Am Acad Child Adolesc Psychiatry
VL - 31
IS - 3
CY - UNITED STATES
N2 - In this study, 11 prepubertal hyperactive boys with tic disorder received placebo and three doses of methylphenidate (0.1, 0.3, and 0.5 mg/kg) for 2 weeks each, under double-blind conditions. Each boy was observed for approximately 20 hours in the school setting (classroom seatwork activities, lunchroom, and playground). Results showed that methylphenidate effectively suppressed hyperactive/disruptive behaviors in the classroom and physical aggression in the lunchroom and on the playground. Methylphenidate also reduced the occurrence of vocal tics in the classroom and the lunchroom. None of the motor tic measures revealed drug effects, but the lowest mean rate of motor tics occurred on the 0.3 mg/kg dose. On an operationally defined minimal effective dose, only one boy experienced motor tic exacerbation
SN - 0890-8567
AD - Department of Psychiatry and Behavioral Science, SUNY Stony Brook 11794-8790
UR - 1592778
ER -

TY - JOUR
ID - 2102
T1 - [Exercise-induced airway obstruction in asthmatic children and adolescents]. [Czech]
A1 - Zapletal,A.
A1 - Zbojan,J.
A1 - Pohanka,V.
Y1 - 1992/03//
N1 - Zapletal, A. Zbojan, J. Pohanka, V
Ceskoslovenska pediatrie
cw3, 0403576

IM
English Abstract. Journal Article
Czech
KW - MEDLINE
KW - Adolescent
KW - Asthma
KW - Exercise-Induced/di [Diagnosis]
KW - Exercise-Induced/pp [Physiopathology]
KW - Child
KW - Exercise Test
KW - Humans
KW - Respiratory Mechanics
RP - NOT IN FILE
SP - 141
EP - 147
JF - Ceskoslovenska Pediatrie
JA - Cesk Pediatr
VL - 47
IS - 3
CY - CZECHOSLOVAKIA
N2 - In 115 asymptomatic asthmatic children and adolescents (age 6-18 years) there was studied the magnitude of airway obstruction, induced by various physical efforts and assessed from the recording of maximum expiratory flow-volume curves and in some patients by "specific" airway conductance measurement in a body plethysmograph. The effects of 5 minutes free running outdoors, 5 minutes of exercise on a bicycle ergometer (2 watts/kg of body weight), routine swimming training in swimming pool and of forced expiration maneuver on the magnitude of airway obstruction were assessed. The most frequent and largest degree of airway obstruction was observed after 5 min. free running outdoors (heart rate after running 160-200/min). The obstruction was revealed in 80-100% asthmatics in various groups. The chosen lung function parameters showed exercise-induced airway obstruction in the same patients in various proportions as well as the magnitude of the obstruction. Following free running outdoors the values of maximum expiratory flow at 25% of vital capacity and "specific" airway conductance were most reduced. Spontaneous retreat of obstruction was observed in the course of 2 hours. The physical exercise on a bicycle ergometer was a small stimulus in inducing of airway obstruction. The swimming in a pool did not provoke any obstruction. In 10% of our asthmatics airway obstruction was observed following forced expiration maneuver. Airway obstruction induced by 5 minutes free running outdoors and assessed best by flow-volume curves appeared as a suitable test in the assessment of airway hyperresponsiveness
SN - 0069-2328
AD - Oddeleni kardiopulmonalni funkcni diagnostiky, Kardiocentrum, Fakultni nemocnice Praha-Motol
UR - 1591810
ER -

TY - JOUR
ID - 2103
T1 - Self-reported safety practices in child care facilities
A1 - O'Connor,M.A.
A1 - Boyle,W.E.,Jr.
A1 - O' Connor,G.T.
A1 - Letellier,R.
Y1 - 1992/01//
N1 - O'Connor, M A. Boyle, W E Jr. O' Connor, G T. Letellier, R
American journal of preventive medicine
8704773, apl
IM
Journal Article. Research Support, U.S. Gov't, P.H.S.
English
KW - MEDLINE
KW - Accidental Falls/pc [Prevention & Control]

KW - Age Factors
KW - Child
KW - Child Day Care Centers/st [Standards]
KW - Preschool
KW - Humans
KW - Interviews as Topic
KW - Licensure/lj [Legislation & Jurisprudence]
KW - New Hampshire
KW - Play and Playthings
KW - Safety
KW - Wounds and Injuries/mo [Mortality]
KW - Wounds and Injuries/pc [Prevention & Control]
RP - NOT IN FILE
SP - 14
EP - 18
JF - American Journal of Preventive Medicine
JA - Am J Prev Med
VL - 8
IS - 1
CY - UNITED STATES
N2 - To determine the prevalence of safety hazards and current injury prevention practices in child care settings, we administered a structured telephone interview to a geographically stratified, randomly selected sample of licensed child care facilities. Representatives of 130 child care facilities responded to questions about current injury prevention practices. Specific hazards assessed were related to burns, falls, poisoning, playgrounds, and emergency telephone numbers. Results indicated that 26.8% of providers who knew the temperature of their tap water stated that it was over 130 degrees F.; 14.1% had space heaters accessible to children; 30.3% of those with stairs accessible to children lacked safety gates; 61.4% of those with playgrounds did not have an impact-absorbing surface under playground equipment; 16.9% of respondents had an unexpired bottle of syrup of ipecac; 55.8% demonstrated that a poison control center telephone number was available to them; and 80% of providers could demonstrate the availability of the telephone number of the local ambulance. We conclude that potential and remedial injury hazards exist in some licensed child care centers and that providers of child care within licensed facilities are a promising target for childhood injury prevention interventions
SN - 0749-3797
AD - Department of Maternal and Child Health, Injury Prevention Resource and Research Center, Dartmouth Medical School, Hanover, NH 03756
UR - 1575995
ER -

TY - JOUR
ID - 2104
T1 - Incidence and prevalence of Lyme disease in a suburban Westchester County community
A1 - Alpert,B.
A1 - Esin,J.
A1 - Sivak,S.L.
A1 - Wormser,G.P.
Y1 - 1992/01//
N1 - Alpert, B. Esin, J. Sivak, S L. Wormser, G P
New York state journal of medicine
oba, 0401064
IM
Comparative Study. Journal Article
English
KW - MEDLINE
KW - Adolescent
KW - Adult
KW - Aged

KW - Animals
KW - Antibodies
KW - Bacterial/an [Analysis]
KW - Arachnid Vectors/mi [Microbiology]
KW - Arachnid Vectors/py [Pathogenicity]
KW - Borrelia burgdorferi Group/im [Immunology]
KW - Borrelia burgdorferi Group/py [Pathogenicity]
KW - Child
KW - Humans
KW - Incidence
KW - Lyme Disease/di [Diagnosis]
KW - Lyme Disease/ep [Epidemiology]
KW - Lyme Disease/et [Etiology]
KW - Middle Aged
KW - New York/ep [Epidemiology]
KW - Prevalence
KW - Questionnaires
KW - Residence Characteristics
KW - Seasons
KW - Suburban Population
KW - Ticks/mi [Microbiology]
KW - Ticks/py [Pathogenicity]
RP - NOT IN FILE
SP - 5
EP - 8
JF - New York State Journal of Medicine
JA - N Y State J Med
VL - 92
IS - 1
CY - UNITED STATES
N2 - This study was designed to determine the incidence and prevalence of Lyme disease in a section of Chappaqua, NY, a residential community in which Lyme disease is epidemic, and to identify risk factors for this disease. On the basis of clinical history and baseline serologic testing, the overall prevalence of Lyme disease for 114 persons entering the study was 8.8%. The incidence during the 5-month study period of May through September 1989 was 2.6%; all three incident cases had erythema migrans (EM). Hours outdoors per week in play or exercise correlated with the occurrence of Lyme disease
SN - 0028-7628
AD - Division of General Internal Medicine, Department of Medicine, Westchester County Medical Center, Valhalla, NY 10595
UR - 1574232
ER -

TY - JOUR
ID - 2105
T1 - After-school child care and self-esteem in school-age children
A1 - Berman,B.D.
A1 - Winkleby,M.
A1 - Chesterman,E.
A1 - Boyce,W.T.
Y1 - 1992/04//
N1 - Berman, B D. Winkleby, M. Chesterman, E. Boyce, W T
Pediatrics
oxv, 0376422
AIM, IM
Journal Article. Research Support, U.S. Gov't, P.H.S.
English
KW - MEDLINE

KW - Age Factors
KW - Body Image
KW - Child
KW - Child Behavior
KW - Child Care
KW - Child Development
KW - Cross-Sectional Studies
KW - Educational Measurement
KW - Employment
KW - Female
KW - Humans
KW - Interpersonal Relations
KW - Male
KW - Mothers
KW - Occupations
KW - Parents
KW - Self Concept
KW - Sibling Relations
KW - Social Desirability
KW - Time Factors
RP - NOT IN FILE
SP - 654
EP - 659
JF - Pediatrics
VL - 89
IS - 4 Pt 1
CY - UNITED STATES
N2 - Although more than 2 million US children are in self-care after school, little is known of the extent to which self-care may adversely affect developmental processes, such as the development of self-esteem. To test the hypothesis that lower self-esteem is associated with being in self-care, 297 subjects in fourth and sixth grades from three ethnically diverse schools in northern California were enrolled in a cross-sectional study during November 1987. Sixty percent of subjects were in adult in-home care, 13% in adult out-of-home care, 19% in self-care, and 8.0% in older sibling care. No significant differences in self-competence scores, as measured by the Harter Self-perception Profile for Children, were observed for children in self-care compared with the three other care groups. However, children who were cared for by older siblings unexpectedly exhibited lower self-competence scores for five of the six self-competence domains, with three domains showing significance at P less than .05. Children in self-care were significantly more isolated socially than children in adult care, reporting fewer opportunities to play outside or have friends visit at their homes. The results indicate that children in sibling care may be at potentially greater risk for negative effects on self-esteem and social development. Children in self-care may also experience more social isolation after school than children in other forms of afterschool care
SN - 0031-4005
AD - Child Development Center, Children's Hospital, Oakland, CA 94609
UR - 1557246
ER -

TY - JOUR
ID - 2106
T1 - Total energy expenditure and spontaneous activity in relation to training in obese boys
A1 - Blaak,E.E.
A1 - Westerterp,K.R.
A1 - Bar-Or,O.
A1 - Wouters,L.J.
A1 - Saris,W.H.
Y1 - 1992/04//
N1 - Blaak, E E. Westerterp, K R. Bar-Or, O. Wouters, L J. Saris, W H
The American journal of clinical nutrition

3ey, 0376027
AIM, IM
Journal Article
English
KW - MEDLINE
KW - Adipose Tissue
KW - Basal Metabolism
KW - Body Composition
KW - Body Water
KW - Body Weight
KW - Child
KW - Deuterium/du [Diagnostic Use]
KW - Energy Metabolism
KW - Exercise/ph [Physiology]
KW - Humans
KW - Male
KW - Obesity/th [Therapy]
KW - Oxygen Consumption
KW - Questionnaires
RP - NOT IN FILE
SP - 777
EP - 782
JF - American Journal of Clinical Nutrition
JA - Am J Clin Nutr
VL - 55
IS - 4
CY - UNITED STATES
N2 - Obese boys [n = 10, aged 10-11 y, average percent body fat (%BF) 32.4] were given a regimented training program (4 wk, 5 1-h sessions, 45 min cycling/wk at 50-60% of predetermined VO2max) to investigate whether they modify their energy expenditure by reducing or augmenting their spontaneous physical activities. No change was observed in mean weight (52.7 vs 52.9 kg), %BF based on 2H- and 18O-dilution and densitometry (32.4 vs 31.7), sleeping metabolic rate (5.83 vs 5.68 MJ/24 h), and spontaneous activity by heart rate recording (percent time of light intensity: 85.3 vs 83.6; medium: 11.2 vs 12.4; and heavy: 3.5 vs 3.9) and activity questionnaires (861 vs 821 min physical activity/wk). There was a 12% increase in average daily metabolic rate by doubly labeled water, half of which can be explained by the energy cost of training and the rest by an increase in energy expenditure outside the training hour. In conclusion, training leads to an appreciable augmentation in the overall energy expenditure of obese children, even with a lack of change in spontaneous physical activity
SN - 0002-9165
AD - Department of Human Biology, University of Limburg, Maastricht, The Netherlands
UR - 1550058
ER -

TY - JOUR
ID - 2107
T1 - Evaluation of an intervention to reduce playground hazards in Atlanta child-care centers
A1 - Sacks,J.J.
A1 - Brantley,M.D.
A1 - Holmgreen,P.
A1 - Rochat,R.W.
Y1 - 1992/03//
N1 - Sacks, J J. Brantley, M D. Holmgreen, P. Rochat, R W
American journal of public health
1254074, 3xw
AIM, IM
Journal Article
English

KW - MEDLINE
KW - Accident Prevention
KW - Administrative Personnel/ed [Education]
KW - Child Day Care Centers/og [Organization & Administration]
KW - Child Day Care Centers/st [Standards]
KW - Child Day Care Centers/sn [Statistics & Numerical Data]
KW - Child
KW - Preschool
KW - Evaluation Studies as Topic
KW - Georgia
KW - Humans
KW - Play and Playthings
KW - Safety
RP - NOT IN FILE
SP - 429
EP - 431
JF - American Journal of Public Health
JA - Am J Public Health
VL - 82
IS - 3
CY - UNITED STATES
N2 - In 1988, we identified playground hazards at 58 child-care centers (CCCs) and intervened by showing the director the hazards and distributing safety information. In 1990, we evaluated the 58 intervention CCCs as well as 71 randomly selected control CCCs. Intervention centers had 9.4 hazards per playground; control centers had 8.0. We conclude that the intervention was ineffective
SN - 0090-0036
AD - Division of Injury Control, Centers for Disease Control, Atlanta, GA 30333
UR - 1536361
ER -

TY - JOUR
ID - 2108
T1 - Oxygen uptake plateau during maximal treadmill exercise in children
A1 - Rowland,T.W.
A1 - Cunningham,L.N.
Y1 - 1992/02//
N1 - Rowland, T W. Cunningham, L N
Chest
0231335, d1c
AIM, IM
Journal Article
English
KW - MEDLINE
KW - Child
KW - Exercise
KW - Female
KW - Heart Rate
KW - Humans
KW - Male
KW - Oxygen Consumption
KW - Physical Endurance
KW - Reference Values
KW - Respiration
RP - NOT IN FILE
SP - 485
EP - 489
JF - Chest

VL - 101
IS - 2
CY - UNITED STATES
N2 - The leveling of oxygen uptake at high work loads has been utilized to define maximal VO2 during progressive exercise testing. Exercise studies in children have indicated, however, that a VO2 plateau can commonly be demonstrated in less than one half of subjects. To examine the potential contributions of subject effort, aerobic fitness, and nonaerobic performance to this variability, 15 children ages 7 to 10 years performed progressive treadmill walking to exhaustion with a ramp protocol. The VO2 plateau was defined as a change in VO2 during the final minute of exercise less than 2 SD below the mean of increases between the previous 4 to 5 submaximal minutes. Five subjects (33.3 percent) demonstrated a plateau. No significant differences in mean peak VO2, heart rate, or respiratory exchange ratio were observed between plateau and nonplateau groups. Testing results of speed (50-yard sprint) and leg power (vertical jump) were also similar. These findings suggest that following: (1) subject effort, aerobic fitness, and nonaerobic factors do not explain the presence or absence of a VO2 plateau during exercise testing of children; and (2) a VO2 plateau should not be used as a requirement for defining VO2max during treadmill testing in this age group
SN - 0012-3692
AD - Department of Pediatrics, Baystate Medical Center, Springfield, Mass. 01199
UR - 1735277
ER -

TY - JOUR
ID - 2109
T1 - Winds of change in Zambia
A1 - Bond,V.
Y1 - 1992/05//
N1 - Bond, V
WorldAIDS
9011394
J
Journal Article
English
KW - MEDLINE
KW - Acquired Immunodeficiency Syndrome
KW - Adolescent
KW - Africa
KW - Africa South of the Sahara
KW - Eastern
KW - Age Factors
KW - Behavior
KW - Biology
KW - Child
KW - Culture
KW - Demography
KW - Developing Countries
KW - Disease
KW - Education
KW - Extramarital Relations
KW - Foster Home Care
KW - HIV Infections
KW - Interpersonal Relations
KW - Population
KW - Population Characteristics
KW - Risk Factors
KW - Sex Education
KW - Sexual Behavior
KW - Sexual Partners
KW - Virus Diseases

KW - Zambia
RP - NOT IN FILE
SP - 3
EP - 4
JF - WorldAIDS
IS - 21
CY - ENGLAND
SN - 0954-6510
UR - 12317434
ER -

TY - JOUR
ID - 2110
T1 - Hypopituitarism and stalk agenesis: a congenital syndrome worsened by breech delivery?
A1 - Maghnie,M.
A1 - Larizza,D.
A1 - Triulzi,F.
A1 - Sampaolo,P.
A1 - Scotti,G.
A1 - Severi,F.
Y1 - 1991///
N1 - Maghnie, M. Larizza, D. Triulzi, F. Sampaolo, P. Scotti, G. Severi, F
Hormone research
gbi, 0366126
IM
Journal Article
English
KW - MEDLINE
KW - Adult
KW - Breech Presentation
KW - Cesarean Section
KW - Child
KW - Female
KW - Growth Disorders/dt [Drug Therapy]
KW - Growth Disorders/pa [Pathology]
KW - Growth Disorders/pp [Physiopathology]
KW - Growth Hormone/df [Deficiency]
KW - Growth Hormone/tu [Therapeutic Use]
KW - Humans
KW - Hypopituitarism/pa [Pathology]
KW - Hypopituitarism/pp [Physiopathology]
KW - Magnetic Resonance Imaging
KW - Male
KW - Pituitary Gland/ab [Abnormalities]
KW - Pituitary Gland/pa [Pathology]
KW - Pituitary Gland
KW - Anterior/ab [Abnormalities]
KW - Posterior/ab [Abnormalities]
KW - Pituitary Hormones
KW - Anterior/bl [Blood]
KW - Anterior/df [Deficiency]
KW - Pregnancy
RP - NOT IN FILE
SP - 104
EP - 108
JF - Hormone Research
JA - Horm Res

VL - 35
IS - 3-4
CY - SWITZERLAND
N2 - Thirty-seven patients with idiopathic hypopituitarism, of whom 12 had multiple pituitary hormone deficiencies (MPHD) and 25 isolated growth hormone deficiency (IGHD), were evaluated by magnetic resonance imaging (MRI). Twenty-two of the 37 showed congenital anterior pituitary hypoplasia, stalk agenesis and ectopic posterior pituitary gland at the infundibular recess (group A), while the remaining 15 presented isolated anterior pituitary hypoplasia (group B). Perinatal histories obtained from all patients demonstrated that 18/22 children of group A (81.81%) had histories of adverse perinatal events, with breech presentation in 15 (68.18%). Twelve of 12 children of group A born by breech delivery developed MPHD; 3 born by cesarean section for breech presentation had only IGHD. Patients of group B had also a high incidence of perinatal insults (12/15, 80%), but breech delivery was markedly less frequent (13.33 vs. 68.18% of group A) and responsible for only IGHD. Group B had also higher percentages of maternal spontaneous abortion and low birth weight. Our study suggests that several factors may play a role in the development of growth hormone deficiency. Some patients had severe perinatal insults apparently leading to hypopituitarism. We were able to define by MRI a group of patients with congenital abnormalities, such as anterior pituitary hypoplasia, stalk agenesis and posterior pituitary ectopia, among whom breech presentation was very common. In this group, breech delivery was always followed by MPHD while cesarean or normal delivery in such patients was followed by IGHD only
SN - 0301-0163
AD - Pediatric Department, University of Pavia, IRCCS Policlinico S. Matteo, Italy
UR - 1806462
ER -

TY - JOUR
ID - 2111
T1 - Quantitative ultraviolet skin exposure in children during selected outdoor activities
A1 - Melville,S.K.
A1 - Rosenthal,F.S.
A1 - Luckmann,R.
A1 - Lew,R.A.
Y1 - 1991/06//
N1 - Melville, S K. Rosenthal, F S. Luckmann, R. Lew, R A
Photodermatology, photoimmunology & photomedicine
awp, 9013641
IM
Journal Article
English
KW - MEDLINE
KW - Adolescent
KW - Child
KW - Environmental Exposure/an [Analysis]
KW - Environmental Monitoring
KW - Female
KW - Film Dosimetry
KW - Humans
KW - Male
KW - Recreation
KW - Skin/re [Radiation Effects]
KW - Skin Neoplasms/et [Etiology]
KW - Ultraviolet Rays/ae [Adverse Effects]
RP - NOT IN FILE
SP - 99
EP - 104
JF - Photodermatology, Photoimmunology & Photomedicine
JA - Photodermatol.Photoimmunol.Photomed.
VL - 8
IS - 3

CY - DENMARK
N2 - We determined the cumulative exposure of 3 body sites to ultraviolet radiation from sunlight for 126 children observed from 1-3 d during a variety of common recreational activities at a girl scout camp, baseball camp and community baseball field. Median arm exposure to children playing baseball at a camp ranged from 27.6% to 33.2% of the possible ambient exposure. These exposures are similar to adult exposures reported for comparable activities. Median exposure to the arm at the girl scout camp during mixed activities ranged from 9.0% to 26.5% of possible ambient exposure. At the girl scout camp, exposure both within and between activity groups varied substantially and were more variable than the baseball players' exposure. Arm exposure was greater than cheek and forehead exposure for all subject groups, with an arm-to-cheek exposure ratio ranging from 1.7 to 2.3. For organized sports, such as baseball, it may be possible to assign a single exposure estimate for use in epidemiologic studies or risk estimates. However, for less uniform outdoor activities, wide variability in exposure makes it more difficult to predict an individual's exposure
SN - 0905-4383
AD - Department of Family and Community Medicine, University of Massachusetts Medical School, Worcester
UR - 1804295
ER -

TY - JOUR
ID - 2112
T1 - Choices at weaning: main factor in ingestive behavior
A1 - Ciampolini,M.
A1 - Vicarelli,D.
A1 - Bini,S.
Y1 - 1991/01//
N1 - Ciampolini, M. Vicarelli, D. Bini, S
Nutrition (Burbank, Los Angeles County, Calif.)
beu, 8802712
IM
Clinical Trial. Journal Article. Randomized Controlled Trial. Research Support, Non-U.S. Gov't
English
KW - MEDLINE
KW - Blood Glucose/me [Metabolism]
KW - Child
KW - Preschool
KW - Diarrhea
KW - Infantile/dh [Diet Therapy]
KW - Eating
KW - Energy Intake
KW - Female
KW - Food
KW - Humans
KW - Infant
KW - Male
KW - Maternal Behavior
KW - Prospective Studies
KW - Vegetables
KW - Weaning
RP - NOT IN FILE
SP - 51
EP - 54
JF - Nutrition
VL - 7
IS - 1
CY - UNITED STATES
N2 - "Spontaneous," i.e., self-promoted, feeding without the promotion of offering of energy-dense food (eating incentives), was maintained for 7 months in a randomized, controlled, prospective, semiblind investigation in 88

2-year-old diarrheic children. This behavior was associated with 30% lower energy intake throughout the investigation period while normal growth and blood assessments were maintained and intestinal functions improved. The dependence of a child's energy intake on maternal administrative behavior at each meal was proposed. The energy content of each meal was thus investigated in relation to a preprandial request for food, glycemia, and acceptance of nonstarchy vegetables (NSV). A sample of 210 meals eaten by 10 children with chronic nonspecific diarrhea in the second year of life before mothers were instructed in spontaneous feeding was compared with 210 meals after 1.5 months of spontaneous feeding. Compliance, food weight, preprandial glycemia, and outdoor activities were reported by the mothers in 7-day diaries. Requests for food by the child before the meal was seen and acceptance of more than two-thirds of the prescribed amount of NSV were associated with significantly lower preprandial glycemia and lower energy intake in the investigation of all meals before and after instructions were given. Feeding children on demand and habitual NSV administration may safely educate children to avoid overeating after weaning
SN - 0899-9007
AD - Dipartimento di Pediatria, Universita di Firenze, Florence, Italy
UR - 1802186
ER -

TY - JOUR
ID - 2113
T1 - Predictive value of intraoperative electrocorticograms in resective epilepsy surgery
A1 - McBride,M.C.
A1 - Binnie,C.D.
A1 - Janota,I.
A1 - Polkey,C.E.
Y1 - 1991/10//
N1 - McBride, M C. Binnie, C D. Janota, I. Polkey, C E
Annals of neurology
6ae, 7707449
IM
Journal Article
English
KW - MEDLINE
KW - Action Potentials/ph [Physiology]
KW - Adolescent
KW - Adult
KW - Brain/pp [Physiopathology]
KW - Child
KW - Preschool
KW - Electroencephalography
KW - Epilepsy/pp [Physiopathology]
KW - Epilepsy/su [Surgery]
KW - Female
KW - Humans
KW - Male
KW - Middle Aged
KW - Monitoring
KW - Intraoperative
KW - Predictive Value of Tests
KW - Prognosis
RP - NOT IN FILE
SP - 526
EP - 532
JF - Annals of Neurology
JA - Ann Neurol
VL - 30
IS - 4
CY - UNITED STATES

N2 - The preresection and postresection intraoperative electrocorticograms of 76 consecutive patients undergoing resective surgery for intractable epilepsy were analyzed to see if location, configuration, and discharge rate of epileptiform activity correlated with type and location of pathology of the resected specimens and outcome in regard to seizure control. The location of the predominant spike focus did not correlate with either type of location of pathology or with seizure outcome from temporal lobe surgery (n = 58). The presence of spontaneous or activated spikes outside the resected area did not correlate with outcome from any surgery type. Positive spikes recorded from the amygdala and anterior hippocampus (n = 37) were not associated with type or location of pathology, but bursts of fast repetitive spikes on these needle recordings tended to associate with mesiotemporal pathology (p = less than 0.02) and with mesial temporal sclerosis (p = less than 0.04). A preresection spike discharge rate of 1 per 4 minutes or less was associated with a poor outcome in 5 of 6 patients (p = 0.03), whereas a rate of 18 or more per minute was associated with a good outcome in 15 of 18 patients (p less than 0.06). Persistence of 50% or more of the preresection epileptiform activity in the postresection electrocorticogram after temporal lobectomy correlated with poor outcome in 80% (p = less than 0.03), although the absolute amount of epileptiform activity remaining in the postresection electrocorticogram did not correlate with outcome. Further studies are needed to define the role of intraoperative electrocorticograms in resective epilepsy surgery
SN - 0364-5134
AD - Maudsley Hospital, London, England
UR - 1789682
ER -

TY - JOUR
ID - 2114
T1 - Children with mild learning difficulties in an integrated and in a special school: comparisons of behaviour, teasing and teachers' attitudes
A1 - Martlew,M.
A1 - Hodson,J.
Y1 - 1991/11//
N1 - Martlew, M. Hodson, J
The British journal of educational psychology
awn, 0370636
IM
Comparative Study. Journal Article
English
KW - MEDLINE
KW - Adaptation
KW - Psychological
KW - Attitude
KW - Child
KW - Education
KW - Special
KW - Female
KW - Humans
KW - Learning Disorders/px [Psychology]
KW - Learning Disorders/th [Therapy]
KW - Mainstreaming (Education)
KW - Male
KW - Peer Group
KW - Play and Playthings
KW - Social Behavior
RP - NOT IN FILE
SP - 355
EP - 372
JF - British Journal of Educational Psychology
JA - Br J Educ Psychol
VL - 61
IS - Pt 3

CY - ENGLAND
N2 - Comparisons were made between children and teachers in two schools, one a mainstream school with an integrated resource unit for children with mild learning difficulties, the other a special school. Seventeen children with mild learning difficulties from the special school and ten children with mild learning difficulties from the mainstream school, matched with ten mainstream children for age and sex, took part in the study. Observations were made on children in the playground and their social proximity and positive and negative behaviours were categorised. Self-reports on teasing, bullying and friendships were also collected from children in the mainstream school. Teachers from both schools completed a questionnaire on their attitudes to integration. There was no difference in amount of social contact children with mild learning difficulties had in the mainstream and special school nor were there significant differences in total positive and negative behaviours. Mainstream children, however, played significantly less frequently with children with mild learning difficulties and this was more marked in the older than the younger children. Self-reports from children in the integrated school indicated that the children with mild learning difficulties were teased/bullied more and made fewer friends than the mainstream children. Teachers in the mainstream were more positive and teachers in the special school were more negative towards integration. The special school teachers' expectations fitted more with the findings of the study than did those of the mainstream teachers
SN - 0007-0998
AD - Department of Psychology, University of Sheffield
UR - 1786214
ER -

TY - JOUR
ID - 2115
T1 - [Family planning and maternal and child care in present-day society]. [Russian]
A1 - Safonova,T.I.
Y1 - 1991///
N1 - Safonova, T Ia
Sovetskoe zdravookhranenie / Ministerstvo zdravookhraneniia SSSR
0404526, uwk
IM, J
English Abstract. Journal Article
Russian
KW - MEDLINE
KW - Abortion
KW - Induced/td [Trends]
KW - Adolescent
KW - Adult
KW - Child
KW - Child Health Services/og [Organization & Administration]
KW - Child Health Services/td [Trends]
KW - Family Characteristics
KW - Family Planning Services/mt [Methods]
KW - Family Planning Services/td [Trends]
KW - Female
KW - Humans
KW - Maternal Health Services/og [Organization & Administration]
KW - Maternal Health Services/td [Trends]
KW - Parity
KW - Pregnancy
KW - USSR
RP - NOT IN FILE
SP - 48
EP - 52
JF - Sovetskoe Zdravookhranenie
JA - Sov.Zdravookhr.
IS - 9
CY - USSR

N2 - The evidence is provided of a close relationship between the features of women's reproductive behaviour, their health status and the health of their children: the lack of rational planning child-birth results in increasing maternal and infant mortality rates, prematurity, birth of unwanted children. The use of abortions as one of the basic means of birth control produces a negative effect, i.e. they are a frequent cause of sterility, gynaecological diseases, complicated future pregnancies and deliveries. There is a description of the priority tasks and conditions for the effective introduction of family planning into the everyday life--the necessity of taking into account sociopsychological, regional aspects of birth control within a family, demographic situation at the local level; identification of women populations which are in need of family planning activities
SN - 0038-5239
UR - 1785071
ER -

TY - JOUR
ID - 2116
T1 - EEG features and their evolution in the acute phase of haemorrhagic shock and encephalopathy syndrome
A1 - Harden,A.
A1 - Boyd,S.G.
A1 - Cole,G.
A1 - Levin,M.
Y1 - 1991/11//
N1 - Harden, A. Boyd, S G. Cole, G. Levin, M
Neuropediatrics
nza, 8101187
IM
Journal Article
English
KW - MEDLINE
KW - Adolescent
KW - Brain/pp [Physiopathology]
KW - Brain Diseases/di [Diagnosis]
KW - Brain Diseases/pp [Physiopathology]
KW - Cerebral Hemorrhage/di [Diagnosis]
KW - Child
KW - Preschool
KW - Disseminated Intravascular Coagulation/di [Diagnosis]
KW - Disseminated Intravascular Coagulation/pp [Physiopathology]
KW - Electroencephalography
KW - Evaluation Studies as Topic
KW - Female
KW - Humans
KW - Infant
KW - Male
KW - Shock
KW - Hemorrhagic/di [Diagnosis]
KW - Hemorrhagic/pp [Physiopathology]
RP - NOT IN FILE
SP - 194
EP - 197
JF - Neuropediatrics
VL - 22
IS - 4
CY - GERMANY
N2 - Serial EEGs have been carried out during the acute phase of haemorrhagic shock and encephalopathy syndrome (HS&E) in 22 infants and children aged 3 months to 14 years. Most patients presented with fits and coma and all had shock with bleeding and disseminated intravascular coagulation (DIC). The initial EEG showed prolonged runs of often rhythmic discharges which fluctuated in amount and amplitude with varying distribution and morphology ("electrical storms"). Over a period of days the "electrical storms" gradually

decreased leaving only low amplitude EEG activities or evolving to electrocerebral silence (7 cases). Fifteen patients died and all five children with multifocal "electrical storms" who survived showed gross neurological handicap. The rather distinctive EEG pattern is unusual in the context of an acute encephalopathy outside the neonatal period although similar "electrical storms" may be seen in a less extreme form in infants and children with other conditions associated with DIC. This EEG pattern presumably reflects changes in the cerebral microcirculation which in HS&E are usually relentlessly progressive and associated with devastating cortical damage
SN - 0174-304X
AD - Department of Clinical Neurophysiology, Hospital for Sick Children, London, U.K
UR - 1775215
ER -

TY - JOUR
ID - 2117
T1 - [Toxicological assessment. A solid basis for preventive health care? Considerations on the preventive character of guidelines for (heavy) metal contamination of children's playgrounds]. [German]
A1 - Muller,L.
Y1 - 1991/11//
N1 - Muller, L
Das Offentliche Gesundheitswesen
ofe, 0107170
IM
English Abstract. Journal Article
German
KW - MEDLINE
KW - Arsenic/an [Analysis]
KW - Cadmium/an [Analysis]
KW - Child
KW - Preschool
KW - Environmental Pollutants/an [Analysis]
KW - Germany
KW - Humans
KW - Infant
KW - Lead/an [Analysis]
KW - Maximum Allowable Concentration
KW - Metals/an [Analysis]
KW - Play and Playthings
KW - Reference Values
KW - Soil Pollutants/an [Analysis]
RP - NOT IN FILE
SP - 736
EP - 745
JF - Offentliche Gesundheitswesen
JA - Offentl.Gesundheitswes.
VL - 53
IS - 11
CY - GERMANY
N2 - The present report discusses the standard limit reference values for metal content of playgrounds, set by the authorities in North-Rhine-Westphalia, Berlin, Hamburg and Bremen. Taking the metals arsenic, lead and cadmium as an example, the causative role of region specific conditions for establishing the limit values for protective measures are described. Essentially, two factors influence the decision: 1. the background concentrations of metals in sand and soils differ between the various states; 2. there is a lack of a uniform concept for the quantitative evaluation of oral intake as a main route of ingestion of metals endangering the health of children on child playgrounds. Due to insufficient data, arbitrary assumptions in respect of intake, relevant age group or playing-time on playgrounds are made. From a toxicological point of view the standard limit values, exceed the span of toxicological tolerance, in an attempt to combine pragmatic and toxicological considerations. However, only few of these values agree with the basic principles of preventive care

SN - 0029-8573
UR - 1837341
ER -

TY - JOUR
ID - 2118
T1 - Asthma in the vicinity of power stations: I. A prevalence study
A1 - Henry,R.L.
A1 - Abramson,R.
A1 - Adler,J.A.
A1 - Wlodarcyzk,J.
A1 - Hensley,M.J.
Y1 - 1991///
N1 - Henry, R L. Abramson, R. Adler, J A. Wlodarcyzk, J. Hensley, M J
Pediatric pulmonology
owh, 8510590
IM
Comment. Journal Article. Research Support, Non-U.S. Gov't
English
KW - MEDLINE
KW - Air Pollution/ae [Adverse Effects]
KW - Asthma/ep [Epidemiology]
KW - Asthma/et [Etiology]
KW - Child
KW - Cross-Sectional Studies
KW - Female
KW - Humans
KW - Incidence
KW - Male
KW - New South Wales/ep [Epidemiology]
KW - Power Plants
KW - Respiratory Hypersensitivity/ep [Epidemiology]
KW - Respiratory Hypersensitivity/et [Etiology]
KW - Respiratory Tract Infections/ep [Epidemiology]
KW - Respiratory Tract Infections/et [Etiology]
KW - Risk Factors
RP - NOT IN FILE
SP - 127
EP - 133
JF - Pediatric Pulmonology
JA - Pediatr Pulmonol
VL - 11
IS - 2
CY - UNITED STATES
N2 - Respiratory symptoms, atopy, and bronchial reactivity were measured in primary school children living in Lake Munmorah (LM), a coastal town near two power stations, and in Nelson Bay (NB), a coastal town free from any possible major sources of outdoor air pollution. A prevalence survey and longitudinal follow-up study were performed 1 year apart. In both studies, the prevalence of ever wheezed, current wheezing, breathlessness, wheezing with exercise, diagnosed asthma, and use of drugs for asthma at LM were all approximately double the prevalence at NB (all P values less than 0.01). The prevalence of bronchial reactivity was significantly greater at LM than NB (P less than 0.01) at the first but not the second survey. By contrast, no significant differences were found between the two areas for skin test atopy or for parental history of allergic disease. Multivariate analysis supported the conclusion from the univariate analysis that there was more wheezing at LM compared to NB at both studies, when adjusted for atopy, smoking in the home, age, and sex. As expected, a positive skin test reaction to house dust mite was the predominant explanatory variable. Asthma was more common in the community near power stations (LM) than in the NB area. The absence of significant differences in skin test atopy and parental history of allergic disease argued against major genetic differences between the

two groups. By contrast, the more common reporting of siblings' chest disease and asthma in Lake Munmorah supported an environmental cause
SN - 8755-6863
AD - Disciplines of Paediatrics, University of Newcastle, New South Wales, Australia
UR - 1758730
ER -

TY - JOUR
ID - 2119
T1 - School injuries. Epidemiology and clinical features of 307 cases registered at hospital during one school year
A1 - Bergstrom,E.
A1 - Bjornstig,U.
Y1 - 1991/09//
N1 - Bergstrom, E. Bjornstig, U
Scandinavian journal of primary health care
8510679, sif
IM
Journal Article
English
KW - MEDLINE
KW - Adolescent
KW - Age Factors
KW - Ankle Injuries/ep [Epidemiology]
KW - Child
KW - Female
KW - Humans
KW - Male
KW - Outpatient Clinics
KW - Hospital
KW - Physical Education and Training
KW - Questionnaires
KW - Risk Factors
KW - School Health Services
KW - Sports
KW - Sweden/ep [Epidemiology]
KW - Wounds and Injuries/cl [Classification]
KW - Wounds and Injuries/ep [Epidemiology]
KW - Wrist Injuries/ep [Epidemiology]
RP - NOT IN FILE
SP - 209
EP - 216
JF - Scandinavian Journal of Primary Health Care
JA - Scand J Prim Health Care
VL - 9
IS - 3
CY - SWEDEN
N2 - Injuries at school were studied for one school year in 57 primary and three secondary schools (13,733 students, aged 7-19 years) at Umea in northern Sweden. The injury rate was 22/1000 student years. There was a wide variation in injury rate between different schools. The boy/girl ratio was 1.1/1. Physical education was the dominating activity at the time of injury for the older students and play in the school yard for the younger. The majority of the students had a minor injury, but 17% had a fracture. The injured students did not seem to have more somatic, psychological or social problems than students in general. Fewer competitive sports and ball games and more adult supervision and organized activities during breaks are suggestions to reduce injuries at school. A hospital-based injury registration system is well fitted for serving as a base for analysing school injuries
SN - 0281-3432

AD - Department of Pediatrics, University of Umea, Sweden
UR - 1754755
ER -

TY - JOUR
ID - 2120
T1 - A one-year prospective ED cohort of pediatric trauma
A1 - Yamamoto,L.G.
A1 - Wiebe,R.A.
A1 - Matthews,W.J.,Jr.
Y1 - 1991/10//
N1 - Yamamoto, L G. Wiebe, R A. Matthews, W J Jr
Pediatric emergency care
pau, 8507560
IM
Journal Article. Research Support, U.S. Gov't, P.H.S.
English
KW - MEDLINE
KW - Accidents
KW - Traffic/mo [Mortality]
KW - Traffic/pc [Prevention & Control]
KW - Adolescent
KW - Child
KW - Preschool
KW - Emergency Service
KW - Hospital
KW - Female
KW - Hawaii/ep [Epidemiology]
KW - Hospitalization
KW - Humans
KW - Infant
KW - Injury Severity Score
KW - Male
KW - Morbidity
KW - Prospective Studies
KW - Risk Factors
KW - Wounds and Injuries/ep [Epidemiology]
KW - Wounds and Injuries/et [Etiology]
KW - Wounds and Injuries/mo [Mortality]
KW - Wounds and Injuries/pc [Prevention & Control]
RP - NOT IN FILE
SP - 267
EP - 274
JF - Pediatric Emergency Care
JA - Pediatr Emerg Care
VL - 7
IS - 5
CY - UNITED STATES
N2 - During a 12-month period ending on November 30, 1988, data were collected on 4623 pediatric patients visiting a pediatric emergency department with trauma (excluding burns). Sixty-one percent were male. Common causes of the trauma included suspected child abuse (4%), organized sports (6%), nonorganized sports (4%), pedestrian motor vehicle accidents (MVAs) (3%), bicycle MVAs (2%), and automobile MVAs (3%). However, most of the incidents had none of the identified associated activities surrounding the trauma (60%). Incidents took place at home (41%), on the street (11%), at school (10%), and at a playground or park (10%). Injuries involved the external body (59%), extremity (26%), head or neck (13%), face (4%), chest (1%), and abdomen (2%) and were more common during the summer. Injury severity scores had a mean of 1.8 and were grouped as: less than 3 (80%), 3-6 (19%), and greater than 6 (1.6%). Factors associated with higher trauma

severity included MVAs, water-related injuries, sports, streets, schools, parks, playgrounds, skateboards, skates, and alcohol
SN - 0749-5161
AD - Department of Pediatrics, University of Hawaii John A. Burns School of Medicine, Honolulu
UR - 1754484
ER -

TY - JOUR
ID - 2121
T1 - Public schools welcome students with disabilities as full members
A1 - Davern,L.
A1 - Schnorr,R.
Y1 - 1991///
N1 - Davern, L. Schnorr, R
Children today
d2e, 0320227, 0320227
IM
Journal Article
English
KW - MEDLINE
KW - Activities of Daily Living/px [Psychology]
KW - Adolescent
KW - Child
KW - Disabled Persons/ed [Education]
KW - Disabled Persons/px [Psychology]
KW - Education of Intellectually Disabled/mt [Methods]
KW - Humans
KW - Individuality
KW - Mainstreaming (Education)/mt [Methods]
KW - Social Adjustment
RP - NOT IN FILE
SP - 21
EP - 25
JF - Children Today
JA - Child Today
VL - 20
IS - 2
CY - UNITED STATES
N2 - As noted in a parent newsletter, the inclusion of children and young adults with disabilities with other students in our schools will result in "an ever widening circle of people who believe that disabilities are a part of life, that people with disabilities are a part of [our] natural environment who should not be isolated, and that people with disabilities can have a positive effect on non-disabled people and the general community". All students can benefit from regular class activities even though the individual goals for some may be quite different. Such efforts are not always easy to implement, and depend on adequate support for both students and school personnel--but the benefits for all students are substantial. As one regular education teacher noted: "Above all, we want our kids to show concern for others, now and in their future days. Typical kids in integrated classrooms benefit because these goals are as much a part of their daily classroom experience as the 'academics'. In reality, I believe the academics are enhanced because of these goals." And this teacher observed: "I believe that all kids have a right to be in inclusive classrooms--to be educated with their peers. When everybody in a class is considered equal, some very great things will happen."
SN - 0361-4336
AD - Inclusive Education Project, Syracuse University, New York
UR - 1835920
ER -

TY - JOUR
ID - 2122

T1 - [Playground accidents among children in greater Arhus]. [Danish]
A1 - Mikkelsen,S.S.
A1 - Lindblad,B.E.
A1 - Nafei,A.
A1 - Teichert,G.
A1 - Kroner,K.
Y1 - 1991/11/11/
N1 - Mikkelsen, S S. Lindblad, B E. Nafei, A. Teichert, G. Kroner, K
Ugeskrift for laeger
0141730, wm8
IM
English Abstract. Journal Article
Danish
KW - MEDLINE
KW - Accident Prevention
KW - Accidents/sn [Statistics & Numerical Data]
KW - Adolescent
KW - Child
KW - Preschool
KW - Denmark/ep [Epidemiology]
KW - Female
KW - Humans
KW - Incidence
KW - Infant
KW - Injury Severity Score
KW - Male
KW - Play and Playthings
RP - NOT IN FILE
SP - 3239
EP - 3242
JF - Ugeskrift for Laeger
JA - Ugeskr.Laeger
VL - 153
IS - 46
CY - DENMARK
N2 - The aim of this study was to determine the incidence rate and to determine the etiology and the severity of accidents caused by playground equipment during the period 1.1.1989-31.12.1989 and to compare the results with the study made by Christensen et al (1-3). The number of children aged below 15 years was 42,635. A total of 270 playground accidents were registered, which resulted in an incidence rate of 6.3 per 1,000 children per year. This is a reduction from 9.3 per 1,000 children per year in 1981. The incidence rates between boys and girls were 7.0 and 5.6 per 1,000 children per year, as compared with respectively. The sex ratio for the population studied was 1.31 to 1 as compared with 1.44 to 1 in 1981. The sex ratio in the background population was 1.05 both in 1981 and 1989. The greatest number of accidents were seen in spring, especially in April and May. Swings, climbing frames, slides, playhouses and playcastles were responsible for 80% of the accidents. The etiologies were fall accidents in 211 cases, being caught between two objects in 17 cases, being kit by an object in 21 cases and different causes in 21 cases. The underlying surfaces were sufficiently soft in 24% of the cases. The severity of the injury was classified according to the Abbreviated Injury Scale and was found to be: no lesions 1.1%, minor lesions 70%, moderate lesions 25.1% and severe lesions 3.7%. A total of 17 patients were admitted and of these 13 patients had fractures. The possibilities of reducing the number of injuries resulting from playground equipment are discussed
SN - 0041-5782
AD - Ortopaedkirurgisk afdeling, Arhus Amtssygehus
UR - 1957375
ER -

TY - JOUR
ID - 2123

T1 - [Cranial injuries among children in the county of Ringkobing. 2. Analysis of accidents]. [Danish]
A1 - Hansen,T.B.
A1 - Pless,S.
A1 - Gravers,M.
Y1 - 1991/10/14/
N1 - Hansen, T B. Pless, S. Gravers, M
Ugeskrift for laeger
0141730, wm8
IM
English Abstract. Journal Article
Danish
KW - MEDLINE
KW - Accident Prevention
KW - Accidents/cl [Classification]
KW - Accidents/sn [Statistics & Numerical Data]
KW - Adolescent
KW - Brain Injuries/ep [Epidemiology]
KW - Brain Injuries/et [Etiology]
KW - Brain Injuries/pc [Prevention & Control]
KW - Child
KW - Preschool
KW - Craniocerebral Trauma/ep [Epidemiology]
KW - Craniocerebral Trauma/et [Etiology]
KW - Craniocerebral Trauma/pc [Prevention & Control]
KW - Denmark/ep [Epidemiology]
KW - Humans
KW - Infant
KW - Questionnaires
KW - Seasons
KW - Time Factors
RP - NOT IN FILE
SP - 2947
EP - 2949
JF - Ugeskrift for Laeger
JA - Ugeskr.Laeger
VL - 153
IS - 42
CY - DENMARK
N2 - Questionnaires were sent concerning all of the patients aged 0-15 years who were admitted to the Ringkobing County Hospitals during the period 1.1.1982 to 31.8.1989 with head injuries. The object of the questionnaires was to elucidate the circumstances of the accident which resulted in hospitalization. Questionnaires concerning 988 patients (67.5%) were returned. Definite accumulation of the accidents was observed during the period 12.00-18.00 with 55.2% of all the accidents. The majority of the accidents (72.3%) occurred out-of-doors and the majority of these on transport areas. Bicycle accidents were most common, most frequently in children over the age of four years. Where younger children were concerned, accidents in the home were commonest. Accidents in playgrounds and during sports constituted only 13% of the total material. A relatively great proportion of the children (42.9%) were under supervision, usually from their own family, when the accident occurred. On the basis of this investigation, the authors recommend that possible prophylactic measures in the County of Ringkobing should be directed towards bicycle accidents among the older children and accidents in the homes where smaller children are concerned
SN - 0041-5782
AD - Holstebro Centralsygehus, ortopaedkirurgisk afdeling
UR - 1949320
ER -

TY - JOUR
ID - 2124

T1 - Faecal indicator bacteria on the hands and the effectiveness of hand-washing in Zimbabwe
A1 - Kaltenthaler,E.
A1 - Waterman,R.
A1 - Cross,P.
Y1 - 1991/10//
N1 - Kaltenthaler, E. Waterman, R. Cross, P
The Journal of tropical medicine and hygiene
kav, 0406044
IM
Comparative Study. Journal Article. Research Support, Non-U.S. Gov't
English
KW - MEDLINE
KW - Adult
KW - Agriculture
KW - Child
KW - Preschool
KW - Colony Count
KW - Microbial
KW - Enterobacteriaceae/gd [Growth & Development]
KW - Enterococcus/gd [Growth & Development]
KW - Female
KW - Hand/mi [Microbiology]
KW - Hand Disinfection/mt [Methods]
KW - Humans
KW - Humidity
KW - Infant
KW - Mothers
KW - Rural Population
KW - Soaps
KW - Socioeconomic Factors
KW - Urban Population
KW - Zimbabwe
RP - NOT IN FILE
SP - 358
EP - 363
JF - Journal of Tropical Medicine & Hygiene
JA - J Trop Med Hyg
VL - 94
IS - 5
CY - ENGLAND
N2 - Two aspects of hand-washing were explored in this study. Mothers and children from 80 families in Zimbabwe were asked to wash their hands in the traditional manner in sterile water. Mothers were asked to answere a socio-economic questionnaire. By using the questionnaire and observations in conjunction with the microbiological data from hand-washing, factors were identified which contributed to high counts on the hands. These were: high relative humidity, living on a commercial farm, and having an infant in the family. Mothers and children 1-5 years of age had higher counts than children of 6-12 years. People recently involved in outdoor physical activities such as farming had higher counts than those involved in other activities. The traditional hand-washing method was also compared with two other hand-washing methods: hand-washing using soap and hand-washing using a container known as the mukombe. Hand-washing with soap was the most effective method closely followed by hand-washing using the mukombe. The least effective method was traditional hand-washing
SN - 0022-5304
AD - Blair Research Laboratory, Ministry of Health, Harare, Zimbabwe
UR - 1942217
ER -

TY - JOUR

ID - 2125
T1 - Playground injuries: a scientific appraisal of popular concerns
A1 - Ball,D.J.
A1 - King,K.L.
Y1 - 1991/08//
N1 - Ball, D J. King, K L
Journal of the Royal Society of Health
jx3, 8303144
IM
Journal Article
English
KW - MEDLINE
KW - Accidents/sn [Statistics & Numerical Data]
KW - Child
KW - Great Britain/ep [Epidemiology]
KW - Humans
KW - Play and Playthings
KW - Public Opinion
KW - Risk Factors
KW - Wounds and Injuries/ep [Epidemiology]
RP - NOT IN FILE
SP - 134
EP - 137
JF - Journal of the Royal Society of Health
JA - J R Soc Health
VL - 111
IS - 4
CY - ENGLAND
N2 - The widespread public concern in Britain over childhood injuries arising from playground accidents is examined using a new national data base on leisure accidents managed by the Department of Trade and Industry. Popular beliefs about the level of risk, the nature and cause of injury, and remedial measures are found to be unsupported by the analysis. Playground risks are comparatively low; accident causes are diverse and most involve long bone injuries and not head injuries as has been widely reported; and the cost of some popular risk reduction measures would seem to be incommensurate with the reasonably-anticipated risk reduction which they might afford. It is suggested that for those who wish to reduce risk further, consideration should be given to increasing public awareness of the many causes of risk, of the opportunities which the individual has to control risk, and of the inevitability of residual risk
SN - 0264-0325
AD - Environmental Risk Assessment Unit, School of Environmental Sciences, University of East Anglia, Norwich
UR - 1941873
ER -

TY - JOUR
ID - 2126
T1 - Epidemiology and prediction of sports-related traumatic injuries. [Review] [61 refs]
A1 - Pinkham,J.R.
A1 - Kohn,D.W.
Y1 - 1991/10//
N1 - Pinkham, J R. Kohn, D W
Dental clinics of North America
e10, 0217440, 0217440
D, IM
Journal Article. Review
English
KW - MEDLINE
KW - Adolescent

KW - Adult
KW - Aged
KW - Athletic Injuries/ep [Epidemiology]
KW - Athletic Injuries/et [Etiology]
KW - Child
KW - Humans
KW - Maxillofacial Injuries/ep [Epidemiology]
KW - Maxillofacial Injuries/et [Etiology]
KW - Middle Aged
KW - Mouth/in [Injuries]
KW - Probability
KW - Risk Factors
KW - Tooth Injuries
KW - United States/ep [Epidemiology]
RP - NOT IN FILE
SP - 609
EP - 626
JF - Dental Clinics of North America
JA - Dent Clin North Am
VL - 35
IS - 4
CY - UNITED STATES
N2 - All sports injuries, and this is certainly true of dentally related sports injuries, occur for predictable reasons. In some instances, these predictors have been well studied and are recognized by participants as well as coaches, dentists, and other responsible parties. In other instances, there is much to be learned about these predictors. There is no question that predictors have direct influence upon the incidence and prevalence data for dental injuries related to each sport. Because of the variability of these predictors from one sport to another, from one sports participant to another, from one age group to another, and even from on playground, field, or court to another, the importance and validity and usefulness of much of the data available currently is debatable. The importance of sports dentistry is not based solely on the urgency afforded by a given number of injuries among a specified number of participants over a certain amount of time. It is the predictable nature of these injuries and the need for the profession to take responsible action in their prevention and treatment that provides the strongest imperative for a sports dentistry movement. [References: 61]
SN - 0011-8532
AD - Pediatric Dentistry, University of Iowa College of Dentistry, Iowa City
UR - 1682167
ER -

TY - JOUR
ID - 2127
T1 - Loss of cellular immune reactivity during acute Plasmodium falciparum malaria
A1 - Hviid,L.
A1 - Theander,T.G.
A1 - bu-Zeid,Y.A.
A1 - Abdulhadi,N.H.
A1 - Jakobsen,P.H.
A1 - Saeed,B.O.
A1 - Jepsen,S.
A1 - Bayoumi,R.A.
A1 - Jensen,J.B.
Y1 - 1991/08//
N1 - Hviid, L. Theander, T G. Abu-Zeid, Y A. Abdulhadi, N H. Jakobsen, P H. Saeed, B O. Jepsen, S. Bayoumi, R A. Jensen, J B
FEMS microbiology immunology
ao3, 8901230
IM

Journal Article. Research Support, Non-U.S. Gov't. Research Support, U.S. Gov't, P.H.S.
English
KW - MEDLINE
KW - Acute Disease
KW - Adolescent
KW - Adult
KW - Aged
KW - Antibodies
KW - Protozoan/an [Analysis]
KW - Child
KW - Humans
KW - Lymphocyte Activation
KW - Malaria
KW - Falciparum/im [Immunology]
KW - Middle Aged
KW - Receptors
KW - Interleukin-2/an [Analysis]
KW - T-Lymphocytes/im [Immunology]
KW - Tuberculin/im [Immunology]
RP - NOT IN FILE
SP - 219
EP - 227
JF - FEMS Microbiology Immunology
JA - FEMS Microbiol Immunol
VL - 3
IS - 4
CY - NETHERLANDS
N2 - Sixteen patients suffering from acute Plasmodium falciparum malaria were studied. All were residents of an area of unstable malaria-transmission in Eastern Sudan. Blood-samples were drawn at diagnosis, and 7 and 30 days later. Blood-samples from thirteen donors, drawn outside the malaria transmission season 5 months prior to the attack, were included in the study. Lymphoproliferative responsiveness to purified soluble malarial antigens and to the unrelated antigen PPD was lost during the acute phase of the disease in most donors, but was regained during convalescence, except in four donors recrudescing or reinfected by day 30. In contrast to the suppression of antigenic responses, cellular responses to phytohaemagglutinin (PHA) remained virtually unaffected. All donors showed elevated plasma-levels of soluble IL-2 receptor during the acute phase of the disease which normalized during convalescence. Five donors examined by fluorescence-activated cell sorting (FACS) showed no increase in surface expression of IL-2 receptor on peripheral lymphocytes. The data indicate that acute P. falciparum malaria causes a depletion of antigen-reactive T-cells from the peripheral circulation, probably due to homing of this cell-population to lymphoid tissues. It was also found that acute-phase plasma was suppressive to PPD-induced proliferative responses, indicating an additional suppressive mechanism operating in vivo
SN - 0920-8534
AD - Department of Infectious Diseases, University Hospital (Rigshospitalet), Copenhagen, Denmark
UR - 1931134
ER -

TY - JOUR
ID - 2128
T1 - Risk factors for Crimean-Congo hemorrhagic fever in rural northern Senegal
A1 - Chapman,L.E.
A1 - Wilson,M.L.
A1 - Hall,D.B.
A1 - LeGuenno,B.
A1 - Dykstra,E.A.
A1 - Ba,K.
A1 - Fisher-Hoch,S.P.
Y1 - 1991/10//

N1 - Chapman, L E. Wilson, M L. Hall, D B. LeGuenno, B. Dykstra, E A. Ba, K. Fisher-Hoch, S P
The Journal of infectious diseases
ih3, 0413675
AIM, IM
Journal Article. Research Support, U.S. Gov't, Non-P.H.S.. Research Support, U.S. Gov't, P.H.S.
English
KW - MEDLINE
KW - Adolescent
KW - Adult
KW - Age Factors
KW - Aged
KW - Animals
KW - Antibodies
KW - Viral/bl [Blood]
KW - Child
KW - Cohort Studies
KW - Cross-Sectional Studies
KW - Female
KW - Hemorrhagic Fever Virus
KW - Crimean-Congo/im [Immunology]
KW - Hemorrhagic Fever
KW - Crimean/ep [Epidemiology]
KW - Crimean/tm [Transmission]
KW - Humans
KW - Immunoglobulin G/an [Analysis]
KW - Insect Bites and Stings/co [Complications]
KW - Male
KW - Middle Aged
KW - Prevalence
KW - Questionnaires
KW - Regression Analysis
KW - Risk Factors
KW - Rural Population
KW - Senegal/ep [Epidemiology]
KW - Ticks
KW - Transients and Migrants
RP - NOT IN FILE
SP - 686
EP - 692
JF - Journal of Infectious Diseases
JA - J Infect Dis
VL - 164
IS - 4
CY - UNITED STATES
N2 - The extent of infection among 722 residents of an enzootic focus of Crimean-Congo hemorrhagic fever (CCHF) virus in rural northern Senegal and putative modes of transmission were studied by a cross-sectional seroprevalence survey done from February through May 1989. Anti-CCHF virus IgG was found in 13.1% of 283 persons who completed a standard questionnaire and provided blood samples. Seropositivity rates were similar between sexes and increased significantly with age among nomadic persons. Behavior patterns providing exposure to multifactorial risk factors were gender-based. Male risk factors, primarily associated with herding activities, included sleeping outside during seasonal migrations (also a risk factor for nomadic women), bite by a tick (adult male Hyalomma truncatum), tick bite during the cool dry season, and contact with sick animals. Human infection of CCHF occurred more frequently or with less mortality in the region studied than has been found elsewhere in Africa; however, the rate of seroconversion-associated illness is undetermined. Hyalomma ticks appear to be the primary transmission mode
SN - 0022-1899
AD - Division of Viral and Rickettsial Diseases, CDC, Atlanta, GA 30333

UR - 1910069
ER -

TY - JOUR
ID - 2129
T1 - School-based assessment of attention deficit-hyperactivity disorder. [Review] [94 refs]
A1 - Atkins,M.S.
A1 - Pelham,W.E.
Y1 - 1991/09//
N1 - Atkins, M S. Pelham, W E
Journal of learning disabilities
0157312, iwo
C
Journal Article. Review
English
KW - MEDLINE
KW - Attention Deficit Disorder with Hyperactivity/di [Diagnosis]
KW - Attention Deficit Disorder with Hyperactivity/px [Psychology]
KW - Child
KW - Education
KW - Special
KW - Humans
KW - Learning Disorders/di [Diagnosis]
KW - Learning Disorders/px [Psychology]
KW - Referral and Consultation
KW - Sociometric Techniques
RP - NOT IN FILE
SP - 197
EP - 204
JF - Journal of Learning Disabilities
JA - J Learn Disabil
VL - 24
IS - 4
CY - UNITED STATES
N2 - Schools are uniquely situated to provide information relevant to the assessment and treatment of Attention Deficit-Hyperactivity Disorder (ADHD) across a variety of tasks, settings, and observers. The importance of the school setting to the assessment and treatment of ADHD has resulted in the development of numerous measures appropriate for such assessment, but few guidelines for their use. The purpose of this article is to describe school-based assessment procedures and their relationship to ADHD. These procedures include teacher rating measures, direct observations in classrooms and on playgrounds, peer rating and sociometric measures, and permanent product measures such as academic performance. It is recommended that school-based assessment of ADHD involve the concurrent use of multiple measures to assess adequately the wide variety of symptoms associated with this disorder. It is further recommended that assessment serve the purpose of determining treatment efficacy to avoid unwarranted preoccupation with diagnostic issues that are as yet unresolved. The components of a comprehensive school-based assessment of ADHD are described, and their relationship to school-based treatment is discussed. [References: 94]
SN - 0022-2194
AD - Department of Pediatrics, Children's Hospital, Philadelphia, PA
UR - 1875154
ER -

TY - JOUR
ID - 2130
T1 - Effects of single- and multiday ozone exposures on respiratory function in active normal children
A1 - Spektor,D.M.
A1 - Thurston,G.D.
A1 - Mao,J.

A1 - He,D.
A1 - Hayes,C.
A1 - Lippmann,M.
Y1 - 1991/08//
N1 - Spektor, D M. Thurston, G D. Mao, J. He, D. Hayes, C. Lippmann, M
Environmental research
ei2, 0147621
IM
Journal Article. Research Support, U.S. Gov't, Non-P.H.S.. Research Support, U.S. Gov't, P.H.S.
English
KW - MEDLINE
KW - Adolescent
KW - Air Pollutants/an [Analysis]
KW - Child
KW - Dose-Response Relationship
KW - Drug
KW - Environmental Exposure
KW - Female
KW - Forced Expiratory Flow Rates/de [Drug Effects]
KW - Forced Expiratory Flow Rates/ph [Physiology]
KW - Forced Expiratory Volume/de [Drug Effects]
KW - Forced Expiratory Volume/ph [Physiology]
KW - Humans
KW - Male
KW - Ozone/pd [Pharmacology]
KW - Regression Analysis
KW - Respiration/de [Drug Effects]
KW - Respiration/ph [Physiology]
KW - Spirometry
KW - Time Factors
KW - Vital Capacity/de [Drug Effects]
KW - Vital Capacity/ph [Physiology]
RP - NOT IN FILE
SP - 107
EP - 122
JF - Environmental Research
JA - Environ Res
VL - 55
IS - 2
CY - UNITED STATES
N2 - Ventilatory function was measured twice daily on 46 healthy children aged 8-14 years on at least 7 days for each child during a 4-week period at a northwestern New Jersey residential summer camp in 1988. The highest 1-hr O3 concentration was 150 ppb, while the highest 12-hr H+ concentration (as H2SO4) was 18.6 micrograms/m3. The highest temperature-humidity index was 81 degrees F. The regressions of FVC, FEV1, FEF25-75, and PEFR on O3 in the hour preceding the afternoon function measurements yielded slopes essentially the same as those measured on other children at the same camp in 1984. Regressions of the changes in function between the late morning and late afternoon function measurements on average O3 concentration between them produced significant, but somewhat smaller effects, while regressions of morning function on O3 during the previous day indicated small but still significant effects. There were no significant correlations with other measured environmental variables including H+. Based on the results of this study and similar previous studies, we conclude that O3 exposures in ambient air produce greater lung function deficits in active young people in natural settings then does pure O3 in controlled chamber exposure studies because of: (1) longer exposures; (2) potentiation by other factors in the ambient exposures; (3) the persistence of effects from prior day's exposures; and (4) the persistence of a transient response associated with the daily peak of exposure. It follows that projections of likely effects in the real world from controlled chamber exposure studies should either have a large margin of safety, or the judgment of the extent of effects likely to occur among populations should be based directly on the effects observed in field studies

SN - 0013-9351
AD - Institute of Environmental Medicine, New York University Medical Center, Tuxedo 10987
UR - 1868814
ER -

TY - JOUR
ID - 2131
T1 - Analysis of US child care safety regulations
A1 - Runyan,C.W.
A1 - Gray,D.E.
A1 - Kotch,J.B.
A1 - Kreuter,M.W.
Y1 - 1991/08//
N1 - Runyan, C W. Gray, D E. Kotch, J B. Kreuter, M W
American journal of public health
1254074, 3xw
AIM, IM
Journal Article. Research Support, U.S. Gov't, P.H.S.
English
KW - MEDLINE
KW - Child Day Care Centers/lj [Legislation & Jurisprudence]
KW - Child Day Care Centers/st [Standards]
KW - Child
KW - Preschool
KW - Data Collection
KW - Humans
KW - Safety
KW - United States
RP - NOT IN FILE
SP - 981
EP - 985
JF - American Journal of Public Health
JA - Am J Public Health
VL - 81
IS - 8
CY - UNITED STATES
N2 - BACKGROUND: With 1.9 million US children cared for in organized group child care, the safety of these children is a public health concern. In the absence of federal policy, each state has developed its own day care safety regulations. METHODS: After creating a set of 36 criteria from three sets of national guidelines, we assessed the safety regulations of 45 states. With a mailed survey of state day care regulatory personnel, we examined the processes of formulating and implementing safety policy in 47 states. RESULTS: For 24 of the 36 items, more than half the states' regulations were below the criteria or failed to mention the topic. Most notable is the inattention to playground safety, choking hazards, and firearms. CONCLUSION: The uneven quality of regulations may be a reflection of a regulatory process that is fragmented, with many different groups sharing authority and with limited involvement of injury prevention specialists
SN - 0090-0036
AD - University of North Carolina School of Public Health, Department of Health Behavior and Health Education, Chapel Hill
UR - 1854015
ER -

TY - JOUR
ID - 2132
T1 - [Perspectives on chest wall mechanics in infants and young children]. [Japanese]
A1 - Katayama,M.
A1 - Miyasaka,K.
Y1 - 1991/03//

N1 - Katayama, M. Miyasaka, K
Nihon Kyobu Shikkan Gakkai zasshi
kqd, 7505737
IM
English Abstract. Journal Article
Japanese
KW - MEDLINE
KW - Biomechanical Phenomena
KW - Child
KW - Preschool
KW - Functional Residual Capacity/ph [Physiology]
KW - Humans
KW - Infant
KW - Newborn
KW - Movement/ph [Physiology]
KW - Thorax/ph [Physiology]
RP - NOT IN FILE
SP - 302
EP - 306
JF - Nihon Kyobu Shikkan Gakkai Zasshi
VL - Japanese Journal of Thoracic Diseases. 29
IS - 3
CY - JAPAN
N2 - The chest wall of neonates and infants is compliant and thus the outward recoil of the chest wall and the inward recoil of the lungs theoretically balance at a low lung volume (FRC) incompatible with gas exchange. The neonates and infants, however, have a number of mechanisms that actively maintain a high FRC: (1) Laryngeal breaking during expiration. (2) Short expiratory time relative to long time expiratory time constant. (3) Sustained inspiratory muscle tone during expiratory phase. When lung recoil is increased as in many disease states, the third mechanism fails due to muscle fatigue. The increased lung recoil pulls in the chest wall and a catastrophic decrease in FRC occurs. The measurement of respiration from the outside through chest wall movement is especially important in infants and young children for these reasons. The other reason is that the breathing pattern remains unaltered, unlike measurement via the airway such as by mask and pneumotachometer
SN - 0301-1542
AD - Department of Anesthesia & ICU National Children's Hospital, Tokyo
UR - 2067150
ER -

TY - JOUR
ID - 2133
T1 - The ecological validity of laboratory and analogue assessment methods of ADHD symptoms. [Review] [115 refs]
A1 - Barkley,R.A.
Y1 - 1991/04//
N1 - Barkley, R A
Journal of abnormal child psychology
0364547, h33
IM
Journal Article. Research Support, U.S. Gov't, P.H.S.. Review
English
KW - MEDLINE
KW - Attention Deficit Disorder with Hyperactivity/di [Diagnosis]
KW - Attention Deficit Disorder with Hyperactivity/px [Psychology]
KW - Child
KW - Child Behavior Disorders/di [Diagnosis]
KW - Child Behavior Disorders/px [Psychology]
KW - Humans
KW - Impulsive Behavior/di [Diagnosis]

KW - Impulsive Behavior/px [Psychology]
KW - Motor Activity
KW - Social Environment
RP - NOT IN FILE
SP - 149
EP - 178
JF - Journal of Abnormal Child Psychology
JA - J Abnorm Child Psychol
VL - 19
IS - 2
CY - UNITED STATES
N2 - An important question in the attempt to generalize laboratory findings on attention deficit-hyperactivity disorder (ADHD) children to clinical issues concerning their assessment, diagnosis, outcome, and treatment response is the ecological validity of the commonly used tasks and analogue behavioral observation procedures. This paper examines the concept of ecological validity and issues inherent in its evaluation. The evidence from a variety of sources is then reviewed on the relationship between laboratory methods of assessing inattention, impulsivity, and overactivity and measures of these same constructs in natural settings. Additional findings pertaining to this issue from a recent study of 140 ADHD and normal children and 159 ADHD and normal adolescents using a multimethod battery of tests are also reported. In general, the ecological validity of most methods is of a low to moderate degree, with some traditional laboratory tasks proving unsatisfactory. A few tasks demonstrated acceptable degrees of ecological validity but even these require improvement. It is concluded that future advances in ecological validity are likely to come from: (a) a greater reliance on assessments of the target behaviors in natural settings and (b) combining several of the more promising tasks and analogue methods into a battery that is taken over longer time intervals than has been customary and averaged across repeated administrations. [References: 115]
SN - 0091-0627
AD - Department of Psychiatry, University of Massachusetts Medical Center, Worcester 01655
UR - 2056161
ER -

TY - JOUR
ID - 2134
T1 - Social interaction among deaf preschoolers. The effects of language ability and age
A1 - Lederberg,A.R.
Y1 - 1991/03//
N1 - Lederberg, A R
American annals of the deaf
0414670, 2ba, 3ba
IM
Journal Article
English
KW - MEDLINE
KW - Age Factors
KW - Analysis of Variance
KW - Child
KW - Preschool
KW - Deafness/px [Psychology]
KW - Female
KW - Humans
KW - Interpersonal Relations
KW - Language Development
KW - Male
KW - Play and Playthings
RP - NOT IN FILE
SP - 53
EP - 59
JF - American Annals of the Deaf

JA - Am Ann Deaf
VL - 136
IS - 1
CY - UNITED STATES
N2 - This study examined the relationship between deaf preschoolers' language abilities and their play behavior. Twenty-nine deaf children aged three to five years were observed during outdoor free play throughout the school year. Their language abilities varied widely and did not correlate with age. On the basis of two language tests, they were divided into three language ability groups--high, middle, and low. Language ability was found to be related to several aspects of play and social interaction. The children with high language ability were more likely than the other children to play with two partners at the same time (i.e., engage in triadic interaction), to interact with teachers, to prefer to play with children of similar language ability, to use language, and to receive language from their partners. Most of these effects seemed to be due solely to differences between the children with high language ability and those in the other two groups. Children in the low and middle language ability groups behaved similarly. Language ability was not related to any other aspect of peer relations. Thus, the impact of language ability seems limited. These results, in conjunction with past research, suggest that, for the most part, deaf preschoolers' language and social skills develop independently from each other
SN - 0002-726X
AD - Department of Educational Foundations, Georgia State University
UR - 2048452
ER -

TY - JOUR
ID - 2135
T1 - Video analysis of playground injury-risk situations
A1 - Coppens,N.M.
A1 - Gentry,L.K.
Y1 - 1991/04//
N1 - Coppens, N M. Gentry, L K
Research in nursing & health
7806136, raq
IM, N
Journal Article. Research Support, Non-U.S. Gov't
English
KW - MEDLINE
KW - Aggression
KW - Child
KW - Female
KW - Humans
KW - Male
KW - New England/ep [Epidemiology]
KW - Play and Playthings
KW - Risk Factors
KW - Schools
KW - Sex Factors
KW - Videotape Recording
KW - Wounds and Injuries/ep [Epidemiology]
KW - Wounds and Injuries/px [Psychology]
RP - NOT IN FILE
SP - 129
EP - 136
JF - Research in Nursing & Health
JA - Res Nurs Health
VL - 14
IS - 2
CY - UNITED STATES

N2 - An observational study was conducted to examine injury-risk situations as they occurred during playground recess periods. A process analysis approach was used to describe antecedent conditions, response, consequence, and reaction depicted in the video scenes. Results indicate that boys were more involved than girls, playground equipment was frequently used improperly, aggressive behaviors were sometimes a factor, situations occurred more frequently with nonteacher than teacher monitors, and children infrequently took action to stop the situations. Other children were more likely to react to the target child than the monitor. Direction is provided for child and monitor safety promotion programs
SN - 0160-6891
AD - Nursing Department, College of Health Professions, University of Lowell, Massachusetts 01854
UR - 2047534
ER -

TY - JOUR
ID - 2136
T1 - [Developmental problems of children, adolescents and young adults in families with an alcohol problem]. [German]
A1 - Jakob,P.
Y1 - 1991/02//
N1 - Jakob, P
Praxis der Kinderpsychologie und Kinderpsychiatrie
pj6, 0404246
IM
English Abstract. Journal Article
German
KW - MEDLINE
KW - Adolescent
KW - Adult
KW - Alcoholism/px [Psychology]
KW - Child
KW - Child of Impaired Parents/px [Psychology]
KW - Family Therapy/mt [Methods]
KW - Humans
KW - Personality Development
KW - Risk Factors
KW - Social Environment
RP - NOT IN FILE
SP - 49
EP - 55
JF - Praxis der Kinderpsychologie und Kinderpsychiatrie
JA - Prax Kinderpsychol.Kinderpsychiatr.
VL - 40
IS - 2
CY - GERMANY
N2 - The situation of children and adolescents in families with an alcohol problem has been neglected in the mental health field. A role model is therapeutically beneficial, because "role" is a metaphor which implies that change can take place within a short period of time. These roles can be considered generalizations on various functions which present a bind to the alcoholism system for the children. Behavior which conforms to these roles impairs the formation of personal identity, individuation from the family of origin, and the development of stable relationships outside of the nuclear family. Therapy should aim at activating resources, rather than focussing on deficits
SN - 0032-7034
AD - Systemische Praxisgemeinschaft Munchen
UR - 2034589
ER -

TY - JOUR
ID - 2137

T1 - Clinical and epidemiologic characteristics of patients referred for evaluation of possible Kawasaki disease. United States Multicenter Kawasaki Disease Study Group
A1 - Burns,J.C.
A1 - Mason,W.H.
A1 - Glode,M.P.
A1 - Shulman,S.T.
A1 - Melish,M.E.
A1 - Meissner,C.
A1 - Bastian,J.
A1 - Beiser,A.S.
A1 - Meyerson,H.M.
A1 - Newburger,J.W.
Y1 - 1991/05//
N1 - Burns, J C. Mason, W H. Glode, M P. Shulman, S T. Melish, M E. Meissner, C. Bastian, J. Beiser, A S. Meyerson, H M. Newburger, J W
The Journal of pediatrics
jlz, 0375410
AIM, IM
Clinical Trial. Comparative Study. Journal Article. Multicenter Study. Research Support, U.S. Gov't, P.H.S.
English
KW - MEDLINE
KW - Age Factors
KW - Child
KW - Diagnosis
KW - Differential
KW - Humans
KW - Measles/di [Diagnosis]
KW - Mucocutaneous Lymph Node Syndrome/di [Diagnosis]
KW - Mucocutaneous Lymph Node Syndrome/ep [Epidemiology]
KW - Physical Examination
KW - Risk Factors
KW - United States/ep [Epidemiology]
RP - NOT IN FILE
SP - 680
EP - 686
JF - Journal of Pediatrics
JA - J Pediatr
VL - 118
IS - 5
CY - UNITED STATES
N2 - STUDY OBJECTIVES: (1) To determine those diseases that most often mimic Kawasaki disease (KD) in the United States. (2) To examine the physical findings and laboratory studies that influenced experienced clinicians to exclude the diagnosis of KD. (3) To compare epidemiologic features of patients with KD and patients referred for evaluation of possible KD in whom alternative diagnoses were established. DESIGN: Case comparison study. SETTING: Seven pediatric tertiary care centers. PATIENTS: Consecutive sample of 280 patients with KD and 42 comparison patients examined within the first 14 days after onset of fever. MEASUREMENTS AND MAIN RESULTS: (1) Infectious diseases, particularly measles and group A beta-hemolytic streptococcal infection, most closely mimicked KD and accounted for 35 (83%) of 42 patients in the comparison group. (2) The standard diagnostic clinical criteria for KD were fulfilled in 18 (46%) of 39 patients in whom other diagnoses were established. (3) Patients with KD were significantly less likely to have exudative conjunctivitis or pharyngitis, generalized adenopathy, and discrete intraoral lesions, and more likely to have a perineal distribution of their rash. The patients with KD were also more likely to have anemia and elevated erythrocyte sedimentation rate; leukocyte count less than 10 X 10(3)/mm3 and platelet count less than 200 X 10(3)/mm3 were significantly less prevalent in patients with KD. (4) Residence within 200 yards of a body of water was more common among KD patients. CONCLUSIONS: (1) Measles and streptococcal infection should be excluded in patients examined for possible KD. (2) Laboratory studies that may be useful in discriminating patients with KD from those with alternative diagnoses include hemoglobin concentration, erythrocyte

sedimentation rate, and serum alanine aminotransferase activity. (3) Residence near a body of water may be a risk factor for the development of KD
SN - 0022-3476
AD - Center for Molecular Genetics, University of California at San Diego School of Medicine, La Jolla 92093
UR - 2019921
ER -

TY - JOUR
ID - 2138
T1 - Care of the abandoned child
A1 - Raghunath,M.
Y1 - 1991/07//
N1 - Raghunath, M
ICCW news bulletin
101084396
J
Journal Article
English
KW - MEDLINE
KW - Adolescent
KW - Adoption
KW - Age Factors
KW - Asia
KW - Automatic Data Processing
KW - Behavior
KW - Child
KW - Child Abuse
KW - Child Care
KW - Child Rearing
KW - Crime
KW - Demography
KW - Developing Countries
KW - Evaluation Studies as Topic
KW - Foster Home Care
KW - India
KW - Legislation as Topic
KW - Population
KW - Population Characteristics
KW - Records as Topic
KW - Social Problems
RP - NOT IN FILE
SP - 23
EP - 29
JF - ICCW News Bulletin
JA - ICCW News Bull
VL - 39
IS - 3-4
CY - INDIA
UR - 12317285
ER -

TY - JOUR
ID - 2139
T1 - [Clinical study on arterio-venous differences in clinical biochemical assay for infants and children at induction of general anesthesia (Enflurane-N2-O-O2). First report: liver function tests]. [Japanese]
A1 - Ikeda,T.
A1 - Yamada,T.

A1 - Yamada,M.
A1 - Okumura,Y.
A1 - Fukuoka,S.
A1 - Fujimoto,M.
A1 - Konagaya,K.
Y1 - 1990/09//
N1 - Ikeda, T. Yamada, T. Yamada, M. Okumura, Y. Fukuoka, S. Fujimoto, M. Konagaya, K
Aichi Gakuin Daigaku Shigakkai shi
7501066, 31y
D
Comparative Study. English Abstract. Journal Article
Japanese
KW - MEDLINE
KW - Alanine Transaminase/bl [Blood]
KW - Analysis of Variance
KW - Anesthesia
KW - General
KW - Arteries
KW - Aspartate Aminotransferases/bl [Blood]
KW - Bilirubin/bl [Blood]
KW - Blood Chemical Analysis/mt [Methods]
KW - Child
KW - Preschool
KW - Enflurane
KW - Humans
KW - Infant
KW - Liver Function Tests/mt [Methods]
KW - Liver Function Tests
KW - Pseudocholinesterase/bl [Blood]
KW - Veins
RP - NOT IN FILE
SP - 887
EP - 893
JF - Aichi Gakuin Daigaku Shigakkai Shi - Aichi-Gakuin Journal of Dental Science
JA - Aichi Gakuin Daigaku Shigakkai Shi
VL - 28
IS - 3
CY - JAPAN
N2 - The arterial blood is often drawn during general anesthesia to measure many biochemical parameters, and, outside the operative period, those parameters are measured mainly in the venous blood. In this way, the A-V differences (the differences between the data from the arterial blood and those from the venous blood) will be found. However, there are few reports about the A-V difference in infants and children. The purpose of this investigation was to compare the arterial blood data with the venous data at the induction of general anesthesia (Enflurane-N2O-O2). In this report, Plasma total bilirubin concentration (by the Michaelson method), GOT activity, GPT activity (by the Karmen method), gamma-GTP activity (by the gamma-glutamyl CPA substrate method) and pseudocholinesterase activity (by the Shibata-Takahashi method) were measured with both the arterial (from the radial Artery) and the venous samples (from the saphena Magna), in 60 cases of infants and children who had no systemic disease. They were divided into three groups according to age. (Group I: three months old to one year old, Group II: one year old to three years old, and Group III: three years old to six years old.) Each group consisted of 20 patients. The difference and correlation coefficients between arterial and venous measurements were analysed with the paired t-test and correlation analysis Fs = [r2(n-2)/(1-r2)]1/2. Additionally, the data was analysed with a one dimensional analysis for all Groups. In the results, the A-V difference was so small that the conclusion was reached that in these liver function tests there would be no problem in regarding arterial measurements as venous measurements
SN - 0044-6912
AD - Department of Anesthesiology, School of Dentistry, Aichi-Gakuin University, Nagoya, Japan
UR - 2135143

ER -

TY - JOUR
ID - 2140
T1 - Children's frequency of participation in moderate to vigorous physical activities
A1 - Simons-Morton,B.G.
A1 - O'Hara,N.M.
A1 - Parcel,G.S.
A1 - Huang,I.W.
A1 - Baranowski,T.
A1 - Wilson,B.
Y1 - 1990/12//
N1 - Simons-Morton, B G. O'Hara, N M. Parcel, G S. Huang, I W. Baranowski, T. Wilson, B
Research quarterly for exercise and sport
r6y, 8006373
IM
Journal Article. Research Support, U.S. Gov't, P.H.S.
English
KW - MEDLINE
KW - Child
KW - Exercise
KW - Female
KW - Humans
KW - Male
KW - Physical Education and Training
KW - Schools
KW - Sex Factors
RP - NOT IN FILE
SP - 307
EP - 314
JF - Research Quarterly for Exercise & Sport
JA - Res Q Exerc Sport
VL - 61
IS - 4
CY - UNITED STATES
N2 - Third and fourth grade boys (n = 422) and girls (n = 390) in four Texas elementary schools reported their participation in moderate to vigorous physical activities (MVPAs) over a 3-day period. Students were surveyed during class on successive days. On a subsample (n = 44), the agreement between reported and observed physical activities during physical education or recess was 86.3%. Running, walking fast, games and sports, and bicycling accounted for 70% of Total MVPAs. Of Total MVPAs reported, 47.0% for boys and 44.6% for girls were 10 min or longer in duration (LMVPA). The average number of LMVPAs per day was 1.7 for both boys and girls. Students reported significantly more occurrences of LMVPAs out of school than during school. Significant interaction between grade and gender indicated that third grade boys reported more Total MVPAs and LMVPAs than third grade girls, but fourth grade boys reported fewer Total MVPAs and LMVPAs than fourth grade girls reported fewer Total MVPAs and LMVPAs than fourth grade girls. During the 3-day reporting period, 12.3% of boys and 13.3% of girls reported no LMVPAs, and 35.6% of boys and girls reported fewer than one LMVPAs per day. While the majority of children reported obtaining at least some activity daily, a substantial proportion of children in this sample reported fewer than one LMVPA daily, indicating that many children may not be obtaining adequate amounts of physical activity
SN - 0270-1367
AD - School of Public Health, University of Texas Health Science Center, Houston 77225
UR - 2132887
ER -

TY - JOUR
ID - 2141
T1 - [Determining guidelines for metals in children's playgrounds in North Rhine-Westphalia]. [German]

A1 - Viereck,L.
A1 - Kramer,M.
A1 - Eikmann,T.
A1 - Konig,W.
A1 - Bertges,W.D.
A1 - Gableske,R.
A1 - Krieger,T.
A1 - Michels,S.
A1 - Exner,M.
A1 - Weber,H.
Y1 - 1990/01//
N1 - Viereck, L. Kramer, M. Eikmann, T. Konig, W. Bertges, W D. Gableske, R. Krieger, T. Michels, S. Exner, M. Weber, H
Das Offentliche Gesundheitswesen
ofe, 0107170
IM
English Abstract. Guideline. Journal Article
German
KW - MEDLINE
KW - Child
KW - Preschool
KW - Germany
KW - Humans
KW - Metals/to [Toxicity]
KW - Play and Playthings
KW - Quality Control
KW - Soil/st [Standards]
KW - Soil Pollutants/to [Toxicity]
RP - NOT IN FILE
SP - 7
EP - 15
JF - Offentliche Gesundheitswesen
JA - Offentl.Gesundheitswes.
VL - 53
IS - 1
CY - GERMANY
N2 - In 1990, the State of North-Rhine-Westphalia established an ordinance on the quality of playground soil and sand. This ordinance includes guideline values for toxic metals (arsenic, lead, cadmium, chromium) in playground soil not covered by vegetation and quality standards for sand to be applied on playgrounds. Additionally guideline values were set for mercury, nickel and thallium. The guideline values include two categories: guideline value I represents the upper limits (95-percentiles) of the background levels of toxic metals generally found in upper soil layers in the State of North-Rhine-Westphalia. Guideline values II ("action levels") were selected on the basis of toxicological considerations. In cases where concentrations of metals above these guideline values are detected, immediate actions (urgent redevelopment measures) are required. Quality standards for playground sand were established to ensure that only noncontaminated sand is applied for playgrounds
SN - 0029-8573
AD - Hygiene-Institut des Ruhrgebiets, Gelsenkirchen, Aachen
UR - 2150550
ER -

TY - JOUR
ID - 2142
T1 - [Health effects of ozone as component of summer smog]. [German]
A1 - Kappos,A.D.
A1 - Koss,G.
Y1 - 1990/01//

N1 - Kappos, A D. Koss, G
Das Offentliche Gesundheitswesen
ofe, 0107170
IM
English Abstract. Journal Article
German
KW - MEDLINE
KW - Adolescent
KW - Adult
KW - Biotransformation
KW - Child
KW - Humans
KW - Male
KW - Ozone/ae [Adverse Effects]
KW - Ozone/pk [Pharmacokinetics]
KW - Respiration/de [Drug Effects]
KW - Respiratory Function Tests
KW - Smog/ae [Adverse Effects]
KW - Time Factors
RP - NOT IN FILE
SP - 16
EP - 22
JF - Offentliche Gesundheitswesen
JA - Offentl.Gesundheitswes.
VL - 53
IS - 1
CY - GERMANY
N2 - During the sunny summers of 1989 and 1990 in Germany the outdoor ozone levels repeatedly exceeded the value of the air quality guidelines proposed by the World Health Organization. Both in multiple sites within industrial polluted and in supposedly clean outside areas the ozone concentrations exceeded the value of 180 micrograms/m3. The following paper is intended to contribute to an understanding of the health effects of ozone. It compiles data and views on the formation and analysis of ozone, on its distribution as ambient ground level pollutant, on respiratory and common symptoms in man and on the toxicokinetics, responses and pathogenesis in experimental animals. Studies on healthy exercising adult volunteers exposed to 240 micrograms/m3 ozone in purified lab air or in ambient air revealed a significant influence on connections between forced vital capacity, forced expiratory volume, flow rate and peak expiratory flow rate and the pollutant. Exposure to lower ozone levels induced the same effects, but they were of smaller magnitude. Children aged 8-15 years exercising under field conditions showed decrements in lung function even at ozone levels well below 200 micrograms/m3. Restitution of lung function needs periods of several days, although the induction of effects is a matter of hours. The relevance of the transient pulmonary responses is widely unclear. Among patients with chronic obstructive pulmonary disease or asthma, the functional responsiveness to ozone is not greater than among healthy subjects. Interindividual differences in responsiveness occur but are not predictable. Information on chronic effects is rather limited.(ABSTRACT TRUNCATED AT 250 WORDS)
SN - 0029-8573
AD - Abteilung Gesundheit und Umwelt, Gesundheit und Soziales, Hamburg
UR - 2150545
ER -

TY - JOUR
ID - 2143
T1 - Epidemiology of playground equipment injuries resulting in hospitalization
A1 - Chalmers,D.J.
A1 - Langley,J.D.
Y1 - 1990/12//
N1 - Chalmers, D J. Langley, J D
Journal of paediatrics and child health
arp, 9005421

IM
Journal Article. Research Support, Non-U.S. Gov't
English
KW - MEDLINE
KW - Adolescent
KW - Child
KW - Preschool
KW - Female
KW - Hospitalization/sn [Statistics & Numerical Data]
KW - Humans
KW - Infant
KW - Male
KW - New Zealand/ep [Epidemiology]
KW - Play and Playthings
KW - Schools
KW - Wounds and Injuries/ep [Epidemiology]
RP - NOT IN FILE
SP - 329
EP - 334
JF - Journal of Paediatrics & Child Health
JA - J Paediatr Child Health
VL - 26
IS - 6
CY - AUSTRALIA
N2 - Preparation of the New Zealand Playground Standard was seriously hampered by a lack of published information on the incidence, nature, and circumstances of playground equipment-related injuries. The present study was aimed at redressing this lack of information. Examination of all discharges from New Zealand public hospitals for 1984 identified 1125 children less than 15 years of age who had been admitted for the treatment of playground equipment-related injury. This gave an incidence rate of 137/100,000 children per year. The mean age was 7.2 years. The commonest injury was fracture of the upper limb (48%), followed by intracranial injury (26%). One-third occurred at school, climbing apparatus (40%) was most often involved, and the great majority involved falls (93%). With this study it has been possible for the first time in any country to report an incidence rate for all causes of playground equipment-related injuries resulting in hospitalization, based on data for an entire population. The findings support the emphasis placed on falls in the Standard
SN - 1034-4810
AD - Department of Preventive and Social Medicine, University of Otago Medical School, Dunedin, New Zealand
UR - 2073418
ER -

TY - JOUR
ID - 2144
T1 - Unintentional injuries to students at school
A1 - Langley,J.D.
A1 - Chalmers,D.
A1 - Collins,B.
Y1 - 1990/12//
N1 - Langley, J D. Chalmers, D. Collins, B
Journal of paediatrics and child health
arp, 9005421
IM
Journal Article. Research Support, Non-U.S. Gov't
English
KW - MEDLINE
KW - Adolescent
KW - Child
KW - Preschool

KW - Female
KW - Hospitalization/sn [Statistics & Numerical Data]
KW - Humans
KW - Male
KW - New Zealand/ep [Epidemiology]
KW - Schools
KW - Wounds and Injuries/ep [Epidemiology]
KW - Wounds and Injuries/mo [Mortality]
KW - Wounds and Injuries/pc [Prevention & Control]
RP - NOT IN FILE
SP - 323
EP - 328
JF - Journal of Paediatrics & Child Health
JA - J Paediatr Child Health
VL - 26
IS - 6
CY - AUSTRALIA
N2 - Unintentional injuries at school have been identified as a significant public health problem. A major barrier to the development of injury prevention policy has been the absence of national data on the circumstances of injuries to students which occur at school. This study sought to determine the incidence, nature, and circumstances of injuries which resulted in death or hospitalization. Fatalities were identified from national mortality data for 1977-86 inclusive. Coroners' files were then examined to obtain details of the circumstances of injury. Hospitalization cases were identified from the national hospital discharge summary for 1986. Fifteen fatalities were identified. The circumstances of the deaths were diverse with the most frequent event being a fall (n = 4). A total of 1013 first admissions to hospital were identified, giving an overall incidence rate of 152/100,000 students/year. Injury rates declined with increasing age, and males had higher rates than females for all ages. Fractures of the upper and lower limbs and intracranial injury represented more than three-quarters of all injury. The two leading causes of injury, falls, and incidents involving striking against or being struck by a person or object, represented 89% of all incidents. The use of playground equipment and involvement in sporting activity were two of the more common aspects of many injury events. The results suggest that prevention policy should place emphasis on those in their first 2 years of schooling, falls from playground equipment, provision of protective equipment for sporting activities, sporting activities designed to minimize physical contact, establishment of standardized injury referral procedures, first-aid training, and a standardized injury reporting system
SN - 1034-4810
AD - Department of Preventive and Social Medicine, University of Otago, Dunedin, New Zealand
UR - 2073417
ER -

TY - JOUR
ID - 2145
T1 - Classroom and playground interaction of students with and without disabilities
A1 - Roberts,C.
A1 - Pratt,C.
A1 - Leach,D.
Y1 - 1990/12/19/
N1 - Roberts, C. Pratt, C. Leach, D
Exceptional children
enx, 0375042
IM
Journal Article. Research Support, Non-U.S. Gov't
English
KW - MEDLINE
KW - Adolescent
KW - Child
KW - Disabled Persons/ed [Education]
KW - Disabled Persons/px [Psychology]

KW - Female
KW - Humans
KW - Intellectual Disability/px [Psychology]
KW - Interpersonal Relations
KW - Learning Disorders/px [Psychology]
KW - Mainstreaming (Education)
KW - Male
KW - Play and Playthings
KW - Social Behavior
RP - NOT IN FILE
SP - 212
EP - 224
JF - Exceptional Children
JA - Except.Child
VL - 57
IS - 3
CY - UNITED STATES
N2 - The classroom and playground behaviors of students with disabilities, in an integrated classroom, are frequently cited as reasons for rejection of these students by their regular-class peers. We compared the classroom and playground behavior of 95 students with mild disabilities with that of 95 students without disabilities, age and sex matched, and enrolled in the same classes. The children, age 8-13 years, attended state government elementary schools. Behaviors were observed using a time-sampling method with nine categories of behavior in each setting. The behavior of both groups of students was in many ways similar, regardless of the presence of disabilities
SN - 0014-4029
UR - 2149552
ER -

TY - JOUR
ID - 2146
T1 - Value of health, incidence of depression, and level of self-esteem in low-income mothers of pre-school children
A1 - Burns,E.I.
A1 - Doremus,P.C.
A1 - Potter,M.B.
Y1 - 1990/04//
N1 - Burns, E I. Doremus, P C. Potter, M B
Issues in comprehensive pediatric nursing
g94, 7702326
N
Journal Article
English
KW - MEDLINE
KW - Adolescent
KW - Adult
KW - Attitude to Health
KW - Child
KW - Preschool
KW - Depressive Disorder/ep [Epidemiology]
KW - Depressive Disorder/nu [Nursing]
KW - Depressive Disorder/px [Psychology]
KW - Female
KW - Health Promotion
KW - Humans
KW - Life Style
KW - Mothers/px [Psychology]
KW - Poverty

KW - Questionnaires
KW - Self Concept
RP - NOT IN FILE
SP - 141
EP - 153
JF - Issues in Comprehensive Pediatric Nursing
JA - Issues Compr Pediatr Nurs
VL - 13
IS - 2
CY - UNITED STATES
N2 - Nurses are continually promoting health and healthy lifestyles. This contribution requires that nurses understand client values and priorities. Traditionally, women have had responsibility for their own and their families' health. Nurses must recognize and understand the complex influences shaping the value women place on health. The literature indicates that women want to participate actively in their own health care by assuming more responsibility for their health and well-being. This research evolved as a result of nursing student and faculty interactions with mothers of pre-school children enrolled in a Headstart program. During these experiences, observations related to the needs of the mothers included poor health practices (smoking, being overweight), lack of motivation, statements of feeling depressed, difficulty making decisions, feelings of being overwhelmed by parenting demands, powerlessness, and disenfranchisement. This descriptive, correlational study was designed to determine the value of health, incidence of depression, and characteristics of self-esteem among low-income mothers of pre-school children. This sample of convenience was comprised of 133 low-income mothers who responded to a mailed survey. Three instruments were used in the study: the Wallston and Wallston Health Values Scale, the Center for Epidemiologic Studies Depression Scale (CES-D), and the Rosenberg Self-Esteem Scale. Data were analyzed by collection of frequency of response, which was then converted to a percentage. To determine if differences existed in respect to demographic variables, ANOVA (self-esteem and depression) and Chi Square (health) measures were used. Participation in activities outside the home accounted for a significant difference in self-esteem. Mothers involved in activities reported higher self-esteem. The findings suggest that participation in activities may increase self-esteem and lower depression. Over three-quarters (85%) of mothers placed a high value on health. Based on their high valuing of health and the potential for increasing self-esteem through activity, it can be concluded that the women in this sample will benefit from planned health activities. It is also anticipated that, because of the strong relationship between self-esteem and depression, women who participate in these activities will demonstrate lower levels of depression
SN - 0146-0862
UR - 2272853
ER -

TY - JOUR
ID - 2147
T1 - The VER as a diagnostic marker for childhood abdominal migraine
A1 - Mortimer,M.J.
A1 - Good,P.A.
Y1 - 1990/10//
N1 - Mortimer, M J. Good, P A
Headache
2985091r, g1n
IM
Comparative Study. Journal Article
English
KW - MEDLINE
KW - Abdominal Pain/et [Etiology]
KW - Child
KW - Preschool
KW - Evoked Potentials
KW - Visual
KW - Female
KW - Humans
KW - Male

KW - Migraine Disorders/co [Complications]
KW - Migraine Disorders/di [Diagnosis]
KW - Migraine Disorders/pp [Physiopathology]
KW - Nausea/et [Etiology]
KW - Vomiting/et [Etiology]
RP - NOT IN FILE
SP - 642
EP - 645
JF - Headache
VL - 30
IS - 10
CY - UNITED STATES
N2 - Abdominal migraine is a common childhood migraine equivalent, for which diagnostic criteria have not been defined. As in other children with migraine equivalents this leads to difficulties in diagnosis and determination of prevalence. By recording the fast wave activity (beta rhythmn) in the visual evoked response (VER) to red and white flash, the pattern stimulation, 27 out of 28 children with clinically diagnosed abdominal migraine revealed significant differences compared with normal controls, outside the attack phase. Comparisons with children diagnosed as migraine with or without aura revealed, from the VER findings of higher amplitude fast wave activity and the presence of paroxysmal sharp wave activity, that abdominal migraine appears to be a specific form of childhood migraine. We found that both clinically and electrophysiologically, abdominal migraine changes with age; older children exhibiting a shorter duration of abdominal pain during attacks, and less evidence of sharp wave activity in the VER
SN - 0017-8748
AD - Birmingham and Midland Eye Hospital, UK
UR - 2272813
ER -

TY - JOUR
ID - 2148
T1 - Normal energy intake range in children with chronic nonspecific diarrhea: association of relapses with the higher level
A1 - Ciampolini,M.
A1 - Vicarelli,D.
A1 - Seminara,S.
Y1 - 1990/10//
N1 - Ciampolini, M. Vicarelli, D. Seminara, S
Journal of pediatric gastroenterology and nutrition
jl6, 8211545
IM
Clinical Trial. Journal Article. Randomized Controlled Trial. Research Support, Non-U.S. Gov't
English
KW - MEDLINE
KW - Child
KW - Preschool
KW - Chronic Disease
KW - Diarrhea/dh [Diet Therapy]
KW - Diet
KW - Energy Intake
KW - Energy Metabolism
KW - Female
KW - Growth
KW - Humans
KW - Infant
KW - Male
KW - Recurrence
RP - NOT IN FILE
SP - 342

EP - 350
JF - Journal of Pediatric Gastroenterology & Nutrition
JA - J Pediatr Gastroenterol Nutr
VL - 11
IS - 3
CY - UNITED STATES
N2 - An increase in energy intake often occurs at weaning. The increase may be due partly to prompting by the caregiver to accelerate the child's weight gain and partly motivated by the palatability of common weaning foods. Increased food intakes initiated during weaning and continued into the second year of life may be associated with chronic, nonspecific diarrhea in selected children. An educational project was designed to reduce intakes augmented by either cause. Reductions were achieved by the regulation of energy-dense foods in the child's diet and reliance on the child's appetite control to determine meal size. The educational intervention was applied prospectively under nonblinded, controlled conditions. Children, 1 to 2 years of age, with chronic nonspecific diarrhea were assigned randomly to either a treatment or control group. Compliance, food consumption, preprandial glycemia, and outdoor activities were reported by the children's mothers in four 7-day diaries; symptoms related to the children's clinical condition and anthropometric and biochemical indices of nutritional status were noted at the beginning and end of a 7-month period. Forty-four of 53 children in the experimental group maintained compliance, and 44 of 47 children in the control group completed the follow-up. Energy intake decreased significantly by almost one-third in the experimental group. Growth, skinfold thickness measurements, and outdoor activities were similar between experimental and control groups over the 7-month period. Diarrheal episodes occurred in 6, 1, and 2 children in the experimental group at 1.5, 3, and 7 months and in 22, 18, and 15 children in the control group, respectively (p less than 0.002).(ABSTRACT TRUNCATED AT 250 WORDS)
SN - 0277-2116
AD - Dipartimento di Pediatria, Universita di Firenze, Florence, Italy
UR - 2246716
ER -

TY - JOUR
ID - 2149
T1 - [Comparative studies of Toxocara infections in Belgium and in other countries]. [French]
A1 - Cauchie,V.
A1 - Chaillet,P.
A1 - Bigaignon,G.
A1 - Tomasi,J.P.
A1 - Vervoort,T.
Y1 - 1990///
N1 - Cauchie, V. Chaillet, P. Bigaignon, G. Tomasi, J P. Vervoort, T
Acta clinica Belgica
0370306
IM
Comparative Study. English Abstract. Journal Article
French
KW - MEDLINE
KW - Adolescent
KW - Animals
KW - Antibodies
KW - Helminth/ip [Isolation & Purification]
KW - Belgium
KW - Child
KW - Preschool
KW - Dog Diseases/ps [Parasitology]
KW - Dogs
KW - Environment
KW - Enzyme-Linked Immunosorbent Assay
KW - Humans
KW - Infant

KW - Parasite Egg Count
KW - Sensitivity and Specificity
KW - Toxocariasis/im [Immunology]
KW - Toxocariasis/tm [Transmission]
KW - Toxocariasis/ve [Veterinary]
RP - NOT IN FILE
SP - 227
EP - 239
JF - Acta Clinica Belgica
JA - Acta Clin Belg
VL - 45
IS - 4
CY - BELGIUM
N2 - An enzyme-linked immunosorbent assay with larval stage antigen was used to measure the concentration of antibodies to Toxocara sp in 2206 human sera. The results led to the determination of the specificity of our method, the experimental cutoff estimation and the Toxocara sp seroprevalence in different human populations. The prevalence of canine toxocariasis in pet dogs and the associated environmental contamination were studied through examination of 107 faecal and 20 sand-pits samples for Toxocara sp eggs. Our immunological studies and environmental research revealed the existence of toxocariasis as a zoonotic disease in Belgium. We compare here our results with those found in the world literature: in spite of some surprising results, the environmental factors are important for toxocaral infection. The urban residence, the restricted number of playgrounds for children and areas for dogs, the socioecological status are preponderant in the spread of infection
SN - 0001-5512
AD - Laboratoire de Serologie, Cliniques Universitaires Saint-Luc, Bruxelles
UR - 2173315
ER -

TY - JOUR
ID - 2150
T1 - Methylphenidate in aggressive-hyperactive boys: I. Effects on peer aggression in public school settings
A1 - Gadow,K.D.
A1 - Nolan,E.E.
A1 - Sverd,J.
A1 - Sprafkin,J.
A1 - Paolicelli,L.
Y1 - 1990/09//
N1 - Gadow, K D. Nolan, E E. Sverd, J. Sprafkin, J. Paolicelli, L
Journal of the American Academy of Child and Adolescent Psychiatry
hg5, 8704565
IM
Clinical Trial. Controlled Clinical Trial. Journal Article
English
KW - MEDLINE
KW - Aggression/de [Drug Effects]
KW - Attention Deficit Disorder with Hyperactivity/dt [Drug Therapy]
KW - Child
KW - Preschool
KW - Dose-Response Relationship
KW - Drug
KW - Double-Blind Method
KW - Humans
KW - Male
KW - Methylphenidate/ad [Administration & Dosage]
KW - Peer Group
KW - Social Environment
RP - NOT IN FILE

SP - 710
EP - 718
JF - Journal of the American Academy of Child & Adolescent Psychiatry
JA - J Am Acad Child Adolesc Psychiatry
VL - 29
IS - 5
CY - UNITED STATES
N2 - One of the least documented "known" effects of methylphenidate in hyperactive children is the suppression of peer aggression. In this study, 11 aggressive-hyperactive children received a low (0.3 mg/kg) and moderate (0.6 mg/kg) dose of methylphenidate and placebo for 2 weeks each under double-blind conditions. Children were observed in public school settings during classroom seatwork activities, lunch, and recess. Results showed that methylphenidate suppressed nonphysical aggression (p = 0.06) in the classroom, and a moderate dose decreased physical aggression (p less than 0.01) and verbal aggression (p = 0.07) on the playground. The effect on the rate of appropriate social interaction was variable. The majority of subjects exhibited either the same or higher levels of appropriate social interaction on the 0.6 mg/kg dose compared with placebo. In the classroom, both doses of methylphenidate also resulted in reduced levels of motor movement, off-task behavior, noncompliance, and disruptiveness. Teacher ratings of hyperactivity and conduct problem symptoms revealed drug effects, whereas parallel parent instruments did not
SN - 0890-8567
AD - Department of Psychiatry and Behavioral Science, State University of New York, Stony Brook, NY 11794-8790
UR - 2228923
ER -

TY - JOUR
ID - 2151
T1 - [Physical exercise among school children. A nation-wide sociomedical study of 1,671 children 11-15 years of age]. [Danish]
A1 - Holstein,B.E.
A1 - Ito,H.
A1 - Due,P.
Y1 - 1990/09/17/
N1 - Holstein, B E. Ito, H. Due, P
Ugeskrift for laeger
0141730, wm8
IM
English Abstract. Journal Article
Danish
KW - MEDLINE
KW - Adolescent
KW - Child
KW - Denmark
KW - Exercise
KW - Female
KW - Health Behavior
KW - Health Promotion
KW - Humans
KW - Leisure Activities
KW - Male
KW - Socioeconomic Factors
RP - NOT IN FILE
SP - 2721
EP - 2727
JF - Ugeskrift for Laeger
JA - Ugeskr.Laeger
VL - 152
IS - 38

CY - DENMARK
N2 - A total of 1,671 children aged 11, 13 and 15 years from a nationwide random sample of schools completed an anonymous questionnaire on their social backgrounds, social networks, health, life satisfaction, and health behaviour. The proportion practising outdoor activities at least three times a week was 57%; 37% did some physical exercise in their leisure time sufficient to make them breathless or sweat at least four times a week; 43% at least four hours a week. Two thirds were active members of a sports club. A minority of 8% did not carry out any physical exercise during their leisure time sufficient to make them breathless or sweat. Boys did more physical exercise than girls irrespective of the measurement of exercise. The most important motives for physical exercise were to improve health, to get in good shape, to have fun, and to make new friends. More than 90% of the children intended to do physical exercise at the age of 20. High intensity of physical activity was associated with high social class, strong social integration in the peer group, easy contact with parents and friends, physical exercise among parents and best friends, high degree of life satisfaction, and good health
SN - 0041-5782
AD - Institut for Social Medicin, Kobenhavns Universitet
UR - 2219503
ER -

TY - JOUR
ID - 2152
T1 - Antibody to hepatitis C virus in multiply transfused patients with thalassaemia major
A1 - Wonke,B.
A1 - Hoffbrand,A.V.
A1 - Brown,D.
A1 - Dusheiko,G.
Y1 - 1990/08//
N1 - Wonke, B. Hoffbrand, A V. Brown, D. Dusheiko, G
Journal of clinical pathology
ht3, 0376601
AIM, IM
Journal Article
English
KW - MEDLINE
KW - Adolescent
KW - Adult
KW - Aspartate Aminotransferases/bl [Blood]
KW - Blood Transfusion
KW - Child
KW - Preschool
KW - Female
KW - Ferritins/bl [Blood]
KW - Hepatitis Antibodies/an [Analysis]
KW - Hepatitis C/im [Immunology]
KW - Hepatitis
KW - Chronic/pa [Pathology]
KW - Viral
KW - Human/im [Immunology]
KW - Humans
KW - Infant
KW - Liver/pa [Pathology]
KW - Male
KW - Thalassemia/im [Immunology]
KW - Thalassemia/pa [Pathology]
KW - Thalassemia/th [Therapy]
RP - NOT IN FILE
SP - 638
EP - 640
JF - Journal of Clinical Pathology

JA - J Clin Pathol
VL - 43
IS - 8
CY - ENGLAND
N2 - Seventeen of 73 (23.3%) multiply transfused patients with thalassaemia major (age range, 1-39 years) tested positive for antibody to hepatitis C virus (anti-HCV). Eleven of the 24 patients regularly transfused in countries outside Britain were anti-HCV seropositive; only six of the 49 regularly transfused in Britain were seropositive. The incidence of anti-HBs and anti-HBc was similar to that of anti-HCV in both the British and foreign patients. The anti-HCV seropositive patients showed significantly higher plasma aspartate aminotransferase activities (AST), mean (SD) 10.2 (70.3) U/l, and serum ferritin concentrations, 4067 (2708) micrograms/l, than the anti-HCV seronegative patients (AST, 33.9 (15.6) U/l; serum ferritin 2051 (2092) U/l), respectively. Among the 36 patients who had earlier undergone liver biopsy 10 of 21 with histological features of chronic active hepatitis or cirrhosis, or both, were seropositive for anti-HCV whereas only one of 15 without histological evidence of chronic viral hepatitis was seropositive for anti-HCV. It is concluded that HCV is a major cause of chronic hepatitis in patients with thalassaemia major and is associated with raised AST activity and serum ferritin concentration compared with patients seronegative for anti-HCV
SN - 0021-9746
AD - Department of Haematology, Royal Free Hospital, London
UR - 2119395
ER -

TY - JOUR
ID - 2153
T1 - Ultraviolet exposure to children from sunlight: a study using personal dosimetry
A1 - Rosenthal,F.S.
A1 - Lew,R.A.
A1 - Rouleau,L.J.
A1 - Thomson,M.
Y1 - 1990/04//
N1 - Rosenthal, F S. Lew, R A. Rouleau, L J. Thomson, M
Photodermatology, photoimmunology & photomedicine
awp, 9013641
IM
Journal Article
English
KW - MEDLINE
KW - Cheek
KW - Child
KW - Environmental Monitoring
KW - Film Dosimetry
KW - Humans
KW - Massachusetts
KW - Risk Factors
KW - Seasons
KW - Skin/re [Radiation Effects]
KW - Skin Neoplasms/et [Etiology]
KW - Sunlight/ae [Adverse Effects]
KW - Ultraviolet Rays/ae [Adverse Effects]
KW - Wrist
RP - NOT IN FILE
SP - 77
EP - 81
JF - Photodermatology, Photoimmunology & Photomedicine
JA - Photodermatol.Photoimmunol.Photomed.
VL - 7
IS - 2
CY - DENMARK

N2 - Childhood exposure to ultraviolet (UV) radiation in sunlight may be an important risk factor for later development of skin diseases. We quantified the UVB radiant exposure on the wrist and face of children performing mixed outdoor activities at a summer camp. Polysulfone film badges were attached directly to the skin for several hours and simultaneously were used to measure ambient UVB levels. The results for the ratio of site-specific exposure to ambient exposure indicate a high degree of intersubject variability, a parity of samples taken in right and left positions, and a significantly higher exposure on the wrist compared with the face. The technique was well accepted by the subjects but some badges could not be read because of loss or damage. Measurement of exposure ratios in additional settings as well as data on childhood activity patterns are needed for an overall assessment of childhood exposure
SN - 0905-4383
AD - Department of Family and Community Medicine, University of Massachusetts Medical School, Worcester
UR - 2400678
ER -

TY - JOUR
ID - 2154
T1 - Airways and air pollution in childhood: state of the art. [Review] [60 refs]
A1 - Hoppenbrouwers,T.
Y1 - 1990///
N1 - Hoppenbrouwers, T
Lung
la2, 7701875
IM
Journal Article. Research Support, Non-U.S. Gov't. Review
English
KW - MEDLINE
KW - Air Pollutants/ae [Adverse Effects]
KW - Asthma/et [Etiology]
KW - Child
KW - Preschool
KW - Female
KW - Humans
KW - Infant
KW - Newborn
KW - Lung Diseases
KW - Obstructive/et [Etiology]
KW - Pregnancy
KW - Respiratory Tract Infections/et [Etiology]
KW - Risk Factors
KW - Sudden Infant Death/et [Etiology]
KW - Tobacco Smoke Pollution/ae [Adverse Effects]
RP - NOT IN FILE
SP - 335
EP - 346
JF - Lung
VL - 168 Suppl
CY - UNITED STATES
N2 - Correlations between ambient pollutants and health effects, such as observed in SIDS, may in reality be to a larger extent the result of indoor sources. These distinctions between indoor and outdoor sources, while important, do not detract from the overall conclusion that pollution affects the airway in children directly and indirectly. Much still needs to be learned about the permanence of these effects, the mechanism by which the effect is mediated, and the conditions under which some of these effects are maximal. Two approaches seem particularly suited to shed further light on these issues. First, identification of biological markers for exposure to pollutants will yield both more accurate measures of exposure to pollutants and information about health consequences. Second, newer modeling techniques promise to predict health outcomes under a variety of environmental conditions. Shumway et al., for instance, describe a promising model predicting an increase in

mortality due to ambient pollutants in the Los Angeles Basin with higher levels under extremes of temperature, especially during cold spells. Time series and factor analyses may further our knowledge as well. In the near future, large cohort studies should begin to reveal the cumulative effects of air pollution on the respiratory system, especially in relation to active smoking. Finally, studies in Black children are virtually unavailable. Given their high risk for respiratory illnesses, such studies are sorely needed. [References: 60]
SN - 0341-2040
AD - LAC/USC Medical Center, Department of Pediatrics
UR - 2117134
ER -

TY - JOUR
ID - 2155
T1 - Quantification of airborne moulds in the homes of children with and without wheeze
A1 - Strachan,D.P.
A1 - Flannigan,B.
A1 - McCabe,E.M.
A1 - McGarry,F.
Y1 - 1990/05//
N1 - Strachan, D P. Flannigan, B. McCabe, E M. McGarry, F
Thorax
vqw, 0417353
IM
Journal Article. Research Support, Non-U.S. Gov't
English
KW - MEDLINE
KW - Bronchi/pp [Physiopathology]
KW - Child
KW - Environmental Exposure
KW - Housing
KW - Humans
KW - Respiratory Hypersensitivity/et [Etiology]
KW - Respiratory Hypersensitivity/pp [Physiopathology]
KW - Respiratory Sounds/pp [Physiopathology]
KW - Respiratory Sounds
KW - Spores
KW - Fungal
RP - NOT IN FILE
SP - 382
EP - 387
JF - Thorax
VL - 45
IS - 5
CY - ENGLAND
N2 - A population survey of 1000 7 year old children found a significant excess of wheeze among children whose homes were reported to be mouldy (odds ratio 3.70, 95% confidence limits 2.22, 6.15). The airborne mould flora was quantified by repeated volumetric sampling during the winter in three rooms of the homes of 88 children. All of these had previously completed spirometric tests before and after a six minute free running exercise challenge. Total airborne mould counts varied from 0 to 41,000 colony forming units (CFU)/m3, but were generally in the range 50-1500 CFU/m3, much lower than the concentrations found outdoors in summer. The principal types of fungi identified are all known to be common out of doors, and most were found on at least one occasion in most of the homes. Median and geometric mean total mould counts were not related to reports of visible mould in the home, or to a history of wheeze in the index child. The heterogeneous group of non-sporing fungi (mycelia sterilia) were the only airborne fungi present at significantly higher concentrations in the homes of wheezy children (geometric mean 2.1 v 0.7 CFU/m3. A non-significant increase in total mould counts was observed in the homes of children with a 10% or greater decline in FEV1 after exercise (geometric mean 354 v 253 CFU/m3). Questionnaire reports of mould in the home may be a poor indicator of exposure to airborne spores. The total burden of inhaled mould spores from indoor sources is probably not an important

determinant of wheeze among children in the general population. Although the association with mycelia sterilia could be a chance finding, these non-sporing isolates may include a potent source of allergen
SN - 0040-6376
AD - Department of Epidemiology and Population Sciences, London School of Hygiene and Tropical Medicine
UR - 2382244
ER -

TY - JOUR
ID - 2156
T1 - Effects of obesity, social interactions, and physical environment on physical activity in preschoolers
A1 - Klesges,R.C.
A1 - Eck,L.H.
A1 - Hanson,C.L.
A1 - Haddock,C.K.
A1 - Klesges,L.M.
Y1 - 1990///
N1 - Klesges, R C. Eck, L H. Hanson, C L. Haddock, C K. Klesges, L M
Health psychology : official journal of the Division of Health Psychology, American Psychological Association
ejl, 8211523
IM
Journal Article. Research Support, Non-U.S. Gov't. Research Support, U.S. Gov't, P.H.S.
English
KW - MEDLINE
KW - Child
KW - Preschool
KW - Female
KW - Humans
KW - Male
KW - Motor Activity
KW - Obesity/ge [Genetics]
KW - Obesity/px [Psychology]
KW - Parent-Child Relations
KW - Play and Playthings
KW - Risk Factors
KW - Sex Factors
KW - Social Environment
RP - NOT IN FILE
SP - 435
EP - 449
JF - Health Psychology
JA - Health Psychol
VL - 9
IS - 4
CY - UNITED STATES
N2 - Examined demographic, environmental, and parent-child interactional correlates of physical activity in a group of 222 preschoolers. Activity levels were assessed with a system that quantified directly observed physical activity in the natural environment. Using regression-modeling procedures, results revealed a significant relationship between (a) child's relative weight, parental weight status, and percentage of time spent outdoors (environment) and (b) children's activity levels. Parental obesity was associated with lower levels of physical activity in children, childhood relative weight was associated with slightly higher levels of physical activity, and more outdoor activity was associated with higher activity levels. Parental participation in children's activities also significantly interacted with levels of parental obesity in predicting activity levels. Those children with a 50% risk for obesity (as defined by both, one, or neither parent being overweight) had small changes in activity across levels of parent-child interaction, whereas those at higher risk for obesity responded with increased activity as parent-child interactions increased. Results are discussed, and the implications of these findings for future intervention efforts are examined
SN - 0278-6133

AD - Department of Psychology, Memphis State University, TN 38152
UR - 2373068
ER -

TY - JOUR
ID - 2157
T1 - Playground hazards in Atlanta child care centers
A1 - Sacks,J.J.
A1 - Holt,K.W.
A1 - Holmgreen,P.
A1 - Colwell,L.S.,Jr.
A1 - Brown,J.M.,Jr.
Y1 - 1990/08//
N1 - Sacks, J J. Holt, K W. Holmgreen, P. Colwell, L S Jr. Brown, J M Jr
American journal of public health
1254074, 3xw
AIM, IM
Journal Article
English
KW - MEDLINE
KW - Child Day Care Centers/st [Standards]
KW - Child
KW - Preschool
KW - Equipment Safety
KW - Georgia/ep [Epidemiology]
KW - Humans
KW - Play and Playthings/st [Standards]
KW - Wounds and Injuries/ep [Epidemiology]
KW - Wounds and Injuries/et [Etiology]
RP - NOT IN FILE
SP - 986
EP - 988
JF - American Journal of Public Health
JA - Am J Public Health
VL - 80
IS - 8
CY - UNITED STATES
N2 - We identified 684 playground hazards in 66 child care centers despite regulations mandating that the grounds be hazard-free. Of 21 centers with less than or equal to 5 hazards, 42.9 percent reported a playground-related injury in the previous year; of 25 centers with 6-11 hazards, 52.0 percent reported a playground-related injury; and of 20 centers with greater than or equal to 12 hazards, 60.0 percent reported a playground-related injury. Climbing equipment greater than or equal to 6 feet tall generally had inadequate impact-absorbing undersurfacing and had over twice the rate of fall injuries as climbing equipment less than 6 feet
SN - 0090-0036
AD - Division of Injury Epidemiology and Control, CDC, Atlanta, GA 30333
UR - 2368864
ER -

TY - JOUR
ID - 2158
T1 - [General environmental pollutants and passive smoking]. [Review] [74 refs] [German]
A1 - Meister,R.
Y1 - 1990/02//
N1 - Meister, R
Pneumologie (Stuttgart, Germany)
pne, 8906641
IM

English Abstract. Journal Article. Review
German
KW - MEDLINE
KW - Adult
KW - Air Pollutants/ae [Adverse Effects]
KW - Child
KW - Humans
KW - Respiratory Tract Diseases/et [Etiology]
KW - Risk Factors
KW - Smog/ae [Adverse Effects]
KW - Tobacco Smoke Pollution/ae [Adverse Effects]
RP - NOT IN FILE
SP - 378
EP - 386
JF - Pneumologie
VL - 44 Suppl 1
CY - GERMANY, WEST
N2 - Epidemiological studies-especially data from smog episodes-indicate that antropogenous outdoor air pollution exercises a deleterious effect on health and particularly on the respiratory organs. Controlled exposure test in animals and man confirm this. The main pollutants are SO2, suspended dust particles (dust aerosols or solid atmospheric condensation nuclei) as well as NO2 (NOx) and O3. The adverse influence of quite a number of meteorological factors such as low temperature and inversion cannot be denied. During smog conditions in January 1985 in the Federal Republic of Germany there was a highly significant negative correlation between atmospheric temperature and the rate of exacerbations of bronchitis. Indoor air pollution is gaining in importance. Airtight sealing of buildings associated with reduced indoor ventilation results in novel health upsets ("sick building syndrome"). Interiors are characterised by an accumulation of CO2, CO, NO2, dust aerosols and various organic substances such as benzene, benzypyrene, formaldehyde, nitrosamines etc. Cigarette smoke is a frequent cause of indoor air pollution. The possible unhealthy effects of passive smoking (mainly the inhalation of sidestream smoke) have been frequently studied. Infants of smoking parents are more often affected by respiratory diseases than non-exposed children. The same applies to schoolchildren: the incidence of bronchial signs and symptoms increases with increasing smoke consumptions of the parents. However, no definitely established effect on lung function has been seen in children, adults and asthmatics. The important question as to whether passive smoking increases lung cancer risk is still a subject of controversial discussion among experts. [References: 74]
SN - 0934-8387
AD - Marienkrankenhaus, Klinik fur Erkrankungen der Atmungsorgane, Asthma und Allergie, Bad Lippspringe
UR - 2195521
ER -

TY - JOUR
ID - 2159
T1 - [Aeromonas species as cause of diarrhea and infections outside the gastrointestinal tract in The Netherlands]. [Dutch]
A1 - Havelaar,A.H.
A1 - Guinee,P.A.
A1 - During,M.
A1 - Peeters,M.F.
Y1 - 1990/05/26/
N1 - Havelaar, A H. Guinee, P A. During, M. Peeters, M F
Nederlands tijdschrift voor geneeskunde
nuk, 0400770
IM
English Abstract. Journal Article. Research Support, Non-U.S. Gov't
Dutch
KW - MEDLINE
KW - Adolescent
KW - Adult

KW - Aeromonas/cl [Classification]
KW - Aeromonas/ip [Isolation & Purification]
KW - Aeromonas/py [Pathogenicity]
KW - Age Factors
KW - Aged
KW - Child
KW - Preschool
KW - Diarrhea/mi [Microbiology]
KW - Feces/mi [Microbiology]
KW - Humans
KW - Middle Aged
KW - Netherlands
KW - Seasons
RP - NOT IN FILE
SP - 1053
EP - 1057
JF - Nederlands Tijdschrift voor Geneeskunde
JA - Ned Tijdschr Geneeskd
VL - 134
IS - 21
CY - NETHERLANDS
N2 - In a national survey in the period May 1986-December 1987, Aeromonas was isolated from 277 out of 16,857 (1.6%) samples of watery, bloody or mucous stool, from patients with diarrhoea. There was a clear seasonal pattern (less than 1% in winter, up to 3% in summer). A. caviae was isolated most frequently (49%), followed by A. sobria (35%) and A. hydrophila (15%). Some non-identifiable strains were isolated as well. Aeromonas were isolated in particular from faeces of patients aged over 70 years (predominantly A. sobria) or under 5 years (predominantly A. caviae). In 67% Aeromonas was isolated as the only possible bacterial cause of diarrhoea, but in 33% other enteropathogenic bacteria were found as well (17% Campylobacter, 14% Salmonella, 2% Shigella). In addition, all Aeromonas isolates were collected which were obtained in normal diagnostic activities in the participating laboratories, among others from blood, from pus or wound fluid, from faecal samples which did not meet the above mentioned criteria. A. caviae was the dominant species in 'other' faeces and 'various' body sites but was not isolated from blood. The results of this study do not indicate that routine examinations for Aeromonas in faeces of patients with diarrhoea are necessary
SN - 0028-2162
AD - Rijksinstituut voor Volksgezondheid en Milieuhygiene, Bilthoven
UR - 2366898
ER -

TY - JOUR
ID - 2160
T1 - Anaerobic threshold alterations caused by interval training in 11-year-olds
A1 - Hoffor,A.S.
A1 - Harrison,A.C.
A1 - Kirk,P.A.
Y1 - 1990/03//
N1 - Hoffor, A S. Harrison, A C. Kirk, P A
The Journal of sports medicine and physical fitness
0376337, k6v
IM
Journal Article
English
KW - MEDLINE
KW - Anaerobic Threshold/ph [Physiology]
KW - Child
KW - Exercise Test
KW - Heart Rate
KW - Humans

KW - Male
KW - Oxygen Consumption
KW - Physical Education and Training
KW - Physical Endurance/ph [Physiology]
RP - NOT IN FILE
SP - 53
EP - 56
JF - Journal of Sports Medicine & Physical Fitness
JA - J Sports Med Phys Fitness
VL - 30
IS - 1
CY - ITALY
N2 - The purpose of this study was to examine the effects of the interval training on metabolic parameters at maximal work and at the anaerobic threshold in 11 year olds. The subjects were five healthy male children. They trained outdoor for 50 min a day, five times a week for six weeks, using interval work at 25 and 50% above their anaerobic threshold. Before and after training program, they performed a progressive exercise test on a cycle ergometer. During the last 15 sec of each power output measurements were made of oxygen uptake (VO2), carbon dioxide output (VCO2), heart rate (HR), ventilation (VE), ventilatory equivalent for oxygen (VEO2) and ventilatory equivalent for carbon dioxide VECO2). Following training, the group increased their anaerobic threshold (expressed as %VO2max) significantly (P less than 0.05) during the progressive exercise test, by 22%. Also at the anaerobic threshold level, increases were observed following training in CO2 output (VCO2-AT) and respiratory exchange ratio (R). Oxygen uptake (VO2, l.min-1) was increased by 19%, but the difference was not significant (P greater than 0.05). Maximal ventilatory equivalent for (VECO2max) decreased significantly (P less than 0.05). Maximal heart rate was reduced significantly (P less than 0.05). We conclude that training led to an increase of both anaerobic and aerobic metabolism, at any submaximal work above the anaerobic threshold, for this specific age group
SN - 0022-4707
AD - Department of Pulmonary Physiology, LAC/University of Southern California-Medical Center
UR - 2366536
ER -

TY - JOUR
ID - 2161
T1 - Recurrent intracranial ependymomas in children. Survival, patterns of failure, and prognostic factors
A1 - Goldwein,J.W.
A1 - Glauser,T.A.
A1 - Packer,R.J.
A1 - Finlay,J.L.
A1 - Sutton,L.N.
A1 - Curran,W.J.
A1 - Laehy,J.M.
A1 - Rorke,L.B.
A1 - Schut,L.
A1 - D'Angio,G.J.
Y1 - 1990/08/01/
N1 - Goldwein, J W. Glauser, T A. Packer, R J. Finlay, J L. Sutton, L N. Curran, W J. Laehy, J M. Rorke, L B. Schut, L. D'Angio, G J
Cancer
0374236, clz
AIM, IM
Journal Article
English
KW - MEDLINE
KW - Actuarial Analysis
KW - Adolescent
KW - Antineoplastic Combined Chemotherapy Protocols/tu [Therapeutic Use]
KW - Brain Neoplasms/mo [Mortality]

KW - Brain Neoplasms/pa [Pathology]
KW - Brain Neoplasms/th [Therapy]
KW - Child
KW - Preschool
KW - Cisplatin/tu [Therapeutic Use]
KW - Combined Modality Therapy
KW - Ependymoma/mo [Mortality]
KW - Ependymoma/pa [Pathology]
KW - Ependymoma/th [Therapy]
KW - Female
KW - Humans
KW - Infant
KW - Male
KW - Neoplasm Recurrence
KW - Local
KW - Prognosis
KW - Radiotherapy Dosage
KW - Retrospective Studies
RP - NOT IN FILE
SP - 557
EP - 563
JF - Cancer
VL - 66
IS - 3
CY - UNITED STATES
N2 - Thirty-six pediatric patients (ages 0.8-16.8 years) with recurrent intracranial ependymoma were treated for a total of 52 separate cases of relapse from 1970 to 1989. Therapy consisted of surgery in 33 cases and chemotherapy in 38 cases. Twelve patients received radiation at the time of first relapse, and five of these 12 who had initially been treated with surgery and chemotherapy alone were irradiated to full dose. The 2-year actuarial survival and progression-free survival (PFS) rates are 29% and 23%, respectively. Two-year survival after treatment of first relapse is 39%. Of the 52 cases, there have been 44 subsequent relapses (and one septic death), three of which have occurred in the five patients treated with definitive radiation. Twenty-seven relapses have occurred exclusively with local disease. Eight patients failed with disease outside as well as in the primary site. Survival was better for patients who had histologically benign lesions at relapse (53% versus 9%, P less than 0.02), and for patients in first versus subsequent relapse (p less than 0.005). Cisplatin and etoposide (VP-16) appeared to be the most active chemotherapeutic agents. The authors conclude that some patients with histologically benign ependymoma at first relapse may benefit from aggressive therapy, with occasional long-term, progression-free survival possible. Patients with malignant lesions, or patients who relapse a second time, are less likely to benefit from conventional therapy for a significant period of time
SN - 0008-543X
AD - Department of Radiation Oncology, University of Pennsylvania, Philadelphia
UR - 2364367
ER -

TY - JOUR
ID - 2162
T1 - General anesthesia does not alter the viscoelastic or transport properties of human respiratory mucus
A1 - Rubin,B.K.
A1 - Finegan,B.
A1 - Ramirez,O.
A1 - King,M.
Y1 - 1990/07//
N1 - Rubin, B K. Finegan, B. Ramirez, O. King, M
Chest
0231335, d1c
AIM, IM

Journal Article. Research Support, Non-U.S. Gov't
English
KW - MEDLINE
KW - Adolescent
KW - Adult
KW - Aged
KW - Anesthesia
KW - General
KW - Child
KW - Preschool
KW - Elasticity
KW - Female
KW - Humans
KW - Infant
KW - Intubation
KW - Intratracheal
KW - Male
KW - Middle Aged
KW - Mucociliary Clearance/de [Drug Effects]
KW - Mucociliary Clearance/ph [Physiology]
KW - Mucus/de [Drug Effects]
KW - Mucus/ph [Physiology]
KW - Respiratory Physiological Phenomena
KW - Respiratory System/de [Drug Effects]
KW - Viscosity
RP - NOT IN FILE
SP - 101
EP - 104
JF - Chest
VL - 98
IS - 1
CY - UNITED STATES
N2 - Observations that mucus transport rates (MTR) are depressed in anesthetized animals and humans have led to speculation that general anesthesia depresses ciliary activity or adversely alters the physical properties of the respiratory mucus (RM). We investigated the possibility that anesthesia changes the physical properties of RM in such a way as to depress ciliary transport. We collected 33 samples of RM from the endotracheal tubes (ETTs) of 25 people aged 1 to 79 years undergoing elective surgery who had no clinical evidence of lung disease. We measured the rigidity, viscoelasticity, spinnability, and the percentage of solid composition of these specimens as well as the transport of the collected RM across the mucus-depleted frog palate. These physical properties were not significantly different from RM collected from awake volunteers using the bronchoscopy brush collection technique. Differences in spinnability, transportability, and solid content of paired mucus samples from the inside and outside of the ETTs are suggestive of altered RM hydration, but this requires further study. The decrease in MTR during general anesthesia is probably due to mechanisms other than alterations in the physical properties of mucus
SN - 0012-3692
AD - University of Alberta, Department of Pediatrics (Pulmonary), Edmonton, Canada
UR - 2361374
ER -

TY - JOUR
ID - 2163
T1 - Aerobics enhances cardiovascular fitness and agility in preschoolers
A1 - Alpert,B.
A1 - Field,T.
A1 - Goldstein,S.
A1 - Perry,S.
Y1 - 1990///

N1 - Alpert, B. Field, T. Goldstein, S. Perry, S
Health psychology : official journal of the Division of Health Psychology, American Psychological Association
ejl, 8211523
IM
Journal Article. Research Support, U.S. Gov't, P.H.S.
English
KW - MEDLINE
KW - Arousal
KW - Attitude to Health
KW - Child Development
KW - Child
KW - Preschool
KW - Exercise
KW - Exercise Test
KW - Humans
KW - Motor Activity
KW - Motor Skills
KW - Physical Fitness
KW - Play and Playthings
KW - Self Concept
RP - NOT IN FILE
SP - 48
EP - 56
JF - Health Psychology
JA - Health Psychol
VL - 9
IS - 1
CY - UNITED STATES
N2 - Investigated effects of aerobic exercise on a sample of 24 preschoolers. Thirty minutes of aerobic exercises were provided daily for a period of 8 weeks for a group of 12 children while the remaining 12 children engaged in freeplay on the school playground. The children were given pretests and posttests on the following measures: a submaximal exercise test on a pediatric bicycle (baseline and three workloads), an agility test, a health knowledge test, a self-esteem scale, and an observational measure of their gross-motor activity. Despite comparability on pretests, significant group X repeated measures effects suggested that the aerobic exercise group showed decreases in heart rate at all three workloads as well as increases in agility and self-esteem following the exercise program. These findings suggest that cardiovascular fitness, agility, and self-esteem can be facilitated in preschoolers by an aerobic exercise program
SN - 0278-6133
AD - University of Miami Medical School, FL 33101
UR - 2323328
ER -

TY - JOUR
ID - 2164
T1 - Integrating children with severe disabilities for intensified outdoor education: focus on feasibility
A1 - Rynders,J.E.
A1 - Schleien,S.J.
A1 - Mustonen,T.
Y1 - 1990/02//
N1 - Rynders, J E. Schleien, S J. Mustonen, T
Mental retardation
mtw, 18640670r
IM
Case Reports. Journal Article. Research Support, U.S. Gov't, Non-P.H.S.
English
KW - MEDLINE
KW - Adolescent

KW - Child
KW - Female
KW - Humans
KW - Intellectual Disability/rh [Rehabilitation]
KW - Interpersonal Relations
KW - Male
KW - Peer Group
KW - Recreation
KW - Reinforcement
KW - Social
KW - Social Perception
RP - NOT IN FILE
SP - 7
EP - 14
JF - Mental Retardation
JA - Ment Retard.
VL - 28
IS - 1
CY - UNITED STATES
N2 - The feasibility of an intensified (i.e., all-day-for-2-weeks) integrated camping experience for children with and without severe disabilities was evaluated as was the impact of integrated programming on camp staff members' attitudes. Procedures employed for promoting social interactions included positive reinforcement and cooperative learning strategies. Task-analytic procedures were used to teach campers with severe disabilities a domestic skill and a life-long leisure activity. By the end of the 2 weeks, children with severe disabilities demonstrated substantially improved skills in targeted activities. Furthermore, campers without disabilities substantially increased their prosocial interaction bids, and ratings reflective of friendship increased significantly. Also, staff members' perceptions of operating an integrated camp versus a segregated one and their perceptions of the presence of participants with disabilities as facilitative of camp operations improved significantly
SN - 0047-6765
AD - Department of Educational Psychology, University of Minnesota, Minneapolis 55455
UR - 2308513
ER -

TY - JOUR
ID - 2165
T1 - Computerized activation sequence mapping of the human atrial septum
A1 - Chang,B.C.
A1 - Schuessler,R.B.
A1 - Stone,C.M.
A1 - Branham,B.H.
A1 - Canavan,T.E.
A1 - Boineau,J.P.
A1 - Cain,M.E.
A1 - Corr,P.B.
A1 - Cox,J.L.
Y1 - 1990/02//
N1 - Chang, B C. Schuessler, R B. Stone, C M. Branham, B H. Canavan, T E. Boineau, J P. Cain, M E. Corr, P B. Cox, J L
The Annals of thoracic surgery
683, 15030100r
AIM, IM
Journal Article. Research Support, U.S. Gov't, P.H.S.
English
KW - MEDLINE
KW - Adolescent
KW - Adult

KW - Arrhythmias
KW - Cardiac/pp [Physiopathology]
KW - Atrial Flutter/pp [Physiopathology]
KW - Cardiac Pacing
KW - Artificial
KW - Child
KW - Electrocardiography/is [Instrumentation]
KW - Electrodes
KW - Equipment Design
KW - Female
KW - Heart Atria
KW - Heart Conduction System/ph [Physiology]
KW - Heart Septum/ph [Physiology]
KW - Humans
KW - Image Processing
KW - Computer-Assisted
KW - Male
KW - Middle Aged
KW - Tachycardia
KW - Atrioventricular Nodal Reentry/pp [Physiopathology]
RP - NOT IN FILE
SP - 231
EP - 241
JF - Annals of Thoracic Surgery
JA - Ann Thorac Surg
VL - 49
IS - 2
CY - UNITED STATES
N2 - To delineate the propagation of electrical activation in the atrial septum, atrial epicardial and atrial septal maps were recorded intraoperatively using a 156-channel computerized mapping system in 12 patients during sinus rhythm (n = 10), supraventricular tachycardia associated with septal pathways in Wolff-Parkinson-White syndrome (n = 3), atrioventricular (AV) node reentrant tachycardia (n = 4), and atrial flutter (n = 5). The epicardial and septal data were recorded simultaneously from 156 atrial electrodes, digitized, analyzed, and displayed as isochronous maps on a two-dimensional diagram of the atria. During sinus rhythm, the activation wave fronts propagated most rapidly along the large muscle bundles of the atrial septum. During supraventricular tachycardia associated with Wolff-Parkinson-White syndrome, the earliest site of retrograde atrial activation usually corresponded to the position of atrial insertion of the septal pathways. However, the earliest site of activation during orthodromic supraventricular tachycardia was different from that during ventricular pacing in 1 patient with a posterior septal accessory pathway localized by the epicardial mapping study. The data document the rationale for dividing the ventricular end of the accessory pathways (ie, the endocardial technique) rather than the atrial end (ie, the epicardial technique) in patients with Wolff-Parkinson-White syndrome. During AV node reentrant tachycardia, atrial activation data suggested that atrial tissue lying outside the confines of the anatomical AV node is a necessary link in this common arrhythmia. Thus, these atrial septal maps explain why surgical dissection, or properly positioned small cryolesions placed in the region of the AV node, can ablate AV node reentrant tachycardia without altering normal AV node function. The maps recorded during atrial flutter suggest the importance of the atrial septum as one limb of a macroreentrant circuit responsible for the arrhythmia, and imply that atrial flutter is amenable to control by surgical techniques. These studies demonstrate the details of normal atrial septal activation, the importance of the atrial septum in a variety of different atrial arrhythmias, and the basis of and potential for surgical ablation of the most common types of supraventricular arrhythmias
SN - 0003-4975
AD - Department of Surgery, Washington University School of Medicine, Barnes Hospital, St. Louis, Missouri
UR - 2306145
ER -

TY - JOUR
ID - 2166

T1 - Lacerations in urban children. A prospective 12-January study
A1 - Baker,M.D.
A1 - Selbst,S.M.
A1 - Lanuti,M.
Y1 - 1990/01//
N1 - Baker, M D. Selbst, S M. Lanuti, M
American journal of diseases of children (1960)
3gs, 0370471
AIM, IM
Journal Article. Research Support, Non-U.S. Gov't
English
KW - MEDLINE
KW - Adolescent
KW - Age Factors
KW - Child
KW - Preschool
KW - Female
KW - Follow-Up Studies
KW - Humans
KW - Infant
KW - Male
KW - Philadelphia
KW - Prospective Studies
KW - Questionnaires
KW - Seasons
KW - Sex Factors
KW - Time Factors
KW - Urban Population
KW - Wounds and Injuries/ep [Epidemiology]
KW - Wounds and Injuries/et [Etiology]
KW - Wounds and Injuries/pc [Prevention & Control]
RP - NOT IN FILE
SP - 87
EP - 92
JF - American Journal of Diseases of Children
JA - Am J Dis Child
VL - 144
IS - 1
CY - UNITED STATES
N2 - We prospectively investigated the epidemiologic characteristics of all lacerations (N = 2834) repaired at the Children's Hospital of Philadelphia (Pa) during 1987 and identified common hazards and possible avenues of intervention. Two-year-old children incurred most injuries; males outnumbered females 2:1. Almost two thirds (61.8%) of all lacerations occurred from May through September, and 62.2% between 3 and 9 PM. Most injuries occurred indoors (47.0%), on the sidewalk or street (22.5%), or in the residential yard (13.0%). Injuries usually occurred during play (42.3%) or daily activity (32.1%); 1247 (44.0%) involved some sort of fall. Vectors most frequently causing injury were broken glass bottles (15.0%), wooden furniture (12.0%), and asphalt or concrete (11.0%). Broken glass bottles also most frequently inflicted injuries resulting in functional impairment (0.2%), hospitalization (0.9%), or both. Complications were seen in 8% of all lacerations. Our data confirm the importance of injury-prevention strategies aimed at reduction of discarded glass objects (ie, recycling legislation), improved furniture design, and improved municipal services (ie, street repair)
SN - 0002-922X
AD - Department of Pediatrics, University of Pennsylvania School of Medicine, Philadelphia
UR - 2294725
ER -

TY - JOUR
ID - 2167

T1 - [Permanent sequelae of injuries in the otorhinolaryngology field]. [Czech]
A1 - Novak,J.
A1 - Ruzicka,J.
Y1 - 1989///
N1 - Novak, J. Ruzicka, J
Sbornik vedeckych praci Lekarske fakulty Karlovy univerzity v Hradci Kralove. Supplementum
uc2, 0414150
IM
English Abstract. Journal Article
Czech
KW - MEDLINE
KW - Adolescent
KW - Adult
KW - Aged
KW - Child
KW - Preschool
KW - Craniocerebral Trauma/co [Complications]
KW - Craniocerebral Trauma/et [Etiology]
KW - Ear/in [Injuries]
KW - Female
KW - Hearing Disorders/et [Etiology]
KW - Humans
KW - Male
KW - Middle Aged
KW - Nose/in [Injuries]
KW - Retrospective Studies
RP - NOT IN FILE
SP - 101
EP - 141
JF - Sbornik Vedeckych Praci Lekarske Fakulty Karlovy Univerzity V Hradci Kralove - Supplementum
JA - Sb.Ved.Pr Lek Fak.Karlovy Univerzity Hradci Kralove Suppl
VL - 32
IS - 2
CY - CZECHOSLOVAKIA
N2 - The present retrospective study was aimed to scrutinize constant traumatic sequelae in otorhinolaryngology on 586 traumatic reports, which have been documented by the Bohemian State Insurance starting from 1965 to 1985. Constant sequelae appeared to be subsequent both to individual damages of otorhinolaryngologic organs and multiple traumas, from them 59.4% affected the ear region. Most frequently were presented the partial hearing loss, deafness, and labyrinth lesions especially in multiple traumas. As to the nasal area, the amount of 22.7 per cent consisted of external nasal shape deformities with the disordered passage, and olfactory disorders in multiple traumas. The remaining 17.9% sequelae was concerned with the anatomical and functional lesions of other otorhinolaryngologic organs. To determine features and the extent of constant sequelae, most current diagnostical approaches and consiliary examinations were used. However, the objective assessment of olfactory disorders and tinnitus aurium is stated to be still diagnostically intricated. Both the simulation and aggravation were found out in 2.5% of insurers. Other relations were also studied, e.g.: insurer vice versa physician. In addition to other causes, the most frequent ones represented an underestimation of traumatic consequences all along with their erroneous treatment, low levelled outpatient care of ORL specialists as to the multiple trauma management outside ORL service. In many instances, the injured subjects were not appropriately recommended to provide them with simple and effective surgical treatment of yet manifested constant sequelae. The proper expertize activity was complicated with the defective trauma documentation, terminology variants and erroneous regulations dealing with the recompensation of injuries
SN - 0049-5522
UR - 2609113
ER -

TY - JOUR
ID - 2168

T1 - Biomicroscopic evaluation and photography of ocular toxoplasmosis
A1 - Hirokawa,H.
A1 - Trempe,C.L.
A1 - Takahashi,M.
A1 - Kado,M.
A1 - Akiba,J.
A1 - Yoshida,A.
Y1 - 1989/10//
N1 - Hirokawa, H. Trempe, C L. Takahashi, M. Kado, M. Akiba, J. Yoshida, A
Annals of ophthalmology
5pa, 0210137
IM
Case Reports. Journal Article
English
KW - MEDLINE
KW - Adolescent
KW - Adult
KW - Aged
KW - Child
KW - Chorioretinitis/et [Etiology]
KW - Female
KW - Humans
KW - Male
KW - Middle Aged
KW - Photography
KW - Toxoplasmosis
KW - Ocular/co [Complications]
KW - Ocular/pa [Pathology]
KW - Visual Acuity
KW - Vitreous Body/pa [Pathology]
RP - NOT IN FILE
SP - 379
EP - 383
JF - Annals of Ophthalmology
JA - Ann Ophthalmol
VL - 21
IS - 10
CY - UNITED STATES
N2 - The vitreous of 39 eyes with ocular toxoplasmosis was studied and photographed using an El Bayadi-Kajiura preset lens. In active cases of toxoplasmosis, the vitreous showed inflammatory opacities and gel shrinkage. In inactive cases, the vitreous was often liquefied. Compared with 39 normal eyes of age- and sex-matched controls, the prevalence of partial posterior vitreous detachment was significantly higher in toxoplasmosis (P less than .001). A higher prevalence of posterior vitreous detachment was found in eyes with the major chorioretinal lesion outside the temporal vascular arcade than in those with lesions inside the temporal vascular arcade (P less than .05). Our results indicated that various vitreous changes may cause posterior vitreous detachment and that location of the inflammatory focus may influence the vitreous condition
SN - 0003-4886
AD - Eye Research Institute of Retina Foundation, Boston, MA
UR - 2589741
ER -

TY - JOUR
ID - 2169
T1 - Health care in Nicaragua
A1 - Britten,S.
Y1 - 1989/07//

N1 - Britten, S
Medicine and war
8508146, m6n
IM, J
Journal Article
English
KW - MEDLINE
KW - Child
KW - Preschool
KW - Delivery of Health Care
KW - Female
KW - Humans
KW - Immunization
KW - Infant
KW - Infant Mortality
KW - Newborn
KW - Male
KW - Nicaragua
KW - Rehabilitation
KW - War
RP - NOT IN FILE
SP - 132
EP - 136
JF - Medicine & War
JA - Med War
VL - 5
IS - 3
CY - ENGLAND
N2 - A two-week health study tour in Nicaragua in 1987 organized by the Health Network of the (British) Nicaragua Solidarity Campaign afforded the author of this report access to health service planning, first-hand observation of the immunization programme, and visits to centres for rehabilitation of the disabled, both from the war and civil causes. The efficacy of the low technology immunization and oral rehydration programmes is contrasted with the struggle to rehabilitate the war disabled with grossly inadequate resources
SN - 0748-8009
UR - 2796849
ER -

TY - JOUR
ID - 2170
T1 - Practice patterns and family life--a survey of Melbourne medical graduates
A1 - Dennerstein,L.
A1 - Lehert,P.
A1 - Orams,R.
A1 - Ewing,J.
A1 - Burrows,G.
Y1 - 1989/10/02/
N1 - Dennerstein, L. Lehert, P. Orams, R. Ewing, J. Burrows, G
The Medical journal of Australia
0400714, m26
IM
Comparative Study. Journal Article. Research Support, Non-U.S. Gov't
English
KW - MEDLINE
KW - Adult
KW - Age Factors
KW - Aged
KW - 80 and over

KW - Child
KW - Child Care
KW - Education
KW - Medical
KW - Continuing
KW - Employment
KW - Family
KW - Female
KW - Humans
KW - Income
KW - Life Style
KW - Male
KW - Middle Aged
KW - Personal Satisfaction
KW - Physicians
KW - Women/px [Psychology]
KW - Women
KW - Professional Practice
KW - Questionnaires
KW - Retirement
KW - Sampling Studies
KW - Sex Factors
KW - Victoria
RP - NOT IN FILE
SP - 386
EP - 390
JF - Medical Journal of Australia
JA - Med J Aust
VL - 151
IS - 7
CY - AUSTRALIA
N2 - To investigate sex differences in the professional achievements and personal life-styles of graduates, a questionnaire survey was conducted. The sample comprised surviving female medical graduates of the University of Melbourne and an equal number of male medical graduates who were matched by year of graduation. The final response rate was 70% (1764 subjects returned questionnaires) and was representative for both age and sex. This article describes the practice patterns and family lives of graduates. Considerable sex-related differences were found in the professional achievements and personal life-styles of the surveyed medical graduates. Women's professional careers tended to be more circumscribed than were those of male colleagues. Women were less involved in areas outside clinical practice such as teaching or lecturing, committees, medical administration, and research and its publication. Female doctors earned significantly (P less than 0.0001) less than did male doctors and were more likely to work as employees, locums or in sessional employment (P less than 0.0001). Women were more involved in all aspects of household activities, especially during midlife (40-60 years of age)--the peak career years for male doctors. The career underachievement of female doctors is likely to continue unless considerable changes are made to current postgraduate training schemes and career structures
SN - 0025-729X
AD - Faculty of Medicine, University of Melbourne, Carlton, Vic
UR - 2796814
ER -

TY - JOUR
ID - 2171
T1 - The differential validity of teacher ratings of inattention/overactivity and aggression
A1 - Atkins,M.S.
A1 - Pelham,W.E.
A1 - Licht,M.H.
Y1 - 1989/08//

N1 - Atkins, M S. Pelham, W E. Licht, M H
Journal of abnormal child psychology
0364547, h33
IM
Journal Article
English
KW - MEDLINE
KW - Achievement
KW - Aggression/px [Psychology]
KW - Attention
KW - Attention Deficit Disorder with Hyperactivity/di [Diagnosis]
KW - Attention Deficit Disorder with Hyperactivity/px [Psychology]
KW - Child
KW - Child Behavior Disorders/di [Diagnosis]
KW - Child Behavior Disorders/px [Psychology]
KW - Humans
KW - Learning Disorders/di [Diagnosis]
KW - Learning Disorders/px [Psychology]
KW - Male
KW - Motor Activity
KW - Psychological Tests
KW - Social Behavior
RP - NOT IN FILE
SP - 423
EP - 435
JF - Journal of Abnormal Child Psychology
JA - J Abnorm Child Psychol
VL - 17
IS - 4
CY - UNITED STATES
N2 - The ability of school-based measures of child behavior to predict differentially the IOWA Conners teacher rating scale Inattention/Overactivity (IO) and Aggression (A) factors was evaluated in a sample of 71 school-aged boys. Multiple measures and multiple methods of assessment were utilized to provide a comprehensive assessment of social and academic behavior. These included direct observations of children in the classroom and on the playground, examination of the organization of children's desks, measures of academic performance, peer nominations of popularity and rejection, and sociometric ratings using the Pupil Evaluation Inventory. Despite moderately high correlations between the teacher rating factors (r = .60), considerable evidence was provided for differential validity on measures of academic performance, peer rating measures, and measures of disruptive or inappropriate classroom and playground behavior. These differences between IO and A factors indicated that the factors were logically independent in important ways, supporting prior work validating separate hyperactivity and aggression dimensions
SN - 0091-0627
AD - Department of Pediatric Psychology, Children's Seashore House, Children's Hospital of Philadelphia, Pennsylvania 19104
UR - 2794255
ER -

TY - JOUR
ID - 2172
T1 - The epidemiology of injuries in Atlanta day-care centers
A1 - Sacks,J.J.
A1 - Smith,J.D.
A1 - Kaplan,K.M.
A1 - Lambert,D.A.
A1 - Sattin,R.W.
A1 - Sikes,R.K.
Y1 - 1989/09/22/

N1 - Sacks, J J. Smith, J D. Kaplan, K M. Lambert, D A. Sattin, R W. Sikes, R K
JAMA : the journal of the American Medical Association
7501160
AIM, IM
Journal Article
English
KW - MEDLINE
KW - Accidental Falls/sn [Statistics & Numerical Data]
KW - Child
KW - Child Day Care Centers
KW - Preschool
KW - Female
KW - Georgia
KW - Humans
KW - Male
KW - Prospective Studies
KW - Seasons
KW - Time Factors
KW - Wounds and Injuries/ep [Epidemiology]
RP - NOT IN FILE
SP - 1641
EP - 1645
JF - JAMA
VL - 262
IS - 12
CY - UNITED STATES
N2 - From June 1987 through May 1988, we collected reports of injuries among 5300 children who attended 71 day-care centers in Atlanta, Ga. One hundred forty-three injuries severe enough for the child to require medical or dental care were reported, including 63 lacerations (44.1%), 23 fractures (16.1%), 5 crush injuries (3.5%), 4 dislocations (2.8%), 2 human bites (1.4%), and 2 concussions (1.4%). The head was the site of 98 (68.5%) injuries. Peak hours for injuries were 11 AM and 4 PM, the peak day was Monday, and the peak season was summer. After adjustment for hours and dates of attendance, the rate was 1.77 injuries per 100,000 child-hours in day care; among preschool-aged children, infants had the lowest rate (0.77) and 2-year-old children had the highest rate (2.26). Almost 47% of injuries occurred on the playground; falls were involved in 70% of such injuries. Because 33% of all injuries resulted from falls on the playground, impact-absorbing playground surfaces may be a possible intervention to reduce injuries
SN - 0098-7484
AD - Division of Injury Epidemiology and Control, Centers for Disease Control, Atlanta, GA 30333
UR - 2769919
ER -

TY - JOUR
ID - 2173
T1 - Cognitive-behavioral therapy and relationship therapy in the treatment of children referred for antisocial behavior
A1 - Kazdin,A.E.
A1 - Bass,D.
A1 - Siegel,T.
A1 - Thomas,C.
Y1 - 1989/08//
N1 - Kazdin, A E. Bass, D. Siegel, T. Thomas, C
Journal of consulting and clinical psychology
0136553, hw3
IM
Clinical Trial. Journal Article. Randomized Controlled Trial. Research Support, U.S. Gov't, P.H.S.
English
KW - MEDLINE

KW - Adolescent
KW - Antisocial Personality Disorder/px [Psychology]
KW - Antisocial Personality Disorder/th [Therapy]
KW - Behavior Therapy/mt [Methods]
KW - Child
KW - Child Behavior Disorders/px [Psychology]
KW - Child Behavior Disorders/th [Therapy]
KW - Preschool
KW - Cognition
KW - Combined Modality Therapy
KW - Female
KW - Humans
KW - Interpersonal Relations
KW - Male
RP - NOT IN FILE
SP - 522
EP - 535
JF - Journal of Consulting & Clinical Psychology
JA - J Consult Clin Psychol
VL - 57
IS - 4
CY - UNITED STATES
N2 - The present study evaluated alternative treatments for children (N = 112, ages 7-13) referred for severe antisocial behavior. Children were randomly assigned to one of three treatments: problem-solving skills training (PSST), problem-solving skills training with in vivo practice (PSST-P), which included therapeutically planned activities to extend training to settings outside of treatment, or client-centered relationship therapy (RT). PSST and PSST-P children showed significantly greater reductions in antisocial behavior and overall behavior problems, and greater increases in prosocial behavior than RT children. These effects were evident on measures obtained immediately after treatment and at a 1-year follow-up, and on measures of child performance at home and at school. PSST-P children showed greater changes than PSST children on measures of functioning at school at posttreatment, but these differences were no longer evident at follow-up. Children in both PSST conditions showed significant reductions in deviant behavior and improvements in prosocial behavior from pretreatment to follow-up, whereas RT children tended to remain at their pretreatment level of functioning. Notwithstanding the significant improvements, comparisons with nonclinic (normative) samples revealed that the majority of youth remained outside of the normal range of deviant behavior. Possible directions for improving treatment for antisocial youth are highlighted
SN - 0022-006X
UR - 2768614
ER -

TY - JOUR
ID - 2174
T1 - Effective strategies for population studies of acute air pollution health effects
A1 - Lippmann,M.
Y1 - 1989/05//
N1 - Lippmann, M
Environmental health perspectives
ei0, 0330411
IM
Journal Article. Research Support, U.S. Gov't, Non-P.H.S.. Research Support, U.S. Gov't, P.H.S.
English
KW - MEDLINE
KW - Adult
KW - Air Pollutants/ae [Adverse Effects]
KW - Child
KW - Epidemiologic Methods
KW - Humans

KW - Respiratory Function Tests
KW - United States
RP - NOT IN FILE
SP - 115
EP - 119
JF - Environmental Health Perspectives
JA - Environ Health Perspect
VL - 81
CY - UNITED STATES
N2 - A series of field studies involving repetitive functional measurements in relatively small populations of healthy children and adults engaged in normal outdoor activities has shown that significant decrements in respiratory function are associated with exposures to ozone (O3) at concentrations below the national ambient air quality standard. The ability to detect such effects can be attributed, at least in part, to the study design criteria used, which emphasized maximization of signal-to-noise ratios. Locations were selected to ensure relatively high exposures to relatively uniformly distributed secondary pollutants, with minimal exposure to local sources of primary pollutants. Populations were selected that would be engaged in active recreation out of doors. Populations of healthy persons were used to minimize variability in baseline function. We found that the magnitude of the O3-associated decrements in respiratory function was dependent on the variability in sensitivity to O3 among the population, the minute ventilation during outdoor activity, and the duration of the outdoor exposure. We also concluded that the O3-associated responses were potentiated by the presence of other air pollutants
SN - 0091-6765
AD - New York University Medical Center, Institute of Environmental Medicine, NY 10016
UR - 2759053
ER -

TY - JOUR
ID - 2175
T1 - Psychological linguistics: implications for a theory of initial development and a method for research
A1 - Bijou,S.W.
Y1 - 1989///
N1 - Bijou, S W
Advances in child development and behavior
2k7, 0370417
IM
Journal Article
English
KW - MEDLINE
KW - Child
KW - Child Language
KW - Preschool
KW - Humans
KW - Infant
KW - Newborn
KW - Intellectual Disability/px [Psychology]
KW - Language Development
KW - Male
KW - Motor Skills
KW - Psycholinguistics
KW - Research Design
RP - NOT IN FILE
SP - 221
EP - 241
JF - Advances in Child Development & Behavior
JA - Adv Child Dev Behav
VL - 21
CY - UNITED STATES

N2 - Presented here are an hypothesis of language development and a description of a method for studying language, both based on Kantor's psychological linguistics. According to our hypothesis, language develops in four stages. In the first, which spans the first 9 to 15 months, random movements evolve into body management, manual, and locomotor skills that enable an infant to engage in play activities, among other things, with the mother and others. Simple gestural communication soon follows. Random babbling progresses beyond the sounds that are more universal in the language to the family's particular pattern of intonations and inflections, and receptive speech begins, that is, the infant learns to react to simple verbal-vocal requests. During the second stage, which coincides with the child's second year, vocalizations evolve into "idiosyncratic expressions" and rough approximations of heard sound patterns. Language is now holophrastic--one-word utterances--mostly of the mediative variety. During the third stage, which extends to about 30 months of age, language skills are described as first-approximation referential interactions: they are longer and more detailed, include some narrative interchanges, and refer to past and future events. Typically the child now talks to him or herself, or engages in expressive referential interactions. In the fourth stage, when the child reaches the age of 52 months or so, second-approximation speech begins. By now the vocal apparatus is sufficiently developed to enable a child to make most of the vowel sounds, and the enhanced language skills set the occasion for more frequent linguistic interchanges. He or she also begins to respond to symbols and other representations of objects and events. In other words, he or she begins to engage in symbolizing behavior. Language obviously continues to develop beyond the fourth stage. Social conditions outside of the family further affect language style and generate specialized forms of speech, notably the jargon of a particular occupation or hobby. The method for studying language interactions involves, through the use of TV tapes, a description of the behavior of a speaker and the listener, the referent, and the setting conditions. The behavior of a speaker is analyzed in terms of the frequency with which he or she begins a language interchange, the average length of the initiation, the modality (verbal-vocal, gestural, or both) and the accompanying behavior. It is also analyzed for content, including time frame, actuality, and persons, animals, objects, activities, and others. Noted, too, are the feeling reactions regarding the referents.(ABSTRACT TRUNCATED AT 400 WORDS)
SN - 0065-2407
AD - Department of Psychology, and University of Arizona, Tucson 85721
UR - 2750574
ER -

TY - JOUR
ID - 2176
T1 - Head injuries in children and implications for their prevention
A1 - MacKellar,A.
Y1 - 1989/06//
N1 - MacKellar, A
Journal of pediatric surgery
jmj, 0052631
IM
Journal Article
English
KW - MEDLINE
KW - Accidental Falls
KW - Accidents
KW - Home/pc [Prevention & Control]
KW - Home/sn [Statistics & Numerical Data]
KW - Traffic/mo [Mortality]
KW - Traffic/pc [Prevention & Control]
KW - Traffic/sn [Statistics & Numerical Data]
KW - Bicycling
KW - Child
KW - Preschool
KW - Craniocerebral Trauma/pc [Prevention & Control]
KW - Female
KW - Head Protective Devices
KW - Humans
KW - Infant

KW - Male
KW - Play and Playthings
KW - Seat Belts
KW - Sex Factors
RP - NOT IN FILE
SP - 577
EP - 579
JF - Journal of Pediatric Surgery
JA - J Pediatr Surg
VL - 24
IS - 6
CY - UNITED STATES
N2 - Between October 1986 and September 1987, 7,967 children presented to the emergency department of the Princess Margaret Hospital for Children for the first time with injuries. Head injuries had been sustained by 3,187 (40%). The data was collected by use of a special form distributed by the nursing staff and completed by parents and medical staff. Information was obtained about the age and sex of the patients, the nature of the injury, the site of occurrence, the activity at that time, and the context in which it took place. The use, or otherwise, of safety devices was also recorded. Injury severity was classified as follows: minor treated in casualty department (80.7%); moderate, admitted to general ward (17.1%); severe, admitted to intensive care (2.2%). Examination of the data showed certain recurring problems, ie, surfaces onto which children fell, bicycles, playgrounds, sporting injuries, vehicle accidents, inadequate supervision. Implications for prevention are (1) improved home design, particularly flooring and furniture; (2) better planning of home surroundings, play areas, and equipment; (3) determination of age at which children are capable of riding bicycles and coping with traffic; (4) extension and enforcement of restraint regulations to protect children in vehicles; (5) better supervision of children engaged in sports and recreational activities, and use of safety devices; (6) increased community awareness of the frequency of head injuries, and knowledge of how the numbers can be reduced and the severity diminished; and (7) improved knowledge of child growth and development, and better supervision by all care-givers
SN - 0022-3468
AD - Department of Paediatric Surgery, Princess Margaret Hospital for Children, Perth, Western Australia
UR - 2738825
ER -

TY - JOUR
ID - 2177
T1 - Consumer product-related injuries in Athens, Ohio, 1980-85: assessment of emergency room-based surveillance
A1 - Hopkins,R.S.
Y1 - 1989/03//
N1 - Hopkins, R S
American journal of preventive medicine
8704773, apl
IM
Journal Article. Research Support, Non-U.S. Gov't
English
KW - MEDLINE
KW - Adolescent
KW - Adult
KW - Age Factors
KW - Aged
KW - Burns/ep [Epidemiology]
KW - Burns/et [Etiology]
KW - Child
KW - Consumer Product Safety
KW - Emergency Service
KW - Hospital
KW - Female

KW - Fractures
KW - Bone/ep [Epidemiology]
KW - Bone/et [Etiology]
KW - Humans
KW - Leg Injuries/ep [Epidemiology]
KW - Leg Injuries/et [Etiology]
KW - Male
KW - Middle Aged
KW - Ohio
KW - Patient Admission
KW - Population Surveillance
KW - Sex Factors
KW - Wounds and Injuries/ep [Epidemiology]
KW - Wounds and Injuries/et [Etiology]
RP - NOT IN FILE
SP - 104
EP - 112
JF - American Journal of Preventive Medicine
JA - Am J Prev Med
VL - 5
IS - 2
CY - UNITED STATES
N2 - Using the National Electronic Injury Surveillance System of the United States Consumer Product Safety Commission, I computed the average annual rates per 1,000 population of consumer product-related injuries in residents of Athens County, Ohio, and seem at one hospital. These rates do not include intentional injuries or injuries to occupants of motor vehicles, to pedestrians hit by motor vehicles, or those occurring on the job or in house fires. Average annual rates for all injuries combined ranged from 102 per 1,000 boys 10-14 years of age to 11.7 per 1.000 men 65-74 years of age. For all males, the annual rate was 53 per 1,000 and for all females, 32 per 1,000. The most common parts of the body injured were the fingers (accounting for 15% of visits), face (12%), ankle (8.5%), hands (8%), and head (7.9%). The most common injury types were lacerations (33%), contusions and abrasions (22%), sprains (16%), and fractures (13%). Hospital admission rates varied by age, sex, type of injury, and part of body injured. Comparison of a sample of NEISS records to emergency room records showed that data were abstracted by clerical personnel with a high degree of accuracy. Review of fractures of the lower leg identified a cluster of injuries in young girls from playground merry-go-rounds. Review of burn injuries identified problems with scald injuries in young adult women and with eye injuries from welding in young men. The system can be used to evaluate injury control measures taken on a local level
SN - 0749-3797
AD - Department of Preventive Medicine, College of Medicine, Ohio State University, Columbus
UR - 2730789
ER -

TY - JOUR
ID - 2178
T1 - Appropriate sex education for black teens
A1 - Banks,I.W.
A1 - Wilson,P.I.
Y1 - 1989///
N1 - Banks, I W. Wilson, P I
Adolescence
2h8, 0123667
IM, J
Journal Article
English
KW - MEDLINE
KW - Adolescent
KW - Adolescent Behavior
KW - African Americans/px [Psychology]

KW - Child
KW - Curriculum
KW - Family
KW - Female
KW - Health Knowledge
KW - Attitudes
KW - Practice
KW - Humans
KW - Male
KW - Sex Education
KW - Sexual Behavior
KW - Social Perception
RP - NOT IN FILE
SP - 233
EP - 245
JF - Adolescence
VL - 24
IS - 93
CY - UNITED STATES
N2 - This article is based on a year-long study of 207 low-income black youths. The major purpose of the research was twofold: (1) to study black teens' perceptions of family relationships in an effort to identify family member(s) who had the most potential for encouraging greater contraceptive responsibility and influencing teens' decisions about being or becoming sexually active, and (2) to study dating and sexual behavior, attitudes toward contraception, and sources of information, in an effort to identify more specific sex education needs of black teens and provide a basis for delineating pertinent characteristics of an effective sex education curriculum
SN - 0001-8449
AD - Department of Curriculum and Instruction, University of Kentucky, Lexington 40506
UR - 2728971
ER -

TY - JOUR
ID - 2179
T1 - Determinants of physical ability in 7th grade schoolchildren
A1 - Halfon,S.T.
A1 - Bronner,S.
Y1 - 1989/03//
N1 - Halfon, S T. Bronner, S
European journal of epidemiology
ere, 8508062
IM
Journal Article. Research Support, Non-U.S. Gov't
English
KW - MEDLINE
KW - Anthropometry
KW - Child
KW - Child Development
KW - Female
KW - Food Habits
KW - Humans
KW - Male
KW - Menarche
KW - Physical Fitness
KW - Running
KW - Sex Factors
RP - NOT IN FILE
SP - 90
EP - 96

JF - European Journal of Epidemiology
JA - Eur J Epidemiol
VL - 5
IS - 1
CY - ITALY
N2 - The aim of the study was to determine factors associated with running time in children aged 12 years, according to sex and sexual maturation of girls. The physical ability was measured, using a 1000 meter run in a group of 942 boys and girls aged 12. In addition, data was collected on family sports activities and the children's sports activities, using questionnaires for parents and children. Before the 1000 meter run, physical examination was carried out. Nutrition habits of the family in the home and outside were self-reported in a questionnaire. The mean time for run in boys was shorter by 40 seconds than that for girls. Premenarche girls ran significantly faster than postmenarche girls (p less than 0.05). The best predictors for short running time were triceps skinfold thickness and sport motivation of the children. Weaker correlation was observed between food variables, family characteristics and running time. The highest prediction of running time by the measured variables was found for postmenarche girls, explained by the high correlation of running time with the behavioral variables. In boys and premenarche girls, the best predictors for running time were the physical and physiological characteristics of the children
SN - 0393-2990
AD - Department of Medical Ecology, Hebrew University, Hadassah Medical School, Jerusalem, Israel
UR - 2707398
ER -

TY - JOUR
ID - 2180
T1 - [Cooperation between East Germany and Czechoslovakia in studying the effect of environmental pollution on school children]. [German]
A1 - Pelech,L.
Y1 - 1989/01//
N1 - Pelech, L
Zeitschrift fur die gesamte Hygiene und ihre Grenzgebiete
0420111, xuo
IM
English Abstract. Journal Article
German
KW - MEDLINE
KW - Air Pollution/ae [Adverse Effects]
KW - Child
KW - Child Development
KW - Czechoslovakia
KW - Germany
KW - East
KW - Humans
KW - International Cooperation
KW - Risk Factors
RP - NOT IN FILE
SP - 50
JF - Zeitschrift fur die Gesamte Hygiene und Ihre Grenzgebiete
JA - Z Gesamte Hyg
VL - 35
IS - 1
CY - GERMANY, EAST
N2 - This report presents key outcomes of joint growth and development studies conducted in children permanently living in areas adversely affected by pollutants in air. Further attention is focussed on the evaluation of effects of some of the so-called compensatory measures applied in Czechoslovakia, namely a 3-week outdoor schooling organized for these children in the relatively clean recreational areas. The beneficial health effects of year's season and specifically of the spontaneous, active and sufficiently long outdoor exercise are documented in graphs. As it is pointed out in this context, children in the place of their permanent residence

tend to have relative little exercise outdoor, may run an increased risk of exposure to indoor pollutants, and may suffer from passive smoking effects if their parents cannot refrain from smoking habits at home
SN - 0049-8610
UR - 2922946
ER -

TY - JOUR
ID - 2181
T1 - Rural maternal, child, and adolescent health. [Review] [28 refs]
A1 - McManus,M.A.
A1 - Newacheck,P.W.
Y1 - 1989/02//
N1 - McManus, M A. Newacheck, P W
Health services research
g2l, 0053006
IM, J
Journal Article. Research Support, Non-U.S. Gov't. Research Support, U.S. Gov't, Non-P.H.S.. Research Support, U.S. Gov't, P.H.S.. Review
English
KW - MEDLINE
KW - Acute Disease/ep [Epidemiology]
KW - Adolescent
KW - Child
KW - Child Health Services/ut [Utilization]
KW - Preschool
KW - Chronic Disease/ep [Epidemiology]
KW - Delivery of Health Care/og [Organization & Administration]
KW - Demography
KW - Female
KW - Humans
KW - Infant
KW - Newborn
KW - Maternal Health Services/ut [Utilization]
KW - Medicaid/sn [Statistics & Numerical Data]
KW - National Center for Health Statistics (U.S.)
KW - Pregnancy
KW - Rural Health/sn [Statistics & Numerical Data]
KW - United States
KW - Vital Statistics
RP - NOT IN FILE
SP - 807
EP - 848
JF - Health Services Research
JA - Health Serv Res
VL - 23
IS - 6
CY - UNITED STATES
N2 - Authors cite recommendations for research in light of a general lack of current literature on health status, health services utilization, organization and delivery of health services, and health care financing in this field. [References: 28]
SN - 0017-9124
AD - McManus Health Policy, Inc., Washington, DC 20016
UR - 2645249
ER -

TY - JOUR
ID - 2182

T1 - The Children's Law Act (No. 61 of 1988), 8 July 1988
A1 - Authors Canada.Newfoundland.
Y1 - 1989///
N1 - Canada. Newfoundland
Annual review of population law
8008840
J
Legislation
English
KW - MEDLINE
KW - Adolescent
KW - Age Factors
KW - Americas
KW - Behavior
KW - Canada
KW - Child
KW - Child Custody
KW - Child Rearing
KW - Demography
KW - Developed Countries
KW - Family Characteristics
KW - Family Relations
KW - Insemination
KW - Artificial
KW - Legislation as Topic
KW - North America
KW - Parents
KW - Population
KW - Population Characteristics
KW - Reproduction
KW - Reproductive Techniques
RP - NOT IN FILE
SP - 46
EP - 52
JF - Annual Review of Population Law
JA - Annu Rev Popul Law
VL - 16
CY - UNITED STATES
SN - 0364-3417
UR - 12344432
ER -

TY - JOUR
ID - 2183
T1 - Children Act 1989 (No 41 of 1989), 16 November 1989
A1 - United Kingdom.
Y1 - 1989///
N1 - United Kingdom
Annual review of population law
8008840
J
Legislation
English
KW - MEDLINE
KW - Adolescent
KW - Age Factors
KW - Behavior

KW - Child
KW - Child Care
KW - Child Custody
KW - Child Rearing
KW - Demography
KW - Developed Countries
KW - Europe
KW - Family Characteristics
KW - Family Relations
KW - Great Britain
KW - Legislation as Topic
KW - Parents
KW - Population
KW - Population Characteristics
KW - Public Policy
RP - NOT IN FILE
SP - 103
JF - Annual Review of Population Law
JA - Annu Rev Popul Law
VL - 16
CY - UNITED STATES
SN - 0364-3417
UR - 12344029
ER -

TY - JOUR
ID - 2184
T1 - Selective exposure of active and sociable children to schistosomiasis
A1 - Kvalsvig,J.D.
A1 - Becker,P.J.
Y1 - 1988/10//
N1 - Kvalsvig, J D. Becker, P J
Annals of tropical medicine and parasitology
68e, 2985178r
IM
Journal Article. Research Support, Non-U.S. Gov't
English
KW - MEDLINE
KW - Child
KW - Child Behavior
KW - Developmental Disabilities/et [Etiology]
KW - Environmental Exposure
KW - Humans
KW - Schistosomiasis haematobia/co [Complications]
KW - Schistosomiasis haematobia/tm [Transmission]
KW - Social Behavior
KW - South Africa
RP - NOT IN FILE
SP - 471
EP - 474
JF - Annals of Tropical Medicine & Parasitology
JA - Ann Trop Med Parasitol
VL - 82
IS - 5
CY - ENGLAND
N2 - Investigations into the impact of schistosomiasis on the physical and psychological development of children have yielded contradictory findings. This may be due to a failure to take account of transmission

factors. In areas where Schistosoma haematobium is endemic, children who bathe regularly in contaminated water are more likely to contract the disease. The harmful effects of the disease may be masked in these more active and gregarious children, particularly when infections are mild and other parasitic infections and nutrional deficiencies are common. The social interaction of schoolchildren in an endemic area was monitored over a period of four months, and the principal finding was that infected children were more sociable than uninfected children in terms of their membership of the main playground groups. This paper discusses the evidence for selective exposure and the implications of the hypothesis for future research
SN - 0003-4983
AD - Department of Psychology, University of Natal, Durban, South Africa
UR - 2476973
ER -

TY - JOUR
ID - 2185
T1 - The social behavior of peer-identified aggressive, withdrawn, and aggressive/withdrawn children
A1 - Lyons,J.
A1 - Serbin,L.A.
A1 - Marchessault,K.
Y1 - 1988/10//
N1 - Lyons, J. Serbin, L A. Marchessault, K
Journal of abnormal child psychology
0364547, h33
IM
Journal Article. Research Support, Non-U.S. Gov't
English
KW - MEDLINE
KW - Aggression/px [Psychology]
KW - Child
KW - Female
KW - Gender Identity
KW - Humans
KW - Internal-External Control
KW - Male
KW - Peer Group
KW - Psychological Tests
KW - Shyness
KW - Social Adjustment
KW - Social Behavior
KW - Videotape Recording
RP - NOT IN FILE
SP - 539
EP - 552
JF - Journal of Abnormal Child Psychology
JA - J Abnorm Child Psychol
VL - 16
IS - 5
CY - UNITED STATES
N2 - The behavioral patterns associated with peer ratings of aggression and withdrawal were explored. First, a discriminant function analysis (N = 74) using seven observational variables was found to significantly identify groups of Aggressive, Withdrawn, and Contrast fourth- and fifth-grade girls and boys. Aggressive/Withdrawn children were not distinguishable from Contrasts. In subsequent analyses comparing the behaviors of children in the four groups at two schools (total N = 117), children in the Aggressive and Withdrawn groups each showed distinctive patterns of social behavior, which were consistent across the two schools. The behavior of the Aggressive/Withdrawn children was not significantly different from that of Contrast children. However, results from one school suggested that Aggressive/Withdrawn children may receive a disproportionate amount of aggression from peers. Finally, the behavior patterns displayed by the deviant groups were similar for girls and boys, allowing for sex differences in base rates of playground behavior. These results confirm the observability

of peer-identified patterns of aggression and withdrawal, and provide a detailed description of the behavior of such children in a free-play situation
SN - 0091-0627
AD - Department of Psychology, Concordia University, Montreal, Quebec, Canada
UR - 3235746
ER -

TY - JOUR
ID - 2186
T1 - Leads from the MMWR. Playground-related injuries in preschool-aged children--United States, 1983-1987
Y1 - 1988/11/18/
N1 - JAMA : the journal of the American Medical Association
7501160
AIM, IM
Journal Article
English
KW - MEDLINE
KW - Accidental Falls/pc [Prevention & Control]
KW - Child Day Care Centers
KW - Child
KW - Preschool
KW - Equipment Safety
KW - Female
KW - Humans
KW - Infant
KW - Male
KW - Play and Playthings
KW - Recreation
KW - Schools
KW - Sex Factors
KW - United States
KW - Wounds and Injuries/ep [Epidemiology]
KW - Wounds and Injuries/et [Etiology]
KW - Wounds and Injuries/pc [Prevention & Control]
RP - NOT IN FILE
SP - 2799
EP - 2800
JF - JAMA
VL - 260
IS - 19
CY - UNITED STATES
SN - 0098-7484
UR - 3184342
ER -

TY - JOUR
ID - 2187
T1 - Playground-related injuries in preschool-aged children--United States, 1983-1987
A1 - Centers for Disease Control (CDC)
Y1 - 1988/10/21/
N1 - Centers for Disease Control (CDC)
MMWR. Morbidity and mortality weekly report
ne8, 7802429
IM
Journal Article
English
KW - MEDLINE

KW - Accidental Falls/pc [Prevention & Control]
KW - Child Day Care Centers
KW - Child
KW - Preschool
KW - Equipment Safety
KW - Female
KW - Humans
KW - Infant
KW - Male
KW - Play and Playthings
KW - Recreation
KW - Schools
KW - Sex Factors
KW - United States
KW - Wounds and Injuries/ep [Epidemiology]
KW - Wounds and Injuries/et [Etiology]
KW - Wounds and Injuries/pc [Prevention & Control]
RP - NOT IN FILE
SP - 629
EP - 632
JF - MMWR - Morbidity & Mortality Weekly Report
JA - MMWR Morb Mortal Wkly Rep
VL - 37
IS - 41
CY - UNITED STATES
SN - 0149-2195
UR - 3139984
ER -

TY - JOUR
ID - 2188
T1 - Modulation of the cell-mediated immune response in kala-azar and post-kala-azar dermal leishmaniasis in relation to chemotherapy
A1 - Neogy,A.B.
A1 - Nandy,A.
A1 - Ghosh,Dastidar B.
A1 - Chowdhury,A.B.
Y1 - 1988/02//
N1 - Neogy, A B. Nandy, A. Ghosh Dastidar, B. Chowdhury, A B
Annals of tropical medicine and parasitology
68e, 2985178r
IM
Journal Article. Research Support, Non-U.S. Gov't
English
KW - MEDLINE
KW - Adolescent
KW - Adult
KW - Antimony Sodium Gluconate/tu [Therapeutic Use]
KW - Cell Migration Inhibition
KW - Child
KW - Female
KW - Humans
KW - Leishmaniasis/dt [Drug Therapy]
KW - Leishmaniasis/im [Immunology]
KW - Leishmaniasis
KW - Visceral/dt [Drug Therapy]
KW - Visceral/im [Immunology]

KW - Leukocyte Count
KW - Leukocytes/im [Immunology]
KW - Lymphocyte Activation
KW - Male
KW - T-Lymphocytes
RP - NOT IN FILE
SP - 27
EP - 34
JF - Annals of Tropical Medicine & Parasitology
JA - Ann Trop Med Parasitol
VL - 82
IS - 1
CY - ENGLAND
N2 - Cell-mediated immune (CMI) response and the concurrent clinical events were studied longitudinally before, during and after treatment in 24 kala-azar (KA) and ten post-kala-azar dermal leishmaniasis (PKADL) patients for a period of six months. The status of specific CMI response was estimated by in vitro tests, viz. lymphocyte transformation and leucocyte migration inhibition in response to L. donovani antigen. The generalized CMI response was assessed by lymphocyte transformation in the presence of mitogen (phytohaemagglutinin) and quantitation of the circulatory T-lymphocyte population. To measure the drug response, the extent of clinical improvement following treatment was used as a yardstick. The results showed that suppression of the CMI response was both specific and generalized in nature during the active stage of KA; but in PKADL, unlike in KA, suppression of the CMI response was found to be associated only at the specific level. With administration of the drug (sodium antimony gluconate), the immunosuppression was gradually eliminated with concomitant clinical improvement in both KA and PKADL patients, although the latter took a longer period of time and a larger amount of drugs compared to the former
SN - 0003-4983
AD - Division of Parasitology, School of Tropical Medicine, Calcutta, India
UR - 2840867
ER -

TY - JOUR
ID - 2189
T1 - Severe anaphylactic reactions outside hospital: etiology, symptoms and treatment
A1 - Soreide,E.
A1 - Buxrud,T.
A1 - Harboe,S.
Y1 - 1988/05//
N1 - Soreide, E. Buxrud, T. Harboe, S
Acta anaesthesiologica Scandinavica
0370270
IM
Journal Article
English
KW - MEDLINE
KW - Adolescent
KW - Adult
KW - Aged
KW - Aircraft
KW - Ambulances
KW - Anaphylaxis/et [Etiology]
KW - Anaphylaxis/th [Therapy]
KW - Child
KW - Drug Hypersensitivity/th [Therapy]
KW - Emergencies
KW - Female
KW - Humans
KW - Insect Bites and Stings/co [Complications]

KW - Insect Bites and Stings/th [Therapy]
KW - Life Support Care
KW - Male
KW - Middle Aged
RP - NOT IN FILE
SP - 339
EP - 342
JF - Acta Anaesthesiologica Scandinavica
JA - Acta Anaesthesiol Scand
VL - 32
IS - 4
CY - DENMARK
N2 - Twenty-seven patients with severe anaphylactic reactions were treated by two anesthesiologist-staffed ambulance helicopters during a 5-year period. All patients had signs of respiratory and/or circulatory failure. Cutaneous symptoms were described in 70% of the patients, gastrointestinal symptoms in 30% and central nervous symptoms in 85%. Wasp sting and drugs were the most common causes. Other etiologies were bee sting, snakebite, nuts and exercise. All patients were provided with advanced pre-hospital medical care. Epinephrine was employed in 78% of the patients. I.v. fluid therapy was started in 77%. Three patients (11%) needed endotracheal intubation. The time-span from onset of symptoms to medical attendance exceeded 45 min in two patients; both died. Of the surviving patients, 23 were hospitalized. The majority had no signs of respiratory or circulatory failure on arrival in the emergency room and needed only observation in the hospital
SN - 0001-5172
AD - Norwegian Air Ambulance, Central Hospital of Akershus, Nordbyhagen
UR - 3394485
ER -

TY - JOUR
ID - 2190
T1 - Prediction of children's blood lead levels on the basis of household-specific soil lead levels
A1 - Schilling,R.J.
A1 - Bain,R.P.
Y1 - 1988/07//
N1 - Schilling, R J. Bain, R P
American journal of epidemiology
3h3, 7910653
IM
Journal Article. Research Support, U.S. Gov't, Non-P.H.S.. Research Support, U.S. Gov't, P.H.S.
English
KW - MEDLINE
KW - Child
KW - Preschool
KW - Humans
KW - Idaho
KW - Infant
KW - Lead/an [Analysis]
KW - Lead/bl [Blood]
KW - Models
KW - Theoretical
KW - Montana
KW - Regression Analysis
KW - Smoking
KW - Soil/an [Analysis]
KW - Soil Pollutants
RP - NOT IN FILE
SP - 197
EP - 205
JF - American Journal of Epidemiology

JA - Am J Epidemiol
VL - 128
IS - 1
CY - UNITED STATES
N2 - To help guide policy decisions about removing lead-contaminated soils, the authors estimated a regression model for predicting a child's blood lead level on the basis of his or her household-specific soil lead level. The data analyzed were blood lead levels (1-45 micrograms/dl) and household-specific soil lead levels (53-20,700 ppm) of 596 children aged 1-5 years who lived in the Helena Valley of Montana and the Silver Valley of Idaho during August 1983. A non-threshold, multiple linear regression model indicated that the estimated mean natural log transformed blood lead level increased by 0.231 micrograms/dl for each unit increase in natural log transformed soil lead level (ppm), after adjusting for the average number of daily outdoor play hours and whether someone in the household smoked. The model predicted that, at a soil lead level of 1,000 ppm, a child who does not play outside and who does not live in a household where someone smokes would be at low risk of lead toxicity (blood lead level between 4 and 24 micrograms/dl)
SN - 0002-9262
AD - Center for Environmental Health and Injury Control, Centers for Disease Control, Atlanta, GA 30333
UR - 3381826
ER -

TY - JOUR
ID - 2191
T1 - Determinants of pediatric injuries
A1 - Horwitz,S.M.
A1 - Morgenstern,H.
A1 - DiPietro,L.
A1 - Morrison,C.L.
Y1 - 1988/06//
N1 - Horwitz, S M. Morgenstern, H. DiPietro, L. Morrison, C L
American journal of diseases of children (1960)
3gs, 0370471
AIM, IM
Journal Article. Research Support, Non-U.S. Gov't
English
KW - MEDLINE
KW - Attitude
KW - Child
KW - Continental Population Groups
KW - Employment
KW - Humans
KW - Longitudinal Studies
KW - Mothers/px [Psychology]
KW - Pediatrics/ut [Utilization]
KW - Prospective Studies
KW - Socioeconomic Factors
KW - Wounds and Injuries/ep [Epidemiology]
RP - NOT IN FILE
SP - 605
EP - 611
JF - American Journal of Diseases of Children
JA - Am J Dis Child
VL - 142
IS - 6
CY - UNITED STATES
N2 - Injuries are an important health issue for children. Previous research, however, has presented confusing and conflicting results on the determinants of childhood injuries, particularly psychosocial predictors, largely due to methodologic problems. The purpose of this analysis, based on a prospective follow-up study of 532 children, was to identify factors related to injuries encountered in a prepaid group practice during a 12-month

period. Using logistic regression, we found four factors independently associated with the risk of at least one treated injury: high activity level, high rate of pediatric utilization for non-injury-related visits during the follow-up period, occurrence of a treated injury during the year preceding the follow-up period, and negative attitude toward medical care providers by the child's mother. In addition, four factors were found to be independent predictors of injuries judged severe enough to always warrant medical care: occurrence of a treated injury in the preceding year, high rate of pediatric utilization for non-injury-related visits during the follow-up period, working more than 15 hours a week outside the home by the child's mother, and more life events reported by the mother for the year preceding the follow-up period. Since family stressors are related specifically to the risk of more severe injuries, which are unlikely to escape medical attention, we conclude that these factors probably are related to the occurrence of common injuries of early childhood and not exclusively to utilization behavior. We therefore suggest that children from families with these characteristics be targeted for injury prevention strategies
SN - 0002-922X
AD - Department of Epidemiology, Yale University School of Medicine, New Haven, CT 06510
UR - 3369398
ER -

TY - JOUR
ID - 2192
T1 - Redecoration of external leaded paintwork and lead-in-dust concentrations in school playgrounds
A1 - Schwar,M.J.
A1 - Alexander,D.J.
Y1 - 1988/01//
N1 - Schwar, M J. Alexander, D J
The Science of the total environment
uj0, 0330500
IM
Journal Article
English
KW - MEDLINE
KW - Child
KW - Dust/an [Analysis]
KW - Environmental Pollution
KW - Humans
KW - Lead/an [Analysis]
KW - Paint
KW - Schools
KW - Time Factors
RP - NOT IN FILE
SP - 45
EP - 59
JF - Science of the Total Environment
JA - Sci Total Environ
VL - 68
CY - NETHERLANDS
N2 - Playground lead-in-dust concentrations have been measured immediately after the external redecoration of 216 schools in 1983/4 and of 63 schools in 1984/5. Schools were divided into four age categories: A, pre-1914; B, 1914-1939; C, 1945-1965; and D, post-1965. The results show that concentrations can vary widely from school to school within a given age category. For each school, dust samples were collected from an area adjacent to the facade which had been redecorated, referred to here as the 'corridor' area, and from the 'remaining' playground area. The highest mean corridor concentrations were found in playgrounds of schools of age categories A and B, the highest mean value being 74,400 micrograms g-1. The highest value for age category D schools was 2600 micrograms g-1. The mean concentration for all schools in age categories A to D, was respectively 4720, 2820, 820 and 630 micrograms g-1 for the corridor area and 1180, 730, 400 and 440 micrograms g-1 for the remaining area. The highest lead-in-dust concentrations tended to occur within 2.5 m of the building facade. A limited number of measurements showed that concentrations in the corridor area tended to reduce with time, falling to half their initial value (after correcting for the local background level) in about

90-170 days. A model is suggested linking lead-in-dust concentrations in the corridor and remaining areas to lead-in-paint debris dust concentrations
SN - 0048-9697
AD - Air Pollution Group, London Scientific Services, United Kingdom
UR - 3363323
ER -

TY - JOUR
ID - 2193
T1 - The social behavior of depressed children: an observational study
A1 - Altmann,E.O.
A1 - Gotlib,I.H.
Y1 - 1988/02//
N1 - Altmann, E O. Gotlib, I H
Journal of abnormal child psychology
0364547, h33
IM
Journal Article. Research Support, Non-U.S. Gov't
English
KW - MEDLINE
KW - Child
KW - Depressive Disorder/px [Psychology]
KW - Female
KW - Humans
KW - Male
KW - Peer Group
KW - Psychological Tests
KW - Self Concept
KW - Social Adjustment
KW - Social Behavior
RP - NOT IN FILE
SP - 29
EP - 44
JF - Journal of Abnormal Child Psychology
JA - J Abnorm Child Psychol
VL - 16
IS - 1
CY - UNITED STATES
N2 - Few investigations have examined directly the psychosocial functioning of depressed children. In the present study, 20 depressed and 20 nondepressed fourth- and fifth-grade children were observed in free play during their recess period at school, and their self-perceptions were assessed in subsequent individual sessions. The 10 boys and 10 girls in each group were selected according to their scores on both the Child Depression Inventory and the Peer Nomination Inventory of Depression. Analyses conducted on the eight categories of behavioral observations revealed significant differences between the social behavior of the depressed and the nondepressed children. Although the depressed children made more overtures for social contact than did the nondepressed children and were approached by other children more frequently, they spent more time alone and engaged in a higher frequency of negative interactions with their peers. Consistent with these results, the depressed children's responses to the Self-Perception Profile for Children indicated that they experienced themselves as less socially competent in general, as well as less competent across several specific domains of functioning. These findings are discussed as they relate to developmental processes, theories of adult depression, and recent studies on socially isolated children, and directions for future research in this area are offered
SN - 0091-0627
AD - Department of Psychology, Boston University
UR - 3361029
ER -

TY - JOUR
ID - 2194
T1 - The effect of paternal social support on maternal disruption caused by childhood asthma
A1 - Wasilewski,Y.
A1 - Clark,N.
A1 - Evans,D.
A1 - Feldman,C.H.
A1 - Kaplan,D.
A1 - Rips,J.
A1 - Mellins,R.B.
Y1 - 1988///
N1 - Wasilewski, Y. Clark, N. Evans, D. Feldman, C H. Kaplan, D. Rips, J. Mellins, R B
Journal of community health
7600747, hut
IM
Journal Article. Research Support, Non-U.S. Gov't. Research Support, U.S. Gov't, P.H.S.
English
KW - MEDLINE
KW - Adult
KW - Age Factors
KW - Asthma/nu [Nursing]
KW - Child
KW - Ethnic Groups
KW - Female
KW - Health Education
KW - Home Nursing/px [Psychology]
KW - Humans
KW - Male
KW - Mothers/px [Psychology]
KW - Parents/ed [Education]
KW - Paternal Behavior
KW - Social Environment
KW - Social Support
RP - NOT IN FILE
SP - 33
EP - 42
JF - Journal of Community Health
JA - J Community Health
VL - 13
IS - 1
CY - UNITED STATES
N2 - Baseline data obtained from a study of 228 low-income Black and Hispanic mothers of children with asthma were analyzed to test the hypothesis that the presence of adults in the household, and paternal involvement in asthma care would be associated with less disruption of mothers' daily activities due to the child's asthma. The data were analyzed using multiple regression techniques. We controlled for other variables that might affect the amount of disruption that mothers reported, including the child's age and severity of asthma. The mere presence of other adults in the household, including the father, was not significantly associated with disruption. The frequency of the father's involvement in the care of the child's asthma was inversely associated with disruption regardless of his presence in the household. Hispanic ethnicity and severity of the child's asthma were directly associated with disruption while the age of the child was inversely associated with disruption. We conclude that it is a specific type of assistance (sharing asthma tasks) rendered by fathers living within or outside of the household that reduces disruption reported by mothers. The study suggests that health care providers can improve education and counseling for families who have a child with asthma by emphasizing to fathers their importance in the care of asthma, and by helping them identify specific actions they can take to manage the child's disease
SN - 0094-5145

AD - Department of Pediatrics, College of Physicians and Surgeons, Columbia University, New York, New York 10032
UR - 3360979
ER -

TY - JOUR
ID - 2195
T1 - [One year after Chernobyl. Cesium-137 levels in premature, newborn and nursing infants and in younger and older children of Bonn and environs]. [German]
A1 - Koch,H.C.
A1 - Knopp,R.
A1 - Niesen,M.
A1 - Halfmann,K.
A1 - Hartmann,C.
A1 - Burmeister,W.
Y1 - 1988/04/22/
N1 - Koch, H C. Knopp, R. Niesen, M. Halfmann, K. Hartmann, C. Burmeister, W
Deutsche medizinische Wochenschrift (1946)
ecl, 0006723
IM
Comparative Study. English Abstract. Journal Article
German
KW - MEDLINE
KW - Accidents
KW - Breast Feeding
KW - Cesium Radioisotopes/an [Analysis]
KW - Child
KW - Preschool
KW - Female
KW - Germany
KW - West
KW - Humans
KW - Infant
KW - Newborn
KW - Premature
KW - Male
KW - Nuclear Reactors
KW - Radioactive Fallout/an [Analysis]
KW - Suburban Population
KW - Ukraine
KW - Urban Population
KW - Whole-Body Counting
RP - NOT IN FILE
SP - 623
EP - 630
JF - Deutsche Medizinische Wochenschrift
JA - Dtsch Med Wochenschr.
VL - 113
IS - 16
CY - GERMANY, WEST
N2 - From May to August 1987, 137Cs activity was measured in a total-body counter (4 pi counter) in 33 premature and mature newborns (group I), 40 infants and young children up to 2 4/12 years of age (group II), and 15 children between 3 and 6 8/12 years (group III), all from Bonn or its environs. The mean of measurements in group II, at 3.7 Bq/kg body-weight, was lower than that of group I (5.8 Bq) and III (9.4 Bq). Mean radiation exposure, calculated from these data, was 1.7 mrem/a for group I, 1.1 mrem/a for group II, and 2.8 mrem/a for group III. A comparison with present Cs values in adults and measurements made at the end of the 1950's and beginning of 60's showed good agreement. There were no significant differences, as regards Cs

activity, between newborns or infants who had been formula-fed or breast-fed. The measured radiation exposure of the three groups is about 1% of natural radiation exposure. Thus, present-day results indicate that there will be no damage to health outside the natural scatter
SN - 0012-0472
AD - Universitats-Kinderklinik und Poliklinik, Bonn
UR - 3129272
ER -

TY - JOUR
ID - 2196
T1 - Efficacy of Haemophilus influenzae type b capsular polysaccharide vaccine
A1 - Black,S.B.
A1 - Shinefield,H.R.
A1 - Hiatt,R.A.
A1 - Fireman,B.H.
Y1 - 1988/03//
N1 - Black, S B. Shinefield, H R. Hiatt, R A. Fireman, B H
The Pediatric infectious disease journal
oxj, 8701858
IM
Journal Article. Research Support, Non-U.S. Gov't
English
KW - MEDLINE
KW - Bacterial Capsules
KW - Bacterial Vaccines
KW - Child
KW - Preschool
KW - Haemophilus Infections/ep [Epidemiology]
KW - Haemophilus Infections/mi [Microbiology]
KW - Haemophilus Infections/pc [Prevention & Control]
KW - Haemophilus Vaccines
KW - Haemophilus influenzae
KW - Humans
KW - Polysaccharides
KW - Bacterial
KW - Retrospective Studies
KW - Risk
KW - Seasons
KW - Socioeconomic Factors
RP - NOT IN FILE
SP - 149
EP - 156
JF - Pediatric Infectious Disease Journal
JA - Pediatr Infect Dis J
VL - 7
IS - 3
CY - UNITED STATES
N2 - The efficacy of the Haemophilus influenzae type b (Hib) polyribose phosphate vaccine was evaluated in a population of 120,000 children from 23 through 71 months of age in the Kaiser Permanente Medical Care Program (KPMCP) in Northern California over the 2-year period from June 1, 1985, through May 31, 1987. Approximately 37% of the population were vaccinated by the end of the first year and 60% were vaccinated by the end of the second year. There were 35 cases of Hib disease, 4 of whom were vaccine failures. Cases of Hib disease were identified by multiple modality case ascertainment, consisting of: (1) active surveillance in KPMCP microbiology laboratories; (2) active surveillance on KPMCP pediatric wards by a study physician; (3) retrospective review of computer-stored hospital discharge diagnoses; and (4) a review of all hospitalizations outside the health plan. The medical records of cases, matched controls and a random sample of the population were reviewed to obtain information on vaccination and related variables. Efficacy was evaluated using two

complementary methods. In a retrospective surveillance approach, efficacy was estimated to be 68% (95% confidence limits of 4, 89%). In a matched case-control analysis, efficacy was estimated to be 69% (95% confidence limits of -13, 91%). Adjustment for day care attendance and parental occupation slightly reduced the efficacy estimate. Other possible confounders including race, parental education and number of siblings were considered. Four cases of Hib disease were observed within 1 week following receipt of vaccine and before the time when immunity could have developed. There are several plausible explanations for the occurrence of these early cases including the possibility of chance alone.(ABSTRACT TRUNCATED AT 250 WORDS)
SN - 0891-3668
AD - Department of Pediatrics, Permanente Medical Group, Inc., Oakland, CA
UR - 3258659
ER -

TY - JOUR
ID - 2197
T1 - Knemometry in childhood: accuracy and standardization of a new technique of lower leg length measurement
A1 - Hermanussen,M.
A1 - Geiger-Benoit,K.
A1 - Burmeister,J.
A1 - Sippell,W.G.
Y1 - 1988/01//
N1 - Hermanussen, M. Geiger-Benoit, K. Burmeister, J. Sippell, W G
Annals of human biology
57r, 0404024
IM
Journal Article
English
KW - MEDLINE
KW - Adolescent
KW - Anthropometry/is [Instrumentation]
KW - Child
KW - Preschool
KW - Female
KW - Humans
KW - Leg/gd [Growth & Development]
KW - Male
KW - Reference Standards
RP - NOT IN FILE
SP - 1
EP - 15
JF - Annals of Human Biology
JA - Ann Hum Biol
VL - 15
IS - 1
CY - ENGLAND
N2 - Recently, a new anthropometric method of accurate lower leg length measurement (knemometry) was introduced. The present study was performed to investigate rigorously its strengths and weaknesses for auxological purposes, and to promote a standardized method of use. The data are based on 6 x 2200 single estimations of the lower leg length in 90 children of both sexes with normal, tall or short stature aged between 2.4 and 17.1 years. The overall technical error of this measurement was found to be 0.16 mm, which is reduced to 0.13 mm if the first estimation within each series of six is ignored. The following sources of systematic error could be detected: intra-daily variation, dependency on physical pressure put on or removed from the leg, vigorous physical activity prior to the measuring procedure, and a slow but significant correlation between day-to-day variation of lower leg length and body weight (r = 0.299, b = 0.372 mm/kg). There was no covariation with atmospheric parameters such as outside temperature, barometric pressure and others. After elimination of a linear trend from the individual growth curves, there was a remaining day-to-day variation of SD = 0.697 mm. This represents real changes of lower leg length and cannot be explained by the technical error of measurement

which contributes to less than 10% of the total variation. Some of this day-to-day variation may be explained by non-linearity of growth. Knemometry appears to be a robust and precise new tool for the investigation of short term kinetics of longitudinal growth. Proposals for a standardized use of this technique are given
SN - 0301-4460
AD - Department of Pediatrics, University of Kiel, FR Germany
UR - 3348587
ER -

TY - JOUR
ID - 2198
T1 - Effects of verapamil on left ventricular diastolic filling in children with hypertrophic cardiomyopathy
A1 - Shaffer,E.M.
A1 - Rocchini,A.P.
A1 - Spicer,R.L.
A1 - Juni,J.
A1 - Snider,R.
A1 - Crowley,D.C.
A1 - Rosenthal,A.
Y1 - 1988/02/15/
N1 - Shaffer, E M. Rocchini, A P. Spicer, R L. Juni, J. Snider, R. Crowley, D C. Rosenthal, A
The American journal of cardiology
3dq, 0207277
AIM, IM
Journal Article. Research Support, U.S. Gov't, P.H.S.
English
KW - MEDLINE
KW - Administration
KW - Oral
KW - Adolescent
KW - Cardiomyopathy
KW - Hypertrophic/dt [Drug Therapy]
KW - Hypertrophic/ri [Radionuclide Imaging]
KW - Child
KW - Exercise Test
KW - Follow-Up Studies
KW - Heart/ri [Radionuclide Imaging]
KW - Humans
KW - Myocardial Contraction/de [Drug Effects]
KW - Radionuclide Angiography
KW - Technetium/du [Diagnostic Use]
KW - Time Factors
KW - Verapamil/tu [Therapeutic Use]
RP - NOT IN FILE
SP - 413
EP - 417
JF - American Journal of Cardiology
JA - Am J Cardiol
VL - 61
IS - 6
CY - UNITED STATES
N2 - The effects of oral verapamil on resting left ventricular (LV) diastolic filling were examined in 10 children and adolescents with hypertrophic cardiomyopathy. Measurements of diastolic filling were made from gated technetium-99m radionuclide angiograms with postbeat rejection of data outside a 5% RR-interval window. LV time-activity curves were generated and the rapid-filling phase fit with a 3 degrees polynomial to calculate the peak filling rate and the time from end-systole to the point of peak filling. All patients had a radionuclide angiogram performed before and after 0.25 to 3 years of oral verapamil therapy. Verapamil did not change the LV ejection fraction but increased the peak filling rate (3.24 +/- 0.15 to 4.62 +/- 1.05 end-diastolic volume/s,p

less than 0.01) and reduced the time to peak filling (217 +/- 57 to 168 +/- 63 ms, p less than 0.01). An increase in exercise endurance as measured by exercise treadmill test and subjective symptomatic improvement were also seen after verapamil therapy. Thus, in children with hypertrophic cardiomyopathy, symptomatic improvement and LV diastolic filling parameters improved with long-term oral verapamil
SN - 0002-9149
AD - Department of Pediatric Cardiology, Mott Children's Hospital, University of Michigan Medical Center, Ann Arbor 48109-0204
UR - 3341224
ER -

TY - JOUR
ID - 2199
T1 - Determinants of indoor NO2-concentration and the personal exposures of children and their mothers in Japanese homes
A1 - Dassen,W.G.
A1 - Matsuki,H.
A1 - Kasuga,H.
A1 - Misawa,K.
A1 - Yokoyama,H.
A1 - Shimizu,Y.
Y1 - 1987/05//
N1 - Dassen, W G. Matsuki, H. Kasuga, H. Misawa, K. Yokoyama, H. Shimizu, Y
The Tokai journal of experimental and clinical medicine
vzm, 7704186
IM
Journal Article
English
KW - MEDLINE
KW - Adult
KW - Child
KW - Environmental Exposure
KW - Environmental Monitoring
KW - Female
KW - Housing
KW - Humans
KW - Male
KW - Nitrogen Dioxide/an [Analysis]
KW - Vehicle Emissions
RP - NOT IN FILE
SP - 83
EP - 95
JF - Tokai Journal of Experimental & Clinical Medicine
JA - Tokai J Exp Clin Med
VL - 12
IS - 2
CY - JAPAN
N2 - In February and in July 1986 the personal exposures to NO2 of 60 children and their mothers were measured and NO2-concentrations in their homes were measured. In the summer also the NO2-concentration in the kitchen was determined during the time that the mother was in the kitchen. The mothers were asked to keep a diary of the activities of her and her child during that day and of the time that the heating appliances, cooking range, etc. were used. In the winter indoor NO2-concentrations and personal exposures of mother and child were much higher than in the summer. In the winter in many cases the Japanese Ambient Air Quality Standard is exceeded. The elevated levels in the winter can be related to the use of NO2-producing heating appliances especially the gas- or oilstove without a flue. Appliance of a flue still causes the indoor NO2-concentrations to be elevated. The division of a study population into two exposure categories on the basis of presence of a non-vented gasstove is therefore inappropriate. An instantaneous gas fired water heater may also contribute to elevated indoor NO2-concentrations and personal exposures, although the present survey could not provide

detailed information about the use of a flue and the location of the geyser. The time that a gasrange is being used may influence the indoor NO2-concentrations, but the results from this survey were not clear. Ventilation in the kitchen reduces the indoor NO2-concentrations, although this seems especially true in case the gas range is being used for a long time. In houses with smokers higher NO2-concentrations were found than in houses with non smokers. In the winter the non-vented gas stove is the most important indoor NO2-source. The present study was not able to demonstrate the effect of outdoor NO2-concentrations on the indoor air concentrations. A longer time span would be necessary. The housestructure, i.e. the construction of the house and the number of rooms influence the indoor NO2-concentrations and personal exposure. In the winter all indoor NO2-concentrations are highly intercorrelated and also highly correlated with the personal exposures of mother and child. Multiple regression models based on either measured or time-weighted NO2-concentrations explain about 85 percent of the variance in the personal exposures of child and mother in the winter. The exposures calculated from the time-weighted NO2-concentrations correlate very well with the measured personal exposures. This was not the case in the summer.(ABSTRACT TRUNCATED AT 400 WORDS)
SN - 0385-0005
AD - Department of Public Health, School of Medicine, Tokai University
UR - 2453087
ER -

TY - JOUR
ID - 2200
T1 - Life threat and posttraumatic stress in school-age children
A1 - Pynoos,R.S.
A1 - Frederick,C.
A1 - Nader,K.
A1 - Arroyo,W.
A1 - Steinberg,A.
A1 - Eth,S.
A1 - Nunez,F.
A1 - Fairbanks,L.
Y1 - 1987/12//
N1 - Pynoos, R S. Frederick, C. Nader, K. Arroyo, W. Steinberg, A. Eth, S. Nunez, F. Fairbanks, L
Archives of general psychiatry
72c, 0372435
AIM, IM
Journal Article. Research Support, Non-U.S. Gov't
English
KW - MEDLINE
KW - Acute Disease
KW - Age Factors
KW - Child
KW - Ethnic Groups
KW - Female
KW - Humans
KW - Life Change Events
KW - Male
KW - Personality Inventory
KW - Psychiatric Status Rating Scales
KW - Sex Factors
KW - Stress Disorders
KW - Post-Traumatic/di [Diagnosis]
KW - Post-Traumatic/et [Etiology]
KW - Post-Traumatic/px [Psychology]
KW - Violence
RP - NOT IN FILE
SP - 1057
EP - 1063
JF - Archives of General Psychiatry

JA - Arch Gen Psychiatry
VL - 44
IS - 12
CY - UNITED STATES
N2 - One hundred fifty-nine children (14.5% of the student body) were sampled after a fatal sniper attack on their elementary school playground. Systematic self-reports of posttraumatic stress disorder (PTSD) symptoms were obtained by use of a child PTSD Reaction Index. Analysis of variance revealed significant differences by exposure but not by sex, ethnicity, or age. Additional analyses were conducted of individual item response, overall severity of PTSD reaction, symptom grouping, and previous life events. The results provide strong evidence that acute PTSD symptoms occur in school-age children with a notable correlation between proximity to the violence and type and number of PTSD symptoms. Sampling at approximately one month after the trauma provided adequate delineation among exposure groups. The symptom profile of highly exposed children lends validity to the diagnosis of acute PTSD in childhood
SN - 0003-990X
AD - Department of Psychiatry and Behavioral Sciences, UCLA
UR - 3689093
ER -

TY - JOUR
ID - 2201
T1 - The assessment of children's physical activity: a comparison of methods
A1 - Klesges,L.M.
A1 - Klesges,R.C.
Y1 - 1987/10//
N1 - Klesges, L M. Klesges, R C
Medicine and science in sports and exercise
8005433, mg8
IM, S
Journal Article. Research Support, Non-U.S. Gov't. Research Support, U.S. Gov't, P.H.S.
English
KW - MEDLINE
KW - Child Behavior
KW - Child
KW - Preschool
KW - Electronics
KW - Female
KW - Humans
KW - Male
KW - Methods
KW - Motion
KW - Physical Exertion
RP - NOT IN FILE
SP - 511
EP - 517
JF - Medicine & Science in Sports & Exercise
JA - Med Sci Sports Exerc
VL - 19
IS - 5
CY - UNITED STATES
N2 - The authors assessed the convergent validity and the sources of error in an electronic single-plane accelerometer (i.e., Caltrac Personal Activity Computer (Hemokinetics, Inc., Vienna, VA). The device was validated against observed all-day physical activity levels of children in their natural environment. Thirty pre-school children were observed in non-structured activity for periods of approximately 9 h while wearing the accelerometer. The results revealed moderately high but variable Spearman rank-order correlations between hourly readings of the accelerometer and the observational system (range of correlations = 0.62 to 0.95). An all-day accelerometer reading significantly correlated with the observational instrument (rho = 0.54). A step-wise regression analysis revealed that the best behavioral predictor of the all-day accelerometer reading was the

observed behavior of walking, explaining 32% of the total variance. Older vs younger children (i.e., greater than 32.5 months), females vs males, and overweight (i.e., 75 percentile or greater) vs normal weight children tended to show higher correlations between direct observation and accelerometer readings. Implications of these findings and the utilization of the accelerometer in epidemiologic research are discussed
SN - 0195-9131
AD - Department of Psychology, Memphis State University, TN 38152
UR - 3683156
ER -

TY - JOUR
ID - 2202
T1 - The role of squirrels in sustaining monkeypox virus transmission
A1 - Khodakevich,L.
A1 - Szczeniowski,M.
A1 - Manbu,ma Disu
A1 - Jezek,Z.
A1 - Marennikova,S.
A1 - Nakano,J.
A1 - Messinger,D.
Y1 - 1987/04//
N1 - Khodakevich, L. Szczeniowski, M. Manbu-ma-Disu. Jezek, Z. Marennikova, S. Nakano, J. Messinger, D
Tropical and geographical medicine
wgj, 0376231
IM
Journal Article
English
KW - MEDLINE
KW - Animals
KW - Wild
KW - Antibodies
KW - Viral/an [Analysis]
KW - Carrier State/ep [Epidemiology]
KW - Carrier State/ve [Veterinary]
KW - Chick Embryo
KW - Child
KW - Preschool
KW - Chiroptera
KW - Democratic Republic of the Congo
KW - Disease Reservoirs
KW - Female
KW - Humans
KW - Infant
KW - Monkeypox virus/im [Immunology]
KW - Monkeypox virus/ip [Isolation & Purification]
KW - Poxviridae Infections/ep [Epidemiology]
KW - Poxviridae Infections/tm [Transmission]
KW - Poxviridae Infections/ve [Veterinary]
KW - Rodentia
KW - Sciuridae
KW - Ticks
KW - Vero Cells
RP - NOT IN FILE
SP - 115
EP - 122
JF - Tropical & Geographical Medicine
JA - Trop Geogr Med
VL - 39

IS - 2
CY - NETHERLANDS
N2 - When the research on the ecology of monkeypox virus entered the latest stage in 1984, three groups of animals were considered priority candidates for maintenance of virus circulations in nature because of their relatively higher population density: terrestrial rodents, squirrels, and gregarious bats. Following the isolation of the virus from a wild squirrel in 1985 another survey was carried out in Bumba zone of Zaire in January-February 1986, which included collection of animal samples, later on tested by WHO collaborating laboratories. No antibodies were found in 233 rodents tested. A high prevalence of monkeypox-specific antibodies (24.7%) found in 320 squirrels of Funisciurus anerythrus species suggested that these animals sustain virus transmission in the areas surrounding human settlements. A consistently high level of antibody prevalence found among squirrels Heliosciurus rufobrachium suggested that this species is also steadily involved in the transmission. The squirrels, the only mammals frequently infected by monkeypox in the areas of human activities, which are frequently trapped by the population, are obviously a major source of infection for human beings. Out of 39 primates collected during the survey from the primary forest, three were found seropositive for monkeypox. At the moment, however, it is difficult to say whether the primates play an important role in sustaining virus transmission in nature, or (like human beings) that they are an occasional host of the virus. Possibilities are discussed of monkeypox virus transmission being sustained by squirrels outside the areas of major human activities
SN - 0041-3232
UR - 2820094
ER -

TY - JOUR
ID - 2203
T1 - Physical exercise program for children with bronchial asthma
A1 - Szentagothai,K.
A1 - Gyene,I.
A1 - Szocska,M.
A1 - Osvath,P.
Y1 - 1987/05//
N1 - Szentagothai, K. Gyene, I. Szocska, M. Osvath, P
Pediatric pulmonology
owh, 8510590
IM
Journal Article. Research Support, Non-U.S. Gov't
English
KW - MEDLINE
KW - Adolescent
KW - Asthma/pp [Physiopathology]
KW - Asthma/rh [Rehabilitation]
KW - Cardiovascular System/pp [Physiopathology]
KW - Child
KW - Preschool
KW - Exercise Therapy
KW - Female
KW - Follow-Up Studies
KW - Gymnastics
KW - Humans
KW - Male
KW - Physical Fitness
KW - Running
KW - Swimming
RP - NOT IN FILE
SP - 166
EP - 172
JF - Pediatric Pulmonology
JA - Pediatr Pulmonol

VL - 3
IS - 3
CY - UNITED STATES
N2 - A long-term physical exercise program was established for a large number of children with bronchial asthma. Asthmatic children were first taught to swim on their backs to prevent breathing problems customary for beginners using other strokes. They concurrently participated in gymnasium exercises, and the program was later completed with outdoor running. Program effectiveness was evaluated by monitoring asthmatic symptoms, changes in medication, and changes in the activity and physical fitness of the children. Data collected from 121 children showed that during the first year in the program the number of days with asthmatic symptoms decreased in a large majority of the patients while medication was decreased. School absenteeism and hospitalization dropped markedly. Parental evaluation of the children indicated much improvement in 51.2%, improvement in 40.5%, unchanged condition in 7.4%, and deterioration of general health was only reported in one child (0.8%). The same extent of improvement continued during the second year. The Cooper test was applied for the first time to such an exercise program and indicated that the participating asthmatic children performed as well as a control group of nonasthmatic children, and the cardiovascular efficiency of the asthmatics was actually better
SN - 8755-6863
UR - 3615039
ER -

TY - JOUR
ID - 2204
T1 - Ethylene oxide (ETO) as a major cause of anaphylactoid reactions in dialysis (a review). [Review] [60 refs]
A1 - Bommer,J.
A1 - Ritz,E.
Y1 - 1987/04//
N1 - Bommer, J. Ritz, E
Artificial organs
8zk, 7802778
IM
Journal Article. Review
English
KW - MEDLINE
KW - Anaphylaxis/ci [Chemically Induced]
KW - Antibodies/im [Immunology]
KW - Child
KW - Complement Activation
KW - Dermatitis
KW - Contact/et [Etiology]
KW - Occupational/ci [Chemically Induced]
KW - Drug Hypersensitivity/et [Etiology]
KW - Ethylene Oxide/ae [Adverse Effects]
KW - Ethylene Oxide/im [Immunology]
KW - Humans
KW - Kidneys
KW - Artificial/ae [Adverse Effects]
KW - Renal Dialysis/ae [Adverse Effects]
KW - Sterilization/mt [Methods]
RP - NOT IN FILE
SP - 111
EP - 117
JF - Artificial Organs
JA - Artif Organs
VL - 11
IS - 2
CY - UNITED STATES

N2 - Ethylene oxide (ETO), an alkylating compound of high chemical reactivity, is widely used for gas sterilization, but recently serious ETO side reactions have been recognized. With chronic ETO exposure, increased spontaneous abortion, sister chromatid exchange, and leukemia are observed. After medical use of ETO outside nephrology, contact dermatitis, cardiopulmonary shock (during cardiopulmonary surgery), allergic local reactions to ETO sterilized lenses, and anaphylactoid reactions to ETO sterilized catheters have been described. In numerous dialysis patients widespread hypersensitivity to ETO has been documented by skin prick test and ETO radioallergosorbent test (RAST). Furthermore an anaphylactoid "first-use reaction" was described in dialyzed patients, most of whom were using hollow-fiber dialyzers. After long discussions whether complement activation versus hypersensitivity is the cause of such acute anaphylactoid reactions, more recent studies using either ETO RAST or basophil degranulation tests implicate ETO hypersensitivity as their major cause. The high prevalence of sensitization to ETO and the frequency, unpredictability, and potential danger of anaphylactoid reactions to ETO lead to the conclusion that ETO sterilization of dialyzers should be discontinued, since alternative modalities of sterilization are currently available. [References: 60]
SN - 0160-564X
UR - 2954522
ER -

TY - JOUR
ID - 2205
T1 - Unprogrammed effects of training high-status peers to interact with severely handicapped children
A1 - Sasso,G.M.
A1 - Rude,H.A.
Y1 - 1987///
N1 - Sasso, G M. Rude, H A
Journal of applied behavior analysis
0174763, hdr
IM
Journal Article
English
KW - MEDLINE
KW - Autistic Disorder/px [Psychology]
KW - Child
KW - Disabled Persons/px [Psychology]
KW - Education
KW - Special
KW - Female
KW - Humans
KW - Intellectual Disability/px [Psychology]
KW - Interpersonal Relations
KW - Male
KW - Peer Group
KW - Social Desirability
RP - NOT IN FILE
SP - 35
EP - 44
JF - Journal of Applied Behavior Analysis
JA - J Appl Behav Anal
VL - 20
IS - 1
CY - UNITED STATES
N2 - We examined the effects of a peer initiation intervention with high- and low-status nonhandicapped students on the behavior of untrained peers toward handicapped students. In the context of a counterbalanced withdrawal design, high- and low-status nonhandicapped students were taught to direct social initiations to eight severely handicapped students during recess activities. The interactions of the high-status students resulted in higher levels of initiations by untrained peers toward the handicapped students than did the interactions of the low-status students. Social response levels were also differentially affected by the status of the peer initiator
SN - 0021-8855

UR - 2953705
ER -

TY - JOUR
ID - 2206
T1 - Clinical prediction of cervical spine injuries in children. Radiographic abnormalities
A1 - Rachesky,I.
A1 - Boyce,W.T.
A1 - Duncan,B.
A1 - Bjelland,J.
A1 - Sibley,B.
Y1 - 1987/02//
N1 - Rachesky, I. Boyce, W T. Duncan, B. Bjelland, J. Sibley, B
American journal of diseases of children (1960)
3gs, 0370471
AIM, IM
Journal Article
English
KW - MEDLINE
KW - Accidents
KW - Traffic
KW - Adolescent
KW - Athletic Injuries/ra [Radiography]
KW - Cervical Vertebrae/in [Injuries]
KW - Cervical Vertebrae/ra [Radiography]
KW - Child
KW - Preschool
KW - Female
KW - Humans
KW - Male
KW - Medical History Taking
KW - Physical Examination
KW - Play and Playthings
KW - Sports
RP - NOT IN FILE
SP - 199
EP - 201
JF - American Journal of Diseases of Children
JA - Am J Dis Child
VL - 141
IS - 2
CY - UNITED STATES
N2 - During the seven-year period from 1976 through 1982, 2133 cervical spine radiographs were obtained for children less than 18 years of age at two hospitals in Tucson. Twenty-five children (1.2%) had x-ray evidence of a cervical spine injury, with a male to female ratio of 4:1. Vehicular accidents accounted for 36% of cervical spine radiographic abnormalities, and sports or playground accidents accounted for an additional 36%. In an attempt to find clinical predictors that might identify x-ray abnormalities, we reviewed the medical records of these 25 children with abnormalities and 713 randomly selected children without x-ray evidence of cervical spine injuries for the following: method of injury, presenting complaints, physical examination findings, therapy, and complications. No single clinical predictor had a sensitivity of 100% when considered in isolation, but clinical assessment consisting of EITHER a complaint of neck pain OR involvement in a vehicular accident with head trauma would have correctly identified all 25 cases of cervical spine injury. If this information had been used prospectively, the number of cervical spine radiographs ordered would have been reduced by 32%. We conclude that the use of this clinical "marker" would have positively identified all children with cervical spine injuries and would have reduced by one third the cost and radiation exposure associated with cervical spine radiographs. Because of the serious consequences of missing a cervical spine injury, we suggest that other studies confirm these results before this information is accepted as a recommendation

SN - 0002-922X
UR - 3812387
ER -

TY - JOUR
ID - 2207
T1 - The pathogenesis of virus-associated encephalopathies: a prospective study of immunological mechanisms
A1 - Kennedy,C.R.
A1 - Bird,D.
A1 - Chrzanowska,K.
A1 - Stephens,S.
A1 - Webster,A.D.
Y1 - 1987/02//
N1 - Kennedy, C R. Bird, D. Chrzanowska, K. Stephens, S. Webster, A D
Clinical immunology and immunopathology
dea, 0356637
IM, X
Journal Article. Research Support, Non-U.S. Gov't
English
KW - MEDLINE
KW - Adolescent
KW - Adult
KW - Antibody Formation
KW - Blood-Brain Barrier
KW - Child
KW - Preschool
KW - Encephalitis/et [Etiology]
KW - Encephalitis/im [Immunology]
KW - Encephalitis/pa [Pathology]
KW - Humans
KW - Immunity
KW - Cellular
KW - Infant
KW - Inflammation
KW - Killer Cells
KW - Natural/im [Immunology]
KW - T-Lymphocytes/cl [Classification]
KW - T-Lymphocytes/im [Immunology]
KW - Virus Diseases/co [Complications]
KW - Virus Diseases/im [Immunology]
KW - Virus Diseases/pa [Pathology]
RP - NOT IN FILE
SP - 218
EP - 228
JF - Clinical Immunology & Immunopathology
JA - Clin Immunol Immunopathol
VL - 42
IS - 2
CY - UNITED STATES
N2 - Thirty-two patients, including 29 children, presenting with acute unexplained encephalopathies were studied prospectively for evidence of virus infection, immunodeficiency, and immunologic involvement in the pathogenesis of their illnesses. Twenty-five of these patients had a clinical diagnosis of encephalitis. Twenty-two of these 25 had laboratory evidence of active virus infection, the majority with viruses usually associated with self-limiting illness outside the central nervous system. In patients with encephalitis, immune competence, as reflected by T-cell numbers and subsets in peripheral blood, in vitro interferon production, natural killer activity, and specific antiviral antibody production, was normal. Transudation of albumin into the cerebrospinal fluid (CSF), a measure of blood-brain barrier breakdown, was seen in 40% of patients. Intrathecal antibody

synthesis was suggested by an elevated IgG index in 9/20 CSF/serum pairs but was confirmed by an elevated specific IgG ratio in only 3. The serum IgG1 and IgG3 subclass levels were significantly elevated at the time of the illness and remained elevated 8 months later; IgG2 and IgG4 levels were normal. IgE levels were elevated in 50% of patients. Serum levels of IgM antibodies against Escherichia coli measured 8 months after the neurological illness were also significantly higher in encephalitis patients than in age-matched healthy controls. Human myelin basic protein did not induce proliferation in peripheral blood lymphocytes in any patient. We conclude that most encephalopathies associated with viral infections are not due to an underlying generalized immunodeficiency, and probably result from an inappropriately vigorous immune response
SN - 0090-1229
UR - 3492319
ER -

TY - JOUR
ID - 2208
T1 - Epidemiology of school injuries: a 2-year experience in a municipal health department
A1 - Sheps,S.B.
A1 - Evans,G.D.
Y1 - 1987/01//
N1 - Sheps, S B. Evans, G D
Pediatrics
oxv, 0376422
AIM, IM
Journal Article
English
KW - MEDLINE
KW - Accidental Falls
KW - Accidents
KW - Adolescent
KW - Athletic Injuries/ep [Epidemiology]
KW - British Columbia
KW - Child
KW - Contusions/ep [Epidemiology]
KW - Craniocerebral Trauma/ep [Epidemiology]
KW - Humans
KW - Population Surveillance
KW - Public Health Administration
KW - School Health Services
KW - Schools
KW - Sports
KW - Wounds and Injuries/ep [Epidemiology]
RP - NOT IN FILE
SP - 69
EP - 75
JF - Pediatrics
VL - 79
IS - 1
CY - UNITED STATES
N2 - School injuries occurring in a municipal school system during a 2-year period were reviewed to identify epidemiologic features of school injuries, to determine data requirements for ongoing injury surveillance, and to identify potential preventive strategies. Overall, 3,009 injuries were reported (2.82/100 students per year). Elementary school students had only a slightly higher rate (2.85) than secondary school students (2.78). However, the cause, nature, school location of injury, and body area injured formed distinct patterns in these two groups. Playgrounds were responsible for the highest overall and elementary school rates, whereas sports areas and classrooms had the highest rates among secondary school students. Falls were the most frequent cause of injury in elementary schools whereas, as expected, sports injuries were the most frequent cause among secondary school students. Contusions and abrasions of the head were the most frequent type of injury for both groups, although more common among elementary school students, whereas fractures, sprains, strains, and

dislocations were more frequent among secondary school students. Although the proportion of severe injuries to secondary school students was slightly higher (39 v 35%), the rate of referral of students to a hospital or physicians among secondary school students (1.21 per 100 student-hours) was almost twice the rate of elementary school students (0.65 per 100 student-hours). Problems with definition of injury severity and the need to explore the social aspects of schools as a factor in injuries emerged as important considerations for future research
SN - 0031-4005
UR - 3797172
ER -

TY - JOUR
ID - 2209
T1 - The time costs of children
A1 - Turchi,B.
Y1 - 1987///
N1 - Turchi, B
Social science
20810190r
J
Journal Article
English
KW - MEDLINE
KW - Adolescent
KW - Age Factors
KW - Americas
KW - Behavior
KW - Child
KW - Child Care
KW - Child Rearing
KW - Demography
KW - Developed Countries
KW - Developing Countries
KW - Economics
KW - Employment
KW - Family Characteristics
KW - Family Relations
KW - Housekeeping
KW - Models
KW - Economic
KW - Theoretical
KW - Mothers
KW - North America
KW - Parents
KW - Population
KW - Population Characteristics
KW - Population Dynamics
KW - Social Behavior
KW - Time Factors
KW - United States
RP - NOT IN FILE
SP - 185
EP - 190
JF - Social Science
JA - Soc Sci (Kans.)
VL - 72
IS - 2-4
CY - UNITED STATES

SN - 0037-7848
UR - 12342049
ER -

TY - JOUR
ID - 2210
T1 - [Therapeutic and corrective work with stammering preschool children in a daytime semi-hospital]. [Russian]
A1 - Mokrovskaia,A.A.
Y1 - 1986///
N1 - Mokrovskaia, A A
Zhurnal nevropatologii i psikhiatrii imeni S.S. Korsakova (Moscow, Russia : 1952)
y9y, 8710066
IM
English Abstract. Journal Article
Russian
KW - MEDLINE
KW - Ambulatory Care Facilities
KW - Child
KW - Preschool
KW - Humans
KW - Moscow
KW - Psychotherapy
KW - Group
KW - Speech Therapy
KW - Speech-Language Pathology
KW - Stuttering/px [Psychology]
KW - Stuttering/th [Therapy]
RP - NOT IN FILE
SP - 1565
EP - 1569
JF - Zhurnal Nevropatologii i Psikhiatrii Imeni S - S - Korsakova
JA - Zh Nevropatol.Psikhiatr.Im S S Korsakova
VL - 86
IS - 10
CY - USSR
N2 - Therapeutic and corrective work with stammering preschool children conducted at a day-time semi-hospital institution presents a complex of measures aimed at improving the speech and the entire psychic activity of the child. A semi-hospital form of the organization makes it possible, along with therapeutic and corrective measures, to constantly train the speech in the most diverse situations of the microsocial environment outside the hospital and thus contributes to the development of social and adaptive behaviour which the child needs for further studying in the general education school
SN - 0044-4588
UR - 3544614
ER -

TY - JOUR
ID - 2211
T1 - Role reversals: an analysis of therapeutic effects achieved with disruptive boys during their appointments as peer monitors
A1 - Fowler,S.A.
A1 - Dougherty,B.S.
A1 - Kirby,K.C.
A1 - Kohler,F.W.
Y1 - 1986///
N1 - Fowler, S A. Dougherty, B S. Kirby, K C. Kohler, F W
Journal of applied behavior analysis

0174763, hdr
IM
Case Reports. Journal Article. Research Support, U.S. Gov't, P.H.S.
English
KW - MEDLINE
KW - Child
KW - Child Behavior Disorders/th [Therapy]
KW - Cues
KW - Environment
KW - Feedback
KW - Generalization (Psychology)
KW - Humans
KW - Interpersonal Relations
KW - Male
KW - Peer Group
KW - Recreation
KW - Role
RP - NOT IN FILE
SP - 437
EP - 444
JF - Journal of Applied Behavior Analysis
JA - J Appl Behav Anal
VL - 19
IS - 4
CY - UNITED STATES
N2 - Three 7-year-old boys with histories of negative interactions were appointed to monitor individual classmates during daily noon recess. As monitors, the boys awarded points to their classmates for playing appropriately and, on rare occasions, withdrew a point for negative interactions. The three boys immediately decreased their own rates of negative interactions during the sessions in which they were appointed as monitors. Two of the boys concomitantly increased their rates of positive interactions. Their reductions in negative interactions were not maintained during reversals and did not clearly generalize to the morning or afternoon recess periods. Subsequent appointment of the boys as peer monitors during the morning recess produced similar improvements in their behavior. Results suggest that appointment to the role of peer monitor may itself function as an intervention
SN - 0021-8855
UR - 3804878
ER -

TY - JOUR
ID - 2212
T1 - Standardized extracts, trees. [Review] [46 refs]
A1 - Belin,L.
Y1 - 1986/11//
N1 - Belin, L
Clinical reviews in allergy
cla, 8308524
IM
Comparative Study. Journal Article. Review
English
KW - MEDLINE
KW - Administration
KW - Oral
KW - Adult
KW - Allergens/ad [Administration & Dosage]
KW - Allergens/im [Immunology]
KW - Allergens/st [Standards]
KW - Child

KW - Cross Reactions
KW - Humans
KW - Immunotherapy
KW - Plant Extracts/ad [Administration & Dosage]
KW - Plant Extracts/im [Immunology]
KW - Plant Extracts/st [Standards]
KW - Pollen/ip [Isolation & Purification]
KW - Respiratory Hypersensitivity/im [Immunology]
KW - Respiratory Hypersensitivity/th [Therapy]
KW - Trees
RP - NOT IN FILE
SP - 389
EP - 404
JF - Clinical Reviews in Allergy
JA - Clin Rev Allergy
VL - 4
IS - 4
CY - UNITED STATES
N2 - Sensitization to pollen from wind-pollinated trees causes significant allergy problems during springtime. The magnitude of prevalence is comparable with and, in some areas, even higher than grass pollen allergy. The symptom periods are often prolonged by cross-reactivity among species and sometimes families as well as by simultaneous sensitivity to several trees, pollinating at different times. There is a need for standardized extracts to improve diagnostic precision and treatment efficacy. A substantial amount of immunochemical data on the allergens from Betulaceae species, particularly Betula (birches), are available. Relevant methods to quantitatively and qualitatively measure the allergens of birch pollen extracts are clearly reproducible in laboratories around the world. By international collaboration, such work has generated an IS that has been accepted by the IUIS Subcommittee on Allergen Standardization. The IS is a yardstick for authorities, manufacturers, and researchers that is carefully specified in terms of 1 major and a few intermediate allergens, and also of total biologic activity by RAST inhibition. A meaningful declaration of biologic activity in vivo is awaited. As yet, only a few clinical trials have been undertaken with standardized tree pollen extracts. These are, however, highly interesting, particularly those that now have started with an oral route of administration. [References: 46]
SN - 0731-8235
UR - 3542164
ER -

TY - JOUR
ID - 2213
T1 - Health risk analysis of human exposures to soil amended with sewage sludge contaminated with polychlorinated dibenzodioxins and dibenzofurans
A1 - Eschenroeder,A.
A1 - Jaeger,R.J.
A1 - Ospital,J.J.
A1 - Doyle,C.P.
Y1 - 1986/10//
N1 - Eschenroeder, A. Jaeger, R J. Ospital, J J. Doyle, C P
Veterinary and human toxicology
xbv, 7704194
IM
Journal Article
English
KW - MEDLINE
KW - Adult
KW - Benzofurans/ae [Adverse Effects]
KW - Child
KW - Dioxins/ae [Adverse Effects]
KW - Health

KW - Humans
KW - Risk
KW - Sewage/ae [Adverse Effects]
KW - Skin Absorption
KW - Soil Pollutants/ae [Adverse Effects]
KW - Tetrachlorodibenzodioxin/ae [Adverse Effects]
RP - NOT IN FILE
SP - 435
EP - 442
JF - Veterinary & Human Toxicology
JA - Vet Hum Toxicol
VL - 28
IS - 5
CY - UNITED STATES
N2 - The risk of cancer to humans exposed to soil treated with wastewater/sewage treatment plant sludge, known to be contaminated with small amounts of polychlorinated dibenzodioxins and dibenzofurans (PCDDs and PCDFs), was evaluated. The particulate-bound PCDDs and PCDFs are found in trace amounts in the effluent from ground water pumping (dewatering) at an abandoned wood preservation facility. The water, which was sent to a water recovery plant, underwent primary and secondary treatment prior to discharge. The residual sludge was added to agricultural soil as a conditioner. The present analysis treats the extreme case of sludge applied near the home of a target individual, a lifetime resident, who is also a farm worker in the area of the application. The successive stages of infancy, childhood and adulthood are treated separately to assess the contributions of typical age-specific indoor and outdoor activities on exposure rates. Five toxicity rating schemes using so-called TCDD equivalents, and two unit risk slopes are applied to the chemical profile in sludge to determine the cancer potency of the soil contaminants. These risk estimates range from 1 X 10-8 to 3 X 10-7
SN - 0145-6296
UR - 3776090
ER -

TY - JOUR
ID - 2214
T1 - Increasing autistic children's spontaneous verbalizations of affection: an assessment of time delay and peer modeling procedures
A1 - Charlop,M.H.
A1 - Walsh,M.E.
Y1 - 1986///
N1 - Charlop, M H. Walsh, M E
Journal of applied behavior analysis
0174763, hdr
IM
Journal Article
English
KW - MEDLINE
KW - Affect
KW - Autistic Disorder/px [Psychology]
KW - Autistic Disorder/th [Therapy]
KW - Behavior Therapy/mt [Methods]
KW - Child
KW - Education of Intellectually Disabled
KW - Humans
KW - Imitative Behavior
KW - Interpersonal Relations
KW - Male
KW - Peer Group
KW - Social Adjustment
KW - Social Behavior

KW - Verbal Behavior
RP - NOT IN FILE
SP - 307
EP - 314
JF - Journal of Applied Behavior Analysis
JA - J Appl Behav Anal
VL - 19
IS - 3
CY - UNITED STATES
N2 - We assessed the efficacy of time delay and peer modeling procedures in increasing autistic children's spontaneous verbalizations of affection. Four autistic children were taught to spontaneously say "I like (love) you" in response to a hug from a familiar person and their mother. Generalization from a free play training setting to free play outdoors and at home was assessed. Ancillary social and affection behaviors were also observed. Results indicated that the time delay was a quick and effective procedure for all the children. Peer modeling was unsuccessful in teaching the target behavior
SN - 0021-8855
UR - 3771424
ER -

TY - JOUR
ID - 2215
T1 - Growth of clinical haematology in South Tees Health District 1983-5
A1 - Summerfield,G.P.
A1 - Chandler,J.E.
Y1 - 1986/10/25/
N1 - Summerfield, G P. Chandler, J E
British medical journal (Clinical research ed.)
b4x, 8302911
AIM, IM
Journal Article
English
KW - MEDLINE
KW - Adult
KW - Child
KW - Day Care
KW - England
KW - Hematologic Diseases/th [Therapy]
KW - Hospitals
KW - District
KW - General
KW - Humans
KW - Laboratories
KW - Outpatient Clinics
KW - Hospital/og [Organization & Administration]
KW - Pathology Department
RP - NOT IN FILE
SP - 1083
EP - 1085
JF - British Medical Journal Clinical Research Ed
JA - Br Med J (Clin Res Ed)
VL - . 293
IS - 6554
CY - ENGLAND
N2 - A review of the workload of two newly appointed clinical haematologists in a health district with a population of 307,000 showed that the clinical case load increased rapidly in the first two years. The management of patients with blood disorders, which had previously been dispersed among many departments both within and outside the district, was now concentrated within a single unit with occasional referral as

appropriate to regional centres. The development of a clinical haematology service in district general hospitals cannot occur without funding and facilities, including suitably located beds for haematology patients and specifically appointed junior medical staff. Highly trained and experienced nursing staff are essential for the management of patients with bone marrow failure; day care facilities and community nursing are also valuable. Changes and developments in the laboratory as a result of these additional clinical activities are also necessary
SN - 0267-0623
UR - 3094783
ER -

TY - JOUR
ID - 2216
T1 - Variability in counseling experiences and training among ESB-accredited programs
A1 - McCarthy,P.
A1 - Culpepper,N.B.
A1 - Lucks,L.
Y1 - 1986/09//
N1 - McCarthy, P. Culpepper, N B. Lucks, L
ASHA
92g, 0370563, 0370563
IM, J
Journal Article
English
KW - MEDLINE
KW - Accreditation
KW - Adult
KW - Audiology/ed [Education]
KW - Child
KW - Communication Disorders/rh [Rehabilitation]
KW - Counseling/td [Trends]
KW - Humans
KW - Speech-Language Pathology/ed [Education]
KW - United States
RP - NOT IN FILE
SP - 49
EP - 52
JF - ASHA
VL - 28
IS - 9
CY - UNITED STATES
SN - 0001-2475
UR - 3533091
ER -

TY - JOUR
ID - 2217
T1 - Outdoor and indoor soccer: injuries among youth players
A1 - Hoff,G.L.
A1 - Martin,T.A.
Y1 - 1986/05//
N1 - Hoff, G L. Martin, T A
The American journal of sports medicine
7609541, 3yr
IM
Comparative Study. Journal Article
English
KW - MEDLINE
KW - Adolescent

KW - Athletic Injuries/ep [Epidemiology]
KW - Child
KW - Female
KW - Humans
KW - Male
KW - Soccer
KW - Sports
RP - NOT IN FILE
SP - 231
EP - 233
JF - American Journal of Sports Medicine
JA - Am J Sports Med
VL - 14
IS - 3
CY - UNITED STATES
N2 - The incidence of injuries among youth soccer players (under age 16) participating in indoor soccer was 4.5 times that of youth players in outdoor soccer when calculated per 100 hours of team play, and 6.1 times greater when calculated per 100 hours of player game participation. Injuries were few among players under age 10 in either game, and the number of injuries increased with age in the older players. Medical assistance was required for 6.5% of the injuries among outdoor players and for 24.3% among indoor players. Overall, 66.6% of the injuries were the result of physical contact between players. No relationship was observed between the risk of injury and playing position, conduct of warm-up exercises, or the team having a licensed coach
SN - 0363-5465
UR - 3752363
ER -

TY - JOUR
ID - 2218
T1 - Effects of exposure to factor concentrates containing donations from identified AIDS patients. A matched cohort study
A1 - Jason,J.
A1 - Holman,R.C.
A1 - Dixon,G.
A1 - Lawrence,D.N.
A1 - Bozeman,L.H.
A1 - Chorba,T.L.
A1 - Tregillus,L.
A1 - Evatt,B.L.
Y1 - 1986/10/03/
N1 - Jason, J. Holman, R C. Dixon, G. Lawrence, D N. Bozeman, L H. Chorba, T L. Tregillus, L. Evatt, B L
JAMA : the journal of the American Medical Association
7501160
AIM, IM, X
Journal Article
English
KW - MEDLINE
KW - Acquired Immunodeficiency Syndrome/im [Immunology]
KW - Acquired Immunodeficiency Syndrome/tm [Transmission]
KW - Adolescent
KW - Adult
KW - Aged
KW - Antibodies
KW - Viral/an [Analysis]
KW - Antigen-Antibody Complex/an [Analysis]
KW - Blood Donors
KW - Centers for Disease Control and Prevention (U.S.)
KW - Child

KW - Preschool
KW - Deltaretrovirus/im [Immunology]
KW - Factor IX/tu [Therapeutic Use]
KW - Factor VIII/tu [Therapeutic Use]
KW - HIV Antibodies
KW - Hemophilia A/th [Therapy]
KW - Humans
KW - Immunoglobulins/an [Analysis]
KW - Leukocyte Count
KW - Lymphocyte Activation/de [Drug Effects]
KW - Middle Aged
KW - Mitogens/pd [Pharmacology]
KW - Risk
KW - T-Lymphocytes/cl [Classification]
KW - United States
RP - NOT IN FILE
SP - 1758
EP - 1762
JF - JAMA
VL - 256
IS - 13
CY - UNITED STATES
N2 - We compared recipients of eight lots of factors VIII and IX voluntarily withdrawn from distribution because one donor was known to have subsequently developed the acquired immunodeficiency syndrome with a nonexposed cohort matched by age, sex, and factor use. The factor VIII recipient cohorts did not differ in prevalence of antibody to human immunodeficiency virus (HIV) (exposed, 75%; nonexposed, 86%), T-cell subset numbers (median: exposed, 619 T-helper cells per cubic millimeter; nonexposed, 659 T-helper cells per cubic millimeter), T-helper to T-suppressor ratios, or immunoglobulin levels. Exposed individuals had higher levels of immune complexes by C1q binding and staphylococcal binding assays and lower responses to phytohemagglutinin and concanavalin A. However, only the staphylococcal binding assay values were outside the normal range for our laboratory. Factor IX recipient cohorts did not differ in HIV antibody prevalence (exposed, 30%; nonexposed, 40%) or any immune tests. Although exposed and nonexposed individuals did not differ from each other in a clinically meaningful fashion at initial testing, both the exposed and nonexposed cohorts had high rates of HIV seroprevalence. Market withdrawals were clearly insufficient means of limiting the spread of HIV in hemophilic patients; however, the currently available methods of donor screening and viral inactivation of blood products will prevent continued exposure within this population
SN - 0098-7484
UR - 3018305
ER -

TY - JOUR
ID - 2219
T1 - Determinants of maladaptive behavior of institutionalized mentally retarded individuals
A1 - Duker,P.C.
A1 - van,Druenen C.
A1 - Jol,K.
A1 - Oud,H.
Y1 - 1986/07//
N1 - Duker, P C. van Druenen, C. Jol, K. Oud, H
American journal of mental deficiency
3ma, 0372647
IM
Journal Article
English
KW - MEDLINE
KW - Adaptation
KW - Psychological

KW - Adolescent
KW - Adult
KW - Aged
KW - Child
KW - Preschool
KW - Disabled Persons/px [Psychology]
KW - Female
KW - Humans
KW - Institutionalization
KW - Intellectual Disability/px [Psychology]
KW - Intelligence
KW - Male
KW - Middle Aged
KW - Professional-Patient Relations
KW - Psychological Tests
KW - Social Adjustment
KW - Social Environment
RP - NOT IN FILE
SP - 51
EP - 56
JF - American Journal of Mental Deficiency
JA - Am J Ment Defic.
VL - 91
IS - 1
CY - UNITED STATES
N2 - The maladaptive behavior of 474 persons from a residential facility for mentally retarded individuals was investigated. Eleven independent variables pertaining to demographic and environmental characteristics were initially related to the frequency and topography of residents' maladaptive behavior. Multiple regression analysis revealed that maladaptive behaviors were related to sex, social age, chronological age, resident's time spent on outside activities, number of residents per direct-care staff member, presence of sensory handicaps, and ambulation. Results were related to existing literature and discussed in terms of their use for intervention efforts and differential assignment of resources
SN - 0002-9351
UR - 2943159
ER -

TY - JOUR
ID - 2220
T1 - Analysis of environmental determinants of aggression and disruption in mentally retarded children
A1 - Mace,F.C.
A1 - Page,T.J.
A1 - Ivancic,M.T.
A1 - O'Brien,S.
Y1 - 1986///
N1 - Mace, F C. Page, T J. Ivancic, M T. O'Brien, S
Applied research in mental retardation
8101119, 6kl
IM
Case Reports. Journal Article. Research Support, U.S. Gov't, P.H.S.
English
KW - MEDLINE
KW - Adolescent
KW - Aggression/px [Psychology]
KW - Attention
KW - Behavior Therapy/mt [Methods]
KW - Child
KW - Child Behavior Disorders/co [Complications]

KW - Child Behavior Disorders/th [Therapy]
KW - Preschool
KW - Environment
KW - Female
KW - Humans
KW - Intellectual Disability/co [Complications]
KW - Intellectual Disability/rh [Rehabilitation]
KW - Male
KW - Play and Playthings
KW - Reinforcement
KW - Social
RP - NOT IN FILE
SP - 203
EP - 221
JF - Applied Research in Mental Retardation
JA - Appl Res Ment Retard.
VL - 7
IS - 2
CY - UNITED STATES
N2 - This study evaluated methods for empirically identifying environmental determinants of aggressive and disruptive behavior in three developmentally delayed children. A variety of sessions conducted on an inpatient treatment unit were designed to stimulate contingencies maintaining aggression and disruption in the subjects' natural environments. The basic analog conditions included: instructional demands, contingent social disapproval, and structured play. In addition, variations of the three basic conditions were developed based on informal observation and parental reports of naturally occurring contingencies. Results showed considerable variability both between and within subjects. However, for each subject a specific analog condition was shown to produce relatively higher rates of aberrant behavior. Subsequently, behavioral treatments based on assessment results were successfully implemented with two of the subjects. Results are discussed in terms of the importance and practical limitations of conducting systematic assessment of aberrant repertoires in developmentally delayed clients
SN - 0270-3092
UR - 3729384
ER -

TY - JOUR
ID - 2221
T1 - Ethnic, gender, and age preferences among deaf and hearing preschool peers
A1 - Lederberg,A.R.
A1 - Chapin,S.L.
A1 - Rosenblatt,V.
A1 - Vandell,D.L.
Y1 - 1986/04//
N1 - Lederberg, A R. Chapin, S L. Rosenblatt, V. Vandell, D L
Child development
0372725, d28
IM
Journal Article
English
KW - MEDLINE
KW - Age Factors
KW - Child
KW - Preschool
KW - Choice Behavior
KW - Deafness/px [Psychology]
KW - Ethnic Groups/px [Psychology]
KW - Female
KW - Humans

KW - Language Development
KW - Male
KW - Peer Group
KW - Play and Playthings
KW - Sex Factors
RP - NOT IN FILE
SP - 375
EP - 386
JF - Child Development
JA - Child Dev
VL - 57
IS - 2
CY - UNITED STATES
N2 - The present study concerns ethnic, age, and gender playmate preferences of deaf and hearing preschoolers who were observed during outdoor free play at their respective schools over a 7-month period. Hispanic, black, and white children were included. It was hypothesized that peer preferences would be less apparent among deaf children than among hearing children if these preferences were based on (a) language differences between ethnic groups (e.g., Spanish-English differences), age groups (e.g., verbal fluency differences), or gender groups; or (b) cultural values communicated by speech. Both deaf and hearing children preferred to play with children of the same ethnicity, gender, and age as themselves. In addition, play among children of the same ethnicity, gender, and age was qualitatively different from play among children of different ethnicity, gender, and age. The only effect of deafness was to decrease the amount of gender segregation that occurred. The results suggest that the development of ethnic, gender, and age playmate preferences is not dependent on intergroup language differences or spoken cultural messages. Several nonlinguistic factors that might contribute to early peer preferences are discussed
SN - 0009-3920
UR - 3956318
ER -

TY - JOUR
ID - 2222
T1 - Programming rapid generalization of correct articulation through self-monitoring procedures
A1 - Koegel,L.K.
A1 - Koegel,R.L.
A1 - Ingham,J.C.
Y1 - 1986/02//
N1 - Koegel, L K. Koegel, R L. Ingham, J C
The Journal of speech and hearing disorders
k5z, 0376335
IM
Journal Article. Research Support, Non-U.S. Gov't. Research Support, U.S. Gov't, Non-P.H.S.. Research Support, U.S. Gov't, P.H.S.
English
KW - MEDLINE
KW - Behavior/ph [Physiology]
KW - Child
KW - Female
KW - Generalization (Psychology)/ph [Physiology]
KW - Humans
KW - Male
KW - Monitoring
KW - Physiologic
KW - Speech/ph [Physiology]
RP - NOT IN FILE
SP - 24
EP - 32
JF - Journal of Speech & Hearing Disorders

JA - J Speech Hear Disord
VL - 51
IS - 1
CY - UNITED STATES
N2 - The purpose of this study was to investigate the use of a self-monitoring activity in the clinical and natural environment as a method of promoting rapid generalization of a target speech sound to beyond-treatment conditions. The subjects were 13 children who lateralized /s/ and /z/, substituted /theta/ for /s/ and /delta/ for /z/, and/or substituted /w/ for /r/. The children attended public school speech therapy, individually or in small groups, twice weekly for 15- to 20-min sessions throughout the entire study. The investigation was conducted in the context of a multiple baseline research design replicated across subjects and behaviors (for 1 subject). The results demonstrated that when the self-monitoring activity was implemented, the children began to generalize the use of the correct speech sound to their spontaneous speech outside of the treatment setting. The results are discussed in terms of their theoretical implications for generalization issues and in relation to their applied value for clinicians with large case loads or otherwise limited amounts of time to spend with individual clients
SN - 0022-4677
UR - 3945056
ER -

TY - JOUR
ID - 2223
T1 - A comparative study on the health effects of smoking and indoor air pollution in summer and winter
A1 - Matsuki,H.
A1 - Kasuga,H.
A1 - Osaka,F.
A1 - Yanagisawa,Y.
A1 - Nishimura,H.
Y1 - 1985/08//
N1 - Matsuki, H. Kasuga, H. Osaka, F. Yanagisawa, Y. Nishimura, H
The Tokai journal of experimental and clinical medicine
vzm, 7704186
IM
Journal Article. Research Support, Non-U.S. Gov't
English
KW - MEDLINE
KW - Air Pollution
KW - Child
KW - Climate
KW - Creatinine/ur [Urine]
KW - Female
KW - Housing
KW - Humans
KW - Hydroxyproline/ur [Urine]
KW - Male
KW - Microclimate
KW - Nitrogen Dioxide/an [Analysis]
KW - Seasons
KW - Smoking
KW - Tobacco Smoke Pollution
KW - Urban Population
RP - NOT IN FILE
SP - 427
EP - 437
JF - Tokai Journal of Experimental & Clinical Medicine
JA - Tokai J Exp Clin Med
VL - 10
IS - 4
CY - JAPAN

N2 - This study was performed by a comparison between summer and winter for the purpose of demonstration the actual conditions of health effects of indoor air pollution with special reference to NO2 and smoking, on the subjects composed of 820 school children and their 546 mothers in the two areas with different ambient NO2 concentrations. In either case, examination was carried out with standardized questionnaire test for respiratory symptoms, personal NO2 exposure measurement using the filter badge by Yanagisawa, and analysis of urinary hydroxyproline and creatinine in two areas with different ambient NO2 levels. Personal NO2 exposure level in winter season was 2-3 times higher than that in summer, particularly NO2 level among residents living in homes with non-vented stove for space heating was substantially higher from those of residents with vented stove. Wives with vented stove had a moderate exposure level in winter season by the contribution of NO2 originated from the kitchen and poor ventilation rate. Since the hydroxyproline to creatinine ratio (HOP-ratio) of children increased more, their household location were nearer to any heavy traffic roads in summer, health effects from automobile exhaust were suggested only in summer season. In summer season, personal NO2 exposure level were almost the same with the ambient NO2 concentrations over both areas. These results suggest that indoor air pollution in winter season may be separated from outdoor air pollution. It was a matter of course that hydroxyproline to creatinine ratio in winter season was higher than that in summer, in any group and in any area, but the range of variation of hydroxyproline to creatinine ratio was smaller by far than that of personal NO2 exposure level. Judging from urinary hydroxyproline to creatinine ratio, health effects of active smoking and passive smoking increased with increasing the number of smoked, dose-dependently in any season. According to stepwise multiple regression analysis, hydroxyproline to creatinine ratio had significant relation to either NO2 and active or passive smoking, but personal NO2 exposure level had no relation to cigarette smoking. NO2 and cigarette smoke were seemed to make hydroxyproline to creatinine ratio increase, independently each other
SN - 0385-0005
UR - 3836525
ER -

TY - JOUR
ID - 2224
T1 - Rheological and transport properties of airway secretions in cystic fibrosis--relationships with the degree of infection and severity of the disease
A1 - Puchelle,E.
A1 - Jacquot,J.
A1 - Beck,G.
A1 - Zahm,J.M.
A1 - Galabert,C.
Y1 - 1985/12//
N1 - Puchelle, E. Jacquot, J. Beck, G. Zahm, J M. Galabert, C
European journal of clinical investigation
en3, 0245331
IM
Journal Article. Research Support, Non-U.S. Gov't
English
KW - MEDLINE
KW - Adolescent
KW - Biological Transport
KW - Active
KW - Child
KW - Cilia/ph [Physiology]
KW - Cystic Fibrosis/co [Complications]
KW - Cystic Fibrosis/pp [Physiopathology]
KW - Female
KW - Humans
KW - Male
KW - Mucus/ph [Physiology]
KW - Respiratory System/pp [Physiopathology]
KW - Respiratory Tract Infections/co [Complications]
KW - Respiratory Tract Infections/pp [Physiopathology]

KW - Rheology
KW - Sputum/ph [Physiology]
KW - Sputum/se [Secretion]
KW - Viscosity
RP - NOT IN FILE
SP - 389
EP - 394
JF - European Journal of Clinical Investigation
JA - Eur J Clin Invest
VL - 15
IS - 6
CY - ENGLAND
N2 - It has recently been suggested that in cystic fibrosis (CF), there is no rheological abnormality of airway secretions other than that associated with purulence, and that the apparent inhibition in the mucociliary transport rate might be partly due to a ciliary inhibitor present in these secretions. In order to ascertain this assumption, expectorated airway secretions were collected without salivary contamination in twenty-four CF patients and the rheological properties were measured. Using a photometric method, the effects of CF sputum samples were analysed on the ciliary beat frequency (Fm) of the frog palate, and we measured their mucociliary transport rate (TR). In all but one CF sputum, TR and Fm were lower than that of the control frog mucus (median TR: 18.7 and 11.6 mm min-1; median Fm: 12.3 and 11.3 Hz, respectively). In the eighteen patients in whom the rheological properties were outside the range for optimal mucociliary transport, the clinical Shwachman score was significantly (P less than 0.05) lower (median score: 66.2 points) than in the six patients with optimal rheologic properties (median score: 73 points). In the eleven CF patients with superinfection, the apparent viscosity (eta o) was significantly higher (P less than 0.01; median eta o: 24.4 Pa. s) and TR, expressed as a percentage of the reference value, was significantly lower (P less than 0.05; median Tr: 54.5%) in comparison with the values obtained for the thirteen non-superinfected CF patients (median eta o: 15 Pa. s and median TR: 66% respectively). The CF patients with markedly hyperviscous sputum (eta o higher than 30 Pa. s) exhibited a low Shwachman score
SN - 0014-2972
UR - 3938409
ER -

TY - JOUR
ID - 2225
T1 - Arthroscopy of the subtalar joint: an experimental approach
A1 - Parisien,J.S.
A1 - Vangsness,T.
Y1 - 1985///
N1 - Parisien, J S. Vangsness, T
Arthroscopy : the journal of arthroscopic & related surgery : official publication of the Arthroscopy Association of North America and the International Arthroscopy Association
abt, 8506498
IM
Journal Article
English
KW - MEDLINE
KW - Adult
KW - Ankle Joint/ah [Anatomy & Histology]
KW - Arthroscopy/mt [Methods]
KW - Child
KW - Humans
KW - Joint Diseases/di [Diagnosis]
KW - Middle Aged
RP - NOT IN FILE
SP - 53
EP - 57
JF - Arthroscopy

VL - 1
IS - 1
CY - UNITED STATES
N2 - Talocalcaneal articulations are relatively complex and functionally very important because they play a major role in the movements of inversion and eversion of the foot. Few reports on arthrography of the subtalar joints are available in the literature, and, similarly, little attention has been paid by arthroscopists to these joints. This preliminary study briefly defines the normal anatomy of the subtalar joints and describes a new technique of arthroscopic examination of the posterior subtalar joint. The distal lower extremities of six fresh cadavers were used in these experiments. All the subtalar joints were supple. A 2.7-mm arthroscope was used to carry out arthroscopic and anatomic examinations. A technique of examination with one anterior portal and one posterior portal is described in detail. When the anterior portal was used, the egress needle was placed posteriorly; when the posterior portal was used, the converse was true. By using the two portals, the following intraarticular structures could be visualized: a major part of the convex posterior calcaneal facet of the talus and the posterior talar facet of the calcaneus; the synovial lining laterally and posteriorly; the posterior aspect of the interosseous talocalcaneal ligament; and the posterior recess of the joint. The results of this experimental study indicate that arthroscopy of the posterior subtalar joint is technically feasible. Clinically, the possible indications for arthroscopy would include state of the articular cartilage in suspected cases of degenerative arthritis, rheumatoid arthritis, and infection; visualization of the joint after intraarticular fracture to evaluate chronic pain syndrome in the hindfoot; biopsy; management of sinus tarsi syndrome; loose body removal
SN - 0749-8063
UR - 4091910
ER -

TY - JOUR
ID - 2226
T1 - Rocky Mountain spotted fever acquired in Florida, 1973-83
A1 - Sacks,J.J.
A1 - Janowski,H.T.
Y1 - 1985/12//
N1 - Sacks, J J. Janowski, H T
American journal of public health
1254074, 3xw
AIM, IM
Journal Article
English
KW - MEDLINE
KW - Adolescent
KW - Adult
KW - Aged
KW - Arachnid Vectors
KW - Child
KW - Preschool
KW - Female
KW - Florida
KW - Humans
KW - Male
KW - Middle Aged
KW - Rocky Mountain Spotted Fever/ep [Epidemiology]
KW - Rocky Mountain Spotted Fever/tm [Transmission]
KW - Seasons
KW - Ticks
RP - NOT IN FILE
SP - 1422
EP - 1424
JF - American Journal of Public Health
JA - Am J Public Health
VL - 75

IS - 12
CY - UNITED STATES
N2 - From 1973 to 1983, 49 Florida residents were reported with confirmed Rocky Mountain spotted fever (RMSF), 25 of whom were considered to have had Florida-acquired disease. Although there was no history of tick exposure for six of these 25 persons, all had contact with dogs or outdoor activities during the incubation period. The tick vectors of RMSF are widely distributed throughout Florida. We conclude that RMSF, although rare in Florida, can be acquired in the state
SN - 0090-0036
UR - 4061716
ER -

TY - JOUR
ID - 2227
T1 - Neurotoxic chemical exposure scenarios and suggested solutions
A1 - Schaumberg,H.H.
A1 - Arezzo,J.
A1 - Otto,D.A.
A1 - Eckerman,D.A.
Y1 - 1985/07//
N1 - Schaumberg, H H. Arezzo, J. Otto, D A. Eckerman, D A
Neurobehavioral toxicology and teratology
8104162, nxx
IM
Journal Article
English
KW - MEDLINE
KW - Adult
KW - Child
KW - Environmental Exposure
KW - Environmental Pollutants/po [Poisoning]
KW - Female
KW - Humans
KW - Male
KW - Mass Screening/mt [Methods]
KW - Models
KW - Theoretical
KW - Nervous System Diseases/ci [Chemically Induced]
KW - Nervous System Diseases/pc [Prevention & Control]
KW - Occupational Diseases/ci [Chemically Induced]
RP - NOT IN FILE
SP - 351
EP - 353
JF - Neurobehavioral Toxicology & Teratology
JA - Neurobehav.Toxicol Teratol.
VL - 7
IS - 4
CY - UNITED STATES
N2 - This manuscript reports the results of a problem-solving exercise presented to participants at a Workshop on Neurotoxicology Testing in Human Populations held in Rougemont, North Carolina in October, 1983. Response recommendations are the consensus of workshop participants. These are not comprehensive or definitive solutions and should be interpreted with caution. Each exposure scenario represents a "real world" situation previously encountered by neurologists and epidemiologists. Ideally, a toxin affects a single, easily tested modality early in the illness before producing a complex neurological syndrome. Unfortunately this rarely happens; difficult choices and compromises about testing are usually necessary. At the end of each scenario likely areas of early subtle dysfunction were discussed and possible testing methods were outlined. Working participants were instructed to comment on the approach to the scenarios and to add to or dispute the proposed tests. It is clear that there are no right or wrong answers; however, the participants were urged to try to reach

some sort of consensus. In each instance participants assumed that they were being notified by the company and not by a state or federal agency, and were to determine what would be the most reasonable and effective way to offer help or obtain outside assistance in these situations
SN - 0275-1380
UR - 4058644
ER -

TY - JOUR
ID - 2228
T1 - Different malaria control activities in an area of Liberia--effects on malariometric parameters
A1 - Bjorkman,A.
A1 - Hedman,P.
A1 - Brohult,J.
A1 - Willcox,M.
A1 - Diamant,I.
A1 - Pehrsson,P.O.
A1 - Rombo,L.
A1 - Bengtsson,E.
Y1 - 1985/06//
N1 - Bjorkman, A. Hedman, P. Brohult, J. Willcox, M. Diamant, I. Pehrsson, P O. Rombo, L. Bengtsson, E
Annals of tropical medicine and parasitology
68e, 2985178r
IM
Journal Article. Research Support, Non-U.S. Gov't
English
KW - MEDLINE
KW - Child
KW - Preschool
KW - Humans
KW - Immunoglobulins/an [Analysis]
KW - Insect Vectors
KW - Liberia
KW - Malaria/ep [Epidemiology]
KW - Malaria/ps [Parasitology]
KW - Malaria/pc [Prevention & Control]
KW - Plasmodium falciparum
KW - Plasmodium malariae
KW - Spleen/pa [Pathology]
RP - NOT IN FILE
SP - 239
EP - 246
JF - Annals of Tropical Medicine & Parasitology
JA - Ann Trop Med Parasitol
VL - 79
IS - 3
CY - ENGLAND
N2 - The epidemiology of malaria was studied in a West African mining town (Yekepa) and three surrounding zones defined as Close, Middle and Far areas. Malariometric parameters were investigated in children two to nine years of age at the end of the rainy season. In Yekepa, vector control measures and intense suppression of malaria with drugs had created an almost hypoendemic situation with a spleen rate of 11%. In Close area, vector control was applied to some extent and malaria drugs were frequently used for treatment; the spleen rate was 40%. In Middle area, a mobile clinic provided sporadic malaria treatment to small children, but the clinic did not reach out to Far area. The spleen rates were 95 and 99%, respectively. Three species of Plasmodium were found in all areas. The prevalences in Far area were P. falciparum 82%, P. malariae 39% and P. ovale 9%. The crude parasite rates increased from 13% in Yekepa to 92% in Far area, whereas haematocrit levels decreased from 37.6 to 35.2, respectively. Plasmodium falciparum seropositivity, as measured by indirect immunofluorescence, was 74% in Yekepa and 99% in Middle and Far areas. Total IgG concentrations ranged

from 18 g1(-1) in Yekepa to 33 g1(-1) in Far area. Three main anopheline species were found in the zones outside Yekepa. Their relative frequencies in Far area were Anopheles funestus 45%, A. hancocki 37%, and A. gambiae 18%. The local inoculation rates gradually increased outwards from Yekepa from less than 0.01 to 0.17 inoculations per man and night at the beginning of the dry season.(ABSTRACT TRUNCATED AT 250 WORDS)
SN - 0003-4983
UR - 3896168
ER -

TY - JOUR
ID - 2229
T1 - Lead in playground dust and on the hands of schoolchildren
A1 - Duggan,M.J.
A1 - Inskip,M.J.
A1 - Rundle,S.A.
A1 - Moorcroft,J.S.
Y1 - 1985/07//
N1 - Duggan, M J. Inskip, M J. Rundle, S A. Moorcroft, J S
The Science of the total environment
uj0, 0330500
IM
Journal Article
English
KW - MEDLINE
KW - Child
KW - Preschool
KW - Dust/an [Analysis]
KW - Female
KW - Hand
KW - Humans
KW - Lead/an [Analysis]
KW - Male
KW - Play and Playthings
KW - Schools
KW - Skin/an [Analysis]
RP - NOT IN FILE
SP - 65
EP - 79
JF - Science of the Total Environment
JA - Sci Total Environ
VL - 44
IS - 1
CY - NETHERLANDS
N2 - Measurements of the quantity of lead on the hands of schoolchildren and of the concentration of lead in dust from school playgrounds have been carried out; the hand-lead was collected by wiping with a moist tissue. When hand-lead and dust-lead values were averaged for each school, a statistically significant relationship between the two sets of means was obtained. Some limited measurements of size distribution of the dust particles on the children's wipes were also made; these indicated that most of the particles were less than 10 micron in diameter
SN - 0048-9697
UR - 4023696
ER -

TY - JOUR
ID - 2230
T1 - The use of peer monitors to reduce negative interaction during recess
A1 - Dougherty,B.S.

A1 - Fowler,S.A.
A1 - Paine,S.C.
Y1 - 1985///
N1 - Dougherty, B S. Fowler, S A. Paine, S C
Journal of applied behavior analysis
0174763, hdr
IM
Case Reports. Journal Article. Research Support, U.S. Gov't, Non-P.H.S.. Research Support, U.S. Gov't, P.H.S.
English
KW - MEDLINE
KW - Aggression
KW - Behavior Therapy/mt [Methods]
KW - Child
KW - Child Behavior Disorders/th [Therapy]
KW - Humans
KW - Intellectual Disability/rh [Rehabilitation]
KW - Male
KW - Peer Group
RP - NOT IN FILE
SP - 141
EP - 153
JF - Journal of Applied Behavior Analysis
JA - J Appl Behav Anal
VL - 18
IS - 2
CY - UNITED STATES
N2 - The negative interactions of a midly retarded child, Dennis, were reduced in three daily recess periods, with the use of a point system. Adult monitors initiated the intervention in the morning recess; reductions achieved during adult monitoring were maintained in that recess during two subsequent conditions: peer monitoring and self-monitoring. Dennis' negative interactions were reduced next in the afternoon recess by peer monitors. Again, reductions were maintained during a subsequent self-monitoring condition. Finally, during the noon recess, Dennis was trained to serve as a peer monitor for Ed, a moderately retarded classmate. Dennis' rate of negative interactions quickly decreased following his appointment as a peer monitor. The results show that a point system, originally designed for adult monitoring, can be adapted without loss of program effectiveness for peer monitoring or self-monitoring. The results also suggest that classmates who serve as peer monitors may benefit significantly from their role. The conditions under which these therapeutic effects occur and the role that treatment order effects may play in this process require further investigation
SN - 0021-8855
UR - 4019350
ER -

TY - JOUR
ID - 2231
T1 - Non-natural deaths in two health districts
A1 - Keeling,J.W.
A1 - Golding,J.
A1 - Millier,H.K.
Y1 - 1985/06//
N1 - Keeling, J W. Golding, J. Millier, H K
Archives of disease in childhood
6xg, 0372434
AIM, IM
Journal Article
English
KW - MEDLINE
KW - Accidents
KW - Home

KW - Traffic
KW - Adolescent
KW - Age Factors
KW - Child
KW - Preschool
KW - England
KW - Female
KW - Humans
KW - Infant
KW - Infant Mortality
KW - Male
KW - Mortality
KW - Sex Factors
KW - Social Class
RP - NOT IN FILE
SP - 525
EP - 529
JF - Archives of Disease in Childhood
JA - Arch Dis Child
VL - 60
IS - 6
CY - ENGLAND
N2 - Details of all deaths in children (less than 16 years) resident in Oxfordshire and West Berkshire from 1970-9 were ascertained; 259 non-natural deaths were identified. Half were due to road traffic accidents, and another quarter occurred during play outside the home. There were differences in sex, age, and social class between the types of accident. A clustering of fatal accidents, particularly to pedestrians, was observed at around the time of the child's birthday. It is suggested that the excitement engendered overrides the child's normal caution. Local action to reduce accidents from specific hazards might complement national education campaigns
SN - 1468-2044
UR - 4015169
ER -

TY - JOUR
ID - 2232
T1 - Age-related changes of human serum antibodies to dietary and colonic bacterial antigens measured by an enzyme-linked immunosorbent assay
A1 - Scott,H.
A1 - Rognum,T.O.
A1 - Midtvedt,T.
A1 - Brandtzaeg,P.
Y1 - 1985/04//
N1 - Scott, H. Rognum, T O. Midtvedt, T. Brandtzaeg, P
Acta pathologica, microbiologica, et immunologica Scandinavica. Section C, Immunology
8206624, 1op
IM
Journal Article. Research Support, Non-U.S. Gov't
English
KW - MEDLINE
KW - Adolescent
KW - Adult
KW - Aged
KW - Aging
KW - Antigens
KW - Bacterial/im [Immunology]
KW - Child
KW - Preschool

KW - Colon/mi [Microbiology]
KW - Enzyme-Linked Immunosorbent Assay
KW - Food Microbiology
KW - Humans
KW - Immunoglobulin A/an [Analysis]
KW - Immunoglobulin G/an [Analysis]
KW - Immunoglobulin M/an [Analysis]
KW - Immunoglobulins/an [Analysis]
KW - Middle Aged
RP - NOT IN FILE
SP - 65
EP - 70
JF - Acta Pathologica, Microbiologica, et Immunologica Scandinavica - Section C, Immunology
JA - Acta Pathol Microbiol Immunol Scand [C]
VL - 93
IS - 2
CY - DENMARK
N2 - IgG, IgA and IgM antibody activities in human serum to six dietary and eight gut-related microbial antigens were measured by an enzyme-linked immunosorbent assay (ELISA). IgG activities to five of the dietary antigens decreased with age; IgM activities to four of them were weaker in old people than in children. Old people showed weaker IgM but stronger IgG activities to some of the microbial antigens than children did. A decline in IgG and IgM antibody titres to most dietary antigens with increasing age is consistent with the development of systemic hyporesponsiveness due to continuous antigenic stimulation of the intestinal immune system. Persistence of microbial antigens in the gut, moreover, may lead to systemic hyporesponsiveness of IgM-producing cells. Concurrently raised IgG titres to three of the bacteria might be explained by antigenic stimulation outside the intestinal immune system
SN - 0108-0202
UR - 4013749
ER -

TY - JOUR
ID - 2233
T1 - Distributions of cardiopulmonary variables in pediatric survivors and nonsurvivors of septic shock
A1 - Pollack,M.M.
A1 - Fields,A.I.
A1 - Ruttimann,U.E.
Y1 - 1985/06//
N1 - Pollack, M M. Fields, A I. Ruttimann, U E
Critical care medicine
dtf, 0355501
AIM, IM
Journal Article
English
KW - MEDLINE
KW - Adolescent
KW - Blood Pressure
KW - Cardiac Output
KW - Child
KW - Preschool
KW - Hemodynamics
KW - Humans
KW - Hydrogen-Ion Concentration
KW - Infant
KW - Oxygen Consumption
KW - Prognosis
KW - Pulmonary Wedge Pressure
KW - Resuscitation

KW - Shock
KW - Septic/bl [Blood]
KW - Septic/mi [Microbiology]
KW - Septic/mo [Mortality]
RP - NOT IN FILE
SP - 454
EP - 459
JF - Critical Care Medicine
JA - Crit Care Med
VL - 13
IS - 6
CY - UNITED STATES
N2 - The association of cardiopulmonary variables with outcome was investigated in 42 pediatric patients (18 survivors) with septic shock. All cardiopulmonary variables were obtained during active BP support. The variable distributions were separated into ranges by two empiric cutoff methods: normal ranges and the survivor median values. The proportion of survivors with normal values of wedge pressure and cardiac index was significantly (p less than .05) higher than the proportion of survivors outside the normal range. The percentage of survival also significantly (p less than .05) increased with above-normal values of oxygen consumption, arteriovenous O2 content difference, O2 extraction, pH, and core temperature. There were significantly (p less than .05) more nonsurvivors with wedge pressure, pulmonary shunt, and pH values below the survivor medians. Therapeutic goals based on the distributions of these eight variables isolated patient groups with survival rates of 59% to 75%, compared to the overall survival rate of 43%
SN - 0090-3493
UR - 3995997
ER -

TY - JOUR
ID - 2234
T1 - Obesity a family matter: creating new behavior. [Review] [63 refs]
A1 - Frankle,R.T.
Y1 - 1985/05//
N1 - Frankle, R T
Journal of the American Dietetic Association
h6f, 7503061
AIM, IM
Journal Article. Review
English
KW - MEDLINE
KW - Adolescent
KW - Adult
KW - Behavior Therapy
KW - Body Weight
KW - Child
KW - Counseling
KW - Family
KW - Female
KW - Humans
KW - Male
KW - Obesity/dh [Diet Therapy]
KW - Obesity/ge [Genetics]
KW - Parents
RP - NOT IN FILE
SP - 597
EP - 602
JF - Journal of the American Dietetic Association
JA - J Am Diet Assoc
VL - 85

IS - 5
CY - UNITED STATES
N2 - The family as culture transmitter plays a role in the development of health maintenance practices. A review of the epidemiological variables associated with obesity suggests that familial factors--genetic and environmental--have a significant effect on the onset, causes, and prognosis of treatment. Chances of success in losing weight may depend upon the extent of family functioning or upon finding a support system within and/or outside the family. The weight control program described is family oriented. Life-style changes learned in the group meetings reach home and affect family members. Four-pronged in its approach (nutrition, behavior change, exercise, and a positive support system), the program uses "skill builders," which are special situational modules that deal with specific family situations. A skill builder offers a step-by-step guided approach to identifying and changing problem behavior in the area of diet and exercise, e.g., eating in social situations, new food shopping patterns, and the role of significant others in the weight loss process. [References: 63]
SN - 0002-8223
UR - 3886764
ER -

TY - JOUR
ID - 2235
T1 - Manometric evaluation of functional upper gut symptoms
A1 - Malagelada,J.R.
A1 - Stanghellini,V.
Y1 - 1985/05//
N1 - Malagelada, J R. Stanghellini, V
Gastroenterology
fh3, 0374630
AIM, IM
Comparative Study. Journal Article. Research Support, Non-U.S. Gov't. Research Support, U.S. Gov't, P.H.S.
English
KW - MEDLINE
KW - Adolescent
KW - Adult
KW - Aged
KW - Child
KW - Preschool
KW - Dyspepsia/cl [Classification]
KW - Dyspepsia/pp [Physiopathology]
KW - Eating
KW - Esophagus/pp [Physiopathology]
KW - Fasting
KW - Female
KW - Gastrointestinal Motility
KW - Humans
KW - Infant
KW - Newborn
KW - Intestine
KW - Small/pp [Physiopathology]
KW - Male
KW - Manometry/mt [Methods]
KW - Middle Aged
KW - Pressure
KW - Stomach/pp [Physiopathology]
KW - Syndrome
KW - Time Factors
RP - NOT IN FILE
SP - 1223
EP - 1231
JF - Gastroenterology

VL - 88
IS - 5 Pt 1
CY - UNITED STATES
N2 - Pressure activity in the stomach and upper intestine was studied in 104 patients referred to the Mayo Clinic for evaluation of functional symptoms (nausea, vomiting, upper abdominal pain, or other dyspeptic symptoms in the absence of structural gut abnormalities). Manometric abnormalities were found in 75 patients. Forty-three of these had gastric abnormalities and 32 patients had both gastric and intestinal abnormalities. In the stomach, decreased antral phasic pressure activity after a solid meal was the most common abnormality. In the upper intestine, unpropagated bursts of phasic and tonic contractile activity were a relatively frequent abnormality but a number of other altered manometric patterns also were observed. Digestive tract symptoms were not good predictors of the presence or site of the gastrointestinal manometric abnormalities. Patients with associated neurologic, urologic, or metabolic (diabetes) disease were more likely to exhibit manometric abnormalities than were those without evidence of disease outside the gut. Almost two-thirds of the patients with symptoms and normal manometry presented features suggestive of psychiatric disease. We conclude that in patients with severe functional-type symptoms gastrointestinal manometry is a useful technique to evidence the underlying gut motor disturbance that is present in a relatively high proportion of these patients
SN - 0016-5085
UR - 3979747
ER -

TY - JOUR
ID - 2236
T1 - Heel pain due to retrocalcaneal bursitis-radiographic diagnosis (with an historical footnote on Sever's disease)
A1 - Heneghan,M.A.
A1 - Wallace,T.
Y1 - 1985///
N1 - Heneghan, M A. Wallace, T
Pediatric radiology
owi, 0365332
IM
Journal Article
English
KW - MEDLINE
KW - Achilles Tendon/ah [Anatomy & Histology]
KW - Achilles Tendon/pa [Pathology]
KW - Adolescent
KW - Bursitis/co [Complications]
KW - Bursitis/ra [Radiography]
KW - Calcaneus/gd [Growth & Development]
KW - Calcaneus/pa [Pathology]
KW - Calcaneus/ra [Radiography]
KW - Child
KW - Diagnosis
KW - Differential
KW - Female
KW - Humans
KW - Male
KW - Pain
RP - NOT IN FILE
SP - 119
EP - 122
JF - Pediatric Radiology
JA - Pediatr Radiol
VL - 15
IS - 2
CY - GERMANY, WEST

N2 - Retrocalcaneal bursitis is a distinct condition causing posterior heel pain in active, healthy children. It appears to result from post-traumatic inflammation of the soft tissues of the posterior heel, and is unrelated to avascular necrosis of the calcaneal apophysis. The diagnosis may be confirmed radiographically by the loss of the lucent retrocalcaneal recess, with a normal Achilles tendon and superficial soft tissue contour, and intact cortex of the underlying os calcis
SN - 0301-0449
UR - 3975103
ER -

TY - JOUR
ID - 2237
T1 - Visceral larva migrans: a risk assessment in Baton Rouge, Louisiana
A1 - Smith,R.E.
A1 - Hagstad,H.V.
A1 - Beard,G.B.
Y1 - 1984/12//
N1 - Smith, R E. Hagstad, H V. Beard, G B
International journal of zoonoses
gtk, 7505008
IM
Journal Article
English
KW - MEDLINE
KW - Animals
KW - Ascariasis/tm [Transmission]
KW - Child
KW - Dog Diseases/mi [Microbiology]
KW - Dog Diseases/tm [Transmission]
KW - Dogs
KW - Female
KW - Humans
KW - Larva Migrans
KW - Visceral/tm [Transmission]
KW - Male
KW - Risk
KW - Toxocariasis/tm [Transmission]
KW - Toxocariasis/ve [Veterinary]
RP - NOT IN FILE
SP - 189
EP - 194
JF - International Journal of Zoonoses
JA - Int J Zoonoses
VL - 11
IS - 2
CY - TAIWAN
N2 - Visceral larva migrans, VLM, is a disease of young children. Due to lack of awareness of the disease as well as its non-specific symptomology, a survey of the potential risk of acquiring the disease was conducted in the Baton Rouge, Louisiana area. The greatest number of dogs showing positive results to tests for the presence of the eggs of Toxocara canis were those less than one year of age. Very few of the soil samples collected from twenty different parks/playgrounds were found to contain eggs of T. canis. A higher percentage of soil samples from individual yards proved to be contaminated with the eggs of the parasite. Even though the incidence of VLM in Baton Rouge is reported to be quite low, the potential for the infection certainly is present. Veterinarians must be prepared to take a leading role in the education of their clients as to the potential of the disease for the children associated with new puppies, and the measures parents can take to reduce the exposure potential
SN - 0377-0168
UR - 6534905

ER -

TY - JOUR
ID - 2238
T1 - Clinical application of a live varicella vaccine (Oka strain) in a hospital
A1 - Naganuma,Y.
A1 - Osawa,S.
A1 - Takahashi,R.
Y1 - 1984/09//
N1 - Naganuma, Y. Osawa, S. Takahashi, R
Biken journal
0373117, 9xs
IM
Journal Article
English
KW - MEDLINE
KW - Antibodies
KW - Viral/bi [Biosynthesis]
KW - Chickenpox/pc [Prevention & Control]
KW - Child
KW - Preschool
KW - Cross Infection/pc [Prevention & Control]
KW - Female
KW - Follow-Up Studies
KW - Herpesvirus 3
KW - Human/im [Immunology]
KW - Humans
KW - Japan
KW - Male
KW - Risk
KW - Vaccination
KW - Vaccines
KW - Attenuated/im [Immunology]
KW - Viral Vaccines/im [Immunology]
RP - NOT IN FILE
SP - 59
EP - 61
JF - Biken Journal
JA - Biken J
VL - 27
IS - 2-3
CY - JAPAN
N2 - A total of 239 children, including 22 high-risk children and 55 non-high risk diseased children have been immunized with a live varicella vaccine (Oka strain) since June, 1978. No clinical reaction attributable to the vaccine has been observed. Of these children, 87 received emergency vaccination. Of 47 children receiving emergency vaccination because they had been in contact with varicella patients either in hospital, school or a playground, only 5 developed varicella and their symptoms were mild. Of 40 children receiving emergency vaccination because of exposure to varicella in their home, 10 developed mild varicella and 30 were protected. Clinical symptoms of varicella when seen seemed to be due to incomplete protection because the vaccine was given too late rather than to clinical reactions to the vaccine. During follow-up period of 6 to 66 months after vaccination, 8 children showed very mild rashes without fever as the result of exogenous varicella infection
SN - 0006-2324
UR - 6100059
ER -

TY - JOUR
ID - 2239

T1 - Evaluation of progress using the context input process and product model
A1 - Sims,M.
A1 - Bridgman,G.
Y1 - 1984/11//
N1 - Sims, M. Bridgman, G
Child: care, health and development
7602632, c9x
IM
Journal Article. Research Support, Non-U.S. Gov't
English
KW - MEDLINE
KW - Activities of Daily Living
KW - Child
KW - Preschool
KW - Cognition
KW - Disabled Persons
KW - Down Syndrome/rh [Rehabilitation]
KW - Education
KW - Special/st [Standards]
KW - Humans
KW - Intellectual Disability/rh [Rehabilitation]
KW - Intelligence Tests
KW - Language
KW - Motor Skills
KW - New Zealand
KW - Parents
KW - Questionnaires
RP - NOT IN FILE
SP - 359
EP - 379
JF - Child: Care, Health & Development
JA - Child Care Health Dev
VL - 10
IS - 6
CY - ENGLAND
N2 - Most evaluations of programme effectiveness are based on process data (the data generated by the programme) and used for internal monitoring and service development. Context (the ecology of therapeutic systems), input (the identification of what is in fact different between two therapeutic systems) and product (the consequence of different inputs, taking account of context) evaluations are rarely, if ever, produced. In this paper (using input and product evaluations) the progress of twelve Down's syndrome and twelve multiply-handicapped children attending, over a period of 18 months, two different therapeutic systems operated by the New Zealand Society for the Intellectually Handicapped (Auckland Branch/outside Auckland Branches) is assessed. The progress of the Auckland children is significantly better in a number of areas than for those outside Auckland. This improvement in progress can be attributed to input differences that reflect the Auckland Branch's policy of service development. The most important determinants of a successful programme are not staff/student ratios, time in class or specialist assistance, all of which favour the outside Auckland groups, but the degree of structure, detail of programmes and assessments, and quality of parent contact in which Auckland seems superior. The results for the Down's syndrome group compare favourably with those from other Down's syndrome programmes
SN - 0305-1862
UR - 6241850
ER -

TY - JOUR
ID - 2240
T1 - The prevalence of Toxocara eggs in the sand in children's playgrounds in Frankfurt/M
A1 - Duwel,D.

Y1 - 1984/12//
N1 - Duwel, D
Annals of tropical medicine and parasitology
68e, 2985178r
IM
Journal Article
English
KW - MEDLINE
KW - Child
KW - Germany
KW - West
KW - Humans
KW - Parasite Egg Count
KW - Play and Playthings
KW - Risk
KW - Soil Microbiology
KW - Toxocara/ip [Isolation & Purification]
RP - NOT IN FILE
SP - 633
EP - 636
JF - Annals of Tropical Medicine & Parasitology
JA - Ann Trop Med Parasitol
VL - 78
IS - 6
CY - ENGLAND
N2 - During an investigation of 31 sandpits in children's playgrounds in and around Frankfurt/M Toxocara eggs were detected in 27. The prevalence in areas with few or no parks (averaged 14.8 eggs 100 g-1 sand) was much higher than in districts where there was more room for the dogs to run about (5.3 eggs 100 g-1 sand). Larval development was complete in 40% of isolated Toxocara eggs after four weeks. The importance of these findings in connection with the risk, to children playing, of a possible infection (larva migrans visceralis) and preventive measures are discussed
SN - 0003-4983
UR - 6532333
ER -

TY - JOUR
ID - 2241
T1 - Reduction of eye gouging using a response interruption procedure
A1 - Slifer,K.J.
A1 - Iwata,B.A.
A1 - Dorsey,M.F.
Y1 - 1984/12//
N1 - Slifer, K J. Iwata, B A. Dorsey, M F
Journal of behavior therapy and experimental psychiatry
htn, 0245075
IM
Case Reports. Journal Article. Research Support, U.S. Gov't, P.H.S.
English
KW - MEDLINE
KW - Arousal
KW - Behavior Therapy/mt [Methods]
KW - Child
KW - Humans
KW - Intellectual Disability/px [Psychology]
KW - Intellectual Disability/th [Therapy]
KW - Male
KW - Reinforcement Schedule

KW - Self Mutilation/px [Psychology]
KW - Self Mutilation/th [Therapy]
KW - Stereotyped Behavior
RP - NOT IN FILE
SP - 369
EP - 375
JF - Journal of Behavior Therapy & Experimental Psychiatry
JA - J Behav Ther Exp Psychiatry
VL - 15
IS - 4
CY - UNITED STATES
N2 - A profoundly retarded male with severe congenital impairment of vision and hearing was treated for self-inflicted eye gouging. Prior to intervention, continuous mechanical restraint was required to prevent the response, precluding participation in educational and play activities. The response topography, the nature of the client's deficits, and a preliminary behavioral and medical assessment suggested that the response functioned as a source of sensory self-stimulation. Presentation of toys plus differential reinforcement of other behavior (DRO) as alternate sources of stimulation during baseline had no impact on eye gouging. The introduction of a contingent response interruption procedure reduced eye gouging and decreased the amount of time spent in restraints. Treatment effects were replicated in a group setting, and in the natural environment. Parents and school personnel were trained to use the treatment, and eye gouging remained infrequent at a 9-month follow-up
SN - 0005-7916
UR - 6526949
ER -

TY - JOUR
ID - 2242
T1 - The relationship between indoor nitrogen dioxide concentration levels and personal exposure: a pilot study
A1 - Hoek,G.
A1 - Meijer,R.
A1 - Scholten,A.
A1 - Noij,D.
A1 - Lebret,E.
Y1 - 1984///
N1 - Hoek, G. Meijer, R. Scholten, A. Noij, D. Lebret, E
International archives of occupational and environmental health
gpn, 7512134
IM
Journal Article
English
KW - MEDLINE
KW - Adult
KW - Air/an [Analysis]
KW - Air Pollutants/an [Analysis]
KW - Child
KW - Preschool
KW - Environmental Exposure
KW - Female
KW - Humans
KW - Monitoring
KW - Physiologic
KW - Netherlands
KW - Nitrogen Dioxide/an [Analysis]
KW - Pilot Projects
RP - NOT IN FILE
SP - 73
EP - 78
JF - International Archives of Occupational & Environmental Health

JA - Int Arch Occup Environ Health
VL - 55
IS - 1
CY - GERMANY, WEST
N2 - A small, personal monitoring study was performed in a subpopulation (14 families) of a case-control study on the relationship between indoor nitrogen-dioxide exposure and respiratory diseases of schoolchildren. Mothers, schoolchildren and pre-schoolchildren were asked to carry duplicate Palmes diffusion tubes during one week. Simultaneously nitrogen-dioxide concentrations were measured in the kitchen, living room, bedroom, outdoors and--for a few participants--at school and at work. Information on time activity patterns was gathered by means of a self administered diary. Several models for estimating exposure were constructed and tested against measured exposure. The personal exposure of the participants could well be explained by models containing indoor concentrations. Models with time-weighted average concentrations did not explain personal exposure better than models containing indoor concentrations. A calculated time-weighted average exposure was found to underestimate measured personal exposure by an average 20%, probably because the average concentration in a location does not necessarily reflect the actual exposure in that location. Personal exposure of mothers and children was very similar and highly correlated, indicating that the personal exposure of the mother might be a reasonable estimate for the exposure of the child
SN - 0340-0131
UR - 6526502
ER -

TY - JOUR
ID - 2243
T1 - Lyme disease in New Jersey, 1978-1982
A1 - Bowen,G.S.
A1 - Schulze,T.L.
A1 - Parkin,W.L.
Y1 - 1984/07//
N1 - Bowen, G S. Schulze, T L. Parkin, W L
The Yale journal of biology and medicine
xr7, 0417414
IM
Journal Article
English
KW - MEDLINE
KW - Adolescent
KW - Adult
KW - Antibodies
KW - Bacterial/an [Analysis]
KW - Child
KW - Preschool
KW - Cross-Sectional Studies
KW - Female
KW - Fluorescent Antibody Technique
KW - Humans
KW - Infant
KW - Lyme Disease/di [Diagnosis]
KW - Lyme Disease/ep [Epidemiology]
KW - Lyme Disease/im [Immunology]
KW - Male
KW - Middle Aged
KW - New Jersey
KW - Seasons
KW - Spirochaetales/im [Immunology]
RP - NOT IN FILE
SP - 661
EP - 668

JF - Yale Journal of Biology & Medicine
JA - Yale J Biol Med
VL - 57
IS - 4
CY - UNITED STATES
N2 - From 1978 to 1982, 117 cases of Lyme disease were reported in New Jersey. The number of cases increased each year from four in 1978 and 1979 to 56 in 1982. Forty-eight percent of cases occurred in a four-township area in central Monmouth County. The proportion of cases with arthritis decreased in 1982 because of early antibiotic treatment and better reporting of milder cases. The proportion of cases with positive serology increased with severity of the clinical syndrome. About 25 percent of patients had exposure to ticks because of occupations that required outdoor activities. Lyme disease is a growing public health problem in New Jersey
SN - 0044-0086
UR - 6393614
ER -

TY - JOUR
ID - 2244
T1 - Social skills training with mentally handicapped people: a review. [Review] [53 refs]
A1 - Robertson,I.
A1 - Richardson,A.M.
A1 - Youngson,S.C.
Y1 - 1984/11//
N1 - Robertson, I. Richardson, A M. Youngson, S C
The British journal of clinical psychology / the British Psychological Society
bok, 8105533
IM
Case Reports. Journal Article. Review
English
KW - MEDLINE
KW - Adolescent
KW - Adult
KW - Behavior Therapy/mt [Methods]
KW - Child
KW - Cues
KW - Environment
KW - Feedback
KW - Female
KW - Generalization (Psychology)
KW - Humans
KW - Imitative Behavior
KW - Intellectual Disability/rh [Rehabilitation]
KW - Interpersonal Relations
KW - Male
KW - Middle Aged
KW - Reinforcement (Psychology)
KW - Role Playing
KW - Social Behavior
KW - Token Economy
KW - Verbal Behavior
RP - NOT IN FILE
SP - 241
EP - 264
JF - British Journal of Clinical Psychology
JA - Br J Clin Psychol
VL - 23
IS - Pt 4
CY - ENGLAND

N2 - Four questions are asked about the use of social skills training procedures with mentally handicapped people. The first is, 'What are social skills in the context of mental handicap?', and it is suggested that they involve a complex array of perceptual, cognitive, motor and motivational processes, all of which can be disrupted due to various problems common among mentally handicapped people. The second question, 'What changes do social skills training programmes aim to achieve?', leads to the conclusion that most programmes have concentrated on motor, and to some extent on motivational processes, at the expense of perceptuo-cognitive ones. In asking, 'Have the programmes been successful in achieving these changes?', the answer is a guarded 'yes', given the limited aims of most investigators. Generalization to the natural environment has only occasionally been established, though it is often not measured. The fourth question is, 'What is the clinical significance of the changes obtained by such programmes and how can future ones be made more clinically relevant?'. The answer to the first part of the question is that, in general, their clinical utility has not been established. Future programmes could be made more clinically relevant if they were integrated with strategies for producing more benign and stimulating environments for mentally handicapped people. [References: 53]
SN - 0144-6657
UR - 6391587
ER -

TY - JOUR
ID - 2245
T1 - Survey of the previous investigation and treatment by private practitioners of patients with pulmonary tuberculosis attending government chest clinics in Hong Kong. Hong Kong Chest Service/British Medical Research Council
Y1 - 1984/09//
N1 - Tubercle
1273730, wh9
IM
Journal Article
English
KW - MEDLINE
KW - Adolescent
KW - Adult
KW - Aged
KW - Ambulatory Care Facilities
KW - Antitubercular Agents/tu [Therapeutic Use]
KW - Child
KW - Preschool
KW - Costs and Cost Analysis
KW - Family Practice
KW - Female
KW - Hong Kong
KW - Hospitals
KW - Proprietary
KW - Humans
KW - Infant
KW - Male
KW - Middle Aged
KW - Outpatient Clinics
KW - Hospital
KW - Patient Acceptance of Health Care
KW - Private Practice
KW - State Medicine
KW - Time Factors
KW - Tuberculosis
KW - Pulmonary/dt [Drug Therapy]
KW - Pulmonary/ec [Economics]
KW - Pulmonary/th [Therapy]
RP - NOT IN FILE

SP - 161
EP - 171
JF - Tubercle
VL - 65
IS - 3
CY - SCOTLAND
N2 - A questionnaire was applied to 159 consecutive patients with smear-positive and 187 with smear-negative pulmonary tuberculosis attending the 8 full-time Government chest clinics in Hong Kong for the first time for their current illness, who had already attended a private practitioner at a private general practice, a private clinic, or an independent hospital, outside the Government or Government-subsidised Service. The aim was to obtain details of management by private practitioners, and in particular of the antituberculosis chemotherapy prescribed. The great majority (86%) of the patients had originally attended a private practitioner because of symptoms. Only 18% had had their sputum examined, although 76% had had a chest radiograph; 65% of the smear-positive and 71% of the smear-negative patients had been told that they had, or might have, tuberculosis. For 40% there was an interval of more than a month between their first attendance at a private practitioner and at a Government chest clinic. Only 11% of the patients were referred without delay to a Government chest clinic, and another 21% once tuberculosis had been diagnosed or suspected. The patients were asked to name the drugs they had been prescribed, to produce samples, or to identify them from a perspex board containing samples of all 49 antituberculosis tablets and capsules known to be available in Hong Kong at the time. In the event, only 11% of the patients could name some or all of their medicaments and only 7% could produce samples, although a further 76% were able to describe them with or without the aid of the perspex board. In all, 19% of the patients were definitely or probably prescribed an antituberculosis regimen, although this was not always an adequate regimen. The findings suggest that there is considerable scope for active case-finding aimed at encouraging patients who are likely to have tuberculosis to attend a Government chest clinic
SN - 0041-3879
UR - 6485112
ER -

TY - JOUR
ID - 2246
T1 - Epidemiology of injuries in a large, urban school district
A1 - Boyce,W.T.
A1 - Sprunger,L.W.
A1 - Sobolewski,S.
A1 - Schaefer,C.
Y1 - 1984/09//
N1 - Boyce, W T. Sprunger, L W. Sobolewski, S. Schaefer, C
Pediatrics
oxv, 0376422
AIM, IM
Journal Article
English
KW - MEDLINE
KW - Adolescent
KW - Arizona
KW - Child
KW - Preschool
KW - Female
KW - Humans
KW - Male
KW - School Health Services
KW - Schools
KW - Sex Factors
KW - Urban Population
KW - Wounds and Injuries/ep [Epidemiology]
RP - NOT IN FILE
SP - 342

EP - 349
JF - Pediatrics
VL - 74
IS - 3
CY - UNITED STATES
N2 - Injuries represent the single greatest threat to the health and well-being of US children. A large number of childhood injuries are sustained in schools, yet little is currently known of the epidemiologic features of school-related injuries. A surveillance of injuries occurring in a large, urban school district during a 2-year period was conducted. Nurses in each of the district's 96 schools completed reporting forms on all injuries meeting standardized criteria, and both principals and nurses completed questionnaires on school characteristics that were judged potentially important to the injury rate in individual schools. A total of 5,379 injuries were reported, among the district's 55,000 students, for an overall injury rate of 49 injuries/1,000 student-years. Injury rates were higher for boys than girls at all age levels. Self-caused and sports-related injuries comprised nearly half of all those reported, and 14% were related to use of playground or sports equipment. Eighteen percent of injuries were severe, and playground- and equipment-related injuries were significantly more likely to be severe (P less than .001). Rates of injury among individual schools varied markedly, with schools at the two extremes separated by a 25-fold difference in rates. Higher overall injury rates were found in schools with longer hours, alternative educational programs, less experienced school nurses, and lower student-to-staff ratios (P less than .0001)
SN - 0031-4005
UR - 6472965
ER -

TY - JOUR
ID - 2247
T1 - [Sports injuries in children. Epidemiologic study]. [French]
A1 - Du Boullay,C.T.
A1 - Bardier,M.
A1 - Cheneau,J.
A1 - Bortolasso,J.
A1 - Gaubert,J.
Y1 - 1984///
N1 - Du Boullay, C T. Bardier, M. Cheneau, J. Bortolasso, J. Gaubert, J
Chirurgie pediatrique
c0a, 7804068
IM
English Abstract. Journal Article
French
KW - MEDLINE
KW - Age Factors
KW - Athletic Injuries/ep [Epidemiology]
KW - Child
KW - Female
KW - France
KW - Humans
KW - Male
RP - NOT IN FILE
SP - 125
EP - 135
JF - Chirurgie Pediatrique
JA - Chir Pediatr
VL - 25
IS - 3
CY - FRANCE
N2 - Among 49 000 cases of infantile emergencies which were received in the BUCI (Bloc d'urgence chirurgical infantile: surgical infantile emergency unit), 5 546 were sport traumas. At an early age, they were caused by outdoor plays; during adolescence, the main cases were caused by team sports. Males are

predominant. The number of cases has been regularly progressing, particularly since 1976. The fashion in sports is influenced by medias (i.e. skate board), and can be opposed to the continuous practice of popular sports (swimming, ball games, bycicle. There are winter, summer, school timed sports (the latter being influenced by the sportive scholar associations). The most frequent sports are cycling, football playing, swimming and horse riding, athleticism skating, Other are occasionnal. Changes in sport fashions, female increasing participation, such as horse riding and skating, democratisation (skiing, riding), the worsening of traumas; the pathology concerning bystanders, are described. Cranial and peripheric pathology are dominant. Trunk traumas are scarce but severe. Each sport has an elective pathologic localisation. Injury mechanisms are found, such as stirrup, saddle, ski baton pathology. There is traumatologic similarities; skate board and roller skating; judo and atheleticism; cycling and horse riding. Sport in children is not a replica of the one among adults. Riding a bike is not cycling. Some sports are dangerous: cycling, horse riding, rugby. A traumatological outline is revealed. Preventive measures should be taken. The socio-economical cost is heavy
SN - 0180-5738
UR - 6467483
ER -

TY - JOUR
ID - 2248
T1 - Playground equipment injuries in a large, urban school district
A1 - Boyce,W.T.
A1 - Sobolewski,S.
A1 - Sprunger,L.W.
A1 - Schaefer,C.
Y1 - 1984/09//
N1 - Boyce, W T. Sobolewski, S. Sprunger, L W. Schaefer, C
American journal of public health
1254074, 3xw
AIM, IM
Journal Article
English
KW - MEDLINE
KW - Arizona
KW - Child
KW - Epidemiologic Methods
KW - Equipment Safety
KW - Female
KW - Humans
KW - Male
KW - Play and Playthings
KW - Schools
KW - Urban Population
KW - Wounds and Injuries/ep [Epidemiology]
RP - NOT IN FILE
SP - 984
EP - 986
JF - American Journal of Public Health
JA - Am J Public Health
VL - 74
IS - 9
CY - UNITED STATES
N2 - We studied the epidemiologic features of playground equipment-related injuries occurring in a large, urban school district over a two-year period. Nurses in each of the district's 68 elementary schools completed self-coded reporting forms on all injuries meeting standardized criteria. A total of 511 equipment-related injuries were reported, an incidence of 8.9 injuries per 1,000 student-years. One-fourth of the injuries were severe, and climbing equipment was disproportionately represented among playground equipment associated with injuries. Extreme variability was found among school-specific rates of equipment injury, with schools at the two extremes separated by as much as a 40-fold difference in incidence. Two school characteristics--smaller

student enrollments and the presence of alternative educational programs--were significantly associated with higher equipment-related injury rates
SN - 0090-0036
UR - 6465412
ER -

TY - JOUR
ID - 2249
T1 - Evaluation of primary health care: approaches, comments and criticisms
A1 - Vaughan,J.P.
A1 - Walt,G.
A1 - Ross,D.
Y1 - 1984/04//
N1 - Vaughan, J P. Walt, G. Ross, D
Tropical doctor
wgc, 1301706
IM, J
Journal Article
English
KW - MEDLINE
KW - Child
KW - Preschool
KW - Community Health Workers/st [Standards]
KW - Costs and Cost Analysis
KW - Evaluation Studies as Topic
KW - Female
KW - Health Status Indicators
KW - Humans
KW - India
KW - Infant
KW - Newborn
KW - Male
KW - Outcome and Process Assessment (Health Care)
KW - Primary Health Care/st [Standards]
RP - NOT IN FILE
SP - 56
EP - 60
JF - Tropical Doctor
JA - Trop Doct.
VL - 14
IS - 2
CY - ENGLAND
SN - 0049-4755
UR - 6729973
ER -

TY - JOUR
ID - 2250
T1 - Biological monitoring of lead effects in a smelter community before and after emission control
A1 - Prpic-Majic,D.
A1 - Meczner,J.
A1 - Telisman,S.
A1 - Kersanc,A.
Y1 - 1984/01/27/
N1 - Prpic-Majic, D. Meczner, J. Telisman, S. Kersanc, A
The Science of the total environment
uj0, 0330500

IM
Comparative Study. Journal Article
English
KW - MEDLINE
KW - Adolescent
KW - Adult
KW - Air Pollution/pc [Prevention & Control]
KW - Child
KW - Preschool
KW - Environmental Exposure
KW - Female
KW - Hemoglobins/an [Analysis]
KW - Humans
KW - Infant
KW - Lead/bl [Blood]
KW - Lead
KW - Lead Poisoning/ep [Epidemiology]
KW - Lead Poisoning/pc [Prevention & Control]
KW - Male
KW - Metallurgy
KW - Porphobilinogen Synthase/bl [Blood]
KW - Protoporphyrins/bl [Blood]
KW - Time Factors
RP - NOT IN FILE
SP - 277
EP - 288
JF - Science of the Total Environment
JA - Sci Total Environ
VL - 32
IS - 3
CY - NETHERLANDS
N2 - Biological monitoring of lead effects in specific population groups living in the vicinity of a lead smelter has been followed up after effective emission control, in order to estimate the length of time necessary before the characteristic indicators of increased lead absorption normalize. The period established could be a realistic basis for predicting the time necessary to elapse before a lead contaminated area can be proclaimed as safe for normal living. Three groups of subjects were examined: preschool children, school children and mothers. The biological monitoring included the measurement of delta-aminolevulinic acid dehydratase activity (ALAD) and the concentration of zinc-protoporphyrin (ZPP), blood lead (Pb-B) and hemoglobin (Hb). The results obtained demonstrated a gradual normalization of the characteristic indicators of lead absorption, although they were still outside corresponding values in the control group even four years after the installation of effective emission control. These findings emphasize the advantages of biological monitoring over environmental for objective assessment of internal and external lead exposure following effective engineering control of the emission source
SN - 0048-9697
UR - 6710127
ER -

TY - JOUR
ID - 2251
T1 - Passive smoking, gas cooking, and respiratory health of children living in six cities
A1 - Ware,J.H.
A1 - Dockery,D.W.
A1 - Spiro,A.,III
A1 - Speizer,F.E.
A1 - Ferris,B.G.,Jr.
Y1 - 1984/03//
N1 - Ware, J H. Dockery, D W. Spiro, A 3rd. Speizer, F E. Ferris, B G Jr
The American review of respiratory disease

426, 0370523
AIM, IM
Comparative Study. Journal Article. Research Support, Non-U.S. Gov't. Research Support, U.S. Gov't, Non-P.H.S.. Research Support, U.S. Gov't, P.H.S.
English
KW - MEDLINE
KW - Activities of Daily Living
KW - Air Pollutants/ae [Adverse Effects]
KW - Child
KW - Cooking
KW - Female
KW - Fossil Fuels/ae [Adverse Effects]
KW - Humans
KW - Male
KW - Parents
KW - Respiratory Function Tests
KW - Respiratory Tract Diseases/ep [Epidemiology]
KW - Respiratory Tract Diseases/et [Etiology]
KW - Tobacco Smoke Pollution/ae [Adverse Effects]
KW - Urban Population
RP - NOT IN FILE
SP - 366
EP - 374
JF - American Review of Respiratory Disease
JA - Am Rev Respir Dis
VL - 129
IS - 3
CY - UNITED STATES
N2 - As part of a longitudinal study of the respiratory health effects of indoor and outdoor air pollutants, pulmonary function, respiratory illness history, and symptom history were recorded at 2 successive annual examinations of 10,106 white children living in 6 cities in the United States. Parental education, illness history, and smoking habits also were recorded, along with the fuel used for cooking in the child's home. Maternal cigarette smoking was associated with increases of 20 to 35% in the rates of 8 respiratory illnesses and symptoms investigated, and paternal smoking was associated with smaller but still substantial increases. Illness and symptom rates were linearly related to the number of cigarettes smoked by the child's mother. Illness rates were higher for children of current smokers than for children of ex-smokers. The associations between maternal smoking status and childhood respiratory illnesses and symptoms were reduced but not eliminated by adjustment for parental illness history. Levels of forced expiratory volume in one second (FEV1) were significantly lower for children of current smokers than for children of nonsmokers at both examinations and highest for children of ex-smokers. Levels of forced vital capacity (FVC) were lower for children of nonsmokers than for children of current smokers at both examinations, but the difference was statistically significant only at the first examination. Both the increase in mean FVC and the decrease in mean FEV1 among children of current smokers were linearly related to daily cigarette consumption. None of the respiratory illnesses and symptoms studied was significantly associated with exposure to gas cooking in the child's home.(ABSTRACT TRUNCATED AT 250 WORDS)
SN - 0003-0805
UR - 6703495
ER -

TY - JOUR
ID - 2252
T1 - A controlled trial of a physical fitness program for fifth grade students
A1 - Duncan,B.
A1 - Boyce,W.T.
A1 - Itami,R.
A1 - Puffenbarger,N.
Y1 - 1983/10//

N1 - Duncan, B. Boyce, W T. Itami, R. Puffenbarger, N
The Journal of school health
k13, 0376370
IM, N
Clinical Trial. Comparative Study. Controlled Clinical Trial. Journal Article. Research Support, Non-U.S. Gov't
English
KW - MEDLINE
KW - Child
KW - Clinical Trials as Topic
KW - Humans
KW - Physical Education and Training/mt [Methods]
KW - Physical Fitness
RP - NOT IN FILE
SP - 467
EP - 471
JF - Journal of School Health
JA - J Sch Health
VL - 53
IS - 8
CY - UNITED STATES
N2 - The relative effectiveness of a structured physical fitness program was compared with that of the customary organized activities for fifth grade students. The level of fitness was compared at the beginning and end of the nine-month academic year as well as after the three-month summer recess during which time no structured program was offered. The experimental group showed significant improvement over the control group in flexibility, strength and endurance during the school year; part of that improvement was maintained over the summer months; the physical fitness program had a positive influence on the general activity level of the students and was adopted by nine of the other 10 classrooms in the experimental school
SN - 0022-4391
UR - 6358700
ER -

TY - JOUR
ID - 2253
T1 - The effects of a good behavior game on the disruptive behavior of Sundanese elementary school students
A1 - Saigh,P.A.
A1 - Umar,A.M.
Y1 - 1983///
N1 - Saigh, P A. Umar, A M
Journal of applied behavior analysis
0174763, hdr
IM
Journal Article
English
KW - MEDLINE
KW - Behavior Therapy/mt [Methods]
KW - Child
KW - Child Behavior Disorders/th [Therapy]
KW - Developing Countries
KW - Humans
KW - Learning Disorders/th [Therapy]
KW - Play and Playthings
KW - Sudan
KW - Token Economy
RP - NOT IN FILE
SP - 339
EP - 344
JF - Journal of Applied Behavior Analysis

JA - J Appl Behav Anal
VL - 16
IS - 3
CY - UNITED STATES
N2 - An endemic version of the Good Behavior Game was applied in a rural Sudanese second-grade classroom. Official letters of commendation, extra time for recess, victory tags, and a winner's chart were used as backup reinforcers. The class was divided into two teams, and the teacher indicated she would place a check on the board after every rule violation. The students were also told that the team with the fewest marks would win the game and receive the aforementioned prizes. After an initial adaptation period, the rate of disruption was charted across four treatment phases: viz., baseline I, introduction of the game, baseline II, and reintroduction of the game. It was observed that the game phases were associated with marked decreases in the rate of seat leaving, talking without permission, and aggression. The teacher, principal, parents, and students were consequently individually interviewed, and their comments spoke strongly for the social validity of the game
SN - 0021-8855
UR - 6643325
ER -

TY - JOUR
ID - 2254
T1 - The Danish oral health care service for children: its achievements
A1 - Schwarz,E.
Y1 - 1983/09//
N1 - Schwarz, E
International dental journal
gpt, 0374714
D, IM
Journal Article
English
KW - MEDLINE
KW - Adolescent
KW - Child
KW - Child Health Services
KW - Preschool
KW - Denmark
KW - Dental Caries/pc [Prevention & Control]
KW - Dental Health Services
KW - Evaluation Studies as Topic
KW - Health Planning
KW - Humans
KW - Infant
KW - Oral Health
RP - NOT IN FILE
SP - 221
EP - 230
JF - International Dental Journal
JA - Int Dent J
VL - 33
IS - 3
CY - ENGLAND
N2 - Child dental services in Denmark were legally defined in 1971 and have functioned under this law for about a decade. Although no operational goals were defined, it is fair to say that the activities and results of the service have lived up to the expectations held at the inception of the law. The evaluation and planning system has made it possible to monitor developments in health as well as the use of resources. There have been consistent trends towards improved dental health in children and at the same time no increases in the use of personnel. There is general agreement in society that child services should remain a public activity. Pressures upon the services in the years to come must be expected to mount due to increased competition for scarce resources, simultaneous improvement in dental health and a reduction in the number of children. These

pressures will stimulate a re-examination of goals within the service so that appropriate short and long term local action can be taken before outside influences force changes with deleterious effects on the structure and function of the service. The success of the service and its ultimate achievements, therefore, will have to be measured against its abilities to adapt to changes in health as well as to an economic situation which will be more demanding than hitherto
SN - 0020-6539
UR - 6579026
ER -

TY - JOUR
ID - 2255
T1 - Reflections on parenting: a multigenerational perspective
A1 - Taylor,D.
Y1 - 1983/09//
N1 - Taylor, D
Family process
esa, 0400666
IM
Journal Article. Research Support, Non-U.S. Gov't
English
KW - MEDLINE
KW - Adaptation
KW - Psychological
KW - Adult
KW - Attitude to Death
KW - Child
KW - Child Rearing
KW - Female
KW - Grief
KW - Humans
KW - Male
KW - Parent-Child Relations
RP - NOT IN FILE
SP - 341
EP - 346
JF - Family Process
JA - Fam Process
VL - 22
IS - 3
CY - UNITED STATES
N2 - This paper presents some of the underlying patterns of parenting that have emerged from six years of ethnographic field research into the role of the family in children's acquisition and use of reading and writing skills. The research has provided some insights into the many complex and interrelated ways that children's literate activities are mediated and affected by multigenerational family patterns and by their personal experience of everyday life both within and outside the family. Two central themes are discussed: the spirit of change, which breaks the patterns of the past and brings new interpretations into the present, and the spirit of conservation, which maintains the continuation of family patterns from one generation to the next. Also described are the important parental attitudes of social flexibility and resiliency that are frequently evident in these processes of conservation and change of multigenerational patterns. One specific family story provides the background for the theoretical issues that are explored
SN - 0014-7370
UR - 6628647
ER -

TY - JOUR
ID - 2256

T1 - T-cell maturation in the human thymus and tonsil: peanut agglutinin binding T lymphocytes in thymus and tonsil differ in maturation stage
A1 - Schuurman,H.J.
A1 - Brekelmans,P.
A1 - Daemen,T.
A1 - Broekhuizen,R.
A1 - Kater,L.
Y1 - 1983/11//
N1 - Schuurman, H J. Brekelmans, P. Daemen, T. Broekhuizen, R. Kater, L
Clinical immunology and immunopathology
dea, 0356637
IM
Journal Article
English
KW - MEDLINE
KW - Cell Separation/mt [Methods]
KW - Child
KW - Preschool
KW - Humans
KW - Infant
KW - Lectins/me [Metabolism]
KW - Lymphocyte Activation
KW - Palatine Tonsil/cy [Cytology]
KW - Peanut Agglutinin
KW - T-Lymphocytes/ph [Physiology]
KW - Thymus Gland/cy [Cytology]
RP - NOT IN FILE
SP - 271
EP - 281
JF - Clinical Immunology & Immunopathology
JA - Clin Immunol Immunopathol
VL - 29
IS - 2
CY - UNITED STATES
N2 - The finding of peanut agglutinin (PNA) binding capacity, supposed to be a marker of immature lymphocytes, within the T-cell population of the human thymus (58%) and tonsil (10%) prompted the comparison of maturation stages of PNA binding (PNA+) and nonbinding (PNA-) T cells in both organs. The proliferative response after mitogenic stimulation of purified PNA+ fractions was significantly less than that of purified PNA- fractions. The results of mitogen dose-response experiments, of variation in time of culture harvest, and of addition of irradiated allogeneic peripheral blood non-T cells indicated the intrinsic mitogen unresponsiveness of cells in the PNA+ fractions. The mitogen response of tonsil fractions was higher than that of thymocyte fractions. Cells with an immature immunologic phenotype were enriched in the thymocyte PNA+ fraction, and almost absent in the tonsil fractions. Both tonsil fractions contained cells with the immunologic phenotype of mature T cells, and showed a purine interconversion enzyme makeup comparable to mature T lymphocytes. It is concluded that the tonsil PNA+ T cell is a functionally immature lymphocyte which is in a further maturation stage than PNA+ or PNA- thymocytes. The presence of PNA+ T cells outside the thymus is of relevance for the clinical evaluation of PNA binding assays and suggests the occurrence of T-cell maturation within the tonsil environment
SN - 0090-1229
UR - 6605224
ER -

TY - JOUR
ID - 2257
T1 - Nutrition and iodine versus genetic factors in endemic goiter
A1 - Sanchez,Franco F.
A1 - Cacicedo,L.

A1 - Morreale de,Escobar G.
A1 - Escobar del,Rey F.
Y1 - 1983/06//
N1 - Sanchez Franco, F. Cacicedo, L. Morreale de Escobar, G. Escobar del Rey, F
Journal of endocrinological investigation
iam, 7806594
IM
Journal Article
English
KW - MEDLINE
KW - Adolescent
KW - Child
KW - Preschool
KW - Consanguinity
KW - Diet
KW - Female
KW - Goiter
KW - Endemic/et [Etiology]
KW - Endemic/ge [Genetics]
KW - Humans
KW - Iodine/df [Deficiency]
KW - Iodine/ur [Urine]
KW - Male
KW - Nitrogen/ur [Urine]
KW - Nutritional Physiological Phenomena
KW - Spain
RP - NOT IN FILE
SP - 185
EP - 188
JF - Journal of Endocrinological Investigation
JA - J Endocrinol Invest
VL - 6
IS - 3
CY - ITALY
N2 - Consanguinity has been considered to be the major factor responsible for the high incidence of goiter in the area of Las Hurdes in Spain. However, iodine deficiency was later found to be severe enough to account for endemic goiter, and the presence of cretins in this area. Children from very similar family and socioeconomic backgrounds were found to be on three different nutritional programs, depending on the schools they were attending, and it appeared of interest to determine the effect of nutrition on the goiter incidence in children from the same population. Total iodine, nitrogen and creatinine concentrations were measured in casual urine samples. All three were found to change in parallel in the different subpopulations. They were low in schoolchildren receiving most of their meals at home. Such meals were composed of locally grown food. They were normal in children living in a boarding school, where all of the food is provided from outside the area. They were intermediate in children from a day care center, who received only some meals from an outside source. In the boarding school, goiter incidence was 21%, as opposed to 87% for schoolchildren fed at home. Such results indicate that in this area consanguinity plays a minor role, if any, in the high incidence of goiter
SN - 0391-4097
UR - 6619528
ER -

TY - JOUR
ID - 2258
T1 - Very young children's memory for the location of objects in a large-scale environment
A1 - DeLoache,J.S.
A1 - Brown,A.L.
Y1 - 1983/08//

N1 - DeLoache, J S. Brown, A L
Child development
0372725, d28
IM
Journal Article. Research Support, Non-U.S. Gov't. Research Support, U.S. Gov't, Non-P.H.S.. Research Support, U.S. Gov't, P.H.S.
English
KW - MEDLINE
KW - Child Development
KW - Child
KW - Preschool
KW - Cognition
KW - Cues
KW - Discrimination Learning
KW - Female
KW - Humans
KW - Infant
KW - Male
KW - Memory
KW - Mental Recall
KW - Orientation
KW - Pattern Recognition
KW - Visual
KW - Social Environment
KW - Space Perception
RP - NOT IN FILE
SP - 888
EP - 897
JF - Child Development
JA - Child Dev
VL - 54
IS - 4
CY - UNITED STATES
N2 - 2 experiments were carried out to investigate 18- to 30-month-old children's memory for the location of a hidden object. In the first experiment, young children were observed in 2 different memory-for-location tasks, both conducted in their own homes. In 1, a toy was hidden in a natural location, and in the other it was hidden in one of a set of boxes with picture cues on top of them. Memory performance was significantly better when the toy was hidden within the natural environment. The effect of different types of hiding locations was examined further in the second experiment. No age differences were found when an object was hidden either in the natural environment or in 1 of a set of unmarked boxes (although performance was better in the former condition). However, in the third condition the older subjects (24-30 months) effectively used a landmark cue (a nearby piece of furniture) to help them remember in which plain box a toy had been hidden, but the young subjects (18-22 months) did not profit from such potential cues. The results, as well as some previous research with delayed-response tasks, were interpreted as reflecting developmental changes in very young children's ability to exploit available cues. The pattern of results suggested the possibility that 2-year-old children are capable of a simple form of mnemonic strategy, actively associating an available cue with the information to be remembered
SN - 0009-3920
UR - 6617310
ER -

TY - JOUR
ID - 2259
T1 - Cultural factors in the epidemiology of filariasis due to Brugia malayi in an endemic community in Malaysia
A1 - Riji,H.M.
Y1 - 1983/03//

N1 - Riji, H M
The Southeast Asian journal of tropical medicine and public health
uvn, 0266303
IM
Journal Article
English
KW - MEDLINE
KW - Adolescent
KW - Adult
KW - Animals
KW - Attitude to Health
KW - Brugia
KW - Child
KW - Preschool
KW - Filariasis/ep [Epidemiology]
KW - Humans
KW - Malaysia
KW - Middle Aged
RP - NOT IN FILE
SP - 34
EP - 39
JF - Southeast Asian Journal of Tropical Medicine & Public Health
JA - Southeast Asian J Trop Med Public Health
VL - 14
IS - 1
CY - THAILAND
N2 - A study was carried out to identify some of the cultural factors in the epidemiology of filariasis in an endemic community in Malaysia. The viewpoint of the community, data an responses on knowledge of illness and filariasis, host related factors, health examination, vector study were analysed and discussed. The observations noted on cultural factors were: Occupational pattern: Different agricultural occupations seemed to related to transmission in terms of body exposure. Activities not related to production of crops: Play groups in late afternoon, bathing of household members near and after sunset, congregations at prayer houses very much exposed the population to mosquito bites in different degree in terms of length of time spent outside the house. Knowledge of filariasis: Filariasis was understood in terms of elephantiasis, the chronic stage of the disease. Other signs of disease-adenolymphangitis, red lines running down one or both legs and abscess were generally recognized but not often annonated with specific disease. Attitude toward disease: Filariasis was not seen as a health problem and the idea of filarial worms was still hard to believe. Knowledge on disease causation: Biological causes were generally recognized, though the idea of bacteria was not widespread
SN - 0125-1562
UR - 6612423
ER -

TY - JOUR
ID - 2260
T1 - Studies on nasopharyngeal carcinoma in the Sudan--I. Epidemiology and Aetiology
A1 - Hidayatalla,A.
A1 - Malik,M.O.
A1 - El Hadi,A.E.
A1 - Osman,A.A.
A1 - Hutt,M.S.
Y1 - 1983/06//
N1 - Hidayatalla, A. Malik, M O. El Hadi, A E. Osman, A A. Hutt, M S
European journal of cancer & clinical oncology
enw, 8112045
IM
Journal Article
English

KW - MEDLINE
KW - Adolescent
KW - Adult
KW - Age Factors
KW - Aged
KW - Antibodies
KW - Viral/an [Analysis]
KW - Blood Group Antigens
KW - Child
KW - Preschool
KW - Ethnic Groups
KW - Female
KW - Herpesvirus 4
KW - Human/im [Immunology]
KW - Humans
KW - Male
KW - Middle Aged
KW - Nasopharyngeal Neoplasms/ep [Epidemiology]
KW - Nasopharyngeal Neoplasms/et [Etiology]
KW - Nasopharyngeal Neoplasms/im [Immunology]
KW - Sex Factors
KW - Sudan
RP - NOT IN FILE
SP - 705
EP - 710
JF - European Journal of Cancer & Clinical Oncology
JA - Eur J Cancer Clin Oncol
VL - 19
IS - 6
CY - ENGLAND
N2 - Three hundred and seventy-four cases of nasopharyngeal carcinoma (NPC) recorded in the Sudan Cancer Registry (SCR) and 512 cases seen at the Radiation and Isotope Centre, Khartoum (RICK) were analysed. NPC formed 5.8% of all cancer cases in the SCR and 7.2% at the RICK; this is the highest frequency so far reported outside the Chinese. The male/female ratio was 3:1; NPC was the commonest tumour in males at the RICK (12.1%) and second commonest in the SCR (9.2%). It tended to occur in younger patients (youngest, 3 yr), with 14 and 12.1% of cases in children 14 yr or under in the SCR and RICK respectively; it is the commonest childhood malignancy in the Sudan. The ethnic and geographical distribution of the cases showed that racial susceptibility played a significant role in the aetiology of NPC in the Sudan whereas environmental factors could be excluded; however, Epstein-Barr virus might be an exciting factor
SN - 0277-5379
UR - 6307705
ER -

TY - JOUR
ID - 2261
T1 - Prospective study of burn wound excision of the hands
A1 - Goodwin,C.W.
A1 - Maguire,M.S.
A1 - McManus,W.F.
A1 - Pruitt,B.A.,Jr.
Y1 - 1983/06//
N1 - Goodwin, C W. Maguire, M S. McManus, W F. Pruitt, B A Jr
The Journal of trauma
kaf, 0376373
AIM, IM
Journal Article
English

KW - MEDLINE
KW - Adolescent
KW - Adult
KW - Burns/pp [Physiopathology]
KW - Burns/su [Surgery]
KW - Burns/th [Therapy]
KW - Child
KW - Preschool
KW - Exercise Therapy
KW - Hand Injuries/pp [Physiopathology]
KW - Hand Injuries/su [Surgery]
KW - Hand Injuries/th [Therapy]
KW - Humans
KW - Middle Aged
KW - Movement
KW - Prospective Studies
KW - Skin Transplantation
KW - Time Factors
RP - NOT IN FILE
SP - 510
EP - 517
JF - Journal of Trauma-Injury Infection & Critical Care
JA - J Trauma
VL - 23
IS - 6
CY - UNITED STATES
N2 - To examine the role of early excision and grafting in the preservation of maximal function of hands with deep dermal burns, we prospectively evaluated 164 burned hands in consecutively admitted patients (mean age, 29 years; mean burn size, 37% of body surface). All hands with burn depths of second degree, deep second degree, or third degree above the level of the tendons and joint capsules were assessed preoperatively, intraoperatively, and at discharge from the hospital. Patients were treated by excision and grafting in the first or second postburn week, by delayed grafting alone, or by allowing primary healing. Total active range of motion measurements were made on the day of discharge (mean, 64th postoperative day). Mean operative blood loss per hand was 1,270 ml. When all (alive and dead) patients undergoing early excision and grafting were examined by a binomial probability model, early surgery was shown to produce no adverse affect on survival. Excision and grafting of hands with deep dermal burns, whether early or late, offered no advantage over physical therapy and primary healing in maintaining hand function. Likewise, hands with more superficial burns responded equally to operative and nonoperative treatment. While early excision and grafting of hands with third-degree burns tended to produce poorer results than did initial nonoperative care and late grafting, the differences are just outside the range of significance. Early excision and grafting of selected third-degree injuries of the hands may be indicated in patients with small total body surface burns in order to shorten hospital stay. However, early surgical intervention in patients with massive burns should be directed toward area coverage, not toward hand excision
SN - 0022-5282
UR - 6345800
ER -

TY - JOUR
ID - 2262
T1 - Signs of capillary formation in the early phase of wound healing in children
A1 - Raekallio,J.
A1 - Viljanto,J.
Y1 - 1983///
N1 - Raekallio, J. Viljanto, J
Experimental pathology
eqr, 8108218
IM

Journal Article. Research Support, Non-U.S. Gov't
English
KW - MEDLINE
KW - Capillaries/ph [Physiology]
KW - Cell Aggregation
KW - Cellulose
KW - Child
KW - Preschool
KW - Drainage/is [Instrumentation]
KW - Electron Transport Complex IV/me [Metabolism]
KW - Endothelium/ph [Physiology]
KW - Female
KW - Fibroblasts/ph [Physiology]
KW - Humans
KW - Infant
KW - Newborn
KW - Male
KW - Neovascularization
KW - Pathologic
KW - Silicone Elastomers
KW - Time Factors
KW - Wound Healing
RP - NOT IN FILE
SP - 67
EP - 72
JF - Experimental Pathology
JA - Exp Pathol
VL - 23
IS - 2
CY - GERMANY, EAST
N2 - Small silicone rubber tubes containing a standard size viscose cellulose sponge (Cellstic) were implanted in wounds of 23 children at the end of routine surgery. The Cellstics were drawn out of the wounds 1-4 days after implantation. Single cells and cell aggregates, called cell aggregation centers (CAC), were washed out of the Cellstic sponges by the retrograde injection technique, centrifuged, studied histologically and histochemically for cytochrome oxidase activity. Small leukocytic CACs and solitary cells, also including some macrophages and a few endothelial cells, were observed on the first postoperative day. On the second postoperative day and thereafter 2-4 endothelial cells were attached to each other surrounding a lumen. These capillary segment-looking formations (CSF) resembled capillary segments and they were seen both inside and outside the (now larger) CACs. Outside the CACs solitary endothelial cells occasionally exhibited ring-like formations. High cytochrome oxidase activity was observed especially in many CSFs but the solitary and the ring-shaped endothelial cells, in- and outside the CACs, also showed intense oxidative metabolism
SN - 0232-1513
UR - 6189737
ER -

TY - JOUR
ID - 2263
T1 - Child rearing in poor urban Jamaica
A1 - Grantham-McGregor,S.
A1 - Landman,J.
A1 - Desai,P.
Y1 - 1983/03//
N1 - Grantham-McGregor, S. Landman, J. Desai, P
Child: care, health and development
7602632, c9x
IM

Journal Article. Research Support, Non-U.S. Gov't
English
KW - MEDLINE
KW - Child Development
KW - Child Rearing
KW - Child
KW - Preschool
KW - Female
KW - Humans
KW - Jamaica
KW - Language Development
KW - Male
KW - Parent-Child Relations
KW - Play and Playthings
KW - Poverty
KW - Social Environment
KW - Urban Population
RP - NOT IN FILE
SP - 57
EP - 71
JF - Child: Care, Health & Development
JA - Child Care Health Dev
VL - 9
IS - 2
CY - ENGLAND
N2 - Child rearing practices and attitudes were investigated using a questionnaire, among 75 families with 31--60-month-old children in poor suburban Kingston. A pattern emerged of many social contacts, outdoor activities and authoritarian discipline. While teaching and preparation for school were highly regarded, there was little conscious effort to foster cognitive and language development through play. Child rearing appears to reflect the influences of an African heritage, Western urbanization and poverty. Items from the questionnaire were used to devise an index of stimulation. Developmental assessments (DQs) were performed on a subgroup of 45 children. The results correlated positively with the index of stimulation
SN - 0305-1862
UR - 6850998
ER -

TY - JOUR
ID - 2264
T1 - Population dynamics among the Totos of West Bengal: a positive response to culture contact
A1 - Sinha,D.
A1 - Pal,B.C.
Y1 - 1983/04//
N1 - Sinha, D. Pal, B C
Journal of biosocial science
hjn, 0177346
IM, J
Journal Article. Research Support, Non-U.S. Gov't
English
KW - MEDLINE
KW - Adolescent
KW - Adult
KW - Aged
KW - Child
KW - Preschool
KW - Emigration and Immigration
KW - Female
KW - Fertility

KW - Humans
KW - India
KW - Infant
KW - Newborn
KW - Male
KW - Middle Aged
KW - Mortality
KW - Population Dynamics
KW - Social Change
RP - NOT IN FILE
SP - 237
EP - 245
JF - Journal of Biosocial Science
JA - J Biosoc Sci
VL - 15
IS - 2
CY - ENGLAND
SN - 0021-9320
UR - 6841387
ER -

TY - JOUR
ID - 2265
T1 - Behavioral school psychology goes outdoors: the effect of organized games on playground aggression
A1 - Murphy,H.A.
A1 - Hutchison,J.M.
A1 - Bailey,J.S.
Y1 - 1983///
N1 - Murphy, H A. Hutchison, J M. Bailey, J S
Journal of applied behavior analysis
0174763, hdr
IM
Journal Article
English
KW - MEDLINE
KW - Aggression/px [Psychology]
KW - Aggression
KW - Behavior Therapy/mt [Methods]
KW - Child
KW - Child Behavior Disorders/pc [Prevention & Control]
KW - Child Behavior Disorders/px [Psychology]
KW - Preschool
KW - Female
KW - Humans
KW - Male
KW - Play and Playthings
KW - Schools
RP - NOT IN FILE
SP - 29
EP - 35
JF - Journal of Applied Behavior Analysis
JA - J Appl Behav Anal
VL - 16
IS - 1
CY - UNITED STATES
N2 - This research focuses on the inappropriate, largely aggressive, behaviors of 344 K-2 children assigned to a playground prior to the beginning of the school day. Initially, a system for observing large numbers of children

freely roaming over a large, open area was developed. This observational method was then used to determine the effectiveness of providing organized games for reducing potentially dangerous playground behaviors. Using a reversal (ABAB) design, it was found that the games, rope jumping, and foot racing, along with an infrequently used time-out procedure, significantly reduced the frequency of inappropriate incidents. It is suggested that when dealing with large groups, antecedent environmental manipulations may be more practical than providing consequences for the behaviors exhibited by identified individuals
SN - 0021-8855
UR - 6833166
ER -

TY - JOUR
ID - 2266
T1 - A family with heterozygous factor X Friuli defect outside Friuli
A1 - Girolami,A.
A1 - Lazzarin,M.
A1 - Procidano,M.
A1 - Luzzatto,G.
Y1 - 1983/03//
N1 - Girolami, A. Lazzarin, M. Procidano, M. Luzzatto, G
Blut
a8w, 0173401
IM
Case Reports. Journal Article. Research Support, Non-U.S. Gov't
English
KW - MEDLINE
KW - Adult
KW - Blood Coagulation Disorders/ep [Epidemiology]
KW - Blood Coagulation Disorders/ge [Genetics]
KW - Child
KW - Factor X
KW - Female
KW - Heterozygote
KW - Humans
KW - Italy
KW - Partial Thromboplastin Time
KW - Pedigree
KW - Prothrombin Time
RP - NOT IN FILE
SP - 149
EP - 154
JF - Blut
VL - 46
IS - 3
CY - GERMANY, WEST
N2 - Three members of the same family were found to have a clotting defect consistent with the diagnosis of heterozygous factor X Friuli disorder. The main features of the defect were a mild prolongation of prothrombin time and partial thromboplastin time, but a normal Stypven-Cephalin clotting time. Factor X activity was 40-50% of normal using tissue thromboplastin, but was perfectly normal using Russell's viper venom and cephalin. Using chromogenic substrate S-2222 the level was 30% of normal. Immunologically, factor X was normal. Bleeding manifestations were mild if any. The hereditary pattern was autosomal. The family comes from an area far away from Friuli and represents the first example of factor X Friuli discovered outside the Friuli
SN - 0006-5242
UR - 6824795
ER -

TY - JOUR
ID - 2267

T1 - Diagnosis of house dust mite allergy in asthmatic children: what constitutes a positive history?
A1 - Murray,A.B.
A1 - Ferguson,A.C.
A1 - Morrison,B.J.
Y1 - 1983/01//
N1 - Murray, A B. Ferguson, A C. Morrison, B J
The Journal of allergy and clinical immunology
h53, 1275002
AIM, IM
Journal Article. Research Support, Non-U.S. Gov't
English
KW - MEDLINE
KW - Adolescent
KW - Allergens/im [Immunology]
KW - Asthma/et [Etiology]
KW - Asthma/im [Immunology]
KW - Bronchial Provocation Tests
KW - Child
KW - Preschool
KW - Dust
KW - Female
KW - Humans
KW - Hypersensitivity/co [Complications]
KW - Hypersensitivity/di [Diagnosis]
KW - Infant
KW - Male
KW - Mites/im [Immunology]
KW - Questionnaires
KW - Skin Tests
RP - NOT IN FILE
SP - 21
EP - 28
JF - Journal of Allergy & Clinical Immunology
JA - J Allergy Clin Immunol
VL - 71
IS - 1 Pt 1
CY - UNITED STATES
N2 - Standardized questions were put to the parents of 530 children, referred consecutively for evaluation of asthma, to determine which features in the history were associated with house-dust mite allergy. Bronchial challenge tests performed on 19 of the children confirmed that there is a highly significant association between a positive skin prick test and a positive bronchial challenge test to Dermatophagoides farinae mite antigen. One hundred and eighteen (23%) of the children had positive prick tests to the mite. There is a highly significant association between a positive prick test to mite and a history that the subject's respiratory symptoms become worse when there is exposure to domestic activity that stirs up house dust (vacuuming, dusting, sweeping, making the bed, or shaking out blankets) or that the symptoms improve when out of doors. Seasonal variation and other features in the history are of little value in distinguishing mite-sensitive from mite-insensitive asthmatics. Although the 4% whose only positive prick test reaction was to mite had significant worsening of asthma during the colder months compared with the remainder, most mite-positive subjects had multiple allergies and had no characteristic seasonal pattern. The presence of a positive prick test to mite was not associated with aggravation of asthma either at night in bed or in the morning on awakening. A history similar to that of mite-sensitive subjects was elicited in those with a positive prick test to house dust. A positive history of house dust or house-dust mite allergy in asthmatics is one in which respiratory symptoms become worse during domestic activity that stirs up house dust or improve when outdoors
SN - 0091-6749
UR - 6822690
ER -

TY - JOUR
ID - 2268
T1 - Fertility behaviour in low-income countries
A1 - Abdullah,A.A.
Y1 - 1983/09//
N1 - Abdullah, A A
Bangladesh Development Studies
9878828, 9878828
J
Journal Article
English
KW - MEDLINE
KW - Asia
KW - Bangladesh
KW - Child
KW - Demography
KW - Developing Countries
KW - Economics
KW - Fertility
KW - India
KW - Population
KW - Population Dynamics
KW - Sexual Behavior
KW - Social Class
KW - Socioeconomic Factors
RP - NOT IN FILE
SP - 1
EP - 139
JF - Bangladesh Development Studies
JA - Bangladesh Dev Stud
VL - 11
IS - 3
CY - BANGLADESH
SN - 0304-095X
UR - 12340705
ER -

TY - JOUR
ID - 2269
T1 - Mechanical determinants of bone modeling. [Review] [45 refs]
A1 - Frost,H.M.
Y1 - 1982///
N1 - Frost, H M
Metabolic bone disease & related research
mue, 7900146
IM
Journal Article. Review
English
KW - MEDLINE
KW - Adult
KW - Age Factors
KW - Bone Development
KW - Bone and Bones/ah [Anatomy & Histology]
KW - Bone and Bones/pa [Pathology]
KW - Child
KW - Female
KW - Humans

KW - Male
KW - Models
KW - Biological
KW - Poliomyelitis/pa [Pathology]
KW - Stress
KW - Mechanical
KW - Tibial Fractures/pa [Pathology]
RP - NOT IN FILE
SP - 217
EP - 229
JF - Metabolic Bone Disease & Related Research
JA - Metab Bone Dis Relat Res
VL - 4
IS - 4
CY - FRANCE
N2 - In angulated long bone malunions in children, bone architectural changes arise which act consistently in kind, location, and direction, while the associated bone strain-stress patterns become definable as qualitative differences from the norm. Comparison of such information reveals that bone architectural adaptations correlate one-to-one with dynamic flexural strain orientation and magnitude but not with any single principal stress. The above plus other facts suggest six axioms which can explain many of the observed architectural adaptations of lamellar bone in response to specific mechanical bone-loading phenomena in normal as well as pathologic states. In essence those axioms state: In growing mammals and under repetitive, uniformly oriented, nontrivial, dynamic flexural strains, all lamellar bone surfaces drift in the concave-tending direction. As a result of these strains, a bone would adopt a size and configuration that minimize its flexural deformation during normal activities. The axioms provide reasonable explanations for inwaisting of vertebral bodies and long bone metaphyses, correction of malunions, increasing outside diameter and cortical thickness during growth, circular, elliptical, and triangular cross-sections, natural curvatures of whole bones, many architectural changes due to neuromotor abnormalities arising in early life, and the more rapid modeling seen in very young children or after recent fractures. [References: 45]
SN - 0221-8747
UR - 6763662
ER -

TY - JOUR
ID - 2270
T1 - The effects of physical exercise on self-stimulation and appropriate responding in autistic children
A1 - Kern,L.
A1 - Koegel,R.L.
A1 - Dyer,K.
A1 - Blew,P.A.
A1 - Fenton,L.R.
Y1 - 1982/12//
N1 - Kern, L. Koegel, R L. Dyer, K. Blew, P A. Fenton, L R
Journal of autism and developmental disorders
7904301, hgw
IM
Journal Article. Research Support, U.S. Gov't, Non-P.H.S.. Research Support, U.S. Gov't, P.H.S.
English
KW - MEDLINE
KW - Adolescent
KW - Autistic Disorder/rh [Rehabilitation]
KW - Child
KW - Child Behavior
KW - Preschool
KW - Education
KW - Special
KW - Female

KW - Humans
KW - Male
KW - Physical Exertion
KW - Play and Playthings
KW - Running
KW - Self Stimulation
RP - NOT IN FILE
SP - 399
EP - 419
JF - Journal of Autism & Developmental Disorders
JA - J Autism Dev Disord
VL - 12
IS - 4
CY - UNITED STATES
N2 - A major problem encountered with autistic children is their characteristic self-stimulatory behavior, which frequently interferes with on-task responding and other appropriate behaviors. However, the experimental literature suggests that with many populations, increased physical activity might positively influence subsequent responding. Therefore, the purpose of this study was to investigate the use of increased physical activity (in this experiment, jogging) as a possible method of decreasing subsequent self-stimulatory behaviors as well as increasing subsequent appropriate responding. Seven autistic children with exceptionally high levels of self-stimulatory behavior participated in the investigation. Self-stimulatory and appropriate behaviors were measured both before and after jogging in a repeated-reversal design. The results demonstrated the following: (1) Brief jogging sessions produced decreases in subsequent levels of self-stimulatory behaviors and also produced increases in appropriate play and academic responding; (2) These changes after jogging were evident in three different experimental settings: during academic responding on preschool level tasks' in a clinic; during ball-playing in an outside play area; and in a quiet room, while no other activity was occurring; (3) Supplementary measures obtained in an applied classroom setting showed a similar relationship with both increases in on-task activity and general interest ratings for school tasks following the jogging sessions
SN - 0162-3257
UR - 7161239
ER -

TY - JOUR
ID - 2271
T1 - [Effectiveness of the examination of the different categories of contacts of pulmonary tuberculosis patients as a part of epidemiological investigations]. [Romanian]
A1 - Dobre,V.
A1 - Silaghi,I.
A1 - Muller,A.
A1 - Barbosu,I.
Y1 - 1982/07//
N1 - Dobre, V. Silaghi, I. Muller, A. Barbosu, I
Revista de igiena, bacteriologie, virusologie, parazitologie, epidemiologie, pneumoftiziologie.
Pneumoftiziologia
7514105
IM
English Abstract. Journal Article
Romanian
KW - MEDLINE
KW - Adolescent
KW - Adult
KW - Aged
KW - Carrier State/di [Diagnosis]
KW - Child
KW - Preschool
KW - Female
KW - Humans

KW - Infant
KW - Male
KW - Middle Aged
KW - Radiography
KW - Thoracic
KW - Romania
KW - Tuberculin Test
KW - Tuberculosis
KW - Pulmonary/di [Diagnosis]
KW - Pulmonary/ep [Epidemiology]
KW - Pulmonary/pc [Prevention & Control]
RP - NOT IN FILE
SP - 187
EP - 194
JF - Revista de Igiena Bacteriologie Virusologie Parazitologie Epidemiologie Pneumoftiziologie Seria Pneumoftiziologia
JA - Rev Ig Bacteriol.Virusol.Parazitol.Epidemiol Pneumoftiziol.Pneumoftiziol.
VL - 31
IS - 3
CY - ROMANIA
N2 - In view of evaluating the global efficiency of the epidemiologic investigation in relation to its area an analysis was carried out of 127 investigations involving 1 252 tb contacts. The contacts were classified in three groups: intrafamilial and intradomiciliary, intrafamilial but outside the home, and extrafamilial and outside the home; the results of the examinations were compared between them on the one hand and with the proportion of allergic children (51.6%) and the instantaneous prevalence of bacilli eliminating cases (0.31%) on the other hand. These were obtained in an area with a high endemic ratio of tuberculosis, following integral biological and radiophotographic case-finding. Children who were tb contacts, belonging to the first group also included the highest proportion of allergies (48.3%) while in the other two groups the number of allergic children was similar to that of allergic children in the locality with a high endemic ratio of tuberculosis. The radiophotographic examination of adults who were contacts of tuberculous patients showed that the lowest proportion of tuberculous patients with active tuberculosis (0.4%), which was found in the third group, was still above the instantaneous prevalence of bacilliferic patients from the area with high ratio of endemic tuberculosis. In conclusion, the present technical structure of epidemiological investigations corresponds to the actual level of the endemy and no group of contacts can be excluded from the investigation
SN - 0377-5011
UR - 6296972
ER -

TY - JOUR
ID - 2272
T1 - Results of attempted cardiopulmonary resuscitation of patients dying suddenly outside the hospital in Reykjavik and the surrounding area, 1976-1979
A1 - Gudjonsson,H.
A1 - Baldvinsson,E.
A1 - Oddsson,G.
A1 - Asgeirsson,E.
A1 - Kristjansson,H.
A1 - Hardarson,T.
Y1 - 1982///
N1 - Gudjonsson, H. Baldvinsson, E. Oddsson, G. Asgeirsson, E. Kristjansson, H. Hardarson, T
Acta medica Scandinavica
0370330
IM
Journal Article
English
KW - MEDLINE
KW - Adolescent

KW - Adult
KW - Aged
KW - Ambulances
KW - Child
KW - Preschool
KW - Female
KW - Heart Arrest/ep [Epidemiology]
KW - Heart Arrest/th [Therapy]
KW - Humans
KW - Iceland
KW - Infant
KW - Newborn
KW - Male
KW - Middle Aged
KW - Prognosis
KW - Resuscitation
KW - Transportation of Patients
RP - NOT IN FILE
SP - 247
EP - 251
JF - Acta Medica Scandinavica
JA - Acta Med Scand
VL - 212
IS - 4
CY - SWEDEN
N2 - Cardiopulmonary resuscitation (CPR) was attempted in 222 cases of sudden death at the City Hospital, Reykjavik, during 1976-79. Of the 68 patients (31%) successfully resuscitated, 47 died in the hospital and 21 (9%) were discharged, 17 in good mental and physical condition. The mean combined response and transport time was 12.1 min and the ambulance mean time of response 7.3 min. The first ECG revealed considerable prognostic indications. Of the 90 patients who had ventricular fibrillation on admission, 42 (47%) were successfully resuscitated and 18 (20%) were subsequently discharged. Among 114 patients with asystole, resuscitation was successful in 23 (20%) and two (2%) were discharged. Immediate first aid in situ had a definite prognostic influence. These results compare favourably with those obtained elsewhere where the organization of first aid and emergency transport is similar. They do not, however, match the results achieved by fully specialized resuscitation teams trained to operate outside the hospital. Results of CPR of patients with cardiac arrest out of hospital in Reykjavik show increasing improvement over the years. This may be partly explained by a considerable public debate on this issue in 1978 and subsequent streamlining of activities
SN - 0001-6101
UR - 7148520
ER -

TY - JOUR
ID - 2273
T1 - [Gradual discharge as part-time inpatient treatment]. [German]
A1 - Poustka,F.
Y1 - 1982/09//
N1 - Poustka, F
Psychiatrische Praxis
qck, 0423204
IM
English Abstract. Journal Article
German
KW - MEDLINE
KW - Adolescent
KW - Adult
KW - Child
KW - Day Care

KW - Germany
KW - West
KW - Halfway Houses
KW - Humans
KW - Intelligence
KW - Mental Disorders/rh [Rehabilitation]
KW - Night Care
KW - Patient Discharge
KW - Prognosis
KW - Rehabilitation
KW - Vocational/mt [Methods]
KW - Residential Treatment/mt [Methods]
KW - Social Adjustment
RP - NOT IN FILE
SP - 155
EP - 159
JF - Psychiatrische Praxis
JA - Psychiatr Prax
VL - 9
IS - 5
CY - GERMANY, WEST
N2 - About 34 p.c. of the patients coming from Mannheim or outskirts treated in the child and adolescent psychiatric clinic situated in the centre of Mannheim attended a kindergarten, a public school or being at work outside the clinic while in part-time in-patient treatment or before release. These patients scored somewhat but not significantly higher on intelligence tests, had better school attainment and consisted of a relatively higher proportion of females compared with patients who were not involved in activities outside the clinic. Diagnostically a somewhat greater number of psychosis, neurotic/emotional disorders were found among the part-time group and they showed also more associations with abnormal familial background, a higher proportion of part-time patients were not discharged to their families and have been treated for a longer time respectively. Not related to the length of treatment a significantly more positive successful treatment could be demonstrated among the part-time group. Particular aspects of a more realistic approach of treatment are discussed
SN - 0303-4259
UR - 7146223
ER -

TY - JOUR
ID - 2274
T1 - Excessive daytime sleepiness: a 1-year study in an unselected inpatient population
A1 - Franceschi,M.
A1 - Zamproni,P.
A1 - Crippa,D.
A1 - Smirne,S.
Y1 - 1982///
N1 - Franceschi, M. Zamproni, P. Crippa, D. Smirne, S
Sleep
sws, 7809084
IM
Journal Article
English
KW - MEDLINE
KW - Adolescent
KW - Adult
KW - Aged
KW - Child
KW - Disorders of Excessive Somnolence/ep [Epidemiology]
KW - Electroencephalography
KW - Female

KW - Humans
KW - Inpatients
KW - Italy
KW - Male
KW - Middle Aged
KW - Narcolepsy/ep [Epidemiology]
KW - Prospective Studies
KW - Sex Factors
KW - Sleep Apnea Syndromes/ep [Epidemiology]
KW - Sleep Disorders/ep [Epidemiology]
RP - NOT IN FILE
SP - 239
EP - 247
JF - Sleep
VL - 5
IS - 3
CY - UNITED STATES
N2 - A prospective epidemiological investigation of excessive daytime sleepiness (EDS) was carried out in an unselected inpatient population admitted to a general hospital during a 1-year period. The study comprised 2518 patients, 1347 female and 1171 male, aged 6-92 years (mean, 55.2). On the basis of histories and clinical and polysomnographic data, EDS was found in 28 cases (1.11%). Of these, 25 (0.99%; 18 female and 7 male; mean age, 61.3) had sleep apnea syndromes (SAS) with predominantly obstructive apnea. Two patients (0.07%; one female and one male; mean age, 65.5) had idiopathic CNS hypersomnia, and one male patient (0.03%) aged 48 years had a combination of narcolepsy and SAS. Differences and agreements of our findings with previous literature data are discussed. The present study shows that in an unselected inpatient population, EDS is a relatively common sleep disorder, usually found in mild to moderate forms. Because it is not severe, it disturbs only relatively domestic activities of elderly retired patients or others who do not work outside the home, and is often masked by other troubles. For these reasons, EDS in the aging population may pass unnoticed in epidemiological studies based only on data from sleep disorder centers
SN - 0161-8105
UR - 7134730
ER -

TY - JOUR
ID - 2275
T1 - Peer popularity and peer communication patterns: hyperactive versus active but normal boys
A1 - King,C.A.
A1 - Young,R.D.
Y1 - 1981/12//
N1 - King, C A. Young, R D
Journal of abnormal child psychology
0364547, h33
IM
Journal Article. Research Support, U.S. Gov't, P.H.S.
English
KW - MEDLINE
KW - Attention Deficit Disorder with Hyperactivity/px [Psychology]
KW - Child
KW - Communication
KW - Humans
KW - Interpersonal Relations
KW - Male
KW - Peer Group
KW - Role
KW - Social Desirability
KW - Social Perception
RP - NOT IN FILE

SP - 465
EP - 482
JF - Journal of Abnormal Child Psychology
JA - J Abnorm Child Psychol
VL - 9
IS - 4
CY - UNITED STATES
N2 - Classroom peer perceptions of 18 teacher-nominated hyperactive and 18 teacher-nominated active but normal elementary school-age boys, as revealed in two sociometric measures (Bower's Class Play, like-dislike nomination), were compared. Results indicated that hyperactives were significantly different from actives on all sociometric measures in that they were perceived more negatively. Peer communication patterns also were assessed. The communicative content, communicative quality, and attention to task instructions of hyperactive boys were compared to those of comparison boys. Limited support was found for the earlier finding that hyperactive boys were less likely than comparison boys to modulate behavior in response to changing external cues and to respond to subtle social learning opportunities. The communication task was found to be highly engaging for both groups of boys, and results suggest that hyperactive children may not lack the interpersonal skills necessary for referential communication, although they may be unable to use them consistently in all settings. The need to continue the study of peer relations of hyperactive children in naturalistic settings is stressed
SN - 0091-0627
UR - 7328227
ER -

TY - JOUR
ID - 2276
T1 - Utilization of factor analysis for the evaluation of ontogenetic and functional changes in the electrical activity of the brain
A1 - Pratusevich,YuM
A1 - Solov'ev,A.V.
A1 - Kvasov,G.I.
Y1 - 1981///
N1 - Pratusevich YuM. Solov'ev, A V. Kvasov, G I
Journal of hygiene, epidemiology, microbiology, and immunology
2985116r, iev
IM
Journal Article
English
KW - MEDLINE
KW - Aging
KW - Child
KW - Electroencephalography
KW - Humans
KW - Mental Fatigue/pp [Physiopathology]
RP - NOT IN FILE
SP - 259
EP - 269
JF - Journal of Hygiene, Epidemiology, Microbiology & Immunology
JA - J Hyg Epidemiol Microbiol Immunol
VL - 25
IS - 3
CY - CZECHOSLOVAKIA
N2 - The tendency of the index, amplitude and its dispersion during the year is opposite to the age-dependent tendency of changes in these indices and points to a chronic component of mental fatigue. It is very important that in the second half of the school year the dispersion of the EEG amplitude increases in all sections of the brain and in all frequency ranges: an increase in EEG amplitude dispersion in parallel with an increase in the amplitude itself indicates that the process of synchronization and the ultraliminal inhibition connected with it in different sections of the brain and in various frequency ranges of the EEG is not stagnant or inert but mobile and

instable and can therefore be relatively easily removed by special hygienic measures. As an example of the latter we can mention the use of a special regime of exercise (Yu. M. Pratusevich et al., 1975), addition of supplementary factors of nutrition to the diet of the time the children spend in the fresh air engaging in outdoor games (Yu. M. Pratusevich, 1964)
SN - 0022-1732
UR - 6117592
ER -

TY - JOUR
ID - 2277
T1 - The consistency of a class of coercive child behaviors across school settings for individual subjects
A1 - Harris,A.
A1 - Reid,J.B.
Y1 - 1981/06//
N1 - Harris, A. Reid, J B
Journal of abnormal child psychology
0364547, h33
IM
Journal Article. Research Support, U.S. Gov't, P.H.S.
English
KW - MEDLINE
KW - Aggression
KW - Child
KW - Child Behavior
KW - Coercion
KW - Family
KW - Humans
KW - Male
KW - Schools
RP - NOT IN FILE
SP - 219
EP - 227
JF - Journal of Abnormal Child Psychology
JA - J Abnorm Child Psychol
VL - 9
IS - 2
CY - UNITED STATES
N2 - This study addressed the problem of the intraindividual consistency of a class of coercive child behaviors across two school settings. Observational data collected on eight coercive behaviors of 53 boys in classrooms and on the playground. Previous Guttman scale analyses had shown that these eight coercive behaviors constituted a response class. The rates of the eight coercive responses in the response class were rank-ordered separately for each of the 53 subjects in each of the two settings. The intraindividual rank-order correlations were all positive, and 37 were statistically reliable. This number of significant correlations was further shown to be significantly greater than chance. It was concluded that there is a substantial amount of cross-situational consistency if intraresponse class analyses are done for individuals
SN - 0091-0627
UR - 7276402
ER -

TY - JOUR
ID - 2278
T1 - Quantitative regional blood-flow analysis and its clinical application during routine bone-scanning
A1 - Deutsch,S.D.
A1 - Gandsman,E.J.
A1 - Spraragen,S.C.
Y1 - 1981/02//

N1 - Deutsch, S D. Gandsman, E J. Spraragen, S C
The Journal of bone and joint surgery. American volume
hjr, 0014030
AIM, IM
Journal Article
English
KW - MEDLINE
KW - Adolescent
KW - Adult
KW - Aged
KW - Arthritis/ri [Radionuclide Imaging]
KW - Bone Diseases/pp [Physiopathology]
KW - Bone Diseases/ri [Radionuclide Imaging]
KW - Bone and Bones/bs [Blood Supply]
KW - Bone and Bones/ri [Radionuclide Imaging]
KW - Child
KW - Preschool
KW - Female
KW - Femur Head Necrosis/ri [Radionuclide Imaging]
KW - Hip Joint
KW - Humans
KW - Infant
KW - Legg-Calve-Perthes Disease/ri [Radionuclide Imaging]
KW - Male
KW - Middle Aged
KW - Osteomyelitis/ri [Radionuclide Imaging]
KW - Regional Blood Flow
KW - Synovitis/ri [Radionuclide Imaging]
KW - Technetium/du [Diagnostic Use]
RP - NOT IN FILE
SP - 295
EP - 305
JF - Journal of Bone & Joint Surgery - American Volume
JA - J Bone Joint Surg Am
VL - 63
IS - 2
CY - UNITED STATES
N2 - A method of combined dynamic and static bone-imaging using technetium 99-labeled phosphonate is described. During the dynamic part of the study, immediately after intravenous injection of the radionuclide, counts per second were recorded over a period of time for analogous regions of normal and diseased bones. The counts per second then were plotted against time to give time-activity curves for each of these regions. The time-activity curves were divided into arterial, venous, and blood-pool phases. Each phase was integrated and then divided by the time-span of the phase to give average total counts per unit of time. The values of these normalized integrals were directly proportional to the blood flow to the regions of bone from which they were calculated. Ratios of normalized integrals from the analogous regions of the diseased bone to the normal bone were calculated to determine whether the blood flow to the diseased bone was decreased or increased. Static images of the bone structures analyzed during the dynamic portion of the study were obtained two hours after the initial injection of the radionuclide and the total number of counts for analogous regions on the static images of the normal and diseased bones were static images of the normal and diseased bones were recorded. The total number of counts for each region was divided by the area of that region to give average total counts. Ratios of the normalized counts were calculated for analogous regions of the diseased to the normal bone to determine whether there was an increase or decrease in concentration of the radionuclide. The method first was used to evaluate hip pain in children and adults. In order to recognize an abnormal result, mean values and two standard deviations were calculated for the dynamic and static ratios in a group of fifty patients who had no clinical complaints about the hips or lower extremities. Any ratios outside of the range of two standard deviations were considered abnormal. The method later was used to evaluate other bones, such as the tibia and humerus. Any value for the ratios beyond 1 +/- 0.20 was considered abnormal for these studies

SN - 0021-9355
UR - 7462285
ER -

TY - JOUR
ID - 2279
T1 - Effects of ball size, ball color, and preferred color on catching by young children
A1 - Isaacs,L.D.
Y1 - 1980/10//
N1 - Isaacs, L D
Perceptual and motor skills
ozb, 0401131
IM
Journal Article
English
KW - MEDLINE
KW - Child
KW - Choice Behavior
KW - Color Perception
KW - Female
KW - Humans
KW - Male
KW - Motor Skills
KW - Size Perception
RP - NOT IN FILE
SP - 583
EP - 586
JF - Perceptual & Motor Skills
JA - Percept.Mot.Skills
VL - 51
IS - 2
CY - UNITED STATES
N2 - 45 males and 45 females between 7 and 8 years of age were acquired to catch rubber playground balls which varied in both size and color. Prior to the administration of the criterion catch test, subjects indicated their choice for a preferred color ball. Analysis indicated that the 6-in. balls (diameter) were caught significantly better than were 10-in. balls. Although the main effect of ball color was nonsignificant, the children caught balls of preferred color significantly better than those of non-preferred colors. Males scored significantly higher than the females on the catching task
SN - 0031-5125
UR - 7443379
ER -

TY - JOUR
ID - 2280
T1 - The social worker's role: a study of private and voluntary hospitals
A1 - Adelson,G.
A1 - Leader,M.A.
Y1 - 1980/11//
N1 - Adelson, G. Leader, M A
Hospital & community psychiatry
gcj, 0040250
IM, Q
Comparative Study. Historical Article. Journal Article
English
KW - MEDLINE
KW - Child
KW - Environment

KW - Family Therapy
KW - Female
KW - History
KW - 20th Century
KW - Hospitals
KW - Proprietary
KW - Voluntary
KW - Humans
KW - Interprofessional Relations
KW - Male
KW - Patient Discharge
KW - Role
KW - Social Work
KW - Psychiatric/hi [History]
KW - Psychiatric
KW - United States
RP - NOT IN FILE
SP - 776
EP - 780
JF - Hospital & Community Psychiatry
JA - Hosp Community Psychiatry
VL - 31
IS - 11
CY - UNITED STATES
N2 - Social workers have played an integral role in the treatment of psychiatric patients in hospitals since the earliest days of the 20th century. At first totally dependent on the support and approval of the medical profession, psychiatric social work later became professionalized and began to take responsibility for a wide variety of duties in the institution. Through a study of private and voluntary hospitals, the authors found that despite decades of acceptance by medical staff, the social worker today functions in an environment where final administrative authority on hiring staff and deciding the need for social work services often falls outside the social work department. The survey also identifies the relative importance of various professional duties and the impact of social work on institution decision-making. Involvement by social workers in patient discharge planning could well ensure the future of social work in hospitals in a time of tight budgets, they conclude
SN - 0022-1597
UR - 7000660
ER -

TY - JOUR
ID - 2281
T1 - Realities for change in child health care: existing patterns and future possibilities
A1 - Stacey,M.
Y1 - 1980/06/21/
N1 - Stacey, M
British medical journal
b4w, 0372673
AIM, IM
Journal Article
English
KW - MEDLINE
KW - Adolescent
KW - Child
KW - Child Development
KW - Child Health Services
KW - Preschool
KW - Diet
KW - Disabled Persons
KW - Environment

KW - Female
KW - Forecasting
KW - Humans
KW - Infant
KW - Infant Mortality
KW - Maternal Behavior
KW - Poverty
KW - Social Class
RP - NOT IN FILE
SP - 1512
EP - 1515
JF - British Medical Journal
JA - Br Med J
VL - 280
IS - 6230
CY - ENGLAND
N2 - In assessing some of the existing patterns and future possibilities in child health care it was found that the continuing large social class differences in morbidity and mortality may be attributed to continued poverty, both of income and therefore of diet, and also to environmental deprivation. The absence of safe places for children to play, for example, is related to the high accident rates experienced by children. Doctors admit to awareness of these social and environmental causes of unnecessary morbidity and mortality among children but have failed to address the causes directly. While the causes are outside the immediate professional provenance of doctors, it is argued that, aware as they are of this aetiology, they have a moral and professional responsibility to act collectively as a pressure group urging improvements on the relevant authorities (as they have done in the case of smoking and clean air, for example)
SN - 0007-1447
UR - 6446349
ER -

TY - JOUR
ID - 2282
T1 - Strangulation in childhood: epidemiology and clinical course
A1 - Feldman,K.W.
A1 - Simms,R.J.
Y1 - 1980/06//
N1 - Feldman, K W. Simms, R J
Pediatrics
oxv, 0376422
AIM, IM
Journal Article
English
KW - MEDLINE
KW - Accident Prevention
KW - Accidents
KW - Home
KW - Adolescent
KW - Asphyxia/co [Complications]
KW - Asphyxia/ep [Epidemiology]
KW - Asphyxia/et [Etiology]
KW - Beds/st [Standards]
KW - Brain Damage
KW - Chronic/et [Etiology]
KW - Child
KW - Preschool
KW - Clothing
KW - Equipment and Supplies/st [Standards]
KW - Female

KW - Government Agencies
KW - Humans
KW - Hypoxia
KW - Brain/et [Etiology]
KW - Infant
KW - Newborn
KW - Male
KW - Play and Playthings
KW - Safety
KW - Sudden Infant Death/et [Etiology]
KW - Suicide
KW - United States
RP - NOT IN FILE
SP - 1079
EP - 1085
JF - Pediatrics
VL - 65
IS - 6
CY - UNITED STATES
N2 - A review of 233 cases of childhood strangulation was made to determine injury epidemiology. Consistent patterns of injury were observed. Crib and playground equipment strangulations are already the subject of Consumer Product Safety Commission (CPSC) preventative regulations. High chair-, playpen mesh-, pacifier cord-, and clothing-related injuries would be amenable to prevention by improved product safety regulation. The CPSC has been petitioned to remedy the first two of these hazards. Many crib and rope strangulations can only be prevented by direct patient counseling. Although Congress empowered and required the CPSC to recognize and correct unsafe products, its surveillance systems may obscure the very design defects that cause injury. Likewise, political considerations may slow enactment of new design regulations. Physicians who treat childhood accident victims are in a position to recognize hazardous products and lobby for their improvement. Of the 233 cases 38 were further evaluated to delineate the clinical course of childhood strangulation. Victims who fail to resume normal cardiopulmonary function by the time of hospitalization have a poor prognosis for neurologic recovery with current modes of therapy. Improved treatment of hypoxic and ischemic cerebral injury might prevent some of the deaths and handicaps resulting from strangulation
SN - 0031-4005
UR - 7375230
ER -

TY - JOUR
ID - 2283
T1 - Behavioral comparisons of hyperactive and normally active children in natural settings
A1 - Zentall,S.S.
Y1 - 1980/03//
N1 - Zentall, S S
Journal of abnormal child psychology
0364547, h33
IM
Journal Article
English
KW - MEDLINE
KW - Attention
KW - Child
KW - Child Behavior
KW - Female
KW - Humans
KW - Hyperkinesis/px [Psychology]
KW - Hyperkinesis/th [Therapy]
KW - Male
KW - Verbal Behavior

RP - NOT IN FILE
SP - 93
EP - 109
JF - Journal of Abnormal Child Psychology
JA - J Abnorm Child Psychol
VL - 8
IS - 1
CY - UNITED STATES
N2 - Matched pairs of hyperactive and normally active children were observed in six natural classroom settings and a number of specific behaviors continuously recorded. Both groups of children showed differences in behavior as a function of settings (selected to vary in amount of external stimulation and structure), but only certain settings differentiated hyperactive from control children. Hyperactive children displayed significantly more noise-vocalization and more disruptive and off-task behavior in the most frequently observed (low stimulation) classroom settings (e.g., seat work). Type of off-task behavior (out-of-seat or visually off-task) depended upon amount of classroom structure (i.e., teacher- vs. self-directed). Results were discussed in terms of their implications for identification and treatment of hyperactive children through the modification of the antecedent conditions of stimulation and structure
SN - 0091-0627
UR - 7372952
ER -

TY - JOUR
ID - 2284
T1 - Caloric intake and expenditure of obese boys
A1 - Waxman,M.
A1 - Stunkard,A.J.
Y1 - 1980/02//
N1 - Waxman, M. Stunkard, A J
The Journal of pediatrics
jlz, 0375410
AIM, IM
Comparative Study. Journal Article. Research Support, U.S. Gov't, P.H.S.
English
KW - MEDLINE
KW - Adolescent
KW - Body Height
KW - Body Weight
KW - Child
KW - Diet
KW - Energy Intake
KW - Energy Metabolism
KW - Family
KW - Feeding Behavior
KW - Humans
KW - Male
KW - Obesity/px [Psychology]
KW - Obesity
KW - Oxygen
KW - Physical Exertion
KW - Time Factors
RP - NOT IN FILE
SP - 187
EP - 193
JF - Journal of Pediatrics
JA - J Pediatr
VL - 96
IS - 2

CY - UNITED STATES
N2 - Caloric intake and expenditure of children in four families were assessed by nonparticipant observations of family dinners and school lunches. In each family there were one obese boy and one nonobese brother whose ages were within two years of each other. For family dinners the nonobese brother served as a control; for school lunches, a nonobese peer served as a control. The obese boys consumed more calories (766 +/- 290) than did their nonobese brothers at dinner (504 +/- 183) and far more (907 +/- 217) than their nonobese peers at lunch (500 +/- 386). The obese boys also ate faster (65.7 +/- 37.0 kcal/minute) than their brothers at dinner (31.7 +/- 13.8 kcal/minute) and far faster (103.5 +/- 40.9 kcal/minute) than their nonobese peers at lunch (46.2 +/- 22.5 kcal/minute). Time-sampled activity assessments showed the obese boys far less active than their controls inside the home, slightly less active outside the home, and equally active at school. When these activity values were converted into energy expenditure by measurement of oxygen consumption, obese boys expended more calories in moving than did their controls; as a result, there was no difference in energy expenditure between obese and nonobese boys at home and greater energy expenditure outside the home and at school. Increased intake, thus, and not decreased caloric output maintained the obesity of these four boys. In this respect, obesity in childhood may differ from obesity in adult life
SN - 0022-3476
UR - 7351577
ER -

TY - JOUR
ID - 2285
T1 - Behavioral epidemiology of food additives
A1 - Weiss,B.
A1 - Cox,C.
A1 - Young,M.
A1 - Margen,S.
A1 - Williams,J.H.
Y1 - 1979///
N1 - Weiss, B. Cox, C. Young, M. Margen, S. Williams, J H
Neurobehavioral toxicology
nx6, 8001482
IM
Journal Article. Research Support, U.S. Gov't, Non-P.H.S.. Research Support, U.S. Gov't, P.H.S.
English
KW - MEDLINE
KW - Child
KW - Preschool
KW - Female
KW - Food Additives/to [Toxicity]
KW - Food Coloring Agents/to [Toxicity]
KW - Humans
KW - Male
KW - Motor Activity/de [Drug Effects]
RP - NOT IN FILE
SP - 149
EP - 155
JF - Neurobehavioral Toxicology
JA - Neurobehav.Toxicol
VL - 1 Suppl 1
CY - UNITED STATES
N2 - Behavioral toxicology in the natural environment can be considered a special branch of epidemiology. Behavioral epidemiology, because it typically relies on complex functional criteria, faces all of the problems of behavior measurement posed by uncontrollable variation, and amplified even further by chemical exposure. Many such issues arose in a study of behavioral responses to artificial food colors in children. Difficulties in employing Applied Behavioral Analysis in such a context run the gamut from selection of retrospective criteria to appropriate statistical models
SN - 0191-3581

AD - Department of Radiation Biology and Biophysics, University of Rochester School of Medicine and Dentistry, NY 14642
UR - 299572
ER -

TY - JOUR
ID - 2286
T1 - Accounting for changes in family life of families with spina bifida children
A1 - Joosten,J.
Y1 - 1979/12//
N1 - Joosten, J
Zeitschrift fur Kinderchirurgie und Grenzgebiete
yfg, 0413634
IM
Journal Article
English
KW - MEDLINE
KW - Activities of Daily Living
KW - Child
KW - Child Care
KW - Preschool
KW - Family
KW - Humans
KW - Sleep Deprivation
KW - Spina Bifida Occulta/px [Psychology]
KW - Stress
KW - Psychological
RP - NOT IN FILE
SP - 412
EP - 417
JF - Zeitschrift fur Kinderchirurgie und Grenzgebiete
JA - Z Kinderchir.Grenzgeb.
VL - 28
IS - 4
CY - GERMANY, WEST
N2 - The use of time, division of household chores, and the use of resources outside the immediate family has been studied in 22 families with a spina bifida child aged between 4-6 years and compared with 22 families having a normal child of the same age group. In the families with a spina bifida child, both parents spent more time in child care, and depended more upon friends and parents and neighours. The mothers experienced lack of sleep and increased stress
SN - 0044-2909
UR - 399415
ER -

TY - JOUR
ID - 2287
T1 - Kala-azar in Ethiopia: survey of south-west Ethiopia. The Leishmanin skin test and epidemiological studies
A1 - Fuller,G.K.
A1 - Lemma,A.
A1 - Haile,T.
A1 - Gemeda,N.
Y1 - 1979/10//
N1 - Fuller, G K. Lemma, A. Haile, T. Gemeda, N
Annals of tropical medicine and parasitology
68e, 2985178r
IM

Journal Article
English
KW - MEDLINE
KW - Adolescent
KW - Adult
KW - Child
KW - Preschool
KW - Ecology
KW - Ethiopia
KW - Female
KW - Hepatomegaly/ep [Epidemiology]
KW - Humans
KW - Leishmaniasis/ep [Epidemiology]
KW - Leishmaniasis
KW - Visceral/ep [Epidemiology]
KW - Visceral/ps [Parasitology]
KW - Male
KW - Middle Aged
KW - Psychodidae
KW - Skin Tests
KW - Splenomegaly/ep [Epidemiology]
RP - NOT IN FILE
SP - 417
EP - 430
JF - Annals of Tropical Medicine & Parasitology
JA - Ann Trop Med Parasitol
VL - 73
IS - 5
CY - ENGLAND
N2 - The Leishmanin skin test was performed on 1353 people in a kala-azar endemic region of south-west Ethiopia. Physical examinations were also carried out on 2723. Two of these individuals, both males, had active visceral leishmaniasis with Leishmania organisms demonstrated by spleen puncture. Two other males, including one member of the research team, had parasitologically proven cutaneous leishmaniasis. Because there was negligible migration and little movement of individuals outside of their tribal territories, the geographical distribution of skin test positivity and clinical findings could be determined and correlated with environmental parameters. The level of positive skin tests for the groups tested ranged from over 64% for the three tribes collectively inhabiting the lower regions of the Omo Valley (altitude approx. 500 m) to 6.4% for the Suri tribe, which lives at 1400 m. Skin test positivity was highest in areas of deeply fissuring montmorillonite soils and where Phlebotomus langeroni orientalis have been collected. Termite mounds of the pipe-organ type seemed to occur independently of the proportion of positive skin tests, possibly because alternative resting and breeding sites for sandflies were available in the cotton clay soil or because of the cultural patterns of the people. Almost always, males had a markedly higher prevalence of positive skin tests than did females. The degree of positivity was strongly correlated with increasing age, most positive conversions occurring in the ten to 20 year olds, the age at which males join cattle camps as part of their herding activities. Splenomegaly reached a prevalence of nearly 50% among the Hamar speaking people to the east of the Omo River, where the pattern of disease suggests malaria as the principal cause. Hepatomegaly, however, was highest in the lower Omo Basin among the Nyangatom, Dassanetch and Kerre, where hydatid disease was a major cause of liver enlargement, but seemed unrelated to the proportion of positive Leishmanin skin tests
SN - 0003-4983
UR - 534446
ER -

TY - JOUR
ID - 2288
T1 - Where people die
A1 - Katz,B.P.
A1 - Zdeb,M.S.

A1 - Therriault,G.D.
Y1 - 1979/11//
N1 - Katz, B P. Zdeb, M S. Therriault, G D
Public health reports (Washington, D.C. : 1974)
9716844, qja
AIM, IM
Journal Article
English
KW - MEDLINE
KW - Adolescent
KW - Adult
KW - Age Factors
KW - Aged
KW - Child
KW - Preschool
KW - Female
KW - Hospitals
KW - Veterans
KW - Humans
KW - Infant
KW - Male
KW - Marriage
KW - Middle Aged
KW - Mortality
KW - New York
KW - Nursing Homes
KW - Sex Factors
RP - NOT IN FILE
SP - 522
EP - 527
JF - Public Health Reports
JA - Public Health Rep
VL - 94
IS - 6
CY - UNITED STATES
N2 - Death certificates for 1977 filed with the New York State Department of Health were studied to determine where people died. Data were examined by the location and cause of death and by the age, sex, race, and marital status of the decedent. Comparisons were made with a similar study in which U.S. data were used for 1958 events. Approximately 60 percent of all the 1977 deaths in upstate New York occurred in hospitals; only 27 percent occurred outside an institution. The location of death varied by all the factors studied. Within all age categories, males had a higher percentage of hospital deaths. In those age categories in which nursing home deaths comprised a significant proportion of total deaths, females had a higher percentage of such deaths than males. Differences in the location of death according to its cause reflect the nature of the cause of death, for example, whether it was of sudden onset or the result of chronic disease. Most people do not consider in advance where they might die. The idea that age, sex, and marital status, as well as the more obvious cause, all play a part in the location may seem surprising. Yet all these factors were found to be associated withe location of deaths in upstate New York, and there is no reason to believe that this association does not hold true for the entire nation. More research, however, needs to be done based on more years and other geographic artal stutus may be instructive as to the present state of health resources
SN - 0033-3549
UR - 515338
ER -

TY - JOUR
ID - 2289
T1 - A field study of the stress response syndrome. Young women after hysterectomy
A1 - Kaltreider,N.B.

A1 - Wallace,A.
A1 - Horowitz,M.J.
Y1 - 1979/10/05/
N1 - Kaltreider, N B. Wallace, A. Horowitz, M J
JAMA : the journal of the American Medical Association
7501160
AIM, IM
Case Reports. Journal Article
English
KW - MEDLINE
KW - Adaptation
KW - Psychological
KW - Adolescent
KW - Adult
KW - Child
KW - Depression/px [Psychology]
KW - Emotions
KW - Female
KW - Humans
KW - Hysterectomy/px [Psychology]
KW - Interview
KW - Life Change Events
KW - Mother-Child Relations
KW - Self Concept
KW - Sexual Behavior
KW - Stress
KW - Psychological/px [Psychology]
RP - NOT IN FILE
SP - 1499
EP - 1503
JF - JAMA
VL - 242
IS - 14
CY - UNITED STATES
N2 - The symptom pattern previously delineated as the stress response syndrome in a mental health setting was hypothesized to be useful in conceptualizing reactions to a traumatic event in a nonpsychiatric patient population. The experience of loss resulting from nonelective hysterectomy for benign disease in women of childbearing age was selected as a relevant field study model. Twenty-eight women were studied one year after hysterectomy, using extensive psychological interviewing by women clinicians and experiential rating scales. Twelve subjects had a mild stress response syndrome, and five subjects had a serious level of intrusive and avoidant symptoms. Increasing severity of response was associated with persisting child-wish, deterioration in sexual functioning, and change in self-concept. Women who did well postoperatively generally had no future wish for children and were actively committed to achievement outside of the home
SN - 0098-7484
UR - 470086
ER -

TY - JOUR
ID - 2290
T1 - An evaluation of elementary students' voluntary physical activity during recess
A1 - Hovell,M.F.
A1 - Bursick,J.H.
A1 - Sharkey,R.
A1 - McClure,J.
Y1 - 1978/12//
N1 - Hovell, M F. Bursick, J H. Sharkey, R. McClure, J
Research quarterly

r6x, 0401301
IM
Journal Article
English
KW - MEDLINE
KW - Adolescent
KW - Adult
KW - Child
KW - Evaluation Studies as Topic
KW - Female
KW - Humans
KW - Hypercholesterolemia/pc [Prevention & Control]
KW - Male
KW - Obesity/pc [Prevention & Control]
KW - Physical Exertion
KW - Physical Fitness
KW - Risk
KW - Students
RP - NOT IN FILE
SP - 460
EP - 474
JF - Research Quarterly
JA - Res Q
VL - 49
IS - 4
CY - UNITED STATES
SN - 0034-5377
UR - 741084
ER -

TY - JOUR
ID - 2291
T1 - Joseph's disease: an autosomal dominant neurological disease in the Portuguese of the United States and the Azores Islands
A1 - Rosenberg,R.N.
A1 - Nyhan,W.L.
A1 - Coutinho,P.
A1 - Bay,C.
Y1 - 1978///
N1 - Rosenberg, R N. Nyhan, W L. Coutinho, P. Bay, C
Advances in neurology
2nx, 0367524
IM
Journal Article
English
KW - MEDLINE
KW - Adolescent
KW - Adult
KW - Aged
KW - Azores
KW - California
KW - Child
KW - Chromosome Aberrations
KW - Chromosome Disorders
KW - Electrophoresis
KW - Polyacrylamide Gel
KW - Female

KW - Fibroblasts/an [Analysis]
KW - Genes
KW - Dominant
KW - HLA Antigens/an [Analysis]
KW - Humans
KW - Male
KW - Massachusetts
KW - Middle Aged
KW - Movement Disorders/di [Diagnosis]
KW - Movement Disorders/ep [Epidemiology]
KW - Movement Disorders/ge [Genetics]
KW - Mutation
KW - Pedigree
KW - Portugal/eh [Ethnology]
KW - Proteins/an [Analysis]
KW - Skin/cy [Cytology]
KW - United States
RP - NOT IN FILE
SP - 33
EP - 57
JF - Advances in Neurology
JA - Adv Neurol
VL - 21
CY - UNITED STATES
N2 - Our objective has been to trace Joseph's disease to its geographic origins and to determine the spectrum of clinical manifestations. This goal we have achieved by documenting type I and II disease within the Joseph and Sousa families. The major neuropathologic findings are a progressive neuronal loss involving the striatum, nigra, dentate nucleus of the cerebellum, and lower motor neurons in the brainstem and spinal cord. The homozygote form of the disease produces type I disease with onset in early childhood of progressive dystonia, athetosis, and spasticity. Type I disease tends to have its onset by age 25 years in heterozygotes and lasts about 15 years on the average. Type II disease, which we consider the result of a single dose of the mutant gene, usually begins somewhat later and runs its course over a 20-year period. Type III disease documented in the Thomas family is the most benign. Its onset is often in the fifth decade, and it progresses slowly into the eighth decade. Patients may benefit from antiparkinson medication including dihydroxyphenylalanine and anticholinergic agents (e.g., amantadine). A molecular marker for the disease is being sought actively, and several interesting patterns have already been documented by means of patient fibroblast cultures and two-dimensional acrylamide gel protein separations. The mutant gene is clearly outside the HLA complex but may be linked to it. The only biochemical change noted thus far is a reduced CSF level of HVA that probably reflects the loss of dopamine-synthesizing neurons in the substantia nigra and is thus a secondary effect of disease. Although the disease is a very old one which we can trace back to the early 19th century on the island of Flores, it may be recurring de novo by new gene mutations at an unstable gene locus in a genetically vulnerable population. Now that the spectrum of clinical expression has been identified and the mode of inheritance established as an autosomal dominant wherever the disease has been found, it is believed that its true incidence will become more evident by virtue of better detection and that the true incidence will actually increase because of increased assimilation of affected persons into other ethnic groups
SN - 0091-3952
UR - 735930
ER -

TY - JOUR
ID - 2292
T1 - Reye's syndrome: preservation of mitochondrial enzymes in brain and muscle compared with liver
A1 - Robinson,B.H.
A1 - Taylor,J.
A1 - Cutz,E.
A1 - Gall,D.G.
Y1 - 1978/11//

N1 - Robinson, B H. Taylor, J. Cutz, E. Gall, D G
Pediatric research
owl, 0100714
IM
Comparative Study. Journal Article
English
KW - MEDLINE
KW - Brain/en [Enzymology]
KW - Brain/ul [Ultrastructure]
KW - Child
KW - Citrate (si)-Synthase/me [Metabolism]
KW - Female
KW - Fructose-Bisphosphatase/me [Metabolism]
KW - Glucose-6-Phosphatase/me [Metabolism]
KW - Glutamate Dehydrogenase/me [Metabolism]
KW - Humans
KW - Male
KW - Mitochondria/en [Enzymology]
KW - Mitochondria
KW - Liver/en [Enzymology]
KW - Liver/ul [Ultrastructure]
KW - Muscle/en [Enzymology]
KW - Muscle/ul [Ultrastructure]
KW - Pyruvate Carboxylase/me [Metabolism]
KW - Pyruvate Dehydrogenase Complex/me [Metabolism]
KW - Reye Syndrome/en [Enzymology]
KW - Reye Syndrome/pa [Pathology]
KW - Succinate Dehydrogenase/me [Metabolism]
RP - NOT IN FILE
SP - 1045
EP - 1047
JF - Pediatric Research
JA - Pediatr Res
VL - 12
IS - 11
CY - UNITED STATES
N2 - The activities of five mitochondrial enzymes tested in liver from patients with Reye's syndrome were measured. Citrate synthase, glutamic dehydrogenase, succinic dehydrogenase, pyruvate carboxylase, and pyruvate dehydrogenase were all outside of the range shown by control samples and well below them in activity. The activity of two extramitochondrial enzymes, glucose-6-phosphatase, which is a microsomal enzyme, and fructose-1,6-diphosphatase, which is a soluble enzyme, were in the normal range in samples from Reye's syndrome patients. In both muscle and brain the activities of the mitochondrial enzyme, citrate synthase, glutamic dehydrogenase, and succinic dehydrogenase were all within the control range. Pyruvate dehydrogenase was found to be normal in muscle from these patients
SN - 0031-3998
UR - 214743
ER -

TY - JOUR
ID - 2293
T1 - Adenosine deaminase and purine nucleoside phosphorylase activities in lymphocytes from patients with lung cancer
A1 - Ogawa,K.
A1 - Tominaga,K.
A1 - Taoka,S.
A1 - Yata,K.
A1 - Tsubura,E.

Y1 - 1978/08//
N1 - Ogawa, K. Tominaga, K. Taoka, S. Yata, K. Tsubura, E
Gann = Gan
fgj, 8214471
IM
Journal Article
English
KW - MEDLINE
KW - Adenosine Deaminase/bl [Blood]
KW - Adolescent
KW - Adult
KW - Child
KW - Humans
KW - Lung Neoplasms/en [Enzymology]
KW - Lymphocytes/en [Enzymology]
KW - Middle Aged
KW - Nucleoside Deaminases/bl [Blood]
KW - Pentosyltransferases/bl [Blood]
KW - Purine-Nucleoside Phosphorylase/bl [Blood]
RP - NOT IN FILE
SP - 471
EP - 475
JF - Gann
VL - 69
IS - 4
CY - JAPAN
N2 - The activity of adenosine deaminase (EC 3.5.4.4.) was significantly lower in lymphocytes from patients with lung cancer than in those from healthy subjects, whereas the activity of purine nucleoside phosphorylase (EC 2.4.2.1.) was significantly higher in lymphocytes from the patients than in those from normal controls. When the one activity was plotted against the other, the plots for patients with lung cancer were all outside the frame formed by the lower and higher limits of the standard deviation of the mean of normal activities of the two enzymes. The ratio of adenosine deaminase activity to purine nucleoside phosphorylase activity was lower in patients with lung cancer than in controls. The possible effect of this ratio on the function of lymphocytes was briefly discussed. These enzyme activities were suggested to be useful measures of the immune responsiveness of patients with lung cancer
SN - 0016-450X
UR - 101412
ER -

TY - JOUR
ID - 2294
T1 - [Osteoarticular tuberculosis in children (author's transl)]. [French]
A1 - Bumbic,S.
A1 - Zegarac,D.
A1 - Lukac,R.
Y1 - 1978///
N1 - Bumbic, S. Zegarac, D. Lukac, R
Chirurgie pediatrique
c0a, 7804068
IM
Case Reports. English Abstract. Journal Article
French
KW - MEDLINE
KW - Age Factors
KW - Child
KW - Preschool
KW - Female

KW - Humans
KW - Infant
KW - Male
KW - Sex Factors
KW - Tuberculosis
KW - Osteoarticular/ep [Epidemiology]
KW - Osteoarticular/ra [Radiography]
KW - Osteoarticular
KW - Spinal/ep [Epidemiology]
KW - Spinal/ra [Radiography]
KW - Yugoslavia
RP - NOT IN FILE
SP - 93
EP - 99
JF - Chirurgie Pediatrique
JA - Chir Pediatr
VL - 19
IS - 2
CY - FRANCE
N2 - A group of sixteen children suffering from osteoarticular tuberculosis were seen over the past five years in the Belgrade Paediatric Surgery Clinic. This disease is now extremely rare and effects chiefly children born outside maternity departments and not receiving BCG vaccination at birth for different reasons. The osteoarticular tuberculosis rate in the Socialist Republic of Serbia over this period was thus one in 500,000 children. The disease is three times as common in male children, most often affecting the hip and presenting above all at about the age of nine. At the time of admission, eight children had tuberculosis lesions affecting the lungs, visible and active or latent, partially or completely. In five children, one of the parents was receiving treatment for active pulmonary tuberculosis at the time of onset of the disease. Conservative treatment (immobilisation, tuberculous bacteriostatic therapy and general measures) was used in fifteen children and only one underwent surgery, in addition to the standard treatment described. In only one child, there was ankylosis of the hip and the end of treatment, the others having less sequelae of returning completely to normal. In one girl, osteoarticular tuberculosis was complicated by exsudative tuberculosis pericarditis
SN - 0180-5738
UR - 709713
ER -

TY - JOUR
ID - 2295
T1 - 225 skateboard injuries in children
A1 - Illingworth,C.M.
A1 - Jay,A.
A1 - Noble,D.
A1 - Collick,M.
Y1 - 1978/10//
N1 - Illingworth, C M. Jay, A. Noble, D. Collick, M
Clinical pediatrics
dhe, 0372606, 8407647
AIM, IM
Journal Article
English
KW - MEDLINE
KW - Adolescent
KW - Athletic Injuries/di [Diagnosis]
KW - Athletic Injuries/ep [Epidemiology]
KW - Child
KW - Preschool
KW - England
KW - Equipment and Supplies/st [Standards]

KW - Female
KW - Health Expenditures
KW - Humans
KW - Male
KW - Play and Playthings
KW - Protective Clothing
KW - Retrospective Studies
KW - Skating
KW - Sports
RP - NOT IN FILE
SP - 781
EP - 782
JF - Clinical Pediatrics
JA - Clin Pediatr (Phila)
VL - 17
IS - 10
CY - UNITED STATES
N2 - Among 225 skateboard accidents treated at the Children's Hospital, Sheffield, in 8 months from August 1977, there were 92 fractures (40.9 per cent), of which 19 required manipulation under general anesthetic. Most involved the upper limb. Next most frequent were lacerations, bruises, abrasions, and injuries of joints and soft tissues. Four children had concussions (one with a fractured skull), 8 others had minor head injury, and 23 had soft tissue injuries of the head and neck. As for the reasons for the accidents, 20 per cent were due to hitting a stone, 10 per cent to a hole or bump in the pavement, 8 per cent to collisions, and three accidents to faults in the skateboards. Sixteen of the children were injured on the first day of their skateboarding. The role of protective clothing was uncertain. The city has no skateboard parks. Injuries due to skateboards were compared to injuries due to playground equipment
SN - 0009-9228
UR - 100266
ER -

TY - JOUR
ID - 2296
T1 - The development of the angle of the anterior chamber in vertebrate eyes
A1 - Barishak,Y.R.
Y1 - 1978/08/01/
N1 - Barishak, Y R
Documenta ophthalmologica. Advances in ophthalmology
ebf, 0370667
IM
Comparative Study. Journal Article
English
KW - MEDLINE
KW - Animals
KW - Newborn
KW - Anterior Chamber/ah [Anatomy & Histology]
KW - Anterior Chamber/em [Embryology]
KW - Anterior Chamber/gd [Growth & Development]
KW - Cattle
KW - Chick Embryo
KW - Child
KW - Preschool
KW - Ciliary Body/ah [Anatomy & Histology]
KW - Ciliary Body/gd [Growth & Development]
KW - Endothelium/cy [Cytology]
KW - Humans
KW - Infant
KW - Low Birth Weight

KW - Premature
KW - Iris/ah [Anatomy & Histology]
KW - Lens
KW - Crystalline/ah [Anatomy & Histology]
KW - Rats
KW - Trabecular Meshwork/ah [Anatomy & Histology]
RP - NOT IN FILE
SP - 329
EP - 360
JF - Documenta Ophthalmologica
JA - Doc Ophthalmol
VL - 45
IS - 2
CY - NETHERLANDS
N2 - The development of the angle of the anterior chamber of the eye in chick embryos (avian eye), in new born rats (rodents), in calf fetuses (ungulates) and in humans (primates) has been studied. The main determinant in the configuration of the angle is the two-layered structure of the mesoderm of the ciliary body: the external muscular layer inserted into the spur and growing with same growth rate as that of the corneoscleral mesoderm and internal fibrovascular layer giving rise to the ciliary processes and growing with same growth rate as that of the neural ciliary and iris epithelium. First the neural epithelium grows faster than the corneoscleral mesoderm and the ciliary processes appear anteriorly positioned and later the corneoscleral mesoderm grows faster than the neuroepithelium and the ciliary processes appear posteriorly positioned. The eyes of premature and low birth weight babies present the different stages of normal embryological development of the angel. The angle in eyes of various embryopathies and developmental diseases presents aspects similar to those of the premature and low birth weight babies. The etiopathogenic mechanism in cause is the same in both groups: arrest of development. During the first post-natal years, the corneoscleral mesoderm grows at a greater rate than the inner neuro-epithelium and the region of the most active growth is located at the ora serrata. That is why, during this period, the ora serrata recedes, giving rise to the formation of retinal teeth, the angle despens and makes a recess and the Lange fold disappears
SN - 0012-4486
UR - 688852
ER -

TY - JOUR
ID - 2297
T1 - The use of physical restraint in the treatment of self-injury and as positive reinforcement
A1 - Favell,J.E.
A1 - McGimsey,J.F.
A1 - Jones,M.L.
Y1 - 1978///
N1 - Favell, J E. McGimsey, J F. Jones, M L
Journal of applied behavior analysis
0174763, hdr
IM
Case Reports. Journal Article
English
KW - MEDLINE
KW - Adolescent
KW - Behavior Therapy
KW - Child
KW - Education of Intellectually Disabled
KW - Female
KW - Humans
KW - Male
KW - Reinforcement (Psychology)
KW - Restraint
KW - Physical/mt [Methods]

KW - Self Mutilation/th [Therapy]
KW - Stereotyped Behavior
RP - NOT IN FILE
SP - 225
EP - 241
JF - Journal of Applied Behavior Analysis
JA - J Appl Behav Anal
VL - 11
IS - 2
CY - UNITED STATES
N2 - Two experiments investigated the effects of a treatment package on the self-injurious behavior of three profoundly retarded persons who appeared to enjoy the physical restraints used to prevent their self-injury. The treatment package included physically restraining subjects contingent on increasing periods of time during which no self-injury occurred, and providing them with toys and attention during intervals between restraints. A reversal and multiple-baseline analysis documented that the rapid and complete reduction in self-injury by all subjects was due to this treatment package. Because these results suggested that physical restraint might function as a positive reinforcer, in a third experiment physical restraint was applied contingent on a marble placement response with one subject. A reversal design demonstrated that toy play systematically increased when each response resulted in restraint. The experiments have implications for the nonaversive remediation of self-injury in individuals who are restrained, as well as for the development and maintenance of self-injury in natural settings
SN - 0021-8855
UR - 670112
ER -

TY - JOUR
ID - 2298
T1 - On supplementing the selenium intake of New Zealanders. 2. Prolonged metabolic experiments with daily supplements of selenomethionine, selenite and fish
A1 - Robinson,M.F.
A1 - Rea,H.M.
A1 - Friend,G.M.
A1 - Stewart,R.D.
A1 - Snow,P.C.
A1 - Thomson,C.D.
Y1 - 1978/05//
N1 - Robinson, M F. Rea, H M. Friend, G M. Stewart, R D. Snow, P C. Thomson, C D
The British journal of nutrition
az4, 0372547
IM
Clinical Trial. Controlled Clinical Trial. Journal Article
English
KW - MEDLINE
KW - Adolescent
KW - Adult
KW - Animals
KW - Child
KW - Female
KW - Fishes
KW - Food
KW - Fortified
KW - Humans
KW - Intestinal Absorption
KW - Male
KW - Middle Aged
KW - Muscular Diseases/me [Metabolism]
KW - New Zealand

KW - Selenium/ad [Administration & Dosage]
KW - Selenium/me [Metabolism]
RP - NOT IN FILE
SP - 589
EP - 600
JF - British Journal of Nutrition
JA - Br J Nutr
VL - 39
IS - 3
CY - ENGLAND
N2 - 1. The daily intake of selenium by three subjects was supplemented with 100 microgram Se as selenomethionine (Semet-Se) or sodium selenite (selenite-Se)/d for 10-11 weeks, or with 65 microgram Se as in mackerel (Scomber japonicus) (fish-Se)/d for 4 weeks. 2. Urinary and faecal excretion of Se was measured and also Se concentration in whole blood, plasma and erythrocytes. Measurements on blood were made at intervals after supplementation had ceased. 3. Selenite-Se was not as well absorbed (0.46 of the intake) during the first 4 weeks as Semet-Se (0.75 of the intake) and fish Se (0.66 of the intake). 4. Blood Se increased steadily with Semet-Se, from 0.08 to 0.18 microgram Se/ml, but more slowly with selenite-Se, reaching a plateau in 7-8 weeks at 0.11 microgram Se/ml. Plasma Se increased more rapidly with Semet-Se than with selenite-Se, so that initially with Semet-Se plasma Se was greater than erythrocyte Se. 5. Daily urinary excretion increased with all forms of supplement, with initially a greater proportion of absorbed selenite-Se being excreted than Semet-Se or fish-Se. A close relationship was found between plasma Se and 24 h urinary excretion. The findings suggested that there was a rapid initial excretion of presumably unbound Se then a slower excretion of residual unbound, loosely bound or bound Se. 6. Total retentions of 3.5 mg selenite-Se and 4.5 mg Semet-Se were large when compared with an estimate of body content of 6 mg Se, derived in another paper (Stewart, Griffiths, Thomson & Robinson, 1978). Retention of Semet-Se and fish-Se appeared to be reflected in blood Se, whereas for selenite-Se, blood Se reflected retention for only a short period after which Se appeared to be retained without altering the blood Se. This suggested that Semet-Se and selenite-Se were metabolized differently. 7. A double blind-dosing trail with 100 microgram Semet-Se was carried out for 12 weeks on twenty-four patients with muscular complaints in Tapanui, a low-Se-soil area. Blood Se increased in the experimental group (from 0.067 to 0.143 microgrm Se/ml); clinical findings were not conclusive and will be presented elsewhere. 8. Bood Se was measured in New Zealand residents before travelling to Europe or to North America. On return their blood Se was increased, and depending upon the period of time spent outside New Zealand some values reached concentrations found in visitors and new settlers to New Zealand. 9. The results from these studies and the earlier studies of single and multiple dosing have been used to look at the various criteria in use for assessing Se status of subjects. It is suggested that plasma Se be used in preference to 24 h urinary excretion, and in addition to whole blood Se and glutathione peroxidase (EC 1.11.1.9) activity
SN - 0007-1145
UR - 565216
ER -

TY - JOUR
ID - 2299
T1 - [Certain data concerning the analysis of the foci of infectious hepatitis in cases of subsequent occurrence of the disease in families]. [Russian]
A1 - Zharikov,A.N.
Y1 - 1978/01//
N1 - Zharikov, A N
Zhurnal mikrobiologii, epidemiologii, i immunobiologii
y9o, 0415217
IM
English Abstract. Journal Article
Russian
KW - MEDLINE
KW - Adolescent
KW - Adult
KW - Age Factors
KW - Child

KW - Hepatitis A/ep [Epidemiology]
KW - Hepatitis A/ge [Genetics]
KW - Hepatitis A/tm [Transmission]
KW - Humans
KW - Interpersonal Relations
KW - Moscow
RP - NOT IN FILE
SP - 84
EP - 87
JF - Zhurnal Mikrobiologii, Epidemiologii i Immunobiologii
JA - Zh Mikrobiol.Epidemiol Immunobiol.
IS - 1
CY - USSR
N2 - Materials of epidemiological analysis of familial infectious hepatitis nidality in 104 families are presented. The data obtained pointed to the preponderant affection of children constituting 97.1% of primary and 87.7% of subsequent cases in familial foci. Infection was brought to the families by schoolchildren in 57.7% of the cases, and by organized preschoolchildren--in 31.7% of the cases. The greatest affection of schoolchildren aged from 7 to 14 years in the foci with subsequent occurrence of the disease (77.7%) was explained by their marked age activity. This led to the conclusion that mass prophylaxis with alpha-globulin of these particular groups of children was of primary importance. The differences of the specific ratio of adults who contracted the disease in familial foci and in the region as a whole pointed to the possibility of infection of adults outside the family and on the prevalence in them of serum hepatitis
SN - 0372-9311
UR - 629131
ER -

TY - JOUR
ID - 2300
T1 - [Epidemiological studies after an outbreak of meningitis (author's transl)]. [German]
A1 - Gartner,H.
A1 - Sonntag,H.G.
Y1 - 1977/12//
N1 - Gartner, H. Sonntag, H G
Zentralblatt fur Bakteriologie, Parasitenkunde, Infektionskrankheiten und Hygiene. Erste Abteilung Originale. Reihe B: Hygiene, praventive Medizin
y53, 1306477
IM
English Abstract. Journal Article
German
KW - MEDLINE
KW - Adolescent
KW - Child
KW - Preschool
KW - Coxsackievirus Infections/ep [Epidemiology]
KW - Echovirus Infections/ep [Epidemiology]
KW - Enterovirus/ip [Isolation & Purification]
KW - Enterovirus B
KW - Human/ip [Isolation & Purification]
KW - Family
KW - Female
KW - Germany
KW - West
KW - Humans
KW - Infant
KW - Male
KW - Meningitis
KW - Viral/ep [Epidemiology]

KW - Viral/mi [Microbiology]
KW - Play and Playthings
KW - Schools
KW - Nursery
KW - Seasons
RP - NOT IN FILE
SP - 548
EP - 556
JF - Zentralblatt fur Bakteriologie, Parasitenkunde, Infektionskrankheiten und Hygiene - Erste Abteilung Originale - Reihe B: Hygiene, Praventive Medizin
JA - Zentralbl.Bakteriol.[Orig.B]
VL - 165
IS - 5-6
CY - GERMANY, WEST
N2 - The outbreak of meningitis during the period from the end of August to the beginning of October 1974 in Kiel affected 45 children, particularly those in the age group between 4 and 9 years. An potential causative agents ECHO viri were identified in 15/45 cases and Coxsackie B viri in 2/45 cases. The patients presented the typical meningitic symptoms (fever, headache, stiffness of the neck). In all cases the disease ran a benign course. - Epidemiologically, it was possible to trace 32/45 cases back to three locally demarcated groups with demonstrable possibilities of contact for the diseased children (family, playground, kindergarden and school). The remaining 13/45 cases occurred isolated over the urban area without any visible connection. - This outbreak of meningitis must be regarded as an accumulation of illnesses resulting from contact infections by enteroviri and lacking or deficient disinfection. Besides the rather limited measures adopted to prevent the spread of such an infection, the correct psychological management of the population especially by the health authorities is of great importance in such epidemics
SN - 0300-9661
UR - 610256
ER -

TY - JOUR
ID - 2301
T1 - Generalization of an operant remediation program for syntax with language delayed children
A1 - Mulac,A.
A1 - Tomlinson,C.N.
Y1 - 1977/09//
N1 - Mulac, A. Tomlinson, C N
Journal of communication disorders
0260316, hur
IM
Clinical Trial. Journal Article. Randomized Controlled Trial
English
KW - MEDLINE
KW - Child
KW - Preschool
KW - Conditioning
KW - Operant
KW - Environment
KW - Female
KW - Generalization (Psychology)
KW - Humans
KW - Language Disorders/th [Therapy]
KW - Male
KW - Methods
RP - NOT IN FILE
SP - 231
EP - 243
JF - Journal of Communication Disorders

JA - J Commun Disord
VL - 10
IS - 3
CY - NETHERLANDS
N2 - Generalization of the Monterey Behavioral Sciences Institute operant language program was assessed. Six male and three female children (4 years, 4 months to 6 years, 3 months) receiving language remediation were randomly assigned to (I) the Monterey program for the syntactic structure "is interrogative" (including the home carryover phase) plus an extended transfer program devised by the investigators (II) the Monterey "is interrogative" program alone (including the home carryover), or (III) a control activity (articulation training). Language samples evoked by a variety of language tasks were collected outside the treatment settling preceding and following treatment. Standard within-clinic measures indicated that groups I and II improved a significant, equal amount. However, the extraclinic language measures showed that group I demonstrated significantly greater improvement than group II; neither group II nor group III showed significant extraclinic improvement. Extraclinic generalization occurred, therefore, only for the group receiving the special extended transfer training
SN - 0021-9924
UR - 903408
ER -

TY - JOUR
ID - 2302
T1 - Spinal cord injury in children and adolescents: diagnostic pitfalls and therapeutic considerations in the acute stage [proceedings]
A1 - Hachen,H.J.
Y1 - 1977/05//
N1 - Hachen, H J
Paraplegia
oqt, 2985038r
IM
Journal Article
English
KW - MEDLINE
KW - Acute Disease
KW - Adolescent
KW - Child
KW - Preschool
KW - Female
KW - Humans
KW - Male
KW - Paraplegia/et [Etiology]
KW - Psychology
KW - Respiratory Insufficiency/et [Etiology]
KW - Respiratory Insufficiency/th [Therapy]
KW - Spinal Cord Injuries/co [Complications]
KW - Spinal Cord Injuries/di [Diagnosis]
KW - Spinal Cord Injuries/th [Therapy]
KW - Thromboembolism/et [Etiology]
KW - Water-Electrolyte Imbalance/et [Etiology]
KW - Water-Electrolyte Imbalance/th [Therapy]
RP - NOT IN FILE
SP - 55
EP - 64
JF - Paraplegia
VL - 15
IS - 1
CY - SCOTLAND
N2 - Traumatic spinal cord lesions in children are infrequent (2 to 5 per cent of all cases admitted to specialised paraplegic centres depending on whether the upper age limit is set at 10 or 15 years). Traffic accidents are

responsible for at least 50 per cent of the lesions; playground accidents and various sports add another 35 per cent. A large proportion of the accidents have been found to be related to the child's normal desire for adventure and exploration. The segment most frequently involved in our own series of 18 cases was the cervical and upper thoracic spine. Histopathological studies have shown that splitting of the cartilaginous end-plate in the growth zone of the vertebrae is a common finding. Radiological signs of spinal trauma are less evident than in adults; they may be totally missing. Precise neurological assessment must rely on repeated examination and close clinical observation, especially in the comatous child with a head injury. Spinal cord involvement must be suspected and the child treated as a paraplegic until definite proof of a normal neurological status is available. Due to a highly labile water electrolyte balance in the early post-traumatic stage and considerable fluctuations in plasma volume and temperature regulation, permanent monitoring of the cardiovascular function, body temperature and diuresis is mandatory. In children below the age of 10, deep vein thrombosis and embolism are exceptional (sepsis creates a high-risk situation requiring anticoagulation). In the initial treatment of spinal injury only conservative measures should be considered; there are no indications for laminectomy, nor for spinal fusion. In the tetraplegic child below the age of 6, skull-traction should be avoided and immobilisation of the cervical segment achieved by bilateral padded head-rests
SN - 0031-1758
UR - 896257
ER -

TY - JOUR
ID - 2303
T1 - Nutrition survey of schoolchildren in greater Winnipeg. I. Descriptive and anthropometric data
A1 - Ellestad-Sayed,J.
A1 - Haworth,J.C.
A1 - Medovy,H.
Y1 - 1977/03/05/
N1 - Ellestad-Sayed, J. Haworth, J C. Medovy, H
Canadian Medical Association journal
0414110, ckw
AIM, IM
Journal Article
English
KW - MEDLINE
KW - Body Height
KW - Body Weight
KW - Child
KW - Female
KW - Humans
KW - Male
KW - Manitoba
KW - Nutrition Surveys
KW - Occupations
KW - Parents
KW - School Health Services
KW - Social Class
RP - NOT IN FILE
SP - 490
EP - 492
JF - Canadian Medical Association Journal
JA - Can Med Assoc J
VL - 116
IS - 5
CY - CANADA
N2 - By sequential random numbering 10 schools in greater Winnipeg were selected for a nutrition survey. Interviews were conducted with 201 grade 3 children and 182 grade 6 children for whom parental consent was obtained. Of these, 48 in grade 3 and 51 in grade 6 were studied in further detail. There were no differences in descriptive data between the general and detailed groups or among the 10 schools. Most fathers were skilled or

unskilled labourers and about 50% of the mothers were homemakers without outside employment; parental occupation did not influence eating patterns. Breakfast was the meal most often missed; 8% of the 383 children had come to school without breakfast. Since many children in grade 3 had prepared their own breakfast and since there was a relative lack of physical activity, school health programs should incorporate more than nutritional supplements and nutrition education. On the basis of body weight and height the nutritional status of the 99 children studied in detail was judged to be generally satisfactory; according to the Boston standards the boys were heavy and tall, and the girls were normal in weight but short
SN - 0008-4409
UR - 837314
ER -

TY - JOUR
ID - 2304
T1 - [Psychohygienic problems of development in early childhood (author's transl)]. [German]
A1 - Pechstein,J.
Y1 - 1976///
N1 - Pechstein, J
Zentralblatt fur Bakteriologie, Parasitenkunde, Infektionskrankheiten und Hygiene. Erste Abteilung Originale. Reihe B: Hygiene, praventive Medizin
y53, 1306477
IM
English Abstract. Journal Article
German
KW - MEDLINE
KW - Child Behavior Disorders/et [Etiology]
KW - Child Care
KW - Child Development
KW - Child
KW - Preschool
KW - Family Characteristics
KW - Housing
KW - Humans
KW - Infant
KW - Mental Health
KW - Social Environment
RP - NOT IN FILE
SP - 211
EP - 228
JF - Zentralblatt fur Bakteriologie, Parasitenkunde, Infektionskrankheiten und Hygiene - Erste Abteilung Originale - Reihe B: Hygiene, Praventive Medizin
JA - Zentralbl.Bakteriol.[Orig.B]
VL - 163
IS - 1-4
CY - GERMANY, WEST
N2 - For a "social environmental protection" as one of the most urgent problems in infantile psychohygiene, the following conclusions should be taken into consideration: 1. In particular during the first years of life the development of the child is, within certain limits, sensitive to influences from the environment and therefore it can be fostered or disturbed from the outside. Congenital, prenatal, perinatal and postnatal injuries to the brain and sensory organs must be given great attention psychohygienically. 2. The social influences exercised on the child by persons of his environment, their availability, their emotional relationships and the development of a durable link predominate psychohygienically. In addition, the material security of young families and their type of accommodation (e.g. granting of loans to help set up a home) must be considered. 3. The possibilities of exercising a positive educational influence on a mentally and psychically sound development of the child as a person and member of society are greatest at the beginning of life and diminish during childhood. The effectiveness of negative educational and social interference factors is also subject to this rule from which again the necessity of adopting psychohygienic measures for the young child is derived. 4. The phenomenon of increasingly imbalanced behaviour of older children and juveniles which cause concern today, are as a rule

symptoms of a chiefly environmentally determined disturbance upsetting the personality, which often originates in early childhood. Psychohygienic measures must be concentrated on this. 5. The inadequate care of young children accounting for one third of the population, a phenomenon which can be read in the statistical records and which has risen sharply during the last few years, makes it possible to forecast a further increase in developmental and behavioral disturbances of both an intellectual and psychological nature as well as in infantile and juvenile criminality in the coming years to an at present unimaginable extent. 6. In contrast to general opinion, the situation calls for an urgent fundamental revaluation of the importance of intrafamiliar social conditions of development and of the parental educational work on the small child, which to date, have been underestimated and neglected. At the same time many of the presently overrated extrafamiliar institutions of education and training for the older child need to be relativised. 7. The advances in developmental biology which allow us to realize the basic dependence of the future social fate of the individual - and thus the extent of disturbed social relationships in society - on the intrafamily conditions must become a political issue, if psychohygiene as ameans to prevent avoidable disturbances is to be taken seriously. Thus the securement of adequate intrafamily conditions with respect to development and upbringing, in particular of the young child, must head the list of social political priorities, also in times when money is in short supply. 8..
SN - 0300-9661
UR - 1015042
ER -

TY - JOUR
ID - 2305
T1 - Reflection-impulsivity and behavioral problems in emotionally disturbed boys
A1 - Finch,A.J.,Jr.
A1 - Nelson,W.M.,III
Y1 - 1976/06//
N1 - Finch, A J Jr. Nelson, W M 3rd
The Journal of genetic psychology
i9z, 2985112r
IM
Journal Article
English
KW - MEDLINE
KW - Achievement
KW - Affective Symptoms/co [Complications]
KW - Aggression
KW - Child
KW - Child Behavior Disorders/co [Complications]
KW - Cognition
KW - Fear
KW - Humans
KW - Impulsive Behavior
KW - Male
KW - Projection
KW - Projective Techniques
KW - Reaction Time
KW - Social Alienation
KW - Verbal Behavior
RP - NOT IN FILE
SP - 271
EP - 274
JF - Journal of Genetic Psychology
JA - J Genet Psychol
VL - 128
IS - 2d Half
CY - UNITED STATES
N2 - In order to investigate the difference in the behavioral problems presented by cognitively impulsive and reflective emotionally disturbed boys, the parents of 17 impulsive and 13 reflective boys in residential treatment

were asked to rate their sons' behavior. Results indicated that in contrast to reflective emotionally disturbed boys, impulsives were more likely to talk of others blaming them unfairly, threaten to harm themselves, hit and bully other children, and be excessively rough in play. On the other hand, reflectives were more unwilling to talk with adults outside the family. Results were discussed in terms of supporting both previous research and Kagan and Kogan's (6) "fear of failure" hypothesis
SN - 0022-1325
UR - 778339
ER -

TY - JOUR
ID - 2306
T1 - Immune function at diagnosis in relation to responses to therapy in acute lymphocytic leukemia of childhood
A1 - Jose,D.G.
A1 - Ekert,H.
A1 - Colebatch,J.
A1 - Waters,K.
A1 - Wilson,F.
A1 - O'Keefe,D.
Y1 - 1976/06//
N1 - Jose, D G. Ekert, H. Colebatch, J. Waters, K. Wilson, F. O'Keefe, D
Blood
a8g, 7603509
AIM, IM
Journal Article
English
KW - MEDLINE
KW - Adolescent
KW - Antibodies
KW - Neoplasm/an [Analysis]
KW - Antibody Specificity
KW - Antigens
KW - Binding Sites
KW - Antibody
KW - Child
KW - Preschool
KW - Drug Therapy
KW - Combination
KW - Female
KW - Humans
KW - Infant
KW - Lectins/pd [Pharmacology]
KW - Leukemia
KW - Lymphoid/di [Diagnosis]
KW - Lymphoid/im [Immunology]
KW - Lymphoid/th [Therapy]
KW - Leukocyte Count
KW - Lymphocyte Activation
KW - Male
KW - T-Lymphocytes
RP - NOT IN FILE
SP - 1011
EP - 1021
JF - Blood
VL - 47
IS - 6
CY - UNITED STATES

N2 - Tests of immune capacity were performed on blood from 49 children with newly diagnosed, untreated acute lymphocytic leukemia, and relation to prognosis was determined. Patients were treated with multiple-drug therapy and prophylactic cranial irradiation. Median follow-up time was 16 mo (range 10--37 mo). Principal unfavorable findings at diagnosis were absolute numbers of T lymphoid cells outside the range 850--2500/mul blood, absence of whole blood responses to phytohemagglutinin in vitro, a low titer of complexed antibody, and the presence in serum of free leukemic blast cell membrane antigen. Fourteen patients showed two or more unfavorable findings at diagnosis. Eleven of these have died. Four of the remaining 35 patients have died. A shorter duration of first remission was found among patients with abnormal numbers of T cells at diagnosis. The findings suggest that the immunologic capacity of the patient at diagnosis is an important determinant in responses to therapy
SN - 0006-4971
UR - 1084177
ER -

TY - JOUR
ID - 2307
T1 - Autonomic nervous system and adaptation to cold in man
A1 - LeBlanc,J.
A1 - Dulac,S.
A1 - Cote,J.
A1 - Girard,B.
Y1 - 1975/08//
N1 - LeBlanc, J. Dulac, S. Cote, J. Girard, B
Journal of applied physiology
0376576, hef
IM
Journal Article
English
KW - MEDLINE
KW - Adaptation
KW - Physiological
KW - Adolescent
KW - Adult
KW - Autonomic Nervous System/ph [Physiology]
KW - Blood Pressure
KW - Cheek/ph [Physiology]
KW - Child
KW - Cold Temperature
KW - Face/ph [Physiology]
KW - Female
KW - Hand/ph [Physiology]
KW - Heart Rate
KW - Humans
KW - Inuits
KW - Male
KW - Skin Temperature
KW - Time Factors
RP - NOT IN FILE
SP - 181
EP - 186
JF - Journal of Applied Physiology
JA - J Appl Physiol
VL - 39
IS - 2
CY - UNITED STATES
N2 - The responses to cold hand test (blood pressure increase and tachycardia) and to a cold face test (blood pressure increase and bradycardia) were used to study the role of the autonomic nevrous system in cold

adaptation in humans. The Eskimos (men, women, children) were shown to have a very weak sympathetic response to cold but the vagal response (bradycardia) was identical to that of white people. A group of mailmen from Quebec city living outdoors approximately 30 h/wk throughout the year was also studied. A significant decline in the cold pressor response and an enhanced bradycardia (cold face test) were observed at the end of the winter. Similarly the fall in skin temperature of the cheek was not as pronounced when the measurements were made in May compared to those made in October. A group of soldiers was also studied before and after an Arctic expedition. It was found that the bradycardia of the cold face test was also more pronounced after sojourning in the cold. These results indicate that repeated exposures to severe cold in men activate some adaptive mechanisms characterized by a diminution of the sympathetic response and a concomitant enhancement of the vagal activation normally observed when the extremities and the face are exposed to cold
SN - 0021-8987
UR - 1176377
ER -

TY - JOUR
ID - 2308
T1 - The effects of load and force on tremor at the normal human elbow joint
A1 - Joyce,G.C.
A1 - Rack,P.M.
Y1 - 1974/07//
N1 - Joyce, G C. Rack, P M
The Journal of physiology
jqv, 0266262
IM
Journal Article
English
KW - MEDLINE
KW - Adolescent
KW - Adult
KW - Biomechanical Phenomena
KW - Child
KW - Elbow/ph [Physiology]
KW - Female
KW - Forearm/ph [Physiology]
KW - Humans
KW - Male
KW - Middle Aged
KW - Muscles/ph [Physiology]
KW - Physical Exertion
KW - Reflex
KW - Wrist
RP - NOT IN FILE
SP - 375
EP - 396
JF - Journal of Physiology
JA - J Physiol (Lond)
VL - 240
IS - 2
CY - ENGLAND
N2 - 1. Spontaneous flexion-extension tremor of the elbow was recorded in normal human subjects while they exerted a flexing force against different loads.2. Increases in the flexing force up to (1/2) or (3/4) of the subject's maximum were accompanied by increases in the amplitude of the tremor. A further increase to very large forces was associated with a decrease in tremor.3. When the subject flexed against a spring, the frequency of the tremor was related to the properties of the spring; with increasingly stiff springs the tremor frequency increased up to about 12 Hz. With the stiffest springs, however, the tremor became irregular and very small in amplitude.4. Weights added to the limb at the wrist reduced the frequency of tremor.5. By using appropriate combinations of spring and mass, the principal tremor frequency could be adjusted between 2 and 12 Hz, higher

in some subjects. Within this range the frequency(2) was approximately proportional to the spring stiffness/mass, just as it is in a spring-mass system, though the forearm provided some of this mass, and some of the spring-like resistance was in the flexor muscles.6. When by suitable loading the tremor frequency was brought between 8 and 12 Hz, and the subject forcibly flexed his elbow, the tremor became regular and large in amplitude. This was a form of limited instability in the stretch reflex which arose because a powerful reflex response to extension of the elbow acted back on the flexor muscles after a delay.7. When the limb was so loaded that its natural frequency was outside this 8-12 Hz range, it behaved as a filter, and in response to any disturbing noise it oscillated mainly at frequencies close to the natural frequencies of either the mechanical system or the stretch reflex.8. This filtering function of the limb with its stretch reflex probably plays an important part in the control of normal movement and tremor.9. The large flexing forces that were here associated with vigorous tremor normally occur when a limb supports a large mass. Such a mass would give the mechanical system within the limb a low natural frequency; inconveniently large oscillations in the 8-12 Hz range would therefore seldom occur
SN - 0022-3751
UR - 4419584
ER -

TY - JOUR
ID - 2309
T1 - New information on lead in dirt and dust as related to the childhood lead problem
A1 - Haar,G.T.
A1 - Aronow,R.
Y1 - 1974/05//
N1 - Haar, G T. Aronow, R
Environmental health perspectives
ei0, 0330411
IM
Journal Article
English
KW - MEDLINE
KW - Air Pollution/an [Analysis]
KW - Child
KW - Preschool
KW - Dust/an [Analysis]
KW - Feces/an [Analysis]
KW - Housing
KW - Humans
KW - Infant
KW - Lead/an [Analysis]
KW - Lead/ur [Urine]
KW - Lead Poisoning/et [Etiology]
KW - Paint/an [Analysis]
KW - Pica
KW - Radioisotopes
KW - Soil/an [Analysis]
RP - NOT IN FILE
SP - 83
EP - 89
JF - Environmental Health Perspectives
JA - Environ Health Perspect
VL - 7
CY - UNITED STATES
N2 - It has been known for many years that the eating of leaded paint is the prime cause of lead poisoning and elevated blood leads of children living in deteriorated housing. Recently, there has been speculation that children may eat dirt and dust contaminated with lead exhausted from cars and that this amount of ingested lead is sufficient to contribute significantly to the childhood lead problem. This paper reports on a twopart study conducted to evaluate the validity of the dirt-and-dust hypotheses. The first part of the study was made to

determine the source of lead in dirt to which children are normally exposed. Dirt samples were taken in old urban areas around 18 painted frame houses and 18 houses of brick construction. Samples also were taken around seven old frame farmhouses remote from traffic. Based on the fact that lead concentrations in the dirt were similar in city and rural yards at corresponding distances from the houses, it is clear that nearly all of the lead in dirt around these houses is due to paint from the houses. Lead antiknock additives are therefore not a significant contributor to the lead content of dirt around houses where children usually play. The second part of the study used a naturally occurring radioactive tracer (210)Pb to determine the relative amounts of dust and other lead-containing materials (e.g., paint) eaten by young children. This tracer is present in very low concentrations in paint and in significantly higher concentrations in fallout dust. Stable lead and (210)Pb were analyzed in fecal material from eight children suspected of having elevated body burdens of lead and ten children living in good housing where lead poisoning is not a problem. The normal children averaged 4 mug Pb/g dry feces, with a range of 2 to 7. Of the eight children suspected of having elevated lead body burdens, two had fecal lead values within the normal range. However, the remaining six were 4 to 400 times as high. Despite these differences in fecal lead between the two groups, the groups were essentially identified in the (210)Pb content of their feces. The "elevated" children averaged 0.040 pCi of (210)Pb dry feces, while the normal group averaged 0.044 pCi/g. The results provide sound evidence that these children suspected of elevated lead body burden were not ingesting dust or air-suspended particulate
SN - 0091-6765
UR - 4831152
ER -

TY - JOUR
ID - 2310
T1 - North American blastomycosis in Central Canada. A review of 36 cases
A1 - Kepron,M.W.
A1 - Schoemperlen
A1 - Hershfield,E.S.
A1 - Zylak,C.J.
A1 - Cherniack,R.M.
Y1 - 1972/02/05/
N1 - Kepron, M W. Schoemperlen. Hershfield, E S. Zylak, C J. Cherniack, R M
Canadian Medical Association journal
0414110, ckw
AIM, IM
Journal Article
English
KW - MEDLINE
KW - Adolescent
KW - Adult
KW - Aged
KW - Amphotericin B/tu [Therapeutic Use]
KW - Blastomycosis/di [Diagnosis]
KW - Blastomycosis/dt [Drug Therapy]
KW - Blastomycosis/ep [Epidemiology]
KW - Blastomycosis/ra [Radiography]
KW - Canada
KW - Child
KW - Female
KW - Humans
KW - Lung Diseases
KW - Fungal/ra [Radiography]
KW - Male
KW - Middle Aged
KW - Occupations
KW - Rural Population
KW - United States
KW - Urban Population

RP - NOT IN FILE
SP - 243
EP - 246
JF - Canadian Medical Association Journal
JA - Can Med Assoc J
VL - 106
IS - 3
CY - CANADA
N2 - Experience with 36 cases of North American blastomycosis in Central Canada is reported. Symptoms referable to the respiratory tract predominated, but no uniform clinical pattern for the disease process was evident. Cutaneous and genitourinary tract involvement was present in only a small proportion of patients. The majority of the patients resided in rural areas or were associated with some form of outdoor occupation or activity. No characteristic radiologic appearance could be identified in this series. Amphotericin B administered intravenously is the treatment of choice for this condition. North American blastomycosis is more widespread on this continent than has been previously demonstrated
SN - 0008-4409
UR - 5057959
ER -

TY - JOUR
ID - 2311
T1 - [Hygienic requirements for the organization of a children's institution playground]. [Russian]
A1 - Luchina,K.I.
Y1 - 1969/01//
N1 - Luchina, K I
Gigiena i sanitariia
fpz, 0412700
IM
Journal Article
Russian
KW - MEDLINE
KW - Child
KW - Preschool
KW - Environmental Exposure
KW - Humans
KW - School Health Services
KW - Schools
KW - Nursery
KW - USSR
RP - NOT IN FILE
SP - 33
EP - 37
JF - Gigiena i Sanitariia
JA - Gig Sanit.
VL - 34
IS - 1
CY - USSR
SN - 0016-9900
UR - 5348984
ER -

TY - JOUR
ID - 2312
T1 - [Medical supervision of adolescents on the playground]. [German]
A1 - Nemessuri,M.
Y1 - 1968/09/01/

N1 - Nemessuri, M
Zeitschrift fur arztliche Fortbildung
xs6, 0414004
IM
Journal Article
German
KW - MEDLINE
KW - Adolescent
KW - Child
KW - Heart Function Tests
KW - Humans
KW - Male
KW - Physical Fitness
KW - Proteinuria/ep [Epidemiology]
KW - Pulse
KW - Reaction Time
KW - Respiratory Function Tests
KW - Sports
KW - Sports Medicine
KW - Time Factors
RP - NOT IN FILE
SP - 963
EP - 969
JF - Zeitschrift fur Arztliche Fortbildung (Jena)
JA - Z Arztl.Fortbild.(Jena)
VL - 62
IS - 17
CY - GERMANY, EAST
SN - 0044-2178
UR - 5712248
ER -

TY - JOUR
ID - 2313
T1 - Rehabilitation of patients with through-knee amputation
A1 - Early,P.F.
Y1 - 1968/11/16/
N1 - Early, P F
British medical journal
b4w, 0372673
AIM, IM
Journal Article
English
KW - MEDLINE
KW - Adult
KW - Aged
KW - Amputation/rh [Rehabilitation]
KW - Amputation Stumps
KW - Artificial Limbs
KW - Bandages
KW - Child
KW - Preschool
KW - Exercise Therapy
KW - Female
KW - Gangrene/su [Surgery]
KW - Humans
KW - Knee/su [Surgery]

KW - Leg/ab [Abnormalities]
KW - Leg Injuries/su [Surgery]
KW - Locomotion
KW - Male
KW - Middle Aged
KW - Poliomyelitis/su [Surgery]
KW - Time Factors
RP - NOT IN FILE
SP - 418
EP - 421
JF - British Medical Journal
JA - Br Med J
VL - 4
IS - 5628
CY - ENGLAND
N2 - One hundred and one patients with through-knee amputations attending the Manchester limb-fitting centre are reviewed. Most amputations were performed for trauma or vascular disease. The interval from amputation to measurement for the first prosthesis averaged 12 weeks in cases of primary healing, and 21 weeks when healing was delayed. Artificial limbs were successfully fitted to 83%, and only 10% failed to use either a limb or a pylon. Three-quarters of those with outdoor mobility returned to work.Disarticulation through the knee has several advantages over above-knee amputation: in particular, the long end-bearing stump facilitates balance and control of the prosthesis. Disadvantages are a tendency to slow healing of the wound, lack of an internal knee mechanism in the artificial limb, and the bulky appearance of the limb. The results of rehabilitation could be improved by careful selection of patients and attention to operative detail; stump bandaging and exercises; earlier attendance at the limb-fitting centre to be measured for pylon or artificial limb; and improvements in design and production of prostheses
SN - 0007-1447
UR - 5687605
ER -

TY - JOUR
ID - 2314
T1 - Studies on man vector contact in some malarious areas in Colombia
A1 - Elliott,R.
Y1 - 1968///
N1 - Elliott, R
Bulletin of the World Health Organization
7507052, c80
IM
Journal Article
English
KW - MEDLINE
KW - Adolescent
KW - Adult
KW - Anopheles
KW - Behavior
KW - Child
KW - Preschool
KW - Colombia
KW - Ecology
KW - Female
KW - Habits
KW - Humans
KW - Infant
KW - Insect Vectors
KW - Malaria/et [Etiology]
KW - Male

RP - NOT IN FILE
SP - 239
EP - 253
JF - Bulletin of the World Health Organization
JA - Bull World Health Organ
VL - 38
IS - 2
CY - SWITZERLAND
N2 - The frequency of man-biting by mosquitos depends upon the amount of contact between man and mosquito, which in turn depends upon the behaviour patterns of both. In order to examine these relationships in an area with a high incidence of malaria, a study was made in 1965 of the nocturnal movements and of mosquito biting habits in five localities in the malarious area of Colombia that were in the thirteenth or fourteenth cycle of biennial DDT spraying.The populations were classed into five age- and sex-groups, i.e., men and women over the age of 15 years, boys and girls from 5 to 15 years, and children under 5. A number of differences in the habits of these groups were discovered in relation to time spent indoors or outside, but close to, the house.The habits of the four main anopheline vector species were studied in relation to human activities. For three of the species (A. albimanus, A. darlingi, A. nuneztovari) it is suggested that a low relative importance of outdoor biting is caused not by a density-dependent factor but by an anopheline gonotrophic cycle or by relative humidity or both. The fourth species (A. punctimacula), common only at one locality, displayed a complex pattern of biting behaviour with, however, a much greater frequency of outdoor biting than in the other species.It is considered that in these localities malaria is probably transmitted mainly inside sprayed houses by vectors that are susceptible to the insecticides in use but which are not sufficiently reduced in numbers or in life-expectancy to interrupt the transmission of the parasite
SN - 0042-9686
UR - 5302300
ER -

TY - JOUR
ID - 2317
T1 - Valuing the invaluable: Effects of individual, school and cultural factors on the environmental values of children
A1 - Boeve-de Pauw,Jelle
Y1 - 2013///
N1 - Peer Reviewed Journal: 2013-15967-012
English
Journal; Peer Reviewed Journal
KW - PsycInfo
KW - Childhood Development
KW - Environmental Attitudes
KW - Schools
KW - Sociocultural Factors
KW - Values
KW - Environmental Education
RP - NOT IN FILE
SP - 249
EP - 250
JF - Environmental Education Research
VL - 19:
IS - 2
N2 - The environmental values (EV) of children are, across disciplines and theories, regarded as essential preconditions of environmental behavior (EB). This study identifies and explains variation in EV and what they tell us about the EB of children. The study makes an important contribution to the design and evaluation of environmental education initiatives. The 2006 PISA data were used in the study (398,750 15-year-olds from 56 countries) as well as about 3000 children ages 10-16 from Flanders, Vietnam and Guatemala. Data treatment methods were quantitative and include multilevel regression models to discern different levels of variation in the data (individuals within schools within cultures) and multi-group confirmatory factor analyses to assess issues of measurement invariance in EV. The key findings include; (1) children's EV are not just individual traits, they

are also accounted for the social and natural context within which individuals operate; (2) gender differences in EV might reflect methodological artifacts rather than genuine differences; (3) schools matter, environmental knowledge can be encouraged through hands-on teaching, EV through school participation in environmental learning activities outside the classroom; (4) schools have a greater impact on their students' environmental knowledge than on their EV; (5) eco-schools matter, they have an effect on their students' EV but might be applying an approach that, in the long run, has little chance of achieving behavior change and (6) EV affect EB and do so in a culturally specific manner; educational efforts should account for this cultural specificity. (PsycINFO Database Record (c) 2014 APA, all rights reserved)
SN - 1350-4622
AD - Boeve-de Pauw, Jelle: University of Antwerp, Institute for Education and Information Sciences, Antwerp, Belgium
ER -

TY - JOUR
ID - 2318
T1 - In search of understanding children's engagement with nature and their learning experiences in one urban kindergarten classroom
A1 - Ghafouri,Farveh
Y1 - 2014///
N1 - Dissertation Abstract: 2014-99030-567
English
Dissertation Abstract
KW - PsycInfo
KW - Classrooms
KW - Kindergarten Students
KW - Learning
KW - Photographs
KW - Urban Environments
KW - Child Attitudes
KW - Digital Video
RP - NOT IN FILE
SP - No
JF - Dissertation Abstracts International Section A: Humanities and Social Sciences
VL - 74:
IS - 8-A(E
N2 - Considering the context of large city schools, this study explores what variables in a kindergarten classroom may impact the process of children's engagement with nature. In particular I examine the central role of children and teacher in co-constructing their own unique understanding, knowledge, and attitude towards the natural world. In this study, I examine nature-child's connection considering the complexity of nature beyond a pre-packaged concept (Louv, 2007) and avoiding a linear identification of a cause and effect relationship between children's learning experiences and nature, (Kellert, 2005). This qualitative case study is based on extensive classroom observations, in which 20 kindergarten children and their teacher participate. The children's direct, indirect, and vicarious experiences with nature are documented using digital photography, video-audio recording, and collection of artifacts. I interview the classroom teacher two times and invite the parents to fill up a questionnaire about their children's experiences with nature outside the school time. I use the techniques and procedure of the grounded theory to analyze the data. A comparative analysis of the five learning episodes demonstrates four major factors that when all woven together encourage and sustain the children's engagement with nature. These factors are: investigating children's meaningful and autotelic questions, encountering and experiencing nature in familiar contexts, developing emotional bonding, and having sufficient time. The findings show the crucial role of the classroom teacher in creating five main conditions to engage the children in the process of each inquiry. She offers the children many opportunities to use their prior skills and knowledge, take responsibility of their own learning, and experiment with learning as a process. She often responds positively to the children's learning endeavours and communicates her high confidence and expectations for them. This study makes an important contribution to the field of early childhood education and environmental education by demonstrating the possibilities and challenges in actively and holistically engaging children with nature in school settings. The findings shed light on our understanding of children and teacher's sense of

ownership and motivation as two driving forces of learning. (PsycINFO Database Record (c) 2014 APA, all rights reserved)
SN - 0419-4209
AD - Ghafouri, Farveh: U Toronto, Canada
ER -

TY - JOUR
ID - 2319
T1 - Measuring "flow" in Michigan youth firearm deer hunters and implications for hunter recruitment
A1 - Everett,Michael Winthrop
Y1 - 2014///
N1 - Dissertation Abstract: 2014-99030-381
English
Dissertation Abstract
KW - PsycInfo
KW - Firearms
KW - Personnel Recruitment
KW - Flow (Consciousness State)
KW - Satisfaction
KW - Support Groups
RP - NOT IN FILE
SP - No
JF - Dissertation Abstracts International Section A: Humanities and Social Sciences
VL - 74:
IS - 8-A(E
N2 - The steady decline of populations taking up recreational hunting is cause for concern among state and federal wildlife agencies. Young hunters are the future of hunting. As the average age of hunters continues to increase, young people participating in hunting activities provide an opportunity for insight to change the current declining trend. Flow theory and hunter satisfaction have both received extensive attention in research literature, however they have been utilized in separate and disconnected settings. An attempt to combine both constructs, as a way to measure intrinsic and extrinsic motivational, mood, and flow indicators of youth hunters has not been attempted to date. Measuring and understanding antecedents of "flow" in youth is an important consideration when determining why youth enjoy hunting as an outdoor recreational experience. This exploratory study utilizes flow theory and the four-channel model of flow to examine relationships among youth hunters' (12 to 16 years of age) experiences and satisfaction during Michigan's Special Youth Firearm White-tailed Deer Hunt (n=43). This research provided a snapshot in time of young hunters, their experiences and the satisfaction of those experiences during the youth hunt. Of the youth participants in this study, 69% indicated that they intend to go back out in the field for Michigan's opening day of firearm deer season (November 15, 2012). Additionally, 97.6% of youth respondents intend to hunt in future years, providing support for implementation of special hunting opportunities where youth and mentors can enjoy the experience of hunting together. This exploratory research suggests that there is a significant and direct relationship between "flow" experiences and young hunters that hear deer and other animals, and see white-tailed deer. Knowledge of congruence between "flow" experiences and hearing deer and other animals, and seeing white-tailed deer offers the opportunity for further research on a much broader scale of licensed young hunters in Michigan. This research also has the potential to inform recruitment and retention personnel of state wildlife agencies about ways to support programs that promote hunting activities within younger populations in the future. (PsycINFO Database Record (c) 2014 APA, all rights reserved)
SN - 0419-4209
AD - Everett, Michael Winthrop: Michigan State U., US
ER -

TY - JOUR
ID - 2320
T1 - Evaluation of motor skills in children participating in social/sports projects. [Portuguese]. [References]
A1 - Santos,A.
Y1 - 2013///

N1 - Peer Reviewed Journal: 2013-35929-003
Portuguese
Journal; Peer Reviewed Journal
KW - PsycInfo
KW - Athletic Participation
KW - Educational Programs
KW - Elementary School Students
KW - Extracurricular Activities
KW - Motor Development
KW - Childhood Development
KW - Motor Processes
KW - Social Interaction
KW - Sports
RP - NOT IN FILE
SP - 50
EP - 60
JF - Motricidade
VL - 9:
IS - 2
N2 - Besides the biological evolutionary process, studies have shown that environmental and social factors can influence the motor development. The aim of this study was to evaluate the motor skills of students participating in social educational projects, sports projects and those not engaged in structured extracurricular activities in Florianopolis-SC-Brazil. Were evaluated 136 children 8-9 years old, both sexes, from 40 public schools. For data collection was used the Motor Development Scale and a structured interview regarding the activities undertaken by the child during the period that they are not in school. Descriptive statistics, ANOVA and the Post-hoc test of Tukey were used to data analysis, with significance level $p < .05$. The results showed higher patterns of motor development in children that participating in social projects with sport character and expressive low coordination patterns in children who do not participate in structured activities or projects outside of school. (PsycINFO Database Record (c) 2014 APA, all rights reserved) (journal abstract)
SN - 1646-107X
AD - Santos, A. M.: Universidade Estadual de Santa Catarina (UDESC), Brazil Neto, F. R.: Universidade Estadual de Santa Catarina (UDESC), Brazil Pimenta, R. A.: Faculdade de Desporto, Universidade do Porto, Portugal
ER -

TY - JOUR
ID - 2321
T1 - Children's performance estimation in mathematics and science tests over a school year: A pilot study. [References]
A1 - Roderer,Thomas
Y1 - 2013///
N1 - Peer Reviewed Journal: 2013-17855-001
English
Journal; Peer Reviewed Journal
KW - PsycInfo
KW - Academic Achievement
KW - Cognitive Ability
KW - Mathematics Achievement
KW - Metacognition
KW - Science Achievement
KW - Childhood Development
KW - Estimation
KW - Performance
KW - Problem Solving
RP - NOT IN FILE
SP - 5
EP - 24

JF - Electronic Journal of Research in Educational Psychology
VL - 11:
IS - 1
N2 - Introduction: The metacognitve ability to accurately estimate ones performance in a test, is assumed to be of central importance for initializing task-oriented effort. In addition activating adequate problem-solving strategies, and engaging in efficient error detection and correction. Although school children's ability to estimate their own performance has been widely investigated, this was mostly done under highly-controlled, experimental set-ups including only one single test occasion. Method: The aim of this study was to investigate this metacognitive ability in the context of real achievement tests in mathematics. Developed and applied by a teacher of a 5th grade class over the course of a school year these tests allowed the exploration of the variability of performance estimation accuracy as a function of test difficulty. Results: Mean performance estimations were generally close to actual performance with somewhat less variability compared to test performance. When grouping the children into three achievement levels, results revealed higher accuracy of performance estimations in the high achievers compared to the low and average achievers. In order to explore the generalization of these findings, analyses were also conducted for the same children's tests in their science classes revealing a very similar pattern of results compared to the domain of mathematics. Discussion and Conclusion: By and large, the present study, in a natural environment, confirmed previous laboratory findings but also offered additional insights into the generalisation and the test dependency of students' performances estimations. (PsycINFO Database Record (c) 2014 APA, all rights reserved) (journal abstract)
SN - 1699-5880
AD - Roderer, Thomas: Department of Psychology, University of Bern, Bern, Switzerland Roebers, Claudia M.: Department of Psychology, University of Bern, Bern, Switzerland
ER -

TY - JOUR
ID - 2322
T1 - A playground social skills intervention for children exhibiting emotional and behavioral problems
A1 - DiFolco,Kelly
Y1 - 2014///
N1 - Dissertation Abstract: 2014-99020-446
English
Dissertation Abstract
KW - PsycInfo
KW - Behavior Problems
KW - Emotional Disturbances
KW - Intervention
KW - Playgrounds
KW - Social Skills Training
KW - Child Psychiatry
RP - NOT IN FILE
SP - No
JF - Dissertation Abstracts International: Section B: The Sciences and Engineering
VL - 74:
IS - 7-B(E
N2 - The author examined the effects of small-group social skills instruction on the playground behaviors of three second-grade students with emerging emotional and behavior problems. Over the course of six weeks, students were taught three social skills lessons that corresponded to problematic behaviors observed on the playground. A multiple baseline design was used to determine the effect of social skills instruction on social behaviors observed on the playground. Teachers also completed a rating scale before and after the intervention to measure any changes in social skills in the classroom. Over the course of the intervention, students moderately increased their percentage of time spent socializing appropriately during recess, but results varied across participants. Mixed results were also found on the social skills rating scale across students. (PsycINFO Database Record (c) 2014 APA, all rights reserved)
SN - 0419-4217
AD - DiFolco, Kelly: U Nebraska at Omaha, US
ER -

TY - JOUR
ID - 2323
T1 - Analyzing the mediators between nature-based outdoor recreation and emotional well-being. [References]
A1 - Korpela,K.
Y1 - 2014///
N1 - Peer Reviewed Journal: 2014-00756-002
English
Journal; Peer Reviewed Journal
KW - PsycInfo
KW - Physical Activity
KW - Recreation
KW - Social Interaction
KW - Well Being
KW - Nature (Environment)
KW - Emotional Adjustment
RP - NOT IN FILE
SP - 1
EP - 7
JF - Journal of Environmental Psychology
VL - 37 Mar 2014, pp. 1-7.
N2 - The evidence concerning the relative importance of physical activity, restorative experiences, and social interaction as mediators between exposure to nature and well-being has been inconsistent. We investigated whether there is a relationship between the average time used for nature-based recreation and emotional well-being and whether it is mediated through restorative experiences, social company and the perceived duration of the most recent nature-based recreation visit. A sample of 3060 Finnish people (38.3% response rate) aged 15-74 years participated in a survey using an internet and a mail questionnaire. Multiple mediation analysis using bootstrapping revealed an association between the self-reported participation in nature-based recreation and emotional well-being through restorative experiences when adjusting for age, gender, household income, the level of leisure time physical activity, and the frequency of active transportation. The amount of social company or the duration of the most recent nature-based recreation visit did not mediate the association between the average time spent on nature-based recreation and emotional well-being. The result accords with the evidence of the restorative and well-being effects of nature exposure but more evidence of causality and studies comparing different mediators in different population groups are needed. (PsycINFO Database Record (c) 2014 APA, all rights reserved) (journal abstract)
SN - 0272-4944
AD - Korpela, K.: School of Social Sciences and Humanities, University of Tampere, Tampere, Finland Borodulin, K.: National Public Health Institute, Department of Health Promotion and Chronic Disease Prevention, Helsinki, Finland Neuvonen, M.: Finnish Forest Research Institute, Vantaa, Finland Paronen, O.: UKK Institute for Health Promotion Research, Tampere, Finland Tyrvainen, L.: Finnish Forest Research Institute, Vantaa, Finland
ER -

TY - JOUR
ID - 2324
T1 - The functional architecture for face-processing expertise: Fmri evidence of the developmental trajectory of the core and the extended face systems. [References]
A1 - Haist,Frank; Adamo,Maha; Han Wazny,Jarnet; Lee,Kang; Stiles,Joan
Y1 - 2013///
N1 - Peer Reviewed Journal: 2013-39880-044
English
Journal; Peer Reviewed Journal
KW - PsycInfo
KW - Face Perception
KW - Neuroimaging
KW - Amygdala
KW - Developmental Measures
KW - Social Interaction

RP - NOT IN FILE
SP - 2893
EP - 2908
JF - Neuropsychologia
VL - 51:
IS - 13
N2 - Expertise in processing faces is a cornerstone of human social interaction. However, the developmental course of many key brain regions supporting face preferential processing in the human brain remains undefined. Here, we present findings from an FMRI study using a simple viewing paradigm of faces and objects in a continuous age sample covering the age range from 6 years through adulthood. These findings are the first to use such a sample paired with whole-brain FMRI analyses to investigate development within the core and extended face networks across the developmental spectrum from middle childhood to adulthood. We found evidence, albeit modest, for a developmental trend in the volume of the right fusiform face area (rFFA) but no developmental change in the intensity of activation. From a spatial perspective, the middle portion of the right fusiform gyrus most commonly found in adult studies of face processing was increasingly likely to be included in the FFA as age increased to adulthood. Outside of the FFA, the most striking finding was that children hyperactivated nearly every aspect of the extended face system relative to adults, including the amygdala, anterior temporal pole, insula, inferior frontal gyrus, anterior cingulate gyrus, and parietal cortex. Overall, the findings suggest that development is best characterized by increasing modulation of face-sensitive regions throughout the brain to engage only those systems necessary for task requirements. (PsycINFO Database Record (c) 2014 APA, all rights reserved) (journal abstract)
SN - 0028-3932
AD - Haist, Frank: Psychiatry Department, University of California, San Diego, La Jolla, CA, US Adamo, Maha: Center for Human Development, University of California, San Diego, La Jolla, CA, US Han Wazny, Jarnet: Center for Human Development, University of California, San Diego, La Jolla, CA, US Lee, Kang: Dr. Eric Jackman Institute of Child Study, University of Toronto, Toronto, ON, Canada Stiles, Joan: Center for Human Development, University of California, San Diego, La Jolla, CA, US
ER -

TY - JOUR
ID - 2325
T1 - A naturalistic study of stereotype threat in young female chess players. [References]
A1 - Rothgerber,Hank
Y1 - 2014///
N1 - Peer Reviewed Journal: 2013-45409-005
English
Journal; Peer Reviewed Journal
KW - PsycInfo
KW - Chess
KW - Environment
KW - Human Females
KW - Stereotyped Attitudes
KW - Threat
KW - Adolescent Development
KW - Childhood Development
RP - NOT IN FILE
SP - 79
EP - 90
JF - Group Processes & Intergroup Relations
VL - 17:
IS - 1
N2 - The present research sought to determine whether young female chess players would demonstrate stereotype threat susceptibility in a naturalistic environment. Data from 12 scholastic chess tournaments indicated that females performed worse than expected when playing against a male opponent, achieving 83% of the expected success based on their own and their opponent's prerating. These effects were strongest for the youngest players in lower elementary school but also present for those in upper elementary. Stereotype threat susceptibility was most pronounced in contexts that could be considered challenging: when playing a strong or

moderate opponent and when playing someone in a higher or the same grade. As evidence of disengagement, those most vulnerable to stereotype threat were less likely to continue playing in future chess tournaments. These results were not found in a matched comparison male group suggesting the outcomes were unique to stereotype threat and not universal to young chess players. (PsycINFO Database Record (c) 2014 APA, all rights reserved) (journal abstract)
SN - 1368-4302
AD - Rothgerber, Hank: Department of Psychology, Bellarmine University, Louisville, KY, US Wolsiefer, Katie: Department of Psychology & Neuroscience, University of Colorado, CO, US
ER -

TY - JOUR
ID - 2326
T1 - Physical activity, self-regulation, and early academic achievement in preschool children. [References]
A1 - Becker,Derek R.
Y1 - 2014///
N1 - Peer Reviewed Journal: 2013-45134-004
English
Journal; Peer Reviewed Journal
KW - PsycInfo
KW - Academic Achievement
KW - Childhood Play Behavior
KW - Physical Activity
KW - Preschool Students
KW - Self Regulation
RP - NOT IN FILE
SP - 56
EP - 70
JF - Early Education and Development
VL - 25:
IS - 1
N2 - Research Findings: The present study investigated whether active play during recess was associated with self-regulation and academic achievement in a prekindergarten sample. A total of 51 children in classes containing approximately half Head Start children were assessed on self-regulation, active play, and early academic achievement. Path analyses indicated that higher active play was associated with better self-regulation, which in turn was associated with higher scores on early reading and math assessments. Practice or Policy: Results point to the benefits of active play for promoting self-regulation and offer insight into possible interventions designed to promote self-regulation and academic achievement. (PsycINFO Database Record (c) 2014 APA, all rights reserved) (journal abstract)
SN - 1040-9289
AD - Becker, Derek R.: Oregon State University, Corvallis, OR, US McClelland, Megan M.: Oregon State University, Corvallis, OR, US Loprinzi, Paul: Bellarmine University, KY, US Trost, Stewart G.: School of Human Movement Studies, University of Queensland, Brisbane, QLD, Australia
ER -

TY - JOUR
ID - 2327
T1 - A favorable built environment is associated with better physical fitness in European adolescents. [References]
A1 - Vanhelst,Jeremy; Beghin,Laurent; Salleron,Julia; Ruiz,Jonatan
Y1 - 2013///
N1 - Peer Reviewed Journal: 2013-41124-019
English
Journal; Peer Reviewed Journal
KW - PsycInfo
KW - Adolescent Development
KW - Exercise
KW - Lifestyle

KW - Physical Activity
KW - Physical Fitness
KW - Built Environment
RP - NOT IN FILE
SP - 844
EP - 849
JF - Preventive Medicine: An International Journal Devoted to Practice and Theory
VL - 57:
IS - 6
N2 - Objective: To assess the association between the built environment and physical fitness and physical activity in adolescents. Methods: The study included 3528 adolescents, aged 12.5-17.5 years, who participated in the Healthy Lifestyle in Europe by Nutrition in Adolescence (HELENA) study. The health-related physical fitness components were assessed using the physical fitness tests. Participants wore a uniaxial accelerometer (ActiGraph) for 7 days to measure physical activity. A specific questionnaire addressing the built environment was used. Potential confounding factors including age, gender, body mass index, body composition, pubertal status, smoking, educational level of parents, and socioeconomic status were analyzed using backward stepwise linear regression analysis. Results: Heavy traffic in the neighborhood was the strongest factor negatively associated with both physical fitness and physical activity (both P < 0.05). Conversely, a secure bicycling or walking route from home to school was positively associated with various components of physical fitness and physical activity (P < 0.01). Outdoor fields and gymnasiums near home were also associated with better physical fitness (P < 0.01), but not with physical activity. Conclusions: A favorable built environment may contribute to health-related physical fitness and physical activity of adolescents and should be considered in future interventions and health promotion strategies. (PsycINFO Database Record (c) 2014 APA, all rights reserved) (journal abstract)
SN - 0091-7435
AD - Vanhelst, Jeremy: INSERM U995, University Lille Nord de France, Lille, France Beghin, Laurent: INSERM U995, University Lille Nord de France, Lille, France Salleron, Julia: Department of Biostatistics, University Lille Nord de France, Lille, France Ruiz, Jonatan R.: Unit for Preventive Nutrition, Department of Biosciences and Nutrition, Karolinska Institutet, Huddinge, Sweden Ortega, Francisco B.: Unit for Preventive Nutrition, Department of Biosciences and Nutrition, Karolinska Institutet, Huddinge, Sweden De Bourdeaudhuij, Ilse: Department of Movement and Sport Sciences, Ghent University, Ghent, Belgium Molnar, Denes: Department of Pediatrics, Pecs University, Pecs, Hungary Manios, Yannis: Department of Nutrition and Dietetics, Harokopio University, Athens, Greece Widhalm, Kurt: Department of Pediatrics, School of Medicine, Vienna University, Vienna, Austria Vicente-Rodriguez, German: GENUD (Growth, Exercise, Nutrition and Development) Research Group, Facultad de Ciencias de la Salud, Zaragoza University, Zaragoza, Spain Mauro, Beatrice: National Research Institute for Food and Nutrition, Rome, Italy Moreno, Luis A.: GENUD (Growth, Exercise, Nutrition and Development) Research Group, Facultad de Ciencias de la Salud, Zaragoza University, Zaragoza, Spain Sjostrom, Michael: Unit for Preventive Nutrition, Department of Biosciences and Nutrition, Karolinska Institutet, Huddinge, Sweden Castillo, Manuel J.: Department of Medical Physiology, School of Medicine, Granada University, Granada, Spain Gottrand, Frederic: INSERM U995, University Lille Nord de France, Lille, France
ER -

TY - JOUR
ID - 2328
T1 - Shalom Sesame: Using media to promote Jewish education and identity. [References]
A1 - Fisch,Shalom M.
Y1 - 2013///
N1 - Peer Reviewed Journal: 2013-33030-007
English
Journal; Peer Reviewed Journal
KW - PsycInfo
KW - Educational Programs
KW - Ethnography
KW - Judaism
KW - Self Concept
KW - Media Exposure

KW - Family
RP - NOT IN FILE
SP - 297
EP - 314
JF - Journal of Jewish Education
VL - 79:
IS - 3
N2 - A family survey, ethnographic study, and quasi-experimental study investigated Shalom Sesame's potential to enhance understanding of Jewish culture and identity among preschool families. Preschoolers demonstrated significant learning, recognizing that people who looked different could be Jewish, and in knowledge about Hebrew words, Jewish holidays, and things they would see in Israel. Learning also extended beyond the screen, via spontaneous family discussions/activities, and an increased desire to celebrate holidays or visit Israel. The videos held particular value for interfaith families and those outside established Jewish communities. Parents valued Shalom Sesame for helping children connect to the broader Jewish community, deepening their own connection, and educating non-Jewish relatives and friends. (PsycINFO Database Record (c) 2014 APA, all rights reserved) (journal abstract)
SN - 1524-4113
AD - Fisch, Shalom M.: MediaKidz Research & Consulting, OH, US Lemish, Dafna: College of Mass Communication and Media Arts, Southern Illinois University, IL, US Spezia, Elizabeth: WSIU Public Broadcasting, IL, US Siegel, Deborah: MediaKidz Research & Consulting, OH, US Fisch, Susan R. D.: MediaKidz Research & Consulting, OH, US Alade, Fashina: MediaKidz Research & Consulting, OH, US Kasdan, Daniel: MediaKidz Research & Consulting, OH, US
ER -

TY - JOUR
ID - 2329
T1 - Facilitating children's use of emotional language
A1 - Ahern,Elizabeth Carole
Y1 - 2013///
N1 - Dissertation Abstract: 2013-99240-027
English
Dissertation Abstract
KW - PsycInfo
KW - Interpersonal Interaction
KW - Language
KW - Parents
RP - NOT IN FILE
SP - No
JF - Dissertation Abstracts International: Section B: The Sciences and Engineering
VL - 74:
IS - 6-B(E
N2 - The study examined the effects of rapport building (emotional, NICHD) and prompt type (what-next, cued-action, cued-emotion, what-think) on 142 4- to 9-year-old maltreated children's spontaneous and prompted emotional language. Children in the emotional rapport building condition narrated the last time they felt good and the last time they felt bad on the playground. Children in the NICHD rapport building condition narrated their last birthday party and what happened yesterday. Following rapport building, all children were presented a series of story stems about positive and negative situations. Emotional rapport building demonstrated some positive albeit temporary effects on children's use of emotional language. Cued-emotion prompts appeared to be the most productive in eliciting emotional language. Overall, there were few effects due to age. Children often produced less emotional language when describing negative events, particularly with respect to their spontaneous utterances, suggesting reluctance. These differences largely disappeared when children were asked additional questions, particularly cued-emotion questions. The results support the utility of emotional rapport building and cued-emotion prompts as a means of increasing children's use of emotional language. (PsycINFO Database Record (c) 2014 APA, all rights reserved)
SN - 0419-4217
AD - Ahern, Elizabeth Carole: U Southern California, US
ER -

TY - JOUR
ID - 2330
T1 - Net time negotiations within the family. [References]
A1 - Robinson,Laura
Y1 - 2013///
N1 - Peer Reviewed Journal: 2013-13818-006
English
Journal; Peer Reviewed Journal
KW - PsycInfo
KW - Family
KW - Negotiation
KW - Internet Usage
KW - High School Students
KW - Social Issues
RP - NOT IN FILE
SP - 542
EP - 560
JF - Information, Communication & Society
VL - 16:
IS - 4
N2 - Drawing on data from one-on-one and focus group interviews with high school students from schools in agricultural California, this research examines how American families negotiate what we call net time. The article explores intra-familial bargaining over time spent on the internet. Analysis pays special attention to families that prioritize capital-enhancing activities such as schoolwork and college applications. In these families, access to resources is guided by implicit social contracts between parents and children, as well as between siblings. The findings illuminate how these social contracts imply particular rights and responsibilities depending on the families' level of wiredness: highly wired, partially wired, and unwired families. Comparing the experiences of students from these three groups reveals that members of each kind of family experience a different form of net time. While youths from highly wired families enjoy individualized net time, members of partially wired families divvy up household net time. The most disadvantaged youth come from unwired families in which family members must make sacrifices for youth to obtain net time outside of the household. The examination illuminates the logics that underpin the familial negotiations over each kind of net time. Ultimately, familial social contracts over net time have the power to encourage or hinder use of net time for capital-enhancing activities. (PsycINFO Database Record (c) 2014 APA, all rights reserved) (journal abstract)
SN - 1369-118X
AD - Robinson, Laura: Department of Sociology, Santa Clara University, Santa Clara, CA, US Schulz, Jeremy: Department of Sociology, University of California, Berkeley, Berkeley, CA, US
ER -

TY - JOUR
ID - 2331
T1 - EMDR as an integrative therapeutic approach for the treatment of separation anxiety disorder. [References]
A1 - Morrissey,Michelle
Y1 - 2013///
N1 - Peer Reviewed Journal: 2013-40366-002
English
Journal; Peer Reviewed Journal
KW - PsycInfo
KW - Eye Movement Desensitization Therapy
KW - Integrative Psychotherapy
KW - Separation Anxiety
KW - Exposure Therapy
KW - Family Therapy
RP - NOT IN FILE
SP - 200

EP - 207
JF - Journal of EMDR Practice and Research
VL - 7:
IS - 4
N2 - This case study reports the use of eye movement desensitization and reprocessing (EMDR) and family therapy for a 10-year-old boy with severe separation anxiety disorder (SAD). It illustrates how the use of the standard EMDR protocol for the boy and his mother combined with family therapy, led to symptom alleviation and restored appropriate developmental functioning as evidenced by behavioral outcomes. The participant initially presented with severe anxiety about separating from his mother, several years after his parents went through a painful divorce. Treatment focused on processing the boy's disturbing memories of past nontraumatic events in 14 EMDR sessions; his mother received 4 EMDR sessions to address her perceived marital failure and guilt about the effects of her ensuing depression on him. Eight family therapy sessions were used to help the family spend positive time together. Prior to treatment, the child had been unable to play outside, checked on his mother frequently, and could not attend activities without her. At the end of treatment, he was able to play with friends outside, ride his bike around town, engage in after school activities, and sleep over at his friends' houses. Gains were maintained at 6-month follow-up. Treatment did not include instruction in parenting skills or psychoeducation for the mother, or any exposure therapy for the child. (PsycINFO Database Record (c) 2014 APA, all rights reserved) (journal abstract)
SN - 1933-3196
AD - Morrissey, Michelle: Spanish Peaks Behavioral Health Center, Pueblo, CO, US
ER -

TY - JOUR
ID - 2332
T1 - Socio-environmental influences on physical activity among young people: A qualitative study. [References]
A1 - Kirby,Joanna
Y1 - 2013///
N1 - Peer Reviewed Journal: 2013-40077-003
English
Journal; Peer Reviewed Journal
KW - PsycInfo
KW - Peer Relations
KW - Physical Activity
KW - School Environment
KW - Socioeconomic Status
RP - NOT IN FILE
SP - 954
EP - 969
JF - Health Education Research
JA - Health Educ Res
VL - 28:
IS - 6
N2 - This multi-methods qualitative study aimed to identify environmental factors that influence physical activity participation among young people in Edinburgh, Scotland. School pupils (aged 11-13 years) took part using photography, computer blogs, maps and focus group discussions (FGDs). Eleven computer sessions (n = 131) and 14 FGDs (n = 63) took place. Factors influencing physical activity behaviour included proximity and access to local facilities, family and peers and the school physical activity environment. A variety of facilitators and barriers to participation were also reported. Most notable was the importance of cost and value for money when choosing physical activities which, although more evident among pupils attending schools in areas of low socio-economic status (SES), was relevant across all SES groups. Reporting easy access to sports facilities was more common among pupils attending schools from high/medium SES. Use of green-space for physical activity was reported among pupils from all schools, but was more common among those from low SES schools. Pupils were, in general, satisfied with the facilities available at school, but felt time for physical education could be increased. Findings may help inform interventions, aimed at promoting physical activity at local level. (PsycINFO Database Record (c) 2014 APA, all rights reserved) (journal abstract)
SN - 0268-1153

AD - Kirby, Joanna: Division of Health Sciences, Warwick Medical School, University of Warwick, Coventry, United Kingdom Levin, Kate A.: Child and Adolescent Health Research Unit (CAHRU), School of Medicine, University of St Andrews, Fife, United Kingdom Inchley, Jo: Child and Adolescent Health Research Unit (CAHRU), School of Medicine, University of St Andrews, Fife, United Kingdom
ER -

TY - JOUR
ID - 2333
T1 - An examination of children's oral storytelling in a first grade storytelling circle
A1 - West,Jean Ann
Y1 - 2013///
N1 - Dissertation Abstract: 2013-99230-387
English
Dissertation Abstract
KW - PsycInfo
KW - Classrooms
KW - Elementary School Students
KW - Knowledge Level
KW - Learning
KW - Storytelling
KW - Teaching
RP - NOT IN FILE
SP - No
JF - Dissertation Abstracts International Section A: Humanities and Social Sciences
VL - 74:
IS - 6-A(E
N2 - The purposes of this naturalistic, descriptive study were to examine the stories that children in one first grade classroom told in a storytelling circle, how the children's knowledge, in-school experiences, and out-of-school experiences were reflected in their stories, and how the children used their knowledge and experiences as tools to make sense of the world through their stories. The study was designed as a naturalistic, descriptive study employing an interpretive approach to examining the children's oral stories. Nine first grade children in one classroom were grouped heterogeneously into three storytelling groups. Data collected from the three groups included a total of 80 story rounds, each of which included an oral story along with preceding and succeeding dialogue from other children and the researcher. The researcher employed participant observation and a discourse analytic method of inquiry to analyze the stories told by children in a storytelling circle. The children proved themselves to be active constructors and co-constructors of meaning who skillfully told their stories for the very authentic purposes of making sense for themselves and for others. The stories were structurally complex and interesting to tellers and listeners. The children flexibly coconstructed stories that included a wide variety of settings, episodes, and codas as well as complex plots that situated people, characters, objects, and actions across time and space in ways that suited the purposes of and were meaningful to the tellers and the listeners. The children also drew upon a wide range of everyday knowledge and experiences, especially from out of school, which they called upon as resources for the co-construction of the stories. They used language in flexible ways that appropriated combinations of genres which they were able to utilize effectively in ways that helped them to organize and relate their experiences through the stories. Listening to the children's stories proved to be an effective way to learn about the children's knowledge, rooted in their experiences both inside and outside of school. This approach broadens current conceptions of children as learners by focusing on their strengths and resources as starting points for teaching and learning. Nine story maps are attached in Appendices E, F, G, H, I, J, K, L, and M. (PsycINFO Database Record (c) 2014 APA, all rights reserved)
SN - 0419-4209
AD - West, Jean Ann: Texas Woman's U., US
ER -

TY - JOUR
ID - 2334
T1 - Child physical activity in relation to school physical activity practices
A1 - Carlson,Jordan A.
Y1 - 2013///

N1 - Dissertation Abstract: 2013-99220-494
English
Dissertation Abstract
KW - PsycInfo
KW - Exercise
KW - Schools
KW - Best Practices
RP - NOT IN FILE
SP - No
JF - Dissertation Abstracts International: Section B: The Sciences and Engineering
VL - 74:
IS - 5-B(E
N2 - Background. Schools provide an important opportunity for children to meet physical activity guidelines. However, physical activity during school is low, and more evidence is needed to support schools to adopt best practices related to physical activity. Objective. The purpose of the present study was to investigate the relation of elementary school physical activity practices related to Physical Education (PE), recess, classroom time, and after-school time to school SES and children's objectively measured physical activity. Methods. Participants were 172 children from 97 elementary schools in the San Diego, CA and Seattle, WA regions. Children wore accelerometers to estimate physical activity for 3.7+1.7 school days. A survey was completed by school informants (PE teachers and principals) to assess physical activity-related school practices (63% response rate). Accelerometer data were scored based on individual school start and end times to derive in-school and after-school minutes/day of MVPA. ANOVAs with LSD post-hoc tests were used to investigate disparities in physical activity-related practices for low-, moderate-, and high-SES schools. Three-level linear mixed effects regression models were used to investigate the association between school practices and children's MVPA. The 5 most important practices, based on their relationship with in-school MVPA, were combined into a summary score. Results. The 5-item school physical activity practice summary score was significantly associated with in-school MVPA, where children had 2.4 more minutes/day of in-school MVPA for every additional practice reflected in the summary score (p = .037). Having a PE teacher was the strongest practice correlated with in-school MVPA, where children at schools with a PE teacher had 6 more minutes/day of in-school MVPA (p = .114). Having someone other than a classroom teacher supervise recess was non-significantly related to 3 more minutes/day of in-school MVPA (p = .363). Conclusions. The present study provides evidence for adopting a package of school physical activity practices to improve children's physical activity during school. Particularly, not having a PE teacher appears to be a leading contributor to low rates of physical activity. Attention should be paid to economically disadvantaged schools, because schools could be contributing to disparities in childhood obesity and inactivity. (PsycINFO Database Record (c) 2013 APA, all rights reserved)
SN - 0419-4217
AD - Carlson, Jordan A.: U California, San Diego, US
ER -

TY - JOUR
ID - 2335
T1 - The Aftercare and School Observation System (ASOS): Reliability and component structure. [References]
A1 - Ingoldsby,Erin M.
Y1 - 2013///
N1 - Peer Reviewed Journal: 2013-33299-002
English
Journal; Peer Reviewed Journal
KW - PsycInfo
KW - Aftercare
KW - Observation Methods
KW - Psychometrics
KW - School Based Intervention
KW - Test Reliability
KW - Test Validity
RP - NOT IN FILE
SP - 893
EP - 902

JF - Journal of Child and Family Studies
VL - 22:
IS - 7
N2 - This study examines the psychometric properties and component structure of a newly developed observational system, the Aftercare and School Observation System (ASOS). Participants included 468 children drawn from a larger longitudinal intervention study. The system was utilized to assess participant children in school lunchrooms and recess and various afterschool environments. Exploratory factor analyses examined whether a core set of component constructs assessing qualities of children's relationships, caregiver involvement and monitoring, and experiences in school and aftercare contexts that have been linked to children's behavior problems would emerge. Construct validity was assessed by examining associations between ASOS constructs and questionnaire measures assessing children's behavior problems and relationship qualities in school and aftercare settings. Across both settings, two factors showed very similar empirical structures and item loadings, reflecting the constructs of a negative/aggressive context and caregiver positive involvement, with one additional unique factor from the school setting reflecting the extent to which caregiver methods used resulted in less negative behavior and two additional unique factors from the aftercare setting reflecting positivity in the child's interactions and general environment and negativity in the child's interactions and setting. Modest correlations between ASOS factors and aftercare provider and teacher ratings of behavior problems, adult-child relationships, and a rating of school climate contributed to our interpretation that the ASOS scores capture meaningful features of children's experiences in these settings. This study represents the first step of establishing that the ASOS reliably and validly captures risk and protective relationships and experiences in extrafamilial settings. (PsycINFO Database Record (c) 2013 APA, all rights reserved) (journal abstract)
SN - 1062-1024
AD - Ingoldsby, Erin M.: OMNI Institute, Denver, CO, US Shelleby, Elizabeth C.: University of Pittsburgh, Pittsburgh, PA, US Lane, Tonya: Department of Psychology, University of Pittsburgh, Pittsburgh, PA, US Shaw, Daniel S.: Department of Psychology, University of Pittsburgh, Pittsburgh, PA, US Dishion, Thomas J.: Department of Psychology, Arizona State University, Phoenix, AZ, US Wilson, Melvin N.: Department of Psychology, University of Virginia, Charlottesville, VA, US
ER -

TY - JOUR
ID - 2336
T1 - Psychological tests in childhood schizophrenia
A1 - des Lauriers,Austin; Halpern,Florence
Y1 - 1947///
N1 - Peer Reviewed Journal: 2013-40428-003
English
Journal; Peer Reviewed Journal
KW - PsycInfo
KW - Childhood Schizophrenia
KW - Intelligence Measures
KW - Psychological Assessment
KW - Psychometrics
KW - Shock Therapy
RP - NOT IN FILE
SP - 57
EP - 67
JF - American Journal of Orthopsychiatry
VL - 17:
IS - 1
N2 - This study examines schizophrenic children who received shock therapy and were given a battery of psychological tests both before and after the treatment. The battery consisted of an intelligence test, either the Stanford-Binet or the Bellevue-Wechsler Intelligence test, depending upon the age of the child and his previous psychometric history; educational tests; the drawing of a man; and the Rorschach. In many cases the Thematic Apperception test, the Vigotsky, a word association test, and play techniques were also used. It was felt that this battery covered those aspects of personality functioning which clinically appeared most affected by the disease process. The age range among the hundred or more children examined, was roughly from four to eleven years. On the basis of the test findings it appears that the schizophrenic child shows many of the personality attributes

found in other clinical groups as well as in normal children. However, the development of these attributes and the uses to which they are put are colored by his specific needs and problems. His behavior is an effort at finding equilibrium, at achieving some unity within himself, and between himself and the outside world. This whole question of anxiety, what is gained by keeping it mobilized or by relieving it, is a point which must be answered at this time with due consideration for the techniques available for treating childhood schizophrenia. (PsycINFO Database Record (c) 2013 APA, all rights reserved)
SN - 0002-9432
AD - des Lauriers, Austin: Bellevue Hospital, Psychiatric Division, New York, NY, US Halpern, Florence: Bellevue Hospital, Psychiatric Division, New York, NY, US
ER -

TY - JOUR
ID - 2337
T1 - A methodological approach to the evaluation of treatment in young nonverbal children. [References]
A1 - Meyer,Lucile R.
Y1 - 1961///
N1 - Peer Reviewed Journal: 2013-39315-007
English
Journal; Peer Reviewed Journal
KW - PsycInfo
KW - Nonverbal Communication
KW - Psychotherapy
KW - Schizophrenia
KW - Treatment Effectiveness Evaluation
KW - Methodology
RP - NOT IN FILE
SP - 748
EP - 756
JF - American Journal of Orthopsychiatry
VL - 31:
IS - 4
N2 - This article presents a methodological approach to the evaluation of treatment in young nonverbal children. The evaluation of treatment is only part of a large project which aims to study the play patterns and ego functions of nonverbal schizophrenic and retarded children between the ages of four and six years. At the beginning of the project one of the main problems encountered was intake. During the terminal conferences, various post project dispositions of children were devised. They tended to fall into two main categories: those children who, in the opinion of the staff, would continue to benefit from living at home with the support of psychotherapy and those who should be hospitalized outside the home. The focus in this communication has been on rather gross time measures. In contrast, one might evaluate sequential changes at three-month intervals from our present data and, to take one additional step, monthly, even weekly observations, might be subjected to scrutiny. However, the yearly index changes provide an opportunity to map out directions of movement in areas or combinations of areas which have led, so one notes retrospectively, to a favorable or unfavorable course of treatment. The next goal in evaluating treatment would be to describe more continuously the behaviors of a child in a specific area or combination of areas in which change has occurred, to analyze again the procured data in terms of change patterns, to note focal points where the child appears to be in transition from one phase to another in the treatment process, and finally to describe the events preceding and following these nodal points. (PsycINFO Database Record (c) 2013 APA, all rights reserved)
SN - 0002-9432
AD - Meyer, Lucile R.: Division of Child Psychiatry, St. Luke's Hospital, New York, NY, US
ER -

TY - JOUR
ID - 2338
T1 - Patterns of victimization locations in elementary school children: Effects of grade level and gender. [References]
A1 - Fite,Paula J.
Y1 - 2013///

N1 - Peer Reviewed Journal: 2013-39271-006
English
Journal; Peer Reviewed Journal
KW - PsycInfo
KW - Child Abuse Reporting
KW - Elementary School Students
KW - Grade Level
KW - Human Sex Differences
KW - Victimization
KW - Peer Relations
KW - Bullying
RP - NOT IN FILE
SP - 585
EP - 597
JF - Child & Youth Care Forum
VL - 42:
IS - 6
N2 - Background: Little research has examined the locations in which youth are victimized, particularly outside the school context. Further, it is not clear if the locations in which youth are victimized vary as a function of grade level or gender. Objective: The goals of the current study were to: (1) Determine the locations inside and outside of the school context in which elementary school students are most likely to report being victimized, and (2) Examine whether the locations in which victimization takes place varies by grade level and gender. Methods: Associations were examined in a sample of 186 2nd thru 5th grade students (52 % male) who reported experiencing victimization. Results: The playground was the most common place in which victimization was reported, followed by home and the neighborhood. Boys were more likely than girls to report being victimized on the bus or during a sporting activity, while girls were more likely than boys to report being victimized at home. No grade level effects were found, suggesting that specific locations of victimization did not become more or less evident at older grade levels. Conclusions: Findings indicate that there are many locations inside and outside the school context that need to be further monitored for the prevention of victimization and that gender differences may need to be considered. (PsycINFO Database Record (c) 2013 APA, all rights reserved) (journal abstract)
SN - 1053-1890
AD - Fite, Paula J.: Clinical Child Psychology Program, University of Kansas, Lawrence, KS, US Williford, Anne: School of Social Welfare, University of Kansas, Lawrence, KS, US Cooley, John L.: Clinical Child Psychology Program, University of Kansas, Lawrence, KS, US DePaolis, Kathryn: School of Social Welfare, University of Kansas, Lawrence, KS, US Rubens, Sonia L.: Clinical Child Psychology Program, University of Kansas, Lawrence, KS, US Vernberg, Eric M.: Clinical Child Psychology Program, University of Kansas, Lawrence, KS, US
ER -

TY - JOUR
ID - 2339
T1 - An experiment in group psychotherapy. [References]
A1 - Hewitt,Helen
Y1 - 1945///
N1 - Peer Reviewed Journal: 2013-37772-008
English
Journal; Peer Reviewed Journal
KW - PsycInfo
KW - Child Psychotherapy
KW - Group Psychotherapy
KW - Nursery School Students
KW - Therapeutic Camps
KW - Emotional Responses
KW - Needs
KW - Social Behavior
RP - NOT IN FILE

SP - 112
EP - 127
JF - American Journal of Orthopsychiatry
VL - 15:
IS - 1
N2 - This article presents a study in the form of an experiment in group psychotherapy with children. In these times when the large group is becoming more of a social necessity with the increased demand for day camps, nursery schools, and after-school playground supervision, the fate of these socially maladjusted children takes on emphatic significance. In an effort to find a solution for the problem this study was undertaken. An attempt was made to analyze the personal needs of a few of these group misfits and lead them into group living through the medium of a small controlled unit built up unobtrusively within the large group. While the present study was carried out under somewhat different conditions than have heretofore been reported, the principles upon which the group was formed and by which it was handled were similar to those laid down by these previous studies. Selection of members for group psychotherapy was made after the camp season had been under way for a week. Through observations made in the small group, the personal needs of each child became apparent and the interactions of the children studied. The group was under the quiet permissive control of an adult who accepted, with friendly emotional response, each child and his attitudes, and whom each child appeared to trust. (PsycINFO Database Record (c) 2013 APA, all rights reserved)
SN - 0002-9432
AD - Hewitt, Helen: Washington University, School of Medicine, St. Louis, MO, US Gildea, Margaret C.- L.: Washington University, School of Medicine, St. Louis, MO, US
ER -

TY - JOUR
ID - 2340
T1 - Learning potential in youth's online networks: A multilevel approach. [References]
A1 - Unlusoy,Asli; de Haan,Mariette; Leander,Kevin; Volker,Beate
Y1 - 2013///
N1 - Peer Reviewed Journal: 2013-34096-048
English
Journal; Peer Reviewed Journal
KW - PsycInfo
KW - Educational Attainment Level
KW - Learning
KW - Online Social Networks
KW - Digital Computers
KW - Educational Administration
KW - Ethnic Identity
KW - Immigration
RP - NOT IN FILE
SP - 522
EP - 533
JF - Computers & Education
VL - 68 Oct 2013, pp. 522-533.
N2 - Networked learning is currently gaining more attention in the study of digital learning. Little attention has been paid to informal networks outside educational institutes. The current study contributes to this knowledge gap by researching the personal online networks of both immigrant and native youth in the Netherlands while focusing on how youth experience these networks in terms of learning. We mapped the online, personal networks of youth from various ethnic backgrounds and education levels living in the Netherlands. We investigated how these networks mediate learning according to participants' perspectives and whether all youth populations make equal use of their networks' learning potential. We applied an ego-network approach and collected information from 1227 participants (aged 12-18) regarding their online network contacts (e.g., age, gender, location) (in total 6135 contacts) and their interactions. Multilevel regression analyses were conducted respectively to measure the impact of individual and network-characteristics on network activities and to see whether network activities predicted perceived learning potential. The results support the relevance of a network perspective for the study of online learning. The divide between the variances of our two dependent variables - network activities and perceived learning potential- underpin that if we want to understand how young people

learn in digital environments, merely looking at the individual level is not enough. Furthermore, the study showed that dense networks, with similar others and strong bonds with online contacts were fostering the perceived learning in networks, although certain patterns also significantly differed per group. (PsycINFO Database Record (c) 2013 APA, all rights reserved) (journal abstract)
SN - 0360-1315
AD - Unlusoy, Asli: Utrecht University, Faculty of Social Sciences, Utrecht, Netherlands de Haan, Mariette: Utrecht University, Faculty of Social Sciences, Utrecht, Netherlands Leander, Kevin: Peabody College, Vanderbilt University, Nashville, TN, US Volker, Beate: Utrecht University, Faculty of Social Sciences, Utrecht, Netherlands
ER -

TY - JOUR
ID - 2341
T1 - The relationships between Turkish adolescents' internet addiction, their perceived social support and family activities. [References]
A1 - Gunuc,Selim
Y1 - 2013///
N1 - Peer Reviewed Journal: 2013-32387-012
English
Journal; Peer Reviewed Journal
KW - PsycInfo
KW - Family Relations
KW - Internet Addiction
KW - Social Support
KW - Adolescent Attitudes
RP - NOT IN FILE
SP - 2197
EP - 2207
JF - Computers in Human Behavior
VL - 29:
IS - 6
N2 - The main purpose of this research is to investigate the relationships between adolescents' Internet addiction, their perceived social support and the activities carried out with the mother, father and family. The study designed with the survey model involved 166 adolescents who applied to Merkez Efendi Public Hospital Moris Sinasi Children's Clinic, Child Development Polyclinic between February 2012 and June 2012 for problems specific to the period of adolescence. The age range of the participants was between 12 and 18 with the average age of 15.5. The findings of the study revealed a medium level of negative relationship (r = -.37) between perceived social support and Internet addiction. The adolescents spending time with their mothers had a higher level of perceived social support and a lower level of Internet addiction. Also, t-test was applied to see whether the adolescents' perceived social support and Internet addiction differed with respect to the activities they carried out with the mother, father and family. It was found out that a number of activities (watching TV, eating meals, chatting, shopping and spending time outside) the adolescents carried out with their mother increased their level of perceived social support although the levels of Internet addiction did not differ with respect to the types of activities carried out only with the mother, only with the father and with the family. (PsycINFO Database Record (c) 2013 APA, all rights reserved) (journal abstract)
SN - 0747-5632
AD - Gunuc, Selim: Anadolu University, Educational Faculty, Eskisehir, Turkey Dogan, Ayten: Anadolu University, Educational Faculty, Eskisehir, Turkey
ER -

TY - JOUR
ID - 2342
T1 - A repeated measures experiment of green exercise to improve self-esteem in UK school children. [References]
A1 - Reed,Katharine; Wood,Carly; Barton,Jo; Pretty,Jules
Y1 - 2013///

N1 - Peer Reviewed Journal: 2013-28945-001
English
Journal; Peer Reviewed Journal
KW - PsycInfo
KW - Exercise
KW - Repeated Measures
KW - Self Esteem
KW - Environmental Planning
KW - Physical Education
RP - NOT IN FILE
JF - PLoS ONE [Electronic Resource]
JA - PLoS ONE
VL - 8:
IS - 7
N2 - Exercising in natural, green environments creates greater improvements in adult's self-esteem than exercise undertaken in urban or indoor settings. No comparable data are available for children. The aim of this study was to determine whether so called 'green exercise' affected changes in self-esteem; enjoyment and perceived exertion in children differently to urban exercise. We assessed cardiorespiratory fitness (20 m shuttle-run) and self-reported physical activity (PAQ-A) in 11 and 12 year olds (n = 75). Each pupil completed two 1.5 mile timed runs, one in an urban and another in a rural environment. Trials were completed one week apart during scheduled physical education lessons allocated using a repeated measures design. Self-esteem was measured before and after each trial, ratings of perceived exertion (RPE) and enjoyment were assessed after completing each trial. We found a significant main effect (F (1,74), = 12.2, p < 0.001), for the increase in self-esteem following exercise but there was no condition by exercise interaction (F (1,74), = 0.13, p = 0.72). There were no significant differences in perceived exertion or enjoyment between conditions. There was a negative correlation (r = -0.26, p = 0.04) between habitual physical activity and RPE during the control condition, which was not evident in the green exercise condition (r = -0.07, p = 0.55). Contrary to previous studies in adults, green exercise did not produce significantly greater increases in self-esteem than the urban exercise condition. Green exercise was enjoyed more equally by children with differing levels of habitual physical activity and has the potential to engage less active children in exercise. (PsycINFO Database Record (c) 2013 APA, all rights reserved) (journal abstract)
SN - 1932-6203
AD - Reed, Katharine: School of Biological Sciences, University of Essex, Colchester, United Kingdom Wood, Carly: School of Biological Sciences, University of Essex, Colchester, United Kingdom Barton, Jo: School of Biological Sciences, University of Essex, Colchester, United Kingdom Pretty, Jules N.: School of Biological Sciences, University of Essex, Colchester, United Kingdom Cohen, Daniel: Instituto de Investigaciones, Escuela de Medicina, Universidad de Santander, Bucaramanga, Colombia Sandercock, Gavin R. H.: School of Biological Sciences, University of Essex, Colchester, United Kingdom
ER -

TY - JOUR
ID - 2343
T1 - 'Where's the bear? Over there!'-Creative thinking and imagination in den making. [References]
A1 - Canning,Natalie
Y1 - 2013///
N1 - Peer Reviewed Journal: 2013-28755-003
English
Journal; Peer Reviewed Journal
KW - PsycInfo
KW - Childhood Play Behavior
KW - Creativity
KW - Imagination
KW - Thinking
KW - Sociocultural Factors
RP - NOT IN FILE
SP - 1042
EP - 1053

JF - Early Child Development and Care
VL - 183:
IS - 8
N2 - This small scale research project examines opportunities for creative thinking and imagination through den making in a rural private day nursery with its own woodland area on the borders of England and Wales in the UK. The research is underpinned by sociocultural theory and is an ethnographic study of non-participant observations of children aged between three and four years old and early years practitioners involved in supporting their play. The focus is on children's creative play in peer social groups and examines the way in which children explore their environment and utilise their play space and resources to sustain imagination and creativity. The research considers how the environment and den-making context provides opportunities for possibility thinking [Craft, A. (2001). Little c creativity. In A. Craft, B. Jeffrey, & M. Liebling (Eds.), Creativity in education (pp. 45-61). London: Continuum], where children are encouraged to explore 'what if?' questions. The research explores the way in which an outdoor environment can support flexible opportunities and resources where children are able to engage in imaginative and creative play, develop their communication skills and build relationships with other children and adults. The research considers children's fascination with the story 'bears in the wood' and how early years practitioners facilitated their creative thinking and imagination. (PsycINFO Database Record (c) 2013 APA, all rights reserved) (journal abstract)
SN - 0300-4430
AD - Canning, Natalie: Department of Education and Language Studies, Open University, Milton Keynes, United Kingdom
ER -

TY - JOUR
ID - 2344
T1 - Social communication disorder outside autism? A diagnostic classification approach to delineating pragmatic language impairment, high functioning autism and specific language impairment. [References]
A1 - Gibson,Jenny; Adams,Catherine; Lockton,Elaine; Green,Jonathan
Y1 - 2013///
N1 - Peer Reviewed Journal: 2013-36361-004
English
Journal; Peer Reviewed Journal
KW - PsycInfo
KW - Diagnosis
KW - Language Disorders
KW - Linguistics
KW - Pragmatics
KW - Social Interaction
KW - Autism
KW - Etiology
RP - NOT IN FILE
SP - 1186
EP - 1197
JF - Journal of Child Psychology and Psychiatry
VL - 54:
IS - 11
N2 - Background: Developmental disorders of language and communication present considerable diagnostic challenges due to overlapping of symptomatology and uncertain aetiology. We aimed to further elucidate the behavioural and linguistic profile associated with impairments of social communication occurring outside of an autism diagnosis. Methods: Six to eleven year olds diagnosed with pragmatic language impairment (PLI), high functioning autism (HFA) or specific language impairment (SLI) were compared on measures of social interaction with peers (PI), restricted and repetitive behaviours/interests (RRBIs) and language ability. Odds ratios (OR) from a multinomial logistic regression were used to determine the importance of each measure to diagnostic grouping. MANOVA was used to investigate differences in subscale scores for the PI measure. Results: Greater degrees of PI difficulties (OR = 1.22, 95% CI = 1.05-1.41), RRBI (OR = 1.23, 95% CI = 1.06-1.42) and expressive language ability (OR = 1.16, 95% CI = 1.03-1.30) discriminated HFA from PLI. PLI was differentiated from SLI by elevated PI difficulties (OR = 0.82, 95% CI = 0.70-0.96) and higher expressive language ability (OR = 0.88, 95% CI = 0.77-0.98), but indistinguishable from SLI using RRBI (OR = 1.01, 95%

CI=0.94-1.09). A significant effect of group on PI subscales was observed (= 1.38, F(4, 56) = 19.26, p < .01) and PLI and HFA groups shared a similar PI subscale profile. Conclusions: Results provide empirical support for a conceptualisation of PLI as a developmental impairment distinguishable from HFA by absence of RRBIs and by the presence of expressive language difficulties. PI difficulties appear elevated in PLI compared with SLI, but may be less pervasive than in HFA. Findings are discussed with reference to the proposed new category of 'social communication disorder' in DSM-5. (PsycINFO Database Record (c) 2013 APA, all rights reserved) (journal abstract)
SN - 0021-9630
AD - Gibson, Jenny: Department of Developmental Psychiatry, University of Cambridge, Cambridge, United Kingdom Adams, Catherine: Institute of Brain, Behaviour and Mental Health, University of Manchester, Manchester, United Kingdom Lockton, Elaine: Institute of Brain, Behaviour and Mental Health, University of Manchester, Manchester, United Kingdom Green, Jonathan: Institute of Brain, Behaviour and Mental Health, University of Manchester, Manchester, United Kingdom
ER -

TY - JOUR
ID - 2347
T1 - Studies in the psychology of death. [References]
A1 - Alexander,Irving E; Adlerstein,Arthur
Y1 - 1960///
N1 - Book: 2013-25223-003
English
Book; Edited Book
KW - PsycInfo
KW - Death and Dying
KW - Psychology
RP - NOT IN FILE
SP - 65
EP - 92
JF - David, Henry P [Ed]; Brengelmann, J
VL - C [Ed]. :
IS - 1960
N2 - (from the chapter) Each man learns early in life that some day he must die. What role does this information play in his development? How does it affect his aims, his wishes, his behavior? At the present time our best sources of information on these questions are outside the field of psychology-in literature, philosophy, religion, and medicine. While such sources have yielded rich insights about the meaning that death has for human beings, there has been little attempt to apply scientific procedures to select among these ideas. Perhaps it is time for this further step to be taken. It is the purpose of this chapter to describe some primitive attempts, utilizing the more traditional techniques and methods of our science, to study some aspects of man's reaction to death. First let us glance briefly at the state of our knowledge about death as it exists in the psychological literature. (PsycINFO Database Record (c) 2013 APA, all rights reserved)
AD - Alexander, Irving E.: Training Branch, National Institute of Mental Health, Bethesda, MD, US Adlerstein, Arthur M.: Neurological Research Center, Children's Hospital, Philadelphia, PA, US
ER -

TY - JOUR
ID - 2348
T1 - Exit plans for students with disabilities attending separate approved special education programs in the private sector. [References]
A1 - Kellner,Millicent H.
Y1 - 2003///
N1 - Peer Reviewed Journal: 2013-35435-006
English
Journal; Peer Reviewed Journal
KW - PsycInfo
KW - Educational Programs
KW - Private Sector

KW - Special Education
KW - Communication Disorders
KW - Disabilities
KW - Pervasive Developmental Disorders
RP - NOT IN FILE
SP - 85
EP - 97
JF - Career Development for Exceptional Individuals
VL - 26:
IS - 1
N2 - The exit plans of 1,632 students from 15 states attending 92 approved, out-of-district special education programs in the private sector were studied, via a survey instrument, including programs for medical disorders, pervasive developmental and/or communication disorders, and emotional and behavioral disorders. Results indicate that students from programs for medical and pervasive developmental/communication disorders were likely to plan transfers to self-contained programs within their districts, whereas students from emotional/behavioral disorders programs tended to plan transfers to other approved, private programs outside their districts. Among the graduates/aged-out students, those leaving medical and emotional/behavioral disorders programs were likely to plan pursuit of mainstream postschool activities, whereas those from pervasive developmental/communication disorders programs tended to make postschool plans to enter vocational rehabilitation programs. Follow-up studies are needed to determine the degree to which these plans were actually carried out over time. (PsycINFO Database Record (c) 2013 APA, all rights reserved) (journal abstract)
SN - 0885-7288
AD - Kellner, Millicent H.: Project Development, CPC Behavioral Healthcare, High Point Schools, Morganville, NJ, US Strickarz, Barbara: CPC Behavioral Healthcare, High Point Schools, Morganville, NJ, US
ER -

TY - JOUR
ID - 2349
T1 - Feasibility and preliminary efficacy of the Fit4Fun intervention for improving physical fitness in a sample of primary school children: A pilot study. [References]
A1 - Eather,Narelle
Y1 - 2013///
N1 - Peer Reviewed Journal: 2013-30621-003
English
Journal; Peer Reviewed Journal
KW - PsycInfo
KW - Physical Fitness
KW - School Based Intervention
KW - Elementary Schools
KW - Exercise
RP - NOT IN FILE
SP - 389
EP - 411
JF - Physical Education and Sport Pedagogy
VL - 18:
IS - 4
N2 - Objective: The primary objective of this study was to evaluate the feasibility and preliminary efficacy of a school-based physical fitness intervention (Fit4Fun) on the physical fitness and physical activity (PA) levels of primary school children. Methods: A group-randomized controlled trial with a 3-month wait-list control group was conducted in two primary schools in the Hunter Region, NSW, Australia. Participants (n = 48 students; mean age = 10.9 years + 0.7) were randomized by the schools into the Fit4Fun intervention (n = 32 students) or the control (n = 17) conditions. Fit4Fun was an 8-week programme that included: 8 x 60 min health and physical education (HPE) lessons, a break-time activity programme (recess and lunch) and a home fitness programme. The control group participated in their usual weekly 60 min HPE lessons. Assessments were taken at baseline and post-intervention (8-week) to determine changes in health-related fitness (HRF) levels, PA and attitudes towards HRF testing. Objectively measured PA (mean steps/day) was assessed using 7 days of

pedometery and HRF was assessed using a battery of tests including: seven-stage sit-up test, push-up test, basketball throw, wall squat, sit and reach, shoulder stretch, 20 m shuttle test, and height and weight measurements. A questionnaire was also administered to assess perceptions of physical fitness and physical fitness testing and changes in attitudes to fitness testing. Intervention effects were assessed using analysis of covariance and Cohen's d effect sizes are reported. Results: Children in the intervention group improved in all HRF measures with significant group x time effects ($p < 0.05$) observed in the seven-stage sit-up test ($d = 0.9$), the sit and reach tests (right leg $d = 1.0$, left leg $d = 0.9$, both legs $d = 1.1$) and the wall squat tests (right leg $d = 0.9$, left leg $d = 0.6$). No significant group x time effect was found in the beep test, basketball throw, PA measure or psychological measures. The control group did not display significant within-group effects for any measure. Conclusions: Results indicate that a multi-component HRF intervention for primary school children that targeted the three areas of a health-promoting school and incorporated social support for participation in physical fitness activities was feasible and efficacious in improving muscular fitness and flexibility levels of children. (PsycINFO Database Record (c) 2013 APA, all rights reserved) (journal abstract)
SN - 1740-8989
AD - Eather, Narelle: School of Education, Faculty of Education and Arts, University of Newcastle, Callaghan, NSW, Australia Morgan, Philip J.: School of Education, Faculty of Education and Arts, University of Newcastle, Callaghan, NSW, Australia Lubans, David R.: School of Education, Faculty of Education and Arts, University of Newcastle, Callaghan, NSW, Australia
ER -

TY - JOUR
ID - 2350
T1 - Provisions for outdoor play and learning in Slovene preschools. [References]
A1 - Kos,Marjanca
Y1 - 2013///
N1 - Peer Reviewed Journal: 2013-28756-003
English
Journal; Peer Reviewed Journal
KW - PsycInfo
KW - Childhood Play Behavior
KW - Learning
KW - Playgrounds
KW - Preschool Students
KW - Environment
RP - NOT IN FILE
SP - 189
EP - 205
JF - Journal of Adventure Education and Outdoor Learning
VL - 13:
IS - 3
N2 - This study examined play and learning in the natural environment and on the playgrounds of Slovene preschools. It included 140 preschool teachers and 264 parents of children who attended preschools in 21 Slovene towns. Data were collected through questionnaires with questions referring to time spent outdoors, children's outdoor activities, barriers to outdoor play and learning, opinions regarding safety as well as parents' perspectives of outdoor play and learning. The results show that, on average, Slovene children spend 23% of their time at preschool outdoors during the warm months and 13% during the cold months, of which they generally spend three hours per week in the natural environment during the warm months and 1.5 hours during the cold. Answers to the question about children's activities in nature and on the playground indicate that the potentials offered by the outdoor environment are not effectively used. Parents' opinions show a positive attitude towards outdoor play and learning. The majority of both parents and preschool teachers thus share the opinion that Slovene preschools should dedicate more attention and time to play and learning in the natural environment. (PsycINFO Database Record (c) 2013 APA, all rights reserved) (journal abstract)
SN - 1472-9679
AD - Kos, Marjanca: Faculty of Education, University of Ljubljana, Ljubljana, Slovenia Jerman, Janez: Faculty of Education, University of Ljubljana, Ljubljana, Slovenia
ER -

TY - JOUR
ID - 2351
T1 - Manhood, rivalry, and the creation of a Canadian "hockey world": Media coverage of early Stanley Cup Hockey challenges, 1894--1907
A1 - Lorenz,Stacy Lyle
Y1 - 2013///
N1 - Dissertation Abstract: 2013-99170-580
English
Dissertation Abstract
KW - PsycInfo
KW - Athletes
KW - Mass Media
KW - Popular Culture
KW - Rivalry
KW - Sports
KW - Masculinity
KW - Newspapers
RP - NOT IN FILE
SP - No
JF - Dissertation Abstracts International Section A: Humanities and Social Sciences
VL - 74:
IS - 3-A(E
N2 - This study examines media narratives of high-level amateur and professional hockey in Canada during the late nineteenth and early twentieth centuries. In particular, this project analyzes English Canadian newspaper coverage of Stanley Cup "challenge" games and championship series between 1894 and 1907. It assesses local and national newspaper reporting on hockey, as well as the telegraph reconstructions that enabled fans to share a simultaneous experience of distant games. Early Stanley Cup matches are valuable case studies for examining the cultural meanings of hockey in Canada. Media reports and experiences of hockey brought Canadians into local and national communities of interest, while constructing narratives of regional identity, civic boosterism, and community rivalry. Press coverage and telegraph re-enactments of Stanley Cup challenges contributed significantly to the growth of a mediated Canadian "hockey world"-and a broader "world of sport"-during this time period. By 1903, Stanley Cup hockey games had become "national" Canadian events, followed by audiences across the country. Hockey also played an important role in the construction of gender and class identities, and in debates about amateurism, professionalism, and community representation in sport. By examining media descriptions of "brutal" and "strenuous" play, this study explores the connections between violence and manhood in Canadian hockey. Narratives of robust and hard-hitting hockey expressed both ideals of respectable, middle-class masculinity and characteristics of rough, working-class masculinity. In addition, this project analyzes how notions of civic identity changed as hockey clubs evolved from amateur teams represented by players who were "members" of their home community to professional aggregations that included paid imports from outside the town. A growing emphasis on securing the professional athletes that could ensure victory led to praise for a team's efforts to please its supporters, or "customers." By investigating key issues related to media, gender, and community identities in early hockey, this research addresses important gaps in the study of sport history and the analysis of sport and Canadian popular culture. More specifically, this thesis answers the need for careful, scholarly examinations of the cultural narratives attached to Canadian hockey-and the development of the Canadian sports media-in a historical context. (PsycINFO Database Record (c) 2013 APA, all rights reserved)
SN - 0419-4209
AD - Lorenz, Stacy Lyle: U Alberta, Canada
ER -

TY - JOUR
ID - 2352
T1 - Facilitating peer engagement between children with autism and their classmates at school
A1 - Kretzmann,Mark
Y1 - 2013///

N1 - Dissertation Abstract: 2013-99170-246
English
Dissertation Abstract
KW - PsycInfo
KW - Autism
KW - Classmates
KW - Elementary Schools
KW - Peer Relations
KW - School Based Intervention
KW - Psychosocial Readjustment
KW - Student Engagement
RP - NOT IN FILE
SP - No
JF - Dissertation Abstracts International Section A: Humanities and Social Sciences
VL - 74:
IS - 3-A(E
N2 - This study examined an effort to improve peer engagement at elementary school for children with an autism spectrum disorder (ASD). Using a wait-list-controlled design, a manualized psychosocial intervention called Engage was delivered to children with ASD during lunchtime and recess. The initial treatment (IT) group consisted of seven (one female) elementary school students with autism and the wait-list (WL) group contained three (one female) children with ASD. The naturalistic intervention aimed to 1) reduce alone time and increase engagement with peers, 2) increase initiations and responses to peers, 3) activate school staff to facilitate peer engagement for children, and 4) demonstrate that children receiving the Engage intervention perceive more peer engagement and rate their overall lunchtime and recess experience higher than the children in the WL condition. Analyses revealed that the proportion of alone or unengaged time was significantly reduced for the IT group. While time spent engaged was not significantly increased for the IT group at exit, there was a significant treatment effect at midpoint. Child initiations and responses to peers were also significantly increased at midpoint but not at exit. School staff at the site where the initial treatment was delivered showed increased responsive and strategic behaviors around participants at lunch and recess compared to staff at the WL school. Lastly, children receiving the intervention reported playing with more peers and having an overall better lunch and recess experience than those in the wait-list group. (PsycINFO Database Record (c) 2013 APA, all rights reserved)
SN - 0419-4209
AD - Kretzmann, Mark: U California, Los Angeles, US
ER -

TY - JOUR
ID - 2353
T1 - Could exposure to everyday green spaces help treat ADHD? Evidence from children's play settings. [References]
A1 - Taylor,Andrea Faber; Kuo,Frances
Y1 - 2011///
N1 - Peer Reviewed Journal: 2013-12592-004
English
Journal; Peer Reviewed Journal
KW - PsycInfo
KW - Attention Deficit Disorder with Hyperactivity
KW - Childhood Play Behavior
KW - Environmental Effects
KW - Treatment
RP - NOT IN FILE
SP - 281
EP - 303
JF - Applied Psychology: Health and Well-Being
VL - 3:
IS - 3

N2 - Background: An estimated 4.4 million children in the United States suffer from Attention Deficit/Hyperactivity Disorder (ADHD), and most would benefit from a low-cost, side-effect-free way of managing their symptoms. Previous research suggests that after isolated exposures to greenspace, children's ADHD symptoms are reduced. This study examined whether routine exposures to greenspace, experienced through children's everyday play settings, might yield ongoing reductions in ADHD symptoms. Methods: Data on 421 children's ADHD symptoms and usual play settings were collected using a national Internet-based survey of parents. Results: Findings suggest that everyday play settings make a difference in overall symptom severity in children with ADHD. Specifically, children with ADHD who play regularly in green play settings have milder symptoms than children who play in built outdoor and indoor settings. This is true for all income groups and for both boys and girls. Interestingly, for hyperactive children, the apparent advantage of green spaces is true only for relatively open green settings. Conclusions: These and previous findings collectively suggest that it is time for randomized clinical trials testing the impacts of regular exposure to greenspace as a treatment for ADHD. (PsycINFO Database Record (c) 2013 APA, all rights reserved) (journal abstract)
SN - 1758-0846
AD - Taylor, Andrea Faber: University of Illinois at Urbana-Champaign, Urbana, IL, US Kuo, Frances E. (Ming): University of Illinois at Urbana-Champaign, Urbana, IL, US
ER -

TY - JOUR
ID - 2354
T1 - Being with difference: Parenting experiences of gay adoptive fathers
A1 - Vinjamuri,Mohan Krishna
Y1 - 2013///
N1 - Dissertation Abstract: 2013-99151-065
English
Dissertation Abstract
KW - PsycInfo
KW - Adoptive Parents
KW - Life Experiences
KW - Parenting
KW - Fathers
RP - NOT IN FILE
SP - No
JF - Dissertation Abstracts International Section A: Humanities and Social Sciences
VL - 74:
IS - 2-A(E
N2 - Increasingly more gay men are becoming parents or desire to become parents. Families headed by openly gay fathers live in environments that are still largely homophobic and heterosexist. This study describes the challenges, opportunities, and rewards gay adoptive fathers experience at home and in their communities. In-depth phenomenological interviews were conducted with gay adoptive fathers from 20 families (18 gay couples and 2 single gay men). Fathers adopted children through both domestic and international routes. The children of the fathers ranged in age from 9 months to 22 years. Using a social constructionist lens and descriptive phenomenological analysis, themes within each interview and across interviews were identified. The men in this study became parents in a society dominated by the beliefs that all children need a female mother, gay men cannot and should not be parents, and homosexuality is morally wrong. Their parenting stories illuminate social landscapes dotted with evolving attitudes towards gay parenting, structural inequities against LGBT communities, and entrenched beliefs about gender, sexuality and family. While some fathers worked with adoption professionals who challenged these attitudes and advocated for same sex parenting, many fathers regularly encountered heteronormative biases in the adoption system. When asked what it was like to parent as gay men, fathers explained that in many respects their day to day experiences were very similar to those of heterosexual parents, and particularly to other adoptive parents. At the same time, they often faced reminders at home and in public spaces that they were not part of a heterosexual order. From sidewalk to airport, hospital to playground, classroom to cafe, gay fathers and their families drew attention. Fathers regularly encountered questions and comments about the nature of their family. They had to decide if and how to explain themselves or correct others' assumptions, while modeling honesty and pride about their families to their children. Research on families headed by same sex parents has largely focused on the "impacts" gay and lesbian parents have on children's social, emotional and psychological adjustment and the degree to which their families are similar to

families with heterosexual parents. The stories shared in this study move beyond such questions and dive into the heart of being with difference and the meanings difference has for gay fathers, their children and those around them. The fathers provide a vivid picture of their emotional bonds with their children and the strengths and resiliencies they and their children develop living in environments that are largely homophobic and heterosexist. With the information provided by this dissertation, practitioners can challenge heteronormative biases in social work practice, education, and public policy. By revealing the insidious ways heteronormativity "shows up", the results prompt social workers to investigate their beliefs about gay male sexuality and intimacy, the primacy of the heterosexual nuclear family, and conventional notions that a child needs both a female mother and a male father. The father's experiences raise provocative practice questions about nurturing and child rearing. The stories urge practitioners to investigate complex and taken for granted notions about gender and parenthood, and help them engage more sensitively with families headed by same sex parents. (PsycINFO Database Record (c) 2013 APA, all rights reserved)
SN - 0419-4209
AD - Vinjamuri, Mohan Krishna: City U New York, US
ER -

TY - JOUR
ID - 2355
T1 - Urgent expectations and silenced knowledge on spontaneous sport space as public health promoter and sport stimulator. [References]
A1 - Fahlen,Josef
Y1 - 2011///
N1 - Peer Reviewed Journal: 2013-19789-003
English
Journal; Peer Reviewed Journal
KW - PsycInfo
KW - Health Promotion
KW - Physical Activity
KW - Public Health
KW - Sports
KW - Adolescent Development
KW - Childhood Development
RP - NOT IN FILE
SP - 167
EP - 191
JF - EJSS European Journal for Sport and Society
VL - 8:
IS - 3
N2 - This paper takes its departure in a nation-wide program for redesigning school-yards and public spaces to stimulate self-organized physical activity among children and teenagers in Sweden. The purpose of the study was to explore if and how a redesigned schoolyard can promote physical activity among the pupils at the school during the school day. Findings are based on data from 29 interviews, 948 questionnaires and 9 observations at a secondary school in a city in the north of Sweden before and after the renovation of the schoolyard. Results show how the facilities attract first and most already physically active pupils with the less active as spectators. Norms and unspoken rules for usage are established to the benefit of the already physically active, boys and older pupils. This phenomenon is further strengthened by the actual design of the facilities which favors previous experience in sport at the expense of the less experienced. The overall conclusion is that the chances of reaching inactive children and teenagers with this kind of intervention are small. (PsycINFO Database Record (c) 2013 APA, all rights reserved) (journal abstract)
SN - 1613-8171
AD - Fahlen, Josef: Umea University, Department of Education, Umea, Sweden
ER -

TY - JOUR
ID - 2356
T1 - Evaluating interventions to increase physical activity in preschool children with and without disabilities
A1 - Herriott,Shane K.

Y1 - 2013///
N1 - Dissertation Abstract: 2013-99150-454
English
Dissertation Abstract
KW - PsycInfo
KW - Disabilities
KW - Exercise
KW - Obesity
KW - School Based Intervention
KW - Environmental Policy
RP - NOT IN FILE
SP - No
JF - Dissertation Abstracts International Section A: Humanities and Social Sciences
VL - 74:
IS - 2-A(E
N2 - Obesity is a worldwide problem occurring at earlier and earlier ages. Although obesity is a complex problem with multiple contributing factors, one potential component that may be relatively easy for educators to impact is the amount of physical activity children engage in during the school day. Currently, studies show that many preschool-aged children with and without disabilities are not reaching the recommended daily guidelines for physical activity. This study investigated two ways to increase physical activity during recess for 15 preschoolers - 8 with disabilities, 7 without - using an alternating treatment design. Effects of teacher directed activities and increased presence of playground materials on physical activity levels were evaluated using an Actigraph accelerometer and observational data was recorded using a modified version of the Observational System for Recording Activity in Children - Preschool Version (OSRAC-P). Results showed that both interventions were effective in increasing moderate to vigorous (MVPA) physical and decreasing sedentary activity for most participants. Individual results as well as group comparisons are discussed. (PsycINFO Database Record (c) 2013 APA, all rights reserved)
SN - 0419-4209
AD - Herriott, Shane K.: U Washington, US
ER -

TY - JOUR
ID - 2357
T1 - The adolescent with cancer's school re-entry experience: Exploration of predictors and successful outcomes
A1 - Brimeyer,Chasity
Y1 - 2013///
N1 - Dissertation Abstract: 2013-99141-193
English
Dissertation Abstract
KW - PsycInfo
KW - Adolescent Development
KW - Chemotherapy
KW - Neoplasms
KW - Radiation Therapy
KW - School Based Intervention
KW - Schools
RP - NOT IN FILE
SP - No
JF - Dissertation Abstracts International: Section B: The Sciences and Engineering
VL - 74:
IS - 1-B(E
N2 - More adolescents are surviving cancer and being treated outside the hospital-setting, allowing them to return to typical activities of development like attending school. Effective preparation for school re-entry is an important aspect of easing the transition back-to-school following a cancer diagnosis. Previous research has used the terms school re-entry, school re-entry preparation, and school reintegration interchangeably. For the purposes of the current study, school re-entry refers to going back-to-school. School re-entry preparation or

interventions refers to the preparatory process implemented for the initial return to school following a cancer diagnosis. Conversely, school reintegration refers to the ongoing or long-term adjustment of the child or adolescent with cancer to the school environment. Research in school re-entry preparation began in the late 1970s, but much is still not well understood about appropriate school re-entry preparation in terms of application, outcome, or the adolescent's needs. The adolescent with cancer's perspective has been grossly neglected in research. Furthermore, current research lacks an operational definition of "successful school re-entry" and knowledge of specific factors associated with positive school re-entry outcomes. Identifying variables that positively impact the school re-entry process is critical to improving and individualizing school re-entry interventions. The current study used an adaption of the Disability-Stress Coping Model (DSC; Wallander & Varni, 1998) to both define school re-entry success and identify potential predictors that may impact school re-entry success. In addition, to obtain a more comprehensive picture of re-entry success, adolescents with cancer rated the success of their back-to-school experiences. The current study asked 85 adolescents with cancer (ages 11 - 19 years) to complete a web-based survey asking about their re-entry experiences and daily functioning. Results provided tentative support for a discrepancy between professional and adolescent re-entry needs/goals. Findings suggest that a subset of adolescents with cancer may be at risk for poor school re-entry/reintegration outcomes, including females, being of lower SES, having certain types of cancer (brain tumors, leukemia), undergoing specific treatments (radiation, chemotherapy), being out of school for longer periods of time, having pre-morbid academic difficulties, and/or having poor social support. Adolescents who demonstrate these risk factors may warrant increased, specialized attention when preparing to return to school. In addition to systemic and within-person factors from the adapted DSC model, findings suggest that developmental characteristics of adolescence must be considered in planning re-entry intervention. Appropriate modifications to school re-entry preparation for adolescents may include utilizing electronic and social media, implementing peer mentors, focusing on reinforcing appropriate social circles, and emphasizing autonomy. More research is needed to understand how to best assist the adolescent with cancer in returning to school. (PsycINFO Database Record (c) 2013 APA, all rights reserved)
SN - 0419-4217
AD - Brimeyer, Chasity: U Iowa, US
ER -

TY - JOUR
ID - 2358
T1 - Exploring the micro-social geography of children's interactions in preschool: A long-term observational study and analysis using geographic information technologies. [References]
A1 - Torrens,Paul M.
Y1 - 2013///
N1 - Peer Reviewed Journal: 2013-16149-002
English
Journal; Peer Reviewed Journal
KW - PsycInfo
KW - Childhood Play Behavior
KW - Physical Activity
KW - Preschool Students
KW - Social Environments
KW - Social Interaction
KW - Classroom Environment
KW - Cluster Analysis
KW - Geography
KW - Playgrounds
KW - Time
RP - NOT IN FILE
SP - 584
EP - 614
JF - Environment and Behavior
VL - 45:
IS - 5
N2 - The authors describe an observational and analytic methodology for recording and interpreting dynamic microprocesses that occur during social interaction, making use of space-time data collection techniques,

spatial-statistical analysis, and visualization. The scheme has three investigative foci: Structure, Activity Composition, and Clustering. In each case, these are associated with either acquiring resources or using socioenvironmental features to influence social intercourse. For each point of focus, the authors provide an analytic strategy and demonstration of its usefulness, using data generated from a 2.5-year observational study of young children's play behavior. Each tool, and its associated concepts, is used to illustrate how early socializing behavior is embedded in time and space. The results show that geography is a significant catalyst for social dynamics in young children: It provides the opportunity for novel interpretations of sociality along with a better understanding of the influence that geographical factors (location, space, place, spatial structure, spatial composition, landmarks, site) have on the evolving reciprocal interplay between individuals and groups. (PsycINFO Database Record (c) 2013 APA, all rights reserved) (journal abstract)
SN - 0013-9165
AD - Torrens, Paul M.: University of Maryland, Department of Geographical Sciences, College Park, MD, US Griffin, William A.: Arizona State University, School of Social and Family Dynamics, Tempe, AZ, US
ER -

TY - JOUR
ID - 2359
T1 - The effect of background music on bullying: A pilot study. [References]
A1 - Ziv,Naomi
Y1 - 2013///
N1 - Peer Reviewed Journal: 2013-17208-003
English
Journal; Peer Reviewed Journal
KW - PsycInfo
KW - Music
KW - Bullying
KW - School Based Intervention
RP - NOT IN FILE
SP - 83
EP - 90
JF - Children & Schools
VL - 35:
IS - 2
N2 - School bullying is a source of growing concern. A number of intervention programs emphasize the importance of a positive school climate in preventing bullying behavior. The aim of the presented pilot study was to examine whether calming background music, through its effect on arousal and mood, could create a pleasant atmosphere and reduce bullying occurrence. On the first week, 56 sixth-grade students from two classes completed a bullying and arousal questionnaire after the big recess on three consecutive days, as a baseline measurement. On the second week, calming background music was played during the big recess on three consecutive days and children completed the same questionnaires. On the final week, music was removed and participants completed the questionnaires again on three consecutive days. Results showed significantly reduced bullying occurrence, lower arousal levels, and higher enjoyment of recess when music was played. Bullying occurrence increased on the third week, though it remained lower than on the first week. Arousal levels returned to baseline level once music was removed. Results suggest calming background music may be used in combination with other intervention actions to create a positive school atmosphere and reduce aggressive behavior. (PsycINFO Database Record (c) 2013 APA, all rights reserved) (journal abstract)
SN - 1532-8759
AD - Ziv, Naomi: College of Management-Academic Studies, Rishon Le-Zion, Israel Dolev, Einat: Max Stern Academic College, Emek Yizre'el, Israel
ER -

TY - JOUR
ID - 2360
T1 - Teachers' beliefs related to activity play in the preschool setting: A phenomenological investigation
A1 - Pisha,Lorelei Emma
Y1 - 2013///

N1 - Dissertation Abstract: 2013-99131-206
English
Dissertation Abstract
KW - PsycInfo
KW - Exercise
KW - Phenomenology
KW - Project Head Start
KW - Teachers
KW - Attitudes
RP - NOT IN FILE
SP - No
JF - Dissertation Abstracts International Section A: Humanities and Social Sciences
VL - 74:
IS - 1-A(E
N2 - The purpose of the present study was to investigate how early childhood teachers working in Head Start programs made meaning of preschool-age children's physical activity play by exploring their personal childhood and professional experiences with physical activity play. The study was conducted to contribute research-based recommendations for promoting evidence-based practices in young children's physical activity play at inclusive preschools serving diverse children and families. Thirteen early childhood educators from Head Start centers serving children who are ability, culturally, linguistically, and socioeconomically diverse in the greater Washington D.C. area participated in the phenomenological investigation. Data was collected via an in-depth phenomenological interview and two questionnaires. Findings provide research-based evidence that teachers' beliefs about physical activity play influence their practices and may be an important factor impacting the implementation of evidence-based practices in young children's physical activity play at preschool. Teachers' beliefs about the following factors influenced their professional practices related to physical activity play: disability, parents'/caregivers' responsibilities, the teacher's role during outdoor recess, interactions with siblings and peers, administrative policies, practices, and expectations, SES, gender, and the changing culture of childhood. Findings inform the design and delivery of effective professional development initiatives, as well as evidence-based recommendations for professional organizations for evidence-based practices for promoting preschoolers' physical activity play. (PsycINFO Database Record (c) 2013 APA, all rights reserved)
SN - 0419-4209
AD - Pisha, Lorelei Emma: The George Washington U., US
ER -

TY - JOUR
ID - 2362
T1 - Out-of-school literacy activities of affluent early adolescents: Selective competencies and hidden needs
A1 - Hester,Amy
Y1 - 2013///
N1 - Dissertation Abstract: 2013-99111-357
English
Dissertation Abstract
KW - PsycInfo
KW - Adolescent Attitudes
KW - Competence
KW - Literacy
KW - Needs
KW - Self Efficacy
RP - NOT IN FILE
SP - No
JF - Dissertation Abstracts International Section A: Humanities and Social Sciences
VL - 73:
IS - 12-A(E
N2 - Few studies have documented the literacy activities in an after-school setting of affluent early adolescents assigned to remedial reading. This may be because these students are not considered to be at risk of academic failure. The out-of-school literacy activities of 3 sixth-grade students were examined in this qualitative research. Multiple data sources included observations, interviews, detailed field notes, audio and video tapes, and literacy

samples. They were analyzed using grounded theory methods for coding data as well as a priori coding. From the data analysis 4 hypotheses were generated: (a) in addition to staying connected to their peers, participants were using social networking to support their academic work; (b) the participants demonstrated more skills and competencies in their out-of-school literacy practices than standardized tests measure; (c) the participants engaged in personally relevant literacy activities outside of school in which they have a high level of self-efficacy; and (d) participants were creating as well as seeking distractions at different times during the study. The participants each presented a unique literacy profile. John demonstrated an ability to locate different forms of media and combining them (Video and Music). However, his visually sophisticated presentations camouflaged his lack of understanding. He has a high level of self-efficacy as a video game player but those skills are not valued in the school setting. John needs support to further develop his skills and competencies particularly for academic contexts. Sally demonstrated a thorough understanding of concepts. She utilized different sources of information for her project. She persevered when encountering difficulties in school related tasks. The study revealed a discrepancy between her test scores and her skills and competencies. Amanda demonstrated an ability to create multimedia presentations. She has a negative perception of herself as a learner. However, she appears to have a high level of self-efficacy related to technology and computers. She is aware of her challenges and seeks assistance. An awareness of the participants' unique literacy profiles outside of school will work towards expanding our understanding them as learners. (PsycINFO Database Record (c) 2013 APA, all rights reserved)
SN - 0419-4209
AD - Hester, Amy: Fordham U., US
ER -

TY - JOUR
ID - 2363
T1 - Physiological and behavioral engagement in social contexts as predictors of adolescent depressive symptoms. [References]
A1 - Morgan,Judith K.
Y1 - 2013///
N1 - Peer Reviewed Journal: 2013-26189-001
English
Journal; Peer Reviewed Journal
KW - PsycInfo
KW - Adolescent Development
KW - Behavior Analysis
KW - Physiological Stress
KW - Psychosocial Factors
KW - Risk Factors
KW - Human Males
KW - Major Depression
KW - Socioeconomic Status
KW - Symptoms
RP - NOT IN FILE
SP - 1117
EP - 1127
JF - Journal of Youth and Adolescence
VL - 42:
IS - 8
N2 - Depressive symptoms are considered to have evolutionary social functions to reduce social risks with peers and family members. However, social processes and their relationship to depressive symptoms have been understudied in adolescent boys. Low engagement in social contexts may predict depressive symptoms in adolescent boys, as it may signify efforts to reduce social risks. To address these issues, this study focused on 160 boys at risk for affective problems based on low socioeconomic status. We evaluated how behavioral and physiological engagement in peer and family contexts, respectively, in late childhood predicted depressive symptoms at age 12 and age 15. Social withdrawal was measured across late childhood (ages 9-12) in a camp setting using a latent variable of teacher ratings of withdrawn behavior, peer nominations of withdrawn behavior, and camp counselor ratings of withdrawn behavior. Physiological reactivity was measured during a provocative parent-child conversation using respiratory sinus arrhythmia (RSA) at age 12. Social withdrawal in

late childhood predicted depressive symptoms at age 12. The combination of high levels of social withdrawal with peers from ages 9-12 and low RSA reactivity with a parent at age 12 predicted higher depressive symptoms at age 15. Withdrawal in multiple social contexts may place boys at risk for depressive symptoms during the vulnerable period of adolescence. (PsycINFO Database Record (c) 2013 APA, all rights reserved) (journal abstract)
SN - 0047-2891
AD - Morgan, Judith K.: Department of Psychiatry, University of Pittsburgh, Pittsburgh, PA, US Shaw, Daniel S.: Department of Psychology, University of Pittsburgh, Pittsburgh, PA, US Forbes, Erika E.: Department of Psychiatry, University of Pittsburgh, Pittsburgh, PA, US
ER -

TY - JOUR
ID - 2364
T1 - The superheroes social skills program: A study examining an evidence-based program for elementary-aged students with autism spectrum disorders who are frequently bullied
A1 - Segura,Brandon P.
Y1 - 2013///
N1 - Dissertation Abstract: 2013-99110-076
English
Dissertation Abstract
KW - PsycInfo
KW - Evidence Based Practice
KW - Pervasive Developmental Disorders
KW - Social Skills
KW - Social Skills Training
KW - Bullying
KW - Elementary School Students
RP - NOT IN FILE
SP - No
JF - Dissertation Abstracts International Section A: Humanities and Social Sciences
VL - 73:
IS - 12-A(E
N2 - The current study investigated the effectiveness of an evidenced-based social skills program, the Superheroes Social Skills program to determine its effectiveness with children who have autism spectrum disorders (ASD) and have been identified by teachers or parents as being highly bullied at school. Three participants with ASD, between the ages of 6 and 10, received socials skills instruction using the Superheroes Social Skills program. Along with the social skills lessons, Superheroes Social Skills includes lessons that specifically address bullying. All participants received instruction three times a week for 12 weeks. There were also eight normally developing students who attended the lessons and served as peer models. Generalization probes of social interaction during free play periods, in both a research and naturalistic setting, were conducted for each participant in order to determine treatment efficacy. After the implementation of the program, effect sizes (ES), Percentage of All Non-Overlapping Data (PAND), and Percentage of Non-Overlapping Data (PND) were calculated to examine differences in the amount of social interaction during the free play periods. The average total social engagement score for the participants showed a moderate ES using PAND (ES=0.34) and the No Assumptions method (ES=0.42). In the naturalistic setting, which was the playground at recess, large ES were found using PAND (ES=0.92) and the No Assumptions method (ES=0.85). In order to assess the program's impact on the victim's response to bullying, the participants with ASD engaged in bullying role-play scenarios during the intervention. The victim's behavioral responses were coded to determine if any changes were made. Increases in appropriate responding to bullying and the use of appropriate body language were observed across participants. Along with the observational data, the participants' responses on pre- and postmeasures of social responsiveness and victimization were compared. The results of the study suggest increases in social skills and decreases in reports of being a victim of bullying. (PsycINFO Database Record (c) 2013 APA, all rights reserved)
SN - 0419-4209
AD - Segura, Brandon P.: U Utah, US
ER -

TY - JOUR
ID - 2366
T1 - Family literacy activities and parental-child interactive reading with preschoolers from Asian immigrant families in the North Texas area of the United States
A1 - Suwannakhae,Yaowaluk
Y1 - 2013///
N1 - Dissertation Abstract: 2013-99090-582
English
Dissertation Abstract
KW - PsycInfo
KW - Family
KW - Immigration
KW - Literacy
KW - Parent Child Communication
KW - Reading
KW - Preschool Students
KW - South Asian Cultural Groups
RP - NOT IN FILE
SP - No
JF - Dissertation Abstracts International Section A: Humanities and Social Sciences
VL - 73:
IS - 11-A(E
N2 - There were two purposes of this descriptive research design. The first purpose of the study was to investigate family literacy activities with preschool-aged children and parental expectations of their children's development and future school success as reported by Asian immigrant families. The second purpose was to observe the interactive reading behaviors of parents and their preschool-aged children. The online Family Literacy Activities Survey (Payne, Whitehurst, & Angell, 1994) and the Adult-Child Interactive Reading Inventory (DeBruin-Parecki, 2007) were employed for data collection. The survey data was coded and analyzed by using the SPSS 19.0 for Windows. Correlations were calculated to determine the relationships between parental reading behavior categories and child behavior categories. In all, 265 respondents took the Family Literacy Online survey and 25 parent-child dyads voluntarily participated in the video recorded observation of interactive reading sessions. The findings revealed that more than half of the parents were not having any reading difficulty when they were in school. Parents reported that they spent time outside home an average of 3-5 hours a day, and they hardly ever went to the library with their preschool aged child. Parents themselves spent from one to three hours watching television per day, and they allowed their preschool aged children to watch television one to three hours daily. Regarding family reading activities, almost fifty percent of the parents reported that they owned approximately 11-20 picture books. The parents reported that they or another family member began to read to the preschooler at 7-12 months on average. One hundred thirty-seven (52.1%) of the respondents reported that their children requested a parent to read with them "once or twice a month," While one hundred thirty-seven of the parents reported that their children looked at books by themselves once or twice a month. When the preschool aged child was read, more than half of parents responded that their children genuinely liked it. Concerning parental expectations for their children's development, and future school successes, the findings reveal that teacher, school, parent, and child equally share responsibility for the preschooler's development in different aspects including health, school success, social skills, and acquiring reading ability. However, in all of these aspects of development, teachers have a slightly larger share of the overall responsibility except in the area of increasing vocabulary. Parent and child behaviors evident in video recordings of shared reading sessions provided findings that revealed for both adults and children, as measured by the Adult-Child Interactive Reading Inventory (ACIRI), the highest means scores were in the category "Enhancing Attention to Text", while the lowest mean scores were in the category" Using Literacy Strategies". In terms of the relationships between parental reading behavior categories and child behavior categories, the findings demonstrated that the relationships between parental reading behavior categories and child behavior categories were found to be significantly correlated in a positive manner. The results of the study will potentially benefit parents, children, and families by providing child development specialists and early childhood educators with information about the home literacy environments and literacy support activities of Asian immigrant families. (PsycINFO Database Record (c) 2013 APA, all rights reserved)
SN - 0419-4209
AD - Suwannakhae, Yaowaluk: Texas Woman's U., US

ER -

TY - JOUR
ID - 2367
T1 - Matthew effects and the reading-writing connection
A1 - Craig,David James
Y1 - 2013///
N1 - Dissertation Abstract: 2013-99091-058
English
Dissertation Abstract
KW - PsycInfo
KW - Elementary School Students
KW - Reading Comprehension
KW - Writing Skills
RP - NOT IN FILE
SP - No
JF - Dissertation Abstracts International Section A: Humanities and Social Sciences
VL - 73:
IS - 11-A(E
N2 - This study examined the relationship between fifth-grade students' out-ofschool reading habits and measures of their reading comprehension and writing abilities. The sample was composed of forty-two students attending an urban school in Northeastern Massachusetts. Each morning, for fifteen consecutive weeks, students recorded an approximation of the number of minutes they spent on designated out-of-school activities for the previous evening. The total amount of minutes they spent reading was recorded on an evening activity-log and the mean amount of nightly reading volume, per student, was calculated. Student reading comprehension ability was measured by percentile scores on two standardized reading assessments. Students produced written responses in a weekly journal, and these were analyzed by way of two qualitative writing rubrics. A Least Squares Regression model was adopted for the analysis of outcome variables. The regression mode Is explicitly sought to investigate Stanovich's Matthew effects theory (2000), where student reading volume operated as the predictor variable. Results confirmed the study's research hypothesis. Student reading volume provided a correlation coefficient of r=.37 for reading comprehension and r=.61 for writing achievement. Moreover, all regression model results proved to be significant at a p=.05 level. Student reading volume proved to be a significant predictor for both reading and writing outcomes. The findings of this study support the overarching argument of the Matthew effects theory. Students who read a great deal outperformed their peers on measures of both reading comprehension and writing ability. This is because the increased amount of print exposure they experienced resulted in an expansion of their cognitive and linguistic knowledge base. In turn, this increased knowledge base reciprocally affected literacy outcomes. The educational implications of the study are that classroom practices which encourage student reading habits, both inside and outside of the classroom, are critical for all aspects of student literacy development. (PsycINFO Database Record (c) 2013 APA, all rights reserved)
SN - 0419-4209
AD - Craig, David James: Boston U., US
ER -

TY - JOUR
ID - 2368
T1 - Self-esteem and emotional well-being of students in academic difficulty: A study in daily life. [French]. [References]
A1 - Salamon,Reda
Y1 - 2013///
N1 - Peer Reviewed Journal: 2013-13032-006
French
Journal; Peer Reviewed Journal
KW - PsycInfo
KW - Academic Underachievement
KW - Anxiety
KW - Emotional States

KW - Self Esteem
KW - Well Being
KW - Depression (Emotion)
KW - Learning Disorders
RP - NOT IN FILE
SP - 24
EP - 30
JF - Journal de Therapie Comportementale et Cognitive
VL - 23:
IS - 1
N2 - Introduction: Learning difficulties at school are characterized by complex causes and serious consequences both socially and psychologically. Various studies have established the link between learning difficulties and low self-esteem [3-7] as well as their fundamental role in determining the pupil's future and their daily experiences. However, some research has also shown that children with learning difficulties often have other emotional problems, notably higher anxiety and depression scores [9-13], which raises the question of the specific role of self-esteem. The objective of this article is to examine the potentially independent role of these psychological variables (self-esteem, anxiety and depression) and their distinct impact on the daily life of pupils with learning difficulties. Method: Participants: two groups of sixth grade pupils were selected based on their results in national tests in French and Math according to their rate of correct responses in the two subjects (TRC2 M): one group of students "highest school performance" (TRC2M > 80%) and the other "poorest school performance" (TRC2M < 50%). These pupils were chosen from ten randomly selected middle schools in the Gironde: "normal" middle schools (n = 5) or middle schools in educational priority zones ("ZEP"; n = 5). In total, 128 adolescents in sixth grade (55% girls) took part and the average age was 11.44 (SD = .62). Sixty-six of these pupils were doing well and 62 were struggling. Procedure: using the Experience Sampling Method (ESM), the participants were equipped with a PSION REVO palmtop computer for a period of 7 consecutive days. Every time the integrated computer alarm emitted a beep, pupils were invited to respond to a series of questions on the screen. These alarms sounded outside school hours and the pupils were questioned about their physical and social environments, their activities and their moods in their daily lives. Alongside this method, pupils had to respond to clinical questionnaires measuring anxiety, depression, self-esteem, and also provide sociodemographic information. The data was analyzed with the help of multilevel models to examine the role of individual differences concerning self-esteem, anxiety and depression on intra-individual variables, such as emotional states, perceived impact of events and emotional reactivity to stress. Results: Children with learning difficulties had lower self-esteem and greater anxiety or depressive symptomatology than students who were doing well. However, with the ESM method, the results showed that self-esteem was not significantly associated with anxious or depressive moods, or with the perceived impact of negative events. In contrast, the level of anxiety and clinical depression was associated with higher daily levels of anxious or depressive affect, even with a higher perceived impact of daily events. Discussion: Thanks to the ESM method, the independent relationships linking self-esteem, anxiety and depression in the pupil's daily life were analyzed. Contrary to what was observed with the anxiety trait and depression, self-esteem was not significantly associated with negative affect in daily life, with perceived impact of events or with emotional reactivity. These results are useful in the prevention or appropriate treatment as they suggest that distinctive strategies are necessary to improve low self-esteem in relation to other emotional problems in this population. To identify possible therapeutic targets, future studies could examine the links between self-esteem and other aspects of daily life, such as positive emotions, social interactions, or other variables not examined in the current study. (PsycINFO Database Record (c) 2013 APA, all rights reserved) (journal abstract)
SN - 1155-1704
AD - Salamon, Reda: INCIA, CNRS, UMR 5287, Universite Bordeaux 2, Bordeaux, France Husky, Mathilde M.: INCIA, CNRS, UMR 5287, Universite Bordeaux 2, Bordeaux, France Swendsen, Joel D.: INCIA, CNRS, UMR 5287, Universite Bordeaux 2, Bordeaux, France
ER -

TY - JOUR
ID - 2369
T1 - Nature trips and traditional methods for food procurement in relation to weight status. [References]
A1 - Bere,Elling
Y1 - 2013///

N1 - Peer Reviewed Journal: 2013-06647-010
English
Journal; Peer Reviewed Journal
KW - PsycInfo
KW - Body Weight
KW - Food
KW - Physical Activity
KW - Nature (Environment)
KW - Body Mass Index
KW - Human Sex Differences
KW - Parent Child Relations
KW - Parent Educational Background
KW - Recreation
KW - Traditions
RP - NOT IN FILE
SP - 180
EP - 184
JF - Scandinavian Journal of Public Health
JA - Scand J Public Health
VL - 41:
IS - 2
N2 - Aims: The purpose of this study is to assess the relationships between trips in nature, gathering of wild plants, fishing and hunting and weight status. Methods: Data from a cross-sectional questionnaire survey of 996 parents of sixth- and seventh-graders from 38 randomly chosen schools in two Norwegian counties. All data are self-reported: Weight and height (participants were considered as overweight if BMI were 25 or higher), family trips in nature (dichotomized into >once a week vs. less than once a week), gathering of wild plants/mushrooms, fishing and hunting (all dichotomized into >sometimes vs. never), sex, family education level and general physical activity level. Multivariate logistic regression analyses were performed with overweight as the dependent variable Results: Adjusting for all outdoor activities; those engaging in nature trips (OR = 0.52; 95% CI = 0.37-0.75) and those engaging in gathering (OR = 0.73; 95% CI = 0.55-0.98) were less frequently overweight, while those fishing (OR = 1.83; 95% CI = 1.35-2.47) were more frequently overweight. After also adjusting for sex, family education level and general physical activity level, nature trips (OR = 0.52; 95% CI = 0.36-0.75) and fishing (OR = 1.53; 95% CI = 1.12-2.10) were still significant, gathering was not. No association between hunting and weight status was observed. Conclusion: Frequent family trips in nature might be an important behaviour in order to reverse the obesity epidemic. (PsycINFO Database Record (c) 2013 APA, all rights reserved) (journal abstract)
SN - 1403-4948
AD - Bere, Elling: Department of Public Health, Sport and Nutrition, University of Agder, Kristiansand, Norway Westersjo, Johne H.: Department of Public Health, Sport and Nutrition, University of Agder, Kristiansand, Norway
ER -

TY - JOUR
ID - 2370
T1 - Effects of an intervention program for supporting school entry by children with developmental disabilities. [Japanese]. [References]
A1 - Yamamoto,Shinya; Kagami,Yuko; Tamura,Yukari; Higashikawa,Hiroaki; Isawa,Shinzo
Y1 - 2012///
N1 - Peer Reviewed Journal: 2012-23397-007
Japanese
Journal; Peer Reviewed Journal
KW - PsycInfo
KW - Developmental Disabilities
KW - School Based Intervention
KW - Special Education
KW - Training
RP - NOT IN FILE

SP - 65
EP - 74
JF - Japanese Journal of Special Education
VL - 50:
IS - 1
N2 - The present study examined effectiveness of a training program for the transition from kindergarten to elementary school by children with developmental disabilities. The purpose of the training program was to teach the children skills needed in order to participate in classes in elementary school. Participants were 5 senior kindergarten students with developmental disabilities. The 4-session training program was conducted in the elementary school that the participants would be entering. In the first session, the children introduced themselves and did exercises, such as with a trampoline and balance beam. The second session consisted of activities such as how to use the nurse's office and the lunch room. The third session consisted of arts and crafts, and the fourth, of language training and stacking blocks. In addition, all sessions included practice on greetings for the start of the class, recess, and free time, including greetings when entering the classroom, greetings at the start of class, raising one's hand for permission to ask a question, watching the presenter, and standing in line. Children who performed a target behavior correctly were praised; if they did not practice the target behavior, the trainer prompted them to do so. The children's target behaviors were evaluated through video recordings of the children and from questionnaires completed by the trainers. In addition, after the children had entered elementary school, their behavior was assessed there. The assessments indicated that after the training, all the children had acquired most of the target behaviors, and did them even after the end of the training program. The present results suggest that the training program was effective in assisting these children in the transition from kindergarten to elementary school. (PsycINFO Database Record (c) 2013 APA, all rights reserved) (journal abstract)
SN - 0387-3374
AD - Yamamoto, Shinya: Hyogo Prefectural Inamino Special Support School, Inami-Cho, Japan Kagami, Yuko: Hyogo Prefectural Awaji Special Support School, Sumoto-Shi, Japan Tamura, Yukari: Kobe Municipal Seiyou-Higashi Special Support School, Kobe-Shi, Japan Higashikawa, Hiroaki: Akashi Municipal Center for Developtnental Support, Akashi-Shi, Japan Isawa, Shinzo: Hyogo University of Teacher Education, Kato-Shi, Japan
ER -

TY - JOUR
ID - 2371
T1 - A person-context assessment of the prevalence and associated social-academic outcomes of self-care for children and adolescents
A1 - Parente,Maria Emanuela
Y1 - 2013///
N1 - Dissertation Abstract: 2013-99060-499
English
Dissertation Abstract
KW - PsycInfo
KW - Academic Aptitude
KW - Adolescent Attitudes
KW - Childhood Development
KW - Self Care Skills
KW - Peers
KW - Personality Measures
KW - Well Being
RP - NOT IN FILE
SP - No
JF - Dissertation Abstracts International: Section B: The Sciences and Engineering
VL - 73:
IS - 9-B(E
N2 - Following a bioecological framework, this dissertation closely examines the prevalence, predictors, and associated outcomes of self care (unsupervised time outside of school). The dissertation utilizes the nationally representative Panel Study of Income Dynamics Child Development Supplement (N=2,570, ages 5-18) and the longitudinal NICHD Study of Early Child Care (N = 959, ages 8-10), two large-scale national datasets that offer

detailed time-diary information as to child and adolescent activities and supervision. Both variable- and pattern-based approaches were used to create self-care types based on three contextual features (i.e., supervision, location, and the presence of peers during self care) in order to elucidate: (A) factors that predict the use of child and adolescent self care, and (B) the circumstances under which child and adolescent self care are associated with study outcomes. Overall results suggest that self care is (1) a common context for children and adolescents, (2) that the type of self care utilized differs according to several characteristics (e.g., amount of time in self care, child age, family income-to-needs, and neighborhood poverty), and (3) that associated outcomes of self care differ by self care contextual features. More specifically, the location of and peer association during self care are associated with several study outcomes, including higher externalizing behavior (adolescents but not for younger groups), lower well-being, and higher body mass index. Relations between self care and academic outcomes were not statistically significant. (PsycINFO Database Record (c) 2013 APA, all rights reserved)
SN - 0419-4217
AD - Parente, Maria Emanuela: U California, Irvine, US
ER -

TY - JOUR
ID - 2372
T1 - Reliability and validity of MoMo-Physical-Activity-Questionnaire for Adolescents (MoMo-AFB). [German]. [References]
A1 - Jekauc,Darko
Y1 - 2013///
N1 - Peer Reviewed Journal: 2013-10388-004
German
Journal; Peer Reviewed Journal
KW - PsycInfo
KW - Adolescent Attitudes
KW - Physical Activity
KW - Psychometrics
KW - Test Reliability
KW - Test Validity
KW - Questionnaires
RP - NOT IN FILE
SP - 100
EP - 110
JF - Diagnostica
VL - 59:
IS - 2
N2 - This paper presents the psychometric properties of the MoMo-AFB (Bos, Worth, Opper, Oberger & Woll, 2009), a questionnaire designed to measure the physical activity of children and adolescents. The MoMo-AFB consists of 28 items, covering the fields of everyday physical activity, sports within and outside of organised clubs, physical education as well as compliance with physical activity guidelines. A study was conducted with 109 boys and 87 girls aged between 9 and 17 years. The MoMo-AFB was given on two separate occasions to evaluate reliability and was compared with accelerometry data of ActiGraph GT1X to evaluate validity. The overall kappa/weighted kappa coefficient for the one-week testretest reliability was .66 (SD = .19) and the overall intraclass correlation (ICC) .68. Self-reported and accelerometer-measured average weekly time spent performing moderate to vigorous physical activity were significantly correlated (Spearman r = .29). Regarding reliability and validity, the MoMo-AFB is comparable with other international published physical activity questionnaires for adolescents. (PsycINFO Database Record (c) 2013 APA, all rights reserved) (journal abstract)
SN - 0012-1924
AD - Jekauc, Darko: Universitat Konstanz, Fachgruppe Sportwissenschaft, Konstanz, Germany Wagner, Matthias O.: Abteilung fur Sport und Bewegung, Fakultat I Institut fur Gesundheitswissenschaften, Padagogische Hochschule Schwabisch Gmund, Schwabisch Gmund, Germany Kahlert, Daniela: Universitat Potsdam, Potsdam, Germany Woll, Alexander: Universitat Konstanz, Fachgruppe Sportwissenschaft, Konstanz, Germany
ER -

TY - JOUR
ID - 2373
T1 - A case study of teaching phonemic awareness to parents and children: Scaffolded preschool tutoring with kinesthetic motions for phonemes
A1 - Kindervater,Terry M.
Y1 - 2013///
N1 - Dissertation Abstract: 2013-99050-254
English
Dissertation Abstract
KW - PsycInfo
KW - Parents
KW - Phonemes
KW - Phonological Awareness
KW - Teaching
KW - Tutoring
KW - Childhood Development
KW - Preschool Students
RP - NOT IN FILE
SP - No
JF - Dissertation Abstracts International Section A: Humanities and Social Sciences
VL - 73:
IS - 9-A(E
N2 - The purpose of this case study was to examine the interactions between parents and children as they participated in weekly tutoring sessions to develop phonemic awareness (PA) with the use of kinesthetic motions for the phoneme (KMP). The study examined how the parents extended the learning and explored the changes in their literacy understandings. In addition, it documented the children's growth in PA. The four major sources of data of the study were: field notes constructed from recordings of the tutoring sessions and dyads reading a poem at the beginning and end of the study; parental logs; interviews; and literacy measures of the children. The study chronicled the change in the parents as they developed the ability to teach PA. The parent-child interactions demonstrated the parents acted as effective tutors. They scaffolded attending to sound with KMPs and developed the following understandings through the use of poetry: to direct their children to attend to print non-verbally by pointing and verbally with the use of specific language for directionality, letters and punctuation. The children developed PA, as evidenced in their use of KMPs outside of the sessions, in their identification of letter sounds and ability to encode phonemes. The parents' guidance of the children to text resulted in growth in concepts about print. The implications of the study include the need to incorporate concrete markers, such as the KMP, with preschoolers in PA activities, the need to teach parents to scaffold interaction with text through pointing, and the need to seriously consider engaging parents in academic tutoring. (PsycINFO Database Record (c) 2013 APA, all rights reserved)
SN - 0419-4209
AD - Kindervater, Terry M.: Kent State U., US
ER -

TY - JOUR
ID - 2374
T1 - The impact of preschoolers' gender and sensory preferences on physical activity levels during play. [References]
A1 - Ismael,Noor Taleb; Lawson,Lisa Mische
Y1 - 2012///
N1 - Peer Reviewed Journal: 2012-27751-005
English
Journal; Peer Reviewed Journal
KW - PsycInfo
KW - Activity Level
KW - Childhood Play Behavior
KW - Human Sex Differences
KW - Physical Activity
KW - Preschool Students

RP - NOT IN FILE
SP - 429
EP - 435
JF - The British Journal of Occupational Therapy
VL - 75:
IS - 9
N2 - Introduction: This study examined the differences between boys and girls in relation to activity levels during play and sensory preferences for activity level, body position and movement. A review of the literature revealed no evidence to explain if boys and girls prefer different body positions while playing or if body position might be used to measure their activity levels. Method: This retrospective study used data from demographic forms, children's Sensory Profiles and observations of children's play in classroom settings. The sample consisted of 53 typically developing children between ages 3-5 years from a suburban preschool in Kansas in the United States. Results: Analysis of data from play observations revealed no significant differences between boys and girls in relation to body position mean (p = 0.33), body position standard deviation (p = 0.19) and total number of change position times (p = 0.28). Analysis of data from children's Sensory Profiles showed no significant differences (p = 0.97) between boys' and girls' seeking preferences reflected by activity level, body position and movement items in the Sensory Profile. Conclusion: Future research should investigate children's play in outdoor playgrounds and implement more rigorous methods in rating activity levels. (PsycINFO Database Record (c) 2013 APA, all rights reserved) (journal abstract)
SN - 0308-0226
AD - Ismael, Noor Taleb: Department of Rehabilitation Sciences, Faculty of Applied Medical Sciences, Jordan University of Science and Technology, Irbid, Jordan Lawson, Lisa Mische: University of Kansas Medical Centre, Kansas City, KS, US
ER -

TY - JOUR
ID - 2375
T1 - A peer-mediated play intervention for preschoolers with social and play deficits
A1 - Klug,Ashley Moeller
Y1 - 2013///
N1 - Dissertation Abstract: 2013-99040-344
English
Dissertation Abstract
KW - PsycInfo
KW - Intervention
KW - Peers
KW - Social Skills
KW - Childhood Play Development
KW - Pretend Play
RP - NOT IN FILE
SP - No
JF - Dissertation Abstracts International: Section B: The Sciences and Engineering
VL - 73:
IS - 8-B(E
N2 - Play constitutes a significant portion of childhood and is a natural setting in which children display their understanding of the world and practice social skills. The current study measured the effects of a play intervention on the development of play and social skills among three four-year-old preschoolers who displayed low-level skills in both domains. The intervention involved 12 30-minute sessions following a plan-play-review structure, adult facilitation of social and pretend play, and inclusion of a peer model to reinforce newly learned skills. All three children improved their play skills according to observational data from the PIECES. Two children increased their time spent in pretend play versus exploratory play, and one child increased time spent involved in play versus nonplay. None of the children increased the frequency of their social interactions with playmates as a result of the intervention, indicating these children may benefit from more direct social skills instruction. (PsycINFO Database Record (c) 2013 APA, all rights reserved)
SN - 0419-4217
AD - Klug, Ashley Moeller: U Nebraska at Omaha, US
ER -

TY - JOUR
ID - 2376
T1 - Self-monitoring as a means to generalize social skills
A1 - Childs,Deborah
Y1 - 2013///
N1 - Dissertation Abstract: 2013-99030-438
English
Dissertation Abstract
KW - PsycInfo
KW - Self Monitoring
KW - Social Skills
RP - NOT IN FILE
SP - No
JF - Dissertation Abstracts International Section A: Humanities and Social Sciences
VL - 73:
IS - 8-A(E
N2 - Social skills are important for success in school as well as in life. Social skills training (SST) has been effective in teaching students required skills within the context of the training site, but often these have not generalized to additional settings (DuPaul & Eckert, 1994). This study researches the effect of adding self-management to an existing training program to determine if it will increase generalization of learned behaviors. Elementary students in a mid-west school were taught social skills in a pull-out program, but those skills did not generalize for many of those students into the classroom or other settings. Five students were then selected for additional intervention by teacher and counselor nominations. Each student had a target social skill that was identified for improvement. The target skill was assessed using multiple measures such as records of student discipline, teacher ratings, student ratings, grades, teacher survey, parent survey, and student survey. Adding a self-management system for four weeks increased generalization of learned social skills to other settings such as classroom, playground, cafeteria, art, and music classes. Implications for practice and future research are discussed. (PsycINFO Database Record (c) 2013 APA, all rights reserved)
SN - 0419-4209
AD - Childs, Deborah: U Missouri - Saint Louis, US
ER -

TY - JOUR
ID - 2377
T1 - English language learners' reading self-efficacy and achievement using 1:1 Mobile learning devices
A1 - Walters,Jennifer L.
Y1 - 2013///
N1 - Dissertation Abstract: 2013-99030-414
English
Dissertation Abstract
KW - PsycInfo
KW - Academic Achievement
KW - Foreign Language Learning
KW - Self Efficacy
KW - Technology
KW - Cellular Phones
KW - English as Second Language
KW - Internet
KW - Language
KW - Reading
RP - NOT IN FILE
SP - No
JF - Dissertation Abstracts International Section A: Humanities and Social Sciences
VL - 73:
IS - 8-A(E

N2 - Handheld technology devices allow users to be mobile and access the Internet, personal data, and third-party content applications in many different environments at the users' convenience. The explosion of these mobile learning devices around the globe has led adults to value them for communication, productivity, and learning. Outside of the school setting, many adolescents and children have access to, or own mobile learning devices. The use of these devices by children on a daily basis in schools is a relatively new phenomenon, with just four percent of elementary students doing so in classrooms in 2010 (Gray, Thomas & Lewis, 2010). This mixed methods study researched a one-to-one implementation of 287 mobile technology devices, the iPod touch, in fourth- and fifth-grade elementary classrooms over eight months. The focus was to explore the mobile learning device's relationship to English language learners' reading achievement levels, to English language learners' self-efficacy in reading English, and to explore the benefits and limitations of the device's daily use as perceived by the students. The hypothesis was that the practice of reading and related literacy activities with the iPod touch would augment English learners' vicarious learning experiences, and thereby effect student cognitive engagement, reading self-efficacy, and reading academic achievement. The study used validated surveys and assessments to measure students' beliefs about reading and their knowledge of reading. Additionally, English language learner interview data were collected and analyzed to uncover perceived benefits and limitations of utilizing 1:1 mobile learning devices for daily literacy activities. Analysis of the data revealed significantly elevated levels of self-efficacy in reading for the experimental group with 1:1 handheld technology, while academic gains in reading for the experimental and control groups were statistically similar. Students in the experimental group described a virtually-enhanced socio-cultural context for communicating and learning with the handheld technology. Implications for practice, policy, leadership, and future research are discussed. (PsycINFO Database Record (c) 2013 APA, all rights reserved)
SN - 0419-4209
AD - Walters, Jennifer L.: U California, San Diego, US
ER -

TY - JOUR
ID - 2379
T1 - "I was born in the hood": Fear of crime, outdoor recreation and physical activity among Mexican-American urban adolescents. [References]
A1 - Stodolska,Monika
Y1 - 2013///
N1 - Peer Reviewed Journal: 2012-35083-001
English
Journal; Peer Reviewed Journal
KW - PsycInfo
KW - Adolescent Development
KW - Crime
KW - Mexican Americans
KW - Physical Activity
KW - Recreation
KW - Environmental Stress
KW - Fear
KW - Urban Environments
RP - NOT IN FILE
SP - 1
EP - 15
JF - Leisure Sciences
VL - 35:
IS - 1
N2 - The study examines how perceptions of crime affect outdoor recreation and physical activity among Mexican-American youth of different ages and how Mexican-American youth negotiate constraints related to fear of crime. Theories of environmental stress and human territorial functioning theory are used to frame the findings of the study. In-depth interviews were conducted with 25 Mexican-American adolescents ages 11-18 residing in Chicago, Illinois. The findings show that crime prevents youth from visiting parks or places that require crossing gang boundaries, and that fear restricts participation in outdoor recreation. Activities that take place in the vicinity of homes and on school property during school hours, as well as activities that are organized and supervised by adults, are considered safer than unorganized and unsupervised activities.

Adolescents use negotiation strategies to foster their participation in outdoor recreation and physical activity. (PsycINFO Database Record (c) 2013 APA, all rights reserved) (journal abstract)
SN - 0149-0400
AD - Stodolska, Monika: Department of Recreation, Sport and Tourism, University of Illinois at Urbana-Champaign, Champaign, IL, US Shinew, Kimberly J.: Department of Recreation, Sport and Tourism, University of Illinois at Urbana-Champaign, Champaign, IL, US Acevedo, Juan Carlos: Department of Recreation, Sport and Tourism, University of Illinois at Urbana-Champaign, Champaign, IL, US Roman, Caterina G.: Department of Criminal Justice, Temple University, Philadelphia, PA, US
ER -

TY - JOUR
ID - 2380
T1 - "Superheroes in the resource room": A study examining implementation of the Superhero Social Skills program by a resource teacher with students with externalizing behavior problems
A1 - Springer,Benjamin James
Y1 - 2013///
N1 - Dissertation Abstract: 2013-99031-164
English
Dissertation Abstract
KW - PsycInfo
KW - Behavior Problems
KW - Behavioral Assessment
KW - Resource Teachers
KW - School Based Intervention
KW - Social Skills Training
KW - Aggressive Behavior
KW - Social Skills
RP - NOT IN FILE
SP - No
JF - Dissertation Abstracts International Section A: Humanities and Social Sciences
VL - 73:
IS - 8-A(E
N2 - The current study evaluated the effectiveness of the Superhero Social Skills program in increasing the social engagement skills and decreasing the aggressive behavior of students with externalizing behavior problems as implemented by a resource teacher. There have been no empirical evaluations of the Superhero Social Skills program as implemented by educators other than graduate students in school psychology familiar with the program. The Superhero Social Skills program was administered to 5 elementary age participants with training occurring for 30 minutes twice per week in a public elementary school. The participating resource teacher taught 11 lessons from the program across 11 weeks. Effects were primarily assessed through a momentary time sampling procedure during both analog (structured free play) and recess (unstructured play) situations, for intervention and follow-up phases. The momentary time sampling recorded each participant's percentage of time spent socially engaged (initiating and responding) and/or engaged in aggression (verbal and physical). Both effect sizes and the percentage of all nonoverlapping data points (PAND) were calculated. Pre- and posttreatment effects were also evaluated for the Social Skills Improvement System (SSIS) and the Behavior Assessment System for Children, 2nd Edition (BASC-2). Consumer satisfaction, social validity, and treatment integrity factors were also examined. Results indicated that the Superhero Social Skills program had a small effect in enhancing participants' social engagement with peers and had a medium effect in reducing aggressive behavior of student participants. Positive social engagement was observed to generalize from the instructional setting to the natural setting of recess. The current study also found that the Superhero Social Skills program was implemented with a high rate of fidelity (85%) by a resource teacher unfamiliar with the program as measured by treatment integrity probes across each lesson. Additionally, the participating resource teacher rated the program as easy to implement and the Superhero Social Skills program was rated as being socially valid, acceptable, and effective by the student participants, parents, and teachers. (PsycINFO Database Record (c) 2013 APA, all rights reserved)
SN - 0419-4209
AD - Springer, Benjamin James: U Utah, US
ER -

TY - JOUR
ID - 2381
T1 - Varencline in autistic disorder: Hypothesis and case report of single-patient crossover. [References]
A1 - Arnold,L.
Y1 - 2013///
N1 - Peer Reviewed Journal: 2013-05755-012
English
Journal; Peer Reviewed Journal
KW - PsycInfo
KW - Autism
KW - Drug Therapy
RP - NOT IN FILE
SP - 61
EP - 64
JF - Journal of Child and Adolescent Psychopharmacology
VL - 23:
IS - 1
N2 - Presents a case report of a 9-year-old boy had an established diagnosis of autistic disorder, made at 3 years of age because of delayed language (he did not speak until he was almost 4 years of age), idiosyncratic language, stereotyped movements and play, sensory intolerances, unresponsiveness to instruction, poor social skills, poor eye contact, emotional distance, disregard of people, heedlessness of real danger (darting, running into street), and irrational fear of harmless situations/objects. After some instruction in behavior modification, his parents trained him to use words and observe some social amenities. Once he attained language, it became obvious that he was high-functioning, with clinical intelligence quotient (IQ) estimate above average. His parents had heard of the nicotinic receptor deficiency hypothesis for autism described previously, and requested a trial of varenicline, knowing the risks. A cautious trial was initiated, with availability of phone consultation; the parents were given a copy of the Ohio Autism Clinical Impressions Scale to plot his progress. At that point (age 9) he weighed ~40 kg. He started with half of a 0.5 mg tablet per day for a week and then was given 0.5 mg/day. Within a month, observers outside the family were spontaneously commenting about the improvement in conversational ability, social interaction, and reasoning/coherence. He was continuing his 10 mg atomoxetine. To our knowledge, this is the first report of an individual with autism treated with varenicline, an 4b2 nicotinic receptor partial agonist. The unintended ABAB treatment experience made an experiment of nature with rather encouraging results. The parents were so impressed with the results that for several months they continued to buy varenicline for him at considerable expense (because the off-label use is not covered by their insurance). They share the prescriber's concerns about long-term safety and volunteered to have this case study published to promote research in a more controlled fashion. (PsycINFO Database Record (c) 2013 APA, all rights reserved)
SN - 1044-5463
AD - Arnold, L. Eugene: Department of Psychiatry, Ohio State University, Columbus, OH, US Anand, Rene: Department of Pharmacology, Ohio State University, Columbus, OH, US Aman, Michael: Departments of Psychiatry, Ohio State University, Columbus, OH, US
ER -

TY - JOUR
ID - 2382
T1 - The intersection of physical activity, self-regulation and academic achievement: Implications for educational success
A1 - El Nokali,Nermeen E.
Y1 - 2013///
N1 - Dissertation Abstract: 2013-99020-455
English
Dissertation Abstract
KW - PsycInfo
KW - Academic Achievement
KW - Age Differences
KW - Educational Programs
KW - Physical Activity

KW - Self Regulation
KW - Childhood Development
KW - Exercise
KW - Obesity
RP - NOT IN FILE
SP - No
JF - Dissertation Abstracts International: Section B: The Sciences and Engineering
VL - 73:
IS - 7-B(E
N2 - In recent years young children have concurrently experienced startling rises in their rates of obesity and stark reductions in their opportunities for physical activity. These trends have potentially serious implications for young children's school performance. However, much of the current physical activity research is concerned with health-related outcomes and the prediction of physical activity. Less work has examined the influence of activity on other domains of child development, such as academic and social skills. The current dissertation is comprised of two studies examining opportunities for and levels of physical activity in two distinct developmental periods (preschool and elementary school) and elucidating associations of physical activity with children's self-regulation and achievement. Examining two independent samples facilitated comparisons of physical activity characteristics and associations across age groups, school settings and demographic contexts. The first study consisted of a within-group analysis of a primarily low-income minority subsample of 4 and 5 year-old preschoolers (N = 104) drawn from the Pitt School Readiness Study, a study of preschoolers from the metropolitan Pittsburgh area. Results from this examination suggest that, although children's moderate to vigorous activity was not related to self-regulation or achievement, opportunities for physical activity seemed important. Specifically, more time in free play predicted worse attention, and more time in recess predicted more externalizing behavior, less self-control, and worse math achievement. In contrast, more physical education time predicted better reading and math skills. The second study consisted of analyses on a large, economically and ethnically diverse sample of third and fifth graders (N = 993) and a low income subsample (n = 297). Across these samples, more physical education emerged as a positive predictor of self-control but more recess time was negatively associated with math achievement. Accelerometry measured physical activity was not predictive across outcomes. Finally, post-hoc examinations revealed that attention, self-control, and externalizing behaviors acted as possible agents of indirect associations between opportunities for physical activity and achievement. Overall, findings across both studies suggest that children benefit most from physical activity that is structured and regularly scheduled within school settings. Furthermore, large quantities of unstructured activity seemed detrimental for self-regulation and achievement. (PsycINFO Database Record (c) 2013 APA, all rights reserved)
SN - 0419-4217
AD - El Nokali, Nermeen E.: U Pittsburgh, US
ER -

TY - JOUR
ID - 2384
T1 - The potential of mobile technologies to connect teaching and learning inside and outside of the classroom. [References]
A1 - Hooft,Mark van't
Y1 - 2013///
N1 - Book: 2012-32391-012
English
Book; Edited Book
KW - PsycInfo
KW - Learning
KW - Teaching
KW - Technology
KW - Mobile Devices
KW - Classrooms
RP - NOT IN FILE
SP - 175
EP - 186
JF - Mouza, Chrystalla [Ed]; Lavigne, Nancy [Ed]

VL - :
IS - 2013
N2 - (from the chapter) Rapid developments in mobile devices and pervasive wireless networks continuously redefine the ways in which we live, work, play, and learn. In fact, wireless mobile devices enable anytime and anywhere access to digital tools and resources in popular and easy-to-use formats and allow instant communication across time and place. As such, they place an ever-increasing amount of control in our hands and enable us to have rich user experiences as we access, aggregate, create, customize, and share digital information in a variety of formats. These unique characteristics and capabilities of wireless mobile devices provide exciting opportunities for teaching and learning that can change what is "pedagogically possible". Specifically, their mobility and connectivity allow students to access information at their fingertips and connect their learning to the outside world across a variety of real and digital contexts. The unique affordances of mobile technologies and their implications for teaching and learning are the focus of this chapter. (PsycINFO Database Record (c) 2013 APA, all rights reserved)
AD - Hooft, Mark van't: Kent State University, Kent, OH, US
ER -

TY - JOUR
ID - 2385
T1 - Beyond the scores: Mathematics identities of African American and Hispanic fifth graders in an urban elementary community school
A1 - Fleshman,Paula Jenniver
Y1 - 2013///
N1 - Dissertation Abstract: 2013-99010-422
English
Dissertation Abstract
KW - PsycInfo
KW - Communities
KW - Elementary School Students
KW - Mathematics
KW - Urban Environments
KW - Latinos/Latinas
KW - Blacks
KW - Schools
RP - NOT IN FILE
SP - No
JF - Dissertation Abstracts International Section A: Humanities and Social Sciences
VL - 73:
IS - 7-A(E
N2 - As mathematics identity affects students' learning and doing of mathematics, it is critical to understand the mathematics identities of African American and Hispanic students as the mathematical performance and pursuits of far too many continue to lag behind. Further, as community schools have been shown to positively impact students in urban communities, it is also critical to understand how mathematics identities are developed within community schools. This study explores the culture, structures, and processes of an urban elementary community school including its afterschool archery program relative to fifth grade students' mathematics identities. It also explores students' math positioning, enactment, and perspectives in the classroom and archery. The theoretical framework encompasses multiple theories and perspectives: identity theory, cultural-historical activity theory, ecological systems theory, and culturally responsive pedagogy. Ethnography of one urban elementary community school was conducted over one school year plus summer camp using mixed methods. In total, 33 fifth graders and 13 adults participated in the study. In addition to school and community agency artifacts collected, observations inside and outside of the classrooms were conducted along with student brainstorming exercises and student and adult interviews. State math assessment scores were collected for 2009 and 2010 and pre- and post-surveys on students' mathematics beliefs and attitudes were conducted. While 7 out of 10 fifth graders favored mathematics and considered themselves as mathematicians, as defined in a broader sense that reflects habits of mind as opposed to simply skills, less than four out of 10 saw themselves in careers considered math- or science-related. Interestingly, students who had heard the word "mathematician" scored significantly higher on state math assessments than their peers who had not. In the classroom, students positioned themselves in different ways relative to their mathematics identity such as leader, helper,

independent, math smart, social learner, and agent of their own learning. Outside of the classroom, the afterschool archery program bore positive relevance in students' mathematics identities, including a student with Attention Deficit/Hyperactivity Disorder, through culturally responsive instruction, a culture of respect, and goal-setting. Study results can inform community school processes, cultures, and structures as well as children's media. (PsycINFO Database Record (c) 2013 APA, all rights reserved)
SN - 0419-4209
AD - Fleshman, Paula Jenniver: City U New York, US
ER -

TY - JOUR
ID - 2386
T1 - An instrument to measure traditional and cyber bullying and victimization behaviors in overseas middle years students
A1 - Blakeney,Kent Matthew
Y1 - 2013///
N1 - Dissertation Abstract: 2013-99010-372
English
Dissertation Abstract
KW - PsycInfo
KW - Behavior Problems
KW - Students
KW - Victimization
KW - Bullying
KW - Cyberbullying
KW - Conservatism
RP - NOT IN FILE
SP - No
JF - Dissertation Abstracts International Section A: Humanities and Social Sciences
VL - 73:
IS - 7-A(E
N2 - The purpose of this study was to test the Overseas Middle Years Student Bullying and Victimization Questionnaire for validity and reliability, then identify statistically significant differences between gender, grade, and national status with four constructs; traditional bullying, traditional victimization, cyber bullying, and cyber victimization. A cross-sectional, self-report, nonexperimental, quantitative research design was employed. Out of a population of 496, the sample included 189 (38.10%) sixth, seventh, and eighth-grade students in two overseas schools in Europe. The results of two reviews by a panel of experts found the instrument was valid. The reliability was tested using Cronbach's for overall correlation (.917), each of the constructs; traditional victimization (.864), cyber victimization (.898), traditional bully (.813), and cyber bully (.916). Furthermore, each construct was tested by removing one item. In 18 of 19 items, the correlation decreased while the nominal increase in the other item was insignificant. The study found that bullying most often occurs in hallways and lockers (60.3%), on the playground (44.4%), in the cafeteria (27.0%), and in classrooms (27.0%). Young men were found to be significantly more likely than young women to be physical victims and verbal bullies. Sixth-grade students were significantly more likely to be verbal victims, eighth-grade students were significantly more likely to be physical bullies, and sixth and seventh-grade students were significantly more likely to be relational bullies. Finally, traditional bullies were 3.27 times more likely than cyber bullies while traditional victims were 2.44 more likely than cyber victims. Limitations included the small sample size and the number of I do not know responses in each of the construct items. (PsycINFO Database Record (c) 2013 APA, all rights reserved)
SN - 0419-4209
AD - Blakeney, Kent Matthew: Capella U., US
ER -

TY - JOUR
ID - 2387
T1 - A pilot study to examine the effect of additional structured outdoor playtime on preschoolers' physical activity levels. [References]
A1 - Alhassan,Sofiya; Nwaokelemeh,Ogechi; Lyden,Kate; Goldsby,TaShauna; Mendoza,Albert
Y1 - 2013///

N1 - Peer Reviewed Journal: 2012-33928-003
English
Journal; Peer Reviewed Journal
KW - PsycInfo
KW - Childhood Play Behavior
KW - Physical Activity
KW - Physical Education
KW - Preschool Students
RP - NOT IN FILE
SP - 23
EP - 35
JF - Child Care in Practice
VL - 19:
IS - 1
N2 - The impact of additional structured outdoor playtime on preschoolers' physical activity (PA) level is unclear. The purpose of this pilot study was to explore the effects of increasing structured outdoor playtime on preschoolers' PA levels. Eight full-day classrooms (n = 134 children) from two preschool programmes were randomised into a treatment (STRUCT, n = 4) or control (CON, n = 4) condition. Both groups received an additional 30 minutes of outdoor playtime three days per week for four weeks. The STRUCT intervention consisted of previously tested structured outdoor playtime activities/games. The CON intervention consisted of free outdoor playtime. Children were individually recruited (n = 75) for the objective assessment of PA levels. The PA levels of 67 children (age, 4.190.8 years; STRUCT, n = 38; CON, n = 29) were assessed at baseline and during week four for seven consecutive days using Actigraph accelerometers. Data were analysed using mixed-model analysis of variance. Time spent in vigorous PA significantly increased during the 30-minute intervention time for the STRUCT group compared with the CON group (group x time interaction: F(1, 36) = 4.91, p = 0.04). Compared with baseline, a significant increase was observed in the STRUCT group's time spent engaged in moderate-to-vigorous PA (MVPA) during the intervention time, but this increase was not significant compared with the CON group (baseline: STRUCT, 1.7 + 2.0 min; CON, 1.9 + 2.4 min; week four: STRUCT, 4.9 + 3.1 min; CON, 3.3 + 2.5 min). Compared with the CON group, the STRUCT group spent a significantly greater percentage of time engaged in MVPA and a significantly lower percentage of time engaged in sedentary activity during the preschool day. In conclusion, this study provides preliminary evidence that increasing preschoolers' exposure to structured activities during outdoor playtime could lead to improvements in their PA levels. The present intervention could be used to help preschoolers meet the recommended levels of PA. (PsycINFO Database Record (c) 2013 APA, all rights reserved) (journal abstract)
SN - 1357-5279
AD - Alhassan, Sofiya: University of Massachusetts Amherst, Amherst, MA, US Nwaokelemeh, Ogechi: University of Massachusetts Amherst, Amherst, MA, US Lyden, Kate: University of Massachusetts Amherst, Amherst, MA, US Goldsby, TaShauna: University of Connecticut, Storrs, CT, US Mendoza, Albert: University of Massachusetts Amherst, Amherst, MA, US
ER -

TY - JOUR
ID - 2388
T1 - An investigation of play: From the voices of fifth- and sixth-grade talented and gifted students. [References]
A1 - Beisser,Sally Rapp; Gillespie,Catherine Wilson; Thacker,Valerie Marsh
Y1 - 2013///
N1 - Peer Reviewed Journal: 2012-32575-003
English
Journal; Peer Reviewed Journal
KW - PsycInfo
KW - Elementary School Students
KW - Gifted
KW - Problem Solving
KW - Recreation
KW - Student Attitudes
KW - Rural Environments

KW - Suburban Environments
KW - Urban Environments
RP - NOT IN FILE
SP - 25
EP - 38
JF - Gifted Child Quarterly
VL - 57:
IS - 1
N2 - The purpose of this study was to explore the concept of play through the eyes of talented and gifted (TAG) fifth- and sixth-grade students. Three focus groups consisting of fifth- and sixth-grade TAG students were conducted in one urban, one suburban, and one rural school district in the Midwest. Students were asked to describe the value of play in the cognitive, physical, and social domains. Additional open-ended questions were asked about the importance of play and its relevance to their lives. From these results, an online survey was created. Data from an additional 162 fifth- and sixth-grade TAG respondents substantiated that play is a significant activity for learning and socializing in the general classroom, gifted programs, and outside their classrooms. Despite its importance, students experienced structural barriers for time to play. Putting the Research to Use: Results of this study hold implications for teachers to acknowledge the value of play as a tool for learning. Children see play as a renewal activity. They see play as motivating and as a way to increase attention, retention, and focus in learning. Children emphasized that play develops teamwork and problem-solving skills. Play enhances meaningful activities in the talented and gifted program as well as the general classroom. Results of this study suggest that parents should acknowledge their children's need for play in all three domains (cognitive, physical, and social) and allow time for self-directed play after school with limits on scheduled activities. (PsycINFO Database Record (c) 2013 APA, all rights reserved) (journal abstract)
SN - 0016-9862
AD - Beisser, Sally Rapp: School of Education, Drake University, Des Moines, IA, US Gillespie, Catherine Wilson: Drake University, Des Moines, IA, US Thacker, Valerie Marsh: School of Education, Drake University, Des Moines, IA, US
ER -

TY - JOUR
ID - 2389
T1 - Psychological assessment in school settings. [References]
A1 - Braden,Jeffery P.
Y1 - 2013///
N1 - Book: 2012-28466-012
English
Book; Edited Book
KW - PsycInfo
KW - Academic Achievement
KW - Learning
KW - Psychoeducation
KW - Psychological Assessment
KW - School Based Intervention
KW - School Environment
KW - School Psychologists
KW - School Psychology
KW - Schools
KW - Trends
RP - NOT IN FILE
SP - 291
EP - 314
JF - Graham, John R [Ed]; Naglieri, Jack A [Ed]; Weiner, Irving B [Ed]
VL - :
IS - 2013
N2 - (from the chapter) The procedures most common to psychological assessment in school settings are relevant to the populations and problems served in schools. Therefore, school-based psychologists emphasize assessment of academic achievement and student learning, use interventions that emphasize educational or

learning approaches, and use consultation to implement interventions. Because children experience problems in classrooms, playgrounds, homes, and other settings that support education, interventions to address problems are generally implemented in the setting where the problem occurs. School-based psychologists generally do not provide direct services (e.g., play therapy) outside of educational settings. Consequently, psychologists in school settings consult with teachers, parents, and other educators to implement interventions. Psychological assessment procedures that address student learning, psychoeducational interventions, and intervention implementation mediated via consultation are emphasized to a greater degree in schools than in other settings. The remainder of this chapter addresses aspects of psychological assessment that distinguish practices in school-based settings from practices in other settings. The chapter is organized into three major sections: assessment purposes, current practices, and future trends of psychological assessment in schools. (PsycINFO Database Record (c) 2014 APA, all rights reserved)
AD - Braden, Jeffery P.: Department of Psychology, North Carolina State University, Raleigh, NC, US
ER -

TY - JOUR
ID - 2390
T1 - Autism: School-based cognitive-behavioral interventions. [References]
A1 - Bolton,Jessica B.
Y1 - 2012///
N1 - Book: 2012-04950-016
English
Book; Edited Book
KW - PsycInfo
KW - Cognitive Behavior Therapy
KW - Pervasive Developmental Disorders
KW - School Based Intervention
KW - Autism
KW - Measurement
KW - Treatment Planning
RP - NOT IN FILE
SP - 469
EP - 501
JF - Mennuti, Rosemary B [Ed]; Christner, Ray W [Ed]; Freeman, Arthur [Ed]
VL - :
IS - 2012
N2 - (from the chapter) Since the first edition of this book in 2006, the concept of using cognitive-behavioral therapy (CBT) techniques with children and adolescents with autism spectrum disorders (ASDs) has gained popularity in terms of available techniques and research. Children and adolescents with ASD, by definition, exhibit social impairments, which occur concurrently with deficits in expressive and/or receptive verbal and nonverbal language and the presentation of perseverative behaviors and a restrictive pattern of interests. These behaviors all present a need for intervention, and may be targeted by speech/language therapy, occupational therapy, classroom management systems, and social-skills training, to name a few. For ASD, each "symptom" is typically treated by differing types of intervention, which requires comprehensive evaluations of the problem behaviors and careful treatment planning. Of the symptoms present, cognitive-behavioral therapy has been used to help in the areas of improving social skills, enhancing perspective-taking, increasing self-monitoring behaviors, and reducing comorbid symptoms of internalizing disorders, specifically anxiety. This chapter aims to highlight some promising treatment options for children and adolescents with ASD, and to help practitioners select and monitor interventions to help establish evidence of effectiveness. It is the hope that this chapter is able to highlight the importance and need for more research in this area. (PsycINFO Database Record (c) 2014 APA, all rights reserved)
AD - Bolton, Jessica B.: South Middleton School District, Boiling Springs, PA, US McPoyle-Callahan, Jennifer E.: South Middleton School District, Boiling Springs, PA, US Christner, Ray W.: Cognitive Health Solutions, LLC, Hanover, PA, US
ER -

TY - JOUR
ID - 2391

T1 - School-based psychoeducational approaches to prevention
A1 - O'Dea,Jennifer A.
Y1 - 2011///
N1 - Book: 2011-20792-049
English
Book; Edited Book
KW - PsycInfo
KW - Body Image
KW - Eating Disorders
KW - Psychoeducation
KW - School Based Intervention
KW - Schools
RP - NOT IN FILE
SP - 334
EP - 441
JF - Cash, Thomas F [Ed]; Smolak, Linda [Ed]
VL - :
IS - 2011
N2 - (from the chapter) School-based programs to prevent eating disorders and improve body image have evolved since the heavy focus on psychoeducation in the late 1980s. Psychoeducational programs were traditionally based on the adaption of clinical psychological counseling techniques to be implemented in school settings, and they also relied heavily on the didactic presentation of knowledge and information. Most school-based programs now include a range of strategies and preventive approaches combined with traditional psychoeducational strategies, including media literacy, cognitive dissonance, small-group work activities, role play, peer-led teaching, and the use of computer and Internet technology. Several challenges for school-based prevention impede progress, including disputes over program delivery and content, the personal attitudes and professional training of teachers, and issues of measurement among "normal" populations in natural environments. More evidence is required in order to produce effective programs that can be implemented in a cost-effective manner, throughout the world, among males and females of all ages. This chapter aims to provide an overview of the research in school-based body image programs and to identify the current trends and future directions in this area. (PsycINFO Database Record (c) 2014 APA, all rights reserved)
AD - O'Dea, Jennifer A.: Faculty of Education and Social Work, University of Sydney, Sydney, NSW, Australia Yager, Zali: Faculty of Education, La Trobe University, VIC, Australia
ER -

TY - JOUR
ID - 2392
T1 - A treatment package for reducing aggression and improving playground behavior. [References]
A1 - Marchant,Michelle
Y1 - 2012///
N1 - Peer Reviewed Journal: 2012-09162-002
English
Journal; Peer Reviewed Journal
KW - PsycInfo
KW - Aggressive Behavior
KW - Playgrounds
KW - School Based Intervention
KW - Elementary School Students
KW - Treatment
RP - NOT IN FILE
SP - 5
EP - 25
JF - Journal of Evidence-Based Practices for Schools
VL - 13:
IS - 1
N2 - Referrals of elementary students who had engaged in aggressive behavior on the playground prompted the implementation of a nonclassroom treatment package as an extension of a schoolwide positive behavior support

program. The package consisted of (1) teaching students new playground rules during physical education class and posting the rules in the gym and on the playground, (2) providing reminders of the rules, (3) modifying the playground to facilitate appropriate play, (4) encouraging playground monitors to take a more active supervisory role, and (5) conducting a self-management program for the students who consistently engaged in aggressive behavior. The effects of the program were evaluated by recording three students' levels of physical and verbal aggression and appropriate play and by asking the playground supervisors to rate the recess behavior of all students during baseline and treatment. (PsycINFO Database Record (c) 2014 APA, all rights reserved) (journal abstract)
SN - 1523-4738
AD - Marchant, Michelle: Special Education Department, Brigham Young University, Provo, UT, US Young, K. Richard: McKay School of Education, Brigham Young University, Provo, UT, US Lindberg, Jana: McKay School of Education, Brigham Young University, Provo, UT, US Fisher, Adam: McKay School of Education, Brigham Young University, Provo, UT, US Solano, Brock: McKay School of Education, Brigham Young University, Provo, UT, US
ER -

TY - JOUR
ID - 2393
T1 - African cognitive ability: Research, results, divergences and recommendations. [References]
A1 - Rindermann,Heiner
Y1 - 2013///
N1 - Peer Reviewed Journal: 2012-25059-001
English
Journal; Peer Reviewed Journal
KW - PsycInfo
KW - Cognitive Ability
KW - Cognitive Development
KW - Daily Activities
KW - Environmental Attitudes
RP - NOT IN FILE
SP - 229
EP - 233
JF - Personality and Individual Differences
VL - 55:
IS - 3
N2 - In the past different researchers have come to diverging cognitive ability estimates for people in Africa and of African descent. The paper tries to check the validity of past results by comparing them with outcomes of two new psychometric test studies from East and South Africa; with results from student assessment studies; with predictions based on those variables which, outside Africa, correlate most strongly with intelligence; and by comparing them with further indicators of cognitive ability (descriptions of everyday life and human accomplishment). Integrating these cognitive ability measures with the application of several corrections (due to the higher age of students in Africa, lower African school enrollment, selectivity of samples and higher African secular IQ rise), the best guess for an African average is IQ 75. Finally, possible environmental and genetic (evolutionary, therefore past environmental) causes are discussed and suggestions are given how to enhance cognitive development in African countries. (PsycINFO Database Record (c) 2013 APA, all rights reserved) (journal abstract)
SN - 0191-8869
AD - Rindermann, Heiner: Department of Psychology, Chemnitz University of Technology, Chemnitz, Germany
ER -

TY - JOUR
ID - 2394
T1 - The influence of experience upon imagery perspectives in adolescent sport performers. [References]
A1 - John,K.
Y1 - 2011///

N1 - Peer Reviewed Journal: 2011-27016-001
English
Journal; Peer Reviewed Journal
KW - PsycInfo
KW - Athletes
KW - Conceptual Imagery
KW - Sports
KW - Adolescent Development
KW - Athletic Participation
KW - Experiences (Events)
KW - Visual Perception
RP - NOT IN FILE
JF - Journal of Imagery Research in Sport and Physical Activity
VL - 6:
IS - 1
N2 - Current imagery literature suggests that imagery perspectives may be subject to a default position (Morris & Spittle, 2001), with experience influencing how successfully individuals can utilize internal and external visual imagery. According to this proposition, the default imagery perspective is an internal 1st person perspective. However, few imagery inventories have been designed to differentiate and accurately measure these imagery perspectives (see Morris, Spittle, & Watt, 2005). Fewer still have considered athletic populations, in particular adolescent cohorts. Consequently, the current research examined the use of internal visual imagery (IVI), external visual imagery (EVI), and kinesthetic imagery (KI) amongst adolescent sport performers and whether the amount of hours engaged in practice outside of competition influenced their adoption. Eighty-seven (36 male, 51 female) county level participants from sports academies in the United Kingdom (M age = 14.0, SD = 1.92), from 6 interactive sports, completed the Vividness of Movement Imagery Questionnaire-2 (VMIQ-2; Roberts et al., 2008). Participants were grouped relative to the amount of practice they had engaged in since playing their current sport competitively. A repeated measures ANOVA recorded significant differences amongst participants vividness of imagery, Wilkes' F (2, 85) = 3.166, $p < 0.05$, 2 = .07, post hoc pairwise comparisons using a Bonferroni adjustment revealed no significant differences between sub-scales. Results to MANOVA recorded no significant differences between VMIQ-2 sub-scales and three accumulated practice groupings, Wilkes' F (3, 82) = 436.14, $p > 0.32$, 2 = .04. The results demonstrate adolescent sport performers possess between clear and reasonably vivid to moderately clear and vivid imagery ability when using IVI, EVI, and KI. Future research should consider the impact of environmental factors that influence the development of these modalities and perspectives. (PsycINFO Database Record (c) 2013 APA, all rights reserved) (journal abstract)
SN - 1932-0191
AD - John, K. Parker: University of Gloucestershire, Cheltenham, United Kingdom Lovell, Geoff: University of the Sunshine Coast, QLD, Australia
ER -

TY - JOUR
ID - 2395
T1 - Parent and child perceptions of grade one children's out of school play. [References]
A1 - Lehrer,Joanne Shari; Petrakos,Hariclia Harriet
Y1 - 2011///
N1 - Peer Reviewed Journal: 2011-27135-007
English
Journal; Peer Reviewed Journal
KW - PsycInfo
KW - Child Attitudes
KW - Childhood Development
KW - Childhood Play Behavior
KW - Parental Attitudes
RP - NOT IN FILE
SP - 74
EP - 92
JF - Exceptionality Education International

VL - 21:
IS - 2-3
N2 - The transition from kindergarten to Grade 1 is marked by a sudden decrease in children's play time (Brostrom, 2005; Hartmann & Rollett, 1994). This study sought to examine 69 Montreal-area Grade 1 children's beliefs about out of school play, as well as their parents' (N = 56) perceptions of their play. Findings indicate that parents value play for their child's development and education, for building relationships and self-esteem, for processing emotional experiences, for relaxation, and for fun. Many parents expressed a preference for active outdoor play, as well as pretend and creative play, board games, and puzzles, explaining that they discouraged television and video games, aggressive play, and play with sexual themes. Children reported enjoying active outdoor play, toys, construction and art activities, and video game play, with parents, siblings, friends, and pets. Limitations to play included lack of neighbourhood children, parents or siblings who did not play, and restrictions to inviting friends over. (PsycINFO Database Record (c) 2013 APA, all rights reserved) (journal abstract)
SN - 1183-322X
AD - Lehrer, Joanne Shari: Concordia University, Montreal, PQ, Canada Petrakos, Hariclia Harriet: Concordia University, Montreal, PQ, Canada
ER -

TY - JOUR
ID - 2396
T1 - Education, the Playground Project and elements of psychodrama. [References]
A1 - Court,Geoffrey; Higley,Jeffrey; Lousada,Olivia
Y1 - 2012///
N1 - Book: 2012-04044-011
English
Book; Edited Book
KW - PsycInfo
KW - Elementary School Students
KW - Playgrounds
KW - Psychodrama
KW - School Based Intervention
KW - Interpersonal Relationships
KW - Behavior
KW - Childhood Development
KW - Elementary Education
KW - Peers
RP - NOT IN FILE
SP - 106
EP - 116
JF - Leigh, Lauraine [Ed]; Gersch, Irvine [Ed]; Dix, Ann [Ed]; Haythorne, Deborah [Ed]
VL - :
IS - 2012
N2 - (from the chapter) The events described in this chapter took place in the early 1980s. It was a time when educational drama (Slade 1954) and drama therapy (Jennings 1973), as well as psychodrama, were taking their place amongst other developments in a movement towards the 'education of the whole person', and around which there was great excitement. Our project still seems to us both universal and timeless. We believe it represents an educational approach still being fought for in today's mainstream classroom. Despite its title, The Playground Project happened in a classroom with a group of 23 children, aged 10-11 years old, and their teacher at a primary school on a deprived inner-city estate. Strong feelings, often reflected in the children's behaviour, were getting in the way of useful learning, and the teacher was looking for ways of addressing the problem. At that time a freelance performer working in the school as actor-in-residence suggested that the psychodramatist Olivia Lousada might be able to help. The Playground Project was the result. Like the project itself, the name originated through Olivia Lousada's experiences both as a professional and as a parent. She had been running a course for teachers who ran nurture groups, in which each session focused on a theme such as trust, power, or status. When the teachers tried out the material in the classroom, they were amazed to discover what was really going on in the peer group of the children: who liked whom, who stuck up for whom, how they felt about the bully in the class who felt he had no friends. This they learnt through the sociometry (the mapping of

relationships). Olivia saw that the same insights might help her own six-year-old son, who was himself having a bad time in the playground. She knew that many children find the playground difficult and lonely, with a culture of its own. What if the energy of this peer group culture could be harnessed and used creatively? The original project took the form of six fortnightly sessions of one and a half hours each, but the richness of the process meant that sessions soon had to be doubled in length, something more easily managed in the days before the National Curriculum. We have chosen two of these sessions to describe in detail: the third session, 'Power', in which we ran into difficulties-from which much was learned-and the fifth session, 'the Magic Shop', which illustrates the access to creativity of this work. (PsycINFO Database Record (c) 2013 APA, all rights reserved)
AD - Court, Geoffrey: Circle Works, United Kingdom Lousada, Olivia: London Psychodrama Network, London, United Kingdom
ER -

TY - JOUR
ID - 2397
T1 - Creative learning environments in education-A systematic literature review. [References]
A1 - Davies,Dan; Jindal-Snape,Divya; Collier,Chris; Digby,Rebecca; Hay,Penny; Howe,Alan
Y1 - 2013///
N1 - Peer Reviewed Journal: 2012-23284-001
English
Journal; Peer Reviewed Journal
KW - PsycInfo
KW - Creativity
KW - Learning Environment
KW - Teaching
KW - Teaching Methods
KW - Professional Development
RP - NOT IN FILE
SP - 80
EP - 91
JF - Thinking Skills and Creativity
VL - 8 Apr 2013, pp. 80-91.
N2 - This paper reports on a systematic review of 210 pieces of educational research, policy and professional literature relating to creative environments for learning in schools, commissioned by Learning and Teaching Scotland (LTS). Despite the volume of academic literature in this field, the team of six reviewers found comparatively few empirical studies published in the period 2005-2011 providing findings addressing the review objectives. There was, however a reasonable weight of research evidence to support the importance of the following factors in supporting creative skills development in children and young people: flexible use of space and time; availability of appropriate materials; working outside the classroom/school; 'playful' or 'games-bases' approaches with a degree of learner autonomy; respectful relationships between teachers and learners; opportunities for peer collaboration; partnerships with outside agencies; awareness of learners' needs; and non-prescriptive planning. The review also found evidence for impact of creative environments on pupil attainment and the development of teacher professionalism. LTS intend to use the review as a basis for recommendations to Scottish schools in promoting creativity within Curriculum for Excellence. However, the findings of the review and methodological gaps in the reviewed studies have implications for policy, practice and research internationally. (PsycINFO Database Record (c) 2013 APA, all rights reserved) (journal abstract)
SN - 1871-1871
AD - Davies, Dan: Centre for Research in Early Scientific Learning, Bath Spa University, Bath, United Kingdom Jindal-Snape, Divya: University of Dundee, Dundee, United Kingdom Collier, Chris: Centre for Research in Early Scientific Learning, Bath Spa University, Bath, United Kingdom Digby, Rebecca: Centre for Research in Early Scientific Learning, Bath Spa University, Bath, United Kingdom Hay, Penny: Centre for Research in Early Scientific Learning, Bath Spa University, Bath, United Kingdom Howe, Alan: Centre for Research in Early Scientific Learning, Bath Spa University, Bath, United Kingdom
ER -

TY - JOUR
ID - 2398
T1 - Teaching children with autism spectrum disorder to mand "where?". [References]

A1 - Marion,Carole
Y1 - 2012///
N1 - Peer Reviewed Journal: 2012-31807-001
English
Journal; Peer Reviewed Journal
KW - PsycInfo
KW - Autism
KW - Behavior Analysis
KW - Operant Conditioning
KW - Teaching
KW - Verbal Communication
KW - Motivation Training
KW - Pervasive Developmental Disorders
RP - NOT IN FILE
SP - 273
EP - 294
JF - Journal of Behavioral Education
VL - 21:
IS - 4
N2 - A modified multiple-baseline design across participants was used to evaluate a procedure for teaching the mand "Where?" to 3 children with autism. The participants were 3 and 5 years old and were participating in an intensive applied behavior analysis program. The participants were able to mand for items they wanted when the items were not in sight but were unable to ask where an item was located. The procedure consisted of a preference assessment for play activities, contrived conditioned motivating operations (CMO's), prompting the children to mand "Where," and consequences for correct and incorrect responding. Each contrived CMO consisted of an opportunity for the child to mand "Where" while playing with a selected activity, prompting the child to mand, and reinforcing a correct response by answering the question "Where." Two of the participants learned to mand "Where" after training with 1 CMO and the mand generalized to novel contrived situations, activities, and the natural environment and was maintained over time (up to 4-weeks), whereas one participant required training with a second CMO before generalization occurred. (PsycINFO Database Record (c) 2013 APA, all rights reserved) (journal abstract)
SN - 1053-0819
AD - Marion, Carole: St. Paul's College, University of Manitoba, Winnipeg, Canada Martin, Garry L.: St. Paul's College, University of Manitoba, Winnipeg, Canada Yu, C. T.: St. Paul's College, University of Manitoba, Winnipeg, Canada Buhler, Charissa: St. Amant Research Centre, Winnipeg, Canada Kerr, Danni: St. Paul's College, University of Manitoba, Winnipeg, Canada
ER -

TY - JOUR
ID - 2399
T1 - Social networks and friendships at school: Comparing children with and without ASD. [References]
A1 - Kasari,Connie; Locke,Jill; Gulsrud,Amanda; Rotheram-Fuller,Erin
Y1 - 2011///
N1 - Peer Reviewed Journal: 2011-07919-001
English
Journal; Peer Reviewed Journal
KW - PsycInfo
KW - Friendship
KW - Pervasive Developmental Disorders
KW - Social Behavior
KW - Social Networks
KW - Elementary School Students
KW - Schools
RP - NOT IN FILE
SP - 533
EP - 544
JF - Journal of Autism and Developmental Disorders

VL - 41:
IS - 5
N2 - Self, peer and teacher reports of social relationships were examined for 60 high-functioning children with ASD. Compared to a matched sample of typical children in the same classroom, children with ASD were more often on the periphery of their social networks, reported poorer quality friendships and had fewer reciprocal friendships. On the playground, children with ASD were mostly unengaged but playground engagement was not associated with peer, self, or teacher reports of social behavior. Twenty percent of children with ASD had a reciprocated friendship and also high social network status. Thus, while the majority of high functioning children with ASD struggle with peer relationships in general education classrooms, a small percentage of them appear to have social success. (PsycINFO Database Record (c) 2013 APA, all rights reserved) (journal abstract)
SN - 0162-3257
AD - Kasari, Connie: Semel Institute, University of California Los Angeles, Los Angeles, CA, US Locke, Jill: Semel Institute, University of California Los Angeles, Los Angeles, CA, US Gulsrud, Amanda: Semel Institute, University of California Los Angeles, Los Angeles, CA, US Rotheram-Fuller, Erin: Temple University, Philadelphia, PA, US
ER -

TY - JOUR
ID - 2400
T1 - Adherence to active play and electronic media guidelines in preschool children: Gender and parental education considerations. [References]
A1 - Loprinzi,Paul D.
Y1 - 2013///
N1 - Peer Reviewed Journal: 2013-03586-010
English
Journal; Peer Reviewed Journal
KW - PsycInfo
KW - Childhood Play Behavior
KW - Mass Media
KW - Parent Educational Background
KW - Preschool Students
RP - NOT IN FILE
SP - 56
EP - 61
JF - Maternal and Child Health Journal
VL - 17:
IS - 1
N2 - The primary purpose of the present study was to examine adherence to current active play and electronic media use guidelines in a sample of US preschool-aged children and to examine whether differences occurred across gender and parental education. 164 parents completed an on-line survey to estimate preschool children's active play and sedentary behaviors. For weekdays, 50% of the sample met the active play guideline with this increasing to 65% during the weekend. With respect to electronic media use, 90% of the sample met guidelines during the week, with the percentage meeting guidelines dropping to 78% during the weekend. A greater percentage of preschool children from high parental education families (83.4 + 3.3) met electronic media use guidelines on the weekends, compared to preschool children from low parental education families (59.4 + 8.1) ($p = 0.002$). Our findings indicate that a substantial portion of preschool children are not meeting active play guidelines and that adherence to active play and electronic media use guidelines may be influenced by parental education. (PsycINFO Database Record (c) 2013 APA, all rights reserved) (journal abstract)
SN - 1092-7875
AD - Loprinzi, Paul D.: Department of Exercise Science, Lansing School of Nursing and Health Sciences, Bellarmine University, Louisville, KY, US Schary, David P.: Program in Exercise and Sport Science, School of Biological and Population Health Sciences, College of Public Health and Human Sciences, Oregon State University, Corvallis, OR, US Cardinal, Bradley J.: Program in Exercise and Sport Science, School of Biological and Population Health Sciences, College of Public Health and Human Sciences, Oregon State University, Corvallis, OR, US
ER -

TY - JOUR
ID - 2401
T1 - Physical risk taking in elementary-school children: Measurement and emotion regulation issues. [References]
A1 - Morrongiello,Barbara A.
Y1 - 2012///
N1 - Peer Reviewed Journal: 2012-02349-008
English
Journal; Peer Reviewed Journal
KW - PsycInfo
KW - Elementary Schools
KW - Emotional Regulation
KW - Risk Taking
RP - NOT IN FILE
SP - 492
EP - 496
JF - Personality and Individual Differences
VL - 52:
IS - 4
N2 - Children who engage in physical risk taking experience more injuries. In the present study children 7-12 years completed laboratory tasks and standardized questionnaires to explore inter-relations between measures of risk taking and to determine if the Balloon Analog Risk Task (BART) is a valid index of physical risk taking. Emotion regulation skills also were assessed to determine if poorer skills are associated with greater risk taking, and what measures of risk taking show these relations. Results revealed that general measures of risk taking propensity predicted both BART performance and physical risk taking. Similarly, poorer emotion regulation skills were associated with greater risk taking scores for both the BART and physical risk taking task. Measures of physical risk taking related to one another, however, none of these scores related to performance on the BART. Implications for measuring physical risk taking and exploring emotional links to risk taking in future research are discussed. (PsycINFO Database Record (c) 2013 APA, all rights reserved) (journal abstract)
SN - 0191-8869
AD - Morrongiello, Barbara A.: Psychology Department, University of Guelph, Guelph, ON, Canada Kane, Alexa: Psychology Department, University of Guelph, Guelph, ON, Canada McArthur, Brae Ann: Psychology Department, University of Guelph, Guelph, ON, Canada Bell, Melissa: Psychology Department, University of Guelph, Guelph, ON, Canada
ER -

TY - JOUR
ID - 2402
T1 - Daily life mechanisms of stress spillover among early adolescents experiencing academic difficulty. [References]
A1 - Salamon,Reda
Y1 - 2011///
N1 - Peer Reviewed Journal: 2012-17701-002
English
Journal; Peer Reviewed Journal
KW - PsycInfo
KW - Stress
KW - Students
KW - Academic Achievement
KW - Anxiety
KW - Depression (Emotion)
KW - Emotional States
RP - NOT IN FILE
SP - 453
EP - 463
JF - European Journal of Psychology of Education
VL - 26:

IS - 4
N2 - Research has suggested that academic stress may "spillover" into other life domains and have negative psychological or social consequences for children and adolescents outside of school settings, but relatively few investigations have examined mediators and moderators of spillover. The current study explored the mediating role of state affect and the moderating roles of prior academic performance and mood disturbances on spillover in a sample of 131 French adolescents. Participants completed clinical measures of anxiety and depression and participated in a 7-day ambulatory monitoring phase that involved multiple daily assessments of mood, behaviors, and activities. Spillover was observed for family events and subsequent school-related events, as well as between family and leisure events. These associations remained significant when controlling for immediate mood responses, suggesting that state affect does not play a salient mediating role. There was no evidence that spillover was moderated by academic difficulty, anxiety, depression, or gender. Results are discussed in terms of the role that emotional processes may play in spillover phenomena as well as the reciprocal influence that academic and non-academic events may exert each other. (PsycINFO Database Record (c) 2013 APA, all rights reserved) (journal abstract)
SN - 0256-2928
AD - Salamon, Reda: Department of Psychology, University of Bordeaux 2, Bordeaux, France Johnson, Elizabeth I.: University of Tennessee, Knoxville, TN, US Swendsen, Joel: National Center for Scientific Research, CNRS UMR 5287, Bordeaux, France
ER -

TY - JOUR
ID - 2403
T1 - Students with parents involved in substance abuse or dependence. [References]
A1 - Devine,Brian R.
Y1 - 2013///
N1 - Book: 2013-00765-008
English
Book; Edited Book
KW - PsycInfo
KW - Children of Alcoholics
KW - Learning Environment
KW - Parent Child Relations
KW - Parental Characteristics
KW - School Based Intervention
KW - Adjustment
KW - Drug Abuse
KW - Parents
KW - Schools
RP - NOT IN FILE
SP - 119
EP - 128
JF - Rossen, Eric [Ed]; Hull, Robert [Ed]
VL - :
IS - 2013
N2 - (from the chapter) Research unequivocally demonstrates the negative effects of parental substance abuse or dependency on their children. For example, children of alcoholics demonstrate higher rates of substance abuse, behavioral problems, aggression, internalizing disorders (e.g., anxiety or depression), attention deficit/hyperactivity disorder (ADHD), and psychiatric distress than their nonaffected peers, and they tend to have lower academic achievement and verbal ability. The most serious threat, however, is to younger children whose fathers are drug abusers and live in the household; in contrast, those with fathers who do not abuse substances have the lowest rates of clinical symptoms. A parent who is addicted or who is abusing alcohol or drugs is often impaired and unable to take proper care of a child, which may lead to a student's unkempt appearance, tardiness to school, falling asleep in class, or incomplete or missing homework (the case for Anthony in the vignette). Parents may drive their children while intoxicated or high. An affected child may have an advanced knowledge of alcohol or drugs, or there may be great discomfort when the subject is mentioned. These children may also get uncomfortable when the topic of parents is discussed, given the potential for feelings of guilt or shame. Other indications may be that the parent is never involved in any of the child's

activities or that the student partakes in various after-school activities as an attempt to avoid having to go home. It is clear that the problem of parental substance abuse is widespread and poses serious challenges to children and adolescents, putting them at risk for a host of problems. It is also clear that many of the solutions to these problems lie outside the reach of educators in schools. Nevertheless, there are strategies that any educator can employ to engage these students more successfully, to provide them with the optimum learning environment, and to support their efforts to meet the challenges with which they are faced. With this understanding in mind, educators can be better prepared to help these students, who are always present in their classrooms and whose success depends on an informed and caring dedication on the part of those who teach them. (PsycINFO Database Record (c) 2013 APA, all rights reserved)
AD - Devine, Brian R.: Parker High School, Janesville School District, US
ER -

TY - JOUR
ID - 2404
T1 - The neighborhood social environment and body mass index among youth: A mediation analysis. [References]
A1 - Veitch,Jenny; van Stralen,Maartje
Y1 - 2012///
N1 - Peer Reviewed Journal: 2012-32169-001
English
Journal; Peer Reviewed Journal
KW - PsycInfo
KW - Body Mass Index
KW - Obesity
KW - Physical Activity
KW - Social Environments
KW - Leisure Time
KW - Mediation
KW - Neighborhoods
RP - NOT IN FILE
JF - The International Journal of Behavioral Nutrition and Physical Activity
VL - 9 Mar 2012, ArtID 31.
N2 - Background: This study aimed to examine associations between aspects of the neighborhood social environment and body mass index (BMI) in youth both cross-sectionally and prospectively; and whether this association was mediated by physical activity, screen-time and sedentary time. Methods: Data were collected in 2004 and 2006 in high and low socio-economic areas of Melbourne, Australia. In 2004, 185 children aged 8-9 years (47% boys) and 359 children aged 13-15 years (45% boys) participated. Parents reported their perceptions of aspects of the social environment (i.e. social networks and social trust/cohesion), and physical activity (i.e. time spent outdoors by their children; and their younger children's walking and cycling trips) and screen-time (i.e. TV viewing, computer use). The older children self-reported their walking and cycling trips and their screen-time. All children wore an accelerometer to objectively assess outside-school hours moderate-to-vigorous physical activity and sedentary time. BMI was calculated from height and weight measured in 2004 and 2006. Multilevel linear regression analyses were conducted to examine associations between the social environment and BMI. Mediation analyses using the products of coefficient method were conducted to determine whether associations between the social environment and BMI were mediated by the time spent in a range of physical activity and sedentary behaviors. Results: Cross-sectional and prospective regression analyses showed that a more positive social network and higher social trust/cohesion was related to lower BMI among children. There was no evidence that time spent in physical activity or sedentary behaviors mediated this relation, despite significant associations between social networks and screen-time and between screen-time and BMI. Conclusions: The findings suggest that the neighborhood social environment may be important for preventing overweight and obesity in children. Further research investigating the mechanisms through which the neighborhood social environment exerts its effect on BMI is needed. (PsycINFO Database Record (c) 2013 APA, all rights reserved) (journal abstract)
SN - 1479-5868
AD - Veitch, Jenny: Centre for Physical Activity and Nutrition Research, Deakin University, Burwood, VIC, Australia van Stralen, Maartje M.: EMGO Institute for Health and Care Research, VU University Medical Center, Amsterdam, Netherlands Chinapaw, Mai J. M.: EMGO Institute for Health and Care Research, VU

University Medical Center, Amsterdam, Netherlands te Velde, Saskia J.: EMGO Institute for Health and Care Research, VU University Medical Center, Amsterdam, Netherlands Crawford, David: Centre for Physical Activity and Nutrition Research, Deakin University, Burwood, VIC, Australia Salmon, Jo: Centre for Physical Activity and Nutrition Research, Deakin University, Burwood, VIC, Australia Timperio, Anna: Centre for Physical Activity and Nutrition Research, Deakin University, Burwood, VIC, Australia
ER -

TY - JOUR
ID - 2405
T1 - Assessing middle school student participation in online vs. face-to-face environments
A1 - Oravetz,Cathleen L.
Y1 - 2012///
N1 - Dissertation Abstract: 2012-99230-211
English
Dissertation Abstract
KW - PsycInfo
KW - Computer Assisted Instruction
KW - Internet
KW - Learning Environment
KW - Middle School Students
KW - Middle Schools
RP - NOT IN FILE
SP - 2117
JF - Dissertation Abstracts International Section A: Humanities and Social Sciences
VL - 73:
IS - 6-A
N2 - Educators have observed reluctance in middle school students to vocally engage in small group learning tasks, the result of which could be a decrease in student learning. The same students have been observed collaborating with peers outside of the classroom when using technology. The purpose of this study is to determine if technology provides a means to increase participation in the classroom, which in turn may increase student learning. Social constructivism and developmental theories guided this study. Eighty eighth-grade middle school students participated in a mixed-method, between-subject quasi-experiment to determine whether there was a difference in participation amount and content by students in small-group, learning-centered discussions using online vs. face-to-face environments. Students in groups of four used either an online chat tool or face-to-face discussion during a four-part activity. Data was coded, guided by Cohen's Kappa inter-rater reliability. The data was analyzed using independent sample t-tests, Fisher exact tests, relative percentage comparisons, Gini Coefficient comparisons, and Mann-Whitney-Wilcoxon testing. Significant findings ($p < .05$, $p < .01$) were demonstrated in participation amount, participation content, male participation amount, male participation content, and female participation content. Technology has the ability to enhance educational systems in order to prepare students to effectively and efficiently communicate and collaborate in today's high-tech environment. Accessing Web 2.0 tools in the classroom is a good first step as students can integrate their current out-of-school technological lifestyle and place it into the hands of educators to mold and form into quality 21st century life skills. (PsycINFO Database Record (c) 2013 APA, all rights reserved)
SN - 0419-4209
AD - Oravetz, Cathleen L.: Northeastern U., US
ER -

TY - JOUR
ID - 2406
T1 - Cognitive aspects of creativity: Science learning through serious educational games. [References]
A1 - Annetta,Leonard A.
Y1 - 2013///
N1 - Book: 2012-34618-004
English
Book; Edited Book
KW - PsycInfo
KW - Computer Games

KW - Creativity
KW - Research and Development
KW - Science Education
KW - Cognition
KW - Psychology
RP - NOT IN FILE
SP - 53
EP - 62
JF - Banks Gregerson, Mary [Ed]; Snyder, Heather T [Ed]; Kaufman, James C [Ed]
VL - :
IS - 2013
N2 - (from the chapter) Over $20 billion game industry and just now educators are seeing the benefit if used for good. Although some innovators have been using educational games for some time, it has only been in recent years. Serious Educational Games (Annetta, 2008) have become a highly sought after initiative by both educators and researchers alike. Gaming has conventionally been viewed as a mindless activity engaged in by young men and women during their free time. As a high school teacher in the mid- 1990s I had tried to assign homework to my students. When no homework came back or homework came back without much evidence of deep thinking, it was apparent students were engaging in something outside of school much more interesting then what I was proposing for them to spend their time doing. It was the mainstreaming of console game systems-such as Nintendo and Sega Genesis. It was then I knew we had to make that bridge. Through several years of research and funding, we have begun to develop a framework for fostering creativity in science learning. We did this by allowing students, and in some respects teachers, the ability to create their own games in a three-dimensional environment. We anticipated that creating games would foster a deeper understanding of content. Our data supported this notion (Annetta, 2010; Annetta & Bronack, 2010; Annetta, Folta, & Klesath, 2010; Annetta & Holmes, 2007; Annetta, Holmes, Cheng, & Folta, 2010; Annetta, Lamb, Bowling, & Cheng, 2011; Annetta, Lamb, & Stone, 2010; Annetta, Mangrum, Holmes, Collazo, & Cheng, 2009; Annetta, Minogue, Holmes, & Cheng, 2009; Cheng, Annetta, & Folta, 2011; Holmes, Thurmond, Annetta, & Sears, 2012). This chapter will focus on the research and development we have been working on and how it is grounded in learning science, psychology, and cognition. (PsycINFO Database Record (c) 2013 APA, all rights reserved)
AD - Annetta, Leonard A.: College of Education and Human Development, George Mason University, Fairfax, VA, US Holmes, Shawn Y.: University of Regina, Regina, SK, Canada Vallett, David: College of Education and Human Development, George Mason University, Fairfax, VA, US Fee, Matthew: College of Education and Human Development, George Mason University, Fairfax, VA, US Cheng, Rebecca: College of Education and Human Development, George Mason University, Fairfax, VA, US Lamb, Richard: College of Education and Human Development, George Mason University, Fairfax, VA, US
ER -

TY - JOUR
ID - 2407
T1 - Environmental exposure to airborne microbial sub-micrometer particles and airway inflammation in children
A1 - Singh,Umesh
Y1 - 2012///
N1 - Dissertation Abstract: 2012-99220-509
English
Dissertation Abstract
KW - PsycInfo
KW - Asthma
KW - Environment
KW - Inflammation
KW - Stress
RP - NOT IN FILE
SP - 2779
JF - Dissertation Abstracts International: Section B: The Sciences and Engineering
VL - 73:
IS - 5-B

N2 - Introduction: Exposures to airborne microbial contaminants in sub-micrometer particles (PM1) have not been well characterized in different environments. Health effects due to these exposures are even more obscure. Methods: Concentrations of airborne size specific (<1, 1-1.8, >1.8 m) microbial contaminants (endotoxin, -glucan) were determined using cyclone samplers in two distinctly different environments: farms and homes. Using inhalable samplers and vacuum cleaners airborne inhalable and dust contaminants from these homes were collected. This approach was used to compare PM1 microbial contaminants in a particular home with those in inhalable particles and in dust inside the same home. Samples were analyzed with Limulus Amebocyte Lysate assays for endotoxin and -glucan. Walkthrough surveys and questionnaires determined home characteristics and other exposures. Airway inflammation was assessed in school-age children by measuring exhaled nitric oxide (eNO) levels with non-invasive nitric oxide monitoring system (NIOX-Flex). Skin-prick test were also employed to determine atopy. Results: Relative proportions of PM1 (<1 m) microbial contaminants from total airborne concentrations were significantly higher in homes despite their significantly higher actual concentrations on farms. In homes, PM1 and inhalable endotoxin concentrations correlated weakly with that in dust and with each other. PM1 endotoxin levels were not significantly associated with eNO levels in asthmatics but had significant inverse association with eNO levels in non-asthmatics. Among non-asthmatics, eNO levels were significantly lower for those children who lived in homes with levels of dog allergens above detection limits and for those with lower parental income. Discussion: Moderate disturbance activities in homes preferentially allow PM1 microbial contaminants to predominate among total airborne particles compared to farming environments that have dynamic activity levels that cause re-suspension of large particles. In addition to dust, airborne PM1 or inhalable microbial contaminants may originate from outdoor sources. High PM1 endotoxin was protective only for non-asthmatics. It is well known that T-helper 1 cells predominantly mediate immune responses in non-asthmatics who do not have pre-existing airway inflammation. PM1 endotoxin exposures, therefore, may have a beneficial effect; along with dog allergens, these could possibly drive naive immune systems away from inflammatory responses. For asthmatics, detectable dog allergens in homes may incite inflammatory response, possibly as irritants, in their already primed allergic immune systems. Among non-asthmatics from low-income parents, an adaptive immunity favoring Th1 immune response through higher microbial contaminant exposures may have favored protection against development of atopic response. This is in contrast to non-asthmatics from highest income parents who had significantly less microbial contaminant exposures. Subsequently, this may have led to predominantly Th2 adaptive immune response that is highly associated with atopy. Because atopy is a significant predictor of airway inflammation, it infers that non-asthmatics from lowest income parents had protective effects against airway inflammation compared to those non-asthmatics from highest income parents. Conclusion: PM1 endotoxin may be a better predictor of health outcome than inhalable or larger particles. Exposures to PM1 endotoxin may protect against airway inflammation in non-asthmatic school-age children. Whether this protection is sustained, needs to be confirmed through follow-up studies. (PsycINFO Database Record (c) 2013 APA, all rights reserved)
SN - 0419-4217
AD - Singh, Umesh: U Cincinnati, US
ER -

TY - JOUR
ID - 2408
T1 - Evaluation of Instant Recess exercise breaks as a means for implementing lausd physical activity policy in elementary schools
A1 - Woods,Carol Denise
Y1 - 2012///
N1 - Dissertation Abstract: 2012-99220-385
English
Dissertation Abstract
KW - PsycInfo
KW - Elementary Schools
KW - Exercise
KW - Health Promotion
KW - Physical Activity
KW - School Based Intervention
KW - Policy Making
RP - NOT IN FILE
SP - 2868

JF - Dissertation Abstracts International: Section B: The Sciences and Engineering
VL - 73:
IS - 5-B
N2 - The Los Angeles County Department of Public Health estimated that the obesity prevalence among Los Angeles County public school children is estimated to be 26%, with another 19% of children at risk of becoming overweight. Consequently, obesity increases the likelihood of developing many diseases, such as hypertension, chronic kidney disease, heart disease, diabetes mellitus, glaucoma, osteoporosis, neurological diseases, certain cancers, and even Alzheimer's disease. This dissertation systematically examines the Instant Recess exercise break intervention implemented in classroom settings, to see if it allows students to get more physical activity throughout the day. This project has implications for school districts around the country, as the childhood obesity epidemic spurs the passage of school physical activity promotion policies with untested efficacy or effectiveness, particularly in low-income environments. This is a classic intervention study in which mixed methods were used to analyze the data, including: accelerometer and pedometer recordings, teacher pre and post surveys, and weekly teacher logs. In conclusion, quantitative results found that students took 1840-1910 more steps with Instant Recess than those without. Qualitative results found that teachers were more likely to implement Instant Recess in their classrooms if they felt their administration was supportive about using Instant Recess as well. Additionally, issues of teacher IT savvy, technical support, and student enthusiasm were factors in Instant Recess uptake. (PsycINFO Database Record (c) 2013 APA, all rights reserved)
SN - 0419-4217
AD - Woods, Carol Denise: U California, Los Angeles, US
ER -

TY - JOUR
ID - 2409
T1 - A qualitative methodological approach toward the understanding of Louisiana's prekindergarten LA 4 quartile I students
A1 - Forsyth,Ouida F.
Y1 - 2012///
N1 - Dissertation Abstract: 2012-99220-088
English
Dissertation Abstract
KW - PsycInfo
KW - Childhood Development
KW - Learning
KW - Social Skills
RP - NOT IN FILE
SP - 2875
JF - Dissertation Abstracts International: Section B: The Sciences and Engineering
VL - 73:
IS - 5-B
N2 - This dissertation focused on the learning and interactional characteristics of LA 4 prekindergarten children who have remained in the first quartile at the end of the school year on the Developmental Skills Checklist. Their data were examined using a qualitative research deign. This design used progress reports, artifacts, child observation and teacher semi-structured interview data. The children were observed during their free play time in learning centers. This observation format was chosen because it represents their most natural, authentic play setting, thus allowing an opportunity to see them through the lens of their own choices. The results of these data were examined using the categorization of artifact teacher progress reports, context of observational interaction, and interactional/learning activities, as well as teacher interviews. Observer reactions were presented to more fully interpret findings. Patterns emerged revealing similarities and differences in the various contexts and activities for both the target group of struggling children and the comparison group who demonstrated progress out of quartile one by the end of the school year. Results characterize the target group as less interactive and likely to initiate conversation, preferences for solitary and parallel play, limited literacy knowledge, and readiness for kindergarten, as well as increased inattention to their chosen play activities and inappropriate social skills. This study reveals the importance of analyzing interactional behaviors of children in natural settings. Important implications regarding the value of qualitative research designs to investigate the actual social interaction, language, and literacy characteristics of children who fail to make progress in a high quality

prekindergarten program are essential to increasing our understanding of their deficits. (PsycINFO Database Record (c) 2013 APA, all rights reserved)
SN - 0419-4217
AD - Forsyth, Ouida F.: U Louisiana at Lafayette, US
ER -

TY - JOUR
ID - 2410
T1 - Pedometers, accelerometers, and observational methods: A comparison of measurements of physical activity in fourth-grade students
A1 - Greene,Amanda
Y1 - 2012///
N1 - Dissertation Abstract: 2012-99210-063
English
Dissertation Abstract
KW - PsycInfo
KW - Exercise
KW - Methodology
KW - Observation Methods
KW - Physical Activity
KW - Schools
KW - Activity Level
KW - Grade Level
KW - Measurement
RP - NOT IN FILE
SP - 1726
JF - Dissertation Abstracts International Section A: Humanities and Social Sciences
VL - 73:
IS - 5-A
N2 - In recent years physical inactivity among students has become a matter of great concern. Nearly 65% of students do not meet the daily recommended level of physical activity, which is 60 minutes or more of moderate to vigorous physical activity each day, with 50% of that time being spent in moderate to vigorous levels of activity (CDC, 2010b). As a result, the 21 st century has shown to be a time of many health problems such as, obesity, diabetes, and heart disease. In fact, nearly one third of all children are considered obese or overweight (Slawta & DeNeui, 2009). Researchers suggest that these health problems are directly related to students' sedentary lifestyles (Pate et al., 2006). Schools play a pivotal role in addressing and increasing physical activity during the school day. The purpose of this research study was to measure levels of physical activity in elementary students during school hours. Specifically, the study sought to discover if there were increased levels of physical activity while students were using a cross-curricular adventure playground, as compared to when they were engaged in free play or physical education class. The study also compared the different measurement types (pedometers, accelerometers, and the observational method) used to assess physical activity, to indicate which measurement types were most feasible in the elementary school setting. Schools are ideal locations for assessing levels of physical activity, as 95% of all children are enrolled in these learning institutions (National Center for Educational Statistics, 2005). The data indicate that physical education provided for the highest levels of moderate to vigorous activity, while both free play and cross-curricular activity still rendered adequate levels of physical activity. After studying the different measurement protocols (pedometers, accelerometers, and the observational method) used in this study, results suggested the pedometer to be the most feasible device to use for measuring children in these types of physical activity settings. One implication for practice was implementing cross-curricular physical activity as a supplement to other physical activities or as an addition to physical education classes in an effort to allow more time for academic instruction while having students engaged in physical activity. Another recommendation for practice was to use pedometers as a cost-effective physical activity measurement device for elementary students. (PsycINFO Database Record (c) 2013 APA, all rights reserved)
SN - 0419-4209
AD - Greene, Amanda: East Tennessee State U., US
ER -

TY - JOUR
ID - 2411
T1 - Elementary schools' response to recommendations for the prevention of childhood obesity: A secondary data analysis using the 2000 and 2006 School Health Policies and Programs Surveys
A1 - Barrett,Edith
Y1 - 2012///
N1 - Dissertation Abstract: 2012-99200-520
English
Dissertation Abstract
KW - PsycInfo
KW - Educational Programs
KW - Elementary Schools
KW - Obesity
KW - Policy Making
KW - Prevention
KW - Data Collection
KW - Surveys
RP - NOT IN FILE
SP - 2108
JF - Dissertation Abstracts International: Section B: The Sciences and Engineering
VL - 73:
IS - 4-B
N2 - The rise of childhood obesity in the past three decades has established this issue as a national health crisis and an epidemic. Schools are primary sites for both the study and the prevention of childhood obesity, because they are environments where most children can be reached, and where children's health habits are influenced (IOM, 2005). The study was a secondary data analysis using data from the 2000 and 2006 School Health Policies and Programs Surveys (SHPPS). The goal of this study was to examine and compare data pertaining to exercise, nutrition, health screening and education in elementary schools by the economic level of the school and by the year of the survey. Chi-square Tests were used to examine the relationship between the economic level and the variables in the study, and between the year of the survey and the variables in the study. The Mantel-Haenszel Test was employed to examine the relationship of the economic level with the study variables while controlling for the year. By year of survey, the results indicated that in 2006, schools were less likely to screen for Body Mass Index/Height and Weight, less likely to provide nutrition education, and less likely to provide nutrition education than in 2000. In 2006, schools were more likely to provide recess in grade four, offer fruit, and not allow junk food at school parties than in 2000. By economic level, the results indicated that poor schools were less likely to provide soda sports drinks not 100% juice, and were more likely to require physical education than the not poor schools. While controlling for the year of the study, the not poor schools were more likely to offer soda/sports drinks not 100% juice, were less likely to require physical education, and were more likely to offer physical education 3 days or less per week than the poor schools. The results of this study showed that although there have been some small changes in the school health environment related to preventing childhood obesity, these changes have not met the recommendations of expert groups in the field of childhood obesity. (PsycINFO Database Record (c) 2012 APA, all rights reserved)
SN - 0419-4217
AD - Barrett, Edith: U Massachusetts Boston, US
ER -

TY - JOUR
ID - 2414
T1 - Ethnic identity and discrimination among children. [References]
A1 - Friesen,Jane; Arifovic,Jasmina; Wright,Stephen
Y1 - 2012///
N1 - Peer Reviewed Journal: 2012-30577-010
English
Journal; Peer Reviewed Journal
KW - PsycInfo
KW - Childhood Development
KW - Ethnic Identity

KW - Games
KW - Race and Ethnic Discrimination
RP - NOT IN FILE
SP - 1156
EP - 1169
JF - Journal of Economic Psychology
VL - 33:
IS - 6
N2 - We engaged over 430 Canadian children in a series of activities designed to reveal their evaluations of three ethnic groups (White, East Asian and South Asian), their identification with these groups, and their behavior towards them in a dictator game. Our experiments took place at the children's schools during their normal school day, allowing us to evaluate the salience and effects of ethnic identities on economically relevant behavior in an important natural setting. We find that children from the dominant White category have a clear sense of White ethnic identity, and tend to favor White recipients in the dictator game relative to East Asian or South Asian recipients. Minority East Asian children reveal a more complex ethnic identity; they perceive themselves to be equally similar to White and East Asian children. Unlike Whites, East Asian children do not favor recipients from their own East Asian category, nor do they favor recipients with whom they tend to identify. If anything, East Asian children show out-group favoritism. (PsycINFO Database Record (c) 2012 APA, all rights reserved) (journal abstract)
SN - 0167-4870
AD - Friesen, Jane: Department of Economics, Simon Fraser University, Burnaby, BC, Canada Arifovic, Jasmina: Department of Economics, Simon Fraser University, Burnaby, BC, Canada Wright, Stephen C.: Department of Psychology, Simon Fraser University, Burnaby, BC, Canada Ludwig, Andreas: Department of Economics, Simon Fraser University, Burnaby, BC, Canada Giamo, Lisa: Department of Psychology, Simon Fraser University, Burnaby, BC, Canada Baray, Gamze: Department of Psychology, Bilkent University, Turkey
ER -

TY - JOUR
ID - 2415
T1 - The effect of team pentathlon on ten- to eleven-year-old childrens' engagement in physical activity. [References]
A1 - Michaud,Valerie; Nadeau,Luc; Martel,Denis; Gagnon,Jocelyn; Godbout,Paul
Y1 - 2012///
N1 - Peer Reviewed Journal: 2012-27397-008
English
Journal; Peer Reviewed Journal
KW - PsycInfo
KW - Exercise
KW - Physical Education
KW - Teachers
KW - Teams
RP - NOT IN FILE
SP - 543
EP - 562
JF - Physical Education and Sport Pedagogy
VL - 17:
IS - 5
N2 - Background: To promote regular physical activity (PA) among children and adolescents, authors recommend that physical education (PE) teachers offer their students programmes that would allow them to be physically active outside PE classes. However, such programmes are rarely rigorously assessed and it is recommended that further studies be undertaken to better understand characteristics of interventions that render children and adolescents more physically active. Aims: The purposes of this study were (a) to examine the effectiveness of a PA intervention, the team pentathlon, and (b) to examine whether the impact of team pentathlon was different for boys and girls. Method: Four primary school PE teachers and eight 5th grade elementary classes (two for each PE teacher: one experimental and one control) took part in the study. Overall, 86 students (44 girls; 42 boys) experienced team pentathlon in addition to their regular PE classes whereas 82 students (41 girls; 41 boys) simply attended their regular PE classes. The research design included: (a) a 3-week

baseline measure of the physical activity volume (PAV) for both the experimental group (EG) and the control group (CG); (b) the implementation of a 5-week intervention programme and weekly PAV measures for the EG (weeks 4 to 8) and periodic PAV measures for the CG (weeks 4, 6 and 8); (c) a 2-week recess for both groups (weeks 9 and 10); (d) PAV measures for both groups during weeks 11 and 12. PAV was computed in terms of pentathlon hours (PH), meaning the actual number of activity hours reported by students, corrected according to the intensity (energy cost) of the practiced physical activities. A repeated measures ANOVA was performed to see if there was a difference between the experimental and control groups, between boys and girls and between the four repeated measures. Non-parametric analyses were performed to see if there was any significant difference between reported volumes of physical activity for each PE teacher's classes. Results: Overall the results showed that control students' level of activity remained stable while experimental students were significantly more physically active at the time of the pentathlon implementation as well as three weeks after the end of the intervention programme. In both experimental and control groups, girls accumulated less pentathlon hours each week than boys. Results also showed that the regular practice of PA, outside of PE classes, varied between schools, both in control and experimental conditions. At times, the weekly volume of physical activity also varied greatly among students from the same classes. Conclusion: Team pentathlon brought about a greater volume of physical exercise among ten- and eleven-year-old children. Although girls reported a slightly larger raw volume of physical activity per week than boys, they accumulated lower pentathlon hour values due to their choice of less intense activities. Finally, the results also showed that regular PA, outside PE classes, may vary from one school to another or even among students from the same class. (PsycINFO Database Record (c) 2012 APA, all rights reserved) (journal abstract)
SN - 1740-8989
AD - Michaud, Valerie: Departement d'Education Physique, Universite Laval, Quebec, PQ, Canada Nadeau, Luc: Departement d'Education Physique, Universite Laval, Quebec, PQ, Canada Martel, Denis: Departement d'Education Physique, Universite Laval, Quebec, PQ, Canada Gagnon, Jocelyn: Departement d'Education Physique, Universite Laval, Quebec, PQ, Canada Godbout, Paul: Departement d'Education Physique, Universite Laval, Quebec, PQ, Canada
ER -

TY - JOUR
ID - 2417
T1 - Correlates of sedentary behaviours in preschool children: A review. [References]
A1 - Hinkley,Trina; Salmon,Jo; Okely,Anthony
Y1 - 2010///
N1 - Peer Reviewed Journal: 2012-32479-001
English
Journal; Peer Reviewed Journal
KW - PsycInfo
KW - Health
KW - Health Behavior
KW - Preschool Students
RP - NOT IN FILE
JF - The International Journal of Behavioral Nutrition and Physical Activity
VL - 7 Sep 2010, ArtID 66.
N2 - Background: Sedentary behaviour has been linked with a number of health outcomes. Preschool-aged children spend significant proportions of their day engaged in sedentary behaviours. Research into the correlates of sedentary behaviours in the preschool population is an emerging field, with most research being published since 2002. Reviews on correlates of sedentary behaviours which include preschool children have previously been published; however, none have reported results specific to the preschool population. This paper reviews articles reporting on correlates of sedentary behaviour in preschool children published between 1993 and 2009. Methods: A literature search was undertaken to identify articles which examined correlates of sedentary behaviours in preschool children. Articles were retrieved and evaluated in 2008 and 2009. Results: Twenty-nine studies were identified which met the inclusion criteria. From those studies, 63 potential correlates were identified. Television viewing was the most commonly examined sedentary behaviour. Findings from the review suggest that child's sex was not associated with television viewing and had an indeterminate association with sedentary behaviour as measured by accelerometry. Age, body mass index, parental education and race had an indeterminate association with television viewing, and outdoor playtime had no association with television viewing. The remaining 57 potential correlates had been investigated too infrequently to be able to draw robust

conclusions about associations. Conclusions: The correlates of preschool children's sedentary behaviours are multi-dimensional and not well established. Further research is required to provide a more comprehensive understanding of the influences on preschool children's sedentary behaviours to better inform the development of interventions. (PsycINFO Database Record (c) 2012 APA, all rights reserved) (journal abstract)
SN - 1479-5868
AD - Hinkley, Trina: Centre for Physical Activity and Nutrition Research, Deakin University, Burwood, VIC, Australia Salmon, Jo: Centre for Physical Activity and Nutrition Research, Deakin University, Burwood, VIC, Australia Okely, Anthony D.: Interdisciplinary Educational Research Institute, University of Wollongong, Wollongong, NSW, Australia Trost, Stewart G.: College of Health & Human Sciences, Oregon State University, Corvallis, OR, US
ER -

TY - JOUR
ID - 2422
T1 - Independent mobility, perceptions of the built environment and children's participation in play, active travel and structured exercise and sport: The PEACH project. [References]
A1 - Page,Angie S; Cooper,Ashley
Y1 - 2010///
N1 - Peer Reviewed Journal: 2012-31862-001
English
Journal; Peer Reviewed Journal
KW - PsycInfo
KW - Built Environment
KW - Childhood Play Behavior
KW - Perception
KW - Physical Mobility
KW - Commuting (Travel)
KW - Exercise
KW - Physical Activity
KW - Sports
RP - NOT IN FILE
JF - The International Journal of Behavioral Nutrition and Physical Activity
VL - 7 Feb 2010, ArtID 17.
N2 - Background: Independent mobility (IM) and perceptions of the built environment may relate differentially to children's participation in various physical activity contexts. This cross-sectional study investigated whether independent mobility and perceptions of the built environment in boys and girls were related to physical activity in three different contexts (outdoor play, structured exercise/sport, active commuting). Methods: Thirteen hundred and seven 10-11 year old boys and girls from 23 schools in a large UK city completed a computerised questionnaire. Independent variables in logistic regression analyses were weekly self-reported frequency of participation in outdoor play, structured exercise/sport and mode of travel home from school. Dependent variables were perceptions of the environment (aesthetics, nuisance, safety, social norm, constraint, play space, accessibility), local and area independent mobility and linear distance from home to school. Analyses were adjusted for body mass index, minutes of daylight after school, level of neighbourhood deprivation and pubertal status. Results: For boys, local independent mobility (Local-IM) was related to an increased likelihood of everyday participation in play (OR 1.58: 95% CI 1.19-2.10), structured exercise/sport (OR 1.42: 1.06-1.89) and active commuting (OR 1.40: 1.07-1.87) but was only related to active commuting for girls (OR 1.49: 1.07-2.07). Boys and girls were more likely to report playing out every day if they had higher scores for Social Norm (Boys: OR 1.63 (1.12-2.37); Girls: OR 1.53 (1.01-2.31)) and, for girls only, more positive perceptions of traffic safety (OR 1.63: 1.14-2.34). Easy access to a range of destinations was the dominant predictor for taking part in structured exercise/sport everyday (Boys: OR 1.62 (1.01-2.66); Girls: OR 1.65 (1.07-2.53)). Shorter distance from home to school (OR 0.99: 0.98-0.99) and, for boys only, greater perceived accessibility (OR 1.87: 1.04-3.36) were significantly related to active commuting to school. Conclusions: Perceptions of the physical environment relate differently to different physical activity contexts and by gender. The only consistent correlate for outdoor play, structured ex/sport and active commuting was higher independent mobility to visit local destinations (Local-IM) for boys. Considering both the physical activity context and its independent correlates should improve the specificity of physical activity interventions in children. (PsycINFO Database Record (c) 2012 APA, all rights reserved)

SN - 1479-5868
AD - Page, Angie S.: Department of Exercise, Nutrition & Health Sciences, University of Bristol, Bristol, United Kingdom Cooper, Ashley R.: Department of Exercise, Nutrition & Health Sciences, University of Bristol, Bristol, United Kingdom Griew, Pippa: Department of Exercise, Nutrition & Health Sciences, University of Bristol, Bristol, United Kingdom Jago, Russell: Department of Exercise, Nutrition & Health Sciences, University of Bristol, Bristol, United Kingdom
ER -

TY - JOUR
ID - 2429
T1 - Children's recess physical activity: Movement patterns and preferences. [References]
A1 - Woods,Amelia Mays; Graber,Kim; Daum,David
Y1 - 2012///
N1 - Peer Reviewed Journal: 2012-09638-004
English
Journal; Peer Reviewed Journal
KW - PsycInfo
KW - Childhood Play Behavior
KW - Individual Differences
KW - Physical Activity
KW - Preferences
KW - Student Characteristics
KW - Social Processes
RP - NOT IN FILE
SP - 146
EP - 162
JF - Journal of Teaching in Physical Education
VL - 31:
IS - 2
N2 - The benefits of recess can be reaped by all students regardless of socioeconomic status, race, or gender and at relatively little cost. The purpose of this study was to examine physical activity (PA) variables related to the recess PA patterns of third and fourth grade children and the social preferences and individuals influencing their PA (friends and parents). Data were collected on students (N = 115) utilizing the System of Observing Children's Activity and Relationships during Play (SOCARP) instrument. In addition, each child was interviewed during the recess period in which SOCARP was completed. Results found that boys spent significantly more time being very active (t (95.64) = 3.252, d = .62, p < .008) than girls and preferred sport activities (t = (73.62) 5.64, d = 1.14, p < .0125) in large groups (t (69.34) = 4.036, d = .83, p < .0125). Meanwhile, girls preferred locomotor activities (t (113) = 3.19, d = .60, p < .0125), sedentary activities (t (113) = 2.829, d = .53, p < .0125) and smaller groups (t (112.63) = 4.259, d = .79, p < .0125). All 115 participants indicated that they wanted to spend time with their friends during recess. (PsycINFO Database Record (c) 2012 APA, all rights reserved) (journal abstract)
SN - 0273-5024
AD - Woods, Amelia Mays: University of Illinois, Urbana, IL, US Graber, Kim: University of Illinois, Urbana, IL, US Daum, David: University of Illinois, Urbana, IL, US
ER -

TY - JOUR
ID - 2430
T1 - The effects of prompts and a group-oriented contingency on out-of-school physical activity in elementary school-aged students. [References]
A1 - Hastie,Peter; van der Mars,Hans; Layne,Todd; Wadsworth,Danielle
Y1 - 2012///
N1 - Peer Reviewed Journal: 2012-09638-003
English
Journal; Peer Reviewed Journal
KW - PsycInfo
KW - Elementary School Students

KW - Physical Activity
KW - Student Characteristics
KW - Contingency Management
RP - NOT IN FILE
SP - 131
EP - 145
JF - Journal of Teaching in Physical Education
VL - 31:
IS - 2
N2 - This study examined the effectiveness of three conditions in which 48 fourth-grade students were prompted to be physically active out of school. Using an alternating treatments design (Cooper, Heron, & Heward, 2007) the three intervention conditions included: (a) Baseline: No prompting of students, (b) Teacher Prompts: Verbal prompt to "remember to do something active after school today", and (c) Teacher Prompts and group-oriented contingencies: Verbal prompts with an index card where students could record their activity to earn bonus points as part of a team challenge. Graphically plotted pedometer data depicting data paths, variability, and trends within and across three conditions showed that students were more active outside of school only when the contingent reinforcement (c) was in place. This suggests that using prompts and group-oriented contingencies within Sport Education appears to be an effective and authentic context for promoting independent (i.e., free play) out-of-school time physical activity. (PsycINFO Database Record (c) 2012 APA, all rights reserved) (journal abstract)
SN - 0273-5024
AD - Hastie, Peter: Auburn University, Auburn, AL, US van der Mars, Hans: Arizona State University, Mesa, AZ, US Layne, Todd: Auburn University, Auburn, AL, US Wadsworth, Danielle: Auburn University, Auburn, AL, US
ER -

TY - JOUR
ID - 2431
T1 - Time management in 12 Chilean kindergarten classrooms: Recess, snack and a little teaching. [Spanish]. [References]
A1 - Strasser,Katherine; Lissi,Maria Rosa; Silva,Macarena
Y1 - 2009///
N1 - Peer Reviewed Journal: 2011-27975-008
Spanish
Journal; Peer Reviewed Journal
KW - PsycInfo
KW - Classroom Management
KW - Classrooms
KW - Kindergartens
KW - Time Management
KW - Teaching
KW - Funding
KW - Socioeconomic Status
RP - NOT IN FILE
SP - 85
EP - 96
JF - Psykhe: Revista de la Escuela de Psicologia
VL - 18:
IS - 1
N2 - This study examined time management in 12 kindergarten classrooms from 9 Chilean schools with diverse sources of funding and socioeconomic status (SES). We conducted 33 observations in the 12 classrooms (average duration 197 minutes), and determined the amount of time devoted to different activities. More than half of the time in these classrooms was spent in non-instructional activities such as recess, snack, and managing the children's behavior. Additionally, the distribution of instructional time is not in accordance with current findings regarding the activities that are more fruitful for children's development. These results are independent of the schools' source of funding and SES. (PsycINFO Database Record (c) 2012 APA, all rights reserved) (journal abstract)

SN - 0717-0297
AD - Strasser, Katherine: Escuela de Psicologia, Pontificia Universidad Catolica de Chile, Santiago, Chile Lissi, Maria Rosa: Escuela de Psicologia, Pontificia Universidad Catolica de Chile, Santiago, Chile Silva, Macarena: Escuela de Psicologia, Pontificia Universidad Catolica de Chile, Santiago, Chile
ER -

TY - JOUR
ID - 2434
T1 - Exploring the leisure experiences of young people with spinal cord injury or disease. [References]
A1 - Hynes,Kimberley; Galvin,Jane; Howie,Linsey
Y1 - 2012///
N1 - Peer Reviewed Journal: 2012-27185-007
English
Journal; Peer Reviewed Journal
KW - PsycInfo
KW - Leisure Time
KW - Narratives
KW - Spinal Cord Injuries
KW - Interpersonal Relationships
RP - NOT IN FILE
SP - 361
EP - 368
JF - Developmental neurorehabilitation
JA - Dev Neurorehabil
VL - 15:
IS - 5
N2 - Purpose: To explore the perceptions of young people with a spinal cord injury or disease (SCI/D) about their leisure participation at school, home and in the community. Methods: The study used narrative enquiry to elicit the unique leisure stories of three participants aged 8-11 years with a SCI/D during two face-to-face, semi-structured interviews. Pictorial data about participants' interpersonal relationships were also gathered using a 'social atom' tool. Results: Thematic analysis of participants' narratives produced three themes. 'Inside school leisure' and 'outside school leisure' detailed participants' lived experiences of leisure. 'A can-do approach to leisure' drew attention to the optimistic attitudes held by the participants and their active engagement in leisure despite their SCI/D. Conclusions: Clinicians, families and educators should explore the leisure preferences of young people with a SCI/D and support their participation in activities, which offer enjoyment, friendship and a sense of achievement. (PsycINFO Database Record (c) 2012 APA, all rights reserved) (journal abstract)
SN - 1751-8423
AD - Hynes, Kimberley: Departmentl of Occupational Therapy, La Trobe University, Melbourne, VIC, Australia Galvin, Jane: Departmentl of Occupational Therapy, La Trobe University, Melbourne, VIC, Australia Howie, Linsey: Departmentl of Occupational Therapy, La Trobe University, Melbourne, VIC, Australia
ER -

TY - JOUR
ID - 2435
T1 - Implementation of a manualized communication intervention for school-aged children with pragmatic and social communication needs in a randomized controlled trial: The Social Communication Intervention Project. [References]
A1 - Adams,Catherine; Lockton,Elaine; Gaile,Jacqueline; Earl,Gillian; Freed,Jenny
Y1 - 2012///
N1 - Peer Reviewed Journal: 2012-26267-002
English
Journal; Peer Reviewed Journal
KW - PsycInfo
KW - School Based Intervention
KW - Treatment Guidelines
KW - Language Therapy
KW - Speech Language Pathology

KW - Communication Skills Training
KW - Early Intervention
KW - Interpersonal Communication
KW - Pragmatics
RP - NOT IN FILE
SP - 245
EP - 256
JF - International Journal of Language & Communication Disorders
VL - 47:
IS - 3
N2 - Background: Speech-language interventions are often complex in nature, involving multiple observations, variable outcomes and individualization in treatment delivery. The accepted procedure associated with randomized controlled trials (RCT) of such complex interventions is to develop and implement a manual of intervention in order that reliable treatment delivery can be achieved. Aims: To present the rationale, structure and content of an intensive manualized intervention as implemented within an RCT for children with complex pragmatic and social communication needs; to investigate factors associated with implementation in a mainstream school environment; and to determine treatment fidelity. Methods & Procedures: The manualized SCIP intervention, including procedures for developing individualized treatment plans, was developed and then implemented within an RCT with 57 school-aged children with complex pragmatic communication needs (CwPLI). The paper describes the delivery protocol, staffing requirements, and content and structure of the intervention. A mapping procedure for individualization of intervention and the implemented components of intervention are presented. The findings from a school-therapy alliance checklist for recording factors affecting implementation in a school context are also reported. Treatment fidelity was carried out using measures of delivered versus planned treatment content and quality of therapy. Outcomes & Results: The manual was effective at detailing intervention procedures and allowing for development of individualized treatment plans whilst maintaining satisfactory treatment fidelity. Treatment planning and delivery required continuous specialist speech and language therapist input with assistants needing substantive training and supervision. Key components of intervention for CwPLI were therapies aimed at improving conversation skills, narrative construction, comprehension monitoring, understanding of social cues and metapragmatic awareness. The school-therapy alliance checklist indicated high rates of therapist-education staff and therapist-parent liaison. Parents were nearly always involved in treatment planning though only half-attended therapy sessions. Learning support provision in schools for participating children was highly variable. Conclusions & Implications: Predetermining theoretical background, structure, and content in a treatment manual contributed towards the quality and reliability of intervention within the context of an RCT. The implementation of SCIP intervention in a broader clinical context is discussed with reference to staff expertise, the therapeutic process and the essential ingredients of social communication intervention. (PsycINFO Database Record (c) 2012 APA, all rights reserved) (journal abstract)
SN - 1368-2822
AD - Adams, Catherine: Human Communication and Deafness Group, University of Manchester, Manchester, United Kingdom Lockton, Elaine: Human Communication and Deafness Group, University of Manchester, Manchester, United Kingdom Gaile, Jacqueline: Human Communication and Deafness Group, University of Manchester, Manchester, United Kingdom Earl, Gillian: NHS Lothian, Scotland Freed, Jenny: Human Communication and Deafness Group, University of Manchester, Manchester, United Kingdom
ER -

TY - JOUR
ID - 2436
T1 - Diana, leaving the cult: Play therapy in childhood and talk therapy in adolescence. [References]
A1 - Goldberg,Lorna
Y1 - 2011///
N1 - Peer Reviewed Journal: 2012-23173-003
English
Journal; Peer Reviewed Journal
KW - PsycInfo
KW - Cultism
KW - Early Experience
KW - Personality Development

KW - Play Therapy
KW - Psychoanalysis
RP - NOT IN FILE
SP - 33
EP - 43
JF - International Journal of Cultic Studies
VL - 2:
IS - 1
N2 - This paper presents clinical material from the psychoanalytically oriented therapy of an 8- year-old girl, who was born and raised in a cult. The author considers how this child's response to the traumatic events of her cult experience influenced her developing personality. Therapy sessions, beginning about thirty years ago, occurred during two time frames: Play therapy lasted for 2 years. Therapy was resumed for 1 year in adolescence. Play therapy focused on helping this client deal with some of her painful cult experiences and aided in the transition to a new world outside. Later, talk therapy during adolescence enhanced her ability to deal with adolescent difficulties that appeared to be impacted by cult experiences. (PsycINFO Database Record (c) 2012 APA, all rights reserved) (journal abstract)
SN - 2154-7270
AD - Goldberg, Lorna: Institute for Psychoanalytic Studies, NJ, US
ER -

TY - JOUR
ID - 2437
T1 - Mobile devices as support rather than distraction for mobile learners: Evaluating guidelines for design. [References]
A1 - Eliasson,Johan; Pargman,Teresa Cerratto; Nouri,Jalal; Spikol,Daniel; Ramberg,Robert
Y1 - 2011///
N1 - Peer Reviewed Journal: 2012-16168-001
English
Journal; Peer Reviewed Journal
KW - PsycInfo
KW - Distraction
KW - Geometry
KW - Human Computer Interaction
KW - Learning Environment
KW - Mobile Devices
KW - Activity Theory
KW - Middle School Students
RP - NOT IN FILE
SP - 1
EP - 15
JF - International Journal of Mobile and Blended Learning
VL - 3:
IS - 2
N2 - This article questions the design of mobile learning activities that lead students to spend time focusing on the mobile devices at the expense of interacting with other students or exploring the environment. This problem is approached from an interaction design perspective, designing and analysing geometry-learning activities. The authors present six guidelines for designing mobile learning activities, where mobile devices support rather than distract students from contents and contexts relevant to the learning goals. The guidelines are developed through video analysis of groups of middle school students doing learning activities outdoors and evaluated using the task model. The guidelines suggest that students (1) assume roles based on a different functionality of each device, (2) use devices as contextual tools, that the activities, (3) include physical interaction with the environment, (4) let teachers assume roles, (5) encourage face-to-face communication, and (6) introduce students to the mobile devices. (PsycINFO Database Record (c) 2012 APA, all rights reserved) (journal abstract)
SN - 1941-8647

AD - Eliasson, Johan: Stockholm University, Stockholm, Sweden Pargman, Teresa Cerratto: Stockholm University, Stockholm, Sweden Nouri, Jalal: Stockholm University, Stockholm, Sweden Spikol, Daniel: Stockholm University, Stockholm, Sweden Ramberg, Robert: Stockholm University, Stockholm, Sweden
ER -

TY - JOUR
ID - 2438
T1 - Exploring the challenges of supporting collaborative mobile learning. [References]
A1 - Nouri,Jalal; Cerratto-Pargman,Teresa; Eliasson,Johan; Ramberg,Robert
Y1 - 2011///
N1 - Peer Reviewed Journal: 2012-16166-004
English
Journal; Peer Reviewed Journal
KW - PsycInfo
KW - Activity Theory
KW - Collaborative Learning
KW - Cooperative Learning
KW - Scaffolding
KW - Mobile Devices
KW - Classrooms
RP - NOT IN FILE
SP - 54
EP - 69
JF - International Journal of Mobile and Blended Learning
VL - 3:
IS - 4
N2 - Mobile technology opens up opportunities for collaborative learning in otherwise remote contexts outside the classroom. A successful realization of these opportunities relies, however, on mobile learning activities providing adequate collaboration structures. This article presents an empirical study aimed at examining the role played by mobile devices, teachers and task structures as a means for collaborative learning in geometry. The study focused on the analysis of the nature of collaboration that unfolded when students measured areas outdoors in the field. The analysis of the mobile learning activity was conducted from an Activity theory perspective. The findings obtained indicate that the collaboration observed may be impaired if: 1) the functionalities needed for collaborative problem-solving are asymmetrically distributed on a number of mobile devices; 2) task-related information is not accessible to all learners; 3) the task structure is not sufficiently complex; 4) teacher scaffolding is too readily available; and 5) necessary collaborative skills are not developed. (PsycINFO Database Record (c) 2012 APA, all rights reserved) (journal abstract)
SN - 1941-8647
AD - Nouri, Jalal: Stockholm University, Stockholm, Sweden Cerratto-Pargman, Teresa: Department of Computer and Systems Sciences (DSV), Stockholm University, Stockholm, Sweden Eliasson, Johan: Department of Computer and System Sciences, Stockholm University, Stockholm, Sweden Ramberg, Robert: Department of Psychology, Stockholm University, Stockholm, Sweden
ER -

TY - JOUR
ID - 2439
T1 - Variables involved in the acquisition of Catalan in linguistic support classes in primary school in Catalonia: An empirical study. [Spanish]
A1 - Siques,Carina; Perera,Santiago; Vila,Ignasi
Y1 - 2012///
N1 - Peer Reviewed Journal: 2012-10073-015
Spanish
Journal; Peer Reviewed Journal
KW - PsycInfo
KW - Elementary Schools
KW - Human Migration
KW - Linguistics

KW - Writing Skills
RP - NOT IN FILE
SP - 444
EP - 456
JF - Anales de Psicologia
VL - 28:
IS - 2
N2 - Linguistic support given to migrant students who incorporate late into the school systems is usually situated in two opposite poles. On one end of the pole is the linguistic support offered in mainstream class-rooms and, on the other end, learning the school language in special class-rooms outside school premises. With the creation of linguistic support classes in schools which receive migrant population, Catalonia has adopted an intermediate position. The research shows the weigh which some explanatory variables have in the learning of Catalan. Two different tests were used: one to measure school adaptation and another to measure linguistic knowledge. The participants were 10415 primary school children who attended linguistic support classes. The results reveal that the variable school adaptation is the one which modifies more the probability of students succeeding in the different linguistic tests used. However, the probability is not the same in the tests assessing oral and writing skills. In the writing tests, the students who have a high school adaptation have nearly twice as many probabilities of being successful whereas in the oral tests the variable length of stay in the country plays an important role as well. The students' family language also modifies the students' linguistic results. (PsycINFO Database Record (c) 2012 APA, all rights reserved) (journal abstract)
SN - 0212-9728
AD - Siques, Carina: Universidad de Girona, Girona, Spain Perera, Santiago: Universidad de Girona, Girona, Spain Vila, Ignasi: Universidad de Girona, Girona, Spain
ER -

TY - JOUR
ID - 2440
T1 - A concept-map integrated dynamic assessment system for improving ecology observation competences in mobile learning activities. [References]
A1 - Hung,Pi-Hsia; Hwang,Gwo-Jen; Su,
Y1 - 2012///
N1 - Peer Reviewed Journal: 2012-24335-002
English
Journal; Peer Reviewed Journal
KW - PsycInfo
KW - Gifted
KW - Observational Learning
KW - School Learning
KW - Science Education
KW - Mobile Devices
KW - Competence
KW - Ecology
KW - Learning
KW - Students
RP - NOT IN FILE
SP - 10
EP - 18
JF - TOJET: The Turkish Online Journal of Educational Technology
VL - 11:
IS - 1
N2 - Observation competence plays a fundamental role in outdoor scientific investigation. The computerized concept mapping technique as a Mindtool has shown the potential for enhancing meaningful learning in science education. The purposes of the present study are to develop a concept map integrated mobile learning design for ecology observation and to examine the implementation effect for elementary school children. Also, the difference in growth slopes between gifted and average students is investigated. Eighteen gifted students and thirty average students were included in this study. A computerized dynamic assessment system which combines a Computerized Ecology Observation Competence Assessment (CEOCA) and a concept map

integrated ecology observation learning design were implemented. The results of the hierarchical linear model (HLM) analysis reveal that the overall growth slope is significant (= 0.27, p < .01). The effect size is 0.53. The growth slope of the gifted students is a little higher than that of the average students. However, the slope difference between ability groups is not significant. The results suggest that a well designed concept map integrated learning system demonstrates very promising potential for enhancing both the gifted and average students' mobile observation competence. The system developed in this study could be a useful resource for elementary school outdoor learning design. (PsycINFO Database Record (c) 2012 APA, all rights reserved) (journal abstract)
SN - 2146-7242
AD - Hung, Pi-Hsia: Graduate Institute of Measurement and Statistics, National University of Tainan, Tainan, Taiwan Hwang, Gwo-Jen: Graduate Institute of Digital Learning and Education, National Taiwan University of Science and Technology, Taipei, Taiwan Su, I-Hsiang: Graduate Institute of Measurement and Statistics, National University of Tainan, Tainan, Taiwan Lin, I-Hua: Graduate Institute of Measurement and Statistics, National University of Tainan, Tainan, Taiwan
ER -

TY - JOUR
ID - 2441
T1 - Attributes of child care centers and outdoor play areas associated with preschoolers' physical activity and sedentary behavior. [References]
A1 - Sugiyama,Takemi
Y1 - 2012///
N1 - Peer Reviewed Journal: 2012-10511-002
English
Journal; Peer Reviewed Journal
KW - PsycInfo
KW - Child Care
KW - Childhood Play Behavior
KW - Physical Activity
KW - Preschool Students
RP - NOT IN FILE
SP - 334
EP - 349
JF - Environment and Behavior
VL - 44:
IS - 3
N2 - This study examined characteristics of child care centers associated with preschoolers' moderate-to-vigorous physical activity and sedentary behavior while in child care (MVPA-C, SB-C), and attributes of outdoor play areas associated with the same behaviors during outdoor time (MVPA-O, SB-O). Participants were 89 children (3 -5 years) recruited from 10 child care centers in Brisbane, Australia. Children's activity was measured by accelerometer over a 3-day period. Center characteristics and outdoor play area attributes were identified through survey and observation. We found that children were mostly sedentary while in child care. Lower child-staff ratios and using indoor play areas for motor activity were associated with more MVPA-C and less SB-C. Fixed play equipment in outdoor areas was conducive to more MVPA-O and less SB-O. This study suggests the possibility of enhancing preschoolers' activity levels through changing these attributes. Further research with more centers from diverse settings is needed to test these findings. (PsycINFO Database Record (c) 2012 APA, all rights reserved) (journal abstract)
SN - 0013-9165
AD - Sugiyama, Takemi: Cancer Prevention Research Centre, University of Queensland, Brisbane, QLD, Australia Okely, Anthony D.: Interdisciplinary Educational Research Institute, University of Wollongong, NSW, Australia Masters, Jane M.: Cancer Prevention Research Centre, University of Queensland, Brisbane, QLD, Australia Moore, Gary T.: University of Sydney, Sydney, NSW, Australia
ER -

TY - JOUR
ID - 2442
T1 - Connection to nature: Children's affective attitude toward nature. [References]

A1 - Cheng,Judith Chen-Hsuan; Monroe,Martha
Y1 - 2012///
N1 - Peer Reviewed Journal: 2012-00003-002
English
Journal; Peer Reviewed Journal
KW - PsycInfo
KW - Environmental Education
KW - Student Attitudes
RP - NOT IN FILE
SP - 31
EP - 49
JF - Environment and Behavior
VL - 44:
IS - 1
N2 - A connection to nature index was developed and tested to measure children's affective attitude toward the natural environment. The index was employed through a survey that investigates students' attitude toward Lagoon Quest, a mandatory environmental education program for all fourth-grade, public school students in Brevard County, Florida. Factor analyses were conducted to explore and confirm different factors in the connection to nature index. A path analysis was conducted to examine the association among variables. The results suggest four dimensions in the children's connection to nature index: (a) enjoyment of nature, (b) empathy for creatures, (c) sense of oneness, and (d) sense of responsibility. Children's connection to nature influences their intention to participate in nature-based activities in the future. Children's connection to nature, their previous experience in nature, their perceived family value toward nature, and their perceived control positively influenced their interest in performing environmentally friendly behaviors. (PsycINFO Database Record (c) 2012 APA, all rights reserved) (journal abstract)
SN - 0013-9165
AD - Cheng, Judith Chen-Hsuan: Department of Tourism and Hospitality Management, Tamkang University, Taipei, Taiwan Monroe, Martha C.: School of Forest Resources and Conservation, University of Florida, Gainesville, FL, US
ER -

TY - JOUR
ID - 2443
T1 - A cross-cultural study of observed conflicts between young children. [References]
A1 - Martinez-Lozano,Virginia; Sanchez-Medina,Jose
Y1 - 2011///
N1 - Peer Reviewed Journal: 2011-15298-001
English
Journal; Peer Reviewed Journal
KW - PsycInfo
KW - Childhood Play Behavior
KW - Collectivism
KW - Conflict
KW - Cross Cultural Differences
KW - Individualism
KW - Childhood Development
RP - NOT IN FILE
SP - 895
EP - 907
JF - Journal of Cross-Cultural Psychology
VL - 42:
IS - 6
N2 - Young children (5 to 6 years old) from an individualistic (the Netherlands) culture and a collectivistic (South of Spain: Andalusia) culture were videotaped during leisure time on their school playground. Based on the perspective of individualism-collectivism (IND-COL), cultural differences were expected with respect to observed conflict behaviors. Conflict episodes were analyzed with respect to conflict issues, strategies, and outcomes. In Andalusia, an unexpected high number of conflicts were observed, about three times higher than in

the Netherlands. As expected, Andalusian children turned out to be more concerned with control of play and behavior and Dutch children more with control of objects and space. With respect to strategy use, Andalusian children used negotiation more often than Dutch children. The latter included more often nonverbal and directive ingredients in their strategies. Dutch children ended their conflicts by means of social or physical rupture much more often than Andalusian children. The latter preferred to continue the interaction, even if this required submission to others' wishes. Results are discussed from the perspective of IND-COL, with particular emphasis on four characteristics of studies of peer conflicts: definition of conflict, method of data collection, age of participants, and social setting of the participants. (PsycINFO Database Record (c) 2012 APA, all rights reserved) (journal abstract)
SN - 0022-0221
AD - Martinez-Lozano, Virginia: University Pablo de Olavide, Seville, Spain Sanchez-Medina, Jose A.: University Pablo de Olavide, Seville, Spain Goudena, Paul P.: Utrecht University, Utrecht, Netherlands
ER -

TY - JOUR
ID - 2444
T1 - An examination of a middle school advisory program as it relates to student and teacher relationships
A1 - Smith,Daniel Wayne Jr
Y1 - 2012///
N1 - Dissertation Abstract: 2012-99150-117
English
Dissertation Abstract
KW - PsycInfo
KW - Academic Achievement
KW - Adolescent Development
KW - Educational Programs
KW - Middle School Students
KW - Student Teachers
KW - Middle Schools
KW - School Based Intervention
KW - Teachers
RP - NOT IN FILE
SP - 439
JF - Dissertation Abstracts International Section A: Humanities and Social Sciences
VL - 73:
IS - 2-A
N2 - It takes a special person to be an educator at the middle school level. Young adolescents during these years are often dazed and confused as they are undergoing more changes in their lives than any other time, outside of infancy. In recognizing these needs, educators began calling for a reform to create "developmentally responsive" schools since the early 1900's. The idea of "developmentally responsive" becomes even more critical in the middle school years as adolescents need advice, models, guidance and direction. In response to these needs, many national organizations have recommended advisory programs as a component of an effective middle school program. While many have given this idea valiant effort, many schools have failed to implement and sustain an effective advisory program. This mixed methods intervention study examines the ways in which a middle school developed and implemented an advisory program. The goal of this program is to establish positive student-teacher relationships which will in turn impact student achievement. While the entire school district participated in the middle school advisory program, this study uses sixth grade teachers at one school as the participants for the study. Ten teachers participated in the study to assess seventeen student-teacher relationships based on the Student Teacher Relationship Scale (STRS) developed by Dr. Robert Pianta. This instrument analyzes the dimensions of Conflict, Closeness and Dependency to determine the overall quality of the relationship. Pre and post test surveys were administered after one semester of the intervention advisory program. In addition, interviews were conducted with teachers and administrators based on the survey results. The findings from this study show that the development, training, implementation and evaluation of the advisory program in this form had a positive impact on 65% of the student-teacher relationships. The Closeness subscale had the largest impact on the relationship as 71% of the studied relationships improved in this area. Teachers who designed lessons and activities geared towards the needs of the students and equally, participated yielded the greatest results. School division leaders, administrators, teachers and legislators should continue to

strive to develop positive relationships with students. The findings from this study should be examined when considering the development of a middle school advisory program. (PsycINFO Database Record (c) 2012 APA, all rights reserved)
SN - 0419-4209
AD - Smith, Daniel Wayne: U Virginia, US
ER -

TY - JOUR
ID - 2445
T1 - Trajectories and patterns of student engagement: Evidence from a longitudinal study. [References]
A1 - Wylie,Cathy
Y1 - 2012///
N1 - Book: 2012-11872-028
English
Book; Edited Book
KW - PsycInfo
KW - Student Engagement
KW - Academic Achievement
KW - Trends
RP - NOT IN FILE
SP - 585
EP - 599
JF - Christenson, Sandra L [Ed]; Reschly, Amy L [Ed]; Wylie, Cathy [Ed]
VL - :
IS - 2012
N2 - (from the chapter) Longitudinal study of student engagement patterns is relatively rare but sheds useful light on the factors that contribute to different levels of student engagement in school and its role in student achievement. This chapter uses data from a New Zealand study to focus on changes in student engagement patterns between the ages of 10 and 16, to show (a) the range of individual trajectories of student engagement that lie behind overall declines, and (b) how these different trajectories are related to differences in competency levels and to activities and relationships outside school in ways that compound the patterns of engagement in learning in the school environment and vice versa. Looking at student engagement longitudinally raises the question of whether decline in student engagement levels overall is related to transitions between schools or occurs more as part of general human development that may be better supported by different learning opportunities than schools currently provide. The chapter ends with the case for more longitudinal research into the nature and role of student engagement across different schooling contexts. (PsycINFO Database Record (c) 2012 APA, all rights reserved)
AD - Wylie, Cathy: New Zealand Council for Educational Research, Wellington, New Zealand Hodgen, Edith: New Zealand Council for Educational Research, Wellington, New Zealand
ER -

TY - JOUR
ID - 2446
T1 - It's our school too: Youth activism as educational reform, 1951--1979
A1 - Ajunwa,Kelechi
Y1 - 2012///
N1 - Dissertation Abstract: 2012-99130-070
English
Dissertation Abstract
KW - PsycInfo
KW - Activism
KW - Educational Reform
KW - High Schools
RP - NOT IN FILE
SP - 104
JF - Dissertation Abstracts International Section A: Humanities and Social Sciences
VL - 73:

IS - 1-A
N2 - Activism has the potential for reform (Howard, 1976). Unlike previous studies on high school activism this study places a primary focus on underground newspapers and argues that underground newspapers allowed high school students to function as activists as well as educational reformers. In order to make this argument, this study examined over 150 underground newspapers and other primary source publications. The goals and tactics of high school activists evolved from the 1950s to the 1970s. During this time there were some shifts in ideologies, strategies, and priorities that were influenced by both an ever increasing student frustration with school leaders and by outside historical events. Underground newspapers captured the shift that occurred in the objectives and tactics of student activists. As a result, the contents of underground newspapers were the primary focus of this study. My study reveals that there were three types of student activists: "incidental" activists who simply wanted to change individual school policies, "intentional" activists who wanted high school students to have greater authority and autonomy in schools, and lastly, "radical" activists who desired an end to oppression of people based on race, class, sex, and age. The evidence overwhelmingly indicates that for the most part incidental, intentional, and radical student activists were all working towards improving their high schools. This common goal was pivotal in the development of a Youth Empowerment social movement, which would be born out of the actions of all three types of high school activists. Incidental activists were the focal point of attention for school administrators in the 1950s, however; intentional and radical activists would take center stage by the late 1960s. Throughout the 1970s intentional and radical activists would overshadow incidental activists and dominate the high school activism scene. (PsycINFO Database Record (c) 2012 APA, all rights reserved)
SN - 0419-4209
AD - Ajunwa, Kelechi: Temple U., US
ER -

TY - JOUR
ID - 2447
T1 - Observations of children's interactions with teachers, peers, and tasks across preschool classroom activity settings. [References]
A1 - Booren,Leslie M.
Y1 - 2012///
N1 - Peer Reviewed Journal: 2012-16933-005
English
Journal; Peer Reviewed Journal
KW - PsycInfo
KW - Classroom Environment
KW - Human Sex Differences
KW - Peer Relations
KW - Preschool Education
KW - Teacher Student Interaction
RP - NOT IN FILE
SP - 517
EP - 538
JF - Early Education and Development
VL - 23:
IS - 4
N2 - Research Findings: This descriptive study examined classroom activity settings in relation to children's observed behavior during classroom interactions, child gender, and basic teacher behavior within the preschool classroom. A total of 145 children were observed for an average of 80 min during 8 occasions across 2 days using the Individualized Classroom Assessment Scoring System, an observational measure that conceptualizes behavior into teacher, peer, task, and conflict interactions. Findings indicated that on average children's interactions with teachers were higher in teacher-structured settings, such as large group. On average, children's interactions with peers and tasks were more positive in child-directed settings, such as free choice. Children experienced more conflict during recess and routines/transitions. Finally, gender differences were observed within small group and meals. Practice or Policy: These findings might encourage teachers to be thoughtful and intentional about what types of support and resources are provided so children can successfully navigate the demands of particular settings. These findings are not meant to discourage certain teacher behaviors or imply the value of certain classroom settings; instead, by providing an evidence-based picture of the conditions under which children display the most positive interactions, they can make teachers more aware of choices within

these settings and assist powerfully in professional development and interventions. (PsycINFO Database Record (c) 2012 APA, all rights reserved) (journal abstract)
SN - 1040-9289
AD - Booren, Leslie M.: Center for the Advanced Study of Teaching and Learning, University of Virginia, Charlottesville, VA, US Downer, Jason T.: Center for the Advanced Study of Teaching and Learning, University of Virginia, Charlottesville, VA, US Vitiello, Virginia E.: Teachstone, Inc., VA, US
ER -

TY - JOUR
ID - 2448
T1 - To bully or not to bully, that is not the question: Western Australian early adolescents' in search of a reputation. [References]
A1 - Houghton,Stephen John; Nathan,Elijah; Taylor,Myra
Y1 - 2012///
N1 - Peer Reviewed Journal: 2012-15625-004
English
Journal; Peer Reviewed Journal
KW - PsycInfo
KW - Adolescent Development
KW - Human Sex Differences
KW - Reputation
KW - Bullying
KW - Daily Activities
RP - NOT IN FILE
SP - 498
EP - 522
JF - Journal of Adolescent Research
VL - 27:
IS - 4
N2 - Twenty-eight early adolescent boys and girls suspended from school for bullying provided accounts of the importance of reputation in their daily lives, specifically how they initiated, promoted, and then maintained their reputation through bullying. Overall, bullying was a deliberate choice perpetrated to attain a nonconforming reputation and was initially promoted through visibility of physical bullying. These actions became more covert, particularly among girls, during the promotion phase. Sex differences were most marked in the maintenance phase. Although both boys and girls used cyber bullying to deliberately induce a sense of apprehension and fear, boys also deliberately damaged their victim's houses and gardens outside of school hours to induce a greater sense of fear and hence maintain their nonconforming reputation. (PsycINFO Database Record (c) 2012 APA, all rights reserved) (journal abstract)
SN - 0743-5584
AD - Houghton, Stephen John: University of Western Australia, Perth, WAU, Australia Nathan, Elijah: University of Western Australia, Perth, WAU, Australia Taylor, Myra: University of Western Australia, Perth, WAU, Australia
ER -

TY - JOUR
ID - 2449
T1 - Cognitive and language proficiencies predict variability in neural activity mediating semantic and syntactic processing in children
A1 - Hampton Wray,Amanda Michelle
Y1 - 2012///
N1 - Dissertation Abstract: 2012-99140-230
English
Dissertation Abstract
KW - PsycInfo
KW - Childhood Development
KW - Cognitive Ability
KW - Evoked Potentials

KW - Language Proficiency
KW - Semantics
KW - Syntax
RP - NOT IN FILE
SP - 640
JF - Dissertation Abstracts International: Section B: The Sciences and Engineering
VL - 73:
IS - 1-B
N2 - Evidence from studies of language development indicates that abilities in specific cognitive and language domains are likely to show different patterns of strengths and weaknesses across individuals. Therefore, we hypothesized that the relationships between distinct cognitive and language proficiencies and the underlying neural activity for specific language functions also differ across individuals. To date, the understanding of relationships between language proficiency and neural indices mediating language processing has been limited to the use of composite measures of language proficiency in adults. Furthermore, very little evidence exists regarding the interactions between various aspects of cognition and event-related brain potentials (ERPs) indexing language processing in children. The current study addresses the following question: How do proficiencies in specific cognitive and language functions impact neural indices mediating language processing in children? Thirty typically developing seven- and eight-year-olds were divided into high-normal and low-normal proficiency groups based on performance on nonverbal IQ, auditory word recall, and grammatical morphology tasks. ERPs were elicited by semantic anomalies and phrase-structure violations in naturally spoken sentences. Consistent with our hypothesis, the proficiencies for each of the specific cognitive and language tasks uniquely contributed to individual variability in neural indices of semantic (N400) and syntactic (P600) processing. These results suggest that distinct aptitudes within broader domains of cognition and language, even within the normal range, influence the neural signatures of semantic and syntactic processing. Furthermore, the current findings have important implications for the design and interpretation of developmental studies of ERPs indexing language processing, and they highlight the need to take into account cognitive abilities both within and outside the classic language domain. (PsycINFO Database Record (c) 2012 APA, all rights reserved)
SN - 0419-4217
AD - Hampton Wray, Amanda Michelle: Purdue U., US
ER -

TY - JOUR
ID - 2450
T1 - A comparison of two social skills interventions for children with autism spectrum disorder
A1 - Mathai,Grace
Y1 - 2012///
N1 - Dissertation Abstract: 2012-99120-029
English
Dissertation Abstract
KW - PsycInfo
KW - Autism
KW - Intervention
KW - Pervasive Developmental Disorders
KW - Risk Factors
KW - Social Skills Training
KW - Childhood Development
RP - NOT IN FILE
SP - 7717
JF - Dissertation Abstracts International: Section B: The Sciences and Engineering
VL - 72:
IS - 12-B
N2 - Social reciprocity deficits are a core feature of the autism spectrum disorders (ASD) and a major source of impairment regardless of cognitive or language ability (Carter, Davis, Klin, & Volkmar, 2005). Since these impairments do not naturally remit with age, it is critical to intervene as early as possible to offset potential risk factors (Tantum, 2003). Group training approaches provide children with teaching opportunities with other children and allow for the direct instruction of skills within a structured environment (Bellini, Peters, Benner, & Hopf, 2007). Research in social skills group research has increased, but several questions remain. This study

evaluated the outcome of a social skills curriculum for 37 children between the ages of 8 to 14 with a diagnosis of ASD within two different treatment contexts, the camp and clinic model. The camp model simulates a natural setting in which children with ASD spend 5 hours each day for 10 days where social skills are taught through engaging activities and interactions with peers both typical and with ASD. The clinic model, on the other hand, is a one hour a week session spread over 10 to 12 weeks where social skills are taught and practiced while parents observe through a one-way mirror and are trained on the intervention methods. Both clinic and camp model treatments are then compared with a third group who experience both treatments within the clinic and camp setting. Results show that the combined context had the highest treatment effects, followed by the camp model and, finally, the clinic model. Analysis of covariance did not indicate the groups differ from each other significantly in terms of treatment gains. The implications of these results are discussed in terms of translation of research into clinical practice, use of appropriate outcome measures, and generalization of skills through parent training and utilization of training programs within the natural context. While the intervention and results are promising, replication with larger samples and use of a control group are needed. (PsycINFO Database Record (c) 2012 APA, all rights reserved)
SN - 0419-4217
AD - Mathai, Grace: U Louisville, US
ER -

TY - JOUR
ID - 2451
T1 - Sensation seeking and narrative transportation: High sensation seeking children's interest in reading outside of school. [References]
A1 - Jensen,Jakob; Imboden,Kristen; Ivic,Rebecca
Y1 - 2011///
N1 - Peer Reviewed Journal: 2011-22329-005
English
Journal; Peer Reviewed Journal
KW - PsycInfo
KW - Elementary School Students
KW - Narratives
KW - Reading
KW - Sensation Seeking
RP - NOT IN FILE
SP - 541
EP - 558
JF - Scientific Studies of Reading
VL - 15:
IS - 6
N2 - High sensation seekers (HSS) prefer messages that allow them to maintain an optimal level of arousal (i.e., highly arousing messages). Transportation theory suggests that narrative immersion in a story may moderate reader arousal, and thus HSS message selection. To test this idea, a survey was administered to 120 fourth and fifth graders. In addition to measuring sensation seeking and transportation, the survey assessed interest in reading outside of school (i.e., leisure reading). HSS were found to be less likely to engage in leisure reading. Consistent with the activation model of information exposure, a moderated mediation analysis revealed that whether HSS engaged in leisure reading hinged on their level of narrative transportation (moderator) and subsequent reader boredom (mediator). HSS who successfully transported while reading were no different than their low sensation seeking peers. From a genre standpoint, high sensation seeking children with high narrative transportation scores were found to be especially interested in science fiction/fantasy books. Strategies for increasing leisure reading as well as constructing narratives that are engaging to high sensation seeking children are discussed. (PsycINFO Database Record (c) 2012 APA, all rights reserved) (journal abstract)
SN - 1088-8438
AD - Jensen, Jakob: Purdue University, West Lafayette, IN, US Imboden, Kristen: Purdue University, West Lafayette, IN, US Ivic, Rebecca: Purdue University, West Lafayette, IN, US
ER -

TY - JOUR
ID - 2452

T1 - Spontaneous disappearance of cerebral convexity arachnoid cyst. [References]
A1 - Matushita,Hamilton
Y1 - 2012///
N1 - Peer Reviewed Journal: 2012-17605-018
English
Journal; Peer Reviewed Journal
KW - PsycInfo
KW - Brain Damage
KW - Cerebral Ischemia
KW - Diagnosis
KW - Magnetic Resonance Imaging
RP - NOT IN FILE
SP - 473
EP - 474
JF - Arquivos de Neuro-Psiquiatria
VL - 70:
IS - 6
N2 - Presents a case report of eight-year-old previously healthy boy fell off a swing in a playground and hit his head on the ground. He presented vomiting but no disturbance of consciousness. He was admitted to the emergency unit of the hospital on the same day. Cranial computed tomography (CT) was then performed, which demonstrated an arachnoid cysts (AC) in the right frontal convexity, with compression and distortion of the frontal lobe, and bulging of adjacent bone, thereby causing cranial asymmetry. There were no neurological deficits. The child was discharged from the emergency unit and referred to the outpatient clinic. Magnetic resonance imaging (MRI) obtained 40 days later confirmed the previous CT findings. The possibility of spontaneous disappearance of ACs may corroborate the use of conservative therapy, especially for incidental findings of ACs. Prophylactic surgery on asymptomatic ACs is not justified, because the course with spontaneous regression has been increasingly reported in the literature. Since asymptomatic ACs may not be diagnosed, the actual rate of spontaneous disappearance of ACs may be higher than it is suggested by the literature. (PsycINFO Database Record (c) 2012 APA, all rights reserved)
SN - 0004-282X
AD - Matushita, Hamilton: Sao Paulo University Medical School, Sao Paulo, Brazil Cardeal, Daniel D.: Hospital das Clinicas, Sao Paulo University Medical School, Sao Paulo, Brazil Monaco, Bernardo: Hospital das Clinicas, Sao Paulo University Medical School, Sao Paulo, Brazil
ER -

TY - JOUR
ID - 2453
T1 - Family dynamics and the educational experience
A1 - Barrett,Muriel
Y1 - 2012///
N1 - Book: 2012-02642-008
English
Book; Edited Book
KW - PsycInfo
KW - Attachment Behavior
KW - Family Relations
KW - Family Therapy
KW - Learning Ability
KW - Countertransference
KW - Psychotherapeutic Transference
RP - NOT IN FILE
SP - 85
EP - 102
JF - High, Helen [Ed]
VL - :
IS - 2012

N2 - (from the chapter) In the author's role as a remedial teacher/Educational Psychotherapist at the Tavistock Clinic, (1973-1986), she saw children individually and in peer and family groups, the latter sometimes with colleagues. All the children were referred for either lack of basic skills, suspended learning (suddenly being unable to continue learning), or showing resistance to learning. Unlike the established mode of family therapy, our work focussed on the family dynamic relating to, and often reflecting, the referred child's capacity and attitude to learning skills and tasks. The dynamic of family behaviour sometimes mirrored their referred child's learning problems, particularly in relation to communication. If there were hidden agendas words became, consciously or unconsciously, misinterpreted. Families sometimes revealed certain attributes similar to those of their child, including: an aggressive stance, bored indifference, low self-esteem, poor temporal sense (e.g. about appointment times), defensive behaviour (blaming others) and an unclear sense of themselves as "competent and active members of the outside world" (Heard & Barrett, 1977). A crucial key to understanding any family interaction is our own understanding of what we are experiencing in the transference or countertransference. Observation offers insight into secure or insecure patterns of attachment behaviour seen in the interaction between family members. Encouraging them to express opinions and feelings about each other either enhances or inhibits their willingness to acknowledge the significance of family loss or transitions. The parents' experience of their own education may affect the extent of their co-operation with us by their choosing: limited acceptance of help for their child, to include parental ongoing participation, to be open to links being made between family interactive behaviour and a referred child's capacity to learn. Although some families are resistant to new experiences, the author thinks that any intervention into family dynamics is likely to bring about some change. In families who are secure in their attachments, losses will be managed in a way that enables the family, and the individual members within it, to mourn, express anger, sadness or joy, and to play, explore, learn, and continue their goal-seeking. For those whose experience has led to anxious attachments, a new way of playing and learning may be dismissed or only partially tolerated. While maintaining homeostasis may feel safer for parents, the author has found that many children recognize and readily respond to the idea of this new way of learning that is being proposed. (PsycINFO Database Record (c) 2012 APA, all rights reserved)
ER -

TY - JOUR
ID - 2454
T1 - Learning in a u-Museum: Developing a context-aware ubiquitous learning environment. [References]
A1 - Chen,Chia-Chen; Huang,Tien-Chi
Y1 - 2012///
N1 - Peer Reviewed Journal: 2012-17714-004
English
Journal; Peer Reviewed Journal
KW - PsycInfo
KW - Awareness
KW - Curriculum
KW - Learning Environment
KW - Teaching Methods
KW - Technology
KW - Classrooms
KW - Museums
RP - NOT IN FILE
SP - 873
EP - 883
JF - Computers & Education
VL - 59:
IS - 3
N2 - Context-awareness techniques can support learners in learning without time or location constraints by using mobile devices and associated learning activities in a real learning environment. Enrichment of context-aware technologies has enabled students to learn in an environment that integrates learning resources from both the real world and the digital world. Although learning outside of the traditional classroom is an innovative teaching approach, the two main problems are the lack of proper learning strategies and the capacity to acquire knowledge on subjects effectively. To manage these problems, this study proposes a context-aware ubiquitous learning system (CAULS) based on radio-frequency identification (RFID), wireless network, embedded handheld device, and database technologies to detect and examine real-world learning behaviors of students. A

case study of an aboriginal education course was conducted in classrooms and at the Atayal u-Museum in Taiwan. Participants included elementary school teachers and students. We also designed and used a questionnaire based on the Unified Theory of Acceptance and Use of Technology (UTAUT) theory to measure the willingness for adoption or usage of the proposed system. The experimental results demonstrated that this innovative approach can enhance their learning intention. Furthermore, the results of a posttest survey revealed that most students' testing scores improved significantly, further indicating the effectiveness of the CAULS. (PsycINFO Database Record (c) 2012 APA, all rights reserved) (journal abstract)
SN - 0360-1315
AD - Chen, Chia-Chen: Department of Information Management, Tunghai University, Taichung, Taiwan Huang, Tien-Chi: Department of Information Management, National Taichung University of Science and Technology, Taichung, Taiwan
ER -

TY - JOUR
ID - 2455
T1 - Cognitive behavioral treatment for trauma symptoms in American Indian youth: Preliminary findings and issues in evidence-based practice and reservation culture. [References]
A1 - Morsette,Aaron; van den Pol,Richard; Schuldberg,David; Swaney,Gyda; Stolle,Darrell
Y1 - 2012///
N1 - Peer Reviewed Journal: 2012-16211-005
English
Journal; Peer Reviewed Journal
KW - PsycInfo
KW - Cognitive Behavior Therapy
KW - Evidence Based Practice
KW - Symptoms
KW - Trauma
KW - American Indians
RP - NOT IN FILE
SP - 51
EP - 62
JF - Advances in School Mental Health Promotion
VL - 5:
IS - 1
N2 - This study reports on the use of Cognitive Behavioral Intervention for Trauma in Schools (CBITS), an evidence-based practice, with 43 students in six schools on three American Indian reservation communities. CBITS was helpful in treating the majority of American Indian children and adolescents who were experiencing symptoms of post-traumatic stress disorder and depression. Using a limited quasi-experimental design, on a measure of traumatic stress, there was symptom reduction with the passage of time; however, symptoms reduced more substantially with treatment. Local cultural experts, that is, Tribal Elders, were invited to provide traditional cultural activities and teachings in the introductory lesson and at graduation. A 3-year follow-up involving school counselors reported on acceptability and additional impacts of the program. They expressed that treating trauma was a priority for their students, CBITS was perceived as an advantageous approach, and counselors said they would use it in the future. Further, counselors observed positive results outside of the treatment setting, and integrating traditional cultural activities was perceived favorably. We discuss some of the issues surrounding attempts to evaluate the nature and contribution of traditional American Indian cultural activities to evidence-based trauma treatment. (PsycINFO Database Record (c) 2012 APA, all rights reserved) (journal abstract)
SN - 1754-730X
AD - Morsette, Aaron: National Native Children's Trauma Center, University of Montana, Missoula, MT, US van den Pol, Richard: National Native Children's Trauma Center, University of Montana, Missoula, MT, US Schuldberg, David: National Native Children's Trauma Center, University of Montana, Missoula, MT, US Swaney, Gyda: National Native Children's Trauma Center, University of Montana, Missoula, MT, US Stolle, Darrell: National Native Children's Trauma Center, University of Montana, Missoula, MT, US
ER -

TY - JOUR

ID - 2456
T1 - Growing minds: The relationship between parental attitude about nature and the development of fine and gross motor skills in children
A1 - McFarland,Amy Lene'
Y1 - 2012///
N1 - Dissertation Abstract: 2012-99100-253
English
Dissertation Abstract
KW - PsycInfo
KW - Childhood Development
KW - Motor Development
KW - Motor Skills
KW - Parental Attitudes
KW - Human Nature
RP - NOT IN FILE
SP - 6451
JF - Dissertation Abstracts International: Section B: The Sciences and Engineering
VL - 72:
IS - 11-B
N2 - Adults associate their childhood with playing outdoors, often in natural settings. This type of free play is valuable to child development. Children tend to use outdoor play areas in physically active ways, and time spent outdoors relates positively to increased physical activity in children. However, children today are spending an increasing amount of time indoors rather than outdoors. Recent research has shown that the amount of time children spend in outdoor play is directly related to parental concerns about their children's exposure to outdoor hazards. The purpose of this project was to investigate the relationship between parental attitude toward nature and the development of fine and gross motor skills in young children. The sample for this study was drawn from parents of children ages 3 to 5 years old enrolled at one of two University Child Development Centers. The assessment tool used was composed of sections that ask parents about their attitudes toward nature, about their young children spending time outdoors, how much time their children actually spend in outdoor and indoor activities, and standard demographic questions. The childcare centers independently assessed children's motor development. Statistical comparisons included correlations and ANOVA. Results from this study indicated that parents had positive views toward nature and towards their child's outdoor recreation. Parents who had more positive views toward nature also tended to have more positive views toward their child's outdoor recreation. Children who participated in certain indoor activities tended to score higher in the area of fine motor skills. However, children who spent more time indoors in free play had lower gross motor skill scores. Parents who scored better on the Parental Attitude toward Their Child's Outdoor Recreation scale reported that their child spent more time in outdoor free play and outdoor organized sports and activities. Parents preferred outdoor play spaces when compared to indoor play spaces and specifically those outdoor spaces that were constructed with more nearby natural components. (PsycINFO Database Record (c) 2012 APA, all rights reserved)
SN - 0419-4217
AD - McFarland, Amy Lene': Texas A&M U., US
ER -

TY - JOUR
ID - 2457
T1 - Violence risk and gang affiliation in youth offenders: A recidivism study. [References]
A1 - Chu,Chi Meng; Daffern,Michael; Thomas,Stuart; Lim,Jia Ying
Y1 - 2012///
N1 - Peer Reviewed Journal: 2012-08905-007
English
Journal; Peer Reviewed Journal
KW - PsycInfo
KW - Juvenile Delinquency
KW - Juvenile Gangs
KW - Recidivism
KW - Violence
KW - Risk Assessment

RP - NOT IN FILE
SP - 299
EP - 315
JF - Psychology, Crime & Law
VL - 18:
IS - 3
N2 - Youth gangs are ubiquitous around the world and have been problematic for the social and criminal justice agencies. Despite widespread public concern, there has been relatively scarce empirical scrutiny of youth gangs internationally and little outside of America and Europe. In particular, the activities of youth gangs, the function of gang membership, the criminogenic needs of gang-affiliated youth, and the risk of criminal recidivism for gang-affiliated youth remain unclear. Against this background, this study explored the sociodemographic characteristics, risk and rate of criminal recidivism in a cohort of 165 male youth offenders in Singapore, of which 58 were gang-affiliated. Multivariate analyses revealed that gang-affiliated youth offenders were significantly more likely to have histories of substance use, weapon use and violence than nongang-affiliated youth offenders. Gang-affiliated offenders also scored higher on measures of risk for recidivism (SAVRY and YLS/CMI), and engaged in violent and other criminal behaviors more frequently during follow-up. These differences indicate a significant relationship between gang affiliation and criminal recidivism in youth offenders. Furthermore, these findings have important clinical and policy implications, indicating an increased requirement for additional and more intensive assessment and tailored interventions for gang-affiliated youth offenders. (PsycINFO Database Record (c) 2012 APA, all rights reserved) (journal abstract)
SN - 1068-316X
AD - Chu, Chi Meng: Monash University, Centre for Forensic Behavioural Science, Melbourne, VIC, Australia Daffern, Michael: Monash University, Centre for Forensic Behavioural Science, Melbourne, VIC, Australia Thomas, Stuart: Monash University, Centre for Forensic Behavioural Science, Melbourne, VIC, Australia Lim, Jia Ying: Ministry of Community Development, Youth and Sports, Clinical and Forensic Psychology Branch, Singapore, Singapore
ER -

TY - JOUR
ID - 2458
T1 - The importance of teacher-student conversations in early literacy
A1 - Krizek,Patricia A.
Y1 - 2012///
N1 - Dissertation Abstract: 2012-99090-528
English
Dissertation Abstract
KW - PsycInfo
KW - Communication Skills
KW - Conversation
KW - Language
KW - Literacy
KW - Teacher Student Interaction
KW - Childhood Development
KW - Mealtimes
KW - Oral Communication
KW - Teachers
RP - NOT IN FILE
SP - 3999
JF - Dissertation Abstracts International Section A: Humanities and Social Sciences
VL - 72:
IS - 11-A
N2 - Oral language is an essential communication skill that is often overlooked when preparing children to read (Miller, 2010). Children in lower-income families typically have fewer conversations with adults and by the time they enter kindergarten they have only heard and are able to understand half of the words that high-income children can (Hart & Risley, 2003, 1995). Boys start school speaking fewer words than girls, which in turn leads to lower average reading scores for boys (Tyre, 2008). This puts boys at risk, as they need critical reading skills to be academically successful (Brozo, 2010). Considering the importance of oral language in the development of

early literacy skills, it is necessary to provide consistent times for conversations to take place in preschool classrooms (Bond & Wasik, 2009). The current quantitative study is guided by the following questions: What is the importance of teacher-student conversations in preschool and if preschool teachers regularly schedule conversations with individual students, will it improve their language and literacy development? Does the preschool teacher's encouraging the children to communicate affect their language and literacy development? Does the amount of years a preschool teacher has been teaching affect the student's language and literacy development? This study examined the developmental growth on five language and literacy Measures from the Desired Results Developmental Profile Revised (DRDP-R) of 54 lower socioeconomic status (SES) state preschool boys in a large urban school district in southern California. The students' 27 state preschool teachers scheduled unscripted conversations with them four times a day for three months-during mealtimes, at recess, at the dramatic play area, and at the library center. Research-based strategies were used to guide the conversations. The teachers' ability to encourage children to communicate and their years of teaching experience was also considered. The results of this study indicated that there was a highly significant difference (p <.01) in the developmental growth by the Scheduled Conversations Treatment Group compared to the Infrequent Conversations Control Group based on the t-test on the DRDP-R: Comprehends meaning (oral language), Expresses self through language, and Uses language in conversation and significant difference (p <.05) developmental growth on Letter and word knowledge. The Pearson product-moment correlation did not show a significant correlation between the student's developmental growth on the measures and the teacher's ability to encourage children to communicate nor on their years of teaching experience. (PsycINFO Database Record (c) 2012 APA, all rights reserved)
SN - 0419-4209
AD - Krizek, Patricia A.: U Redlands, US
ER -

TY - JOUR
ID - 2459
T1 - Nature connection, outdoor play, and environmental stewardship in residential environmental education
A1 - Andrejewski,Robert G.
Y1 - 2012///
N1 - Dissertation Abstract: 2012-99090-184
English
Dissertation Abstract
KW - PsycInfo
KW - Childhood Development
KW - Childhood Play Behavior
KW - Environmental Education
KW - Playgrounds
RP - NOT IN FILE
SP - 4300
JF - Dissertation Abstracts International Section A: Humanities and Social Sciences
VL - 72:
IS - 11-A
N2 - A lack of exposure to the natural world has led to a generation of children disconnected from nature. This phenomenon has profound negative implications for the physical and psychological well being of today's youth. Residential environmental education provides one avenue to connect children to nature. One purpose of this study was to investigate the role of Outdoor School, a residential environmental education program, on ecological knowledge, children's connection to nature, school belonging, outdoor play attitude, environmental stewardship attitude, outdoor play behavior, and environmental stewardship behavior, as reported by participants. A quasi-experimental research design was utilized in the study. A total of 228 fifth grade students (156 treatment, 72 control) from central Pennsylvania participated. The results of the program evaluation indicated that Outdoor School was successful in achieving significant, positive gains in the areas of ecological knowledge, connection to nature, outdoor play behavior, and environmental stewardship behavior. No change was found from pretest to post-test in outdoor play attitudes, environmental stewardship attitudes, and school belonging. Additionally, the study addressed gaps in the literature regarding the relationship between connection to nature, environmental stewardship, and outdoor play using two different approaches. An adaptation of the Theory of Planned Behavior (TPB) was used to predict outdoor play behavior in children. In this model, favorable attitudes, subjective norms, and perceived behavioral control lead to intentions to perform a given

behavior. Intention to perform the behavior is the best predictor for behavior performance. For this study, participants' feeling of connection to nature was added as an affective independent variable. This model explained 45% of the variance in outdoor play. The hypothesis that a connection to nature would be a significant predictor of both attitudes toward outdoor play was supported by testing of the model. Finally, nature connection was tested as a full mediator of the relationship between outdoor play and environmental stewardship. There is support for the idea that direct experience in the outdoors facilitates environmental behaviors, but more research is needed to understand this relationship. Testing of the model failed to demonstrate that nature connection fully mediated the relationship between outdoor play and environmental stewardship; however, a feeling of connectedness to nature augmented the influence that outdoor play behavior exerts on environmental stewardship behavior. (PsycINFO Database Record (c) 2012 APA, all rights reserved)
SN - 0419-4209
AD - Andrejewski, Robert G.: The Pennsylvania State U., US
ER -

TY - JOUR
ID - 2460
T1 - Shared parent-child leisure time in four countries. [References]
A1 - Craig,Lyn
Y1 - 2012///
N1 - Peer Reviewed Journal: 2012-11219-007
English
Journal; Peer Reviewed Journal
KW - PsycInfo
KW - Childrearing Practices
KW - Family Work Relationship
KW - Leisure Time
KW - Mothers
KW - Parent Child Relations
KW - Child Care
KW - Childrearing Attitudes
KW - Countries
KW - Parental Attitudes
RP - NOT IN FILE
SP - 211
EP - 229
JF - Leisure Studies
VL - 31:
IS - 2
N2 - Sharing leisure experiences is thought to promote family bonding and communication, and foster children's intellectual, social and psychological development. Such time is 'purposive', and requires planning, organisation and maintenance, usually from the mother. Do parents and children spend more time in shared leisure together in countries where mothers do more childcare and spend longer each day with children? Using time use data we compare shared parent-child shared leisure time in four countries with different maternal work-force participation and attitudes to substitute childcare and child-raising: USA, Australia, Denmark and France. We create harmonised measures of leisure time mothers are with children, and time both parents are with children engaging in leisure in the same location (except in the USA, which has information from only one parent). To isolate shared interactive activities, time is further subdivided into (a) watching TV, DVDs or videos with children, (b) non-TV leisure time with children and (c) leisure time with children outside the family home. We find that overall time with children varies substantially between countries, reflecting mothers' average time in paid work, but time in shared parent-child leisure, particularly outside the family home, does not. (PsycINFO Database Record (c) 2012 APA, all rights reserved) (journal abstract)
SN - 0261-4367
AD - Craig, Lyn: University of New South Wales, Social Policy Research Centre, Sydney, NSW, Australia
Mullan, Killian: University of New South Wales, Social Policy Research Centre, Sydney, NSW, Australia
ER -

TY - JOUR

ID - 2461
T1 - 'Can we play on the swings and roundabouts?': Creating inclusive play spaces for disabled young people and their families. [References]
A1 - Jeanes,Ruth
Y1 - 2012///
N1 - Peer Reviewed Journal: 2012-11219-006
English
Journal; Peer Reviewed Journal
KW - PsycInfo
KW - Disabilities
KW - Facilities
KW - Leisure Time
KW - Playgrounds
KW - Recreation Areas
KW - Family Members
RP - NOT IN FILE
SP - 193
EP - 210
JF - Leisure Studies
VL - 31:
IS - 2
N2 - The importance of play for children's development and wellbeing has been well established. However, disabled children and their families frequently find accessing play sites challenging due to a range of physical and social barriers and are therefore unable to experience many of the benefits play in this setting provides. Increasingly, United Kingdom policy has recognised this with the 2008 Play Strategy making an explicit commitment supported by funding to ensure that more inclusive public play facilities are developed. Using a case study example of a newly developed 'inclusive' play facility, this paper examines the views of young disabled people and their families regarding what makes a play facility inclusive. Using a socio-spatial analysis, the paper identifies the importance of not only addressing physical constraints but also creating a space where disability is viewed positively and able-bodied discourses are not privileged at the expense of others. The importance of consulting with families, location of facilities and the role of play workers are all analysed. The paper concludes by critically examining the implications of the findings for the delivery of the Play Strategy particularly within the context of public spending cuts imposed by the new Coalition Government, and considers whether inclusive play spaces can become a standard and embedded part of community facilities. (PsycINFO Database Record (c) 2012 APA, all rights reserved) (journal abstract)
SN - 0261-4367
AD - Jeanes, Ruth: Monash University, Sport and Outdoor Recreation, Faculty of Education Peninsula Campus, Frankston, VIC, Australia Magee, Jonathan: University of Central Lancashire, School of Sport, Tourism and the Outdoors, Preston, United Kingdom
ER -

TY - JOUR
ID - 2462
T1 - Teaching joint attention to children with autism through a sibling-mediated behavioral intervention. [References]
A1 - Ferraioli,Suzannah J.
Y1 - 2011///
N1 - Peer Reviewed Journal: 2011-25065-002
English
Journal; Peer Reviewed Journal
KW - PsycInfo
KW - Attention
KW - Autism
KW - Behavior Therapy
KW - Intervention
KW - Sibling Relations
KW - Siblings

RP - NOT IN FILE
SP - 261
EP - 281
JF - Behavioral Interventions
VL - 26:
IS - 4
N2 - Joint attention is pivotal to the development of complex social skills and language, and many individuals with Autism Spectrum Disorders display deficits in this domain. Behavioral interventions targeting joint attention are evidenced to be effective in teaching these skills to young children with autism, but these treatments have traditionally been implemented by adults in structured settings. Concerns regarding the generalizability of skills acquired under such conditions have been raised. Four typically developing children were trained to implement a joint attention intervention to their siblings with autism in the home. Joint attention was measured pre-treatment and post-treatment during play sessions and during a structured, adult-mediated assessment to evaluate maintenance and generalization. Gains in responding to joint attention were observed for all four participants; gains in initiations were observed in three participants. Rates of imitation and behavioral requests also increased in structured and naturalistic settings. This study supports the efficacy of siblings as interventionists to target complex social skills in the natural environment. The implications of these findings for treatments targeting joint attention and for siblings as interventionists are discussed. (PsycINFO Database Record (c) 2012 APA, all rights reserved) (journal abstract)
SN - 1072-0847
AD - Ferraioli, Suzannah J.: Douglass Developmental Disabilities Center, Rutgers, State University of New Jersey, New Brunswick, NJ, US Harris, Sandra L.: Douglass Developmental Disabilities Center, Rutgers, State University of New Jersey, New Brunswick, NJ, US
ER -

TY - JOUR
ID - 2463
T1 - Neighborhood poverty and maternal fears of children's outdoor play. [References]
A1 - Kimbro,Rachel Tolbert; Schachter,Ariela
Y1 - 2011///
N1 - Peer Reviewed Journal: 2011-20023-008
English
Journal; Peer Reviewed Journal
KW - PsycInfo
KW - Fear
KW - Mother Child Relations
KW - Neighborhoods
KW - Poverty
KW - Safety
RP - NOT IN FILE
SP - 461
EP - 475
JF - Family Relations: An Interdisciplinary Journal of Applied Family Studies
VL - 60:
IS - 4
N2 - Investigating children's outdoor play unites scholarship on neighborhoods, parental perceptions of safety, and children's health. Utilizing the Fragile Families and Child Well-being Study (N = 3,448), we examine mothers' fear of their 5-year-old children playing outdoors, testing associations with neighborhood social characteristics, city-level crime rates, maternal mental health, and social support. Living in public housing, perceptions of low neighborhood collective efficacy, and living in a Census tract with a higher proportion of Blacks and households in poverty are associated with higher odds of maternal fear, but crime rates are not a significant predictor of fear. We also demonstrate that not being depressed-but not social support or collective efficacy-buffers the influence of neighborhood poverty on maternal fears of outdoor play. (PsycINFO Database Record (c) 2012 APA, all rights reserved) (journal abstract)
SN - 0197-6664
AD - Kimbro, Rachel Tolbert: Department of Sociology, Rice University, Houston, TX, US Schachter, Ariela: Department of Sociology, Stanford University, Stanford, CA, US

ER -

TY - JOUR
ID - 2464
T1 - Beyond family and ethnic culture: Understanding the preconditions for the potential realization of social capital. [References]
A1 - Oseguera,Leticia
Y1 - 2011///
N1 - Peer Reviewed Journal: 2011-17735-013
English
Journal; Peer Reviewed Journal
KW - PsycInfo
KW - Academic Achievement
KW - Racial and Ethnic Differences
KW - Social Capital
KW - Student Engagement
RP - NOT IN FILE
SP - 1136
EP - 1166
JF - Youth & Society
VL - 43:
IS - 3
N2 - This article extends our conceptual understanding of social capital and school achievement through a comparative race and ethnic approach. Using the National Educational Longitudinal Study (NELS) 1988-1990 panel, this article develops a more comprehensive understanding of school achievement by exploring circumstances, which the authors call "preconditions," leading to the potential for the realization of social capital. These "preconditions" are used to explain academic engagement disparities between Southeast Asian, Black, Mexican, and White high school youth. Whereas previous research on social capital leaves the mechanism through which social capital influences school outcomes unspecified, this study focuses on a behavior associated with positive educational outcomes-time per week spent on homework outside of school. Although preconditions for parental capital appear to have some influence on students' study behavior, so too do preconditions between and within schools, such as peers and teachers. This research shows that relationships outside the family-that is, within and between school opportunities for social capital-play a significant role in explaining variation between the four ethnic groups. (PsycINFO Database Record (c) 2012 APA, all rights reserved) (journal abstract)
SN - 0044-118X
AD - Oseguera, Leticia: Pennsylvania State University, Department of Education Policy Studies, University Park, PA, US Conchas, Gilberto Q.: University of California, Department of Education, Irvine, CA, US Mosqueda, Eduardo: University of California, Santa Cruz, CA, US
ER -

TY - JOUR
ID - 2465
T1 - Availability of commercial physical activity facilities and physical activity outside of school among high school students. [References]
A1 - Isgor,Zeynep
Y1 - 2011///
N1 - Peer Reviewed Journal: 2011-13555-009
English
Journal; Peer Reviewed Journal
KW - PsycInfo
KW - Facilities
KW - High School Students
KW - Physical Activity
KW - After School Programs
RP - NOT IN FILE
SP - 707

EP - 715
JF - Journal of Physical Activity & Health
JA - J Phys Act Health
VL - 8:
IS - 5
N2 - Background: Environmental factors may play an important role in the determination of physical activity behaviors. Methods: This study used the Child Development Supplement of the Panel Study of Income Dynamics to examine the association between the availability of objectively measured commercial physical activity-related instruction facilities and weekly physical activity participation among high school students outside of school physical education classes. A Negative Binomial count model was used to examine the number of days of vigorous physical activity (at least 30 minutes/day) per week and a Probit model was used to examine the probability of frequent (4 or more days/week) vigorous physical activity participation. Results: The results indicated that an additional instruction school per 10,000 capita per 10 square miles was associated with an 8-percent increase in the weekly number of days of vigorous physical activity participation and a 4 percentage point increase in the likelihood of frequent physical activity participation for female adolescents only. By income, associations were larger for low- versus high-income female youths. Conclusion: Increased availability of local area physical activity-related instruction facilities may help to increase female high school students' physical activity levels, particularly among low-income female students. (PsycINFO Database Record (c) 2012 APA, all rights reserved) (journal abstract)
SN - 1543-3080
AD - Isgor, Zeynep: Dept of Economics, University of Illinois at Chicago, Chicago, IL, US Powell, Lisa M.: Institute for Health Research and Policy, University of Illinois at Chicago, Chicago, IL, US
ER -

TY - JOUR
ID - 2466
T1 - Students' motivation to study music: The finnish context. [References]
A1 - Juvonen,Antti
Y1 - 2011///
N1 - Peer Reviewed Journal: 2011-12414-005
English
Journal; Peer Reviewed Journal
KW - PsycInfo
KW - Exercise
KW - Motivation
KW - Music
KW - Secondary Education
KW - Schools
KW - Students
RP - NOT IN FILE
SP - 73
EP - 88
JF - Research Studies in Music Education
VL - 33:
IS - 1
N2 - This article reports the Finnish component of an international mapping exercise concentrating on students' motivation to study music as compared to other school subjects. The study was based on expectancy-value theoretical framework, and the data was collected using a questionnaire. The questions focused on students' competence beliefs, values and perceptions of task difficulty. A sample of 1654 students (grades 5-12) was taken from the Finnish elementary (lower and higher levels) and upper secondary schools. The questionnaire was sent to all Finnish schools, with the principals acting as gatekeepers. The data was generated at schools from all over Finland, and it may be considered representative. The results show that Finnish children like music education, although they do not consider it among the most valuable school subjects. Those students who learned music also outside school seemed to be more interested in all schools subjects than were other students. (PsycINFO Database Record (c) 2012 APA, all rights reserved) (journal abstract)
SN - 1321-103X
AD - Juvonen, Antti: University of Eastern Finland, Finland

ER -

TY - JOUR
ID - 2467
T1 - Maternal mental health, neighborhood characteristics, and time investments in children. [References]
A1 - Frech,Adrianne; Kimbro,Rachel Tolbert
Y1 - 2011///
N1 - Peer Reviewed Journal: 2011-10730-006
English
Journal; Peer Reviewed Journal
KW - PsycInfo
KW - Family
KW - Mental Health
KW - Mother Child Relations
KW - Neighborhoods
KW - Fragile X Syndrome
KW - Mothers
KW - Time
KW - Well Being
RP - NOT IN FILE
SP - 605
EP - 620
JF - Journal of Marriage and Family
VL - 73:
IS - 3
N2 - We use data from the Fragile Families and Child Wellbeing Study (N = 3,572) to examine relationships between maternal depression and mothers' time investments with their 5-year-old children in outings, trips to playgrounds or parks, time spent reading with the child, and time spent playing indoors with the child. We also examine whether mothers' self-assessments of neighborhoods are associated with time investments in children. Findings indicate that persistent maternal depression is associated with fewer time investments in all four activities with 5-year-old children. Mothers' fear of children playing outdoors and self-assessments of neighborhood collective efficacy are associated with indoor and outdoor activities with children, but do not mediate the relationships between maternal depression and maternal time investments. In sum, maternal depression and neighborhood context play significant but largely independent roles in regulating mothers' time spent in primary child-care activities. (PsycINFO Database Record (c) 2012 APA, all rights reserved) (journal abstract)
SN - 0022-2445
AD - Frech, Adrianne: University of Akron, Akron, OH, US Kimbro, Rachel Tolbert: Rice University, Houston, TX, US
ER -

TY - JOUR
ID - 2468
T1 - Can young children make active choices? Outcomes of a feasibility trial in seven-year-old children. [References]
A1 - Hands,Beth; Larkin,Dawne; Rose,Elizabeth; Parker,Helen; Smith,Anne
Y1 - 2011///
N1 - Peer Reviewed Journal: 2011-09757-005
English
Journal; Peer Reviewed Journal
KW - PsycInfo
KW - Choice Behavior
KW - Health Education
KW - Physical Activity
KW - School Based Intervention
KW - Exercise
KW - Intervention

RP - NOT IN FILE
SP - 625
EP - 637
JF - Early Child Development and Care
VL - 181:
IS - 5
N2 - This project evaluated the feasibility of a child-centred 24-week physical activity intervention. The Play5 strategy encourages children to choose to play vigorously five times a day for about as long as school recess. The seven-year-old children were randomised to control (n = 176) or intervention groups (n = 132). Data were collected at baseline and post-intervention. Programme evaluation included parent questionnaire, teacher records and a seven-day pedometer record with a sub-sample of children. The programme was successful in increasing vigorous activity (p = 0.04), but did not impact on moderate or sedentary activities. Young children were able to choose to be active without parent or teacher direction. (PsycINFO Database Record (c) 2012 APA, all rights reserved) (journal abstract)
SN - 0300-4430
AD - Hands, Beth: Institute for Health and Rehabilitation Research, University of Notre Dame Australia, Fremantle, WA, Australia Larkin, Dawne: School of Sport Science, Exercise and Health, University of Western Australia, Crawley, WA, Australia Rose, Elizabeth: School of Health Sciences, University of Notre Dame Australia, Fremantle, WA, Australia Parker, Helen: School of Health Sciences, University of Notre Dame Australia, Fremantle, WA, Australia Smith, Anne: School of Physiotherapy, Curtin University of Technology, Bentley, WA, Australia
ER -

TY - JOUR
ID - 2469
T1 - Children in the neighbourhood: Sense of safety and well-being. [References]
A1 - Migliorini,Laura
Y1 - 2011///
N1 - Book: 2011-04970-018
English
Book; Edited Book
KW - PsycInfo
KW - Fear
KW - Safety
KW - Self Esteem
KW - Urban Environments
KW - Well Being
KW - Child Attitudes
KW - Crime
KW - Neighborhoods
RP - NOT IN FILE
SP - 215
EP - 225
JF - Bonaiuto, Marino [Ed]; Bonnes, Mirilia [Ed]; Nenci, Anna Maria [Ed]; Carrus, Giuseppe [Ed]
VL - :
IS - 2011
N2 - (from the chapter) A considerable body of research addresses the role of physical environment in child development. Children's experience of their environment is connected with their perception of safety and with the feelings of fear. Safety is an essential resource for everyday life that could influence the sense of well-being. The first purpose of this chapter is to analyse the feelings of safety and fear of crime that arise from living as a child in urban environments that differ in structural and social features; afterward to investigate children's self-esteem and well-being and their relationship with sense of security. 518 Italian students (average age 10 years) from primary schools of three different urban neighbourhoods participated in this study. The questionnaire was composed by demographic information, measures of urban sense of safety and self-esteem, and well-being scale that includes measures of outdoor and indoor activities. The children's personal sense of safety does not seem to be related to structural elements of neighbourhood. These data underline that more research is needed to analyse this construct in relation to neighbourhoods that are different in structural features. Furthermore, children who

participate in more outdoor activities report higher levels of well-being and self-esteem. (PsycINFO Database Record (c) 2012 APA, all rights reserved)
AD - Migliorini, Laura: Department of Anthropological Sciences (DISA), University of Genova, Italy
Cardinali, Paola: Department of Human Study (DISTUM), University of Genova, Italy
ER -

TY - JOUR
ID - 2470
T1 - Gender as a practical concern in children's management of play participation
A1 - Cromdal,Jakob
Y1 - 2011///
N1 - Book: 2011-04798-014
English
Book; Edited Book
KW - PsycInfo
KW - Childhood Play Behavior
KW - Classification (Cognitive Process)
KW - Human Sex Differences
KW - Male Female Relations
KW - Peer Relations
KW - Sex Role Attitudes
KW - Sex Roles
KW - Social Identity
KW - Sociocultural Factors
RP - NOT IN FILE
SP - 294
EP - 309
JF - Speer, Susan A [Ed]; Stokoe, Elizabeth [Ed]
VL - :
IS - 2011
N2 - (from the chapter) I examine in this chapter how children invoke cultural conceptions of gender by categorizing persons and their actions in certain ways, and how this practice serves to deal with local matters of participation in peer group activities. Drawing on a corpus of video-recorded recess activities of two groups of students attending grades 2 and 4 respectively (roughly corresponding to 8 and 10 years of age) at an English school in Sweden, I present two episodes of interaction in which the participants are pursuing different interactional projects with regard to play participation. In the first episode, two girls are collaboratively building their actions so as to enforce their territorial rights. The second extract shows how a group of girls strive to engage two boys in an upcoming play event. The analysis explores how, in pursuing these interactional projects, the participants invoke and exploit some culturally distributed notions of gendered behaviour. By examining the participants' categorization work, I demonstrate how gender stereotypes are brought about in the interaction to accomplish social action. In other words, the present chapter points at some ways through which gender relations as well as social and moral orders are invoked and locally produced in children's mundane interaction. By fleshing out the methods through which the participants accomplish this, the analysis adds to the existing body of work in ethnomethodology on gender-in-interaction. More generally, by analysing the interactional workings of membership categories, this chapter contributes to our understanding of how young people draw upon and exploit local orders-as well as those of society at large-to organize their ordinary activities. (PsycINFO Database Record (c) 2012 APA, all rights reserved)
ER -

TY - JOUR
ID - 2471
T1 - Meaning-making through musical play: Cultural psychology of the playground. [References]
A1 - Marsh,Kathryn
Y1 - 2011///
N1 - Book: 2011-04025-003
English
Book; Edited Book

KW - PsycInfo
KW - Childhood Play Behavior
KW - Cross Cultural Psychology
KW - Interpersonal Interaction
KW - Learning
KW - Music
KW - Childhood Development
KW - Meaningfulness
KW - Playgrounds
KW - Sociocultural Factors
KW - Teaching
RP - NOT IN FILE
SP - 41
EP - 60
JF - Barrett, Margaret S [Ed]
VL - :
IS - 2011
N2 - (from the chapter) For many children, play of varying kinds provides a means for engaging with the world around them. Children can observe and play out patterns of behaviour and other social and cultural phenomena, sometimes as a solitary pursuit, but frequently in the company of others. In this chapter, I examine the ways in which school-aged children construct meaning through the generation and performance of musical play forms, both drawing on and transforming cultural influences. Issues of appropriation, transculturation and identity as manifested in musical play are addressed. The interactive pedagogy of the playground, through which children's agency is fully realized in processes of mutual learning and teaching, is also demonstrated. (PsycINFO Database Record (c) 2012 APA, all rights reserved)
AD - Marsh, Kathryn: University of Sydney, Sydney Conservatorium of Music, Sydney, NSW, Australia
ER -

TY - JOUR
ID - 2472
T1 - Performance and interaction during 'reading hour' in a Spanish secondary school. [References]
A1 - Poveda,David
Y1 - 2011///
N1 - Peer Reviewed Journal: 2011-03663-009
English
Journal; Peer Reviewed Journal
KW - PsycInfo
KW - Peer Relations
KW - Reading
KW - Secondary Education
KW - Self Concept
KW - Multiculturalism
KW - Literacy
KW - Performance
KW - Schools
KW - Student Characteristics
RP - NOT IN FILE
SP - 79
EP - 92
JF - Linguistics and Education
VL - 22:
IS - 1
N2 - This paper examines students' activity during reading hour in a multicultural secondary school in Madrid (Spain). It discusses two dimensions of the event: (1) How elements such as body posture, reading volume, reading speed and clarity were used by students to position themselves in relation to their linguistic competencies, the social order of the classroom and the institutional arrangements they participate in; and (2) how interactions around the text were used by students for peer play by drawing from identities formed outside

the classroom. The data illustrates how reading hour was taken by students, especially by some Latin American students, to temporarily redefine aspects of the institutional order they were placed in. These results also invite complicating the outside/inside distinction by showing the multiple layers of institutional and out-of-school life that are collapsed into these categories. (PsycINFO Database Record (c) 2012 APA, all rights reserved) (journal abstract)
SN - 0898-5898
AD - Poveda, David: Departamento Interfacultativo de Psicologia Evolutiva y de la Educacion, Facultad de Psicologia, Universidad Autonoma de Madrid, Madrid, Spain
ER -

TY - JOUR
ID - 2473
T1 - The implications of future time perspective and planning ability for children's emotion regulation
A1 - Puddester,Leah M.
Y1 - 2012///
N1 - Dissertation Abstract: 2012-99080-291
English
Dissertation Abstract
KW - PsycInfo
KW - Ability
KW - Childhood Development
KW - Cognitive Ability
KW - Emotional Control
KW - Time Perspective
RP - NOT IN FILE
SP - 6396
JF - Dissertation Abstracts International: Section B: The Sciences and Engineering
VL - 72:
IS - 10-B
N2 - Studying emotion regulation in childhood has particular importance for understanding the developmental trajectory of these abilities, as well as for informing preventative work that could offset later psychopathology. Gross (1998a) has distinguished between antecedent- and response focused emotion regulation strategies, and has shown that emotion regulation strategies that occur earlier in the emotion generative process have more favourable outcomes. The first step in antecedent emotion regulation is situation selection, which involves seeking out or avoiding certain situations or environments in order to regulate emotion. In fact, there is a growing psychological literature on children's environmental choices, or environmental niche picking, and how they may affect self-regulatory processes such as emotion regulation. To date, most research has focused on environmental choices for preferred (favourite) environments, and little attention has been focused on non-preferred environments, namely the school playground - the predominant social environment in which children spend their formative years. One's ability to plan, think about the future, and delay immediate gratification are also thought to be important factors in antecedent focused emotion regulation, as such skills allow an individual to move beyond immediate concerns. Few studies have investigated the relationship between emotion regulation and children's future time perspective and planning ability. The present study sought to add to existing research on emotion regulation by investigating the mechanisms by which children develop healthy emotion regulation skills. It was hypothesized that children who scored higher on future time perspective and planning ability would demonstrate better emotion regulation and be rated by peers as less aggressive. It was also hypothesized that any relationship between emotion regulation and aggression would be mediated by future time perspective and/or planning ability. Participants were 82 elementary school students in grades 3 (13 females, 15 males), 4 (17 females, 13 males), and 5 (17 females, 7 males). Parents and homeroom teachers of children also participated by completing the Lability/Negativity scale of the Emotion Regulation Checklist (Shields and Cicchetti, 1997). Children completed peer nomination measures of social behaviour using the Revised Class Play (Masten, Morrison, and Pellegrini, 1985) and one measure of emotion regulation, the Environmental Choices task (Ledingham, Rafter, & Genot, 1995). Children also completed two measures of means-end thinking: the Children's Time Perspective Inventory (Myers, 2000), and the Woodcock-Johnson III Test of Cognitive Abilities - Planning subtest. Results indicated that children as young as the age of 7 could be classified as having a future time perspective, and that future time perspective significantly predicted peer nominations of children's aggression. In addition, children's planning ability, as measured by the Woodcock-

Johnson, played a significant role not only in predicting children's self-reported emotion regulation reasons for environmental choices, but also in accounting for parent ratings of children's emotion regulation. This study also highlighted the importance of environmental niche picking for emotion regulation, with a majority of children indicating that they actively used different locations on the playground to help them regulate their negative emotional states. Contrary to hypotheses, there was no evidence that future time perspective or planning ability mediated the relationship between emotion regulation and aggression. Limitations and implications of the current findings are discussed. (PsycINFO Database Record (c) 2012 APA, all rights reserved)
SN - 0419-4217
AD - Puddester, Leah M.: U Ottawa, Canada
ER -

TY - JOUR
ID - 2474
T1 - The mathematical competencies of toddlers expressed in their play and daily life activities in Norwegian kindergartens. [References]
A1 - Reikeras,Elin; Loge,Inger Kristine; Knivsberg,Ann-Mari
Y1 - 2012///
N1 - Peer Reviewed Journal: 2012-10172-006
English
Journal; Peer Reviewed Journal
KW - PsycInfo
KW - Daily Activities
KW - Kindergartens
KW - Mathematical Ability
KW - Recreation
KW - Competence
KW - Early Childhood Development
RP - NOT IN FILE
SP - 91
EP - 114
JF - International Journal of Early Childhood
VL - 44:
IS - 1
N2 - Research on toddlers' mathematical knowledge is sparse. Studies on children's mathematical competencies before school age have mostly focused on older children. Few of the previous studies have included large groups of toddlers, few have been conducted in natural settings, and few have been directed at a broad field of mathematical knowledge. The objective of this study was to investigate which mathematical competencies a large group of toddlers' in Norwegian kindergartens expressed through play and daily life activities. A total of 1,003 children participated. Their competencies were registered when they were between 30 and 33 months. The assessment material consisted of 36 items, divided into three main areas: number and counting, geometry and problem solving. The information was collected through authentic assessment; the staff in the kindergartens observed the toddlers' competencies in play and daily life activities. The competencies were registered as mastered, partly mastered or mastering not yet observed. The toddlers showed mathematical competencies in all areas observed. A wide dispersion was found; both for the total score and the subareas' scores. The largest variance was found in number and counting. Our participants displayed lower levels of competencies in using number words and reciting number sequences than reported from previous studies and higher competencies in puzzle-making and following instructions on spatial words. The results indicate that the assessment material may be a valuable tool for the preschool teachers in identifying the variety of competencies mastered by the children in kindergarten. The need for future research is highlighted and discussed. (PsycINFO Database Record (c) 2012 APA, all rights reserved) (journal abstract)
SN - 0020-7187
AD - Reikeras, Elin: Faculty of Arts and Education, National Centre for Reading Education and Research, University of Stavanger, Stavanger, Norway Loge, Inger Kristine: Faculty of Arts and Education, Centre for Behavioural Research, University of Stavanger, Stavanger, Norway Knivsberg, Ann-Mari: Faculty of Arts and Education, National Centre for Reading Education and Research, University of Stavanger, Stavanger, Norway
ER -

TY - JOUR
ID - 2475
T1 - Preparing the room
A1 - Waldburg,Camilla
Y1 - 2012///
N1 - Book: 2011-17968-008
English
Book; Edited Book
KW - PsycInfo
KW - Psychotherapeutic Processes
KW - Psychotherapy
KW - School Based Intervention
KW - Symbolism
KW - Working Space
KW - Psychotherapeutic Transference
KW - Schools
RP - NOT IN FILE
SP - 79
EP - 89
JF - French, Lyn [Ed]; Klein, Reva [Ed]
VL - :
IS - 2012
N2 - (from the chapter) This chapter discusses the space within which therapy in schools occurs. Before looking at the practical aspects of the room-what's involved in booking and preparing it for sessions-it's useful to think about the symbolic significance of the space, both on a conscious and unconscious level. Using the same room each week communicates to the child that the physical boundaries framing the work are reliable and dependable, echoing the consistency and availability of the therapist for the child. Ensuring that the same room is available each week contributes to creating a therapeutic culture both within the room and outside, in the school at large. Staff and pupils come to know what the room is used for and when sessions take place. This should result in fewer disruptions, especially once the school therapist has established the service. As the therapeutic relationship unfolds, the room in which the ongoing narrative takes shape acts as a symbolic container. The room itself holds the client's story as well as the emotional history of past and present relationships, including both the real and the transference relationship between client and therapist. On a deeply unconscious level, the room comes to stand for the outer receptacle holding the contents of the client's mind, a concrete manifestation of the therapist's mind, which contains and processes what is revealed, both verbally and non-verbally, as well as all that is fantasised about in that particular space. Because of the symbolic power of the room, most practitioners choose to arrange it in the same way each week and have a fixed set of art and play resources available. This conveys the message that in the face of powerful primitive feelings, fantasies and mental states, the room-and, by association, the therapist's mind-can function as a robust enough container. It goes without saying, however, that the most important component of this process is the therapist's mind: her capacity to go on thinking and not be overwhelmed by the material presented. So, while a dedicated and secure room is the ideal, many therapists do their work day in and day out in less than perfect circumstances ranging from different rooms every week in the school setting to providing out-of-door sessions in refugee camps. (PsycINFO Database Record (c) 2012 APA, all rights reserved)
AD - Waldburg, Camilla: A Space for Creative Learning and Support, Hackney, East London, United Kingdom
ER -

TY - JOUR
ID - 2476
T1 - Peer exclusion in preschool children's play: Naturalistic observations in a playground setting. [References]
A1 - Fanger,Suzanne Marie; Frankel,Leslie Ann; Hazen,Nancy
Y1 - 2012///
N1 - Peer Reviewed Journal: 2012-10344-004
English
Journal; Peer Reviewed Journal
KW - PsycInfo
KW - Childhood Play Behavior

KW - Peer Relations
KW - Playgrounds
KW - Social Acceptance
KW - Preschool Students
RP - NOT IN FILE
SP - 224
EP - 254
JF - Merrill-Palmer Quarterly
VL - 58:
IS - 2
N2 - To better understand peer exclusion in young children, 42 four- to six-year-olds were observed in an outdoor setting, and their verbalizations were recorded using wireless microphones. The frequency of peer exclusion and social aggression, the various techniques children used to exclude their peers (unmitigated, mitigated, ignoring, and planning exclusion) and the relation of gender and social status (acceptance, rejection, and dominance scores) to children's use of exclusion were examined. Peer exclusion occurred frequently, whereas nonexclusionary forms of social aggression were rare. More dominant boys, less rejected boys, and more rejected girls excluded peers more frequently than did other children. Children who were more socially accepted tended to more frequently ignore their peers to exclude them. All girls and boys who were more socially accepted used higher rates of mitigated, subtle forms of exclusion. In addition, boys who were more dominant used higher rates of unmitigated, direct peer exclusion. (PsycINFO Database Record (c) 2012 APA, all rights reserved) (journal abstract)
SN - 0272-930X
AD - Fanger, Suzanne Marie: Department of Human Development and Family Sciences, University of Texas, Austin, TX, US Frankel, Leslie Ann: Department of Human Development and Family Sciences, University of Texas, Austin, TX, US Hazen, Nancy: Department of Human Development and Family Sciences, University of Texas, Austin, TX, US
ER -

TY - JOUR
ID - 2477
T1 - The 'land in-between': A comparative European study of the victimisation of young people travelling to and from school. [References]
A1 - Moore,Stephen; Maclean,Rachel; Jefford,Tom
Y1 - 2011///
N1 - Peer Reviewed Journal: 2011-25767-002
English
Journal; Peer Reviewed Journal
KW - PsycInfo
KW - Antisocial Behavior
KW - Friendship
KW - Safety
KW - Students
KW - Victimization
RP - NOT IN FILE
SP - 246
EP - 259
JF - Crime Prevention and Community Safety
VL - 13:
IS - 4
N2 - This article draws upon the initial results of a survey of school students from eight European countries regarding their experiences of victimisation and offending while travelling to and from school. This is the first such comparative survey to be undertaken. This article provides evidence from the research that travel between the perceived safety of home and school is a risky activity for a significant proportion of young people across Europe. Utilising the notion of anti-social behaviour as the most useful measure for comparative research of victimisation of young people, this article suggests that almost one-fifth of the young people are regularly subjected to behaviour they consider anti-social. However, the majority of young people show surprisingly high levels of self-confidence and security. The key is the existence of friendship groups, which provide security

from victimisation, and if something negative does occur, the friendship group also provides a place to share concerns. Teachers, police officers, youth workers and even parents are far less important in dealing with incidents of anti-social behaviour. This article concludes by arguing that this points to a move away from increasing 'formal' interventions, such as CCTV, adults travelling on buses with young people, or police officers outside schools, towards supporting the, already existing, informal methods used by young people to limit the possibility of victimisation. (PsycINFO Database Record (c) 2012 APA, all rights reserved) (journal abstract)
SN - 1460-3780
AD - Moore, Stephen: Department of Family and Community Studies, Anglia Ruskin University, Cambridge, United Kingdom Maclean, Rachel: Department of Family and Community Studies, Anglia Ruskin University, Cambridge, United Kingdom Jefford, Tom: Head of Youth Support Services, Cambridgeshire County Council, United Kingdom
ER -

TY - JOUR
ID - 2478
T1 - Positioning and identity in the academic literacy experiences of elementary English language learners
A1 - Hickey,Pamela J.
Y1 - 2012///
N1 - Dissertation Abstract: 2012-99050-389
English
Dissertation Abstract
KW - PsycInfo
KW - Academic Environment
KW - Elementary Education
KW - English as Second Language
KW - Literacy
KW - Language
RP - NOT IN FILE
SP - 3116
JF - Dissertation Abstracts International Section A: Humanities and Social Sciences
VL - 72:
IS - 9-A
N2 - This study investigates the academic literacy experiences of elementary English Language Learners (ELLs) in first grade, fourth grade, and sixth grade. Participants included students as well as their reading/language arts mainstream teachers and their English for Speakers of Other Languages (ESOL) teachers. Informed by both cross-sectional cross-case study and narrative inquiry methodology, this study used positioning theory and identity theory as complementary analytic lenses. Students' positionings, both reflexive self-positioning and interactive positioning by others, were identified and named through analyses of their interactions in academic literacy events during reading/language arts. In order to consider the ways that students' positionings may afford or constrain their access to and engagement with academic literacy events, the researcher created an analytic framework naming student positions. Additionally, positions were considered in light of the ways that they mediated students' levels of engagement as literacy events unfolded. To investigate the construction of students' literate identities, the researcher examined students' patterns of positioning during literacy events and considered interview data from students and teachers as well as field notes that documented conversations with participants. The researcher also gathered two self-portraits from student participants, including one self-portrait showing the student engaged in an academic literacy task at school and one showing the student engaged in a fun activity outside of the school context. The study demonstrated that students' positionings, both positive and constraining, may work to construct and re-construct students' literate identities even as students' literate identities may inform the ways that students take on and negotiate positions in a recursive process. The study also found that students with strong literate identities bridging home and school contexts took on more positive positions thus engaging more deeply with academic literacy tasks than students with striving literate identities. Students with striving literate identities often took on positions of constraint in strategic moves that allowed them to get through literacy tasks without engaging deeply. Finally, this study demonstrated the powerful ways that teachers may support students' deep engagement with literacy tasks through positive positioning and following through on their lesson implementation by offering opportunities for re-positioning and the use of scaffolds. (PsycINFO Database Record (c) 2012 APA, all rights reserved)
SN - 0419-4209

AD - Hickey, Pamela J.: U Maryland, Coll Park, US
ER -

TY - JOUR
ID - 2479
T1 - "Thinking outside the pack": Examining physically active smokers and implications for practice among Ontario residents. [References]
A1 - Gauthier,Alain P.
Y1 - 2012///
N1 - Peer Reviewed Journal: 2012-11574-017
English
Journal; Peer Reviewed Journal
KW - PsycInfo
KW - Health Promotion
KW - Physical Activity
KW - Smoking Cessation
KW - Tobacco Smoking
RP - NOT IN FILE
SP - 395
EP - 403
JF - Health Promotion Practice
JA - HEALTH PROMOT PRACT
VL - 13:
IS - 3
N2 - Introduction: It is estimated that tobacco use kills more than 5 million people annually; it is the leading cause of preventable deaths. Recent public health interventions have likely contributed to a steady decline in rates of smoking over the past decade. Nevertheless, innovative and cost-effective approaches to smoking cessation remain a public health priority. The purpose of this study was to profile physically active smokers. Method: Data from the Canadian Community Health Survey 2007-2008-Ontario Sharing File were used. Responses from 41,800 persons aged 12 years and older were assessed to compare (a) the sociodemographic characteristics of physically active smokers to physically active nonsmokers in Ontario and (b) the types of leisure-time physical activities that are more commonly practiced among active Ontario smokers to active nonsmokers. Results: Pearson 2 and independent samples t tests revealed that active smokers were more likely to be male, younger, single, and less educated and to have lower income than active nonsmokers. Active smokers were also more likely to report inexpensive, low-intensity, and solitary leisure-time physical activities. Conclusion. Our findings have important implications for physical activity promotion among smokers. Physical activity interventions for smokers need to be tailored differently than for nonsmokers. (PsycINFO Database Record (c) 2012 APA, all rights reserved) (journal abstract)
SN - 1524-8399
AD - Gauthier, Alain P.: Laurentian University, Sudbury, ON, Canada Snelling, Susan J.: Sudbury & District Health Unit, Sudbury, ON, Canada King, Michael: Sudbury & District Health Unit, Sudbury, ON, Canada
ER -

TY - JOUR
ID - 2480
T1 - The joint engagement skills of children at risk for Autism Spectrum Disorder
A1 - Richmond,Alisha S.
Y1 - 2012///
N1 - Dissertation Abstract: 2012-99060-334
English
Dissertation Abstract
KW - PsycInfo
KW - At Risk Populations
KW - Diagnosis
KW - Pervasive Developmental Disorders
KW - Ability
RP - NOT IN FILE

SP - 5266
JF - Dissertation Abstracts International: Section B: The Sciences and Engineering
VL - 72:
IS - 9-B
N2 - Joint engagement is the child's ability to share an experience involving an object with social partners. This skill is critical to the social and communication development of young children. Previous semi-structured investigations in clinical settings revealed that children with Autism Spectrum Disorder (ASD) present with deficits in joint engagement. Additionally, findings from retrospective studies of home videotapes of children later diagnosed with ASD indicate that ASD symptoms can be observed within naturally occurring activities prior to a diagnosis. Therefore a systematic method of examining the joint engagement skills of children at risk for ASD within everyday activities is warranted. Children between 17 and 34 months (N=42) were recruited from the FIRST WORDS Project prospective study based on risk for ASD. Participants were videotaped during everyday activities and interactions with maternal caregivers. Consistent with the results of retrospective home video studies of children diagnosed with ASD and previous joint engagement investigations, the participants in the current study spent more time playing with toys than in other everyday activities, such as book reading and playing with people. Results indicated that the participants spent the majority of their time in object engagement without symbols and rarely participated in supported or coordinated joint engagement. Children who spent more time in coordinated joint engagement received lower autism severity ratings in the social affect domain of the Autism Diagnostic Observation Schedule (ADOS; Lord, Rutter, DiLvore, & Risi, 2002). Additionally, object engagement with and without symbols was associated with autism severity. The understanding and use of symbols during various engagement states was associated with fewer autism symptoms and higher verbal ability. Overall, these findings indicated that systematic home observations of children's joint engagement skills could contribute to the early identification of ASD symptoms in infants and toddlers. These observations also provide detailed information on children's use and understanding of symbols within everyday activities. Examining the joint engagement skills of children at risk for ASD in natural environments could validate caregivers' concerns of ASD symptoms and help build consensus with a diagnostic evaluation in a clinical setting. The current findings also have important implications for promoting the education of caregivers and professionals on the importance of this pivotal skill. (PsycINFO Database Record (c) 2012 APA, all rights reserved)
SN - 0419-4217
AD - Richmond, Alisha S.: The Florida State U., US
ER -

TY - JOUR
ID - 2481
T1 - The role of policy and the built environment on children's in-school physical activity in the Mississippi Delta
A1 - Gamble,Abigail
Y1 - 2012///
N1 - Dissertation Abstract: 2012-99060-245
English
Dissertation Abstract
KW - PsycInfo
KW - Active Living
KW - Exercise
KW - Physical Activity
KW - School Environment
KW - Student Attitudes
RP - NOT IN FILE
SP - 5251
JF - Dissertation Abstracts International: Section B: The Sciences and Engineering
VL - 72:
IS - 9-B
N2 - School environments provide an ideal setting for children to develop and adopt active living behaviors as a way of life. The primary purpose of this investigation was to describe state, district and school level policy regarding in-school physical activity, the school built environment and in-school physical activity of children ages 6 through 11 years in the Mississippi Delta. A mixed-methods approach was used to garner a rich

understanding of how current policy and the built environment influence in-school physical activity and weight status. Eleven public schools in three districts representing two counties in the Mississippi Delta participated in this investigation. The mean physical education class time was 39.2 minutes +8.13 (range 38.33; 95% CI = 37.66-40.75). The mean percent of physical education class time spent in moderate-to-vigorous physical activity (MVPA) was 36.43% + 1.5% (95% CI = 33.57-39.28), a mean of 13.99 minutes + 5.78 (range 27.0; 95% CI = 12.89-15.08). A significant relationship was found between children's in-school physical activity and their BMI (r = 0.629; p = 0.05). There were significant inverse relationships between the presence and quality of amenities to the school built environment and the students BMI (r = -0.619; p = 0.04), waist-to-height ratio (r = -0.819; p = 0.002) and total body weight (r = -0.615; p = 0.044). There was also a significant inverse relationship between the presence and quality of built environment features and waist-to-height ratio (r = -0.713; p = 0.014). There was no significant relationship between children's in-school physical activity and aspects of the school built environment. If students are given the opportunity for unstructured daily physical activity it is likely they will meet current physical activity guidelines. Two of the schools in this investigation provided little opportunity for physical activity through recess and physical education classes and had the highest BMI scores. Not surprising, when students in these schools were given the opportunity to be physically activity they were among the most physically active students in this study. Despite Mississippi State law, school districts and schools are failing to adhere to policies that provide students the opportunity to engage in 150-minutes of weekly physical activity and are in violation of current state law. (PsycINFO Database Record (c) 2012 APA, all rights reserved)
SN - 0419-4217
AD - Gamble, Abigail: U Mississippi, US
ER -

TY - JOUR
ID - 2482
T1 - Project WetKids: An out-of-school program fostering citizen scientists in the middle years. [References]
A1 - Cwikla,Julie
Y1 - 2012///
N1 - Book: 2011-30521-006
English
Book; Edited Book
KW - PsycInfo
KW - Ecology
KW - Educational Programs
KW - Environmental Education
KW - Science Education
KW - Wilderness Experience
KW - Awareness
KW - Family
KW - Middle School Students
KW - Occupational Aspirations
KW - Nature (Environment)
RP - NOT IN FILE
SP - 87
EP - 98
JF - Oxford, Rebecca L [Ed]; Lin, Jing [Ed]
VL - :
IS - 2012
N2 - (from the chapter) Project WetKids provides Pascagoula, Mississippi middle school students and their families with wetland, environmental, estuary, and watershed experiences with local scientists, engineers, and naturalists. Extensive activities provide participants (1) real-world, locally relevant science-based events; (2) meaningful scientific experiences and interactions with field scientists; and (3) exposure to science, technology, engineering, and mathematics career opportunities in the Gulf Coast region. This chapter will detail the bi-monthly out-of-school experiences, weekend family events, and Summer Academy provided by Project WetKids. Participants' science content knowledge, their environmental awareness and actions, and their career aspirations were (and continue to be) monitored during the program. Results of these analyses as well as the activities, events, and inspiration that precipitated their growth are described in the chapter. Environmentally rich activities, students' exposure and interaction with scientists, and family-based events provide the

scaffolding to move the community toward awareness and then action of the importance of wetlands education specifically and eco-education more broadly. In addition to the benefits for individual students, there is great potential for a broader impact for an increased science, technology, engineering, and mathematics (STEM) workforce if students gain that sense of excitement from science. As stated by Saunders (2009), "If America hopes to effectively address the 'STEM pipeline' problem, we must find ways of developing young learners' interest in STEM education and must sustain that interest throughout their remaining school years." In this chapter, we describe how Project WetKids seeks to make such an impact. (PsycINFO Database Record (c) 2012 APA, all rights reserved)
AD - Cwikla, Julie: University of Southern Mississippi Gulf Coast, MS, US Barry, Christopher: University of Southern Mississippi, Department of Psychology, MS, US
ER -

TY - JOUR
ID - 2483
T1 - Predisposed to participate? The influence of family socio-economic background on children's sports participation and daily amount of physical activity. [References]
A1 - Nielsen,Glen; Gronfeldt,Vivian; Toftegaard-Stockel,Jan; Andersen,Lars Bo
Y1 - 2012///
N1 - Peer Reviewed Journal: 2011-29858-001
English
Journal; Peer Reviewed Journal
KW - PsycInfo
KW - Athletic Participation
KW - Family
KW - Physical Activity
KW - Socioeconomic Status
KW - Sports
KW - Activity Level
RP - NOT IN FILE
SP - 1
EP - 27
JF - Sport in Society
VL - 15:
IS - 1
N2 - From a Bourdieu-inspired understanding of how personal resources ('capitals') enable certain practices in certain contexts, the links between families' cultural, social and economic capitals, and children's daily physical activity were investigated in 500 suburban Danish schoolchildren using questionnaire data and accelerometer measures. Family socio-economic position (SEP) was found to be positively associated with children's participation in organized sport, which could be explained by differences in family capitals. By contrast, this study found no relationship between families' SEP and the amounts of general physical activity in children. This reflected the tendencies for club-organized sport to contribute a relatively small amount to the overall amount of physical activity in children, and for children of low SEP to be equally active in other settings such as school-breaks, day care and neighbourhood playgrounds. (PsycINFO Database Record (c) 2012 APA, all rights reserved) (journal abstract)
SN - 1743-0437
AD - Nielsen, Glen: Department of Exercise and Sports Sciences, University of Copenhagen, Copenhagen, Denmark Gronfeldt, Vivian: Department of Exercise and Sports Sciences, University of Copenhagen, Copenhagen, Denmark Toftegaard-Stockel, Jan: Center for Research in Childhood Health, University of Southern Denmark, Denmark Andersen, Lars Bo: Center for Research in Childhood Health, University of Southern Denmark, Denmark
ER -

TY - JOUR
ID - 2484
T1 - Student attitudes toward and recall of outside day: An environmental science field trip. [References]
A1 - Nadelson,Louis S; Jordan,
Y1 - 2012///

N1 - Peer Reviewed Journal: 2012-09041-006
English
Journal; Peer Reviewed Journal
KW - PsycInfo
KW - Educational Field Trips
KW - Environmental Education
KW - Free Recall
KW - Retention
KW - Student Attitudes
RP - NOT IN FILE
SP - 220
EP - 231
JF - The Journal of Educational Research
VL - 105:
IS - 3
N2 - Field trips are effective because they situate learning and facilitate knowledge transfer, thereby influencing students learning attitudes, interests, and motivation. Variations in field trip configurations and the subsequent affective and cognitive influences provided the motivation for this study of Outside Day-an environmental education field trip for 6th-grade students. The participants were immediately postassessed on their attitudes toward the event and 1 month later assessed on their event activity recall. Results indicate the students held positive attitudes toward the field trip and recalled a hands-on orienteering activity most frequently. The discussion provides explanations and implications of findings. (PsycINFO Database Record (c) 2012 APA, all rights reserved) (journal abstract)
SN - 0022-0671
AD - Nadelson, Louis S.: College of Education, Boise State University, Boise, ID, US Jordan, J. Richard: Timberline High School, ID, US
ER -

TY - JOUR
ID - 2485
T1 - Preschooler's physical activity levels and associations with lesson context, teacher's behavior, and environment during preschool physical education. [References]
A1 - Eveline,Van Cauwenberghe; Valery,Labarque; Jessica,Gubbels; Ilse,De Bourdeaudhuij; Greet,Cardon
Y1 - 2012///
N1 - Peer Reviewed Journal: 2011-24169-001
English
Journal; Peer Reviewed Journal
KW - PsycInfo
KW - Activity Level
KW - Physical Activity
KW - Physical Education
KW - Preschool Students
KW - Behavior
KW - Preschool Education
KW - School Environment
KW - Teachers
RP - NOT IN FILE
SP - 221
EP - 230
JF - Early Childhood Research Quarterly
VL - 27:
IS - 2
N2 - Preschools offer two main opportunities, recess and physical education (PE), for preschool children to participate in physical activity (PA) and are considered a suitable institution for PA promotion given the large number of children enrolled. Although PE can have a meaningful effect on preschoolers' PA levels, preschool PE characteristics associated with increased PA have, to the best of our knowledge, not been identified. Therefore, the purpose of the present study was to examine preschooler's PA levels and the associations with

lesson context, teacher behavior, and environment during preschool physical education. A random sample of 573 preschoolers (288 boys; Mage = 5.4 years, SD = 0.4) from 35 preschools was examined during one PE class. Findings indicated that preschoolers accumulated 12 min (33%) of moderate-to-vigorous PA (MVPA), 5 min (13%) of light PA, and 20 min (54%) of sedentary behavior during PE. Forty-seven percent of the variance in pupils' MVPA may be attributed to differences between PE classes. Less knowledge content, less promotion, less management, less preschoolers per 100 m2, using obstruction material, and not using throwing equipment were significantly associated with higher MVPA levels. These predictors explained 56% of the variance in MVPA at the PE class-level. To conclude, PE in its current format contributes only a small amount to the PA requirements of preschoolers. Preschoolers' MVPA levels were related to modifiable PE characteristics indicating that preschool PE can be restructured to increase MVPA. (PsycINFO Database Record (c) 2012 APA, all rights reserved) (journal abstract)
SN - 0885-2006
AD - Eveline, Van Cauwenberghe: Department of Movement and Sport Sciences, University of Ghent, Ghent, Belgium Valery, Labarque: HUBrussel (part of association KULeuven), Research Centre PRAGODI, Brussels, Belgium Jessica, Gubbels: Department of Health Promotion, Maastricht University Medical Centre, Maastricht, Netherlands Ilse, De Bourdeaudhuij: Department of Movement and Sport Sciences, University of Ghent, Ghent, Belgium Greet, Cardon: Department of Movement and Sport Sciences, University of Ghent, Ghent, Belgium
ER -

TY - JOUR
ID - 2486
T1 - Changing perceptions of middle school students regarding bullying: The impact of student-centered interventions
A1 - Dare,Susan
Y1 - 2012///
N1 - Dissertation Abstract: 2012-99031-021
English
Dissertation Abstract
KW - PsycInfo
KW - Middle School Students
KW - School Based Intervention
KW - Student Attitudes
KW - Bullying
RP - NOT IN FILE
SP - 2743
JF - Dissertation Abstracts International Section A: Humanities and Social Sciences
VL - 72:
IS - 8-A
N2 - Previous research suggests that bullying is a serious problem with long-term consequences for both the bullies and the victims. These consequences run the gamut from school maladjustment to criminal behavior to suicidal ideation. This longitudinal study utilized ex post facto data collected by one Burlington County school district. Perceptions and behaviors of students in the district's two middle schools were examined by comparing survey responses collected in October 2007 with survey responses collected in October 2009. In addition, survey responses collected in October 2009 were compared with May/June 2010 responses to see if students' perceptions and behaviors changed over the course of one school year. Finally, survey responses from the district's October 2009 seventh graders were compared with survey responses from the district's May/June 2010 sixth graders. The incoming seventh graders had not participated in any of the district's planned interventions while the outgoing sixth graders had participated in the interventions throughout the 2009-2010 school year. The groups were relatively similar in age and middle school experiences so it was believed that the October 2009 seventh graders could represent the pre-intervention group and the May/June 2010 sixth graders could represent the post-intervention group. Results were compared to examine the effectiveness of the school's ongoing bullying interventions. Grade level and gender comparisons suggested that most students felt less safe during the course of one school year. Sixth graders were most likely to be fearful of bullying and were most likely to perceive bullying as a serious problem. All forms of bullying increased as students moved from sixth to seventh and from seventh to eighth grade. All forms of bullying except verbal bullying increased during one school year. The most problematic areas where bullying was most likely to occur were outdoor recess areas, hallways and hall locker areas. Gender comparisons suggested that boys were more likely to bully physically and verbally and

girls were more likely to bully indirectly or through cyber-bullying. When students' ethnicities were considered one interesting finding was that African American students at the two latter survey dates and Hispanic students at the final survey date were more likely to believe that they were bullied due to their race, whereas all other groups reported that the most likely reason for bullying was appearance or dress. Two-year comparisons of October 2007 and October 2009 responses suggested that students in 2007 felt safer and students in 2009 were more likely to perceive bullying as a serious problem. The 2007 group was more likely to bully physically, verbally or indirectly whereas the 2009 group was more likely to cyber-bully. The only exception to this was cyber-bullying through instant messaging, which was more prevalent in 2007. Pre- and post-intervention comparisons suggested that the district's interventions may have been at least partially successful. While outdoor recess continued to be the most likely place where bullying would occur, the percentage of students who reported this dropped precipitously when these two groups were compared. Verbal bullying also declined suggesting that although the interventions did not specifically target this behavior they may have played a role in the decline. Unfortunately it is impossible to know with any degree of certainty whether these declines were due to the interventions or due to some other unknown influence. Recommendations for the school district and suggestions for future research were also discussed. (PsycINFO Database Record (c) 2012 APA, all rights reserved)
SN - 0419-4209
AD - Dare, Susan: Wilmington U. (Delaware), US
ER -

TY - JOUR
ID - 2487
T1 - A social ecological study examining individual, household, and neighborhood influences on children's physical activity and active commuting to/from school
A1 - Salomonsen-Sautel,Stacy
Y1 - 2012///
N1 - Dissertation Abstract: 2012-99040-252
English
Dissertation Abstract
KW - PsycInfo
KW - Living Arrangements
KW - Neighborhoods
KW - Physical Activity
KW - Social Environments
KW - Risk Factors
KW - Schools
RP - NOT IN FILE
SP - 4606
JF - Dissertation Abstracts International: Section B: The Sciences and Engineering
VL - 72:
IS - 8-B
N2 - Children need to develop active living behaviors that will continue into adolescence and adulthood as well as increase their physical activity to reduce the risk of obesity and related chronic illnesses. Using a multilevel social ecological approach, the overall aim of this study was to identify neighborhood correlates of children's physical activity and active commuting to/from school. Methods: A cross-sectional research design was employed to acquire and link secondary data from several sources at the individual and neighborhood level. The sample included 863 children, ages 5 to 14, and a household member living in 89 neighborhoods/ZIP codes in the Denver metropolitan area. Child data were obtained from the 2005 and 2007 Child Health Survey (CHS), which was directly linked to family members' data from the 2005 and 2007 Behavioral Risk Factor Surveillance System (BRFSS). Neighborhood data were linked to child and household data. Results: The combined partial proportional odds model revealed that mean neighborhood safety (for five or one to four days versus zero days) and percentage of minority residents were significantly related to children's active commuting to/from school. For boys, the individual level hierarchical linear model found that family members' physical activity influenced boys' physical activity, and the separate neighborhood models revealed three neighborhood variables (percentage of individuals with at least a BA degree, reported incidents for rape, and reported incidents for all violent offenses) that directly influenced boys' physical activity. For girls, the individual level multiple linear regression model revealed that family members' physical activity predicted girls' physical activity, and the

separate neighborhood models found four neighborhood variables (population density, reported incidents for murder, reported incidents for robbery, and number of school playgrounds) that directly influenced girls' physical activity. Contrary to the hypothesis, parental perceptions of neighborhood safety did not mediate the effect of total violent crime on boys or girls' physical activity or active commuting to/from school. In addition, children's physical activity was not correlated with their active commuting. Conclusion: Neighborhood safety is an important factor for discretionary active commuters. Future research should explore in greater detail the influence of crime on boys and girls' physical activity. (PsycINFO Database Record (c) 2012 APA, all rights reserved)
SN - 0419-4217
AD - Salomonsen-Sautel, Stacy: U Colorado at Denver, US
ER -

TY - JOUR
ID - 2488
T1 - Assessment of intra-urban traffic-related air pollution on asthmatic children's exposure at schools in the Paso del Norte region
A1 - Raysoni,Amit U.
Y1 - 2012///
N1 - Dissertation Abstract: 2012-99040-035
English
Dissertation Abstract
KW - PsycInfo
KW - Asthma
KW - Elementary Schools
KW - Pollution
KW - Public Health
KW - Urban Environments
RP - NOT IN FILE
SP - 4525
JF - Dissertation Abstracts International: Section B: The Sciences and Engineering
VL - 72:
IS - 8-B
N2 - Traffic-related air pollution can be a major public health concern in any urban area. This problem is compounded in the Paso del Norte region that has experienced rapid economic growth, and a substantial number of people living in close vicinity of major roadways. The desert surroundings, arid weather, frequent temperature inversions, heavy border traffic at the international ports of entry between El Paso and Ciudad Juarez, and poorly maintained vehicle fleet further exacerbates this problem. A growing body of air quality and epidemiologic research has discerned the linkage between traffic emissions and respiratory disorders. Initial health investigations in the Paso del Norte region have found associations between various air pollutant indicators and adverse respiratory outcomes. However, monitoring traffic pollution as an indicator of population health and the subsequent ability to accurately reflect changes in the respiratory health of sensitive populations like school-going asthmatic children has been challenging in this region. Given the substantial amount of time children spend within school microenvironments, assessing children's school-based exposures is essential for preventing children's health risks to air pollutants. Indeed, the importance of characterizing children's exposures in schools corresponds with the US Environmental Protection Agency's recent initiative to promote outdoor air monitoring network near schools. As part of a binational health effects study investigating the impact of traffic air pollution on asthmatic children, paired indoor and outdoor concentrations of fine and coarse PM (PM2.5 and PM10-2.5), black carbon, and NO2 were determined for 16 weeks in 2008 at four elementary schools in the international community of El Paso - Ciudad Juarez on the U.S.-Mexico border. Fifty-eight asthmatic subjects from these four schools were recruited. Health outcomes (weekly exhaled nitric oxide [eNO] measurements and daily respiratory symptoms) were recorded for the study period. Two schools (one in each city) were located in high traffic density zones and the other two in zones of low traffic density. Strong spatial heterogeneity in air pollutant concentrations existed in the region with all outdoor pollutant concentrations, in general, higher in Ciudad Juarez than in El Paso by two-fold or more. Concentrations of all pollutants, except PM in Ciudad Juarez, at high traffic density zones in both cities were higher than those measured at their respective low traffic density zones. Traffic-related PM pollution in Ciudad Juarez was confounded by the ubiquitous fugitive dust emissions from unpaved roads and regional unprotected bare soil such that both PM2.5 and PM10-2.5 measured

in the low traffic density zone surpassed those measured in the high traffic density zone. Aided by inter-site and intra-pollutant analyses, concentrations of NO2 and black carbon were confirmed to be better indicators for traffic-related pollution. Indoor air pollution was found to be well associated with outdoor air pollution, although differences existed among all schools in student activities, building tightness, use of ventilation system, temperature control devices, and building materials. Routine cooking and food preparation in classrooms could result in high level of indoor NO 2 which pose unintended health risks to school children. Significant associations between the weekly (96-hr) averages of several measured pollutants and eNO with effects estimates ranging from 1 to 3% increases in eNO per interquartile range increases in pollutant concentrations were observed. Effect estimates from models using indoor pollutant school concentrations were generally more robust than corresponding models using outdoor school or ambient concentrations. This research characterizes the intra-urban variability (PsycINFO Database Record (c) 2012 APA, all rights reserved)
SN - 0419-4217
AD - Raysoni, Amit U.: U Texas at El Paso, US
ER -

TY - JOUR
ID - 2489
T1 - Playgrounds found superior to school polices for increasing physical activity. [References]
A1 - No,authorship indicated
Y1 - 2012///
N1 - Peer Reviewed Journal: 2012-04962-012
English
Journal; Peer Reviewed Journal
KW - PsycInfo
KW - Physical Activity
KW - Physical Education
KW - Playgrounds
KW - Policy Making
KW - School Facilities
RP - NOT IN FILE
SP - 148
EP - 149
JF - Journal of Sport & Exercise Psychology
JA - J Sport Exerc Psychol
VL - 34:
IS - 1
N2 - Presents a study which aims to examine two ways that schools may encourage physical activity behavior, one, number of permanent play facilities and second, school policies related to physical activity like quality of physical education programs, promotion of physical activity, safety issues, and the adequacy of school facilities. The amount of permanent playground equipment at the school was significantly linked to physical activity levels, although school policies were not. Specifically, for every 10 additional playground pieces, children engaged in 3.2% more average physical activity and 8.3% more moderate-to-vigorous physical activity (MVPA) during recess. Physical activity outside of school hours was 5.6% higher on average and 10.5% higher for MVPA for every 10 additional pieces of play equipment. Building more permanent playgrounds and structures appeared to be a relatively cost-effective strategy for increasing physical activity behaviors both during recess and after school hours. (PsycINFO Database Record (c) 2012 APA, all rights reserved)
SN - 0895-2779
ER -

TY - JOUR
ID - 2490
T1 - Variation in children's classroom engagement throughout a day in preschool: Relations to classroom and child factors. [References]
A1 - Vitiello,Virginia E; Booren,Leslie
Y1 - 2012///

N1 - Peer Reviewed Journal: 2011-23255-001
English
Journal; Peer Reviewed Journal
KW - PsycInfo
KW - Classroom Environment
KW - Peers
KW - Preschool Students
KW - Student Engagement
KW - Teachers
KW - Age Differences
KW - Human Sex Differences
RP - NOT IN FILE
SP - 210
EP - 220
JF - Early Childhood Research Quarterly
VL - 27:
IS - 2
N2 - This study examined sources of variability in preschool children's positive and negative engagement with teachers, peers, and tasks, and how that variability was related to both classroom activity settings (e.g., teacher-structured time, outdoor time, transitions) and child factors (age, gender). Participants were 283 socioeconomically and linguistically diverse children drawn from 84 classrooms, 34-63 months old (M = 50.8, SD = 6.5). Each child's engagement was observed and rated multiple times within a single day. Results suggested that children's engagement varied significantly across the preschool day. Activity settings that provided children with a greater degree of choice (free choice and outdoor time) were associated with more positive engagement with peers and tasks, while teacher-structured activities were associated with more positive engagement with teachers. Transitions emerged as a difficult part of the day, associated with less positive engagement with teachers and tasks. Older children were rated higher on peer and task engagement. These findings, together with previous research, suggest that both characteristics of the classroom setting and child factors are associated with children's classroom engagement throughout a day in preschool. (PsycINFO Database Record (c) 2012 APA, all rights reserved) (journal abstract)
SN - 0885-2006
AD - Vitiello, Virginia E.: University of Virginia, Charlottesville, VA, US Booren, Leslie M.: University of Virginia, Charlottesville, VA, US Downer, Jason T.: University of Virginia, Charlottesville, VA, US Williford, Amanda P.: University of Virginia, Charlottesville, VA, US
ER -

TY - JOUR
ID - 2491
T1 - School participation and social networks of children with complex communication needs, physical disabilities, and typically developing peers. [References]
A1 - Raghavendra,Parimala; Olsson,Catherine; Sampson,Janelle; Mcinerney,Rachael; Connell,Timothy
Y1 - 2012///
N1 - Peer Reviewed Journal: 2012-05273-005
English
Journal; Peer Reviewed Journal
KW - PsycInfo
KW - Communication Disorders
KW - Participation
KW - Physical Disorders
KW - Social Interaction
KW - Social Networks
KW - Peer Relations
RP - NOT IN FILE
SP - 33
EP - 43
JF - AAC: Augmentative and Alternative Communication
VL - 28:

IS - 1
N2 - The aim of the study was to describe and compare the school participation and social networks of children with physical disabilities and complex communication needs (Group CCN), children with physical disabilities only (Group PD), and children with typical development (Group TD). The 39 participants, 10-15 years of age, were observed for 4 hours at school. School staff and the parent and/or child provided information on children's social networks. A striking observation was that, while participants in Group TD continuously conversed and socialized with peers inside and outside classrooms; those in Group CCN rarely used aided AAC, were provided with limited communication opportunities at school, and had fewer acquaintances and friends. Findings warrant intervention at the participation level at school and in the community. (PsycINFO Database Record (c) 2012 APA, all rights reserved) (journal abstract)
SN - 0743-4618
AD - Raghavendra, Parimala: Novita Children's Services, Adelaide, SA, Australia Olsson, Catherine: Novita Children's Services, Adelaide, SA, Australia Sampson, Janelle: Novita Children's Services, Adelaide, SA, Australia Mcinerney, Rachael: Novita Children's Services, Adelaide, SA, Australia Connell, Timothy: Department for Communities and Social Inclusion-Disability Services, Adelaide, SA, Australia
ER -

TY - JOUR
ID - 2492
T1 - "Don't ruin my pretend": Kids sustaining play interactions in out-of-school settings
A1 - Eyerman,Suzanne
Y1 - 2012///
N1 - Dissertation Abstract: 2012-99010-211
English
Dissertation Abstract
KW - PsycInfo
KW - Childhood Play Behavior
KW - Elementary School Students
KW - Peer Relations
KW - Schools
RP - NOT IN FILE
SP - 2260
JF - Dissertation Abstracts International Section A: Humanities and Social Sciences
VL - 72:
IS - 7-A
N2 - As much as play is researched and discussed by people interested in children and childhood, studies often fail to examine closely the ways that kids accomplish their play. This study sought to answer the question of how children sustain their play interactions. By making use of qualitative methods to collect and analyze data, the play of elementary-age children was studied in the summer camp and school recess settings. In these settings, children were less closely supervised by adults and were freer to make their own decisions about their interactions, in comparison to more structured settings such as school classrooms or organized sports. Findings from this study revealed that kids worked to sustain their play interactions by creating a participation framework that included one participant being in charge within each interaction. The kid in charge played the role of the boss and managed the play activity. The other kids followed and took on support roles. In addition, findings revealed that unwritten rules around social interactions were important to the ways kids interacted with each other. Play activities were found to be made up of nuanced communication that was evidence of the work kids did to sustain their interactions. By managing their own play activity, kids worked to sustain interactions without the guidance of adults in the moment. (PsycINFO Database Record (c) 2012 APA, all rights reserved)
SN - 0419-4209
AD - Eyerman, Suzanne: U Colorado at Boulder, US
ER -

TY - JOUR
ID - 2493
T1 - Parent-child treatment: Searching for a lost mother
A1 - Harrison,Alexandra M.
Y1 - 2012///

N1 - Book: 2011-30604-006
English
Book; Edited Book
KW - PsycInfo
KW - Mother Child Relations
KW - Psychoanalysis
KW - Psychotherapeutic Techniques
KW - Videotapes
KW - Child Psychotherapy
KW - Clients
KW - Dyads
KW - Early Childhood Development
KW - Mothers
KW - Psychoanalysts
KW - Psychotherapeutic Processes
RP - NOT IN FILE
SP - 79
EP - 92
JF - Akhtar, Salman [Ed]
VL - :
IS - 2012
N2 - (from the chapter) Psychoanalysts listen and reflect on what they hear from their patients and on what they do with them. Child analysts also attend to the activity of the play with their young patients and to the symbolic representations in the play. Rarely have analysts-adult or child-learned from observational data in the meticulous manner of scientific researchers, and rarer still are attempts to gather this observational data and make it available for others studying the analytic process. All psychoanalysts would agree that what is communicated outside of language in the analytic hour has great value. How do other disciplines teach about what happens outside of language and symbols? One way is through watching videos. A tennis player can look at videos of Roger Federer and watch his strokes, yet eventually the player will have to develop 'muscle memory' to be able to implement the moves in a match. This is the focus of the current chapter. Here, I will use the case of a dyadic (mother-child) treatment to illustrate how videotape and particularly the microanalysis of the videotape can enhance our understanding of what goes on in psychotherapy and give us insights into therapeutic technique. In this chapter I will use the case of "Jose," a 3-year old to 4-year old boy, to demonstrate the value of videotape and microanalytic technique to the understanding of therapeutic change. The treatment was a dyadic treatment, in which the author saw mother and child together, using videotape in several different modes during the therapy. In each of the three examples of videotape from the case, a moment of new meaning created by mother and child together was accompanied by the 'music' of highly coordinated rhythms of vocal and action turns. (PsycINFO Database Record (c) 2012 APA, all rights reserved)
AD - Harrison, Alexandra M.: Harvard Medical School, Boston, MA, US
ER -

TY - JOUR
ID - 2494
T1 - The use of bibliotherapy in natural environments to develop social skills in young children
A1 - Chai,Angie Yuyoung
Y1 - 2012///
N1 - Dissertation Abstract: 2012-99020-387
English
Dissertation Abstract
KW - PsycInfo
KW - Bibliotherapy
KW - Childhood Development
KW - Curriculum
KW - School Based Intervention
KW - Social Skills
KW - Classrooms
KW - Problem Solving

KW - Social Issues
RP - NOT IN FILE
SP - 4314
JF - Dissertation Abstracts International: Section B: The Sciences and Engineering
VL - 72:
IS - 7-B
N2 - The purpose of this study was to explore the use of bibliotherapy as an intervention to increase social problem-solving skills in young children, delivered by classroom teachers within a natural classroom setting. Previous research in this area has been limited to bibliotherapy interventions delivered by clinicians in small group settings. However, guidelines for delivering interventions in natural environments suggest that teacher-led, classroom-based, and child-initiated activities would provide the most natural, school-based setting for young children. The intervention for this study was delivered by classroom teachers and integrated into the classroom curriculum. The participants included 96 students from three kindergarten and three first grade classrooms in a demographically diverse elementary school located in a large urban school district. Classes were randomly assigned to either a treatment group or wait-list control group. The treatment group received the bibliotherapy intervention with reinforcement activities while the wait-list control group received no intervention during the intervention period. A one-way ANOVA, ANCOVAs, and correlations were used to analyze results across groups within a pre-post experimental design. After the intervention, the treatment group demonstrated significantly higher social problem-solving skills than the wait-list control group. Also, the results suggest that children who participated in the bibliotherapy intervention were able to maintain their social problem-solving skills and generalize their skills they to other situations. (PsycINFO Database Record (c) 2012 APA, all rights reserved)
SN - 0419-4217
AD - Chai, Angie Yuyoung: Fordham U., US
ER -

TY - JOUR
ID - 2495
T1 - Participation in play activities of children with cerebral palsy
A1 - Malkawi,Somaya H.
Y1 - 2011///
N1 - Dissertation Abstract: 2011-99220-040
English
Dissertation Abstract
KW - PsycInfo
KW - Ability
KW - Cerebral Palsy
KW - Childhood Play Behavior
KW - Motor Processes
KW - At Risk Populations
RP - NOT IN FILE
SP - 2715
JF - Dissertation Abstracts International: Section B: The Sciences and Engineering
VL - 72:
IS - 5-B
N2 - The aim of this study was to describe the overall play participation of children with cerebral palsy (CP) aged 9-13 years by measuring what activities they participate in using the Preteen Play Profile (Henry, 2000); and to explore variations in participation in different play categories based on each child's manual ability, gross motor function, CP distribution, age and gender. A mail-based survey was conducted of children with CP from three facilities in the United States. Of 252 surveys mailed, 15 were undeliverable because of the wrong addresses and 93 completed surveys were returned (39.2%). The children (58 males, 35 females) were aged between 9 and 13 years (mean age 11 years 1 month, SD= 1.06). Findings showed that children with CP participated in a wide range of activities in the past year. The majority of play activities were indoor rather than outdoor, sedentary rather than active, and lack structure. Watching television and listening to music was by far the children's major play activity. Play categories were ranked from highest to lowest in play participation as follows: indoor, creative, social, summer, outdoor, winter, sports and lessons. It was found that children with quadriplegia who are in Levels IV and V on both the MACS and GMFCS are the most at risk groups of

decreased play participation. The children's manual ability and gross motor function were the main predictors of play participation indicating the need for using both classification systems in practice and research. Children of both genders participated in similar play activities but when analyzing separate play categories, females achieved significantly higher scores than males in creative, lessons and social activities. The results of this study pointed to the need for integration of more outdoor, sports, and social play into the daily routines and curricula of centers, and schools that serve children with CP. The study concluded with some recommendations on how deficiencies and gaps in play participation can be filled through several proactive approaches that pediatric occupational therapists can take in conjunction with caregivers and community members to improve the lives of children with CP. (PsycINFO Database Record (c) 2012 APA, all rights reserved)
SN - 0419-4217
AD - Malkawi, Somaya H.: U Kentucky, US
ER -

TY - JOUR
ID - 2496
T1 - Forty-five minutes of physical activity at school each day? Curricular promotion of physical activity in grades one to four. [References]
A1 - Liersch,Sebastian; Henze,Vicky; Robl,Markus; Schnitzerling,Jorg; Suermann,Thomas; Mayr,Eckart; Krauth,Christian; Walter,Ulla
Y1 - 2011///
N1 - Peer Reviewed Journal: 2011-15345-006
English
Journal; Peer Reviewed Journal
KW - PsycInfo
KW - Curriculum Development
KW - Physical Activity
KW - Prevention
KW - School Based Intervention
RP - NOT IN FILE
SP - 329
EP - 338
JF - Journal of Public Health
VL - 19:
IS - 4
N2 - Aim: According to the US Department of Health and Human Services (2008) and the World Health Organization (2008), regular physical activity is essential to the healthy development of children and contributes to the reduction of chronic diseases throughout their life span. However, coordinated comprehensive national and international programs to promote physical activity and sports participation are lacking. According to the German Sports Federation (2006), physical education (PE) classes at German schools are increasingly being canceled or taught outside the school. "fit for pisa" is a German intervention developed in response to the demand for scientific evaluation of interventions like daily physical education. Its goal is to provide quality management-secured, structured and standardized PE instruction by qualified instructors. Subjects and methods: The "fit for pisa" intervention has been implemented at five primary schools in Gottingen, Germany, since 2003. This daily physical education intervention is based on quality standards. At the five primary schools participating in "fit for pisa," students receive a supplementary 3 class hours of PE/week in addition to the mandatory 2 class hours/week for the entire school term. In other words, the children now receive one 45-min session of PE each school day. Daily physical education is now a part of their mandatory school curriculum. The curriculum content and teaching methods have been continuously developed and optimized based on the state guidelines for Lower Saxony. Results: Teacher training is held at the participating schools once a month to promote standardized implementation and quality management of the intervention program. Additional monthly meetings are held so that the teachers can discuss any problems, deviations or other issues occurring in the classes. Checklists are provided for the teachers and external trainers to document the content and objectives of each lesson. Supervisors evaluate the checklists at regular intervals. Using these instruments, the results of the 4-year intervention program were evaluated at the five participating primary schools. Conclusions: This study provides important data for recommendations to implement additional physical education in primary schools, for the corresponding quality assurance instruments and for the optimization of physical education guidelines for primary schools. (PsycINFO Database Record (c) 2012 APA, all rights reserved) (journal abstract)

SN - 0943-1853
AD - Liersch, Sebastian: Institute for Epidemiology, Social Medicine and Health System Research, Hannover Medical School, Hanover, Germany Henze, Vicky: ASC Gottingen von 1846 e.V, Gottingen, Germany Robl, Markus: Center for Child and Adolescent Health Gottingen, Georg August University of Gottingen, Gottingen, Germany Schnitzerling, Jorg: ASC Gottingen von 1846 e.V, Gottingen, Germany Suermann, Thomas: Association of Statutory Health Insurance, Gottingen, Germany Mayr, Eckart: Public Health Authorities of Gottingen, Gottingen, Germany Krauth, Christian: Institute for Epidemiology, Social Medicine and Health System Research, Hannover Medical School, Hanover, Germany Walter, Ulla: Institute for Epidemiology, Social Medicine and Health System Research, Hannover Medical School, Hanover, Germany
ER -

TY - JOUR
ID - 2497
T1 - Huts and heartache: The affordance of playground huts for legal debate in early childhood social organisation. [References]
A1 - Bateman,Amanda
Y1 - 2011///
N1 - Peer Reviewed Journal: 2011-20337-002
English
Journal; Peer Reviewed Journal
KW - PsycInfo
KW - Elementary School Students
KW - Playgrounds
KW - Social Interaction
RP - NOT IN FILE
SP - 3111
EP - 3121
JF - Journal of Pragmatics
VL - 43:
IS - 13
N2 - This article discusses the way in which four-year-old children engaged in the complex co-production of rules and social governance in their primary school playground in Wales, UK. Through an inductive investigation into children's everyday social interactions at morning break time the child's view of their spatial affordances can be revealed. These affordances are the spaces the children talk into being (Heritage, 1978) and to what end. By making explicit reference to the wooden huts in their co-production of playground rules and governance the children talked those spaces into being and made them noticeable as important places for the practice of such agency in their everyday social organisation process. (PsycINFO Database Record (c) 2012 APA, all rights reserved) (journal abstract)
SN - 0378-2166
AD - Bateman, Amanda: Faculty of Education, University of Waikato, Hamilton, New Zealand
ER -

TY - JOUR
ID - 2498
T1 - Preschoolers' social skills: Advances in assessment for intervention using social behavior ratings. [References]
A1 - Frey,Jennifer R.
Y1 - 2011///
N1 - Peer Reviewed Journal: 2011-27780-001
English
Journal; Peer Reviewed Journal
KW - PsycInfo
KW - School Based Intervention
KW - Social Behavior
KW - Social Skills
KW - Preschool Students
RP - NOT IN FILE

SP - 179
EP - 190
JF - School Mental Health
VL - 3:
IS - 4
N2 - Social skills play an important role in young children's successes in and outside of school. For two decades, educators have used the Social Skills Rating System (SSRS; Gresham & Elliott, 1990) as a tool for describing children's social behaviors and for planning interventions to improve social skills. Although widely used, some researchers have criticized certain aspects of the preschool version of the SSRS. In 2008, a revision of the instrument, the Social Skills Improvement System-Rating Scales (SSiS-RS; Gresham & Elliott), was published. In this article, we examine the development of the preschool versions of SSRS and SSiS-RS and provide a comparison of the two rating systems, focusing on the content, structure, psychometric properties, and intended use of the instruments. We then describe the SSiS-RS within an assessment for intervention model. We conclude that the SSiS-RS is an improved assessment instrument over the preschool SSRS and provide recommendations for future studies that will contribute to the evolving validity evidence for the resulting scores and decisions made from them. (PsycINFO Database Record (c) 2012 APA, all rights reserved) (journal abstract)
SN - 1866-2625
AD - Frey, Jennifer R.: Vanderbilt University, Nashville, TN, US Elliott, Stephen N.: Learning Sciences Institute, Arizona State University, Tempe, AZ, US Gresham, Frank M.: Louisiana State University, Baton Rouge, LA, US
ER -

TY - JOUR
ID - 2499
T1 - Examining the effectiveness and efficiency of two delivery models to teach children abduction prevention skills
A1 - Seckinger-Bancroft,Kimberly E.
Y1 - 2011///
N1 - Dissertation Abstract: 2011-99170-276
English
Dissertation Abstract
KW - PsycInfo
KW - Computer Assisted Instruction
KW - Prevention
KW - Training
KW - Treatment Effectiveness Evaluation
RP - NOT IN FILE
SP - 827
JF - Dissertation Abstracts International Section A: Humanities and Social Sciences
VL - 72:
IS - 3-A
N2 - Nearly all children receive abduction prevention training. Most traditional education programs increase the learner's knowledge, but often fail to produce concomitant behavior change. Behavioral Skills Training (BST) is a multi-component, behavior-based training strategy with empirical support demonstrating its effectiveness in teaching children safety skills, behavioral generalization and maintenance over time. BST, however, is restricted by financial, human and time costs and limited resources to implement the training protocol. These factors likely limit widespread adoption of the training model. This study examined the use of computer-based instruction that emphasized active responding and mastery level performance requirements to teach school-aged children abduction prevention skills. Computer-Based Instruction (CBI) was compared against traditional BST (instructions, modeling, rehearsal, feedback and in situ training) on measures of training effectiveness and efficiency. Forty children (Mage = 10 years, 2 months) were randomly assigned to the CBI experimental group or BST control group. Evaluation was conducted via in situ assessments in laboratory and naturalistic settings at baseline, post-training, two weeks and one month following training. Results revealed clinically and statistically significant improvements in child performance of target safety behaviors after training for both groups. Behavioral generalization to naturalistic settings and skill maintenance was demonstrated at follow-up assessments. Differences in child performance were not observed between training delivery models. Training

time and number of training trials to program/skill mastery was less for BST compared to CBI. Costs and resource needs were greater during the program development phase for CBI. Program implementation expenses associated with CBI were minimal and cost per unit of delivery decreased exponentially with successive implementation of the intervention. Per unit of delivery costs for BST were fixed and total investment associated with this model increased across successive implementations. Taken as a whole, CBI was as effective as BST in teaching children abduction prevention skills. Across multiple learners, the computer-based instruction program becomes a more economical delivery model. Findings are also discussed relating to child emotional response during training and assessment sessions and correspondence between the learner's verbal report of behavior during simulated abduction situations and behavioral performance during live assessments. (PsycINFO Database Record (c) 2012 APA, all rights reserved)
SN - 0419-4209
AD - Seckinger-Bancroft, Kimberly E.: Western Michigan U., US
ER -

TY - JOUR
ID - 2500
T1 - Cyber bullying: Practices to face digital aggression. [References]
A1 - Wong-Lo,Mickie; Bullock,Lyndal
Y1 - 2011///
N1 - Peer Reviewed Journal: 2011-15824-008
English
Journal; Peer Reviewed Journal
KW - PsycInfo
KW - Aggressive Behavior
KW - Internet
KW - Bullying
RP - NOT IN FILE
SP - 317
EP - 325
JF - Emotional & Behavioural Difficulties
VL - 16:
IS - 3
N2 - The display of aggressive behaviours among the digital generation has modernized the traditional attributes of bullying. Cases of bullying are no longer confined to physical altercations between peers in school hallways or hostile verbal exchanges during recess periods. With the progression of technology, educators must recognize the changes of our students' behaviours as lives become modified by the digital era. The issues of cyber bullying have gained global visibility due to the disheartening publicized cases of the victims. While the families of victims mourn the loss of their children and earnestly seek to understand the cause of each account, researchers actively examine and implement preventative measures in hopes of educating individuals (e.g., general and special educators, parents, students, community professionals) to distinguish the warning signs of aggressive behaviours in digital forms. (PsycINFO Database Record (c) 2012 APA, all rights reserved) (journal abstract)
SN - 1363-2752
AD - Wong-Lo, Mickie: Department of Special Education, Northeastern Illinois University, Chicago, IL, US Bullock, Lyndal M.: Department of Educational Psychology-Special Education, University of North Texas, Denton, TX, US Gable, Robert A.: Department of Education, Old Dominion University, Norfolk, VA, US
ER -

TY - JOUR
ID - 2501
T1 - Location and gender as variables affecting social networks in a children's multi-user virtual environment
A1 - Job-Sluder,Kirk
Y1 - 2011///
N1 - Dissertation Abstract: 2011-99170-071
English
Dissertation Abstract
KW - PsycInfo

KW - Computer Assisted Instruction
KW - Learning Environment
KW - Social Learning
KW - Social Networks
KW - Virtual Classrooms
KW - Social Interaction
RP - NOT IN FILE
SP - 911
JF - Dissertation Abstracts International Section A: Humanities and Social Sciences
VL - 72:
IS - 3-A
N2 - A central argument for the creation of Online Learning Environments is they permit learners to engage in social learning freed from the constraints of shared space and time (Hara, Bonk, & Angeli, 2000). However, other research suggests that location and work relationships (Hampton & Wellman, 2003; Koku & Wellman, 2004), as well as race, ethnicity and gender (McPherson, Smith-Lovin, & Cook, 2001), have an influence on the shape of social networks. Studies of late-elementary school children suggest that they have strong preferences for same-gendered interaction in classroom and playground settings. This research uses Social Network Analysis and Discourse Analysis to evaluate the effects of shared school location and gender on the social networks created by late-elementary students in Quest Atlantis, an international game-like social learning environment. Three different modes of computer-mediated communication interaction were sampled: synchronous public chat, asynchronous private e-mail, and asynchronous private telegrams. This study found that participants sent disproportionate numbers of messages to participants located at the same geographic site. This effect was most pronounced for mail messages, which were almost exclusively sent to same-site participants. Participants also disproportionately sent e-mail and telegrams, although not chat messages, to members of the same gender. Designers and managers of online social learning environments should consider that effective communication across sites separated by geography may require active mediation and team building beyond the existence of CMC utilities. Social network analysis can provide an useful tool for evaluating efforts to build online social learning environments. (PsycINFO Database Record (c) 2012 APA, all rights reserved)
SN - 0419-4209
AD - Job-Sluder, Kirk: Indiana U., US
ER -

TY - JOUR
ID - 2502
T1 - Parental perceptions of the effects of the high-stakes taks test on the home lives of at-risk fifth grade students
A1 - Westfall,Dawn M.
Y1 - 2011///
N1 - Dissertation Abstract: 2011-99150-523
English
Dissertation Abstract
KW - PsycInfo
KW - Academic Achievement
KW - At Risk Populations
KW - Homework
KW - Parental Attitudes
KW - Schools
RP - NOT IN FILE
SP - 576
JF - Dissertation Abstracts International Section A: Humanities and Social Sciences
VL - 72:
IS - 2-A
N2 - In Texas, fifth grade students are required to pass both the reading and math sections of the Texas Assessment of Knowledge and Skills, or TAKS test, in order to be promoted to the next grade level. The purpose of this study is to describe parents' perceptions of the influence of the high-stakes TAKS test on the family lives of at-risk fifth grade students. Parents of students identified as at-risk for failure on the TAKS test

by their schools were given a 12-item survey with three components: the effects of TAKS on the student and family, the effects of TAKS on how students spend time outside of school, and parent attitudes about TAKS as a fair measure of achievement. A series of three one-way ANOVAS was used, comparing each independent variable (family, time, and fairness) to a series of dependent variables (gender, race, and attendance at a Title I school) to look for variability between these groups in their attitudes towards the independent variables. The results indicated that many parents perceive that the TAKS affects their families by causing their child and other family members to express concerns about passing the test and by causing the parent to worry about how their child is reacting to the pressures of the test. Parents perceived that the TAKS test affects how much time students spend playing with friends as well as watching television or movies. Many parents did not agree that TAKS is a fair measure of student achievement for their child or other children. The ANOVAs indicated statistically significant findings among race groups and their scores on "family" and "fairness." Asian/Pacific Islander parents indicated significantly less effect of TAKS on their student and family than did white parents. Asian/Pacific Islander parents also perceived TAKS as fairer measure of student achievement than did white parents. As well, Hispanic parents also perceived TAKS as a fairer measure of student achievement than did both white and Black/African American parents. Findings indicate that perhaps schools and teachers would be surprised to discover the amount of stress TAKS is causing families and students, particularly those at risk for failure as well as those groups that might not have previously been thought to "care" about school. The level of negativity caused by TAKS appears to be an undesirable unintended consequence of the assessment system, so educators may want to reconsider their policies and practices for TAKS-related parent engagement, homework, and test preparation. (PsycINFO Database Record (c) 2012 APA, all rights reserved)
SN - 0419-4209
AD - Westfall, Dawn M.: U Houston, US
ER -

TY - JOUR
ID - 2503
T1 - Impact of increased technology use on the free-time choices of fifth and seventh grade students in a southern school district
A1 - Miller,Cynthia Susan
Y1 - 2011///
N1 - Dissertation Abstract: 2011-99191-013
English
Dissertation Abstract
KW - PsycInfo
KW - Choice Behavior
KW - Grade Level
KW - Schools
KW - Students
KW - Technology
KW - Educational Programs
RP - NOT IN FILE
SP - 1268
JF - Dissertation Abstracts International Section A: Humanities and Social Sciences
VL - 72:
IS - 4-A
N2 - The study sought to determine if increased technology use affects the free-time choices of students. While technology options have grown exponentially, time remains a fixed commodity. Therefore, it is suggested that students who increasingly use technology must draw time from more traditional childhood activities. Students' free-time activities were examined to document discernable patterns among the activities valued by students who use technology extensively and the activities valued by students who use technology less frequently. Study participants were fifth and seventh grade students in a semi-rural suburban county in the southeastern United States. The data collection instrument was a self-reporting survey in which students were asked to specify their relative interest in six traditional activities in comparison to specific technology-based alternatives. Students were also asked to estimate the number of minutes per week they spent on traditional and technology-related activities. Based on their time estimates, high and low quartiles of technology use were established. The forced-choice responses of students in the upper quartile of technology use were compared to the forced-choice responses of students in the lower quartile of technology use to determine if there were differences in their

expressed preferences for the six traditional activities included in the study. Although findings revealed that students in the upper quartile of technology use were less interested in all six traditional activities studied than were students in the lower quartile of technology use, reading for fun, supervised activities, outdoor activities, and having a hobby were activities more readily relinquished than were spending time with family and playing with friends, indicating their relative value among the two groups. As students abandon traditional childhood activities to pursue technology-driven options, adults who are concerned about childhood development might explore alternative means for obtaining the benefits those six activities once provided. Recommendations are made for replicating the study among different populations. Although gender and grade level were two variables that were examined in this study, it would be beneficial to determine if findings would be similar among students with more or less access to technology, with divergent socio-economic means, and from diverse ethnic backgrounds. (PsycINFO Database Record (c) 2012 APA, all rights reserved)
SN - 0419-4209
AD - Miller, Cynthia Susan: U Tennessee at Chattanooga, US
ER -

TY - JOUR
ID - 2504
T1 - Gender. [References]
A1 - Leaper,Campbell
Y1 - 2011///
N1 - Book: 2011-26924-012
English
Book; Edited Book
KW - PsycInfo
KW - Gender Identity
KW - Human Sex Differences
KW - Psychosocial Development
KW - Sex Role Attitudes
KW - Sex Roles
KW - Adolescent Development
KW - Behavior
KW - Childhood Development
KW - Cognitions
KW - Emotions
RP - NOT IN FILE
SP - 289
EP - 315
JF - Underwood, Marion K [Ed]; Rosen, Lisa H [Ed]
VL - :
IS - 2011
N2 - (from the chapter) Gender shapes human lives in large and small ways. Whether a person is female or male influences her or his clothing, hobbies, social interactions, occupations, and incomes. To consider the pervasiveness of gender in our daily lives, imagine what it would be like if a person tried to live outside of the categories "male" and "female." That is the premise of Lois Gould's fictional children's story about a child named X who was raised by its parents without revealing its gender to others. In the story, X encounters resistance from peers, teachers, and other parents who want to treat X as a girl or a boy. In the story's happy ending, the other children begin to emulate X once they realize that X is having twice as much fun as they are. That is, without being constrained by traditional gender categories, X has the flexibility to pursue a greater range of play activities. The idea of not revealing one's gender to other people may seem like something that could only happen in a children's story. However, a Swedish couple is not revealing the gender of their 2 & 1/2-year-old child named Pop. According to the mother, "We want Pop to grow up more freely and avoid being forced into a specific gender mould at the outset". The fictional story of X, as well as the real cases of Pop and Shane Whalley, bring into stark relief society's reliance on gender to define and constrain human experience. In the present chapter, we review some of the ways in which this occurs in children's lives. We describe children's gender development during three age periods: infancy and early childhood (approximately birth-5 years), middle childhood (approximately 6-12 years), and adolescence (approximately 13-18 years). Because children construct their own beliefs about gender and use these beliefs to guide their behavior, we focus on the role of children's

cognitions, affect, and behavior in shaping their gender development. At the same time, because children are born into a world in which gender has significant meaning, we highlight the ways in which other people's gender-stereotyped expectations and behaviors differentially affect girls' and boys' beliefs, goals, and behaviors. However, because gender affects virtually every facet of individuals' lives, we are unable to review every important aspect of gender development. We concentrate on subjects most closely linked to social development and hope that readers will be inspired to read more broadly on other related topics. (PsycINFO Database Record (c) 2012 APA, all rights reserved)
AD - Leaper, Campbell: Department of Psychology, University of California, Santa Cruz, Santa Cruz, CA, US Bigler, Rebecca S.: Department of Psychology, University of Texas at Austin, Austin, TX, US
ER -

TY - JOUR
ID - 2505
T1 - The IDEFICS validation study on field methods for assessing physical activity and body composition in children: Design and data collection. [References]
A1 - Bammann,K.
Y1 - 2011///
N1 - Peer Reviewed Journal: 2011-18468-010
English
Journal; Peer Reviewed Journal
KW - PsycInfo
KW - Diets
KW - Human Body
KW - Lifestyle
KW - Physical Activity
KW - Anthropometry
RP - NOT IN FILE
SP - S79
EP - S87
JF - International Journal of Obesity
JA - Int J Obes (Lond)
VL - 35:
IS - Suppl 1
N2 - Objective: To describe the design, measurements and fieldwork of the IDEFICS (Identification and prevention of dietary- and lifestyle-induced health effects in children and infants) physical activity and body composition validation study, and to determine the potential and limitations of the data obtained. Design: Multicentre validation study. Subjects: A total of 98 children from four different European countries (age: 4-10 years). Methods: An 8-day measurement protocol was carried out in all children using a collaborative protocol. Reference methods were the doubly labelled water method for physical activity, and a three- and a four-compartment model for body composition. Investigated field methods were accelerometers, a physical activity questionnaire and various anthropometric measurements. Results: For the validation of physical activity field methods, it was possible to gather data from 83 to 89 children, laying the basis for age- and sex-specific results. The validation of body composition field methods is possible in 64-80 children and allows sex-specific analyses but has only limited statistical power in the youngest age group (<6 years). The amount of activity energy expenditure (AEE) varied between centres, sexes and age groups, with boys and older children having higher estimates of AEE. After normalisation of AEE by body weight, most group-specific differences diminished, except for country-specific differences. Conclusion: The IDEFICS validation study will allow age- and sex-specific investigation of questions pertaining to the validity of several field methods of body composition and physical activity, using established reference methods in four different European countries. From the participant analyses it can be concluded that the compliance for the investigated field methods was higher than that for the reference methods used in this validation study. (PsycINFO Database Record (c) 2012 APA, all rights reserved) (journal abstract)
SN - 0307-0565
AD - Bammann, K.: Department of Biometry, Bremen Institute for Prevention Research and Social Medicine, University of Bremen, Bremen, Germany Sioen, I.: Department of Public Health, Ghent University, Ghent, Belgium Huybrechts, I.: Department of Public Health, Ghent University, Ghent, Belgium Casajus, J. A.: Growth, Exercise, Nutrition and Development Research Group, University of Zaragoza, Zaragoza, Spain

Vicente-Rodriguez, G.: Growth, Exercise, Nutrition and Development Research Group, University of Zaragoza, Zaragoza, Spain Cuthill, R.: Faculty of Biomedical and Life Sciences, Integrative and Systems Biology, University of Glasgow, Glasgow, United Kingdom Konstabel, K.: Department of Chronic Diseases, National Institute for Health Development, Tallinn, Estonia Tubic, B.: Department of Paediatrics, Institute for Clinical Sciences, Queen Silvia Children's Hospital, Sahlgrenska Academy, University of Gothenburg, Gothenburg, Sweden Wawro, N.: Department of Biometry, Bremen Institute for Prevention Research and Social Medicine, University of Bremen, Bremen, Germany Rayson, M.: BioTel Ltd, Bristol, United Kingdom Westerterp, K.: Department of Human Biology, Maastricht University Medical Centre, Maastricht, Netherlands Marild, S.: Department of Paediatrics, Institute for Clinical Sciences, Queen Silvia Children's Hospital, Sahlgrenska Academy, University of Gothenburg, Gothenburg, Sweden Pitsiladis, Y. P.: Faculty of Biomedical and Life Sciences, Integrative and Systems Biology, University of Glasgow, Glasgow, United Kingdom Reilly, J. J.: Physical Activity for Health Research Group, School of Psychological Sciences and Health, University of Strathclyde, Glasgow, United Kingdom Moreno, L. A.: Growth, Exercise, Nutrition and Development Research Group, University of Zaragoza, Zaragoza, Spain De Henauw, S.: Department of Public Health, Ghent University, Ghent, Belgium
ER -

TY - JOUR
ID - 2506
T1 - Football pitches and Barbie dolls: Young children's perceptions of their school playground. [References]
A1 - Pearce,Gemma
Y1 - 2011///
N1 - Peer Reviewed Journal: 2011-25781-006
English
Journal; Peer Reviewed Journal
KW - PsycInfo
KW - Childhood Development
KW - Childhood Play Behavior
KW - Playgrounds
KW - Football
RP - NOT IN FILE
SP - 1361
EP - 1379
JF - Early Child Development and Care
VL - 181:
IS - 10
N2 - Playgrounds and play times offer valuable contexts for children to explore and learn about themselves and their social lives. This study sought to gather evidence of children's perceptions of their playgrounds and play times, specifically whether the playgrounds were seen positively or negatively and the types of activities in which they engaged. Child-oriented methods were adopted inspired by the Mosaic approach. The main themes from the focus group interviews were social play (friendship, loneliness/solitude and fair play); physical activity play (activities and rationale); risk (injuries and bullying); and gender (action/stillness and gendered roles). Research suggests that segregation declines when adult supervision supports shared play, and our experiences suggest that such intervention is the most likely solution to the 'problem' of gender play in this setting. (PsycINFO Database Record (c) 2012 APA, all rights reserved) (journal abstract)
SN - 0300-4430
AD - Pearce, Gemma: School of Sport and Exercise Sciences, University of Birmingham, Birmingham, United Kingdom Bailey, Richard P.: RBES Ltd., Birmingham, United Kingdom
ER -

TY - JOUR
ID - 2507
T1 - High-ability students' time spent outside the classroom. [References]
A1 - Makel,Matthew C.
Y1 - 2011///

N1 - Peer Reviewed Journal: 2011-25255-004
English
Journal; Peer Reviewed Journal
KW - PsycInfo
KW - Ability Level
KW - Academic Achievement
KW - Extracurricular Activities
KW - Classrooms
KW - Homework
KW - Human Sex Differences
KW - Time
RP - NOT IN FILE
SP - 720
EP - 749
JF - Journal of Advanced Academics
VL - 22:
IS - 5
N2 - This study considered how three groups of academically talented high school students-those who attended an academic summer program (TIP), those who qualified for the program but chose not to attend (QNA), and those who did not qualify (DNQ)-spent time outside the classroom. These groupings differentiated students by ability (QNA vs. DNQ) and attendance (TIP vs. QNA). Male-female comparisons were also conducted. By comparing participation rates across a variety of activities and by sex, the current study helps explain the lives of high-ability students outside the arena by which they are defined: their academic ability. Results reveal numerous group and sex differences based on how high-ability students spend their time outside the classroom. Females tended to participate more than males in activities that were generally positively associated with academic achievement, while also participating in more types of activities. Males, however, reported watching more TV and were less likely to participate in any activity. QNA students reported spending more time on academic-related activities, such as homework and academic clubs, than did DNQ students, indicating a generally higher interest in academic endeavors. However, the QNA and TIP groups differed only in their service club participation rates, indicating that attending a summer program is not associated with spending time outside the classroom differently during the school year. This research underscores the heterogeneity of different groups of high-ability students and suggests some caution when generalizing from research findings based only on program participants. Knowing how students spend their time can help parents, educators, and researchers understand and foster adolescent development. (PsycINFO Database Record (c) 2012 APA, all rights reserved) (journal abstract)
SN - 1932-202X
AD - Makel, Matthew C.: Duke University, Durham, NC, US Li, Yan: DePaul University, Chicago, IL, US Putallaz, Martha: Duke University, Durham, NC, US Wai, Jonathan: Duke University, Durham, NC, US
ER -

TY - JOUR
ID - 2508
T1 - Assessment of early childcare programs in Jordan. [References]
A1 - Al-Omari,Aieman A.
Y1 - 2011///
N1 - Peer Reviewed Journal: 2011-02551-006
English
Journal; Peer Reviewed Journal
KW - PsycInfo
KW - Caregivers
KW - Child Day Care
KW - Parents
RP - NOT IN FILE
SP - 128
EP - 150
JF - Early Education and Development
VL - 22:

IS - 1
N2 - Research Findings: Forty-five child caregivers and 120 parents participated in this study to examine perceptions of childcare programs in Jordan. The researchers developed a questionnaire that consisted of 6 dimensions: health, education, parent-caregiver relationship, facilities, building/landscape, and playground. Moreover, interviews with 10 child caregivers and 20 parents were conducted. Results indicated that child caregivers expressed moderate satisfaction with the programs. In contrast, parents expressed lower satisfaction with the childcare programs. The results also revealed that caregivers and parents perceived the playground area as effective but found health and the parent-caregiver relationship ineffective. Practice or Policy: This study highlights the need to supervise childcare programs effectively and the importance of fostering a strong partnership between child caregivers and parents. (PsycINFO Database Record (c) 2012 APA, all rights reserved) (journal abstract)
SN - 1040-9289
AD - Al-Omari, Aieman A.: Educational Administration Department, Hashemite University, Zarqa, Jordan Ihmeideh, Fathi M.: Child Education Department, Hashemite University, Zarqa, Jordan Al-Dababneh, Khouloud A.: Child Education Department, Hashemite University, Zarqa, Jordan
ER -

TY - JOUR
ID - 2509
T1 - The effects of an enhanced fluency intervention on fourth and fifth grade struggling readers
A1 - Miller,Janice A.
Y1 - 2011///
N1 - Dissertation Abstract: 2011-99010-196
English
Dissertation Abstract
KW - PsycInfo
KW - Comprehension
KW - Grade Level
KW - Reading
KW - School Based Intervention
KW - Verbal Fluency
KW - Elementary School Students
RP - NOT IN FILE
SP - 2402
JF - Dissertation Abstracts International Section A: Humanities and Social Sciences
VL - 71:
IS - 7-A
N2 - This case study followed struggling readers as they moved through an enhanced fluency intervention which included explicit instruction in fluency, comprehension, and "good reading" behaviors. There were fourteen students in fourth and fifth grades who participated in small group reading classes outside their general reading class. The intervention used the commercially available text of Read Naturally, Master's Edition (Matsoff, 2006). The teacher provided explicit sequential instruction with each small group. Enhancements in fluency included instruction in text markings and prosody, teacher modeling, and a variety of readings. Enhancements in comprehension included explicit instruction in the reading strategies of predicting, clarifying, questioning, and retelling. Instruction on what "good reading behaviors" used "good reader" slogans and guided practice. Achievement data were collected using pre and post intervention measures. Student interviews and questionnaires were administered pre and post intervention to determine changes in the student's perception of him/herself as a reader. Questionnaires and interviews examined the student use of "good reading" behaviors. Parent and teacher observations were elicited through questionnaires to assess these behaviors. The enhanced fluency intervention had a positive effect on the struggling readers as the group means increased on standardized and curriculum-based assessments. Changes were noted as the students used the vocabulary of "good reading" behaviors on final student interviews. Responses on the final student and adult questionnaires reported "good reading" behaviors at home and at school but responses indicated students did not use the majority of "good reading" behaviors in their independent reading activities. (PsycINFO Database Record (c) 2012 APA, all rights reserved)
SN - 0419-4209
AD - Miller, Janice A.: Widener U., US

ER -

TY - JOUR
ID - 2510
T1 - Rock Up: An initiative supporting students' wellbeing in their transition to secondary school. [References]
A1 - Carmen,Brenda; Waycott,Louise; Smith,Ken
Y1 - 2011///
N1 - Peer Reviewed Journal: 2010-25669-011
English
Journal; Peer Reviewed Journal
KW - PsycInfo
KW - Elementary Schools
KW - Learning
KW - Risk Factors
KW - School Transition
KW - Well Being
KW - Education
RP - NOT IN FILE
SP - 167
EP - 172
JF - Children and Youth Services Review
VL - 33:
IS - 1
N2 - This paper reports the background and findings of the community-based pilot program 'Rock Up' to school. The Rock Up program was directed at a niche section of young students in Grades 5, 6, and 7 to respond to identified risk factors associated with disengagement from formal education. The Rock Up approach was to energise these students' connection to learning as they approach the difficult stage of transition from primary school to secondary school. Ultimately, Rock Up aims to assist those students in their development of wellbeing, within and outside of the school environment. The operational measures of wellbeing covered; academic, social, emotional, behavioural, and school absence. Thirteen students, who had been identified by their respective teachers, were invited to participate in individual and/or group activities that focused on their wellbeing and developing their readiness for secondary school. Written and verbal feedbacks from students, teachers, parents, and the program facilitator, were in the main very positive. Analysis of the data of the first round of the program using a three-wave questionnaire and a participatory action research approach indicated the need to proceed to a new round using more refined processes. When fully developed, Rock Up is intended to be the vanguard for collaborative practice between the education and community sectors throughout Victoria and to be an example of an efficient and effective model of transition for this age-group. (PsycINFO Database Record (c) 2012 APA, all rights reserved) (journal abstract)
SN - 0190-7409
AD - Carmen, Brenda: MacKillop Family Services, Footscray, VIC, Australia Waycott, Louise: MacKillop Family Services, Footscray, VIC, Australia Smith, Ken: Australian Catholic University, Faculty of Education, Melbourne, VIC, Australia
ER -

TY - JOUR
ID - 2511
T1 - Developmental coordination disorder: Function, participation, and assessment. [References]
A1 - Clark,Jane E; Whitall,Jill
Y1 - 2011///
N1 - Peer Reviewed Journal: 2011-11616-003
English
Journal; Peer Reviewed Journal
KW - PsycInfo
KW - Dyspraxia
KW - Ability Level
KW - Measurement
KW - Participation

RP - NOT IN FILE
SP - 1243
EP - 1244
JF - Research in Developmental Disabilities
JA - Res Dev Disabil
VL - 32:
IS - 4
N2 - Imagine what it is like to be a child whose clumsy actions make it difficult to keep up with peers on the playground or to accomplish everyday tasks of daily living such as tying shoes or buttoning a coat. This special issue of Research in Developmental Disabilities is focused on the disorder that defines these children, known as Developmental Coordination Disorder. The papers presented here are selected from the VIIIth International Conference on Developmental Disorder which was held, for the first time in the United States, in Baltimore, MD, 2009. We have grouped the contributions into three sections. The first two sections represent the International Classification of Functioning, Disability and Health framework (WHO, 2001) - (1) Body Functions: Psychological, Cognitive and Neuromotor Function; and (2) Activities and Participation. The third section is titled - (3) Assessment and Measurement. (PsycINFO Database Record (c) 2012 APA, all rights reserved)
SN - 0891-4222
AD - Clark, Jane E.: Department of Kinesiology, School of Public Health, University of Maryland, College Park, MD, US Whitall, Jill: Department of Physical Therapy & Rehabilitation Science, University of Maryland, Baltimore, MD, US
ER -

TY - JOUR
ID - 2512
T1 - Handclapping songs: A spontaneous platform for child development among 5-10-year-old children. [References]
A1 - Brodsky,Warren
Y1 - 2011///
N1 - Peer Reviewed Journal: 2011-18364-007
English
Journal; Peer Reviewed Journal
KW - PsycInfo
KW - Childhood Development
KW - Cognitive Ability
KW - Music Education
KW - Motor Processes
KW - Music
RP - NOT IN FILE
SP - 1111
EP - 1136
JF - Early Child Development and Care
VL - 181:
IS - 8
N2 - The impact of music activity on children's motor and cognitive skills has been investigated with music learning, instrument lessons and classroom music. While none have employed natural utterances, singing games or playground/street songs, these musical experiences of childhood are acknowledged as a major platform for child development. The current study isolated handclapping songs exploring the association of performance quality with classroom academic achievement and examined whether children who spontaneously engage in handclapping songs activity demonstrate improved motor or cognitive abilities. Finally, the study investigated the outcome of a two-group eight-week classroom intervention. The study found that: (1) children who were more skillful at performing handclapping songs were more efficient First Graders; (2) Second Graders who spontaneously engage in handclapping songs were advantaged in bimanual coupling patterns, verbal memory and handwriting; and (3) classroom handclapping songs training was more efficient than music appreciation classes in developing non-music skills among Second and Third Graders. (PsycINFO Database Record (c) 2012 APA, all rights reserved) (journal abstract)
SN - 0300-4430

AD - Brodsky, Warren: Music Science Lab, Department of the Arts, Ben-Gurion University of Negev, Beer-Sheva, Israel Sulkin, Idit: Music Science Lab, Department of the Arts, Ben-Gurion University of Negev, Beer-Sheva, Israel
ER -

TY - JOUR
ID - 2513
T1 - Positive behavior support through family-school collaboration for young children with autism. [References]
A1 - Blair,Kwang-Sun Cho; Lee,In-Suk; Cho,Su-Je; Dunlap,Glen
Y1 - 2011///
N1 - Peer Reviewed Journal: 2011-07290-003
English
Journal; Peer Reviewed Journal
KW - PsycInfo
KW - Autism
KW - Behavior Modification
KW - Behavior Problems
KW - Collaboration
KW - Intervention
KW - Mothers
KW - Teachers
RP - NOT IN FILE
SP - 22
EP - 36
JF - Topics in Early Childhood Special Education
VL - 31:
IS - 1
N2 - In this study, a multiple baseline design across three young children with autism was used to assess the impact of individualized behavior support, implemented through family-school collaboration, on the children's appropriate and problem behaviors and adult-child interactions. A positive behavior support process was used to promote family-school collaboration and to design the individualized intervention. Data indicated that the children's target behaviors improved with the intervention during circle time at school and play time at home, and their behaviors generalized to nontargeted center time at school and play at the community playground. Teacher and mother positive interactions with the children increased while negative interactions decreased. Social validity indicated that the interventions provided through family-school collaboration were acceptable and effective. (PsycINFO Database Record (c) 2012 APA, all rights reserved) (journal abstract)
SN - 0271-1214
AD - Blair, Kwang-Sun Cho: Department of Child and Family Studies, University of South Florida, Tampa, FL, US Lee, In-Suk: Department of Special Education, Kongju National University, Gongju, Korea Cho, Su-Je: Graduate School of Education, Fordham University, New York City, NY, US Dunlap, Glen: Department of Child and Family Studies, University of South Florida, Tampa, FL, US
ER -

TY - JOUR
ID - 2514
T1 - Promising practices for family engagement in out-of-school time
A1 - Kreider,Holly
Y1 - 2011///
N1 - Book: 2011-11784-000
English
Book; Edited Book
(Created by PsycINFO) Foreward Acknowledgments Section I: Conceptualizing family engagement in out-of-school time (OST) * Engaging families in out-of-school time programs / Suzanne M. Bouffard, Helen Westmoreland, Kelley O'Carroll and Priscilla M. Little / 3-19 * A typology of family engagement in youth development settings / Holly Kreider and Shayna Cunningham / 21-29 * Developmental assets as a framework for engaging families in out-of-school time / Andrew J. Schneider-Munoz / 31-41 Section II: Families' selection

of and advocacy through OST programs * The role of parents in students' structured and unstructured out-of-school-time activity participation / Amanda Sommerfeld / 45-53 * Faith-based out-of-school-time opportunities: Impacts of faith-based initiatives on children and families / S. Russell Vaden / 55-67 * Empowering families to guide and advocate for their children's education: Lessons from the Higher Achievement Program / Richard A. Tagle / 69-80 Section III: Using OST to help families support children's learning process * Engaging families in student homework: Action steps for educators / Frances L. Van Voorhis / 83-96 * Promoting language and literacy outcomes through shared reading at home / Holly Kreider, Georganne Morin, Gabrielle E. Miller and Angie Bush / 97-107 Section IV: Reaching families through nontraditional OST settings and partnerships * Family engagement through health clinics / Kathryn Nakagawa, Martha Cocchiarella, Emily Earl, Stacy Moreno and Rebecca Birr / 111-120 * It takes a village: Community schools and family engagement / Michael P. Evans and Darlene Kamine / 121-134 * Developing university and community partnerships that support family engagement in out-of-school time settings / Barbara C. Jentleson / 135-147 List of contributors
KW - PsycInfo
KW - Academic Achievement
KW - After School Programs
KW - Educational Programs
KW - Family
KW - Involvement
KW - Childhood Development
KW - Schools
RP - NOT IN FILE
SP - Charlotte, NC, US
JF - (2011)
VL - Promising practices for family engagement in out-of-school time. xi, 150 pp. Charlotte, NC, US: IAP Information Age Publishing; US.
N2 - (from the cover) This monograph addresses the expanding field of family involvement to out-of-school time (OST). OST may be defined as time outside of state required time limits for compulsory school attendance but time in which students are engaged in planned academic or enrichment activities. During the past decade, OST programs have burgeoned across the US. OST programs are offered to children and youth, elementary through high school, as structured and safe venues to increase student academic achievement, and extend students' interests. (from the foreword) Family engagement in OST programs is vitally important because, as during state-required schooling time, family engagement in OST is linked to student academic achievement. Prior to the turn of the century, families had to secure privately funded OST opportunities for their children. Thus, OST programs were dependent on for-profit or nonprofit organizations and not available for all children and youth. In recent years, well-known sports and entertainment personalities publicly attributed OST to their current success. Increasingly, federal, state and local funds have been allocated to the development of a variety of afterschool programs and other OST learning opportunities across the United States. Thus, during the past decade, educational evaluators and researchers within the United States have expanded their attention to study OST learning opportunities. (PsycINFO Database Record (c) 2012 APA, all rights reserved)
ER -

TY - JOUR
ID - 2515
T1 - Social representations of wellness among 9-11 year old youth: A participatory action research study
A1 - Bolzak,Christine Taggart
Y1 - 2011///
N1 - Dissertation Abstract: 2011-99120-302
English
Dissertation Abstract
KW - PsycInfo
KW - Daily Activities
KW - Impression Formation
KW - Predelinquent Youth
KW - Health Care Policy
KW - Concept Formation
KW - Social Demonstrations
RP - NOT IN FILE

SP - 7384
JF - Dissertation Abstracts International: Section B: The Sciences and Engineering
VL - 71:
IS - 12-B
N2 - The Child Nutrition Act of 2004 mandated the creation of wellness policies in all school districts and encouraged youth participation in their creation. Initial reports show a lack of youth participation in community wellness discussions. This participatory action research study used qualitative methods (Photovoice and Literacy Through Photography), and aimed to demonstrate: the socially constructed meanings, or social representations, a purposive sample of youth (ages 9-11) gave to the concept "wellness" in their everyday lives, how their conceptualization of wellness changed over time using the methodologies; their personal impressions and shared understandings of how their settings affect their wellness; and their recommendations for the enhancement of their settings in order to promote wellness. For the purpose of data analysis, some a priori categories were utilized since wellness is a construct that already has dimensions defined in the literature. The participants' conceptualization of wellness fit with Prilleltensky & Fox's (2007) dimensions of wellness: personal, relational, and collective, and included: environment, physical activity, safety, food, freedom, self-directed learning opportunities, transportation, inner life/spirituality, and positive social relationships. Participants made recommendations to enhance their settings' ability to promote wellness. For example, participants stressed the need for access to nature, especially green space for physical activity, and active transportation opportunities. Recess, physical education, and healthy food options were identified as important in the school setting. Community settings and resources that create opportunities for self-directed learning and physical activity in a group setting were seen as critical. Participants acknowledged that wellness can be a social justice issue when their peers live in areas that promote violence rather than wellness-enhancing community assets. The results from this study demonstrate that youth view wellness as more than just nutrition and physical activity, the primary components of most school wellness policies. Consequently, any comprehensive wellness policy should be developed utilizing a more wholistic conceptualization of the term. Participants expressed that their settings can have an impact on wellness; a concept congruent with the social ecological approach in health promotion. This study's findings demonstrate that youth are able to conceptualize wellness and make a significant contribution to their community's wellness discussions. (PsycINFO Database Record (c) 2012 APA, all rights reserved)
SN - 0419-4217
AD - Bolzak, Christine Taggart: U Illinois at Chicago, Health Sciences Center, US
ER -

TY - JOUR
ID - 2516
T1 - Multicultural gifted education (2nd ed.). [References]
A1 - Ford,Donna Y.
Y1 - 2011///
N1 - Book: 2011-17539-000
English
Book; Authored Book
(Created by PsycINFO) Acknowledgements Preface Racially and culturally different students Historical and legal perspectives: Educating gifted and racially and culturally different students Educator preparation in gifted and multicultural education Gifted and multicultural education: Goals, objectives, and models Assessment: Multicultural considerations Multicultural curriculum Multicultural instruction Counseling students from a multicultural perspective Racially and culturally different families of gifted students Research and evaluation from a multicultural perspective Case studies and scenarios for reflection Afterword References Appendix A: Sample multicultural activities: Preinstruction or reinforcement exercises Appendix B: Multicultural education program evaluation guidelines and checklist About the author
KW - PsycInfo
KW - Gifted
KW - Multicultural Education
KW - Special Education
KW - Curriculum
KW - Educational Measurement
KW - Experimentation
KW - Racial and Ethnic Differences

KW - School Counseling
KW - Students
KW - Teaching
RP - NOT IN FILE
SP - Waco, TX, US
JF - (2011)
VL - Multicultural gifted education :
IS - 2nd ed.
N2 - (from the preface) In seeking to create gifted education programs and Advanced Placement (AP) classes that are multicultural, I address the following questions throughout this book: Why do all students need an education that is responsive in general and multicultural in particular? Why is a multicultural education essential for White and racially and culturally different students? What is an education that is multicultural? Which components are most important? When is a multicultural education needed? In what school settings is a multicultural education needed? How can educators implement multicultural education so that it does not become an add-on but instead becomes an integral and integrated aspect of the educational process for gifted and advanced learners? Finally, I focus not only on students, but also on educators by offering recommendations to help educators of gifted students to become more culturally competent-aware, knowledgeable, skilled, and responsive. I believe that being culturally competent is indeed a survival skill, both inside and outside the school walls. An important feature of the book is its use of vignettes, scenarios, and brief case studies. This book addresses what I think is needed in the two programs often used to serve high-ability students (i.e., gifted education and AP classes) if they are to truly become multicultural-if education is to affirm the dignity and humanity of traditionally disenfranchised students and open the minds and hearts of White students and educators about those who are different from them. School personnel in gifted education and AP classes must explore the need(s) and rationale for multicultural perspectives in assessment, research, curriculum, instruction, and counseling. Just as we address academic and intellectual diversity, educators of gifted students and other advanced learners must attend to racial and cultural differences. We must seek excellence and equity for all students, acknowledging in the process that all students are the future, worth educating, and deserving of an education that meets their interests and needs. Educators of gifted and AP students must be proactive rather than reactive in addressing this increasing diversity and difference(s); they must seek both excellence and equity-the two are inextricably bound! (PsycINFO Database Record (c) 2012 APA, all rights reserved)
AD - Ford, Donna Y.: Vanderbilt University, Nashville, TN, US
ER -

TY - JOUR
ID - 2517
T1 - An 11-year-old boy with ongoing psychotic symptoms. [References]
A1 - Mayanil,Tushita
Y1 - 2011///
N1 - Peer Reviewed Journal: 2011-13300-006
English
Journal; Peer Reviewed Journal
KW - PsycInfo
KW - Cerebrospinal Fluid
KW - Drug Therapy
KW - Neuroleptic Drugs
KW - Psychiatric Symptoms
KW - Psychosis
KW - Automated Information Retrieval
KW - Lexical Access
KW - Reading
KW - Treatment
KW - Words (Phonetic Units)
RP - NOT IN FILE
SP - 319
EP - 321
JF - Psychiatric Annals
VL - 41:

IS - 6
N2 - Presents a case report of an 11-year-old, right-handed male brought by his mother for treatment of ongoing psychotic symptoms. He began to have significant difficulties in reading and word retrieval. A few months later, he began talking and laughing to himself and was evaluated in the emergency room of a local hospital to rule out organic causes for these behavioral changes. He had a normal magnetic resonance imaging (MRI) scan of his brain, cerebrospinal fluid (CSF) exam, and electroencephalogram (EEG). He was diagnosed with depression and prescribed sertraline, which was not helpful. The patient was hospitalized for 7 weeks after he attempted to jump out of a car in response to auditory hallucinations. He was discharged on a combination of olanzapine, risperidone and lithium. He showed a gradual decrease in his psychotic symptoms and began reintegrating slowly back into the school and the community. Our patient is currently in partial remission on a combination of olanzapine, risperidone and lithium. He is attending therapeutic day school and is able to enjoy outdoor activities. (PsycINFO Database Record (c) 2012 APA, all rights reserved)
SN - 0048-5713
AD - Mayanil, Tushita: Pediatric Brain Research and Intervention Center, University of Illinois at Chicago, Chicago, IL, US Pavuluri, Mani: Pediatric Brain Research and Intervention Center, University of Illinois at Chicago, Chicago, IL, US
ER -

TY - JOUR
ID - 2518
T1 - A study of the relationship between physical fitness and academic performance
A1 - Rodenroth,Karen
Y1 - 2011///
N1 - Dissertation Abstract: 2011-99090-350
English
Dissertation Abstract
KW - PsycInfo
KW - Academic Achievement
KW - Exercise
KW - Health
KW - Physical Fitness
RP - NOT IN FILE
SP - 3891
JF - Dissertation Abstracts International Section A: Humanities and Social Sciences
VL - 71:
IS - 11-A
N2 - In this study, fourth and fifth grade students' physical fitness levels were compared to their academic achievement based on the idea that health and physical fitness have an impact on the ability to achieve academically. Because of the recent pressures of No Child Left Behind, many schools have opted to limit the amount of time students spend in physical education classes and recess. With the increased percentages of students who suffer from diabetes and other health related risks, eliminating or reducing physical activity from the school day is not the answer. Data was collected from 90 students (46 males and 44 females) during the 2009-2010 school year by using the President's Challenge Physical Fitness Test, STAR Reading Percentile scores, and Grade Point Averages (GPA's). Through multiple regressions, the researcher did not find statistically significant relationships between physical fitness levels and STAR Reading Percentiles or between physical fitness levels and Grade Point Averages. When physical fitness levels were combined with STAR Reading percentile scores, a significant correlation was found between these two variables and Grade Point Averages. A significant correlation was found between physical fitness levels and mathematics. Lastly, another significant correlation was found between STAR, Grade Point Averages, and sit ups. With these varying results, the researcher decided to retain the following null hypotheses of this study: there will not be a significant relationship between physical fitness scores based on the President's Challenge Physical Fitness Test and academic performance based on STAR Reading Percentile scores for fourth and fifth graders at the participating school, and there will not be a significant relationship between physical fitness scores based on the President's Challenge Physical Fitness Test and academic performance based on Grade Point Averages for fourth and fifth graders at the participating school. This study does not prove causality; therefore, the results should be interpreted with caution. (PsycINFO Database Record (c) 2012 APA, all rights reserved)
SN - 0419-4209

AD - Rodenroth, Karen: Liberty U., US
ER -

TY - JOUR
ID - 2519
T1 - Agency and structure in the life-world of Aboriginal children in Central Australia. [References]
A1 - Eickelkamp,Ute
Y1 - 2011///
N1 - Peer Reviewed Journal: 2010-17746-001
English
Journal; Peer Reviewed Journal
KW - PsycInfo
KW - Ethnography
KW - Indigenous Populations
KW - Psychosocial Development
KW - Socialization
KW - Agency
RP - NOT IN FILE
SP - 502
EP - 508
JF - Children and Youth Services Review
VL - 33:
IS - 4
N2 - This paper offers ethnographic observations on the place of "agency" in the lives of Aboriginal children in Central Australia. The focus is on children's play and adult-child interaction in the remote community of Ernabella, the oldest settlement in the Anangu Pitjantjatjara Yankunytjatjara (APY) Lands, northern South Australia. The circumstance of Aboriginal children in remote areas is introduced as a contrasting picture: on the one hand, the life of Aboriginal families is shaped by their marginalised socio-economic position and dependency on the state, which has become especially evident in the recently intensified efforts by the government to "mainstream" Aboriginal communities deemed in "social chaos" in order to regulate and thereby improve children's lives; on the other there is the social fact that children enjoy a comparatively high level of autonomy within the Aboriginal domain. The much-observed "freedom" from parental discipline, however, does not simply mean that children assert their will without regard for certain social rules. Rather, it is suggested that it is precisely the relatively low level of direct instruction and reprimand by adults that fosters children's ability to pattern their behaviour in relation to one another and to structure their social world and understandings according to the meanings that they co-create. Agency of this kind often occurs through imaginative play and spontaneously. This paper does not address policy issues directly. However, it is relevant as a background paper on Aboriginal children's lives that contributes to the understanding and recognition of existing social capacities outside institutional settings. (PsycINFO Database Record (c) 2012 APA, all rights reserved) (journal abstract)
SN - 0190-7409
AD - Eickelkamp, Ute: Department of Anthropology, Sydney University, Sydney, NSW, Australia
ER -

TY - JOUR
ID - 2520
T1 - Long-term effects of childhood maltreatment on adult sexual risk behavior and sexually transmitted infections among black females in the united states: A latent variable analysis
A1 - Regan,Rotrease S.
Y1 - 2011///
N1 - Dissertation Abstract: 2011-99080-242
English
Dissertation Abstract
KW - PsycInfo
KW - Blacks
KW - Child Abuse
KW - Infectious Disorders
KW - Sexual Risk Taking

KW - Sexually Transmitted Diseases
KW - Human Females
RP - NOT IN FILE
SP - 6074
JF - Dissertation Abstracts International: Section B: The Sciences and Engineering
VL - 71:
IS - 10-B
N2 - Recent, research has pointed to childhood maltreatment as a risk factor for adolescent and young adult sexual risk behaviors and sexually transmitted infections (STIs). Although several prospective studies have examined the long term effects of childhood maltreatment on adult HIV risk behaviors or sexual health outcomes, few have conducted research among representative samples of black women in the United States. Because of the disproportionately high rates of both childhood maltreatment and STI/HIV infection in this population, it is important to examine this association as well as the pathways by which these constructs are associated. Using data from The National Longitudinal Study of Adolescent Health (Add Health), a nationally representative cohort of adolescent black females (N = 1,552) who were in grades 7-12 during 1994-5 and followed into young adulthood, the overarching goal of this study was to develop and test two structural equation models of the simultaneous interrelationships amongst theoretically-derived variables as they relate to childhood maltreatment, and 1) adult sexual risk behavior and 2) number of sexually transmitted infections in young adulthood. The research for this dissertation was grounded in a conceptual approach that posits that factors outside the individual's control are associated with sexual risk behavior. Further it is posited that childhood experiences have long-term effects on the individual, and involve a cascade of influences culminating in risk behavior or acquisition of an STI. The primary finding of this study was that childhood maltreatment is significantly associated with adult sexual risk behavior and STI among black women living in the United States. These associations were found to be only partially mediated by adolescent and adult characteristics (emotional distress, alcohol use, age at first sex, adolescent attachment and religiosity). Lower adolescent attachment, early age at first intercourse, and adult alcohol use were each separate significant mediators of the relationship between childhood maltreatment and adult sexual risk behavior. Significant mediators of the relationship between childhood maltreatment and adult STI diagnosis were: lower age at first sex, and a path via higher adolescent and adult emotional distress. It was hypothesized that religiosity would be an important mediator in this study, providing protection against engagement in risky behaviors and also STIs. However, religiosity was not a significant mediator of either association, and was significantly directly associated with lower sexual risk behaviors, but not significantly associated with STI diagnoses. This was an unexpected finding and suggests that even if religiosity as a form of social control, plays a role in reducing risky behaviors, it may not be enough to protect black women from acquiring sexually transmitted infections. This finding contributes to the literature that suggests that high rates of STI/HIV are not associated with risky behaviors among black women. Public health interventions that address behavioral modification, should also include components that screen for and address past abuse histories. While some hypothesized pathways did not reach statistical significance in this study, variables included, such as emotional distress, alcohol use, and gang involvement and early sexual debut were associated with childhood maltreatment and also associated with sexual risk behaviors. Addressing the trauma from childhood maltreatment provides an opportunity to deal with the underlying reasons for risky activities, and may enhance the effectiveness of behavioral interventions. More research is needed to understand the burden of STI/HIV among black women. Interpersonal relationship characteristics, social networks and macro-level contextual variables should be included in future research addressing this crisis. (PsycINFO Database Record (c) 2012 APA, all rights reserved)
SN - 0419-4217
AD - Regan, Rotrease S.: U California, Los Angeles, US
ER -

TY - JOUR
ID - 2521
T1 - Empowering aspects of transition from kindergarten to first grade through children's voices. [References]
A1 - Loizou,Eleni
Y1 - 2011///
N1 - Peer Reviewed Journal: 2011-05022-005
English
Journal; Peer Reviewed Journal
KW - PsycInfo

KW - Empowerment
KW - Kindergartens
KW - School Transition
KW - Voice
RP - NOT IN FILE
SP - 43
EP - 55
JF - Early Years: An International Journal of Research and Development
VL - 31:
IS - 1
N2 - This study was designed to investigate the reflective comments of 55 first grade children regarding their experiences in kindergarten and first grade. Data collection involved a conversational interview during which children voiced their reflections and comparisons on specific issues (e.g. friends, teacher, learning) they had encountered during kindergarten and were currently experiencing in first grade. The data was analyzed through Foucault's tenets of power. The aspects of the transition that empowered children were noted and further discussed, as well as aspects that were perceived as limiting. Specifically, children seem to find some things empowering: the newness of the experience; the challenging and hard work; the independence they have in a spacious school playground; the access to a canteen and separate bathroom areas and the opportunity to make more friends. On the other hand, children noted several limiting elements: the structure of the day and specifically the constant lesson-work model; the teacher's more stern behaviors and the scarcity of fun. (PsycINFO Database Record (c) 2012 APA, all rights reserved) (journal abstract)
SN - 0957-5146
AD - Loizou, Eleni: Department of Education, University of Cyprus, Nicosia, Cyprus
ER -

TY - JOUR
ID - 2522
T1 - Decreasing stereotypy in preschoolers with autism spectrum disorder: The role of increased physical activity and function
A1 - McLaughlin,Constance Ann Hylton
Y1 - 2011///
N1 - Dissertation Abstract: 2011-99070-206
English
Dissertation Abstract
KW - PsycInfo
KW - Early Intervention
KW - Pervasive Developmental Disorders
KW - Physical Activity
KW - Preschool Students
KW - Stereotyped Behavior
KW - Autism
KW - Exercise
RP - NOT IN FILE
SP - 3619
JF - Dissertation Abstracts International Section A: Humanities and Social Sciences
VL - 71:
IS - 10-A
N2 - This study used increased physical activity during recess to reduce stereotypy in preschoolers with Autism Spectrum Disorder. Results indicate increasing physical activity can be used as an intervention to reduce automatically maintained stereotypy in preschoolers with ASD. The intervention had a lesser effect on a preschooler whose stereotypy was maintained by attention. Results show topographies of the stereotypy were differentially affected for each individual. Not all motor stereotypies were affected equally within the same individual, and for one child, vocal stereotypies appeared to become less variable. Social validity measures indicate teachers would be willing to use this intervention in the future. Implications for using physical activity as an intervention for preschoolers with stereotypy are discussed. (PsycINFO Database Record (c) 2012 APA, all rights reserved)
SN - 0419-4209

AD - McLaughlin, Constance Ann Hylton: U Washington, US
ER -

TY - JOUR
ID - 2523
T1 - Children's motivation to engage in physical activity during recess
A1 - Efrat,Merav W.
Y1 - 2011///
N1 - Dissertation Abstract: 2011-99050-255
English
Dissertation Abstract
KW - PsycInfo
KW - Childhood Development
KW - Motivation
KW - Physical Activity
KW - Schools
KW - Exercise
KW - Self Efficacy
RP - NOT IN FILE
SP - 3140
JF - Dissertation Abstracts International Section A: Humanities and Social Sciences
VL - 71:
IS - 9-A
N2 - The purpose of this study was to explore strategies that may increase children's motivation to engage in physical activity during unstructured in-school free-time, such as recess. One hundred and sixty-two students, recruited from three elementary schools and nine fourth grade classrooms, were randomly assigned to one of three groups: (1) a teacher's daily verbal persuasion to engage in physical activity during recess; (2) a modeling of active recess time games by a competent adult; and (3) a comparison group that received no treatment. Pre- and post-treatment self-efficacy and effort data were collected utilizing a recess time physical activity self-efficacy instrument and accelerometers. A Spearman's rank correlation analysis determined that, in the context of recess, self-efficacy and effort are not related. A series of 2 x 3 ANCOVAs with pre-treatment scores as covariates determined that following treatment, the verbal persuasion group participants, compared to the modeling group participants, had significantly greater self-efficacy and effort mean scores. A borderline significant gender by treatment interaction effect on effort indicated that verbal persuasion was slightly more effective at increasing males, compared to female's effort. A series of 3 x 2 repeated measures ANOVAs determined that from pre- to post-treatment only participants in the verbal persuasion group spent a significantly higher percent of their recess time engaged in moderate to vigorous physical activity. Lastly, a principal component analysis with varimax rotation indicated that the barrier self-efficacy scale utilized in this study consisted of two types of barrier self-efficacy: adult verbal persuasion barrier self-efficacy and other barrier self-efficacy. Given school districts current budget and curriculum constrains, this study provides strong evidence that one cost effective strategy for increasing children's daily physical activity levels is a small dose of social prompting in the form of a teacher's verbal persuasion. In light of evidence suggesting that physical activity is linked to lower obesity rates, and substantial health and academic benefits, social prompting may be a useful strategy for addressing the childhood obesity epidemic, and improving students' academic and health outcomes. (PsycINFO Database Record (c) 2012 APA, all rights reserved)
SN - 0419-4209
AD - Efrat, Merav W.: U Southern California, US
ER -

TY - JOUR
ID - 2524
T1 - Child care and early education. [References]
A1 - DeBey,Mary
Y1 - 2009///
N1 - Book: 2009-08424-022
English
Book; Edited Book

KW - PsycInfo
KW - Child Care
KW - Educational Program Evaluation
KW - Educational Programs
KW - Learning Environment
KW - Preschool Education
KW - Project Head Start
RP - NOT IN FILE
SP - 641
EP - 666
JF - Mowder, Barbara A [Ed]; Rubinson, Florence [Ed]; Yasik, Anastasia E [Ed]
VL - :
IS - 2009
N2 - (from the chapter) Early childhood education refers to all education and child care programs that serve young children from birth through age 8. As adults quickly learn when a young child surprisingly mimics a word intended only for adults to hear, children learn in all environments. Young children do not distinguish learning from playtime, so for young children all environments are learning environments. Therefore, whether a child is in child care for long hours each day or in a preschool a few hours a week, the quality of the experience is critical. Many young children are in some type of center-based early childhood program prior to kindergarten. The U.S. Department of Education estimated that 43% of 3-year-olds and 69% of 4-year-olds were enrolled in a center-based early childhood program in 2005. Outside of the home, prekindergarten children are found in care by relatives and family friends, family and center-based child care, public and private preschools such as Head Start, and Early Head Start, and early childhood special education programs. Unlike elementary and secondary education, which guarantees a free public education, parents with prekindergarten children are primarily responsible for finding and financing their child's education and care. The goal of early childhood care and education is to provide safe language-rich environments where infants, toddlers, and young children explore their world; deeply connect with materials, other children and adults; and develop healthy bodies and minds. This chapter surveys options for early childhood education and child care and addresses assessment of quality for programs serving young children. (PsycINFO Database Record (c) 2014 APA, all rights reserved)
AD - DeBey, Mary: Brooklyn College of the City University of New York, New York, NY, US
ER -

TY - JOUR
ID - 2525
T1 - Patterns of activity of Irish children aged five to eight years: City living in Ireland today. [References]
A1 - Lynch,Helen
Y1 - 2009///
N1 - Peer Reviewed Journal: 2009-05651-004
English
Journal; Peer Reviewed Journal
KW - PsycInfo
KW - Childhood Development
KW - Daily Activities
KW - Occupations
KW - Urban Environments
RP - NOT IN FILE
SP - 44
EP - 49
JF - Journal of Occupational Science
VL - 16:
IS - 1
N2 - The study of children's occupation is receiving more attention in the occupational science literature. This trend corresponds with a global interest on child health and well-being. Research into the activity patterns of children in natural environments offers to advance knowledge about children's participation, health and occupational development. This study was conducted using time-use methods to explore and identify activities and environments of Irish children aged between 5 and 8 years in 2007. Findings support the need for environmental contexts to be taken into account when considering occupational development. Further study of

time-use methods is warranted for use in examining the children-environment-occupation relationship. (PsycINFO Database Record (c) 2013 APA, all rights reserved) (journal abstract)
SN - 1442-7591
AD - Lynch, Helen: Department of Occupational Science and Occupational Therapy, University College Cork, Cork, Ireland
ER -

TY - JOUR
ID - 2526
T1 - Health behavior interests of adolescents with unhealthy diet and exercise: Implications for weight management. [References]
A1 - Thunfors,Peter
Y1 - 2009///
N1 - Peer Reviewed Journal: 2009-10452-008
English
Journal; Peer Reviewed Journal
KW - PsycInfo
KW - Adolescent Attitudes
KW - Diets
KW - Eating Behavior
KW - Health Behavior
KW - Weight Control
KW - Exercise
KW - Lifestyle
KW - Obesity
RP - NOT IN FILE
SP - 634
EP - 645
JF - Health Education Research
JA - Health Educ Res
VL - 24:
IS - 4
N2 - This study sought to determine individual factors that may influence adolescents' interests in various health behaviors and, by extension, their potential interest in programs that promote healthy lifestyles and reduce obesity. The sample consisted of 737 rural Pennsylvania (United States) middle and high school students not involved in either healthy exercise or dietary behaviors (a target group for health-promoting interventions). Participants completed a self-report measure of their general health functioning, including their interests in sports programs, outdoor recreation programs, weightlifting, weight loss and healthy eating/cooking. Nurses measured body mass indices (BMIs). The vast majority of the sample endorsed self-efficacy in healthy eating and physical activity, and this self-efficacy was associated with interest in a healthy diet and outdoor recreation. Interest in healthy activities was consistently higher among 7th graders (age mean 5 12.6 years) than 11th graders (age mean 5 16.3 years). Females were more interested in weight loss and healthy eating/cooking, whereas males were more interested in weightlifting. Higher BMI only predicted interest in weight loss. These results indicate that adolescent health interests vary on the basis of their gender, grade level, BMI and self-efficacy. These trends are potentially important to consider when seeking to match intervention programs to adolescent interests. (PsycINFO Database Record (c) 2013 APA, all rights reserved) (journal abstract)
SN - 0268-1153
AD - Thunfors, Peter: Child Behavioral Health, Baystate Medical Center, Springfield, MA, US Collins, Bradley N.: Department of Public Health, College of Health Professions, Temple University, Philadelphia, PA, US Hanlon, Alexandra L.: Department of Public Health, College of Health Professions, Temple University, Philadelphia, PA, US
ER -

TY - JOUR
ID - 2527
T1 - Wellness appraisal among adolescents in Jordan: A model from a developing country: A cross-sectional questionnaire survey. [References]

A1 - Haddad,Linda G.
Y1 - 2009///
N1 - Peer Reviewed Journal: 2009-07324-003
English
Journal; Peer Reviewed Journal
KW - PsycInfo
KW - Adolescent Attitudes
KW - Developing Countries
KW - Health
KW - Health Behavior
KW - Questionnaires
KW - Models
RP - NOT IN FILE
SP - 130
EP - 139
JF - Health Promotion International
JA - Health Promot Internation.
VL - 24:
IS - 2
N2 - The demographic and economic transition that many developing countries, including Jordan, are undergoing is producing important changes in diet and lifestyle that greatly impact the development of chronic illness. The health behavior of adolescents in developing countries constitutes one of the most serious global challenges we face. The purpose of this study was to explore the wellness appraisal of Jordanian adolescents. It specifically describes the (i) self-care and health history pattern, (ii) to assess physical activity and nutrition appraisal, (iii) quality of life appraisal and (iv) school and outside activities appraisal. A self-administrated questionnaire collected the data from adolescent groups (boys and girls.). A multi-stage stratified random sample was obtained from six public schools by first selecting the educational directorate located in the city of Irbid, which is located in the north of Jordan. A random sub sample of six individual schools was then selected. Individual classes were then selected. A total of 269 boys and 261 girls (12-17-year old) were included in the analysis. The results showed that the students engaged in risky health behaviors which could lead to short- and long-term health problems. The most alarming finding of this study was students' nutritional habits, including less than the daily requirements of fruits, vegetables, milk and meat, while the intake of fast food, soft drinks and sweets were higher than recommended. School-based health promotion and wellness programs should be established in Jordan to influence the health behaviors of adolescents and parents and to avoid further deterioration of their health. Jordanian school curriculum needs to integrate more precise health education programs about diet, exercise, self care and other life style behaviors. More detailed studies are needed with more elaborate instruments about food habits, physical activities and psycho social life. (PsycINFO Database Record (c) 2013 APA, all rights reserved) (journal abstract)
SN - 0957-4824
AD - Haddad, Linda G.: Virginia Commonwealth University School of Nursing, Richmond, VA, US Owies, Arwa: Faculty of Nursing, Jordan University of Science and Technology, Irbid, Jordan Mansour, Amani: Ministry of Health, Irbid, Jordan
ER -

TY - JOUR
ID - 2528
T1 - Individual differences in children's risk perception and appraisals in outdoor play environments. [References]
A1 - Little,Helen
Y1 - 2010///
N1 - Peer Reviewed Journal: 2010-26649-004
English
Journal; Peer Reviewed Journal
KW - PsycInfo
KW - Childhood Play Behavior
KW - Individual Differences
KW - Risk Perception

KW - Sensation Seeking
KW - Risk Taking
RP - NOT IN FILE
SP - 297
EP - 313
JF - International Journal of Early Years Education
VL - 18:
IS - 4
N2 - Child characteristics including age, gender, risk-taking behaviour and sensation seeking are thought to influence children's ability to appraise risks. The present study investigated children's risk perceptions and appraisals in the context of common outdoor physical play activities. Risk perceptions and appraisal of four- and five-year olds were assessed using a pictorial risk appraisal measure and through individual semi-structured interviews examining children's decision-making. Sensation seeking and risk-taking behaviour were assessed using self-and parent-report measures. Results revealed that the children were able to identify injury risk behaviours however differentiating the severity of the potential injury was less accurate. Furthermore, the children appeared to use these judgements in their play to inform their behaviour whilst using playground equipment. Whilst they actively sought out those activities that offered challenge and excitement, they were aware of their abilities and showed caution in engaging in activities that were beyond their current capabilities. The results have implications for the provision of calculated risk-taking in outdoor play and for adult responses to the risk-taking behaviour of children. (PsycINFO Database Record (c) 2013 APA, all rights reserved) (journal abstract)
SN - 0966-9760
AD - Little, Helen: Institute of Early Childhood, Macquarie University, Sydney, NSW, Australia Wyver, Shirley: Institute of Early Childhood, Macquarie University, Sydney, NSW, Australia
ER -

TY - JOUR
ID - 2529
T1 - Towards studies of giftedness. [Lithuanian]. [References]
A1 - Misiuniene,Jurga
Y1 - 2010///
N1 - Peer Reviewed Journal: 2011-19430-003
Lithuanian
Journal; Peer Reviewed Journal
KW - PsycInfo
KW - Attention
KW - Childhood Development
KW - Gifted
KW - Individual Differences
KW - School Learning
KW - Elementary School Students
KW - Preschool Students
RP - NOT IN FILE
SP - 37
EP - 58
JF - International Journal of Psychology: A Biopsychosocial Approach / Tarptautinis psichilogijos zurnalas: Biopsichosocialinis poziuris
VL - 5 2010, pp. 37-58.
N2 - Background: This article presents the results of two studies related to successful learning at school. Successful passing from pre-school to school activities depends on the mental maturity of children. Peculiarity of the development of the attention is important to successful learning at primary school. Aims and methods: The first study was aimed for evaluation of differences of learning different educational contents, and the Control Drawing Observation method was used. The second study had the aim to investigate the characteristics of attention of primary schoolchildren using the Black-Red Numbers Table method and interview with teachers. Results: Pupils learning different educational contents had the same mental maturity levels. But higher mental maturity level was related to concentration ability and work self-sustaining. There were found no gender or school motivation relationship with mental maturity, but girls and boys differed in school motivation. The main

problems for pre-school-aged children were related to the memorization and carrying out of instructions. It was found that intensity, concentration and displacement of attention and both ability to stand from the outside stimulus and the ability to listen and follow verbal instructions were significantly related to respondents' age. The intensity and concentration was also related to the level of progressiveness at school. The results didn't confirm the hypothesis that concentration of attention is related to the educational content. Conclusions: The mental maturity level is not related to the educational content. The progressiveness at school is connected to the intensity and concentration of the attention. All its features (intensity, concentration and displacement) develop according to the age of primary pupils. (PsycINFO Database Record (c) 2013 APA, all rights reserved) (journal abstract)
SN - 1941-7233
AD - Misiuniene, Jurga: Vytauto Didziojo universitetas, Lithuania Grigaite, Bronislava: Vytauto Didziojo universitetas, Lithuania
ER -

TY - JOUR
ID - 2530
T1 - Review of Talk and social interaction in the playground
A1 - Mills,Carol Bishop
Y1 - 2010///
N1 - Peer Reviewed Journal: 2010-10479-007
English
Journal; Peer Reviewed Journal
KW - PsycInfo
KW - Oral Communication
KW - Playgrounds
KW - Pragmatics
KW - Social Interaction
RP - NOT IN FILE
SP - 245
EP - 247
JF - Journal of Language and Social Psychology
VL - 29:
IS - 2
N2 - Reviews the book Talk and social interaction in the playground by Carly W. Butler (2008). Scholars who investigate the social lives of children can choose from among multiple lenses to frame their studies. Butler's work is based on 30 hours of recorded talk of 6- and 7-year-old children in an elementary school over a period of 1 month, but analysis in this work focuses on the interactions of 11 children on the playground at school during lunch. As Butler clearly articulates, these 3 days provide a rich and coherent corpus of material that allows for analysis by looking for recurring practices in the fairy club, as well as the detailed analyses of particular episodes of interaction in the club. This book adds to the work on children's social interaction and highlights the practices that allow children to engage in coordinated action. In this book, children's play is respected and honored on the terms of the children and never dismissed as mere child's play. Overall, Butler makes a significant contribution to a growing and important body of work in children's social studies. This book will be of interest to conversation analysts, ethnomethodologists, and children's communication researchers, but other scholars who are looking for clear and detailed discussions of research methods should also take a serious look at this book. (PsycINFO Database Record (c) 2012 APA, all rights reserved)
SN - 0261-927X
AD - Mills, Carol Bishop: University of Alabama, Tuscaloosa, AL, US
ER -

TY - JOUR
ID - 2531
T1 - Measuring childhood materialism: Refining and validating Schor's Consumer Involvement Scale. [References]
A1 - Bottomley,Paul A.
Y1 - 2010///

N1 - Peer Reviewed Journal: 2010-13786-005
English
Journal; Peer Reviewed Journal
KW - PsycInfo
KW - Brand Preferences
KW - Consumer Attitudes
KW - Psychometrics
KW - Test Validity
KW - Materialism
KW - Awareness
KW - Dissatisfaction
KW - Involvement
RP - NOT IN FILE
SP - 717
EP - 740
JF - Psychology & Marketing
VL - 27:
IS - 7
N2 - This paper examines the measurement of childhood materialism using Schor's ([2004]) Consumer Involvement Scale. Schor treated consumer involvement empirically as a unidimensional construct, though she suggested that conceptually it may be multidimensional. Using confirmatory factor analysis procedures on data collected from children in the U.S. and U.K., the psychometric superiority of a three-factor structure is established, comprising dissatisfaction, consumer orientation, and brand awareness components. Additional analyses demonstrate distinct associations between each of these components and other constructs, including self-esteem, outside school activities, and child-parent relations. The scale's generalizability across boys and girls is also confirmed. The results suggest that Schor's Consumer Involvement Scale will be useful for researchers interested in studying the important topic of materialism in children. (PsycINFO Database Record (c) 2012 APA, all rights reserved) (journal abstract)
SN - 0742-6046
AD - Bottomley, Paul A.: Cardiff University, Cardiff, United Kingdom Nairn, Agnes: Lyon Business School, Lyon, France Kasser, Tim: Knox College, IL, US Ferguson, Yuna L.: University of Missouri-Columbia, Columbia, MO, US
ER -

TY - JOUR
ID - 2532
T1 - Obesity in early childhood and working in pre-school settings. [References]
A1 - de Silva-Sanigorski,Andrea M.
Y1 - 2010///
N1 - Book: 2010-10942-029
English
Book; Edited Book
KW - PsycInfo
KW - Childhood Development
KW - Health Promotion
KW - Obesity
KW - Prevention
KW - Weight Gain
KW - Critical Period
KW - Preschool Education
KW - Risk Factors
RP - NOT IN FILE
SP - 253
EP - 262
JF - Waters, Elizabeth [Ed]; Swinburn, Boyd [Ed]; Seidell, Jacob [Ed]; Uauy, Ricardo [Ed]
VL - :
IS - 2010

N2 - (from the chapter) This chapter examines several issues on obesity in early childhood that have direct practical relevance to supporting healthy weight gain in this critical period of life. The first fundamental issue for child health nursing practice is the definition of normal growth in early childhood, which leads directly into an examination of the assessment of at-risk growth patterns and other risk factors for later childhood (and adult) obesity. Appropriate feeding practices are fundamental to healthy weight gain in early childhood and these recommendations are reviewed as well as the importance of active play, motor skill development and reduced television viewing time. Patterns for all human behaviors are highly influenced by environments, and this is most true in the early childhood years. Thus, the many preschool settings outside the home environment are also examined as opportunities for implementing the policies and practices needed to promote healthy eating and active play. (PsycINFO Database Record (c) 2012 APA, all rights reserved)
AD - de Silva-Sanigorski, Andrea M.: WHO Collaborating Centre for Obesity Prevention, Deakin University, Geelong, VIC, Australia Corvalan, Camila: School of Public Health, Faculty of Medicine, University of Chile, Santiago, Chile Uauy, Ricardo: London School of Hygiene and Tropical Medicine, London, England
ER -

TY - JOUR
ID - 2533
T1 - Is there an association between spatial access to parks/green space and childhood overweight/obesity in Calgary, Canada? [References]
A1 - Potestio,Melissa L.
Y1 - 2009///
N1 - Peer Reviewed Journal: 2009-24916-001
English
Journal; Peer Reviewed Journal
KW - PsycInfo
KW - Epidemiology
KW - Obesity
KW - Overweight
KW - Recreation Areas
KW - Childhood Development
KW - Spatial Ability
RP - NOT IN FILE
JF - The International Journal of Behavioral Nutrition and Physical Activity
VL - 6 Nov 2009, ArtID 77.
N2 - Background: The recent increase in childhood obesity is expected to add significantly to the prevalence of chronic diseases. We used multivariate multilevel analysis to examine associations between parks/green space and childhood overweight/obesity across communities in Calgary, Canada, a city characterized by intensified urban sprawl and high car use. Methods: Body Mass Index was calculated from measured height and weight data obtained from 6,772 children (mean age = 4.95 years) attending public health clinics for pre-school vaccinations. Each child's home postal code was geocoded using ESRI ArcGIS 9.2. We examined four measures of spatial access to parks/green space (based on Geographic Information Systems): 1) the number of parks/green spaces per 10,000 residents, 2) the area of parks/green space as a proportion of the total area within a community, 3) average distance to a park/green space, and 4) the proportion of parks/green space service area as a proportion of the total area within a community. Analyses were adjusted for dissemination area median family income (as a proxy for an individual child's family income) community-level education, and community-level proportion of visible minorities. Results: In general, parks/green space at the community level was not associated with overweight/ obesity in Calgary, with the exception of a marginally significant effect whereby a moderate number of parks/green spaces per 10,000 residents was associated with lower odds of overweight/obesity. This effect was non-significant in adjusted analyses. Conclusion: Our null findings may reflect the popularity of car travel in Calgary, Canada and suggest that the role built environment characteristics play in explaining health outcomes may differ depending on the type of urban environment being studied. (PsycINFO Database Record (c) 2012 APA, all rights reserved) (journal abstract)
SN - 1479-5868
AD - Potestio, Melissa L.: University of Calgary, Department of Community Health Sciences, Faculty of Medicine, Calgary, AB, Canada Patel, Alka B.: University of Calgary, Department of Community Health Sciences, Faculty of Medicine, Calgary, AB, Canada Powell, Christopher D.: University of Calgary, Bachelor of Health Sciences Program, Faculty of Medicine, Calgary, AB, Canada McNeil, Deborah A.: University of

Calgary, Faculty of Nursing and Population and Public Health, Alberta Health Services, Calgary, AB, Canada Jacobson, R. Daniel: University of Calgary, Department of Geography, Faculty of Social Sciences, Calgary, AB, Canada McLaren, Lindsay: University of Calgary, Department of Community Health Sciences, Faculty of Medicine, Calgary, AB, Canada
ER -

TY - JOUR
ID - 2535
T1 - About Belgians, Turks and mothers-in-law. Metalinguistic abilities in early adolescence. [Dutch]. [References]
A1 - de Milliano,I.
Y1 - 2010///
N1 - Peer Reviewed Journal: 2010-24208-002
Dutch
Journal; Peer Reviewed Journal
KW - PsycInfo
KW - Age Differences
KW - Grade Level
KW - Knowledge Level
KW - Language Development
KW - Metalinguistics
KW - Jokes
RP - NOT IN FILE
SP - 334
EP - 349
JF - Pedagogische Studien
VL - 87:
IS - 5
N2 - This study investigated the metalinguistic abilities of 91 prevocational and pre-academic students from 7th and 9th grade. To prevent educational influences, we measured comprehension of joking riddles, 'language of the playground', rather than typical academic language performance. Preacademic students performed better than prevocational students, 9th graders better than 7th graders and riddles which require linguistic knowledge to resolve the joke were more difficult than riddles requiring world knowledge. The study also revealed that vocabulary was an important predictor of metalinguistic abilities, and that riddle comprehension and appreciation were weakly correlated. Furthermore, metalinguistic abilities and vocabulary size of pre-academic 9th graders was larger than of their peers from 7th grade, but this was not the case for prevocational students. Because vocabulary, grade and school track were related, it can be concluded that linguistic riddle comprehension is a valid and appropriate measurement of metalinguistic abilities of young adolescents. (PsycINFO Database Record (c) 2012 APA, all rights reserved) (journal abstract)
SN - 0165-0645
AD - de Milliano, I. I. C. M.: Universiteit van Tilburg, Tilburg, Netherlands Kurvers, J. J. H.: Faculteit Maatschappij- en Gedragswetenschappen, Universiteit van Amsterdam, Amsterdam, Netherlands
ER -

TY - JOUR
ID - 2536
T1 - Routines in a kindergarten: Narrative sequence of an educational course. [Italian]. [References]
A1 - la Stella,Rossana
Y1 - 2010///
N1 - Peer Reviewed Journal: 2010-19131-006
Italian
Journal; Peer Reviewed Journal
KW - PsycInfo
KW - Education
KW - Educational Programs
KW - Kindergartens
KW - Narratives

RP - NOT IN FILE
SP - 269
EP - 278
JF - Psicologia dell'educazione
VL - 4:
IS - 2
N2 - The authoress makes reference to an educational practice realized in a day nursey, in order to manage a cognitive research about the children's routines in the framework of an educational path. In this institutional context occurs a series of experiences, which involve the body as well as its espressive language, articulated in a sequence of paradoxical messages of opposite value. Inside/outside, near/far, narrow/large etc... are topological dimensions drawn by childish gesture and by the educator that is "naturally" prepared to receive the projective movement. It is just in the orderly steadiness of an institutional structure that it is possibile the flexible modulation of child's impulses. If it will be given to the child the opportunity to meet someone able to "play" with him in a "frontier territory", inhabited by rapidly changing of emotions and sensations. (PsycINFO Database Record (c) 2012 APA, all rights reserved) (journal abstract)
SN - 1971-3711
ER -

TY - JOUR
ID - 2537
T1 - Fifth-, sixth-, and seventh- grade students' use of non-classroom spaces during recess: The case of three private schools in Izmir, Turkey. [References]
A1 - Kasal,Altu; Do an,Fehmi
Y1 - 2010///
N1 - Peer Reviewed Journal: 2010-10136-001
English
Journal; Peer Reviewed Journal
KW - PsycInfo
KW - Age Differences
KW - Childhood Play Behavior
KW - Elementary Schools
KW - Physical Activity
KW - School Environment
KW - Classrooms
KW - Private School Education
RP - NOT IN FILE
SP - 518
EP - 532
JF - Journal of Environmental Psychology
VL - 30:
IS - 4
N2 - This study investigates fifth, sixth, and seventh grade students' place preferences between indoor and outdoor non-classroom spaces during recess and their activity patterns in these spaces in three private elementary schools. The study explores whether differences in the variety and organization of the spaces of school facilities have an impact on the place preferences of students and whether students are aware of the reasons for their preferences. Students' place preferences and their activities were determined with field observations and a 30-item questionnaire with Yes/No and open-ended questions. A total of 173 students (n = 51 School 1; n = 70 School 2; and n = 52 School 3) participated in the questionnaire. The Chi-Square test, a non-parametric statistical analysis test, was used to analyze the students' answers to the questionnaire. The results indicate that students prefer places which offer variety and which are large enough to avoid congestion and that, in general, students are aware of the spatial features of their environments and make choices accordingly. When students are given a choice of outdoors or indoors, they tend to choose according to which is more conducive to their activities. If both outdoor and indoor spaces are conducive, students tend to use both. If neither is conducive to their activities, students either alter their behavior patterns, for example, developing a preference for stationary activities or staying inside the classroom, or they convert available spaces to accommodate their activities. It is concluded that students are good sources of information in the design and

planning of the environments they occupy. (PsycINFO Database Record (c) 2012 APA, all rights reserved) (journal abstract)
SN - 0272-4944
AD - Kasal, Altu: Izmir Institute of Technology, Department of Architecture, Izmir, Turkey Do an, Fehmi: Izmir Institute of Technology, Department of Architecture, Izmir, Turkey
ER -

TY - JOUR
ID - 2538
T1 - After school care arrangements and student academic performance and misbehavior in middle school
A1 - Baisley,Barbara Ann
Y1 - 2010///
N1 - Dissertation Abstract: 2010-99210-288
English
Dissertation Abstract
KW - PsycInfo
KW - Academic Achievement
KW - After School Programs
KW - Behavior Problems
KW - Child Day Care
KW - Middle Schools
KW - Students
RP - NOT IN FILE
SP - 1529
JF - Dissertation Abstracts International Section A: Humanities and Social Sciences
VL - 71:
IS - 5-A
N2 - The present study examined the types of settings in which students receive care after school and the activities in which students engage in these particular settings. Data were extracted from the National Household for Education Statistics 2005 data set using students enrolled only in 6th, 7 th or 8th grade (N = 4,659). The variables of interest included student academic misbehavior and student academic performance, the type of care arrangement relative, non-relative, parent, center and self as well as the activities homework/school-related, computers, art, chores/work, outdoor play/sports, indoor play/sports, and television/videos/music, in which students are engaged within each after school care arrangement. Chi-square analyses were conducted to assess any differences in the activities in which students were engaged while at after school care arrangements to determine to what extent these activities contribute to students' academic performance and misbehavior. An over-all pattern in the data regarding the care arrangements showed a positive relationship between the parent care arrangement and the occurrence of student misbehavior. In regards to the activities in which students were engaged after school, a positive relationship was found between homework activities and student academic performance and misbehavior. These findings provide some information to assist parents in selecting the best possible environments for their children after school. (PsycINFO Database Record (c) 2012 APA, all rights reserved)
SN - 0419-4209
AD - Baisley, Barbara Ann: George Mason U., US
ER -

TY - JOUR
ID - 2539
T1 - Removing interictal fast ripples on electrocorticography linked with seizure freedom in children. [References]
A1 - Wu,J.
Y1 - 2010///
N1 - Peer Reviewed Journal: 2010-24194-006
English
Journal; Peer Reviewed Journal
KW - PsycInfo
KW - Electrodes

KW - Measurement
KW - Pediatrics
KW - Seizures
KW - Electrophysiology
KW - Methodology
RP - NOT IN FILE
SP - 1686
EP - 1694
JF - Neurology
VL - 75:
IS - 19
N2 - Background: Fast ripples (FR, 250-500 Hz) detected with chronic intracranial electrodes are proposed biomarkers of epileptogenesis. This study determined whether resection of FR-containing neocortex recorded during intraoperative electrocorticography (ECoG) was associated with postoperative seizure freedom in pediatric patients with mostly extratemporal lesions. Methods: FRs were retrospectively reviewed in 30 consecutive pediatric cases. ECoGs were recorded at 2,000 Hz sampling rate and visually inspected for FR, with reviewer blinded to the resection and outcome. Results: Average age at surgery was 9.1 + 6.7 years, ECoG duration was 11.8 + 8.1 minutes, and postoperative follow-up was 27 + 4 months. FRs were undetected in 6 ECoGs with remote or extensive lesions. FR episodes (n = 273) were identified in ECoGs from 24 patients, and in 64% FRs were independent of spikes, sharp waves, voltage attenuation, and paroxysmal fast activity. Of these 24 children, FR-containing cortex was removed in 19 and all became seizure-free, including 1 child after a second surgery. The remaining 5 children had incomplete FR resection and all continued with seizures postoperatively. In 2 ECoGs, the location of electrographic seizures matched FR location. FR-containing cortex was found outside of MRI and FDG-PET abnormalities in 6 children. Conclusion: FRs were detected during intraoperative ECoG in 80% of pediatric epilepsy cases, and complete resection of FR cortex correlated with postoperative seizure freedom. These findings support the view that interictal FRs are excellent surrogate markers of epileptogenesis, can be recorded during brief ECoG, and could be used to guide future surgical resections in children. (PsycINFO Database Record (c) 2012 APA, all rights reserved) (journal abstract)
SN - 0028-3878
AD - Wu, J. Y.: Division of Pediatric Neurology, Mattel Children's Hospital, UCLA, Los Angeles, CA, US Sankar, R.: Division of Pediatric Neurology, Mattel Children's Hospital, UCLA, Los Angeles, CA, US Lerner, J. T.: Division of Pediatric Neurology, Mattel Children's Hospital, UCLA, Los Angeles, CA, US Matsumoto, J. H.: Division of Pediatric Neurology, Mattel Children's Hospital, UCLA, Los Angeles, CA, US Vinters, H. V.: Division of Neurology, David Geffen School of Medicine, University of California Los Angeles, Los Angeles, CA, US Mathern, G. W.: Department of Neurosurgery, David Geffen School of Medicine, University of California Los Angeles, Los Angeles, CA, US
ER -

TY - JOUR
ID - 2540
T1 - "A"-kids: Activity kcal intervention daily study effects of 100-kcal daily energy expenditure on total moderate-to-vigorous physical activity in 3rd grade children
A1 - Howe,Cheryl A.
Y1 - 2010///
N1 - Dissertation Abstract: 2010-99200-245
English
Dissertation Abstract
KW - PsycInfo
KW - Energy Expenditure
KW - Games
KW - Grade Level
KW - Intervention
KW - Physical Activity
KW - Exercise
KW - Obesity
RP - NOT IN FILE
SP - 2350

JF - Dissertation Abstracts International: Section B: The Sciences and Engineering
VL - 71:
IS - 4-B
N2 - A selection of common children's games were measured in a laboratory-based study to be enjoyable and to elicit sufficient physical activity energy expenditure (PAEE) in 3rd grade children to combat the purported chronic energy surplus of childhood obesity (~100 kcalday -1). PAEE during the games was similar for boys and girls, yet overweight children expended greater PAEE relative to body weight than healthy weight children. During a subsequent simulated recess program, the enjoyment declined over the 10-session program with no significant decline in PAEE. Using the enjoyable games of known energy cost in a structured recess program for 9 weeks successfully increased total daily PA compared to the control school who reported substantially greater amount of free-play time. The greater amount of acquired PA in the intervention school children did not affect the amount of time spent in sedentary pursuits but it did result in a smaller increase in body weight after 9 weeks. More research is needed to expand on this initial list of games that reduce the excessive weight gain in children when incorporated into a structured recess intervention. (PsycINFO Database Record (c) 2012 APA, all rights reserved)
SN - 0419-4217
AD - Howe, Cheryl A.: U Massachusetts Amherst, US
ER -

TY - JOUR
ID - 2541
T1 - A study of the associations between childhood obesity and three forms of social capital
A1 - Bala-Brusilow,Cynthia
Y1 - 2010///
N1 - Dissertation Abstract: 2010-99190-141
English
Dissertation Abstract
KW - PsycInfo
KW - Childhood Development
KW - Comprehension
KW - Obesity
KW - Social Capital
KW - Academic Achievement
RP - NOT IN FILE
SP - 1447
JF - Dissertation Abstracts International Section A: Humanities and Social Sciences
VL - 71:
IS - 4-A
N2 - The purpose of the study was to expand the understanding of childhood obesity in American children by examining the associations between obesity in children and measures of social capital. Persons between 2 and 20 years of age are categorized as "obese" if their BMI is in 95th percentile or above for their age and sex using the Center for Disease Control and Prevention (CDC) BMI-for-age growth charts. Obesity prevalence has more than quadrupled in the last 40 years in the United States for children. The prevalence rate is 17.0% for children ages 6-11 years. Social capital, in the study of health, can be defined as resources accrued and/or accessed from social relationships/social bonds at multiple levels including the individual, family, neighborhood, community or nation. The research quantitatively analyzed the associations between the likelihood of childhood obesity and BMI with personal social capital, family social capital and neighborhood social capital. The research was conducted with a public use dataset from the 2003 National Survey of Children's Health. This survey is part of the State and Local Area Integrated Telephone Survey Program (SLAITS) conducted by the CDC's National Center for Health Statistics (NCHS) and was funded by the Maternal and Child Health Bureau (MCHB). A dataset for the study was created for the 10,018 10 and 11 year olds for whom height and weight was available. The study population had an obesity prevalence of 20.4. Logistic and OLS multiple regression models were employed for hypotheses testing. Eleven indicators were categorized as measures of personal social capital, family social capital or neighborhood social capital. The regression models clearly identified many individually significant measures of social capital but their (the regression models) were weak in their predictive power. Five individual indicators of social capital were particularly noteworthy for having consistently significant associations with the likelihood of obesity and BMI throughout the research. These include type of school

(private or public), moving, number of siblings, parents knowing friends and participating in activities outside of school. The research supports the idea that the study of children's social capital (personal, family and neighborhood) is a viable way to expand the understanding of the pathways behind the social patterning of childhood obesity in the United States. (PsycINFO Database Record (c) 2012 APA, all rights reserved)
SN - 0419-4209
AD - Bala-Brusilow, Cynthia: Wayne State U., US
ER -

TY - JOUR
ID - 2542
T1 - The role of parafoveal information in rapid letter naming
A1 - Logan,Jessica A.
Y1 - 2010///
N1 - Dissertation Abstract: 2010-99180-187
English
Dissertation Abstract
KW - PsycInfo
KW - Cognitive Ability
KW - Foveal Vision
KW - Letters (Alphabet)
KW - Naming
KW - Serial Learning
KW - Reading Ability
KW - Visual Acuity
RP - NOT IN FILE
SP - 2076
JF - Dissertation Abstracts International: Section B: The Sciences and Engineering
VL - 71:
IS - 3-B
N2 - Several cognitive components are thought to explain the relation of rapid serial naming and reading ability including the constructs of phonological access, attention, automaticity, articulation, global processing speed, and visual acuity. One cognitive component that has gone understudied when investigating rapid serial naming is parafoveal processing. The goal of the present study was to examine the role that parafoveal information, information outside the direct visual focal point, plays in the rapid serial naming task. Two experiments explicitly varied the amount and type of information available to the right of the focal point in manipulated versions of the rapid serial naming task. The performance of forty-one first-grade students were examined for differences between manipulated conditions and relations with reading outcomes at three levels of processing, low-level visual processing, initial lexical access, and higher-order cognition. Results suggested that initial visual processing occurs for the letter directly in the focal area, as well as the letter immediately following it, and that children who were better at rapid naming were significantly more impaired by the lack of parafoveal preview than were the children at the lowest end of the distribution of rapid naming performance. (PsycINFO Database Record (c) 2012 APA, all rights reserved)
SN - 0419-4217
AD - Logan, Jessica A. R.: The Florida State U., US
ER -

TY - JOUR
ID - 2543
T1 - Early red flags for autism spectrum disorders in toddlers in the home environment
A1 - Book,Lorraine Adair
Y1 - 2010///
N1 - Dissertation Abstract: 2010-99180-185
English
Dissertation Abstract
KW - PsycInfo
KW - Caregivers
KW - Daily Activities

KW - Home Environment
KW - Pervasive Developmental Disorders
RP - NOT IN FILE
SP - 1662
JF - Dissertation Abstracts International: Section B: The Sciences and Engineering
VL - 71:
IS - 3-B
N2 - Early detection of autism spectrum disorders (ASD) is crucial to optimizing child and family outcomes. Most research on the early diagnostic features of ASD has been done in the clinic environment; therefore there is a need for research on diagnostic features displayed in a natural environment. Observation of behaviors of young children in their home environment is a possible way to assist in earlier diagnosis in a less time consuming, less stressful, and more cost-effective manner for families. The primary purpose of this study was to explore and quantify red flags for ASD in the behaviors of young children between 17 and 36 months of age (N = 60) demonstrated in the home environment. Measures on the Systematic Observation of Red Flags for Autism Spectrum Disorders in Young Children at Home (SORF-Home; Wetherby & Woods, 2009) were coded from video recorded home observation samples of the child interacting with a parent or caregiver during everyday activities. Overall, the results of this study demonstrated that early red flags of ASD were evident in the home environment. Seven of the red flags identified by previous researchers to be evident in the clinic environment (lack of warm, joyful expression with directed gaze, lack of coordination of gaze, facial expression, gestures and sounds, lack of response to name, lack of communicative vocalizations with consonants, inappropriate eye gaze, lack of response to contextual cues, and lack of sharing interest or enjoyment) were also found to be demonstrated by a majority of children during the home observations. Examination of the relationship between the early red flags and developmental level revealed no significant correlations between the red flags and nonverbal cognitive developmental level and medium to large negative significant correlations between some red flags and verbal developmental level. Significant correlations were observed between many of the early red flags and ASD symptom severity on the Autism Diagnostic Observation Schedule (ADOS; Lord, Rutter, DiLavore, & Risi, 2002). No significant correlations were observed between measures of parent report of red flags and the early red flags measured in the home environment. The results of this exploratory study extend knowledge on the nature of red flags for ASD in young children to the home environment which adds valuable information for both practicing clinicians and families. The findings also provide promising information on the utility of the SORF-Home as an interactive screening measure for ASD and offer important implications for early identification of ASD. (PsycINFO Database Record (c) 2012 APA, all rights reserved)
SN - 0419-4217
AD - Book, Lorraine Adair: The Florida State U., US
ER -

TY - JOUR
ID - 2544
T1 - Students' motivation to study music: The United States of America. [References]
A1 - McPherson,Gary E; Hendricks,Karin
Y1 - 2010///
N1 - Peer Reviewed Journal: 2010-25174-007
English
Journal; Peer Reviewed Journal
KW - PsycInfo
KW - Academic Achievement Motivation
KW - Music Education
KW - Learning
KW - Students
RP - NOT IN FILE
SP - 201
EP - 213
JF - Research Studies in Music Education
VL - 32:
IS - 2
N2 - As a part of a larger international mapping exercise to examine students' motivation to study music as compared to other school subjects, this article draws upon data from a sample of 3037 students in the USA to

observe perceptions of values, competence and interest in music study (in school versus outside of school) among music learners and non-music learners. Students were grouped into three grade levels: (a) 6, (b) 7-9, and (c) 10-12. Music learners in the USA had significantly higher motivational profiles for music and some other school subjects as compared to non-music learners. Music interest inside of school was ranked significantly lower than for any other subject, while music interest outside of school was ranked second highest for any subject in grades 6 and 7-9, and highest of all subjects in grades 10-12. This article addresses cultural and contextual issues in the USA to consider how music advocates might better demonstrate the importance and usefulness of music study as an academic course. Practical recommendations include encouraging a broader emphasis beyond performance and competition, and promoting opportunities for autonomous music learning within the school setting. (PsycINFO Database Record (c) 2012 APA, all rights reserved) (journal abstract)
SN - 1321-103X
AD - McPherson, Gary E.: University of Melbourne, Melbourne, VIC, Australia Hendricks, Karin S.: University of Illinois, IL, US
ER -

TY - JOUR
ID - 2545
T1 - Peer-mediated social skills instruction and self-management strategies for students with autism
A1 - Labbe-Poisson,Kimberly A.
Y1 - 2010///
N1 - Dissertation Abstract: 2010-99160-311
English
Dissertation Abstract
KW - PsycInfo
KW - Autism
KW - Peer Tutoring
KW - Self Management
KW - Social Behavior
KW - Social Skills Training
KW - Social Skills
KW - Strategies
RP - NOT IN FILE
SP - 1329
JF - Dissertation Abstracts International: Section B: The Sciences and Engineering
VL - 71:
IS - 2-B
N2 - Increasing socialization opportunities for individuals with autism has been an ongoing educational process. There continues to be a need for a systematic approach to facilitate increases in social skills among students with autism in general education settings. One such model of instruction that has promising results is Pivotal Response Training (PRT) (Koegel & Koegel, 1995). PRT is a comprehensive service delivery model that uses both a developmental approach and applied behavior analysis (ABA) procedures, to provide opportunities for learning within the context of the child's natural environment (Koegel, Koegel, Harrower, & Carter, 1999). It focuses on providing opportunities for learning social behavior within the context of the individual's natural environment. The current study utilized peer-mediated PRT combined with self-management procedures, to support the independent use of social behaviors among students with autism. Three elementary-age students with autism from a self contained classroom participated. These students were trained to implement self-management procedures to assist these students. Six elementary-age general education students were trained in PRT. The effectiveness of these treatments in increasing social behaviors was evaluated using a multiple baseline across subjects designs. Probes were obtained during general education activities (e.g., lunch, gym) and in the school playground to assess generalization effects. Data were obtained via videotaped 10 minute sessions using 10-s partial interval recording. External validity was measured by the Social Responsiveness Scale (SRS) which was completed by the special education and general education teachers. The results of this study demonstrated that peer-mediated PRT combined with self-management procedures increased the social behaviors, maintained interactions, and initiated conversations by students with autism. It also had a collateral effect on the attention behaviors of supported and coordinated joint attention, and the strength of these results suggests that they can be generalized to general education settings (e.g., playground, lunch room, gymnasium). (PsycINFO Database Record (c) 2012 APA, all rights reserved)

SN - 0419-4217
AD - Labbe-Poisson, Kimberly A.: U Southern Maine, US
ER -

TY - JOUR
ID - 2546
T1 - A qualitative analysis of the bullying prevention and intervention recommendations of students in Grades 5 to 8. [References]
A1 - Cunningham,Charles E; Cunningham,Lesley
Y1 - 2010///
N1 - Peer Reviewed Journal: 2010-21124-001
English
Journal; Peer Reviewed Journal
KW - PsycInfo
KW - Age Differences
KW - Prevention
KW - Social Isolation
KW - Students
KW - Bullying
KW - Intervention
KW - Middle School Students
RP - NOT IN FILE
SP - 321
EP - 338
JF - Journal of School Violence
VL - 9:
IS - 4
N2 - Focus groups explored the bullying prevention suggestions of 62 Grade 5 to 8 students. Discussions were transcribed and analyzed thematically. Students advocated a comprehensive approach including uniforms, increased supervision, playground activities, group restructuring to prevent social isolation, influential presenters, prevention skills training, solution-focused posters, and meaningful consequences. In addition, students suggested that parents should improve relationships with their children, respond to aggression, limit exposure to media violence, and support school-based discipline. The failure to respond effectively to students who bully in defiance of antibullying presentations, and who retalitate when reported or disciplined, undermines prevention programs by reducing the willingness of bystanders to intervene or report bullying, and influencing the attitudes of younger pupils. The approach advocated by students is supported by meta-analyses of the effective components of bullying prevention trials. (PsycINFO Database Record (c) 2012 APA, all rights reserved) (journal abstract)
SN - 1538-8220
AD - Cunningham, Charles E.: Department of Psychiatry and Behavioural Neurosciences, McMaster University, Hamilton, ON, Canada Cunningham, Lesley J.: Hamilton-Wentworth District School Board, Hamilton, ON, Canada Ratcliffe, Jenna: Department of Psychiatry and Behavioural Neurosciences, McMaster University, Hamilton, ON, Canada Vaillancourt, Tracy: Faculty of Education, University of Ottawa, Ottawa, ON, Canada
ER -

TY - JOUR
ID - 2547
T1 - Children without toys: How home computer use impacts school achievement, behavior and attitudes
A1 - Marquis,Justin W.
Y1 - 2010///
N1 - Dissertation Abstract: 2010-99150-036
English
Dissertation Abstract
KW - PsycInfo
KW - Academic Achievement
KW - Attitudes

KW - Computers
KW - Technology
KW - Toys
KW - Middle Schools
KW - Middle School Students
RP - NOT IN FILE
SP - 538
JF - Dissertation Abstracts International Section A: Humanities and Social Sciences
VL - 71:
IS - 2-A
N2 - This study chronicles the home and school technology practices of a group of 7th grade middle school students representing varying social and economic backgrounds for the purpose of exploring possible ways in which exposure to computer technology in the home helps to inform the ways in which children demonstrate technology literacy when using computers in an academic setting. Further, this study seeks to illustrate that differences in the quality of access to technology and familial support at home are reflected in the behavior, attitude and achievement levels that these children exhibit in school when engaged in technology-related academic activities. For the purpose of illustrating the exhibited differences in technological literacy skills of the students under consideration, the study describes in detail both the type and depth of computer use in the home; including familial support, parental and sibling knowledge and attitudes and other non-academic influences outside of school. The findings of the study indicate that students without home computer access are at a significant disadvantage in relation to their peers in terms of developing technological literacy. (PsycINFO Database Record (c) 2012 APA, all rights reserved)
SN - 0419-4209
AD - Marquis, Justin W.: Indiana U., US
ER -

TY - JOUR
ID - 2548
T1 - A longitudinal analysis of the physical skills of students entering kindergarten
A1 - Johnson,Britton Thaddeus
Y1 - 2010///
N1 - Dissertation Abstract: 2010-99170-311
English
Dissertation Abstract
KW - PsycInfo
KW - Childhood Development
KW - Kindergartens
KW - Obesity
KW - Physical Activity
KW - Students
KW - Physique
RP - NOT IN FILE
SP - 888
JF - Dissertation Abstracts International Section A: Humanities and Social Sciences
VL - 71:
IS - 3-A
N2 - Concern about the level of physical skill development of young children has surfaced since the late 1900s due to the increase of childhood obesity and the decrease in opportunities for children to play freely outside. Increased usages of technology and changes in the way people live have also contributed to this concern. Project First Step began in 1991 to measure the physical skills of beginning kindergarten children to provide teachers with information on strengths and deficiencies in this domain. The focus of this research goes beyond a descriptive quantitative study looking at trends over the past 17 years of children's physical skills and includes aspects of a comparative descriptive design. In addition, an analysis of the physical skills that children possess during the final time period (2006-2008) will be addressed to assist teachers in developing strategies for young children's optimal learning. The works of Piaget and Gesell provide the theoretical base for this study. The results of this study showed that physical skill levels have dropped significantly from the 1991-1992 time period until the 2006-2008 time period. Significant drops occurred in 52 of the 66 t-tests, indicating that physical skill

levels are decreasing in all 10 areas screened and for overall skills as well. The scores were significantly lower in all areas in the 2006-2008 time period than in the 1991-1992 time period. The results of this study will help parents and educators to address the declining opportunities for children's physical skill development. (PsycINFO Database Record (c) 2012 APA, all rights reserved)
SN - 0419-4209
AD - Johnson, Britton Thaddeus: Walden U., US
ER -

TY - JOUR
ID - 2549
T1 - Exploring how experience with planning impacts first grade students' planning and solutions to engineering design problems
A1 - Portsmore,Merredith D.
Y1 - 2010///
N1 - Dissertation Abstract: 2010-99170-226
English
Dissertation Abstract
KW - PsycInfo
KW - Curriculum
KW - Engineering Psychology
KW - Experience Level
KW - Grade Level
KW - Teaching
KW - Cognitive Psychology
KW - Problem Solving
RP - NOT IN FILE
SP - 901
JF - Dissertation Abstracts International Section A: Humanities and Social Sciences
VL - 71:
IS - 3-A
N2 - This dissertation presents research that investigated how first grade students' ability to construct solutions and to plan through drawing for engineering design problems is related to their participation in a LEGO-based engineering curriculum with two variations on the instruction for planning. The quasi-experimental design engaged two first grade classrooms in an urban K-6 Science and Technology elementary school outside of Boston, MA in a set of activities that asked students to construct solutions to engineering design problems inspired by the story of Goldilocks and the Three Bears. The planning classroom was provided with instructions on how to plan and students were required to plan through drawing prior to constructing their solution to engineering design problems. Students in the spontaneous classroom were not given instruction in planning and were allowed immediate access to materials to construct their solution. Students' drawings and constructed artifacts for engineering design problems during pre and post assessments as well as during the classroom were collected. The analysis of the classroom data found that students were able to successfully construct solutions to engineering design problems with increasingly number of requirements. Pre and post comparisons of students' performance on problems with materials they had had extensive experience with (LEGO) and craft materials (non-LEGO) found that students only made gains in constructing solutions to engineering design problems with materials they had prior experience with. The planning intervention appeared to have no relationship with students' ability to construct solutions that addressed requirements that were clearly presented to the students. However, there may be a relationship with less obvious requirements regarding aesthetics (as measured by symmetry of the artifacts) and material selection. In general, the findings suggest that planning through drawing may help students preserve their ideas but that first grade students do not use drawings for planning in the same manner as adults do. Implications for engineering education for early elementary are also discussed. (PsycINFO Database Record (c) 2012 APA, all rights reserved)
SN - 0419-4209
AD - Portsmore, Merredith D.: Tufts U., US
ER -

TY - JOUR
ID - 2550

T1 - Making the margin visible: Out-of-school literacy practices among Mexican heritage English learners in an English-only district
A1 - Burrows-Goodwill,Shivani Linda
Y1 - 2010///
N1 - Dissertation Abstract: 2010-99111-086
English
Dissertation Abstract
KW - PsycInfo
KW - Cohort Analysis
KW - English as Second Language
KW - Foreign Language Learning
KW - Literacy
KW - Urban Environments
KW - Family
KW - Summer Camps (Recreation)
RP - NOT IN FILE
SP - 4553
JF - Dissertation Abstracts International Section A: Humanities and Social Sciences
VL - 70:
IS - 12-A
N2 - The Hispanic academic achievement gap in California is often attributed to language difference, low economic status, and assumed cultural deficit within families (Neufeld & Fitzgerald, 2001; Saracho, 2007). When students who fit these descriptors achieve academic scores of proficiency and above, we can benefit from examining their repertoires of family literacy practices. This qualitative study examined the summer literacy practices within the nine Spanish-speaking families of a second grade cohort attending a small Title 1 urban school in Southern California. At home adult support was in Spanish, and material means were limited by low-income status, yet six of the nine achieved above average on state tests in English. In this descriptive, two-phase study I used semi-structured interviews and video elicitation to answer these questions: (1)What do Mexican-heritage children do during the summer vacation? (2)What funds of literacy are available to them? (3)How do home and community ecological and cultural-linguistic contexts influence their engagement with these funds of literacy? The goal was to identify patterns of literacy engagement with local resources as they emerged through daily family routines within an apparently homogeneous sample. I used the framework of ecocultural activity settings to capture literacy events involving adults and children in their naturalistic settings. Inductive analysis of multiple data sources revealed a wide range of practices and an abundance of literacy resources within each home. Four of the six higher achieving children were found to be involved in church or bible study community activities that required literacy in Spanish. Adults in these cases provided structure and engagement to summer practices, with the purpose of moral education. In two of the cases fathers were the dominant agents of literacy. In all cases older siblings mediated practices in English. These findings complicate the deficit assumptions associated with low-income, linguistic minorities by providing illustrating how families leverage local funds of literacy to support their children's overall literacy development. The study claims the need for a new framework of literacy research that integrates the role out-of-school literacy activities in overall literacy development, and the need to acknowledge the literacy resources Mexican-heritage students bring to the classroom. (PsycINFO Database Record (c) 2012 APA, all rights reserved)
SN - 0419-4209
AD - Burrows-Goodwill, Shivani Linda: U California, San Diego, US
ER -

TY - JOUR
ID - 2551
T1 - An investigation of the effects of portable play equipment to increase physical activity levels of preschool children
A1 - Quinones Padovani,Carlos E.
Y1 - 2010///
N1 - Dissertation Abstract: 2010-99110-326
English
Dissertation Abstract
KW - PsycInfo

KW - Apparatus
KW - Childhood Play Behavior
KW - Early Childhood Development
KW - Physical Activity
KW - Preschool Students
KW - Activity Level
KW - Exercise
RP - NOT IN FILE
SP - 4568
JF - Dissertation Abstracts International Section A: Humanities and Social Sciences
VL - 70:
IS - 12-A
N2 - The purpose of this study was to investigate the effects of a portable play equipment intervention intended to increase the PA levels of preschool children during outdoor classroom time. The following hypotheses were formulated: (a) the intervention using portable play equipment will increase the PA of all children during their outdoor classroom period as measured by pedometer steps. (b) boys will be more physically active as measured by pedometer steps than girls during the preschool outdoor classroom period. The research questions addressed in the study were: (a) what types of activities do preschool children participate in during their outdoor classroom period? (b) what type of portable play equipment is used most often by preschool children during their outdoor classroom period? and (c) is there a difference in the activities and equipment participated in between preschool boys and girls during their outdoor classroom period? An ABAB reversal design was used to study the physical activity measured by pedometers (Yamax New Lifestyles Digi-Walker SW - 701) in preschool children when the portable play equipment was introduced to the outside classroom. The first intervention phase was using portable play equipment and the second intervention phase used a teacher to encourage active play for four randomly selected children. A modified version of the Observational System for Recording Physical Activity in Children-Preschool Version (OSRAC-P) (Brown et al., 2006) was used to code the activities and the intensity of the PA the children did during outside classroom time. A repeated-measure by time analysis was completed to see the differences between baseline phase and intervention phase data. The same analysis was done to see the differences between the teacher intervention phase and baseline phase. The significance level was set to $p< 0.05$. The PA change between the first baseline and the first intervention was statistically significant. The PA levels significantly increased when the portable equipment was introduced the first time to the outside classroom time. However, the change between the second baseline and the second intervention was not statistically significant. Finally the teacher intervention for the four randomly selected students was not statistically significant either. The results of the study do not support the first hypothesis even though there were statistical differences between the first baseline and the first intervention. The intervention using portable play equipment did not increase PA of all children during their outdoor classroom period as measured by pedometer steps. The second hypothesis is not supported by this study. Boys were not more physically active than girls as measured by pedometer steps. The amount of steps for boys and girls were similar throughout the study. The results indicate that portable play equipment with teacher encouragement has the potential to increase the physical activity levels of preschool children but more research is necessary to understand the complexity of behavior of this age group. (PsycINFO Database Record (c) 2012 APA, all rights reserved)
SN - 0419-4209
AD - Quinones Padovani, Carlos E.: The Florida State U., US
ER -

TY - JOUR
ID - 2552
T1 - Perceptions of at-risk middle school students participating in a support program to foster school connectedness and the ethic of care
A1 - Rigby,Gerard F.
Y1 - 2010///
N1 - Dissertation Abstract: 2010-99110-205
English
Dissertation Abstract
KW - PsycInfo
KW - At Risk Populations
KW - Ethics

KW - Life Experiences
KW - Middle School Students
KW - Perception
KW - Middle Schools
RP - NOT IN FILE
SP - 4576
JF - Dissertation Abstracts International Section A: Humanities and Social Sciences
VL - 70:
IS - 12-A
N2 - Although much of the violence prevention literature has focused on evaluating intervention programs, few studies have included the perceptions of students in the effort to help them. The goal of this study was to deepen our understanding of their lived experiences and consider the implications for more effective school policies and programs. It is critical to understand what helps at-risk students feel connected to school and want to actively participate in the effort to establish safe leaning communities. This study is based on a qualitative research design to include the voice of students on the problems they have experienced in traditional classes, as well as the supports to help them achieve success. Students were considered at-risk because they had experienced difficulty in the past with academics, behavior and their social interactions at school. The decision to focus on middle school students was made given the extent of bullying behaviors at that level and the need to understand what helps them care about their education and want to do well in their classes. The methods used to collect data included individual and group interviews, visual data in the form of photographs taken by students and two direct observations of their support program classes. All of the data in the study was managed and analyzed through the use of a qualitative data analysis software, specifically NVivo 2.0. It was then organized into an electronic project, called the E-Project , which contained all of the documents generated within the study, including raw data and interpretative memos, as well as specific codes and links within the data utilized for analysis. Each of the students initially provided examples as to why they had become so disconnected in their traditional classes and no longer wanted to achieve success. They repeatedly emphasized a lack of care as the most significant problem they experienced and the failure of teachers to establish meaningful connections with them at school. Although academic issues were at the inner core of their concerns, they expressed a significant problem with how their behavior was managed and the wider social dynamics that impacted all of their experiences. Students focused on the beliefs and actions of teachers who allowed them to experience a pattern of failure and utilized punitive discipline that further isolated them in their classes. They especially voiced frustration and anger at the hypocrisy of teachers who said they cared, yet blamed and humiliated them in front of peers without regard for their fundamental social and emotional needs. As all of the students in the study were participating in a support program, they were also able to identify the academic, behavioral and social experiences that helped them actively participate and become involved at school. Students emphasized the need for teachers to believe it is their responsibility to find solutions to the problems they experienced and take the actions necessary to make a positive difference in their lives. They wanted teachers to genuinely care about them and not accept failure as a choice they can make under any conditions. It was important for teachers to stop the bullying behaviors that prevented students from taking academic risks without the fear of being embarrassed or shamed in front of their peers. It was essential for teachers to establish a safe classroom so they could enjoy working together with a sense of community. Students consistently voiced throughout the data the importance of having teachers not only help them achieve academic success, but also recognize their lives outside of the classroom. They emphasized the profound need to be included and valued as important members of the school community and explained how their academic, behavioral and social needs are connected to each other. It was important to celebrate their accomplishments with traditions. (PsycINFO Database Record (c) 2012 APA, all rights reserved)
SN - 0419-4209
AD - Rigby, Gerard F.: U Massachusetts Lowell, US
ER -

TY - JOUR
ID - 2553
T1 - The design of a lunchtime relational aggression prevention program targeting elementary school aged females
A1 - Elkinson,Lauren Brooke
Y1 - 2010///

N1 - Dissertation Abstract: 2010-99090-300
English
Dissertation Abstract
KW - PsycInfo
KW - Elementary School Students
KW - Elementary Schools
KW - Problem Solving
KW - Aggressive Behavior
KW - Prevention
RP - NOT IN FILE
SP - 4176
JF - Dissertation Abstracts International Section A: Humanities and Social Sciences
VL - 70:
IS - 11-A
N2 - Aggression, in all of its forms, is a growing problem in today's schools. Relational aggression, the attempt to harm another person through manipulation and damage of relationships with others, is a specific form of aggression that is not only increasingly prevalent, but also undertreated (Crick & Gropeter, 1995). Children gossip about peers, exclude each other from activities, and engage in name-calling, among other relationally aggressive behaviors. Females are more likely to engage in this behavior; males tend to engage in more physically aggressive behaviors (Yoon, Barton, & Taiariol, 2004). While frequently dismissed by teachers and administrators as "typical" childhood behavior, studies have shown that relational aggression can be as harmful as physical aggression (Bauman & Del Rio, 2006; Yoon et al., 2004). The purpose of this dissertation is to design and develop a classroom program for a central New Jersey public school to be implemented with third to fifth grade female students during lunch, an unstructured time of day during which children often engage in relational aggression with peers. The program is based on a thorough needs assessment conducted in the middle school for which the program is being designed (Maher, 2000). The program design also relies on Dodge's (1986) Information Processing Model as a foundation for its design (Lochman & Dunn, 1993). The needs assessment examined students' peer relationships and students' bullying behaviors through student and teacher questionnaires. The program consists of ten classroom-based lessons that address bullying, relationship skills, anger management, and problem solving. The other part of the program is a teambuilding component for implementation during lunch and recess. The dissertation includes detailed lesson plans as well as session outlines for teambuilding activities. Also included are introductory lessons, which provide school personnel with a clear lesson for introducing each component of the program to students Finally, the limitations of the program, implications for the program's use, and future directions are also discussed. (PsycINFO Database Record (c) 2012 APA, all rights reserved)
SN - 0419-4209
AD - Elkinson, Lauren Brooke: Rutgers The State U New Jersey, Graduate School of Applied and Professional Psychology, US
ER -

TY - JOUR
ID - 2554
T1 - Relationships between students' fitness levels and academic achievement
A1 - Roberts,Theresa Linam
Y1 - 2010///
N1 - Dissertation Abstract: 2010-99070-246
English
Dissertation Abstract
KW - PsycInfo
KW - Academic Achievement
KW - Health
KW - Physical Fitness
KW - Educational Psychology
RP - NOT IN FILE
SP - 3794
JF - Dissertation Abstracts International Section A: Humanities and Social Sciences
VL - 70:

IS - 10-A
N2 - This study compared fifth grade students' physical fitness levels to academic achievement based on the premise that health and physical fitness has an effect on one's ability to learn and achieve academically. Due to No Child Left Behind and the mounting pressures to reach Adequate Yearly Progress, many school officials view non-assessed activities like physical education and recess as unnecessary, consequently creating a case for the elimination of any subject that is not directly measured through standardized testing. Finding a link between fitness and academic achievement may cause educational leaders to reevaluate time spent during the school day. Data was collected for 113 students during the 2008-2009 school year by using the FitnessGram, STAR Reading and Math Percentiles, and Grade Point Averages (GPA's). Through multiple regression, the researcher found statistically significant relationships between physical fitness and two of the three measures used for academic achievement: STAR Math Percentiles ($p = 0.0063 < 0.05$; $R = 0.26 > 0.195$) and GPA's ($p = 0.0124 < 0.05$; $R = 0.23 > 0.195$). Therefore, the hypothesis was accepted, validating a link between fitness and academic achievement. This study does not prove causality; it is more probable that physical fitness and academic achievement influence each other in ways that are still vague. (PsycINFO Database Record (c) 2012 APA, all rights reserved)
SN - 0419-4209
AD - Roberts, Theresa Linam: Liberty U., US
ER -

TY - JOUR
ID - 2555
T1 - Interpretations of bullying: How students, teachers, and principals perceive negative peer interactions in elementary schools
A1 - Harger,Brent
Y1 - 2010///
N1 - Dissertation Abstract: 2010-99070-129
English
Dissertation Abstract
KW - PsycInfo
KW - Elementary Schools
KW - Peer Relations
KW - School Violence
KW - Teachers
KW - Bullying
KW - Peers
KW - School Principals
RP - NOT IN FILE
SP - 4076
JF - Dissertation Abstracts International Section A: Humanities and Social Sciences
VL - 70:
IS - 10-A
N2 - Despite increased media attention following high-profile school shootings, bullying continues to be defined as a nationwide problem. While researchers in a number of fields have studied this problem, much of the existing literature ignores information about how individuals in schools actually define and interpret bullying. In order to better understand these interactions, my dissertation is a multi-method study combining interviews with 53 students and 10 adults and over 430 hours of participant observation with fifth grade students at two rural elementary schools. Drawing on the sociological tradition of symbolic interactionism, these methods allow me to understand bullying from the perspectives of those in the schools. I argue that bullying is socially constructed by students and adults who take social contexts into account when determining whether or not an action should be defined as bullying and how they will respond. While adults in this study focused on outcomes, students focused on intentions and included a number of caveats in their definitions, stating that those who were joking, retaliating, or making fun of younger students were not engaging in bullying. Further, the images associated with the word "bully" in popular culture led a number of participants to hold views of bullying that focused on people rather than actions, thinking of bullies as those who were always mean. These person-centered definitions allowed them to "define away" bullying as a problem in their schools, despite the continued presence of interactions that fit typical definitions of bullying. Because they could not directly observe all of the interactions taking place in a classroom, at lunch, or on the playground, bullying often went

unobserved by adults. As a result, students were able to use the school rules as weapons against each other, selectively reporting rule violations by peers that they disliked, whether or not their peers were guilty of those violations. These findings demonstrate the ways that bullying is a part of the culture in these elementary schools. As such, solutions to this problem demand an understanding of and consideration for the larger school culture in order to improve students' daily experiences at school. (PsycINFO Database Record (c) 2012 APA, all rights reserved)
SN - 0419-4209
AD - Harger, Brent: Indiana U., US
ER -

TY - JOUR
ID - 2556
T1 - Self-control and perceived control as mediators of the relationship between molecular family stability and adjustment
A1 - Sokolowski,Karen L.
Y1 - 2010///
N1 - Dissertation Abstract: 2010-99060-160
English
Dissertation Abstract
KW - PsycInfo
KW - Child Attitudes
KW - Daily Activities
KW - Family Relations
KW - Self Control
KW - Adjustment
RP - NOT IN FILE
SP - 5848
JF - Dissertation Abstracts International: Section B: The Sciences and Engineering
VL - 70:
IS - 9-B
N2 - Molecular family stability can be defined as the predictability and consistency of the daily activities and routines of the family, including activities in the home as well as activities occurring outside the home that are arranged and supported by the family. Previous research has demonstrated a relationship between the constructs of molecular family stability and child adjustment, with greater family stability associated with better child adjustment. The current study evaluates several control-related constructs as potential mechanisms through which the relationship between family stability and child adjustment may operate. Self-control and aspects of perceived control were assessed as possible mediators of this relationship. The specific aspects of perceived control included were perceived primary, secondary, and anxiety control. It was hypothesized that self-control and perceived control would mediate the relationship between family stability and child adjustment. Participants were 67 children in grades 3 through 8 and their parents. Children and parents were each asked to complete measures of family stability, self-control, perceived control, and adjustment. Multiple regression analyses were used to evaluate the hypothesized mediational model. Results generally support the hypotheses that self-control and perceived primary control mediate the relationship between molecular family stability and adjustment; however, the hypotheses that perceived secondary and anxiety control mediate the relationship between family stability and adjustment were not supported. Additionally, evidence emerged suggesting a suppression situation in which perceived primary control, child age, and family income appear to serve as suppressors revealing a significant and positive relationship between child-reported molecular family stability and one particular aspect of adjustment, anxiety symptoms. Results are discussed in terms of avenues for further research and potential clinical applications of the construct of molecular family stability. (PsycINFO Database Record (c) 2012 APA, all rights reserved)
SN - 0419-4217
AD - Sokolowski, Karen L.: State U New York at Albany, US
ER -

TY - JOUR
ID - 2557

T1 - Effects of peer-mediated intervention on social competence in children with autism spectrum disorders (ASD)
A1 - Cunningham,Megan P.
Y1 - 2010///
N1 - Dissertation Abstract: 2010-99020-272
English
Dissertation Abstract
KW - PsycInfo
KW - Intervention
KW - Peer Relations
KW - Pervasive Developmental Disorders
KW - Social Skills
RP - NOT IN FILE
SP - 4478
JF - Dissertation Abstracts International: Section B: The Sciences and Engineering
VL - 70:
IS - 7-B
N2 - Children with Autism Spectrum Disorders (ASD) manifest a triad of deficits, in the areas of socialization, communication, and repetitive and stereotyped behaviors (American Psychiatric Association, 2000; Volkmar, 2005). Tailoring interventions to address these issues is important when working with children with ASD. It is critical to remember that although children with ASD share common deficits, each child is entirely unique. Interventions must be tailored to each child's specific needs and interests. The current study assessed the effectiveness of peer-mediated intervention on increasing social skills in a sample of 6, 5 to 9 year old children with ASD. The intervention was run class-wide, during students' recess time. Although perhaps the most naturalized setting to practice socialization goals, recess remains one of the least utilized limes to address goals during the school day. Peer-mediated intervention addresses goals in the most naturalistic of settings, actual peer interactions, while minimizing cost in teacher time and expense to the district. Participants were paired and instructed to play with typically developing buddies. Buddy pairs were rotated to promote exposure to multiple peer exemplars. Intervention occurred in 3 weekly sessions over 6 weeks, for a total of 18 sessions. Prior to intervention, 2 weeks of baseline data was collected on participants' social bids. Social bids were adapted from those assessed in Baumringer's (2002) study. Doctoral students were trained in the fidelity of treatment, and once 80% reliable on the operationally defined behaviors, collected data on the target dyads. Data was collected on frequency of social bids and percent of time engaged with peers during baseline, intervention, and at follow-up. Social bids were defined as any behaviors that functioned to maintain or initiate peer interaction (Baumringer, 2002). In addition to direct observational data, teachers and parents completed The Social Skills Rating System (SSRS; Gresham & Elliot, 1990) prior to and following intervention. The study utilized a single subject (N=6), ABA design. Results were assessed via visual inspection of data. Busk and Serlin's (1992) techniques were also used to monitor effect sizes for each participant. It was hypothesized that following the intervention, participants would demonstrate: (1) an increased frequency of social bids; (2) an increase in percent of time spent engagement with peers; and (3) increased levels of overall social skills from pre to post assessment as measured by the SSRS-Teacher Form. Results demonstrated strong support for Hypothesis 1. Five of the 6 participants demonstrated increases in the frequency of bids. Results also demonstrated strong support for Hypothesis 2. All participants demonstrated significant increases in percent engagement with peers. Hypothesis 3 was not supported; analysis of teacher ratings did not indicate consistent gains in social skills. Implications for future research are discussed. (PsycINFO Database Record (c) 2012 APA, all rights reserved)
SN - 0419-4217
AD - Cunningham, Megan P.: Hofstra U., US
ER -

TY - JOUR
ID - 2558
T1 - Parental adherence to a brief intervention for managing bedtime problems in preschoolers
A1 - Seng,Alexandra Watkins
Y1 - 2010///
N1 - Dissertation Abstract: 2010-99020-231
English
Dissertation Abstract

KW - PsycInfo
KW - Behavior Modification
KW - Behavior Problems
KW - Intervention
KW - Parents
RP - NOT IN FILE
SP - 4496
JF - Dissertation Abstracts International: Section B: The Sciences and Engineering
VL - 70:
IS - 7-B
N2 - The effectiveness of an intervention is dependent on one's active implementation of key intervention elements. In the child mental health field, an intervention often requires parents to implement certain behavior modification strategies in the home. There are a number of factors that could influence whether intervention strategies are used outside of initial training. However, little research has examined the factors related to parenting adherence to treatment strategies. This study explored whether the same factors that are related to initial parental engagement (i.e., child, parent and family contextual factors) also influence subsequent use of intervention strategies presented in a one-time workshop. Participants were parents of children (3-5 years old) who reported difficulties at bedtime. Participants attended a one time workshop, completed pre and post workshop measures and were contacted two and four weeks post-workshop to assess their level of adherence to strategies and the degree of bedtime problems their child was experiencing. Cognitive variables, adapted from the Theory of Planned Behavior and The Health Belief Model, were expected to mediate the influence of child, parent and family factors on adherence. Contrary to hypotheses, perceived benefits, barriers and parent expectations did not significantly mediate the path to adherence. A decrease in bedtime problems was seen across groups. Those parents who most closely adhered to the evidence-based strategies saw the greatest amount of improvement four weeks after the workshop. Limitations of the current study and suggestions for future research are discussed. (PsycINFO Database Record (c) 2012 APA, all rights reserved)
SN - 0419-4217
AD - Seng, Alexandra Watkins: U South Carolina, US
ER -

TY - JOUR
ID - 2559
T1 - A sciencing programme and young children's exploratory play in the sandpit. [References]
A1 - van Schijndel,Tessa J.
Y1 - 2010///
N1 - Peer Reviewed Journal: 2010-19140-005
English
Journal; Peer Reviewed Journal
KW - PsycInfo
KW - Childhood Play Behavior
KW - Childhood Play Development
KW - Educational Programs
KW - Playgrounds
RP - NOT IN FILE
SP - 603
EP - 617
JF - European Journal of Developmental Psychology
VL - 7:
IS - 5
N2 - A six-week sciencing programme, directed at stimulating exploratory play, was implemented with 2- and 3-year-olds in a day-care centre. The core of the programme consisted of guided play with children in the centre's sandpit. The effectiveness of the programme was determined with ecologically valid methods consisting of pre- and post-observations of children's exploratory behaviour during free sandpit play in the experimental group as well as in a control group. A systematic observation scheme for exploratory play, the Exploratory Play Scale, was used for this purpose. The experimental group showed an increase in level of exploratory play from pre- to post-observations, while the control group did not. This study shows that a small-scale sciencing

programme can have an effect on children's level of free exploratory play. (PsycINFO Database Record (c) 2012 APA, all rights reserved) (journal abstract)
SN - 1740-5629
AD - van Schijndel, Tessa J. P.: University of Amsterdam, Amsterdam, Netherlands Singer, Elly: University of Amsterdam, Amsterdam, Netherlands van der Maas, Han L. J.: University of Amsterdam, Amsterdam, Netherlands Raijmakers, Maartje E. J.: University of Amsterdam, Amsterdam, Netherlands
ER -

TY - JOUR
ID - 2560
T1 - Stepping toward physical activity requirements: Integrating pedometers into early childhood settings. [References]
A1 - Robinson,Leah E; Wadsworth,Danielle
Y1 - 2010///
N1 - Peer Reviewed Journal: 2010-14776-003
English
Journal; Peer Reviewed Journal
KW - PsycInfo
KW - Early Childhood Development
KW - Health
KW - Monitoring
KW - Physical Activity
KW - Quality of Life
KW - Apparatus
KW - Obesity
RP - NOT IN FILE
SP - 95
EP - 102
JF - Early Childhood Education Journal
VL - 38:
IS - 2
N2 - Physical activity is an essential component for lifelong wellness and the quality of life. Over the past years, childhood obesity has dramatically increased. Data supports that young children are adopting sedentary behaviors within and outside of school hours that may contribute to obesity and other health-related diseases. This paper provides early childhood practitioners with an overview of physical activity recommendations for young children between the ages of three to five and cost-effective means to assess physical activity through step count pedometers. Finally, some pedometer activities are provided along with a description of the children's reactions and behaviors to the integration of pedometers. Both the activities and pedometer appear to be easily incorporated into any classroom or recess settings to get your children moving and active. (PsycINFO Database Record (c) 2012 APA, all rights reserved) (journal abstract)
SN - 1082-3301
AD - Robinson, Leah E.: Auburn University, Auburn, AL, US Wadsworth, Danielle D.: Auburn University, Auburn, AL, US
ER -

TY - JOUR
ID - 2561
T1 - Playing for keeps: Life and learning on a public school playground. [References]
A1 - Meier,Deborah; Engel,Brenda S; Taylor,Beth
Y1 - 2010///
N1 - Book: 2010-13714-000
English
Book; Authored Book
(Created by PsycINFO) (Abbreviated) Preface Acknowledgments Note on authorship Introduction Part I: Sources of play Domesticity Scary stuff The environment The curriculum: "Long ago and far away" Part II: Aspects of play "Wishful thinking" Laws, rules, and understandings Playing games Age, gender, and race Influence of the media: TV, comics, toys, films, and video games Epilogue: Wondering about the future

Appendix A: Mission Hill school, a Boston pilot school Appendix B: The Mission Hill newsletter References Further reading Index About the authors
KW - PsycInfo
KW - Childhood Play Behavior
KW - Playgrounds
KW - School Facilities
RP - NOT IN FILE
SP - New
JF - (2010)
VL - Playing for keeps: Life and learning on a public school playground. xiii, 127 pp. New York, NY, US: Teachers College Press; US.
N2 - (from the cover) Why is play important in the lives of children? What crucial aspects of learning are being neglected in the current near-elimination of recess time in public schools? Playing for Keeps, coauthored by the well-known writer and educational leader Deborah Meier and two colleagues with equally long experience in schools, explores these questions. Based on close observations on a public school playground, the book shows children at play in a relatively natural, unstructured environment. The reader is virtually there, seeing, listening in, able to appreciate the children's curiosity, humor, intelligence, and inventiveness. Readers will recognize the children's voices and ways of thinking, and perhaps be reminded of their own childhood, their own children, or the children they teach. The authors comment on the observations, adding to the reader's own perceptions. This lively, engaging book makes a strong case for the importance of free exploration, wonder, imagination, and play to the learning and growth of children. (PsycINFO Database Record (c) 2012 APA, all rights reserved)
AD - Meier, Deborah: Steinhardt School of Education, New York University, New York, NY, US Engel, Brenda S.: Lesley University, Cambridge, MA, US Taylor, Beth: Mission Hill School, Boston, MA, US
ER -

TY - JOUR
ID - 2562
T1 - Physical activity and sedentary behaviours in Hong Kong primary school children: Prevalence and gender differences. [References]
A1 - Lam,Jessica W.
Y1 - 2010///
N1 - Peer Reviewed Journal: 2010-12656-022
English
Journal; Peer Reviewed Journal
KW - PsycInfo
KW - Activity Level
KW - Childhood Development
KW - Epidemiology
KW - Human Sex Differences
KW - Physical Activity
KW - Primary School Students
RP - NOT IN FILE
SP - 96
EP - 97
JF - Preventive Medicine: An International Journal Devoted to Practice and Theory
VL - 51:
IS - 1
N2 - Low levels of physical activity (PA) and high amounts of sedentary behavior (SB) contribute to the onset and maintenance of various chronic diseases. we report on both PA and SB data in a representative population-based sample in Hong Kong children. Using self-reported data on weekly frequency and average duration of participation in Moderate-to-vigorous physical activity (MVPA) in and outside-school, weekly minutes spent on in-school, outside-school, and total MVPA were computed for each study participant. For sedentary activities,we computed total weekly minutes spent on: TV/video viewing; homework and other educational activities; computer use; electronic sedentary games; non-electronic sedentary games; and all types of sedentary activities. Children reported spending more time on outside-school than in-school MVPA, with the total amount of MVPA falling short of the PA recommendations for children of 420 min per week. A greater proportion of boys than girls were found to invest more time in computer use and electronic and non-electronic sedentary

games, while girls were likely to engage in homework/educational activities. The prevalence of SB associated with electronic media use is very high, with Hong Kong children spending nearly twice the time on such activities than on MVPA. Intervention should focus on the reduction of SB related to the use of electronic media. (PsycINFO Database Record (c) 2012 APA, all rights reserved)
SN - 0091-7435
AD - Lam, Jessica W. K.: University of Hong Kong, Institute of Human Performance, Pokfulam, Hong Kong Sit, Cindy H. P.: University of Hong Kong, Institute of Human Performance, Pokfulam, Hong Kong Cerin, Ester: University of Hong Kong, Institute of Human Performance, Pokfulam, Hong Kong
ER -

TY - JOUR
ID - 2563
T1 - Social and emotional learning in the classroom: Promoting mental health and academic success. [References]
A1 - Merrell,Kenneth W.
Y1 - 2010///
N1 - Book: 2010-09986-000
English
Book; Authored Book
(Created by PsycINFO) (Abbreviated) Social and emotional learning: What it is, and what it can do for your students Social and emotional learning curricula: A review of selected programs The essentials of using social and emotional learning in the classroom Using social and emotional learning to foster academic learning One size does not fit all: Adapting social and emotional learning for use in our multicultural world When social and emotional learning in the classroom is not enough: Linking students to mental health services Assessment and evaluation strategies in social and emotional learning Using social and emotional learning within school systems: Organizational dynamics and strategic planning References Index
KW - PsycInfo
KW - Classrooms
KW - Emotional Development
KW - Psychosocial Development
KW - School Learning
KW - Social Learning
KW - Academic Achievement
KW - Health Promotion
KW - Mental Health
RP - NOT IN FILE
SP - New
JF - (2010)
VL - Social and emotional learning in the classroom: Promoting mental health and academic success. xviii, 174 pp. New York, NY, US: Guilford Press; US.
N2 - (from the book) Social and emotional learning (SEL) in the classroom is our message in this book for promoting mental health and academic success. Although the specific techniques, strategies, and concepts of SEL are not new, it has only been the past few years-perhaps since the mid-1990s-that SEL has been articulated as a cohesive framework and has gradually evolved into a field with a huge potential reach and influence. Through the efforts of individual prevention scientists and practitioners, such as those who founded the Collaborative for Academic, Social, and Emotional Learning (www.casel.org), SEL has matured and expanded to the point where it is beginning to make a significant difference in our schools and communities. As you will see in the research cited, the potential impact of SEL is most impressive, although still in its nascent stages. Hence, we see this book as an "A-to-Z" guide to spur educators and mental health professionals to start using SEL in a cohesive and structured way in schools and related settings. The eight chapters of this book are not exhaustive by any means, but they will take you through almost every aspect of using SEL, from planning and getting support for it, to selecting curriculum tools, to delivering content in a way that fits your students and communities, to measuring and evaluating the outcomes that you produce. Chapter 1 introduces and defines SEL and realistically assesses what it can offer you and your students. Chapter 2 includes an overview of several of the most widely used and researched SEL programs currently available for adoption. Chapter 3 covers the essentials of using SEL in the classroom, including a wide variety of practical material ranging from preparing your students and selecting materials to developing your own plans for SEL activities if you are not

using a packaged program. Chapter 4 explores the link between SEL and academic learning in schools and contains many practical examples and suggestions for how SEL programs and strategies can be woven into the fabric of the curriculum in a manner that supports academic learning. Recognizing that delivery of SEL programming in schools occurs in a complex and increasingly diverse and pluralistic social context, Chapter 5 addresses the need for adapting SEL content to make it culturally appropriate in specific settings and includes a practical, step-by-step guide for doing so. Although we obviously highly value SEL as a mental health promotion tool in schools, we recognize that basic classroom-based SEL programming alone is not enough to fully meet the mental health needs of our students with the most severe and complex problems. Thus, Chapter 6 focuses on enhancing SEL to maximize its benefit to the most distressed students and examines practical ways to help link those students with expanded mental health programming in the broader community. Chapter 7 is a basic primer on using social-emotional assessment, measurement, and evaluation strategies within SEL and briefly overviews some exciting new tools and innovations in this area, some of which are literally "just out the door." And finally, Chapter 8 delves into the intricacies of using SEL within complex organizations such as schools, providing a solid foundation for understanding how systems change, as well as practical strategies for planning and gaining support for using SEL in your own organization. (PsycINFO Database Record (c) 2012 APA, all rights reserved)
AD - Merrell, Kenneth W.: University of Oregon, Eugene, OR, US Gueldner, Barbara A.: Parker Pediatrics and Adolescents, Denver, CO, US
ER -

TY - JOUR
ID - 2564
T1 - Technology and young children: How 4-7 year olds perceive their own use of computers. [References]
A1 - McKenney,Susan
Y1 - 2010///
N1 - Peer Reviewed Journal: 2010-08998-022
English
Journal; Peer Reviewed Journal
KW - PsycInfo
KW - Computer Attitudes
KW - Ethnic Values
KW - School Environment
KW - Socioeconomic Status
KW - Technology
RP - NOT IN FILE
SP - 656
EP - 664
JF - Computers in Human Behavior
VL - 26:
IS - 4
N2 - This study examined young children's access, perceptions and use of technology within and outside of school settings. One hundred sixty seven children from varied ethnic and socio-economic backgrounds participated in the study. Regardless of gender, socio-economic status or ethnic group, most children had access to computers in and outside of schools. In both settings, pre-K through grade 2 children report that they primarily use computers to play games. Children's attitudes toward computers are positive. Older children tend to use the computer more often, are better able to operate the computer and their attitudes towards computers are more positive. Boys' attitudes towards computers are more positive than the attitudes of girls, but no gender differences were found for computer use nor ability level. While no significant differences were found between the attitudes of Dutch and immigrant children, the latter group indicated more frequent use. Also, children from a lower socio-economic neighborhood had more positive attitudes towards computers and used computers slightly more often than middle class children. The findings of this study inform the debate on the desirability of young children's exposure to computers at home as well as in educational settings. Further, these findings may help educators and parents to both critically assess their current practices (e.g. the relative value of the most frequently used applications-games), and strive to integrate developmentally appropriate uses of technology at home and in classrooms. (PsycINFO Database Record (c) 2012 APA, all rights reserved) (journal abstract)
SN - 0747-5632

AD - McKenney, Susan: Department of Curriculum Design & Educational Innovation, University of Twente, Enschede, Netherlands Voogt, Joke: Department of Curriculum Design & Educational Innovation, University of Twente, Enschede, Netherlands
ER -

TY - JOUR
ID - 2566
T1 - Places to avoid: Population-based study of student reports of unsafe and high bullying areas at school. [References]
A1 - Vaillancourt,Tracy; Brittain,Heather; Bennett,Lindsay; Arnocky,Steven; McDougall,Patricia; Hymel,Shelley; Short,Kathy; Sunderani,Shafik; Scott,Carol; Mackenzie,Meredith; Cunningham,Lesley
Y1 - 2010///
N1 - Peer Reviewed Journal: 2010-04757-004
English
Journal; Peer Reviewed Journal
KW - PsycInfo
KW - Safety
KW - School Environment
KW - Student Attitudes
KW - Bullying
RP - NOT IN FILE
SP - 40
EP - 54
JF - Canadian Journal of School Psychology
VL - 25:
IS - 1
N2 - Students' perceptions of school safety and experiences with bullying were examined in a large Canadian cohort of 5,493 girls and 5,659 boys in Grades 4 to 12. Results indicate notable differences in when and where students felt safe based on their own perceptions of safety and their own experiences with bullying, particularly across elementary and secondary schools. For elementary students, especially those involved in bullying, the playground/school yard and outside recess/break time were particularly hazardous, whereas for secondary students involved in bullying, the hallways, school lunchroom/cafeteria, and outside recess/break were considered especially dangerous. The commonality across student-identified unsafe areas is that they tend to not be well supervised by school personnel. Accordingly, the present results underscore the need to increase adult supervision in areas in which an overwhelming majority of students report feeling unsafe. (PsycINFO Database Record (c) 2012 APA, all rights reserved) (journal abstract)
SN - 0829-5735
AD - Vaillancourt, Tracy: Children's Mental Health and Violence Prevention, University of Ottawa, Ottawa, ON, Canada Brittain, Heather: McMaster University, Hamilton, ON, Canada Bennett, Lindsay: McMaster University, Hamilton, ON, Canada Arnocky, Steven: Department of Psychology, Neuroscience, & Behaviour, McMaster University, Hamilton, ON, Canada McDougall, Patricia: Department of Psychology, St. Thomas More College (STM), University of Saskatchewan, Saskatoon, SK, Canada Hymel, Shelley: Faculty of Education, University of British Columbia, Vancouver, BC, Canada Short, Kathy: Hamilton-Wentworth District School Board, Hamilton, ON, Canada Sunderani, Shafik: Department of Psychology, Neuroscience, & Behaviour, McMaster University, Hamilton, ON, Canada Scott, Carol: McMaster University, Hamilton, ON, Canada Mackenzie, Meredith: McMaster University, Hamilton, ON, Canada Cunningham, Lesley: Hamilton Wentworth District School Board, Hamilton, ON, Canada
ER -

TY - JOUR
ID - 2567
T1 - Understanding and generalizing communication patterns in children with selective mutism. [References]
A1 - Zelinger,Laurie
Y1 - 2010///
N1 - Book: 2010-04666-017
English
Book; Edited Book

KW - PsycInfo
KW - Communication
KW - Elective Mutism
KW - Play Therapy
KW - School Based Intervention
KW - Psychotherapeutic Techniques
KW - Therapists
RP - NOT IN FILE
SP - 359
EP - 377
JF - Drewes, Athena A [Ed]; Schaefer, Charles E [Ed]
VL - :
IS - 2010
N2 - (from the chapter) Working with a child with selective mutism requires knowledge of the characteristics of the disorder, training in appropriate therapeutic techniques, the ability of the therapist to project personal qualities of patience and acceptance, and the therapist's ability to work as part of a team within the school. Behavioral contracting, use of classroom and curriculum modifications, determination of test accommodations, provision of parent and teacher training, and coordination of related services are all likely elements in an overall plan at one time or another and may fall within the play therapist's arena if she takes on a consulting role. Likewise, information from outside sources (i.e., psychotherapy or psychopharmacology) may filter through her. The play therapist has the unique position of understanding the student and gaining his or her trust, as they work to minimize the impact of the disorder and facilitate typical social patterns. She will be the conduit for change in the child as well as the facilitator of generalization of these behaviors to the classroom setting. The play therapist's application of unique skills, position within the school team, knowledge of selective mutism, and personal involvement, will be the variables responsible for increasing mainstream social behaviors in the child who could not do it alone. (PsycINFO Database Record (c) 2012 APA, all rights reserved)
AD - Zelinger, Laurie: Oceanside Public Schools, Oceanside, NY, US
ER -

TY - JOUR
ID - 2568
T1 - Group sandplay in elementary schools. [References]
A1 - Kestly,Theresa
Y1 - 2010///
N1 - Book: 2010-04666-013
English
Book; Edited Book
KW - PsycInfo
KW - Friendship
KW - Group Psychotherapy
KW - Play Therapy
KW - School Based Intervention
KW - Elementary School Students
KW - Elementary Schools
RP - NOT IN FILE
SP - 257
EP - 281
JF - Drewes, Athena A [Ed]; Schaefer, Charles E [Ed]
VL - :
IS - 2010
N2 - (from the chapter) "You mean you're just going to let us play?" asks a fifth-grade boy who is just beginning his first session in a sand tray friendship group at school. He and five other boys are going to play together for an hour using small trays of sand and miniature figurines. They will meet for the next 12 weeks to create scenes in their sand trays and then tell stories about their worlds if they choose. It is hard for the boys to believe they are actually going to play during school time. Unless it is recess time, the idea of play with other children during school hours is a surprise for many children and for adults as well. Even more surprising is the idea that fundamental behavior changes can occur in children in a relatively short period of time through playing

together and telling stories to one another. Several months after one sand tray friendship group ended, the school principal noticed that the boys from the group had not been referred to her office since they had participated in the group. She was surprised. This group of fifth-grade boys, referred primarily for fighting on the playground and disruptive behavior in the classroom, had been frequent visitors to her office before they started the sand tray group play. The principal was especially impressed by their ability to maintain their behavior changes several months after the group ended, and as a result, she requested a significant increase in the number of friendship groups for the following school year. Once again, the principal noted a dramatic decrease in referrals to her office among the children who participated in the expanded group sandplay program. Although the structure of the friendship groups is simple in form and function, the underlying concepts are grounded in current neuroscience and theoretical principles. Why and how these sand tray friendship groups work is the focus of this chapter. (PsycINFO Database Record (c) 2012 APA, all rights reserved)
AD - Kestly, Theresa: Sand Tray Training Institute of New Mexico, Corrales, NM, US
ER -

TY - JOUR
ID - 2569
T1 - Using play therapy assessment in an elementary and intermediate school setting. [References]
A1 - Schmidt,Mary May
Y1 - 2010///
N1 - Book: 2010-04666-005
English
Book; Edited Book
KW - PsycInfo
KW - Educational Measurement
KW - Elementary Schools
KW - Play Therapy
KW - School Based Intervention
KW - School Environment
KW - Elementary School Students
KW - Intermediate School Students
RP - NOT IN FILE
SP - 107
EP - 122
JF - Drewes, Athena A [Ed]; Schaefer, Charles E [Ed]
VL - :
IS - 2010
N2 - (from the chapter) Any school-based assessment, whether it is time limited for a specific purpose or ongoing, is an inescapable feature of education. Assessment occurs daily within the classroom as children work through the curricula. Teachers monitor growth using multidimensional curriculum tools such as Rubrics to measure acquisition of readiness skills and then the academic skills themselves. When learning is not occurring at the hoped-for rate, staff teams, often called Child Study Teams, meet to suggest and design individualized remediation models. Sometimes, the interference with learning is related to social-emotional-behavioral problems of the target child, the child in his or her classroom setting, and/or the child in his or her family. The tools of play therapy can be a more informative venue than that of typical social-emotional assessment tools. The principles and techniques of play assessment are widely applicable and developmentally appropriate to use to observe the target child across multiple settings in the elementary school, including the playground; to assist the Child Study Team in developing recommendations for remediation; for more formal processes such as developing a functional behavioral assessment and a Behavior Intervention Plan, a school intervention plan (now called Academic Intervention Services), a Section 504 Plan, or for developing a Committee on Special Education Individualized Education Plan. The focus of this chapter will be on using a play assessment process for the Committee on Special Education (CSE) because these evaluations are the most comprehensive of the school-based assessments. (PsycINFO Database Record (c) 2012 APA, all rights reserved)
AD - Schmidt, Mary May: American Board of School Neuropsychology, Monticello, NY, US
ER -

TY - JOUR
ID - 2570

T1 - Implicit beliefs about self and nature: Evidence from an IAT game. [References]
A1 - Bruni,Coral M.
Y1 - 2010///
N1 - Peer Reviewed Journal: 2010-03651-004
English
Journal; Peer Reviewed Journal
KW - PsycInfo
KW - Attitudes
KW - Games
KW - Self Concept
KW - Nature (Environment)
RP - NOT IN FILE
SP - 95
EP - 102
JF - Journal of Environmental Psychology
VL - 30:
IS - 1
N2 - Recent psychological research has examined the beliefs that individuals hold about their relationship with the natural world. This article builds on the previous literature on connectedness with nature and we introduce a new game version of the Implicit Association Test (IAT), known as FlexiTwins. The game is intended to facilitate research using the IAT and provides a flexible platform for measuring implicit associations. In this paper, we report three studies in which we use the game to measure self-nature associations. Study 1 reports the development and validation of FlexiTwins with a college sample. Study 2 further validates the game using a known-groups sample of environmental activists. Finally, Study 3 examines the implicit connectedness of a sample of 30 grade school children. Across all three studies, we find good evidence for reliability, as well as theoretically meaningful results. (PsycINFO Database Record (c) 2012 APA, all rights reserved) (journal abstract)
SN - 0272-4944
AD - Bruni, Coral M.: California State University, San Marcos, CA, US Schultz, P. Wesley: California State University, San Marcos, CA, US
ER -

TY - JOUR
ID - 2571
T1 - The kinesthetic classroom: Teaching and learning through movement. [References]
A1 - Lengel,Traci
Y1 - 2010///
N1 - Book: 2010-03469-000
English
Book; Authored Book
(Created by PsycINFO) Foreword [by] Jean Blaydes Madigan Preface Acknowledgments About the authors Introduction to movement with purpose The brain-body connection Classroom management Preparing the brain Providing brain breaks Supporting exercise and fitness Developing class cohesion Reviewing content Teaching content What matters most Resources References Index
KW - PsycInfo
KW - Academic Achievement
KW - Learning
KW - Physical Activity
KW - Teaching
KW - Curriculum
KW - Student Attitudes
KW - Well Being
RP - NOT IN FILE
SP - Thousand
JF - (2010)
VL - The kinesthetic classroom: Teaching and learning through movement. xv, 156 pp. Thousand Oaks, CA, US: Corwin Press; US.

N2 - (from the cover) Current research shows that regular physical activity helps children and teenagers perform better in school. Taking physical activity beyond gym class and recess, this inspiring book shows how to integrate movement with classroom teaching and learning. Drawing on cutting-edge educational research, the authors describe how regular physical movement increases attention span and helps the brain master new information. Readers will learn how to use short activity breaks to refocus students and movement-based games to reinforce the academic curriculum. This resource includes (1) User-friendly information on the influence of physical activity on the brain and how combining it with academics contributes to student achievement Hundreds of movement activities that can be easily implemented in the classroom, many requiring two minutes or less; (2) Discussion of how movement can contribute to classroom management and community; and (3) Illustrations of action sequences for gross motor activities. With an emphasis on teaching the whole child, The Kinesthetic Classroom shows how movement can improve students' mental and physical well-being and contribute to their joy in learning. (PsycINFO Database Record (c) 2012 APA, all rights reserved)
AD - Lengel, Traci: Pocono Mountain School District, PA, US Kuczala, Mike: Regional Training Center, Randolph, NJ, US
ER -

TY - JOUR
ID - 2572
T1 - World of Whyville: An introduction to tween virtual life. [References]
A1 - Kafai,Yasmin B.
Y1 - 2010///
N1 - Peer Reviewed Journal: 2010-00116-001
English
Journal; Peer Reviewed Journal
KW - PsycInfo
KW - Computer Games
KW - Internet
KW - Virtual Reality
RP - NOT IN FILE
SP - 3
EP - 22
JF - Games and Culture: A Journal of Interactive Media
VL - 5:
IS - 1
N2 - Virtual worlds have become the new playground for millions of tweens but we know little what it takes to become a player in a virtual community. This paper focuses on one virtual world, called Whyville.net, which at the time of our study hosted over 1.2 million players ages 8 to 16 years, with the average player 12.3 years old. Girls represent over 68% of all players. A guided tour to the various places in Whyville, the different types of science games, and the range of players' activities provides insights into what players talk about, with whom they socialize, what they like to play, and how they engage in investigations. In 2005, a group of researchers set out to study Whyville by recruiting hundreds of participants online and offline in classrooms and after school clubs and by conducting observations, surveys, and interviews about their online activities. In addition, we collected log files that captured their online movements and chat interaction during a six month period. We discuss ethical issues of conducting research as adults in tween virtual communities and limitations of capturing the complexities of interactions simultaneously in online and offline spaces. The paper concludes with an overview of following papers in the special issue that cover players' avatar designs, representation of race, cheating practices, learning how to throw virtual mudballs, and participation in virtual epidemic. (PsycINFO Database Record (c) 2012 APA, all rights reserved) (journal abstract)
SN - 1555-4120
AD - Kafai, Yasmin B.: Graduate School of Education, University of Pennsylvania, Philadelphia, PA, US
ER -

TY - JOUR
ID - 2573
T1 - Creative and playful learning: Learning through game co-creation and games in a playful learning environment. [References]
A1 - Kangas,Marjaana

Y1 - 2010///
N1 - Peer Reviewed Journal: 2009-24961-001
English
Journal; Peer Reviewed Journal
KW - PsycInfo
KW - Creativity
KW - Curriculum
KW - Games
KW - Learning Environment
KW - Recreation
KW - Learning
RP - NOT IN FILE
SP - 1
EP - 15
JF - Thinking Skills and Creativity
VL - 5:
IS - 1
N2 - This paper reports on a pilot study in which children aged 7-12 (N = 68) had an opportunity to study in a novel formal and informal learning setting. The learning activities were extended from the classroom to the playful learning environment (PLE), an innovative playground enriched by technological tools. Curriculum-based learning was intertwined with game co-creation, play, and computer games in the PLE. The results indicate that the children considered learning in groups, through co-creation and turning fact into fiction, to be a rewarding way to learn, practice group work and use their imagination for a common goal. Teachers felt their role was important and challenging, especially in terms of the amount of tutoring and lesson planning. The study shows that one way to foster activity, creativity, imagination, and group work skills-along with academic achievement-is to integrate fact and fiction and a playful learning environment in teaching, studying and learning. (PsycINFO Database Record (c) 2012 APA, all rights reserved) (journal abstract)
SN - 1871-1871
AD - Kangas, Marjaana: Centre for Media Pedagogy, Faculty of Education, University of Lapland, Rovaniemi, Finland
ER -

TY - JOUR
ID - 2574
T1 - Literacy motivation and school/non-school literacies among students enrolled in a middle-school ESOL program. [References]
A1 - Sturtevant,Elizabeth G.
Y1 - 2010///
N1 - Peer Reviewed Journal: 2009-24597-005
English
Journal; Peer Reviewed Journal
KW - PsycInfo
KW - English as Second Language
KW - Literacy
KW - Motivation
KW - Reading
KW - Writing Skills
KW - Academic Achievement Motivation
KW - Middle School Students
KW - Schools
KW - Self Concept
RP - NOT IN FILE
SP - 68
EP - 85
JF - Literacy Research and Instruction
VL - 49:
IS - 1

N2 - This study examined literacy motivation among middle-school students in English for speakers of other languages (ESOL) classes by using a 20-question survey and semi-structured interview. The survey results identified similarities among students of different genders, but differences between the beginning ESOL group and the more advanced groups, with the beginning group exhibiting higher motivation in the subsection of "valuing" of reading. Multiple linear regression with gender, ESOL group, and self-concept as reader predicted value of reading, with ESOL group having the highest prediction power. The eight students interviewed generally reported a strong interest in reading and writing, particularly outside of school, and a wide array of literacy activities within the family in which the students were both learners and teachers. (PsycINFO Database Record (c) 2012 APA, all rights reserved) (journal abstract)
SN - 1938-8071
AD - Sturtevant, Elizabeth G.: George Mason University, Fairfax, VA, US Kim, Grace S.: George Mason University, Fairfax, VA, US
ER -

TY - JOUR
ID - 2575
T1 - Perspective taking. [References]
A1 - Fireman,Gary D.
Y1 - 2010///
N1 - Book: 2009-22409-005
English
Book; Edited Book
KW - PsycInfo
KW - Childhood Development
KW - Cognitive Development
KW - Role Taking
KW - Theory of Mind
RP - NOT IN FILE
SP - 85
EP - 100
JF - Sandberg, Elisabeth Hollister [Ed]; Spritz, Becky L [Ed]
VL - :
IS - 2010
N2 - (from the chapter) Consider how much time we spend thinking of others. Almost every expressive act of communication (except maybe for exclamations!) requires that we think of the listeners or readers of our messages. All forms of social interaction demand some thought of those around us; we need to consider their expectations and anticipate their reactions. Failure to think of others is often problematic. And, when it comes to analyzing events or situations, a complete understanding often involves looking at a problem from different points of view. Considering different points of view is so common that many of us may take perspective-taking skills for granted. Yet, the ability to take the perspective of others is not always an obvious part of our psychological response, and very young children often show marked limitations in being able to see the world from another's point of view. The game of hide-and-seek is a consummate example of the need for perspective taking. One child covers her eyes while a second child finds a hiding place; after a prearranged time (say, until the count of 10), the first child seeks the second child, who should be hidden out of view. In order to successfully hide you need to think of what the seeker can and cannot see; in order to successfully seek, you need to imagine where someone might hide outside of your perspective. When very young children play hide-and-seek, sometimes their lack of perspective taking is obvious. (PsycINFO Database Record (c) 2012 APA, all rights reserved)
AD - Fireman, Gary D.: Department of Psychology, Suffolk University, Boston, MA, US Kose, Gary: Department of Psychology, Long Island University, Brooklyn Campus, Brooklyn, NY, US
ER -

TY - JOUR
ID - 2576
T1 - Resilient playgrounds. [References]
A1 - Doll,Beth; Brehm,Katherine [Col]
Y1 - 2010///

N1 - Book: 2009-22408-000
English
Book; Authored Book
(Created by PsycINFO) List of figures and tables Series editors' foreword Introduction to resilient playgrounds Students' peer relationships School playground facilities Data-based problem solving Playground interventions [by] Katherine Brehm and Beth Doll Documenting the impact of resilient playgrounds projects Case examples Top 10 valuable resources for resilient playgrounds Appendix A: Resilient playgrounds playground safety checklist Appendix B: Resilient playgrounds playground supervision checklist Appendix C: Resilient playgrounds promoting playground play checklist Appendix D: ClassMaps survey 2007 Appendix E: Resilient playgrounds plan for change form Appendix F: Teasing form Appendix G: Teasing thermometer Appendix H: The parents' friendship meeting: A parent information curriculum for parents of children without friends References Index CD contents: Practical resources
KW - PsycInfo
KW - Peer Relations
KW - Playgrounds
KW - School Facilities
KW - Social Interaction
KW - Social Skills
KW - Measurement
KW - Monitoring
KW - School Based Intervention
KW - Students
RP - NOT IN FILE
SP - New
JF - (2010)
VL - Resilient playgrounds. x, 173 pp. New York, NY, US: Routledge/Taylor & Francis Group; US.
N2 - (from the introduction) A special connection exists between students' social competence and the playgrounds that schools provide for them. Some of the social success that students experience can be attributed to the physical setting of the playground, the social climate created with their peers, and the caretaking environments that are fostered by school recess practices. Strengthening playground settings can be an important first step toward fostering students' social success and ultimately their social competence. These, then, are my two overarching goals for this book, Resilient playgrounds: (a) to provide a conceptual framework for understanding playground contexts and their relation to students' social success, and (b) to articulate a strategy for assessing playgrounds, identifying features that might disrupt students' social interactions, intervening to modify these features, and monitoring to ensure that the interventions have led to more successful peer relationships. (from the cover) This book provides tools and strategies for school mental health professionals, teachers, and administrators to evaluate and improve the recess experience in order to ensure that children benefit as much as possible from this important time. An accompanying CD contains forms, examples, PowerPoint presentations, and other resources to support the procedures discussed throughout the book. (PsycINFO Database Record (c) 2012 APA, all rights reserved)
AD - Doll, Beth: School Psychology Program, University of Nebraska, Lincoln, NE, US Brehm, Katherine: Ysleta Independent School District, TX, US
ER -

TY - JOUR
ID - 2577
T1 - System thinking skills at the elementary school level. [References]
A1 - Assaraf,Orit Ben-Zvi; Orion,Nir
Y1 - 2010///
N1 - Peer Reviewed Journal: 2010-08509-003
English
Journal; Peer Reviewed Journal
KW - PsycInfo
KW - Curriculum
KW - Elementary Education
KW - Elementary School Students
KW - Systems

KW - Thinking
KW - Elementary Schools
RP - NOT IN FILE
SP - 540
EP - 563
JF - Journal of Research in Science Teaching
VL - 47:
IS - 5
N2 - This study deals with the development of system thinking skills at the elementary school level. It addresses the question of whether elementary school students can deal with complex systems. The sample included 40 4th grade students from one school in a small town in Israel. The students studied an inquiry-based earth systems curriculum that focuses on the hydro-cycle. The program involved lab simulations and experiments, direct interaction with components and processes of the water cycle in the outdoor learning environment and knowledge integration activities. Despite the students' minimal initial system thinking abilities, most of them made significant progress with their ability to analyze the hydrological earth system to its components and processes. As a result, they recognized interconnections between components of a system. Some of the students reached higher system thinking abilities, such as identifying interrelationships among several earth systems and identifying hidden parts of the hydrological system. The direct contact with real phenomena and processes in small scale scenarios enabled these students to create a concrete local water cycle, which could later be expanded into large scale abstract global cycles. The incorporation of outdoor inquiry-based learning with lab inquiry-based activities and knowledge integration assignments contributed to the 4th grade students' capacity to develop basic system thinking abilities at their young age. This suggests that although system thinking is regarded as a high order thinking skill, it can be developed to a certain extent in elementary school. With a proper long-term curriculum, these abilities can serve as the basis for the development of higher stages of system thinking at the junior-high/middle school level. (PsycINFO Database Record (c) 2012 APA, all rights reserved) (journal abstract)
SN - 0022-4308
AD - Assaraf, Orit Ben-Zvi: Science and Technology Education Department, Ben-Gurion University of the Negev, Be'er-Sheva, Israel Orion, Nir: Science Teaching Department, Weizmann Institute of Science, Rehovot, Israel
ER -

TY - JOUR
ID - 2578
T1 - Guest editorial. [References]
A1 - Munn,Penny; van der Aalsvoort,Diny; Lauchlan,Fraser
Y1 - 2010///
N1 - Peer Reviewed Journal: 2011-04408-001
English
Journal; Peer Reviewed Journal
KW - PsycInfo
KW - Communication
KW - Early Childhood Development
KW - Education
KW - Preschool Students
KW - School Learning
KW - Academic Achievement
KW - Childhood Play Behavior
KW - Early Experience
KW - Family
KW - Preschool Teachers
RP - NOT IN FILE
SP - 6
EP - 9
JF - Educational and Child Psychology
VL - 27:
IS - 4

N2 - British children start school at a very early age - they are among the youngest in Europe when they begin formal schooling as 4-year-olds. Today, the majority of pre-school children spend at least part of their day away from their families, and early education outside of the home is increasingly being offered to children under the age of 3. In recent years the UK Government has begun to see Children's Centres and early years policies as a way to reduce the impact of social disadvantage before school entry. Partly as a result of policy and practice changes over the last decade and a half, early years education in the UK is now a rapidly growing academic field which draws on multiple disciplines - sociology, education, philosophy, psychology, and linguistics among others. Most of the papers in this edition address three main issues; the views that teachers and early years practitioners hold of children's play activity, the nature of learning in the early years, and the psychology of young children's communication. (PsycINFO Database Record (c) 2012 APA, all rights reserved)
SN - 0267-1611
ER -

TY - JOUR
ID - 2580
T1 - A design based research of an earth systems based environmental curriculum. [References]
A1 - Assaraf,Orit Ben-Zvi; Orion,Nir
Y1 - 2009///
N1 - Peer Reviewed Journal: 2010-17893-005
English
Journal; Peer Reviewed Journal
KW - PsycInfo
KW - Curriculum Development
KW - Environmental Education
KW - Junior High School Students
KW - Science Education
KW - Literacy
RP - NOT IN FILE
SP - 47
EP - 62
JF - Eurasia Journal of Mathematics, Science & Technology Education
VL - 5:
IS - 1
N2 - This article presents a model for the development of an environmentally oriented unit designed to be implemented as an integral part of the science core curriculum. The program's main goal is encouraging students at the junior high-school level to develop systems-thinking and environmental insight as a basis for environmental literacy. A design-based research was employed in order to construct the learning program and improve it in consecutive cycles. The findings indicate that junior high school students who were involved in the learning process-through knowledge integration activities, scientific inquiry, and outdoor learning-achieved a meaningful improvement of their cyclic and systemic understanding of the water cycle. The article concludes with a summary of the program's design elements we recommend using in other programs seeking to foster students' understanding of natural cycles within the context of their influence on people's daily lives, rather than in the isolation of their specific scientific domains. It is suggested that an environmentally based science curriculum should involve authentic, real environmental topics, and at the same time, it should emphasize the role of scientific knowledge and skills that are needed for the development of environmental literacy. (PsycINFO Database Record (c) 2012 APA, all rights reserved) (journal abstract)
SN - 1305-8223
AD - Assaraf, Orit Ben-Zvi: Ben Gurion University of Negev, Beer-Sheva, Israel Orion, Nir: Weizmann Institute of Science, Israel
ER -

TY - JOUR
ID - 2581
T1 - School location and gender differences in personal, social, and environmental correlates of physical activity in Cypriot middle school children. [References]
A1 - Loucaides,Constantinos A.
Y1 - 2009///

N1 - Peer Reviewed Journal: 2010-04076-007
English
Journal; Peer Reviewed Journal
KW - PsycInfo
KW - Human Sex Differences
KW - Middle School Students
KW - Neighborhoods
KW - Physical Activity
KW - Rural Environments
KW - Urban Environments
RP - NOT IN FILE
SP - 722
EP - 730
JF - Journal of Physical Activity & Health
JA - J Phys Act Health
VL - 6:
IS - 6
N2 - Background: A number of studies indicate higher prevalence of overweight and obesity among rural school children. The purpose of this study was to examine the differences in personal, social, and environmental correlates of physical activity between school location (urban versus rural) and gender. Methods: Middle school children (N = 676) from different districts in Cyprus completed questionnaires assessing physical activity and potential correlates. Results: Children from rural schools reported higher friend support for physical activity and more ease of walk to a bus station from their home. Urban school children reported higher presence of sidewalks in their neighborhood. Boys reported more hours per day playing outside and higher enjoyment and friend support for physical activity than girls, whereas girls reported higher means in the variable 'I see a lot of people walking or being physically active in my neighborhood'. Significant two-way interactions between gender and school location were noted with rural school girls having less favorable scores in a number of correlates of physical activity. Conclusions: More studies are needed to further understand the higher incidence of overweight and obesity observed among rural youth. Girls from rural areas may be targeted as a priority group for promoting physical activity. (PsycINFO Database Record (c) 2012 APA, all rights reserved) (journal abstract)
SN - 1543-3080
AD - Loucaides, Constantinos A.: Centre of Educational Research and Evaluation, Cyprus Ministry of Education and Culture, Nicosia, Cyprus
ER -

TY - JOUR
ID - 2582
T1 - Preschool assessment: The diagnostic classroom model. [References]
A1 - Clingenpeel,Beth T.
Y1 - 2009///
N1 - Peer Reviewed Journal: 2010-01989-009
English
Journal; Peer Reviewed Journal
KW - PsycInfo
KW - Developmental Measures
KW - Diagnosis
KW - Learning Environment
KW - Models
KW - Preschool Students
KW - Classrooms
KW - Early Childhood Development
KW - Public School Education
RP - NOT IN FILE
SP - 177
EP - 191
JF - Journal of Early Childhood and Infant Psychology

VL - 5 2009, pp. 177-191.
N2 - Early childhood assessment has evolved to an environmental model encompassing not only child attributes, but ecological factors relating to child functioning within systems as well. The purpose of this article is to describe the successful use of a diagnostic classroom model for preschool assessment in a public school district in southeastern Virginia. The diagnostic classroom model is a structured learning environment where dynamic assessments of children aged 3 through 5 can occur by allowing children to be observed in a more natural setting over time. As a mode for assessment, the diagnostic classroom addresses two key features of best practices of early childhood assessment: families play a vital role in the process and methods accommodate children's developmental status. Descriptions of the classroom and stakeholder satisfaction data are discussed. (PsycINFO Database Record (c) 2012 APA, all rights reserved) (journal abstract)
SN - 1554-6144
AD - Clingenpeel, Beth T.: Virginia Beach City Public Schools, Virginia Beach, VA, US Good, Kelli R.: Virginia Beach City Public Schools, Virginia Beach, VA, US McCleu-Jackson, Karen J.: Virginia Beach City Public Schools, Virginia Beach, VA, US
ER -

TY - JOUR
ID - 2583
T1 - The relationship between self-esteem, campus climate and parental involvement on academic performance in african american boys
A1 - Price,Bobby D.J.
Y1 - 2009///
N1 - Dissertation Abstract: 2009-99231-199
English
Dissertation Abstract
KW - PsycInfo
KW - Academic Achievement
KW - Blacks
KW - Campuses
KW - Parental Involvement
KW - Self Esteem
RP - NOT IN FILE
SP - 1940
JF - Dissertation Abstracts International Section A: Humanities and Social Sciences
VL - 70:
IS - 6-A
N2 - The primary purpose of this study is to examine the relationship between the independent variables self-esteem, campus climate and parental involvement compared to the dependent variables grades and academic performance in African American Boys. This study will include Private school and Public school students from a large city. African American boys (age 11-16) will be selected to participate in the study, also previous academic performance will be obtained. Additionally, this examination will include the use of the follow instrument. (1) Stanford 10 Achievement Test (Stat 10) (Klein, 1993) achievement test which provides information on student performance; (2) Rosenberg Self-Esteem Scale (SES) (Rosenberg, 1965) for the purpose of obtaining a measurement of global self-esteem, and the Campus Climate Survey (McBrien, & Brandt, 1997). Self-esteem has become a household word. Teachers, parents, therapists, and others have focused efforts that may cause many positive outcomes and benefits. Self-esteem is literally defined by how much value people place on themselves (Baffle, 1991). It is the evaluative component of self-knowledge. High self-esteem refers to a highly favorable global evaluation of the self (Baffle, 1990). Low self-esteem, by definition, refers to an unfavorable definition of the self. Self-esteem does not carry any definitional requirement of accuracy whatsoever. School culture and climate refers to the sum of the values, cultures, safety practices, and organizational structures within a school that cause it to function and react in particular ways. Some schools are said to have a nurturing environment that recognizes children and treats them as individuals; others may have the feel of authoritarian structures where rides are strictly enforced and hierarchical control is strong. Teaching practices, diversity, and the relationships among administrators, teachers, parents, and students contribute to school climate. Although the two terms are somewhat interchangeable, school climate refers mostly to the school's effects on students, while school culture refers more to the way teachers and other staff members work together. Parental involvement is linked to children's school readiness. Research shows that greater parent

involvement in children's learning positively affects the child's school performance, including higher academic achievement (McNea1, 1999) and greater social and emotional development (Bredekamp, & Copple, 1997). Simple interactions, such as reading to young children may lead to greater reading knowledge and skills (Snow, Burns, & Griffin, P., 1998). And, children with richer home literacy environments demonstrate higher levels of reading knowledge and skills at kindergarten entry. Parent involvement outside of home, such as participation in extracurricular activities, relates to their reading, general knowledge, and mathematics knowledge and skills (Reaney, 3 Denton, & West, 2002). However, more information is needed to examine what specific types of parent involvement are related to kindergartners' early literacy and how the relationship varies for children from diverse backgrounds. Procedures. Academic data was obtained from participating schools in a large city. Volunteer African American boys who participate in this research will be selected from two schools, one private and one charter. Test and surveys will be administered by researcher to students selected from the sixth grade class. The research analyses will be performed using SPSS Graduate Pack 11.0.1 for Microsoft Windows. The statistical analysis which will be utilized, is the T test and Spearman Rank Correlation Test. This test will determine if a relationship exist for questions 1-3. Question 4, 5, and 6 will be analyzed using a T Test for independent groups, Question 7 will be analyzed using the appropriate multiple correlation technique. (PsycINFO Database Record (c) 2012 APA, all rights reserved)
SN - 0419-4209
AD - Price, Bobby D.: Texas Southern U., US
ER -

TY - JOUR
ID - 2584
T1 - Youth and nature: Assessing the impact of an integrated wellness curriculum on nature based play and nature appreciation for youth in out-of-school time recreation programming
A1 - Lewis,Terence Grant Jr
Y1 - 2009///
N1 - Dissertation Abstract: 2009-99230-008
English
Dissertation Abstract
KW - PsycInfo
KW - Curriculum
KW - Health
KW - Recreation
KW - Schools
KW - Exercise
RP - NOT IN FILE
SP - 2235
JF - Dissertation Abstracts International Section A: Humanities and Social Sciences
VL - 70:
IS - 6-A
N2 - The United States is facing a dilemma related to the wellbeing of today's children and adolescents. Youth, on a broader scale, are increasingly afflicted with what author Richard Louv, in his book Last Child in the Woods (2005), has labeled "nature deficit disorder" (NDD). The NDD phenomenon is considered to have emerged as a result of children and adolescents not having as many direct experiences with or exposure to nature. Estimations are that from the years 1997 to 2003 there was a 50% drop in the number of youth who participated in outdoor activities, including walking, hiking, and fishing ("Mother Nature Knows Best", 2007). Furthermore, only six percent of children age 9 to 13 plays outside on their own (Louv, 2007). Play, however, is considered an essential part of the learning process throughout life and should not be neglected (Rieber, Smith, & Noah, 1998). In addition, there is growing evidence indicating that there are healthy outcomes associated with unstructured play in nature and that although children only play outdoors for limited amounts of time, they enjoy playing outdoors, and given more choice and/or opportunity, many would play outdoors more than they currently do (British Market Research Bureau, 2005; Ericson, 2001; White & Stoecklin, 1998). The purpose of the study was to further investigate the premise that structured integrative health and wellness programs in an out-of-school setting can play a significant role in helping young people adopt physical activity habits and behaviors, as well as influencing dietary choices. This study focused specifically on the evaluation of the Sajai Wise Kids Outdoors program to gain an increased comprehension of the effects that may result from intentional out-of-school time programming with wellness and outdoor exploration focus areas. The program was designed

to be fun, experiential, and educational in promoting nature based play and nature appreciation in youth ages 6 to11 years of age while teaching them basic wellness concepts. The research design used to evaluate the program's effects is best described as a mixed-methods approach. The study is based on a quasi-experimental, repeated measures design that utilized a collective (multi-site) case study technique with pre-, post- and follow-up assessments. Multiple sources of evidence were used in the study, including surveys, observations, and focus groups with youth and staff. The Theory of Planned Behavior (TPB) served as the theoretical framework as the latent constructs of the TPB have been indicated in the literature as being predictive of behavior related to physical activity and dietary choices. Although program enrollment in the cities of St. Paul and San Francisco totaled 170 participants, 67 youth ages 6 to 13 from diverse socio-economic and cultural backgrounds completed all three phases of data collection. Quantitative measures were administered to assess change in adolescent outcomes related to the latent constructs of the TPB. While results of the quantitative analysis did not show significant differences across the three measures of the study, youth were found to display significantly higher than average means at baseline. This supports the possibility that youth who showed a higher affiliation towards the constructs measured at baseline, as well as at the end of implementation, will have a continued or greater desire or intention to spend time in the outdoors, thus leading to increased physical activity. Triangulation of qualitative and quantitative findings provided further evidence that out-of-school time programs that are intentionally designed to engage youth with concepts focused on wellness and the natural environment, such as Wise Kids Outdoors, have the ability to serve as a gateway of opportunity for youth to explore and discover a relationship with nature. (PsycINFO Database Record (c) 2012 APA, all rights reserved)
SN - 0419-4209
AD - Lewis, Terence Grant: U Minnesota, US
ER -

TY - JOUR
ID - 2585
T1 - An exploration of emotion language use by preschool-aged children and their parents: Naturalistic and lab settings
A1 - Fellows,Michelle Dyan
Y1 - 2009///
N1 - Dissertation Abstract: 2009-99220-344
English
Dissertation Abstract
KW - PsycInfo
KW - Childhood Development
KW - Emotions
KW - Language
KW - Parent Child Communication
KW - Parents
RP - NOT IN FILE
SP - 3203
JF - Dissertation Abstracts International: Section B: The Sciences and Engineering
VL - 70:
IS - 5-B
N2 - Emotion language use provides insight into a person's emotional landscape. However, little is known about how preschool aged children and their parents use emotion language in their real world interactions. To address the shortcomings of the current body of empirical work on naturally occurring emotion language, this dissertation asks the following four research questions: (1) How do children and parents use emotion words in their daily lives?; (2) How is children's emotion language related to parents' emotion language?; (3) How is emotion language use related to emotional functioning?; and (4) How does emotion language in a lab setting compare to a natural setting? The first question relates to descriptive information that is unknown about emotion language use in real world interactions of young children and parents. Points of interest include whether family members differ in rates of emotion language use, whether use of positive and negative emotion words are related within a family member, and how stable emotion language use is over a one year interval. The second question addressed by this dissertation relates to the relationship between parent and child emotion language use. From a social learning theory perspective, it would be expected that children's emotion language use will be related to parents' emotion language use. However, gender plays a large role in emotion socialization so will likely impact the correspondence between parent and child emotion language. The third goal is to examine what

the emotion language used by an individual illuminates about his/her emotional world. Two competing models are possible: emotion language as a reflection of emotion experience or emotion language as an emotion regulation strategy. The reflection model posits that emotion word use simply mirrors an individual's emotional experience, leading to the prediction that individuals who use more emotion language, particularly negative emotion language, demonstrate more emotional dysfunction. The regulation model, on the other hand, suggests that using emotion words acts as an emotion regulation technique, leading to the prediction that people who use more emotion language would actually be expected to show less emotional dysfunction. The last goal of this dissertation is to evaluate the ecological validity of emotion language measurement in traditional lab settings. The vast majority of research on parent-child emotion language comes from laboratory based paradigms. To date, no empirical work has measured emotion language in naturally occurring parent-child interactions to see if use in the lab corresponds to use in everyday life. The methodology of this project will be able to answer this question because emotion language use by families is measured in both settings. This dissertation implements a naturalistic methodology tool to answer the above questions. Thirty-five preschool aged children and their parents were recruited to participate in a two-wave longitudinal study in which the children wore a digital recording device for one day at each of the time points to capture acoustic information about the emotion language and behaviors they and their parents use in their daily lives. Additionally, participants completed a traditional laboratory based paradigm used to study emotion language within families. Parents also completed self-report measures related to emotion functioning for themselves and their child. Results indicate that children and their parents use high rates of positive emotion but very low rates of negative emotion in their naturally occurring interactions. This is different from lab based paradigms that elicit high rates of both positive and negative emotion language from children and parents. (PsycINFO Database Record (c) 2012 APA, all rights reserved)
SN - 0419-4217
AD - Fellows, Michelle Dyan: U Texas at Austin, US
ER -

TY - JOUR
ID - 2586
T1 - Factors influencing the identity of Chinese English language learners: A case study of Chinese high school students in the American Southwest
A1 - Zhao,Xun
Y1 - 2009///
N1 - Dissertation Abstract: 2009-99170-083
English
Dissertation Abstract
KW - PsycInfo
KW - English as Second Language
KW - High School Students
KW - Self Concept
KW - Social Environments
RP - NOT IN FILE
SP - 792
JF - Dissertation Abstracts International Section A: Humanities and Social Sciences
VL - 70:
IS - 3-A
N2 - Research in the field TESOL has focused on the identity issues for a decade. Chinese English language learners have been regarded as high achievers in U.S. school traditionally. The neglected view has been the perspectives of Chinese English language learners themselves and their relationship with social environments in their learning process. My purpose of this case study is to conceptualization the factors influencing act Chinese English language learners' identity. Through the perspectives of students, parents, and their school teachers and counselors, conceptualization of learning English as struggle over identity, meaning, and social position, and being accepted was accomplished. I studied with four participants who had been in the U.S. for no more than five and half years. They had finished their elementary schools in China and came to the U.S. with their parents. Six parents agreed to talk with me about their parental involvement in both China and the U.S. Twelve school teachers and two counselors share their perspectives with me. I observed Chinese English language learners seven days each in their own ethnic community after they finished school or at weekends. I observed them three days of each in their school environment such as classroom, library, gym, hallway, cafeteria and playground.

My field notes and reflective journal were my important source of data. I interpreted and presented my findings in terms of themes. There were six themes evolved from data: learning English in China and U.S. Secondary School, Being Pressured to School Success, Societal Status of Chinese ELLs in Secondary school, Acquiring Societal Status in the U.S., Maintaining identity in Chinese Community, Linguistic and Cultural Navigation in the Borderlands. Through these themes, the linguistic, cultural, societal, community, and geographical factors were found that influence the identity of Chinese English language learners. (PsycINFO Database Record (c) 2012 APA, all rights reserved)
SN - 0419-4209
AD - Zhao, Xun: New Mexico State U., US
ER -

TY - JOUR
ID - 2587
T1 - Physical activity intervention in early care and education settings
A1 - Qiu,Wei
Y1 - 2009///
N1 - Dissertation Abstract: 2009-99110-169
English
Dissertation Abstract
KW - PsycInfo
KW - Child Care
KW - Childhood Development
KW - Physical Activity
KW - Social Learning
RP - NOT IN FILE
SP - 4887
JF - Dissertation Abstracts International Section A: Humanities and Social Sciences
VL - 69:
IS - 12-A
N2 - This study examined the effectiveness of an eight-week intervention program that was developed to increase children's physical activity in early care and education settings. Social learning theory and the dynamics systems approach were used as the guiding theoretical frameworks in this study. Seven hypotheses were made to guide the evaluation of the intervention program. The intervention took place from November 2006 through January 2007 in the three-, four-year-old, and kindergarten classes at the University of Delaware Laboratory Preschool. One component of the intervention was physical activity curriculum for teachers to implement in children's daily routines. The other component was short energy burst activities also known as Planned Energetic Play (PEP) that teachers could embed in their existing curriculum. For the evaluation of the intervention program, data were obtained from the participants on four occasions: baseline (T1), mid-point of the intervention (T2), immediately following the completion of the intervention (T3), and four months following the completion of intervention (T4). The Actical accelerometer was used to measure children's daily, indoor, and outdoor physical activity at the four times. Repeated Measures Analysis of Variance (ANOVA) was performed to compare children's activity energy expenditure (AEE) and moderate and vigorous physical activity (MVPA) obtained across the four times of measurement. In addition, class, gender, and children's BMI at the beginning of the intervention were entered as between-subjects factors in the Repeated Measures ANOVA. Children who had the lowest level of physical activity at baseline were compared with the rest of the participants regarding the impact of the intervention program. The results showed that this center based intervention appeared to have a capacity for having an effect on increasing children's physical activity in the early care and education setting. The effectiveness was associated with class and the participant's activity level at the beginning of the intervention. Children's gender and weight status were not significant predictors of the outcome of the intervention. The implementation and effectiveness of this physical activity intervention program had implication on many factors related to early childhood education. Factors at the micro level included teacher attitudes and values, early childhood curriculum, and facilities of early care and education programs. At the macro level, early care policies and regulations had a strong influence on children's physical activity and their participation in programs that aimed at preventing childhood obesity. (PsycINFO Database Record (c) 2012 APA, all rights reserved)
SN - 0419-4209
AD - Qiu, Wei: U Delaware, US

ER -

TY - JOUR
ID - 2588
T1 - Quantitative analysis of a school-based intervention for elementary school children
A1 - Hall,Barbara Ann
Y1 - 2009///
N1 - Dissertation Abstract: 2009-99040-464
English
Dissertation Abstract
KW - PsycInfo
KW - Elementary School Students
KW - Health Promotion
KW - Mental Health
KW - Mental Health Services
KW - School Based Intervention
RP - NOT IN FILE
SP - 5075
JF - Dissertation Abstracts International: Section B: The Sciences and Engineering
VL - 69:
IS - 8-B
N2 - This paper explores the effectiveness of a school-based mental health project. Research has identified limitations in mental health care for children in the U.S. The provision of services in naturalistic settings geared toward preventing illness and promoting health is currently gaining support across political, social, and educational sectors. Therefore, the purpose of this study was to evaluate the effectiveness of the Primary Project, a school-based indicated prevention program grounded in developmental ecological theory, designed to increase the capacity of schools to identify and provide intervention for at risk children. The research question focused on determining whether or not participation in the program resulted in a decrease in targeted behavioral problems and an increase in competencies. The study addressed limitations of an earlier multi-year study of the Primary Project and included semi-structured resiliency activities in an attempt to strengthen the intervention for children with externalizing behavior problems. Children in the treatment group participated in 12 individual play sessions conducted by paraprofessionals. Archival pre-post test data for 54 treatment and 54 control-wait group children enrolled in grades K through 3 was analyzed using a MANOVA with repeated measures. Results did not support hypotheses; no significant overall condition x time effect between group difference was found. The current study has important implications for prevention research, and social change. Program evaluation is critical for social-policy development. The rigorous design of this study provides a replicable model for future program evaluation research useful for psychologists searching for direction in the investigation of cost-effective school-based mental health programs for at risk students. (PsycINFO Database Record (c) 2012 APA, all rights reserved)
SN - 0419-4217
AD - Hall, Barbara-Ann P.: Walden U., US
ER -

TY - JOUR
ID - 2590
T1 - Motivation and achievement in physical education. [References]
A1 - Chen,Ang
Y1 - 2009///
N1 - Book: 2009-24219-025
English
Book; Edited Book
KW - PsycInfo
KW - Academic Achievement
KW - Academic Achievement Motivation
KW - Physical Education
KW - School Learning
RP - NOT IN FILE

SP - 553
EP - 574
JF - Wenzel, Kathryn R [Ed]; Wigfield, Allan [Ed]
VL - :
IS - 2009
N2 - (from the chapter) Theories of achievement and motivation in physical education have experienced a significant transformation during the past two decades. Learning achievement in physical education, traditionally characterized by proficiency in playing sports and developing sport-related skills, is being replaced gradually by a demonstrated mastery of knowledge and skills related to healthful living. Similar to the study of motivation in learning academic subject matter, learner motivation in physical education has been viewed through multiple theoretical lenses beyond the behaviorist perspective (e.g., using win/loss as rewards for motivation). Currently, researchers are examining how achievement and motivation strategies can be used to help learners achieve the goals of developing and sustaining a physically active lifestyle. In this chapter, we will frame our discussion of learners' achievement and motivation in physical education in the broad context of curriculum and student learning to explore the critical relationships among content, motivation, and learning achievement. We will (a) discuss the impact of major achievement motivation theories on research in physical education, (b) review and critique major research findings, and (c) discuss possible challenges for future research in physical education. We have focused this review on research conducted with K-12 students in physical education, rather than physical activity associated with sport or recess. Although all three are beneficial to students in school, the meaningfulness of achievement in sport is limited to a small group of highly skilled students, while recess does not represent a meaningful achievement setting. The emphasis on physical education allows us to examine achievement in physical education learning environments within a domain specific context. Thus, the studies reviewed in this chapter were conducted in physical education settings, rather than in youth sport or recess. For sport-related motivation issues, we refer the reader to an excellent collection of reviews on motivation edited by Roberts (2001). (PsycINFO Database Record (c) 2012 APA, all rights reserved)
AD - Chen, Ang: Department of Exercise & Sport Science, University of North Carolina at Greensboro, Greensboro, NC, US Ennis, Catherine D.: Department of Exercise & Sport Science, University of North Carolina at Greensboro, Greensboro, NC, US
ER -

TY - JOUR
ID - 2591
T1 - Active living Logan Square: Joining together to create opportunities for physical activity. [References]
A1 - Gomez-Feliciano,Lucy; McCreary,Linda
Y1 - 2009///
N1 - Peer Reviewed Journal: 2009-23216-011
English
Journal; Peer Reviewed Journal
KW - PsycInfo
KW - Active Living
KW - Communities
KW - Physical Activity
RP - NOT IN FILE
SP - S361
EP - S367
JF - American Journal of Preventive Medicine
JA - Am J Prev Med
VL - 37:
IS - 6, Suppl 2
N2 - Background: The Active Living Logan Square target audience is the community of the southwest corner of Logan Square, an urban Chicago community of 84,466 residents, mostly Latinos. Through the Active Living by Design (ALbD) initiative, the Logan Square Neighborhood Association leveraged its 48 years of existence in the neighborhood to create opportunities and build partnerships. Intervention: Activities addressed three primary goals: (1) enhance school environments and practices to support physical activity before, during, and after the school day; (2) encourage individuals and families to enjoy outdoor activities in their own communities; and (3) create safe, inviting places for activity that connect to surrounding communities. The partnership's participatory

approach involved a variety of community stakeholders in developing and implementing affordable, accessible, culturally acceptable, and sustainable physical activities for children and their families. Results: The partnership successfully piloted Open Streets (temporary street closures) and advocated for development of the Bloomingdale Trail, an elevated rails-to-trails project. In schools, the partnership changed the culture at McAuliffe Elementary to support healthy behaviors through new policies, physical projects, and programs. Lessons learned: Vital components of the project's success included a full-time coordinator with strong community ties; time to build healthy relationships within the partnership and community; the use of culturally relevant strategies; and flexibility to welcome complementary opportunities. Conclusions: The partnership intentionally did not produce a single community-recognized campaign; instead, it chose to use limited resources to promote tangible programs and projects that led to sustainable and replicable changes that promote physical activity. (PsycINFO Database Record (c) 2012 APA, all rights reserved) (journal abstract)
SN - 0749-3797
AD - Gomez-Feliciano, Lucy: Logan Square Neighborhood Association, University of Illinois at Chicago, Chicago, IL, US McCreary, Linda L.: University of Illinois at Chicago, College of Nursin, Chicago, IL, US Sadowsky, Rob: Active Transportation Alliance, Chicago, IL, US Peterson, Serena: Chicago Public Schools, McAuliffe Elementary, Chicago, IL, US Hernandez, Adolfo: Active Transportation Alliance, Chicago, IL, US McElmurry, Beverly J.: University of Illinois at Chicago, College of Nursin, Chicago, IL, US Park, Chang Gi: University of Illinois at Chicago, College of Nursin, Chicago, IL, US
ER -

TY - JOUR
ID - 2592
T1 - Vegetation and outdoor recess time at elementary schools: What are the connections? [References]
A1 - Arbogast,Kelley L.
Y1 - 2009///
N1 - Peer Reviewed Journal: 2009-23109-004
English
Journal; Peer Reviewed Journal
KW - PsycInfo
KW - Elementary Schools
KW - Environmental Effects
KW - Plants (Botanical)
KW - School Environment
KW - Childhood Play Behavior
KW - Elementary School Students
RP - NOT IN FILE
SP - 450
EP - 456
JF - Journal of Environmental Psychology
VL - 29:
IS - 4
N2 - Empirical and anecdotal evidence suggests that landscapes with more vegetation have a positive impact on children's focus, attention, and cognitive development. In school, children are able to regain focus, suppress impulses, and pay attention in class longer after exposure to natural settings. Because children spend much of their time in school, the amount and types of vegetation on school grounds may influence their development. Public elementary schools in the Commonwealth of Virginia (N =988) were surveyed to examine correlations between school ground vegetation and outside recess. The number of trees on school grounds, the size of the school grounds, and the presence of sports fields were modestly correlated with greater outside recess time. These correlations support common sense because sports fields facilitate supervised play and larger school grounds provide space for sports fields and playgrounds and additional opportunities for free play. More trees on school grounds provide a welcoming environment for students and teachers, and encourage outside play. These results may help school personnel design and maintain school grounds that increase outdoor recess time. (PsycINFO Database Record (c) 2012 APA, all rights reserved) (journal abstract)
SN - 0272-4944
AD - Arbogast, Kelley L.: Virginia Tech, VA, US Kane, Brian C. P.: University of Massachusetts, MA, US Kirwan, Jeffrey L.: Virginia Tech, VA, US Hertel, Bradley R.: Virginia Tech, VA, US
ER -

TY - JOUR
ID - 2593
T1 - Preschool children's outdoor play area preferences. [References]
A1 - Holmes,Robyn M.
Y1 - 2009///
N1 - Peer Reviewed Journal: 2009-22406-009
English
Journal; Peer Reviewed Journal
KW - PsycInfo
KW - Childhood Play Behavior
KW - Playgrounds
KW - Preferences
KW - Preschool Students
RP - NOT IN FILE
SP - 1103
EP - 1112
JF - Early Child Development and Care
VL - 179:
IS - 8
N2 - This study explores preschool children's outdoor play preferences. The sample was 40 (20 male, 20 female) primarily European-American three and four year olds. Data were collected via naturalistic observation and analyzed using repeated measures ANOVAs and MANOVAs. The independent variable was sex of child; dependent variable was play space (riding, sandbox, slide, jungle gym, open space, castle/play house, swings). Findings revealed a significant effect of sex of child on choice of play space-boys preferred to play on the jungle gym and swings, whereas girls preferred the sandbox more than boys. These findings both support and contradict existing literature. Future research should pursue children's use of micro spaces in their play environments. (PsycINFO Database Record (c) 2012 APA, all rights reserved) (journal abstract)
SN - 0300-4430
AD - Holmes, Robyn M.: Monmouth University, West Long Branch, NJ, US Procaccino, Jill K.: Monmouth University, West Long Branch, NJ, US
ER -

TY - JOUR
ID - 2594
T1 - Home environment and family resources to support literacy interaction: Examples from families of children with disabilities. [References]
A1 - Dolezal-Sams,Juli M.
Y1 - 2009///
N1 - Peer Reviewed Journal: 2009-12055-003
English
Journal; Peer Reviewed Journal
KW - PsycInfo
KW - Childhood Development
KW - Disabilities
KW - Family Socioeconomic Level
KW - Home Environment
KW - Parent Child Relations
KW - Responsibility
RP - NOT IN FILE
SP - 603
EP - 630
JF - Early Education and Development
VL - 20:
IS - 4
N2 - Research Findings: Research on early literacy development within the family focuses primarily on parent-child interactions as they use literacy materials, typically books. However, features of the home environment

and organization of family life, which provide the framework within which these interactions occur, are rarely investigated. These variables include space; time; family members' roles, responsibilities, and relationships; whether literacy activities are routines; and influences of community and culture. In this exploratory study, these physical, social, and symbolic resources of the family environment were measured using a home tour, photographs, book-reading observation, and an interview during two visits to six families who had children with mild to moderate disabilities. The data generated by these methods were then used to complete a summary measure, the Home Literacy Resource Checklist, for each family. The results indicated that the families who had previously reported reading to their child 1 to 3 times per week differed from those who had reported reading daily in resources such as help from people outside of the immediate family, existence of book-reading routines, a predictable daily schedule, use of the library, and presence of literacy materials. Practice or Policy: Further development of these measures and their use with a larger and more diverse sample are warranted so that practical knowledge about home and family life can contribute to interventions designed to improve the quantity and quality of family book reading. (PsycINFO Database Record (c) 2012 APA, all rights reserved) (journal abstract)
SN - 1040-9289
AD - Dolezal-Sams, Juli M.: Department of Child and Family Studies, University of Tennessee, Knoxville, TN, US Nordquist, Vey M.: Department of Child and Family Studies, University of Tennessee, Knoxville, TN, US Twardosz, Sandra: Department of Child and Family Studies, University of Tennessee, Knoxville, TN, US
ER -

TY - JOUR
ID - 2595
T1 - The toy bag: An examination of its history & use in early intervention for infants and toddlers with special needs. [References]
A1 - Nwokah,Eva
Y1 - 2009///
N1 - Book: 2009-10203-009
English
Book; Edited Book
KW - PsycInfo
KW - Childhood Play Behavior
KW - Early Intervention
KW - Special Needs
KW - Toys
KW - Early Childhood Development
KW - History
KW - Infant Development
RP - NOT IN FILE
SP - 166
EP - 182
JF - Clark, Cindy Dell [Ed]
VL - :
IS - 2009
N2 - (from the chapter) The use of toys as an integral and essential part of therapy for young children with delay and disabilities has stimulated published resources that describe or advertise toys suitable for early intervention. Such resources show how toys may be used in both assessment and treatment (Schwartz, 2004). How toys are altered, adapted or used depends on the child and the nature of the disability or disorder. Toys may help infants with special needs "tune in", "reach out", "make discoveries", or increase "sensory awareness" (Segal, 1988). Some toys may be adapted for children with physical or visual challenges, for instance, through the use of activation switches, modified knobs and handles, or securing a toy to a stationary surface (Musselwhite, 1986). Early intervention services to children aged 0-3 years support the child and the family in enhancing the child's skill development. Intervention builds on the child's strengths and improves those developmental domains (such as communication or locomotion) to help the child reach full potential. As early intervention services became federally mandated to be provided in the child's familiar or "natural environment," professionals typically carried toys and materials in a "toy bag" to a home or day-care. The theoretical rationale for the role of toys as part of play in early intervention, the history of this aspect of the material culture of early

intervention, and the changed focus of early intervention services related to the use of toys are the topic of this article. (PsycINFO Database Record (c) 2012 APA, all rights reserved)
AD - Nwokah, Eva: Department of Communication Sciences & Disorders, University of North Carolina at Greensboro, Greensboro, NC, US
ER -

TY - JOUR
ID - 2596
T1 - Media use vs. work and play in middle childhood. [References]
A1 - Hofferth,Sandra L.
Y1 - 2009///
N1 - Peer Reviewed Journal: 2009-09783-006
English
Journal; Peer Reviewed Journal
KW - PsycInfo
KW - Childhood Development
KW - Childhood Play Behavior
KW - Daily Activities
KW - Mass Media
RP - NOT IN FILE
SP - 127
EP - 129
JF - Social Indicators Research
VL - 93:
IS - 1
N2 - As new media continue to be introduced, they become especially troubling for educators, who fear that children will grow up as zombies, lacking creativity, inspiration, and literacy-and becoming sedentary and overweight. This brief article analyzes how media use is nested in children's everyday lives. In particular, it focuses on the hypothesis of media displacement of other activities. According to the displacement hypothesis, media displace creative activities (such as free play), achievement-related activities (like reading and studying), and physical activities. In this article, "play" refers to unstructured noncomputer leisure-time activities not directly related to school, including indoor and outdoor play. Achievement-related activities (such as studying and reading) are the "work" of children in middle childhood. Physical activities include time in structured sports and in walking/hiking outdoors. Time spent in a new activity has to come from another previous activity, leading to the expectation that many other activities will decline. The results support the concerns of those who fear that media interfere with other activities. Of particular concern is that in both years, children's nonmedia play hours and their sleep hours are shorter with greater media use. Play and sleep both seem to offer large blocks of children's time from which new activities can be carved out. Video game playing and television viewing are negatively associated with reading, study and outdoor activities, again suggesting potential negative effects on achievement and physical activity of extensive media use. The large amount of time spent watching television provides a large pool of available time from which to expand new media and may reflect a medium that will decline in the future. (PsycINFO Database Record (c) 2012 APA, all rights reserved)
SN - 0303-8300
AD - Hofferth, Sandra L.: Department of Family Science, University of Maryland, College Park, MD, US
ER -

TY - JOUR
ID - 2597
T1 - The impact of lifestyles and food knowledge on the food patterns of German children. [References]
A1 - von Normann,Konstantin
Y1 - 2009///
N1 - Peer Reviewed Journal: 2009-09502-004
English
Journal; Peer Reviewed Journal
KW - PsycInfo
KW - Child Attitudes
KW - Eating Behavior

KW - Food
KW - Leisure Time
KW - Lifestyle
KW - Knowledge Level
RP - NOT IN FILE
SP - 382
EP - 391
JF - International Journal of Consumer Studies
VL - 33:
IS - 4
N2 - The German youth is relatively well informed about healthy eating, but does not act in accordance with this knowledge. It is also known from empirical studies that there is an association between lifestyles, food knowledge and the eating patterns of older teenagers. However, is there a similar correlation in regard to younger children? This article shows results of a study addressing this issue by empirically investigating leisure time activities, food intake and food knowledge. The study conducted with 907 children, on average 12 years old, out of an urban region in northwest Germany using a written questionnaire, implies that food patterns and food knowledge have to be improved because they are-on average-only marginally acceptable. On this level, they are not in line with a healthy future. For the first time in Germany, food patterns and food knowledge of seventh grade pupils were analysed in relation to their lifestyles. The study revealed four lifestyles. One family-oriented lifestyle and three non-family oriented lifestyles: outdoor and sociable youth, culture-oriented youth, and multimedia kids. The family-oriented lifestyle influences the children's food patterns in a positive way; the three found non-family-oriented lifestyles lead to less preferable food patterns. The results imply that there is a phase of unhealthy food patterns at the beginning of the second life decade, which seems to be linked to the detachment from the parents and the gained independency, expressed by the first self-chosen lifestyles. As lifestyles have such a vital influence on healthy eating, and family is no longer the place where children get their everyday competencies, school has to enable pupils to make healthier food and lifestyle choices. (PsycINFO Database Record (c) 2012 APA, all rights reserved) (journal abstract)
SN - 1470-6423
AD - von Normann, Konstantin: Abteilung Haushaltswissenschaft und ihre Didaktik, Westfalische Wilhelms-Universitat, Munster, Germany
ER -

TY - JOUR
ID - 2598
T1 - Is recess an achievement context? An application of expectancy-value theory to playground choices. [References]
A1 - Spencer-Cavaliere,Nancy; Dunn,Janice Causgrove; Watkinson,
Y1 - 2009///
N1 - Peer Reviewed Journal: 2009-08502-003
English
Journal; Peer Reviewed Journal
KW - PsycInfo
KW - Academic Achievement
KW - Childhood Play Behavior
KW - Decision Making
KW - Playgrounds
RP - NOT IN FILE
SP - 40
EP - 53
JF - Alberta Journal of Educational Research
VL - 55:
IS - 1
N2 - This study investigated the application of an expectancy-value model to children's activity choices on the playground at recess. The purpose was to test the prediction that expectancies for success and subjective task values are related to decisions to engage in specific recess activities such as climbing, playing soccer, or skipping rope. Participants included 242 children in grades IA from five schools. Participation in playground activities was assessed using the Activities for Daily Living in Physical Play (ADL-PP) (Watkinson et al, 2001).

Task-specific expectancies and values were measured on the playground activities contained in the ADL-PP. The results indicated that children's perceptions of competence were significantly higher on playground skills that they had identified as most important compared with least important (p < .001). Multivariate prediction analyses revealed that the data supported the predictions of the model (p < .0001) suggesting that recess can be an achievement context. (PsycINFO Database Record (c) 2012 APA, all rights reserved) (journal abstract)
SN - 0002-4805
AD - Spencer-Cavaliere, Nancy: Faculty of Physical Education and Recreation, University of Alberta, Edmonton, AB, Canada Dunn, Janice Causgrove: Faculty of Physical Education and Recreation, University of Alberta, Edmonton, AB, Canada Watkinson, E. Jane: Faculty of Kinesiology and Recreation Management, University of Manitoba, Winnipeg, MB, Canada
ER -

TY - JOUR
ID - 2599
T1 - Affordances for risky play in preschool: The importance of features in the play environment. [References]
A1 - Sandseter,Ellen Beate Hansen
Y1 - 2009///
N1 - Peer Reviewed Journal: 2009-05876-009
English
Journal; Peer Reviewed Journal
KW - PsycInfo
KW - Childhood Play Behavior
KW - Playgrounds
KW - Preschool Education
KW - Preschool Students
KW - Risk Taking
RP - NOT IN FILE
SP - 439
EP - 446
JF - Early Childhood Education Journal
VL - 36:
IS - 5
N2 - The purpose of this article is to qualitatively explore the affordances for risky play in two different preschool outdoor environments, an ordinary preschool playground and a nature playground, based on Gibson (The ecological approach to visual perception, 1979) theory of affordances and Heft's and Kyttea's (Heft in Children's Environ Qual 5(3) 29-37, 1988; Kytta in J Environ Psychol 22:109-123, 2002, Kytta in J Environ Psychol 24:179-198, 2004) extended work on this theory. Observations of risky play in two Norwegian preschools, one ordinary preschool (where play took place on an ordinary playground) and one nature and outdoor preschool (where play took place in a nature area) were conducted. In addition, the children were interviewed about their actualized affordances of risky play, their mobility license, and the constraints on risky play. The results show that both play environments afford an extensive amount of risky play among the children, and that the degree of mobility license tolerated by the staff is an important factor for the children to actualize these affordances. Differences in the qualities and features in the two play environments were found to have an impact on the degree of riskiness in the play situations. As such, the nature playground afforded a higher degree of risk in children's risky play. (PsycINFO Database Record (c) 2012 APA, all rights reserved) (journal abstract)
SN - 1082-3301
AD - Sandseter, Ellen Beate Hansen: Department of Physical Education, Queen Maud University, College of Early Childhood Education, Trondheim, Norway
ER -

TY - JOUR
ID - 2600
T1 - Bilingual and biliteracy practices: Japanese adolescents living in the United States. [References]
A1 - Haneda,Mari
Y1 - 2009///

N1 - Peer Reviewed Journal: 2009-05128-002
English
Journal; Peer Reviewed Journal
KW - PsycInfo
KW - Bilingualism
KW - English as Second Language
KW - Expatriates
KW - Japanese Cultural Groups
KW - Literacy
RP - NOT IN FILE
SP - 7
EP - 29
JF - Journal of Asian Pacific Communication
VL - 19:
IS - 1
N2 - In this paper, we report the findings of our qualitative inquiry conducted with two male and two female sojourner students in their early teens living in the United States. Sojourner students, an under-researched population in literacy studies, refers to expatriate children who reside and study abroad for a number of years because of their parents' jobs and who anticipate eventual return to their home country. Our participants were Japanese sojourner students. Drawing on multiple sources of data, including the students' literacy logs that documented their reading and writing activities in Japanese and English, interview transcripts, and literacy artifacts, we investigated what kind of literacy practices they engaged in outside school and what developing bilingual and biliterate competences meant to them as individuals. Our findings indicate that (a) although the four students spent much time on academic literacy in Japanese and English outside school, they also had active literate lives of their own; and (b) gender affected not only how they perceived their competencies in the two languages but also how they allocated their time outside school to engage in literacy practices in each language. While there is little investigation of this student population from the perspective of gender, we suggest that it is an important issue to take into account in future research. (PsycINFO Database Record (c) 2012 APA, all rights reserved) (journal abstract)
SN - 0957-6851
AD - Haneda, Mari: School of Teaching and Learning, Ohio State University, Columbus, OH, US Monobe, Gumiko: School of Teaching and Learning, Ohio State University, Columbus, OH, US
ER -

TY - JOUR
ID - 2602
T1 - Make books, not war: Workshops at a summer camp in Bosnia. [References]
A1 - Darvin,Jacqueline
Y1 - 2009///
N1 - Peer Reviewed Journal: 2009-03995-008
English
Journal; Peer Reviewed Journal
KW - PsycInfo
KW - Creative Arts Therapy
KW - Creative Writing
KW - Literacy
KW - Storytelling
KW - Summer Camps (Recreation)
KW - Cross Cultural Communication
KW - Ethnic Identity
KW - Nonprofit Organizations
KW - Social Integration
RP - NOT IN FILE
SP - 50
EP - 59
JF - Literacy
VL - 43:

IS - 1
N2 - This article focuses on a bookmaking project that was conducted with pre-adolescent Serbian and Bosnian girls at a summer camp outside Sarajevo, Bosnia in 2005. During this camp, children from Bosnia, Serbia and Croatia were brought together by the Global Children's Organisation to engage in a variety of activities, including conflict resolution, art, athletics and academics. The literacy workshops that were offered gave the children the opportunity to write, illustrate and construct their own books about topics such as peace, friendship and the preservation of nature in Bosnia. The workshops were conducted in English and Serbo-Croatian, using a variety of pedagogical strategies. Upon completion of their books, the girls hosted a 'story hour' for the younger children at the camp and their stories were read aloud and discussed. This article explores the story telling of children in post-war Bosnia (through writing samples) and discusses the powerful social component of literacy events and their ability to unite and heal disparate groups. Additionally, this article explores writing as a means of imagining a more hopeful future and how as writers, children are shapers of their own cultures and of their individual and collaborative identities within those cultures. (PsycINFO Database Record (c) 2012 APA, all rights reserved) (journal abstract)
SN - 1741-4350
AD - Darvin, Jacqueline: Queens College, City University of New York, Flushing, NY, US
ER -

TY - JOUR
ID - 2603
T1 - Learning outdoors: The Forest School approach. [References]
A1 - O'Brien,Liz
Y1 - 2009///
N1 - Peer Reviewed Journal: 2009-03966-004
English
Journal; Peer Reviewed Journal
KW - PsycInfo
KW - Childhood Development
KW - Learning
KW - Schools
KW - Learning Environment
KW - Teaching Methods
RP - NOT IN FILE
SP - 45
EP - 60
JF - Education 3-13
VL - 37:
IS - 1
N2 - This paper outlines the role that Forest School can play in children's development. With over 100 Forest Schools in England, 20 in Scotland and 20 in Wales, this concept is growing across Britain. Forest School involves children having regular contact with woodland over an extended period of time; it allows them to become familiar, and have contact, with the natural environment. The recent Learning outside the classroom manifesto highlights the importance of children and young people gaining experience of the world beyond the classroom. Twenty-four children from seven schools in Oxfordshire, Shropshire and Worcestershire were observed over an eight-month period as they attended Forest School. Improvements in the children's confidence, motivation and concentration, language and communication and physical skills were recorded by teachers and Forest School leaders. Changes took time to occur, highlighting the need for repeated and regular contact with the natural environment, especially for children who do not have access to nature as part of their everyday lives. (PsycINFO Database Record (c) 2012 APA, all rights reserved) (journal abstract)
SN - 0300-4279
AD - O'Brien, Liz: Forest Research, Surrey, United Kingdom
ER -

TY - JOUR
ID - 2604
T1 - Where is the imbalance? [References]
A1 - Chan,John H.

Y1 - 2009///
N1 - Peer Reviewed Journal: 2009-03480-006
English
Journal; Peer Reviewed Journal
KW - PsycInfo
KW - Crime Victims
KW - Bullying
RP - NOT IN FILE
SP - 177
EP - 190
JF - Journal of School Violence
VL - 8:
IS - 2
N2 - For many researchers, the concept of a power imbalance is central to the understanding of bullying, and its presence in the bully-victim relationship is a prerequisite condition that needs to be fulfilled before bullying is deemed to have taken place. Despite the concept's central importance in many definitions of bullying, the nature of the power imbalance in many types of interpersonal aggression invariably labelled as bullying remains unexplored and unclear, and the concept itself has not received much empirical investigation. One major reason for the absence of experimental data on this important issue in bullying can be attributable to the over-reliance on anonymous questionnaires in the extant bullying literature. From the data collected using a newly developed non-anonymous instrument, Chan (2002) found that in the elementary school sample surveyed, a large percentage of bullying was carried out by one's peers or age-equals, and not by someone outside of one's class. The importance of these findings was examined in the context of the conceptual difficulties ensuing from the power imbalance assumption, as well as the implications for school management (e.g., play-time arrangements) and teacher training. (PsycINFO Database Record (c) 2012 APA, all rights reserved) (journal abstract)
SN - 1538-8220
AD - Chan, John H. F.: Psychological Services, Toronto Catholic District School Board, North York, ON, Canada
ER -

TY - JOUR
ID - 2605
T1 - Children with attention deficits concentrate better after walk in the park. [References]
A1 - Taylor,Andrea Faber; Kuo,Frances
Y1 - 2009///
N1 - Peer Reviewed Journal: 2009-03131-003
English
Journal; Peer Reviewed Journal
KW - PsycInfo
KW - Attention Deficit Disorder with Hyperactivity
KW - Concentration
KW - Exercise
KW - Walking
KW - Nature (Environment)
RP - NOT IN FILE
SP - 402
EP - 409
JF - Journal of Attention Disorders
JA - J Atten Disord
VL - 12:
IS - 5
N2 - Objective: In the general population, attention is reliably enhanced after exposure to certain physical environments, particularly natural environments. This study examined the impacts of environments on attention in children with ADHD. Method: In this within subjects design, each participant experienced each of three treatments (environments) in single blind controlled trials. Seventeen children 7 to 12 years old professionally diagnosed with ADHD experienced each of three environments-a city park and two other well-kept urban settings-via individually guided 20-minute walks. Environments were experienced 1 week apart, with

randomized assignment to treatment order. After each walk, concentration was measured using Digit Span Backwards. Results: Children with ADHD concentrated better after the walk in the park than after the downtown walk (p = .0229) or the neighborhood walk (p = .0072). Effect sizes were substantial (Cohen's d =.52 and .77, respectively) and comparable to those reported for recent formulations of methylphenidate. Conclusion: Twenty minutes in a park setting was sufficient to elevate attention performance relative to the same amount of time in other settings. These findings indicate that environments can enhance attention not only in the general population but also in ADHD populations. "Doses of nature" might serve as a safe, inexpensive, widely accessible new tool in the tool kit for managing ADHD symptoms. (PsycINFO Database Record (c) 2012 APA, all rights reserved) (journal abstract)
SN - 1087-0547
AD - Taylor, Andrea Faber: Department of Natural Resources and Environmental Sciences, University of Illinois, Urbana-Champaign, Urbana, IL, US Kuo, Frances E.: Department of Natural Resources and Environmental Sciences, University of Illinois, Urbana-Champaign, Urbana, IL, US
ER -

TY - JOUR
ID - 2606
T1 - Physical and social contexts of physical activities among adolescent girls. [References]
A1 - Kuo,JoAnn
Y1 - 2009///
N1 - Peer Reviewed Journal: 2009-02443-002
English
Journal; Peer Reviewed Journal
KW - PsycInfo
KW - Exercise
KW - Human Females
KW - Physical Activity
KW - Physical Fitness
KW - Social Influences
KW - Adolescent Development
KW - Environment
RP - NOT IN FILE
SP - 144
EP - 152
JF - Journal of Physical Activity & Health
JA - J Phys Act Health
VL - 6:
IS - 2
N2 - Background: With limited opportunities for physical activity during school hours, it is important to understand the contexts of physical activities done outside of school time. Given the importance of physical and social aspects of environments, the purpose of this study was to describe where and with whom girls participate in physical activities outside of school. Methods: Participants were 1925 sixth-grade girls in the Trial of Activity for Adolescent Girls (TAAG). At baseline, they completed a 3-day physical activity recall (3DPAR), reporting the main activity performed during 30-minute intervals and the physical and social contexts of physical activities. Results: The most frequently reported physical activities done outside of school time were house chores, walking (for transportation or exercise), dance, basketball, playing with younger children, and running or jogging. The most common location for these activities was at home or in the neighborhood. With the exception of household chores, these activities were typically done with at least one other person. Conclusions: Interventions that promote physical activities that can be done at or around home or developing supportive social networks for physical activity would be consistent with the current physical activity contexts of adolescent girls. (PsycINFO Database Record (c) 2012 APA, all rights reserved) (journal abstract)
SN - 1543-3080
AD - Kuo, JoAnn: Dept of Kinesiology, University of Maryland, College Park, MD, US Schmitz, Kathryn H.: Center for Clinical Epidemiology and Biostatistics, University of Pennsylvania, Philadelphia, PA, US Evenson, Kelly R.: Dept of Epidemiology, University of North Carolina at Chapel Hill, Chapel Hill, NC, US McKenzie, Thomas L.: School of Exercise and Nutritional Sciences, San Diego State University, San Diego, CA, US Jobe, Jared B.: Division of Prevention and Population Sciences, National Heart, Lung, and Blood Institute, Bethesda,

MD, US Rung, Ariane L.: Epidemiology Program, Louisiana State University School of Public Health, New Orleans, LA, US Gittelsohn, Joel: Dept of International Health, Johns Hopkins Bloomberg School of Public Health, Baltimore, MD, US Pate, Russell R.: Dept of Exercise Science, University of South Carolina, Columbia, SC, US
ER -

TY - JOUR
ID - 2607
T1 - Spotting altruistic dictator game players and mingling with them: The elective assortation of classmates. [References]
A1 - Pradel,Julia
Y1 - 2009///
N1 - Peer Reviewed Journal: 2009-01590-004
English
Journal; Peer Reviewed Journal
KW - PsycInfo
KW - Altruism
KW - Classmates
KW - Egotism
KW - Games
KW - Schools
KW - Cooperation
KW - Selfishness
RP - NOT IN FILE
SP - 103
EP - 113
JF - Evolution and Human Behavior
VL - 30:
IS - 2
N2 - Altruism can evolve through assortation if the selfish advantage of egoistic individuals is outcompeted by the benefits of mutual cooperation between altruists. This selection process is possible if (a) individuals can distinguish altruists from egoists and (b) altruists cooperate electively with other altruists, leaving egoists no chance but to mingle with each other. This study investigates whether these two conditions are fulfilled in a natural setting. One hundred twenty-two students of six secondary school classes (age 10 to 19 years) played an anonymous dictator game, which functioned as a measure of altruism. Afterwards and unannounced, the students had to estimate their classmates' decisions and did so better than chance. Sociometry revealed that the accuracy of predictions depended on social closeness. Friends and disliked classmates were judged more accurately than liked classmates or those met with indifference. Moreover, altruists were friends with more altruistic persons than were egoists. The results confirm the existence of the two prerequisites for the evolution of altruism through assortation: the predictability of altruistic behavior and the association of altruists. (PsycINFO Database Record (c) 2012 APA, all rights reserved) (journal abstract)
SN - 1090-5138
AD - Pradel, Julia: Department of Economic and Social Psychology, University of Cologne, Cologne, Germany Euler, Harald A.: Department of Economics, Institute of Psychology, University of Kassel, Germany Fetchenhauer, Detlef: Department of Economic and Social Psychology, University of Cologne, Cologne, Germany
ER -

TY - JOUR
ID - 2608
T1 - Introduction
A1 - Lawton,Millicent; Berns,Barbara Brauner; Sandler,Judith Opert
Y1 - 2009///
N1 - Book: 2008-12224-001
English
Book; Edited Book
KW - PsycInfo

KW - Curriculum Development
KW - Educational Reform
KW - Instructional Media
KW - Science Education
KW - Teaching Methods
KW - Mathematics
KW - Teaching
RP - NOT IN FILE
SP - 1
EP - 5
JF - Berns, Barbara Brauner [Ed]; Sandler, Judith Opert [Ed]
VL - :
IS - 2009
N2 - (from the chapter) Without question, the education of America's students in science, from kindergarten through Grade 12, stands at a critical crossroads. The generally inadequate outcomes from science teaching and learning in recent years are well documented on both national and international testing yardsticks: too many students have neither the skills nor the understanding in science to enable them to compete in the increasingly scientific and technically oriented global workplace or to successfully pursue higher education in science or science education. This book represents the collective experiences of half of the eight "dissemination and implementation" centers that were funded by the National Science Foundation (NSF), begun between 1997 and 1999, to foster the understanding and use of exemplary mathematics and science instructional materials. Four centers were created to focus on math instructional materials exclusively, three were funded to do the same for science materials only, and one dealt with both subject areas. This book covers the activities of the latter four centers' work in science. (PsycINFO Database Record (c) 2012 APA, all rights reserved)
AD - Lawton, Millicent: Educational Development Center, Inc., US Berns, Barbara Brauner: Educational Development Center, Inc., US Sandler, Judith Opert: Educational Development Center, Inc., US
ER -

TY - JOUR
ID - 2609
T1 - Using Embedded Music Therapy Interventions to Support Outdoor Play of Young Children with Autism in an Inclusive Community-Based Child Care Program. [References]
A1 - Kern,Petra
Y1 - 2006///
N1 - Peer Reviewed Journal: 2007-02814-001
English
Journal; Peer Reviewed Journal
KW - PsycInfo
KW - Autism
KW - Child Day Care
KW - Childhood Play Behavior
KW - Music Therapy
KW - Peer Relations
KW - Community Services
KW - Peers
KW - Social Interaction
RP - NOT IN FILE
SP - 270
EP - 294
JF - Journal of Music Therapy
JA - J Music Ther
VL - 43:
IS - 4
N2 - For young children with autism enrolled in community-based inclusive child care programs, outdoor play can be a major challenge. The aim of this music therapy intervention was to improve peer interactions and meaningful play on the playground for four boys with autism by adding an outdoor music center and using original songs composed for each participant. A collaborative approach was used to support the implementation

of the intervention by the children's teachers, engaging classroom peers as formal and informal helpers. The effects of the interventions were examined using a multiple baseline design with four conditions replicated across the four children. The results indicate that the musical adaptation of the playground itself did not improve social interactions of children with autism significantly, but it facilitated their play and involvement with peers by attraction to the sound and opportunity to use the instruments. The song interventions produced desirable peer interaction outcomes, and the collaborative consultative approach enabled teachers to implement interventions successfully in ongoing playground routines. In addition, peer-mediated strategies increased peer interactions and meaningful play on the playground. (PsycINFO Database Record (c) 2014 APA, all rights reserved) (journal abstract)
SN - 0022-2917
AD - Kern, Petra: Frank Porter Graham Child Development Institute, University of North Carolina at Chapel Hill, Chapel Hill, NC, US Aldridge, David: University of Witten-Herdecke, Germany
ER -

TY - JOUR
ID - 2610
T1 - Social information processing and aggression in understanding school violence: An application of Crick and Dodge's model. [References]
A1 - Nigoff,Amy
Y1 - 2008///
N1 - Book: 2008-02298-005
English
Book; Edited Book
KW - PsycInfo
KW - Aggressive Behavior
KW - Prevention
KW - School Violence
KW - Social Cognition
KW - Social Interaction
KW - Mental Models
KW - Peer Relations
KW - Playgrounds
KW - Schools
KW - Social Processes
RP - NOT IN FILE
SP - 79
EP - 93
JF - Miller, Thomas W [Ed]
VL - :
IS - 2008
N2 - (from the chapter) The current chapter will review a theory of how children interpret and process social situations and how these processes can be biased in a way that leads the child to aggression. Children are in school for 8 hours of their day. Most of their social interactions occur there, when they are in classrooms or on the playground with other children. Mistakes and biases in the social information processing steps often manifest at school. By possessing an understanding of these steps, one would be in a better position to prevent aggression from happening. The current examination will consist of a review of a theory of social information processing and research connecting biases in processing to aggression. Finally, we will present a scenario exploring how social information processing theories can be used to treat and prevent school violence. (PsycINFO Database Record (c) 2013 APA, all rights reserved)
ER -

TY - JOUR
ID - 2611
T1 - Prevention strategies for parents on adolescent smoking: A gap between what they know and what they practice. [References]
A1 - Holub,Christina K.
Y1 - 2006///

N1 - Book: 2006-22344-007
English
Book; Edited Book
KW - PsycInfo
KW - Intervention
KW - Parent Child Relations
KW - Parent Training
KW - Parental Attitudes
KW - Tobacco Smoking
KW - Parental Characteristics
KW - Prevention
KW - Smoking Cessation
RP - NOT IN FILE
SP - 147
EP - 162
JF - Devore, Dorothy M [Ed]
VL - :
IS - 2006
N2 - (from the chapter) More than 6 million adolescents who have become addicted to tobacco will die of tobacco-related illness as adults. As tobacco use remains the leading preventable cause of morbidity and mortality in the United States, developing even more vigorous prevention programs is an important public health priority. Each day about 4,000 youths, age 12-17, will try a cigarette for the first time and about half will become daily smokers. From a socioecological standpoint, there are individual as well as contextual factors related to adolescent smoking. However, there are two important factors that deserve particular attention for their strong influence: peer and parental influence. While there is debate as to whether parents or peers have more influence on smoking (especially in late adolescence), parenting behaviors can affect affiliation with smoking peers. Previous research has identified several parental characteristics associated with adolescent's smoking such as perceived parental influence, smoking status, smoking-related prompting, parental monitoring, and expectations not to smoke. Parents who smoke are encouraged to quit while their children are young; nevertheless, children of smoking parents have lower rates of smoking when parents communicate strong anti-smoking messages and establish household smoking ban rules. Information and guidelines available to parents about smoking prevention typically emphasize the importance of teaching children how to say no or walk away from cigarette offers, ensuring child involvement with sports or other extracurricular activities, talking with children about tobacco in the media, establishing household rules, and being aware of with whom and where children spend their time outside of the home. Healthy Generations, a study examining parental influences and adolescent smoking, found that parents are aware of some smoking prevention strategies (e.g., talking to children about smoking and involving them in extracurricular activities), but not all (e.g., knowing who their friends are). Our research has also shown that what parents do know about prevention does not always translate into the actions they actually take against adolescent smoking. Despite the majority of parents in our study reporting that they discouraged their children from smoking by talking to them about its harmful effects on the body, less than 10% of the parents mentioned teaching their children to say no, knowing who their friends are, talking about family experiences, and keeping them occupied with sports. The majority of parents in our study were willing to participate in smoking intervention programs; however the biggest barrier in being able to do activities with their children was lack of time due to work. Future research on factors that can help facilitate implementation of smoking prevention strategies by parents is important for developing parent-related program components to reduce incidence and prevalence of adolescent smoking. (PsycINFO Database Record (c) 2013 APA, all rights reserved)
AD - Holub, Christina K.: University of North Carolina at Chapel Hill, Chapel Hill, NC, US Candelaria, Jeanette I.: San Diego State University, San Diego, CA, US Laniado-Laborin, Rafael: Universidad Autonoma de Baja California, Mexico
ER -

TY - JOUR
ID - 2613
T1 - Dog attack: Physical trauma with associated brain injury. [References]
A1 - Apps,Jennifer Niskala
Y1 - 2008///

N1 - Book: 2008-17086-013
English
Book; Edited Book
KW - PsycInfo
KW - Cerebrovascular Accidents
KW - Cognitive Ability
KW - Neuropsychological Assessment
KW - Dogs
KW - Injuries
KW - Motor Coordination
KW - Physical Therapy
KW - Physique
KW - Vision
RP - NOT IN FILE
SP - 121
EP - 128
JF - Apps, Jennifer Niskala [Ed]; Newby, Robert F [Ed]; Roberts, Laura Weiss [Ed]
VL - :
IS - 2008
N2 - (from the chapter) This chapter describes a neuropsychological assessment case report of an 8 year old girl who was very seriously mauled by two large dogs. She had been to her mother's friend's house many times and was familiar with her mother's friend's dogs. When the women stepped outside during one visit, Anna thought the two dogs might like to play together, so she took down the gate that separated them from each other. She remembers reaching for the rope toy, thinking she would play with them. She remembers the sounds as first the male full-grown dog attacked her and then the adult female joined in, biting her severely, mauling her head and neck. She remembers being dragged across the room and calling out for help. During 9 hours of surgical intervention to stabilize her injuries, Anna experienced pulmonary distress and required resuscitation. The attack had focused on the right side of her face and neck. Her injuries included nearly complete occlusion of the right internal carotid artery and focal injuries to both vertebral arteries. Her right ear was severed, the facial nerves on the right side of her face were injured, and her right eye was dislodged from its socket. Facial bones required reconstruction, and plates were placed in the right eye socket and across the bridge of her nose. Anna also experienced two cerebral infarcts. The first was during the attack itself. Emergency computerized tomography (CT) and angiogram revealed an embolic stroke at the distribution of the middle cerebral artery on the right. The second stroke came immediately following the completion of her surgery in the right mid-parietal lobe. Three years later, Anna was demonstrating problems with coordinated motor movements. Physical therapy had been completed some time ago; however, like most adolescents, Anna began having trouble remaining compliant with her home exercises. Her parents described her as "sassy" at times, with some "normal" adolescent defiance and occasional giddy behavior. They portrayed her as an affectionate girl, invested in her family, friends, teachers, and church. At the time of evaluation, they stated that Anna had continued concerns regarding her vision and her difficulty using her left hand. However, they clearly stated that they and Anna had no problems with her scars, her physical appearance, or remorse about the injury. The family was interested in seeking neuropsychological evaluation only to determine whether there were any "permanent" cognitive sequelae. (PsycINFO Database Record (c) 2013 APA, all rights reserved)
AD - Apps, Jennifer Niskala: Department of Psychiatry & Behavioral Medicine, Medical College of Wisconsin, Milwaukee, WI, US
ER -

TY - JOUR
ID - 2614
T1 - Body composition and physical activity like health parameters in children in a rural Sevillian population. [Spanish]. [References]
A1 - de Hoyo Lora,Moises; Corrales,Borja Sanudo
Y1 - 2007///
N1 - Peer Reviewed Journal: 2008-18166-004
Spanish
Journal; Peer Reviewed Journal
KW - PsycInfo

KW - Activity Level
KW - Body Size
KW - Health
KW - Quality of Life
KW - Rural Environments
KW - Conscience
KW - Exercise
KW - Habits
RP - NOT IN FILE
SP - 52
EP - 62
JF - RICYDE
VL - Revista internacional de Ciencias del Deporte / The International Journal of Sport Science. 3:
IS - 6
N2 - Introduction: At the moment we can find a great conscience on the benefits that the regular practice of physical activity contribute, together with healthy feeding habits, can help to avoid or slow down the manifestation of certain diseases. In this sense the analysis of the body composition and the physical activity levels in the childhood will allow, if we are able to act consequently, to define the styles and the quality of life in the adult stage. Method: A total of 211 subjects were analyzed, ages between 8 and 12 years (n1 = 96 boys; n2 = 115 girls) of the Sevillian population of Fuentes de Andalucia. Once grouped based on the physical activity level, the kinanthropometric variables and body composition were measured. Results and Discussion: Of the analyzed sample only 34,60% were physically active. The average fat percentage of the masculine group was 15,77% (SD = 8,23), whereas for the girls it is of 22,37% (SD = 6,79). On the other hand, 46,92% of the analyzed sample shows overweight or obesity. Conclusion: The children population of Fuentes de Andalucia that are regularly active outside the physical education classes have less body fat and also less overweight than those who don't practice any physical activity. (PsycINFO Database Record (c) 2013 APA, all rights reserved) (journal abstract)
SN - 1885-3137
AD - de Hoyo Lora, Moises: Universidad de Sevilla, Sevilla, Spain Corrales, Borja Sanudo: Universidad de Sevilla, Sevilla, Spain
ER -

TY - JOUR
ID - 2615
T1 - Patterns of Engagement in Young Children with and without Developmental Delay. [References]
A1 - Almqvist,Lena
Y1 - 2006///
N1 - Peer Reviewed Journal: 2006-06828-009
English
Journal; Peer Reviewed Journal
KW - PsycInfo
KW - Delayed Development
KW - Developmental Disabilities
KW - Environment
KW - Health
KW - Well Being
KW - Experiences (Events)
RP - NOT IN FILE
SP - 65
EP - 75
JF - Journal of Policy and Practice in Intellectual Disabilities
VL - 3:
IS - 1
N2 - The purpose of this study was to identify patterns of process characteristics capturing the essence of children's experiences in their natural environment and their possible association with health and well-being operationalized as engagement for young children with and without developmental delay. Data were gathered from 1035 children between 1 and 3 years in community-based preschools. Teachers and parents responded to

questionnaires concerning interaction, activity, and engagement, as well as demographic and biopsychosocial information. A cluster analysis was conducted to find homogenous patterns related to engagement. Five distinct patterns were identified, all related to different levels of engagement. Several factors, within both the child and the environment, were associated with high levels of engagement. Interaction skills and availability of activities appear to be strong predictors of high-level engagement, regardless whether or not the child has been identified as developmentally delayed. (PsycINFO Database Record (c) 2013 APA, all rights reserved) (journal abstract)
SN - 1741-1122
AD - Almqvist, Lena: Department of Social Science, University of Malardalen, Vasteras, Sweden
ER -

TY - JOUR
ID - 2616
T1 - Patterns and correlates of drinking in offspring from the San Diego Prospective Study. [References]
A1 - Schuckit,Marc A.
Y1 - 2007///
N1 - Peer Reviewed Journal: 2007-13517-009
English
Journal; Peer Reviewed Journal
KW - PsycInfo
KW - Age Differences
KW - Alcohol Abuse
KW - Alcoholism
KW - Children of Alcoholics
KW - Offspring
RP - NOT IN FILE
SP - 1681
EP - 1691
JF - Alcoholism: Clinical and Experimental Research
VL - 31:
IS - 10
N2 - Background: The decision among young people to drink is complex and reflects multiple domains of influence. This paper applies the results from a previous test of an externalizing-based model predicting heavy drinking and alcohol problems in the probands from the San Diego Prospective Study to evaluate how similar characteristics relate to the decision to drink in their offspring. Methods: Data were generated from multiple sources for 152 offspring with a mean age of 17.2 years. Information on the family histories, personal alcohol and other substance use, socioeconomic stratum, the child's gestational problems, and additional characteristics were gathered from face-to-face interviews with a parent approximately every 5 years between their mid-20s and mid-40s. Data regarding the drinking status and additional variables applicable to the offspring were extracted from the 25-year (T25) epoch of the family evaluations using data supplied directly by the offspring. The relationships of variables to the drinking status in that generation were determined through correlations, regression analyses, and an AMOS-based structural equation model (SEM). Results: Significant correlations to the drinking status in offspring were observed for age, but not for sex. Using age-adjusted backgrounds and data supplied by the offspring at T25, the most robust correlations to the drinking status were seen for a disinhibition measure, peer drinking, expectations of the effects of alcohol, and the history of having worked outside the home. When placed into an SEM, the former 3 variables performed in a manner similar to that observed in the original probands in the prediction of the drinking status, in a model with good fit characteristics. Conclusions: These data from a prospective study support the importance of similar domains across 2 generations in characterizing age-appropriate alcohol-related outcomes. (PsycINFO Database Record (c) 2012 APA, all rights reserved) (journal abstract)
SN - 0145-6008
AD - Schuckit, Marc A.: Department of Psychiatry, University of California, San Diego, CA, US Smith, Tom L.: Department of Psychiatry, University of California, San Diego, CA, US Pierson, Juliann: Department of Psychiatry, University of California, San Diego, CA, US Danko, George P.: Department of Psychiatry, University of California, San Diego, CA, US Allen, Rhonda C.: Department of Psychiatry, University of California, San Diego, CA, US Kreikebaum, Sara: Department of Psychiatry, University of California, San Diego, CA, US
ER -

TY - JOUR
ID - 2617
T1 - Race, parental socioeconomic status, and computer use time outside of school among young American children, 1997 to 2003. [References]
A1 - Ono,Hiromi; Tsai,Hsin-Jen
Y1 - 2008///
N1 - Peer Reviewed Journal: 2008-16668-005
English
Journal; Peer Reviewed Journal
KW - PsycInfo
KW - Computers
KW - Parental Characteristics
KW - Racial and Ethnic Differences
KW - Socioeconomic Status
RP - NOT IN FILE
SP - 1650
EP - 1672
JF - Journal of Family Issues
VL - 29:
IS - 12
N2 - This article investigates the role that parental socioeconomic status plays in forming the racial gap in home computer use among young school-age children. Descriptive statistics from time diary data of 6- to 11-year-olds in the Panel Study of Income Dynamics, Child Development Supplement, reveal that Black American children spend substantially fewer minutes on the computer than do non-Hispanic White children; however, the racial gap in percentage terms has narrowed between 1997 and 2003. Multivariate analyses indicate that the extent to which parental socioeconomic status explains the racial gap declined between 1997 and 2003-in 2003, less than 10% of the gap is explained by family income and household head's education. The pattern of decline is not observed when analyzing the racial gap in reading time. As home computer ownership approaches saturation, the racial gap in computer use time may increasingly reflect the underlying racial boundaries rather than parental status differentials. (PsycINFO Database Record (c) 2012 APA, all rights reserved) (journal abstract)
SN - 0192-513X
AD - Ono, Hiromi: Washington State University, Pullman, WA, US Tsai, Hsin-Jen: I-Shou University, Taiwan
ER -

TY - JOUR
ID - 2618
T1 - Using computer-presented social stories and video models to increase the social communication skills of children with High-Functioning Autism Spectrum Disorders. [References]
A1 - Sansosti,Frank J.
Y1 - 2008///
N1 - Peer Reviewed Journal: 2008-09347-004
English
Journal; Peer Reviewed Journal
KW - PsycInfo
KW - Computer Assisted Instruction
KW - Pervasive Developmental Disorders
KW - School Based Intervention
KW - Social Skills
KW - Videotape Instruction
KW - Ability Level
KW - Childhood Development
KW - Communication Skills
KW - Social Skills Training
RP - NOT IN FILE
SP - 162
EP - 178

JF - Journal of Positive Behavior Interventions
VL - 10:
IS - 3
N2 - The purpose of this study was to investigate the effects of computer-presented Social Stories and video models on the social communication skills of three children with High-Functioning Autism/Asperger's Syndrome (HFA/AS). Using a multiple-baseline across-participants design, computer-presented Social Stories and video models were implemented and direct observations of the participants' identified target behaviors were collected two times per week during unstructured school activities (e.g., recess). Overall, data demonstrated that the combined treatment package was effective for improving the rates of social communication for the participants, although modifications to allow access to social reinforcement were needed in two cases. In addition, all three participants demonstrated maintenance of skills at a 2-week follow-up. However, generalization of skills was only observed for one participant. This research adds evidence that a combined intervention presented via computer may be a beneficial method for remediating social skill difficulties for individuals with HFA/AS. (PsycINFO Database Record (c) 2012 APA, all rights reserved) (journal abstract)
SN - 1098-3007
AD - Sansosti, Frank J.: Kent State University, Kent, OH, US Powell-Smith, Kelly A.: Dynamic Measurement Group, US
ER -

TY - JOUR
ID - 2619
T1 - Using an observational framework to investigate adult language input to young children in a naturalistic environment. [References]
A1 - Andersen,Claire Elizabeth; Marinac,Julie
Y1 - 2007///
N1 - Peer Reviewed Journal: 2007-15043-004
English
Journal; Peer Reviewed Journal
KW - PsycInfo
KW - Communication
KW - Language Development
KW - Mothers
KW - Parent Child Relations
KW - Parental Expectations
RP - NOT IN FILE
SP - 307
EP - 324
JF - Child Language Teaching and Therapy
VL - 23:
IS - 3
N2 - The correlation between the communicative intent of parents, in terms of their expectation of a response and the response patterns of young children aged 23-25 months during parent-child interactions, was investigated. An Observational Framework was used to code these parameters in interactions between 36 children and their mothers. The children were assigned by cluster analysis to 'advanced', 'typical' and 'delayed' language groups and their responses were coded with respect to the degree of correctness or appropriateness within the interaction. Differences in both the parental response expectations and the children's response patterns across the three clusters are discussed. (PsycINFO Database Record (c) 2012 APA, all rights reserved) (journal abstract)
SN - 0265-6590
AD - Andersen, Claire Elizabeth: School of Health and Rehabilitation Sciences, University of Queensland, Brisbane, QLD, Australia Marinac, Julie V.: School of Health and Rehabilitation Sciences, University of Queensland, Brisbane, QLD, Australia
ER -

TY - JOUR
ID - 2620

T1 - What difference does it make? Assessing outcomes from participation in a residential environmental education program. [References]
A1 - Stern,Marc J.
Y1 - 2008///
N1 - Peer Reviewed Journal: 2008-13400-003
English
Journal; Peer Reviewed Journal
KW - PsycInfo
KW - Educational Programs
KW - Environmental Attitudes
KW - Environmental Education
KW - Student Attitudes
KW - Awareness
KW - Interests
KW - Learning
KW - Participation
RP - NOT IN FILE
SP - 31
EP - 43
JF - The Journal of Environmental Education
VL - 39:
IS - 4
N2 - The authors explored the influences of 3- and 5-day residential environmental education programs at the Great Smoky Mountains Institute at Tremont (TN) on participants' connections with nature, environmental stewardship, interest in learning and discovery, and awareness of the Great Smoky Mountains National Park and biodiversity. The authors found significant positive, short-term effects on all outcomes of interest. Also, 3-month delayed posttests indicated retention of significant gains in environmental stewardship and awareness, whereas other gains faded. Longer stays and active engagement of visiting teachers in on-site instruction enhanced most outcomes. The authors discuss the influences of previsit preparation and group size on participants. (PsycINFO Database Record (c) 2012 APA, all rights reserved) (journal abstract)
SN - 0095-8964
AD - Stern, Marc J.: Department of Forestry, Virginia Tech, VA, US Powell, Robert B.: Department of Parks, Recreation, and Tourism Management, Clemson University, Clemson, SC, US Ardoin, Nicole M.: Stanford University, Stanford, CA, US
ER -

TY - JOUR
ID - 2621
T1 - A positive psychology approach to tackling bullying in secondary schools: A comparative evaluation. [References]
A1 - Richards,Andrew; Rivers,Ian; Akhurst,Jacqui
Y1 - 2008///
N1 - Peer Reviewed Journal: 2009-06944-007
English
Journal; Peer Reviewed Journal
KW - PsycInfo
KW - High Schools
KW - Positive Psychology
KW - Bullying
KW - Secondary Education
RP - NOT IN FILE
SP - 72
EP - 81
JF - Educational and Child Psychology
VL - 25:
IS - 2

N2 - Anti-bullying interventions in schools favour approaches that practically tackle the problems in the classroom as well as the playground. However, the effectiveness of curriculum-based interventions is often context specific. A Positive Psychology (PP) approach to tackle bullying focuses upon the individual strengths of pupils rather than behaviours. It foregrounds the greater involvement of pupils in problem-solving the issue of bullying, and promotes development of personal qualities that are valued both socially and individually. In this study, a positive psychology intervention programme was designed for implementation in a school's year seven Personal, Social & Health Education (PSHE) lessons, with a control group recruited from another school. The effectiveness of the programme was measured both pre- and post-intervention using self-report questionnaires which included items on bullying behaviour, general well-being and mental health. Results indicated that, among those pupils who experienced the PP intervention programme, levels of bullying reduced and they scored marginally better in terms of general well-being but not mental health. Further developments in the programme are underway. (PsycINFO Database Record (c) 2012 APA, all rights reserved) (journal abstract)
SN - 0267-1611
AD - Richards, Andrew: Social Work Research and Development Unit, University of York, York, United Kingdom Rivers, Ian: Queen Margaret University, Edinburgh, United Kingdom Akhurst, Jacqui: Faculty of Health and Life Sciences, York St John University College, United Kingdom
ER -

TY - JOUR
ID - 2622
T1 - Boys' perception and experience of relational aggression
A1 - Paskewich,Brooke Suzanne
Y1 - 2008///
N1 - Dissertation Abstract: 2008-99220-333
English
Dissertation Abstract
KW - PsycInfo
KW - Aggressive Behavior
KW - Experience Level
KW - Management Personnel
KW - Perception
KW - Training
KW - Relational Aggression
RP - NOT IN FILE
SP - 3274
JF - Dissertation Abstracts International: Section B: The Sciences and Engineering
VL - 69:
IS - 5-B
N2 - Research on aggression commonly cites that boys engage primarily in physical aggression, as characterized by the pursuit of dominance via hitting, kicking, and threatening while girls engage primarily in relational aggression, which includes a plethora of behaviors geared toward manipulating others' social interactions and inflicting harm on peers so as to cause damage to peer relationships. Although most research suggests that girls engage in relational aggression more than boys, contradictory findings suggest that there is no significant difference between boys' and girls' perpetration of, or victimization by, relational aggression. In fact, some studies indicate that boys engage in relational aggression at a higher rate than girls. Further, it is found that boys involved in relationally aggressive acts exhibit significantly higher degrees of social-psychological maladjustment than girls involved in relational aggression. Despite these findings, however, there has been relatively little focus on the experiences of relational aggression in boys. This is perhaps due to the attention given to overt physical aggression in boys and their tendency to ignore a relational aggressor or respond with a physically aggressive act in return, both of which lead the relationally aggressive act to go unnoticed. In any case, given that boys are involved in relational aggression and suffer its consequences, studying their perception and experience of relational aggression is warranted and is the goal of the current research. As a sub-project of grant-funded research at The Children's Hospital of Philadelphia, a focus group methodology was employed to study boys' perceptions and experiences of relational aggression. Twelve 5th grade children (6 males in one group and 6 females in another group) at a West Philadelphia elementary school participated in the focus group study over the course of seven weeks. All participants were of low socioeconomic status, African American, and from regular education classes. Boys in this study provided initial evidence that they experience some aspects of

relational aggression in similar ways as girls. For example, for both boys and girls, relational aggression includes gossip, rumors, and exclusionary behaviors, is a common problem, is typically enacted in unstructured and unsupervised settings, and is carried over from one location to another. For both sexes, there is a link between physical and relational aggression. Finally, adults' feelings toward males and females involved in relational aggression tend to vacillate and their responses to relationally aggressive behaviors are inconsistent at best. On the other hand, this study provides critical data on the ways in which boys and girls perceive and experience relational aggression differently. For instance, the primary functions of relational aggression in boys are to show off and to expel anger, while for girls relational aggression tends to be enacted out of jealousy or revenge. Second, male perpetrators of relational aggression are not popular, while female perpetrators are popular. This may be due to a gender-based stigma that exists for boys who are involved in gender non-normative behaviors, but not for girls. Finally, although both boys and girls perceive relational aggression as a problem and can identify its negative implications, boys do not see as much of a need for help as girls do. Results from the study enhance the literature on girls and relational aggression, provide critical information on boys' perceptions and experiences of relational aggression and treatment needs, and can contribute to aggression prevention programming for youth, as well as parent, teacher, and playground supervisor training programs. (PsycINFO Database Record (c) 2012 APA, all rights reserved)
SN - 0419-4217
AD - Paskewich, Brooke Suzanne: Chestnut Hill Coll., US
ER -

TY - JOUR
ID - 2623
T1 - Performing self and society: Growth and maturity at a Japanese junior high school
A1 - Grimes-Maclellan,Dawn Marie
Y1 - 2008///
N1 - Dissertation Abstract: 2008-99211-221
English
Dissertation Abstract
KW - PsycInfo
KW - Junior High Schools
KW - Learning
KW - Society
RP - NOT IN FILE
SP - 1851
JF - Dissertation Abstracts International Section A: Humanities and Social Sciences
VL - 69:
IS - 5-A
N2 - This study brings us inside a contemporary public junior high school in Kobe, Japan, providing a window on students' everyday experiences and struggles with growing up and learning the principles and responsibilities of becoming a member of Japanese adult society. On the one hand demonstrating teachers' ethnopedagogical schema of curriculum and pedagogy that is strongly linked to cultural notions of developmental readiness, this study on the other hand explores how student participation in an increasingly complex series of lessons, activities, and practices shape their identities over time as junior high school students and as members of Japanese society. Social discourses and conditions in broader society emerge in teacher practice and surface in students' conversations and writings that offer glimpses of how they see themselves and their futures vis-a-vis these received views. As they navigate between the worlds of school and of broader society, school events such as school excursions and "Let's Try Week" challenge students to extend concepts of selfhood by providing opportunities for performing emerging concepts of self and society outside the school perimeter. (PsycINFO Database Record (c) 2012 APA, all rights reserved)
SN - 0419-4209
AD - Grimes-Maclellan, Dawn Marie: U Illinois At Urbana-Champaign, US
ER -

TY - JOUR
ID - 2624
T1 - "U got 2 move it" pilot study: Impact of an after-school interactive video exertainment program for underserved children

A1 - Young,Tammy L.
Y1 - 2008///
N1 - Dissertation Abstract: 2008-99200-200
English
Dissertation Abstract
KW - PsycInfo
KW - Childhood Development
KW - Digital Video
KW - Obesity
KW - Overweight
RP - NOT IN FILE
SP - 2281
JF - Dissertation Abstracts International: Section B: The Sciences and Engineering
VL - 69:
IS - 4-B
N2 - Background. The prevalence of childhood overweight in the United States has increased by 50% in the last two decades (Schumann, Nichols X Lingston, 2002) and 25% to 30% of U.S. children between the ages of 6 and 17 are obese (Moran, 1999). Several studies show that sedentary activities such as television viewing, using a computer, and playing video games increase the risk of childhood obesity (Ebbeling, Pawlak & Ludwig, 2002; Dennison, Erb & Jenkins, 2002; Yackel, 2003). An innovative means of reducing sedentary activity in children through the use of video "exergames" or "exertainment" is becoming a popular means of increasing physical activity in children. A limited number of studies show that "exergames" can make a positive contribution to players' stress management, weight management, fitness, and health (Lieberman, 2006). More research is needed to determine if and how these interactive video games improve physical activity and overall health in children at high risk for obesity. Purpose. The purpose of this pilot study was to assess the effects of a four-week interactive video "exertainment" program in underserved children, as part of an after-school program based on social cognitive theory. Method. Participants consisted of 58 children recruited from an existing after-school program in the San Bernardino City Unified School District. The participants were children ages 7-12 years of lower socioeconomic status living in Southern California. Pre- and post-assessments were made to see if there were improvements in attitude, self-efficacy, outcome expectations, body composition, cardiovascular endurance, body image, physical activity outside the intervention, absenteeism, and academic achievement. Analyses. Results from this study showed that there was a 16% decrease in resting heart rate from baseline to post-intervention for males (p=le;.001) and a 12.9% decrease in resting heart rate for females (p=le;.001). Average active heart rate decreased for all subjects post-intervention representing at 7.16% decrease for males (p=041) and 5.2% decrease for females (p=053). Absenteeism rates improved significantly for students participating in the intervention. The mean number of days students were absent during the intervention (3.73, p=020) decreased compared to prior to starting the intervention (000, p=000). Academic scores showed significant improvements in the students' mean math performance while participating in the intervention (t=3.63, p=001) compared to non-participants (t=2.16, p=.042). The average percent body fat in males showed a non-significant decrease by less than 1% (-.89, p=.295). There were no significant changes on the other study variables. Application to Health Education. The "U Got 2 Move It" program focused on promoting healthy lifestyle modifications in America's youth as a means of reducing the risk of obesity in underserved children by introducing an appealing format for activity-interactive video games in after-school programs. The results from this pilot test will serve as a model for health educators to partner with schools and community-based organizations to develop future health-related programs focusing on preventing or reducing the threat of obesity and related health problems in high risk children. (PsycINFO Database Record (c) 2012 APA, all rights reserved)
SN - 0419-4217
AD - Young, Tammy L.: Loma Linda U., US
ER -

TY - JOUR
ID - 2625
T1 - The effect of online quizzes on student achievement in high school chemistry
A1 - Deeter,Christopher L.
Y1 - 2008///

N1 - Dissertation Abstract: 2008-99190-423
English
Dissertation Abstract
KW - PsycInfo
KW - Academic Achievement
KW - Chemistry
KW - High School Education
KW - Internet
KW - Students
RP - NOT IN FILE
SP - 1319
JF - Dissertation Abstracts International Section A: Humanities and Social Sciences
VL - 69:
IS - 4-A
N2 - The idea of student engagement has come to the forefront of the United States educational system over the past decade. Student engagement requires learners to be actively involved in all stages of the learning process. This study focuses on the use of online quizzes in the chemistry classroom as a means to help students become more engaged in their learning outside of the classroom. Students were given three different types of online quizzes over the course of a chemistry chapter. Student scores on end of the chapter examinations was used to determine whether there was a significant difference in the amount of learning that occurred when a student took each of the three types of online quizzes. Students in a private parochial high school chemistry class completed online quizzes over the course of a semester. The quizzes were taken after completing assigned readings from the chemistry text. After each reading, a third of the students took online multiple-choice quizzes, a third took a paragraph quiz, and a third took no quiz. Scores received from end of chapter tests were evaluated to determine if the impact each of the quiz types had on the learning. All statistical analysis was done using SPSS using two-way split plot ANOVA with condition (paragraph, multiple-choice, nothing) as the within subject factor and group (A, B, C) as between subject factor. The data indicates that there was no significance within the condition F (1.877, 90.087) =.996, p>.05, or the interaction results. F (3.754, 90.087) =.509, p>.05. The data indicated that the effect of group was not significant either. F (2, 48) =.981, p>.05. Interviews undertaken to explain this outcome discovered that students did not become engaged with the content until the night before each test. When they did so, they used a teacher-provided study guide as their primary learning tool. (PsycINFO Database Record (c) 2012 APA, all rights reserved)
SN - 0419-4209
AD - Deeter, Christopher L.: U Nebraska - Lincoln, US
ER -

TY - JOUR
ID - 2626
T1 - Parents' ratings of resilience and leadership scores recorded on the Roets Rating Scale for Leadership by girls in grades 6 through 8
A1 - Fernandez Fernandez,Susana
Y1 - 2008///
N1 - Dissertation Abstract: 2008-99171-013
English
Dissertation Abstract
KW - PsycInfo
KW - Grade Level
KW - Leadership
KW - Parents
KW - Resilience (Psychological)
KW - Rating Scales
RP - NOT IN FILE
SP - 872
JF - Dissertation Abstracts International Section A: Humanities and Social Sciences
VL - 69:
IS - 3-A

N2 - Using the concept of resilience, as well as its processes and mechanisms, this study proposed to investigate the possible relationship between resilience and leadership. More specifically, the purpose of this research was to explore the possible relationship between parents' perceptions of resilience and their daughters' standardized leadership scores per the Roets Rating Scale for Leadership (RRSL) instrument. This research utilized a correlational design to determine whether, and to what degree, the resilience variable was related to leadership. The possibility of a relationship between the two variables was suggested by research conducted on leadership (French & Stright, 1991; Jablin, 1997; Landau & Weissler, 1991; Thompson, 2000; Trawick-Smith, 1988), parenting (Arntson & Turner, 1987; Bellinger & Gleason, 1982; Hartup & Moore, 1990; Huang, 1999; Teichner, Ames, & Kerig, 1997; Whorton & Karnes, 1992), and resilience (Fraser, 1997; Krovetz, 1999; Lugg & Boyd, 1993; Masten, 2001; Masten & Coatsworth, 1998; Smith & Prior, 1995) as well as derived from experience and observation. Method. The research design selected for this research study was a correlational design with the quantitative paradigm as its primary methodology. This methodology was chosen to explore the magnitude and the direction of the relationship between the variables of leadership and resilience in the hopes of helping the field of leadership to solve practical problems such as closing the gap between the "theories that integrate the process of leader development with that of leader performance" (Chan & Drasgow, 2001, p. 481). Additionally, the study was designed as correlational because this quasi-analytic design provided the greater ecological validity by being more easily applicable outside laboratory settings. The procedures for this study indicated the need to obtain two group scores, one for each variable of interest. Resilience ratings were used as the independent variables and the total leadership score served as the dependent variable. The resilience measures included four resilience behavior factors and a total resilience behavior score as well as the four social support factors and total social support score. Mediating variables of age, GPA, and type of parents' biological relationship, and mother vs. father ratings were also addressed. Scores for the narrative section of the RRSL were calculated by assigning a code to the girls' responses. 153 participants were recruited from four different public schools in Regions I, III, and IV of Miami-Dade County Public Schools. The data collection for the leadership variable (RRSL) occurred in a natural setting, while the data collection for the resilience variable (RRAPRS) occurred in the participant's home. The mother and the father, or the two primary caregivers, of each participant were asked to complete the rating scale separately. Both an English version and a Spanish version of the RRAPRS instrument was sent together for parents to choose the one they preferred. Major findings. Although a significant relationship between the concept of leadership and resilience could not be established across the border, the findings regarding father and male caregiver influence, and the variables of academic achievement, resilience, and grade, seem to pose interesting implications for families and educators. More specifically, when correlating the constructs of leadership and resilience, the literature suggests strong environmental or situational components affecting both of them. While there was a lack of statistical significance when correlating leadership and resilience, this could have been due to both constructs been difficult to measure objectively since so many factors play a role in their development and manifestation. (PsycINFO Database Record (c) 2012 APA, all rights reserved)
SN - 0419-4209
AD - Fernandez Fernandez, Susana: Barry U - Adrian Dominican School Of Education, US
ER -

TY - JOUR
ID - 2627
T1 - Pedometer-determined physical activity patterns of fourth and fifth grade school children
A1 - Brusseau,Timothy A.J.
Y1 - 2008///
N1 - Dissertation Abstract: 2008-99151-140
English
Dissertation Abstract
KW - PsycInfo
KW - Childhood Development
KW - Elementary Schools
KW - Grade Level
KW - Physical Activity
KW - Physical Education
RP - NOT IN FILE
SP - 548
JF - Dissertation Abstracts International Section A: Humanities and Social Sciences

VL - 69:
IS - 2-A
N2 - The purpose of this study was to describe the daily pedometer-determined Physical Activity (PA) patterns of 4th and 5th grade children during the segmented school week (i.e., recess, Physical Education, lunchtime, school day, outside of school, weekdays, and weekends). Eight hundred and twenty-nine children from six Southwestern United States elementary schools participated by wearing pedometers (Yamax Digiwalker SW-200) for seven consecutive days (5 school days and two weekend days). Students recorded their steps at arrival and departure from school as well as before and after Physical Edcuation, recess, and lunchtime. They used a recording sheet independently over the weekend. Results showed that boys took significantly more total steps/weekday than girls, outside of school steps, steps during lunchtime, steps during recess, and steps during school. During Physical Education, both boys and girls accumulated a similar number of steps. For boys, lunchtime represented the largest single source of daily PA (13.4%) obtained during school, followed by PE (12.7%) and recess (9.5%). For girls Physical Education was the largest single source (14.3%), followed by lunchtime (11.7%) and recess (8.3%). Significant differences were also found across ethnicity/racial groups, Body Mass Index cut points, and schools. A similar number of steps were also accumulated on weekend days. This study is critical, as it was the first with a large diverse sample investigating students' PA patterns across various activity segments. The daily steps/day patterns of fourth and fifth grade children are variable; however, an understanding of the contributions of the in-school segments can serve as baseline measures for practitioner and researchers to use in school based PA interventions. (PsycINFO Database Record (c) 2012 APA, all rights reserved)
SN - 0419-4209
AD - Brusseau, Timothy A.: Arizona State U., US
ER -

TY - JOUR
ID - 2628
T1 - Attitudes, beliefs, and behaviors of academically talented and well-abled middle school children in their use of the Internet
A1 - Geyer,Roger W.
Y1 - 2008///
N1 - Dissertation Abstract: 2008-99150-491
English
Dissertation Abstract
KW - PsycInfo
KW - Attitudes
KW - Classrooms
KW - Internet
KW - Middle School Education
KW - Academic Achievement
RP - NOT IN FILE
SP - 577
JF - Dissertation Abstracts International Section A: Humanities and Social Sciences
VL - 69:
IS - 2-A
N2 - Using the Internet as a source for learning is rapidly transforming how we work and live. Internet-savvy children are vigorously participating in this new activity-largely outside of the classroom. A better understanding of there students' attitudes, beliefs, and behaviors will help guide educators in making the necessary pedagogical changes to meet 21st century social, cultural, and economic change. This study investigated the construct of Internet-savviness exhibited by academically talented and well-abled youth, ages eight through fourteen. Grounded in learning theories of social constructivism and distributed intelligence, a survey scale was developed to measure Internet-savviness and to elicit these children's Internet-related attitudes, beliefs, and behaviors. Reliability and validity analyses of the scales revealed satisfactory levels of internal consistency. An exploratory factor analysis revealed a clear, underlying structure of the following dimensions: (1) computer mediated communication, (2) creative expression, (3) information gathering, (4) Internet fluency, (5) Internet-self efficacy, and (6) social collaboration. ANOVA, MANOVA, and Regression analyses along with correlation and descriptive statistics analyses were applied to other variables of interest including Internet access speed, age, gender, frequency of Internet use, and type and location of access. Internet-savvy scores

corresponded to self reports of Beginner, Intermediate and Advanced Internet users. Thirty-three percent of youth rated themselves as Advanced users which aligns with previous research on Internet-savvy teens. Although females and males differed in Internet activities and scored below males on Internet-savviness, they closed the gap by age 12. Regarding gender, there were no statistical differences on dimension or total IS scores in this study. Doing something creative, exchanging images, access speed, age and access at home and at a friend's house were statistically significant predictors of IS scores. Effect sizes were reported. Narrative data was collected from the participants, analyzed and summarized as a way to identify central themes regarding Internet use in and outside of school and to triangulate on the multidimensional nature of Internet-savviness. (PsycINFO Database Record (c) 2012 APA, all rights reserved)
SN - 0419-4209
AD - Geyer, Roger W.: U Virginia, US
ER -

TY - JOUR
ID - 2629
T1 - Anxiety disorders in children with williams syndrome, their mothers, and their siblings: Implications for the etiology of anxiety disorders
A1 - Leyfer,Ovsanna
Y1 - 2008///
N1 - Dissertation Abstract: 2008-99100-332
English
Dissertation Abstract
KW - PsycInfo
KW - Anxiety Disorders
KW - Epidemiology
KW - Etiology
KW - Siblings
KW - Williams Syndrome
KW - Genetics
KW - Mothers
RP - NOT IN FILE
SP - 7670
JF - Dissertation Abstracts International: Section B: The Sciences and Engineering
VL - 68:
IS - 11-B
N2 - Genetic factors play an important role in the etiology of anxiety disorders. Williams syndrome (WS), a genetic disorder caused by a deletion on chromosome 7q11.23 and associated with increased prevalence of anxiety disorders relative to the general population and other genetic disorders associated with intellectual disability, can be used in the search for susceptibility genes for anxiety disorders. This study examines the prevalence of anxiety disorders in children with WS, their mothers, and their siblings as well as predictors of anxiety in these groups, in order to facilitate the use of WS in studies of genetics of anxiety disorders. Anxiety disorders were assessed in 109 children with WS ages 4-16 years, 106 mothers, and 72 siblings in the same age range. Prevalence was compared to that in the general population. Children with WS had a significantly higher prevalence of specific phobia, generalized anxiety disorder (GAD), and separation anxiety in comparison to children in the general population. The mothers had a significantly higher prevalence of GAD than women in the general population, but the prevalence rate for GAD in this group prior to the birth of the child with WS was comparable to that for women in the general population. The siblings had a significantly higher prevalence of specific phobia in comparison to children in the general population, but it was similar to the rates reported in other studies of specific phobia in typically developing children. The odds of a child with WS having an anxiety disorder increased with age and the severity of maternal anxiety. The odds of mothers having an anxiety disorder increased if the mother was not employed outside of home and if the child with WS was a male. The elevated prevalence rates of anxiety disorders in children with WS suggest a connection between the deletion found in WS and anxiety disorders. Given the increased prevalence of anxiety disorders in children with WS, genetic studies examining possible links between particular gene(s) deleted in WS and anxiety are warranted. It would also be worthwhile to investigate relations between genes deleted in WS and genes previously implicated in anxiety disorders. (PsycINFO Database Record (c) 2012 APA, all rights reserved)
SN - 0419-4217

AD - Leyfer, Ovsanna: U Louisville, US
ER -

TY - JOUR
ID - 2630
T1 - The impact of resource allocation on professional development for the improvement of teaching and student learning within a site-based managed elementary school: A case study
A1 - Cain,Lisa Marie
Y1 - 2008///
N1 - Dissertation Abstract: 2008-99071-050
English
Dissertation Abstract
KW - PsycInfo
KW - Learning
KW - Professional Development
KW - Resource Allocation
KW - Teaching
KW - Elementary School Students
KW - Program Development
RP - NOT IN FILE
SP - 4147
JF - Dissertation Abstracts International Section A: Humanities and Social Sciences
VL - 68:
IS - 10-A
N2 - The purpose of this study was to investigate how much funding a site-based managed school allocated toward promoting effective professional development at an elementary school level. Additionally, the study examined a district and school's practice of allocating resources for professional development and determined how funding and where the funding for professional development originated. Furthermore, an evaluation process was used to identify the strengths and weaknesses of the school's professional development programs as they compared to an evidence-based professional development model. This study used conceptual frameworks adapted from the research literature to guide data collection and analysis for four research questions: (a) How are personnel used for the delivery of professional development? (b) How are resource allocation decisions for professional development determined? (c) How much funding is directed toward professional development and from what sources do these funds originate? (d) How is professional development used to promote organizational capacity and student achievement? This descriptive-analytic case study was conducted in one elementary school, Sutherland Elementary, in a decentralized unified school district, Glenn Park Unified, located just 30 miles outside of Los Angeles' metropolitan area in Southern California. The district and school were selected on the basis of: (a) implementation of innovative professional development; (b) evidence of administrative freedom to allocate funds to professional development; and (c) significant gains in the school's Academic Performance Index (API) over the last 5 years. Data analysis revealed that Sutherland Elementary and Glenn Park Unified School District employed several research-based strategies pertaining to professional development that aimed at building the capacity of the organization and the individuals. Additionally, the data uncovered that while Sutherland Elementary had the autonomy to decide upon professional development activities, the district supported the school site. An evaluation of Sutherland's allocation practices for professional development revealed a disconnect with the research provided by the evidence-based model. However, the data also highlighted that Sutherland Elementary and Glenn Park Unified School District received a greater amount of professional development resources than could be accounted for in the school and district's expenditures due to partnerships with universities and educational foundations. The study provided conclusions addressing the six core strategies of the conceptual framework, the role of the district office in a decentralized setting, and the analysis between the decentralized setting of Glenn Park Unified as compared to a centralized management style. Five major themes surrounding professional development practices appeared to illustrate the overall success of the school and district: (a) a vision for learning; (b) data-driven decision making; (c) effective utilization of resources; (d) collaboration; and (e) site autonomy. Recommendations for successful professional development practices at other schools and districts included: (a) establishing a clear vision and goals for teaching and learning; (b) utilizing data to make professional development decisions; (c) establishing the needed access to ongoing, on-site, fulltime coaching; (d) adopting a financial plan that mirrors those used in the private sector; (e) collaboration across the school and district; and (f) encouraging site autonomy for professional

development decisions coupled with district support as needed. (PsycINFO Database Record (c) 2012 APA, all rights reserved)
SN - 0419-4209
AD - Cain, Lisa Marie: U Southern California, US
ER -

TY - JOUR
ID - 2631
T1 - Subjective and objective ratings of neighborhood conditions: Do neighborhoods matter to children's health?
A1 - Schaefer-Mcdaniel,Nicole
Y1 - 2008///
N1 - Dissertation Abstract: 2008-99060-529
English
Dissertation Abstract
KW - PsycInfo
KW - Neighborhoods
KW - Physical Health
KW - Social Environments
KW - After School Programs
RP - NOT IN FILE
SP - 6363
JF - Dissertation Abstracts International: Section B: The Sciences and Engineering
VL - 68:
IS - 9-B
N2 - Neighborhood research, the body of work exploring the link between the neighborhood context and residents' behaviors, has received growing attention in recent years. Although this literature is rapidly expanding, there is currently no consensus on how researchers should measure the neighborhood space. Many researchers rely on census data that have been shown to be problematic, while others conduct independent observations of neighborhoods and interview residents about their perceptions of neighborhood conditions. Unfortunately, the few studies that incorporate residents' perceptions of neighborhoods generally rely on adult ratings even if the outcome of interest is concerned with children. As this poses a potentially serious methodological issue in neighborhood research, this dissertation gave voice to children using a mixed methods design. First, ten preadolescent children enrolled in after-school programs in two New York City neighborhoods participated in open-ended interviews and walking tours of their neighborhoods. Qualitative findings revealed that children frequently discussed the following elements: neighborhood quality, safety, social, and physical disorder. These findings informed the design of a survey assessing children's and parents' perceptions of their neighborhoods and children's mental and physical health. 126 children, aged nine to 13, enrolled in after-school programs in three neighborhoods and 117 parents completed this survey. The children also listed five blocks around their home that constituted their neighborhoods. These blocks were observed by two outside raters and census data were collected at the block group level of aggregation. Results from the quantitative study phase suggest that children and parents showed some overlap in their perceptions of neighborhood conditions but these subjective ratings did not relate to the neighborhood observations and census data. Subjective ratings of neighborhood safety mediated the relationship between neighborhood disorder and neighborhood quality and child-rated perceptions of neighborhood conditions predicted their ratings of mental health. Neither parents' neighborhood perceptions, neighborhood observations, nor the census data directly related to children's mental health. On the other hand, observed physical disorder and child-rated neighborhood safety predicted physical health. The dissertation supports the hypothesis that subjective perceptions of neighborhood conditions related best to children's health and that children should be actively recognized in research concerning them. (PsycINFO Database Record (c) 2012 APA, all rights reserved)
SN - 0419-4217
AD - Schaefer-Mcdaniel, Nicole: City U New York, US
ER -

TY - JOUR
ID - 2632

T1 - The impact of ethnicity, age and cue word type on autobiographical memories of African American and European American adults
A1 - Gabston,Mattie Sheherrazzade
Y1 - 2008///
N1 - Dissertation Abstract: 2008-99060-496
English
Dissertation Abstract
KW - PsycInfo
KW - Autobiographical Memory
KW - Blacks
KW - Ethnic Identity
KW - Reminiscence
RP - NOT IN FILE
SP - 6348
JF - Dissertation Abstracts International: Section B: The Sciences and Engineering
VL - 68:
IS - 9-B
N2 - The growing ethnic diversity of the United States has created a need to reinvestigate older concepts, such as the relations between autobiographical memory and the reminiscence bump, in new ways shaped by the shifting demographics of the American population. In keeping with these shifts, an investigation of the autobiographical memories produced by adult participants in terms of the ratings they provided for the level of positive affect, emotional intensity, and identity salience for each memory, and how these factors are impacted by cue word type (Civil Rights Targeted or Era Neutral), participant ethnicity (African American or European American) and participant age (child versus adolescent during the Civil Rights Movement) was carried out. It was found that African American participants reported memories that differed significantly in both their timing and their ratings from those provided by European Americans. African Americans tended to provide memories from an earlier point in their lives, with an emphasis on the first half of the reminiscence bump (ages 11-20). European Americans tended to place an equal emphasis on each half of the entire reminiscence bump (ages 11-30). There were also variations in their memory ratings. With the exception of ratings of affect, ratings by African Americans of the emotional intensity and identity salience of their memories were higher than those of the European Americans. It was concluded that ethnic identity can play a major role in how we perceive our autobiographical memories, as well as what period of life those memories are drawn from. An exploratory structural equation model was also run to investigate the relations among the ratings of affect, emotional intensity, and identity salience for the memories that were located either within the reminiscence bump or outside of this time period. This was performed in order to investigate which, if any, of these factors was the best predictor of the type of memories represented in the reminiscence bump. The generally weak relations between location relative to the reminiscence bump and the participants' ratings of their memories indicated that the model should be revisited and that other factors should be considered. (PsycINFO Database Record (c) 2012 APA, all rights reserved)
SN - 0419-4217
AD - Gabston, Mattie Sheherrazzade: U California, Davis, US
ER -

TY - JOUR
ID - 2633
T1 - An investigation of the validity of the Computer Assisted Child Interview (CACI) as a self-report measure of children's academic performance and school experience
A1 - Chow,Chi Ping
Y1 - 2008///
N1 - Dissertation Abstract: 2008-99030-129
English
Dissertation Abstract
KW - PsycInfo
KW - Academic Achievement
KW - Computer Assisted Testing
KW - Experience Level
KW - Self Report

KW - Test Validity
KW - Childhood Development
KW - Performance
KW - Psychometrics
KW - Schools
KW - Test Reliability
RP - NOT IN FILE
SP - 3359
JF - Dissertation Abstracts International Section A: Humanities and Social Sciences
VL - 68:
IS - 8-A
N2 - Collecting assessment information on child characteristics and social emotional behaviors has predominantly relied on the use of adult informants. However, there has been an increase in the recognition of the importance of obtaining information directly from the child to supplement other assessment methods. This increase has been due in part to the improvement of the technical adequacy of recent self-report measures, and the understanding of the important role of self-perceptions on children's behavior through motivation. Limited self-report measures have been available in the past for assessing the concept of young children age 7 and below. This study investigated the Computer Assisted Child Interview (CACI) as a self-report measure of children's academic performance and school performance to determine its validity. The CACI is a computer assisted self-report assessment tool for children 4 to 10 years of age. It is used to obtain child perception of two broad areas: their perceptions of parenting behavior and strategies, and their own behavior and experiences. This study utilized a non-experimental within-subjects correlational design. The reliability of the CACI was established from a prior pilot project. The main focus of this study was to determine the relations between the predictor variables, particularly the CACI subscales of School Experience and Education Support, which solicited child input on their school difficulties and performance and the dependent variables such as parent and teacher reports. The Child Behavior Checklist (CBCL), the Teacher Report Form (TRF), the Peabody Picture Vocabulary Test-III (PPVT-III), and playground observation were used as criterion measures. Results indicated significant relations between child report on CACI School Experience subscale with the CBCL School Competence, and three TRF academic subscales. The CACI School Experience also correlated significantly with the Peer Rejection and Antisocial subscales of the Teacher Perception of Risk measure. No significant correlation was found between the CACI School Experience and the PPVT-III. Education Support scores predicted PPVT-III and positive playground behavior in the opposite direction. Scores from the CACI School Experience significantly predicted a combined construct of parent and teacher perception of student academic performance. Finally, hierarchical regression analyses indicated that the CACI school subscales contributed unique variance in predicting different models of outcome construct. (PsycINFO Database Record (c) 2012 APA, all rights reserved)
SN - 0419-4209
AD - Chow, Chi Ping: U Oregon, US
ER -

TY - JOUR
ID - 2634
T1 - Review of Volume I songbook: Songs & laughter on the playground
A1 - Schwartz,Elizabeth K.
Y1 - 2008///
N1 - Peer Reviewed Journal: 2008-19359-010
English
Journal; Peer Reviewed Journal
KW - PsycInfo
KW - Childhood Play Behavior
KW - Laughter
KW - Music
KW - Music Therapy
KW - Musical Instruments
RP - NOT IN FILE
SP - 134
EP - 135

JF - Music Therapy Perspectives
VL - 26:
IS - 2
N2 - Reviews the book, Volume I Songbook: Songs & Laughter on the Playground by P. Kern and A. M. Snell (2007). True to its title, this volume shares songs but also detailed information on strategies and interventions for turning playground time into a fun-filled as well as therapeutic music time. The book is a continuation of the work begun by author Petra Kern on incorporating music, music therapy and music instruments on the playground. The beginning Preface and Introduction are short statements but should be read very carefully for they contain the fundamental philosophy of practice of the two authors. The focus is in keeping with current trends in education which emphasize least restrictive environment and opportunities for inclusion and generalization. The music composed by both Kern and Snell is appropriate to the age and abilities of young and early school age children. The songs are simple, but not simplistic, with catchy melodies and rhythms. Each uses language that is functional outside of the song and can be generalized for use in everyday communication. Within this simplicity though, many of the songs contain some rhythmic or harmonic surprise that will appeal to the adults who work with children-supporting the author's stated purpose of encouraging others "to make music a part of their everyday lives". (PsycINFO Database Record (c) 2012 APA, all rights reserved)
SN - 0734-6875
AD - Schwartz, Elizabeth K.: Alternatives for Children, Setauket, NY, US
ER -

TY - JOUR
ID - 2635
T1 - Factors distinguishing most and least effective schools in terms of reading achievement: A residual approach. [References]
A1 - Papanastasiou,Constantinos
Y1 - 2008///
N1 - Peer Reviewed Journal: 2008-18787-005
English
Journal; Peer Reviewed Journal
KW - PsycInfo
KW - Academic Achievement
KW - Evaluation
KW - Reading Achievement
KW - School Learning
KW - Schools
KW - Literacy
KW - School Environment
RP - NOT IN FILE
SP - 539
EP - 549
JF - Educational Research and Evaluation
VL - 14:
IS - 6
N2 - This study was based on the Cyprus data from both the student questionnaire and the tests of the Progress in International Reading Literacy Study (PIRLS), undertaken in 2001 under the auspices of the International Association for the Evaluation of Educational Achievement (IEA). The sample included 3,001 fourth-grade students from Cyprus, whose average age was 9.7 at the time of testing. The statistical analysis used to distinguish the more and less effective schools was based on the residuals. From this analysis, 6 factors explaining school differences in reading achievement were identified. The 1st factor, which is the strongest one, is related to class activities subsequent to the reading class. The 2nd most important factor is related to reading outside school. The 3rd factor is related to the time spent on homework. The 4th factor relates to attitudes toward reading. The 5th and 6th factors are activities during teaching and school climate. (PsycINFO Database Record (c) 2012 APA, all rights reserved) (journal abstract)
SN - 1380-3611
AD - Papanastasiou, Constantinos: Department of Education, University of Cyprus, Nicosia, Cyprus
ER -

TY - JOUR
ID - 2636
T1 - Discourses on family time: The cultural interpretation of family togetherness in Los Angeles and Rome. [References]
A1 - Kremer-Sadlik,Tamar; Fatigante,Marilena; Fasulo,Alessandra
Y1 - 2008///
N1 - Peer Reviewed Journal: 2008-14899-002
English
Journal; Peer Reviewed Journal
KW - PsycInfo
KW - Family Relations
KW - Family Structure
KW - Family
KW - Parents
KW - Discourse Analysis
RP - NOT IN FILE
SP - 283
EP - 309
JF - Ethos
VL - 36:
IS - 3
N2 - In this article, we examine U.S. and Italian parents' discourses on family time in parent-filled weekly activity charts and interviews with parents. Analysis indicates that in Los Angeles, California, parents talk about sheltering and isolating their nuclear family from the outside world and from everyday routine by creating special times and special activities for the nuclear family. In contrast, Roman parents' discourse allows for spontaneous times with the family that are diffused within routines and merged with other community members, institutions, and social spaces. We argue that differences displayed in parents' discourses are shaped by culturally specific orientations toward time, family, and individual versus shared responsibility. Through this cross-cultural comparison we contribute to the understanding of how local cultural models shape different ways in which parents interpret time spent in family and influence individuals' perceptions of their everyday lives. (PsycINFO Database Record (c) 2012 APA, all rights reserved) (journal abstract)
SN - 0091-2131
AD - Kremer-Sadlik, Tamar: Center on Everyday Lives of Family, Department of Anthropology, UCLA, Los Angles, CA, US Fatigante, Marilena: Department of Developmental and Social Psychology, Sapienza University of Rome, Rome, Italy Fasulo, Alessandra: Department of Developmental and Social Psychology, Sapienza University of Rome, Rome, Italy
ER -

TY - JOUR
ID - 2637
T1 - Physical activity of children ages 6-8: The beginning of school attendance. [References]
A1 - Fromel,Karel; Stelzer,Jiri; Groffik,Dorota; Ernest,James
Y1 - 2008///
N1 - Peer Reviewed Journal: 2008-14885-003
English
Journal; Peer Reviewed Journal
KW - PsycInfo
KW - Elementary School Students
KW - Kindergarten Students
KW - Leisure Time
KW - Physical Activity
KW - Physical Education
RP - NOT IN FILE
SP - 29
EP - 40
JF - Journal of Research in Childhood Education
VL - 23:

IS - 1
N2 - This study investigated the physical activity (PA) levels of 6- to 8-year-old children over a seven-day period. The participants consisted of 35 girls and 36 boys in kindergarten and 113 girls and 131 boys in 1st grade. Physical activity (PA) is defined as "any body movement produced by skeletal muscles resulting in energy expenditure" and measured in this study using a Caltrac accelerometer. In addition to the data produced by the accelerometer, a detailed activity log was kept for each participant. The results revealed no statistically significant difference in PA levels between kindergarten and 1st-grade participants, F(1,311) = 1.16, p = .282. However, boys were more physically active than girls F(1, 311) = 66.10, p < .001, 2 = .17, and the level of PA was higher on school days than on free days for both sexes, F(1, 311) = 20.45, p < .001, 2 = .06. Finally, the data on the children's PA at school strongly supports the importance of physical education lessons (PE lessons) and recess periods in the daily school routine. (PsycINFO Database Record (c) 2012 APA, all rights reserved) (journal abstract)
SN - 0256-8543
AD - Fromel, Karel: Academy of Physical Education, Katowice, Poland Stelzer, Jiri: Valdosta State University, Valdosta, GA, US Groffik, Dorota: Academy of Physical Education, Katowice, Poland Ernest, James: Valdosta State University, Valdosta, GA, US
ER -

TY - JOUR
ID - 2638
T1 - Assessment of outdoor school environments and physical activity in Ankara's primary schools. [References]
A1 - Ozdemir,Aydin
Y1 - 2008///
N1 - Peer Reviewed Journal: 2008-12862-008
English
Journal; Peer Reviewed Journal
KW - PsycInfo
KW - Educational Measurement
KW - Elementary Schools
KW - Physical Activity
KW - School Environment
RP - NOT IN FILE
SP - 287
EP - 300
JF - Journal of Environmental Psychology
VL - 28:
IS - 3
N2 - Outdoor school environments are sites for play and physical activity for many children, and shortcomings within these environments are considered significant factors that contribute to children's inactive lifestyles and high levels of childhood obesity. This study explores the associations between the physical characteristics of schoolyards and the physical activity of third and fourth year students in five Turkish primary schools. Data were collected through multiple methods, including behavior mapping of student activities during recess, physical assessments of schoolyards, and interviews with students, teachers and administrations. The findings show similarities in the landscape features and physical qualities of schoolyards, particularly in the types of play and activities in which students engage. Results indicated that active students who walk to and from schools have lower body mass index (BMI) values than passive students, and students in schools with larger yards have lower BMI values. Most of the students prefer spacious and vegetated yards. A major concern is the crowdedness of the yards during recess that limit children's activity. Schoolyards with advanced landscape features are preferred more, and this in turn affects students' positive satisfaction. Outdoor school environments have a correlation to health outcomes and should be designed to promote more activity. Improving the physical and landscape qualities of the public schoolyards should be the primary concern of the designers in order to increase awareness of natural environment and more important, increase the health of children. (PsycINFO Database Record (c) 2012 APA, all rights reserved) (journal abstract)
SN - 0272-4944
AD - Ozdemir, Aydin: Department of Landscape Architecture, Ankara University, Ankara, Turkey Yilmaz, Oguz: Department of Landscape Architecture, Ankara University, Ankara, Turkey

ER -

TY - JOUR
ID - 2639
T1 - Entertainment modeling through physiology in physical play. [References]
A1 - Yannakakis,Georgios N.
Y1 - 2008///
N1 - Peer Reviewed Journal: 2008-12394-005
English
Journal; Peer Reviewed Journal
KW - PsycInfo
KW - Childhood Play Behavior
KW - Games
KW - Playgrounds
KW - Pleasure
KW - Simulation
KW - Cardiovascular Reactivity
KW - Physiological Arousal
KW - Psychophysiology
RP - NOT IN FILE
SP - 741
EP - 755
JF - International Journal of Human-Computer Studies
VL - 66:
IS - 10
N2 - This paper is an extension of previous work on capturing and modeling the affective state of entertainment ('fun') grounded on children's physiological state during physical game play. The goal is to construct, using representative statistics computed from children's physiological signals, an estimator of the degree to which games provided by the playground engage the players. Previous studies have identified the difficulties of isolating elements of physical activity attributed to reported entertainment derived (solely) from heart rate (HR) recordings. In the present article, a survey experiment on a larger scale and a physical activity control experiment for surmounting those difficulties are devised. In these experiments, children's HR, blood volume pulse (BVP) and skin conductance (SC) signals, as well as their expressed preferences of how much 'fun' particular game variants are, are obtained using games implemented on the Playware physical interactive playground. Given effective data collection, a set of numerical features is computed from these measurements of the child's physiological state. A comprehensive statistical analysis shows that children's reported entertainment preferences correlate well with specific features of the recorded signals. Preference learning techniques combined with feature set selection methods permit the construction of user models that predict reported entertainment preferences given suitable signal features. The most accurate models are obtained through evolving artificial neural networks and are demonstrated and evaluated on a Playware game and a control task requiring physical activity. The best network is able to correctly match expressed preferences in 69.64% of cases on previously unseen data (p-value=0.0022) and indicates two dissimilar classes of children: those that prefer constantly energetic play of low mental/emotional load; and those that report as fun a dynamic play that involves high mental/emotional load independently of physical effort. The generality of the methodology, its limitations, its usability as a real-time feedback mechanism for entertainment augmentation and as a validation tool are discussed. (PsycINFO Database Record (c) 2012 APA, all rights reserved) (journal abstract)
SN - 1071-5819
AD - Yannakakis, Georgios N.: Center for Computer Games Research, IT-University of Copenhagen, Copenhagen S, Denmark Hallam, John: Maersk Mc-Kinney Moller Institute, University of Southern Denmark, Odense M, Denmark
ER -

TY - JOUR
ID - 2640
T1 - Vulnerability of orphan caregivers vs. non-orphan caregivers in KwaZulu-Natal
A1 - DeSilva,M.
Y1 - 2008///

N1 - Peer Reviewed Journal: 2009-12420-003
English
Journal; Peer Reviewed Journal
KW - PsycInfo
KW - Caregiver Burden
KW - Caregivers
KW - Demographic Characteristics
KW - Orphans
KW - Social Capital
KW - Susceptibility (Disorders)
RP - NOT IN FILE
SP - 102
EP - 111
JF - Vulnerable Children and Youth Studies
VL - 3:
IS - 2
N2 - Using data from a cohort of school going children, their households and caregivers (the Amajuba Child Health and Wellbeing Project, ACHWRP) in KwaZulu-Natal, South Africa, we compared demographic characteristics, care burden, health, physical function and social capital of caregivers of orphans (n = 300) with those of non-orphans (n = 298). This analysis presents cross-sectional findings from questions for caregivers that were added for the study's second round, conducted between September 2005 and June 2006. Caregivers of all children were overwhelmingly women (87%). Compared to non-orphan caregivers, caregivers of orphans were on average older (49.5 vs. 45.2 years, p = 0.0002), had fewer years of education (6.7 vs. 7.8 years, p = 0.0042) and were less likely to be married or cohabiting (29% vs. 46%; p < 0.0001). Caregivers of orphans reported caring for more children than those of non-orphans (4.8 vs. 3.8 children, p < 0.0001). A larger proportion of orphan caregivers reported having cared for an ill adult in the previous year (28% vs. 19%), and for that adult to have been a biological child (p = 0.0531). There was a high prevalence (55%) of self-reported poor health among all caregivers; caregivers of orphans were more likely to report poor general health and chronic illness. Although there was a high prevalence of functional impairment (self-reported inability to perform primary activity) among all caregivers (59%), there was no difference between groups. In terms of social capital, although similar proportions of orphan and non-orphan caregivers reported having friends outside the household, orphan caregivers were less likely to have a source of a small emergency loan [51% vs. 63%; adjusted odds ratio: 0.60 (0.41-0.88)]. Our results suggest that orphan caregivers are indeed more vulnerable, and that their particular limitations and needs must be considered when developing strategies for assisting vulnerable households to ensure better support for both caregivers and the children in their care. (PsycINFO Database Record (c) 2012 APA, all rights reserved) (journal abstract)
SN - 1745-0128
AD - DeSilva, M. Bachman: Boston University School of Public Health, Center for International Health and Development (CIHD), Boston, MA, US Beard, J.: Boston University School of Public Health, Center for International Health and Development (CIHD), Boston, MA, US Cakwe, M.: University of KwaZulu-Natal, Health Economics and AIDS Research Division (HEARD), Durban, South Africa McCoy, K.: Boston University School of Public Health, Center for International Health and Development (CIHD), Boston, MA, US Nkosi, B.: University of KwaZulu-Natal, Health Economics and AIDS Research Division (HEARD), Durban, South Africa Parikh, A.: University of KwaZulu-Natal, Health Economics and AIDS Research Division (HEARD), Durban, South Africa Quinlan, T.: University of KwaZulu-Natal, Health Economics and AIDS Research Division (HEARD), Durban, South Africa Skalicky, A.: Boston University School of Public Health, Center for International Health and Development (CIHD), Boston, MA, US Tshabangu-Soko, S.: Boston University School of Public Health, Center for International Health and Development (CIHD), Boston, MA, US Zhuwau, T.: University of KwaZulu-Natal, Health Economics and AIDS Research Division (HEARD), Durban, South Africa Simon, J.: Boston University School of Public Health, Center for International Health and Development (CIHD), Boston, MA, US
ER -

TY - JOUR
ID - 2641
T1 - Reflections on the behavior of children in schools outdoor area: What we have learned from observing children's activities. [Portuguese]

A1 - de Sa Fernandes,Odara; Elali,Gleice Azambuja
Y1 - 2008///
N1 - Peer Reviewed Journal: 2009-11496-002
Portuguese
Journal; Peer Reviewed Journal
KW - PsycInfo
KW - Behavior Analysis
KW - Childhood Play Behavior
KW - Quality of Life
KW - School Environment
KW - Environmental Psychology
KW - Recreation
RP - NOT IN FILE
SP - 41
EP - 52
JF - Paideia: Cadernos de Psicologia e Educacao
VL - 18:
IS - 39
N2 - To understand the different relations children establish with the environment, an investigation on the use of several areas of a school patio and the interaction between children, (three to seven years old), during recreation time was performed. Data were collected through systematic observation, and place-centered mapping, which is an observational technique used in Environmental Psychology. According to the observed: playing in group was predominant, specially in areas with multiple-utility equipments, sports court, and in large open shady areas; differences in the use of the spaces were found related to children's age and gender. The implications of these results appoint to the need of planning outdoor areas that allow diverse activities, and promote quality of life for children regardless their age or gender. (PsycINFO Database Record (c) 2012 APA, all rights reserved) (journal abstract)
SN - 0103-863X
AD - de Sa Fernandes, Odara: Universidade Federal do Rio Grande do Norte, Natal, Brazil Elali, Gleice Azambuja: Centro de Tecnologia, Universidade Federal do Rio Grande do Norte, Natal, Brazil
ER -

TY - JOUR
ID - 2642
T1 - Influence of socio-demographics and residential environment on leisure activity participation. [References]
A1 - Kemperman,Astrid D.
Y1 - 2008///
N1 - Peer Reviewed Journal: 2008-10011-003
English
Journal; Peer Reviewed Journal
KW - PsycInfo
KW - Demographic Characteristics
KW - Leisure Time
KW - Recreation
KW - Social Environments
KW - Urban Environments
RP - NOT IN FILE
SP - 306
EP - 324
JF - Leisure Sciences
VL - 30:
IS - 4
N2 - Participating in leisure activities provides important benefits for all people. However, there is a substantial reduction of (green) leisure opportunities in cities, and the green spaces that are available often are poorly accessible. The purpose of this study is to analyze the diversity of participation in various types of leisure activities and to determine the relationship between leisure activity participation and characteristics of the residential environment depending on socio-demographic characteristics. Diary data from 803 respondents

living in the Eindhoven region in the Netherlands were used. Four segments were identified that differ regarding their leisure activity participation, and a modest impact of green space accessibility was found. (PsycINFO Database Record (c) 2012 APA, all rights reserved) (journal abstract)
SN - 0149-0400
AD - Kemperman, Astrid D. A. M.: Urban Planning Group, Eindhoven University of Technology, Eindhoven, Netherlands Timmermans, Harry J. P.: Urban Planning Group, Eindhoven University of Technology, Eindhoven, Netherlands
ER -

TY - JOUR
ID - 2643
T1 - A health passport to promote children's regular practice of physical activity outside of school. [References]
A1 - Blais,Mireille
Y1 - 2008///
N1 - Peer Reviewed Journal: 2008-09515-007
English
Journal; Peer Reviewed Journal
KW - PsycInfo
KW - Accountability
KW - Elementary School Students
KW - Home Environment
KW - Physical Activity
KW - Physical Education
KW - Teachers
RP - NOT IN FILE
SP - 416
EP - 433
JF - Journal of Teaching in Physical Education
VL - 27:
IS - 3
N2 - Most of the existing ecological studies have been conducted during class instructional time. The purpose of this qualitative study was to describe (a) how students (N = 102; grade 2-4) engaged in a strategy named Health Passport taking place mainly outside of school time and (b) how four physical education teachers held students accountable for their involvement in physical activity during a long period of time (3-7 months). An inductive approach guided the data analysis, based on observational notes, interviews, and the content of the students' Health Passport. The results indicated that children displayed five different profiles of involvement in the completion of the tasks related to their passport. Physical education teachers chose to trust students' self-management capacity instead of using a formal evaluation to hold them accountable. The experiment of the Health Passport showed that physical education teachers can put together and implement accountability strategies to support students' regular practice of physical activity at home. (PsycINFO Database Record (c) 2012 APA, all rights reserved) (journal abstract)
SN - 0273-5024
AD - Blais, Mireille: Departement des Sciences de l'Activite Physique, Universite du Quebec a Trois-Rivieres, Trois-Rivieres, PQ, Canada
ER -

TY - JOUR
ID - 2644
T1 - The utilization and effects of positive behavior support strategies on an urban school playground. [References]
A1 - Franzen,Kate
Y1 - 2008///
N1 - Peer Reviewed Journal: 2008-09347-003
English
Journal; Peer Reviewed Journal
KW - PsycInfo
KW - Behavior Modification

KW - Behavior Problems
KW - Elementary Schools
KW - Playgrounds
KW - School Based Intervention
KW - Urban Environments
RP - NOT IN FILE
SP - 150
EP - 161
JF - Journal of Positive Behavior Interventions
VL - 10:
IS - 3
N2 - Rates of problem behavior at urban elementary school playgrounds are of growing concern. The purpose of this study was to examine how the implementation of a recess intervention within the context of School-wide Positive Behavior Support (SwPBS), a systemwide, team-driven, data-based decision-making continuum of support, affected disruptive student behavior and teacher supervision on the playground in an urban elementary school. Specifically, this study replicated investigations conducted by Lewis and colleagues through teaching recess-related behaviors to students and using group contingencies to reinforce appropriate student behaviors. A multiple baseline design was used to assess the effects of SwPBS on the frequency of five target behaviors. Results indicated decreases in disruptive behaviors across three grade levels and increases in active teacher supervision. (PsycINFO Database Record (c) 2012 APA, all rights reserved) (journal abstract)
SN - 1098-3007
AD - Franzen, Kate: Kansas State Department of Education, KS, US Kamps, Debra: Juniper Gardens Children's Project, University of Kansas, Kansas City, KS, US
ER -

TY - JOUR
ID - 2645
T1 - Daily physical activity levels of elementary school-aged children with and without mental retardation. [References]
A1 - Foley,John T.
Y1 - 2008///
N1 - Peer Reviewed Journal: 2008-08034-005
English
Journal; Peer Reviewed Journal
KW - PsycInfo
KW - Elementary School Students
KW - Physical Activity
KW - Intellectual Development Disorder
RP - NOT IN FILE
SP - 365
EP - 378
JF - Journal of Developmental and Physical Disabilities
VL - 20:
IS - 4
N2 - One of the goals of Healthy People 2010 is to eliminate health disparities of people with disabilities. However, there is a deficit of information on physical activity levels in children with mental retardation (MR) in both the school and out of school environments. The purpose of this study was to investigate the physical activity levels of elementary school-aged children with and without MR in both school, after-school and weekend environments. Accelerometers were used to record physical activity levels of nine children with mild MR and 33 children without (NMR). Four multiple regressions were performed to identify the difference between groups while accounting for age, gender and BMI. While at school, physical activity levels for children with MR were significantly lower during both recess and physical education, $p < 0.001$, $p < 0.001$ respectively, After school and weekend physical activity levels for children with MR were significantly lower, $p < 0.001$, $p = 0.006$ respectively. The findings of this study indicate that the disparity in physical activity in individuals with MR begins as early as the elementary school years and is measurable across environments. (PsycINFO Database Record (c) 2012 APA, all rights reserved) (journal abstract)
SN - 1056-263X

AD - Foley, John T.: Department of Physical Education, UNY Cortland, Cortland, NY, US Bryan, Rebecca R.: Department of Nutrition and Exercise Sciences, Oregon State University, Corvallis, OR, US McCubbin, Jeffrey A.: Department of Nutrition and Exercise Sciences, Oregon State University, Corvallis, OR, US
ER -

TY - JOUR
ID - 2646
T1 - Shared family activities and the transition from childhood into adolescence. [References]
A1 - Crosnoe,Robert
Y1 - 2008///
N1 - Peer Reviewed Journal: 2008-02385-002
English
Journal; Peer Reviewed Journal
KW - PsycInfo
KW - Childhood Development
KW - Family
KW - Life Changes
KW - Socioeconomic Status
KW - Television Viewing
RP - NOT IN FILE
SP - 23
EP - 48
JF - Journal of Research on Adolescence
VL - 18:
IS - 1
N2 - Drawing on time use data from the Child Development Supplement of the Panel Study of Income Dynamics, this study identified five different profiles of shared time between parents and young people at different stages of development. In childhood, all profiles had high rates of shared television viewing, but some were oriented toward in-home activities and others toward activities outside the home (e.g., cultural events). These latter profiles tended to be higher in socioeconomic advantage, and the young people in them tended to demonstrate greater gains in math, but not reading, achievement across the transition into adolescence. In adolescence, shared activity profiles favored low amounts of shared time between parents and adolescents across activities and disfavored shared time in public domains. (PsycINFO Database Record (c) 2012 APA, all rights reserved) (journal abstract)
SN - 1050-8392
AD - Crosnoe, Robert: University of Texas at Austin, Austin, TX, US Trinitapoli, Jenny: University of Texas at Austin, Austin, TX, US
ER -

TY - JOUR
ID - 2647
T1 - Social skills intervention for children with autism during interactive play at a public elementary school. [References]
A1 - Licciardello,Cristina C.
Y1 - 2008///
N1 - Peer Reviewed Journal: 2008-02275-002
English
Journal; Peer Reviewed Journal
KW - PsycInfo
KW - Autism
KW - Peer Relations
KW - School Based Intervention
KW - Social Interaction
KW - Social Skills
KW - Childhood Play Behavior
KW - Peers
KW - Social Skills Training

RP - NOT IN FILE
SP - 27
EP - 37
JF - Education & Treatment of Children
VL - 31:
IS - 1
N2 - We evaluated a social skills intervention with four children who had autism during interactive play with typical peers at a public elementary school. Paraprofessional staff (classroom assistants) used preteaching, rewards, and prompting to increase social initiations by the children toward peers. The children's social responses to peer initiations also were measured and rewarded. Intervention was introduced in a multiple baseline design and produced increases in social interactions between the children and peers. The application of social skills training in naturalistic settings and topics for future research are discussed. (PsycINFO Database Record (c) 2012 APA, all rights reserved) (journal abstract)
SN - 0748-8491
AD - Licciardello, Cristina C.: University of Massachusetts, MA, US Harchik, Alan E.: May Institute, Randolph, MA, US Luiselli, James K.: May Institute, Randolph, MA, US
ER -

TY - JOUR
ID - 2648
T1 - The social and emotional development of knee children. [References]
A1 - Henderson,Bruce B.
Y1 - 2008///
N1 - Electronic Collection: 2008-01455-001
English
Electronic Collection
KW - PsycInfo
KW - Emotional Development
KW - Psychosocial Development
RP - NOT IN FILE
JF - PsycCRITIQUES
VL - 53:
IS - 15
N2 - Reviews the book, Socioemotional development in the toddler years: Transitions and transformations, edited by Celia A. Brownell and Claire B. Kopp (see record 2007-15136-000). Many might argue that toddlerhood, like middle childhood, has been relatively neglected by developmentalists. While infants, preschoolers, and adolescents have been the focus of broad developmental theory and research for a long time, toddlers (one- and two-year-olds, called "knee babies" or "knee children" by Margaret Mead) have received attention only in specific domains, such as language development and attachment. However, it is clear with this book that their time has come. Clever experimenters have found ways to tap the toddler mind, and modern theories make what goes on from ages one to three important. Sociocultural theories, with their emphasis on language and communication, and epigenetic and dynamic systems theories, with an emphasis on behavioral organization within a biological perspective, see these early years as crucial. Most of the contributors to this book use some version of sociocultural or epigenetic theory to explain what is going on during the toddler years. The contributors are good about organizing their chapters by telling readers what they have to say, saying it, and telling them what they said. However, the chapter authors clearly did not read each other's contributions, so there is considerable unnoted redundancy throughout. The editors weave the contributions together nicely in their introductory chapter, but that integration occurs before the reader has seen the chapters. Other concerns include the lack of an author index and too little use of descriptions of toddler activity in natural environments. Finally, several authors allude to the role of objects and the physical environment in the interactions with others. Yet the picture of toddlerhood presented in this book is peculiarly underrepresented by key toddler activities such as object play, exploration, and problem solving. Despite these quibbles, this is an excellent book. The authors collectively make a convincing case for the importance of toddlerhood in social and emotional development. (PsycINFO Database Record (c) 2012 APA, all rights reserved)
SN - 1554-0138
ER -

TY - JOUR
ID - 2649
T1 - Overview of behavioral assessment with children and adolescents. [References]
A1 - Reitman,David; Christiansen,Heather; Snyder,Julie
Y1 - 2008///
N1 - Book: 2007-19009-001
English
Book; Edited Book
KW - PsycInfo
KW - Adolescent Development
KW - Behavioral Assessment
KW - Childhood Development
KW - Clinical Psychology
KW - Mental Disorders
KW - Psychodiagnosis
RP - NOT IN FILE
SP - 3
EP - 24
JF - Hersen, Michel [Ed]; Reitman, David [Ed]
VL - :
IS - 2008
N2 - (from the chapter) Behavioral assessment has evolved rapidly since Hersen and Bellack (1976) first surveyed the field over 30 years ago. Child behavioral assessment (CBA), in particular, has grown increasingly complex. Whereas the earliest treatments of behavioral assessment focused on broad areas of concern such as "behavioral excesses" and "behavioral deficits," contemporary efforts "suggest a field that is becoming more inclusive, and at the same time more highly specialized" (Reitman, 2006, p.3). Much of the growing specialization in behavioral assessment has been fueled by the Diagnostic and Statistical Manual of Mental Disorders (DSM; American Psychiatric Association, 2000). Indeed, Child Behavioral Assessment (Ollendick & Hersen, 1984) devoted only a single chapter to diagnostic issues. By contrast, a recent special section of the Journal of Clinical Child and Adolescent Psychology (see Mash & Hunsley, 2005) emphasizes the DSM taxonomy yet reveals limitations in DSM-focused assessment that has broad implications for CBA (see Kazdin, 2005; Pelham, Fabiano, & Massetti, 2005). In this overview, we discuss CBA past and present and offer some perspectives on the future of research and practice in this ever-developing field. Behavior therapy has undergone many changes since its inception in the 1950s. During its formative years, behavior therapy focused heavily on the development of more effective treatments, but the importance of behavioral assessment practices was relatively unappreciated until the late 1970s and early 1980s. Since the early 1980s, trends in therapy have continued to drive changes in assessment practices. Most recently, concerns have arisen over the merits of some forms of behavioral assessment. Specifically, questions have arisen about diagnostic assessment, especially in the realm of treatment monitoring and outcome evaluation. As a result, the next decade may well feature more research evaluating the utility of functional behavioral assessment and greater appreciation for the diverse functions of behavioral assessment in child behavior therapy. Finally, as with the evidence-based treatment movement, there appears to be a large gap between the assessment practices utilized by university and grant-funded researchers and assessment activities in clinical practice settings. The greater empirical scrutiny that is being brought to bear on assessment in clinical practice appears consistent with societal trends emphasizing demands for accountability outside of and within the mental health system (Lambert & Hawkins, 2004). Although preliminary data suggest the need for changes in assessment practices to accommodate the realities of clinical practice, there are only limited data concerning guidance on empirically based assessment as it should be practiced in the field. (PsycINFO Database Record (c) 2012 APA, all rights reserved)
AD - Reitman, David: Center for Psychological Studies, Nova Southeastern University, Fort Lauderdale, FL, US Christiansen, Heather: Center for Psychological Studies, Nova Southeastern University, Fort Lauderdale, FL, US Snyder, Julie: Center for Psychological Studies, Nova Southeastern University, Fort Lauderdale, FL, US
ER -

TY - JOUR
ID - 2650
T1 - Girls on the sidelines: "Gendered" development in early childhood classrooms. [References]
A1 - de Groot Kim,Sonja

Y1 - 2008///
N1 - Book: 2007-14754-021
English
Book; Edited Book
KW - PsycInfo
KW - Early Childhood Development
KW - Physical Education
KW - Sex Roles
KW - Socialization
KW - Sociocultural Factors
KW - Exercise
KW - Human Sex Differences
KW - Physical Activity
KW - Physical Development
KW - Teachers
RP - NOT IN FILE
SP - 358
EP - 369
JF - van Oers, Bert [Ed]; Wardekker, Wim [Ed]; Elbers, Ed [Ed]; Van der Veer, Rene [Ed]
VL - :
IS - 2008
N2 - (from the chapter) In this chapter, I focus on the physical activity patterns of some of the two- and three-year-old boys during transition times while they waited in the classroom to go play outside or in the hallway. It appeared that these boys regularly sent their teachers nonverbal, gender-related messages. I will discuss (1) how socialization of two- and three-year-old boys and girls into their gender roles seemed to be influenced by the sociocultural context of their particular setting, a classroom in a childcare center; (2) how a small group of toddler boys seemed to transform unintentional physical body contact activity into intentional body contact activity, which resulted in gaining earlier access to space and resources and receiving more teacher attention; and (3) how a year later, in response to a different set of sociocultural expectations, some of the three-year-old boys seemed to transform body contact activity into a more socially acceptable, noncontact activity, aided by voice and hand gestures. They similarly gained earlier access to space and resources and received more teacher attention. (PsycINFO Database Record (c) 2012 APA, all rights reserved)
AD - de Groot Kim, Sonja: Kean University, Union, NJ, US
ER -

TY - JOUR
ID - 2651
T1 - Virtual playgrounds: Children's multi-user virtual environments for playing and learning with science. [References]
A1 - Kafai,Yasmin B.
Y1 - 2008///
N1 - Book: 2007-09150-008
English
Book; Edited Book
KW - PsycInfo
KW - Computer Assisted Instruction
KW - Computer Games
KW - Learning
KW - Science Education
KW - Virtual Reality
KW - Technology
RP - NOT IN FILE
SP - 196
EP - 217
JF - Willoughby, Teena [Ed]; Wood, Eileen [Ed]
VL - :
IS - 2008

N2 - (from the chapter) Our chapter will present an analysis of different activities available in Whyville, in particular casual, collaborative, and community science-related games, and the potential they hold for science inquiry in informal settings. In our evaluation, we were interested to find out where players spend their time and to what extent they become engaged in Whyville's various science games. We worked with two classes of sixth-grade students aged 10-12 and asked them about their activity preferences in Whyville at different time points during their three months online at school and at home. Our analyses have shown that multi-user virtual environments (MUVEs) such as Whyville offer a promising informal place for children to learn and play with science in multiple ways. The categorization of different science game genres helped us to identify Whypox as a distinct feature of MUVEs because it facilitated the immersion of the whole community rather than individual players, it is clear that these immersive features in MUVEs deserve further design efforts and study in how they can lead to greater engagement in science and technology that possibly might lead to improved understanding of science and technology ideas. (PsycINFO Database Record (c) 2012 APA, all rights reserved)
AD - Kafai, Yasmin B.: U California, Graduate School of Education and Information Studies, Los Angeles, CA, US Giang, Michael T.: U California, Graduate School of Education and Information Studies, Psychological Studies in Education Program, Los Angeles, CA, US
ER -

TY - JOUR
ID - 2652
T1 - The acquisition process of children's game songs during peer teaching: A semi-structured experiment with pre-school children. [References]
A1 - Ogawa,Yoko
Y1 - 2007///
N1 - Peer Reviewed Journal: 2009-10419-007
English
Journal; Peer Reviewed Journal
KW - PsycInfo
KW - Music
KW - Peers
KW - Playgrounds
KW - Strategies
KW - Teachers
RP - NOT IN FILE
SP - 69
EP - 78
JF - Research Studies in Music Education
VL - 29:
IS - 1
N2 - A semi-structured experiment was conducted to identify children's acquisition processes when learning playground game songs. Participants in the study were 20 pairs of children aged 4, 5, and 6 years, with each pair consisting of a younger child-learner and a senior child-teacher. The pairs were divided into two groups; Group 1 was observed by a researcher and a video-recorder, while Group 2 was observed using only a video-recorder. All child-teachers were well-acquainted with how to play the games, however the games were unfamiliar to the child-learners. Results suggest that child-teachers change their strategy of teaching depending on the conditions of instruction. Child-teachers in Group 1 used a variety of strategies including: demonstrating; playing and singing with the learner; slowing down; and, instructing. In contrast, child-teachers in Group 2: demonstrated; frequently played with the learner; sang alone; and, increased the tempo and rate of repetition even though the child-learners did not understand. In Group 1 over 50% of the time was devoted to demonstration by child-teachers, 40% to children playing together, and 5% to dialogue. In Group 2, over 90% of the time was devoted to the children playing together, and approximately 10% to demonstration by child-teachers. The article suggests that this latter instruction mode is not only a common feature of child-teachers' teaching in Group 2, it is also reflective of the teaching strategies of the masters of Japanese traditional music. (PsycINFO Database Record (c) 2012 APA, all rights reserved) (journal abstract)
SN - 1321-103X
AD - Ogawa, Yoko: Tottori University, Tottori, Japan Murao, Tadahiro: Aichi University of Education, Japan
ER -

TY - JOUR
ID - 2653
T1 - Using a tactile map with a 5-year-old child in a large-scale outdoor environment. [References]
A1 - Renshaw,Rebecca L.
Y1 - 2007///
N1 - Peer Reviewed Journal: 2008-11283-002
English
Journal; Peer Reviewed Journal
KW - PsycInfo
KW - Blind
KW - Childhood Play Behavior
KW - Mobility Aids
KW - Pediatrics
RP - NOT IN FILE
SP - 113
EP - 120
JF - RE:view: Rehabilitation and Education for Blindness and Visual Impairment
VL - 39:
IS - 3
N2 - The authors constructed an 11 x 17 in. tactile map to teach routes in an outdoor play environment to a 5-year-old blind child. After learning the routes on the tactile map, the child independently navigated them in the outdoor play environment. With practice, the child reduced the time necessary to learn and navigate the routes. The authors identify some concerns about the design of the project and suggest ways in which future researchers could resolve those issues. (PsycINFO Database Record (c) 2012 APA, all rights reserved) (journal abstract)
SN - 0899-1510
AD - Renshaw, Rebecca L.: University of Pittsburgh, Pittsburgh, PA, US Zimmerman, George J.: University of Pittsburgh, Pittsburgh, PA, US
ER -

TY - JOUR
ID - 2654
T1 - Parental bookreading in low-income families: Differences and similarities between fathers and mothers in frequency and style of reading and the impact on children's language and literacy development
A1 - Duursma,A.
Y1 - 2007///
N1 - Dissertation Abstract: 2007-99230-509
English
Dissertation Abstract
KW - PsycInfo
KW - Childhood Development
KW - Family
KW - Literacy
KW - Lower Income Level
KW - Reading
KW - Fathers
KW - Language
KW - Mothers
RP - NOT IN FILE
SP - 2310
JF - Dissertation Abstracts International Section A: Humanities and Social Sciences
VL - 68:
IS - 6-A
N2 - This thesis presents three studies of parental bookreading in low-income families. The first study examined differences between fathers and mothers in reported frequency of bookreading at child ages 24 and 36 months and in the summer before children entered Kindergarten. This study also looked at differences (e.g., education, race/ethnicity, language) between families where both parents report reading frequently to their toddlers compared to families where only mother reports reading frequently. The second study investigated

whether paternal bookreading makes an additional contribution to children's language and literacy, over and above maternal bookreading. The third study examined differences and similarities in style between fathers and mothers when reading to their toddlers. Results indicated that mothers reported reading more frequently to their toddlers than fathers. Furthermore, fathers in families where both parents reported reading regularly to their children were likely to have higher levels of education than fathers in families where only the mother did so. Children in families where two parents read to them had higher scores on measures of cognition and language. Paternal bookreading, in addition to maternal bookreading, predicted children's emergent literacy skills and oral language skills assessed prior to kindergarten entry. Both fathers and mothers actively engaged their children in the bookreading interaction. Fathers used more non-immediate talk (talk related to objects and events outside the book) than mothers did. Fathers were also more likely to help their child decode the text at preK. Mothers on the other hand were more likely to engage their children by eliciting text segments. Both parents engaged in talk about the book itself. The findings in this thesis emphasize the importance of including fathers in research studies when examining child development in a family context. Most of the research on bookreading has focused on mothers since they are often still the primary caregiver of the child. The results from these studies demonstrate that fathers do make a difference in children's language and literacy development. More research is needed to investigate fathers' unique contributions to child development. (PsycINFO Database Record (c) 2012 APA, all rights reserved)
SN - 0419-4209
AD - Duursma, A. Elisabeth: Harvard U., US
ER -

TY - JOUR
ID - 2655
T1 - Mexican-American adolescents and metabolic syndrome: Deciphering the role of acculturation
A1 - Ruiz,Rafael Enrique
Y1 - 2007///
N1 - Dissertation Abstract: 2007-99200-432
English
Dissertation Abstract
KW - PsycInfo
KW - Acculturation
KW - Metabolic Syndrome
KW - Mexican Americans
KW - Diets
KW - Food Intake
KW - Physical Activity
RP - NOT IN FILE
SP - 2300
JF - Dissertation Abstracts International: Section B: The Sciences and Engineering
VL - 68:
IS - 4-B
N2 - Metabolic syndrome (defined by three or more of the following traits: elevated triglycerides, low HDL, abdominal overweight, elevated fasting plasma glucose, and elevated blood pressure) has been linked to an increased risk of type-2 diabetes and cardiovascular disease. Cook and colleagues (2003) demonstrated that Mexican American have the highest prevalence of metabolic syndrome among adolescents. Thus, understanding the factors associated with an elevated risk for metabolic syndrome in this population is paramount. This study examines the role of acculturation on the risk of metabolic syndrome and its antecedents, physical activity and dietary intake, in Mexican-American adolescents. A pooled cross-section (N=1831) of adolescents (12-19 yrs.) was taken from the NHANES 1999-2002. Logistic and linear regression models were used to examine the relationships between acculturation and metabolic syndrome, physical activity, and dietary intake controlling for other known correlates. The overall prevalence of metabolic syndrome was 4.8% or approximately 1.5 million U.S. adolescents. Mexican American ethnicity, overweight, gender, and inactivity, were associated with a higher prevalence of metabolic syndrome. Mexican-American adolescents were more likely to be overweight and inactive compared to Caucasians. Acculturation did not have a direct association with metabolic syndrome, but higher levels of acculturation were associated with increased physical activity and increased daily intake of calories, carbohydrates, saturated fat, and the number of times eating meals outside the home. Mexican American males were the most likely to have metabolic syndrome. Acculturation seems to be playing a role in

the risk profile of Mexican-American adolescents, which increases their daily dietary intake that may contribute to metabolic syndrome via overweight. Acculturation also increased the likelihood of physical activity, but this association may be insufficient to override the other risk factors. These results suggest that acculturation should be considered when designing and implementing interventions or policies aimed at Mexican-American adolescents to reduce overweight, increase physical activity, and promote healthy dietary options. This may help to reduce the treatment costs associated with overweight and type-2 diabetes. (PsycINFO Database Record (c) 2012 APA, all rights reserved)
SN - 0419-4217
AD - Ruiz, Rafael Enrique: U North Carolina At Chapel Hill, US
ER -

TY - JOUR
ID - 2656
T1 - Theory of mind ability in the preadolescent language broker: Connections between language brokering, social cognition, and academic achievement
A1 - Love,Julia Anne
Y1 - 2007///
N1 - Dissertation Abstract: 2007-99014-009
English
Dissertation Abstract
KW - PsycInfo
KW - Academic Achievement
KW - Cognitive Ability
KW - Language
KW - Social Cognition
KW - Human Sex Differences
RP - NOT IN FILE
SP - 651
JF - Dissertation Abstracts International: Section B: The Sciences and Engineering
VL - 68:
IS - 1-B
N2 - This study examined the connections between language brokering, social cognition, and academic achievement for preadolescent Latinos. Gender differences in language brokering activity, theory of mind ability, and academic achievement were also examined. There is a paucity of research on theory of mind development in older children from low SES and ethnic minority communities, as well as children from cultures outside the United States and Western Europe. There is no research on the relation between language brokering, theory of mind ability, and academic achievement. This study uses both a cultural approach and a theory of mind approach to study the socio-cognitive development of Latino preadolescents from immigrant families. Participants were 117 Mexican American 6th graders (M = 11.33 years; 56 girls, 61 boys) from a semi-rural agricultural community in Oregon. Each participant completed a questionnaire consisting of the Language Brokering Scale, the Short Acculturation Scale for Hispanic Youth, the Parent-Child Bonding Scale, and demographic information. Participants also completed tests of receptive English vocabulary (PPVT-III), receptive Spanish vocabulary (TVIP), and responded to two theory of mind vignettes developed to assess higher order theory of mind ability. Participants who reported being chosen as the primary language broker out-performed non-primary language brokers on theory of mind ability, things brokered, GPA, and reading achievement test scores. Girls out-performed boys on theory of mind ability, biculturalism, Spanish vocabulary, and GPA. Non-primary girls language brokered for more people than primary girls, and non-primary boys language brokered for less people than primary boys. Non-primary girls also reported more biculturalism than any other sub-group. English vocabulary, language broker order, and feelings about brokering were positively related to theory of mind ability for boys. Language broker order and biculturalism were positively related to theory of mind ability for girls. The relationship between theory of mind ability and academic achievement was non-existent for girls, and seemed to be masked by language brokering variables for boys. These results are discussed in relation to cultural variation in theory of mind, and traditional Latino gender socialization goals. Implications for the classroom and suggestions for future research are also discussed. (PsycINFO Database Record (c) 2012 APA, all rights reserved)
SN - 0419-4217
AD - Love, Julia Anne: The Claremont Graduate U., US

ER -

TY - JOUR
ID - 2657
T1 - Perceived classroom climate and motivation in physical education during the move to middle school
A1 - Cox,Anne E.
Y1 - 2007///
N1 - Dissertation Abstract: 2007-99005-093
English
Dissertation Abstract
KW - PsycInfo
KW - Classroom Environment
KW - Middle Schools
KW - Motivation
KW - Physical Education
RP - NOT IN FILE
SP - 3344
JF - Dissertation Abstracts International Section A: Humanities and Social Sciences
VL - 67:
IS - 9-A
N2 - Declines in physical activity-related beliefs and behaviors have been observed in the physical education setting as students move through middle school (Parish & Treasure, 2003). According to the stage-environment fit hypothesis (Eccles & Midgley, 1989) and self-determination theory (Deci & Ryan, 1985) these declines may be due in part to change in students' perceptions of the physical education context. Certain changes in the social context may undermine key individual perceptions that impact self-determined motivation, affect, and behavior. The purposes of this dissertation were to: (a) examine the relationships among and change in students' perceptions of the classroom climate, individual perceptions (competence, autonomy, relatedness), self-determined motivation, and enjoyment in physical education and physical activity outside of school and (b) explain change in these variables as students move into the first two years of middle school. Students completed a multi-form questionnaire with psychometrically sound measures of study variables as 5th and 6th graders (n = 518) and again the next school year (n = 449). Results from structural equation modeling analyses revealed that perceived teacher support mediated the positive relationships between perceived mastery climate and individual perceptions. Perceived competence, autonomy, and relatedness partially mediated the positive relationship between perceived teacher support and self determined motivation. Further, students' enjoyment in physical education mediated the positive relationship between self-determined motivation and physical activity outside of school. Group difference analyses showed that students' perceptions of teacher support (older cohort who changed teachers only), enjoyment in physical education, and physical activity behavior declined from one school year to the next. Finally, results of structural models of change in study variables supported cross-sectional analyses. This study extends past research by including perceived teacher support in an examination of the physical education classroom climate, providing a description of motivation-related variables in physical education as students move into middle school and demonstrating the link between students' physical education experiences and their physical activity behavior outside of school. Developing strategies that help students feel supported by their teacher and socially connected in physical education may promote self-determined motivation in physical education and, in turn, greater physical activity outside of school. (PsycINFO Database Record (c) 2012 APA, all rights reserved)
SN - 0419-4209
AD - Cox, Anne E.: Purdue U., US
ER -

TY - JOUR
ID - 2658
T1 - Play and joint attention of children with autism in the preschool classroom
A1 - Wong,Connie Shu-Ya
Y1 - 2007///
N1 - Dissertation Abstract: 2007-99001-089
English
Dissertation Abstract

KW - PsycInfo
KW - Attention
KW - Autism
KW - Childhood Play Behavior
KW - Classroom Environment
KW - Preschool Education
RP - NOT IN FILE
SP - 2534
JF - Dissertation Abstracts International Section A: Humanities and Social Sciences
VL - 67:
IS - 7-A
N2 - Young children learn by actively interacting with their environment. They explore their surroundings to play with objects and with other people. Through these interactions, they gain new knowledge and understanding which contributes to their social and cognitive development. Young children with autism may have difficulties interacting with their environment because of their specific deficits in symbolic play and joint attention. The purpose this study was to provide information on play and joint attention behaviors in a naturalistic environment, the preschool special education classroom, for children with autism (N=27) as compared to children with mental retardation or other developmental delays (N=28). The participants were observed in their classroom environment for approximately two hours over three separate days. The children were also assessed individually on visual, motor, receptive, and expressive language skills as well on play and joint attention. The children ranged in age from three to five years old and their mental ages ranged from 18.5 to 59 months. Child characteristics and demographics were not significantly different between the two groups. Results show that children with autism spent more of their time in an unengaged state than in any other engagement state, which directly translated into fewer symbolic play and joint attention behaviors. Additionally, children were not showing their optimal levels of symbolic play at school, nor were their teachers targeting those levels. Compared to children with mental retardation or other developmental delays, children with autism displayed fewer responses to and initiations of joint attention. Finally, the findings indicate that although teachers seldom focused on symbolic play and joint attention in their teaching, when they did, it was rarely for the purposes of increasing symbolic play and joint attention skills. These findings suggest the importance of teachers targeting play and joint attention skills in their preschool special education classes, specifically for children with autism. (PsycINFO Database Record (c) 2012 APA, all rights reserved)
SN - 0419-4209
AD - Wong, Connie Shu-Ya: U California, Los Angeles, US
ER -

TY - JOUR
ID - 2659
T1 - An evaluation of school-wide positive behavior support in middle schools with the Oregon Healthy Teens Student Survey
A1 - Smolkowski,Keith
Y1 - 2007///
N1 - Dissertation Abstract: 2007-99001-031
English
Dissertation Abstract
KW - PsycInfo
KW - Middle Schools
KW - Positivism
KW - Schools
KW - Student Attitudes
KW - Support Groups
RP - NOT IN FILE
SP - 2532
JF - Dissertation Abstracts International Section A: Humanities and Social Sciences
VL - 67:
IS - 7-A
N2 - Schools have long sought measures to improve student behavior. Positive Behavior Support (PBS) aims to provide students with clearly defined, carefully taught, and consistently rewarded behavioral expectations, as

well as punishers consistent with the level and function of misbehavior. School-wide PBS addresses student behavior in the public areas of the school, such as the hallways, cafeteria, playground, and so on, through PBS practices and the use of office discipline referrals for evaluation and decision making. This study assessed the influence of school-wide PBS in middle schools using student surveys collected by the Oregon Healthy Teens project. The research hypotheses posited that (a) school-wide PBS would improve school context measures, such as teacher praise; (b) school-wide PBS would improve student target behaviors, such as delinquency and truancy; (c) school-wide PBS would not necessarily change measures of student competence, such as social skills; (d) student competence would moderate the effect of school-wide PBS on student target behaviors; and (e) if the target behaviors were associated with school-wide PBS, then school context measures would mediate those effects. The analyses compared student reports of school context, target behaviors, and competence in a quasi-experimental design with 103 randomly selected middle schools across three years, among which 32 received training in school-wide PBS. Student survey data and office discipline referrals were also correlated with measures of school-wide PBS implementation collected in 19 schools. The longitudinal, multilevel statistical models accounted for the nested structure of the data, which included student, cohort, and school levels. The results failed to support the research hypotheses, as no tests of context, target behaviors, competence, or moderator effects obtained statistical significance. With no differences in target behaviors, mediational analyses were not applicable. A thorough appraisal of this investigation revealed that the lack of support for the research hypotheses likely arose from limitations of the quasi-experimental design as well as survey questions that inadequately measured the changes in behavior expected from the implementation of school-wide PBS. (PsycINFO Database Record (c) 2012 APA, all rights reserved)
SN - 0419-4209
AD - Smolkowski, Keith: U Oregon, US
ER -

TY - JOUR
ID - 2660
T1 - Using bullying incident density to evaluate the Olweus Bullying Prevention Programme. [References]
A1 - Black,Sally A.
Y1 - 2007///
N1 - Peer Reviewed Journal: 2007-19048-007
English
Journal; Peer Reviewed Journal
KW - PsycInfo
KW - Program Evaluation
KW - Racial and Ethnic Groups
KW - School Based Intervention
KW - Urban Environments
KW - Bullying
RP - NOT IN FILE
SP - 623
EP - 638
JF - School Psychology International
VL - 28:
IS - 5
N2 - Bullying negatively impacts the mental and physical health of student victims, bullies and bystanders. The Olweus Bullying Prevention Programme is an internationally recognized school based programme demonstrated effective in research. The purpose of this study was to determine if the Bullying Prevention Programme was effective for urban youth from diverse ethnic backgrounds. The programme was implemented in one large urban school district in the United States. Bullying incident density was used to evaluate programme outcomes. An independent evaluator performed 319 observation sets, 7,589 minutes. Data were standardized to incident density and reported back to school committees who developed and implemented culturally and developmentally relevant interventions based on the Olweus model. At baseline, incident density was 65 incidents per 100 student hours. After four years, incident density decreased by 45 percent to 36 incidents per 100 student hours. Programme components associated with decreased incidents were posting of rules, consistent enforcement of positive and negative consequences and training adult monitors to engage students in activities. Supplementary strategies were socialized recess, providing pro-social activities, reorganization of lunches and recess and separation of grades or genders. Results suggest that the Bullying Prevention Programme reduces

incident density by up to 65 percent. (PsycINFO Database Record (c) 2012 APA, all rights reserved) (journal abstract)
SN - 0143-0343
AD - Black, Sally A.: Health Services Department, Saint Joseph's University, Philadelphia, PA, US Jackson, Ericka: School District of Philadelphia's Office of School Climate, Philadelphia, PA, US
ER -

TY - JOUR
ID - 2662
T1 - Mapping the social and physical contexts of physical activity across adolescence using ecological momentary assessment. [References]
A1 - Dunton,Genevieve Fridlund; Whalen,Carol
Y1 - 2007///
N1 - Peer Reviewed Journal: 2007-15284-004
English
Journal; Peer Reviewed Journal
KW - PsycInfo
KW - Adolescent Development
KW - Environmental Effects
KW - Exercise
KW - Physical Activity
KW - Walking
KW - Psychosocial Factors
RP - NOT IN FILE
SP - 144
EP - 153
JF - Annals of Behavioral Medicine
JA - Ann Behav Med
VL - 34:
IS - 2
N2 - Background: Research has sought to understand how environmental factors influence adolescent physical activity, yet little is known about where and with whom adolescents are physically active. Purpose: This study used electronic ecological momentary assessment (e.EMA) to map the social and physical contexts of exercise and walking across adolescence. Differences in physical activity contexts by gender, grade in school, day of the week, and season were examined. Methods: Twice a year between 9th and 12th grade, 502 adolescents (51% female) of mixed ethnicity (55% White) participated in 4-day e.EMA intervals (Thursday-Sunday) where their primary activity (e.g., exercise, TV, homework), social company (e.g., friends, family, class), and physical location (e.g., home, school, outdoors) were assessed every 30 (+10) min during waking hours. Results: Overall, greater proportions of exercise and walking were reported with friends, outdoors, and at school. However, boys were more likely to report exercising and walking in outdoor locations than girls. Exercising with classmates, family, and at school decreased across high school. Walking with family, friends, and outdoors also decreased. On weekdays compared to weekends, students reported a greater proportion of their exercise and walking at school. Students were more likely to report exercising and walking outdoors in the fall and the spring than in the winter. Conclusion: e.EMA showed that the social and physical contexts of adolescent exercise and walking vary as a function of gender, grade in school, day of the week, and season. Understanding the contexts of physical activity during the high school years can be helpful in designing interventions during adolescence. (PsycINFO Database Record (c) 2012 APA, all rights reserved) (journal abstract)
SN - 0883-6612
AD - Dunton, Genevieve Fridlund: Department of Psychology and Social Behavior, University of California, Irvine, Irvine, CA, US Whalen, Carol K.: Department of Psychology and Social Behavior, University of California, Irvine, Irvine, CA, US Jamner, Larry D.: Department of Psychology and Social Behavior, University of California, Irvine, Irvine, CA, US Floro, Josh N.: Department of Psychology and Social Behavior, University of California, Irvine, Irvine, CA, US
ER -

TY - JOUR
ID - 2663

T1 - Modifying socially withdrawn behavior: A playground intervention for students with internalizing behaviors. [References]
A1 - Marchant,Michelle R.
Y1 - 2007///
N1 - Peer Reviewed Journal: 2007-15238-003
English
Journal; Peer Reviewed Journal
KW - PsycInfo
KW - Internalization
KW - Playgrounds
KW - School Based Intervention
KW - Students
KW - Withdrawal (Defense Mechanism)
RP - NOT IN FILE
SP - 779
EP - 794
JF - Psychology in the Schools
VL - 44:
IS - 8
N2 - There is little research regarding interventions for children with internalizing behaviors in schools, both within classrooms and in nonclassroom environments. In response to this need, a nonclassroom treatment package, consisting of (a) social skills instruction, (b) mediated self-management, and (c) a reinforcement system, was implemented to modify the socially withdrawn behavior of 3 elementary students. The effects of this treatment package were evaluated on the school playground--during recess--by recording both the number of communicative acts and the total time spent engaged in appropriate peer play for each target student. All target students showed marked improvement in their playground, social interaction. Future research should be conducted with similar populations, using variations of the described methods in other school settings. (PsycINFO Database Record (c) 2012 APA, all rights reserved) (journal abstract)
SN - 0033-3085
AD - Marchant, Michelle R.: Brigham Young University, Provo, UT, US Solano, Brock R.: Brigham Young University, Provo, UT, US Fisher, Adam K.: BYU-PBS Initiative, Provo, UT, US Caldarella, Paul: BYU-PBS Initiative, Provo, UT, US Young, K. Richard: Brigham Young University, Provo, UT, US Renshaw, Tyler L.: Brigham Young University, Provo, UT, US
ER -

TY - JOUR
ID - 2664
T1 - Socioemotional development in the toddler years: Transitions and transformations
A1 - Brownell,Celia A.
Y1 - 2007///
N1 - Book: 2007-15136-000
English
Book; Edited Book
(Created by PsycINFO) * Transitions in toddler socioemotional development: Behavior, understanding, relationships / Celia A. Brownell and Claire B. Kopp / 1-39 Part I: Understanding self and others * Understanding self and others in the second year / Chris Moore / 43-65 * The codevelopment of self and sociomoral emotions during the toddler years / Michael E. Mascolo and Kurt W. Fischer / 66-99 * The transformation of prosocial behavior from infancy to childhood / Dale E. Hay and Kaye V. Cook / 100-131 * The origins of empathic morality in toddlerhood / Martin L. Hoffman / 132-145 Part II: Play and communication * Pretend play in toddlers / Angeline Lillard / 149-176 * Children's relationships with other children / Claire Hughes and Judy Dunn / 177-200 * Intentional communication / Nameera Akhtar and Carmen Martinez-Sussmann / 201-220 * Becoming a language user: Entering a symbolic world / Katherine Nelson / 221-240 * Revisiting A toddler's life for the toddler years: Conversational participation as a tool for learning across knowledge domains / Marilyn Shatz / 241-257 Part III: Self-regulation * The emergence of self-regulation: Biological and behavioral control mechanisms supporting toddler competencies / Susan D. Calkins / 261-284 * Autonomy, compliance, and internalization / David R. Forman / 285-319 * Taming the tempest in the teapot: Emotion regulation in toddlers / Ross A. Thompson and Rebecca Goodvin / 320-341 Part IV: Biological

and cultural perspectives * The cognitive neuroscience of early socioemotional development / Martha Ann Bell and Christy D. Wolfe / 345-369 * Biobehavioral approaches to early socioemotional development / Kristin A. Buss and H. Hill Goldsmith / 370-395 * The sociocultural context of transitions in early socioemotional development / Susan M. Perez and Mary Gauvain / 396-419 Part V: Individual differences and applications * Social relations, self-awareness, and symbolizing: A perspective from autism / R. Peter Hobson / 423-450 * A systemic approach to assessment of normative and atypical socioemotional function in toddlers / Hiram E. Fitzgerald, Jessica Barnes and Jason Almerigi / 451-481 Index
KW - PsycInfo
KW - Early Childhood Development
KW - Emotional Development
KW - Psychosocial Development
KW - Social Skills
KW - Individual Differences
RP - NOT IN FILE
SP - New
JF - (2007)
VL - Socioemotional development in the toddler years: Transitions and transformations. xi, 497 pp. New York, NY, US: Guilford Press; US.
N2 - (from the jacket) The second and third years of life are a time of momentous growth and change. Leaving infancy behind, toddlers become increasingly independent as they begin to speak, walk, and participate actively in their widening social world. This volume brings together leading investigators to examine the key social and emotional transitions that characterize this pivotal period. Illuminating the pathways by which children develop socioemotional competence, how specific abilities emerge, and the nature and significance of individual differences, this is an authoritative reference for anyone studying or working with very young children. The introductory chapter provides an integrative overview of socioemotional functioning in toddlerhood and identifies basic issues and questions to be addressed. Subsequent sections scrutinize typical and atypical developmental processes on multiple levels, from evolutionary foundations and neurobiology to attachment and peer relationships. Tightly written chapters yield compelling insights into toddlers' emotions, pretend play, communication, prosocial behavior, self-control, autonomy, and attempts to understand themselves and others. Current empirical literature is reviewed; innovative research methods and findings are explained, including concrete examples; and implications for early intervention and prevention are considered. Throughout, the book emphasizes that no single behavior can be used as a yardstick of socioemotional competence and that it is the continuing interplay among codeveloping systems that moves the toddler forward. The final chapter presents a systematic framework for socioemotional assessment. Accessible and thought provoking, this timely work offers state-of-the-art information and ideas for developmental psychologists and early childhood educators, as well as graduate-level students. It will also be of interest to child mental health practitioners. (PsycINFO Database Record (c) 2012 APA, all rights reserved)
AD - Brownell, Celia A.: University of Pittsburgh, Early Social Development Laboratory, Pittsburgh, PA, US Kopp, Claire B.: Private practice, Los Angeles, CA, US
ER -

TY - JOUR
ID - 2665
T1 - Dangerous activities within an invisible playground: A study of emergent male football play and teachers' perspectives of outdoor free play in the early years of primary school. [References]
A1 - Jarvis,Pam
Y1 - 2007///
N1 - Peer Reviewed Journal: 2007-12897-002
English
Journal; Peer Reviewed Journal
KW - PsycInfo
KW - Childhood Play Behavior
KW - Human Males
KW - Primary School Students
KW - Soccer
KW - Teacher Attitudes
RP - NOT IN FILE

SP - 245
EP - 259
JF - International Journal of Early Years Education
VL - 15:
IS - 3
N2 - This research was carried out during a study which focused upon the rough and tumble play of children in the early years department of a suburban primary school in northern England. The child sample's playtime activities were ethnographically observed over a period of 18 calendar months, during which time interviews were also carried out with the children's class teachers and several other adults taking part in the children's daily school routines. It was found that the narratives created by a cohort of four-and-a-half- to six-and-a-half-year-old boys around their football (soccer) play were socially and symbolically complex, providing evidence that such play is a highly valuable developmental activity. However, the interview data indicated that owing principally to very low adult:child ratios in playground supervision, the focus of the adults was strongly directed towards negative aspects of outdoor free play. (PsycINFO Database Record (c) 2012 APA, all rights reserved) (journal abstract)
SN - 0966-9760
AD - Jarvis, Pam: Bradford College, Bradford, WYK, United Kingdom
ER -

TY - JOUR
ID - 2666
T1 - Ribbons, racism, and a placenta: The challenges and surprises of culturally relevant teaching. [References]
A1 - Lyman,Kate
Y1 - 2007///
N1 - Book: 2007-12861-009
English
Book; Edited Book
KW - PsycInfo
KW - Cultural Sensitivity
KW - Curriculum Development
KW - Students
KW - Teaching
KW - Classrooms
KW - Curriculum
KW - Teachers
RP - NOT IN FILE
SP - 170
EP - 201
JF - Caro-Bruce, Cathy [Ed]; Flessner, Ryan [Ed]; Klehr, Mary [Ed]; Zeichner, Kenneth [Ed]
VL - :
IS - 2007
N2 - (from the chapter) Building on the work of scholars in the area of culturally relevant teaching, Lyman's study captures the power of attending to knowledge students bring with them as they enter her second and third-grade classroom. Capitalizing on conversations she overhears on the playground and in the classroom, Lyman builds a unique curriculum that attends to the needs and concerns of her students while meeting--and often exceeding--the standards those same students are expected to master. By examining recordings of classroom interactions, field notes, student work, and district measures, Lyman shows the power of a curriculum that has direct relevance to her students' lives. (PsycINFO Database Record (c) 2012 APA, all rights reserved)
AD - Lyman, Kate: Hawthorne Elementary School, Madison, WI, US
ER -

TY - JOUR
ID - 2667
T1 - Walking the talk in bullying prevention: Teacher implementation variables related to initial impact of the Steps to Respect program. [References]
A1 - Hirschstein,Miriam K.
Y1 - 2007///

N1 - Peer Reviewed Journal: 2007-10861-001
English
Journal; Peer Reviewed Journal
KW - PsycInfo
KW - Counseling
KW - Prevention
KW - Social Skills
KW - Teaching
KW - Bullying
KW - Teachers
KW - Victimization
RP - NOT IN FILE
SP - 3
EP - 21
JF - School Psychology Review
VL - 36:
IS - 1
N2 - This study examined relationships between teacher implementation of a comprehensive bullying prevention program and student outcomes. Implementation in third- through sixth-grade classrooms (N = 36) was measured by observation and teacher report. Student outcomes were measured by student surveys and teacher ratings of peer social skills (N = 549) and observations of playground behaviors (n = 298). Multilevel modeling showed that teacher coaching of students involved in bullying was associated with less observed victimization and destructive bystander behavior among students engaged in these problems at pretest, and less observed aggression among fifth- and sixth-grade students. Support for skill generalization related to reductions in observed aggression and victimization among older students. Adherence to lessons was associated with higher ratings of peer social skills. Quality of lesson instruction corresponded to greater self-reported victimization, as well as more perceived difficulty responding assertively to bullying. Implications for school-based practice and future research directions are discussed. (PsycINFO Database Record (c) 2012 APA, all rights reserved) (journal abstract)
SN - 0279-6015
AD - Hirschstein, Miriam K.: Committee for Children, Seattle, WA, US Van Schoiack Edstrom, Leihua: Committee for Children, Seattle, WA, US Frey, Karin S.: Committee for Children, Seattle, WA, US Snell, Jennie L.: Committee for Children, Seattle, WA, US MacKenzie, Elizabeth P.: Committee for Children, Seattle, WA, US
ER -

TY - JOUR
ID - 2668
T1 - The impact of social-behavioral learning strategy training on the social interaction skills of four students with Asperger syndrome. [References]
A1 - Bock,Marjorie A.
Y1 - 2007///
N1 - Peer Reviewed Journal: 2007-09909-004
English
Journal; Peer Reviewed Journal
KW - PsycInfo
KW - Aspergers Syndrome
KW - Social Behavior
KW - Social Interaction
KW - Social Skills Training
RP - NOT IN FILE
SP - 88
EP - 95
JF - Focus on Autism and Other Developmental Disabilities
VL - 22:
IS - 2

N2 - This study examined the effect of a social-behavioral learning strategy intervention (SODA) on the social interaction skills of 4 elementary school children with Asperger syndrome (AS). More specifically, the study investigated the effect of SODA training on the abilities of 4 children with AS to participate in cooperative learning activities, play organized sport games, and visit with their peers during lunch. A multiple-baseline-across-settings design (Tawney & Gast, 1984) was used to analyze social behavior without SODA (baseline) and with SODA (intervention) during fourth- or fifth-grade social studies cooperative learning activities, noon recess, and lunch. Maintenance probes occurred once a month for 5 months following completion of the intervention. The participants benefited from the SODA intervention. They presented increased percentages of time spent learning cooperatively, playing organized sport games, and visiting during lunch when SODA training began. When SODA training discontinued, they maintained high performance across all study conditions, nearly matching those achieved by 4 peers without disabilities. In addition, the participants presented long-term memory of SODA 1 month after maintenance. (PsycINFO Database Record (c) 2012 APA, all rights reserved) (journal abstract)
SN - 1088-3576
AD - Bock, Marjorie A.: Associated Colleges of Central Kansas, McPherson, KS, US
ER -

TY - JOUR
ID - 2669
T1 - Becoming whole again through critical thought: A recipe
A1 - Brock,Rochelle
Y1 - 2007///
N1 - Book: 2007-07840-015
English
Book; Edited Book
KW - PsycInfo
KW - Blacks
KW - Critical Thinking
KW - Teaching Methods
KW - Theatre
KW - Urban Environments
KW - Social Justice
KW - Writing Skills
RP - NOT IN FILE
SP - 223
EP - 231
JF - Kincheloe, Joe L [Ed]; hayes, kecia [Ed]
VL - :
IS - 2007
N2 - (create) I believe a requirement of any effective Urban Education program is instilling in students the ability and knowledge to critically deconstruct the world that constructs them. Students in Urban environments are subjected to a myriad of issues that they do not always understand. Although they are aware of the poverty that surrounds them, the knowledge to analyze the reasons of the poverty may not be in their immediate grasp. Without such knowledge they mistakenly believe poverty is only the fault of the individual because they are not aware that in 2005 the Black unemployment was 10.8% compared to 4.8% for White unemployment or that the causes of high unemployment are due in part to outsourcing, systemic racism that attempts to ensure that even if qualified they will not be hired, poor schools that either do not adequately prepare students for the job market or are so problematic that students drop out before graduation. The student is not aware of the governmental policies that create and maintain the condition under which they live/survive. As a teacher committed to social justice for Black students, I work to make my curriculum such that it forces students to think in new ways. I know the phrase "thinking outside the box" is overused, but it provides the perfect conceptual intent I want my students to visualize. The box is that place that confines the mind and body of the students, subverts their drive toward realizing their full humanity. When students think outside of the box, their thinking is not at the margins but at the center. They succeed in turning the box inside out. I so wish I could provide a fail-safe lesson plan to be used in any and every situation. But of course that is impossible if we understand and accept the changing nature and fluidity of critical thought. Instead, I offer an assignment I have given my students, which provides the space to question and reflect on a specific issue utilizing critical thought/critical cognition as the vehicle to

understanding. When working with my students on issues that are difficult to understand, I ask them to write a dialogical play, complete with stage directions. The play is written in the questioning Socratic method and the characters (preferably only two) represent the confusion the student feels about the subject matter. Writing in dialogue allows the students to talk to themselves, question what they have just written, think in deeper ways about the parts that make up the issues they are writing about. Like a two-year-old, the dialogical play provides them the opportunity to constantly ask 'why'? Besides that, it's fun and allows the students to stretch their creative juices by tackling sensitive issues as another person. Inform your students that the stage directions should convey in a literal and metaphorical way both the mystification and enlightenment the characters (and by extension the students) experience. The process of conceptualizing and writing the play allows students to question themselves, constantly delving deeper into obscure meanings. As a conclusion for this exercise, you can either have certain students perform their play for the entire class, or depending on class size and time available, all of the plays can be performed in a culminating event for the class. (PsycINFO Database Record (c) 2012 APA, all rights reserved)
SN - 1058-1634
AD - Brock, Rochelle: Early Childhood Education Department, Georgia State University, Atlanta, GA, US
ER -

TY - JOUR
ID - 2670
T1 - Parent's social support for children's outdoor physical activity: Do weekdays and weekends matter? [References]
A1 - Beets,Michael W.
Y1 - 2007///
N1 - Peer Reviewed Journal: 2007-07610-012
English
Journal; Peer Reviewed Journal
KW - PsycInfo
KW - Childhood Play Behavior
KW - Exercise
KW - Parental Attitudes
KW - Physical Activity
KW - Human Sex Differences
RP - NOT IN FILE
SP - 125
EP - 131
JF - Sex Roles
VL - 56:
IS - 1-2
N2 - In this study we examined the relationships among parental social supportive behaviors for children's weekday and weekend outdoor physical activity (OPA). Thirty-nine girls and 29 boys 8 to 11 years wore pedometers for 7 days. Sixty-five mothers and 50 fathers responded to a social support questionnaire about OPA that was comprised of four dimensions: encouragement, playing with, use of activity as family recreation, and watch. After controlling for age and sport participation, regression analyses indicated that mothers' use of activity as recreation was positively associated with girls' weekday OPA. On the weekend, fathers' play with son was positively related to OPA. The effectiveness of family-based activity interventions depends on when parents have opportunities to be present for their child's activity and gender differences in the support provided. (PsycINFO Database Record (c) 2012 APA, all rights reserved) (journal abstract)
SN - 0360-0025
AD - Beets, Michael W.: Department of Public Health, Oregon State University, Corvallis, OR, US Vogel, Randy: USD 264, Clearwater, KS, US Chapman, Stanley: Wichita State University, Wichita, KS, US Pitetti, Kenneth H.: Wichita State University, Wichita, KS, US Cardinal, Bradley J.: Department of Public Health, Oregon State University, Corvallis, OR, US
ER -

TY - JOUR
ID - 2671

T1 - Are social competence difficulties caused by performance or acquisition deficits? The importance of self-regulatory mechanisms. [References]
A1 - Gumpel,Thomas P.
Y1 - 2007///
N1 - Peer Reviewed Journal: 2007-05329-003
English
Journal; Peer Reviewed Journal
KW - PsycInfo
KW - Emotional Disturbances
KW - Noncontingent Reinforcement
KW - Performance
KW - Self Regulation
KW - Social Skills
KW - Behavior Disorders
KW - Learning Disabilities
KW - Intellectual Development Disorder
RP - NOT IN FILE
SP - 351
EP - 372
JF - Psychology in the Schools
VL - 44:
IS - 4
N2 - We conducted three studies which examined the performance vs. skill acquisition model of social skills deficits. In Study 1, baseline social behaviors for a random sample of 12 boys with comorbid emotional and behavioral disorders (EBD), learning disabilities (LD), language delays, and Attention Deficit Hyperactivity Disorder (ADHD) revealed that prosocial behaviors as well as inappropriate behaviors exist comorbidly in behavioral repertoires, supporting a performance rather than acquisition model of social competence difficulties. In Study 2, an ABAC design was used to examine the efficacy of a self-management intervention with noncontingent (B) and contingent (C) reinforcement for three elementary aged boys with EBD. Generalization was demonstrated in natural settings for the contingent reinforcement phase only, but was not observed over time. Study 3 replicated Study 2's procedures using an ABAC multiple baseline across participants design with a sample of adolescents with varying degrees of mental retardation. Two of the three participants responded favorably to the self-monitoring training and showed marked improvements in prosocial play skills during recess; for the third participant, no behavioral changes were observed. Results from all three studies are discussed from a social learning theory perspective. The efficacy of the data collecting procedure and implications of the results are discussed. (PsycINFO Database Record (c) 2012 APA, all rights reserved) (journal abstract)
SN - 0033-3085
AD - Gumpel, Thomas P.: Hebrew University of Jerusalem, Jerusalem, Israel
ER -

TY - JOUR
ID - 2672
T1 - Evaluation of a Spanish version of the Buss and Perry aggression questionnaire: Some personal and situational factors related to the aggression scores of young subjects. [References]
A1 - Santisteban,Carmen
Y1 - 2007///
N1 - Peer Reviewed Journal: 2007-05166-002
English
Journal; Peer Reviewed Journal
KW - PsycInfo
KW - Adolescent Attitudes
KW - Aggressive Behavior
KW - Extracurricular Activities
KW - Psychometrics
KW - Test Construction
KW - Evaluation

KW - Reading
KW - Test Scores
RP - NOT IN FILE
SP - 1453
EP - 1465
JF - Personality and Individual Differences
VL - 42:
IS - 8
N2 - The psychometric properties of a Spanish translation of the Buss and Perry aggression questionnaire adapted for pre-adolescents and adolescents have been studied. A maximum-likelihood confirmatory factor analysis corroborates Buss and Perry's four-factor structure. The internal consistency of each factor and of the total score has proved to be satisfactory. The four factors are more closely interrelated in pre-adolescents than in adolescents. A second aim of this work has been to further our understanding of the possible relationship between aggression scores and certain outside-school activities of the subjects studied. Two thousand two hundred and eight students filled in the questionnaire together with a self-report concerning their habits. The results show that the aggression scores rise concomitantly with time spent watching television and playing video games and decrease the greater the time devoted to extra-curricular reading or moderate amounts of homework. Statistically significant differences relating to age and gender were also found. (PsycINFO Database Record (c) 2012 APA, all rights reserved) (journal abstract)
SN - 0191-8869
AD - Santisteban, Carmen: Instituto de Estudios Biofuncionales, Universidad Complutense de Madrid, Madrid, Spain Alvarado, Jesus M.: Instituto de Estudios Biofuncionales, Universidad Complutense de Madrid, Madrid, Spain Recio, Patricia: Instituto de Estudios Biofuncionales, Universidad Complutense de Madrid, Madrid, Spain
ER -

TY - JOUR
ID - 2673
T1 - Promoting social-emotional development in young children: Mental health supports in early childhood environments. [References]
A1 - Donahue,Paul J.
Y1 - 2007///
N1 - Book: 2007-03642-013
English
Book; Edited Book
KW - PsycInfo
KW - Child Care
KW - Early Childhood Development
KW - Emotional Development
KW - Mental Health
KW - Psychosocial Development
RP - NOT IN FILE
SP - 281
EP - 312
JF - Perry, Deborah F [Ed]; Kaufmann, Roxane K [Ed]; Knitzer, Jane [Ed]
VL - :
IS - 2007
N2 - (from the chapter) Raising young children in this country has become an increasingly challenging endeavor. Many families across the economic spectrum do not live in communities with established child-rearing networks. The mobility of families, which often reduces access to extended kin, and the financial realities, which often require both parents to work outside the home, are just two of the factors that have limited the transfer of child-rearing techniques and strategies across generations and across town. In many places, the pressures to focus on achievement-oriented activities at a young age have limited parents' time and desire to attend to more basic developmental concerns. Early childhood centers are in a unique position to bridge this gap. Once viewed as playful and optional additions to family life, preschools and child care centers fill important educational and economic demands in this country. Young children are especially vulnerable to the disruptions caused by traumatic events because they do not have well-developed physical or psychological resources to defend against them. They depend on adults to help them make sense of the trauma, heighten their

resilience, and shield them from its ill effects. When their primary caregivers are also affected, they are less available to provide reassurance to their young children and to help them re-establish their sense of safety and security. As a result, the burden of care for these children often shifts, at least in part, to caregivers outside their home. The child care center often takes on added significance for children affected by trauma who crave consistency and nurturance and are looking for a safe haven where they can play and learn and focus on more age-appropriate tasks of development. (PsycINFO Database Record (c) 2012 APA, all rights reserved)
AD - Donahue, Paul J.: Child Development Associates, Scarsdale, NY, US Provet, Anne Gersony: Westchester Medical Center, Valhalla, NY, US
ER -

TY - JOUR
ID - 2674
T1 - Telling themselves who they are: What one out-of-school time study revealed about underachieving readers. [References]
A1 - Alvermann,Donna E; Hagood,Margaret
Y1 - 2007///
N1 - Peer Reviewed Journal: 2007-03507-004
English
Journal; Peer Reviewed Journal
KW - PsycInfo
KW - Academic Underachievement
KW - Extracurricular Activities
KW - Mass Media
KW - Popular Culture
KW - Reading
KW - Motivation
KW - Reading Ability
RP - NOT IN FILE
SP - 31
EP - 50
JF - Reading Psychology
VL - 28:
IS - 1
N2 - The purpose of this study was to explore whether or not adolescents who are deemed underachievers and who struggle to read school-assigned textbooks will engage with popular culture texts of their own choosing (e.g., magazines, comics, TV, video games, music CDs, graffiti, e-mail, and other Internet-mediated texts). The 60 student participants, who were enrolled in grades 7-9 in a small city school district in the southeastern U.S., self-identified mostly as not being interested in reading. Thirty attended weekly meetings of an out-of-school time media club and kept a daily out-of-school time activity log for 14 weeks (the intervention group); the other 30 were assigned to a comparison group and did not attend the weekly media club meetings but did keep a daily out-of-school time activity log for the same 14-week period. Independent t-tests applied to data from the daily activity logs revealed several interesting contrasts between the two groups. One unexpected finding was the relatively large amount of time that participants in both groups reported they spent reading outside of school. (PsycINFO Database Record (c) 2012 APA, all rights reserved) (journal abstract)
SN - 0270-2711
AD - Alvermann, Donna E.: University of Georgia, Athens, GA, US Hagood, Margaret C.: College of Charleston, Charleston, SC, US Heron-Hruby, Alison: Utah State University, Logan, UT, US Hughes, Preston: University of Georgia, Athens, GA, US Williams, Kevin B.: University of Georgia, Athens, GA, US Yoon, Jun-Chae: Ewha Womans University, Seoul, South Korea
ER -

TY - JOUR
ID - 2675
T1 - "Do you like the sun?"--Encountering a child outside of psychoanalysis. [German]. [References]
A1 - Schacht,Lore
Y1 - 2007///

N1 - Peer Reviewed Journal: 2007-03315-003
German
Journal; Peer Reviewed Journal
KW - PsycInfo
KW - Childhood Neurosis
KW - Psychoanalysis
RP - NOT IN FILE
SP - 41
EP - 60
JF - Kinderanalyse
VL - 15:
IS - 1
N2 - During a flight of 50 minutes in a non-analytic relational field, a sequence of scenes occurred between the 6-year-old Farah and the author, increasingly taking the shape of a message. Diverse vulnerabilities--whether belonging to infantile neurosis in a wider sense or not--were just as clearly observable in the playful dialogue as the unswerving integrative efforts of the child. This event motivated the author instead of considering as usual the chasm between pathology and normality to look this time into the opposite direction onto the continuum between normality and pathology. Beside theoretical preoccupations it was the emotional effect of the long-term aesthetic presence of this experience which led to its publication. (PsycINFO Database Record (c) 2012 APA, all rights reserved) (journal abstract)
SN - 0942-6051
ER -

TY - JOUR
ID - 2676
T1 - The relationship of process and product performance of the two-handed sidearm strike. [References]
A1 - Miller,Judith; Vine,Ken; Larkin,Dawne
Y1 - 2007///
N1 - Peer Reviewed Journal: 2007-02648-006
English
Journal; Peer Reviewed Journal
KW - PsycInfo
KW - Motor Skills
KW - Physical Activity
KW - Sports
RP - NOT IN FILE
SP - 61
EP - 75
JF - Physical Education and Sport Pedagogy
VL - 12:
IS - 1
N2 - Background: Many researchers are concerned with the proficiency of children in movement education. They are expressing this concern through the assessment of fundamental motor skills, owing to the established links between the proficiency of fundamental motor skills and subsequent involvement in sport and physical activity. The assessment of fundamental motor skills has predominantly employed a qualitative approach. Another form of assessment involves quantitative measurement; however, the relationship between process and product assessment paradigms is largely unexplored. Purpose: To investigate the relationship between the movement process and product measures of fundamental motor skill performances for primary school-aged children. The relationship between process and product assessment of fundamental motor skills is at the centre of this research. Participants: included 161 six to ten year-old children including 86 girls and 75 boys. The primary school-aged children participated in the study after parental permission and university ethics approval for the research were granted. Research design: involved a cross-sectional design which involved collecting data in an 'ecologically valid' environment--a school playground. Children were withdrawn from class three at a time and individually asked to strike the ball from a batting tee for three warm-up trials. Six trials for each child were measured in terms of process and product performance. Data collection: occurred in the school. The students were video recorded as they performed six trials of tee-ball striking, responding to the task goal of 'hit the ball as far as you can into the batting V'. These recordings were later coded using a (process) amalgamated striking

instrument that is comprised of three levels of efficiency for 10 components of the strike. The components of the process instrument were derived from both the Component Approach and the Total Body Configuration models. The distance or product scores were measured from the batting tee to the resting place of the ball. Data analysis: The six trials for the 161 children were analysed by firstly taking the process observations and coding them using the amalgamated striking instrument. These process codes were analysed using the partial credit form of Rasch (Quest) analysis. Subsequently, Quest provided case estimates, transforming ordinal observations to interval data. These process data were then compared to other interval measures--the distance measured in metres represented the product of the performance. Process scores (case estimates) were compared to product (metres) data to find the relationship. Findings: A significant correlation between the process and product measures for each of the six trials (r = .51 - .66, p < .0001) indicated there is a positive relationship between the process and product measures of the fundamental motor skill performance of the strike for each of the six trials. Conclusion: The application of the Rasch model allows for investigation of two different forms of data (ordinal and interval). The exploration of the relationship between process and product performance indicates the significant correlation between the two performances for these data. Choice of assessment technique now is more open, with some confidence in the association established for these two techniques. With considerable variance still unaccounted for, further exploration of this type of investigation would be prudent. (PsycINFO Database Record (c) 2012 APA, all rights reserved) (journal abstract)
SN - 1740-8989
AD - Miller, Judith: University of New England, Armidale, NSW, Australia Vine, Ken: University of New England, Armidale, NSW, Australia Larkin, Dawne: University of Western Australia, WAU, Australia
ER -

TY - JOUR
ID - 2677
T1 - Cross-sectional comparison of physical activity and inactivity patterns in Chinese and Filipino youth. [References]
A1 - Tudor-Locke,C.
Y1 - 2007///
N1 - Peer Reviewed Journal: 2006-23252-009
English
Journal; Peer Reviewed Journal
KW - PsycInfo
KW - Adolescent Development
KW - Childhood Development
KW - Cross Cultural Differences
KW - Motor Processes
RP - NOT IN FILE
SP - 59
EP - 66
JF - Child: Care, Health and Development
VL - 33:
IS - 1
N2 - Background: To compare and contrast youth physical activity (PA) and inactivity patterns in two developing Asian countries: the Philippines and China. Methods: Comparative analysis of 1997-1999 Cebu Longitudinal Health and Nutrition Survey and the 1997 China Health and Nutrition Survey, large-scale surveys that included questions on type, frequency and duration of: commuting mode to school, sports/exercise in and outside of school, select sedentary activities and chores. Results: Filipino data included 760 males and 872 females aged 14-16 years. The comparable Chinese sample consisted of 202 males and 197 females. Active commuting is proportionately high in both countries (70-71% in the Philippines vs. 77-90% in China), although commuting by bicycling is rare in the Philippines (<1%) vs. China (35%). Patterns of school sport/exercise participation differ between countries by gender; more Filipino males report school sport/exercise than females (63 vs. 49%) vs. China, where more females participate than males (75 vs. 69%). Sport/exercise outside of school is proportionately low (6-12%) for youth from both countries with a single exception: 74% of Filipino males participate in extra-curricular sport/exercise. Although a higher percentage of Filipino youth report watching television > 4 h/day (< 10%) vs. Chinese youth (< 1%), both are lower than comparable US reports. Conclusion: In the Philippines, continued modernization augurs a decrease in local primary PA sources (chores and active commuting). In China, where youth already are not expected to perform chores, shifts to more

passive commuting modes (i.e. increased motorized transportation) are anticipated. (PsycINFO Database Record (c) 2012 APA, all rights reserved) (journal abstract)
SN - 0305-1862
AD - Tudor-Locke, C.: Walking Research Laboratory, Department of Exercise and Wellness, Arizona State University East, Mesa, AZ, US Ainsworth, B. E.: Prevention Research Centre, Department of Epidemiology and Biostatistics, Norman J. Arnold School of Public Health, University of South Carolina, Columbia, SC, US Adair, L. S.: Carolina Population Center, Department of Nutrition, University of North Carolina, Chapel Hill, NC, US Du, S.: Institute of Nutrition and Food Safety, Chinese Centers for Disease Control and Prevention, Beijing, China Lee, N.: Carolina Population Center, Department of Nutrition, University of North Carolina, Chapel Hill, NC, US Popkin, B. M.: Carolina Population Center, Department of Nutrition, University of North Carolina, Chapel Hill, NC, US
ER -

TY - JOUR
ID - 2678
T1 - Issues in preschool assessment. [References]
A1 - Nagle,Richard J.
Y1 - 2007///
N1 - Book: 2006-20171-002
English
Book; Edited Book
KW - PsycInfo
KW - Early Childhood Development
KW - Educational Measurement
KW - Preschool Students
KW - Early Intervention
KW - School Psychologists
RP - NOT IN FILE
SP - 29
EP - 48
JF - Bracken, Bruce A [Ed]; Nagle, Richard J [Ed]
VL - :
IS - 2007
N2 - (from the chapter) The National Association of School Psychologists (NASP), recognizing the importance of early identification and intervention for young children's psychological and developmental difficulties, adopted a position statement on early childhood assessment to guide the fair and accurate identification of the developmental needs of young children (Bracken, Bagnato, & Barnett, 1991). This position statement was later revised in 2005 (National Association of School Psychologists, 2005). The current NASP position endorses multidisciplinary team assessments within an ecological model that includes multiple procedures, multiple sources of information, across multiple settings, and across time in order to yield a comprehensive viewpoint of the child's abilities. These multidimensional assessments should be linked to intervention strategies and should be conceptualized using more than a single methodology or theoretical framework. Furthermore, the position also underscores the importance of the full integration of parents and/or caretakers into the assessment process, including systematic data gathering in the natural environment. The foundation of the NASP statement on early childhood assessment practices is based on evidence garnered through research and professional practice. This chapter will discuss several critical issues that have emerged as psychologists in preschool settings strive to promote best practice during assessment activities. (PsycINFO Database Record (c) 2012 APA, all rights reserved)
AD - Nagle, Richard J.: Appalachian State University, Boone, NC, US
ER -

TY - JOUR
ID - 2679
T1 - The neurobehavioral and social-emotional development of infants and children. [References]
A1 - Tronick,Ed
Y1 - 2007///

N1 - Book: 2006-13259-000
English
Book; Authored Book
(Created by PsycINFO) Acknowledgments Introduction Part I: Neurobehavior The neonatal behavioral assessment scale as a biomarker of the effects of environmental agents on the newborn Behavioral assessment scales: The NICU network neurobehavioral scale, the neonatal behavioral assessment scale, and the assessment of the preterm infant's behavior Kicking coordination captures differences between full-term and premature infants with white matter disorder Late dose-response effects of prenatal cocaine exposure on newborn neurobehavioral performance Similar and functionally typical kinematic reaching parameters in 7- and 15-month-old in utero cocaine-exposed and unexposed infants Part II: Culture Introduction: Cross-cultural studies of development The role of culture in brain organization, child development, and parenting Multiple caretaking in the context of human evolution: Why don't the efe know the western prescription for child care? The manta pouch: A regulatory system for Peruvian infants at high altitude Mother-Infant interaction among the Gusii of Kenya Part III: infant social-emotional interaction Interactive mismatch and repair: Challenges to the coping infant Emotions and emotional communication in infants The mutual regulation model: The infant's self and interactive regulation and coping and defensive capacities Infant-mother face-to-face interaction: Age and gender differences in coordination and the occurrence of miscoordination The transfer of affect between mothers and infants Mother-infant face-to-face interaction: Influence is bidirectional and unrelated to periodic cycles in either partner's behavior Beyond the face: An empirical study of infant affective configurations of facial, vocal, gestural, and regulatory behaviors Part IV: Perturbations: Natural and experimental The primacy of social skills in infancy The infant's response to entrapment between contradictory messages in face-to-face interaction Depressed mothers and infants: The failure to form dyadic states of consciousness Specificity of infants' response to mothers' affective behavior The impact of maternal psychiatric illness on infant development Making up is hard to do, especially for mothers with high levels of depressive symptoms and their infant sons Gender differences and their relation to maternal depression Infant moods and the chronicity of depressive symptoms: The cocreation of unique ways of being together for good or ill, paper 1: The normal process of development and the formation of moods Infant moods and the chronicity of depressive symptoms: the cocreation of unique ways of being together for good or ill, paper 2: The formation of negative moods in infants and children of depressed mothers The stress of normal development and interaction leads to the development of resilience and variation in resilience Part V: Dyadic expansion of consciousness and meaning making Infant responses to impending collision: Optical and real Dyadically expanded states of consciousness and the process of therapeutic change Implicit relational knowing: Its role in development and psychoanalytic treatment Noninterpretive mechanisms in psychoanalytic therapy: The "something more" than interpretation Emotional connections and dyadic consciousness in infant-mother and patient-therapist interactions: Commentary on a paper by Frank Lachmann A model of infant mood states and sandarian affective waves "Of course all relationships are unique": How cocreative processes generate unique mother-infant and patient-therapist relationships and change other relationships Why is connection with others so critical? The formation of dyadic states of consciousness and the expansion of individuals' states of consciousness Contributions to understanding therapeutic change: Now we have a playground Credits References Index
KW - PsycInfo
KW - Childhood Development
KW - Emotional Development
KW - Meaning
KW - Parent Child Relations
KW - Psychosocial Development
KW - Consciousness States
KW - Sociocultural Factors
RP - NOT IN FILE
SP - New
JF - (2007)
VL - The neurobehavioral and social-emotional development of infants and children. xii, 571 pp. New York, NY, US: W W Norton & Co; US.
N2 - (from the jacket) Over the course of his esteemed career, author Ed Tronick has received funding for hundreds of key studies in the US and abroad on normal and abnormal infant and child development--including his Mutual Regulation Model and Still-Face Paradigm, which revolutionized our understanding of infants' emotional capacities and coping--all of which led to critical contributions in the field. Organized into five parts--(I) Neurobehavior, (II) Culture, (III) Infant Social-Emotional Interaction, (IV) Perturbations: Natural and

Experimental, and (V) Dyadic Expansion of Consciousness and Meaning Making--this book represents Tronick's major ideas and studies regarding infant-adult interactions, developmental processes, and mutual regulation, carefully addressing such questions as: (a) What is a state of consciousness? (b) What are the developing infant's capacities for neurobehavioral self-organization? (c) How are early infant-adult interactions organized? (d) How can we understand the nature of normal versus abnormal development? (e) How do self- and mutual regulation relate to developmental processes? (e) Is meaning making purely a function of the brain, or is it in our bodies as well? In an introduction to this compilation of work, Tronick writes, '...the focus of my research has been on the nature of how people live in the world and how they change both themselves and their relation to the world over moments, hours, days, and years." The neurobehavioral and social-emotional development of infants and children demonstrates these aims in one simple volume, and offers readers a first-ever look at the important repertoire of a highly respected clinician and researcher. (PsycINFO Database Record (c) 2012 APA, all rights reserved)
AD - Tronick, Ed: Harvard Medical School, Boston, MA, US
ER -

TY - JOUR
ID - 2680
T1 - Gender-linked differences in the toys, television shows, computer games, and outdoor activities of 5- to 13-year-old children. [References]
A1 - Cherney,Isabelle D.
Y1 - 2006///
N1 - Peer Reviewed Journal: 2007-07674-012
English
Journal; Peer Reviewed Journal
KW - PsycInfo
KW - Computer Games
KW - Human Sex Differences
KW - Recreation
KW - Television
KW - Toys
KW - Leisure Time
RP - NOT IN FILE
SP - 717
EP - 726
JF - Sex Roles
VL - 54:
IS - 9-10
N2 - This study was designed to compare how 5- to 13-year-old children's leisure activity preferences differ with age and gender. Responses from 60 boys and 60 girls about their favorite toys, television shows, computer games, and outdoor activities were compared across leisure categories. The results showed that gender was a significant factor. Overall, boys spent more time in these leisure activities than girls did. They spent the most time engaged in sports, watching television, and playing computer games, whereas girls spent the most time watching television. Results from a gender index for all activities indicated that boys' leisure preferences became slightly more masculine with age. For girls, preferences for television shows became more feminine with age, but preferences for toys, computer games, and sports became less feminine. These self-chosen preferences may provide differential opportunities for the development of visual-spatial skills, achievement, initiative, self-regulation, and social skills. (PsycINFO Database Record (c) 2012 APA, all rights reserved) (journal abstract)
SN - 0360-0025
AD - Cherney, Isabelle D.: Department of Psychology, Creighton University, Omaha, NE, US London, Kamala: University of Toledo, Toledo, OH, US
ER -

TY - JOUR
ID - 2681
T1 - A comparison of posttraumatic distress related to seasonal natural disasters in exposed and non-exposed children

A1 - Lack,Caleb W.
Y1 - 2006///
N1 - Dissertation Abstract: 2006-99024-279
English
Dissertation Abstract
KW - PsycInfo
KW - Distress
KW - Emotions
KW - Natural Disasters
KW - Posttraumatic Stress Disorder
RP - NOT IN FILE
SP - 3456
JF - Dissertation Abstracts International: Section B: The Sciences and Engineering
VL - 67:
IS - 6-B
N2 - Scope and method of study. This study was designed to assess and follow the presence of posttraumatic stress disorder symptomology in children exposed to a tornado and compare that to the same symptomology in demographically similar children who had not been exposed to a tornado. This was done to examine the differences between these groups, both of whom live in a disaster-prevalent area, inside and outside of tornado season. The study also examined the roles that re-exposure to environmental cues, exposure to disaster-related media, attributions, and other factors play in maintaining a child's level of distress, as well as the children's general behavioral and emotional functioning. Participants in the study were 95 exposed children and 70 non-exposed children ages 8-12. The exposed children had at least one tornado strike their hometown in late May or early June 2004, while the non-exposed children's hometown had not had a tornado within 10 miles of it in the past five years. Each participant and their parent completed a series of questionnaires designed to measure the above variables in November 2004 (6 months post tornado and outside tornado season) and May 2005 (12 months post tornado and inside tornado season). Findings and conclusions. The current study supported the proposed stability of posttraumatic distress symptoms in tornado-exposed children, showing no decrease from six to 12 months post tornado. Also supported was the difference in posttraumatic distress symptom level between exposed and non-exposed children, with non-exposed children displaying significantly lower amounts of tornado-related PTSD symptoms both outside and inside tornado season than exposed children. The degree of difference between these two groups, while statistically significant, was not as large as expected, as the non-exposed children did show mild levels of posttraumatic distress. In contrast to expectations, results showed that tornado-specific distress was not related to more global behavioral or emotional functioning. The current study also supported the relationship between the factors of exposure, attributions, and distress. (PsycINFO Database Record (c) 2012 APA, all rights reserved)
SN - 0419-4217
AD - Lack, Caleb W.: Oklahoma State U., US
ER -

TY - JOUR
ID - 2682
T1 - Toddlers' experimental usage of novel verbs: Relations with naturalistic talk
A1 - Smith,Melissa Anne
Y1 - 2006///
N1 - Dissertation Abstract: 2006-99018-238
English
Dissertation Abstract
KW - PsycInfo
KW - Childhood Development
KW - Language Development
KW - Verbs
RP - NOT IN FILE
SP - 1734
JF - Dissertation Abstracts International: Section B: The Sciences and Engineering
VL - 67:
IS - 3-B

N2 - Building on a number of recent studies examining verb development in natural and experimental contexts, this dissertation is the first to have examined in the same population children's naturalistic verb performance and their ability to generalize novel verbs in an experimental setting. In addition, this study investigated the role of discourse context on children's novel verb usage. Twenty-three 2- to 4-year-old children (eight 24-29 month-olds, eight 30-35 month-olds, seven 36-43 month-olds) and their primary caregivers participated in two observation and novel verb training sessions over the course of one week at their homes. Caregiver and child dyads first were observed in naturalistic play and subsequently each dyad engaged in a novel verb training task with the experimenter. At the end of the second visit all children participated in an elicited production task. Analysis of the naturalistic productions indicated that as the children became older they were more able to use transitive constructions in diverse ways (measured in terms of the number of verb types and tokens used, as well as the range of grammatical frames employed). In contrast, intransitive verbs were used in restricted ways across age groups. An examination of age differences in use of novel verbs in the experimental setting provided evidence for age differences in the contexts (training vs. elicitation) where children produced novel verbs, Nevertheless, in contrast to predictions, there was little evidence of children (regardless of age group) being able to produce a novel verb in a way it was not modeled in the input, suggesting that the children lacked full generalization abilities. A comparative analysis of the relation between verb productivity in natural and experimental contexts indicated a relationship between the ability to use verb constructions diversely in natural settings and ability to generalize novel verbs. Discussion focuses on how an understanding of a child's natural language diversity may affect interpretation of novel verb training studies and why so few instances of generalization were found in the present study in contrast to prior research. (PsycINFO Database Record (c) 2012 APA, all rights reserved)
SN - 0419-4217
AD - Smith, Melissa Anne: Clark U., US
ER -

TY - JOUR
ID - 2683
T1 - Children's aggression at recess: Examining the relationship between the playground environment, aggressive behavior, and reports of worry
A1 - Siemers,Erin E.
Y1 - 2006///
N1 - Dissertation Abstract: 2006-99017-093
English
Dissertation Abstract
KW - PsycInfo
KW - Aggressive Behavior
KW - Anxiety
KW - Child Attitudes
KW - Childhood Play Behavior
RP - NOT IN FILE
SP - 839
JF - Dissertation Abstracts International Section A: Humanities and Social Sciences
VL - 67:
IS - 3-A
N2 - Aggression negatively impacts children's psychological and academic well being. The playground environment is more susceptible to aggressive behavior than more structured academic settings like the classroom. In this study, the relation between staff and student reports of student playground aggression was examined. Then, the relation between environmental playground factors (i.e., playground activities, playground supervisor ratios, active supervision, and playground rules) and student reports of aggression was examined. Finally, the relation of playground aggression to student self-reports of playground worries was evaluated. School Climate Theory provided the conceptual framework for evaluating playground characteristics, student aggression, and worry. Participants included 767 third, fourth, and fifth grade students and 57 playground supervisors from 10 Midwest elementary schools. Participants completed reports of aggression (students and staff), playground worries (students only), and playground environmental factors (staff only). Bivariate correlations were used to examine the relation between staff and student reports of aggression. Hierarchical Linear Modeling analyses were conducted to examine the relation among playground factors, aggression, and playground worries. This study used a novel approach to measuring the predictive relation of aggression on

children's worries through student self-report measures. Results of this study showed that staff and students reports of playground aggression were inconsistently correlated. Although overt physical and verbal playground aggression scores were significantly correlated, relational playground aggression and playground conflict were not associated. Additionally, four playground characteristics were linked to playground aggression: cooperative games, supervisor ratios, active monitoring, and playground rules. Finally, when students reported more playground aggression, their reports of playground worry were higher. (PsycINFO Database Record (c) 2012 APA, all rights reserved)
SN - 0419-4209
AD - Siemers, Erin E.: U Nebraska - Lincoln, US
ER -

TY - JOUR
ID - 2684
T1 - Something's happening: Emergence of social worlds on the school playground
A1 - Huecker,Esther Molder
Y1 - 2006///
N1 - Dissertation Abstract: 2006-99010-280
English
Dissertation Abstract
KW - PsycInfo
KW - Childhood Development
KW - Playgrounds
KW - Psychosocial Development
KW - Schools
KW - Social Interaction
KW - Friendship
KW - Morality
RP - NOT IN FILE
SP - 6334
JF - Dissertation Abstracts International: Section B: The Sciences and Engineering
VL - 66:
IS - 11-B
N2 - This study explores the lives of children on the school playground to examine what happens and how children's social worlds emerge. Recess is an important part of the school day when social and moral actions are constructed in the midst of activity (Goodwin, 2002). Based on a 10-month school year, this ethnographic study of 5th-and-6th-grade children in a private Christian school in Southern California focused on peer interactions, especially when something unanticipated happens and moral dilemmas ensue. By attending to significant and situated social, cultural and historical events of mediated action (Wertsch, 1998), this study addressed children socially occupied (Lawlor, 2003, 2004) in collaborative activities. Observations, fieldnotes, video tapes as well as student interviews and journaling provided a rich basis to capture the multiple perspectives involved when complex and dynamic occupations emerge as meaningful social activities. Centered primarily on the interactions of 7 girls in the 5th grade, ages 10 and 11, fine-grained microanalysis of key events generate insights into how individuals negotiate times of uncertainty, make sense of their experiences, and shape their identities over time. Moral dilemmas reflect each individual's emotional vulnerability of struggles with fairness and responsibility toward others when pulled between personal desires and collective expectations. As powerful everyday encounters, moral dilemmas propel actions and present opportunities for students to find new ways to act through improvised occupations. When several girls organized a band to write and perform songs, meanings of membership and positions are negotiated at multiple levels of group engagement. Other girls created occupations of songwriting to voice resistance to frustrations with peer pressures, express reshaped identities (Holland, Lachicotte, Skinner, & Cain, 1998) and construct future possibilities for friendships. Changes occur within and between peer groups as the girls on the periphery of practice (Wenger, 1998) shift positions within and between these groups. These findings support occupations as dynamic endeavors with others. Building on the research in occupational science, this study contributes to what happens on the school playground and how occupations emerge as joint improvisations when students attempt to resolve moral dilemmas and transform identities in complex social interactions and relationships. (PsycINFO Database Record (c) 2012 APA, all rights reserved)
SN - 0419-4217

AD - Huecker, Esther Molder: U Southern California, US
ER -

TY - JOUR
ID - 2685
T1 - A grant proposal for an outdoor behavioral healthcare summer camp for boys at risk for developing externalizing behaviors
A1 - Barnhart,Catherine R.
Y1 - 2006///
N1 - Dissertation Abstract: 2006-99010-175
English
Dissertation Abstract
KW - PsycInfo
KW - Adolescent Development
KW - At Risk Populations
KW - Health Care Services
KW - Therapeutic Camps
KW - Violence
KW - Curriculum
RP - NOT IN FILE
SP - 6260
JF - Dissertation Abstracts International: Section B: The Sciences and Engineering
VL - 66:
IS - 11-B
N2 - Violence in America is an epidemic that service providers need to address with the growing statistics of youth violence and aggression. There are cost benefits to intervening early, when risk factors are becoming evident. Prevention works on a continuum and there are many types of programs that are successful in targeting violence. The National Institute of Mental Health (NIMH) and the Surgeon General have released reports that identify risk factors. Prevention research shows that secondary prevention is successful by targeting peer relations, social skills, and problem-solving skills. The Center for Disease Control and Prevention (CDC) identifies that social-cognitive problem-solving prevention programs are model programs. They identify, and developmental research supports, that middle childhood (ages 6-12) is an ideal time to target these risk factors with programs that teach social-cognitive problem-solving skills. Presently, Outdoor Behavioral Healthcare (OBH), which, through weak research, has shown benefits with a general population, have developed programs that seem effective in working with at-risk youth. The research is still minimal. The research exists, but only in the last five or ten years has the research begun to operationally define variables and utilize quantitative measures and research designs. Promoting Alternative Thinking Strategies (PATHS) is a curriculum that is empirically validated to successfully prevent violent behaviors by teaching emotional, social, and problem-solving skills in elementary school classrooms. It is also identified by violence prevention research as a model program. The curriculum has been replicated in other settings. The goal of this proposal is to provide an innovative approach to violence prevention with at-risk youth (ages 8-12) by combining the OBH programming and activities with PATHS curriculum lessons. The program will adopt the themed lessons from the PATHS curriculum and teach them while instructing campers on outdoor activities. By doing so, the outdoor activities will have specific aims in their lessons learned and the PATHS lessons will have immediate and real life applications. The program will be implemented and evaluated to add to the research in the areas of violence prevention, PATHS, and OBH. The eight-week outdoor program will incorporate a simple interrupted time series design utilizing screening questionnaire to aid in inclusion criteria, the Kusche Affective Interview-Revised (KAI-R), the Child Behavior Checklist (CBCL), the Youth Outcome Questionnaire (YO-Q), semi-structured interviews, and module evaluations. The participants will be 64 males between the ages of 8 and 12 years. The goal of the evaluation is to and to research in the field, but also ensure program quality. Through the research, the hypothesis is that results with this innovative approach, combining the OBH theory and programming with PATHS theory and curriculum, will demonstrate success in reducing the probability that the participants will develop violent behaviors in adolescence. (PsycINFO Database Record (c) 2012 APA, all rights reserved)
SN - 0419-4217
AD - Barnhart, Catherine R.: The Chicago School Of Professional Psychology, US
ER -

TY - JOUR
ID - 2686
T1 - Effectiveness of music therapy interventions on joint attention in children diagnosed with autism: A pilot study
A1 - Reitman,Michelle Renee
Y1 - 2006///
N1 - Dissertation Abstract: 2006-99010-034
English
Dissertation Abstract
KW - PsycInfo
KW - Autism
KW - Childhood Development
KW - Music Therapy
KW - Parents
KW - Language Development
RP - NOT IN FILE
SP - 6315
JF - Dissertation Abstracts International: Section B: The Sciences and Engineering
VL - 66:
IS - 11-B
N2 - Joint attention, the ability to selectively attend to an object of mutual interest, is a critical skill that develops early in life and consequentially affects future social-cognitive skills, social-emotional skills, and language development. It is widely known and accepted that children with autism have difficulties with joint attention. Children with autism need types of intervention that will systematically facilitate as much cooperative, negotiated and culturally relevant learning as possible. Interventions need to be deliberately monitored with attention to the learner's moment-to-moment motivational and interpersonal responses. Music therapy is one effective method for increasing interpersonal understanding, joint motivation, and cooperative skills. Music therapy interventions have been used with children diagnosed with autistic disorder for the past forty years. The present study examines the effectiveness of music therapy intervention on development of joint attention behaviors in children diagnosed with autistic spectrum disorder both within treatment and as reported by parents outside of treatment. Participants were 3 to 5 year old non-verbal males diagnosed with autism spectrum disorder. All participants fell in the Severely Autistic range of functioning as measured by the Childhood Autism Rating Scale (CARS) (Schopler, Reichler, and Renner, 1988). Participants received eight 30-minute individual music therapy sessions, twice weekly. Sessions consisted of five categories of musical experiences: greeting song, seated imitation activity, instrument playing, gross motor musical movement/imitation activity, and closing/goodbye song. Both live and recorded music was used. Musical improvisations were often created during sessions based upon the child's vocal and motor output with intent to musically join, reflect, and expand the child in his experience. Most parents noted improvements in responsivity to social cues and social approach behaviors as measured by the Parent Version of the Pervasive Developmental Disorder - Behavioral Inventory (PDDBI) (Cohen and Sudhalter, 1999). Parents noted improvement ranging between 8% and 40%. Positive changes were found in other areas measured on the PDDBI as well (e.g. decreased Ritualisms/Resistance to Change, Arousal Regulation Problems, and Specific Fears, and increased Phonological Skills). Video analysis of initial and final music therapy sessions showed that approximately 70% of participants had noticeable increases in joint attention behaviors. The range of improvement was between 36% and 200%. Results indicate that the addition of music therapy intervention to a child's treatment program can have positive outcomes and may be an effective method for increasing joint attention skills in some children with autism. (PsycINFO Database Record (c) 2012 APA, all rights reserved)
SN - 0419-4217
AD - Reitman, Michelle Renee: Carlos Albizu U., US
ER -

TY - JOUR
ID - 2687
T1 - The family represented: Mother- and father-child co-constructed narratives about families
A1 - Dilallo,Meaghan E.
Y1 - 2006///

N1 - Dissertation Abstract: 2006-99008-213
English
Dissertation Abstract
KW - PsycInfo
KW - Cognitive Ability
KW - Emotions
KW - Family Relations
KW - Narratives
KW - Parent Child Relations
KW - Metaphor
RP - NOT IN FILE
SP - 5679
JF - Dissertation Abstracts International: Section B: The Sciences and Engineering
VL - 66:
IS - 10-B
N2 - The recent expansion of research guided by the metaphor of co-construction of family narratives has significantly contributed to the child and parent development literature (Bearison & Dorval, 2002; Wang, Leichtman, & Davies, 2000). Researchers have borrowed from various theories, including Vygotskian notions of how young children internalize thought and language, to capture the way parents scaffold their children's growing cognitive abilities (Rogoff, 1991; Wertsch, 1992). Similarly, potential also exists for using narratives to capture the manner in which parents help children develop an understanding of family dynamics and emotion (Oppenheim, Nir, Warren, & Emde, 1997). This report contributes to the field's knowledge base, by introducing a new method for assessing co-constructed narrative representations of family and emotion and toddlers' interactions with their parents. This approach builds upon two complementary premises: (1) parents are essential agents in socializing their children's knowledge of emotions (Denham, 1998; Hughes, & Dunn, 1997), and (2) parents can modify the impact of family process through the ways they portray relationships to their children through narratives (McHale, 1997). Eighty-seven families with 30-month old sons (n = 44) or daughters (n = 43) comprised the sample. In separate sessions, mothers and fathers joined their preschooler at a dollhouse with a family of dolls matching their own family structure, and were asked to narrate and enact three stories about a happy, a sad, and a mad family. The study evaluated the co-constructions by examining both (1) the process of the sessions, and (2) the content of the stories. Further, parents rated their child's behaviors on standardized surveys and teachers likewise rated the child's adaptive skills outside of the home. Additionally, the whole family took part in a semi-structured family play session, which was rated for overall levels of family warmth and conflict. Results documented several associations between both mother-child and father-child co-constructed narrative representations and process measures and observed family-level dynamics. Father-child, but not mother-child, co-constructed measures were also associated with children's socioemotional adjustment. Analyses established that father-child dyad representations in general did not mediate the relationship between family functioning and toddlers socioemotional behaviors, except in one instance. (PsycINFO Database Record (c) 2012 APA, all rights reserved)
SN - 0419-4217
AD - Dilallo, Meaghan E.: Clark U., US
ER -

TY - JOUR
ID - 2688
T1 - Using video modeled social stories to increase the social communication skills of children with high functioning autism/asperger's syndrome
A1 - Sansosti,Frank J.
Y1 - 2006///
N1 - Dissertation Abstract: 2006-99006-201
English
Dissertation Abstract
KW - PsycInfo
KW - Aspergers Syndrome
KW - Autism
KW - Intervention
KW - Storytelling

KW - Videotapes
KW - Childhood Development
KW - Communication Skills
KW - Social Reinforcement
KW - Social Skills
RP - NOT IN FILE
SP - 5104
JF - Dissertation Abstracts International: Section B: The Sciences and Engineering
VL - 66:
IS - 9-B
N2 - The purpose of this study was to investigate the effects of individualized video-modeled Social Story interventions on the social communication skills of three children with High Functioning Autism/Asperger's Syndrome (HFA/AS). Using a multiple-baseline across participants design, video-modeled Social Stories were implemented and direct observations of the participants' identified target behaviors were collected two times per week during unstructured school activities (e.g., recess). Overall, data demonstrated that video modeled Social Stories were effective for improving the rates of social communication for the participants, though modifications to allow access to social reinforcement was needed in two cases. In addition, all three participants demonstrated maintenance of skills at a two-week follow-up. However, generalization of skills was only observed for one participant. The present research provides no definitive claims of the effectiveness of a combined intervention for children with HFA/AS. Rather, it adds preliminary evidence that a combined intervention that teaches social communication skills and provides for a model of appropriate behavior may be a beneficial method of remediating social skill difficulties for many children and youth with HFA/AS. (PsycINFO Database Record (c) 2012 APA, all rights reserved)
SN - 0419-4217
AD - Sansosti, Frank J.: U South Florida, US
ER -

TY - JOUR
ID - 2689
T1 - Impact of school context on the relations between deviant peer affiliations and problem behaviors during the middle school years: An exploratory analysis using hierarchical linear modeling
A1 - Comeau,Colleen
Y1 - 2006///
N1 - Dissertation Abstract: 2006-99005-075
English
Dissertation Abstract
KW - PsycInfo
KW - Adolescent Attitudes
KW - Behavior Problems
KW - Middle Schools
KW - Peers
KW - School Environment
KW - Resilience (Psychological)
RP - NOT IN FILE
SP - 3208
JF - Dissertation Abstracts International Section A: Humanities and Social Sciences
VL - 66:
IS - 9-A
N2 - Educators face a considerable challenge in addressing youth at risk for serious problem behaviors. Particularly during the middle school years, the school environment may play a vital role in shaping pathways of risk or resilience for preadolescents. However, contextual factors within the school environment that influence student outcomes have not been adequately assessed via traditional methods (e.g., students' self-report questionnaires). Using a novel observational assessment of the school environment, the current study explored how school contextual factors are associated with a range of student outcomes. Participants in this study are 1076 preadolescent students from 8 middle schools in the Pacific Northwest (76% European-American; 54% female). Unobtrusive observational data was collected in each of the 8 local middle schools. Observed factors within the school environment included the physical environment (e.g., noise level, adequacy of lighting in the

hallways), management clarity (e.g., rules and expectations clearly posted), the emotional environment (e.g., student affect), and staff behaviors (e.g., quality of staff monitoring). Non-classroom settings were observed (e.g., hallways, outside corridors) during school transition times. One of the primary aims of this study was to examine the utility of the school observational measure, including the measure's reliability and validity. Using hierarchical linear modeling, both student report of school climate and observations of school context were examined as predictors of deviant peer affiliations and the student outcome variables (substance use, antisocial behavior, and positive engagement). There is initial support for the validity of the observational measure. The results suggest that student outcomes are associated with indicators of the school environment, particularly the school-level factors of a positive social-emotional environment and staff monitoring. There is limited evidence of a moderating effect of school context in the relations between deviant peer affiliations and the student outcomes. Sex differences emerged, in which the presence of certain school context factors was more protective for the female students. The observational assessment tool used in this study may provide future school-based research and intervention programs with a more cost- and time-effective data collection alternative. (PsycINFO Database Record (c) 2012 APA, all rights reserved)
SN - 0419-4209
AD - Comeau, Colleen: U Oregon, US
ER -

TY - JOUR
ID - 2690
T1 - The effects of different recess timing regimens on preschoolers' classroom attention. [References]
A1 - Holmes,Robyn M.
Y1 - 2006///
N1 - Peer Reviewed Journal: 2006-20004-005
English
Journal; Peer Reviewed Journal
KW - PsycInfo
KW - Attention
KW - Classrooms
KW - Preschool Students
RP - NOT IN FILE
SP - 735
EP - 743
JF - Early Child Development and Care
VL - 176:
IS - 7
N2 - This study examined the effects of different recess timing regimens on preschoolers' classroom attention. Using cognitive immaturity theory, we predicted that attention to a classroom task would be greater after a recess break. We also examined the extent to which different recess timing regimens related to post-recess attention. Participants were 27 European American children (18 girls and nine boys; mean = 55.56 months, standard deviation = 4.01) from a northeastern US preschool. Observations were made in the classroom before and after recess and outdoors on the playground during recess. Findings revealed that post-recess attention was greater following sustained outdoor play periods. Gender differences emerged. Girls were more attentive to classroom tasks than boys were. Our findings support and parallel empirical findings with primary school children on the role of recess in children's cognitive performance. It seems reasonable that outdoor recess breaks rejuvenate young children and help them attend to classroom tasks. (PsycINFO Database Record (c) 2012 APA, all rights reserved) (journal abstract)
SN - 0300-4430
AD - Holmes, Robyn M.: Monmouth University, West Long Branch, NJ, US Pellegrini, Anthony D.: University of Minnesota, MN, US Schmidt, Susan L.: Monmouth University, West Long Branch, NJ, US
ER -

TY - JOUR
ID - 2691
T1 - Practical projects: Birth to 5 years
A1 - Spratt,Jenny
Y1 - 2006///

N1 - Book: 2006-12910-007
English
Book; Edited Book
KW - PsycInfo
KW - Arts
KW - Childhood Play Behavior
KW - Observation Methods
KW - Theatre
RP - NOT IN FILE
SP - 52
EP - 56
JF - Bruce, Tina [Ed]
VL - :
IS - 2006
N2 - (from the chapter) This chapter considers how observations of children's play can be undertaken at any time--indoors, outdoors and in more unusual situations, such as a visit to a theatre. It is often during situations that we are not expecting, that observations provide the richest moments in children's play. If practitioners are not tuning into the child, these moments are easily missed. Aims of the chapter are to develop an understanding of (1) ways of tuning into children; (2) ways of observing children's pauses and silences; and (3) ways of engaging with theatre and the arts. (PsycINFO Database Record (c) 2012 APA, all rights reserved)
AD - Spratt, Jenny: Peterborough City Council, Early Years & Childcare Services, United Kingdom
ER -

TY - JOUR
ID - 2692
T1 - Children's neighborhood place as a psychological and behavioral domain. [References]
A1 - Min,Byungho
Y1 - 2006///
N1 - Peer Reviewed Journal: 2006-11681-005
English
Journal; Peer Reviewed Journal
KW - PsycInfo
KW - Behavior
KW - Childhood Development
KW - Environment
KW - Neighborhoods
KW - Social Interaction
KW - Psychology
RP - NOT IN FILE
SP - 51
EP - 71
JF - Journal of Environmental Psychology
VL - 26:
IS - 1
N2 - Interviews of 91 children living in a large high-rise, high-density planned neighborhood of 5277 families reveal that children find most of their psychologically valued settings within the neighborhood outdoor spaces such as designated play areas and developed parks. They are aware of differences in experience between such valued 'places' and other 'spaces'. They choose the places because such places offer some key environmental attributes--spatial, physical, and social--that support behaviors that children want to engage in. Due to these attributes, behaviors in places are different from behaviors in other spaces, with the former being more purposive, social, creative, and dependent on particular affordances of the setting. This can be confirmed in a follow-up behavioral analysis of three pairs of most popular places and their counterpart settings. This observational analysis shows that settings children mention to be important to them are utilized more often (about twice as much) than their counterparts, showing a variety of behaviors, incorporating more intentional activities (as opposed to transient, short-lived ones), encouraging group behaviors (as opposed to single-person activities), and providing children with a sense of their own territorial play area. The study demonstrates that in children's neighborhood environment, emergence of such important places is related to use pattern and there are

some key attributes and core behaviors greatly contributing to children's place experience. (PsycINFO Database Record (c) 2012 APA, all rights reserved) (journal abstract)
SN - 0272-4944
AD - Min, Byungho: Faculty of Architecture, Ajou University, Suwon, South Korea Lee, Jongmin: Faculty of Architecture, Ajou University, Suwon, South Korea
ER -

TY - JOUR
ID - 2693
T1 - Maternal involvement in preschool children's education in Japan: Relation to parenting beliefs and socioeconomic status. [References]
A1 - Yamamoto,Yoko
Y1 - 2006///
N1 - Peer Reviewed Journal: 2006-11665-006
English
Journal; Peer Reviewed Journal
KW - PsycInfo
KW - Mother Child Relations
KW - Parental Attitudes
KW - Parental Involvement
KW - Preschool Education
KW - Socioeconomic Status
KW - Mothers
KW - Preschool Students
RP - NOT IN FILE
SP - 332
EP - 346
JF - Early Childhood Research Quarterly
VL - 21:
IS - 3
N2 - Studies conducted in the US consistently demonstrate that parenting self-efficacy and construction of the parent role are critical elements associated with parents' involvement in their children's elementary school education. Less is known about the dynamics of parent involvement during the preschool period, or in nations outside the US. This study examined the relation of maternal beliefs and family socioeconomic status (SES) to three dimensions of parent involvement in Japan: preschool selection strategies, engagement in reading at home, and involvement in activities at the preschool. Interview and questionnaire data were obtained from 108 Japanese mothers, all of whom had a child in the last year of preschool. Consistent with theory and findings in the US, parenting self-efficacy and family role construction were associated with Japanese mothers' strategies for selecting preschools and frequency of engaging in home reading. Findings regarding family SES demonstrated a culturally specific pattern; mothers of higher SES background were more likely to access formal sources of information and to engage in daily home reading but less likely to participate at the school site. (PsycINFO Database Record (c) 2012 APA, all rights reserved)
SN - 0885-2006
AD - Yamamoto, Yoko: University of California, Berkeley, Berkeley, CA, US Holloway, Susan D.: University of California, Berkeley, Berkeley, CA, US Suzuki, Sawako: University of California, Berkeley, Berkeley, CA, US
ER -

TY - JOUR
ID - 2694
T1 - Motivation and Physical Activity in Adolescents With Visual Impairments. [References]
A1 - Kozub,Francis M.
Y1 - 2006///
N1 - Peer Reviewed Journal: 2006-10699-001
English
Journal; Peer Reviewed Journal
KW - PsycInfo

KW - Adolescent Development
KW - Motivation
KW - Motor Processes
KW - Vision Disorders
KW - Residential Care Institutions
RP - NOT IN FILE
SP - 149
EP - 160
JF - RE:view: Rehabilitation and Education for Blindness and Visual Impairment
VL - 37:
IS - 4
N2 - To identify appropriate free-time experiences, it is important to know what motivational factors predict physical activity decision making. In this study, I explore differences in free-time motivation scores between adolescents with visual impairments from a residential setting who are at criterion levels of body mass indexes (BMIs) and their fellow students who score outside the healthy zones. Participants in the study were 31 students (11 women and 20 men), 12 to 21 years old, who were involved in educational and after-school residential programs at a midwestern school for the blind. All participants had residual sight but were classified as visually impaired with vision deficits that affected their educational performance. None of the participants had any coexisting cognitive or physical disabilities. The resulting data revealed motivational differences among these participants. Physical activity continues to be a recommended course of action to alleviate obesity and improve physical functioning, and the data from this inquiry present interesting results that need further study. (PsycINFO Database Record (c) 2012 APA, all rights reserved)
SN - 0899-1510
AD - Kozub, Francis M.: Department of Kinesology, Indiana University, Bloomington, IN, US
ER -

TY - JOUR
ID - 2695
T1 - Teaching motor skills to children with cerebral palsy and similar movement disorders: A guide for parents and professionals. [References]
A1 - Martin,Sieglinde
Y1 - 2006///
N1 - Book: 2006-09267-000
English
Book; Authored Book
(Created by PsycINFO) (Abbreviated) Preface and Acknowledgments Introduction Developmental Delay and Cerebral Palsy Gross Motor Development Obstacles to Motor Development Helping Your Child Learn Motor Skills Flexible Muscles and Joints Head-up Happy Baby in Back-lying Tummy Time Guarding Against Falls Sitting Pretty Getting Up and Crawling Leg Exercises and Standing with Arm Support Balance Standing without Arm Support and Walking Walking and More Extra Strengthening and Having Fun Additional Interventions for Children with Cerebral Palsy By Lisa Barnett, DPT Appendix References Index
KW - PsycInfo
KW - Cerebral Palsy
KW - Exercise
KW - Motor Skills
KW - Physical Therapy
KW - Teaching
KW - Caregivers
KW - Intervention
KW - Motor Performance
KW - Movement Disorders
KW - Self Care Skills
RP - NOT IN FILE
SP - Bethesda, MD, US
JF - (2006)
VL - Teaching motor skills to children with cerebral palsy and similar movement disorders: A guide for parents and professionals. xviii, 237 pp. Bethesda, MD, US: Woodbine House; US.

N2 - (from the cover) While most families of young children with cerebral palsy and similar conditions work in conjunction with a physical therapist a few times a week, it takes daily intervention to help a child reach his motor potential and become more independent. Teaching Motor Skills is the resource that parents, therapists, and other caregivers can consult to help young children with gross motor delays learn and practice motor skills outside of therapy sessions. Written by an experienced physical therapist who is also the mother of a child with cerebral palsy, this comprehensive guide examines the physical characteristics of cerebral palsy and similar conditions--muscle tightness and weakness, increased or decreased flexibility, abnormal reflexes, impaired sensory perception--that affect a child's ability to sit, crawl, stand, and walk. Teaching Motor Skills offers dozens of easy-to-follow exercises with accompanying photos that parents may incorporate into many daily routines at home with the guidance and support of their child's physical therapist. Exercises address: head control; protective reactions; proper positioning; independent sitting with and without arm support; transitional movements; daily stretching; improving muscle strength and coordination; balance training; and gait training. Full of anecdotes and frequently-asked-questions, with a chapter on medical interventions and bracing, and an appendix of equipment and supplies, this user-friendly guide helps parents and professionals coordinate their efforts to achieve the best possible outcome for the child. (PsycINFO Database Record (c) 2012 APA, all rights reserved)
AD - Martin, Sieglinde: Children's Close To Home Health Care Center, Dublin, OH, US
ER -

TY - JOUR
ID - 2696
T1 - Imitative Interaction Increases Social Interest and Elicited Imitation in Non-verbal Children with Autism. [References]
A1 - Heimann,Mikael; Laberg,Kari E; Nordoen,Bodil
Y1 - 2006///
N1 - Peer Reviewed Journal: 2006-08875-008
English
Journal; Peer Reviewed Journal
KW - PsycInfo
KW - Contingency Management
KW - Early Intervention
KW - Imitation (Learning)
KW - Pervasive Developmental Disorders
KW - Social Interaction
KW - Autism
KW - Interests
KW - Nonverbal Ability
KW - Oral Communication
RP - NOT IN FILE
SP - 297
EP - 309
JF - Infant and Child Development
VL - 15:
IS - 3
N2 - Recent studies indicate that being intensely imitated for a brief period of time increases social interest among children with autism. The aim of this study was to replicate and extend these findings. Twenty children with an autism spectrum disorder (ASD) were randomly assigned to one of two interaction strategies: imitation (n = 10) or contingent (n = 10). The children had little or no functional speech, and their developmental age averaged 25 months (mean chronological age = 6:5 years). Both conditions were presented with repeated sessions of a modified version of Nadel's 'still-face' paradigm (still-face/intervention/ still-face/spontaneous play). The analysis revealed a significant increase of both proximal and distal social behaviours (touch and look at person) for the imitation condition, which confirms previous reports. In addition, an increase in elicited imitation, as measured with the PEP-R developmental assessment procedure, was also observed for children in the imitation condition, but not in the contingent condition. This finding extends earlier reports in that it suggests that the social expectancies unlocked by imitation also spread to tasks outside the experimental setting. (PsycINFO Database Record (c) 2012 APA, all rights reserved) (journal abstract)
SN - 1522-7227

AD - Heimann, Mikael: University of Bergen, Bergen, Norway Laberg, Kari E.: Eikelund Resource Centre, Bergen, Norway Nordoen, Bodil: Eikelund Resource Centre, Bergen, Norway
ER -

TY - JOUR
ID - 2697
T1 - A Dominating Imaginary Friend, Cruelty to Animals, Social Withdrawal, and Growth Deficiency in a 7-year-old Girl with Parents with Schizophrenia
A1 - Shapiro,Henry L.
Y1 - 2006///
N1 - Peer Reviewed Journal: 2006-08601-008
English
Journal; Peer Reviewed Journal
KW - PsycInfo
KW - Antisocial Behavior
KW - Parent Child Relations
KW - Pretend Play
KW - Schizophrenia
KW - Aggressive Behavior
KW - Eating Behavior
KW - Family Background
KW - Offspring
KW - Social Isolation
RP - NOT IN FILE
SP - 231
EP - 236
JF - Journal of Developmental and Behavioral Pediatrics
VL - 27:
IS - 3
N2 - Tessa is a 7-year-old girl with odd behaviors, worsening over the preceding year. She spends a significant amount of time alone in her room, talking to "Richard." Her father observed that Tessa is "in her own world." In school, she often glares at the teacher and refuses to work. She is alone at recess without any real friends. Her father reports that she eats little and only after he tastes the food. She is increasingly cruel to both real and toy animals. She tied a string around her cat's neck and swung it around, saying she wanted to punish it. She was heard to say to her stuffed cat, "Die, b....; die." Richard told her to do this and other "bad" things. Tessa has not had chronic health problems or developmental concerns. During the first 18 months of life, her height was between the 5th and 10th percentiles; it declined to below the 3rd percentile over the past 2 years. Weight has been consistently between the 3rd and 5th percentiles. Tessa's parents both have been diagnosed with schizophrenia. There are at least 7 first- and second-degree relatives with the same diagnosis. Both parents cared for Tessa for 4 years with a lot of support. Then, her father left the home, but he was in contact with her while managing his own illness. When the mother's illness and compliance worsened, Tessa was removed for neglect at 5 1/2 years old. Two foster homes preceded Dad's award of sole custody 13 months ago. Mother's weekly visitations are quite upsetting to Tessa. Tessa is a verbal, solemn, small, well-dressed girl with no physical abnormalities. She talks with advanced vocabulary and syntax, with normal prosody and good conversational skills. She says Richard is a bad boy who gets her into trouble at school. She equivocates when asked if he is real or imagined or if others can see him. She says that she misses her mother. In this article, several doctors offer their professional opinions on diagnosis and treatment of this challenging case. (PsycINFO Database Record (c) 2012 APA, all rights reserved)
SN - 0196-206X
AD - Shapiro, Henry L.: Developmental-Behavioral Pediatrics, All Children's Hospital, St. Petersburg, FL, US Prince, Jefferson B.: Department of Child Psychiatry, North Shore Medical Center, Salem, MA, US Ireland, Rachel: Developmental and Behavioral Pediatrics, University of California, San Diego, CA, US Stein, Martin T.: University of California, San Diego, CA, US
ER -

TY - JOUR
ID - 2698

T1 - The environmental conditions, health and development of children and adolescents. [References]
A1 - Sevcikova,L.'
Y1 - 2006///
N1 - Peer Reviewed Journal: 2006-08063-010
English
Journal; Peer Reviewed Journal
KW - PsycInfo
KW - Adolescent Development
KW - Childhood Development
KW - Chronicity (Disorders)
KW - Environment
KW - Lifestyle
KW - Health
KW - Risk Factors
RP - NOT IN FILE
SP - 65
EP - 68
JF - Homeostasis in Health and Disease
VL - 44:
IS - 1-2
N2 - The present study evaluates the trend of morbidity and growth of children and adolescents--the most vulnerable part of population. Negative trends in health of Slovak children and adolescents reflect probably increasing social differences, changes in life style and non-systematic preventive health care during certain periods of life--in school children, students, and working adolescents. A significant increase in chronic diseases and disorders in children and adolescents during the last decade are probably the results of many genetic, nutritional, environmental and also psychosocial factors related to changing life style patterns. Routine health statistics in Slovak Republic did not include some serious preventable diseases and disorders, which have an increasing trend in the young population and may have serious consequences in adulthood. Increase in chronic diseases prevalence is in contrast with trends of acceleration and shows the impact of present big social disparities. Prevention requires identification and elimination of known environmental risk factors in children and adolescents, and reinstitution of systematic intensive preventive health care. The health educational activities should become materialized in healthy life psychosocial conditions and environment of houses, schools, and playgrounds. Our results require further comprehensive analysis and international comparison. (PsycINFO Database Record (c) 2012 APA, all rights reserved)
SN - 0960-7560
AD - Sevcikova, L'.: Institute of Hygiene, Faculty of Medicine, Comenius University, Bratislava, Slovak Republic Jurkovicova, J.: Institute of Hygiene, Faculty of Medicine, Comenius University, Bratislava, Slovak Republic Stefanikova, Z.: Institute of Hygiene, Faculty of Medicine, Comenius University, Bratislava, Slovak Republic Machacova, E.: Institute of Epidemiology, Faculty of Medicine, Comenius University, Bratislava, Slovak Republic Sobotova, L'.: Institute of Hygiene, Faculty of Medicine, Comenius University, Bratislava, Slovak Republic Aghova, L'.: Institute of Hygiene, Faculty of Medicine, Comenius University, Bratislava, Slovak Republic
ER -

TY - JOUR
ID - 2699
T1 - Motor Function and Social Participation in Kindergarten Children. [References]
A1 - Bar-Haim,Yair; Bart,Orit
Y1 - 2006///
N1 - Peer Reviewed Journal: 2006-04983-007
English
Journal; Peer Reviewed Journal
KW - PsycInfo
KW - Childhood Play Behavior
KW - Group Participation
KW - Kindergarten Students
KW - Motor Processes

KW - Social Interaction
KW - Individual Differences
RP - NOT IN FILE
SP - 296
EP - 310
JF - Social Development
VL - 15:
IS - 2
N2 - This study focused on the associations between individual variations in children's motor abilities and individual differences in social participation and play behavior. Indoor and outdoor play behavior patterns of 88 kindergarten children were observed, and a battery of standard assessments of basic motor functions was administered. The findings indicate significant associations between children's motor abilities and social and nonsocial forms of play. Results are discussed in relation to existing conceptual models of the underlying causes for nonsocial behavior. (PsycINFO Database Record (c) 2012 APA, all rights reserved) (journal abstract)
SN - 0961-205X
AD - Bar-Haim, Yair: Tel-Aviv University, Tel-Aviv, Israel Bart, Orit: Tel-Aviv University, Tel-Aviv, Israel
ER -

TY - JOUR
ID - 2700
T1 - The Educator's Role in Child and Adolescent Mental Health. [References]
A1 - Waller,Raymond J.
Y1 - 2006///
N1 - Book: 2006-03928-001
English
Book; Edited Book
KW - PsycInfo
KW - Adolescent Development
KW - Childhood Development
KW - Mental Health
KW - Roles
KW - School Based Intervention
KW - Classroom Management
KW - Mental Disorders
KW - Resilience (Psychological)
KW - Teachers
RP - NOT IN FILE
SP - 3
EP - 13
JF - Waller, Raymond J [Ed]
VL - :
IS - 2006
N2 - (from the chapter) Teachers face more challenges today than during any other time. Students present with more challenges than ever before, particularly in the area of mental health needs. Clear evidence suggests that a variety of mental problems are increasing in frequency, and access to and availability of services for students with mental health needs have not improved for decades. In the face of such need, it is easy for educators to accept the responsibility of this burden borne by so many of today's students. But teachers should resist the propensity to shoulder a burden that is outside the purview of their professional training and focus. Educators should acknowledge, however, that they can have a positive impact on children's mental health. Teachers can play an incalculable role in fostering psychological resilience in children, which can serve to protect them from exposure to a variety of risk factors. The strategies suggested in this chapter are classroom techniques that can assist in the development of these protective factors. They are techniques that have been found to be effective, while actually tending to decrease, rather than increase, one's workload. In addition, the impact of protective factors seems to be cumulative so that the more of these strategies a teacher implements, the more protection a student may cultivate. Teachers are among the most involved adults in the lives of children. In our society, physicians maintain a high level of professional prestige, whereas teaching has lost credibility. Ironically, a child may go through life and rarely, if ever, see a physician. The same cannot be said of educators. If a child

begins to show symptoms of a mental health problem, teachers are likely to be the first professionals to see them. Thus, teachers can also play an indispensable role in recognition of problems, referral, participation in interdisciplinary planning, advocacy, and monitoring of their students and can do so without making a difficult job impossible. To assist in this, and to fill a void in most teacher training programs, this book is devoted to helping teachers recognize the most common mental health issues seen in children and adolescents and to offer strategies on how they can help encourage and support all their students. (PsycINFO Database Record (c) 2012 APA, all rights reserved)
AD - Waller, Raymond J.: Piedmont College, Demorest, GA, US Bresson, Diane J.: J. H. House Elementary School, Conyers, GA, US Waller, Katherine S.: Piedmont College, Demorest, GA, US
ER -

TY - JOUR
ID - 2701
T1 - Play behaviours and play object preferences of young children with autistic disorder in a clinical play environment. [References]
A1 - Dominguez,Anna; Ziviani,Jenny; Rodger,Sylvia
Y1 - 2006///
N1 - Peer Reviewed Journal: 2006-03162-005
English
Journal; Peer Reviewed Journal
KW - PsycInfo
KW - Autism
KW - Childhood Play Behavior
KW - Childhood Play Development
KW - Recreation Areas
KW - Playgrounds
RP - NOT IN FILE
SP - 53
EP - 69
JF - Autism
VL - 10:
IS - 1
N2 - Play is the primary occupation of childhood and provides a potentially powerful means of assessing and treating children with autistic disorder. This study utilized a cross-sectional comparison design to investigate the nature of play engagement in children with AD (n = 24), relative to typically developing children (n = 34) matched for chronological age. Play behaviours were recorded in a clinical play environment. Videotapes comprising 15 minutes of the children's spontaneous play behaviour were analysed using time-interval analysis. The particular play behaviours observed and play objects used were coded. Differences in play behaviours ($p < 0.0001$) and play object preferences ($p < 0.0001$) were identified between the groups. Findings regarding play behaviour contribute to contention in the literature surrounding functional and symbolic play. Explanations for play object preferences are postulated. Recommendations are made regarding clinical application of findings in terms of enhancing assessment and intervention by augmenting motivation. (PsycINFO Database Record (c) 2012 APA, all rights reserved) (journal abstract)
SN - 1362-3613
AD - Dominguez, Anna: University of Queensland, St Lucia, QLD, Australia Ziviani, Jenny: University of Queensland, St Lucia, QLD, Australia Rodger, Sylvia: University of Queensland, St Lucia, QLD, Australia
ER -

TY - JOUR
ID - 2702
T1 - Using social stories to improve the social behavior of children with Asperger syndrome. [References]
A1 - Sansosti,Frank J.
Y1 - 2006///
N1 - Peer Reviewed Journal: 2006-01113-006
English
Journal; Peer Reviewed Journal
KW - PsycInfo

KW - Aspergers Syndrome
KW - Social Behavior
KW - Social Skills
KW - Intervention
RP - NOT IN FILE
SP - 43
EP - 57
JF - Journal of Positive Behavior Interventions
VL - 8:
IS - 1
N2 - To date, the empirical support for the use of social story interventions for children with Asperger syndrome (AS) is small. The purpose of this study was to examine the effects of individualized social story interventions on the social behavior of three children with AS. Using a multiple-baseline-across-participants design, social stories were implemented, and direct observations of the participants' identified target behaviors were conducted three times per week during unstructured school activities (e.g., recess). Data revealed an increase in the social behavior of two of the three participants when the treatment was implemented. Unfortunately, maintenance of target behaviors was not observed. These data provide some initial support for the use of social stories to teach social skills to children diagnosed with AS. However, failure to demonstrate skill maintenance and poor results for one participant highlight possible limitations of the social story intervention and suggest a strong need for further research. Recommendations for future research endeavors and the potential benefits of social story interventions are discussed. (PsycINFO Database Record (c) 2012 APA, all rights reserved) (journal abstract)
SN - 1098-3007
AD - Sansosti, Frank J.: District School Board of uPasco County, FL, US Powell-Smith, Kelly A.: University of South Florida, Tampa, FL, US
ER -

TY - JOUR
ID - 2703
T1 - Where Is the Mathematics? Everywhere! [References]
A1 - Masarik,Kate
Y1 - 2006///
N1 - Book: 2005-15169-007
English
Book; Edited Book
KW - PsycInfo
KW - Mathematics Education
KW - Nontraditional Education
KW - Geometry
KW - Schools
KW - Teachers
RP - NOT IN FILE
SP - 97
EP - 108
JF - Pumpian, Ian [Ed]; Fisher, Douglas [Ed]; Wachowiak, Susan [Ed]
VL - :
IS - 2006
N2 - (from the chapter) A part of mathematics is learning how to work with numbers, but mathematical knowledge also includes knowing what the numbers represent; for example, understanding the amount off a price (discount); using patterns to explain natural phenomenon and business trends, etc. Mathematics reform stresses the contextualization of the mathematics. As teachers, we can bring real mathematics into the classroom; however, we cannot forget to include the mathematics outside of the classroom, such as a geometry nature hike to look at the patterns of the veins in leaves and a similar pattern in the branching out of tree limbs; being able to determine the area and perimeter of the layout of safety mats under the playground equipment; or comparing the angles of slides for different age groups. The 'School in the Park' (SITP) program, developed by the combined efforts of the staff at Balboa Park and Rosa Parks Elementary (under the direction of the City Heights Educational Pilot), provides the students multiple opportunities to study mathematics in the world

outside the classroom. Using the students' experiences in the SITP, I want to point out not only the obvious mathematics being used, but also the not--so--obvious mathematical experiences that teachers can tap into as they continue working with the students in the classroom across grade levels. (PsycINFO Database Record (c) 2012 APA, all rights reserved)
ER -

TY - JOUR
ID - 2704
T1 - School in the Park-A Unique Learning Experience for Children and Teachers. [References]
A1 - Pumpian,Ian; Wachowiak,Susan; Fisher,Douglas
Y1 - 2006///
N1 - Book: 2005-15169-001
English
Book; Edited Book
KW - PsycInfo
KW - Community Facilities
KW - Curricular Field Experience
KW - Experiential Learning
KW - Learning Environment
KW - Budgets
KW - Curriculum
KW - Schools
KW - Teachers
KW - Teams
RP - NOT IN FILE
SP - 1
EP - 14
JF - Pumpian, Ian [Ed]; Fisher, Douglas [Ed]; Wachowiak, Susan [Ed]
VL - :
IS - 2006
N2 - (from the chapter) We believe 'School in the Park' is a unique and bold educational endeavor implemented during a time of shrinking education budgets. Few schools and districts may have access to the resources necessary to operate a program on the scale of 'School in the Park'. However, the practical lessons learned are useful for understanding how community--based learning experiences can enhance educational outcomes for children. In addition, conditions can be created in which students will be motivated, focused, and excited about engaging in challenging learning tasks that most would consider out of their reach. Provided the right conditions exist, we would be thrilled if this book facilitated every school being able to offer multiple opportunities to apply the principles of 'School in the Park'. At the very least, understanding the principles and practices of 'School in the Park' will result in rethinking and reorganizing traditional field trip resources and moving instruction beyond: 1. Grade--level standards must drive the selection of environments, activities, materials, and assessments. 2. Grade--level standards can be taught and reinforced via hands--on, interdisciplinary, and experiential learning. 3. Formal (directed) and informal (constructive) learning need not be polar opposites. 4. Nontraditional classroom environments, when carefully selected, can provide motivation, purpose, and a depth of understanding for a standards--based curriculum. 5. If nontraditional classroom environments are to be effective in supporting a standards--based curriculum, then the instructional program must coordinate classroom and nonclassroom learning. The curriculum must be mapped and integrated into a year--long subject--specific pacing chart that identifies key standards that will be taught and assessed. 6. The number of nontraditional classroom environments accessible to most schools is limitless and can be inventoried via an environmental scan. Not all of these environments will require extensive logistical arrangements and costs. 7. Extending learning outside of traditional classroom settings creates an opportunity to expand instructors and create teaching teams. Time to engage all of the teaching adults in common planning is time well spent and can serve to support multiple classes and programs. 8. Extending learning outside of traditional classroom settings can increase the prior knowledge students bring to future learning environments and thereby increase their cultural capital. These principles and practices are described and exemplified throughout this book. We also hope that this book prompts new public and private interest in creating partnerships that continue to promote adding new environments and experiences to excite and enhance learning outcomes for students. Readers are challenged to begin to create their own asset maps of facilities and locations in their immediate community that are readily

available to provide educational opportunities that will extend and expand 'School in the Park'-type teaching and learning. (PsycINFO Database Record (c) 2012 APA, all rights reserved)
AD - Pumpian, Ian: San Diego State University, San Diego, CA, US Wachowiak, Susan: Director of School in the Park, US Fisher, Douglas: San Diego State University, San Diego, CA, US
ER -

TY - JOUR
ID - 2705
T1 - Quality Improvement Activities and Expanded School Mental Health Services. [References]
A1 - Nabors,Laura A.
Y1 - 2004///
N1 - Peer Reviewed Journal: 2004-15260-009
English
Journal; Peer Reviewed Journal
KW - PsycInfo
KW - Accountability
KW - Mental Health Program Evaluation
KW - Mental Health Programs
KW - Quality of Care
KW - School Based Intervention
KW - Program Evaluation
RP - NOT IN FILE
SP - 596
EP - 616
JF - Behavior Modification
JA - Behav Modif
VL - 28:
IS - 4
N2 - Quality improvement (QI) activities, designed to enhance program evaluation efforts and to improve quality of care, have become increasingly important in children's services research. QI activities examine outcomes and provide accountability data that can be very useful for telling the story of the development, implementation, and effectiveness of expanded school mental health (ESMH) programs. This article discusses the implementation and results of QI activities for two ESMH programs, one relatively new program, and one with 10 years of experience. When presenting each case example, the authors review key QI activities such as developing new measures and interventions, evaluating costs of care, and disseminating results to clinicians, program administrators, and funders. The article concludes with a discussion of the importance of QI in school-based mental health and reviews directions for future research and program development. (PsycINFO Database Record (c) 2012 APA, all rights reserved) (journal abstract)
SN - 0145-4455
AD - Nabors, Laura A.: Department of Psychology, University of Cincinnati, Cincinnati, OH, US Leff, Stephen S.: Department of Pediatrics, University of Pennsylvania School of Medicine, Philadelphia, PA, US Power, Thomas J.: Department of Pediatrics, University of Pennsylvania School of Medicine, Philadelphia, PA, US
ER -

TY - JOUR
ID - 2706
T1 - Gender at play: Fourth-grade girls and boys on the playground. [References]
A1 - Boyle,D.
Y1 - 2003///
N1 - Peer Reviewed Journal: 2003-00864-004
English
Journal; Peer Reviewed Journal
KW - PsycInfo
KW - Elementary School Students
KW - Human Sex Differences
KW - Leisure Time
KW - Social Interaction

KW - Playgrounds
KW - Schools
RP - NOT IN FILE
SP - 1326
EP - 1345
JF - American Behavioral Scientist
VL - 46:
IS - 10
N2 - This study explores the complicated social interactions related to gender that take place at school recess. Using grounded theory methodology and a social constructionist perspective, the researchers observed 67 recesses in New England and then examined the data to determine which themes emerged. Results demonstrate that there is great complexity in preadolescent gender relations and that their incidence and significance is influx during recess time. Among the results noted were ways in which children reinforce their gender identities; the wide intragender variability in choice of activities especially for girls; and clear evidence of borderwork that is, those interactions where boys and girls play together but there is something about the play that actually reinforces the gender divisions rather than diminishes them. Future research should examine ways adults can support children in developing relationships based on mutual collegiality and liking, thereby building their sense of competence and self-esteem. (PsycINFO Database Record (c) 2012 APA, all rights reserved) (journal abstract)
SN - 0002-7642
AD - Boyle, D. Ellen: Center for Research on Women, Wellesley College, Wellesley, MA, US Marshall, Nancy L.: Center for Research on Women, Wellesley College, Wellesley, MA, US Robeson, Wendy W.: Center for Research on Women, Wellesley College, Wellesley, MA, US
ER -

TY - JOUR
ID - 2707
T1 - Psychotherapy for Anxiety Disorders in Children. [References]
A1 - Muratori,Filippo
Y1 - 2005///
N1 - Book: 2005-06999-010
English
Book; Edited Book
KW - PsycInfo
KW - Anxiety Disorders
KW - Child Psychotherapy
KW - Childhood Neurosis
KW - Psychodynamic Psychotherapy
KW - Treatment Effectiveness Evaluation
KW - Parent Child Relations
KW - Parents
RP - NOT IN FILE
SP - 233
EP - 262
JF - Velotis, Calvin M [Ed]
VL - :
IS - 2005
N2 - (from the chapter) Epidemiological works suggest that anxiety disorders (AD) are the most prevalent problem in infancy and childhood. The clinical picture varies according to the particular disorder, developmental stage and comorbidity. Moreover cross sectional and longitudinal studies point out AD as a malleable but more persistent disorder than once thought. Psychotherapy is quite widely used in the treatment of children with AD and research in this field has advanced considerably in the last few decades. A large body of studies found short-term efficacy of behavioral treatments. Some studies have shown long term effects over time, and several studies tend to support that outcomes are better for brief child psychotherapy where specific problems are targeted. Greater therapeutic precision and a careful sculpturing of treatments to children's clinical needs are also necessary for psychodynamic psychotherapy, which is widely used in mental services. After the description of a a time-limited combined individual and parent-focused psychodynamic psychotherapy for

children in a clinical, naturalistic setting, a research project to evaluate its short and long term efficacy is presented. The treatment timing, phases and strategy are described as well as the role of intrapsychic conflicts and parental representations which are the focus of the treatment. Fifty-eight children meeting DSM-IV criteria for pure or mixed AD were assigned to either active treatment or community services; all subjects were measured at baseline, after six months and at a two-year follow-up, by C-GAS and CBCL. The benefits of treatment manifest both immediately and with delayed onset (sleeper effect): patients sought mental health services at a significantly lower rate than the compared condition. These findings permit to propose psychodynamic psychotherapy as a useful therapeutic tool in AD and illustrates the importance of developing empirical research on psychodynamic-oriented treatment widely applied in clinical practice but less frequently studied through careful long-term outcome evaluation. The role of the intensive involvement of the parents in our combined psychodynamic psychotherapy is proposed as an area of specific research for this type of treatment. (PsycINFO Database Record (c) 2012 APA, all rights reserved)
AD - Muratori, Filippo: Department of Developmental Neuroscience, Scientific Institute Stella Maris, University of Pisa, Pisa, Italy Picchi, Lara: Department of Developmental Neuroscience, Scientific Institute Stella Maris, University of Pisa, Pisa, Italy
ER -

TY - JOUR
ID - 2708
T1 - Leisure activities, alcohol, and nicotine consumption in people with a hypomanic/hyperthymic temperament. [References]
A1 - Krumm-Merabet,Christiane; Meyer,Thomas
Y1 - 2005///
N1 - Peer Reviewed Journal: 2005-01603-017
English
Journal; Peer Reviewed Journal
KW - PsycInfo
KW - Hypomania
KW - Leisure Time
KW - Recreation
KW - Time
KW - Alcohol Drinking Patterns
KW - Peer Relations
KW - Sports
KW - Tobacco Smoking
RP - NOT IN FILE
SP - 701
EP - 712
JF - Personality and Individual Differences
VL - 38:
IS - 3
N2 - Several studies show an association between the Hypomanic Personality scale (HPS; Eckblad & Chapman, 1986) and variables related to bipolar disorders. In theory, people with a hypomanic temperament are thought to be more active than others. The purpose of this study was to examine empirically which kinds of activities students with a hypomanic temperament typically pursue outside of the school context and especially how much time they engage in these activities. We hypothesized that students would report a high proportion of activities that are fun- and reward-related, also including problematic ones (e.g. alcohol abuse). Teenage students (n = 4045) completed several questionnaires, including the HPS, CES-D, questions about performance in school, future success, and leisure activities. Using the HPS, a high-risk group (n = 300) was denned and compared to a control group (n = 1709). As hypothesized, "hypomanic" adolescents spent most of their time outside of school socializing with friends and pursuing sports rather than attending to duties such as homework, smoked more often, drank alcohol more often, and were more often involved in physical fights. The results are discussed within the context of a model that regards dysregulation of the Behavioral Activation System as a vulnerability factor in bipolar disorders. (PsycINFO Database Record (c) 2012 APA, all rights reserved) (journal abstract)
SN - 0191-8869

AD - Krumm-Merabet, Christiane: Department of Clinical and Physiological Psychology, Psychological Institute, Eberhard Karls University, Tubingen, Germany Meyer, Thomas D.: Department of Clinical and Physiological Psychology, Psychological Institute, Eberhard Karls University, Tubingen, Germany
ER -

TY - JOUR
ID - 2709
T1 - The introduction of the Webster-Stratton Incredible Years Classroom Dinosaur School Programme in Gwynedd, North Wales: A pilot study. [References]
A1 - Hutchings,Judy; Lane,Eleanor; Owen,Richard Ellis; Gwyn,Rhiain
Y1 - 2004///
N1 - Peer Reviewed Journal: 2005-01044-001
English
Journal; Peer Reviewed Journal
KW - PsycInfo
KW - Behavior Problems
KW - Classroom Environment
KW - Educational Programs
KW - Problem Solving
KW - Therapeutic Processes
RP - NOT IN FILE
SP - 4
EP - 15
JF - Educational and Child Psychology
VL - 21:
IS - 4
N2 - The Webster-Stratton Classroom Dinosaur School Programme was developed as a therapeutic intervention to teach social and problem-solving skills and to reduce behavioural problems in young children. It has subsequently been developed as a classroom programme for use in schools (Webster-Stratton et al., 2001a). In 2001 Gwynedd Education Service in North West Wales undertook a pilot delivery of the classroom programme in the reception class of one primary school. This paper describes the programme and the detail that went into ensuring that the plan would be effective. It also reports on some outcomes from the first year of the programme in terms of pre- and post-intervention measures of child behaviour and use of social and problem solving skills. Interviews were also conducted with school staff and parents and their perceptions of the programme's effectiveness are reported. Results demonstrated positive effects on academic performance, social and emotional development and reductions in behavioural problems. There was evidence that improvements had generalised to other settings, playground and home, and to other children in the school. The success of the programme in the first year helped to ensure the continuation of the programme, which is now in fourteen schools. The paper concludes with discussion of why the programme has been successful and describes the way in which the plan has evolved since this first year. (PsycINFO Database Record (c) 2012 APA, all rights reserved) (journal abstract)
SN - 0267-1611
AD - Hutchings, Judy: Incredible Years Wales Centre, University of Wales, Bangor, United Kingdom Lane, Eleanor: Incredible Years Wales Centre, University of Wales, Bangor, United Kingdom Owen, Richard Ellis: Conwy Council Education Department, United Kingdom Gwyn, Rhiain: Ysgol Babanod, Bangor, United Kingdom
ER -

TY - JOUR
ID - 2710
T1 - Outcome Measures to Assess the Effectiveness of Bullying-prevention Programs in the Schools. [References]
A1 - Left,Stephen S; Power,Thomas
Y1 - 2004///
N1 - Book: 2004-00070-014
English
Book; Edited Book

KW - PsycInfo
KW - Educational Program Evaluation
KW - Measurement
KW - School Based Intervention
KW - Treatment Outcomes
KW - Bullying
KW - Questionnaires
KW - Schools
RP - NOT IN FILE
SP - 269
EP - 293
JF - Espelage, Dorothy L [Ed]; Swearer, Susan M [Ed]
VL - :
IS - 2004
N2 - (from the chapter) There are numerous innovative bullying and aggression-prevention programs being used in schools across the country and worldwide. This chapter presents a review of the most commonly used sets of measures to identify aggressors and bullies and to determine the effectiveness of bullying and aggression prevention programs. The review will focus on the strengths and limitations of each set of measures. In addition, the chapter describes how our research team has collaborated with the adults who supervise students on the playground and in the lunchroom to develop and validate several innovative community-responsive questionnaires that can be used in conjunction with other methods to evaluate the effectiveness of bullying prevention and intervention efforts. The chapter concludes by discussing how to translate research to practice in the development outcome measures designed to evaluate the effectiveness of school-based bullying prevention programs. (PsycINFO Database Record (c) 2012 APA, all rights reserved)
AD - Left, Stephen S.: Children's Hospital of Philadelphia, Department of Pediatric Psychology, Philadelphia, PA, US Power, Thomas J.: Children's Hospital of Philadelphia, University of Pennsylvania School of Medicine, Philadelphia, PA, US Goldstein, Amy B.: Children's Hospital of Philadelphia, Department of Pediatric Psychology, Philadelphia, PA, US
ER -

TY - JOUR
ID - 2711
T1 - Phonological training in children with dyslexia using temporally modified speech: A three-step pilot investigation. [References]
A1 - Habib,Michel; Rey,Veronique; Daffaure,Virginie; Camps,Roselyne; Espesser,Robert; Joly-Pottuz,Barbara; Demonet,Jean-Francois
Y1 - 2002///
N1 - Peer Reviewed Journal: 2002-15837-003
English
Journal; Peer Reviewed Journal
KW - PsycInfo
KW - Cognitive Processes
KW - Dyslexia
KW - Phonology
KW - Speech Rate
KW - Speech Therapy
KW - Auditory Perception
KW - Theories
RP - NOT IN FILE
SP - 289
EP - 308
JF - International Journal of Language & Communication Disorders
VL - 37:
IS - 3
N2 - Investigated the usefulness of intensively training children with dyslexia with daily exercises based on the temporal processing theory of dyslexia. In the first study, 12 children, aged 10-12 yrs, received training for 1 hr a day, 5 days a week, over 5 weeks, and they were assessed on phonological tasks before, during and after

training. In a second study, 29 children with dyslexia, aged 5-12 yrs, received a similar training, but for only 15 min a day, 7 days a week, over 6 weeks, part at the speech therapist office, part in their own homes. The finding of comparable improvement in a more 'natural' environment and in children over a wider age range was found. However, this study also showed that 1 of 4 children did not improve as expected, prompting a third study where 23 children (mean age 9.5 yrs) underwent specific tasks presumably exploring various aspects of temporal processing in order to find predictors of training efficacy. A 'temporal order judgement' task was found to be best correlated with post-training improvement, suggesting that one could use this task for selecting the best candidates for temporo-phonological training. These studies provide further justification for a rational, indication-based temporo-phonological treatment of dyslexia. (PsycINFO Database Record (c) 2012 APA, all rights reserved)
SN - 1368-2822
AD - Habib, Michel: CHU Timone, Faculty of MEdicine, Marseille, France Rey, Veronique: U Provence, Lab Parole et Language, Aix-en-Provence, France Daffaure, Virginie: CHU Timone, Faculty of MEdicine, Marseille, France Camps, Roselyne: CHU Timone, Faculty of MEdicine, Marseille, France Espesser, Robert: CHU Purpan, INSERM U455, Toulouse, France Joly-Pottuz, Barbara: CHU Purpan, INSERM U455, Toulouse, France Demonet, Jean-Francois: CHU Purpan, INSERM U455, Toulouse, France
ER -

TY - JOUR
ID - 2713
T1 - Defiant Behavior in Two- and Three-Year-Olds: A Vygotskian Approach. [References]
A1 - Reefer,Laura Reynolds
Y1 - 2005///
N1 - Peer Reviewed Journal: 2006-09435-007
English
Journal; Peer Reviewed Journal
KW - PsycInfo
KW - Childhood Play Behavior
KW - Early Childhood Development
KW - Psychosocial Development
KW - Vygotsky (Lev)
KW - Child Care
RP - NOT IN FILE
SP - 105
EP - 111
JF - Early Childhood Education Journal
VL - 33:
IS - 2
N2 - Defiant behavior in two- and three-year-olds is a natural expression of a young child's growing autonomy, and, although frustrating to adults, is a natural phase of a child's socio-emotional growth. L.S. Vygotsky's socio-historical theory of the development included a discussion of "crisis" periods, one of which he felt occurred at age three. The crisis that Vygotsky asserted occurred at three is characterized by a group of seven "behaviors", all of which indicate a struggle with the child's position in relation to family social structures. This study examined the occurrences and characteristics of defiant behavior in two-and three-year-old children during outdoor play at a child development center, and the existence and context of defiant behaviors the modern childcare setting. Patterns in defiant behaviors during play in different age groups were apparent, and suggested an instrument to facilitate the documentation and analysis of defiant behavior in modern group-care settings. (PsycINFO Database Record (c) 2012 APA, all rights reserved) (journal abstract)
SN - 1082-3301
AD - Reefer, Laura Reynolds: Department of Education, University of South Carolina, Charleston, SC, US
ER -

TY - JOUR
ID - 2714
T1 - The degraded. Outside, to earth, died. The trauma transmitted and inflicted in the analysis of a six-year-old child. [Italian]. [References]
A1 - Herzog,James M.

Y1 - 2005///
N1 - Peer Reviewed Journal: 2006-05654-010
Italian
Journal; Peer Reviewed Journal
KW - PsycInfo
KW - Family Relations
KW - Psychoanalysis
KW - Transgenerational Patterns
KW - Trauma
RP - NOT IN FILE
SP - 537
EP - 563
JF - Rivista di Psicoanalisi
VL - 51:
IS - 2
N2 - The author presents the analysis of a precocious traumatized little girl, which reveals the ways in which historical trauma is transmitted and intra-familial trauma is both disguised and represented. The play as it evolves is seen to simultaneously communicate what the child struggles with and to resolutely try to hide what has actually happened. Analyst and child together participate in play which utilizes displacement, enactment and interactive enactment, the latter play mode being the very hallmark of profound traumatic experience. Carlotta, the child, helps the analyst to follow her quest for meaning making even as the interaction between them adheres to and departs from the deepening pentimenti of traumatic experience, which needs to be unraveled and reconstructed in order that her own developmental progression can be rejoined. The analysis facilitates Carlotta's capacity to play in a more unfettered fashion and to assist her family's recovery as well. (PsycINFO Database Record (c) 2012 APA, all rights reserved) (journal abstract)
SN - 0035-6492
ER -

TY - JOUR
ID - 2715
T1 - Needs assessment and development of a bullying prevention program for elementary school students
A1 - Wilcox,Cloey
Y1 - 2005///
N1 - Dissertation Abstract: 2005-99024-401
English
Dissertation Abstract
KW - PsycInfo
KW - Elementary School Students
KW - Needs Assessment
KW - Program Development
KW - School Based Intervention
KW - Bullying
RP - NOT IN FILE
SP - 3471
JF - Dissertation Abstracts International: Section B: The Sciences and Engineering
VL - 66:
IS - 6-B
N2 - Bullying affects between fifteen and twenty percent of students in the United States (Batsche and Knoff, 1994) and up to eighty-eight percent of students witness bullying in school (Quiroz, 2002). A needs assessment was conducted to investigate the prevalence and patterns of bullying in a suburban elementary school. The Olweus Bully/Victim Questionnaire was used to survey 134 third through fifth grade students. This survey ascertained the percentage of bullies and victims as well as students' views about bullying. A bullying prevention program was designed to fit the needs of students and staff. Key staff members were interviewed to determine what character education programs the school already had in place and what resources would be available for a new bullying prevention program. Results of student surveys indicated that 49.3% of students were bullied and 10.4% of students reported bullying others. There was no significant difference in the prevalence of bullying by gender or grade. Students were most likely to be bullied on the playground and in the

lunchroom. The majority of victims reported that the bullying had lasted for at least a year. The majority of students reported feeling sorry for victims; however it was also reported that students rarely tried to stop bullying. Results from staff interviews revealed that the school has a variety of social programs in place at the primary, secondary and tertiary levels of prevention. A program was developed that consists of four components; a playground and lunchroom program, bullying lesson plans, a school-wide anti-bullying policy and a method for anonymous reporting. This program was designed to complement existing programs and utilize the limited resources available. Results are discussed and further areas of research are suggested. (PsycINFO Database Record (c) 2012 APA, all rights reserved)
SN - 0419-4217
AD - Wilcox, Cloey: Rutgers The State U New Jersey, Graduate School Of Applied And Professional Psychology, US
ER -

TY - JOUR
ID - 2716
T1 - Long and short-term effects of video games on impulsive responses in boys: An alternative to content-based research
A1 - Schleifer,Eric
Y1 - 2005///
N1 - Dissertation Abstract: 2005-99022-129
English
Dissertation Abstract
KW - PsycInfo
KW - Computer Games
KW - Human Males
KW - Impulsiveness
KW - Response Latency
RP - NOT IN FILE
SP - 2862
JF - Dissertation Abstracts International: Section B: The Sciences and Engineering
VL - 66:
IS - 5-B
N2 - Prior research in the field of video games has focused primarily on how violent content affects aggressive behavior. The current study was designed to show that game-users' ability to press a button and elicit an immediate and fixed response from the game will have an effect on impulsive reactions outside the gaming environment. Forty boys, age ten to fourteen, were given two questionnaires that gathered information regarding their video gaming habits (past and present) and their propensity for disinhibition or impulsivity in certain situations as measured by the Behavioral Activation System (BAS) Sensitivity Scale (Carver & White, 1994). Before and after engaging in a reward-based activity (playing video games vs. shooting baskets for a prize), participants were asked to perform a Time Perception Task as a measure of cognitive impulsivity. There were no significant differences between the experimental conditions. Participants who reported playing more video games per day, however, showed greater cognitive impulsivity following cessation of the reward-based activity regardless of which condition they were in. Results concerning the BAS Sensitivity Scale yielded findings in two of the three sub-scales of this measure. Participants who reported playing more video games per day reported a more impulsive, persistent pursuit of desired goals (Drive scale). Results also indicated that these same participants tended to respond more positively to the occurrence and anticipation of rewards (Reward Responsiveness scale). When viewed collectively, these findings indicate some support for the model proposed in this study, which attempted to link video game play with Patterson and Newman's (1993) theory of disinhibition. Also discussed are possible applications of the proposed model for future studies correlating high video game play and high BAS ratings with affect regulation, capacity for fantasy/imagination, clinical populations (ADHD, ODD, and Bi-polar), agency/locus of control, academics, and gender differences. (PsycINFO Database Record (c) 2012 APA, all rights reserved)
SN - 0419-4217
AD - Schleifer, Eric: Adelphi U, Inst Advanced Psychological Studies, US
ER -

TY - JOUR

ID - 2717
T1 - The induction of observational learning repertoires in preschool children with developmental disabilities as a function of peer-yoked contingencies
A1 - Stolfi,Lauren
Y1 - 2005///
N1 - Dissertation Abstract: 2005-99022-108
English
Dissertation Abstract
KW - PsycInfo
KW - Contingency Management
KW - Developmental Disabilities
KW - Observational Learning
KW - Peers
KW - Preschool Students
RP - NOT IN FILE
SP - 2807
JF - Dissertation Abstracts International: Section B: The Sciences and Engineering
VL - 66:
IS - 5-B
N2 - Two experiments tested the effects of "peer-yoked contingencies" on the establishment of observational learning repertoires using a time-lagged multiple probe design across participants. The three participants in each experiment were preschoolers with mild developmental delays who did not have an observational learning repertoire prior to this study. Both experiments were conducted in a small tutorial room in a preschool for students with disabilities located outside of a major metropolitan area. In Experiment 1, a baseline probe for observational learning was first conducted in which a participant was seated next to a peer while the experimenter taught the peer to vocally identify a set of pictures. Once the peer mastered that set of pictures, the participant was probed for the same set of responses. This procedure was then repeated with the same participant and a different peer. The peer-yoked contingencies were then implemented in which a participant and one of the peers (the "game peer") played a game in which the participant was required to observe the peer's responses to a pre-set criterion in order to win the game for the team. Once the participant mastered the game, another probe for observational learning was conducted. The results of Experiment 1 showed that, following the peer-yoked contingencies, all of the participants showed an increase in the number of responses learned through observation. However, the degree of this increase varied across the three participants, and one participant showed only a very slight increase in observational learning. Experiment 2 was a direct replication of the previous experiment, with the major difference being that participants were exposed to additional sessions of the peer-yoked contingencies if they did not acquire observational learning after a single exposure. The results of Experiment 2 also showed that the peer-yoked contingencies were effective in teaching observational learning repertoires to these preschoolers and that the participants required different numbers of sessions of peer-yoked contingencies in order to establish this repertoire. (PsycINFO Database Record (c) 2012 APA, all rights reserved)
SN - 0419-4217
AD - Stolfi, Lauren: Columbia U., US
ER -

TY - JOUR
ID - 2718
T1 - Effects of reinforcement schedules on intrinsic motivation and the overjustification effect
A1 - Harrington,Scott Winslow
Y1 - 2005///
N1 - Dissertation Abstract: 2005-99016-183
English
Dissertation Abstract
KW - PsycInfo
KW - Intrinsic Motivation
KW - Reinforcement Schedules
KW - External Rewards
RP - NOT IN FILE

SP - 1156
JF - Dissertation Abstracts International: Section B: The Sciences and Engineering
VL - 66:
IS - 2-B
N2 - The effect of rewards on intrinsic motivation has been evaluated extensively over the last 30 years, and is still fervently debated. Some researchers claim that when extrinsic rewards are terminated while engaging in an activity that is intrinsically motivating and previously reinforced, the rates decrease below baseline levels (viz., the "Overjustification effect"). Other researchers claim rewards have no detrimental effect on intrinsic motivation; if reductions in behavior are reported, the behavior eventually returns to baseline levels. This investigation evaluated the effects of rewards on intrinisically motivating playground activities with three children. The participants were 2 girls and 1 boy, ranging from ages 8-11. The experiment utilized a multielement, multiple baseline design across participants, and provides data on percent of session engaged, as well as scores on an 8-item attitudinal self-report. Reinforcement was provided on fixed-ratio 4 and variable ratio 4 schedules. Discriminative stimuli are associated with each condition to facilitate discrimination. Other methodological advances include preference assessments on the intrinsically motivating playground activity and stimulus (reinforcer) preference-assessments to identify actual reinforcers rather than rewards. Moreover, a time-series design was utilized to allow for a comprehensive analysis of the data across conditions and a 2-week follow-up was also conducted. Results indicated reinforcement has no detrimental effect on intrinsic motivation for all 3 participants; conversely, increases in behavior in the postsession and 2-week follow-up data were observed. The 8-item self-report measure also showed no detrimental effects from the delivery of rewards. These results extend the behavioral literature on rewards and intrinsic motivation. (PsycINFO Database Record (c) 2012 APA, all rights reserved)
SN - 0419-4217
AD - Harrington, Scott Winslow: U Nevada, Reno, US
ER -

TY - JOUR
ID - 2719
T1 - Naturalistic observations of girls' and boys' playground aggression
A1 - Smith,Carla E.
Y1 - 2005///
N1 - Dissertation Abstract: 2005-99014-373
English
Dissertation Abstract
KW - PsycInfo
KW - Aggressive Behavior
KW - Elementary School Students
KW - Playgrounds
KW - Human Sex Differences
KW - Bullying
RP - NOT IN FILE
SP - 576
JF - Dissertation Abstracts International: Section B: The Sciences and Engineering
VL - 66:
IS - 1-B
N2 - The purpose of the present study was to explore relations among the forms and social dynamics of girls' playground aggression in comparison to boys' playground aggression. Physical, verbal, and social forms of aggression were assessed, as well as the extent to which the focal child was in a position of social power relative to peers, and the extent to which peers aligned with the focal child during the aggressive episode. Also, peer ratings of popularity and aggression were examined. The observational and survey data were drawn from a large, longitudinal study of bullying and victimisation among elementary school students (grades 1 through 6) attending two schools in metropolitan Toronto, Canada. The subsample of 39 girls and 39 boys were observed engaging in aggressive behaviour during episodes in which they were focal children (i.e., children who were the focus of the observations). The focal children were 5 to 12 years of age (M = 8.77, SD = 1.76). Naturalistic observations of playground interactions revealed that a larger proportion of girls than boys used indirect forms of aggression (i.e., social aggression), whereas a smaller proportion of girls than boys used direct forms of aggression (i.e., physical and verbal aggression). In addition, abstinence from physical aggression and reliance

on social aggression was associated with higher levels of social power among girls in comparison to boys. Theoretical implications and implications for research methodology, risk identification, and intervention are discussed. (PsycINFO Database Record (c) 2012 APA, all rights reserved)
SN - 0419-4217
AD - Smith, Carla E.: York U., Canada
ER -

TY - JOUR
ID - 2720
T1 - Teacher-child relationships and preschool children's cortisol fluctuations
A1 - Lisonbee,Jared A.
Y1 - 2005///
N1 - Dissertation Abstract: 2005-99010-024
English
Dissertation Abstract
KW - PsycInfo
KW - Child Care
KW - Classrooms
KW - Hydrocortisone
KW - Preschool Students
KW - Teacher Student Interaction
RP - NOT IN FILE
SP - 6086
JF - Dissertation Abstracts International: Section B: The Sciences and Engineering
VL - 65:
IS - 11-B
N2 - Teacher-child relationships were examined as possible predictors of children's cortisol change in a preschool setting. Cortisol fluctuations across the day (daily change) for children (n = 186) in 17 full-day childcare classrooms were assessed in the morning and again in the afternoon on two days (one in Winter and one in Spring). In order to isolate the effects of the teacher-child relationships on cortisol, each child and his or her teacher took part in a one-on-one interaction episode outside of the classroom, and cortisol change was measured before and after the interaction episode (interaction change). Hierarchical Multivariate Linear Modeling (HMLM) was used to assess associations among teacher-child relationship qualities and cortisol change while taking into account or controlling for nested measurement (e.g., time since waking), child (e.g., sex and temperament), and classroom/teacher (e.g., group size and observed teacher negativity) effects. Morning to afternoon changes in cortisol replicated findings from previous research, with 51.2% and 45.8% of children showing increases in cortisol across the day, rather than the typical circadian pattern of decreases across the day. Modest stability was found for a.m. to p.m. cortisol change across the Winter and Spring assessments ($r = .20$, $p < .05$) with morning values showing the greatest consistency ($r = .30$, $p < .001$). Cortisol levels were associated with elapsed time since wake-up, but not with duration of sample collection. Teachers' reports of clingy relationships were associated with children's cortisol increases from morning to afternoon in the Winter classroom assessment. Teachers' reports of relationship conflict were associated with increases in cortisol across the teacher-child interaction task for boys but not girls. Child reports of relationship negativity were also marginally related to cortisol increases across the teacher-child interaction. Although the influence of teacher-child relationships on children's cortisol activity were not consistent across all measurement periods, the results provided a stringent test of the hypotheses and evidence that children's relationships with their teachers in the childcare setting may influence cortisol fluctuations, even after controlling for the effects of child, classroom, and measurement factors known to influence cortisol level. (PsycINFO Database Record (c) 2012 APA, all rights reserved)
SN - 0419-4217
AD - Lisonbee, Jared A.: Auburn U., US
ER -

TY - JOUR
ID - 2721
T1 - The relationship between personal factors and task specific teacher efficacy judgments: Implications for student reading gains (Ohio)

A1 - Sokol,Matthew P.
Y1 - 2005///
N1 - Dissertation Abstract: 2005-99009-064
English
Dissertation Abstract
KW - PsycInfo
KW - Academic Achievement
KW - Classroom Management
KW - Reading Comprehension
KW - Teacher Characteristics
KW - Teaching Methods
KW - Judgment
KW - Reading
KW - Self Efficacy
KW - Teachers
RP - NOT IN FILE
SP - 4106
JF - Dissertation Abstracts International Section A: Humanities and Social Sciences
VL - 65:
IS - 11-A
N2 - Recent literature has conceptualized teacher efficacy as context and task specific. Little is known, however, as to variables that predict such task specific teacher efficacy judgments, and there have been no investigations into the potential relationship between such efficacy judgments and student academic outcomes. The purpose of this study was to identify personal and organizational perceptual variables, as well as demographic variables predictive of teacher efficacy judgments in the areas of classroom management, student engagement, and instructional strategies. This study then assessed the relationships and predictive ability of the three task specific teacher efficacy judgments with student gain in the area of reading comprehension. Utilizing the recently developed Ohio State Teacher Efficacy Scale with 136 general education-upper elementary teachers (3rd through 6th grade), the current investigation revealed that both hardiness personality disposition and having a perceived high percentage of actively engaged students highly correlated with, and significantly contributed to, elevated teacher efficacy judgments in the three assessed tasks. Additional analyses revealed that supportive principal leadership accounted for significant variance in classroom management efficacy only. The reading performances of 2,011 general education students were deemed admissible for this study and included 699 third graders, 722 fourth graders, and 590 fifth graders. Analyses of the task specific teacher efficacy/student academic gain relationship revealed no significant correlations in the predicted direction. Subsequent regression analyses also revealed that none of the three efficacy judgments accounted for significant variance in student reading comprehension gain in the predicted direction. Recommendations for future research include the need for a case study approach of teachers who possess a hardy personality disposition, report a high number of actively engaged students, and supportive principal leadership to better understand how they differ from others in the way they process sources of efficacy information. Inclusion of both regular education and "at-risk" students when assessing educational outcomes in teacher efficacy research is also critical. The current study excluded students who received remedial reading support outside of the classroom setting, and previous research suggests that this "at risk" group may benefit the most from having highly efficacious instructors. Expanding the scope of assessed educational outcomes would also appear appropriate and include both academic and affective domains. Finally, the current study attempted to extend previous findings by utilizing a pre-post academic measure that was highly correlated with the students' actual curriculum. Recommendations for improvement include the use of pre-post measures that are curriculum based, standardized, and normed on the local population, along with use of cross-validation procedures that may include the use of nation ally-normed assessments, grades, or other curriculum based measures. (PsycINFO Database Record (c) 2012 APA, all rights reserved)
SN - 0419-4209
AD - Sokol, Matthew P.: Wayne State U., US
ER -

TY - JOUR
ID - 2722

T1 - Childhoods in place and placeless childhoods: An historical geography of young people in Yorkville and East Harlem, 1940--2000 (New York City)
A1 - Wridt,Pamela J.
Y1 - 2005///
N1 - Dissertation Abstract: 2005-99003-098
English
Dissertation Abstract
KW - PsycInfo
KW - Child Attitudes
KW - Experiences (Events)
KW - Geography
KW - History
KW - Urban Environments
KW - Leisure Time
RP - NOT IN FILE
SP - 3182
JF - Dissertation Abstracts International Section A: Humanities and Social Sciences
VL - 65:
IS - 8-A
N2 - This dissertation provides an analysis of how young people's everyday lives outside of school in Yorkville and East Harlem have changed from the 1940s until present time, and what factors contribute to consistencies or differences in young people's use and experience of their local environment. This research seeks to contribute to the limited academic literature on the historical geography of childhood in urban communities. The focus of this investigation is upon the period of middle childhood (roughly the period of childhood between ages 11 and 13), a time when most young people are able to actively and autonomously explore their communities. The emphasis of the research is on changes in children's geographies, or how children use, think about, and make sense of place in their everyday life. I compare children's geographies over three different time periods: (1) the 1940s, working with seniors in their 60s and 70s; (2) the 1970s, working with adults in their 30s; and (3) present time (2000s), working with young people aged 11-13. Topics explored in the research include young people's sense of place, their interactions with peers and adults in the community, their leisure time activities, their use of public space and their geographic territories. (PsycINFO Database Record (c) 2012 APA, all rights reserved)
SN - 0419-4209
AD - Wridt, Pamela J.: City U New York, US
ER -

TY - JOUR
ID - 2723
T1 - The athletic profile of people from the Island of Majorca in accordance with sociodemographic variables and levels of participation. [Spanish]. [References]
A1 - Sampol,Pere Palou; Verdaguer,Francesc Xavier Ponseti; Rotger,Pere
Y1 - 2005///
N1 - Peer Reviewed Journal: 2005-16635-004
Spanish
Journal; Peer Reviewed Journal
KW - PsycInfo
KW - Athletic Participation
KW - Human Sex Differences
KW - Leisure Time
KW - Motor Processes
KW - Sports
KW - Profiles (Measurement)
RP - NOT IN FILE
SP - 225
EP - 236
JF - Revista de Psicologia del Deporte
VL - 14:
IS - 2

N2 - Participating in sports is one of people's favourite leisure time activities, but athletes are not a homogeneous group at any time, owing to obvious differences in gender and socio-demographic factors. Maximum participation in physical and sport activities occurs during a child's school years. On the other hand, participation in sports drops massively when children leave school, owing to a multitude of factors that depend on psychological and socio-personal factors. The objective of this article is to identify the athletic profile of people from the island of Majorca in accordance with sociodemographic variables and levels of participation. This paper is part of research conducted during 2001 that aimed to study and identify the athletic habits of the Majorcan population between 10 and 14 years of age. A sample of 2661 subjects was studied and a previously validated questionnaire based on authors' work was used for evaluation. The results obtained in this study demonstrate that taking part in sports has been consolidated as a life style in our society and that it is a favourite occupation for Majorcan pre-adolescents during free time. It is also significant to emphasize the obvious differences between males and females in the age groups studied in connection with taking part in sports outside physical education classes. (PsycINFO Database Record (c) 2012 APA, all rights reserved) (journal abstract)
SN - 1132-239X
AD - Sampol, Pere Palou: Dep. Ciencias de la Educacion y Didacticas Especificas, Universitat de les Illes Balears, Palma de Mallorca, Spain
ER -

TY - JOUR
ID - 2724
T1 - Achieving learning goals through play: Teaching young children with special needs (2nd ed.). [References]
A1 - Widerstrom,Anne H.
Y1 - 2005///
N1 - Book: 2005-15297-000
English
Book; Authored Book
(Created by PsycINFO) About the Author Preface Acknowledgments I: The Role of Play in Early Childhood Education Why Is Play Important? Changing Perceptions of Play in Early Childhood Education Adapting Play for Children with Special Needs II: Planning and Writing Goals and Objectives for Children with Disabilities A Planning Matrix for Play Individualizing Goals for Play III: Let's Play: Embedding Techniques and Learning Goals in Play Activities Block Play: How High Is Our Tower? Sand and Water Play: Mud Pies Are Delicious! Pretend Play: I'll Be the Mommy and You Be the Daddy Manipulative Play: Real Materials Are Best Outdoor Games and Play: See How Fast I Can Go! Play with Music and Rhythm: Beating Our Drums and Tooting Our Horns Play with Creative Materials: Getting Messy with Glue and Paint Emergent Literacy: Please Read Me a Story Catalog of Play Activities: Embedding Learning Goals in Play IV: Playing Together and Making Friends Big Groups, Little Groups: Let's All Learn Together Learning Social Skills Through Play Connecting Home to School and Beyond: Enabling Families to Participate in the Classroom and Community Achieving Jasmine's Learning Goals Through Play References Appendix A: Sources for Adaptive Equipment and Learning Materials Appendix B: Guidelines for Developmentally Appropriate Practice: Adaptations for Preschool Children with Disabilities Appendix C: Resources for Including Children with Special Needs Appendix D: Play Checklists Appendix E: Reproducible Planning Matrices and Play Quiz Index
KW - PsycInfo
KW - Childhood Play Behavior
KW - Educational Objectives
KW - Learning Strategies
KW - Special Needs
KW - Teaching
KW - Disabilities
RP - NOT IN FILE
SP - Baltimore, MD, US
JF - (2005)
VL - Achieving learning goals through play: Teaching young children with special needs :
IS - 2nd ed.
N2 - (from the cover) Play is more than fun; it's a powerful teaching tool that helps young children learn. The second edition of this practical guide gives early childhood educators creative, ready-to-use strategies for weaving individual learning goals into play activities throughout the school day. Developed for use with young

children who have special needs--but equally effective for typically developing children--Achieving Learning Goals through Play helps teachers (a) foster children's cognitive, communication, motor, social, and preliteracy skills during group activities and free play; (b) use existing classroom play centers and materials to create age-appropriate learning opportunities; (c) support individual and group learning goals during familiar types of play, such as outdoor activities and music and rhythm play; (d) work play activities into the classroom schedule with straightforward planning sheets; and (e) adapt activities for children with special needs. Helpful appendixes offer guidelines for developmentally appropriate practice, resources for including children with disabilities, and photocopiable planning matrices. With this strategy-filled handbook, early childhood educators will help all their students reach their individual learning goals in fun, creative ways. (PsycINFO Database Record (c) 2012 APA, all rights reserved)
AD - Widerstrom, Anne H.: San Francisco State U, Dept of Special Education, School of Education, San Francisco, CA, US
ER -

TY - JOUR
ID - 2725
T1 - First step to success early intervention program: A study of effectiveness with Native-American children. [References]
A1 - Diken,Ibrahim H.
Y1 - 2005///
N1 - Peer Reviewed Journal: 2005-15017-009
English
Journal; Peer Reviewed Journal
KW - PsycInfo
KW - American Indians
KW - Childhood Play Behavior
KW - Early Intervention
RP - NOT IN FILE
SP - 444
EP - 465
JF - Education & Treatment of Children
VL - 28:
IS - 4
N2 - This study examined the effectiveness of the First Step to Success (FSS) early intervention program with four Native-American students, their teachers, and their parents on (a) targeted students' problem behaviors, (b) class-wide student behaviors, and (c) teacher behaviors. Participant teachers and parents were also interviewed to gather their perceptions of the FSS program. The results of direct observations of targeted students' play behaviors on the playground revealed that the FSS program had a significant positive affect on all participant students' social play behaviors. As soon as the intervention started, all participant students' social play behaviors significantly increased and their nonsocial behaviors decreased. All participant students showed higher levels of social play behaviors as soon as the intervention was initiated. Substantial decreases in problem behaviors were also reported by two teachers. Some positive changes in class-wide student behaviors and teacher behaviors were reported by the participant teachers. All but one parent reported significant changes in problem behaviors of targeted students. They all were highly satisfied with the program and rated it as easy to use. Limitations of the study and directions for future research are discussed. (PsycINFO Database Record (c) 2012 APA, all rights reserved) (journal abstract)
SN - 0748-8491
AD - Diken, Ibrahim H.: Anadolu University, Turkey Rutherford, Robert B.: Arizona State University, Tempe, AZ, US
ER -

TY - JOUR
ID - 2726
T1 - Preventing trouble: Making schools safer places using positive behavior supports. [References]
A1 - Oswald,Karen; Safran,Stephen; Johanson,George
Y1 - 2005///

N1 - Peer Reviewed Journal: 2005-14010-005
English
Journal; Peer Reviewed Journal
KW - PsycInfo
KW - Behavior Problems
KW - Middle School Students
KW - Prevention
KW - Safety
KW - School Based Intervention
KW - Middle Schools
RP - NOT IN FILE
SP - 265
EP - 278
JF - Education & Treatment of Children
VL - 28:
IS - 3
N2 - Effective management of disruptive behaviors in schools is a national concern. While substantial resources are often allocated toward individual students who exhibit challenging behavior, less emphasis is placed on preventative interventions in common areas such as hallways, cafeterias and playgrounds. The purpose of this study was to determine whether a multi-component Positive Behavior Support (PBS) intervention consisting of positive practice, pre-correction, verbal praise, reinforcement, correction of inappropriate behavior, active supervision, discussion of behavior with students and on-time dismissal could improve middle school students' problematic hallway behaviors. The 5-week intervention phase resulted in a very large reduction between baseline and treatment phases of 42.36% across 950 students. This study provides evidence that readily available and cost-effective techniques can be used to make school common areas safer and more orderly. Implications for the use of school-based PBS practices are discussed. (PsycINFO Database Record (c) 2012 APA, all rights reserved) (journal abstract)
SN - 0748-8491
AD - Oswald, Karen: Ohio University, Athens, OH, US Safran, Stephen: Ohio University, Athens, OH, US Johanson, George: Ohio University, Athens, OH, US
ER -

TY - JOUR
ID - 2727
T1 - A safe place to grow: A group treatment manual for children in conflicted, violent, and separating homes
A1 - Roseby,Vivienne; Johnston,Janet; Gentner,Bettina; Moore,Erin
Y1 - 2005///
N1 - Book: 2005-12510-000
English
Book; Authored Book
(Created by PsycINFO) Acknowledgments Introduction Part I: Little Kids' (LK) Manual LK Session 1. Saying Hello and Making Me Feel Safe in Group LK Session 2. How I am Feeling: Same and Different LK Session 3. Feeling Words and Faces: Who I Am LK Session 4. Exploring Levels of Feeling LK Session 5. Managing My Good and Bad Feelings at One Time LK Session 6. Feeling and Being Me: Separate and Together LK Session 7. It's Okay to Be Different LK Session 8. Going Back and Forth: Managing Transitions and Change LK Session 9. Managing When Parents Fight LK Session 10. Safety Planning and Ways of Coping LK Session 11. Masking Feelings: My Inside and Outside Self LK Session 12. Remembering and Saying Good-Bye LK Session Tips and Guidelines Part II: Big Kids' (BK) Manual BK Session 1. Saying Hello and Making a Safe Place to Work Together BK Session 2. Exploring Levels of Feelings, Actions, and Points of View BK Session 3. Making a Safe Inside Place and Learning the Rules of Role-Play BK Session 4. Exploring a Safe Inside Place and Back to Reality BK Session 5. Defining Wishes for Yourself and Rules That Work for Your Family and Relationships BK Session 6. Exploring the Mirror: Who You Are and How You Show Your Feelings BK Session 7. Exploring Your Inside Self and Your Outside Self BK Session 8. Making a Family Sculpture: Who We Are and Who We Could Be BK Session 9. Becoming the Experts on Living with Change, Loss, and Conflict BK Session 10. Telling Your Story So Far, Thinking About Who You Want to Be in the Future, and Saying Good-Bye Part III: Appendixes Appendix A. Troubleshooting for Both LK and BK Groups Appendix B. Psychoeducational Groups for Parents Appendix C. An Evaluation Study of the Group Model Notes Bibliography Index

KW - PsycInfo
KW - Domestic Violence
KW - Experiences (Events)
KW - Family Conflict
KW - Group Counseling
KW - Marital Separation
RP - NOT IN FILE
SP - Binghamton, NY, US
JF - (2005)
VL - A safe place to grow: A group treatment manual for children in conflicted, violent, and separating homes. ix, 220 pp. Binghamton, NY, US: Haworth Maltreatment and Trauma Press/The Haworth Press; US.
N2 - (from the introduction) This manual presents a unique, step-by-step model for treating children who have experienced chronic conflict or violence within their families. The first edition was developed specifically for children of highly conflicted separating and divorced families who were referred by family courts to the Judith Wallerstein Center for the Family in Transition, which has been one of the country's foremost centers for divorce research. The second edition includes changes that have been made to the model during the past seven years as it has been applied more broadly in multiethnic, multicultural neighborhoods, schools, and family agencies to help children who are living in a whole range of stressful situations related to family conflict and violence. Whereas the first edition focused solely on older school-age children, the second edition includes a section specifically designed for younger school-age children as well. Now the manual provides developmentally appropriate treatment curricula for children ranging from about five to fourteen years. (PsycINFO Database Record (c) 2012 APA, all rights reserved)
AD - Roseby, Vivienne: Families First Residential Treatment Center, Davis, CA, US
ER -

TY - JOUR
ID - 2728
T1 - Factors Influencing the Physically Active Leisure of Children and Youth: A Qualitative Study. [References]
A1 - Thompson,Angela M.
Y1 - 2005///
N1 - Peer Reviewed Journal: 2005-11165-006
English
Journal; Peer Reviewed Journal
KW - PsycInfo
KW - Academic Achievement
KW - Leisure Time
KW - Motor Processes
KW - Physical Education
KW - Student Attitudes
KW - Data Collection
KW - Phenomenology
KW - Schools
RP - NOT IN FILE
SP - 421
EP - 438
JF - Leisure Sciences
VL - 27:
IS - 5
N2 - A phenomenological framework guided data collection and analysis of the prevalent influences on attitudes and behaviors of elementary, junior, and secondary school students towards their physically active leisure. Twenty-two students participated in individual in-depth interviews. Parental influence and outdoor play effected choice and level of physically active leisure for elementary students. Opportunities for organized and non-structured physically active leisure and friends were significant for junior students. Academic responsibilities and organized physically active leisure were influential for secondary students. Common threads were parental influence and the belief that the students were too old to start new activities. These findings highlight the importance of providing opportunities in safe environments for children and youth to participate in

a variety of structured and non-structured forms of physically active leisure. (PsycINFO Database Record (c) 2012 APA, all rights reserved) (journal abstract)
SN - 0149-0400
AD - Thompson, Angela M.: Department of Human Kinetics, St. Francis Xavier University, Antigonish, NS, Canada Rehman, Laurene A.: School of Health and Human Performance, Dalhousie University, Halifax, NS, Canada Humbert, M. Louise: College of Kinesiology, University of Saskatchewan, Saskatoon, SK, Canada
ER -

TY - JOUR
ID - 2729
T1 - Predicting changes in children's self-perceptions from playground social activities and interactions. [References]
A1 - Boulton,Michael J.
Y1 - 2005///
N1 - Peer Reviewed Journal: 2005-09367-004
English
Journal; Peer Reviewed Journal
KW - PsycInfo
KW - Human Sex Differences
KW - Playgrounds
KW - Self Perception
KW - Social Acceptance
KW - Social Interaction
KW - Childhood Play Behavior
KW - Physical Appearance
KW - Psychosocial Development
RP - NOT IN FILE
SP - 209
EP - 226
JF - British Journal of Developmental Psychology
JA - BR J DEV PSYCHOL
VL - 23:
IS - 2
N2 - The general aim of this study was to examine the concurrent and longitudinal (6 month) associations between 8- to 9-year-old children's (N = 75) social activities and interactions during recess and their self-perceptions, and to test for gender differences in those associations. The underlying rationale was that recess provides an important, and hitherto unstudied, context in which children's experiences can impact on their views of themselves. Consistent with this proposition, several of the playground variables were significantly correlated concurrently with participants' self-perceptions regarding social acceptance, and, particularly, athletic competence. Even stronger evidence came from the longitudinal analyses which indicated that group size positively predicted changes in social acceptance and global self-worth scores; network positively predicted changes in physical appearance and global self-worth scores; rule games positively predicted changes in athletic competence, physical appearance and global self-worth scores; conversation negatively predicted changes in athletic competence, and alone negatively predicted changes in physical appearance and global self-worth scores. Several significant gender differences were obtained: the association between rule games and changes in social acceptance was negative for girls but positive for boys; a positive association between conversation and social acceptance was evident among girls but not boys; and a negative association between conversation and changes in athletic competence was evident among boys but not girls. The theoretical and practical implications of these results are discussed. (PsycINFO Database Record (c) 2012 APA, all rights reserved) (journal abstract)
SN - 0261-510X
AD - Boulton, Michael J.: University College Chester, United Kingdom
ER -

TY - JOUR
ID - 2730
T1 - Emotional Availability: Differential Predictions to Infant Attachment and Kindergarten Adjustment Based on Observation Time and Context. [References]

A1 - Biringen,Zeynep; Damon,Jamie; Grigg,Wendy; Mone,Jen; Pipp-Siegel,Sandra; Skillern,Shauna; Stratton,Janie
Y1 - 2005///
N1 - Peer Reviewed Journal: 2005-08606-002
English
Journal; Peer Reviewed Journal
KW - PsycInfo
KW - Attachment Behavior
KW - Kindergarten Students
KW - Mother Child Relations
KW - School Adjustment
KW - Early Childhood Development
KW - Emotions
KW - Infant Development
KW - Prediction
KW - Time
RP - NOT IN FILE
SP - 295
EP - 308
JF - Infant Mental Health Journal
VL - 26:
IS - 4
N2 - Two studies are used to illustrate the importance of context and length of time in the use of the Emotional Availability Scales (EAS) to predict aspects of child development. The purpose of the first study was to examine whether prediction of attachment by the EAS is better with increasing amounts of time. We scored emotional availability (EA) every 15 min for a total of 2 hr, with correlations showing an increasingly stronger relation with attachment with increasing time. In addition, difference scores were calculated between the first and the last 15 min for each EAS dimension. The difference score was significantly higher for the insecure group, suggesting that we need more observation time for the prediction of insecure attachments than is the case for the prediction of secure attachments. The second study investigated whether EA is differentially predictive based on context. We explored play contexts versus reunion contexts. We also explored the relations with other indices of child development. Results revealed that some dimensions of EA (e.g., maternal nonhostility) are difficult to detect outside of a stress context. Maternal nonhostility during the reunion (but not the play situation) was correlated with child aggression in the kindergarten classroom. In addition, most dimensions of EA assessed in the reunion context were better predictors of teacher reports of kindergarten adjustment than was EA assessed in the play situation. (PsycINFO Database Record (c) 2012 APA, all rights reserved) (journal abstract)
SN - 0163-9641
AD - Biringen, Zeynep: Colorado State University, Ft Collins, CO, US Damon, Jamie: Colorado State University, Ft Collins, CO, US Grigg, Wendy: Colorado State University, Ft Collins, CO, US Mone, Jen: Colorado State University, Ft Collins, CO, US Pipp-Siegel, Sandra: Colorado State University, Ft Collins, CO, US Skillern, Shauna: Colorado State University, Ft Collins, CO, US Stratton, Janie: Colorado State University, Ft Collins, CO, US
ER -

TY - JOUR
ID - 2731
T1 - Access Nature
A1 - Stout,Jessica
Y1 - 2005///
N1 - Peer Reviewed Journal: 2005-08011-005
English
Journal; Peer Reviewed Journal
KW - PsycInfo
KW - Curriculum
KW - Disabilities
KW - Environmental Attitudes
KW - Environmental Education

RP - NOT IN FILE
SP - 158
EP - 159
JF - Therapeutic Recreation Journal
VL - 39:
IS - 2
N2 - Access Nature (by Bethe Gilbert Almeras and David Heath,2001) is an inclusive curriculum created by the National Wildlife Federation. The main premise for designing this curriculum was to offer educators a tool that would aid in formulating quality environmental education programs that would reach and stimulate all participants with and without disabilities. This curriculum is designed around objectives in outdoor and environmental education with a main theme of habitat. Each of the activities in this sixty-hour curriculum is geared towards youth and teens ages 6-14. Specific adaptations are incorporated that address four categories of disabilities: hearing, learning/cognition, motor, and visual. invaluable. This manual is a very useful resource for gaining awareness into the little things that can be done to create a safe and stimulating environment for all participants regardless of their abilities. The suggestions and guidelines for including participants with disabilities transcend the scope of the listed activities to programming in general. The Access Nature curriculum encourages facilitators to increase their comfort level working with inclusive audiences by offering all students the opportunity to explore nature. (PsycINFO Database Record (c) 2012 APA, all rights reserved) (journal abstract)
SN - 0040-5914
AD - Stout, Jessica: Children's Hospital of Philadelphia, Philadelphia, PA, US
ER -

TY - JOUR
ID - 2733
T1 - African American Father Involvement and Preschool Children's School Readiness. [References]
A1 - Downer,Jason T.
Y1 - 2005///
N1 - Peer Reviewed Journal: 2005-07982-002
English
Journal; Peer Reviewed Journal
KW - PsycInfo
KW - Blacks
KW - Father Child Relations
KW - Project Head Start
KW - School Readiness
KW - Family Relations
KW - Involvement
KW - Parental Characteristics
KW - Preschool Students
RP - NOT IN FILE
SP - 317
EP - 340
JF - Early Education and Development
VL - 16:
IS - 3
N2 - A developmental ecological model was used to identify child attributes, father characteristics, and familial factors associated with multidimensional father involvement with preschool children enrolled in Head Start. The relations between father involvement and children's school readiness were also investigated. Eighty-five African American fathers and father figures were surveyed about their involvement in child care, home-based educational and school-based educational activities. Children's school readiness competencies were evaluated via teacher report or direct assessment. Father involvement in child care and home-based educational activities were predicted by different contextual factors and child attributes. Fathers were more involved in child care activities when they lived in a child's home and when a child was highly emotional. Fathers who perceived the existence of a strong parenting alliance reported more involvement in home-based educational activities. Father involvement in child care and home-based educational activities was associated with higher levels of children's emotion regulation. Findings are consistent with a contextual, multidimensional perspective of African

American fathering and hold policy implications for fatherhood initiatives in the early childhood education field. Efforts to increase father involvement may be most effective when addressing the multitude of influences on fathering behavior and focusing on father-child activities that occur outside of the preschool setting. (PsycINFO Database Record (c) 2012 APA, all rights reserved) (journal abstract)
SN - 1040-9289
AD - Downer, Jason T.: University of Virginia, Charlottesville, VA, US Mendez, Julia L.: Temple University, Philadelphia, PA, US
ER -

TY - JOUR
ID - 2734
T1 - Recess: Its role in education and development. [References]
A1 - Pellegrini,Anthony D.
Y1 - 2005///
N1 - Book: 2005-07528-000
English
Book; Authored Book
(Created by PsycINFO) Preface Acknowledgments The Debate Over Recess: A Sad Tale of the Disjunctive Between Educational Policy and Scientific Research A Brief History of the Place of Play and Recess in American Schools The School Playground as a Venue for Children's Social Development The Two Worlds of the Playground: Gender Segregation at Recess Gender Differences in Preference for Outdoor Recess Children's Play and Rough-and-Tumble Play on the Playground Children's Games on the Playground at Recess The Role of Recess in Children's Cognitive Performance in Classrooms Summing Up: What Are the Implications of Recess for Children in School? References Author Index Subject Index
KW - PsycInfo
KW - Childhood Development
KW - Childhood Play Behavior
KW - Educational Programs
KW - Playgrounds
KW - Recreation
KW - Elementary Schools
RP - NOT IN FILE
SP - Mahwah, NJ, US
JF - (2005)
VL - Recess: Its role in education and development. xii, 202 pp. Mahwah, NJ, US: Lawrence Erlbaum Associates Publishers; US.
N2 - (from the preface) Writing a book about recess could be a very questionable endeavor for a serious academic psychologist. At first blush it seems to be a pretty trivial topic. It's the time during the school day when there's a break from what's typically considered the most serious work of the day--reading, writing, and arithmetic. Reflecting this trivial sort of tenor, it's also that time that kids will, perhaps only half-jokingly, say is their favorite part of the school day. There's even a cartoon TV show for kids called Recess! All of this reinforces the popular notion that recess is indeed trivial and certainly not worthy of serious academic and scientific attention. This perception has, I think, led to many schools questioning the role of recess in the school day. This orientation, I think, has done much more harm than good. If studying the role of recess during the school day seems questionable, so too, some people think, is the idea of taking recess away from kids in primary school. When people first hear of this state of affairs, a frequent initial reaction is disbelief--"You've got to be kidding!" This book attempts to broach these two views of recess; the perceived value of recess and the movement to eliminate or reduce the school recess period from the primary school day. Over the past 10 years, I have been contacted by a number of folk concerned with this trend. They know, in their hearts, that something is wrong here. When they speak with me, they ask for advice on how to counter the trend. I hope that this book helps them in this important job. (PsycINFO Database Record (c) 2012 APA, all rights reserved)
AD - Pellegrini, Anthony D.: University of Minnesota, MN, US
ER -

TY - JOUR
ID - 2735
T1 - Adolescents' involvement in non-romantic sexual activity. [References]

A1 - Manning,Wendy D.
Y1 - 2005///
N1 - Peer Reviewed Journal: 2005-06093-006
English
Journal; Peer Reviewed Journal
KW - PsycInfo
KW - Adolescent Development
KW - Romance
KW - Sexual Development
KW - Sexual Intercourse (Human)
KW - Social Dating
KW - Risk Factors
RP - NOT IN FILE
SP - 384
EP - 407
JF - Social Science Research
VL - 34:
IS - 2
N2 - The majority of teens are having sex, however, we know little about sex that occurs outside the traditional dating context and the factors associated with these non-romantic sexual experiences. Prior work indicates that healthy sexual behaviors depend on the relationship context of sexual intercourse, but research on the characteristics of teens who engage in non-romantic sex is limited. We use two waves of the National Longitudinal Study of Adolescent Health to analyze adolescents' reports of non-romantic sexual intercourse, and whether key protective and risk factors as well as normative orientations distinguish the context within which sexual activity occurs. We find that the majority of sexually active teens have had some sexual experience outside of a romantic relationship. We conclude that teenagers' sexual experiences are not static over time, because 60% of sexually active teenagers have had sex in both romantic and non-romantic contexts. Our multivariate analyses indicate that the teen's normative beliefs have significant effects on teenage non-romantic sexual activity. The traditional risk and protective factors often are not related to teens' decisions about sexual partners. These results have implications for our understanding and encouragement of healthy adolescent sexual behaviors. (PsycINFO Database Record (c) 2012 APA, all rights reserved) (journal abstract)
SN - 0049-089X
AD - Manning, Wendy D.: Department of Sociology, Bowling Green University, Bowling Green, OH, US
Longmore, Monica A.: Department of Sociology, Bowling Green University, Bowling Green, OH, US
Giordano, Peggy C.: Department of Sociology, Bowling Green University, Bowling Green, OH, US
ER -

TY - JOUR
ID - 2736
T1 - Interaction of Social and Play Behaviors in Preschoolers With and Without Pervasive Developmental Disorder. [References]
A1 - Pierce-Jordan,Sandra; Lifter,Karin
Y1 - 2005///
N1 - Peer Reviewed Journal: 2005-05385-004
English
Journal; Peer Reviewed Journal
KW - PsycInfo
KW - Childhood Play Behavior
KW - Pervasive Developmental Disorders
KW - Preschool Students
KW - Social Behavior
KW - Social Interaction
RP - NOT IN FILE
SP - 34
EP - 47
JF - Topics in Early Childhood Special Education
VL - 25:

IS - 1
N2 - This study investigated the relationship between the social and play behaviors of young children with pervasive developmental disorder (PDD) and without PDD. Videotaped observations of 21 preschool children (12 with PDD and 9 without PDD) were examined independently for (a) social complexity and (b) play complexity that was assessed on an individualized basis for each child. The researchers used log-linear analyses to analyze the data. The results supported an inverse relationship between play and social interaction: The children's play within a social interaction tended to be less complex than their play outside of the interaction. All children, regardless of diagnosis, demonstrated similar behavioral patterns, although the children without PDD engaged in social interaction to a greater extent. These results suggest that social interventions should be implemented in the context of play activities that the child has mastered, as opposed to those activities the child is in the process of learning. (PsycINFO Database Record (c) 2012 APA, all rights reserved) (journal abstract)
SN - 0271-1214
AD - Pierce-Jordan, Sandra: May Institute, US Lifter, Karin: Northeastern University, Boston, MA, US
ER -

TY - JOUR
ID - 2737
T1 - Helping students fix problems and avoid crises: An easy-to-use intervention resource for grades 1-4
A1 - Greene,Lawrence J.
Y1 - 2005///
N1 - Book: 2005-05018-000
English
Book; Authored Book
(Created by PsycINFO) (Abbreviated) Introduction About the Author Unit 1: The Child Who Doesn't Have Friends For Educators: Examining the Dynamics and Implications of Social Rejection For Students: The Child Who Doesn't Have Friends Unit 2: The Child Who Has Difficulty Learning For Educators: Examining the Emotional Implications of Learning Problems For Students: The Child Who Has Difficulty Learning Unit 3: The Child Who Steals For Educators: Examining the Dynamics and Implications of Stealing For Students: The Child Who Steals Unit 4: The Child Who Tells Lies For Educators: Examining the Dynamics and Implications of Lying For Students: The Child Who Tells Lies Unit 5: The Child Who Is a Bully For Educators: Examining the Dynamics and Implications of Bullying For Students: The Child Who Is a Bully Unit 6: The Child Who Does Poorly in Sports For Educators: Examining the Implications of Being Poorly Coordinated For Students: The Child Who Does Poorly In Sports Appendix 1: Psychological Overlay and Learning Differences Appendix 2: Psychological Problems Index
KW - PsycInfo
KW - Childhood Development
KW - Emotional Adjustment
KW - School Based Intervention
KW - Peer Relations
KW - Social Skills Training
RP - NOT IN FILE
SP - Thousand
JF - (2005)
VL - Helping students fix problems and avoid crises: An easy-to-use intervention resource for grades 1-4. xv, 182 pp. Thousand Oaks, CA, US: Corwin Press; US.
N2 - (from the introduction) All children experience problems from time to time. These dilemmas go with the territory of being a child and are part of the developmental learning curve. In the long run, the vast majority of the predicaments prove inconsequential, and most children cannot even remember the previous day's dilemma. Having an argument with a friend, being kept in the classroom during recess for misbehavior, or getting a poor grade on a homework assignment are unlikely to leave a permanent imprint on a child's psyche, unless, of course, the scenarios are recurring and scripted. Of far greater concern are the more intractable problems that are often directly attributable to the student's attitudes and behavior. Children who chronically lie, tease, steal, bully, cheat, spread rumors, or act in socially inappropriate ways are transmitting a clear message that they are unhappy campers. Their actions are certain to generate undesirable repercussions that typically include peer rejection, alienation, and repeated admonitions and punishments from teachers and parents. Youngsters who are socially rejected, bullied, belittled, teased for looking or acting different, disparaged for having learning problems or other disabilities, or scorned for poor athletic skills are clearly at psychological risk. Their traumatic

experiences in the classroom and on the playground are all but certain to profoundly, and perhaps indelibly, warp their perceptions about themselves and their world. (from the cover) For the child who doesn't have friends, does poorly in sports, has difficulty learning, or who steals, lies, or bullies, Greene provides solid, proven methods to help these learners discover and practice better life skills. Helping Students Fix Problems and Avoid Crises includes: insightful stories for teachers and students illustrating each problem; practical activities and exercises illustrating techniques to help students address each problem; supplemental reproducible exercises; and encouraging follow-up and application exercises. The author divides each problematic issue into two sections: for educators, he looks at the dynamics and implications of each problem, and for students, he provides classroom scripts and exercises to bring their own awareness to each problem. This essential classroom resource also includes illuminating ten-minute read-aloud stories, strategic thinking questions, optional activities, and reproducible exercises! (PsycINFO Database Record (c) 2012 APA, all rights reserved)
ER -

TY - JOUR
ID - 2738
T1 - Development of System Thinking Skills in the Context of Earth System Education. [References]
A1 - Assaraf,Orit Ben-Zvi; Orion,Nir
Y1 - 2005///
N1 - Peer Reviewed Journal: 2005-04339-002
English
Journal; Peer Reviewed Journal
KW - PsycInfo
KW - Cognitive Ability
KW - Science Education
KW - Systems Theory
KW - Thinking
KW - High School Education
RP - NOT IN FILE
SP - 518
EP - 560
JF - Journal of Research in Science Teaching
VL - 42:
IS - 5
N2 - The current study deals with the development of system thinking skills at the junior high school level. The sample population included about 50 eighth-grade students from two different classes of an urban Israeli junior high school who studied an earth systems-based curriculum that focused on the hydro cycle. The study addressed the following research questions: (a) Could the students deal with complex systems?; (b) What has influenced the students' ability to deal with system perception?; and (c) What are the relationship among the cognitive components of system thinking? The research combined qualitative and quantitative methods and involved various research tools, which were implemented in order to collect the data concerning the students' knowledge and understanding before, during, and following the learning process. The findings indicated that the development of system thinking in the context of the earth systems consists of several sequential stages arranged in a hierarchical structure. The cognitive skills that are developed in each stage serve as the basis for the development of the next higher-order thinking skills. The research showed that in spite of the minimal initial system thinking abilities of the students most of them made some meaningful progress in their system thinking skills, and a third of them reached the highest level of system thinking in the context of the hydro cycle. Two main factors were found to be the source of the differential progress of the students: (a) the students' individual cognitive abilities, and (b) their level of involvement in the knowledge integration activities during their inquiry-based learning both indoors and outdoors. (PsycINFO Database Record (c) 2012 APA, all rights reserved) (journal abstract)
SN - 0022-4308
AD - Assaraf, Orit Ben-Zvi: Weizmann Institute of Science, Science Teaching Department, Rehovot, Israel
Orion, Nir: Weizmann Institute of Science, Science Teaching Department, Rehovot, Israel
ER -

TY - JOUR
ID - 2739

T1 - After-School and Summer Enrichment Programs
A1 - Jonas,Sarah
Y1 - 2005///
N1 - Book: 2005-03917-005
English
Book; Edited Book
KW - PsycInfo
KW - Academic Achievement
KW - After School Programs
KW - Communities
KW - Educational Programs
KW - Schools
KW - Urban Environments
RP - NOT IN FILE
SP - 57
EP - 71
JF - Dryfoos, Joy G [Ed]; Quinn, Jane [Ed]; Barkin, Carol [Ed]
VL - :
IS - 2005
N2 - (from the chapter) Research has demonstrated that nonschool hours provide a powerful opportunity for community-based organizations to help children and schools reach their academic goals. For example, Reginald Clark says, "Youngsters who engage in constructive learning activities outside of school are more likely to excel in school and in life than those who do not. By participating in a well-rounded array of activities, they are able to practice and thoroughly learn skills required for academic success." Based on this knowledge, The Children's Aid Society (CAS) aims to integrate after-school and summer enrichment programs with classroom instruction through a model that both supports the school's academic mission and promotes healthy youth development. The vast majority of students in CAS's 10 community schools, which are located in New York City, struggle with basic skills; in addition, more than half are Spanish speakers grappling with English as a second language. Their parents, teachers, and principals look to after-school and summer programs to help address their needs. The challenge for CAS is to answer this call in a way that is fun and engaging for children and achievable for program staff. (PsycINFO Database Record (c) 2012 APA, all rights reserved)
AD - Jonas, Sarah: Children's Aid Society, New York, NY, US
ER -

TY - JOUR
ID - 2740
T1 - Keeping All Children Healthy: Challenges to Leading an Active Lifestyle for Preschool Children Qualifying for At-Risk Programs. [References]
A1 - Goodway,Jacqueline D.
Y1 - 2005///
N1 - Peer Reviewed Journal: 2005-03879-004
English
Journal; Peer Reviewed Journal
KW - PsycInfo
KW - Activity Level
KW - At Risk Populations
KW - Lifestyle
KW - Motor Processes
KW - Preschool Students
KW - Urban Environments
RP - NOT IN FILE
SP - 142
EP - 155
JF - Family & Community Health: The Journal of Health Promotion & Maintenance
VL - 28:
IS - 2

N2 - This study examined contextual factors associated with physical activity of urban African American preschoolers (N = 59). Qualitative research methodologies utilized data from home visits, caregiver and child interviews, field notes, and document collection. The primary finding suggested, "In underserved communities fundamental barriers exist that obstruct young children's ability to be physically active." Four themes were developed revealing that outside environments limited opportunities for physical activity, home environments resulted in sedentary behaviors, and communities had limited physical activity role models. Despite this, young children enjoyed being physically active. (PsycINFO Database Record (c) 2012 APA, all rights reserved) (journal abstract)
SN - 0160-6379
AD - Goodway, Jacqueline D.: School of Physical Activity and Educational Services, Ohio State University, Columbus, OH, US Smith, Dennis W.: Department of Health and Human Performance, University of Houston, Houston, TX, US
ER -

TY - JOUR
ID - 2741
T1 - Review of Fostering Adolescents
A1 - Sellick,Clive
Y1 - 2005///
N1 - Peer Reviewed Journal: 2005-03807-009
English
Journal; Peer Reviewed Journal
KW - PsycInfo
KW - At Risk Populations
KW - Foster Care
KW - Parenting Skills
KW - Parents
RP - NOT IN FILE
SP - 180
EP - 181
JF - Child & Family Social Work
VL - 10:
IS - 2
N2 - Reviews the book Fostering Adolescents by E. Farmer et al (2004). This book is the published account of a Department of Health-funded research study that forms part of the Supporting Parents research series published by Jessica Kingsley. Its focus, therefore, includes the parenting skills required by the foster carers of adolescents and the support they and the young people require to ensure stable and successful placements. In this study two main outcome measures were used: placement disruption and placement quality in respect of 68 young people aged between 11 and 17 who were with foster carers from 14 local authorities and 2 independent fostering agencies. Results showed that fewer disruptions occurred when the young people were helped to talk about their past and present lives with their foster carers or where carers are supported and encouraged to communicate with a young person according to their emotional rather than actual age and understanding. Placements are more likely to be successful when foster carers were skilful at monitoring the activities of the young people outside their house or where they took a role in preparing them for life after placement. The book is timely as further government money and attention are currently being paid towards the development of treatment foster care across the country, for young people such as those in this study. Its findings are also of major importance and relevance to fostering managers of both local authorities and independent fostering providers who continue to grapple with the challenges of fostering teenagers in ways that are safe, secure and successful. (PsycINFO Database Record (c) 2012 APA, all rights reserved)
SN - 1356-7500
AD - Sellick, Clive: U East Anglia, United Kingdom
ER -

TY - JOUR
ID - 2742
T1 - Structure of Childrens Temperament Aged 3 to 9 based on Teachers Descriptions. [Chinese]. [References]
A1 - Wen,Liu

Y1 - 2005///
N1 - Peer Reviewed Journal: 2005-02994-010
Chinese
Journal; Peer Reviewed Journal
KW - PsycInfo
KW - Developmental Measures
KW - Personality Development
KW - Personality
KW - Test Construction
KW - Test Reliability
KW - Test Validity
RP - NOT IN FILE
SP - 67
EP - 72
JF - Acta Psychologica Sinica
VL - 37:
IS - 1
N2 - Temperament is often regarded as biologically based psychological tendencies with intrinsic paths of development. The existing research findings reveal that the development of temperament plays a unique role in protesting and understanding children's personality and socialization in the future. With emphasis on the enhancement of people's quality in the 21st century, research on children's temperament will be of much value in developing developmental theories and promoting children's healthy personality development. Since the 1980s, developmental psychologists outside China have conducted a large quantity of research, both empirical and theoretical, in this area of children's temperament. This has made it one of the most active and important research areas in child psychology. In contrast, systematic research efforts have been rare in China's developmental psychology, and the general tendencies of the development of children's temperament in Chinese culture remain a big area to be explored. A comprehensive literature review is made with regard to the important empirical findings, major theoretical models as well as the research deficiency. The purpose of this study is to reveal the Structure of Chinese children's temperament aged 3 to 9, to develop indigenous instrument. Based on the open questionnaires that Chinese teachers provided on their students' characteristics, one temperament questionnaires for ages 3 to 9 was developed. Confirmatory Factor Analysis was conducted using AMOS and SPSS in attempt to testify the temperament structure of Chinese children. The major findings in the study are as follows: 1) The questionnaire of children's temperament has high reliability and validity so that it can be used by teachers and researchers to evaluate children's temperament. 2) The structure of Chinese children aged 3-9 is a multifactor, which involves five aspects; emotionality, activity, reaction, social inhibition and attention. (PsycINFO Database Record (c) 2012 APA, all rights reserved) (journal abstract)
SN - 0439-755X
AD - Wen, Liu: Department of Psychology, Liaoning Normal University, Dalian, China Lizhu, Yang: Department of Psychology, Liaoning Normal University, Dalian, China
ER -

TY - JOUR
ID - 2743
T1 - Mother-child conversations about gender: Understanding the acquisition of essentialist beliefs: II. Methods and preliminary results
A1 - Gelman,Susan A.
Y1 - 2004///
N1 - Peer Reviewed Journal: 2009-23551-002
English
Journal; Peer Reviewed Journal
KW - PsycInfo
KW - Child Attitudes
KW - Human Sex Differences
KW - Mother Child Communication
KW - Sex Role Attitudes
KW - Concept Formation
KW - Mothers

RP - NOT IN FILE
SP - 15
EP - 32
JF - Monographs of the Society for Research in Child Development
VL - 69:
IS - 1
N2 - Part II of the monograph Mother-child conversations about gender: Understanding the acquisition of essentialist beliefs, by Gelman et al (see records 2004-18660-001, 2009-23551-001, 2009-23551-003, 2009-23551-004, 2009-23551-005, 2009-23551-006, and 2004-18660-002). Presents a description of the methods used in this study and preliminary findings. To examine gender talk in mother-child conversations, we videotaped mothers and their young children discussing a picture book that depicted stereotypical and counter-stereotypical gendered activities (e.g., a boy playing football; a boy sewing). These interactions were transcribed, coded, and analyzed in fine-grained detail. Mothers and children also completed tasks that measured gender stereotyping and/ or gender constancy. Participants were 72 mother-child dyads, 24 at each of three child ages: 2 years (range 2.4-3.0; mean age 2.7), 4 years (range 4.0-5.0; mean age 4.7), and 6 years (range 6.1-7.0; mean age 6.7). Half the children at each age were girls, half were boys. The majority of the participants were white (94% of mothers, 92% of children); the remaining were ethnic or racial minorities (Asian, African-American, or other). Approximately one-third of the mothers indicated that they did not work outside the home (N57 at age 2 years, 10 at age 4 years, and 9 at age 6 years). Twenty university undergraduates provided ratings to help pre-select the activities that would be depicted in the picture book. (PsycINFO Database Record (c) 2012 APA, all rights reserved)
SN - 0037-976X
AD - Gelman, Susan A.: Department of Psychology, University of Michigan, Ann Arbor, MI, US Taylor, Marianne G.: Psychology Department, Pacific Lutheran University, Tacoma, WA, US Nguyen, Simone P.: Department of Psychology, University of North Carolina, Wilmington, NC, US
ER -

TY - JOUR
ID - 2744
T1 - Constructing adolescents differently: On the value of listening to Singapore youngsters talking popular culture texts. [References]
A1 - Kramer-Dahl,Anneliese
Y1 - 2004///
N1 - Peer Reviewed Journal: 2005-12635-002
English
Journal; Peer Reviewed Journal
KW - PsycInfo
KW - Academic Achievement
KW - Adolescent Attitudes
KW - Popular Culture
KW - Reading Skills
KW - Sociocultural Factors
RP - NOT IN FILE
SP - 217
EP - 241
JF - Linguistics and Education
VL - 15:
IS - 3
N2 - In contrast to the commonsense discourse of youth-as-unruly widely circulating in the West, the kind of discourse which has become part of the Singapore public and academic imagination is one that mobilizes constructions of youth as narrowly achievement-oriented, as "exam-smart muggers", who "lack an enquiring mind". This study attempts to complicate this picture of Singaporean adolescents, showing them to be sophisticated meaning-makers who employ texts of different modes to construct shifting subjectivities in their everyday lives. It draws on selected focus-group data my students and I have collected over the past two years from Singaporean 10- to 12-year-olds as they talk with their peers and us about the texts they like to read and watch in their spare time [Lim, L. E. (2002). Fast cars and magical cards: A study of gendered choices in cartoons. Singapore: Nanyang Technological University, Honours Academic Exercise; Seah, H. L. (2003).

Gender differences in the reading habits and attitudes of primary pupils in single-sex government-aided schools in Singapore. MA (applied linguistics) thesis: Nanyang Technological University]. Building on Moss' [Moss, G. (2000). Informal literacies and pedagogic discourse. Linguistics and Education, 11, 47-64; Moss, G. (2001). On literacy and the social organisation of knowledge inside and outside school. Language and Education", 15, 146-161] research on knowledge about informal literacies as a "horizontal discourse" (Bernstein), in the discussion of this data I argue that the youngsters whose voices we hear are not entirely trapped within monochrome schooled literacy practices, even though much of what goes on at home may reinforce these; rather, in the spaces they make for themselves, they can be seen engaged in an array of out-of-school activities around texts, displaying special competencies and taking up multiple reading strategies and positions as they navigate them. Having a better understanding of the kinds of "impr; oper" knowledge about literacy that is generated alongside the privileged pedagogized version in many Singapore homes can not only help complicate our current image of Singaporean youth but also help re-envision literacy education in schools in ways that the students' unsanctioned experiences and competencies with texts are recognized and built upon. (PsycINFO Database Record (c) 2012 APA, all rights reserved) (journal abstract)
SN - 0898-5898
AD - Kramer-Dahl, Anneliese: National Institute of Education, Singapore, Singapore
ER -

TY - JOUR
ID - 2745
T1 - Implications of a relationship-focused intervention targeting aggression, violence and resilience among middle school adolescents
A1 - Spears,Beverly Ann
Y1 - 2004///
N1 - Dissertation Abstract: 2004-99023-081
English
Dissertation Abstract
KW - PsycInfo
KW - Aggressive Behavior
KW - Early Intervention
KW - Resilience (Psychological)
KW - School Environment
KW - Violence
KW - Middle School Students
KW - Safety
RP - NOT IN FILE
SP - 2365
JF - Dissertation Abstracts International Section A: Humanities and Social Sciences
VL - 65:
IS - 6-A
N2 - The purpose of this study was two fold. First, to examine the changes in levels of aggression, delinquency and the three domains of resilience in middle school adolescents who participated in a relationship-focused intervention compared to those who did not participate. Second, to determine adolescents' perception of school safety, (how safe they felt at school and how much power they perceived they had to influence or change their school environment). A total of 164 middle school students met all study requirements which included completing both a pre-test and post-test. The relationship-focused intervention took place at two inner city middle schools located in a city enhanced enterprise zone. For a twelve-week period, students met with a program facilitator during one hour weekly classroom instruction sessions and two hours weekly outdoor/field activities. The relationship-focused intervention used a recently developed curriculum-based program for which the investigator served as lead writer. The study used a theoretical framework of resilience that links social learning and attachment theories. This framework illustrated that resilience can be strengthened in adolescents when they model the prosocial behaviors of an attached figure and are positively reinforced. The study used a quasi-experimental, pre-test/post-test comparison design with experimental and comparison groups. The resilience domains were measured using the Individual Protective Factor Index (IPFI): Social Bonding, (school, family, pro-social norms), Personal Competencies (self-concept, self-control, self-efficacy, and positive outlook) and Social Competencies (assertiveness, confidence and cooperation). Aggression and delinquency were measured using syndrome scales from the Youth Self Report (YSR). For hypotheses testing, repeated-

measures Multivariate Analysis (MANOVA/MANCOVA) procedures were conducted. Three qualitative questions were used to obtain adolescents' perceptions about their safety in school and their power to make a difference in their school environment. The results of the program found that mean scores were moving toward changes in levels of behavior for those who participated in the relationship-focused intervention. Qualitative data showed differences in adolescents' attitudes and intended behaviors after participating in the intervention. The investigator concludes that the relationship-focused intervention was promising but needed continuous rigorous evaluation including testing of a methodology of a post to pre-test comparison design with adolescents. (PsycINFO Database Record (c) 2012 APA, all rights reserved)
SN - 0419-4209
AD - Spears, Beverly Ann: U Houston, US
ER -

TY - JOUR
ID - 2746
T1 - The involvement of motor function in social play and solitary behavior and its relation to child behavior problems during the kindergarten years
A1 - Goldenberg Bart,Orit
Y1 - 2004///
N1 - Dissertation Abstract: 2004-99018-246
English
Dissertation Abstract
KW - PsycInfo
KW - Behavior Problems
KW - Childhood Play Behavior
KW - Social Behavior
RP - NOT IN FILE
SP - 1577
JF - Dissertation Abstracts International: Section B: The Sciences and Engineering
VL - 65:
IS - 3-B
N2 - This study focused on the potential contribution of individual variations in children's motor functions to individual differences in social participation and play behavior. The study further examined whether the interaction of social behavior with peers and children's motor abilities was related to the occurrence of behavior problems. Indoors and outdoors play behavior patterns of 88 kindergarten children (53 girls) were observed, and a battery of standard assessments of basic motor functions was administered. Maternal reports on children's behavior problems were collected. The findings indicate modest but significant associations between typically developed children's play behavior and various domains of motor functioning. High frequency of social play was associated with better motor planning and execution, posture control, and visual-motor coordination. In contrast, high frequencies of solitary behavior were associated with poor motor function. The combinations of high frequency of social play or low frequency of solitary behavior and high motor ability appear to serve as a protective factor that reduces child behavior problems. It is concluded that motor functions appear to play a significant role in children's social adaptation. Possible links between motor functions, their underlying brain's structures, and the extant neurobehavioral models of social withdrawal are discussed. (PsycINFO Database Record (c) 2012 APA, all rights reserved)
SN - 0419-4217
AD - Goldenberg Bart, Orit: Touro U International, US
ER -

TY - JOUR
ID - 2747
T1 - A comparison of the play performance of boys with autism and that of boys without disabilities in Taiwan
A1 - Lee,I.Ching
Y1 - 2004///
N1 - Dissertation Abstract: 2004-99012-241
English
Dissertation Abstract
KW - PsycInfo

KW - Autism
KW - Childhood Play Behavior
KW - Cognitive Ability
KW - Human Males
RP - NOT IN FILE
SP - 6048
JF - Dissertation Abstracts International: Section B: The Sciences and Engineering
VL - 64:
IS - 12-B
N2 - This study assessed the play performance of 37 boys with autism and 42 boys without disabilities, aged 3 to 5 years, in Taiwan. Play performance was determined from videotaped observation of boys playing in the naturalistic environment, and rated using the standardized instrument of the Revised Knox Preschool Play Scale (PPS). Results indicated that the autistic boys scored significantly lower than the normal boys on PPS and its four dimensions. Analyses demonstrated a significantly higher correlation between the PPS age scores and the chronological age in the normal boys than in the autistic boys. Discriminant analyses revealed that the age scores from PPS and its dimension of Space Management, Pretense/Symbolic, and Participation, each correctly classified the subjects into their actual groups with a high degree of precision (i.e., the classification rate ranged from .84 to .98). The score from Material Management dimension only correctly classified 67% of the subjects. The Chinese Version of Peabody Picture Vocabulary Test-Revised (PPVT-R) was administered to estimate subjects' cognitive capability. Of 37 subjects with autism, only 15 subjects completed the test independently with a score over 70. An additional statistic was applied to compare the performance of this subgroup of autistic boys with their normal peers. Analysis indicated that this subgroup of autistic boys scored significantly lower on PPS and three of its four dimensions (Material Management, Pretense/Symbolic, and Participation) than their normal peers did. However, caution should be given to the interpretation of these results since the cognitive functioning (as measured by PPVT-R) was significantly lower in this subgroup of autistic boys, as compared to the normal controls. This study broadened understanding of play performance in the Taiwanese population and was informative of the potential play deficiencies that may help explain a wide range of the impairments associated with autism. It also raised a number of concerns that demand future investigation. Some comments regarding the implication of the findings and the application of PPS in the Taiwanese population were directed to Taiwanese practitioners. (PsycINFO Database Record (c) 2012 APA, all rights reserved)
SN - 0419-4217
AD - Lee, I-Ching: U Southern California, US
ER -

TY - JOUR
ID - 2748
T1 - The effects of caring adults, religiosity, and resiliency on African American middle school girls' school attachment, self-concept, and participation in out-of-school time programs
A1 - Trammel,Ming Shi
Y1 - 2004///
N1 - Dissertation Abstract: 2004-99011-026
English
Dissertation Abstract
KW - PsycInfo
KW - Religiosity
KW - Resilience (Psychological)
KW - Self Concept
KW - Academic Achievement
KW - Anger Control
KW - Middle School Students
KW - Schools
RP - NOT IN FILE
SP - 4361
JF - Dissertation Abstracts International Section A: Humanities and Social Sciences
VL - 64:
IS - 12-A

N2 - The study examined the effects of caring adults, religiosity, and resiliency on African American middle school girls' self-concept, school attachment, and participation in out-of-school time programs. The study was conducted in two phases. In Phase I, African American girls (n = 130), grades 6-8, who resided in Columbus, Ohio, were asked to complete a survey to obtain data on the specific variables of interest. A sample of the girls reported on the survey that they participated in out-of-school time (OST) programs took part in a series of focus groups (Phase II). The focus groups were done to gather in-depth information regarding girls perceptions of the role of OST activities on their development. Overall, the girls in this study had moderated to fairly high levels of self-concept, school attachment, and resiliency. They also perceived that there were caring adults (other than parents) in their lives. The overwhelming majority of girls reported involvement in at least two OST activities. A positive correlation emerged between girls resiliency and religiosity. The girls scores on the school attachment and self-concept measures were positively correlated. MANOVA analyses revealed that African American girls who had higher levels of caring adults in their lives (e.g., the degree of nurturing relationships with adults outside of their parents) and resiliency scores had significantly higher self-concept and school attachment scores than girls who had significantly lower caring adults and resiliency scores. Girls who reported higher levels of religiosity had significantly higher self-concept scores than girls who reported lower levels of religiosity. However, there was no difference for reported levels of religiosity on school-attachment scores. Regression analyses found that girls' levels of caring adults and religiosity accounted for a significant amount of the variance in their self-concept scores. Results also showed that girls' levels of caring adults and resiliency accounted for a significant amount of the variance in their school attachment scores. However, caring adults, resiliency, and religiosity did not account for a significant amount of the variance in the number of OST programs. The results from the qualitative portion of the study supplemented the findings from the Phase I examination. In particular, reports from the various focus groups indicated that the majority of the girls perceived that participation in OST activities has an influence on various aspects of their development (e.g., anger management and academic achievement). Participation in OST activities also provided the girls with a set of caring adults other than parents who provided guidance and support to them. (PsycINFO Database Record (c) 2012 APA, all rights reserved)
SN - 0419-4209
AD - Trammel, Ming Shi: Howard U., US
ER -

TY - JOUR
ID - 2749
T1 - Links between marital conflict and child social withdrawal: Mediating effects of parenting behaviors and moderating effect of parental anxiety and depression
A1 - Robinson,George Canby
Y1 - 2004///
N1 - Dissertation Abstract: 2004-99010-102
English
Dissertation Abstract
KW - PsycInfo
KW - Family Relations
KW - Marital Conflict
KW - Parental Characteristics
KW - Physical Abuse
KW - Anxiety
KW - Mediation
KW - Parental Attitudes
KW - Teachers
RP - NOT IN FILE
SP - 5799
JF - Dissertation Abstracts International: Section B: The Sciences and Engineering
VL - 64:
IS - 11-B
N2 - This study utilized a mediation/moderation mixed model to examine the direct and indirect links between marital conflict and children's asocial and excluded behaviors as rated by teachers. The ecological validity of this study was enhanced by using independent raters of children's behavior and direct observations of parenting behavior. The participants (n = 72) in this study were a combination of families with substantiated cases of

physical abuse that were referred by a state human services agency and families recruited from the community. Participating children had a mean age of 7.3 years and ranged between 4 and 9 years old. To determine direct effects, marital conflict was assessed using a modified version of the Conflict Tactics Scales. Children's social behaviors were measured by two subscales on the Social Behavior Scale. To calculate indirect effects mediated through parenting behavior, parents and children participated in three 10-minute vignettes that were videotaped and coded. Overall, results supported a direct effects model in which marital conflict predicted 5% of the variance in children's asocial behavior. Asocial behaviors in the classroom were also significantly associated with peer rejection and low engagement on the playground. Although moderation of the relation between marital conflict and the three parenting behavior domains by parental anxiety, depression, and stress was not supported, substantial support was found for the negative impact of parent psychopathology and stress had on parenting behavior. Prevention/intervention efforts for children and parents using a family systems perspective were emphasized. (PsycINFO Database Record (c) 2012 APA, all rights reserved)
SN - 0419-4217
AD - Robinson, George Canby: North Carolina State U., US
ER -

TY - JOUR
ID - 2750
T1 - Beyond situational specificity and other hypothetical constructs: A practical approach to general outcome measurement of preschool social skill development
A1 - Silberglitt,Benjamin
Y1 - 2004///
N1 - Dissertation Abstract: 2004-99007-074
English
Dissertation Abstract
KW - PsycInfo
KW - Personality Measures
KW - Social Interaction
KW - Social Skills
KW - Social Skills Training
KW - Test Validity
RP - NOT IN FILE
SP - 3595
JF - Dissertation Abstracts International Section A: Humanities and Social Sciences
VL - 64:
IS - 10-A
N2 - The purpose of this paper is to investigate current developments and controversies in the assessment of early childhood social skill, and to explore an alternative approach, focusing on the "practical utility" of assessment. The paper begins with an introduction to assessment of preschool social skill development; a review of the situational specificity debate in personality assessment and its implications for early childhood social skill assessment; a presentation of General Outcome Measurement (G.O.M.) as an alternative assessment approach; and a discussion of needed research to create a practical and useful general outcome measure of preschool social skill development. A standard of utility is emphasized as an important approach to developing an assessment tool. Three major issues are identified, facing the further development of social turns as a general outcome measure of early childhood social interaction: (1) The effect of situational variation in the data collection format on G.O.M. of social interaction skill; (2) The effect of situational variation on the validity of G.O.M.; and (3) The need to determine factors outside of the child that can account for variance in a G.O.M. Thirty-five children between the ages of 40 and 66 months participated in the study, 19 males and 16 females. Participants were predominantly from families with middle to high SES, and the ethnicity of participants was predominantly White, with 2 Asian-American and 2 African-American participants. Three children in the study were receiving special education services under an Individualized Education Plan (IEP), with social/behavior goals on their IEP. Participants played in dyadic play situations, with one of two specific toys and either a familiar or unfamiliar playmate. A repeated-measures design was used, in which all target children played in all four possible situations. Results indicate that setting has a significant effect on G.O.M. of preschooler's social skills, and must be accounted for in developing a practical measure. In addition, a significant portion of the measure's variance can be accounted for by the behavior of the peer, namely, the number of times the peer attempted to

initiate social interaction. Implications for implementing general outcome measurement of social interaction skill are discussed. (PsycINFO Database Record (c) 2012 APA, all rights reserved)
SN - 0419-4209
AD - Silberglitt, Benjamin: U Minnesota, US
ER -

TY - JOUR
ID - 2751
T1 - Executive Function in Preschool Children: Examination Through Everyday Behavior. [References]
A1 - Isquith,Peter K.
Y1 - 2004///
N1 - Peer Reviewed Journal: 2004-16917-003
English
Journal; Peer Reviewed Journal
KW - PsycInfo
KW - Cognitive Ability
KW - Cognitive Assessment
KW - Daily Activities
KW - Early Childhood Development
KW - Test Construction
KW - Factor Structure
KW - Executive Function
RP - NOT IN FILE
SP - 403
EP - 422
JF - Developmental Neuropsychology
VL - 26:
IS - 1
N2 - Clinical assessment of executive function in preschool-age children is challenging given limited availability of standardized tasks and preschoolers' variable ability to participate in lengthy formal evaluation procedures. Given the benefits of ecological validity of measuring behavior by rating scales, the Behavior Rating Inventory of Executive Function (Gioia, Isquith, Guy, & Kenworthy, 2000) was modified for use with children ages 2 through 5 years to assess executive functions in an everyday context. The scale development process, based on samples of 460 parents and 302 teachers, yielded a single 63-item measure with 5 related, but nonoverlapping, scales, with good internal consistency and temporal stability. Exploratory factor analyses identified 3 consistent factors: Emergent Metacognition, Flexibility, and Inhibitory Self-Control across parent and teacher samples. In a second study with a mixed sample of preschool children with various developmental disorders, parents and teachers rated these preschool children as having greater executive difficulties in most domains than matched controls. Such rating-scale methodology may be a useful complementary tool by which to reliably assess executive functions in preschool children via everyday behaviors in the natural environment. (PsycINFO Database Record (c) 2012 APA, all rights reserved) (journal abstract)
SN - 8756-5641
AD - Isquith, Peter K.: Department of Psychiatry, Dartmouth Hitchcock Medical School, Lebanon, NH, US Gioia, Gerard A.: Pediatric Neuropsychology Program, Center for Neurosciences and Behavioral Medicine, Children's National Medical Center, Washington, DC, US Espy, Kimberly Andrews: Department of Family and Community Medicine, Southern Illinois University School of Medicine, Carbondale, IL, US
ER -

TY - JOUR
ID - 2752
T1 - Toddlers' emerging symbolic play: A first-born advantage? [References]
A1 - Kowalski,Helen S; Wyver,Shirley
Y1 - 2004///
N1 - Peer Reviewed Journal: 2004-16664-008
English
Journal; Peer Reviewed Journal
KW - PsycInfo

KW - Age Differences
KW - Birth Order
KW - Childhood Play Behavior
KW - Peer Relations
KW - Child Day Care
KW - Childhood Play Development
RP - NOT IN FILE
SP - 389
EP - 400
JF - Early Child Development and Care
VL - 174:
IS - 4
N2 - The influence of older peers on younger children's emerging symbolic play was examined for 24 young children in eight participating long-day child-care centres. Toddler participants (aged from 17 to 31 months) were observed and video-taped in three conditions in free play in the outdoor environment as follows: condition one, with their same-age peers; condition two, with their older, preschool-aged peers; and condition three, in a dyad situation with a familiar often 'self-chosen' preschool-aged peer. Fourteen of the participants were first-borns and 10 were later-borns. The toddlers' symbolic play was coded in the four dimensions of symbolic play ('decontextualization', 'thematic content', 'organization of themes', and 'self-other relationships') in accordance with Westby's (1991) 'Symbolic play scale'. First-born participants exhibited higher levels of symbolic play in all four dimensions as identified by Westby (1991) in mixed-age free play sessions. Additionally, in mixed-age free play, first-borns scored significantly higher in three dimensions in the most frequently occurring levels of symbolic play than did later-born participants. (PsycINFO Database Record (c) 2012 APA, all rights reserved) (journal abstract)
SN - 0300-4430
AD - Kowalski, Helen S.: Institute of Early Childhood, Macquarie University, North Ryde, NSW, Australia Wyver, Shirley R.: Institute of Early Childhood, Macquarie University, North Ryde, NSW, Australia Masselos, Grace: Faculty of Education, University of Wollongong, Australia De Lacey, Philip: Faculty of Education, University of Western Sydney, SA, Australia
ER -

TY - JOUR
ID - 2753
T1 - EatFit: A Goal-Oriented Intervention that Challenges Adolescents to Improve Their Eating and Fitness Choices. [References]
A1 - Horowitz,Marcel; Shilts,Mical Kay; Townsend,Marilyn
Y1 - 2004///
N1 - Peer Reviewed Journal: 2004-15503-006
English
Journal; Peer Reviewed Journal
KW - PsycInfo
KW - Adolescent Attitudes
KW - Exercise
KW - Health Behavior
KW - Physical Fitness
KW - School Based Intervention
KW - Diets
KW - Middle Schools
KW - Self Efficacy
RP - NOT IN FILE
SP - 43
EP - 44
JF - Journal of Nutrition Education and Behavior
VL - 36:
IS - 1
N2 - Surveys show that many young people do not engage in the recommended levels of physical activity, nor do they have diets meeting the US Dietary Guidelines. These health behaviors contribute to obesity, now at an

all-time high among adolescents. With this in mind, we developed "EatFit" to improve the dietary and physical activity behaviors of middle school students. The physical activity assessment has students examine their current fitness activity behaviors and guides them through goal setting and contracting. Each week, students track their eating and fitness goal progress. Many skill-building activities are included in the EatFit curriculum. Recipe preparation and tasting provide students with the opportunity to increase their self-efficacy for choosing and preparing foods that support their nutrition goal. Among the fitness lessons, activities are included that allow students to practice physical activity skills (eg, leg lunges, stretches). Practicing the skills in the classroom will increase students' self-efficacy so that they will be more likely to perform the physical activities outside the classroom. This education intervention was pilot tested with 34 middle school students who were Expanded Food and Nutrition Education Program (EFNEP) participants. Data from self-reported recall instruments indicated that students made positive changes in dietary behaviors (41%), dietary self-efficacy (29%), physical activity behaviors (56%), and physical activity self-efficacy (26%). Seventy-four percent and 79% of the students rated themselves as making one lasting improvement in their dietary and physical activity behaviors, respectively. (PsycINFO Database Record (c) 2012 APA, all rights reserved)
SN - 1499-4046
AD - Horowitz, Marcel: University of California-Davis, Davis, CA, US Shilts, Mical Kay: University of California-Davis, Davis, CA, US Townsend, Marilyn S.: University of California-Davis, Davis, CA, US
ER -

TY - JOUR
ID - 2754
T1 - 'What do you notice?' Using posters containing questions and general instructions to guide preschoolers' science and mathematics learning. [References]
A1 - Ramsey,Jennifer L.
Y1 - 2004///
N1 - Peer Reviewed Journal: 2004-11507-003
English
Journal; Peer Reviewed Journal
KW - PsycInfo
KW - Mathematics Education
KW - Preschool Students
KW - School Learning
KW - Science Education
KW - Teaching Methods
KW - Visual Displays
RP - NOT IN FILE
SP - 31
EP - 45
JF - Early Child Development and Care
VL - 174:
IS - 1
N2 - To increase preschoolers' science and mathematics learning experiences both within and outside the classroom, we developed age-appropriate materials containing general questions and instructions that teachers and parents could use when interacting with their students and children. The questions and instructions were related to a specific science or mathematics skill (e.g., classifying), but were general enough so that they could be applied to a variety of situations. Observations of three classrooms and interviews with the classroom teachers and aides, and the parents of the children in the classrooms suggested that use of these materials was beneficial to the children, teachers, and parents, and resulted in increasing the amount of science and mathematics activities and discussions in two of the classrooms as well as outside the classrooms. (PsycINFO Database Record (c) 2012 APA, all rights reserved) (journal abstract)
SN - 0300-4430
AD - Ramsey, Jennifer L.: Charles A. Dana Center, University of Texas, Austin, TX, US Fowler, Marilyn L.: Charles A. Dana Center, University of Texas, Austin, TX, US
ER -

TY - JOUR
ID - 2755

T1 - Friendship and Loneliness Among Bullies and Victims: Data From Seven Countries. [References]
A1 - Eslea,Mike; Menesini,Ersilia; Morita,Yohji; O'Moore,Mona; Mora-Merchan,Joaquin
Y1 - 2004///
N1 - Peer Reviewed Journal: 2004-10868-006
English
Journal; Peer Reviewed Journal
KW - PsycInfo
KW - Cross Cultural Differences
KW - Friendship
KW - Loneliness
KW - Victimization
KW - Bullying
KW - Human Females
KW - Human Males
KW - Human Sex Differences
KW - Peer Relations
RP - NOT IN FILE
SP - 71
EP - 83
JF - Aggressive Behavior
JA - Aggress.Behav
VL - 30:
IS - 1
N2 - Research suggests that the relationship between school bullying and its various risk factors should be clearer among girls than boys, and should become stronger with age, as roles within the peer group stabilise. This paper tests this theory by comparing sex, school type, and bully /victim status differences in friendships and playground social interactions, using data from nine surveys in seven countries: China, England, Ireland, Italy, Japan, Portugal, and Spain. A total of approximately 48,000 children completed various translations of the Olweus Bullying Questionnaire. Small but generally consistent main effects were found for sex and school type (boys and primary pupils enjoyed playtimes more and had more friends, but were also more likely to spend playtimes alone). Larger effects were consistently found for bully/victim status (victims were significantly worse off on all the measures in all the samples where a difference was found, while bullies and neutrals did not differ consistently), but the interactions between these factors varied widely between samples and there were few consistent patterns. It is concluded that bullying is a universal phenomenon with many negative correlates for victims and few (if any) for bullies, but that there are cultural variations in the... (PsycINFO Database Record (c) 2012 APA, all rights reserved)
SN - 0096-140X
AD - Eslea, Mike: U Central Lancashire, Dept of Psychology, Preston, US Menesini, Ersilia: U Florence, Dept of Psychology, Florence, Italy Morita, Yohji: Osaka City U, Dept of Sociology, Osaka, Japan O'Moore, Mona: Trinity Coll, Education Dept, Dublin, Ireland Mora-Merchan, Joaquin A.: U Seville, Dept of Developmental and Educational Psychology, Seville, Spain Pereira, Beatriz: U Minho, Institute of Childhood Studies, Minho, Portugal Smith, Peter K.: U of London, Goldsmiths Coll, Dept of Psychology, London, United Kingdom
ER -

TY - JOUR
ID - 2756
T1 - A short-term longitudinal study of children's playground games in primary school: Implications for adjustment to school and social adjustment in the USA and the UK. [References]
A1 - Pellegrini,Anthony D.
Y1 - 2004///
N1 - Peer Reviewed Journal: 2004-10349-006
English
Journal; Peer Reviewed Journal
KW - PsycInfo
KW - Games
KW - Human Sex Differences
KW - Playgrounds

KW - School Adjustment
KW - Social Adjustment
KW - Childhood Play Behavior
KW - Childhood Play Development
KW - Elementary School Students
KW - Elementary Schools
KW - Prediction
KW - Trends
RP - NOT IN FILE
SP - 107
EP - 123
JF - Social Development
VL - 13:
IS - 1
N2 - In this study we described development of boys' and girls' games on the school playground at recess as they progressed across the first year of primary school in London (UK) and Minneapolis (USA) in order to document age-related trends in games during this period and to examine the predictive role of games in children's adjustment to school and more general social adjustment. Consistent with expectations, games accounted for a significant portion of children's, but especially boys', behavior during recess. American children played games more frequently, especially simple games, relative to English children. Consistent with expectation, the complexity of games increased, especially for boys, across the school year for children in both countries. We also examined the extent to which facility with games forecasted adjustment to school and social adjustment. Games predicted both, but only in the USA sample. Results are discussed in terms of games being an important developmental task for children of this age. Implications for future research and educational policy are discussed. (PsycINFO Database Record (c) 2012 APA, all rights reserved) (journal abstract)
SN - 0961-205X
AD - Pellegrini, Anthony D.: University of Minnesota, MN, US Blatchford, Peter: Institute of Education, University of London, London, England Kato, Kentaro: University of Minnesota, MN, US Baines, Ed: Institute of Education, University of London, London, England
ER -

TY - JOUR
ID - 2757
T1 - Social Skills Intervention during Elementary School Recess: A Visual Analysis. [References]
A1 - nderson-Butcher,Dawn
Y1 - 2003///
N1 - Peer Reviewed Journal: 2005-07471-002
English
Journal; Peer Reviewed Journal
KW - PsycInfo
KW - Behavior Problems
KW - Elementary School Students
KW - Playgrounds
KW - School Based Intervention
KW - Social Skills Training
KW - Elementary Schools
KW - School Environment
KW - School Facilities
KW - School Violence
KW - Bullying
RP - NOT IN FILE
SP - 135
EP - 146
JF - Children & Schools
VL - 25:
IS - 3

N2 - Given the prevalence of and growing concern about school violence perpetuated by bullies on school playgrounds, the need for progressive state-of-the-art interventions that promote the psychosocial development of children who are most susceptible to school violence during the school day must be acknowledged. The most vulnerable time for many children is during recess on the school playground. Programs during recess hold great potential for promoting positive playground and school experiences among children. The study discussed in this article builds on two studies that explored the effect of a social skills intervention on problem behaviors displayed by elementary school children during recess. Findings conclude that social skills intervention significantly decreased problem behaviors among school children at recess. Implications for behavioral management and healthy school environments are explored. (PsycINFO Database Record (c) 2012 APA, all rights reserved) (journal abstract)
SN - 1532-8759
AD - Anderson-Butcher, Dawn: College of Social Work, Ohio State University, Columbus, OH, US Newsome, W. Sean: College of Social Work, University of Illinois, Chicago, IL, US Nay, Stephanie: Utah Heart Clinic, UT, US
ER -

TY - JOUR
ID - 2758
T1 - Representing the Mental World in Children's Social Behavior: Playing Hide-and-Seek and Keeping a Secret. [References]
A1 - Peskin,Joan
Y1 - 2003///
N1 - Peer Reviewed Journal: 2004-20813-003
English
Journal; Peer Reviewed Journal
KW - PsycInfo
KW - Childhood Development
KW - Childhood Play Behavior
KW - Cognitive Development
KW - Social Behavior
KW - Theory of Mind
KW - Social Cognition
KW - Social Skills
RP - NOT IN FILE
SP - 496
EP - 512
JF - Social Development
VL - 12:
IS - 4
N2 - This study examined the relationship between children's developing theory of mind and their ability to engage in two social behaviors which have, as their cognitive underpinning, the representation that what one knows may not be accessible to others. Children of 3, 4, and 5 years, in a quasi-naturalistic setting, played hide-and-seek and also were required to keep a secret about a surprise. The ability to play hide-and-seek was significantly related to children's ability to refrain from disclosing the secret, and there was a significant relationship between these behaviors and children's social cognition, as measured by theory of mind tasks. The relationship between these social behaviors and tasks measuring executive function was not significant once age was taken into account. With regard to the development of these social behaviors, few 3- year-olds, but most 4-year-olds, and almost all 5-year-olds could successfully play hide-and-seek and keep a secret. This study demonstrates the importance of the conceptual understanding of mental states in the young child's social world. (PsycINFO Database Record (c) 2012 APA, all rights reserved) (journal abstract)
SN - 0961-205X
AD - Peskin, Joan: University of Toronto, Toronto, ON, Canada Ardino, Vittoria: Universita Cattolica, Milan, Italy
ER -

TY - JOUR
ID - 2759

T1 - Pointing accuracy in children is dependent on age, sex and experience. [References]
A1 - Lehnung,Maria; Leplow,Bernd; Haaland,Vegard Oksendal; Mehdorn,Maximilian; Ferstl,Roman
Y1 - 2003///
N1 - Peer Reviewed Journal: 2004-18615-007
English
Journal; Peer Reviewed Journal
KW - PsycInfo
KW - Age Differences
KW - Familiarity
KW - Human Sex Differences
KW - Spatial Ability
KW - Spatial Perception
KW - Environment
KW - Experiences (Events)
RP - NOT IN FILE
SP - 419
EP - 425
JF - Journal of Environmental Psychology
VL - 23:
IS - 4
N2 - We investigated the influence of age, sex and familiarity on spatial performance assessed with a pointing task in 84 children: 21 kindergartners, 21 first-graders, 21 fifth-graders and 21 sixth-graders. Spatial performance was assessed in the children's natural environment, their respective school or kindergarten. The children had to indicate the direction of prominent landmarks on the school campus while sitting in their classroom, drawing an arrow on a sheet of paper. Since the children had spent different amounts of time at their respective schools, experience with the environment was regarded as a covariate. Data analysis revealed significant effects for age, sex and experience. Fifth-graders aged around 10 years outperformed the younger children. Sex differences revealed male superiority at all ages. Experience with the environment was also shown to play a vital role. Children who had spent a longer time at the school were able to demonstrate better spatial knowledge by pointing more accurately towards unseen landmarks. (PsycINFO Database Record (c) 2012 APA, all rights reserved) (journal abstract)
SN - 0272-4944
AD - Lehnung, Maria: Department of Psychology, Christian-Albrechts-University of Kiel, Kiel, Germany Leplow, Bernd: Department of Psychology, Martin-Luther-University of Halle-Wittenberg, Halle-Wittenberg, Germany Haaland, Vegard Oksendal: Department of Psychology, University of Oslo, Oslo, Norway Mehdorn, Maximilian: Clinic for Neurosurgery, Christian-Albrechts-University of Kiel, Kiel, Germany Ferstl, Roman: Department of Psychology, Christian-Albrechts-University of Kiel, Kiel, Germany
ER -

TY - JOUR
ID - 2760
T1 - Differentiating Parent Practices Related to Adolescent Behavior in the Free Time Context. [References]
A1 - Hutchinson,Susan L.
Y1 - 2003///
N1 - Peer Reviewed Journal: 2004-12214-003
English
Journal; Peer Reviewed Journal
KW - PsycInfo
KW - Adolescent Development
KW - Leisure Time
KW - Parent Child Communication
KW - Parenting Style
RP - NOT IN FILE
SP - 396
EP - 422
JF - Journal of Leisure Research
VL - 35:

IS - 4
N2 - The purpose of this study was to examine parenting practices associated with adolescent free time use. Interviews (n = 17) were conducted with parents of adolescents (12-14 years) in two communities. Eight were from a university-based community in the northern U.S., and nine were recruited from rural communities outside a large metropolitan center in Eastern Canada. The parents in this study used a number of practices to structure, regulate and support their adolescents' behavior in the free time domain. These practices extended from: (a) parents' beliefs and expectations of the free time context, (b) the ways in which parents communicated and enforced these beliefs and expectations, (c) parents' actions to direct their adolescents' activity engagement, (d) strategies used to monitor the adolescents' activities, (e) the provision of resources to support preferred activity engagement, and (f) parents' efforts to support autonomous behavior in die free time context. The results are discussed in terms of extant literature on parental structuring and support of adolescents' free time behavior and leisure interests. (PsycINFO Database Record (c) 2012 APA, all rights reserved) (journal abstract)
SN - 0022-2216
AD - Hutchinson, Susan L.: School of Hotel, Restaurant and Recreation Management, Pennsylvania State University, University Park, PA, US Baldwin, Cheryl K.: Human Services Program, Aurora University, Aurora, IL, US Caldwell, Linda L.: School of Hotel, Restaurant and Recreation Management, Pennsylvania State University, University Park, PA, US
ER -

TY - JOUR
ID - 2761
T1 - Health actions and health promotion among a sample of rural Black South African children. [References]
A1 - Promtussananon,Supa
Y1 - 2003///
N1 - Peer Reviewed Journal: 2004-11523-006
English
Journal; Peer Reviewed Journal
KW - PsycInfo
KW - Blacks
KW - Daily Activities
KW - Health Behavior
KW - Health Promotion
KW - Hygiene
KW - Health
KW - Rural Environments
RP - NOT IN FILE
SP - 39
EP - 44
JF - Journal of Child and Adolescent Mental Health
VL - 15:
IS - 1
N2 - The purpose of this study was to investigate the daily activities that young South African children perceived as health related activities. The sample included 117 children (33, ages 5-7 years; 44, ages 8-10 years; and 40, ages 11-12 years) chosen by two-stage cluster sampling in a rural community of the central region of the Northern Province. Results indicated that the most important health actions around healthy eating were, 'washing hands'; around dressing, 'wearing warm clothes'; around keeping clean, 'washing body and hair'; going to sleep, 'washing body'; activity at school, 'read and write'; recess and leisure time, 'playing football or netball'. Personal hygiene (washing, brushing teeth, etc.) was the most common health action of the children around healthy eating, keeping clean and going to sleep. Some health actions were insufficiently mentioned, brushing teeth for example and some wrongly mentioned such as eating porridge before going to sleep. This study showed that children have insufficient knowledge and understanding about health related activities. Results suggest that health professionals and teachers should consider providing more information about health related activities for young children. (PsycINFO Database Record (c) 2012 APA, all rights reserved) (journal abstract)
SN - 1728-0583
AD - Promtussananon, Supa: Department of Community Health, University of Venda for Science and Technology, Thohoyandu, South Africa Peltzer, Karl: Department of Community Health, University of Venda for Science and Technology, Thohoyandu, South Africa

ER -

TY - JOUR
ID - 2762
T1 - Friendship formation in inclusive early childhood classrooms: What is the teacher's role? [References]
A1 - Buysse,Virginia; Goldman,Barbara Davis; Skinner,Martie
Y1 - 2003///
N1 - Peer Reviewed Journal: 2004-10062-006
English
Journal; Peer Reviewed Journal
KW - PsycInfo
KW - Friendship
KW - Mainstreaming (Educational)
KW - Roles
KW - Strategies
KW - Teachers
KW - Classroom Environment
RP - NOT IN FILE
SP - 485
EP - 501
JF - Early Childhood Research Quarterly
VL - 18:
IS - 4
N2 - This study examined strategies teachers reported using to support friendships among young children in inclusive early childhood classrooms. The teachers included 25 general early childhood educators from 9 child care centers and 20 early childhood special educators from 9 specialized programs. The Playmates and Friends Questionnaire for Teachers documented the number and nature of children's friendships and teachers' reported use of 11 different strategies to support individual friendships. Overall, the friendship strategies that teachers reported using most frequently included providing sufficient free choice time, allowing children to form their own friendships, and commenting on the play between friends. The majority of teachers reported rarely using two strategies: arranging for children to play together outside of the classroom and allowing two friends to exclude other children from their play. Teachers reported using more active friendship strategies to support dyads in which one or both children had special needs. Implications of these findings to support friendship formation in inclusive early childhood settings and directions for future research are presented. (PsycINFO Database Record (c) 2012 APA, all rights reserved) (journal abstract)
SN - 0885-2006
AD - Buysse, Virginia: University of North Carolina at Chapel Hill, FPG Child Development Institute, Chapel Hill, NC, US Goldman, Barbara Davis: University of North Carolina at Chapel Hill, FPG Child Development Institute, Chapel Hill, NC, US Skinner, Martie L.: University of North Carolina at Chapel Hill, FPG Child Development Institute, Chapel Hill, NC, US
ER -

TY - JOUR
ID - 2763
T1 - An analysis of the effects and methodological concerns of ability and sex on children's peer acceptance and friendships
A1 - Leblanc,Catherine Anne
Y1 - 2003///
N1 - Dissertation Abstract: 2003-95020-208
English
Dissertation Abstract
KW - PsycInfo
KW - Cooperative Learning
KW - Friendship
KW - Human Sex Differences
KW - Learning Disabilities
KW - Peer Relations

KW - Social Skills
RP - NOT IN FILE
SP - 1926
JF - Dissertation Abstracts International: Section B: The Sciences and Engineering
VL - 64:
IS - 4-B
N2 - The study explored the effects of ability (i.e., learning disabled [LD] versus nonlearning disabled [NLD]) and sex (i.e., male/female) on children's peer acceptance ratings and friendships when working in groups with cooperative learning goals. Methodological concerns in measuring peer acceptance and friendship were also explored. Finally, the correlation between students' peer acceptance/rejection ratings and their frequency of solitary lunchtime interactions and solitary outside play was investigated. Sixty fifth graders from two New York State school districts that used student work groups with cooperative goals for instruction participated in the study. Thirty LD (15 girls and 15 boys) and thirty NLD (15 girls and 15 boys) students assigned to typically achieving, inclusion classrooms were asked to list who they liked most and liked least in their classroom. On a scale of 1-5, students were also asked to rate how much they liked to play with their classmates on the playground. To assess the correlation between students' peer acceptance/rejection rates and their frequency of solitary behavior, children's unstructured social interaction time at lunch and recess was recorded. The results showed that contrary to expectations, there were no significant differences between LD and NLD children's peer acceptance/rejection scores. However, when LD girls and boys were directly compared, NLD children preferred LD boys to LD girls. Apart from ability, peer acceptance varied by sex. Moreover, regardless of sex, LD children engaged in significantly higher instances of solitary lunchtime and outside play behavior. No correlation between sociometric scales and frequency of solitary lunchtime interactions or solitary play was noted. These results are discussed in terms of their implications for future research and educational practice. (PsycINFO Database Record (c) 2012 APA, all rights reserved)
SN - 0419-4217
AD - Leblanc, Catherine Anne: Columbia U., US
ER -

TY - JOUR
ID - 2764
T1 - Parental emotion processes, family risk status, and patterns of emotionality in children
A1 - Root,Carol Ann
Y1 - 2003///
N1 - Dissertation Abstract: 2003-95020-112
English
Dissertation Abstract
KW - PsycInfo
KW - At Risk Populations
KW - Emotional Responses
KW - Family Background
KW - Emotional Control
KW - Family
KW - Hostility
KW - Mothers
RP - NOT IN FILE
SP - 1889
JF - Dissertation Abstracts International: Section B: The Sciences and Engineering
VL - 64:
IS - 4-B
N2 - The present study investigated relations amongst parental emotion processes, family risk status, and emotion biases in children. Participants included 94 mothers, 39 fathers, and their children between 3-6 years of age (41 girls, 53 boys; M age = 4 years, 4 months) from diverse socio-economic backgrounds. Eighty-three families were followed up one year later. Parental appraisal styles were assessed via a naturalistic story-reading and explanation method, and maternal anger response and emotion regulation were measured by semi-structured interview and self-report questionnaire, respectively. Children's Time 1 and Time 2 emotion biases included teacher and mother ratings of emotion, peer ratings of emotion, as well as classroom and playground observations of emotion. Mothers using a disproportionate number of hostile appraisals over prosocial

appraisals when speaking with children was associated with children's sad/fearful biases at school. Responding angrily to their children's negative emotions was associated with both anger and sad/fearful biases at school. On bivariate correlation, paternal hostile appraisal styles were associated with children's sad/fearful biases at home. As predicted, mothers in high-risk families utilized fewer prosocial appraisals than low-risk mothers when explaining events to their children, and they tended to use a higher level of hostile appraisals than mothers in low-risk families. Similar patterns were found for fathers' hostile appraisals. Mothers in the high-risk group also demonstrated significantly more angry responses and less emotion regulation to their children's sadness than mothers in the low-risk group. Finally, longitudinal analyses showed a strong continuity of children emotion biases over a one year period, particularly with regard to anger biases. Maternal anger responses and emotion regulation at Time 1 had no predictive effect on a change in children's emotion biases over time; however, a hostile balance of maternal appraisals was found to predict change in children's anger biases in the home and school, with different effects in high- and low-risk families. These findings and future directions are discussed in the context of current theoretical, empirical, and clinical work. (PsycINFO Database Record (c) 2012 APA, all rights reserved)
SN - 0419-4217
AD - Root, Carol Ann: U Toronto, Canada
ER -

TY - JOUR
ID - 2765
T1 - The effects of prior information on social interactions between children with disabilities and their typical peers
A1 - Esteban-Elie,Sheri Elizabeth
Y1 - 2003///
N1 - Dissertation Abstract: 2003-95012-111
English
Dissertation Abstract
KW - PsycInfo
KW - Developmental Disabilities
KW - Peers
KW - Social Behavior
KW - Social Interaction
RP - NOT IN FILE
SP - 6091
JF - Dissertation Abstracts International: Section B: The Sciences and Engineering
VL - 63:
IS - 12-B
N2 - Integration of children with disabilities into mainstream settings has become increasingly common in recent years. Research into interventions to enhance the success of such efforts have primarily focused on interventions after the child with a disability has been introduced. One area of intervention that has been relatively neglected in the literature is that of intervention before the child enters the classroom. In a computer analog study, Esteban and Romanczyk (2000) investigated the influence of various types of prior information on typical peers' willingness to interact with a novel peer. Their results showed that combining information about similarities with information about characteristics of disability was superior to simply providing information about the aspects of disability alone. In addition to the focus on intervention prior to meeting a novel peer, Esteban and Romanczyk (2000) used the pervasive developmental disorders (PDDs) as the model for the disability information, in contrast to most other studies of attitudes toward disability that have focused primarily on physical disabilities or mental retardation. The current study was conducted as an extension of that research to a quasi-naturalistic setting. Seventy-eight third- and fourth-grade boys participated. After being given information about one of four boys with PDD, each participant then interacted with that child for two 5 minute segments. One segment consisted of a structured puzzle activity, while the other was unstructured. After each segment, the participant completed a questionnaire regarding the segment, which included six behavioral intentions questions. At the end of the session, the participant was given the choice of playing with the other child or playing alone. Social behaviors displayed by both children were measured. The effect of information was not replicated for most outcome measures. A main effect of activity type was found for many measures such that the unstructured activity was superior to the structured activity, contrary to hypotheses. Possible explanations of these unexpected findings are discussed. In addition, exploratory analyses regarding the effects

of specific behaviors displayed by the children with PDD and the correspondence between self-report and actual behavior were conducted and are discussed. (PsycINFO Database Record (c) 2012 APA, all rights reserved)
SN - 0419-4217
AD - Esteban-Elie, Sheri Elizabeth: State U New York At Binghamton, US
ER -

TY - JOUR
ID - 2766
T1 - Physical characteristics as determinants of trait attribution and forgiveness
A1 - Knight,Jennifer
Y1 - 2003///
N1 - Dissertation Abstract: 2003-95008-152
English
Dissertation Abstract
KW - PsycInfo
KW - Forgiveness
KW - Personality Traits
KW - Racial and Ethnic Attitudes
KW - Conservation (Concept)
KW - Interpersonal Interaction
RP - NOT IN FILE
SP - 4965
JF - Dissertation Abstracts International: Section B: The Sciences and Engineering
VL - 63:
IS - 10-B
N2 - Psychologists who have studied the development of racial attitudes have offered support for the sociocognitive theory of Aboud (1988). For example, children's reports of own-group preferences have been found to decline around age 7 (Doyle & Aboud, 1995), which has been associated with the attainment of conservation skills and increased cognitive flexibility in these children. The present study sought to explore how participants' racial attitudes were related to their attribution of traits to characters in a story. In addition, the relationship between racial attitudes and children's willingness to forgive an antagonist was explored. Forty-seven Caucasian participants between the ages of 7 and 10 years participated in this study. All were found to score predominately in the nonstereotyped category of the modified Preschool Racial Attitude Measure-II (PRAM-II) (Bigler & Liben, 1993). Children were asked to rate a narrative describing an interaction between two children, one an antagonist and the other a victim in a playground incident. Participants were systematically assigned to one of two experimental conditions. In one condition, the antagonist was Caucasian and the victim was African American. In the other, the antagonist was African American and the victim was Caucasian. Children then completed the vignette PRAM-II and the Enright Forgiveness Inventory for Children-Revised (EFIC-R). Results revealed no significant differences between the two experimental conditions on either measure. Based on the results of previous studies (Bigler & Liben, 1993; Doyle, Beaudet, & Aboud, 1988), greater variability was anticipated in the children's responses on the modified PRAM-II. Possible reasons (e.g., geographical differences, developmental age, transparency of the measure) are provided to account for the limited range of scores obtained in the present study. Accordingly, recommendations are given for future research on the development of racial attitudes in children. (PsycINFO Database Record (c) 2012 APA, all rights reserved)
SN - 0419-4217
AD - Knight, Jennifer: U Southern Mississippi, US
ER -

TY - JOUR
ID - 2767
T1 - Effects of home- and school-based interventions for students with and at-risk for antisocial behavior
A1 - Olson-Tinker,Heidi Kristine
Y1 - 2003///
N1 - Dissertation Abstract: 2003-95004-383
English
Dissertation Abstract

KW - PsycInfo
KW - Antisocial Behavior
KW - At Risk Populations
RP - NOT IN FILE
SP - 3899
JF - Dissertation Abstracts International: Section B: The Sciences and Engineering
VL - 63:
IS - 8-B
N2 - Antisocial behavior is defined as "recurrent violations of socially prescribed patterns of behavior" (Simcha-Fagan, Langner, Gersten, & Eisenberg, 1975, p. 7). The purpose of the current study was to investigate the effects of home- and school-based interventions for students with an at-risk for antisocial patterns of behavior. Prospective participants were identified using a modified multiple-gating screening procedure (Walker & Severson, 1990), wherein three consenting Kindergarten and first grade teachers nominated students whose behavior pattern most closely matched an operationalized definition of antisocial behavior. Thereafter, students were screened for eligibility using parent- and teacher-rated social behaviors. Finally, students were required to demonstrate at least average intellectual ability. Three Kindergarten and two first grade students met criteria for inclusion in the study and received home and school-based interventions. Two intervention sequences were utilized based upon the First Steps to Intervention program designed by Walker and colleagues (Walker, Stiller, Golly, Kavanagh, Severson, & Feil, 1997) using a multiple baseline-multiple sequence design (Noell & Gresham, 2001). Pre- and post-test ratings were obtained on the Social Skills Rating System (SSRS; Gresham & Elliott, 1990) and Critical Events Index (CEI; Walker & Severson, 1990). Observations were conducted across four behaviors: total disruptive behavior, academic engaged time, negative social interaction, and solitary play during recess. Results showed that both home and school interventions resulted in high effect sizes and that larger decreases in disruptive behavior were demonstrated from baseline to phase one intervention than from phase one to phase two intervention. Collateral effects on academic engaged time were demonstrated for all participants across phases. Optimal treatment sequences were noted for disruptive behavior and academic engaged time but were confounded by order effects. Independent ratings conducted by two behavior analysts showed low-to-moderate interrater agreement. Moreover, ratings based on interocular inspection showed little correspondence to standardized difference effect sizes. Additional results regarding the interventions' effect on unstructured play behavior are presented and discussed, along with study strengths, weaknesses, and implications for future research. (PsycINFO Database Record (c) 2012 APA, all rights reserved)
SN - 0419-4217
AD - Olson-Tinker, Heidi Kristine: U California, Riverside, US
ER -

TY - JOUR
ID - 2768
T1 - Childhood Depressive Symptoms, Physical Activity and Health Related Fitness. [References]
A1 - Tomson,Lois Michaud; Pangrazi,Robert
Y1 - 2003///
N1 - Peer Reviewed Journal: 2003-10604-001
English
Journal; Peer Reviewed Journal
KW - PsycInfo
KW - Exercise
KW - Health
KW - Major Depression
KW - Physical Fitness
KW - Symptoms
KW - Physical Activity
KW - Sports
RP - NOT IN FILE
SP - 419
EP - 439
JF - Journal of Sport & Exercise Psychology
JA - J Sport Exerc Psychol
VL - 25:

IS - 4
N2 - While research has confirmed a negative relationship between adult depression and physical activity, there is little evidence for children. This study examined the relationship of being classified as physically active or inactive by a parent or a teacher to depressive symptoms in children 8 to 12 years of age (N= 933). It also assessed the relationship of playing sports outside of school, and of meeting health related fitness standards, to symptoms of depression. Relative risk of depressive symptoms for inactive classification was 2.8 to 3.4 times higher than it was for active, 1.3 to 2.4 times higher for children not playing sports outside of school, and 1.5 to 4.0 times higher for those not meeting health related fitness goals. (PsycINFO Database Record (c) 2012 APA, all rights reserved) (journal abstract)
SN - 0895-2779
AD - Tomson, Lois Michaud: School of Curriculum, Teaching and Learning, Griffith University, Brisbane, QLD, Australia Pangrazi, Robert P.: Department of Kinesiology, Arizona State University, Tempe, AZ, US Friedman, Glenn: Health Evaluation & Longevity Planning, Paradise Valley, AZ, US Hutchison, Ned: Health Evaluation & Longevity Planning, Paradise Valley, AZ, US
ER -

TY - JOUR
ID - 2769
T1 - The social context of school playground games: Sex and ethnic differences, and changes over time after entry to junior school. [References]
A1 - Blatchford,Peter; Baines,Ed; Pellegrini,Anthony
Y1 - 2003///
N1 - Peer Reviewed Journal: 2003-09724-002
English
Journal; Peer Reviewed Journal
KW - PsycInfo
KW - Childhood Play Behavior
KW - Peer Relations
KW - Playgrounds
KW - Racial and Ethnic Differences
KW - Social Interaction
KW - Games
KW - Human Sex Differences
RP - NOT IN FILE
SP - 481
EP - 505
JF - British Journal of Developmental Psychology
JA - BR J DEV PSYCHOL
VL - 21:
IS - 4
N2 - This short-term longitudinal study examined activities at recess and peer relations. We were interested in changes over the school year, and the sex and ethnic mix of groups. Data came from systematic observations of 129 pupils (61 boys and 68 girls) aged 7-8 years. Results showed that peer interaction dominated recess. Ball games increased over the year, and chasing games decreased. Aggression was most common during vigorous play and conversation, but not ball games. Cleavage in boys' and girls' play and activity was common but not inevitable. Mixed-sex play was not supported by particular game types. Boys' game networks were larger than those of girls but there were no sex differences in active networks. There was little ethnic group segregation on playgrounds, and games became more integrated with time. The results indicate that playground activities can have a positive role in social relations between different ethnic groups. (PsycINFO Database Record (c) 2012 APA, all rights reserved) (journal abstract)
SN - 0261-510X
AD - Blatchford, Peter: U London, Inst of Education, London, United Kingdom Baines, Ed: U London, Inst of Education, London, United Kingdom Pellegrini, Anthony: U Minnesota, Dept of Educational Psychology, MN, US
ER -

TY - JOUR

ID - 2770
T1 - The Evaluation of Preschoolers' Interaction in the Playground: An Environmental Psychology Approach. [Portuguese]. [References]
A1 - Sager,Fabio; Sperb,Tania Mara; Roazzi,Antonio; Martins,Fernanda Marques
Y1 - 2003///
N1 - Peer Reviewed Journal: 2003-08273-019
Portuguese
Journal; Peer Reviewed Journal
KW - PsycInfo
KW - Interpersonal Interaction
KW - Playgrounds
KW - Preschool Students
RP - NOT IN FILE
SP - 203
EP - 215
JF - Psicologia: Reflexao e Critica
VL - 16:
IS - 1
N2 - The physical-spatial aspects of environments and their relation to subjectivity and human behavior have been increasingly studied by psychologists. Thus, this study investigated the relationship between playgrounds of two public preschools of Porto Alegre and the interaction of 50 5 to 6 year-old children who were enrolled in these schools. Children were observed at the playgrounds, which presented differences in terms of area (size), density and materials. The interaction states observed were related to types of play and toys used. Results drawn from quantitative analyses showed that children established more associative and parallel interaction states in the large playground, and unoccupied and solitary in the small playground. As for the type of toy, the association of this aspect with the interaction in the large playground was smaller than in the small one. A higher association between interactions and types of play in the small playground was found. The study shows that concerning environmental aspects the large playground favors a variety of interactions states. (PsycINFO Database Record (c) 2012 APA, all rights reserved) (journal abstract)
SN - 0102-7972
AD - Sager, Fabio: Universidade Federal do Rio Grande do Sul, Porto Alegre, Brazil Sperb, Tania Mara: Universidade Federal do Rio Grande do Sul, Porto Alegre, Brazil Roazzi, Antonio: Universidade Federal de Pernambuco, Brazil Martins, Fernanda Marques: Universidade Federal do Rio Grande do Sul, Porto Alegre, Brazil
ER -

TY - JOUR
ID - 2771
T1 - Effects of Friendship Circles on the Social Interactions of Elementary Age Students with Mild Disabilities. [References]
A1 - Miller,Monica Campbell; Cooke,Nancy
Y1 - 2003///
N1 - Peer Reviewed Journal: 2003-07831-002
English
Journal; Peer Reviewed Journal
KW - PsycInfo
KW - Disabilities
KW - Friendship
KW - Social Interaction
KW - Students
KW - Classroom Environment
RP - NOT IN FILE
SP - 167
EP - 184
JF - Journal of Behavioral Education
VL - 12:
IS - 3

N2 - This study assessed the effects of Friendship Circles on the social interactions between students with mild disabilities and their nondisabled peers. A multiple probe design was used to analyze the social interactions during lunch. Data also were collected in the form of sociograms and anecdotal records. In addition, generalization was assessed during recess observations. Results indicated that the Friendship Circle intervention produced increases in appropriate interactions at lunch for all three students and for two there were increases in friendly play at recess. Anecdotal data suggested improved social interactions for two of the students throughout the day and improved social behaviors in the general education classroom such as cooperation during group activities. Implications for future research are discussed. (PsycINFO Database Record (c) 2012 APA, all rights reserved) (journal abstract)
SN - 1053-0819
AD - Miller, Monica Campbell: Department of Counseling, Special Education, and Child Development, University of North Carolina at Charlotte, Charlotte, NC, US Cooke, Nancy L.: Department of Counseling, Special Education, and Child Development, University of North Carolina at Charlotte, Charlotte, NC, US Test, David W.: Department of Counseling, Special Education, and Child Development, University of North Carolina at Charlotte, Charlotte, NC, US White, Richard: Department of Counseling, Special Education, and Child Development, University of North Carolina at Charlotte, Charlotte, NC, US
ER -

TY - JOUR
ID - 2772
T1 - Teacher-child interactions and preschool children's perceptions of self and peers. [References]
A1 - Colwell,Malinda J.
Y1 - 2003///
N1 - Peer Reviewed Journal: 2003-06742-008
English
Journal; Peer Reviewed Journal
KW - PsycInfo
KW - Child Attitudes
KW - Peer Relations
KW - Preschool Students
KW - Self Perception
KW - Teacher Student Interaction
KW - Peers
KW - Preschool Teachers
RP - NOT IN FILE
SP - 249
EP - 258
JF - Early Child Development and Care
VL - 173:
IS - 2-3
N2 - This study examined connections between teacher-child interaction and children's perceptions of self and peers. 47 predominately white, middle-class preschoolers (27 boys, twenty girls; 40 European-American, 2 Hispanic, 2 African-American, 3 Asian) were observed during indoor and outdoor activity at their preschool over a period of four months. Children's time spent interacting with teachers, as well as children's cooperative behavior, emotional expressions, and aggression toward teachers was assessed. In addition, children participated in interviews designed to assess their self-perceptions and perceptions of peers. Results revealed that girls spent more time interacting with teachers than boys, and had more cooperative and positive interactions with teachers than did boys. Correlation analyses indicated that the quality of teacher-child interaction was differentially linked to boys' and girls' self-perceptions and perceptions of peers. The implications of interactions between teacher and child for children's cognitions about self and others are discussed. (PsycINFO Database Record (c) 2012 APA, all rights reserved)
SN - 0300-4430
AD - Colwell, Malinda J.: Texas Tech U, Lubbock, TX, US Lindsey, Eric W.: Texas Tech U, Lubbock, TX, US
ER -

TY - JOUR
ID - 2773

T1 - Social skills instruction for students at risk for antisocial behavior: The effects of small-group instruction. [References]
A1 - Lane,Kathleen L.
Y1 - 2003///
N1 - Peer Reviewed Journal: 2003-06719-012
English
Journal; Peer Reviewed Journal
KW - PsycInfo
KW - Antisocial Behavior
KW - At Risk Populations
KW - Group Instruction
KW - Social Skills Training
KW - Teaching Methods
KW - Elementary School Students
RP - NOT IN FILE
SP - 229
EP - 248
JF - Behavioral Disorders
VL - 28:
IS - 3
N2 - This study examined the effectiveness of social skills instruction for seven elementary-age students (aged 8.05 yrs-9.74 yrs) at risk for antisocial behavior who were unresponsive to a schoolwide primary intervention program. Students received instruction in social skills in a small-group setting over a 10-week period. A comprehensive list of acquisition deficits was generated for each student using an empirically validated assessment tool. These acquisition deficits became the core content of each intervention group. Results revealed lasting decreases in both disruptive behaviors in the classroom and negative social interactions on the playground. Students also experienced increases in percentage of academic engaged time. Findings from a social validity measure indicated that students viewed the intervention procedures as acceptable and reportedly used the newly acquired skills in multiple settings. Limitations are discussed, and directions for future research are provided. (PsycINFO Database Record (c) 2012 APA, all rights reserved)
SN - 0198-7429
AD - Lane, Kathleen L.: Vanderbilt U, Nashville, TN, US Wehby, Joseph: Vanderbilt U, Nashville, TN, US Menzies, Holly M.: California State U, Los Angeles, CA, US Doukas, Georgia L.: U California, Riverside, CA, US Munton, Sarah M.: U California, Riverside, CA, US Gregg, Rebecca M.: U California, Riverside, CA, US
ER -

TY - JOUR
ID - 2774
T1 - A multimethod, multi-informant, and multidimensional perspective on psychosocial adjustment in preadolescents with spina bifida. [References]
A1 - Holmbeck,Grayson N.
Y1 - 2003///
N1 - Peer Reviewed Journal: 2003-06685-020
English
Journal; Peer Reviewed Journal
KW - PsycInfo
KW - Emotional Adjustment
KW - Psychosocial Development
KW - Social Adjustment
KW - Spina Bifida
KW - Measurement
RP - NOT IN FILE
SP - 782
EP - 796
JF - Journal of Consulting and Clinical Psychology
VL - 71:
IS - 4

N2 - This study examined the psychosocial adjustment of preadolescents with spina bifida in relation to a comparison sample of able-bodied preadolescents (8- and 9-year-olds; n = 68 in each sample). The study also examined the potential clinical utility of a narrowband multimethod, multi-informant, and multidimensional perspective on the assessment of psychosocial functioning in children and adolescents with pediatric conditions. Findings revealed that children with spina bifida tended to be socially immature and passive, less likely to have social contacts outside of school, more dependent on adults for guidance, less competent scholastically, less physically active, less likely to make independent decisions, and more likely to exhibit attention and concentration difficulties. No group differences were found for externalizing symptoms, affective functioning, or global self-worth, suggesting resilience in these domains for the spina bifida sample. Findings also suggest that low socioeconomic status and the presence of a physical disability may be additive risk factors for certain psychosocial adjustment difficulties. (PsycINFO Database Record (c) 2012 APA, all rights reserved) (journal abstract)
SN - 0022-006X
AD - Holmbeck, Grayson N.: Loyola U Chicago, Dept of Psychology, Chicago, IL, US Westhoven, Venette C.: Loyola U Chicago, Dept of Psychology, Chicago, IL, US Phillips, Wendy Shapera: Loyola U Chicago, Dept of Psychology, Chicago, IL, US Bowers, Rachael: Loyola U Chicago, Dept of Psychology, Chicago, IL, US Gruse, Christine: Loyola U Chicago, Dept of Psychology, Chicago, IL, US Nikolopoulos, Tina: Loyola U Chicago, Dept of Psychology, Chicago, IL, US Totura, Christine M. Wienke: Loyola U Chicago, Dept of Psychology, Chicago, IL, US
ER -

TY - JOUR
ID - 2775
T1 - Recess in middle school: What do the students do? [References]
A1 - Jarrett,Olga S; Duckett-Hedgebeth,MIchelle
Y1 - 2003///
N1 - Book: 2003-06677-013
English
Book; Edited Book
KW - PsycInfo
KW - Childhood Play Behavior
KW - Middle School Students
KW - Peer Relations
KW - Physical Education
RP - NOT IN FILE
SP - 227
EP - 241
JF - Lytle, Donald E [Ed]
VL - :
IS - 2003
N2 - (create) Studied how middle school students spend time during recess. Of specific interest was how adolescents interact, how much of the playground behavior can be considered physically active, to what extent recess behaviors can be considered "play," and how much negative behavior occurred. These questions were explored by systematically observing the play area during recess. A secondary interest emerged while conducting the observational research: Why do some students not go out for recess? How do students view recess, and what suggestions do they have for improving it? These questions were explored through a student survey. Findings suggest that recess provides valuable opportunities for students to interact with their peers, engage in physical activity, and play games. The observational coding system and the quick count used can be employed by other researchers, teachers, or parents to gather data on what behaviors occur during recess. This study suggests that recess is important to students and allows for play and other social behaviors that would not occur in structured physical education classes. Any school system considering the substitution of physical education for recess should learn what actually occurs during recess and consult with students before making a decision. (PsycINFO Database Record (c) 2012 APA, all rights reserved)
AD - Jarrett, Olga S.: Georgia State University, Department of Early Childhood Education, Atlanta, GA, US Duckett-Hedgebeth, MIchelle: Atlanta Technical College, Atlanta, GA, US
ER -

TY - JOUR
ID - 2776
T1 - The role of pauses in family interactions at the table with children aged 3-4 years. [Italian]. [References]
A1 - Maroni,Barbara; Gnisci,Augusto; Pontecorvo,Clotilde
Y1 - 2003///
N1 - Peer Reviewed Journal: 2003-05348-005
Italian
Journal; Peer Reviewed Journal
KW - PsycInfo
KW - Childhood Development
KW - Conversation
KW - Family Relations
KW - Socialization
KW - Speech Pauses
KW - Family
RP - NOT IN FILE
SP - 129
EP - 155
JF - Giornale Italiano di Psicologia
VL - 30:
IS - 1
N2 - Pauses and silence in family conversation has been investigated as a process implied in children's development and socialization. We grounded our study on the international literature about discourse's uses in natural settings. Nine hours of videotaped recordings of 6 dinners of 3 different families, consisting of father, mother, young child (3-4 years) and an older child, have been systematically observed in two different periods (8 months between the two measurements). Results have been analysed with log-linear and show that: 1) in the management of pauses there are shared rules and also a variability between families; 2) the person who speaks before the pause is usually the same one speaking after it (self-transition); however when the other-transition is taken into consideration, the patterns of association between familiar roles can be understood with a developmental explanation; 3) few differences and more similarities are identified among the two different periods. Overall results are discussed from the perspective of the role played by pause management within the family system at dinner table. (PsycINFO Database Record (c) 2012 APA, all rights reserved)
SN - 0390-5349
AD - Maroni, Barbara: Universita di Roma "La Sapienza", Roma, Italy
ER -

TY - JOUR
ID - 2777
T1 - Time budgets of the mothers of pre-school children: An analysis of mother-child joint activities. [References]
A1 - Galboda-Liyanage,Kumari Chandani; Scott,Stephen; Prince,Martin James
Y1 - 2003///
N1 - Peer Reviewed Journal: 2003-05148-007
English
Journal; Peer Reviewed Journal
KW - PsycInfo
KW - Daily Activities
KW - Mother Child Relations
KW - Mothers
KW - Preschool Students
KW - Time
KW - Budgets
RP - NOT IN FILE
SP - 273
EP - 283
JF - British Journal of Developmental Psychology
JA - BR J DEV PSYCHOL

VL - 21:
IS - 2
N2 - The objective of this research was to describe how a group of mothers of pre-school children spend their days in relation to the index pre-school child and in general. We examined time use by means of the Yesterday's interview for Mothers of Pre-schoolers (YIMP), a semi-structured interview, with 31 mothers recruited from mother and toddler groups. Detailed time budgets were obtained on three full days covering the content, duration and environmental context of mother-child joint activities. Reading was the most common joint activity. A substantial proportion of activities took place outside the home. Research implications in relation to the environmental context of joint activities are discussed. (PsycINFO Database Record (c) 2012 APA, all rights reserved) (journal abstract)
SN - 0261-510X
AD - Galboda-Liyanage, Kumari Chandani: London School of Hygiene & Tropical Medicine, London, United Kingdom Scott, Stephen: Inst of Psychiatry, London, United Kingdom Prince, Martin James: London School of Hygiene & Tropical Medicine, London, United Kingdom
ER -

TY - JOUR
ID - 2778
T1 - Social development and social relationships in middle childhood. [References]
A1 - McHale,Susan M.
Y1 - 2003///
N1 - Book: 2003-04683-010
English
Book; Edited Book
KW - PsycInfo
KW - Adaptive Behavior
KW - Childhood Development
KW - Interpersonal Interaction
KW - Psychosocial Development
KW - Social Behavior
KW - Social Skills
RP - NOT IN FILE
SP - 241
EP - 265
JF - Lerner, Richard M [Ed]; Easterbrooks, M
VL - Ann [Ed]; Mistry, Jayanthi [Ed]. :
IS - 2003
N2 - (from the chapter) Middle childhood is a time of considerable change in children's social competencies and interpersonal relationships. Descriptions of the salient features of this developmental period highlight an expansion of the social world as children spend increasing amounts of time outside their homes, away from their families, at school, with peers, and in extracurricular activities. Learning to interact effectively in these new and diverse social settings is at the core of social development in middle childhood. Building on conceptual models that emphasize adaptation to interpersonal and setting demands as fundamental to social behavior and social competence, in this chapter the authors describe the nature of and influences on social development between ages 6 and 12 yrs. (PsycINFO Database Record (c) 2012 APA, all rights reserved)
AD - McHale, Susan M.: Pennsylvania State U, Human Development & Family Studies, University Park, PA, US Dariotis, Jacinda K.: Pennsylvania State U, Human Development & Family Studies, University Park, PA, US Kauh, Tina J.: Pennsylvania State U, Human Development & Family Studies, University Park, PA, US
ER -

TY - JOUR
ID - 2779
T1 - Review of Assessment, Evaluation, and Programming System (AEPSTM) for Infants and Children, Second Edition. [References]
A1 - Horn,Eva M.
Y1 - 2003///

N1 - Peer Reviewed Journal: 2003-03816-006
English
Journal; Peer Reviewed Journal
KW - PsycInfo
KW - Early Intervention
KW - Evaluation
KW - Measurement
KW - Special Education
KW - Special Needs
KW - Early Childhood Development
RP - NOT IN FILE
SP - 41
EP - 42
JF - Topics in Early Childhood Special Education
VL - 23:
IS - 1
N2 - The second edition of the AEPS (2002) is a comprehensive, curriculum-embedded measurement system for young children with special needs ages birth through 6 years. According to the reviewer, the AEPS scaffolds the design of activity-based interventions in child-centered natural environments and guides an ongoing evaluation process to ensure that these interventions are working and that children are making meaningful, developmental progress. (PsycINFO Database Record (c) 2012 APA, all rights reserved)
SN - 0271-1214
AD - Horn, Eva M.: U Kansas, Dept of Special Education, KS, US
ER -

TY - JOUR
ID - 2780
T1 - The use of pedometry to evaluate the physical activity levels among preschool children in Hong Kong. [References]
A1 - Louie,Lobo
Y1 - 2003///
N1 - Peer Reviewed Journal: 2003-02683-005
English
Journal; Peer Reviewed Journal
KW - PsycInfo
KW - Activity Level
KW - Aerobic Exercise
KW - Childhood Play Behavior
KW - Exercise
KW - Preschool Students
KW - Early Childhood Development
KW - Human Sex Differences
KW - Physical Education
RP - NOT IN FILE
SP - 97
EP - 107
JF - Early Child Development and Care
VL - 173:
IS - 1
N2 - The present study investigated physical activity trends among young children aged three, four, and five years old in Hong Kong preschools using pedometry. Subjects were 86 boys and 62 girls. The Digi-walker sw-200 electronic pedometers were utilized to collect activity data during daily regular physical activities in three preschools. School A was located in a rural area, whereas Schools B and C were in urban areas. An observation inventory. Children Activity Rating's Scale (CARS), was used to categorize the intensity level of the physical activities and to validate the pedometer counts. In line with overseas studies, significant age and gender differences in physical activity levels were also observed among the Hong Kong preschool children. A two (gender) x three (age groups) analysis of variance indicated that both main effects were statistically significant.

Older children in the school (School A) with a larger outdoor play space, in a rural district, were much more active than their counterparts in urban schools (Schools B and C) with limited indoor play space. The pedometer counts correlated significantly with the CARS scores, which suggested that pedometry can be a reliable tool to measure the intensity of physical activity level of preschool children. (PsycINFO Database Record (c) 2012 APA, all rights reserved)
SN - 0300-4430
AD - Louie, Lobo: Hong Kong Baptist U, Dept of Physical Education, Hong Kong, China Chan, Lily: Hong Kong Inst of Education, School of Early Childhood Education, Hong Kong, China
ER -

TY - JOUR
ID - 2781
T1 - What Happens During the School Day? Time Diaries from a National Sample of Elementary School Teachers. [References]
A1 - Roth,Jodie L.
Y1 - 2003///
N1 - Peer Reviewed Journal: 2003-02555-001
English
Journal; Peer Reviewed Journal
KW - PsycInfo
KW - Academic Achievement
KW - Racial and Ethnic Differences
KW - Recreation
KW - Student Characteristics
KW - Time On Task
KW - Blacks
KW - Elementary School Students
KW - Whites
RP - NOT IN FILE
SP - 317
EP - 343
JF - Teachers College Record
VL - 105:
IS - 3
N2 - This study presents data collected from a nationally-representative sample of teachers of first through fifth graders. Teachers completed a time diary, recording exact beginning and ending times for all the target student's school activities for a randomly selected day. We examined students' total time in school and their activities while there. Students attending school for the longest day were significantly more likely to be White and have fewer special needs, and to have smaller classes with a larger percentage of White students and a smaller percentage of students of other races than students attending for less time daily. We grouped students' activities at school into four categories that accounted for all but 9 minutes of the school day: academic, enrichment, recess, and maintenance activities. We found variations in how students spent their time based on student, family, and classroom characteristics. Teachers of African American students reported spending more time on academic subjects, and less time on enrichment and recess activities than teachers of white students. The same pattern emerged for teachers of more advantaged students, and classrooms with a larger percentage of White students. Results are discussed in terms of school reform efforts and inequality issues. (PsycINFO Database Record (c) 2012 APA, all rights reserved)
SN - 0161-4681
AD - Roth, Jodie L.: National Center for Children and Families, Teachers College, Columbia University, New York, NY, US Brooks-Gunn, Jeanne: National Center for Children and Families, Teachers College, Columbia University, New York, NY, US Linver, Miriam R.: National Center for Children and Families, Teachers College, Columbia University, New York, NY, US Hofferth, Sandra L.: Department of Family Studies, University of Maryland, College Park, MD, US
ER -

TY - JOUR
ID - 2782

T1 - Serious youth gun offenders and the epidemic of youth violence in Boston. [References]
A1 - Braga,Anthony A.
Y1 - 2003///
N1 - Peer Reviewed Journal: 2003-02278-002
English
Journal; Peer Reviewed Journal
KW - PsycInfo
KW - Epidemics
KW - Juvenile Delinquency
KW - Violence
KW - Criminal Profiling
KW - Homicide
RP - NOT IN FILE
SP - 33
EP - 54
JF - Journal of Quantitative Criminology
VL - 19:
IS - 1
N2 - Boston, like many other major cities, experienced a sudden increase in youth homicides during the late 1980s and early 1990s. Research evidence suggests that the epidemic of urban youth violence was intensely concentrated among criminally active young Black males residing in disadvantaged urban neighborhoods rather than all young Black males residing in disadvantaged Black neighborhoods. Other researchers, however, suggest that there was a diffusion of guns and gun violence from youth involved in street crack markets to youth outside the drug trade who armed themselves primarily for self-protection against the armed criminally active youth. In this paper, criminal history data are analyzed to determine whether the criminal profile of Boston arrested youth gun offenders (aged 12-24 yrs) changed over time and micro-level data on youth gun assault incidents in Boston are examined to unravel whether there were noteworthy changes in the nature of these violent events over time. The results of these analyses suggest that the youth violence epidemic in Boston was highly concentrated among serious youth gun offenders rather than a diffusion of guns away from the street drug trade, gangs, and criminally active youth. (PsycINFO Database Record (c) 2012 APA, all rights reserved)
SN - 0748-4518
AD - Braga, Anthony A.: Harvard U, John F. Kennedy School of Government, Program in Criminal Justice Policy & Management, Cambridge, MA, US
ER -

TY - JOUR
ID - 2783
T1 - "Errorless" Rehabilitation: Strategies of Proactive Intervention for Individuals with Brain Injury and Their Children. [References]
A1 - Ducharme,Joseph M.
Y1 - 2003///
N1 - Peer Reviewed Journal: 2003-01684-005
English
Journal; Peer Reviewed Journal
KW - PsycInfo
KW - Behavior Problems
KW - Brain Damage
KW - Models
KW - Parent Child Relations
KW - Rehabilitation
KW - Distress
KW - Offspring
KW - Quality of Life
RP - NOT IN FILE
SP - 88
EP - 104
JF - The Journal of Head Trauma Rehabilitation

VL - 18:
IS - 1
N2 - Background: Individuals with brain injury are prone to severe behavior problems related to various postinjury variables, including confusion, emotional distress, fear of challenging activity, and loss of skills to access desired outcomes. Objectives: This article proposes the use of a new model, errorless rehabilitation, to improve rehabilitation outcomes by systematically focusing on graduated and success-based exposure of clients with brain injury to increasingly demanding circumstances, to provide them with the ability to tolerate and manage the natural environment. Main Outcome Measures: Research on difficulties parents with brain injury experience with their children is subsequently reviewed and a new approach (based on the same principles as Errorless Rehabilitation) to treatment of parent-child relationship issues after brain injury is described. Conclusions: A case study of a father with brain injury and his oppositional son is presented to demonstrate a specific application of this model for enhancing the quality of life of parents with such impairments. (PsycINFO Database Record (c) 2012 APA, all rights reserved) (journal abstract)
SN - 0885-9701
AD - Ducharme, Joseph M.: University of Toronto and Hamilton Health Sciences, Toronto, ON, Canada
ER -

TY - JOUR
ID - 2784
T1 - Accessibility or obstacles? Children's independent mobility and valuation of the outdoor environment. [References]
A1 - Heurlin-Norinder,Mia
Y1 - 2003///
N1 - Book: 2003-00929-012
English
Book; Edited Book
KW - PsycInfo
KW - Aesthetic Preferences
KW - Community Development
KW - Commuting (Travel)
KW - Psychosocial Factors
KW - Spatial Organization
KW - Child Attitudes
KW - Safety
RP - NOT IN FILE
SP - 161
EP - 172
JF - Moser, Gabriel [Ed]; Pol, Enric [Ed]; Bernard, Yvonne [Ed]; Bonnes, Mirilia [Ed]; Corraliza, Jose Antonio [Ed]; Giuliani, Maria Vittoria [Ed]
VL - :
IS - 2003
N2 - (from the chapter) The author shows that children's design preferences for neighbourhoods are related to their degree of independent mobility and how they evaluate their environment. Analyses point to differences concerning the interaction with the physical environment and social aspects of the neighbourhood environment, in terms of affordances and individual evaluations. In the first study 732 children (8 and 11 years old) in 9 schools filled in questionnaires concerning, for example, how they had come to school on that particular day, how they used to travel to activities after school, if they were allowed to visit friends and go out and play without being followed by any adults. The children also made drawings showing their way to school. The aim of the second, qualitative study was to get a deeper understanding of differences that were found in the first study, but also the meaning of the neighbourhood's planning and characteristics related to children's independent mobility. Traffic-separated areas seem to be very positive for children's independent mobility, but they are not enough if you want the neighbourhood to be stimulating, developing and safe. There is more to it. (PsycINFO Database Record (c) 2012 APA, all rights reserved)
AD - Heurlin-Norinder, Mia: Stockholm Inst of Education, The Dept of Society, Culture & Learning, Research Group in Environmental Psychology & Education, Stockholm, Sweden
ER -

TY - JOUR
ID - 2785
T1 - 'I forgot the sky!' children's stories contained within their drawings. [References]
A1 - Coates,Elizabeth
Y1 - 2002///
N1 - Peer Reviewed Journal: 2004-17069-002
English
Journal; Peer Reviewed Journal
KW - PsycInfo
KW - Drawing
KW - Narratives
KW - Storytelling
KW - Symbolism
RP - NOT IN FILE
SP - 21
EP - 35
JF - International Journal of Early Years Education
VL - 10:
IS - 1
N2 - What stories do young children's drawings contain? So often children's free drawings are not held in high regard but are seen as an activity which is undertaken outside lesson time or as an illustration to some prescribed task. However, the concentration and care with which some children undertake a drawing suggests that the content has real significance and has not been undertaken lightly. This small-scale study arose from observations in a school setting of young children talking to themselves as they drew pictures. Often it seemed that this talk was a rehearsal for the drawing and it was this link that the project set out to explore. Children aged from 3 to 7 years were studied in their classrooms where they were participating in a free choice of activities. This paper examines the drawings collected together with transcriptions of the children's descriptions of their drawings. It looks at the symbolism contained within the pictures and the stories woven within each one. The study is intended as a pilot for a longitudinal research project focusing not only on children's drawings and accompanying narrative but extending to an exploration of emergent writing. (PsycINFO Database Record (c) 2012 APA, all rights reserved) (journal abstract)
SN - 0966-9760
AD - Coates, Elizabeth: Institute of Education, University of Warwick, Coventry, United Kingdom
ER -

TY - JOUR
ID - 2786
T1 - Hanging out: Community-based after-school program for children. [References]
A1 - Garner,Ruth
Y1 - 2002///
N1 - Book: 2004-12814-000
English
Book; Edited Book
(Created by PsycINFO) Introduction: Hanging Out [by] Ruth Garner * An Alternative to Self-Care in a Small Midwestern Town / Ruth Garner, Yong Zliao, and Mark Gillingham / 1-17 * After-School Literacy Clubs: A Mix of Media, Books, and Desires / Donna E. Alvermann, Margaret C. Hagood, Alison Heron, Josephine Peyton Young, and Beth Jones Ricks / 19-40 * University Students Promoting Science in the Community / Susan P. Bruce and Bertram C. Bruce / 41-57 * The Persistence of Vision: A Reflective Examination and Narrative of Sustainability and Fulfillment in a Small After-School Program / Dean J. Grosshandler and Elizabeth Niswander Grosshandler / 59-74 * Experiments with Design in an After-School Asian Literature Club / Sapna Vyas and Punyashloke Mishra / 75-91 * After-School Tutoring and Children at Risk / Barbara A. Wasik, Mary Alice Bond, and Annemarie Hindman / 95-112 * Working at Play in Four Chicago Computer Clubs / Ellen Youniss, Mark Gillingham, Donna Nowatzki, and Erin Roche / 113-135 * Cultural Practices in Fifth Dimension Sites Around the World / Margaret A. Gallego and William E. Blanton / 137-147 * Commentary: Ingredients of Successful After-School Programs-The Experience of KLICK / Yong Zhao and Mark Gillingham / 149-152 * Commentary: Crossing Lines / Patricia A. Alexander and Jonna M. Kulikowich /

153-166 * Commentary: After-School Programs and Structured Activities that Support Children's Development / Deborah Lowe Vandell and Kim M. Pierce / 167-178 Index About the Editors and Contributors
KW - PsycInfo
KW - After School Programs
KW - Child Care
KW - Community Facilities
KW - Educational Programs
KW - School Facilities
RP - NOT IN FILE
SP - Westport, CT, US
JF - (2002)
VL - Hanging out: Community-based after-school program for children. ix, 186 pp. Westport, CT, US: Bergin & Garvey; US.
N2 - (from the introduction) This book is a collection of accounts of what children do after school, both outside and inside (especially inside) after-school centers. Centers are affiliated with communities, and because different communities have different values, different ways of doing things, centers differ. Readers will encounter centers in public schools, in libraries, and in labs. They'll find them in a small town, in small cities near large universities, and in big cities. They'll read about one cluster of centers that began in an urban setting but migrated to other communities, both in the United States and elsewhere. Readers will encounter academic and nonacademic centers, centers for young children and for older children, centers that provide activities for 5 children at a time and for 50. They'll see that some centers, like Baldwin's, are technology-oriented, whereas others emphasize reading, writing, or science activities. They'll note that some large multicenter programs have large multiyear grants, whereas some small operations just get by. In reading the accounts, readers will notice that the centers described by contributing authors, varied though they might be, have informality in common. Contributing authors have been invited to say something about how we should judge the work of an after-school center. (PsycINFO Database Record (c) 2012 APA, all rights reserved)
AD - Zhao, Yong: Michigan State University, MI, US Gillingham, Mark: Great Books Foundation, Chicago, IL, US
ER -

TY - JOUR
ID - 2787
T1 - Changing antisocial behavior patterns in young boys: A structured cooperative learning approach. [References]
A1 - Quinn,Mary Magee
Y1 - 2002///
N1 - Peer Reviewed Journal: 2003-07473-002
English
Journal; Peer Reviewed Journal
KW - PsycInfo
KW - Antisocial Behavior
KW - At Risk Populations
KW - Behavior Change
KW - Cooperative Learning
KW - School Based Intervention
KW - Elementary School Students
KW - Human Males
RP - NOT IN FILE
SP - 380
EP - 395
JF - Education & Treatment of Children
VL - 25:
IS - 4
N2 - This study examined the effectiveness of using a structured cooperative learning approach to increase appropriate behaviors of young boys who were identified as being at risk for the development of antisocial behavior patterns. Students received a six-week cooperative learning intervention using positive peer role models to teach interpersonal problem-solving skills through the combined use of cognitive and behavioral

techniques. Results show a significant increase in academic engaged time; however, the intervention failed to produce significantly lower rates of externalizing antisocial behaviors or negative playground interactions. (PsycINFO Database Record (c) 2012 APA, all rights reserved) (journal abstract)
SN - 0748-8491
AD - Quinn, Mary Magee: American Insts for Research, Washington, DC, US
ER -

TY - JOUR
ID - 2788
T1 - Activities and staffing patterns in therapeutic wilderness camps: A national survey. [References]
A1 - Fuentes,Angel Ilarraza; Burns,Ronald
Y1 - 2002///
N1 - Peer Reviewed Journal: 2003-06868-008
English
Journal; Peer Reviewed Journal
KW - PsycInfo
KW - Criminal Justice
KW - Juvenile Delinquency
KW - Organizational Characteristics
KW - Organizations
KW - Wilderness Experience
KW - Goals
KW - Management Personnel
RP - NOT IN FILE
SP - 41
EP - 62
JF - Journal of Offender Rehabilitation
VL - 35:
IS - 1
N2 - Describes therapeutic wilderness programming (TWP) as it is currently being used within the context of American Juvenile Justice. Based upon a review of the TWP literature and their personal familiarity with these programs, the authors developed a series of questions designed to provide additional insight regarding the structure, characteristics, and goals of TWP. The surveys were addressed to the chief administrative officer or executive director at each program. 35 of the original 106 surveys were returned for a 33.0 completion rate. Among other things, it was found that such programs are primarily intended to provide an alternative to punitive confinement, with the main goal of rehabilitating offenders. It was found that academic education, backpacking, and canoeing are the most popular means to achieve this goal. Findings are presented with regard to camp size, and camp staff/personnel issues are discussed. Finally, it is also noted that most of the literature regarding wilderness programs is found outside traditional criminal justice-, juvenile justice-, and criminology journals, despite the finding that states are heavily relying upon wilderness types of programs within their respective juvenile justice systems. (PsycINFO Database Record (c) 2012 APA, all rights reserved)
SN - 1050-9674
AD - Fuentes, Angel Ilarraza: Texas Christian U, Ft Worth, TX, US Burns, Ronald: Texas Christian U, Ft Worth, TX, US
ER -

TY - JOUR
ID - 2789
T1 - Influences and Mediators of the Effect of Poverty on Young Adolescent Depressive Symptoms. [References]
A1 - Eamon,Mary Keegan
Y1 - 2002///
N1 - Peer Reviewed Journal: 2003-06226-007
English
Journal; Peer Reviewed Journal
KW - PsycInfo
KW - Major Depression

KW - Mothers
KW - Parenting Style
KW - Poverty
RP - NOT IN FILE
SP - 231
EP - 242
JF - Journal of Youth and Adolescence
VL - 31:
IS - 3
N2 - Data from a sample of young adolescents between the ages of 10 and 12 years (N = 898) from the mother-child data set of the National Longitudinal Survey of Youth were analyzed in a study of influences that explain the relation between poverty and depressive symptoms measured 2 years later. Other variables that predicted youth depressive symptoms were also identified. Results indicated that neighborhood problems, nonparticipation in outside school and neighborhood activities, residing with mothers who exhibited depressive symptoms, and mother's use of physical punishment were partial mediators of the effect of poverty on depressive symptoms 2 years later. Youth health status, lower levels of school satisfaction, marital-partner conflict, and father's emotional support also predicted depressive symptoms. The findings indicate that youth depressive symptoms are multiply determined and that poverty can adversely affect young adolescents in many ways. (PsycINFO Database Record (c) 2012 APA, all rights reserved) (journal abstract)
SN - 0047-2891
AD - Eamon, Mary Keegan: U Illinois at Urbana-Champaign, School of Social Work, Urbana, IL, US
ER -

TY - JOUR
ID - 2790
T1 - Formative assessment of social competence: Indicators of success in authentic elementary school tasks
A1 - Doyle,Joleen Marie
Y1 - 2002///
N1 - Dissertation Abstract: 2002-95023-062
English
Dissertation Abstract
KW - PsycInfo
KW - Academic Achievement
KW - Educational Measurement
KW - Social Skills
RP - NOT IN FILE
SP - 2128
JF - Dissertation Abstracts International Section A: Humanities and Social Sciences
VL - 63:
IS - 6-A
N2 - Student outcomes are improved when ongoing intervention decisions are based upon formative assessment data. In the area of social competence, existing assessment instruments do not meet all criteria for optimal formative assessment tools. A brief, simple observation tool (ISC: Indicators of Social Competence) was designed around broad indicator behaviors that could reflect overall student performance. Research questions centered on reliability (stability and representativeness) and concurrent criterion-related validity of ISC data. ISC observations were conducted across days with 48 third-grade students during authentic school tasks (independent seatwork and unstructured free play on the playground). Parent and teacher ratings of student social competence served as criterion measures. Reliability. Most correlations reflecting stability of ISC data across days were statistically significant, but in the weak to moderate range. ISC data from the first half of a task period (i.e., seatwork or recess) adequately represented ISC data obtained from the entire task period. The stability and representativeness results suggest that collecting several brief samples will produce the highest quality data per investment of time. Validity. Three of four ISC behaviors correlated significantly with teacher judgments of social competence. None of the correlations between ISC data and parent judgments of social competence reached statistical significance. These results suggest that the ISC measure adequately reflects the social behaviors that are important for success in academic and free play tasks. Practitioners could consider using the ISC measure for formative assessment purposes, after reviewing its strengths and limitations, their data needs, and other available social assessment instruments. Researchers are encouraged to review the

challenges and outcomes of this project in their efforts to advance our assessment technology. (PsycINFO Database Record (c) 2012 APA, all rights reserved)
SN - 0419-4209
AD - Doyle, Joleen Marie: U Oregon, US
ER -

TY - JOUR
ID - 2791
T1 - "Everybody get page by page": How children use peer reading talk and interaction to construct and to cross lines of gender and popularity
A1 - Bower,Aviva Donahue
Y1 - 2002///
N1 - Dissertation Abstract: 2002-95021-035
English
Dissertation Abstract
KW - PsycInfo
KW - Elementary School Students
KW - Interpersonal Interaction
KW - Peer Relations
KW - Popularity
KW - Reading
RP - NOT IN FILE
SP - 1705
JF - Dissertation Abstracts International Section A: Humanities and Social Sciences
VL - 63:
IS - 5-A
N2 - This qualitative study of an ethnically diverse urban third grade classroom asks how children used their peer structured talk and interactions during reading activities to negotiate peer identities and relationships. The study addresses the broad issue of how official literacy practices and children's unofficial social worlds are interwoven, drawing upon and mutually transforming one another. Theories of language socialization (Ochs, 1996), interactional sociolinguistics (Goffman, 1981), and interpretive approaches to child socialization (Gaskins, Miller, & Corsaro, 1992) frame the study; methods from ethnography of communication and ethnography (Erickson, 1992; Walcott, 1994, 1995) are used to create an analytic framework. Children's talk and interaction are analyzed as agentive social work during two reading activities that involved peer structured discussion of text: guided reading and self-selected reading. Data collected over a five month period include field notes, audio taped interviews with teacher and children, and video taped data. The findings suggest that reading activities provided opportunities to both maintain and to rework salient social categories of gender and popularity. The analysis of four children's talk during one guided reading activity shows how a less popular boy was able to reshape the official literacy activity so that it more closely resembled a unofficial peer interaction that had taken place earlier. He used the literacy activity to rework his social alignment to the girls at his table, and to ease gender and popularity divides. The analysis of self-selected reading activities shows that partner reading during self-selected reading was a girl gendered activity. Boys and girls could partner read together, but this was not easy to organize. There were, however, socially meaningful alternatives to partner reading during self-selected reading. Boys and less popular girls used talk during self-selected reading time outside of partner reads to affiliate with peers and to reshape the constraints of gender and popularity divides. The study suggests that peer structured reading talks may be a particularly rich arena in which children can work and rework constructions of peer identity, and that more research is needed to understand how to support children's efforts to use peer reading in this way. (PsycINFO Database Record (c) 2012 APA, all rights reserved)
SN - 0419-4209
AD - Bower, Aviva Donahue: State U New York At Buffalo, US
ER -

TY - JOUR
ID - 2792
T1 - Gender differences in social acceptance and play behavior
A1 - Sondell,Sandra Kuperman
Y1 - 2002///

N1 - Dissertation Abstract: 2002-95020-252
English
Dissertation Abstract
KW - PsycInfo
KW - Childhood Play Behavior
KW - Human Sex Differences
KW - Preferences
KW - Social Acceptance
RP - NOT IN FILE
SP - 2076
JF - Dissertation Abstracts International: Section B: The Sciences and Engineering
VL - 63:
IS - 4-B
N2 - Kindergarten, first, and second grade children and their teachers were recruited from an elementary school in Oak Park, Illinois to examine social preferences and play behavior. Past research (e.g. Ladd, 1983; Pellegrini, Blatchford, & Kato, 2001) has found gender differences in children's behavior on the playground. These studies have also suggested that boys rejected by their classmates are often found to be playing with girls. At this age, where gender is a key factor in children's thoughts and actions, these findings led us to question what happens when children are rejected by their same-sex peers, but accepted by their opposite-sex peers. This study examined the differences in play behavior across gender and social status. To add to the existing literature, it explored the behavior profiles of children who are accepted by opposite-gender peers. Finally, it evaluated how teachers perceive the children who are accepted by opposite-gender peers. Findings indicated distinct behavior profiles for boys and girls at this young age: Boys played in cooperative, rule-governed activities, while girls engaged in associative, social conversation behaviors. Boys who were rejected by their same-gender peers ("Non-normal Boys"), but accepted by opposite gender peers did not show differences in play behavior when compared to the group of "Normal Boys." "Non-normal Girls" were found to have a distinct play profile compared to the other groups of children, marked by a significantly higher frequency of solitary play. No differences were found in teacher ratings across the groups of children. (PsycINFO Database Record (c) 2012 APA, all rights reserved)
SN - 0419-4217
AD - Sondell, Sandra Kuperman: Illinois Inst Technology, US
ER -

TY - JOUR
ID - 2793
T1 - Play behavior and peer status of five to seven year old children as a function of cognitive development
A1 - Azzi,Lorenzo George
Y1 - 2002///
N1 - Dissertation Abstract: 2002-95020-248
English
Dissertation Abstract
KW - PsycInfo
KW - Childhood Development
KW - Childhood Play Behavior
KW - Cognitive Development
KW - Peer Relations
KW - Status
RP - NOT IN FILE
SP - 2087
JF - Dissertation Abstracts International: Section B: The Sciences and Engineering
VL - 63:
IS - 4-B
N2 - A great deal of changes occur in the lives of children between the ages of five to seven. Foremost in these changes is entry into a formal school environment and the shift from preoperational thought to concrete operational thought. During this time, a child also has the important task of negotiating social exchanges with same-aged peers, a relatively new task for many children. A child's social competence is closely related to a wide variety of factors that determine success in social and academic circles. Further understanding of how

children acquire their social status may be an instrumental first step in assisting children to have more social success and avoid the pitfalls associated with rejected peer status. The results of this study demonstrate the importance of cognitive developmental level when examining the social behavior of five to seven year old children. Results indicate that the social play behavior of children on playgrounds serves as a partial mediator in the relationship between cognitive developmental level and social status. (PsycINFO Database Record (c) 2012 APA, all rights reserved)
SN - 0419-4217
AD - Azzi, Lorenzo George: Illinois Inst Technology, US
ER -

TY - JOUR
ID - 2794
T1 - The effect of classroom meetings on the reduction of recess problems: A single case design
A1 - Murphy,Patrick Sean
Y1 - 2002///
N1 - Dissertation Abstract: 2002-95019-070
English
Dissertation Abstract
KW - PsycInfo
KW - Classroom Behavior
KW - Elementary School Students
RP - NOT IN FILE
SP - 1256
JF - Dissertation Abstracts International Section A: Humanities and Social Sciences
VL - 63:
IS - 4-A
N2 - This paper will establish classroom meetings as an effective problem-solving intervention in the reduction of recess problems. A multiple baseline across groups analysis was conducted of problems related to recess in three 4/5 th grade classrooms. Social problem-solving classroom meetings were introduced in each class and staggered every two weeks following two weeks of baseline for each class. This study was conducted over 9 weeks with daily data collection of recess reports and teacher surveys. Classroom meetings were effective in reducing the overall reports of recess problems. They were most effective in reducing problems of inclusion, playing alone and not joining others' play. Classroom meetings had a lesser effect on reports of Conflict problems, especially arguing. Implications of the study, including recommendations and suggestions for further study are addressed. (PsycINFO Database Record (c) 2012 APA, all rights reserved)
SN - 0419-4209
AD - Murphy, Patrick Sean: U Denver, US
ER -

TY - JOUR
ID - 2795
T1 - The virtual classroom: An application in the assessment of attention deficit-hyperactivity disorder (ADHD)
A1 - Bowerly,Todd David
Y1 - 2002///
N1 - Dissertation Abstract: 2002-95018-192
English
Dissertation Abstract
KW - PsycInfo
KW - Attention Deficit Disorder with Hyperactivity
KW - Neuropsychological Assessment
KW - Virtual Classrooms
KW - Virtual Reality
RP - NOT IN FILE
SP - 1554
JF - Dissertation Abstracts International: Section B: The Sciences and Engineering
VL - 63:

IS - 3-B
N2 - Virtual reality (VR) technology applications targeting neuropsychological assessment and rehabilitation are now feasible and offer advantages that are not available using existing methods. The pilot study evaluated a VR Head-Mounted Display "classroom environment" designed for the assessment of Attention Deficit-Hyperactivity Disorder (ADHD). The Virtual Classroom scenario presents a standard classroom environment containing desks, plants, a teacher, blackboard, pictures on the walls, a large window looking out onto a street with vehicles and a playground, and a pair of doorways through which activity occurs. Within The Virtual Classroom, participants are presented with a traditional AX continuous performance test (CPT) on the blackboard and attention performance (RT) and other motor activity (head-turning, arm and leg-shaking) are recorded while a series of typical classroom distracters (i.e., activity and sounds) are systematically presented. Data were collected on 10 ADHD diagnosed children (aged 8 to 12) and 10 undiagnosed normal control children, comparing performance on the AX task and a basic listen-look-respond task, with and without distractions. VR exposure consisted of three conditions presented for 10 minutes each. Condition 1 measured performance on the AX task without distractions. Condition 2 was the same task with distractions included. Condition 3 consisted of a more realistic "ecologically valid" attention task requiring the integration of audio and visual attention processes. A battery of standard psychometric tests was administered to all participants for comparison with findings from the VR scenario. It was found that The Virtual Classroom scenario was able to differentiate between ADHD children and normal controls and in the expected direction. Within The Virtual Classroom, ADHD children responded with more omission and commission errors and greater levels of hyperactivity than normal controls among all conditions. ADHD children displayed more distractibility in their response to distractions than normal controls. Virtual Classroom measures were highly correlated with tests traditionally used to diagnose ADHD, the behavior checklist and CPT. The Virtual Classroom brings specific assets and improvements to the existing procedures and protocol for the assessment of ADHD. These assets and improvements strongly support the continued development and refinement of this virtual assessment tool. Suggestions for future research and clinical implications are discussed. (PsycINFO Database Record (c) 2012 APA, all rights reserved)
SN - 0419-4217
AD - Bowerly, Todd David: Fuller Theological Seminary, School Of Psychology, US
ER -

TY - JOUR
ID - 2796
T1 - Rough-and-tumble between parents and children and children's social competence
A1 - Mellen,Henry S.
Y1 - 2002///
N1 - Dissertation Abstract: 2002-95018-125
English
Dissertation Abstract
KW - PsycInfo
KW - Childhood Play Behavior
KW - Parent Child Relations
KW - Social Skills
RP - NOT IN FILE
SP - 1588
JF - Dissertation Abstracts International: Section B: The Sciences and Engineering
VL - 63:
IS - 3-B
N2 - The purpose of this study was to determine whether rough-and-tumble was a dimension of parent-child physical play and whether parent-child rough-and-tumble was related to preschool children's social competence. Participants were 157 married parents and 56 preschool teachers from the suburbs of a large northeastern metropolis. The core hypothesis was that father-son rough-and-tumble would be related to sons' social competence. It was predicated on the example of playground rough-and-tumble, a predominantly male activity that has been linked with the social competence of schoolboys. Both parents completed the Physical Play Questionnaire. The PPQ was a measure developed by the researcher to quantify frequencies of parent-child physical play. Fathers, mothers, and teachers rated preschool children with the Social Competence and Behavior Evaluation. Additionally, fathers and mothers filled out the Ray Directiveness Scale. Parental directive behavior was believed to be a mediating variable. Rough-and-tumble emerged as one of four principal factors of physical

play between parents and preschool children. Fathers and sons rough-and-tumbled approximately twice as much as mothers and daughters, respectively. Father-son rough-and-tumble was not associated with son's social competence, but father-son intimate physical play was. (Parental directiveness had no material effect on any relation). Additional analyses revealed that father-son rough-and-tumble behaviors that involved direct body contact were related moderately with sons' social competence and very strongly with 3-year-old sons' social competence. No kind of parental physical play was linked with daughters' social competence. The results of this study confirm that rough-and-tumble was both an intergenerational and domestic phenomenon. The quality of father-son rough-and-tumble was as critical to the contribution to sons' social competence as was the frequency. Because its benefits were both age- and gender-specific, rough-and-tumble emerged as an important factor for future research into the developmental course of parent-child play and parental gender-role socialization of children. (PsycINFO Database Record (c) 2012 APA, all rights reserved)
SN - 0419-4217
AD - Mellen, Henry S.: Adelphi U, Inst Advanced Psychological Studies, US
ER -

TY - JOUR
ID - 2797
T1 - Effects of adventure education utilizing a portable climbing wall activity on children's self-efficacy
A1 - Newhouse,Gretchen C.
Y1 - 2002///
N1 - Dissertation Abstract: 2002-95016-376
English
Dissertation Abstract
KW - PsycInfo
KW - Self Efficacy
KW - Wilderness Experience
RP - NOT IN FILE
SP - 763
JF - Dissertation Abstracts International: Section B: The Sciences and Engineering
VL - 63:
IS - 2-B
N2 - The purpose of this study was to compare the effectiveness of an intervention consisting of an adventure education activity using a climbing wall (Treadwall) activity with no intervention as a means for enhancing children's general self-efficacy. A total of five fourth-grade and five fifth-grade classes were involved in the study, including 84 fourth-grade and 87 fifth-grade students. The researcher utilized Cowen's et al. (1991) Perceived Self-Efficacy Scale in assessing experimental and comparison group participants' general self-efficacy at a pretest and posttest, with one week between the tests. The 20-item Perceived Self-Efficacy Scale contains three subscales that consist of: Difficult Situations, New Experiences, and Problems with People. The data was analyzed using the t-test of independent means to compare pretest and posttest scores on the Perceived Self-efficacy Scale, Difficult Situations Subscale, New Experiences Subscale, and Problems with People Subscale across the experimental and comparison groups. ANCOVA (Analysis of Covariance) was used when initial differences in groups were determined to potentially influence the posttest results. Based upon data analysis, findings revealed that significant differences were found in the Problems with People Subscale between fourth- and fifth-graders who participated in the three-hour Treadwall activity, and those who were assigned to the comparison group. No significant differences were found between the experimental and comparison groups for the Perceived Self-efficacy Scale, Difficult Situations Subscale and New Experiences Subscale. (PsycINFO Database Record (c) 2012 APA, all rights reserved)
SN - 0419-4217
AD - Newhouse, Gretchen C.: U New Mexico, US
ER -

TY - JOUR
ID - 2798
T1 - Organizational features as facilitators of youth gang aggression and antisocial behavior
A1 - Wyrick,Phelan Alexander
Y1 - 2002///

N1 - Dissertation Abstract: 2002-95014-116
English
Dissertation Abstract
KW - PsycInfo
KW - Aggressive Behavior
KW - Antisocial Behavior
KW - Juvenile Gangs
RP - NOT IN FILE
SP - 593
JF - Dissertation Abstracts International: Section B: The Sciences and Engineering
VL - 63:
IS - 1-B
N2 - Youth gangs are the subject of widespread fear and fascination in large part because of the high levels of violent and illegal behavior that gang members commit. Recent longitudinal studies have demonstrated that elevated levels of gang member offending are the consequence of group processes that facilitate such offending, rather than selection processes in which gangs recruit youth who are already high level offenders. Group cohesiveness and norms supporting aggression and antisocial behavior have been discussed as processes likely to play a role in facilitating offending. However, the importance and sophistication of youth gang organizational characteristics has been a topic of considerable debate among gang researchers and practitioners. Many experts view youth gangs as loosely affiliated groups with low levels of organization. But this view stands in contrast to evidence that many gangs possess characteristics suggestive of organization (e.g., leaders, identifiable symbols, regular meetings). In the current study, a conceptual framework is developed for considering the role of organizational features in facilitating gang member offending, and the relationship between these features and four categories of offending is tested using hierarchical multiple regression. Data are drawn from open ended interviews with 140 gang members in Denver, Colorado, Aurora, Colorado, and Broward County, Florida. Results show that organizational features, including the presence of gang leadership, role differentiation, codes of conduct, initiation rituals, subgroups, and regular meetings, account for a significant amount of variance in gang member self-reported violent, property, and weapons offending, but not drug sales. Organizational features that appear to make the most important individual contributions are regular meetings and codes of conduct. The findings demonstrate that organizational features of youth gangs should not be dismissed in trying to understand gang member offending. Although youth gangs may not reflect high levels of organizational sophistication by outside or objective standards, the presence of organizational features is related to higher levels of offending among gang members. It is likely that organizational features serve to bring individual gang members into closer adherence with group norms that support aggressive and antisocial behavior. (PsycINFO Database Record (c) 2012 APA, all rights reserved)
SN - 0419-4217
AD - Wyrick, Phelan Alexander: The Claremont Graduate U., US
ER -

TY - JOUR
ID - 2799
T1 - Behavioral transitions in children with autism compared to children with Down syndrome in their natural habitats
A1 - Brown,Shannon Ann
Y1 - 2002///
N1 - Dissertation Abstract: 2002-95013-137
English
Dissertation Abstract
KW - PsycInfo
KW - Autism
KW - Behavior Change
KW - Down's Syndrome
RP - NOT IN FILE
SP - 79
JF - Dissertation Abstracts International Section A: Humanities and Social Sciences
VL - 63:
IS - 1-A

N2 - It has been assumed that children with autism have difficulty transitioning from one activity to another, but there has been little systematic investigation of the transitioning behavior of children with autism in their natural environments. The methods of ecological psychology were used in this study to explore how children with autism transition from one activity to another in their natural environments and how the transitions made by children with autism compare to those made by children with Down syndrome. A new construct, the behavioral transition unit (BTU), was developed for use in this study. Eight boys with autism and 8 boys with Down syndrome between the ages of 6 and 10 years were observed in their homes using the methods of ecological psychology. The structure of the transitions and the behavior of the children and others in their homes during transitions were examined. It was found that the boys with autism spent more time transitioning and had more frequent BTUs, which indicates a more immature pattern of behavior than that exhibited by the boys with Down syndrome. The boys with autism also took more time beginning activities and less time ending activities than did the boys with Down syndrome. This may reflect particular cognitive deficits seen in people with autism. Family members and caregivers were more likely to become involved in the activities of the autism group. In addition, the children with Down syndrome had the goal of getting attention or interacting with others more often then did the children with autism. There were some interesting similarities between the groups also. The relationship of these results to other research and theory in autism is discussed. (PsycINFO Database Record (c) 2012 APA, all rights reserved)
SN - 0419-4209
AD - Brown, Shannon Ann: Indiana U., US
ER -

TY - JOUR
ID - 2800
T1 - Relational aggression: An analysis of early childhood indicators
A1 - Suarez,Mariann
Y1 - 2002///
N1 - Dissertation Abstract: 2002-95010-248
English
Dissertation Abstract
KW - PsycInfo
KW - Aggressive Behavior
KW - Interpersonal Interaction
KW - Verbal Communication
KW - Relational Aggression
RP - NOT IN FILE
SP - 5395
JF - Dissertation Abstracts International: Section B: The Sciences and Engineering
VL - 62:
IS - 11-B
N2 - The study investigated the development of verbal/physical and relational aggression in a community sample of five to seven year old children (n = 161). Playground behavioral observations and peer nominations were used to assess gender differences and continuity in levels of perpetrating and being victimized by verbal/physical and relational aggression across a 12-month period, as well as to examine the convergence between measures (i.e. self-report and behavioral observation). Results showed significant gender differences in displays of aggression. Males were more often perpetrators and victims of verbal/physical and relational aggression in comparison to same aged females. Males and females were found to engage in icing amounts of verbal/physical aggression with age, with males showing a higher level of verbal/physical aggression at younger ages and continuing with this trend as they mature. In contrast, males and females did not demonstrate similar age-related change in relational aggression. As males matured, they engaged in increased displays of relational aggression, while females evidenced a decrease relational aggression. Modest levels of cross-time continuity were observed in the perpetration and being victimized by verbal/physical and relational aggression for both genders on the two measures. The covariation between verbal/physical and relational aggression for both genders was significant but greater for males. Convergence between measures was found for verbal/physical aggression, with the exception of females at the spring of kindergarten. In contrast, convergence between instruments was not found for relational aggression. For both genders, the covariation between perpetration and being victims of verbal/physical aggression was significant, with associations strengthening as children aged. The covariation between perpetration and being victims of relational aggression for both genders was less strong

than for verbal/physical aggression, and appeared to diminish with age. Results suggest that young males and females engage in verbal/physical aggression at increasing rates from age 5 to 7, with males showing rates of relational aggression that surpass those of females. Findings provide initial evidence for the importance of studying relational aggression in younger samples, as well as for the utility in incorporating observational methods in studies of aggression. (PsycINFO Database Record (c) 2012 APA, all rights reserved)
SN - 0419-4217
AD - Suarez, Mariann: Wichita State U., US
ER -

TY - JOUR
ID - 2801
T1 - No words necessary: An ethnography of daily activities with young children who don't talk. -+
A1 - Spitzer,Susan Lanell
Y1 - 2002///
N1 - Dissertation Abstract: 2002-95010-001
English
Dissertation Abstract
KW - PsycInfo
KW - Autism
KW - Daily Activities
KW - Meaning
RP - NOT IN FILE
SP - 5075
JF - Dissertation Abstracts International: Section B: The Sciences and Engineering
VL - 62:
IS - 11-B
N2 - Understanding and participating in the activities of young children, especially children with developmental disabilities such as autism, can be difficult for adults. In this qualitative study, the guiding question was "How do young children with developmental disabilities create and convey meaning through their daily activities?". The participants were a total of 5 three- and four-year-old children, with a diagnosis of autism, who did not consistently use language to communicate spontaneously. Participant-observation was used to collect data in the home and other natural settings in which the child engaged in daily activities. Interviews with key adults in the child's daily life provided supplemental information. Analysis of fieldnotes and transcripts was focused on the children's occupations, that is, meaningful, directed activities that occupied their time. Unique patterns and preferences were identified and described for each child and his or her activities. The meaning, from the child's perspective, was interpreted with close attention to how a child did the activity and its relationship to the child's needs, desires, and abilities. Findings were related to the following conceptual topics: defining occupation, framing an occupation, the nature of changes within an occupation over time, temporal links to past occupations, occupational structure, self-direction, and reframing of required activities into more satisfying occupations. Although they shared a diagnosis, through their engagement in activities, the children were seen as unique individuals with their own particular interests and abilities. Categories of the children's activities with others were identified as being equipped (accepting materials from another), guided (accepting assistance from another), mentored (incorporating another's suggestion), and shared (maintaining reciprocal interaction). The participants used their bodies, sensory perception, objects, and scripted action instead of words to mediate and focus their interaction on an activity with shared meaning. There was an implicit agreement about the shared meaning of the activity-what they were doing, why, and how. Through these shared activities, the children seemed to be connecting and building relationships with other people. Suggestions were made for balancing person-centered and family-centered services for children with developmental disabilities. (PsycINFO Database Record (c) 2012 APA, all rights reserved)
SN - 0419-4217
AD - Spitzer, Susan Lanell: U Southern California, US
ER -

TY - JOUR
ID - 2802
T1 - Children, attention, and nature: Evidence from AD/HD and poor urban populations
A1 - Faber Taylor,Andrea Jean

Y1 - 2002///
N1 - Dissertation Abstract: 2002-95004-237
English
Dissertation Abstract
KW - PsycInfo
KW - Attention
KW - Attention Deficit Disorder with Hyperactivity
KW - Ecology
KW - Urban Environments
RP - NOT IN FILE
SP - 3827
JF - Dissertation Abstracts International: Section B: The Sciences and Engineering
VL - 62:
IS - 8-B
N2 - Children's attentional functioning impacts both their ability to perform everyday tasks and their potential for success in life. For example, children need a robust capacity to direct their attention for academic and social success. Can nature support children's directed attention? Attention Restoration Theory (Kaplan & Kaplan, 1989; Kaplan 1995) proposes that natural environments contain qualities that foster attentional restoration by allowing directed attention to rest. A growing body of evidence with adults indicates that nature does indeed support attention. We know little, however, about nature's potential for supporting children's attention. This dissertation examines whether nature in children's everyday environments is related to their attentional functioning. Two studies were conducted, each focusing on a distinct population of 7-12 year old children. One study examined 96 children with Attention Deficit Disorder living in predominately middle and upper-class neighborhoods with relatively high levels of nature. Data were collected through a written survey and follow-up focus group. The other study examined 169 children with normal attentional functioning, living in an inner city setting with relatively low levels of nature. In this study, data were collected through one-on-one interviews and objective performance measures. Is children's contact with everyday nature linked to their attentional functioning? This question was addressed through analyses involving both within and between-subjects comparisons, and multiple forms of everyday nature-activity setting, playspace, and residential nature. Numerous findings indicated that contact with everyday nature is related to better attentional functioning. In addition, some gender differences in the nature-attention relationship were discovered. The studies here are among the first to provide evidence for a systematic positive link between nature and attention in children. This work also contributes to Attention Restoration Theory, and the literature on the benefits of views of nature. Implications for design of children's spaces are discussed. (PsycINFO Database Record (c) 2012 APA, all rights reserved)
SN - 0419-4217
AD - Faber Taylor, Andrea Jean: U Illinois At Urbana-Champaign, US
ER -

TY - JOUR
ID - 2803
T1 - Views of nature and self-discipline: Evidence from inner city children. [References]
A1 - Taylor,Andrea Faber; Kuo,Frances E; Sullivan,William
Y1 - 2002///
N1 - Peer Reviewed Journal: 2002-17166-006
English
Journal; Peer Reviewed Journal
KW - PsycInfo
KW - At Risk Populations
KW - Childhood Development
KW - Home Environment
KW - Self Control
KW - Urban Environments
KW - Academic Underachievement
KW - Adolescent Pregnancy
KW - Juvenile Delinquency
RP - NOT IN FILE

SP - 49
EP - 63
JF - Journal of Environmental Psychology
VL - 22:
IS - 1-2
N2 - Children growing up in the inner city are at risk of academic underachievement, juvenile delinquency, teenage pregnancy, and other important negative outcomes. Avoiding these outcomes requires self-discipline (SDP). SDP, in turn, may draw on directed attention, a limited resource that can be renewed through contact with nature. This study examined the relationship between near-home nature and 3 forms of SDP in 169 inner city girls and boys (aged 7-12 yrs) randomly assigned to 12 architecturally identical high-rise buildings with varying levels of nearby nature. Parent ratings of the naturalness of the view from home were used to predict children's performance on tests of concentration, impulse inhibition, and delay of gratification. Regressions indicate that, on average, the more natural a girls view from home, the better her performance at SDP. For girls, view accounted for 20% of the variance in scores on the combined SDP index. For boys, who typically spend less time playing in and around their homes, view from home showed no relationship to performance on any measure. These findings suggest that, for girls, green space immediately outside the home can help them lead more effective, self-disciplined lives. For boys, perhaps more distant green spaces are equally important. (PsycINFO Database Record (c) 2012 APA, all rights reserved)
SN - 0272-4944
AD - Taylor, Andrea Faber: U Illinois Urbana-Champaign, IL, US Kuo, Frances E.: U Illinois Urbana-Champaign, IL, US Sullivan, William C.: U Illinois Urbana-Champaign, IL, US
ER -

TY - JOUR
ID - 2804
T1 - Reducing problem behaviors on the playground: An investigation of the application of schoolwide positive behavior supports. [References]
A1 - Lewis,Timothy J.
Y1 - 2002///
N1 - Peer Reviewed Journal: 2002-12068-005
English
Journal; Peer Reviewed Journal
KW - PsycInfo
KW - Behavior Modification
KW - Behavior Problems
KW - Childhood Play Behavior
KW - Early Intervention
KW - Elementary School Students
KW - Childhood Development
KW - Playgrounds
KW - School Environment
RP - NOT IN FILE
SP - 181
EP - 190
JF - Psychology in the Schools
VL - 39:
IS - 2
N2 - In an effort to reduce problem behavior, it is recommended that schools develop a continuum of positive behavior support (PBS) that focuses on prevention/early intervention as well as individualized student support systems for those students who display chronic problem behaviors. While there is a large database to support the use of PBS practices at the individual student level, the knowledge base supporting prevention/early intervention strategies is characterized as emerging. The purpose of this study was to investigate the efficacy of PBS prevention/early intervention strategies on the rate of problem behavior displayed by 450 elementary school students during recess. Specifically, this study examined the effects of directly teaching playground-related behaviors and the use of a group contingency to reinforce mastery in an elementary school. Results indicated that the intervention reduced the frequency of problem behavior across three recess periods. Results

are discussed with respect to support of an emerging empirical database and implications for practice. (PsycINFO Database Record (c) 2012 APA, all rights reserved)
SN - 0033-3085
AD - Lewis, Timothy J.: U Missouri--Columbia, Columbia, MO, US Powers, Lisa J.: U Missouri--Columbia, Columbia, MO, US Kelk, Michele J.: U Missouri--Columbia, Columbia, MO, US Newcomer, Lori L.: U Missouri--Columbia, Columbia, MO, US
ER -

TY - JOUR
ID - 2805
T1 - An observational study of preschooler's aggressive behavior in free play, in relation to gender and peer group status. [Japanese]. [References]
A1 - Hatakeyama,Miho
Y1 - 2002///
N1 - Peer Reviewed Journal: 2002-11631-004
Japanese
Journal; Peer Reviewed Journal
KW - PsycInfo
KW - Aggressive Behavior
KW - Childhood Play Behavior
KW - Human Sex Differences
KW - Peer Relations
KW - Status
KW - Social Isolation
RP - NOT IN FILE
SP - 252
EP - 260
JF - Japanese Journal of Developmental Psychology
VL - 13:
IS - 3
N2 - Examined aggressive behaviors of preschool children according to the context in which they occur and examined subtypes of aggressive behavior in relation to gender, peer group status, and numbers of aggressive children. Four-and 5-yr-old preschoolers (16 boys and 18 girls) were observed in a natural setting for 1 yr. A total of 160 aggressive behaviors were observed and classified into 3 categories: Proactive/overt aggression, instrumental/bullying, and relational aggression. Results show that boys show more proactive/overt aggression and instrumental/bullying aggression than girls, while girls show more relational aggressive behaviors than boys. In addition, children were aggressive according to their peer group status. Children who were classified as "nuclear" in their peer group had higher frequencies of relational aggressive behavior than children who were otherwise classified. Children who were classified as "isolated" suffered more than others from relational aggression. (PsycINFO Database Record (c) 2012 APA, all rights reserved)
SN - 0915-9029
AD - Hatakeyama, Miho: Hiroshima U, Faculty of Education, Japan Yamazaki, Akira: Hiroshima U, Faculty of Education, Japan
ER -

TY - JOUR
ID - 2806
T1 - Peer relationships of preadolescent students with disabilities who attend a separate school. [References]
A1 - Hall,Laura J.
Y1 - 2002///
N1 - Peer Reviewed Journal: 2002-11075-006
English
Journal; Peer Reviewed Journal
KW - PsycInfo
KW - Middle School Students
KW - Peer Relations
KW - Schools

KW - Special Education
KW - Special Education Students
KW - Peers
RP - NOT IN FILE
SP - 399
EP - 409
JF - Education & Training in Mental Retardation & Developmental Disabilities
VL - 37:
IS - 4
N2 - Research describing peer relationships of students with disabilities has focused on younger children and students attending inclusive schools. The following study incorporated multiple measures that included: peer nominations, peer interviews, and direct observations in the playground, as a means of describing the peer relationships of 7 middle-school students (aged 5-18 yrs) who attend a separate school for students with disabilities. Results were compared to those obtained using the same measures in a nearby inclusive school, for one child who attended both schools, and for typical middle-school students. Results reveal that students with disabilities who attend a separate school experience a variety of peer relationships that included reciprocal positive nominations as preferred peers as well as low social status, lack of social interaction during recess and engagement in age-inappropriate activities. Results suggest that strategies to facilitate peer relationships that include students with disabilities are needed to foster the development of positive interactions between peers during unstructured periods at school. (PsycINFO Database Record (c) 2012 APA, all rights reserved)
SN - 1079-3917
AD - Hall, Laura J.: San Diego State U, San Diego, CA, US
ER -

TY - JOUR
ID - 2807
T1 - Exclusion in girls' peer groups: Ethnographic analysis of language practices on the playground. [References]
A1 - Goodwin,Marjorie Harness
Y1 - 2002///
N1 - Peer Reviewed Journal: 2002-08292-003
English
Journal; Peer Reviewed Journal
KW - PsycInfo
KW - Childhood Play Behavior
KW - Human Females
KW - Language
KW - Peer Relations
KW - Social Acceptance
KW - Criticism
RP - NOT IN FILE
SP - 392
EP - 415
JF - Human Development
VL - 45:
IS - 6
N2 - Documents the linguistic and nonverbal resources through which girls practice forms of social exclusion and ridicule in their spontaneous play, focusing on an age group (10-12 yr olds). The role of dispute and exclusion in children's groups are considered. The author discusses 3 groups of 10-12 yr olds that have been studied ethnographically. Psychological studies of aggressive behavior are reviewed that differentiate female forms of 'relational aggression' from other forms. Play patterns of different gender groups are discussed. How social class is made relevant in girls' everyday conversations is explored by discussing forms of comparisons that girls make. The article also deals with forms of exclusion within a girls' group, both among status equals as well as with respect to a 'tagalong' girl, a person defined by her marginal status. Through attention to language practices, the author demonstrates how girls articulate for each other their concern or disdain for one another, and position themselves relative to other girls. (PsycINFO Database Record (c) 2012 APA, all rights reserved)
SN - 0018-716X

AD - Goodwin, Marjorie Harness: U California, Dept of Anthropology, Los Angeles, CA, US
ER -

TY - JOUR
ID - 2808
T1 - Prevention of victimization: Survival Skills for urban youth. [References]
A1 - Mikalsen,Elena
Y1 - 2002///
N1 - Peer Reviewed Journal: 2002-06924-003
English
Journal; Peer Reviewed Journal
KW - PsycInfo
KW - Behavior Problems
KW - Conflict Resolution
KW - Prevention
KW - Urban Environments
KW - Violence
KW - Age Differences
KW - Human Sex Differences
KW - Self Esteem
KW - Victimization
RP - NOT IN FILE
SP - 33
EP - 44
JF - Journal of Prevention & Intervention in the Community
VL - 24:
IS - 2
N2 - Evaluated the unique prevention program implemented in inner city parks designed to prevent the development of short- and long-term consequences of exposure to chronic violence. 55 children and adolescents (aged 5-15 yrs) attending inner city Houston parks and playgrounds were recruited for participation. Results indicated that participants benefited significantly from the program, as evidenced by a decrease in endorsement of submissive conflict resolution strategies and behavioral problems. Higher self-esteem was related to increased program gains. Gender emerged as another moderator, while age was unrelated to improvement. (PsycINFO Database Record (c) 2012 APA, all rights reserved)
SN - 1085-2352
AD - Mikalsen, Elena: U Houston, Houston, TX, US Vincent, John P.: U Houston, Houston, TX, US Harris, Gerald E.: U Houston, Houston, TX, US
ER -

TY - JOUR
ID - 2809
T1 - Activities useful for fostering creativity: Singaporean children views. [References]
A1 - Tan,Ai-girl; Law,Lai-Chong
Y1 - 2002///
N1 - Peer Reviewed Journal: 2002-04826-005
English
Journal; Peer Reviewed Journal
KW - PsycInfo
KW - Classroom Behavior
KW - Creativity
KW - Daily Activities
KW - Social Learning
KW - Student Attitudes
KW - Participation
KW - Students
RP - NOT IN FILE
SP - 59

EP - 74
JF - Korean Journal of Thinking & Problem Solving
VL - 12:
IS - 2
N2 - 385 children (aged 9-12 yrs) in Singapore rated the degree of usefulness of the activities for fostering creativity on a 5-Likert scale (1=not very useful, 5=very useful). They comprised Grade 4 (29.9%), Grade 5 (31.9%), and Grade 6 (38.2%) students of Singapore schools. Three clusters (C) were elicited from their responses. Cluster 1 (C1) was featured by 15 "student-centered participatory activities." Children belonging to this cluster (26%) seemed to hold relatively similar conceptions of creativity with the experts. They seemed to understand that creativity challenges an individual's multiple competencies. Cluster 2 (C2) was nearly characterized by all items, except verbal presentation, recess, listening to jokes, and video show with a final cluster center "3." Cluster 3 (C3) focuses on teacher guidance, recitation, and independent learning. 40% of the children belonged to a cluster (C3) resembling learning activities of the Singaporean everyday classroom of the upper elementary level. Singaporean children sit for 2 streaming examinations at the age of 10 yrs (Grade 4) and at the age of 12 yrs (Grade 6). Half of the 4th graders (n=58, 50.4%) belonged to this cluster, indicating possible influences of the national streaming examination on their responses. (PsycINFO Database Record (c) 2012 APA, all rights reserved)
SN - 1598-723X
AD - Tan, Ai-girl: Nanyang Technological U, Singapore Law, Lai-Chong: Swiss Federal Inst for Techology, Zurich, Switzerland
ER -

TY - JOUR
ID - 2810
T1 - The social skills and social status of mainstreamed students with intellectual disabilities. [References]
A1 - Kemp,Coral
Y1 - 2002///
N1 - Peer Reviewed Journal: 2002-04396-003
English
Journal; Peer Reviewed Journal
KW - PsycInfo
KW - Mainstreaming (Educational)
KW - Social Skills
KW - Status
KW - Intellectual Development Disorder
RP - NOT IN FILE
SP - 391
EP - 411
JF - Educational Psychology
VL - 22:
IS - 4
N2 - Reports on the social skills and social status of 22 students (aged 7.5-11.3 yrs) with mainly moderate intellectual disabilities who had received an inclusive preschool intervention and were subsequently followed up from 18 months to more than 5 years later in their mainstream classrooms. Measures included direct assessment of social interaction in the playground, social status obtained by interviewing classmates, and the ratings of classroom teachers, parents and school principals. Large differences were found between the students with disabilities and their typical peers for amount of time spent interacting with peers and amount of time spent in isolation, with a moderate difference found for interactions with teachers. However, no difference was found between the social status of the 2 groups, and the students with disabilities were still spending more than half their time in the playground interacting with typically developing peers. Parents generally rated their children as having better social skills than did principals or teachers. A moderate relationship was found between the direct measures of peer interactions and teachers' perceptions of peer interaction skills. (PsycINFO Database Record (c) 2012 APA, all rights reserved)
SN - 0144-3410
AD - Kemp, Coral: Macquarie U Special Education Ctr, Sydney, NSW, Australia Carter, Mark: Macquarie U Special Education Ctr, Sydney, NSW, Australia
ER -

TY - JOUR
ID - 2811
T1 - Maternal socialization of safety practice among Mexican American children. [References]
A1 - Power,Thomas G.
Y1 - 2002///
N1 - Peer Reviewed Journal: 2002-02435-003
English
Journal; Peer Reviewed Journal
KW - PsycInfo
KW - Home Environment
KW - Mother Child Relations
KW - Safety
KW - Socialization
KW - Strategies
KW - Behavior
KW - Knowledge Level
KW - Latinos/Latinas
RP - NOT IN FILE
SP - 83
EP - 97
JF - Journal of Applied Developmental Psychology
VL - 23:
IS - 1
N2 - The relationship between maternal socialization strategies and children's safety practices was examined in a sample of 80 low-income Mexican American mothers (mean age 31.8 yrs) and their 4- to 8-yr-old children. Mothers were interviewed about the socialization strategies they used to influence their child's safety practices and about their children's safety behaviors in the home. Observations of the home environment yielded a measure of the family's use of proactive safety strategies. Children's safety knowledge was assessed in a structured play situation conducted in a simulated home environment. Results show that although mothers uniformly set safety rules regarding child behavior inside and outside the home, a significant number of mothers engaged in relatively few proactive practices to ensure child safety. Children who showed the greatest degree of safety knowledge and/or behavior tended to have mothers who used proactive safety strategies and who relied on consequence explanations versus commands or unelaborated explanations to teach child safety. (PsycINFO Database Record (c) 2012 APA, all rights reserved)
SN - 0193-3973
AD - Power, Thomas G.: Washington State U, Dept of Human Development, Pullman, WA, US Olvera, Norma: U Houston, Dept of Health & Human Performance, Houston, TX, US Hays, Jennifer: Baylor Coll of Medicine, Dept of Health Promotion, Houston, TX, US
ER -

TY - JOUR
ID - 2812
T1 - Children's social behavior before and after the availability of broadcast television: Findings from three studies in a naturalistic setting
A1 - Charlton,Tony; Davie,Ronald; Gunter,Barrie; Thomas,Cilla
Y1 - 2002///
N1 - Book: 2001-10129-006
English
Book; Edited Book
KW - PsycInfo
KW - Social Behavior
KW - Television Viewing
RP - NOT IN FILE
SP - 107
EP - 134
JF - Charlton, Tony [Ed]; Gunter, Barrie [Ed]; Hannan, Andrew [Ed]

VL - :
IS - 2002
N2 - (from the chapter) This chapter reports on 3 research studies, each using separate and independent measures of behavior to monitor young children's behavior across the availability of broadcast television on the island of St. Helena. Study 1 involved cohorts of nursery class children (aged 3-5 yrs) and incorporated teachers' ratings of children's behavior around school. Study 2 investigated 3- to 8-yr-olds' freeplay behavior in 2 school playgrounds in 1994 (before the availability of broadcast TV), and on two post-TV occasions in 1997, and 1998. Study 3 centered on a focus group discussion with 16- to 18-yr-old students from a secondary school on the island. The students' discussion threw light on potentially mitigating environmental factors. Findings on the whole showed little change in children's social behavior following the introduction of broadcast TV on the island. (PsycINFO Database Record (c) 2012 APA, all rights reserved)
AD - Charlton, Tony: Cheltenham & Gloucester Coll of Higher Education, Cheltenham, United Kingdom
ER -

TY - JOUR
ID - 2813
T1 - The impact of television on children's leisure
A1 - Hannan,Andrew
Y1 - 2002///
N1 - Book: 2001-10129-004
English
Book; Edited Book
KW - PsycInfo
KW - Leisure Time
KW - Recreation
KW - Television Viewing
RP - NOT IN FILE
SP - 65
EP - 87
JF - Charlton, Tony [Ed]; Gunter, Barrie [Ed]; Hannan, Andrew [Ed]
VL - :
IS - 2002
N2 - (create) Investigated the effect of the introduction of television to the leisure activities of children (aged 9-12 yrs) on St. Helena. The project team conducted a series of surveys based on children's diary entries that attempted to record children's "leisure" behavior and other activities undertaken outside school including "work" in or outside the home and daily tasks such as washing and eating. The first survey took place in October 1994 (before television), the second in 1995 after the introduction of the Cable News Network, the third in 1997 and the fourth in 1998, both following the addition of other cable networks. Results indicate that the arrival of television had a considerable impact on the leisure activities of these children, however television has not (for the great majority) dominated their lives, even when interest was at its height in 1997. The children have continued to engage in a wide range of out-of-home and in-home activities and have not dramatically shifted their behaviors. Although changes are taking place in the pattern of leisure activities of these children and the arrival of television has clearly been an important influence, the authors attribute these changes to the process of growing up, of which television has been another aspect of this experience. (PsycINFO Database Record (c) 2012 APA, all rights reserved)
AD - Hannan, Andrew: U Plymouth, Plymouth, United Kingdom
ER -

TY - JOUR
ID - 2814
T1 - A naturalistic approach to child social behaviour: Exchange of resources in preschoolers. [Spanish]
A1 - Gaviria,Elena
Y1 - 1994///
N1 - Peer Reviewed Journal: 1995-86171-001
Spanish
Journal; Peer Reviewed Journal
KW - PsycInfo

KW - Childhood Play Behavior
KW - Dominance
KW - Social Interaction
RP - NOT IN FILE
SP - 115
EP - 128
JF - Revista de Psicologia Social
VL - 9:
IS - 2
N2 - Studied spontaneous social interaction, social dominance, and resource exchange during free play among preschool children. 28 preschool students engaged in controlled free play while 28 (all aged 4-6 yrs) participated in passive vigilance free play. Ss' behavior during recess was recorded; Each S was monitored for a minimum of 40 min. The results were evaluated according to friendly contact, interchange of material resources, antagonistic dominance, authority, leadership, fighting, disobedience, symmetry of dyadic interchanges, equity, group participation, and type of supervision. Statistical tests were used. (English abstract) (PsycINFO Database Record (c) 2014 APA, all rights reserved)
SN - 0213-4748
AD - Gaviria, Elena: U Nacional de Educacion a Distancia, Facultad de Psicologia, Madrid, Spain
ER -

TY - JOUR
ID - 2815
T1 - Leisure time and drug consumption. [Spanish]
A1 - Alonso Sanz,Carlos; del Barrio Gandara,Victoria
Y1 - 1994///
N1 - Peer Reviewed Journal: 1995-85633-001
Spanish
Journal; Peer Reviewed Journal
KW - PsycInfo
KW - Drug Usage
KW - Leisure Time
RP - NOT IN FILE
SP - 71
EP - 93
JF - Revista de Psicologia Social
VL - 9:
IS - 1
N2 - Studied the relationship between the use of leisure time and the consumption of legal drugs. Ss were 277 7th-grade students (aged 11-13 yrs). Information on personal factors, use of leisure time, history of consumption of tobacco or alcohol, and intent to use tobacco or alcohol in the future was obtained by questionnaire. The results were evaluated according to participation in sports, and social or educational activities outside of school. (English abstract) (PsycINFO Database Record (c) 2014 APA, all rights reserved)
SN - 0213-4748
AD - Alonso Sanz, Carlos: U Nacional de Educacion a Distancia, Facultad de Psicologia, Madrid, Spain
ER -

TY - JOUR
ID - 2816
T1 - Child care and education for Spanish children younger than six years. [Spanish]
A1 - Palacios,Jesus
Y1 - 1989///
N1 - Peer Reviewed Journal: 1990-79183-001
Spanish
Journal; Peer Reviewed Journal
KW - PsycInfo
KW - Child Day Care
KW - Preschool Education

RP - NOT IN FILE
SP - 83
EP - 116
JF - Infancia y Aprendizaje / Journal for the Study of Education and Development
VL - 46 1989, pp. 83-116.
N2 - Examines from a macroscopic perspective the preschool education available in Spain for children younger than 6 yrs. In contrast to most other countries, Spain offers few choices for preschool education; almost 98% of public preschool programs are provided by the Ministry of Education or autonomous communities, and serve four- or five-year-olds, while over 94% of private programs (mostly for those under four) are provided by private sources or the Catholic church. Almost 65% of early childhood educators work in the public sector, and over two-thirds are trained for that age level. Finally, 52% of pupils rely on school transportation; 89% of programs operate from 9 AM to 5 PM; 52% of children attend from 4 to 5 hours a day; 47% of programs are housed in school buildings; 4% lack outside playgrounds; and 95% offer an educational program. (English abstract) (PsycINFO Database Record (c) 2014 APA, all rights reserved)
SN - 0210-3702
AD - Palacios, Jesus: U Sevilla, Spain
ER -

TY - JOUR
ID - 2817
T1 - The home dreams and waking fantasies of boys and girls between ages 9 and 15: A longitudinal study. [References]
A1 - Strauch,Inge
Y1 - 1999///
N1 - Peer Reviewed Journal: 1999-11074-003
English
Journal; Peer Reviewed Journal
KW - PsycInfo
KW - Age Differences
KW - Dream Content
KW - Dreaming
KW - Fantasy
KW - Human Sex Differences
KW - Content Analysis
KW - Daydreaming
KW - Fantasy (Defense Mechanism)
RP - NOT IN FILE
SP - 153
EP - 161
JF - Dreaming
VL - 9:
IS - 2-3
N2 - 12 boys and 12 girls provided home dreams and waking fantasies (WFs) at 3 age levels: 9-11, 11-13, and 13-15 yrs. A total of 299 dreams and 286 WFs were coded by 2 independent raters using C. S. Hall and R. Van de Castle (1966) content categories. There were few changes in dreams or WFs of either boys or girls, but dream reports were longer at ages 13-15 yrs, aggression/friendliness percent increased over time, joint-sex peer groups became more frequent, and girls showed a decline in animal percent. The tendency for men to dream mostly about other men and for women to dream equally of women and men was found in both dreams and WFs. Dreams and WFs differed markedly, with dreams containing more outdoor and unfamiliar settings and more bizarreness. In dreams, Ss tended to portray themselves as victims of aggression and recipients of friendliness, but in WFs they took a more active role as aggressors and befrienders. (PsycINFO Database Record (c) 2013 APA, all rights reserved)
SN - 1053-0797
AD - Strauch, Inge: U Zurich, Abteilung Klinische Psychologie, Zurich, Switzerland
ER -

TY - JOUR

ID - 2818
T1 - Infant day care facilitates preschool social behavior
A1 - Field,Tiffany; Masi,Wendy; Goldstein,Sheri; Perry,Susan; Parl,Silke
Y1 - 1988///
N1 - Peer Reviewed Journal: 1989-29126-001
English
Journal; Peer Reviewed Journal
KW - PsycInfo
KW - Attachment Behavior
KW - Child Day Care
KW - Childhood Play Behavior
KW - Social Behavior
RP - NOT IN FILE
SP - 341
EP - 359
JF - Early Childhood Research Quarterly
VL - 3:
IS - 4
N2 - 71 children (aged 23-68 mo) entering infant day care at varying times and receiving varying amounts of care were compared on their reunion-with-parent behavior, teacher and parent ratings of their behavior, and playground play interactions. Age of entry into day care (<6 mo vs >6 mo) had no significant effect on attachment to mother as measured by reunion behaviors or on play and socialization skills as measured by observation and behavior rating scales in a full-time day-care setting. Ss with more hours and months of day care engaged in less watching, solitary play, and teacher comfort-seeking behavior and they showed more cooperative play, positive affect, peer interaction, and positive verbal interaction. (PsycINFO Database Record (c) 2013 APA, all rights reserved)
SN - 0885-2006
AD - Field, Tiffany: U Miami School of Medicine, Mailman Ctr for Child Development, FL, US
ER -

TY - JOUR
ID - 2819
T1 - Effects of preschool environments on nonverbal social behavior: Toddlers' interpersonal distances to teachers and classmates change with environmental density, classroom design, and parent-child interactions
A1 - Burgess,J.
Y1 - 1989///
N1 - Peer Reviewed Journal: 1989-31147-001
English
Journal; Peer Reviewed Journal
KW - PsycInfo
KW - Classroom Environment
KW - Interpersonal Interaction
KW - Parent Child Relations
KW - Personal Space
KW - Preschool Students
KW - Childhood Play Behavior
KW - Preschool Teachers
RP - NOT IN FILE
SP - 261
EP - 276
JF - Child Psychology & Psychiatry & Allied Disciplines
VL - 30:
IS - 2
N2 - 12 toddlers' (aged 22-32 mo) interpersonal spacing patterns were studied in preschool environments of different density and design. Results show that an apparently spacious open classroom may produce behavioral changes reminiscent of crowding in young children. When more space was available on an outdoor playground children (1) increased interpersonal distances overall and (2) aggregated more with classmates and teachers,

fragmenting into subgroups that were separated from the class overall. With visual dividers added to the classroom to facilitate subgrouping, toddlers formed larger subgroups with teachers. Parent/child behavior during separation also affected distances: longer hand-holding and verbalization were found in children who stayed further from others. (PsycINFO Database Record (c) 2013 APA, all rights reserved)
SN - 0021-9630
AD - Burgess, J. Wesley: Stanford U School of Medicine, CA, US
ER -

TY - JOUR
ID - 2820
T1 - Grief reactions in school age children following a sniper attack at school
A1 - Pynoos,Robert S; Nader,
Y1 - 1987///
N1 - Peer Reviewed Journal: 1989-05452-001
English
Journal; Peer Reviewed Journal
KW - PsycInfo
KW - Death and Dying
KW - Grief
KW - Violence
KW - Followup Studies
RP - NOT IN FILE
SP - 53
EP - 63
JF - Israel Journal of Psychiatry and Related Sciences
VL - 24:
IS - 1-2
N2 - Investigated the reactions of 251 elementary school children (aged 6-13 yrs) 1 yr subsequent to a sniper attack on their school playground in which a 10-yr-old was killed and 14 others were wounded. A childhood grief reaction inventory was developed to collect data. Scores increased with degree of acquaintance with the deceased schoolmate and with degree of exposure to the violence. Results show that children responded similarly to adults (as previously reported in a number of studies), both in the nature and frequency of grief reactions and in that normal grieving was still ongoing after 1 yr. There is also evidence that while at times there was an interplay between grief and posttraumatic stress reactions in children, at other times the 2 reactions occurred independently. (PsycINFO Database Record (c) 2013 APA, all rights reserved)
SN - 0333-7308
AD - Pynoos, Robert S.: U California, Neuropsychiatric Inst, Los Angeles, US
ER -

TY - JOUR
ID - 2822
T1 - Momentary time sampling as an estimate of percentage time: A field validation
A1 - Saudargas,Richard A.
Y1 - 1990///
N1 - Peer Reviewed Journal: 1991-11456-001
English
Journal; Peer Reviewed Journal
KW - PsycInfo
KW - Sampling (Experimental)
KW - Time Estimation
KW - Elementary School Students
RP - NOT IN FILE
SP - 533
EP - 537
JF - Journal of Applied Behavior Analysis
JA - J Appl Behav Anal
VL - 23:

IS - 4
N2 - Examined the percentage time estimates of momentary time sampling (MTS) against the real time obtained with handheld computers among 16 1st-4th graders in a natural setting. Concurrent observations were conducted by 1 observer who used 15-sec MTS and a 2nd who used a handheld computer. Results for 6 behaviors (school work, looking, other activity, child interaction, teacher interaction, and out of seat) show a close correspondence between the MTS percentage observation intervals and the real-time percentage observation time. However, 15-sec MTS tended not to sample low-frequency short-duration behaviors. Short-interval MTS estimated percentage time accurately for a wide range of behavior frequencies and durations, and observers using MTS in a natural setting are able to obtain accurate data. (PsycINFO Database Record (c) 2013 APA, all rights reserved)
SN - 0021-8855
AD - Saudargas, Richard A.: U Tennessee, Knoxville, US
ER -

TY - JOUR
ID - 2823
T1 - School injuries in Athens: Socioeconomic and family risk factors
A1 - Petridou,Eleni; Kouri,Nicoletta; Trichopoulos,Dimitrios; Revinthi,Katharine; Skalkidis,Yannis; Tong,Donald
Y1 - 1994///
N1 - Peer Reviewed Journal: 1995-19336-001
English
Journal; Peer Reviewed Journal
KW - PsycInfo
KW - Family Structure
KW - Injuries
KW - Socioeconomic Status
KW - Elementary School Students
KW - Junior High School Students
KW - Preschool Students
RP - NOT IN FILE
SP - 490
EP - 491
JF - Journal of Epidemiology and Community Health
VL - 48:
IS - 5
N2 - Examined the importance of family and social risk factors for injuries at school in Athens, Greece. During study on 80 nonconsecutive days, 101 injuries occurred, 49 on the playground, 39 in classrooms or corridors, 10 during sporting activities, and 3 during transportation to or from school. Results indicated that low SES increases the risk for injuries, not only by creating adverse environmental conditions, but through mechanisms related to behavior and education that can operate even in the school environment. Children from single parent families were 5 times more likely to sustain a school injury. (PsycINFO Database Record (c) 2013 APA, all rights reserved)
SN - 0143-005X
AD - Petridou, Eleni: Athens U Medical School, Dept of Hygiene & Epidemiology, Ctr for Research & Prevention of Injuries, Greece
ER -

TY - JOUR
ID - 2824
T1 - A social learning model of peer choice in the natural environment
A1 - Snyder,James; West,Lisa; Stockemer,Vanessa; Gibbons,Shannon; Almquist-Parks,Laura
Y1 - 1996///
N1 - Peer Reviewed Journal: 1996-01776-004
English
Journal; Peer Reviewed Journal
KW - PsycInfo

KW - Childhood Play Behavior
KW - Choice Behavior
KW - Classroom Behavior
KW - Peer Relations
KW - Social Processes
KW - Blacks
KW - Preschool Students
RP - NOT IN FILE
SP - 215
EP - 237
JF - Journal of Applied Developmental Psychology
VL - 17:
IS - 2
N2 - Examined the social processes used by 74 African American children (aged 49-62 mo) to choose peer affiliates in a natural classroom setting. Preference ratings for each classmate were collected individually from each S, and the amount of time each S spent with peers during free play periods in the classroom was observed and coded. Results indicate that Ss were selective in their affiliation with peers; they spent substantial social time with a small number of peers and little time with remaining peers. Ss' initial affiliation with each of an array of same- and opposite-gender Ss and temporal changes in those affiliations were strongly related to the relative proportion of positive consequences the Ss experienced during social interaction with those peers. Strong mutual affiliations or friendships were established between Ss who provided each other with the highest levels of positive social consequences available from peers in the classroom. (PsycINFO Database Record (c) 2013 APA, all rights reserved)
SN - 0193-3973
AD - Snyder, James: Wichita State U, Dept of Psychology, Wichita, KS, US
ER -

TY - JOUR
ID - 2825
T1 - Writing as a social process
A1 - McLane,Joan B.
Y1 - 1992///
N1 - Book: 1991-97794-013
English
Book; Edited Book
KW - PsycInfo
KW - Community Services
KW - Interpersonal Interaction
KW - Written Communication
KW - Language Development
RP - NOT IN FILE
SP - 304
EP - 318
JF - Moll, Luis C [Ed]
VL - :
IS - 1992
N2 - (from the introduction) McLane presents her work with students at a community-based, after-school program / as part of a broader research effort to analyze children's writing outside of school, she describes her efforts to facilitate and support a broad range of writing for the students at the after-school day-care program building on Vygotskian notions about developing writing as a complex cultural activity, she devloped activities, in collaboration with teachers, within which print would play a prominent role in communicating with others / as students acquired control over the situations for writing, they began to explore what they could do with print, using writing to play, to experiment, to develop or maintain social relationships, and even to fight with one another (PsycINFO Database Record (c) 2013 APA, all rights reserved)
AD - McLane, Joan B.: Erikson Inst, Chicago, IL, US
ER -

TY - JOUR
ID - 2826
T1 - Effectiveness of using a computer to improve attention to visual analysis activities of five preschool children with disabilities. [References]
A1 - Del Pilar Cardona,Maria; Martinez,Alba Lucia; Hinojosa,Jim
Y1 - 2000///
N1 - Peer Reviewed Journal: 2005-05360-003
English
Journal; Peer Reviewed Journal
KW - PsycInfo
KW - Computer Applications
KW - Disabilities
KW - Preschool Students
KW - Special Education
KW - Visual Attention
RP - NOT IN FILE
SP - 42
EP - 56
JF - Occupational Therapy International
VL - 7:
IS - 1
N2 - This study used a single-subject reversal research design to examine the effectiveness of using a computer to improve attention to visual analysis activities in five preschool children with disabilities in a special education programme. Baseline levels of attention to task behaviours were measured during the performance of fine motor tabletop visual analysis activities of matching, requiring shape recognition, counting, free drawing, and size and colour discrimination. During the intervention phase, computer-based visual analysis activities were introduced. Results suggest that each child's attention to task performance improved during the computer-based activities, when measured by the number of distractions, although sitting tolerance and visual attention to the task did not change. The findings of this study, although limited to five participants with developmental disabilities, suggest that the students were interested and motivated to engage in computer-based activities. More research is needed to examine a longer intervention phase and the effectiveness of computer-based intervention in natural settings such as a classroom. (PsycINFO Database Record (c) 2013 APA, all rights reserved) (journal abstract)
SN - 0966-7903
AD - Hinojosa, Jim: New York University, New York, NY, US
ER -

TY - JOUR
ID - 2827
T1 - Children adopted to Finland from countries outside Europe. [Swedish]
A1 - Kvist,Beatrice; Viemero,Vappu; Forsten,Nina
Y1 - 1989///
N1 - Peer Reviewed Journal: 1991-70234-001
Swedish
Journal; Peer Reviewed Journal
KW - PsycInfo
KW - Adoption (Child)
KW - Cross Cultural Differences
KW - Immigration
KW - Aggressiveness
KW - Anxiety
KW - Ego Identity
KW - Leisure Time
KW - Self Concept
RP - NOT IN FILE
SP - 97
EP - 108
JF - Nordisk Psykologi

VL - 41:
IS - 2
N2 - Studied the self-image, self-ideal, ego strength, aggressiveness, anxiety, and leisure activities of adopted vs nonadopted Ss. Human subjects: 14 normal schoolage children (aged 10-12 yrs) (adoptees from Bangladesh, the Philippines, Thailand, and Vietnam). 13 normal Finnish schoolage children (aged 10-22 yrs). Psychological assessment and rating scales were used. (English abstract) Developmental. (PsycINFO Database Record (c) 2012 APA, all rights reserved)
SN - 0029-1463
AD - Kvist, Beatrice: Abo Akademi, Psykologiska Inst, Finland
ER -

TY - JOUR
ID - 2828
T1 - Scheme for Observing Activity Level: Reliability and convergent validity
A1 - Eaton,Warren O.
Y1 - 1987///
N1 - Peer Reviewed Journal: 1989-07099-001
English
Journal; Peer Reviewed Journal
KW - PsycInfo
KW - Activity Level
KW - Observation Methods
KW - Rating Scales
KW - Statistical Reliability
KW - Statistical Validity
RP - NOT IN FILE
SP - 273
EP - 280
JF - Journal of Psychoeducational Assessment
VL - 5:
IS - 3
N2 - Investigated reliability and convergent validity of the Scheme for Observing Activity Level (SOAL) developed by the present authors, using data from 3 studies. In Exp I, 12 male and 10 female preschoolers, (mean age 33.3 mo) were observed indoors and on a playground for 2-min intervals. In Exp II, 15 boys and 24 girls (mean age 44.4 mo) were observed while wearing an actometer, and results were correlated with the SOAL. In Exp III, 62 Ss (mean age 51.7 mo) were observed on indoor climbing structures for 1-min intervals. Correlations between Exp I and Exp III were .5. The SOAL displayed moderate convergence with alternative measures of activity level. (PsycINFO Database Record (c) 2012 APA, all rights reserved)
SN - 0734-2829
AD - Eaton, Warren O.: U Manitoba, Winnipeg, Canada
ER -

TY - JOUR
ID - 2829
T1 - At home with nature: Effects of "greenness" on children's cognitive functioning
A1 - Wells,Nancy M.
Y1 - 2000///
N1 - Peer Reviewed Journal: 2000-16123-003
English
Journal; Peer Reviewed Journal
KW - PsycInfo
KW - Cognitive Development
KW - Home Environment
KW - Housing
KW - Lower Income Level
KW - Childhood Development
KW - Urban Environments

RP - NOT IN FILE
SP - 775
EP - 795
JF - Environment and Behavior
VL - 32:
IS - 6
N2 - The nearby natural environment plays a far more significant role in the well-being of children residing in poor urban environments than has previously been recognized. Using a premove/postmove longitudinal design, this research explores the linkage between the naturalness or restorativeness of the home environment and the cognitive functioning of 17 low-income urban children (aged 7-12 yrs). Both before and after relocation, objective measures of naturalness were used along with a standardized instrument (the Attention Deficit Disorders Evaluation Scale) measuring the children's cognitive functioning. Results show that children whose homes improved the most in terms of greenness following relocation also tended to have the highest levels of cognitive functioning following the move. The implications with respect to policy and design are also discussed. (PsycINFO Database Record (c) 2012 APA, all rights reserved)
SN - 0013-9165
AD - Wells, Nancy M.: U California, School of Social Ecology, Irvine, CA, US
ER -

TY - JOUR
ID - 2830
T1 - Gender and territoriality in games played by nine-to-twelve-year-old schoolchildren
A1 - Kelle,Helga
Y1 - 2000///
N1 - Peer Reviewed Journal: 2000-12609-002
English
Journal; Peer Reviewed Journal
KW - PsycInfo
KW - Childrens Recreational Games
KW - Exercise
KW - Sex Roles
KW - Teams
KW - Territoriality
KW - Elementary School Students
KW - Human Sex Differences
KW - Secondary Education
RP - NOT IN FILE
SP - 164
EP - 197
JF - Journal of Contemporary Ethnography
VL - 29:
IS - 2
N2 - Taking recent ethnomethodological insights into gender as an ongoing interactional accomplishment as a starting point, a research group conducted an ethnographic exploration of the practices of gender differentiation among nine- to twelve-year-old schoolchildren in Germany. From 1993-1997, the two researchers observed two classes of 20 children each at the Laborschule in Bielefeld in their school years 4, 5, and 6. This article deals with a small segment of these gender differentiation practices: spontaneous territorial games, characterized by a high degree of physical activity in which the children split up into gender-homogeneous teams. Based on the detailed analysis of two recess situations, the article explores how gender-defined territories are achieved interactionally and how gender polarity is thus staged. The concluding play-theoretical reflections in particular show that territorial games simplify, alienate, and aestheticize gender arrangements. (PsycINFO Database Record (c) 2012 APA, all rights reserved)
SN - 0891-2416
AD - Kelle, Helga: U Bielefeld, Germany
ER -

TY - JOUR

ID - 2831
T1 - Family therapists and school counselors: A collaborative endeavor
A1 - Rotter,Joseph C.
Y1 - 1999///
N1 - Peer Reviewed Journal: 1999-01615-011
English
Journal; Peer Reviewed Journal
KW - PsycInfo
KW - Behavior Problems
KW - Cotherapy
KW - Family Therapy
KW - School Counseling
KW - Models
RP - NOT IN FILE
SP - 276
EP - 279
JF - The Family Journal
VL - 7:
IS - 3
N2 - Presents the case study of Robert to demonstrate an interaction collaboration model for family therapists and school counselors. When family therapy originates outside the school setting, the school is likely to be ignored, but communication is often poor between school and therapists even in cases where therapy originates through school referral. Robert (aged 9 yrs), repeatedly demonstrated acting-out school behavior. To assist him in gaining control of his emotions, implemented procedures and activities included Robert's unconditionally sanctioned attendance at social events, setting up a routine contact schedule with his distant father, minor classroom changes, and school group counseling sessions. All family members set aside quality time. The school counselor coordinated classroom arrangements with Robert's teachers. (PsycINFO Database Record (c) 2012 APA, all rights reserved)
SN - 1066-4807
AD - Rotter, Joseph C.: U South Carolina, Dept of Educational Psychology, Columbia, SC, US
ER -

TY - JOUR
ID - 2832
T1 - Physiological, behavioral, and psychological effects associated with television viewing in schoolboys: An exploratory study
A1 - Myrtek,Michael; Scharff,Christian; Brugner,Georg; Muller,Wolfgang
Y1 - 1996///
N1 - Peer Reviewed Journal: 1996-05720-002
English
Journal; Peer Reviewed Journal
KW - PsycInfo
KW - Behavioral Assessment
KW - Computer Applications
KW - Psychophysiology
KW - Television Viewing
KW - Emotional Responses
KW - Monitoring
RP - NOT IN FILE
SP - 301
EP - 323
JF - The Journal of Early Adolescence
VL - 16:
IS - 3
N2 - 50 German schoolboys (aged 10-13 yrs) demonstrate a new procedure for gathering physiological, psychological, and behavioral data on TV viewing in the home environment on a normal school day, using a special ambulatory monitoring device.The mean time spent watching TV was 123 min per day, during which the

Ss watched mostly entertainment and action shows. Results indicate that different types of TV programs had differing physiological effects and more specifically, heart rate (HR) as compared to other programs was low for entertainment and high for action shows. Compared to school and leisure time, additional HR during TV viewing was much higher. Boys with high TV consumption read fewer books, showed diminished activities outside home, a tendency for reduced school-related homework, and reduced interest in hobbies. Findings show that psychophysiological effects of TV viewing can be monitored in the home environment. (PsycINFO Database Record (c) 2012 APA, all rights reserved)
SN - 0272-4316
AD - Myrtek, Michael: U Freiburg, Psychologisches Inst, Forschungsgruppe Psychophysiologie, Freiburg, Germany
ER -

TY - JOUR
ID - 2833
T1 - School/nonschool friendship patterns in early adolescence
A1 - DuBois,David L.
Y1 - 1993///
N1 - Peer Reviewed Journal: 1993-37108-001
English
Journal; Peer Reviewed Journal
KW - PsycInfo
KW - Environment
KW - Friendship
KW - Racial and Ethnic Differences
KW - Social Skills
KW - Blacks
KW - Whites
RP - NOT IN FILE
SP - 102
EP - 122
JF - The Journal of Early Adolescence
VL - 13:
IS - 1
N2 - Examined the school/nonschool ecology of early adolescent friendship activity using data from 115 female and 95 male White students and 39 male and 33 female Black students. Principal components analysis of friendship measures revealed distinct dimensions for activities that occur during and outside school with the best school friend. Social skill and environment-related obstacles to seeing school friends outside school were also identified, particularly among Black Ss. Activity with the best school friend was more frequent during school and environmental obstacles interfered to a greater extent with seeing school friends outside school than did social skill problems. Activity with the best school friends during and outside school was related to self-esteem among boys but not among girls. (PsycINFO Database Record (c) 2012 APA, all rights reserved)
SN - 0272-4316
AD - DuBois, David L.: U Missouri, Columbia, US
ER -

TY - JOUR
ID - 2834
T1 - Factor structure of the School Function Assessment
A1 - Coster,Wendy J.
Y1 - 1999///
N1 - Peer Reviewed Journal: 2000-05366-008
English
Journal; Peer Reviewed Journal
KW - PsycInfo
KW - Criterion Referenced Tests
KW - Disorders
KW - Factor Structure

KW - Statistical Validity
KW - Student Characteristics
KW - Educational Measurement
KW - Individualized Instruction
KW - Mainstreaming (Educational)
KW - Peer Relations
KW - Self Care Skills
RP - NOT IN FILE
SP - 665
EP - 677
JF - Educational and Psychological Measurement
VL - 59:
IS - 4
N2 - The School Function Assessment (SFA) was developed to provide information on students' abilities to meet functional demands of the elementary school program. This judgment-based, criterion-referenced assessment supports a comprehensive, detailed examination of the extent to which students with a variety of disabilities are performing important school-related functional tasks and activities such as moving around the school, using classroom materials, interacting with peers, and caring for personal needs. The factor structure underlying the SFA Activity Performance scales was investigated using data from two heterogeneous national samples of students with disabilities (266 and 341 Ss aged 5-14 yrs). Analysis with the principal axis technique and oblique rotation identified two factors, a Cognitive/Behavioral Function dimension and a Physical Function dimension. These factors were moderately correlated, which is congruent with definitions of the function construct that emphasize the integration of physical, cognitive, and social abilities in the performance of daily activities in the natural environment. (PsycINFO Database Record (c) 2012 APA, all rights reserved)
SN - 0013-1644
AD - Coster, Wendy J.: Boston U, Sargent Coll of Health & Rehabilitation Sciences, Dept of Occupational Therapy, Boston, MA, US
ER -

TY - JOUR
ID - 2835
T1 - Pathways to delinquency in Hong Kong and Guangzhou (South China). [References]
A1 - Wong,Dennis S.
Y1 - 2001///
N1 - Peer Reviewed Journal: 2002-00137-005
English
Journal; Peer Reviewed Journal
KW - PsycInfo
KW - Academic Achievement
KW - Adolescent Attitudes
KW - Juvenile Delinquency
KW - Parent Child Relations
KW - Peer Relations
KW - Extracurricular Activities
KW - Religiosity
KW - Sports
KW - Teacher Student Interaction
RP - NOT IN FILE
SP - 91
EP - 115
JF - International Journal of Adolescence and Youth
VL - 10:
IS - 1-2
N2 - Examined the interactive effects of criminogenic factors on delinquency among youth in China and Hong Kong. Ss in the retrospective study comprised 63 male youth (aged 12-18 yrs) residing in Hong Kong and Guangzhou, China, 32 of whom were convicted delinquents. Ss completed interviews concerning parental closeness and supervision, reaction to parental supervision, academic performance, relationships with

classmates and teachers, reactions to teaching practices, and time spent in sports activities, youth clubs, and religious activities. Results show that 65% of Hong Kong delinquent Ss, and 40% of Guangzhou delinquent Ss came from broken homes. Nearly all delinquent Ss reported that they were ignored by their parents and seldom felt understood. Delinquent Ss exhibited higher school drop-out rates, which they attributed to their own behavioral problems, academic difficulties, or poor teacher-student relationships. Non-delinquent Ss associated with friends from school; in contrast, delinquent Ss met their peers in video arcades, street corners, or playgrounds and had close ties to neighborhood gangs. (PsycINFO Database Record (c) 2012 APA, all rights reserved)
SN - 0267-3843
AD - Wong, Dennis S. W.: City U of Hong Kong, Dept of Applied Social Studies, Kowloon, Hong Kong
ER -

TY - JOUR
ID - 2836
T1 - High-risk Behavior by Boys in Traffic Situations in Belem. [Portuguese]. [References]
A1 - de Assis Rocha,Joao Bosco
Y1 - 2001///
N1 - Peer Reviewed Journal: 2003-07965-002
Portuguese
Journal; Peer Reviewed Journal
KW - PsycInfo
KW - At Risk Populations
KW - Human Males
KW - Motor Traffic Accidents
KW - Risk Factors
KW - Risk Taking
KW - Drivers
KW - Parents
KW - Witnesses
RP - NOT IN FILE
SP - 23
EP - 33
JF - Arquivos Brasileiros de Psicologia
VL - 53:
IS - 3
N2 - Every year thousands of Brazilian children die or get injured from traffic accidents. To know the inherent factors to the dangerous behavior at the moment accidents happen, we studied twenty non-lethal cases involving children from 5 to 10 years old. The study included not only the interview of the victims, their parents, witness and drivers but also the use of psychological tests, questionnaires and drawings which aimed the reconstruction of the accident scene and the better understanding of the behavior of the subjects. Secondary factors, such as the type of road, climatic conditions, day and time of accident, and the reason for the subject's presence at the scene, were also evalued. The results indicated that 1) lower-income boys, due to their precarious living conditions were more exposed to high-risk traffic conditions. The following aspects were characteristic of accident causality: a) they were busy with some outdoor activity, b) were distracted, c) had poor schooling, and d) seemed unaware of traffic-related dangers. 2) With reference to their psychological profiles, the boys did not differ significantly from peer controls, which led to the conclusion that available psychological test are not predictive of accident proneness. In conclusion, there is an urgent need for restructuring the Brazilian transit system. (PsycINFO Database Record (c) 2012 APA, all rights reserved) (journal abstract)
SN - 0100-8692
AD - de Assis Rocha, Joao Bosco: Universidade D. Bosco, Campo Grande, Brazil
ER -

TY - JOUR
ID - 2837
T1 - Free play use of space by preschoolers from diverse backgrounds: Factors influencing activity choices
A1 - Harper,Lawrence V.
Y1 - 1998///

N1 - Peer Reviewed Journal: 1998-10856-008
English
Journal; Peer Reviewed Journal
KW - PsycInfo
KW - Childhood Play Behavior
KW - Psychosocial Development
KW - Racial and Ethnic Differences
KW - Socioeconomic Status
KW - Day Care Centers
KW - Playgrounds
KW - Preschool Students
RP - NOT IN FILE
SP - 423
EP - 446
JF - Merrill-Palmer Quarterly
VL - 44:
IS - 3
N2 - Analyses were made of the location and quality of free play of 244 3- to 5-year-olds from 6 different preschool and day-care centers. Although SES and ethnicity were confounded with amount of space and the range of choices available, 13 play sites/activities could account for at least half the children's time in every center. There was no simple relation between the amount of space allocated to a setting and the proportion of time that the children used it. Patterns of use varied as a function of weather-related accessibility in spacious facilities. Differences across samples drawn from the same SES/ethnic pool indicated that the particular make-up of a group (the "cohort") in the same physical context can affect specific patterns of usage. Among children drawn from all 6 centers, there were sex differences in time spent in 5 of 9 common play sites. Moreover, across centers and a broader spectrum of areas, certain play sites consistently yielded different patterns of social exchanges with peers and adults. (PsycINFO Database Record (c) 2012 APA, all rights reserved)
SN - 0272-930X
AD - Harper, Lawrence V.: U California, Dept of Human & Community Development, Davis, CA, US
ER -

TY - JOUR
ID - 2838
T1 - Observations of aggressive and nonaggressive children on the school playground
A1 - Pepler,Debra J.
Y1 - 1998///
N1 - Peer Reviewed Journal: 1997-39002-004
English
Journal; Peer Reviewed Journal
KW - PsycInfo
KW - Aggressiveness
KW - Antisocial Behavior
KW - Childhood Play Behavior
KW - Peer Relations
KW - Prosocial Behavior
RP - NOT IN FILE
SP - 55
EP - 76
JF - Merrill-Palmer Quarterly
VL - 44:
IS - 1
N2 - Naturalistic observations were made of 17 aggressive and 22 nonaggressive children in Grades 1 to 6, filmed with video cameras and remote microphones on school playgrounds. Observers coded interactive behaviors, affective valence, and play states. Aggressive children displayed more verbal and physical aggression, more prosocial behaviors, and higher rates of interaction than did nonaggressive children. The 2 groups spent similar time in solitary and group activities. Sequential analyses indicated that peers made similar initiations to aggressive and nonaggressive children, but aggressive children were more likely to respond

antisocially. Aggressive children initiated more mixed behaviors (prosocial and antisocial) than did nonaggressive children. (PsycINFO Database Record (c) 2012 APA, all rights reserved)
SN - 0272-930X
AD - Pepler, Debra J.: York U, LaMarsh Ctr for Research on Violence & Conflict Resolution, North York, ON, Canada
ER -

TY - JOUR
ID - 2839
T1 - Temporary and long-term friendships in hearing and deaf preschoolers
A1 - Lederberg,Amy R.
Y1 - 1987///
N1 - Peer Reviewed Journal: 1988-14334-001
English
Journal; Peer Reviewed Journal
KW - PsycInfo
KW - Deaf
KW - Friendship
KW - Peer Relations
KW - Hearing Disorders
KW - Longitudinal Studies
RP - NOT IN FILE
SP - 515
EP - 533
JF - Merrill-Palmer Quarterly
VL - 33:
IS - 4
N2 - Observed 33 hearing and 29 deaf 3-5 yr olds during outdoor play at their schools over 7 mo. Three types of peer relationships were examined: nonfriends, temporary friends, and long-term friends. Both deaf and hearing Ss typically had 1 temporary friend and 1 long-term friend at a given time. The 2 types of friends differed from the nonfriends in the affective tone of their interactions. Compared to nonfriends, friends were less likely to behave negatively and were more likely to be of the same sex and ethnicity. Long-term friends engaged in more social than parallel play and were more similar in age and ethnicity. Except for differences in stability, the pattern of friendship was similar for deaf and hearing preschoolers. (PsycINFO Database Record (c) 2012 APA, all rights reserved)
SN - 0272-930X
AD - Lederberg, Amy R.: U Texas-Dallas, Richardson, US
ER -

TY - JOUR
ID - 2840
T1 - Disabled children's integration in school: From social representation to subjectivity
A1 - Donfrancesco,R.
Y1 - 1996///
N1 - Peer Reviewed Journal: 1997-03747-002
English
Journal; Peer Reviewed Journal
KW - PsycInfo
KW - Disorders
KW - Educational Programs
KW - Mainstreaming (Educational)
KW - Verbal Memory
KW - Visual Memory
KW - Classroom Behavior
RP - NOT IN FILE
SP - 283
EP - 295

JF - Giornale di Neuropsichiatria dell'Eta Evolutiva
VL - 16:
IS - 4
N2 - Evaluated integration in school of 14 disabled children (aged 7.6-13.11 yrs) during the experience of a stage for memory improvement that was carried out during class time. Ss were divided into 3 working groups; all Ss attended primary school, except one child who attended Italian middle school. All Ss willingly accepted the stage and actively took part in the proposed work; at the end of the stage (3 mo; a total of 60 hrs) Ss' visual memory and verbal memory were improved (p < 0.005 and < 0.001 respectively). The children's behavior after integration in school was studied using the Social Emotional Dimension Scale (J. B. Hutton and G. T. Roberts, 1986); similar values were obtained before and after the stage. Results suggest that selected Ss may be able to work outside general class during some hours a week without detriment to their capacity to socialize and to integrate in school. An Italian translation of this article precedes the English version abstracted here. (PsycINFO Database Record (c) 2012 APA, all rights reserved)
SN - 0392-4483
AD - Donfrancesco, R.: USL 10, Child Neuropsychiatry, Firenze, Italy
ER -

TY - JOUR
ID - 2841
T1 - Early detection of social anxiety: Reliability and validity of a teacher questionnaire for the identification of social anxiety in young children. [References]
A1 - Bokhorst,Koos
Y1 - 2001///
N1 - Peer Reviewed Journal: 2001-09407-006
English
Journal; Peer Reviewed Journal
KW - PsycInfo
KW - Rating Scales
KW - Social Anxiety
KW - Test Reliability
KW - Test Validity
KW - Teachers
RP - NOT IN FILE
SP - 787
EP - 798
JF - Social Behavior and Personality
VL - 29:
IS - 8
N2 - In order to enable teachers to detect social anxiety in preschoolers the authors developed a scale. The TRSA-"junior" (Teacher Rating Scale of Social Anxiety-"junior") is a brief Likert-type questionnaire for the identification of social anxiety in 5-to 6-year-old children. Reliability (internal consistency plus test-retest correlation) collected in 2 samples (N=173, N=285) was high. Convergent and divergent validity of this newly developed rating-scale were also studied. A high level of social anxiety correlated with a low level of social participation, as observed on the playground. The TRSA-"junior" score correlated with "internalizing" as measured by the Behavior Questionnaire for Toddlers and Preschoolers (BQTP) and the Child Behavior Check List (CBCL), and with the subscales "anxious-depressed," "thought problems," "social problems" and "withdrawn behavior" of the CBCL. In the second study a high score on social anxiety appeared to be related to less positive sociometric judgments by peers. The authors conclude that the TRSA-"junior" may aid in the identification and prevention of social anxiety at an early age. In addition, the scale would appear to be a useful instrument in research, given its sound psychometric qualities. (PsycINFO Database Record (c) 2012 APA, all rights reserved)
SN - 0301-2212
AD - Bokhorst, Koos: Free U Amsterdam, Faculty of Psychology & Education, Dept of Education, Amsterdam, Netherlands
ER -

TY - JOUR

ID - 2842
T1 - Outdoor activities as a basis for environmental responsibility
A1 - Palmberg,Irmeli E; Kuru,Jari
Y1 - 2000///
N1 - Peer Reviewed Journal: 2000-05494-003
English
Journal; Peer Reviewed Journal
KW - PsycInfo
KW - Curriculum
KW - Educational Field Trips
KW - Environmental Attitudes
KW - Responsibility
KW - Elementary School Students
KW - Emotional Adjustment
KW - Personal Values
KW - Personality Traits
RP - NOT IN FILE
SP - 32
EP - 36
JF - The Journal of Environmental Education
VL - 31:
IS - 4
N2 - Examined the effects of outdoor environmental education activities involving school children. 36 students (aged 11-12 yrs) residing in Finland were categorized as experiencing high, moderate, or low levels of outdoor education, which included field trips, hiking, camps, canoeing, and sailing. Collected data included questionnaire responses, interviews, drawings, landscape photographs, and S observations during outdoor activities. Results show that nature experiences developed Ss' self-confidence and feelings of safety, which in turn increased their willingness to participate in future outdoor activities. In comparison to Ss experienced with outdoor activities, those who were inexperienced were more uncertain and anxious about trying new things, were quicker to express their inability, and had more difficulties in working as a group. Experienced Ss showed a strongly empathic relationship to nature, better social behavior, and higher moral judgements. (PsycINFO Database Record (c) 2012 APA, all rights reserved)
SN - 0095-8964
AD - Palmberg, Irmeli E.: Abo Akademi U, Dept of Teacher Education, Vaasa, Finland
ER -

TY - JOUR
ID - 2843
T1 - Musical learning: Differences between boys and girls in Hong Kong Chinese co-educational secondary schools. [References]
A1 - Ho,Wai chung
Y1 - 2001///
N1 - Peer Reviewed Journal: 2002-13630-003
English
Journal; Peer Reviewed Journal
KW - PsycInfo
KW - Human Sex Differences
KW - Music
KW - Music Education
KW - Stereotyped Attitudes
KW - Student Attitudes
KW - School Environment
RP - NOT IN FILE
SP - 41
EP - 54
JF - British Journal of Music Education
VL - 18:

IS - 1
N2 - Presents an overview of boys' and girls' musical learning inside and outside school. Ss were 877 pupils (414 boys and 463 girls) in 9 Chinese secondary schools. Most of the Ss were aged 12-16 yrs. The paper argues that patterns of gender stereotyping associated with music among Hong Kong students have some similarities with those in the Western world. The impact of gender beliefs was most evident in types of instrumental learning, types of music activities, and listening and singing preferences. The Ss' attitudes toward the promotion of popular and Western classical music in school emerged as statistically significant, while their attitude toward Chinese classical music was nonsignificant. (PsycINFO Database Record (c) 2012 APA, all rights reserved)
SN - 0265-0517
AD - Ho, Wai-chung: Hong Kong Baptist U, Hong Kong
ER -

TY - JOUR
ID - 2844
T1 - Treatment of child aggression: Development and assessment of multi-agent programs of behavioral intervention. [Spanish]. [References]
A1 - Velazquez,Hector Ayala; Caso-Lopez,Alicia Chaparro; Juarez,Monica Fulgencio; Cabrera,Francisco Pedroza; Chaine,Silvia Moralez; Trejo,Ayme Pacheco; Gonzalez,Brenda Mendoza; Sanchez,Aura Ortiz; Sandoval,Eloisa Vargas; Torres,Noemi Barragan
Y1 - 2001///
N1 - Peer Reviewed Journal: 2002-10326-001
Spanish
Journal; Peer Reviewed Journal
KW - PsycInfo
KW - Aggressive Behavior
KW - Behavior Modification
RP - NOT IN FILE
SP - 1
EP - 34
JF - Revista Mexicana de Analisis de la Conducta
VL - 27:
IS - 1
N2 - Evaluated the effectiveness of 8 behavioral intervention programs directed at modifying aggressive behavior in children. These interventions were designed based on those factors that research in the field has identified as being directly linked to the development and maintenance of childhood aggression. They were offered to the three groups of change agents that have been shown to be related to these patterns of behavior: the child itself, it's parents and teachers. 84 children participated in this study and were exposed to one of several to the following intervention programs: Parent Training for young children (6-9 yrs), Parent Training older children (10-13 yrs); Classroom Behavior management; Playground Behavior management; Self-control training in anger management for young children (6-9 yrs); Self-control training in anger management for older children (10-13 yrs), Problem Solving training with peers, and Social Skills training. For each of the intervention programs, a series of measures were obtained before and after completing the training, which indicate an important level of effectiveness in the reduction of aggressive behavior, suggesting the viability of its application and their pertinence in the development of multimodal intervention strategies for this population. (PsycINFO Database Record (c) 2012 APA, all rights reserved)
SN - 0185-4534
AD - Velazquez, Hector Ayala: U Nacional Autonoma de Mexico, Mexico
ER -

TY - JOUR
ID - 2845
T1 - Psycho-sensory static load and physical activity in daily regimen of Slovak school children. [References]
A1 - Sevcikova,L.'
Y1 - 2001///
N1 - Peer Reviewed Journal: 2002-08703-011
English
Journal; Peer Reviewed Journal

KW - PsycInfo
KW - Activity Level
KW - Daily Activities
KW - Exercise
KW - Leisure Time
KW - Physical Activity
KW - Elementary School Students
KW - Junior High School Students
KW - Sleep
KW - Sports
RP - NOT IN FILE
SP - 211
EP - 213
JF - Homeostasis in Health and Disease
VL - 41:
IS - 5
N2 - Examined psychosensory and static load and physical activity among Slovak school children. 2,108 1st-9th grade Slovak schoolchildren reported daily regimen activities in1982; 2,339 1st-9th grade Slovak schoolchildren did so in 1998. Data were compared concerning: (1) psycho-sensory and static load; (2) leisure time sports activities; (3) sleep; and (4) outdoor activities. Results show that significantly more younger subjects (Ss) attended optional and private lessons and other educational activities in 1998, compared to 1982. New daily sedentary and psycho-sensory activities with personal computers were reported by 36% of Ss. Watching television significantly increased in all Ss, and was correlated with a significant shortening of sleep. Regular sports activity increased in younger Ss. Physical and sport activity was lower in females than in males. 84% of younger Ss relaxed outdoors <3 hrs daily. (PsycINFO Database Record (c) 2012 APA, all rights reserved)
SN - 0960-7560
AD - Sevcikova, L'.: Comenius U, Medical Faculty, Inst of Hygiene, Bratislava, Slovak Republic Stefanikova, Z.: Comenius U, Medical Faculty, Inst of Hygiene, Bratislava, Slovak Republic Jurkovicova, J.: Comenius U, Medical Faculty, Inst of Hygiene, Bratislava, Slovak Republic Ruzanska, S.: Comenius U, Medical Faculty, Inst of Hygiene, Bratislava, Slovak Republic Sabolova, M.: Comenius U, Medical Faculty, Inst of Hygiene, Bratislava, Slovak Republic Aghova, L'.: Comenius U, Medical Faculty, Inst of Hygiene, Bratislava, Slovak Republic
ER -

TY - JOUR
ID - 2846
T1 - An elementary school-based prevention program targeting modifiable antecedents of youth delinquency and violence: Linking the Interests of Families and Teachers (LIFT). [References]
A1 - Eddy,J.
Y1 - 2001///
N1 - Book: 2002-01577-012
English
Book; Edited Book
KW - PsycInfo
KW - Crime Prevention
KW - Educational Programs
KW - Juvenile Delinquency
KW - Urban Environments
KW - Violence
KW - Elementary School Teachers
KW - Parents
RP - NOT IN FILE
SP - 128
EP - 139
JF - Walker, Hill M [Ed]; Epstein, Michael H [Ed]
VL - :
IS - 2001

N2 - (create) This reprinted chapter originally appeared in Journal of Emotional & Behavioral Disorders, 2000(Fal), Vol 8(3), 165-176. (The following abstract of the original article appeared in record 2000-02433-004). Describes Linking the Interests of Families and Teachers (LIFT), a prevention program designed for delivery to children and parents within the elementary school setting. The LIFT targets for change those child and parent behaviors thought to be most relevant to the development of adolescent delinquent and violent behaviors, namely child oppositional, defiant, and socially inept behavior and parent discipline and monitoring. The 3 major components of the LIFT are (1) classroom-based child social and problem skills training, (2) playground-based behavior modification, and (3) group-delivered parent training. The results of a randomized controlled evaluation of the LIFT, using 1st and 5th graders and parents from high juvenile crime neighborhoods, are reviewed. To date, the program has positively impacted the targeted antecedents. Most importantly, during the 3 yrs following the program, the LIFT delayed the time that participants first became involved with antisocial peers during middle school, as well as the time to first patterned alcohol use, to first marijuana use... (PsycINFO Database Record (c) 2012 APA, all rights reserved)
AD - Eddy, J. Mark: Oregon Social Learning Ctr, Oregon Prevention Research Ctr, Eugene, OR, US Reid, John B.: Oregon Social Learning Ctr, Eugene, OR, US Fetrow, Rebecca A.: Oregon Social Learning Ctr, Eugene, OR, US
ER -

TY - JOUR
ID - 2847
T1 - The development of explanations for biological phenomena: Children's and adults' understanding of inheritance
A1 - Korpan,Connie Anna
Y1 - 2001///
N1 - Dissertation Abstract: 2001-95022-231
English
Dissertation Abstract
KW - PsycInfo
KW - Age Differences
KW - Genetics
KW - Transgenerational Patterns
RP - NOT IN FILE
SP - 2517
JF - Dissertation Abstracts International: Section B: The Sciences and Engineering
VL - 62:
IS - 5-B
N2 - This research concerns biological understanding of inheritance. Among other things, biological understanding entails knowing that biological processes operate outside the forces of mechanical and intentional causes. A question that has been the center of much debate is: When do children possess such understanding? One view is that before the age of approximately 7 years, children interpret biological phenomena in terms of psychological forces such as beliefs and intentions and that between 7- and 10-years of age, they undergo something akin to paradigm shift. The other view is that children possess an autonomous theoretical framework related to biology at a much earlier age. Of particular interest in the current study is the types of explanations individuals endorse or generate when thinking about biological inheritance. In this study, students in Grades 2, 4, 6, and university were systematically and intensively interviewed on the topic of inheritance. They were presented with three tasks. In the Generation Task, participants were presented with a variety of items intended to elicit explanations about how physical traits are inherited across one or two generations. In the Endorsement Task, participants were asked to render an opinion concerning seven explanations for the color of an animal, a plant, and an artifact. Finally, in the Background Knowledge Task, participants were asked questions that were designed to determine their knowledge about genes and the role they play in transmission of traits, their knowledge about the environment and how it influences the expression of traits, and their intuitive statistical knowledge regarding natural variability and homogeneity. In the Generation Task, participants in all age groups generated approximately six types of explanations, suggesting that participants have at hand a variety of explanations from which to choose. Adults tended to generate explanations that were congruous with biological theories of inheritance but included a clear causal mechanism in their explanations. Children's explanations were also mostly congruous with biological theories of inheritance, but frequently lacked a clear causal mechanism. Implications of evaluating biological understanding and its development from the perspective of multiple,

overlapping explanations are discussed. (Abstract shortened by UMI.) (PsycINFO Database Record (c) 2012 APA, all rights reserved)
SN - 0419-4217
AD - Korpan, Connie Anna: U Alberta, Canada
ER -

TY - JOUR
ID - 2848
T1 - An interplay-based social skills group for children with Asperger's syndrome
A1 - Doyle,Melissa M.
Y1 - 2001///
N1 - Dissertation Abstract: 2001-95019-112
English
Dissertation Abstract
KW - PsycInfo
KW - Aspergers Syndrome
KW - Group Psychotherapy
KW - Social Skills
RP - NOT IN FILE
SP - 1583
JF - Dissertation Abstracts International Section A: Humanities and Social Sciences
VL - 62:
IS - 4-A
N2 - Asperger's Syndrome is a cluster of cognitive, social and affective deficits that falls in the continuum of Pervasive Developmental Disorders. Current methods of intervention have met with limited success and have not been replicated outside of the treatment setting. In this study, an Interplay based brief group treatment, utilized a metaphor of acting skills to teach social skills to a group of six latency aged boys with Asperger's Syndrome. A measurement instrument, designed for this study, assessed play skills, social skills, Theory of Minds Skills, and verbal and non-verbal communication skills. Pre- and post-intervention results were measured using separate single case research designs. Only minor gains in skills were noted, although qualitative report of group members and parents were positive. Recommendations for future treatment and research include small group size, increased parent involvement, and a high child to facilitator ratio that permits both participation and reflection on the group process. (PsycINFO Database Record (c) 2012 APA, all rights reserved)
SN - 0419-4209
AD - Doyle, Melissa M.: Smith Coll School For Social Work, US
ER -

TY - JOUR
ID - 2849
T1 - Group entry strategies of socially excluded children as a function of sex, ethnicity, and sociometric status
A1 - Bradley,Kathy Denise
Y1 - 2001///
N1 - Dissertation Abstract: 2001-95018-183
English
Dissertation Abstract
KW - PsycInfo
KW - Human Sex Differences
KW - Racial and Ethnic Differences
KW - Social Acceptance
RP - NOT IN FILE
SP - 1613
JF - Dissertation Abstracts International: Section B: The Sciences and Engineering
VL - 62:
IS - 3-B
N2 - Understanding the process of social exclusion is necessary to develop effective intervention programs. The effects of peer relationship problems are far reaching, and include academic difficulties, problems with parental relationships, and even a variety of mental disorders. Researchers have a good understanding of the antecedents

of social exclusion and its deleterious effects on the outcast child; however, much less is known about the process of social exclusion in the context of peer group entry. It has been determined that peer group entry attempts is one of the most difficult social situations for young children, and a child's performance, or lack thereof, in this domain is significantly correlated with the peers' behavior toward that child. Of the studies which have focused on peer group entry, most have been conducted in laboratory settings. The present study examines naturally-occurring group entry attempts of rejected and neglected children, focusing on the types of group entry attempts made, how the group responds to these children, and whether these two findings are a function of subgroup differences. Participants for this study came from a larger study examining the effects of a social exclusion intervention on the social and emotional experiences of kindergarten children in the classroom: The sample for this study was 41 kindergarten-aged children who were identified from the original study at risk of peer group exclusion based on sociometric status. These children were observed during freeplay in the classroom and on the playground for 12, 5-minute observations across at least 5 different days. Observations were conducted during the first half of the school year, and again during the second half. Behaviors were coded on the basis of group entry attempts made and responses of the group. The different patterns observed help explain the strategies used, processes, and outcomes of peer group entry for socially excluded children, and, therefore, provide knowledge that can be applied for use by teachers, and also to increase the effectiveness of interventions focusing on childhood social exclusion. (PsycINFO Database Record (c) 2012 APA, all rights reserved)
SN - 0419-4217
AD - Bradley, Kathy Denise: U Texas At Austin, US
ER -

TY - JOUR
ID - 2850
T1 - Modifiability of the psychomotor domain
A1 - Kamps,Paulene Helen
Y1 - 2001///
N1 - Dissertation Abstract: 2001-95011-005
English
Dissertation Abstract
KW - PsycInfo
KW - Constructivism
KW - Down's Syndrome
KW - Gross Motor Skill Learning
KW - Perceptual Motor Learning
RP - NOT IN FILE
SP - 4672
JF - Dissertation Abstracts International Section A: Humanities and Social Sciences
VL - 61:
IS - 12-A
N2 - A case study design was used to determine if a cognitive approach, based on socio-constructivist theory, could facilitate gross motor skill acquisition in preschool aged children with Down Syndrome (Ds). After a pre-assessment, seven children, ranging in age from 2 years 10 months, to 5 years 9 months, participated in six trials each for sliding and jumping activities. Two intervention formats were used with each child. The command and practice (C&P) format focused primarily on the physical aspects of motor skill acquisition while the graduated prompt (GP) format was designed to influence the cognitive domain. After each intervention session, tasks measuring children's declarative knowledge of the motor skills were presented. Procedural knowledge was measured by observing the child in gross motor play at a large playground one day and one week after the intervention session. Information about each child's sliding and jumping related behaviours was collected using video observations, floor plan/maps, anecdotal notes, parent interviews, and journaling. Qualitative and quantitative information was reported in a case by case approach and compared using cross-case analysis. Patterns of improved and more independent psychomotor behaviours emerged during the intervention session, were observed later in free play sessions, and confirmed by parents. For the most part, current theories of motor learning and control were unable to explain the changed motor behaviours of the children. Therefore, a hypothetical description which blends motor learning with Vygotsky's socio-historical theory, is presented as the explanation for why and how psychomotor skill improvements were made. This project determined that the psychomotor domain is modifiable. Implications are that the GP format is a useful assessment model for the

psychomotor domain, and therefore, when teaching children with Ds gross motor skills, people should use verbal, visual, gestural/kinesthetic prompts, demonstrations of skills displayed with dolls and models, and explanations aimed at influencing cognitive understandings of motor skills. Future research options include motor theory development with Vygotsky's proposals, finding new ways to assess psychomotor knowledge, and using declarative and procedural knowledge when teaching motor skills to children with movement disorders/motor delays. (PsycINFO Database Record (c) 2012 APA, all rights reserved)
SN - 0419-4209
AD - Kamps, Paulene Helen: U Calgary, Canada
ER -

TY - JOUR
ID - 2851
T1 - Play in a working-class Taiwanese preschool
A1 - Liang,Chung Hui
Y1 - 2001///
N1 - Dissertation Abstract: 2001-95007-171
English
Dissertation Abstract
KW - PsycInfo
KW - Childhood Play Behavior
RP - NOT IN FILE
SP - 3891
JF - Dissertation Abstracts International Section A: Humanities and Social Sciences
VL - 61:
IS - 10-A
N2 - The goal of this study was to investigate children's everyday play in a working class Taiwanese preschool. Because Taiwan is currently rethinking the direction of early childhood education, it is timely to look closely at play in a preschool setting. Since recent debates in the U.S. have led some American schools to sacrifice free-play time in order to increase time for teaching academic subjects, investigation of children's play in this academic-oriented cultural case will be illuminating to American educators as well. This study was ethnographic in approach, involving thorough observation and investigation of children's play at regular intervals over a period of three years. The researcher was able to establish a special role in the preschool to access the children's peer groups that were not often accessible to adults. The findings indicate that children constantly engaged in play, and their play fitted well into the daily routine; in their L year (equivalent to the kindergarten year in the U.S.) the children earned more official play time by behaving well and concentrating on assigned tasks than they were in the M year (equivalent to the final preschool year in the U.S.); the children displayed a complex way of exploring male/female relationships both inside and outside of play. These findings suggest a strong cultural influence of Confucian ideas to the children's preschool life and their play environment. Children's hard work was rewarded by more official play time when they were in the L year. The issue of gender was crucial to the understanding of children's friendships and daily interaction with peers. Overall, this study provides a basic understanding of the Taiwanese preschool children's life, which is essential for educational reform in Taiwan. The fact that the children were flexible to fit play into their daily routine, and their way of earning more play time by good conduct, challenges to American educators that increasing academic time does not necessarily equal decreasing play time. (PsycINFO Database Record (c) 2012 APA, all rights reserved)
SN - 0419-4209
AD - Liang, Chung-Hui: U Illinois At Urbana-Champaign, US
ER -

TY - JOUR
ID - 2852
T1 - Social behaviors of children with language impairment on the playground: A pilot study. [References]
A1 - Fujiki,Martin; Brinton,Bonnie; Isaacson,Ted; Summers,Connie
Y1 - 2001///
N1 - Peer Reviewed Journal: 2001-17249-004
English
Journal; Peer Reviewed Journal

KW - PsycInfo
KW - Childhood Play Behavior
KW - Language Disorders
KW - Peer Relations
KW - Social Interaction
RP - NOT IN FILE
SP - 101
EP - 113
JF - Language, Speech, and Hearing Services in Schools
VL - 32:
IS - 2
N2 - The purpose of this pilot investigation was to examine playground interactions of eight children (aged 6-10 years) with learning impairments (LI) and their typical peers in order to clarify further the social behaviors of these children. The participants were videotaped during the morning and lunch recesses. Their behavior was coded into one of the 37 subcategories and the subcategories were grouped into 6 general categories. Children with LI demonstrated significantly more withdrawn behaviors than did their peers. Typical children spent considerably more time interacting with peers than did children with LI. No other significant differences were observed. (PsycINFO Database Record (c) 2012 APA, all rights reserved)
SN - 0161-1461
AD - Fujiki, Martin: Brigham Young U, Dept of Audiology & Speech Language Pathology, Provo, UT, US
ER -

TY - JOUR
ID - 2853
T1 - Child-centered group play therapy. [References]
A1 - Landreth,Garry L.
Y1 - 2001///
N1 - Book: 2001-16195-012
English
Book; Edited Book
KW - PsycInfo
KW - Group Psychotherapy
KW - Play Therapy
KW - Therapeutic Processes
KW - Child Psychotherapy
KW - Diagnosis
KW - Treatment Planning
RP - NOT IN FILE
SP - 181
EP - 202
JF - Landreth, Garry L [Ed]
VL - :
IS - 2001
N2 - (from the chapter) In the child-centered group play therapy (GPT) process, children experience an environment that is marked by warmth and acceptance and promotes safety and growth. This environment allows children the opportunity to discover that their peers may be struggling too, which helps to deconstruct the barriers that children have of feeling along in their pain. A sense of belonging develops, and children appropriate ways to relate within and outside the playroom. In the child-centered GPT process, children experience a consistent and accepting response from the therapist, regardless of their presenting problems, degree of normality, or extent of personal adjustment. The group experience allows children to communicate about these issues on both an intrapersonal and interpersonal level. The child-centered therapy process takes the focus off the therapist and allows the children to experience self-exploration, self-discovery, and self-realization. It is the actualization of empowerment. This chapter discusses issues related to diagnosis and treatment planning, therapeutic limiting setting, the rationale for utilizing group play therapy, and group structure and logistics. A case example is presented of child-centered GPT involving 3 children (7 and 8 yrs old). (PsycINFO Database Record (c) 2012 APA, all rights reserved)
AD - Landreth, Garry L.: U North Texas, Counselor Education Dept, Denton, TX, US

ER -

TY - JOUR
ID - 2854
T1 - Behavior sampling and ethnography: Complementary methods for understanding home-school connections among Latino immigrant families. [References]
A1 - Weisner,Thomas
Y1 - 2001///
N1 - Peer Reviewed Journal: 2001-14176-002
English
Journal; Peer Reviewed Journal
KW - PsycInfo
KW - Academic Achievement
KW - Daily Activities
KW - Ethnography
KW - Home Environment
KW - Sampling (Experimental)
KW - Immigration
KW - Lower Income Level
KW - Methodology
KW - Latinos/Latinas
RP - NOT IN FILE
SP - 20
EP - 46
JF - Field Methods
VL - 13:
IS - 1
N2 - Both ethnography and experience-sampling methods (ESMs) are effective for assessing children's home activities. The authors combined them to examine home activities that were school-like, complementary to school, or unrelated to school. The activities were then related to children's school achievement in a sample of 8 low-income Latino immigrant families and their 10-11 yr old children at risk for low school achievement. Children reported a wide variety of types of activities in their evening routines. Children with higher school achievement were engaged in chores; homework; monitored outside activities; family social activities and hobbies; and self-directed, goal-oriented activities. Children with lower levels of school achievement tended to be engaged in more television, video games, peer and solitary play, and resting. ESMs are a valuable and effective complement to ethnographic and school achievement data in the study of home activities and home-school relationships. (PsycINFO Database Record (c) 2012 APA, all rights reserved)
SN - 1525-822X
AD - Weisner, Thomas: U California, Dept of Psychiatry & Anthropology, Los Angeles, CA, US
ER -

TY - JOUR
ID - 2855
T1 - Connecting school and community with science learning: Real world problems and school-community partnerships as contextual scaffolds. [References]
A1 - Bouillion,Lisa M.
Y1 - 2001///
N1 - Peer Reviewed Journal: 2001-11906-002
English
Journal; Peer Reviewed Journal
KW - PsycInfo
KW - Communities
KW - Elementary Education
KW - Environmental Education
KW - Pollution
KW - Science Education
KW - Elementary School Teachers

KW - Language Arts Education
KW - Mathematics
KW - Parents
RP - NOT IN FILE
SP - 878
EP - 898
JF - Journal of Research in Science Teaching
VL - 38:
IS - 8
N2 - Discusses an approach to helping children see and discover meaning in their science learning beyond the classroom. Two 5th grade teachers, their students, a science teacher, a technology coordinator, and a bilingual instructor participated in a student-identified problem of pollution along a nearby river as an interdisciplinary anchor for teaching science, math, language arts, and civics. The resources, knowledge, and experience of students, parents, and teachers were combined to clean up a riverbank site and transform it into a community green space. Explored areas included the kinds of activities students were engaged in, how they evolved over time, and the kinds of supporting knowledge and experience that were brought together. The real-world problems were interdisciplinary in nature, were relevant to both the curriculum and students' lives, and were highly visible and accessible. (PsycINFO Database Record (c) 2012 APA, all rights reserved)
SN - 0022-4308
AD - Bouillion, Lisa M.: Northwestern U, Evanston, IL, US
ER -

TY - JOUR
ID - 2856
T1 - Football participation in the primary school playground: The role of coordination impairments. [References]
A1 - Smyth,Mary M.
Y1 - 2001///
N1 - Peer Reviewed Journal: 2001-11814-005
English
Journal; Peer Reviewed Journal
KW - PsycInfo
KW - Football
KW - Measurement
KW - Movement Disorders
KW - Playgrounds
RP - NOT IN FILE
SP - 369
EP - 379
JF - British Journal of Developmental Psychology
JA - BR J DEV PSYCHOL
VL - 19:
IS - 3
N2 - The goal of the present investigation was to examine whether early coordination impairments were related to later football participation in the school playground. The participants were 64 boys (aged 6-10 yrs), 32 in a movement impaired group and 32 in a non-impaired group, who were divided into groups of those who were often alone and those who were not. The not-alone group was further subdivided into those who played football for considerable periods and those who did not. There were 10 boys with poor scores on the Movement Assessment Battery for Children (Movement ABC) who were not often alone and who played football for considerable amounts of time. Analyses of the subscales of the ABC indicated that the balance subscale was significantly related to participation in football, but that some boys with relatively poor balance scores did play football. Only extremely poor performance on the balance tasks of the Movement ABC was related to non-participation in football. Some of the key differences between groups of children with movement impairments in terms of their inclusion in social and physical games like football may not relate to hand/eye coordination and manual control, but to the ability to maintain posture while carrying out other movements, particularly when balance skills are extremely poor. (PsycINFO Database Record (c) 2012 APA, all rights reserved)
SN - 0261-510X

AD - Smyth, Mary M.: Lancaster U, Dept of Psychology, Lancaster, United Kingdom
ER -

TY - JOUR
ID - 2857
T1 - Overlap in bilingual play: Some implications of code-switching for overlap resolution. [References]
A1 - Cromdal,Jakob
Y1 - 2001///
N1 - Peer Reviewed Journal: 2001-11714-002
English
Journal; Peer Reviewed Journal
KW - PsycInfo
KW - Bilingualism
KW - Childhood Play Behavior
KW - Code Switching
RP - NOT IN FILE
SP - 421
EP - 451
JF - Research on Language and Social Interaction
VL - 34:
IS - 4
N2 - Examines children's procedures for dealing with simultaneous bilingual speech as it arises in multiparty play episodes. Sequential analyses of more than 10 hr of videorecorded recess activities at an English school in Sweden revealed that Swedish-English bilingual children in grades 2 and 4 use code-switching and an array of methods to minimize the overlapping passage. Exceptions to this may be found in exchanges that are demonstrably competitive with regard to turn taking, in which participants' actions strive to gain exclusive rights to the floor, often resulting in stretched overlaps. Moreover, the sequential location of bilingual overlap onset proved relevant for its resolution: Whereas in interjacent onset participants would use different methods to deal with simultaneity, resulting in various outcomes of overlap negotiation, instances of overlapping turn beginnings occasioned by multiple self-selection were always resolved the same way, with the speaker diverging from the language of previous turn(s) keeping the floor. It is therefore suggested that the linguistic contrast arising with the code-switch may enhance second speakers' chances to acquire the floor and that the effectiveness of this "turn security device" is strongly dependent on its sequential placement. (PsycINFO Database Record (c) 2012 APA, all rights reserved)
SN - 0835-1813
AD - Cromdal, Jakob: Linkoping U, Dept of Child Studies, Linkoping, Sweden
ER -

TY - JOUR
ID - 2858
T1 - Students' use of time outside of school: A case for after school programs for urban middle school youth. [References]
A1 - Shann,Mary H.
Y1 - 2001///
N1 - Peer Reviewed Journal: 2001-09614-003
English
Journal; Peer Reviewed Journal
KW - PsycInfo
KW - After School Programs
KW - At Risk Populations
KW - Extracurricular Activities
KW - Leisure Time
KW - Disadvantaged
KW - Middle School Students
KW - Minority Groups
KW - Program Development
RP - NOT IN FILE

SP - 339
EP - 356
JF - The Urban Review
VL - 33:
IS - 4
N2 - Students in 4 inner-city middle schools serving mostly (90.9%) economically disadvantaged, minority youth were asked how they spend their time after school and on weekends. Analysis of responses from 1,583 students revealed that the vast majority did not participate in after-school programs (77.2%) or lessons of any kind (86.5%). Students reported extensive time spent watching television or hanging out with friends. Weekends presented a similar pattern of unstructured social activity with even more television viewing and considerably less homework. Implications for designing after school programs are discussed in light of the students' highly unproductive use of time outside school. (PsycINFO Database Record (c) 2012 APA, all rights reserved)
SN - 0042-0972
AD - Shann, Mary H.: Boston U, School of Education, Boston, MA, US
ER -

TY - JOUR
ID - 2859
T1 - The natural environment as a playground for children: The impact of outdoor play activities in pre-primary school children. [References]
A1 - Fjortoft,Ingunn
Y1 - 2001///
N1 - Peer Reviewed Journal: 2001-09550-006
English
Journal; Peer Reviewed Journal
KW - PsycInfo
KW - Childhood Play Behavior
KW - Motor Development
KW - Playgrounds
KW - School Attendance
KW - School Environment
KW - Preschool Students
RP - NOT IN FILE
SP - 111
EP - 117
JF - Early Childhood Education Journal
VL - 29:
IS - 2
N2 - In Scandinavia it has become popular for kindergartners to spend more time outdoors in the natural environment. Some kindergartens are organized as outdoor schools, where the children, aged 3-6 yrs, spend all or most of the day outdoors in a natural environment. Playing in a natural environment seems to have positive effects on children; they become more creative in their play, and play activities and play forms are increasing. It is also indicated that absence due to sicknesses is lower among children in outdoor kindergartens than in the traditional ones. At the very least it is evident that children's motor fitness is improved in such an environment. They move easily around in a rugged terrain and cope with physical challenges, which improve their motor ability. Although few in number, these studies indicate that the natural environment is a stimulating arena for learning in general, and for motor fitness training in particular. The present research corroborates the main findings. (PsycINFO Database Record (c) 2012 APA, all rights reserved)
SN - 1082-3301
AD - Fjortoft, Ingunn: Telemark U Coll, Dept of Teacher Training, Notodden, Norway
ER -

TY - JOUR
ID - 2860
T1 - The educational benefits of a ten-week home-based wild bird feeding program for children. [References]
A1 - Beck,Alan M.
Y1 - 2001///

N1 - Peer Reviewed Journal: 2001-07609-002
English
Journal; Peer Reviewed Journal
KW - PsycInfo
KW - Birds
KW - Education
KW - Knowledge Level
KW - Teaching Methods
KW - Elementary School Students
RP - NOT IN FILE
SP - 19
EP - 28
JF - Anthrozoos
VL - 14:
IS - 1
N2 - Evaluated a 10-wk educational home-based program for feeding wild birds, intended to increase elementary school age children's knowledge about birds, especially those wild birds commonly encountered at outdoor home feeders. The authors measured changes from pre- to post-program in 65 7-12-yr-old children's knowledge about wild birds, as well as in environmental attitudes. The goal was not only to increase bird knowledge in the target child but also to involve other family members in home-based nature education activities. After the program, 7-9-yr-old boys and girls showed significant gains in bird knowledge, but older children (10-12 yrs) did not significantly improve. Children's increased knowledge was positively associated with parental education. There was no systematic change in environmental attitudes. Parents identified family involvement as a particularly beneficial aspect of the program, and 90% of contacted families were still feeding birds one year after program termination. (PsycINFO Database Record (c) 2012 APA, all rights reserved)
SN - 0892-7936
AD - Beck, Alan M.: Purdue U, School of Veterinary Medicine, Dept of Veterinary Pathobiology, West Lafayette, IN, US
ER -

TY - JOUR
ID - 2861
T1 - Compliance with home rehabilitation therapy by parents of children with disabilities in Jews and Bedouin in Israel. [References]
A1 - Galil,Aharon; Carmel,Sara; Lubetzky,Hasi; Vered,Stavia; Heiman,Nurit
Y1 - 2001///
N1 - Peer Reviewed Journal: 2001-06374-005
English
Journal; Peer Reviewed Journal
KW - PsycInfo
KW - Arabs
KW - Exercise
KW - Home Care
KW - Jews
KW - Treatment Compliance
KW - Caregiver Burden
KW - Client Satisfaction
KW - Disorders
KW - Family Relations
KW - Socioeconomic Status
RP - NOT IN FILE
SP - 261
EP - 268
JF - Developmental Medicine & Child Neurology
JA - Dev Med Child Neurol
VL - 43:
IS - 4

N2 - Evaluated factors influencing compliance with a home therapeutic program for children with disabilities in Jewish and Bedouin Israeli populations. Members of 83 Bedouin and 110 Jewish families with disabled children (mean ages 6.8 and 9.54 mo) completed questionnaires concerning compliance with suggested home exercise, disability status and medical diagnoses, belief in outcome, satisfaction with service, and economic burden. Results show that Bedouin Ss had more severe disabilities than did Jewish Ss. Bedouin families had lower levels of education, more mothers working outside the home, more children, and stronger feeling of burden from caring for Ss. Compliance was significantly lower among Bedouin families. Families with higher levels of intensity of destiny-questioning were less compliant with therapeutic regimes. A trial intervention program for Bedouin families, involving telephone contact, translation facilities, and detailed explanations during visits to the center, increased the compliance rate of the Bedouin to 76%, compared to 81% for Jewish families. It is concluded that increased attention through strengthened communication can greatly improve compliance with home therapeutic programs. (PsycINFO Database Record (c) 2012 APA, all rights reserved)
SN - 0012-1622
AD - Galil, Aharon: Soroka U Medical Ctr, Zusman Child Development Ctr, Beer Sheva, Israel
ER -

TY - JOUR
ID - 2862
T1 - Spontaneous sonorous productions during playtime. [French]. [References]
A1 - Lapoujade,Christine
Y1 - 2001///
N1 - Peer Reviewed Journal: 2001-01944-003
French
Journal; Peer Reviewed Journal
KW - PsycInfo
KW - Childhood Play Behavior
KW - School Environment
KW - Acoustics
KW - Elementary School Students
KW - Music
KW - Singing
RP - NOT IN FILE
SP - 167
EP - 179
JF - Enfance
VL - 53:
IS - 2
N2 - Studied the relation of spontaneous sonorous productions during play time and environmental factors in play areas at 8 nursery schools and 12 primary schools in France. Ss included 50-100 children (aged 3-6 yrs) per nursery school play area and 60-200 children (aged 6-12 yrs) per primary school play area. Children's sounds and environmental sounds were tape-recorded during 30 min intervals and scored using the Repertoire of Sound Effects method (J. -F. Augoyard, 1987). Musical and other sound productions of children were evaluated according to timbre, scale changes, audibility, rhythm, and melodic elements; environmental factors were evaluated according to background sounds, sound uniformity, number and spatial arrangement of children, movement of children, and tension. The results show that children's spontaneous productions tend to be school-specific. The results also suggest that disturbing demographic and acoustic conditions at some playgrounds may increase tension via sound overload. (PsycINFO Database Record (c) 2012 APA, all rights reserved)
SN - 0013-7545
AD - Lapoujade, Christine: U Rene Descartes-Paris V, Lab de Psychologie Clinique et Psychopathologique, Paris, France
ER -

TY - JOUR
ID - 2863
T1 - Physical activity for preschool children: Growing up fit-Together. [References]
A1 - Bosch,Ann; Glatzmeier,Darlene; Servais,Lisa; Reicks,Marla
Y1 - 2000///

N1 - Peer Reviewed Journal: 2008-09795-009
English
Journal; Peer Reviewed Journal
KW - PsycInfo
KW - Health Education
KW - Health Promotion
KW - Physical Activity
KW - Physical Education
KW - Eating Behavior
KW - Lower Income Level
KW - Mothers
KW - Nutrition
KW - Overweight
KW - Preschool Students
RP - NOT IN FILE
SP - 64
EP - 65
JF - Journal of Nutrition Education
VL - 32:
IS - 1
N2 - There is an increasing prevalence of overweight in preschool children among the U.S. low-income population. Although research indicates that important correlates of physical activity in preschoolers are time spent outdoors and prompts to be active by adults, there are few educational materials available for parents that build on this research. Nutrition educators with the University Extension Service developed materials to increase physical activity and improve eating habits of low-income families with preschool-aged children in a mid-state cluster of counties. To learn more about the physical activity and eating habits of families, focus group interviews were held with three groups of Hispanic and Anglo mothers attending WIC clinics. Information was obtained about needs, barriers, and motivators for physical activity and reactions to ideas about proposed types of educational materials. Results indicated that barriers to being active with preschoolers were lack of time and energy and limited resources. Educational materials were developed based on needs suggested by focus group participants. Materials included an activity calendar, reminder refrigerator magnet, poster, motivational videotape, and brochures. Most women responded favorably when asked if they used the calendar; and more than half indicated that it helped them be more active with their preschooler by serving as a reminder and a source of ideas for activities. The materials are currently being used by county staff, with future plans to make them available statewide. (PsycINFO Database Record (c) 2012 APA, all rights reserved)
SN - 0022-3182
AD - Bosch, Ann: University of Minnesota Extension Service, St. Paul, MN, US Glatzmeier, Darlene: University of Minnesota Extension Service, St. Paul, MN, US Servais, Lisa: School of Public Health, University of Minnesota, St. Paul, MN, US Reicks, Marla: Department of Food Science and Nutrition, University of Minnesota, St. Paul, MN, US
ER -

TY - JOUR
ID - 2864
T1 - Physical activity of children with and without mental retardation in inclusive recess settings. [References]
A1 - Lorenzi,David G.
Y1 - 2000///
N1 - Peer Reviewed Journal: 2001-17960-004
English
Journal; Peer Reviewed Journal
KW - PsycInfo
KW - Childhood Play Behavior
KW - Group Differences
KW - Physical Activity
KW - Physical Endurance
KW - Heart Rate
KW - Intellectual Development Disorder

RP - NOT IN FILE
SP - 160
EP - 167
JF - Education & Training in Mental Retardation & Developmental Disabilities
VL - 35:
IS - 2
N2 - Compared physical activity levels of children with and without mental retardation. 34 children (aged 5.5-12 yrs) were assessed in an inclusive recess setting and monitored for heart rate, activity counts and observational activity levels. Based on the data analysis, boys demonstrated significantly higher values on all comparisons. Boys with mental retardation demonstrated higher activity counts and heart rates than boys without mental retardation while no group differences were apparent on the observational checklist. It was concluded that an inclusive, non-structured recess setting appears to encourage and facilitate physical activity for children with mental retardation. (PsycINFO Database Record (c) 2012 APA, all rights reserved)
SN - 1079-3917
AD - Lorenzi, David G.: U Georgia, Athens, GA, US
ER -

TY - JOUR
ID - 2865
T1 - Inclusion of students with moderate or severe disabilities in educational and community settings: Perspectives from parents and siblings. [References]
A1 - Gallagher,Peggy A.
Y1 - 2000///
N1 - Peer Reviewed Journal: 2001-17960-002
English
Journal; Peer Reviewed Journal
KW - PsycInfo
KW - Disorders
KW - Imitation (Learning)
KW - Parental Attitudes
KW - School Environment
KW - Siblings
KW - Communities
RP - NOT IN FILE
SP - 135
EP - 147
JF - Education & Training in Mental Retardation & Developmental Disabilities
VL - 35:
IS - 2
N2 - Investigated parent (PA) and sibling (SI) perspectives on the educational and community inclusion of students (aged 3-20 yrs) with moderate or severe disabilities. Interviews with PAs from 21 families identified the type and extent of inclusive educational and community settings in which the student and his/her PAs and SIs were involved. Eight of these target SIs were also interviewed to provide insight on their experience of inclusion. Mothers reported involvement in an average of almost 4 community activities, with fathers averaging 3 activities, siblings averaging 1.33 activities each, and the student usually engaged in only one activity outside of school. Themes that emerged included: 1) the belief that the child with a disability can function and achieve in the real world; 2) the desire to have the child with a disability around typical children as much as possible in order to serve as models of skill and behavior; 3) the PAs' view that regular education teachers are unwilling or untrained to accommodate children with disabilities; 4) the general satisfaction with the amount and type of inclusion both at school and in the community; and 5) the view that the student with disabilities models the typical sibling. SIs' perceptions tended to mirror those of the PAs. (PsycINFO Database Record (c) 2012 APA, all rights reserved)
SN - 1079-3917
AD - Gallagher, Peggy A.: Georgia State U, Dept of Educational Psychology & Special Education, Atlanta, GA, US
ER -

TY - JOUR
ID - 2866
T1 - The play interactions of young children with and without disabilities: Individual and environmental influences
A1 - Hestenes,Linda L.
Y1 - 2000///
N1 - Peer Reviewed Journal: 2001-03185-004
English
Journal; Peer Reviewed Journal
KW - PsycInfo
KW - Childhood Play Behavior
KW - Classroom Environment
KW - Developmental Disabilities
KW - Social Interaction
KW - Preschool Students
RP - NOT IN FILE
SP - 229
EP - 246
JF - Early Childhood Research Quarterly
VL - 15:
IS - 2
N2 - Examined preschool children's play interactions and beliefs in inclusive preschool settings in order to better understand the experience of inclusive classrooms for children with and without disabilities. Individual interviews of 21 typically developing children and observations of 29 children with and without disabilities in the classroom and on the playground provided data for the study. Summaries of children's play patterns showed a tendency for children without disabilities to engage in more cooperative play and less solitary play and onlooking behavior than did their peers with disabilities. Descriptive comparisons of activity choice showed a high level of similarity between the types of activities that children with and without disabilities selected during free play. Typically developing children spent less time interacting with their peers with disabilities than was expected, and children with disabilities interacted less with their typically developing peers than was expected. An understanding of disability was predictive of stated preference to play with hypothetical peers with disabilities. Actual interactions with peers with disabilities were predicted by children's age and teacher presence, but not by an understanding of disability or stated playmate preference. (PsycINFO Database Record (c) 2012 APA, all rights reserved)
SN - 0885-2006
AD - Hestenes, Linda L.: U North Carolina, Dept of Human Development & Family, Greensboro, NC, US
ER -

TY - JOUR
ID - 2867
T1 - The relationship between physical activity and stress response of children. [Japanese]
A1 - Uechi,Hiroaki; Takenaka,Koji; Oka,Koichiro
Y1 - 2000///
N1 - Peer Reviewed Journal: 2001-00888-001
Japanese
Journal; Peer Reviewed Journal
KW - PsycInfo
KW - Activity Level
KW - Exercise
KW - Rating Scales
KW - Stress Reactions
KW - Test Construction
KW - Elementary School Students
KW - Physical Activity
RP - NOT IN FILE
SP - 1
EP - 8

JF - Japanese Journal of Health Psychology
VL - 13:
IS - 2
N2 - Two experiments developed a physical activity scale for elementary school children, and examined the relationship between their daily physical activity and stress responses. In Exp 1, 234 pupils in grades 4-6 completed a battery of questionnaires in the classroom. Factor analysis revealed that the physical activity scale had a single-factor structure that consisted of 5 factors: physical education class, recess, lunch, after school, and the evening. 306 Ss in grades 4-6 participated in Exp 2, where results of a correlation analysis of the relationship between daily physical activities and stress responses indicated that the amount of daily physical activity was consistently correlated with the stress response subscales: physical states, depressive-anxious feeling, irritated-angry feeling, and helplessness. It was concluded that the increase of physical activity improves children's mental health. (PsycINFO Database Record (c) 2012 APA, all rights reserved)
SN - 0917-3323
AD - Uechi, Hiroaki: Waseda U, Graduate School of Human Sciences, Saitama, Japan
ER -

TY - JOUR
ID - 2868
T1 - Social behaviour during the preschool years in relation to: Theory of mind, future-oriented thinking and language
A1 - Angelopoulos,Maria
Y1 - 2000///
N1 - Dissertation Abstract: 2000-95022-384
English
Dissertation Abstract
KW - PsycInfo
KW - Future
KW - Language
KW - Social Behavior
KW - Theory of Mind
KW - Thinking
RP - NOT IN FILE
SP - 2740
JF - Dissertation Abstracts International: Section B: The Sciences and Engineering
VL - 61:
IS - 5-B
N2 - The present study examined the relationship between peer social behaviours in naturalistic settings and lab-based measures of social understanding. A sample of 120 3- and 4-year-old children were presented with tasks assessing theory of mind, future-oriented self-control and sharing, peer-rated likability and empathic concern. They were also given a standard measure of general language ability. Children were also videotaped during a 10-minute naturalistic peer play session. Parents and teachers completed questionnaires rating children's global social skills, problem behaviours, and mental state concepts. It was hypothesized that older children would perform more successfully on lab-based measures and that their peer social behaviours would be more sophisticated. Results indicated that most of the lab measures, questionnaires, and naturalistic observations discriminated between the 3- and 4-year-olds in the expected direction. Compared to the 3-year-olds, the 4-year-olds achieved higher scores on lab-based measures of social understanding and these abilities enabled them to interact in meaningfully different ways with others. Four-year-olds interactive abilities were also seen as more sophisticated than 3-year-olds by peers, parents, and teachers. Three-year-olds used their restricted abilities in an indiscriminate fashion and were periodically successful in their interactions. In contrast, 4-year-olds showed more purposeful social behaviour. The patterns of data provide evidence of clear developmental differences between age-groups. These findings are discussed in terms of their implications for children's social development. (PsycINFO Database Record (c) 2012 APA, all rights reserved)
SN - 0419-4217
AD - Angelopoulos, Maria: Dalhousie U., Canada
ER -

TY - JOUR

ID - 2869
T1 - The role of challenge/initiative recreation games as a therapeutic regimen in fostering a positive self-concept for inner-city preschool children
A1 - Uzomah,Celestina Nnenna
Y1 - 2000///
N1 - Dissertation Abstract: 2000-95020-060
English
Dissertation Abstract
KW - PsycInfo
KW - Childhood Play Behavior
KW - Childrens Recreational Games
KW - Competition
KW - Initiative
KW - Urban Environments
RP - NOT IN FILE
SP - 1916
JF - Dissertation Abstracts International: Section B: The Sciences and Engineering
VL - 61:
IS - 4-B
N2 - The purpose of this research was to determine whether a challenge/initiative program conducted in an existing recreation setting that was not in an outdoor wilderness could be used as a treatment technique in enhancing a positive self-concept of low-income inner-city children. Data were collected using a pre-test-post-test control group design with the Piers-Harris Children's Self-Concept Scale (1996), (the standard and the modified scale version) administered from mid-April to late May of 1999. The dependent variables for this study were the post-test and the pre-test self concept scores as measured by the Piers-Harris Children's Self-Concept Scale (Piers & Harris, 1996). The independent variables were the types of treatment program experimental or control/ or challenge/initiative games or traditional games. Also sex, age, and ethnic background were considered independent variables. A total of 96 three-, four- and five-year old inner-city pre-schoolers were randomly assigned to experimental and control groups. The treatment group received challenge/initiative game treatment; the control group received traditional playground type competitive games for a duration of six weeks. Analysis of Variance (ANOVA) was used to determine whether there was a significant difference in overall self-concept as measured by the Piers-Harris Children's Self-Concept Scale (Piers and Harris, 1996) of post-test scores of low-income children who participated in the challenge/initiative games (experimental group) when compared with the low-income children who were involved in traditional recreational games (control group). Multivariate analysis of variance (MANOVA) was also used to test for differences in self-concept scores, pre-test scores (dependent variables), and the independent variables of sex, age and ethnic background. Results showed that there were significant and practical differences between the treatment and control groups when measured with the modified scale. The results of this study indicated that there is a need to address the development of children's self-concept within the pre-school setting of the inner city. Future investigations are needed, however, to identify other recreation programs to be included in the school curriculum to allow the children opportunities to explore their feelings and perform tasks successfully, thereby increasing their sense of competence, causing significant increases in their self-concept. Happy, cooperative, well-behaved children are more likely to make the pre-school environment a better work setting for the adults, and pre-school environments that promote the widespread use of cooperative games may have a positive impact on future social behavior. (PsycINFO Database Record (c) 2012 APA, all rights reserved)
SN - 0419-4217
AD - Uzomah, Celestina Nnenna: U Maryland Coll Park, US
ER -

TY - JOUR
ID - 2870
T1 - Effects of empathy training on the peer approval of third graders
A1 - Otfinowski,Giovanna A.
Y1 - 2000///
N1 - Dissertation Abstract: 2000-95018-327
English
Dissertation Abstract

KW - PsycInfo
KW - Empathy
KW - Peer Relations
KW - Social Approval
RP - NOT IN FILE
SP - 1621
JF - Dissertation Abstracts International: Section B: The Sciences and Engineering
VL - 61:
IS - 3-B
N2 - The effects of empathy training on the peer approval of students who had the most least-liked votes on a class sociogram and were considered the least empathic by their classmates was investigated. Peer approval was assessed through the administration of a pre, post, and follow-up classroom sociogram, Structured playground observations were utilized to determine if the empathy training resulted in an increase in positive peer interactions and a decrease in negative peer interactions. Peer perceptions of classmates' empathy were determined through the use of a sociometric format Students were asked to name the classmates they would like to be with if they felt sad and which they would not like to be with when they felt sad. Acquisition of empathy skills was measured by the pre, post, and follow-up administration of Bryant's Index of Empathy for Children, and Adolescents (1982). A multiple baseline across subjects design was utilized, to determine if empathy training increased the three participants' playground peer approval. Study results indicated that teaching children empathic responses increased peer approval as reflected by playground observations. These observations indicated that peers positively approached participants more and that the approaches of the participants were accepted more frequently. Participants also displayed a decrease in aggressive behavior. A six month follow-up of participants' playground behavior demonstrated that behavioral improvements had been maintained. Peer approval, as measured by a classroom sociogram administered immediately after the intervention's, completion, revealed minimal improvement in the participant's peer status. The minimal gains made by the participants on the classroom's pre- and post-intervention sociogram and empathy measure were not maintained six months later. These findings are consistent with previous results which indicate that empathy training contributes to an increase in prosocial skills and a decrease in aggressive behavior. (PsycINFO Database Record (c) 2012 APA, all rights reserved)
SN - 0419-4217
AD - Otfinowski, Giovanna A. I.: U San Francisco, US
ER -

TY - JOUR
ID - 2871
T1 - A descriptive study of the body use enculturation of typically developing first graders in the school environment
A1 - Matthews,Pamela S.
Y1 - 2000///
N1 - Dissertation Abstract: 2000-95011-130
English
Dissertation Abstract
KW - PsycInfo
KW - Body Size
KW - Motor Performance
KW - Posture
KW - School Environment
KW - Classroom Environment
KW - Elementary School Students
RP - NOT IN FILE
SP - 4319
JF - Dissertation Abstracts International Section A: Humanities and Social Sciences
VL - 60:
IS - 12-A
N2 - The purpose of this study was to explore how, in the culture of an elementary school, children gain knowledge about moving, sitting still, and using their bodies in response to the daily demands of schooling. It describes the body use of a class of typically developing first graders (while viewing their learning as a step in

the larger enculturation process that prepares them for adult work roles in our society). Using qualitative research methods, the children and their teachers were observed and interviewed over an entire school year. The children also drew pictures and told stories about their perceptions and understanding of classroom and playground environment. Their skills in the areas of body concept and body use, and their physical size were assessed using quantitative research methods. After examining the school culture, four factors (program structure, the physical environment, and the behaviors of the children and their teachers) were found to have both overt and covert influences on body use. Two contradictory movement agendas (one expansive, the other limiting) were observed to operate simultaneously in the school setting. Body use factors identified as important in the school environment included sensory and perceptual processing, movement, neuromuscular integrity, cognition, and psychosocial. skills. The children's performance on a repeated measure of body concept did not change significantly over the school year as had been anticipated. Their body use and physical size did increase significantly between pre and posttesting. Detailed descriptions of posture and movement (gross motor, upper extremity, and fine motor), based on observational data, were developed. From the children's stories, six themes relative to the body use enculturation process were identified. They included teaching and learning, work and play, understanding rules and routines, pleasing adults, and pleasing yourself. Recommendations for school personnel include advocating for more space, greater variability in classroom environments, and enriched playground environments. The development of teacher training materials in the areas of body use learning and classroom management of movement is also suggested. (PsycINFO Database Record (c) 2012 APA, all rights reserved)
SN - 0419-4209
AD - Matthews, Pamela S.: U New Mexico, US
ER -

TY - JOUR
ID - 2872
T1 - A study of group entry techniques and equivalent social interactions of prekindergarten children
A1 - Ross,Theodosia Barkley
Y1 - 2000///
N1 - Dissertation Abstract: 2000-95011-018
English
Dissertation Abstract
KW - PsycInfo
KW - Childhood Play Behavior
KW - Human Sex Differences
KW - Social Acceptance
KW - Social Interaction
RP - NOT IN FILE
SP - 4313
JF - Dissertation Abstracts International Section A: Humanities and Social Sciences
VL - 60:
IS - 12-A
N2 - The purposes of this study are: (1) observe ways in which prekindergarten children initiate social interactions among peers to gain group entry and (2) examine the equivalent interactions from the children already in the group and (3) examine whether boys and girls use different social strategies and behaviors in their play. The research was conducted in a prekindergarten center located in a public elementary school located in a large urban community. The school served predominantly black middle class children. The center had four to five year to five year old children attending the extended day prekindergarten program five days a week. The children's uninhibited group play with their peers in centers and on the playground was examined through time sampling Written descriptive observations of the children's activities during 10-15-minute-time observations were recorded on social interaction checklists and through field notes. Each child was observed according to a prearranged list, over a period of 15 weeks yielding to a total of 200 minutes of observations per child. The researcher served as an on-site participant observer three to four hours per day, four days a week. The methodology of participant observation consists of principles, strategies, procedures, methods, and techniques of research. Through participant observations, it is possible to describe what goes on, who goes on, who or what is involved, when and whether things happen, how they occur, and why-at-least from the standpoint of participants-things happen as they do in particular situations. The findings revealed: (1) prekindergarten children are accepted into social context by their peers more often than they are rejected. (2) When prekindergarten

children use verbal statements or commands when entering a social context, they are usually accepted by the peers than when attempting to enter non verbally. (3) Peers we more likely to non verbally accept or reject children who attempt to enter a social context and (4) Prekindergarten children rarely engage in solitary activities or avoid making bids for entrance into social contexts. The results from this study indicate that prekindergarten children who acquire social skills and verbal communication strategies are more likely to be accepted by their peers when attempting entrance into social context. (PsycINFO Database Record (c) 2012 APA, all rights reserved)
SN - 0419-4209
AD - Ross, Theodosia Barkley: U Sarasota, US
ER -

TY - JOUR
ID - 2873
T1 - Father-child interaction and its relation to children's interactions with peers
A1 - Carrillo,Sonia
Y1 - 2000///
N1 - Dissertation Abstract: 2000-95006-257
English
Dissertation Abstract
KW - PsycInfo
KW - Father Child Relations
KW - Peer Relations
RP - NOT IN FILE
SP - 4926
JF - Dissertation Abstracts International: Section B: The Sciences and Engineering
VL - 60:
IS - 9-B
N2 - The first relationships a child establishes are within the family context. As children grow older, their social interactions outside the family environment increase, and peers become more influential in their socialization. A very important research question in social development is how the family and the peer systems are related. Do children's interactions within the family influence children's interactions with peers? Although it is frequently argued that family interactions serve as a base for the establishment of interactions with peers, the nature of the processes and mechanisms by which the family and peer systems are linked have not been elucidated. More specifically, evidence of the role of family members other than the mother in children's peer interactions has been limited. The main purpose of the present research was to extend the studies of family-peer linkages to one of these neglected family members: the father. Thirty-five 7-year-old children (18 girls and 17 boys) were videotaped behind a one-way mirror in two 24-minute play sessions, one with their father, and one with an unfamiliar same-sex peer. Videotapes were scored using rating scales that assessed father individual behaviors with children, father-child dyadic interactions and children individual behaviors with peers. The results showed that some father individual behaviors were correlated with children individual social behaviors with peers. Similarly, dyadic synchrony was found to be significantly related to children's social behaviors with peers. However, the two types of measures (individual vs. dyadic) did not correlate with the same children's behaviors. Further statistical analyses showed that dyadic synchrony contributed to the prediction of children's social behavior with peers above and beyond father individual behaviors. These results suggest that both individual and dyadic measures of father-child interaction are necessary for an understanding and prediction of children's social interactions out of the family context. (PsycINFO Database Record (c) 2012 APA, all rights reserved)
SN - 0419-4217
AD - Carrillo, Sonia: U Texas At Austin, US
ER -

TY - JOUR
ID - 2874
T1 - Does the matching law explain how the allocation of teacher attention relates to the allocation of children's engagement with toys?
A1 - Parra,Aida Margarita
Y1 - 2000///

N1 - Dissertation Abstract: 2000-95004-299
English
Dissertation Abstract
KW - PsycInfo
KW - Attention
KW - Child Day Care
KW - Childhood Play Behavior
KW - Resource Allocation
KW - Toys
KW - Student Teachers
KW - Teacher Student Interaction
RP - NOT IN FILE
SP - 4197
JF - Dissertation Abstracts International: Section B: The Sciences and Engineering
VL - 60:
IS - 8-B
N2 - The matching law (Herrnstein, 1961) is a mathematical expression that predicts how individuals distribute their behavior between alternative sources of reinforcement. In general, the law states that the proportion of behavior given to a source will match the proportion of reinforcement received from that source. Tests of the matching law in experimental and natural settings have suggested applications to educational settings. The present study asked how well the matching law can predict from a teacher's allocation of attention to children's engagement with toys how the children will allocate their time to engagement or nonengagement. The participants were three very young, typically developing girls (ages 19, 21, and 24 months) who attended a university-based infant and toddler full-daycare program; and three teachers, who at the time were undergraduate students enrolled in a practicum in early-childhood education. After an analysis of how the teachers and the children allocated their time during a baseline condition, four subsequent experimental conditions were programmed in an ABC1DC2 design for two of the children, and three subsequent experimental conditions were programmed in an A 1BCA2 design for the third child. In each condition, different ratios of teacher attention (sometimes combinations of talk, laughs, and touches; and sometimes only talk) were prescribed for the children's engagement and nonengagement. When the relative values of teacher attention for engagement were compared to the relative values of child engagement, the results overall suggested that the matching law was predictive: Across conditions, the proportion of teacher attention to the children's engagement predicted a roughly matching proportion of the children's engagement, relative to their nonengagement. All three participants, however, showed some degree of undermatching and bias. (PsycINFO Database Record (c) 2012 APA, all rights reserved)
SN - 0419-4217
AD - Parra, Aida Margarita: U Kansas, US
ER -

TY - JOUR
ID - 2875
T1 - Peer processes and bullying: Naturalistic observation on the playground
A1 - O'Connell,Paul Desmond
Y1 - 2000///
N1 - Dissertation Abstract: 2000-95004-265
English
Dissertation Abstract
KW - PsycInfo
KW - Aggressive Behavior
KW - Childhood Play Behavior
KW - Distress
KW - Emotional Responses
KW - Peer Relations
KW - Playgrounds
KW - Victimization
KW - Bullying
RP - NOT IN FILE

SP - 4306
JF - Dissertation Abstracts International: Section B: The Sciences and Engineering
VL - 60:
IS - 8-B
N2 - The purpose of this research was to examine the peer processes that occur during episodes of bullying on the school playground. These processes were examined within a social learning theory perspective, while also drawing on emotional contagion theory, an ethological approach to studying dominance hierarchies, and dynamic systems theory. Fifty-three segments of videotape that contained a peer group viewing bullying on the school playground were examined. Peers were coded for actively supporting the bully, passively watching the bullying, and for actively supporting the victim. Also coded were the levels of affect, aggression, and distress of bullies, victims, and peers, throughout each episode. On average, four peers viewed the schoolyard bullying, with a range from two to 14 peers. Averaged across all episodes, peers spent 53% of their time passively viewing bullying, 25% of their time actively supporting victims, and 22% of their time actively supporting bullies. Older boys were more likely to spend time helping the bully, and less likely to spend time helping the victim, than were younger boys, or girls of either grade level. Sequential analyses were used to examine, over time, peer influence on bully affect and aggression ratings. Peer active support for the bully was related to subsequent decreases in bully negative affect ratings, and increases in aggression ratings. Other types of peer interaction with the bully or victim (i.e., passively watching the bullying, intervening to help the victim) were not associated with changes in bully affect or aggression ratings. Chi-square analyses indicated few relationships between the number of peers present and intervention on the victim's behalf. Regression analyses of peer social status indicated a relationship to intervention on the victim's behalf: overall, the trend was for less disliked children to intervene more, while more disliked children were less likely to intervene. Taken together, the data suggest that peers are often aware of playground bullying, but may feel powerless to effectively intervene. The viability of a dynamic systems framework for examining peer influences on playground bullying is considered, and the results of the study are discussed with regard to possible intervention strategies. (PsycINFO Database Record (c) 2012 APA, all rights reserved)
SN - 0419-4217
AD - O'Connell, Paul Desmond: York U., Canada
ER -

TY - JOUR
ID - 2876
T1 - A comparative study of the motivational patterns and achievement measures of Korean and Caucasian American fifth and sixth graders
A1 - Chun,Joan J.
Y1 - 2000///
N1 - Dissertation Abstract: 2000-95003-175
English
Dissertation Abstract
KW - PsycInfo
KW - Academic Achievement
KW - Academic Achievement Motivation
KW - Elementary School Students
KW - Racial and Ethnic Differences
KW - Korean Cultural Groups
KW - Whites
RP - NOT IN FILE
SP - 2796
JF - Dissertation Abstracts International Section A: Humanities and Social Sciences
VL - 60:
IS - 8-A
N2 - To date, a small number of researchers have identified cultural factors that appear to explain the high academic achievement of Asian American students. In their explanations for achievement, some have acknowledged motivation by citing related constructs such as high achievement standards, effort attributions, and perseverance. This study was undertaken to determine if the motivational patterns of Korean and Caucasian American students vary significantly, assuming differences in cultural values and socialization practices at home. Additionally, in light of the intensive focus on the math success of Asians, patterns of motivation were

assessed with respect to both social studies and math. Questionnaires were administered to fifth and sixth graders (n = 275) attending schools in predominantly upper-middle SES neighborhoods. Students answered questions tapping various motivational constructs such as goal orientations and achievement standards. Their parents were asked questions designed to capture thoughts about child-rearing and academic expectations. Data were subjected to race by sex analyses of variance and regression analyses. Students tended to take the same approach to learning math and social studies. Moreover, with respect to patterns of motivation, little distinguished Korean American students from Caucasians. However, as reported in existing literature, Korean students tended to hold higher academic standards for themselves than their Caucasian peers and in fact felt subject to higher parental expectations. They believed themselves to be more able in math than social studies, but did not significantly differ from Caucasians with respect to achievement measures. A greater percent of Korean students than Caucasian participated in activities outside of school. As for parents, the Koreans expected their children to maintain set grades. Caucasian parents were not so inclined. Finally, Korean parents, like their children, appeared to view math and social studies differently. Not only did they believe their children to be better at math, they tended to feel that their children work harder at it than social studies. To better understand patterns of motivation and achievement as well as differing views toward various school subjects, future studies should examine more diverse groups of students, socioeconomic and otherwise. (PsycINFO Database Record (c) 2012 APA, all rights reserved)
SN - 0419-4209
AD - Chun, Joan J.: U Chicago, US
ER -

TY - JOUR
ID - 2877
T1 - A comparison of the effect of developmentally-oriented low-organized games and free play recess on the self-esteem of young children in a psychiatric partial hospital
A1 - White,Gene Dampier Jr
Y1 - 2000///
N1 - Dissertation Abstract: 2000-95001-137
English
Dissertation Abstract
KW - PsycInfo
KW - Childhood Development
KW - Childhood Play Behavior
KW - Childrens Recreational Games
KW - Psychiatric Patients
KW - Self Esteem
RP - NOT IN FILE
SP - 2428
JF - Dissertation Abstracts International Section A: Humanities and Social Sciences
VL - 60:
IS - 7-A
N2 - The purpose of this study was to compare the effects of Leonard Gordon Institute developmentally-oriented, low-organized games and free play recess on the self-esteem of young children (N = 20). The subjects were 5 to 8 year old patients in a psychiatric partial hospital. All 20 of the young children had been diagnosed with some type of psychiatric disorder according to the Diagnostic and Statistical Manual (DSM IV). Most of the young children also suffered from Attention Deficit/Hyperactive Disorder, with accompanying cognitive developmental delays. The intervention was a program of 25, 30-min sessions of Leonard Gordon Institute developmentally-oriented, low-organized games. The young children were evaluated four times, over a period of 15 weeks, using the Behavior Rating Scale of Presented Self-Esteem in Young Children. The intervention took place during the 6th and 10th weeks. A within-subjects design was used to allow for the maximum number of young children to participate in the intervention. The data were examined with a one-way analysis of variance for repeated measures with four levels. Based upon the findings of this analysis, young children did not increase their self-esteem, at the $p < .05$ level of significance. In an effort to elicit qualitative feedback about the participants' experience in the game program, the young children were interviewed, individually, following their participation in the intervention. Questions and participant responses are included and discussed. (PsycINFO Database Record (c) 2012 APA, all rights reserved)
SN - 0419-4209

AD - White, Gene Dampier: Temple U., US
ER -

TY - JOUR
ID - 2878
T1 - Exploring the nature of giftedness in preschool children. [References]
A1 - Hodge,Kerry A.
Y1 - 2000///
N1 - Peer Reviewed Journal: 2000-16980-002
English
Journal; Peer Reviewed Journal
KW - PsycInfo
KW - Academic Achievement Prediction
KW - Achievement Measures
KW - Development
KW - Gifted
RP - NOT IN FILE
SP - 46
EP - 73
JF - Journal for the Education of the Gifted
VL - 24:
IS - 1
N2 - Utilized qualitative and quantitative measures to explore the abilities of 11 young children (36-49 mo old) nominated by their parents as gifted. A part-time preschool enrichment program provided a naturalistic setting in which to investigate measures that might reflect potential giftedness, predict later achievement, and reveal individual profiles of development. The curriculum invited children to display any differences from typical preschoolers. Characteristics nominated by parents as indicators of their child's giftedness were consistent with indicators in the research literature and were generally supported by norm-referenced test results and teacher observations. In spite of test limitations, both quantitative and qualitative measures played a valuable role in revealing advanced abilities amidst diverse profiles of abilities, skills, and interests. Longitudinal data would help to confirm the potential apparent in some of these children as giftedness. (PsycINFO Database Record (c) 2012 APA, all rights reserved)
SN - 0162-3532
AD - Hodge, Kerry A.: Macquarie U, Special Education Ctr, Early Intervention Network, Sydney, NSW, Australia
ER -

TY - JOUR
ID - 2879
T1 - Observations of bullying in the playground and in the classroom
A1 - Craig,Wendy M.
Y1 - 2000///
N1 - Peer Reviewed Journal: 2000-13568-002
English
Journal; Peer Reviewed Journal
KW - PsycInfo
KW - Aggressive Behavior
KW - Classroom Environment
KW - Perpetrators
KW - Playgrounds
KW - Victimization
KW - Peer Relations
KW - Bullying
RP - NOT IN FILE
SP - 22
EP - 36
JF - School Psychology International

VL - 21:
IS - 1
N2 - Employed naturalistic observations to compare 185 school children's bullying and victimization in the playground and in the classroom. The results indicated that there were more opportunities to observe aggression and receive and initiate aggression in the playground than in the classroom. The frequency of bullying was higher in the playground (4.5 episodes per hour) than in the classroom (2.4 episodes per hour). The nature of bullying reflected the constraints of the context (i.e., direct bullying was more prevalent in the playground and indirect bullying was more prevalent in the classroom). Being at the receiving end of aggression was more likely to occur in the playground as compared to the classroom. Nonaggressive children were more likely to bully in the playground, whereas aggressive children were more likely to bully in the classroom. There was no difference across context in the proportion of episodes of reinforcement with peers present or in the rate of peer and teacher intervention. The results highlight the necessity of a systemic intervention program that addresses not only the individual characteristics of bullies and victims, but also the roles of the peer group, teachers and the school. (PsycINFO Database Record (c) 2012 APA, all rights reserved)
SN - 0143-0343
AD - Craig, Wendy M.: Queen's U, Dept of Psychology, Kingston, ON, Canada
ER -

TY - JOUR
ID - 2880
T1 - A curricular frame for physics education: Development, comparison with students' interests, and impact on students' achievement and self-concept
A1 - Haussler,Peter
Y1 - 2000///
N1 - Peer Reviewed Journal: 2000-12285-001
English
Journal; Peer Reviewed Journal
KW - PsycInfo
KW - Curriculum Development
KW - Physics
KW - Science Achievement
KW - Science Education
KW - Student Attitudes
KW - Professional Personnel
KW - Self Concept
KW - Students
RP - NOT IN FILE
SP - 689
EP - 705
JF - Science Education
VL - 84:
IS - 6
N2 - Presents 3 interlinked studies aimed at (1) developing a curricular frame for physics education; (2) assessing student interest in the contents, contexts, and activities that are suggested by that frame; and (3) developing a curriculum in line with that frame and measuring its cognitive and emotional effects on students. The curricular frame was developed by drawing on the expertise of 73 persons from the fields of physics and other sciences as well as education. Data of some 8,000 11-16 yr old students and information of the presently taught physics curriculum were sampled longitudinally and cross-sectionally. The 3rd study comprised 23 experimental and 7 control classes. As a result of the comparison between the features of the curricular frame, the interest structure of students, and the current physics curriculum, there was a remarkable congruency between students' interest in physics and the kind of physics education identified by the science and education professionals as being relevant. The data suggest physics be taught so that students have a chance to develop a positive physics-related self-concept and to link physics with situations they encounter outside the classroom. A curriculum based on these principles proved superior compared to a traditional curriculum. (PsycINFO Database Record (c) 2012 APA, all rights reserved)
SN - 0036-8326
AD - Haussler, Peter: U Kiel, Inst for Science Education, Germany

ER -

TY - JOUR
ID - 2881
T1 - Internet usage by students in an Israeli high school
A1 - Nachmias,Rafi; Mioduser,David; Shemla,Anat
Y1 - 2000///
N1 - Peer Reviewed Journal: 2000-08588-003
English
Journal; Peer Reviewed Journal
KW - PsycInfo
KW - Computer Applications
KW - Human Computer Interaction
KW - Internet
KW - Student Characteristics
KW - Internet Usage
KW - High School Students
KW - Junior High School Students
RP - NOT IN FILE
SP - 55
EP - 73
JF - Journal of Educational Computing Research
VL - 22:
IS - 1
N2 - Examined the purpose and patterns of Internet usage among 384 junior-high and high school students (aged 12-18 yrs) in Israel, with a focus on the linkage between Internet usage and school activities. The findings show that the Internet was being used by about half of the students, with the main use being for communication. Search for information was the second reason. A low degree of connection was found between Internet usage and school practice. The results show significant sex differences. In general, more boys were using the Internet more extensively than girls. Only a few differences among age groups were found. The findings are discussed in terms of the range of Internet use among youngsters and its emerging role as an alternative knowledge resource outside school. (PsycINFO Database Record (c) 2012 APA, all rights reserved)
SN - 0735-6331
AD - Nachmias, Rafi: Tel-Aviv U, School of Education, Tel Aviv, Israel
ER -

TY - JOUR
ID - 2882
T1 - The self-regulatory nature of preschool children's private speech in a naturalistic setting
A1 - Patrick,Elizabeth
Y1 - 2000///
N1 - Peer Reviewed Journal: 2000-08387-003
English
Journal; Peer Reviewed Journal
KW - PsycInfo
KW - Age Differences
KW - Home Environment
KW - Self Management
KW - Self Talk
KW - Speech Development
KW - Psycholinguistics
KW - Speech Characteristics
RP - NOT IN FILE
SP - 45
EP - 61
JF - Applied Psycholinguistics
VL - 21:

IS - 1
N2 - Private speech (PS)-or speech for the self-was studied in 25 preschool-age children to determine how widely and with what characteristics it occurs when examined in the familiar home setting. Activities were selected that required several steps and that were intended to engage working memory or longer term recall. Both quantitative (numbers of words and utterances produced) and qualitative (utterance type) analyses were conducted on the children's PS. Across the age range from 4.5 to 6 yrs, younger children had higher frequencies of PS than older children. Increased information loads resulted in more PS, indicating a relation between PS and task difficulty; however, total quantities of PS were not correlated with degree of task success. Self-regulatory utterances accounted for 89% of the PS produced; the majority of these utterances involved repetitions of the instructions that appeared to enhance recall, to help pace the children's activity, or to enable deliberation with respect to making choices. There was a strong age trend in which the amount of audible PS decreased with age. Qualitative differences across tasks are presented and considered in relation to how PS functions for different activities and possibly achieves adaptive significance. (PsycINFO Database Record (c) 2012 APA, all rights reserved)
SN - 0142-7164
AD - Patrick, Elizabeth: George Washington U, Washington, DC, US
ER -

TY - JOUR
ID - 2883
T1 - Phenomenological and functional diversity of violence among children. [German]
A1 - Oswald,Hans
Y1 - 2000///
N1 - Peer Reviewed Journal: 2000-08101-001
German
Journal; Peer Reviewed Journal
KW - PsycInfo
KW - Conflict
KW - Frustration
KW - Peer Relations
KW - Psychosocial Development
KW - Violence
KW - Elementary School Students
RP - NOT IN FILE
SP - 3
EP - 15
JF - Praxis der Kinderpsychologie und Kinderpsychiatrie
JA - Prax Kinderpsychol.Kinderpsychiatr.
VL - 49:
IS - 1
N2 - Examined everyday interactions with peers of children observed in natural settings of a school. Ss were 122 grade school children (aged 10 yrs) in Germany. Ss were observed engaging in a poetry writing task and playground activities to determine which kind of activity caused the most frustration and conflict. This article discusses the diverse functions of violence in children's interactions, such as affirmation of reciprocity and justice, experience of agency and control, preservation of territories against intruders, clear messages, compensations of lacking capacities, and self-defense. Results show that quiet activities, such as reading and writing, yields higher frustration levels, but lower incidences of physical violence; whereas, rough-and-tumble play yields more physical confrontations. (PsycINFO Database Record (c) 2012 APA, all rights reserved)
SN - 0032-7034
AD - Oswald, Hans: U Potsdam, Inst fur Padagogik, Potsdam, Germany
ER -

TY - JOUR
ID - 2884
T1 - Childhood social development: The essential readings
A1 - Craig,Wendy
Y1 - 2000///

N1 - Book: 2000-07089-000
English
Book; Edited Book
(Created by PsycINFO) Preface Acknowledgments Introduction: What is social development? [by] Wendy Craig Part I: Social relationships Introduction to Part I * Attachments: "Infant-mother attachment" / M. D. S. Ainsworth * Parenting: "Supportive parenting, ecological context, and children's adjustment: A seven-year longitudinal study" / G. S. Pettit, J. E. Bates and K. A. Dodge * Friendships: "The company they keep: Friendships and their developmental significance" / W. W. Hartup * Peer relations: "Preadolescent friendship and peer rejection as predictors of adult adjustment" / C. L. Bagwell, A. F. Newcomb and W. M. Bukowski Part II: The school context Introduction to Part II * Bullying and victimization: "Observations of bullying and victimization in the school yard" / W. M. Craig and D. J. Pepler * Friendship and school adjustment: "Friendship quality as a predictor of young children's early school adjustment" / G. W. Ladd, B. J. Kochenderfer and C. C. Coleman * Extracurricular activities: "Do extracurricular activities protect against early school dropout?" / J. L. Mahoney and R. B. Cairns Part III: Gender identity, the self, and moral development Introduction to Part III * Gender differences: "Gender and relationships: A developmental account" / E. E. Maccoby * Self-concept: "Structure, stability, and development of young children's self-concepts: A multicohort-multioccasion study" / H. W. Marsh, R. Craven and R. Debus * Moral reasoning: "Children's reasoning about interpersonal and moral conflicts" / J. G. Smetana, M. Killen and E. Turiel Part IV: Aggression Introduction to Part IV * Forms of aggression: "Engagement in gender normative versus nonnormative forms of aggression: Links to social-psychological adjustment" / N. R. Crick * Development of antisocial behavior: "A developmental perspective on antisocial behavior" / G. R. Patterson, B. D. DeBaryshe and E. Ramsey * Prevention of conduct disorders: "The role of social context in the prevention of conduct disorder" / J. D. Coie and M. R. Jacobs Index
KW - PsycInfo
KW - Experimentation
KW - Methodology
KW - Psychosocial Development
KW - Theories
KW - Aggressive Behavior
KW - Human Sex Differences
KW - Interpersonal Interaction
KW - Moral Development
KW - School Environment
KW - Self Concept
RP - NOT IN FILE
SP - Malden
JF - (2000)
VL - Childhood social development: The essential readings. xi, 375 pp. Malden: Blackwell Publishing.
N2 - (from the preface) Introduces students to the field of childhood social development and is intended for readers who are newcomers to the field of social development research and are interested in reading current and original articles by developmental theorists and researchers on various aspects of social development. This book also is designed as a supplementary text for undergraduate and graduate courses in developmental psychology and social development. The selection of articles is based on several criteria. (1) The articles should represent the current state of research into social development: thus, 11 of the 13 articles have been published in the 1990s. (2) The articles should be representative of current theoretical and methodological approaches in social development, as well as illustrating elegant empirical research. (3) The articles should be accessible, in the sense of being understandable to students with limited backgrounds in psychology. (4) Finally, because this book is one of a series, the authors have tried to avoid repeating the same article in different readers. (create) The chapters are journal reprints that were covered by PsycINFO at the time of their original publication and can be found in the PsycLIT journal database. (PsycINFO Database Record (c) 2012 APA, all rights reserved)
AD - Craig, Wendy: Queen's U, Kingston, ON, Canada
ER -

TY - JOUR
ID - 2885
T1 - The diachronic approach and solutions to interpersonal conflicts
A1 - Montangero,Jacques; Pons,Francisco; Cattin,Jean-Pierre

Y1 - 2000///
N1 - Peer Reviewed Journal: 2000-05949-006
English
Journal; Peer Reviewed Journal
KW - PsycInfo
KW - Conflict
KW - Conflict Resolution
KW - Thinking
KW - Time Perspective
RP - NOT IN FILE
SP - 415
EP - 429
JF - British Journal of Developmental Psychology
JA - BR J DEV PSYCHOL
VL - 18:
IS - 3
N2 - Examined the relationships between levels of the diachronic approach in children and their ability to imagine solutions to a conflict between children. 60 children (aged 8-12 yrs) were presented 1st with tasks assessing their level of diachronic tendency (propensity to evoke past or future stages of a present situation), of dissociation of 2 temporal sequences (absence of covariation of a cause and its delayed effect) and of dynamic synthesis (ability to describe a set of pictures representing the successive steps of an event by a single noun phrase). During a 2nd session, the participants were presented with a drawing representing 2 boys fighting in the playground and they were asked to explain the reasons for the fight. Participants then had to imagine several solutions to prevent the 2 boys fighting. Results showed a development in the ability to imagine solutions to a conflict and a significant correlation between the levels of this development and the levels of 2 aspects of the diachronic approach, temporal dissociation and dynamic synthesis. (PsycINFO Database Record (c) 2012 APA, all rights reserved)
SN - 0261-510X
AD - Montangero, Jacques: U Geneva, Faculty of Psychology & Education Sciences, Geneva, Switzerland
ER -

TY - JOUR
ID - 2886
T1 - Coping with clumsiness in the school playground: Social and physical play in children with coordination impairments
A1 - Smyth,Mary M.
Y1 - 2000///
N1 - Peer Reviewed Journal: 2000-05949-005
English
Journal; Peer Reviewed Journal
KW - PsycInfo
KW - Childhood Play Behavior
KW - Motor Coordination
KW - Movement Disorders
KW - Physical Contact
KW - Social Interaction
KW - Childhood Play Development
KW - Playgrounds
RP - NOT IN FILE
SP - 389
EP - 413
JF - British Journal of Developmental Psychology
JA - BR J DEV PSYCHOL
VL - 18:
IS - 3
N2 - Examined the physical and social play of children, with a focus on types of motor play and whether children played alone or in groups. The playground activity of 110 children (aged 6-10 yrs) was observed for 10

5-min periods. 55 children were assigned to a developmental coordination disorder (DCD) group and 55 to a control group, on the basis of their scores on the Movement Assessment Battery for Children. Results indicate that Ss in the DCD group spent more time alone, were onlookers more often, and played formal games in large groups less often if they were boys and informal games in large groups less often if they were girls. It was found that social fantasy play did not differentiate between the 2 groups but social physical play did, particularly in the older age groups. Play performance in the DCD group was more variable overall with some boys taking an active part in team games while others never took part in them. It is suggested that as social non-physical play decreases with age, some children with impaired coordination may not become involved in social physical play and that children with impaired coordination can become isolated and solitary in the school playground. Various routes to social exclusion for these children are proposed. (PsycINFO Database Record (c) 2012 APA, all rights reserved)
SN - 0261-510X
AD - Smyth, Mary M.: Lancaster U, Dept of Psychology, Lancaster, England
ER -

TY - JOUR
ID - 2887
T1 - Changes in attitudes among children and elderly adults in intergenerational group work
A1 - Pinquart,Martin; Wenzel,Silka; Sorensen,Silvia
Y1 - 2000///
N1 - Peer Reviewed Journal: 2000-02792-002
English
Journal; Peer Reviewed Journal
KW - PsycInfo
KW - Attitudes
KW - Intergenerational Relations
RP - NOT IN FILE
SP - 523
EP - 540
JF - Educational Gerontology
VL - 26:
IS - 6
N2 - Changes in intergenerational attitudes in 32 children (aged 8-11 yrs) and 20 elderly adults (mean age 71.7 yrs) were investigated using activity groups. Applying the theory of realistic intergroup conflict, the authors varied the amount of joint activity between groups. In the experimental group, joint activities (e.g., writing stories, making glove puppets, performing a puppet show) were organized by the researcher. In the control group, although there was visual contact among them, the children and elderly participants worked alone. The elderly adults' ratings of the participating children became more positive only in the experimental group. However, children's ratings of the elderly participants improved regardless of the experimental condition. Seven wks after the end of the intervention, only the children's changes in attitudes toward the elderly participants remained significant. Improvements in intergenerational attitudes during group activities were associated with increased frequency of intergenerational contact outside the group. (PsycINFO Database Record (c) 2012 APA, all rights reserved)
SN - 0360-1277
AD - Pinquart, Martin: Friedrich Schiller U, Dept of Developmental Psychology, Jena, Germany
ER -

TY - JOUR
ID - 2889
T1 - Managing work and family: The decision to outsource child care in families engaged in family-owned businesses
A1 - Avery,Rosemary J.
Y1 - 2000///
N1 - Peer Reviewed Journal: 2000-00753-001
English
Journal; Peer Reviewed Journal
KW - PsycInfo

KW - Child Day Care
KW - Family Structure
KW - Household Management
KW - Outsourcing
KW - Working Conditions
KW - Business Organizations
KW - Home Environment
RP - NOT IN FILE
SP - 227
EP - 258
JF - Journal of Family and Economic Issues
VL - 21:
IS - 3
N2 - Investigated the decision to outsource child care among 103 families involved in family-owned businesses who had at least 1 child under 5 yrs of age. A management framework is used to examine the impact of inputs to the decision (i.e., goals, resources) and level of management activity (i.e., planning, implementing) in these families as predictors of the choice to outsource child care. Data are from a subsample of the data from a project by M. Winter et al (1998) undertaken in 1997. Household managers who worked in the family business were less likely to outsource child care than were those who worked outside the family business. More educated household managers purchased more and managers in larger families purchased fewer hours of child care. Female household managers purchased significantly fewer hours of child care than did their male counterparts. (PsycINFO Database Record (c) 2012 APA, all rights reserved)
SN - 1058-0476
AD - Avery, Rosemary J.: Cornell U, Dept of Policy Analysis & Management, Ithaca, NY, US
ER -

TY - JOUR
ID - 2890
T1 - Kid customer? Commercialization of playspace and the commodification of childhood
A1 - McKendrick,John H.
Y1 - 2000///
N1 - Peer Reviewed Journal: 2000-00675-003
English
Journal; Peer Reviewed Journal
KW - PsycInfo
KW - Childhood Play Behavior
KW - Commercialization
KW - Consumer Attitudes
KW - Playgrounds
RP - NOT IN FILE
SP - 295
EP - 314
JF - Childhood: A Global Journal of Child Research
VL - 7:
IS - 3
N2 - Considers the commercialization of playgrounds and its significance to the commodification of childhood. J. McNeal's (1992) theory of the social group of children as a market is introduced as a framework through which commercial playgrounds may be understood. An introduction to commercial playgrounds and the project from which the article is derived sets the context for an analysis of how parents and children perceive and experience these environments, how they are marketed to them and how they consume them. Data was collected as part of a project in which research was conducted in 10 field sites, 872 families were surveyed, of which 30 Ss (adults and 5-12 yr olds) were interviewed, and, in turn, 7 participated in a family day-visit/postvisit video-based interview. It was found that it is overly simplistic to suggest that these developments are testimony to the new-found consumer power of children. Children play a marginal role in the production of these play environments; in contributing to parents' information field prior to decision-making; and in the visit decision-making process. Some groups of children were found to be more active consumers of these spaces. (PsycINFO Database Record (c) 2012 APA, all rights reserved)

SN - 0907-5682
AD - McKendrick, John H.: Glasgow Caledonia U, School of Social Sciences, Glasgow, United Kingdom
ER -

TY - JOUR
ID - 2891
T1 - A longitudinal exploration of the continuity of children's social participation and social withdrawal across socioeconomic status levels and social settings
A1 - Schneider,Barry H.
Y1 - 2000///
N1 - Peer Reviewed Journal: 2000-00512-002
English
Journal; Peer Reviewed Journal
KW - PsycInfo
KW - Social Environments
KW - Social Interaction
KW - Social Isolation
KW - Socioeconomic Status
KW - Communities
KW - Home Environment
KW - Psychosocial Development
KW - School Environment
RP - NOT IN FILE
SP - 497
EP - 519
JF - European Journal of Social Psychology
VL - 30:
IS - 4
N2 - The main purpose of this study was to determine the stability across time and social setting of children's social participation and social withdrawal. The authors followed a sample of 98 Canadian children, aged 5 yrs at the beginning of the study, for 2 yrs. Data were collected through home visits, interviews with parents, and direct observation of Ss. Children from lower-SES homes were less involved than those from the higher-SES in group play after school and during outdoor recess. Across the full sample, social withdrawal was not very stable. There was, however, considerable stability across setting and time among extreme groups of withdrawn participants. Social withdrawal was largely unrelated to parents' initiation of peer contacts for their children. (PsycINFO Database Record (c) 2012 APA, all rights reserved)
SN - 0046-2772
AD - Schneider, Barry H.: U Ottawa, School of Psychology, Ottawa, ON, Canada
ER -

TY - JOUR
ID - 2892
T1 - Using the school environment to promote physical activity and healthy eating
A1 - Wechsler,Howell; Devereaux,Randolph S; Davis,Margarett; Collins,Janet
Y1 - 2000///
N1 - Peer Reviewed Journal: 2000-00505-006
English
Journal; Peer Reviewed Journal
KW - PsycInfo
KW - Exercise
KW - Health Promotion
KW - Nutrition
KW - Physical Activity
KW - School Environment
KW - Diets
KW - Health Behavior
KW - Students

RP - NOT IN FILE
SP - S121
EP - S137
JF - Preventive Medicine: An International Journal Devoted to Practice and Theory
VL - 31:
IS - 2,Pt.2
N2 - The role of the community environment in shaping dietary and physical activity (PA) behaviors has received increasing attention in recent years. Although schools are a key part of the community environment, interventions that promote PA and healthy eating among students through changes in the school environment have received relatively little attention. After reviewing the role of environmental factors in shaping health behavior, this paper describes the various aspects of the school environment that influence PA and nutrition behaviors. Relevant research is described and new directions are proposed for 5 key environmental influences: recess periods, intramural sports and PA programs, PA facilities, foods and beverages available outside of the school meals program, and psychosocial support for PA and healthy eating. Recess, intramural programs, and access to school PA facilities outside of school hours can provide opportunities for health-enhancing PA. States, school districts, and schools can establish strong policies and implement creative interventions to promote healthy eating through the foods and beverages offered at school. Schools can offer psychosocial support for PA and healthy eating through school policies, role modeling by staff, and the use of cues and incentives. (PsycINFO Database Record (c) 2012 APA, all rights reserved)
SN - 0091-7435
AD - Wechsler, Howell: US Ctrs for Disease Control & Prevention, National Ctr for Chronic Disease Prevention & Health Promotion, Div of Adolescent & School Health, Atlanta, GA, US
ER -

TY - JOUR
ID - 2893
T1 - The effects of pre-correction and active supervision on the recess behavior of elementary students. [References]
A1 - Lewis,Timothy J.
Y1 - 2000///
N1 - Peer Reviewed Journal: 2000-00404-001
English
Journal; Peer Reviewed Journal
KW - PsycInfo
KW - Behavior Problems
KW - Child Discipline
KW - Childhood Play Behavior
KW - Prompting
KW - Social Skills
KW - Elementary School Students
KW - Playgrounds
RP - NOT IN FILE
SP - 109
EP - 121
JF - Education & Treatment of Children
VL - 23:
IS - 2
N2 - Increasing a school's capacity to effectively deal with challenging behavior on a systemwide basis has increasingly been advocated as a promising practice. However, there is limited empirical support for large scale interventions focusing on specific school settings. The purpose of this study was to examine the effectiveness of implementing a pre-correction and active supervision strategy on the rate of problem behavior observed during recess on an elementary school playground. Participants were 475 students and 42 staff in an elementary school serving kindergarten through 5th grade. Using a multiple baseline across groups design, results indicate that the intervention reduced rates of problem behavior exhibited by elementary students, but was not effective in increasing rates of active supervision on the part of playground monitors. (PsycINFO Database Record (c) 2012 APA, all rights reserved)
SN - 0748-8491

AD - Lewis, Timothy J.: U Missouri, Dept of Special Education, Columbia, MO, US
ER -

TY - JOUR
ID - 2894
T1 - Developing portfolios with learning disabled students
A1 - Boerum,Lisa J.
Y1 - 2000///
N1 - Peer Reviewed Journal: 2000-00036-003
English
Journal; Peer Reviewed Journal
KW - PsycInfo
KW - Expectations
KW - Junior High School Students
KW - Learning Disabilities
KW - Measurement
KW - Performance
RP - NOT IN FILE
SP - 211
EP - 238
JF - Reading & Writing Quarterly: Overcoming Learning Difficulties
VL - 16:
IS - 3
N2 - Investigated the ways in which alternative and authentic assessments would result in students of varying learning abilities increasing their self-expectations and performance levels. During the 1995-96 school year, 25 6th-grade students engaged in authentic and alternative learning experiences, reflection, goal setting, and self-assessment. A portfolio design was used that would allow Ss to give evidence of their growth as learners to an outside audience. Four Ss were targeted and their work collected. Presented are a synopsis of findings including the goals they set for the different quarters and reflections or writing products of selected units of instruction. Evidence was collected demonstrating that when given the opportunity to engage in performance-based activities and assessments, students with learning disabilities can begin to clarify their strengths and weaknesses as learners in order to set goals for improvement. (PsycINFO Database Record (c) 2012 APA, all rights reserved)
SN - 1057-3569
ER -

TY - JOUR
ID - 2895
T1 - Taste of food, fun, and fitness: A community-based program to teach young girls to feel better about their bodies. [References]
A1 - Coller,Tanya G.
Y1 - 1999///
N1 - Peer Reviewed Journal: 2008-09797-007
English
Journal; Peer Reviewed Journal
KW - PsycInfo
KW - Eating Behavior
KW - Health Education
KW - Health Promotion
KW - Human Females
KW - Physical Activity
KW - Body Image
KW - Community Services
KW - Food
KW - Health Attitudes
KW - Physical Fitness
KW - Weight Control

RP - NOT IN FILE
SP - 292
EP - 293
JF - Journal of Nutrition Education
VL - 31:
IS - 5
N2 - The purpose of this study was to develop, implement, and evaluate an after-school program aimed at preventing unhealthy dieting and excessive weight preoccupation. This study targeted preadolescents (ages 10-12) and took the prevention program outside of the classroom, incorporating the curriculum into the Girl Scout program. This program used a nonacademic approach to teaching concepts similar to those in programs that have previously been targeted at older adolescents within school setting. The primary objectives of the program were to develop healthful attitudes toward eating, physical activity, body image, and weight control in order to prevent unhealthy behaviors. The program in this study consisted of six weekly, 90-minute sessions that focused on three health messages: "Enjoy a variety of foods," "Physical activity is fun," and "Be proud of yourself-everyone is different". Results showed that changes in eating attitudes and behaviors were minimal. The parents completed written evaluation and focus group questions. They reported that they enjoyed the program but found that they had little time to read the parent tip sheets. The minimal impact of the intervention on eating attitudes and behaviors has led to the conclusion that programs of this nature (i.e., short duration, low intensity) may need to take a more focused approach. (PsycINFO Database Record (c) 2012 APA, all rights reserved)
SN - 0022-3182
AD - Coller, Tanya G.: Division of Epidemiology, School of Public Health, University of Minnesota, Minneapolis, MN, US Neumark-Sztainer, Dianne: Division of Epidemiology, School of Public Health, University of Minnesota, Minneapolis, MN, US Bulfer, Joan: City of Bloomington Health Division, Bloomington, MN, US Engebretson, Jan: City of Bloomington Health Division, Bloomington, MN, US
ER -

TY - JOUR
ID - 2896
T1 - The role of participation in in-school and outside-of-school activities in the talent development of gifted students. [References]
A1 - Olszewski-Kubilius,Paula; Lee,Seon-Young
Y1 - 1999///
N1 - Peer Reviewed Journal: 2004-95234-003
English
Journal; Peer Reviewed Journal
KW - PsycInfo
KW - Ability
KW - After School Programs
KW - Extracurricular Activities
KW - Gifted
KW - Educational Programs
RP - NOT IN FILE
SP - 107
EP - 123
JF - Journal of Secondary Gifted Education
VL - 10:
IS - 3
N2 - Based on survey responses from 230 students enrolled in a summer gifted program at a university, this study gives a description of gifted students' participation in extracurricular activities in and outside of school. Findings show that gifted students were more involved in competitions, clubs, or other extracurricular activities in mathematics than in other subject areas and were the least involved in computer science activities. Sports were the most frequent extracurricular and outside-of-school activities, as well as playing and working with computers. The data reveal some gender-stereotypical tendencies regarding participation in and outside-of-school activities and gender-typical patterns of support from parents. Grade and course differences were also found. Contributions this study makes to the existing literature are to assess the consonance of children's participation in outside-of-school and extracurricular activities with their talent area and to document

empirically parental involvement and independent home study for gifted adolescents. (PsycINFO Database Record (c) 2012 APA, all rights reserved) (journal abstract)
SN - 1077-4610
AD - Olszewski-Kubilius, Paula: Center for Talent Development, Northwestern University, Evanston, IL, US
Lee, Seon-Young: Center for Talent Development, Northwestern University, Evanston, IL, US
ER -

TY - JOUR
ID - 2897
T1 - Draw the line/respect the line: A curriculum to prevent sexual risk. [References]
A1 - Marin,Barbara Van Oss
Y1 - 1999///
N1 - Book: 2000-07441-004
English
Book; Edited Book
KW - PsycInfo
KW - At Risk Populations
KW - Curriculum Development
KW - Prevention
KW - Psychosexual Behavior
KW - Sex Education
KW - Middle School Students
RP - NOT IN FILE
SP - 171
EP - 186
JF - Interamerican Society of Psychology
VL - :
IS - 1999
N2 - (from the chapter) The epidemics of pregnancy and sexually transmitted diseases (including HIV) that currently affect so many youth must be addressed. Despite adequate knowledge about how pregnancy and HIV transmission may occur and how to prevent them, many youth put themselves at risk through sexual behavior. When questioned, youth will often comment that sex "just happened." Interventions must take into account the cultural, social, and contextual aspects of sexual behavior. A curriculum is presented that was developed to assist 6th, 7th, and 8th grade students in postponing sexual activity and using protection if they are sexually active. Focus groups and practice sessions were used extensively in development. Evaluation of the curriculum involves a 36-mo longitudinal randomized trial following 2,892 middle school students. The curriculum has many characteristics that are shared by successful HIV prevention and sex education curricula: theoretical foundation, focus on social pressures of sexual behaviors, and modeling and practicing communication and negotiation skills. Specific activities from the 20-lesson curriculum and activities of the educators outside the classroom (during office hours) are discussed. (PsycINFO Database Record (c) 2012 APA, all rights reserved)
AD - Marin, Barbara Van Oss: U California, Ctr for AIDS Prevention Studies, San Francisco, CA, US
ER -

TY - JOUR
ID - 2898
T1 - The relationship of participation in a low-element challenge course to adolescents' self-reported perceptions of group cohesion
A1 - Glass,Joseph Scott
Y1 - 1999///
N1 - Dissertation Abstract: 1999-95023-102
English
Dissertation Abstract
KW - PsycInfo
KW - Age Differences
KW - At Risk Populations
KW - Group Cohesion
KW - Human Sex Differences

KW - Racial and Ethnic Differences
KW - Teams
RP - NOT IN FILE
SP - 1920
JF - Dissertation Abstracts International Section A: Humanities and Social Sciences
VL - 60:
IS - 6-A
N2 - During recent years there has been an increase in the use of challenge course programs as a method of building teams (Springett, 1987). Although participation in these activities is growing, research in this area remains limited (Johnson, 1992). There is a need for studies to further investigate the use of challenge course programs and examine possible outcomes of their implementation. One of the key factors in the development of a group is the idea of cohesiveness (Griffin & Pennscott, 1991). A setting in which group cohesion is desired is with challenge courses (Davis, Ray, & Sayles, 1995). The use of outdoor activities as a team-building technique has become increasingly popular (Springett, 1987). Generally, participants who have been removed from their normal social context engage in a number of physical activities that often are not directly related to the group's primary purpose, with the aim of attaining new goals, both as individuals and as a group (Martin & Davids, 1995). The label "challenge course" has been used to encompass a wide array of programs used with diverse populations. For the purposes of this study, the term was used to describe a low-element (close to the ground) experiential program designed for groups rather than individuals. These group exercises require participants to share responsibility and to solve problems as a team. Successful solutions to these problems depend upon the extent to which group members cooperate, trust and communicate with one another. In addition, these group exercises impart "lessons," which participants will later apply to problems in their personal lives (Harris, Mealy, Matthews, Lucas, & Moczygemba, 1993). A review of related literature provided no study that investigated whether one-day low-element challenge course programs increased the perception of group cohesion among adolescents ages 11 to 14. This research was designed to measure the perception of group cohesion among adolescents in this age range who participated in a low-element challenge course experience. The variables of gender, race, age, and being labeled "at risk" were included to determine their effect on the outcome of the study. With limited amounts of previous research in this field, this study sought to broaden the knowledge about the impact of challenge course programs, and add credibility to activities which have long claimed to produce favorable results. This study revealed that participating in a low-element challenge course program did increase perceptions of group cohesion among adolescents 11-14 years old. Furthermore the study showed that the variables of gender, race, age and being labeled "at risk" had no significant effect on the results. In addition, this research provided an instrument created specifically for use in measuring perceptions of group cohesion among adolescents. (PsycINFO Database Record (c) 2012 APA, all rights reserved)
SN - 0419-4209
AD - Glass, Joseph Scott: U North Carolina at Greensboro, US
ER -

TY - JOUR
ID - 2899
T1 - Metacommunication and problem solving in a collaborative task of young children. (cognitive development)
A1 - Wang,Younghee
Y1 - 1999///
N1 - Dissertation Abstract: 1999-95021-207
English
Dissertation Abstract
KW - PsycInfo
KW - Cooperation
KW - Interpersonal Communication
KW - Metalinguistics
KW - Problem Solving
RP - NOT IN FILE
SP - 1446
JF - Dissertation Abstracts International Section A: Humanities and Social Sciences
VL - 60:
IS - 5-A

N2 - The present research attempted to address how metacommunication of young children relates to the products in a collaborative problem solving, along with the three main purposes: first, to investigate the relationship between metacommunicative talk in a dyad and the proper movement of the robot; second, to compare metacommunicative output by the two roles in the robot game, which are Operator and Witness; and last, to examine the increase of metacommunicative talk over the course of the games. To answer the question above, using a small battery-operated robot, 10 kindergartners and 12 first-second graders were asked to play games, which were a collaborative task that requires two children; Operator and Witness. Each dyad participated in three sessions, playing both roles during one session. After quantitative analysis of the data administered to address the three purposes, qualitative analysis was done to detect the in-depth processes between metacommunication and the collaborative problem solving. The relationships between metacommunicative talk in a dyad and the proper movement of the robot were complicated, indicating the relationships are more task-specific. It was found that there is a fair amount of role-dependent variability. Frequencies of metacommunicative output vary from the subcategories of metacommunication. Usage of the subcategories is related to the role that a child plays in the robot games. Adversely to prediction about the last purpose, the metacommunicative usage decreased as the sessions continue. The plausible accounts were addressed about the findings. Qualitative analysis revealed that the children utilized metacommunication for various reasons. They are to clarify statements, to retell previous statements, to prompt the games, to expatiate insufficient instructions voluntarily or with input from the outside, to be aware of linguistic references, to repeat other's statement, to regulate other person's statement in order to stop partner's talk or to start own talking, and finally to integrate a previous statement to the present communication. In coda, the benefits of using metacommunication were acquisition of a correct concept on the instruction given and advancement of communication skills as the game proceeded. (PsycINFO Database Record (c) 2012 APA, all rights reserved)
SN - 0419-4209
AD - Wang, Younghee: U Massachusetts, US
ER -

TY - JOUR
ID - 2900
T1 - The social interaction patterns of depressed and nondepressed children within structured and unstructured school contexts
A1 - Xydas,Maria
Y1 - 1999///
N1 - Dissertation Abstract: 1999-95018-136
English
Dissertation Abstract
KW - PsycInfo
KW - Childhood Development
KW - Depression (Emotion)
KW - School Environment
KW - Social Interaction
RP - NOT IN FILE
SP - 1353
JF - Dissertation Abstracts International: Section B: The Sciences and Engineering
VL - 60:
IS - 3-B
N2 - Depression in children has been associated with social skills deficits and limited and negative social interactions with others. The purpose of this study was to apply the interpersonal model of depression to depressed and nondepressed children and to examine their temporal interaction sequences. This study compared the behavior of 21 depressed and 25 nondepressed children, from third to sixth grade classes, in a local elementary school within structured gym class and unstructured free play contexts. A real time direct observation method was utilized to code the children's behaviors within a time-sampled interval coding system. Children were observed for six ten-minute intervals, for a total of 60 minutes, distributed equally and randomly across both settings. Children's behaviors were coded into positive, depressive, aggressive, noncompliant and solitary behavior categories by trained observers. Within each 20 second interval, the observers first coded the target child's behavior (A), followed by reciprocal responses (B) from his/her partner, peer or teacher, and finally the consequent behavior displayed by the target child (C). Results indicated that the depressed group exhibited a lower propensity to display positive behaviors than the nondepressed group. This group also spent

more time engaged in solitary activity: (a) alone in isolation from others; (b) alone displaying depressive affect; and (c) alone but in proximity of others. Although they were not able to maintain them, depressed children initiated as many interactions with partners as nondepressed children. Their interactions, whether positive or negative, were significantly shorter in duration compared to those of nondepressed children. Negative behaviors for both groups occurred with significantly greater frequency in the playground than the classroom. Gender differences indicate that boys displayed significantly more aggressive initiations and exchanges than girls, whereas girls exhibited a higher frequency of isolative behaviors with depressive affect than boys. Results support an interpersonal model of depression for children. Depressed children demonstrated difficulty in maintaining their interactions despite their efforts to initiate contact with others. (PsycINFO Database Record (c) 2012 APA, all rights reserved)
SN - 0419-4217
AD - Xydas, Maria: Hofstra U, US
ER -

TY - JOUR
ID - 2901
T1 - Social competence and social cognition of children with attention deficit hyperactivity disorder
A1 - Rajagopalan,Vaidehi R.
Y1 - 1999///
N1 - Dissertation Abstract: 1999-95010-098
English
Dissertation Abstract
KW - PsycInfo
KW - Attention Deficit Disorder
KW - Behavior Problems
KW - Hyperkinesis
KW - Social Cognition
KW - Social Skills
RP - NOT IN FILE
SP - 6095
JF - Dissertation Abstracts International: Section B: The Sciences and Engineering
VL - 59:
IS - 11-B
N2 - This study was designed to obtain a comprehensive assessment of social competence and social functioning problems of elementary school children with Attention Deficit/Hyperactivity Disorder (ADHD) using multirater rating scales and social cognitive measures. The sample consisted of 83 children, both boys and girls, between the ages of 8 to 12. There were 41 children in the ADHD group and 42 in the comparison group, which consisted of typically developing children. Social competence was measured using the Social Skills Rating Scales (SSRS). The scale was administered to parents, teachers, and children. The social cognitive measures consisted of six stories depicting children in three conditions (intentional, non-intentional, and ambiguous) and in two contexts (classroom and playground). The children listened to the stories and identified the intent of the story characters, generated behavioral responses for both self and the story character, and assigned a punishment level. The findings indicated that teachers and parents rated ADHD children lower than the typically developing children on social skills, and higher on the problem behaviors domains, whereas the ADHD children rated themselves to be comparable to the typically developing children in the self-control and empathy subscales. However, when standard scores were utilized for comparison, both the groups were comparable in social skills, problem behaviors and academic competence. In social cognitive measures, the girls in the ADHD group assigned higher levels of punishment in the intentional-playground context than did those in the comparison group. This suggests that the ADHD girls may be at risk for negative social consequences since they were more punitive than the typically developing girls. Although the relationship between social competence and social cognition was not established for all variables, nevertheless, the findings do show that the ADHD children may not have serious deficits in overall social skills functioning, social knowledge or social competence. (PsycINFO Database Record (c) 2012 APA, all rights reserved)
SN - 0419-4217
AD - Rajagopalan, Vaidehi R.: Saint Louis U., US
ER -

TY - JOUR
ID - 2902
T1 - Optimism as a factor that promotes resilience in inner-city middle-school students exposed to high levels of community violence
A1 - Egger,Sukey Merry Suzanne
Y1 - 1999///
N1 - Dissertation Abstract: 1999-95006-237
English
Dissertation Abstract
KW - PsycInfo
KW - Adjustment
KW - Optimism
KW - Resilience (Psychological)
KW - Urban Environments
KW - Violence
RP - NOT IN FILE
SP - 5078
JF - Dissertation Abstracts International: Section B: The Sciences and Engineering
VL - 59:
IS - 9-B
N2 - Research on resilience to urban stress has focused on family factors, social support outside the family, and personality factors as important determinants of adjustment. The current study examined optimism as a personality factor that promotes positive adjustment in inner-city middle-school students exposed to high levels of community violence. In phase one, optimism and comparative optimism were investigated, with particular attention to optimism about controllable and uncontrollable events. Three models were examined, testing a mediating, additive, and moderating relationship among exposure to community violence, optimism, and emotional adjustment. In phase two, two mediating models of the relationship between optimism and adjustment were tested. The first of these was based on Sroufe's (1990) organizational model of self, with self-system functioning as a mediator. Self-system functioning comprises a set of expectations about self, others, and interactions with others. The second model was based on Scheier and Carver's (1982) work in which behavioral self-regulation is a mediator. Behavioral self-regulation involves use of active coping strategies, rather than withdrawal tactics. Participants were 250 (133 boys, 117 girls) 11-16-year-olds recruited from two public middle schools in primarily African-American and Latino/a, low-income communities. In phase one, consistent with the adult literature, adolescents evidenced comparative optimism, believing that they were less likely to experience negative events than was the average person their age. Participants believed they were more likely to experience uncontrollable events (e.g., being shot or killed) than controllable events (e.g., drinking alcohol). Adolescents exposed to higher levels of community violence experienced more emotional distress and were less optimistic, particularly about uncontrollable events, than were those exposed to lower levels. Support was found for an additive model, in which exposure to community violence and optimism both contribute independently to emotional distress. Phase two data indicated that optimism influences both the development of self-system functioning and positive adjustment. Behavioral self-regulation appeared to mediate the relationship between optimism and adjustment for students who were doing well in school, but not for students referred for counseling groups due to referred students' use of behavioral withdrawal as a coping strategy. Longitudinal research is needed to verify the directionality of the current findings. Interventions that target optimistic beliefs by encouraging mastery of challenging situations may provide a means to improve adjustment and self-system functioning in adolescents. (PsycINFO Database Record (c) 2012 APA, all rights reserved)
SN - 0419-4217
AD - Egger, Sukey Merry Suzanne: U California, Los Angeles, US
ER -

TY - JOUR
ID - 2903
T1 - The role of play in the development of social competence of at-risk preschoolers: An observational study
A1 - Phillip,Germaine Joan
Y1 - 1999///

N1 - Dissertation Abstract: 1999-95006-163
English
Dissertation Abstract
KW - PsycInfo
KW - At Risk Populations
KW - Childhood Play Behavior
KW - Human Sex Differences
KW - Racial and Ethnic Groups
KW - Social Skills
RP - NOT IN FILE
SP - 5170
JF - Dissertation Abstracts International: Section B: The Sciences and Engineering
VL - 59:
IS - 9-B
N2 - The purpose of this study was to explore and describe the role of play in the development of social competence of at-risk preschoolers. The study was guided by four research questions: What is the role of play? How does play advance the development of social competence in at-risk preschoolers? What are the components of play? How does the classroom influence play? Initially there were 19 preschool children in the study. Their ages ranged from 48 months to 66 months. The group was comprised of eleven girls and eight boys. The girls ranged in age from four years to five years and three months, having a mean age of four years and nine months. The boys ranged in age from four years to four years and eleven months, having a mean age of four years and seven months. Thirteen of the children were African-American, five were white, and one was Latino. Three of the children were classified as having speech and language problems, and seven of the children were enrolled in the program for other unspecified circumstances. By the end of the school year, there were 20 children in the study. All of the children were from low income families, all had been recruited from the county's Head Start waiting list, and all had been enrolled in the Head Start program on a full-time basis. All of the children were considered to be at risk of failure. Observation, interview, documents, and artifacts were used to address the four research questions. The children's social behaviors were observed as they interacted in the course of indoor and outdoor play. Social problem solving skills were assessed in an interview with each child. Hypothetical play situations were presented to the children in photographic representations and discussion. The children were questioned as to their interpretations of each play situation and what ideas they had to resolve the problem. The collected data were examined using domain analysis procedures. Vygotsky's theory of play provided the framework for interpreting the findings. The study's findings indicated that, for at-risk preschoolers, social information processing is fundamental to social competence; aggressive strategies lead to non-compliance and conflict situations; mixed-age collaborative play enhance problem solving strategies and prosocial skills; components of play advance total development, the social context of play influence peer interaction; making choices promotes self control and an acceptance of responsibility for one's actions; and caring and supportive adults in the play environment promote positive peer interaction and enhance social competence. (PsycINFO Database Record (c) 2012 APA, all rights reserved)
SN - 0419-4217
AD - Phillip, Germaine Joan: U Florida, US
ER -

TY - JOUR
ID - 2904
T1 - A comparative analysis of playground peer interactions of children with Down syndrome
A1 - Terry-Gage,Susann
Y1 - 1999///
N1 - Dissertation Abstract: 1999-95005-099
English
Dissertation Abstract
KW - PsycInfo
KW - Childhood Play Behavior
KW - Down's Syndrome
KW - Educational Placement
KW - Peer Relations
KW - Special Education

RP - NOT IN FILE
SP - 3406
JF - Dissertation Abstracts International Section A: Humanities and Social Sciences
VL - 59:
IS - 9-A
N2 - The purpose of this study was to examine the peer interactions of children with Down syndrome during recess. Twenty-nine children aged 6 to 11 years were observed during recess in two different educational placements, a special education placement (N = 14) and an included placement (N = 15). Two behavior measures were employed in this study. One was used to directly assess peer interactions on the playground (Howes, 1980). The other was used by teachers to assess the social competence of their students (Guralnick, 1990a). Each child was observed two times during their recess for a total of 30 minutes of observation time. Play categories that were coded included entry behavior, response to entry behavior, agonistic behavior, agonistic response, involvement, play behavior, affect and interactions with adults. There were group differences (special education versus included) in the categories of involvement (onlooker behavior), and positive adult interactions. Children with Down syndrome in the general education placement spent less time in onlooker behavior during recess than children in the special education placement. Children in the special education placement had adults managing their behavior at recess in a positive manner approximately three times as often as children in the included placement. There were also gender differences in the categories of unoccupied behavior, neutral affect and negative adult interactions. Girls were engaged in unoccupied behavior nearly three times as often as boys. Boys displayed neutral affect twice as frequently as girls and were observed in negative adult interactions while girls had no observations of negative adult interactions during recess. In spite of differences in observed playground behaviors of children with Down syndrome, teachers rated the social abilities of the children similarly. Overall the results suggest educational context had little effect on the peer interactions of children with Down syndrome. Gender influences and particular child characteristics (etiology specific) may be important contributors to peer interactions for school-aged children. (PsycINFO Database Record (c) 2012 APA, all rights reserved)
SN - 0419-4209
AD - Terry-Gage, Susann: U California, Los Angeles, US
ER -

TY - JOUR
ID - 2905
T1 - Teaching conflict resolution skills to children: A comparison between a curriculum based and a modified peer mediation program
A1 - Harris,Peggy Aline
Y1 - 1999///
N1 - Dissertation Abstract: 1999-95005-055
English
Dissertation Abstract
KW - PsycInfo
KW - Conflict Resolution
KW - Curriculum
KW - Knowledge Level
KW - Peers
KW - Teaching Methods
RP - NOT IN FILE
SP - 3397
JF - Dissertation Abstracts International Section A: Humanities and Social Sciences
VL - 59:
IS - 9-A
N2 - The purpose of this study was to compare the effectiveness of two approaches to conflict resolution instruction on the knowledge and behavior of elementary school children. One approach, the modified peer mediation program, employed a class meeting procedure for addressing student conflicts. The second approach, the curriculum based program, utilized the Second Step Violence Prevention Curriculum. Two fourth grade classes and one third grade class were randomly assigned to treatment groups. A remaining third grade class served as the control group and received no treatment. Classroom teachers led instructional sessions in conflict resolution strategies for the course of a semester. An analysis of covariance (ANCOVA) was conducted on

Matson's Evaluation of Social Skills with Youngsters (MESSY), Teacher and Student versions and "How I Solve Problems" test. A repeated measures analysis of variance was used to analyze scores on the Conflict Report Forms, Self Report. A Chi Square analysis was conducted on the number of office referrals. The playground observation provided a naturalistic component to the study and results were described nonstatisticaily. Initial examination of the results indicated grade level differences. For this reason, results were analyzed separately along grade levels. The results of the MESSY, Teacher Version, indicated teacher perception of growth was significantly greater for the curricular group of third graders and the class meeting group of fourth graders. The results of the MESSY, Student Version, indicated a treatment by gender interaction. The third grade boys in the class meeting group scored significantly better than the girls in the same group, and the third grade girls in the curriculum group scored significantly better than the boys in the same group. None of the other analyses indicated significant differences between the groups. This study represented the first attempt to compare conflict resolution programs for elementary school children. The results suggest that a combination program, which relies primarily on teacher directed lessons for younger children with increased student responsibilities for children over third grade, may provide the type of eclectic approach necessary to benefit all children. (PsycINFO Database Record (c) 2012 APA, all rights reserved)
SN - 0419-4209
AD - Harris, Peggy Aline: U Kansas, US
ER -

TY - JOUR
ID - 2906
T1 - The influence of guided interactive story telling (GISTT) on children's playground behavior-event knowledge, emotions, and actions
A1 - Toscano,Mary Ellen
Y1 - 1999///
N1 - Dissertation Abstract: 1999-95003-263
English
Dissertation Abstract
KW - PsycInfo
KW - Childhood Play Behavior
KW - Emotional States
KW - Interpersonal Interaction
KW - Storytelling
RP - NOT IN FILE
SP - 2846
JF - Dissertation Abstracts International Section A: Humanities and Social Sciences
VL - 59:
IS - 8-A
N2 - This case study involves a change in the method of interaction between an elementary school principal (participant-observer) and students (informants) who have been involved in playground misbehavior incidents. The site is a suburban school in a primarily white, middle socioeconomic community. The grade levels of the school span nursery to grade three. Prior to the beginning of this study, the school had lost its playing fields and much of its playground structure due to district building and safety initiatives. The purpose of the study is to describe, explain, and evaluate the "Guided Interactive Story Telling" (GIStT) process. This method of interaction was developed to enable young school children, ages 5-10, to recount playground misbehavior events to the school principal. The study evaluated the influence of GIStT on the understandings, emotions, and subsequent actions of its participants. The study compared the effectiveness of GIStT with the more traditional approach formerly used at the school. The latter was an interview-type of interaction based on due process and assertive discipline (DuAD). The results of the study followed the "effect" pattern: z-scores of the cognitive and emotional quality of the scripts were above the critical value; and the time-series graph for repeated misbehaviors showed a decline after GIStT was introduced. The problem-finding character of the interaction was noted to be an essential element in predicting the students' success. (PsycINFO Database Record (c) 2012 APA, all rights reserved)
SN - 0419-4209
AD - Toscano, Mary-Ellen: U Lowell, US
ER -

TY - JOUR
ID - 2907
T1 - The case study of a Taiwanese young child's emergent literacy development
A1 - Lin,Pei Yu
Y1 - 1999///
N1 - Dissertation Abstract: 1999-95003-128
English
Dissertation Abstract
KW - PsycInfo
KW - Early Childhood Development
KW - Home Environment
KW - Literacy
KW - School Environment
RP - NOT IN FILE
SP - 2845
JF - Dissertation Abstracts International Section A: Humanities and Social Sciences
VL - 59:
IS - 8-A
N2 - The purpose of this case study was to portray the literate behavior of a Taiwanese young child in naturalistic settings at home and the preschool. A further purpose was to explore the socio-cultural characteristics embedded in the literacy environment the young child experienced. A qualitative case study method was implemented to systematically gather descriptive data in the naturalistic field and to inductively derive common features grounded in one young child's literate behavior and the literacy environment the child experienced. The field work conducted for this study lasted about ten months, during which time the child aged from four years seven months to five years five months. The findings of this study suggest that the participant constructed her emergent literacy knowledge through consistent social and functional engagement in literacy activities at home and the preschool. The literate behavior patterns demonstrated by the participant are illustrated in the aspects of knowledge about print, use of print, story reenactment, and the attitudinal behavior toward literacy. In addition, a varied range of adult attitudes toward literacy learning, literacy activities held at home and school, literacy props, as well as literacy facilitation are identified in this study. Concluding remarks in the perspective of the investigator and the directions for further research are provided. (PsycINFO Database Record (c) 2012 APA, all rights reserved)
SN - 0419-4209
AD - Lin, Pei-Yu: U Northern Colorado, US
ER -

TY - JOUR
ID - 2908
T1 - Relations between activities and cooperative playground interactions for preschool-age children with special needs. [References]
A1 - Nabors,Laura; Willoughby,Jennifer; Badawi,Mohamed
Y1 - 1999///
N1 - Peer Reviewed Journal: 1999-15317-004
English
Journal; Peer Reviewed Journal
KW - PsycInfo
KW - Cooperative Learning
KW - Playgrounds
KW - Social Interaction
KW - Special Needs
RP - NOT IN FILE
SP - 339
EP - 352
JF - Journal of Developmental and Physical Disabilities
VL - 11:
IS - 4

N2 - Investigated the relations among cooperative interactions of 70 children with or without special needs (aged 3.2-5.6 yrs) in groups of typically developing peers, peers having special needs, and in inclusive groups, as well as the complexity of the activities in which they were engaged in. Complex activities were those involving fine motor skills as well as higher levels of cognitive, social, and language abilities. The associations among child and program level factors and the children's involvement in different cooperative activities also were examined. Findings indicated that children with or without special needs were likely to engage in cooperative play only with other typically developing children during complex activities (e.g., playing with small toys in the sand or talking). Results also indicated that children with or without special needs tended to engage in cooperative play in inclusive groups (composed of children with and without special needs) during less complex or low-demand activities involving predominantly gross motor skills (e.g., sliding or running). Interventions are needed to facilitate involvement of children with special needs in complex activities on the playground. This is an important area for future research and educational practice efforts. (PsycINFO Database Record (c) 2012 APA, all rights reserved)
SN - 1056-263X
AD - Nabors, Laura: U Maryland, School of Medicine, Dept of Psychiatry, Div of Child & Adolescent Pyschiatry, Baltimore, MD, US
ER -

TY - JOUR
ID - 2909
T1 - Recreational activities of students with mental retardation in three types of schoolyards. [French]. [References]
A1 - Dore,Robert; Dion,Eric; Chapdelaine,Therese; Brunet,Jean-Pierre; Wagner,Serge
Y1 - 1999///
N1 - Peer Reviewed Journal: 1999-11887-003
French
Journal; Peer Reviewed Journal
KW - PsycInfo
KW - Childhood Play Behavior
KW - Mainstreaming (Educational)
KW - Playgrounds
KW - Special Education
KW - Elementary School Students
KW - Intellectual Development Disorder
RP - NOT IN FILE
SP - 39
EP - 53
JF - Revue Francophone de la Deficience Intellectuelle
VL - 10:
IS - 1
N2 - Studied the effect of the elementary school playground on the social and solitary activities of students with mental retardation who learn in regular classrooms, special classrooms in a regular school, or a special school. 26 moderately mentally retarded elementary school students (mean age 9 yrs) who attended regular classes (10 Ss), a special class in a regular school (8 Ss), or a special school (8 Ss) in Canada were observed during at least 1 recreational period lasting at least 15 min. One O was present in about 85% of cases, and 2 Os were present in about 15% of cases. Compared with Ss from special classrooms or a special school, Ss from regular classrooms had more opportunities to interact with normal peers during recess (although their school playground offered less play-related materials than playgrounds designed for mentally retarded students), were more involved in group play, and demonstrated less rough play and less solitary play. However, Ss in all 3 environments often were alone and unoccupied during recess. (PsycINFO Database Record (c) 2012 APA, all rights reserved)
SN - 0847-5733
AD - Dore, Robert: U Quebec a Montreal, Dept des Sciences de l'Education, Montreal, Canada
ER -

TY - JOUR
ID - 2910
T1 - Prevention strategies for at-risk students and students with EBD in urban elementary schools

A1 - Kamps,Debra; Kravits,Tammy; Stolze,Julie; Swaggart,Brenda
Y1 - 1999///
N1 - Peer Reviewed Journal: 1999-11175-006
English
Journal; Peer Reviewed Journal
KW - PsycInfo
KW - Behavior Disorders
KW - Classroom Behavior Modification
KW - Emotional Disturbances
KW - Prevention
KW - Elementary School Students
KW - Peer Tutoring
KW - Social Skills Training
KW - Urban Environments
RP - NOT IN FILE
SP - 178
EP - 188
JF - Journal of Emotional and Behavioral Disorders
VL - 7:
IS - 3
N2 - Used a multilevel universal intervention program for at-risk students and those identified as having emotional and behavioral problems in urban schools. The program addressed classroom management, social skills, and peer tutoring. Students at risk or with emotional and behavioral disorders (EBD) were selected based on the Systematic Screening for Behaviors Disorder test. This sample included 21 boys and 3 girls in K-7th grade, with 16 African American and 8 Caucasian students. An experimental-control, posttest design was used to analyze data from a target group and a control group. Direct observations revealed differences in on-task, positive recess interaction and play, aggression and, out-of-seat behaviors.Teacher reports indicated significant differences for appropriately requesting attention, following directions, and reducing disruptive behaviors for the target group.Implications for early prevention and behavioral support systems for students with or at risk for EBD are discussed. (PsycINFO Database Record (c) 2012 APA, all rights reserved)
SN - 1063-4266
AD - Kamps, Debra: U Kansas, Inst for Life Span Studies, Lawrence, KS, US
ER -

TY - JOUR
ID - 2911
T1 - Stability and change in paternal involvement among urban African American fathers. [References]
A1 - Coley,Rebekah Levine; Chase-Lansdale,
Y1 - 1999///
N1 - Peer Reviewed Journal: 1999-11039-008
English
Journal; Peer Reviewed Journal
KW - PsycInfo
KW - Blacks
KW - Father Child Relations
KW - Involvement
KW - Parental Investment
KW - Urban Environments
KW - Fathers
KW - Mothers
RP - NOT IN FILE
SP - 416
EP - 435
JF - Journal of Family Psychology
JA - J Fam Psychol
VL - 13:
IS - 3

N2 - Urban African American fathers' involvement with their children born outside of marriage is assessed through maternal reports. Multivariable composites of paternal involvement indicate that about half of unmarried fathers are highly involved at the time of birth and when children are preschool age, and half are uninvolved. However, nearly 40% of fathers move into or out of active parenting during this time. Multinomial logit analyses indicate that paternal education and employment increase the likelihood that fathers will be highly involved as children age and decrease the odds of a loss of paternal involvement. Strong or harmonious mother-father relations (romantic or not) increase the odds of fathers' being highly involved and mediate the impact of fathers' residential and marital status. Finally, neither new maternal partners nor highly involved grandmothers deter paternal involvement. (PsycINFO Database Record (c) 2012 APA, all rights reserved) (journal abstract)
SN - 0893-3200
AD - Coley, Rebekah Levine: Harvard U, John F. Kennedy School of Government, Malcolm Wiener Ctr for Social Policy, Cambridge, MA, US
ER -

TY - JOUR
ID - 2912
T1 - Cooperative games: A pathway to improving health
A1 - Carlson,J.
Y1 - 1999///
N1 - Peer Reviewed Journal: 1999-10338-011
English
Journal; Peer Reviewed Journal
KW - PsycInfo
KW - Classroom Behavior Modification
KW - Classroom Environment
KW - Cooperation
KW - Games
RP - NOT IN FILE
SP - 230
EP - 236
JF - Professional School Counseling
VL - 2:
IS - 3
N2 - Conducted a 7-session, cooperative games program with a 2nd grade class of 24 students who had demonstrated trouble with working together cooperatively. The session goals were to have fun, provide 30 min of activity, and to learn about cooperation and teamwork. The classroom teacher used this intervention as a springboard for a unit on cooperation in the classroom. The program was evaluated following each session by a question and answer period with the children and by evaluatory comments by the classroom teacher. Direct observation of the games and the students by the counselor provided further opportunity for evaluation. following the intervention, some of the formerly excluded children said that they were now being included in activities during recess and outside of school activities. A shift in attitudes from the first to the last session was observed, with more helping behaviors noticed towards the end. The class had to learn to incorporate everyone to reach individual and group goals. Over the course of the sessions, cooperation and group cohesion when participating in these games increased. The teacher was also able to translate the cooperative game rules and philosophy to reading groups and other in-class activities. (PsycINFO Database Record (c) 2012 APA, all rights reserved)
SN - 1096-2409
AD - Carlson, J. Matthew: Pennsylvania State U, State College, PA, US
ER -

TY - JOUR
ID - 2913
T1 - "And now I'm addicted!": A counselor's plan to teach kids about addiction. [References]
A1 - Halverson,Susan
Y1 - 1999///

N1 - Peer Reviewed Journal: 1999-08258-011
English
Journal; Peer Reviewed Journal
KW - PsycInfo
KW - Drug Abuse Prevention
KW - Drug Education
KW - Drug Usage Attitudes
KW - Student Attitudes
KW - Tobacco Smoking
KW - Elementary School Students
KW - School Counselors
RP - NOT IN FILE
SP - 147
EP - 149
JF - Professional School Counseling
VL - 3:
IS - 2
N2 - This article discusses an elementary guidance counselor's plan to help students understand the physical and social consequences of smoking as well as what it means to be addicted. The counselor implemented his plan on November 21, the Great American Smokeout Day, to 4th-grade classes. Every 30 minutes the students would have to go outside for 5 minutes, to represent how often a "two pack a day" smoker needs to smoke. Each student was given a Chinese Finger Trap to keep his or her hands occupied during these periods. Teacher's evaluation sheets were filled out immediately after the experiment and student surveys were conducted 4 mo later. Student surveys consisted of questions about nicotine and addiction as well as if they planned to smoke when they were old enough. All students said they would never smoke. It is concluded that counselors need to give students as much information as possible so that they are better equipped to make good choices. (PsycINFO Database Record (c) 2012 APA, all rights reserved)
SN - 1096-2409
AD - Halverson, Susan: Portland State U, School of Education, Special & Counselor Education, Portland, OR, US
ER -

TY - JOUR
ID - 2914
T1 - Children, advertising, and the Internet: An exploratory study
A1 - Henke,Lucy L.
Y1 - 1999///
N1 - Book: 1999-02408-001
English
Book; Edited Book
KW - PsycInfo
KW - Advertising
KW - Child Attitudes
KW - Computer Applications
KW - Leisure Time
KW - Preferences
KW - Communications Media
KW - Daily Activities
KW - Mass Media
KW - Persuasive Communication
RP - NOT IN FILE
SP - 73
EP - 80
JF - Schumann, David W [Ed]; Thorson, Esther [Ed]
VL - :
IS - 1999

N2 - (from the chapter) Investigates children's perceptions of advertising on the World Wide Web (WWW) and examines the potential for displacement of media and of nonmedia-related activities by the WWW. The study assesses children's perceptions of the persuasive intent of favorite Web sites and identifies their preferences for WWW surfing vs other favorite media-related and nonmedia-related after-school activities. Based on previous research, the following questions are addressed: 1. does the WWW have the potential to displace other advertising media, 2. does the WWW have the potential to displace other non-media related activities, and 3. do children ages 9-11 yrs demonstrate the ability to identify the persuasive intent of advertising on the WWW? Fourth graders visited the authors' university to 'surf the Net' and completed pretest and posttest questionnaires and an introductory hands-on Internet session. 39% of Ss said they would prefer to surf the Net than to engage in their favorite after-school activity. Ss were most willing to give up playing with friends or siblings, inside (79%) or outside (89%). 67% of Ss naming TV viewing as a favorite activity would prefer to surf the Net than to watch TV. Only 13% of Ss thought the purpose of their favorite site was to advertise. (PsycINFO Database Record (c) 2012 APA, all rights reserved)
AD - Henke, Lucy L.: U New Hampshire, Durham, NH, US
ER -

TY - JOUR
ID - 2915
T1 - The effect of school size upon educational and environmental factors. [References]
A1 - Al-Soliman,Tarik
Y1 - 1999///
N1 - Peer Reviewed Journal: 1999-01295-001
English
Journal; Peer Reviewed Journal
KW - PsycInfo
KW - School Environment
KW - School Facilities
KW - Schools
KW - Size
KW - Classrooms
KW - Crowding
KW - Educational Administration
KW - Educational Objectives
KW - School Principals
RP - NOT IN FILE
SP - 181
EP - 204
JF - Journal of Architectural and Planning Research
VL - 16:
IS - 3
N2 - Investigated the effect of school size (number of students and classrooms, class size, perceived crowdedness) on various educational and environmental factors in Saudi Arabia, based on a 1990 survey of 1,031 boys' schools. The survey included site observations, personal interviews, and a questionnaire for school principals. Factors investigated included school level (elementary, intermediate, secondary), ownership, spatial elements (library, cafeteria, laboratories, lecture hall, art room, playground), availability of educational technology, building attributes (room sizes, climate control, etc.), school maintenance, principals' authority in running schools, and educational goal achievement. Results show that large schools were more satisfying and had higher levels of achievement. However, large schools were associated with problems of maintenance, daily supervision, and less authority available to principals as school administrators. (PsycINFO Database Record (c) 2012 APA, all rights reserved)
SN - 0738-0895
AD - Al-Soliman, Tarik: King Saud U, Coll of Architecture & Planning, Riyadh, Saudi Arabia
ER -

TY - JOUR
ID - 2916

T1 - Interactions on an elementary school playground: Variations by age, gender, race, group size, and playground area
A1 - Lewis,Tracey E; Phillipsen,Leslie
Y1 - 1998///
N1 - Peer Reviewed Journal: 1999-13035-004
English
Journal; Peer Reviewed Journal
KW - PsycInfo
KW - Childhood Play Behavior
KW - Interpersonal Interaction
KW - Playgrounds
KW - Age Differences
KW - Elementary School Students
KW - Group Size
KW - Human Sex Differences
KW - Racial and Ethnic Differences
RP - NOT IN FILE
SP - 309
EP - 320
JF - Child Study Journal
VL - 28:
IS - 4
N2 - Children in two age groups (88 first- and second graders and 76 fifth- and sixth graders) were observed in a study of elementary school playground interactions. A time-sampling procedure was used to record the gender, race, and group size of all interactive play in seven playground areas during each age group's recess period. Older children played in same-gender, larger groups more than the younger children did. Girls and boys were equally likely to play in same- and mixed-race groupings. While both same- and mixed-gender interaction was more likely to be mixed- rather than same-race, mixed-gender interaction was much more likely to include more than one race than all girls' or all boys' interactions. Mixed-race interactions also included more children than same-race interactions. Other analyses examined variations in playground area by age, gender, race, and group size. (PsycINFO Database Record (c) 2012 APA, all rights reserved)
SN - 0009-4005
AD - Lewis, Tracey E.: U Memphis, Ctr for Research in Educational Policy, Memphis, TN, US
ER -

TY - JOUR
ID - 2917
T1 - The relative cost of children's physical play. [References]
A1 - Pellegrini,Anthony D.
Y1 - 1998///
N1 - Peer Reviewed Journal: 1999-11976-024
English
Journal; Peer Reviewed Journal
KW - PsycInfo
KW - Childhood Play Behavior
KW - Energy Expenditure
KW - Calories
RP - NOT IN FILE
SP - 1053
EP - 1061
JF - Animal Behaviour
VL - 55:
IS - 4
N2 - Analyzed energetic costs (expressed in terms of caloric expenditure) associated with physical play in 4 field experiments of play in 30 5.3-11.5 yr old primary school children. Exp 1 established the concurrent validity of an observational checklist to estimate caloric expenditure of children's physical play. Exp 2 compared caloric expenditure of the play (defined as all behavior exhibited during play time) for 2 age groups of children

(7.0-7.7 vs 9.1-9.9 yr olds) during playtime outdoors and during indoor sedentary activity. Results show that caloric expenditure of outdoor activity was greater and was significantly correlated with ambient temperature. In Exp 3, children were observed during indoor play to control for the influence of ambient temperature. Outdoor physical play was more energetically costly than indoor physical play. In Exp 4, children's behavior was observed outdoors and caloric expenditure for play, games and other activities was compared. Physical play was more costly than other forms of behavior and games. Estimates of total energetic costs of play ranged from 6 to 15%. Results are discussed in terms of the relatively low caloric costs of play. (PsycINFO Database Record (c) 2012 APA, all rights reserved)
SN - 0003-3472
AD - Pellegrini, Anthony D.: U Minnesota, Dept of Educational Psychology, Minneapolis, MN, US
ER -

TY - JOUR
ID - 2918
T1 - Comparative study of the natural habitat behaviors of children with autism and children with down syndrome: An ecological approach
A1 - Ruble,Lisa Ann
Y1 - 1998///
N1 - Dissertation Abstract: 1998-95022-324
English
Dissertation Abstract
KW - PsycInfo
KW - Autism
KW - Down's Syndrome
KW - Ecological Psychology
RP - NOT IN FILE
SP - 2459
JF - Dissertation Abstracts International: Section B: The Sciences and Engineering
VL - 59:
IS - 5-B
N2 - Little is known about children with autism in their natural environment, and children with autism who have more limited cognitive and verbal abilities. This study is the first to apply methods from ecological psychology to increase the understanding of autism. A better understanding of behavior that occurs in the natural habitat will aid diagnostic and assessment efforts and the identification of ecologically-relevant treatment variables. The ecological approach described by Scott (1980) was applied to study the in-depth structure of the behavior stream. Participants included 8 boys with autism and 8 boys with Down syndrome between the ages of 6 and 10 of similar adaptive communication levels. Narrative records were collected and analyzed based on duration and frequency of behaviors. Each record was analyzed by marking the behavior stream into activity units, which are naturally occurring chunks of behavior from the perspective of the child and occurring along a constant psychological direction. Each activity unit was then coded. Findings based on the structure of the activity units revealed that children with autism exhibited activity units of shorter duration and with less overlap. No differences in features related to the setting or in qualitative aspects of activity units were found. Partners of children with Down syndrome were more likely to be rated as being involved with high duration. Children with autism were more likely to use physical mechanism (e.g., physical contact, physical assistance); children with Down syndrome used more gestures (e.g., pointing, nodding). Both groups of children spent most of their time playing and watching TV. Analysis of the duration of intention of activity units revealed that children spent similar amounts of time participating in all but two types of activities. Children with autism spent more time readying their environment; children with Down syndrome spent more time eating and drinking. Subcategory analysis revealed that children with autism were four times more likely to fail to respond. In summary, findings that children with autism behaved in a more sequential manner, engaged in one activity at a time, shifted frequently from one activity to another, and persisted in a given activity for a relatively short time suggest behavior of more immature children then expected based on mental age. (PsycINFO Database Record (c) 2012 APA, all rights reserved)
SN - 0419-4217
AD - Ruble, Lisa Ann: Indiana U, US
ER -

TY - JOUR
ID - 2919
T1 - The effectiveness of the cares program in increasing academic and social skills for children at risk in the elementary school
A1 - Steele,Margaret Joan
Y1 - 1998///
N1 - Dissertation Abstract: 1998-95021-141
English
Dissertation Abstract
KW - PsycInfo
KW - Educational Program Evaluation
KW - Mathematics Achievement
KW - Reading Skills
KW - Social Skills
KW - Spelling
KW - At Risk Populations
KW - Elementary School Students
KW - Program Evaluation
RP - NOT IN FILE
SP - 1462
JF - Dissertation Abstracts International Section A: Humanities and Social Sciences
VL - 59:
IS - 5-A
N2 - School personnel search for ways to help behavior-disordered students maximize their potential in the classroom. One school district administration took primary responsibility for student success by developing and funding a specially-designed program for children at risk in the elementary school. This study examined: the effectiveness of the CARES program in increasing reading, mathematics, and spelling skills, as well as, increasing appropriate social skills; and how any increase in skills compared to increases in social and academic skills of similar students on a waiting list for the CARES program who stayed in their regular classroom without further intervention. The CARES program contained seven components designed to: (a) elicit voluntary cooperation, (b) intervene early, (c) teach positive social skills, (d) meet individual academic needs, (e) offer personal counseling, (f) encourage regular parental participation, and (g) network as active partners with parents and outside social service agencies. The experimental group consisted of 18 third through fifth grade students and the control group totalled 13. The differences between the two groups' pre and post-test scores on the Burks' Behavior Rating Scale and the Behavior Assessment System for Children, as measured by the Mann Whitney U test on the small sample, determined if the students' social skills showed a significant gain. Pre and post-tests of students' academic growth were assessed using the Wide Range Achievement Test-Revised and the Wechsler Individual Achievement Test. Statistically significant improvement was seen after 3 months for the experimental subjects compared to control group participants in increasing reading, mathematics, and spelling skills, as well as, increasing appropriate social skills. From the results of this study, the data appear to support a relationship between increased academic and social skill levels of the experimental subjects and the CARES program. Previous studies included one or more of its seven components, but none with all seven combined in one program like CARES. (PsycINFO Database Record (c) 2012 APA, all rights reserved)
SN - 0419-4209
AD - Steele, Margaret Joan: U San Francisco, US
ER -

TY - JOUR
ID - 2920
T1 - Thought processes and strategies of eight, eleven, and fourteen-year-old students while engaged in music composition
A1 - Younker,Betty Anne
Y1 - 1998///
N1 - Dissertation Abstract: 1998-95009-036
English
Dissertation Abstract
KW - PsycInfo

KW - Age Differences
KW - Cognitive Processes
KW - Cognitive Techniques
KW - Music
KW - Technology
RP - NOT IN FILE
SP - 4217
JF - Dissertation Abstracts International Section A: Humanities and Social Sciences
VL - 58:
IS - 11-A
N2 - The purpose of the study was to explore thought processes and strategies of three 8-, three 11-, and three 14-year-old students that occurred while composing with technology. In this descriptive study, the following data were analyzed: (1) students' thoughts, responses, and behaviors that occurred during three composing sessions; (2) students' verbal responses to questions, given after each composing session, about their composing activities; and (3) students' verbal responses to questions, given before and after all composing sessions, about composing in general. The findings from the above three analyses were examined to discern what developmental patterns, if any, emerged across ages. Nine students were involved in seven 1-hour sessions. In two age-specific information sessions, students were introduced to a software program. In the third session, each student met individually to review what was covered during the information sessions and was asked questions about composing in general. In sessions four, five, and six, each student was asked to compose, think aloud while composing, and respond to questions via an unstructured interview technique. Each session ended with the student responding to questions about the composing activities. In the seventh session, the students presented their compositions to each other. Analysis of data for research question 1 revealed nine themes: elements of music, concepts, expressive gestures, composing processes, incorporation of known material, non-composing processes, technological problems, outlier verbal reports, and outside influences. Analysis of data for research question 2 generated five categories: awareness of activities, ability to recall activities, occurrence of mental activities between sessions, ability to recall interaction with musical elements, and range of evaluative comments. For research question 3, data were organized around: changes in content of specific responses, relationships between descriptions and experiences, descriptions of beginning composing activities, movement from third to first person, and perceptions about composing and listening. Developmentally, patterns emerged in the composing behaviors and in the treatment of timbre, beat, melody, and harmony. (PsycINFO Database Record (c) 2012 APA, all rights reserved)
SN - 0419-4209
AD - Younker, Betty Anne: Northwestern U, US
ER -

TY - JOUR
ID - 2921
T1 - Prosocial entry behaviors used by preschoolers to enter play groups in the natural setting of the classroom
A1 - Wilburn,Reudene Elizabeth
Y1 - 1998///
N1 - Dissertation Abstract: 1998-95005-110
English
Dissertation Abstract
KW - PsycInfo
KW - Childhood Play Behavior
KW - Classroom Environment
KW - Interpersonal Interaction
KW - Prosocial Behavior
KW - Preschool Students
RP - NOT IN FILE
SP - 3411
JF - Dissertation Abstracts International Section A: Humanities and Social Sciences
VL - 58:
IS - 9-A
N2 - The purpose of this research was to identify specific entry behaviors used by preschoolers ranging in age from 4 3 to 5.2 as attempts are made into play groups in the natural setting of the classroom and play yard.

When successful entry behaviors are displayed by preschoolers and are identified by researchers or teachers, those behaviors can possibly be taught to preschoolers who exhibit a need for more control over their entry strategies. Potentially successful entry bid strategies might be hovering near the group while observing their activities, making statements and displaying body language relevant to the group's activities. The design of this research is sociological and ethnographic in terms of the researcher having observed patterns of behaviors as they emerged during play in the natural setting. The basis of this observation, analysis, and evaluation is predicated on related research and literature in the fields of educational psychology, sociology, and educational studies. (PsycINFO Database Record (c) 2012 APA, all rights reserved)
SN - 0419-4209
AD - Wilburn, Reudene Elizabeth: Fordham U, US
ER -

TY - JOUR
ID - 2922
T1 - Children's language and sociodramatic play with multicultural materials
A1 - Huber,Linda K.
Y1 - 1998///
N1 - Dissertation Abstract: 1998-95001-019
English
Dissertation Abstract
KW - PsycInfo
KW - Childhood Play Behavior
KW - Drama
KW - Interpersonal Communication
KW - Multicultural Education
KW - Preschool Students
KW - Concept Formation
KW - Familiarity
KW - Language Development
RP - NOT IN FILE
SP - 2525
JF - Dissertation Abstracts International Section A: Humanities and Social Sciences
VL - 58:
IS - 7-A
N2 - The purpose of this study was to examine children's play and language when a variety of unfamiliar multicultural pretend foods and cooking utensils not typically found in dramatic play centers were introduced. The study was conducted in a laboratory preschool setting with three multiage heterogeneous groups of children over a period of 7 weeks. Three methods of data collection were employed: (a) videotapes of children in the dramatic play center, (b) a notebook of observations made by the teachers when the researcher was not present, and (c) interviews with 18 children. The children who chose to play in the dramatic play center were videotaped daily during indoor play time for 1 week prior to the introduction of new materials. The new multicultural pretend foods and cooking utensils were introduced during the second week of the study. The children were then videotaped daily for 2 more weeks and then once each week for the next 4 weeks. The data collected from the videotapes and teacher notes were used to develop coding categories. Categories of children's play were: (a) time spent playing with unfamiliar materials, (b) conventional use of materials, and (c) unconventional use of materials. Categories of children's language were: (a) labeling, (b) other conversation in dramatic play, (c) questions, and (d) conversation about the materials outside of the dramatic play center. The data were interpreted to discover how children interacted with and talked about the multicultural pretend foods and cooking utensils. It was concluded that children interacted with the unfamiliar multicultural pretend foods and cooking utensils in much the same manner as familiar materials. Labeling items or being able to put words with the foods and cooking utensils appeared to be important to the children. Children used unfamiliar materials unconventionally more often than they used familiar materials unconventionally. Furthermore, children did not see a relationship between the items in the dramatic play center and materials in other parts of the preschool. (PsycINFO Database Record (c) 2012 APA, all rights reserved)
SN - 0419-4209
AD - Huber, Linda K.: Ball State U, US
ER -

TY - JOUR
ID - 2923
T1 - A qualitative analysis of metaplay in the preschool years
A1 - Trawick-Smith,Jeffrey
Y1 - 1998///
N1 - Peer Reviewed Journal: 1998-11681-002
English
Journal; Peer Reviewed Journal
KW - PsycInfo
KW - Childhood Play Behavior
KW - Taxonomies
RP - NOT IN FILE
SP - 433
EP - 452
JF - Early Childhood Research Quarterly
VL - 13:
IS - 3
N2 - Previous play research has focused on social or representational processes occurring within make believe. This study examined young children's metaplay-the process of suspending actual role playing to think or communicate about pretend themes from outside of the play frame. 12 preschool Ss were videotaped during 8 separate half-hour sessions, as they engaged in spontaneous free play with a same-aged peer. Videotapes were transcribed and analyzed using the constant comparative method-a naturalistic data processing procedure. The primary researcher and a 2nd observer unitized all metaplay behaviors, categorized these units, named the resulting categories, and wrote rules for inclusion. The outcome was a typology of metaplay behaviors, comprised of 3 broad categories-initiations, responses, and constructions-and 38 subcategories. Each specific metaplay behavior was described and working hypotheses were formulated regarding its cognitive, metacognitive, and social significance. It is concluded that a rich array of developmentally useful interactions-which increase in frequency and complexity with age-transpire outside of actual role playing. (PsycINFO Database Record (c) 2012 APA, all rights reserved)
SN - 0885-2006
AD - Trawick-Smith, Jeffrey: Eastern Connecticut State U, Education Dept, Willimantic, CT, US
ER -

TY - JOUR
ID - 2924
T1 - Leisure preferences and open space needs in an urban Chinese American community
A1 - Zhang,Tingwei
Y1 - 1998///
N1 - Peer Reviewed Journal: 1998-11616-003
English
Journal; Peer Reviewed Journal
KW - PsycInfo
KW - Chinese Cultural Groups
KW - Environmental Planning
KW - Leisure Time
KW - Preferences
KW - Recreation Areas
KW - Racial and Ethnic Attitudes
KW - Recreation
RP - NOT IN FILE
SP - 338
EP - 355
JF - Journal of Architectural and Planning Research
VL - 15:
IS - 4

N2 - Explored leisure preferences and open space needs of 12-60 yr old Chinese Americans of Chicago's Chinatown. 203 face-to-face interviews and focus group discussions were used to identify outdoor leisure patterns and preferences, in general, and with respect to new park development being planned for the community. Findings show that although some popular activities were no different from what might be expected for the mainstream Anglo American population, the meaning and significance of these activities had clear and unique ties to Chinese culture. Preferences for the new Chinatown park development mirrored activity preferences, emphasizing facilities that enhance the natural environment for passive activities. Notable differences in activity preferences were found within the sample of respondents according to age, generational status, and other factors. (PsycINFO Database Record (c) 2012 APA, all rights reserved)
SN - 0738-0895
AD - Zhang, Tingwei: U Illinois, Coll of Urban Planning & Public Affairs, Chicago, IL, US
ER -

TY - JOUR
ID - 2925
T1 - Children's health actions within the context of daily living
A1 - Kalnins,Ilze; Jutras,Sylvie; Normandeau,Sylvie; Morin,Pauline
Y1 - 1998///
N1 - Peer Reviewed Journal: 1998-11043-006
English
Journal; Peer Reviewed Journal
KW - PsycInfo
KW - Childhood Development
KW - Daily Activities
KW - Health Behavior
RP - NOT IN FILE
SP - 460
EP - 472
JF - American Journal of Health Behavior
JA - Am J Health Behav
VL - 22:
IS - 6
N2 - Examined the health actions young children say they take in contexts that structure their day including eating, dressing, keeping clean, going to sleep, schoolwork, recess, leisure time, and going places. Structured interviews were conducted with 1,674 5-12 yr old children. Children mentioned actions related to eating (food choices, eating behaviors), engaging in physical activity, avoiding illness or injury, being clean, getting fresh air and mental health (fostering interpersonal relationships, helping others, and making oneself feel good or relaxed). The authors conclude that the report of these these actions by large numbers of children, and across different contexts, supports their inclusion as key topics in developmentally appropriate health promotion. (PsycINFO Database Record (c) 2012 APA, all rights reserved)
SN - 1087-3244
AD - Kalnins, Ilze: U Toronto, Dept of Public Health Sciences, Toronto, ON, Canada
ER -

TY - JOUR
ID - 2926
T1 - Sex differences in the games children play
A1 - Lever,Janet
Y1 - 1998///
N1 - Book: 1998-07661-002
English
Book; Edited Book
This reprinted chapter originally appeared in Social Problems, 1976, Vol 4, pp. 478-487)
KW - PsycInfo
KW - Childrens Recreational Games
KW - Human Sex Differences
KW - Sex Roles

KW - Socialization
KW - School Adjustment
RP - NOT IN FILE
SP - 102
EP - 112
JF - Myers, Kristen A [Ed]; Anderson, Cynthia D [Ed]; Risman, Barbara J [Ed]
VL - :
IS - 1998
N2 - (from the chapter) This reprinted article originally appeared in (Social Problems, 1976, Vol 23[4], 478-487). The following abstract of the original article appeared in record 1977-08033-001.) Studied the role of the world of play and games in childhood socialization. Data from observations of school-yards, interviews, questionnaires, and playtime activity analysis provided information about 181 5th graders, half of whom were from suburban and half from city schools. Sex differences were observed in "real" play activities: (a) Boys played outdoors more than girls; (b) Although boys and girls played alone about the same proportion of the time, when they were in social play, boys more often played in larger groups; (c) Boys' play occurred in more heterogeneous groups; (d) Girls more often played in predominantly male games than boys played in girls' games; (e) Boys played competitive games more often than girls; (f) Girls played more than boys and boys "gamed" more than girls. It is suggested that play and games contribute to the preservation of traditional sex-role divisions in society by equipping boys with the social skills needed for occupational careers while equipping girls with the social skills better suited for family careers. (PsycINFO Database Record (c) 2012 APA, all rights reserved)
AD - Lever, Janet: Northwestern U, Evanston, IL, US
ER -

TY - JOUR
ID - 2927
T1 - Comparison of the quality of preschoolers' play in housekeeping and thematic sociodramatic play centers
A1 - Bagley,Donna M.
Y1 - 1998///
N1 - Peer Reviewed Journal: 1998-04199-007
English
Journal; Peer Reviewed Journal
KW - PsycInfo
KW - Childhood Play Behavior
KW - Classroom Environment
KW - Role Playing
KW - Preschool Students
RP - NOT IN FILE
SP - 71
EP - 77
JF - Journal of Research in Childhood Education
VL - 12:
IS - 1
N2 - Differences in the quality of play in thematic and housekeeping organizational patterns of the sociodramatic play center in preschool classrooms were documented by biweekly videotaping children's play during free choice time in 18 classrooms of an early childhood program in a moderately sized Midwest city. Videotapes for each classroom group were divided into the 4 quarters of the academic year. Of these, 68 were randomly selected and evaluated for play quality. The thematic organization resulted in higher quality sociodramatic play in which the children enacted more roles outside the home, utilized more aspects of their roles, demonstrated higher levels of symbolic prop use, and played longer. Early childhood educators wishing to utilize the thematic organization of the sociodramatic play center will find the practice supported, leading to longer play episodes, increased symbolic prop use, and higher quality make-believe. (PsycINFO Database Record (c) 2012 APA, all rights reserved)
SN - 0256-8543
ER -

TY - JOUR

ID - 2928
T1 - Using simultaneous prompting within an activity-based format to teach dressing skills to preschoolers with developmental delays
A1 - Sewell,Teena J.
Y1 - 1998///
N1 - Peer Reviewed Journal: 1998-01993-003
English
Journal; Peer Reviewed Journal
KW - PsycInfo
KW - Activities of Daily Living
KW - Developmental Disabilities
KW - Prompting
KW - Self Care Skills
KW - Skill Learning
RP - NOT IN FILE
SP - 132
EP - 145
JF - Journal of Early Intervention
VL - 21:
IS - 2
N2 - A multiple probe across skills single-S research design was used to evaluate the effectiveness of a simultaneous prompting procedure with a physical guidance controlling prompt to teach 3 dressing skills each to 2 preschoolers (aged 28 and 25 mo) with disabilities. The format was activity-based in that both probe and instructional trials were embedded within activities that occurred throughout the day when dressing would be a natural activity (e.g., putting on a jacket to go outside to play) in the preschool setting. Both students maintained the skills with 90% accuracy up to 6 wks following acquisition. (PsycINFO Database Record (c) 2012 APA, all rights reserved)
SN - 1053-8151
AD - Sewell, Teena J.: Cumberland River Comprehensive Care, US
ER -

TY - JOUR
ID - 2929
T1 - Applying the BeSafe method to product safety evaluation
A1 - Benedyk,Rachel
Y1 - 1998///
N1 - Peer Reviewed Journal: 1997-42652-001
English
Journal; Peer Reviewed Journal
KW - PsycInfo
KW - Human Factors Engineering
KW - Playgrounds
KW - Risk Management
KW - Safety
KW - Risk Assessment
KW - Consumer Behavior
KW - Evaluation
RP - NOT IN FILE
SP - 5
EP - 13
JF - Applied Ergonomics
VL - 29:
IS - 1
N2 - The BeSafe (Behavioural Safety) Method, has been developed by International Mining Consultants Ltd for assessing and reducing risk in industrial contexts. BeSafe is an ergonomic method of risk analysis and reduction, targeted at prevention of accidents due to human errors and violations, and emphasizing management strategies for action. Using the BeSafe Method, it may be possible to structure safety assessments of the use of

products and target improvement measures. The authors tested the relevant parts of the method on children's play equipment in 24 playgrounds, and questionnaires were also administered to 348 7-11 yr olds and 15 adults. Useful results were obtained, suggesting that BeSafe could be adapted for other consumer products in a similar way. (PsycINFO Database Record (c) 2012 APA, all rights reserved)
SN - 0003-6870
AD - Benedyk, Rachel: U London, University Coll, Ergonomics & HCI Unit, London, England
ER -

TY - JOUR
ID - 2930
T1 - Preliminary results of psychodynamic treatment of child autism. [Italian]
A1 - Zirilli,Maria; Cocconi,Rossella; Torelli,Cristina
Y1 - 1997///
N1 - Peer-Reviewed Status-Unknown: 1999-00274-005
Italian
Journal; Peer-Reviewed Status-Unknown
KW - PsycInfo
KW - Autism
KW - Psychoanalysis
RP - NOT IN FILE
SP - 49
EP - 59
JF - Psichiatria dell'infanzia e dell'adolescenza
VL - 64:
IS - 1
N2 - Studied the outcome of psychodynamic treatment in young children lacking language and relational play. Human Ss: Nine Italian preschool children (aged 2-5 yrs) (autism). The Italian translation of the Peabody Picture Vocabulary Test (PPVT) was administered to 2 Ss. Treatment efficacy was measured periodically in all Ss. Results are discussed in relation to the important role of the natural environment (the family and school settings) in the treatment of autistic children. Treatments: Game therapy and psychoanalytic family therapy were used, and educational support was provided. Treatment lasted from 6 mo to 4 yrs. (English abstract) (PsycINFO Database Record (c) 2012 APA, all rights reserved)
SN - 0393-361X
AD - Zirilli, Maria: Azienda USL di Parma, Servizio di Psicoterapia per l'Infanzia e l'Adolescenza, Parma, Italy
ER -

TY - JOUR
ID - 2931
T1 - Collaboration and flexible care in school-based mental health services to children who are severely emotionally disturbed: An evaluation of program components and outcomes
A1 - Heisler-Scott,Claudette
Y1 - 1997///
N1 - Dissertation Abstract: 1997-95022-330
English
Dissertation Abstract
KW - PsycInfo
KW - Employee Efficiency
KW - Mental Health Programs
KW - Schools
KW - Cooperation
RP - NOT IN FILE
SP - 2677
JF - Dissertation Abstracts International: Section B: The Sciences and Engineering
VL - 58:
IS - 5-B
N2 - This is an evaluation of the Partnership, a school-based mental health service program for children labelled "Seriously Emotionally Disturbed (SED)." Two characteristics emerge as priorities in the literature

regarding the needs of emotionally disturbed children. One is the development of models of care that offer services that can be adapted to the needs of each child or family. The other vital component is collaborative interaction among adult caregivers. To date, neither education nor mental health services have shown consistent success in meeting the complex needs of SED children. The Partnership is a school-based mental health service program that strives to incorporate recommendations for flexible care and collaborative relationships. Forty-three children, first through fifth graders, from five SED special day classrooms located in three San Francisco public schools were included in the study. Also included were their adult providers and caregivers. Process and outcome evaluations were undertaken. Outcomes were assessed based on student academic and social functioning ratings which were collected from a variety of sources. Program components were assessed using the Partnership Survey which was developed for the purpose of this evaluation. This was a single measure that captured the unique perceptions of professionals, parents, and caregivers alike. Findings indicated that the different adult collaborator groups (parents/caregivers, teaching staff, outside providers) rated the program very highly. Some exceptions were thought to be related to phases of development in collaborative relationships. The consistently high ratings of the program by parents and caregivers in this study are noteworthy, in that the literature has shown that this group is typically dissatisfied with mental health services to their SED children. A striking finding related to the characteristics of the population of SED students was the large number of stressful or traumatic events in their lives. These included frequent home placement changes coupled with a very high number of school placement changes. Although correlations were not found between the stressful events and the students' school functioning, dramatic increases in school stability were found among Partnership students. Relationships found between collaborators' ratings of the Partnership and the students' social functioning suggest that adult collaborators play a key role in students' success. (PsycINFO Database Record (c) 2012 APA, all rights reserved)
SN - 0419-4217
AD - Heisler-Scott, Claudette: California School of Professional Psychology - Berkeley/alameda, US
ER -

TY - JOUR
ID - 2932
T1 - Play behaviors and peer interactions of preschoolers in classroom and playground settings
A1 - Shim,Sook Young
Y1 - 1997///
N1 - Dissertation Abstract: 1997-95017-144
English
Dissertation Abstract
KW - PsycInfo
KW - Childhood Play Behavior
KW - Classroom Environment
KW - Peer Relations
KW - Playgrounds
KW - Age Differences
KW - Child Day Care
RP - NOT IN FILE
SP - 0731
JF - Dissertation Abstracts International Section A: Humanities and Social Sciences
VL - 58:
IS - 3-A
N2 - The relationship among different settings for young children's play behaviors and peer interactions was examined. The participants were forty-one children from 2 to 5 years of age (21 2- and 3-year-olds and 20 4- and 5-year-olds) enrolled in three child care programs. The children were videotaped for 5 minutes each on 4 different days both indoors and outdoors. Program measures described the quality of the child care program (the Assessment Profile for Early Childhood Programs) and additional measures described the playground setting. Children's play behaviors were categorized using the Parten-Smilansky Scale, which combines social play categories and cognitive play categories (Rubin, Watson, & Jambor, 1978) using 16 categories of play behavior. Children's peer interactions were categorized using the Peer Play Scale (Howes & Matheson, 1992). Results indicated that all six classrooms and their related playgrounds were mediocre child care programs. Children were more likely to engage in parallel functional play and the highest form of peer play (i.e., social pretend play) and play behavior (i.e., interactive dramatic play) outdoors than indoors. The younger age group was more

likely to be engaged in playing alone or interacting with adults, whereas the older age group was more likely to interact with peers. These findings reinforce the importance of both the indoor and the outdoor environment for promoting more complementary play behaviors and peer interactions. Finally, additional findings of relationships between the Parten-Smilansky Scale and the Peer Play Scale showed that the combination of the two scales provide a more valid and comprehensive assessment of children's social interactions with peers in naturally occurring contexts. (PsycINFO Database Record (c) 2012 APA, all rights reserved)
SN - 0419-4209
AD - Shim, Sook-Young: Iowa State U, US
ER -

TY - JOUR
ID - 2933
T1 - Social-cognitive perspective taking in student mediators
A1 - Lane-Garon,Pamela S.
Y1 - 1997///
N1 - Dissertation Abstract: 1997-95017-133
English
Dissertation Abstract
KW - PsycInfo
KW - Conflict Resolution
KW - Mediation
KW - Role Taking
KW - Social Cognition
KW - Elementary School Students
KW - Human Sex Differences
KW - Peers
RP - NOT IN FILE
SP - 0738
JF - Dissertation Abstracts International Section A: Humanities and Social Sciences
VL - 58:
IS - 3-A
N2 - Social-cognitive perspective taking of elementary school students was examined in the context of a schoolwide conflict resolution program. The study compared perspective taking of children trained as mediators with untrained participants over the course of an academic year. Dependent variables were perspective taking and interpersonal problem-solving strategy choice. Independent variables were mediation training/practice and gender. Dependent variables included cognitive, affective and projective aspects of perspective taking. One hundred twelve students in 4th through 8th grades in a bicultural school community participated. Sixty-two students were selected and trained to mediate the disputes of their peers on the playground. Another 50 students served as a comparison group for purposes of evaluation. The 50 nonmediators were aware of the program and sometimes participated as disputants in mediations on the playground. Training was assisted by the Arizona State Attorney General's Office of Community Relations. The interval between pre and post intervention assessment was six months. Results of repeated measures of analyses of variance showed that perspective taking scores on the cognitive and affective perspective taking scales of the Davis Interpersonal Reactivity Index increased over time for all participants, with the students trained as mediators showing greater increases than the untrained students. Pre-to-post increases in perspective taking indicate that the cognitive and affective perspective taking of mediators may be enhanced by mediation training and playground mediation practice. Further, the finding that perspective taking scores of untrained participants increased substantially suggests that observational learning and participation in mediation as disputants may also have a favorable influence on perspective taking. Females scored slightly higher than males on perspective taking measures, although not significantly so. Boys and girls showed different (PsycINFO Database Record (c) 2012 APA, all rights reserved)
SN - 0419-4209
AD - Lane-Garon, Pamela S.: Arizona State U, US
ER -

TY - JOUR
ID - 2934

T1 - Making friends at school: The social interaction patterns of young children with physical disabilities
A1 - Richardson,Pamela Kellund
Y1 - 1997///
N1 - Dissertation Abstract: 1997-95011-133
English
Dissertation Abstract
KW - PsycInfo
KW - Childhood Play Behavior
KW - Classroom Behavior
KW - Peer Relations
KW - Physical Disorders
KW - Interpersonal Interaction
RP - NOT IN FILE
SP - 5114
JF - Dissertation Abstracts International Section A: Humanities and Social Sciences
VL - 57:
IS - 12-A
N2 - This study compared the peer interaction patterns, peer social networks, and interactions patterns with adults of young children with and without physical disabilities. Three pairs of classmates ranging in age from five to eight years participated. Each pair consisted of one child with a physical disability and normal cognition and a typically developing classmate who were matched for demographic variables. Momentary time sampling techniques were used to record children's interactions with peers on the playground during recess. Semi-structured behavioral observations were used to record qualitative aspects of children's interactions with peers and teachers. Teacher interviews provided additional information regarding children's educational programs, social adjustment, and peer relationships. Analysis of quantitative data revealed that children with physical disabilities had cooperative play episodes of shorter duration, spent less time in interactive behaviors of any kind, had more interruptions in cooperative play episodes, and had less intensity of interaction than their peers. Analysis of qualitative data revealed that children with physical disabilities had a strong desire to engage in social interactions, but difficulty initiating and sustaining interactions. Teachers often interrupted the work and play interactions of children with physical disabilities, limiting the amount of peer interaction time available. Children with physical disabilities actively initiated peer interactions, but had more unsuccessful interaction attempts than their peers, and approached adults for social interactions much more frequently than their typically developing peers. Children with physical disabilities demonstrated a strong desire to be helpful and involved in classroom activities, but were marginalized as classroom contributors both as a result of physical limitations and because of poor social initiation skills. (PsycINFO Database Record (c) 2012 APA, all rights reserved)
SN - 0419-4209
AD - Richardson, Pamela Kellund: U Washington, US
ER -

TY - JOUR
ID - 2935
T1 - The expression of care in the rough and tumble play of pre-adolescent boys
A1 - Reed,Thomas L.
Y1 - 1997///
N1 - Dissertation Abstract: 1997-95009-052
English
Dissertation Abstract
KW - PsycInfo
KW - Childhood Play Behavior
KW - Human Males
KW - Interpersonal Interaction
RP - NOT IN FILE
SP - 4649
JF - Dissertation Abstracts International Section A: Humanities and Social Sciences
VL - 57:
IS - 11-A

N2 - Rough and tumble (R&T) play is widely researched in relationship to social affiliation and the cognitive benefits associated with participation in this type of play. One less researched aspect of R&T play is the affective dimension, more specifically, the way in which boys care for one another through R&T play. The first part of this research examines the various ways in which boys participate in R&T play, how the R&T scenario is set up, who plays, what the rules are and how they are enforced. Boys were video taped while engaging in R&T play and during a game called Smear the Queer (Smear). Data gathered from observing the video taped segments of R&T play clearly suggests that R&T play is a way in which boys express care for one another, that Smear is a camouflage for intimacy, and that R&T play is more like a dance among the players than a helter-skelter mix of running, chasing, and tackling. The second part of this study involved interviewing the players while they watched selected video segments of R&T play and expressions of care exhibited during R&T play. Analysis of the interview transcripts established that the boys were able to clearly delineate the difference between R&T play and aggression. Furthermore, the participants were able to validate the game of Smear as a way in which boys are able to express care for one another. Finally, the participants described R&T play as the vehicle in which they are able to experience physical intimacy with one another. It is concluded that R&T play is clearly misunderstood by the uninformed observer. The players are keenly aware that engagement in R&T play is a time that it is acceptable to hug, hold hands, and otherwise experience bodily contact not permitted outside the play arena. (PsycINFO Database Record (c) 2012 APA, all rights reserved)
SN - 0419-4209
AD - Reed, Thomas L.: U South Carolina, US
ER -

TY - JOUR
ID - 2936
T1 - Are sibling roles altered when one child has diabetes?
A1 - Colvin,Cheryl Halpern
Y1 - 1997///
N1 - Dissertation Abstract: 1997-95008-351
English
Dissertation Abstract
KW - PsycInfo
KW - Birth Order
KW - Diabetes Mellitus
KW - Role Expectations
KW - Siblings
KW - Activity Level
KW - Age Differences
KW - Household Management
KW - Human Sex Differences
RP - NOT IN FILE
SP - 6562
JF - Dissertation Abstracts International: Section B: The Sciences and Engineering
VL - 57:
IS - 10-B
N2 - This study addressed sibling roles in families where one child has diabetes. Subjects consisted of 48 families who had second-borns with diabetes and 48 families who had second-borns without diabetes. Parents and first-borns were interviewed about how many hours per week first- and second-borns spent performing household chores and outside of home activities. The amount of time spent doing chores and activities was examined based on respondent (parent or child), health status of the second-born, and the child's age, birth status, and gender. Children engaged in approximately 10.3 hours of work per week. Respondents had similar perceptions of how much work was performed by first-borns. However, parents felt that second-borns performed more work than was reported by first-borns. Older siblings' performance of chores was not affected by having a younger sibling with diabetes. Children with diabetes performed more chores than healthy peers and appeared to be "super" capable. Excluding diabetes care, age was positively associated with the amount of chores children performed. One exception was that parents perceived adolescent and preadolescent first-borns who had a younger sibling with diabetes to have equal responsibilities. Controlling for age, sibling care was the only type of work that was associated with birth order. Gender was not as predictive of household work as in previous studies. The amount of outside of home activities engaged in by first-borns was not affected by their

sibling's health status. Male second-borns who had diabetes performed more outside of home activities than female second-borns with diabetes. The amount of activities a child performed was affected by their own health status but not their sibling's health status. (PsycINFO Database Record (c) 2012 APA, all rights reserved)
SN - 0419-4217
AD - Colvin, Cheryl Halpern: U Florida, US
ER -

TY - JOUR
ID - 2937
T1 - Assessing the effects of a peer mediation training program on skill acquisition, maintenance and generalization
A1 - Winston,Markay Larraine
Y1 - 1997///
N1 - Dissertation Abstract: 1997-95001-092
English
Dissertation Abstract
KW - PsycInfo
KW - Generalization (Learning)
KW - Mediation
KW - Peer Relations
KW - Skill Learning
RP - NOT IN FILE
SP - 2863
JF - Dissertation Abstracts International Section A: Humanities and Social Sciences
VL - 57:
IS - 7-A
N2 - The purpose of the study, conducted in a Midwestern, suburban intermediate school with a diverse student population, was to evaluate the effectiveness of a peer mediation training program on the skill acquisition, maintenance, and generalization of trained students. Co-mediator skill proficiency was assessed for 10 pairs of students in the training group and 7 pairs in the control group. A mixed method, pretest-posttest control group design was employed. Analogue role-play tests, naturalistic observation, self-report data, and parental observations were utilized to evaluate skill acquisition, treatment integrity, social acceptability, and generalization of student skills. Statistically significant results (p <) indicated that trained students demonstrated appropriate skills across mediation steps during analogue assessment and within the natural setting. Further, treatment integrity measures demonstrated proper implementation of mediation skills during actual mediation sessions. Social acceptability data, although inconclusive, revealed that the program was positively perceived. Finally, parental observations showed that active mediators successfully demonstrated learned skills in the home. (PsycINFO Database Record (c) 2012 APA, all rights reserved)
SN - 0419-4209
AD - Winston, Markay Larraine: U Cincinnati, US
ER -

TY - JOUR
ID - 2938
T1 - What did you learn outside of school today? Using structured interviews to document home and community activities related to science and technology
A1 - Korpan,Connie A.
Y1 - 1997///
N1 - Peer Reviewed Journal: 1997-42231-002
English
Journal; Peer Reviewed Journal
KW - PsycInfo
KW - Daily Activities
KW - Interviews
KW - Learning
KW - Science Education
KW - Technology

KW - Community Facilities
KW - Elementary School Students
KW - Interests
KW - Kindergarten Students
KW - Leisure Time
KW - Mothers
RP - NOT IN FILE
SP - 651
EP - 662
JF - Science Education
VL - 81:
IS - 6
N2 - Describes the development 2 structured interview procedures: The Community and Home Activities Related to Technology and Science for parents of Preschool Children (CHARTS/PS) and for School-Age Children (CHARTS/SA). These interviews can be used to construct profiles of children's exposure to science activities outside of school, such as watching TV, reading, attending exhibits or events at community facilities, participating in experiments or demonstrations at home, and asking questions of parents. The CHARTS/PS interview was conducted with the mothers of 29 kindergarten children, while the CHARTS/SA interview was conducted with 35 5th and 6th graders. Focusing primarily on the CHARTS/PS interview, the article describes how these interviews were developed, what kinds of information this type of research enabled the authors to obtain, and what lessons they have learned in the process. The level of extracurricular participation reported in a wide range of science-related activities was very high. Structured interviews can help teachers gain information about students' exposure to science-related learning activities in their home and community. This information can be used as a platform on which classroom instruction can be built. (PsycINFO Database Record (c) 2012 APA, all rights reserved)
SN - 0036-8326
AD - Korpan, Connie A.: U Alberta, Dept of Psychology, Edmonton, AB, Canada
ER -

TY - JOUR
ID - 2939
T1 - Learning and teaching mathematics: An international perspective
A1 - Nunes,Terezinha
Y1 - 1997///
N1 - Book: 1997-36375-000
English
Book; Edited Book
(Created by PsycINFO) (Abbreviated) Contributors About this book: A brief overview [by] T. Nunes and P. Bryant Part I: Mathematics and intelligence * The nature of mathematical concepts / Gerard Vergnaud / 5-28 * Systems of signs and mathematical reasoning / Terezinha Nunes / 29-44 Part II: The development of mathematical understandings * Mathematical understanding in the nursery school years / Peter Bryant / 53-67 * Word problems: A vehicle for promoting authentic mathematical understanding and problem solving in the primary school? / L. Verschaffel and E. De Corte / 69-97 * Children's understandings of turn and angle / Sandra Magina and Celia Hoyles / 99-114 * Knowing about proportions in different contexts / Janice A. Singer, Amy S. Kohn and Lauren B. Resnick / 115-132 * Mathematical concepts at the secondary school level: The learning of algebra and functions / Carolyn Kieran / 133-158 Part III: Social and cultural influences on mathematics learning * Happy birthday to you: Early mathematical thinking of Asian, South American, and US children / Herbert P. Ginsberg, Y. Elsie Choy, Luz Stella Lopez, Rebecca Netley and Chi Chao-Yuan / 163-207 * Learning arithmetic with an abacus / Giyoo Hatano / 209-231 * What children and teachers count as mathematics / Guida de Abreu, Alan J. Bishop and Geraldo Pompeu, Jr. / 233-264 * Social interactions and mathematics learning / M. L. Schubauer-Leoni and A.-N. Perret-Clermont / 265-283 Part IV: Constructing knowledge in the classroom * Meaning mathematically with computers / Richard Noss / 289-314 * Mediating between concrete and abstract / Koeno Gravemeijer / 315-345 * Charming fractions or fractions being charmed? / L. Streefland / 347-371 * Didactic engineering / Regine Douady / 373-401 References Author index Subject index
KW - PsycInfo
KW - Constructivism

KW - Mathematics Education
KW - School Learning
KW - Teaching Methods
RP - NOT IN FILE
JF - (1997)
VL - Learning and teaching mathematics: An international perspective. xiv, 443 pp. Hove, England: Psychology Press/Erlbaum :
IS - UK
N2 - (from the cover) The contributors in this book on teaching and learning mathematics all hold the view that mathematics is a form of intelligent problem solving which plays an important part in children's lives outside the classroom as well as in it. One theme of the chapters is that an important part of teaching mathematics at school is to make explicit for children mathematical ideas which they have already picked up and used in more informal settings. The authors also stress the social construction of children's mathematical knowledge at school through the acquisition of new modes of representation which increases the power of their mathematical understanding. Two major contributions of the book are to seek an integration of social and cognitive aspects of learning and to bring together for the first time in English a comprehensive description of teaching methods based on the idea of social construction which have been developed in Holland, France and Switzerland. (PsycINFO Database Record (c) 2012 APA, all rights reserved)
AD - Nunes, Terezinha: U London, Inst of Education, London, England
ER -

TY - JOUR
ID - 2940
T1 - Social skilling through cooperative learning
A1 - Jordan,Don W.
Y1 - 1997///
N1 - Peer Reviewed Journal: 1997-07480-001
English
Journal; Peer Reviewed Journal
KW - PsycInfo
KW - Classroom Behavior
KW - Classroom Behavior Modification
KW - Cooperative Learning
KW - Social Behavior
KW - Social Skills
KW - Elementary School Students
KW - Longitudinal Studies
RP - NOT IN FILE
SP - 3
EP - 21
JF - Educational Research
VL - 39:
IS - 1
N2 - Examined the effects of cooperative learning on the social skills of students and their behavior both in and out of the classroom. The 1st author implemented a 10-wk cooperative learning program in a class of 10-12 yr olds, to develop social and academic skills. Classroom activities provided specific training in, and required Ss to use and monitor the use of, identified social skills. Task-oriented skills included sharing, persuading and managing time. Person-oriented skills covered being positive, valuing others and conflict resolution. Ss worked in pairs and larger groups, with each S responsible for a specific academic or social task. Findings show evidence of social growth even though the study lasted only 10 wks. Social interactions became noticeably more varied and students agreed to work in groups, including members they did not like. These changes carried over into playground activities. The interpersonal relationships of previously isolated Ss improved and there were also improvements in student behavior. (PsycINFO Database Record (c) 2012 APA, all rights reserved)
SN - 0013-1881
AD - Jordan, Don W.: Woodbridge District High School, Woodbridge, TAS, Australia
ER -

TY - JOUR
ID - 2941
T1 - The effects of pre-school experience on reading attainment: A four year cross-sectional study
A1 - Davies,Julie
Y1 - 1997///
N1 - Peer Reviewed Journal: 1997-06048-002
English
Journal; Peer Reviewed Journal
KW - PsycInfo
KW - Educational Background
KW - Preschool Education
KW - Reading Achievement
KW - Longitudinal Studies
RP - NOT IN FILE
SP - 255
EP - 266
JF - Educational Psychology
VL - 17:
IS - 3
N2 - Using a 4-yr cross-sectional study, the reading attainments of a sample of 834 Year 2 children who had one of 3 types of preschool experiences (nursery school attendance, playgroup attendance, or no preschool provision outside the home) were examined. Ss' scores on the Primary Reading Test (PRT) were compared over the 4 yrs, as were the reading Standardised Assessment Task (SAT) scores of the last 2 cohorts. Results are not clear cut. For 3 out of the 4 yrs, the group with no-preschool experience performed significantly less well on the PRT than did at least 1 of the other 2 groups. The SAT levels of Ss who attended nursery school were higher than those of the other 2 groups. It appears that preschool provision had an effect on children's reading attainments and that this effect was still significant in Year 2. The validity of the SAT is considered as a factor in the relative reading attainments of the 3 groups. (PsycINFO Database Record (c) 2012 APA, all rights reserved)
SN - 0144-3410
AD - Davies, Julie: Manchester Victoria U, School of Education, Manchester, England
ER -

TY - JOUR
ID - 2942
T1 - Children's relationships with adults and peers: An examination of elementary and junior high school students
A1 - Lynch,Michael
Y1 - 1997///
N1 - Peer Reviewed Journal: 1997-04139-005
English
Journal; Peer Reviewed Journal
KW - PsycInfo
KW - Mother Child Relations
KW - Peer Relations
KW - School Adjustment
KW - Teacher Student Interaction
KW - Classmates
KW - Elementary School Students
KW - Middle School Students
RP - NOT IN FILE
SP - 81
EP - 99
JF - Journal of School Psychology
VL - 35:
IS - 1

N2 - Social and affective processes connected to children's relationships inside and outside the school setting are important factors in children's successful adaptation to school. This study examines the relationships of 1,226 low-risk elementary and middle school children across a variety of relationship partners. Descriptive data on the profile of these school-aged children's patterns of relatedness with others are presented. Developmental trends are explored as well. There is a shift in the self-reported quality of children's relationships with adults (mothers and teachers) and peers (best friends and classmates) from elementary school to middle school. In middle school, children report more positive perceptions of their relationships with peers and less positive perceptions of their relationships with adults than do elementary school children. These findings are discussed in terms of the implications that children's interpersonal relationships have for facilitating readiness to learn and active engagement in school. Limitations of the present study and methodological issues connected to the assessment of relationships are discussed as well. (PsycINFO Database Record (c) 2012 APA, all rights reserved) (journal abstract)
SN - 0022-4405
AD - Lynch, Michael: U Rochester, Mt Hope Family Ctr, Rochester, NY, US
ER -

TY - JOUR
ID - 2943
T1 - The role of recess in children's cognitive performance
A1 - Pellegrini,Anthony D.
Y1 - 1997///
N1 - Peer Reviewed Journal: 1997-03594-003
English
Journal; Peer Reviewed Journal
KW - PsycInfo
KW - Leisure Time
KW - Recreation
KW - School Learning
KW - Primary School Students
RP - NOT IN FILE
SP - 35
EP - 40
JF - Educational Psychologist
VL - 32:
IS - 1
N2 - Presents a rationale for the importance of recess in primary school curriculum. The authors propose that children's immature cognitions are well adapted for their particular phases of development and that children can get the most out of school when educators take advantage of children's immaturity to foster attention to classroom work. Because young children are not able to focus their attention for extended periods of time as well as older children, their cognitive performance will be hindered if they persist at tasks requiring focused attention for prolonged periods. The authors argue that by giving them frequent breaks during and between highly focused cognitive and academic tasks, their performance on later tasks should be enhanced relative to children who are not given such breaks. Also, performance on repeated tasks requiring focused attention decreases over time and trials, until rest periods are given. Rest periods in the form of recess enhances subsequent performance, although the nature of the recess activity may affect the extent of the subsequent benefits. (PsycINFO Database Record (c) 2012 APA, all rights reserved)
SN - 0046-1520
AD - Pellegrini, Anthony D.: U Georgia, Inst for Behavioral Research, Athens, GA, US
ER -

TY - JOUR
ID - 2944
T1 - Evaluation of an individualized, context-based social skills training program for children with learning disabilities
A1 - Wiener,Judith
Y1 - 1997///

N1 - Peer Reviewed Journal: 1997-03105-004
English
Journal; Peer Reviewed Journal
KW - PsycInfo
KW - Behavior Problems
KW - Learning Disabilities
KW - Problem Solving
KW - Social Acceptance
KW - Social Skills Training
KW - Classroom Environment
KW - Elementary School Students
RP - NOT IN FILE
SP - 40
EP - 53
JF - Learning Disabilities Research & Practice
VL - 12:
IS - 1
N2 - An individualized, classroom-based social skills training program, which used a combination of coaching and social problem-solving approaches, was developed and evaluated with a sample of 45 children, 9 to 12 years old, with learning disabilities, in self-contained special education classes. Thirteen children received a 6-week intervention (Intervention 1), 7 received a 12-week intervention (Intervention 2), and 25 were in a no-treatment control group. We found modest gains in social skills and decreases in problem behaviors in the Intervention 1 group compared to the control group as measured by self-report, teacher rating scales, peer nominations, and classroom and playground observations. The peer acceptance of the Intervention 1 children remained stable over the school year, whereas the peer acceptance of the children in the control group deteriorated. Equivalent changes were not observed in the Intervention 2 group, largely due to dynamics specific to that classroom. (PsycINFO Database Record (c) 2012 APA, all rights reserved) (journal abstract)
SN - 0938-8982
AD - Wiener, Judith: Ontario Inst for Studies in Education, Dept of Applied Psychology, Toronto, Canada
ER -

TY - JOUR
ID - 2945
T1 - An examination of parent-child shared time
A1 - Bryant,W.
Y1 - 1996///
N1 - Peer Reviewed Journal: 1997-41405-019
English
Journal; Peer Reviewed Journal
KW - PsycInfo
KW - Family
KW - Human Sex Differences
KW - Leisure Time
KW - Parent Child Relations
KW - Working Women
RP - NOT IN FILE
SP - 227
EP - 237
JF - Journal of Marriage and the Family
VL - 58:
IS - 1
N2 - Data from time diaries kept by parents in 2-parent, 2-child families in 4 states in 1977 and 1978 were used to examine time shared by parents and children, as well as parent solitary times, in a number of household activities. The analysis focused on how the mother's employment time affected shared parent-child time and whether the time was sex-typed. In addition, a weak test for whether parent-child shared time stimulated children's human capital development was devised and the hypothesis confirmed. Mothers who spent more time in market work shared less traditionally defined child-care time, but only with the older child. In contrast, as a

mother's time in market work increased, parent-child shared housework and shared leisure time increased. Household activities shared by the parent and the child were sex-typed. Mothers tended to share more time with daughters in meal preparation and family-care activities, and fathers tended to share more time with their sons in activities involving the home, yard, car, and pet maintenance and in shopping activities. (PsycINFO Database Record (c) 2012 APA, all rights reserved)
SN - 0022-2445
AD - Bryant, W. Keith: Cornell U, Ithaca, NY, US
ER -

TY - JOUR
ID - 2946
T1 - Popularity and rejection in children's peer groups: A South African perspective
A1 - Bonn,Marta
Y1 - 1996///
N1 - Peer Reviewed Journal: 1997-43792-001
English
Journal; Peer Reviewed Journal
KW - PsycInfo
KW - Childhood Play Behavior
KW - Classroom Behavior
KW - Peer Relations
KW - Popularity
KW - Social Acceptance
KW - Blacks
KW - Playgrounds
RP - NOT IN FILE
SP - 1
EP - 14
JF - Early Child Development and Care
VL - 125 Nov 1996, pp. 1-14.
N2 - Observed and compared the behavior of 2 groups of Black South African children who had been identified as either popular or rejected by their peers. 631 children (aged 8 yrs to 12 yrs 3 mo) nominated classmates they liked most and least. 29 rejected (RCs) and 29 popular children (PCs) were identified, and were observed in their school playground during recess and in their classroom. Findings indicate that social rejection was related to aggression, disruptiveness, and rule violations; popularity was associated with prosocial behavior among peers. Characteristics of PCs were that they were seldom aggressive, they reminded others of the rules of the group, they provided guidance in difficult circumstances, they were caring and affectionate, and they were the instigators and recipients of positive behaviors. RCs initiated more negative interactions with others, and were also the recipients of more negative and aversive interactions. Additionally, RCs were more frequently observed in interactive activities on the playground, but PCs engaged in these for longer periods. (PsycINFO Database Record (c) 2012 APA, all rights reserved)
SN - 0300-4430
AD - Bonn, Marta: U South Africa, Inst for Behavioural Sciences, Pretoria, South Africa
ER -

TY - JOUR
ID - 2947
T1 - Playtime--using a systems' perspective
A1 - Rennie,Ewen F.
Y1 - 1996///
N1 - Peer Reviewed Journal: 1997-02655-004
English
Journal; Peer Reviewed Journal
KW - PsycInfo
KW - Childhood Play Behavior
KW - Preschool Students
KW - Primary School Students

KW - Recreation
KW - Student Attitudes
KW - Playgrounds
RP - NOT IN FILE
SP - 161
EP - 165
JF - Educational Psychology in Practice
VL - 12:
IS - 3
N2 - For children at school, playtime occupies more time than that recommended to be allocated to any formal curriculum area. Educational psychologists (EPs) can have a role in encouraging schools to consider ways of structuring playtime to increase the quality of children's social experiences and preventing troubling behavior. To inform such a role better, this study sets out to gain an impression of the Scottish playground from the perspective of 290 preschool and primary school students through the use of a questionnaire. (PsycINFO Database Record (c) 2012 APA, all rights reserved)
SN - 0266-7363
ER -

TY - JOUR
ID - 2948
T1 - Promoting academic and social behaviors of children in integrated kindergarten classes: The effects of cooperative learning
A1 - Nowak,Therese Anne
Y1 - 1996///
N1 - Dissertation Abstract: 1996-95022-016
English
Dissertation Abstract
KW - PsycInfo
KW - Academic Achievement
KW - Cooperation
KW - Cooperative Learning
KW - Interpersonal Interaction
KW - Prosocial Behavior
KW - Childhood Play Behavior
KW - Kindergarten Students
RP - NOT IN FILE
SP - 1964
JF - Dissertation Abstracts International Section A: Humanities and Social Sciences
VL - 57:
IS - 5-A
N2 - This project explored the effects of a cooperative learning program implemented in integrated kindergarten classrooms. Academic performance, cooperative interactions during lessons, and prosocial behaviors during play activities were examined. Scores on curriculum-based mathematics probes and direct observations of cooperative interactions during the intervention served as the primary dependent measures. Additionally, direct observations during a play period following the cooperative learning lesson and weekly observations in a free play setting outside the classroom served to assess generalization of prosocial behaviors in natural settings. A multiple probe design across classrooms combined with a quasi-experimental group design was employed. Pretest and posttest measures on standardized basic readiness scales and student sociometric nominations/ratings were statistically compared at the conclusion of the study. Social validity measures, procedural integrity, and one-month maintenance data also were collected. Results indicated that the cooperative learning procedures lead to improved academic growth as measured by both the curriculum-based mathematics probes and the mathematics section of the standardized group readiness test. Levels of cooperative behaviors increased substantially for all participants during the instructional period when cooperative learning was in effect. Furthermore, prosocial skills learned and practiced during the intervention generalized to the free play settings in and outside of the classroom environments. An increase in interactive play behaviors also was observed for all participants during these time periods. However, for cooperative behaviors, the generality of effects with respect to maintenance over time was not supported. No statistically significant differences were

noted between experimental and control groups on the sociometric nomination measure, but an improvement was seen for both work and play measures on the rating scales. S (PsycINFO Database Record (c) 2012 APA, all rights reserved)
SN - 0419-4209
AD - Nowak, Therese Anne: Lehigh U, US
ER -

TY - JOUR
ID - 2949
T1 - Kindergartners who become friends: Classroom influences on interactions and patterns of stability and change
A1 - Winterhoff,Paul Andrew
Y1 - 1996///
N1 - Dissertation Abstract: 1996-95015-180
English
Dissertation Abstract
KW - PsycInfo
KW - Friendship
KW - Peer Relations
KW - Social Interaction
KW - School Environment
RP - NOT IN FILE
SP - 1472
JF - Dissertation Abstracts International: Section B: The Sciences and Engineering
VL - 57:
IS - 2-B
N2 - This research was a year-long longitudinal study examining the friend making activity of kindergarten children. The subjects were a diverse sample of 38 children, attending two kindergarten classes in a suburban public school in North Carolina. The sample included 15 girls (mean age 65 months), and 23 boys (mean age 67.6 months). Median income of subjects' families was $40,000. Spot observational methods were used to record children's social interactions. Observational data were aggregated by seven coding periods to identify frequent playmates. Teacher and child nominations of individual's common playmates were recorded at the end of each coding period. The three methods of playmate identification were compared to determine the concordance between them. It was hypothesized that concordance between all methods would exist. Individual children were identified as friends by the frequency, stability, and quality of their interactions, and the concordance between at least two identification methods. It was hypothesized that the individuals who became stable friends with classmates would engage in more interactions outside of the proximity of teachers, and during free activity times. It was also hypothesized that boys would be more likely than girls to be identified as stable friends. By using observational data, three stability-change types of individuals were identified. Marginals were relative social isolates. Changers were children who changed play partners. Consistents had one or more frequent and lasting play partners. Data signifying the level of joint activity and positive reciprocal interactions by types were compared. Graphs were constructed showing the variability of these interactions by types across the year under varying conditions of the proximal setting variables, teacher presence and classroom structure. Results showed a lack of concordance between assessment methods during the first semester (average agreement level 48.8%). Teacher and child nominatio (PsycINFO Database Record (c) 2012 APA, all rights reserved)
SN - 0419-4217
AD - Winterhoff, Paul Andrew: U North Carolina at Greensboro, US
ER -

TY - JOUR
ID - 2950
T1 - Childhood depressive symptoms, physical activity and health-related fitness
A1 - Michaud-Tomson,Lois
Y1 - 1996///

N1 - Dissertation Abstract: 1996-95012-125
English
Dissertation Abstract
KW - PsycInfo
KW - Exercise
KW - Major Depression
KW - Physical Fitness
KW - Cardiovascular System
KW - Physical Endurance
RP - NOT IN FILE
SP - 4700
JF - Dissertation Abstracts International Section A: Humanities and Social Sciences
VL - 56:
IS - 12-A
N2 - Depression is a major public health concern, with a robust continuity from childhood through adulthood. This study had six objectives: first and second, to examine the relationship of being classified as physically inactive by a parent or a teacher to depressive symptoms in 8 to 12 year old children (n = 933); third, to assess the relationship of playing sports outside of school, to depressive symptoms; fourth and fifth, to determine whether meeting Fitnessgram health-related fitness standards for body composition and cardiovascular endurance were related to depressive symptoms, and; sixth, to examine the prevalence of depressive symptoms in this population. Using the "Dimensions of Depression Profile" and applying DSM-IV criteria, symptoms of major depression were identified in.48 to.65% of children, while symptoms of minor depression were noted in 8.4% of girls and 6.3% of boys. Chi squares revealed significant (p <) differences for girls classified as inactive by a teacher and for boys classified as inactive by a parent (p <). Differences were also noted for boys not playing sports outside of school (p <) and for those not meeting body composition standards (p <), as well as for girls not meeting cardiovascular standards (p <). Relative risk of depressive symptoms for inactive classification was 2.8 to 3.4 times higher than for active, 1.3 to 2.4 times higher for children not playing sports, and 1.5 to 4.0 times higher for those not meeting health goals. (PsycINFO Database Record (c) 2012 APA, all rights reserved)
SN - 0419-4209
AD - Michaud-Tomson, Lois: Arizona State U, US
ER -

TY - JOUR
ID - 2951
T1 - The impact of maternal employment and mother/child involvement on children's adaptive functioning in school
A1 - Morris,Elizabeth Ware
Y1 - 1996///
N1 - Dissertation Abstract: 1996-95006-190
English
Dissertation Abstract
KW - PsycInfo
KW - Employment Status
KW - Mother Child Relations
KW - School Adjustment
KW - Working Women
KW - Mothers
RP - NOT IN FILE
SP - 3512
JF - Dissertation Abstracts International Section A: Humanities and Social Sciences
VL - 56:
IS - 9-A
N2 - This research investigates the impact of maternal employment and mother/child involvement on children's adaptive functioning in school. Data were collected from mothers (n = 97) on a daily basis over a period of 37 days to assess their level of involvement in leisure activities with their children. The mothers were categorized on the basis of their self-reported employment status: unemployed outside the home, employed part-time, or employed full-time. Teacher reports of child functioning at school were collected on 89 of the children whose

mothers completed home reports of involvement. Analyses revealed no statistically significant relationship between shared mother/child leisure activities and school performance as a function of mothers' level of employment. In general, the mother's level of employment had no impact on the level of involvement in daily activities between mother and child as reported by the mother. However, increased involvement on the part of a mother employed full-time had a modest negative statistical association with the child's adaptive functioning at school. Future studies might collect data from the child as a corroborative reporter on involvement in parent/child activities in the home. It is recommended that the involvement of the father be examined to learn something more of the changing roles of mothers and fathers in the nuclear family. The evolution of more equally shared parenting responsibilities warrants further study of how differing family environments impact children's social and cognitive functioning at school. (PsycINFO Database Record (c) 2012 APA, all rights reserved)
SN - 0419-4209
AD - Morris, Elizabeth Ware: U Southern California, US
ER -

TY - JOUR
ID - 2952
T1 - The effects of combined self-management strategies on the generalization of social behavior changes in children with social skills deficits
A1 - Kim,Hwangyong
Y1 - 1996///
N1 - Dissertation Abstract: 1996-95004-109
English
Dissertation Abstract
KW - PsycInfo
KW - Child Psychology
KW - Cognitive Generalization
KW - Self Management
KW - Social Skills
KW - Behavior Modification
KW - Interpersonal Interaction
KW - Social Behavior
RP - NOT IN FILE
SP - 3079
JF - Dissertation Abstracts International Section A: Humanities and Social Sciences
VL - 56:
IS - 8-A
N2 - Despite their efficacy in skill acquisition, externally managed behavioral interventions have been criticized widely for poor generalization and maintenance effects. In the past two decades, various self-management strategies have been developed to involve students in their own behavior change process and to increase the generalization and maintenance of behavior changes. Among self-management strategies, self-recording, self-evaluation, and self-graphing are considered useful, especially when they are combined systematically. Because findings of self-management studies to date have been inconsistent, additional self-management research must be conducted to support claims about achieving generalization and maintenance. This study examined the relative effects of a combination of self-management strategies to a combination of externally managed treatments on the generalization and maintenance of playground social behavior changes. A multiple baseline across subjects design was used. All seven subjects in this study were taught specific playground social skills. During the generalization intervention phase, subjects in the external treatment condition spent extra time practicing their newly learned social skills through role-playing. Immediately after each morning recess, the subjects received positive feedback from the investigator for their behavioral performance on the playgrounds. In contrast, subjects in the self-management intervention were asked to monitor their own behaviors by using a golf-counter during morning recess to count the frequency with which they used learned social skills. At the end of recess, they self-evaluated and self-graphed their performance according to pre-established criteria on a self-management observation sheet developed for this study. A partial interval time sampling method was used to collect daily observation data for each subject during morning recess and after-lunch-free time on the playgrounds. The results indicated that neithe (PsycINFO Database Record (c) 2012 APA, all rights reserved)
SN - 0419-4209

AD - Kim, Hwangyong: U Oregon, US
ER -

TY - JOUR
ID - 2953
T1 - Old battles, new frontiers: A study of television violence and social work with children
A1 - Lazar,Bonnie A.
Y1 - 1996///
N1 - Peer Reviewed Journal: 1996-07019-004
English
Journal; Peer Reviewed Journal
KW - PsycInfo
KW - Social Cognition
KW - Social Interaction
KW - Social Workers
KW - Television Viewing
KW - Violence
RP - NOT IN FILE
SP - 527
EP - 540
JF - Child & Adolescent Social Work Journal
VL - 13:
IS - 6
N2 - Described the place of TV violence in one type of social exchange, children's interactions with social workers. 21 social workers who worked primarily with children in schools, in community mental health agencies, and in private practice were interviewed, using grounded theory methodology. Social workers were asked to recount a time when they believed that a child between 6-12 yrs old had drawn, played, or talked about themes or characters related to violence witnessed on TV. Results confirm the assumptions that children recreate what they see on TV and share their experience of TV with others. In addition, social workers do observe TV-related themes and characters in offices and on the playground and are aware that children use TV violence specifically and thematically in interaction with others. TV violence appears to be a largely unacknowledged presence in social work practice with children. (PsycINFO Database Record (c) 2012 APA, all rights reserved)
SN - 0738-0151
AD - Lazar, Bonnie A.: U Southern Maine, Portland, ME, US
ER -

TY - JOUR
ID - 2954
T1 - Self-recording during unsupervised academic activity: Effects on time spent out of class
A1 - Stecker,Pamela M.
Y1 - 1996///
N1 - Peer Reviewed Journal: 1996-06626-001
English
Journal; Peer Reviewed Journal
KW - PsycInfo
KW - Learning Disabilities
KW - Performance
KW - Self Monitoring
KW - Special Education Students
KW - Time Out
KW - Self Report
RP - NOT IN FILE
SP - 133
EP - 147
JF - Exceptionality
VL - 6:
IS - 3

N2 - Investigated the effects of a self-monitoring technique, including self-recording and graphing of performance, on length of time spent out of class. Two similar multiple baseline studies were used to assess the effectiveness of the interventions with 5 students with mild disabilities (4 with learning disabilities and 1 with serious emotional disturbance), who were participating in an ongoing computer assessment project that required them to spend time outside of the classroom without direct teacher supervision. Results indicate that the self-recording procedures were effective in reducing the total length of time spent outside of the classroom (during the required activities) for all participants. The accuracy of student self-recordings and the social validity of the intervention are discussed. (PsycINFO Database Record (c) 2012 APA, all rights reserved)
SN - 0936-2835
AD - Stecker, Pamela M.: Clemson U, Dept of Educational Foundations & Special Education, Clemson, SC, US
ER -

TY - JOUR
ID - 2955
T1 - The Norwegian perspective on issues of quality in day care
A1 - Borge,Anne I.
Y1 - 1996///
N1 - Peer Reviewed Journal: 1996-05006-002
English
Journal; Peer Reviewed Journal
KW - PsycInfo
KW - Child Day Care
KW - Costs and Cost Analysis
KW - Day Care Centers
KW - Quality of Services
KW - Disorders
KW - Special Needs
RP - NOT IN FILE
SP - 129
EP - 137
JF - European Journal of Psychology of Education
VL - 11:
IS - 2
N2 - Since 1953, Norwegian national standards regulate ratios of children to staff, training requirements, group sizes, leadership and subsidies. Both national and local subsidies support the management and quality level of day care centers. In Norway a current viewpoint on quality in day care is to stimulate innovation in day care activities by rewarding local initiative and creativity. Outdoor activities and giving the children freedom to learn are highly valued. Preschool teacher education, which is three years at college, shares a common frame of a governmental curricular guideline as well as a nationwide curriculum for day care. A responsive and warm interaction between staff, children and parents is a definite moral value of the preschool teacher profession. Current issues of quality are related to the shortage of highly qualified directors and leaders in public day care as well as the expansion of private centers with questionable quality. Quality control of day care centers is now discussed as a necessary surveillance system of the quality of contexts and interactions in children's daily life. (PsycINFO Database Record (c) 2012 APA, all rights reserved) (journal abstract)
SN - 0256-2928
AD - Borge, Anne I. H.: U Oslo, Dept of Special Education, Oslo, Norway
ER -

TY - JOUR
ID - 2956
T1 - Sexual Abuse in Preschools: Context, Aftermath, and Controversy
A1 - Bottoms,Bette L.
Y1 - 1995///
N1 - Electronic Collection: 2004-17636-020
English
Electronic Collection

KW - PsycInfo
KW - Child Abuse
KW - Preschool Students
KW - Sexual Abuse
RP - NOT IN FILE
JF - PsycCRITIQUES
VL - 40:
IS - 11
N2 - Originally published in Contemporary Psychology: APA Review of Books, 1995, Vol 40(11), 1067-1068. This is a review of the book, "Behind the Playground Walls: Sexual Abuse in Preschools" (see record 1993-97375-000). The authors of "Behind the Playground Walls" detail their research into the circumstances and clinical aftermath of several preschool sexual abuse cases, including the nation's most prominent case of alleged ritualistic child abuse, the McMartin Daycare case in Manhattan Beach, California. Three groups of children are compared: (a) alleged victims from highly publicized and legally unsubstantiated ritualistic abuse cases in southern California; (b) nonritualistic sexual abuse victims from a less controversial, legally confirmed day-care case in Nevada; and (c) nonabused control children attending day care. The authors are to be commended for their longitudinal design, for testing theory in a field sometimes lacking it, for conducting the research in spite of many pragmatic difficulties, and for producing a book that is readable by laypersons but that includes enough detail about method and analysis to be useful for serious researchers. The book has enough merit to transcend the controversy inherent in its topic. Child abuse researchers and therapists and families of children involved in sexual abuse cases will benefit from the findings of this research. (PsycINFO Database Record (c) 2012 APA, all rights reserved)
SN - 1554-0138
AD - Bottoms, Bette L.: University of Illinois at Chicago, Chicago, IL, US
ER -

TY - JOUR
ID - 2957
T1 - The great outdoors: Restoring children's right to play outside
A1 - Rivkin,Mary S.
Y1 - 1995///
N1 - Book: 2001-14076-000
English
Book; Authored Book
(Created by PsycINFO) (Abbreviated) Preface Acknowledgments Vanishing habitats and access Considerations in designing play areas Great school grounds Safety outdoors Peaceful playgrounds Broader community efforts Restoring the birthright Notes Selected Bibliography Appendixes
KW - PsycInfo
KW - Childhood Development
KW - Childhood Play Behavior
KW - Primary School Students
KW - Recreation Areas
KW - Wilderness Experience
RP - NOT IN FILE
SP - Washington, DC, US
JF - (1995)
VL - The great outdoors: Restoring children's right to play outside. v, 106 pp. Washington, DC, US: National Association for the Education of Young Children; US.
N2 - (from the preface) Argues the importance and benefits of children spending time outdoors. The book offers guidelines on how to restore and build children's outdoor play opportunities. It also provides inspiring examples of play spaces, and offers practical ideas for bringing the great outdoors to primary schools. (PsycINFO Database Record (c) 2012 APA, all rights reserved)
AD - Rivkin, Mary S.: U Maryland Baltimore County, Catonsville, MD, US
ER -

TY - JOUR
ID - 2958

T1 - Conflicts in creativity
A1 - Freeman,Joan
Y1 - 1995///
N1 - Peer Reviewed Journal: 1998-02273-007
English
Journal; Peer Reviewed Journal
KW - PsycInfo
KW - Academic Achievement
KW - Creativity
KW - Education
KW - Gifted
RP - NOT IN FILE
SP - 188
EP - 200
JF - European Journal for High Ability
VL - 6:
IS - 2
N2 - Creative thinking involves both emotion and personality, including enough confidence and courage to consider new approaches to problems, rather than hiding in the security of familiar and accepted ways of thinking. There is a conflict in education between the need for emotional control for academic excellence and the need for a more free and open minded approach. Students who were extremely successful academically are often found to be creatively inhibited because of the narrow focus and pressure of their school education. The problem for teachers is to enable intellectually gifted pupils, who have the ability for high academic achievement, to keep a playful, creative approach to their work and general outlook. An environment in which the exceptionally able child can prosper all round must be balanced. This implies enough time with other people to make good social relationships and develop interests outside study areas. Topics discussed include: where creativity starts, the creative cost of high academic achievement, and education for creativity (imaginative play). (PsycINFO Database Record (c) 2012 APA, all rights reserved)
SN - 0937-4450
ER -

TY - JOUR
ID - 2959
T1 - Young children's friendships: Development and assessment of a new checklist. [Italian]
A1 - Baumgartner,Emma
Y1 - 1995///
N1 - Peer Reviewed Journal: 1997-85307-001
Italian
Journal; Peer Reviewed Journal
KW - PsycInfo
KW - Childhood Play Behavior
KW - Friendship
KW - Observation Methods
RP - NOT IN FILE
SP - 21
EP - 33
JF - Eta Evolutiva
VL - 52 Oct 1995, pp. 21-33.
N2 - Conducted an observational study to identify different kinds of relationships among preschool children in natural settings. Human Ss: 17 normal Italian preschool children (aged 27-46 mo) (Ss from 1 day care center) (Group 1). 28 normal Italian preschool children (aged 33-41 mo) (Ss from 2 different day care centers) (Group 2). Group 1 Ss were observed in their day care center upon arrival, during mealtime, and during free play. Group 2 Ss were observed twice, about 1 mo apart, during mealtime and free play. Ss' individual behavior, proximity to other Ss, degree of intimacy with other Ss, sharing, conflicts, and prosocial behavior were assessed using a checklist. (English abstract) (PsycINFO Database Record (c) 2012 APA, all rights reserved)
SN - 0392-0658
AD - Baumgartner, Emma: U degli Studi Roma "La Sapienza", Italy

ER -

TY - JOUR
ID - 2960
T1 - Differential diagnosis of ADHD: Are objective measures of attention, impulsivity, and activity level helpful?
A1 - Matier-Sharma,Kristin; Perachio,Nancy; Newcorn,Jeffrey
Y1 - 1995///
N1 - Peer Reviewed Journal: 1997-06129-003
English
Journal; Peer Reviewed Journal
KW - PsycInfo
KW - Attention Deficit Disorder
KW - Differential Diagnosis
KW - Hyperkinesis
KW - Measurement
KW - Test Validity
RP - NOT IN FILE
SP - 118
EP - 127
JF - Child Neuropsychology
VL - 1:
IS - 2
N2 - Assessed the degree to which performance on continuous performance tests (CPT) and a solid-state actigraph could be used clinically to distinguish between attention deficit hyperactivity disorder (ADHD) and other psychiatric symptoms in 97 patients (aged 6.5-13 yrs) from a child psychiatry outpatient clinic (40 with ADHD symptoms and 57 without ADHD symptoms) and in 18 normal controls. Discriminant function analyses correctly classified 72.4% of Ss in the ADHD vs normal control comparison, and 66.2% in the ADHD vs non-ADHD comparison. Poor performance on objective measures was generally indicative of ADHD relative to normal controls, but "average" performance could not be used to rule out ADHD. Diagnostic classification indices were much lower in the ADHD vs non-ADHD patient discrimination. CPT inattention scores had moderate sensitivity, but low specificity; CPT impulsivity and actigraph scores had low sensitivity but high specificity. Results suggest that examination of CPT performance and objectively assessed activity level are not diagnostic in and of themselves, and should not be used outside the context of a full clinical examination. (PsycINFO Database Record (c) 2012 APA, all rights reserved)
SN - 0929-7049
AD - Matier-Sharma, Kristin: City U New York, Queens Coll, Flushing, NY, US
ER -

TY - JOUR
ID - 2961
T1 - Computer games, self-esteem and gratification of needs in adolescents
A1 - Colwell,John; Grady,Clare; Rhaiti,Sarah
Y1 - 1995///
N1 - Peer Reviewed Journal: 1996-33730-001
English
Journal; Peer Reviewed Journal
KW - PsycInfo
KW - Computer Games
KW - Need Satisfaction
KW - Self Esteem
RP - NOT IN FILE
SP - 195
EP - 206
JF - Journal of Community & Applied Social Psychology
VL - 5:
IS - 3

N2 - Examined the relationship between gratification of needs, self-esteem, and computer games. 120 children (aged 11-17 yrs) from North London were given a questionnaire concerning computer game use, companionship, and solitude/escape. Results show that 92.5% of Ss played regularly. Playing computer games was equally popular with males and females, but males spent more time on it. Positive correlations between playing and items on the needs scales were obtained. Males who were heavy players scored highly on the preference to friends' need, but interestingly they were also likely to see their friends more often outside school, thus providing no support for the theory that computer games are taking the place of normal social interaction. For females there was evidence of a negative relationship between self-esteem and need gratification through playing computer games. (PsycINFO Database Record (c) 2012 APA, all rights reserved)
SN - 1052-9284
AD - Colwell, John: Middlesex U, School of Psychology, London, England
ER -

TY - JOUR
ID - 2962
T1 - The effects of recess timing on children's playground and classroom behaviors
A1 - Pellegrini,A.
Y1 - 1995///
N1 - Peer Reviewed Journal: 1996-28699-001
English
Journal; Peer Reviewed Journal
KW - PsycInfo
KW - Childhood Play Behavior
KW - Classroom Behavior
KW - Human Sex Differences
KW - Playgrounds
KW - Elementary School Students
KW - Kindergarten Students
RP - NOT IN FILE
SP - 845
EP - 864
JF - American Educational Research Journal
VL - 32:
IS - 4
N2 - Examined the effects of different recess timing regimens on elementary school students' classroom and recess behaviors. In Exp 1, recess timing for 34 boys and 28 girls in grades K, 2, and 4 was varied by 30 min. An analysis of pre- and postrecess behavior and of outdoor recess behavior showed that prerecess inattention varied as a function of deprivation duration. Boys in particular were more socially interactive on the playground following a long deprivation compared to a short deprivation. Recess behaviors did not relate significantly to postrecess inattention. However, inattention rates were higher before recess than after. Exp 2 utilized a similar paradigm with a sample of 2nd and 4th graders. In general, results from Exp 1 were replicated. In Exp 3, Ss' recess was manipulated, but the recess period was indoors. Results of the 2 samples replicated each other and the preceding experiments. (PsycINFO Database Record (c) 2012 APA, all rights reserved)
SN - 0002-8312
AD - Pellegrini, A. D.: U Georgia, Dept of Elementary Education, Athens, US
ER -

TY - JOUR
ID - 2963
T1 - Bully and victim problems in elementary schools and students' beliefs about aggression
A1 - Bentley,Karen M.
Y1 - 1995///
N1 - Peer Reviewed Journal: 1996-28685-001
English
Journal; Peer Reviewed Journal
KW - PsycInfo
KW - Aggressive Behavior

KW - Elementary School Students
KW - Student Attitudes
KW - Victimization
RP - NOT IN FILE
SP - 153
EP - 165
JF - Canadian Journal of School Psychology
VL - 11:
IS - 2
N2 - Studied the prevalence, general trends and nature of bully/victim problems in elementary school students, and determined whether bullies and victims endorsed certain aggression-supporting beliefs to other peers. 379 4th-6th graders (aged 8-22 yrs) in Canada were assessed on the Bully/Victim Questionnaire (D. Olweus, 1989) and Belief Measures (R. G. Slaby & N. G. Guerra, 1988). Results show that 21.3% of the Ss were bullied and 11.6% bullied others. Victims tended to be the youngest Ss and were at risk for being bullied by both age mates and older Ss. Verbal abuse was the most common form of bullying, and the playground was the most common location. There was no significant association between gender and direct or indirect bullying. Bullies tended to be in the older grades and were mainly boys. They were more likely than victims, and students who were neither bullies nor victims, to endorse aggression-supporting beliefs. (PsycINFO Database Record (c) 2012 APA, all rights reserved)
SN - 0829-5735
AD - Bentley, Karen M.: Maple Ridge-Pitt Meadows School District, BC, Canada
ER -

TY - JOUR
ID - 2964
T1 - From barnyards to backyards: An exploration through adult memories and children's narratives in search of an ideal playscape
A1 - Raymund,Judi Ferrel
Y1 - 1995///
N1 - Peer Reviewed Journal: 1996-15978-001
English
Journal; Peer Reviewed Journal
KW - PsycInfo
KW - Associative Processes
KW - Autobiographical Memory
KW - Childhood Play Behavior
KW - Environmental Psychology
RP - NOT IN FILE
SP - 362
EP - 380
JF - Children's Environments
VL - 12:
IS - 3
N2 - To explore changes in the residential outdoor play landscape, this study surveyed 40 adults on their memories of middle-childhood experiences and interviewed 14 children (aged 6-11 yrs). The physical landscape as well as personal meanings attached to childhood places were studied for both adults and children. Though both groups showed an unquestionable preference for the outdoors, it appeared as though the amount of imaginary, creative, and non-equipment game play was reduced. Probably the largest changes occurred in the extended home yard environments, where fields, woods, and orchards were replaced by formal city parks and busy streets. These landscape changes, as well as personal meanings of place, address important interdisciplinary issues. (PsycINFO Database Record (c) 2012 APA, all rights reserved)
SN - 0886-0505
ER -

TY - JOUR
ID - 2965
T1 - Early identification of antisocial boys: A multi-method approach

A1 - Quinn,Mary Maggie; Mathur,Sarup
Y1 - 1995///
N1 - Peer Reviewed Journal: 1996-14654-001
English
Journal; Peer Reviewed Journal
KW - PsycInfo
KW - Aggressive Behavior
KW - Antisocial Behavior
KW - At Risk Populations
KW - Behavioral Assessment
KW - Peer Relations
RP - NOT IN FILE
SP - 272
EP - 281
JF - Education & Treatment of Children
VL - 18:
IS - 3
N2 - Sought to identify the critical factors that differentiated 56 1st grade boys, at-risk for developing antisocial behavior patterns, from a randomly chosen comparison group. Through the use of a multi-method approach (consisting of teacher nominations, teacher ratings, and direct observation of behavior) 3 areas of significance emerged. Boys nominated as at-risk for developing antisocial behavior patterns were found to have higher teacher ratings of aggression, more negative peer interactions on the playground, and lower levels of academic engaged time than their comparison peers. These findings have implications for designing interventions to prevent the escalation of antisocial behavior patterns as well as to increase the pace and complexity of classroom instructional delivery. (PsycINFO Database Record (c) 2012 APA, all rights reserved)
SN - 0748-8491
AD - Quinn, Mary Maggie: Arizona State U, Special Education Program, Tempe, AZ, US
ER -

TY - JOUR
ID - 2966
T1 - Children's voices and the construction of children's spaces: The example of playcare centers in the United Kingdom
A1 - Smith,Fiona
Y1 - 1995///
N1 - Peer Reviewed Journal: 1996-14082-001
English
Journal; Peer Reviewed Journal
KW - PsycInfo
KW - Child Care
KW - Childhood Play Behavior
KW - Environmental Planning
KW - Recreation Areas
RP - NOT IN FILE
SP - 389
EP - 396
JF - Children's Environments
VL - 12:
IS - 3
N2 - Examined playcare provision, defined as providing care for children aged 5-12 yrs in communal settings which explicitly offer creative play opportunities. The role of children in determining the sort of playcare environment they spend time in after school and during school holidays was assessed. Data were obtained from 229 interviews with children. Results suggest that children are increasingly excluded from the independent use of public space which has important implications for the kinds of play opportunities available to them. The paper shows 1 possible way of enabling children to exercise their right to play through the use of playcare centers. The research was designed to provide a public forum for the voices of children, to allow them to

express their own views and opinions about what happens to them outside school hours. (PsycINFO Database Record (c) 2012 APA, all rights reserved)
SN - 0886-0505
AD - Smith, Fiona: Brunel University Coll, Dept of Geography & Geology, Osterley Campus, Isleworth, England
ER -

TY - JOUR
ID - 2967
T1 - Coping with children's temperament: A guide for professionals
A1 - Carey,William B.
Y1 - 1995///
N1 - Book: 1995-98287-000
English
Book; Authored Book
(Created by PsycINFO) Foreword by Stella Chess and Alexander Thomas Preface Reorienting professionals to the new understanding of temperament differences in children The nature and practical relevance of temperament: What do we know? The nature and practical relevance of temperament: What can we do? The newborn period General parental care: Infants, toddlers, and preschoolers Day care: Preschool child care outside the home Physical health care Developmental variation and behavioral adjustment problems Middle childhood and school performance Adolescence Environmental stressors and crises Conclusion: An opportunity to improve the care of our children Appendixes References Index
KW - PsycInfo
KW - Childhood Development
KW - Individual Differences
KW - Personality
KW - Behavior Problems
RP - NOT IN FILE
SP - New
JF - (1995)
VL - Coping with children's temperament: A guide for professionals. xiv, 235 pp. New York, NY, US: Basic Books; US.
N2 - (from the jacket) Children's temperaments make a substantial contribution to their environments and their interactions with them. Certain of these largely inborn behavioral style differences, which are not abnormal in themselves, may lead to clinical problems by predisposing children to abrasive, incompatible relationships with caregivers. [The authors] present a . . . summary of the recent clinical research on temperament, in particular from the last 10 yrs, coupled with practical suggestions on how to use this information in a variety of clinical and educational settings. . . . They take the professional-psychologist, physician, nurse, teacher, day care worker, and social worker-through all the stages of a child's life to show how the effects of temperament play out in infancy, the preschool years, middle childhood, and adolescence. [The] book brings theory and practice to life and shows how all those who work with children can improve their care by understanding these important differences. (PsycINFO Database Record (c) 2012 APA, all rights reserved)
AD - Carey, William B.: U Pennsylvania, School of Medicine, Philadelphia, PA, US
ER -

TY - JOUR
ID - 2968
T1 - Behavior with peers and perceptions of self: Correlates of attachment
A1 - Demulder,Elizabeth Kyle
Y1 - 1995///
N1 - Dissertation Abstract: 1995-95023-076
English
Dissertation Abstract
KW - PsycInfo
KW - Attachment Behavior
KW - Mother Child Relations
KW - Peer Relations

KW - Self Perception
RP - NOT IN FILE
SP - 3473
JF - Dissertation Abstracts International: Section B: The Sciences and Engineering
VL - 56:
IS - 6-B
N2 - Relations between patterns of attachment with mother and subsequent behaviour with peers and perceptions of self were examined in a sample of 39 five year-old children (22 girls, 17 boys). Attachment classifications and ratings had been determined when the children were 4 1/2 years old, using procedures and coding systems originally developed for infants by Ainsworth (Ainsworth, Blehar, Waters, & Wall, 1978) and modified for 3-4 year-old children by Cassidy and Marvin (1988). When each child was five years old, behaviour with peers was assessed through direct observation on the school playground. A continuous commentary of interactions was made into a hand-held microphone, while a radio microphone concealed on the child picked up the child's speech and speech directed toward him/her. Tapes were transcribed using a coding system based on that used by Hinde, Easton, Meller and Tamplin (1983). Perceptions concerning perceived competence and social acceptance (Harter & Pike, 1984), self-efficacy (Wheeler & Ladd, 1982), perceived popularity with and liking of peers and interpersonal problem-solving ability (Shure & Spivack, 1974) were assessed in a series of interview sessions. Analyses revealed meaningful relations between patterns of attachment and behaviour and perceptions. Insecure-ambivalent children exhibited more negative behaviour toward peers and sought the attention of peers more than did secure and insecure-avoidant children, and they complied to control attempts by peers less than did secure children. Insecure-avoidant children tended to engage in more neutral, less involved behaviour with peers. In addition, insecure-avoidant children generally reported negative self-perceptions while insecure-ambivalent children reported very positive perceptions concerning competence, social acceptance and peer relationships. Analyses indicated different patterns of relations for girls and boys. The results provide support for the predictive validity of the modified attac (PsycINFO Database Record (c) 2012 APA, all rights reserved)
SN - 0419-4217
AD - Demulder, Elizabeth Kyle: U Cambridge (United Kingdom), US
ER -

TY - JOUR
ID - 2969
T1 - "Laro tayo.": Parent-child and peer play activities of Filipino children and related variables
A1 - Bernardo,Marita Depante
Y1 - 1995///
N1 - Dissertation Abstract: 1995-95011-125
English
Dissertation Abstract
KW - PsycInfo
KW - Age Differences
KW - Childhood Play Behavior
KW - Human Sex Differences
KW - Parent Child Relations
KW - Socioeconomic Status
KW - Peer Relations
KW - Popularity
KW - Self Concept
KW - Social Behavior
RP - NOT IN FILE
SP - 5580
JF - Dissertation Abstracts International: Section B: The Sciences and Engineering
VL - 55:
IS - 12-B
N2 - This study identified differences in play activities engaged in by children varying in sex, age and socioeconomic status. Frequency of parent-child play was correlated with parental acceptance and child's self-concept. Sex, age and socioeconomic differences in parent-child play activities were described. Lastly, differences in play and other social behaviors of children varying in popularity and self-concept were identified.

Four hundred thirty-seven first and fourth graders from two public and two private coeducational schools completed a Home Play Survey, a sociometric instrument, the Child Parental-Acceptance Rejection Questionnaire and the Pasao Self-Concept Scale. Based on the results of the sociometric measure, 32 selected children were observed during recess. Results of the Home Play Survey showed that there were age, sex and socioeconomic differences in play activities at home. These differences could be accounted for by social and lifestyle variations among families and by cognitive and social development of children. Among the fourth graders, more frequent parent-child play was associated with more parental acceptance indicating that parent-child play could be a cause or an indication of positive parent-child relations. Among the first graders, more frequent parent-child play was associated with more parental rejection and higher self-concept. The latter indicates that this interaction can enhance competencies and relationships. The PARQ scores could have a different interpretation for younger children and that moderate parental acceptance is associated with more frequent parent-child play. Different models for predicting self-concept, with parent-child play and parental rejection as predictors were separately presented for Grade One and Grade Four children. Sex-typing was observed in parent-child play activities. There were fewer play activities among the lower class older children than among the middle class and the lower class younger children. The dat (PsycINFO Database Record (c) 2012 APA, all rights reserved)
SN - 0419-4217
AD - Bernardo, Marita Depante: Michigan State U, US
ER -

TY - JOUR
ID - 2970
T1 - Family emotional expressiveness as a mediator of children's social competence
A1 - Boyum,Lisa Ann
Y1 - 1995///
N1 - Dissertation Abstract: 1995-95005-191
English
Dissertation Abstract
KW - PsycInfo
KW - Expressed Emotion
KW - Family Relations
KW - Peer Relations
KW - Social Skills
RP - NOT IN FILE
SP - 4112
JF - Dissertation Abstracts International: Section B: The Sciences and Engineering
VL - 55:
IS - 9-B
N2 - The purpose of this study was to explore the connection between emotional expression in the child's family environment and children's social competence with peers at school. Previous research was extended in two ways: by direct observation and quantification of affective dimensions characterizing family expressiveness, and by extending prior laboratory-based work to the naturalistic environment. Fifty families of kindergarten girls and boys, varying by sociometric status, were videotaped in spontaneous dinner-table interactions. The parents completed questionnaires concerning emotional expressiveness of self and spouse, rating the frequency, intensity, and clarity of expressive behaviors. Videotapes were coded for frequency, intensity, clarity, and type of affect exchanged between parent-parent and parent-child dyads. Results indicated that family affective interactions previously observed in structured parent-child play interactions are generalizable to the unstructured home environment. Both parental expressiveness and observed parental affect were found to be meaningful predictors of children's sociometric ratings. Specific observed affect measurement validated the concept of positive expressiveness and clarified questions regarding negative expressiveness. The predictiveness of expressiveness ratings to children's social status and social competence with peers was improved by the addition of intensity and clarity ratings. Differences were found in parental sensitivity to negative expressiveness and in the effects of negative affect on boys and girls. The results of this study have implications for understanding the role of emotion as a mediator linking the child's family and peer systems, and for developing and implementing family-based interventions aimed at remediating children's social competence deficits and related problems. (PsycINFO Database Record (c) 2012 APA, all rights reserved)
SN - 0419-4217

AD - Boyum, Lisa Ann: U Illinois at Urbana-Champaign, US
ER -

TY - JOUR
ID - 2971
T1 - Naturalistic observations of bullies and victims in the school yard
A1 - Craig,Wendy Marion
Y1 - 1995///
N1 - Dissertation Abstract: 1995-95005-020
English
Dissertation Abstract
KW - PsycInfo
KW - Aggressive Behavior
KW - Peer Relations
KW - School Environment
KW - Social Discrimination
KW - Victimization
KW - Bullying
RP - NOT IN FILE
SP - 4139
JF - Dissertation Abstracts International: Section B: The Sciences and Engineering
VL - 55:
IS - 9-B
N2 - The objective of this research was to describe and examine the bullying on the school playground. The subjects were children observed either bullying or being victimized on videotapes of playground interaction gathered in a previous study on peer relations. Data were derived from two sources, questionnaires and observations of bullying interactions. The questionnaire measures collected were: Marsh Self Description Questionnaire (Marsh & Smith, 1982), Social Intentional Problem Solving Inventory (Bream, Hymel, & Rubin, 1986), Teacher's Report Form of the Child Behavior Checklist (Achenbach & Edelbrock, 1986), Teacher Version of the Marsh Self Concept Questionnaire (Marsh, Smith & Barnes, 1986), Revised Class Play (Masten, Morison, & Pellegrini, 1985), and a measure of sociometric status (Coie & Dodge, 1983). A total of 52 hours of video and remote audio recordings of playground behaviour were collected at two schools, in the winter and spring. The results indicated that bullying occurs regularly on the playground, approximately once every seven minutes and is of short duration, 37 seconds. Three percent of the episodes were racist. Bullies used weapons in 4% of the episodes, while only one victim used a weapon to defend himself. The majority of bullying episodes (68%) occurred within 120 feet of the school building. Adults intervened in 4% of the episodes, while peers intervened in 11% of the episodes. Adults, however, were more likely to intervene than peers it they were present. While some peers intervened in bullying, others participated in a variety of capacities that may have contributed to the continuation of these episodes, either by observing, or participating. Peers were involved in some capacity in 85% of the episodes. Bullies and victims were equally likely to be involved in bullying and victimization from the winter to the spring. Contrary to expectations, males and females were equally likely to be involved in bullying. Males were more likely than femal (PsycINFO Database Record (c) 2012 APA, all rights reserved)
SN - 0419-4217
AD - Craig, Wendy Marion: York U, Canada
ER -

TY - JOUR
ID - 2972
T1 - God or Darwin? The development of beliefs about the origin of species
A1 - Evans,Evelyn Margaret
Y1 - 1995///
N1 - Dissertation Abstract: 1995-95004-152
English
Dissertation Abstract
KW - PsycInfo
KW - Child Attitudes

KW - Comprehension
KW - Darwinism
KW - God Concepts
KW - Theory of Evolution
KW - Elementary School Students
RP - NOT IN FILE
SP - 2335
JF - Dissertation Abstracts International Section A: Humanities and Social Sciences
VL - 55:
IS - 8-A
N2 - In two studies I address the question of how an understanding of the origins of species is achieved. Study One is an investigation of when and how children come to adopt natural-evolutionary explanations for biological origins, as opposed to psychological-creationist explanations. Forty-nine elementary school children (6- to 12-years old) were asked about their natural history knowledge and about the origins of biological entities. A coherent pattern was demonstrated, with systematic age-related shifts from a primitive natural explanation, spontaneous generationism, to an intentional explanation, creationism, and from there, for some, to a more sophisticated natural explanation, evolutionism. The presence of evolutionism was positively related to subjects' natural history knowledge, independently of age. In Study Two, both creationist and evolutionist beliefs were investigated in more detail. Subjects were 175 children and their parents, from fundamentalist and non-fundamentalist elementary schools. In the non-fundamentalist community, a replication of the pattern of results found in Study One, was achieved. For the fundamentalist community, creationist explanations dominated at all ages, and all natural explanations, including knowledge of natural-history, were suppressed. Additional measures across the two communities revealed comparable parent educational levels, and comparable child interests in neutral areas, such as outdoor activities and reading, but divergent interests and knowledge in religious activities, fossils and adaptation. It was not until early adolescence (10- to 12-years) that the children's pattern of responses converged on the pattern found in the adults of their respective communities. It is argued that the divergent developmental pattern across the two communities can be optimally explained with a model of constructive interactionism: Children generate both natural and intentional beliefs about origins, while the communities privilege certai (PsycINFO Database Record (c) 2012 APA, all rights reserved)
SN - 0419-4209
AD - Evans, Evelyn Margaret: U Michigan, US
ER -

TY - JOUR
ID - 2973
T1 - Closeness between African-American father-figures and children as correlates of the children's achievement and psychosocial functioning: An African psychology perspective
A1 - Whitmore,Gerald Patrick
Y1 - 1995///
N1 - Dissertation Abstract: 1995-95001-174
English
Dissertation Abstract
KW - PsycInfo
KW - Academic Achievement
KW - Blacks
KW - Fathers
KW - Psychosocial Development
RP - NOT IN FILE
SP - 3032
JF - Dissertation Abstracts International: Section B: The Sciences and Engineering
VL - 55:
IS - 7-B
N2 - Using an African Psychology paradigm, this study examined whether African-American children's academic achievement and psychosocial functioning are related to the closeness they feel with their biological fathers and with other male parenting figures in their lives. The sample included 95 African-American, inner-city, fourth through sixth grade children. Fifty-one percent of the children (n = 48) had a father or father-figure living in the home. The Inventory of Family Feelings (IFF) was used to measure closeness between children and

their fathers, and between children and other male parenting figures living in the home. The IFF demonstrated outstanding internal consistency reliability with this sample. The Comprehensive Test of Basic Skills was used as a standardized measure of achievement. The Child Behavior Checklist was used to assess children's School Competence (grades), Total Competence, Internalizing Behavior Problems, Externalizing Behavior Problems, and Total Behavior Problems. In addition, qualitative interviews were conducted with five of the children and their primary caregivers to explore the children's significant relationships with male parenting figures (other than biological fathers) outside of the home. This expanded definition of significant male parenting figures was consistent with an African Psychology perspective. Results of the quantitative analyses showed: (1) School grades correlate significantly with children's closeness to their biological fathers; (2) achievement test scores in math correlate significantly with children's closeness to male parenting figures living in the home (regardless of whether those male parenting figures are biological fathers or other father-figures); and (3) children's aggressive and delinquent behavior problems correlate with less closeness to their biological fathers. The qualitative interviews revealed that a wide variety of men in the extended family and in the community were playing significant father (PsycINFO Database Record (c) 2012 APA, all rights reserved)
SN - 0419-4217
AD - Whitmore, Gerald Patrick: California School of Professional Psychology - Berkeley/alameda, US
ER -

TY - JOUR
ID - 2974
T1 - Theory of mind development and social understanding
A1 - Astington,Janet Wilde; Jenkins,Jennifer
Y1 - 1995///
N1 - Peer Reviewed Journal: 1995-43290-001
English
Journal; Peer Reviewed Journal
KW - PsycInfo
KW - Concept Formation
KW - Mind
KW - Social Interaction
KW - Social Perception
RP - NOT IN FILE
SP - 151
EP - 165
JF - Cognition and Emotion
VL - 9:
IS - 2-3
N2 - A cross-sectional, correlational study of 30 children (aged 3-5 yrs) investigated relations between their theory of mind development and social interaction, controlling for age and general language ability; the effect of sex was examined as well. All Ss were native English speakers, although for 10, it was a second language. Ss's overall performance on 4 standard false belief tasks was associated with their production of joint proposals and explicit role assignments during a 10-min session of pretend play. False belief task performance was not associated with the child's total amount of pretend play or with a measure of empathic concern. The significance of an association is discussed between a laboratory measure of theory of mind development and children's behavior observed in a naturalistic setting. (PsycINFO Database Record (c) 2012 APA, all rights reserved)
SN - 0269-9931
AD - Astington, Janet Wilde: U Toronto, Inst of Child Study, ON, Canada
ER -

TY - JOUR
ID - 2975
T1 - Enhancement of the social interaction and status of neglected children: A peer-pairing approach
A1 - Morris,Tracy L.
Y1 - 1995///
N1 - Peer Reviewed Journal: 1995-31100-001
English
Journal; Peer Reviewed Journal

KW - PsycInfo
KW - Elementary School Students
KW - Peer Relations
KW - Popularity
KW - Social Interaction
KW - Followup Studies
RP - NOT IN FILE
SP - 11
EP - 20
JF - Journal of Clinical Child Psychology
VL - 24:
IS - 1
N2 - Assessed the utility of a peer-pairing procedure for enhancing the sociometric status and positive social interaction of peer-neglected children. Sociometric nominations for 229 1st and 2nd graders (mean age 7.6 yrs) were obtained and used to identify 24 peer-neglected, 24 popular, and 24 average children. Peer-neglected and popular Ss were randomly assigned to peer-pairing or to control conditions. Each neglected S in the pairing condition was paired with a popular S from her or his own classroom, and the pairs participated in 12 15-min play sessions over 4 wks. Behavioral observations of social interaction were conducted for Ss during recess. Sociometric nominations and behavioral observations were reconducted after intervention and 1 mo after treatment. The paired group experienced significant improvement in both sociometric status and positive interaction rate compared with controls, and improvement was maintained at 1-mo follow-up. (PsycINFO Database Record (c) 2012 APA, all rights reserved)
SN - 0047-228X
AD - Morris, Tracy L.: West Virginia U, Dept of Psychology, Morgantown, US
ER -

TY - JOUR
ID - 2976
T1 - The effects of a peer-mediated self-evaluation procedure on the recess behavior of students with behavior problems
A1 - Nelson,J.
Y1 - 1995///
N1 - Peer Reviewed Journal: 1995-25513-001
English
Journal; Peer Reviewed Journal
KW - PsycInfo
KW - Behavior Problems
KW - Childhood Play Behavior
KW - Peer Relations
KW - Self Evaluation
KW - Human Males
RP - NOT IN FILE
SP - 117
EP - 126
JF - Remedial and Special Education
VL - 16:
IS - 2
N2 - Investigated the effects of a self-evaluation procedure on the recess behavior of 3 7-yr-olds with behavior problems, and determined whether the use of 3 age-matched peers in the procedure would facilitate the generalization of behavioral gains. Following the baseline, a peer-mediated self-evaluation procedure was introduced in the morning recess period according to the time-lagged procedures required by a multiple baseline across Ss design. The procedure produced clear improvements in the recess behavior of the target students. These improvements were maintained throughout all experimental conditions as the self-evaluation procedure was systematically faded. In addition, the treatment effects generalized to an afternoon recess period. (PsycINFO Database Record (c) 2012 APA, all rights reserved)
SN - 0741-9325
AD - Nelson, J. Ron: Eastern Washington U, Dept of Applied Psychology, Cheney, US

ER -

TY - JOUR
ID - 2977
T1 - Antisocial behavior in school: Strategies and best practices
A1 - Walker,Hill M.
Y1 - 1995///
N1 - Book: 1994-98933-000
English
Book; Authored Book
(Created by PsycINFO) Antisocial behavior patterns in children and youth: Characteristics, causes, and outcomes Issues and procedural recommendations regarding effective interventions for antisocial behavior in school The acting-out behavior cycle of antisocial students in the classroom: A conceptual model Strategies for managing the phases of acting-out behavior Establishing a schoolwide discipline plan Instructing and managing the classroom environment An instructional approach to teaching adaptive behavior patterns Instructing and managing the antisocial student on the playground Social skills: Importance and assessment Social skills: Implementation and generalization Parent involvement in the schooling of antisocial students Case-study applications of best practices with antisocial students School violence, gangs, and safety: Toward proactive strategies Epilogue Appendix A: Empirical foundations in discriminating, predicting, and changing aggressive, antisocial behavior in school and at home Appendix B: The case of Judge Hargreaves: Society is failing its children, forsaking its future Appendix C: Generals in the war on school violence
KW - PsycInfo
KW - Antisocial Behavior
KW - Classroom Behavior Modification
KW - Classroom Discipline
KW - At Risk Populations
KW - Elementary School Students
KW - High School Students
KW - Junior High School Students
KW - Prevention
KW - Social Learning
RP - NOT IN FILE
SP - Belmont, CA, US
JF - (1995)
VL - Antisocial behavior in school: Strategies and best practices. xxiii, 481 pp. Belmont, CA, US: Thomson Brooks/Cole Publishing Co; US.
N2 - (from the cover) Designed to enhance educators' understanding of the nature, origins, and causes of antisocial behavior, this book offers interventions and model programs that can be used in preventing or remediating this growing problem. Inside you'll find: a comprehensive approach to intervention involving teachers, peers, and parents of students with antisocial behavior patterns; methods for early identification of students at risk for antisocial behavior patterns; ways of designing an optimally effective classroom environment; case studies that illustrate the application of intervention procedures; [and] steps you can take to prevent and remediate antisocial behavior patterns. (from the preface) This book is written for the educator-practitioner who must cope with students who either already have or are at high risk for developing antisocial behavior patterns. This book is based upon principles of social learning that govern how diverse forms of behavior are acquired, maintained, and reduced or eliminated. (PsycINFO Database Record (c) 2012 APA, all rights reserved)
AD - Walker, Hill M.: U Oregon, Eugene, OR, US
ER -

TY - JOUR
ID - 2978
T1 - School recess and playground behavior: Educational and developmental roles
A1 - Pellegrini,Anthony D.
Y1 - 1995///
N1 - Book: 1994-98891-000
English

Book; Authored Book
(Created by PsycINFO) Acknowledgments Children on the playground at recess: What's so important? Playgrounds and children's behavior at recess Theory and method Dimensions of the playground and children's behavior: Implications for social cognition development The effects of recess timing on elementary school children's recess and classroom behavior Longitudinal relations between playground behavior and cognition: Explorations in social dimensions of cognition The rough-and-tumble play of primary school children: Contemporaneous and longitudinal relations Preference for outdoor play during early adolescence Longitudinal relations between social networks and adjustment to middle school Adolescent boys' rough-and-tumble play Conclusions and implications Index
KW - PsycInfo
KW - Childhood Development
KW - Childhood Play Behavior
KW - Playgrounds
KW - Cognitive Development
KW - Middle School Students
KW - Peer Relations
KW - Primary School Students
KW - Psychosocial Development
KW - School Environment
KW - Social Cognition
RP - NOT IN FILE
SP - Albany, NY, US
JF - (1995)
VL - School recess and playground behavior: Educational and developmental roles. xii, 186 pp. Albany, NY, US: State University of New York Press; US.
N2 - (from the cover) The recess period represents a unique part of the school day. It is one of the few times when children can interact with their peers on their own terms with minimal adult intervention. Consequently, it represents one of the few places in primary and middle schools to observe spontaneous peer interaction. "School Recess and Playground Behavior" offers a programmatic examination of a neglected aspect of children's behavior and informs related literatures such as the educational, social-developmental, and cognitive-developmental literatures. (PsycINFO Database Record (c) 2012 APA, all rights reserved)
AD - Pellegrini, Anthony D.: U Georgia, Athens, GA, US
ER -

TY - JOUR
ID - 2979
T1 - Children who have no breakfast. [References]
A1 - Box,Val
Y1 - 1994///
N1 - Peer Reviewed Journal: 2009-18747-002
English
Journal; Peer Reviewed Journal
KW - PsycInfo
KW - Classrooms
KW - Eating Behavior
KW - Elementary Schools
KW - Human Sex Differences
RP - NOT IN FILE
SP - 10
EP - 13
JF - Health Education
VL - 94:
IS - 4
N2 - The aim of this study is to investigate young children in primary schools not having had any breakfast; and without being provided with a snack for the morning break. To collect the data, researchers went into classrooms, usually between the beginning of morning school and break time, while the children could still remember what they had eaten before school. One of the researchers would address the whole class and explain

that they wanted to collect very special and secret information. They asked the children to take the piece of paper which would be given to them, put their name and class on it, divide it into four, and draw all the things they did before going to school. The findings suggest that out of the entire study sample, 15 out of each 100 children had a snack on the way to school or in the playground before school started, but this often consisted only of sweets. Only eight out of 100 had a drink. We found that more girls (six in 100) went without breakfast than boys. Worryingly, the rate was highest in four-and five year-olds; among eight-year-olds, the rate was four in 100. It is known that going without breakfast is even more common among older adolescents-but they may be able to compensate more easily during the rest of the day by buying or bringing snacks or food of their own choice. (PsycINFO Database Record (c) 2012 APA, all rights reserved)
SN - 0965-4283
AD - Box, Val: Health Education Unit, University of Southampton, Southampton, United Kingdom Landman, Jackie: Queen Margaret College, Edinburgh, United Kingdom
ER -

TY - JOUR
ID - 2980
T1 - A descriptive assessment of social relationships in integrated classrooms
A1 - Hall,Laura J.
Y1 - 1994///
N1 - Peer Reviewed Journal: 1995-31129-001
English
Journal; Peer Reviewed Journal
KW - PsycInfo
KW - Mainstreaming (Educational)
KW - Social Interaction
KW - Special Education Students
KW - Elementary School Students
KW - Preschool Students
RP - NOT IN FILE
SP - 302
EP - 313
JF - Journal of the Association for Persons with Severe Handicaps
VL - 19:
IS - 4
N2 - Used multiple measures in 4 integrated classes of disabled and nondisabled young children to assess social relationships. Nine 10-min observational recordings during indoor and outdoor free play activities, peer nominations, and teacher and peer interviews provided information about social status and strength of association between children with disabilities and their classmates. Results revealed that reciprocal, positive relationships were found between children with disabilities and classmates in all classes. Individual differences in social status and profile of children with disabilities indicate that varied patterns of social relationships are found in integrated classrooms. Brief interviews with the children and teaching staff provided valuable information about relationships based on helping or pity, shared interest in activities, and friendship. (PsycINFO Database Record (c) 2012 APA, all rights reserved)
SN - 0274-9483
AD - Hall, Laura J.: Deakin U, School of Studies in Disability, Burwood, Vict, Australia
ER -

TY - JOUR
ID - 2981
T1 - Effects of conflict resolution training on elementary school students
A1 - Johnson,David W.
Y1 - 1994///
N1 - Peer Reviewed Journal: 1995-31017-001
English
Journal; Peer Reviewed Journal
KW - PsycInfo
KW - Conflict Resolution

KW - Elementary School Students
KW - Negotiation
KW - Peer Relations
KW - Followup Studies
RP - NOT IN FILE
SP - 803
EP - 817
JF - The Journal of Social Psychology
VL - 134:
IS - 6
N2 - A peer-mediation training program was conducted in 4 multi-age classrooms in an American, suburban, middle-class elementary school. 92 students in the 1st-6th grades received 30 min of training per day for 6 wks, focusing on negotiation and mediation procedures and skills. Prior to the training program, frequent conflicts involving physical aggression, playground activities, access to or possession of objects, turn taking, put-downs and teasing, and academic work were reported. Ss were able to transfer the procedures and skills and apply them in real conflicts among classmates. Careful observation of hallways, the lunchroom, the playground, and the gymnasium revealed that 4 mo after training, Ss seriously and carefully used these procedures to resolve highly emotional and prolonged conflicts with fellow students. Discipline problems were eliminated as Ss became more autonomous in managing their conflicts constructively. (PsycINFO Database Record (c) 2012 APA, all rights reserved)
SN - 0022-4545
AD - Johnson, David W.: U Minnesota, Cooperative Learning Ctr, Minneapolis, US
ER -

TY - JOUR
ID - 2982
T1 - How children spend their time: A sample survey for use in exposure and risk assessments
A1 - Silvers,Abraham
Y1 - 1994///
N1 - Peer Reviewed Journal: 1995-28598-001
English
Journal; Peer Reviewed Journal
KW - PsycInfo
KW - Daily Activities
KW - Risk Assessment
RP - NOT IN FILE
SP - 931
EP - 944
JF - Risk Analysis
JA - Risk Anal
VL - 14:
IS - 6
N2 - Assessed the nature and environment of children's (aged 5-12 yrs) activities in telephone surveys of 1,000 households in 6 states. Children's behavior was sampled on both weekdays and weekends over all 4 seasons of the year using a retrospective time diary to allocate time to activities during the previous 24 hrs. Information was obtained on the kinds and locations of activities, the nature of the microenvironments of the locations, and the time spent in the different environments. Measures of variability in addition to mean hours per day are reported. Results of this study closely match those of J. A. Wiley (1991) on California children's activities, particularly TV viewing, sleeping, eating, and school attendance. Also, the present Ss spent most of their time indoors at home, indicating that risk assessments should focus on indoor, on-site hazards. (PsycINFO Database Record (c) 2012 APA, all rights reserved)
SN - 0272-4332
AD - Silvers, Abraham: Electric Power Research Inst, Palo Alto, CA, US
ER -

TY - JOUR
ID - 2983

T1 - Development of social maturity during infancy and contributing influences of environmental components including motor and mental development: An exploratory study
A1 - Phatak,Pramila
Y1 - 1994///
N1 - Peer Reviewed Journal: 1995-24693-001
English
Journal; Peer Reviewed Journal
KW - PsycInfo
KW - Cognitive Development
KW - Emotional Maturity
KW - Motor Development
KW - Psychosocial Development
KW - Longitudinal Studies
RP - NOT IN FILE
SP - 66
EP - 72
JF - Psychological Studies
VL - 39:
IS - 2-3
N2 - Motor development, mental development, and 15 environmental components were the independent variables and social maturity (SCM) was the dependent variable in a study of 89 infants assessed at 3, 6, 9, 12, 18, 24, and 30 mo of age. Ss' total sleep hours, toys, interactions, and type of activities were repeatedly recorded. Independent variables were studied for their contribution in the variance of social maturity. The components were grouped under development, child's life at home, parental characteristics, family characteristics, and physical environment. The highest contributing component under each group was used to develop a descriptive picture of congenial environment for development of SCM during infancy. A child having educated parents, an elderly mother with commitment for outside work, and a father occupied in work related to learning or skills with managerial freedom was in an environment contributing positively to development of SCM. (PsycINFO Database Record (c) 2012 APA, all rights reserved)
SN - 0033-2968
AD - Phatak, Pramila: KEM Hosp, Pune, India
ER -

TY - JOUR
ID - 2984
T1 - A longitudinal study of school peer networks and adjustment to middle school
A1 - Pellegrini,A.
Y1 - 1994///
N1 - Peer Reviewed Journal: 1995-19335-001
English
Journal; Peer Reviewed Journal
KW - PsycInfo
KW - Middle School Students
KW - Peer Relations
KW - School Adjustment
KW - Social Networks
KW - Human Sex Differences
KW - Longitudinal Studies
RP - NOT IN FILE
SP - 403
EP - 412
JF - Educational Psychology
VL - 14:
IS - 4
N2 - Examined longitudinal changes in boys' and girls' school peer networks in Grades 6 and 7 and determined the extent to which network measures in the 1st yr of middle school predicted school adjustment in the 2nd yr. 56 White Ss were observed on the playground for 22 mo, and Ss and their teachers completed measures of Ss'

popularity, physical attractiveness, ability, achievement, and behavior. While certain personality and behavioral measures were stable across the 1st 2 yrs of middle school, networks became more gender integrated across the period. Sixth-grade network measures were predictors of 7th-grade adjustment, and Ss' adjustment could be explained in terms of learning and exhibiting gender-stereotyped behavior. (PsycINFO Database Record (c) 2012 APA, all rights reserved)
SN - 0144-3410
AD - Pellegrini, A. D.: U Georgia, Dept of Elementary Education, Athens, US
ER -

TY - JOUR
ID - 2985
T1 - Follow up study of children of 5-8 years using child protection programmes in Australia and New Zealand
A1 - Briggs,Freda
Y1 - 1994///
N1 - Peer Reviewed Journal: 1995-07514-001
English
Journal; Peer Reviewed Journal
KW - PsycInfo
KW - Educational Programs
KW - Elementary School Students
KW - Prevention
KW - Safety
KW - Sexual Abuse
KW - Followup Studies
KW - Kindergarten Students
RP - NOT IN FILE
SP - 111
EP - 117
JF - Early Child Development and Care
VL - 100 Jun 1994, pp. 111-117.
N2 - Reinterviewed 126 Australian and 117 New Zealand children 1 yr after exposure to sexual abuse prevention programs. These included Protective Behaviors in Australia, aimed at developing assertiveness skills and empowering the children, and Keeping Ourselves Safe, developed by New Zealand educational and police experts to meet the unique needs of the Maori, European, and Pacific Islander populations. The survey used problem-solving methods to assess whether Ss could suggest safe strategies for dealing with situations such as becoming separated from parents, facing a babysitter who wants to play undressing games, being approached by a strange woman who meets them outside of school, and being touched inappropriately by an adult. There was no marked improvement in the quality of the Australian Ss' responses. However, all the New Zealand Ss showed some gains in safety skills; significant predictors of success were teacher commitment and socioeconomic class. (PsycINFO Database Record (c) 2012 APA, all rights reserved)
SN - 0300-4430
AD - Briggs, Freda: U South Australia, De Lissa Inst of Early Childhood & Family Studies, Magill, Australia
ER -

TY - JOUR
ID - 2986
T1 - How well preschool children know their friends
A1 - Field,Tory; Miller,Jennifer; Field,Tiffany
Y1 - 1994///
N1 - Peer Reviewed Journal: 1995-04999-001
English
Journal; Peer Reviewed Journal
KW - PsycInfo
KW - Friendship
RP - NOT IN FILE
SP - 101
EP - 109

JF - Early Child Development and Care
VL - 100 Jun 1994, pp. 101-109.
N2 - Used a sample of 16 preschoolers (10 boys and 6 girls; aged 27-62 mo) to determine if children accurately report their best friends, know their best friends' physical characteristics, and understand the function of friends. Data were collected through classroom and playground free time sociogram observations, teacher responses, and interviews with the children. Ss' self-report of best friends corresponded with teachers' reports, however Ss were not accurate in reporting with whom they had played that day. Ss were accurate in reporting friends' age, hair color, and height, but often forgot their eye color and last name. Preschoolers cited playing with each other and liking each other most frequently as the reason why they liked their best friend. (PsycINFO Database Record (c) 2012 APA, all rights reserved)
SN - 0300-4430
AD - Field, Tory: U Miami School of Medicine, Touch Research Inst, FL, US
ER -

TY - JOUR
ID - 2987
T1 - The effects of a probation intervention on juvenile offenders' self-concepts, loci of control, and perceptions of juvenile justice
A1 - Minor,Kevin I.
Y1 - 1994///
N1 - Peer Reviewed Journal: 1994-46769-001
English
Journal; Peer Reviewed Journal
KW - PsycInfo
KW - Internal External Locus of Control
KW - Justice
KW - Juvenile Delinquency
KW - Probation
KW - Self Concept
KW - Family Therapy
KW - Juvenile Justice
KW - Occupational Guidance
KW - Wilderness Experience
RP - NOT IN FILE
SP - 490
EP - 511
JF - Youth & Society
VL - 25:
IS - 4
N2 - Examined the impact of job preparation, short-term outdoor adventure, and family relationships programming on juvenile probationers' self-concepts, loci of control (LOC), and perceptions of juvenile justice (PJJ). 45 probationers (aged 12-17 yrs), of whom 15 had been placed on intensive probation, participated in the study. Seven Ss from intensive and 15 Ss from moderate probation were put in the experimental-moderate group, and 8 Ss from intensive and 15 Ss from moderate probation were put in the control-moderate group. The experimental intervention proved to be no more effective than the standard probation services offered in reducing Ss' offense activities or in modifying their self-concepts, LOC, and PJJ. Although the PJJ displayed by all groups improved somewhat in the pre- to posttest interval, the PJJ measured at posttesting were moderate and did not significantly differ between groups. (PsycINFO Database Record (c) 2012 APA, all rights reserved)
SN - 0044-118X
AD - Minor, Kevin I.: Eastern Kentucky U, Dept of Correctional Services, Richmond, US
ER -

TY - JOUR
ID - 2988
T1 - Do voluntary changes in inspiratoryxpiratory ratio prevent exercise-induced asthma?
A1 - Ceugniet,Francois; Cauchefer,Francoise; Gallego,Jorge
Y1 - 1994///

N1 - Peer Reviewed Journal: 1994-45735-001
English
Journal; Peer Reviewed Journal
KW - PsycInfo
KW - Asthma
KW - Respiration
KW - Running
RP - NOT IN FILE
SP - 181
EP - 188
JF - Biofeedback & Self Regulation
VL - 19:
IS - 2
N2 - 24 asthmatic boys (aged 12-18 yrs), divided into 3 groups corresponding to 3 instructions on breathing pattern, underwent 2 exercise provocation tests, 24 hrs apart (outdoor running for 6 min). The 1st test was identical for all the Ss. In the 2nd test, the 1st group did not receive any instruction concerning breathing pattern. The 2nd group was instructed to adopt equal inspiratory and expiratory times (TI/TE = 1). The 3rd group had to adopt an expiratory time 3 times longer than inspiratory time (TI/TE = 1:3). The 3 groups displayed similar pulmonary function tests (FEV1 and forced vital capacity), cardiac frequency, and running performances. However, FEV1 significantly improved in the 2nd session. (PsycINFO Database Record (c) 2012 APA, all rights reserved)
SN - 0363-3586
AD - Ceugniet, Francois: Le Balcon de Cerdagne, Font-Romeu, France
ER -

TY - JOUR
ID - 2989
T1 - Transition to school: Developmental trajectories and school experiences
A1 - Belsky,Jay
Y1 - 1994///
N1 - Peer Reviewed Journal: 1994-39250-001
English
Journal; Peer Reviewed Journal
KW - PsycInfo
KW - Academic Underachievement
KW - Childhood Development
KW - School Adjustment
KW - Student Characteristics
KW - At Risk Populations
KW - Child Care
KW - Elementary School Students
KW - Family Background
KW - Kindergarten Students
KW - Preschool Students
RP - NOT IN FILE
SP - 106
EP - 119
JF - Early Education and Development
VL - 5:
IS - 2
N2 - Argues that since difficulties experienced by children are traceable to problems in underachievement that begin during the first years of early schooling, a thorough understanding of the transition-to-school process is needed. Two principle assumptions in examining this process are suggested. First, multiple risk factors associated with certain child, family, and child care characteristics are expected to predict early school experience both in and out of the classroom, and, thereby, social and academic success and failure in the early school years. Secondly, it is expected that day-to-day experiences in the classroom and on the playground will

exacerbate or mitigate risk, resulting in some children performing better than would otherwise be expected and others functioning worse than anticipated. (PsycINFO Database Record (c) 2012 APA, all rights reserved)
SN - 1040-9289
AD - Belsky, Jay: Pennsylvania State U, Dept of Human Development & Family Studies, US
ER -

TY - JOUR
ID - 2990
T1 - Relation between ratings and observations of stimulant drug response in hyperactive children
A1 - Nolan,Edith E; Gadow,Kenneth
Y1 - 1994///
N1 - Peer Reviewed Journal: 1994-33939-001
English
Journal; Peer Reviewed Journal
KW - PsycInfo
KW - Behavior Problems
KW - Drug Therapy
KW - Hyperkinesis
KW - Observation Methods
KW - Rating Scales
KW - CNS Stimulating Drugs
RP - NOT IN FILE
SP - 78
EP - 90
JF - Journal of Clinical Child Psychology
VL - 23:
IS - 1
N2 - Examined the relation between ratings and observations of 34 hyperactive children (aged 5-13 yrs) in public schools in placebo-controlled, double-blind stimulant drug evaluations. The Ss received either 2 or 3 doses of methylphenidate and placebo for 2 wks. Findings indicate that rating scale scores were significantly correlated with classroom, lunchroom, and playground observations of negativistic (e.g., aggression, noncompliance, interference) but not hyperactive (inattention, motor movement) behaviors. However, when the data were analyzed controlling for the variance accounted for by the other dimension (partial correlations), there was evidence supporting the differential validity of hyperactivity and negativistic behavior scales of the IOWA Conners Teacher's Rating Scale and the Abbreviated Teacher Rating Scales across settings and as measures of drug response. (PsycINFO Database Record (c) 2012 APA, all rights reserved)
SN - 0047-228X
AD - Nolan, Edith E.: State U New York, Dept of Psychiatry & Behavioral Science, Stony Brook, US
ER -

TY - JOUR
ID - 2991
T1 - The play of peer cultures in a city school yard: "reeling," "writhing," and "a rhythmic kick."
A1 - Beresin,Ann Richman
Y1 - 1993///
N1 - Dissertation Abstract: 1996-70688-001
English
Dissertation Abstract
KW - PsycInfo
KW - Childhood Play Behavior
KW - Ethnography
KW - Peer Relations
KW - Folklore
RP - NOT IN FILE
SP - 2275
JF - Dissertation Abstracts International
VL - 54:

IS - 6-A
SN - 0419-4217
AD - Beresin, Ann Richman: U Pennsylvania, US
ER -

TY - JOUR
ID - 2992
T1 - The authority of cultural tools in a sociocultural approach to mediated agency
A1 - Wertsch,James V.
Y1 - 1993///
N1 - Peer Reviewed Journal: 1995-01351-001
English
Journal; Peer Reviewed Journal
KW - PsycInfo
KW - Language
KW - Oral Communication
KW - Social Behavior
KW - Social Environments
KW - Sociocultural Factors
RP - NOT IN FILE
SP - 227
EP - 239
JF - Cognition and Instruction
VL - 11:
IS - 3-4
N2 - Focuses on the importance of task situation definition in naturalistic settings where adults and children interact together, and the children play a more active role and contribute actively to the flow of discourse. Naturalistic data taken from 1 9-yr-old girl's activities and discourse during an entire day in the home, at school, and in other social settings are presented and analyzed. These data provide evidence for the heterogeneity of speech events and for the use of cultural tools in order to change the authoritative structure of the discourse. An expanded interpretation of the sociocultural approach requires that authority and value should be assumed as essential properties of both intermental and intramental functioning. (PsycINFO Database Record (c) 2012 APA, all rights reserved)
SN - 0737-0008
AD - Wertsch, James V.: Clark U, France L. Hiatt School of Psychology, Dept of Psychology, Worcester, MA, US
ER -

TY - JOUR
ID - 2993
T1 - Effectiveness of self-modeling as a social skills training and status improvement technique for neglected children
A1 - Mehaffey,Joyce Irene
Y1 - 1993///
N1 - Dissertation Abstract: 1994-74368-001
English
Dissertation Abstract
KW - PsycInfo
KW - Interpersonal Interaction
KW - Popularity
KW - Self Monitoring (Personality)
KW - Social Isolation
KW - Social Skills Training
KW - Elementary School Students
RP - NOT IN FILE
SP - 3445
JF - Dissertation Abstracts International

VL - 53:
IS - 10-A
SN - 0419-4217
AD - Mehaffey, Joyce Irene: U Massachusetts, US
ER -

TY - JOUR
ID - 2994
T1 - Socialization of children's playground skill
A1 - Butcher,Janice
Y1 - 1993///
N1 - Peer Reviewed Journal: 1994-29101-001
English
Journal; Peer Reviewed Journal
KW - PsycInfo
KW - Childhood Play Behavior
KW - Parental Attitudes
KW - Playgrounds
KW - Practice
KW - Risk Taking
RP - NOT IN FILE
SP - 731
EP - 738
JF - Perceptual and Motor Skills
VL - 77:
IS - 3, Pt 1
N2 - Analyzed the relationship between playground skill (measured on the Playground Skills Test) and several socialization variables, including opportunities for practice and parental involvement with playground play. Data were collected for 64 children (aged 7-9 yrs). Descriptive statistics are presented for availability of playground equipment, frequency of playground play outside of school hours, and amount of parental involvement. Playground proficiency was not related to frequency of playground play or to direct parental support (attendance and assistance while child was at a playground). Parental perceptions of a child's skill and attempts at risk-taking were significantly correlated with playground proficiency. A regression analysis of all socialization variables showed that the only significant predictor of playground proficiency was a child's risk-taking attempts. (PsycINFO Database Record (c) 2012 APA, all rights reserved)
SN - 0031-5125
AD - Butcher, Janice: U Manitoba, Faculty of Physical Education & Recreation Studies, Winnipeg, Canada
ER -

TY - JOUR
ID - 2995
T1 - Multimethod comparison of similarity in school adjustment of siblings and unrelated children. [References]
A1 - Lewin,Lewis M.
Y1 - 1993///
N1 - Peer Reviewed Journal: 1994-17024-001
English
Journal; Peer Reviewed Journal
KW - PsycInfo
KW - School Adjustment
KW - Siblings
KW - Social Adjustment
RP - NOT IN FILE
SP - 963
EP - 969
JF - Developmental Psychology
JA - Dev Psychol

VL - 29:
IS - 6
N2 - Examined similarities among 45 sibling pairs in Grades 2-5 in their social and academic adaptation to the school setting. Measures included teacher ratings and ranking of academic skills, social behavior, and peer acceptance; peer sociometric ratings; and direct observations on the playground with peers and in the classroom with teachers and peers. Comparisons were made with randomly selected, unrelated S pairs matched on sex, grade, and classroom. Significant correlations were found only among sibling pairs on peer ratings of social preference, teachers' judgments of academic competence, popularity, social behavior and school adjustment, positive behavior with peers on the playground, and teachers' disapproving behavior in the classroom. Results underscore the need for more multiagent and multimethod research on sibling concordant-discordant adjustment regardless of theoretical orientation. (PsycINFO Database Record (c) 2012 APA, all rights reserved)
SN - 0012-1649
AD - Lewin, Lewis M.: Oregon Research Inst, Eugene, US
ER -

TY - JOUR
ID - 2996
T1 - Girls' and boys' use of directives in pretend play
A1 - Lloyd,Barbara
Y1 - 1993///
N1 - Peer Reviewed Journal: 1994-12934-001
English
Journal; Peer Reviewed Journal
KW - PsycInfo
KW - Childhood Play Behavior
KW - Human Sex Differences
KW - Language Development
KW - Oral Communication
KW - Pretend Play
KW - Followup Studies
RP - NOT IN FILE
SP - 122
EP - 130
JF - Social Development
VL - 2:
IS - 2
N2 - Children (aged 4-5 yrs) were recorded in everyday classroom interaction at the beginning and the end of their 1st year at school. Groups of 8-20 children were invited to come into the classroom during the lunch playground break and 12 recordings were made. The children's use of language in pretend play was analyzed from transcriptions of the video record. Although girls directed a greater proportion of their speech to controlling others, there were no consistent differences between girls and boys in the use of directives or instructions to pretend. On their entry to school these children displayed few distinct gender-related patterns in their spontaneous speech. (PsycINFO Database Record (c) 2012 APA, all rights reserved)
SN - 0961-205X
AD - Lloyd, Barbara: U Sussex, School of Social Sciences, Brighton, England
ER -

TY - JOUR
ID - 2997
T1 - Boys' rough-and-tumble play, social competence and group composition
A1 - Pellegrini,A.
Y1 - 1993///
N1 - Peer Reviewed Journal: 1994-07550-001
English
Journal; Peer Reviewed Journal
KW - PsycInfo
KW - Childhood Play Behavior

KW - Peer Relations
KW - Personality
KW - Popularity
KW - Elementary School Students
KW - Problem Solving
KW - Social Interaction
RP - NOT IN FILE
SP - 237
EP - 248
JF - British Journal of Developmental Psychology
JA - BR J DEV PSYCHOL
VL - 11:
IS - 3
N2 - Examined relations between aspects of 42 male 5th graders' rough-and-tumble play (RTP) and social competence. Ss were observed on the school playground for 20 wks and were asked to nominate peers whom they liked most and least, rank-order peers in terms of dominance, and solve hypothetical social problems. Teachers completed a temperament questionnaire for each S. Neither relative frequency of RTP nor the vigor dimension of RTP accounted for significant variance in Ss' popularity beyond other forms of reciprocal social interactions. Further, relative frequency of RTP was negatively, but not significantly, related to popularity. The flexibility dimension of RTP did account for significant variance in social problem solving. Both aspects (i.e., activity and flexibility) of temperament measured correlated significantly with the vigor level of RTP. (PsycINFO Database Record (c) 2012 APA, all rights reserved)
SN - 0261-510X
AD - Pellegrini, A. D.: U Georgia, Inst for Behavioral Research, Athens, US
ER -

TY - JOUR
ID - 2998
T1 - The emotional life of the toddler
A1 - Lieberman,Alicia F.
Y1 - 1993///
N1 - Book: 1993-98477-000
English
Book; Authored Book
(Created by PsycINFO) (Abbreviated) Acknowledgments The emotional importance of early relationships Who is the toddler? The challenges of being (and raising) a toddler The question of temperament The active toddler: Racing ahead The shy toddler: Taking one's time Early anxieties Issues to negotiate When parents divorce The toddler in child care Conclusion: Staying close and letting go References Index
KW - PsycInfo
KW - Childrearing Practices
KW - Early Childhood Development
KW - Emotional Development
KW - Anxiety
KW - Infant Development
KW - Stress
RP - NOT IN FILE
SP - New
JF - (1993)
VL - The emotional life of the toddler. ix, 244 pp. New York, NY, US: Free Press; US.
N2 - (from the jacket) [This book offers] an in-depth examination of the varied and intense emotional life of children from ages one to three. Drawing on her lifelong research, [the author] addresses commonly asked questions and issues [raised by the parents of these children]. [The author] gives new insights on how best to handle common toddler problems, such as sleep disturbances, sibling rivalry, and temper tantrums. She also provides invaluable information on the normal anxieties of this age and how to recognize when anxiety is excessive, needing special attention. Finally, she gives us a fresh, insightful picture of how toddlers typically handle the stress of their parents' divorce and outside child care, and provides sensitive practical advice on

easing the toddler through these transitions. [This book] is an invaluable resource for parents, clinicians, researchers, and child-care workers alike. (PsycINFO Database Record (c) 2012 APA, all rights reserved)
AD - Lieberman, Alicia F.: U California, Professor of Psychology, San Francisco, CA, US
ER -

TY - JOUR
ID - 2999
T1 - Play, dream, fantasy, and enactment in Bornstein's "Obsessional Child," then and now
A1 - Ostow,Mortimer
Y1 - 1993///
N1 - Book: 1993-98417-012
English
Book; Edited Book
KW - PsycInfo
KW - Obsessions
KW - Psychoanalysis
KW - Case Report
KW - Childhood Play Behavior
KW - Dreaming
KW - Emotions
KW - Imagination
RP - NOT IN FILE
SP - 201
EP - 218
JF - Solnit, Albert J [Ed]; Cohen, Donald J [Ed]; Neubauer, Peter B [Ed]
VL - :
IS - 1993
N2 - (create) Compares the affects, the pattern of affect regulation and the organizing fantasies in dream, fantasy, play, and enactment. The author presents material from [B.] Bornstein's (1953) case report of Sherry, an obsessional eight-yr-old, as well as material from his own work with the same patient as an adult, to illustrate the difference between play in analysis and play outside analysis and to demonstrate a continuity in manner of expression at any given time and a continuity in time. (PsycINFO Database Record (c) 2012 APA, all rights reserved)
AD - Ostow, Mortimer: Jewish Theological Seminary of America, Emeritus Professor of Pastoral Psychiatry, New York, NY, US
ER -

TY - JOUR
ID - 3000
T1 - Patterns of social behavior on the playground in 9- to 11-year-old girls and boys: Relation to teacher perceptions and to peer ratings of aggression, withdrawal, and likability
A1 - Serbin,Lisa A.
Y1 - 1993///
N1 - Book: 1993-98114-005
English
Book; Edited Book
KW - PsycInfo
KW - Aggressive Behavior
KW - Childhood Play Behavior
KW - Social Isolation
KW - Elementary School Students
KW - Human Sex Differences
KW - Peer Evaluation
KW - Playgrounds
KW - Teacher Attitudes
RP - NOT IN FILE
SP - 162

EP - 183
JF - Hart, Craig H [Ed]
VL - :
IS - 1993
N2 - (from the chapter) contains an overview of two observational studies: one using a sample of 117 peer-identified aggressive, withdrawn, aggressive/withdrawn, and contrast children aged 9-11 [grades 4-6], the second involving a randomly selected sample of 60 girls and boys the same age, observed over the school year under the same conditions / [present] brief descriptions of the play patterns of each sample, focusing on aggressive and withdrawn behavior / explore and compare the relation between teacher and peer perceptions and aggressive and withdrawn play behavior in the two samples / discuss differences in the significance aggressive playground behavior appears to have for boys and girls (PsycINFO Database Record (c) 2012 APA, all rights reserved)
AD - Serbin, Lisa A.: Concordia U, Ctr for Research in Human Development, Professor, Montreal, PQ, Canada
ER -

TY - JOUR
ID - 3001
T1 - Bullies on the playground: The role of victimization
A1 - Olweus,Dan
Y1 - 1993///
N1 - Book: 1993-98114-003
English
Book; Edited Book
KW - PsycInfo
KW - Aggressive Behavior
KW - Playgrounds
KW - Victimization
KW - Educational Programs
KW - Elementary School Students
KW - Junior High School Students
RP - NOT IN FILE
SP - 85
EP - 128
JF - Hart, Craig H [Ed]
VL - :
IS - 1993
N2 - (from the introduction) considers bully/victim problems / situations involving bullies and their victims are prominent in playground environments / to assist in dealing with these problems, [the author] provides a wealth of information derived from data collected in two recent large-scale studies that were conducted in Norway and Sweden [with students in primary and junior high school (grades 2-9)] describes what bullying is and is not, how frequently bully/victim problems occur, family background and personality characteristics of bullies and victims, and age and sex differences regarding bully/victim problems / presents data suggesting that parents and teachers are generally aware of bully/victim problems and do relatively little to stop them additional data suggest that most bullying occurs on the school playground and that the size of the class or school has little relation to the relative frequency of bully/victim problems / [discusses] ways that supervision practices during recess promote or discourage bullying behavior / [presents] an intervention program designed to assist bullies and their victims / specific, practical guidelines for helping bullies and victims are also presented (PsycINFO Database Record (c) 2012 APA, all rights reserved)
AD - Olweus, Dan: U Bergen, Dept of Personality Psychology, Professor, Bergen, Norway
ER -

TY - JOUR
ID - 3002
T1 - Behind the playground walls: Sexual abuse in preschools
A1 - Waterman,Jill
Y1 - 1993///

N1 - Book: 1993-97375-000
English
Book; Authored Book
(Created by PsycINFO) Part I: Scope of the problem of ritualistic and nonritualistic sexual abuse in preschools A tale of two communities / Jane McCord Background literature / Jill Waterman and Sharon Ben-Meir Study methods and procedures / Robert J. Kelly Limitations of the study / Robert J. Kelly Part II: Content and process of disclosures of sexual abuse in preschools Perspectives on what happened to the children / Jill Waterman Disclosure patterns in psychotherapy / Jane McCord and Lauren Shapiro Gonzalez Part III: Effects on children Overall level of distress / Robert J. Kelly Cognitive and school-related effects / Robert Lusk Emotional effects / Robert J. Kelly and Sharon Ben-Meir Effects on sexuality / Robert J. Kelly Effects on interpersonal relationships / Mary Kay Oliveri Comparison of effects of reported ritualistic and nonritualistic sexual abuse / Jill Waterman Part IV: Effects on others Impact on parents / Jane McCord Impact on family relationships / Jill Waterman Impact on therapists / Mary Kay Oliveri and Jill Waterman Part V: Understanding the effects: Coping patterns and mediators Parental reactions and coping patterns / Jane McCord Mediators of effects on children: What enhances optimal functioning and promotes healing? / Jill Waterman and Robert J. Kelly Clinical implications / Mary Kay Oliveri, Martha Cockriel and Michele Dugan Part VI: Final considerations Uses and abuses of research: Cross-currents of community exploitation / Roland C. Summit The research perspective / David Finkelhor and Kathleen Kendall-Tackett Conclusions and recommendations / Jill Waterman, Robert J. Kelly, Mary Kay Oliveri and Jane McCord References Index
KW - PsycInfo
KW - Child Abuse
KW - Sexual Abuse
KW - Coping Behavior
KW - Preschool Students
RP - NOT IN FILE
SP - New
JF - (1993)
VL - Behind the playground walls: Sexual abuse in preschools. xii, 308 pp. New York, NY, US: Guilford Press; US.
N2 - (from the jacket) Representing over 6 years of research with children reporting nonritualistic and ritualistic sexual abuse in preschool settings, "Behind the Playground Walls" is a clear, comprehensive examination of the effects of reported molestation on young children and their families. Based on data gathered from the children, parents, and therapists in a comparison study, the book covers the effects on children's behavior, fears, PTSD [posttraumatic stress disorder] symptoms, sexuality, achievement, school performance, and self-concept. Moreover, the ways in which the children and their families coped with reports of abuse and the factors associated with positive short- and long-term outcome are detailed. "Behind the Playground Walls" is valuable reading for psychologists, psychiatrists, social workers, pediatricians, teachers, and day care center personnel. Written in an accessible style, it also serves as a text for graduate and advanced undergraduate courses on child abuse, child psychopathology and intervention, children and the law, and family problems. (PsycINFO Database Record (c) 2012 APA, all rights reserved)
AD - Waterman, Jill: U California, Dept of Psychology, Adjunct Professor, Los Angeles, CA, US
ER -

TY - JOUR
ID - 3003
T1 - Aggressive fighting in British middle school children
A1 - Boulton,Michael J.
Y1 - 1993///
N1 - Peer Reviewed Journal: 1993-47243-001
English
Journal; Peer Reviewed Journal
KW - PsycInfo
KW - Aggressive Behavior
KW - Human Sex Differences
KW - Middle School Students
KW - Playgrounds
RP - NOT IN FILE

SP - 19
EP - 39
JF - Educational Studies
VL - 19:
IS - 1
N2 - In Study 1, the time when aggressive fighting involving 86 8- and 11-yr-old children took place was examined by direct playground observations during recess. There was a tendency, significant in the younger group, for more fights in the last quarter of recess. In Study 2, the causes of fights, the sex of the participants, the proportion of fights that were escalated by other children joining in in a nonconciliatory way, and the proportion in which children intervened to stop the fights were investigated. The most common causes of fights were disputes over some aspect of a rule-governed game, teasing, retaliations to accidental hurt/injury, and disputes over space. Most of the fights involved boys only, were not escalated by other children, and did not involve attempts to stop them by other children. In Study 3, many children expressed generally negative attitudes toward fighting. (PsycINFO Database Record (c) 2012 APA, all rights reserved)
SN - 0305-5698
AD - Boulton, Michael J.: Sheffield Hallam U, School of Health & Community Studies Psychology Section, England
ER -

TY - JOUR
ID - 3004
T1 - School recess: Implications for education and development
A1 - Pellegrini,A.
Y1 - 1993///
N1 - Peer Reviewed Journal: 1993-39230-001
English
Journal; Peer Reviewed Journal
KW - PsycInfo
KW - Childhood Play Behavior
KW - Curriculum
KW - Recreation
KW - Student Characteristics
KW - Age Differences
KW - Elementary School Students
KW - Human Sex Differences
KW - Kindergarten Students
KW - Personality
KW - Preschool Students
RP - NOT IN FILE
SP - 51
EP - 67
JF - Review of Educational Research
VL - 63:
IS - 1
N2 - Explores the effects of child-level variables (e.g., gender, age, and temperament) and school-level variables (e.g., recess timing and duration) on children's recess behavior. The implications of recess are discussed in terms of impact on classroom behavior and on measures of social and cognitive competence. Recess has important educational and developmental implications. Timing and duration of recess relate to playground activity, and possibly to classroom behavior, in ways that interact with age, gender, and temperament. (PsycINFO Database Record (c) 2012 APA, all rights reserved)
SN - 0034-6543
AD - Pellegrini, A. D.: U Georgia, Athens, US
ER -

TY - JOUR
ID - 3005
T1 - Social interaction in same and cross gender pre-school peer groups: A participant observation study

A1 - Smith,Anne B.
Y1 - 1993///
N1 - Peer Reviewed Journal: 1993-35419-001
English
Journal; Peer Reviewed Journal
KW - PsycInfo
KW - Childhood Play Behavior
KW - Group Dynamics
KW - Human Sex Differences
KW - Social Interaction
KW - Kindergarten Students
KW - Male Female Relations
KW - Peers
KW - Preschool Students
RP - NOT IN FILE
SP - 29
EP - 42
JF - Educational Psychology
VL - 13:
IS - 1
N2 - Investigated styles of interaction and gender cleavage in children's (N = 64) play groups at the pre-school level. About a third of play groups observed in a part-time and full-time early childhood center were of mixed gender and two-thirds were same gender. Mixed groups were larger than same gender groups and kindergarten (part-time) groups were larger than childcare (full-time) center groups. In the kindergarten, outdoor play was much more common and there was a significant difference in boys preferring to play outdoors, followed by mixed groups, and then girls. In both centers boisterous play was more likely in boys' groups than girls' groups, with mixed groups more similar to boys' groups in the predominance of boisterous play. While there were examples of successful cross-gender co-operative play, there were also examples of the breakdown of reciprocity due to contrasting styles of play. (PsycINFO Database Record (c) 2012 APA, all rights reserved)
SN - 0144-3410
AD - Smith, Anne B.: U Otago, Dunedin, New Zealand
ER -

TY - JOUR
ID - 3006
T1 - Linkages among preschoolers' playground behavior, outcome expectations, and parental disciplinary strategies
A1 - Hart,Craig H.
Y1 - 1992///
N1 - Peer Reviewed Journal: 2000-05448-001
English
Journal; Peer Reviewed Journal
KW - PsycInfo
KW - Child Discipline
KW - Childhood Play Behavior
KW - Expectations
KW - Parental Attitudes
KW - Mothers
KW - Preschool Students
RP - NOT IN FILE
SP - 265
EP - 283
JF - Early Education and Development
VL - 3:
IS - 4
N2 - To explore relationships among parent's self-reported disciplinary strategies, preschoolers' outcome expectations, and playground behavior, 136 mothers of preschool-aged children (aged 39-71 mo) participated in

home disciplinary style interviews. Measures of preschoolers' outcome expectations and observations of children's prosocial, antisocial/disruptive, and nonsocial/withdrawn playground behavior in preschool settings were also obtained. Results indicate that power-assertive mothers had preschoolers who engaged in more antisocial/disruptive behavior and who expected successful instrumental outcomes for hostile methods of resolving peer conflict. Preschoolers with such outcome expectations also participated in more antisocial playground behavior. Older preschoolers of inductive mothers engaged in more prosocial behavior and expected prosocial behavior to lead to both instrumental gains and enhanced relations with peers. Preschoolers who were more prosocial envisioned friendly-assertive strategies as leading to instrumental gains and, in the case of less nonsocial behavior, to enhanced relations with peers. Children's outcome expectations were also found to be different for older vs younger preschoolers. (PsycINFO Database Record (c) 2012 APA, all rights reserved)
SN - 1040-9289
AD - Hart, Craig H.: Brigham Young U, Dept of Family Sciences, Provo, UT, US
ER -

TY - JOUR
ID - 3007
T1 - The existential crisis of the children of Calcutta slums and red light areas
A1 - Deb,Sibnath
Y1 - 1992///
N1 - Peer Reviewed Journal: 1999-00379-001
English
Journal; Peer Reviewed Journal
KW - PsycInfo
KW - Child Abuse
KW - Poverty Areas
RP - NOT IN FILE
SP - 1
EP - 14
JF - Journal of Behavioural Sciences
VL - 3:
IS - 2
N2 - In order to assess the magnitude and types of child abuse among 6-16 yrs children, four slums and two red light areas of Calcutta were purposively selected. 165 slum and 100 red light area households participated in this study. One third of the house holds were below poverty line and half of the head of the households were illiterate. Only 50.2 % of 6-16 yrs children were found attending school. The majority of the children were involved in non-economic activities both inside and outside the house. Children in the red light areas were very often used to entertain their mother's customers by bringing cigarettes and alcoholic drinks. Significantly higher numbers of children in the slums were involved in economic activities than the children in the red light areas. Slum children had to work for 6-10 hrs a day for a meagre earning of Rs. 4-10 per day. Child abuse in terms of deprivation from the right to an education and involvement in various deviant activities was more common in the slums than in the red light areas where girls were more vulnerable of commercialized vice. (PsycINFO Database Record (c) 2012 APA, all rights reserved)
SN - 1028-9097
AD - Deb, Sibnath: Cini-child In Need Inst, Calcutta, India
ER -

TY - JOUR
ID - 3008
T1 - Kindergarten children's social-cognitive status as a predictor of first-grade success
A1 - Pellegrini,Anthony D.
Y1 - 1992///
N1 - Peer Reviewed Journal: 1993-31320-001
English
Journal; Peer Reviewed Journal
KW - PsycInfo
KW - Academic Achievement
KW - Achievement

KW - Adjustment
KW - Preschool Education
KW - Social Interaction
KW - Elementary School Students
KW - Kindergarten Students
KW - Longitudinal Studies
KW - Peer Relations
RP - NOT IN FILE
SP - 565
EP - 577
JF - Early Childhood Research Quarterly
VL - 7:
IS - 4
N2 - Examined the extent to which social-cognitive aspects of children's kindergarten experience predict success in 1st grade. Success was defined in terms of achievement, a personality measure, and behavior with peers. 24 5-yr-olds under adult supervision were observed on their school playground during recess for 2 yrs. 75% of the variance in their 1st-grade achievement was predicted with measures of kindergarten achievement, object play, and peer interaction. Peer interaction was positively related to achievement, whereas adult-directed behaviors were negatively related to achievement. Aspects of 1st-grade children's personality disorders and aggressive behavior were predicted by kindergarten popularity. (PsycINFO Database Record (c) 2012 APA, all rights reserved)
SN - 0885-2006
AD - Pellegrini, Anthony D.: U Georgia, Inst for Behavioral Research, Athens, US
ER -

TY - JOUR
ID - 3009
T1 - The relation between the length of play period and the frequency of reported conflicts by preschool children
A1 - Boisen,Melissa A.
Y1 - 1992///
N1 - Peer Reviewed Journal: 1993-28990-001
English
Journal; Peer Reviewed Journal
KW - PsycInfo
KW - Child Day Care
KW - Childhood Play Behavior
KW - Conflict
KW - Preschool Students
RP - NOT IN FILE
SP - 310
EP - 319
JF - Education & Treatment of Children
VL - 15:
IS - 4
N2 - Investigated the relation between length of play period and the frequency of reported conflicts (RCs) by preschool children. 23 children (aged 2.5-5 yrs) participated in the study, which encompassed 64 days of data collection and was conducted in the natural setting of a day care center. The rate of RCs for the class during 30 min of a 60-min play period was compared to the rate during a 30-min period broken into 2 15-min play periods. When the 30-min play periods were scheduled, the mean numbers of RCs were 4.0 and 6.9, respectively. When 2 15-min play periods were scheduled, the mean numbers of RCs decreased to 1.8 and 1.37, respectively. (PsycINFO Database Record (c) 2012 APA, all rights reserved)
SN - 0748-8491
AD - Boisen, Melissa A.: U Kansas, Lawrence, US
ER -

TY - JOUR

ID - 3010
T1 - Consistency of behaviors within a naturalistic setting: An examination of the impact of context and repeated observations on mother-child interactions
A1 - Pett,Marjorie A.
Y1 - 1992///
N1 - Peer-Reviewed Status-Unknown: 1993-25246-001
English
Journal; Peer-Reviewed Status-Unknown
KW - PsycInfo
KW - Mother Child Relations
KW - Observation Methods
KW - Social Environments
KW - Mothers
RP - NOT IN FILE
SP - 367
EP - 385
JF - Behavioral Assessment
VL - 14:
IS - 3-4
N2 - Examined the impact of context and repeated observations on mother-child interactions with 36 mothers and their preschool children (aged 3-5 yrs). Families were videotaped during both dinner and a structured block play sequence on 2 occasions. A within-Ss repeated measures design examined the impact of context (dinner and blocks) and time (1st and 2nd videotapings) on the observed interactions. Findings indicate that change in context consistently had a stronger impact on the dyad's controlling and supportive behaviors and their reciprocity than did repeated observations. Significant time x context x marital status (divorced vs married) effects were observed for mothers' supportive behaviors. (PsycINFO Database Record (c) 2012 APA, all rights reserved)
SN - 0191-5401
AD - Pett, Marjorie A.: U Utah Coll of Nursing, Salt Lake City, US
ER -

TY - JOUR
ID - 3011
T1 - Participation in playground activities at middle school
A1 - Boulton,Michael J.
Y1 - 1992///
N1 - Peer Reviewed Journal: 1993-25198-001
English
Journal; Peer Reviewed Journal
KW - PsycInfo
KW - Age Differences
KW - Childhood Play Behavior
KW - Human Sex Differences
KW - Social Interaction
KW - Playgrounds
RP - NOT IN FILE
SP - 167
EP - 182
JF - Educational Research
VL - 34:
IS - 3
N2 - Examined the participation of 43 8-yr-old and 43 11-yr-old children (39 girls and 47 boys) in playground activities in Study 1. At both ages, the 2 most common categories of behavior engaged in were sociable activities and rule games. The older Ss spent significantly more time in rule games and significantly less time alone than the younger Ss. Girls spent significantly more time in sociable activities and significantly less time alone than boys. Middle school children's feelings at being excluded by other groups were examined by interview in Study 2. It appeared that the older boys were largely responsible for the lack of mixed age and

mixed sex play on the playground. Molar categories of behavior coded in the study are appended. (PsycINFO Database Record (c) 2012 APA, all rights reserved)
SN - 0013-1881
AD - Boulton, Michael J.: Sheffield City Polytechnic, School of Health & Community Studies, England
ER -

TY - JOUR
ID - 3012
T1 - Playground behaviors of school-age children in relation to nutrition, schooling, and family characteristics. [References]
A1 - Espinosa,Michael P.
Y1 - 1992///
N1 - Peer Reviewed Journal: 1993-09237-001
English
Journal; Peer Reviewed Journal
KW - PsycInfo
KW - Activity Level
KW - Educational Background
KW - Family Background
KW - Nutrition
KW - Social Interaction
KW - Childhood Play Behavior
KW - Emotions
KW - Food Intake
KW - Longitudinal Studies
KW - Playgrounds
KW - Socioeconomic Status
RP - NOT IN FILE
SP - 1188
EP - 1195
JF - Developmental Psychology
JA - Dev Psychol
VL - 28:
IS - 6
N2 - Food intake measured over the course of a year, duration of schooling, and family SES were related to playground behaviors in a sample of 111 Kenyan school-age children. Better nourished children were more active, happy, and showed more leadership behavior, whereas poorly nourished children appeared more anxious on the school playground. Children who had attended less school were more solitary and inactive than children with more school experience. Aggression on the playground was related only to family background. The relations between food intake and activity level persisted even when family education, SES, and school participation were considered. (PsycINFO Database Record (c) 2012 APA, all rights reserved)
SN - 0012-1649
AD - Espinosa, Michael P.: U California, Neuropsychiatric Inst, Los Angeles, US
ER -

TY - JOUR
ID - 3013
T1 - The ecology of parent-child communications about daily experiences in preschool and day care
A1 - Bradbard,Marilyn R.
Y1 - 1992///
N1 - Peer Reviewed Journal: 1993-07484-001
English
Journal; Peer Reviewed Journal
KW - PsycInfo
KW - Child Day Care
KW - Experiences (Events)
KW - Parent Child Communication

KW - Preschool Education
KW - Preschool Students
RP - NOT IN FILE
SP - 131
EP - 141
JF - Journal of Research in Childhood Education
VL - 6:
IS - 2
N2 - 90 parents whose children attended preschool/day care programs completed a 50-item checklist to assess the frequency, content, and context of parent-child communication about school. Parents and children communicated most frequently on weekdays (particularly when the parent picked the child up from school), at meal times, and when the other parent returned home from work. Topics of conversation focused on the child and his/her friends, outside play, learning activities, and special events. Children, particularly girls, often communicated by describing, asking questions, and performing. Mothers initiated conversations most frequently, particularly with younger children, though fathers and children did so quite often. Conversations were most often triggered by daily routines and parental observations of children at school. (PsycINFO Database Record (c) 2012 APA, all rights reserved)
SN - 0256-8543
AD - Bradbard, Marilyn R.: Auburn U, AL, US
ER -

TY - JOUR
ID - 3014
T1 - Rough-and-tumble play and social problem solving flexibility
A1 - Pellegrini,A.
Y1 - 1992///
N1 - Peer Reviewed Journal: 1993-05203-001
English
Journal; Peer Reviewed Journal
KW - PsycInfo
KW - Adaptability (Personality)
KW - Childhood Play Behavior
KW - Human Males
KW - Personality
KW - Problem Solving
KW - Social Interaction
RP - NOT IN FILE
SP - 13
EP - 26
JF - Creativity Research Journal
VL - 5:
IS - 1
N2 - Investigated the relationship between boys" temperament and rough-and-tumble (R&T) play and determined the group composition of R&T, games, and other forms of reciprocal social interaction. The extent to which the vigor and flexibility components of R&T are related to social affiliation and social problem solving flexibility was explored. 13 sociometrically defined popular boys (aged 107-138 mo) were observed on their school playground during recess for 20 sessions. They nominated peers they liked most and least, ordered peers in terms of dominance, and solved hypothetical social problems. Teachers completed temperament questionnaires. R&T groups were similar to other social groupings in terms of number and dominance symmetry. The flexibility dimension of R&T was related to affiliation and social problem solving. Temperament was minimally associated with measures of behavior. (PsycINFO Database Record (c) 2012 APA, all rights reserved)
SN - 1040-0419
AD - Pellegrini, A. D.: U Georgia, Inst for Behavioral Research, Athens, US
ER -

TY - JOUR

ID - 3015
T1 - "Mom lets me go there": The role of environment and supervision in children's minor injuries
A1 - Peterson,Lizette
Y1 - 1991///
N1 - Peer Reviewed Journal: 1993-10246-001
English
Journal; Peer Reviewed Journal
KW - PsycInfo
KW - Environment
KW - Injuries
KW - Permissive Parenting
RP - NOT IN FILE
SP - 15
EP - 23
JF - Children's Environments Quarterly
VL - 8:
IS - 3-4
N2 - Examined descriptions of children's injury types within specific environments, with level of supervision considered explicitly. 61 mothers recorded each injury occurrence and reported bi-weekly on their 8-9 yr old children's minor injuries. Children had relatively few injuries in environments usually thought to involve risk (e.g., bathrooms, stairs); instead, children were injured in environments in which they spent the most time (e.g., their own yard, living room, bedroom). Two exceptions were playgrounds and roadways, which constituted the 2 largest injury categories and which also resulted in the highest proportion of potentially serious injuries. Level of supervision was found to vary by location, with vigilant supervision present in the majority of injuries in the kitchen, playing field, car, and bodies of water. (PsycINFO Database Record (c) 2012 APA, all rights reserved)
SN - 0886-0505
AD - Peterson, Lizette: U Missouri, Columbia, US
ER -

TY - JOUR
ID - 3016
T1 - Ways of increasing of mental efficiency of the deaf pupils. [Russian]
A1 - Bekmuratov,N.
Y1 - 1991///
N1 - Peer-Reviewed Status-Unknown: 1992-87225-001
Russian
Journal; Peer-Reviewed Status-Unknown
KW - PsycInfo
KW - Cognitive Ability
KW - Deaf
KW - Exercise
KW - Special Education
KW - Elementary School Students
KW - Physical Education
RP - NOT IN FILE
SP - 51
EP - 56
JF - Defektologiya
VL - 5 1991, pp. 51-56.
N2 - Studied the effect of a daily program of physical activity on mental performance. Ss were 50 Russian school-age children (deafness) (1st and 5th graders). Control Ss followed the traditional school day. Experimental Ss participated in a program consisting of the following: 10-22 min of morning gymnastics (8-22 exercises), a 15-20-min morning walk outside before classes, and 5-7 min of gymnastics before each class. The diagnostic part of the study was conducted in October, the experimental regimen was conducted throughout the year, and the results were veried in April-May. (PsycINFO Database Record (c) 2012 APA, all rights reserved)
SN - 0130-3074

AD - Bekmuratov, N. Sh.: USSR Academy of Pedagogical Sciences, Scientific Research Inst of Defectology, Moscow, USSR
ER -

TY - JOUR
ID - 3017
T1 - The play preferences of preschool girls and boys: The effects of programme structure and teacher participation
A1 - Swiecicka,Ewa M.
Y1 - 1991///
N1 - Peer Reviewed Journal: 1992-33057-001
English
Journal; Peer Reviewed Journal
KW - PsycInfo
KW - Childhood Play Behavior
KW - Preferences
KW - Preschool Education
KW - Teacher Student Interaction
KW - Classroom Behavior
KW - Preschool Students
RP - NOT IN FILE
SP - 109
EP - 121
JF - Early Child Development and Care
VL - 74 1991, pp. 109-121.
N2 - Examined differences in the play activities of preschool children under conditions of relatively high and low program structure. The Ss were 259 girls and 286 boys (aged 4-5 yrs) from 26 Australian preschools and 72 of their teachers. Results indicate that (1) children in high-structure programs spend more time on what teachers would perceive as "educational" activities; (2) children in high-structure programs are slightly more oriented to adult organized and led activities, while children in low-structure programs are more oriented to self-initiated activities and peer interactions; and (3) children (especially boys) in low-structure programs are more involved in outdoor play, much of a gross-motor kind. (PsycINFO Database Record (c) 2012 APA, all rights reserved)
SN - 0300-4430
AD - Swiecicka, Ewa M.: Children's Services Office, Salisbury, SA, Australia
ER -

TY - JOUR
ID - 3018
T1 - Boredom in the middle school years: Blaming schools versus blaming students
A1 - Larson,Reed W.
Y1 - 1991///
N1 - Peer Reviewed Journal: 1992-14618-001
English
Journal; Peer Reviewed Journal
KW - PsycInfo
KW - Boredom
KW - Contextual Associations
KW - Individual Differences
KW - Middle School Students
KW - Personality Traits
RP - NOT IN FILE
SP - 418
EP - 443
JF - American Journal of Education
VL - 99:
IS - 4

N2 - Used time-sampling data to evaluate experiences of boredom of students in Grades 5-9, both as a state (i.e., related to specific school and nonschool activities) and as a trait (i.e., related to individual dispositions) that students bring to school. 392 Ss carried pagers and reported on their activities and emotions at random times over 1 wk when signaled. While boredom was reported frequently during schoolwork, it was also prevalent outside school, and the same persons report boredom across these contexts. High rates of boredom were correlated with high ability and, when ability was controlled, with oppositional behavior, but not with the onset of adolescence. Data suggest that individual dispositions are an important contributor to boredom. Nonetheless, variations in rates of boredom across classroom situations indicate that schools should be structured to reduce student boredom. (PsycINFO Database Record (c) 2012 APA, all rights reserved)
SN - 0195-6744
AD - Larson, Reed W.: U Illinois, Urbana-Champaign, US
ER -

TY - JOUR
ID - 3019
T1 - Partner preferences in middle school children's playful fighting and chasing: A test of some competing functional hypotheses
A1 - Boulton,Michael J.
Y1 - 1991///
N1 - Peer Reviewed Journal: 1992-01056-001
English
Journal; Peer Reviewed Journal
KW - PsycInfo
KW - Affection
KW - Age Differences
KW - Childhood Play Behavior
KW - Physical Strength
KW - Preferences
RP - NOT IN FILE
SP - 177
EP - 193
JF - Ethology & Sociobiology
VL - 12:
IS - 3
N2 - Observations of 86 middle school children (younger Ss with a mean age of 8.5 yrs and older Ss with a mean age of 11.5 yrs) participating in rough-and-tumble play (r/t) were related to sociometric data for liking and strength. Partners in r/t liked each other more than chance predicted, and tended to be closely matched for strength. Neither initiators of r/t episodes nor recipients were consistently perceived to be the strongest partner. Ss were also found to like their partners in a wide range of non-r/t activities significantly more than chance would predict. Thus children could simply choose to spend time with peers that they like, rather than r/t representing a special case. This view is supported by the finding that Ss had the same partners in a range of playground activities as they had for r/t. (PsycINFO Database Record (c) 2012 APA, all rights reserved)
SN - 0162-3095
AD - Boulton, Michael J.: U Sheffield, England
ER -

TY - JOUR
ID - 3020
T1 - The crisis of acute hospitalization: Case of Seth, age 7
A1 - Wojtasik,Susan
Y1 - 1991///
N1 - Book: 1991-98575-015
English
Book; Edited Book
KW - PsycInfo
KW - Hospitalized Patients
KW - Play Therapy

KW - Spinal Cord Injuries
KW - Case Report
RP - NOT IN FILE
SP - 295
EP - 309
JF - Webb, Nancy Boyd [Ed]
VL - :
IS - 1991
N2 - (from the chapter) [discusses the case of Seth] a 7-year-old boy admitted to a major metropolitan trauma center as a result of a fall from playground equipment / he suffered a life-threatening injury to his spinal cord / Seth was admitted to the pediatric intensive care unit and spent the first 2 months of his hospitalization in this setting / play interventions were begun on the day of his admission and continued on a daily basis throughout his 9-month hospital stay (PsycINFO Database Record (c) 2012 APA, all rights reserved)
AD - Wojtasik, Susan: Bellevue Hosp Ctr, Child Life Dept, Director, New York, NY, US
ER -

TY - JOUR
ID - 3021
T1 - The development of cooperative behaviour in children. [Italian]
A1 - Tani,Franca
Y1 - 1991///
N1 - Peer Reviewed Journal: 1991-78578-001
Italian
Journal; Peer Reviewed Journal
KW - PsycInfo
KW - Cooperation
KW - Psychosocial Development
RP - NOT IN FILE
SP - 45
EP - 53
JF - Eta Evolutiva
VL - 39 Jun 1991, pp. 45-53.
N2 - Studied the development of relational and instrumental cooperation among school-age children. Human Ss: 102 male and female Italian school-age children (aged 6-11 yrs). Ss were observed daily (5 min/day for 2 wks) during recess when contact with the teacher was minimal, and Ss could engage in spontaneous activity. Behavioral interactions were coded on a previously prepared observation grid. The results were evaluated according to each S's sex and grade. Statistical tests were used. (English abstract) (PsycINFO Database Record (c) 2012 APA, all rights reserved)
SN - 0392-0658
AD - Tani, Franca: U degli Studi, Pisa, Italy
ER -

TY - JOUR
ID - 3022
T1 - A comparison of structural and contextual features of middle school children's playful and aggressive fighting
A1 - Boulton,Michael J.
Y1 - 1991///
N1 - Peer Reviewed Journal: 1991-30089-001
English
Journal; Peer Reviewed Journal
KW - PsycInfo
KW - Age Differences
KW - Aggressive Behavior
KW - Childhood Play Behavior
KW - Human Sex Differences
RP - NOT IN FILE

SP - 119
EP - 145
JF - Ethology & Sociobiology
VL - 12:
IS - 2
N2 - Compared features of rough-and-tumble of play (RTP) and aggressive fighting (AF) based on direct playground observations of 2 classes each of 8- and 11-yr-old children. Most bouts of RTP and AF were dyadic, but significantly more bouts of the former involved 3 or more participants. RTP contained more individual action patterns than AF, but the latter was more varied in form. Both wrestling and chasing were seen more in RTP than AF, but restraint in bouts of chasing was not more common in playful than aggressive chasing. Positive and neutral facial expressions were more characteristic of RTP, and negative facial expressions were more characteristic of AF. RTP initiations were more likely to receive an RTP response and less likely to receive an aggressive response than were aggressive initiations. (PsycINFO Database Record (c) 2012 APA, all rights reserved)
SN - 0162-3095
AD - Boulton, Michael J.: U Sheffield, England
ER -

TY - JOUR
ID - 3023
T1 - Development of a Playground Skills Test
A1 - Butcher,Janice
Y1 - 1991///
N1 - Peer Reviewed Journal: 1991-26165-001
English
Journal; Peer Reviewed Journal
KW - PsycInfo
KW - Childhood Play Behavior
KW - Measurement
KW - Test Construction
KW - Statistical Validity
KW - Test Reliability
RP - NOT IN FILE
SP - 259
EP - 266
JF - Perceptual and Motor Skills
VL - 72:
IS - 1
N2 - Developed the 6-item Playground Skills Test, designed to measure elementary-school-age children's motor skill in moving on playground structures. Responses to the test, analyzed for test-retest reliability, objectivity, internal consistency, and content and construct validity, indicate the test is both reliable and valid. Initial normative data are reported for 63 7-9 yr olds. (PsycINFO Database Record (c) 2012 APA, all rights reserved)
SN - 0031-5125
AD - Butcher, Janice: U Manitoba, Faculty of Physical Education & Recreation Studies, Winnipeg, Canada
ER -

TY - JOUR
ID - 3024
T1 - Middle school behavioral profiles of antisocial and at-risk control boys: Descriptive and predictive outcomes
A1 - Walker,Hill M.
Y1 - 1990///
N1 - Peer Reviewed Journal: 1997-72966-001
English
Journal; Peer Reviewed Journal
KW - PsycInfo
KW - Academic Achievement

KW - Academic Failure
KW - Antisocial Behavior
KW - School Adjustment
KW - Middle School Students
RP - NOT IN FILE
SP - 61
EP - 77
JF - Exceptionality
VL - 1:
IS - 1
N2 - Conducted 2 studies on the school behavior adjustment status of 2 groups of boys: an antisocial group (n = 39) and an at-risk control group (n = 41). In Study 1, the authors compared the groups on a series of behavioral measures across Grades 5, 6, and 7 that included (1) teacher ratings of social skills, (2) classroom observations, (3) playground observations, and (4) school archival records. Results indicate extremely problematic behavioral profiles for the antisocial Ss and much more favorable profiles for the at- risk controls. The behavior profiles for the groups appeared to be quite consistent and stable across the middle school years. In Study 2, the authors used a series of selected 5th-grade variables, derived from the 4 major clusters of study measures, as predictors in regression analyses of Ss' status of 7th grade criterion measures of school success or failure. The criterion measures predicted were (1) reading and math achievement, (2) school discipline contacts, (3) attendance, and (4) time spent within a nonregular classroom. There were multiple Rs for reading achievement and time spent in a nonregular classroom. Multiple Rs for math achievement, school discipline contacts, and attendance were moderate to moderately high. (PsycINFO Database Record (c) 2012 APA, all rights reserved)
SN - 0936-2835
AD - Walker, Hill M.: U Oregon, Ctr on Human Development, Eugene, US
ER -

TY - JOUR
ID - 3025
T1 - Differential treatment of students by middle school science teachers: Unintended cultural bias
A1 - Contreras,A.
Y1 - 1990///
N1 - Peer Reviewed Journal: 1992-37078-001
English
Journal; Peer Reviewed Journal
KW - PsycInfo
KW - Minority Groups
KW - Racial and Ethnic Differences
KW - Science Education
KW - Teacher Characteristics
KW - Teacher Student Interaction
KW - Blacks
KW - Middle School Students
KW - Teachers
KW - Whites
RP - NOT IN FILE
SP - 433
EP - 444
JF - Science Education
VL - 74:
IS - 4
N2 - Described differential treatment (DT) of minority students by 2 middle school science teachers (Teacher 1 [White] and Teacher 2 [Black]) and explained patterns of DT within the framework of cultural perspective. Four classes taught by the teachers were examined. Two major aspects of DT were discussed for each teacher: how science content was presented in daily instructional activities and how school policies and classroom practices on citizenship grades operated concerning students' participation in outdoor science activities. Teacher 1 provided learning environments for enriched students (mostly White), but used nonverbal gestures and verbal expressions that led to disengagement in science learning in his regular class (mostly non-White). Teacher 2

treated her 2 classes equitably. Findings suggest significant implications for classroom practices, policy-making, and further research. (PsycINFO Database Record (c) 2012 APA, all rights reserved)
SN - 0036-8326
AD - Contreras, A.: U Los Andes, Nucleo U Rafael Rangel, Trujillo, Venezuela
ER -

TY - JOUR
ID - 3026
T1 - A cross-situational analysis of antisocial behavior exhibited by boys in three settings
A1 - Spira,Deborah A.
Y1 - 1990///
N1 - Dissertation Abstract: 1991-51809-001
English
Dissertation Abstract
KW - PsycInfo
KW - Antisocial Behavior
KW - Childhood Play Behavior
KW - Classroom Environment
KW - Home Environment
KW - Early Intervention
RP - NOT IN FILE
SP - 1524
JF - Dissertation Abstracts International
VL - 51:
IS - 3-B
SN - 0419-4217
AD - Spira, Deborah A.: U Oregon, US
ER -

TY - JOUR
ID - 3027
T1 - Separation anxiety in pre-school children
A1 - Brar,S; Brar,
Y1 - 1990///
N1 - Peer Reviewed Journal: 1991-27470-001
English
Journal; Peer Reviewed Journal
KW - PsycInfo
KW - Childrearing Practices
KW - Separation Anxiety
RP - NOT IN FILE
SP - 13
EP - 17
JF - Journal of Personality and Clinical Studies
VL - 6:
IS - 1
N2 - Explored the effect of child rearing (CR) practices on separation anxiety (SA) in 40 preschool children. The Seguine Form Board Test (G. Arthur, 1947) and an SA rating check list were used to measure Ss' intelligence and SA. CR attitude of their mothers was measured with the Parental Attitude Research Instrument. Less intelligent Ss had more SA, and their mothers scored high on dimensions of CR attitudes like fostering dependency, encouraging verbalization, marital conflicts, exclusion of outside influences, approval of activity, and acceleration of development. (PsycINFO Database Record (c) 2012 APA, all rights reserved)
SN - 0970-1206
AD - Brar, S.: Punjab Agricultural U, Laboratory Nursery School, Ludhiana, India
ER -

TY - JOUR

ID - 3028
T1 - Elementary school children's playground behavior: Implications for children's social-cognitive development
A1 - Pellegrini,A.
Y1 - 1990///
N1 - Peer Reviewed Journal: 1991-22673-001
English
Journal; Peer Reviewed Journal
KW - PsycInfo
KW - Age Differences
KW - Childhood Play Behavior
KW - Human Sex Differences
KW - Playgrounds
KW - Cognitive Development
KW - Elementary School Students
KW - Kindergarten Students
KW - Social Behavior
RP - NOT IN FILE
SP - 8
EP - 16
JF - Children's Environments Quarterly
VL - 7:
IS - 2
N2 - 18 boys and 17 girls in kindergarten, 16 boys and 14 girls in 2nd grade, and 14 boys and 15 girls in 4th grade were observed for 8 mo during their recess periods on the school's playground. The observers used scan-sampling techniques and the children were administered measures of socio-cognitive status. Data indicate that behavior varied by location on the playground and by gender. Further, boys and girls self-selected themselves into different environments to engage in preferred forms of play. Only boys' behavior appear strongly related to measures of both adaptation and maladaptation. Results are discussed in terms of boys' propensity for active, outdoor play, and the usefulness of such behavioral data for gauging children's social-cognitive status. (PsycINFO Database Record (c) 2012 APA, all rights reserved)
SN - 0886-0505
AD - Pellegrini, A. D.: U Georgia, Inst for Behavioral Research, Athens, US
ER -

TY - JOUR
ID - 3029
T1 - Time on educational activities at home and educational progress in infant school
A1 - Plewis,Ian; Mooney,Ann; Creeser,Rosemary
Y1 - 1990///
N1 - Peer Reviewed Journal: 1991-19802-001
English
Journal; Peer Reviewed Journal
KW - PsycInfo
KW - Academic Achievement
KW - Demographic Characteristics
KW - Homework
KW - Parent Child Relations
KW - Blacks
KW - Elementary School Students
KW - Racial and Ethnic Differences
KW - Whites
RP - NOT IN FILE
SP - 330
EP - 337
JF - British Journal of Educational Psychology
JA - Br J Educ Psychol

VL - 60:
IS - 3
N2 - Examined the amount of time 196 6-yr-olds in inner London engage in educational activities at home, and linked these results with earlier research (e.g., B. Tizard et al, 1988) on the association between parental help with reading and children's reading progress. The median child in this sample read aloud at home about 20 min per week, and received about 40 min per week total teaching in reading, writing, and mathematics outside school. Consistent with the results of Tizard et al, Black parents heard their children read more than White parents and girls spent more time with books than boys. The more educational qualifications the mother had, the more time the children spent on all the measured educational activities. (PsycINFO Database Record (c) 2012 APA, all rights reserved)
SN - 0007-0998
AD - Plewis, Ian: U London Inst of Education, Thomas Coram Research Unit, England
ER -

TY - JOUR
ID - 3030
T1 - A comparison of social interactions using a friendship awareness activity
A1 - Fritz,Marie F.
Y1 - 1990///
N1 - Peer Reviewed Journal: 1991-13911-001
English
Journal; Peer Reviewed Journal
KW - PsycInfo
KW - Friendship
KW - Social Interaction
KW - Social Skills Training
KW - Intellectual Development Disorder
KW - Elementary School Students
RP - NOT IN FILE
SP - 352
EP - 359
JF - Education & Training in Mental Retardation
VL - 25:
IS - 4
N2 - Determined the effectiveness of a friendship awareness activity as an unstructured intervention strategy for affecting the frequency and type of social interactions between the students with mental retardation and their nonhandicapped peers. Four 8-20 yr old students with disabilities were investigated using a modified multiple baseline research design. Observational data were collected using a time sampling technique during recess and lunch periods. The friendship awareness activity alone was not enough to produce changes in social interaction between students with and without disabilities. When interactions between disabled and nondisabled peers did occur, they were brief. (PsycINFO Database Record (c) 2012 APA, all rights reserved)
SN - 1042-9859
AD - Fritz, Marie F.: Indiana State U, School of Education, Terre Haute, US
ER -

TY - JOUR
ID - 3032
T1 - Children's PTSD reactions one year after a sniper attack at their school
A1 - Nader,Kathi; Pynoos,Robert; Fairbanks,Lynn; Frederick,Calvin
Y1 - 1990///
N1 - Peer Reviewed Journal: 1991-07485-001
English
Journal; Peer Reviewed Journal
KW - PsycInfo
KW - Grief
KW - Posttraumatic Stress Disorder
KW - Violence

RP - NOT IN FILE
SP - 1526
EP - 1530
JF - The American Journal of Psychiatry
VL - 147:
IS - 11
N2 - Explored the impact of a sniper attack on elementary school children: 19 Ss who were on the playground during the attack, 21 Ss who were in school but not on the playground, 28 Ss absent from school that day, and 32 Ss who were on a 3-wk vacation from the year-round school. A children's grief inventory was used to examine the relation between Ss grief reactions and their posttraumatic stress reaction. 14 mo after the attack, level of exposure to that event remained the primary predictor of ongoing posttraumatic stress disorder (PTSD) in the Ss. Guilt feelings and knowing the child who was killed were associated with a greater number of symptoms. Grief reactions occurred independent of degree of exposure to the event. (PsycINFO Database Record (c) 2012 APA, all rights reserved)
SN - 0002-953X
AD - Nader, Kathi: U California Neuropsychiatric Inst, Los Angeles, US
ER -

TY - JOUR
ID - 3033
T1 - Preschoolers' behavioral orientations and patterns of peer contact: Predictive of peer status?
A1 - Ladd,Gary W.
Y1 - 1990///
N1 - Book: 1990-97775-003
English
Book; Edited Book
KW - PsycInfo
KW - Peer Relations
KW - Popularity
KW - Social Behavior
KW - Interpersonal Interaction
KW - Longitudinal Studies
KW - Playgrounds
KW - Preschool Students
RP - NOT IN FILE
SP - 90
EP - 115
JF - Asher, Steven R [Ed]; Coie, John D [Ed]
VL - :
IS - 1990
N2 - (from the introduction) show the benefits of focusing on the playground setting and also show the benefits of studying [preschool] children longitudinally within the school year their research indicates that popular and unpopular children differ not only in the content of their social behavior but in how they distribute their interaction among different group members across the school year (from the chapter) specifically, the stability of children's behavioral orientations and contact patterns is examined over the course of a school year, and relationships between these measures and children's classroom peer status are explored in terms of both change over time and direction of effect / findings concerning temporal trends and sex differences in the interpersonal context of children's interactions are presented / conclude with a discussion of the implications of these findings for intervention research with low-status children (PsycINFO Database Record (c) 2012 APA, all rights reserved)
AD - Ladd, Gary W.: U Illinois Urbana-Champaign, Dept of Educational Psychology, Urbana, IL, US
ER -

TY - JOUR
ID - 3034
T1 - Physically abused preschoolers' responses to peers' distress. [References]
A1 - Klimes-Dougan,Bonnie; Kistner,Janet

Y1 - 1990///
N1 - Peer Reviewed Journal: 1990-28546-001
English
Journal; Peer Reviewed Journal
KW - PsycInfo
KW - Child Abuse
KW - Distress
KW - Emotional Responses
KW - Peers
RP - NOT IN FILE
SP - 599
EP - 602
JF - Developmental Psychology
JA - Dev Psychol
VL - 26:
IS - 4
N2 - Response to peers' distress for which they were (target-caused) or were not (bystander) the cause were coded for 11 abused and nonabused preschoolers between the ages of 35 and 67 months. Each child was observed on a playground for an average of 115 min. Consistent with results of research with toddlers, abused preschoolers exhibited more inappropriate responses (aggression and withdrawal) toward distressed peers (bystander incidents) than did nonabused preschoolers. Also, abused children were more likely to cause distress of peers. These findings suggest that even among abused children who have had opportunities to interact with nonabusive caregivers and peers (i.e., regularly attended day care), differences in responses to peers' distress are obtained. (PsycINFO Database Record (c) 2012 APA, all rights reserved)
SN - 0012-1649
AD - Klimes-Dougan, Bonnie: Florida State U, US
ER -

TY - JOUR
ID - 3035
T1 - Interracial friendship in a Zimbabwean primary school
A1 - Wilson,David
Y1 - 1990///
N1 - Peer Reviewed Journal: 1990-22250-001
English
Journal; Peer Reviewed Journal
KW - PsycInfo
KW - Blacks
KW - Friendship
KW - Racial and Ethnic Relations
KW - Whites
RP - NOT IN FILE
SP - 111
EP - 113
JF - The Journal of Social Psychology
VL - 130:
IS - 1
N2 - A study of 94 Black and 110 White primary school children (aged 7-14 yrs) in Zimbabwe did not show a gradation of in-group friendship (IGF) patterns as Ss moved from classroom companions to playmates in school and outside school. These findings were contrary to those obtained by J. Davies and I. F. Turner (see record 1985-14144-001) in the UK. In general, there were no significant IGF patterns for Blacks and Whites. (PsycINFO Database Record (c) 2012 APA, all rights reserved)
SN - 0022-4545
AD - Wilson, David: U Zimbabwe, Harare, Zimbabwe
ER -

TY - JOUR

ID - 3036
T1 - A review of research on playgrounds
A1 - Lloyd,Eva; Melhuish,Edward; Moss,Peter; Owen,Charlie
Y1 - 1989///
N1 - Peer Reviewed Journal: 1998-11208-008
English
Journal; Peer Reviewed Journal
KW - PsycInfo
KW - Childhood Play Behavior
KW - Childhood Play Development
KW - Parent Child Relations
KW - Demographic Characteristics
KW - Social Interaction
RP - NOT IN FILE
SP - 77
EP - 99
JF - Early Child Development and Care
VL - 43 1989, pp. 77-99.
N2 - This review researches on pre-school playgroups in the UK. For this review, we located some 60 references providing research findings or statistical information on playgroups in the UK. The research areas covered in this paper include: geographical distribution of playgroups; the family background; concept and practices of parental involvement; and the experiences of children in playgroups. In conclusion, playgroups provide for more children than any other form of pre-school service: most children who use playgroups attend for 2 or 3 half-day sessions a week. Registered playgroups are varied in many respects. A majority involve parents in management, although many do not conform to the model of a "parent run" service. Also, there are large variations in the geographical distribution of playgroups. Provision is highest in the South and in rural and suburban areas. There is however a strong inverse relationship between levels of playgroups and nursery education provision. (PsycINFO Database Record (c) 2012 APA, all rights reserved)
SN - 0300-4430
AD - Lloyd, Eva: U London, Inst of Education, Thomas Coram Research Unit, London, England
ER -

TY - JOUR
ID - 3037
T1 - A study of classroom scripts. [French]
A1 - Loranger,Michel; Arsenault,Rodolphe; Plante,Chantal
Y1 - 1989///
N1 - Peer Reviewed Journal: 1991-71914-001
French
Journal; Peer Reviewed Journal
KW - PsycInfo
KW - Classroom Behavior
KW - Classroom Environment
KW - Elementary School Students
KW - High School Students
RP - NOT IN FILE
SP - 277
EP - 294
JF - Canadian Journal of Education
VL - 14:
IS - 3
N2 - Studied the structural features of classroom scripts of elementary and secondary school students. Human subjects: 64 male and female schoolage children and adults (4th- and 6th-grade primary school students and 2nd- and 5th-level secondary school students). Ss were interviewed individually and asked to relate how they would explain what happens in a classroom setting to a child who has never been to school. Transcripts of the conversations were analyzed using the grill technique of G. H. Bower et al (1979). The numbers and types of goals, activities/events inside and outside the classroom, student and teacher roles, and school accessories

identified by the Ss were evaluated according to sex and class level. Scripts for entering a classroom, working in a classroom, and leaving a classroom; and scripts for recess were also determined. Statistical tests were used. (English abstract) (PsycINFO Database Record (c) 2012 APA, all rights reserved)
SN - 0380-2361
AD - Loranger, Michel: U Laval, Quebec, Canada
ER -

TY - JOUR
ID - 3038
T1 - Foursquare: A glossary and "native" taxonomy of game rules
A1 - Hughes,Linda A.
Y1 - 1989///
N1 - Peer-Reviewed Status-Unknown: 1991-09635-001
English
Journal; Peer-Reviewed Status-Unknown
KW - PsycInfo
KW - Childrens Recreational Games
KW - Human Females
KW - Taxonomies
KW - Longitudinal Studies
RP - NOT IN FILE
SP - 103
EP - 136
JF - Play & Culture
VL - 2:
IS - 2
N2 - Observed 27 female and 12 male 4th and 5th graders who regularly played the game Foursquare (a ball-bouncing game) at recess over 2 yrs. A subsample of 10 female players was interviewed. Analysis focused on (1) differential use of and appeal to rules at different junctures in the game and(2) the relationship between stated rules and observed action in the game. Four interlocking criteria consistently arose in Ss' discussions of similarities and differences among rules: (1) what kinds of actions rules governed, (2) how rules governed those actions, (3) whether rules were supposed to be changed, and (4) the primary purpose of the actions rules governed. A taxonomy of the Ss' rules is elaborated. (PsycINFO Database Record (c) 2012 APA, all rights reserved)
SN - 0894-4253
AD - Hughes, Linda A.: U Delaware Coll of Education, Newark, US
ER -

TY - JOUR
ID - 3039
T1 - "Taking care": Maintaining the self and the home in early adolescence
A1 - Duckett,Elena; Raffaelli,Marcela; Richards,Maryse
Y1 - 1989///
N1 - Peer Reviewed Journal: 1990-19503-001
English
Journal; Peer Reviewed Journal
KW - PsycInfo
KW - Experiences (Events)
KW - Household Management
KW - Psychosocial Development
RP - NOT IN FILE
SP - 549
EP - 565
JF - Journal of Youth and Adolescence
VL - 18:
IS - 6

N2 - Explored 401 5th-9th graders' experiences of the basic daily tasks of personal care (e.g., grooming, eating) and household maintenance (e.g., chores, errands), using the experience sampling method (M. Csikszentmihalyi and R. Larson; see record 1988-03173-001). Quantity of time, companionship, and subjective states in these activities were examined during 1 week. These tasks were particularly subject to sex typing. Girls engaged in grooming more often than boys; time spent grooming increased with age for girls but not for boys. Boys did more outside chores, while girls did more indoor chores. Girls were also more likely to carry out household tasks with family, while boys did more chores alone. Older girls reported a greater sense of motivation and choice than younger girls. (PsycINFO Database Record (c) 2012 APA, all rights reserved)
SN - 0047-2891
AD - Duckett, Elena: Loyola U, Chicago, IL, US
ER -

TY - JOUR
ID - 3040
T1 - Environmental education, children, and animals
A1 - Margadant-Van Arcken,Marjan
Y1 - 1989///
N1 - Peer Reviewed Journal: 1990-07126-001
English
Journal; Peer Reviewed Journal
KW - PsycInfo
KW - Child Attitudes
KW - Environmental Attitudes
KW - Pets
KW - Animals
RP - NOT IN FILE
SP - 14
EP - 19
JF - Anthrozoos
VL - 3:
IS - 1
N2 - 26 open interviews were conducted with about 250 kindergarten children (aged 6-12 yrs) who were asked about their pet animals, experiences with wild animals, and activities in natural environments. Results are described in light of the way in which children try to form a consistent concept of their world, using a synthetical way of thinking. The development of child-animal relationships is discussed as following a pattern in which the child moves from a somewhat fearful initial contact to a trusting relationship in which a fusion of horizons occurs. Letting children play freely with animals is strongly recommended if environmental education is to transfer awareness and knowledge. (PsycINFO Database Record (c) 2012 APA, all rights reserved)
SN - 0892-7936
ER -

TY - JOUR
ID - 3041
T1 - Children's attitudes toward play: An investigation of their context specificity and relationship to organized sport experiences
A1 - Greer,Donald L.
Y1 - 1989///
N1 - Peer Reviewed Journal: 1990-04234-001
English
Journal; Peer Reviewed Journal
KW - PsycInfo
KW - Child Attitudes
KW - Childhood Play Behavior
KW - Experience Level
KW - Sports
KW - Sports (Attitudes Toward)
RP - NOT IN FILE

SP - 336
EP - 342
JF - Journal of Sport & Exercise Psychology
JA - J Sport Exerc Psychol
VL - 11:
IS - 3
N2 - 585 4th and 5th graders completed a questionnaire on attitudes toward play (ATP) in 3 contexts (neighborhood play, recess at school, and organized youth sports). The relationship between the amount of experience in organized sports and ATP was also examined. Results are consistent with the conclusion that treating ATP as a generalized disposition may be a questionable practice with regard to developing an understanding of attitudinal transformations in response to childhood play experiences. Ss reported more professionalized ATP in some social contexts than in others, and many Ss reported that winning was more important in organized sports than in neighborhood play. (PsycINFO Database Record (c) 2012 APA, all rights reserved)
SN - 0895-2779
AD - Greer, Donald L.: U Nebraska School of Health, Physical Education & Recreation, Omaha, US
ER -

TY - JOUR
ID - 3042
T1 - Elementary school children's rough-and-tumble play
A1 - Pellegrini,A.
Y1 - 1989///
N1 - Peer Reviewed Journal: 1990-02917-001
English
Journal; Peer Reviewed Journal
KW - PsycInfo
KW - Age Differences
KW - Aggressive Behavior
KW - Childhood Play Behavior
KW - Elementary School Students
KW - Kindergarten Students
RP - NOT IN FILE
SP - 245
EP - 260
JF - Early Childhood Research Quarterly
VL - 4:
IS - 2
N2 - Four observers rated 48 male and 46 female kindergartners', 2nd graders', and 4th graders' rough-and-tumble play (RTP) with respect to aggression over an 8-mo period. Ss were observed during playground school recess periods. Ss' sociometric status, social problem solving, antisocial behavior, and ability to discriminate between RTP and aggression were measured. The incidence of RTP varied according to sex and playground location; RTP accounted for 11% of play behaviors. Boys engaged in significantly more RTP than did girls. Aggression rarely occurred and did not vary by age, sex, or playground location. RTP led to games with rules and was positively correlated, for boys, with social competence. Girls' RTP was positively related to antisocial behavior. (PsycINFO Database Record (c) 2012 APA, all rights reserved)
SN - 0885-2006
AD - Pellegrini, A. D.: U Georgia Inst for Behavioral Research, Athens, US
ER -

TY - JOUR
ID - 3043
T1 - Biosocial bases of representational activity during early childhood
A1 - Strayer,Floyd Francis; Moss,Ellen; Blicharski,Teresa
Y1 - 1989///

N1 - Book: 1989-98706-002
English
Book; Edited Book
KW - PsycInfo
KW - Cognitive Development
KW - Psychosocial Development
KW - Social Interaction
KW - Child Day Care
KW - Home Environment
RP - NOT IN FILE
SP - 21
EP - 44
JF - Winegar, Lucien T [Ed]
VL - :
IS - 1989
N2 - (from the book) reports and discusses the implications of a study examining . . . home-reared and day-care children (from the chapter) instead of emphasizing the fundamental role of inherent intellectual abilities, a biosocial model of cognitive growth places primary emphasis upon variations in social contexts that orient individual growth and produce phenotypic variability in children's representational activity examined how the quality of primary bonding and the diversity of early social relations outside of the family setting contribute to the child's use of specific representational tactics in common, everyday problem-solving situations / research focused upon problem solving during structured dyadic play at home with mother / Attachment Q-Sort Questionaire / was completed by 93 middle-class, French-speaking mothers within a few weeks of their child's second birthday (PsycINFO Database Record (c) 2012 APA, all rights reserved)
AD - Strayer, Floyd Francis: Lab of Human Ethology, Montreal, PQ, Canada
ER -

TY - JOUR
ID - 3044
T1 - Children's social networks and social supports
A1 - Belle,Deborah
Y1 - 1989///
N1 - Book: 1989-97450-000
English
Book; Edited Book
(Created by PsycINFO) Introduction: Studying children's social networks and social supports [by] Deborah Belle Part 1 Children's social networks and supports in context * Historical changes in children's networks in the family and community / Tamara K. Hareven / 15-36 * The ecology of children's social support networks / Anne Marie Tietjen / 37-69 * Cultural and universal aspects of social support for children: Evidence from the Abaluyia of Kenya / Thomas S. Weisner / 70-90 * Work, play, and social relationships among children in a Giriama community / Martha Wenger / 91-115 Part 2 Characteristics of children's social networks * The social networks of girls and boys from early through middle childhood / Candice Feiring and Michael Lewis / 119-150 * The development of children's social networks / Wyndol Furman / 151-172 * Gender differences in children's social networks and supports / Deborah Belle / 173-188 Part 3 Measurement issues * Mapping children's support networks: Conceptual and methodological issues / Sharlene A. Wolchik, Janette Beals and Irwin N. Sandler / 191-220 * Parents and children as informants concerning children's social networks / Phyllis Zelkowitz / 221-237 Part 4 Building supportive networks * Parents as managers of children's peer relationships / Ross D. Parke and Navaz P. Bhavnagri / 241-259 * The school-nonschool ecology of early adolescent friendships / Barton J. Hirsch and David L. Dubois / 260-274 Implications of supportive involvements * Social support as a protective factor for children in stress / Irwin N. Sandler, Paul Miller, Jerome Short and Sharlene A. Wolchik / 277-307 * Obtaining support from friends during childhood and adolescence / Thomas J. Berndt / 308-331 * The need for support in relation to the need for autonomy / Brenda K. Bryant / 332-351 Author index Subject index
KW - PsycInfo
KW - Childhood Development
KW - Social Support
KW - Sibling Relations

KW - Social Interaction
RP - NOT IN FILE
SP - Oxford, England
JF - (1989)
VL - Children's social networks and social supports. x, 373 pp. Oxford, England: John Wiley & Sons; England.
N2 - (from the preface) The book attempts to synthesize this new knowledge [current theorizing and research on children's networks and sources of support], to suggest implications from this research for supportive interventions for children, and to raise new questions and provide new tools for further study. The volume takes an inclusive view of childhood, with some chapters focusing on a particular developmental period and others spanning the entire age range from infancy to adolescence. The book also takes an inclusive view of social networks, including under this heading individuals both inside and outside the child's household, institutional connections, and even pets. The book is meant for researchers, clinicians, and those with public-policy concerns about the well-being of children, as well as for students of these topics. (PsycINFO Database Record (c) 2012 APA, all rights reserved)
AD - Belle, Deborah: Boston U, Dept of Psychology, Assistant Professor, Boston, MA, US
ER -

TY - JOUR
ID - 3045
T1 - Children's social problem solving: Comparison of the Open Middle Interview and Children's Assertive Behavior Scale
A1 - Waas,Gregory A.
Y1 - 1989///
N1 - Peer-Reviewed Status-Unknown: 1989-38679-001
English
Journal; Peer-Reviewed Status-Unknown
KW - PsycInfo
KW - Interviews
KW - Problem Solving
KW - Rating Scales
KW - Social Skills
KW - Test Validity
KW - Assertiveness
KW - Childhood Play Behavior
KW - Peer Relations
RP - NOT IN FILE
SP - 219
EP - 230
JF - Behavioral Assessment
VL - 11:
IS - 2
N2 - The Open Middle Interview (OMI) and the Children's Assertive Behavior Scale-Self-Report were compared for their ability to discriminate among 56 4th-grade boys known to differ in social competence, to predict behavior in a naturalistic setting, and to correlate with peer ratings of likability. Although both procedures measure similar constructs, significant differences were found. Each scale differentiated between socially rejected and popular boys. Only the OMI, however, consistently correlated with observed playground behavior. Neither measure was consistently related to peer ratings. (PsycINFO Database Record (c) 2012 APA, all rights reserved)
SN - 0191-5401
AD - Waas, Gregory A.: Northern Illinois U, De Kalb, US
ER -

TY - JOUR
ID - 3046
T1 - Children's naturalistic entry behavior and sociometric status: A developmental perspective. [References]
A1 - Putallaz,Martha
Y1 - 1989///

N1 - Peer Reviewed Journal: 1989-22052-001
English
Journal; Peer Reviewed Journal
KW - PsycInfo
KW - Childhood Play Development
KW - Group Dynamics
KW - Peer Relations
KW - Popularity
KW - Social Skills
KW - Age Differences
KW - Psychosocial Development
RP - NOT IN FILE
SP - 297
EP - 305
JF - Developmental Psychology
JA - Dev Psychol
VL - 25:
IS - 2
N2 - Extended previous research by examining the relation between children's entry behavior and sociometric status under more naturalistic conditions. First, 3rd, and 5th graders (N = 72) of high, low, and average status were observed during recess. Observers coded Ss' entry and noninteractive behaviors, peers' responses, and the size of groups with whom Ss interacted. Low-status Ss engaged in more passive entry attempts and less sustained group interaction than high-status Ss, and were accepted less and ignored more. Compared with 5th graders, 1st graders attempted entry proportionately more, were alone more, joined smaller groups, and sustained interaction with groups a smaller proportion of time. Group size influenced both the choice of entry bids and the group's response to an entering peer. (PsycINFO Database Record (c) 2012 APA, all rights reserved)
SN - 0012-1649
AD - Putallaz, Martha: Duke U, Durham, NC, US
ER -

TY - JOUR
ID - 3047
T1 - The displacement effect: Assessing the relation between television viewing and reading performance
A1 - Neuman,Susan B.
Y1 - 1988///
N1 - Peer Reviewed Journal: 1989-11416-001
English
Journal; Peer Reviewed Journal
KW - PsycInfo
KW - Leisure Time
KW - Reading Skills
KW - Television Viewing
KW - Intellectual Development
RP - NOT IN FILE
SP - 414
EP - 440
JF - Reading Research Quarterly
VL - 23:
IS - 4
N2 - A synthesis of 8 statewide reading assessments and a secondary analysis of the 1984 National Assessment of Educational Progress, which included measures of scholastic achievement, out-of-school activities, and reported TV viewing exposure, were used to analyze 4 theories of how TV viewing displaces other activities in school-age children. Results indicate that differences in reading scores for Ss watching 2-4 hrs/day were small beyond 4 hrs of viewing, the effects were negative and increasingly deleterious. Analysis of outside activities suggested that TV viewing was tied to a different set of needs and gratifications than leisure reading, sports, or spending time with friends. For most Ss, however, time spent on TV viewing was tempered by the increasing

demands of schooling and the onset of social activities. (French, Spanish & German abstracts) (PsycINFO Database Record (c) 2012 APA, all rights reserved)
SN - 0034-0553
AD - Neuman, Susan B.: U Lowell, Coll of Education, MA, US
ER -

TY - JOUR
ID - 3048
T1 - The structure of politically relevant behaviours in pre-school peer groups
A1 - Barner-Barry,Carol
Y1 - 1988///
N1 - Book: 1989-97415-011
English
Book; Edited Book
KW - PsycInfo
KW - Group Cohesion
KW - Political Socialization
KW - Leadership
RP - NOT IN FILE
SP - 261
EP - 276
JF - Chance, Michael R
VL - A [Ed]; Omark, Donald R [Ed]. :
IS - 1988
N2 - (from the chapter) group cohesion / within any naturally occurring group that remains relatively stable over a significant period of time, certain functions must be performed that can be regarded as "governance functions" / a leader . . . in these functional areas might then be regarded as one who is, in fact, governing the group / this analysis will focus on such behaviours, using terminology and some general conceptual frameworks borrowed from the discipline of Political Science [reported study results of] 38 children attending a 4-week summer playground programme / three-and-a-half to six-and-a-half years / 20 children who were attending nursery school (PsycINFO Database Record (c) 2012 APA, all rights reserved)
AD - Barner-Barry, Carol: U of Maryland, Dept of Political Science, Catonsville, MD, US
ER -

TY - JOUR
ID - 3049
T1 - Friends' and classmates' interactions on academic tasks. [References]
A1 - Berndt,Thomas J.
Y1 - 1988///
N1 - Peer Reviewed Journal: 1989-17208-001
English
Journal; Peer Reviewed Journal
KW - PsycInfo
KW - Academic Achievement
KW - Classmates
KW - Cooperation
KW - Friendship
KW - Peer Relations
KW - Elementary School Students
KW - Junior High School Students
RP - NOT IN FILE
SP - 506
EP - 513
JF - Journal of Educational Psychology
VL - 80:
IS - 4

N2 - As they worked on two academic tasks, pairs of friends and pairs of other classmates were observed to determine the effects of existing social relationships on interactions during cooperative learning. A total of 130 third graders and seventh graders were paired either with a close friend or with another classmate who was not a close friend. Each pair of students was videotaped, first, as they discussed topics from social studies texts and, second, as they took turns asking each other questions about the topics. Measures of the content of the students' conversations and of interactional processes (e.g., agreements and disagreements) showed no significant differences between friends' and other classmates' interactions. On a posttask questionnaire, however, the pairs of friends said that they engaged in more academic and nonacademic activities outside the experimental setting than did the pairs of other classmates. The implications of the results for the assignment of students to cooperative learning groups are discussed. (PsycINFO Database Record (c) 2012 APA, all rights reserved)
SN - 0022-0663
AD - Berndt, Thomas J.: Purdue U, West Lafayette, IN, US
ER -

TY - JOUR
ID - 3050
T1 - Growth in reading and how children spend their time outside of school
A1 - Anderson,Richard C.
Y1 - 1988///
N1 - Peer Reviewed Journal: 1989-13521-001
English
Journal; Peer Reviewed Journal
KW - PsycInfo
KW - Leisure Time
KW - Reading Achievement
KW - Elementary School Students
KW - Reading Ability
RP - NOT IN FILE
SP - 285
EP - 303
JF - Reading Research Quarterly
VL - 23:
IS - 3
N2 - 155 5th-graders wrote down every day on activity forms how many minutes they spent on a wide range of out-of-school activities. Forms were completed for periods of 8-26 wks. The distribution of times for most activities was positively skewed. Among all the ways Ss spent their time, reading books was the best predictor of measures of reading achievement, including gains in reading achievement between 2nd and 5th grade. However, on most days most Ss did little or no book reading. (French, Spanish & German abstracts) (PsycINFO Database Record (c) 2012 APA, all rights reserved)
SN - 0034-0553
AD - Anderson, Richard C.: U Illinois, Ctr for the Study of Reading, Urbana-Champaign, US
ER -

TY - JOUR
ID - 3051
T1 - Elementary-school children's rough-and-tumble play and social competence. [References]
A1 - Pellegrini,A.
Y1 - 1988///
N1 - Peer Reviewed Journal: 1989-08147-001
English
Journal; Peer Reviewed Journal
KW - PsycInfo
KW - Aggressive Behavior
KW - Childhood Play Behavior
KW - Social Skills
RP - NOT IN FILE
SP - 802

EP - 806
JF - Developmental Psychology
JA - Dev Psychol
VL - 24:
IS - 6
N2 - The intent of this study was to describe relations between elementary-school children's rough-and-tumble play and their social competence. Elementary-school children (Grades K, 2, and 4) were observed on the school playground during their recess periods. Results suggested that rough-and-tumble play for popular children led to games-with-rules, whereas it led to aggression for rejected children. Furthermore, popular children's rough-and-tumble was positively correlated with measures of social competence. These results are discussed in terms of the possible effects of the sociometric composition of rough-and-tumble play groups. (PsycINFO Database Record (c) 2012 APA, all rights reserved)
SN - 0012-1649
AD - Pellegrini, A. D.: U Georgia, Inst for Behavioral Research, Athens, US
ER -

TY - JOUR
ID - 3052
T1 - The relationship of day care quality to children's free-play behavior and social problem-solving skills
A1 - Holloway,Susan D.
Y1 - 1988///
N1 - Peer Reviewed Journal: 1989-08106-001
English
Journal; Peer Reviewed Journal
KW - PsycInfo
KW - Child Day Care
KW - Childhood Play Behavior
KW - Problem Solving
KW - Quality of Care
KW - Social Skills
RP - NOT IN FILE
SP - 39
EP - 53
JF - Early Childhood Research Quarterly
VL - 3:
IS - 1
N2 - Examined the relationship of daycare quality to the activities of 55 4-yr-olds during free play and their knowledge of social problem solving. Indicators of daycare quality included quality of interaction with teachers, arrangement of the physical space, spaciousness of the environment, appropriateness and variety of the materials provided for play, quality of the outdoor area, group size, and child-teacher ratio. Relationships were found between these dimensions and Ss' absorption in solitary play and knowledge of social problem solving. Most relationships held up after the effects of social class were statistically removed. (PsycINFO Database Record (c) 2012 APA, all rights reserved)
SN - 0885-2006
AD - Holloway, Susan D.: U Maryland, Coll of Education, College Park, US
ER -

TY - JOUR
ID - 3053
T1 - Predicting preschoolers' peer status from their playground behaviors
A1 - Ladd,Gary W.
Y1 - 1988///
N1 - Peer Reviewed Journal: 1989-04702-001
English
Journal; Peer Reviewed Journal
KW - PsycInfo
KW - Childhood Play Behavior

KW - Peer Relations
KW - Status
KW - Longitudinal Studies
RP - NOT IN FILE
SP - 986
EP - 992
JF - Child Development
JA - Child Dev
VL - 59:
IS - 4
N2 - Investigated the relation between preschoolers' peer behavior and status over a school year by exploring whether early behaviors predicted changes in peer status or vice versa. 28 White preschoolers' playground behaviors and peer status were assessed at 3 times during the school year (fall, winter, and spring). Analysis of the behavioral antecedents of status produced some findings that were consistent with those reported for grade school samples (e.g., J. D. Coie and J. Kupersmidt; see record 1984-11902-001). Higher levels of cooperative play at the outset of the school year predicted gains in peer acceptance by the end of the year. Arguing forecasted increases in peer rejection at both the middle and end of the school year. In contrast, early peer status was not predictive of changes in preschoolers' later social or nonsocial behavior. (PsycINFO Database Record (c) 2012 APA, all rights reserved)
SN - 0009-3920
AD - Ladd, Gary W.: Purdue U, West Lafayette, IN, US
ER -

TY - JOUR
ID - 3054
T1 - Differentiation of fantasy and reality: Preschoolers' reactions to interruptions in their play. [References]
A1 - DiLalla,Lisabeth F.
Y1 - 1988///
N1 - Peer Reviewed Journal: 1988-26105-001
English
Journal; Peer Reviewed Journal
KW - PsycInfo
KW - Childhood Play Development
KW - Cognitive Development
KW - Imagination
KW - Reality Testing
KW - Age Differences
RP - NOT IN FILE
SP - 286
EP - 291
JF - Developmental Psychology
JA - Dev Psychol
VL - 24:
IS - 2
N2 - In a study of the development of the differentiation of fantasy from reality, it was hypothesized that preschool children would progress through a stepwise sequence of developing ever clearer boundaries between fantasy and reality. Evidence for this development was based on children's reactions to various kinds of interruptions to their fantasy play that were initiated by an experimenter as she engaged in role playing with each of 39 children. The children were 21/2 to 61/2 years of age. The results indicated that as age increased, children showed higher steps in the sequence, until at 5 and 6 years they could incorporate an interruption into the fantasy scenario without stepping outside the fantasy and without having the interruption upset them. (PsycINFO Database Record (c) 2012 APA, all rights reserved)
SN - 0012-1649
AD - DiLalla, Lisabeth F.: U Colorado, Inst for Behavioral Genetics, US
ER -

TY - JOUR

ID - 3055
T1 - A longitudinal assessment of the development of antisocial behavior in boys: Rationale, methodology, and first-year results
A1 - Walker,Hill M.
Y1 - 1987///
N1 - Peer Reviewed Journal: 1988-04733-001
English
Journal; Peer Reviewed Journal
KW - PsycInfo
KW - Academic Achievement Motivation
KW - Antisocial Behavior
KW - At Risk Populations
KW - Interpersonal Interaction
KW - Social Skills
KW - Child Discipline
KW - Teacher Student Interaction
RP - NOT IN FILE
SP - 7
EP - 16
JF - RASE: Remedial & Special Education
VL - 8:
IS - 4
N2 - Conducted a 5-yr longitudinal study of the development of antisocial behaviors in 80 high-risk 5th-grade boys. Analyzed were selection criteria, demographic data, and measures related to academics and social behavior. Results from the 1st yr of the study regarding 35 Ss of Cohort I indicate that Ss in the group more at risk for engaging in antisocial behavior (n = 16) were significantly different from Ss in the control group (n = 19) in their amounts of academic engaged time, frequencies of negative interactions with peers in playground situations, frequency of discipline contacts, and in teacher ratings of their social skills. (PsycINFO Database Record (c) 2012 APA, all rights reserved)
SN - 0741-9325
AD - Walker, Hill M.: U Oregon Coll of Education, Div of Special Education & Rehabilitation
ER -

TY - JOUR
ID - 3056
T1 - Electively mute children: A therapeutic approach
A1 - Weininger,Otto
Y1 - 1987///
N1 - Peer Reviewed Journal: 1990-05374-001
English
Journal; Peer Reviewed Journal
KW - PsycInfo
KW - Elective Mutism
KW - Individual Psychotherapy
KW - Play Therapy
KW - Case Report
RP - NOT IN FILE
SP - 25
EP - 42
JF - Journal of the Melanie Klein Society
VL - 5:
IS - 1
N2 - Focuses on a theoretical position of elective mutism (EM) derived from clinical experience with a 5-yr-old girl and a 6-yr-old girl whose EM was present for 3 yrs. In both cases, individual play psychotherapy was the chosen intervention. Different theories of EM focus on passive-aggressive behavior, fear/anxiety reduction, controlling/withholding aspects, and family dynamics. EM is viewed as a distortion, at a point of crisis, of a basic process of psychic development. Therapeutic approaches must adjust to particularities, since each case

will reveal its own special pattern of interconnection that links the child's experience of self, family, and outside world. (PsycINFO Database Record (c) 2012 APA, all rights reserved)
AD - Weininger, Otto: U Toronto, Ontario Inst for Studies in Education, Canada
ER -

TY - JOUR
ID - 3057
T1 - Home use and evaluation by suburban youth: Gender differences
A1 - Schiavo,R.
Y1 - 1987///
N1 - Peer Reviewed Journal: 1989-18531-001
English
Journal; Peer Reviewed Journal
KW - PsycInfo
KW - Home Environment
KW - Human Sex Differences
KW - Suburban Environments
RP - NOT IN FILE
SP - 8
EP - 12
JF - Children's Environments Quarterly
VL - 4:
IS - 4
N2 - Interviewed 59 middle-class public-school suburban children (aged 8-18 yrs) about their use of places inside the house and outside on household property. Ss also photographed all places important to them. Bedrooms were particularly salient in the Ss' daily lives and evaluations. Female Ss evaluated their homes more positively, and the sexes differed in terms of which places were important and how places in the home were used for personal activities and social interactions. It is concluded that these different patterns of home use were related to gender differences in attachment. (PsycINFO Database Record (c) 2012 APA, all rights reserved)
SN - 0886-0505
AD - Schiavo, R. Steven: Wellesley Coll, MA, US
ER -

TY - JOUR
ID - 3058
T1 - Rural children's time in household activities
A1 - Lawrence,Frances C.
Y1 - 1987///
N1 - Peer Reviewed Journal: 1989-01098-001
English
Journal; Peer Reviewed Journal
KW - PsycInfo
KW - Household Management
KW - Rural Environments
KW - Demographic Characteristics
RP - NOT IN FILE
SP - 927
EP - 937
JF - Psychological Reports
JA - Psychol Rep
VL - 61:
IS - 3
N2 - Examined the amount of time spent by 1,045 rural children (aged 6-17 yrs) in household activities and several demographic factors related to their time use, using data from a survey of family use of time in which home interviews were conducted with families from rural areas of 10 states. Findings indicate that Ss averaged 65 min/day in household tasks. Ss spent the most time in shopping and in maintenance of home, yard, car, and pets. Age and sex of the child, education and employment of the mother, state of residence, and season of the

year significantly affected time spent on tasks. Results are interpreted in terms of E. H. Erikson's (1968) stages of development. (PsycINFO Database Record (c) 2012 APA, all rights reserved)
SN - 0033-2941
AD - Lawrence, Frances C.: Louisiana State U & A&M Coll, School of Home Economics, Baton Rouge, US
ER -

TY - JOUR
ID - 3059
T1 - Effects of individual and group contingencies on disruptive playground behavior
A1 - Thomas,Jerry R.
Y1 - 1987///
N1 - Peer Reviewed Journal: 1988-21682-001
English
Journal; Peer Reviewed Journal
KW - PsycInfo
KW - Behavior Modification
KW - Behavior Problems
KW - Childhood Play Behavior
KW - Cognitive Development
KW - Friendship
KW - Elementary School Students
RP - NOT IN FILE
SP - 66
EP - 76
JF - Journal of Research & Development in Education
VL - 20:
IS - 3
N2 - Studied the effect of behavior modification techniques on 191 2nd- and 5th-grade students' playground behavior and cognitive knowledge of friendship. An event recorder instrument was developed, and misbehavior on the playground was recorded. Ss were identified as being more and less disruptive from the baseline measures, and the knowledge of friendship was determined. Two behavioral treatments were administered with continuous recording of misbehaviors. Baseline measures were continued when treatments were withdrawn. At the conclusion of the study, the knowledge of friendship was measured again. Results indicate that for 5th graders, both treatments were equally effective in reducing disruptive playground behaviors. For 2nd graders, behavior games were not effective when used as the 1st treatment, but individual behavior contracts were very effective. (PsycINFO Database Record (c) 2012 APA, all rights reserved)
SN - 0022-426X
AD - Thomas, Jerry R.: Louisiana State U, US
ER -

TY - JOUR
ID - 3060
T1 - Children's, teachers', and parents' perceptions of play
A1 - Rothlein,Liz
Y1 - 1987///
N1 - Peer Reviewed Journal: 1988-19800-001
English
Journal; Peer Reviewed Journal
KW - PsycInfo
KW - Child Attitudes
KW - Childhood Play Behavior
KW - Parental Attitudes
KW - Teacher Attitudes
KW - Preschool Teachers
RP - NOT IN FILE
SP - 45
EP - 53

JF - Early Childhood Research Quarterly
VL - 2:
IS - 1
N2 - Interviewed 103 children (aged 2-6 yrs) and surveyed 73 of their parents and 60 preschool teachers about their perceptions of play. Most parents defined play as something for fun or amusement, while teachers saw it both as fun and as an opportunity for cognitive and social development. The majority of teachers did not view play as an integral part of the curriculum, but instead described a learn-play dichotomy. Parents did not support having their children spend a large amount of time in play during preschool. Children's favorite play activities according to children and their parents and teachers were outdoor play, dramatic play, blocks, and art activities. (PsycINFO Database Record (c) 2012 APA, all rights reserved)
SN - 0885-2006
AD - Rothlein, Liz: U Miami, School of Education & Allied Professions, Coral Gables, FL, US
ER -

TY - JOUR
ID - 3061
T1 - Social-cognitive and behavioral characteristics of peer-rejected boys. [References]
A1 - French,Doran C.
Y1 - 1987///
N1 - Peer Reviewed Journal: 1988-19752-001
English
Journal; Peer Reviewed Journal
KW - PsycInfo
KW - Aggressive Behavior
KW - Peer Relations
KW - Problem Solving
KW - Social Approval
KW - Social Skills
KW - Social Interaction
RP - NOT IN FILE
SP - 103
EP - 112
JF - Professional School Psychology
VL - 2:
IS - 2
N2 - Identified 13 rejected and 13 popular 3rd- and 4th-grade boys using sociometric nomination measures. Playground observations and interview assessments of social problem solving were administered during the winter and spring. Rejected Ss both exhibited and received more aversive behavior than popular Ss. Although no status differences in the overall rate of interaction were found, rejected Ss exhibited more onlooker behavior and solitary play than the popular ones. No differences in the frequency of alternative solutions to hypothetical social problems were found between status groups. The solutions of rejected Ss were, however, less effective and more aggressive than those of populars. Chosen solutions of rejected Ss were also more likely to be ineffective and aggressive than those of populars. (PsycINFO Database Record (c) 2012 APA, all rights reserved)
SN - 0886-3016
AD - French, Doran C.: Lewis & Clark Coll, Portland, OR, US
ER -

TY - JOUR
ID - 3062
T1 - Expression of affection in day care
A1 - Twardosz,Sandra; Botkin,Darla; Cunningham,Jo
Y1 - 1987///
N1 - Peer Reviewed Journal: 1988-12203-001
English
Journal; Peer Reviewed Journal
KW - PsycInfo

KW - Affection
KW - Day Care Centers
KW - Preschool Students
KW - Preschool Teachers
KW - Teacher Student Interaction
KW - Facial Expressions
KW - Physical Contact
RP - NOT IN FILE
SP - 133
EP - 151
JF - Child Study Journal
VL - 17:
IS - 2
N2 - Measured 65 teachers' and 123 children's facial, verbal, and physical affectionate behaviors in 7 daycare centers during a variety of daily routines. Affectionate behavior occurred much less frequently than other social interaction, and smiling occurred more frequently than verbal or physical affection. Children almost never expressed affection verbally, although their teachers did. Physical affection occurred most frequently during unstructured indoor and outdoor freeplay periods; verbal affection occurred most frequently during structured small group activities. Analyses of behavioral patterns across centers indicated that centers that ranked high on the expression of some types of affectionate behaviors during particular daily routines did not necessarily rank high on all affectionate behaviors. (PsycINFO Database Record (c) 2012 APA, all rights reserved)
SN - 0009-4005
AD - Twardosz, Sandra: U Tennessee, Knoxville, US
ER -

TY - JOUR
ID - 3063
T1 - A move from hospital to community: Evaluation of community contacts
A1 - Firth,Hugh
Y1 - 1987///
N1 - Peer Reviewed Journal: 1988-08567-001
English
Journal; Peer Reviewed Journal
KW - PsycInfo
KW - Activity Level
KW - Deinstitutionalization
KW - Interpersonal Interaction
KW - Physical Disorders
KW - Residential Care Institutions
RP - NOT IN FILE
SP - 341
EP - 354
JF - Child: Care, Health and Development
VL - 13:
IS - 5
N2 - Evaluated the activities and social contacts of a group of 5 severely mentally handicapped people (aged 10-18 yrs) who left a hospital to live in a residential setting. Results show that the Ss experienced increased contact with their families and spent more time in a wider variety of activities outside the house. The degree of handicap had little effect on results. Activities depended almost entirely on the staff. The nature of the Ss' social contacts is examined, and conclusions are drawn about staff selection and training and attention to the maintenance as well as the provision of social contact. (PsycINFO Database Record (c) 2012 APA, all rights reserved)
SN - 0305-1862
AD - Firth, Hugh: Prudhoe Hosp, Northumberland District Psychology Service, England
ER -

TY - JOUR

ID - 3064
T1 - Individualizing social skills training for behavior-disordered children. [References]
A1 - Schneider,Barry H.
Y1 - 1987///
N1 - Peer Reviewed Journal: 1987-32144-001
English
Journal; Peer Reviewed Journal
KW - PsycInfo
KW - Behavior Disorders
KW - Social Skills Training
RP - NOT IN FILE
SP - 444
EP - 445
JF - Journal of Consulting and Clinical Psychology
VL - 55:
IS - 3
N2 - Individualized social skills training was compared with nonindividualized social skills training and wait-list control conditions. Participants were 35 behavior-disordered children at a treatment center. Role-play tests indicated that children in both the individualized and nonindividualized conditions mastered the content of the training sessions. Observations of recess play indicated that the increase in cooperative play after treatment was significantly higher for the individualized training group. There were no significant differences in observed aggression. (PsycINFO Database Record (c) 2012 APA, all rights reserved)
SN - 0022-006X
AD - Schneider, Barry H.: U Ottawa, Child Study Ctr, Canada
ER -

TY - JOUR
ID - 3065
T1 - Natural social behaviors in hyperactive children: Dose effects of methylphenidate. [References]
A1 - Whalen,Carol K.
Y1 - 1987///
N1 - Peer Reviewed Journal: 1987-29071-001
English
Journal; Peer Reviewed Journal
KW - PsycInfo
KW - Drug Dosages
KW - Drug Therapy
KW - Hyperkinesis
KW - Methylphenidate
KW - Social Behavior
RP - NOT IN FILE
SP - 187
EP - 193
JF - Journal of Consulting and Clinical Psychology
VL - 55:
IS - 2
N2 - The effects of methylphenidate on the social behaviors of hyperactive children between the ages of 6 and 11 were examined during relatively unstructured activities in outdoor settings. The 12 younger (mean age = 7-8 years) and 12 older (mean age = 9-21 years) children received placebo, a low (0.3 mg/kg), and a moderate (0.6 mg/kg) dose of methylphenidate. During recess, lunch, and exercise sessions, trained observers coded the children's actions as either appropriate social, negative social, or nonsocial behavior. Both age groups showed decrements in negative social behaviors when placebo was compared with the low dose of methylphenidate. Only the younger group showed incremental improvement between the low and moderate doses. There were no significant age or dosage effects on the rates of nonsocial behaviors, which remained low throughout the study. Several implications for the treatment of hyperactive children are discussed, including (a) the finding that disruptive behaviors can be reduced successfully without decreasing overall sociability, (b) the importance of interpersonal heterogeneity and the distinction between group and individual patterns of response, and (c) the

need to study relationships and friendships as well as social actions. (PsycINFO Database Record (c) 2012 APA, all rights reserved)
SN - 0022-006X
AD - Whalen, Carol K.: U California, Program in Social Ecology, Irvine
ER -

TY - JOUR
ID - 3066
T1 - Grade and gender differences in and the stability and correlates of the structural complexity of children's playground games
A1 - Borman,Kathryn M.
Y1 - 1987///
N1 - Peer Reviewed Journal: 1987-27818-001
English
Journal; Peer Reviewed Journal
KW - PsycInfo
KW - Age Differences
KW - Childhood Play Behavior
KW - Human Sex Differences
KW - Cognitive Development
KW - Longitudinal Studies
RP - NOT IN FILE
SP - 241
EP - 251
JF - International Journal of Behavioral Development
VL - 10:
IS - 2
N2 - 25 2nd graders' (12 males, 13 females) and 24 5th graders' (10 males, 14 females) playground activities during school recess were studied with a 1-yr follow-up. At the 2nd time of assessment, measures were also taken of logical reasoning, interpersonal understanding, and understanding game rules. Both grade and gender differences were found in the complexity of Ss' activities. Generally, older Ss participated in more complex activities than younger Ss, and boys participated in more complex activities than girls. The complexity of children's activities increased over the 1-yr period, especially for boys. The correlates of game complexity differed for boys and girls. For boys, game complexity was negatively related to interpersonal understanding and positively related to understanding game rules. For girls, game complexity was positively related to interpersonal understanding. (PsycINFO Database Record (c) 2012 APA, all rights reserved)
SN - 0165-0254
AD - Borman, Kathryn M.: U Cincinnati Coll of Education
ER -

TY - JOUR
ID - 3067
T1 - Mothers, fathers, and children in an urban park playground: A comparison of dyads and triads. [References]
A1 - Liddell,Christine
Y1 - 1987///
N1 - Peer Reviewed Journal: 1987-18434-001
English
Journal; Peer Reviewed Journal
KW - PsycInfo
KW - Father Child Relations
KW - Mother Child Relations
KW - Parent Child Relations
KW - Playgrounds
RP - NOT IN FILE
SP - 262
EP - 266

JF - Developmental Psychology
JA - Dev Psychol
VL - 23:
IS - 2
N2 - Observations were made of mother-child, father-child, and mother-father-child groups in an urban park playground. Results show great similarity between father-child dyads and mother-father-child triads, with mother-child dyads being consistently different. It is suggested that the similarities between triads and father-child dyads may occur because fathers and children rarely spend time alone together. Their dyadic rates of interaction and activity thus resemble those found in the triadic setting they usually experience. Group size and roles and relationships appear to exert a complex interactive effect on family-interaction patterns. The degree to which results might be situation-specific is considered, and implications for our understanding of family-interaction processes are discussed. (PsycINFO Database Record (c) 2012 APA, all rights reserved)
SN - 0012-1649
AD - Liddell, Christine: National Inst for Personnel Research, Human Sciences Research Council, Johannesburg, South Africa
ER -

TY - JOUR
ID - 3068
T1 - Rough and tumble, friendship, and dominance in schoolchildren: Evidence for continuity and change with age
A1 - Humphreys,Anne P.
Y1 - 1987///
N1 - Peer Reviewed Journal: 1987-18420-001
English
Journal; Peer Reviewed Journal
KW - PsycInfo
KW - Age Differences
KW - Childhood Play Behavior
KW - Dominance
KW - Friendship
KW - Interpersonal Attraction
RP - NOT IN FILE
SP - 201
EP - 212
JF - Child Development
JA - Child Dev
VL - 58:
IS - 1
N2 - Examined play-fighting and chasing activity among 7-, 9-, and 11-yr-olds (n = 29, 29, 36, respectively) in their school playground, where it took up some 10% of their time. Judging by characteristics of initiations and terminations, it tended to occur in a friendly, nonaggressive context. Sociometric information supported this interpretation. Observed rough-and-tumble partners liked each other better at all ages than chance predicted. With increasing age, the importance of strength relations in partner choice increased. 11-yr-olds tended to choose partners who were similar in strength to, but weaker than, themselves, while neither factor influenced 7- and 9-yr-olds' choices significantly. Findings suggest that rough-and-tumble activity performs an affiliative function in young children, becoming important for dominance and fighting-skill development in older children, although immediate motivation remains nonaggressive. (PsycINFO Database Record (c) 2012 APA, all rights reserved)
SN - 0009-3920
AD - Humphreys, Anne P.: U Sheffield, England
ER -

TY - JOUR
ID - 3069
T1 - Problem-solving skills training and relationship therapy in the treatment of antisocial child behavior. [References]

A1 - Kazdin,Alan E; Esveldt-Dawson,Karen; French,Nancy
Y1 - 1987///
N1 - Peer Reviewed Journal: 1987-16684-001
English
Journal; Peer Reviewed Journal
KW - PsycInfo
KW - Antisocial Behavior
KW - Cognitive Therapy
KW - Problem Solving
KW - Psychiatric Patients
KW - Relationship Therapy
KW - Psychiatric Hospital Programs
KW - Social Skills Training
RP - NOT IN FILE
SP - 76
EP - 85
JF - Journal of Consulting and Clinical Psychology
VL - 55:
IS - 1
N2 - The present investigation evaluated the effects of cognitive-behavioral problem-solving skills training (PSST) and nondirective relationship therapy (RT) for the treatment of antisocial child behavior. Psychiatric inpatient children (N = 56, ages 7-13) were assigned randomly either to PSST, RT, or to a treatment-contact control condition (in which children met individually with a therapist but did not engage in specific activities designed to alter antisocial behavior). Children were hospitalized during the period in which treatment was administered and discharged thereafter. The PSST condition led to significantly greater decreases in externalizing and aggressive behaviors and in overall behavioral problems at home and at school and to increases in prosocial behaviors and in overall adjustment than the RT and contact-control conditions. These effects were evident immediately after treatment and at a 1-year follow-up. The RT and control children did not consistently improve over the treatment and follow-up periods. Comparisons with nonclinical (normative) levels of functioning revealed that a significantly higher proportion of PSST children, compared with those in other conditions, fell within the normative range for prosocial behavior at posttreatment and at follow-up. Even so, the majority of PSST children and almost all RT and control children remained outside the normative range of deviant behavior. The implications of the results for further research for antisocial youth are highlighted. (31 ref) (PsycINFO Database Record (c) 2012 APA, all rights reserved)
SN - 0022-006X
AD - Kazdin, Alan E.: U Pittsburgh School of Medicine, Western Psychiatric Inst & Clinic
ER -

TY - JOUR
ID - 3070
T1 - Observational validation of a peer nomination technique for identifying aggressive, withdrawn, and aggressive/withdrawn children. [References]
A1 - Serbin,Lisa A.
Y1 - 1987///
N1 - Peer Reviewed Journal: 1987-15264-001
English
Journal; Peer Reviewed Journal
KW - PsycInfo
KW - Aggressive Behavior
KW - Peer Evaluation
KW - Social Isolation
KW - Observation Methods
KW - Peer Relations
RP - NOT IN FILE
SP - 109
EP - 110
JF - Journal of Consulting and Clinical Psychology

VL - 55:
IS - 1
N2 - Membership in peer-identified aggressive, withdrawn, or aggressive and withdrawn groups and in a nondeviant contrast group was predicted based on quantitative observations of playground behavior. Subjects were selected from a pool of 174 fifth and sixth graders who had completed the Pupil Evaluation Inventory. Of 43 selected aggressive, withdrawn, aggressive and withdrawn, and contrast subjects, 65% were correctly classified, based on their behavior on the playground. These results confirm the ability of peers to identify children who show specific patterns of atypical social interaction. (6 ref) (PsycINFO Database Record (c) 2012 APA, all rights reserved)
SN - 0022-006X
AD - Serbin, Lisa A.: Concordia U, Ctr for Research in Human Development, Montreal, Canada
ER -

TY - JOUR
ID - 3071
T1 - The effects of total body movement on reading achievement
A1 - Van Osdol,Bob M.
Y1 - 1974///
N1 - Peer Reviewed Journal: 1975-01928-001
English
Journal; Peer Reviewed Journal
KW - PsycInfo
KW - Elementary School Students
KW - Exercise
KW - Reading Achievement
KW - Teaching Methods
RP - NOT IN FILE
SP - 16
EP - 19
JF - Australian Journal of Mental Retardation
VL - 3:
IS - 1
N2 - Describes 4 of 38 grid games that were used to determine if total body movement would help improve the reading ability of 26 6-14 yr olds in elementary school who were experiencing reading problems. The Ss had been diagnosed as having learning disabilities on the basis of reading levels that were below grade levels and the Ss' inability to perform at a passing level in a regular classroom. For 16 wks an experimental and a control group were taught with the same instructional materials, content sequences, and time allotments. The experimental group was also given the total body movement exercises for 20 min each day, while the control group was given a placebo treatment that consisted of small group reading with assignments outside the regular classroom. Comparison of pre- and posttreatment scores on the Wide Range Achievement Tests indicate that the experimental group demonstrated a higher reading level than the control group at the end of the testing period. Results suggest that the total body movement treatment did enhance the reading ability of the students beyond the increase evident when traditional classroom methods are used. (PsycINFO Database Record (c) 2013 APA, all rights reserved)
SN - 0045-0634
AD - Van Osdol, Bob M.: U Tulsa
ER -

TY - JOUR
ID - 3072
T1 - Play in pre-school centres: II. Effects on play of the child's social class and of the educational orientation of the centre
A1 - Tizard,Barbara; Philips,Janet; Plewis,Ian
Y1 - 1976///
N1 - Peer Reviewed Journal: 1977-05613-001
English
Journal; Peer Reviewed Journal

KW - PsycInfo
KW - Childhood Play Development
KW - Preschool Education
KW - Social Class
RP - NOT IN FILE
SP - 265
EP - 274
JF - Child Psychology & Psychiatry & Allied Disciplines
VL - 17:
IS - 4
N2 - Observed the free play of 109 working- and middle-class 3-4 yr olds to examine the effects of social class and the educational orientation of the center on the children's play. Data indicate that both social class and the type of preschool center affected aspects of the Ss' play. There was no support for the belief that the presence of trained teachers improved the quality of the play. Working class Ss were more than twice as likely as middle-class Ss to play outside, particularly with wheeled vehicles, while middle-class Ss more often chose paints an pattern-making. Results also show that the working-class Ss were less often involved in symbolic play than their middle-class counterparts, and more often made only partial use of play materials, both characteristic of the play of younger children. (PsycINFO Database Record (c) 2013 APA, all rights reserved)
SN - 0021-9630
AD - Tizard, Barbara: U London Inst of Education, England
ER -

TY - JOUR
ID - 3073
T1 - Self-reports of children in treatment: Is assertiveness in the eyes of the beholder?
A1 - Schneider,Barry H.
Y1 - 1984///
N1 - Peer Reviewed Journal: 1984-22218-001
English
Journal; Peer Reviewed Journal
KW - PsycInfo
KW - Assertiveness
KW - Behavior Disorders
KW - Inventories
KW - Self Report
KW - Test Validity
RP - NOT IN FILE
SP - 70
EP - 73
JF - Journal of Clinical Child Psychology
VL - 13:
IS - 1
N2 - Examined the validity of the Children's Assertiveness Inventory (CAI) with 28 male and 8 female 7-13 yr old behavior-disordered children. The CAI was cross-validated with peer ratings, teacher ratings (Conners Teacher Rating Scale and a social behavior assessment checklist), and direct observations of Ss at recess play. Results show that Ss who rated themselves as assertive were rated as aggressive by their peers and less responsible academically and socially by their teachers. There was no relationship between CAI scores and observation categories. Results suggest that further investigation is necessary to validate the CAI. (20 ref) (PsycINFO Database Record (c) 2013 APA, all rights reserved)
SN - 0047-228X
AD - Schneider, Barry H.: U Ottawa School of Psychology, Child Study Ctr, Canada
ER -

TY - JOUR
ID - 3074
T1 - Initial analysis of the accepts curriculum: Efficacy of instructional and behavior management procedures for improving the social adjustment of handicapped children

A1 - Walker,Hill M.
Y1 - 1983///
N1 - Peer-Reviewed Status-Unknown: 1983-29086-001
English
Journal; Peer-Reviewed Status-Unknown
KW - PsycInfo
KW - Classroom Behavior Modification
KW - Contingency Management
KW - Elementary School Students
KW - Social Skills Training
KW - Disorders
KW - Peer Relations
KW - Social Adjustment
RP - NOT IN FILE
SP - 105
EP - 127
JF - Analysis & Intervention in Developmental Disabilities
VL - 3:
IS - 1
N2 - 26 mentally handicapped 6-12 yr olds participated in a study of a social skills training (SST) procedure. Ss were randomly assigned to 1 of 3 groups: SST plus contingency management procedures (Group 1), SST only (Group 2), and control. Ss in Group 1 were exposed to an SST curriculum that taught critical classroom behaviors facilitative of classroom adjustment and peer-to-peer social skills. For this group, behavior management procedures were applied in the classroom and on the playground to strengthen specific skills taught by the curriculum. Training required 4-7 wks and was conducted using small-group teaching formats. Teacher ratings, a criterion role-play test (CRPT), and behavioral observation data recorded in classroom and playground were used to assess changes in Ss' behavior. Results favored the 2 experimental groups over the control on all dependent measures. However, significant differences were obtained only on the CRPT and classroom observation data. One of 3 interactive measures derived from playground observation data approached significance. Group 1 was favored on ratings of classroom and peer-to-peer skills as well as playground observations. Results are discussed in terms of study limitations, ways of improving intervention procedures, and needed research in the social skills area vis-a-vis mainstreaming. (47 ref) (PsycINFO Database Record (c) 2013 APA, all rights reserved)
SN - 0270-4684
AD - Walker, Hill M.: U Oregon Coll of Education, Ctr on Human Development, Eugene
ER -

TY - JOUR
ID - 3075
T1 - A Behavioral Strategy for Language Training of A Child With Autistic Behaviors. [References]
A1 - Sulzbacher,Stephen I.
Y1 - 1973///
N1 - Book: 2006-11488-026
English
Book; Edited Book
KW - PsycInfo
KW - Autism
KW - Behavior Therapy
KW - Social Skills Training
RP - NOT IN FILE
SP - 327
EP - 351
JF - Stedman, James M [Ed]; Patton, William F [Ed]; Walton, Kay F [Ed]
VL - :
IS - 1973
N2 - (create) This reprinted article originally appeared in (Journal of Speech and Hearing Disorders, 1970, Vol 35, pp. 256-276). The case of Teddy illustrates the strategy of the experimental analysis of behavior in treating

the communication deficits of a child with autistic behaviors. The speech clinician coordinated the efforts of parents, teacher, and psychologist to generate and maintain appropriate behavior of the child in various settings. Consistently applying the same procedures to the child's behavior at school, at home, and in the clinic is of critical importance in maintaining and generalizing newly acquired vocal responses and nonvocal behavior to all areas in the child's environment (Lovaas, 1968). Teddy was born after a full-term pregnancy and his birth history was essentially normal. His acquisition of gross motor skills followed a somewhat delayed pattern, and medical reports, when Teddy was six, listed several "soft" signs of neurological dysfunction. Multidisciplinary evaluations between ages four and five indicated a poor prognosis with diagnoses of mental retardation, infantile autism, organic brain damage, emotional disturbance, and arrested hydrocephalus. later it was discovered that he had a bilateral conductive hearing loss. Teddy was six years, five months old at the beginning of the program. Three overall, long-range goals for Teddy's treatment were formulated: (1) deceleration of certain undesired vocal and nonvocal behaviors; (2) acquisition of the language and social skills required for success in school; and (3) generalization of these skills to Teddy's environment away from the clinic. After five months of language and behavior training, both in the clinic and when extended to the home, the initial behavioral objectives of treatment were achieved. Steps were taken to find an appropriate school placement for Teddy. After the treatments were extended to the school environment the effects of the training procedures were beginning to show in his ability to learn appropriate responses quickly and in the spontaneous generalization of his responses. During the early treatment there had been several unsuccessful attempts to teach Teddy some play skills and appropriate social behavior. Although Teddy would try out his responses on peers in these situations, his repertoire was not sufficient for him to maintain communication. Reports indicate during his first two years of school he still preferred to play alone at recess. During the last year, however, he began to acquire social skills appropriate for his age and routinely played with other children in school and in his neighborhood. Teddy's language repertoire was greatly expanded and he was able to routinely converse in sentences in an essentially normal manner. His general articulation was what one would expect from a child his age. The only reported difficulty was that he occasionally spoke too fast when excited, but this is not uncommon for children his age. The question of which of the procedures in force was related to changes in Teddy's behavior cannot be answered with the data available on Teddy. However, the intent of this study was pragmatic: to demonstrate how procedures, whose effects on behavior have already been rigorously demonstrated (for example, Girardeau and Spradlin, 1970), can be applied clinically without sacrificing scientific rigor. (PsycINFO Database Record (c) 2013 APA, all rights reserved)
ER -

TY - JOUR
ID - 3076
T1 - Social skills training with children: Proceed with caution
A1 - Berler,Ellen S; Gross,Alan
Y1 - 1982///
N1 - Peer Reviewed Journal: 1982-26273-001
English
Journal; Peer Reviewed Journal
KW - PsycInfo
KW - Learning Disabilities
KW - Role Playing
KW - Social Skills Training
RP - NOT IN FILE
SP - 41
EP - 53
JF - Journal of Applied Behavior Analysis
JA - J Appl Behav Anal
VL - 15:
IS - 1
N2 - Three learning-disabled (LD) males between the ages of 8 yrs 2 mo and 10 yrs 10 mo, selected on the basis of peer sociometric ratings and teacher referral, received social-skills training. A group training procedure consisting of coaching, modeling, behavior rehearsal, and feedback was used to teach Ss the target behaviors of eye contact and appropriate verbal responses. The following measures were obtained during baseline, posttreatment, and 1-mo follow-up for the experimental Ss and 3 control Ss: (a) performance on role-play scenes not trained during treatment sessions, (b) behavioral observations in a free play setting, and (c)

sociometric ratings. In addition, the trained and untrained role-play scenes were administered by novel experimenters following treatment. Results indicate that socially unskilled, LD children can be taught to respond appropriately to role-play situations. However, improved performance did not generalize to the natural school setting, and treatment did not affect ratings of peer acceptance. (17 ref) (PsycINFO Database Record (c) 2013 APA, all rights reserved)
SN - 0021-8855
AD - Berler, Ellen S.: West Virginia U, Morgantown
ER -

TY - JOUR
ID - 3077
T1 - Educational provision, parental expectation and physical disability
A1 - O'Hagan,Frank J.
Y1 - 1984///
N1 - Peer Reviewed Journal: 1984-27274-001
English
Journal; Peer Reviewed Journal
KW - PsycInfo
KW - Parental Attitudes
KW - Physical Disorders
KW - Social Support
KW - Special Education
KW - Student Characteristics
KW - Expectations
RP - NOT IN FILE
SP - 31
EP - 38
JF - Child: Care, Health and Development
VL - 10:
IS - 1
N2 - Interviewed parents of 24 5-12 yr old physically disabled children attending primary schools who had been referred to child guidance for various reasons. Data were collected to determine their characteristics and the external and educational support they received. Results show that Ss received medical, social work, and education attention. Most parents regarded their initial contact with professionals as poor or very poor; the majority believed they needed more help from outside agencies. Most parents expressed satisfaction with the response of teachers to the needs of their children. Most parents felt that they had played an important part in the decision to place their child in a regular school, and they were satisfied with that placement. Data show that measured IQ for spina bifida Ss was of questionable value and that there was a lack of interest in measures of social integration and adjustment for these Ss on the part of psychologists. (6 ref) (PsycINFO Database Record (c) 2012 APA, all rights reserved)
SN - 0305-1862
AD - O'Hagan, Frank J.: U Strathclyde, Glasgow, Scotland
ER -

TY - JOUR
ID - 3078
T1 - Involved and uninvolved student perceptions in indoor and outdoor school settings
A1 - Shaw,Terry J.
Y1 - 1981///
N1 - Peer Reviewed Journal: 1983-06747-001
English
Journal; Peer Reviewed Journal
KW - PsycInfo
KW - Elementary School Students
KW - Involvement
KW - School Environment
KW - Self Perception

KW - Student Attitudes
KW - Classroom Environment
RP - NOT IN FILE
SP - 135
EP - 145
JF - The Journal of Early Adolescence
VL - 1:
IS - 2
N2 - Used a 10-item attitudinal survey to assess 57 involved and 44 uninvolved 6th graders' perceptions of teachers, school, and self in 3 settings: (1) the regular classroom situation 2 wks before an outdoor education experience, (2) immediately following a 2-day outdoor education experience, and (3) 2 wks after the outing in the regular classroom. The activities for the outing were designed to encourage a high degree of physical, mental, and at times, emotional involvement. The outdoor experience was rated as significantly more positive than the regular classroom experience by involved and uninvolved Ss as well as the total population of 213 6th graders. During the outing the uninvolved Ss' perception of teacher trust improved significantly more than the involved Ss' perception. Ss had significantly more positive perception of the regular classroom 2 wks after the outing than 2 wks before. Findings indicate that outdoor education is a highly appropriate mode of instruction for improving uninvolved and involved students' perceptions of school, teachers, and self. (18 ref) (PsycINFO Database Record (c) 2012 APA, all rights reserved)
SN - 0272-4316
AD - Shaw, Terry J.: Kansas State U, Manhattan
ER -

TY - JOUR
ID - 3079
T1 - Enhancing classroom attentiveness by preselection of back-up reinforcers in a token economy
A1 - Kazdin,Alan E; Geesey,Sally
Y1 - 1980///
N1 - Peer Reviewed Journal: 1981-06392-001
English
Journal; Peer Reviewed Journal
KW - PsycInfo
KW - Classroom Discipline
KW - Secondary Reinforcement
KW - Special Education
KW - Token Economy Programs
KW - Intellectual Development Disorder
RP - NOT IN FILE
SP - 98
EP - 114
JF - Behavior Modification
JA - Behav Modif
VL - 4:
IS - 1
N2 - Examined the effects of 2 methods of selecting and exchanging back-up reinforcers with 2 mentally retarded 6- and 7-yr-old boys participating in a token economy in a special education class. In separate experiments, each S received tokens contingent upon attentive behavior. Methods of selecting and exchanging back-up reinforcers (i.e., free time, a recess, selection of a small toy, playing with a valued toy) consisted of preselecting back-up events that were to be purchased in advance of earning the tokens vs selecting back-up events only after tokens had been earned. The 2 methods were compared in separate simultaneous-treatment designs for each child. In each experiment, preselecting back-up reinforcers led to higher levels of attentive behavior than selecting back-up events after the tokens had been earned. The possible influence of making back-up events less remote in relation to the token-earning behaviors and the prompting function of the preselected procedure are discussed. (19 ref) (PsycINFO Database Record (c) 2012 APA, all rights reserved)
SN - 0145-4455
AD - Kazdin, Alan E.: Pennsylvania State U, University Park
ER -

TY - JOUR
ID - 3080
T1 - The relationship of egocentrism and measures of socialization in a sample of elementary school children
A1 - Karmos,Joseph S.
Y1 - 1978///
N1 - Peer Reviewed Journal: 1980-20829-001
English
Journal; Peer Reviewed Journal
KW - PsycInfo
KW - Egocentrism
KW - Socialization
RP - NOT IN FILE
SP - 1219
EP - 1224
JF - Educational and Psychological Measurement
VL - 38:
IS - 4
N2 - Studied egocentrism and its relationship to certain socialization variables. The 219 Ss in Grades 1-5 were from 2 rural communities in southern Illinois. It was expected that the study would support Piaget's theory on egocentrism. Data were gathered through (a) Ss' ratings of peers, (b) teachers' ratings of pupil popularity, (c) an egocentrism test derived from the writings of Piaget, and (d) observation of playground behavior. High correlations of ratings and test results to playground behavior were found. (7 ref) (PsycINFO Database Record (c) 2012 APA, all rights reserved)
SN - 0013-1644
AD - Karmos, Joseph S.: Southern Illinois U
ER -

TY - JOUR
ID - 3081
T1 - The modification and validation of the Behavior Coding System for school settings
A1 - Harris,Alice; Kreil,Dennis; Orpet,Russel
Y1 - 1977///
N1 - Peer Reviewed Journal: 1978-32728-001
English
Journal; Peer Reviewed Journal
KW - PsycInfo
KW - Aggressive Behavior
KW - Educational Measurement
KW - Elementary School Students
KW - Human Males
KW - Test Validity
RP - NOT IN FILE
SP - 1121
EP - 1126
JF - Educational and Psychological Measurement
VL - 37:
IS - 4
N2 - Reports validation of the noxious codes of the Behavior Coding System for the measurement of aggressive behavior in schools. Ss were 20 5th- and 6th-grade boys identified as either aggressive or prosocial. Six 10-min observations were made of the Ss in both classroom and playground settings. The noxious codes were found to discriminate between aggressive and prosocial Ss and to discriminate between playground and classroom settings for the aggressive Ss. (PsycINFO Database Record (c) 2012 APA, all rights reserved)
SN - 0013-1644
AD - Harris, Alice: California State U, Long Beach
ER -

TY - JOUR
ID - 3082
T1 - Preschool play activities and reading achievement
A1 - Wells,Marian E.
Y1 - 1970///
N1 - Peer Reviewed Journal: 1972-03767-001
English
Journal; Peer Reviewed Journal
KW - PsycInfo
KW - Academic Achievement
KW - Elementary School Students
KW - Recreation
KW - Social Behavior
RP - NOT IN FILE
SP - 214
EP - 219
JF - Journal of Learning Disabilities
JA - J Learn Disabil
VL - 3:
IS - 4
N2 - Studied 14 achieving and 14 underachieving 3rd graders to discover if there was a relationship between selected preschool play activities and achievement in reading. Underachievers spent significantly more time than achievers outdoors in the winter and associated with the mother and father during outdoor play. No significant differences were found in the kinds of activities participated in or amount of time spent in each. Data suggest that factors other than the preschool play activities investigated account for level of achievement in reading. (PsycINFO Database Record (c) 2012 APA, all rights reserved)
SN - 0022-2194
AD - Wells, Marian E.: Unified School District No. 1, Lincoln Reading Center, Kenosha, Wis
ER -

TY - JOUR
ID - 3083
T1 - Parent-child attitudes and communication processes in families with stutterers and families with non-stutterers
A1 - Moore,Margaret
Y1 - 1979///
N1 - Peer Reviewed Journal: 1981-28290-001
English
Journal; Peer Reviewed Journal
KW - PsycInfo
KW - Father Child Relations
KW - Mother Child Relations
KW - Parental Attitudes
KW - Parental Role
KW - Stuttering
KW - Parent Child Communication
KW - Psychological Stress
RP - NOT IN FILE
SP - 173
EP - 180
JF - British Journal of Disorders of Communication
VL - 14:
IS - 3
N2 - Ss were 28-50 yr old White parents of nonstuttering (NS; 14 mother and 13 fathers) and stuttering (ST; 14 mothers and 13 fathers) children-12 male and 2 female 4-13 yr old stutterers. Parents of NSs were matched with the ST group by virtue (a) of having a child of the same sex and age as the stuttering child and (b) the same number of children in the family. All parents completed the Parent Attitude Research Instrument and a measure

of attitudes toward the freedom of children; STs completed the Family Relations Test. Results indicate that the fathers of STs were more rigid and conventional concerning children's natural expressions than fathers of NSs. Mothers of STs were more accepting of the parent role, exercised more democratic principles within the family, and were more protective of their children with regard to outside influences. Although it was not determined whether parental attitudes of the mother and father directly caused the child to stutter, it is suggested that the stern, intolerant position of the father could create a stressful environment conducive to an impediment of speech development. (16 ref) (PsycINFO Database Record (c) 2012 APA, all rights reserved)
SN - 0007-098X
AD - Moore, Margaret: U Queensland, Brisbane, Australia
ER -

TY - JOUR
ID - 3084
T1 - Sex and age differences in aggressive behavior in two- to five-year-old children. [Portuguese]
A1 - Maciel,Vania M.
Y1 - 1985///
N1 - Peer Reviewed Journal: 1987-00855-001
Portuguese
Journal; Peer Reviewed Journal
KW - PsycInfo
KW - Age Differences
KW - Aggressive Behavior
KW - Human Sex Differences
RP - NOT IN FILE
SP - 52
EP - 77
JF - Arquivos Brasileiros de Psicologia
VL - 37:
IS - 2
N2 - Studied age and sex differences in the aggressive behavior of a total of 84 2-5 yr olds from different schools during 63 hrs of observation in a 3-phase investigation. Eight categories of aggressive behavior (5 inspired by the work of M. Manning [1972] and 3 used for the first time in studies with children), were defined and tested. Behavior was observed by direct techniques on the school playground during free play. Aggression as defined by Manning was reassessed in Phase 1, different types of aggression were categorized and tested in Phase 2, and the frequency of these types of aggression and the relationship between incidence, sex, and age were studied in Phase 3. Boys and younger children, independent of sex, had the highest frequencies of aggressive behavior. Most aggression involved 2 children. Ss were classified as initiators, receivers, and intermediaries and into high, middle, and low aggression subgroups. The implications of the findings for preschool education are discussed. (English abstract) (16 ref) (PsycINFO Database Record (c) 2012 APA, all rights reserved)
SN - 0100-8692
AD - Maciel, Vania M.: Ministerio do Interior, Brasilia, Brazil
ER -

TY - JOUR
ID - 3085
T1 - Social networks of popular, average, and rejected children in school settings
A1 - Ladd,Gary W.
Y1 - 1983///
N1 - Peer Reviewed Journal: 1984-02522-001
English
Journal; Peer Reviewed Journal
KW - PsycInfo
KW - Classroom Behavior
KW - Elementary School Students
KW - Peer Relations
KW - Social Approval

KW - Status
KW - Friendship
RP - NOT IN FILE
SP - 283
EP - 307
JF - Merrill-Palmer Quarterly
VL - 29:
IS - 3
N2 - Evidence from studies conducted in classrooms and analogue settings suggests that popular and unpopular children form their own unique subsystems and that their interactions with peers differ in quality. The present study assessed the behaviors and peer networks of popular, average, and rejected children in a larger and more diverse social setting. Based on sociometric measures administered in each classroom at 2 elementary schools, 48 3rd and 4th graders were selected to represent each sociometric membership group and then observed during mixed-grade recess periods. Analysis of children's behaviors indicated that rejected Ss spend less time in prosocial interactions and more time in agonistic and unoccupied behaviors than did popular or average Ss and paralleled average Ss in time spent watching others play. Analyses of various network measures revealed that a larger proportion of rejected Ss' interactions, as compared to those of popular and average Ss, was conducted in small groups and distributed among younger and/or unpopular companions. Popular and average Ss were also named as friends by a greater proportion of their frequent playground companions; they also tended to have networks that were more cliquish or comprised of mutual friends. (26 ref) (PsycINFO Database Record (c) 2012 APA, all rights reserved)
SN - 0272-930X
AD - Ladd, Gary W.: Purdue U, West Lafayette
ER -

TY - JOUR
ID - 3086
T1 - Behavior patterns of socially rejected and neglected preadolescents: The roles of social approach and aggression
A1 - Dodge,Kenneth A.
Y1 - 1982///
N1 - Peer Reviewed Journal: 1983-03195-001
English
Journal; Peer Reviewed Journal
KW - PsycInfo
KW - Childhood Play Behavior
KW - Peer Relations
KW - Social Acceptance
KW - Social Isolation
RP - NOT IN FILE
SP - 389
EP - 409
JF - Journal of Abnormal Child Psychology
JA - J Abnorm Child Psychol
VL - 10:
IS - 3
N2 - Sociometric nominations were used to select groups of popular, average, rejected, and neglected 3rd- and 5th-grade children (N = 320) in 2 studies. Ss' peer-interactive behaviors were naturalistically observed in their classrooms and on the playground. In contrast to popular Ss, rejected Ss displayed fewer task-appropriate behaviors and more task-inappropriate and aggressive behaviors. Whereas rejected Ss prosocially approached peers as frequently as did popular ones, peer responses to the approaches of rejected Ss were more likely to be negative. Neglected Ss, on the other hand, displayed relatively few task-inappropriate and aggressive behaviors, and socially approached peers infrequently. Their approaches also met with frequent rebuff by peers. Findings are discussed in terms of the behavioral bases of sociometric status. Suggestions for clinical researchers interested in behavioral change with rejected and neglected children are included. (28 ref) (PsycINFO Database Record (c) 2012 APA, all rights reserved)
SN - 0091-0627

AD - Dodge, Kenneth A.: Indiana U, Bloomington
ER -

TY - JOUR
ID - 3087
T1 - Affecting environmental attitudes through outdoor education
A1 - Shepard,Clinton L.
Y1 - 1985///
N1 - Peer Reviewed Journal: 1987-17017-001
English
Journal; Peer Reviewed Journal
KW - PsycInfo
KW - Conservation (Ecological Behavior)
KW - Elementary School Students
KW - Environmental Attitudes
KW - Junior High School Students
KW - Summer Camps (Recreation)
RP - NOT IN FILE
SP - 20
EP - 23
JF - The Journal of Environmental Education
VL - 17:
IS - 2
N2 - Examined whether participation in outdoor education programs had a measurable impact on the environmental attitudes of 613 9-14 yr olds attending summer camp. Results indicate that conceptually a positive learning experience took place. Findings suggest that (1) there is a relationship between program length and conservation attitude development; (2) an initial acclimatization period for urban campers is desirable before implementing conceptual activities; and, (3) there is greater opportunity to develop positive conservation attitudes in 1st-time campers. (11 ref) (PsycINFO Database Record (c) 2012 APA, all rights reserved)
SN - 0095-8964
AD - Shepard, Clinton L.: Ohio State U, School of Natural Resources
ER -

TY - JOUR
ID - 3088
T1 - The effects of a tire playground on children's attitude toward play time
A1 - Pinciotti,Patricia
Y1 - 1986///
N1 - Peer Reviewed Journal: 1989-18528-001
English
Journal; Peer Reviewed Journal
KW - PsycInfo
KW - Child Attitudes
KW - Childhood Play Behavior
KW - Environmental Attitudes
KW - Environmental Planning
KW - Playgrounds
RP - NOT IN FILE
SP - 30
EP - 39
JF - Children's Environments Quarterly
VL - 3:
IS - 3
N2 - In a study of 187 children in Grades kindergarten through 3, Ss (1) were observed before and after construction of a new playground; (2) were administered a questionnaire at the beginning and end of the study to assess Ss' attitudes about the new and old playgrounds, how much they liked recess, and their favorite activities; (3) made drawings of themselves playing in both playgrounds; and (4) were interviewed. Results are

discussed in terms of the new playground's success in meeting the Ss' needs for movement, competence, social interaction, privacy and retreat, and imaginative play. (PsycINFO Database Record (c) 2012 APA, all rights reserved)
SN - 0886-0505
AD - Pinciotti, Patricia: East Stroudsburg U, PA, US
ER -

TY - JOUR
ID - 3089
T1 - The power of nature orientations of girls and boys toward biotic and abiotic play settings on a reconstructed schoolyard
A1 - Moore,Robin C.
Y1 - 1986///
N1 - Peer Reviewed Journal: 1989-18523-001
English
Journal; Peer Reviewed Journal
KW - PsycInfo
KW - Child Attitudes
KW - Childhood Play Behavior
KW - Environmental Planning
KW - Human Sex Differences
KW - Playgrounds
KW - School Environment
RP - NOT IN FILE
SP - 52
EP - 69
JF - Children's Environments Quarterly
VL - 3:
IS - 3
N2 - Describes responses of elementary school children to a redesigned school playground. Behavior mapping, preference and attitude measurements, and perception data indicate that abiotic settings generated more conflict and stress than biotic settings, which engendered more harmonious relationships among children. Data on types of play activities, sex differences in play, and play values are presented. (PsycINFO Database Record (c) 2012 APA, all rights reserved)
SN - 0886-0505
AD - Moore, Robin C.: North Carolina State U School of Design, Ctr for Design Research & Service, US
ER -

TY - JOUR
ID - 3090
T1 - Alone at home: The home without parents as a territory without security
A1 - Sebba,Rachel
Y1 - 1986///
N1 - Peer Reviewed Journal: 1988-26274-001
English
Journal; Peer Reviewed Journal
KW - PsycInfo
KW - Child Self Care
KW - Emotional Responses
KW - Home Environment
KW - Parent Child Relations
RP - NOT IN FILE
SP - 28
EP - 34
JF - Children's Environments Quarterly
VL - 3:
IS - 2

N2 - Analyzed essays by 75 Israeli 7-11 yr olds on their experiences when suddenly left at home without parental supervision. Ss generally described the experience of being left home alone as forced on them and fearful. Younger Ss perceived the environment as an active entity and attributed their fears to outside sources. Older Ss, with a more rational approach to their environment, viewed their fears as inconvenient feelings derived from inside and stimulated by the situation of loneliness. Younger Ss felt more challenged by the circumstances than older Ss. Findings emphasize the importance of the social component of the home environment in determining children's emotional states. (PsycINFO Database Record (c) 2012 APA, all rights reserved)
SN - 0886-0505
AD - Sebba, Rachel: Technion--Israel Inst of Technology, Faculty of Architecture & Town Planning, Haifa, Israel
ER -

TY - JOUR
ID - 3091
T1 - Preschoolers' play behavior in outdoor environments: Effects of traditional and contemporary playgrounds
A1 - Hart,Craig H.
Y1 - 1986///
N1 - Peer Reviewed Journal: 1988-16565-001
English
Journal; Peer Reviewed Journal
KW - PsycInfo
KW - Childhood Play Behavior
KW - Environment
KW - Interpersonal Interaction
KW - Motor Performance
KW - Playgrounds
KW - Activity Level
RP - NOT IN FILE
SP - 668
EP - 678
JF - American Educational Research Journal
VL - 23:
IS - 4
N2 - Studied the effect of traditional playgrounds (TPs) and contemporary playgrounds (CPs) on play behaviors (PBs) of 40 preschool children in 2 age groups (aged 2-3 yrs and 4-5 yrs), videotaped during their 30-min outdoor play period at a nursery school. Each videotaped PB category was coded. Chi-square analyses and a repeated measures analysis of variance (ANOVA) were done. No significant differences were found between the 2 playgrounds concerning the cognitive play categories. More unoccupied behavior occurred on the CP. Less solitary play occurred on the TP. Findings concerning locomotion, verbal interaction, balance-posture, receipt propulsion, fine motor, and body image categories showed marginally more sitting behavior on the CP. More passive physical activity occurred in the CP setting. The decrease in these behaviors appeared to be supported by an increase in active climbing on the TP. The CP reduced physical play activity slightly. (PsycINFO Database Record (c) 2012 APA, all rights reserved)
SN - 0002-8312
AD - Hart, Craig H.: Purdue U, West Lafayette, IN, US
ER -

TY - JOUR
ID - 3092
T1 - Students and their writing: Perceptions, motivations, and behaviors
A1 - Rasinski,Timothy V.
Y1 - 1986///
N1 - Peer Reviewed Journal: 1988-12196-001
English
Journal; Peer Reviewed Journal
KW - PsycInfo

KW - Elementary School Students
KW - School Environment
KW - Student Attitudes
KW - Written Communication
KW - Followup Studies
RP - NOT IN FILE
SP - 294
EP - 299
JF - National Reading Conference Yearbook
VL - 35 1986, pp. 294-299.
N2 - Administered a questionnaire on perceptions, interests, and behaviors regarding writing to 96 3rd- and 4th-grade students who were under 2 different educational regimens-1 program embodied a traditional and mainstream approach, while the other followed a more informal and open approach. Completed questionnaires were summarized by classroom and returned to the teacher for inspection and feedback. The study was initially performed in October and repeated in June of the following year. Ss in the informal program appeared to have an advantage over their counterparts in the traditional classrooms across all measures employed in the study. Results are discussed for the following factors: definition of writing, affective attitude toward writing, purpose for writing, frequency of writing outside school, and duration of writing activity. (PsycINFO Database Record (c) 2012 APA, all rights reserved)
AD - Rasinski, Timothy V.: U Georgia, US
ER -

TY - JOUR
ID - 3093
T1 - What nursery school teachers ask us about: Psychoanalytic consultations in preschools: Living with Spiderman et al: Mastering aggression and excitement
A1 - Hall,Ruth
Y1 - 1986///
N1 - Peer-Reviewed Status-Unknown: 1988-04086-001
English
Journal; Peer-Reviewed Status-Unknown
KW - PsycInfo
KW - Aggressive Behavior
KW - Childhood Play Behavior
KW - Emotional Development
KW - Television Viewing
RP - NOT IN FILE
SP - 89
EP - 99
JF - Emotions & Behavior Monographs
VL - Mo 5 1986, pp. 89-99.
N2 - Discusses the mastering of feelings of aggression and excitement by the preschool child. The problem of TV-influenced aggressive play has persisted for generations. Conflicting forces act outside the child and shape the future adult. The forces inside the child vary with age. With repeated experience, children learn that it is possible to be angry without endangering themselves or loved ones. A young child is very vulnerable to the example of magic people on TV who indulge their urges but fight off danger. Excitement and aggression, not created by TV, are stimulated and given a false measure of reality and adult sanction. If TV is limited, children invent their own monsters and aggressive play, but these feelings are easier to master without outside stimulation. Self-control results in inner safety and self-esteem, which is suggested as the best approach to monsters and superhuman beings in the classroom. (0 ref) (PsycINFO Database Record (c) 2012 APA, all rights reserved)
SN - 0734-9890
AD - Hall, Ruth: Cleveland Psychoanalytic Inst, Cleveland Ctr for Research in Child Development, OH
ER -

TY - JOUR
ID - 3094

T1 - Some personality and motivational correlates of children's prosocial behavior
A1 - Larrieu,Julie
Y1 - 1986///
N1 - Peer Reviewed Journal: 1987-21565-001
English
Journal; Peer Reviewed Journal
KW - PsycInfo
KW - Assistance (Social Behavior)
KW - Empathy
KW - Personality Correlates
KW - Sharing (Social Behavior)
RP - NOT IN FILE
SP - 529
EP - 542
JF - The Journal of Genetic Psychology: Research and Theory on Human Development
VL - 147:
IS - 4
N2 - Investigated the prosocial behavior of 76 4th-graders by observing their sharing, caring, and helping actions on the playground and by obtaining sociometric measures (peer nomination) of a variety of prosocial actions. Personal characteristics such as assertiveness, empathy, self-concept, value orientation, and moral-motivational reasoning based on role taking were also assessed. Prosocial responding was moderately coherent for girls, but caring, sharing, and helping were relatively independent domains for boys. Associations between prosocial behavior and personal characteristics were observed. Sex differences in these relationships suggest that females' predominant prosocial responses reflect expressiveness and caring, whereas males' most salient prosocial responses are more active and instrumental. (PsycINFO Database Record (c) 2012 APA, all rights reserved)
SN - 0022-1325
AD - Larrieu, Julie: U Puget Sound
ER -

TY - JOUR
ID - 3095
T1 - Different cooperative learning procedures and cross-handicap relationships
A1 - Johnson,David W.
Y1 - 1986///
N1 - Peer Reviewed Journal: 1987-08802-001
English
Journal; Peer Reviewed Journal
KW - PsycInfo
KW - Behavior Problems
KW - Emotional Disturbances
KW - Home Environment
KW - Learning Disabilities
KW - Social Interaction
KW - Cooperation
KW - Elementary School Students
KW - School Environment
RP - NOT IN FILE
SP - 247
EP - 252
JF - Exceptional Children
JA - Except.Child
VL - 53:
IS - 3
N2 - Compared the effects of different levels of cooperation on cross-handicap interaction in 2 studies. In Study 1, 27 handicapped (i.e., with severe learning and behavioral problems) and 45 nonhandicapped 6th graders were randomly assigned to 3 conditions (cooperative controversy, cooperative debate, and individualistic) stratifying

for sex, ability level, and handicap. Ss participated in the study for 55 min/day for 11 instructional days. In Study 2, 15 handicapped (i.e., learning disabled and emotionally disturbed) and 36 nonhandicapped 4th graders were randomly assigned to 2 conditions (intergroup cooperation and intergroup competition) stratifying for sex, ability, and handicap. Ss participated in the study for 55 min/day for 10 instructional days. An activity report scale was given to Ss to determine who they interacted with in structured class activities, unstructured class activities, school activities outside of class, and activities in the home. Results indicate that both cooperative conditions promoted more constructive cross-handicap interaction than the individualistic condition; pure cooperation promoted more frequent cross-handicap interaction than did a mixture of cooperation and competition. The interaction patterns formed within cooperative learning situations generalized into unstructured class and school activities. (6 ref) (PsycINFO Database Record (c) 2012 APA, all rights reserved)
SN - 0014-4029
AD - Johnson, David W.: U Minnesota, Coll of Education, Minneapolis
ER -

TY - JOUR
ID - 3096
T1 - Social reasoning and social behavior in reflective and impulsive children
A1 - Bernfeld,Gary A.
Y1 - 1986///
N1 - Peer Reviewed Journal: 1986-29667-001
English
Journal; Peer Reviewed Journal
KW - PsycInfo
KW - Impulsiveness
KW - Motivation
KW - Reasoning
KW - Social Behavior
KW - Human Males
RP - NOT IN FILE
SP - 221
EP - 227
JF - Journal of Clinical Child Psychology
VL - 15:
IS - 3
N2 - 86 3rd-grade boys were given measures of cognitive style, social reasoning, and IQ, along with nonsocial tasks. Teachers completed measures of the Ss' motivation and behavior; each S was also rated by 3 female and 3 male peers. Results indicate that impulsive Ss (n = 45) chose assertive responses to conflicts less often than reflective Ss (n = 41). However, no style group differences were found for other types of self-rated responses or for teacher and male peer ratings of social behavior. Impulsives were rated by their female peers as more physically aggressive on the playground and as more verbally aggressive, likely to leave social conflicts, and assertive in the classroom. Highly reliable group differences were found for the teacher, peer, and experimenter ratings of motivation, with impulsives being rated as less motivated than reflectives. Impulsives demonstrated maladaptive social behavior and inadequate motivation, in spite of their adequate social reasoning. Findings suggest that the traditional social cognitive skill deficit model of maladaptive behavior is inadequate and that control deficits (e.g., impulsivity) and production deficits (e.g., low motivation) merit consideration in the treatment of maladaptive behavior in general and impulsivity in particular. (26 ref) (PsycINFO Database Record (c) 2012 APA, all rights reserved)
SN - 0047-228X
AD - Bernfeld, Gary A.: William Roper Hull Home, Calgary, Canada
ER -

TY - JOUR
ID - 3097
T1 - Television as a talking picture book: A prop for language acquisition
A1 - Lemish,Dafna
Y1 - 1986///

N1 - Peer Reviewed Journal: 1986-29622-001
English
Journal; Peer Reviewed Journal
KW - PsycInfo
KW - Language Development
KW - Parent Child Communication
KW - Television Viewing
KW - Longitudinal Studies
RP - NOT IN FILE
SP - 251
EP - 274
JF - Journal of Child Language
JA - J Child Lang
VL - 13:
IS - 2
N2 - Observed the behaviors of 16 children (aged 6 mo to 2 yrs 5 mo at 1st observation) while viewing TV in their own homes for a period of 6-8 mo at a time when Ss were actively involved in the language acquisition process. At the last observation, Ss ranged in age from 1 yr 2 mo to 3 yrs. Findings reveal an overwhelming and consistent occurrence of language-related behaviors among Ss and their parents in the viewing situation. Four main categories of Ss' verbalizations were identified, including designating objects, characters, animals, and other things on the screen; questioning about TV content; repetition of TV dialog; and description of TV content. Four main categories of parental verbal behavior paralleled the kinds of child behavior and included designation, questions, responses to child comments, and description. Within each category of child or parent behavior there were identifiable subtypes, and examples are presented. The categories are compared with those reported for parent-child interactions outside the viewing experience, particularly joint book-reading. A model of TV as a talking picture book is proposed. It is argued that TV has the potential to serve as a facilitator of children's language acquisition. Transcripts of observations of 2 Ss are appended. (36 ref) (PsycINFO Database Record (c) 2012 APA, all rights reserved)
SN - 0305-0009
AD - Lemish, Dafna: Haifa U, Israel
ER -

TY - JOUR
ID - 3098
T1 - Imitation in a naturalistic setting as a function of response uncertainty
A1 - Miller,David J.
Y1 - 1986///
N1 - Peer Reviewed Journal: 1986-26924-001
English
Journal; Peer Reviewed Journal
KW - PsycInfo
KW - Imitation (Learning)
KW - Social Behavior
KW - Classroom Behavior
RP - NOT IN FILE
SP - 113
EP - 121
JF - The Journal of Genetic Psychology: Research and Theory on Human Development
VL - 147:
IS - 1
N2 - Examined the notion that individuals uncertain about the appropriateness of their behavior may imitate to reduce their uncertainty by observing 24 preschool boys and girls (aged 3-5 yrs) in their regular classroom. 12 Ss in the experimental condition were observed for 48 min/day (during free play) for 1 wk. Subsequently, they were moved into a different classroom and observed for another week. The teacher, peers, and surroundings were unfamiliar to the Ss, and it was predicted that this manipulation would create a sense of response uncertainty and, hence, increase imitative behavior. 12 control Ss were observed in their original classrooms for both 1-wk periods. Imitation was scored by frequency of occurrence. Additionally, the type of social interaction

engaged in by the S was recorded using the scan-sampling technique at 30-sec intervals. Results indicate that mode of social interaction did not change for Ss in either condition from Week 1 to Week 2. The experimental Ss, however, imitated significantly more in Week 2 than in Week 1 and more in Week 2 than did the control Ss in Week 1 or Week 2. (13 ref) (PsycINFO Database Record (c) 2012 APA, all rights reserved)
SN - 0022-1325
AD - Miller, David J.: U Missouri, Columbia
ER -

TY - JOUR
ID - 3099
T1 - The effects of classroom organization on mainstreamed preschool children
A1 - Burstein,Nancy D.
Y1 - 1986///
N1 - Peer Reviewed Journal: 1986-15822-001
English
Journal; Peer Reviewed Journal
KW - PsycInfo
KW - Classroom Environment
KW - Mainstreaming (Educational)
KW - Autism
KW - Delayed Development
KW - Disorders
KW - Down's Syndrome
RP - NOT IN FILE
SP - 425
EP - 434
JF - Exceptional Children
JA - Except.Child
VL - 52:
IS - 5
N2 - Investigated the relationship between classroom organization and the experiences of 9 preschool handicapped (HC) children (aged 2.8-6 yrs) and 9 non-HC children (aged 2.1-5.3 yrs) in mainstreamed settings. Six HC Ss were developmentally delayed, 1 had Down's syndrome, 1 was cognitively and hearing impaired, and 1 was autistic. Ss were observed in 3 settings that differed in grouping, supervision, and teacher direction: center time, rug time, and outdoor play. Findings indicate, as predicted, that HC Ss differed from non-HC Ss in their classroom experiences. HC Ss spent less time on-task, interacted more frequently with adults and less frequently with peers than did non-HC Ss. Furthermore, HC and non-HC Ss' experiences were differentially affected by the setting. HC Ss spent less time on-task in rug time than in center time while HC Ss' time on-task was similar in each setting. HC Ss' interaction with peers was similar in all settings while non-HC Ss interacted most frequently with peers in outdoor play. (23 ref) (PsycINFO Database Record (c) 2012 APA, all rights reserved)
SN - 0014-4029
AD - Burstein, Nancy D.: California State U, Training Project, Northridge
ER -

TY - JOUR
ID - 3100
T1 - Evaluation of toy preference and associated movement behaviors of preschool orthopedically handicapped children
A1 - Loovis,E.
Y1 - 1985///
N1 - Peer Reviewed Journal: 1987-04541-001
English
Journal; Peer Reviewed Journal
KW - PsycInfo
KW - Childhood Play Behavior
KW - Motor Skills
KW - Physical Disorders

KW - Toy Selection
RP - NOT IN FILE
SP - 117
EP - 126
JF - Adapted Physical Activity Quarterly
JA - Adapt Phys Act Q
VL - 2:
IS - 2
N2 - Conducted a 7-wk study to evaluate toy preference and associated gross and fine motor movements of 15 preschool orthopedically handicapped children (aged 3-6 yrs) in a free-play situation. Observation sessions were scheduled 2 times/wk, each lasting 1 hr. 20 toys were evaluated using a modified version of the procedure developed by H. R. Quilitch et al (1972, 1977). Measurement of movement behavior associated with toy play involved application of a movement glossary developed by the experimenter. A Wilcoxon 2-sample rank test revealed no significant differences for either gender, age, or ambulation in relation to toy preference or nature of movement demonstrated. Analysis revealed that Ss spent considerable time using toys in a manner that did not correspond to their design. The observation that Ss more often sat either in, on, or next to a toy and made fewer attempts to move that toy in a purposeful manner suggests that, in a natural setting, the actual vs expected outcomes of organized, informal toy play may be less than desirable. It was recommended that orthopedically handicapped children might benefit from learning how to play under the direction of a parent, teacher, or similar individual. (16 ref) (PsycINFO Database Record (c) 2012 APA, all rights reserved)
SN - 0736-5829
AD - Loovis, E. Michael: Cleveland State U
ER -

TY - JOUR
ID - 3101
T1 - The role of the occupational therapist in child psychiatry: A case illustration
A1 - Davis,Susan
Y1 - 1985///
N1 - Peer Reviewed Journal: 1986-25541-001
English
Journal; Peer Reviewed Journal
KW - PsycInfo
KW - Child Psychiatry
KW - Occupational Therapy
KW - Psychotherapeutic Techniques
KW - Sex Offenses
KW - Therapist Role
KW - Case Report
RP - NOT IN FILE
SP - 266
EP - 268
JF - The British Journal of Occupational Therapy
VL - 48:
IS - 9
N2 - Presents the case report of a girl (aged 6 yrs at time of initial psychiatric referral) who was the victim of multiple sexual assaults by individuals outside of the family to illustrate how occupational therapy techniques can be used for individual therapy with children. The therapist's use of painting and body-image exercises to relax the S and encourage expression of emotions in a nonrestrictive and supportive environment is described. (12 ref) (PsycINFO Database Record (c) 2012 APA, all rights reserved)
SN - 0308-0226
AD - Davis, Susan: Sunderland District General Hosp, Dept of Child, Adolescent & Family Psychiatry, England
ER -

TY - JOUR
ID - 3102

T1 - Imaginativeness in preschoolers and single parent families
A1 - Cornelius,Georgianna M.
Y1 - 1985///
N1 - Peer Reviewed Journal: 1986-14210-001
English
Journal; Peer Reviewed Journal
KW - PsycInfo
KW - Creativity
KW - Family Structure
KW - Imagination
KW - Single Parents
RP - NOT IN FILE
SP - 56
EP - 66
JF - The Journal of Creative Behavior
VL - 19:
IS - 1
N2 - Synthesized selected research results of studies focusing on imaginativeness, creative behaviors, and single parent families. The finding of T. D. Yawkey and M. C. Yawkey (in press) that children in single-parent families display higher imaginative scores, have more imaginative companions and more imaginative talk with these companions, play more imaginative games when alone, and engage in more imaginative outdoor games than those in dual-parent families is discussed along with similar results from other studies that show significant correlates between high degrees of imaginativeness and single-parent families. Suggestions for single parents to use to develop their children's imaginativeness are presented with regard to the following parental roles: present but unengaged, attentive but uninvolved, engaged and responsive, and facilitator/modeler. (18 ref) (PsycINFO Database Record (c) 2012 APA, all rights reserved)
SN - 0022-0175
AD - Cornelius, Georgianna M.: Pennsylvania State U, University Park
ER -

TY - JOUR
ID - 3103
T1 - Assessment of popularity and sociability in preschool children: The relationship between teachers' ratings and behaviors in a naturalistic setting
A1 - Factor,David C.
Y1 - 1985///
N1 - Peer Reviewed Journal: 1986-13383-001
English
Journal; Peer Reviewed Journal
KW - PsycInfo
KW - Childhood Play Behavior
KW - Measurement
KW - Rating
KW - Sociability
KW - Social Approval
RP - NOT IN FILE
SP - 271
EP - 282
JF - Child Study Journal
VL - 15:
IS - 4
N2 - Assessed the relationship of 2 approaches (teacher's ratings and observation of behaviors in a naturalistic setting) used to measure children's social behavior that are frequently used to select children for social-skill training. 26 preschool children (aged 31-71 mo) were observed during free-play activity times. Behaviors were coded for the most prominent type of play for each 15-sec time unit. The Ss' playmates were also coded to obtain 2 popularity measures. Ss were observed randomly for 2 min each day on consecutive school days until 12 min of observational data were obtained for each S. The 2 classroom teachers rated each S on a form

measuring the S's social behavior and nominated the 5 most popular and 5 most isolated children in the classroom. Correlations among the behavior categories indicated that group positive behavior was negatively related to solitary, parallel, and teacher-directed behaviors. Furthermore, group positive behavior was positively related to group positive popularity and group negative behavior was positively related to group negative popularity. Group positive popularity was also positively related to group negative popularity. The 6 items on the teacher rating form tended to be correlated more with group negative behaviors than isolate or group positive behaviors. Also there was little relationship between the teacher-nominated Ss and the ranking of those Ss according to their behavior in a naturalistic setting. (22 ref) (PsycINFO Database Record (c) 2012 APA, all rights reserved)
SN - 0009-4005
AD - Factor, David C.: Thistletown Regional Ctr, Rexdale, Canada
ER -

TY - JOUR
ID - 3104
T1 - Mediation essays vs. time out: Behavior management on elementary school playgrounds
A1 - Marshall,Albert J.
Y1 - 1985///
N1 - Dissertation Abstract: 1985-55171-001
English
Dissertation Abstract
KW - PsycInfo
KW - Behavior Problems
KW - Classroom Behavior Modification
KW - Elementary School Students
RP - NOT IN FILE
SP - 2807
JF - Dissertation Abstracts International
VL - 45:
IS - 9-A
SN - 0419-4217
AD - Marshall, Albert J.: U Iowa
ER -

TY - JOUR
ID - 3105
T1 - Dual-career couples and the psychological adjustment of offspring: A review
A1 - Bennett,Bert
Y1 - 1985///
N1 - Peer-Reviewed Status-Unknown: 1985-25169-001
English
Journal; Peer-Reviewed Status-Unknown
KW - PsycInfo
KW - Dual Careers
KW - Emotional Adjustment
KW - Parental Occupation
RP - NOT IN FILE
SP - 287
EP - 295
JF - School Counselor
VL - 32:
IS - 4
N2 - Defines the dual-career (DC) concept in terms of a relationship in which both the woman and the man work outside the home and are actively involved in developing a professional lifestyle, which demands intense involvement, long hours, and much psychic energy. Views regarding maternal employment prior to the 1960's and during the 1960's and 1970's are presented. The literature on the effects of maternal employment on preschoolers is summarized in terms of attachment, sex roles, IQ, academic achievement, and psychosocial

adjustment. Several issues pertaining to the adjustment of school-aged children of DC couples are addressed; these include time spent with children, role models, academic achievement and IQ, adequacy of substitute care, psychosocial adjustment, child's perceived rejection, and career aspirations. It is acknowledged that some factors are specific to SES levels. The characteristics of DC couples are delineated, and some factors that tend to decrease the negative effects of a DC lifestyle are discussed. (28 ref) (PsycINFO Database Record (c) 2012 APA, all rights reserved)
SN - 0036-6536
AD - Bennett, Bert: Florida State U, Tallahassee
ER -

TY - JOUR
ID - 3106
T1 - An observational study of the social adjustment of spina bifida children in integrated settings
A1 - Low,Guat Tin
Y1 - 1985///
N1 - Peer Reviewed Journal: 1985-21424-001
English
Journal; Peer Reviewed Journal
KW - PsycInfo
KW - Mainstreaming (Educational)
KW - Peer Relations
KW - Social Adjustment
KW - Spina Bifida
KW - Elementary School Students
RP - NOT IN FILE
SP - 81
EP - 83
JF - British Journal of Educational Psychology
JA - Br J Educ Psychol
VL - 55:
IS - 1
N2 - Observed 8 5-7 yr old spina bifida children of average academic ability attending regular primary schools in Australia and 8 matched controls in the playground during 2 recess and 2 lunch breaks. Analysis showed that disabled Ss, 5 of whom were in wheelchairs or on crutches and 3 with no need of aid for mobility, had significantly fewer interactions overall, interacted with fewer people, and spent more time alone or in dyads. Peers initiated significantly fewer interactions with disabled Ss, suggesting lower levels of acceptance. Underlying patterns of interaction differed, especially in terms of setting and length. The disabled also interacted less with opposite-sex peers. Findings raise doubts about the presumed benefits of integration. (6 ref) (PsycINFO Database Record (c) 2012 APA, all rights reserved)
SN - 0007-0998
AD - Low Guat Tin: Inst of Education, Singapore
ER -

TY - JOUR
ID - 3107
T1 - Impact of different types of cooperative learning on cross-ethnic and cross-sex relationships. [References]
A1 - Warring,Douglas
Y1 - 1985///
N1 - Peer Reviewed Journal: 1985-18606-001
English
Journal; Peer Reviewed Journal
KW - PsycInfo
KW - Cooperation
KW - Elementary School Students
KW - Interpersonal Influences
KW - Racial and Ethnic Relations
KW - Teaching Methods

KW - Human Females
KW - Human Males
RP - NOT IN FILE
SP - 53
EP - 59
JF - Journal of Educational Psychology
VL - 77:
IS - 1
N2 - Hypothesized that the effects of cooperative experiences on cross-ethnic and cross-sex relationships would be strongest for structured class activities, next strongest for unstructured class activities in the broader school environment, and least strong for activities in students' homes. In Study 1, 74 6th graders were randomly assigned to 3 conditions (cooperative controversy, cooperative debate, and individualistic) stratifying for sex, ability level, and ethnic membership. Ss participated in the study for 55 min/day for 11 instructional days. In Study 2, 51 4th graders were randomly assigned to 2 conditions (intergroup cooperation and intergroup competition) stratifying for sex, ability, and ethnic membership. Ss participated in the study for 55 min/day for 10 instructional days. An activity report scale was administered to Ss to determine with whom they interacted in structured class activities, unstructured class activities, school activities outside of class, and activities in the home. Results show that in Study 1, both the cooperative controversy and cooperative debate conditions promoted more positive cross-sex and cross-ethnic relationships than did individualistic learning. In Study 2, intergroup cooperation promoted more positive cross-sex and cross-ethnic relationships than intergroup competition. Relationships formed within cooperative learning situations did not generalize into unstructured class, school, and home activities. (18 ref) (PsycINFO Database Record (c) 2012 APA, all rights reserved)
SN - 0022-0663
AD - Warring, Douglas: U Minnesota, Coll of Education
ER -

TY - JOUR
ID - 3108
T1 - Learning disabled and non-LD children at play
A1 - Levy,Linda
Y1 - 1984///
N1 - Peer Reviewed Journal: 1985-09846-001
English
Journal; Peer Reviewed Journal
KW - PsycInfo
KW - Childhood Play Behavior
KW - Learning Disabilities
KW - Social Interaction
RP - NOT IN FILE
SP - 43
EP - 50
JF - RASE: Remedial & Special Education
VL - 5:
IS - 6
N2 - Sociometric measures were used in conjunction with observational techniques to study the social integration of learning disabled (LD) elementary school children. 34 LD and 34 non-LD 3rd-5th graders were observed on the school playground for a total of 90 min each. Few substantive differences between the LD and non-LD groups were observed, although LD Ss tended to play alone more often. Sociometric questionnaires administered to Ss' classmates indicated that LD Ss had lower status than the non-LD Ss, and that the non-LD Ss played with about half of the LD Ss for whom they expressed liking. (55 ref) (PsycINFO Database Record (c) 2012 APA, all rights reserved)
SN - 0741-9325
AD - Levy, Linda: East Ramapo Central School District, Spring Valley, NY
ER -

TY - JOUR
ID - 3109

T1 - Investigation of factors which produce the mental overload of schoolchildren in the educational process and outside school activities. [Romanian]
A1 - Voicu,C.
Y1 - 1984///
N1 - Peer Reviewed Journal: 1987-08682-001
Romanian
Journal; Peer Reviewed Journal
KW - PsycInfo
KW - Elementary School Students
KW - Extracurricular Activities
KW - Fatigue
KW - Junior High School Students
KW - Work Load
KW - Curriculum Development
RP - NOT IN FILE
SP - 18
EP - 31
JF - Revista de Psihologie
VL - 30:
IS - 1
N2 - Investigated the causes of mental overload in 126 schoolchildren in Grades 5-8. Questionnaires completed by teachers, graphs demonstrating weekly fatigue patterns, and students' class schedules were analyzed. The number of hours devoted to schoolwork, extracurricular activities, and free time was calculated. It was found that the weekly school workload ranged from 48 hrs 54 min for 5th graders to 57 hrs for 8th graders. The excess burden was attributed mainly to extracurricular activities. Several proposals for preventing mental overload of schoolchildren are provided, including reducing the frequency and duration of extracurricular activities and scheduling them right after the end of regular morning classes. Recommendations are made to help teachers prevent mental overload of their students. (English abstract) (PsycINFO Database Record (c) 2012 APA, all rights reserved)
SN - 0034-8759
ER -

TY - JOUR
ID - 3110
T1 - Prosocial behavior: A study of preschoolers. [Portuguese]
A1 - Banco,Angela U.
Y1 - 1984///
N1 - Peer-Reviewed Status-Unknown: 1987-06915-001
Portuguese
Journal; Peer-Reviewed Status-Unknown
KW - PsycInfo
KW - Human Sex Differences
KW - Peer Relations
KW - Prosocial Behavior
KW - Psychosocial Development
KW - Classroom Behavior
RP - NOT IN FILE
SP - 43
EP - 61
JF - Psicologia
VL - 10:
IS - 1
N2 - Studied the interactive behavior of 35 preschoolers (most ages 4 yrs 4 mo to 5 yrs 5 mo) in the classroom during free play and lunch recess over a period of 39.22 hrs. Observational data were collected using the categorical running commentary technique. The behavioral events identified with prosocial behavior were categorized and coded. 946 episodes of prosocial behavior were identified. Boys were responsible for a significantly greater number of categories relating to objects, place, privileges, and cooperation. There were no

sex differences with regard to the categories "help" and "empathy." 68% of the episodes occurred during the lunch break, without any inducement. Most events following episodes of prosocial behavior were positive. The acquisition and development of prosocial behavior are discussed. (English abstract) (22 ref) (PsycINFO Database Record (c) 2012 APA, all rights reserved)
SN - 0101-6016
AD - Banco, Angela U.: U Federal da Paraiba, Joaio Pessoa, Brazil
ER -

TY - JOUR
ID - 3111
T1 - A Playground Movement Confidence Inventory for elementary school children
A1 - Crawford,Michael E.
Y1 - 1984///
N1 - Dissertation Abstract: 1985-51657-001
English
Dissertation Abstract
KW - PsycInfo
KW - Educational Measurement
KW - Elementary School Students
KW - Playgrounds
RP - NOT IN FILE
SP - 1090
JF - Dissertation Abstracts International
VL - 45:
IS - 4-A
SN - 0419-4217
AD - Crawford, Michael E.: Indiana U
ER -

TY - JOUR
ID - 3112
T1 - Block construction: Children's developmental landmarks in representation of space
A1 - Reifel,Stuart
Y1 - 1984///
N1 - Peer-Reviewed Status-Unknown: 1985-25139-001
English
Journal; Peer-Reviewed Status-Unknown
KW - PsycInfo
KW - Childhood Play Development
KW - Perceptual Development
KW - Spatial Perception
RP - NOT IN FILE
SP - 61
EP - 67
JF - Young Children
VL - 40:
IS - 1
N2 - Discusses the advantages of building with blocks for young children and how they can learn about space and spatial relationships from positioning blocks. Developmental changes in the symbolic representation of space with blocks are reviewed: As children mature, they make use of a wider range of spatial forms to represent spatial configurations. Basic spatial representations (e.g., on, by, in) with blocks are generally mastered before 4 yrs of age, after which further spatial elaborations (e.g., enclosure) begin to emerge. Also emergent at this time are the differentiation of objects within a construction, clarification of indoor and outdoor space, and coordination of landmarks. Suggested activities for increasing children's awareness of spatial concepts are presented, and implications for classroom teachers are noted. To promote spatial representation by children with blocks, teachers should provide a sufficient number of blocks and ample space, help children notice and explore spatial features of their world, and pay attention to the configurations in children's block

constructions and how they relate to their development of spatial concepts. (17 ref) (PsycINFO Database Record (c) 2012 APA, all rights reserved)
SN - 0044-0728
AD - Reifel, Stuart: U Texas, Austin
ER -

TY - JOUR
ID - 3113
T1 - Friendship choices in an integrated primary school in Northern Ireland
A1 - Davies,J.
Y1 - 1984///
N1 - Peer Reviewed Journal: 1985-14144-001
English
Journal; Peer Reviewed Journal
KW - PsycInfo
KW - Friendship
KW - Protestantism
KW - Roman Catholicism
KW - Social Integration
RP - NOT IN FILE
SP - 185
EP - 186
JF - British Journal of Social Psychology
VL - 23:
IS - 2
N2 - Examined, via sociometric measures, the friendship choices of 100 Protestant and Roman Catholic 7-11 yr olds. Little religious bias emerged from their choices of friends in class and in the playground. Outside school, however, Protestants tended to play with other Protestants. This finding may reflect demographic factors rather than religious in-group bias. (3 ref) (PsycINFO Database Record (c) 2012 APA, all rights reserved)
SN - 0144-6665
AD - Davies, J.: Queen's U of Belfast, Northern Ireland
ER -

TY - JOUR
ID - 3114
T1 - Teaching cooperative play to behavior-problem preschool children
A1 - Wolfe,Vicky V.
Y1 - 1983///
N1 - Peer Reviewed Journal: 1987-22800-001
English
Journal; Peer Reviewed Journal
KW - PsycInfo
KW - Behavior Problems
KW - Childhood Play Behavior
KW - Cooperation
KW - Praise
KW - Token Economy Programs
KW - Teaching Methods
RP - NOT IN FILE
SP - 1
EP - 9
JF - Education & Treatment of Children
VL - 6:
IS - 1
N2 - Examined the effects of a reinforcement program on social behaviors of 3 preschool children (aged 3.5-4 yrs) who displayed inappropriate behaviors during peer interactions. A multiple-baseline design across the 3 Ss and 2 settings (morning and afternoon classrooms) was employed to determine the effects of reinforcement on

each S's rate of cooperative play and on intervals spent in time-out due to aggression. For 15 min each session, Ss were rewarded a token for each minute that they engaged in cooperative play. Tokens were exchanged for outside time. Once cooperative play exceeded an 80% criterion, tokens were faded out but verbal praise continued. The program was effective in increasing cooperative play among these Ss by at least 50% over baseline, and the behavior changes were maintained when tokens were withdrawn. (PsycINFO Database Record (c) 2012 APA, all rights reserved)
SN - 0748-8491
AD - Wolfe, Vicky V.: West Virginia U, Morgantown
ER -

TY - JOUR
ID - 3115
T1 - School-age child care: In support of development and learning
A1 - Nieting,Peggy L.
Y1 - 1983///
N1 - Peer Reviewed Journal: 1984-17610-001
English
Journal; Peer Reviewed Journal
KW - PsycInfo
KW - Child Day Care
RP - NOT IN FILE
SP - 6
EP - 11
JF - Childhood Education
VL - 60:
IS - 1
N2 - Argues that childcare programs for school-age children promote commitment to care essential for their best physical, social, emotional, and cognitive development. There is a gap between need and availability of adequate care. Single-parent families require institutions outside the home, and the acceptable expression of parent priorities for work increases the need for accommodations. Childcare by the school can focus on social and emotional development and can complement, support, and extend the school's educational purposes. Recreation departments, libraries, museums, scouts, boys' and girls' clubs, and 4-H have provided childcare in the past, and they continue to play an important role in supplementing both family and school opportunities for a child's healthy growth and development. A quality childcare program is flexible with appropriate activities to meet developmental needs. The child needs peer group interaction, with appropriate limits set by adults. The child needs opportunities to experience success, a sense of industry, and a sense of competence at physical and intellectual levels. Awareness of needs is basic in planning. (18 ref) (PsycINFO Database Record (c) 2012 APA, all rights reserved)
SN - 0009-4056
AD - Nieting, Peggy L.: Coastal Academy, Myrtle Beach, SC
ER -

TY - JOUR
ID - 3116
T1 - Changes in the frequency of language use by Down's syndrome children interacting with nonretarded peers
A1 - Knox,Marie
Y1 - 1983///
N1 - Peer Reviewed Journal: 1984-12559-001
English
Journal; Peer Reviewed Journal
KW - PsycInfo
KW - Down's Syndrome
KW - Language Development
KW - Peer Relations
RP - NOT IN FILE
SP - 185
EP - 190

JF - Education & Training of the Mentally Retarded
VL - 18:
IS - 3
N2 - Investigated the changes in frequency of language used by 6 Down's syndrome children as they interacted with nonretarded peers within a normal school playground setting. Two Ss were 3.5-4 yrs of age, 4 Ss were 6-7 yrs old. Each S was observed once a week over a 9-wk period. Recordings were made of the percentage of the observation period engaged in interaction, frequency of initiation of an interaction, and frequency of responding to peers' verbal directions. As a group, the Down's syndrome Ss demonstrated a positive relationship between percentage of time engaged in interaction and the increasing frequency of language use in interaction. However, a less consistent relationship emerged when scores were examined on an individual basis. Implications are drawn regarding the benefits that may accrue to a retarded child from interaction with his/her nonretarded peers. (18 ref) (PsycINFO Database Record (c) 2012 APA, all rights reserved)
SN - 0013-1237
AD - Knox, Marie: Macquarie U School of Education, Unit for Rehabilitation Studies, North Ryde, Australia
ER -

TY - JOUR
ID - 3117
T1 - The role of siblings during pediatric hospitalization
A1 - Knafl,Kathleen A.
Y1 - 1983///
N1 - Peer Reviewed Journal: 1984-10370-001
English
Journal; Peer Reviewed Journal
KW - PsycInfo
KW - Hospitalized Patients
KW - Siblings
RP - NOT IN FILE
SP - 13
EP - 22
JF - Issues in Comprehensive Pediatric Nursing
JA - Issues Compr Pediatr Nurs
VL - 6:
IS - 1
N2 - Interviewed 56 families who had a hospitalized 5-12 yr old and 1 or more children (n = 104) living at home who were under 18 yrs old. Results show that most siblings maintained regular, direct contact with the hospitalized child. Almost half the siblings participated actively in accommodating family life to the hospitalization by assuming additional responsibilities during this time. Parents reported negative responses to the hospitalization in only 16% of the siblings. Negative responses clustered in those siblings cared for outside their own home during the hospitalization and in the 4-21 yr age range. It is suggested that visits to the hospital do not harm and may be beneficial to siblings. (8 ref) (PsycINFO Database Record (c) 2012 APA, all rights reserved)
SN - 0146-0862
AD - Knafl, Kathleen A.: U Illinois, Chicago
ER -

TY - JOUR
ID - 3118
T1 - Development of social competencies: Preschool influences upon perspective taking and listening skill development
A1 - DeMarsh,Joseph P.
Y1 - 1983///
N1 - Peer Reviewed Journal: 1983-33556-001
English
Journal; Peer Reviewed Journal
KW - PsycInfo
KW - Group Discussion

KW - Listening Comprehension
KW - Preschool Students
KW - Role Taking
KW - Social Skills
KW - Imitation (Learning)
KW - Role Playing
RP - NOT IN FILE
SP - 75
EP - 84
JF - Child Study Journal
VL - 13:
IS - 2
N2 - Hypothesized that a highly structured adult-directed preschool program that used discussions, role playing, and social modeling would, in contrast to control-group comparisons, positively increase listening skills and perspective-taking competencies. 36 3-5 yr olds were assigned to experimental and control groups; Ss in the experimental group were placed in the 10-wk university preschool program. Ss were administered measures of affective and perceptual perspective taking and the Pre-Primary Auditory Screening Test pre- and postprogram. Comparison of scores indicated that affective perspective taking and listening skills increased with time. Perceptual perspective taking failed to demonstrate significant gains for either experimentals or controls. However, listening skills and affective perspective taking showed significant increases between pre- and posttest measures for the experimental group, while affective perspective taking showed significant increases for controls. It is suggested that either maturation or a common experience outside of the preschool education accounted for intraindividual changes. (22 ref) (PsycINFO Database Record (c) 2012 APA, all rights reserved)
SN - 0009-4005
AD - DeMarsh, Joseph P.: Utah State U
ER -

TY - JOUR
ID - 3119
T1 - Muscular endurance and experience with playground apparatus
A1 - Gabbard,Carl
Y1 - 1983///
N1 - Peer Reviewed Journal: 1983-32376-001
English
Journal; Peer Reviewed Journal
KW - PsycInfo
KW - Apparatus
KW - Childhood Play Behavior
KW - Physical Fitness
KW - Playgrounds
RP - NOT IN FILE
SP - 538
JF - Perceptual and Motor Skills
VL - 56:
IS - 2
N2 - Results of a study of 83 49-83 mo olds indicate that Ss in a free-play condition, with the opportunity to use specific apparatus, produced sufficient activity to significantly increase their level of upper-body muscular endurance, as measured by a straight-arm hang. It is suggested that a specially designed play environment contributes to children's physical fitness. (2 ref) (PsycINFO Database Record (c) 2012 APA, all rights reserved)
SN - 0031-5125
AD - Gabbard, Carl: Texas A&M U, College Station
ER -

TY - JOUR
ID - 3120
T1 - Kindergarten children prefer same-color peers
A1 - Finkelstein,Neal W.

Y1 - 1983///
N1 - Peer Reviewed Journal: 1983-25428-001
English
Journal; Peer Reviewed Journal
KW - PsycInfo
KW - Blacks
KW - Peer Relations
KW - Preferences
KW - Racial and Ethnic Differences
KW - Whites
RP - NOT IN FILE
SP - 502
EP - 508
JF - Child Development
JA - Child Dev
VL - 54:
IS - 2
N2 - 38 Black and 25 White kindergartners were observed during classroom instruction and during recess on school playgrounds in both the fall and spring. As compared with a random model of selecting same-color peers for social interaction, both Blacks and Whites showed same-color preferences in their social behavior. This tendency was greater during recess than during classroom instruction and was greater in spring (after the 1st 7-9 mo of schooling) than in the fall. Although neither Blacks nor Whites behaved differently toward other-color as compared with same-color peers, Whites talked relatively more than Blacks, and Blacks emitted relatively more negative behavior and commands than Whites. It is concluded that Black and White children indicated a preference for same-color peers at the beginning of kindergarten, that this preference increased over the school year, and that differences in behavioral style between Blacks and Whites may play a role in same-color preferences. (17 ref) (PsycINFO Database Record (c) 2012 APA, all rights reserved)
SN - 0009-3920
AD - Finkelstein, Neal W.: U North Carolina, Chapel Hill
ER -

TY - JOUR
ID - 3121
T1 - Exploratory study of the influence of urban or rural environment on the time-budgets of 10-yr-olds. [French]
A1 - Touzard,Hubert; Dufour-Lefort,Florence
Y1 - 1983///
N1 - Peer Reviewed Journal: 1985-16897-001
French
Journal; Peer Reviewed Journal
KW - PsycInfo
KW - Family Socioeconomic Level
KW - Rural Environments
KW - Self Management
KW - Urban Environments
KW - Housing
KW - Leisure Time
RP - NOT IN FILE
SP - 779
EP - 787
JF - Bulletin de Psychologie
VL - 37:
IS - 15-17
N2 - Surveyed 160 10-yr-olds from 2 geographic locations (rural, urban), 2 types of residences (single family homes, apartments), and 3 socioeconomic levels (blue collar, white collar, managerial). Data were gathered on 10 activity categories: daily activities (e.g., meals), family tasks, scholarly activities, indoor leisure, sports, outdoor play, walks, TV viewing, exceptional activities, and social center activities. Geographic location

differentiated time-budgets on several dimensions: Time spent outdoors was more important in rural locations, as were outdoor play and walks. No differences were apparent among the other categories; TV viewing was equal across categories. Type of residence and socioeconomic level appeared to have little influence on the manner in which 10-yr-olds budgeted their time. (5 ref) (PsycINFO Database Record (c) 2012 APA, all rights reserved)
SN - 0007-4403
AD - Touzard, Hubert: U Lille III, Ctr de Recherches en Psychologie Sociale, France
ER -

TY - JOUR
ID - 3122
T1 - Movement therapy and the specific use of polarities with developmentally disabled children
A1 - Thompson,Laurel
Y1 - 1982///
N1 - Peer Reviewed Journal: 1984-31892-001
English
Journal; Peer Reviewed Journal
KW - PsycInfo
KW - Attention Span
KW - Developmental Disabilities
KW - Eye Contact
KW - Treatment
KW - Verbal Communication
KW - Case Report
KW - Motor Performance
RP - NOT IN FILE
SP - 33
EP - 40
JF - Pratt Institute Creative Arts Therapy Review
VL - 3 1982, pp. 33-40.
N2 - Outlines the use of creative arts therapy with a boy (age 5 yrs 10 mo; IQ 52) who attended a school for the developmentally disabled. Movement therapy was inaugurated to help him reach the level of psychic organization that would allow him to make eye contact and relate his verbalizations to reality. The therapist began by mirroring the self-stimulation of S and introducing structured activities (e.g., play with hoop and balanced eye contact in a "hello-goodbye" game). Increasing mastery of movements initiated by the therapist helped S explore passivity and/or activity. S needed reassurance at this time that his body integration was being maintained; tracing lines of his body reinforced body part connections. When S began to introduce various prohibitions into the sessions, the 3rd and final level of organization of polarities had been reached. A "superhero" sequence of strength was used in the termination process; some regression occurred but outside of sessions S progressed in most activities, especially in his concentration and attention span. It is suggested that for polarities to be balanced, both opposing elements must be developed so that they present an appropriate pull for each other. (PsycINFO Database Record (c) 2012 APA, all rights reserved)
SN - 0196-8459
ER -

TY - JOUR
ID - 3123
T1 - Nature of characteristics of the children enrolled in laboratory nursery school from 1975-1978
A1 - Singh,M.
Y1 - 1982///
N1 - Peer-Reviewed Status-Unknown: 1984-22873-001
English
Journal; Peer-Reviewed Status-Unknown
KW - PsycInfo
KW - Child Discipline
KW - Childhood Play Behavior
KW - Nursery School Students

KW - Student Characteristics
KW - Parent Training
RP - NOT IN FILE
SP - 33
EP - 39
JF - Asian Journal of Psychology & Education
VL - 10:
IS - 1-4, Spec Iss
N2 - Administered a questionnaire to parents of 54 preschool children attending a laboratory nursery school during the period 1975-1978; parents completed the questionnaire at the time of their children's admission. Results show that 88.8% of the children had one sibling or were only children. Almost all were full-term babies and had been immunized against disease. Most were right-handed and most had habits of nail-biting, thumb sucking, and nose picking. Their most common fears were of darkness and loud sounds. Milk, cereals, and pulses were liked by most children. Mothers selected toys for their children who liked to play indoors and outdoors; most preferred to play with siblings and friends. Interests in music, dance, clothes, and pets were commonly reported. The mother was the primary disciplinary agent. It is suggested that parent education programs are needed to help parents understand the needs of their children. (5 ref) (PsycINFO Database Record (c) 2012 APA, all rights reserved)
AD - Singh, M. B.: Punjab Agricultural U, Coll of Home Science, Ludhiana, India
ER -

TY - JOUR
ID - 3124
T1 - Family life space: Environmental effects on the development of children's sociability
A1 - Robinson,JoAnn L.
Y1 - 1982///
N1 - Dissertation Abstract: 1983-53582-001
English
Dissertation Abstract
KW - PsycInfo
KW - Child Day Care
KW - Crowding
KW - Home Environment
KW - Neighborhoods
KW - Peer Relations
KW - Psychosocial Development
RP - NOT IN FILE
SP - 1307
EP - 1308
JF - Dissertation Abstracts International
VL - 43:
IS - 4-A
SN - 0419-4217
AD - Robinson, JoAnn L.: Cornell U
ER -

TY - JOUR
ID - 3125
T1 - Sex differences in play at kindergarten
A1 - Halliday,Jan
Y1 - 1982///
N1 - Peer Reviewed Journal: 1983-29023-001
English
Journal; Peer Reviewed Journal
KW - PsycInfo
KW - Childhood Play Behavior
KW - Human Sex Differences

KW - Kindergarten Students
KW - Preferences
KW - Teacher Student Interaction
RP - NOT IN FILE
SP - 161
EP - 170
JF - New Zealand Journal of Educational Studies
VL - 17:
IS - 2
N2 - Examined sex differences in the preferred play activities of 11 boys and 11 girls (aged 4-5 yrs) who attended an urban kindergarten and the influence of teacher participation and location of activities on such differences. Ss were observed during 9 play sessions, and play activity was recorded using a time-sampling method. Sex differences in play generally conformed to previous research. Boys had a high preference for sandpit play and other energetic outdoor activities, while girls preferred quieter, indoor activities. However, there were more similarities between the activities chosen and avoided by each sex than has previously been reported. Also, boys showed a relatively low frequency of aggression and rough play. While girls tended to choose activities in which their teachers were involved, their most-preferred activity was climbing. Although the presence of female teachers seemed to maintain many of the girls' stereotyped choices of activity, this same presence appeared to extend the range of boys' choices beyond activities that were stereotypically male. (12 ref) (PsycINFO Database Record (c) 2012 APA, all rights reserved)
SN - 0028-8276
AD - Halliday, Jan: U Auckland, New Zealand
ER -

TY - JOUR
ID - 3126
T1 - Questions about the relationship between social cognition and social behaviour: The search for the missing link
A1 - Lefebvre-Pinard,Monique
Y1 - 1982///
N1 - Peer Reviewed Journal: 1983-27682-001
English
Journal; Peer Reviewed Journal
KW - PsycInfo
KW - Cognitive Development
KW - Experimental Design
KW - Methodology
KW - Psychosocial Development
KW - Social Behavior
KW - Literature Review
KW - Social Perception
KW - Theory Formulation
RP - NOT IN FILE
SP - 323
EP - 337
JF - Canadian Journal of Behavioural Science/Revue canadienne des sciences du comportement
VL - 14:
IS - 4
N2 - Describes the research context in which the question of the relationship between cognition and behavior has arisen within the field of social-cognitive development. A selective review of some of the studies emanating from this author's research program concerning the specific issue of the relations between the level of social cognition in the child and the adequacy of the child's social interactions in a naturalistic setting are presented. Studies included (1) the identification of the influence of children's level of social cognitive skills on the frequency and adequacy of their dyadic verbal communications during free play, (2) the examination of the relationships between preschooler's performance on laboratory measures of role playing and communication skills, and (3) measures of their request performance incorporating criteria similar to those in social-cognitive tasks. The potential flaws and limits of the original ways of measuring and conceptualizing these relations are

examined. Finally, a sketch of the conditions that need to be considered for specific types of social behavior to be regulated by particular aspects of cognition is presented. (French abstract) (46 ref) (PsycINFO Database Record (c) 2012 APA, all rights reserved)
SN - 0008-400X
AD - Lefebvre-Pinard, Monique: U Quebec, Montreal, Canada
ER -

TY - JOUR
ID - 3127
T1 - Locus of control and task persistence in preschool children
A1 - Galejs,Irma
Y1 - 1982///
N1 - Peer Reviewed Journal: 1983-06728-001
English
Journal; Peer Reviewed Journal
KW - PsycInfo
KW - Internal External Locus of Control
KW - Persistence
KW - Preschool Students
RP - NOT IN FILE
SP - 227
EP - 231
JF - The Journal of Social Psychology
VL - 117:
IS - 2
N2 - 16 preschool children were identified as having an internal or external locus of control based on the Stephens-Delys Reinforcement Contingency Interview and were observed interacting with peers, teachers, and classroom materials. It was predicted that internal Ss would show greater task persistence by spending more time in physical-motor, cognitive, and social activities, while external Ss would spend more time in non-task-involved behaviors, such as watching others and moving around. Results do not confirm this hypothesis. Findings are discussed in terms of a distinction between experimental and natural settings. (10 ref) (PsycINFO Database Record (c) 2012 APA, all rights reserved)
SN - 0022-4545
AD - Galejs, Irma: Iowa State U, Ames
ER -

TY - JOUR
ID - 3128
T1 - Activities and interactions of severely handicapped and nonhandicapped students during recess at two integrated elementary schools
A1 - Baumgart,Diane J.
Y1 - 1982///
N1 - Dissertation Abstract: 1982-74130-001
English
Dissertation Abstract
KW - PsycInfo
KW - Childhood Play Behavior
KW - Elementary School Students
KW - Interpersonal Interaction
KW - Disorders
RP - NOT IN FILE
SP - 3099
EP - 4000
JF - Dissertation Abstracts International
VL - 42:
IS - 7-A
SN - 0419-4217

AD - Baumgart, Diane J.: U Wisconsin, Madison
ER -

TY - JOUR
ID - 3129
T1 - Imitation of peers and adults in a natural setting: A functional analysis
A1 - Grusec,Joan E; Abramovitch,Rona
Y1 - 1982///
N1 - Peer Reviewed Journal: 1982-27943-001
English
Journal; Peer Reviewed Journal
KW - PsycInfo
KW - Imitation (Learning)
KW - Peer Relations
KW - Social Interaction
RP - NOT IN FILE
SP - 636
EP - 642
JF - Child Development
JA - Child Dev
VL - 53:
IS - 3
N2 - In Study 1, immediate imitation of peers was observed during free play in 4 groups of preschoolers (N = 67) ranging in age from 23 mo to 5 yrs. Amount of imitation decreased with age. A majority of imitations were followed by positive consequences in the form of maintained or increased social interaction, including counterimitation. Ss whose imitations were successful in this sense imitated more, while those who responded positively to being imitated were imitated more. Generally, there was a positive relationship between dominance and being imitated. There were no sex differences in imitating or being imitated. In Study 2, immediate imitation of both peers and adults was observed in 6 groups of preschoolers (N = 64) ranging in age from 27 mo to 5 yrs. The results for peer imitation replicated those of the 1st study. In addition, most of the Ss' imitations of adults were followed by positive consequences, although the content of peer and adult imitation differed. (10 ref) (PsycINFO Database Record (c) 2012 APA, all rights reserved)
SN - 0009-3920
AD - Grusec, Joan E.: U Toronto, Canada
ER -

TY - JOUR
ID - 3130
T1 - Measurement of sex-typed play: A comparison between laboratory and naturalistic observation procedures
A1 - Serbin,Lisa A.
Y1 - 1982///
N1 - Peer-Reviewed Status-Unknown: 1982-24828-001
English
Journal; Peer-Reviewed Status-Unknown
KW - PsycInfo
KW - Behavioral Assessment
KW - Observation Methods
KW - Preference Measures
KW - Statistical Reliability
KW - Statistical Validity
KW - Childhood Play Behavior
KW - Sex Roles
KW - Toy Selection
RP - NOT IN FILE
SP - 225
EP - 235
JF - Behavioral Assessment

VL - 4:
IS - 2
N2 - The sex-typed play behavior of 48 preschoolers (37-60 mo old) was assessed using (a) a behavioral sampling procedure within the laboratory, (b) the Toy Preference Test, and (c) observation within a classroom. All 3 measures proved to be internally consistent and/or stable over time, although the responses of males showed less consistency than those of females. Correlations for data from all Ss found that both laboratory measures correlated significantly with rates of "masculine" play in the classroom. When within-sex correlations were examined, however, neither laboratory measure consistently predicted Ss' classroom behavior. Results suggest that normal sex-typed play behavior in a naturalistic setting cannot be accurately predicted relative to their same-sex peers by means of the laboratory-based procedures typically employed in the literature. (26 ref) (PsycINFO Database Record (c) 2012 APA, all rights reserved)
SN - 0191-5401
AD - Serbin, Lisa A.: Concordia U, Ctr for Research in Human Development, Montreal, Canada
ER -

TY - JOUR
ID - 3131
T1 - Development of social spacing in normal and mentally retarded children
A1 - Burgess,J.
Y1 - 1981///
N1 - Peer Reviewed Journal: 1984-20788-001
English
Journal; Peer Reviewed Journal
KW - PsycInfo
KW - Childhood Play Behavior
KW - Personal Space
KW - Physical Contact
KW - Intellectual Development Disorder
KW - Age Differences
KW - Cognitive Development
RP - NOT IN FILE
SP - 89
EP - 95
JF - Journal of Nonverbal Behavior
VL - 6:
IS - 2
N2 - Measured distances between closest playmates during free play in 50 mentally retarded and 48 normal children. Ss in both groups were in either Grades 1-3 or 4-6. Unobtrusive observation techniques minimized interference and a spacious playground environment was chosen to avoid effects of confining walls on spatial behavior. Younger Ss (Grades 1-3) kept closer distances than older Ss (Grades 4-6), independent of clinical history or sex. Parallels are drawn with findings of previous observational studies in adults and nonhuman primates. Two hypotheses, based on cognitive development and social disturbance, are proposed to explain the findings. One hypothesis proposes that closer distances and increased touching occur under conditions of social disturbance; the other suggests that younger children may stay closer to a single playmate because their cognitive development does not facilitate simultaneous interaction with many members in a group. (38 ref) (PsycINFO Database Record (c) 2012 APA, all rights reserved)
SN - 0191-5886
AD - Burgess, J. Wesley: U California School of Medicine, Neuropsychiatric Inst Mental Retardation Research Ctr, Los Angeles
ER -

TY - JOUR
ID - 3132
T1 - Increasing coping behavior in children with aggressive behavior: Evaluation of the relative efficacy of the components of a treatment package
A1 - Blue,Susan W.
Y1 - 1981///

N1 - Peer-Reviewed Status-Unknown: 1982-24056-001
English
Journal; Peer-Reviewed Status-Unknown
KW - PsycInfo
KW - Aggressive Behavior
KW - Coping Behavior
KW - Imitation (Learning)
KW - Secondary Reinforcement
RP - NOT IN FILE
SP - 51
EP - 60
JF - Child Behavior Therapy
VL - 3:
IS - 1
N2 - Evaluated the relative efficacy of the components of a treatment package for aggressive behavior. 25 males (6-9 yrs old) were selected on the basis of observation and teacher and peer ratings of aggressive behavior. Five Ss were assigned to each of 5 groups: waiting list control, attention placebo, contingent reward, modeling, and modeling plus rehearsal. Five-day baseline and posttreatment phases consisted of daily participation in a verbal taunting game. Then, for 10 days, each treatment group participated in a verbal taunting game receiving token reinforcement for coping. The modeling group viewed a 3-min videotape of a coping model before participation, and the modeling plus rehearsal group viewed the videotape and verbally rehearsed coping statements before participation. Generalization measures on the school playground were conducted throughout the study. Results indicate the effectiveness of contingent reward, modeling, and modeling plus rehearsal procedures, and further indicate that the addition of modeling and rehearsal components to the contingent reward procedure may not be necessary. Suggestions for systematic replication are discussed. (11 ref) (PsycINFO Database Record (c) 2012 APA, all rights reserved)
SN - 0162-1416
AD - Blue, Susan W.: Florida State U, Tallahassee
ER -

TY - JOUR
ID - 3133
T1 - Cooperative play socialization among preschool children
A1 - Orlick,Terry
Y1 - 1981///
N1 - Peer-Reviewed Status-Unknown: 1982-09633-001
English
Journal; Peer-Reviewed Status-Unknown
KW - PsycInfo
KW - Childhood Play Behavior
KW - Childrens Recreational Games
KW - Cooperation
KW - Socialization
RP - NOT IN FILE
SP - 54
EP - 63
JF - Journal of Individual Psychology
VL - 37:
IS - 1
N2 - Studied the effects of cooperative games on the cooperative behavior of 4-yr-olds. Ss were randomly assigned to 1 of 3 groups, each group evenly divided between the sexes: 10 were exposed to a cooperative games (CG) program, 8 to an individual games program, and 10 to free play (FP). Each group engaged in its program for 16 wks. Before and after this treatment, observations were made of the cooperative and noncooperative task behavior, physical contact, and verbal behavior of Ss during outdoor and indoor recess. Cooperative behavior outdoors increased significantly for CG Ss. Unexpectedly, noncooperative behavior increased significantly among FP Ss when playing indoors. There was a general tendency for all Ss to be more cooperative when playing outside. (21 ref) (PsycINFO Database Record (c) 2012 APA, all rights reserved)

SN - 0022-1805
AD - Orlick, Terry: U Ottawa, School of Human Kinetics & Leisure Studies, Canada
ER -

TY - JOUR
ID - 3134
T1 - Social interaction and social organization of Israeli five- to seven-year-olds
A1 - Hertz-Lazarowitz,Rachel; Feitelson,Dina; Zahavi,Shoshana; Hartup,Willard
Y1 - 1981///
N1 - Peer Reviewed Journal: 1982-05561-001
English
Journal; Peer Reviewed Journal
KW - PsycInfo
KW - Childhood Play Behavior
KW - Social Interaction
RP - NOT IN FILE
SP - 143
EP - 155
JF - International Journal of Behavioral Development
VL - 4:
IS - 2
N2 - Observed 408 kindergartners and 1st graders in outdoor free play, 40 min each day, for 3 continuous wks. Social interactions and social organization were recorded using a modified version of M. B. Parten's (1932) system of categorization. Findings show that Ss engaged mainly in positive-active behaviors. Solitary play and onlooker behavior accounted for 30% of Ss' social behavior. Parallel play occurred less frequently than reported earlier for younger children, although no age differences were observed between kindergartners and 1st graders. Boys were significantly more active than girls and exhibited negative social interaction more frequently. Social organization was balanced. Ss were observed 57% in dyads, 27% in groups of 3 and 4, and 14% in groups with more than 5 Ss. Kindergartners participated in significantly smaller groups than 1st graders. (29 ref) (PsycINFO Database Record (c) 2012 APA, all rights reserved)
SN - 0165-0254
AD - Hertz-Lazarowitz, Rachel: U Haifa, School of Education, Israel
ER -

TY - JOUR
ID - 3135
T1 - Play behaviors of handicapped preschool children in the presence and absence of nonhandicapped peers
A1 - Field,Tiffany; Roseman,Scott; de Stefano,Louis; Koewler,John
Y1 - 1981///
N1 - Peer Reviewed Journal: 1981-31282-001
English
Journal; Peer Reviewed Journal
KW - PsycInfo
KW - Childhood Play Behavior
KW - Mainstreaming (Educational)
KW - Nursery School Students
KW - Preschool Education
KW - Disorders
KW - Peer Relations
RP - NOT IN FILE
SP - 49
EP - 58
JF - Journal of Applied Developmental Psychology
VL - 2:
IS - 1
N2 - 16 minimally handicapped and 18 nonhandicapped children (CA 2.5-4 yrs, handicapped Ss were on the average 10 mo younger developmentally) were observed playing as separate classes and as a combined group on

their preschool playground. Play behaviors directed toward self, toys, teachers, and peers were recorded using a time sampling procedure. Repeated measures analyses revealed that handicapped Ss engaged in less peer-directed and more teacher-directed behaviors than the similar CA but developmentally more advanced normal Ss. During the combined group play situation, normal Ss directed more behaviors to their own classmates, while handicapped Ss directed approximately equal amounts of behavior to their own classmates and to their normal peers. These and other results suggest that normal Ss were not negatively affected and the handicapped Ss were positively affected by the integrated play situation. (21 ref) (PsycINFO Database Record (c) 2012 APA, all rights reserved)
SN - 0193-3973
AD - Field, Tiffany: U Miami School of Medicine, Mailman Ctr for Child Development, FL
ER -

TY - JOUR
ID - 3136
T1 - Positive socialization via cooperative games. [References]
A1 - Orlick,Terry D.
Y1 - 1981///
N1 - Peer Reviewed Journal: 1981-28893-001
English
Journal; Peer Reviewed Journal
KW - PsycInfo
KW - Cooperation
KW - Games
KW - Kindergarten Students
KW - Sharing (Social Behavior)
KW - Teaching Methods
KW - Happiness
KW - Socialization
RP - NOT IN FILE
SP - 426
EP - 429
JF - Developmental Psychology
JA - Dev Psychol
VL - 17:
IS - 4
N2 - Assessed the effects of a cooperatively structured games program on sharing and on happiness when playing games among 71 5-yr-olds. Ss consisted of 4 intact, half-day kindergarten classes from 2 schools. Each school had 1 experimental group and 1 control group, both of which were taught by the same classroom teacher. Experimental groups were exposed to an 18-wk cooperative games program, and control groups were exposed to a traditional games program of equal duration. Pre- and postmeasures on a candy-sharing task revealed that Ss in the cooperative games program increased their sharing significantly more than did those in the traditional games program. Overall happiness when playing games outside of school increased for all groups. (12 ref) (PsycINFO Database Record (c) 2012 APA, all rights reserved)
SN - 0012-1649
AD - Orlick, Terry D.: U Ottawa, Canada
ER -

TY - JOUR
ID - 3137
T1 - Social relations among 21/2 to 3 year old children: Role of imitation and study of interactions. [French]
A1 - Winnykamen,F.
Y1 - 1980///
N1 - Peer Reviewed Journal: 1983-00897-001
French
Journal; Peer Reviewed Journal
KW - PsycInfo
KW - Imitation (Learning)

KW - Interpersonal Interaction
RP - NOT IN FILE
SP - 77
EP - 84
JF - Bulletin de Psychologie
VL - 34:
IS - 1-4
N2 - Data are presented for 5 Ss from a larger investigation that included 20 21/2-3 yr olds from 3 daycare centers. 15-min filmed observations were made during 4 types of activities characteristic of the centers: meals, indoor and outdoor free play, seat work, and directed play. Four categories of behaviors were coded: imitation, coaction, initiation, and response. Ss' behaviors can generally be described as socialized. Imitation, which appeared as a positive social behavior, was used to establish contact. Response behaviors were more numerous than initiation behaviors. (31 ref) (PsycINFO Database Record (c) 2012 APA, all rights reserved)
SN - 0007-4403
AD - Winnykamen, F.: U Paris V, Lab de Psychologie Genetique, France
ER -

TY - JOUR
ID - 3138
T1 - Effects of deprivation on exercise play in nursery school children
A1 - Smith,Peter K.
Y1 - 1980///
N1 - Peer Reviewed Journal: 1981-25584-001
English
Journal; Peer Reviewed Journal
KW - PsycInfo
KW - Childhood Play Behavior
KW - Deprivation
KW - Exercise
RP - NOT IN FILE
SP - 922
EP - 928
JF - Animal Behaviour
VL - 28:
IS - 3
N2 - Observed the incidence of exercise play outdoors in nursery school children aged 2.10-4.9 yrs after a short or long period of confinement indoors. For both boys (18 Ss) and girls (18 Ss) there was a greater incidence of exercise play after the long period of confinement, the levels decreasing with time. No sex differences were obtained. Results are considered in relation to theories of the functional significance of exercise play. (26 ref) (PsycINFO Database Record (c) 2012 APA, all rights reserved)
SN - 0003-3472
AD - Smith, Peter K.: U Sheffield, England
ER -

TY - JOUR
ID - 3139
T1 - Engagement style (agent vs. patient) in childhood and adolescence
A1 - McKinney,John P.
Y1 - 1980///
N1 - Peer Reviewed Journal: 1980-20835-001
English
Journal; Peer Reviewed Journal
KW - PsycInfo
KW - Age Differences
KW - Family Size
KW - Human Sex Differences
KW - Personality Traits

KW - Self Perception
KW - Adolescent Development
KW - Self Concept
RP - NOT IN FILE
SP - 192
EP - 209
JF - Human Development
VL - 23:
IS - 3
N2 - Investigated in 3 studies the active vs reactive dimension of development as a personality variable called engagement style. Ss were 51 male undergraduates, who completed Rotter's Internal-External Locus of Control Scale, and 403 2nd-12th graders. The dimension of development refers to the perception one has of oneself either as doing (agent) or being done to (patient). The dimension is distinguished from similar constructs, such as locus of control and locus of causality. A semi-projective measure of engagement style was developed and was demonstrated to yield high reliability coefficients. In studies using the Test of Engagement Style, the following hypotheses were confirmed. Boys from large families had more extreme scores (i.e., more agent or more patient) than those from small families. Agency scores declined significantly with age from childhood to adolescence. Boys from entrepreneurial family settings had higher agency scores than those from bureaucratic settings, while girls whose mothers work outside the home were more agent than those whose mothers are not so employed. Just as the active-reactive dimension is currently a focal point in developmental theorizing, engagement style is seen as an important analogous personal construct. (50 ref) (PsycINFO Database Record (c) 2012 APA, all rights reserved)
SN - 0018-716X
AD - McKinney, John P.: Michigan State U
ER -

TY - JOUR
ID - 3140
T1 - The acquisition of genderlect
A1 - Hass,Adelaide
Y1 - 1979///
N1 - Peer-Reviewed Status-Unknown: 1981-20973-001
English
Journal; Peer-Reviewed Status-Unknown
KW - PsycInfo
KW - Dialect
KW - Human Sex Differences
KW - Language Development
KW - Sex Roles
RP - NOT IN FILE
SP - 101
EP - 113
JF - Annals of the New York Academy of Sciences
JA - Ann N Y Acad Sci
VL - 327 1979, pp. 101-113.
N2 - Investigated the hypothesis that children of both sexes initially learn the mother's language, but that boys shift to the use of male dialect with contact outside the home. 12 males and 12 females (aged 4-12 yrs) were observed playing in same-sex and mixed-sex dyads. Of the 45 language features investigated, only 8 showed sex differences that held across both age and type of dyad. Two of these features showed an increase in sex-typing with age. Three other features were typical of boys or girls at the youngest age but practically dropped out at the older ages. Five additional features varied with the sex of the play partner, but in all cases the variation occurred in the girls' speech. In general, the patterns of development were similar for boys and girls. However, those features that emerged as sex-associated fit traditional sex stereotypes. Perhaps more important is the variability in girls' speech as a function of the sex of the listener. Results indicate that the concept of "genderlect" must be broadened to include variation across contexts and conversational partners. (15 ref) (PsycINFO Database Record (c) 2012 APA, all rights reserved)
SN - 0077-8923

AD - Hass, Adelaide: State U New York, Coll at New Paltz
ER -

TY - JOUR
ID - 3141
T1 - The effects of sex, structure and social interaction on preschoolers' play behaviors in a naturalistic setting
A1 - Brenner,Mark
Y1 - 1979///
N1 - Peer Reviewed Journal: 1980-20812-001
English
Journal; Peer Reviewed Journal
KW - PsycInfo
KW - Childhood Play Behavior
KW - Childhood Play Development
KW - Human Sex Differences
KW - Recreation Areas
KW - Social Interaction
RP - NOT IN FILE
SP - 91
EP - 105
JF - Instructional Science
VL - 8:
IS - 1
N2 - Observed play behavior of 9 male and 9 female preschool children in their natural environment. The study focused on the general demographics of free-play behavior, sex differences in the uses of various play areas, the effect of different play areas on the incidence of make-believe vs exploratory play, and the differential influence of play area on social interaction. Results indicate that both boys and girls used areas that had traditionally been thought of as sex-typed. For example, both sexes appeared with equal frequencies in the household area and the block and truck area. Within these areas, the sexes apportioned their time equally in terms of play activities. Overall, the household area elicited more make-believe play and social interaction than did the other play areas. (40 ref) (PsycINFO Database Record (c) 2012 APA, all rights reserved)
SN - 0020-4277
AD - Brenner, Mark: U Illinois Inst for Child Behavior & Development, Champaign
ER -

TY - JOUR
ID - 3142
T1 - Development of search procedures in real-life spatial environments. [References]
A1 - Wellman,Henry W.
Y1 - 1979///
N1 - Peer Reviewed Journal: 1980-00781-001
English
Journal; Peer Reviewed Journal
KW - PsycInfo
KW - Age Differences
KW - Cognitive Development
KW - Logical Thinking
KW - Memory
KW - Spatial Orientation (Perception)
RP - NOT IN FILE
SP - 530
EP - 542
JF - Developmental Psychology
JA - Dev Psychol
VL - 15:
IS - 5

N2 - Children's abilities to search for missing objects in real-life environments were investigated in 2 studies with a total of 70 2 yr 7 mo-6 yr 2 mo old Ss. Exp I involved searches of 8 locations on a school playground; Exp II involved searches of 8 large cupboards. In each study, Ss performed a free search followed by a logical search. In free search, the S was instructed to search the 8 locations to find a missing item. In logical search a critical search area within the 8 locations was defined for the Ss on the basis of (a) where the item was last seen and (b) where it was first discovered missing. In free search, more older than younger Ss searched each location at least once and searched all locations sequentially. Detailed comparisons of search patterns under the free and logical conditions revealed that older Ss understood the critical search area. In contrast, younger Ss' searches under the logical condition depended on one salient association of a location with the missing item. The developmental trends indicated that several memory and logical processes determine search procedures. The development and integration of these processes are discussed. (8 ref) (PsycINFO Database Record (c) 2012 APA, all rights reserved)
SN - 0012-1649
AD - Wellman, Henry W.: U Michigan Ctr for Human Growth & Development, Ann Arbor
ER -

TY - JOUR
ID - 3143
T1 - Difference and similarity in perceptual structure of parental leadership between parents and their children. [Japanese]
A1 - Fujita,Ayako
Y1 - 1978///
N1 - Peer Reviewed Journal: 1980-20891-001
Japanese
Journal; Peer Reviewed Journal
KW - PsycInfo
KW - Childrearing Practices
KW - Parent Child Relations
KW - Parents
KW - Social Perception
KW - Attitudes
KW - Parental Attitudes
RP - NOT IN FILE
SP - 111
EP - 119
JF - Japanese Journal of Experimental Social Psychology
VL - 17:
IS - 2
N2 - Analyzed similarities and discrepancies in the cognitive structures and attitudes of parents and children using a questionnaire and interviews. Ss were the members and parents of 5 classes of 2nd-yr students (75 boys and 65 girls) at a public middle school in Japan. The questionnaire used was constructed from H. Kojima's version of the Parental Behavior Inventory (PBI). From the 192 categories of the PBI, 16 categories weighted to measure psychological control/autonomy and 10 categories weighted for acceptance/rejection were selected. Items relating to acceptance, child-centeredness, positive involvement, and acceptive individuation were used to assess emotional support; and items relating to control, enforcement, control through guilt, hostile control, instilling persistent anxiety, extreme autonomy, and intrusiveness were used to assess the control/autonomy relationship. The children were interviewed during a morality class at school, and the parents were each sent a questionnaire. The 4 dyadic relationships-father/son, father/daughter, mother/son, and mother/daughter-were separately analyzed. Results show several areas of discrepancy. Fathers rated questioning their sons about outside activities as showing a personal interest in their son's behavior and thereby offering emotional support; the sons viewed this questioning as a control mechanism. A complex qualitative difference was noted in the cognitive perceptions of girls in relating to both of their parents. (English summary) (17 ref) (PsycINFO Database Record (c) 2012 APA, all rights reserved)
SN - 0387-7973
AD - Fujita, Ayako: Osaka U, Japan
ER -

TY - JOUR
ID - 3144
T1 - Extraversion and outdoor play in middle childhood
A1 - Roberts,Alasdair
Y1 - 1978///
N1 - Peer Reviewed Journal: 1980-11804-001
English
Journal; Peer Reviewed Journal
KW - PsycInfo
KW - Childhood Play Behavior
KW - Childrens Recreational Games
KW - Environmental Attitudes
KW - Extraversion
RP - NOT IN FILE
SP - 37
EP - 42
JF - Educational Research
VL - 21:
IS - 1
N2 - Administered the Junior Eysenck Personality Inventory to 334 children ages 11-13 yrs. Ss' familiarity with the area's traditional outdoor games had previously been tested by multiple-choice questions and by asking them to list games from memory. A "home orientation" rating was also obtained from their reported attachment to indoor activities, including reading, hobbies, and TV. No relationships were found between extraversion, rejection of indoor activities, and familiarity with outdoor games. Daily exposure to school playgrounds was the most important factor in Ss' knowledge of traditional games, rather than personality or out-of-school habits. (19 ref) (PsycINFO Database Record (c) 2012 APA, all rights reserved)
SN - 0013-1881
AD - Roberts, Alasdair: Aberdeen Coll of Education, Scotland
ER -

TY - JOUR
ID - 3145
T1 - An examination of the indoor and outdoor play patterns of urban and rural preschool children
A1 - Ostro,Joel M.
Y1 - 1978///
N1 - Dissertation Abstract: 1979-25805-001
English
Dissertation Abstract
KW - PsycInfo
KW - Childhood Play Behavior
KW - Rural Environments
KW - Urban Environments
RP - NOT IN FILE
SP - 6247
EP - 6248
JF - Dissertation Abstracts International
VL - 38:
IS - 12-B
SN - 0419-4217
AD - Ostro, Joel M.: Pennsylvania State U
ER -

TY - JOUR
ID - 3146
T1 - Interpersonal touch among preschool children at play
A1 - Williams,Sharon J.
Y1 - 1978///

N1 - Peer Reviewed Journal: 1979-23585-001
English
Journal; Peer Reviewed Journal
KW - PsycInfo
KW - Blacks
KW - Dyads
KW - Human Sex Differences
KW - Racial and Ethnic Differences
KW - Whites
KW - Body Language
KW - Intimacy
KW - Peer Relations
KW - Social Class
RP - NOT IN FILE
SP - 501
EP - 508
JF - The Psychological Record
VL - 28:
IS - 4
N2 - Previous research has indicated that interpersonal touch is more likely for female than for male children, more likely for same-gender dyads, and more likely for Black than for White children. The effects of race and social class on touch have not been observed separately. In the present study, a total of 274 3-5 yr old children were observed at play in Black low-income, Black middle-income, White low-income, and White middle-income preschools. Rates of touch were higher for low-income children in inside play areas and higher for same-gender pairs. In outside play areas, touch was higher for Black same-gender pairs, but there was no race difference in touch for different-gender pairs. In general, rates of touch were higher than those previously reported for older American children and adults, but lower than those for both children and adults from some other cultures. (13 ref) (PsycINFO Database Record (c) 2012 APA, all rights reserved)
SN - 0033-2933
AD - Williams, Sharon J.: U Missouri, Kansas City
ER -

TY - JOUR
ID - 3147
T1 - Peer imitation in a natural setting
A1 - Abramovitch,Rona
Y1 - 1978///
N1 - Peer Reviewed Journal: 1979-20813-001
English
Journal; Peer Reviewed Journal
KW - PsycInfo
KW - Age Differences
KW - Childhood Play Behavior
KW - Childhood Play Development
KW - Imitation (Learning)
KW - Peers
KW - Dominance
RP - NOT IN FILE
SP - 60
EP - 65
JF - Child Development
JA - Child Dev
VL - 49:
IS - 1
N2 - 55 4-11 yr olds, in 5 age-groups, were observed for instances of immediate imitation of peers in a free-play setting. Imitation decreased with age, from an average of 14.82 to an average of 1.80 imitative acts per S per hour, with more verbal than motor acts being imitated for all age groups. Judges were not able to

discriminate between those acts imitated by the younger and those imitated by the older Ss. Dominant Ss (assessed by the amount of time an S was looked at and by teachers' ratings) were imitated more; in 4 of the 5 groups dominant Ss imitated more than nondominant ones. Among preschoolers, boys imitated more and were imitated more than girls. (PsycINFO Database Record (c) 2012 APA, all rights reserved)
SN - 0009-3920
AD - Abramovitch, Rona: U Toronto, Erindale Coll, Mississauga, Canada
ER -

TY - JOUR
ID - 3148
T1 - Crowding and aggressive behavior among school children. [French]
A1 - Desportes,Michele; Lemaine,Jean-Marie
Y1 - 1978///
N1 - Peer Reviewed Journal: 1981-21057-001
French
Journal; Peer Reviewed Journal
KW - PsycInfo
KW - Aggressive Behavior
KW - Crowding
RP - NOT IN FILE
SP - 937
EP - 944
JF - Bulletin de Psychologie
VL - 32:
IS - 18
N2 - Tested the hypothesis that crowded conditions increase aggressive behaviors. 10 male and 8 female elementary school students were observed twice weekly for 8 consecutive weeks during noon recreation on a crowded and an uncrowded day on the school playground. The 45-min recreation period was divided into 9 segments of 5 min each, and each S was observed once during each segment. Observed aggressive behaviors were coded into 5 categories: moderate verbal attack, menacing verbal attack, physical teasing, menacing gestures, and physical attack. Conditions of crowding in the home were reported from a parent questionnaire. No interaction effects were obtained between aggressive behaviors and density of crowding on the playground; however, Ss who displayed most aggressive behaviors tended to live in crowded home conditions. (9 ref) (PsycINFO Database Record (c) 2012 APA, all rights reserved)
SN - 0007-4403
ER -

TY - JOUR
ID - 3149
T1 - Behaviorally based masculine- and feminine-activity-preference scales for preschoolers: Correlates with other classroom behaviors and cognitive tests. [References]
A1 - Connor,Jane M.
Y1 - 1977///
N1 - Peer Reviewed Journal: 2007-17572-027
English
Journal; Peer Reviewed Journal
KW - PsycInfo
KW - Childhood Play Behavior
KW - Classroom Behavior
KW - Preference Measures
KW - Sex Roles
KW - Test Construction
KW - Femininity
KW - Human Sex Differences
KW - Intelligence
KW - Masculinity
KW - Preschool Students

KW - Visuospatial Ability
RP - NOT IN FILE
SP - 1411
EP - 1416
JF - Child Development
JA - Child Dev
VL - 48:
IS - 4
N2 - 2 sets of scales were developed designed to measure the strength of sex typing in children's play patterns in a naturalistic setting. Over a 12-week observational period, the scales based upon those activities showing a sex difference in play preferences appeared to be more stable than those scales based upon adult ratings of masculine and feminine activities. Masculine and feminine activity preferences, as measured by the more stable scales, were correlated with observational measures of other classroom behavior and performance on 3 cognitive tests. These results suggested that (a) many children have already learned to avoid opposite-sex activities by the time they enter nursery school; (b) sex-role learning during the preschool period appears to involve increasing attention to same-sex activities; and (c) the development of visual-spatial ability in boys is related to involvement in masculine activities. The advantages of a behaviorally based definition of masculine and feminine activity preference are discussed. (PsycINFO Database Record (c) 2012 APA, all rights reserved) (journal abstract)
SN - 0009-3920
AD - Connor, Jane M.: State University of New York at Binghamton, Binghamton, NY, US Serbin, Lisa A.: State University of New York at Binghamton, Binghamton, NY, US
ER -

TY - JOUR
ID - 3150
T1 - The non-English speaking child in your classroom
A1 - Allen,Virginia G.
Y1 - 1977///
N1 - Peer-Reviewed Status-Unknown: 1980-04053-001
English
Journal; Peer-Reviewed Status-Unknown
KW - PsycInfo
KW - Elementary School Students
KW - Elementary School Teachers
KW - Instructional Media
KW - Teacher Attitudes
KW - Teaching Methods
KW - Foreign Language Learning
RP - NOT IN FILE
SP - 504
EP - 508
JF - Reading Teacher
VL - 30:
IS - 5
N2 - Suggests specific attitudes and activities to help teachers meet the needs of non-English-speaking children. A series of questions with analysis of errors, exchange lessons with other classmates, dialogs with puppets, use of apparatus and materials, and physical education classes and recess may aid in evaluating and helping these children. Needed are (a) simple diagnostic tools to assess language abilities, (b) interesting teaching-English-as-a-Second-Language materials, and (c) materials that relate specifically to a child's particular language group and culture. (PsycINFO Database Record (c) 2012 APA, all rights reserved)
SN - 0034-0561
AD - Allen, Virginia G.: Ohio State U, Marion
ER -

TY - JOUR
ID - 3151

T1 - Some empirical studies concerning social integration in the "Orientierungsstufe." [German]
A1 - Charlton,Michael; Marx,Rita; Rudolph,Horst; Sievers,Beate
Y1 - 1977///
N1 - Peer-Reviewed Status-Unknown: 1980-02018-001
German
Journal; Peer-Reviewed Status-Unknown
KW - PsycInfo
KW - Elementary School Students
KW - Parental Occupation
KW - Social Class
KW - Social Interaction
KW - Student Attitudes
KW - Peer Relations
KW - Socioeconomic Class Attitudes
RP - NOT IN FILE
SP - 47
EP - 68
JF - Zeitschrift fur Empirische Padagogik
VL - 1:
IS - 4
N2 - In the 1st 2 classes of a pilot program for an integrated "Orientierungsstufe" (orientation classes for later choice of schools, Grades 5 and 6) in a Hamburg school, samples were taken for an "Ideal-Real" comparison of the extent of social integration in these classes. The following methods were used for verification: (a) a sociometric rating system: changes in sociometrical status during the 2-yr coeducational period (126 Ss); (b) behavioral observation: association frequency during recess (181 Ss); and (c) an attitude questionnaire: stereotype expectancies concerning members of the different social classes and their influence on the choice of friends (156 Ss). Certain classrooms showed class preferences when choosing a playmate. The expectancies regarding a doctor's son vs a dock worker's son differed significantly; these expectancies, however, were highly differentiated. On the whole, the extent of actual social integration in the school tested could be regarded as satisfactory. (34 ref) (PsycINFO Database Record (c) 2012 APA, all rights reserved)
AD - Charlton, Michael: Albert-Ludwigs-U Freiburg, Psychology Inst, West Germany
ER -

TY - JOUR
ID - 3152
T1 - Altruism in children: The significance of nonverbal behavior
A1 - Ginsburg,Harvey J.
Y1 - 1977///
N1 - Peer Reviewed Journal: 1979-30770-001
English
Journal; Peer Reviewed Journal
KW - PsycInfo
KW - Aggressive Behavior
KW - Altruism
KW - Cues
KW - Gestures
KW - Judgment
RP - NOT IN FILE
SP - 82
EP - 86
JF - Journal of Communication
VL - 27:
IS - 4
N2 - Observed 108 boys in Grades 3-6 for 12 wks during their recess hour on the school playground and videotaped incidents of fighting between 2 boys. Of 281 fights, 46 ended as a result of aid given by a 3rd boy. Analysis of the videotapes suggested that such acts of altruism had a high probability of occurrence when the aggressor failed to heed gestures communicating submission by the child under attack. In a 2nd study, 30 boys

and girls in Grades 4 and 5 were asked to predict whether a videotaped segment of a fight would be terminated by the intervention of a 3rd child or of its own accord. Although Ss had difficulty verbalizing the reasons for their judgments, the children could predict an act of altruism on the basis of nonverbal cues that took place between participants in a fight. Implications of the miscommunication of nonverbal cues between aggressor and the child under attack are discussed in terms of the "bystander effect." (PsycINFO Database Record (c) 2012 APA, all rights reserved)
SN - 0021-9916
AD - Ginsburg, Harvey J.: Southwest Texas State U
ER -

TY - JOUR
ID - 3153
T1 - The outdoor behavior of Chicano children in Colorado
A1 - Auslander,Nathan
Y1 - 1977///
N1 - Peer-Reviewed Status-Unknown: 1979-07494-001
English
Journal; Peer-Reviewed Status-Unknown
KW - PsycInfo
KW - Poverty Areas
KW - Recreation Areas
KW - Rural Environments
KW - Sex Linked Developmental Differences
KW - Urban Environments
KW - Childhood Play Behavior
KW - Mexican Americans
KW - Social Behavior
RP - NOT IN FILE
SP - 214
EP - 216
JF - Man-Environment Systems
VL - 7:
IS - 4
N2 - Describes a study of Chicano children 3-12 yrs old, of both sexes, living in 3 poverty areas in Colorado, 1 rural and 2 urban. Observations were made in early and late afternoon, 7 days/wk, in each of the 4 seasons. Results are summarized briefly as they show differences according to site, sex, and ages of the Ss. A troublesome finding in the urban areas is that the girls do not get a fair share of outdoor activity. They are more closely supervised and learn a more passive role than boys. At all sites different children use different spaces, and for different activities. The implications of the study for the design of schools for children are outlined. (PsycINFO Database Record (c) 2012 APA, all rights reserved)
SN - 0025-1550
AD - Auslander, Nathan: U Colorado
ER -

TY - JOUR
ID - 3154
T1 - Children's playgrounds: Survey of accidents
Y1 - 1977///
N1 - Peer-Reviewed Status-Unknown: 1978-28452-001
English
Journal; Peer-Reviewed Status-Unknown
KW - PsycInfo
KW - Accidents
KW - Playgrounds
KW - Preferences
RP - NOT IN FILE
SP - 8

EP - 13
JF - International Playground Association Newsletter
VL - 6:
IS - 5
N2 - The British Research Institute for Consumer Affairs conducted research on children's preferences, use, and accidents on different types of conventional play equipment. Methods included inspections of all playgrounds in 4 parliamentary constituencies, and surveys of parents, 7-12 yr old children, and hospital accident and emergency department reports in 3 additional communities. Preferences and use patterns ranged from high on flat swings and big combination slides to low for climbing frames, and various rocking horses and boats. All measures indicated that accidents were most pronounced on swings (23-51% of all accidents), then roundabouts (10-32%), slides (10-23%), and climbing frames (1-28%). The most common causes of injuries were (a) the surfaces on which the child fell, and (b) moving equipment. The Design Council of Great Britain estimates that at least 20,000 playground accidents a year need hospital treatment; the Research Institute for Consumer Affairs estimates that all accidents treated either by a doctor or a hospital approximate 150,000/yr. (PsycINFO Database Record (c) 2012 APA, all rights reserved)
ER -

TY - JOUR
ID - 3155
T1 - Effects of auditory stimulation upon the motor test performance of severely retarded children
A1 - Booth,G.
Y1 - 1977///
N1 - Peer Reviewed Journal: 1978-08099-001
English
Journal; Peer Reviewed Journal
KW - PsycInfo
KW - Attention
KW - Auditory Stimulation
KW - Motor Performance
KW - Intellectual Development Disorder
RP - NOT IN FILE
SP - 19
EP - 24
JF - AEP (Association of Educational Psychologists) Journal
VL - 4:
IS - 4
N2 - Exposed 9 retarded, overactive Ss age 5-14 to successive testing on 2 simple form boards. On the easier of the 2 tasks, mean performance time increased significantly when a controlled "soothing"" auditory input could be heard rather than normal playground noises. However, this difference disappeared on the more difficult task. Possible implications regarding attention and learning in retarded children are discussed. (PsycINFO Database Record (c) 2012 APA, all rights reserved)
ER -

TY - JOUR
ID - 3156
T1 - An ethological analysis of nonverbal inhibitors of aggressive behavior in male elementary school children. [References]
A1 - Ginsburg,Harvey J.
Y1 - 1977///
N1 - Peer Reviewed Journal: 1978-07723-001
English
Journal; Peer Reviewed Journal
KW - PsycInfo
KW - Aggressive Behavior
KW - Body Language
KW - Childhood Play Behavior
KW - Nonverbal Communication

RP - NOT IN FILE
SP - 417
EP - 418
JF - Developmental Psychology
JA - Dev Psychol
VL - 13:
IS - 4
N2 - Tested the hypothesis that a child's displays of human submission involving diminished body stature have a higher probability of terminating attack by an aggressor than other behaviors exhibited by the child during an agonistic encounter. Groups of 28-34 8-12 yr old male elementary school students were observed in 24 40-min sessions over a 6-wk period. A videotape unit was activated whenever the initiation of agonistic interaction was observed. Results indicate that behaviors involving a diminution of body stature (kneeling, bowing, shoe tying) were found to precede the cessation of agonistic activity between the students significantly more frequently than other forms of behavior exhibited by the student under attack (e.g., threat displays, face-to-face interaction). It is suggested that to the extent that the aggressive encounters of children during play activity represent at least one form of human agonistic activity, a constellation of behaviors exist which serve an appeasement function. (PsycINFO Database Record (c) 2012 APA, all rights reserved)
SN - 0012-1649
AD - Ginsburg, Harvey J.: Southwest Texas U
ER -

TY - JOUR
ID - 3157
T1 - An application of operant principles to instruction
A1 - Brigham,Thomas A.
Y1 - 1976///
N1 - Peer-Reviewed Status-Unknown: 1979-02011-001
English
Journal; Peer-Reviewed Status-Unknown
KW - PsycInfo
KW - Classroom Behavior Modification
KW - Elementary School Students
KW - Positive Reinforcement
KW - Sentence Structure
KW - Written Language
KW - Teaching Methods
RP - NOT IN FILE
SP - 45
EP - 48
JF - Educational Technology
VL - 16:
IS - 3
N2 - Most behavior modification programs that have been used in an educational setting have been concerned primarily with classroom discipline. In an application of operant procedures to the instructional setting, the present study analyzed the effects of antecedent stimuli (e.g., prompts, instructions, lessons) and contingent positive reinforcers on pupils' use of prepositional phrases in their written compositions. Ss were 24 middle-class pupils in a combined 3rd-4th grade classroom. A simple point system was utilized in which extra points for written assignments could be exchanged for extra recess or activity time. Instructions and differential reinforcement were used singly and in combination to assess their effectiveness. The sequence of experimental manipulations, and a summary of ANOVA values are given in tabular form. Results indicate that the condition combining classical components of instruction with the operant reinforcement procedure was far superior to the other conditions. It is suggested that an integrated program involving both antecedent and consequent components will be more effective than programs using either component alone. (PsycINFO Database Record (c) 2012 APA, all rights reserved)
SN - 0013-1962
AD - Brigham, Thomas A.: Washington State U, Ctr for Applied Behavioral Research
ER -

TY - JOUR
ID - 3158
T1 - Children's evaluation of the colors black and white and their interracial play behavior
A1 - Stabler,John R.
Y1 - 1976///
N1 - Peer Reviewed Journal: 1977-27292-001
English
Journal; Peer Reviewed Journal
KW - PsycInfo
KW - Aesthetic Preferences
KW - Attitudes
KW - Blacks
KW - Color
KW - Whites
KW - Interpersonal Interaction
RP - NOT IN FILE
SP - 191
EP - 197
JF - Child Study Journal
VL - 6:
IS - 4
N2 - Previous research has demonstrated that the connotations of the color white are positive and the connotations of the color black are negative. The present study investigated the relationship between children's response to these colors and their interracial behavior. 20 Black and White male and female 4-5 yr olds were observed in a free play situation on a school playground. Color preference and color attitudes were measured in 3 different ways. A correlation was found between playground behavior (i.e., positive or negative play with peers) and response to the colors black and white as measured in a situation in which Ss guessed the locus of origin of positive and negative self-evaluative statements broadcast simultaneously from black and white boxes. The correlation was substantially higher for females than for males. The high correlation for females is discussed in terms of a difference between the sexes in rate of perceptual and linguistic development. (PsycINFO Database Record (c) 2012 APA, all rights reserved)
SN - 0009-4005
AD - Stabler, John R.: Georgia State U
ER -

TY - JOUR
ID - 3159
T1 - Nursery school rooms and their effect on children's involvement
A1 - Hoffman,Marjorie
Y1 - 1976///
N1 - Peer-Reviewed Status-Unknown: 1977-22134-001
English
Journal; Peer-Reviewed Status-Unknown
KW - PsycInfo
KW - Architecture
KW - Classroom Environment
KW - Nursery Schools
KW - Participation
KW - Recreation
KW - Nursery School Students
RP - NOT IN FILE
SP - 54
EP - 87
JF - Graduate Research in Urban Education & Related Disciplines
VL - 8:
IS - 2

N2 - Attempted to answer the question, "Can a preschool classroom be designed in which children function at a high level of involvement without being directed and restricted by adults?" The 2-fold approach to this question involved studying 5 private nursery school classrooms and measuring the degree of involvement exhibited by the children in those rooms during free play. The classrooms were rated for quality through the use of a modified form of an instrument designed by Prescott, Jones, and Kritchevsky for studying playgrounds. The rooms with the higher ratings for space quality had fewer uninvolved children. The findings indicate a relationship between the quality of a play area, as defined and measured by the instrument, and young children's involvement there during free play. It is concluded that the instrument used is potentially useful for designing and evaluating classrooms. (19 ref) (PsycINFO Database Record (c) 2012 APA, all rights reserved)
SN - 0017-2839
ER -

TY - JOUR
ID - 3160
T1 - Free-play fantasy behavior in preschool children: Relations among gender, age, season, and location
A1 - Sanders,Karen M.
Y1 - 1976///
N1 - Peer Reviewed Journal: 1977-20907-001
English
Journal; Peer Reviewed Journal
KW - PsycInfo
KW - Age Differences
KW - Childhood Play Development
KW - Environment
KW - Seasonal Variations
KW - Sex Linked Developmental Differences
KW - Imagination
RP - NOT IN FILE
SP - 1182
EP - 1185
JF - Child Development
JA - Child Dev
VL - 47:
IS - 4
N2 - Time-sampled observations were made of the free-play fantasy behavior of 4 groups of 10 3-5 yr olds in a preschool setting. Males displayed more, and more active, fantasy play than females; older Ss engaged in more make-believe than younger ones. Increases in fantasy play over the year depended on age, sex, and the nature of the activity. Males and older Ss performed more make-believe play outdoors than females and younger Ss. Whereas interactive fantasy play was shown more often by males and older Ss, cooperative role taking was more common among females. The greater role-taking fantasy play of females as compared with males was not simply a function of the females greater use of a "homemaking" play area. The opportunity for outdoor play may be related to the greater overall fantasy play of males. (PsycINFO Database Record (c) 2012 APA, all rights reserved)
SN - 0009-3920
AD - Sanders, Karen M.: U California, Davis
ER -

TY - JOUR
ID - 3162
T1 - Levels of aggression in a traditional and a pluralistic school
A1 - Johnston,Kathleen D.
Y1 - 1976///
N1 - Peer Reviewed Journal: 1977-04397-001
English
Journal; Peer Reviewed Journal
KW - PsycInfo
KW - Aggressive Behavior

KW - Elementary School Students
KW - Open Classroom Method
KW - Recreation
KW - School Environment
KW - Verbal Communication
RP - NOT IN FILE
SP - 146
EP - 151
JF - Educational Research
VL - 18:
IS - 2
N2 - Tested the hypothesis that less aggression would be observed on the playground of a pluralistic school than on the playground of a traditional school. This hypothesis is based on the notion that the pluralistic school environment would be less frustrating to the student, and therefore students would be less likely to aggress during recess. Two undergraduates, naive to the purpose of the study, observed and tabulated 1st-3rd graders' aggressive verbalizations and behaviors during morning recesses at a pluralistic and a traditional school. The majority of pupils at each school were lower class. The traditional school was 30% Chicano, 5% Black, and 63% White, while the pluralistic school was 31% Chicano, 27% Black, and 38% White. Results indicate that significantly less physical and verbal aggression occurred on the playground at the pluralistic school. It is suggested that the implications of these results may be particularly valuable to the education of lower class and minority children. (PsycINFO Database Record (c) 2012 APA, all rights reserved)
SN - 0013-1881
AD - Johnston, Kathleen D.: Scripps Coll
ER -

TY - JOUR
ID - 3163
T1 - Preference for and achievement in elementary school subjects and associated personality traits
A1 - Mosak,Harold H.
Y1 - 1975///
N1 - Peer Reviewed Journal: 1980-04152-001
English
Journal; Peer Reviewed Journal
KW - PsycInfo
KW - Academic Achievement
KW - Elementary School Students
KW - Personality Traits
KW - Preferences
KW - Student Attitudes
RP - NOT IN FILE
SP - 23
EP - 26
JF - Individual Psychologist
VL - 12:
IS - 2
N2 - Suggests that spelling and penmanship are done well by children who wish to conform and are done poorly by rebels and "free spirits," and that when poor performance is attributed to such things as perceptual difficulties rather than attitudes, the children use it as an excuse in future troubles. Problem-solving subjects, such as mathematics and grammar, are done well by self-reliant children. The social myth that girls are not gifted in mathematics discourages them. Poor reading may be the result of rebellion against the entire educational and social system, of the poor reading material used in classes, or of social attitudes (e.g., reading is a "sissy" activity). Children who have recess as their favorite subject indicate that they have no use for schooling at all and find little enjoyment or excitement in it. After having eliminated medical and other factors, personality should be considered as a source of failure in various school subjects. (14 ref) (PsycINFO Database Record (c) 2012 APA, all rights reserved)
SN - 0019-7149
AD - Mosak, Harold H.: Private practice, Chicago, IL

ER -

TY - JOUR
ID - 3164
T1 - Intensive and extensive peer behavior: Longitudinal and cross-sectional analyses
A1 - Waldrop,Mary F.
Y1 - 1975///
N1 - Peer Reviewed Journal: 1977-01026-001
English
Journal; Peer Reviewed Journal
KW - PsycInfo
KW - Friendship
KW - Human Sex Differences
KW - Peer Relations
KW - Psychosocial Development
RP - NOT IN FILE
SP - 19
EP - 26
JF - Child Development
JA - Child Dev
VL - 46:
IS - 1
N2 - Data on peer relations were obtained when 35 males and 27 females were 21/2 yrs old and again when they were 71/2 yrs old. Ss who at age 21/2 were friendly, involved with their peers, and able to cope with aggressive peers were likely, at age 71/2, to spend many hours outside school with peers, to be socially at ease, and to be the ones who decided with whom they would play and what they would play. In other words, sociability at 21/2 was positively related to sociability at 71/2. Social behavior at 71/2 had very different meanings for males than for females. The highly social males, when with peers, tended to have extensive peer relations; that is, they usually played with groups of males. The highly social females, when with peers, tended to have intensive peer relations; that is, they usually played with only 1 other female. (28 ref) (PsycINFO Database Record (c) 2012 APA, all rights reserved)
SN - 0009-3920
AD - Waldrop, Mary F.: NIMH, Child Research Branch, Bethesda, MD
ER -

TY - JOUR
ID - 3165
T1 - The teacher as researcher: On moral development
A1 - Budin,Howard R.
Y1 - 1975///
N1 - Peer Reviewed Journal: 1976-29711-001
English
Journal; Peer Reviewed Journal
KW - PsycInfo
KW - Age Differences
KW - Childhood Development
KW - Moral Development
KW - Observation Methods
KW - Teacher Student Interaction
KW - Classroom Behavior
RP - NOT IN FILE
SP - 181
EP - 190
JF - The Urban Review
VL - 8:
IS - 3

N2 - Observed 2 classes of 20 children each, one group aged 5 and another aged 6-8, in accordance with beliefs that (a) spontaneous language and action reflect the thoughts of children more realistically than answers to the interview questions, that have traditionally, been used by most child development researchers; and (b) specially trained, outside researchers and evaluators are unnecessary for the valid documentation of social and moral development within schools, since observation, record keeping, and reflection upon child development constitute half of teaching. 307 children-to-teacher comments collected over 14 school days shed light on (a) children's concepts of school rules; (b) the effects on children of time schedules and of activity type and procedures; and (c) behavioral differences between ages and individuals. Explanations are suggested for findings. The relationship of the study to J. Piaget's views on environmental and social interaction effects on child development is discussed. Limitations of the study and its implications for further research are detailed. (PsycINFO Database Record (c) 2012 APA, all rights reserved)
SN - 0042-0972
AD - Budin, Howard R.: Central Park East School, New York, NY
ER -

TY - JOUR
ID - 3166
T1 - Programming response maintenance after withdrawing token reinforcement
A1 - Jones,Russell T.
Y1 - 1975///
N1 - Peer Reviewed Journal: 1975-21946-001
English
Journal; Peer Reviewed Journal
KW - PsycInfo
KW - Classroom Behavior Modification
KW - Special Education
KW - Token Economy Programs
KW - Intellectual Development Disorder
RP - NOT IN FILE
SP - 153
EP - 164
JF - Behavior Therapy
VL - 6:
IS - 2
N2 - Evaluated a strategy designed to maintain behavior after withdrawing a token reinforcement program. In a special education classroom, 4 6-8 yr old educable retarded children received token reinforcement for attentive classroom behavior throughout the day. Using a multiple-baseline design across morning and afternoon class sessions, the following phases were presented: Baseline, Token Reinforcement, Tokens plus Peer Praise and Delayed Reinforcement, and Teacher and Peer Praise with a Group Contingency. Phases after the initial token program were designed to gradually fade tokens and substitute "natural" reinforcers (peer praise and activities available in the setting) without a loss of behavior gains. In the last phase, tokens were no longer used in the classroom. 2 follow-up phases, conducted 2 and 9 wks after all contingencies had been withdrawn, showed that inappropriate behavior had been maintained at the low levels achieved during the initial program. Data at the end of the last follow-up phase (approximately 12 wks after any contingency had been in effect) failed to show recovery of inappropriate behavior. (37 ref) (PsycINFO Database Record (c) 2012 APA, all rights reserved)
SN - 0005-7894
AD - Jones, Russell T.: Pennsylvania State U
ER -

TY - JOUR
ID - 3167
T1 - Preschool children's use of space: Sex differences in outdoor play. [References]
A1 - Harper,Lawrence V.
Y1 - 1975///
N1 - Peer Reviewed Journal: 1975-07099-001
English
Journal; Peer Reviewed Journal

KW - PsycInfo
KW - Childhood Play Development
KW - Human Sex Differences
KW - Sex Roles
RP - NOT IN FILE
SP - 119
JF - Developmental Psychology
JA - Dev Psychol
VL - 11:
IS - 1
N2 - Observed 3-5 yr old children's free-play activities over a 2-yr period. Boys played outdoors consistently more than did girls, and girls spent more time indoors at craft tables and in the kitchen. (PsycINFO Database Record (c) 2012 APA, all rights reserved)
SN - 0012-1649
AD - Harper, Lawrence V.: U California, Davis
ER -

TY - JOUR
ID - 3168
T1 - Cognitive effects of playgroup attendance
A1 - Turner,Irene F.
Y1 - 1974///
N1 - Peer-Reviewed Status-Unknown: 1978-08729-001
English
Journal; Peer-Reviewed Status-Unknown
KW - PsycInfo
KW - Childhood Play Behavior
KW - Cognitive Development
KW - Disadvantaged
KW - Playgrounds
KW - Preschool Education
RP - NOT IN FILE
SP - 30
EP - 35
JF - Irish Journal of Education
VL - 8:
IS - 1-2
N2 - Compared 72 children who attended 8 preschool playgrounds for disadvantaged children in Belfast with 72 who attended the same playgrounds exactly twelve months later and with a control group of 72 nonattenders. Retests 6 mo after initial testing on the Peabody Picture Vocabulary Test and the Columbia Mental Maturity Scale, when the mean ages of the 3 groups were 50, 49, and 49 mo respectively, revealed significant differences between attenders and nonattenders on both measures. Between-playgroup and between-cohort differences were not significant. These effects, which occurred in the context of a traditional nursery curriculum, are attributed to the variety of activities in which the children were encouraged to engage and the warmth, interest, and participation of the playgroup leaders. (17 ref) (PsycINFO Database Record (c) 2012 APA, all rights reserved)
AD - Turner, Irene F.: Queen's U, Belfast, Northern Ireland
ER -

TY - JOUR
ID - 3169
T1 - The relations of mechanically recorded activity level to varieties of preschool play behavior
A1 - Halverson,Charles F.
Y1 - 1973///
N1 - Peer Reviewed Journal: 1975-00818-001
English
Journal; Peer Reviewed Journal
KW - PsycInfo

KW - Childhood Play Development
KW - Measurement
RP - NOT IN FILE
SP - 678
EP - 681
JF - Child Development
JA - Child Dev
VL - 44:
IS - 3
N2 - Reported relations between data from a simple and reliable activity recorder, O data of vigorous play in outdoor and indoor settings, and teachers' ratings of 58 21/2-yr-old children. The recorder consists of 3 small manual wristwatches placed in a small packet so that each watch records movement in 1 of 3 dimensions (horizontal, vertical, or lateral). Any movement across 1 dimension results in a movement of the sweep-hand of the appropriate watch. Data from the recorder were highly related to other objective data for both sexes outdoors, but only males showed generality of play across situations. Recorder data showed males more active than females and were related to nearly all teachers' ratings for both sexes. (15 ref) (PsycINFO Database Record (c) 2012 APA, all rights reserved)
SN - 0009-3920
AD - Halverson, Charles F.: National Inst. of Mental Health, Child Research Branch, Bethesda, Md
ER -

TY - JOUR
ID - 3170
T1 - Assessment of disadvantaged minority group children
A1 - Torrance,E.
Y1 - 1973///
N1 - Peer Reviewed Journal: 1974-24124-001
English
Journal; Peer Reviewed Journal
KW - PsycInfo
KW - Cultural Test Bias
KW - Disadvantaged
KW - Educational Measurement
KW - Minority Groups
RP - NOT IN FILE
SP - 3
EP - 10
JF - School Psychology Digest
VL - 2:
IS - 4
N2 - Presents some of the constructive approaches used to assess more adequately than do standard tests the status of disadvantaged minority group children. Most standard tests exhibit both racial and socioeconomic bias in their questions. Research supporting this contention is described. It is suggested that tests biased in favor of disadvantaged minorities could give useful information about what might be expected of these children. Attempts to create such instruments are detailed. Other alternatives suggested are improved test administration and nontest kinds of assessment, e.g., observations made in the classroom or playground. (PsycINFO Database Record (c) 2012 APA, all rights reserved)
SN - 0160-5569
AD - Torrance, E. Paul: U. Georgia
ER -

TY - JOUR
ID - 3171
T1 - Children's choice of playground equipment: Development of methodology for integrating user preferences into environmental engineering. [References]
A1 - Peterson,George L.
Y1 - 1973///

N1 - Peer Reviewed Journal: 1974-12129-001
English
Journal; Peer Reviewed Journal
KW - PsycInfo
KW - Human Factors Engineering
KW - Playgrounds
KW - Preference Measures
KW - Recreation
KW - Toy Selection
RP - NOT IN FILE
SP - 233
EP - 238
JF - Journal of Applied Psychology
VL - 58:
IS - 2
N2 - Determined whether preference scaling techniques could be used to aid engineering system design. Children's play equipment was selected as the design area. Paired comparison and rank-order methods were used to determine whether 48 3rd-4th graders had preferences for different types of play equipment; whether these preferences were reliable over time; and whether actual use of play equipment was predictable from the preference scales developed. Photographic stimuli were used for the scaling and were presented to Ss. The frequency of use of play equipment was also determined using time-lapse photography. Results indicate that (a) Ss did have stable and reliable preferences for play equipment and (b) these preferences were correlated with actual usage of the equipment. (PsycINFO Database Record (c) 2012 APA, all rights reserved)
SN - 0021-9010
AD - Peterson, George L.: Northwestern U
ER -

TY - JOUR
ID - 3172
T1 - Children's playground activities in nursery school and in primary school: Modifications in content and concepts of play. [French]
A1 - Hurtig,Marie-Claude; Hurtig,Michel; Paillard,Monique
Y1 - 1972///
N1 - Peer-Reviewed Status-Unknown: 1975-04025-001
French
Journal; Peer-Reviewed Status-Unknown
KW - PsycInfo
KW - Childhood Play Development
KW - Elementary School Students
KW - Nursery School Students
RP - NOT IN FILE
SP - 185
EP - 200
JF - Cahiers de Psychologie
VL - 15:
IS - 3-4
N2 - Compared play activities of children in nursery and primary school in terms of social participation and play content. 12 boys and 12 girls were observed in upper nursery school and 5 boys and 5 girls in primary school. 6 boys and 6 girls were followed from nursery to primary school, and 20 boys and 20 girls through the 1st 3 mo of primary school. Nursery school play was characterized by greater variety, less structure, and a greater proportion of nonplay activity than primary school play (37% vs 14%). In the transition from nursery to primary school, differences in amount of structured play were more marked in girls than in boys. In semidirective interviews with 30 children, nursery children described their play without giving it a label. Primary school children would simply name the game, implying a commonly understood play culture. (English summary) (PsycINFO Database Record (c) 2012 APA, all rights reserved)
SN - 0373-8965
AD - Hurtig, Marie-Claude: U Provence, Lab de Psychologie de l'Enfant et de l'Education scolaire, France

ER -

TY - JOUR
ID - 3173
T1 - Play preferences of nursery school children
A1 - Muralidharan,Rajalakshmi
Y1 - 1972///
N1 - Peer-Reviewed Status-Unknown: 1974-21749-001
English
Journal; Peer-Reviewed Status-Unknown
KW - PsycInfo
KW - Childhood Play Development
KW - Kindergarten Students
KW - Nursery School Students
KW - Recreation
RP - NOT IN FILE
SP - 66
EP - 77
JF - Indian Educational Review
VL - 7:
IS - 2
N2 - Investigated the play preferences of 68 junior, senior nursery, and kindergarten school children in Delhi. Ages ranged from 4 yrs, 2 mo to 5 yrs, 3 mo. Ss were drawn from all socioeconomic status categories. Each child was observed 45 min daily for 5 days continuously by the classroom teacher during play situations. Ranks based on total preference scores were used to indicate the preference of the group for different play equipments. Slide, sound pit, nesting frames, and crayon drawing were found to be the preferred play activities. No relationship was seen in the play preference between boys and girls in kindergarten for outdoor games. The trend for indoor games at the junior nursery level was similar. Girls showed contrasting play preferences across educational levels. Results are interpreted in terms of maturational factors. (PsycINFO Database Record (c) 2012 APA, all rights reserved)
SN - 0019-4700
AD - Muralidharan, Rajalakshmi: National Inst. of Education, New Delhi, India
ER -

TY - JOUR
ID - 3174
T1 - Effects of social modeling on classroom performance
A1 - Heffernan,Michael S; Forness,Steven
Y1 - 1972///
N1 - Peer-Reviewed Status-Unknown: 1973-25728-001
English
Journal; Peer-Reviewed Status-Unknown
KW - PsycInfo
KW - Attention
KW - Elementary School Students
KW - Imitation (Learning)
KW - Mathematics Education
RP - NOT IN FILE
SP - 3
EP - 9
JF - Florida Journal of Educational Research
VL - 1972, pp. 3-9.
N2 - Used a single-S research design to examine the effects of peer modeling on arithmetic-task attention in 3 males 9, 10, and 11 yr. old. Models were chosen on the basis of playground observations and introduced for brief periods in the classroom near the target Ss. Time spent looking at assignments increased for 2 Ss, and produced adverse effects in the 3rd. Differential uses of modeling techniques in the classroom are discussed. (PsycINFO Database Record (c) 2012 APA, all rights reserved)

SN - 0428-7355
ER -

TY - JOUR
ID - 3175
T1 - Relationships of space utilization by children with selected aspects of behavior
A1 - Mulhauser,Frederick A.
Y1 - 1972///
N1 - Peer-Reviewed Status-Unknown: 1973-24602-001
English
Journal; Peer-Reviewed Status-Unknown
KW - PsycInfo
KW - Motor Development
KW - Motor Processes
KW - Perceptual Development
KW - Playgrounds
KW - Recreation
RP - NOT IN FILE
SP - 47
EP - 54
JF - Research Quarterly
JA - Res Q
VL - 43:
IS - 1
N2 - Filmed 54 kindergartners while at play on the school playground. Space utilization was assessed by 10-min film segments taken on each of 6 randomly selected days covering a 7-wk period. Ss were also given the Bender Visual-Motor Gestalt Test, 3 performance subtests of the Wechsler Preschool Primary Scale of Intelligence, a body image test, a body-size concept test, and a self-drawing task (to measure space utilization in graphic production). Results lend support to the concept that young children can be differentiated by the amount of space each uses while engaged in self-initiated nonstructured locomotor movement. Also, relationships were found between space utilization by children and selected aspects of behavior. (PsycINFO Database Record (c) 2012 APA, all rights reserved)
AD - Mulhauser, Frederick A.: Wayne State U
ER -

TY - JOUR
ID - 3176
T1 - The activity-interaction group: A process for short-term counseling with elementary school children
A1 - Komechak,Marilyn G.
Y1 - 1971///
N1 - Peer-Reviewed Status-Unknown: 1973-07869-001
English
Journal; Peer-Reviewed Status-Unknown
KW - PsycInfo
KW - Educational Counseling
KW - Elementary School Students
KW - Group Counseling
RP - NOT IN FILE
SP - 13
EP - 20
JF - Elementary School Guidance & Counseling
VL - 6:
IS - 1
N2 - Presents a model for counseling groups of elementary school children which synthesizes theory and practice derived from T-group theory, developmental psychology, perceptual psychology, client-centered counseling, behaviorism, and the humanistic-existential orientation. The rationale for the use of this model in an educational setting is discussed. Group structure and guidelines for counselor behavior are described.

Descriptions and interpretations of 4 sessions with 1 female and 2 male 5-6 yr. olds are presented. It is concluded that the activity-interaction model allows for the arrangement of conditions required for learning and functioning both within and outside of the school. (PsycINFO Database Record (c) 2012 APA, all rights reserved)
SN - 0013-5976
AD - Komechak, Marilyn G.: Child Study Center, Ft. Worth, Tex
ER -

TY - JOUR
ID - 3177
T1 - Improved classroom performance by reinforcement outside of the classroom
A1 - McIntire,Roger; Davis,Gayle; Pumroy,Donald
Y1 - 1970///
N1 - Peer-Reviewed Status-Unknown: 1970-19514-001
English
Journal; Peer-Reviewed Status-Unknown
KW - PsycInfo
KW - Academic Achievement
KW - Classrooms
KW - Elementary School Students
KW - Reinforcement
KW - School Learning
RP - NOT IN FILE
SP - 747
EP - 748
JF - Proceedings of the Annual Convention of the American Psychological Association
VL - 5:
IS - Pt. 2
N2 - Each day, percentage scores on classroom and homework papers determined assignment of 26 5th and 27 6th grade students into 3 groups. Children who were above 90% or had 10% improvement were given access to all activities in a project room. Children with less than 10% improvement were given limited access to the activities. Children who decreased more than 10% were restricted to teaching machines. When spelling scores were no longer included in percentage scores, spelling scores decreased. When spelling was again counted but math was not, spelling scores increased and math scores decreased. Individual performances are presented for these reversals. (PsycINFO Database Record (c) 2012 APA, all rights reserved)
AD - McIntire, Roger: U. Maryland
ER -

TY - JOUR
ID - 3178
T1 - The effect of problem-solving training on the creative art work of sixth grade students
A1 - Slapo,Daniel
Y1 - 1969///
N1 - Peer-Reviewed Status-Unknown: 1971-31736-001
English
Journal; Peer-Reviewed Status-Unknown
KW - PsycInfo
KW - Cognitive Processes
KW - Creativity
KW - Drawing
KW - Elementary School Students
RP - NOT IN FILE
SP - 42
EP - 62
JF - Graduate Research in Urban Education & Related Disciplines
VL - 4:
IS - 2

N2 - Studied how to help children incorporate more original ideas into their art production. Ss were 30 6th graders. The instructional material was a series of problem-solving tasks presented in detective stories with cartoon illustrations in booklet form. There were 16 lessons with an average of 35 pages each. An opaque projector was used in order for the trainer to read to the entire class at 1 time and was followed up by oral discussions of the problems. The test was a framed 6" * 9 1/2" paper on which the S drew a picture appropriate to children playing in a school yard. Ss' drawings were evaluated by 4 art teachers as to whether they were stimulus-free or stimulus-bound, and on originality. Results indicate that the experimental group of 15 did significantly better on these variables than did the 15 controls. Findings suggest that schoolchildren are trained to give responses similar to teachers' directions. Training by projecting the lessons on a screen to the entire class encouraged oral discussion and produced enthusiasm and involvement. Further study is needed to see if this creative expression remains with these Ss or if continuous training is necessary. The study indicates that creative expression can be stifled or developed in accord with atmosphere and curriculum variables. (34 ref.) (PsycINFO Database Record (c) 2012 APA, all rights reserved)
SN - 0017-2839
AD - Slapo, Daniel: City Coll., City U. New York, School of Education
ER -

TY - JOUR
ID - 3179
T1 - Perception of prejudice in Mexican-American preschool children
A1 - Werner,Norma E; Evans,Idella
Y1 - 1968///
N1 - Peer Reviewed Journal: 1969-09543-001
English
Journal; Peer Reviewed Journal
KW - PsycInfo
KW - Color Perception
KW - Prejudice
KW - Visual Discrimination
RP - NOT IN FILE
SP - 1039
EP - 1046
JF - Perceptual and Motor Skills
VL - 27:
IS - 3, Pt. 2
N2 - Conducted structured doll-play interviews with 40 Mexican-American 4- and 5-yr-olds, to explore where and when skin color discrimination and the evaluation of color differences occur. The study attempted to determine whether discrimination and evaluation occur simultaneously and whether they were functions of the S's age and sex, or exposure to group participation outside the home. The Ss tended to group the dolls by sex and size before grouping them by skin color. Evaluation on the basis of skin color occurred at the same time the discrimination was made, "good" dolls being white and "bad" dolls dark. After exposure to school the doll with which the child identified was white, with a tendency for boys to perceive the white adult male doll as larger than the dark one of the same size. Major dimensions of good and bad parents and good and bad children were inferred from the S's descriptions of their behavior. Results have been related to comparable studies with regard to Negro and Oriental children and the similarities and differences discussed. (PsycINFO Database Record (c) 2012 APA, all rights reserved)
SN - 0031-5125
AD - Werner, Norma E.: San Jose State Coll
ER -

TY - JOUR
ID - 3180
T1 - Training of behavior in games with rules. [NonEnglish]
A1 - Artemova,L.
Y1 - 1968///

N1 - Peer-Reviewed Status-Unknown: 1969-04526-001
NonEnglish
Journal; Peer-Reviewed Status-Unknown
KW - PsycInfo
KW - Games
KW - Social Behavior
KW - Teaching Methods
RP - NOT IN FILE
SP - 26
EP - 29
JF - Doshkol'Noe Vospitanie
VL - 41:
IS - 9
N2 - Reports on observations made in the course of a study of the possibilities of teaching the principles of social behavior in older preschool children by means of collective games (both didactic and outdoor) where rules are observed. Desired outcomes are not immediate and require a long time to develop under the guidance of the teacher, since in the beginning the decisive influence on the behavior of children is not so much the rules as the behavior of play-partners, their attitude toward the rules, and the ability to influence playmates. (PsycINFO Database Record (c) 2012 APA, all rights reserved)
ER -

TY - JOUR
ID - 3181
T1 - SOCIAL BEHAVIOR OF FOUR-YEAR-OLD CHILDREN DURING OUTDOOR PLAY IN DAY CARE CENTERS
A1 - HUGGINS,JOYCE M.
Y1 - 1966///
N1 - Dissertation Abstract: 1967-03278-001
English
Dissertation Abstract
KW - PsycInfo
KW - Recreation
KW - Social Interaction
KW - Teachers
RP - NOT IN FILE
SP - 993
EP - 994
JF - Dissertation Abstracts International
VL - 27:
IS - 4-A
SN - 0419-4217
AD - HUGGINS, JOYCE M.: ARIZONA STATE U
ER -

TY - JOUR
ID - 3182
T1 - The case for a transitional first grade
A1 - Oberlin,Diana S.
Y1 - 1965///
N1 - Peer Reviewed Journal: 2011-17088-008
English
Journal; Peer Reviewed Journal
KW - PsycInfo
KW - Academic Underachievement
KW - Educational Programs
KW - School Based Intervention
KW - School Readiness

KW - School Transition
KW - Childhood Development
KW - Elementary School Students
KW - Kindergarten Students
KW - Parental Attitudes
RP - NOT IN FILE
SP - 129
EP - 133
JF - Psychology in the Schools
VL - 2:
IS - 2
N2 - The following report is a summary of what the Mountainside system has been attempting by way of early identification of a child's developmental level and needs, readiness for learning, and the ways learning can be fostered. In the spring of 1963, on reviewing the number of underachievers in the upper grades who were recognized as early as 1955-1957, but for whom no recommendation was made, and in recognizing the large number of immature and/or anxious children in the kindergarten and considering the competitive situation which such children encounter at the first grade level, it was decided by the administration to form a special first grade, which for want of a better term was named the Transitional First Grade. The major purpose of this transitional first grade was to allow children who needed extra growing and learning time to have the opportunity for a more individualized program, extra and special materials, a specialist teacher who has worked with nursery and kindergarten level children, plus the socialization opportunities of playground, music, special programs with the other children in more formal first grades. Twenty children were found to be eligible for the transitional first grade. The twenty sets of parents were then separately interviewed by the psychologist and the principal of the school the child attended. Of the twenty sets of parents interviewed, four elected to send the child to a private school first grade, and one family was so emotionally disturbed by the whole situation that its child was placed in a formal first grade. The transitional class was thus stared in September 1963 with fifteen pupils. Advantages and disadvantages of the transitional first grade are discussed. (PsycINFO Database Record (c) 2012 APA, all rights reserved)
SN - 0033-3085
AD - Oberlin, Diana S.: Mountainside Public Schools, Mountainside, NJ, US
ER -

TY - JOUR
ID - 3183
T1 - Psychological ecology of a nursery school
A1 - Shure,Myrna Beth
Y1 - 1963///
N1 - Peer Reviewed Journal: 1964-09945-001
English
Journal; Peer Reviewed Journal
KW - PsycInfo
KW - Childhood Development
KW - Ecology
KW - Games
KW - Nursery Schools
KW - Psychosocial Development
RP - NOT IN FILE
SP - 979
EP - 992
JF - Child Development
JA - Child Dev
VL - 34:
IS - 4
N2 - The patterns of behavior of 7 boys and 7 girls, 4 years old, were observed in the naturalistic environment of 5 indoor areas (art, book, doll, games, block) of a nursery school. 200 behavioral samples at 5-minute intervals were recorded in each area during the free play period. Interest centered on the number of appearances in each area, their mobility, whether activity was relevant to an area, the children's affect, the constructiveness

of behavior, and the complexity of social participation. (PsycINFO Database Record (c) 2012 APA, all rights reserved)
SN - 0009-3920
ER -

TY - JOUR
ID - 3184
T1 - Recording a child's behavior during routines
A1 - Cohen,Dorothy H.
Y1 - 1958///
N1 - Book: 2008-18848-003
English
Book; Authored Book
KW - PsycInfo
KW - Behavior
KW - Daily Activities
KW - Early Childhood Development
KW - Observation Methods
RP - NOT IN FILE
SP - 9
EP - 25
JF - Cohen, Dorothy H; Stern, Virginia
VL - :
IS - 1958
N2 - (from the chapter) Since we need a starting point, let us start with observing a child at tasks and behavior that make up so much a part of a young child's life--the routines. At school we generally think of these as clean-up, toileting, snack-time, lunch, rest, etc. These are the "uncreatrve" but necessary aspects of the program that are repeated day after day, the activities around which many a program revolves. Let us look at a child about to become involved in a routine--for example, getting dressed for outdoors. Although this seems to be a simple and obvious activity, let us look at a child with the following questions in mind: What is the stimulus for the activity? What is the setting? What seem to be the child's reactions? It sounds as though each of these questions requires an answer, as in a questionnaire. On the contrary, the questions are only reminders of things to be aware of as you are observing. (PsycINFO Database Record (c) 2012 APA, all rights reserved)
AD - Cohen, Dorothy H.: Paterson State College, NJ, US Stern, Virginia: Bank Street College of Education, NY, US
ER -

TY - JOUR
ID - 3185
T1 - Situational differences in leader behavior. [References]
A1 - Hare,A.
Y1 - 1957///
N1 - Peer Reviewed Journal: 2005-14242-002
English
Journal; Peer Reviewed Journal
KW - PsycInfo
KW - Childhood Play Behavior
KW - Leadership Style
KW - Social Interaction
KW - Playgrounds
RP - NOT IN FILE
SP - 132
EP - 135
JF - The Journal of Abnormal and Social Psychology
VL - 55:
IS - 1

N2 - Two types of leaders have been contrasted in the child study literature since the early 1930's, one group-oriented, the other, self-oriented. In the present research, systematic observations of boys of each type are made on the school playground, where these patterns have been observed in the past, and in the more intimate, less competitive situation of the back yard. Six group-oriented leaders and six self-oriented leaders were selected from the third grades of four New England public schools on the basis of a teacher's rating and ratings by two previous observers of the boys. During the winter and spring of 1954 these twelve nine-year-old boys were observed on the playground during a free play period and in the neighborhood, using the set of categories for interaction process analysis developed by Bales. The clinical judgments of teachers and social scientists are not entirely validated since boys of both leader types show the same amount of aggression on the school playground, although the self-oriented leader is more aggressive in the neighborhood. Other expected differences appear since the self-oriented leader gives fewer suggestions and shows more tension in both situations. The major finding of this research is that there are more differences between situations than between the two leader styles. (PsycINFO Database Record (c) 2012 APA, all rights reserved)
SN - 0096-851X
AD - Hare, A. Paul: Harvard U, Cambridge, MA, US
ER -

TY - JOUR
ID - 3186
T1 - A simultaneous observation-and-recording method with specimen records of activity patterns in young children. [References]
A1 - Washburn,Ruth W.
Y1 - 1936///
N1 - Peer Reviewed Journal: 2011-15702-012
English
Journal; Peer Reviewed Journal
KW - PsycInfo
KW - Activity Level
KW - Childhood Development
KW - Observation Methods
RP - NOT IN FILE
SP - 74
EP - 82
JF - Psychological Monographs
VL - 47:
IS - 2
N2 - For a number of years one of the primary concerns of those interested in the children who make up what is known as the Nursery Group at the Clinic of Child Development has been objectification of the records of observations of the spontaneous behavior of these young children during free play periods. The important question of method in the study of the social behavior of young children has recently been summarized and evaluated elsewhere. In this study, the 15 subjects were drawn from a group of normal, healthy children between the ages of 2 and 5 years. Three of the 15 children were in daily attendance in the Nursery during the academic year. Seventy-five observations were made, 5 observations of the activity patterns of each child. At least 2 of the 5 observations were outdoors. Each observation was 5 min. in length. The records obtained by the use of the device not only enable one to account relatively accurately for each second of time during which a child is under observation, but they also have a cinematographic quality. It is increasingly evident that this recording device can be used in making observations of many categories of behavior. It tends to increase the observer's objectivity and to decrease the tendency to interpret observations. The results show that if several observers, each one interested in a different behavior category, could make their observations of a child's behavior during the same 5-min. period, and then "pool" their observations, a relatively complete picture might result. (PsycINFO Database Record (c) 2012 APA, all rights reserved)
SN - 0096-9753
AD - Washburn, Ruth W.: Yale University, New Haven, CT, US
ER -

TY - JOUR
ID - 3187

T1 - The child's day in the preschool
Y1 - 1934///
N1 - Book: 2008-18814-004
English
Book; Authored Book
KW - PsycInfo
KW - Nursery Schools
KW - Preschool Education
KW - Age Differences
KW - Recreation Areas
KW - School Nurses
RP - NOT IN FILE
SP - 25
EP - 26
JF - Iowa Child Welfare Research Station
VL - :
IS - 1934
N2 - (create) This chapter discusses the preschool schedule of activities. Similarities and differences between the age groups (age 2-5) are discussed. Specific topics include daily inspection by the school nurse, and outdoor play. (PsycINFO Database Record (c) 2012 APA, all rights reserved)
ER -

TY - JOUR
ID - 3188
T1 - Playground behavior differentiating artistic from non-artistic children. [References]
A1 - Dow,Mildred
Y1 - 1933///
N1 - Peer Reviewed Journal: 2011-15504-009
English
Journal; Peer Reviewed Journal
KW - PsycInfo
KW - Artistic Ability
KW - Childhood Development
KW - Childhood Play Behavior
KW - Personality
RP - NOT IN FILE
SP - 82
EP - 94
JF - Psychological Monographs
VL - 45:
IS - 1
N2 - The question as to whether differences in personality distinguish individuals of one profession from those of another has received considerable attention in both popular and psychological thought. In the present study the following questions were considered: (a) is there a difference in the reaction patterns of an artistically superior group of children as compared to an artistically inferior group of children; (b) is there a difference in the amount of progressive activity of this artistically superior group as compared to the amount of progressive activity of this artistically inferior group. The subjects used for this comparison were children selected from the Preschool of the Iowa Child Welfare Research Station and the Iowa University Elementary School. We find that the Z group, both boys and girl, excel the X group in progressive activity on equipped playgrounds. The boys of the Z group are considerably more active than the girl, but the Z girl is considerably more active than the X boys. In general we conclude that probably the primary difference between artistic and inartistic groups is revealed in the greater interest of the artistic group and the correspondingly greater response to play materials. According to the above results because of their greater inactivity, girls may devote more time to play with materials of which art materials are a part. (PsycINFO Database Record (c) 2012 APA, all rights reserved)
SN - 0096-9753
ER -

TY - JOUR
ID - 3189
T1 - Concerning the magnitude of the behavior sample for the study of behavior traits in children
A1 - Smith,M.
Y1 - 1931///
N1 - Peer Reviewed Journal: 1933-04787-001
English
Journal; Peer Reviewed Journal
KW - PsycInfo
KW - Childhood Development
KW - Personality Traits
RP - NOT IN FILE
SP - 480
EP - 485
JF - Journal of Applied Psychology
VL - 15:
IS - 5
N2 - Two boys between six and seven years of age were observed for a total of 500 minutes in periods of approximately five minutes' duration. The observations were made in the playroom and on the playground of a child placing institution during regular play periods. The material on each child was analyzed and classified into 42 categories in such a manner as to have mutually exclusive groupings of 50 minutes each for intercorrelations. The instances of occurrence of certain types of behavior, such as facial movements, contact with others, walking, etc., were counted. Examination of data indicates that large samples of observed behavior show a higher reliability than small samples. However, even in 50-minute groupings, when conditions of observation are carefully controlled and short periodic observations are made, the mean reliability is well above .80, while 100-minute groupings are slightly below .90 and 150-min-minute groupings are about .92. (PsycINFO Database Record (c) 2012 APA, all rights reserved)
SN - 0021-9010
ER -

TY - JOUR
ID - 3190
T1 - The aim and development of child-psychology
A1 - Stern,William; Barwell,Anna [Trans]
Y1 - 1924///
N1 - Book: 2007-11827-001
English
Book; Authored Book
KW - PsycInfo
KW - Child Psychology
KW - Developmental Stages
KW - Early Childhood Development
KW - Montessori Method
KW - Psychoanalysis
KW - Trends
RP - NOT IN FILE
SP - 23
EP - 34
JF - Stern, William; Barwell, Anna [Trans]
VL - :
IS - 1924
N2 - (from the chapter) The entire youth of a human being falls quite naturally into three periods, each of six to seven years, and everyone of these periods may be looked upon as a whole, complete in itself. The distinguishing feature of the first period is its character of play. The human being develops his natural powers by play; by it the child, through his senses, comes to a realisation of the world outside himself, gains control over his own movements, and learns to express and communicate his ideas in speech. The student of the first period has to do with the child whose world is the nursery and the kindergarten, and the difference between the

psychology of early childhood and the psychology of school-age is no less marked than that between first education and school pedagogics. The problems of the first period are, primarily, of a genetic order concerned with the gradual unfolding of the mental powers from their very earliest beginning, whilst in school-psychology, the all-important point of interest is the existent psychic condition at fixed stages of childhood. And this difference makes itself felt even in the methods of scientific research; in early childhood by far the most important point is the continuous observation of as many sides as possible of the individual child, whilst at school-age we have to combine a wide observation of the child with experimental testing of separate powers and with statistic results of examination of great numbers. We will now attempt to give a short resume of the work done in child-psychology up to the present time, and, in so doing, follow first the main-trend development and then two remarkable off-shoots, viz. psychoanalysis and the Montessori movement. (PsycINFO Database Record (c) 2012 APA, all rights reserved)
ER -

TY - JOUR
ID - 3191
T1 - Pre-adolescence
A1 - Hall,G.
Y1 - 1907///
N1 - Book: 2009-04889-001
English
Book; Authored Book
KW - PsycInfo
KW - Childhood Development
KW - Developmental Age Groups
RP - NOT IN FILE
SP - 1
EP - 6
JF - Hall, G
VL - Stanley. :
IS - 1907, reprinted 1912
N2 - (from the chapter) The years from about eight to twelve constitute a unique period of human life. The acute stage of teething is passing, the brain has acquired nearly its adult size and weight, health is almost at its best, activity is greater and more varied than it ever was before or ever will be again, and there is peculiar endurance, vitality, and resistance to fatigue. The child develops a life of its own outside the home circle, and its natural interests are never so independent of adult influence. Perception is very acute, and there is great immunity to exposure, danger, accident, as well as to temptation. Reason, true morality, religion, sympathy, love, and esthetic enjoyment are but very slightly developed. Everything, in short, suggests that this period may represent in the individual what was once for a very protracted and relatively stationary period an age of maturity in the remote ancestors of our race, when the young of our species, who were perhaps pygmoid, shifted for themselves independently of further parental aid. Rousseau would leave prepubescent years to nature and to these primal hereditary impulsions and allow the fundamental traits of savagery their fling till twelve. Biological psychology finds many and cogent reasons to confirm this view if only a proper environment could be provided. The child revels in savagery, and if its tribal, predatory, hunting, fishing, fighting, roving, idle, playing proclivities could be indulged in the country and under conditions that now, alas! seem hopelessly ideal, they could conceivably be so organized and directed as to be far more truly humanistic and liberal than all that the best modern school can provide. We should transplant the human sapling, I concede reluctantly, as early as eight, but not before, to the schoolhouse. Even if it be prematurely, he must be subjected to special disciplines and be apprenticed to the higher qualities of adulthood; for he is not only a product of nature, but a candidate for a highly developed humanity. (PsycINFO Database Record (c) 2012 APA, all rights reserved)
AD - Hall, G. Stanley: Clark University, Worcester, MA, US
ER -

TY - JOUR
ID - 3192
T1 - Der Stundenplan
A1 - Jones,Guernsey
Y1 - 2005///

N1 - Peer Reviewed Journal: 2005-13206-011
English
Journal; Peer Reviewed Journal
KW - PsycInfo
KW - Education
KW - Fatigue
KW - Learning
KW - Schools
RP - NOT IN FILE
SP - 666
EP - 668
JF - Psychological Review
VL - 4:
IS - 6
N2 - Reviews the article "Der Studenplan" by H. Schiller (1897). The reviewer discusses the topic of fatigue and how it relates to conditions in the school room. The concept of designing opening and closing hours of school as well as the distribution of pauses for recreation based on fatigue is all discussed. In the article by Schiller, it is proposed that the first hour be devoted to the most difficult subject, followed by subjects of more general interest. The second hour should be followed by a somewhat longer recess, after which the child is prepared for another difficult subject. It is suggested that the last hour be devoted to subjects such as writing, singing, and gymnastic drill. Lastly, it is suggested that when only two hours a week are devoted to a subject, it gives much better results if the exercises come on consecutive days, or even on consecutive hours. (PsycINFO Database Record (c) 2012 APA, all rights reserved)
SN - 0033-295X
AD - Jones, Guernsey: University of Nebraska, NE, US
ER -

TY - JOUR
ID - 3193
T1 - Vitamin D status and childhood health
A1 - Shin,Y.H.
A1 - Shin,H.J.
A1 - Lee,Y.-J.
Y1 - 2013///
N1 - 2013661885
English
Journal: Review
KW - EMBASE
KW - age
KW - allergy
KW - Asthma/dt [Drug Therapy]
KW - bone mineralization
KW - Calcification
KW - calcium absorption
KW - cardiovascular disease
KW - Child
KW - child health
KW - ethnicity
KW - gender
KW - growth plate
KW - Human
KW - Insulin Resistance
KW - meta analysis (topic)
KW - milk
KW - neoplasm
KW - Obesity
KW - osteomalacia

KW - osteoporosis
KW - phosphate blood level
KW - Pregnancy
KW - Prevalence
KW - randomized controlled trial (topic)
KW - recommended drug dose
KW - respiratory tract disease
KW - respiratory tract infection
KW - review
KW - rickets
KW - risk factor
KW - season
KW - seasonal influenza/dt [Drug Therapy]
KW - Sunlight
KW - Vitamin D Deficiency/di [Diagnosis]
KW - vitamin D deficiency/dt [Drug Therapy]
KW - Vitamin D Deficiency/ep [Epidemiology]
KW - 25 hydroxyvitamin D/dt [Drug Therapy]
KW - colecalciferol/ct [Clinical Trial]
KW - colecalciferol/dt [Drug Therapy]
KW - placebo
RP - NOT IN FILE
SP - 417
EP - 423
JF - Korean Journal of Pediatrics
VL - 56
IS - 10
CY - South Korea
N2 - Vitamin D is an essential component of bone and mineral metabolism; its deficiency causes growth retardation and skeletal deformities in children and osteomalacia and osteoporosis in adults. Hypovi-taminosis D (vitamin D insufficiency or deficiency) is observed not only in adults but also in infants, children, and adolescents. Previous studies suggest that sufficient serum vitamin D levels should be maintained in order to enhance normal calcification of the growth plate and bone mineralization. Moreover, emerging evidence supports an association between 25-hydroxyvitamin D (25(OH)D) levels and immune function, respiratory disease, obesity, metabolic syndrome, insulin resistance, infection, allergy, cancers, and cardiovascular diseases in pediatric and adolescent populations. The risk factors for vitamin D deficiency in the pediatric population are season (winter), insufficient time spent outdoors, ethnicity, older age, more advanced stage of puberty, obesity, low milk consumption, low socioeconomic status, and female gender. It is recommended that all infants, children, and adolescents have a minimum daily intake of 400 IU (10 mug) of vitamin D. Since the vitamin D status of the newborn is highly related to maternal vitamin D levels, optimal vitamin D levels in the mother during pregnancy should be maintained. In conclusion, given the important role of vitamin D in childhood health, more time spent in outdoor activity (for sunlight exposure) and vitamin D supplementation may be necessary for optimal health in infants, children, and adolescents. 2013 by The Korean Pediatric Society
SN - 1738-1061
AD - (Shin) Department of Pediatrics, CHA Gangnam Medical Center, CHA University College of Medicine, Seoul, South Korea (Shin) Department of Pediatrics, National Medical Center, Seoul, South Korea (Lee) Department of Family Medicine, Yongin Severance Hospital, Yonsei University College of Medicine, Seoul, South Korea
ER -

TY - JOUR
ID - 3195
T1 - Is the epidemiology of alkhurma hemorrhagic fever changing?: A three-year overview in Saudi Arabia
A1 - Memish,Z.A.
A1 - Fagbo,S.F.
A1 - Ali,A.O.
A1 - AlHakeem,R.

A1 - Elnagi,F.M.
A1 - Bamgboye,E.A.
Y1 - 2014///
N1 - 2014163637
English
Journal: Article
KW - EMBASE
KW - Adolescent
KW - Adult
KW - Age Distribution
KW - Alkhurma hemorrhagic fever disease/ep [Epidemiology]
KW - article
KW - Child
KW - clinical feature
KW - disease surveillance
KW - fatality
KW - Female
KW - Flavivirus infection/ep [Epidemiology]
KW - gastrointestinal symptom
KW - Human
KW - Incidence
KW - Infant
KW - laboratory diagnosis
KW - major clinical study
KW - Male
KW - Prevalence
KW - preventive medicine
KW - real time polymerase chain reaction
KW - retrospective study
KW - reverse transcription polymerase chain reaction
KW - Saudi Arabia
KW - Sex Ratio
KW - symptomatology
KW - tick borne disease/ep [Epidemiology]
KW - virus detection
KW - virus isolation
KW - virus transmission
RP - NOT IN FILE
JF - PLoS ONE [Electronic Resource]
JA - PLoS ONE
VL - 9
IS - 2
CY - United States
N2 - Background: The epidemiology of Alkhurma hemorrhagic fever disease is yet to be fully understood since the virus was isolated in 1994 in the Kingdom of Saudi Arabia. Setting: Preventive Medicine department, Ministry of Health, Kingdom of Saudi Arabia. Design: Retrospective analysis of all laboratory confirmed cases of Alkhurma hemorrhagic fever disease collected through active and passive surveillance from 1st-January 2009 to December, 31, 2011. Results: Alkhurma hemorrhagic fever (AHFV) disease increased from 59 cases in 2009 to 93 cases in 2011. Cases are being discovered outside of the region where it was initially diagnosed in Saudi Arabia. About a third of cases had no direct contact with animals or its products. Almost all cases had gastro-intestinal symptoms. Case fatality rate was less than 1%. Conclusions: Findings in this study showed the mode of transmission of AHFV virus may not be limited to direct contact with animals or its products. Gastro-intestinal symptoms were not previously documented. Observed low case fatality rate contradicted earlier reports. Close monitoring of the epidemiology of AHFV is recommended to aid appropriate diagnosis. Housewives are advised to wear gloves when handling animals and animal products as a preventive measure. 2014 Memish et al

AD - (Memish, Fagbo, Ali, AlHakeem, Bamgboye) Public Health Directorate, Ministry of Health, Riyadh, Saudi Arabia (Elnagi) Ministry of Health, Najran, Saudi Arabia (Memish) College of Medicine, Al-Faisal University, Riyadh, Saudi Arabia (Bamgboye) Department of Medical Statistics and Epidemiology, University of Ibadan, Ibadan, Nigeria
ER -

TY - JOUR
ID - 3196
T1 - Methylphenidate treatment for child with narcolepsy-cataplexy syndrome: a case report
A1 - Dogru,H.
A1 - Gencoglan,S.
A1 - Ozatalay,E.
Y1 - 2013///
N1 - 75004804
English
Journal: Conference Abstract
KW - EMBASE
KW - Human
KW - Child
KW - psychopharmacology
KW - narcolepsy
KW - Adolescent
KW - Case Report
KW - cataplexy
KW - Sleep
KW - muscle tone
KW - daytime somnolence
KW - patient
KW - Hospital
KW - Male
KW - lifespan
KW - adulthood
KW - neuropsychological test
KW - nerve cell
KW - hypothalamus
KW - drop attack
KW - adolescence
KW - electromyogram
KW - somnolence
KW - Weight Gain
KW - nuclear magnetic resonance imaging
KW - Electroencephalography
KW - Blood
KW - Academic Achievement
KW - Physical Examination
KW - hallucination
KW - Wakefulness
KW - teacher
KW - parasomnia
KW - diseases
KW - allele
KW - Cerebrospinal Fluid
KW - clinical examination
KW - medical history
KW - Diagnosis
KW - Social Behavior
KW - Facial Expression

KW - mental instability
KW - brain
KW - Methylphenidate
KW - central stimulant agent
KW - orexin
KW - antidepressant agent
KW - modafinil
KW - neurotransmitter
KW - hydroxybutyric acid
RP - NOT IN FILE
SP - S181
EP - S182
JF - Bulletin of Clinical Psychopharmacology
VL - Conference: 5th International Congress on Psychopharmacology and International Symposium on Child and Adolescent Psychopharmacology
IS - 5
N2 - Narcolepsy-cataplexy syndrome is characterized by excessive, severe sleepiness during the day, sudden loss of muscle tone with emotional lability (cataplexy), sleep paralysis, hypnagogic or hypnopompic hallucinations and weight gain. Although none of the major symptoms is exclusive to narcolepsy, cataplexy is the most specific symptom and is rarely present outside of narcolepsy. For the first time as a clinical picture shaped by a sudden loss of muscle tone in 1877 was reported by Westphal. The onset of narcolepsy with cataplexy is usually during teenage and young adulthood (10-15 ages) and persists throughout the lifetime. We purposed to report a case with narcolepsy diagnosed. A-11-year-old male was brought to our hospital due to drop attacks, which had occurred two-three times in a week since three months, when he was playing. It was learned that the patient had sudden loss of muscle tone with emotional changes such as smiling, while he was playing, and after these attacks he had continued to play again. He was sleeping all day long. His teachers recognised the decreased academic performance. Routine blood biochemistry and hematological investigations were normal. Brain Magnetic Resonance Imaging (MRI) and Electroencephalography (EEG), and electromyogram (EMG) were normal. He was subjected to Multiple Sleep Latency Test (MSLT) resulting in the mean sleep latency of 1 minute with presence of excessive daytime sleepiness and demonstrated the presence of sleep-onset REM periods (SOREMP). Neuropsychological tests were normal. After the reevaluation of the history and physical examination, he was diagnosed as narcolepsy. Methylphenidate 10 mg/day treatment was started and the dose was increased to 20 mg/day after two weeks. After two months of methylphenidate treatment, the symptoms were resolved. Narcolepsy is a syndrome characterized by excessive sleeping. Pathophysiological studies have shown that the disease is caused by the early loss of neurons in the hypothalamus that produce hypocretin, a wakefulness-associated neurotransmitter present in cerebrospinal fluid. The cause of neural loss could be autoimmune since most patients have the HLA DQB1 allele that predisposes individuals to the disorder. Treatment is with stimulant drugs (modafinil or the other stimulants; methylphenidate etc.,) to suppress daytime sleepiness, antidepressants for cataplexy, and hydroxybutyrate for both symptoms. A clinical examination and exhaustive medical history are essential for diagnosis and treatment. Narcolepsy is not rare, but it is an underrecognized and underdiagnosed condition at younger age. If left undiagnosed and untreated, early onset narcolepsy can interfere with psychological, social, and cognitive functioning and development andundermine academic and social activities
SN - 1017-7833
AD - (Dogru, Gencoglan, Ozatalay) Akdeniz University, Faculty of Medicine, Department of Child and Adolescent Psychiatry, Antalya, Turkey
ER -

TY - JOUR
ID - 3197
T1 - The roles of vitamin D and cathelicidin in the development of acute asthma attacks in children
A1 - Batmaz,S.B.
A1 - Arikoglu,T.
A1 - Karaismailoglu,E.
A1 - Kuyucu,S.
Y1 - 2013///

N1 - 71368602
English
Journal: Conference Abstract
KW - EMBASE
KW - allergy
KW - Asthma
KW - Child
KW - clinical immunology
KW - Human
KW - serum
KW - Multivariate Analysis
KW - Risk
KW - blood level
KW - risk factor
KW - infectious complication
KW - innate immunity
KW - questionnaire
KW - Drug Therapy
KW - serology
KW - mite
KW - season
KW - sun exposure
KW - sampling
KW - Infection
KW - patient
KW - allergic disease
KW - adaptive immunity
KW - cathelicidin
KW - vitamin D
KW - marker
KW - vitamin D binding protein
KW - polypeptide antibiotic agent
KW - steroid
RP - NOT IN FILE
SP - 223
JF - Allergy: European Journal of Allergy and Clinical Immunology
VL - Conference: European Academy of Allergy and Clinical Immunology and World Allergy Organization World Allergy and Asthma Congress 2013 Milan Italy. Conference Start: 20130622 Conference End: 20130622. Conference Publication:
IS - var.pagings
N2 - Introduction: Recent evidence, about the various effects of vitamin D (vit D) on innate and adaptive immunity has led to search for its role in asthma and allergic diseases. The deffects in the innate immune system, such as the capacity to increase the production of broad spectrum antimicrobial peptides like cathelicidin may predispose to infectious complications. The aim of this study was to determine the role of vit D and cathelicidin in the development of acute asthma attacks among 7-17 years old allergic asthmatic-children. Material and Methods: The study included 35 patients with acute asthma exacerbation triggered by an infection, 32 children with controlled asthma and 21 healthy children, all matched by sampling season and for asthma subgroups, matched by mono-mite sensitisation and previous severity and medication score of asthma. In all groups, a comprehensive questionnaire, serum 25- OH vit D, vitamin D-binding protein (VDBP) and cathelicidin levels, markers of allergy, viral serology and spirometric indices were employed. Factors that influence serum vit D levels and the development of asthma attacks were evaluated with multivariate linear and logistic analysis. Results: The mean serum vit D level was 14.09 + 5.75 in the attack group, 28.47 + 13.88 in the stable asthma group and 12.95 + 7.15 in healthy controls. The differences between attack and stable asthma groups were highly significant ($P < 0.001$). On the contrary, mean cathelicidin level was significantly higher in acute asthma group than controlled asthmatics ($P = 0.002$). Cathelicidin levels showed a negative correlation with vit D levels ($P = 0.002$, -0.380 spearman c.c). Furthermore, there was a positive correlation between the spirometric indices and the level of vit D among asthmatics ($P < 0.05$). Multivariate analysis of risk factors that

may influence vit D levels revealed that younger age (P = 0.046), high BMI (P = 0.025), longer duration of sun exposure (P < 0.001), and high amount of dietary vit D (P < 0.001) independently increased serum vit D levels. Furthermore, multivariate analysis of risk factors that may result in acute asthma vs controlled asthmatics showed that the increase in serum levels of vitamin D significantly reduced the risk of asthma attacks (P = 0.030, adjusted odds ratio 0.862) independent of age, sex, allergic markers, use of inhaled steroids, BMI, time spent outside (as a marker of activity level), serum levels of cathelicidin and VDBP
SN - 0105-4538
AD - (Batmaz, Arikoglu, Kuyucu) Department of Pediatric Allergy and Immunology, Mersin University, Faculty of Medicine, Mersin, Turkey (Karaismailoglu) Department of Biostatistics, Hacettepe University, Faculty of Medicine, Ankara, Turkey
ER -

TY - JOUR
ID - 3198
T1 - The European survey on adverse systemic reactions due to allergen immunotherapy: The 'EASSI' pilot survey
A1 - Calderon,M.A.
A1 - Rodriguez Del,Rio P.
A1 - Vidal,C.
A1 - Pfaar,O.
A1 - Just,J.
A1 - Worm,M.
A1 - Linneberg,A.
A1 - Passalacqua,G.
A1 - Demoly,P.
Y1 - 2013///
N1 - 71368103
English
Journal: Conference Abstract
KW - EMBASE
KW - allergy
KW - Immunotherapy
KW - clinical immunology
KW - Asthma
KW - Human
KW - adverse drug reaction
KW - patient
KW - physician
KW - questionnaire
KW - Italy
KW - Dermatophagoides
KW - France
KW - Epidemiology
KW - drug surveillance program
KW - Medical Dictionary for Regulatory Activities
KW - Safety
KW - Child
KW - Pollen
KW - community
KW - Europe
KW - error
KW - Adult
KW - book
KW - Haplorhini
KW - respiratory tract allergy
KW - Ethics
KW - dander

KW - Spain
KW - register
KW - Alternaria
KW - Germany
KW - medical specialist
KW - clinical trial (topic)
KW - allergen
KW - immunoglobulin E
RP - NOT IN FILE
SP - 4
JF - Allergy: European Journal of Allergy and Clinical Immunology
VL - Conference: European Academy of Allergy and Clinical Immunology and World Allergy Organization World Allergy and Asthma Congress 2013 Milan Italy. Conference Start: 20130622 Conference End: 20130622. Conference Publication:
IS - var.pagings
N2 - Background: At present, there is no European report on clinically relevant systemic reactions due to the regular use of subcutaneous or sublingual allergen immunotherapy (SCIT and SLIT, respectively) outside clinical trials. Using an electronic survey and a 'harmonised terminology' according to MedDRA we aimed to prospectively collect systemic adverse reactions due to (allergen immunotherapy) AIT from real life clinical settings. Methods: Under the framework of the EAACI, a team of European specialists in AIT, pharmacovigilance, epidemiology and drugs regulation, set up a survey to be conducted, first as a pilot, in four countries (France, Italy, Germany and Spain). A designated 'country coordinator' is responsible for following specific country ethics requirements and to select at least 30 doctors per country. Patients have been recruited the same day they received their first dose of either SCIT or SLIT. The survey started on 01/09/12 and is planned to be finished on 31/01/14. Patient inclusion criteria are: adults and children, with IgE mediated pollen and/or house dust mite, Alternaria, and/or animal dander respiratory allergies who will initiate AIT. The Survey Monkey online survey instrument is used by participant doctors to submit information directly into a central base via a blinded coded programme which allows to follow any adverse reaction due to AIT without compromising patients' and doctors' anonymity. A list of symptoms and their definition was selected from the Med- DRA dictionary in order to report all adverse reactions in this survey. Results: Three questionnaires have been generated: the Doctor Questionnaire, the Patient Questionnaire and the Reaction questionnaire; as well as a handbook and a mistake report form. A list of 30 terms for single symptoms has been selected to report adverse reactions. As of Jan 15th 2013, a total of 144 doctors have been recruited, 1233 patients included and 15 adverse reactions due to AIT have been reported. Conclusion: This is the first report in Europe collecting systemic adverse reactions due to AIT in a prospective real life setting. This survey will bring interesting data to the scientific community and could provide the platform for a future pan-European registry on AIT safety
SN - 0105-4538
AD - (Calderon) Imperial College, National Heart and Lung Institute, London, United Kingdom (Rodriguez Del Rio) Hospital Universitario Nino Jesus, Allergy, Madrid, Spain (Vidal) Hospital Clinico, Universitario de Santiago de Compostela, Allergy, Santiago de Compostela, Spain (Pfaar) Zentrum fur Rhinologie und Allergologie, Universitats HNO-Klinik Mannheim, Wiesbaden, Germany (Just) Centre de l'Asthme et des Allergies, Hopital d'Enfant Armand Trousseau, Paris, France (Worm) Allergy-Center-Charite, Charite-Universitatsmedizin Berlin, Department of Dermatology and Allergology, Berlin, Germany (Linneberg) Glostrup University Hospital, Copenhagen University Hospital, Research Centre for Prevention and Health, Glostrup, Denmark (Passalacqua) Allergy and Respiratory Diseases, University of Genoa, Genoa, Italy (Demoly) Hospital Arnaud de Villeneuve, University Hospital of Montpellier and INSERM U657, Montpellier, France
ER -

TY - JOUR
ID - 3201
T1 - Study on air pollution and respiratory health of children in Delhi, India
A1 - Mathew,J.
A1 - Goyal,R.
A1 - Taneja,K.K.
A1 - Arora,N.
Y1 - 2014///

N1 - 71351075
English
Journal: Conference Abstract
KW - EMBASE
KW - Air Pollution
KW - Health
KW - Child
KW - Human
KW - India
KW - allergic asthma
KW - immunology
KW - questionnaire
KW - student
KW - Spirometry
KW - respiratory tract disease
KW - Pollution
KW - obstruction
KW - maximum permissible dose
KW - adolescence
KW - air pollutant
KW - Monitoring
KW - Traffic
KW - sampler
KW - Particulate Matter
KW - wheezing
KW - logistic regression analysis
KW - coughing
KW - school
KW - long term exposure
KW - Human Activities
RP - NOT IN FILE
SP - AB97
JF - Journal of Allergy and Clinical Immunology
VL - Conference: 2014 Annual Meeting of the American Academy of Allergy, Asthma and Immunology, AAAAI 2014 San Diego, CA United States. Conference Start: 20140228 Conference End: 20140304. Conference Publication:
IS - var.pagings
N2 - RATIONALE: Air pollutants may cause respiratory problems in children during adolescence. The study was aimed at assessing the respiratory health of children exposed to pollution in different areas of Delhi. METHODS: Schools were selected from commercial-Chandni Chowk (CC), industrial- Mayapuri (MP) and residential -Sarojini Nagar (SN) areas. Indoor and outdoor levels of SO₂, NO_x and Particulate matter (PM) were estimated using samplers in classrooms and at monitoring stations. Questionnaire was designed based on internationally valid questionnaires for respiratory illness. Students completed the questionnaire and performed spirometry test. RESULTS: Indoor and outdoor PM₁₀ concentration was highest in CC (815+354.45 & 337+85 mug/m²) and lower in MP (694.6+322.9 & 274+78mg/m²) and SN (534.3+94.22 & 197+48 mg/m²). These were too high & above the permissible limits of 100mg/m². However, levels of SO₂ and NO_xwere below the limit. Students (1814) aged between 12-16 years participated in the survey. Questionnaire data showed that "wheeze, cough and cold" were most prevalent in CC (29.6%) panel of students followed by MP (15.9%). Spirometry tests demonstrated that 19% of CC & 14% of MP subjects suffered mild obstruction whereas 16% of CC & 32% of MP subjects had severe obstruction. Conditional logistic regression analysis of the association between chronic exposure to PM and respiratory symptoms showed a significant positive relationship in these areas. CONCLUSIONS: Commercial and industrial zones with high traffic movement and human activities contribute more PM which affects the pulmonary health. The study indicates that high pollution levels may lead to respiratory illness in children
SN - 0091-6749

AD - (Mathew) CSIR, Institute of Genomics and Integrative Biology, Delhi, India (Goyal) CSIR, National Environmental Engineering Research Institute, Delhi, India (Taneja) CSIR, Istitute of Genomics and Integrative Biology, Delhi, India (Arora) CSIR, Institute of Genomics and Integrative Biology, New Delhi, India
ER -

TY - JOUR
ID - 3202
T1 - Educational activities to support compliance in inherited metabolic disorders (IMD) patients with protein/aminoacids restricted diet
A1 - Lavorgna,S.
A1 - Vinuesa,M.V.
A1 - Blasi,S.
Y1 - 2013///
N1 - 71346087
English
Journal: Conference Abstract
KW - EMBASE
KW - patient
KW - Human
KW - diet restriction
KW - Metabolism
KW - metabolic disorder
KW - Food
KW - workshop
KW - Diet
KW - dietary compliance
KW - prescription
KW - Eating
KW - metabolic regulation
KW - Self Care
KW - protein intake
KW - Patient Compliance
KW - Child
KW - acid
RP - NOT IN FILE
SP - S311
EP - S312
JF - Journal of Inherited Metabolic Disease
VL - Conference: 12th International Congress of Inborn Errors of Metabolism, ICIEM 2013 Barcelona Spain. Conference Start: 20130903 Conference End: 20130906. Conference Publication:
IS - var.pagings
N2 - In diets that require the calculation of protein/amino acids from natural food it is a constant demanding task to turn the dietary prescription into a "meal". Difficulties in dietary compliance are frequent and can be solved with educational strategies involving the child and family, to anticipate everyday situations and problems. Participatory multithematic workshops were designed based on the real needs. The aim of the study was to achieve nutritional treatment compliance through the implementation of educational workshops for IMD patients and their families. Seventeen patients (6m-14y) and 26 relatives attended 3 workshops planned according their needs, including educational and culinary activities. The topics discussed were qualitative (understanding IMD and diet, allowed/prohibited foods, use of medical/low-protein foods, handling special situations, self-care, eating outside the home) and quantitative (weigh-measure food, calculations, food records, exchanges). Recipes were designed to encourage participation and consumption of foods allowed, expanding to a variety of menus. Educational workshops encourage dietary compliance in a practical way, improve conventional instruction, and provide the opportunity to share ideas and experiences in the diet management. The continuation of these activities is important to measure and evaluate the impact of changes on adherence and hopefully improve the longterm metabolic control
SN - 0141-8955
AD - (Lavorgna, Vinuesa, Blasi) Hospital Garrahan. Area de Alimentacion, Buenos Aires, Argentina

ER -

TY - JOUR
ID - 3203
T1 - Use of miglustat in four children with infantile-onset Niemann-Pick disease type C
A1 - Hasanotlu,A.
A1 - Kucukcongar,A.
A1 - Tumer,L.
A1 - Ezgu,F.S.
A1 - Kasapkara,C.S.
Y1 - 2013///
N1 - 71345951
English
Journal: Conference Abstract
KW - EMBASE
KW - Niemann Pick disease
KW - Human
KW - Child
KW - Metabolism
KW - Therapy
KW - patient
KW - Female
KW - Speech
KW - girl
KW - Walking
KW - saccadic eye movement
KW - motor retardation
KW - gaze paralysis
KW - eye movement disorder
KW - disability
KW - Male
KW - miglustat
KW - chitotriosidase
RP - NOT IN FILE
SP - S277
JF - Journal of Inherited Metabolic Disease
VL - Conference: 12th International Congress of Inborn Errors of Metabolism, ICIEM 2013 Barcelona Spain. Conference Start: 20130903 Conference End: 20130906. Conference Publication:
IS - var.pagings
N2 - Miglustat is the only therapy options in NPC patients. Here discussed the effect of miglustat therapy in four children with NPC disease and initial symptoms of visceromegaly. Case 1 A 10 days old female infant was diagnosed NPC. Due to her vertical gaze palsy, motor retardation, the miglustat therapy was started at the age of 3 years. She can walk with support outside, start to speak at the first year of the therapy. Case 2 A 10 months old female was diagnosed NPC. Neurological development was completely normal until the age of five years. The abnormal saccadic eye movement was noted and she was started on miglustat therapy. Case 3 A 3 years old male patient was diagnosed NPC. Because of his walking, speech disabilities, abnormal eye movements, miglustat therapy was started at 4 years old. His walking condition was stable, his speech was improved. Case 4 A 2 months old girl patient was diagnosed NPC. The miglustat therapy was started at the age of one year because of her neurodevelopmental delay. But we had to stop the treatment at the three months of the therapy, because of the intractable diahrrea. The level of chitotriosidase activity was decreased in all patients
SN - 0141-8955
AD - (Hasanotlu, Kucukcongar, Tumer, Ezgu, Kasapkara) Div Metab Dis, Univ Gazi Hosp, Ankara, Turkey
ER -

TY - JOUR
ID - 3204

T1 - Improvement in social deficits in autism spectrum disorders using a theatre-based, peer-mediated intervention
A1 - Corbett,B.A.
A1 - Swain,D.M.
A1 - Coke,C.
A1 - Simon,D.
A1 - Newsom,C.
A1 - Houchins-Juarez,N.
A1 - Jenson,A.
A1 - Wang,L.
A1 - Song,Y.
Y1 - 2014///
N1 - 2014135009
English
Journal: Article
KW - EMBASE
KW - Adolescent
KW - Arousal
KW - article
KW - autism/th [Therapy]
KW - Child
KW - child parent relation
KW - clinical article
KW - Cognitive Therapy
KW - comparative study
KW - disease association
KW - Female
KW - Human
KW - Male
KW - neuropsychological test
KW - Peer Group
KW - Perception
KW - preschool child
KW - pretest posttest design
KW - priority journal
KW - school child
KW - Social Behavior
KW - Social Cognition
KW - Social Interaction
KW - summer camp model
KW - hydrocortisone/ec [Endogenous Compound]
RP - NOT IN FILE
SP - 4
EP - 16
JF - Autism Research
VL - 7
IS - 1
CY - United States
N2 - Social Emotional NeuroScience Endocrinology Theatre is a novel intervention program aimed at improving reciprocal social interaction in youth with autism spectrum disorder (ASD) using behavioral strategies and theatrical techniques in a peer-mediated model. Previous research using a 3-month model showed improvement in face perception, social interaction, and reductions in stress. The current study assessed a 2-week summer camp model. Typically developing peers were trained and paired with ASD youth (8-17 years). Social perception and interaction skills were measured before and after treatment using neuropsychological and parental measures. Behavioral coding by reliable, independent raters was conducted within the treatment context (theatre) and outside the setting (playground). Salivary cortisol levels to assess physiological arousal were measured across contexts (home, theatre, and playground). A pretest-posttest design for within-group

comparisons was used, and prespecified pairwise comparisons were achieved using a nonparametric Wilcoxon signed-rank test. Significant differences were observed in face processing, social awareness, and social cognition (P<0.05). Duration of interaction with familiar peers increased significantly over the course of treatment (P<0.05), while engagement with novel peers outside the treatment setting remained stable. Cortisol levels rose on the first day of camp compared with home values yet declined by the end of treatment and further reduced during posttreatment play with peers. Results corroborate previous findings that the peer-mediated theatre program contributes to improvement in core social deficits in ASD using a short-term, summer camp treatment model. Future studies will explore treatment length and peer familiarity to optimize and generalize gains. Autism Res 2014,7: 4-16. 2013 International Society for Autism Research, Wiley Periodicals, Inc
SN - 1939-3792
AD - (Corbett, Swain, Simon, Newsom, Jenson) Department of Psychiatry, Vanderbilt University, Nashville, TN, United States (Corbett, Swain, Simon, Newsom, Houchins-Juarez, Wang) Vanderbilt Kennedy Center, Nashville, TN, United States (Coke) University School of Nashville, Nashville, TN, United States (Wang, Song) Department of Biostatistics, Vanderbilt University, Nashville, TN, United States
ER -

TY - JOUR
ID - 3206
T1 - Non-overweight and overweight children's physical activity during school recess
A1 - Ridgers,N.D.
A1 - Saint-Maurice,P.F.
A1 - Welk,G.J.
A1 - Siahpush,M.
A1 - Huberty,J.L.
Y1 - 2014///
N1 - 2014111180
English
Journal: Article
KW - EMBASE
KW - accelerometer
KW - article
KW - Child
KW - cross-sectional study
KW - Female
KW - Human
KW - major clinical study
KW - Male
KW - Obesity
KW - Physical Activity
KW - priority journal
KW - school
KW - school child
KW - school recess
KW - United States
RP - NOT IN FILE
SP - 129
EP - 136
JF - Health Education Journal
VL - 73
IS - 2
CY - United Kingdom
N2 - Objective: Little research has investigated children's physical activity levels during school recess and the contribution of recess to school day physical activity levels by weight status. The aims of this study were to examine non-overweight and overweight children's physical activity levels during school recess, and examine the contribution of recess to school day physical activity. Design: Cross-sectional. Setting: Four elementary schools located in Nebraska, United States of America (USA). Methods: Two hundred and seventeen children (99 boys, 118 girls; 47.9% overweight) wore a uni-axial accelerometer for five consecutive school days during

autumn 2009. The proportion of time spent engaged in sedentary (SED), light (LPA), moderate (MPA) and vigorous (VPA) intensity physical activity during recess was determined using age-specific accelerometer thresholds. Results: Overweight children engaged in more %MPA and less %VPA than non-overweight children, respectively. No differences were found between overweight and healthy weight children's moderateto- vigorous physical activity (MVPA). Recess contributed 16.9% and 16.3% towards non-overweight and overweight children's school day %MVPA, respectively. Conclusion: Examining %MVPA as an outcome variable may mask differences in recess physical activity levels between non-overweight and overweight children. Future research is needed to establish why healthy weight and overweight children engage in differing levels of %MPA and %VPA during recess. 2013 The Author(s)
SN - 0017-8969
AD - (Ridgers) Centre for Physical Activity and Nutrition Research, Deakin University, 221 Burwood Highway, Burwood, VIC 3125, Australia (Saint-Maurice, Welk) Department of Kinesiology, Iowa State University, United States (Siahpush) Department of Health Promotion, Social and Behavioral Health, College of Public Health, University of Nebraska Medical Center, United States (Huberty) College of Education, University of Nebraska, Omaha, United States
ER -

TY - JOUR
ID - 3207
T1 - Children with generalized joint hypermobility and musculoskeletal complaints: State of the art on diagnostics, clinical characteristics and treatment
A1 - Engelbert,R.
A1 - Scheper,M.
A1 - Rameckers,E.
A1 - Verbunt,J.
A1 - Remvig,L.
A1 - Juul-Kristensen,B.
Y1 - 2013///
N1 - 71334936
English
Journal: Conference Abstract
KW - EMBASE
KW - Human
KW - joint laxity
KW - musculoskeletal disease
KW - Diagnosis
KW - rheumatology
KW - rheumatic disease
KW - Child
KW - fitness
KW - environmental factor
KW - narrative
KW - disability
KW - Physical Activity
KW - Muscle Strength
KW - proprioception
KW - sport
KW - data base
KW - synthesis
KW - osteogenesis imperfecta
KW - systematic review (topic)
KW - longitudinal study
KW - MEDLINE
KW - systematic review
KW - Motor Development
KW - bicycle
KW - Young Adult

KW - connective tissue
KW - Prevalence
KW - arthralgia
KW - distress syndrome
KW - Rehabilitation
KW - skin blood vessel
KW - Exercise
KW - Pain
KW - collagen disease
KW - intervention study
KW - chronic pain
RP - NOT IN FILE
JF - Annals of the Rheumatic Diseases
JA - Ann Rheum Dis
VL - Conference: Annual European Congress of Rheumatology of the European League Against Rheumatism, EULAR 2013 Madrid Spain. Conference Start: 20130612 Conference End: 20130615. Conference Publication:
IS - var.pagings
N2 - Background For children the prevalence of generalized joint hypermobility (GJH) varies from 7-36%, primarily depending on the tests and criteria used for diagnosing GJH. Children experience a great variety of impairments as a result of increased laxity of connective tissues. This not only affects physical fitness, motor development, and proprioception, but also include problems with organsystems (skin, vessel and internal organs) and psychological distress. As a result, children experience functional disability, which often presents difficulties in normal daily life. Objectives To provide a state of the art on diagnostics, clinical characteristics and treatment of GJH and JHS in children and young adults. Methods A narrative review was performed regarding diagnostics and clinical characteristics. A systematic review with a critical appraisal and best evidence synthesis was performed regarding interventions. Searches of databases Medline and Central were performed. The search terms used were joint hypermobility in GJH, JHS, as well as collagen diseases with joint hypermobility, child, treatment and rehabilitation. Results Transition literature was explored by ICF domains body function and structure, activity, participation and environmental factors. Knowledge on function and activity in GJH and JHS showed joint hypermobility (based on the Beighton scale with different cut-off points) associated with joint pain, reduced muscle strength and proprioception while children with GJH did not experience functional limitations in daily activities. Children with GJH did not spend fewer weekly hours of physical activity than healthy reference groups. In children with JHS and exercise-induced pain reduced aerobic fitness was found. Knowledge on participation showed a significantly decreased participation in housework, riding a bicycle, taking part in sport or outdoor games, and a higher frequency for non-sporting games. The initial search on interventions identified 1318 studies, eventually leading to three studies (JHS: n=2, Osteogenesis Imperfecta: n=1) available for critical appraisal. These studies showed significant benefits of enhancing physical fitness in terms of relieving musculoskeletal complaints and reducing disability. Conclusions Based on the narrative and a systematic reviews, function and activity in GJH and JHS were negatively influenced, especially in JHS. Regarding participation, personal and environmental factors was significantly decreased for participation in housework, taking part in sport or outdoor games, as well as a higher frequency for non-sporting games. Based on the sparsely available knowledge of intervention studies, future longitudinal studies should focus on the effect of physical activity and fitness interventions, as well as muscle strength and stabilization training in general, in particular in relation to the hypermobile joints in particular. In JHS and chronic pain, the effectiveness of a multidisciplinary approach should be investigated
SN - 0003-4967
AD - (Engelbert, Scheper) Rehabilitation, University Hospital Amsterdam (AMC), Amsterdam, Netherlands (Engelbert, Scheper) Education for Physical therapy, University of Applied Sciences, Amsterdam, Netherlands (Rameckers, Verbunt) Rehabilitation Medicine, Maastricht University Hospital, Maastricht, Netherlands (Remvig) Rheumatology, Rigshospitalet, Copenhagen, Denmark (Juul-Kristensen) Research Unit for Musculoskeletal Function and Physiotherapy, Institute of Sports Science and Clinical Biomechanics, Odense, Denmark
ER -

TY - JOUR
ID - 3208
T1 - The use of mobile computing devices in anesthesia resident education: A cross-sectional survey study

A1 - Sun,J.S.
A1 - Clemenson,A.M.
A1 - Garbin,M.C.
A1 - Chu,L.F.
Y1 - 2013///
N1 - 71325606
English
Journal: Conference Abstract
KW - EMBASE
KW - Anesthesia
KW - Education
KW - Society
KW - Technology
KW - devices
KW - Human
KW - medical education
KW - operating room
KW - Pain
KW - Child
KW - Environment
KW - intensive care
KW - Reading
KW - computer
KW - student
KW - anesthesiology
KW - United States
KW - Voice
KW - procedures
KW - regional anesthesia
KW - general hospital
KW - coffee
KW - e-mail
KW - data analysis
KW - analgesia
KW - Infant
KW - obstetric anesthesia
KW - simulator
KW - gender
KW - questionnaire
KW - multiple choice test
KW - book
KW - reproducibility
KW - Newborn
KW - survival
KW - case study
KW - Intubation
KW - fiber optics
KW - residency education
KW - ether
RP - NOT IN FILE
SP - 68
EP - 69
JF - Anesthesia and Analgesia
VL - Conference: 2013 Annual Meeting of the Society for Technology in Anesthesia, STA 2013 Scottsdale, AZ United States. Conference Start: 20130109 Conference End: 20130112. Conference Publication:
IS - var.pagings

N2 - Introduction: Recently, there has been an increased interest in integrating mobile computing devices, such as the iPad, into medical education. Five months ago, the Stanford Anesthesia residency program provided mobile computing devices (iPad 3, Apple, Cupertino, CA) to 63 of our residents for the duration of their residency. A cross-sectional survey study was conducted in order to understand how these residents are using mobile computing devices for anesthesia education. Specifically, we sought to understand how often the residents were using the mobile computing devices, in what ways they were using the devices, and how this has impacted their anesthesia residency training. Methods: iPad Information: We provided our residents with 64 GB wifi iPad 3 units, which were preloaded with Anesthesiology Journals (Anesthesia & Analgesia, Anesthesiology, NEJM Journal), Crisis Code (an application to teach crisis management principles in the context of ACLS, iLarynx (a fiberoptic intubation simulator), ePocrates (a drug reference guide), ComunicatOR (an operating room translation application), StanMed (a critical care medicine application) and Ether Mobile (a paging application). Textbooks installed on the iPads included: Miller's Anesthesia, Seventh Edition, Obstetric Anesthesia Handbook, Fifth Edition, Datta, Smith's Anesthesia for Infants and Children, Eighth Edition, Handbook of Neuroanesthesia, Fourth Edition, Handbook of Ambulatory Anesthesia, Second Edition, Essentials of Pain Medicine, 3rd Edition, Complications of Regional Anesthesia, Second Edition, Clinical Anesthesia Procedures of the Massachusetts General Hospital, Eighth Edition, Case Studies of Near Misses in Clinical Anesthesia, Brock-Utne, Anesthesia Student Survival Guide, and Anesthesia Intensive Care and Pain in Neonates and Children. Survey Design: After testing for reproducibility and clarity, we deployed our survey to our 63 anesthesia residents. The survey asked the residents questions about how they used the iPad for studying and residency tasks. The survey included 9 open-ended questions, and 17 multiple-choice questions. We organized the questionnaire into three general domains: * General Information: We collected demographic information from the students such as gender, age, and previous experience with mobile technology. * iPad Usage: We collected information about when the residents were using the iPads, and the impact on their desktop computer use. * iPad Applications: We collected information about individual applications that we preloaded onto the iPad. Data Analysis: After we received our results, we analyzed them using a mixed-method. The open-ended collected data was entered into NVivo 10 and the questions were analyzed using qualitative methods. Results: 90.2% of Stanford Anesthesia Residents agree or strongly agree that an iPad would enhance their ability to learn anesthesia. 85.7% of residents surveyed also agreed or strongly agreed that iPads were under-utilized in anesthesia education. After receiving the iPad, 64.3% of our residents said that they were using their personal desktop computer less. When asked what frequency the residents used their iPad, 46.4% used them daily, 39.3% used them every other day, and the remaining 14.3% used them once a week. The two main activities that the residents used the iPads for most frequently are e-mail and reading medical books or papers. The places that the residents are using the iPad most often are at home, followed by inside the OR, and then inside coffee places. The main reason stated for using it at home was because the iPad was too bulky to carry with them at all times. We also asked the residents how useful the iPad provided by Stanford was for specific activities. 85.7% of residents said that the iPads were useful or very useful for accessing medical papers and 95% said that the iPads were useful or very useful for studying. 71.4% thought the iPads were useful or very useful for clinical issues. Out of the individual applications that we provided to the residents, the applications rated most useful were the books and journals. One of our residents stated: "[the iPad] has revolutionized access to texts and primary literature for studying and preparing every day." Conclusions: We found that a majority of our residents reported that the iPad is underutilized in medical education. Our survey has shown us that when given iPads, the majority of our residents use their iPads almost daily, and that the iPads have been reported to be useful for studying and accessing medical books and journals. The residents have also reported that they are reading more, chiefly because the iPad is an excellent resource for studying at home and outside of the operating room clinical environment. However, the residents did voice concerns that they would lose the iPad because of its small size and inability to securely store the device in the clinical environment. Some residents also reported that the iPads were too bulky to carry around in the operating room. Future work in exploring the use of mobile computing devices may focus on implementation of the iPad Mini as a more portable option for in anesthesia medical education

SN - 0003-2999

AD - (Sun, Clemenson) Research Assistant, Stanford Anesthesia Informatics, Media (AIM) Lab, United States (Garbin) UNICAMP Brazil, Brazil (Chu) Dep. of Stanford Anesthesia Informatics, Media (AIM) Lab, United States

ER -

TY - JOUR

ID - 3209

T1 - Where the needs of mothers with rheumatoid arthritis meet arthritis-related health care
A1 - Prodinger,B.
A1 - Shaw,L.
A1 - Laliberte-Rudman,D.
A1 - Stamm,T.
Y1 - 2013///
N1 - 71330011
English
Journal: Conference Abstract
KW - EMBASE
KW - Human
KW - mother
KW - rheumatoid arthritis
KW - Arthritis
KW - Health Care
KW - rheumatology
KW - rheumatic disease
KW - Female
KW - Health
KW - patient
KW - Interview
KW - information processing
KW - recipient
KW - pension
KW - Child
KW - Employment
KW - Reading
KW - invalidity
KW - outpatient
KW - medical record
KW - friend
KW - parttime employment
KW - parameters
KW - Austria
KW - diseases
RP - NOT IN FILE
JF - Annals of the Rheumatic Disease
VL - Conference: Annual European Congress of Rheumatology of the European League Against Rheumatism, EULAR 2012 Berlin Germany. Conference Start: 20120606 Conference End: 20120609. Conference Publication:
IS - var.pagings
N2 - Background People with rheumatoid arthritis (RA) are provided comprehensive health care most commonly in institutionalized settings. Critique has been raised that such settings are organized primarily to treat illness and disease-related symptoms of people rather than maintaining and promoting people's health and full participation in their daily lives. Inadequacy in addressing particularly women's health issues has also been described. Objectives The aim of this study was to understand how Austrian mothers with RA manage their everyday lives and how their needs are attended in arthritis-related health care in Austria. Methods An institutional ethnographic study was conducted. Seven Austrian mothers with RA, aged between 28 and 41, and varying working and living conditions (being on maternity leave, part-time employment, social assistance and invalidity pension recipient) were recruited. Data collection included interviews and participation observations. Women's health care records were included into analysis as they are exemplary for the interaction between the patient and health care over time. Institutional ethnographic analysis comprised first inductive reading of the data to understand what the women are actually doing in their daily lives; an analysis of the hospital records to understand the organization of and relations within arthritis-related health care in an outpatient rheumatology department; and consequently an analysis of how women's daily lives are met within these relations was explored. Results The ethnographic account of this study reveals how participants try to satisfy and meet the needs of their families, put effort into maintaining paid employment and continuing (outdoor-) activities with

family and friends, engage in informal caregiving for their children and significant others, while also integrate their disease-related symptoms. Given the multiple demands due to family and work, the women highlighted that challenges in daily life are not necessarily due to disease-related symptoms or limitations in performing a particular task, but in the coordination of multiple tasks simultaneously as they unfold throughout the entire day. The analysis of the documents revealed that arthritis-related health care is ruled by relations based on biomedical concepts that focus predominantly on disease parameters and measurable, objectifiable dimensions of people's functioning. The complexity of participants' daily life remains likely unattended in specialized arthritis-related health care as its multidimensionality cannot be easily reduced into distinct measurable concepts and outcomes. Conclusions This study underscores the importance of interdisciplinary treatment in specialized arthritis-related health care based on a comprehensive understanding of and attendance to how the disease might affect people's health and participation in daily life
SN - 0003-4967
AD - (Prodinger, Stamm) Medical University of Vienna, Vienna, Austria (Prodinger, Shaw, Laliberte-Rudman) Graduate Program in Health and Rehabilitation Sciences, Field of Occupational Science, University of Western Ontario, Canada (Shaw, Laliberte-Rudman) School of Occupational Therapy, University of Western Ontario, Canada
ER -

TY - JOUR
ID - 3210
T1 - Development of the united kingdom evaluation of daily activities questionnaire in rheumatoid arthritis: Psychometric testing
A1 - Hammond,A.
A1 - Tyson,S.
A1 - Tennant,A.
A1 - Nordenskiold,U.
A1 - Greenhill,Y.
Y1 - 2013///
N1 - 71329922
English
Journal: Conference Abstract
KW - EMBASE
KW - questionnaire
KW - rheumatoid arthritis
KW - rheumatology
KW - rheumatic disease
KW - United Kingdom
KW - Human
KW - validity
KW - test retest reliability
KW - Pain
KW - internal consistency
KW - reliability
KW - Fatigue
KW - Cronbach alpha coefficient
KW - rating scale
KW - bath
KW - hand pain
KW - Hospital
KW - Female
KW - clinical practice
KW - patient
KW - laundry
KW - Child
KW - Cooking
RP - NOT IN FILE
JF - Annals of the Rheumatic Disease

VL - Conference: Annual European Congress of Rheumatology of the European League Against Rheumatism, EULAR 2012 Berlin Germany. Conference Start: 20120606 Conference End: 20120609. Conference Publication:
IS - var.pagings
N2 - Background The Evaluation of Daily Activity Questionnaire (EDAQ) is a patient reported measure of activity limitations in Rheumatoid Arthritis (RA) (1,2) which we have revised and updated for use in the UK. This version includes 138 items in 14 "domains" (Eating/Drinking; Personal Care; Dressing; Bathing; Cooking; Moving Indoors; House Cleaning; Laundry; Moving and Transfers; Moving Outdoors; Gardening/Household Maintenance; Caring; and Leisure/Social Activities). Each domain is split into two sections: one (A) scores whether activities can be performed without aids, alternate methods or help; and another (B) which scores whether the activities can be performed with aids or alternate methods. All items are scored on a 0-3 scale (no difficulty to unable to do). Objectives To test the validity, reliability and minimal detectable difference of the EDAQ in people with RA in the UK. Methods Participants from 14 Rheumatology clinics completed postal questionnaires of demographic questions, the EDAQ (UK version), HAQ, SF36v2, RAQOL, pain, hand pain and fatigue numeric rating scales (NRS) and a current condition severity scale. Three weeks later, the EDAQ was mailed again to evaluate test-retest reliability: using kappa co-efficients for individual items; and non-parametric correlations for domain scores. Non-parametric correlations assessed the validity of the 14 domains of the EDAQ against the other measures. Cronbach's alpha evaluated internal consistency. Results 383 people participated: 286 women; age =60.38 (SD 11.18) years; RA duration =13.2 years (SD 10.72). 118 (31%) were employed; 65 had children <18y at home. Average pain score =4.99 (SD 2.59) and fatigue =5.61 (SD 2.53). All domains of the EDAQ correlated significantly (p<0.0005) with; An exception was the "Caring" domain; most (59%) felt this domain inapplicable to them and validity was lower although still significant (rs=0.22 to 0.35). Internal consistency was high in all domains (Cronbach's alpha=0.89-0.97 for Section A and 0.88-0.98 for Section B). 309 (81%) completed Test 2 and test-retest reliability was moderate for most (n=108) items (0.41-0.60); substantial for 28 (0.61-0.80) and fair (0.21-0.40) for two. Reliability of total scores was excellent for 11 domains (rs=0.81 -0.88) and moderate-substantial for three (rs=0.54-0.73. Minimal detectable differences was 1.60 to 4.61. Conclusions The UK version of the EDAQ is a unidimensional, valid, reliable, sensitive measure of daily activity in people with RA. It can therefore be used in both clinical practice and research. (Table Presented)
SN - 0003-4967
AD - (Hammond, Tyson, Greenhill) Centre for Health Sciences Research, University of Salford, Salford, Sweden (Tennant) Rehabilitation Medicine, University of Leeds, Leeds, United Kingdom (Nordenskiold) Rehabilitation Medicine, Sahlgrenska Academy, Goteborg, Sweden
ER -

TY - JOUR
ID - 3211
T1 - Simplifying prognostic testing in cystic fibrosis: How does heart rate recovery after sub-maximal aerobic exercise correlate with maximal exercise testing results?
A1 - Cohen,S.
A1 - Orenstein,D.
Y1 - 2013///
N1 - 71321771
English
Journal: Conference Abstract
KW - EMBASE
KW - cystic fibrosis
KW - Aerobic Exercise
KW - Heart Rate
KW - Exercise Test
KW - Human
KW - Child
KW - resting heart rate
KW - Exercise
KW - Adult
KW - Mortality
KW - Population

KW - patient
KW - Oxygen Consumption
KW - correlation coefficient
KW - lung function test
KW - hypothesis
KW - exercise physiology
KW - exercise tolerance
KW - lung disease
KW - Male
KW - genetic disorder
KW - pediatric hospital
RP - NOT IN FILE
SP - 362
JF - Pediatric Pulmonology
JA - Pediatr Pulmonol
VL - Conference: 27th Annual North American Cystic Fibrosis Conference Salt Lake City, UT United States. Conference Start: 20131017 Conference End: 20131019. Conference Publication:
IS - var.pagings
N2 - Background: Cystic fibrosis (CF) is the most common profoundly lifeshortening genetic disease among Caucasians. Poor exercise capacity, as represented by low maximal oxygen consumption (VO₂max), has been shown to be a powerful independent predictor of mortality in pediatric and adult CF patients. However, the testing required to measure VO₂max is uncomfortable and resource-intensive. Submaximal exercise tests that can be performed outside an exercise physiology lab have been developed to address these concerns. A 3-minute step test at a cadence of 30 steps per minute has been verified as a feasible submaximal field exercise test for use in children and adults with CF. Also, heart rate recovery after sub-maximal exercise correlates closely to all-cause mortality in adult non-CF populations. Hypothesis: In children 8 and older with CF, a shorter time to return to resting heart rate (HR) and a greater percent change from maximum HR one minute following a submaximal exercise test will positively correlate with greater percent predicted VO₂max as measured by a maximal exercise test. Methods: Twenty-two children 8 years of age and older (range 8-19, 59% male) with CF were recruited from the CF center at Children's Hospital of Pittsburgh. Each subject performed a 3-minute stepping exercise on a 15 cm step at a cadence of 30 complete steps per minute (STEP). HR and SpO₂ were measured at rest, during, and after STEP. Subjects then performed a maximal oxygen consumption test. Data from routine pulmonary function tests were also collected. Percent of maximal HR at one minute post-STEP and time in minutes to reach resting HR were compared with VO₂max by calculating Pearson's Correlation Coefficients. Results: All subjects were able to complete STEP, with no adverse events. Average percent predicted FEV₁ was 86% (range 36%-111%) and average percent predicted VO₂max was 110% (range 83%-137%). Maximum HR on STEP was on average significantly lower than maximum HR from the maximal test (139 vs. 190, p<0.01). Fifty percent of subjects returned to their resting HR less than one minute after completing STEP. Altogether, neither shorter time to resting HR after STEP nor change in percent of maximal HR at one minute post-STEP correlated significantly with percent predicted VO₂max. However, lower peak HR during STEP correlated significantly with percent predicted VO₂max (R = 0.5, p<0.02). Also, in children with mild CF (FEV₁ > 80%), shorter time to resting HR correlated significantly with percent predicted VO₂max (R = 0.5, p<0.05). Conclusions: All subjects completed STEP without any adverse events. STEP is a submaximal test in this patient population. As fifty percent of subjects returned to resting heart rate within one minute of completing STEP, change in percent of maximal heart rate at one minute post-STEP is not a useful measure of heart rate recovery after STEP in children with CF. Lower peak heart rate during STEP correlated with percent predicted VO₂max, as did shorter time to resting heart rate in children with mild lung disease. HR during and after STEP may reflect maximal exercise tolerance in children with CF
SN - 8755-6863
AD - (Cohen) University of Pittsburgh, School of Medicine, Pittsburgh, PA, United States (Orenstein) Pediatric Pulmonology, Children's Hospital of Pittsburgh, Pittsburgh, PA, United States
ER -

TY - JOUR
ID - 3212

T1 - Anaesthesia techniques in IR: A practical approach
A1 - Batistaki,C.
Y1 - 2013///
N1 - 71323116
English
Journal: Conference Abstract
KW - EMBASE
KW - Society
KW - Europe
KW - Anesthesia
KW - Human
KW - procedures
KW - Female
KW - patient
KW - general anesthesia
KW - Monitoring
KW - airway
KW - operating room
KW - immobility
KW - nuclear magnetic resonance imaging
KW - medical history
KW - analgesia
KW - Imaging
KW - anesthesist
KW - interventional radiology
KW - Blood Pressure
KW - Risk
KW - pharmacokinetics
KW - Deep Sedation
KW - ascites
KW - respiration control
KW - Diagnosis
KW - recovery room
KW - Safety
KW - endotracheal intubation
KW - pulse oximetry
KW - Pain
KW - electrocardiogram
KW - capnometry
KW - gold standard
KW - varicosis
KW - Resuscitation
KW - metabolite
KW - sedation
KW - radiology
KW - acid aspiration
KW - stimulation
KW - inhalational drug administration
KW - patient comfort
KW - Morbidity
KW - diet restriction
KW - thermography
KW - brain artery aneurysm
KW - hypotension
KW - Wakefulness
KW - central venous pressure
KW - brain protection

KW - surgery
KW - radiologist
KW - planning
KW - severe hepatic impairment
KW - radiology department
KW - Interpersonal Communication
KW - Physical Examination
KW - transjugular intrahepatic portosystemic shunt
KW - implant
KW - machine
KW - side effect
KW - Pregnancy
KW - allergy
KW - critically ill patient
KW - Child
KW - implantable pump
KW - informed consent
KW - defibrillator
KW - Decision Making
KW - magnet
KW - electrode
KW - thermal injury
KW - heating
KW - stent
KW - air conditioning
KW - pacemaker
KW - metastasis
KW - monitor
KW - Drug Therapy
KW - tumor ablation
KW - breathing circuit
KW - Personnel
KW - liver disease
KW - ureter stent
KW - injury
KW - radiofrequency ablation
KW - preoperative evaluation
KW - Heart Rate
KW - artificial embolism
KW - coil embolization
KW - angioplasty
KW - hypocapnia
KW - abdominal pressure
KW - Rupture
KW - pressure measurement
KW - parameters
KW - neurosurgery
KW - central nervous system
KW - anesthetic agent
KW - rocuronium
KW - neuromuscular blocking agent
KW - opiate
KW - remifentanil
KW - cisatracurium
KW - suxamethonium
KW - alfentanil
KW - sufentanil

KW - fentanyl
KW - etomidate
KW - propofol
KW - desflurane
KW - sevoflurane
KW - midazolam
KW - mivacurium
KW - sugammadex
KW - contrast medium
KW - musk
RP - NOT IN FILE
SP - S95
EP - S96
JF - CardioVascular and Interventional Radiology
VL - Conference: Cardiovascular and Interventional Radiological Society of Europe, CIRSE 2013 Barcelona Spain. Conference Start: 20130914 Conference End: 20130918. Conference Publication:
IS - var.pagings
N2 - Continuous development of interventional radiology (IR) techniques has led to an increased demand of high-quality anaesthetic services to support patients undergoing diagnostic or therapeutic procedures. In most cases, IR techniques are accompanied by minor stimulation and pain, requiring light or deep sedation. However, in some instances, general anaesthesia with endotracheal intubation or laryngeal musk is required. Such cases include procedures necessitating complete immobility to obtain improved images, special operations (neuroradiological, interventions near vital organs or vessels, lengthy or painful procedures, procedures in children), or involve uncooperative patients. In addition, general anaesthesia is preferred in patients who require adequate airway control or have a risk of aspiration of gastric contents (i.e. severe ascites, risk of oesophageal varices rupture, increased intra-abdominal pressure of any cause). The choice of the anaesthetic technique and the drugs used are selected on the basis of the type, duration and demands of the procedure to be performed, age and the medical history of the patient, medications administered and the facilities of each IR suite. One of the biggest problems for anaesthesiologists during IR procedures is the administration of anaesthesia in places outside the operating theatre, with a large distance with the operating rooms and the post-anaesthesia care unit, without the ability to obtain immediate help when required. This makes decision-making in IR anaesthesia rather difficult, especially in highly demanding procedures or in critically ill patients or patients with airway difficulties. Early communication and planning of the procedure with the radiologists is essential to obtain safe and high-quality anaesthesia. The American Society of Anesthesiologists (ASA) has provided guidelines on administration of anaesthesia outside the operating theatre, which should be strictly followed. Radiology rooms where patients are under sedation or anaesthesia should have all the necessary equipment and drugs for patient's resuscitation. The same applies to adequate monitoring, which should include pulse oximetry, capnography and non-invasive blood pressure, in addition to continuous electrocardiogram and a neurostimulator in cases where neuromuscular blocking agents are administered. All monitoring equipment and intravenous lines should be adequately distanced to be easily reached and observed by the anaesthesiologist. It is of great importance that all patients that are going to receive general anaesthesia are adequately assessed pre-operatively and an informed consent is obtained. A full medical history (including possible contrast media allergies, pregnancy, implants, comorbidities) is essential, in addition to physical examination and airway evaluation. Patients with difficult airways, whether anticipated or not, can be problematic in a setting outside the operating theatre; if a difficulty is suspected, endotracheal intubation should be performed in the operating suite because of the greater availability of help and equipment. However, equipment for difficult airway control should be available also in the radiology department to manage the unanticipated difficult airway. Fasting should follow the guidelines for all general anaesthesia procedures. The anaesthetic technique varies according to the procedure and patients' requirements and co-morbidities. Inhalational (sevoflurane, desflurane) and intravenous agents (propofol, midazolam, etomidate) may be used, selected according to their pharmacokinetic properties on an individualised basis. Drugs of short duration, with no active metabolites and no residual post-operative action, are the best option for the IR patient. Opioids with this profile include small doses of fentanyl, sufentanil or alfentanil, but mainly remifentanil, a strong opioid with rapid initiation and termination of action. In cases where a neuromuscular blocking agent is required to facilitate immobility, intermediate acting drugs may be used (rocuronium, cisatracurium, mivacurium). Succinylcholine is the gold standard for rapid sequence induction, but its side effects and contraindications may not allow its use. Rocuronium is a great alternative in this setting; it can be rapidly reversed because of the presence of a specific reversal agent sugammadex. Post-operative care

should be performed in a post-anaesthesia care unit, as in all cases of general anaesthesia. Patients should be observed for signs of haemodynamic or respiratory instability, with control of blood pressure, heart rate and post-operative pain. Paediatric patients require special consideration, with early preoperative evaluation and involvement of specialised anaesthesia personnel familiar with paediatric anaesthesia and peri-operative management. Special considerations CT-guided procedures: CT-guided procedures under general anaesthesia require attention with intravenous lines and breathing circuit to prevent their dislodged by movement of the patient during the scan. Tumour ablation may require advanced analgesia because most of these procedures are painful, especially when they involve bony metastases. Magnetic Resonance Imaging: The most important issues in MRI diagnostic studies are the necessity of monitoring and ventilation equipment appropriate for use in an MRI suite; the need for keeping the patient immobile in to obtain the best quality of MRI scan; the problem of patient distance and inaccessibility, especially during prolonged scanning; the extended length of most MRI procedures; the heating of monitoring wires that can cause thermal injury underneath the ECG electrodes and pulse oximetry probe; the effect of the magnet on ferrous materials (stents, cardioverters-defibrillators, pacemakers, implanted pumps and neurostimulators, all of which should be identified from patient history). The anaesthesia provider should be present in the MRI suite or watch the patient from the control room, if mirroring of the monitors and direct observation of the anaesthesia machine is adequate. Transjugular intrahepatic portosystemic shunt (TIPS): The procedure is usually performed under general anaesthesia to achieve immobility and safety because patients have severe liver failure, requiring total control of airway (with rapid sequence induction in cases of ascites) and advanced monitoring (cardiovascular and respiratory). Anaesthetic drugs used should be carefully selected in the course of pharmacokinetic changes that accompany severe liver disease. Interventional radiology/neuroradiology procedures: Angiography/embolisation, ureteral stents, trauma interventional procedures, thermography or radiofrequency ablation of tumours are more painful than simple imaging, requiring deeper levels of analgesia to achieve patient comfort. Interventional neuroradiologic procedures (such as endovascular embolisation of AVMs, coil embolisation of cerebral aneurysms, angioplasty, thromboplasty) are being performed under various CNS pathological states and most of the times general anaesthesia is required to achieve the optimal imaging. These procedures are highly-demanding and may require deliberate hypotension, hypocapnia, control of endocranial pressure or rapid transition between deep sedation and awake states. Monitoring in this case may be advanced, with intra-arterial and/or central venous pressure measurement, or may include neurophysiological parameters. These procedures follow the principles of neurosurgery as for brain protection and optimisation. In conclusion, the minimally-invasive nature of interventional radiology procedures puts the necessity of development of anaesthetic techniques, towards optimisation of anaesthesia, analgesia and immobility, in addition to minimal or no residual action of anaesthetic drugs after surgery and provision of safety throughout the procedure
SN - 0174-1551
AD - (Batistaki) Second Department of Anaesthesiology, University of Athens, Attikon Hospital, Athens, Greece
ER -

TY - JOUR
ID - 3213
T1 - Peer mediation to increase communication and interaction at recess for students with autism spectrum disorders
A1 - Mason,R.
A1 - Kamps,D.
A1 - Turcotte,A.
A1 - Cox,S.
A1 - Feldmiller,S.
A1 - Miller,T.
Y1 - 2014///
N1 - 2014084217
English
Journal: Article
KW - EMBASE
KW - article
KW - Autism
KW - Case Report
KW - Child

KW - Evidence Based Practice
KW - Human
KW - Interpersonal Communication
KW - Learning Environment
KW - Male
KW - Peer Group
KW - preschool child
KW - priority journal
KW - school child
KW - social adaptation
KW - Social Interaction
KW - social validity
RP - NOT IN FILE
SP - 334
EP - 344
JF - Research in Autism Spectrum Disorders
VL - 8
IS - 3
CY - United Kingdom
N2 - Recess plays an integral role in the social and emotional development of children given the time provided to engage in interactions with others and practice important social skills. Students with ASD, however, typically fail to achieve even minimal benefit from recess due to social and communication impairments as well as a tendency to withdraw. Implementation of evidence-based interventions such as peer-mediated social skills groups, are necessary to ensure recess is an advantageous learning environment for students with ASD. A multiple-baseline design across participants was used to determine if a functional relationship exists between a social skills instructional program combined with peer networks with school staff as implementers and increases in level of communicative acts for participants with ASD at recess. Results indicate all participants demonstrated an immediate increase in the number of communicative acts with the introduction of the intervention. Implications for practice are discussed. 2013 Elsevier B.V. All rights reserved
SN - 1750-9467
AD - (Mason, Kamps, Turcotte, Cox, Feldmiller, Miller) Juniper Gardens Children's Project, University of Kansas, Kansas City, KS 66101, United States
ER -

TY - JOUR
ID - 3214
T1 - Factors associated with early sexual activity among chilean adolescents
A1 - Electra,G.A.
A1 - Temistocles,M.G.
A1 - Adela,M.V.
A1 - Vania,M.N.
Y1 - 2013///
N1 - 23900321
English, Spanish
Journal: Article
KW - EMBASE
KW - Adolescent
KW - Adolescent Behavior
KW - age
KW - article
KW - Child
KW - Chile/ep [Epidemiology]
KW - Coitus
KW - cross-sectional study
KW - family relation
KW - Female
KW - Human

KW - Male
KW - psychological aspect
KW - risk factor
KW - socioeconomics
KW - Young Adult
RP - NOT IN FILE
SP - 313
EP - 319
JF - Revista Medica de Chile
JA - Rev Med Chil.
VL - 141
IS - 3
CY - Chile
N2 - Background: Nowadays, adolescent population begins sexual activity earlier, a behavior with negative psychological and social consequences. Aim: To determine the association between family factors and early sexual activity in adolescents of low and middle socioeconomic level. Material and Methods: A sample of 3,210 adolescents, who confidentially requested care in a sexual and reproductive health university center, between 2000 and 2007, was analyzed. Adolescents who started sexual activity before 15 years of age, and those who started sexual activity after 15 years of age, were compared. Data was collected from structured interviews conducted at the adolescent's first visit. Logistic regression was used to identify family factors associated with early sexual initiating in both, men and women. Results: Family factors associated with early sexual activity start were not being raised by both parents, a poor parent-child relationship, a poor family communication, mothers with a history of adolescent motherhood, mothers employed outside the home and family dysfunction. Among women, the variables associated with a higher risk of early sexual debut were not married parents and history of adolescent fatherhood record among parents. Among men, these variables were permissions without restriction during the week and punishment when family rules were broken. Conclusions: Family factors must be considered in the design of strategies to prevent early sexual activity
SN - 0034-9887
AD - (Electra, Temistocles, Adela, Vania) Centro de Medicina Reproductiva y Desarrollo Integral de la Adolescencia, Universidad de Chile, Chile
ER -

TY - JOUR
ID - 3215
T1 - Evaluation of the physical activity of children in grades 1-3 attending the primary schools in the Myslenice Poviat and in the City of Krakow
A1 - Zwirska,J.
A1 - Jagielski,P.
A1 - Jablonska,W.
A1 - Bajer,M.
A1 - Styczen,A.
A1 - Schlegel-Zawadzka,M.
Y1 - 2013///
N1 - 2014079939
English
Journal: Article
KW - EMBASE
KW - Academic Achievement
KW - article
KW - Child
KW - Exercise
KW - Female
KW - Human
KW - Male
KW - Physical Activity
KW - primary school
KW - rural area

KW - school child
KW - sex difference
KW - Television Viewing
RP - NOT IN FILE
SP - 182
EP - 187
JF - Medicina Sportiva
VL - 17
IS - 4
CY - Poland
N2 - Objective: To assess the physical activity of children attending grades 1-3 of the primary schools in the region of southern-central Poland (the Malopolska Region). Methods: The study was conducted in selected primary schools in the years 2009-2012. These included 1,140 students aged 7-9 years, coming from rural areas (658), small towns (284), and one large city (198) - the regional capital: Krakow. The parents of the children completed questionnaires, which included questions pertaining to their children's performance of physical activities at school and outside of school. Results: Among the 1,140 children studied were 560 (49.1%) boys and 580 (50.9%) girls. Both boys and girls living in Krakow spent the least time (less than one hour aday) in front of aTV or computer screen as compared with the other children. Children living in rural areas would spend 2-3 hours aday on those activities. The largest percentage of children who attended PE classes regularly was from Krakow (100% of the boys and 97.6% of the girls). The case was similar with extracurricular activities that required alot of exercise - 84.7% of the boys and 85.7% of the girls attended those. Children living in smaller towns were significantly less likely to undertake additional physical activity ($P < 0.05$). Parents of boys were less likely to decide to excuse their children from participation in PE classes than parents of girls ($P < 0.05$). Conclusions: The place of residence and the gender of the child both have an effect on his or her participation in exercises and recreational activities. Copyright 2013 Medicina Sportiva
AD - (Zwirska, Jablonska, Schlegel-Zawadzka) Department of Human Nutrition, Institute of Public Health, Faculty of Health Sciences, ul. Grzegorzecka 20, 31-531 Krakow, Poland (Jagielski) Department of Genetic Diagnosis and Nutrigenomics, Clinical Biochemistry, Jagiellonian University Medical College, Krakow, Poland (Bajer) Department of Health Promotion and Protection, Myslenice Poviat Starosty, Myslenice, Poland (Styczen) Independent Public Health Care Centre in Myslenice, Myslenice, Poland
ER -

TY - JOUR
ID - 3216
T1 - Analysis of the status and influence factors of vision development in preschool children
A1 - Wu,G.-Q.
A1 - Tong,M.-L.
A1 - Wei,N.
A1 - Wu,X.-X.
A1 - Li,Z.
Y1 - 2013///
N1 - 2014066170
Chinese
Journal: Article
KW - EMBASE
KW - article
KW - Birth Weight
KW - Child
KW - Child Development
KW - eye examination
KW - eye refraction
KW - Gestational Age
KW - Human
KW - illumination
KW - mastication
KW - Nursery
KW - preschool child

KW - questionnaire
KW - Television Viewing
KW - Vision
KW - Visual Acuity
KW - visual disorder
RP - NOT IN FILE
SP - 2495
EP - 2498
JF - International Eye Science
VL - 13
IS - 12
CY - China
N2 - AIM: To investigate the visual acuity, refraction and influence factors of preschool children in some nurseries in Nanjing. METHODS: Children from 4 to 6 years old in Nanjing attended the visual acuity examination, and filled in factors questionnaire. The results were analyzed. RESULTS: Visual acuity at P₅ was 0.4 of 4 years, 0.5 of 5 years, and 0.6 of 6 years. Refraction, gestational age, birth weight, closely using eyes, daily watching television time, daily outdoor activity time, picky eaters situation, chewing situation, parents diopter, home lighting status were associated with vision disorders. Factors score at P₅ is 5. CONCLUSION: The visual acuity less than P₅ at each ages was abnormal vision. Child whose factors score of less than 5 had high risk of abnormal vision, and need to pay close attention to visual conditions
SN - 1672-5123
AD - (Wu, Li) School of Public Health Nanjing Medical University, Nanjing 210000, Jiangsu Province, China (Wu, Tong, Wei, Wu) Department of Children's Eye Health, Nanjing Maternity and Child Health Care Hospital, Affiliated to Nanjing Medical University, Nanjing 210000, Jiangsu Province, China
ER -

TY - JOUR
ID - 3217
T1 - Shoulder MR arthography: Tips for the general pediatric radiologist
A1 - Dahmoush,H.
A1 - Jaramillo,D.
A1 - Cahill,A.M.
A1 - Chauvin,N.
Y1 - 2013///
N1 - 71311767
English
Journal: Conference Abstract
KW - EMBASE
KW - nuclear magnetic resonance
KW - radiologist
KW - Human
KW - Society
KW - radiology
KW - Shoulder
KW - pathology
KW - injury
KW - Diagnosis
KW - intraarticular drug administration
KW - ligament
KW - arthrography
KW - patient
KW - planning
KW - Morbidity
KW - tendon
KW - Pain
KW - bone injury

KW - lacrimal fluid
KW - athlete
KW - Population
KW - Adolescent
KW - Bone
KW - cartilage injury
KW - surgery
KW - recurrent dislocation
KW - shoulder dislocation
KW - patient positioning
KW - injection
KW - Imaging
KW - parameters
KW - artifact
KW - periosteum
KW - orthopedic surgeon
KW - Child
KW - Case Report
KW - stabilizing agent
RP - NOT IN FILE
SP - S401
EP - S402
JF - Pediatric Radiology
JA - Pediatr Radiol
VL - Conference: 56th Annual Meeting of the Society for Pediatric Radiology San Antonio, TX United States. Conference Start: 20130514 Conference End: 20130518. Conference Publication:
IS - var.pagings
N2 - Purpose or Case Report: Shoulder dislocations are relatively common in adolescents, with 40% of all cases occurring in patients less than 22 years of age. Structural damage ensued can lead to significant pain and morbidity and predispose children to re-injury. When the static stabilizers of the shoulder are disrupted, patients will often experience recurrent dislocation. With surgical correction, young athletes are afforded the best opportunity to achieve long-term stability and return to play. MR arthrography (MRA) of the shoulder offers detailed joint anatomy; enabling depiction of ligament, tendon, bone and cartilage injury and is used to assist the orthopedic surgeons in surgical planning. This exhibit will serve to provide the general pediatric radiologist with a practical approach to shoulder MR arthrography from intraarticular injection to image interpretation. Methods & Materials: Proper intraarticular injection technique will be demonstrated including the authors' experience using a posterior shoulder injection approach used because most abnormalities are found in the anterior labrum. Optimal MR patient positioning, sequence selection and imaging parameters will be described. The normal glenohumeral joint anatomy will be depicted as well as tips to depict normal variants (coracoid physis, sublabral recess, Buford complex and the bare spot) and image artifacts that should not be mistaken for pathology. Common pathologies involving the glenohumeral ligaments and anteroinferior glenoid will be explored including hints to differentiate between cartilaginous Bankart injuries, Bony Bankart injuries, Perthes lesions and Anterior Labroligamentous Periosteal Sleeve Avlusions (ALPSA). In addition, strategies for accurate diagnosis of superior labral anteropeosterior (SLAP) tears will be highlighted. Results: A good knowledge of the MR appearance of normal shoulder anatomy and anatomic variants is crucial in order to accurately evaluate for pathology. With proper intraarticular injection, MR acquisition and diagnostic experience; MRA is effective method for the detection of labroligamentous abnormalities, bone injuries and musculotendinous pathology. Conclusions: When performed correctly, shoulder MRA is an important diagnostic modality that can aid with preoperative planning in the pediatric population
SN - 0301-0449
AD - (Dahmoush, Jaramillo, Cahill, Chauvin) Children's Hospital of Philadelphia, Philadelphia, PA, United States
ER -

TY - JOUR
ID - 3219
T1 - An assessment of knowledge gaps in paediatric liver transplant recipients and their care-givers

A1 - Tan,L.N.
A1 - Liwanag,M.J.
A1 - Tan,P.S.Y.
A1 - Quak,S.H.
A1 - Aw,M.M.
Y1 - 2013///
N1 - 71309289
English
Journal: Conference Abstract
KW - EMBASE
KW - liver graft
KW - graft recipient
KW - caregiver
KW - Human
KW - Asian
KW - liver
KW - Adolescent
KW - parent
KW - Drug Therapy
KW - Transplantation
KW - Child
KW - questionnaire
KW - custody
KW - Education
KW - patient
KW - liver function
KW - Safety
KW - graft dysfunction
KW - mother
KW - Female
KW - bile duct atresia
KW - lifestyle modification
KW - managed care
KW - medical care
KW - Adult
KW - exposure
KW - diseases
KW - pet animal
KW - physician
KW - sport
KW - liver disease
RP - NOT IN FILE
SP - S664
EP - S665
JF - Hepatology International
VL - Conference: 23rd Conference of the Asian Pacific Association for the Study of the Liver, APASL 2013 Singapore Singapore. Conference Start: 20130606 Conference End: 20130609. Conference Publication:
IS - var.pagings
N2 - Introduction: Education of transplant recipients and their care-givers is an important aspect of post-transplant care. As biliary atresia is the main indication for liver transplant in children, education is given to their parents. Often, physicians may neglect to "educate" these children as they grow older. This may have implications for adolescents and transition to adult service, where the shift in medical care is from parent-led to self-managed care. Aim: To assess the knowledge of the transplant recipients or their care-givers regarding various aspects of care, in order to identify knowledge gaps. Method: A questionnaire was administered over a period of 24 months (2009-2011). The questionnaire was completed by a caregiver proxy for children<12 years, and self-administered by adolescents > 12 years. These questions focused mainly on understanding of liver graft, post-transplant medications and lifestyle modifications. Results: These patients were aged 1-25 (mean

9.69) years and between 0.03-13 (mean 4.67) years post-transplant. All adolescent questionnaires (n = 9) were self-administered while parent proxy for children<12 years (n = 20) were mostly mothers (60%, n = 12). Results showed that parents were significantly more knowledgeable than the adolescents in terms of liver function, p = .001; graft dysfunction, p = .037; and dietary/environmental safety practices, p = .000. There was no statistically significant difference between parent-proxy and adolescent knowledge of; and attitude towards taking medications, adherence, sports and outdoor safety, pet-keeping and illness exposure post-transplant. Conclusion: Although adolescent patients did not receive active education regarding medications, they were aware of their medications, indications and dose timings. They may have learnt it from their parents or picked it up after being on the medications for some time. However, they had poor knowledge of liver function and implications of liver disease. This may imply that adolescents who are non-compliant may have a lack of insight to the implications of graft dysfunction
SN - 1936-0533
AD - (Tan, Liwanag, Tan, Quak, Aw) Paediatrics, Khoo Teck Puat - National University, National University Hospital, Singapore, Singapore (Quak, Aw) Department of Paediatrics, Yong Loo Lin School of Medicine, National University of Singapore, Singapore, Singapore
ER -

TY - JOUR
ID - 3220
T1 - Does parental perception of neighborhood safety predicts PA in children from different SES
A1 - Pizarro,A.N.
A1 - Ribeiro,J.
A1 - Teixeira,J.
A1 - Santos,M.P.
Y1 - 2012///
N1 - 71303283
English
Journal: Conference Abstract
KW - EMBASE
KW - Epidemiology
KW - Human
KW - Child
KW - Society
KW - Safety
KW - parent
KW - Education
KW - Physical Activity
KW - Environment
KW - girl
KW - Regression Analysis
KW - Portugal
KW - Adolescent
KW - childhood
KW - gender
KW - Crime
KW - Female
KW - questionnaire
KW - model
KW - Health
KW - accelerometer
KW - community
RP - NOT IN FILE
SP - S192
EP - S193
JF - European Journal of Epidemiology
JA - Eur J Epidemiol

VL - Conference: IEA-EEF European Congress of Epidemiology 2012: Epidemiology for a Fair and Healthy Society Porto Portugal. Conference Start: 20120905 Conference End: 20120908. Conference Publication:
IS - var.pagings
N2 - Background Achieving daily recommendations of 60 min of moderate- to-vigorous physical activity (PA) during childhood has well documented benefits. Despite this knowledge many children do not engage in sufficient PA and children from disadvantage families are known to have even lower levels of PA. Time spent outdoor is highly correlated to higher levels of PA, however safety concerns within disadvantage communities may be limiting children's outdoor play. Therefore the aim of this study is to examine the association between parents' safety perceptions of the neighbourhood environment and children's physical activity according to socio economic status (SES). Methods Participants were 298 adolescents (164 girls) with mean age of 11.59 (+0.80) years old from Porto, Portugal. PA was obtained with Actigraph accelerometer model GT1M. Parent's safety perceptions of the neighbourhood environment were obtained by a selfreport questionnaire with 11 items on road safety, crime and stranger danger; SES was obtained based on parent's educational level and was collapsed into two categories: B9 years of education (low) and >10 years of education (high). Results Adjusted (gender, age) binary logistics regression analysis showed that parental overall safety perception is associated with achieving PA recommendations only in children from low SES. In low SES families children from parents perceiving more "Safety for outdoor playing during the day" have higher odds (OR = 3.50 CI: 1.55-7.91) of achieving PA recommendations than children whose parents recognize less safety. Conclusions Parent's perceptions of safety in the neighbourhood appear to be an important correlate of PA in low SES families. Therefore interventions to promote neighbourhood safety within these families may be of particular relevance from a health perspective, since they may rely more on the neighbourhood to get their daily PA, than children from high SES
SN - 0393-2990
AD - (Pizarro, Ribeiro, Teixeira, Santos) Porto University, Faculty of Sports, Portugal
ER -

TY - JOUR
ID - 3221
T1 - Parents' perceptions of residential neighborhood, children's sedentary behaviors, and outdoor play in school children
A1 - Padez,C.
A1 - Ferrao,M.M.
A1 - Freitas,F.
A1 - Gama,A.
A1 - Mourao,I.
A1 - Nogueira,H.
A1 - Rosado,V.
A1 - Santana,A.
Y1 - 2012///
N1 - 71303172
English
Journal: Conference Abstract
KW - EMBASE
KW - Epidemiology
KW - Human
KW - Society
KW - school child
KW - Child
KW - parent
KW - boy
KW - Male
KW - Environment
KW - computer
KW - girl
KW - Television
KW - Female
KW - Obesity

KW - school
KW - Safety
KW - Social Environment
KW - questionnaire
KW - Prevalence
KW - Physical Activity
KW - Child Behavior
KW - Risk
KW - land use
KW - cross-sectional study
KW - environmental factor
KW - epidemic
KW - Health
KW - multivariate logistic regression analysis
KW - Education
KW - public health problem
KW - Electron
KW - Portugal
RP - NOT IN FILE
SP - S150
JF - European Journal of Epidemiology
JA - Eur J Epidemiol
VL - Conference: IEA-EEF European Congress of Epidemiology 2012: Epidemiology for a Fair and Healthy Society Porto Portugal. Conference Start: 20120905 Conference End: 20120908. Conference Publication:
IS - var.pagings
N2 - Background Despite trends indicating a recent stabilization in the upward obesity trend in children in some countries such as Portugal, the present values remains a huge public health problem. Although the causes of this epidemic are multifactorial, environmental factors such as unsafe neighborhoods are one factor that has been thought to increase obesity risk in younger children by limiting their outdoor play and increasing sedentary indoor activities such as television, computer use and play electronic games. Recent studies have pointed to associations between parental perceptions of neighborhood and their children's health. Objectives The main purpose of this study is to analyze the association between sedentary behaviors, children's outdoor play and parents' perceptions of some characteristics of the built environment. Method A cross-sectional study was done in a sample of 1,466 children, aged 6.0-10.0 years living in Oporto. Private and public schools were randomly selected. Parents filled out a questionnaire about family and child behaviours such as TV viewing, PC use, electronic games and outdoor play during weekdays and at the weekend. They also filled out the "Environmental Module" standard questionnaire of the International Physical Activity Prevalence Study (IPS, 2002). Environmental characteristics were combined into two neighborhood dimensions- factor 1-neighborhood built environment, factor 2-neighborhood social environment and safety. A Multivariate Logistic Regression analysis adjusted for age, parental education and the cluster of schools was used. Results A negative parental perception of the residential environment was positively associated with the following behaviors: more time watching Television, among boys (> 2 h/day, OR = 1.66, CI 1.18-2.33, p<0.05); more time spent using the computer, in boys and girls respectively: (> 1 h/day, OR = 2.17, CI 1.31-3.6, p<0.05; OR = 1.13, CI 0.84-1.9, p<0.05); more time spent playing electronic games, in boys and girls: (> 1 h/day, OR = 2.97, CI 1.56-5.6, p<0.01; OR = 1.66, CI 1.12-2.45, p<0.01); less time playing outdoors, in boys and girls: (activity outside<1 h/day, OR = 1.69, CI 1.22-2.33, p<0.01; OR = 1.31, CI = 1.01-1.7, p<0.05). Conclusion When parents have a negative perception of their residential environment the children tend to spend more time inside house watching television, using the computer and playing electron games and less time playing outside. Our results point out for a need to improve the urban design and promoting mixed land uses-as well as the social environment, especially, safety
SN - 0393-2990
AD - (Padez, Ferrao, Freitas, Gama, Mourao, Nogueira, Rosado, Santana) Department of Life Sciences, Research Centre for Anthropology and Health, University of Coimbra, Portugal; Department of Animal Biology, University of Lisbon, Research Centre for Anthropology and Health, University of Coimbra, Portugal; Department of Sports, University of Tras-os-Montes e Alto Douro, Vila Real, Portugal; Department of Geography, University of Coimbra, Research Centre for Anthropology and Health, University of Coimbra,

Portugal; Center of Anthropobiology, Tropical Research Institute, Lisbon, Research Centre for Anthropology and Health, University of Coimbra, Portugal
ER -

TY - JOUR
ID - 3222
T1 - Association of obesity with sedentary beahaviors and play outdoor among children
A1 - Padez,C.
A1 - Ferrao,M.M.
A1 - Freitas,F.
A1 - Gama,A.
A1 - Mourao,I.
A1 - Nogueira,H.
A1 - Rosado,V.
A1 - Santana,A.
Y1 - 2012///
N1 - 71303088
English
Journal: Conference Abstract
KW - EMBASE
KW - Epidemiology
KW - Child
KW - Human
KW - Obesity
KW - Society
KW - childhood obesity
KW - Television Viewing
KW - school
KW - sampling
KW - multivariate logistic regression analysis
KW - weight
KW - kindergarten
KW - chi square test
KW - questionnaire
KW - Risk
KW - parent
KW - Prevalence
KW - height
KW - cross-sectional study
RP - NOT IN FILE
SP - S118
JF - European Journal of Epidemiology
JA - Eur J Epidemiol
VL - Conference: IEA-EEF European Congress of Epidemiology 2012: Epidemiology for a Fair and Healthy Society Porto Portugal. Conference Start: 20120905 Conference End: 20120908. Conference Publication:
IS - var.pagings
N2 - Background Sedentary behaviors such as television viewing is one of the major factors contributing to childhood obesity. Objectives This study aims to analyze the association between sedentary behaviors such television viewing, passive play inside house, outdoor play and children obesity. Methods A cross-sectional study was done in a sample of 11,554 children, aged 6.0-10.0 y. Private and public kindergartens and schools were randomly selected. Sampling was based on a sex and age-specific proportionate stratified random design with the district are the primary sampling unit. Weight and height were measured, and parents filled out a questionnaire about family and child characteristics: TV viewing, passive play inside house and outdoor play. Overweight and obesity, using cut-off points as defined by the IOTF, were used. Data was analyzed using the Chi-square test and multivariate logistic regression analysis adjusted for age, sex and the cluster of schools. Results We found 21.1 % of overweight and 9.0 % of obese children. The prevalence of obesity increased by time of TV viewing:<2 h, 26.9 %, 2-4 h, 30.2 %, and +4 h 34.2 %, p<0.001; decreased by time spent with

passive play inside house: <2 h, 32.6 %, 2-4 h, 30.3 %, and +4 h 27.7 %, p<0.001 and decreased by active play outdoor: <1 h/day 37.4 %, 2-4 h/day 30.1 %, >3 h/day 26.4, p<0.001. The odds ratio (OR) for childhood obesity increased by television viewing (reference <2 h; 2-4 h: 1.18; [4 h: 1.39), decreased by time spent in passive play inside house (reference<2 h; 2-4 h: 0.72;[4 h: 0.58). Conclusions We conclude that TV viewing is positive associated with childhood obesity and either passive play inside house or active play outdoor are both effective ways to decrease childhood obesity
SN - 0393-2990
AD - (Padez, Ferrao, Freitas, Gama, Mourao, Nogueira, Rosado, Santana) Department of Life Sciences, Research Centre for Anthropology and Health, University of Coimbra, Portugal; Department of Animal Biology, University of Lisbon, Research Centre for Anthropology and Health, University of Coimbra, Portugal; Department of Sports, University of Tras-os-Montes e Alto Douro, Vila Real, Portugal; Department of Geography, University of Coimbra, Research Centre for Anthropology and Health, University of Coimbra, Portugal; Center of Anthropobiology, Tropical Research Institute, Lisbon, Research Centre for Anthropology and Health, University of Coimbra, Portugal
ER -

TY - JOUR
ID - 3223
T1 - Active travel to school and independent mobility in Portuguese children
A1 - Marques,E.A.
A1 - Pizarro,A.N.
A1 - Teixeira,J.
A1 - Ribeiro,J.C.
A1 - Santos,M.P.
Y1 - 2012///
N1 - 71303085
English
Journal: Conference Abstract
KW - EMBASE
KW - Epidemiology
KW - Child
KW - Human
KW - school
KW - Society
KW - Travel
KW - Physical Activity
KW - active transport
KW - bicycle
KW - questionnaire
KW - car
KW - Male
KW - boy
KW - logistic regression analysis
KW - cross-sectional study
KW - Sedentary Lifestyle
KW - Cronbach alpha coefficient
KW - independent variable
KW - Health
KW - city
KW - passive transport
KW - Walking
KW - Principal Component Analysis
KW - girl
KW - Adult
KW - friend
KW - dependent variable
KW - Female

KW - accelerometer
KW - gender
KW - Cognitive Development
KW - environmental factor
RP - NOT IN FILE
SP - S116
EP - S117
JF - European Journal of Epidemiology
JA - Eur J Epidemiol
VL - Conference: IEA-EEF European Congress of Epidemiology 2012: Epidemiology for a Fair and Healthy Society Porto Portugal. Conference Start: 20120905 Conference End: 20120908. Conference Publication:
IS - var.pagings
N2 - Background Compelling evidence suggests that a sedentary lifestyle is being encouraged, at least in part, by environmental factors that affect individuals' physical activity choices. Encouraging physical activity early in life is crucial to positively influence current and future health. Active travel to school is one possible routine to assist children increasing daily physical activity. However, previous studies demonstrated that children may be spending less time unsupervised outside the home. Thus, children's independent mobility has been declining, which may in part explain the reported declines in active commuting to school Objectives This cross-sectional study investigated whether independent mobility was related to active travel to school. Methods six hundred and twenty-five 10-14 years old boys (n = 292) and girls (n = 333) from 10 schools in a large Portuguese city were included. The independent variable in logistic regression analysis was mode of travel home to school measured by a selfreported questionnaire, asking "How do you usually travel to school". Options were walk, bicycle, car, bus or train; those travelling by car/ motocycle and bus/train were merged into a single "passive transport" group and those travelling by bicycle or walking were merged into an "active transport" group. Dependent variable was independent mobility assessed using the stem "How often are you allowed to go to the following places on your own or with friends (without an adult)"; from the eleven questions, which were part of the self-completed questionnaire, one dimension (scale) was derived using categorical principal components analysis (Cronbach alpha = 0.901). Minutes of moderate to vigorous physical activity (MVPA) were objectively measured using accelerometers. The analysis was adjusted for gender, age, and MVPA. Results Independent mobility was significantly associated with active transport to school: i.e., children with high independent mobility were 1.44 times more likely to walk or cycle (95 % CI 1.12-1.86). Conclusions Independent mobility appears to be an important independent correlate of active transport to school. Conversely, low level of independent mobility can negatively influence physical activity, which may lead to decreased levels of social, emotional, and cognitive development
SN - 0393-2990
AD - (Marques, Pizarro, Teixeira, Ribeiro, Santos) Research Centre in Physical Activity, Health and Leisure Faculty of Sport, University of Porto, Portugal
ER -

TY - JOUR
ID - 3224
T1 - The role of the school in the promotion of physical activity in Italian school-children
A1 - Nardone,P.
A1 - Buoncristiano,M.
A1 - Lamberti,A.
A1 - Spinelli,A.
A1 - Baglio,G.
A1 - Bonciani,M.
Y1 - 2012///
N1 - 71303007
English
Journal: Conference Abstract
KW - EMBASE
KW - Epidemiology
KW - school child
KW - Human
KW - Physical Activity

KW - Society
KW - school
KW - Child
KW - sport
KW - Risk
KW - parent
KW - multivariate logistic regression analysis
KW - Regression Analysis
KW - Cluster Analysis
KW - procedures
KW - sampling
KW - primary school
KW - Physical Education
KW - citizenship
KW - University
KW - Education
KW - high school
KW - Lifestyle
KW - Recreation
KW - Health
RP - NOT IN FILE
SP - S86
JF - European Journal of Epidemiology
JA - Eur J Epidemiol
VL - Conference: IEA-EEF European Congress of Epidemiology 2012: Epidemiology for a Fair and Healthy Society Porto Portugal. Conference Start: 20120905 Conference End: 20120908. Conference Publication:
IS - var.pagings
N2 - Background Physical activity, both as formal sports and free play, has an important role in the cognitive, physical and relational development of the child and also contributes to the maintenance of good health and ideal bodyweight. For these reasons, it is suggested that children should participate in physical activity (including that at school, sports training and active recreation) for at least one hour per day. The Italian child surveillance system, OKkio alla SALUTE, has collected data relating to the physical activity of children aged 8-9 years, and the contribution made by the school. Objectives To investigate the association between the physical activity of children and their family background, and to assess the contribution of the schools. Methods The survey has been conducted on a representative sample of children from the 3rd year of primary school using a standardized procedure. The children were classified as "active" if in the day before the survey they had participated in physical activity at school, or sports or played outside. The study uses multiple logistic regression to investigate the effects of environmental, familial and scholastic variables on the risk of being classified "active". The analysis uses appropriate methods to take account of complex sampling scheme used to collect the data (stratified cluster sample design and representativeness at local or regional level). Results In the 2010 survey, 42,549 children participated with only 3 % who refused. The percentage of children not classified as active was 18.4 % (95 % CI: 17.5-19.3 %). The regression analysis revealed a statistically significant association with the educational level and the citizenship of the parents. The odds ratio (OR) associated with high school education was 0.88 (95 % CI: 0.80-0.97), while for a university degree the OR was 0.84 (95 % CI: 0.73-0.96). For children of foreign parents the OR was 1.22 (95 % CI: 1.05-1.41). The presence of initiatives to improve physical activity in the schools was protective, OR = 0.74 (95 % CI: 0.61-0.90), as was the inclusion of two hours per week of physical education in the children's timetable, OR = 0.83 (95 % CI: 0.72-0.95). Conclusion The data collected in 2010 has revealed that about 1 out of 5 of Italian children aged 8-9 is not physically active, especially in the South of the country. The schools can make an important contribution to promote a healthy lifestyle, including adequate physical activity, in Italian schoolchildren
SN - 0393-2990
AD - (Nardone, Buoncristiano, Lamberti, Spinelli, Baglio, Bonciani) Italian National Institute of Health, Italy
ER -

TY - JOUR
ID - 3225
T1 - Audio-visual rear-projection systeminmagnetic resonance for pediatric population

A1 - Sanchez-Montanez,A.
A1 - Delgado,I.
A1 - Salazar,J.P.
A1 - Corral,J.F.
A1 - Vazquez,E.
A1 - Enriquez,G.
Y1 - 2013///
N1 - 71301412
English
Journal: Conference Abstract
KW - EMBASE
KW - procedures
KW - sedation
KW - Pediatrics
KW - Population
KW - Society
KW - neuroradiology
KW - comfort
KW - Human
KW - patient
KW - nuclear magnetic resonance
KW - Child
KW - nuclear magnetic resonance imaging
KW - image quality
KW - Lung
KW - Anxiety
KW - parental consent
KW - anesthesist
KW - patient comfort
KW - clinical practice
KW - audiovisual equipment
KW - university hospital
KW - Functional Magnetic Resonance Imaging
KW - ionizing radiation
KW - brain
KW - Personnel
RP - NOT IN FILE
SP - S53
JF - Neuroradiology
VL - Conference: 37th European Society of Neuroradiology Annual Meeting Frankfurt Germany. Conference Start: 20130928 Conference End: 20131001. Conference Publication:
IS - var.pagings
N2 - PURPOSE -To reduce movements of children improving image quality -To reduce scan times allowing a greater number of explorations -To avoid or minimize number of sedations reducing costs -To allow performing MR in non-candidates patients for sedation -To improve patient comfort by reducing the claustrophobic feeling -Availability for visual and auditory functional MRI METHODS We have been studying the benefits of our developed audio-visual rearprojection (AVRP) system that works in 1.5 and 3 T Magnetic Resonance for two years in our institution at The Vall d'Hebron University Hospital. The system consists of: -DVD-DVD player, Movies, Projector (outside the room) -Display and Cranial coil-mirrors (inside the room) -Audio-system (both outside and inside the room) RESULTS In our clinical practice, the group of patients considered 'uncooperative' represents about 50 %. These patients require sedation depending on the anesthesiologist's availability, patient's respiratory status, and parental consent, among other factors. AVRP system has been demonstrated as a very useful tool in the 1.5 and 3 T MRI to improve image quality, as patients remain still over the duration of the scan, with less need to repeat sequences, reducing scanning times. It also minimizes claustrophobic feelings of anxiety and discomfort, avoiding to practice sedation for MRI in these cases. Furthermore, it has been observed a significant reduction in the number of sedation in children age limit. Similarly, patients with medical contraindications for sedation such as patients with lung problems who had to be studied with CT, now take

advantage of MRI thus, preventing the undesirable intrinsic effect of ionizing radiation. Additionally AVRP system opens the door to functional MR evaluation of primary auditory and visual areas. Finally, AVRP system has a very low cost of implementation, an ease of use and involves a not inconsiderable cost reductions enabling the potential realization of a larger number of studies in a lesser time, requiring less personnel and equipment for sedation. CONCLUSIONS The AVRP system has proven very useful for the practice of MRI in pediatric population. It has demonstrated significant benefit to children as young as 3 years of age in completing an interpretable brain MR exam without sedation/anesthesia
SN - 0028-3940
AD - (Sanchez-Montanez, Delgado, Salazar, Corral, Vazquez, Enriquez) Vall D'Hebron University Hospital, Diagnostic Imaging, Barcelona, Spain
ER -

TY - JOUR
ID - 3226
T1 - Outdoors environments are important for health enhancing physical activity in deprived environments
A1 - Eyre,E.
A1 - Duncan,M.
A1 - Birch,S.
A1 - Cox,V.
Y1 - 2013///
N1 - 71300514
English
Journal: Conference Abstract
KW - EMBASE
KW - Environment
KW - Physical Activity
KW - Obesity
KW - Health
KW - Child
KW - Human
KW - weight
KW - Safety
KW - Sunlight
KW - thematic analysis
KW - Interview
KW - information processing
KW - seasonal variation
KW - Weather
KW - United Kingdom
KW - ward
RP - NOT IN FILE
SP - 169
JF - Obesity Facts
JA - Obes Facts
VL - Conference: 20th European Congress on Obesity, ECO 2013 Liverpool United Kingdom. Conference Start: 20130512 Conference End: 20130515. Conference Publication:
IS - var.pagings
N2 - Introduction: Physical activity (PA) is important for weight management but PA may be restricted by environmental influences (obesogenic environments). The study aims to assess the importance of outdoors environments in deprived areas on PA engagement. Methods: 46 children (8.6 + 0.55years;BMI:17.7 + 4.4kg/m², BF% 21 + 9) from the two most deprived wards of Coventry, UK wore a combined HR and GPS monitor (Garmin 406) for 4 days from which time spent in moderate to vigorous physical activity (MVPA) was determined. Children took part in focus group interviews exploring environmental facilitators and barriers to PA which were analysed using thematic analysis. Results: Children spent more time in MVPA in outdoor (greenspace, street) than indoor environments (outside = 49 + 35% vs. inside 27 + 25%, P = 0.00) for both weekdays and weekends (P=0.01). Children reported that they were more active and preferred PA in outdoors environments. Children spent more significantly more time in MVPA outdoors on weekdays during

increased hours of sunlight (spring/summer) compared to reduced hours of sunlight (autumn/winter) (69 + 30mins vs 44 + 60mins , P=0.01). Children reported that weather, parental constraints and safety were barriers to PA. There was no difference in time in MVPA outside or inside between weight status groups (P<0.05). Conclusion: Outdoor environments are important for health enhancing PA but interactions with outdoor environments is limited by seasonality, parental constraints and safety in deprived environments. Interaction with outdoor environments was not influenced by weight status
SN - 1662-4025
AD - (Eyre, Duncan, Birch, Cox) Coventry University, Coventry, United Kingdom
ER -

TY - JOUR
ID - 3227
T1 - Increasing physical activity in primary schools in an english county
A1 - Huber,J.W.
A1 - Sixsmith,J.A.
A1 - Browne,J.
A1 - Doe,E.
A1 - Ryder,R.
Y1 - 2013///
N1 - 71300472
English
Journal: Conference Abstract
KW - EMBASE
KW - primary school
KW - Obesity
KW - Physical Activity
KW - Human
KW - Child
KW - school
KW - questionnaire
KW - Internet
KW - Walking
KW - Swimming
KW - Football
KW - boy
KW - Male
KW - parent
KW - procedures
KW - teacher
KW - epidemic
KW - school child
RP - NOT IN FILE
SP - 157
JF - Obesity Facts
JA - Obes Facts
VL - Conference: 20th European Congress on Obesity, ECO 2013 Liverpool United Kingdom. Conference Start: 20130512 Conference End: 20130515. Conference Publication:
IS - var.pagings
N2 - Introduction: Lack of physical activity in primary school children is considered to be a major driver of the obesity epidemic. In an English county with slightly above average obesity levels, an intervention to increase physical activity in and outside school in 7 to 10 year olds placed facilitators in schools to help five schools to achieve this aim. Methods: A pre and after design (baseline and post intervention) collects questionnaire data from children. Schools were selected to represent a range of areas in the county with a range of deprivation levels as measured by the index of multiple deprivation. Questionnaires ask about the type and intensity of activities children engage in and their attitudes towards physical activity. Children completed the internet survey in class at school. In addition qualitative data will be collected from children, teachers and facilitators. An opt-out consent procedure was used with parents and children. Results: At baseline 419 children have taken part in

the study. Mean age was 8 years (SD 0.95); 51% of the children were boys. The most popular physical activities were football, cycling, swimming and walking. Attitudes towards physical activity were generally positive. Conclusion: The baseline data indicate that the pupils are active and have a positive attitude to physical activity. It will be interesting to see whether the facilitators' tailored interventions will improve on these baseline data
SN - 1662-4025
AD - (Huber, Sixsmith, Doe, Ryder) University of Northampton, Northampton, United Kingdom (Browne) Northamptonshire Partnership, Northampton, United Kingdom
ER -

TY - JOUR
ID - 3228
T1 - Prevention of overweight from birth till age 3 years; preliminary results
A1 - Beltman,M.
A1 - Vlasblom,E.
A1 - L'Hoir,M.P.
A1 - Raat,H.
A1 - Boere-Boonekamp,M.M.
Y1 - 2013///
N1 - 71300423
English
Journal: Conference Abstract
KW - EMBASE
KW - Obesity
KW - Prevention
KW - Human
KW - Child
KW - parent
KW - soft drink
KW - meal
KW - Sleep
KW - habit
KW - Prevalence
KW - Child Rearing
KW - Lifestyle
KW - questionnaire
KW - childhood
KW - juvenile
KW - Exercise
KW - Health Care
KW - night
KW - weight
KW - univariate analysis
KW - sleep time
KW - Reinforcement
RP - NOT IN FILE
SP - 143
JF - Obesity Facts
JA - Obes Facts
VL - Conference: 20th European Congress on Obesity, ECO 2013 Liverpool United Kingdom. Conference Start: 20130512 Conference End: 20130515. Conference Publication:
IS - var.pagings
N2 - Background: The prevalence of overweight among children is rising rapidly and childhood overweight increasingly occurs at a younger age. The 'BBOFT+' intervention aims at implementing healthy lifestyle habits through focusing on effective child rearing from birth: sleeping, breastfeeding, (outdoor) activity, breakfast, soft drinks and TV time. This study aims to evaluate the effects on the prevention of overweight of the BBOFT+ intervention, compared to care-as-usual (CAU), as applied to children of 0-3 years and their parents. Method: In a cluster-randomized control trial, 68 Youth Health Care teams participated. Parents of more than 2500 children

filled out questionnaires at the age of 2 weeks, 6, 15 and 36 months. Parent-reported child's weight and length are used. Results: The first univariate analyses show no difference in nightly sleep duration between the BOFT+ and the CAU group at the age of 6 months. However, at the age of 15 months, children of the CAU group slept at night on average 6.6 minutes shorter than children in the BBOFT+ group ($p < 0.05$). At age 15 months, no difference in BMISDS, parental control, reinforcement or warmth was found between the BBOFT+ and the CAU group. Conclusion: The BBOFT+ intervention seem to have an effect on sleep at the age of 15 months. Further analyses will be conducted for the topics outdoor exercise, activity and play, breakfast, soft drinks and TV time. Furthermore the differences in BMI between BBOFT+ and CAU will be evaluated. These will be presented during the congress
SN - 1662-4025
AD - (Beltman, Vlasblom, L'Hoir, Boere-Boonekamp) TNO, Leiden, Netherlands (Raat) Erasmus MC, Rotterdam, Netherlands (Boere-Boonekamp) University Twente, Enschede, Netherlands
ER -

TY - JOUR
ID - 3229
T1 - Review analysis of the association between the prevalence of activated brown adipose tissue and outdoor temperature
A1 - Huang,Y.-C.
A1 - Hsu,C.-C.
A1 - Wang,P.-W.
A1 - Chang,Y.-H.
A1 - Chen,T.-B.
A1 - Lee,B.-F.
A1 - Chiu,N.-T.
Y1 - 2012///
N1 - 2012279141
English
Journal: Review
KW - EMBASE
KW - Adolescent
KW - Adult
KW - Body Weight
KW - brown adipose tissue
KW - Child
KW - cold exposure
KW - computer assisted emission tomography
KW - diet restriction
KW - environmental temperature
KW - Female
KW - Human
KW - major clinical study
KW - Male
KW - review
KW - school child
KW - stimulus response
KW - systematic review
KW - systematic review (topic)
KW - Taiwan
KW - temperature measurement
KW - thermogenesis
KW - tropic climate
KW - fluorodeoxyglucose f 18
RP - NOT IN FILE
JF - The Scientific World Journal
VL - 2012 , 2012. Article Number: 793039. Date of Publication: 2012.
CY - United Kingdom

N2 - Brown adipose tissue (BAT) is important for regulating body weight. Environmental temperature influences BAT activation. Activated BAT is identifiable using F 18 -fluorodeoxyglucose positron emission tomography/computed tomography (F 18 -FDG PET/CT). F 18 -FDG PET/CT scans done between June 2005 and May 2009 in our institution in tropical southern Taiwan and BAT studies from PubMed (2002-2011) were reviewed, and the average outdoor temperatures during the study periods were obtained. A simple linear regression was used to analyze the association between the prevalence of activated BAT (P) and the average outdoor temperature (T). The review analysis for 9 BAT studies (n = 16, 765) showed a significant negative correlation (r = - 0.741, P = 0.022) between the prevalence of activated BAT and the average outdoor temperature. The equation of the regression line is P () = 6.99 - 0.20 T (Cdegree). The prevalence of activated BAT decreased by 1 for each 5 Cdegreeincrease in average outdoor temperature. In a neutral ambient temperature, the prevalence of activated BAT is low and especially rare in the tropics. There is a significant linear negative correlation between the prevalence of activated BAT and the average outdoor temperature. Copyright 2012 Yung-Cheng Huang et al
AD - (Huang, Hsu, Wang, Chang) Department of Nuclear Medicine, Kaohsiung Chang Gung Memorial Hospital, Chang Gung University, Kaohsiung 83301, Taiwan (Republic of China) (Huang) Department of Information Engineering, I-Shou University, Kaohsiung, Taiwan (Republic of China) (Chen) Department of Medical Imaging and Radiological Sciences, I-Shou University, Kaohsiung 82445, Taiwan (Republic of China) (Lee, Chiu) Department of Nuclear Medicine, College of Medicine, National Cheng Kung University Hospital, Tainan 70428, Taiwan (Republic of China)
ER -

TY - JOUR
ID - 3230
T1 - Playgrounds in Novi Sad (Serbia) and their influences on children's health and development
A1 - Krkljes,M.
A1 - Jevtic,M.
Y1 - 2012///
N1 - 2012343289
English
Journal: Article
KW - EMBASE
KW - article
KW - Child
KW - Child Development
KW - child health
KW - child safety
KW - equipment
KW - Government
KW - health hazard
KW - Human
KW - major clinical study
KW - play
KW - Recreation
KW - Serbia
KW - urban area
RP - NOT IN FILE
SP - 864
EP - 874
JF - HealthMED
VL - 6
IS - 3
CY - Bosnia and Herzegovina
N2 - Playgrounds are spaces in urban environments where children spend most of their free time. Continuous and various games in the open, with numerous physical and emotional reactions, have great significance for their health. The importance of playgrounds is based on possibilities for children to realize different needs for their proper psychophysical development. The aim of this research was to explore what kinds of playgrounds are adequate for children according to their development levels. Focusing on playgrounds in the urban area of

Novi Sad (Serbia), the paper analyzes different playgrounds and sport fields. The analyzed examples are differently positioned, shaped and various in used elements, as well as in the overall quality of surroundings. Play opportunities and problems within playgrounds were identified and documented in order to determine possible strategies and recommendations to improve their quality and provide children with healthy and secure environments. Upon analyzing playgrounds in Novi Sad, conclusions were made that the unified need of children for play does exist in diversified places and that all spaces have certain influence on children's development and health. There is an obvious lack of adequate playgrounds, which would fully satisfy modern children's needs
SN - 1840-2291
AD - (Krkljes) University of Novi Sad, Faculty of Technical Sciences, Serbia (Jevtic) University of Novi Sad, Faculty of Medicine, Serbia
ER -

TY - JOUR
ID - 3231
T1 - Vitamin D status in a sample of preschool children aged from 1 to 6 years visiting the pediatrics clinic at Jordan University Hospital
A1 - Jazar,A.S.
A1 - Takruri,H.R.
A1 - Khuri-Bulos,N.A.
Y1 - 2011///
N1 - 2012373157
English
Journal: Article
KW - EMBASE
KW - article
KW - artificial milk
KW - body mass
KW - Body Weight
KW - Breast Feeding
KW - Child
KW - controlled study
KW - Female
KW - Human
KW - Jordan
KW - Lifestyle
KW - major clinical study
KW - Male
KW - Nutritional Status
KW - pediatric hospital
KW - Physical Activity
KW - preschool child
KW - university hospital
KW - vitamin blood level
KW - vitamin D deficiency
KW - vitamin intake
KW - calcifediol
KW - Calcium
KW - vitamin D
RP - NOT IN FILE
SP - 308
EP - 316
JF - Jordan Medical Journal
VL - 45
IS - 4
CY - Jordan

N2 - Objectives: Vitamin D status was investigated in 200 children (100 males and 100 females) visiting the Pediatrics Clinic at Jordan University Hospital. Methods: Subjects were categorized into two age groups: 1-3 years (toddlers) and 3-6 years (preschool children). Data were collected on children's lifestyle factors, dietary intake, height, weight and BMI. Serum 25-hydroxyvitamin D ₃ (25 (OH) D₃) concentration was determined. Results: 16.5% of the children had vitamin D deficiency (serum 25 (OH) D₃ < 15 ng/mL), while 15.5% had vitamin D insufficiency (serum 25 (OH) D₃ from 15 to 20 ng/mL). Serum 25 (OH) D₃ levels for the toddlers (26.25 + 1.16) were significantly higher (P<0.05) than those of the preschool children (21.49 + 1.16). Serum 25 (OH) D₃ was significantly higher (P<0.05) for exclusively formula-fed children (27.42 + 0.86) or children with mixed feeding (26.69 + 0.92) than for those who were exclusively breast-fed (17.02 + 0.90). Also, serum 25 (OH) D₃ levels were positively correlated with the duration of formula feeding (r = 0.4849, P<0.0001), duration of outdoor physical activity (r = 0.3940, P<0.0001), monthly frequency of outdoor physical activity (r = 0.61087, P<0.0001), and vitamin D (r = 0.4678, p<0.0001) and calcium (r =0.4951, p<0.0001) intakes. However, serum 25 (OH) D₃ levels were negatively correlated with duration of breast feeding (r = -0.3554, P<0.0001) and BMI (r = -0.4235; P<0.0001). Conclusions: It is concluded that hypovitaminosis D is common in preschool children and that lifestyle conditions influence vitamin D status. 2011 DAR Publishers/University of Jordan. All Rights Reserved
SN - 0446-9283
AD - (Jazar, Takruri) Department of Nutrition and Food Technology, Faculty of Agriculture, University of Jordan, Amman, Jordan (Khuri-Bulos) Department of Pediatrics, Faculty of Medicine, University of Jordan, Amman, Jordan
ER -

TY - JOUR
ID - 3233
T1 - An uncommon cause of inflammation
A1 - Saung,M.T.
A1 - Catanese,L.
A1 - Joshi,P.
Y1 - 2012///
N1 - 71297140
English
Journal: Conference Abstract
KW - EMBASE
KW - Society
KW - Internal Medicine
KW - Inflammation
KW - Human
KW - patient
KW - Diagnosis
KW - erythrophagocytosis
KW - Hospital
KW - histiocyte
KW - hypertransaminasemia
KW - Hepatitis
KW - fever
KW - sepsis
KW - cytopenia
KW - Infection
KW - hypertriglyceridemia
KW - Therapy
KW - natural killer cell
KW - Epstein Barr virus
KW - community acquired pneumonia
KW - leukopenia
KW - survival
KW - Kidney Failure

KW - Adult
KW - Survival Rate
KW - autoimmune disease
KW - immune deficiency
KW - Transplantation
KW - pathophysiology
KW - immune response
KW - fluid resuscitation
KW - Child
KW - autosomal recessive inheritance
KW - lymph node
KW - erythroid precursor cell
KW - hemophagocytic syndrome
KW - spleen
KW - bone marrow
KW - bone marrow biopsy
KW - thorax radiography
KW - faintness
KW - blood cell
KW - smear
KW - Flow Cytometry
KW - splenomegaly
KW - myalgia
KW - clinical feature
KW - neutropenia
KW - cell activity
KW - central nervous system disease
KW - Female
KW - T lymphocyte
KW - health care quality
KW - pancytopenia
KW - virus infection
KW - Cell Transplantation
KW - cytokine storm
KW - diseases
KW - outpatient
KW - follow up
KW - laboratory
KW - hematopoietic cell
KW - central nervous system
KW - adult respiratory distress syndrome
KW - dexamethasone
KW - etoposide
KW - antihypertensive agent
KW - autoantibody
KW - antibiotic agent
KW - interleukin 2
KW - Receptor
KW - cyclosporin
KW - immunoglobulin
KW - DNA
KW - lactate dehydrogenase
RP - NOT IN FILE
SP - S390
JF - Journal of General Internal Medicine
VL - Conference: 35th Annual Meeting of the Society of General Internal Medicine, SGIM 2012 Orlando, FL United States. Conference Start: 20120509 Conference End: 20120512. Conference Publication:

IS - var.pagings
N2 - LEARNING OBJECTIVE 1: Recognize clinical features of hemophagocytic lymphohistiocytosis (HLH) LEARNING OBJECTIVE 2: Treat HLH CASE: A 53 year-old woman presented to an outside hospital with complaints of 1 week of fevers, myalgias, URI symptoms, and 1 episode of "fainting." She was hypotensive, but responded to fluid resuscitation. Marked hematologic derangements were seen, including leukopenia of 1.3 with 15% bands, transaminitis AST 397/ALT 239, ferritinemia 65,000 and LDH 2,000. A chest radiograph showed a possible infiltrate, leading to the diagnosis of presumed severe sepsis secondary to community-acquired pneumonia, but pancytopenia and transaminitis worsened despite broadspectrum antibiotics, prompting the transfer to our hospital. Additional labs at our hospital showed neutropenia of 0.5, hypertriglyceridemia 379, and a positive DIC panel. Flow cytometry and a peripheral smear did not indicate malignancy. All infectious and rheumatologic studies were negative except for a positive EBV DNA PCR. A bone marrow biopsy showed hemophagocytosis with numerous nucleated erythroid precursors engulfed by histiocytes, indicative of HLH. DISCUSSION: There are two types of HLH which present similarly. Primary HLH is due to an autosomal recessive trait and is predominantly seen in children. Secondary HLH is seen more often in adults, and is caused by the body's reaction to another condition such as infections (EBV is the most commonly associated infection), autoimmune disease, malignancies, immune deficiencies or suppression, and post-transplantation. The central pathophysiology of HLH is an excessive immune response. A cytokine storm leads multi-organ dysfunction via infiltration of activated T-lymphocytes, natural killer (NK) cells and histiocytes. Autoantibodies may also contribute to the cytopenia. The key diagnostic finding is hemophagocytosis of blood cells in the bone marrow, spleen and lymph nodes. HLH can typically be diagnosed if at least five of the following criteria are fulfilled: fever, splenomegaly, cytopenia, hypertriglyceridemia or hypofibrinogemia, hemophagocytosis, hepatitis, low or absent NK cell activity, marked ferritinemia and elevated IL-2 receptor. Though HLH is a rare condition, incidence may be higher given the need for complex diagnostic workup and is commonly misdiagnosed as severe sepsis (as in this patient's case), FUO, hepatitis, renal failure, ARDS, and various CNS diseases to name a few. Prompt diagnosis is crucial, since without treatment, median survival is 1 to 2 months. To address this, the first international treatment protocol, HLH-94, was developed and studies have shown a significant improvement in survival rates. The HLH-2004 protocol (Grade 1B) is the current standard of care: immediate immunochemotherapy with dexamethasone and etoposide, aggressive supportive therapy and for selected patients, hematopoietic cell transplantation (HCT). Whether cyclosporine should be administered upfront as proposed by HLH-94 or later is controversial. This patient was treated with dexamethasone and intravenous immunoglobulin since the latter is an appropriate adjunct for most viral infections. The patient improved following 2 days of therapy and supportive care, and etoposide was never started. EBVassociated HLH can range from spontaneously resolving inflammation to persisting illness requiring HCT, and the patient's presentation is closer to the former. During her outpatient follow-up visit, her laboratory values normalized except for a slight down-trending ferritinemia
SN - 0884-8734
AD - (Saung, Catanese, Joshi) Boston Medical Center, Boston, MA, United States
ER -

TY - JOUR
ID - 3234
T1 - Pattern of physical activity and its differences among school girls and boys in Pakistan
A1 - Khawaja,S.
A1 - Khoja,A.A.
A1 - Otwani,K.
A1 - Khuwaj,A.K.
Y1 - 2010///
N1 - 71294908
English
Journal: Conference Abstract
KW - EMBASE
KW - Human
KW - Female
KW - student
KW - boy
KW - Male
KW - Pakistan

KW - school child
KW - scientist
KW - Physical Activity
KW - Adolescent
KW - girl
KW - school
KW - questionnaire
KW - Health
KW - childhood obesity
KW - fitness
KW - Child
KW - cardiovascular disease
KW - computer
KW - Bone
KW - Physical Education
KW - Responsibility
KW - Public Health
KW - psychological well being
KW - physical inactivity
KW - Television
KW - Energy Expenditure
KW - Obesity
KW - chi square test
KW - caloric intake
KW - Graduate
KW - Social Class
KW - high school
KW - city
KW - sitting
KW - parent
KW - adolescent health
KW - ion
RP - NOT IN FILE
SP - 55
JF - European Journal of Medical Research
JA - Eur J Med Res
VL - Conference: 21st European Students' Conference. Promising Medical Scientists Willing to Look Beyond Berlin Germany. Conference Start: 20101013 Conference End: 20101017. Conference Publication:
IS - var.pagings
N2 - Introduction: Obesity among school going children is one of the major global health challenge. Childhood obesity is the result of a long lasting imbalance between energy intake and energy expenditure. A major contributing factor is physical inactivity which is also closely linked to bone health, cardiovascular disease, overall body fitness and psychological well being .The objectives of this study were to identify the pattern of physical activity and its difference among adolescent girls and boys. Aim: Level of physical activity is poor amongst adolescents in Pakistan. Method: A cross-sectional, questionnaire based survey was conducted in five inner city secondary schools serving predominantly middle and lower socioeconomic class in Karachi and Quetta. In all, 314 adolescents (13 to 17 years) completely responded to the questionnaire. All the data was collected and managed by trained medical graduates. Chi-square test was used to calculate the differences of physical activity pattern among girls and boys. Results: Questionnaire was administered to 159 boys (50.6%) and 155 girls (49.4%). Large majority (83.7%) of adolescents were using vehicle to and from school and over half (55.1%) of respondents spent three hours or more on watching television, playing computer games or doing other sitting activities. Only 35.7% of adolescents were involved in vigorous activities; more boys compared to girls (52.2% vs. 18.7%; p<0.001). Similarly, boys were found to be more physically active for at least thirty minutes per day as compared to girls (45.3% vs. 27.7%; p=0.001). More than half of the study participants (55.7%) were taught about physical education and 61.2% of them had accessibility of play ground other than school. Conclus ion: Substantially large proportion of adolescents particularly girls were physically inactive in this study. There is a need for those with responsibility for adolescent's health, including parents, schools, and

community health providers, to consider and address the need for effective interventions to encourage increased physical activity level among adolescents
SN - 0949-2321
AD - (Khawaja, Khoja, Otwani, Khuwaj) Dow University of Health Sciences, Karachi, Pakistan, MEDICINE, Pakistan
ER -

TY - JOUR
ID - 3235
T1 - Implementation of intensive interventions in treatment across an organization
A1 - Flinn,N.A.
A1 - Pieper,K.M.
Y1 - 2013///
N1 - 71291542
English
Journal: Conference Abstract
KW - EMBASE
KW - Rehabilitation
KW - Evidence Based Practice
KW - rehabilitation medicine
KW - rehabilitation research
KW - Human
KW - patient
KW - fitness
KW - intensive care
KW - transference
KW - clinical practice
KW - courage
KW - Therapy
KW - Self Evaluation
KW - planning
KW - speech language pathologist
KW - physiotherapist
KW - Population
KW - occupation
KW - Speech Therapy
KW - Diagnosis
KW - wolf
KW - cerebrovascular accident
KW - spinal cord injury
KW - outpatient
KW - hospital patient
KW - United States
KW - Child
KW - disability
KW - fox
KW - community based rehabilitation
KW - Parkinson disease
KW - Adult
KW - movement therapy
KW - chlorpromazine
RP - NOT IN FILE
SP - e58
EP - e59
JF - Archives of Physical Medicine and Rehabilitation

VL - Conference: American Congress of Rehabilitation Medicine Annual Conference, Progress in Rehabilitation Research, ACRM 2013 Orlando, FL United States. Conference Start: 20131112 Conference End: 20131116. Conference Publication:
IS - var.pagings
N2 - Objective: Intensive therapy, such as constraint-induced movement therapy and BIG and LOUD, have been shown to improve outcomes better than standard care rehabilitation for a wide range of client groups, including adults and children with stroke (Wolf, et al., 2006; Duncan, 2011; Szaflarski, 2008; Lo, 2010), Parkinsons Disease (Fox, 2006), and individuals with spinal cord injury (Dobkin, 2006). In September of 2012 Courage Center started an intensive therapy intervention for all therapy and fitness programs to help therapists change their practice and incorporate higher intensity interventions to improve outcomes. The evaluation plan includes two patient and staff surveys of patient effort (1 month prior, and 2 months into the project). At baseline, clients reported 40% of sessions outside targeted effort range, and staff reported 29% of sessions outside the targeted range of effort. Two months into the project, clients reported 36% of sessions outside targeted effort range, and staff reported 20% of sessions outside the targeted range of effort. A survey of staff about implementation of intensive intervention revealed that 95% of staff said the implementation had gone well. The most common strategies implemented included "use it or lose it" and increased repetition. The most common strategies they were planning to implement next were "use it and improve it" and transference. Staff report that patients are making faster improvements than expected, and early results from outcome measures are promising. Design: This was a quasi-experimental prospective cohort design, examining comparisons of staff and patient reports of effort during sessions collected prior to and after implementation of the intensive intervention project. Setting: Courage Center is a community-based rehabilitation and resource center for persons with disabilities located in Minneapolis, Minnesota. The Center has both inpatient and outpatient physical, occupational, and speech therapy, as well as an extensive fitness programs, and serves a population with a wide spectrum of rehabilitation needs. Participants: All staff in therapy and fitness began implementing intensive strategies beginning in November 2012. This included 28 occupational, 49 physical therapists, 15 speech language pathologists, as well as 25 fitness staff. Clients prior to this date received standard care treatment and are the historical comparisons for this project. Interventions: Intensive therapy and behavioral techniques that encourage greater patient engagement and effort in rehabilitation were used. These techniques were modeled after and Kleim-s (2008) review, and involve specific 10 strategies to increase intensity, regardless of the intervention. Representatives from all departments, all professions and all sites made up an Intensive Intervention Implementation Team. They developed training materials, a self-assessment, and reminder materials that could be used during sessions to remind staff to incorporate more intensity into the session. Reminders to implement the new strategies were incorporated into all staff and department meetings, as well as into diagnostic specific clinical practice groups that met monthly. The members of the Intensive Intervention Implementation Team and the clinical practice groups were asked to practice intensive interventions, and became local leaders in the process. All staff were asked to complete the self-assessment, and to select 2 strategies to implement across all clients for the following month. All other activities in the facility, including Journal Club and the quality committee focused on this project. Main Outcome Measure(s): Outcomes for this project were patient and staff ratings of session intensity, measured at two points (1 month prior and 2 months into the project) as well as a staff survey about the implementation process and practice change. Results: As of February 2012, all staff had been charged with implementing intensive interventions for 2 months. At baseline, clients reported 40% of sessions outside targeted effort range, and staff reported 29% of sessions outside the targeted range of effort. Two months into the project, clients reported 36% of sessions outside targeted effort range, and staff reported 20% of sessions outside the targeted range of effort. A survey about implementation of intensive intervention revealed that 95% of staff said the implementation had gone well. The most common strategies implemented included "use it or lose it" and increased repetition. The most common strategies they were planning to implement next were "use it and improve it" and transference. This survey was responded to by 49% of the staff. Conclusions: The findings from this study provide additional support for the importance of increased patient effort early in the rehabilitation, and speak to the value of incorporating intensive intervention techniques in post-acute rehabilitation settings
SN - 0003-9993
AD - (Flinn) Courage Center, United States
ER -

TY - JOUR
ID - 3236

T1 - The cross-cultural adaptation and psychometric evaluation of the children's assessment of participation and enjoyment-Chinese version
A1 - Huang,Y.-Y.
A1 - Li,C.-Y.
A1 - Huang,I.-C.
A1 - Bendixen,R.
A1 - Chen,K.-L.
A1 - Weng,W.-C.
Y1 - 2013///
N1 - 71291418
English
Journal: Conference Abstract
KW - EMBASE
KW - Human
KW - Child
KW - rehabilitation research
KW - rehabilitation medicine
KW - Adaptation
KW - validity
KW - test retest reliability
KW - internal consistency
KW - skill
KW - neuromuscular disease
KW - parent
KW - Speech
KW - reliability
KW - Duchenne muscular dystrophy
KW - Cerebral Palsy
KW - questionnaire
KW - construct validity
KW - Diagnosis
KW - Sample Size
KW - Evidence Based Practice
KW - boy
KW - girl
KW - Male
KW - content validity
KW - Female
KW - Interview
RP - NOT IN FILE
SP - e13
EP - e14
JF - Archives of Physical Medicine and Rehabilitation
VL - Conference: American Congress of Rehabilitation Medicine Annual Conference, Progress in Rehabilitation Research, ACRM 2013 Orlando, FL United States. Conference Start: 20131112 Conference End: 20131116. Conference Publication:
IS - var.pagings
N2 - Objective: This study is to develop the Chinese-version of the Children's Assessment of Participation and Enjoyment (CAPE-C) and examine its psychometric properties for Chinese speaking children with and without neuromuscular disorders. Design: The CAPE was translated and culturally adapted into a Chinese version (CAPE-C). All the parents assisted their children to complete the CAPE-C. The content and response process validity were established by an expert panel and the cognitive interviews for six participants. The internal consistency, item discrimination, omega coefficient reliability, and known group validity were evaluated through a crosssectional design. The test-retest reliability was examined by a repeatedmeasures design. Setting: All the questionnaires were completed by mail. Participants: A total of 201 parents of 157 unaffected children, and 44 children with cerebral palsy and Duchenne muscular dystrophy aged 6-15 years were included. Interventions: No intervention in this project. Main Outcome Measure(s): The content validity, response process

validity, internal consistency, item discrimination, omega coefficient reliability, test-retest reliability, and known group validity were evaluated. Results: For the CAPE-C overall and five domain items, the internal consistency was 0.55 to 0.87, and the test-retest reliability was 0.82-0.97. Six items with insufficient discrimination were found in both overall and domain scores. The omega coefficient was 0.831, excluding skill-based domain items. The unaffected children significantly participated more in overall and domain activities than affected children (except for skill-based activities). Apart from self-improvement activities, the participation intensity of CAPE-C activities was significantly higher in younger (6-10 years) children than older (11-15 years) ones. Girls significantly took more skill-based activities than boys. Conclusions: Consequently, to enhance children's participation outcome generalization across cultures, this newly developed and standardized CAPE-C provides an evidence-based tool to measure outside-class activity participation for children with and without neuromuscular disorders of Chinese speaking background. Future studies with larger sample size and different diagnosis samples are recommended to further support different types of construct validity of the CAPE-C
SN - 0003-9993
AD - (Huang) University of Florida, United States
ER -

TY - JOUR
ID - 3237
T1 - Helpful vs. harmful: Social networks and their association with weight loss
A1 - Winston,G.J.
A1 - Phillips,E.
A1 - Charlson,M.
Y1 - 2013///
N1 - 71292794
English
Journal: Conference Abstract
KW - EMBASE
KW - social network
KW - weight reduction
KW - Society
KW - Internal Medicine
KW - Human
KW - eating habit
KW - Eating
KW - Hispanic
KW - Adult
KW - model
KW - friend
KW - Feeding Behavior
KW - Female
KW - Child
KW - weight
KW - Social Support
KW - coworker
KW - Behavior Change
KW - Physical Activity
KW - Population
KW - Obesity
KW - United States
KW - Analysis of Variance
RP - NOT IN FILE
SP - S86
EP - S87
JF - Journal of General Internal Medicine
VL - Conference: 36th Annual Meeting of the Society of General Internal Medicine, SGIM 2013 Denver, CO United States. Conference Start: 20130424 Conference End: 20130427. Conference Publication:
IS - var.pagings

N2 - BACKGROUND: Obesity has been shown to spread through social networks. However, further data are needed on social network structure and function in weight loss behaviors, particularly in race/ethnic minority populations. The aim of this analysis was to examine social network structure, its influence on eating habits and relationship to weight loss among adults enrolled in a behavior change weight loss study. METHODS: The Small Changes and Lasting Effects (SCALE) trial is an ongoing 1 year weight loss intervention among non-Hispanic black and Hispanic adults, BMI>25 kg/m², living primarily in Harlem and the South Bronx, New York. Participants made small changes in their eating behaviors and set individual physical activity goals. Social network members were defined as people important in the participant's life; for example family, friends, coworkers. Data on participants' social network structure were collected at study close-out using the convoy model of social support. The convoy model consists of 3 concentric circles (Fig. 1); the closest network members were listed in the inner circle (circle 1), members not as close in the middle circle, and least close in the outer circle. Participants identified the network members who influenced their eating habits and if they helped or hindered their eating goals in SCALE. Weight loss was calculated as the difference between weight measured at study enrollment and close-out. Data were analyzed using chi-square and ANOVA tests as appropriate. RESULTS: There were 45 index participants included in the analysis (mean age 51 years, 38 % female, 40 % non-Hispanic black, 60 % Hispanic) and 469 social network members. The average network size was 10.4 members, with the largest percent of network members in circle 1 (60 %) followed by circle 2 (25 %) and circle 3 (15 %). The majority of network members were women (62 % circle 1, 77 % circle 2 and 74 % circle 3). 77 % of participants indicated that at least one social network member influenced their eating habits, these members were primarily in circle 1 (74 %). Among the social network members who influenced eating habits, 80 % helped participants with their SCALE eating goals, 8 % made it more difficult, and 11 % had no impact (p<0.0001). Network members who helped participants with their eating goals were primarily in circle 1 (74 %). Children and friends were identified as being more helpful than partners/spouses (25 %, 25 % and 8 % respectively). Network members identified as helpful lived primarily outside the home (70 %). There was a trend towards greater weight loss among participants who reported having social network members that helped them with their eating goals compared to those without help (-3.4 lbs vs. + 3.8 lbs, p=0.06). CONCLUSIONS: In SCALE, the majority of participants indicated that social network members influenced their eating habits. There was a trend towards greater weight loss among participants who had social network help in their eating behavior goals compared to those without help. These data provide information on the structure of social networks among overweight/obese adults residing in an urban setting and provide evidence that social networks can be helpful in weight loss efforts
SN - 0884-8734
AD - (Winston, Phillips, Charlson) Weill Cornell Medical College, New York, NY, United States
ER -

TY - JOUR
ID - 3238
T1 - Particulate air pollution and susceptibility to the development of pulmonary tuberculosis disease in North Carolina: An ecological study
A1 - Smith,G.S.
A1 - Schoenbach,V.J.
A1 - Richardson,D.B.
A1 - Gammon,M.D.
Y1 - 2014///
N1 - 2014022345
English
Journal: Article
KW - EMBASE
KW - Adolescent
KW - Adult
KW - Aged
KW - Air Pollution
KW - ambient air
KW - article
KW - Child
KW - disease association
KW - disease course

KW - disease predisposition
KW - Female
KW - Human
KW - Infant
KW - long term exposure
KW - lung tuberculosis
KW - Male
KW - Middle Aged
KW - Newborn
KW - Particulate Matter
KW - preschool child
KW - resident
KW - school child
KW - United States
KW - Young Adult
RP - NOT IN FILE
SP - 103
EP - 112
JF - International Journal of Environmental Health Research
JA - Int J Environ Health Res
VL - 24
IS - 2
CY - United Kingdom
N2 - Although Mycobacterium tuberculosis is the causative agent of pulmonary tuberculosis (PTB), environmental factors may influence disease progression. Ecologic studies conducted in countries outside the USA with high levels of air pollution and PTB have suggested a link between active disease and ambient air pollution. The present investigation is the first to examine the ambient air pollution-PTB association in a country, where air pollution levels are comparatively lower. We used Poisson regression models to examine the association of outdoor air pollutants, PM10 and PM2.5 with rates of PTB in North Carolina residents during 1993-2007. Results suggest a potential association between long-term exposure to particulate matter (PM) and PTB disease. In view of the high levels of air pollution and high rates of PTB worldwide, a potential association between ambient air pollution and tuberculosis warrants further study. 2013 2013 Taylor & Francis
SN - 0960-3123
AD - (Smith, Schoenbach, Richardson, Gammon) UNC Chapel Hill School of Public Health, Epidemiology, Chapel Hill, NC, United States
ER -

TY - JOUR
ID - 3239
T1 - Global and local cancer risks after the Fukushima Nuclear Power Plant accident as seen from Chernobyl: A modeling study for radiocaesium (134Cs & 137Cs)
A1 - Evangeliou,N.
A1 - Balkanski,Y.
A1 - Cozic,A.
A1 - Moller,A.P.
Y1 - 2014///
N1 - 2013812141
English
Journal: Article
KW - EMBASE
KW - Adult
KW - article
KW - atmospheric deposition
KW - atmospheric transport
KW - cancer incidence
KW - cancer risk
KW - Cause of Death

KW - chemical analysis
KW - Chernobyl accident
KW - Child
KW - concentration (parameters)
KW - controlled study
KW - dosimetry
KW - Female
KW - Fukushima Nuclear Accident
KW - Human
KW - Infant
KW - Japan
KW - Male
KW - molecular model
KW - nonhuman
KW - nuclear power plant
KW - priority journal
KW - radiation exposure
KW - radioactivity
KW - radiosensitivity
KW - Risk Assessment
KW - risk factor
KW - school child
KW - sensitivity analysis
KW - sex difference
KW - solid tumor
KW - cesium 134
KW - cesium 137
RP - NOT IN FILE
EP - 27
JF - Environment International
JA - Environ Int
VL - 64
IS - pp 17-27
CY - United Kingdom
N2 - The accident at the Fukushima Daiichi Nuclear Power Plant (NPP) in Japan resulted in the release of a large number of fission products that were transported worldwide. We study the effects of two of the most dangerous radionuclides emitted, ¹³⁷Cs (half-life: 30.2years) and ¹³⁴Cs (half-life: 2.06years), which were transported across the world constituting the global fallout (together with iodine isotopes and noble gasses) after nuclear releases. The main purpose is to provide preliminary cancer risk estimates after the Fukushima NPP accident, in terms of excess lifetime incident and death risks, prior to epidemiology, and compare them with those occurred after the Chernobyl accident. Moreover, cancer risks are presented for the local population in the form of high-resolution risk maps for 3 population classes and for both sexes. The atmospheric transport model LMDZORINCA was used to simulate the global dispersion of radiocaesium after the accident. Air and ground activity concentrations have been incorporated with monitoring data as input to the LNT-model (Linear Non-Threshold) frequently used in risk assessments of all solid cancers. Cancer risks were estimated to be small for the global population in regions outside Japan. Women are more sensitive to radiation than men, although the largest risks were recorded for infants; the risk is not depended on the sex at the age-at-exposure. Radiation risks from Fukushima were more enhanced near the plant, while the evacuation measures were crucial for its reduction. According to our estimations, 730-1700 excess cancer incidents are expected of which around 65% may be fatal, which are very close to what has been already published (see references therein). Finally, we applied the same calculations using the DDREF (Dose and Dose Rate Effectiveness Factor), which is recommended by the ICRP, UNSCEAR and EPA as an alternative reduction factor instead of using a threshold value (which is still unknown). Excess lifetime cancer incidents were estimated to be between 360 and 850, whereas 220-520 of them will be fatal. Nevertheless, these numbers are expected to be even smaller, as the response of the Japanese official authorities to the accident was rapid. The projected cancer incidents are much lower than the casualties occurred from the earthquake itself (>20,000) and also smaller than the accident of Chernobyl. 2013 Elsevier Ltd

SN - 0160-4120
AD - (Evangeliou, Balkanski, Cozic) Laboratoire des Sciences du Climat et de l'Environnement (LSCE), CEA-UVSQ-CNRS UMR 8212, Institut Pierre et Simon Laplace, L'Orme des Merisiers, F-91191 Gif sur Yvette Cedex, France (Moller) Laboratoire d'Ecologie, Systematique et Evolution, CNRS UMR 8079, Universite Paris-Sud, Batiment 362, F-91405 Orsay Cedex, France
ER -

TY - JOUR
ID - 3240
T1 - Children's exposure to arsenic from cca-treated wood staircases
A1 - Gress,J.
Y1 - 2012///
N1 - 71290015
English
Journal: Conference Abstract
KW - EMBASE
KW - Human
KW - Child
KW - exposure
KW - Epidemiology
KW - Society
KW - Walking
KW - Soil
KW - sitting
KW - United States
KW - arsenic
RP - NOT IN FILE
SP - S674
JF - Epidemiology
VL - Conference: 24th Annual Conference of the International Society for Environmental Epidemiology, ISEE 2012 Columbia, SC United States. Conference Start: 20120826 Conference End: 20120830. Conference Publication:
IS - var.pagings
N2 - Two-story apartment complexes in Florida commonly have a large exterior staircase made from CCAtreated wood. Children living in these residences have frequent contact with steps and handrails while walking, sitting and playing on the staircases outside their homes Soil arsenic concentrations under twostory staircases at 6 large apartment complexes, where over 1,500 people reside, ranged from 6 to over 160 ppm, representing a source of arsenic exposure to children playing under and around these structures. Currently, we are measuring arsenic concentrations in the surface residue of these staircases and on objects stored under them, including grills, toys and children's bikes. These results will be used to identify variables that may lead to increased exposure to arsenic by children living in these types of apartment complexes and provide evidence in support of mitigation strategies
SN - 1044-3983
AD - (Gress) University of Florida, United States
ER -

TY - JOUR
ID - 3241
T1 - Indoor air sampling for particulate matter and nitrogen dioxide in pittsburgh communities near active steel mills
A1 - Tunno,B.
A1 - Cambal,L.
A1 - Howell,J.
A1 - Michanowicz,D.
A1 - Carr,J.
A1 - Gillooly,S.
A1 - Shields,K.

A1 - Holguin,F.
A1 - Clougherty,J.
Y1 - 2012///
N1 - 71289982
English
Journal: Conference Abstract
KW - EMBASE
KW - air sampling
KW - Particulate Matter
KW - ambient air
KW - community
KW - Asthma
KW - Epidemiology
KW - Society
KW - Human
KW - Child
KW - sampling
KW - summer
KW - monitor
KW - Monitoring
KW - exposure
KW - winter
KW - air quality standard
KW - Smoking
KW - Temperature
KW - Spectrophotometry
KW - filter
KW - Cooking
KW - city
KW - iron and steel industry
KW - United States
KW - nitrogen dioxide
KW - steel
KW - politef
KW - carbon
KW - trace metal
RP - NOT IN FILE
SP - S641
JF - Epidemiology
VL - Conference: 24th Annual Conference of the International Society for Environmental Epidemiology, ISEE 2012 Columbia, SC United States. Conference Start: 20120826 Conference End: 20120830. Conference Publication:
IS - var.pagings
N2 - Background and Aims: Braddock and Clairton, Pennsylvania, in the eastern suburbs of Pittsburgh, are home to the Edgar Thomson Steel Works and Clairton Coke Works, which are two active steel mills. These towns exceed average annual (>15 mug/m3) and daily (>35 mug/m3) National Ambient Air Quality Standards (NAAQS) for particulate matter (PM2.5). Within the past few decades, asthma rates have been increasing in the Pittsburgh region. Methods: A subset of 14 homes from an asthmatic cohort was selected for a 1 week sampling session of indoor air during the summer 2011, which was repeated for an additional week in the winter of 2012. Personal Exposure Monitors (PEMs) were used to sample particulate matter (PM2.5) and passive Ogawa badges were used to sample NO2. Teflon filters were pre- and post-weighed to calculate PM2.5 concentration, NO2 badges were analysed by UV-VIS spectrophotometry, and EC and trace metal analysis were performed. Results: Within the 21 homes for summer sampling, a mean PM2.5 concentration of 25.8 mug/m3 (SD = 22.7 mug/m3) and a mean NO2 concentration of 13.9ppb (SD = 7.2ppb) were detected. Homes with an asthmatic child had a mean concentration of 30.4 mug/m3 for PM2.5 and 13.9ppb for NO2, whereas the other 7 homes with no asthmatic child had a mean concentration of 16.6 mug/m3 for PM2.5 and 9.7ppb for NO2. For elemental carbon, a mean of 2.8 absorbance units (SD = 1.2 abs) was found. A seasonal comparison will be made.

Temperature, number of cigarettes smoked, and time spent cooking appear to have strong correlations to PM. Conclusions: This pilot data was used to detect PM, EC, and NO2 concentrations in homes that have an asthmatic child and are located in communities near an active steel mill. Indoor results will be compared to an outdoor stationary monitoring campaign throughout Pittsburgh
SN - 1044-3983
AD - (Tunno, Cambal, Howell, Michanowicz, Carr, Gillooly, Shields, Clougherty) University of Pittsburgh, Graduate School of Public Health, Department of Environmental and Occupational Health, United States (Holguin) University of Pittsburgh Medical Center, Department of Pediatric Pulmonology, Pediatric Environmental Medicine Center, United States
ER -

TY - JOUR
ID - 3242
T1 - The global burden of disease attributable to household air pollution from cooking with solid fuels: Estimates from the GBD 2010
A1 - Smith,K.R.
A1 - Bruce,K.
Y1 - 2012///
N1 - 71289732
English
Journal: Conference Abstract
KW - EMBASE
KW - indoor air pollution
KW - biomass
KW - household
KW - Air Pollution
KW - Population
KW - Cooking
KW - solid
KW - Society
KW - Epidemiology
KW - Human
KW - exposure
KW - cataract
KW - Child
KW - cerebrovascular accident
KW - Asia
KW - pneumonia
KW - Female
KW - diseases
KW - heart disease
KW - Pollution
KW - passive smoking
KW - Africa South of the Sahara
KW - gas
KW - meta analysis
KW - systematic review (topic)
KW - Death
KW - lung cancer
KW - Risk Assessment
KW - ischemic heart disease
KW - Risk
KW - Monitoring
KW - Indian
KW - Male
KW - model
KW - fuel

KW - coal
RP - NOT IN FILE
SP - S389
JF - Epidemiology
VL - Conference: 24th Annual Conference of the International Society for Environmental Epidemiology, ISEE 2012 Columbia, SC United States. Conference Start: 20120826 Conference End: 20120830. Conference Publication:
IS - var.pagings
N2 - Background The Global Burden of Diseases Study 2010 updates the first international Comparative Risk Assessment as part of the Global Burden of Disease Project, which estimated that the annual burden attributable to household air pollution (HAP) from use of solid fuels in 2000 was 1.6 million premature deaths and 54 million lost years of healthy life (DALYs), largely due to child pneumonia and COPD in women in sub-Saharan Africa and Asia. Objectives We quantify for 1990, 2005, and 2010 the revised burden attributable to HAP based on additional epidemiologic studies and new approaches for estimating exposure and exposure-response functions. Methods We estimated world-wide household exposure to fine particulate air pollution, PM2.5, by combining 500+ nationally representative household energy surveys with a model of household PM2.5 levels based on data from Indian monitoring studies. We derived risk functions for ischemic heart disease, stroke, COPD, lung cancer, cataracts, and child pneumonia using recent systematic reviews and meta-analyses and for some endpoints drawing on Generalized Exposure Response relationships derived along with evidence from the literature on active and passive smoking and outdoor air pollution. Since household sources contribute substantially to outdoor pollution, we also include a portion of the outdoor air pollution burden in the HAP estimates for Asia. Conclusions Preliminary estimates suggest the burden attributable to HAP is considerably higher than the previous estimate because evidence supports 1) impacts on men as well as women, 2) inclusion of additional diseases (heart disease, cataract, and stroke); 3) a portion of outdoor air pollution impacts are now included under HAP; and 4) a lower counterfactual level is applied, equivalent to cooking with gas fuels
SN - 1044-3983
AD - (Smith) University of California Berkeley, United States (Bruce) University of Liverpool, United Kingdom
ER -

TY - JOUR
ID - 3243
T1 - A chemical risk reduction program for residents living near the koppers superfund site: The eco-health festival!
A1 - Gress,J.
Y1 - 2012///
N1 - 71289482
English
Journal: Conference Abstract
KW - EMBASE
KW - contamination
KW - community
KW - Empowerment
KW - risk reduction
KW - Health
KW - Society
KW - Epidemiology
KW - Human
KW - Child
KW - United States
KW - exposure
KW - Justice
KW - sampling
KW - Dust
KW - water quality
KW - soil pollution
KW - Soil

KW - lowest income group
KW - organization
KW - dangerous goods
KW - gardening
KW - chicken
KW - Music
KW - University
KW - Health Education
KW - Social Justice
KW - medical education
KW - Environment
KW - musician
KW - Adult
KW - river
KW - assay
KW - Education
KW - parent
KW - Infant
KW - dioxin
RP - NOT IN FILE
SP - S139
JF - Epidemiology
VL - Conference: 24th Annual Conference of the International Society for Environmental Epidemiology, ISEE 2012 Columbia, SC United States. Conference Start: 20120826 Conference End: 20120830. Conference Publication:
IS - var.pagings
N2 - The Koppers Superfund site, in Gainesville, Florida, operated as a wood treatment facility for nearly 100 years before closing in 2009. At that time, soil testing in residential yards around the site found elevated levels of dioxin, directly impacting hundreds of residents. Sampling of indoor dust from 200 homes using the CALUX assay detected high levels of dioxin-like chemicals in homes up to .5 mile from the site. To address the concern and confusion generated by these findings, Suwannee River Area Health Education Center developed the Gainesville Area Health Education Project and received a $25,000 USEPA Environmental Justice small grant to implement the project. Community outreach activities over the year will culminate in the Eco-Health Festival, to be held in the center of the impacted neighborhood on Earth Day, 2012. Local experts from the University of Florida and other organizations will speak on over 30 topics identified by residents as areas where they desire more information related to the soil contamination, such as impacts on water quality, gardening, raising chickens, keeping children safe and reducing overall chemical exposures in the home. A Superfund Art Show will highlight related work from adults and children in the neighborhood. Local musicians will perform social justice and environment-related music. A mini-film festival will highlight environmental justice struggles experienced by other communities around the world. The overall goal of the festival is to strengthen the resiliency of this low income neighborhood through building community ties and empowering residents with accessible scientific information related to the contamination of their homes and yards. The results from this project will be used to develop future family-friendly environmental education forums in similarly impacted communities around the country, with an emphasis on providing parents with easyto- understand information on ways to protect infants and children from exposure to hazardous chemicals in and around the home
SN - 1044-3983
AD - (Gress) University of Florida, United States
ER -

TY - JOUR
ID - 3244
T1 - Health risk assessment of aldehydes in the activities space of young children-focused on children's facilities and indoor playgrounds
A1 - Lee,C.S.
A1 - Yang,J.Y.
A1 - Kim,H.-H.
A1 - Yang,S.H.

A1 - Kim,S.D.
A1 - Shin,D.C.
A1 - Lim,Y.W.
Y1 - 2011///
N1 - 71291051
English
Journal: Conference Abstract
KW - EMBASE
KW - Human
KW - Child
KW - Risk Assessment
KW - health hazard
KW - Society
KW - Epidemiology
KW - cancer risk
KW - ambient air
KW - hazard
KW - Health
KW - lifespan
KW - exposure
KW - neoplasm
KW - Child Care
KW - kindergarten
KW - Day Care
KW - summer
KW - Environment
KW - winter
KW - high performance liquid chromatography
KW - aldehyde
KW - formaldehyde
KW - carcinogen
KW - acetaldehyde
KW - benzaldehyde
RP - NOT IN FILE
SP - S282
JF - Epidemiology
VL - Conference: 22nd Annual Conference of the International Society for Environmental Epidemiology, ISEE 2010 Seoul South Korea. Conference Start: 20100828 Conference End: 20100901. Conference Publication:
IS - var.pagings
N2 - Background/Aims: Children's facilities are an important indoor environment for children and exposure area with potential harmful health effects for children who spend most of their daily life. This study was performed to investigate concentration of aldehydes in indoor air at children's facilities. Methods: The samples of indoor air were collected at various children's facilities (40 day-care houses, 42 child-care centers, 44 kindergartens, and 42 indoor playgrounds) in summer (July-September, 2007), winter (January-February, 2008), and spring (March-April, 2008) periods, and analyzed by HPLC. Indoor air measurement after that assessed the lifetime cancer and non-cancer risk of aldehydes exposure of young children. We estimated the lifetime excess cancer risks of formaldehyde, acetaldehyde, and the hazard quotients of non-carcinogens (benzaldehyde and formaldehyde). Formaldehyde evaluated both cancer and non-cancer risk. Results: The indoor and outdoor (I/O) ratio of aldehydes exceeds 1.0. It appeared the interior materials have indoor source. Also, the relationship between childcare facilities and formaldehyde level was statistically significant. Aldehydes of health assessment result, formaldehyde average excess cancer risks of young children were 1 x 10⁻⁴-1 x 10⁻⁵ level in all facilities. And hazard quotients of 4 non-carcinogens did not exceed 1.0 for all subjects in all facilities. Conclusion: To better understand results, investigators need to account for the variability of contaminants both within and between children's facilities
SN - 1044-3983

AD - (Lee, Yang, Kim, Yang, Kim, Shin, Lim) Institute for Environmental Research, Yonsei University, Seoul, South Korea (Shin) Department of Preventive Medicine, College of Medicine, Yonsei University, Seoul, South Korea
ER -

TY - JOUR
ID - 3245
T1 - Influence of environmental exposure on blood lead levels of young children in Seoul, Korea: A pilot study
A1 - Seo,S.C.
A1 - Cho,Y.
A1 - Choi,S.H.
A1 - Kim,D.H.
A1 - Yeon,K.
A1 - Kim,S.
A1 - Choi,J.W.
Y1 - 2011///
N1 - 71291041
English
Journal: Conference Abstract
KW - EMBASE
KW - Environmental Exposure
KW - lead blood level
KW - Child
KW - Human
KW - Korea
KW - pilot study
KW - Society
KW - Epidemiology
KW - residential area
KW - exposure
KW - environmental factor
KW - blood sampling
KW - atomic absorption spectrometry
KW - Blood
KW - statistical analysis
KW - Risk
KW - Nervous System
KW - furnace
KW - soil pollution
KW - laboratory
KW - multivariate logistic regression analysis
KW - Lead Poisoning
KW - Smoking
KW - Food
KW - house dust
KW - triton x 100
KW - drinking water
KW - graphite
KW - Paint
RP - NOT IN FILE
SP - S279
JF - Epidemiology
VL - Conference: 22nd Annual Conference of the International Society for Environmental Epidemiology, ISEE 2010 Seoul South Korea. Conference Start: 20100828 Conference End: 20100901. Conference Publication:
IS - var.pagings
N2 - Background/Aims: Lead exposure is associated with impairment of nerve system. In particular, children are at the greatest risk due to their proximity to the floor and nature for placing hands in their mouths. Further

research is necessary for better understanding of lead exposure in children. However, little is known about blood lead levels of younger children in Korea. The objective of this study was to characterize lead levels in blood of children; and to investigate the environmental factors associated with blood lead levels. Methods: We took blood samples from 68 children (5-9 years) who resided in an industrial complex (n = 19) and residential areas (n = 49) in Seoul, and did not have any disease and clinical symptoms related to lead poisoning. Frozen samples were moved to the laboratory and kept in freezer before analysis. Graphite furnace atomic absorption spectrometry was used for analysis followed by pretreatment with 0.1% Triton X-100 and standard solution. Statistical analyses (SPSS, version 12.0) were performed to compare lead concentrations between an industrial complex and residential areas. The multiple logistic regression was performed to investigate the influence of environmental factors (house types, distance between house and vehicle road, types of drinking water, smoking of housemate, time spent outdoor, and ground types in the playground) on blood lead levels. Results: Mean age of subjects was 74.6 months. Blood lead levels ranged from 0.3 to 2.2 mug/dL, and mean was 1.1 + 0.4 mug/dL. Environmental variables and sex were not significantly associated with blood lead levels in this study. However, lead levels for residential areas (mean: 1.2 mug/dL [range: 0.5-2.2]) were significantly higher than those for an industrial complex (mean: 0.8 mug/dL [0.3-1.4]) (P < 0.01). Conclusion: This result may suggest that exposure to lead may often occur through lead-contained materials (eg, toys, food, and house dust originated from lead-based paint), as well as polluted soil and air
SN - 1044-3983
AD - (Seo, Cho, Choi, Yeon, Kim, Choi) Institute for Occupational and Environmental Health, Korea University, Seoul, South Korea (Kim) Department of Health Policy and Management, University of North Carolina, Chapel Hill, NC, United States
ER -

TY - JOUR
ID - 3246
T1 - Child-specific health risk assessment of 25 indoor air pollutants in child-care facilities and indoor playgrounds
A1 - Lim,Y.W.
A1 - Kim,H.H.
A1 - Lee,C.S.
A1 - Yang,S.H.
A1 - Kim,S.D.
A1 - Jeon,J.M.
A1 - Lee,J.Y.
A1 - Sohn,J.R.
A1 - Chang,Y.S.
A1 - Shin,D.C.
A1 - Yang,J.Y.
Y1 - 2011///
N1 - 71291036
English
Journal: Conference Abstract
KW - EMBASE
KW - Human
KW - Risk Assessment
KW - health hazard
KW - Child Care
KW - ambient air
KW - air pollutant
KW - Society
KW - Epidemiology
KW - Child
KW - Environment
KW - pollutant
KW - exposure
KW - cancer risk
KW - summer

KW - Inhalation
KW - winter
KW - risk reduction
KW - mass fragmentography
KW - skin
KW - hazard
KW - kindergarten
KW - Adult
KW - Day Care
KW - vapor
KW - Dust
KW - seasonal variation
KW - gas
KW - plasma
KW - Health
KW - pesticide
KW - dichlorvos
KW - metal
KW - formaldehyde
KW - polybrominated diphenyl ether
KW - aldehyde
KW - carcinogen
KW - volatile organic compound
RP - NOT IN FILE
SP - S277
JF - Epidemiology
VL - Conference: 22nd Annual Conference of the International Society for Environmental Epidemiology, ISEE 2010 Seoul South Korea. Conference Start: 20100828 Conference End: 20100901. Conference Publication:
IS - var.pagings
N2 - Background/Aims: Children's exposures to environmental contaminants are expected to be different and, in many cases, much higher than adults. Differences in the behavior of children, particularly the way in which children interact with their environment, may also have a profound effect on the magnitude of exposures to contaminants. Especially, children's facilities are an important indoor environment for children and exposure area with potential harmful health effects for children who spend most of their daily life. Therefore, the present study was performed to assess the levels and characteristics of several pollutants in indoor environments, and to predict the child-specific health risk of indoor pollutants at children's facilities. Methods: The levels of 25 indoor pollutants (8 volatile organic compound [VOC], 3 aldehydes, 5 metal, 2 pesticides, 4 retardants, 3 phthalates) were assessed in indoor samples (dust, respiratory particulates, vapor, surface) at various children's facilities (40 day-care houses, 42 child-care centers, 44 kindergartens, and 42 indoor playgrounds) in summer (July-September, 2007), winter (January and February, 2008), and spring (March-April, 2008) periods. We analyzed using gas chromatography-mass spectrometry for VOCs, pesticides, inductively coupled plasma for metals, and phthalates, and high-resolution gas chromatography/high-resolution mass spectrometryfor polybrominated diphenyl ethers. Results: The levels of VOCs, aldehydes, polybrominated diphenyl ethers, and pesticides showed the seasonal variations, but the levels of phthalates and metals in summer period were similar to those in winter periods. The excess cancer risks of 9 carcinogens for children were 10-3-10-5 level in all facilities and excess cancer risks of dichlorvos and formaldehyde were more than 10-4 level, respectively. The 14% in the subject facilities were exceed to 1.0 of hazard index for 16 noncarcinogens. The exposure of dichlorvos could be done through various routes such as inhalation, handto- mouth, and skin contact, and major route of formaldehyde was inhalation for child in indoor environment. Conclusion: The dichlorvos and formaldehyde were selected as the major concern pollutants and need the risk reduction management plan at children's facilities
SN - 1044-3983
AD - (Lim, Kim, Lee, Yang, Kim, Shin, Yang) Institute for Environmental Research, College of Medicine, Yonsei University, Seoul, South Korea (Jeon) Department of Civil and Environmental Engineering, Suncheon First College, Suncheon, South Korea (Lee) Institute of Life Science and Resources, Kyung Hee University, Yongin, South Korea (Sohn) College of Health Science, Korea University, Seoul, South Korea (Chang) School

of Environmental Engineering, Pohang University of Science and Technology, Pohang, South Korea (Shin) Department of Preventive Medicine, College of Medicine, Yonsei University, Seoul, South Korea
ER -

TY - JOUR
ID - 3247
T1 - Geographical information system time-activity and population exposure modeling
A1 - Beale,L.
A1 - Wyatt,D.
A1 - Fabbri,F.
A1 - Davies,G.
A1 - Briggs,D.
Y1 - 2011///
N1 - 71291013
English
Journal: Conference Abstract
KW - EMBASE
KW - geographic information system
KW - population exposure
KW - model
KW - Society
KW - Epidemiology
KW - exposure
KW - Population
KW - Air Pollution
KW - pollutant
KW - Human
KW - velocity
KW - Technology
KW - Traffic
KW - Travel
KW - school
KW - school child
KW - night
KW - global positioning system
RP - NOT IN FILE
SP - S270
JF - Epidemiology
VL - Conference: 22nd Annual Conference of the International Society for Environmental Epidemiology, ISEE 2010 Seoul South Korea. Conference Start: 20100828 Conference End: 20100901. Conference Publication:
IS - var.pagings
N2 - Background/Aims: Although it is increasingly recognized that environmental concentrations do not necessarily equate to individual exposure, concentrations at place of residence are often used to approximate exposure. However, individual exposure can vary, depending on circumstances such as the amount of time spent indoors and outdoors (Jarup 2004). Furthermore, environmental pollutants and exposures vary spatially and temporally with, for example, different levels during day and night (Briggs 2005). A better approximation of exposure can be obtained through time-activity modeling, using a mixture of individual level data and statistical (aggregated) data from time-activity surveys. Methods: A model has been programmed in a geographical information system (ESRI ArcGIS) using network analysis tools. The model can use geocoded data for start and end locations of each trip (X/Y), start and end times of each trip, trip mode, travel speed, route, and destination type. Using these data, the model is able to reproduce the time-activity sequence. However, in many cases much, if not all, of these data will not be known and so the model can probabilistically impute, on the basis of available statistical information, any of these data. These statistical data comprise generalized distributions for the study population (eg, national surveys). Results: Using data collected as part of a study to analyze the effect of traffic-related air pollution on the journey to school (Walker et al, 2009), model results have been validated. Results using 31 time-activity diaries, including actual routes taken by school children (captured using global positioning system technology), showed the model to be a reasonable predictor of routes. Conclusion: This

research demonstrates the difference obtained in exposure values from fixed home location in comparison with modeled spatio-temporal data. These results allow some quantification of exposure misclassification that may be introduced into small-scale spatial epidemiological analysis
SN - 1044-3983
AD - (Beale, Fabbri, Briggs) SAHSU, MRC Centre for Environment and Health, Imperial College London, London, United Kingdom (Wyatt, Davies) Lancaster Environment Centre, Lancaster University, Lancaster, United Kingdom
ER -

TY - JOUR
ID - 3248
T1 - A case-control study: Exposure assessment of volatile organic compounds and formaldehyde for asthma in children
A1 - Choi,J.
A1 - Hwang,G.
Y1 - 2011///
N1 - 71290918
English
Journal: Conference Abstract
KW - EMBASE
KW - Human
KW - exposure
KW - Asthma
KW - Epidemiology
KW - Child
KW - Society
KW - case control study
KW - control group
KW - student
KW - gas chromatography
KW - parent
KW - sampling
KW - spectrophotometer
KW - questionnaire
KW - Behavior
KW - sampler
KW - informed consent
KW - Traffic
KW - Content Analysis
KW - patient
KW - primary school
KW - Prevention
KW - volatile organic compound
KW - formaldehyde
KW - benzene
KW - toluene
KW - methylcyclohexane
KW - ortho xylene
RP - NOT IN FILE
SP - S242
JF - Epidemiology
VL - Conference: 22nd Annual Conference of the International Society for Environmental Epidemiology, ISEE 2010 Seoul South Korea. Conference Start: 20100828 Conference End: 20100901. Conference Publication:
IS - var.pagings
N2 - Background/Aims: On the basis of results of a questionnaire distributed to the parents of students in an elementary school located in Seongbuk-gu, Seoul, an asthma case group and a control group were selected. Methods: Through written messages sent to the parents of students, informed consent was obtained; 33 and 40

students were enrolled in the case and control groups, respectively. To determine the volatile organic compounds (VOCs) and formaldehyde levels of exposure, we performed personal, indoor, and outdoor measurements using a passive sampler for 3 days. In addition, the behavior patterns for the 3 days in the case and control groups were determined through daily time-activity pattern-diaries. The VOCs were analyzed by gas chromatography with flame ionization detection, and formaldehyde was analyzed by a UV spectrophotometer. Results: In comparing the results of the levels of VOCs and formaldehyde exposure between the case and control groups, it was shown that toluene and o-xylene were higher in personal sampling, and the benzene concentration was higher in the case group in indoor measurements. In outdoor measurements, benzene and methylcyclohexane were higher in the patient group ($P < 0.05$), the asthma ORs were 1.1 and 32.9, respectively. Based on the activity content analysis by time, it was shown that the students spent 88.0%, 7.2%, and 4.9% of their time indoors, outdoors, and in traffic, respectively. Conclusion: In conclusion, it was shown that the childhood asthma case group was exposed to higher VOCs than the control group, and it was found by measurement methods that VOCs in personal sampling were higher than the values obtained in indoor and outdoor measurements, and the formaldehyde value was the highest in indoor measurements. Therefore, management of VOCs and formaldehyde is required for the prevention of childhood asthma, and personal measurement should be considered for proper evaluation
SN - 1044-3983
AD - (Choi, Hwang) IOH Korea University, Seoul, South Korea
ER -

TY - JOUR
ID - 3249
T1 - Passive air sampling: Advantages, limitations, and challenges
A1 - Fan,Z.-H.
Y1 - 2011///
N1 - 71290561
English
Journal: Conference Abstract
KW - EMBASE
KW - air sampling
KW - Society
KW - Epidemiology
KW - sampler
KW - exposure
KW - air pollutant
KW - Human
KW - adsorption
KW - Health
KW - Monitoring
KW - sampling
KW - species
KW - Child
KW - Air Pollution
KW - diffusion
KW - velocity
KW - pollutant
KW - Temperature
KW - community
KW - pregnant woman
KW - reproducibility
KW - Female
KW - volatile organic compound
KW - polybrominated diphenyl ether
KW - polycyclic aromatic hydrocarbon
KW - organic compound
RP - NOT IN FILE
SP - S132

JF - Epidemiology
VL - Conference: 22nd Annual Conference of the International Society for Environmental Epidemiology, ISEE 2010 Seoul South Korea. Conference Start: 20100828 Conference End: 20100901. Conference Publication:
IS - var.pagings
N2 - Background/Aims: Accurate measurement of air pollutants in outdoor, indoor, and or personal air is critical for assessing exposure to air pollution and potential health effects. Passive sampler is becoming more and more an effective alternative for conventional active sampler in exposure and health effects studies, given its simplicity and low cost. Also, many passive samplers are capable of providing comparable performance to active samplers in terms of sensitivity and reproducibility. Methods: A variety types of passive samplers with different adsorption media, trapping principles, geometric designs, and the techniques used for the release of the trapped analytes and their final determination techniques have been developed to measure different air pollutants, including single pollutant, such as O3 and SO2, and a group of chemicals, such as Volatile organic compounds, carbonyls, semi-volatile organic compounds (such as polycyclic aromatic hydrocarbons and polybrominated diphenyl ethers). Those samplers have been widely used in exposure and epidemiological studies and found very desirable in many situations, such as monitoring of small children, senior people, and pregnant women, as well as synchronic monitoring air pollutants at multiple locations and microenvironments in community exposure studies. However, limitations still exist for passive samplers. Results: Many samplers are subject to effects of temperature, sampling duration, wind speed, and air concentrations. Also, a long sampling time is often required in order to get enough mass for detection. Further, it is a challenge to measure non-volatile species by passive sampler due to the low diffusion of particle to the adsorption medium. Thus, there is a need to develop a sensitive, reliable, inexpensive, and friendly use passive sampler for the species primarily distributed in particle phase. Conclusion: Last, a suitable and simple validation system needs to be developed for the evaluation of passive samplers performances for semivolatile organic compounds and particles
SN - 1044-3983
AD - (Fan) University of Medicine, Dentistry of New Jersey, Piscataway, NJ, United States
ER -

TY - JOUR
ID - 3250
T1 - Multimedia and multipathway aggregate exposure assessment for polycyclic aromatic hydrocarbons in korean volunteers
A1 - Lee,J.-H.
A1 - Kim,S.-K.
A1 - Kim,C.-K.
A1 - Park,P.-S.
A1 - Park,J.-H.
A1 - Lee,T.-W.
A1 - Seol,H.-S.
Y1 - 2011///
N1 - 71290445
English
Journal: Conference Abstract
KW - EMBASE
KW - Multimedia
KW - exposure
KW - volunteer
KW - Human
KW - Society
KW - Epidemiology
KW - groups by age
KW - Dust
KW - air sampling
KW - toddler
KW - ambient air
KW - Food Intake
KW - Soil
KW - kitchen

KW - school
KW - toxicity
KW - office worker
KW - Adult
KW - Adolescent
KW - lifespan
KW - Inhalation
KW - ingestion
KW - Child
KW - polycyclic aromatic hydrocarbon
RP - NOT IN FILE
SP - S95
EP - S96
JF - Epidemiology
VL - Conference: 22nd Annual Conference of the International Society for Environmental Epidemiology, ISEE 2010 Seoul South Korea. Conference Start: 20100828 Conference End: 20100901. Conference Publication:
IS - var.pagings
N2 - Background/Aims: Multimedia and multipathway aggregate exposure assessment for polycyclic aromatic hydrocarbons (PAHs) was conducted for 180 individuals in different age groups. Methods: PAHs were analyzed in several types of samples for inhalation exposure (personal air, indoor air [at house, school, office, and kitchen], outdoor air) and oral exposure (indoor floor dust, dust adhering to hand, soil, and food/beverage) routes. The 180 persons were divided into 5 groups: toddler (0-6 years), children and teenagers (7-18), and 3 adult groups including housekeeper (19-60), office worker (19-60) and the elder (>60). The concentrations of PAHs were expressed as sum of toxicity equivalent quotients (TEQs) for the 15 individual PAH concentrations in the samples. Results: The median concentrations (~15 PAHs) ranged from 0.073 to 11 ng-TEQ/m3 for personal air samples, 0.073 to 2.2 ng-TEQ/m3 for indoor air samples, 0.073 to 4.1 ng-TEQ/m3 for outdoor air samples, 0.0029 to 41 ng-TEQ/g for dust and soil samples, and 0.32 to 2.6 ng-TEQ/g for food/beverage samples. Average daily dose in toddler group was 27.5 ng-TEQ/kg/d (median), which was 2.8-4.1 times higher than those in the other age groups (6.7-9.7 ng-TEQ/kg/d). Lifetime average daily dose for all the subjects was 16.9 ng-TEQ/kg/d. Conclusion: Food intake for all age groups was found as a major exposure route in all age groups (96.0%-99.5%). Except for food ingestion, dust ingestion by mouthing activities, such as hand-to-mouth activity, was the most important exposure route in toddler group (76.8%). In other age groups, inhalation of gaseous and particulate PAHs is the most important exposure route (92.9%-97.1%)
SN - 1044-3983
AD - (Lee, Kim, Park, Park, Lee, Seol) NeoEnBiz Co., Bucheon, Seoul, South Korea (Kim, Kim) Seoul National University, Seoul, South Korea
ER -

TY - JOUR
ID - 3252
T1 - The effect of between blood manganese level and attention deficit/hyperactivity disorder index in early school age children
A1 - Chung,S.E.
A1 - Cheong,H.-K.
A1 - Kwon,H.-J.
A1 - Ha,M.
A1 - Kang,D.
A1 - Hong,Y.-C.
A1 - Leem,J.-H.
Y1 - 2011///
N1 - 71290362
English
Journal: Conference Abstract
KW - EMBASE
KW - diseases
KW - school
KW - Child

KW - Human
KW - Society
KW - Epidemiology
KW - manganese blood level
KW - Blood
KW - primary school
KW - computer
KW - Food
KW - questionnaire
KW - exposure
KW - Attention Deficit Disorder
KW - calorie
KW - skill
KW - nerve cell differentiation
KW - screening
KW - Population
KW - Korea
KW - Education
KW - Female
KW - Maternal Age
KW - Male
KW - city
KW - Birth Order
KW - gender
KW - parent
KW - Health
KW - Smoking
KW - Environmental Exposure
KW - manganese
KW - tobacco smoke
KW - Water
RP - NOT IN FILE
SP - S69
EP - S70
JF - Epidemiology
VL - Conference: 22nd Annual Conference of the International Society for Environmental Epidemiology, ISEE 2010 Seoul South Korea. Conference Start: 20100828 Conference End: 20100901. Conference Publication:
IS - var.pagings
N2 - Background/Aims: Manganese (Mn) is a naturally occurring element, and presence of Mn in water, food, and air low level are ubiquitous. Excessive exposure to Mn, in spite of its essentiality, in earlier life can affect neurodevelopment and could possibly be associated with development of neurodegenerative disorders in later life. The goal of this study was to examine the association between low levels of Mn in blood and symptoms of attention-deficit hyperactivity disorder (ADHD) among Korean school age children. Methods: Subjects were recruited from a nationwide school age children's cohort, Children's Health and Environmental Research (CHEER) on the first graders of 22 elementary schools from 10 Korean cities in the year from 2005 to 2007. We have selected 1045 children (543 males and 497 females) between 6 and 8 years old (mean 6.9 + 0.6 years) from 6 elementary schools in 3 metropolitan areas of Korea. We measured the blood Mn level and conducted DuPaul scale questionnaire for screening ADHD. Questionnaire on the general characteristics, developmental history, disease history, current behavioral features, computer using skill, environmental exposures including environmental tobacco smoking, and food frequency was done on the parents of the children. Results: The mean blood Mn level was 14.9 mug/L (SD: 4.0 mug/L). The mean scores of sum of DuPaul scales were 8.87 + 7.4. Mean DuPaul score of group categorized by children's blood Mn levels of <12.0, 12.0 -18.0,18.0 -24.0, and 24.0 mug/L was 9.17, 8.84, 7.70, and 10.56, respectively (P < 0.05). After adjusting for potential confounders: age, area of residence, gender, birth order, maternal age at delivery, education level, familiarity with computer, environmental tobacco smoke, calories of total intake, and frequency of outdoor activities; DuPaul scale level was significantly related with the blood Mn level group. Conclusion: This study suggests that blood Mn level was significantly associated with ADHD scale improvement. Relatively narrow range of blood Mn levels in

children without prominent environmental sources of Mn might have been a factor for this less strong relationship. Further study in a population with higher level of exposure may provide a different relationship
SN - 1044-3983
AD - (Chung, Cheong) Department of Social and Preventive Medicine, Sungkyunkwan University, School of Medicine, Suwon, South Korea (Chung, Cheong) Center for Molecular Medicine, Samsung Biomedical Research Institute, Suwon, South Korea (Kwon, Ha) Department of Preventive Medicine, Dankook University, College of Medicine, Cheonan, South Korea (Kang) Department of Preventive Medicine, Pusan National University, School of Medicine, Pusan, South Korea (Hong) Department of Preventive Medicine, Seoul National University, College of Medicine, Seoul, South Korea (Leem) Department of Occupational Medicine, Inha University, College of Medicine, Incheon, South Korea
ER -

TY - JOUR
ID - 3253
T1 - Indoor air pollution in different Mongolian dwellings and children's health in ulaanbaatar (Mongolia)
A1 - Gombojav,E.
A1 - Purev,E.
A1 - Badrakh,B.
A1 - Shinee,E.
Y1 - 2011///
N1 - 71290274
English
Journal: Conference Abstract
KW - EMBASE
KW - indoor air pollution
KW - Human
KW - Health
KW - Society
KW - Epidemiology
KW - Child
KW - Mongolia
KW - Smoking
KW - bronchitis
KW - air pollutant
KW - Prevalence
KW - ambient air
KW - household
KW - Cooking
KW - heating
KW - winter
KW - air conditioning
KW - parent
KW - questionnaire
KW - Air Pollution
KW - city
KW - social medicine
KW - maternal smoking
KW - Asthma
KW - population growth
KW - Health Status
KW - Income
KW - sibling
KW - Female
KW - Urbanization
KW - Education
KW - gender
KW - environmental factor

KW - respiratory tract disease
KW - cross-sectional study
KW - Electricity
KW - air quality
KW - respiratory function
KW - fossil fuel
KW - cigarette smoke
KW - nitrous oxide
KW - fuel
KW - coal
RP - NOT IN FILE
SP - S42
JF - Epidemiology
VL - Conference: 22nd Annual Conference of the International Society for Environmental Epidemiology, ISEE 2010 Seoul South Korea. Conference Start: 20100828 Conference End: 20100901. Conference Publication:
IS - var.pagings
N2 - Background/Aims: The capital city of Mongolia, Ulaanbaatar, has experienced a recent period of urbanization and population growth, which has resulted in increased pressure on the environment and public health, and on the city to provide necessary infrastructure. Survey data indicated that air pollution in Ulaanbaatar is higher in the winter months. High levels of emissions from the increased burning of fossil fuels over the winter, contribute to the outdoor and indoor air pollution. Objective: The aim of the study was to determine the prevalence of asthma, bronchitis, and other respiratory symptoms in children, and to identify factors that may affect the indoor air quality in different types of dwellings. Methods: A cross-sectional study was designed to determine the prevalence of respiratory diseases in children, and describe some of the environmental factors to which those children are exposed (for example, type of residence, type of heating used, levels of indoor smoking). A modified questionnaire of the American Thoracic Society 3 was used with questions including age, gender of the child, education of parents, family income, health status of parents and siblings, parental and maternal smoking, ventilation, and type of heating and cooking. Results: The study recorded high levels of diagnosed bronchitis (19%) and reported respiratory symptoms (77.8%). Results indicated that more than 50% of households' burnt fossil fuels on a daily basis and almost 60% reported regular smoking indoors. There were 59.2% of households with at least one smoker; of these, 86% smoked every day. Almost onethird of households had smoking visitors at home. Despite major differences in the use of fossil fuels, respiratory symptoms were similar across all dwelling types. The high prevalence of respiratory symptoms and bronchitis in this study suggests that indoor air pollutants are probably having a detrimental effect on the respiratory health of children in Ulaanbaatar. Wood and coal were the main source of cooking and heating fuels in Mongolian dwellings, electricity was used for cooking in 38% of ghers and houses. Conclusion: The rates of diagnosed bronchitis and reported respiratory symptoms reported in this study indicate that these are major health problems in Ulaanbaatar. Questionnaire results indicated that more than 50% of households burnt fossil fuels on a daily basis, almost 60% of dwellings had regular cigarette smoking and almost all dwellings had no active ventilation in winter time. All of these factors can contribute to the production of air pollutants such as SO2, CO, PM₁₀, and nitrous oxides. The major health effects of these common indoor air pollutants, expected from the burning of fossil fuels and cigarette smoke, are respiratory effects. The high prevalence of bronchitis and respiratory symptoms in this study suggests that indoor air pollutants may be having a detrimental effect on the respiratory health of children in Ulaanbaatar. The level of bronchitis and respiratory symptoms in apartments indicates that factors other than the burning of fossil fuels within the dwelling, such as smoking and outdoor air pollution, need to be considered
SN - 1044-3983
AD - (Gombojav) School of Public Health, Health Sciences University, Ulaanbaatar, Mongolia (Purev) Public Health Institute, Ulaanbaatar, Mongolia (Badrakh) Ministry of Health, Ulaanbaatar, Mongolia (Shinee) World Health Organization, Representative Office, Ulaanbaatar, Mongolia
ER -

TY - JOUR
ID - 3254
T1 - Correlates of physical activity of children and adolescents: A systematic review of reviews
A1 - Sterdt,E.
A1 - Liersch,S.

A1 - Walter,U.
Y1 - 2014///
N1 - 2014004123
English
Journal: Article
KW - EMBASE
KW - Adolescent
KW - article
KW - Child
KW - Child Behavior
KW - Competence
KW - cultural factor
KW - Demography
KW - Educational Status
KW - emotion
KW - ethnicity
KW - Human
KW - Income
KW - internal validity
KW - Motivation
KW - Physical Activity
KW - priority journal
KW - psychological aspect
KW - social aspect
KW - social status
KW - Social Support
KW - sport
KW - systematic review
RP - NOT IN FILE
SP - 72
EP - 89
JF - Health Education Journal
VL - 73
IS - 1
CY - United Kingdom
N2 - Objective: The aim of this study was to identify promoting and inhibiting correlates associated with the physical activity (PA) of children and adolescents (aged 3-18). The intention was to demonstrate the complexity of correlates of PA and to determine possible influencing factors. Design: A systematic review of reviews. Methods: Systematic database research was carried out in Medline, Cochrane Library, EMBASE, PsycInfo, Springer Link and Thieme Connect. Inclusion criteria were that the study: (a) was classified as a systematic review with or without meta-analysis; (b) was published between 2000 and 2009; (c) dealt with children and adolescents aged 3-18; (d) had as its dependent variable any measure of overall PA; and (e) reviewed associations between quantitatively measured variables and PA. The internal validity of the systematic reviews thus identified was evaluated using a validated quality instrument. Results: Nine systematic reviews without meta-analysis and one systematic review with meta-analysis were selected. Altogether 16 correlates were identified which were consistently associated with PA of children and/or adolescents: sex, age, ethnicity, parental education, family income, socioeconomic status, perceived competence, self-efficacy, goal orientation/motivation, perceived barriers, participation in community sports, parental support, support from significant others, access to sport/recreational facilities und time outdoors. Conclusions: Although the findings of the reviews covered are to some extent heterogeneous, it is possible to identify consistent correlates of PA in children and adolescents. The results show that PA is a complex and multi-dimensional behaviour determined by numerous biological, psychological, sociocultural and environmental factors. The Author(s) 2013
SN - 0017-8969
AD - (Sterdt, Liersch, Walter) Institute for Epidemiology, Social Medicine and Health Systems Research, Hanover Medical School, Carl-Neuberg-Str.1, Hanover, D-30625, Germany
ER -

TY - JOUR
ID - 3255
T1 - PI3K pathway activation provides a novel therapeutic target for pediatric ependymoma and is an independent marker of progression-free survival
A1 - Rogers,H.A.
A1 - Mayne,C.
A1 - Chapman,R.J.
A1 - Kilday,J.-P.
A1 - Coyle,B.
A1 - Grundy,R.G.
Y1 - 2013///
N1 - 2013790516
English
Journal: Article
KW - EMBASE
KW - article
KW - brain tumor/di [Diagnosis]
KW - brain tumor/et [Etiology]
KW - cell proliferation
KW - Child
KW - disease marker
KW - drug targeting
KW - Enzyme Activation
KW - ependymoma/di [Diagnosis]
KW - ependymoma/et [Etiology]
KW - gene
KW - Gene Expression
KW - gene mutation
KW - Human
KW - human cell
KW - human tissue
KW - Immunohistochemistry
KW - in vitro study
KW - major clinical study
KW - pik3ca gene
KW - preschool child
KW - priority journal
KW - progression free survival
KW - protein expression
KW - spinal cord tumor/di [Diagnosis]
KW - spinal cord tumor/et [Etiology]
KW - tumor localization
KW - cyclin D1/ec [Endogenous Compound]
KW - Ki 67 antigen/ec [Endogenous Compound]
KW - phosphatidylinositol 3 kinase/ec [Endogenous Compound]
KW - phosphatidylinositol 3,4,5 trisphosphate 3 phosphatase/ec [Endogenous Compound]
KW - phosphoprotein/ec [Endogenous Compound]
KW - protein kinase B/ec [Endogenous Compound]
KW - tumor marker/ec [Endogenous Compound]
RP - NOT IN FILE
SP - 6450
EP - 6460
JF - Clinical Cancer Research
JA - Clin Cancer Res
VL - 19
IS - 23
CY - United States

N2 - Abstract Purpose: Currently, there are few effective adjuvant therapies for pediatric ependymoma outside confocal radiation, and prognosis remains poor. The phosphoinositide 3-kinase (PI3K) pathway is one of the most commonly activated pathways in cancer. PI3Ks transduce signals from growth factors and cytokines, resulting in the phosphorylation and activation of AKT, which in turn induces changes in cell growth, proliferation, and apoptosis. Experimental Design: PI3K pathway status was analyzed in ependymoma using gene expression data and immunohistochemical analysis of phosphorylated AKT (P-AKT). The effect of the PI3K pathway on cell proliferation was investigated by immunohistochemical analysis of cyclin D1 and Ki67, plus in vitro functional analysis. To identify a potential mechanism of PI3K pathway activation, PTEN protein expression and the mutation status of PI3K catalytic subunit a-isoform gene (PIK3CA) was investigated. Results: Genes in the pathway displayed significantly higher expression in supratentorial than in posterior fossa and spinal ependymomas. P-AKT protein expression, indicating pathway activation, was seen in 72% of tumors (n =169) and P-AKT expression was found to be an independent marker of a poorer progression-free survival. A significant association between PI3K pathway activation and cell proliferation was identified, suggesting that pathway activation was influencing this process. PTEN protein loss was not associated with P-AKT staining and no mutations were identified in PIK3CA. Conclusions: Our results suggest that the PI3K pathway could act as a biomarker, not only identifying patients with a worse prognosis but also those that could be treated with therapies targeted against the pathway, a strategy potentially effective in a high percentage of ependymoma patients. 2013 American Association for Cancer Research
SN - 1078-0432
AD - (Rogers, Mayne, Chapman, Kilday, Coyle, Grundy) Children's Brain Tumour Research Centre, Queen's Medical Centre, University of Nottingham, Nottingham NG7 2UH, United Kingdom
ER -

TY - JOUR
ID - 3257
T1 - Effectiveness of a fall-prevention training program for persons with lower extremity amputations: Initial results
A1 - Wyatt,M.
A1 - Sessoms,P.
A1 - Grabiner,M.
A1 - Kingsbury,T.
A1 - Thesing,N.
A1 - Sturdy,J.
A1 - Unfried,B.
A1 - Kaufman,K.
Y1 - 2013///
N1 - 71274457
English
Journal: Conference Abstract
KW - EMBASE
KW - Human
KW - Society
KW - leg amputation
KW - Child
KW - Adult
KW - Training
KW - Prevention
KW - Amputation
KW - patient
KW - treadmill
KW - Environment
KW - Rehabilitation
KW - Walking
KW - prosthesis
KW - limb prosthesis
KW - Fear
KW - War

KW - microprocessor
KW - Government
KW - navy
KW - leg injury
KW - Lebanon
KW - computer
KW - Reaction Time
KW - questionnaire
KW - velocity
KW - disabled person
KW - community
KW - devices
KW - Funding
KW - Leg
KW - surgery
KW - terrorism
KW - Male
RP - NOT IN FILE
SP - S102
JF - Gait and Posture
VL - Conference: 21st Annual Meeting of the European Society of Movement Analysis for Adults and Children, ESMAC 2012 Stockholm Sweden. Conference Start: 20120913 Conference End: 20120915. Conference Publication:
IS - var.pagings
N2 - Introduction: The global war on terrorism has resulted in many warfighters sustaining lower extremity injuries including amputations. The key factors that limit the ability of patients with amputations to achieve maximum functional capabilities are falls and fear of falling. This research program was designed to rehabilitate lower extremity amputees to increase trust in their prosthesis and reduce falls by using a novel training method. Patients/materials and methods: Four service members with unilateral traumatic transtibial amputations were recruited at the Naval Medical Center San Diego (NMCSD). A perturbation testing protocol was developed utilizing a Computer Assisted Rehabilitation Environment (CAREN). This immersive virtual environment contains a motion platform and instrumented dual belt treadmill. The testing protocol delivered a perturbation simulating a trip in the natural environment and was used as a pre-test and post-test assessment of the rehabilitative program. Select biomechanical data using motion analysis methods were collected and calculated to determine effectiveness of the subjects' ability to recover from these disturbances. The fall prevention training program utilized a microprocessor-controlled Active-Step treadmill (Simbex, Lebanon, NH) designed to deliver task specific training perturbations. Three types of perturbations were used during six, 30 min training sessions: static and static walk, during which the belt moved while the patient was standing still and to which the patient responded with one or multiple forward steps, respectively; and eTRIP perturbations, during which the perturbation was delivered at a random time while the subject was walking and required the subject to recover with multiple forward steps. Results: These preliminary data report on four male subjects, 24.3+2.1 years of age, who had been walking without an assistive device for 7.1+1.7 months. After participating in the fall prevention program, all subjects successfully avoided falling during the post-training perturbation test. The mean maximum trunk flexion angle from pre to post-test improved (38+7degree vs. 25+7degree on prosthetic limb perturbation; 32+13degree vs. 20+3 degree on non-prosthetic limb perturbation) and trunk velocities at time of recovery step also improved. Questionnaire responses indicated improved reaction times, increased confidence and heightened awareness of their ability to recover from a stumble or trip in the community. Discussion and conclusions: This novel rehabilitation method uses an innovative treadmill training method aimed at increasing the ability of the subjects with amputations to rely on their prostheses during challenging perturbations and thus improving their functional capabilities. This type of training has been shown to reduce falls in older adults [1]. The preliminary results indicate that this rehabilitation method will also be effective for those with amputations by increasing their ability to recover from a major perturbation as well as their functional performance. Funding: DoD Grant No. DM090896; The Navy Bureau of Medicine and Surgery, Wounded Ill and Injured. Views in the article are those of the authors and not of the DoN, DoD, or the US Government
SN - 0966-6362

AD - (Wyatt, Kingsbury, Thesing, Unfried) Naval Medical Center San Diego, San Diego, United States (Sessoms, Sturdy) Naval Health Research Center, San Diego, United States (Grabiner) University of Illinois, Chicago, United States (Kaufman) Mayo Clinic, Rochester, United States
ER -

TY - JOUR
ID - 3258
T1 - Moving on and burning out. Reasons youth discontinue sport participation
A1 - Baker,R.
A1 - DiFiori,J.P.
A1 - Gray,A.
Y1 - 2012///
N1 - 71270588
English
Journal: Conference Abstract
KW - EMBASE
KW - sport
KW - medical society
KW - Sports Medicine
KW - juvenile
KW - Human
KW - burnout
KW - athlete
KW - Male
KW - Female
KW - fitness
KW - girl
KW - boy
KW - childhood
KW - Adolescent
KW - Health
KW - student
KW - questionnaire
KW - Leadership
KW - injury
KW - parent
KW - study design
KW - Child
KW - Self Esteem
KW - Training
RP - NOT IN FILE
SP - 189
JF - Clinical Journal of Sport Medicine
JA - Clin J Sport Med
VL - Conference: 21st Annual Meeting of the American Medical Society for Sports Medicine Atlanta, GA United States. Conference Start: 20120421 Conference End: 20120425. Conference Publication:
IS - var.pagings
N2 - Purpose: Participation in youth athletics provides an opportunity for developing self-esteem and leadership qualities and socializing with peers and promotes general health and fitness. Despite these important benefits, there is little data describing why youngsters withdraw from sports. The purpose of this study was to assess the reasons youth discontinue sport participation. Methods and Study Design: A questionnaire was administered to National Collegiate Athletic Association Division 1 student-athletes at a single institution. The survey included information on overall sport participation during childhood and asked subjects to cite specific reasons as to why they ended participation in a given sport. Subjects were also asked to rate their feelings of burnout during different stages of participation. Results: Two hundred ninety-six subjects (162 women, 134 men, average age 19 years) representing 22 sports completed the survey. The 3 most common reasons for discontinuing a sport were: subjects felt they were better at another sport (35%), loss of interest/burnout (26%), and too busy with

other activities (19%). Between 14 to 17 years, discontinuation due to an injury or a poor relationship with a coach markedly increased. Only 1% cited too much pressure from a parent or coach for ending sport participation. Subjects rating their level of burnout from moderate to high more than doubled between 12 to 18 years (26.8%) versus 6 to 11 years (10.4%). Reported levels of burnout were higher among adolescent girls (37%) versus boys (24.5%). Conclusions: This study demonstrates that for successful athletes who participated in multiple sports as children, choosing to focus on another sport or preserving time for interests outside of sports accounted for the vast majority (54%) of reasons for sport discontinuation. Feelings of burnout affected more than one-fourth of athletes beyond 12 years of age
SN - 1050-642X
AD - (Baker, DiFiori, Gray) UCLA, Division of Sports Medicine, Los Angeles, CA, United States
ER -

TY - JOUR
ID - 3259
T1 - Traumatic injuries of primary teeth: Analysis of types and causes
A1 - Gorseta,K.
A1 - Vranic,D.N.
A1 - Skrinjaric,T.
A1 - Glavina,D.
Y1 - 2010///
N1 - 2010648274
Croatian, English
Journal: Article
KW - EMBASE
KW - Age Distribution
KW - article
KW - bicycle
KW - Child
KW - deciduous tooth
KW - dentin
KW - disease classification
KW - disease severity
KW - enamel
KW - Female
KW - fracture
KW - Human
KW - incisor
KW - major clinical study
KW - Male
KW - pathogenesis
KW - preschool child
KW - Risk Assessment
KW - risk factor
KW - sex difference
KW - sport
KW - tooth crown
KW - tooth injury/di [Diagnosis]
KW - tooth injury/et [Etiology]
RP - NOT IN FILE
SP - 47
EP - 52
JF - Acta Stomatologica Croatica
VL - 44
IS - 1
CY - Croatia
N2 - Introduction: The purpose of this study was to identify some risk factors related to the occurrence of dental trauma to the deciduous teeth and to identify particular type of dental injuries in children referred to Department

of Paediatric Dentistry, School of Dental Medicine, University of Zagreb. Methods: The study was carried out in the sample of 97 children, aged 1 to 5 years, with injured 166 primary teeth. The sample of 97 patients included 63 boys and 34 girls. The epidemiological model "agent-host-environment" has been applied in the study. Data have been entered into a data base program (Microsoft Access) designed for this project. Results: The analysis has shown that the highest frequency of injuries occurred in children between 2 and 4 years of age. The frequency ratio between boys and girls was 1.8:1. The most frequently affected teeth were maxillary central incisors (in 79 %). Lateral luxation as the most common type of injury was observed in 34.9 % of cases. Most of the injuries occurred at home (62.8%) or on the playground (15.1%). Crashes against hard objects (49.5%) and falls (36.1%) were the main causes of trauma. Conclusion: Predominant types of traumatic injuries to the primary teeth are luxations and subluxations (66.2%). The most of injuries to the primary teeth occurred during children's play and were consequences of falls. Since the most common place of incident occurrence is the home (62.8%). The parents and caregivers should be informed about possibilities for prevention of traumatic injuries in young children
SN - 0001-7019
AD - (Gorseta, Vranic, Skrinjaric, Glavina) Department of Pediatric and Preventive Dentistry, School of Dental Medicine University of Zagreb, Croatia
ER -

TY - JOUR
ID - 3260
T1 - Population-based analysis of High Hospital Utilization (HHU) among children and adolescents with Sickle Cell Disease (SCD)
A1 - Theodore,R.S.
A1 - Quarmyne,M.-O.
A1 - Bost,J.
A1 - Dampier,C.
A1 - Lane,P.A.
Y1 - 2013///
N1 - 71265341
English
Journal: Conference Abstract
KW - EMBASE
KW - Human
KW - hospital utilization
KW - Child
KW - sickle cell anemia
KW - hematology
KW - Adolescent
KW - Society
KW - Population
KW - Genotype
KW - hospital patient
KW - patient
KW - Insurance
KW - Medicaid
KW - Hospitalization
KW - data base
KW - Diagnosis
KW - clinical study
KW - emergency care
KW - outpatient
KW - Health Care
KW - health care cost
KW - health service
KW - managed care
KW - disease severity
KW - Hospital

KW - school
KW - emergency ward
KW - outpatient department
KW - Electrophoresis
KW - hemoglobin
KW - hydroxyurea
RP - NOT IN FILE
JF - Blood
VL - Conference: 55th Annual Meeting of the American Society of Hematology, ASH 2013 New Orleans, LA United States. Conference Start: 20131207 Conference End: 20131210. Conference Publication:
IS - var.pagings
N2 - Background Sickle cell disease (SCD) is characterized by marked heterogeneity in clinical manifestations, severity and utilization of health care services. This heterogeneity is particularly evident with regards to utilization of inpatient services, which account for a large percentage of total health care costs and significant absence from school and work. Previous studies of utilization, based on analysis of administrative datasets, have been limited by inaccuracies in ICD-coding, inability to differentiate among SCD genotypes, and/or inability to capture individuals never hospitalized. Single center studies have been limited by potential disease severity referral bias, inability to capture service utilization at outside facilities, and relatively small numbers. Objectives We sought to identify children and adolescents with SCD with high hospital utilization (HHU) and to determine the relationship of HHU with age, SCD genotype, and insurance coverage. We also sought to examine trends over a three year period (2010-2012) and explore the relationship of any trends in hospital utilization over time. Methods The clinical database of the SCD Program at Children's Healthcare of Atlanta (CHOA) was used to identify all patients with SCD, ages > 5 years, who received comprehensive outpatient, acute care, and inpatient services at three CHOA facilities in metro Atlanta during 2010-2012. SCD genotype was confirmed for each patient by review of hematologic and clinical data, including results of diagnostic hemoglobin electrophoresis. All outpatient clinic, emergency department, and inpatient utilization was captured as well as insurance (payer) status at each encounter. HHU was defined as >3 admissions during a calendar year. Data were analyzed for significance by Chi-square. The extent to which the data were population-based was determined from the Georgia Hospital Association database with includes DRG-level data for all inpatient hospitalizations in Georgia. Results Hospitalizations at CHOA accounted for 96.1% of all SCD admissions for children within the 28-county greater metro Atlanta area. During 2010-2012, 1348 individual children and adolescents > 5yr of age with SCD received comprehensive care at CHOA (1076 in 2010, 1112 in 2011, 1159 in 2012): 928 (68.8%) had SS/S betadegreethal, 334 (24.8%) SC, 86 (6.4%) S beta+thal. Of 1159 active patients in 2012, 441 (38.1%) were 5-9 yr of age, 419 (36.2%) 10-14 yr, 299 (25.8%) > 15 yr; 474 (40.9%) had traditional medicaid (MC), 205 (17.7%) managed-care medicaid (MMC), 374 (32.3%) private payer (PP), and 105 (9.1%) multiple payers (MP). HHU (> 3 admissions/yr) decreased from 119 of 1076 (11.1%) in 2010 to 96 of 1112 (8.6%) in 2011 and 95 of 1159 (8.2%) in 2012 (p <.01). The Table shows percentage HHU for each year by age, genotype, and payer. Conclusions For children and adolescents with SCD, HHU decreased during the three year period. HHU was significantly associated with older age and with MC and MP insurance coverage during all three years. HHU was significantly associated with the SS/S betadegreethal genotype in 2010, but not in 2011-12. The extent to which the decline of HHU among patients with SS/S betadegreethal genotype was related to increasing use of hydroxyurea is being investigated. (Table Presented)
SN - 0006-4971
AD - (Theodore, Quarmyne, Dampier, Lane) Aflac Cancer and Blood Disorders Center, Children's Healthcare of Atlanta, Emory University, Atlanta, GA, United States (Bost) Center for Outcomes and Quality Measurement, Children's Healthcare of Atlanta, Atlanta, GA, United States
ER -

TY - JOUR
ID - 3261
T1 - Serum high-density lipoprotein cholesterol level and lifestyle habits among Japanese junior high school students
A1 - Hongo,M.
A1 - Hidaka,H.
A1 - Sakaguchi,S.
A1 - Nakanishi,K.
A1 - Terasawa,F.

A1 - Izawa,A.
A1 - Yazaki,Y.
A1 - Ikeda,U.
Y1 - 2013///
N1 - 71258684
English
Journal: Conference Abstract
KW - EMBASE
KW - serum
KW - Lifestyle
KW - habit
KW - Japanese
KW - high school student
KW - Human
KW - Society
KW - cardiology
KW - student
KW - Male
KW - boy
KW - girl
KW - Female
KW - school
KW - Physical Activity
KW - Prevention
KW - diet restriction
KW - gender
KW - diseases
KW - calorie
KW - sport
KW - Exercise
KW - car
KW - abdominal obesity
KW - medical examination
KW - fish
KW - Prevalence
KW - school health service
KW - risk factor
KW - cardiometabolic risk
KW - questionnaire
KW - Diet
KW - Adult
KW - blood sampling
KW - venous blood
KW - weight
KW - cross-sectional study
KW - school child
KW - glucose blood level
KW - Waist Circumference
KW - screening
KW - Blood Pressure
KW - Television
KW - meal
KW - Population
KW - Plant
KW - ischemic heart disease
KW - Risk
KW - high density lipoprotein cholesterol

KW - high density lipoprotein
RP - NOT IN FILE
SP - 314
JF - European Heart Journal
VL - Conference: European Society of Cardiology, ESC Congress 2013 Amsterdam Netherlands. Conference Start: 20130831 Conference End: 20130904. Conference Publication:
IS - var.pagings
N2 - Purpose: Many epidemiological studies have revealed a consistent inverse association between High-Density Lipoprotein Cholesterol (HDL-C) level and the risk of coronary heart disease in adults. Frequent Japanese style diet, which is characterized by adequate total calories, increased intake of fish and plant foods, and decreased intake of animal fat, has been shown to cause an elevation of HDL-C level. The present study was designed to examine the relationship between serum HDL-C level and dietary patterns and habits and physical activity in a sample of healthy Japanese junior high school students. Methods: Between April 2006 and March 2009, we conducted a cross-sectional study of 1064 Japanese junior high school students (570 boys and 494 girls, aged 12.1 to 15.0 years) who had annual school health examinations and received care programs for prevention of lifestyle-related diseases. The students underwent measurements of resting blood pressure, waist circumference, and weight and provided overnight fasting venous blood samples. Each participant was then asked to complete a simple self-administered questionnaire addressing lifestyle factors, such as dietary patterns and habits and physical activity, as well as the co-existence of cardiometabolic risk factors. Results: Students in the lowest quartile of HDL-C (< 55 mg/dL for boys and < 56 mg/dL for girls) showed increased prevalence of abdominal obesity, hypertriglyc-eridemia, and increased fasting plasma glucose compared with the highest quar-tile (> 74 mg/dL for boys and > 75 mg/dL for girls). In both genders, the ratios of students who were often commuting to and from school by car were significantly higher and who were liking exercise and doing sports outside of school were lower in the lowest quartile of the students compared with those who showed HDL-C level more than these values. The ratio of students who were watching television during meals was significantly higher in boys with lower HDL-C level than those without. There was no significant difference in other dietary patterns or habits between students with and without low HDL-C level in both genders. Conclusions: Serum lower HDL-C level was strongly associated with reduced daily physical activity among junior high school students. This study may provide insights into the role of HDL-C in the school screening system for the development of more effective educational programs on prevention of lifestyle-related diseases in the Japanese population of school children
SN - 0195-668X
AD - (Hongo, Hidaka, Sakaguchi, Nakanishi, Terasawa, Izawa, Yazaki, Ikeda) Shinshu University, Matsumoto, Japan
ER -

TY - JOUR
ID - 3262
T1 - Myopia: From bushwalks to cram schools-the environment has it
A1 - Rose,K.A.
Y1 - 2013///
N1 - 71257481
English
Journal: Conference Abstract
KW - EMBASE
KW - myopia
KW - school
KW - Environment
KW - New Zealand
KW - college
KW - ophthalmologist
KW - Human
KW - Education
KW - Prevalence
KW - Population
KW - childhood
KW - high myopia

KW - refraction error
KW - Health
KW - Policy
KW - Sunlight
KW - examination
KW - Mathematics
KW - Asian
KW - computer
KW - Child
KW - visual impairment
KW - pathology
KW - Asia
RP - NOT IN FILE
SP - 108
EP - 109
JF - Clinical and Experimental Ophthalmology
VL - Conference: 45th Annual Scientific Congress of the Royal Australian and New Zealand College of Ophthalmologists Hobart, TAS Australia. Conference Start: 20131102 Conference End: 20131106. Conference Publication:
IS - var.pagings
N2 - In a matter of decades, the prevalence of myopia has reached exceptionally high levels in urban East Asia, with some locations reporting levels as high as 70% or more in school leavers, with the prevalence of high myopia escalating to around 20%. While there is an assumption that myopic refraction is associated with a genetic propensity among East Asian populations, rises in myopia prevalence have also been reported in Western countries including the USA. This widespread rapid increase in myopia between generations indicates that there is a strong environmental component in the development of refractive error. While it is intuitive to link this rise in prevalence with excessive levels of near work inducing high and sustained accommodative effort and/or the rise in the use of electronic media including computers, after years of scientific investigation there is little evidence to support these contentions. More recently studies, including longitudinal population-based studies, have shown that time spent outdoors in childhood is a factor that may prevent the development of myopia and a plausible biological mechanism for this effect has been suggested. However, this factor alone does not explain all the variance in refractive error in a population. Despite the lack of strong association with near work, there has been a long established association between number of years of education and/or high levels of educational attainment and the development of myopia. Systematic investigation of global education measures provided by the Organisation for Economic Cooperation and Development (OECD) Program in Secondary Assessment (PISA) and the Trends in International Mathematics and Science Study (TIMSS) predominantly demonstrate on a country-to-country basis, the known link between education performance and myopia, yet there are notable exceptions and variations. Closer examination of the available data, however, implicates the combination of high educational outcomes and high education load, the latter largely associated with attendance at out-of-school educational tutorials or cram schools. The mechanism of this effect is only speculative but may involve limitations on the time available for school aged children to spend outdoors during sunlight hours. These investigations suggest that policy initiatives in relation to education and childhood activities may be needed to reverse the trend to towards high rates of myopia and associated health costs, as well as limiting the potential for greater levels of visual impairment through ocular pathology associated with high myopia
SN - 1442-6404
AD - (Rose) University of Sydney, Sydney, NSW, Australia
ER -

TY - JOUR
ID - 3263
T1 - Eye injuries in children-a worldwide perspective
A1 - De Souza,C.F.
A1 - Philip,S.
A1 - Clayfield-Hoskin,A.
A1 - Mackey,D.A.
Y1 - 2013///

N1 - 71257301
English
Journal: Conference Abstract
KW - EMBASE
KW - Human
KW - eye injury
KW - Child
KW - New Zealand
KW - college
KW - ophthalmologist
KW - MEDLINE
KW - Education
KW - hazard
KW - exposure
KW - traffic accident
KW - Environment
KW - boy
KW - Male
KW - data base
KW - Incidence
KW - Accident Prevention
KW - Prevention
KW - Adult
KW - eye protection
KW - developing country
KW - accident
KW - War
KW - Language
KW - sport
KW - eye
KW - contact sport
KW - weapon
KW - terrorism
KW - Cochrane Library
KW - law
KW - Safety
KW - medical care
KW - investment
KW - systematic review
RP - NOT IN FILE
SP - 30
JF - Clinical and Experimental Ophthalmology
VL - Conference: 45th Annual Scientific Congress of the Royal Australian and New Zealand College of Ophthalmologists Hobart, TAS Australia. Conference Start: 20131102 Conference End: 20131106. Conference Publication:
IS - var.pagings
N2 - Purpose: To conduct a systematic review of the clinico-epidemiological patterns of paediatric ocular trauma reported worldwide. Method: The literature search included nine databases (PubMed, Medline, Embase, Google Scholar, Cochrane Library, Scopus, Science Direct, Web of Science, Web of Knowledge) including articles with key words related with eye injuries in children and synonyms. No limitations of language were made. Results: Paediatric ocular trauma has incidence rates of 0.746 to 9.9 per 10,000 worldwide. Global similarities are found in the higher involvement of boys and poor or absent adult supervision in the domestic environment. Eye injuries occur in developing countries in road accidents and working places due to inexistence or poor compliance to safety legislation and exposure to activities incommensurate with their abilities. Seasonal trends for eye injuries exist, as they are associated with outdoors recreational activities and exposure to hazards such as game weapons or unprotected contact sports. Likewise, festive events include serious eye hazards such as fireworks. Sports account for 10% of severe eye injuries in children. Children are particularly vulnerable to

severe ocular trauma in warfare scenarios and terrorist attacks, representing up to near 40% of all casualties. Conclusion: Adoption of specific measures for eye protection has dramatically reduced the incidence of ocular trauma. Globally, a great need for education concerning eye injury prevention continues to exist. Parental level of education and access to specialized medical assistance are associated with better outcomes. Ocular trauma prevention and optimum management also relies on investment in research/data collection and surveillance resources
SN - 1442-6404
AD - (De Souza, Philip, Clayfield-Hoskin, Mackey) Centre for Ophthalmology and Visual Science, University of Western Australia, Lions Eye Institute, Perth, Australia
ER -

TY - JOUR
ID - 3264
T1 - Active surveillance of sudden cardiac death in young athletes by periodic internet searches
A1 - Choi,K.
A1 - Pan,Y.P.
A1 - Pock,M.
A1 - Chang,R.-K.
Y1 - 2013///
N1 - 2013771659
English
Journal: Article
KW - EMBASE
KW - Adolescent
KW - Adult
KW - aorta rupture
KW - aorta stenosis
KW - article
KW - automated external defibrillator
KW - Autopsy
KW - Child
KW - congestive cardiomyopathy
KW - coronary artery anomaly
KW - coronary artery disease
KW - death certificate
KW - disease surveillance
KW - Female
KW - heart arrest
KW - heart arrhythmia
KW - heart disease
KW - heart infarction
KW - Human
KW - hypertrophic cardiomyopathy
KW - Internet
KW - major clinical study
KW - Male
KW - mitral valve prolapse
KW - myocarditis
KW - Physical Activity
KW - prospective study
KW - Resuscitation
KW - school child
KW - sport injury
KW - sudden cardiac death
RP - NOT IN FILE
SP - 1816
EP - 1822

JF - Pediatric Cardiology
JA - Pediatr Cardiol
VL - 34
IS - 8
CY - United States
N2 - The authors hypothesized that prospective, systematic Internet searches could identify occurrences of sudden cardiac death (SCD) in athletes and would be useful for establishing a system of active surveillance. Weekly advanced Google searches of the Internet were conducted for cases of SCD in young athletes during a 12-month period (2007-2008). Athletes ages 11-30 years who collapsed during a game, practice, or within an hour of exercise were included in the study. Individuals with known histories of cardiac issues and events occurring outside the United States were excluded. Verification of SCD was by autopsy reports and death certificates from county coroner offices and vital record agencies. Initially, 71 events were identified. Verification for the cause of death by coroner reports was possible in 45 cases, 43 (96 %) of which were confirmed to be SCDs. A total of 69 individuals 11-30 years of age (mean 17 + 5 years) died suddenly of cardiovascular causes while participating in 15 different organized sports and a variety of nonorganized physical activities. The most common cause of death was hypertrophic cardiomyopathy (30 %), followed by coronary artery anomalies (9 %), and myocarditis (9 %). The incidence of athlete SCD, the types of sports involved, and the cardiac causes of death in our study were comparable with those of previous reports. Readily available Internet searches have the potential to be a powerful tool for identifying occurrences of athlete SCD. An active surveillance system using Google searches followed by coroner report verification can provide important epidemiologic and clinical information. 2013 Springer Science+Business Media New York
SN - 0172-0643
AD - (Choi, Pock, Chang) Division of Cardiology, Department of Pediatrics, Harbor-UCLA Medical Center, 1000 West Carson Street, Torrance CA 90509, United States (Pan) Department of Family Medicine, Southern California Kaiser Permanente Medical Group, Diamond Bar CA, United States
ER -

TY - JOUR
ID - 3265
T1 - Proactive infection control measures to prevent nosocomial transmission of carbapenem-resistant Enterobacteriaceae in a non-endemic area
A1 - Cheng,V.C.-C.
A1 - Chan,J.F.-W.
A1 - Wong,S.C.-Y.
A1 - Chen,J.H.-K.
A1 - Tai,J.W.-M.
A1 - Yan,M.-K.
A1 - Kwan,G.S.-W.
A1 - Tse,H.
A1 - To,K.K.-W.
A1 - Ho,P.-L.
A1 - Yuen,K.-Y.
Y1 - 2013///
N1 - 2013746532
English
Journal: Article
KW - EMBASE
KW - Adolescent
KW - Adult
KW - Aged
KW - article
KW - bacterial colonization
KW - bacterial load
KW - carbapenem resistant enterobacteriaceae infection/di [Diagnosis]
KW - Child
KW - disease surveillance
KW - Disease Transmission

KW - disk diffusion
KW - Enterobacteriaceae
KW - Enterobacteriaceae infection/di [Diagnosis]
KW - feces analysis
KW - Female
KW - hospital infection
KW - Hospitalization
KW - Human
KW - Infant
KW - Infection Control
KW - major clinical study
KW - Male
KW - preschool child
KW - risk factor
KW - beta lactamase inhibitor
KW - carbapenem
KW - cephalosporin
KW - quinolone derivative
RP - NOT IN FILE
SP - 4504
EP - 4509
JF - Chinese Medical Journal
VL - 126
IS - 23
CY - China
N2 - Background Identification of hospitalized carbapenem-resistant Enterobacteriaceae (CRE)-positive patient is important in preventing nosocomial transmission. The objective of this study was to illustrate the implementation of proactive infection control measures in preventing nosocomial transmission of CRE in a healthcare region of over 3200 beds in Hong Kong between October 1, 2010 and December 31, 2011. Methods The program included active surveillance culture in patients with history of medical tourism with hospitalization and surgical operation outside Hong Kong within 12 months before admission, and "added test" as an opportunistic CRE screening in all fecal specimens submitted to the laboratory. Outbreak investigation and contact tracing were conducted for CRE-positive patients. Serial quantitative culture was performed on CRE-positive patients and the duration of fecal carriage of CRE was analyzed. Results During the study period, a total of 6533 patients were screened for CRE, of which 76 patients were positive (10 from active surveillance culture, 65 from "added test", and 1 secondary case from contact tracing of 223 patients with no nosocomial outbreak), resulting in an overall rate of CRE fecal carriage of 1.2%. The median time of fecal carriage of CRE was 43 days (range, 13-119 days). Beta-lactam-beta-lactamase-inhibitors, cephalosporins, and fluoroquinolones were associated significantly with high fecal bacterial load when used 90 days before CRE detection, while use of cephalosporins, carbapenems, and fluoroquinolones after CRE detection are significantly associated with longer duration of carriage. The duration of fecal carriage of CRE also correlates significantly with the initial fecal bacterial load (Pearson correlation: 0.53; P=0.02). Conclusion Proactive infection control measures by enhanced surveillance program identify CRE-positive patients and data obtained are useful for the planning of and resource allocation for CRE control
SN - 0366-6999
AD - (Cheng, Chan, Wong, Chen, Yan, Kwan, Tse, To, Ho, Yuen) Department of Microbiology, Queen Mary Hospital, Hong Kong (Cheng, Chan, Wong, Tai) Infection Control Team, Queen Mary Hospital, Hong Kong
ER -

TY - JOUR
ID - 3266
T1 - Vitamin D supply in infancy, childhood, and adolescence
A1 - Mos,A.
Y1 - 2012///
N1 - 71255018
English
Journal: Conference Abstract

KW - EMBASE
KW - laboratory
KW - clinical chemistry
KW - infancy
KW - Human
KW - childhood
KW - adolescence
KW - Society
KW - exposure
KW - Child
KW - Sunlight
KW - endocrinology
KW - serum
KW - Medicine
KW - vegetarian diet
KW - high risk population
KW - Pediatrics
KW - Physical Activity
KW - Germany
KW - Nutrition
KW - chronic patient
KW - Adolescent
KW - cost benefit analysis
KW - reference value
KW - Infant
KW - groups by age
KW - vitamin D
KW - 25 hydroxyvitamin D
RP - NOT IN FILE
SP - A214
JF - Clinical Chemistry and Laboratory Medicine
VL - Conference: Congress of Clinical Chemistry and Laboratory Medicine 9th Annual Meeting of the German Society for Clinical Chemistry and Laboratory Medicine, DGKL 2012 Mannheim Germany. Conference Start: 20120926 Conference End: 20120929. Conference Publication:
IS - var.pagings
N2 - Current national and international publications describe a suboptimal vitamin D intake or an inadequate vitamin D status in all age groups. The aim of this statement issued by the Nutrition Committee of the German Society of Paediatrics and Adolescent Medicine (DGKJ) in cooperation with the working group of paediatric endocrinology (APE) is to summarize the available literature and evidence for reference values of vitamin D intake and vitamin D supply as well as for the current consumption. On this basis, recommendations for infants, children, and adolescents living in Germany were developed. Reasonable measures for achieving an improved vitamin D status are: protected sunlight exposure, intensive physical activity (at least 1 h daily) outside, and increase of vitamin D intake via supplements. Special attention is given to risk groups (children nourished on a vegetarian diet, migrants, persons with limited sunlight exposure, and chronically ill persons) for which regular control of serum 25-hydroxyvitamin D levels has to be considered. A systematic supervision of vitamin D supply in childhood and adolescence and a cost-benefit analysis of recommended or conducted measures should be performed
SN - 1434-6621
AD - (Mos) Sektion Padiatrische Endokrinologie und Diabetologie, Universitat Ulm, Abt. Kinder- und Jugendmedizin, Ulm, Germany
ER -

TY - JOUR
ID - 3267
T1 - Risk factors and preventative strategies for adolescent sexual violence-a cross-national urban perspective
A1 - Goddard,A.
A1 - Khadr,S.

A1 - Webb,K.O.
Y1 - 2013///
N1 - 71254075
English
Journal: Conference Abstract
KW - EMBASE
KW - Adolescent
KW - Human
KW - sexual violence
KW - adolescent health
KW - risk factor
KW - Child
KW - gang
KW - Male
KW - United Kingdom
KW - boy
KW - sexual exploitation
KW - partner violence
KW - girl
KW - Female
KW - victim
KW - Violence
KW - sexual assault
KW - Health
KW - Risk
KW - offender
KW - population research
KW - social care
KW - school
KW - Policy
KW - workshop
KW - city
KW - juvenile
KW - gender
KW - friend
KW - sexuality
KW - Prevention
KW - Society
KW - Clothing
KW - Population
KW - Crime
KW - Education
KW - learning disorder
KW - marker
RP - NOT IN FILE
SP - 6
JF - Turk Pediatri Arsivi
VL - Conference: 10th International Congress on Adolescent Health Istanbul Turkey. Conference Start: 20130611 Conference End: 20130613. Conference Publication:
IS - var.pagings
N2 - OBJECTIVES: 1.Compare and discuss the nature of and risk factors for the various forms of adolescent sexual violence in culturally diverse/multinational urban settings 2.Explore known preventative strategies and discuss novel approaches to identification and intervention. METHODS: Sexual violence affects individuals of all ages, cultures, gender, orientation and abilities. Some groups may be disproportionately affected. 30% of under 18s in London, England, are non-white but in the years 2008-2011, 50% of 13- 17 year olds attending the London Havens Sexual Assault Referral Centres came from census groups other than White British, White Irish or White Other. Ten percent had learning difficulties compared with a population average of 2.5%. Based on

evidence submitted to the current CSEGG (Child Sexual Exploitation In Gangs* and Groups*) Inquiry in England, at least 16,500 children under 18 years were identified as being at risk of child sexual exploitation during one year and 2,409 children were confirmed as victims of sexual exploitation in gangs and groups during the 14-month period from August 2010 to October 2011. There was a higher rate of victimisation amongst black and minority ethnic (BME) children and young people than has been previously identified. (* where gangs mainly comprise men and boys aged 13 - 25 years old, who take part in many forms of criminal activity, such as knife crime or robbery, who can engage in violence against other gangs, and who have identifiable markers such as territory, a name, sometimes clothing etc. While children can be sexually exploited by a gang, this is not the reason why a gang is formed. Groups involves people who come together in person or online for the purpose of setting up, co-ordinating and/or taking part in the sexual exploitation of children in either an organised or opportunistic way.) In terms of intimate partner violence in adolescent relationships, a recent National Society for the Prevention of Cruelty to Children (NSPCC) study in the UK echoed findings in the USA in that high numbers of adolescents experience physical, emotional and sexual violence in their intimate partner relationships (a quarter of girls and 18 per cent of boys reported some form of physical partner violence, three-quarters of girls and half of boys reported some form of emotional partner violence. One in three girls and 16 per cent of boys reported some form of sexual partner violence). The incidence, frequency and level of negative impacts rates for all forms of violence were greater for girls. The majority of young people either told a friend about the violence or told no one. Most respondents in this study were white. We propose an interactive session to: *compare and contrast risk factors for adolescent sexual assault among urban youth in major cities of different countries with attention given to particular characteristics and vulnerabilities associated with adolescents who are trafficked, homeless, unaccompanied minors run-aways and/or in the care of the state *share experiences of preventative strategies among workshop attenders- including government/public health preventative policies and measures, school-based educational measures or those targeted at preventing revictimisation *identify and discuss obstacles to interventions: particularly those related to culture, social marginalisation and legal frameworks *examine and discuss efficacy of interventions CONCLUSIONS:Large numbers of adolescents are victims of sexual violence and exploitation in settings outside the family: in intimate partnerships and by gangs and groups. There are high rates of under-identification. Better understanding of the settings, susceptible groups, risk factors and behavioural identifiers that might increase the likelihood that an adolescent is a victim of sexual violence is needed. In many countries existing health, judicial and social care structures have started but struggle to address these emerging problems. Effective strategies are needed for education and early identification of at-risk adolescents and treatment of those already affected by sexual violence
SN - 1306-0015
AD - (Goddard) Imperial College Healthcare NHS Trust, London Haven Sexual Assault Referral Centres, United Kingdom (Khadr) University College London, London Haven Sexual Assault Referral Centres, United Kingdom (Webb) Sexual Exploitation Service, United Kingdom
ER -

TY - JOUR
ID - 3268
T1 - Environmental and occupational respiratory diseases-1057. Correlation between air pollution and respiratory health of school children in Delhi
A1 - Mathew,J.
A1 - Goyal,R.
A1 - Taneja,K.K.
A1 - Arora,N.
Y1 - 2013///
N1 - 71252271
English
Journal: Conference Abstract
KW - EMBASE
KW - Health
KW - Air Pollution
KW - respiratory tract disease
KW - school child
KW - Human
KW - Wechsler Intelligence Scale

KW - school
KW - questionnaire
KW - Spirometry
KW - student
KW - maximum permissible dose
KW - Child
KW - ambient air
KW - Population
KW - exposure
KW - Urban Population
KW - Education
KW - health survey
KW - health hazard
KW - air pollutant
KW - obstruction
KW - Traffic
KW - diseases
KW - pollutant
KW - Human Activities
RP - NOT IN FILE
JF - World Allergy Organization Journal
VL - Conference: 2nd WAO International Scientific Conference, WISC 2012 Hyderabad India. Conference Start: 20121206 Conference End: 20121209. Conference Publication:
IS - var.pagings
N2 - Background: Urban air pollution (indoor and outdoor) poses a major health hazard for city dwellers. Exposure to ambient air pollution may result in respiratory health disorders. The aim of this study was to correlate air pollutant levels to the respiratory health of school children in Delhi. Methods: The questionnaire was designed based on the internationally valid questionnaires for respiratory illness. The respiratory health survey was approved by the Directorate of Education-Delhi and schools were selected based on land usage pattern i.e. commercial (Chandni Chowk), industrial (Mayapuri) and residential (Sarojini Nagar) areas. Approximately 1800 students (600 / zone) of age 10-14 years participated in the survey which included spirometry tests also. Indoor and outdoor levels of SO₂, NO₂ and PM were also measured in the school premises. Results: The questionnaire data showed that the students having respiratory disorder symptoms was maximum in Chandni Chowk (66%) followed by Mayapuri (59%) and Sarojini Nagar (46%). Spirometry test results demonstrated that a significant population of subjects in Chandni Chowk (19%) had mild to severe pulmonary obstruction. However the percentage of subjects with such conditions was comparatively less in Maypuri (17%) and Sarojini Nagar (14%) area. Indoor and outdoor PM₁₀ concentration at schools located in Chandni Chowk, a commercial zone, was observed to be 815+354.45 mug/m³ & 337+85 mug/m³ respectively, which is ten times above the permissible limits. The PM10 concentration was lower in Mayapuri (694.6+322.9 mug/m³ & 274+78 mug/m³), an industrial zone, and was least in Sarojini Nagar (534.3+94.22 mug/m³ & 197+48 mug/m³) which is a residential zone. However, levels of SO2 and NO2 were under the permissible limits in all three areas. Conclusions: There is a correlation between pollutant levels and symptoms of respiratory illness in children. Commercial zones like Chandni Chowk with high traffic movement and human activities contribute more PM which affects the pulmonary health of children
SN - 1939-4551
AD - (Mathew, Arora) Allergy and Immunology Section, Institute of Genomics and Integrative Biology, Delhi, India (Goyal) Air Pollution National Environmental Engineering Research Institute, Delhi Zonal Lab, India (Taneja) Lipid Biochemistry, Institute of Genomics and Integrative Biology, Delhi, India
ER -

TY - JOUR
ID - 3269
T1 - A living donor liver transplantation for uncorrectable intra-and extrahepatic biloma after transcatheter arterial chemoembolization
A1 - Chang,S.-H.
A1 - Hong,Y.-S.

A1 - Kang,B.J.
A1 - Nam,S.E.
A1 - Lee,E.K.
A1 - Sim,J.H.
Y1 - 2012///
N1 - 71251098
English
Journal: Conference Abstract
KW - EMBASE
KW - living donor
KW - Liver Transplantation
KW - biloma
KW - chemoembolization
KW - Transplantation
KW - Society
KW - liver
KW - Infection
KW - Human
KW - Cholangitis
KW - abscess
KW - cholestasis
KW - bile duct stone
KW - Biopsy
KW - outpatient department
KW - operation duration
KW - procedures
KW - liver cell carcinoma
KW - abdominal wall
KW - peritoneal cavity
KW - Inflammation
KW - patient
KW - adhesion
KW - recurrent infection
KW - ascites
KW - Klebsiella pneumoniae
KW - pus
KW - Child
KW - peritoneum
KW - recipient
KW - transfusion
KW - percutaneous drainage
KW - biliary tract drainage
KW - Case Report
KW - Risk
KW - erythrocyte
KW - antibiotic agent
KW - hepatitis B surface antigen
KW - meropenem
KW - imipenem
RP - NOT IN FILE
SP - 663
JF - Transplantation
VL - Conference: 24th International Congress of the Transplantation Society Berlin Germany. Conference Start: 20120715 Conference End: 20120719. Conference Publication:
IS - var.pagings
N2 - Introduction: We experienced a living liver transplantation of an unusual indication of uncorrectable intra- and extrahepatic biloma after transcatheter arterial chemoembolization (TACE) which could be a relative

contraindication so that we are reporting it. Case report: A 45 year old patient was a carrier of HBsAg for more than 25 years. He was diagnosed to be hepatocellular carcinoma (HCC) in 2004. He then underwent TACE 6 times and had a complication of biliary stricture in Nov. 2007. At that time the problem was solved with an interventional procedure. In Apr. 2008, he underwent another TACE for HCC, and after that a long segment of biliary stricture was developed. With recurrent cholangitis, there developed two huge biloma, one in the liver and one outside the liver. Percuatenous transhepatic biliary drainage (PTBD) for the intrahepatic biloma and percutaneous drainage (PCD) for the extrahepatic biloma with long standing antibiotics administration were done, but with all effort there was no improvement. The Child- Turcote-Pugh score was 6 and Klebsiella pneumoniae (sensitive with only imipenem and meropenem) was detected in culture for the drain discharge. The CT shows two large biloma, one in the liver and one outside the liver. The only well looking portion was the posterior section of the liver. There was massive adhesion due to recurrent infection and inflammation and about 300cc of ascites was in the abdominal cavity. Despite the effort not to spill the pus from cavity, it was inevitable. The extrahepatic biloma was found to be abdominal wall abscess which was covered with thick peritoneum. The total recipient operative time was 12 hours and 25 minutes and required transfusion was 8 units of packed RBC and 4 units of FFP. The biopsy showed HCC of 3.0 x 1.5 x 1.2 cm in the caudate lobe. Hepatolithiasis with chronic and active cholangitis and multiple abscess formation was also seen. He is now visiting outpatient clinic without a problem. Usually active and uncontrolled infection outside of the hepatobliliary system is thought to be an absolute contraindication to liver transplantation and chronic and refractory infection is thought to be a relative contraindication. But in this case, we concluded the infection is localized and controlled under drain and antibiotic administration. So with this conclusion and the situation with no other options we can think of, we decided to do a living donor liver transplantation with the risk of infection and turned out with a good result
SN - 0041-1337
AD - (Chang, Hong, Kang, Nam, Lee, Sim) Konkuk University, School of Medicine, Surgery, Seoul, South Korea
ER -

TY - JOUR
ID - 3270
T1 - The effectiveness of a fundamental motor skill intervention in pre-schoolers with motor problems depends on gender but not environmental context
A1 - Bardid,F.
A1 - Deconinck,F.J.A.
A1 - Descamps,S.
A1 - Verhoeven,L.
A1 - De,Pooter G.
A1 - Lenoir,M.
A1 - D'hondt,E.
Y1 - 2013///
N1 - 2013718311
English
Journal: Article
KW - EMBASE
KW - article
KW - Child
KW - controlled study
KW - Curriculum
KW - environmental factor
KW - environmental impact
KW - Female
KW - Human
KW - Locomotion
KW - Male
KW - Motor Development
KW - motor dysfunction
KW - Motor Performance
KW - Physical Activity

KW - Physical Education
KW - preschool child
KW - questionnaire
KW - scoring system
KW - sex difference
KW - social status
RP - NOT IN FILE
SP - 4571
EP - 4581
JF - Research in Developmental Disabilities
JA - Res Dev Disabil
VL - 34
IS - 12
CY - United States
N2 - This study evaluated the effect of a 10-week fundamental motor skill programme in pre-schoolers with motor problems. Alongside the general effect of the intervention, we also explored possible gender differences and the role of the environmental context (living community, socio-economic status, and recreational space inside/outside the house). The intervention group (n=47; 20 and 27) received twenty 60-min motor skill sessions (2 per week) in addition to the regular physical education curriculum for pre-schoolers; the control group (n=46; 21 and 25) did not receive additional practice. General motor competence, and locomotor and object control subscales, were assessed before and after the intervention using the Test of Gross Motor Development 2nd edition (TGMD-2). Data regarding environmental factors were gathered through a questionnaire. A Group. x. Gender. x. Time ANOVA revealed that the intervention group benefited significantly from the intervention and scored better than the control group at the post-test for general motor competence and both sub-categories (locomotor and object control skill). Moreover, the intervention programme was found to be effective in helping 49% of the intervention group to achieve an average motor skill level, according to the TGMD-2 norms, while a further decline in motor competence was observed in the control group. Interestingly, the effect appeared to be gender-specific, since object control skill improved only in girls of the intervention group. Considering the environmental context, none of the above-mentioned factors was found to have an influence on the effectiveness of the intervention. The present study highlights the need for an early motor skill programme with a gender-specific approach in order to help low skilled boys and girls master a diverse set of motor skills. 2013 Elsevier Ltd
SN - 0891-4222
AD - (Bardid, Deconinck, Descamps, Lenoir, D'Hondt) Department of Movement and Sports Sciences, Ghent University, Belgium (Deconinck) School of Healthcare Science, Manchester Metropolitan University, United Kingdom (Verhoeven, De Pooter) Department of Teacher Education, Karel de Grote Hogeschool, Belgium (D'Hondt) Faculty of Physical Education, Vrije Universiteit Brussel, Belgium
ER -

TY - JOUR
ID - 3271
T1 - A newly emerged cutaneous leishmaniasis focus in central Iran
A1 - Nateghi,Rostami M.
A1 - Saghafipour,A.
A1 - Vesali,E.
Y1 - 2013///
N1 - 2013754146
English
Journal: Article
KW - EMBASE
KW - Adolescent
KW - Adult
KW - Age Distribution
KW - article
KW - autumn
KW - Child
KW - controlled study

KW - cryotherapy
KW - Diabetes Mellitus
KW - disease surveillance
KW - drug effect
KW - drug safety
KW - endemic disease
KW - Female
KW - health center
KW - Human
KW - Infant
KW - inflammation/si [Side Effect]
KW - Iran
KW - Leishmania major
KW - lupus erythematosus/di [Diagnosis]
KW - major clinical study
KW - malaise/si [Side Effect]
KW - Male
KW - medical history
KW - nausea/si [Side Effect]
KW - parasite incidence
KW - patient information
KW - preschool child
KW - risk factor
KW - school child
KW - skin defect
KW - skin leishmaniasis/dt [Drug Therapy]
KW - skin leishmaniasis/ep [Epidemiology]
KW - skin leishmaniasis/et [Etiology]
KW - spring
KW - summer
KW - Treatment Outcome
KW - unspecified side effect/si [Side Effect]
KW - vomiting/si [Side Effect]
KW - winter
KW - Work
KW - internal transcribed spacer 1/ec [Endogenous Compound]
KW - meglumine antimonate/ae [Adverse Drug Reaction]
KW - meglumine antimonate/dt [Drug Therapy]
KW - meglumine antimonate/il [Intralesional Drug Administration]
KW - ribosome DNA/ec [Endogenous Compound]
RP - NOT IN FILE
SP - e1198
EP - e1206
JF - International Journal of Infectious Diseases
VL - 17
IS - 12
CY - Netherlands
N2 - Objectives: This study was performed to evaluate the epidemiological status of cutaneous leishmaniasis (CL) in the most important endemic foci of Qom Province, central Iran. The city of Qom is the largest center for Shi'a scholarship in the world and is a significant pilgrimage destination. Methods: During 2006-2011, all suspected CL patients with skin lesion(s) referred to regional health centers of Ghomrood and Ghanavat regions, and all actively detected cases, were examined clinically and parasitologically for CL. Patient information was recorded and patients were categorized based on the number and size of the lesions. Odds ratios (OR) of different risk factors were calculated. Results: A total of 849 (59.2% male, 40.8% female) confirmed cases of CL were enrolled; the average incidence rate of the disease was 14.9 per 100. 000 people. During the study period 2006-2011, the trend in CL incidence showed no sudden variations in the areas studied, except for an outbreak of CL in 2009. Leishmania major was identified as the causative agent based on internal transcribed

spacer 1 (ITS1) ribosomal DNA PCR analysis. During the study period, the age distribution of CL cases was relatively stable, with the majority (50%) of patients aged 1-25 years. Most cases (n = 468; 55.1%) had a single lesion and 82 (9.6%) patients had four or more lesions (range 1-29). The risk of developing multiple lesions was significantly increased in patients with seasonal jobs (summer workers) (p = 0.023; OR 1.516) and significantly decreased in patients who were affected in winter (p = 0.010; OR 0.398). The risk of developing large-sized lesions (>1. cm) was significantly increased in patients in the age groups >25 years (p = 0.001-0.015; OR 2.5-3.5) and decreased in patients with seasonal jobs (summer workers) (p = 0.005; OR 0.570). Conclusions: The present data show the importance of CL as a health problem in suburban areas of Qom Province. In order to identify other epidemiological aspects of leishmaniasis in this area, studies on vectors and reservoirs are recommended. Since leishmaniasis caused by L. major is typically zoonotic, control measures should focus on rodents as the main reservoirs and Phlebotomus papatasi as the main vector. Awareness should be raised in the high-risk populations comprising people with diabetes, young adults (<25 years old), and those who work outdoors during the summer. 2013 International Society for Infectious Diseases
SN - 1201-9712
AD - (Nateghi Rostami) Department of Public Health, Faculty of Health, Qom University of Medical Sciences, Ruhollah Sq., 14739-79966, Qom, Iran, Islamic Republic of (Saghafipour) Department of Medical Entomology and Vector Control, School of Public Health, Tehran University of Medical Sciences, Tehran, Iran, Islamic Republic of (Vesali) Qom Provincial Health Center, Qom University of Medical Sciences, Qom, Iran, Islamic Republic of
ER -

TY - JOUR
ID - 3272
T1 - Clinical risk factors and impact of vitamin D deficiency in children
A1 - Weber,G.
Y1 - 2013///
N1 - 71246296
English
Journal: Conference Abstract
KW - EMBASE
KW - vitamin D deficiency
KW - Child
KW - Human
KW - endocrinology
KW - risk factor
KW - Risk
KW - supplementation
KW - Health
KW - Chronic Disease
KW - Infant
KW - Diet
KW - skin
KW - Clothing
KW - Absorption
KW - sun exposure
KW - bone mineralization
KW - Lifestyle
KW - Prevalence
KW - Female
KW - Hospitalization
KW - Sunlight
KW - patient
KW - pathology
KW - season
KW - malabsorption
KW - egg yolk
KW - nut

KW - Air Pollution
KW - fish
KW - diet supplementation
KW - Europe
KW - Food
KW - Skin Pigmentation
KW - pregnant woman
KW - kidney disease
KW - Bone
KW - enteropathy
KW - exposure
KW - developing country
KW - vitamin D
KW - sunscreen
RP - NOT IN FILE
SP - 8
JF - Hormone Research in Paediatrics
VL - Conference: 9th Joint Meeting of Paediatric Endocrinology ESPE - PES - APEG - APPES - ASPAE - JSPE - SLEP Milan Italy. Conference Start: 20130919 Conference End: 20130922. Conference Publication:
IS - var.pagings
N2 - During the past two decades, low vitamin D status has re-emerged as a major paediatric health issue both in developed and in developing countries. First of all, the concern is related to increased prevalence in paediatric age, due mainly to modification in lifestyle, cultural attitudes (less time spent outdoors, use of veils or covering clothes, sunscreen), air pollution and incorrect diet. In addition, recent studies suggest vitamin D, besides the well-known role in bone mineralization, is linked to several extra-skeletal effects. Most of the vitamin D requirements are provided by sun exposure. However, sunlight is not risk-free, and vitamin D absorption varies a lot according to different seasons, degree of latitude, time of day, area of skin exposure and skin pigmentation. Natural content of vitamin D in paediatric diet is mostly found in non staple food such as fatty fish, nuts and egg yolk; alternative sources are fortified foods, which however are not very common in Europe. A major risk for infants is maternal vitamin D deficiency, in particular in dark skinned or veiled pregnant women and in infants breastfed for a long period. In older children risks of deficiency include long term hospitalizations due to chronic illnesses or patients with skin pathologies. A debated topic is if lower levels of 25(OH)D found in obese children have negative consequences for bone health. Children suffering from various severe intestinal diseases may present vitamin D deficiency due to malabsorption. Interference with the activation of vitamin D may be caused by severe chronic hepatic or renal diseases, or by the chronic use of several drugs. The supplementation of vitamin D is a subject of active debate among authors. While supplementation is univocally accepted for all children with chronic diseases or other vitamin D-deficiency risk factors, supplementation is still under discussion for healthy children
SN - 1663-2818
AD - (Weber) San Raffaele University, Paediatrics, Milan, Italy
ER -

TY - JOUR
ID - 3273
T1 - Sustaining quality while scaling-up adolescent ART: Findings from Zimbabwe?s largest adolescent cohort
A1 - Shroufi,A.
A1 - Dixon,M.
A1 - Gunguwo,H.
A1 - Nyathi,M.
A1 - Ndlovu,M.
A1 - Saint-Sauveur,J.F.
A1 - Taziwa,F.
A1 - Ndebele,W.
A1 - Ferreyra,C.
Y1 - 2012///

N1 - 71248278
English
Journal: Conference Abstract
KW - EMBASE
KW - Human
KW - Adolescent
KW - scale up
KW - Zimbabwe
KW - acquired immune deficiency syndrome
KW - Adult
KW - hazard
KW - Hospital
KW - survival
KW - Social Behavior
KW - proportional hazards model
KW - juvenile
KW - Cohort Analysis
KW - Counseling
KW - Therapy
KW - follow up
KW - log rank test
KW - Death
KW - Child
KW - adolescence
KW - Human immunodeficiency virus
RP - NOT IN FILE
SP - 264
JF - Journal of the International AIDS Society
JA - J Int AIDS Soc
VL - Conference: 19th International AIDS Conference Washington, DC United States. Conference Start: 20120722 Conference End: 20120727. Conference Publication:
IS - var.pagings
N2 - Background: As Anti Retroviral Treatment (ART) programmes improve survival in early life the number of adolescents in need of ART will continue to increase. Programmes will need to scale up services which address the particular needs of adolescents. Here we assess the experience of such scale up in a large urban adolescent cohort. Methods: Between 2004 and 2010 9,360 adults and 1,776 adolescents commenced antiretroviral therapy (ART) at Mpilo OI ART clinic, Bulawayo. A package of specific activities for adolescents was implemented, to provide comprehensive care including active defaulter tracing, a comprehensive, dedicated counselling programme and psycho-social activities operating both inside and outside the clinic, such as a youth club. Adolescents were engaged in decisions regarding their care. In this retrospective cohort analysis adolescents were defined as those > 10 and <19 years old at ART initiation date. Cox's proportional hazards model was used to calculate hazards and the log rank test to assess significance. Results: Between 2004 and 2010 a six-fold increase in adolescent ART initiations occurred (Figure 1). 12 month adolescent loss to follow increased in the first 3 years of the programme, peaking at 7%, subsequently falling to below 5% by the end of the study period. There was no significant difference between adult and adolescent (Figer presented) hazard of death. (HR=0.92, p=0.3793) Loss to follow up was significantly higher in adults than adolescents. (HR=1.92, p<0.0005) (Figure 2). Conclusion: These results contrast other research, which generally show adolescent outcomes to be worse than those in adults. Outcomes in expanding programmes have also been shown to suffer elsewhere. As more HIV positive children survive into adolescence, ART programmes must scale up comprehensive services for this group; we show that good results are feasible with dedicated clinical and psychosocial resources in resource poor settings
SN - 1758-2652
AD - (Shroufi, Saint-Sauveur, Taziwa) Medecins Sans Frontieres, Medical Department, Harare, Zimbabwe (Dixon, Gunguwo, Nyathi, Ndlovu, Ndebele) Mpilo Central Hospital, Bulawayo, Zimbabwe (Ferreyra) Medecins Sans Frontieres, Barcelona, Spain
ER -

TY - JOUR
ID - 3274
T1 - Knowledge and practice of asthmatic children's parents about daily air quality
A1 - Yazdanparast,T.
A1 - Mehdi,S.M.S.
A1 - Khalilzadeh,S.
A1 - Salehpour,S.
A1 - Boloursaz,M.R.
A1 - Baghaie,N.
A1 - Hassanzad,M.
A1 - Velayati,A.A.
Y1 - 2013///
N1 - 2013686148
English
Journal: Article
KW - EMBASE
KW - Air Pollution
KW - air quality
KW - article
KW - Asthma
KW - Child
KW - clinical practice
KW - cross-sectional study
KW - Environmental Exposure
KW - Human
KW - Iran
KW - knowledge
KW - major clinical study
KW - parental attitude
KW - questionnaire
KW - respiratory function
KW - school child
KW - scoring system
KW - surgical mask
RP - NOT IN FILE
SP - 23
EP - 28
JF - Tanaffos
VL - 12
IS - 3
CY - Iran, Islamic Republic of
N2 - Background: Knowledge and practice about air pollution are essential subjects in special groups such as cardio-pulmonary patients. For children with air pollution-related diseases, knowledge and attitude of parents play a determining role in this respect. Since providing a coherent curriculum needs evidence-based information, this survey was conducted to assess the knowledge and practice of asthmatic children's parents about daily air quality since asthmatic children are among the most vulnerable at-risk groups when it comes to air pollution. Materials and Methods: All parents of asthmatic children referred to the Pediatric Clinic of Masih Daneshvari Hospital during one year period (250 people) completed knowledge and practice questionnaire on air pollution. Knowledge questions consisted of familiarity with pollution standard index (PSI), ways to find out about it, respiratory effects of air pollution and etc. Practice questions consisted of reducing outdoor presence and activity of children and actions taken to reduce air pollution in polluted days. Results: In general, 3.2% of parents were familiar with PSI, 12.5% were aware of ways to find out about daily air quality, 65.2% were aware of air pollution respiratory effects, 65.6% were aware of air pollution effects on asthmatic children and 4.4% were aware of ineffectiveness of surgical masks in prevention of air pollution health effects. The obtained practice score ranged from 4 to 16, and the participants' mean score was equal to 11.79. Conclusion: This study revealed that parents of asthmatic children were aware of air pollution hazards for their children and wanted to

prevent them but they did not know how. Therefore, asthmatic children in Tehran are still exposed to risks of air pollution. 2013 NRITLD, National Research Institute of Tuberculosis and Lung Disease, Iran
SN - 1735-0344
AD - (Yazdanparast, Mehdi, Salehpour) Chronic Respiratory Diseases Research Center, National Research Institute of Tuberculosis and Lung Diseases (NRITLD), Shahid Beheshti University of Medical Sciences, Tehran, Iran, Islamic Republic of (Khalilzadeh, Boloursaz, Baghaie, Hassanzad) Pediatric Respiratory Diseases Research Center, NRITLD, Shahid Beheshti University of Medical Sciences, Tehran, Iran, Islamic Republic of (Velayati) Clinical Tuberculosis and Epidemiology Research Center, NRITLD, Shahid Beheshti University of Medical Sciences, Tehran, Iran, Islamic Republic of
ER -

TY - JOUR
ID - 3275
T1 - Drug-food-and food-drug-interactions relevant for patients under PPI and aromatase inhibitor treatments: A shared responsibility for personalized nutritional and pharmacotherapy
A1 - Jenzer,H.
A1 - Sadeghi,L.
A1 - Krause,C.
A1 - Stute,P.
A1 - Stanga,Z.
Y1 - 2013///
N1 - 71239736
English
Journal: Conference Abstract
KW - EMBASE
KW - food drug interaction
KW - patient
KW - Human
KW - Responsibility
KW - Drug Therapy
KW - clinical pharmacy
KW - Therapy
KW - Food
KW - Female
KW - drug bioavailability
KW - risk factor
KW - bacterial overgrowth
KW - Absorption
KW - breast cancer
KW - medication therapy management
KW - malabsorption
KW - Male
KW - Prevention
KW - acidity
KW - stomach acid secretion
KW - stimulation
KW - broccoli
KW - grape
KW - Glycyrrhiza
KW - degeneration
KW - cabbage
KW - dissociation
KW - peristalsis
KW - community
KW - Pregnancy
KW - rice
KW - mother

KW - stomach pH
KW - Child
KW - buckwheat
KW - Asthma
KW - millet
KW - vegetable
KW - information processing
KW - grain
KW - Risk
KW - cauliflower
KW - grapefruit
KW - hospital acquired pneumonia
KW - mushroom
KW - Nutrition
KW - testosterone blood level
KW - ovulation
KW - bioavailability
KW - pKa
KW - mammary gland
KW - carcinogenesis
KW - cancer patient
KW - aromatase inhibitor
KW - aromatase
KW - pepsin A
KW - carboxylic acid
KW - erythromycin
KW - peptide
KW - digoxin
KW - nifedipine
KW - penicillin derivative
KW - alendronic acid
KW - estradiol
KW - estrogen
KW - vegetable oil
KW - alcohol
KW - melatonin
KW - Adjuvant
KW - isoenzyme
KW - glycyrrhizic acid
RP - NOT IN FILE
SP - 981
JF - International Journal of Clinical Pharmacy
JA - Int J Clin Pharm
VL - Conference: 41st ESCP Symposium on Clinical Pharmacy: Personalised and Safe Therapy Barcelona Spain. Conference Start: 20121029 Conference End: 20121031. Conference Publication:
IS - var.pagings
N2 - Background and objectives: Food-food and food-drug interactions are emerging topics in literature and risk factors for ADE in practice. Substrates may modify absorption or interact with the 57 metabolizing CYP450 isoenzymes. The aim of this work is to describe relevant interactions outside the current interaction tables on absorption (PPI) and on CYP19A1 (aromatase inhibitors). Settings and method: 188 references froma systematic online literature research were evaluated. Nutritional and/or medication reconciliation options rely on a multidisciplinary focus group approach. Main outcome measures: Around 130 ingredients have been retrieved and listed. Results: PPIs change gastric pH permanently to>4.0, thus suppress gastric acid secretion and impair pepsin activation for cleavage of peptide bonds. This loss of acidity brings with it bacterial overgrowth, community or hospital acquired pneumonia (increased risk ratio of 1.89 in PPI patients), increase of asthma among children from mothers treated with PPI in pregnancy, mucosal degeneration/transmucosal leak/hyperpermeability, thus increase of drug availability (e.g. nifedipine, digoxin, penicillin's, erythromycin, or

alendronate), and malabsorption, thus reduction of drug bioavailability (e.g. carboxylic or vinylogous carboxylic acids as a result of altered dissociation). Prevention options comprise allergenic food avoidance, buffering, pepsin replacement, peristalsis stimulation, and melatonin. Intervention may focus on nutrition and medication reconciliation. Inhibition of aromatase has been described for tea and soy, whole grains from buckwheat, millet, or brown and purple rice, cruciferous vegetables (cabbage, broccoli, cauliflower), grapefruit, grape seed oil, and mushrooms. Induction of aromatase can be associated with ethanol. Liquorice (glycyrrhizin) reduces serum testosterone in men and women and supports regular ovulation by induction of aromatase. Conclusions: Concise interaction tables do not explain extra-hepatic interactions. Inappropriate long-term PPI treatment is a documented risk factor for bacterial overgrowth, mucosal degeneration, hyperpermeability, and altered bioavailability. Care should be given to ingredients having pKa<4.5. Breast cancer develops mainly post-menopausally, when estradiol levels have decreased by 90 % due to ovarian exhaustion. Therefore, endogenous production in the mammary gland catalyzed by aromatase is determinant for tumor genesis rather than estrogen uptake. However, a practical benefit arising from adjuvant nutritional treatment of breast cancer patients remains to be elucidated
SN - 2210-7703
AD - (Jenzer, Sadeghi, Krause) R and D Nutrition and Dietetics, Bern University of Applied Sciences, Bern, Switzerland (Stute) Department of Gynecologic Endocrinology and Reproductive Medicine, Bern University Women's Hospital, Bern, Switzerland (Stanga) Division of Endocrinology, Diabetes and Clinical Nutrition, Bern, Switzerland (Stanga) Department of Internal Medicine, Bern University Hospital, Bern, Switzerland
ER -

TY - JOUR
ID - 3276
T1 - Off-label drug use in neonatal setting in Slovak Republic: A crosssectional survey
A1 - Slazneva,J.
A1 - Kakosova,V.
A1 - Dolnikova,D.
A1 - Kuzelova,M.
Y1 - 2013///
N1 - 71239692
English
Journal: Conference Abstract
KW - EMBASE
KW - off label drug use
KW - Slovakia
KW - clinical pharmacy
KW - Therapy
KW - Newborn
KW - Human
KW - Gestational Age
KW - Child
KW - pathology
KW - adverse drug reaction
KW - weight
KW - drug dosage form
KW - patient
KW - therapy effect
KW - pediatrician
KW - Female
KW - ward
KW - retrolental fibroplasia
KW - Low Birth Weight
KW - Male
KW - digestive system
KW - gender
KW - Blood
KW - medical record

KW - Adult
KW - prematurity
KW - diet supplementation
KW - drug dose
KW - Classification
KW - Metabolism
KW - hematologic disease
KW - prescription
KW - Drug Therapy
KW - university hospital
KW - Safety
KW - research priority
KW - drug formulation
KW - clotrimazole
KW - diclofenac
KW - tramadol
RP - NOT IN FILE
SP - 964
JF - International Journal of Clinical Pharmacy
JA - Int J Clin Pharm
VL - Conference: 41st ESCP Symposium on Clinical Pharmacy: Personalised and Safe Therapy Barcelona Spain. Conference Start: 20121029 Conference End: 20121031. Conference Publication:
IS - var.pagings
N2 - Background and objectives: Paediatricians are often forced to extrapolate drug doses from older children and adults, to manipulate with drug's formulations or to change indicated route of administration resulting in off-label drug use. In general, off-label drug use may result in benefit, no therapeutic effect, or adverse reaction. The aim of this survey was to determine the extent and pattern of off-label used medicinal products in newborns and to highlight the therapeutic areas with the highest need. Settings and method: The research was performed over 3 month period in April-June 2012, and involved all newborns admitted to Neonatal Pathology Unit of Children's University Hospital in Bratislava who received a pharmacotherapy. From the medical records at the ward, data about patients' gender, gestational age, weight and diagnose were collected. Dose, frequency, intended route of administration and dosage form were recorded for each prescribed drug. In line with other studies, intravenous replacement solutions, blood products and dietary supplements were excluded. Each recorded medicine was reviewed for off-label use status, based on the information in the approved Summary of Product Characteristics (SPC). A medicinal product was regarded as off-label if it was used outside the age range specified in SPC. Main outcome measures: Proportion of off-label administered medicinal products, identification of the most frequently off-label used drugs in newborns. Results and conclusion: In total, 389 medicinal products containing 67 different active substances were administered to 88 hospitalised neonates (48 male, 40 female). The median gestational age was 34.9 + 4.8 weeks and 46 % were preterm newborns. Retinopathy of prematurity (n = 17) and low birth weight (n = 11) were the most common reasons for hospitalisation. According to the ATC classification the most prescribed medicinal products were drugs for the alimentary tract and metabolism (30 %), drugs for blood disorders (22.3 %), ophthalmologicals (10.5 %) and anti-infectives for systemic use (7.9 %). Of all drug prescriptions, 20.5 % were used in off-label manner. Fifty percent of the newborns received at least one off-label drug. The most frequently off-label prescribed drugs were clotrimazole, ofloxacine, diclofenac and tramadol. Our results indicate that off-label prescribing is common in neonatological field in Slovak Republic, and we believe that our data will stimulate national regulatory authorities to monitor safety concerns associated with off-label drug use in newborns as well as identify research priorities in this field
SN - 2210-7703
AD - (Slazneva, Kuzelova) Pharmacology and Toxicology, Faculty of Pharmacy, Comenius University, Bratislava, Slovakia (Kakosova) Hospital Pharmacy, Children's University Hospital, Bratislava, Slovakia (Dolnikova) Neonatal Pathology Unit, Children's University Hospital, Bratislava, Slovakia
ER -

TY - JOUR
ID - 3277
T1 - Fibre type-specific satellite cell content in two models of muscle disease

A1 - Bankole,L.-C.
A1 - Feasson,L.
A1 - Ponsot,E.
A1 - Kadi,F.
Y1 - 2013///
N1 - 2013723782
English
Journal: Article
KW - EMBASE
KW - Adolescent
KW - Adult
KW - article
KW - basement membrane
KW - Child
KW - clinical article
KW - controlled study
KW - dermatomyositis/et [Etiology]
KW - Duchenne muscular dystrophy/et [Etiology]
KW - fiber
KW - histopathology
KW - Human
KW - human tissue
KW - muscle biopsy
KW - muscle disease
KW - muscle injury
KW - polymyositis
KW - priority journal
KW - satellite cell
KW - school child
KW - skeletal muscle
KW - Ki 67 antigen/ec [Endogenous Compound]
KW - transcription factor PAX7/ec [Endogenous Compound]
RP - NOT IN FILE
SP - 826
EP - 832
JF - Histopathology
VL - 63
IS - 6
CY - United Kingdom
N2 - Aims: Muscle satellite cells (SCs) are responsible for the regenerative events following muscle fibre injury. This study aimed to improve our understanding of SC behaviour in two models of muscle disorder with different pathological mechanisms and onset of disease. Methods and results: Pax7⁺SC content was assessed in types I and II fibres of patients with Duchenne muscular dystrophy (DMD; n = 9; age 13 + 2 years), polymyositis/dermatomyositis (PM/DM; n = 9; age 52 + 12 years) and in controls (n = 5; age 26 + 5 years). Pax7⁺SCs number in type I and II fibres was higher (P < 0.05) in DMD and in PM/DM compared to controls. Type I fibres were associated with a higher number of Pax7⁺SCs compared to type II fibres only in DMD; Pax7⁺SCs number in type I fibres was about threefold higher in DMD compared to PM/DM (P < 0.05). In DMD, Pax7⁺SC content in small regenerating fibres (0.09 + 0.09 SCs/fibre) was similar to that in fibres from healthy skeletal muscle. The proportion of activated SCs (Ki-67⁺SCs) was fivefold lower in DMD (0.4 + 0.4%) compared to PM/DM (2.8 + 2%). Pax7⁺ cells located outside the basal lamina were observed in DMD muscles only. Conclusion: The capacity to generate new SCs is increased even in severely impaired muscles and a fibre type-specific enhancement of SC occurs in type I muscle fibres in DMD. 2013 John Wiley & Sons Ltd
SN - 0309-0167
AD - (Bankole, Ponsot, Kadi) Muscle and Exercise Physiology Research Group (MEP-RG), School of Health and Medical Sciences, Orebro University, Orebro, Sweden (Bankole, Feasson) Laboratory of Exercise

Physiology (LPE EA 4338), University of Lyon, Saint-Etienne, France (Bankole, Feasson) Unit of Myology, University Hospital of Saint-Etienne, Saint-Etienne, France
ER -

TY - JOUR
ID - 3279
T1 - The Incubation Period of Buruli Ulcer (Mycobacterium ulcerans Infection)
A1 - Trubiano,J.A.
A1 - Lavender,C.J.
A1 - Fyfe,J.A.M.
A1 - Bittmann,S.
A1 - Johnson,P.D.R.
Y1 - 2013///
N1 - 2013707671
English
Journal: Review
KW - EMBASE
KW - Adolescent
KW - Adult
KW - Aged
KW - Buruli Ulcer/di [Diagnosis]
KW - Buruli ulcer/dt [Drug Therapy]
KW - Buruli ulcer/su [Surgery]
KW - Child
KW - Environmental Exposure
KW - Female
KW - Human
KW - incubation time
KW - Infant
KW - limb injury
KW - major clinical study
KW - Male
KW - mosquito
KW - Newborn
KW - Polymerase Chain Reaction
KW - preschool child
KW - retrospective study
KW - review
KW - school child
KW - structured questionnaire
KW - telephone interview
KW - clarithromycin/dt [Drug Therapy]
KW - moxifloxacin/dt [Drug Therapy]
KW - rifampicin/dt [Drug Therapy]
RP - NOT IN FILE
JF - PLoS Neglected Tropical Diseases
VL - 7
IS - 10
CY - United States
N2 - Introduction:Buruli Ulcer (BU) is caused by the environmental microbe Mycobacterium ulcerans. Despite unclear transmission, contact with a BU endemic region is the key known risk factor. In Victoria, Australia, where endemic areas have been carefully mapped, we aimed to estimate the Incubation Period (IP) of BU by interviewing patients who reported defined periods of contact with an endemic area prior to BU diagnosis.Method:A retrospective review was undertaken of 408 notifications of BU in Victoria from 2002 to 2012. Telephone interviews using a structured questionnaire and review of notification records were performed. Patients with a single visit exposure to a defined endemic area were included and the period from exposure to disease onset determined (IP).Results:We identified 111 of 408 notified patients (27%) who had a residential

address outside a known endemic area, of whom 23 (6%) reported a single visit exposure within the previous 24 months. The median age of included patients was 30 years (range: 6 to 73) and 65% were male. 61% had visited the Bellarine Peninsula, currently the most active endemic area. The median time from symptom onset to diagnosis was 71 days (range: 34-204 days). The midpoint of the reported IP range was utilized to calculate a point estimate of the IP for each case. Subsequently, the mean IP for the cohort was calculated at 135 days (IQR: 109-160; CI 95%: 113.9-156), corresponding to 4.5 months or 19.2 weeks. The shortest IP recorded was 32 days and longest 264 days (Figure 1 & 2). IP did not vary for variables investigated.Conclusions:The estimated mean IP of BU in Victoria is 135 days (IQR: 109-160 days), 4.5 months. The shortest recorded was IP 34 days and longest 264 days. A greater understanding of BU IP will aid clinical risk assessment and future research. 2013 Trubiano et al
SN - 1935-2727
AD - (Trubiano, Johnson) Department of Infectious Diseases, Austin Health, Heidelberg, VIC, Australia (Trubiano, Bittmann) Victorian Department of Health, Melbourne, VIC, Australia (Lavender, Fyfe) Victorian Infectious Disease References Laboratory (VIDRL), North Melbourne, VIC, Australia (Lavender, Fyfe, Johnson) World Health Organization Collaborating Centre for Mycobacterium ulcerans (Western Pacific Region), VIDRL, North Melbourne, VIC, Australia (Johnson) Department of Medicine, Austin Health, University of Melbourne, Heidelberg, VIC, Australia (Johnson) Department of Microbiology and Immunology, University of Melbourne, Parkville, VIC, Australia
ER -

TY - JOUR
ID - 3280
T1 - High seroprevalence of human herpes virus 8 (HHV-8) antibodies among vertically HIV-infected pediatric patients living in Germany
A1 - Feiterna-Sperling,C.
A1 - Konigs,C.
A1 - Notheis,G.
A1 - Buchholz,B.
A1 - Weizsaecker,K.
A1 - Eberle,J.
A1 - Hofmann,J.
Y1 - 2012///
N1 - 71227741
English
Journal: Conference Abstract
KW - EMBASE
KW - seroprevalence
KW - Human herpesvirus 8
KW - patient
KW - Human
KW - Germany
KW - Drug Therapy
KW - Human immunodeficiency virus infection
KW - Human immunodeficiency virus
KW - Child
KW - Western Europe
KW - Infection
KW - gender
KW - Adolescent
KW - Female
KW - ethnicity
KW - United States
KW - Kaposi sarcoma
KW - Prevalence
KW - virus load
KW - highly active antiretroviral therapy
KW - cell count

KW - Asia
KW - Population
KW - Gammaherpesvirinae
KW - Demography
KW - Risk
KW - immune deficiency
KW - Adult
KW - medical history
KW - serum
KW - mother
KW - university hospital
KW - cross-sectional study
KW - Africa
KW - Eastern Europe
KW - Male
KW - Antibody
KW - CD4 antigen
KW - antigen
RP - NOT IN FILE
SP - 77
EP - 78
JF - Journal of the International AIDS Society
JA - J Int AIDS Soc
VL - Conference: 11th International Congress on Drug Therapy in HIV Infection Glasgow United Kingdom. Conference Start: 20121111 Conference End: 20121115. Conference Publication:
IS - var.pagings
N2 - Background: Human herpes virus 8 (HHV-8), a gamma herpes virus, is the etiological agent for Kaposi sarcoma (KS). HIV-infected adults with advanced immunodeficiency are at risk. Prevalence data of HHV-8 infection in HIV-infected children living in non-endemic areas are limited. Serologic studies indicate low seroprevalence rates of 3-4% for healthy children living in United States and Germany [1]. Purpose of the study: The aim of the study was to determine the seroprevalence of HHV-8 antibodies among vertically HIV-infected pediatric patients in Germany and to evaluate their association with age, gender, ethnicity, and other demographic factors. Methods: In 2012, a multi-center cross-sectional study was conducted in four University Hospitals in Germany. Stored frozen serum specimens obtained from vertically HIV-infected children and adolescents were tested for antibodies against lytic and latent HHV-8 antigens. Data on patients' demographic characteristics and medical history were recorded. Results: A total of 214 HIV-infected children and adolescents (105 males, 109 females) were included. The median age was 10.2 years (range 1 months-22.6 years). A high proportion of these children (62%) was born in Western Europe, whereas 65% (139/214) of their mothers were born in countries outside Western Europe. The majoritiy (91%) of the children had been treated with highly active antiretroviral therapy and 55.2% (116/210) had a HIV-viral load<50 copies/mL. The median CD4 cell count was 1000/L (range 3-4400). The overall seroprevalence of HHV-8 antibodies was 23.8% (51/214). Seroprevalence rates did not show significant differences between age or gender. In the group of young children aged 1 month to 35 months, 19.4% (46/31) had HHV-8 antibodies, compared to 25% (25/100) in children aged 36 months to 11 years, and 24.1% (20/83) children 12 years and older. In the study group, seroprevalence rates were significantly lower in children who were born in Western Europe (p<0.01) compared to those born in Africa, Asia, or Eastern Europe. Clinical symptoms of HHV-8 infection were reported to be uncommon; only one child had a history of KS at 2 years of age. Conclusions: Vertically HIV-infected pediatric patients living in Germany showed a high HHV-8 seroprevalence of 23.8%. These rates were higher as expected in the normal pediatric population. The findings suggest that HHV-8 infection occurred already in the first years of life
SN - 1758-2652
AD - (Feiterna-Sperling) Charite Universitatsmedizin Berlin, Pediatric Pneumology and Immunology, Berlin, Germany (Konigs) Goethe University, Department of Pediatrics, Frankfurt, Germany (Notheis) Ludwig-Maximilians Universitat Munchen, Dr. von Haunersches Kinderspital, Munich, Germany (Buchholz) University Children's Hospital, Medical Faculty Mannheim, Mannheim, Germany (Weizsaecker) Charite Universitatsmedizin Berlin, Department of Obstetrics, Berlin, Germany (Eberle) Ludwig-Maximilians Universitat Munchen, Max von Pettenkofer-Institut, Munich, Germany (Hofmann) Charite Universitatsmedizin Berlin, Institute of Medical Virology, Berlin, Germany

ER -

TY - JOUR
ID - 3281
T1 - The use of self-modeling to promote social interactions among young children
A1 - Buggey,T.
A1 - Ogle,L.
Y1 - 2013///
N1 - 2013700379
English
Journal: Article
KW - EMBASE
KW - article
KW - Autism
KW - Case Report
KW - Child
KW - Child Behavior
KW - clinical observation
KW - Female
KW - groups by age
KW - Human
KW - Interpersonal Communication
KW - Male
KW - play
KW - preschool child
KW - priority journal
KW - Self Concept
KW - social adaptation
KW - Social Interaction
KW - Socialization
KW - video self modeling
RP - NOT IN FILE
SP - 202
EP - 211
JF - Focus on Autism and Other Developmental Disabilities
VL - 28
IS - 4
CY - United States
N2 - Video self-modeling (VSM) has been used to teach social skills to children with autism older than 4 years of age. Attempts to use VSM with younger children with disabilities have produced less than favorable results; however, it is unclear whether VSM could be used to promote social initiations by typically developing children. Thirty minutes of staged filming, in which the four typically developing participants were prompted to interact with a peer with autism on the playground and inside during center time, was edited into 2.5- to 3-min clips. Each clip took less than 2 hr to edit. Data were collected on frequency of solitary play, initiations, parallel play, and engaged play and were then analyzed in a multiple-baseline-across-participants single-case design. Visual analysis led to the conclusion that VSM did not affect the typically developing children's behavior. Limitations of the study and cautions for using VSM with very young children are discussed. Hammill Institute on Disabilities 2012
SN - 1088-3576
AD - (Buggey, Ogle) University of Tennessee at Chattanooga, 205 Hunter Hall, Chattanooga, TN 37402, United States
ER -

TY - JOUR
ID - 3283
T1 - Examining family demographic and environmental effects on sleep in children aged 2-5 years with and without autism spectrum disorder

A1 - Richdale,A.
Y1 - 2013///
N1 - 71226578
English
Journal: Conference Abstract
KW - EMBASE
KW - Sleep
KW - Autism
KW - Human
KW - Child
KW - questionnaire
KW - Environment
KW - parent
KW - meal
KW - caregiver
KW - environmental factor
KW - heart atrium septum defect
KW - Income
KW - girl
KW - cultural factor
KW - boy
KW - Temperature
KW - habit
KW - e-mail
KW - register
KW - Hygiene
KW - Male
KW - computer
KW - Female
KW - Fear
KW - University
KW - Television
KW - Child Care
KW - gas
KW - tablet
KW - toddler
KW - preschool child
KW - developmental disorder
KW - Education
KW - Employment
KW - night
RP - NOT IN FILE
SP - 8
JF - Sleep and Biological Rhythms
VL - Conference: 25th ASM of Australasian Sleep Association and Australasian Sleep Technologists Association, Sleep DownUnder 2013 Brisbane, QLD Australia. Conference Start: 20131017 Conference End: 20131019. Conference Publication:
IS - var.pagings
N2 - Introduction: Parents of children < 6 years frequently report that their child has sleep problems. Poor sleep is often related to bedtime associations and routines, behavioural issues surrounding sleep, or night time fears. Sleep hygiene factors, including the sleep environment, screen time, and meals prior to bed, may also impact on sleep and socio-cultural factors and parent knowledge about child sleep may also affect family sleep practices. This study investigated relationships between family demographics and environmental factors in relation to reported sleep difficulties in children aged 2-5 years. Method: Questionnaire packages were given to 204 families attending a childcare centre and 38 families (56%) of children aged 2-5 years on a university participant registry responded to an email. The primary caregivers (n = 96) of 101 children, mean age 47.5 months (SD = 12.9 months) returned completed packages which consisted of a demographic questionnaire, an environment

and sleep questionnaire and the Children's Sleep Habits Questionnaire (CSHQ). There were 63 boys and 34 girls; 28 of the children had ASD and 2 children had a global developmental delay. Results: On parent report 55.4% of the children had a sleep problem (79% of the children with ASD); the average CSHQ total score was significantly higher in these children than in children without a reported sleep problem (p < .001). CSHQ scores did not differ for children with and without ASD. Results showed that higher CSHQ scores were related to family income, and primary caregiver employment and education. CSHQ score was not related to timing of meals prior to bed, or to meal type or drinks consumed, or outside play. CSHQ score was related to the child feeling too hot or cold during sleep, use of electric or gas heaters, time spent watching television and time spent on computers or tablets. Discussion: These results indicate the importance of considering the impact of family and environmental factors on sleep problems in toddlers and pre-school children, regardless of whether the child has a developmental disability. In particular the temperature associated with the child's sleep environment and screen time appear to be important factors. It was surprising that even at this young age screen time is impacting on children's sleep
SN - 1446-9235
AD - (Richdale) La Trobe University, Bundoora, VIC, Australia
ER -

TY - JOUR
ID - 3284
T1 - 10 Years waldpiratencamp in Germany
A1 - Schmid,D.
Y1 - 2013///
N1 - 71215940
English
Journal: Conference Abstract
KW - EMBASE
KW - Society
KW - oncology
KW - Germany
KW - Human
KW - survivor
KW - sibling
KW - neoplasm
KW - patient
KW - parent
KW - courage
KW - cancer therapy
KW - Child
KW - Fear
KW - disabled person
KW - pediatric nurse
KW - helplessness
KW - non profit organization
KW - community
KW - Government
KW - rowing
KW - Therapy
KW - childhood cancer
KW - rock climbing
KW - Responsibility
KW - Ireland
KW - climbing
KW - wheelchair
KW - France
KW - Hungary
KW - Italy
KW - Canada

KW - United States
KW - Aftercare
KW - cancer patient
KW - Adult
KW - Adolescent
RP - NOT IN FILE
SP - 53
JF - Pediatric Blood and Cancer
VL - Conference: 45th Congress of the International Society of Paediatric Oncology, SIOP 2013 Hong Kong China. Conference Start: 20130925 Conference End: 20130928. Conference Publication:
IS - var.pagings
N2 - Purpose/Objective: The Waldpiratencamp is a camp for kids, teenagers and young adults with and after cancer and their siblings. The camp of the Deutsche Kinderkrebsstiftung (German Childhood Cancer Foundation) was established in August 2003 in Heidelberg, Germany. Main goal is to support the young survivors in their return to everyday life. They should relax after the strenuous cancer therapy and gain strength and new courage in a cautious way. Materials and Methods: The Waldpiratencamp offers 8-day camps for survivors and siblings as well as weekends for different kind of groups, e.g. bereaved parents. The program is always best suited for the needs of the participants. The staff of professional educators and pediatric nurses offers various activities in nature like hiking, rock climbing and canoeing. Collective experiences in outdoor activities should facilitate the exchange of experiences within the survivors / siblings community. Results: Patients suffer from helplessness and passiveness during their therapy. The camp wants to retrieve the patients from this distressed situation. Every child gets individual support to rediscover his still existing abilities and his power for life. Fears are recognized as 'normal' and self-doubt experienced as surmountable. Group activities consolidate selfesteem and self-confidence. The participants take individual responsibility and consideration for handicapped survivors at the climbing wall and on the high rope course (latter is also suitable for wheelchair users). Conclusions: There are camps for kids with and after cancer in France, Ireland, Hungary, Italy, Canada and in the United States. TheWaldpiratencamp is the only camp of this kind in Germany. In 2008 the Waldpiratencamp was awarded with a price for aftercare for young cancer patients. In 2009 it was elected from the Federal Government of Germany as one of 365 outstanding places in Germany
SN - 1545-5009
AD - (Schmid) Deutsche Kinderkrebsstiftung, German Childhood Cancer Foundation, Bonn, Germany
ER -

TY - JOUR
ID - 3285
T1 - Physical activity in a group of school children in mexico city: Associated factors and cutoff points measured by accelerometry
A1 - Hernandez,M.T.J.M.
A1 - Pineda,C.M.D.
A1 - Ibanez,N.R.
A1 - Ortiz-Hernandez,L.
Y1 - 2013///
N1 - 2013671248
English, Spanish
Journal: Article
KW - EMBASE
KW - Accelerometry
KW - Aerobic Exercise
KW - article
KW - body fat
KW - Child
KW - disease association
KW - Female
KW - fitness
KW - Human
KW - Male
KW - Nutritional Status

KW - Physical Activity
RP - NOT IN FILE
SP - 372
EP - 379
JF - Boletin Medico del Hospital Infantil de Mexico
JA - Bol.Med Hosp Infant Mex
VL - 70
IS - 5
CY - Mexico
N2 - Background. At any age, physical activity (PA) is critical to maintaining health. Different PA guidelines have been formulated to improve the health of children and adolescents. We undertook this study to analyze the difference in physical activity levels in school children during the day and week and to determine whether two recommendations of physical activity (frequency and duration vs. accumulated duration) in school children identify difference in physical fitness and fatness. Methods. Seventy-one school children participated. A tri-axis accelerometer (ActiGraph) was used for 7 consecutive days. Minutes spent on moderate or vigorous physical activity (MVPA) were estimated. Two criteria were used to define whether a child is active: >60 min of MVPA/day and 300 min of MVPA/week. Nutritional status was assessed using the body mass index (BMI) and physical fitness with the FITNESSGRAM protocol. Results. It was shown that 12.5% of school children were active according to 60 min MVPF/day criterion and 55.5% with 300 min MVPF/week criteria. School children were more active during school recess and afternoons on weekdays and afternoons and nights on weekends. Boys were more actives than girls. The time spent on vigorous and moderate activities was positively correlated with aerobic fitness (r =0.23, p =0.057 and r =0.32, p =0.007, respectively). There was a positive correlation between the time spent on sedentary activities and body fat (r =0.343, p =0.003) and negative with light and moderate activities (r =-0.249, p =0.003; r =-0.249, p =0.013, respectively). School children classified as active according to 300 min of MVPA/week criteria showed better aerobic capacity. Conculsion: The proportion of active school children differs according to the physcial activity criterion. it was shown that physical actvity varied according to gender and time of day. The time spent on high intentity activities appears to have an influence on aerobic fitness of school children
SN - 1665-1146
AD - (Hernandez, Pineda, Ibanez, Ortiz-Hernandez) Departamento de Atencion a la Salud, Universidad Autonoma Metropolitana Unidad Xochimilco, Mexico, DF, Mexico
ER -

TY - JOUR
ID - 3286
T1 - Risk Factors and Preventative Strategies for Adolescent Sexual Violence - A Cross-National Urban Perspective
A1 - Andrea Goddard Sophie Khadr Katriona Ogilvy Webb
Y1 - 2013///
N1 - 75000156
English
Journal: Conference Abstract
KW - EMBASE
KW - Adolescent
KW - Human
KW - sexual violence
KW - adolescent health
KW - risk factor
KW - Child
KW - gang
KW - Male
KW - United Kingdom
KW - boy
KW - sexual exploitation
KW - partner violence
KW - girl
KW - Female

KW - victim
KW - Violence
KW - sexual assault
KW - Health
KW - Risk
KW - offender
KW - population research
KW - social care
KW - school
KW - Policy
KW - workshop
KW - city
KW - juvenile
KW - gender
KW - friend
KW - sexuality
KW - Prevention
KW - Society
KW - Clothing
KW - Population
KW - Crime
KW - Education
KW - learning disorder
KW - marker
RP - NOT IN FILE
SP - 6
JF - Turkish Archives of Pediatrics
VL - Conference: 10th International Congress on Adolescent Health
IS - 10th
N2 - OBJECTIVES: 1.Compare and discuss the nature of and risk factors for the various forms of adolescent sexual violence in culturally diverse/multinational urban settings 2.Explore known preventative strategies and discuss novel approaches to identification and intervention. METHODS: Sexual violence affects individuals of all ages, cultures, gender, orientation and abilities. Some groups may be disproportionately affected. 30% of under 18s in London, England, are non-white but in the years 2008-2011, 50% of 13- 17 year olds attending the London Havens Sexual Assault Referral Centres came from census groups other than White British, White Irish or White Other. Ten percent had learning difficulties compared with a population average of 2.5%. Based on evidence submitted to the current CSEGG (Child Sexual Exploitation In Gangs* and Groups*) Inquiry in England, at least 16,500 children under 18 years were identified as being at risk of child sexual exploitation during one year and 2,409 children were confirmed as victims of sexual exploitation in gangs and groups during the 14-month period from August 2010 to October 2011. There was a higher rate of victimisation amongst black and minority ethnic (BME) children and young people than has been previously identified. (* where gangs mainly comprise men and boys aged 13 - 25 years old, who take part in many forms of criminal activity, such as knife crime or robbery, who can engage in violence against other gangs, and who have identifiable markers such as territory, a name, sometimes clothing etc. While children can be sexually exploited by a gang, this is not the reason why a gang is formed. Groups involves people who come together in person or online for the purpose of setting up, co-ordinating and/or taking part in the sexual exploitation of children in either an organised or opportunistic way.) In terms of intimate partner violence in adolescent relationships, a recent National Society for the Prevention of Cruelty to Children (NSPCC) study in the UK echoed findings in the USA in that high numbers of adolescents experience physical, emotional and sexual violence in their intimate partner relationships (a quarter of girls and 18 per cent of boys reported some form of physical partner violence, three-quarters of girls and half of boys reported some form of emotional partner violence. One in three girls and 16 per cent of boys reported some form of sexual partner violence). The incidence, frequency and level of negative impacts rates for all forms of violence were greater for girls. The majority of young people either told a friend about the violence or told no one. Most respondents in this study were white. We propose an interactive session to: *compare and contrast risk factors for adolescent sexual assault among urban youth in major cities of different countries with attention given to particular characteristics and vulnerabilities associated with adolescents who are trafficked, homeless, unaccompanied minors run-aways and/or in the care of the state

*share experiences of preventative strategies among workshop attenders- including government/public health preventative policies and measures, school-based educational measures or those targeted at preventing revictimisation *identify and discuss obstacles to interventions: particularly those related to culture, social marginalisation and legal frameworks *examine and discuss efficacy of interventions CONCLUSIONS:Large numbers of adolescents are victims of sexual violence and exploitation in settings outside the family: in intimate partnerships and by gangs and groups. There are high rates of under-identification. Better understanding of the settings, susceptible groups, risk factors and behavioural identifiers that might increase the likelihood that an adolescent is a victim of sexual violence is needed. In many countries existing health, judicial and social care structures have started but struggle to address these emerging problems. Effective strategies are needed for education and early identification of at-risk adolescents and treatment of those already affected by sexual violence
SN - 1306-0015
AD - (Goddard) Imperial College Healthcare NHS Trust, London Haven Sexual Assault Referral Centres, United Kingdom (Khadr) University College London, London Haven Sexual Assault Referral Centres, United Kingdom (Webb) Sexual Exploitation Service, United Kingdom
ER -

TY - JOUR
ID - 3287
T1 - Toxocariasis a rare parasitosis in county mures, Romania
A1 - Barabas,E.C.
A1 - Cretu,C.M.
A1 - Mihilescu,P.
A1 - Satan,E.
Y1 - 2013///
N1 - 71210956
English
Journal: Conference Abstract
KW - EMBASE
KW - toxocariasis
KW - parasitosis
KW - Romania
KW - Society
KW - microbiology
KW - Human
KW - Child
KW - dog
KW - Toxocara
KW - rural area
KW - coughing
KW - Soil
KW - central nervous system
KW - Toxocara canis
KW - nematode
KW - ingestion
KW - visceral larva migrans syndrome
KW - Infection
KW - Anemia
KW - Diagnosis
KW - Larva Migrans
KW - Western blotting
KW - zygote
KW - eosinophil count
KW - liver
KW - cat
KW - Toxocara cati
KW - erythrocyte count

KW - hypereosinophilia
KW - Leukocyte Count
KW - laboratory
KW - pet animal
KW - meal
KW - vegetable
KW - Visual Acuity
KW - personal hygiene
KW - contamination
KW - Fruit
KW - Lung
KW - genus
KW - enzyme linked immunosorbent assay
KW - albendazole
KW - immunoglobulin E
KW - immunoglobulin G
KW - anthelmintic agent
RP - NOT IN FILE
SP - 4
EP - 5
JF - Acta Microbiologica et Immunologica Hungarica
VL - Conference: Annual Meeting of the Hungarian Society for Microbiology 2012 Keszthely Hungary. Conference Start: 20121024 Conference End: 20121026. Conference Publication:
IS - var.pagings
N2 - The larval forms of nematodes belonging to the genus Toxocara causing infection in humans are: Toxocara canis (specific to dogs), Toxocara cati (specific to cats). They lead to syndromes called Larva migrans visceralis (LMV) or Larva migrans ocularis (LMO), due to ingestion of viable, embryonated eggs transmitted by soil. The LMV syndrome appears in children under 5 years and the most affected organs are the liver, lungs and Central Nervous System (CNS). The LMO is present in children aged between 5- 10 years, and is characterised by unilateral ocular impairment. We review the cases of children suspected of Toxocara infection. Each case came from rural area and they all were less than 8 years old. Two cases were associated with decreasing of the sight and three with cough and expectoration. One child was detected accidentally, without having any symptoms. No CNS implication was evidenced. The clinical findings were not specific, laboratory results showed increased white blood cell counts with hypereosinophilia and lower red blood cell counts. In one case severe anaemia was detected. The increased IgE titers were guiding us to the diagnosis of toxocariasis, wich was confirmed by specific IgG anti- Toxocara ELISA and by Western Blot. Using the ELISA test, titers over 1,1 UI/ml were considered as positive results. Appropriate treatment was based on Albendazol, with good compliance. The normalised eosinophil counts and disappearing of symptoms showed improving of their status. In conclusion, toxocariasis has to be considered in children from rural area with coughing and expectoration or visual acuity problems. This disease can be prevented by strictly respecting personal hygiene measures, by avoiding contamination of soil and playgrounds designated for children, by washing fruits and vegetables before use, by thorough hand wash before meals, by avoiding children's geophagy, applying regulate anthelminthic treatment of pets, especially for dogs under 6 month
SN - 1217-8950
AD - (Barabas) Department of Pharmacology, Clinical Farmacology and Microbiology, University for Medicine and Pharmacy, Targu Mures, Romania (Cretu) Parasitology Department, University Carol Davila, Bucharest, Romania (Mihilescu) Parasitology, Eco-Para-Diagnostic Ltd., Bucharest, Romania (Satan) Bacteriology Unit, Emergency County Hospital, Targu Mures, Romania
ER -

TY - JOUR
ID - 3288
T1 - Regional implementation of a pediatric cardiology chest pain guideline using SCAMPs methodology
A1 - Angoff,G.H.
A1 - Kane,D.A.
A1 - Giddins,N.
A1 - Paris,Y.M.

A1 - Moran,A.M.
A1 - Tantengco,V.
A1 - Rotondo,K.M.
A1 - Arnold,L.
A1 - Toro-Salazar,O.H.
A1 - Gauthier,N.S.
A1 - Kanevsky,E.
A1 - Renaud,A.
A1 - Geggel,R.L.
A1 - Brown,D.W.
A1 - Fulton,D.R.
Y1 - 2013///
N1 - 2013632591
English
Journal: Article
KW - EMBASE
KW - Adolescent
KW - Adult
KW - algorithm
KW - Ambulatory Care
KW - anamnesis
KW - aorta valve regurgitation/di [Diagnosis]
KW - article
KW - blood analysis
KW - cardiopulmonary exercise test
KW - Child
KW - clinical effectiveness
KW - clinical practice
KW - controlled study
KW - coronary artery anomaly
KW - dizziness
KW - dyspnea
KW - electrocardiogram
KW - faintness
KW - family history
KW - Female
KW - fever
KW - health care planning
KW - heart atrium septum defect/di [Diagnosis]
KW - heart left ventricle hypertrophy/di [Diagnosis]
KW - heart murmur
KW - heart palpitation
KW - heart right ventricle hypertrophy/di [Diagnosis]
KW - Human
KW - incidental finding
KW - major clinical study
KW - Male
KW - medical practice
KW - Methodology
KW - mitral valve prolapse/di [Diagnosis]
KW - patent foramen ovale/di [Diagnosis]
KW - patient care
KW - Patient Selection
KW - pericarditis
KW - Physical Examination
KW - practice guideline
KW - priority journal

KW - school child
KW - standardized clinical assessment and management plans
KW - symptomatology
KW - thorax pain
KW - thorax radiography
KW - urinalysis
KW - C reactive protein/ec [Endogenous Compound]
RP - NOT IN FILE
SP - e1010
EP - e1017
JF - Pediatrics
VL - 132
IS - 4
CY - United States
N2 - Background and objectives: Chest pain is a complaint for which children are frequently evaluated. Cardiac causes are rarely found despite expenditure of considerable time and resources. We describe validation throughout New England of a clinical guideline for cost-effective evaluation of pediatric patients first seen by a cardiologist for chest pain using a unique methodology termed the Standardized Clinical Assessment and Management Plans (SCAMPs). Methods: A total of 1016 ambulatory patients, ages 7 to 21 years initially seen for chest pain at Boston Children's Hospital (BCH) or the New England Congenital Cardiology Association (NECCA) practices, were evaluated by using a SCAMPs chest pain guideline. Findings were analyzed for diagnostic elements, patterns of care, and compliance with the guideline. Results from the NECCA practices were compared with those of Boston Children's Hospital, a regional core academic center. Results: Two patients had chest pain due to a cardiac etiology, 1 with pericarditis and 1 with an anomalous coronary artery origin. Testing performed outside of guideline recommendations demonstrated only incidental findings. Patients returning for persistent symptoms did not have cardiac disease. The pattern of care for the NECCA practices and BCH differed minimally. Conclusions: By using SCAMPs methodology, we have demonstrated that chest pain in children is rarely caused by heart disease and can be evaluated in the ambulatory setting efficiently and effectively using minimal resources. The methodology can be implemented regionally across a wide range of clinical practice settings and its approach can overcome a number of barriers that often limit clinical practice guideline implementation. 2013 by the American Academy of Pediatrics
SN - 0031-4005
AD - (Angoff, Gauthier) Department of Pediatrics, Dartmouth-Hitchcock Medical Center, Geisel School of Medicine at Dartmouth, Lebanon, NH, United States (Kane, Tantengco) Department of Pediatrics, UMass Medical Center, University of Massachusetts Medical School, Worcester, MA, United States (Giddins) Department of Pediatrics, Fletcher Allen Health Care, University of Vermont Medical School, Burlington, VT, United States (Paris) Department of Pediatrics, Baystate Medical Center, Springfield, MA, United States (Paris, Moran) Department of Pediatrics, Tufts University School of Medicine, Boston, MA, United States (Moran) Department of Pediatrics, Maine Medical Center, Portland, ME, United States (Rotondo) Department of Pediatrics, Hasbro Children's Hospital, Warren Alpert Medical School at Brown University, Providence, RI, United States (Arnold) Department of Pediatrics, Harvard Vanguard Medical Associates, Boston, MA, United States (Toro-Salazar) Department of Pediatrics, Connecticut Children's Medical Center, University of Connecticut, Hartford, CT, United States (Kanevsky, Renaud, Geggel, Brown, Fulton) Department of Pediatrics, Division of Pediatric Cardiology, Boston Children's Hospital, Boston, MA, United States (Geggel, Brown, Fulton) Department of Pediatrics, Harvard Medical School, Boston, MA, United States (Fulton) Cardiology Outpatient Services, Department of Cardiology, Children's Hospital Boston, 300 Longwood Ave, Boston, MA 02115, United States
ER -

TY - JOUR
ID - 3290
T1 - Comparing bodily pain in caregivers of children with cerebral palsy affecting arms and legs versus predominantly legs
A1 - Luo,R.
A1 - Semel,J.
A1 - Youssef,J.
Y1 - 2013///

N1 - 71205858
English
Journal: Conference Abstract
KW - EMBASE
KW - Human
KW - Leg
KW - Cerebral Palsy
KW - Rehabilitation
KW - Child
KW - Arm
KW - physical medicine
KW - caregiver
KW - Pain
KW - pain assessment
KW - health survey
KW - Health
KW - wellbeing
KW - Education
KW - health care need
KW - anticipatory guidance
KW - patient
KW - disease course
KW - Autism
KW - physiotherapy
KW - university hospital
KW - outpatient
KW - community hospital
KW - developmental disorder
RP - NOT IN FILE
SP - S193
EP - S194
JF - PM and R
VL - Conference: 2013 Annual Assembly of the American Academy of Physical Medicine and Rehabilitation National Harbor, MD United States. Conference Start: 20131003 Conference End: 20131006. Conference Publication:
IS - var.pagings
N2 - of caregivers of children with developmental disabilities. Design: 200 caregivers were surveyed using the SF36 health survey and demographic questions from the National Survey of Children with Special Healthcare Needs. The SF36 Health Survey employs 36 questions to assess a subject's functional health and wellbeing in 8 health domains. The SF-36 bodily pain scale was used to assess the magnitude and interference of bodily pain. How much bodily pain have you had during the past 4 weeks? (1-none, 2-very mild, 3-mild, 4-moderate, 5-severe, 6-very severe) During the past 4 weeks how much did pain interfere with your normal work (Including both work outside the home and housework)? (1- not at all, 2-a little bit, 3-moderately, 4-quite a bit, 5-extremely) Setting: Two specialty care centers - one in a community hospital and a second in an outpatient university hospital setting Participants: 200 caregivers of children with predominantly autism or cerebral palsy participated. Interventions: n/a. Main Outcome Measures: SF-36 bodily pain scale. Results or Clinical Course: How much bodily pain have you had during the past 4 weeks? (1-none, 2-very mild, 3-mild, 4- moderate, 5-severe, 6-very severe) Average score caregivers of children with CP affecting arms and legs = 3.0; legs only 2.3. During the past 4 weeks how much did pain interfere with your normal work (Including both work outside the home and housework)? (1-not at all, 2-a little bit, 3-moderately, 4-quite a bit, 5- extremely) Average score caregivers of children with CP affecting arms and legs = 2.3; legs only 1.7. Conclusions: Our results indicate that caregivers of patients with CP affecting both arms and legs have more bodily pain when compared to CP affecting legs-only. We propose that this is likely due to the necessity of active repetitive lifting of these children, over many years versus having the option of providing support through less strenuous means, as is the case with CP affecting only the legs. We propose that anticipatory guidance through physical therapy and education in proper lifting technique from an early age for caregivers would improve their pain. Further studies should validate our findings

SN - 1934-1482
AD - (Luo) Stony Brook University, School of Medicine, Stony Brook, NY, United States
ER -

TY - JOUR
ID - 3291
T1 - Fecal impaction or infection? Baclofen pump infection without fever: A case report
A1 - Shieh,E.
A1 - Dalal,P.B.
A1 - McManus,M.
Y1 - 2013///
N1 - 71205854
English
Journal: Conference Abstract
KW - EMBASE
KW - Infection
KW - fever
KW - pump
KW - Human
KW - Case Report
KW - physical medicine
KW - Rehabilitation
KW - feces impaction
KW - patient
KW - Therapy
KW - spasticity
KW - implantation
KW - Rett syndrome
KW - physician
KW - Female
KW - rib
KW - book
KW - Streptococcus agalactiae
KW - stomach tube
KW - aspiration
KW - egg
KW - intrathecal drug administration
KW - catheter
KW - needle
KW - computer assisted tomography
KW - diseases
KW - abdominal swelling
KW - Liquid
KW - pediatrician
KW - Hospital
KW - disease course
KW - pediatric hospital
KW - tertiary health care
KW - urine
KW - reimplantation
KW - abscess
KW - dystonia
KW - liquorrhea
KW - Pain
KW - Child
KW - abdominal radiography
KW - baclofen

KW - diazepam
KW - ampicillin
RP - NOT IN FILE
SP - S192
JF - PM and R
VL - Conference: 2013 Annual Assembly of the American Academy of Physical Medicine and Rehabilitation National Harbor, MD United States. Conference Start: 20131003 Conference End: 20131006. Conference Publication:
IS - var.pagings
N2 - Case Description: An 18-year-old female with a history of Rett Syndrome and secondary spasticity with baclofen pump placement in January 2007. She presented to her pediatrician with 1.5 months of abdominal swelling without fever, change in tone, or recent illness. An abdominal x-ray was ordered and showed a moderate stool burden. The patient received multiple clean-outs and manual disimpaction without any improvement. She was seen by numerous subspecialists in the subsequent weeks without any additional symptoms. At her scheduled baclofen pump refill, the patient had an unchanged exam aside from a small egg sized mass under her right ribs. During the refill, the physician was unable to access the pump even with the longest needle. An abdominal CT scan was obtained, which showed a 6.5 x 8.0 x 10.0 cm abscess surrounding the baclofen pump. Setting: Tertiary care pediatric hospital Results or Clinical Course: Upon admission to the hospital, surgical exploration of the site revealed a large purulent fluid collection, requiring removal of the baclofen pump. Post-operatively, baclofen and diazepam were administered via her gastrostomy tube to prevent acute withdrawal. Labs eventually revealed group B streptococcus in her aspirate, urine, and vaginal swab cultures. She was treated with ampicillin and gradually improved to her baseline. The patient was discharged home on oral baclofen and has been scheduled for pump reimplantation this month. Discussion: Intrathecal baclofen (ITB) therapy has been a common treatment for spasticity and dystonia for many years. We are continuing to understand the complications of ITB including cerebrospinal fluid leakage, catheter malfunction, and infection. The incidence of infection has decreased significantly over the last 10 years as implantation techniques have improved. Despite the relatively low incidence of infection, particularly outside of the initial implantation time window, it must be considered as part of any differential when patients present with abdominal distention or pain. This case is especially unusual because the child did not demonstrate any additional infectious symptoms such as change in activity, fever, or increased tone. Conclusions: Caution must be taken when evaluating patients with ITB therapy and abdominal distention, as infection must always be on the differential
SN - 1934-1482
AD - (Shieh) TJUH/A.I. DuPont Hospital for Children, Philadelphia, PA, United States
ER -

TY - JOUR
ID - 3292
T1 - Adolescents and caffeine containing beverages: To take or not to take energy drinks?
A1 - Santangelo,B.
A1 - Lapolla,R.
A1 - D'Altilia,N.
A1 - Di,Ninno F.
A1 - Guida,A.
A1 - Merla,R.
A1 - Pizzolorusso,I.
A1 - Gorgoglione,S.
A1 - Calabrese,C.
A1 - Pacilio,A.
A1 - Lotti,F.
A1 - Pettoello-Mantovani,M.
A1 - Campanozzi,A.
Y1 - 2013///
N1 - 71204598
English
Journal: Conference Abstract
KW - EMBASE

KW - Human
KW - beverage
KW - energy drink
KW - Adolescent
KW - coffee
KW - athletic performance
KW - Child
KW - meal
KW - seizure
KW - mania
KW - flavor
KW - student
KW - questionnaire
KW - Drinking
KW - Self Report
KW - pediatrician
KW - weight reduction
KW - recommended drug dose
KW - Population
KW - sudden death
KW - school
KW - Exercise
KW - consumer
KW - maximum permissible dose
KW - cerebrovascular accident
KW - caffeine
RP - NOT IN FILE
SP - e275
EP - e276
JF - Digestive and Liver Disease
VL - Conference: 20th National SIGENP Congress Ancona Italy. Conference Start: 20131010 Conference End: 20131012. Conference Publication:
IS - var.pagings
N2 - Energy drinks (EDs) are beverages marketed to improve energy, weight loss, attention, athletic performance, and concentration. Caffeine is the main active ingredient in EDs; many of them contain 70-80mg of caffeine for a 33 cl serving. Heavy caffeine consumption, such as drinking EDs, has been associated with serious consequences such as seizures, mania, stroke, and sudden death. Adolescent's and child's caffeine consumption should not exceed 100 mg/day and 2.5 mg/kg/day, respectively. The daily caffeine's intake should take account of all sources of this substance (coffee, tea, soft and energy drinks). Objective: (1) To investigate the extent of energy drink consumption in adolescents; (2) to understand the reasons why teens' consumption is widespread; (3) to estimate the amount of daily caffeine taken from coffee and beverages containing caffeine (including EDs). Methods: A self-report questionnaire related to EDs' consumption has been developed and then proposed to 1232 students aged 12-19 years old (mean age 14.8+3.8). The data have been collected and then analyzed. Results: According to our surveys, EDs are consumed by 378/1232 (30.7%) of subjects; among them 27/378 (7.1%) drink these beverages 1 time/day at least, 122/378 (32.3%) 1-2 times/week and 179/378 (47.3%) 1-2 times/month. Most of them, 341/378 (90.2%) admit to consume EDs for the flavour, 147/378 (38.9%) find them refreshing, 68/378 (18%) and 39/378 (10.3%) declare to achieve an improvement in sports performance and intellectual activity, respectively. 270/378 (71.4%) of participants declare to consume these beverages when outside, 106/378 (28%) at meals, 74/378 (19.6%) in front of TV/PC, only 39/378 (10.3%) at school and 38/378 (10%) during or after physical exercise. We have also evaluated the amount (in mg) of caffeine (from coffee and caffeinated beverages) taken daily. Among consumers of caffeine (891/1232 - 72.3%), 495/891 (55.5%) consume less than the daily maximum level allowed, 265/891 (29.7%) consume between 100 and 200 mg/day of caffeine and 131/891 (14.7%) exceed the maximum dose, consuming daily more than 200mg and up to 300 mg/day. Conclusions: Our data show that, among a pediatric population consuming caffeine, 396/891 of subjects (44.4%) exceeded the maximum recommended dose. Pediatricians should be aware of the possible effects of EDs and screen for their consumption to educate children and their families to decrease or eliminate the inappropriate use of these beverages

SN - 1590-8658
AD - (Santangelo, Lapolla, D'Altilia, Di Ninno, Guida, Merla, Pizzolorusso, Gorgoglione, Calabrese, Pacilio, Lotti, Pettoello-Mantovani, Campanozzi) Department of Pediatrics, University of Foggia, Foggia, Italy
ER -

TY - JOUR
ID - 3293
T1 - Heavy metal burdens of public primary school children in Ibadan north-west local government area, Nigeria related to playground soils and classroom dusts
A1 - Ademuyiwa,O.
A1 - Akinwunmi,F.
A1 - Atobatele,Z.
A1 - Adewole,O.
A1 - Odekunle,K.
A1 - Arogundade,L.
A1 - Odukoya,O.O.
Y1 - 2013///
N1 - 71201160
English
Journal: Conference Abstract
KW - EMBASE
KW - primary school
KW - school child
KW - Human
KW - Government
KW - Nigeria
KW - Soil
KW - Dust
KW - organization
KW - toxicology
KW - Blood
KW - Child
KW - school
KW - urinalysis
KW - urine
KW - rural area
KW - urban area
KW - atomic absorption spectrometry
KW - heavy metal
KW - iron
KW - zinc
KW - metal
RP - NOT IN FILE
SP - S107
EP - S108
JF - Toxicology Letters
JA - Toxicol Lett
VL - Conference: 49th Congress of the European Societies of Toxicology, EUROTOX 2013 Interlaken Switzerland. Conference Start: 20130901 Conference End: 20130904. Conference Publication:
IS - var.pagings
N2 - Information about heavy metal intake of children in Nigeria related to playground soils and classroom dusts compared to economically advanced countries is lacking. Playground soil and classroom dust samples were therefore collected from 8 public primary schools - 6 urban and 2 semi-rural - located within Ibadan North-West Local Government Area of Oyo State in Nigeria. Blood and spot urine samples were also collected from the pupils of the selected schools (n = 253). The samples were analysed for Pb, Cu, Zn, Fe and Mn by flame atomic absorption spectrophotometry. Higher Pb levels were detected in blood of the children (mean blood Pb level of 57.40+28.56mug/dl) as well as in dust and soil samples from the 6 schools located in urban areas while

lower levels were obtained in the other 2 schools located in semi-rural area (mean blood Pb of 17.43+8.54mug/dl). Mean metal concentrations in the samples were as follows: classroom dusts - Pb 54.71+36.91, Cu 26.71+29.67, Zn 413.07+324.33, Fe 7828.30+2852.83, Mn 96.46+10.88 mg/kg; playground soils - Pb 45.76+36.23, Cu 18.29+16.69, Zn 368.97+425.73, Fe 7633.40+4191.23, Mn 92.70+7.87 mg/kg; blood - Pb 36.23+29.43mug/dl, Cu 0.67+0.21, Zn 3.50+1.42, Fe 272.09+115.87, Mn 0.07+0.05mug/ml and urine - Pb 0.42+0.36, Cu 0.07+0.05, Zn 0.37+0.38, Fe 0.32+0.33, Mn 0.01+0.02mug/ml. No correlation was observed between blood and dust metals of these children. A positive correlation (r = 0.168, p = 0.008)washowever observed between blood Pb and playground soil. These suggest that heavy metal accumulation in these children might be from their schools' playgrounds and other sources
SN - 0378-4274
AD - (Ademuyiwa, Atobatele, Adewole, Odekunle) Department of Biochemistry, Abeokuta, Nigeria (Akinwunmi, Arogundade, Odukoya) Department of Chemistry, Federal University of Agriculture, Abeokuta, Nigeria
ER -

TY - JOUR
ID - 3294
T1 - School-a safe place to learn and play?
A1 - Thomas,E.
A1 - Dyas,J.
A1 - Krishna,C.V.
A1 - Thompson,J.P.
Y1 - 2012///
N1 - 71197406
English
Journal: Conference Abstract
KW - EMBASE
KW - school
KW - Human
KW - Child
KW - exposure
KW - Plant
KW - Learning
KW - Hospital
KW - Education
KW - United Kingdom
KW - Learning Environment
KW - nut
KW - plant seed
KW - primary school
KW - plant leaf
KW - boy
KW - Male
KW - Government
KW - flower
KW - Risk Assessment
KW - toxicity
KW - girl
KW - Female
KW - rat
KW - student
KW - intoxication
KW - Prevention
KW - non profit organization
KW - Food
KW - poison
KW - sodium hydroxide

KW - toxic substance
KW - adrenalin
RP - NOT IN FILE
SP - 298
JF - Clinical Toxicology
VL - Conference: 2012 International Congress of the European Association of Poisons Centres and Clinical Toxicologists, EAPCCT 2012 London United Kingdom. Conference Start: 20120525 Conference End: 20120601. Conference Publication:
IS - var.pagings
N2 - Objective: The foundation stage/phase was introduced into the primary schools of England and Wales in September 2008. Government guidelines recommend a whole school approach (ages 3 - 11) to an ethos of learning which includes using the outdoors as a learning environment alongside traditional classroom-based education. Students are encouraged to learn by direct, hands-on experience through play and pupil-led investigations and consequently they have access to a much larger variety of potentially toxic agents and plants. Methods: Enquiries to NPIS between 1st September 2009 and 31st August 2011 relating to exposures of children (ages 3 - 11) to potentially toxic materials in schools were analysed with regard to type of material involved and severity of symptoms. Results: A total of 574 enquiries were received during the study period, 37.5% involving girls and 61.5% involving boys. Children aged five were the subject of the highest number (17.6%) of enquiries. The most common agents involved in exposures to pupils were plants (leaves, flowers, seeds and nuts), low toxicity miscellaneous products, chemicals, ice packs (food), cleaning products and pharmaceuticals. Although the majority of exposures resulted in minimal symptoms only, 12.7% of enquiries from schools required referral to hospital for treatment and a further 24% of enquiries were from hospital where the child had already been admitted. Some of the more serious cases involved exposure to agents such as isopropyl methyl sodium hydroxide, unused EpiPens (epinephrine auto-injector) and rat bait. Conclusion: Schools are encouraged to provide a new learning experience for pupils but should ensure that a comprehensive risk assessment is carried out on all materials, including plants, to which children might become exposed. Do NPIS centre staff have a role to play in providing expert poisoning-prevention education to schools
SN - 1556-3650
AD - (Thomas, Dyas, Krishna, Thompson) National Poisons Information Service (Cardiff), Cardiff and Vale University Health Board, Cardiff, United Kingdom
ER -

TY - JOUR
ID - 3295
T1 - Environmental testing for lead in Accra, Ghana
A1 - Mysliwiec,R.
A1 - Altman,N.
A1 - Moye,E.
A1 - Youssouf,A.
A1 - Hoffman,R.S.
A1 - Soghoian,S.
Y1 - 2012///
N1 - 71197383
English
Journal: Conference Abstract
KW - EMBASE
KW - Ghana
KW - exposure
KW - Human
KW - Soil
KW - Risk
KW - Population
KW - Child
KW - Food
KW - Color
KW - Lead Poisoning
KW - inspector

KW - devices
KW - Mass Screening
KW - convenience sample
KW - gas
KW - Adult
KW - health hazard
KW - market
KW - Eating
KW - jewelry
KW - Environmental Health
KW - Environment
KW - herbal medicine
KW - Liquid
KW - risk reduction
KW - intoxication
KW - Risk Assessment
KW - hope
KW - poison
KW - Paint
KW - metal
KW - Gasoline
KW - sulfur
KW - toxin
RP - NOT IN FILE
SP - 292
JF - Clinical Toxicology
VL - Conference: 2012 International Congress of the European Association of Poisons Centres and Clinical Toxicologists, EAPCCT 2012 London United Kingdom. Conference Start: 20120525 Conference End: 20120601. Conference Publication:
IS - var.pagings
N2 - Objective: Lead is an established environmental toxin and the most common metal responsible for poisoning. Through the combination of environmental regulation and whole population screening, the incidence of lead poisoning has been greatly reduced in many countries world-wide. Similar approaches are now being adopted in West African countries like Ghana, which banned the sale of leaded gasoline in 2003. However such regulations are far from institutionalized and many adults and children remain at risk. Methods: This research was undertaken as a first step to identify common environmental sources of lead in Accra, Ghana, with the goal of developing testing and risk reduction strategies. Samples of soil, pottery, paint, and other objects from thoroughfares, playgrounds, markets, and gas stations were collected. Samples were taken from areas easily accessed by passersby, including children, in a convenience sample of neighborhoods suspected of being at greatest risk of lead exposure. Coordinates for each sample were determined using a handheld Global Position Satellite device and were marked on a standard map. Each sample was tested in an identical manner using Lab Inspector Lead Test Kits from Abotex, which uses a sulfur-based solution to produce a semiquantitative color change in the presence of lead. Results: Out of a total of 332 samples, there were 75 positive Results: 33 soil, 10 eating utensils, 14 building tiles that had fallen onto the road, 5 cans that had previously contained foodstuffs, 2 bowls with chipped paint, 5 pieces of metal jewelry, 2 paint chips scraped from walls, 2 pieces of solder/wire, one discarded battery, and one sample of liquid herbal medicine. Thirteen of the positive soil samples were taken from the sites of gasoline retailers. The majority of the positive samples were collected in less-developed residential neighborhoods and lower socioeconomic areas. Conclusion: The data presented above indicates that lead is likely present in the environment in Accra, Ghana, and exposure to the population is a concern. We advocate consideration of lead exposure in both individual and population-based environmental health risk assessments, and hope to begin widespread testing in areas where risks of exposure seem highest
SN - 1556-3650
AD - (Mysliwiec, Altman, Moye, Youssouf, Hoffman, Soghoian) Department of Emergency Medicine, NYU School of Medicine, Bellevue Hospital Center, New York, United States
ER -

TY - JOUR

ID - 3297
T1 - Twisted sisters: The carbon monoxide toxicity that wasn't
A1 - Olives,T.
Y1 - 2013///
N1 - 71192087
English
Journal: Conference Abstract
KW - EMBASE
KW - emergency health service
KW - toxicity
KW - toxicology
KW - Human
KW - Child
KW - exposure
KW - Diagnosis
KW - patient
KW - Hospital
KW - seizure
KW - vomiting
KW - mother
KW - headache
KW - Female
KW - leukocytosis
KW - intoxication
KW - nausea
KW - pill
KW - neurologic disease
KW - parent
KW - gait
KW - urine
KW - blood sampling
KW - feeding
KW - intensive care unit
KW - poison center
KW - Drug Therapy
KW - Case Report
KW - carbon monoxide
KW - antidepressant agent
KW - amfebutamone
KW - carboxyhemoglobin
KW - prochlorperazine
KW - radafaxine
KW - toxin
KW - mydriatic agent
RP - NOT IN FILE
SP - 647
JF - Clinical Toxicology
VL - Conference: 2013 Annual Meeting of the North American Congress of Clinical Toxicology, NACCT 2013 Atlanta, GA United States. Conference Start: 20130927 Conference End: 20131002. Conference Publication:
IS - var.pagings
N2 - Background: Clustered symptoms, such as headache, vomiting, and vague neurologic symptoms in carbon monoxide (CO) exposure, suggest a common toxin. Carboxyhemoglobin levels are often unavailable, and clinical suspicion often guides management. In this series, we report three sisters transferred from a rural hospital for CO toxicity. Their history betrayed the true exposure. Case report: 3 sisters ages 3, 5 and 5, were transferred from a rural hospital for CO toxicity. The mother reported 1 week of headaches in her children. The children described acute nausea and intractable vomiting on arrival to an aunt's home that afternoon, unchanged

by prochlorperazine. Relatives sharing space with the children denied similar symptoms. Outside workup revealed moderate leukocytosis and normal metabolic panels in all three patients. The elder children suffered brief seizures prior to transfer; additional medication exposures were denied. Accompanying blood samples were sent for carboxyhemoglobin content. On arrival, the patients were tachycardic but otherwise vitally normal. Both elder children were incontinent of urine. All were markedly mydriatic and variably agitated. All had gait instability; one child actively hallucinated. All remained altered until admission, nearly 10 hours past presumed exposure. Parents were pressed separately regarding additional history. The mother's fianceconfided that she was "on the highest dose" of bupropion XL; she admitted to finding this out of place. One of the 5-year olds endorsed finding the pills and feeding them to herself and her sisters. The children were admitted to the pediatric intensive care unit with child protective services and poison center input. Carboxyhemoglobin levels were normal, and the three were discharged the next day. 9 days later, bupropion and hydroxybupropion levels returned markedly elevated (Table). Case discussion: This cluster of pediatric bupropion overdoses was complicated by seizure activity and missed diagnosis. The initial diagnosis of CO toxicity led to transfer to a hyperbaric center, but serial inquiries by staff in light of an incongruous clinical setting led to the diagnosis. Early discordance between symptomatic patients and asymptomatic relatives was highly suggestive of an exposure other than CO, and subsequent history gathering made the diagnosis. Conclusion: History matters in the workup of potential exposures. This case demonstrates both the importance of accurate historytaking and the effect of premature closure in clinically significant bupropion toxicity. (Table Presented)
SN - 1556-3650
AD - (Olives) Hennepin County Medical Center, Minneapolis, MN, United States
ER -

TY - JOUR
ID - 3298
T1 - Death due to crotalid envenomation in a child treated with polyvalent immune Fab
A1 - Obafemi,A.I.
A1 - Gardner,M.
A1 - Young,A.C.
A1 - Morrissey,R.P.
Y1 - 2011///
N1 - 71192425
English
Journal: Conference Abstract
KW - EMBASE
KW - envenomation
KW - Child
KW - Human
KW - toxicology
KW - Death
KW - Blood
KW - patient
KW - snake
KW - laboratory
KW - petechia
KW - girl
KW - ankle
KW - Female
KW - pH
KW - puncture
KW - capillary
KW - stridor
KW - skin
KW - Resuscitation
KW - acute hemorrhagic pancreatitis
KW - cryoprecipitate
KW - Crotalus
KW - Case Report

KW - abnormal respiratory sound
KW - thrombocyte
KW - Mortality
KW - deterioration
KW - plasma
KW - fatality
KW - parent
KW - hematology
KW - consultation
KW - smear
KW - bleeding tendency
KW - hemolysis
KW - pediatric hospital
KW - hematocrit
KW - gastritis
KW - Cause of Death
KW - snakebite
KW - examination
KW - wound
KW - blood vessel
KW - anaphylaxis
KW - traffic and transport
KW - convulsion
KW - international normalized ratio
KW - hypertensive factor
KW - blood clotting factor 7
KW - poison
KW - Oxygen
KW - D dimer
KW - antihypertensive agent
KW - fibrinogen
KW - fructose bisphosphate
RP - NOT IN FILE
SP - 575
EP - 576
JF - Clinical Toxicology
VL - Conference: 2011 North American Congress of Clinical Toxicology Annual Meeting Washington, DC United States. Conference Start: 20110921 Conference End: 20110926. Conference Publication:
IS - var.pagings
N2 - Background: Crotalid bite fatalities are rare in the US: about 10 deaths per 7000 bites annually. No pediatric deaths have been reported since the advent of crotaline Fab. We report the death of a 23 month old girl due to crotalid envenomation. Case Report: A 23 month old girl screamed at a playground. Her parents found her near a small rattlesnake with blood on her left ankle. Within one minute she vomited, became unresponsive, and had generalized convulsions. EMS transported her to a pediatric hospital within two hours, receiving O 2 and IV NS en route. Initial VS were: BP, 106/85 mmHg; HR, 184/min; RR, 46/min; SpO₂, 100% on NRB; and GCS, 11 (E4 V3 M4). The patient had blood in her nares and mouth. Respirations were labored with coarse breath sounds and stridor. Capillary refill was sluggish. Skin was cool and pale with petechiae. Her medial left ankle had four puncture wounds. She was intubated within 20 minutes. Fresh blood was noted in the ETT. Laboratory studies revealed: WBC 33 k/mcL; Hct 22%; Plt 10 k/mcL; hemolysis on peripheral smear; INR 19; PTT > 300 s; fibrinogen < 70mcg/ml; D-dimer > 22 mcg/ml; FDP 320 mcg/ml; ABG: 7.09/31/116/-19. IV crotaline Fab was begun 3 hours post bite. The patient received 6 vials as well as 4 units PRBC, 5 units plasma, 2 units platelets, and 10 units cryoprecipitate. In PICU at 3.5 hours post bite she received factor VII per hematology consultation. She became hypotensive and acidotic (pH 6.9), requiring resuscitation with multiple vasopressors. Poison control was consulted 4.5 hours post bite and advocated repeat doses of crotaline Fab. Prior to a second dose the patient became pulseless. She was pronounced dead less than five hours post bite. Postmortem findings included: four fang marks anterior to the medial left malleolus; cutaneous and serous petechiae; and acute hemorrhagic pancreatitis and gastritis. The cause of death was listed as hemorrhagic

diathesis due to snake bites. Discussion: Pediatric death from snake envenomation is extremely rare in the US. We found no case reported in the literature since the advent of crotaline Fab. In this case the snake was not formally identified, but the history, examination and laboratory findings were most consistent with crotalid envenomation. The rapidity of systemic symptoms and clinical deterioration suggested envenomation directly into a blood vessel. The possibility of an anaphylactoid reaction could not be ruled out. Extended transportation time may have contributed to mortality, but it is unclear whether earlier treatment would have prevented death given the extreme presentation. Conclusion: Children who quickly develop systemic effects may succumb to crotalid envenomation despite comprehensive management including crotaline Fab
SN - 1556-3650
AD - (Obafemi, Gardner) North Texas Poison Center, Dallas, TX, United States (Young) UT SouthWestern Medical School, Dallas, TX, United States (Morrissey) Central Texas Poison Center at Scott and White, Temple, TX, United States
ER -

TY - JOUR
ID - 3299
T1 - A humanistic approach to paediatric radiation therapy - One family's journey
A1 - Collier,D.
Y1 - 2013///
N1 - 2013614894
English
Journal: Article
KW - EMBASE
KW - anaplastic ependymoma tumor/rt [Radiotherapy]
KW - anaplastic ependymoma tumor/su [Surgery]
KW - article
KW - brain tumor/rt [Radiotherapy]
KW - brain tumor/su [Surgery]
KW - cancer patient
KW - cancer radiotherapy
KW - cancer size
KW - cancer surgery
KW - Case Report
KW - Child
KW - childhood cancer/rt [Radiotherapy]
KW - childhood cancer/su [Surgery]
KW - clinical decision making
KW - clinical effectiveness
KW - Comprehension
KW - Counseling
KW - ependymoma
KW - Fatigue
KW - Female
KW - hormone substitution
KW - Human
KW - hypopituitarism
KW - Interpersonal Communication
KW - medical specialist
KW - medication compliance
KW - multimodality cancer therapy
KW - neurologist
KW - neurosurgeon
KW - nuclear magnetic resonance imaging
KW - occipital lobe
KW - patient care
KW - pediatric surgery
KW - Peer Group

KW - practice guideline
KW - priority journal
KW - psychologist
KW - radiation oncologist
KW - school child
KW - short term memory
KW - thyroxine blood level
KW - tumor cell
KW - Hydrocortisone
KW - thyroxine
RP - NOT IN FILE
SP - 306
EP - 310
JF - Radiography
VL - 19
IS - 4
CY - United Kingdom
N2 - Purpose: To present a patient view of a treatment journey, highlighting the strengths and the weaknesses from that experience and recognising a potential for Advanced Practice on behalf of Radiation Therapists. The patient was aged 7 when diagnosed in December 2007 with an anaplastic ependymona tumour in the occipital lobe and had surgery to effect maximal tumour resection. The pathology of the tumour upon removal indicated that it was a Grade III anaplastic ependymoma some 3cm across. In early 2008 the patient embarked on radiation therapy receiving 59.4Gy over 33 treatments. This case was an inaugural patient for a novel intervention which aimed at reducing anxiety and helping the patient to cope with paediatric radiotherapy. The intervention was outstandingly successful and the diversionary activities within the treatment regime undoubtedly added to the effectiveness of the treatment. The patient maintained near normal daily activities missing only the first couple of hours of school each day during the treatment period. The paper will cover this from that patient's perspective and then report on the post treatment impact of that treatment. It will present the patient's view and outcomes in the evolution of the patient management process and improved healthcare. Results: The paper identifies the important role of communication and understanding amongst all members of the team, including the patient. It comments on the need to recognise the full range of options available and the best practice guidelines which could be adopted. Above it touches on the impact the adoption of all of these has on the patient treatment journey. It opens up the question of the appropriate mechanisms of involvement for the patient in this journey and the associated decision making surrounding this involvement, and this paper specifically comments on this. It identifies other professionals' world-wide who are working on elements of this approach. Conclusion: The paper will highlight some significant areas of patient need following the weeks of treatment as the longer term impact and effects of treatment emerged. It focuses on recognition of the patient's place and how best to use multidisciplinary care as a two-way communication tool. It asks the profession to translate what appears to be a practice based outside of core resources into one which is central to effective support for the patient journey. It is a report on the patient feedback, on what is a successful and highly desirable programme and offers further comment on the longer term issues surrounding post treatment events and experience. 2013 The College of Radiographers
SN - 1078-8174
AD - (Collier) Australian Institute of Radiography, Australia
ER -

TY - JOUR
ID - 3301
T1 - Nutritional assessment of children and teenagers with overweight and/or obesity to metabolic syndrome predisposition
A1 - Freiberg,C.
A1 - Nascimento,D.
Y1 - 2013///
N1 - 71180715
English
Journal: Conference Abstract
KW - EMBASE

KW - Obesity
KW - metabolic syndrome X
KW - Human
KW - disease predisposition
KW - Adolescent
KW - Nutrition
KW - Child
KW - nutritional assessment
KW - Nutritional Status
KW - Physical Activity
KW - meal
KW - Population
KW - mother
KW - questionnaire
KW - gender
KW - nutrition education
KW - Health
KW - eating disorder
KW - eating habit
KW - vomiting
KW - family history
KW - patient
KW - Female
KW - adulthood
KW - Food Intake
KW - school
KW - cardiovascular disease
KW - Prevention
KW - Weight Control
KW - Prevalence
KW - Diabetes Mellitus
KW - habit
KW - hypercholesterolemia
KW - risk factor
KW - Cholesterol
RP - NOT IN FILE
SP - 1541
JF - Annals of Nutrition and Metabolism
VL - Conference: 20th International Congress of Nutrition Granada Spain. Conference Start: 20130915 Conference End: 20130920. Conference Publication:
IS - var.pagings
N2 - Background and objectives: The prevalence of metabolic syndrome is increasing worldwide, and has followed the global trend to obesity. It is essential to identify and control all the factors that make up metabolic syndrome both in adolescents and in children for the prevention of cardiovascular disease in adulthood. The aim of the study was to assess the nutritional status of children and adolescents who are overweight and/or obese and their predisposition to metabolic syndrome. Methods: We evaluated 100 patients, age 5 to 18 years, of both genders, in routine clinical care and that were diagnosed with overweight (BMI p 85-95) or obese (BMI p 95) second WHO/2007. We carried out anthropometric measurements, biochemical tests and questionnaires with the mothers. Results: The study showed that 87% had family history of disease related to nutritional status like hypercholesterolemia, diabetes mellitus and overweight/obesity; 42% started the overweight/obesity under the age of 5 years; 72% don't make any treatment for weight control; 32% eat their meals everyday in front of TV/computer, of these, 45% for more than 4 hours daily; 69% do not do any kind of physical activity outside of school; 57% of the population has no control food intake, consuming them anytime even without being hungry, and 38% eats until feeling sick; 11% have a habit of vomiting inducing after meals showing signs of eating disorders; 39% have high daily consumption of sweets; 74% have cholesterol levels increased considering that this is a population of minors and that many factors are present characteristic of the metabolic syndrome. Conclusions: To improve the health and nutritional status of children and adolescents is necessary to develop

strategies and activities that promote nutrition education, good eating habits and encourage regular physical activity. These actions contribute to the reduction of risk factors for metabolic syndrome
SN - 0250-6807
AD - (Freiberg, Nascimento) Universidade Guarulhos, Sao Paulo, Brazil
ER -

TY - JOUR
ID - 3302
T1 - Vitamin d status among families in Denmark: Baseline data from the vitmaD study
A1 - Madsen,K.H.
A1 - Mejborn,H.
A1 - Tetens,I.
A1 - Andersen,E.W.
A1 - Molgaard,C.
A1 - Andersen,R.
A1 - Rasmussen,L.B.
Y1 - 2013///
N1 - 71180515
English
Journal: Conference Abstract
KW - EMBASE
KW - Nutrition
KW - Denmark
KW - serum
KW - Prevalence
KW - leisure
KW - Chronic Disease
KW - model
KW - Physical Activity
KW - questionnaire
KW - Lifestyle
KW - blood sampling
KW - Adult
KW - Human
KW - Child
KW - cross-sectional study
KW - Health
KW - gender
KW - Bone
KW - vitamin D
KW - vitamin
KW - 25 hydroxyvitamin D
RP - NOT IN FILE
SP - 1446
JF - Annals of Nutrition and Metabolism
VL - Conference: 20th International Congress of Nutrition Granada Spain. Conference Start: 20130915 Conference End: 20130920. Conference Publication:
IS - var.pagings
N2 - Background and objectives: The beneficial effect of vitamin D in bone health is acknowledged and the vitamin has also been associated with several chronic diseases. It is therefore relevant to determine the prevalence of vitamin D insufficiency in different groups, and vitamin D statuses within families have not been studied previously. The objective of the present study was to evaluate serum 25-hydroxyvitamin D (25(OH)D) concentrations among families in Denmark (56 degreeN) after seasonal UVB peak and to ascertain determining factors. Methods: Cross-sectional study with 755 children and adults (4-60 y) recruited as families in the VitmaD study. Blood samples were collected in September-October 2010, and vitamin D status was measured as serum 25(OH)D concentration by LC-MS/MS. Vitamin D intake and life style factors were assessed in self-administered questionnaires. Determinants of vitamin D status were identified in a linear mixed model with

family as a random variable. Results: Mean (+SD) serum 25(OH)D concentration was 75 + 20 nmol/l (range 9-162 nmol/l) and only 10 % had 25(OH) D <50 nmol/l. Determinants of serum 25(OH)D were age (p=0.036), BMI class (p=0.001), multi vitamin use (p=0.033), sun behaviour (p=0.005), outdoor stay (p=0.033), sun vacation (p<0.001), and physical activity (p=0.040). Gender (p=0.692) and vitamin D intake (p=0.238) were not associated to serum 25(OH)D. Conclusions: The prevalence of vitamin D insufficiency among families in Denmark was low after seasonal UVB peak. Sun vacation was the strongest determinant for vitamin D status at this time of the year
SN - 0250-6807
AD - (Madsen, Mejborn, Tetens, Andersen, Rasmussen) Division of Nutrition, National Food Institute, Technical University of Denmark, Kongens Lyngby, Denmark (Andersen) Department of Applied Mathematics and Computer Science, Technical University of Denmark, Kongens Lyngby, Denmark (Molgaard) Department of Nutrition, Exercise and Sports, Faculty of Science, University of Copenhagen, Copenhagen, Denmark
ER -

TY - JOUR
ID - 3303
T1 - The importance of having interest in cooking and vegetables and fruit intake for chewing consciousness in primary schoolchildren
A1 - Tanaka,N.
A1 - Okuda,T.
A1 - Shinohara,H.
A1 - Hamaguchi,I.
A1 - Asano,Y.
A1 - Higashine,Y.
A1 - Kang,J.
A1 - Wakimoto,K.
A1 - Hirano,N.
Y1 - 2013///
N1 - 71179390
English
Journal: Conference Abstract
KW - EMBASE
KW - Cooking
KW - mastication
KW - Consciousness
KW - vegetable
KW - Human
KW - school child
KW - Fruit
KW - Nutrition
KW - habit
KW - Learning
KW - questionnaire
KW - Child
KW - Motivation
KW - multiple regression
KW - chi square test
KW - contingency table
KW - statistical analysis
KW - Health
KW - Japan
KW - Eating
KW - primary school
KW - Female
KW - meal
KW - Self Concept
KW - sport

KW - dependent variable
KW - Male
RP - NOT IN FILE
SP - 898
EP - 899
JF - Annals of Nutrition and Metabolism
VL - Conference: 20th International Congress of Nutrition Granada Spain. Conference Start: 20130915 Conference End: 20130920. Conference Publication:
IS - var.pagings
N2 - Background and objectives: The chewing ability is one of important factors for good health and thus for developing and educating dietary good habits. Recently, the decrease in chewing abilities of children is discussed. We examined their chewing consciousness and its relation to daily and dietary habits through questionnaire survey. Methods: Questionnaire survey about daily and dietary habits was done in 592 children (294 males and 298 females, 11- 12 years old) from four primary schools at the Kansai area in Japan. When high score was allotted in their better habits, there were significantly positive correlations between gCC: chewing consciousness and gHIC: having interest in cooking h or gFVFI: frequency of vegetables and fruit intake h. According to each category score, they were divided into high and low score groups. Statistical analysis in contingency tables was done by chi-square test. And then the multiple regression analysis, as CC or gLMLA: learning motivation and learning attitude h with a dependent variable, was done. Results: In the higher score group of HIC, scores of FVFI, CC, and LMLA were significantly higher than those in the lower score group of HIC. In the higher score of FVFI, scores of HIC, g playing sports and studying well h, and also CC were significantly higher than those in its lower score group of FVFI. The multiple regression analysis, as CC with a dependent va riable, indicated that CC was affected by HIC and FVFI with 27 % coefficient of determination. Also, LMLA was affected by FVFI, g self-efficacy h, g refrain from between meals and eating outside h, and CC with 23% coefficient of determination. Conclusions: The present analytical results indicated that the chewing consciousness was affected by having interest in cooking and frequency of vegetables and fruit intake, and the learning motivation and learning attitude was affected by FVFI and CC
SN - 0250-6807
AD - (Tanaka) Faculty of Home Economics, Kobe Women's University, Kobe, Japan (Okuda) Faculty of Human Sciences, Tezukayamagakuin University, Sakai, Japan (Shinohara) Faculty of Education and Culture, University of Miyazaki, Miyazaki, Japan (Hamaguchi) Faculty of Human Sciences, Konan Women's University, Kobe, Japan (Asano) Faculty of Health Science, Kio University, Nara Pref, Japan (Higashine) Department of Health and Nutrition, Osaka Aoyama University, Minoo, Japan (Kang) Department of Nutrition, Hyogo NCC College, Nishinomiya, Japan (Wakimoto) Nishitani Primary School, Takarazuka, Japan (Hirano) Department of Food and Nutrition Science, Kobe Women's Junior College, Kobe, Japan
ER -

TY - JOUR
ID - 3304
T1 - The informas framework for monitoring food marketing to children
A1 - Kelly,B.
A1 - King,L.
A1 - Baur,L.
A1 - Rayner,M.
A1 - Lobstein,T.
A1 - Monteiro,C.
A1 - Macmullan,J.
A1 - Mohan,S.
Y1 - 2013///
N1 - 71179367
English
Journal: Conference Abstract
KW - EMBASE
KW - Monitoring
KW - Marketing
KW - Food
KW - Public Health

KW - Child
KW - Nutrition
KW - Human
KW - alcoholic beverage
KW - Television
KW - Malaysia
KW - Philippines
KW - China
KW - Income
KW - exposure
KW - advertizing
KW - Internet
KW - Mongolia
KW - World Health Organization
KW - Environment
RP - NOT IN FILE
SP - 887
JF - Annals of Nutrition and Metabolism
VL - Conference: 20th International Congress of Nutrition Granada Spain. Conference Start: 20130915 Conference End: 20130920. Conference Publication:
IS - var.pagings
N2 - Background and objectives: The International Network for Food and Obesity/NCD Research, Monitoring and Action Support (INFORMAS) is setting benchmarks for creating healthy food environments. This paper describes the framework for assessing the extent of children's exposure to food marketing and the nature of these promotions. A standardised framework is needed to allow comparisons between countries, between companies and between different time periods. Methods: A review of studies measuring the nature and extent of food marketing was undertaken to identify approaches to monitoring across media platforms. This was supplemented with detailed methods used by the authors in national and international monitoring studies since 2007 to describe food marketing across multiple media, including television, print, internet and outdoor advertising. Results: Food marketing studies indicate children in both high and low/middle income countries are exposed to large volumes of unhealthy food promotions on different media. Active monitoring using a standard assessment tools is currently underway in a range of countries, including China, Mongolia, Malaysia, Philippines and these 2012/3 data will be released. A step-wise approach to monitoring food marketing is proposed, suitable for differing levels of engagement and resources. This framework includes monitoring activities that assess the frequency of food promotions across different media and time points, and measures of persuasive appeal of promotions. Conclusions: Globally, children are exposed to high volumes of unhealthy food promotions. The monitoring framework proposed here will assist in the implementation of the World Health Organization's set of recommendations on the marketing of foods and non-alcoholic beverages to children, which recognise the need for reducing the impact of unhealthy food marketing to children
SN - 0250-6807
AD - (Kelly) School of Molecular Bioscience, University of Sydney, Sydney, Australia (King, Baur) Prevention Research Collaboration, University of Sydney, Sydney, Australia (Rayner) British Heart Foundation Health Promotion Research Group, University of Oxford, Oxford, United Kingdom (Lobstein) International Association for the Study of Obesity, London, United Kingdom (Monteiro) School of Public Health, University of Sao Paulo, Sao Paulo, Brazil (Macmullan) Consumers International, London, United Kingdom (Mohan) Public Health Foundation of India, New Delhi, India
ER -

TY - JOUR
ID - 3305
T1 - Adherence to a new nordic diet in pregnancy is associated with reduced risk of excessive gestational weight gain
A1 - Hillesund,E.R.
A1 - Bere,E.
A1 - Haugen,M.I.
A1 - Overby,N.C.
Y1 - 2013///

N1 - 71178767
English
Journal: Conference Abstract
KW - EMBASE
KW - Diet
KW - Weight Gain
KW - Risk
KW - Nutrition
KW - Pregnancy
KW - Female
KW - Human
KW - Food
KW - bread
KW - pregnant woman
KW - kale
KW - Child
KW - root vegetable
KW - Fruit
KW - mother
KW - logistic regression analysis
KW - caloric intake
KW - milk
KW - potato
KW - berry
KW - fish
KW - food frequency questionnaire
KW - Cohort Analysis
KW - Wilderness
KW - grain
KW - oat
KW - risk factor
KW - model
KW - Eating
KW - Physical Activity
KW - Health
KW - Water
RP - NOT IN FILE
SP - 590
JF - Annals of Nutrition and Metabolism
VL - Conference: 20th International Congress of Nutrition Granada Spain. Conference Start: 20130915 Conference End: 20130920. Conference Publication:
IS - var.pagings
N2 - Background and objectives: The health properties of the New Nordic Diet (NND) are increasingly being investigated in response to awareness of sustainability and public interest in eating local and traditional foods. The purpose of the present study was to investigate the association of NND adherence with risk of excessive gestational weight gain (EGWG) in the Norwegian Mother and Child Cohort Study (MoBa). Methods: A diet score was constructed from relevant items of the food frequency questionnaire (FFQ) completed by pregnant women in the MoBa study in week 22 of pregnancy. The score comprises ten subscales summarizing meal pattern, intake of fruits, root vegetables, kale, potatoes, oatmeal porridge, whole grain bread versus white bread, foods from the wilderness (fish, game and berries), milk versus juice, and water versus soft drinks. Each subscale was dichotomized by the median and assigned a value of "0" or "1". Adding the subscales yielded a score from 0 to 10 with increasing score indicating higher adherence to the NND. EGWG was defined as gestational weight gain in excess of the range considered optimal and recommended by the Institute of Medicine (2009) according to pre pregnancy BMI. For analysis participants were grouped into "low", "medium" and "high" NND-adherence, and the relative risk of EGWG across groups was analyzed with multi-nominal logistic regression. Results: 56771 pregnant women were included in the study. Moving from low to high NND-adherence was associated with reduced odds of experiencing EGWG in the crude model (OR: 0.837; 95%CI:

0.795, 0.882) and after adjusting for energy intake, pre pregnancy BMI, length of gestation and physical activity level (OR: 0.875; 95%CI: 0.825, 0.928). Conclusions: Higher adherence to this a priori defined New Nordic Diet was associated with reduced risk of experiencing EGWG
SN - 0250-6807
AD - (Hillesund, Bere, Overby) Department of Public Health, Sports and Nutrition, University of Agder, Kristiansand, Norway (Haugen) Division of Environmental Medicine, Department of Food Toxicology, Norwegian Institute of Public Health, Oslo, Norway
ER -

TY - JOUR
ID - 3306
T1 - Recommendations for Vitamin D supplementation in infants and young children
A1 - De,Ronne N.
A1 - De,Schepper J.
Y1 - 2013///
N1 - 2013590309
French
Journal: Review
KW - EMBASE
KW - autoimmune disease/et [Etiology]
KW - Breast Feeding
KW - calcium metabolism
KW - Child
KW - controlled study
KW - disease association
KW - health care personnel
KW - high risk population
KW - Human
KW - Infant
KW - Lifestyle
KW - Nutrition
KW - pathophysiology
KW - phosphate metabolism
KW - pregnant woman
KW - prematurity
KW - preschool child
KW - review
KW - skin color
KW - sun exposure
KW - vitamin D deficiency/dt [Drug Therapy]
KW - Vitamin D Deficiency/et [Etiology]
KW - vitamin D deficiency/pc [Prevention]
KW - vitamin D deficiency/th [Therapy]
KW - vitamin intake
KW - vitamin supplementation
KW - Calcium
KW - phosphorus
KW - vitamin D/dt [Drug Therapy]
KW - vitamin D/po [Oral Drug Administration]
RP - NOT IN FILE
SP - 12
EP - 21
JF - Journal de Pharmacie de Belgique
IS - 3
CY - Belgium
N2 - Vitamin D deficiency occurs rather commonly among healthy pregnant women, newborns and young children, especially in certain risk groups. Since vitamin D plays a role in calcium and phosphor metabolism

essential for bone health and in the physiopathology of some autoimmune diseases it seems important to provide recommendations for prevention of vitamin D deficiency. Risk factors include maternal vitamin D deficiency, low intake of fortified food, eg. breastfeeding, low compliance of supplementation, dark skin, inadequate sun exposure, premature birth, overweight, living at high latitude. The aim of this paper is to summarize available data of vitamin D sources, known situations in which deficiency is common and published guidelines on vitamin D supplementation, and translate this information in recommendations for prevention of vitamin D deficiency in healthy paediatric population in Flanders. Infants should receive an oral supplementation of 400 IU/day of vitamin D from birth and this should be continued till the age of 6 years. In cases of dark skin the dose should be 600 IU/day. An healthy life style with outdoor activities and associated sun exposure and intake of fortified nutrition should be advised. The implementation should be promoted by all healthcare professionals working with young children
SN - 0047-2166
AD - (De Ronne) Medische Beleidscel Kind and Gezin, Avenue de la Porte de Hal 27, 1060 Bruxelles, Belgium (De Schepper) Endocrinologie Pediatrique, UZ Brussel, UZ Gent, Belgium
ER -

TY - JOUR
ID - 3307
T1 - Developing a new comprehensive psychosocial care program specifically tailored for gynecological oncology patients and their families
A1 - O'Meara,Y.
A1 - Coulter,J.
Y1 - 2012///
N1 - 71173850
English
Journal: Conference Abstract
KW - EMBASE
KW - psychosocial care
KW - oncology
KW - patient
KW - Human
KW - gynecologic cancer
KW - Society
KW - Female
KW - Ireland
KW - psychotherapist
KW - psychologist
KW - organization
KW - survival
KW - oncologist
KW - gynecologist
KW - cancer patient
KW - Hospital
KW - Anxiety
KW - city
KW - Child
KW - neoplasm
KW - Quality of Life
KW - national health service
RP - NOT IN FILE
SP - E445
JF - International Journal of Gynecological Cancer
VL - Conference: 14th Biennial Meeting of the International Gynecologic Cancer Society, IGCS 2012 Vancouver, BC Canada. Conference Start: 20121013 Conference End: 20121016. Conference Publication:
IS - var.pagings
N2 - Ireland currently has no access to an established psycho oncology service outside of the capital city, Dublin. Patients who attend their gynecologist or oncologist for treatment outside of Dublin depend on

voluntary organizations for their therapeutic support. These services work outside of the National Health Service, and have major limitations on what they can offer. Gynecological cancers have a direct impact on the lives of thousands of Irish women and their families. Annually this number is increasing. It has been well documented internationally that cancer patients who receive the appropriate emotional support are less likely to attend the hospital, feel psychologically stronger, higher compliance rate to treatment and thus increasing survival (Carlson, Bultz, 2002, 2003, 2004). A. Mitchell (2011) highlights the importance of a psychosocial intervention in directly improving anxiety, depression and quality of life in women with gynaecological cancer. We describe the establishment of a comprehensive psycho oncology service in the southern part of the country but unique to Ireland. It specifically meets the needs of gynaecological oncology patients, their families and significant others. We discuss the sourcing of therapists from a broad therapeutic background (systemic psychotherapists, psychologists, psychotherapist, psycho-sexual therapist, child psychologist, art therapists and play therapists). We explain the geographic challenges in meeting the patients' needs group. We outline a specific training module facilitated by clinicians from an interdisciplinary background addressing clinical, practical and emotional needs of this patient group. We examine the need for protocols and the logistics for those embarking on a similar journey
SN - 1048-891X
AD - (O'Meara) Department of Gynaecology, Mater University Hospital, Dublin, Ireland (O'Meara) Systemic Psychotherapy, Family Therapy Association of Ireland, Dublin, Ireland (Coulter) Obstetrics and Gynecology, South Infirmary Victoria University Hospital, Cork, Ireland (Coulter) Obstetrics and Gynecology, University College Cork, Cork, Ireland
ER -

TY - JOUR
ID - 3309
T1 - The radiation footprint on the pediatric trauma patient
A1 - Farzal,Z.
A1 - Wilson,S.
A1 - Brown,E.
A1 - Burkhalter,L.
A1 - Fischer,A.C.
Y1 - 2013///
N1 - 71167349
English
Journal: Conference Abstract
KW - EMBASE
KW - Human
KW - patient
KW - surgeon
KW - college
KW - childhood injury
KW - Radiation
KW - injury
KW - Risk
KW - Imaging
KW - radiation exposure
KW - Child
KW - Diagnostic Imaging
KW - neoplasm
KW - dosimetry
KW - neck
KW - gender
KW - radiation dose
KW - injury scale
KW - pediatric hospital
RP - NOT IN FILE
SP - S78
JF - Journal of the American College of Surgeons

VL - Conference: 99th Annual Clinical Congress of the American College of Surgeons Washington, DC United States. Conference Start: 20131006 Conference End: 20131010. Conference Publication:
IS - var.pagings
N2 - INTRODUCTION: The overuse of medical imaging has recently started an initiative to diminish radiation exposure particularly in children, given the risk of developing cancer. Our goal was to identify the current amount of radiation used for a pediatric trauma evaluation and correlate the levels of radiation with trauma activation status in order to identify the subset of patients most at risk. METHODS: We retrospectively evaluated pediatric trauma patients (N=1050) at a Level I independent children's hospital for all levels of trauma activation (Stats, Alerts, Consults) from June 2010 to January 2011. 215 patients had full dosimetry data available and were analyzed for demographics, mechanism of injury, injury severity score (ISS), imaging modalities, and radiation dosages in milliSieverts (mSv). RESULTS: The demographics include: gender (143M, 72F); age 5.5 yrs [<1-16]; average ISS of Consults, 7.7 [1,16]; Alerts, 8.8 [1,17]; and Stats, 17 [3,38]. Per Stat, the number of CTs ranged from 0-10 with 2-3 CTs in 35% and 4-10 CTs in 40% for one admission. The studies most often repeated were head CTs (45%), face/sinus CTs (13.8%), and neck CTs (10%). The average non-accidental trauma (NAT) Consult patient had four times as many CTs as the average trauma Consult patient. The majority (66%) of outside CTs delivered more radiation, of which 50.0% of the doses were at least double the pediatric dosage. CONCLUSIONS: The identified factors associated with the most radiation include suspected NATs, Stat activations, and outside imaging. This study is the first to correlate the amount of radiation exposure with trauma activation status. (Table Presented)
SN - 1072-7515
AD - (Farzal, Farzal, Wilson, Brown, Burkhalter, Fischer) Children's Medical Center, University of Texas-Southwestern, Dallas, TX, United States
ER -

TY - JOUR
ID - 3310
T1 - Power to the paediatricians! improving research into common child health conditions through the Australian paediatric research network
A1 - Hiscock,H.
Y1 - 2013///
N1 - 71166785
English
Journal: Conference Abstract
KW - EMBASE
KW - pediatrician
KW - Human
KW - child health
KW - Health
KW - Child
KW - Health Care
KW - Hospital
KW - secondary health care
KW - Evidence Based Practice
KW - allergic asthma
KW - Obesity
KW - Medical Audit
KW - Attention Deficit Disorder
KW - Autism
RP - NOT IN FILE
SP - 25
JF - Internal Medicine Journal
VL - Conference: RACP Future Directions in Health Congress 2013 Perth, WA Australia. Conference Start: 20130526 Conference End: 20130529. Conference Publication:
IS - var.pagings
N2 - Background:While Australian paediatricians also manage rare conditions, the bulk of their time is spent on common, chronic conditions such as obesity, autism, attention deficit hyperactivity disorder, allergy, asthma, and developmental problems. This comprises a significant proportion of the estimated $4.5 billion per annum

spent providing health care to 0-14 year old Australian children. Despite the cost, there has been very little research into how best to systematically and consistently manage chronic child health conditions outside tertiary settings. Addressing the need to develop evidence-based interventions in real-life health care settings requires new research platforms that can engage and support paediatricians to participate in large-scale multi-site research. The Australian Paediatric Research Network is one such platform. Aims/Objectives: Formed in 2007, the APRN aims to improve the quality and quantity of research into common child health conditions managed in secondary care settings. Two core problems drive our Network: (1) the substantial cost of secondary care, in the face of a weak evidence base as to its effectiveness; and (2) an under-utilised capacity of Australian paediatricians to participate in research that could improve this evidence base. Methods: With our 400-strong membership, we have conducted a suite of activities that inform practice, training and further research. This includes our annual multi-topic surveys, the first ever national audit of practice outside hospital settings (n = 8,500 children), and establishing our members' research priorities.The coming 18 months will see a repeat of the last two activities, five years on, and a forum of multi-disciplinary experts to shape the APRN's future research. Findings and Conclusions: The bulk of care for children with chronic health conditions takes place outside of hospitals but there is very little research into its effectiveness. The APRN provides an exciting vehicle for such research and is engaging paediatricians in the very research which will change their practice
SN - 1444-0903
AD - (Hiscock) Murdoch Children's Research Institute, Melbourne, VIC, Australia (Hiscock) Royal Children's Hospital, Melbourne, VIC, Australia (Hiscock) Department of Paediatrics, University of Melbourne, Melbourne, VIC, Australia
ER -

TY - JOUR
ID - 3312
T1 - Annual family celebration day
A1 - Getchell,K.N.
A1 - O'Melia,L.
Y1 - 2013///
N1 - 71163549
English
Journal: Conference Abstract
KW - EMBASE
KW - Transplantation
KW - Human
KW - Hospital
KW - recipient
KW - patient
KW - Child
KW - United States
KW - Socialization
KW - medical specialist
KW - Clothing
KW - nurse
KW - pediatric hospital
KW - Golf
KW - Food
KW - physician
KW - feedback system
KW - surgery
KW - sibling
KW - solid
KW - outpatient
KW - graft recipient
KW - scavenger
RP - NOT IN FILE
SP - 76
EP - 77

JF - Pediatric Transplantation
JA - PEDIATR TRANSPLANT
VL - Conference: IPTA 7th Congress on Pediatric Transplantation Warsaw Poland. Conference Start: 20130713 Conference End: 20130716. Conference Publication:
IS - var.pagings
N2 - PURPOSE: At Boston Children's Hospital Pediatric Transplant Center, we aim to provide quality care and support throughout the transplant experience, not only while in the hospital but also out-patient. For the past 6 years, we have offered solid organ transplant (SOT) recipients and their families an opportunity to connect with other SOT recipients, their families, and staff outside of the hospital at our annual Family Celebration Day. Patients, families, and staff are encouraged to share experiences while socializing and participating in activities. METHOD: Upon arrival, attendees sign in, obtain a name tag (identifying what organ he/she received), and review the activity and food schedule. Activies such as bumper boats, miniature golf, a driving range, arcade games, a batting cage and more are offered at the facility. Child Life Specialists provide an opportunity for SOT recipients and their siblings perform a "check-up" at Transplant Clinic on stuffed animals given to them upon arrival. Kids of all ages participate in medical play with their stuffed animal and even perform surgeries (similar to what they have been through). Besides medical play and art, organized games occur including transplant bingo and a scavenger hunt to encourage socialization and educate patients about transplant. RESULTS: Feedback is collected each year which has always been overwhelmingly positive. Families report they appreciate the opportunity to meet and connect with other SOT recipients. They often share experiences and stories, and stay connected after the event. Families also report they look forward to socializing with staff outside of the hospital, seeing their doctors and nurses in "normal" clothes. Several attendees have started fundraising efforts to help support this free event so it can continue for years to come. CONCLUSION: The Pediatric Transplant Center at Boston Children's Hospital's Annual Family Celebration Day is an event that recipients, their families, and staff look forward to year after year. It provides an opportunity for participation in normalizing activities and socialization with peers and families that have similar experiences
SN - 1397-3142
AD - (Getchell) Child Life Services, Boston Children's Hospital, Boston, MA, United States (O'Melia) Pediatric Transplant Center, Boston Children's Hospital, Boston, MA, United States
ER -

TY - JOUR
ID - 3315
T1 - Nasopharyngeal carcinoma in indonesia: Epidemiology, incidence, signs, and symptoms at presentation
A1 - Adham,M.
A1 - Kurniawan,A.N.
A1 - Muhtadi,A.I.
A1 - Roezin,A.
A1 - Hermani,B.
A1 - Gondhowiardjo,S.
A1 - Bing,Tan,I
A1 - Middeldorp,J.M.
Y1 - 2012///
N1 - 2012235638
English
Journal: Article
KW - EMBASE
KW - Adult
KW - advanced cancer/di [Diagnosis]
KW - advanced cancer/ep [Epidemiology]
KW - advanced cancer/et [Etiology]
KW - Age Distribution
KW - Aged
KW - anosmia
KW - article
KW - cancer epidemiology
KW - cancer incidence
KW - cancer staging

KW - cancer survival
KW - carcinogenic activity
KW - Child
KW - clinical feature
KW - controlled study
KW - diplopia
KW - dysphagia
KW - dysphonia
KW - Environmental Exposure
KW - Epstein Barr virus
KW - Epstein Barr virus infection
KW - ethnic difference
KW - eye pain
KW - Female
KW - histopathology
KW - Human
KW - human tissue
KW - Indonesia
KW - LMP1 gene
KW - major clinical study
KW - Male
KW - nasopharynx carcinoma/di [Diagnosis]
KW - nasopharynx carcinoma/ep [Epidemiology]
KW - nasopharynx carcinoma/et [Etiology]
KW - nose obstruction
KW - oncogene
KW - preschool child
KW - school child
KW - serous otitis media
KW - sex difference
KW - squamous cell carcinoma/di [Diagnosis]
KW - squamous cell carcinoma/ep [Epidemiology]
KW - squamous cell carcinoma/et [Etiology]
KW - tinnitus
KW - tumor biopsy
KW - undifferentiated carcinoma
KW - virus reactivation
KW - latent membrane protein 1
KW - tumor marker/ec [Endogenous Compound]
KW - virus DNA
RP - NOT IN FILE
SP - 185
EP - 196
JF - Chinese Journal of Cancer
VL - 31
IS - 4
CY - United States
N2 - Among all head and neck (H&N) cancers, nasopharyngeal carcinoma (NPC) represents a distinct entity regarding epidemiology, clinical presentation, biological markers, carcinogenic risk factors, and prognostic factors. NPC is endemic in certain regions of the world, especially in Southeast Asia, and has a poor prognosis. In Indonesia, the recorded mean prevalence is 6.2/100 000, with 13 000 yearly new NPC cases, but otherwise little is documented on NPC in Indonesia. Here, we report on a group of 1121 NPC patients diagnosed and treated at Dr. Cipto Mangunkusumo Hospital, Jakarta, Indonesia between 1996 and 2005. We studied NPC incidence among all H&N cancer cases (n =6000) observed in that period, focusing on age and gender distribution, the ethnic background of patients, and the disease etiology. We also analyzed most prevalent signs and symptoms and staging of NPC patients at first presentation. In this study population, NPC was the most frequent H&N cancer (28.4%), with a male-to- female ratio of 2.4, and was endemic in the Javanese population.

Interestingly, NPC appeared to affect patients at a relatively young age (20% juvenile cases) without a bimodal age distribution. Mostly, NPC initiated in the fossa of Rosenmuller and spreaded intracranially or locally as a mass in the head. Occasionally, NPC developed at the submucosal level spreading outside the anatomic limits of the nasopharynx. At presentation, NPC associated with hearing problems, serous otitis media, tinnitus, nasal obstruction, anosmia, bleeding, difficulty in swallowing and dysphonia, and even eye symptoms with diplopia and pain. The initial diagnosis is difficult to make because early signs and symptoms of NPC are not specific to the disease. Early-age Epstein-Barr virus (EBV) infection combined with frequent exposure to environmental carcinogenic co-factors is suggested to cause NPC development. Undifferentiated NPC is the most frequent histological type and is closely associated with EBV. Expression of the EBV-encoded latent membrane protein 1(LMP1) oncogene in biopsy material was compared between NPC patients of < 30 years old and those of >30 years old, matched for sex and tumor stage. Higher LMP1 expression in patients of <30 years old was observed, which was related to more locoregional progressivity. Increased medical awareness of prevailing early stage signs and symptoms coupled to use of EBV-related diagnostic tumor markers may lead to down-staging and timely treatment to improve survival of patients with this aggressive disease
SN - 1000-467X
AD - (Adham, Muhtadi, Roezin, Hermani) Department of Otorhinolaryngology, Faculty of Medicine, University of Indonesia, Dr. Cipto Mangunkusumo Hospital, Jakarta, Indonesia (Kurniawan) Department of Anatomy Pathology, Faculty of Medicine, University of Indonesia, Dr. Cipto Mangunkusumo Hospital, Jakarta, Indonesia (Gondhowiardjo) Department of Radiotherapy, Faculty of Medicine, University of Indonesia, Dr. Cipto Mangunkusumo Hospital, Jakarta, Indonesia (Bing Tan) Antoni van Leeuwenhoek Hospital, Netherlands Cancer Institute, Netherlands (Middeldorp) Department of Pathology, VU University Medical Center, Amsterdam, Netherlands
ER -

TY - JOUR
ID - 3316
T1 - Environmental barriers to children's summer outdoor play
A1 - Worobey,J.
A1 - Lelah,L.
A1 - Gaugler,R.
Y1 - 2013///
N1 - 71154470
English
Journal: Conference Abstract
KW - EMBASE
KW - Human
KW - summer
KW - Child
KW - Weather
KW - school
KW - Physical Activity
KW - epidemic
KW - Obesity
KW - Weight Gain
KW - Motor Activity
KW - mosquito
KW - Traffic
KW - heat
KW - Fear
KW - Safety
KW - Health
RP - NOT IN FILE
JF - FASEB Journal
VL - Conference: Experimental Biology 2013, EB Boston, MA United States. Conference Start: 20130420 Conference End: 20130424. Conference Publication:
IS - var.pagings

N2 - Although the health benefits of physical activity are well documented, it appears that even children may exhibit levels of physical activity that are insufficient to prevent or reduce excess weight gain. In light of the current child obesity epidemic, our objective was to survey children to identify the main reasons they might give for not engaging in outdoor play in the summer, ostensibly a time when school is out and weather is most favorable. For this study, 300 children (age 6-14) were surveyed at four county/4-H fairs and asked to indicate how important various factors (e.g., heat, traffic, mosquitoes) were in preventing them from playing outside. The reasons ranked highest were bad weather, bullies/gangs, fear of strangers, no street lights, and it being too hot. These results are somewhat consistent with studies that indicate neighborhood safety as a concern (Carver et al., 2008), but add weather conditions as a factor to be considered when seeking to promote outdoor activity. Allowing access to unused school gyms might be a useful strategy for facilitating large motor activity in summer months
SN - 1530-6860
AD - (Worobey, Lelah) Nutritional Sciences, Rutgers University, New Brunswick, NJ, United States (Gaugler) Vector Biology, Rutgers University, New Brunswick, NJ, United States
ER -

TY - JOUR
ID - 3317
T1 - Process evaluation of a community-based pilot intervention for chronic disease prevention in Guatemalan school-aged children
A1 - Letona,P.
A1 - Gittelsohn,J.
A1 - Chacon,V.
A1 - Ramirez-Zea,M.
A1 - Caballero,B.
Y1 - 2013///
N1 - 71154288
English
Journal: Conference Abstract
KW - EMBASE
KW - Chronic Disease
KW - prophylaxis
KW - school
KW - Child
KW - Human
KW - community
KW - Food
KW - Guatemala
KW - city
KW - low drug dose
KW - Immobilization
KW - prescription
KW - Diet
KW - worker
KW - Government
KW - teacher
KW - Tobacco
KW - randomized controlled trial
KW - Marketing
KW - Interpersonal Communication
KW - risk factor
KW - alcohol
RP - NOT IN FILE
JF - FASEB Journal
VL - Conference: Experimental Biology 2013, EB Boston, MA United States. Conference Start: 20130420 Conference End: 20130424. Conference Publication:
IS - var.pagings

N2 - We examined the implementation of Pilas!, a community-based intervention aimed to reduce risk factors for chronic diseases (unhealthy diet, physical inactivity, alcohol and tobacco use) in school-age children. The intervention consisted of three components (school, food kiosk and community) that were implemented by teachers, children, food vendors, religious leaders, and government workers in two poor communities and two public schools in Guatemala City. Intervention activities included a classroom-based program (4th-6th grade), active games during school recess, healthy recipes and marketing strategies to promote healthy foods in kiosks, and community events. Quantitative and qualitative methods (structured observations, surveys, logs) were used to examine process indicators, which included the extent of delivery (reach), proportion received by target audience (dose), fidelity of intervention protocol, and context. The school component was implemented with high reach, dose and fidelity. The kiosk component was implemented with moderate dose and fidelity, and low reach. The community component was implemented with low dose and reach. Lack of communication and organizational difficulties were identified as barriers to implementation in the kiosk and community components. These findings will be used to plan a randomized controlled trial in Guatemala City
SN - 1530-6860
AD - (Letona, Chacon, Ramirez-Zea) INCAP Research Center for the Prevention of Chronic Disease, Institute of Nutrition of Central America and Panama, Guatemala, Guatemala (Gittelsohn, Caballero) Center for Human Nutrition, Johns Hopkins Bloomberg School of Public Health, Baltimore, MD, United States
ER -

TY - JOUR
ID - 3318
T1 - Physical activity policies at childcare centers and impact on children's physical activity and screen-time behaviors
A1 - Erinosho,T.O.
A1 - Hales,D.P.
A1 - Vaughn,A.E.
A1 - Mazzucca,S.
A1 - Ward,D.S.
Y1 - 2013///
N1 - 71153014
English
Journal: Conference Abstract
KW - EMBASE
KW - Human
KW - Physical Activity
KW - Child
KW - Child Care
KW - Policy
KW - teacher
KW - sitting
KW - Television
KW - preschool child
KW - computer
KW - general practice
KW - Environment
RP - NOT IN FILE
JF - FASEB Journal
VL - Conference: Experimental Biology 2013, EB Boston, MA United States. Conference Start: 20130420 Conference End: 20130424. Conference Publication:
IS - var.pagings
N2 - This study evaluated the association between childcare center policies about physical activity (PA) and children's PA and screen-time behaviors at 50 childcare centers. Data were collected in 2008-2009 using the Environment and Policy Assessment and Observation (EPAO) tool. Directors reported center PA policies (written, unwritten but general practice, no policy). Preschool children's PA and screen-time behaviors were assessed via 4-day observation at each center. Time spent per day in outdoor play, teacher-led PA, sedentary activities, and screen-time were 67.7+33.6, 16.5+11.3, 52.2+28.8, and 39.7+47.9 minutes, respectively. Children engaged in more minutes of outdoor play at centers with a written policy about amount of active playtime; and a

written/unwritten policy about amount of time spent outdoors. Written policies about amount of active playtime and time spent outdoors were also associated with more minutes of sedentary activity. Children engaged in more minutes of teacher-led PA at centers with written policy about amount of time in teacher-led activities. Children spent fewer minutes watching television and using computers at centers with written policies about such behaviors and written policy about staff supervision of media use. Results suggest that written and unwritten policies may be effective at increasing PA, but may be insufficient to help children achieve recommended levels of PA
SN - 1530-6860
AD - (Erinosho, Hales, Vaughn, Mazzucca, Ward) University of North Carolina at Chapel Hill, Chapel Hilll, NC, United States
ER -

TY - JOUR
ID - 3319
T1 - Heightened amygdala activation in maltreated children during pre-attentive emotional processing
A1 - McCrory,E.
Y1 - 2013///
N1 - 71149039
English
Journal: Conference Abstract
KW - EMBASE
KW - amygdaloid nucleus
KW - Human
KW - Child Abuse
KW - Child
KW - Society
KW - processing
KW - psychiatry
KW - nerve potential
KW - Risk
KW - mental disease
KW - Functional Magnetic Resonance Imaging
KW - onset age
KW - intelligence quotient
KW - gender
KW - Facial Expression
KW - community sample
KW - childhood
KW - Institutionalization
KW - anxiety disorder
KW - emotional abuse
KW - abuse
KW - marker
RP - NOT IN FILE
SP - 190S
JF - Biological Psychiatry
JA - Biol Psychiatry
VL - Conference: 68th Annual Scientific Convention and Meeting of the Society of Biological Psychiatry, SOBP 2013 San Francisco, CA United States. Conference Start: 20130516 Conference End: 20130518. Conference Publication:
IS - var.pagings
N2 - Background: Childhood adversity is associated with significantly increased risk of psychiatric disorder. To date, functional magnetic resonance imaging studies of children have mainly focused on the impact of institutionalization rather maltreatment at home and investigated conscious processing of affect. The aim of the current study was to investigate neural response to pre-attentively presented affect cues in a community sample of children with documented experiences of familial maltreatment. Methods: A masked dot-probe paradigm involving pre-attentive presentation of angry, happy and neutral facial expressions was employed using FMRI.

Eighteen maltreated children aged between 10 and 14 years of age, were compared with 23 non-maltreated peers, matched for age, gender, SES, Tanner stage and IQ. Results: Increased neural response was observed in the right amygdala for preattentively presented angry and happy faces in maltreated versus non-maltreated children. Significant negative associations were found between degree of amgydala response and age of onset / duration of abuse for measures of emotional abuse and neglect. Conclusions: We demonstrate for the first time that maltreatment in children is associated with increased amygdala activation to pre-attentively presented positive and threatening faces. In other words, our findings suggest that maltreated children show a general pattern of atypical affect processing, even outside of conscious awareness. Given the evidence for pre-attentive affect processing is also altered in individuals with anxiety disorders, we suggest that this pattern of heightened amygdala activation may constitute a latent neural risk marker, associated with increased vulnerability to future psychiatric disorder
SN - 0006-3223
AD - (McCrory) University College London, London, United Kingdom
ER -

TY - JOUR
ID - 3320
T1 - Sedentarism as a stress generator in a young population of campinas, sp-Brazil
A1 - Saraiva,D.J.B.
A1 - Avezum,A.
A1 - Timerman,A.
A1 - Cesar,L.A.M.
A1 - Mendes,J.H.Z.
A1 - Magalhaes,C.C.
A1 - Saraiva,J.F.K.
Y1 - 2013///
N1 - 71146571
English
Journal: Conference Abstract
KW - EMBASE
KW - Population
KW - Brazil
KW - cardiology
KW - heart disease
KW - generator
KW - Human
KW - school
KW - habit
KW - Physical Activity
KW - Child
KW - Obesity
KW - Television
KW - computer
KW - Lifestyle
KW - city
KW - Anthropometry
KW - Prevalence
KW - structured questionnaire
KW - weight
KW - pathology
KW - cardiovascular risk
KW - sampling
KW - Male
KW - Weight Gain
KW - student
KW - devices
KW - diet restriction

KW - Adolescent
KW - Recreation
KW - Home Environment
KW - Adult
KW - lipid
RP - NOT IN FILE
SP - 477
JF - Cardiology (Switzerland)
VL - Conference: International Academy of Cardiology 18th World Congress on Heart Disease Annual Scientific Sessions 2013 Vancouver, BC Canada. Conference Start: 20130726 Conference End: 20130729. Conference Publication:
IS - var.pagings
N2 - Objective: To evaluate the repercussion of sedentary habits in the lifestyle of children and adolescents, 7 to 18 years old, both sexes, from public schools of the city of Campinas, Brasil. Methods: A cross-sectional epidemiological study with sampling from a school population pool was carried out. Eleven schools were randomly selected in central and peripheral city areas. The assessment protocol comprised a structured questionnaire, anthropometry, and a non fasting lipid profile. In 2010, 4699 students (47,14% of the male sex; mean age, 11.07+2.9 years) were evaluated. Results: Reports showed that 67.25% of the children that practiced outside school physical activities did not present weight excess. Overweight (>+1SD) and obesity (>+2SD) prevalence rates were 15.7% and 16.0%, respectively. Sedentary habits (television, videogames and computers) corresponded to 3.29 hours daily. Low and moderate time for interaction with electronic devices (television, computers and video games) was up to 3,5 hours daily and moderate, intense and exaggerated was considered for more than 4 hours daily. Children that spent more time in front of screens also report having significant stress in the home environment (p<0,0001). A significant correlation between time spent in front of screens and stress in school was also true. (p<0,0001). Conclusion: Physical activity is associated to a healthy life style in a young population, in opposition to sedentary habits generating stress and weight gain. Interventions are required to improve physical activities and reverse the unfavorable tendency of future cardiovascular risk and potential chronic degenerative pathologies in the adult population
SN - 0008-6312
AD - (Saraiva, Avezum, Timerman, Cesar, Mendes, Magalhaes, Saraiva) Sociedade de Cardiologia do Estado de Sao Paulo, Secretaria Municipal de Saude de Campinas, Sao Paulo, Brazil
ER -

TY - JOUR
ID - 3321
T1 - Effect of a governmentally-led physical activity program on motor skills in young children attending child care centers: A cluster randomized controlled trial
A1 - Bonvin,A.
A1 - Barral,J.
A1 - Kakebeeke,T.H.
A1 - Kriemler,S.
A1 - Longchamp,A.
A1 - Schindler,C.
A1 - Marques-Vidal,P.
A1 - Puder,J.J.
Y1 - 2013///
N1 - 2013510238
English
Journal: Article
KW - EMBASE
KW - article
KW - body mass
KW - Child
KW - Child Care
KW - childhood obesity
KW - controlled study
KW - Female

KW - health program
KW - Human
KW - major clinical study
KW - Male
KW - Motor Performance
KW - outcome assessment
KW - Physical Activity
KW - preschool child
KW - Quality of Life
KW - randomized controlled trial
KW - Satisfaction
KW - single blind procedure
RP - NOT IN FILE
JF - International Journal of Behavioral Nutrition and Physical Activity
VL - 10 , 2013. Article Number: 90. Date of Publication: 08 Jul 2013.
CY - United Kingdom
N2 - Objective: To assess the effect of a governmentally-led center based child care physical activity program (Youp'la Bouge) on child motor skills.Patients and methods: We conducted a single blinded cluster randomized controlled trial in 58 Swiss child care centers. Centers were randomly selected and 1:1 assigned to a control or intervention group. The intervention lasted from September 2009 to June 2010 and included training of the educators, adaptation of the child care built environment, parental involvement and daily physical activity. Motor skill was the primary outcome and body mass index (BMI), physical activity and quality of life secondary outcomes. The intervention implementation was also assessed.Results: At baseline, 648 children present on the motor test day were included (age 3.3 + 0.6, BMI 16.3 + 1.3 kg/m², 13.2% overweight, 49% girls) and 313 received the intervention. Relative to children in the control group (n = 201), children in the intervention group (n = 187) showed no significant increase in motor skills (delta of mean change (95% confidence interval: -0.2 (-0.8 to 0.3), p = 0.43) or in any of the secondary outcomes. Not all child care centers implemented all the intervention components. Within the intervention group, several predictors were positively associated with trial outcomes: 1) free-access to a movement space and parental information session for motor skills 2) highly motivated and trained educators for BMI 3) free-access to a movement space and purchase of mobile equipment for physical activity (all p < 0.05).Conclusion: This " real-life" physical activity program in child care centers confirms the complexity of implementing an intervention outside a study setting and identified potentially relevant predictors that could improve future programs. 2013 Bonvin et al.; licensee BioMed Central Ltd
AD - (Bonvin, Barral) Institute of Sport Sciences, University of Lausanne, Quartier Unil - Mouline, Batiment Geopolis, Lausanne 1015, Switzerland (Kakebeeke) Child Development Center, University Children's Hospital, Zurich 8032, Switzerland (Kriemler) Swiss Tropical and Public Health Institute, University of Basel, Socinstrasse 57, Basel 4002, Switzerland (Longchamp) Health League, Rue de la Mouline 8, Chavannes-pres-Renens 1022, Switzerland (Schindler) Institute of Social and Preventive Medicine, University of Basel, Basel, Switzerland (Marques-Vidal, Puder) Service of Endocrinology, Diabetes and Metabolism, Centre Hospitalier Universitaire Vaudois, University of Lausanne, Rue du Bugnon 46, Lausanne 1011, Switzerland
ER -

TY - JOUR
ID - 3322
T1 - Discovery and follow-up of a lead-poisoning outbreak in a shantytown of Le Port, Reunion Island
A1 - Solet,J.-L.
A1 - Renault,P.
A1 - Denys,J.-C.
A1 - Teule,G.
A1 - Dennemont,R.-M.
A1 - Domonte,F.
A1 - Garnier,C.
A1 - Aubert,L.
A1 - Filleul,L.
A1 - Polycarpe,D.
Y1 - 2013///

N1 - 2013486021
English
Journal: Article
KW - EMBASE
KW - Adolescent
KW - Age Distribution
KW - article
KW - Child
KW - disease surveillance
KW - Environmental Exposure
KW - Environmental Monitoring
KW - epidemic/ep [Epidemiology]
KW - follow up
KW - Human
KW - Infant
KW - lead blood level
KW - Lead Poisoning/ep [Epidemiology]
KW - major clinical study
KW - metal recovery
KW - onset age
KW - preschool child
KW - school child
KW - screening test
KW - soil analysis
KW - soil pollution
KW - lead/to [Drug Toxicity]
RP - NOT IN FILE
SP - 329
EP - 337
JF - Revue d'Epidemiologie et de Sante Publique
VL - 61
IS - 4
CY - France
N2 - Background: A national survey conducted in 2008-2009by the French Institute for Public Health Surveillance for detection of lead impact in childhood identified a high blood lead level in a young boy living in the town of "Le Port", Reunion Island. Previously, cases of lead-poisoning on the island had been exceptional; only a dozen cases were reported in the 1980s in adults, related to the use of lead-containing instruments for food preparations. Methods: The family of the index case was invited to participate in screening tests and an environmental investigation was conducted using a standardized questionnaire. Screening was then broadened to the neighborhood of the index case and samples of soil outside the home and in the immediate vicinity were taken. The environmental survey was then extended with soil samples taken from the entire geographical area. Information was then provided to local inhabitants (87families and 287people) in order to encourage lead blood testing for all children under six years and all pregnant women living in the area. Results: The index case lived in the neighborhood of "The Oasis", a shantytown of Le Port. The results of soil analysis revealed heterogeneous pollution of superficial soils by lead throughout the area of the shantytown, the highest level recorded (5200. mg/kg) reached more than 300times the background level of the natural soils of the island. The screening identified 76cases of childhood lead-poisoning (blood lead level greater or equal to 100. mug/L) among 148samples (51%). All cases of blood poisoning involved children under the age of 15years. The median age of children with a positive test was 5.6years; the median blood lead level was 196. mug/L [102-392. mug/L]. Conclusion: The main hypothesis to explain the contamination of the soil in the area of the shantytown is the presence of waste deposits (car batteries) and diffuse activities of metal recovery. The authorities managed to remove all the families from the environmental exposure to lead by rapidly ensuring rehousing outside the contaminated area. 2013 Elsevier Masson SAS
SN - 0398-7620
AD - (Solet, Aubert, Filleul) Cellule de l'Institut de veille sanitaire en region Ocean Indien, Departement de coordination des alertes et regions, Institut de veille sanitaire, 2bis, avenue Georges-Brassens, 97408 Saint-Denis, cedex 9, Reunion (Renault, Domonte, Garnier) Cellule de veille d'alerte et de gestion sanitaires de la

Reunion, Direction de la veille et securite sanitaires, Agence de sante ocean Indien, Saint-Denis, Reunion (Denys, Teule, Dennemont) Service sante-environnement, Delegation de l'Ile de la Reunion, Agence de sante ocean Indien, Saint-Denis, Reunion (Polycarpe) Direction de la veille et securite sanitaires, Agence de sante ocean Indien, Saint-Denis, Reunion
ER -

TY - JOUR
ID - 3323
T1 - 6. Jahrestagung der Osterreichischen Gesellschaft fur Geschlechtsspezifische Medizin, OGGSM 2013
Y1 - 2013///
N1 - 71142291
German, English
Journal: Conference Review
KW - EMBASE
KW - Austria
KW - gender
KW - Pregnancy
KW - Male
KW - Female
KW - Human
KW - Public Health
KW - capillary
KW - venous blood
KW - cardiovascular risk
KW - Exercise
KW - Child
KW - sport
KW - army
KW - athlete
KW - leather industry
KW - glucose blood level
KW - oral glucose tolerance test
KW - sex difference
KW - opiate
KW - endostatin
RP - NOT IN FILE
EP - 8
JF - Wiener Medizinische Wochenschrift
JA - Wien Med Wochenschr.
VL - Conference: 6. Jahrestagung der Osterreichischen Gesellschaft fur Geschlechtsspezifische Medizin, OGGSM 2013 Wien Austria. Conference Start: 20130419 Conference End: 20130419. Conference Publication:
IS - var.pagings
N2 - The proceedings contain 10 papers. The topics discussed include: gender differences in tanning and sun protective behavior - public (skin) health perspectives in Austria; perceived relevance of educative information on public (skin) health in Austria: aspects of preventive gender medicine; opioid maintenance during pregnancy and related gender-specific long-term consequences for the children; awareness of cardiovascular risk factors in Austrian female and male civil and military servants of the ministry of defense and sports; endostatin and physical exercise in young female and male athletes and controls; and comparability of capillary and venous blood glucose measurements during an oral glucose tolerance test in- and outside pregnancy
SN - 0043-5341
ER -

TY - JOUR
ID - 3324
T1 - Post-concussion convulsions and epilepsy
A1 - Cvitanovic-Sojat,L.
A1 - Kukuruzovic,M.

A1 - Malenica,M.
A1 - Kuznik,K.
A1 - Zigman,T.
A1 - Katavic,M.
Y1 - 2013///
N1 - 71137248
English
Journal: Conference Abstract
KW - EMBASE
KW - Epilepsy
KW - convulsion
KW - concussion
KW - Human
KW - Child
KW - contact sport
KW - Consciousness
KW - sport
KW - seizure
KW - muscle cramp
KW - headache
KW - outpatient
KW - Soccer
KW - patient
KW - Football
KW - martial art
KW - data analysis
KW - head injury
KW - Motor Coordination
KW - vomiting
KW - medical history
KW - tremor
KW - Diagnosis
KW - anticonvulsant therapy
KW - diagnostic procedure
KW - brain disease
KW - brain
KW - neurologic disease
KW - dizziness
KW - Risk
KW - Hospital
RP - NOT IN FILE
SP - 95
JF - Epilepsia
VL - Conference: 30th International Epilepsy Congress Montreal, QC Canada. Conference Start: 20130623 Conference End: 20130627. Conference Publication:
IS - var.pagings
N2 - Purpose: To present children admitted to the hospital due to loss of consciousness and convulsions related to sports. Sports commonly leading to concussions include contact martial arts sports, football, soccer and others. Main post-concussive symptoms are headache, followed by convulsions, dizziness, vomiting, lack of motor coordination, cognitive and emotional problems. Repeated concussions can lead to persistent neurological problems. Method: Retrospective data analysis of patients with symptoms related to loss of consciousness and suspected epilepsy, playing different contact sports treated at our Neuropediatric Department during 16 months was done. Results: Fifty four children (mean age 12.5 years) playing different contact sports were admitted to our Department. Epilepsy was suspected in 8 urgently admitted children (3 from playground): 3 had epileptic fits, 3 loss of consciousness, 2 confusion with unilateral cramps; after an outpatient visit 2 came with cramps and 1 with tremor. All children had on average training during 2-7 years, 2-3 times a week, and nearly each weekend. Three children had the diagnosis of epilepsy followed by antiepileptic treatment with a good control

of seizures. Conclusion: Concussions cause different neurological symptoms and convulsions if rest is not long enough to allow the brain to fully heal afterwards. Repeated concussions may lead to epilepsy and chronic encephalopathy. A neuropediatrician must monitor children involved in contact sports, perform diagnostic procedures according to medical history and clinical presentation, advise a child to avoid the activities that may put them at risk for future head injuries and to discontinue contact sports if necessary
SN - 0013-9580
AD - (Cvitanovic-Sojat, Kukuruzovic, Malenica, Kuznik, Zigman, Katavic) Department of Pediatrics, Neuropediatric Unit, UHC Sestre Milosrdnice, Zagreb, Croatia
ER -

TY - JOUR
ID - 3325
T1 - Did Media Attention of the 2009 A(H1N1) Influenza Epidemic Increase Outpatient Antibiotic Use in France?: A Time-Series Analysis
A1 - Bernier,A.
A1 - Ligier,C.
A1 - Guillemot,D.
A1 - Watier,L.
Y1 - 2013///
N1 - 2013476155
English
Journal: Article
KW - EMBASE
KW - 2009 H1N1 influenza
KW - Adolescent
KW - Adult
KW - antibiotic therapy
KW - article
KW - Child
KW - controlled study
KW - drug use
KW - epidemic
KW - France
KW - Human
KW - Infant
KW - information dissemination
KW - major clinical study
KW - mass medium
KW - outpatient
KW - pandemic
KW - preschool child
KW - time series analysis
KW - antibiotic agent
RP - NOT IN FILE
JF - PLoS ONE [Electronic Resource]
JA - PLoS ONE
VL - 8
IS - 7
CY - United States
N2 - Background:In France, the 2009 A(H1N1) influenza epidemic occurred between September 2009 and January 2010. Sparking widespread controversy, it was intensely reported in the media. Despite therapeutic inefficacy, antibiotic consumption and viral respiratory infections are positively correlated, particularly in France, where antibiotic overconsumption is well-known. We first determined the period when media coverage was high, and then compared, during this period, observed outpatient antibiotic consumption to estimated outpatient antibiotic consumption "without media attention".Materials and Methods:To evaluate media coverage, two online databases were consulted: Factiva and Europresse. To quantify outpatient antibiotic consumption, we used data on reimbursements of outpatient systemic antibiotics from the computerized

databases of the two main National Health Insurance agencies. Influenza-like syndromes data came from the French GPs Sentinelles Network. Weekly time-series of antibiotic consumption were modeled by autoregressive moving-average models with exogenous inputs and interventions. Analyses were computed for the entire series and by age group (0-5, 6-15, 16-60, and >60 years).Results:Media coverage was intense between April 2009 and January 2010. No effect on total outpatient antibiotic consumption was observed during the whole mediatic period. However, during the epidemic in France (September 2009-January 2010), we found an antibiotic underconsumption for the entire series, 0-5 and >60 years. Additionally, at the beginning of the pandemic, when cases were still outside France (June 2009-August 2009), we found an antibiotic overconsumption for patients >16 years.Conclusion:The early period of A(H1N1) virus circulation compared with seasonal influenza or an overdeclaration of ILS cases might explain the antibiotic underconsumption observed during the period of active A(H1N1) virus transmission in France. At the pandemic onset, when uncertainty was high, the overconsumption observed for individuals >16 years might have been caused by alarmist media reporting. Additional analyses are needed to understand the determinants of antibiotic consumption during this period. 2013 Bernier et al
AD - (Bernier, Ligier, Guillemot, Watier) INSERM, U 657, Paris, France (Bernier, Ligier, Guillemot, Watier) Institut Pasteur, PhEMI, Paris, France (Bernier, Ligier, Guillemot, Watier) Universite de Versailles Saint Quentin, Faculte de Medecine, Paris Ile de France Ouest, France (Guillemot) AP-HP, Hopital Raymond Poincare, Unite Fonctionnelle de Sante Publique, Garches, France
ER -

TY - JOUR
ID - 3326
T1 - Lung function in children with sickle cell disease
A1 - Kirkby,J.
A1 - Bonner,R.
A1 - Bates,P.
A1 - Strunk,R.
A1 - Kirkham,F.
A1 - Stocks,J.
A1 - Sonnappa,S.
Y1 - 2012///
N1 - 71126075
English
Journal: Conference Abstract
KW - EMBASE
KW - Child
KW - Human
KW - sickle cell anemia
KW - Society
KW - winter
KW - lung function
KW - Spirometry
KW - lung disease
KW - medical specialist
KW - Morbidity
KW - coughing
KW - United Kingdom
KW - African Caribbean
KW - airway
KW - oscillometry
KW - lung volume
KW - questionnaire
KW - Health
KW - consultation
KW - total lung capacity
KW - lung function test
KW - genetic disorder
KW - bronchodilating agent

RP - NOT IN FILE
SP - A18
EP - A19
JF - Thorax
VL - Conference: British Thoracic Society Winter Meeting 2012 London United Kingdom. Conference Start: 20121205 Conference End: 20121207. Conference Publication:
IS - var.pagings
N2 - Introduction Sickle Cell Disease (SCD) is one of the most prevalent genetic diseases with an incidence of ~1 in 200 Afro-Caribbean children in the UK (WHO; 2006). Since SCD can result in significant respiratory morbidity,[1] lung function tests (LFTs) could play an important role in the clinical management of children with SCD. Aim To determine the extent to which LFTs identify differences in children with SCD when compared with healthy Black children. Methods A respiratory health questionnaire was administered, and four commercially available LFTs (Impulse oscillometry (IOS), specific effective airways resistance (sReff), plethysmographic lung volumes, and spirometry) were undertaken in up to 214 healthy Black children and 85 children with SCD aged 4-12y. Results Amongst children with SCD, 50% reported cough on most days, and 25% had been reviewed by a specialist respiratory consultant within 3 months prior to the assessments. When compared with healthy children, 20% had a reduced total lung capacity (TLC), with concurrent reductions in FEV1 and FVC No differences in sReff were observed and IOS outcomes proved to be of limited value, due to poorly defined limits of normality and large between-subject variability. No significant group differences in bronchodilator responsiveness in SCD or healthy children were observed regardless of the outcome measured (Table 1). Conclusion Despite the high proportion of respiratory symptoms reported, the number of children with LFTs falling outside the limits of normal was relatively small. Results suggest a pattern of restrictive lung disease in children with SCD. Of the outcomes assessed, baseline spirometry appears to be the most useful for routine assessment of lung disease in young children with SCD. (Table presented)
SN - 0040-6376
AD - (Kirkby, Bonner, Kirkham, Stocks, Sonnappa) UCL, Institute of Child Health, London, United Kingdom (Bates, Strunk) Washington University, MO, United States
ER -

TY - JOUR
ID - 3327
T1 - Environment-student-playing areas interactions in Aydi{dotless}n, Turkey
A1 - Deniz,B.
A1 - Kilicaslan,C.
A1 - Kara,B.
A1 - Polat,Z.
A1 - Goktug,T.H.
Y1 - 2013///
N1 - 2013449763
English
Journal: Article
KW - EMBASE
KW - article
KW - Basketball
KW - Child
KW - Female
KW - Football
KW - groups by age
KW - Human
KW - Male
KW - preschool child
KW - questionnaire
KW - Recreation
KW - school
KW - school child
KW - Social Environment
KW - Turkey (republic)

RP - NOT IN FILE
SP - 886
EP - 895
JF - Journal of Food, Agriculture and Environment
VL - 11
IS - 2
CY - Finland
N2 - Students generally spend their time in their nearby environment. This environment consists playing areas in school grounds and playgrounds on their way to school. By considering this hypothesis, the aim of the study is to determine whether school grounds and neighbourhood playgrounds on their accessible routes in three different parts of Aydi{dotless}n which are characterized by different patterns of environmental development meet students' needs. Observation and questionnaire forms are used to determine the interaction between environment-student-playing areas and the study is conducted in nine schools and thirty-one playgrounds in their proximity. The results indicated that the interactions of students with the near environment, school grounds and neighbourhood playgrounds can partially meet the needs of children, but address different age groups. School grounds are designed for older children, but playgrounds for younger children. In distorted settlements among the studied areas, playgrounds were limited in their usefulness for group games, while schoolyards were suitable for these games. Because of this situation, 81.1% of the 12-15 age group and all of the 16-18 age group preferred schoolyards. The relationship between school and play was relatively ignored for older age groups, while for younger groups this relationship was taken into account at a rate of 87.2% in all the settlements. It was consequently found that schoolyards and playgrounds do not show differences in their quality and in meeting children's needs for the three settlements studied. Students preferred games related to school. The results show that environmental quality of playing areas should be developed to meet the expectations of different ages of students
SN - 1459-0255
AD - (Deniz, Kilicaslan, Kara, Polat, Goktug) Adnan Menderes University, Department of Landscape Architecture, South Campus, 09100, Aydin, Turkey
ER -

TY - JOUR
ID - 3329
T1 - The evaluation of the effects of passive smoking on children's health with detection of urine cotinine levels
A1 - Kahvecioglu,D.
A1 - Dallar,Y.
A1 - Bostanci,I.
A1 - Tasar,M.
A1 - Dindar,N.
A1 - Ozdilekcan,C.
A1 - Duman,H.
Y1 - 2012///
N1 - 71111283
English
Journal: Conference Abstract
KW - EMBASE
KW - Child
KW - Human
KW - Health
KW - urine
KW - allergy
KW - clinical immunology
KW - passive smoking
KW - Smoking
KW - exposure
KW - Female
KW - wheezing
KW - metabolite
KW - bronchiolitis

KW - pneumonia
KW - dental caries
KW - Otitis Media
KW - prematurity
KW - parameters
KW - Hospital
KW - outpatient department
KW - parent
KW - public health problem
KW - analyzer
KW - Asthma
KW - immunoassay
KW - solid
KW - devices
KW - Diagnosis
KW - diseases
KW - smoking habit
KW - questionnaire
KW - mother
KW - Pregnancy
KW - patient
KW - control group
KW - cotinine
KW - nicotine
RP - NOT IN FILE
SP - 575
EP - 576
JF - Allergy: European Journal of Allergy and Clinical Immunology
VL - Conference: 31st Congress of the European Academy of Allergy and Clinical Immunology Geneva Switzerland. Conference Start: 20120616 Conference End: 20120620. Conference Publication:
IS - var.pagings
N2 - Background: Passive smoke exposure is an important public health problem which has causal relationship between pneumonia, bronchiolitis, broncopneumonia, asthma execarbation and development, otitis media, tooth decay and growth reterdation in children. In this study we aimed evaluate the effects of passive smoke exposure on children's health by urinary cotinin levels with objective parameters. Method: A total number of 80 children whose family reported at least one active smoker in the house and 80 control subjects with no active smoker who admitted to Ankara Training and Research Hospital, Children's Outpatient Clinic were enrolled to study. Parents of children were administered a questionnaire involving sociodemographic characteristics of children and families, smoking habits and diseases within a year. First morning urine was collected to assess passive smoking exposure of children objectively and cotinine level which is the main metabolite of nicotine in the urine was measured by the method of solid-phase competitive immunoassay kemoluminesans with Nicotine Metabolite Immulite 2000 Analyzer (PIL2KNM-10) (DPC, USA) device. The 'cut-off' value of cotinine was recognized as 10 ng/ml for the diagnosis of passive smoking. Subjects with cotinine levels above this value were considered as 'are exposed to passive cigarette smoke'. Result: The number of the disease and frequency of wheezing within the last year was found to be increased in the passive smoking exposure group. Children whose mothers smoked during pregnancy, had a significantly increased incidence of prematurity and wheezing ($P < 0.05$). The median cotinine level was 10 ng/ml in the nonpassive smoking exposure group while, in the exposure group was 20.8 ng/ml, respectively ($P < 0.01$). The cotinine level was over threshold value, 10 ng/ml, in 68.7% of patients in the passive smoking exposure group and 25% of the control group. Conclusion: Twenty-five percent of children who have not active cigarette smokers in their home was shown to be passive exposed smokers. This finding shows families with sick children are biased for reporting the frequency of active smoking and children are exposed to cigarette in places outside the home
SN - 0105-4538
AD - (Kahvecioglu, Dallar, Tasar) Ankara Education and Research Hospital, Pediatrics, Ankara, Turkey (Bostanci, Duman) Dr. Sami Ulus Research and Training Hospital of Women's and Children's Health and Diseases, Pediatric Allergy, Ankara, Turkey (Dindar) Ankara Education and Research Hospital, Biochemistry,

Ankara, Turkey (Ozdilekcan) Dr. Abdurrahman Yurtaslan Ankara Oncology Education and Research Hospital, Pulmonology, Ankara, Turkey
ER -

TY - JOUR
ID - 3330
T1 - Nature and outdoor activity improves atopic dermatitis in children
A1 - Kim,W.
A1 - Kim,H.
A1 - Yoon,H.
Y1 - 2012///
N1 - 71110109
English
Journal: Conference Abstract
KW - EMBASE
KW - atopic dermatitis
KW - Child
KW - Human
KW - allergy
KW - clinical immunology
KW - forest
KW - serum
KW - Therapy
KW - control group
KW - egg
KW - milk
KW - South Korea
KW - eosinophil count
KW - blood sampling
KW - health center
KW - cat
KW - model
KW - cockroach
KW - dog
KW - atopy
KW - Hydrocortisone
KW - vitamin D
KW - immunoglobulin E
KW - vitamin
RP - NOT IN FILE
SP - 141
JF - Allergy: European Journal of Allergy and Clinical Immunology
VL - Conference: 31st Congress of the European Academy of Allergy and Clinical Immunology Geneva Switzerland. Conference Start: 20120616 Conference End: 20120620. Conference Publication:
IS - var.pagings
N2 - Background: Vitamin D has also been inconsistently associated with atopic diseases, although large-scale prospective and randomised studies are lacking. Among the existingstudiesweregiving thesupplements of vitamin D. And, there is no research on these outdoor activities (forest). The role of vitamin D in atopic dermatitis is uncertain. We have analysed the association between serum 25(OH)-D3 levels in atopic dermatitis children. Method: A total of 35 children were enrolled for this study, being referred from local health centers in Republic of Korea, from April to October 2009. They were conveniently assigned to either 'forest group' participating in the forest therapy program or control group. Measurements of salivary cortisol level and QoL were done at initial visits and at 8-week final visits. And also the serum samples were collected and check for 25(OH)-D3 levels. From the blood samples we measured eosinophil counts, total IgE, specific IgE of D. pteronyssinus, D. farinae, Milk, Egg, Cat, Dog, and Cockroach. (Pharmacia & Upjohn Uni-CAP FEIA system) Result: Both groups did not differ significantly in baseline clinical characteristics. The salivary cortisol level change at week 4 and week 8 did not differ between the two groups. But SCORAD index reduction was

significantly and QoL measures slightly improved in the forest group at week 8 compared with the control group. The mean 25(OH)2-D3 levels significantly increased after the outdoor activities, the level were25.82i3/44.81 and 49.90i3/419.77 pg/ml (P = 0.000). Conclusion: The forest therapy program did not induce salivary cortisol level reduction. However, considering the significant decrease in SCORAD index andimprovement in vitamin D level, this may be a useful model of atopic dermatitis management program
SN - 0105-4538
AD - (Kim) Pediatrics, Allergy and Respiratory Research Laboratory, Inje University, Seoul, South Korea
(Kim, Yoon) Allergy and Respiratory Research Laboratory, Inje University, Seoul, South Korea
ER -

TY - JOUR
ID - 3332
T1 - Epidemiological description of rabies reservoir in bats in the Metropolitan Region. Chile. 2000-2009
A1 - Myriam,Favi C.
A1 - Angela,Bassaletti C.
A1 - Javier,Lopez D.
A1 - Luis,Rodriguez A.
A1 - Veronica,Yung P.
Y1 - 2011///
N1 - 21879147
English, Spanish
Journal: Article
KW - EMBASE
KW - Adult
KW - Animal
KW - animal disease
KW - article
KW - bat
KW - Child
KW - Chile/ep [Epidemiology]
KW - disease carrier
KW - Disease Transmission
KW - Female
KW - Human
KW - isolation and purification
KW - Male
KW - Middle Aged
KW - Rabies/ep [Epidemiology]
KW - Rabies virus
KW - Urban Population
KW - virology
RP - NOT IN FILE
SP - 223
EP - 228
JF - Revista Chilena de Infectologia
VL - 28
IS - 3
CY - Chile
N2 - In Chile, in 1985 rabies was detected in insectivorous bats. Since then the epidemiological surveillance activities was extended to these species and the epidemiological pattern was characterized as endemic in bats. In this study we analyzed positive rabies cases in the Metropolitan Region between 2000 and 2009. We identified 325 cases of rabies in 11.472 analyzed samples. We determined an increase of positivity cases in bats between the years of the study. Places with the highest number of positives cases were Las Condes, Santiago, Providencia, Puente Alto and Nunoa. In 147 cases we identify the circumstances in which the bat was found: 89 inside, 45 outside the building and 13 in public spaces. In 39 cases there was contact with humans or animals. These results reinforce the relevance of educating the population against the contact with bats and allow health authorities to take early surveillance and control measures

SN - 0716-1018
AD - (Myriam Favi, Luis Rodriguez, Veronica Yung) Instituto de Salud Publica de Chile, Chile (Angela Bassaletti, Javier Lopez) Universidad Santo Tomas, Facultad de Medicina Veterinaria, Chile
ER -

TY - JOUR
ID - 3334
T1 - The role of community conversations in facilitating local HIV competence: case study from rural Zimbabwe
A1 - Campbell,C.
A1 - Nhamo,M.
A1 - Scott,K.
A1 - Madanhire,C.
A1 - Nyamukapa,C.
A1 - Skovdal,M.
A1 - Gregson,S.
Y1 - 2013///
N1 - 23590640
English
Journal: Article
KW - EMBASE
KW - Adolescent
KW - Adult
KW - Age Distribution
KW - article
KW - automutilation
KW - Child
KW - Female
KW - Human
KW - intoxication/co [Complication]
KW - intoxication/ep [Epidemiology]
KW - Male
KW - Sex Ratio
KW - Sri Lanka/ep [Epidemiology]
RP - NOT IN FILE
JF - BMC Public Health
VL - 13
IS - pp 354
CY - United Kingdom
N2 - This paper examines the potential for community conversations to strengthen positive responses to HIV in resource-poor environments. Community conversations are an intervention method through which local people work with a facilitator to collectively identify local strengths and challenges and brainstorm potential strategies for solving local problems. We conducted 18 community conversations (with six groups at three points in time) with a total of 77 participants in rural Zimbabwe (20% HIV positive). Participants were invited to reflect on how they were responding to the challenges of HIV, both as individuals and in community groups, and to think of ways to better support openness about HIV, kindness towards people living with HIV and greater community uptake of HIV prevention and treatment. Community conversations contributed to local HIV competence through (1) enabling participants to brainstorm concrete action plans for responding to HIV, (2) providing a forum to develop a sense of common purpose in relation to implementing these, (3) encouraging and challenging participants to overcome fear, denial and passivity, (4) providing an opportunity for participants to move from seeing themselves as passive recipients of information to active problem solvers, and (5) reducing silence and stigma surrounding HIV. Our discussion cautions that community conversations, while holding great potential to help communities recognize their potential strengths and capacities for responding more effectively to HIV, are not a magic bullet. Poverty, poor harvests and political instability frustrated and limited many participants' efforts to put their plans into action. On the other hand, support from outside the community, in this case the increasing availability of antiretroviral treatment, played a vital role in enabling communities to challenge stigma and envision new, more positive, ways of responding to the epidemic

AD - (Campbell) Institute of Social Psychology, London School of Economics and Political Science, Houghton Street, WC2A 2AE London, UK
ER -

TY - JOUR
ID - 3335
T1 - Early communicative development in two cultures: A comparison of the communicative environments of children from two cultures
A1 - Lieven,E.
A1 - Stoll,S.
Y1 - 2013///
N1 - 2013385563
English
Journal: Article
KW - EMBASE
KW - Adult
KW - article
KW - Child
KW - Cognitive Development
KW - cultural anthropology
KW - environmental factor
KW - German Federal Republic
KW - Human
KW - imitation
KW - Infant
KW - Interpersonal Communication
KW - Language Development
KW - Nepal
KW - preschool child
KW - priority journal
KW - recording
KW - rural area
KW - Rural Population
KW - videorecording
RP - NOT IN FILE
SP - 178
EP - 206
JF - Human Development
VL - 56
IS - 3
CY - Switzerland
N2 - The nature of young children's communicative environment has been central to theoretical debates about the importance of innate and environmental factors in the development of communication and language. In this paper, we explore aspects of the communicative development and environment of young children growing up in two very different cultures, one in a village of eastern Nepal and the other in a rural area of Western Germany. We analysed longitudinal video recordings of 6 children from each culture in naturalistic settings, at age-matched time points over a period of 8 months. Four children were 8 months old at the outset of the study, 4 were 2 years and 2 months old, and 4 were 3 years old. There were major differences between cultures in the number of adults and children present during the recordings, with other children playing an increasingly important role for the older children in the Nepal recordings. We found no difference between cultures in the onset of pointing and imitation or of reaching, requesting and offering, indicating that these behaviours may be part of a human-specific timetable for socio-cognitive development. We also found that imitation by both the target children and those around them was strictly limited to the youngest group in both cultures. This suggests that imitation may be very important for early development in the prelinguistic phase, while around the age of 2, with the child's developing competence, other ways of interacting take over. The theoretical implications of our results are discussed with reference to the roles of child-intrinsic and environmental factors in the developmental process. Copyright 2013 S. Karger AG, Basel

SN - 0018-716X
AD - (Lieven) School of Psychological Sciences, University of Manchester, Oxford Road, Manchester M13 9PL, United Kingdom (Lieven) Max Planck Institute for Evolutionary Anthropology, Leipzig, Germany (Stoll) University of Zurich, Zurich, Switzerland
ER -

TY - JOUR
ID - 3336
T1 - Hyperdopaminergic crises in familial dysautonomia: A randomized trial of carbidopa
A1 - Norcliffe-Kaufmann,L.
A1 - Martinez,J.
A1 - Axelrod,F.
A1 - Kaufmann,H.
Y1 - 2013///
N1 - 2013383396
English
Journal: Article
KW - EMBASE
KW - Adolescent
KW - Adult
KW - anemia/si [Side Effect]
KW - article
KW - Child
KW - clinical article
KW - controlled study
KW - dopamine urine level
KW - dopaminergic activity
KW - dose response
KW - double blind procedure
KW - drug dose escalation
KW - drug dose titration
KW - drug efficacy
KW - drug safety
KW - dysautonomia
KW - Female
KW - general condition improvement
KW - Human
KW - hyperdopaminergic crisis
KW - Male
KW - neurologic disease
KW - open study
KW - priority journal
KW - randomized controlled trial
KW - retching
KW - school child
KW - symptom
KW - treatment duration
KW - urinary excretion
KW - weakness/si [Side Effect]
KW - alpha tocotrienol
KW - benzodiazepine derivative
KW - carbidopa/ae [Adverse Drug Reaction]
KW - carbidopa/ct [Clinical Trial]
KW - carbidopa/pd [Pharmacology]
KW - clonazepam
KW - clonidine
KW - diazepam

KW - dopamine/ec [Endogenous Compound]
KW - paroxetine
KW - pregabalin
KW - propranolol
RP - NOT IN FILE
SP - 1611
EP - 1617
JF - Neurology
VL - 80
IS - 17
CY - United States
N2 - Objective: The purpose of this study was to determine whether carbidopa (Lodosyn), an inhibitor of dopa-decarboxylase that blocks the synthesis of dopamine outside the brain, is an effective antiemetic in patients with familial dysautonomia (FD) and hyperdopaminergic nausea/retching/vomiting attacks. Methods: We enrolled 12 patients with FD in an open-label titration and treatment study to assess the safety of carbidopa. We then conducted a randomized, double-blind, placebo-controlled, crossover study to evaluate its antiemetic efficacy. Results: Previous fundoplication surgery in each patient studied prevented vomiting, but all of the subjects experienced severe cyclical nausea and uncontrollable retching that was refractory to standard treatments. Carbidopa at an average daily dose of 480 mg (range 325-600 mg/day) was well tolerated. In the double-blind phase, patients experienced significantly less nausea and retching while on carbidopa than on placebo (p < 0.03 and p < 0.02, respectively). Twenty-fourhour urinary dopamine excretion was significantly lower while on carbidopa (147 + 32 mg/gCr) than while on placebo (222 + 41|xg/gCr, p < 0.05). Conclusions: Carbidopa is a safe and effective antiemetic in patients with FD, likely by reducing the formation of dopamine outside the brain. Classification of evidence: This study provides Class II evidence that carbidopa is effective in reducing nausea/retching/ vomiting in patients with FD. 2013 American Academy of Neurology
SN - 0028-3878
AD - (Norcliffe-Kaufmann, Martinez, Axelrod, Kaufmann) Dysautonomia Center, New York University, School of Medicine, New York, United States
ER -

TY - JOUR
ID - 3337
T1 - Health and physical activity: The effect of outdoor play on children's motor development
A1 - Gastaldi,F.G.M.
A1 - Prino,L.E.
A1 - Quaglia,R.
A1 - Longobardi,C.
Y1 - 2013///
N1 - 71088178
English
Journal: Conference Abstract
KW - EMBASE
KW - Child
KW - Human
KW - Motor Development
KW - Health Behavior
KW - juvenile
KW - Physical Activity
KW - Health Education
KW - Health
KW - Obesity
KW - wellbeing
KW - Policy
KW - Blood Pressure
KW - Social Interaction
KW - Risk
KW - osteoporosis

KW - Society
KW - Prevention
KW - urban area
KW - Exercise
KW - Cholesterol
RP - NOT IN FILE
SP - 171
EP - 172
JF - Atencion Primaria
JA - Aten.Primaria
VL - Conference: 1st World Congress of Children and Youth Health Behaviors / 4th National Congress on Health Education Viseu Portugal. Conference Start: 20130523 Conference End: 20130525. Conference Publication:
IS - var.pagings
N2 - Introduction: This literature review examines the link between outdoor activities and children healthy development and well-being. So far, only a few studies have directly examined the influence of children's outdoor activities, physical exercise and motor development. State of the art: Children are more and more growing up disconnected from nature and the outdoors; this is a worldwide phenomenon, occurring in industrialized nations in every setting and in all urban environments throughout the world. As we face contemporary health challenges, such as a growth in level of obesity and stress there is an emerging research and policy interest in the health and wellbeing outcomes associated with use of outdoor spaces. Theoretical and practical implication: To summarize the body of research there is evidence to suggest that outdoor activities are benficial for health. Time spent in outdoor settings, for example, is associated with adequate blood pressure and cholesterol levels; prevention of depression; it is also effective in tackling obesity, preventing osteoporosis; better motor development (strength, balance and coordination); development of children's understanding of risk; increase social interaction; better levels of cognitive functioning and developing creative play activities. Conclusions: This literature review shows that there is general agreement about a positive relationship between outdoor activity and health, in particular there is a link between use of the outdoors and increased physical activity levels bringing also physiological benefits. Encouraging use of the outdoors for positive health benefits requires an understanding of the myriad of influences on children's and of the role of society for policy and interventions
SN - 0212-6567
AD - (Gastaldi, Prino, Quaglia, Longobardi) Department of Psychology, University of Turin, Italy
ER -

TY - JOUR
ID - 3338
T1 - Obesity, diet and physical activity: The reality of our teenagers
A1 - Neves,C.
A1 - Brito,N.
A1 - Fernandes,L.
Y1 - 2013///
N1 - 71087877
English
Journal: Conference Abstract
KW - EMBASE
KW - Human
KW - Diet
KW - Adolescent
KW - Physical Activity
KW - Obesity
KW - Child
KW - juvenile
KW - Health Behavior
KW - Health Education
KW - school
KW - meal

KW - Population
KW - student
KW - fast food
KW - Bone
KW - eating habit
KW - Diabetes Mellitus
KW - childhood
KW - Hypertension
KW - cardiovascular disease
KW - leisure
KW - vegetable
KW - cross-sectional study
KW - Prevalence
KW - Male
KW - risk factor
KW - Statistics
KW - Blood Pressure
KW - sleep disordered breathing
KW - fish
KW - body mass
KW - Food
KW - Recreation
KW - sport
KW - city
KW - childhood obesity
RP - NOT IN FILE
SP - 84
JF - Atencion Primaria
JA - Aten.Primaria
VL - Conference: 1st World Congress of Children and Youth Health Behaviors / 4th National Congress on Health Education Viseu Portugal. Conference Start: 20130523 Conference End: 20130525. Conference Publication:
IS - var.pagings
N2 - Introduction: Childhood obesity (OB) has tripled in adolescents in the past 30 years. Obese youth are more likely to have risk factors for cardiovascular disease, diabetes, bone and joint problems, sleep apnoea and psychological problems. Objectives: To determine the prevalence of overweight (OW), OB and hypertension (HT) in an adolescent school population and to correlate with eating habits (EH) and physical activity (PA). Methods: Analytical cross-sectional study with students from the 5th and 7th grades of 5 schools of 2 different cities based on surveys about EH and PA, determination of Body Mass Index (BMI) and blood pressure (BP). Statistics: SPSS 18 ($p < 0.05$). Results: The study included 501 students, aged from 10 to 17 (median 12 years), 51% males and 60% from the 7th grade, 16% were OW and 12% obese. BP was measured in 492 students, 23% had HT, 18% of those being OW and 22% OB. There was a statistical significant relationship between BMI and BP ($p = 0.004$). Regarding EH: 56% had less than 5 meals/day, 47% had multiple snacks between meals, 5% didn't take breakfast every day, 52% didn't eat soup for lunch and dinner, 91% ate fish less than 5 times/week, 7% always ate sauces at meals, 6% never ate vegetables, 48% ate fried food more than 1 time/week and 21% ingested fast food regularly. Regarding PA: 13% spent more than 2 hours/day watched TV and played video games during school time and 48% during holidays; 32% didn't practice sports outside school. Conclusions: This study showed a high percentage of children with OW/OB and HT, similar to literature values for the Portuguese population. Incorrect EH and PA are present in most cases even in those with normal BMI, which reinforces the need for educational intervention in childhood and adolescent school population
SN - 0212-6567
AD - (Neves) Hospital Pediatrico Carmona da Mota, CHUC, Coimbra, Portugal (Neves, Brito) Hospital Distrital da Figueira da Foz, Figueira da Foz, Portugal (Fernandes) Centro de Saude de Sao Martinho do Bispo, Coimbra, Portugal
ER -

TY - JOUR

ID - 3339
T1 - Home environment-related factors associated with the family-dog biting a child living in the same home
A1 - McV Messam,L.L.
A1 - Kass,P.H.
A1 - Chomel,B.B.
A1 - Hart,L.A.
Y1 - 2013///
N1 - 71079184
English
Journal: Conference Abstract
KW - EMBASE
KW - Human
KW - dog
KW - bite
KW - Child
KW - Society
KW - Epidemiology
KW - Home Environment
KW - Risk
KW - city
KW - dog bite
KW - exposure
KW - Hospital
KW - risk factor
KW - United States
KW - waiting room
KW - questionnaire
KW - Sample Size
KW - Sleep
KW - Cohort Analysis
KW - procedures
KW - Environment
KW - Jamaica
RP - NOT IN FILE
SP - S15
JF - American Journal of Epidemiology
JA - Am J Epidemiol
VL - Conference: 46th Annual Society for Epidemiologic Research, SER Meeting Boston, MA United States. Conference Start: 20130618 Conference End: 20130621. Conference Publication:
IS - var.pagings
N2 - Few studies have examined risk factors for bites by the family dog to persons living in the same home. This veterinary clinic-based retrospective cohort study was aimed at identifying canine environmental risk factors for bites by the family dog to children aged five to fifteen years and living in the same home as the dog. Data were collected by interviewer-administered questionnaire from veterinary clients in the waiting room of clinics in Kingston, Jamaica (236) and San Francisco, USA (61). Exposures of interest were factors relevant to the dog's living environment. Dog bites, in the two-year study period, included those occurring both while the children were (20) and were not playing (9) with the dog and exposure information pertained to the period preceding the dog bite incident. Data were analyzed using binomial regression in SPSS version 20, with confounders selected using directed acyclic graphs and the change-in-estimate procedure. No heterogeneity by city of origin was found and thus data from both cities were pooled for final analyses. Dogs living in homes having no outside yard space (4%) were at higher risk (RR = 3.0; 95% CI: 0.9-9.8) for biting than dogs living in homes that had. Additionally, dogs that were allowed inside (64%) the home (RR = 3.0; 95% CI: 1.0-8.6) and dogs routinely allowed to sleep in the bedroom (23%) of a family member (RR = 3.9; 95% CI: 2.0- 7.9) were also at higher risk for biting than those that were not. Finally, dogs that could leave the premises unaccompanied (16%) were at higher risk (RR = 2.8; 95% CI: 1.4-5.7) for biting than those that could not. While a study with a larger sample size is necessary to detect possible heterogeneity by city, these elevated RRs generally suggest

that family dogs living in closer physical proximity to members of the family may be at elevated risk for biting children living in the same home
SN - 0002-9262
AD - (McV Messam, Kass, Chomel, Hart) University of California Davis, Davis, CA 95616, United States
ER -

TY - JOUR
ID - 3340
T1 - Frontal lobe EEG recorded during the performance of saccadic eye movement tasks in childrenwith FASD
A1 - Hemington,K.S.
A1 - Reynolds,J.N.
Y1 - 2013///
N1 - 71067982
English
Journal: Conference Abstract
KW - EMBASE
KW - Society
KW - frontal lobe
KW - saccadic eye movement
KW - Alcoholism
KW - electroencephalogram
KW - Human
KW - Child
KW - stimulus
KW - control group
KW - devices
KW - eye movement
KW - Memory
KW - laboratory
KW - Executive Function
KW - theta rhythm
KW - electrode
KW - Diagnosis
KW - cognitive defect
KW - brain computer interface
KW - fetal alcohol syndrome
KW - Environment
KW - recording
KW - working memory
KW - comfort
KW - sensor
KW - analysis of covariance
KW - Analysis of Variance
RP - NOT IN FILE
SP - 45A
JF - Alcoholism: Clinical and Experimental Research
VL - Conference: 36th Annual Scientific Meeting of the Research Society on Alcoholism, RSA 2013 Orlando, FL United States. Conference Start: 20130622 Conference End: 20130626. Conference Publication:
IS - var.pagings
N2 - Brain-computer interface devices have recently been developed that record electroencephalogram (EEG) activity using a single dry-sensor electrode, and provide data comparable to that obtained from multi-channel EEG systems at the F3 electrode location. Our lab has previously shown that structured saccadic eye movement tasks probe executive functions and are a sensitive measure of cognitive dysfunction in children Fetal Alcohol Spectrum Disorder (FASD). Our goals are to: 1) quantify the changes in frontal lobe EEG activity that result from manipulating cognitive load during the performance of eye movement tasks; and 2) determine whether dierences in frontal lobe EEG activity correlate to deficits in the performance of eye movement tasks in children with FASD compared with typically developing children. Children aged 8-18 with (clinical group) or without

(control group) an FASD diagnosis performed a memory-guided saccadic eye movement task with one, two or three target stimuli, and EEG was recorded at the FP1 position using the NeuroskyMindwave. In the two or three stimuli conditions, children with an FASD made errors in remembering the sequence of target presentation more frequently than control children (two-way ANOVA, $p < 0.05$).When children made sequence or timing errors during trials in which they were required to remember the location of three stimuli, the FASD group exhibitedmore relative theta band (3-8 Hz) activity in the frontal lobes compared to the control group. Baseline relative theta activity was also dierent between the two groups (two-way ANCOVA, $p < 0.05$). Children with FASD exhibit performance deficits in saccadic eyemovement tasks that probe executive functions (e.g., spatial working memory, response inhibition) involving frontal lobe circuitry. The results of this study suggest that single-channel EEG recording can be used to help identify underlying neuralmechanisms of these deficits. For example, previous studies in the literature have shown that greater relative theta activity is correlated tomemory impairment. Additionally, portable single-channel devices oer greater user comfort than typical EEGrecording equipment as well as flexibility for use outside the laboratory. This will greatly facilitate the study of children with FASD, who may be less tolerant of typical laboratory environments
SN - 0145-6008
AD - (Hemington, Reynolds) Centre for Neuroscience Studies, Queen's University, Kingston, ON K7L 3N6, Canada
ER -

TY - JOUR
ID - 3341
T1 - Variability in the implementation of rapid response teams at american pediatric hospitals
A1 - Sen,A.
A1 - Morgan,R.
A1 - Morris,M.
Y1 - 2012///
N1 - 71065724
English
Journal: Conference Abstract
KW - EMBASE
KW - intensive care
KW - Hospital
KW - rapid response team
KW - Society
KW - Human
KW - physician
KW - hospital bed
KW - compression
KW - financial management
KW - Western Hemisphere
KW - Telephone
KW - Health
KW - Child
KW - thorax
KW - patient transport
KW - Intubation
KW - health care quality
KW - hypothesis
RP - NOT IN FILE
SP - 144
EP - 145
JF - Critical Care Medicine
JA - Crit Care Med
VL - Conference: 42nd Critical Care Congress of the Society of Critical Care Medicine, SCCM 2013 San Juan Puerto Rico. Conference Start: 20130119 Conference End: 20130123. Conference Publication:
IS - var.pagings

N2 - Introduction: Rapid response teams (RRTs) have become standard in American pediatric hospitals, but there is little guidance about their optimal structure and function. Hypothesis: We gathered data characterizing the standard of care across the country to identify factors that correlate with a reduced arrest rate. We hypothesized that an increased RRT dose (# RRTs/# hospital beds) and larger hospital size would correlate with a lower arrest rate. Methods: 62 question telephone survey, including a description of arrest and rapid response team composition and function, and number of events for 2011. Results: We collected data from 30 top American pediatric hospitals, all of which provide ECMO support, have a PICU fellowship, and 24 hour RRTs. A PICU doctor is part of 93% of arrest teams and 73% of RRTs. Of the 17 hospitals with only 1 PICU doctor (fellow or attending) in house overnight, 59% have the single PICU doctor on the RRT. 77% of institutions receive no additional staffing or financial support to run the RRT. 77% of responders have a family activated RRT program, but only 60% report having any family activated calls in 2011. 90% of institutions list "Respiratory" as the reason for most RRT calls. 55% of RRT calls result in a patient transfer to the PICU. The definition for "code event" varies drastically between hospitals, including hospitals belonging to the Children's Health Corporation of America, which has provided a standard definition for codes outside the ICU. Only 60% of responders thought the presence of RRTs had decreased the number of non-ICU chest compressions or emergent intubations. For the 27 hospitals who tracked this data, the median number of RRT calls in 2011 was 130 (range 11 to 664). We found no correlation between RRT dose and arrest rate (r = 0.04). We found a weak correlation showing a decreased arrest rate in the hospitals with more total beds (r = 0.3). Conclusions: This study demonstrates the wide variability in RRT practice in American pediatric hospitals. Although larger hospitals show a weak correlation with lower arrest rates, RRT variability makes it difficult to identify which RRT characteristics are most important in realizing the ultimate goal of preventing floor arrests
SN - 0090-3493
AD - (Sen, Morris) Columbia University, United States (Morgan) Morgan Stanley Children's Hospital of New York Presbyterian, United States
ER -

TY - JOUR
ID - 3342
T1 - Prevalence, Mass, and Glucose-Uptake Activity of 18F-FDG-Detected Brown Adipose Tissue in Humans Living in a Temperate Zone of Italy
A1 - Persichetti,A.
A1 - Sciuto,R.
A1 - Rea,S.
A1 - Basciani,S.
A1 - Lubrano,C.
A1 - Mariani,S.
A1 - Ulisse,S.
A1 - Nofroni,I.
A1 - Maini,C.L.
A1 - Gnessi,L.
Y1 - 2013///
N1 - 2013295926
English
Journal: Article
KW - EMBASE
KW - Adolescent
KW - Adult
KW - age
KW - Aged
KW - article
KW - body build
KW - body mass
KW - brown adipose tissue
KW - Child
KW - Climate
KW - Cohort Analysis
KW - computer assisted tomography

KW - controlled study
KW - environmental parameters
KW - environmental temperature
KW - Female
KW - glucose blood level
KW - glucose transport
KW - Human
KW - Italy
KW - major clinical study
KW - Male
KW - photoperiodicity
KW - population research
KW - positron emission tomography
KW - Prevalence
KW - retrospective study
KW - sex difference
KW - temperate zone
KW - fluorodeoxyglucose f 18
KW - glucose
RP - NOT IN FILE
JF - PLoS ONE [Electronic Resource]
JA - PLoS ONE
VL - 8
IS - 5
CY - United States
N2 - Background:The ¹⁸F-fluorodeoxyglucose (¹⁸F-FDG)-detected brown adipose tissue (BAT), is enhanced by cold stimulus and modulated by other factors that still have to be disentangled. We investigated the prevalence, mass, and glucose-uptake activity of ¹⁸F-FDG-detected BAT in a population of adults living in the temperate climatic zone of the Rome area.Methods and Findings:We retrospectively analyzed 6454 patients who underwent¹⁸F-FDG positron emission tomography/computed tomography (PET/CT) examinations. We found ¹⁸F-FDG BAT in 217 of the 6454 patients (3.36%). Some of them underwent more than one scan and the positive scans were 278 among 8004 (3.47%). The prevalence of patients with at least one positive scan was lower in men (1.77%; 56 of 3161) compared with women (4.88%; 161 of 3293). The BAT positive patients were most frequently younger, thinner and with lower plasma glucose levels compared with BAT negative patients. The amount of BAT in the defined region of interest, the activity of BAT and the number of positive sites of active BAT were similar in both sexes. The prevalence of patients with ¹⁸F-FDG positive PET/CT was highest in December-February, lower in March-May and September-November, and lowest in June-August and was positively correlated with night length and negatively correlated with ambient temperature. Changes in day length and variations of temperature, associated with the prevalence of positive BAT patients. Among the patients who had multiple scans, outdoor temperature was significantly lower and day length was shorter on the occasion when BAT was detected.Conclusions:This study identifies day length, outdoor temperature, age, sex, BMI, and plasma glucose levels as major determinants of the prevalence, mass, and activity of ¹⁸F-FDG-detected BAT. 2013 Persichetti et al
AD - (Persichetti, Basciani, Lubrano, Mariani, Ulisse, Gnessi) Section of Medical Pathophysiology, Food Science and Endocrinology, Department of Experimental Medicine, University of Rome La Sapienza, Rome, Italy (Sciuto, Rea, Maini) Department of Nuclear Medicine, Regina Elena National Cancer Institute, Rome, Italy (Nofroni) Department of Public Health and Infectious Diseases, Medical Statistics and Biometry, University of Rome La Sapienza, Rome, Italy
ER -

TY - JOUR
ID - 3343
T1 - How safe is 'safe' playground equipment?
A1 - Shapiro,M.
A1 - Shapiro,R.
Y1 - 2013///

N1 - 71048363
English
Journal: Conference Abstract
KW - EMBASE
KW - Human
KW - Temperature
KW - Child
KW - sand
KW - parent
KW - mulch
KW - climbing
KW - shoe
KW - Safety
KW - burn
KW - mother
KW - Weather
KW - Humidity
KW - injury
KW - air temperature
KW - composite material
KW - baby
KW - thermometer
KW - laser
KW - Reading
KW - bark
KW - shade
KW - infrared radiation
KW - toddler
KW - Female
KW - heat
KW - thermal injury
KW - summer
KW - Rubber
KW - Water
KW - metal
KW - Rain
KW - plastic
RP - NOT IN FILE
SP - S90
JF - Journal of Burn Care and Research
VL - Conference: 45th Annual Meeting of the American Burn Association Palm Springs, CA United States. Conference Start: 20130423 Conference End: 20130426. Conference Publication:
IS - var.pagings
N2 - Introduction: Most parents think that taking their children to a playground is a safe activity on a hot summer day. The newer equipment and surfaces are marketed as being made of 'safe' materials to help prevent injuries from falls and hidden objects that might be found in gravel or sand. Parents seldom think of burn injuries when they take their children to the park, especially parks with water features and sprinklers. A child can become injured in a matter of seconds when a surface heats up to 140 degrees. With temperatures reaching record highs this year, and with a lack of rain, these safe materials and structures can hold hidden dangers for children. Methods: During June, July and August a study was conducted to determine the surface temperature of various playground equipment and surfaces. Using an infrared laser thermometer, serial temperatures were taken of playground equipment and surrounding surfaces in a Midwest neighborhood playground. Air temperature, humidity and general weather conditions were also documented. Surfaces scanned included rubber mats under equipment, sand, wood mulch, benches, slides, swings and various climbing equipment both plastic and metal. Results: The most significant readings were taken at 3pm when the temperature was 97 degrees with sunny skies. Of 12 surfaces scanned, only 2 were under 120 degrees. The hottest was a dark rubber safety mat (175), light mat (160), baby swing (126) ,H2O fountain (131), metal climbing gym (131), and bark mulch (136).

A bench made of composite material registered 133 degrees. The concrete walkways were 132 degrees and the concrete in the water play area was 104. The other 'safe' play area was the sand play area which had some shade. On another day at 6pm with the temperature at 82 degrees and overcast skies, the rubber mats were each 142 degrees. On all visits to the park there were many families with young children, mostly without shoes. One toddler walked onto the dark rubber mat as I was scanning at 175 degrees and started screaming. I told his mother to place him in the water and warned the families about the high surface temperatures. Conclusions: Parents need to be made aware of the hidden dangers of thermal injuries from playground equipment and surfaces. They also need to check the equipment and surfaces themselves, and make sure their children wear shoes when not in the water play areas. The goal of this project was to have data to show the local park commissions the need for proper warning signs related to park safety issues and potential burn injuries, especially when temperatures make the playground equipment unsafe for children
SN - 1559-047X
AD - (Shapiro, Shapiro) Miami Valley Hospital, Dayton, OH, United States
ER -

TY - JOUR
ID - 3344
T1 - Risk assessment of poisonous plants
A1 - Maren,H.-C.
A1 - Koch,I.-E.
A1 - Hilke,A.-S.
A1 - Pietsch,J.
A1 - Hahn,A.
A1 - Begemann,K.
Y1 - 2013///
N1 - 71050228
English
Journal: Conference Abstract
KW - EMBASE
KW - poisonous plant
KW - Risk Assessment
KW - Human
KW - Plant
KW - exposure
KW - intoxication
KW - Child
KW - toxicity
KW - Germany
KW - Lantana camara
KW - kindergarten
KW - Case Report
KW - Health
KW - Risk
KW - eye
KW - Chelidonium
KW - ingestion
KW - Euonymus
KW - Ilex
KW - poison center
KW - poison
KW - plant toxin
RP - NOT IN FILE
SP - 263
EP - 264
JF - Clinical Toxicology

VL - Conference: 33rd International Congress of the European Association of Poisons Centres and Clinical Toxicologists, EAPCCT 2013 Copenhagen Denmark. Conference Start: 20130528 Conference End: 20130531. Conference Publication:
IS - var.pagings
N2 - Background: Of the 90,000 exposures involving children younger than 6 years of age reported to German poison centers (PCs) every year a fifth are caused by plants. A list of especially poisonous plants, which should not be planted in children's playgrounds, was published in the "Bundesanzeiger" (Federal Gazette) in 2000. The BfR-Committee "Assessment of Intoxications" founded a working group to re-assess the toxicity of plants to protect especially children from severe plant poisoning. Methods: The members of the working group defined criteria for risk assessment of plants in the close proximity of children's playgrounds. Human exposure data, provided by the German PCs in Berlin, Freiburg and Erfurt and by the Swiss PC Zurich were reviewed as well as scientific publications about human exposures and about toxicity of ingredients of plants. Following the assessment of the toxicity of chemicals in analogy to the German Regulations on Dangerous Substances, poisonous plants were re-classified into three categories, namely plants which could lead to minor poisoning, moderate poisoning and severe or deadly poisoning. Results: Out of 43,000 confirmed accidental plant exposures from PCs in Freiburg and Berlin moderate or severe poisoning was experienced in 1.3% restricted to 39 plants. Altogether, 280 plants were re-evaluated. Risk assessment changed for some of the especially poisonous plants of the 2000 list. For example the formerly moderate poisonous plants Lantana camara, Ilex spec., and Euonymus spec. were re-assessed as minor poisonous, and the formerly minor toxic Chelidonium majus was re-assessed as moderately toxic for the eyes. Conclusion: Human data about the risk of health damage after accidental ingestion of small amounts or by accidental dermal or ocular contact are mainly restricted to published case reports. The toxicity of ingredients and of preparations of plants for medical or psychoactive purposes may be completely different. The exposure data of PCs are very useful for risk assessment of plants present in the close proximity of children's playgrounds, kindergartens, etc. The re-assessment and the final table of especially poisonous plants will be published again in the "Bundesanzeiger"
SN - 1556-3650
AD - (Maren) Poisons Information Center VIZ-Freiburg, Center for Pediatrics, Adolescent Medicine, Freiburg, Germany (Koch) Poisons Centre (PC) Charite, University Hospital Berlin, Germany (Hilke) Department of Legal Medicine, University Medical Center, Hamburg, Germany (Pietsch) Institute of Forensic Medicine, Technische Universitat Dresden, Germany (Hahn, Begemann) Federal Institute for Risk Assessment (BfR), Berlin, Germany
ER -

TY - JOUR
ID - 3345
T1 - An observational assessment of physical activity levels and social behaviour during elementary school recess
A1 - Roberts,S.J.
A1 - Fairclough,S.J.
A1 - Ridgers,N.D.
A1 - Porteous,C.
Y1 - 2013///
N1 - 2013265851
English
Journal: Article
KW - EMBASE
KW - article
KW - Child
KW - cross-sectional study
KW - Female
KW - Human
KW - major clinical study
KW - Male
KW - observational study
KW - Physical Activity
KW - play
KW - primary school

KW - priority journal
KW - school child
KW - Social Behavior
KW - Social Interaction
KW - United Kingdom
RP - NOT IN FILE
SP - 254
EP - 262
JF - Health Education Journal
VL - 72
IS - 3
CY - United Kingdom
N2 - Objective: The purpose of the present study was to assess children's physical activity, social play behaviour, activity type and social interactions during elementary school recess using a pre-validated systematic observation system. Design: Cross-sectional. Setting: Two elementary schools located in Merseyside, England. Method: Fifty-six elementary school children (27 boys, 29 girls) were systematically observed during recess over a three-month period using a standardized physical activity observation instrument. Results: The results revealed the boys were engaged in significantly higher levels of physical activity during recess than girls. The boys were also involved in more sport-specific activities and participated in larger group sizes. Positive associations between physical activity and availability of equipment during recess were also reported. Conclusion: This study has confirmed recess as a valuable opportunity for elementary school children to increase their levels of physical activity. However, further studies with larger sample sizes need to identify and explore the relationship between physical activity (PA) and availability of equipment. The Author(s) 2012
SN - 0017-8969
AD - (Roberts, Porteous) Faculty of Education, Community and Leisure, Liverpool John Moores University, United Kingdom (Fairclough) Research Institute for Sport and Exercise Sciences, Liverpool John Moores University, United Kingdom (Ridgers) Centre for Physical Activity and Nutrition, Deakin University, Australia
ER -

TY - JOUR
ID - 3346
T1 - Children's perception of neighborhood safety buffers the effect of neighborhood disorder on sleep
A1 - McGrath,J.
A1 - Jarrin,D.C.
Y1 - 2013///
N1 - 71047165
English
Journal: Conference Abstract
KW - EMBASE
KW - Human
KW - Safety
KW - diseases
KW - Sleep
KW - psychosomatics
KW - Society
KW - Child
KW - Income
KW - household
KW - Poverty
KW - Adult
KW - Crime
KW - population research
KW - parent
KW - unemployment
KW - Housing
KW - Education
KW - Regression Analysis

KW - questionnaire
KW - habit
KW - heart
KW - Crowding
KW - Arousal
KW - Violence
KW - sleep quality
KW - latent period
KW - daytime somnolence
KW - sleep time
KW - Drinking
KW - hazard
KW - Walking
KW - Population
KW - Policy
KW - high school
KW - social status
KW - sleep disorder
KW - Health
KW - phosphorus 33
RP - NOT IN FILE
SP - A
EP - 8
JF - Psychosomatic Medicine
VL - Conference: 71st Annual Scientific Meeting of the American Psychosomatic Society Miami, FL United States. Conference Start: 20130313 Conference End: 20130316. Conference Publication:
IS - var.pagings
N2 - Similar to myriad health conditions, sleep disturbances evidence a socioeconomic gradient. Poorer sleep (latency, efficiency, awakenings) is more prevalent among adults with lower household income, less educational attainment, and unemployment. Individuals of lower socioeconomic status (SES) often live in neighborhoods with more crime, violence, and crowding. Yet, neighborhood disorder does not affect all people equally. In adults, perceived neighborhood safety is protective against the effect of neighborhood disorder on sleep. The present aim was to evaluate whether children's perceived neighborhood safety moderated the association between neighborhood disorder and sleep. Children (N=91, M=10.8yrs) and their parents participated in the Healthy Heart Project. The Canadian Census and Montreal Crime Index were used to derive neighborhood data. SES was measured for family (parent education, household income) and neighborhood (unemployment, poverty, median income, high school degree or less; by census tract). Neighborhood Disorder was measured objectively (violent incidents, total offenses, mobility status, population, persons per household). Neighborhood Safety was based on children's perceptions of safety playing outside or walking alone after dark, as well as neighborhood cleanliness and dangerousness (litter, drinking in public, burglary). Children's sleep was based on self-reported sleep quality and Pediatric Daytime Sleepiness Scale, as well as parentreported sleep duration and Children Sleep Habits Questionnaire. Regression analyses using latent factors indicated greater perceived neighborhood safety moderated the effect of neighborhood disorder on sleep, even after controlling for age, sex, family SES, and neighborhood SES (interaction: Fisher's Z = .33, p<.05). Thus, children's perceived neighborhood safety buffered the association between neighbourhood disorder and sleep, suggesting perceived safety is protective and contributes to resiliency. Future research should examine objective sleep measures. These findings have potential public policy implications for housing and the concentration of disorder and poverty, which contribute to perceived safety and adversely impact children's sleep
SN - 0033-3174
AD - (McGrath) Pediatric Public Health Psychology Laboratory, Concordia University, Montreal, QC, Canada (Jarrin) Ecole de psychologie, Centre de recherche Universite Laval Robert-Giffard, Universite Laval, Quebec City, Canada
ER -

TY - JOUR
ID - 3347

T1 - Is active malaria case detection in the community able to inhibit low-level focal malaria transmission in Zanzibar?
A1 - Molteni,F.
A1 - Anderegg,C.
A1 - Ali,A.O.
A1 - Ali,A.S.
A1 - McElroy,P.D.
A1 - Al-Mafazy,A.-W.
Y1 - 2011///
N1 - 71042846
English
Journal: Conference Abstract
KW - EMBASE
KW - Malaria
KW - tropical medicine
KW - Society
KW - Tanzania
KW - Hygiene
KW - community
KW - screening
KW - Population
KW - Hospital
KW - bed net
KW - Prevalence
KW - diagnostic test
KW - malaria control
KW - Therapy
KW - parasite prevalence
KW - fever
KW - Child
KW - Human
KW - procedures
KW - artemisinin
KW - amodiaquine
KW - artesunate
RP - NOT IN FILE
SP - 207
EP - 208
JF - American Journal of Tropical Medicine and Hygiene
VL - Conference: 60th Annual Meeting of the American Society of Tropical Medicine and Hygiene, ASTMH 2011 Philadelphia, PA United States. Conference Start: 20111204 Conference End: 20111208. Conference Publication:
IS - var.pagings
N2 - Intensive malaria control interventions in Zanzibar, including indoor residual spraying, long-lasting insecticidal nets and artemisinin -based combination therapy have resulted in malaria pre elimination phase (prevalence below 1% in 2011). However, a number of transmission foci have been identifed after implementation of a clinic-based passive surveillance system to gather weekly malaria notifcations. In 2011 Zanzibar introduced a proactive case detection (pACD) effort to screen the population in transmission foci with the aim to fnd and treat asymptomatic malaria cases and reduce transmission by decreasing the parasite prevalence in the population. Two small geographic areas were selected, Area-1 with high seasonal transmission (64 km2) and Area-2 with sustained perennial transmission (28 km2). In Area-1 the entire population was tested using a HRP2-based rapid diagnostic test (RDT) and assessed for current fever. In Area-2 all children <15 yrs were tested using the same procedures. Screening posts were positioned within the targeted villages over a 3-day period in mid-May 2011, just prior to the predicted increase in seasonal transmission. Confrmed malaria cases were treated with artesunate-amodiaquine according to national guidelines. A total of 6,276 (83%) of the targeted population was screened (83.4% in Area-1 and 83.3% in Area-2). Screening in Area-1and -2 yielded 13 and 64 RDT-positive cases, respectively, with a positivity rate of 0.1% among residents <5 years of age in both

areas. Residents older than fve years had a positivity rate of 0.4% and 1.9% in Area-1 and -2, respectively. Variation in village-level positivity rates was detected (0.2-0.6% Area-1 and 1.0-2.7% Area-2). Data regarding clinical symptoms are being analyzed. A second screening session is planned in the frst week of June 2011. The community participation in Zanzibar's frst pACD effort was high and yielded 77 malaria cases outside of a clinic setting. Malaria cases with potential to perpetuate transmission were identifed and treated successfully. Effects of the frst and second screening activity on reducing malaria incidence in these communities will be carefully monitored through the existing weekly surveillance system
SN - 0002-9637
AD - (Molteni, Anderegg) Research Triangle Institute, Dar es Salaam, Tanzania (Ali, Ali, Al-Mafazy) Zanzibar Malaria Control Programme, Zanzibar, Tanzania (McElroy) Centers for Disease Control and Prevention, President's Malaria Initiative, Dar es Salaam, Tanzania
ER -

TY - JOUR
ID - 3348
T1 - Accounting for biases in a household water treatment intervention trial in the Congo
A1 - Enger,K.S.
A1 - Nelson,K.L.
A1 - Rose,J.B.
A1 - Eisenberg,J.N.
Y1 - 2011///
N1 - 71042738
English
Journal: Conference Abstract
KW - EMBASE
KW - water treatment
KW - intervention study
KW - Congo
KW - Society
KW - tropical medicine
KW - Hygiene
KW - household
KW - Human
KW - Child
KW - model
KW - pathogenesis
KW - Infection
KW - devices
KW - dose response
KW - fluid intake
KW - controlled study
KW - Rotavirus
KW - Giardia
KW - diarrheagenic Escherichia coli
KW - Risk Assessment
KW - Morbidity
KW - antimicrobial activity
KW - Population
KW - placebo
KW - Water
KW - drinking water
KW - marker
RP - NOT IN FILE
SP - 174
JF - American Journal of Tropical Medicine and Hygiene

VL - Conference: 60th Annual Meeting of the American Society of Tropical Medicine and Hygiene, ASTMH 2011 Philadelphia, PA United States. Conference Start: 20111204 Conference End: 20111208. Conference Publication:
IS - var.pagings
N2 - The effectiveness of household water treatment (HWT) devices against diarrheal disease is often measured with intervention trials. It can be diffcult to generalize these results outside the study population, and compliance with HWT is often incomplete. We constructed a quantitative microbial risk assessment (QMRA) model to address these issues. The QMRA model simulates a placebo-controlled trial (Boisson et al. 2010, PLoS One 5(9):e12613) of an HWT flter, and accounts for bias due to: A) incomplete compliance with fltration, and B) unexpected antimicrobial activity by the placebo flter. The QMRA model simulates a chain of events in children < 5 years over 12 months with a time unit of 1 day: 1) determine concentrations of 3 marker pathogens (diarrheagenic E. coli, Giardia, and rotavirus) in drinking water; 2) calculate daily doses of pathogens, using pathogen concentrations and water consumption; 3) convert doses to probabilities of infection, using a different dose response function for each pathogen; 4) assign infection to individuals, using probabilities of infection; 5) assign disease, using morbidity ratios. After calibrating the QMRA model to the results of the published trial, the model was used to estimate device effectiveness under different compliance scenarios. Four levels of compliance were considered: low, 65% of children treating 33% of their water; medium, 65% of children treating 67% of their water; high, 65% of children treating 100% of their water; and perfect, 100% of children treating 100% of their water. Compliance was a major driver of effectiveness. Assuming a perfect placebo and low, medium, high, or perfect compliance, the median preventable fraction of reported disease was 14%, 30%, 50%, and 87%, respectively. Adjustment for the imperfect placebo increased the median preventable fraction of disease by 8 percentage points assuming low compliance, but by 22 percentage points assuming medium compliance. The precise level of compliance can greatly affect measurements of HWT effectiveness; such trials should carefully measure compliance
SN - 0002-9637
AD - (Enger, Rose) Michigan State University, East Lansing, MI, United States (Nelson) University of California, Berkeley, CA, United States (Eisenberg) University of Michigan, Ann Arbor, MI, United States
ER -

TY - JOUR
ID - 3349
T1 - Globalization and indigenous health: A case study of the social and environmental context of Achuar child health in an oil extraction zone in the Peruvian Amazon
A1 - Willis,R.N.
Y1 - 2011///
N1 - 71042273
English
Journal: Conference Abstract
KW - EMBASE
KW - case study
KW - child health
KW - extraction
KW - Society
KW - tropical medicine
KW - Hygiene
KW - Health
KW - Human
KW - community
KW - Child
KW - Population
KW - Food
KW - household
KW - diseases
KW - mosquito
KW - river
KW - environmental change
KW - ethnicity

KW - exposure
KW - health service
KW - Social Environment
KW - health belief
KW - Environment
KW - Malaria
KW - Health Status
KW - protection
KW - Policy
KW - quantitative analysis
KW - river basin
KW - indigenous people
KW - World Health Organization
RP - NOT IN FILE
SP - 33
JF - American Journal of Tropical Medicine and Hygiene
VL - Conference: 60th Annual Meeting of the American Society of Tropical Medicine and Hygiene, ASTMH 2011 Philadelphia, PA United States. Conference Start: 20111204 Conference End: 20111208. Conference Publication:
IS - var.pagings
N2 - Indigenous peoples internationally have poorer health status than non-indigenous populations. In the Peruvian Amazon, remote indigenous populations face dual challenges of isolation from health services and increasing exposure to rapid social and environmental change caused by recent expansion of resource extraction activities in their territories. This research uses interdisciplinary methods to explore social and environmental determinants of health for children aged <5yrs in a case study of indigenous Achuar communities in the Corrientes river basin. Malaria and diarrhoeal diseases are endemic in the region. The communities' living, fshing and hunting territories straddle two internationally run oil concessions and oil extraction activities contribute to community members' daily environments. Indigenous Amazonian populations have distinct health belief systems based on their relationship with their physical and social environment. Aiming to respect and learn from local conceptualisation of health and illness, this research addresses determinants of young children's health from multiple perspectives. Firstly, qualitative methods were used to explore community members' (n=30) and local stakeholders' (n=30) perspectives of factors infuencing young children's health. Secondly, quantitative social epidemiological methods were used to characterise structural (e.g. ethnicity) and proximal (e.g. material circumstances) determinants of health based on the framework proposed by the WHO Commission on Social Determinants of Health (n=138 children aged <5 yrs in 97 households). Qualitative results found that respondents across groups identifed food - access to suffcient and appropriate kinds of food - as a key child health determinant. 96% of households receive donated food. Also highlighted were; the biological and spiritual importance of the river, protection from mosquitoes, and access to economic resources. Quantitative analysis compared socio-economic and biomedical child health indicators in the case study setting with other indigenous and non-indigenous poor rural Peruvian communities outside oil extraction zones. Research fndings have implications for policy development to protect children's health in indigenous communities increasingly exposed to large scale extraction activities across the Peruvian Amazon
SN - 0002-9637
AD - (Willis) London School of Hygiene and Tropical Medicine, London, United Kingdom
ER -

TY - JOUR
ID - 3350
T1 - Previously uncharacterized biological activity of the macrolide toxin from mycobacterium ulcerans, mycolactone
A1 - Dickerson,T.J.
A1 - Gale,T.V.
Y1 - 2012///
N1 - 71041224
English
Journal: Conference Abstract
KW - EMBASE

KW - Mycobacterium ulcerans
KW - Society
KW - tropical medicine
KW - Hygiene
KW - biological activity
KW - Human
KW - ulcer
KW - secondary infection
KW - Buruli ulcer
KW - tropical disease
KW - necrosis
KW - Africa
KW - Australia
KW - bacterium
KW - Inflammation
KW - Environment
KW - apoptosis
KW - plasmid
KW - laboratory
KW - exposure
KW - bacterial strain
KW - Staphylococcus epidermidis
KW - consensus
KW - synthesis
KW - Child
KW - cell type
KW - Case Report
KW - macrolide
KW - toxin
KW - virulence factor
KW - enzyme
RP - NOT IN FILE
SP - 179
JF - American Journal of Tropical Medicine and Hygiene
VL - Conference: 61st Annual Meeting of the American Society of Tropical Medicine and Hygiene, ASTMH 2012 Atlanta, GA United States. Conference Start: 20121111 Conference End: 20121115. Conference Publication:
IS - var.pagings
N2 - Mycobacterium ulcerans is the etiological agent causing the neglected tropical disease, Buruli ulcer. This disease is endemic to sub-Saharan Africa and Australia, with case reports primarily occurring in children. Clinically, the disease initially presents as a painless nodule, which deteriorates radially to a necrotic ulcer with characteristic undermined edges. There is a pronounced lack of inflammatory response at the site of ulcers, with only anecdotal mention of secondary infection in the literature. The macrolide toxin produced by M. ulcerans, mycolactone, is accepted as the sole virulence factor and has been shown to cause apoptosis and necrosis in vivo, as well as possess immunomodulatory properties. We hypothesized that the notable lack of secondary infection may also be a phenomenon mediated by mycolactone. Results in our laboratory have shown that exposure to mycolactone arrests the growth of specific bacterial strains, including S. aureus and S. epidermidis. Interestingly, there are no reports of clinically isolated mycolactone-deficient bacteria, and the common consensus is that selection pressure must be present to maintain the otherwise genetically unstable megaplasmid that encodes enzymes for mycolactone synthesis. Our results demonstrate an example of this selection. We have found that recovery of pMUM001 plasmid could be achieved simply by inclusion of lysates from arrested cell types. Given this data, we speculate that mycolactone is an "accidental toxin" whose intended function is to provide M. ulcerans a competitive advantage in its natural environment
SN - 0002-9637
AD - (Dickerson, Gale) Scripps Research Institute, San Diego, CA, United States
ER -

TY - JOUR
ID - 3351
T1 - Shift in dynamics in eastern equine encephalitis virus activity in central New York
A1 - Kramer,L.D.
A1 - Jones,S.A.
A1 - Dupuis,A.P.
A1 - Maffei,J.G.
A1 - Oliver,J.A.
A1 - Howard,J.
A1 - Sherwood,J.A.
A1 - Backenson,B.
Y1 - 2012///
N1 - 71041193
English
Journal: Conference Abstract
KW - EMBASE
KW - Eastern equine encephalomyelitis virus
KW - United States
KW - Society
KW - tropical medicine
KW - Hygiene
KW - dynamics
KW - Human
KW - deer
KW - virus
KW - horse
KW - mosquito
KW - Male
KW - Encephalitis
KW - Alpha virus
KW - Temperature
KW - Genotype
KW - Togavirus
KW - Infection
KW - Prevalence
KW - songbird
KW - Population
KW - Child
KW - bird
KW - Phylogeny
KW - dog
KW - lake
KW - Canada
RP - NOT IN FILE
SP - 169
EP - 170
JF - American Journal of Tropical Medicine and Hygiene
VL - Conference: 61st Annual Meeting of the American Society of Tropical Medicine and Hygiene, ASTMH 2012 Atlanta, GA United States. Conference Start: 20121111 Conference End: 20121115. Conference Publication:
IS - var.pagings
N2 - Eastern equine encephalitis virus (EEEV;Togaviridae:Alphavirus) is a highly pathogenic mosquito-borne virus that produces severe or fatal encephalitis in 30-50% of infected humans and horses. Recently, EEEV has undergone a regime shift in dynamics in the northeastern US and Canada. For three decades (1970-2000) periodic activity was detected in central NY, specifically in counties surrounding Oneida Lake, with 1-2 years of activity in mosquitoes and other hosts, followed by 1-6 years with no detectable activity. However, since 2003, there have been 9 consecutive years with active EEEV transmission, and a significantly higher prevalence of

infection than in previous outbreaks (p = 0.009). This string of 9 consecutive years is a highly unlikely event to occur by chance (p = 0.007 with 15 of 28 years with EEEV presence). The current epizootic coincides with the arrival of a novel genotype of EEEV, the invasion of WNV to the region, and occurs within the backdrop of long term trends in temperature and declines in key songbird populations. The first human case in 26 years occurred in Oswego County in 2009 in a 70yo male, then again in 2010 in a 77yo male, and in 2011 in a 4yo child. EEEV-infected neurologic deer were found in 2008 and 2009. Serosurveys of hunter killed deer < 2yo also have proven useful in determining the range of EEEV activity with 9 EEEV positive deer out of 179 tested from central NY 2007-2009. In 2011, 46 Culiseta melanura pools were found positive (MIR 4.2); the previous 3 year average was 43 (MIR 4.1). Similarly, in 2011,11 horses died from EEEV, while the previous 3 year average was 7 equines. In addition, two neurologic dogs died in 2011. Phylogenetic analysis of isolates indicates the virus is periodically introduced, most likely by migratory birds, but also overwinters in the central NY focus. However, we identified a genetic change in the virus in 2007, the year preceding the first human cases seen since 1983. In summary, although the level of EEEV activity does not appear to be increasing significantly in the enzootic focus in central NY, it appears to be extending its range and intensity outside this historic focus
SN - 0002-9637
AD - (Kramer, Jones, Dupuis, Maffei) Wadsworth Center, Albany, NY, United States (Oliver, Howard, Sherwood, Backenson) New York State Department of Health, Albany, NY, United States
ER -

TY - JOUR
ID - 3353
T1 - Camp courage: Campers and caregivers perspectives
A1 - Sarcone,S.
A1 - Routhieaux,J.
A1 - Seal,A.
A1 - Mehrhof,A.
A1 - Woelk,A.
A1 - Kalpatthi,R.
A1 - Woods,G.
Y1 - 2012///
N1 - 71030902
English
Journal: Conference Abstract
KW - EMBASE
KW - sickle cell anemia
KW - Human
KW - caregiver
KW - courage
KW - patient
KW - sibling
KW - volunteer
KW - Child
KW - sickle cell
KW - physician
KW - profit
KW - social worker
KW - nurse
KW - healing
KW - hope
KW - Health Care
KW - boy
KW - girl
KW - medical specialist
KW - Hospital
KW - psychosocial care
KW - Education
KW - medical care

KW - feedback system
KW - psychologist
KW - advanced practice nurse
KW - Male
KW - Female
RP - NOT IN FILE
SP - E40
EP - E41
JF - American Journal of Hematology
VL - Conference: 6th Annual Sickle Cell Disease Research and Educational Symposium and Annual National Sickle Cell Disease Scientific Meeting Hollywood, FL United States. Conference Start: 20120229 Conference End: 20120302. Conference Publication:
IS - var.pagings
N2 - Background: Camp Courage is a non-profit overnight camp for patients with Sickle Cell Disease and their siblings. This camp was started in 1991 and has been held yearly since then. The camp was initially developed by a sickle cell advanced practice nurse (APN). The initial camp involved a small staff ofa sickle cell physician, the organizing APN and sickle cell social worker. The camp was initially "sponsored" by Children's Mercy Hospital with involvement of volunteer counselors and group-sponsored activities. The camp has been conducted on six different camp sites and serves children ages 6-14 years with all types of Sickle Cell Disease. Siblings are then offered a spot at camp on a first come, first serve basis depending on space availability. The camp has evolved to include multiple health care disciplines and is currently sponsored by an outside non-profit agency, Turning Point: The Center for Hope and Healing. The most recent camp in July 2011 included 56 campers, 5 physicians, 4 nurses, 2 social workers, 2 child life specialists, 1 psychologist, 1 data coordinator, 16 counselors and 38 volunteers. Physicians and APNs were present 24 hours a day to provide medical care, supervision and disease education. Other disciplines and volunteers provided activity and psychosocial support on a daily basis. Volunteer counselors were present during the entire camp experience including nighttime supervision and support in the campers' cabins. Objective: Camp has long been thought by staff to be a positive experience for patients and families, however formal feedback has never been collected. Our goal is to use the information collected to identify positive aspects of camp and also identify areas for improvement, both recreational and educational. Method: Surveys were distributed via mail to 36 families (this included 36 patients, 20 siblings [32 girls and 24 boys], 36 caregivers) to assess possible benefits and negative experiences of the camp. Results: Preliminary results suggest Camp Courage is a positive experience for patients, siblings and caregivers. We will report a complete analysis and evaluation of the survey data returned at the meeting. Conclusion: The information will help us to identify campers and caregivers' overall perspective of the camp experience
SN - 0361-8609
AD - (Sarcone, Routhieaux, Seal, Mehrhof, Woelk, Kalpatthi, Woods) Children's Mercy Hospitals and Clinics, United States
ER -

TY - JOUR
ID - 3354
T1 - Challenges of asthma control in developing and developed countries
A1 - Moin,M.
Y1 - 2013///
N1 - 71027574
English
Journal: Conference Abstract
KW - EMBASE
KW - Asthma
KW - developed country
KW - Prevalence
KW - immunology
KW - allergy
KW - developing country
KW - Human
KW - Population

KW - Iran
KW - Child
KW - gene
KW - risk factor
KW - Mortality
KW - Lifestyle
KW - meta analysis
KW - electronic medical record
KW - Diet
KW - Adult
KW - Income
KW - sensitization
KW - virus infection
KW - Drug Therapy
KW - Chronic Disease
KW - Obesity
KW - Primary Health Care
KW - prevention and control
KW - exposure
KW - Finland
KW - Schizophrenia
KW - Male
KW - atopy
KW - Air Pollution
KW - Diabetes Mellitus
KW - disability
KW - Environment
KW - Smoking
KW - lung function
KW - corticosteroid therapy
KW - Hospitalization
KW - family life
KW - Adolescent
KW - health care cost
KW - epidemic
KW - Society
KW - school
KW - Aged
KW - Liver Cirrhosis
KW - Traffic
KW - city
KW - physician
KW - register
KW - Health
KW - World Health Organization
KW - tobacco smoke
KW - allergen
KW - dipyridamole
RP - NOT IN FILE
SP - S89
JF - Iranian Journal of Allergy, Asthma and Immunology
VL - Conference: 2nd International Congress of Immunology, Asthma and Allergy Tehran Iran, Islamic Republic of. Conference Start: 20130219 Conference End: 20130221. Conference Publication:
IS - var.pagings
N2 - Asthma is the most common and sometime fatal chronic disease of children and a serious public and global health problem. The prevalence of asthma has increased continuously following changes to a modern urban lifestyle during the past three decades especially among children and adolescents. It has a higher

prevalence in developed nations more among poor populations, and in affluent populations compared to poor populations in developing nations. Increasing asthma prevalence in some developed countries has reached a Plato in past few years and its mortality declined recently due to better medications, guidelines for asthma management and measuring outcomes through electronic medical records systems. The recent meta-analysis of the epidemiological studies done in Iran also showed that the prevalence of pediatric asthma symptoms continues increasing at the national level (Average prevalence of 13.14%) and increasing seriously in Tehran megacity (35.4%) and thus increasing the burden of asthma to individuals and society. Asthma intervention programmes also have led to a remarkable decrease in death rates and hospitalizations in developed, developing and deprived countries. A complex gene-gene and gene by environment interactions including epigenetic mechanisms supposed to be related to the worldwide increasing trend of prevalence and burden of asthma. Atopy, male sex, younger age, obesity, allergen exposure, viral infections, occupational sensitizers, tobacco smoke, indoor/outdoor air pollution, diet, stress, and western lifestyle are the main genetic, environmental, socioeconomic, and susceptibility risk factors for development and expression of asthma. About 25 percent of asthmatic adults in low and middle income countries are active smokers and have more severe asthma symptoms, decline in lung function and reduced response to corticosteroid therapy. Large and crowded cities of most developing countries with a heavy traffic are severely polluted which is another environmental risk factor especially for children and elderly. This asthma epidemic has caused a great burden, not only in terms of health care costs but also of reduced participation in family life, of lost school days and days lost from work. The burden of asthma assessed by disability adjusted life years (DALYs) ranks 22 worldwide, which is similar to diabetes, liver cirrhosis and schizophrenia. The surveillance of asthma as part of a global WHO programme is essential for controlling asthma prevalence and burden. Different international and national guidelines are designed and implemented in past 1-2 decades but current level of asthma control still falls short of published guidelines. The experience of the 10 years Asthma Control Programme of Finland showed considerable reduction in the burden of asthma. In Iran the National Asthma Guideline was published under the supervision of the National Committee on Asthma and Allergy in 2009 to be implemented especially at the primary health care physicians level. the First National Asthma Control Programme of our country is expected to be launched in near future too. The First Asthma Registry Programme too is established in Immunology, Asthma and Allergy Research Institute (IAARI) since 2008. In this article an overview of the challenges and strategies of asthma prevention and control in Iran, developing and developed countries are presented
SN - 1735-1502
AD - (Moin) Immunology, Asthma and Allergy Research Institute (IAARI), Tehran University of Medical Sciences, Iran, Islamic Republic of
ER -

TY - JOUR
ID - 3355
T1 - A multi-component intervention increased physical activity in preschool children
A1 - Pate,R.R.
A1 - Brown,W.H.
A1 - Saunders,R.
A1 - Pfeiffer,K.A.
A1 - Dowda,M.
Y1 - 2013///
N1 - 71026537
English
Journal: Conference Abstract
KW - EMBASE
KW - Public Health
KW - Physical Activity
KW - preschool child
KW - Human
KW - medical society
KW - Epidemiology
KW - Nutrition
KW - Metabolism
KW - Exercise
KW - Child

KW - school
KW - parent
KW - teacher
KW - boy
KW - Environment
KW - Male
KW - girl
KW - Education
KW - analysis of covariance
KW - control group
KW - questionnaire
KW - Accelerometry
KW - model
KW - sitting
KW - weight
KW - hypothesis
KW - Female
KW - height
KW - Child Care
RP - NOT IN FILE
JF - Circulation
VL - Conference: American Heart Association's Epidemiology and Prevention/Physical Activity, Nutrition and Metabolism 2013 Scientific Sessions New Orleans, LA United States. Conference Start: 20130319 Conference End: 20130322. Conference Publication:
IS - var.pagings
N2 - Introduction: A majority of preschool-age children spend a significant portion of every weekday in a preschool or child care setting. Studies have shown that children in these settings spend a majority of their time in sedentary behavior and very little time in physical activity. Hypothesis: A multi-component preschool physical activity intervention will increase the amount of time young children spend in moderate-to-vigorous physical activity (MVPA) and decrease the time they spend in sedentary activities, compared to children in control preschools. Methods: The study included 16 preschools with 4-year-old classrooms, paired and randomly assigned to intervention and control groups. The intervention focused on increasing children's physical activity by changing instructional practices and the classroom environment. Research staff trained preschool teachers to engage children in physical activity throughout the school day and provided physical activity materials, ongoing support and technical assistance. Intervention components included physical activity opportunities in the classroom, physical activity outdoors, and active lessons. The intervention was designed to be flexible, and research staff encouraged teachers to adapt it to their classroom environment. Four-year-old children and their parents were recruited in two waves at each school. Measures included child height and weight, child physical activity by accelerometry, and parent questionnaires, measured during the fall and spring of each wave. Mixed model analysis of covariance with preschool as a random variable tested the effects of the intervention on physical activity in the total group and by sex. Results: A total of 379 children (188 intervention and 191 control) were measured at baseline. Children were 4.5+0.4 years of age, and 50% were boys. The intervention schools included a higher percentage of white children compared to the control schools (44.2% versus 39.3%). After adjusting for wave, parent education and length of the school day (half day vs. full day), children in the intervention schools engaged in significantly more MVPA than children in the control schools (7.4+0.2 and 6.7+0.2 minutes/hour, respectively). In the sex-specific analyses, differences between intervention and control were significant for girls (6.9+02 vs. 6.1+0.2 minutes/hour of MVPA, respectively) but not for boys (7.8+03 vs. 7.3+0.3 minutes/hour, respectively). No differences in time spent in sedentary behavior were observed between intervention and control preschools. Conclusions: In conclusion, a flexible, multi-component physical activity intervention that trains teachers to provide children with opportunities to be active throughout the school day increased MVPA in preschool children
SN - 0009-7322
AD - (Pate, Brown, Saunders, Dowda) Univ of South Carolina, Columbia, SC, United States (Pfeiffer) Michigan State, East Lansing, MI, United States
ER -

TY - JOUR

ID - 3356
T1 - UV exposure of tennis players
A1 - Maier,H.
A1 - Grabenhofer,M.
A1 - Maier,C.
A1 - Schiefer,A.
A1 - Maier,B.
A1 - Schmalwieser,A.
Y1 - 2013///
N1 - 71022470
English
Journal: Conference Abstract
KW - EMBASE
KW - tennis
KW - exposure
KW - Human
KW - dosimeter
KW - Child
KW - Radiation
KW - Adult
KW - sensor
KW - Adolescent
KW - Temperature
KW - Germany
KW - sand
KW - optics
KW - Physical Activity
KW - ultraviolet radiation
KW - environmental temperature
KW - field study
KW - cultural anthropology
KW - forehead
KW - erythema
KW - leisure
KW - Risk
KW - summer
KW - sport
KW - sunscreen
RP - NOT IN FILE
SP - e37
JF - Experimental Dermatology
VL - Conference: 40th Annual Meeting of the Arbeitsgemeinschaft Dermatologische Forschung, ADF 2013 Dessau Germany. Conference Start: 20130314 Conference End: 20130316. Conference Publication:
IS - var.pagings
N2 - Outdoor sport is part of leisure time behaviour in the western world which contributes significantly to the annual ultraviolet (UV) exposure. One of the most popular outdoor activities, both in adults and children is tennis. In our field study we measured UV exposure during a training match between two adults and - for the first time - the UV exposure of children playing tennis. The players/parents of the players gave their consent to wear/that their children wear two electronic UV dosimeters (X 2000-10, Gigahertz Optics, Germany; UVA and UVB/C sensor, temperature, 1 min measuring interval), one on the forehead fixed to a shielded headband, the other fixed to the calf by an elastic band with Velcro fastening. We calibrated the dosimeters prior to the field test. The training match between the adult test persons A (43 years) and B (51 years) took place on June 30th from 1:00 to 2:36 PM, the two adolescents C and D, both 17 years of age, played on August 23rd between 1:45 and 14:52 PM on an almost north-south directed tennis court in Vienna. The players were observed by two members of the study team. One of the observers took photos of the players and at 15 min interval standardized photos of the sky. We measured ambient radiation with the help of two dosimeters fixed to a tripod in horizontal position: one dosimeter with the UV sensors directed upward, the second dosimeter with the sensors directed to

the tennis ground in order to measure the reflectance. Sky was absolutely clear on June 30th with an ambient temperature of 35degreeC, whereas on August 26th there was variable cloudiness. The temperature on that day was around 30degreeC. The test persons were asked to behave as usual especially with respect to physical activity and photo protective measures. All our test persons wore similar tennis kits: T-shirt, shorts or short skirt, and tennis socks. All of them applied sunscreens with SPF 30. Three of them used sunglasses. The UV exposures (standard erythema dose = SED) on June 30th were: test person A (head 2.82/calf 2.58), B (head 5.09/calf 1.41) which represent 20.05% to 72.3% of ambient radiation (7.05 SED). The respective values for the training match on August 26th were: test person C (1.54/0.63), test person D (1.98/0.62) which is equivalent to 32% to 100.15% of ambient (1.98 SED). 2.6% of the ambient radiation was reflected from the ground (red sand). The adult tennis players played two sets, our adolescent test persons, however, played only one set. Children are at high risk especially when playing in spring and early in summer when ambient radiation is highest. This should be considered by organizers of tennis tournaments and tennis trainers when scheduling their training lessons
SN - 0906-6705
AD - (Maier, Grabenhofer, Maier, Schiefer, Maier) Department of Dermatology, Medical University of Vienna, Vienna 1090, Austria (Schmalwieser) Institute for Molecular Physiology and Biophysics, University of Veterinary Medicine, Vienna 1220, Austria
ER -

TY - JOUR
ID - 3357
T1 - Team's positive experience with a longitudinal mentoring program for chronic hemodialysis children
A1 - Barrera,J.A.
A1 - Chen,E.C.
A1 - Kanak,M.M.
A1 - Kim,D.S.
A1 - Liu,J.
A1 - Wang,A.
A1 - Wong,C.
A1 - Nguyen,L.
Y1 - 2013///
N1 - 71022189
English
Journal: Conference Abstract
KW - EMBASE
KW - dialysis
KW - Human
KW - Child
KW - hemodialysis
KW - patient
KW - teacher
KW - medical student
KW - nurse
KW - Hospital
KW - school
KW - Morbidity
KW - hospital planning
KW - social psychology
KW - University
KW - outpatient
KW - student
KW - Social Support
KW - chronic patient
KW - parent
KW - pediatric hospital
KW - hemodialysis patient
RP - NOT IN FILE

SP - 156
EP - 157
JF - Hemodialysis International
VL - Conference: 33rd Annual Dialysis Conference and 24th Annual Symposium on Pediatric Dialysis Seattle, WA United States. Conference Start: 20130309 Conference End: 20130312. Conference Publication:
IS - var.pagings
N2 - Background: Pediatric hemodialysis (HD) patients face many psychosocial factors that affect adherence and morbidity. Social interventions could benefit these patients, but very few structured programs currently exist within the hospital system. The PALS program at Stanford University School of Medicine (SUSOM) pairs medical students with chronically ill children to provide social support. We present our PALS experience in the pediatric HD unit. Methods: Five pairs of SUSOM students (Mentors) and Lucile Packard Children's Hospital's outpatient pediatric HD patients (Pals) met weekly or biweekly in the dialysis unit. Activities included: arts and crafts, board games, and homework help. Group field trips facilitated interactions outside of the hospital. Pals and families, Mentors, and HD unit nurses completed surveys regarding their PALS experience. Results: (1) Pals greatly enjoyed doing projects and going on field trips with Mentors. Parents noted a less isolating experience for the child and the family. (2) Medical students valued the opportunity to share the HD experience alongside patients and their families. (3) Nurses felt Mentors supported Pals emotionally and medically, helping patients complete three-hour HD runs with fewer complications. More consistent visits were suggested as a possible improvement to the program. Discussion/ Conclusion: The PALS program achieved its goal of providing support to chronic HD children and families. Important factors in a successful mentoring system include an established program with institutional support, one-to-one matches, longitudinal engagement, and consistent, frequent, and planned interactions
SN - 1492-7535
AD - (Barrera, Chen, Kanak, Kim, Liu, Wang) Stanford University, School of Medicine, Stanford, CA, United States (Wong, Nguyen) Stanford University, School of Medicine, Pediatric Nephrology, Stanford, CA, United States
ER -

TY - JOUR
ID - 3359
T1 - Ultrapure soft water improves the skin barrier function of child atopic dermatitis: The first report
A1 - Togawa,Y.
A1 - Kambe,N.
A1 - Shimojo,N.
A1 - Mochizuki,H.
A1 - Matsuda,H.
A1 - Tanaka,A.
A1 - Matsue,H.
Y1 - 2012///
N1 - 71015115
English
Journal: Conference Abstract
KW - EMBASE
KW - skin
KW - Child
KW - Human
KW - atopic dermatitis
KW - Society
KW - hardness
KW - patient
KW - pruritus
KW - Japan
KW - bath
KW - machine
KW - clinical trial
KW - university hospital
KW - Dermatitis

KW - eczema
KW - skin care
KW - dry skin
KW - serum
KW - Water
KW - soap
KW - tap water
KW - mineral
KW - cation exchange resin
KW - magnesium
KW - Calcium
KW - dye
KW - ion
KW - thymus and activation regulated chemokine
KW - tartrazine
RP - NOT IN FILE
SP - S68
JF - Journal of Investigative Dermatology
VL - Conference: 42nd Annual Meeting of the European Society for Dermatological Research, ESDR 2012 Venice Italy. Conference Start: 20120919 Conference End: 20120922. Conference Publication:
IS - var.pagings
N2 - The presence of mineral contents, mostly those of calcium (Ca++) and magnesium (Mg++) decides hardness in water. In Chiba, where our university hospital is located, the hardness in home tap water is 92 mg/L and it is the second highest area in Japan. The mineral ions react with some components of soap to form an insoluble precipitate known as "soap scum". Since soap scum remains tightly on the skin and can not be easily rinsed away, it may become one of causes that exacerbate dermatitis. In this study, we used cation-exchange resin to prepare ultra-pure soft water (UPSW) almost completely removing both Ca++ and Mg++, and investigated clinical and objective improvement of skin barrier functions in children with atopic dermatitis (AD). Our clinical trial targets 30 child patients of AD from 3 to 6 years old. After installing a UPSW machine (Bihadakko; Miura CO., LTD, Japan) in the bathroom of each home, child patients were randomly assigned to two groups depending on whether they received shower treatment with UPSW or ordinal tap water for 6 weeks. After a washout period of 2 weeks, the treatment protocol was switched each other. The pre- and posttreatment values of the following were compared: the eczema area and severity indexdermatitis score (EASI), TEWL and the outside to inside skin transparency of yellow dye (tartrazine) measured by a colorimeter. In addition, serum TARC was measured and itch was evaluated using a VAS. After shower treatment with UPSW for 6 weeks, most child AD patients improved their dry skin and itch in addition to their EASI. TEWL and the outside to inside skin transparency were slightly reduced. Based on our study, the use of UPSW is considered to be beneficial for skin care of children with AD, especially in the area where the hardness in water is high
SN - 0022-202X
AD - (Togawa, Kambe, Matsue) Dermatology, Chiba University, Chiba, Japan (Shimojo) Pediatrics, Chiba University, Chiba, Japan (Mochizuki) Pediatrics, Tokai University, Kanagawa, Japan (Matsuda, Tanaka) Animal Life Science, Tokyo University of Agriculture and Technology, Tokyo, Japan
ER -

TY - JOUR
ID - 3360
T1 - Bleeding and Thrombotic Emergencies in Pediatric Cardiac Intensive Care: Unchecked Balances
A1 - Giglia,T.M.
A1 - DiNardo,J.
A1 - Ghanayem,N.S.
A1 - Ichord,R.
A1 - Niebler,R.A.
A1 - Odegard,K.C.
A1 - Massicotte,M.P.
A1 - Yates,A.R.
A1 - Laussen,P.C.
A1 - Tweddell,J.S.

Y1 - 2012///
N1 - 2013128073
English
Journal: Review
KW - EMBASE
KW - anticoagulant therapy
KW - blood clot lysis
KW - brain ischemia/co [Complication]
KW - brain ischemia/di [Diagnosis]
KW - catheter thrombosis/co [Complication]
KW - catheter thrombosis/dt [Drug Therapy]
KW - causal attribution
KW - cerebrovascular accident/co [Complication]
KW - cerebrovascular accident/di [Diagnosis]
KW - Child
KW - continuous infusion
KW - cryoprecipitate
KW - cyanotic heart disease/su [Surgery]
KW - disease predisposition
KW - drug bioavailability
KW - drug distribution
KW - drug efficacy
KW - drug half life
KW - drug mechanism
KW - drug safety
KW - fibrinolytic therapy
KW - Fontan Procedure
KW - hemostasis
KW - heparin induced thrombocytopenia/dt [Drug Therapy]
KW - Human
KW - Incidence
KW - intensive care
KW - intensive care unit
KW - lung embolism/dt [Drug Therapy]
KW - Neuroimaging
KW - nuclear magnetic resonance imaging
KW - Partial Thromboplastin Time
KW - perioperative period
KW - peroperative complication/co [Complication]
KW - pharmacological blocking
KW - postoperative complication/co [Complication]
KW - postoperative hemorrhage/co [Complication]
KW - postoperative hemorrhage/dt [Drug Therapy]
KW - postoperative hemorrhage/et [Etiology]
KW - postoperative thrombosis/co [Complication]
KW - postoperative thrombosis/di [Diagnosis]
KW - postoperative thrombosis/dt [Drug Therapy]
KW - postoperative thrombosis/su [Surgery]
KW - priority journal
KW - prosthetic valve thrombosis/co [Complication]
KW - prosthetic valve thrombosis/di [Diagnosis]
KW - prosthetic valve thrombosis/dt [Drug Therapy]
KW - prosthetic valve thrombosis/pc [Prevention]
KW - Prothrombin Time
KW - review
KW - risk factor
KW - systemic pulmonary shunt

KW - thrombocyte transfusion
KW - thromboelastography
KW - thrombophilia
KW - transthoracic echocardiography
KW - treatment duration
KW - treatment indication
KW - treatment response
KW - aminocaproic acid/cm [Drug Comparison]
KW - aminocaproic acid/dt [Drug Therapy]
KW - aminocaproic acid/pd [Pharmacology]
KW - anticoagulant agent/cb [Drug Combination]
KW - anticoagulant agent/dt [Drug Therapy]
KW - antivitamin K/cb [Drug Combination]
KW - antivitamin K/dt [Drug Therapy]
KW - aprotinin/cr [Drug Concentration]
KW - aprotinin/dt [Drug Therapy]
KW - aprotinin/pd [Pharmacology]
KW - argatroban/dt [Drug Therapy]
KW - argatroban/pk [Pharmacokinetics]
KW - heparin/dt [Drug Therapy]
KW - heparin/iv [Intravenous Drug Administration]
KW - heparin/pk [Pharmacokinetics]
KW - Heparin/pd [Pharmacology]
KW - heparin/sc [Subcutaneous Drug Administration]
KW - heparin lyase/dt [Drug Therapy]
KW - hirulog/dt [Drug Therapy]
KW - hirulog/pk [Pharmacokinetics]
KW - low molecular weight heparin/ct [Clinical Trial]
KW - low molecular weight heparin/dt [Drug Therapy]
KW - lysine derivative/dt [Drug Therapy]
KW - lysine derivative/pd [Pharmacology]
KW - protamine/dt [Drug Therapy]
KW - recombinant blood clotting factor 7a/dt [Drug Therapy]
KW - recombinant blood clotting factor 7a/pd [Pharmacology]
KW - thrombin inhibitor/dt [Drug Therapy]
KW - tissue plasminogen activator/dt [Drug Therapy]
KW - tranexamic acid/cm [Drug Comparison]
KW - tranexamic acid/dt [Drug Therapy]
KW - tranexamic acid/pd [Pharmacology]
RP - NOT IN FILE
SP - 470
EP - 491
JF - World Journal for Pediatric and Congenital Hearth Surgery
VL - 3
IS - 4
CY - United States
N2 - Children in the cardiac intensive care unit (CICU) with congenital or acquired heart disease are at risk for hematologic complications, both hemorrhage and thrombosis. The overall incidence of hematologic complications in the CICU is unknown, but risk factors and target groups have been identified where the essential physiologic balance between bleeding and clotting has been disrupted. Although the best management of life-threatening bleeding and clotting is prevention, the cardiac intensivist is often faced with managing life-threatening hematologic events involving patients from within the unit or those who present from outside. Part I of this review deals with the propensity of children with congenital and acquired heart disease to complications of both bleeding and clotting, and includes discussions of perioperative bleeding, thromboses in single-ventricle patients, clotting of Blalock-Taussig shunts and thrombotic complications of mechanical valves. Part II deals with the subject of stroke in children with heart disease. Part III reviews monitoring the effectiveness of

anticoagulation and thrombolysis in the CICU. Currently available diagnostics modalities, medications and management strategies are reviewed and future directions discussed. The Author(s) 2012
SN - 2150-1351
AD - (Giglia) Division of Cardiology, University of Pennsylvania School of Medicine, The Children's Hospital of Philadelphia, 8NW-62, 34th and Civic Center Blvd, Philadelphia, PA 19104, United States (DiNardo) Division of Cardiac Anesthesia, Children's Hospital, Harvard Medical School, Boston, MA, United States (Ghanayem, Tweddell) Division of Critical Care, Children's Hospital of Wisconsin, Milwaukee, WI, United States (Ichord) Division of Neurology, Children's Hospital of Philadelphia, University of Pennsylvania School of Medicine, Philadelphia, PA, United States (Niebler) Division of Critical Care, Children's Hospital of Wisconsin, Milwaukee, WI, United States (Odegard) Division of Cardiovascular Critical Care, Children's Hospital, Harvard Medical School, Boston, MA, United States (Massicotte) Department of Pediatrics, Stoller Children's Hospital, University of Alberta, Edmonton, AB, Canada (Yates) Sections of Cardiology and Critical Care Medicine, Nationwide Children's Hospital, The Ohio State University College of Medicine, Columbus, OH, United States (Laussen) Division of Cardiovascular Critical Care, Children's Hospital, Harvard Medical School, Boston, MA, United States
ER -

TY - JOUR
ID - 3362
T1 - Fentanyl-associated Fatalities Among Illicit Drug Users in Wayne County, Michigan (July 2005-May 2006)
A1 - Algren,D.A.
A1 - Monteilh,C.P.
A1 - Punja,M.
A1 - Schier,J.G.
A1 - Belson,M.
A1 - Hepler,B.R.
A1 - Schmidt,C.J.
A1 - Miller,C.E.
A1 - Patel,M.
A1 - Paulozzi,L.J.
A1 - Straetemans,M.
A1 - Rubin,C.
Y1 - 2013///
N1 - 2013125624
English
Journal: Article
KW - EMBASE
KW - Adolescent
KW - Adult
KW - article
KW - Autopsy
KW - Cause of Death
KW - Child
KW - coroner
KW - data base
KW - Drug Abuse
KW - drug fatality
KW - enzyme linked immunosorbent assay
KW - Female
KW - forensic toxicology
KW - Human
KW - human tissue
KW - Male
KW - mass fragmentography
KW - preschool child
KW - school child

KW - United States
KW - benzoylecgonine
KW - cocaine
KW - diamorphine/to [Drug Toxicity]
KW - drug metabolite
KW - fentanyl/to [Drug Toxicity]
KW - illicit drug
KW - morphine
KW - morphine 6 acetate
KW - norfentanyl
KW - unclassified drug
RP - NOT IN FILE
SP - 106
EP - 115
JF - Journal of Medical Toxicology
VL - 9
IS - 1
CY - United States
N2 - Background: During the summer of 2005, multiple cities in the United States began to report outbreaks of fentanyl-associated fatalities among illicit drug users. The objectives of this study were to (1) determine if an outbreak of fentanyl-associated fatalities occurred in mid-2005 to mid-2006 and (2) to examine trends and compare features of fentanyl-contaminated heroin-associated fatalities (FHFs) with non-fentanyl, heroin-associated fatalities (NFHFs) among illicit drug users. Methods: Baseline prevalence of fentanyl- and heroin-associated deaths was estimated from January to May 2005 based on recorded cause of death (determined by the medical examiner (ME)) using the Wayne County, MI, USA toxicology database. The database was then queried for both FHFs and NFHFs between July 1, 2005 and May 12, 2006. A FHF was defined as having fentanyl or norfentanyl (metabolite) detected in any postmortem biological sample and either (1) detection of heroin or its metabolite (6-acetylmorphine) and/or cocaine or its metabolite (benzoylecgonine) in a postmortem biological specimen or (2) confirmation of fentanyl abuse as the cause of death by the ME or a medical history available sufficient enough to exclude prescription fentanyl or other therapeutic opioid use. A NFHF was defined as detection of heroin, 6-acetylmorphine (heroin metabolite) or morphine in any postmortem biological specimen, heroin overdose listed as the cause of death by the ME, and absence of fentanyl detection on postmortem laboratory testing. Information was systematically collected, trended for each group and then compared between the two groups with regard to demographic, exposure, autopsy, and toxicology data. Logistic regression was performed using SAS v 9. 1 examining the effects of age, gender, and marital status with fentanyl group status. Results: Monthly prevalence of fentanyl-associated fatalities among illicit drug users increased from an average of two in early 2005 to a peak of 24 in May, 2006. In total, 101 FHFs and 90 NFHFs were analyzed. The median age of decedents was 46 and 45 years for the fentanyl and non-fentanyl groups, respectively. Fentanyl-contaminated heroin-associated fatalities (FHFs) were more likely to be female (p = 0. 003). Women aged over 44 years (OR = 4. 67;95 % CI = 1. 29-16. 96) and divorced/widowed women (OR = 14. 18;95 % CI = 1. 59-127. 01) were more likely to be FHFs when compared to women aged less than 44 years and single, respectively. A significant interaction occurred between gender and age, and gender and marital status. Most FHFs had central (heart) blood samples available for fentanyl testing (n = 96; 95 %): fentanyl was detected in most (n = 91; 95 %). Of these, close to half had no detectable heroin (or 6-acetylmorphine) concentrations (n = 37; 40. 7 %). About half of these samples had detectable cocaine concentrations (n = 20; 54 %). Median fentanyl concentration in central blood samples was 0. 02 mug/ml (n = 91, range <0. 002-0. 051 mug/ml) and 0. 02 mug/ml (n = 32, range <0. 004-0. 069 mug/ml) in peripheral blood samples. The geometric mean of the ratio of central to peripheral values was 2. 10 (median C/P = 1. 75). At autopsy, pulmonary edema was the most frequently encountered finding for both groups (77 %). Conclusion: Illicit drugs may contain undeclared ingredients that may increase the likelihood of fatality in users. Gender differences in fentanyl-related mortality may be modified by age and/or marital status. These findings may help inform public health and prevention activities if fatalities associated with fentanyl-contaminated illicit drugs reoccur. 2013 American College of Medical Toxicology (outside the USA)
SN - 1556-9039
AD - (Algren, Monteilh, Punja, Schier, Belson, Patel, Straetemans, Rubin) Centers for Disease Control and Prevention, National Center for Environmental Health, Division of Environmental Hazards and Health Effects, Health Studies Branch, Atlanta, GA, United States (Hepler, Schmidt) Office of the Wayne County Medical

Examiner, Detroit, MI, United States (Miller) Michigan Department of Community Health, Bureau of Epidemiology, Lansing, MI, United States (Paulozzi) Division of Unintentional Injury Prevention, Centers for Disease Control and Prevention, National Center for Injury Prevention and Control, Atlanta, GA, United States
ER -

TY - JOUR
ID - 3363
T1 - To love and to hold: Men describe parenting in the presence of inflammatory arthritis
A1 - Backman,C.L.
A1 - Longson,A.
Y1 - 2012///
N1 - 70998867
English
Journal: Conference Abstract
KW - EMBASE
KW - Human
KW - rheumatology
KW - love
KW - Arthritis
KW - health practitioner
KW - Male
KW - child parent relation
KW - college
KW - Child
KW - Satisfaction
KW - Female
KW - father
KW - psoriatic arthritis
KW - pilot study
KW - ankylosing spondylitis
KW - night
KW - task performance
KW - cross-sectional study
KW - Health Assessment Questionnaire
KW - Content Analysis
KW - Health Status
KW - Quality of Life
KW - Social Support
KW - household
KW - Employment
KW - disability
KW - volunteer
KW - Statistics
KW - Diagnosis
KW - Education
KW - publication
KW - patient
KW - Pain
KW - Hospital
KW - questionnaire
KW - consumer
KW - mother
RP - NOT IN FILE
SP - S379
EP - S380
JF - Arthritis and Rheumatism

VL - Conference: Annual Scientific Meeting of the American College of Rheumatology and Association of Rheumatology Health Professionals 2012 Washington, DC United States. Conference Start: 20121109 Conference End: 20121114. Conference Publication:
IS - var.pagings
N2 - Background/Purpose: Inflammatory arthritis (IA) may limit participation in life roles such as parenting. Surprisingly little research has investigated the impact of IA on parenting tasks and experiences of fathers. This descriptive pilot study adapted a mail survey used in a cross-sectional study of mothers to an online format to (a) examine feasibility of the tool and items for use with men, and (b) assess self-reported performance of parenting tasks, parenting satisfaction, and parenting efficacy in fathers with rheumatoid arthritis (RA), psoriatic arthritis (PsA), or ankylosing spondylitis (AS). Methods: Men with IA were recruited through rheumatologists' offices, arthritis consumer newsletters and web sites, and public education forums on arthritis. Eligibility criteria included a diagnosis of IA confirmed by a rheumatologist and at least one child >18 yrs living with them. Volunteers were sent a web link and password to access the survey. The Parenting Disability Index (PDI) and Parenting Sense of Competency Scale (PSOC) were used to measure parenting outcomes. Surveys also inquired about employment, household work, social support, family quality of life, health status, demographics, and survey feasibility questions. Descriptive statistics were used to assess numeric responses and thematic content analysis used to examine text responses. Results: Twelve men volunteered and 10 submitted complete surveys, and reported taking 15 to 60 minutes to do so. They ranged in age from 38 to 59 years, and had 1 to 5 children ranging in age from 4 months to late 20s. Eight were married, 2 were separated/divorced. Four had RA, 4 had AS, and 2 had PsA, from 1 to 32 years in duration (mean = 17.5). Seven were employed. Health Assessment Questionnaire II scores ranged from 0 to 1.5 (mean =.53, median =.40); the common functional limitation was lifting and moving heavy objects. They reported few limitations in parenting tasks, with PDI scores ranging from.20 to 1.26 (0 to 3 scale), mostly related to having energy to be patient with their child, getting up during the night or early morning, and playing (on the floor or outdoors). PSOC total scores ranged from 2.0 to 2.82 (0 to 6 scale; mean = 2.28, SD =.29), while parenting sense of efficacy subscale ranged from 1.67 to 3.56 (mean = 2.32, SD =.60) and parenting satisfaction subscale ranged from 1.50 to 2.75 (mean = 2.22, SD =.38). Men reported many joys in parenting ("to love and to hold" their children), and several challenges ("communicating the fact I'm in pain in a way that doesn't make my 7 year old worried or overly protective; I also don't want to downplay it"). A key motivator for participating in the survey was "most seminars and clinics are directed at or attended by women, so a men's questionnaire is a must!" Conclusion: The online parenting survey was relevant to the participating men. Although this sample is too small to generalize, findings suggest men with IA experience specific limitations in parenting, but experience great satisfaction with this role. Assessment of parenting task performance may be important to assess in both practice and research settings when selecting outcomes that are meaningful to people living with arthritis
SN - 0004-3591
AD - (Backman) University of British Columbia, Vancouver, BC, Canada (Longson) Arthritis Research Centre of Canada, Vancouver, BC, Canada
ER -

TY - JOUR
ID - 3364
T1 - Interactive nutritional education at the boys & girls club in ferndale, washington
A1 - Mast,B.
Y1 - 2013///
N1 - 70993642
English
Journal: Conference Abstract
KW - EMBASE
KW - Human
KW - boy
KW - medical research
KW - girl
KW - Male
KW - Female
KW - United States
KW - nutrition education
KW - Child

KW - Nutrition
KW - Health
KW - fast food
KW - Food
KW - childhood obesity
KW - community
KW - Physical Activity
KW - Obesity
KW - Curriculum
KW - Hypertension
KW - Population
KW - Teaching
KW - education program
KW - Social Work
KW - positive feedback
KW - Creativity
KW - computer
KW - cardiovascular disease
KW - Risk
RP - NOT IN FILE
SP - 164
JF - Journal of Investigative Medicine
VL - Conference: American Federation for Medical Research Western Regional Meeting, AFMR 2013 Carmel, CA United States. Conference Start: 20130123 Conference End: 20130126. Conference Publication:
IS - var.pagings
N2 - Purpose of Study: Obesity is endemic in the United States and an increasing problem worldwide. Childhood obesity leads to increased risk for T2DM, hypertension, and cardiovascular disease. It is linked to depression and low selfesteem in children. These issues are a major concern in Ferndale, Washington. The goal of the project was to create an interactive and reproducible nutritional curriculum for after school/summer programs like the Boys & Girls Club. Methods Used: Key social service leaders provided input on the health concerns of Ferndale's diverse community. All interviewees identified obesity, T2DM and hypertension as the major health concerns. The Ferndale Boys & Girls Club director indicated their programs covered physical activity, but lacked healthy nutrition. Through collaboration, an hour and a half nutritional education program focused on 8-10 year olds was developed. The professional literature was reviewed for the best teaching methods indicated for the target population. MyPlate.gov and other websites were activity sources. The curriculum produced included: an interactive educational component, outdoor physical activity reinforcing discussion points, "snack art" with healthy foods, and nutrition computer games. Summary of Results: 15 children attended the activity. All were female. Most recognized the nutrition plate and its components, but had trouble identifying to which category food belonged. The range of activities maintained interest and covered concepts repetitively. "Food art" was a favorite and included a healthy snack, creativity, and category identification.Many children saved both their snack and designed plates.Most were eager to know when this session would recur. Conclusions: Childhood obesity is a widespread problem that leads to devastating long-term health consequences. It will not disappear quickly, and it is important that children are educated in a multitude of ways how to make good choices about what they put in their body. Although children are not meal preparers, evidence shows they play a key role in directing family consumption. Positive feedback was received from participants, Boys & Girls Club administration, and the community. The program is inexpensive and reproducible if the club choses to continue nutritional education
SN - 1081-5589
AD - (Mast) University of Washington, School of Medicine, Seattle, WA, United States
ER -

TY - JOUR
ID - 3365
T1 - Have "sun" sense: Sun safetyawareness for youth in miles city, MT
A1 - Herron,K.J.
Y1 - 2013///

N1 - 70993628
English
Journal: Conference Abstract
KW - EMBASE
KW - city
KW - medical research
KW - juvenile
KW - Human
KW - Safety
KW - Child
KW - sun exposure
KW - Curriculum
KW - community
KW - Education
KW - Recreation
KW - Learning
KW - Risk
KW - positive feedback
KW - exposure
KW - protection
KW - skin cancer
KW - skin function
KW - summer
KW - Behavior Modification
KW - adulthood
KW - Health Behavior
KW - skin defect
KW - Evidence Based Practice
KW - Health
KW - community program
KW - ultraviolet radiation
KW - sunscreen
RP - NOT IN FILE
SP - 159
JF - Journal of Investigative Medicine
VL - Conference: American Federation for Medical Research Western Regional Meeting, AFMR 2013 Carmel, CA United States. Conference Start: 20130123 Conference End: 20130126. Conference Publication:
IS - var.pagings
N2 - Purpose of Study: The Miles City community is largely agriculturally based and has many opportunities for outdoor recreation. Both of these factors result in prolonged sun exposure for community participants. Evidence suggests that ultra-violet radiation from sun exposure during early life is associated with increased risk for various adulthood skin cancers. The project's purpose was to implement a sun safety curriculum that teaches youth how to limit sun exposure and prevent skin damage. Methods Used: Studies suggest optimum health behavior modification occurs where a learning infrastructure already subsists. I partnered with a youth summer enrichment program (ROCKS) to implement an evidencebased curriculum. It encompassed sun safety knowledge and behaviors from current literature including: the sun's benefits and harmful effects, healthy skin functions, components of sun protection, and proper sunscreen use. The program was conducted in a multistep fashion that included interactive learning. The first activity was a sun safe relay that reinforced the effectiveness of wearing sun protective articles. In the second activity, children constructed UV sensitive bead bracelets that enhance exposure awareness to damaging UV rays and promote sunscreen use. Summary of Results: The project conveyed sun safety behaviors to 48 children (grades 1-6). Education material was interactive, succinct, and age appropriate. The attendance level, enthusiastic participation, and positive feedback from the program's staff suggest that the project was well received. Children were able to acknowledge appropriate sun safety methods in a Q&A session post-activity. Access to materials and the curriculum used were given to staff for future use. Conclusions: Agricultural and outdoor recreation based communities like Miles City increase children's risk to sun exposure. Fortunately, community programs like ROCKS take interest in youth health and education. They provided great support and guidance throughout the development of this project. By

introducing an evidence-based curriculum for youth members, it seems reasonable to expect promising results related to sun protective methods and awareness. Further studies should be conducted to see if the project leads to increased sun safety behavior among Miles City youth participants
SN - 1081-5589
AD - (Herron) University of Washington, School of Medicine, Seattle, WA, United States
ER -

TY - JOUR
ID - 3366
T1 - Child abuse and mental health
A1 - Yadav,J.S.
A1 - Kaur,S.
A1 - Tiwari,S.C.
A1 - Chandrima,Das S.
A1 - Tripathi,M.N.
A1 - Ajay,Panditt B.
Y1 - 2013///
N1 - 70990994
English
Journal: Conference Abstract
KW - EMBASE
KW - Child Abuse
KW - Human
KW - Mental Health
KW - Indian
KW - medical society
KW - Child
KW - India
KW - abuse
KW - brain
KW - Population
KW - Grief
KW - protection
KW - Atmosphere
KW - Germany
KW - China
KW - Malaysia
KW - Europe
KW - Sexual Abuse
KW - Pain
KW - health practitioner
KW - substance abuse
KW - psychiatry
KW - victim
KW - Achievement
KW - Education
RP - NOT IN FILE
SP - S18
JF - Indian Journal of Psychiatry
VL - Conference: 65th Annual National Conference of the Indian Psychiatric Society, ANCIPS 2013 Bangalore India. Conference Start: 20130110 Conference End: 20130113. Conference Publication:
IS - var.pagings
N2 - Each child is a future of nation, so it is our duty to protect and safeguard that but unfortunately they are not safe in their home, education center, playground and social places. We are living in a scientific era and proud about our progress, new achievements, post and position but we are fail and unable to provide a safe atmosphere to the children. Nineteen percent of the world's children live in India, which constitutes 42% of India's total population. It is shakeable for us 69% of Indian children are victims of physical, emotional and

sexual abuse. Higher rate of abuse is also in Srilanka, Malaysia, China, Germany and Europe. The National Commission for Protection of Child Rights has received 75 cases of child abuse up to October 2011 in Delhi. Any type of abuse gives an unforgettable grief and pain. So we are discussing in the reference of these topics: Child Abuse an Overview: A picture presentation. Biological changes in brain of abused child Impacts of child abuse in late life. Child abuse relation with substance abuse. Child abuse: Psychiatry Aspect Preventive measure of mental health abused children. Child Abuse: Role of mental health professional Legal rights of child abuse
SN - 0019-5545
ER -

TY - JOUR
ID - 3367
T1 - I cut my thumb on a dolly:The story of an octogenarian's reluctant journey to the emergency department (ED) for repair of a laceration and her surprising rise to "interesting patient" status
A1 - South,H.
A1 - Lands,R.
Y1 - 2011///
N1 - 70989687
English
Journal: Conference Abstract
KW - EMBASE
KW - emergency ward
KW - laceration
KW - patient
KW - Human
KW - Geriatrics
KW - Society
KW - thumb
KW - Therapy
KW - toe
KW - Diagnosis
KW - pruritus
KW - Female
KW - weight reduction
KW - widow
KW - lethargy
KW - Hypertension
KW - anorexia
KW - hypothyroidism
KW - laboratory
KW - dyslipidemia
KW - coronary artery disease
KW - Hepatitis
KW - vital sign
KW - sclera
KW - skin
KW - urine
KW - nail bed
KW - serum
KW - spleen
KW - tissues
KW - hallux
KW - multiple myeloma
KW - follow up
KW - Leukocyte Count
KW - thrombocyte
KW - eye
KW - Biopsy

KW - primary biliary cirrhosis
KW - jaundice
KW - melanoma
KW - Anemia
KW - antibody titer
KW - spike
KW - liver
KW - physician
KW - Child
KW - Amputation
KW - myeloma
KW - Fatigue
KW - faintness
KW - Aged
KW - independence
KW - functional status
KW - Quality of Life
KW - Hospital
KW - Facial Expression
KW - Health
KW - hemoglobin
KW - albumin
KW - protein
KW - bilirubin
KW - alkaline phosphatase
KW - mitochondrion antibody
KW - immunoglobulin A
KW - ursodeoxycholic acid
RP - NOT IN FILE
SP - S21
JF - Journal of the American Geriatrics Society
VL - Conference: 2011 Annual Scientific Meeting of the American Geriatrics Society National Harbor, MD United States. Conference Start: 20110511 Conference End: 20110514. Conference Publication:
IS - var.pagings
N2 - An 87-year-old female came to the ED for a laceration on her thumb. While waiting, she experienced near syncope and confusion. She was admitted for evaluation. She described anorexia without weight loss, six months of lethargy, fatigue, and pruritis. She was not aware of yellow eyes or dark urine. She had well controlled hypothyroidism, dyslipidemia, hypertension and coronary artery disease. She was a widow who lived alone. She managed her own affairs and mowed her own yard with a riding mower. She was afebrile. Vital signs were normal. Her sclera were icteric and her skin had a yellow hue. She had no enlargement of liver or spleen. There was a flat, 2X3 cm black lesion with irregular borders involving the nail bed and surrounding tissue of the right great toe. Laboratory: Albumin 2.2g/dL (3.4-4.8), total protein 6.9g/dL (6.4-8.3g/dL), total bilirubin 4.4 mg/dL, direct=3.5mg/dL, Alkaline Phosphatase 293 uL (53-148), AST 556 uL (5-34), ALT 319uL (0-55), hemoglobin 9.6gm/dL, white blood count 4.6x103 and platelets 163x103. Hepatitis panel was negative. Anti-mitochondrial antibody titer was 1:320 (NL <1:20) Her jaundice proved to be due to Primary Biliary Cirrhosis (PBC). Biopsy of the toe revealed acral lentiginous melanoma. The anemia with abnormal total protein/albumen ratio prompted evaluation with serum immunofixation, which identified a monoclonal spike, which proved to be an IgA multiple myeloma. After collaboration with her doctors and her children, she accepted ursodeoxycholic acid for the PBC, which provided relief of her pruritis. She reluctantly allowed amputation of her toe. She declined therapy for the myeloma though she did agree to follow up, realizing at some point she will need symptom care. This case is intriguing because it addresses a confluence of three simultaneous "interesting" diagnoses that a proud and independent elderly lady had dealt with for many months without complaints or requests for intervention. After diagnosis, she still exercised her independence, accepting therapy based on her assessment of its potential affect on her functional status and her quality of life. At each of her clinic visits, she continues to profess regret at her decision to visit the ED for the laceration, noting with a smile that her health has taken a decidedly downward trend ever since
SN - 0002-8614

AD - (South, Lands) Department of Medicine, University of Tennessee, Knoxville, TN, United States
ER -

TY - JOUR
ID - 3368
T1 - Exposure to cricket and its popularity among Trinidadian school boys
A1 - Mansingh,A.
A1 - Seenath,B.
A1 - Palmer,W.
A1 - Singh,P.
Y1 - 2012///
N1 - 70985252
English
Journal: Conference Abstract
KW - EMBASE
KW - Ensifera
KW - school child
KW - Male
KW - Human
KW - communicable disease
KW - economic aspect
KW - exposure
KW - school
KW - Television
KW - student
KW - Football
KW - primary school
KW - ethnicity
KW - high school
KW - sport
KW - questionnaire
KW - Physical Education
KW - sampling
KW - Indian
KW - cross-sectional study
RP - NOT IN FILE
SP - 41
JF - West Indian Medical Journal
VL - Conference: 21st Annual Research Conference on the Non-Communicable Diseases - A Threat to Life and the Economy Mona Jamaica. Conference Start: 20121107 Conference End: 20121109. Conference Publication:
IS - var.pagings
N2 - Objective: To assess the exposure and popularity of cricket among Trinidadian school boys. Method: A cross-sectional study with stratified random sampling of public secondary schools was done in Trinidad, utilizing a questionnaire to assess exposure and participation in cricket. Data were analysed using SPSS version 16.0. Results: The mean age of 509 school boys from 17 schools was 15.3 years (range 12-19 years, SD 0.8), with ethnicity: East Indian (52%), African (24%), Chinese (2%) and other (22%). Football was the favourite sport of 39% and cricket 29%, compared to 34% and 50% respectively when they were in primary school. Football was favoured 2.3 times more than cricket among Afro-Trinidadians, but equally among Indo-Trinidadians. Since primary school, 130 students discontinued cricket, 45% played football instead and 36% played no sport. Half participated in physical education, down from 88% in primary school. Only 38% participated in cricket at school. This was unrelated to ethnicity ($p = 0.124$). However, 73% played cricket outside of school. Students with access to a cricket pitch ($p = 0.011$) or to protective gear ($p = 0.004$) were more likely to participate in cricket at school. Those who did not participate cited lack of interest (50%) or no time (32%) as the main reasons. Students with cable television (70%) were less likely to watch cricket on television. Twenty-twenty cricket was preferred by 73%. Conclusions: In spite of the high level of exposure to cricket

among school boys in Trinidad, its popularity decreases in secondary school. Lack of facilities and access to cable television decreases participation
SN - 0043-3144
AD - (Mansingh, Seenath, Palmer, Singh) Division of Sports Medicine, Faculty of Medical Sciences, University of the West Indies, Kingston 7, Jamaica
ER -

TY - JOUR
ID - 3369
T1 - Mxi1 and n-myc induce neuroblastoma cell apoptosis via distinct pathways
A1 - Armstrong,M.
A1 - Hill,A.
A1 - Wechsler,D.
Y1 - 2012///
N1 - 70986601
English
Journal: Conference Abstract
KW - EMBASE
KW - oncogene c myb
KW - neuroblastoma cell
KW - apoptosis
KW - Society
KW - cell proliferation
KW - neuroblastoma
KW - exposure
KW - serum
KW - Cell Line
KW - Child
KW - Therapy
KW - Human
KW - cell death
KW - Immunohistochemistry
KW - gene
KW - childhood
KW - Prognosis
KW - assay
KW - staining
KW - pathogenesis
KW - stimulation
KW - survival
KW - cell growth
KW - caspase 9
KW - caspase 8
KW - doxycycline
KW - protein
KW - caspase
KW - regulator protein
KW - caspase 3
KW - propidium iodide
KW - broxuridine
RP - NOT IN FILE
SP - 1077
JF - Pediatric Blood and Cancer
VL - Conference: 25th Annual Meeting of the American Society of Pediatric Hematology/Oncology, ASPHO 2012 New Orleans, LA United States. Conference Start: 20120509 Conference End: 20120512. Conference Publication:
IS - var.pagings

N2 - Background: Neuroblastoma is the most common extracranial malignancy of childhood. Myc family proteins regulate cell growth and proliferation in response to mitogenic stimulation, and N-Myc plays a role in the pathogenesis of a subset of highrisk neuroblastoma. In particular, MYCN amplified neuroblastoma is associated with a poor prognosis. Mxi1, a member of the MAD family of transcriptional regulatory proteins, counteracts Myc by repressing transcription of Myc target genes. We hypothesize that Mxi1 antagonizes N-Myc activity in neuroblastoma by inhibiting cell proliferation and viability. Objectives: To determine the impact of modulating Mxi1 expression on N-Mycmediated neuroblastoma cell proliferation and apoptosis.
Design/Method: We constitutively expressed Mxi1 in non-MYCN-amplified SHEP neuroblastoma cells, and also SHEP cells stably transfected to express high levels of MYCN (SHEP/MYCN). We also created neuroblastoma cell lines (Non-MYCN-amplified SH-SY5Y and MYCN-amplified IMR32) that inducibly express Mxi1 upon exposure to doxycycline. Cell proliferation and survival were quantified using BrdU and MTT assays, respectively. Apoptosis was measured by propidium iodide staining and caspase-3, caspase-8 and caspase-9 immunohistochemistry. Results: In non-MYCN-amplified SHEP cells, Mxi1 overexpression independently inhibits cell proliferation and induces cell apoptosis. In the context of MYCN overexpression in SHEP/MYCN or IMR32 cells,Mxi1 inhibits N-Myc-dependent cell proliferation and blocks N-Myc-dependent apoptosis. Outside of MYCN expression, Mxi1 induces cell apoptosis via the caspase 8 pathway. In contrast, in SHEP/MYCN or IMR32 cells that undergo N-Myc-dependent apoptosis upon exposure to low serum, the caspase 9 pathway is active. Conclusion: Overexpression of Mxi1 in MYCN-expressing neuroblastoma cell lines leads to inhibition of N-Myc-dependent cell proliferation and low serum-induced apoptosis. Furthermore, Mxi1 expression decreases cell proliferation and induction of apoptosis independent of N-Myc. The use of distinct caspase pathways by N-Myc and Mxi1 highlights the complexity of cell death regulation in neuroblastoma. The interaction between Mxi1 and N-Myc in neuroblastoma must be considered in the development of novel targeted therapies to improve outcomes in children with neuroblastoma
SN - 1545-5009
AD - (Armstrong, Hill, Wechsler) Duke University, Durham, NC, United States
ER -

TY - JOUR
ID - 3370
T1 - Targeting the bone marrow microenvironment to overcome chemotherapy resistance in ALL
A1 - Allan,Sison E.
A1 - Magoon,D.
A1 - Li,L.
A1 - Rau,R.
A1 - Annesley,C.
A1 - Small,D.
A1 - Brown,P.
Y1 - 2012///
N1 - 70986399
English
Journal: Conference Abstract
KW - EMBASE
KW - bone marrow
KW - microenvironment
KW - Chemotherapy
KW - Society
KW - Human
KW - leukemia cell
KW - in vitro study
KW - Leukemia
KW - relapse
KW - mouse
KW - Child
KW - stroma cell
KW - cell migration
KW - pre B lymphocyte
KW - upregulation

KW - adhesion
KW - chemosensitivity
KW - protection
KW - stroma
KW - patient
KW - apoptosis
KW - Infant
KW - model
KW - xenograft
KW - Cell Line
KW - hypothesis
KW - plerixafor
KW - stromal cell derived factor 1
KW - cell surface receptor
KW - integrin
KW - Receptor
KW - chemokine
KW - biological marker
KW - very late activation antigen 4
RP - NOT IN FILE
SP - 1017
JF - Pediatric Blood and Cancer
VL - Conference: 25th Annual Meeting of the American Society of Pediatric Hematology/Oncology, ASPHO 2012 New Orleans, LA United States. Conference Start: 20120509 Conference End: 20120512. Conference Publication:
IS - var.pagings
N2 - Background: A significant number of children with ALL relapse due to the persistence of chemotherapy-resistant leukemia cells. CXCR4, a cell surface receptor expressed by various leukemias, is activated by the chemokine stromal-derived factor- 1 (SDF-1 or CXCL12), causing migration of leukemia cells to bone marrow niches that may contribute to chemoresistance and relapse. Objectives: We hypothesized that the CXCR4 inhibitor plerixafor would sensitize leukemias to chemotherapy through the interruption of leukemia-stromal cell signaling. Because B-precursor ALL are known to highly express CXCR4, we tested this hypothesis in vitro using ALL cell lines and in vivo using a xenograft model of MLL-rearranged (MLL-r) infant ALL. Design/Method: In vitro, we treated ALL cells with dose ranges of chemotherapy using various culture conditions with or without bone marrow stromal cells to highlight the importance of CXCR4/SDF-1 signaling in chemoresistance. In vivo, we transplanted primary samples of MLL-r ALL into immunodeficient mice and treated cohorts with chemotherapy alone, plerixafor and chemotherapy, or vehicle control. After treatment, we measured leukemic burden and surface CXCR4 (s-CXCR4) expression by organ to determine the effects of our treatment regimen. Results: ALL cells that upregulated s-CXCR4 in response to chemotherapy treatment were protected from chemotherapy-induced apoptosis when co-cultured with bone marrow stroma. Plerixafor diminished stromal protection and conferred chemosensitivity. Mice treated with plerixafor and chemotherapy had decreased leukemic burden, compared to those receiving chemotherapy alone or vehicle control. S-CXCR4 expression was higher in blasts located outside the bone marrow, suggesting that blasts migrating out of the marrow upregulate s-CXCR4 as they home to new niches. Plerixafor also induced increased expression of the SDF-1 receptor CXCR7 and the integrin VLA-4, suggesting that CXCR4 inhibition may lead to recruitment of other molecules critical to leukemia cell trafficking and adhesion. Finally, the efficacy of plerixafor as a chemosensitizer both in vitro and in vivo was predicted by the degree of chemotherapy-induced s-CXCR4 upregulation, identifying a biomarker with the potential to select optimal patients for CXCR4 inhibition. Conclusion: The bone marrow microenvironment is an important mediator of chemotherapy resistance. Inhibition of the CXCR4/SDF-1 axis may result in improved outcomes in children with ALL
SN - 1545-5009
AD - (Allan Sison, Magoon, Li, Rau, Annesley, Small, Brown) Johns Hopkins University, School of Medicine, Baltimore, MD, United States
ER -

TY - JOUR
ID - 3371

T1 - Pediatric palliative care services in Kuwait: A unique experience from middle east countries
A1 - Mittal,R.
A1 - Khalifa,N.M.
A1 - Khalifa,S.O.
A1 - Bedair,R.
A1 - Al,Sayer M.
Y1 - 2011///
N1 - 70987327
English
Journal: Conference Abstract
KW - EMBASE
KW - Kuwait
KW - Middle East
KW - Society
KW - oncology
KW - palliative therapy
KW - Human
KW - Child
KW - Hospital
KW - medical specialist
KW - Bereavement
KW - Pain
KW - Home Care
KW - blood sampling
KW - Drug Therapy
KW - Professional Practice
KW - patient
KW - physiotherapist
KW - nurse
KW - Social Support
KW - terminal care
KW - adrenal cortex carcinoma
KW - analgesia
KW - Blood Transfusion
KW - parent
KW - holistic care
KW - bone tumor
KW - brain tumor
KW - hospitalized child
KW - oxygen breathing
KW - feedback system
KW - physiotherapy
KW - neoplasm
RP - NOT IN FILE
SP - 869
EP - 870
JF - Pediatric Blood and Cancer
VL - Conference: 43rd Congress of the International Society of Paediatric Oncology, SIOP 2011 Auckland New Zealand. Conference Start: 20111028 Conference End: 20111030. Conference Publication:
IS - var.pagings
N2 - Purpose: Pediatric palliative care (PPC) is being recognized as an important aspect of holistic care for children with cancer. PPC can offer a wide range of services including symptom management of dying child, support for the parents, spiritual services, home care, bereavement services etc. Method: Kuwait Association of Care of Children in Hospital (KACCH) is a NGO, which provides support to hospitalized children through Child Life Specialists (CLSs). In November 2005, this NGO started PPC in Kuwait and made a PPC team, which consist of pediatric oncologist, pain management specialist, CLSs, nurses, and physiotherapist. One CLS is assigned for each child. CLSs are key contact person with child and family. The first contact between CLS

and family is made in the hospital. Subsequently home visits are made by CLS to establish better rapport with child and family. The home care services include pain medications, blood sampling, blood transfusion, dressing, care of Hickman's line, physiotherapy, oxygen inhalation etc. The children are also taken out for outdoor activities. The emphasis is on keeping the child at home as long as possible. The family members are encouraged to contact CLS at any hour of the day. Results: Till date 58 children have been registered for palliative care. They suffered from various malignancies including brain tumors, bone tumors, neuroblastomas, adrenocortical carcinoma etc. Out of these 58 children, 50 have died of their disease. They were under care of palliative care team ranging from few weeks to more than 18 months. Majority of the children died in the hospital, but few died at home also. In our assessment and feedback, the patients and their families got tremendous benefit from palliative care services in terms of pain control, social support, and bereavement services. Conclusion: Hospital and home based PPC services make a tremendous impact at "end of life care"
SN - 1545-5009
AD - (Mittal) Ministry of Health Kuwait, Pediatric Oncology, Hawally, Kuwait (Khalifa, Khalifa, Bedair) Kuwait Cancer Centre, Medical Oncology, Shuwaikh, Kuwait (Al Sayer) Kuwait Association for Care of Children in Hospital, Pediatric Palliative Care, Shuwaikh, Kuwait
ER -

TY - JOUR
ID - 3372
T1 - Efficacy and safety of tocilizumab in european children with systemic onset juvenile idiopathic arthritis
A1 - Nemiche,O.
A1 - Dagner,R.
A1 - Quartier,P.
A1 - Cimaz,R.
A1 - Richer,O.
A1 - Pillet,P.
A1 - Hofer,M.
Y1 - 2011///
N1 - 70985491
English
Journal: Conference Abstract
KW - EMBASE
KW - Child
KW - Human
KW - juvenile rheumatoid arthritis
KW - rheumatology
KW - Society
KW - Safety
KW - patient
KW - erythrocyte sedimentation rate
KW - fever
KW - off label drug use
KW - follow up
KW - anaphylaxis
KW - vasculitis
KW - clinical trial
KW - Japanese
KW - disease duration
KW - skin
KW - tocilizumab
KW - enalapril maleate
KW - steroid
KW - C reactive protein
KW - canakinumab
KW - monoclonal antibody
KW - recombinant interleukin 1 receptor blocking agent
KW - tumor necrosis factor alpha

KW - prednisone
KW - Receptor
KW - interleukin 6
KW - methotrexate
RP - NOT IN FILE
JF - Pediatric Rheumatology
VL - Conference: 18th Pediatric Rheumatology European Society, PReS Congress Bruges Belgium. Conference Start: 20110914 Conference End: 20110918. Conference Publication:
IS - var.pagings
N2 - Background: The anti- IL-6 Receptor monoclonal antibody Tocilizumab (TCZ) has demonstrated its efficacy in Japanese children with systemic onset juvenile idiopathic arthritis (SJIA). Aim: To evaluate the efficacy and safety of TCZ in European patients with active SJIA outside a clinical trial (off-label use). Patients and methods: Retrospective review of the files of the patients treated by TCZ in two French, one Swiss and one Italian center. Response to treatment was defined as control of the fever and systemic features for at least 7 days, Improvement of the pediatric ACR score and normalization of erythrocyte sedimentation rate (ESR) and C-reactive protein (CRP) were also considered. Results: 18 patients aged 4-15 years were included. The median disease duration at treatment onset was 4.7 years (0.4-8.8). The median dose of prednisone at TCZ onset was 0.63mg/kg/d. Four patients were also on MTX. Fourteen patients had previously failed to respond one or several biologics, including anti-TNF alpha in 10 cases, anakinra in 14 and canakinumab, in 4. All patients but 4 had active systemic and polyarticular features at TCZ onset. The doses of TCZ ranged between 6 and 12 mg/kg every other week at treatment onset. The mean follow-up on TCZ was 18.2 months (range0.5-48). 90% improvement of the pediatric ACR score was achieved by 9 patients after 3 months. The dose of steroids was tapered in most cases (11/14) within 3 months, and five patients could discontinue steroid treatment after 12 months. TCZ treatment was withdrawn in 5 children for adverse events: anaphylactic reaction in 3 cases, skin vasculitis in 2 other cases. Conclusion: In this study TCZ treatment was effective in most patients with SJIA who previously failed one or several biologics. Adverse events require particular attention since almost one third of patients had to discontinue treatment
SN - 1546-0096
AD - (Nemiche, Dagner, Quartier) Department of Immuno-Hemato-Rhumatology, Necker Enfants Malades Hospital, Paris, France (Cimaz) Service of Pediatric Rheumatology, Florence, Italy (Richer, Pillet) Service of Pediatric Rheumatology, CHU, Bordeaux, France (Hofer) Service of Pediatric Rheumatology, CHU, Vaudois, Lausanne, Geneva, Switzerland
ER -

TY - JOUR
ID - 3373
T1 - Lifestyle risk factors for allergic rhinitis in schoolchildren: Are sports activities a negative factor?
A1 - Takeuchi,J.
A1 - Kusunoki,T.
A1 - Morimoto,T.
A1 - Sakuma,M.
A1 - Mukaida,K.
A1 - Yasumi,T.
A1 - Nishikomori,R.
A1 - Heike,T.
Y1 - 2013///
N1 - 70984466
English
Journal: Conference Abstract
KW - EMBASE
KW - risk factor
KW - allergic rhinitis
KW - school child
KW - Human
KW - sport
KW - allergic asthma
KW - immunology

KW - Lifestyle
KW - Child
KW - questionnaire
KW - parent
KW - Prevalence
KW - primary school
KW - school
KW - statistical significance
KW - logistic regression analysis
KW - Pollen
KW - exposure
KW - Statistics
KW - childhood
KW - Obesity
KW - Rhinitis
KW - allergy
KW - Asthma
KW - passive smoking
KW - eating habit
KW - model
KW - Japan
KW - city
KW - allergen
KW - chlorpheniramine maleate
RP - NOT IN FILE
SP - AB112
JF - Journal of Allergy and Clinical Immunology
VL - Conference: 2013 Annual Meeting of the American Academy of Allergy, Asthma and Immunology, AAAAI 2013 San Antonio, TX United States. Conference Start: 20130222 Conference End: 20130226. Conference Publication:
IS - var.pagings
N2 - RATIONALE: This study aimed to investigate various lifestyle factors and their association with symptoms of allergic rhinitis in schoolchildren. METHODS: A questionnaire regarding lifestyle factors and allergic rhinitis was distributed to the parents of 7-year-old schoolchildren at every primary school in Ohmi-Hachiman City, Shiga Prefecture, Japan. The International Collaborative Study of Asthma and Allergies in Childhood questionnaire provided information about the prevalence of allergic rhinitis. Following the descriptive statistics, we developed univariate and multivariate logistic regression models to evaluate the association between lifestyle factors and allergic rhinitis. P values <0.05 were considered to in-]dicate statistical significance and Pvalues <0.1 were considered to indicate a tendency. RESULTS: Among the parents of 759 7-year-old children who attended primary school, 640 (84.3%) answered the questionnaire. Of these children, 223 (34.8%) had allergic rhinitis symptoms. Among the lifestyle factors investigated, children who did not participate in any sports activities after school had significantly fewer allergic rhinitis symptoms than those who did (OR50.69, P=0.03). Similarly, children whose com-]muting time to school was less than 30 minutes tended to have fewer aller-]gic rhinitis symptoms than those whose commuting time was 30 minutes or more (OR50.72, P=0.07). No significant association between other lifestyle factors (passive smoking, obesity, bedtime, wake-up time, and eating habits) and allergic rhinitis symptoms was found. CONCLUSIONS: Participating in sports activities and commuting were associated with a higher prevalence of allergic rhinitis. Increased exposure to pollen or other outdoor allergens may contribute to these associations
SN - 0091-6749
AD - (Takeuchi, Kusunoki, Morimoto, Sakuma, Mukaida, Yasumi, Nishikomori, Heike) Shiga LAKE Study Group, Japan (Takeuchi) Osaka Saiseikai Nakatsu Hospital, Osaka, Japan (Takeuchi) Kyoto University Health Service, Kyoto, Japan (Kusunoki, Mukaida) Department of Pediatrics, Shiga Medical Center for Children, Shiga, Japan (Morimoto, Sakuma) Center for General Internal Medicine and Emergency Care, Kinki University School of Medicine, Osaka, Japan (Yasumi) Kumiko Allergy Clinic, Kyoto, Japan (Nishikomori, Heike) Department of Pediatrics, Graduate School of Medicine, Kyoto University, Kyoto, Japan
ER -

TY - JOUR
ID - 3374
T1 - Promoting physical activity with frisbee golf in delta junction, Alaska
A1 - Werner,S.
Y1 - 2012///
N1 - 70983302
English
Journal: Conference Abstract
KW - EMBASE
KW - sport
KW - United States
KW - medical research
KW - Physical Activity
KW - Human
KW - Obesity
KW - community
KW - Health
KW - city
KW - Lifestyle
KW - Temperature
KW - medical information
KW - survival
KW - Sunlight
KW - winter
KW - Rural Population
KW - medical care
KW - Learning
KW - urban area
KW - Traffic
KW - Child
KW - Adolescent
RP - NOT IN FILE
SP - 227
EP - 228
JF - Journal of Investigative Medicine
VL - Conference: American Federation for Medical Research Western Regional Meeting, AFMR 2012 Carmel, CA United States. Conference Start: 20120125 Conference End: 20120128. Conference Publication:
IS - var.pagings
N2 - Purpose of Study: National studies estimate that over 60 percent of Americans are overweight. The CDC reports that obesity rates in adolescents aged 12-19 and children aged 6-11 are 18% and 20% respectively. In 2008, the medical care costs related to obesity were $147 billion. Many rural Alaskan communities endure 9-month winters that feature limited daylight and temperatures of sixty below zero, making it difficult to maintain an active lifestyle. 67% of rural Alaskans are overweight, which is significantly higher than the national average as well as urban areas of the state. The purpose of the project was to promote physical activity in the rural community of Delta Junction, Alaska by increasing public interest in the town's new Frisbee Golf course. Methods Used: In order to increase community interest in Frisbee Golf, an event was held at the town's new Frisbee Golf course. The event was free and featured an overview of the game rules, a tour of the course, and group lessons from members of the Professional Disc Golf Association. People who attended also received a free Frisbee Golf disc and a brief lecture on the health benefits of physical activity. Promotion for the event was accomplished with the use of flyers and the recruited help of city hall and the course creator. The local outdoor survival store was also convinced to begin selling Frisbee Golf discs to the community. Summary of Results: People of all ages spend the afternoon in the park learning about Frisbee golf. Over 25 Frisbees were given away. Many members of the community who had never heard of Frisbee Golf learned the rules as well as valuable health information. The local outdoor store sold-out of Frisbee Golf discs within the week and the traffic on the local course increased. Conclusions: Obesity has become one of the most significant health concerns for Americans, especially those living in rural Alaska. Studies have indicated the importance of regular physical activity in preventing the multitude of health issues associated with obesity. Promoting an interest in

Frisbee Golf, a sport that is affordable, easy to participate in, and accessible to all ages, will hopefully encourage people in Delta Junction to engage in a more active lifestyle, thus improving the health of the community
SN - 1081-5589
AD - (Werner) University of Washington, School of Medicine, Seattle, WA, United States
ER -

TY - JOUR
ID - 3375
T1 - Tracking the speech signal - Time-locked MEG signals during perception of ultra-fast and moderately fast speech in blind and in sighted listeners
A1 - Hertrich,I.
A1 - Dietrich,S.
A1 - Ackermann,H.
Y1 - 2013///
N1 - 2013051882
English
Journal: Article
KW - EMBASE
KW - Adolescent
KW - Adult
KW - article
KW - auditory cortex
KW - blindness
KW - Child
KW - clinical article
KW - Comprehension
KW - controlled study
KW - hemispheric dominance
KW - Human
KW - inferior frontal gyrus
KW - Magnetoencephalography
KW - neurological equipment
KW - pitch
KW - right hemisphere
KW - school child
KW - Signal Processing
KW - speech analysis
KW - speech discrimination
KW - Speech Perception
KW - Speech Rate
KW - visual cortex
RP - NOT IN FILE
SP - 9
EP - 21
JF - Brain and Language
VL - 124
IS - 1
CY - United States
N2 - Blind people can learn to understand speech at ultra-high syllable rates (ca. 20. syllables/s), a capability associated with hemodynamic activation of the central-visual system. To further elucidate the neural mechanisms underlying this skill, magnetoencephalographic (MEG) measurements during listening to sentence utterances were cross-correlated with time courses derived from the speech signal (envelope, syllable onsets and pitch periodicity) to capture phase-locked MEG components (14 blind, 12 sighted subjects; speech rate. =. 8 or 16. syllables/s, pre-defined source regions: auditory and visual cortex, inferior frontal gyrus). Blind individuals showed stronger phase locking in auditory cortex than sighted controls, and right-hemisphere visual cortex activity correlated with syllable onsets in case of ultra-fast speech. Furthermore, inferior-frontal MEG components time-locked to pitch periodicity displayed opposite lateralization effects in sighted (towards right

hemisphere) and blind subjects (left). Thus, ultra-fast speech comprehension in blind individuals appears associated with changes in early signal-related processing mechanisms both within and outside the central-auditory terrain. 2012 Elsevier Inc
SN - 0093-934X
AD - (Hertrich, Dietrich, Ackermann) Department of General Neurology, Hertie Institute for Clinical Brain Research, University of Tubingen, Germany (Hertrich) Department of Linguistics, Program of General and Theoretical Linguistics, University of Tubingen, Germany
ER -

TY - JOUR
ID - 3376
T1 - "I try to talk to my children": Sexual health discussions and using natural settings to initiate conversations about sex between African American and latina mothers and their children
A1 - Castellanos,T.J.
A1 - Gaul,Z.
A1 - Willis,L.
A1 - Sutton,M.
A1 - Sneed,C.
Y1 - 2013///
N1 - 70979621
English
Journal: Conference Abstract
KW - EMBASE
KW - Human
KW - Child
KW - Conversation
KW - African American
KW - Society
KW - Female
KW - mother
KW - sexual health
KW - adolescent health
KW - son
KW - parent
KW - daughter
KW - Male
KW - juvenile
KW - Interpersonal Communication
KW - Sexual Behavior
KW - gender
KW - school
KW - Pregnancy
KW - Adolescent
KW - scientist
KW - information processing
KW - United States
KW - Hispanic
KW - Color
KW - Prevention
KW - Television
KW - boy
KW - community
KW - Sexual Development
KW - father
KW - protection
KW - Risk
KW - Human immunodeficiency virus

KW - acquired immune deficiency syndrome
RP - NOT IN FILE
SP - S63
JF - Journal of Adolescent Health
JA - J Adolesc Health
VL - Conference: 2013 Annual Meeting of the Society for Adolescent Health and Medicine, SAHM 2013 Atlanta, GA United States. Conference Start: 20130313 Conference End: 20130316. Conference Publication:
IS - var.pagings
N2 - Purpose: African American and Latino adolescents have higher reported rates of HIV/STD compared to other racial/ethnic groups. Although prior research has identified parental-adolescent communication as important in reducing youth HIV/STD risk, specific strategies used by African American and Latina parents to talk to their children about sex, including when and where to have these conversations, have been understudied. We examined approaches used by African American and Latina mothers for sex-related discussions with their children to inform HIV/STI intervention development efforts. Methods: We recruited mother-child (children ages 12-16 years) pairs from an after-school, community-based organization in Southern California. Fourteen maternal-only focus groups were conducted; 8 African American (n = 31) and 6 Latina (n = 24). Data were transcribed, coded for most common themes by 4 independent scientists and reviewed for differences by race/ethnicity and gender of child. Results: Four key themes emerged when focusing on parent-child discussions about sex: 1) sexual activity discussions varied for sons and daughters, 2) protection from HIV/STI and pregnancy were key topics, 3) using a direct, honest approach, and 2) seizing the moment. For sexual activity discussions, Latina mothers were more likely to talk directly to their daughters ("don't have sex early, like me") but preferred to have father figures speak to their sons ("if he doesn't ask me . . . I would never mention it"). African American mothers were more likely to talk directly to both daughters ("I got pregnant the first time, because my mom did not have that talk . . . I try to talk to my children") and sons ("you can get AIDS the first time you lay down with someone") or ask another male figure to speak to their sons. For the direct approach theme, African American mothers were three times as likely to engage in direct communication about sex, sexual development and the consequences of sex (e.g. STDs, pregnancy and loss of opportunities) with their children, than were Latina mothers. Latina mothers used direct approaches more frequently with female than male children. For the seizing the moment theme, Latina motherdaughter pairs and African American mother-son pairs engaged in "seizing the moment of opportunity" to talk about sex more frequently than other parent-child pairs. Mothers intentionally used natural setting opportunities to initiate discussions about sex with their child, such as when driving to school, when participating in an activity together, or when sexual content was shown in a television program. Conclusions: These data help broaden our understanding about the strategies used by African American and Latina mothers for sex discussions with their sons and daughters; evaluations for impact on youth sexual health outcomes are warranted. These data can also contribute to the development of future culturally-tailored and gender- specific parent-child communication efforts and HIV prevention interventions focusing on youth of color
SN - 1054-139X
AD - (Castellanos, Gaul, Willis, Sutton) Centers for Disease Control and Prevention, United States (Sneed) California State University, Dominguez Hills, United States
ER -

TY - JOUR
ID - 3377
T1 - Informing outdoor smokefree policy: Methods for measuring the proportion of people smoking in outdoor public areas
A1 - Thomson,G.
A1 - Russell,M.
A1 - Jenkin,G.
A1 - Patel,V.
A1 - Wilson,N.
Y1 - 2013///
N1 - 2013044351
English
Journal: Article
KW - EMBASE
KW - Adolescent

KW - Adult
KW - article
KW - Child
KW - controlled study
KW - cost control
KW - Human
KW - intermethod comparison
KW - New Zealand
KW - Observation
KW - play
KW - Policy
KW - priority journal
KW - Risk Assessment
KW - school child
KW - Smoking
KW - United Kingdom
RP - NOT IN FILE
EP - 24
JF - Health and Place
VL - 20
IS - pp 19-24
CY - United Kingdom
N2 - Introduction: To advance the design and implementation of outdoor smokefree area policies, we aimed to develop simple, low-cost methods for measuring smoking in a variety of public places. Methods: Two methods were developed and were used by solo observers during March 2011-February 2012 to measure the proportion of people smoking at a variety of sites. Results: Both methods performed well (n=5553 people observed); the first at 58 sites in the UK and New Zealand (n=3191 observed); the second at 33 sites in New Zealand (n=2362 observed), with significant differences found between the smoking at types of sites and between countries. For the two countries combined, the proportions of people smoking (amongst those over 12 years) in children's play areas was significantly lower compared to all the other sites combined (risk ratio=0.39; 95%CI: 0.20 to 0.76; p=0.002). Conclusions: Solo observers can establish the proportion of people smoking in a range of outdoor sites. Such methods can inform outdoor smokefree area policymaking by providing baseline and post-policy data to enable location targeting and policy evaluation. 2012 Elsevier Ltd
SN - 1353-8292
AD - (Thomson, Russell, Jenkin, Patel, Wilson) University of Otago, Wellington Mein St., Wellington, New Zealand
ER -

TY - JOUR
ID - 3378
T1 - Cytokine status in Ukrainian children with irritable bowel syndrome residing in a radioactive contaminated area
A1 - Sajjadieh,M.R.S.
A1 - Kuznetsova,L.
A1 - Bojenko,V.
Y1 - 2012///
N1 - 2013056570
English
Journal: Article
KW - EMBASE
KW - Adolescent
KW - Adult
KW - article
KW - CD4+ T lymphocyte
KW - Child
KW - controlled study
KW - enzyme linked immunosorbent assay

KW - ethnic group
KW - gamma spectrometry
KW - Human
KW - immune response
KW - irritable colon
KW - major clinical study
KW - preschool child
KW - protein expression
KW - radiation exposure
KW - radioactive contamination
KW - radioactivity
KW - rural area
KW - school child
KW - Th1 cell
KW - Th2 cell
KW - Ukrainian
KW - CD4 antigen/ec [Endogenous Compound]
KW - gamma interferon/ec [Endogenous Compound]
KW - interleukin 4/ec [Endogenous Compound]
RP - NOT IN FILE
SP - 248
EP - 253
JF - Iranian Journal of Immunology
VL - 9
IS - 4
CY - Iran, Islamic Republic of
N2 - Background: The effect of low dose radiation on immune system is shown. Ionizing radiation can affect cytokine production and polarization of T helper cells. Objective: The current study focused on ionizing radiation in Ukrainian children residing in a contaminated area with clinical irritable bowel syndrome. Method: Our study included 75 rural children population aged 4-18 yrs, who lived in a contaminated area exposed to natural environmental radiation with clinical irritable bowel syndrome (categorized in three groups) and 20 rural children participants aged 5-15 yrs who were living in areas with similar levels of radioactive contamination without clinical irritable bowel syndrome as control group. Internal radiation activity was measured by gamma-ray spectrometry. Serum levels of IL-4 and IFN- were measured by enzyme linked immunosorbent assay. Results: A trend towards increased levels of IL-4 was observed in children with clinical irritable bowel syndrome. In these children, IFN- levels were lower than that of the control group. Conclusion: The IBS symptoms in Ukrainian children residing in a contaminated area may have stemmed from Th1 to Th2 immune deviation and differential expression of IL-4 and IFN-
SN - 1735-365X
AD - (Sajjadieh, Kuznetsova) Department of Clinical Immunology and Allergology, National Medical Academy for Post Graduate Education, Kiev, Ukraine (Sajjadieh, Bojenko) Ukrainian Specialized Dispensaries of Radiation Defend Population, Kiev, Ukraine
ER -

TY - JOUR
ID - 3379
T1 - Sun protection counseling by pediatricians has little effect on parent and child sun protection behavior
A1 - Cohen,L.
A1 - Brown,J.
A1 - Haukness,H.
A1 - Walsh,L.
A1 - Robinson,J.K.
Y1 - 2013///
N1 - 2013037549
English
Journal: Article
KW - EMBASE

KW - Adolescent
KW - article
KW - cancer risk
KW - Child
KW - Child Behavior
KW - ethnicity
KW - Exercise
KW - Female
KW - Human
KW - major clinical study
KW - Male
KW - parental attitude
KW - patient counseling
KW - pediatrician
KW - Perception
KW - Physical Activity
KW - priority journal
KW - school child
KW - Self Report
KW - skin cancer
KW - skin color
KW - sport
KW - structured interview
KW - summer
KW - sun exposure
KW - sunburn/pc [Prevention]
KW - sunscreen
RP - NOT IN FILE
SP - 381
EP - 386
JF - Journal of Pediatrics
JA - J Pediatr
VL - 162
IS - 2
CY - United States
N2 - Objective: To compare counseling concerning sun protection and outdoor exercise with the parent's report of the behavior of a child aged 9-16 years old. Study design: Structured interviews of medical personnel in 3 Chicago area practices elicited information about counseling methods and recommendations. In each practice, a convenience sample of parents completed a self-reported survey of their and their child's behavior. Results: Sun protection counseling occurred more frequently than exercise counseling in all practices (P = .014). Sun protection counseling was associated with parental prompting (P = .004), performing a summer camp physical (P = .002), and the child having a sunburn (P = .003). After controlling for the child's age, sex, and skin tone, sun protection counseling was not associated with the child's use of sun protection. In multivariate analysis of the child's sun protection behavior, parental sunburns, indoor tanning in the last 12 months, perception of skin cancer risk, and sun protection self-efficacy were significant (P = .02). Children who pursued outdoor sports were twice as likely to use inadequate sun protection and sustain sunburns (CI 1.3-1.7). Conclusions: The child's sun protection behavior was influenced by parental sun protection, parental perception of skin cancer risk, and parental sun protection self-efficacy; therefore, sun protection for children needs to be aimed at parents as well as children. Communication with parents in a way that incorporates the principles of motivational interviewing may be more effective in promoting behavioral change than admonitions to use sunscreen. Copyright 2013 Mosby Inc
SN - 0022-3476
AD - (Cohen, Robinson) Department of Dermatology, Northwestern University, Feinberg School of Medicine, 132 E Delaware Place, Chicago, IL 60611, United States (Brown, Haukness, Walsh) Department of Pediatrics, Northwestern University, Feinberg School of Medicine, Chicago, IL, United States (Brown, Haukness, Walsh) Pediatric Practice Research Group, Chicago, IL, United States
ER -

TY - JOUR
ID - 3382
T1 - Walk&WorkSpain: Participants, perspectives and experiences on reducing occupational sitting time
A1 - Bort,Roig J.
A1 - Martin,Horcajo M.
A1 - Puig,Ribera A.
A1 - Gonzalez,A.
A1 - Martinez,Lemos,I
Y1 - 2012///
N1 - 70968832
English
Journal: Conference Abstract
KW - EMBASE
KW - Human
KW - sitting
KW - employee
KW - Walking
KW - Workplace
KW - Physical Activity
KW - Male
KW - Female
KW - University
KW - Technology
KW - Interview
KW - Health Promotion
KW - Work
KW - personal experience
KW - pedometer
KW - semi structured interview
KW - meal
KW - car
KW - quantitative analysis
KW - school
KW - Child
KW - Health
RP - NOT IN FILE
SP - S303
JF - Journal of Science and Medicine in Sport
VL - Conference: Be Active 2012 Sydney, NSW Australia. Conference Start: 20121031 Conference End: 20121103. Conference Publication:
IS - var.pagings
N2 - Introduction: Emerging evidence suggests that sedentary behaviour is negatively associated with health. Workplaces are convenient settings for delivering health promotion interventions and reduce occupational sitting time. However, most studies have focused on quantitative analyses to understand the impact of workplace physical activity interventions on reducing occupational sedentary time rather than employees, experiences. We assessed employees, perspectives who undertook a 20-week pedometer-based programme (Walk&WorkSpain)-based on Web technology-that aimed at reducing daily sitting time and increasing daily step counts at work. Method: Two-hundred and sixty-four inactive employees from 4 Spanish universities engaged in Walk&WorkSpain. The intervention group (n = 129) accessed gradually to different strategies through a Webpage: "incidental walking" (active work tasks, i.e. walk talk meetings), "short and long Campus walking routes" (10 minutes, i.e. parking the car a bit far; 20 minutes, i.e. walking at lunch time respectively). Semi-structured interviews were conducted with 8 employees from the intervention to gather qualitative data on personal experiences. The most inactive employees who volunteered were recruited. Participants were evenly divided between men/women and job roles; academic-male (n = 2), academic-female (n = 2), administrative-male (n = 2) and administrative-female (n = 2). Each participant was interviewed three times at the beginning (baseline), middle (8 weeks) and two months after completing the intervention. Interviews were transcribed

verbatim and subjected to inductive coding within the major themes of opinions on sitting reduction strategies and experiences of success when implementing them. Results: At baseline, participants had little awareness about the need of reducing sitting time. Most employees were on the contemplation stage for changing this behavior. Eight weeks later, most participants perceived to implement the different strategies successfully, being incidental walking the mostly widely used followed but short walking routes. However, they could not implement them as regularly as they wanted. At this stage, participants were on preparation for reducing sedentary behavior. The long walks around the Campus could not be implemented successfully at work but most employees put these into practice outside work by the end of the program (i.e. walking to school to pick up their children). At this point, all participants reached the "action stage". Discussion: Walk@WorkSpain was perceived to be a feasible program to promote sustained reductions on sitting time and increases on physical activity both inside and outside work. The program was perceived to be successful not only in improving participants, awareness but also in gradually introducing changes to reduce sitting time in employees, lives
SN - 1440-2440
AD - (Bort Roig, Martin Horcajo, Puig Ribera) Universitat de Vic, Spain (Gonzalez) Universidad del Pais Vasco, Spain (Martinez Lemos) Universidad de Vigo, Spain
ER -

TY - JOUR
ID - 3383
T1 - An anthropological analysis of students, activities during recess in relation to the physical and organizational surroundings
A1 - Olesen,E.
A1 - Troelsen,J.
Y1 - 2012///
N1 - 70968788
English
Journal: Conference Abstract
KW - EMBASE
KW - Human
KW - student
KW - school
KW - Physical Activity
KW - field work
KW - Child
KW - social status
KW - participant observation
KW - Interview
KW - Adolescent
KW - adolescence
KW - Speech
KW - teacher
KW - Denmark
RP - NOT IN FILE
SP - S285
JF - Journal of Science and Medicine in Sport
VL - Conference: Be Active 2012 Sydney, NSW Australia. Conference Start: 20121031 Conference End: 20121103. Conference Publication:
IS - var.pagings
N2 - Introduction: Several studies show that as children grow older and enter their teenage years their physical activity level decreases. The study 'SPACE-Space for physical activity, seek to prevent this decrease in everyday physical activity through a combination of physical and organizational interventions. The Danish public school is the basis for the SPACE-study, almost 90% of children in Denmark attend public school. My PhD. is part of the SPACE-study and explores the connections between the students, social identities, their physical and organizational surroundings and their patterns of movement i.e. during recess. The anthropological fieldwork has been conducted over a three year period making it possible to follow the children from the 5th to the 7th grade; during this period of time the student go from the age of 11 to 14 years old, a transformation from being a child to becoming an adolescent. Methods: The analysis is based upon an anthropological fieldwork

conducted at two Danish public schools. At each school the fieldwork focused primarily upon one class of approximately 25 students. The fieldwork consists of participant observation during the school day both during periods and breaks, participant observation during after school activities, and in depth interviews with the students. All in all I spend 4 months at each school. Results: The norms for movement and physical activity among the students in the 5th to the 7th grade are created and influenced crosswise by the different places and spaces that the students stay and move between. The school yard can be compartmentalized in areas on the basis of the activities that are going on, the school yard elements, the group of students engaged in the activity, the proximity to the school building and the visibility to others. In this presentation the starting point will be the proximity to the school building and the visibility of the area to other students and the teachers on playground duty. The students engaged in physical activities close to the school building where others can see them generally speaking are students with high social status whereas the students engaged in activities on the outskirts of the school grounds have a lower social status. Discussion: The anthropological approach to this area of research contributes with a different type of knowledge and thus a new angle to understanding the processes regarding growing up related to students level of physical activity
SN - 1440-2440
AD - (Olesen, Troelsen) University of Southern Denmark, Institute of Sports Science and Clinical Biomechanics, Denmark
ER -

TY - JOUR
ID - 3384
T1 - Children physical activity correlates and parent physical activity do not have a strong association with physical activity amongst adolescents
A1 - Rachele,J.
A1 - Washington,T.
A1 - Cuddihy,T.
A1 - McPhail,S.
Y1 - 2012///
N1 - 70968770
English
Journal: Conference Abstract
KW - EMBASE
KW - Physical Activity
KW - Human
KW - Adolescent
KW - parent
KW - Child
KW - model
KW - Exercise
KW - sport
KW - questionnaire
KW - adolescence
KW - Socialization
KW - juvenile
KW - Competence
KW - childhood
KW - Lifestyle
KW - Chronic Disease
KW - child parent relation
KW - Peer Group
KW - Self Esteem
KW - independent variable
KW - Health
RP - NOT IN FILE
SP - S278
JF - Journal of Science and Medicine in Sport

VL - Conference: Be Active 2012 Sydney, NSW Australia. Conference Start: 20121031 Conference End: 20121103. Conference Publication:
IS - var.pagings
N2 - Background: Physical activity is a key modifiable behavior impacting a number of important health outcomes. The path to developing chronic diseases commonly commences with lifestyle patterns developed during childhood and adolescence. This study examined whether parent physical activity and other factors correlated with physical activity amongst children are associated with self-reported physical activity in adolescents. Methods: A total of 115 adolescents (aged 12-14) and their parents completed questionnaire assessments. Self-reported physical activity was measured amongst adolescents and their parents using the International Physical Activity Questionnaire for Adolescents (IPAQ-A), and the International Physical Activity Questionnaire (IPAQ) respectively. Adolescents also completed the Children,s Physical Activity Correlates (CPAC), which measured factors that have previously demonstrated association with physical activity amongst children. To examine whether parent physical activity or items from the CPAC were associated with self-reported adolescent physical activity, backward step-wise regression was undertaken. One item was removed at each step in descending order of significance (until two tailed item alpha = 0.05 was achieved). Results: A total of 93 (80.9%) adolescents and their parents had complete data sets and were included in the analysis. Independent variables were removed in the order: perceptions of parental role modeling; importance of exercise; perceptions of parental encouragement; peer acceptance; fun of physical exertion; perceived competence; parent physical activity; self-esteem; liking of exercise; and parental influence. The only variable remaining in the model was 'liking of games and sport, (p = 0.003, adjusted rsquared = 0.085). Discussion: These findings indicate that factors associated with self-reported physical activity in adolescents are not necessarily the same as younger children (aged 8-11). While 'liking of games and sport, was included in the final model, the r-squared value did not indicate a strong association. Interestingly, parent self-reported physical activity was not included in the final model. It is likely that adolescent physical activity may be influenced by a variety of direct and indirect forms of socialization. These findings do support the view that intrinsically motivated themes such as the liking of games and sport take precedence over outside influences, like those presented by parents, in determining youth physical activity behaviors. These findings do not suggest that parents have no influence on adolescent physical activity patterns, but rather, the influence is likely to be more complex than physical activity behavior modeling perceived by the adolescent. Further research in this field is warranted in order to better understand potential contributors to successful physical activity promotion interventions amongst young adolescents
SN - 1440-2440
AD - (Rachele, Washington, Cuddihy, McPhail) Queensland University of Techonlogy, Australia
ER -

TY - JOUR
ID - 3385
T1 - Understanding the relationship between dog ownership and children,s physical activity and sedentary behaviour
A1 - Christian,H.
A1 - Trapp,G.
Y1 - 2012///
N1 - 70968762
English
Journal: Conference Abstract
KW - EMBASE
KW - dog
KW - Human
KW - organization and management
KW - Child
KW - Physical Activity
KW - Environment
KW - Walking
KW - boy
KW - girl
KW - Male
KW - Female

KW - weight
KW - pedometer
KW - parent
KW - Self Report
KW - school
KW - Travel
KW - model
KW - Obesity
KW - height
KW - gender
KW - questionnaire
KW - household
KW - Adult
RP - NOT IN FILE
SP - S275
JF - Journal of Science and Medicine in Sport
VL - Conference: Be Active 2012 Sydney, NSW Australia. Conference Start: 20121031 Conference End: 20121103. Conference Publication:
IS - var.pagings
N2 - Introduction: While evidence of the relationship between dog ownership and physical activity in adults is emerging, the nature of this relationship in children requires further investigation. Given 60-80% of Australian and US households with children have a dog, dog-facilitated physical activity may be an effective way to increase physical activity and decrease child obesity. The study aims were to: 1) examine the individual and environment factors associated with dog ownership and 2) after adjusting for significant individual and environment factors, examine the association between dog ownership and physical activity, walking and sedentary behaviour. Methods: Cross-sectional data from the Western Australian TRavel, Environment, and Kids project (TREK) were analyzed for 1218 children aged 10-12 years. Individual socio-demographic, intrapersonal, social and physical environment factors, child physical activity (walking; physical activity outside of school) and sedentary behavior (screen use) and dog ownership status was collected from child and parent questionnaires. Children,s weekly pedometer steps, height and weight were measured. Regression models progressively adjusted for significant socio-demographic, intrapersonal and environment factors. Results: Approximately 60% of children had a family dog. Dog ownership was associated with, on average, 29 more minutes of walking and 142 more minutes of physical activity/week (p<.01). After adjustment, children who had a dog were 49% more likely to be sufficiently active (420mins) and 32% more likely to have walked in their neighborhood in the last week, compared with non-dog owners (p<.05). Boy, but not girl, dog owners walked more minutes/ week and were more likely to walk in their neighbourhood than non-dog owners (p<.05). Girl, but not boy, dog owners were more likely to be sufficiently active and did more minutes of physical activity than non-dog owners (p<.05). Dog ownership was not associated with screen use, pedometer steps or weight status. Discussion: These results highlight the potential for dog ownership to significantly impact children,s physical activity levels. It is likely that the nature of the relationship and interaction that boys and girls have with their dog may influence physical activity measures differently. Further research is needed to examine how the relationship between (self-report and objective) measures of physical activity and dog ownership and dog walking varies by child age and gender. To optimize the benefits of dog ownership in terms of increased physical activity, there is a need to encourage children (and their parents) to walk with and actively play with their dogs
SN - 1440-2440
AD - (Christian, Trapp) University of Western Australia, Australia
ER -

TY - JOUR
ID - 3386
T1 - Space for active play, designing play spaces for older children (8-12 years)
A1 - Carlisle,R.
Y1 - 2012///
N1 - 70968738
English
Journal: Conference Abstract

KW - EMBASE
KW - Human
KW - Child
KW - consultation
KW - Physical Activity
KW - follow up
KW - Methodology
KW - Voice
KW - community
KW - appetite
KW - Funding
KW - parent
KW - Photography
KW - sport
KW - Experiential Learning
KW - heart
KW - non profit organization
KW - city
KW - Exercise
KW - Australia
RP - NOT IN FILE
SP - S265
JF - Journal of Science and Medicine in Sport
VL - Conference: Be Active 2012 Sydney, NSW Australia. Conference Start: 20121031 Conference End: 20121103. Conference Publication:
IS - var.pagings
N2 - Introduction: The national recommendation for physical activity for children in Australia is a minimum of one hour and up to several hours of moderate to vigorous activity per day. This project focused on play space for children aged 8-12 years and asked whether providing enticing, attractive play spaces would result in more children getting active outdoors. The Space for active play project featured children,s voices briefing the designers of four play spaces in metropolitan Melbourne. Three of the play spaces developed were then evaluated to see if they did in fact attract the target audience identified. Methods: A four method children,s consultation exercise involving experiential learning, reflective notebooks, photography and discussion was developed. The results of this consultation were then validated by classroom based children of the same age, community members and a range of professionals. Once design was completed a pre-construction site evaluation was developed and undertaken in three of the sites, involving the Most Significant Change (MSC) methodology with a small group of children, a classroom based survey, a parents survey and park observation. Following completion of the construction phase follow up evaluation was done in all three sites. Results: The children provided an abundance of ideas and activities that they would like to see and do in their local park. Their ideas went well beyond the traditional play equipment and sports opportunities offered in many municipal parks. These ideas influenced the play space designers in their approach to designing the new play spaces. The evaluation of the actual sites will be completed in June 2012, with early analysis of results suggesting that increased physical activity and outdoor play has been achieved. Discussion: This project has recorded what older children want of their play spaces, and it reveals a strong appetite for a much broader range of play experience than is currently on offer. This information has been documented in order to influence planners and designers of park space and others about older children,s needs. This paper will provide insights into what the children desire in a play space and to what extent the resulting play spaces were successful in attracting older children to engage in active outdoor play. The Space for active play project is an initiative of the Heart Foundation in partnership with Brimbank, Frankston and Moonee Valley city councils and Parks Victoria, with funding from Parks Victoria and VicHealth
SN - 1440-2440
AD - (Carlisle) Heart Foundation (Victoria), Australia
ER -

TY - JOUR
ID - 3387
T1 - Correlates of physical activity among Indigenous and nonindigenous adolescents

A1 - Macniven,R.
A1 - Hearn,S.
A1 - Grunseit,A.
A1 - Richards,J.
A1 - Nutbeam,D.
A1 - Bauman,A.
Y1 - 2012///
N1 - 70968724
English
Journal: Conference Abstract
KW - EMBASE
KW - Adolescent
KW - Human
KW - Physical Activity
KW - school
KW - Lifestyle
KW - Health
KW - Employment Status
KW - model
KW - Australia
KW - sport
KW - Population
KW - community
KW - Child
KW - Female
KW - adolescence
KW - student
KW - Regression Analysis
KW - adulthood
KW - logistic regression analysis
KW - Chronic Disease
KW - Life Expectancy
KW - bivariate analysis
KW - Television
KW - gender
KW - girl
KW - Policy
KW - Male
KW - Adult
KW - Risk
KW - Immobilization
KW - alcohol
RP - NOT IN FILE
SP - S259
JF - Journal of Science and Medicine in Sport
VL - Conference: Be Active 2012 Sydney, NSW Australia. Conference Start: 20121031 Conference End: 20121103. Conference Publication:
IS - var.pagings
N2 - Introduction: In Australia, life expectancy of people of Indigenous descent is 16-17 years lower than their non-Indigenous counterparts. Physical inactivity is the third leading cause of the burden disease for Indigenous adults. However, in children the situation is more positive with almost three quarters (74%) of Indigenous children aged 4-14 years meeting national physical activity recommendations. Adolescence is typically a period where physical activity declines and is an important target point for interventions. This study describes frequency and duration of out-of-school physical activity in a sample of Indigenous and non-Indigenous Australian adolescents and explores relationships between physical activity participation and demographic, health and lifestyle factors. Methods: The Health and Lifestyle of NSW School Students Survey was developed using established measures from surveys conducted in adolescents from similar populations. The survey was

completed by 348 Indigenous and 633 non-Indigenous students aged 13-17 years residing in rural New South Wales (NSW). The survey included questions on frequency and duration of physical activity out of school. These items were combined to establish whether national physical activity recommendations were being achieved. Bivariate analyses stratified by Indigenous status identified demographic, health and lifestyle behaviours statistically related to meeting physical activity recommendations. These variables were included in multiple binary logistic regression models conducting including the total sample and stratified by Indigenous status. Results: Indigenous adolescents were less likely to meet physical activity recommendations through out of school activity than non-Indigenous adolescents (21% versus 28%; p = 0.010). In the stratified regression analyses, Indigenous females had a higher odds of meeting physical activity recommendations than males (OR = 0.39, 95% CI 0.20-0.77) after adjusting for age, membership of a sports team, parental employment status, feeling confident and community involvement. In the non-Indigenous model, adolescents involved in a sports team had higher odds of meeting physical activity recommendations than those not involved in a team (OR = 2.41, 95% CI 1.58-3.67), after adjusting for age, gender, time spent watching television, maternal employment status, alcohol behavior, feeling lonely, feeling confident and community involvement. Discussion: These results provide an indication of physical activity levels in rural Indigenous and non-Indigenous adolescents outside of the school setting where less than a quarter of this sample were meeting physical activity recommendations. Indigenous adolescents, particularly girls, are a specific target group for intervention. Increased policy and practical efforts to reverse the decline in physical activity during adolescence is essential to reduce population disparities in chronic disease risk in adulthood among Indigenous Australians
SN - 1440-2440
AD - (Macniven, Grunseit, Bauman) University of Sydney, Australia (Hearn) Cox Inall Ridgeway, Australia (Richards) University of Oxford, United Kingdom (Nutbeam) University of Southampton, United Kingdom
ER -

TY - JOUR
ID - 3388
T1 - Features of public open spaces: Are there differences between urban and rural areas?
A1 - Veitch,J.
A1 - Salmon,J.
A1 - Ball,K.
A1 - Crawford,D.
A1 - Timperio,A.
Y1 - 2012///
N1 - 70968717
English
Journal: Conference Abstract
KW - EMBASE
KW - rural area
KW - Human
KW - Physical Activity
KW - Child
KW - Medical Audit
KW - Obesity
KW - dog
KW - Walking
KW - landscaping
KW - Australia
KW - Basketball
KW - potential difference
KW - shade
KW - groups by age
KW - Drinking
KW - urban area
KW - Risk
KW - Water
RP - NOT IN FILE
SP - S256

JF - Journal of Science and Medicine in Sport
VL - Conference: Be Active 2012 Sydney, NSW Australia. Conference Start: 20121031 Conference End: 20121103. Conference Publication:
IS - var.pagings
N2 - Introduction: Parks are important settings for physical activity. Park features have been shown to be associated with park visitation and physical activity within parks. There is some evidence that children living in rural locations are at an increased risk of overweight and obesity compared with children living in urban areas and this may be a result of decreased opportunities to be physically active such as having fewer facilities in parks. The aim of this study was to examine potential differences in the features and amenities within parks located in urban compared with rural areas. Methods: An audit tool was designed to identify and evaluate features of parks that may be important for children,s physical activity including: aesthetics (e.g. landscaping, water features, litter and graffiti), amenities (e.g. toilets, drinking fountains, shade, benches, bike racks), activity areas (e.g. open spaces and basketball courts), playgrounds (e.g. equipment and suitability for different age groups), and the presence of courts/ovals, paths and dog signage. Field observers visited 628 parks across Victoria, Australia during 2009-2010 and completed this paper and pencil audit. Results: Park audits were completed at 434 urban and 194 rural parks. The parks located in rural areas scored significantly highly on aesthetic features (3.5 vs 3.2) and the number of activity areas (0.9 vs 0.7), while urban parks scored higher on playground equipment (8.5 vs 6.8), and the number of courts/ovals (7.1 vs 2.7). A higher percentage of urban parks had walking paths (59% vs 42%) and signage regarding dogs (26% vs 14%). Conclusions: The findings demonstrate differences in park features in urban compared with rural areas. Further research is required to examine whether these differences in park features are associated with park-based physical activity, overall physical activity, or time spent in sedentary behaviours among children in urban and rural areas
SN - 1440-2440
AD - (Veitch, Salmon, Ball, Crawford, Timperio) Deakin University, Australia
ER -

TY - JOUR
ID - 3390
T1 - Locating schoolyard physical activity-Using GPS, accelerometerty and GIS
A1 - Schipperijn,J.
A1 - Bondo,Andersen H.
A1 - Demant,Klinker C.
A1 - Rask,S.
A1 - Pawlowski,C.
A1 - Sylvest,C.
A1 - Troelsen,J.
Y1 - 2012///
N1 - 70968715
English
Journal: Conference Abstract
KW - EMBASE
KW - Physical Activity
KW - geographic information system
KW - Human
KW - Child
KW - school child
KW - grass
KW - intervention study
KW - accelerometer
KW - accuracy
KW - groups by age
KW - school
KW - University
KW - United States
KW - computer program
KW - Health Care
KW - Population

KW - climbing
KW - gender
KW - Policy
KW - Rubber
RP - NOT IN FILE
SP - S255
JF - Journal of Science and Medicine in Sport
VL - Conference: Be Active 2012 Sydney, NSW Australia. Conference Start: 20121031 Conference End: 20121103. Conference Publication:
IS - var.pagings
N2 - Introduction: A number of recent studies have found associations between the characteristics of schoolyards and the level of PA of schoolchildren using the schoolyards. Based on these findings, it seems likely that making schoolyards more attractive will help to increase the total amount of PA among schoolchildren. Before starting our 'Activating Schoolyards, intervention study, we carried out an exploratory study with the objective to describe activity patterns and localize places for physical activity on 6 schoolyards varying in size and content, located in different types of neighborhoods. Methods: 745 children, 6-16 years old, enrolled at six schools were asked to wear an accelerometer (ActiGraph GT3X) and a GPS (Qstarz BT-Q1000X) for 5 schooldays to determine their level of activity and movement patterns. GPS positions were recorded every 15 seconds and activity levels were recorded every 2 seconds. GPS and accelerometer data were compiled using the Physical Activity Location Measurement System (PALMS), developed by the Center for Wireless and Population Health Systems at the University of California, San Diego. All elements of each of the 6 schoolyards were mapped in detail using a high-precision GPS (Trimble GeoExplorer XT). ArcGIS software was used to combine PALMSoutput with the schoolyard maps. For each participant, average activity counts per schoolyard element were calculated, and associations between schoolyard elements and average levels of physical activity were calculated. Results: Multi-courts with an artificial grass or rubber surface, lawn areas, grass slopes and other more natural elements were associated with higher than average levels of physical activity in schoolyards during recess. Playground equipment such as climbing frames, slides or swings, was not associated with higher levels of physical activity. Our results also show age, gender and time differences with different schoolyard elements being popular for different age groups, at different times of the day. Discussion: In future schoolyard renovations, or when designing new schoolyards, policy-makers and designers should keep our findings in mind. However, the causal relations between these schoolyard elements and higher levels of physical activity will need to be tested in our 'Activating Schoolyards, intervention study, that will commence in 2013. This study will focus on schoolyard characteristics that are likely to influence the least active children
SN - 1440-2440
AD - (Schipperijn, Bondo Andersen, Demant Klinker, Pawlowski, Troelsen) University of Southern Denmark, Institute of Sports Science and Clinical Biomechanics, Denmark (Rask, Sylvest) Danish Cancer Society, Denmark
ER -

TY - JOUR
ID - 3392
T1 - Physically active at school-It's child's play. The Sydney Playground Project
A1 - Engelen,L.
A1 - Naughton,G.
A1 - Bundy,A.
A1 - Wyver,S.
A1 - Bauman,A.
A1 - Baur,L.
A1 - Perry,G.
A1 - Jessup,G.
A1 - Ragen,J.
Y1 - 2012///
N1 - 70968383
English
Journal: Conference Abstract
KW - EMBASE
KW - Child

KW - Human
KW - school
KW - Physical Activity
KW - Social Interaction
KW - primary school
KW - girl
KW - boy
KW - Male
KW - effect size
KW - weight
KW - Female
KW - height
KW - accelerometer
KW - sport
KW - school child
KW - social adaptation
RP - NOT IN FILE
SP - S117
JF - Journal of Science and Medicine in Sport
VL - Conference: Be Active 2012 Sydney, NSW Australia. Conference Start: 20121031 Conference End: 20121103. Conference Publication:
IS - var.pagings
N2 - Introduction: This research was part of a larger study known as the Sydney Playground Project (SPP), which aims to increase physical activity and social skills in primary school children by means of a low-cost innovative playground intervention. For many children pure sports interventions are not the answer, hence this study engaged children's playful nature to promote physical activity and social interactions through play. The purpose of this part of the studywasto study the effect of the interventiononphysical activity. Methods: 214 (115 boys, 99 girls) 5-7 year old (mean: 6.0 years at baseline) children from 12 Sydney primary schools participated in this CRCT. Baseline data were collected prior to a 14-week playground intervention or recess as normal, followed by post-testing during the last week of intervention. The playground intervention: unstructured items, consisting of or made from recycled material (tyres, balance beams, crash mats) introduced to the school playgrounds for the children to play with freely. Physical activity was measured by accelerometers during school days (0900-1500 for 5 consecutive days at baseline and post-test. Height, weight and BMI were measured. Mixed-effect ML regression (STATA/IC 12), taking clustering and repeated measures into account were used to examine net change from baseline values between groups. Results: Over time, MVPA increased (1.82 min, p = 0.006), and sedentary time decreased (-2.13 min, p = 0.01) significantly during recess in the intervention group, however effect sizes were small. Activity increased independent of BMI. Anecdotally, schools and children were enthusiastic about the playground intervention, quoting less playground demeanour and more fun. Discussion: This play intervention has the potential to be a costeffective way to engage children in increased physical activity and social interactions and can potentially be implemented in a large range of settings
SN - 1440-2440
AD - (Engelen, Bundy, Perry, Jessup, Ragen) Faculty of Health Sciences, University of Sydney, Australia (Naughton) Centre of Physical Activity Across the Lifespan, Australian Catholic University, Australia (Wyver) Institute of Early Childhood, Macquarie University, Australia (Bauman) Public Health, University of Sydney, Australia (Baur) Paediatrics and Child Health, University of Sydney, Australia
ER -

TY - JOUR
ID - 3393
T1 - Review of recess-based interventions on physical activity levels of school aged children and adolescents
A1 - Parrish,A.
A1 - Okely,A.
A1 - Ridgers,N.
A1 - Stanley,R.
A1 - Salmon,J.
Y1 - 2012///

N1 - 70968382
English
Journal: Conference Abstract
KW - EMBASE
KW - Human
KW - Child
KW - school
KW - Adolescent
KW - Physical Activity
KW - randomization
KW - controlled study
KW - Population
KW - randomized controlled trial
KW - follow up
KW - juvenile
KW - procedures
KW - Environment
KW - Checklist
KW - wellbeing
KW - Health
RP - NOT IN FILE
SP - S116
JF - Journal of Science and Medicine in Sport
VL - Conference: Be Active 2012 Sydney, NSW Australia. Conference Start: 20121031 Conference End: 20121103. Conference Publication:
IS - var.pagings
N2 - Introduction: The benefits of physical activity to maintain optimal health and wellbeing in children and adolescents are undisputed. The school environment offers opportunities for children to be physically active. The aim of this review was to systematically examine the effects of recess-based interventions on the physical activity levels of school-aged children and adolescents. Methods: Two authors independently searched the literature using the same search strategies to identify papers reporting interventions which promote playground physical activity during school recess and lunchtime periods. Methodological quality was assessed using an adapted eight item assessment scale. The effects of the interventions were assessed with a rating system used in a recent review of interventions in youth. Results: The search originally retrieved 2265 articles. Nine published peer reviewed journal articles met the inclusion criteria for this review. Eight studies used randomised controlled trials and one was a controlled trial, though none of the studies adequately described the randomisation process. Three studies demonstrated high methodological quality (33%). None of the studies adequately reported the randomisation procedure or used power calculations. Few studies reported potential confounders and three studies had less than a six week follow-up. Seven studies demonstrated a positive intervention effect on children's physical activity levels, with three reporting statistical significant increases and two significant decreases in recess physical activity. The summary of the levels of evidence for intervention effects found inconclusive results for all intervention types, though promising strategies that require further investigation were identified. Discussion: A strength of all of the studies was the use of objective measures to assess physical activity outcomes, though several criteria were consistently absent from the studies. The summary of the levels of evidence for intervention effects found inconclusive results for all intervention types. This could be largely due to the small number of large high quality studies. The use of methodological checklists as a guide when designing intervention research could assist to improve intervention methodological quality in school recess physical activity research. There is an urgent need for higher quality intervention research to strengthen published findings to inform recess physical activity playground interventions. Intervention research is needed in adolescents due to the absence of school recess intervention research in this population
SN - 1440-2440
AD - (Parrish) Faculty of Health and Behavioural Sciences, University of Wollongong, Australia (Parrish, Okely) Interdisciplinary Educational Research Institute, University of Wollongong, Australia (Ridgers, Salmon) Centre for Physical Activity and Nutrition Research, Deakin University, Australia (Stanley) School of Health Sciences, University of South Australia, Australia
ER -

TY - JOUR
ID - 3394
T1 - Improving health-related fitness in children: The Fit-4-Fun randomized controlled trial
A1 - Eather,N.
A1 - Morgan,P.
A1 - Lubans,D.
Y1 - 2012///
N1 - 70968379
English
Journal: Conference Abstract
KW - EMBASE
KW - Human
KW - fitness
KW - Child
KW - randomized controlled trial
KW - Health
KW - Physical Activity
KW - school
KW - primary school
KW - randomized controlled trial (topic)
KW - Body Composition
KW - Curriculum
KW - Education
KW - school child
KW - control group
KW - follow up
KW - Behavior Change
KW - Evidence Based Practice
KW - juvenile
KW - Health Status
KW - Physical Education
KW - Male
KW - Australia
KW - Basketball
RP - NOT IN FILE
SP - S115
JF - Journal of Science and Medicine in Sport
VL - Conference: Be Active 2012 Sydney, NSW Australia. Conference Start: 20121031 Conference End: 20121103. Conference Publication:
IS - var.pagings
N2 - Introduction: Physical fitness is a strong predictor of health status in youth. However, a large proportion of children do not participate in sufficient physical activity of moderate-to-vigorous intensity to achieve the associated health benefits. The aim of this study was to evaluate the impact of a multi-component schoolbased physical fitness education intervention (Fit-4-Fun) on the physical fitness and activity levels of primary school children. Methods: The study involved a group randomized controlled trial with a wait-list control group. Four primary schools in the Hunter Region, NSW, Australia, were randomised by school into the Fit-4-Fun intervention or the control conditions. The study sample included 226 primary school children aged 10-13 years (mean age 10.72 years+0.6, 47.8% male). Fit-4-Fun was a multicomponent school-based physical fitness education intervention delivered as part of the Health and Physical Education (HPE) curriculum and targeted fitness improvements. The program utilised a range of evidence-based behavior change strategies to promote and support physical activity of adequate intensity, duration and type, needed to improve health-related fitness. The 8-week program includes three specific components based on the Health Promoting School Framework, a HPE curriculum program (1x60 min/week), a daily break-time activity program (recess/lunch) and a home fitness program (3x20 min/week). The control group participated in their usual weekly 60 min HPE lessons. Cardio-respiratory fitness (CRF) was the primary outcome and secondary outcomes included body composition, muscular fitness, flexibility and physical activity. Assessments were taken at baseline, 3- and 6-month follow-

up, to determine changes in health-related fitness and physical activity levels. Results: After 6-months, significant intervention effects were evident in cardio-respiratory fitness (beep test adjusted mean difference [95% CI] = 1.14 levels [0.74, 1.55]), body composition (BMI adjusted mean difference [95% CI] = -0.96 kg/m² [-1.42, -0.5] and BMI z-score adjusted mean difference [95% CI] = -0.47 z-scores [- 0.70, -0.25]), flexibility (sit & reach adjusted mean difference [95% CI] = 1.52cm [-0.65, 3.68]), muscular fitness (sit ups mean difference [95% CI] = 0.62 stages [-0.97, -0.26]) and physical activity (steps adjusted mean difference [95% CI] = 3253 steps/day [1776,4730]). There were no group by time effects for three measures of muscular fitness (basketball throw, push-ups and standing jump). Discussion: The Fit-4-Fun program was successful in improving several measures of fitness in primary school-aged children. These findings suggest that well designed HPE programs that promote vigorous activity within and beyond the school day have the potential to improve health-related fitness in children
SN - 1440-2440
AD - (Eather, Morgan, Lubans) University of Newcastle, Physical Activity and Nutrition Priority Research Centre, Australia
ER -

TY - JOUR
ID - 3395
T1 - Strategies to promote children's school based physical activity: Transform-Us! Mid-intervention findings
A1 - Arundell,L.
A1 - Salmon,J.
A1 - Yildirim,M.
A1 - Cerin,E.
A1 - Hesketh,K.
A1 - Ball,K.
A1 - Brown,H.
A1 - China,Paw M.
A1 - Hume,C.
A1 - Crawford,D.
Y1 - 2012///
N1 - 70968378
English
Journal: Conference Abstract
KW - EMBASE
KW - Human
KW - school
KW - Physical Activity
KW - Child
KW - Arm
KW - teacher
KW - Environment
KW - Social Support
KW - sport
KW - Sports Equipment
KW - health hazard
KW - Self Report
KW - Female
KW - Likert scale
KW - meal
KW - examination
KW - asphalt
RP - NOT IN FILE
SP - S114
EP - S115
JF - Journal of Science and Medicine in Sport
VL - Conference: Be Active 2012 Sydney, NSW Australia. Conference Start: 20121031 Conference End: 20121103. Conference Publication:

IS - var.pagings
N2 - Many children engage in suboptimal levels of PA despite the associated health risks. Schools (n = 20) in the Transform-Us! program were randomized to one of four intervention arms that target increases in children's PA (PA), reductions in sedentary behavior (SB), both behaviors (SB + PA) or control current practice (C). This examination focuses on the PA promotion strategies employed in the PA, SB + PA arms compared with the C arm. To promote children's PA each PA, SB + PA class was provided with sporting, circus equipment; asphalt line markings were installed at the school, teachers were asked to encourage PA. Grade 3 children at participating schools were invited to take part in evaluation assessments including the completion of a self-report survey. Findings from the PA promotion strategy questions at baseline (Feb-June 2010), T2 (Nov/Dec) are reported here. Children (n = 425, 55% female) were asked to respond (yes/no) to five items asking about social support for PA from their class teacher, which were then summed to create a 'teacher social support' scale. Children were also asked to indicate if 'there are markings on the walls or on the school playground to help us play games' (perceived availability of line markings); if they are 'allowed to use school sports equipment during recess, lunch breaks' (perceived accessibility of sports equipment), how much they like 'the areas to play in at school' using a 5-point Likert scale (perceived school environment). Between baseline, T2, teacher social support increased in both PA (1.7[1.4] vs 2.1[1.4]), SB arms (1.9[1.4] vs 2.4[1.4) but declined in C (2.0[1.4] vs 1.7[1.5]). For all three arms, increases were seen in perceived availability of line markings (PA: 53.2% vs 69.4%; SB + PA: 59.3% vs 71.4%; C: 60.0% vs 69.8%); perceived accessibility of sport equipment (PA: 87.7% vs 97.2%: SB + PA: 80.7 vs 94%; C: 85.4% vs 97.6%), perceived school environment (PA: 1.44[0.9] vs 1.42[0.8]; SB + PA: 1.5[0.8] vs 1.6[0.7]; C: 1.4[0.9] vs 1.5[0.7]) between baseline, T2. All findings were significant at a 1% probability level. At the mid-intervention time point, findings suggest that PA strategies have increased perceived availability of line markings, accessibility of sport equipment, perceived school environment in children allocated to the PA, SB + PA arms. However, there were also unexpected increases in the C arm for three out of four items. Post-intervention findings will add to these preliminary findings
SN - 1440-2440
AD - (Arundell, Salmon, Hesketh, Ball, Brown, Hume, Crawford) Deakin University, Australia (Yildirim, China Paw) VU University Medical Center, Netherlands (Cerin) University of Hong Kong, Hong Kong
ER -

TY - JOUR
ID - 3396
T1 - Dog ownership, dog walking and children's independent mobility
A1 - Trapp,G.
A1 - Christian,H.
A1 - Villanueva,K.
Y1 - 2012///
N1 - 70968373
English
Journal: Conference Abstract
KW - EMBASE
KW - dog
KW - Human
KW - organization and management
KW - Walking
KW - Child
KW - Physical Activity
KW - boy
KW - Male
KW - Adult
KW - walker
KW - pedometer
KW - parent
KW - school
KW - girl
KW - Environment
KW - Travel
KW - Female

KW - model
KW - questionnaire
KW - household
KW - catalyst
RP - NOT IN FILE
SP - S112
JF - Journal of Science and Medicine in Sport
VL - Conference: Be Active 2012 Sydney, NSW Australia. Conference Start: 20121031 Conference End: 20121103. Conference Publication:
IS - var.pagings
N2 - Introduction: Dog ownership is a catalyst for physical activity in adults. Given 60-80% of Australian and US households with children have a dog, dog-facilitated physical activity may be an effective way to increase children's physical activity. Few studies have examined the relationship between dog ownership and children's physical activity. Importantly, the relationship between dog walking and children's independent mobility has not been investigated. This study aimed to: 1) examine the association between dog walking, physical activity and walking in children and 2) examine if children who report walking their dog are more independently mobile than those who do not walk their dog. Methods: Cross-sectional data from the Western Australian Travel, Environment, and Kids project (TREK) were analyzed for 729 children aged 10-12 years who had a family dog. Sociodemographic information, walking, walking with a dog and physical activity outside of school was collected from child and parent questionnaires. Weekly pedometer steps were also measured. Children reported their independent mobility to 15 neighbourhood destinations. Socio-demographic factors were adjusted for in models. Results: Approximately 60% of children had a family dog. Of the children who owned a dog 55% reported walking their dog in the last week and 45% of these did so without an adult. Children who walked their dog were more independently mobile than those who did not walk their dog (all children, boys & girls $p < 0.01$). Boys who walked their dog without an adult had greater independent mobility than boys who walked their dog with an adult ($p < 0.05$). Dog walkers who were independently mobile did more walking/week (184mins) than those dog walkers who were not independently mobile (128mins; all children $p < 0.01$). For boys, dog walkers who were independently mobile were also more likely to be sufficiently active (72% vs 59%;$p < 0.01$).Dogwalking and independent mobility was not associated with pedometer steps. Discussion: Children who walk their dog are more independently mobile than children who own a dog but do not walk it. Dog walking and independent mobility are associated with children's walking and, for boys, are associated with achieving the recommended level of physical activity. These results highlight that dog walking provides opportunities for children to be independently mobile. Strategies aimed at encouraging older children to walk their dog may assist in increasing children's physical activity. Future research should examine if the promotion of dog walking in families is a viable intervention strategy for increasing children's physical activity
SN - 1440-2440
AD - (Trapp, Christian, Villanueva) University of Western Australia, Australia
ER -

TY - JOUR
ID - 3397
T1 - Predictors of preschool age children's physical activity at long day care
A1 - Bell,C.
A1 - Finch,M.
A1 - Wolfenden,L.
A1 - Morgan,P.
A1 - Freund,M.
A1 - Jones,J.
A1 - Wiggers,J.
Y1 - 2012///
N1 - 70968370
English
Journal: Conference Abstract
KW - EMBASE
KW - Human
KW - Physical Activity
KW - Day Care

KW - Child
KW - Obesity
KW - Australia
KW - Policy
KW - Medical Audit
KW - preschool child
KW - questionnaire
KW - skill
KW - staff training
KW - pedometer
KW - Risk
RP - NOT IN FILE
SP - S111
JF - Journal of Science and Medicine in Sport
VL - Conference: Be Active 2012 Sydney, NSW Australia. Conference Start: 20121031 Conference End: 20121103. Conference Publication:
IS - var.pagings
N2 - Introduction: Understanding predictors of physical activity in centre based care is important for developing interventions to help ensure young children achieve recommended levels of physical activity and minimise their risk of overweight and obesity. The aim of this study was to describe children's levels of physical activity and modifiable centre-based associated with physical activity in long day care. Methods: Pedometers were used to assess physical activity levels of 328 children aged between 3-5 years in 20 randomly selected long day care services in the Hunter Region of New South Wales, Australia. Staff questionnaires and observational audits were used to assess staff and centre-based characteristics including: staff qualification level, staff training in physical activity; staff participating with children in free active play or prompting children to increase physical activity, time spent playing computer/video games, time spent in seated activity, time spent in outdoor play, staff led physical activity, outdoor play area, portable and fixed playground equipment to encourage physical activity, and opportunities for children to learn and practice fundamental movement skills. Results: Over the 6-hour observation period of 0900-1500, the average step count was 5,466 (+2,383) or 15.8 (+6.8) steps/minute. Step counts were significantly higher in centres that had a written physical activity policy (+3.8 steps/minute, p = 0.03), included staff led structured physical activity (+3.7 steps/minute, p < 0.001) and staff participation in active play (+2.9 steps/minute, p = 0.057). Discussion: It is recommended that Australian preschool children obtain 3-hours a day of active play. For children spending a large part of the day in care, much of this activity will need to be obtained during this time. Interventions to increase children's physical activity while in care should support long day care centres to develop and implement physical activity policies and build the capacity of staff to encourage physical activity through delivery of structured activities and participation in physically active play
SN - 1440-2440
AD - (Bell, Finch, Wolfenden, Wiggers) School of Medicine and Public Health, University of Newcastle, Australia (Finch, Wolfenden, Jones, Wiggers) Hunter New England Population Health, Hunter New England Health District, New Zealand (Morgan) School of Education, University of Newcastle, Australia (Freund, Wiggers) Hunter Medical Research Institute, Australia
ER -

TY - JOUR
ID - 3398
T1 - Multidimensional correlates of preschool children's physical activity*
A1 - Hinkley,T.
A1 - Salmon,J.
A1 - Okely,A.
A1 - Hesketh,K.
A1 - Crawford,D.
Y1 - 2012///
N1 - 70968369
English
Journal: Conference Abstract
KW - EMBASE

KW - Human
KW - Physical Activity
KW - preschool child
KW - girl
KW - Child
KW - Male
KW - Female
KW - boy
KW - model
KW - compliance (physical)
KW - parent
KW - outcome variable
KW - accelerometer
KW - sleep time
KW - Environment
KW - health program
KW - Public Health
KW - Health
RP - NOT IN FILE
SP - S110
EP - S111
JF - Journal of Science and Medicine in Sport
VL - Conference: Be Active 2012 Sydney, NSW Australia. Conference Start: 20121031 Conference End: 20121103. Conference Publication:
IS - var.pagings
N2 - Introduction: Physical activity is important for young children's health yet compliance with recommendations is low. Identifying factors associated with young children's physical activity is necessary to inform development of future interventions and public health programs. This study sought to identify multidimensional correlates of preschool children's physical activity. Methods: The social ecological model (SEM)was used to identify constructs potentially associated with preschool children's physical activity across each of the three domains-individual, social, physical environment. Cross-sectional data were collected from 1004 preschool children, aged between three and five years, and their parents in 2008-2009. Physical activity was measured over eight days using ActiGraph accelerometers. Parents completed a comprehensive survey. Generalized linear modeling was used to assess bivariable and multivariable associations between potential correlates and percent of time in each of three physical activity outcome variables (weekly [which included both week and weekend days], week day, weekend day) for boys and girls separately. Results: Correlates of physical activity were found across all the domains of the SEM and varied between boys and girls and between week and weekend days for each of the sexes. Age was the only consistent correlate, with children spending approximately 10% less time in physical activity for each advancing year of age. Some modifiable correlates which were related to more than one physical activity outcome were rules restricting rough games inside and usual daily sleep time for boys (both inversely associated). Spending time outside on weekends and visiting active play spaces supported boys to be more active. For girls, a preference to play inside/draw/do craft rather than be active and child constraints such as lack of time or someone to play with were inversely associated with more than one of the physical activity outcomes. Paternal provision of logistic support was associated with girls spending a greater percent of their time being active on weekends. Discussion: As correlates of preschool children's physical activity are multidimensional, future interventions and programs should ensure they address factors across all levels of the SEM. Potential strategies for promoting preschool children's physical activity should seek to influence identified correlates. Programs may need to consider whether they address week or weekend day physical activity as different correlates would need to be targeted. Sex-specific strategies may also be warranted
SN - 1440-2440
AD - (Hinkley, Salmon, Hesketh, Crawford) Deakin University, Australia (Okely) University of Wollongong, Australia
ER -

TY - JOUR
ID - 3399

T1 - Moderate and vigorous physical activity among 10-16 year old children living in a diverse ethnic minority community: Does the built environment matter?
A1 - Demant,C.
A1 - Schipperijn,J.
A1 - Troelsen,J.
Y1 - 2012///
N1 - 70968348
English
Journal: Conference Abstract
KW - EMBASE
KW - Human
KW - community
KW - ethnic group
KW - Child
KW - Environment
KW - Physical Activity
KW - Male
KW - boy
KW - girl
KW - Female
KW - model
KW - accelerometer
KW - Denmark
KW - Social Support
KW - exposure variable
KW - city
KW - minority group
KW - questionnaire
KW - register
KW - Crowding
KW - hypothesis
KW - parent
KW - school
KW - leisure
KW - sport
KW - Documentation
KW - Health
KW - recombinant erythropoietin
RP - NOT IN FILE
SP - S102
EP - S103
JF - Journal of Science and Medicine in Sport
VL - Conference: Be Active 2012 Sydney, NSW Australia. Conference Start: 20121031 Conference End: 20121103. Conference Publication:
IS - var.pagings
N2 - Introduction: Moderate to vigorous physical activity (MVPA) has a range of social and health benefits among children. Ethnic minority children and children with low Socio Economic Status (SES) often engage in less organized sports than Danish children with high SES. However little is known about objectively measured MVPA among ethnic minority groups in Denmark. Based on baseline data from the When Cities Move Children study, this study investigates the association of the built environment, socio-demographic and social support variables on children's daily physical activity. Methods: Participants were 291 children from four schools in a diverse ethnic minority community in Copenhagen, Denmark (10-16yrs). Physical activity data were obtained using accelerometers (Actigraph GT3X, 30 sec epoch) and Evenson cutpoints were used to determine mean daily time in MVPA. Exposure variables were based on questionnaire data, registry data, GPS/GIS derived variables and accelerometer data. Data were analyzed in STATAmp 12 using a 3-level mixed multilevel model to examine both unadjusted and adjusted models. Results: Among the participants 45% were boys, 42.5% had at least one parentwhodid not work and 64.3% had at least one parent with an ethnic minority background. The

adjusted analyses showed that boys engage in significantly more minutes of daily MVPA than girls (boys: 57.8, girls: 36.9, $p < 0.001$) and that boys from a Danish ethnic family accumulated significantly less minutes of daily MVPA compared to boys from an ethnic minority family ($p < 0.001$). Girls in grade 7 (13-14yrs) accumulated the least minutes of MVPA ($p < 0.05$). Being in a class with a high average mean MVPA was significantly associated with higher individual MVPA among both boys ($p < 0.001$) and girls ($p < 0.05$). None of the included built environment variables were significantly associated with MVPA in the adjusted model. Discussion: This study contradicts the notion that ethnic minority boys are less physically active than Danish boys. Anecdotal work so far confirms the hypothesis that these boys are bound to spend the majority of their leisure time outdoors in their local community due to crowding in their home while girls are confined to the home to stay out of trouble and help in the home. Further analyses based on available GPS data on these participants will provide more insight, leading to new documentation on the association on outdoor time and physical activity level as well as new knowledge on which built environment variables may be relevant for this group of children
SN - 1440-2440
AD - (Demant, Schipperijn, Troelsen) University of Southern Denmark, Institute of Sports Science and Clinical Biomech, Denmark
ER -

TY - JOUR
ID - 3400
T1 - After school hours activities of young Australian school children: Low levels of outdoor play and peer interaction
A1 - Engelen,L.
A1 - Wyver,S.
A1 - Bundy,A.
A1 - Naughton,G.
Y1 - 2012///
N1 - 70968320
English
Journal: Conference Abstract
KW - EMBASE
KW - school child
KW - Human
KW - school
KW - Child
KW - Physical Activity
KW - parent
KW - girl
KW - Male
KW - primary school
KW - Risk
KW - boy
KW - computer
KW - Female
KW - Television
KW - sampling
KW - Mental Health
KW - social adaptation
RP - NOT IN FILE
SP - S91
JF - Journal of Science and Medicine in Sport
VL - Conference: Be Active 2012 Sydney, NSW Australia. Conference Start: 20121031 Conference End: 20121103. Conference Publication:
IS - var.pagings
N2 - Introduction: This research was part of a larger study known as the Sydney Playground Project (SPP), which aims to increase physical activity and social skills in primary school children by means of a low-cost innovative playground intervention. The aim of this part of the project was to investigate what children aged 5-7

years in Sydney do after school hours. Our focus was on whether children spent time indoors or outdoors, who they were with, and the parents' perceptions of the children's levels of physical movement, intensity and involvement. Methods: Experience sampling method (ESM) was used to obtain the data.Weasked parents/carers of 221 children, (119 boys, 102 girls; mean age 6.0 years) to keep a palm pilot close by for four week days. On those days, the palm pilot delivered three brief surveys at random times between 1530-1900. The intention of the survey was to find out 'What your child is doing now' and the details of these activities (full details of the protocol can be found at http://www.biomedcentral.com/1471-2458/11/680). Results: Baseline survey data relating to after school activities indicated 55% of the time was spent indoors in pastimes involving low levels of physical activity. Children were engaged in higher levels of physical activity when outdoors and/or with peers, but these respectively accounted for < 20% and < 10% of survey occasions. The major contributor to children's pastimes was screen time (television or computer, 22%). Discussion: Our findings are considered in the context of opportunities for children to play outdoors with peers after school hours. We examine some of the pressures on parents and others to keep children safe, which may, paradoxically, lead to children engaging in sedentary pastimes with limited peer interaction, hence increasing the risk of later physical and mental health problems
SN - 1440-2440
AD - (Engelen, Bundy) Faculty of Health Sciences, University of Sydney, Australia (Wyver) Institute of Early Childhood, Macquarie University, Australia (Naughton) Centre of Physical Activity Across the Lifespan, Australian Catholic University, Australia
ER -

TY - JOUR
ID - 3401
T1 - Correlates of break-time physical activity in South African primary school learners: SOPLAY
A1 - Uys,M.
A1 - Hendricks,S.
A1 - Draper,C.
A1 - de,Villiers A.
A1 - Steyn,N.
A1 - Fourie,J.
A1 - April,M.
A1 - Lambert,E.
Y1 - 2012///
N1 - 70968318
English
Journal: Conference Abstract
KW - EMBASE
KW - primary school
KW - Physical Activity
KW - school
KW - density
KW - Eating
KW - Human
KW - Environment
KW - planning
KW - teacher
KW - control group
KW - Crowding
KW - lowest income group
KW - meal
KW - Walking
KW - sitting
KW - juvenile
KW - leisure
KW - school child
KW - Policy
KW - Physical Education

RP - NOT IN FILE
SP - S90
EP - S91
JF - Journal of Science and Medicine in Sport
VL - Conference: Be Active 2012 Sydney, NSW Australia. Conference Start: 20121031 Conference End: 20121103. Conference Publication:
IS - var.pagings
N2 - Introduction: There are concerning trends for a decline in time spent in physical activity and physical education and increased sedentary time in South African schoolchildren. 'HealthKick' is a primary school-based intervention for low-income settings that aims to promote healthy eating and physical activity through Action Planning. We were particularly interested in school breaktime, as a possible target for intervention. Factors that have previously been shown to positively influence physical activity during break-time include: playground markings and renovations, teacher supervision and the availability of loose equipment. Aims: This study was a quasi-experimental, post-test only, control-group design and aimed to test whether or not physical activity during break-time was greater in intervention schools that had participated in Action Planning. A secondary aim was to determine the extent to which the school environment influenced physical activity during break-time in South African schools. Methods: The System for Observing Play and Leisure Activity in Youth (SOPLAY) was carried out in eight of the HealthKick schools (4 intervention and 4 control). Visual scans were conducted by multiple observers in pre-selected target areas, noting the activity of each individual within a target area, (sitting, standing, eating, walking, or playing). Observers also noted information about the school environment, including whether the area was usable, accessible, and supervised, if organised activity was taking place, and the availability of equipment. Results: There were no differences between intervention and control schools. Approximately 31% of learners were sedentary, 14% were eating, with 29% and 26% involved in moderate or vigorous play, respectively. Supervision during break was associated with a significantly lower proportion of learners engaged in vigorous play ($P < 0.04$, 24% vs 28%) and a greater number of learners eating their lunch (17% vs 11%). Finally, learner density (number of learners per area scanned) was significantly and inversely associated with physical activity. Only 17.6% of learners in low density areas were sedentary compared to 49.6% of those in high density areas; conversely 28.2% of learners engaged in vigorous play in low density areas, compared to only 13.5% in high density areas ($P < 0.001$). Conclusions: Physical activity during break-time in these South African primary schools was adversely affected by over-crowding and teacher supervision. The results suggest that educators were more involved in 'crowd control' than the promotion of physical activity during break-time, and that interventions may be targeted at the school policy environment to reduce these barriers to physical activity
SN - 1440-2440
AD - (Uys, Hendricks, Draper, April, Lambert) UCT/MRC Research Unit for Exercise Science and Sports Medicine, Faculty of Health Sciences, University of Cape Town, South Africa (De Villiers, Fourie) MRC Chronic Diseases of Lifestyle Unit, Medical Research Council of South Africa, South Africa (Steyn) Cent. for the Stu. of Soc. and Environ. Determ. of Nutr. Popul. Health, Health Sys. and Innov., Human Sciences Research Council, South Africa (April) Sports Science Institute of South Africa, South Africa
ER -

TY - JOUR
ID - 3402
T1 - A cross cultural comparison of sedentary behavior of African and European youth
A1 - Prista,A.
A1 - Lopes,V.
A1 - Saranga,S.
A1 - Nhantumbo,L.
A1 - Santos,F.
A1 - Maia,J.
Y1 - 2012///
N1 - 70968122
English
Journal: Conference Abstract
KW - EMBASE
KW - cultural factor
KW - juvenile

KW - Human
KW - school
KW - Male
KW - Energy Expenditure
KW - groups by age
KW - boy
KW - girl
KW - gender
KW - Female
KW - Population
KW - observational study
KW - model
KW - sitting
KW - Portugal
KW - metabolic equivalent
KW - Child
KW - Physical Activity
KW - screening
KW - Adolescent
KW - Africa
KW - survival
KW - household
KW - rural area
RP - NOT IN FILE
SP - S8
JF - Journal of Science and Medicine in Sport
VL - Conference: Be Active 2012 Sydney, NSW Australia. Conference Start: 20121031 Conference End: 20121103. Conference Publication:
IS - var.pagings
N2 - Introduction: The study of sedentary behavior in rural Africa children and adolescents is scarce, and the goal of this research was to compare the sedentary behavior and light physical activities (LPA) between a rural sample from Mozambican school aged youth (6 to 16 years old) with their European peers, namely from Portugal. Methods: The sample comprises 146 Mozambican (MZ) and 195 Portuguese (PT) youth of both gender, and was split in 3 age groups (6-8yrs; 9-11yrs; 12-16yrs). The Actigraph model 7164 was used to obtain information marking daily PA and sedentary behaviours during 7 consecutive days in PT, and 1 only day in MZ (due to operational and logistic problems). Data files from all participants were screened by detecting blocks of consecutive zeros. Periods with 60 minutes of consecutive zeros were detected and flagged as times in which the monitor was not worn. Participants had to have at least 10 hours of data to count as a valid day. After complete screening, the raw activity "counts" were processed for time spent in the different PA intensities computations. Sedentary behavior was defined as PA with energy expenditure at the level of 1.0-1.5 metabolic equivalent units (METs). LPA was defined as PA with energy expenditure at the level of 1.6-2.9 METs. Puyau regression equation was used to determine the cut-points for PA intensities. Sedentary behavior was identified using a cut-point of <670 counts.min-1, and LPA using a cut-point of >670 and <3003 counts.min-1 Results: Mozambican rural school aged sample showed, at all age-groups and gender, an accentuated lower time spent in daily sedentary activities with a magnitude of difference around 1.5 higher for PT (Boys: MZ=370.9+78.2 vs PT=590.8+81.9; Girls: MZ=389.4+90.5 vs PT=596.2+86.2; F=493.7; p=0.0000). Using country, age and sex as factors, no statistically significant interactions were found. By turn, time spent in light activities were higher in MZ (Boys: MZ=197.5+47.9 vs PT=157.9+42.7; Girls: MZ=211.8+56.0 vs PT=158.9+47.7; F=66.7; p<0.001) in a magnitude of 1.3 higher for MZ. No significant interactions (p>0.05) were observed. Conclusions: Based on simultaneous observational studies, a significant higher time spent in light activities and less in sedentary behavior from the MZ rural school aged population may to be related to survival households activities, long distance walks and outdoor games still present in African rural areas
SN - 1440-2440
AD - (Prista, Saranga, Nhantumbo) Faculty of Physical Education and Sports, Universidade Pedagogica, Mozambique (Lopes) Instituto Politecnico de Braganca, Portugal (Santos, Maia) Faculty of Sports, Oporto University, Portugal
ER -

TY - JOUR
ID - 3403
T1 - Sitting at Home: A review of the influence of the home physical environment on children's sedentary and activity behaviour
A1 - Maitland,C.
A1 - Rosenberg,M.
A1 - Foster,S.
A1 - Braham,R.
A1 - Stratton,G.
Y1 - 2012///
N1 - 70968120
English
Journal: Conference Abstract
KW - EMBASE
KW - Environment
KW - Child
KW - Human
KW - sitting
KW - Recreation
KW - devices
KW - Physical Activity
KW - experimental study
KW - control group
KW - Adolescent
KW - Language
KW - household
KW - Technology
KW - observational study
KW - data base
KW - PsycInfo
KW - New Zealand
KW - Australia
KW - United Kingdom
KW - Netherlands
KW - Income
KW - MEDLINE
KW - Health Promotion
KW - intervention study
RP - NOT IN FILE
SP - S7
JF - Journal of Science and Medicine in Sport
VL - Conference: Be Active 2012 Sydney, NSW Australia. Conference Start: 20121031 Conference End: 20121103. Conference Publication:
IS - var.pagings
N2 - Introduction: The home setting is an important sphere of influence on the sedentary behaviour and activity of children and younger adolescents. Whilst there has been much investigation of the neighbourhood level physical environment, the home physical environment has received less attention. The purpose of this study was to systematically review recent literature on the influence of the home physical environment on sedentary behaviour and physical activity of children and adolescents aged 8-14 years. Methods: Medline, Web of Science, PsycINFO and SPORTDiscus databases were searched for English language peer reviewed journal articles published between 2005 and 2011. Thirty-six observational and 11 experimental studies met the inclusion criteria and were reviewed. Results: Most studies were conducted in high income countries including USA (n=14), Australia (n=8), New Zealand (n=4), UK (n=4) and The Netherlands (n=4). Twenty-one observational studies measured physical activity outcomes, with over half including objective measures; while 20 measured sedentary behaviour outcomes, with three using objective measures. The most commonmeasures of the home physical environment were electronic media devices (n=27), physical activity equipment (n=14)

and yard/gardens (n=5). Inmore than half of studies, positive associations were found between household electronic media devices and sedentary behaviours. Amongst the experimental studies reviewed, the two main strategies to change the home physical environment were, introducing a TV limiting device or an active video game. Introducing a TV limiting device resulted in a decrease in TV viewing in intervention groups compared to control groups. Introducing an active video game showed an increase in active video game play and a decrease in sedentary video game play in intervention groups compared to no intervention control groups in RCT studies. Discussion: In an era of changing home and technology environments, this review provides an up to date summary of the evidence related to the influence of the home physical environment on children's sedentary and activity behaviours. Health promotion interventions should promote strategies to limit electronic media equipment in the home including in children's bedrooms. TV limiting devices and active video games show some promise for decreasing sedentary screen time and increasing children's activity at home, although maintenance remains an issue. Exploration of potential influences in this domain outside of physical activity and media equipment, and further intervention studies, are recommended. The relationship of sedentary time with the physical, social and virtual elements of thehomeenvironment is of particular interest
SN - 1440-2440
AD - (Maitland, Rosenberg, Braham) School of Sports Science,Exercise and Health, University of Western Australia, Australia (Foster) School of Population Health, University of Western Australia, Australia (Stratton) Swansea University, United Kingdom
ER -

TY - JOUR
ID - 3404
T1 - Determinants of change in children's objectively measured sedentary time
A1 - Atkin,A.
A1 - Van,Sluijs E.
A1 - Corder,K.
A1 - Ekelund,U.
A1 - Wijndaele,K.
A1 - Griffin,S.
Y1 - 2012///
N1 - 70968118
English
Journal: Conference Abstract
KW - EMBASE
KW - Human
KW - Child
KW - Male
KW - school
KW - sport
KW - boy
KW - Physical Activity
KW - father
KW - parent
KW - girl
KW - model
KW - Female
KW - linear regression analysis
KW - Population
KW - outcome variable
KW - sibling
KW - body mass
KW - accelerometer
KW - Eating
KW - Recreation
KW - Health
RP - NOT IN FILE
SP - S6

EP - S7
JF - Journal of Science and Medicine in Sport
VL - Conference: Be Active 2012 Sydney, NSW Australia. Conference Start: 20121031 Conference End: 20121103. Conference Publication:
IS - var.pagings
N2 - Introduction: Sedentary behaviours are highly prevalent amongst children and may be adversely associated with cardiometabolic health, independently of participation in physical activity. To inform intervention development, this study examined parental and home-environmental determinants of 1-year change in children's objectively measured sedentary time after-school (Monday-Friday, 1500-2300) and at the weekend (Saturday-Sunday, 0600-2300). Methods: Data are from the Sport, Physical Activity, and Eating Behaviour: Environmental Determinants in Young People (SPEEDY) study. Participants wore accelerometers at baseline and 1 year later. Longitudinal data for the after-school and weekend analyses were available for 854 (41.5% male, mean+SD age 10.2+0.3years) and 718 (41.8% male, age 10.2+0.3years) participants respectively. Information on 26 candidate predictors, including socio-economic status (SES), availability of electronic media and parental rules for active and sedentary behaviours was self-reported by children and/or their parents at baseline. Change in the proportion of registered time spent sedentary (<100cpm) was used as the outcome variable in multi-level linear regression models, adjusted for age, sex, body mass index and baseline sedentary time. Simple and multiple models were run and interactions with sex explored. Results: In absolute terms, sedentary time increased over 1 year (after-school: 4.9+45.1 min/day; weekend 8.0+89.3 min/day). SES was positively associated with change in after-school (beta; 95% CI: 1.02; 0.37, 1.66) and weekend (1.42; 0.65, 2.18) sedentary time. Negative associations with change in after-school sedentary time were observed for having more siblings (-1.00; -1.69, -0.30), greater availability of electronic media (-0.81; -1.29, -0.33), and, for boys, more frequent family visits to the park (-1.89; -3.28, -0.51) and family participation in sport (-1.28; -2.54, -0.02). Maternal weekend screen-time (0.45; 0.08, 0.83) and, in girls, greater parental restriction on playing outside (0.91; 0.08, 1.74) were positively associated with change in weekend sedentary time. For participants with a (step-) father living at home, father's weekend screen-time was positively associated with change in weekend sedentary time (0.40; 0.01, 0.78). Father's physical activity was positively associated with change in boys' after-school sedentary time (1.34; 0.53, 2.15). Discussion: Nine determinants of change in sedentary time were identified, some of which were time- or sex-specific. Higher SES children exhibited greater increases in sedentary time afterschool and at weekends compared to children of low SES, highlighting a potential target population. Intervention strategies that aim to reduce parents' weekend screen-time, increase family participation in sports or recreation (for boys) and promote freedom to play outside (for girls) may contribute towards preventing the age-related increase in sedentary time
SN - 1440-2440
AD - (Atkin, Corder) UKCRC Centre For Diet And Activity Research (CEDAR), United Kingdom (Van Sluijs, Ekelund, Wijndaele, Griffin) Medical Research Council, Epidemiology Unit, United Kingdom (Ekelund) Department of Sports Medicine, Norwegian School of Sports Sciences, Norway
ER -

TY - JOUR
ID - 3405
T1 - Settings for physical activity-Developing a site-specific physical activity behavior model based on multi-level intervention studies
A1 - Troelsen,J.
A1 - Klinker,C.
A1 - Pawlowski,C.
A1 - Christiansen,L.
A1 - Toftager,M.
A1 - Olesen,E.
A1 - Linke,B.
A1 - Schipperijn,J.
Y1 - 2012///
N1 - 70968110
English
Journal: Conference Abstract
KW - EMBASE
KW - Physical Activity

KW - intervention study
KW - model
KW - Human
KW - community
KW - social psychology
KW - Adolescent
KW - Denmark
KW - Child
KW - city
KW - school
KW - conceptual framework
KW - empiricism
KW - Health Behavior
RP - NOT IN FILE
SP - S3
JF - Journal of Science and Medicine in Sport
VL - Conference: Be Active 2012 Sydney, NSW Australia. Conference Start: 20121031 Conference End: 20121103. Conference Publication:
IS - var.pagings
N2 - Introduction: Ecological models of health behavior have potential as theoretical framework to comprehend the multiple levels of factors influencing physical activity (PA). The potential is shown by the fact that there has been a dramatic increase in application of ecological models in research and practice. One proposed core principle is that an ecological model is most powerful if the model is behavior-specific. However, based on multi-level interventions in a Danish context, it must be considered that ecological models also should be site-specific to capture local and cultural aspects related to PA. Methods: Three major multi-level intervention studies are carried out in Denmark with the objective to promote PA in 17 local communities. 1) The SPACE-study is a comprehensive intervention in 7 local school districts (N = 1,348). 2) The When Cities Move Children-study is investigating the effects of urban refurbishment on adolescents' movement patterns (N = 653) and 3) The Valuable Detours for PA-project is a intervention study in 9 municipalities measuring the effect of new established outdoor facilities for PA among 12-85 years old citizens (N = 10,434). The SPACE and the WCMC study used objective measurements of PA combined with e-surveys, the VDPA study is based on self-reported e-survey data. Results: Merging the data from the three intervention studies clarifies different local and cultural aspects with specific effect on PA behavior. This finding is closely related to the fact that the new outdoor PA facilities in the 17 local communities have different size, composition and are targeted to different subgroups with different organizational setup to support the use of the facilities. Despite the conceptual and contextual differences PA behavior is also affected by cultural and social values related to the specific site which not alone can be explained by intrapersonal, interpersonal or organizational factors. Discussion: The Ecological Model of Four Domains of Active Living specifies that factors at multiple levels can influence PA behavior, and emphasizes the importance of behavior-specific models. On this theoretical basis the three mentioned multi-level interventions were planned and implemented. Based on the empirical studies we argue that site-specific factors have to be taken into consideration. A theoretical implication of this finding is to develop a site-specific physical activity behavior model adding a layered structure to the ecological model representing the determinants related to the specific site
SN - 1440-2440
AD - (Troelsen, Klinker, Pawlowski, Christiansen, Toftager, Olesen, Schipperijn) University of Southern Denmark, Denmark (Linke) Region of Southern Denmark, Denmark
ER -

TY - JOUR
ID - 3406
T1 - Ten-year canadian national prospective data on utilization of anti-hemophilic concentrates: Indications and trends
A1 - Walker,I.
A1 - Traore,A.N.
A1 - Iorio,A.
A1 - Ritchie,B.
A1 - Heddle,N.

A1 - Webert,K.E.
A1 - St
A1 - Louis,J.
A1 - Lillicrap,D.
A1 - Teitel,J.M.
A1 - Chan,A.K.C.
Y1 - 2012///
N1 - 70963941
English
Journal: Conference Abstract
KW - EMBASE
KW - hematology
KW - Society
KW - Human
KW - patient
KW - hemophilia
KW - Hospital
KW - prophylaxis
KW - infusion
KW - information processing
KW - Blood
KW - Canada
KW - hospital patient
KW - bleeding
KW - bleeding disorder
KW - register
KW - resource management
KW - Monitoring
KW - transfusion
KW - planning
KW - Child
KW - epidemic
KW - immunological tolerance
KW - surgery
KW - registration
KW - data base
KW - blood bank
KW - computer program
KW - acquired immune deficiency syndrome
KW - blood clotting factor 9
KW - blood clotting factor 8
KW - recombinant blood clotting factor 7a
KW - polymacon
RP - NOT IN FILE
JF - Blood
VL - Conference: 54th Annual Meeting of the American Society of Hematology, ASH 2012 Atlanta, GA United States. Conference Start: 20121208 Conference End: 20121211. Conference Publication:
IS - var.pagings
N2 - Background: In response to the transfusion-transmitted AIDS epidemic the Commission of Inquiry on the Blood System in Canada recommended the development of a monitoring and tracking system of product usage. The Association of Hemophilia Clinic Directors of Canada (AHCDC) implemented the Canadian Hemophilia Assessment and Resource Management System (CHARMS) to track factor concentrates (FC) from the sole suppliers, Canadian Blood Services (CBS) and Hema-Quebec (HQ), to hospitals and to patients' homes. Objective: To identify the indications and locations for infusions of factor VIII (FVIII), factor IX (FIX), recombinant factor VIIa (rFVIIa) and FVIII inhibitor bypass activity (FEIBA) and review utilization during 2000-2009. Methods: All Canadian patients with hemophilia attend the 26 Canadian Hemophilia Treatment Centres (HTCs) and are registered with the Canadian Hemophilia Registry (CHR; ahcdc.ca) from which they

receive a unique number suitable for anonymizing data. HTCs receive product and infusion data from the blood suppliers (CBS, HQ), regional hospitals and patients. HTCs enter these data into their clinic-based CHARMS software program and then export the data to the national database (CentrePoint) where data are validated. Results: From 2000 to 2009, 2,904 patients received, predominantly, FVIII (902 million Units) and FIX (240 million Units). A total of 2,386(82%) had congenital bleeding disorders listed in the Canadian Hemophilia Registry. The remaining 518 were not registered because they either did not have inherited factor deficiency or had registrations pending. Most of FVIII and FIX was infused by patients at home: 90% to 95% for FVIII and 85% to 94% for FIX. The yearly amount of FVIII infused increased over time; this was accounted for by an increase in the number of patients who were treated shown by positive and significant correlation(r=0.89; p=0.04), not so by amount infused per patient which remained constant (p=0.57). Furthermore, there was a linear relationship between an increase in patients and increased utilization, each additional patient predicting for an increased utilization of 96,217 units (P=.04). The highest proportional utilization of both FVIII and FIX was for prophylaxis, and this proportion increased over time, while the proportion used for bleeding remained steady. In 2009, the proportions used for prophylaxis were 73% and 60% for factor VIII and factor IX respectively; for bleeding the proportions were 16% and 30%, for surgery 1% and 4%, for immune tolerance induction 3% and 1%, and unclassified use was 6% and 5% respectively. Among inhibitor by-passing products, rFVIIa was the product most used by patients without bleeding disorders, 20% of VIIa being used by these patients but only 6% of FIEBA. FIEBA was predominant over rFVIIa for prophylaxis in children. Improvements in data collection included a notable decrease in the proportion of Unclassified usage; for FVIII from 12-36% each year from 2000-2004 to 6-16% from 2005-2009; for FIX from 14-41% each year before 2005 to 4-14% after 2004. Specifically, the decrease in Unclassified infusions of FVIII from 16% in 2005 to 6% in 2009 showed a statistically significant trend (p=.02). Conclusions: 1) Data collection became reliable after 2004 when the proportion of Unclassified indications and the annual increase in patient numbers fell to low levels; hence subsequent trends should be compared with the period 2005-2009. 2) The increase in total annual FVIII concentrate use is accounted for by the annual increase in patient numbers, not by the annual use per patient, 3) FC are increasingly being infused for prophylaxis. 4) The major site of infusion is at home, which is outside the scope of the existing tracking system and which comprises only blood suppliers and hospitals; hence, HTCs and a comprehensive data collection system, CHARMS in this case, are both required components of an effective national tracking system and 5) a tracking system involving the entire supply and treatment chain is essential for predicting utilization and planning for future needs
SN - 0006-4971
AD - (Walker) Medicine, McMaster University, Hamilton, ON, Canada (Traore) Medicine, McMaster University, Edmonton, Canada (Iorio) McMaster University, Edmonton, Canada (Ritchie) University of Alberta, Edmonton, Canada (Heddle) Department of Medicine, McMaster University, Hamilton, ON, Canada (Webert) Medicine, Pathology and Molecular Medicine, McMaster University, Hamilton, ON, Canada (St. Louis) Hematology, University of Montreal, Kingston, ON, Canada (Lillicrap) Pathology and Molecular Medicine, Queen's University, Kingston, ON, Canada (Teitel) St. Michael's Hospital, Toronto, ON, Canada (Chan) Pediatrics, McMaster University, Hamilton, ON, Canada
ER -

TY - JOUR
ID - 3407
T1 - Investigation of community and home factors in child physical activity levels
A1 - Graham,M.
A1 - Shannon,V.
A1 - Ice,C.
A1 - Cottrell,L.
Y1 - 2012///
N1 - 70951075
English
Journal: Conference Abstract
KW - EMBASE
KW - Physical Activity
KW - Behavior Modification
KW - Epidemiology
KW - Child
KW - Human

KW - community
KW - Nutrition
KW - Metabolism
KW - Public Health
KW - parent
KW - model
KW - Environment
KW - screening
KW - cardiovascular risk
KW - weight
KW - Home Environment
KW - Self Report
KW - dependent variable
KW - hypothesis
RP - NOT IN FILE
JF - Circulation
VL - Conference: Epidemiology and Prevention/Physical Activity, Nutrition and Metabolism 2012 San Deigo, CA United States. Conference Start: 20120313 Conference End: 20120316. Conference Publication:
IS - var.pagings
N2 - It is known that physical activity (PA) behavior is influenced by many factors within the social ecological model. Using results from parent surveys distributed after their children's completion of a cardiovascular risk screening program, we explored the relationship between home and community environments on the amount of PA in which children engaged. Our hypothesis was that more immediate factors such as parent activity would have a greater impact on child activity than factors in their community environments. A large sample (n=450) of children (ages 5-10 years) were examined. Children's physical activity was assessed by adding the total minutes of active time weekly. Parent physical activity was measured with two self report items regarding the number of days per week they were active. Two scales were constructed to evaluate to effects of the home (9 items; alpha = .829) and community (16 items; alpha = .868). The home environment scale measured elements related to activity opportunities and home schedules; the community scale assessed presence of playgrounds, or safe sidewalks, for example. To assess associations between factors and children's PA, we conducted a linear stepwise regression with child age, parent PA, Home scale, and Community scale as predictors and the log transformed total weekly activity time as the dependent variable. Sixteen percent of children's PA was explained by the tested model. Figure 1 provides specific information about each variable. The home scale had the greatest weight (beta=0.360, p<.001), and proved to have a larger predictive effect than parent PA. These findings are significant for identifying which aspects of a child's surrounding to intervene for maximum impact on physical activity. Figure 1. R² Change between model levels (Figure presented)
SN - 0009-7322
AD - (Graham, Shannon, Ice, Cottrell) West Virginia Univ., Morgantown, WV, United States
ER -

TY - JOUR
ID - 3408
T1 - Parents play significant role in the gender differences associated with physical activity in elementary students
A1 - Blankenship,C.R.
A1 - Ice,C.
A1 - Shannon,V.
A1 - Cottrell,L.
Y1 - 2012///
N1 - 70951073
English
Journal: Conference Abstract
KW - EMBASE
KW - Physical Activity
KW - Exercise
KW - Human
KW - sex difference

KW - elementary student
KW - parent
KW - Epidemiology
KW - Nutrition
KW - Metabolism
KW - gender
KW - Child
KW - Female
KW - Male
KW - model
KW - child parent relation
KW - boy
KW - girl
KW - cardiovascular risk
KW - Adolescent
KW - linear regression analysis
KW - primary school
KW - screening
KW - childhood
RP - NOT IN FILE
JF - Circulation
VL - Conference: Epidemiology and Prevention/Physical Activity, Nutrition and Metabolism 2012 San Deigo, CA United States. Conference Start: 20120313 Conference End: 20120316. Conference Publication:
IS - var.pagings
N2 - BACKGROUND It has been proven that males engage in physical activity more than females starting at a young age. The goal of this study was to investigate gender differences based on parenting behaviors such as the frequency with which parents send their children out to play. METHODS Parents of children participating in a free cardiovascular risk-screening program (CARDIAC Project) completed a behavioral survey. Children (n =472; 43.3% female) who were enrolled in elementary school participated in the study. Parents provided information about children's physical activity and their own ways in which they provided support of activity at home. Children's participation in 12 activities was captured by total number of minutes per week. A combined mean activity score was calculated by summing the children's total time across all of the activities. Parent behavior, specifically the frequency at which parents send their children outside to play was captured using a Likert scale where "1" represented never and "5" represented daily. Child gender and the amount of time allowed outside for play were then entered into a linear regression model to examine their role as potential predictors of child activity. RESULTS A significant gender difference (p<.001) was noted in children's total activity time with boys (n = 237; 751 minutes/week) exhibiting higher mean activity each week than girls (n = 229; 521 minutes/week). Child gender and the amount of time allowed outside to play significantly predicted children's activity levels overall. This model explained 28.9% of the variance in children's activity levels (F = 20.25 (2); p<.001). Specifically, girls had less opportunity (3.75) than boys (4.05) to play outside. CONCLUSION A factor behind the lesser physical activity in female adolescents is significantly related to the decreased opportunity to play outside. Additional parenting practices will be shared if presented. In conclusion, parent behavior plays a significant role in the proven gender differences with childhood physical activity
SN - 0009-7322
AD - (Blankenship, Ice, Shannon, Cottrell) West Virginia Univ., Morgantown, WV, United States
ER -

TY - JOUR
ID - 3409
T1 - Emotional face encoding in youth at risk for bipolar disorder: A preliminary FMRI study
A1 - Brotman,M.A.
A1 - Bones,B.L.
A1 - Olsavsky,A.K.
A1 - Adleman,N.E.
A1 - Deveney,C.M.
A1 - Dickstein,D.P.
A1 - Pine,D.S.

A1 - Leibenluft,E.
Y1 - 2012///
N1 - 70948331
English
Journal: Conference Abstract
KW - EMBASE
KW - bipolar disorder
KW - Risk
KW - juvenile
KW - college
KW - Memory
KW - psychopharmacology
KW - Human
KW - emotion
KW - Functional Magnetic Resonance Imaging
KW - Child
KW - nose
KW - patient
KW - processing
KW - Fear
KW - Hostility
KW - endophenotype
KW - intelligence quotient
KW - brain
KW - Prevalence
KW - Executive Function
KW - pathophysiology
KW - prefrontal cortex
KW - temporal lobe
KW - middle temporal gyrus
KW - hyperactivity
KW - superior temporal gyrus
KW - Reaction Time
KW - diseases
KW - hypothesis
KW - Sex Ratio
KW - response time
KW - scientist
RP - NOT IN FILE
SP - S259
EP - S260
JF - Neuropsychopharmacology
VL - Conference: 51st Annual Meeting of the American College of Neuropsychopharmacology, ACNP 2012 Hollywood, FL United States. Conference Start: 20121202 Conference End: 20121206. Conference Publication:
IS - var.pagings
N2 - Background: Face memory has been proposed as a candidate endophenotype for bipolar disorder (BD) (Glahn et al 2010). However,researchers have yet to examine the neural correlates mediating facememory and encoding in youth. Both euthymic BD youth and childrenat familial risk show deficits in face emotion labeling (Brotman et al2008), and demonstrate amygdala/parahippocampal gyrus hyperactivationduring face processing (Olsavsky et al 2012). Pediatric BDpatients and youth at-risk also show deficits in cognitive control(Brotman et al 2009), and prefrontal dysfunction during cognitiveflexibility tasks (Kim et al 2012). Here, we compare neural activationduring emotional face encoding in pediatric BD patients, children atfamilial risk with a first-degree BD relative, and healthy comparisonyouth. Behaviorally, we expect that pediatric BD patients and at riskyouth will demonstrate deficits in face memory (Glahn et al 2010) andincreased intrasubject variability in response time (ISV-RT) (Brotmanet al 2009). During successful and unsuccessful encoding of faces, wehypothesize that both BD and at risk youth will demonstrate similardysfunction in parahippocampal (Olsavsky et al 2012) and prefrontal(Kim et al 2012) regions mediating face emotion processing.Methods: Functional magnetic resonance

imaging (fMRI) data wereacquired from 77 participants (8-18 years old), including 27 pediatricBD patients, 13 unaffected children at familial risk, and 37 healthycomparison youth. During the encoding phase of the study, fMRI datawere acquired while subjects viewed a series of emotional faces in anevent-related design. During separate blocks, subjects engaged in anexplicit (rating hostility, subjective fear) or implicit (nose width orpassive viewing) face viewing task. Using a subsequent-memoryparadigm (Dickstein et al 2007), memory for faces was tested in asurprise recognition test outside the scanner. The memory testconsisted of viewing neutral faces that included both previouslyviewedactors who had displayed emotions in-scanner and novelactors. A d' score was calculated to measure performance during thepost-scan memory task; higher d' indicates better performance andmemory discrimination. The main contrast of interest was activationduring the two implicit processing tasks (nose width or passiveviewing) while viewing faces remembered accurately (hit) vs. not(miss). We conducted two whole-brain ANOVAs, one for each implicitencoding task, using a p<0.005 threshold with clusters >45 voxels.Results: Groups did not differ on age, IQ, or sex distribution. Therewere no between group differences in face memory as measured by d'.However, consistent with our hypothesis, behavioral analyses revealedthat BD (p<.05) and at risk (p<.005) youth demonstrated deficits inattention as measured by higher ISV-RT relative to healthy comparisonyouth during face encoding ratings (hostility, subjective fear, nosewidth). Groups did not differ in reaction time or face ratings. WholebrainANOVAs on the hit vs. miss contrast revealed two patterns ofactivity, with BD and at risk youth differing from healthy comparisonyouth. During the nose width encoding task, BD (ps<.05) and at risk (ps<.005) youth demonstrated left superior temporal gyrus and leftparahippocampal gyrus hyperactivity relative to healthy comparisonyouth. However, during the passive viewing encoding task, BD(ps<.005) and at risk (p<.05; p=.10) demonstrated hypoactivity inthe right middle temporal gyrus and inferior frontal gyrus.Conclusions: While memory for faces has been proposed as a potentialbehavioral BD endophenotype (Glahn et al 2010), this is the first studyto examine the neural correlates of this deficit in youth. Unexpectedly,groups did not differ behaviorally in face memory discrimination.However, consistent with prior work, relative to healthy comparisonyouth, BD and at risk children demonstrated behavioral deficits inattention, manifest as higher ISV-RT. BD and at risk youth also showedsimilar patterns of neural dysfunction. Together, these findings suggeststhat when BD and at risk youth are able to successfully remember faces,the attentional and neural mechanisms mediating encoding differ fromhealthy comparison youth. Specifically, we observed hyperactivation inparahippocampal and superior temporal regions, and hypoactivation inmiddle temporal and inferior frontal areas. Dysfunction in theparahippocampal gyrus and prefrontal cortex is consistent with priorwork in at risk and BD youth (Kim et al 2012; Olsavsky et al 2012). Thesuperior temporal area is involved in face and emotion perception (Saidet al 2010), and is associated with the pathophysiology of pediatric BD(Pavuluri et al 2010). Our results build on work identifying behavioraldeficits in face memory (Glahn et al 2010) by suggesting neural deficitsassociated with emotional face encoding. Future studies should includelarger samples and a longitudinal design to determine whether theneural deficits associated with emotional face encoding predict the onsetof BD in youth at risk for the illness. With further study, riskstratification and preventive interventions could be used to potentiallymitigate the development and prevalence of BD
SN - 0893-133X
AD - (Brotman, Bones, Olsavsky, Adleman, Deveney, Dickstein, Pine, Leibenluft) National Institute of Mental Health, Bethesda, MD, United States
ER -

TY - JOUR
ID - 3410
T1 - Links between anxiety and activity in nonhuman primates
A1 - Cameron,J.
Y1 - 2012///
N1 - 70947822
English
Journal: Conference Abstract
KW - EMBASE
KW - Anxiety
KW - nonhuman
KW - primate
KW - college
KW - psychopharmacology
KW - Human

KW - Haplorhini
KW - Child
KW - infancy
KW - model
KW - stimulus
KW - Physical Activity
KW - Infant
KW - adolescence
KW - childhood
KW - factorial analysis
KW - Genetics
KW - accelerometer
KW - neuroanatomy
KW - mother
KW - mental disease
KW - Female
KW - Sleep
KW - Heart Rate
KW - rhesus monkey
KW - Cerebrospinal Fluid
KW - alcohol
KW - monoamine
RP - NOT IN FILE
SP - S9
JF - Neuropsychopharmacology
VL - Conference: 51st Annual Meeting of the American College of Neuropsychopharmacology, ACNP 2012 Hollywood, FL United States. Conference Start: 20121202 Conference End: 20121206. Conference Publication:
IS - var.pagings
N2 - Background: Nonhuman primates (NHPs) serve as excellent models for human psychopathology as they share many similarities in behavior, neuroanatomy, and genetics. To determine whether infant monkeys reared in a naturalistic setting could serve as useful models for the development of normally-occurring childhood anxious behaviors, we adapted assessments designed to test anxiety in children. To begin to understand other physiological links to anxious behavior we measured level of physical activity in infancy through pubertal development. Methods: We studied infant rhesus macaques (n=640) raised in a naturalistic outdoor setting. Between 3 and 6 months of age, monkeys were given modified assessments that are routinely used to evaluate behavioral inhibition in children. When monkeys were 3 years of age (just post-pubertal), anxiety tests were again performed and activity was measured by accelerometer. Results: Factor analysis revealed three factors related to anxiety (behavioral inhibition, reticence, impassivity) accounting for 52.8% of variance in observed behaviors. Behavioral inhibition was decreased exploration in the presence of the mother (as defined in children). Reticence was decreased exploration when alone and impassivity was decreased behavioral expression in the face of a threatening or frightening stimulus. Activity measured in infancy was correlated with reticence (r=0.685, p<0.001) and impassivity (r=0.728, p<0.001), and remained correlated with these anxious behaviors in late adolescence, such that monkeys that were more reticent to explore and showed greater impassivity when confronted with novel or frightening stimuli were less active. Both anxious behavior and activity measures were significantly heritable. Anxiety traits were strongly associated with physiological correlates of anxiety (i.e., responsiveness to alcohol, CSF monoamine levels, heart rate and sleep). Conclusions: These findings show that monkeys reared in a naturalistic setting exhibit a spectrum of anxious behaviors that parallel what is observed in clinical settings. Level of physical activity is strongly correlated with the display of several anxious behaviors
SN - 0893-133X
AD - (Cameron) University of Pittsburgh, Pittsburgh, PA, United States
ER -

TY - JOUR
ID - 3411
T1 - PI monotherapy: Helpful in paediatric HIV management?
A1 - Barrow,G.

A1 - Tan,A.
A1 - Wilkins,E.
Y1 - 2011///
N1 - 70565594
English
Journal: Conference Abstract
KW - EMBASE
KW - monotherapy
KW - Human immunodeficiency virus
KW - Human
KW - patient
KW - virus load
KW - Therapy
KW - Drug Therapy
KW - Child
KW - Diagnosis
KW - toxicity
KW - school
KW - peer pressure
KW - laboratory
KW - pharmacy
KW - data base
KW - consultation
KW - lipodystrophy
KW - Drug Resistance
KW - Adult
KW - parent
KW - Health
KW - open study
KW - Male
KW - CD4 antigen
KW - ritonavir
KW - protein
KW - creatinine
KW - tenofovir
KW - darunavir
KW - atazanavir
RP - NOT IN FILE
SP - 35
JF - HIV Medicine
VL - Conference: 17th Annual Conference of the British HIV Association, BHIVA 2011 Bournemouth United Kingdom. Conference Start: 20110406 Conference End: 20110408. Conference Publication:
IS - var.pagings
N2 - Background: Current PENTA guidelines recommend lifelong cART for children. Switching to boosted PI monotherapy in adults with suppressed VL has been shown in randomised open trials to be a viable strategic option with switch/intensification in the occurrence of viral rebound. Outside of studies, boosted PI monotherapy is usually reserved for particular circumstances such as renal dysfunction, simplification, toxicity, or where adherence and drug-selected resistance is a concern. This report describes our experience with children where additional disruptive factors in family, school and home as well as peer pressure and pubertal development are evident. Methods: Patient, pharmacy and laboratory results were reviewed and a database constructed for analysis. Adherence was measured in consultations with patients and their families. Results: Four patients were identified, three patients on PI monotherapy, one on patient on PI double therapy. All patients were male, median age: 14 years (range 11-16 years). Average age at diagnosis of HIV was 6 years (range 2-10 years). All were commenced on combination ARV therapy at diagnosis and had been treated for over 6 years (range 6-10 years). At PI initiation: average CD4 count 677 (range 503-753), 3 patients had undetectable viral loads, one patient whose compliance was poor due to family circumstance had a viral load was 9, 217. Reasons for changing to PI monotherapy included reducing the number of medications (100%),

lipodystrophy(50%),increased protein: creatinine ratio(25%)and drug resistance. The patient prescribed double PI therapy had genotypic resistance to all NRTIs including intermediate resistance to Tenofovir and all NNRTIs. PI prescribed: Loponavir and ritonavir (25%) darunavir with ritonovir (50%) Loponavir, ritonavir and atazanavir (25%) The average CD4 count increase on PI monotherapy was 82 (range 21- 150). All patients had undetectable viral loads after PI monotherapy including the patient with an initial viral load of 9, 217. Conclusion: Paediatric HIV treatment is a challenge in which boosted PI monotherapy may play a helpful role. Patients showed increased CD4 counts and decreased viral load. PI monotherapy allows adherence issues to be worked through without further resistance, simplifies therapy where there is NNRTI/NRTI resistance. All medication changes have to be made with the child and parents on board until they are able to take control of their own health and medications
SN - 1464-2662
AD - (Barrow, Tan, Wilkins) North Manchester General Hospital, Manchester, United Kingdom
ER -

TY - JOUR
ID - 3412
T1 - The "magic of movement": Efficacy of using magic in intensive Hand-Arm Bimanual Intensive Therapy (HABIT) programme
A1 - Green,D.
Y1 - 2011///
N1 - 70565382
English
Journal: Conference Abstract
KW - EMBASE
KW - magic
KW - Arm
KW - disability
KW - childhood
KW - intensive care
KW - Human
KW - Child
KW - hemiplegia
KW - Therapy
KW - spasticity
KW - Exercise
KW - brain injury
KW - Motor Performance
KW - arm movement
KW - Experimental Design
KW - book
KW - questionnaire
KW - skill
KW - summer
KW - motor dysfunction
RP - NOT IN FILE
SP - 28
JF - Developmental Medicine and Child Neurology
VL - Conference: 23rd Annual Meeting of the European Academy of Childhood Disability Rome Italy. Conference Start: 20110608 Conference End: 20110611. Conference Publication:
IS - var.pagings
N2 - Background: Children with motor disorders such as hemiplegia or acquired brain injury, have particular difficulty performing hand and arm movements, especially those involving bimanual control. The Hand-Arm Bimanual Intensive Therapy (HABIT) programme has been found to be beneficial in improving bimanual function in some children with hemiplegia. However the need for significant repetition and massed practice (hours and intensity of therapy contact) is difficult to achieve in clinical settings and thus may limit outcomes. Incorporating magic 'hand' tricks as a motivator to pursue exercises outside of direct therapy was meant to overcome this limitation. Objective: The aim of this study was to examine the efficacy of a modified HABIT

programme-using Magic Tricks to improve upper limb motor function in children with spastic hemiplegia. Method:Movement exercises designed as 'magic' tricks were incorporated into a HABIT protocol. Children between 7 and 15 years (n = 23) participated in a 2-week summer camp involving 60 hours of intervention in a clinical setting. A within (n = 23) and between groups (treated vs treatment wait) experimental design was used to contrast performance across manual tasks pre and post intervention. Measurements of bimanual performance in daily tasks were made using the Assisting Hand Assessment and Children's Hand Experience Questionnaire (CHEQ). The results of the CHEQ as a measure of generalisation will be reported here. Results: Results on the CHEQ within participants show a significant increase in the number of activities performed independently (F(df 2,20) 16,00, p = 0.001) and number of activities performed successfully with two hands (F(df 2,20)17.71, p < 0.001) following participation in the camp. No significant loss of skills was noted at 3 month review. Conclusions: Preliminary results suggest that a modified HABIT programme using magic as a motivator may enhance therapeutic outcomes in children with spastic hemiplegia
SN - 0012-1622
AD - (Green) Tel Aviv University, United Kingdom
ER -

TY - JOUR
ID - 3413
T1 - An unusual cause of constipation in the elderly: Anal sphincter achalasia
A1 - Yazici,C.
A1 - Mutlu,E.
Y1 - 2011///
N1 - 70556765
English
Journal: Conference Abstract
KW - EMBASE
KW - Human
KW - esophagus achalasia
KW - constipation
KW - Aged
KW - anus sphincter
KW - college
KW - gastroenterology
KW - patient
KW - tube
KW - Abdomen
KW - thyroid disease
KW - Hospital
KW - Decompression
KW - sphincter
KW - Differential Diagnosis
KW - dementia
KW - rectum
KW - sigmoidoscopy
KW - Etiology
KW - electrolyte disturbance
KW - colonoscopy
KW - sigmoid volvulus
KW - Diagnosis
KW - Diet
KW - Intestine
KW - suppository
KW - injection
KW - Risk
KW - feces incontinence
KW - Child
KW - early diagnosis

KW - medical record review
KW - Female
KW - medical history
KW - insomnia
KW - recurrent cancer
KW - nonhodgkin lymphoma
KW - colon resection
KW - Ogilvie syndrome
KW - colon cancer
KW - abdominal pain
KW - nausea
KW - vomiting
KW - hematochezia
KW - fever
KW - examination
KW - Anemia
KW - Leukocyte Count
KW - large intestine
KW - ileus
KW - gastrointestinal motility disorder
KW - Hematoma
KW - ascites
KW - sigmoid
KW - heart block
KW - antibiotic agent
KW - soap
KW - enema
KW - macrogol 3350
KW - glycerol
KW - botulinum toxin A
KW - nifedipine
KW - electrolyte
KW - erythromycin
RP - NOT IN FILE
SP - S368
EP - S369
JF - American Journal of Gastroenterology
JA - Am J Gastroenterol
VL - Conference: 76th Annual Scientific Meeting of the American College of Gastroenterology Washington, DC United States. Conference Start: 20111028 Conference End: 20111102. Conference Publication:
IS - var.pagings
N2 - Purpose: To highlight the importance of high suspicion and early diagnosis of IASA in elderly patients with chronic constipation. Methods: Chart review. Results: We present a case of IASA in an 88 year-old female with past medical history of dementia, insomnia, falls, heart block, non-Hodgkin lymphoma, and CHF. The patient had a history of transverse colectomy for colon cancer 16 years prior. She was admitted to the hospital because of worsening abdominal distention and constipation ongoing intermittently for 10 years. She did not have abdominal pain, nausea, vomiting, hematomesis, hematochezia, or fever and her vitals were normal. Examination revealed a soft but markedly distended abdomen without rebound or guarding. Rectal exam did not show impaction or active bleed, but sphincter tone was significantly increased. Labs revealed a normocytic anemia and normal WBC count. KUB showed markedly distended small and large bowel loops and a dilated rectum measuring 2222 cm. Differential diagnoses considered were Ogilvie's syndrome, ileus due to gastrointestinal dysmotility aggravated by thyroid dysfunction or electrolyte abnormalities. CT abdomen showed resolving right ileopsoas hematoma from a prior fall, mild ascites, absence of pneumatosis, and presence of a markedly dilated sigmoid colon and rectum. The patient had a soft rectal tube placed with which her abdomen very rapidly decompressed. She was also started on intravenous erythromycin, empiric antibiotics, and scheduled soap suds enemas. She had multiple bowel movements and did not required flexible sigmoidoscopy for decompression. Further lab investigations excluded thyroid dysfunction, infectious etiologies

such as C. diff, UTI, and significant electrolyte imbalances. Colonoscopy from a prior admission at an outside hospital was negative for sigmoid volvulus or recurrent cancer. Alternative diagnosis of IASA was made. After improvement, she was started on diet, oral Miralax, and glycerin suppositories and the rectal tube was removed. The patient and her family were informed about treatment options such as anal sphincteroctomy and botox injection to the anal sphincter. Given her dementia and the risk of fecal incontinence they decided against these options. The patient was placed on nifedipine cream. Conclusion: Although rarely seen, IASA has been primarily described in children. However, it should be in the differential diagnosis in elderly patients with chronic constipation with increased sphincter tones. High level of suspicion and early treatment with rectal tube decompression leads to rapid resolution of symptoms
SN - 0002-9270
AD - (Yazici) Medicine Mercy Hospital, Chicago, IL, United States (Mutlu) Rush Presbyterian St. Luke's Medical Center, Chicago, IL, United States
ER -

TY - JOUR
ID - 3414
T1 - TSH bioactivity in children with isolated central hypothyroidism and macroorchidism
A1 - Del Blanco,D.G.
A1 - Hokken-Koelega,A.
A1 - Visser,J.A.
A1 - De Graaff,L.C.G.
A1 - De,Rijke Y.
A1 - De Jong,F.H.
A1 - Visser,T.J.
A1 - Barrio,R.
A1 - Moreno,J.C.
Y1 - 2010///
N1 - 70461888
English
Journal: Conference Abstract
KW - EMBASE
KW - drug activity
KW - Society
KW - macroorchidism
KW - Child
KW - hypothyroidism
KW - endocrinology
KW - patient
KW - stimulation
KW - serum
KW - thyroid gland
KW - Phenotype
KW - Puberty
KW - gene
KW - gonadorelin test
KW - secretion
KW - Glycosylation
KW - assay
KW - promoter region
KW - luminescence
KW - genetic transfection
KW - Mutation
KW - protirelin test
KW - hypothesis
KW - Sertoli cell
KW - hospital patient
KW - congenital hypothyroidism

KW - rare disease
KW - boy
KW - Pedigree
KW - testicular enlargement
KW - hypophysis
KW - thyrotropin
KW - inhibin B
KW - protirelin
KW - follitropin
KW - luciferase
KW - follitropin receptor
KW - sex hormone
KW - adenosine phosphate
KW - gonadorelin
KW - Muellerian inhibiting factor
KW - testosterone
KW - thyrotropin receptor
RP - NOT IN FILE
SP - 46
JF - Hormone Research in Paediatrics
VL - Conference: 49th Annual Meeting of the European Society for Paediatric Endocrinology, ESPE 2010 Prague Czech Republic. Conference Start: 20100922 Conference End: 20100925. Conference Publication:
IS - var.pagings
N2 - Isolated central congenital hypothyroidism (ICCH) is a rare disease with so far undescribed associations with peripheral features outside the thyroid axis. Here we present three boys, from two separate pedigrees, with ICCH and progressive testicular enlargement under prepubertal levels of FSH, LH and testosterone. We hypothesized that the secretion of an abnormal TSH molecule with low bioactivity towards the TSH receptor (TSHR) but aberrant stimulation of testicular FSH receptor (FSHR), could explain the phenotype. Methods: Thyroid and sex hormone axes were monitored in these children, including TRH- and GnRH-tests, a-subunit, inhibin-B and anti-mullerian hormone (AMH). Bioactivity of patients' sera was studied against TSHR- and FSHR-transfected cells in separate luciferase reporter assays. Cells were stimulated with hrTSH, rhFSH or patients' sera. Luciferase expression under control of a promoter containing 6 CRE (cyclic-AMP-Responsive-Element) sites was measured by luminescence and normalized for transfection efficiency. Likely candidate genes for the phenotype were fully screened for mutations. Results: All patients had a type 2 (pituitary) response to TRH-test, and pre- pubertal responses to GnRH. They started puberty at 12.5 years with testicular volumes of 8-12 ml, reaching 30-37 ml after puberty. a-subunit is normal, but inhibin-B (351-505 ng/L; n: 138-196) and AMH (18.3-48 ug/L, n: 5-9) were strongly elevated. Patient 1 showed decreased TSH bioactivity for TSHR (n=3) and increased stimulation of FSHR-cells (n=1), compatible with initial hypothesis. Patients 2+3 showed normal serum bioactivity. TSHB, CGA and TRHR genes were wildtype. Conclusions: We present an undescribed, genetically "orphan" association of TSH deficiency and macroorchidism. Macroorchidism is due to increased Sertoli cell mass, secreting inhibin-B and AMH. In patient 1, reduced stimulation of TSHR and increased stimulation of FSHR suggest a posttranslational (glycosylation) defect of TSH. Normal serum bioactivity of patients 2+3 towards TSHR/FSHR suggests a mechanistically different entity
SN - 1663-2818
AD - (Del Blanco, Hokken-Koelega, De Graaff) Erasmus MC, Pediatric Endocrinology, Rotterdam, Netherlands (Visser, De Rijke, De Jong, Visser) Erasmus MC, Internal Medicine, Rotterdam, Netherlands (Barrio) Ramon y Cajal University Hospital, Pediatric Endocrinology, Madrid, Spain (Moreno) La Paz University Hospital, INGEMM, Institute for Medical and Molecular Genetics, Madrid, Spain
ER -

TY - JOUR
ID - 3415
T1 - Adverse effect of outdoor air pollution on cardiorespiratory fitness in Chinese children
A1 - Gao,Y.
A1 - Chan,E.Y.Y.
A1 - Zhu,Y.
A1 - Wong,T.W.

Y1 - 2013///
N1 - 2012699872
English
Journal: Article
KW - EMBASE
KW - adverse outcome
KW - air monitoring
KW - air pollutant
KW - Air Pollution
KW - air quality
KW - ambient air
KW - article
KW - cardiopulmonary function
KW - Child
KW - child health
KW - Chinese
KW - Environmental Exposure
KW - environmental impact assessment
KW - Exercise
KW - Female
KW - fitness
KW - Hong Kong
KW - Human
KW - major clinical study
KW - Male
KW - Oxygen Consumption
KW - Particulate Matter
KW - Physical Activity
KW - priority journal
KW - school child
KW - sex difference
KW - nitrogen dioxide
KW - Ozone
KW - sulfur dioxide
RP - NOT IN FILE
EP - 17
JF - Atmospheric Environment
VL - 64
IS - pp 10-17
CY - United Kingdom
N2 - Little is known about the health impact of air pollution on children's cardiovascular health. A cross-sectional study was conducted and data was analysed in 2048 Chinese schoolchildren (aged 8-10 years) in three districts of Hong Kong to examine the association between exposure to outdoor air pollution and cardiorespiratory fitness. Annual means of ambient PM₁₀, SO₂, NO₂ and O₃ from 1996 to 2003 were used to estimate individual exposure of the subjects. Cardiorespiratory fitness was measured for maximal oxygen uptake (VO_{2max}), predicted by the multistage fitness test (MFT). Height and weight were measured and other potential confounders were collected with questionnaires. Analysis of covariance was performed to estimate the impact of air pollution on complete speed in the MFT and predicted VO_{2max}. The results showed that children in high-pollution district had significantly lower complete speed and predicted VO_{2max} compared to those in low- and moderate-pollution districts. Complete speed and predicted VO_{2max} was estimated to reduce 0.327 km h⁻¹ and 1.53 ml kg⁻¹ min⁻¹ per 10 mug m⁻³ increase in PM₁₀ annual mean respectively, with those in girls being greater than in boys. Being physically active could not significantly result in improved cardiorespiratory fitness in polluted districts. The adverse effect seems to be independent of short-term exposure to air pollution. We concluded that long-term exposure to higher outdoor air pollution levels was negatively associated with cardiorespiratory fitness in Chinese schoolchildren, especially for girls. PM₁₀ is the most relevant pollutant of the adverse

effect. Elevated cardiorespiratory fitness observed in physically activate children could be negated by increased amount of inhaled pollutants during exercise. 2012 Elsevier Ltd
SN - 1352-2310
AD - (Gao, Chan, Zhu, Wong) School of Public Health and Primary Care, The Chinese University of Hong Kong, Hong Kong
ER -

TY - JOUR
ID - 3416
T1 - Geographical differences and patterns in the recreational and leisure participation of children /youth with cerebral palsy: A CAPE International Collaborative Network Study
A1 - King,G.
A1 - Imms,C.
A1 - Palisano,R.
A1 - Majnemer,A.
A1 - Chiarello,L.
A1 - Orlin,M.
A1 - Law,M.
Y1 - 2012///
N1 - 70936258
English
Journal: Conference Abstract
KW - EMBASE
KW - Cerebral Palsy
KW - Human
KW - juvenile
KW - Child
KW - leisure
KW - Canada
KW - groups by age
KW - gender
KW - United States
KW - Gross Motor Function Classification System
KW - Australia
KW - Income
KW - Education
KW - health care system
KW - rehabilitation center
KW - Physical Activity
KW - Female
KW - model
KW - covariance
KW - social adaptation
KW - community
KW - Male
KW - secondary analysis
KW - school
RP - NOT IN FILE
SP - 48
EP - 49
JF - Developmental Medicine and Child Neurology
VL - Conference: 66th Annual Meeting of the American Academy for Cerebral Palsy and Developmental Medicine, AACPDM 2012 Toronto, ON Canada. Conference Start: 20120912 Conference End: 20120915. Conference Publication:
IS - var.pagings
N2 - Background/Objectives: Few investigations have examined cross-cultural or geographical differences in children's outside of school activity participation. The objective of this CAPE International Collaborative

Network study was to conduct secondary analyses of datasets from Canada (Ontario and Quebec), Australia, and the U.S. to examine geographic variation in recreational and leisure participation as a function of region, age group, and gender. Design: Descriptive study (survey). Participants and Setting: Data from 1076 children/youth with cerebral palsy (CP) were included, ages 6 to 20 years (114 from Australia, 217 from Ontario, 245 from Quebec, 500 from the U.S.). There were 611 males and 465 females; 317 were ages 6 to 10, 383 ages 10+ to 13, 233 ages 13+ to 16, and 143 ages 16+ to 21; 358 at GMFCS level I, 405 at level II/III, and 313 at level IV/V. These children and youth had participated in research studies involving the Children's Assessment of Participation and Enjoyment (CAPE) in the four regions. Materials/Methods: Analyses involved CAPE diversity scores for five types of activities (recreational, active physical, social, skill-based, and self-improvement). Three-way analyses of covariance were conducted to examine participation patterns as a function of region, age group, and gender, controlling for family income, education, and GMFCS level. Non-significant interaction effects and covariates were removed fromthemodels, and homogeneity of variance was tested.Effect sizes (partial eta-squared; g2)were calculated. Results: F-tests indicated the significance of factors or covariates, controlling for all other factors and covariates in the model. Region, age group, gender, income, education, and GMFCS level all significantly affected the number of activities engaged in. The largest effects were for region on active physical, F₂₆₈₈=30.6, P<0.0001, ²=0.08), and selfimprovement diversity, F2725=48.4, P<0.0001, ²=0.12; age group on recreational diversity, F (3913)=62.4, P<0.0001, ²=0.17); and GMFCS level on recreational, F (2913)= 53.3, P<0.0001, ²=0.10, active physical, F (2688)=45.1, P<0.0001, ²=0.12, and self-improvement diversity F (2725)=30.0, P<0.0001, ²=0.08. Conclusions/Significance: Children/youth from the U.S. took part in the fewest active physical activities; those from Ontario took part in the most self-improvement activities. The youngest age group took part in the most recreational activities, and those in GMFCS level IV/V had the lowest levels of participation in recreational, active physical, and self-improvement activities, confirming previous research findings. The differences in participation across regions may have important implications for rehabilitation services, health care systems, and community-based services for children with CP
SN - 0012-1622
AD - (King) Bloorview Research Institute, Toronto, ON, Canada (Imms) Australian Catholic University, Melbourne, VIC, Australia (Palisano, Chiarello, Orlin) Drexel University, Philadelphia, PA, United States (Majnemer) McGill University, Montreal, QC, Canada (Law) McMaster University, Hamilton, ON, Canada
ER -

TY - JOUR
ID - 3417
T1 - A direct observation measure of environmental qualities of activity settings (MEQAS)
A1 - King,G.
A1 - Rigby,P.
A1 - Batorowicz,B.
A1 - main-Klein,M.
A1 - Thompson,L.
A1 - Petrenchik,T.
Y1 - 2012///
N1 - 70936232
English
Journal: Conference Abstract
KW - EMBASE
KW - Cerebral Palsy
KW - juvenile
KW - Human
KW - test retest reliability
KW - disability
KW - Occupational Therapy
KW - consultation
KW - Environment
KW - Adult
KW - community
KW - Social Behavior
KW - sampling

KW - psychiatry
KW - Principal Component Analysis
KW - speech disorder
KW - validation study
KW - questionnaire
KW - Social Environment
KW - Psychology
KW - leisure
KW - parent
KW - Rehabilitation
KW - internal consistency
KW - Physical Activity
KW - reliability
KW - Child
KW - wellbeing
KW - Canada
KW - Cronbach alpha coefficient
RP - NOT IN FILE
SP - 30
EP - 31
JF - Developmental Medicine and Child Neurology
VL - Conference: 66th Annual Meeting of the American Academy for Cerebral Palsy and Developmental Medicine, AACPDM 2012 Toronto, ON Canada. Conference Start: 20120912 Conference End: 20120915. Conference Publication:
IS - var.pagings
N2 - Background/Objectives: There is increasing interest in measures of the physical and social environment. The 32-item MEQAS is an observer-rated measure providing a global assessment of aesthetic, physical, social, and opportunityrelated qualities of leisure activity settings for youth with disabilities (e.g. playing ping pong at a community centre, playing piano in the living room). To describe the measure's development and psychometric properties. Design: Instrument Development and Validation Study. Participants and Setting: Six content experts took part in the consultant review phase, with backgrounds in psychology, AAC, speech-language pathology, occupational therapy, inclusive design, and child/adolescent psychiatry. The pretesting phase involved two adult users of AAC and an architect. The psychometric phase involved four trained raters with backgrounds in occupational therapy. The MEQAS was developed at a children's rehabilitation centre in Toronto, Canada. Materials/Methods: Development involved: (1) item generation based on a literature review and existing environmental assessment tools; (2) consultations with experts, including youth with disabilities, parents, and scholars; (3) pretesting; and (4) psychometric testing. Eighty MEQAS questionnaires were completed by sets of raters who observed and independently rated 22 home and community activity settings (representing active, passive, outdoor, indoor, formal, informal, group, and solitary activity settings). Scales were determined using principal components analyses. Test-retest reliability was determined for eight activity settings, rated twice (an interval of 4-6wks) by a trained rater. Results: The factor structure accounted for 79.5% of the variance. The KMO Measure of Sampling Adequacy was 0.73 and Bartlett's Test of Sphericity was significant (P<0.0001). Cronbach's alphas for the scales (Opportunities for Social Activities, Opportunities for Physical Activities, Pleasant Physical Environment, Opportunities for Choice, Opportunities for Personal Growth, and Opportunities to Interact with Adults) ranged from 0.76 to 0.96. Inter-rater reliability of the scale scores (absolute agreement ICCs) ranged from 0.60 to 0.93. Test-retest reliability ranged from 0.70 to 0.90. Conclusions/Significance: Results suggest that theMEQAS has a sound factor structure and preliminary evidence of internal consistency, inter-rater, and test-retest reliability. The MEQAS is the first observer-completed measure of qualities of environmental activity settings. The measure will be useful for the design of social ecological programs for youth with disabilities, and for research on associations between environmental qualities of activity settings and youths' experiences. The MEQAS can be used to promote youth wellness through the design of settings and environments that provide appropriate developmental opportunities
SN - 0012-1622
AD - (King, Rigby) Bloorview Research Institute, Toronto, ON, Canada (King, Rigby, Mcmain-Klein) University of Toronto, Toronto, ON, Canada (Batorowicz, Petrenchik) McMaster University, Hamilton, ON, Canada (Thompson) Holland Bloorview Kids Rehabilitation Hospital, Toronto, ON, Canada
ER -

TY - JOUR
ID - 3418
T1 - Children and adolescents with type 1 diabetes in Nepal: Beyond glycaemic control
A1 - Shrestha,S.
A1 - Kansakar,P.
A1 - Poudyal,B.
A1 - Ogle,G.
A1 - Holley,A.
Y1 - 2012///
N1 - 70933047
English
Journal: Conference Abstract
KW - EMBASE
KW - Human
KW - Adolescent
KW - insulin dependent diabetes mellitus
KW - Diabetes Mellitus
KW - Nepal
KW - Society
KW - Child
KW - Diagnosis
KW - health care facility
KW - parent
KW - school
KW - observational study
KW - Education
KW - disease course
KW - Reading
KW - private hospital
KW - medical record
KW - alternative medicine
KW - Health
KW - questionnaire
KW - Hospital
KW - glucose blood level
KW - Southeast Asia
KW - developing country
KW - hemoglobin A1c
KW - insulin
RP - NOT IN FILE
SP - 136
JF - Pediatric Diabetes
JA - Pediatr Diabetes
VL - Conference: 38th Annual Meeting of the International Society for Pediatric and Adolescent Diabetes, ISPAD 2012 Istanbul Turkey. Conference Start: 20121010 Conference End: 20121013. Conference Publication:
IS - var.pagings
N2 - Nepal is a developing country in Southeast Asia which has limited heath care facilities and low literacy rates. A descriptive observational study assessing social and economic issues impacting diagnosis and subsequent care in 47 children and adolescents enrolled into the Life for a Child (LFC) program at Patan Hospital was undertaken using some questionnaires and reviewing the patient records. The mean age of our children is 15.5 years and the mean age at diagnosis was 11.8 years. The duration of symptoms before diagnosis ranged from a few days to 2 years with a mean duration of 3.3 months. Most children had several visits to the local level health facilities and private clinic visits before a diagnosis of Type I diabetes was made. More than one third of the families went to alternative medicine practices early in the disease course. Eighteen of the children enrolled are from Katmandu valley while 3 families moved to Katmandu after their child's diagnosis. For children from outside Katmandu, the distances to the nearest health facility ranged between 15 minutes to 2

hours. Few of the parents of these children had education beyond grade 10. Many were illiterate and some were literate but with no formal schooling. HbA1c values were lower in children with educated parents and so was the duration of symptoms to diagnosis. Less than a half of the children and/or parents were able to adjust the insulin dosage on their own. Among children and adolescents between 2 to 20 years 36% had a BMI of <5%ile at the time of enrollment. Most children are continuing their studies, though some were at grades not appropriate for their age and a few left schools due to financial constraints. Lack of awareness, unavailability of blood glucose testing at local level health facilities, delay in diagnosis, poor health support system and non-affordability to lifelong treatment are the main challenges. The support from LFC program has played an important role in the lives of these children
SN - 1399-543X
AD - (Shrestha, Kansakar) Patan Academy of Health Sciences, Paediatrics, Lalitpur, Nepal (Poudyal) Patan Academy of Health Sciences, Patan Hospital, Internal Medicine, Lalitpur, Nepal (Ogle) International Diabetes Federation (IDF) Life for a Child Programme, Sydney, Australia (Holley) Baystate Medical Center, Springfield, United States
ER -

TY - JOUR
ID - 3419
T1 - Evaluation and Characterization of Environmental Risks and Asthma Management Knowledge and Awareness Through Surveys of Coaches, Athlete Parents/Guardians, and Athletes
A1 - Therkorn,J.H.
A1 - Shendell,D.G.
Y1 - 2012///
N1 - 2012658183
English
Journal: Article
KW - EMBASE
KW - Adolescent
KW - Adult
KW - article
KW - Asthma
KW - athlete
KW - athlete training intensity
KW - attitude to illness
KW - Awareness
KW - Child
KW - coach
KW - Disease Management
KW - endurance
KW - Environmental Exposure
KW - environmental factor
KW - Female
KW - Health Education
KW - Health Status
KW - health survey
KW - Human
KW - Humidity
KW - Male
KW - nonmedical occupations
KW - nose allergy
KW - parent
KW - patient education
KW - Pollen
KW - priority journal
KW - Risk Assessment
KW - school child
KW - Self Report

KW - sport
KW - Training
KW - wheezing
RP - NOT IN FILE
SP - 106
EP - 116
JF - Journal of Asthma and Allergy Educators
VL - 3
IS - 3
CY - United Kingdom
N2 - Objective. Athletes may face unique barriers to asthma management and are exposed to many different environmental factors while playing sports. The authors' goal was to study participation in sports and perceptions of environmental exposures (health risks) and barriers to asthma management for adolescent and young adult athletes. The need for asthma education among coaches was also investigated. Methods. This study targeted 3 populations: (a) coaches of middle school through college level athletes, (b) parents/guardians of middle school through college level athletes, and (c) college athletes. Online surveys were conducted anonymously. Questions addressed sport-specific asthma management barriers, perceived environmental exposures (health risks), athlete training intensities, and asthma knowledge and awareness among surveyed coaches. Results. There were 120 participants (26 coaches, 37 college athletes, 57 athlete parents/guardians). Most reported perceived environmental health concerns and potential barriers to asthma management for athletes, with 94% and 84% of participants reporting at least one, respectively. There were observed differences in the numbers of environmental health concerns and potential barriers to asthma management reported per person across surveyed populations depending on sport, training intensity, and athlete asthma status. The percentage of correct responses by coaches to 5 asthma knowledge questions ranged from 12% to 88%. Conclusions. Research should further investigate how to prioritize and develop interventions to address potential barriers to asthma management for adolescent athletes in both school and community parks and recreation facility settings. Additionally, solutions are needed to mitigate outdoor and indoor environmental health concerns of athletes. Coaches may benefit from increased asthma awareness and educational interventions aimed at improving asthma knowledge specific to athletics. 2012 The Author(s)
SN - 2150-1297
AD - (Therkorn, Shendell) UMDNJ-School of Public Health, New Brunswick, NJ, United States
ER -

TY - JOUR
ID - 3420
T1 - Three-year epidemiological evaluation of cutaneous leishmaniasis in Qom province (2007-2009)
A1 - Khodadadi,S.
A1 - Dakhili,M.
A1 - Akhlaghi,L.
A1 - Haeri,M.R.
A1 - Tabatabaie,F.
Y1 - 2012///
N1 - 2012684888
English
Journal: Article
KW - EMBASE
KW - Adolescent
KW - Adult
KW - Aged
KW - article
KW - Child
KW - clinical examination
KW - cross-sectional study
KW - disease surveillance
KW - Disease Transmission
KW - Female
KW - groups by age

KW - Human
KW - Incidence
KW - Iran
KW - major clinical study
KW - Male
KW - occupation
KW - preschool child
KW - rural area
KW - school child
KW - skin leishmaniasis/ep [Epidemiology]
KW - urban area
KW - wound fluid
RP - NOT IN FILE
SP - 3056
EP - 3060
JF - HealthMED
VL - 6
IS - 9
CY - Bosnia and Herzegovina
N2 - Background: Cutaneous leishmaniasis is an important parasitic disease of tropical and semi-tropical areas of the world. The current study is carried out to evaluate the epidemiology of cutaneous leishmaniasis in Qom province during 2007-2009. Methods: The study was performed on patients referred to nine health centers in Qom province. The patients were included in the study by random sampling. This descriptive cross-sectional study was performed during 2007-2009. Diagnosis of the disease was based upon the clinical examination and specimens taken from wound serosity, which then underwent Giemsa staining. The demographic data and results of clinical and laboratory evaluations were recorded in patients' datasheet. Results: From 2007 to 2008, the prevalence of leishmaniasis had a decreasing trend, while it had an increasing trend in 2009. The highest prevalence rates were observed in October and November, and the trend was decreasing in winter. The disease occurred mostly in Iranian males, in the age range of 16-20. In 2007, it was reported mostly in rural population, while in 2008 and 2009, it occurred mostly in urban population. In the years studied, most patients had one lesion in arms, legs, face, and then other regions of the body. Conclusion: The causes of the higher prevalence of the infection in men above 15 can be attributed to their less covering, and spending more time outdoor for daily activity and work, both of which cause higher probability of contact with sandflies. Authorities should pay attention to the importance and priority of leishmaniasis control, as well as allocating adequate funds for control measures
SN - 1840-2291
AD - (Khodadadi, Dakhili) Qom Islamic Azad university, QOM Azad university of medical science, Iran, Islamic Republic of (Akhlaghi, Tabatabaie) Parasitology and Mycology Department, School of medicine Tehran university of medical sciences, Tehran, Iran, Islamic Republic of (Haeri) School of medicine, Qom university of medical sciences, Qom, Iran, Islamic Republic of
ER -

TY - JOUR
ID - 3421
T1 - Prevelance of postural disorders in children from Copper Basin in Poland
A1 - Pokrywka,J.
A1 - Fugiel,J.
A1 - Posluszny,P.
Y1 - 2011///
N1 - 2012673856
Polish, English
Journal: Article
KW - EMBASE
KW - article
KW - body posture
KW - Child
KW - Female

KW - Human
KW - kyphosis
KW - lordosis
KW - major clinical study
KW - Male
KW - musculoskeletal disease
KW - Photography
KW - Physical Activity
KW - Poland
KW - postural disorder
KW - Prevalence
KW - questionnaire
KW - Recreation
KW - school child
KW - Television Viewing
RP - NOT IN FILE
SP - 3
EP - 10
JF - Fizjoterapia
VL - 19
IS - 4
CY - Poland
N2 - Unhealthy lifestyle and limited physical activity tend to be main factors adversely affecting the development of the young generation. The aim of this study was to evaluate the prevalence of postural disorders in the sagittal plane as well as to compare posture types taking into consideration physical activity of the examined children. The data was gathered in 2002 and 2008 in Polkowice. The examined group consisted of 2398 children - boys and girls aged 7 to 11. Body posture was measured by means of the photogrammetric method with the use of Moire pattern. A questionnaire was used to gather information concerning the amount of time outside of school spent on physical activity, watching TV or playing computer games. While comparing the prevalence of postural disorders in 2002 and 2008 an overall increase was observed - higher in girls than in boys. The posture types distribution was comparable in both studies. Kyphotic and balanced posture types were predominant in boys, whereas in girls lordotic and balanced types were more frequent. The questionnaire showed that between 2002 and 2008 physical activity after school decreased
SN - 1230-8323
AD - (Pokrywka, Fugiel, Posluszny) Department of Biostructure, University of Physical Education, Wroclaw, Poland
ER -

TY - JOUR
ID - 3422
T1 - Epidemiology of tick-borne encephalitis in the Czech Republic 1970-2008
A1 - Kriz,B.
A1 - Maly,M.
A1 - Benes,C.
A1 - Daniel,M.
Y1 - 2012///
N1 - 2012662015
English
Journal: Article
KW - EMBASE
KW - Adolescent
KW - Adult
KW - Age Distribution
KW - Aged
KW - Altitude
KW - article
KW - Child

KW - climate change
KW - Czech Republic
KW - disease association
KW - fatality
KW - Female
KW - geographic distribution
KW - Human
KW - Incidence
KW - Infant
KW - infection rate
KW - major clinical study
KW - Male
KW - meteorology
KW - preschool child
KW - priority journal
KW - school child
KW - seasonal variation
KW - sex difference
KW - tick borne encephalitis/ep [Epidemiology]
RP - NOT IN FILE
SP - 994
EP - 999
JF - Vector-Borne and Zoonotic Diseases
VL - 12
IS - 11
CY - United States
N2 - This article presents major epidemiologic features of tick-borne encephalitis (TBE) in the Czech Republic, using data of laboratory-confirmed cases since 1970. A total of 17,053 cases of TBE were reported in the Czech Republic (population 10 million) in 1970-2008. The data show several important features. First, the pattern of TBE incidence changed over time. Until the end of the 1970s, TBE was characterized by periods of alternately higher and lower incidence (between 180 and 595 cases per year); the 1980s were a period of low incidence with minimum variability; since the beginning of the 1990s, there has been a steep rise in incidence, with marked year-to-year variation (e.g., 745 cases were registered in 1995, and a maximum of 1029 cases were registered in 2006). Second, the age distribution of TBE incidence has changed. Until the end of 1990s, incidence peaked among those 15-19 years of age, with a gradual decline with age. In the 2000s, however, TBE incidence has been rising in those aged 60-64 years, with a sharp decline in those older than 65 years. Third, the seasonal pattern of TBE has changed markedly over time. In the earlier period, incidence had a clear peak in July/August; since the 1990s, more cases have occurred in earlier and later months of the year. The proportion of cases occurring in April, May, October, and November increased from 9% in the 1970s to 23% in 2000-2008. Fourth, the geographical distribution of TBE also changed over time, with TBE increasingly occurring in the mountainous districts at higher altitudes. These changes in incidence patterns appear to be linked with changes in climatic and meteorological conditions. The link between climate change and TBE incidence is plausible, since TBE is a recreation-related infection associated with outdoor activities, and since climatic changes affect the life cycle of the vector. Copyright 2012, Mary Ann Liebert, Inc. 2012
SN - 1530-3667
AD - (Kriz, Maly, Benes, Daniel) National Institute of Public Health, Srobarova 48, 100 42 Praha 10, Czech Republic
ER -

TY - JOUR
ID - 3424
T1 - Tularemia as a result of outdoor activities for children in the countryside
A1 - Gurcan,S.
A1 - Varol,Saracoglu G.
A1 - Karadenizli,A.
A1 - Ozkayin,E.N.
A1 - Ozturk,S.Z.

A1 - Cicek,C.
A1 - Vatansever,B.
Y1 - 2012///
N1 - 2012659007
English
Journal: Article
KW - EMBASE
KW - article
KW - bacterium culture
KW - bacterium isolate
KW - Child
KW - child health
KW - clinical article
KW - environmental factor
KW - environmental sanitation
KW - epidemic
KW - Female
KW - Francisella tularensis
KW - geographic distribution
KW - Human
KW - lymph node biopsy
KW - Male
KW - molecular diagnosis
KW - Polymerase Chain Reaction
KW - Risk Assessment
KW - risk factor
KW - school child
KW - serology
KW - serum
KW - swimming pool
KW - throat culture
KW - tularemia/di [Diagnosis]
KW - tularemia/et [Etiology]
KW - Turkey (republic)
KW - tap water
RP - NOT IN FILE
SP - 1044
EP - 1049
JF - Turkish Journal of Medical Sciences
VL - 42
IS - 6
CY - Turkey
N2 - Aim: To investigate the features of a new tularemia outbreak that occurred in the Thrace region. Materials and methods: The research team visited the village after the identification of the index case. Serum and throat samples were taken from 41 villagers who were examined, and environmental samples were taken in order to identify the source of the outbreak. Culture, serology, and molecular methods were used to search for Francisella tularensis in these samples. Results: A total of 8 children were diagnosed with tularemia. The adults and all of the other children were seronegative for tularemia. All of the patients had a history of swimming in a pool filled with water from a local stream, and contact with stream water was calculated to increase the risk of developing the disease 9.3-fold. Polymerase chain reaction analysis was positive in a lymph node aspirate of the index case and in the home tap water of 3 patients as well as in the spring water and stream water in the village. Francisella tularensis could not be isolated from any culture of samples. Interestingly, the waterborne tularemia outbreak affected only children. Conclusion: Although tularemia has been not reported from Tekirdag Province for 74 years, the disease reemerged in the region due to the removal of hygienic measures. These clues may signify that the agent had maintained its presence in the region for many years. TUBITAK
SN - 1300-0144

AD - (Gurcan, Cicek) Department of Medical Microbiology, Faculty of Medicine, Trakya University, Edirne, Turkey (Varol Saracoglu) Department of Public Health, Faculty of Medicine, Namik Kemal University, Tekirdag, Turkey (Karadenizli) Department of Medical Microbiology, Faculty of Medicine, Kocaeli University, Kocaeli, Turkey (Ozkayin, Vatansever) Department of Pediatric Infectious Diseases, Faculty of Medicine, Trakya University, Edirne, Turkey (Ozturk) Provincial Health Directorate, Tekirdag, Turkey
ER -

TY - JOUR
ID - 3425
T1 - Why we make ourselves sick and how to make ourselves healthy: the importance of nutrition, exercise and sunlight
A1 - Siever,D.
A1 - Swatzyna,R.
Y1 - 2011///
N1 - 70918441
English
Journal: Conference Abstract
KW - EMBASE
KW - Exercise
KW - Sunlight
KW - Society
KW - neurofeedback
KW - Nutrition
KW - brain
KW - homeostasis
KW - Human
KW - intelligence quotient
KW - model
KW - meal
KW - vitamin D deficiency
KW - winter
KW - electroencephalogram
KW - mood
KW - rhythm
KW - eye
KW - stimulation
KW - pineal body
KW - mental function
KW - supplementation
KW - mental performance
KW - Population
KW - neurologic disease
KW - mental deficiency
KW - dementia
KW - fibromyalgia
KW - seizure
KW - Child
KW - migraine
KW - Multiple Sclerosis
KW - seasonal affective disorder
KW - insomnia
KW - Anxiety
KW - basic needs
KW - Environment
KW - iodine
KW - magnesium
KW - selenium

KW - omega 3 fatty acid
KW - vitamin
KW - Receptor
KW - vitamin D
KW - neurotransmitter
RP - NOT IN FILE
SP - 426
JF - Journal of Neurotherapy
VL - Conference: 19th Annual Conference of the International Society for Neurofeedback and Research, ISNR 2011 Phoenix, AZ United States. Conference Start: 20110914 Conference End: 20110918. Conference Publication:
IS - var.pagings
N2 - E. Roy John and Leslie Prichep (2006) proposed an EEG Homeostatic Model for the brain. In this model, neurotransmitters mediate neuroanatomical structures that generate behavior regulated by emotional experience in the moment of the event. They proposed that "psychological and neurological disorders produce and are caused by deviations from homeostasis" (p. 135). If an environment or psychological challenge continues for an extended period, the set point for homeostasis adapts to a new point. This short course provides information about the importance of nutrition, exercise, and sunlight in the process of reestablishing EEG homeostasis. David Siever will review the literature concerning the effects of nutrition, exercise and sunlight on the brain, and Ron Swatzyna will present his EEG/QEEG study on the impact of breakfast patterns in children. Nutrition affects oscillatory rhythms and neuronal functioning. Iodine boosts IQ and helps prevent mental retardation. Omega 3 s, selenium, and magnesium all improve mental function and reduce depression. Exercise has been shown to be highly effective for improving IQ, math ability, and reducing depression. Exercise should be a mandatory part of any academic program. Preliminary findings using QEEG suggest that what is eaten for breakfast has an effect on both mood and mental performance. As for the importance of sunlight, as much as 6% of northern populations are believed to develop winter blues. Depression, anxiety, insomnia, multiple sclerosis, migraine, seizure, fibromyalgia, and dementia are well correlated with a deficiency in vitamin D. Previous research has always assumed that winter blues was the result of seasonal affective disorder, which comes about as a lack of optic stimulation to UV receptors in the eyes and, ultimately, the pineal gland. There are several similarities in the symptoms of SAD and vitamin D deficiency, and therefore it is possible that SAD has been misdiagnosed since its inception. A recent study on vitamin D deficiency has shown that vitamin D supplementation eliminates the symptoms of SAD in SAD sufferers. The human brain was never designed to be healthy; it was designed to survive. Despite the robustness of the brain, we are not sharp and productive, nor social, lively, and joyful unless we address good nutrition and exercise. We have to work at being mentally healthy. As long as we are ignoring our basic needs, the brain will do what it has to along a predictive pathological course to survive, and we will suffer. Active voluntary participation in good nutritional decisions, exercise routine, and outside activities (if possible) are necessary for EEG Homeostasis
SN - 1087-4208
AD - (Siever) Mind Alive, Inc., Canada (Swatzyna) Tarnow Center for Self-Management, United States
ER -

TY - JOUR
ID - 3427
T1 - Changes in anopheles funestus biting behavior following universal coverage of long-lasting insecticidal nets in benin
A1 - Moiroux,N.
A1 - Gomez,M.B.
A1 - Pennetier,C.
A1 - Elanga,E.
A1 - Djenontin,A.
A1 - Chandre,F.
A1 - Djegbe,I.
A1 - Guis,H.
A1 - Corbel,V.
Y1 - 2012///

N1 - 2012616688
English
Journal: Article
KW - EMBASE
KW - animal behavior
KW - animal experiment
KW - animal tissue
KW - Anopheles funestus
KW - article
KW - bed net
KW - Behavior Change
KW - Benin
KW - bioassay
KW - biological monitoring
KW - Child
KW - controlled study
KW - Female
KW - Human
KW - insect bite
KW - insecticidal activity
KW - insecticide resistance
KW - malaria control
KW - malaria falciparum/pc [Prevention]
KW - nonhuman
KW - parasite transmission
KW - parasite vector
KW - Plasmodium falciparum
KW - preschool child
KW - priority journal
KW - sporozoite
KW - circumsporozoite protein
KW - deltamethrin
RP - NOT IN FILE
SP - 1622
EP - 1629
JF - Journal of Infectious Diseases
JA - J Infect Dis
VL - 206
IS - 10
CY - United Kingdom
N2 - Background. Behavioral modification of malaria vectors in response to vector control methods is of great concern. We investigated whether full coverage of long-lasting insecticide-treated mosquito nets (LLINs) may induce a switch in biting behavior in Anopheles funestus, a major malaria vector in Africa.Methods.Human-landing collections were conducted indoor and outdoor in 2 villages (Lokohoue and Tokoli) in Benin before and 1 year and 3 years after implementation of universal LLIN coverage. Proportion of outdoor biting (POB) and median catching times (MCT) were compared. The resistance of A. funestus to deltamethrin was monitored using bioassays.Results.MCT of A. funestus switched from 2 am in Lokohoue and 3 am in Tokoli to 5 am after 3 years (Mann-Whitney U test, P <. 0001). In Tokoli, POB increased from 45 to 68.1 (odds ratio = 2.55; 95 confidence interval = 1.72-3.78; P <. 0001) 1 year after the universal coverage, whereas POB was unchanged in Lokohoue. In Lokohoue, however, the proportion of A. funestus that bites after 6 am was 26. Bioassays showed no resistance to deltamethrin.Conclusions.This study provides evidence for a switch in malaria vectors' biting behavior after the implementation of LLIN at universal coverage. These findings might have direct consequences for malaria control in Africa and highlighted the need for alternative strategies for better targeting malaria vectors. 2012 The Author
SN - 0022-1899
AD - (Moiroux, Chandre) MIVEGEC (IRD 224-CNRS 5290-UM1-UM2), Institut de Recherche Pour le Developpement, Montpellier, France (Guis) UMR CMAEE, CIRAD, Montpellier, France (Moiroux, Gomez,

Pennetier, Elanga, Djenontin, Djegbe, Corbel) MIVEGEC (IRD 224-CNRS 5290-UM1-UM2), Institut de Recherche Pour le Developpement, Cotonou, Benin (Gomez, Pennetier, Elanga, Djenontin, Djegbe, Corbel) Centre de Recherche en Entomologie de Cotonou, Ministere de la Sante, Cotonou Benin, Benin
ER -

TY - JOUR
ID - 3429
T1 - Psychosocial services utilized by older adolescents and young adults at a comprehensive cancer center
A1 - Folbrecht,J.
A1 - Mayorga,L.
A1 - Cabanillas,C.
A1 - Tafel,A.
A1 - Namm,J.
A1 - Carreras-Irwin,T.
A1 - Trujillo,M.
Y1 - 2012///
N1 - 70910990
English
Journal: Conference Abstract
KW - EMBASE
KW - Human
KW - Adult
KW - cancer center
KW - oncology
KW - Adolescent
KW - patient
KW - Coping Behavior
KW - Social Work
KW - school
KW - Socialization
KW - Child
KW - identity
KW - psychiatry
KW - Body Image
KW - medical education
KW - side effect
KW - neoplasm
KW - Achievement
KW - United States
KW - cancer diagnosis
KW - psychosocial care
KW - screening
KW - independence
KW - Pediatrics
KW - Clinical Psychology
KW - groups by age
KW - Anxiety
KW - clergy
KW - palliative therapy
KW - medical specialist
KW - Social Interaction
KW - social evolution
KW - Diagnosis
RP - NOT IN FILE
SP - 262
JF - Asia-Pacific Journal of Clinical Oncology

VL - Conference: COSA 39th Annual Scientific Meeting and IPOS 14th World Congress of Psycho-Oncology Brisbane, QLD Australia. Conference Start: 20121111 Conference End: 20121115. Conference Publication:
IS - var.pagings
N2 - Background: To an adolescent or young adult (AYA), a diagnosis of cancer poses unique challenges to personal and social development: interrupting social relationships, halting the move towards independence, and postponing achievement of educational and occupational goals. Isolation, side effects of treatment, dependence upon family members, and changes in body image lead to an interruption of identity development and often results in emotional symptoms including anxiety and depression. The goal of this research was to measure the psychosocial services utilized by AYAs in a pediatric program at a comprehensive cancer center. Methods: The Pediatric Psychosocial Support team at a comprehensive cancer center in the United States collected data on services (emotional coping, family conference, social services, medical education, psychosocial screening, socialization, school, symptom management) rendered by the psychosocial team (psychiatry, psychology, clinical social work, child life, palliative care, chaplain) to all patients over a 2 month period. Quantity and types of services utilized were examined contrasting the Pediatric (0 to 15) and AYA (16+) age groups. Results: A total of 1403 encounters were collected amongst 149 unique patients. Services were provided to 56 AYA patients with an average of 14 encounters per patient. Services were provided to 97 pediatric patients with 6 encounters on average. AYAs had a significantly higher number of encounters per patient than Pediatric patients. ($p < 0.05$). The top interventions provided to AYAs were emotional/coping support (44.7%), school (15.3%), socialization (15.1%), and social services (15%). The top interventions for pediatrics were emotional coping (57.3%), socialization/developmental activities (17.3%) and school (9.9). While AYAs required slightly less emotional/ coping support, they needed more support in the area of social service (financial assistance, accessing resources, and advocacy). Finally, AYAs received emotional/coping resources primary from Clinical Social Work professionals, while Pediatric patients received emotional and coping support primarily from Child Life Specialists. Conclusion: The results suggest that AYA oncology patients continue to need outside support to address the needs that arise from a cancer diagnosis. AYA patients like pediatric patients, need support in the area of coping, socialization, and schooling. Unlike pediatric patients, however, they have many more concerns with regards to navigating financial, resource, and advocacy concerns than pediatric patients and their families
SN - 1743-7555
AD - (Folbrecht, Mayorga) City of Hope Cancer Center, Duarte, CA, United States
ER -

TY - JOUR
ID - 3432
T1 - Factors affecting the timing of adiposity rebound
A1 - Satomi,K.
A1 - Katsura,K.
A1 - Go,I.
A1 - Naoto,S.
A1 - Toshimi,S.
A1 - Osamu,A.
Y1 - 2012///
N1 - 70896563
English
Journal: Conference Abstract
KW - EMBASE
KW - Obesity
KW - Society
KW - endocrinology
KW - Human
KW - feeding
KW - Child
KW - Birth Weight
KW - Eating
KW - Breast Feeding
KW - weight
KW - family history
KW - Exercise

KW - Weight Gain
KW - receiver operating characteristic
KW - parent
KW - bottle feeding
KW - infancy
KW - feeding apparatus
KW - beverage
KW - Drinking
KW - habit
KW - kindergarten
KW - baby
KW - father
KW - Male
KW - meal
KW - community
KW - body mass
RP - NOT IN FILE
SP - 172
JF - Hormone Research in Paediatrics
VL - Conference: 51st Annual Meeting of the European Society for Paediatric Endocrinology, ESPE 2012 Leipzig Germany. Conference Start: 20120920 Conference End: 20120923. Conference Publication:
IS - var.pagings
N2 - Background: The age of adiposity rebound (AR), when body mass index (BMI) starts to rise after infancy, is thought to be an origin of obesity in later life. We have already reported that children who exhibited an earlier AR were associated with the higher BMI value and atherogenic metabolic status at 12 years of age. We investigated which factors influenced on an earlier AR, birth weight, initial feeding, family history, meals or exercise. Methods: A total of 533 children in the community were enrolled in the study. Serial measurements of BMI from 4 months to 12 years were carried out prospectively. We calculated the age of AR, defined as the age which the lowest BMI occurred during this period. The subjects were divided into 2 groups according to BMI at 3 years is bigger than at 1.5 years (earlier AR group) or not (later AR group). We asked the answering to the question sheet about weight at birth, initial feeding (breast-feeding, bottle-feeding or mixed feeding), family history, meals, and exercises of their parents when children were at 3 years old. We also analyzed which BMI predicted the obesity at 12 years old, 4, 8, 12, 18 month or 2, 3, 4, 5 or 6 years by using ROC analysis. Results: Weight at birth was associated with earlier AR if birth weight was over 3500g, but was not associated with the timing of AR if it was between 1500g and 3000g. Initial feeding was not related to the timing of AR and the frequency of obesity at 2 years old. None of the breast-feeding subjects showed severe obesity at 12 years old. The factors as follows were associated with later AR; eating breakfast everyday, not eating snacks, non-obese father, the first baby, going to kindergarten. Contrary to expectation the habits of drinking sweet beverages and playing outside were not related to earlier AR. BMI at over 2 years old predicted to the obesity at 12 years old, but BMI in the infantile periods did not. Conclusions: This study showed that obesity at 12 years old was associated with weight gain over 2 years old, but not with the weight gain during infantile period
SN - 1663-2818
AD - (Satomi, Katsura, Go) Dokkyo Medical University, Pediatrics, Mibu, Tochigi, Japan (Naoto, Osamu) Dokkyo Medical University, Pediatrics, Tochigi, Japan (Toshimi) Dokkyo Medical University, Public Health, Tochigi, Japan
ER -

TY - JOUR
ID - 3435
T1 - Proposal of a histopathological predictive rule for the differential diagnosis between American tegumentary leishmaniasis and sporotrichosis skin lesions
A1 - Quintella,L.P.
A1 - Passos,S.R.L.
A1 - De Miranda,L.H.M.
A1 - Cuzzi,T.
A1 - Barros,M.B.D.
A1 - Francesconi-Do-Vale,A.C.

A1 - Galhardo,M.C.G.
A1 - Madeira,M.D.F.
A1 - Figueiredo De Carvalho,M.H.
A1 - Schubach,A.D.O.
Y1 - 2012///
N1 - 2012580905
English
Journal: Article
KW - EMBASE
KW - Adolescent
KW - Adult
KW - Aged
KW - american tegumentary leishmaniasis/di [Diagnosis]
KW - article
KW - Child
KW - cross-sectional study
KW - diagnostic accuracy
KW - diagnostic value
KW - Differential Diagnosis
KW - disease association
KW - Female
KW - histopathology
KW - Human
KW - major clinical study
KW - Male
KW - predictive value
KW - preschool child
KW - priority journal
KW - school child
KW - Sensitivity and Specificity
KW - skin leishmaniasis/di [Diagnosis]
KW - sporotrichosis/di [Diagnosis]
RP - NOT IN FILE
SP - 837
EP - 846
JF - British Journal of Dermatology
JA - Br J Dermatol
VL - 167
IS - 4
CY - United Kingdom
N2 - Background American tegumentary leishmaniasis (ATL) and sporotrichosis exhibit similar histopathology and low frequencies of microorganism detection. Objectives This study seeks to identify microscopic alterations that can distinguish between these diseases. Methods Haematoxylin and eosin stained slides of 171 ATL and 97 sporotrichosis samples from active cutaneous lesions were examined for histopathological alterations. The lesions were diagnosed by isolating the agent (which was not visible) in culture. An intuitive diagnosis was assigned to each slide. The strength of the association between the histopathological findings and the diagnosis was estimated by an odds ratio, and each finding was graded according to a regression model. A score was assigned to each sample based on the histopathological findings. A study of the interobserver reliability was performed by calculating kappa coefficients of the histopathological findings and intuitive diagnoses. Results The markers 'macrophage concentration', 'tuberculoid granuloma' and 'extracellular matrix degeneration' were associated with ATL. 'Suppurative granuloma', 'stellate granuloma', 'different types of giant cells', 'granulomas in granulation tissue' and 'abscess outside the granuloma' were associated with a diagnosis of sporotrichosis. 'Macrophage concentration' and 'suppurative granuloma' had the highest (substantial and almost perfect, respectively) reliability. The regression model score indicated 920% accuracy. The intuitive diagnosis had 825% diagnostic accuracy and substantial reliability. Conclusions Taking into account the clinical and epidemiological context, some histopathological alterations might be useful for the differential diagnosis between ATL and

sporotrichosis cutaneous lesions in cases in which the aetiological agent is not visible. 2012 British Association of Dermatologists
SN - 0007-0963
AD - (Quintella, Passos, De Miranda, Cuzzi, Barros, Francesconi-Do-Vale, Galhardo, Madeira, Figueiredo De Carvalho, Schubach) FIOCRUZ - Instituto de Pesquisa Clinica Evandro Chagas, Servico de Anatomia Patologica, Pavilhao Gaspar Vianna Terreo, Av. Brasil, 4365 IPEC, Rio de Janeiro 21040-360, Brazil
ER -

TY - JOUR
ID - 3436
T1 - Telemedicine to improve access to specialized care for patients with cystic fibrosis
A1 - ppiah-Kubi,G.
A1 - Bhide,R.
A1 - Flewelling,C.
A1 - Tsang,A.
A1 - Tullis,E.
A1 - Westell,S.
A1 - Zinger,S.
Y1 - 2012///
N1 - 70892351
English
Journal: Conference Abstract
KW - EMBASE
KW - cystic fibrosis
KW - telemedicine
KW - Human
KW - patient
KW - Hospital
KW - telehealth
KW - physician
KW - community
KW - Transplantation
KW - videorecording
KW - patient care
KW - Adult
KW - Education
KW - Canada
KW - North America
KW - Weather
KW - lung clearance
KW - Responsibility
KW - Exercise
KW - life sustaining treatment
KW - videoconferencing
KW - physiotherapist
KW - school
KW - Female
KW - nurse practitioner
KW - reimbursement
KW - home intravenous therapy
KW - diseases
KW - follow up
KW - Spirometry
KW - pathophysiology
KW - nurse
KW - mother
KW - Child

KW - Travel
KW - enzyme
RP - NOT IN FILE
SP - 444
EP - 445
JF - Pediatric Pulmonology
JA - Pediatr Pulmonol
VL - Conference: 26th Annual North American Cystic Fibrosis Conference Orlando, FL United States. Conference Start: 20121011 Conference End: 20121013. Conference Publication:
IS - var.pagings
N2 - The Toronto Adult Cystic Fibrosis (CF) Program at St Michael's Hospital was established in 1992 and is North America's largest adult CF program. Two satellite CF clinics were established for patients in northern Ontario communities hundreds of miles away. Frequent follow up in multidisciplinary CF clinics is known to improve patient outcomes. However, satellite clinics do not allow access to the entire multidisciplinary team, as reimbursement is limited for expenses related to physician visits. For appointments at the host hospital scheduling limitations such as accommodating patients' work or school responsibilities, availability of space, the CF physician's schedule, and inclement weather frequently led to last minute clinic cancellations. Objectives: The main goals of this initiative were to improve patient care and education by utilizing videoconferencing. Patients would come to Toronto once a year, attend satellite clinics twice a year and have a telemedicine appointment annually. Methods: In July 2010, monthly telemedicine multidisciplinary CF clinics were established to address the barriers that impede access to care. Patients were interviewed by the local nurse and spirometry and bloodwork were done. Results were sent by FAX to the Toronto clinic and the patients were able to see team members by video link. Results: The telemedicine conference visit was used in two major ways - for routine clinic visits and for episodic illness. To date, there have been 71 patients seen by telemedicine conference with 22 monthly clinics held and 10 patients seen outside of the monthly clinics. As time passed clinicians became more experienced with the format and used the telemedicine link in increasingly diverse ways. Several telemedicine clients in remote locations were cared for on home intravenous therapy. One ventilated client was being considered for transplant while hospitalized 800 miles away and was monitored and coached via telemedicine by both the CF and transplant physicians. For a newly diagnosed client the CF doctor used telehealth to explain how to use enzymes. The nurse practitioner was able to present diagrams of the pathophysiology of cystic fibrosis by video for the patient to see. The physiotherapist used telemedicine to demonstrate correct technique for airway clearance. The mother of a CF child commented that the drive to the telehealth studio took 15 minutes compared with a 10 hour trip to the Toronto clinic. One patient explained that the trip to Toronto meant interrupting her life-sustaining therapies including masks, physio and exercise. Discussion: Telemedicine improved patient care, reduced patient costs and improved inter-professional collaboration. We were able to strengthen relationships with community partners by incorporating visits with local care teams. The telemedicine visits express our client-centred approach to care by maintaining interprofessional collaboration with the CF team while saving the client time and travel costs. Future plans include continuing to promote attendance at telehealth and possibly considering specialized clinics such as CFRD clinics that could be held via telehealth
SN - 8755-6863
AD - (Appiah-Kubi, Tsang, Tullis, Westell) Adult Cystic Fibrosis, St. Michael's Hospital, Toronto, ON, Canada (Bhide, Flewelling) Telemedicine, St. Michael's Hospital, Toronto, ON, Canada (Zinger) Cystic Fibrosis, Health Sciences North, Sudbury, ON, Canada
ER -

TY - JOUR
ID - 3437
T1 - Adherence to the Mediterranean diet and albuminuria levels in adolescents: Emerging data from the Lyceum Leontio Albuminuria (3L) Study
A1 - Dimitriadis,K.
A1 - Tsioufis,C.
A1 - Tsiachris,D.
A1 - Kasiakogias,A.
A1 - Psaltopoulou,T.
A1 - Tzamou,V.
A1 - Mazaraki,A.

A1 - Andrikou,I.
A1 - Pitsavos,C.
A1 - Stefanadis,C.
Y1 - 2012///
N1 - 70882497
English
Journal: Conference Abstract
KW - EMBASE
KW - albuminuria
KW - Adolescent
KW - Human
KW - Mediterranean diet
KW - body mass
KW - sport
KW - excretion
KW - atherosclerosis
KW - Lifestyle
KW - Obesity
KW - questionnaire
KW - urine
KW - Blood Pressure
KW - Child
KW - Heart Rate
KW - Waist Circumference
KW - Television
KW - school
KW - Food
KW - Male
KW - Population
KW - habit
KW - Cardiovascular System
KW - albumin
KW - creatinine
KW - marker
RP - NOT IN FILE
SP - 24
JF - European Heart Journal
VL - Conference: ESC Congress 2012 Munchen Germany. Conference Start: 20120825 Conference End: 20120829. Conference Publication:
IS - var.pagings
N2 - Purpose: Mediterranean diet has favorable effects on the cardiovascular system, while albuminuria is associated with atherosclerosis progression. The aim of the study was to investigate the relationship of dietary habits with urinary albumin excretion, expressed as the albumin to creatinine ratio (ACR), in a cohort of adolescents Methods: A total of 365 adolescents 12-17 years of age [212 males, aged 13.9 years, office blood pressure (BP)=115/67 mmHg] that were included in the Lyceum Leontio Albuminuria (3L) study were considered for analysis. In all participants ACR values were determined in a morning spot urine and for each adolescent a questionnaire was completed on lifestyle and socio-economic characteristics. Moreover, the Mediterranean Diet Quality Index for children and adolescents (KIDMED) was estimated and accordingly subjects were divided into those with optimal (>7), average (4-7) and low (<4) score. Results: Only 6.8% of the participants had optimal KIDMED score, whereas 51.2% had an average and 42% had a low score. Participants with at least average KIDMED score (n=187) compared to those with low KIDMED score (n=153) had higher body mass index (22.2 vs 21.4 kg/m2, p=0.043) and waist circumference (77.6 vs 75.4 cm, p=0.044), spent more frequently time for sports activities outside school (75.2% vs 58%, p=0.001), reported less consumption of foods outside home (3% vs 14%, p<0.001) and less hours of watching television (1.75 vs 2.05 hours, p=0.013). Moreover, those with at least average compared to those with low KIDMED score exhibited higher systolic BP (117 vs 114 mmHg, p=0.039), whereas had lower heart rate (84 vs 87 bpm, p=0.014) and ACR levels (12.6 vs 20.5 mg/g, p=0.015). In the total population, ACR was associated with age (r=-0.11, p=0.044), body mass index

(r=-0.131, p=0.016), systolic BP (r=-0.144, p=0.008) and KIDMED score (r=-0.111, p=0.041). Conclusion: In adolescents there is an inverse relation of KIDMED score with albuminuria and those who adhere to the Mediterranean diet exhibit lower levels of ACR. However, the paradoxical associations of both ACR and KIDMED score with obesity markers and BP levels suggest distinct mechanisms of albuminuria development in adolescents
SN - 0195-668X
AD - (Dimitriadis, Tsioufis, Tsiachris, Kasiakogias, Psaltopoulou, Tzamou, Mazaraki, Andrikou, Pitsavos, Stefanadis) First Cardiology Clinic, University of Athens, Hippokration Hospital, Athens, Greece
ER -

TY - JOUR
ID - 3438
T1 - Hydraulic driven fast and precise nonmagnetic tactile stimulator for neurophysiological and MEG measurements
A1 - Broser,P.J.
A1 - Braun,C.
Y1 - 2012///
N1 - 2012561801
English
Journal: Article
KW - EMBASE
KW - article
KW - Child
KW - controlled study
KW - electric activity
KW - electrostimulation
KW - gold standard
KW - Human
KW - index finger
KW - Magnetoencephalography
KW - needle
KW - neurophysiology
KW - peripheral nerve
KW - phantom
KW - primary somatosensory cortex
KW - somatosensory stimulation
KW - somatosensory system
KW - tactile stimulation
KW - Time
KW - Water
RP - NOT IN FILE
SP - 2852
EP - 2858
JF - IEEE Transactions on Biomedical Engineering
JA - IEEE Trans Biomed Eng
VL - 59
IS - 10
CY - United States
N2 - Electric stimulation of the peripheral nerves is well established as a diagnostic and research tool to analyze the somatosensory system. However, electric stimulation has some disadvantages. Electric stimulation of the median nerve triggers action potentials in all fiber populations of the nerve. Electric stimulation further creates artifacts and courses discomfort which is usually not well tolerated in the awake child. Therefore, the development of a more specific stimulation has constantly been a goal in recent years. There have been several approaches in the past to deliver somatic stimulation. However, all of them failed short in some aspects. In this study, a new type of somatosensory stimulator device was developed and compared against the gold standard of electric stimulation. The stimulation is achieved by repetitive tactile stimulation of the index finger using a blunt needle. In contrast to all previous approaches, we use a hydraulic system to move the needle up and downward.

Given that water is very well suited to conduct pressure pulses it is possible to place the tactile stimulator device holding the needle close to the subject and the hydraulic driving system outside a critical area. Using a phantom, we showed that our stimulator is capable of delivering a stimulus precise on the submillisecond time scale. In addition, we test our stimulator on a healthy adult and compare the results against the electric stimulation. We can show the feasibility of measuring the electric responses of the peripheral nerve and while using MEG also the response of the primary somatosensory cortex. The tactile stimulation showed a more spatial focuses activation of the primary somatosensory cortex when compared against the electric stimulation. The proposed high-precision tactile stimulator will make it possible to analyze the somatosensory system noninvasively in children in the future. 1964-2012 IEEE
SN - 0018-9294
AD - (Broser) Kinderklinik Tuebingen, Abteilung III Neuropaediatrie, 72076 Tuebingen, Germany (Braun) Department of Clinical Psychology, MEG Center, University of Tuebingen, 72076 Tuebingen, Germany
ER -

TY - JOUR
ID - 3439
T1 - Epidemiology of malaria in the Papua New Guinean highlands
A1 - Betuela,I.
A1 - Maraga,S.
A1 - Hetzel,M.W.
A1 - Tandrapah,T.
A1 - Sie,A.
A1 - Yala,S.
A1 - Kundi,J.
A1 - Siba,P.
A1 - Reeder,J.C.
A1 - Mueller,I.
Y1 - 2012///
N1 - 2012547137
English
Journal: Article
KW - EMBASE
KW - Adolescent
KW - Adult
KW - Altitude
KW - article
KW - bed net
KW - Child
KW - endemic disease
KW - epidemic
KW - Evidence Based Practice
KW - Female
KW - fever
KW - health survey
KW - household
KW - Human
KW - major clinical study
KW - malaria/pc [Prevention]
KW - malaria control
KW - malaria falciparum
KW - Male
KW - Microscopy
KW - Papua New Guinea
KW - Plasmodium vivax malaria
KW - preschool child
KW - Prevalence
KW - Risk

KW - Risk Assessment
KW - risk factor
KW - Rural Population
KW - school child
KW - statistical significance
KW - structured questionnaire
KW - Travel
RP - NOT IN FILE
SP - 1181
EP - 1191
JF - Tropical Medicine and International Health
VL - 17
IS - 10
CY - United Kingdom
N2 - Objectives To conduct an in-depth investigation of the epidemiology of malaria in the Papua New Guinea (PNG) highlands and provide a basis for evidence-based planning and monitoring of intensified malaria control activities. Methods Between December 2000 and July 2005, 153 household-based, rapid malaria population surveys were conducted in 112 villages throughout the central PNG highlands. The presence of malaria infections was determined by light microscopy and risk factors assessed using a structured questionnaire. The combined dataset from all individually published surveys was reanalysed. Results The prevalence of malaria infections in the different surveys ranged from 0.0% to 41.8% (median 4.3%) in non-epidemic surveys and 6.6% to 63.2% (median 21.2%, P<0.001) during epidemics. Plasmodium falciparum was the predominant infection below 1400m and during epidemics, Plasmodium vivax at altitudes >1600m. Outside epidemics, prevalence decreased significantly with altitude, was reduced in people using bed nets [odds ratio (OR)=0.8, P<0.001] but increased in those sleeping in garden houses (OR=1.34, P<0.001) and travelling to highly endemic lowlands (OR=1.80, P<0.001). Below 1400m, malaria was a significant source of febrile illness. At higher altitudes, malaria was only a significant source of febrile illness during epidemic outbreaks, but asymptomatic malaria infections were common in non-epidemic times. Conclusions Malaria is once again endemic throughout the PNG highlands in areas below 1400-1500m of altitude with a significant risk of seasonal malaria outbreaks in most area between 1400-1650m. Ongoing control efforts are likely to result in a substantial reduction in malaria transmission and may even result in local elimination of malaria in higher lying areas. 2012 Blackwell Publishing Ltd
SN - 1360-2276
AD - (Betuela, Maraga, Hetzel, Tandrapah, Sie, Siba, Mueller) PNG Institute of Medical Research, Goroka, Papua New Guinea (Betuela, Mueller) Barcelona Centre for International Health Research (CRESIB, Hospital Clinic-Universitat de Barcelona), Barcelona, Spain (Hetzel) The University of Queensland, School of Population Health, Herston, QLD, Australia (Yala, Kundi) Malaria Surveillance and Control Unit, Goroka, Papua New Guinea (Reeder) The Burnet Institute of Medical Research and Public Health, Prahan, VIC, Australia (Mueller) Walter and Eliza Hall Institute, Parkville, VIC, Australia
ER -

TY - JOUR
ID - 3440
T1 - Investigation of obesity and overweight; Impact of eating habits in school children: Constantine 2010-2011
A1 - Sayed,A.
A1 - Dalichaouche,S.
A1 - Rouabah,A.
A1 - Tebbani,F.
A1 - Kabouche,S.
A1 - Rouabah,L.
Y1 - 2012///
N1 - 70866613
English
Journal: Conference Abstract
KW - EMBASE
KW - Society
KW - Obesity

KW - Human
KW - drug surveillance program
KW - school child
KW - pharmacology
KW - Therapy
KW - physiology
KW - eating habit
KW - Child
KW - Prevalence
KW - calorie
KW - Immobilization
KW - weight reduction
KW - body mass
KW - procedures
KW - parent
KW - questionnaire
KW - city
KW - public health problem
KW - boy
KW - breast
KW - Male
KW - Food
KW - girl
KW - Weight Gain
KW - beverage
KW - Physical Activity
KW - epidemic
KW - Diet
KW - Prevention
KW - Female
KW - childhood obesity
RP - NOT IN FILE
SP - 53
JF - Fundamental and Clinical Pharmacology
VL - Conference: 16th Annual Meeting of French Society of Pharmacology and Therapeutics, the 79th Annual Meeting of Society of Physiology, the 33th Pharmacovigilance Meeting, the 13th APNET Seminar and the 10th CHU CIC Meeting Dijon France. Conference Start: 20120404 Conference End: 20120406. Conference Publication:
IS - var.pagings
N2 - Now Childhood obesity is recognized as a public health problem due to prevalence, and rapid development in recent decades. The aim of study was to estimate the prevalence of overweight and obesity among school children 8-9 years living in the region of Constantine and clearly identify their eating habits. An epidemiological study was conducted in 2011 involving 325 school children (165 girls and 160 boys) aged 8-9 years in the town of Constantine. We distributed an anonymous questionnaire, it is reset to be completed by the parents at home, given were treated with EPI info version 6.4. The measurements were performed according to standard procedures (OMS 2007), body mass index (BMI) shows that 3.10% of children are in a state of emaciation, 66.80% are normal, 18.20% are overweight, 12% of children are obese, sex was significantly associated with obesity ($P < 0.001$). Afternoon snack for children recognized as a factor promoting weight gain and found that 50.80% of children who take the afternoon snack are overweight and 48.70% are obese. 93.20% of children are foods high in calories and outside the main meals. 97.40% of obese and overweight children practice saying snacking. Decision-sweetened beverages were significantly correlated with obesity. The results of our survey show that 83.4% of children do not practice physical activity. The relationship between physical inactivity and obesity is significant ($P < 0.001$). The correlation between the type of breastfeeding (breast, bottle, or mixed) and BMI in obese children and not significant ($P > 0.05$). These results suggest that obesity and overweight are a growing epidemic in progress, the unbalanced diet and physical inactivity are key factors that must be controlled by the establishment of a program of early prevention of this silent disease
SN - 0767-3981

AD - (Sayed, Dalichaouche, Rouabah, Tebbani, Kabouche, Rouabah) Biologie Cellulaire et Moleculaire, Constantine, Algeria
ER -

TY - JOUR
ID - 3441
T1 - Effective management of vaccines in primary care
A1 - McGovern,E.M.
A1 - Pawelczyk,K.
A1 - Reilly,V.
A1 - Ahmed,S.
A1 - Bryson,S.M.
Y1 - 2010///
N1 - 70863932
English
Journal: Conference Abstract
KW - EMBASE
KW - primary medical care
KW - United Kingdom
KW - clinical pharmacy
KW - Health Care
KW - pharmacist
KW - Human
KW - Medical Audit
KW - Temperature
KW - storage
KW - feedback system
KW - Risk Management
KW - follow up
KW - Exercise
KW - logging
KW - temperature measurement
KW - surgery
KW - public health service
KW - Public Health
KW - prophylaxis
KW - Risk
KW - Government
KW - cost control
KW - Quality Control
KW - Child
KW - revaccination
KW - Prevention
KW - Safety
KW - Methodology
KW - Funding
KW - patient
KW - patient safety
KW - vaccine
RP - NOT IN FILE
SP - S28
EP - S29
JF - Clinical Pharmacist
VL - Conference: 2010 Joint National Conference of the United Kingdom Clinical Pharmacy Association and the Guild of Healthcare Pharmacists Leeds United Kingdom. Conference Start: 20100514 Conference End: 20100516. Conference Publication:
IS - var.pagings

N2 - Vaccines are biological substances and storage outside of the recommended temperature range may accelerate loss of potency, which cannot be reversed.1 Maintenance of the cold chain for vaccine supply, storage and administration is essential to ensure product quality and patient safety, thereby avoiding serious consequences for both the patient and the NHS, the importance of which was raised in a recent safety report.2 Following the identification of a vaccine incident in Grampian, the Scottish Government Health Department prompted a review of vaccine storage in GP surgeries across NHS Scotland. In this Board a detailed review including a criteria based inspection (audit) of all GP practices was undertaken during May to November 2007. All required improve - ments in their practice, usually in the areas of temperature monitoring or equipment. Subsequently, funding for a one year 'spend to save' project was obtained to establish improved quality assurance processes for vaccine manage - ment in primary care, commencing January 2009. OBJECTIVE To establish a programme of self audit for all GP premises and identify the optimal approach to effective management of vaccines METHODS 1. The previous project had established support for a self audit approach and the previous criteria based tool was adapted for self audit (42 criteria). Engagement with Public Health Facilitators, prescribing leads and lead clinical pharmacists encouraged practices to undertake self audit. In addition practices were asked to place an electronic temperature logger in each fridge to monitor hourly temperatures for 48 hours. Practices could also exceptionally request a practice visit. Following the visits, practices received individualised feedback reports and were asked to return a signed copy to confirm acceptance of the recommendations. A Steering Group was established to monitor the project and review any practices referred for risk management. The project team reviewed individual audit results and provided individualized feedback to practices 2. A retrospective review of all incidents reported during 2008 was undertaken to assess whether these might have been preventable, number of repeat incidences and associated costs. After March 2009 an individualised report for each affected GP practice was generated detailing the incident reported, potential reasons, recommendations to minimise the risk of recurrence and an invitation to undertake self audit where appropriate. This report was copied to the appropriate Clinical Director and lead clinical pharmacist. 3. Guidance and standards relating to vaccine handling and storage is available nationally.1,3 Previous local guidance circulated to practices in May 2007 was reviewed. RESULTS 1. To the end of January 2010, 188 practices (73%) had undertaken audit activity (173 self audits, 15 audit visits, 317 fridges), submitted one month's practice records and undertaken a 48 hour temperature logging exercise of all fridges. The main area for improvement was aspects of temperature monitoring (Figure 1). Where necessary, follow-up actions were arranged e.g. further submission of temperature records. This was required for 76 practices (40%). The Steering Group advised revaccination for three children in one practice. 2. A reduction in the extent and cost associated with incidents was observed (Table 1). 3. The previous local guidance was revised to provide additional background, information on suitable fridges, supplier's details and NHS discount code. (Table Presented) DISCUSSION The project was successful in motivating practices to improve their processes, as indicated by their active participation in self audit, followup actions and return of signed summary reports. Limitations were identified to the self audit process and review of the submitted records and logging exercise revealed some inconsistencies. Prior to March 2009, no attempt was made to identify contributory factors, provide advice on prevention of future incidents or investigate if repeated incidents occur in the same practice. This feedback process is having a positive effect on the number of incidents overall and GPs reporting repeat occurrences, resulting in cost savings of 70k as a result of reducing preventable incidents (from 106.5k in 2008 to 36.5k in 2009). The project methodology and results were successful in raising awareness of the importance of the cold chain, establishing a self audit programme and identifying the priority areas for improvement. Our experience has prompted an application for recurring NHS staffing resource to meet the ongoing service needs. CONCLUSION This project has addressed SGHD concerns and has brought fundamental local change to vaccine management, thereby strengthening health protection, improving risk management and reducing vaccine wastage in GP practices
SN - 1758-9061
AD - (McGovern, Pawelczyk, Reilly, Bryson) Pharmacy Prescribing Support Unit, Public Health Protection Unit, United Kingdom (Ahmed) NHS Greater Glasgow and Clyde (GG and C), United Kingdom
ER -

TY - JOUR
ID - 3442
T1 - The effects of conducting a functional analysis on problem behavior in other settings
A1 - Call,N.A.
A1 - Findley,A.J.
A1 - Reavis,A.R.

Y1 - 2012///
N1 - 2012510777
English
Journal: Article
KW - EMBASE
KW - Aggression
KW - article
KW - automutilation
KW - behavior disorder
KW - Case Report
KW - Child
KW - daily life activity
KW - disruptive behavior
KW - flopping
KW - functional assessment
KW - Human
KW - Male
KW - Methodology
KW - mouthing
KW - patient attitude
KW - preschool child
KW - Reinforcement
KW - school child
KW - spitting
KW - Topography
RP - NOT IN FILE
SP - 1990
EP - 1995
JF - Research in Developmental Disabilities
JA - Res Dev Disabil
VL - 33
IS - 6
CY - United States
N2 - It has been suggested that reinforcing problem behavior during functional analyses (FAs) may be unethical (e.g., Carr, 1977), the implication being that doing so may result in an increase in problem behavior outside of FA sessions. The current study assessed whether conducting a FA resulted in increases in problem behavior outside of the FA setting for 4 participants. The rate of problem behavior was measured outside the FA setting prior to and during a FA. Idiosyncratic results suggest that problem behavior outside of the FA setting may increase, decrease, or be unaffected by conducting a FA. 2012 Elsevier Ltd
SN - 0891-4222
AD - (Call, Findley, Reavis) The Marcus Autism Center, United States (Call) Emory University School of Medicine, United States
ER -

TY - JOUR
ID - 3443
T1 - Association of serum 25-hydroxyvitamin D [25(OH)D] concentrations with dietary patterns in US children
A1 - Martineau,B.
A1 - Van,Fleit B.
A1 - Ganji,V.
Y1 - 2012///
N1 - 70853743
English
Journal: Conference Abstract
KW - EMBASE
KW - serum
KW - Child

KW - Human
KW - Diet
KW - exposure
KW - Sunlight
KW - Multivariate Analysis
KW - supplementation
KW - assay
KW - factorial analysis
KW - food frequency questionnaire
KW - examination
KW - Nutrition
KW - Public Health
KW - 25 hydroxyvitamin D
KW - fenticlor
KW - vitamin D
RP - NOT IN FILE
JF - FASEB Journal
VL - Conference: Experimental Biology 2012, EB San Diego, CA United States. Conference Start: 20120421 Conference End: 20120425. Conference Publication:
IS - var.pagings
N2 - Contribution of dietary sources to vitamin D status is not clearly known. Although sunlight exposure is a major source of circulating serum 25(OH)D, diet is an important contributor in absence of or reduced exposure. Objective of this study was to determine relation between dietary patterns and serum 25(OH)D concentrations of US children using assay-adjusted serum 25(OH)D data. Data from 2 cycles of the National Health and Nutrition Examination Survey 2003-2004 and 2005-2006 for individuals aged < 19 y, were used to study the association between dietary patterns and serum 25(OH)D. Dietary patterns were established using factor analysis based on food-frequency questionnaire data. Eigenvalues and Scree plot were used to derive 2 major principle factors. They were labeled as Western and Healthy dietary patterns. Serum 25(OH)D was significantly lower in Healthy dietary pattern group compared to Western dietary pattern group (61.9 vs 66.1 nmol/L; P<.002). In multivariate analysis, children with higher Western dietary contribution scores to overall diet showed a significant positive relation with serum 25(OH)D (beta=33.8, P=.046). Serum 25(OH)D is associated with Western dietary pattern but not with Healthy pattern. Children consuming healthy pattern diet may benefit from vitamin D supplementation and sunlight exposure (outdoor activities)
SN - 0892-6638
AD - (Martineau, Ganji) Division of Nutrition, Georgia State University, Atlanta, GA, United States (Van Fleit) Department of Mathematics and Statistics, Georgia State University, Atlanta, GA, United States
ER -

TY - JOUR
ID - 3444
T1 - Blastomycosis: An unusual cause of pleural effusion complicating community-acquired pneumonia
A1 - Buu,M.C.
A1 - Conrad,C.K.
Y1 - 2011///
N1 - 70851161
English
Journal: Conference Abstract
KW - EMBASE
KW - blastomycosis
KW - pleura effusion
KW - community acquired pneumonia
KW - Society
KW - Human
KW - pneumonia
KW - Blastomyces
KW - serum
KW - Diagnosis

KW - Lung
KW - lymph
KW - Fatigue
KW - fever
KW - effusion
KW - abnormal respiratory sound
KW - breathing
KW - boy
KW - patient
KW - Male
KW - serology
KW - Child
KW - stain
KW - outpatient
KW - intravenous administration
KW - antibiotic therapy
KW - oxygen saturation
KW - lymphadenopathy
KW - Coccidioides
KW - United States
KW - Histoplasma
KW - lake
KW - dilution
KW - diseases
KW - Bronchoscopy
KW - Therapy
KW - Travel
KW - ultrasound
KW - thorax
KW - anorexia
KW - Hospital
KW - video assisted thoracoscopic surgery
KW - Antibody
KW - ceftriaxone
KW - Azithromycin
KW - cefdinir
KW - amphotericin
KW - pyrrole
KW - lake water
KW - vancomycin
KW - itraconazole
KW - antigen
RP - NOT IN FILE
JF - American Journal of Respiratory and Critical Care Medicine
VL - Conference: American Thoracic Society International Conference, ATS 2011 Denver, CO United States. Conference Start: 20110513 Conference End: 20110518. Conference Publication:
IS - var.pagings
N2 - Pneumonia complicated by effusion that does not improve with antibiotic treatment warrants further investigation to avoid development of lung restriction due to entrapment. This case demonstrates that thorough history is vital and highlights the importance of selecting the proper array of serologic diagnostic tools to aid in rapid, accurate diagnosis of Blastomycosis pneumonia in children. A 31-month-old previously healthy boy was referred to our center for worsening unilateral pneumonia with a large pleural effusion. 8 weeks prior, he developed low-grade fevers, fatigue, and anorexia. CXR revealed a right middle and lower lobe pneumonia with large right-sided pleural effusion. Shortly before the illness, he was vacationing near Lake Huron, Michigan, during which he choked on some lake water while playing. Despite outpatient treatment with 2 doses of intramuscular ceftriaxone and 10 days of oral cefdinir, his symptoms persisted and the CXR worsened (Image 1). He was admitted to an outside hospital for VATS. 150mL of hypocellular transudate was drained. Cell

differential was 1% PMN's, 91% lymphs, and 4% eos. Bacterial, fungal, and AFB stains and cultures were negative. Serum ID Blastomyces antibody was negative at that time. No further serologies were obtained. He received azithromycin, vancomycin, and ceftriaxone for 10 days, then discharged home. For three weeks, the symptoms persisted. CXR was unchanged, CRP and ESR remained elevated, so the patient was referred to our center for further evaluation. On exam, he had normal oxygen saturation and was breathing comfortably with diminished breath sounds in the right lung base. There was no notable lymphadenopathy. Chest ultrasound redemonstrated large right-sided pleural effusion. Bronchoscopy was normal and BAL differential: 3% PMNs, 72% lymphs, 22% monos 22%, and 2% eos. Repeat cultures were negative. Serum Blastomyces antibody CF, which has moderate sensitivity and low specificity, was positive to 1:64 dilutions. ID, which is moderately sensitive and highly specific, was positive (Table 2). Histoplasma and Coccidioides antibody ID and Blastomyces serum antigen were negative. Oral itraconazole was initiated. Fever, fatigue improved. Within 3 weeks of therapy, the effusion, CRP, and ESR nearly resolved. Conclusion: Pulmonary blastomycosis evolves insidiously and can be fatal if not treated quickly. Complicated pleural effusions are rare in pediatric pulmonary blastomycosis. Clinical suspicion is necessary for rapid diagnosis, and the history of travel to an endemic area guided our evaluation. Oral -azole antifungals are highly efficacious with few complications and are an alternative to toxic intravenous therapy with amphotericin B. (Figure presented)
SN - 1073-449X
AD - (Buu, Conrad) Stanford University, School of Medicine, Palo Alto, CA, United States
ER -

TY - JOUR
ID - 3445
T1 - Does engagement in health behaviors explain differences in the prevalence of asthma between urban and rural areas
A1 - Lawson,J.A.
A1 - Pickett,W.
A1 - Janssen,I.
A1 - Bruner,M.
A1 - Madani,K.
Y1 - 2011///
N1 - 70849740
English
Journal: Conference Abstract
KW - EMBASE
KW - Prevalence
KW - Asthma
KW - rural area
KW - Society
KW - Health Behavior
KW - Risk
KW - Obesity
KW - juvenile
KW - Human
KW - Physical Activity
KW - model
KW - passive smoking
KW - multivariate logistic regression analysis
KW - Child
KW - Health Care
KW - Diagnosis
KW - school
KW - Population
KW - Environmental Exposure
KW - physician
KW - vegetable
KW - milk
KW - exposure

KW - sampling
KW - Diet
RP - NOT IN FILE
JF - American Journal of Respiratory and Critical Care Medicine
VL - Conference: American Thoracic Society International Conference, ATS 2011 Denver, CO United States. Conference Start: 20110513 Conference End: 20110518. Conference Publication:
IS - var.pagings
N2 - Rationale and objective: Past research has demonstrated a lower prevalence of childhood asthma in rural areas. The mechanism(s) by which this occurs remain unknown. Our objective was to confirm the existence of a geographic gradient in the prevalence of asthma among Canadian youths and to evaluate whether this gradient is mediated by health behaviors. Methods: Cross-sectional data from 4726 youth (grades 6 to 10) representative of the Canadian population were collected during the 2001-02 Health Behavior in School-Aged Children survey. Study variables included: demographics, health behaviors (physical activity, diet, active and passive smoking), and adiposity status. Geographic region was assessed using Beale codes and categorized as metro, non-metro but adjacent to metro, and rural. Asthma was defined via doctors' diagnoses and at least one of: asthma symptoms or a health care visit for asthma in the past year. Analyses involved multiple logistic regression with generalized estimating equations to account for the clustered sampling design. Results: Asthma prevalence was lower outside of metro regions (metro=17.7%, non-metro-adjacent=15.6%, rural=14.8%; p<0.05). Compared to the metro region, there was reduced risk of asthma in the non-metro-adjacent (OR=0.81, 95%CI=0.65-1.01) and rural (OR=0.76, 95%CI=0.61-0.95) regions in the fully adjusted model. As potential mediators (adiposity status; each of six health behaviors) were removed from the full model, there was minimal change in the association between geographic region and asthma. Being obese or overweight, high physical activity, and exposure to passive smoking increased the risk of asthma while consumption of whole milk and vegetables were protective. Conclusions: We confirmed a reduced risk of asthma in rural vs. metro areas using a national, representative sample of Canadian youth. This association was not mediated by health behaviors. Speculatively, other factors such as environmental exposures, may be the mechanisms through which rural region is related to a lower prevalence of asthma
SN - 1073-449X
AD - (Lawson, Madani) University of Saskatchewan, Saskatoon, Canada (Pickett, Janssen) Queen's University, Kingston, ON, Canada (Bruner) Nipissing University, North Bay, ON, Canada
ER -

TY - JOUR
ID - 3446
T1 - Risk factors for asthma in farming and non-farming environments in children
A1 - Lawson,J.A.
A1 - Walker,A.
A1 - Rennie,D.C.
A1 - Senthilselvan,A.
A1 - Dosman,J.A.
Y1 - 2011///
N1 - 70849739
English
Journal: Conference Abstract
KW - EMBASE
KW - Human
KW - agricultural worker
KW - Asthma
KW - Society
KW - Environment
KW - Child
KW - risk factor
KW - Male
KW - city
KW - exposure
KW - Risk
KW - Prevalence

KW - physician
KW - boy
KW - girl
KW - Canada
KW - Female
KW - Health
KW - father
KW - allergy
KW - emergency ward
KW - Drug Therapy
KW - wheezing
KW - Diagnosis
KW - Environmental Exposure
KW - Health Behavior
KW - household
KW - Smoking
KW - winter
KW - questionnaire
KW - parent
RP - NOT IN FILE
JF - American Journal of Respiratory and Critical Care Medicine
VL - Conference: American Thoracic Society International Conference, ATS 2011 Denver, CO United States. Conference Start: 20110513 Conference End: 20110518. Conference Publication:
IS - var.pagings
N2 - Rationale and objective: Farming environments have been associated with lower childhood asthma prevalence. However, the exposures responsible for this effect are unknown. The objective of this study was to investigate potential risk factors for childhood asthma and to assess whether these associations were consistent between farming and non-farming environments. Methods: Parents of 898 children aged 6-12 years living in an agricultural region of Saskatchewan, Canada, participated in a cross-sectional survey of their children's health. Questionnaires were completed during the winter months between 2004 and 2006, and assessed respiratory health, exposure to farming and household environments health behaviors, and home location. Home location was classified as farm, acreage or town. Asthma was considered present if there was a report of a history of a doctor's diagnosis and at least one of the following in the past year: wheeze, medication use for asthma, or an emergency room or doctor's visit for asthma. Results: Asthma prevalence was the highest in town (16.7%) followed by acreage (15.6%) and farm (14.0%). A parental history of asthma and personal history of allergy were strongly associated with asthma (odds ratios > 3; p<0.01) and were consistent between home locations. Significant interactions (p<0.05) were observed between home location and exposure to cleaning barns as well as sex and father's history of smoking. Children who lived in town but not on farms or acreages and had spent time cleaning or playing in barns in the past 12 months had reduced risk of asthma. Girls, but not boys, whose father smoked at home were at higher risk of having asthma regardless of location. Conclusion: Host characteristics were the strongest predictors of asthma and were consistent between farm, acreage and town locations. However, children's outdoor activities and specific environmental exposures in each home location should be considered in studies investigating risk factors for asthma. Similarly, boys and girls may have different levels of susceptibility to the environment
SN - 1073-449X
AD - (Lawson, Walker, Rennie, Dosman) University of Saskatchewan, Saskatoon, SK, Canada (Senthilselvan) University of Alberta, Edmonton, Canada
ER -

TY - JOUR
ID - 3447
T1 - Research-based methods and outreach approaches for improving respiratory health in pre-school children
A1 - Heck,D.E.
A1 - Shakarjian,M.P.
A1 - Fan,Z.
A1 - Laumbach,R.J.
Y1 - 2011///

N1 - 70849247
English
Journal: Conference Abstract
KW - EMBASE
KW - preschool child
KW - Human
KW - Society
KW - Health
KW - Traffic
KW - Asthma
KW - community
KW - school
KW - student
KW - air quality
KW - Air Pollution
KW - car
KW - ambient air
KW - Child
KW - Policy
KW - follow up
KW - wheezing
KW - Dust
KW - primary school
KW - groups by age
KW - Prevention
KW - population research
KW - translational research
KW - United States
KW - nitrogen dioxide
RP - NOT IN FILE
JF - American Journal of Respiratory and Critical Care Medicine
VL - Conference: American Thoracic Society International Conference, ATS 2011 Denver, CO United States. Conference Start: 20110513 Conference End: 20110518. Conference Publication:
IS - var.pagings
N2 - Vehicle exhaust is the leading source of toxic air pollution for most communities in the Lower Hudson Valley (LHV) of New York State, and a single vehicle used to commute children to school contributes 3 pounds of air pollution monthly. These findings led to the enactment of vehicle idling restrictions around elementary schools in many states including NY. Particulates and contaminants found in dust from traffic have been found to cause persistent wheezing in preschool children, a response associated with the development of asthma and other pulmonary conditions. In this regard studies demonstrate that initial incidents of asthma develop before age 5, suggesting that preschool years mark the critical period for development of the disease and that prevention should be focused at this age group. However little is known about the impact of no idling ordinances on ambient air quality near schools. We hypothesized that measuring levels of traffic related asthma triggers, and subsequently altering traffic flow to lower asthma trigger levels, will have a significant impact on the quality of outdoor and indoor ambient air at school facilities, In the present studies we have analyzed the baseline levels of asthma triggering contaminants of automobile and bus exhaust (nitrogen dioxide, and particulates) at three preschool facilities in Orange County, NY, to develop a community-based effort to incorporate ano idling policy for preschools as part of their environmental program. At the school entrances and in the outdoor play areas on weekdays when students were present, NO2 levels ranging from 22-24 parts/billion were observed. When children were absent, on the weekends and in the evening, NO2 levels ranged from 14-19 parts/billion. Similarly traffic related particle numbers exhibited dramatic increases when vehicular traffic increased during the times of student arrival and departure. Plans to change traffic flow during student arrival and departure and to raise awareness of the effects of car idling through advocacy have been developed. A follow-up study measuring changes in exhaust levels and community compliance as a result of the no idling policies at these three sites is planned. Our goal is to incorporate translational research components of the air quality and moving population studies into the community research process in the longer term. This effort will

not only contribute to the existing research, but will be available as a template for other communities to apply towards reducing vehicle exhaust
SN - 1073-449X
AD - (Heck, Shakarjian) New York Medical College, Valhalla, NY, United States (Fan) UMDNJ, Robert Wood Johnson Medical School, Piscataway, NJ, United States (Laumbach) EOHSI, Piscataway, United States
ER -

TY - JOUR
ID - 3448
T1 - Impact of exercise-related respiratory symptoms on physical activities in children with asthma: Results from the Eib landmark survey
A1 - Stoloff,S.
A1 - Ostrom,N.
A1 - Eid,N.
A1 - Colice,G.
A1 - Craig,T.
A1 - Hayden,M.
A1 - Parsons,J.P.
Y1 - 2011///
N1 - 70848722
English
Journal: Conference Abstract
KW - EMBASE
KW - Physical Activity
KW - Child
KW - Human
KW - Asthma
KW - Society
KW - Exercise
KW - Drug Therapy
KW - wheezing
KW - parent
KW - breathing
KW - coughing
KW - dyspnea
KW - Adolescent
KW - patient
KW - Prevalence
KW - sport
KW - chest tightness
KW - Therapy
KW - Health
KW - Prevention
KW - Adult
KW - salbutamol
RP - NOT IN FILE
JF - American Journal of Respiratory and Critical Care Medicine
VL - Conference: American Thoracic Society International Conference, ATS 2011 Denver, CO United States. Conference Start: 20110513 Conference End: 20110518. Conference Publication:
IS - var.pagings
N2 - Rationale: It is estimated that the majority of people (adults and children) with asthma experience exercise-related respiratory symptoms. However, despite their high prevalence, there has not been a large analysis that has focused on the impact of exercise-related respiratory symptoms on physical activities of children with asthma. Methods: The national EIB Landmark Survey was conducted among parents of 516 children (4-17 years) with asthma or taking medications for asthma in the prior year. Responses assessed whether up to 6 exercise-related respiratory symptoms (shortness of breath, wheezing, coughing, difficulty taking deep breaths, noisy breathing, chest tightness) influenced participation in physical activities. Subgroup

analyses (4-12 and 13-17 years) were also conducted. The survey was sponsored by Teva Respiratory LLC. Results: The majority (80%) of parents with children with asthma reported that their child experienced >1 exercise-related respiratory symptom, and 47% reported >4 symptoms. The most commonly reported symptoms were coughing, shortness of breath, and wheezing. Older children were more likely to avoid activities because of exercise-related respiratory symptoms compared with younger children (31.9% vs 22.9%). Of respondents whose child experienced >1 exercise-related symptom, 37% reported that asthma limited their child's ability to participate "a lot" or "some" in sports/recreation, 26% in normal physical exertion, and 33% in other outdoor activities. A higher percentage of older children were reported to be limited "a lot" in sports participation compared with younger children (13% vs 9%). Only 23% of children with asthma and exercise-related symptoms were reported to take quick-relief medications such as albuterol "always" or "most of the time" prior to exercising; this proportion was higher in older than in younger children (30% vs 19%). Similarly, only 28% of children with asthma with exercise-related symptoms were reported to take quick-relief medications during or after exercise; this proportion was also higher in older vs younger children (37% vs 22%). Conclusions: This large national survey suggests that exercise-related respiratory symptoms among pediatric asthma patients are common. This may reflect uncontrolled asthma, including lack of preventative treatment for these symptoms. The symptoms reported in this study limited the ability of children to participate normally in physical activities, with adolescents compromised to a greater extent than younger children. Importantly, many children do not use short-acting bronchodilators before exercise as per national guidelines. Adherence to controller therapy and prevention of exercise-related symptoms with short-acting bronchodilators should be optimized to support optimal health in children
SN - 1073-449X
AD - (Stoloff) University of Nevada, Carson City, NV, United States (Ostrom) Allergy and Asthma Medical Group and Research Center, San Diego, CA, United States (Eid) Kosair Children's Hospital, Louisville, United States (Colice) Washington Hospital Center, Washington, DC, United States (Craig) Penn State University, Hershey, PA, United States (Hayden) University of Virginia, Charlottesville, VA, United States (Parsons) Ohio State University, Columbus, OH, United States
ER -

TY - JOUR
ID - 3449
T1 - The role of early-life fungi & childhood home dampness in the development of wheeze
A1 - Behbod,B.
A1 - Sordillo,J.E.
A1 - Hoffman,E.B.
A1 - Gold,D.R.
Y1 - 2011///
N1 - 70848214
English
Journal: Conference Abstract
KW - EMBASE
KW - childhood
KW - wheezing
KW - Society
KW - fungus
KW - Cladosporium
KW - Human
KW - Child
KW - yeast
KW - air sampling
KW - Dust
KW - Risk
KW - Alternaria
KW - Telephone
KW - freezing
KW - ambient air
KW - Temperature
KW - United States

KW - parent
KW - exposure
KW - questionnaire
KW - sensitization
KW - cell wall
KW - colony forming unit
KW - vacuum
KW - Exercise
KW - cold air
KW - health care management
KW - mite
KW - Water
KW - glucan
RP - NOT IN FILE
JF - American Journal of Respiratory and Critical Care Medicine
VL - Conference: American Thoracic Society International Conference, ATS 2011 Denver, CO United States. Conference Start: 20110513 Conference End: 20110518. Conference Publication:
IS - var.pagings
N2 - RATIONALE: We explored the role of early-life (2-3months) fungal exposures and annually repeated reports of visible mold, water damage, or dampness in children's homes experiencing wheeze by age 13. METHODS: 498 children of asthmatic/allergic parents in Boston had early-life indoor air and bedroom floor dust samples collected using Burkard culture plates and Eureka Mighty-Mite canister vacuum cleaners, respectively. 383 children also had outdoor air samples when temperatures were sufficiently warm to avoid culture plates freezing. Telephone questionnaires were administered to primary caretakers to assess annual reports of mold, water damage, or dampness in the home, as well as any reports of wheeze (including with cold air or exercise after age 2). Generalized estimating equations with exchangeable working correlation structures included independent predictors of wheeze and potential confounders. Odds ratios (OR) were estimated for an interquartile increase in fungal log-concentrations (colony forming units per cubic meter of air or gram of dust), and for the positive report of visible home dampness. RESULTS: Independent predictors of longitudinally assessed wheeze included early-life dustborne Cladosporium [OR = 1.14 (1.01, 1.29)] and yeasts [OR = 0.91 (0.82, 1.00)], and repeated reports of visible home dampness [OR = 1.32 (1.12, 1.56)]. In subset analyses including outdoor air samples, predictors of wheeze included outdoor airborne Alternaria [OR = 1.33 (0.95, 1.85)] and Cladosporium [OR = 1.27 (0.92, 1.75)], dustborne Cladosporium [OR = 1.18 (1.01, 1.38)], dustborne yeasts [OR = 0.87 (0.78, 0.97)], and annually repeated reports of visible mold, water damage, or home dampness [OR = 1.24 (1.03, 1.50)]. CONCLUSIONS: Accounting for the correlation in repeated measures, early-life Cladosporium and Alternaria, and longitudinally assessed visible reports of mold, water damage, or home dampness increased the odds of wheeze up to the age of 13. The potential protective role of dustborne yeasts may be related to higher levels of cell wall (1-3)-beta-D-glucan, or other correlated unmeasured factors. Whether these long-term effects of early life mold on the risk of wheeze are due, in part, to an increase in sensitization to mold merits further evaluation
SN - 1073-449X
AD - (Behbod, Hoffman) Harvard School of Public Health, Boston, MA, United States (Sordillo, Gold) Harvard Medical School, Boston, MA, United States
ER -

TY - JOUR
ID - 3451
T1 - The educational BfR-garden of poisonous plants - A public show of plants based on the German classifi cation of poisonous plants
A1 - Hahn,A.
A1 - Begemann,K.
A1 - Keipert,R.
A1 - Graefe,A.
Y1 - 2012///
N1 - 70843741
English
Journal: Conference Abstract

KW - EMBASE
KW - Plant
KW - poisonous plant
KW - Public Health
KW - toxicology
KW - Education
KW - intoxication
KW - Human
KW - toxicity
KW - aconite
KW - Child
KW - fatality
KW - species
KW - Clematis
KW - Convallaria
KW - Europe
KW - forest
KW - habitat
KW - Competence
KW - Food
KW - ingestion
KW - summer
KW - Cyclamen
KW - school
KW - kindergarten
KW - Adult
KW - error
KW - Climate
KW - cation
KW - poison
KW - Oxygen
RP - NOT IN FILE
SP - 622
JF - Clinical Toxicology
VL - Conference: 2012 Annual Meeting of the North American Congress of Clinical Toxicology, NACCT 2012 Las Vegas, NV United States. Conference Start: 20121001 Conference End: 20121006. Conference Publication:
IS - var.pagings
N2 - Objectives: Plants delight us in forests and gardens, they produce oxygen, provide comfortable climatic conditions and constitute the most important fraction of human food. Findings made by mankind have resulted in the knowledge that only a defined share of the plants is inedible or poisonous. Deadly poisonous in Middle Europe are only few plants like e.g. Aconitum napellus. Most of the fatal cases refer to mistakes or misuse in adults, whereas fatalities among children are extremely rare. To get an objective assessment of plants, the BfR run a Federal research project which led to the first German Classifi cation of Poisonous Plants (GCPP) in 2000. After a review of data, provided by German poison centres, completed by new literature and under the competence of a working group of the German "Poison Committee", we are about to publish the first review. To refresh the knowledge around poisonous plants, we are now under the construction of a public garden for poisonous plants. Method: Following the assessment of the toxicity of chemicals in analogy to the German Regulations on Dangerous Substances, we re-classifi ed more than 80 plants into three categories, namely plants which could lead to (-) minor poisoning, (-) moderate poisoning and (-) severe or deadly poisoning. For all plants, the habitat (garden, park, great outdoors) and a comparable amount of a possible ingestion have been borne in mind. Based on the first GCPP, we listed plants for the garden under educational reasons into two garden beds: 1) plants with moderate poisoning and severe or deadly poisoning 2) plants belonging to the category minor poisoning. Results: The garden is situated in a protected institute's area, but with a public access for an interested audience. The raised garden beds will have small trails and visit points, where informative signboards - close to the plants - show details about the name, family, toxic parts of the plant, the category of toxicity, etc. For the garden bed (ca. 4 times 6 meter) of the moderate, severe or deadly poisoning plants, we

chose 15 plants from Aconitum napellus to Convallaria majalis. For the counterpart, the garden bed (ca. 4 times 4 meter) of the minor or non-poisoned plants, we chose another 15 plants from Cyclamen to Clematis. Conclusions: The German Classifi cation of Poisonous Plants contains useful information more than 150 different plant species. The fi nal table of poisonous plants will fi nd its counterpart in a list of more than 60 non-poisonous plants appropriate to be used in the close proximity of children's playgrounds, kindergartens, schools, public parks etc. The design of the garden is for educational purpose and will be opened in summer 2012
SN - 1556-3650
AD - (Hahn, Begemann, Keipert) Federal Institute for Risk Assessment (BfR), Berlin, Germany (Graefe) Gymnasium St. Augustin zu Grimma, Grimma, Germany
ER -

TY - JOUR
ID - 3452
T1 - A case of asthma or pneumonia - Maybe not!
A1 - Ratkiewicz,M.M.
A1 - Radford,P.
A1 - Woodward,J.
Y1 - 2010///
N1 - 70845123
English
Journal: Conference Abstract
KW - EMBASE
KW - Asthma
KW - pneumonia
KW - Society
KW - oxygen saturation
KW - ambient air
KW - Human
KW - Hospital
KW - thorax
KW - dyspnea
KW - cyanosis
KW - arteriovenous fistula
KW - Lung
KW - Child
KW - coccidioidomycosis
KW - headache
KW - pediatrician
KW - sinusitis
KW - Altitude
KW - Female
KW - thorax pain
KW - Diagnosis
KW - neutrophil
KW - Pediatrics
KW - pH
KW - coil embolization
KW - disease course
KW - thorax radiography
KW - interventional radiology
KW - lymphadenopathy
KW - pulmonary artery
KW - arteriovenous malformation
KW - sore throat
KW - sweat
KW - lung function test

KW - fever
KW - pulmonary vein
KW - abnormal respiratory sound
KW - tachypnea
KW - emergency ward
KW - coughing
KW - night
KW - serology
KW - parietal cortex
KW - Rendu Osler Weber disease
KW - vein
KW - thin layer chromatography
KW - Forced Expiratory Volume
KW - pediatric hospital
KW - Oxygen
KW - salbutamol
KW - antibiotic agent
KW - hemoglobin
KW - chloride
KW - bronchodilating agent
KW - steroid
KW - fluticasone propionate plus salmeterol
KW - amoxicillin plus clavulanic acid
RP - NOT IN FILE
JF - American Journal of Respiratory and Critical Care Medicine
VL - Conference: American Thoracic Society International Conference, ATS 2010 New Orleans, LA United States. Conference Start: 20100514 Conference End: 20100519. Conference Publication:
IS - var.pagings
N2 - There is a very large arteriovenous fistula (4.8x2.2x3.2) that appears to originate off the left main pulmonary artery abutting the left major fissure in the superior segment of the left lower lobe. There is a very large draining vein into the left inferior pulmonary vein. There appears to be a smaller AV fistula posteriormedially in the left lung base. Asthma and pneumonia are frequent problems in pediatrics. There may be instances when common findings fail to respond to usual care necessitating a search for alternative explanations. This 9 year old female with a history of asthma presented to her pediatrician's office with a 2 day history of cough, fever, sore throat, chest pain and difficulty breathing. She exercised regularly without limitation. When vacationing at high altitude, she complained of headaches, difficulty breathing, and had perioral cyanosis. Family and social history were unremarkable. When she failed to respond to nebulized albuterol in the office, she was sent to a local emergency room. Chest x-ray revealed a left-sided infiltrate. She was discharged on antibiotics, albuterol and oral steroids. She was admitted to an outside hospital two days later when she was not improved. On admission she was hypoxic with room air oxygen saturations in the mid 80's. CBG showed a PaCO2 of 35 and a pH of 7.43. CBC revealed WBC of 12,400 with slightly elevated neutrophils and hemoglobin of 16.9. Chemistry panel was normal. Chest x-ray confirmed a left lower lobe infiltrate and minimal left hilar adenopathy. CT of the sinuses showed pansinusitis. Coccidiomycosis serologies were negative. She remained hospitalized for 6 nights and received IV antibiotics for pneumonia. She was discharged home, despite room air oxygen saturations of 85%, on Augmentin and Advair with a referral to a pediatric pulmonologist in 1 week. She presented to the Pulmonary Clinic with central cyanosis, mild tachypnea, and room air oxygen saturations of 78%. Findings on physical exam included coarse breath sounds on the left and mild clubbing. Pulmonary function tests showed a FVC of 78%, FEV of 84%, FEF25-75 of 105% and TLC of 158% with a significant response after nebulized bronchodilator. With supplemental oxygen at 5 liters per minute, her oxygen saturation was 88%. She was admitted to Phoenix Children's Hospital. Sweat Chloride was normal. Chest CT angiogram showed a large arteriovenous malformation (AVM) in the superior aspect of the left lower lobe and a smaller AVM in the left lung base. Interventional radiology performed coil embolization with significant clinical improvement. A small AVM was also discovered in the left parietal cortex. She was diagnosed with hereditary hemorrhagic telangiectasia. AVMs in children are rare and easily mistaken for other pulmonary problems. This case is a reminder to re-evaluate the diagnosis when the clinical course is not as anticipated. (Figure presented)
SN - 1073-449X

AD - (Ratkiewicz, Radford, Woodward) Phoenix Children's Hospital, Phoenix, AZ, United States
ER -

TY - JOUR
ID - 3453
T1 - Breastfeeding, aeroallergen sensitization and environmental exposures during infancy are determinants of childhood allergic rhinitis at age three
A1 - Codispoti,C.D.
A1 - Levin,L.
A1 - LeMasters,G.
A1 - Ryan,P.
A1 - Reponen,T.
A1 - Villareal,M.
A1 - Burkle,J.
A1 - Lockey,J.E.
A1 - Khurana Hershey,G.K.
A1 - Bernstein,D.
Y1 - 2010///
N1 - 70845037
English
Journal: Conference Abstract
KW - EMBASE
KW - Environmental Exposure
KW - infancy
KW - childhood
KW - allergic rhinitis
KW - Society
KW - sensitization
KW - Human
KW - exposure
KW - Child
KW - Risk
KW - tree
KW - African American
KW - Food
KW - skin
KW - nose
KW - sneezing
KW - Cohort Analysis
KW - prick test
KW - model
KW - Traffic
KW - Monitoring
KW - Air Pollution
KW - land use
KW - Particulate Matter
KW - house dust
KW - Dust
KW - questionnaire
KW - allergy
KW - allergen
KW - contrast medium
KW - carbon
KW - glucan
KW - endotoxin
RP - NOT IN FILE
JF - American Journal of Respiratory and Critical Care Medicine

VL - Conference: American Thoracic Society International Conference, ATS 2010 New Orleans, LA United States. Conference Start: 20100514 Conference End: 20100519. Conference Publication:
IS - var.pagings
N2 - Rationale: Early life predictors of childhood allergic rhinitis (AR) are not well defined. Few studies have addressed host characteristics and environmental exposure determinants of AR in three year old children. The objective of this study is to identify host characteristics, indoor and outdoor environmental exposures during infancy at age one that predict AR at age three in a longitudinal birth cohort. Methods: High risk children from Greater Cincinnati in the Cincinnati Childhood Allergy and Air Pollution Study (CCAAPS) were followed annually in a large birth cohort study from birth to age three. The primary outcome, allergic rhinitis (AR) was defined by parental report of sneezing, runny or blocked nose in the prior 12 months and a positive skin prick test (SPT) to at least one of 15 aeroallergens. AR children were compared to non-atopic, non-symptomatic children. Environmental and standardized medical questionnaires determined exposures and clinical outcomes. Primary activity area dust samples were analyzed for house dust endotoxin (HDE) and (1-3)-beta-D-glucan. Fine particulate matter (PM_{2.5}) sampled at 27 monitoring stations were used to estimate personal elemental carbon attributable to traffic (ECAT) exposure by land use regression model. Results: Of 361 children in this analysis, 116 had AR and 245 were non-atopic, non-symptomatic. Prolonged breastfeeding in African-American children (aOR 0.8; 95% CI 0.6-0.9) and multiple children in the home during infancy were protective of AR (aOR 0.4; 95% CI 0.2- 0.8). Food SPT positivity and tree SPT positivity at age one increased the risk of AR at age three (aOR 4.4; 95% CI 2.1- 9.2) and (aOR 6.8; 95% CI 2.5- 18.7), respectively. HDE exposure was associated with AR, with low and high HDE exposure being protective, (aOR, 0.5; 95% CI, 0.3-0.8) and (aOR, 0.002; 95% CI, <0.001-0.1), respectively. In contrast, medium HDE exposure was associated with an increased risk of AR (aOR, 6.3; 95% CI, 2.3-17.2). ECAT and ETS exposure showed no effect on AR. Conclusions: Prolonged breastfeeding in African-Americans and multiple children in the home during infancy reduced the risk of AR at age three whereas percutaneous reactivity to food and tree allergens enhanced risk. HDE exposure modified the risk of AR bi-directionally depending on the level of HDE exposure
SN - 1073-449X
AD - (Codispoti, Levin, LeMasters, Ryan, Reponen, Villareal, Burkle, Lockey, Bernstein) University of Cincinnati, Cincinnati, OH, United States (Khurana Hershey) Cincinnati Children's Hospital Medical Center, Cincinnati, OH, United States
ER -

TY - JOUR
ID - 3454
T1 - Myocarditis and severe myositis in a 14-year-old female with 2009 H1N1 influenza virus infection
A1 - Silverman,M.A.
A1 - Sandora,T.
A1 - Sharara-Chami,R.
Y1 - 2010///
N1 - 70844370
English
Journal: Conference Abstract
KW - EMBASE
KW - myocarditis
KW - myositis
KW - Female
KW - Influenza virus
KW - virus infection
KW - Society
KW - Human
KW - United States
KW - virus
KW - Echocardiography
KW - pandemic influenza
KW - seasonal influenza
KW - influenza A
KW - laboratory
KW - diseases

KW - Influenza
KW - emergency ward
KW - Hospital
KW - Child
KW - ST segment elevation
KW - anesthesiology
KW - sinus tachycardia
KW - physiology
KW - sepsis
KW - intensive care
KW - immunoassay
KW - Mexico
KW - Swine
KW - Infection
KW - thorax radiography
KW - Rana
KW - Influenza virus A
KW - cardiogenic shock
KW - heart left ventricle failure
KW - Diagnosis
KW - swine influenza
KW - tube
KW - exposure
KW - Intubation
KW - pleura effusion
KW - weakness
KW - Muscle
KW - inotropism
KW - Hospitalization
KW - patient
KW - Drug Therapy
KW - Inflammation
KW - fever
KW - World Health Organization
KW - pediatric hospital
KW - oseltamivir
KW - cefepime
KW - vancomycin
KW - infusion fluid
KW - antivirus agent
KW - antibiotic agent
KW - immunoglobulin
KW - troponin T
RP - NOT IN FILE
JF - American Journal of Respiratory and Critical Care Medicine
VL - Conference: American Thoracic Society International Conference, ATS 2010 New Orleans, LA United States. Conference Start: 20100514 Conference End: 20100519. Conference Publication:
IS - var.pagings
N2 - Myocarditis and Severe Myositis in a 14-year-old Female with 2009 H1N1 Influenza Virus Infection Michael A. Silverman, MD PhD, Thomas J. Sandora, MD MPH, and Rana I. Sharara-Chami, MD Department of Medicine, Division of Infectious Diseases, Departments of Medicine and Laboratory Medicine, Division of Critical Care Medicine, Department of Anesthesiology, Children's Hospital Boston, Boston Massachusetts. Introduction 2009 H1N1 influenza virus, a novel influenza A subtype of swine origin, was first detected in humans in Mexico in March 2009. This virus spread rapidly and the WHO declared an influenza pandemic on June 11, 2009. Myocarditis and severe myositis are uncommon but potentially severe manifestations of seasonal influenza. We present the first reported case of myocarditis and severe myositis in association with 2009 H1N1 virus infection. Case Description In June 2009, a previously healthy 14-year-old female presented to an outside

hospital emergency department in shock after two days of fever, muscle aches and diffuse weakness. She reported exposure to "swine flu". Her chest radiograph and bedside echocardiogram were normal. She received intravenous fluids, oseltamivir, vancomycin and cefepime. A nasopharyngeal swab was positive for influenza A by rapid immunoassay and was subsequently subtyped as 2009 H1N1 virus. In our emergency department, she had sepsis physiology and an S3 gallop. EKG demonstrated sinus tachycardia with ST elevation in the lateral leads. Bedside echocardiogram showed mild to moderate left ventricular dysfunction. Troponin T level was initially 0.04 ng/mL and peaked at 0.8 ng/mL. During the course of her hospitalization, she received inotropic support, intravenous immunoglobulin, oseltamivir and broad-spectrum antibiotics. Her illness was complicated by severe myositis (maximum CK 86,900 U/mL) and bilateral transudative pleural effusions requiring intubation and chest tube drainage. She recovered fully from her illness. Discussion A 14-year-old female presented with shock and 2009 H1N1 influenza virus infection. Echocardiogram and laboratory studies indicated myocarditis and severe myositis. 2009 H1N1 influenza infection should be considered in children with shock, myocarditis or severe myositis. Myocarditis and myositis are important complications of both seasonal influenza and 2009 H1N1 virus, and they require a high clinical suspicion for diagnosis. Aggressive management of cardiogenic shock and renal protective strategies are important aspects of care for patients with influenza myocarditis and myositis, and treatment with IVIG and appropriate antiviral medication to inhibit viral activity and suppress inflammation may have added benefit
SN - 1073-449X
AD - (Silverman, Sandora, Sharara-Chami) Children's Hospital Boston, Boston, MA, United States
ER -

TY - JOUR
ID - 3455
T1 - The impact of pet ownership on microbial communities in settled house dust
A1 - Fujimura,K.
A1 - Johnson,C.C.
A1 - Ownby,D.R.
A1 - Cox,M.J.
A1 - Brodie,E.
A1 - Zoratti,E.
A1 - Woodcroft,K.
A1 - Bobbitt,K.R.
A1 - Wegienka,G.
A1 - Boushey,H.
A1 - Lynch,S.V.
Y1 - 2010///
N1 - 70843081
English
Journal: Conference Abstract
KW - EMBASE
KW - organization and management
KW - microbial community
KW - house dust
KW - Society
KW - pet animal
KW - household
KW - dog
KW - cat
KW - exposure
KW - allergic disease
KW - community structure
KW - fungal community
KW - taxon
KW - Infant
KW - Human
KW - Cluster Analysis
KW - Child

KW - disease course
KW - hypothesis
KW - density
KW - Asthma
KW - plant disease
KW - Student t test
KW - species
KW - Proteobacteria
KW - Dust
KW - allergy
KW - fungus
KW - atopy
KW - spacer DNA
KW - ribosome RNA
KW - RNA 16S
RP - NOT IN FILE
JF - American Journal of Respiratory and Critical Care Medicine
VL - Conference: American Thoracic Society International Conference, ATS 2010 New Orleans, LA United States. Conference Start: 20100514 Conference End: 20100519. Conference Publication:
IS - var.pagings
N2 - RATIONALE: High rates of allergic disease in Westernized countries have led to the hypothesis that lack of microbial exposures early in life may play a key role in subsequent allergy and asthma development. Furred pet ownership has been shown in many studies to be protective against allergic disease development in children which we hypothesize is due to the distinct household microbial communities associated with pet ownership. METHODS: Dust samples from seventeen households with dog(s), cat(s) or no pets were examined. The 16S rRNA PhyloChip, a high-density bacterial microarray and automated rRNA intergenic spacer analysis and sequencing were used to profile bacterial and fungal communities. Hierarchical cluster analysis (HCA) was used to examine community structure. Wilcoxon or Welch's t-tests were performed to determine differences in community structure, metrics (diversity, richness, and evenness) and taxonomic abundances across groups. RESULTS: Bacterial communities from dog owning households were significantly richer ($p<0.036$), more diverse ($p<0.036$) and possessed 45 taxa in significantly higher abundance compared to those with no pets. The majority of these taxa were Proteobacteria, typically detected in early stages of infant GI colonization. Parallel analysis of fungal communities demonstrated significantly lower fungal richness in dog-owning households compared to cat ($p<0.010$) or no pet homes ($p<0.031$). Fungal species detected primarily belonged to the Dothideomycetidaceae and Eurotiomycetidae, which include plant pathogens and allergenic fungi. HCA demonstrated two clearly distinct groups of samples (G1 and G2). Compared with G2 samples, G1 bacterial communities were significantly richer ($p<0.008$), more even ($p<0.002$), more diverse ($p<0.001$) and were from dog and cat-owning households. In comparison, G2 samples included all of the no pet households and a number of dog- and cat-owning homes which possessed animals that were either exclusively indoor or outdoor pets or were very young and recently introduced to the home. CONCLUSION: These data suggest that homes in which pets are permitted both outdoors and indoors possess significantly higher bacterial diversity that coincide with reduced fungal richness. We speculate that infant exposure to this milieu may shape immune development by a number of potential mechanisms that reduce development of atopic disease
SN - 1073-449X
AD - (Fujimura) UCSF, San Francisco, CA, United States (Johnson, Zoratti, Woodcroft, Bobbitt, Wegienka) Henry Ford Hospital, Detroit, MI, United States (Ownby) Medical College of Georgia, Augusta, GA, United States (Cox, Boushey, Lynch) University of California, San Francisco, CA, United States (Brodie) University of California, Berkeley, United States
ER -

TY - JOUR
ID - 3456
T1 - Environmental effects on exercise-induced bronchospasm in healthy children
A1 - Singhal,S.
A1 - Vedal,S.
Y1 - 2010///

N1 - 70842075
English
Journal: Conference Abstract
KW - EMBASE
KW - bronchospasm
KW - Child
KW - Human
KW - Society
KW - Exercise
KW - Humidity
KW - Temperature
KW - Risk
KW - Air Pollution
KW - Spirometry
KW - Asthma
KW - environmental factor
KW - community sample
KW - Population
KW - Forced Expiratory Volume
KW - Canada
KW - exposure
KW - seashore
KW - weight
KW - city
KW - pulp mill
KW - logistic regression analysis
KW - school child
KW - laboratory
KW - population research
KW - Prevalence
KW - Female
KW - Male
KW - Diagnosis
KW - physician
KW - questionnaire
KW - Exercise Test
KW - height
KW - Particulate Matter
KW - Monitoring
KW - independent variable
KW - meteorology
RP - NOT IN FILE
JF - American Journal of Respiratory and Critical Care Medicine
VL - Conference: American Thoracic Society International Conference, ATS 2010 New Orleans, LA United States. Conference Start: 20100514 Conference End: 20100519. Conference Publication:
IS - var.pagings
N2 - Rationale: There is little information on the effects of environmental factors, such as meteorological conditions and air pollution, on exercise-induced bronchospasm (EIB) in population studies. While controlled laboratory exposure studies show that cold, dry air has a greater effect on EIB than warm, humidified air, there is little demonstration of this in real-world settings. Also, while epidemiological studies of asthmatic children have shown an association between air pollution and EIB, few studies have assessed air pollution effects on EIB in healthy children. In this cross-sectional analysis based on a large population community sample, we assess the short-term relationship between environmental factors such as temperature, humidity, and air pollution and EIB in healthy children. Methods: The community sample consisted of all 2078 school children from grades one through six in the pulp-mill town of Port Alberni on the west coast of Vancouver Island, Canada. Each child underwent pre-exercise spirometry, then post-exercise spirometry 10 minutes after a 5-minute supervised run outdoors. A central monitoring station measured daily environmental factors including temperature, humidity,

and airborne particulate matter (PM₁₀) and total suspended particles (TSP) concentrations. The final sample consisted of children who completed the supervised run, met ATS acceptability criteria for spirometry at both the pre- and post-exercise test, and had PM₁₀ measured on the day of testing. Based on parental questionnaire, children with doctor's diagnosis of current asthma were excluded. EIB was defined in three ways: (1) the difference between pre- and post-exercise FEV₁ (FEV-absolute), (2) the ratio (%) of FEV-absolute over baseline FEV₁ (FEV-relative), and (3) a decrease in post-exercise FEV₁ relative to pre-exercise exceeding 9% (EIB-positive). Results: The final population sample consisted of 1,544 children aged 6 to 13 (774 males, 770 females). Median FEV-absolute was 0.010L (interquartile range [IQR] -0.003, 0.070), median FEV-relative was 0.63% (IQR -1.81%, 3.47%), and EIB-positive prevalence was 6.4%. Median PM₁₀ concentration was 17mg/m (IQR 17, 33). Multiple linear and logistic regression analyses that included age, sex, race, height, 3 and weight, and same-day temperature, humidity, and PM₁₀ showed consistent relationship between decreasing temperature and increasing EIB (Table 1). Lower humidity also tended to be associated with increased EIB. PM₁₀ had no association with risk of EIB. Results were unchanged with baseline FEV₁ included as an independent variable. Conclusions: In children without asthma, lower temperature increases risk of EIB. Lower humidity contributes marginally to increasing risk of EIB. Increasing PM₁₀ concentrations does not increase risk of EIB. (Table Presented)
SN - 1073-449X
AD - (Singhal, Vedal) University of Washington, Seattle, WA, United States
ER -

TY - JOUR
ID - 3457
T1 - The relationship between vitamin D status and body composition in pre-or early pubertal nonobese children
A1 - Lee,Y.A.
A1 - Kim,J.Y.
A1 - Kang,M.J.
A1 - Kim,S.M.
A1 - Chung,S.J.
A1 - Shin,C.H.
A1 - Yang,S.W.
Y1 - 2012///
N1 - 70834563
English
Journal: Conference Abstract
KW - EMBASE
KW - Body Composition
KW - Child
KW - Human
KW - Society
KW - vitamin D deficiency
KW - childhood
KW - Prevalence
KW - Puberty
KW - Physical Activity
KW - winter
KW - spine
KW - bone mineral
KW - risk factor
KW - prepuberty
KW - Korea
KW - dietary reference intake
KW - tissues
KW - calcium intake
KW - boy
KW - Male

KW - questionnaire
KW - blood level
KW - Bone Density
KW - dietitian
KW - dual energy X ray absorptiometry
KW - Bone
KW - Health
KW - vitamin D
KW - vitamin
KW - Calcium
RP - NOT IN FILE
JF - Endocrine Reviews
VL - Conference: 94th Annual Meeting and Expo of the Endocrine Society, ENDO 2012 Houston, TX United States. Conference Start: 20120623 Conference End: 20120626. Conference Publication:
IS - var.pagings
N2 - Background: The 2010 Dietary reference intakes for Koreans lowered adequate intake (AI) of vitamin D to 200 IU/day in childhood due to lack of evidence. We evaluated the prevalence of vitamin D deficiency and risk factors for low vitamin D status. We also investigated the relationship between vitamin D status and body composition and bone mineral density (BMD) in pre- or early pubertal nonobese children. We purposed to provide evidence for determining Korean AI of vitamin D. Methods: A total 100 nonobese children (9.3 + 1.9yrs, 45 boys, 71 prepuberty) living in Seoul, Korea were enrolled in wintertime (n = 38, December, 2010 to March, 2011) and summertime (n = 62, June to September, 2011). Z score of the bone mineral content (Z-BMC), fat mass (Z-FM), lean tissue mass (Z-LTM), and total body (Z-TB) and L-spine 1-4 (Z-L1-4) BMD using Dual-energy X-ray absorptiometry were calculated according to the reference for Korean children. Daily calcium (mg/day) and vitamin D intake (U/day) were evaluated by dietitian. The serum level of 25(OH)D below 20ng/mL was defined as vitamin D deficiency. Questionnaires were used to assess regular physical activity and outdoor activities (hrs/week). Results: The prevalence of vitamin D deficiency was 47.4% in winter and 17.7% in summertime. In wintertime, vitamin D status negatively associated with vitamin D intake (P = 0.019) and less outdoor activities (P = 0.015), after adjusting for sex and puberty. About half of children with vitamin D intake below 800 IU/day were vitamin D deficient in winter. The level of 25(OH)D negatively correlated with Z-BMC (P = 0.023), Z-TB (P = 0.018), and Z-L1-4 (P = 0.043), after controlling for sex, puberty, Z-FM, physical activity and the percentage of the adequate calcium intake. However, the Z-FM and Z-LTM had no significant relationship with the level of 25(OH)D. Conclusion s: About half of pre- and early pubertal nonobese children were vitamin D deficient in wintertime. Considering the effect of vitamin D status on bone health in childhood, the importance of adequate vitamin D intake and outdoor activities needs to be emphasized. Current recommendation for AI of vitamin D (200IU/day) in Korean children seems not to be enough to prevent vitamin D deficiency, especially in wintertime
SN - 0163-769X
AD - (Lee, Kim, Kang, Chung, Shin, Yang) Pediatircs, Seoul National University, Children's Hospital, Seoul, South Korea (Kim) Pediatrics, Seoul Red Cross Hospital, Seoul, South Korea
ER -

TY - JOUR
ID - 3458
T1 - Profound ictal apnea in PCDH19 related epilepsy: 2 cases with novel phenotypic and genetic findings
A1 - Nash,K.B.
A1 - Ahronowitz,I.
A1 - Sullivan,J.
Y1 - 2011///
N1 - 70831221
English
Journal: Conference Abstract
KW - EMBASE
KW - Epilepsy
KW - apnea
KW - Society
KW - seizure

KW - gene
KW - Female
KW - Human
KW - girl
KW - fatty acid desaturation
KW - symptom
KW - brain
KW - theta rhythm
KW - eye
KW - Arm
KW - electroencephalogram
KW - inheritance
KW - model
KW - exon
KW - Sequence Analysis
KW - family history
KW - X chromosome
KW - nuclear magnetic resonance imaging
KW - Child
KW - newborn period
KW - heterozygosity
KW - DNA microarray
KW - Genome
KW - mental deficiency
KW - phenobarbital
KW - topiramate
RP - NOT IN FILE
JF - Epilepsy Currents
VL - Conference: 64th Annual Meeting of the American Epilepsy Society, AES and 3rd Biennial North American Regional Epilepsy Congress San Antonio, TX United States. Conference Start: 20101203 Conference End: 20101207. Conference Publication:
IS - var.pagings
N2 - Rationale: Epilepsy and Mental Retardation Limited to Females (EFMR) is a rare condition in which otherwise normal female infants develop sudden onset of seizures. The clinical spectrum is broad, including a Dravet-like syndrome with multiple seizure types. The disease-causing gene was recently identified as PCDH19, which resides on the X chromosome and encodes protocadherin 19. Ictal apnea (IA) has not been reported in this syndrome, and the genetic mechanism by which alteration of PCDH19 results an atypical X-linked inheritance pattern is uncertain. Methods: Case 1: Whole genome array CGH was performed using the SignatureChip 105K oligonucleotide array (10-35 Kb spatial resolution). Case 2: Standard targeted sequence analysis of exons 1-6 of the PCDH19 gene was performed. Results: Case 1: An otherwise healthy 19 mo old girl presented with new onset seizures at age 11 mos. Initial seizure semiology was bilateral upper extremity clonic movements and unresponsiveness. Seizures were refractory to multiple AED trials but finally stopped with topiramate. At age 18 mos she had another seizure cluster with eye opening, upper extremity flexion, and profound apnea with desaturation to SaO2 of 10%. Ictal EEG revealed biposterior onset of rhythmic sharp theta activity with rapid secondary generalization. Inter-ictal EEG was normal. Brain MRI was normal. Family history was negative. CGH microarray detected a deletion in Xq21.33-Xq22.1 (estimated size 2.89-3.01 Mb), which contains the PCDH19 gene. Case 2: An otherwise healthy 22 mo girl presented with new onset seizures at age 8 mos. Initial seizure semiology was eye opening, variable extremity movements, unresponsiveness, and apnea with desaturation to a SaO2 of 50%. Seizures were refractory to multiple AED trials but ultimately controlled on phenobarbital. At age 20 months, she had another seizure cluster with similar semiology and profound ictal apnea with desaturation to SaO2 of 6%. Ictal EEG showed biposterior onset of rhythmic sharp theta activity with rapid secondary generalization. Interictal EEG was normal. Brain MRI was normal. Family history was negative. PCDH19 sequence analysis revealed heterozygosity for c.434-435insG alteration in exon 1 of the PCDH19 gene, which has not been previously reported. Conclusions: Our findings have two fundamental implications. First, profound IA is unusual and has not been described in the setting of a specific epilepsy syndrome outside of the neonatal period. These two cases suggest IA may be a unique seizure type in children with PCDH19 related epilepsy. Additional cases are necessary to determine the strength of this

association. Second, previous authors have proposed a dominant negative model to explain the atypical X-linked inheritance pattern. Case #1 is the first affected female with a deletion of the PCDH19 gene, which represents the most compelling evidence to date that the dominant negative model is incorrect
SN - 1535-7597
AD - (Nash, Sullivan) Neurology, University of California, San Francisco, CA, United States (Ahronowitz) School of Medicine, University of California San Francisco, San Francisco, CA, United States
ER -

TY - JOUR
ID - 3459
T1 - Metabolic and molecular imaging characteristics of epileptogenic dysembryoplastic neuroepithelial tumors (DNETS)
A1 - Alkonyi,B.
A1 - Muzik,O.
A1 - Chugani,D.C.
A1 - Zitron,I.
A1 - Chugani,H.T.
A1 - Sood,S.
A1 - Mittal,S.
A1 - Chakraborty,P.K.
A1 - Juhasz,C.
Y1 - 2011///
N1 - 70831084
English
Journal: Conference Abstract
KW - EMBASE
KW - Epilepsy
KW - neuroepithelioma
KW - Society
KW - molecular imaging
KW - Tumor
KW - Human
KW - seizure
KW - Child
KW - metabolic rate
KW - Arousal
KW - tumor volume
KW - patient
KW - glucose metabolism
KW - Kinetics
KW - surgery
KW - positron emission tomography
KW - Metabolism
KW - metabolite
KW - Immunity
KW - tryptophan metabolism
KW - staining
KW - congenital malformation
KW - tryptophan
KW - kynurenine
KW - tracer
KW - glucose
KW - indoleamine 2,3 dioxygenase
KW - amino acid transporter
KW - carbon 11
KW - fluorine 18
KW - enzyme

RP - NOT IN FILE
JF - Epilepsy Currents
VL - Conference: 64th Annual Meeting of the American Epilepsy Society, AES and 3rd Biennial North American Regional Epilepsy Congress San Antonio, TX United States. Conference Start: 20101203 Conference End: 20101207. Conference Publication:
IS - var.pagings
N2 - Rationale: DNETs are low-grade, epileptogenic tumors with low glucose metabolism on PET. Our recent studies showed accumulation of a[11C]methyl-L-tryptophan (AMT), a PET tracer for the immunomodulatory kynurenine pathway, in epileptogenic malformations and some DNETs. In this study we used 2-deoxy-2[18F]fluoro-D-glucose (FDG) and dynamic AMT PET scanning to evaluate the relation between glucose metabolism, AMT transport and metabolism as well as clinical seizure variables in children with epileptogenic DNETs. Resected DNET specimens were also studied for the presence of Ltype amino acid transporter 1 (LAT1) and indoleamine 2,3-dioxygenase (IDO; a key enzyme of the kynurenine pathway). Methods: Twelve children (age: 2.5-17.5 years) who underwent resective surgery due to epilepsy associated with a DNET were included in the study. Tumors were outlined on MRIs and tumor volumes were calculated. The same tumor regions were superimposed on co-registered FDG and AMT PET images to obtain standardized uptake values (SUVs) and SUV ratios (tumor/contralateral cortex). Furthermore, kinetic parameters of AMT uptake characterizing the tracer transport (by the volume of distribution [VD]) and metabolic rate (by the unidirectional tracer uptake [K-complex]), as well as corresponding tumor/cortex ratios were also calculated in 10 patients and correlated with seizure variables. Resected tumor specimens were immuno-stained for IDO and LAT1. Results: Tumor volumes varied from 0.8 to 51.4 cm3 (median=4.2 cm3). All tumors showed glucose hypometabolism on FDG PET scans (SUV ratio: 0.29-0.64). However, tumor AMT SUV values were above cortical values in 8/12 cases (0.87-1.44) (Figure). Two patients showed increased AMT SUV in additional ipsilateral cortical areas. Kinetic analysis of AMT PET showed increased VD ratios in 8/10 children and high K-complex ratios in 3. Age at seizure onset showed a positive association with FDG SUV of the tumors (r=0.68; p=0.016, Spearman's correlation). Longer epilepsy duration was associated with higher AMT K-complex values in DNETs (p=0.036). Tumor volumes did not correlate with any clinical and PET variables. Nine children had seizure-free outcome, while 3 had recurrent seizures, including the two cases with preoperative cortical AMT increases in areas which were not resected. All tumor specimens showed moderate to intense IDO and LAT1 staining. Conclusions: Despite low glucose metabolic rates and variable sizes, DNETs commonly show high AMT accumulation on PET, which is mostly driven by increased tryptophan transport but also due to tryptophan metabolism. Activation of kynurenine pathway may contribute to immune-resistance of these tumors but can also produce neurotoxic/epileptogenic metabolites. Association of higher tryptophan metabolic rates with longer epilepsy duration suggests a progressive activation of this pathway providing a potential therapeutic target for these lesions. In addition, AMT PET may detect potentially epileptogenic cortex outside DNETs in some cases; this may help optimize surgical resection to facilitate seizure-free outcome
SN - 1535-7597
AD - (Alkonyi, Chugani, Chugani, Juhasz) Division of Pediatric Neurology, Wayne State University, Detroit, MI, United States (Alkonyi, Muzik, Chugani, Chugani, Chakraborty, Juhasz) PET Center, Children's Hospital of Michigan, Detroit, MI, United States (Muzik, Chakraborty) Department of Radiology, Wayne State University, Detroit, MI, United States (Sood, Mittal) Department of Neurosurgery, Wayne State University, Detroit, MI, United States (Zitron, Mittal) Karmanos Cancer Institute, Detroit, MI, United States
ER -

TY - JOUR
ID - 3460
T1 - Invasive intracranial monitoring in pediatric epilepsy surgery: When useful and when not
A1 - Brna,P.
A1 - Jayakar,P.
A1 - Dunoyer,C.
A1 - Duchowny,M.
A1 - Resnick,T.
Y1 - 2011///
N1 - 70830791
English
Journal: Conference Abstract
KW - EMBASE

KW - Epilepsy
KW - Monitoring
KW - surgery
KW - Society
KW - Human
KW - implantation
KW - electroencephalogram
KW - scalp
KW - nuclear magnetic resonance imaging
KW - temporal lobectomy
KW - patient
KW - Child
KW - Imaging
KW - functional neuroimaging
KW - brain
KW - temporal lobe epilepsy
KW - Adult
KW - epileptic focus
KW - motor cortex
KW - temporal lobe
KW - data base
KW - hemisphere
KW - Language
KW - pediatric hospital
KW - nitrogen 15
RP - NOT IN FILE
JF - Epilepsy Currents
VL - Conference: 64th Annual Meeting of the American Epilepsy Society, AES and 3rd Biennial North American Regional Epilepsy Congress San Antonio, TX United States. Conference Start: 20101203 Conference End: 20101207. Conference Publication:
IS - var.pagings
N2 - Rationale: Indications for invasive subdural monitoring in pediatric epilepsy surgery are poorly defined.We sought to critically review our experience with pediatric invasive intracranial monitoring to better delineate when invasive EEG is useful. Methods: Patients who had subdural invasive EEG monitoring for epilepsy surgery were identified from an epilepsy surgery database at Miami Children's Hospital Brain Institute. Exclusion criteria were inadequate subdural data and children with infantile spasms/myoclonic epilepsy. Pre-invasive evaluations were retrospectively reviewed including history, interictal/ictal scalp EEG,MRI and functional imaging in selected cases. Indications for subdural implantation were categorized based on pre-invasive data as :1) Mapping of eloquent cortex 2) Poorly localized epileptogenic zone(EZ)3) Discordant data.Subdural EEG reports,surgical diagrams and functional mapping were reviewed for localization of the EZ and compared with pre-invasive data for congruence. Data on surgical resection, completeness, complications and outcome were analyzed. Results: 102 patients met inclusion criteria.12 were implanted for mapping eloquent cortex,76 for non-localized EZ and 14 for divergent data. In the non-localized group 45% had negative MRIs. A focal area for resection was identified in 99%(68% incomplete). Subdural monitoring impacted resection in 84%. Implantation for functional mapping facilitated incomplete resections in 42% due to proximity to eloquent cortex. Of cases implanted solely for language mapping in a presumed dominant hemisphere,5 were redundant as all underwent anterior left temporal lobectomy. Implantations to determine the extent of involvement beyond the mesial/anterior temporal region were rarely useful particularly if there was a focal MRI lesion.90% resulted in anterior temporal lobectomies. Most poorly localized cases were multilobar (paracentral n=21, frontotemporal n=15, posterior quadrant n=9,other multilobar n=15). 1 paracentral case resulted in explantation.The EZ was outside motor cortex in 38% and only partially overlapped motor in several cases resulting in 5 complete resections. Frontotemporal implantations resulted in lobar resections in 73%. Posterior quadrant and other multilobar implantations usually resulted in more restricted resections than anticipated by non-invasive data(79%)and no quadrantectomies. 14 cases were implanted for discordant data with 13 resolving the discordance. With divergent scalp EEG and MRI subdural EEG more often was concordant with MRI than scalp EEG(71% vs 28%). Conclusions: There is limited pediatric data guiding the use of intracranial monitoring to localize an epileptogenic focus for resective surgery. This study provides an overview of current indications

at an active pediatric epilepsy surgery centre and examines the utility of those practices. We confirmed the adult experience that subdural monitoring is rarely helpful for MRI-lesional temporal lobe epilepsy. Routine use of functional neuroimaging modalities may obviate the need for many invasive cases previously deemed poorly localized
SN - 1535-7597
AD - (Brna) Pediatrics, IWK Health Centre, Halifax, NS, Canada (Jayakar, Dunoyer) Brain Institute, Miami Children's Hospital, Miami, FL, United States (Duchowny, Resnick) Neurology, Miami Children's Hospital, Miami, FL, United States
ER -

TY - JOUR
ID - 3462
T1 - Methods of sleep deprivation in children undergoing sleep-deprived EEG
A1 - Sharma,S.
A1 - Nair,R.R.
Y1 - 2011///
N1 - 70830588
English
Journal: Conference Abstract
KW - EMBASE
KW - Epilepsy
KW - Human
KW - Child
KW - Sleep Deprivation
KW - Sleep
KW - Society
KW - electroencephalogram
KW - parent
KW - patient
KW - Hospital
KW - night
KW - seizure
KW - questionnaire
KW - Female
KW - Male
KW - Anxiety
KW - groups by age
KW - summer
KW - clinical practice
KW - season
KW - diagnostic procedure
KW - outpatient department
KW - Pediatrics
KW - neurology
KW - ethnic group
KW - Diagnosis
KW - pediatric hospital
RP - NOT IN FILE
JF - Epilepsy Currents
VL - Conference: 64th Annual Meeting of the American Epilepsy Society, AES and 3rd Biennial North American Regional Epilepsy Congress San Antonio, TX United States. Conference Start: 20101203 Conference End: 20101207. Conference Publication:
IS - var.pagings
N2 - Rationale: Sleep-deprived EEGs (SDEEG) are commonly used in clinical practice to detect sleep-induced epileptiform abnormalities, which are diagnostic indicators of specific types of epilepsy. While this is a very useful diagnostic technique, studies have shown that the SDEEG places a notable burden on both parents and children. One study reported that nearly fifty percent of parents found it difficult to keep their child awake at

night and thirty percent of parents found it difficult to wake their child in the morning (1). Objective: To prospectively study the specific strategies used by parents and/or children to ensure sleep deprivation before undergoing a SDEEG. The findings of this study will be used to provide guidance for parents and children on how to adopt a convenient method of sleep deprivation. Methods: Inclusion Criteria: 1) Children between 1-17 years who either had seizures or were suspected to have seizures. 2) Male and female participants were included, with no restrictions based on race or ethnic origin. Children referred from the neurology and general pediatrics outpatient clinics at McMaster Children's Hospital for a SDEEG during May 2008-ongoing, fulfilling the inclusion criteria, were studied. All participants were interviewed by either an EEG technologist or co-investigator and were administered a qualitative questionnaire. The questionnaire collected data about the patient's demographic details, previous EEG history, and sleep-deprivation strategies. The data collected was analyzed for trends and main themes. Results: 93 patients (aged 16mos-17 yrs; mean 8.32 yrs) were interviewed (Table 1), to date. 82.8% of children had a previous EEG, with 24.7% of all patients having had a previous SDEEG. Main themes identified for the patients were: 1) Instruction on amount of sleep deprivation; 52.7% of the patients were instructed on how many hours their child should sleep; 2) Parents' reactions to SDEEG; "Will my child fall asleep during EEG?" was the most common reaction (59.1%) and "I do not want to keep myself awake" was the least common (14.0%); 3) Strategies used to keep child awake at night; most common strategy (77.4%) was to watch TV; 4) Strategies used to keep child awake on trip to the hospital on the day of SDEEG; most common strategy (68.8%) was to constantly talk to child in the vehicle; 5) Success of strategies; 90.3% of children did not fall asleep on the trip to the hospital. Conclusions: Certain sleep-deprivation strategies appear to be used across all seasonal and age-specific subgroups (watching TV, playing computer/video games, constantly talking). Indoor activities are largely preferred over outdoor activities, across all seasons. Outdoor activities for sleep-deprivation are most preferred during the summer months. Similarly, parents, across all patient age groups, are primarily concerned with whether their child will fall asleep during the SDEEG. The data collected can be used to help parents and children better cope with sleep-deprivation before SDEEG and to lessen their anxiety regarding adequate sleep deprivation
SN - 1535-7597
AD - (Sharma) BHSc Program, McMaster University, Hamilton, ON, Canada (Nair) Pediatrics, Neurology, McMaster University, Hamilton, ON, Canada
ER -

TY - JOUR
ID - 3463
T1 - The accuracy of hyperventilation and parental report in assessing the activity of absence seizures in children
A1 - O'Mahony,E.
A1 - Libenson,M.H.
Y1 - 2011///
N1 - 70830308
English
Journal: Conference Abstract
KW - EMBASE
KW - Epilepsy
KW - seizure
KW - Child
KW - Human
KW - hyperventilation
KW - Society
KW - parent
KW - patient
KW - electroencephalogram
KW - gold standard
KW - spike wave
KW - Diagnosis
KW - childhood
KW - United States
KW - recording
KW - Drug Therapy

KW - absence
KW - juvenile
KW - pediatric hospital
RP - NOT IN FILE
JF - Epilepsy Currents
VL - Conference: 64th Annual Meeting of the American Epilepsy Society, AES and 3rd Biennial North American Regional Epilepsy Congress San Antonio, TX United States. Conference Start: 20101203 Conference End: 20101207. Conference Publication:
IS - var.pagings
N2 - Rationale: Hyperventilation (HV) is a convenient "bedside" method for eliciting absence seizures (AS) and is often used both to establish a new diagnosis of AS and to monitor the effectiveness of ongoing drug therapy of AS. We set out to measure the sensitivity of HV in two types of "gold standard" groups presumed to have active AS by different criteria: 1) a "high confidence group" of children who manifest spontaneous AS on routine EEG testing (outside of HV) 2) a "lower confidence group" of children whose AS was judged active by parental report. Methods: The EEG diagnosis log at Children's Hospital Boston was screened from January 2006 to June 2010 for all patients with 3 Hz generalized spike-wave discharges or absence seizures. The subset of these patients matching ILAE criteria for childhood or juvenile absence epilepsy formed the study group (n=68). Two comparative "gold standard" groups of EEGs (all with adequate HV) in children with presumed active AS were formed: 1) those EEGs which showed spontaneous AS (outside of HV) 2) those EEGs done at a time that the child's parents reported active AS in their child. Hyperventilation was carried out for a minimum of 3 minutes. An AS was defined as an episode of generalized spike-wave discharges associated with clinical change or unresponsiveness. Results: 53 EEGs that captured spontaneous AS were found in which HV was also done (in 44 unique patients). In 41 (77%) of these EEGs HV also elicited an episode of AS (71% of those untreated [n=28] and 84% of those treated [n=25] at the time of the EEG). 95 EEGs were identified (in 55 unique patients) whose parents reported active AS at the time of the EEG. Of these, HV elicited AS in 59 (62%) EEGs (71% of those untreated [n=41] and 56% of those treated [n=54]). Of the 84 EEGs done at a time that parents reported no active AS, AS was seen spontaneously or during HV in 30 (36%) tracings. Conclusions: Even in children with active AS by the strict criterion of the occurrence of spontaneous AS in the EEG, only 77% had an AS during HV, implying that this technique will miss approximately one quarter of children with active AS. When positive parental report was used as the "gold standard" for presumed active AS, only 62% of children manifested AS during HV; the different rates imply that parents may overestimate the activity of AS approximately one quarter of the time in children who are effectively treated. Conversely, in patients in whom AS was definitely still active by EEG criteria, parents underreported AS activity 36% of the time. This percentage represents a minimum estimate since some children with active AS may not have exhibited their seizures during the period of the EEG recording. These findings imply that parental report has an approximate false-positive rate of one quarter and an approximate false-negative rate of more than one third
SN - 1535-7597
AD - (O'Mahony, Libenson) Division of Epilepsy and Clinical Neurophysiology, Dept of Neurology, Children's Hospital Boston, Boston, MA, United States
ER -

TY - JOUR
ID - 3466
T1 - Impact of the comprehensive epilepsy program in puerto rico: Two-year experience
A1 - Pita,I.
A1 - Arias,J.
A1 - Lopez,A.
A1 - Rios,M.
Y1 - 2012///
N1 - 70829950
English
Journal: Conference Abstract
KW - EMBASE
KW - Epilepsy
KW - Society
KW - Puerto Rico
KW - Human

KW - patient
KW - Monitoring
KW - Diagnosis
KW - Adult
KW - Population
KW - surgery
KW - generalized epilepsy
KW - videorecording
KW - United States
KW - vagus nerve stimulation
KW - diagnostic error
KW - Infant
KW - myoclonus epilepsy
KW - childhood absence epilepsy
KW - frontal lobe epilepsy
KW - Child
KW - temporal lobe
KW - Aged
KW - intractable epilepsy
KW - Female
KW - electroencephalogram
RP - NOT IN FILE
JF - Epilepsy Currents
VL - Conference: 65th Annual Meeting of the American Epilepsy Society, AES Baltimore, MD United States. Conference Start: 20111202 Conference End: 20111206. Conference Publication:
IS - var.pagings
N2 - Rationale: In the puertorrican population, there is scarce information regarding the relative frequency of various types and characteristics of patients with intractable epilepsy. A comprehensive epilepsy program was established in April 2009. We report our data in a group of children and adults who underwent prolonged video-electroencephalographic monitoring in the only Epilepsy Monitoring Unit in Puerto Rico. Methods: A retrospective evaluation of all epilepsy monitoring studies performed from April 2009-April 2011 performed. 153 consecutive patients were analyzed. Results: The majority of patients were females (61%). Two-thirds (66%) of evaluated subjects were adults; infants and elderly patients comprised only 1% of the patients. The most common diagnoses were psychogenic nonepileptic events (39%), localization-related epilepsies (47%) and generalized idiopathic epilepsies (13%). Of the localizationrelated epilepsies, roughly half (47%) were reliably traced to the temporal lobe and frontal epilepsies represented 17% of the evaluated cases. Childhood absence epilepsy was established in half (50%) of the cases showing generalized epileptiform activity and juvenile myoclonic epilepsy represented only 10% of the idiopathic generalized epilepsies. Conclusions: This study illustrates the wide variety of diagnoses encountered in a population of puertorrican patients evaluated at our epilepsy monitoring unit. More than 40 % of patients had a misdiagnosis. Psychogenic nonepileptic events represent at least one-third of the evaluated cases, a relative frequency that is consistent with results obtained in comprehensive epilepsy centers from other countries. Approximately 1/3 of patients are surgical candidates. Two mesial temporal resection were recently performed. Nine percent of patient had vagus nerve stimulation placed and approximately 10% of patients were referred for epilepsy surgery in level four centers in the United States. Through prolonged video-EEG monitoring, a reliable diagnosis can be established and appropriate therapeutic decisions can be started. The comprehensive epilepsy program in Puerto Rico has reduced need for outside referral to 10-12 %
SN - 1535-7597
AD - (Pita, Arias, Rios) University of Puerto Rico, San Juan, Puerto Rico (Pita, Lopez, Rios) Comprehensive Epilepsy Center, HIMA San Pablo, Caguas, Puerto Rico
ER -

TY - JOUR
ID - 3467
T1 - Hemispherectomy in 184 children: Longitudinal seizure outcome and functional status
A1 - Valappil,A.M.N.
A1 - Jehi,L.

A1 - Marashly,A.
A1 - Cosmo,G.
A1 - Lachhwani,D.
A1 - Kotagal,P.
A1 - Wyllie,E.
A1 - Bingaman,W.
A1 - Gupta,A.
Y1 - 2012///
N1 - 70829749
English
Journal: Conference Abstract
KW - EMBASE
KW - Child
KW - Human
KW - seizure
KW - functional status
KW - Epilepsy
KW - Society
KW - hemispherectomy
KW - Language
KW - Reading
KW - patient
KW - surgery
KW - follow up
KW - school
KW - mobilization
KW - questionnaire
KW - chi square test
KW - Statistics
KW - medical record
KW - Counseling
KW - Hospital
KW - Speech
KW - skill
KW - Home Environment
KW - social life
KW - Etiology
KW - grand mal seizure
KW - survival
KW - Male
KW - Adult
KW - structured questionnaire
KW - non profit organization
KW - nuclear magnetic resonance imaging
RP - NOT IN FILE
JF - Epilepsy Currents
VL - Conference: 65th Annual Meeting of the American Epilepsy Society, AES Baltimore, MD United States. Conference Start: 20111202 Conference End: 20111206. Conference Publication:
IS - var.pagings
N2 - Rationale: Data on motor, language, and behavior outcome of children after hemispherectomy is limited to small series with short follow up. We studied the long term functional and longitudinal seizure outcome in a large cohort of hemispherectomy patients from a single center. Methods: 187 children who had hemispherectomy (Jan 1997 - May 2009) at the Cleveland Clinic Foundation were studied. Using a structured questionnaire (17 questions), we contacted families by phone or mail to obtain data on seizure and functional status such as ambulation, visual symptoms, spoken language, reading, schooling and behavior. Questionnaire was drafted to assess function meaningful to the activities of daily life. Medical records were reviewed for pre-operative data. Kaplan- Meier statistics was used to estimate longitudinal seizure outcome. Categorical variables

were analyzed with Chi-square test. Results: Of 187 children (110 males), 104 had right hemispherectomy. Median age for seizure onset was 8 months (1 day-14 yrs) and for surgery was 5 yrs (2 months-18 yrs). At a median follow up of 4.8 yrs (mean 5.3 + 3.3 yrs), 112 of 184 children (61%) were seizure free; additional 13 (7%) had questionable minor spells without habitual pre-operative seizures. 59 (32%) had seizure recurrence, 8 being seizure for 1 year and 16 had >90% reduction in seizures. Overall 149 children (81%) were either seizure free or had major improvement after surgery. On survival analysis, the estimated probability of seizure freedom was 76% at 1 yr, 64% at 3 yrs, 61% at 5 yrs, and stabilized at 56% at 6 years and beyond. Prior hemispheric surgery (n-29, p-0.001), bilateral PET changes (n-23, p- 0.003), presence of generalized seizures (n-67, p- <0.05), and bilateral motor deficits (n-8, p- <0.05) correlated with poor seizure outcome. Etiology and bilateral MRI abnormalities (n-64, 34%) did not influence the seizure outcome. Functional outcome using questionnaire was obtained in 124 children. 102 (83%) patients ambulated independently, 10 walked with assistance and 11 were unable to walk. 31 (25%) patients reported new visual symptoms. Significant behavior issues often requiring constant supervision was reported in 34 (27%), modest behavior issues not affecting social life outside home environment in 23 (19%) and 67 (54%) reported minor or no behavioral problems. 37 (40%) patients had severe spoken language deficits, 42 (34%) had language skills below age level, while 45 (36%) reported no speech issues. 65 (57%) had poor or no reading skills, 31 (27%) read significantly below their age level, and 18 (16%) had age appropriate reading skills. Of 88 children, 64 (73%) were in mainstream school with special services. Only, 6/26 (23%) adults were gainfully employed. Conclusions: The long-term seizure freedom rate following hemispherectomy was 61% at 5 years. After hemispherectomy, functional deficits in spoken language, reading skills, and behavior were more disabling over long term than ambulation and visual deficits. The findings of our study will assist in counseling families prior to hemispherectomy
SN - 1535-7597
AD - (Valappil, Jehi, Marashly, Cosmo, Lachhwani, Kotagal, Wyllie, Bingaman, Gupta) Epilepsy Center/Neurology, Cleveland Clinic, Cleveland, OH, United States (Bingaman) Neurosurgery, Cleveland Clinic, Cleveland, OH, United States
ER -

TY - JOUR
ID - 3468
T1 - Distinctive mri features of the epileptogenic zone in children with tuberous sclerosis
A1 - Krsek,P.
A1 - Kyncl,M.
A1 - Jahodova,A.
A1 - Kudr,M.
A1 - Jayakar,P.
A1 - Dunoyer,C.
A1 - Resnick,T.
A1 - Altman,N.
A1 - Duchowny,M.
Y1 - 2012///
N1 - 70829335
English
Journal: Conference Abstract
KW - EMBASE
KW - Human
KW - tuberous sclerosis
KW - Epilepsy
KW - Society
KW - Child
KW - surgery
KW - patient
KW - nuclear magnetic resonance imaging
KW - thickness
KW - Calcification
KW - seizure
KW - Diagnosis
KW - planning

KW - cortical dysplasia
KW - intractable epilepsy
KW - white matter
KW - Infant
KW - brain
KW - electroencephalogram
KW - pediatric hospital
KW - nitrogen 15
RP - NOT IN FILE
JF - Epilepsy Currents
VL - Conference: 65th Annual Meeting of the American Epilepsy Society, AES Baltimore, MD United States. Conference Start: 20111202 Conference End: 20111206. Conference Publication:
IS - var.pagings
N2 - Rationale: Localization of the epileptogenic zone may be challenging in patients with Tuberous Sclerosis Complex (TSC). Previous experience at our institution has identified the single largest calcified tuber as most likely to be epileptogenic. The aim of our study was to analyze whether additional features of the brain MRI could identify the epileptogenic zone in TSC patients independently of other diagnostic and surgical data. Methods: 31 children with TSC who underwent resective epilepsy surgery at Miami Children's Hospital between 1994 and 2010 were retrospectively studied. Surgical planning had been based on multimodal data with emphasis on electrophysiological investigations. MRI's were independently reevaluated by two experts blinded to all other clinical and diagnostic data. Changes typical of both TSC (tubers, calcifications and cystic lesions) and focal cortical dysplasia (increased cortical thickness, abnormal gyration, gray/white matter blurring, transmantle changes) were identified. Localization of the findings was compared with the location of the resection cavity on the postoperative MRI. We then compared the occurrence of individual MRI changes inside and outside the resection site in 23 subjects with favorable postsurgical seizure outcome, and analyzed reasons for surgical failures in 8 subjects with unfavorable outcome. Results: MRI information alone correctly localized the resection cavity in all 23 TSC patients with favorable seizure outcomes. Significant MRI features in the resection site of these subjects included: largest tuber (n=21), calcification (n=15), cystic lesions (n=2), increased cortical thickness (n=14), abnormal gyration (n=11), transmantle changes (n=7) and gray/white matter blurring in 19/21 cases (two infants had unmyelinated white matter). Increased cortical thickness and abnormal gyration occurred only in the resection site. The relation of other MRI features to the resection site was as follows: largest tubers 11/10 (ratio of subjects with the finding only in the resection site to patients with the finding also occurring outside the resection); calcifications 12/3; transmantle changes 6/1 and gray/white matter blurring 7/12. The resection site was localized correctly using MRI in five other patients with unfavorable postsurgical outcomes; all had incomplete resections of the epileptogenic zone due to overlap with eloquent cortex. The resection site was not localized using MRI in three patients with unfavorable seizure outcomes. One of these patients had overlap of the epileptogenic zone with eloquent cortex; two subjects had multifocal epileptiform activity identified by intracranial EEG. Conclusions: Thorough MRI evaluation is able to accurately localize the epileptogenic zone in a significant proportion of TSC patients. Epileptogenic regions were characterized by "FCD-like" changes outside cortical tubers, especially increased cortical thickness and abnormal gyration. The findings could have important practical consequences for surgical planning in patients with intractable epilepsy due to TSC
SN - 1535-7597
AD - (Krsek, Jahodova, Kudr) Department of Pediatric Neurology, Motol University Hospital, Prague 5, Czech Republic (Kyncl) Department of Radiology, Motol University Hospital, Prague 5, Czech Republic (Jayakar, Dunoyer, Resnick, Duchowny) Department of Neurology and Comprehensive Epilepsy Program, Brain Institute, Miami Children's Hospital, Miami, FL, United States (Resnick, Duchowny) Department of Neurology, University of Miami Miller, School of Medicine, Miami, FL, United States (Altman) Department of Radiology, Miami Children's Hospital, Miami, FL, United States
ER -

TY - JOUR
ID - 3469
T1 - Social, recreation and leisure participation of families who have a child with a severe physical disability: Parents' perspectives
A1 - Davey,H.
A1 - Imms,C.

A1 - Fossey,E.
A1 - Bourketaylor,H.
Y1 - 2012///
N1 - 70828835
English
Journal: Conference Abstract
KW - EMBASE
KW - leisure
KW - Child
KW - Human
KW - physical disability
KW - parent
KW - Cerebral Palsy
KW - Recreation
KW - Social Participation
KW - Interview
KW - narrative
KW - planning
KW - Employment
KW - volunteer
KW - disability
KW - thematic analysis
KW - dynamics
KW - medical specialist
KW - wellbeing
KW - school
KW - caregiver
KW - Risk
KW - primary medical care
KW - wheelchair
KW - Child Rearing
KW - juvenile
KW - Gross Motor Function Classification System
KW - pediatric hospital
RP - NOT IN FILE
SP - 73
EP - 74
JF - Developmental Medicine and Child Neurology
VL - Conference: 6th Biennial Conference of the Australasian Academy of Cerebral Palsy and Developmental Medicine Brisbane, QLD Australia. Conference Start: 20120530 Conference End: 20120602. Conference Publication:
IS - var.pagings
N2 - Objective: Children with severe physical disabilities have high care needs. Consequently they and their families are at risk of restricted opportunities to participate in activities that support their wellbeing, social connections with others, and family cohesion. The child is more likely to rely on family support for their own social participation, rather than on participating in activities with peers, which may further alter the dynamics of the family's social participation. Our objective was to gain an 'insiders' view of family social participation, so as to better understand the enabling and restricting factors involved. Design: Narrative Inquiry. Method: Participants were 10 parents or primary care-givers of youths aged 10-18 years with severe physical disabilities (identified as GMFCS & MACS Levels IV or V) recruited primarily through the Royal Children's Hospital and Specialist schools. Parents were interviewed using the Occupational Performance History Interview-II1 to elicit accounts of daily life within families as well as changes that had occurred over time with their children's growth. Each interview was audio recorded and transcribed verbatim. Narrative analysis was used to construct a core story from each interview within which key participation issues were identified. Secondly thematic analysis was used to compare themes across participant's stories. Results: Parents characterised daily life as involving highly structured routines that were demanding of their time, energy and resources. Routines were dependent to some extent on service provision that was often described as inflexible and not meeting the needs of the family.

Parents prioritised the needs of their child with a disability. Their other roles, including volunteer or paid employment and maintaining relationships both inside and outside the family, were dependent on the suitability and flexibility of care arrangements that could be made for their child. Participating in activities outside the home required parents to access detailed information and plan carefully to cater for their child's needs, particularly concerning toileting and wheelchair accessibility. The personal and financial resources required, and the physical and attitudinal barriers that families faced, meant that 'going out' relied heavily on parents' energy and resources. Parents therefore considered carefully whether social participation was worth the effort. Conclusions: Daily life was demanding of parents' time, energy and resources. Participation outside of core routines required planning and the means to overcome environmental barriers. Family social participation relied on parents' resourcefulness in facing these barriers. Being knowledgeable and mindful of these issues will assist planning feasible family centered interventions
SN - 0012-1622
AD - (Davey, Imms, Fossey) La Trobe University, Melbourne, Australia (Bourketaylor) Monash University, Melbourne, Australia
ER -

TY - JOUR
ID - 3470
T1 - Participation patterns of children with and without physical disabilities in activities outside school
A1 - Black,M.
A1 - Imms,C.
A1 - King,M.
A1 - Shields,N.
Y1 - 2012///
N1 - 70828810
English
Journal: Conference Abstract
KW - EMBASE
KW - Human
KW - physical disability
KW - school
KW - Cerebral Palsy
KW - Child
KW - disability
KW - community
KW - gender
KW - Spina Bifida
KW - Male
KW - girl
KW - intellectual impairment
KW - Female
KW - parent
KW - boy
KW - Physical Activity
KW - Government
KW - convenience sample
KW - questionnaire
KW - Social Behavior
KW - rank sum test
KW - scientist
KW - Satisfaction
RP - NOT IN FILE
SP - 58
JF - Developmental Medicine and Child Neurology
VL - Conference: 6th Biennial Conference of the Australasian Academy of Cerebral Palsy and Developmental Medicine Brisbane, QLD Australia. Conference Start: 20120530 Conference End: 20120602. Conference Publication:

IS - var.pagings
N2 - Objective: This study compared the participation patterns of children with physical disabilities (PD) with their peers with typical development (TD) in activities outside of school. The aim was to describe similarities and differences in the extent (number of activities and frequency), context (location and companionship) and experience (enjoyment and satisfaction) of participation. Design: A two-group matched pairs design, using survey methods. Method: Data were extracted for children with PD (e.g. spina bifida or cerebral palsy) who lived in regional or metropolitan Victoria, from a previous participation study of children with disabilities. Children with PD were rated by parents as having mild (n=14), moderate (n=19), and severe (n=12) disabilities. Children with intellectual disability were excluded. Forty-five children with PD (15 girls, 30 boys; median age of 11 years [range: 6-17y]) were matched according to age, gender, socio-economic background and location (regional or metropolitan living situation) to 45 children with TD. Children with TD were recruited through 17 government and private schools selected by stratified random (n=12) and convenience sampling (n=5). Participation was measured using the Children's Assessment of Participation and Enjoyment (CAPE) and the Preferences for Activity of Children (PAC) questionnaires. Data were collected via mail-outs or with the support of a researcher. As the data were not normally distributed, Mann-Whitney U-tests were used to assess between-group differences. Results: There was no evidence that the participation patterns of children with PD were different to those with TD in extent or context of participation in Recreational, Skillbased or Informal activities (all P>0.05). Of the 20 activities in which most children participated, 17 activities were consistent between groups. There was evidence that children with PD participated less frequently in Formal (P=0.028) and Social activities (P=0.013). Although children with PD participated in fewer Physical activities (P=0.005) there was no evidence of a difference in their frequency of Physical participation (P=0.066). Conclusion: Participation patterns of children with PD and children with TD were similar, although community-based participation of children with PD was lower in comparison to children with TD. There are few recommendations about how much participation is optimal, or what dimensions of participation are most important. Working with individual children and families to facilitate activity within the community in enjoyed and preferred activities may assist to develop healthy participation patterns for life. Future research that investigates engagement in activity will enhance knowledge about how to support high quality participation
SN - 0012-1622
AD - (Black, Imms, King, Shields) La Trobe University, Melbourne, Australia
ER -

TY - JOUR
ID - 3471
T1 - Evaluation of skin changes using infrared thermography
A1 - Kalicki,B.
A1 - Jung,A.
A1 - Rustecka,A.
A1 - Zylak,A.
A1 - Zuber,J.
Y1 - 2012///
N1 - 70824527
English
Journal: Conference Abstract
KW - EMBASE
KW - infrared photography
KW - skin
KW - hemangioma
KW - Temperature
KW - Human
KW - infrared radiation
KW - Therapy
KW - Imaging
KW - capillary hemangioma
KW - retina image
KW - skin surface
KW - Doppler flowmetry
KW - subcutaneous tissue

KW - urticaria
KW - tissues
KW - Diagnosis
KW - skin defect
KW - nose
KW - surgeon
KW - Inflammation
KW - surgery
KW - thermography
KW - Atrophy
KW - healing
KW - camera
KW - Environment
KW - skin test
KW - involution
KW - lymph node
KW - microscope
KW - procedures
KW - squamous cell carcinoma
KW - allergic urticaria
KW - physical disease by body function
KW - temporal lobe
KW - scar
KW - Male
KW - computer program
KW - Child
KW - boy
KW - blood flow
KW - girl
KW - Female
KW - Forearm
KW - skin carcinoma
KW - body regions
KW - Color
KW - diagnostic procedure
KW - abscess
KW - propranolol
KW - allergen
KW - cosmetic
RP - NOT IN FILE
SP - 69
EP - 70
JF - Thermology International
VL - Conference: 16th Congress of the Polish Association of Thermology Zakopane Poland. Conference Start: 20120316 Conference End: 20120318. Conference Publication:
IS - var.pagings
N2 - Thermographic imaging of the skin can be widely used to assess ongoing processes of the skin surface as well as processes within the subcutaneous tissue. The possibility to apply visual images to thermographic images of skin changes allows to gain additional information which can be of great importance for their clinical interpretation. The role of infrared thermography in the diagnosis of inflammatory processes (abscesses, periodontal infiltrations, inflammatory processes with submandibular lymph node involvement) of the skin and surrounding tissues was previously described. Thermographic imaging is an objective diagnostic tool in the interpretation of skin tests with allergens, especially in cases when the skin is covered with other lesions or has a different color. The purpose of our study is to present new opportunities of using thermographic imaging in the evaluation of ongoing processes of the skin surface and within the subcutaneous tissue. 1 The appearance of skin lesions described as allergic urticaria is characterized by great diversity. Typical lesions (wheals) usually show up a few minutes after contact with the allergen and can resolve in a short period of time. Infrared images

of the lesions show, that they are far more expressed than in the visual assessment. Infrared images can also become valuable clues for clinicians, because they describe the activity of the continuing process and thereby can influence the treatment. (Figure presented) In figure 1 a rectangular area, outside the wheal (A4), was selected. After applying a thermographic image we can see a clearly visible area of increased temperature induced by the wheal, with a far greater range than seen in the visual image. The thermografic image was taken in a standard environment using the infrared camera FLIR T-640, which allows applying visual images to thermographic images 2. Skin carcinomas require careful analysis of the surgical field range to determine appropriate and safe surgical margins. On the other hand the cosmetic effect after surgery is very important, that is why surgeons try to minimize the scar. Infrared thermography allows to precisely determine the range of the neoplastic lesion, which very often has a greater size than primary observed. (Figure presented) A skin lesion in the temporal region (figure 3) represents a squamous cell carcinoma, which was qualified for surgical treatment. The surgical field range is intuitively set by the surgeon during the procedure. A histopathologic section is examined under the microscope to ensure safe surgical margins. Infrared images can be helpful in determining the surgical field range.³ The clinical presentation of infantile hemangiomas is characterized by great diversity. Their range in spatial dimension can be difficult to assess in routinely performed Doppler ultrasound. Recent studies have shown a good effect in treating infantile hemangiomas with prolonged propranolol therapy. The involution of hemangiomas during propranolol therapy is associated with formation of atrophic foci. Infrared thermography allows to assess the temperature of hemangiomas, their size in metric units (by using special software). Because of the diversity of hemangiomas, their location, the age of children, the progress of the healing process (atrophy of the hemangioma) it seems that the comparison of average temperatures between an area registered within the hemangioma and an area registered within a symmetrical body part is the most reliable method to describe the treatment efficacy. In cases of an unfavorable location of the hemangioma (i.e. nose area, forearm), it is more objective to compare temperatures between the hemangioma and an adjacent, healthy tissue. (Figure presented) In the thermogram of a 12-months old girl with an infantile hemangioma 2 areas were marked: A1 is an area with high blood flow within the hemangioma determined by Doppler ultrasound, A2 is an area with marked atrophy of a highly vascularized region due to propranolol therapy. The average temperature in the area Al= 36.8degreeC, in the area A2 36.2degreeC. Delta of average temperatures (Figures 5,6)= 0,6degreeC (Figure presented) A 7-months old boy with an inflamed and tender infantile hemangioma of the nose, after 4 months of propranolol therapy. The tenderness of the hemangioma limited the use of Doppler ultrasound. Visual and thermographic evaluation allows to fully control the healing process. CONCLUSION: The presented opportunities of using infrared imaging in the evaluation and interpretation of ongoing pathologic processes of the skin surface and the subcutaneous tissue indicate the high value of thermography as a diagnostic method
SN - 1560-604X
AD - (Kalicki, Jung, Rustecka, Zylak, Zuber) Department of Pediatrics, Pediatric Nephrology and Allergology, Military Institute of Medicine, Warsaw, Poland
ER -

TY - JOUR
ID - 3472
T1 - Impact of recurrent headache on school performance, sleep pattern and socioeconomic norms among Bangladeshi school children
A1 - Saha,N.C.
A1 - Mohammad,S.
A1 - Kazi,S.
A1 - Rahman,E.
A1 - Mollah,A.
Y1 - 2012///
N1 - 70826552
English
Journal: Conference Abstract
KW - EMBASE
KW - Human
KW - neurology
KW - Child
KW - sleep pattern
KW - Academic Achievement

KW - school child
KW - headache
KW - Asian
KW - disability
KW - migraine
KW - Sleep
KW - Social Behavior
KW - Drug Therapy
KW - Arousal
KW - controlled study
KW - sleep disorder
KW - Bangladesh
KW - Social Interaction
KW - headache and facial pain
KW - Classification
KW - structured questionnaire
KW - high school
KW - Government
KW - Quality of Life
RP - NOT IN FILE
SP - 89
EP - 90
JF - Developmental Medicine and Child Neurology
VL - Conference: 12th International Child Neurology Congress and the 11th Asian and Oceanian Congress of Child Neurology Brisbane, QLD Australia. Conference Start: 20120527 Conference End: 20120601. Conference Publication:
IS - var.pagings
N2 - Objective: Recurrent headache (RHA) results in socio-economic burden, adversely affects quality of life, school performance, social interaction and sleep patterns. This study aimed to determine the impacts of different types of RHA as this has not been previously reported in Bangladesh. Design: Cross-sectional cohort controlled study. Method: Children from 24 government-run secondary schools in Dhaka, were selected randomly through a structured questionnaires consisting of (1) information on migraine/tension-type headache (TTH) using the International Classification of Headache Disorder II, (2) evaluation of headache disability using PedMIDAS (paediatric migraine disabilities assessment score) for cases and modified PedMIDAS for controls and impact on economic status and sleep patterns. Results: In RHA-cases, mean disability score (DS) for school performance, inability to perform usual home-work including chores, restricted outdoor play and compromised social activities were higher (more disability) than controls (8.3, 2.4, 4.9, 1 vs 2.6, 0.4, 1.1, 0.2 respectively, $P<0.001$). Average DS was higher (19.57) in RHA than controls (5.63, $P<0.001$) but was also higher in migraine than TTH (21.02 vs 16.27, $P<0.001$). Sleep disturbances, such as difficulties in initiation and maintenance of sleep (61% vs 49%, $P<0.001$), number of awakenings per week (32% vs 19%, $P<0.001$) and fragmentary sleep (16% vs 8%, $P<0.001$) were more common in RHA. No significant difference was observed in sleep measures between migraine and TTH. Medication requirement and monthly cost for headache treatment was higher in RHA and medication was higher for those with migraine than with TTH, $P<0.04$. Conclusion: RHA had greater disability in relation to school performance, home work, social activities, sleep pattern and more treatment cost than controls and disability was higher among migraine than TTH
SN - 0012-1622
AD - (Saha, Mohammad, Kazi, Rahman, Mollah) Dhaka Medical College, Dhaka, Bangladesh
ER -

TY - JOUR
ID - 3474
T1 - Relationships between lifestyle and motor fitness in early-school children
A1 - Podstawski,R.
A1 - Skibniewska,K.A.
A1 - Mroczkowska,A.
Y1 - 2012///

N1 - 2012433089
English
Journal: Article
KW - EMBASE
KW - alcohol consumption
KW - article
KW - Child
KW - child growth
KW - eating habit
KW - Female
KW - Food Intake
KW - Health Behavior
KW - Health Promotion
KW - Health Status
KW - Human
KW - leisure
KW - Lifestyle
KW - major clinical study
KW - Male
KW - Motor Performance
KW - Nutritional Status
KW - Physical Activity
KW - Rural Population
KW - school child
KW - Smoking
RP - NOT IN FILE
SP - 21
EP - 26
JF - Polish Annals of Medicine
VL - 19
IS - 1
CY - Poland
N2 - Introduction: Motor fitness is considered as an individual health measure. Its impact on human organism is extremely important during the early school years due to the intensive child's growth at that time. Aim: The aim of this study was to assess the relationship between the health behavior of early-school children and their motor fitness. Materials and methods: A group of 60 school children attending I-III classes of a rural school was examined in June 2008. The children's motor fitness was determined by employing motor ability tests and interview questionnaires to categorize the children's lifestyles and nutritional patterns. Results were analyzed statistically. Results and discussion: The majority of these school children presented a medium level of motor fitness. Children's opinions concerning their health status and nutritional patterns did not influence their motor fitness. However, a higher percentage of children with a high level of motor fitness was observed among those children with a positive attitude towards health promoting behaviors. A high level of motor fitness was found in about 50% of children spending their free time outdoors 2-3 times a week and a low level in over 57% of children seldom spending their free time outdoors. A higher percentage of children with a low fitness level was detected among those declaring contact with cigarettes and alcohol. Conclusions: Such factors as physical activities during leisure time, children's opinions on their health status and nutritional patterns had only a slight influence on the levels of motor fitness. 2012 Warminsko-Mazurska Izba Lekarska w Olsztynie
SN - 1230-8013
AD - (Podstawski, Mroczkowska) Study Center of Physical Education and Sport, University of Warmia and Mazury in Olsztyn, Poland (Skibniewska) Department of Foundations of Safety, University of Warmia and Mazury in Olsztyn, Poland (Podstawski) Study Center of Physical Education and Sport, University of Warmia and Mazury in Olsztyn, Prawocheriskiego 7, 10-720 Olsztyn, Poland
ER -

TY - JOUR
ID - 3475
T1 - Petrous bone imaging in children

A1 - Rossi,A.
Y1 - 2012///
N1 - 70821435
English
Journal: Conference Abstract
KW - EMBASE
KW - Human
KW - Imaging
KW - Child
KW - petrous bone
KW - postgraduate student
KW - Society
KW - radiology
KW - nuclear magnetic resonance imaging
KW - inner ear
KW - congenital malformation
KW - middle ear
KW - aplasia
KW - mastoiditis
KW - Cholesteatoma
KW - vestibule aqueduct
KW - otitis
KW - follow up
KW - branchial arch
KW - planning
KW - pons angle
KW - Vestibule
KW - Neuroimaging
KW - Inflammation
KW - Infection
KW - Diagnosis
KW - Adult
KW - abscess
KW - external ear
KW - stapes
KW - facial nerve
KW - capillary hemangioma
KW - histology
KW - perception deafness
KW - stenosis
KW - neurofibromatosis
KW - occlusion
KW - Lemierre syndrome
KW - atresia
KW - external auditory canal
KW - Amputation
KW - mastoid
KW - radiologist
KW - conduction deafness
KW - endolymphatic sac
KW - injury
KW - orbit
KW - syndrome CHARGE
KW - cochlear nerve
KW - sigmoid sinus
KW - ionizing radiation
KW - hospital patient

KW - Bone
KW - temporal lobe
KW - patient
KW - antibiotic therapy
KW - cochlea
KW - immune system
KW - disease course
KW - semicircular canal
KW - cranial nerve
KW - auditory tube
KW - eardrum
KW - chronic inflammation
KW - embryology
KW - surgery
KW - bone cell
KW - osteolysis
KW - anamnesis
KW - demineralization
KW - fistula
KW - pathology
KW - diffusion
KW - Meningitis
KW - incus
KW - Hearing Loss
KW - contrast medium
RP - NOT IN FILE
SP - S451
EP - S452
JF - Pediatric Radiology
JA - Pediatr Radiol
VL - Conference: 49th Annual Meeting and 35th Postgraduate Course of the European Society of Paediatric Radiology, ESPR 2012 Athens Greece. Conference Start: 20120528 Conference End: 20120601. Conference Publication:
IS - var.pagings
N2 - Background: Hearing loss (HL) is a frequent reason for CT and MR imaging studies performed in children. However, a generic request of neuroimaging studes for "HL" is not sufficient. The radiologist should require the clinician to specify whether HL is unilateral (and which side is involved) or bilateral, and whether it is sensorineural (SNHL), conductive (CHL), or mixed. This will influence the imaging approach, which is primarily based on a combination of CT and MRI for SNHL or mixed HL, and on CT for CHL. Sensorineural hearing loss (SNHL) In SNHL, CT detects only approximately 20% of causes. Thus, MRI is often the first-line imaging study. The MRI protocol should always include thin-slice (1 mm or less), heavily T2-weighted images obtained with adequate techniques (ie T2 DRIVE, CISS, FIESTA), which allow for a superb delineation of the membranous labyrinth of the inner ear, inner acoustic canal (IAC) with the VIIVIII cranial nerve complex, and cerebellopontine angle cistern. These sequences can be reformatted on any plane, but direct inplane acquisition offers significant advantages in terms of spatial resolution. Because the acoustic lemniscus projects to the Heschl's gyrus in the temporal lobe, MRI studies in patient with SHNL should not focus on the inner ear only, but include the whole pathway. Contrast material may be administered where applicable. Unilateral SNHL is usually caused by acoustic schwannomas in adults. However, this lesion is uncommon in children except for bilateral schwannomas in the setting of neurofibromatosis type II. Unilateral enhancing mass lesions involving the pediatric IAC are rare and usually of other histologies, including capillary hemangiomas in PHACE syndrome. Most cases of uni- or bilateral SNHL in children are caused by malformations of the inner ear. These, in turn, depend on the timing of the derangement in the development of the inner ear, which starts at 23 days with the formation of the otic placode, proceeds with the otic pit (24 days) and otic vesicle (27 days) stages, and subsequently with the appearance of the vestibular and cochlear pouches and, finally, of the semicircular canals, of which the lateral is the last one to appear. The adult size and configuration is reached by the middle of the fetal period (20-22 weeks), and any derangement at intermediate stages will result in a different kind of malformation. Early stage derangement, including the Michel aplasia (complete aplasia of the inner ear) and the

common cavity, are relatively rare. Midstage derangements are more common and include the incomplete partition types I (cystic appearance of cochlea and vestibule) and II, formerly known as Mondini malformation (1.5 cochlear turns, dilated vestibule and vestibular aqueduct). Although CT adequately depicts these malformations, MRI has the advantage of the absence of ionizing radiation and the possibility to assess the presence of the cochlear nerve, a critical issue for management planning which cannot be resolved by CT alone. Moreover, MRI must be used to look for associated abnormalities and syndromic complexes, in particular the CHARGE association which it also comprises. Among other abnormalities, orbital colobomas which must be scrutinized during the same MRI study. Among causes of SNHL in children, the large vestibular aqueduct syndrome (LVAS) is the most common. In LVAS, HL is initially mild, worsens stepwise, and deteriorates with trauma or intercurrent disease, CT and MRI show a markedly enlarged vestibular aqueduct, mono-or bilaterally; MRI also shows the dilated endolymphatic sac protruding into the cerebellopontine angle cistern. Conductive hearing loss (CHL) Causes of CHL can be categorized into congenital (i.e. malformations) and acquired (i.e. otitis and otomastoiditis). Congenital aural aplasia (CAA) is the main cause of congenital CHL. It is characterized by stenosis (types 1 and 2) or atresia (types 3 and 4) of the external acoustic canal, and it is very commonly associated with anomalies of the middle ear and mastoid. This is due to the common embryological origin of the anterior part of the external ear, incus and malleus from the first branchial arch, and the posterior part of the external ear and the stapes from the second branchial arch. CAA is unilateral in 70% of cases, and bilateral in the remainder; it can be isolated or syndromic (Goldenhar, Klippelfeil, Treacher-Collins, and other syndromes). In CAA, CT must be used to carefully scrutinize for associated ossicular anomalies which include incudomalleal fixation and stapes dislocation. The course of the facial nerve must also be carefully evaluated, especially looking for dehiscent courses which may cause problems during ontological surgery. Acquired CHL is mainly cause by otitis, otomastoiditis, and related complications. Acute otitis/otomastoiditis is caused by pharyngeal germs which colonize the middle ear through the Eustachian tuba. Acute otitis/otomastoiditis is a clinical diagnosis that does not require imaging studies for confirmation; the clinical course is usually short, and the process terminates because of the host's immune system, the infection-resistant properties of the mucosal linings, and antibiotic treatment. However, a small proportion of untreated or inadequately treated patients may experience complications which can be a threatening disease and must be diagnosed promptly. Intratemporal complications include incipient mastoiditis, in which infection is restricted to the mucoperiosteal compartment, and coalescent mastoiditis, in which the bone is involved. From this stage, extratemporal complications may ensue, including Bezold's abscesses, sigmoid sinus thormbophlebitis, cerebellar abscess, and Lemierre syndrome. Contrast-enhanced CT is required in the study of complicated mastoiditis to rule out these potentially life-threatening complications; MRI plays a complementary role for confirmation of the diagnosis. Chronic otomastoiditis is caused by the persistence of inflammation in the middle ear cavity leading to mucosal thickening and occlusion of the Eustachian tube. This, in turn, causes retraction of the tympanic membrane at the pars flaccida with development of chronic inflammation which, eventually, may lead to the formation of a cholesteatoma. The Prussak space at the anterior epitympanic recess is the most common location for acquired cholesteatomas, although other locations in the tympanic cavity and petrous bone cells are also possible. Cholesteatomas are characterized on CT as discrete masses causing bone resorption (typicallywith amputation of the scutum) and ossicular demineralization. Labrynthine or facial nerve fistulae must be carefully ruled out since they may be the source for recurrent meningitides. In doubtful cases, MRI is useful to discriminate a cholesteatoma from aspecific inflammation; a cholesteatoma gives restricted diffusion and does not enhance with contrast material administration. MRI is also useful in the post-surgical follow-up in operated cases. Conclusions In conclusion, neuroimaging is a powerful tool for the characterization, management planning, and follow-up of both congenital and acquired petrous bone pathology in children. A careful history-taking and correlation with the clinician are of paramount importance to correctly choose the imaging technique and recognize the various conditions. Profound knowledge of anatomy and embryology is necessary to properly address this difficult, fascinating area
SN - 0301-0449
AD - (Rossi) Neuroradiology Department, G. Gaslini Children's Hospital, Genoa, Italy
ER -

TY - JOUR
ID - 3476
T1 - Approach to the epidemiology of venomous bites in Spain
A1 - Saz-Parkinson,Z.
A1 - del Pino,Luengo M.
A1 - Lopez-Cuadrado,T.

A1 - Andujar,D.
A1 - Carmona-Alferez,R.
A1 - Flores,R.M.
A1 - Amate,J.M.
Y1 - 2012///
N1 - 2012407496
English
Journal: Article
KW - EMBASE
KW - Adolescent
KW - Adult
KW - Aged
KW - agricultural worker
KW - article
KW - Child
KW - Death
KW - Demography
KW - descriptive research
KW - economic evaluation
KW - envenomation/ep [Epidemiology]
KW - Female
KW - geographic distribution
KW - hospital care
KW - hospital cost
KW - Human
KW - leisure
KW - major clinical study
KW - Male
KW - poisonous snake
KW - preschool child
KW - priority journal
KW - Rural Population
KW - school child
KW - snakebite/ep [Epidemiology]
KW - Spain
KW - species habitat
KW - Vipera aspid
KW - Vipera latasti
KW - Vipera seoianei
RP - NOT IN FILE
SP - 706
EP - 711
JF - Toxicon
VL - 60
IS - 4
CY - United Kingdom
N2 - This review presents a geographic distribution of the three autochthonous venomous snake species, which are the only viperids present in Spain, among the Iberian fauna: Vipera aspid; Vipera seoianei and Vipera latasti.This is followed by a detailed descriptive analysis of hospital care provided to patients admitted into hospital due to venomous bites, in the period from 1997 to 2009, using the data from the Spanish hospital discharge registry database.This analysis reveals that in Spain, during this period, 1649 cases were recorded, which means that hospital care was required for more than one hundred cases per year, of which nearly 1% of the cases resulted in death.Cases were recorded in all the Autonomous communities, but more than half (54, 14%) were concentrated in the following four regions: Cataluna, Castilla and Leon, Galicia and Andalucia. It is notable that this concentration of cases is not associated only with the population demographics of the community, but is also the result of the concurrence of very diverse factors of exposure including: habitat of venomous fauna, volume of rural population, farming activities, and practice of outdoor leisure activities.We

also carried out a gross economic calculation for the use of hospital resources by each snakebite case requiring hospital care in Spain, which provided us with an approximate figure of 2000 per case. 2012 Elsevier Ltd
SN - 0041-0101
AD - (Saz-Parkinson, Carmona-Alferez, Flores, Amate) Agencia de Evaluacion de Tecnologias Sanitarias, Instituto de Salud Carlos III, Avenida Monforte de Lemos 5, 28029 Madrid, Spain (del Pino Luengo) I.E.S Eijo y Garay, Madrid, Spain (Lopez-Cuadrado) Centro Nacional de Epidemiologia, Instituto de Salud Carlos III, Avenida Monforte de Lemos 5, 28029 Madrid, Spain (Andujar) Universitat Hohenheim, Institute Phytomedice, Dept Weed Science 360B, D-70599 Stuttgart, Germany
ER -

TY - JOUR
ID - 3477
T1 - Association between contamination of public squares and seropositivity for Toxocara spp. in children
A1 - Manini,M.P.
A1 - Marchioro,A.A.
A1 - Colli,C.M.
A1 - Nishi,L.
A1 - Falavigna-Guilherme,A.L.
Y1 - 2012///
N1 - 2012380307
English
Journal: Article
KW - EMBASE
KW - analytic method
KW - animal parasitosis
KW - article
KW - Asthma
KW - bronchitis
KW - Child
KW - child health
KW - controlled study
KW - Environmental Exposure
KW - enzyme linked immunosorbent assay
KW - eosinophilia
KW - feces analysis
KW - Female
KW - Geography
KW - Human
KW - immunoglobulin blood level
KW - Male
KW - microbial contamination
KW - nonhuman
KW - parasite examination
KW - parasite identification
KW - parasite load
KW - parasite transmission
KW - pet animal
KW - preschool child
KW - public square
KW - school
KW - school child
KW - serology
KW - seroprevalence
KW - skin allergy
KW - society and environment
KW - Toxocara canis
KW - toxocariasis

KW - urban area
KW - water sedimentation technique
KW - immunoglobulin G antibody/ec [Endogenous Compound]
RP - NOT IN FILE
SP - 48
EP - 52
JF - Veterinary Parasitology
VL - 188
IS - 1-2
CY - Netherlands
N2 - A concomitant study was carried out, of the association of positive serology for Toxocara spp. in 90 children who played in public squares used for leisure, with the frequency with which each child used these areas, and the presence of eggs of Toxocara spp. in the sand or grass in these locations. The sand and grass of their peridomiciles and school playgrounds, as well as the feces of their dogs were also analyzed for Toxocara. Serum samples were tested for IgG antibodies to Toxocara canis excreted-secreted larval antigens by ELISA, and blood samples for eosinophilia. The water-sedimentation technique was used to evaluate the presence of parasite eggs in the sand and grass turfs, and in feces of the dogs that also frequented these locations. 16/90 (17.8%) of the children were seropositive for Toxocara spp. There was a positive association between seropositivity in children who played in the public squares six or seven times a week, with a parasite load above 1.1. eggs/g of sand, as well as with contamination of the peridomicile, even at less than 1.0. egg/g of sand. Eosinophilia, the habit of geophagy, age from one to four years, and the presence of parasitized pet dogs were also positively correlated with seropositivity in the children. Eggs were found in 15/15 (100%) of the public squares, 17/90 (18.9%) of the peridomiciles, 3/13 (23.1%) of the schools, and 12/41 (29.3%) of the dogs living in the peridomiciles investigated. 2012 Elsevier B.V
SN - 0304-4017
AD - (Manini) Laboratory of General and Clinical Parasitology, University of Parana (UNIPAR), Praca Mascarenhas de Moraes s/n 87501-000, Umuarama, PR, Brazil (Marchioro, Colli, Nishi, Falavigna-Guilherme) Environmental Parasitology Laboratory, State University of Maringa (UEM), Av Colombo, 5790 - Bloco I-90 sala 11, CEP 87020-900 Maringa, PR, Brazil
ER -

TY - JOUR
ID - 3478
T1 - Healthy people 2020 physical activity objectives: Children and adolescents
A1 - Ryskulova,A.
A1 - Klein,R.
A1 - Hines,R.
A1 - Fulton,J.
A1 - Wargo,J.
Y1 - 2012///
N1 - 70810156
English
Journal: Conference Abstract
KW - EMBASE
KW - Human
KW - Child
KW - Physical Activity
KW - Society
KW - Adolescent
KW - normal human
KW - Epidemiology
KW - computer
KW - videorecording
KW - school
KW - student
KW - Health
KW - examination

KW - Nutrition
KW - Television
KW - Public Health
KW - Obesity
KW - Physical Education
KW - Male
KW - Risk
KW - juvenile
KW - United States
KW - Immobilization
RP - NOT IN FILE
SP - S22
JF - American Journal of Epidemiology
JA - Am J Epidemiol
VL - Conference: 45th Annual Meeting of the Society for Epidemiologic Research, SER 2012 Minneapolis, MN United States. Conference Start: 20120627 Conference End: 20120630. Conference Publication:
IS - var.pagings
N2 - Physical inactivity is an important health issue in the U.S. and more children today are overweight or obese than ever before. Reflecting the importance of physical activity (PA) among children and adolescents Healthy People 2020 included several objectives on PA guidelines compliance and screen time reduction. Multiple data sources were used to provide baselines for the specified HP2020 objectives: 2005-08 National Health and Nutrition Examination Survey, 2009 Youth Risk Behavior Survey, and 2007 National Survey of Children's Health. About 18% of adolescents met current Federal PA guidelines in 2009. Males, white adolescents, and 9th grade students were more active than their counterparts. About 33% of adolescents participated in daily school physical education and 9th graders were more likely to participate in it than older students. Among children ages 0-2 40.6% viewed no television or videos on an average weekday, about 76% of children ages 2-5 and 6-14 viewed TV, videos or played videogames for no more than 2 hours a day, among adolescents the percentage of those who did not exceed 2-hour a day limit was 67.2%. More than 90% of children ages 6-14 and 75% of adolescents in grades 9-12 used a computer or played computer games outside of school (for nonschool work) for no more than 2 hours a day. Healthy People 2020 was launched on December 2, 2010. National data for the PA topic area are currently available for public use. The PA objectives will be tracked during the decade to measure their progress toward 2020 targets
SN - 0002-9262
AD - (Ryskulova, Klein, Hines, Fulton, Wargo) President's Council on Fitness, Sports, Nutrition, CDC, Atlanta, GA, United States
ER -

TY - JOUR
ID - 3479
T1 - Strategies to overcome epidemic of vitamin D deficiency in asia
A1 - Mithal,A.
Y1 - 2011///
N1 - 70806709
English
Journal: Conference Abstract
KW - EMBASE
KW - Asia
KW - vitamin D deficiency
KW - Bone
KW - osteoporosis
KW - epidemic
KW - Human
KW - Population
KW - Food
KW - sun exposure
KW - Aged
KW - Child

KW - India
KW - Asian
KW - China
KW - Skin Pigmentation
KW - South Asia
KW - pregnant woman
KW - Prevalence
KW - serum
KW - Female
KW - Risk
KW - Clothing
KW - Adult
KW - Monitoring
KW - Policy
KW - social worker
KW - exposure
KW - skin
KW - school child
KW - Newborn
KW - Health
KW - vitamin D
KW - colecalciferol
RP - NOT IN FILE
SP - S501
EP - S502
JF - Osteoporosis International
JA - Osteoporos.Int
VL - Conference: IOF Regionals, 2nd Asia-Pacific Osteoporosis and Bone Meeting, ANZBMS Annual Scientific Meeting, with JSBMR Gold Coast, QLD Australia. Conference Start: 20110904 Conference End: 20110908. Conference Publication:
IS - var.pagings
N2 - Vitamin D is an integral determinant of musculoskeletal health. Reports from across the world indicate the presence of widespread vitamin D deficiency. A 2009 IOF review of global vitamin D status highlights the prevalence and severity of vitamin D deficiency in Asia. Despite abundant sunshine, populations frommany countries in Asian region, in particular in South Asia and possibly China are severely affected by vitamin D deficiency. In India, for example, all ages (newborns, children, adults, pregnant women and elderly) have been shown to be affected. Among factors that have been blamed for this epidemic in Asia is skin pigmentation, cultural practices like avoidance of sun exposure, and traditional clothing that allows limited skin exposure. Fortification of food products with vitamin D is not widely practiced in Asia. The majority of the world's population lives in this region of the world, and, given the diverse nature of the countries in the region, each with their own set of unique issues and problems, it is difficult to envisage a uniform strategy for the region as a whole. However, the approaches can broadly be divided into 3 parts: 1. Increasing awareness about importance of sun exposure and outdoor activities in the population, particularly in children. 2. Designing and implementing vitamin D fortification programs. This requires combined expertise of 'vitamin D' experts, nutritionists, chemists, food technologists and social workers, so that a comprehensive plan can be presented to policy makers. Choice of the appropriate food items to be fortified and monitoring strategies are essential to the success of such a program. 3. Selective and judicious use of vitamin D supplements in populations 'at risk'. Typical examples include schoolchildren and the elderly. The dose required to achieve adequate 25(OH) D levels may be much higher than internationally recommended, due to low baseline levels. Studies from India show that at least 2000 IU of cholecalciferol is required daily to achieve optimum serum 25(OH)D levels. There is an urgent need for Asian countries to formulate strategies for combating this epidemic, based on country specific needs and situations
SN - 0937-941X
AD - (Mithal) Endocrinology and Diabetes Division, Medanta, Haryana, India
ER -

TY - JOUR

ID - 3480
T1 - Clinical assessment in a cohort of children with tics and Tourette syndrome
A1 - Perez-Duenas,B.
A1 - Caurin,B.
A1 - Serrano,M.
A1 - Fernandez,E.
Y1 - 2012///
N1 - 70801561
English
Journal: Conference Abstract
KW - EMBASE
KW - tic
KW - Human
KW - Child
KW - clinical assessment
KW - Gilles de la Tourette syndrome
KW - motor dysfunction
KW - Parkinson disease
KW - patient
KW - boy
KW - Academic Achievement
KW - Male
KW - daily life activity
KW - Morbidity
KW - Diagnosis
KW - Attention Deficit Disorder
KW - pharyngitis
KW - sport
KW - Music
KW - painting
KW - Dancing
KW - Recreation
KW - school
KW - risk factor
KW - coughing
KW - Anxiety
KW - compulsion
KW - sniffing
KW - obsession
KW - Noise
RP - NOT IN FILE
SP - S68
JF - Movement Disorders
VL - Conference: 16th International Congress of Parkinson's Disease and Movement Disorders Dublin Ireland. Conference Start: 20120617 Conference End: 20120621. Conference Publication:
IS - var.pagings
N2 - Objective: To analyze risk factors and morbidity associated with tics in children. Background: Tics are the most common movement disorders in children. Methods: Ninety-two children (73% boys, mean age 9 years, range 2-17) were prospectively evaluated with a standardarized protocol for tics. Yale Global Tic Severity Scale (YGTSS) was applied to 65 patients. Results: Children were classified as follows: Tourette syndrome (56%), chronic motor or phonic tics (24%) and tics of <12-month duration (20%). Overall, 97% of the patients had motor simple tics and 73% had complex tics. Tics were limited to the face/neck in 30% of children and were generalized in the rest. Simple (throatclearing, shouting, nonspecific noises, sniffing and coughing) and complex (coprolalia and palilalia) phonic tics affected 75% and 30% of the cohort, respectively. Motor tics showed an earlier onset than phonic tics (6.2 vs 7.2 yr; p<.05).Stressful situations and certain daily-life activities (school exams or homework), but also playing video-games and watching TV, aggravated tics in one-third to one half of patients. As opposite, artistic activities (theatre, dancing, painting, listening to music), playing sports and other

outdoor activities improved them. Throat infections were identified as a trigger factor or as an improving event in 4% and 8% of cases, respectively. Tic-associated pain/thraumatic lesions and disabling tics affected 40% and 20% of patients overtime. The following comorbidities were present in children with tics: obsessions and compulsions (44%), ADHD (36%), anxiety (38%). ADHD was significantly more frequent in boys (44% vs 14%; p=.01). YGTSS scores correlated positively with time of evolution (r5.273, p=.026), and Tourette patients obtained higher scores than other diagnosis (36.7 vs 16.7; p<.001). Poor school performance was associated with higher scores in the YGTSS (37.8 vs 24.5; p=.018) and with the diagnosis of Tourette (33% vs 15%; p=.043) and ADHD (46% vs 18%; p=.007). Conclusions: Several daily-life activities were identified as modifying factors of tic severity and may be important clues for tic management. In a small proportion of children, tics were associated with significant morbidity and poor school performance
SN - 0885-3185
AD - (Perez-Duenas, Caurin, Serrano, Fernandez) EspluguesSpain
ER -

TY - JOUR
ID - 3481
T1 - Technical feasibility and accepta bility of sms based sun protection recommendations for adolescents after organ transplantation
A1 - Sachse,M.M.
A1 - Bottcher,S.
A1 - Zeeb,H.
A1 - Pape,L.
A1 - Wagner,G.
A1 - Wehowsky,F.
A1 - Mehls,O.
A1 - Klaus,G.
A1 - Barz,M.
A1 - Jahn,I.
Y1 - 2012///
N1 - 70795533
English
Journal: Conference Abstract
KW - EMBASE
KW - organ transplantation
KW - protection
KW - Society
KW - Human
KW - dermatology
KW - Adolescent
KW - ultraviolet radiation
KW - Male
KW - Female
KW - light exposure
KW - text messaging
KW - mobile phone
KW - skin cancer
KW - questionnaire
KW - patient
KW - Transplantation
KW - teleconsultation
KW - childhood
KW - follow up
KW - exposure
KW - juvenile
KW - Risk
KW - Child
KW - carcinogen

KW - sunscreen
RP - NOT IN FILE
SP - 83
EP - 84
JF - European Journal of Pediatric Dermatology
VL - Conference: 11th Congress of European Society for Pediatric Dermatology, ESPD 2012 Istanbul Turkey. Conference Start: 20120516 Conference End: 20120519. Conference Publication:
IS - var.pagings
N2 - Introduction. Children and adolescents with organ transplants have an increased risk of skin cancer. The cumulative and intermittent UV exposures are considered main carcinogens for the development of skin cancer. Consequently it is very important to increase sun protection knowledge and to decrease UV light exposure in childhood. Objective. To evaluate the technical feasibility and acceptability of short message service (SMS) based sun protection recommendations for adolescents after organ transplantation following a special sun protection program. Methods. Adolescents after organ transplantation were invited to participate in a special sun protection program. Thereafter the participants received sun protection recommendations on a daily basis for 4 weeks sent to their mobile phones via SMS, depending on the regional UV index. Baseline survey consisted of a questionnaire which was carried out before the sun protection program took place. The follow-up survey (telephone interview, questionnaire) was conducted by trained interviewers after text messaging intervention. Results. A total of 25 (female 8, male 17) adolescent organ transplant patients were enrolled. 19 (female 6, male 13) of these 25 patients took part in the text messaging intervention. According to an interim analysis 84% of the participants (16/19) confirmed the daily receipt of sun protection recommendations on their mobile phones. All participants reported that text messages helped them to remember the most important information of the sun protection training. 11 of the 19 participating youth (females 3, males 8) mentioned that both sun protection training and text messages would have changed their sun protection behavior (e.g. increased application of sunscreen, use of fabric UV-light protection, decrease of outdoor activities during the lunch time). Conclusions. This study demonstrates that SMS based sun protection recommendations are technically feasible and accepted by adolescents after organ transplantation. In addition to the sun protection program this intervention may help on one side to increase sun protection knowledge and on the other side also help to reduce UV light exposure. Sex/gender differences and similarities should be taken into account in further investigations
SN - 1122-7672
AD - (Sachse, Wagner, Barz) Department of Dermatology, Allergology und Phlebology, Hospital Bremerhaven, Hannover, Germany (Bottcher, Zeeb, Wehowsky, Jahn) BIPS-Institute for Epidemiology and Prevention Research GmbH, Hannover, Germany (Pape) Department of Pediatric Nephrology, Hannover Medical School, Hannover, Germany (Mehls) Division of Pediatric Nephrology, Center for Pediatric and Adolescent Medicine, INF 430, University of Heidelberg, Germany (Klaus) KfH Pediatric Kidney Center, Marburg, Germany
ER -

TY - JOUR
ID - 3482
T1 - The challenges of being a father with systemic sclerosis
A1 - Poole,J.L.
A1 - Haygood,D.
A1 - Mendelson,C.F.
Y1 - 2011///
N1 - 70786907
English
Journal: Conference Abstract
KW - EMBASE
KW - Human
KW - rheumatology
KW - father
KW - systemic sclerosis
KW - college
KW - health practitioner
KW - Male
KW - diseases

KW - Child
KW - Scleroderma
KW - child parent relation
KW - parent
KW - Interview
KW - Female
KW - rare disease
KW - Muscle
KW - Qualitative Research
KW - disease duration
KW - questionnaire
KW - Fatigue
KW - Mortality
KW - Fear
KW - Content Analysis
KW - Chronic Disease
KW - Telephone
KW - sport
KW - skin
KW - connective tissue disease
RP - NOT IN FILE
JF - Arthritis and Rheumatism
VL - Conference: Annual Scientific Meeting of the American College of Rheumatology and Association of Rheumatology Health Professionals 2011 Chicago, IL United States. Conference Start: 20111104 Conference End: 20111109. Conference Publication:
IS - var.pagings
N2 - Background/Purpose: Systemic sclerosis (SSc) is an autoimmune connective tissue disease that affects multiple systems and results in thickening of the skin, vascular insufficiency and fibrotic changes in the muscles, joints and internal organs. SSc is a rare disease primarily affecting women. However, men also have SSc. The mean onset of SSc is between 30-50 years of age which are peak years when men are becoming or being fathers. The role of a father can be impacted in many ways by this chronic illness and its unpredictability. Despite the severity of SSc, little is known about the impact this illness has on parenting especially being a father. Therefore, the purpose of this qualitative study is to describe the impact of scleroderma on the role of a father. Methods: Ten fathers with scleroderma that had children 18 years of age and younger living with them at least 50% of the time, participated in this study. The mean age of the fathers was 44.80 years; mean disease duration was 3.76 years. Ninety percent were married, 60% worked full time, and the mean number of children was two. Fathers completed demographic questionnaires and were interviewed over the telephone. The interviews consisted of questions regarding the aspects of SSc that interfered with their ability to parent, situations in which SSc interfered with their ability to parent, what would make parenting easier, advice for other fathers, and support that makes being a father easier. The interviews were tape-recorded and transcribed verbatim. Content analysis was used to determine the primary codes and a summary statement generated for each code. Working from the summary statements the data was aggregated into two key themes with a single overarching theme. Results: The overarching theme, I'm still Dad described the feelings of the fathers that scleroderma does not negate the fact or importance of parenting. Daily tasks and daily routines change but the fathering role stays constant. The two key themes that emerged from the findings related to the emotional impact of the illness and the day to day realities of the illness. The unpredictability and rareness of the illness lead to ongoing feeling of isolation and the fear of the mortality of the illness. The daily activities fathers participated with their children changed related to fatigue, vascular and musculoskeletal physical changes. Even though the men had physical challenges, support systems in place helped them to complete day to day routines. Conclusion: Being a father with scleroderma has positive and negative influences. The negative effects are the inability of the fathers to participate in all of the physically activities the children enjoyed such as outdoor sports and throwing a ball. Being able to take time to spend quality time with the child was a positive influence of the illness
SN - 0004-3591
AD - (Poole, Haygood, Mendelson) University of New Mexico, Albuquerque, NM, United States (Haygood) Bernalillo Public Schools, Bernalillo, NM, United States
ER -

TY - JOUR
ID - 3483
T1 - Milk volume, vitamin D supplementation, outdoor activity and season are important predictors of vitamin D serum level in preschoolers: A target kids! Study
A1 - Maguire,J.L.
A1 - Birken,C.S.
A1 - Mamdani,M.
A1 - Khovratovich,M.
A1 - Wu,Y.Y.
A1 - Vieth,R.
Y1 - 2011///
N1 - 70784111
English
Journal: Conference Abstract
KW - EMBASE
KW - milk
KW - blood level
KW - Human
KW - supplementation
KW - season
KW - preschool child
KW - Society
KW - Child
KW - calcium metabolism
KW - Skin Pigmentation
KW - multiple linear regression analysis
KW - skin
KW - Male
KW - secondary analysis
KW - Canada
KW - Population
KW - serum
KW - Pediatrics
KW - community
KW - cross-sectional study
KW - vitamin D
KW - parathyroid hormone
KW - alkaline phosphatase
KW - Calcium
KW - phosphate
RP - NOT IN FILE
SP - 47A
EP - 48A
JF - Paediatrics and Child Health
VL - Conference: 88th Annual Conference Abstracts of the Canadian Paediatric Society Quebec City, QC Canada. Conference Start: 20110615 Conference End: 20110618. Conference Publication:
IS - var.pagings
N2 - Background: NHANES data have revealed important clinical predictors of vitamin D serum level. However, for preschoolers, significant gaps remain. Objective: To examine the relationship between 25-OH vitamin D serum level and volume of milk, outdoor activity, season, BMI, vitamin D supplementation and skin pigmentation. Our secondary objective was to examine whether biochemical indices of calcium metabolism could define the physiologically normal 25-OH vitamin D level. Design/Methods: A cross-sectional study was performed. Healthy children aged 1-5 years were recruited from TARGet Kids! community-based pediatric practices in Toronto, Canada (43degreeN) during well child visits. The primary analysis examined the direction and magnitude of potential clinical predictors of 25-OH vitamin D level. The secondary analysis examined associations between 25-OH vitamin D level and biochemical measures of calcium metabolism. Results: 849

children were recruited between December 2008 and October 2010. The mean age was 35 months (SD 17), 52% were male, 51% had white skin, and 56% were vitamin D supplemented. Mean 25-OH vitamin D level was 92 nmol/L (SD 33), 31% were below 75 nmol/L and 5% were below 50 nmol/L. Univariable analysis revealed cow's milk intake (p<0.0001), vitamin D supplementation (p<0.0001), outdoor play (p=0.0005) and season (p<0.0001) were associated with 25-OH vitamin D level with a trend towards skin pigmentation (p=0.07) and BMI (p=0.09) but not screen time (p=0.8). Multiple linear regression revealed that 25-OH vitamin D level increased by 5 nmol/L (95% CI 3-7) per 250 mL cup of cow's milk per day, 11 nmol/L (95% CI 6-16) in vitamin D supplemented children, 4.5 nmol/L (95% CI 2-7) per hour of outdoor play per day and 10 nmol/L (95% CI 5-15) May through September. Using these 4 variables for predicting 25-OH vitamin D level <75 nmol/L resulted in an area under the receiver operating curve of 0.81. There was no significant association between 25-OH vitamin D level, PTH, phosphate, calcium or alkaline phosphatase. Conclusions: Serum 25-OH vitamin D in urban 1-5 year old children at 43degreeN is influenced by daily volume of cow's milk, vitamin D supplementation, outdoor play and season. Biochemical measures of calcium metabolism, including PTH, are not useful in determining physiologically normal 25-OH vitamin D levels in this population
SN - 1751-7222
AD - (Maguire, Birken, Mamdani, Khovratovich, Wu, Vieth) St Michael's Hospital, Toronto, ON, Canada
ER -

TY - JOUR
ID - 3484
T1 - Vitamin d and cardiometabolic risk factors in preschoolers: A target kids! Study
A1 - Maguire,J.L.
A1 - Birken,C.S.
A1 - Mamdani,M.
A1 - Khovratovich,M.
A1 - Wu,Y.Y.
A1 - Vieth,R.
A1 - McCrindle,B.W.
A1 - Parkin,P.C.
Y1 - 2011///
N1 - 70784105
English
Journal: Conference Abstract
KW - EMBASE
KW - cardiometabolic risk
KW - risk factor
KW - preschool child
KW - Human
KW - Society
KW - Child
KW - cholesterol blood level
KW - multiple linear regression analysis
KW - exposure
KW - blood level
KW - Population
KW - community
KW - serum
KW - cross-sectional study
KW - supplementation
KW - Skin Pigmentation
KW - secondary analysis
KW - milk
KW - questionnaire
KW - Anthropometry
KW - Blood
KW - season
KW - Obesity

KW - skin
KW - Male
KW - Canada
KW - Pediatrics
KW - Public Health
KW - vitamin D
KW - triacylglycerol
KW - high density lipoprotein
KW - low density lipoprotein
RP - NOT IN FILE
SP - 44A
EP - July
JF - Paediatrics and Child Health
VL - Conference: 88th Annual Conference Abstracts of the Canadian Paediatric Society Quebec City, QC Canada. Conference Start: 20110615 Conference End: 20110618. Conference Publication:
IS - var.pagings
N2 - Background: Little is known about the relationship between vitamin D serum levels and cardiometabolic risk factors in preschoolers. With a potentially long duration of exposure to low vitamin D levels, identifying whether such relationships exist in preschoolers could have important long- term public health implications. Objective: To examine the relationship between 25-OH vitamin D serum level and cardiometabolic risk factors: total cholesterol, triglycerides, HDL, LDL and non-HDL. Design/Methods: A cross-sectional study was performed. Healthy children aged 1-5 years were recruited from TARGet Kids! participating community-based pediatric practices in Toronto, Canada (43degreeN) during well child visits with concurrent measurement of 25-OH vitamin D and cardio- metabolic factors by blood test as well as anthropometrics and dietary exposures by questionnaire. Our primary analysis examined associations between 25-OH vitamin D level and cardiometabolic risk factors. Our secondary analysis used multiple linear regression to examine whether such relationships were independent of BMI and other known factors which influence 25-OH vitamin D level. Results: 849 children were recruited between December 2008 and October 2010. The mean age of the population was 35 months (SD 17), 52% were male, 51% had white skin, 22% were overweight and 6% were obese. Mean 25-OH vitamin D level was 92 nmol/L (SD 33), 31% were below 75 nmol/L and 5% were below 50 nmol/L. Univariable analysis revealed associations between higher 25-OH vitamin D level and lower total cholesterol (p=0.002), lower triglycerides (p<0.0001), lower non-HDL (p=0.02), and a trend towards higher HDL (p=0.07) but not LDL (p=0.5). Multiple linear regression adjusted for BMI z-score, age, milk intake, outdoor activity, season, skin pigmentation and vitamin D supplementation revealed that for each 1 nmol/L increase in 25-OH vitamin D, total cholesterol decreased by 0.17 mmol/L (95% CI 0.11-0.39), triglycerides decreased by 0.15 mmol/L (95% CI 0.10-0.30) and non-HDL decreased by 0.19 mmol/L (95% CI 0.110.56). Conclusions: Serum 25-OH vitamin D in urban 1-5 year old children at 43degreeN is associated with cardiometabolic risk factors. This association appears to be independent of BMI and other clinical variables that are associated with 25-OH vitamin D level
SN - 1751-7222
AD - (Maguire, Birken, Mamdani, Khovratovich, Wu, Vieth, McCrindle, Parkin) St Michael's Hospital, Toronto, ON, Canada
ER -

TY - JOUR
ID - 3485
T1 - Inactive transport: Stroller use and physical activity in preschool children: A target kids! Study
A1 - Birken,C.S.
A1 - Maguire,J.
A1 - Khovratovich,M.
A1 - Wu,Y.Y.
A1 - Mamdani,M.
A1 - McCrindle,B.W.
A1 - Parkin,P.C.
Y1 - 2011///
N1 - 70784040
English
Journal: Conference Abstract

KW - EMBASE
KW - preschool child
KW - Human
KW - Society
KW - Physical Activity
KW - Child
KW - parent
KW - Risk
KW - Temperament
KW - Obesity
KW - Canada
KW - multiple linear regression analysis
KW - Waist Circumference
KW - primary medical care
KW - community
KW - Birth Weight
KW - Child Behavior Checklist
KW - cross-sectional study
KW - stimulus
KW - Pleasure
KW - Family Size
KW - Day Care
KW - Male
KW - model
KW - anticipatory guidance
KW - groups by age
KW - traffic and transport
RP - NOT IN FILE
SP - 21A
EP - July
JF - Paediatrics and Child Health
VL - Conference: 88th Annual Conference Abstracts of the Canadian Paediatric Society Quebec City, QC Canada. Conference Start: 20110615 Conference End: 20110618. Conference Publication:
IS - var.pagings
N2 - Background: Guidelines for physical activity for preschool children promote at least 60 minutes of daily unstructured physical activity (PA). Reduction of sedentary transportation by stroller is encouraged. Objective: To determine if stroller use in healthy children ages 1-5 is associated with PA levels, and adiposity. Modifiable factors associated with increased stroller use were examined. Design/Methods: A cross-sectional study was performed. Healthy children age 1-5 years were recruited at well-child visits from TARGet Kids! community primary care practices in Toronto, Canada with parent reported measures of child and family characteristics including daily PA, nutritional risk (using the NutriSTEP), and child temperament (using the Child Behaviour Checklist). zBMI (WHO) and z-waist circumference (zWC) were used to measure adiposity. Parents reported the proportion of time per week that their child used a stroller or wagon when going for a walk. Multiple linear regression models were used to estimate effects and adjust for potential confounders. Results: 1070 children 1-5 years old were included. The mean age was 35 months; 51% were male. 18% had a zBMI >1 and 6% had zBMI >2. 80% of children 1-2 years of age, compared to 25% of children 3-5 years of age reported using a stroller at least 50% of the time; odds ratio 12.4 (95% CI 9.2, 16.9). Increased stroller use was associated with decreased unstructured PA per week. Adjusted for daycare use, child temperament, and parent perception of child PA level, always using a stroller, compared to never using a stroller, was associated with a decrease in days per week of unstructured physical activity of almost 1 day; -0.72 (-1.2, -0.3). Factors associated with increased stroller use included younger age (p<0.001), and use of a bottle (p=0.004). High effortful control temperament score (pleasure with low stimulus intensity, high attentional focus) was associated with lower stroller use (p=0.03). When adjusted for age, birth weight, nutritional risk, maternal BMI, and maternal PA, there was no association between stroller use and zBMI (p=0.50) or z-WC (p=0.23). Conclusions: Preschool children are spending a large proportion of their outdoor time inactive in a stroller. Anticipatory guidance for parents on appropriate stroller use may be one strategy to improve PA in this age group
SN - 1751-7222

AD - (Birken, Maguire, Khovratovich, Wu, Mamdani, McCrindle, Parkin) Hospital for Sick Children, Toronto, ON, Canada
ER -

TY - JOUR
ID - 3486
T1 - Child care arrangement and BMI/activity levels in preschoolers
A1 - Brouwer,R.
A1 - Ostbye,T.
A1 - jamin-Neelon,S.
Y1 - 2012///
N1 - 70782539
English
Journal: Conference Abstract
KW - EMBASE
KW - Human
KW - Obesity
KW - preschool child
KW - Child Care
KW - Child
KW - mother
KW - Female
KW - Accelerometry
KW - prevention study
KW - sitting
KW - Prevention
KW - Physical Activity
KW - Income
KW - gender
KW - epidemic
RP - NOT IN FILE
SP - 257
JF - Obesity Facts
JA - Obes Facts
VL - Conference: 19th European Congress on Obesity, ECO2012 Lyon France. Conference Start: 20120509 Conference End: 20120512. Conference Publication:
IS - var.pagings
N2 - Introduction: Understanding factors associated with BMI and activity levels in young children can help address the obesity epidemic. An important consideration may be where and with whom children spend time. We explored the relationship between childcare arrangement (CCA) and 1) moderate-to-vigorous physical activity (MVPA), 2) sedentary activity (SED), and 3) BMI in preschoolers.Methods: We assessed CCA at baseline and 1-year later for children (N=229; age 55 months) enrolled in an obesity prevention trial. CCA groups were defined as "mother only" (MO; exclusive mother-care at both timepoints), "outside home" (OH; exclusive care outside the home at both timepoints) and "mixed" (MX; includes part-time care, and multiple CCAs). MVPA/SED was measured via 7-day accelerometry; BMIz-score was calculated from standardized measurements. Results: OH was more likely to be black (29.2% vs. 12.7% in MO, 17.9% in MX), and have income >$l60,000/year (MO: 80% vs. 51.3%, MX: 63.9%). MO was youngest (39.5 months vs. 41 in FC, 46.3 in MX). In unadjusted analysis, MO had significantly lower BMIz (.24) compared to others (MX = .51, p=.05, OH =.58, p=.03), and less SED than FC (369 vs. 395 min/day, p=.05). CCA was not associated with MVPA. After adjusting for child gender, race, and age, MO and OH remained significantly different for BMIz (p=.04). Conclusions: Preschool children cared for exclusively by their mothers may have healthier BMIs and less sedentary time than children in other care. Obesity prevention efforts are important for all preschoolers, and the biggest need may be with arrangements outside of mother-care
SN - 1662-4025
AD - (Brouwer, Ostbye, Benjamin-Neelon) Duke University, Community and Family Medicine, Durham, United States
ER -

TY - JOUR
ID - 3487
T1 - Physical activity and obesity in european children - Objective measurement by accelerometry and The impact of The built environment in The idefics study
A1 - Ahrens,W.
A1 - Buck,C.
A1 - Pohlabeln,H.
A1 - De,Bourdeaudhuij,I
A1 - De,Henauw S.
A1 - Marild,S.
A1 - Molnar,D.
A1 - Moreno,L.A.
A1 - Pitsiladis,Y.
A1 - Reisch,L.
A1 - Siani,A.
A1 - Tornaritis,M.
A1 - Veidebaum,T.
A1 - Konstabel,K.
A1 - Pigeot,I.
Y1 - 2012///
N1 - 70782494
English
Journal: Conference Abstract
KW - EMBASE
KW - Obesity
KW - Human
KW - Accelerometry
KW - Child
KW - Environment
KW - Physical Activity
KW - density
KW - Risk
KW - school
KW - logistic regression analysis
KW - confidence interval
KW - parent
KW - Walking
KW - school child
KW - model
KW - Travel
KW - Europe
KW - Anthropometry
KW - examination
KW - preschool child
KW - recombinant erythropoietin
RP - NOT IN FILE
SP - 244
JF - Obesity Facts
JA - Obes Facts
VL - Conference: 19th European Congress on Obesity, ECO2012 Lyon France. Conference Start: 20120509 Conference End: 20120512. Conference Publication:
IS - var.pagings
N2 - IntroductionReported associations between physical activity (PA) and overweight/obesity in children may be weak due to imprecise measurements and inappropriate cut-offs defining moderate-to-vigorous PA (MVPA). The degree to which children's living environment shapes their PA is unknown.MethodsIn 2007/08, 16,223 children (2-9 years) from 8 European countries participated in extensive examinations including anthropometry

and PA (accelerometry, 60-second epochs, n=7,451). Parents reported socio-demographic, behavioural, medical and nutritional data. Adjusted odds ratios (OR; 95%-confidence intervals [CI]) were estimated by logistic regression. BMI was categorized according to IOTF. We assessed opportunities for PA by a moveability index considering multiple urban forms (footpaths, intersections, playgrounds) which was linked with reported PA (n=460) and MVPA (n=275) in German school children. Using multilevel adjusted regression models, we investigated The impact of The moveability index on PA and travel mode to school.ResultsMVPA varied dramatically depending on The definition used. Using Evenson's criteria, 9.7% (18.3%) of preschool (school) children spent 60+ minutes in MVPA/day. This percentage varied substantially across Europe. Compared to children with 60+ minutes MVPA/day The risk for overweight/obesity was 1.26 (CI 1.04-1.53) and 1.56 (CI 1.29-1.90) in children with 30-60 minutes and <30 minutes MVPA, respectively. Footpath density was positively associated with walking to school (OR=2.17, p=0.034). Density of recreational facilities (beta=0.22, p=002) and The moveability index (beta=0.25, p<0.001) showed a positive effect on MVPA/day.ConclusionMVPA was strongly associated with overweight/obesity in children. The moveability index was a positive predictor of PA, where recreational facilities were The main factor influencing PA
SN - 1662-4025
AD - (Ahrens) BIPS, Institute for Epidemiology and Prevention Research, Epidemiological Methods and Etiologic Research, Bremen, Germany (Buck, Pohlabeln, Pigeot) BIPS, Institute for Epidemiology and Prevention Research, Bremen, Germany (De Bourdeaudhuij) Ghent University, Faculty of Medicine and Health Sciences, Ghent, Belgium (De Henauw) Ghent University, Faculty of Medicine and Health Sciences, Belgium (Marild) Gothenburg University, Gotehnburg, Sweden (Molnar) University of Pecs, Pecs, Hungary (Moreno) Genud (Growth, Exercise, Nutrition and Development) Research Group, University of Zaragoza, Zaragoza, Spain (Pitsiladis) University of Glasgow, Glasgow, United Kingdom (Reisch) Copenhagen Business School, Denmark (Siani) Institute of Food Sciences, Unit of Epidemiology and Population Genetics, National Research Council, Avellino, Italy (Tornaritis) Research and Education Institute of Child Health, Italy (Veidebaum, Konstabel) National Institute for Health Development, Tallinn, Estonia
ER -

TY - JOUR
ID - 3488
T1 - Effects of a physical activity intervention in children attending child care (youp'la bouge program): A cluster-randomized controlled trial
A1 - Antoine,B.
A1 - Jerome,B.
A1 - Susi,K.
A1 - Pedro,M.V.
A1 - Tanja,K.
A1 - Jardena,P.
Y1 - 2012///
N1 - 70781873
English
Journal: Conference Abstract
KW - EMBASE
KW - Human
KW - randomized controlled trial
KW - Child Care
KW - Child
KW - Obesity
KW - Physical Activity
KW - Motor Performance
KW - Quality of Life
KW - parent
KW - Environment
KW - workshop
KW - girl
KW - Female
KW - Health Promotion
KW - accelerometer

RP - NOT IN FILE
SP - 71
JF - Obesity Facts
JA - Obes Facts
VL - Conference: 19th European Congress on Obesity, ECO2012 Lyon France. Conference Start: 20120509 Conference End: 20120512. Conference Publication:
IS - var.pagings
N2 - Introduction: Physical activity (PA) is of high importance for health promotion in young children and child care centers represent ideal settings. To fill the existing gap, we conducted a cluster RCT assessing the effectiveness of Youp'luge, a real-life program in child care centers implemented by governmental agenciesMethods: 58 child care centers were randomly selected and 1:1 assigned to a control and an intervention group. The Youp'luge program supports unstructured PA by providing workshops for educators, adapting the built environment of the child care centers, providing time for PA and involving parents. Motor Skills was the primary and BMI, PA (accelerometers) and Quality of Life secondary outcomes.Results: Of the 1616 children, 1467 (age 3.3 +0.7 yrs, 46% girls) participated in the study. 648 children (BMI 16.2 + 1.2 kg/m², 12% overweight & obese,) were present on the motor test day at baseline (mean attendance at child care 48%). There were no intervention effects in the measured outcomes. However, within the intervention group, associated predictors were identified for improvements in motor skills (free-access movement corner, parental evenings), BMI (motivated and additionally formed educators) and PA (type of equipment).Conclusion: This real-life PA program did not improve motor skills or the secondary outcomes. As many programs have to balance between effectiveness and feasibility outside of a research setting, taking the found predictors into account may improve outcomes in such programs
SN - 1662-4025
AD - (Antoine, Jerome) University of Lausanne, Institut of Sport Sciences, Lausanne, Switzerland (Susi) Swiss Tph, Sweden (Pedro, Jardena) University of Lausanne, Faculty of Biology and Medicine, CHUV, Lausanne, Switzerland (Tanja) University of Zurich, Child Development Center, Switzerland
ER -

TY - JOUR
ID - 3490
T1 - Double jeopardy for childhood obesity: Early childhood risk factors and lack of perception in socially disadvantaged areas
A1 - Gibbons,K.
A1 - Naughton,G.
Y1 - 2012///
N1 - 70781773
English
Journal: Conference Abstract
KW - EMBASE
KW - childhood obesity
KW - childhood
KW - risk factor
KW - Obesity
KW - Human
KW - Child
KW - parent
KW - Food
KW - preschool child
KW - weight
KW - Physical Activity
KW - Television Viewing
KW - Nutrition
KW - Risk
KW - Prevalence
KW - Australia
KW - Government
KW - Interview

KW - Prevention
KW - computer
KW - questionnaire
KW - Education
KW - community
RP - NOT IN FILE
SP - 42
JF - Obesity Facts
JA - Obes Facts
VL - Conference: 19th European Congress on Obesity, ECO2012 Lyon France. Conference Start: 20120509 Conference End: 20120512. Conference Publication:
IS - var.pagings
N2 - Introduction: Childhood obesity is over-represented in socially disadvantaged communities. Families living in these areas in Australia have high proportions of parents with low levels of education, government supported needs, and culturally and linguistically diverse backgrounds. Methods: To understand more about early risk factors for childhood obesity in socially disadvantaged areas, we asked parents (n = 796) and health/education professionals (n=45) of children aged 0 to 8 years about children's nutrition and physical activity practices, and perceptions of their child's weight status. Parents and carers completed questionnaires and a small number of carers also consented to interviews. Local interpreters assisted parents of pre-school children, where appropriate.Results: Despite a national prevalence of childhood overweight and obesity of 20 to 25% among children 4 to 8 years of age, only 5% of parents reported concerns about excessive weight in their child. More carers than parents expressed greater concerns about children's risk of obesity. Approximately three in every four children aged 2-6 years averaged less than two hours of outdoor active play. The recommended one hour of 'screen' time for preschool children was exceeded by 80% of children. In children 6-8 years, television viewing doubled and computer time tripled on weekends. Reports of food insecurity, consumption of take away foods and requests for lower cost healthy foods, indicated complexities facing some families.Conclusion: Early obesity prevention strategies may be a low priority for socially disadvantaged families. Broader base supports and strategies assisting families to translate knowledge into practice are required
SN - 1662-4025
AD - (Gibbons) Murdoch Childrens Research Institute, Royal Children's Hospital, Nutrition, Australia (Naughton) Australian Catholic University, Exercise Science, Melbourne, Australia
ER -

TY - JOUR
ID - 3491
T1 - The who childhood obesity surveillance initiative (COSI) in Greece
A1 - Hassapidou,M.
Y1 - 2012///
N1 - 70781677
English
Journal: Conference Abstract
KW - EMBASE
KW - childhood obesity
KW - Greece
KW - Obesity
KW - Human
KW - school
KW - Child
KW - questionnaire
KW - Female
KW - girl
KW - primary school
KW - boy
KW - Male
KW - Prevalence
KW - Fruit
KW - Lifestyle

KW - Physical Education
KW - sport
KW - groups by age
KW - underweight
KW - student
KW - Eating
KW - childhood
KW - milk
KW - dietitian
KW - Physical Activity
RP - NOT IN FILE
SP - 14
JF - Obesity Facts
JA - Obes Facts
VL - Conference: 19th European Congress on Obesity, ECO2012 Lyon France. Conference Start: 20120509 Conference End: 20120512. Conference Publication:
IS - var.pagings
N2 - The Childhood obesity surveillance initiative in Greece was carried out from November 2010 to March 2011. 5701 children, 7-8 and 9-10 years old (2nd and 4th elementary school grade) from 150 primary schools randomly selected and nationally representative, participated in the study. Anthropometric measurements were performed by experienced dietitians who also completed the student and school questionnaires. Family questionnaires were completed by the family and returned to schools. In the second grade 23% of the children were overweight (OW) and 13,6% obese (OB) whereas in the forth grade 28,9% were overweight and 13,4% obese. No differences were found for both grades in the OW and OB prevalence between boys and girls. Percentage of underweight girls was higher than boys in both age groups. A negative correlation was found between rates of OW and OB and participation in sports. Only 20% of schools reported to provide physical education lessons more than three hours per week. 98% of schools reported to have outside playgrounds or inside play areas where children can play during school breaks and about half schools had organized initiatives/projects to promote a healthy lifestyle. Only 21% of schools had fresh fruits in their canteens and 31% milk. The results showed that childhood overweight and obesity prevalence is very high in Greece. Care should be taken to promote healthy eating and increase physical activity in schools
SN - 1662-4025
AD - (Hassapidou) Alexander Technological Educational Institute, Department of Nutrition and Dietetics, Thessaloniki, Greece
ER -

TY - JOUR
ID - 3492
T1 - Practicing what we preach part 2: A folow-up loo k at healthy active living policy and practice in canadian academic paediatric health centres (CAPHC)-has anything changed?
A1 - Leblanc,C.M.A.
A1 - Chui,N.
A1 - Ambler,K.
A1 - Adamo,K.B.
A1 - Spence,G.
A1 - Orrbine,E.
A1 - Cummings,E.
Y1 - 2010///
N1 - 70780393
English
Journal: Conference Abstract
KW - EMBASE
KW - health center
KW - Society
KW - Policy
KW - Human
KW - Child

KW - Hospital
KW - fast food
KW - machine
KW - Walking
KW - Health
KW - worker
KW - Nutrition
KW - patient
KW - follow up
KW - planning
KW - Physical Activity
KW - sitting
KW - prophylaxis
KW - Health Care Policy
KW - hospital administrator
KW - Prevalence
KW - market
KW - outpatient
KW - fitness
KW - agricultural worker
KW - advertizing
KW - catering service
KW - waiting room
KW - pediatric hospital
KW - budget
RP - NOT IN FILE
SP - 73A
EP - 74A
JF - Paediatrics and Child Health
VL - Conference: 87th Annual Conference of the Canadian Paediatric Society Vancouver, BC Canada. Conference Start: 20100622 Conference End: 20100626. Conference Publication:
IS - var.pagings
N2 - BACKGROUND: Twenty six percent of Canadian children/youth are overweight/obese. Public perception indicates hospitals should promote health and disease-prevention through institutional policy, long-range planning and resource budgeting. In 2007, we performed a survey of all Canadian Paediatric Academic Health Centres [CAPHC] demonstrating inadequate healthy active living (HAL) policy and practices. Fast food vending and patient sedentary activities were prevalent. A strong policy framework was recommended to create and implement HAL guidelines. OBJECTIVES: i) To perform a follow-up survey of all CAPHC to detect changes in HAL policy/practice since 2007. ii) To host a multi-disciplinary, pan-Canadian task force to create a compendium of HAL strategies. METHODS: In 2009, a modified HAL survey was completed by all 16 CAPHC. Local CAPHC champions collected and submitted data. A multi-disciplinary, pan-Canadian task force met to share best HAL practices and create novel hospital-based HAL strategies through several small group sessions. RESULTS : Survey 2009: Health Promoting Committees were similar at 56%. Although nonsmoking policy prevalence was unchanged, only partial adherence was reported by 93% of CAPHC. A mere 19% of CAPHC have written nutrition policy. Most CAPHC cafeterias had limited hours of operation. Sixty two percent of CAPHC had fast food/coffee franchises. In 81% of centres, the vendor determined vending machine content; 38% of CAPHC increased vending machine numbers. No CAPHC had Physical Activity (PA) promotion policies. Outdoor walking trails increased 27-50%. Thirty eight percent of CAPHC children's playrooms offered Wii fit. Thirty one percent of CAPHC had outpatient Child Life workers. None had incentives for staff PA other than discounted fitness club memberships. Suggested Strategies from Taskforce: Unique nutrition initiatives: Dial for dining room service; phase out fast food restaurants; restriction of fast food advertisements; hospital-based farmers markets. Unique PA initiatives: Staff walking challenges; patient treasure hunts; active play in waiting rooms; Active living passports. CONCLUSIONS: Canadian paediatric hospitals have demonstrated some improvement in HAL promotion since the first survey in 2007 but considerable effort is required to develop a more comprehensive and systematic approach. Urgent attention must be given to the common practice of fast food kiosks and vending machines with nutrition-poor content. The deficit of Child Life workers and lack of active play areas for children and teens must also be addressed. The

taskforce developed a compendium of HAL strategies that will be published and shared with all Canadian Pediatric Hospital administrators to help facilitate improvements in HAL practice and policy
SN - 1751-7222
AD - (Leblanc, Chui, Ambler, Adamo, Spence, Orrbine, Cummings) Department of Pediatrics, Stollery Children's Hospital, Edmonton, AB, Canada
ER -

TY - JOUR
ID - 3493
T1 - Pain in infants and young children with sickle cell disease-a prospective longitudinal analysis
A1 - Dampier,C.
A1 - Ely,E.
A1 - Aertker,L.
A1 - Brodecki,D.
A1 - Kesler,K.
A1 - Stuart,M.
Y1 - 2010///
N1 - 70774478
English
Journal: Conference Abstract
KW - EMBASE
KW - Infant
KW - Child
KW - Human
KW - sickle cell anemia
KW - Society
KW - hematology
KW - Pain
KW - dactylitis
KW - sickle cell
KW - emergency care
KW - newborn screening
KW - patient
KW - pain assessment
KW - school
KW - Diagnosis
KW - Hospitalization
KW - swelling
KW - thalassemia
KW - childhood
KW - Education
KW - informed consent
KW - Genotype
KW - Male
KW - surgery
KW - acute chest syndrome
KW - transfusion
KW - astronomy
KW - information processing
KW - consensus
KW - Telephone
KW - parent
KW - Adult
KW - hemoglobinopathy
KW - Adolescent
KW - occlusion
KW - hydroxyurea

RP - NOT IN FILE
JF - Blood
VL - Conference: 52nd Annual Meeting of the American Society of Hematology, ASH 2010 Orlando, FL United States. Conference Start: 20101204 Conference End: 20101207. Conference Publication:
IS - var.pagings
N2 - Pain is the hallmark symptom of vaso-occlusion in sickle cell disease (SCD), but has not been well studied, particularly in children. Previous studies of pain in very young children with SCD have largely been limited to episodes sufficiently severe as to require acute care visits or hospitalizations. Our previous studies in school-aged children and adolescents (Dampier CD et al. J Pediatr Hematol Oncol 2004), as well as those in adults (Smith W et al Ann Inter Med 2009) suggest that vaso-occlusive pain is frequently managed at home outside of acute care medical settings. We sought to obtain similar data about the home pain experience of infants and young children with SCD as reported by parents or guardians, and to explore the relationship between the pain experience and hematological biomarkers. Enrollment and data collection was conducted from Jan 1999-Mar 2008 at the Marian Anderson Sickle Cell Center as part of the Comprehensive Sickle Cell Centers program. Families with SCD infants identified by newborn screening were first approached about the study after completion of confirmatory diagnosis and initial SCD-related education, usually between 4 to 6 months of age. After informed consent, parents/guardians were trained in pain assessment and daily reporting. When sickle cell related pain events occurred, parents/guardians reported pain occurrence, location, associated symptoms and the treatment that they provided. Daily paper diaries were generally used in the first year of life to familiarize families with pain assessment and reporting. Subsequently a novel daily pager system was used to provide a method allowed daily reporting, but with reduced participant burden. A monthly telephone system in conjunction with a daily calendar was used for those families unable to effectively comply with providing daily reports. All pain reports were adjudicated by consensus of study staff and PI/Co-PI. Children were removed from the study for any period of chronic transfusion for clinical events (splenic sequestration, recurrent acute chest syndrome, surgery). Over the study period, 103 children (58% male) were enrolled beginning at a median age of 7.3 months (range 1.5, 65.2 months). This represented about 50% of SCD children referred to the Center by newborn screening during these time periods; the most common reasons for refusal were inability to complete daily reporting and geographic distance from the Center. An SS genotype was present in 50 children (48.5%), SC in 32 (31.1%), SB0thalassemia in 6 (5.8%), SB+thalassemia in 15 (14.6%). Children were actively followed for a median of 3.8 years (range 0.3-7.6 years). The total number of days children were assessed for pain was 141,197 days with an additional 28,079 days of missing data (16%). The total number of days that children had reported pain was 2,288 days (1.6%), which represented 768 distinct episodes of pain. Over 80% of children reported to have pain in the 0-12 month age interval had pain locations (hands/feet) and characteristics (swelling or tenderness) consistent with dactylitis, which became progressively less prevalent in older age intervals. Significantly more SS/SB0 patients (58%) had >2 days of average dactylitis pain during 0-12 months or 12-24 months (53%) intervals compared to SC/SB+ patients (0%) (p=0.04). The timing of the first reported dactylitis event (<2 years or 2 years) significantly predicted the frequency of SCD pain events per person year, with earlier onset associated with more frequent pain events during the study period (p=0.02), for both the SS/SB0 and SC/SB+ (P=0.03) groups. Our study demonstrates the feasibility of initial recruitment and subsequent daily reporting of clinical events by families of infants and young children with SCD over many years, particularly when careful consideration is given to enhance family support and minimizing respondent burden. The onset of an initial dactylitis episode prior 2 years of age, even if treated at home, was associated with more frequent pain throughout childhood, irrespective of hemoglobinopathy type. Such children may be appropriate for interventions, such as oral hydroxyurea, that reduce excessive pain frequency
SN - 0006-4971
AD - (Dampier) Emory University, Atlanta, GA, United States (Ely, Brodecki) Children's Hospital of Philadelphia, United States (Aertker, Kesler) Rho, Inc, United States (Stuart) Pediatrics, Thomas Jefferson University, Philadelphia, PA, United States
ER -

TY - JOUR
ID - 3494
T1 - LEF1 gene mutations in childhood T-cell acute lymphoblastic leukemia and T cell non-hodgkin's lymphoma
A1 - Park,M.-J.
A1 - Kiyokawa,N.
A1 - Kato,M.

A1 - Suzuki,N.
A1 - Oda,M.
A1 - Hara,J.
A1 - Kobayashi,R.
A1 - Horibe,K.
A1 - Ogawa,S.
A1 - Hayashi,Y.
Y1 - 2010///
N1 - 70773838
English
Journal: Conference Abstract
KW - EMBASE
KW - T lymphocyte
KW - hematology
KW - acute lymphoblastic leukemia
KW - gene mutation
KW - Society
KW - childhood
KW - nonhodgkin lymphoma
KW - Human
KW - Mutation
KW - patient
KW - exon
KW - Diagnosis
KW - Child
KW - gene
KW - skin tumor
KW - DNA binding
KW - clinical study
KW - Molecular Weight
KW - comparative genomic hybridization
KW - pleura effusion
KW - lymph node
KW - mutant
KW - blood cell
KW - Blood
KW - bone marrow
KW - Cell Differentiation
KW - cell surface
KW - childhood leukemia
KW - Male
KW - mediastinum mass
KW - Leukocyte Count
KW - Japan
KW - event free survival
KW - missense mutation
KW - enzyme active site
KW - computer program
KW - allelic imbalance
KW - Female
KW - Genome
KW - central nervous system
KW - microarray analysis
KW - Immunophenotyping
KW - upregulation
KW - Wnt signaling pathway
KW - beta catenin

KW - DNA
KW - transcription factor
KW - nucleotide
KW - genomic DNA
RP - NOT IN FILE
JF - Blood
VL - Conference: 52nd Annual Meeting of the American Society of Hematology, ASH 2010 Orlando, FL United States. Conference Start: 20101204 Conference End: 20101207. Conference Publication:
IS - var.pagings
N2 - LEF1 is a member of the LEF/TCF family of DNA-binding transcription factors, which interact with beta-catenin in the WNT signaling pathway. N-terminal LEF1 mutations that impair beta-catenin binding are commonly found inhuman sebaceous skin tumors, and expression of an N-terminal-deleted Lef1 mutant that lacks the beta-catenin binding domain leads to sebaceous skin tumors. The intracellular domain of NOTCH1 has also been shown to function as a co-activator of LEF1, leading to the up-regulation of target genes distinct from those activated by beta-catenin binding. Recently, inactivating mutations of LEF gene have been reported in T-cell acute lymphoblastic leukemia (T-ALL) cases. In this study, we analyzed the frequencies and clinical significance of LEF1 mutations in pediatric T-ALL and T-cell non-Hodgkin's lymphoma (T-NHL). Mutation analysis was performed on 138 of the primary T-ALL and T-NHL patient samples. The clinical data were available 55 newly diagnosed T-ALL and 14 T-NHL patients. These children, aged under 15 years were enrolled into the Japan Association of Childhood Leukemia Study (JACLS) protocol ALL-97 between 1997-2001 and JACLS trial NHL-T98 between 1998-2002. At the time of diagnosis, bone marrow and/or peripheral blood cells were obtained from T-ALL patients and lymph nodes and/or pleural effusions were obtained from T-NHL patients. A total of 69 patients were included in the present study; 49 were male and 20 female; 55 were children diagnosed with T-ALL (median age of 9.5 years; range: 2.0 - 15.0 years) and 14 with T-NHL (median age of 11.0 years; range: 3.7 - 15.0 years). We performed high-resolution array comparative genomic hybridization (array CGH) and sequencing was performed on the entire coding region of LEF1. High molecular weight genomic DNA was used for microarray analysis using Affymetrix GeneChip 50K XbaI, HindIII or 250K NspI, according to the manufacturer's instructions. Genome-wide detection of allelic imbalances was performed using CNAG/AsCNAR software. We identified mono or biallelic LEF1 deletions in 7.5% (5 of 67) of these primary samples. An additional 11.6% (16 of 138) of the cases harbored sequence alterations of LEF1 gene. Twelve of the single nucleotide alterations were found in exons 1, 2 and 3, and four of the frame-shift mutations were found in exons 3, 7, and 8. Frame-shift mutations were novel LEF1 mutations, and mutations in exons 3 and 8 were positioned outside catalytic domain and DNA binding region. Missense mutations in exon 1 were located highly conservative lesion of beta-catenin binding domain of LEF1. Of the 8 LEF1 alterations detected in 16 cases also had NOTCH1 mutations. Analysis of the available clinical data showed that LEF1 gene alteration was not a significant predictor of event-free survival in children with T-ALL treated in JACLS ALL-97 and NHL-T98 protocol. However, LEF1 inactivation was associated with a younger age at the time of diagnosis, but not with sex, white blood cell count, central nervous system involvement, or the presence of an anterior mediastinal mass at the time of diagnosis. Furthermore, analysis of the T-ALL cell surface immunophenotype obtained at the time of diagnosis showed that all LEF1-alterd cases were characterized by developmental arrest at a cortical stage of T-cell differentiation. The clinical significance of these gene alterations and detailed data is discussed
SN - 0006-4971
AD - (Park) Dept. of Hematology/Oncology, Gunma Children's Medical Center, Shibukawa, Japan (Kiyokawa) National Cemter of Child Health and Development, Tokyo, Japan (Kato, Ogawa) Cancer Genomics Project, University of Tokyo, Tokyo, Japan (Suzuki) School of Medicine, Dept. of Pediatrics, Sapporo Medical University, Sapporo, Japan (Oda) Health Sciences, Okayama University, Graduate School of Medicine, Okayama, Japan (Hara) Hematology/Oncology, Medical Children's Center, Osaka City General Hospital, Osaka, Japan (Kobayashi) Depatment of Pediatrics, Sapporo Hokuyu Hospital, Sapporo, Japan (Horibe) Clinical Research Center, National Hospital Organization, Nagoya Medical Center, Japan (Hayashi) Dept. of Hematology/Oncology, Gunma Children's Medical Center, Gunma, Japan
ER -

TY - JOUR
ID - 3495
T1 - RNAi screen reveals TYK2 as a novel therapeutic gene target in T-cell acute lymphoblastic leukemia
A1 - Tyner,J.W.

A1 - Sanda,T.
A1 - Gutierrez,A.
A1 - Glover,J.M.
A1 - Chang,B.H.
A1 - Willis,S.G.
A1 - Ahn,Y.
A1 - Look,A.T.
A1 - Druker,B.J.
Y1 - 2010///
N1 - 70773354
English
Journal: Conference Abstract
KW - EMBASE
KW - T lymphocyte
KW - acute lymphoblastic leukemia
KW - Society
KW - hematology
KW - gene
KW - Human
KW - patient
KW - Therapy
KW - Cell Line
KW - cell viability
KW - mutant
KW - Phosphorylation
KW - cell transformation
KW - Point Mutation
KW - Sequence Analysis
KW - Prognosis
KW - Adult
KW - Child
KW - cancer cell
KW - assay
KW - multigene family
KW - library
KW - oncogene
KW - tumor cell
KW - informed consent
KW - cancer patient
KW - hematologic malignancy
KW - cancer inhibition
KW - Diagnosis
KW - whole cell
KW - Mutation
KW - STAT1 protein
KW - phosphotransferase inhibitor
KW - protein tyrosine kinase
KW - STAT3 protein
KW - protein tyrosine kinase inhibitor
KW - Antibody
KW - small interfering RNA
KW - ficoll
KW - cell extract
KW - phosphotransferase
KW - DNA
RP - NOT IN FILE
JF - Blood

VL - Conference: 52nd Annual Meeting of the American Society of Hematology, ASH 2010 Orlando, FL United States. Conference Start: 20101204 Conference End: 20101207. Conference Publication:
IS - var.pagings
N2 - Background: Tyrosine kinases are widely implicated in the genesis of hematologic malignancies and tyrosine kinase inhibitors have achieved remarkable clinical outcomes. However, most cancer patients are ineligible for this form of therapy because we lack knowledge of the oncogenes driving the growth of their malignant cells. In particular, first-line therapy for T-cell acute lymphoblastic leukemia (T-ALL) fails in 25% of children and more than 50% of adults despite improvements in clinical outcomes due to intensification of therapy, and these relapsed patients have a very poor prognosis. Most current T-ALL therapy regimens lack tyrosine kinase inhibitors suggesting that addition of effective kinase inhibitors to T-ALL therapy may improve clinical outcomes. We report that tyrosine kinase 2 (TYK2), signaling through signal transducer and activator of transcription 1 (STAT1), is vital for the maintenance of T-ALL growth and viability, suggesting that incorporation of TYK2 inhibitors into T-ALL therapy regimens may improve patient outcomes. Methods: T-ALL diagnostic specimens were collected with informed consent and neoplastic cells were isolated on a Ficoll gradient. Cells were treated with an siRNA library that individually silences each member of the tyrosine kinase gene family and cultured for 4 days at which time an MTS assay was used to assess cell viability. DNA was isolated from 16 T-ALL cell lines as well as 50 T-ALL patient specimens. TYK2 was sequenced and identified point mutant were cloned and introduced into Ba/F3 cells for assessment of transformative capacity. Transformed Ba/F3 cells were lysed and immunoblotted with antibodies specific for total or phospho-TYK2, STAT1, STAT3, and ERK1/2. Results: RNAi functional profiling of cells from a T-ALL patient specimen demonstrated dependence on TYK2 for cell viability. Sequence analysis of TYK2 in T-ALL cell lines and patient samples revealed a diversity of TYK2 point mutations. These mutations were located just outside of or within the FERM domain (V15A, G36D, G36R, S47N, R425H), the pseudokinase domain (V731I), and the kinase domain (E957D, and R1027H). Introduction of these point mutants into Ba/F3 cells revealed that 7/8 (all except R425H) transformed these cells to factor-independent growth. Analysis of whole cell extracts from transformed cells revealed constitutive phosphorylation of TYK2, STAT1, STAT3, and ERK1/2. Treatment of transformed cells with JAK Inhibitor I revealed extreme sensitivity of cells to this TYK2 kinase inhibitor as well as reduction of TYK2 AND STAT1 phosphorylation. Conclusions: We demonstrate for the first time that the TYK2-STAT1 pathway is constitutively active and required for viability in the setting of T-ALL, sometimes due to gain-of-function point mutations in TYK2. Small-molecule kinase inhibitors with activity against TYK2 reduce the growth of these TYK2-dependent cells. Hence, we have defined a new, oncogenic signaling pathway in T-ALL cells that can be readily modulated by kinase inhibitors. Interestingly, this pathway has previously been associated with tumor suppression, therefore, our findings indicate that activation of this pathway can have varying effects depending on cellular context. Cumulatively, these data offer new therapeutic options for T-ALL patients while simultaneously providing novel insights into the biology of T-cells and the TYK2-STAT1 signaling cascade
SN - 0006-4971
AD - (Tyner, Glover, Chang, Willis, Druker) Oregon Health and Science University, Knight Cancer Institute, Portland, OR, United States (Sanda, Gutierrez, Ahn, Look) Dana-Farber Cancer Institute, Boston, MA, United States
ER -

TY - JOUR
ID - 3496
T1 - Activated protein C and septic shock: A propensity-matched cohort study
A1 - Rimmer,E.K.
A1 - Kumar,A.
A1 - Doucette,S.
A1 - Marshall,J.
A1 - Dial,S.
A1 - Gurka,D.
A1 - Dellinger,P.
A1 - Sharma,S.
A1 - Penner,C.
A1 - Kramer,A.
A1 - Wood,K.
A1 - Ronald,J.

A1 - Zarychanski,R.
Y1 - 2011///
N1 - 70770290
English
Journal: Conference Abstract
KW - EMBASE
KW - septic shock
KW - Cohort Analysis
KW - Society
KW - hematology
KW - Human
KW - Mortality
KW - patient
KW - controlled clinical trial (topic)
KW - clinical trial (topic)
KW - Hospital
KW - ventilator
KW - dialysis
KW - Length of Stay
KW - APACHE
KW - diseases
KW - Death
KW - Risk
KW - proportional hazards model
KW - sepsis
KW - Propensity Score
KW - intensive care unit
KW - community
KW - clinical trial
KW - statistical significance
KW - Child
KW - controlled clinical trial
KW - activated protein C
KW - hypertensive factor
KW - drotrecogin
KW - coagulating agent
RP - NOT IN FILE
JF - Blood
VL - Conference: 53rd Annual Meeting of the American Society of Hematology, ASH 2011 San Diego, CA United States. Conference Start: 20111210 Conference End: 20111213. Conference Publication:
IS - var.pagings
N2 - Background: Septic shock is a highly inflammatory and pro-coagulant state associated with high mortality. In a single randomized controlled clinical trial, recombinant human activated protein C (rhAPC, drotrecogin alfa) reduced mortality in patients with severe sepsis at high risk of death. Further clinical trials in patients with a lower severity of illness and in children have failed to reproduce these results, and the effectiveness of rhAPC remains the subject of ongoing debate. Insufficient data exists regarding the use of rhAPC outside of a clinical trial. Objective: To evaluate the effectiveness of rhAPC on mortality in a cohort of patients with septic shock. Methods: We conducted a retrospective, propensity-matched, multi-center cohort study in 28 academic and community intensive care units in 3 countries. Propensity scores were based more 33 variables that were either known or suspected to be associated with rhAPC use. Our primary outcomes were mortality over 30 days and 30-day mortality stratified by APACHE II quartile. Secondary outcomes were hospital and ICU mortality and length of stay, ventilator free and vasopressor free days, and the use of acute dialysis. Results: Using a propensity matched Cox proportional hazard model we observed a 5.9% absolute reduction in 30-day mortality associated with activated protein C use [115/342 (33.6%) vs. 135/342 (39.5%), HR 0.83, 95% CI 0.65-1.07, p=0.15]; however this reduction failed to reach statistical significance. Similarly, we observed non-significant reductions in mortality among the 3 lowest APACHE II quartiles (APACHE II <20: 12.5% vs. 18.2%, HR 0.66, 95%CI 0.29 - 1.49, p=0.32; APACHE II 21-25: 20% vs 33.3%, HR 0.55, 95% CI 0.30-1.02, p=0.06; and

APACHE II 26-30: 36.7% vs. 48.7%, HR 0.73, 95% CI 0.45 - 1.19, p=0.21). Hospital mortality (40.6% vs. 46.8%, HR 0.83, 95%CI 0.66-1.04, p=0.10) and ICU mortality (31.0 vs. 35.1%, HR 0.87, 95%CI 0.67-1.13, p=0.28) were not significantly reduced among patients who received rhAPC compared to matched controls. The use of rhAPC was not associated with significant differences in ICU or hospital length of stay, ventilator free days, vasopressor free days or the use of acute dialysis. Conclusion: In this retrospective, multi-center propensity matched cohort study, the use of rhAPC in patients with septic shock was not associated with statistically significant reductions in 30-day mortality. However, the absolute reduction in 30-day mortality was in similar to that observed in previously published clinical trials. Prospective clinical trials evaluating the efficacy of rhAPC are currently ongoing and will serve to define the role of rhAPC in patients with septic shock
SN - 0006-4971
AD - (Rimmer, Kumar) Department of Internal Medicine, University of Manitoba, Winnipeg, MB, Canada (Doucette) Capital District Health Authority, United States (Marshall) Section of Critical Care Medicine, St. Michael's Hospital, Canada (Dial) Section of Critical Care Medicine, Jewish General Hospital, Canada (Gurka) Section of Critical Care Medicine, Rush Medical Center, Rush University, United States (Dellinger) Section of Critical Care Medicine, University of Medicine and Dentistry of New Jersey, United States (Sharma) Section of Critical Care Medicine, Unveristy of Manitoba, Canada (Penner, Kramer) Section of Critical Care Medicine, Brandon Regional Health Authority, Canada (Wood) Section of Pulmonary and Critical Care Medicine, University of Wisconsin Hospital and Clinics, United States (Ronald) Section of Critical Care Medicine, Nanaimo Regional Hospital, Canada (Zarychanski) University of Manitoba, Department of Hematology, Cancercare Manitoba, Winnipeg, MB, Canada
ER -

TY - JOUR
ID - 3497
T1 - Playground behaviors of children with and without sensory processing disorders
A1 - Cosbey,J.
A1 - Johnston,S.S.
A1 - Dunn,M.L.
A1 - Bauman,M.
Y1 - 2012///
N1 - 2012313452
English
Journal: Article
KW - EMBASE
KW - Aggression
KW - article
KW - Child
KW - Child Behavior
KW - childhood disease
KW - clinical article
KW - Conflict
KW - Conversation
KW - Female
KW - Human
KW - Male
KW - play
KW - school child
KW - sensory dysfunction
KW - sensory processing disorder
KW - Social Behavior
KW - Social Interaction
RP - NOT IN FILE
SP - 39
EP - 47
JF - OTJR Occupation, Participation and Health
VL - 32
IS - 2

CY - United States
N2 - It has been well established that participation in social activities is essential to children's development and that disability status can affect a child's participation in everyday activities. However, little research has been done on the impact of sensory processing disorders (SPDs) on social participation and play behaviors. This study is part of a larger study examining the social participation of children with SPD and specifically compares the playground play behaviors of children with SPD and those of their typically developing peers. Both groups of children were observed over multiple sessions during unstructured recess activity and their behaviors were coded and analyzed. Statistically, results show that the play patterns of the two groups were generally similar. However, there were qualitative differences in the play behaviors of the two groups, including conflict, social play, access to play opportunities, and awareness of social cues. Implications for practice and future research are discussed. Copyright American Occupational Therapy Foundation
SN - 1539-4492
AD - (Cosbey) Department of Special Education, Albuquerque, NM, United States (Bauman) University of New Mexico, Albuquerque, NM, United States (Johnston) Department of Special Education, Salt Lake City, UT, United States (Dunn) Department of Occupational Therapy, University of Utah, Salt Lake City, UT, United States
ER -

TY - JOUR
ID - 3499
T1 - Efficacy and safety of lisdexamfetamine dimesylate in children and adolescents with ADHD: A phase 3, randomized, double-blind, multicenter, parallel-group, placebo-and active-controlled, dose-optimized study in Europe
A1 - Gasior,M.
A1 - Coghill,D.
A1 - Soutullo,C.
A1 - Lyne,A.
A1 - Johnson,M.
Y1 - 2012///
N1 - 70760142
English
Journal: Conference Abstract
KW - EMBASE
KW - Human
KW - Safety
KW - Child
KW - psychopharmacology
KW - Adolescent
KW - Europe
KW - college
KW - Attention Deficit Disorder
KW - vital sign
KW - Regression Analysis
KW - Risk Assessment
KW - randomization
KW - Clinical Global Impression scale
KW - diseases
KW - rating scale
KW - headache
KW - decreased appetite
KW - Arm
KW - effect size
KW - confidence interval
KW - insomnia
KW - Drug Therapy
KW - lisdexamfetamine
KW - placebo

KW - Methylphenidate
KW - central stimulant agent
KW - prodrug
KW - amphetamine
RP - NOT IN FILE
SP - 24
JF - Acta Neuropsychiatrica
VL - Conference: 53rd Annual Meeting of the Scandinavian College of Neuropsychopharmacology, SCNP 2012 Copenhagen Denmark. Conference Start: 20120425 Conference End: 20120427. Conference Publication:
IS - var.pagings
N2 - Background: Stimulant medications are commonly used as first-line pharmacotherapies for attention-deficit/hyperactivity disorder (ADHD), but prescribing practices vary greatly across different countries. Several long-acting methylphenidate-and amfetamine-based products are licensed in the US, but fewer treatment options are available in Europe. Lisdexamfetamine dimesylate (LDX) is the first long-acting, prodrug stimulant approved for the treatment of ADHD. To date, all LDX trials have been conducted outside of Europe. Objectives: To investigate the efficacy and safety of LDX, compared with placebo in children and adolescents with at least moderately symptomatic ADHD in Europe. This study also included a reference arm of osmotic-release oral-system methylphenidate (OROS-MPH). Methods: Children (6'C12 years) and adolescents (13C17 years) with an ADHD Rating Scale IV (ADHD-RS-IV) total score 28 were randomized to LDX, OROS-MPH or placebo over 7 weeks at 48 European sites. Primary and key secondary efficacy measures were the change from baseline at endpoint in ADHD-RS-IV total score and Clinical Global Impressions-Global Improvement (CGI-I) score, respectively. Endpoint was defined as the last on-treatment, post-randomization visit at which a valid ADHD-RS-IV total score was observed. Safety assessments included treatment-emergent adverse events (TEAEs) and vital signs. Results: Of 336 subjects randomized, 196 completed the study. The least squares mean change from baseline at endpoint (95% confidence intervals [CI]) in ADHD-RS-IV total score was C24.3 (C26.6, C22.0) for LDX, C18.7 (C21.0, C16.5) for OROS-MPH and C5.7 (C7.9, C3.5) for placebo (p < 0.001 for each active treatment vs placebo). Effect sizes based on the difference (active -placebo) in LS mean change in ADHD-RS-IV total score from baseline to endpoint were 1.804 for LDX and 1.263 for OROS-MPH. The percentage (95% CI) of subjects showing improvement (CGI-I of 1 or 2) at endpoint was 78% (70, 86) for LDX, 61% (51, 70) for OROS-MPH and 14% (8, 21) for placebo (p < 0.001 for each active treatment vs placebo). The most common TEAEs for LDX were decreased appetite (25%), headache (14%) and insomnia (14%). Mean changes in vital signs were small and consistent with the known profile of LDX. Conclusions: LDX was effective and generally well tolerated in children and adolescents with ADHD
SN - 0924-2708
AD - (Gasior, Coghill, Soutullo, Lyne, Johnson) Shire Development LLC, Wayne, PA, United States
ER -

TY - JOUR
ID - 3500
T1 - Risk factors for cardiovascular disease in school age children and teenagers
A1 - Doros,G.
A1 - Popoiu,A.
A1 - Gafencu,M.
A1 - Jurca-Simina,I.E.
A1 - Leahu,L.
A1 - But,A.
Y1 - 2012///
N1 - 70749966
English
Journal: Conference Abstract
KW - EMBASE
KW - Human
KW - school
KW - Society
KW - heart surgery
KW - Adolescent
KW - Japanese

KW - cardiology
KW - Child
KW - pediatric cardiology
KW - cardiovascular disease
KW - risk factor
KW - boy
KW - Male
KW - girl
KW - Female
KW - Obesity
KW - Hypertension
KW - city
KW - Physical Activity
KW - cardiovascular risk
KW - Eating
KW - fast food
KW - urban area
KW - questionnaire
KW - Blood Pressure
KW - Hospital
KW - Prevention
KW - height
KW - weight
RP - NOT IN FILE
SP - S111
EP - S112
JF - Cardiology in the Young
JA - Cardiol Young
VL - Conference: 46th Annual Meeting of the Association for European Paediatric and Congenital Cardiology, AEPC with Joint Sessions with the Japanese Society of Pediatric Cardiology and Cardiac Surgery Istanbul Turkey. Conference Start: 20120523 Conference End: 20120526. Conference Publication:
IS - var.pagings
N2 - Objectives: To detect and to prevent cardiovascular risk factors as: overweight, obesity and hypertension in school age children and teenagers. Methods: We investigated 3747 pupils, 6-18 yo., from different schools from Timisoara, measuring: weight, height, BMI and blood pressure. A questionnaire regarding nutritional behavior and physical activity was completed. Results: There were 2150 girls and 1532 boys in the study, 2744 (73%) from the city and 1003 (27%) from the village. Overweight was found in 20.66% boys and 17.6% girls. Obesity was found in 16.7% boys and 9.16% girls. Overweight and obese boys were equal in both areas, but obese girls were less in town girls. Hypertension was detected in 399 urban children (10.65%) and only in 40 (3.98%) of rural. From the urban area, 47.6% were girls and 52.4% were boys with hypertension. From the village area, 32.5% were girls and 67.5% were boys with hypertension. Increased BMI was found in 172 hypertensive children from town, and in only 17 hypertensive children from village. Overweight in hypertensive children was found in 61 boys and 35 urban girls, in comparison with only 8 boys and 2 village girls. Obesity was present in 51 hypertensive boys and 25 town girls, in comparison with only 1 hypertensive boy and 6 hypertensive girls from the village. The values of hypertension were analyzed with charts. Fast food eating, sandwiches, snacks and sweet drinks were preferred by children from town. Playing outside the house was more frequent in the village group. PC and static play was more frequent in the town group. Conclusions: Obese boys were dominant in urban boys group. Girls were more interested in better look than boys. Hypertension was detected especially in urban children. Children from village eat healthier and do more physical activity. Risk factors for cardiovascular disease are dramatically high in urban children, especially boys, because of fast food eating, snacks, sweet drinks and sedentariness. After this study, a web site for informing and instructing the children and their families, regarding the cardiovascular risk prevention was promoted by our hospital in the schools from Timisoara
SN - 1047-9511
AD - (Doros, Popoiu, Gafencu, Leahu) IIIrd Pediatric Clinic, Dpt. of Pediatric Cardiology, University of Medicine and Pharmacy, Timisoara, Romania (Jurca-Simina, But) Emergency Children Hospital "louis Turcanu", Timisoara, Romania

ER -

TY - JOUR
ID - 3501
T1 - Use of early-tips for high-risk variceal bleeding. Results of a post-RCT surveillance study
A1 - Di,Pascoli M.
A1 - Garcia-Pagan,J.C.
A1 - Caca,K.
A1 - Laleman,W.
A1 - Bureau,C.
A1 - Appenrodt,B.
A1 - Luca,A.
A1 - Zipprich,A.
A1 - Abraldes,J.G.
A1 - Nevens,F.
A1 - Vinel,J.P.
A1 - Bosch,J.
Y1 - 2012///
N1 - 70748786
English
Journal: Conference Abstract
KW - EMBASE
KW - liver
KW - bleeding
KW - Risk
KW - Human
KW - patient
KW - survival
KW - Child
KW - treatment failure
KW - clinical practice
KW - Mortality
KW - clinical trial
KW - follow up
KW - Liver Cirrhosis
KW - Hospital
KW - confidence interval
KW - observational study
KW - Therapy
RP - NOT IN FILE
SP - S262
JF - Journal of Hepatology
VL - Conference: 47th Annual Meeting of the European Association for the Study of the Liver, International Liver Congress 2012 Barcelona Spain. Conference Start: 20120418 Conference End: 20120422. Conference Publication:
IS - var.pagings
N2 - Background: In a recent randomized international clinical trial (RCT) in high-risk cirrhotic patients with acute variceal bleeding, the early use of TIPS was associated with marked and significant reductions both in treatment failure and in mortality. The aim of the present study was to assess whether these excellent results are reproduced in clinical practice outside RCTs. Methods: Retrospective review of patients admitted after inclusion of the last patient in the RCT for acute variceal bleeding and high risk of treatment failure (Child C <14 or Child B plus active bleeding) at the same centres of the original RCT study. All patients treated with early-TIPS were included (n = 45); patients receiving medical therapy with drugs+EBL were included until the moment when the hospital adopted the strategy of using Early-TIPS for all comers (n = 30). Results: TIPS was done within 24 hours in 28 patients; 24-48 h in 11 and 48-72 h in 6. There were no differences in the baseline characteristics of the patients treated with TIPS or Drug+EBL. Median follow-up was 11.2 months. Patients treated with early-TIPS had a much lower incidence of failure to control bleeding or rebleeding than patients receiving Drug+EBL

(3 vs 15; p < 0.001). The 1-year actuarial probability of remaining free of this composite end point was 93% vs 53% (p < 0.001). The same was observed in mortality (1-year actuarial survival was 86% vs 70% respectively (p = 0.056). More important, actuarial curves of failure to control bleeding + rebleeding and of survival in this observational study were well within the confidence intervals of those observed in the RCT. Conclusions: In experienced centres, the application of the early use of TIPS in patients with cirrhosis and a high-risk variceal bleeding offers results similar to those previously observed in the RCT, supporting its use in clinical practice
SN - 0168-8278
AD - (Di Pascoli, Garcia-Pagan, Abraldes, Bosch) Hepatic Hemodynamic Laboratory, Institut de Malalties Digestives i Metaboliques, University of Barcelona, Barcelona, Spain (Caca) Medizinische Klinik I, Klinikum Ludwigsburg, Ludwigsburg, Germany (Laleman, Nevens) Department of Hepatology, University Hospital Gasthuisberg, Katholieke, Universiteit Leuven, Leuven, Belgium (Bureau, Vinel) Service d'Hepatogastro-Enterologi, Centre Hospitalie Universitaire de Purpan, Toulouse, France (Appenrodt) Medical Department I, University of Bonn, Bonn, Germany (Luca) Istituto Mediterraneo per i Trapianti e Terapie ad Alta Specializzazione, Palermo, Italy (Zipprich) Innere Medizin I, Martin Luther University, Halle-Wittenberg, Germany
ER -

TY - JOUR
ID - 3503
T1 - Adherence to the Mediterranean diet in Italian children: Association with having lunch at school
A1 - Roccaldo,R.
A1 - Angelini,V.
A1 - D'Addesa,D.
A1 - Bevilacqua,N.
A1 - Martone,D.
A1 - Toti,E.
A1 - Catasta,G.
A1 - Fabbri,I.
A1 - Galfo,M.
A1 - Censi,L.
Y1 - 2011///
N1 - 70740758
English
Journal: Conference Abstract
KW - EMBASE
KW - Mediterranean diet
KW - school
KW - Nutrition
KW - Human
KW - Child
KW - questionnaire
KW - school child
KW - Cluster Analysis
KW - Wakefulness
KW - Personnel
KW - Body Weight
KW - Italy
KW - Physical Activity
KW - Lifestyle
KW - parent
KW - Food
KW - height
KW - Education
KW - Eating
RP - NOT IN FILE
SP - 248
JF - Annals of Nutrition and Metabolism

VL - Conference: 11th European Nutrition Conference, FENS 2011 Madrid Spain. Conference Start: 20111026 Conference End: 20111029. Conference Publication:
IS - var.pagings
N2 - Introduction: Few data from studies in pediatric samples exploring adherence to the Mediterranean Diet (MD) are available. Children spend approximately half of their waking hours in school. Schools provide 1 to 2 meals daily and are a natural setting for education about healthy food choices. Objectives: The aim of this study was to investigate the association of adherence to the MD with having lunch provided by the school. Method/Design: A cluster sample of 1740 school children, 8-9 years old, selected in Italy was investigated (ZOOM8 study). Parents were given two self-administered questionnaires: a semi-quantitative FFQ and a questionnaire on their child's lifestyle and physical activity. Body weight and height were measured by standardized personnel. Adherence to the MD was assessed by the KIDMED index. Results: 5. 1 % of the sample was classified as "high" adherers of MD, while 61. 1% and 33. 9% had an "average" and a "poor" score respectively. When considering the geographical area a higher frequency of "high" adherence to the MD was observed in the northern regions (6. 0%) compared with the central (4. 9%) and the southern (4. 2%) ones. When children had lunch at school "average" adherence increased up to 63. 7% and "poor" adherence decreased to 31. 7%. Lunch was provided to 61. 4% of the children by the school. Most schools in the southern regions involved in the study did not supply any lunch for lack of facilities. 62. 9% of the children eating lunch at school liked it. When associating "like lunch" with adherence a further slight increase of "high" (5. 4%) and "average" (65. 5%) was noticed, "low" adherence decreased further to 29. 0%. Conclusions: Low adherence rates to the MD were observed in Italian children. Schools with the provision of lunch could play an important role in encouraging the adoption of the MD with its benefits so further investigation is desirable
SN - 0250-6807
AD - (Roccaldo, Angelini, D'Addesa, Bevilacqua, Martone, Toti, Catasta, Fabbri, Galfo, Censi) National Institute for Research on Food and Nutrition, Rome, Italy
ER -

TY - JOUR
ID - 3504
T1 - Cadence control system for paediatric functional electrical stimulation cycling
A1 - Pennycott,A.
A1 - Hunt,K.J.
Y1 - 2010///
N1 - 2012253299
English
Journal: Article
KW - EMBASE
KW - article
KW - Biomedical Engineering
KW - cadence control system
KW - Child
KW - control system
KW - cycling
KW - Exercise
KW - feedback system
KW - functional electrical stimulation
KW - handicapped child
KW - Human
KW - priority journal
KW - spinal cord injury/rh [Rehabilitation]
RP - NOT IN FILE
SP - 237
EP - 242
JF - Biomedical Signal Processing and Control
VL - 5
IS - 3
CY - United Kingdom

N2 - Functional electrical stimulation can be used to enable spinal cord injured children to participate in cycling training as part of a fitness programme or exercise testing procedure. Exercise can reduce the impact of secondary health conditions due to the injury. Functional electrical stimulation has shown promising results in adults with a spinal cord injury, but additional considerations are needed to realise the method for the child with spinal cord injury, partly owing to their limited force producing capacity. An electric motor provides a practical means of performing cycling at controlled cadences, exercising for longer durations and can also be used for recreational outdoor cycling. Here, a novel real-time control technique is developed for cadence regulation during cycling. A feedback control structure is developed based on an empirical model derived from separate identification tests and pole placement and then verified in a series of reference-tracking tests. The system produced cadence responses in close agreement with reference values in all cases and demonstrated satisfactory robustness of stability characteristics. This approach moves towards the practical application of the technology as a training tool for paediatric spinal cord injured subjects. 2010 Elsevier Ltd. All rights reserved
SN - 1746-8094
AD - (Pennycott) Ship Safety Research Centre, University of Strathclyde, United Kingdom (Hunt) Institute for Mechatronic Systems, Bern University of Applied Sciences, Switzerland
ER -

TY - JOUR
ID - 3505
T1 - Evaluation of a family camp intervention in pediatric heart transplant
A1 - Urschel,S.
A1 - Nicholas,D.
A1 - Dodd,B.
A1 - Simard,K.
A1 - Dicke,F.
A1 - West,L.J.
Y1 - 2012///
N1 - 70734324
English
Journal: Conference Abstract
KW - EMBASE
KW - heart graft
KW - Society
KW - heart
KW - lung transplantation
KW - Human
KW - Child
KW - Social Support
KW - parent
KW - Adolescent
KW - Transplantation
KW - sibling
KW - Satisfaction
KW - juvenile
KW - patient
KW - Interview
KW - Motivation
KW - Learning
KW - Adolescent Coping Orientation for Problem Experiences
KW - health care personnel
KW - social network
KW - support group
KW - questionnaire
KW - Education
RP - NOT IN FILE
SP - S82
JF - Journal of Heart and Lung Transplantation

VL - Conference: 32nd Annual Meeting and Scientific Sessions of the International Society for Heart and Lung Transplantation Prague Czech Republic. Conference Start: 20120418 Conference End: 20120421. Conference Publication:
IS - var.pagings
N2 - Purpose: To evaluate a support and education-based weekend camp for children post heart transplant and their families. The intervention with 60 attendants comprised recreational, social and educational activities for children, youth, parents and siblings. Methods and Materials: Evaluation comprised: (I) pre- and post-camp measures: adapted Children's Inventory of Social Support;evaluating sources and need for support, perceived and available support network, actual or utilized support, and satisfaction with support, and the Adolescent Coping Orientation for Problem Experiences on behaviours used in managing difficult situations and life changes; (II) knowledge and satisfaction assessments (III) post-camp qualitative interviews. Results: Participants consistently appreciated the recreational, supportive and educational components of the camp. On average, participants rated the camp 4.21/5, suggesting that the camp met or exceeded expectations (parents: 4.45; patients: 4.33). Key outcomes include increased social support and transplantrelated knowledge. All teenagers who completed the social support questionnaire showed improvement in all domains of experienced support. All but one noted clinical gains in all social support domains; the remaining adolescent reported gains in 3 of 4. Participants liked the variety of activity choices, the expansion of their transplant-related social network, and having fun in a natural setting. They strongly valued the opportunity to develop relationships with peers and health care providers, and actively engaged in support sharing. All participants are interested in a future camp. Recommendations included increased learning modules, including topics of transition and adolescent motivation, and mentoring opportunities for children by older youth. Conclusions: The transplant family camp was found to be an effective and strongly appreciated intervention. Evaluation triggered various improvements in our program including continuous educational events, a sibling support group, an online parent support network, and annually repeated family camps
SN - 1053-2498
AD - (Urschel, Dodd, Simard, West) Pediatric Cardiology, University of Alberta, Edmonton, AB, Canada (Nicholas) Faculty of Social Work, University of Calgary, Calgary, AB, Canada (Dicke) Pediatric Cardiology, Alberta Children's Hospital, Calgary, AB, Canada
ER -

TY - JOUR
ID - 3506
T1 - The Susy Safe project overview after the first four years of activity
A1 - Gregori,D.
A1 - Foltran,F.
A1 - Ballali,S.
A1 - Berchialla,P.
A1 - Zaupa,P.
A1 - Spitzer,P.
A1 - Demetriades,C.
A1 - Sokolova,L.
A1 - Jane Buzarov,Petridou E.
A1 - D'Alessandro,A.
A1 - Pais Clemente,M.A.C.
A1 - Jakubikova,J.
A1 - Van,As S.
A1 - De,Koning T.
A1 - Passali,D.
A1 - Chinsky,A.
A1 - Rodriguez,H.
A1 - Brkic,F.
A1 - Mladina,R.
A1 - Kalakouta,O.
A1 - Melis,A.
A1 - Machalova,M.
A1 - Thomasen,P.C.
A1 - Elsheikh,E.

A1 - Ragab,A.
A1 - Pitkaranta,A.
A1 - Necker,P.C.
A1 - Derelle,J.
A1 - Duwelz,M.
A1 - Francois,M.
A1 - Pezzettigotta,S.
A1 - Pezzettigotta Stephane,Righini C.
A1 - Bernhard,R.
A1 - Jahnke,V.
A1 - Goktas Onder,Zieriacks P.
A1 - Kalampoki,V.
A1 - Simasko,N.
A1 - Skoulakis,C.
A1 - Camaioni,A.
A1 - Cutrone,C.
A1 - Gaudini,E.
A1 - Grasso,D.
A1 - Messi,G.
A1 - Mansi,N.
A1 - Orlando,C.
A1 - Preziosi,S.
A1 - Sorrentini,I.
A1 - Villari,G.
A1 - Trozzi,M.
A1 - Vigo,A.
A1 - Passali,G.C.
A1 - Passali,F.M.
A1 - Nishida,Y.
A1 - Ussatayeva,G.
A1 - De,Hoyos R.
A1 - Ologe,F.E.
A1 - Nasrullah,M.
A1 - Melendez,A.
A1 - Chmielik,M.
A1 - Belchior,T.
A1 - Cobzeanu,M.D.
A1 - Gheorghe,D.C.
A1 - Iorgulescu,A.
A1 - Codrut,C.
A1 - Toader,M.
A1 - Barkociova,J.
A1 - Havelkova,B.
A1 - Zargi,M.
A1 - Pumarola,F.
A1 - Rubio,L.
A1 - Stierna,P.
A1 - Hsu,W.-C.
A1 - rj-Ong,S.
A1 - Chomchai,C.
A1 - Hoep,L.
A1 - Rinkel,R.
A1 - Baskent,E.A.
A1 - Erikci,V.S.
A1 - Onerci,M.
A1 - Graham,J.
A1 - Khwaja,S.

A1 - Raine,C.
A1 - Slapak,I.
Y1 - 2012///
N1 - 2012237669
English
Journal: Article
KW - EMBASE
KW - Adolescent
KW - Age Distribution
KW - article
KW - asthma/co [Complication]
KW - bronchus perforation
KW - Child
KW - child hospitalization
KW - child safety
KW - disease registry
KW - disease surveillance
KW - endoscopic surgery
KW - esophagus foreign body
KW - Europe
KW - Female
KW - food foreign body
KW - foreign body/ep [Epidemiology]
KW - foreign body/su [Surgery]
KW - foreign body aspiration
KW - health hazard
KW - health survey
KW - Human
KW - Incidence
KW - Infant
KW - lung infection/co [Complication]
KW - major clinical study
KW - Male
KW - non food foreign body
KW - pneumonia/co [Complication]
KW - preschool child
KW - priority journal
KW - product safety
KW - register
KW - Risk Assessment
KW - Sex Ratio
RP - NOT IN FILE
SP - S3
EP - S11
JF - International Journal of Pediatric Otorhinolaryngology
JA - Int J Pediatr Otorhinolaryngol
VL - 76
IS - SUPPL. 1
CY - Ireland
N2 - Objectives: to collect relevant, up-to-date, representative, accurate, systematic information, related to foreign bodies (FB) injuries. Methods: The "Susy Safe" registry, a DG SANCO co-funded project gathering data on choking in all EU Countries and beyond, was established in order to create surveillance systems for suffocation injuries able to provide a risk-analysis profile for each of the products causing the injury. Main findings after 4 years of activities are resumed here. Results: 16,878 FB injuries occurred in children aged 0-14 years have been recorded in the SUSY SAFE databases; 8046 cases have been reported from countries outside EU. Almost one quart of the cases involving very young children (less than one year of age) presented a FB located in bronchial tract, thus representing a major threat to their health. Esophageal foreign bodies are still

characterizing injuries occurred to children younger than one year, in older children the most common locations are the ears and the nose. FB type was specified in 10,564 cases. Food objects represented the 26% of the cases, whereas non-food objects were the remaining 74%. Among food objects, the most common were bones, nuts and seed, whereas for the non-food objects pearls, balls and marbles were observed most commonly (29%). Coins were involved in 15% of the non-food injuries and toys represented the 4% of the cases. Conclusions: this data collection system should be been taken into consideration for the calculation of the risk of injuries in order to provide the EU Commission with all the relevant estimates on FB injuries. 2012 Elsevier Ireland Ltd
SN - 0165-5876
AD - (Gregori, Foltran) University of Padova, Italy (Ballali) PROCHILD ONLUS, Italy (Berchialla) University of Torino, Italy (Rodriguez) Hospital De Pediatria Juan P. Garrahan, Argentina (Zaupa, Spitzer) Grosse schutzen Kleine, Austria (Demetriades) Ministry of Commerce, Industry and Tourism, Cyprus (Sokolova, Jane Buzarov) Institute for Respiratory Diseases in Children, FYROM, Greece (Petridou) Athens University, Medical School, Department of Hygiene and Epidemiology, Greece (D'Alessandro) Ministero dello Sviluppo Economico, Italy (Pais Clemente) Instituto Portugues de Tabacologia, Portugal (Jakubikova) Children's University Hospital, Slovakia (Van As) Red Cross War Memorial Children's Hospital, South Africa (De Koning) Voedsel en Waren Autoriteit, Netherlands (Passali) University of Siena, Italy (Chinsky, Rodriguez) Children's Hospital Juan P. Garrahan, Argentina (Brkic) University Clinical Center, Bosnia and Herzegovina (Mladina) University Hospital Salata, Croatia (Kalakouta) Medical and Public Health Services, Ministry of Health, Cyprus (Melis) Aretaeion Hospital, Cyprus (Machalova) Childrens University Hospital, Czech Republic (Thomasen) Gentofte University Hospital of Copenhagen, Denmark (Elsheikh) Suez Canal University, Egypt (Ragab) Menoufiya University Hospital, Egypt (Pitkaranta) Helsinki University Central Hospital, Finland (Necker) Enfants Malades Hospital, France (Derelle) CHU Nancy, France (Duwelz) SOS Benjamin, Observaoire National d'Etudes des Conduites a Risques, France (Francois) Robert Debre Hospital, France (Pezzettigotta, Pezzettigotta Stephane) Armand Trousseau Hospital, France (Righini) CHU A. Michallon, France (Bernhard) St. Joseph Hospital, Germany (Jahnke, Goktas Onder) Charite Campus Virchow, Germany (Zieriacks) Kinderheilkunde und Jugendmedizin, Naturheilverfahren und Akupunktur, Germany (Kalampoki) Athens University, Department of Hygiene and Epidemiology, Greece (Simasko) Democritus University School of Medicine, Greece (Skoulakis) General Hospital of Volos, Greece (Camaioni) San Giovanni Addolorata Calvary Hospital, Italy (Cutrone) University Hospital of Padova, Italy (Gaudini) Ear-Nose-Throat Department, Policlinico Le Scotte, Italy (Grasso, Messi) Burlo Garofolo Pediatric Institute, Italy (Mansi, Orlando) Santobono Pausilipon Pediatric Hospital, Italy (Preziosi) Elisoccorso ospedale Ravenna, Italy (Sorrentini, Villari) G. Rummo Hospital, Italy (Trozzi) Bambino Gesu Pediatric Hospital, Italy (Vigo) Sant'Anna Pediatric Hospital, Italy (Passali) Ear, Nose, and Throat Clinic, University Tor Vergata, Rome, Italy (Passali) ENT Department, Catholic University The Sacred Heart of Rome, Italy (Nishida) National Institute of Advanced Industrial Science and Technology (AIST), Japan (Ussatayeva) Kazakhstan School of Public Health, Kazakhstan (De Hoyos) San Jose-Tec de Monterrrey Hospital, Mexico (Ologe) University of Ilorin Teaching Hospital, Nigeria (Nasrullah) Services Hospital, Paediatric Ward, Pakistan (Melendez) Santo Tomas Hospital, Panama (Chmielik) Medical University of Warsaw, Poland (Belchior) Deco Proteste, Portugal (Cobzeanu) Sf. Spiridon Hospital, Romania (Gheorghe) Maria Sklodowska Curie Hospital, Romania (Iorgulescu) Grigore Alexandrescu Pediatric Hospital, Romania (Codrut, Toader) Sarafoleanu Sf. Maria Hospital, Romania (Barkociova) Children University Hospital, Slovakia (Havelkova) Public Health Authority of the Slovak Republic, Slovakia (Zargi) University Medical Centre, Slovenia (Pumarola) Vall d'Hebron University Hospital, Spain (Rubio) Ruber International Hospital, Spain (Stierna) Huddinge University Hospital, Sweden (Hsu) National Taiwan University Hospital, Taiwan (Republic of China) (Arj-Ong) Ramathibodi Hospital, Thailand (Chomchai) Siriraj Hospital, Thailand (Hoep, Rinkel) VU Medical Center, Netherlands (Baskent) University Ankara Hospital, Turkey (Erikci) Behcet Uz Children Hospital, Turkey (Onerci) Hacettepe University, Turkey (Graham) Royal Free Hampstead NHS Trust, United Kingdom (Khwaja) Royal Manchester Children's Hospital, United Kingdom (Raine) Bradford Royal Innfirmary, United Kingdom (Slapak) Masaryk University, Czech Republic
ER -

TY - JOUR
ID - 3507
T1 - Pragmatic difficulties and compensatory instruments in children with traumatic brain injury: Communication training in small groups
A1 - Ventura,M.
A1 - Quarin,F.
A1 - Massaccesi,E.

Y1 - 2012///
N1 - 70732159
English
Journal: Conference Abstract
KW - EMBASE
KW - brain injury
KW - traumatic brain injury
KW - Human
KW - Interpersonal Communication
KW - Child
KW - Speech
KW - videorecording
KW - skill
KW - Rehabilitation
KW - Language
KW - Monitoring
KW - dynamics
KW - model
KW - social adaptation
KW - frontal lobe
KW - Coping Behavior
KW - Methodology
KW - injury
KW - imitation
KW - brain
RP - NOT IN FILE
SP - 721
EP - 722
JF - Brain Injury
JA - Brain Inj
VL - Conference: 9th World Congress on Brain Injury of the International Brain Injury Association Edinburgh United Kingdom. Conference Start: 20120321 Conference End: 20120325. Conference Publication:
IS - var.pagings
N2 - Introduction: Pragmatic skills, also called social use of language, are among those most frequently affected in brain traumatic injury (TBI) in children.Literature refers that social skills are affected in TBI, but little attention, in respect of rehabilitative intervention, is paid to tasks, contexts, and materials. Professionals involved in rehabilitation of pragmatic skills, meet specific difficulties to structure a "natural" setting that recreates the characteristics of communicative interaction. The small group, with its particular dynamics and communicative interactions, may be regarded as the context closest to natural ones. However, to use it effectively as a rehabilitation tool, there is the need to define its important variables, to assess their progress, and eventually to intervene on them, depending on the targets.Objectives: Monitoring children's and speech therapist's behavior during the interaction in a small group (according to established variables) using a specifically constructed instrument (grid for the analysis of video recordings)Assessing the practical utilization of the evaluation tool and its effectiveness in directing and modulating the rehabilitative interventionMethods: It has been called an evaluation grid of the main communicative, interactive and pragmatic skills that are activated during the small group interaction.Small groups of 3 - 5 children (7.3-10.9 years; mean age 9,1 years) were managed by two speech therapists, one of them laeding the activity, while the other serving as a role model to promote imitation of coping strategies.The training was video recorded and subsequently the material was analyzed using the evaluation grid. In some cases the analysis led to change the behavior of the speech therapists and / or to introduce different activities.Inclusion criteria: presence of frontal lobe damage q.i. =/> 70The activities have been longitudinally evaluated to notice changes in children's behaviors and their consistency with the objectives of rehabilitative goals.Results: The evaluation of small group activities revealed a positive trend in the interactive behaviors of children.In this sense, we suppose that contextual changes gradually introduced by the speech therapists and based on the analysis of video recordings have had a positive effect on the consistency of children's behaviors.The analysis tool has proved to be useful to check dysfunctional behaviors within the workgroup. It can aid to modify and organize current rehabilitative interventions. An appropriate amount of time is required so that speech therapists get used with it and to analyze

the video recordings. Further studies would be needed in order to longitudinally extend the evaluation methodology and to verify the long-term maintenance of positive changes induced
SN - 0269-9052
AD - (Ventura, Quarin, Massaccesi) Bambino Gesu Children Hospital, Roma, Italy
ER -

TY - JOUR
ID - 3508
T1 - The proportion of risk for TBI and persisting symptomatology in cypriot school-age children
A1 - Constantinidou,F.
A1 - Argyrou,K.
Y1 - 2012///
N1 - 70731595
English
Journal: Conference Abstract
KW - EMBASE
KW - brain injury
KW - Human
KW - school
KW - Child
KW - symptomatology
KW - Risk
KW - questionnaire
KW - parent
KW - Greece
KW - student
KW - screening
KW - Cyprus
KW - Population
KW - Mental Health
KW - groups by age
KW - Diagnosis
KW - Male
KW - primary school
KW - Female
KW - laboratory
KW - rehabilitation center
KW - error
KW - Spelling
KW - sport
KW - community
KW - Methodology
RP - NOT IN FILE
SP - 397
EP - May
JF - Brain Injury
JA - Brain Inj
VL - Conference: 9th World Congress on Brain Injury of the International Brain Injury Association Edinburgh United Kingdom. Conference Start: 20120321 Conference End: 20120325. Conference Publication:
IS - var.pagings
N2 - Objectives: This is the first study investigating the proportion of risk for TBI in school-age children attending rural and urban public schools in the Republic of Cyprus. The study identified the number of blows to the head and presence of symptoms using the Greek version of the Brain Injury Screening Questionnaire (BISQ). Causes of TBI and persisting symptomatology are also reported. Methods: Primary Research Design: Survey research Setting: Public school facilities, community, research laboratory Participants: The BISQ was sent out to the parents of 2088 children (ages 6-12) attending rural and urban elementary schools from varied socioeconomic backgrounds. Thirty three percent (33.8%) or 706 parents completed and returned the

questionnaires and participated in the study. Main Outcome Measure: The Greek version of the Brain Injury Screening Questionnaire (BISQ) Results: Out of the 706 valid questionnaires, 41 (5.8%) children were identified at risk for TBI. Twenty two were male students and nineteen were female. Children of the ages 11, 7 and 8 were found to match the criteria of TBI diagnosis at a higher rate than the rest of the age groups. Based on the BISQ, students were identified at risk if they have sustained alteration of mental status and had 5 symptoms specific to TBI. Blows to the head were common within this population. Analysis indicates that 34 out of the 41 parents who completed the questionnaire reported a blow to the head. Most common causes of blows to the head were: falling while biking (12.2%), falling down stairs (10.2%), while in playground (9%), during sports (8.2%) and falling from high place (7.3%). Most frequent symptoms reported by the parents of children identified at risk for TBI were: making spelling mistakes (69.2%), feeling angry (59.0%), being easily distracted (56.4%), screaming or yelling, having temper outbursts (51.3%), arguing (48.7%), talking too much (43.2%) and feeling tired (42.1%). Conclusion: A proportion of unidentified children at risk for TBI attend public schools in Cyprus. These children are at risk to sustain the psychosocial and cognitive manifestations of TBI. The Greek version of the BISQ is a useful tool to be used for the identification of children at risk. These children should be followed in order to assess their overall cognitive and academic status, ensure that they reach age-appropriate milestones, and receive appropriate rehabilitation services and support
SN - 0269-9052
AD - (Constantinidou, Argyrou) University of Cyprus, Nicosia, Cyprus
ER -

TY - JOUR
ID - 3509
T1 - Sexual healthcare among female sex workers working outside of the main entertainment district in Tijuana, Mexico
A1 - Rusch,M.
A1 - Gallardo,M.
A1 - Lozada,R.
A1 - Lindsay,R.
A1 - Mercer,V.J.
A1 - Zuniga,M.L.
A1 - Patterson,T.L.
A1 - Strathdee,S.A.
Y1 - 2011///
N1 - 70729050
English
Journal: Conference Abstract
KW - EMBASE
KW - Female
KW - Prostitution
KW - Mexico
KW - Society
KW - sexually transmitted disease
KW - Health Care
KW - Human
KW - Papanicolaou test
KW - Health
KW - condom
KW - Statistics
KW - logistic regression analysis
KW - registration
KW - Income
KW - uterine cervix cancer
KW - Child
KW - Prevalence
KW - model
KW - Infection
KW - Risk

KW - health care practice
KW - sampling
KW - red light
KW - sexual health
KW - Health Education
KW - cancer screening
KW - high risk population
KW - health service
KW - Human immunodeficiency virus
RP - NOT IN FILE
SP - A338
EP - A339
JF - Sexually Transmitted Infections
VL - Conference: 19th Biennial Conference of the International Society for Sexually Transmitted Diseases Research Quebec City, QC Canada. Conference Start: 20110710 Conference End: 20110713. Conference Publication:
IS - var.pagings
N2 - Background: Sex work is quasi-legal in Tijuana, with a zone of tolerance (Zona Roja, or Red Light district), and a registration card system whereby female sex workers (FSW) pay an annual cost to cover HIV and STI testing. As healthcare efforts are concentrated in the Zona Roja, we set out to assess the frequency of Pap testing among FSW working outside this zone. Methods: Proyecto Amantes de la Salud (Lovers of Health Project) conducted baseline surveys among 403 FSW working in bars outside of Tijuana's Zona Roja using time-location sampling. Surveys included demographics, sexual and substance use behaviour, sex work characteristics and sexual healthcare practices. Average number of annual Pap tests over the past 5 years was calculated using left censoring for the denominator based on: (1) years sexually active and (2) years in sex trade. Descriptive statistics and logistic regression were used to assess factors associated with Pap testing in the past year. Results: Participants ranged in age from 18 to 55 (Median: 28; IQR: 23-32); all were born in Mexico and 98% reported being registered as an FSW. Sex partners included regular, non-client (25%), regular clients (79%) and non-regular clients (99%). Overall, 85% of women reported a pap test in the past year; however, only 35% of sexually active women and 52% of FSW had an average of at least one Pap test per year over the past 5 years see Abstract P5-S6.31 table 1. A (Table presented) small proportion (4%) indicated they had never had a Pap test. In multivariate models, having a Pap test in the past year was negatively associated with income >3500 pesos/month, more years in the sex trade and having regular clients. Marginal positive associations remained with older age, reporting condom use less than half the time with non-regular clients and having any children. Discussion Prevalence of pap tests in the past year was higher than expected and may be attributed to recent efforts by the Tijuana Municipal Health Services to increase outreach to FSWs in these areas. However, since initiating sex work, only half reported the recommended yearly Pap testing which is concerning given the increased risk for HPV infection and cervical cancer among FSW. Sexual health education, including where access services, is needed to encourage regular cancer screening among this high risk population, especially among younger women and women who have been working in the sex trade for longer durations
SN - 1368-4973
AD - (Rusch, Mercer, Zuniga, Patterson, Strathdee) UCSD, San Diego, United States (Gallardo, Lozada) PrevenCasa A.C., Mexico (Lindsay) SDSU-UCSD, United States
ER -

TY - JOUR
ID - 3511
T1 - Pediatric genitourinary injuries treated in us emergency departments from 2002 - 2010: Results from the national electronic injury surveillance system database
A1 - Tasian,G.
A1 - Bagga,H.
A1 - Fisher,P.
A1 - Cinman,N.
A1 - McCulloch,C.
A1 - McAninch,J.
A1 - Breyer,B.
Y1 - 2012///

N1 - 70721040
English
Journal: Conference Abstract
KW - EMBASE
KW - injury
KW - data base
KW - emergency ward
KW - Human
KW - Child
KW - patient
KW - genital system
KW - Risk
KW - Female
KW - United States
KW - Male
KW - testis injury
KW - follow up
KW - boy
KW - Hospital
KW - testis
KW - probability sample
KW - Epidemiology
KW - Clothing
KW - penis
KW - Diagnosis
KW - Exercise
KW - laceration
KW - contusion
KW - Morbidity
KW - hazard
KW - hope
KW - Safety
RP - NOT IN FILE
SP - e485
EP - e486
JF - Journal of Urology
VL - Conference: 2012 Annual Meeting of the American Urological Association, AUA Atlanta, GA United States. Conference Start: 20120519 Conference End: 20120523. Conference Publication:
IS - var.pagings
N2 - INTRODUCTION AND OBJECTIVES: The epidemiology of pediatric genitourinary injuries in the United States (US) is unknown. We describe the epidemiologic features of genitourinary injuries sustained by children that presented to US emergency departments (EDs). METHODS: We analyzed the National Electronic Injury Surveillance System (NEISS) to identify and characterize genitourinary injuries sustained by children 18 years and younger from 2002 to 2010 (n = 10,130). Estimates and standard errors were calculated using data included in NEISS, which is a stratified probability sample of hospital EDs in the United States and its territories validated to produce national estimates of all patients who present to US EDs with an injury. RESULTS: Between 2002 and 2010, an estimated 252,392 children (95% CI 205,579 - 299,194) presented to US EDs with genitourinary injuries. These children had a mean age of 7.4 years (median 6 years, IQR 4 - 10 years). Forty-five percent of the injured children were boys. The yearly incidence of genitourinary injuries was stable across the time period studied. The most commonly injured organs were female external genitalia (37.7%), penises (21.6%), and testicles (12%). The rates of female external genitalia, penile, and testicular injuries per 1000 person years of follow-up were 0.13, 0.08, and 0.04, respectively. The most common diagnoses were contusions (35.5%) and lacerations (32.9%). Fall was the most common mechanism of injury (n=114,017), accounting for 45.2% of the reported injuries. Genitourinary injuries were most commonly associated with sporting and exercise equipment (35.7%), clothing items (15.5%), and playground equipment (6%). Ninety-one percent of patients were treated in the ED and then discharged. CONCLUSIONS: Genitourinary injuries are an important cause of pediatric morbidity. The stability of the injuries across the time

period studied suggests that potential preventative measures to decrease risk of genitourinary injury have not been identified or implemented. Further studies should be conducted to identify which products pose the greatest hazard to children in hopes of increasing awareness and developing safety mechanisms to decrease risk of traumatic genitourinary injury
SN - 0022-5347
AD - (Tasian) PhiladelphiaPAUnited States (Bagga, Fisher, Cinman, McCulloch, McAninch, Breyer) San FranciscoCAUnited States
ER -

TY - JOUR
ID - 3513
T1 - Development and application of a moveability index to quantify possibilites for physical activity in the built environment of children
A1 - Pigeot,I.
A1 - Buck,C.
A1 - Pohlabeln,H.
A1 - Hyubrechts,I.
A1 - Pitsiladis,Y.
A1 - De,Bourdeaudhuij,I
A1 - Reisch,L.
Y1 - 2011///
N1 - 70699851
English
Journal: Conference Abstract
KW - EMBASE
KW - Epidemiology
KW - Human
KW - Environment
KW - Child
KW - Physical Activity
KW - density
KW - Australia
KW - Risk
KW - parameters
KW - Travel
KW - school
KW - parent
KW - Education
KW - Europe
KW - urban area
KW - city
KW - Germany
KW - Income
KW - school child
KW - model
KW - geographic information system
RP - NOT IN FILE
SP - S265
JF - American Journal of Epidemiology
JA - Am J Epidemiol
VL - Conference: 3rd North American Congress of Epidemiology Montreal, QC Canada. Conference Start: 20110621 Conference End: 20110624. Conference Publication:
IS - var.pagings
N2 - Many studies showed evidence of urban forms being environmental correlates of physical activity (PA). Studies that used geographic information systems (GIS) to objectively assess urban forms are mostly carried out in the US or Australia, but rarely in Europe. Findings from these studies cannot be adopted in a straightforward manner, because the environments in the US and Australia differ from urban areas in many European cities.

Built environments do have a different impact on PA across different countries. We adapted the concept of walkability and included recreational facilities offering possibilities for PA particularly for children to investigate the impact of the built environment on PA of children in Germany. Considering multiple urban forms, e.g. footpaths, intersections, and playgrounds, we used a kernel density approach and combined standardized density measures of urban form to develop a moveability index that assesses the opportunities for PA in the German intervention region of the IDEFICS study. The index was applied to PA data of 344 school children reported in the IDEFICS baseline survey conducted in 2007/08. Using multilevel regression models, odds ratios (OR) and regression parameters with 95% confidence limits were estimated to investigate the impact of the built environment on measures of PA and childrens' travel mode to school. After adjusting for sex, age, parents' education and income, the moveability index showed a significant positive effect (= 0.18, p < 0.005) on children's reported hours of PA per day
SN - 0002-9262
AD - (Pigeot, Buck, Pohlabeln, Hyubrechts, Pitsiladis, De Bourdeaudhuij, Reisch) BIPS, University Bremen, Bremen 28359, Germany
ER -

TY - JOUR
ID - 3514
T1 - Reducing unnecessary utilization in acute bronchiolitis care: Results from the value in inpatient pediatrics network
A1 - Ralston,S.
A1 - Shen,M.
A1 - Garber,M.
A1 - Narang,S.
A1 - Pate,B.
Y1 - 2012///
N1 - 70697999
English
Journal: Conference Abstract
KW - EMBASE
KW - bronchiolitis
KW - hospital patient
KW - Pediatrics
KW - Society
KW - Hospital
KW - Human
KW - patient
KW - Hospitalization
KW - thorax radiography
KW - Evidence Based Practice
KW - Therapy
KW - cost control
KW - waste
KW - volunteer
KW - breathing exercise
KW - Peer Group
KW - Child
KW - Chronic Disease
KW - publication
KW - community
KW - university hospital
KW - total quality management
KW - steroid
KW - beta adrenergic receptor stimulating agent
KW - bronchodilating agent
RP - NOT IN FILE
SP - S4

EP - S5
JF - Journal of Hospital Medicine
VL - Conference: 2012 Annual Meeting of the Society of Hospital Medicine, SHM 2012 San Diego, CA United States. Conference Start: 20120401 Conference End: 20120404. Conference Publication:
IS - var.pagings
N2 - Background: Bronchiolitis is the current leading cause of hospitalization in children. Based on the available literature, waste and unnecessary variation in bronchiolitis care are common. Significant work has gone into developing evidence- based guidelines for inpatient bronchiolitis management, though successful strategies for large-scale dissemination outside of academic medical centers are rarely reported. Methods: We formed a voluntary quality improvement collaborative called the Value in Inpatient Pediatrics Network. Our specific aim was to reduce utilization of nonevidence- based therapies in the inpatient care of bronchiolitis. Using the 2006 AAP bronchiolitis guideline as the basis for determining "unnecessary" therapy, we benchmarked the use of bronchodilators, steroids, chest radiography, chest physiotherapy, and viral testing using administrative data supplied by hospitals for the calendar years 2007 through 2010. Inclusion criteria were all non-ICU hospitalizations for bronchiolitis as the primary code at discharge (specifically including patients in observation status). Exclusion criteria were specific chronic illness codes. We then shared specific resources within the network on a voluntary basis, including protocols, scores, order sets and key bibliographies. We (Table presented) provided coaching and advice to participating centers when requested. We met yearly and honored high performing centers at this meeting. Results: A total of 17 centers completed the three active years of the project and are included in this report (Table 1). Aggregate data on 11,568 hospitalizations for bronchiolitis was analyzed. Demographics of participating centers are provided as Table 1. By 2010, the network achieved a 12% absolute decrease (95% CI, 5%-25%) in the number of patients receiving any dose of bronchodilator. We also saw a 3.4 dose per patient (95% CI, 1.4-5.8) absolute reduction in volume of beta-agonist utilized. Overall CPT usage declined 10% (95% CI, 2.6%-17.8%). Decreases in steroids, chest radiography or viral testing in the network were not statistically significant. The data were also analyzed on an institutional level for intra-institutional change. 88% of institutions achieved significant decreases in any bronchodilator usage and 80% did so for doses per patient. Direct cost savings related to decreased beta-agonist usage alone amount to $449,000. Conclusions: Real world effectiveness in dissemination of evidence-based guidelines may be achieved with a volunteer, peer group collaborative bridging community and academic institutions
SN - 1553-5592
AD - (Ralston) University of Texas, Health Science Center, San Antonio, TX, United States (Shen) Dell Children's Hospital, Austin, TX, United States (Garber) Univeristy of South Carolina, College of Medicine, Columbia, SC, United States (Narang) Banner Health System, Phoenix, AZ, United States (Pate) Children's Mercy Hospital, Kansas City, MO, United States
ER -

TY - JOUR
ID - 3515
T1 - Performance of a microenviromental model for estimating personal NO2 exposure in children
A1 - Molter,A.
A1 - Lindley,S.
A1 - de,Vocht F.
A1 - Agius,R.
A1 - Kerry,G.
A1 - Johnson,K.
A1 - Ashmore,M.
A1 - Terry,A.
A1 - Dimitroulopoulou,S.
A1 - Simpson,A.
Y1 - 2012///
N1 - 2012130323
English
Journal: Article
KW - EMBASE
KW - Air Pollution
KW - article

KW - Child
KW - Environmental Exposure
KW - Female
KW - Home
KW - home outdoor modelled exposure
KW - Human
KW - Male
KW - microenvironment
KW - microenvironmental exposure model
KW - model
KW - nearest urban monitoring station
KW - personal monitoring
KW - priority journal
KW - questionnaire
KW - school child
KW - systematic error
KW - urban area
KW - nitrogen dioxide
RP - NOT IN FILE
EP - 233
JF - Atmospheric Environment
VL - 51
IS - pp 225-233
CY - United Kingdom
N2 - A common problem in epidemiological studies on air pollution is exposure misclassification, because investigators often assume exposure is equivalent to outdoor concentrations at participants' homes or at the nearest urban monitor.The aims of this study were: (1) to develop a new microenvironmental exposure model (MEEM), combining time-activity data with modelled outdoor and indoor NO₂ concentrations; (2) to evaluate MEEM against data collected with Ogawa personal samplers (OPS); (3) to compare its performance against datasets typically used in epidemiological studies.Schoolchildren wore a personal NO₂ sampler, kept a time-activity diary and completed a questionnaire. This information was used by MEEM to estimate individuals' exposures. These were then compared against concentrations measured by OPS, modelled outdoor concentrations at the children's home (HOME) and concentrations measured at the nearest urban monitoring station (NUM).The mean exposure predicted by MEEM (mean = 19.6 mug m⁻³) was slightly lower than the mean exposure measured by OPS (mean = 20.4 mug m⁻³). The normalised mean bias factor (0.01) and normalised mean absolute error factor (0.25) suggested good agreement. In contrast, the HOME (mean = 31.2 mug m⁻³) and NUM (mean = 28.6 mug m⁻³) methods overpredicted exposure and showed systematic errors.The results indicate that personal exposure can be modelled by MEEM with an acceptable level of agreement, while methods such as HOME and NUM show a poorer performance. 2012 Elsevier Ltd
SN - 1352-2310
AD - (Molter, de Vocht, Agius) Centre for Occupational and Environmental Health, Health Sciences Group, School of Community Based Medicine, Manchester Academic Health Sciences Centre, The University of Manchester, Oxford Road, Manchester M13 9PL, United Kingdom (Lindley) School of Environment and Development (Geography), The University of Manchester, Oxford Road, Manchester M13 9PL, United Kingdom (Kerry, Johnson, Simpson) The University of Manchester, Manchester Academic Health Science Centre, NIHR Translational Research Facility in Respiratory Medicine, University Hospital of South Manchester NHS Foundation Trust, Wythenshawe Hospital, Southmoor Road, Manchester M23 9LT, United Kingdom (Ashmore, Terry) Environment Department, University of York, Heslington, York YO10 5DD, United Kingdom (Dimitroulopoulou) Department of Mechanical Engineering, University of Western Macedonia, 50100 Kozani, Greece
ER -

TY - JOUR
ID - 3516
T1 - Implementation of the go slow whoa meal pattern in schools through the winning with wellness project: Food service workers' perceptions of implementation feasibility and impact

A1 - Southerland,J.
A1 - Slawson,D.L.
A1 - Schetzina,K.E.
A1 - Dalton,W.T.
Y1 - 2011///
N1 - 70680957
English
Journal: Conference Abstract
KW - EMBASE
KW - school
KW - Human
KW - Obesity
KW - worker
KW - wellbeing
KW - Society
KW - catering service
KW - student
KW - Food
KW - nutrient
KW - nutrition service
KW - teacher
KW - Administrative Personnel
KW - Nutrition
KW - Prevention
KW - nutrition education
KW - Eating
KW - Environment
KW - Learning
KW - information processing
KW - juvenile
KW - Child
RP - NOT IN FILE
SP - S150
JF - Obesity
JA - Obesity (Silver Spring)
VL - Conference: 29th Annual Scientific Meeting of the Obesity Society, Obesity 2011 Orlando, FL United States. Conference Start: 20111001 Conference End: 20111005. Conference Publication:
IS - var.pagings
N2 - Background: School-based interventions hold promise for obesity prevention among youth, with programs implemented via school nutrition services offering a way to both positively influence students' food choices and reinforce nutrition education provided in the classroom. The "Go Slow Whoa" stop light eating pattern is designed to aid students in identifying nutrient-dense food choices and learning moderation for less nutrient-dense foods. Methods: Focus groups were held with 43 cafeteria staff from among eight schools that participated in the "Go Slow Whoa" component of the Winning with Wellness obesity prevention project implemented across rural Central Appalachia. Results: Participants recognized the importance of the role that school nutrition services can play in influencing student choices, yet many indicated that lack of administrative support for cafeteria-based interventions, minimal interaction between staff and teachers and lack of ongoing multi-disciplinary collaboration were barriers to change. Further, the role of family influence on children's food choices was underscored, with a common concern being that students choose less nutritious options due to the patterns learned outside school. Collaboration with teachers and administrators was viewed as a useful mechanism for improved implementation feasibility and overall impact of school-based nutrition programs. According to participants, program effectiveness could also be facilitated by a supportive administrative environment and ongoing technical support. Conclusions: The findings of this qualitative investigation suggest that a primary component of effective school-based nutrition programs is ongoing collaboration between cafeteria staff and school staff and administrators
SN - 1930-7381
AD - (Southerland, Slawson, Schetzina, Dalton) Johnson CityTNUnited States

ER -

TY - JOUR
ID - 3517
T1 - Parental weight perception in ethnically diverse preschool-age children
A1 - Natale,R.
A1 - Lopez-Mitnik,G.
A1 - Messiah,S.E.
A1 - Scott,S.H.
A1 - Uhlhorn,S.B.
A1 - Sanders,L.M.
A1 - Delamater,A.
Y1 - 2011///
N1 - 70680942
English
Journal: Conference Abstract
KW - EMBASE
KW - Child
KW - Human
KW - Obesity
KW - Society
KW - Perception
KW - parent
KW - Hispanic
KW - weight
KW - randomized controlled trial
KW - Eating
KW - Physical Activity
KW - United States
KW - cultural factor
KW - lowest income group
KW - Nutrition
KW - Prevalence
KW - Population
RP - NOT IN FILE
SP - S147
JF - Obesity
JA - Obesity (Silver Spring)
VL - Conference: 29th Annual Scientific Meeting of the Obesity Society, Obesity 2011 Orlando, FL United States. Conference Start: 20111001 Conference End: 20111005. Conference Publication:
IS - var.pagings
N2 - Background: One in four children in the United States under age five are overweight/obese, yet little is known about the association between acculturation, parental weight perception, and weight status in young children. Methods: Parents who consented to have their children participate in a randomized-controlled trial among 28 low-income preschools completed a baseline survey measuring mediating factors related to nutrition. BMI data was collected on all participating children (N = 774). Results: The sample consisted of 60% Hispanic, 13.3% Haitian, and 24% non-Hispanic Black/ Mixed race. 34.55% of children were overweight or obese. Children whose parents were born outside of the US were significantly more likely to report their child's weight as normal when they were overweight/obese (OR=0.61, 95% CI, 0.44-0.85, P=0.003). Children whose parents did not speak English at all or spoke very poorly were also significantly less likely to have an accurate perception of their child's weight versus parents who reported they spoke English well (OR=0.54, 95% CI, 0.40-0.73, p=<0.0001). Non Hispanic parents were significantly more likely to accurately identify their child's weight versus Hispanic parents (Central American/Mexican in particular), (OR=1.49, 95% CI, 1.10-2.03, p=0.010). Conclusions: Despite the high prevalence of overweight children, few parents of overweight/ obese children recognized that their child was overweight. This was more common in thoes who did not speak English proficiently, were born outside of the US, and were of Hispanic origin. Methods for increasing parental

awareness of children's overweight status in these populations are needed and may help engage these families in practices related to healthier eating and increased physical activity
SN - 1930-7381
AD - (Natale, Lopez-Mitnik, Messiah, Scott, Uhlhorn, Sanders, Delamater) MiamiFLUnited States
ER -

TY - JOUR
ID - 3518
T1 - Barriers to physical activity among Chinese children and adolescents, 2000-2009
A1 - Du,S.
A1 - Popkin,B.M.
Y1 - 2011///
N1 - 70680834
English
Journal: Conference Abstract
KW - EMBASE
KW - Human
KW - Physical Activity
KW - Society
KW - Child
KW - Adolescent
KW - Obesity
KW - school
KW - Health
KW - China
KW - student
KW - Female
KW - mother
KW - United States
KW - Government
KW - Nutrition
KW - weight
KW - height
KW - Interview
KW - metabolic equivalent
KW - model
KW - community
RP - NOT IN FILE
SP - S124
EP - S125
JF - Obesity
JA - Obesity (Silver Spring)
VL - Conference: 29th Annual Scientific Meeting of the Obesity Society, Obesity 2011 Orlando, FL United States. Conference Start: 20111001 Conference End: 20111005. Conference Publication:
IS - var.pagings
N2 - Background: Physical activity was reported decreasing among children and adolescents in the U.S. There is limited longitudinal research on physical activity and its determinants in China. Methods: To explore the trends and determinants of physical activities in Chinese children and adolescents after a government campaign to promote children's physical activity and health, we used a subgroup data of 4,117 children and adolescents in school (aged 6-18 yrs) from four waves of the China Health and Nutrition Survey enrolled in 2000 and followed up through 2009. IOTF standards were used on measured weight and height. Direct interviews recorded detailed activities in and out of school as well as culture-specific activities at home. Both time spent in physical activities and metabolic equivalent task (MET, hours per week) were included. Longitudinal mixed models examined physical activity determinants. Results: Overweight and obesity among Chinese children and adolescents doubled from 7.2% in 2000 to 15.1% in 2009. Time spent in physical activities were stable (~30 minutes per day), while homework time significantly increased from 57 minutes to 98 minutes per day. Students in communities without playgrounds or facilities were less likely to participate in any physical activities compared

to their peers. Doing more homework, being discouraged by mothers to engage in physical activities, and lack of facilities were the major determinants of physical activity levels among students. Conclusions: Decreasing homework time, increasing physical activity classes in schools and building more facilities in neighborhoods may promote physical activities among Chinese children and adolescents
SN - 1930-7381
AD - (Du, Popkin) Chapel HillNCUnited States
ER -

TY - JOUR
ID - 3519
T1 - Behavioral outcomes following a pilot study to test the USA version of the mind, exercise, nutrition, do it! (MEND) program to low-income 7-14 year old obese children
A1 - Byrd-Williams,C.
A1 - Hoelscher,D.M.
A1 - Butte,N.F.
A1 - Barlow,S.E.
A1 - Wilson,T.A.
A1 - Sacher,P.M.
A1 - Radley,D.
Y1 - 2011///
N1 - 70680752
English
Journal: Conference Abstract
KW - EMBASE
KW - Human
KW - Child
KW - Obesity
KW - Society
KW - lowest income group
KW - Exercise
KW - pilot study
KW - Nutrition
KW - parent
KW - community
KW - Behavior Modification
KW - skill
KW - Female
KW - Hispanic
KW - Eating
KW - Food
KW - meal
KW - wheat
KW - bread
KW - medical specialist
KW - secondary prevention
KW - United Kingdom
KW - Adaptation
KW - Social Cognitive Theory
KW - Self Concept
RP - NOT IN FILE
SP - S107
JF - Obesity
JA - Obesity (Silver Spring)
VL - Conference: 29th Annual Scientific Meeting of the Obesity Society, Obesity 2011 Orlando, FL United States. Conference Start: 20111001 Conference End: 20111005. Conference Publication:
IS - var.pagings

N2 - Background: MEND is a multicomponent, behaviorally-based obesity secondary prevention program developed and trialed in the UK. This pilot study is the first to test the USA adaptation of MEND when delivered in low-income communities in Austin and Houston, TX. The purpose of this study is to evaluate the effects of an adapted 10-wk intervention on obesity-related behaviors and beliefs. Methods: Children (age 7-14yr) and their parents attended MEND classes at YMCAs. The intervention, based on Social Cognitive Theory, consists of two weekly, 2-h sessions: 60-m behavior modification and nutrition sessions for children and parents followed by 60-m of fun skill-based aerobic activities for children and group sessions for parents. Measurements included validated surveys, and multi-level regressions were conducted to test pre- and post-intervention differences. Results: 85 families participated: children were 45% female, 89% Hispanic, mean age 10.2 +1.55y. At post-test, 89% (33/37) of beliefs and behaviors measured changed in the expected directions. Participants reported consuming less soft drinks, watching less TV, and having higher self-efficacy for healthy eating and being physically active ($p<0.05$). Participants also reported consuming less chips, fried food, punch, and frozen desserts ($p<0.08$) and consuming more breakfast and wheat bread and playing more outdoors ($p<0.08$). Conclusions: Preliminary results indicate MEND positively influenced children's obesity-related beliefs and behaviors, with most changing in the anticipated direction. Behaivors that changed in an unexpected direction provide an opportunity to refine the intervention. The adapted MEND program was well received and is feasible to be delivered by non-specialists at YMCA's in low-income, underserved communities
SN - 1930-7381
AD - (Byrd-Williams, Hoelscher) AustinTXUnited States (Butte, Barlow, Wilson) HoustonTXUnited States (Sacher, Radley) LondonUnited Kingdom
ER -

TY - JOUR
ID - 3521
T1 - Neighborhood greenness and physical activity among preschool-aged children
A1 - Grigsby-Toussaint,D.S.
A1 - Chi,S.-H.
A1 - Fiese,B.H.
Y1 - 2011///
N1 - 70680445
English
Journal: Conference Abstract
KW - EMBASE
KW - Society
KW - Child
KW - Physical Activity
KW - Human
KW - Obesity
KW - exposure
KW - Day Care
KW - United States
KW - vegetation
KW - linear regression analysis
KW - logistic regression analysis
KW - parent
KW - Environment
KW - imagery
RP - NOT IN FILE
SP - S204
JF - Obesity
JA - Obesity (Silver Spring)
VL - Conference: 29th Annual Scientific Meeting of the Obesity Society, Obesity 2011 Orlando, FL United States. Conference Start: 20111001 Conference End: 20111005. Conference Publication:
IS - var.pagings
N2 - Background: Exposure to "green" environments may encourage higher levels of physical activity among children. Few studies, however, have explored this association exclusively in preschool-aged children in the US. Methods: In 2009, children aged 2-3 years (n=365) were recruited from licensed day care centers located in four

counties in Central Illinois. Neighborhood greenness was measured using National Aeronautics and Space (NASA) satellite imagery of vegetation. Outdoor physical activity levels and behaviors were ascertained from parental survey responses. Linear regression was used to examine the relationship between exposure to neighborhood greenness and overall outdoor physical activity levels among children. Logistic regression was then used to explore associations between physical activity behavior and neighborhood greenness while controlling for other covariates. Results: Children living in neighborhoods with higher levels of neighborhood greenness were significantly more likely to engage in outdoor physical activity. Children whose parents spent more time playing with them or transporting them to sporting activities were also more likely to engage in outdoor physical activity. Conclusions: Neighborhood greenness influences physical activity levels among preschool-aged children. Parental involvement, however, attenuates this relationship
SN - 1930-7381
AD - (Grigsby-Toussaint, Chi, Fiese) ChampaignILUnited States
ER -

TY - JOUR
ID - 3522
T1 - Combining gps and accelerometers to determine the locations of physical activity in children
A1 - Oreskovic,N.M.
A1 - Blossom,J.
A1 - Field,A.E.
A1 - Chiang,S.R.
A1 - Winickoff,J.P.
A1 - Kleinman,R.E.
Y1 - 2011///
N1 - 70680433
English
Journal: Conference Abstract
KW - EMBASE
KW - Human
KW - Child
KW - accelerometer
KW - Physical Activity
KW - Obesity
KW - Society
KW - season
KW - summer
KW - school
KW - data collection method
KW - childhood
KW - global positioning system
KW - middle school
KW - United States
KW - winter
KW - scientist
KW - teacher
KW - school child
KW - geographic information system
RP - NOT IN FILE
SP - S202
JF - Obesity
JA - Obesity (Silver Spring)
VL - Conference: 29th Annual Scientific Meeting of the Obesity Society, Obesity 2011 Orlando, FL United States. Conference Start: 20111001 Conference End: 20111005. Conference Publication:
IS - var.pagings
N2 - Background: Children are not achieving sufficient levels of physical activity. We aimed to combine accelerometer and global positioning systems (GPS) use to collect objective information on childhood physical activity (PA). We sought to use these methods to identify the locations where children spend their time and the

locations where children exert the most PA. We tested three different data collection methods to identify the one returning the richest data. Methods: Twenty-four middle-school children from Massachusetts wore accelerometers (ActiGraph GT1M) and GPS units (Garmin Forerunner-201) to quantify and locate childhood PA over 5 weekdays and 2 weekend days. Data were collected in 3 waves (winter, spring, summer) in 2009-2010, each with different study staff (research assistant, teacher, counselor) and collection methods (before-school, after-school, camp). Data points were matched by time, and children's locations throughout the day were mapped using GIS. We mapped the locations where all PA, as well as where moderate-to-vigorous physical activity (MVPA), occurred. Results: A total of 26,406 matched datapoints were collected. Teacherprovided pre-charged equipment yielded the most matched (30.1%, range 10.1-52.3%) and greatest average days (6.1) of data. Children spent most of their time at home, regardless of season. Children spent equal amounts of PA time at home and on streets, and were most likely to be engaged in MVPA on streets. Outdoor use patterns varied by season, with street use increasing in spring, and park and playground use increasing in summer. Conclusions: Using GPS and accelerometers to collect objective information on children's PA locations is feasible. All combined data collection method proved similar data return. Children spent most of their time at home with no or light PA. Children's use of indoor and outdoor spaces may by season
SN - 1930-7381
AD - (Oreskovic, Field, Winickoff, Kleinman) BostonMAUnited States (Blossom) CambridgeMAUnited States (Chiang) RevereMAUnited States
ER -

TY - JOUR
ID - 3523
T1 - Children's mental health: Pattern of referral, distribution of disorders and service use in child psychiatry outpatient setting
A1 - Imran,N.
A1 - Bhatti,M.R.
A1 - Anwar,A.
A1 - Najmi,F.
A1 - Haider,I.I.
Y1 - 2012///
N1 - 2012096516
English
Journal: Article
KW - EMBASE
KW - Adolescent
KW - anxiety disorder
KW - article
KW - Attention Deficit Disorder
KW - Autism
KW - behavior disorder/th [Therapy]
KW - Child
KW - Child Psychiatry
KW - Comorbidity
KW - cross-sectional study
KW - Demography
KW - depression
KW - dissociative disorder
KW - Epilepsy
KW - family history
KW - Family Therapy
KW - Female
KW - health care utilization
KW - health survey
KW - Human
KW - informed consent
KW - language disability/th [Therapy]
KW - learning disorder

KW - major clinical study
KW - Male
KW - mental disease/dt [Drug Therapy]
KW - mental disease/th [Therapy]
KW - Mental Health
KW - outpatient care
KW - Pakistan
KW - parent
KW - patient referral
KW - Play Therapy
KW - preschool child
KW - psychiatric department
KW - psychiatric diagnosis
KW - Psychosis
KW - Sample Size
KW - school child
KW - sex difference
KW - speech articulation
KW - speech disorder/th [Therapy]
KW - Speech Therapy
KW - tertiary health care
KW - tic
KW - anticonvulsive agent/dt [Drug Therapy]
KW - antidepressant agent/dt [Drug Therapy]
KW - benzodiazepine derivative/dt [Drug Therapy]
KW - neuroleptic agent/dt [Drug Therapy]
KW - neurotropic agent/dt [Drug Therapy]
RP - NOT IN FILE
SP - 22
EP - 26
JF - Pakistan Journal of Medical Sciences
VL - 28
IS - 1
CY - Pakistan
N2 - Objective: To determine the pattern of referrals, socio-demographic factors, frequency of psychiatric diagnosis, and help offered to children presenting in Child Psychiatry Department outpatient clinic of a tertiary care hospital in Lahore. Methodology: A cross sectional study of children attending child psychiatry outdoor in a tertiary care hospital in Lahore was conducted. Following informed consent from the parent/ legal guardian, 1000 consecutive new referral to the department were assessed by interviewing the parent and the child. A Structured proforma was used for data collection. ICD-10 criteria were used for the diagnosis of psychiatric illness. Record was made of the interventions offered to the child & family. Results: Total sample size was 1000 children with predominant male gender (65%) and mean age of 8.46 (S.D 4.51).More than half (54.5%) of the children in our sample were not enrolled in school and 19% had family history of psychiatric illness. Among the various sources of referral, self-referral was the highest (84.7%) followed by referral from Pediatrics (8.4%). Psychiatric diagnosis observed were Speech Related Difficulties {including speech delay, articulation problems and specific developmental delays of speech & language} (46.5%), Learning Disability (43.3%), Behavior Problems (26.3), Epilepsy (12.6%), Dissociative Disorders (10.1%), Attention Deficit Hyperactivity Disorder (4.5%), Autism (3.2%), Depression(2.4%), Anxiety(1.7%), Psychosis (1%) and Tics (1%). Co-morbidities were found in 44.7 percent of the children. The various interventions offered included medications, referral to psychologist (for family therapy and individual work), play therapist and speech therapist. Conclusion: Majority of children presenting to the child psychiatry clinic have multiple and complex needs due to high frequency of learning disabilities and co morbidities. The findings illustrate the importance of multidisciplinary approach and to assess the different dimensions of psychopathology in children for future service planning
SN - 1682-024X
AD - (Imran) Child and Family Psychiatry Department, King Edward Medical University/Mayo Hospital, Lahore, Pakistan (Bhatti) Department of Psychiatry, Behavioral Sciences, King Edward Medical University, Mayo Hospital, Lahore, Pakistan (Anwar) Nasar Trust Hospital Shahdra, Lahore, Pakistan (Najmi) King

Edward Medical University, Mayo Hospital, Lahore, Pakistan (Haider) Department of Psychiatry, Fatima Memorial Hospital, Lahore, Pakistan
ER -

TY - JOUR
ID - 3524
T1 - A phase II study of O6-benzylguanine and temozolomide in pediatric patients with recurrent or progressive high-grade gliomas and brainstem gliomas: A Pediatric Brain Tumor Consortium study
A1 - Warren,K.E.
A1 - Gururangan,S.
A1 - Geyer,J.R.
A1 - McLendon,R.E.
A1 - Poussaint,T.Y.
A1 - Wallace,D.
A1 - Balis,F.M.
A1 - Berg,S.L.
A1 - Packer,R.J.
A1 - Goldman,S.
A1 - Minturn,J.E.
A1 - Pollack,I.F.
A1 - Boyett,J.M.
A1 - Kun,L.E.
Y1 - 2012///
N1 - 2012091143
English
Journal: Article
KW - EMBASE
KW - abdominal pain/si [Side Effect]
KW - Adolescent
KW - Adult
KW - alanine aminotransferase blood level
KW - alopecia/si [Side Effect]
KW - anemia/si [Side Effect]
KW - article
KW - aspartate aminotransferase blood level
KW - bone marrow suppression/si [Side Effect]
KW - brain stem tumor/dt [Drug Therapy]
KW - cancer grading
KW - cancer growth
KW - cancer size
KW - cancer survival
KW - chemotherapy induced emesis/si [Side Effect]
KW - Child
KW - childhood cancer/dt [Drug Therapy]
KW - clinical article
KW - clinical evaluation
KW - constipation/si [Side Effect]
KW - controlled study
KW - diarrhea/si [Side Effect]
KW - dose response
KW - drug dose escalation
KW - drug dose reduction
KW - drug efficacy
KW - drug tolerability
KW - fatigue/si [Side Effect]
KW - flushing
KW - gastrointestinal symptom/si [Side Effect]

KW - glioma/dt [Drug Therapy]
KW - headache/si [Side Effect]
KW - Human
KW - infection/si [Side Effect]
KW - leukopenia/si [Side Effect]
KW - lymphocytopenia/si [Side Effect]
KW - maximum tolerated dose
KW - multiple cycle treatment
KW - nausea/si [Side Effect]
KW - neutropenia/si [Side Effect]
KW - nuclear magnetic resonance spectroscopy
KW - phase 2 clinical trial
KW - preschool child
KW - progression free survival
KW - recurrent cancer/dt [Drug Therapy]
KW - school child
KW - side effect/si [Side Effect]
KW - thrombocytopenia/si [Side Effect]
KW - treatment duration
KW - urine retention/si [Side Effect]
KW - 6 o benzylguanine/ae [Adverse Drug Reaction]
KW - 6 o benzylguanine/ct [Clinical Trial]
KW - 6 o benzylguanine/cb [Drug Combination]
KW - 6 o benzylguanine/dt [Drug Therapy]
KW - 6 o benzylguanine/iv [Intravenous Drug Administration]
KW - Ki 67 antigen/ec [Endogenous Compound]
KW - temozolomide/ae [Adverse Drug Reaction]
KW - temozolomide/ct [Clinical Trial]
KW - temozolomide/cb [Drug Combination]
KW - temozolomide/do [Drug Dose]
KW - temozolomide/dt [Drug Therapy]
KW - temozolomide/po [Oral Drug Administration]
RP - NOT IN FILE
SP - 643
EP - 649
JF - Journal of Neuro-Oncology
VL - 106
IS - 3
CY - United States
N2 - To estimate the sustained (>8 weeks) objective response rate in pediatric patients with recurrent or progressive high-grade gliomas (HGG, Stratum A) or brainstem gliomas (BSG, Stratum B) treated with the combination of O⁶-benzylguanine (O6BG) and temozolomide (TMZ). Patients received O6BG 120 mg/m²/d IV followed by TMZ 75 mg/m²/d orally daily for 5 consecutive days of each 28-day course. The target objective response rate to consider the combination active was 17%. A two-stage design was employed. Forty-three patients were enrolled; 41 were evaluable for response, including 25 patients with HGG and 16 patients with BSG. The combination of O6BG and TMZ was tolerable, and the primary toxicities were myelosuppression and gastrointestinal symptoms. One sustained (>8 weeks) partial response was observed in the HGG cohort; no sustained objective responses were observed in the BSG cohort. Long-term (>6 courses) stable disease (SD) was observed in 4 patients in Stratum A and 1 patient in Stratum B. Of the 5 patients with objective response or longterm SD, 3 underwent central review with 2 reclassified as low-grade gliomas. The combination of O6BG and TMZ did not achieve the target response rate for activity in pediatric patients with recurrent or progressive HGG and BSG. 2011 Springer Science+Business Media, LLC. (outside the USA)
SN - 0167-594X
AD - (Warren) Pediatric Oncology Branch, National Cancer Institute, Building 10 CRC, Bethesda, MD 20892, United States (Gururangan, McLendon) Duke University Medical Center, Durham, United States (Geyer) Seattle Children's Hospital, Seattle, United States (Poussaint) Children's Hospital Boston, Boston, United States

(Wallace, Boyett) Operations and Biostatistics Center for the Pediatric Brain Tumor Consortium, Memphis, TN, United States (Balis, Minturn) Children's Hospital of Philadelphia, Philadelphia, United States (Berg) Texas Children's Cancer Center, Houston, United States (Packer) Children's National Medical Center, WA, United States (Goldman) Children's Memorial Hospital, Chicago, United States (Pollack) Children's Hospital of Pittsburgh, Pittsburgh, United States (Kun) St. Jude Children's Research Hospital, Memphis, TN, United States
ER -

TY - JOUR
ID - 3525
T1 - Relationship between physical activity level and demographic characteristics, eating habits and sedantary behaviors of turkish school children aged 11, 13 and 15 years
A1 - Erginoz,E.
A1 - Alikasifoglu,M.
A1 - Ercan,O.
A1 - Uysal,O.
A1 - Ekici,B.
A1 - Kaymak,D.A.
A1 - Gulsah,O.
A1 - Yucel,I.K.
A1 - Ocak,S.
Y1 - 2011///
N1 - 2012076984
English, Turkish
Journal: Article
KW - EMBASE
KW - Adolescent
KW - article
KW - Attitude to Health
KW - body mass
KW - Child
KW - correlational study
KW - cross-sectional study
KW - eating habit
KW - Evaluation
KW - Female
KW - Human
KW - major clinical study
KW - Male
KW - Physical Activity
KW - primary school
KW - school child
KW - Sedentary Lifestyle
KW - Turkey (republic)
RP - NOT IN FILE
SP - 12
EP - 19
JF - Turk Pediatri Arsivi
VL - 46
IS - 1
CY - Turkey
N2 - Aim: The aim of this study was to determine the relationships between physical activity level and demographic characteristics, body mass index and some other health behaviors in Turkish adolescents. Material and Method: The data presented in this study were a part of the Turkish data collected for the cross-sectional and international "Health Behavior in School-aged Children 2005/2006 study" which aimed to evaluate adolescents' health and health behavior around Europe and North America. The data were collected from 3884 11 and 13 years of age (1946 males, 1938 females) students who attended 180 classes of 82 primary schools in 26 cities, and 1668 15 years of age (901 male, 767 female) students who attended 88 classes of 83 high schools

in 26 cities. Statistical analyses were made by using chi square test, factor analysis and logistic regression analysis. Results: Twenty percent of the students reported that they were physically active at least 60 minutes a day every day a week. In logistic regression analysis being 11 and 13 years old, being male, spending four or more evenings with friends outside the home were found to be associated with being physically active. When the "eating both healthy and unhealthy foods less frequently group" was taken as the referans group, the other three groups (healthy foods frequently + unhealty foods less frequently; unhealthy foods frequently + healty foods less frequently; eating both healthy and unhealthy foods frequently) were more likely to be physically active than the reference group. Conclusions: The results of this study are important to show that physical inactivity is common among Turkish adolescents especially females. These results are also important to show that both eating habits and time spent with friends are related to physical activity of the adolescents. Effective health promotion programme, should be especially focused on females and emphasize the importance of adolescents' eating habits. It should also be remembered that peers could play an important role to improve adolescents' physical activity level
SN - 1306-0015
AD - (Erginoz) Istanbul Universitesi Cerrahpasa Tip Fakultesi, Halk Sagligi Anabilim Dali, Istanbul, Turkey (Alikasifoglu, Ercan, Ekici, Gulsah, Yucel, Ocak) Istanbul Universitesi Cerrahpasa Tip Fakultesi, Cocuk Sagligi ve Hastaliklari Anabilim Dali, Istanbul, Turkey (Uysal) Bezm-i Alem Vakif Universitesi Tip Fakultesi, Tibbi Istatistik Tibbi Biliflim Anabilim Dali, Istanbul, Turkey (Kaymak) Bogazici Universitesi Egitim Fakultesi, Istanbul, Turkey
ER -

TY - JOUR
ID - 3526
T1 - Successful bone marrow transplant in a patient with MHC class II deficiency and rubella viremia post-vaccination
A1 - Uygungil,B.
A1 - Bleesing,J.J.
Y1 - 2011///
N1 - 70663677
English
Journal: Conference Abstract
KW - EMBASE
KW - viremia
KW - rubella
KW - Human
KW - Society
KW - patient
KW - bare lymphocyte syndrome
KW - Vaccination
KW - bone marrow transplantation
KW - clinical immunology
KW - T lymphocyte
KW - Infection
KW - rash
KW - immune deficiency
KW - Diagnosis
KW - hand foot and mouth disease
KW - Transplantation
KW - prophylaxis
KW - Risk
KW - serum
KW - engraftment
KW - Child
KW - screening
KW - excision
KW - survival
KW - recipient

KW - epithelium
KW - diseases
KW - procedures
KW - boy
KW - Male
KW - pediatrician
KW - consultation
KW - examination
KW - papule
KW - tonsil
KW - lymphadenopathy
KW - respiratory tract infection
KW - bronchiolitis
KW - Hospitalization
KW - Hypoxia
KW - failure to thrive
KW - chronic diarrhea
KW - endoscopy
KW - graft failure
KW - duodenum
KW - pneumonia
KW - Haemophilus influenzae type b
KW - tetanus
KW - severe combined immunodeficiency
KW - B lymphocyte
KW - monocyte
KW - Flow Cytometry
KW - mumps
KW - nose smear
KW - Blood
KW - unrelated donors
KW - lymphocytic infiltration
KW - CD8 antigen
KW - CD4 antigen
KW - corticosteroid
KW - vaccine
KW - live vaccine
KW - trypsin
KW - immunoglobulin G
KW - immunoglobulin A
KW - immunoglobulin M
KW - immunoglobulin E
KW - CD3 antigen
KW - atovaquone
KW - aciclovir
RP - NOT IN FILE
SP - S24
EP - S25
JF - Journal of Clinical Immunology
VL - Conference: 2011 Clinical Immunology Society Annual Meeting, CIS 2011 Chicago, IL United States. Conference Start: 20110519 Conference End: 20110522. Conference Publication:
IS - var.pagings
N2 - Vaccination programs have successfully minimized the risk from many diseases; however, patients with immune deficiencies remain vulnerable to live vaccines. Infections prior to bone marrow transplantation (BMT) increase the risks of the procedure. Case Presentation: The case presented here is a 2- year-old boy who was referred to our institution for BMT for an immune deficiency. He presented to his pediatrician after his first live vaccinations with MMR and VZV at the age of 1 year with a persistent rash still present at the time of our

consultation. On examination, he was playful and welldeveloped with normal growth. Faint violaceous and coalesced papules were apparent on his face, trunk and extremities. He also had small tonsils and no lymphadenopathy but was otherwise unremarkable. His infectious history was significant for hand foot and mouth disease and two episodes of respiratory infections (pneumonia and RSV bronchiolitis) both requiring hospitalization for hypoxia. Three months prior to his diagnosis, he was admitted with failure to thrive and chronic diarrhea. Endoscopy showed lymphocytic infiltration in the duodenum but was otherwise normal. Infectious stool studies were negative and alpha-1- anti-trypsin was normal. An immune work-up revealed low IgG (<60 mg/dL) and IgA (<7 mg/dL) and normal IgM (32 mg/dL) and IgE (0.2 IU/mL). Haemophilus influenzae b, pneumococcal and tetanus titers were low to undetectable. He had reduced T cells (1,340 K/muL) with low CD4+ (80 cells/muL) and CD8+ (123 cells/muL) cells and a reversed CD4: CD8 ratio (0.7), normal CD19+ (880 cells/muL), CD16/56+ (544 cells/muL), and iNKT cells. The percentage of CD45RA+ T cells was reduced. TCR Vbeta repertoire was normal. A hypomorphic severe combined immune deficiency (SCID) was suspected but genetic studies ruled out defects in IL-7R, CD3, IL2R-, PNP, ADA as well as 22q 11.2 deletion. HLADR on his T cells and APCs was undetectable at the outside institution. We determined that his B cells and monocytes were missing HLA-DR by flow cytometry confirming a diagnosis ofMHC II deficiency. Because of the persistent rash after live vaccineswe sent studies formeasles,mumps, rubella and VZV which revealed detectable rubella by PCR on nasal swab and peripheral blood. Other viral studies were negative except for HSV II by PCR. He continued IVIG, atovaquone prophylaxis and started acyclovir to effectively clear HSV prior to BMT. He had an unrelated donor BMT with an uncomplicated course to date despite prior rubella viremia. Rubella is now undetectable in both nasal and serum samples five months post-transplant. He continues on standard infectious and GVHD prophylaxis with 100% engraftment, no serious infections or GVHD. A mild intermittent rash is being treated with topical corticosteroids. Conclusion: This is the first case to describe successful BMT with clearance of prior chronic vaccine-associated rubella viremia in a child with MHC II deficiency. This case demonstrates several important lessons. First, a widely used screening method for primary immune deficiencies, such as evaluating T-cell excision circles (TRECs), is urgently needed to avoid live vaccination in undiagnosed cases. This is of paramount importance for pre- and posttransplant survival. Immune reconstitution and outcomes in patients undergoing BMT with MHC II deficiency are historically worse than in patients with other forms of SCID presumably due to severe infections prior to transplant, graft failure due to the presence of the recipients alloreactive and normal T cell repertoire and the possible impact on T cell reconstitution due to the absence of MHC on thymic epithelium. The long term outcomes for this patient remain to be seen but are optimistic
SN - 0271-9142
AD - (Uygungil, Bleesing) Cincinnati Children's Hospital Medical Center, Cincinnati, OH, United States
ER -

TY - JOUR
ID - 3527
T1 - Playgrounds without borders: Methods for a playground environmental intervention among U.S. schoolchildren
A1 - LeMaster,J.W.
A1 - Matisziw,T.
A1 - McElroy,J.
A1 - Nilon,C.
A1 - Sayers,S.P.
A1 - Stanis,S.W.
Y1 - 2011///
N1 - 2012068136
English
Journal: Article
KW - EMBASE
KW - accelerometer
KW - article
KW - Child
KW - Child Behavior
KW - controlled study
KW - cross-sectional study
KW - Health Promotion

KW - Human
KW - intervention study
KW - longitudinal study
KW - major clinical study
KW - obesity/pc [Prevention]
KW - Obesity/th [Therapy]
KW - outcome assessment
KW - Physical Activity
KW - Play Therapy
KW - process development
KW - quasi experimental study
KW - school child
KW - school health service
KW - Social Interaction
KW - therapy effect
KW - United States
KW - Walking
RP - NOT IN FILE
JF - Internet Journal of Epidemiology
VL - 10
IS - 1
CY - United States
N2 - Background: Playground environmental interventions that increase children's moderate-to-vigorous physical activity (MVPA) have not been adequately tested among low-income and minority children. Schools are key venues for environmental interventions because they reach children from all socioeconomic groups. Some issues remain unclear: whether these interventions benefit low-income and minority U.S. children; whether increases in MVPA at school are matched by increases outside school; whether benefits last beyond the immediate post-intervention period; and how children's school and neighborhood environments affect their response to the intervention.Methods: Evaluation/study design: quasi-experimental intervention study, using both longitudinal and cross-sectional measurements during seven-day measurement sessions pre-intervention, immediately after intervention inception and every six months thereafter.Intervention: Walking/nature trails enhanced with fixed play equipment designed to stimulate activity directly on playgrounds and indirectly in local parks, trails and neighborhoods. Two schools will receive intervention early (Fall 2011) and two late (Spring 2012).Setting and population: 283 fourth-grade children and their parents (>50% of whom will be from low-income families) from four under-resourced elementary schools in a mid-western, mid-sized city. Measures: Pre- and post-intervention: MVPA measured using geographically-linked accelerometers (indicating location and intensity of MVPA) and cross-sectionally using SOPARC methods to quantify the proportion of 4th graders playing actively on school playgrounds; qualitative interviews with children (cognitive mapping, photo-elicitation); questionnaires including secondary aim outcomes, family socioeconomic data and parent perceptions regarding neighborhood environments; children's height and weight Post-intervention only: focus group discussions with children and parents Analysis: Quantitative outcomes: hierarchical longitudinal data analysis, testing intervention effects as the interaction between study condition and time; Qualitative outcomes: identification and iterative review of codes and themes, using a grounded theory approach to identify cross-cutting concepts that characterize interview and discussion responses. Conclusion: This study will contribute to the prevention of childhood obesity nationally by developing, implementing and documenting an approach to assess the effects of community-developed environmental playground interventions using geographically attributed, longitudinal accelerometer data, cross-sectional SOPARC place-based data and qualitative interview data
AD - (LeMaster, Nilon, Stanis) Family Medicine Research Division, India (Matisziw) University of Missouri, India (McElroy) University of Missouri, School of Medicine, India (Sayers) University of Missouri, School of Health Professions, India
ER -

TY - JOUR
ID - 3528
T1 - Prevalence of overweight and obesity in a private school of Orissa, India
A1 - Patnaik,L.

A1 - Patnaik,S.
A1 - Hussain,M.A.
Y1 - 2011///
N1 - 2012068135
English
Journal: Article
KW - EMBASE
KW - Adolescent
KW - Age Distribution
KW - article
KW - Child
KW - controlled study
KW - cross-sectional study
KW - disease association
KW - family history
KW - Female
KW - Food Intake
KW - health care need
KW - health disparity
KW - health survey
KW - Human
KW - India
KW - major clinical study
KW - Male
KW - Obesity
KW - patient assessment
KW - preschool child
KW - Prevalence
KW - Risk Assessment
KW - risk factor
KW - school child
KW - screening test
KW - Sedentary Lifestyle
RP - NOT IN FILE
JF - Internet Journal of Epidemiology
VL - 10
IS - 1
CY - United States
N2 - Obesity has become a major epidemic causing serious public health concern and contributes to 2.6 million deaths worldwide every year. In developing countries such as India, especially in urban populations and affluent children, obesity is emerging as a major health problem. Objectives: 1. To study the prevalence of overweight & obesity among school children of an affluent school. 2. To assess the risk factors related to obesity. Materials and methods: The present study was a cross sectional study conducted in an affluent English Medium School of Bhubaneswar during Jan. - Feb. 2011. After getting permission from school authorities, the data was collected from students of Class 1 to 10. One section from each class is randomly selected and all the students present during the survey were screened for overweight and obesity. In this way, data was collected from 468 children. Complete data of each child were collected using a pre-designed, pre-tested questionnaire. Result: Out of total 468 school children, 41.9% were boys and 58.1% were girls. 44.4% children belong to 5-10 years age group while the 55.6% children belong to 10-15 years age group. The overall prevalence of overweight and obesity in school children of 5-15 years was found to be 28.63% (overweight - 14.1% and obesity - 14.53%). Maximum prevalence i.e. 36.54% was found in children of 5-10 years age group and 33.65% in boys. Overweight and Obesity was found significantly higher in Children of 5-10 years age group, with family H/O obesity, not playing outdoor games, not doing regular exercise, watching TV, Computer more than 2 hours daily and consuming junk food regularly. Conclusion: Periodic screening for overweight and obesity should be done in schools followed by counseling of parents of overweight and obese children. Counseling of adolescent children on lifestyle modification should be emphasized

AD - (Patnaik, Patnaik) Department of Community Medicine, Institute of Medical Sciences and SUM Hospital, India (Patnaik) Dept. of pediatrics, Kalinga Institute of Medical Sciences, India (Hussain) Indian Institute of Public Health, Bhubaneswar, India
ER -

TY - JOUR
ID - 3530
T1 - Correlates of overweight and obesity among urban school going children of Nagpur city
A1 - Thakre,S.B.
A1 - Mohane,S.P.
A1 - Ughade,S.M.
A1 - Thakre,S.S.
A1 - Morey,S.S.
A1 - Humne,A.Y.
Y1 - 2011///
N1 - 2012053245
English
Journal: Article
KW - EMBASE
KW - Adolescent
KW - Age Distribution
KW - article
KW - caloric intake
KW - Child
KW - controlled study
KW - cross-sectional study
KW - Dancing
KW - Female
KW - Human
KW - India
KW - leisure
KW - major clinical study
KW - Male
KW - nutritional requirement
KW - Obesity/ep [Epidemiology]
KW - Obesity/et [Etiology]
KW - preschool child
KW - Prevalence
KW - Risk Assessment
KW - risk factor
KW - school child
KW - sleep time
KW - sport
KW - Swimming
KW - Television Viewing
KW - traffic and transport
KW - urban area
KW - vegetarian
RP - NOT IN FILE
SP - 1593
EP - 1597
JF - Journal of Clinical and Diagnostic Research
VL - 5
IS - 8
CY - India
N2 - Introduction: Overweight and obesity during childhood are a matter of growing concern in India. Most individuals develop their eating and activity patterns during childhood. Obesity in children and adolescents is

gradually becoming a major public health problem in many developing countries, including India. The objective of the present study is to estimate the prevalence of overweight and obesity among school children and to identify the factors which influenced childhood overweight and obesity. Materials and Methods: A cross-sectional study was carried out from July 2009 to January 2011 on school children who were aged between 5 and 16 years. Results: The prevalence of overweight and obesity among children was 9.00% and 5.5% respectively. Overall, the prevalence of overweight/obesity was 14.52%. The risk of overweight/obesity was significantly (p<0.0120) higher among children who belonged to the upper SES and to the age group of > 10 years, who viewed television for > 1 hour, who slept for only > 7 hours (OR=1.87; 95% CI=1.87-2.53) and who used vehicles to go to school (OR=1.59;95%CI =1.14-2.24). The prevalence of overweight and obesity was significantly (p<0.0032) lower among children who swam, danced regularly (p<0.0353) and played outdoor games (p<0.0323) than their counterparts. Its association was found to be protective against overweight/ obesity. The risk of overweight /obesity was significantly higher among children who were non-vegetarians (OR=1.88; 95% CI=1.39-2.55, p<0.0001), who regularly consumed junk food (OR=2.54; 95% CI=1.82-3.53, p<0.0001), and in whom the calorie intake was more than the recommended daily allowances (OR=1.88; 95% CI=1.39-2.55, p<0.0001). Conclusion: This study confirmed the findings of earlier studies which were carried out in the other parts of the country and it emphasized that regular physical exercise, regulated television viewing, and a healthy eating behaviour could contribute to control overweight and obesity
SN - 2249-782X
AD - (Thakre, Mohane, Ughade, Morey) Preventive and Social Medicine, Government Medical College, Nagpur, India (Thakre, Humne) Preventive and Social Medicine, Indira Gandhi Government Medical College, Nagpur, India
ER -

TY - JOUR
ID - 3531
T1 - Transient pulmonary hypertension in a case of uncomplicated malaria
A1 - Srinivasa,S.
Y1 - 2011///
N1 - 70654169
English
Journal: Conference Abstract
KW - EMBASE
KW - Malaria
KW - pulmonary hypertension
KW - Internal Medicine
KW - Society
KW - Human
KW - patient
KW - Hospital
KW - Echocardiography
KW - Infection
KW - lung artery pressure
KW - blood smear
KW - Plasmodium falciparum
KW - Male
KW - World Health Organization
KW - idiopathic disease
KW - parasite
KW - schistosomiasis
KW - intravascular hemolysis
KW - sickle cell anemia
KW - hemolysis
KW - Immunity
KW - Mortality
KW - lung pressure
KW - wall stress
KW - Mali

KW - fever
KW - vomiting
KW - abdominal pain
KW - United States
KW - thrombocyte
KW - HIV test
KW - parasitemia
KW - computer assisted tomography
KW - Abdomen
KW - pelvis
KW - Human immunodeficiency virus
KW - infarction
KW - Acceleration
KW - thorax pain
KW - dyspnea
KW - exercise tolerance
KW - vital sign
KW - respiratory distress
KW - central venous pressure
KW - Leg
KW - oxygen saturation
KW - ambient air
KW - heart ejection fraction
KW - follow up
KW - heart right ventricle failure
KW - Child
KW - doxycycline
KW - quinine
KW - Nitric Oxide
KW - hemoglobin
KW - lactate dehydrogenase
KW - creatinine
RP - NOT IN FILE
SP - S485
EP - S486
JF - Journal of General Internal Medicine
VL - Conference: 34th Annual Meeting of the Society of General Internal Medicine Phoenix, AZ United States. Conference Start: 20110504 Conference End: 20110507. Conference Publication:
IS - var.pagings
N2 - LEARNING OBJECTIVES: 1. Recognize malaria as an uncommon underlying cause of pulmonary hypertension. 2. Recognize that treatment of uncomplicated malaria may prevent further sequelae of pulmonary hypertension. CASE INFORMATION: A 18 year-old man from Mali with recurrent episodes of malaria initially presented to an outside hospital with three days of fever, vomiting, and diffuse abdominal pain followed by a presyncopal episode. The patient arrived in the United States one month prior to this presentation. Labs were notable for hemoglobin 12 g/dL, platelets 88 K/uL, AST 68 U/L, ALT 104 U/L, LDH 284 U/L, and creatinine 1.3 mg/dL. HIV test was negative. A blood smear on admission was positive for plasmodium falciparum with 0.5% parasitemia. Cat scan of the abdomen and pelvis with contrast showed a splenic lesion consistent with infarction. The patient was treated with a seven day course of doxycycline and quinine for uncomplicated malaria. A transthoracic echocardiogram was performed as part of the presyncopal workup and revealed severe pulmonary hypertension based on decreased pulmonary acceleration time and systolic notching. On hospital day 7, the patient was transferred to the present hospital for further evaluation of his pulmonary hypertension. He denied chest pain, dyspnea, and decreased exercise tolerance. Vital signs were unremarkable. Physical exam was significant for a well appearing male in no acute respiratory distress; elevated jugular venous pressure, a right ventricular heave, a loud P2, and lower extremity edemawere absent.A six minutewalk test revealed oxygen saturations of 98-100% on room air. A repeat transthoracic echocardiogram on hospital day 11 revealed normal pulmonary arterial pressures with a preserved ejection fraction and no chamber enlargement. Two repeat blood smears were negative for plasmodium falciparum. The patient had improved clinically and

was discharged home with scheduled follow up of a serial transthoracic echocardiogram. IMPLICATIONS/DISCUSSION: Pulmonary hypertension (pHTN) is an elevation in the pulmonary arterial pressure, which if severe enough can lead to right ventricular failure. The World Health Organization proposed a classificationofpHTNinto five groups baseduponpathophysiology.Group 1 describes idiopathic disease and the remaining groups focus on an underlying cause. Although parasites, such as schistosomiasis, have been classified in Group 4 as causing pHTN by embolic disease, little is understood about the mechanism by which malaria causes pHTN. One study proposed that malaria causes pHTN by intravascular hemolysis in a manner similar to sickle cell disease. During the hemolysis process, nitric oxide may be depleted leading to increased pulmonary arterial pressures. [1] In patients living in endemic areas of malaria, repeated infections may aid in the development of partial immunity and subsequently lead to less severe infections but, pulmonary hypertension is an important disease sequela that can contribute to increased mortality. In this case, it seems that prompt treatment of uncomplicated malaria with doxycycline and quinine led to the resolution of pulmonary hypertension and presyncopal symptoms. Further studies are needed to fully understand the relationship between malaria and pHTN. [1] Janka J, et al. Increased Pulmonary Pressures and Myocardial Wall Stress in Children with Severe Malaria. The Journal of Infectious Disease. 2010; 202(5):791-800
SN - 0884-8734
AD - (Srinivasa) New York University, School of Medicine, New York, NY, United States
ER -

TY - JOUR
ID - 3532
T1 - Falling off the wagon: The need for a paradigm shift in tackling the obesity epidemic
A1 - Phillips,E.
A1 - Vazquez,E.
A1 - Peterson,J.
A1 - Falk,L.W.
A1 - Boutin-Foster,C.
A1 - Devine,C.
A1 - Wethington,E.
A1 - Wansink,B.
A1 - Charlson,M.
Y1 - 2011///
N1 - 70653770
English
Journal: Conference Abstract
KW - EMBASE
KW - Obesity
KW - Internal Medicine
KW - epidemic
KW - Society
KW - Human
KW - Eating
KW - weight
KW - weight reduction
KW - Hispanic
KW - Health
KW - Feeding Behavior
KW - Adult
KW - health care personnel
KW - Behavior Change
KW - meal
KW - Physical Activity
KW - community
KW - United States
KW - social network
KW - vegetable
KW - Interview

KW - beverage
KW - Energy Expenditure
KW - caloric intake
KW - lifespan
KW - Weight Gain
KW - African American
KW - lifestyle modification
KW - Religion
KW - information processing
KW - informed consent
KW - Female
KW - Population
KW - Health Status
KW - Diabetes Mellitus
KW - computer program
KW - grounded theory
KW - consensus
KW - college
KW - Education
KW - Child
KW - Fruit
KW - household
KW - Environment
KW - Diet
KW - Decision Making
KW - Self Concept
KW - patient
KW - Social Environment
KW - planning
KW - Lifestyle
KW - Food
KW - drinking water
RP - NOT IN FILE
SP - S289
EP - S290
JF - Journal of General Internal Medicine
VL - Conference: 34th Annual Meeting of the Society of General Internal Medicine Phoenix, AZ United States. Conference Start: 20110504 Conference End: 20110507. Conference Publication:
IS - var.pagings
N2 - BACKGROUND: Minority populations are disproportionately affected by the obesity epidemic. In recent years experts in the field have recommended that social and behavioral interventions should be focused on promoting small lifestyle changes that will eliminate or reduce the gradual excess weight gain that occurs in people of all ages over the life span. This "small change" approach is based on analyses that demonstrate that people gradually gain weight over time because of a small average daily difference between energy intake and energy expenditure known as the "energy gap." Based on theory and experimental research on small change strategies we developed a novel behavioral intervention aimed at achieving weight loss among Black and Hispanic adults in two New York City communities through small changes in eating behavior and physical activity. Prior to the implementation of the trial we sought to develop a better understanding of how different experimentally-based small change strategies would translate across different cultural groups. Qualitative group interviews were used to culturally tailor the future intervention. METHODS: Black or Hispanic adults > 21 years of age with a BMI > 25 and a previous weight loss attempt were recruited at two clinical and two church sites in Harlem and the South Bronx, New York. Six focus groups, three in Spanish and three in English were conducted by two moderators using a standardized interview guide following informed consent. Participants were asked to describe previous attempts at changing their eating behaviors to lose weight. Participants were then presented with thirteen experimentally-validated small change eating strategies known to reduce the "energy gap"(i.e. using a 10 inch plate for main meal, not eating when the TV is on, eating breakfast everyday, eating dinner at home at least 6 days a week). Participants voted for the top six strategies they felt could be

easily adopted by themselves and their family or social network. Group discussion explored potential advantages and challenges of adopting each of the six strategies. Sessions were tape-recorded and transcribed verbatim. Responses were entered into Ethnograph qualitative software and systematically analyzed using grounded theory methods. Through an iterative process, concepts were grouped into categories based on similar properties and dimensions. Overarching themes were then developed by three trained qualitative experts.Three additional trained corroborators independently reviewed the transcripts, and consensus was reached with the final themes reported here. RESULTS: 67 participants mean age 54 years+13, 72% women and 60% Hispanic were enrolled. More than half of participants had some college education (58%), 30% were employed, 42% were married and had one or more children. 74% described themselves as weighing too much and being advised by a medical provider to lose weight. The mean BMI was 34+7.2. 52% of participants described their general health as fair or poor. Only 36% of participants met the daily recommendations of eating five or more servings of fruit and vegetables. We identified three primary themes: 1) The Wake Up Call- an interaction with a health care provider or their own personal decision-making (based on psychologic or physiologic reasons) would prompt a self-awareness that obesity was negatively impacting their health and that it was time to initiate a behavior change; 2) Taking Control - preferences for a particular weight loss strategy were chosen based on which aspect of the built and/or social environment participants believe they could gain control over ; and 3) Falling Off theWagon -all participants described the experience of not being able to maintain newly adopted behavior/s long term as a result of either unrealistic weight loss planning and goals or negative environmental influences such as stress. Participants selected the following six small change strategies; 1) Using a smaller plate for the main meal; 2) Making half of the main meal vegetables; 3)Putting snacks out of sight; 4) Eating breakfast everyday; 5) Drinking water instead of sweetened beverages; and 6) Eating dinner at home at least 6 days a week. Common reported advantages of the strategies were: 1) Do not require a sacrifice -"you don't feel like you are giving something up;" 2) Cost-effective -"it's cheaper than eating out all the time"; and 3) Health benefits - "there are lots of health benefits from drinking water". CONCLUSION: Our study demonstrates that among community dwelling African-American and Hispanic adults the need to lose weight is often triggered by outside influences such as a health care provider or change in health status (i.e. new onset diabetes). While individuals are able to select strategies that results in short tem weight loss they reported that they are unable to maintain these efforts long term. Some strategies may conflict with family and cultural meal practices and values, others with daily schedules or household food environments. Participants voted for six out of thirteen small change eating strategies that they believe could be easily adopted and maintained long term by themselves or other members of their family and social network. Our results may also have important implications for translating basic experimental research on small change strategies for diverse cultural groups. The development of obesity interventions utilizing small environmental and behavioral changes may be more sustainable over time in comparison to more robust dieting efforts that yield positive results but cannot be maintained long term. The translation of the small change strategies into more culturally and environmentally relevant forms may also increase the success of interventions. The results of this study will be used to refine and tailor a large scale randomized behavioral intervention targeted at achieving a > 7% within-patient reduction in weight through small sustained changes in eating behavior coupled with sustained increases in lifestyle physical activity
SN - 0884-8734
AD - (Phillips, Peterson, Boutin-Foster, Charlson) Weill Cornell Medical College, New York, NY, United States (Vazquez, Falk) Lincoln Medical Center, Bronx, NY, United States (Devine, Wethington, Wansink) Cornell University, Ithaca, NY, United States
ER -

TY - JOUR
ID - 3533
T1 - Atypical automatic visual change detection in children with autism: An electrophysiological study
A1 - Bonnet-Brilhault,F.
A1 - Clery,H.
A1 - Roux,S.
A1 - Lenoir,P.
A1 - Barthelemy,C.
A1 - Bruneau,N.
A1 - Gomot,M.
Y1 - 2011///

N1 - 70653113
English
Journal: Conference Abstract
KW - EMBASE
KW - pharmacology
KW - drug surveillance program
KW - Human
KW - Child
KW - Society
KW - Autism
KW - physiology
KW - Therapy
KW - morphology
KW - processing
KW - latent period
KW - mismatch negativity
KW - brain
KW - stimulus
KW - visual stimulation
KW - Reaction Time
KW - clinical observation
KW - patient
KW - Environment
RP - NOT IN FILE
SP - 87
JF - Fundamental and Clinical Pharmacology
VL - Conference: 15th Annual Meeting of the French Society of Pharmacology and Therapeutics, the 78th Annual Meeting of the Society of Physiology, the 32nd Pharmacovigilance Meeting, the 12th APNET Seminar and the 9th CHU CIC Meeting Grenoble France. Conference Start: 20110322 Conference End: 20110324. Conference Publication:
IS - var.pagings
N2 - Introduction: The clinical observations of autistic patients show that they react in an unusual way to unattended changes that appear in the environment. Several studies have investigated novelty active detection in children with autism (CWA) and have highlighted visual atypical change processing. However little is known about the automatic change detection, phenomenon that can be study throughout the analysis of the mismatch negativity (MMN). The aim of the present study was to investigate the visual MMN in CWA. ERPs mapping was used to localize in both time and space the brain activations elicited by unattended visual change. Method: Twelve healthy children (aged 8-14 years) and 12 CWA (aged 8- 14 years) participated in the experiment. We used a visual passive oddball paradigm in which the stimuli consisted in the deformation of a circle into an ellipse in the horizontal direction (standard) or in the vertical (deviant). In order to present the visual stimuli outside the focus of attention, a concurrent task was required in which participants had to stare at the central fixation cross and to respond to its disappearance. Results: Behavioral measures showed that CWA displayed longer reaction time than Controls but did not differ in the number of omissions. ERPs analysis revealed several groups differences. Compared to controls CWA showed a typical morphology of response but with a significantly delayed latency. Moreover vMMN displayed shorter latency and atypical morphology in CWA than in controls. Conclusion: This study allowed to evidence an atypical visual automatic processing of change in CWA. These results suggest that abnormalities in change detection in CWA are independent of the sensory modality and might contribute to explain their characteristic behaviours in response to change
SN - 0767-3981
AD - (Bonnet-Brilhault, Clery, Roux, Lenoir, Barthelemy, Bruneau, Gomot) CHRU de Tours, INSERM U930, Universite Francois Rabelais de Tours, Tours, France
ER -

TY - JOUR
ID - 3534
T1 - A study of community design, greenness, and physical activity in children using satellite, GPS and accelerometer data

A1 - Almanza,E.
A1 - Jerrett,M.
A1 - Dunton,G.
A1 - Seto,E.
A1 - Ann,Pentz M.
Y1 - 2012///
N1 - 2012027743
English
Journal: Article
KW - EMBASE
KW - accelerometer
KW - Adolescent
KW - age
KW - analytic method
KW - article
KW - Child
KW - Child Behavior
KW - community
KW - community design
KW - controlled clinical trial
KW - controlled study
KW - cross-sectional study
KW - Environmental Exposure
KW - Environmental Planning
KW - Female
KW - global positioning system
KW - greenness exposure
KW - Human
KW - Income
KW - Male
KW - moderate to vigorous physical activity
KW - normalized difference vegetation index
KW - Physical Activity
KW - preschool child
KW - priority journal
KW - quasi experimental study
KW - school child
KW - Sex Ratio
KW - smart growth community
KW - spatial autocorrelation analysis
RP - NOT IN FILE
SP - 46
EP - 54
JF - Health and Place
VL - 18
IS - 1
CY - United Kingdom
N2 - This study examined relationships between greenness exposure and free-living physical activity behavior of children in smart growth and conventionally designed communities. Normalized Difference Vegetation Index (NDVI) was used to quantify children's (n=208) greenness exposure at 30-s epoch accelerometer and GPS data points. A generalized linear mixed model with a kernel density smoothing term for addressing spatial autocorrelation was fit to analyze residential neighborhood activity data. Excluding activity at home and during school-hours, an epoch-level analysis found momentary greenness exposure was positively associated with the likelihood of contemporaneous moderate-to-vigorous physical activity (MVPA). This association was stronger for smart growth residents who experienced a 39% increase in odds of MVPA for a 10th to 90th percentile increase in exposure to greenness (OR=1.39, 95% CI 1.36-1.44). An individual-level analysis found children who experienced >20. min of daily exposure to greener spaces (>90th percentile) engaged in nearly 5 times the

daily rate of MVPA of children with nearly zero daily exposure to greener spaces (95% CI 3.09-7.20). 2011 Elsevier Ltd
SN - 1353-8292
AD - (Almanza, Jerrett, Seto) Division of Environmental Health Sciences, School of Public Health, University of California, Berkeley, CA 94720-7360, United States (Dunton, Ann Pentz) Department of Preventive Medicine, Keck School of Medicine, University of Southern California, Los Angeles, CA 90089-9031, United States
ER -

TY - JOUR
ID - 3535
T1 - Pediatric blunt splenic trauma: Changing etiopathogenesis
A1 - Sreedher,G.
A1 - McKenna,C.
A1 - Gaines,B.
A1 - Abdalla,W.
A1 - Tadros,S.
Y1 - 2011///
N1 - 70647101
English
Journal: Conference Abstract
KW - EMBASE
KW - spleen injury
KW - radiology
KW - Etiology
KW - injury
KW - sport
KW - Human
KW - motor vehicle
KW - patient
KW - spleen
KW - Child
KW - traffic accident
KW - abdominal injury
KW - retrospective study
KW - emergency health service
KW - hazard
KW - solid
KW - Safety
RP - NOT IN FILE
SP - S347
JF - Pediatric Radiology
JA - Pediatr Radiol
VL - Conference: International Congress of Pediatric Radiology, IPR 2011 London United Kingdom. Conference Start: 20110528 Conference End: 20110531. Conference Publication:
IS - var.pagings
N2 - Purpose: 1. To compare and review the spectrum and severity of blunt splenic trauma in children associated with motor vehicle accidents and sports and related activities 2. To assess severity of associated intra-abdominal injuries in both categories. 3. To compare the AAST trauma grading scores and review image based severity grading of blunt pediatric splenic trauma. Materials and methods: 220 consecutive cases of blunt pediatric splenic trauma cases were analysed in a retrospective study. Patients presented to our Level 1 trauma center over a period of 4 years (2004-2008). Splenic Injury was graded according to AAST grading on CECT in all patients.Associated intra and extraabdominal injuries were categorized. Patients were categorized according to mechanism of injury into Motor vehicle related trauma, Outdoor Sports related trauma and biking related trauma. Results: All grades and severity of splenic injuries were seen in all three categories in our population.However the severity of associated intra and extraabdominal injuries was highest in the motor vehicle group as expected. Biking related injuries were found to be more severe in grade than other outdoor

sports. Handle bar injuries to spleen are a serious hazard and constitute a whopping 22% of all blunt splenic trauma. Frequency of associated abdominal solid visceral trauma is low with sports related trauma. Conclusion: Traditionally motor vehicle trauma is considered as the major culprit in pediatric splenic trauma, however in our study we found a far exceeding number of sports related and biking related trauma to the spleen. This is cause for concern and calls for a need for improved safety measures during pediatric recreational activities
SN - 0301-0449
AD - (Sreedher, McKenna, Gaines, Abdalla, Tadros) Children's Hospital of Pittsburgh, Pittsburgh, United States
ER -

TY - JOUR
ID - 3536
T1 - Use of dietary supplements and anabolic-androgenic steroids among Finnish adolescents in 1991-2005
A1 - Mattila,V.M.
A1 - Parkkari,J.
A1 - Laakso,L.
A1 - Pihlajamaki,H.
A1 - Rimpela,A.
Y1 - 2010///
N1 - 19710236
English
Journal: Article
KW - EMBASE
KW - addiction/ep [Epidemiology]
KW - Adolescent
KW - article
KW - Child
KW - Child Behavior
KW - diet supplementation
KW - doping
KW - Female
KW - Finland/ep [Epidemiology]
KW - Human
KW - Male
KW - Prevalence
KW - protein intake
KW - questionnaire
KW - risk factor
KW - Sex Ratio
KW - socioeconomics
KW - statistical model
KW - Utilization Review
KW - anabolic agent
KW - steroid
KW - vitamin
RP - NOT IN FILE
SP - 306
EP - 311
JF - European Journal of Public Health
JA - Eur J Public Health
VL - 20
IS - 3
CY - United Kingdom
N2 - The aim of the study was to describe the prevalence, trends and associated factors of dietary supplements (DS) and anabolic-androgenic steroids (AAS) use among Finnish adolescents. The sample comprised 30 511 adolescents aged 12-18 years, of which 22 519 (74%) answered a questionnaire. We also studied associations between 14 socioeconomic, health and health behavioural variables and DS and AAS use by logistic regression.

The proportion of respondents using DS was 45% during the past year and it increased linearly by age. Vitamins (37%) and herbal products (13%) were the most common DSs. In 1991, 9% of the boys aged 16-18 years reported protein use, while the frequency in 2005 was 17% (P < 0.001). AAS use was uncommon; only 53 boys (0.5%) and 20 girls (0.2%) reported AAS use. The strongest factors associated with DS use in multivariate model were physical exercise outside sports clubs (OR 1.9; 95% CI: 1.6-2.2), and in sports clubs (OR 1.7; 95% CI: 1.5-1.9). Recurrent drunkenness (OR 5.8; 95% CI: 1.5-21.6) and peer drug use in boys (OR 2.1; 95% CI: 1.2-3.7) were the risk factors for AAS use, whereas physical exercise outside sports clubs (OR 0.3; 95% CI: 0.1-0.5) was a protecting factor. Although the overall use of DS remained at the same level during the study period, there was a slight trend towards increasing use of vitamin and protein supplements. DS use is associated with frequent sports participation and poorer than average health, while AAS use is associated with health-compromising behaviours
AD - (Mattila) The School of Public Health, University of Tampere, Finland
ER -

TY - JOUR
ID - 3537
T1 - Perceived somatic symptoms and aspects of asthma management by analyzing coloured drawings made by chronic asthmatic children
A1 - Pascuet,E.
A1 - Vaillancourt,R.
A1 - Moore,A.-M.
A1 - Scoular,D.
A1 - Stewart,C.
Y1 - 2010///
N1 - 70638421
English
Journal: Conference Abstract
KW - EMBASE
KW - Asthma
KW - Human
KW - Child
KW - summer
KW - patient
KW - Lung
KW - pediatric hospital
KW - coughing
KW - sport
KW - Interpersonal Communication
KW - groups by age
KW - Comprehension
KW - recall
KW - Disease Management
KW - medical information
KW - study design
KW - hospital patient
KW - outpatient
KW - health literacy
RP - NOT IN FILE
SP - 340
JF - Canadian Journal of Hospital Pharmacy
VL - Conference: CSHP Summer Educational Sessions, SES 2010 Halifax, NS Canada. Conference Start: 20100807 Conference End: 20100810. Conference Publication:
IS - var.pagings
N2 - Rationale: The addition of pictures to health information can improve comprehension, recall and treatment adherence, all of which can lead to improved disease management. Objectives: To evaluate self-drawn illustrations made by chronic asthmatic pediatric patients to identify their perception of somatic symptoms and aspects of asthma management. Secondly, to use the emergent themes to develop child-specific pictograms to be

used as a supplementary aid to improve communication for use in the management of chronic asthma in pediatric patients. Study Design and Methods: Chronic inpatient and outpatient asthmatic children recruited at a Canadian pediatric hospital. Participants were asked to draw how they feel when their asthma is under control and during an attack. A multi-disciplinary team determined key drawing features from each individual picture to incorporate these identified themes as pictogram components of an asthma action plan. Results: Drawings from 53 participants analyzed. When asked to draw how they felt when their symptoms were well controlled, emerging themes included images of clear lungs, happy expressions, sunny skies and children playing sports and other outdoor activities. When asked how the children felt during an asthma attack, images of coughing, lung pain/tightness, sad expressions, and confinement or inability to play were common. No differences in common drawing features found between age groups. Conclusion: The next step will be to validate the pictograms with pediatric patients. This development of a validated easy to use asthma action plan can lead to improved asthma outcomes for asthmatic patients with a wide range of health literacy
SN - 0008-4123
AD - (Pascuet, Vaillancourt, Moore, Scoular, Stewart) Children's Hospital of Eastern Ontario (CHEO), Ottawa, ON, Canada
ER -

TY - JOUR
ID - 3538
T1 - Vitamin D deficiency in the pediatric tuberculosis clinic in Winnipeg, Manitoba
A1 - Bouhasan,L.
A1 - Consunji-Araneta,R.
Y1 - 2011///
N1 - 70634707
English
Journal: Conference Abstract
KW - EMBASE
KW - Hospital
KW - Canada
KW - vitamin D deficiency
KW - Tuberculosis
KW - Human
KW - Aborigine
KW - patient
KW - Population
KW - Prevalence
KW - ethnicity
KW - Risk
KW - Child
KW - computer program
KW - blood level
KW - gender
KW - groups by age
KW - seasonal variation
KW - winter
KW - Environment
KW - Analysis of Variance
KW - immigrant
KW - Genetics
KW - Diet
KW - Prevention
KW - Tuberculin Test
KW - Drug Therapy
KW - treatment response
KW - Immunity
KW - Blood
KW - screening

KW - reference value
KW - vitamin D
KW - parathyroid hormone
RP - NOT IN FILE
JF - Chest
VL - Conference: CHEST 2011 Honolulu, HI United States. Conference Start: 20111022 Conference End: 20111026. Conference Publication:
IS - var.pagings
N2 - PURPOSE: Vitamin D (VD) plays an important role in inflammatory and immune reactions and may be critical in the human tuberculosis (TB) response. With the increased prevalence of TB in the aboriginal (A) population, especially those residing in Northern Manitoba (NM) we sought to identify how frequently children seen in the TB Clinic were VD deficient (VDD). METHODS: VD levels were drawn (as part of routine baseline blood work as per clinic protocol after few patients failed to exhibit inadequate response to usual treatment) from patients referred to the TB clinic for contact investigation, school/immigration screening or treatment of primary/pulmonary disease from July 1, 2006 to June 30, 2008. The normal reference value used was 75-250nmol/L. VD insufficiency (VDI) was when the level was 25-75nmol/L and VDD was diagnosed in those with levels <25nmol/L. Results were analyzed using chisquare test and one way ANOVA. Data were analyzed by using SAS 9.2 software. Significance level considered at < 0.05. Log 10 transformations were done for the variables that were not Gaussian. RESULTS: 358 children had blood levels drawn during the 24 month period. The prevalence of VDD as to gender, ethnicity (aboriginal population versus other ethnicities) were similar but was significantly higher in the older age group(>14 years), and in those residing outside of Winnipeg. There was marginally significant difference in levels associated with seasonal variation, being more prevalent in the fall to winter. As expected, PTH levels were significantly higher in those with VDD. CONCLUSIONS: VDD is common in the aboriginal and immigrant populations seen in our clinic. What role and contribution genetics, diet and environment play require further study, especially as to whether the prevention of VDD may reduce the risk of developing TB. Analysis of VD levels in patients subsequently found to be tuberculin negative will provide more information. CLINICAL IMPLICATIONS: VD as an addition to anti-TB medications may be indicated to reduce risk and to facilitate treatment response
SN - 0012-3692
AD - (Bouhasan, Consunji-Araneta) University of Manitoba, Winnipeg, MB, Canada
ER -

TY - JOUR
ID - 3539
T1 - Continual infusion of intrathecal baclofen (ITB): Long term effect on spasticity
A1 - Khudhairi,D.A.
A1 - Aldin,A.S.
A1 - Hamdan,Y.
A1 - Rababah,A.
A1 - Matthana,M.H.
A1 - Pazdirek,J.
A1 - Abdulsalam,J.
Y1 - 2010///
N1 - 21526672
English
Journal: Article
KW - EMBASE
KW - Adolescent
KW - Adult
KW - article
KW - Child
KW - Female
KW - Human
KW - infusion pump
KW - intraspinal drug administration
KW - Male
KW - Middle Aged

KW - spasticity/dt [Drug Therapy]
KW - Time
KW - Treatment Outcome
KW - baclofen/ad [Drug Administration]
KW - central muscle relaxant/ad [Drug Administration]
RP - NOT IN FILE
SP - 851
EP - 855
JF - Middle East Journal of Anesthesiology
JA - Middle East J Anesthesiol
VL - 20
IS - 6
CY - Lebanon
N2 - Background: Spasticity is a disorder of muscular function causing muscular tightness or spasm which occurs when there is damage to the central nervous system whether it is of spinal cord or brain origin. This insult could be either pathological or traumatic. Method: Thirty-three patients had intrathecal Baclofen pumps implanted for severe spasticity. These patients either did not respond to or tolerate oral medications. Results: All patients showed significant improvement in their spasms following the procedure. Improvement was noted not only in spasticity, but also in pain, management of sleep disturbance, activities of daily living, indoor and outdoor mobility and behavior. Conclusion: The number of complications was acceptable, and generally were not lifethreatening. Infection was the significant complication in two patients and this led to explantation of their pumps. Patient satisfaction was very high and was related to improvement in the quality of life for the patients
SN - 0544-0440
AD - (Khudhairi) Faculty of Anaesthesia of the Royal College of Surgeons of Ireland, Prince Sultan Cardiac Centre Consultant Chronic Pain Management, Riyadh Military Hospital Sultan Bin Abdulaziz Humanitarian City, Saudi Arabia (Aldin) Doctor of Medicine, Saudi Arabia (Hamdan, Rababah, Matthana) Consultant Physiatrist, Saudi Arabia (Pazdirek) Board in Physical Medicine and Rehabilitation Consultant Physiatrist, Saudi Arabia (Abdulsalam) Ms in Physical Medicine and Rehabilitation, Saudi Arabia
ER -

TY - JOUR
ID - 3540
T1 - Life+ project: Population exposure to PAHs (EXPAH)
A1 - Gherardi,M.
A1 - Gariazzo,C.
A1 - Gatto,M.P.
A1 - Forastiere,F.
A1 - Cecinato,A.
A1 - Finardi,S.
A1 - Hanninen,O.
A1 - Sozzi,R.
Y1 - 2011///
N1 - 70632192
English
Journal: Conference Abstract
KW - EMBASE
KW - population exposure
KW - toxicology
KW - health care organization
KW - model
KW - exposure
KW - Human
KW - Population
KW - city
KW - sampling
KW - Child
KW - Health

KW - Aged
KW - volunteer
KW - Italy
KW - Air Pollution
KW - device
KW - Particulate Matter
KW - European Union
KW - school
RP - NOT IN FILE
SP - S124
JF - Toxicology Letters
JA - Toxicol Lett
VL - Conference: 47th Congress of the European Societies of Toxicology, EUROTOX 2011 Paris France. Conference Start: 20110828 Conference End: 20110831. Conference Publication:
IS - var.pagings
N2 - Purpose: EXPAH project financed by European Community with LIFE+ funds aims at identifying and quantifying population exposure among children and elderly people to carcinogenic PAHs in particulate matter in highly urbanized areas. The main goal of the project will be the health impact assessment of PAHs. An integrated approach, based on measurements and modeling techniques, will be used to estimate the spatial distribution of population exposure to PAHs, to identify key determinants of high exposures and to estimate potential health effects on the target population. In this paper, we summarize the component of the project consisting of in field campaigns for estimating the actual concentration of PAHs in different areas of the city and for assessing the actual human exposure in different living places (microenvironments). Methods:Measurements will be performed by means of low volume PM2.5 sampling devices in stationary outdoor and indoor locations, such as houses and schools, and by means of PM2.5 personal exposure measurements of volunteers selected between children and elderly people. The sampling will be performed in the city of Rome on a daily period during two seasonal weeks. Results of the study: Results of the in field campaigns will be addressed to: develop an outdoor-indoor infiltration model; estimate the mean exposure of the target populations to PAHs using data from the air pollution model, infiltration models and population time-activity patterns through an exposure model; evaluate the extent of outdoor-indoor PAHs infiltration;evaluate the population exposure to PAHs
SN - 0378-4274
AD - (Gherardi, Gariazzo, Gatto) INAIL Research (formerly ISPESL), Monte Porzio Catone (Rome), Italy (Forastiere) Department of Epidemiology, ASL RME (Healt Localy Agency of Rome), Rome, Italy (Cecinato) Institute for Atmospheric Pollution, National Research Council, Montelibretti (Rome), Italy (Finardi) ARIANET - Environmental Modelling, Milan, Italy (Hanninen) THL (National Institute for Health and Welfare), Helsinky, Finland (Sozzi) Arpalazio (Regional Agency for Environmental Protection), Rome, Italy
ER -

TY - JOUR
ID - 3541
T1 - "Footprints in the Bathroom": The role of spirituality in patient diagnosis
A1 - Meneses,V.
A1 - Vanderbilt,D.
A1 - Barnes,L.
A1 - Augustyn,M.
Y1 - 2011///
N1 - 21217403
English
Journal: Review
KW - EMBASE
KW - anxiety disorder/di [Diagnosis]
KW - Case Report
KW - Child
KW - ethnic group
KW - ethnology
KW - Female

KW - Hispanic
KW - Human
KW - psychological aspect
KW - Religion
KW - review
RP - NOT IN FILE
SP - 169
EP - 171
JF - Journal of Developmental and Behavioral Pediatrics
VL - 32
IS - 2
CY - United States
N2 - CASE: Lucy is a 12-year-old Latina whose mother brought her to the pediatric clinic for help with her anxiety symptoms, "hearing voices," and episodes of "spacing out." Lucy did not cry at birth secondary to vocal cord paralysis and still has the tracheostomy performed at a few days of life. In addition to her neonatal intensive care unit stay, she has been hospitalized for pneumonia. Her mother says she is "sickly" and has a weak immune system due to her asthma symptoms. Lucy also experiences somatic complaints related to gastroesophageal reflux and constipation.Lucy is described as being "in another world" and must be called several times before she responds. Lucy states she is nervous "all the time" and often experiences a fast heartbeat, chest pain, and headache. Her attention and internalizing problems began at age 4 years and have worsened since she entered adolescence. When she is alone in the dark, she sees an unknown person and becomes frightened. She hears the voice of her paternal uncle telling her something will harm her mother and runs crying to "check" on her. Lucy also says she hears voices of paternal family members fighting. She has dreamed that her paternal uncle stabbed her in an alley. Lucy reports experiencing sadness and feelings of wanting to die. She has not tried to harm herself and has no plan to do this. She states that she does not know when she is awake or asleep and "hate people talking in my head." She does not report drug use.Lucy's family is bilingual, English and Spanish. Her mother works part-time as a nutritionist, and her father is a machinist. Her father receives counseling for anger management, and there have been previous unsubstantiated child abuse reports involving Lucy and her younger brother. The maternal uncle has been involved in a gang and attempts to phone Lucy's mother, who does not wish to have contact with him. Lucy's mother expresses strong Christian religious and spiritual beliefs, including demons and spirits that may enter her home and can be cast off with prayer. Recently, the family purchased a "cuadro" (picture) at a yard sale, and Lucy's mother said this resulted in footprints in the bathroom that are disappearing with prayer, guidance from the church minister, and discarding of the "cuadro."The primary pediatrician ordered a home school program, given Lucy's frequent illnesses. She is progressing well with a special education teacher. Her recent psychological evaluation at school showed a below average nonverbal intelligence quotient, with strengths in the areas of perceptual, logical, and abstract relationships. Lucy's mother in the past has been hesitant to follow through with psychiatric evaluation because "it is up to God."In the clinic, she is pale and slender with an ethereal look. She is awake and alert with appropriate affect and mentation. Her tracheostomy is intact without any respiratory distress noted. There are no signs of drug use or active hallucinations. You are left wondering whether these voices need psychiatric management. 2011 Lippincott Williams & Wilkins
SN - 0196-206X
AD - (Meneses, Vanderbilt) Center for Excellence in Developmental Disabilities, Children's Hospital Los Angeles, University of Southern California, Los Angeles, CA, United States (Barnes) Department of Family Medicine, Boston University School of Medicine, Boston, MA, United States (Augustyn) Department of Pediatrics, Boston University School of Medicine, Boston, MA, United States
ER -

TY - JOUR
ID - 3542
T1 - Secondhand tobacco smoke exposure among school-aged youth enrolled in school-based asthma management programs: A mixed methods analysis
A1 - Schlueter,D.F.
A1 - Rasberry,C.N.
A1 - Buckley,R.
A1 - Mast,D.K.
A1 - Cheung,K.

A1 - Luna,P.J.
A1 - Robin,L.
A1 - Greenberg,C.
A1 - Langenfeld,N.A.
Y1 - 2011///
N1 - 2011582402
English
Journal: Article
KW - EMBASE
KW - article
KW - Asthma
KW - Attitude to Health
KW - Child
KW - controlled study
KW - Coping Behavior
KW - disease association
KW - elementary student
KW - Environmental Exposure
KW - health program
KW - health survey
KW - Human
KW - medical information
KW - parental smoking
KW - passive smoking
KW - personality test
KW - primary school
KW - priority journal
KW - qualitative analysis
KW - quantitative analysis
KW - Risk Assessment
KW - risk factor
KW - school child
KW - school health service
KW - strategic planning
KW - tobacco smoke
RP - NOT IN FILE
SP - 173
EP - 180
JF - Journal of Asthma and Allergy Educators
VL - 2
IS - 4
CY - United Kingdom
N2 - The high prevalence of asthma among school-aged youth places a significant burden on students, families, and communities. Secondhand smoke (SHS) exacerbates asthma symptoms and attacks. Parental smoking is likely the most common and recurring source of SHS exposure among children. School-based asthma management programs can play a major role in educating students and their families about how to reduce, eliminate, and cope with SHS exposure. Between January 2007 and May 2008, a total of 121 middle and 248 elementary school students who participated in school-based asthma management programs in elementary school completed pretest and posttest surveys assessing asthma behaviors and management difficulties. Subgroups of 40 middle and 54 elementary school students participated in 15 focus groups. Quantitative data were analyzed using descriptive statistics and independent sample t tests. Thematic analysis was used to identify and interpret prominent themes within qualitative data. Quantitative analysis of elementary school student data revealed students exposed to SHS had significantly higher asthma management difficulties at pretest (2.79 vs 1.98, respectively; $t = 3.4$, $P = .001$) and posttest (2.56 vs 1.74, respectively; $t = 3.8$, $P < .001$) compared with those without SHS exposure. Qualitative results from elementary and middle school students revealed that many students reported SHS exposure resulting from family members smoking at home or in cars. Students and their families reported using ineffective methods (eg, opening a window, drinking water, holding their breath) for

reducing, eliminating, and coping with SHS exposure. School-based asthma management programs should provide education to students and their families on the lasting effects of SHS on asthma symptoms and effective strategies for reducing, eliminating, and coping with SHS exposure in homes and cars. A focus on parent and community involvement can play a key role in addressing students' asthma management needs outside of school. 2011 The Author(s)
SN - 2150-1297
AD - (Schlueter, Luna, Robin, Greenberg, Langenfeld) ICF Macro, 3 Corporate Square NE, Suite 370, Atlanta, GA 30329, United States (Rasberry) Centers for Disease Control and Prevention, Atlanta, GA, United States (Buckley) First 5 Riverside/Riverside County Children and Families Commission, Riverside, CA, United States (Mast) New Mexico Department of Health, Albuquerque, NM, United States (Cheung) Charlotte-Mecklenburg Schools, Charlotte, NC, United States
ER -

TY - JOUR
ID - 3543
T1 - Policy statement - Climatic heat stress and exercising children and adolescents
A1 - McCambridge,T.M.
A1 - Brenner,J.S.
A1 - Benjamin,H.J.
A1 - Cappetta,C.T.
A1 - Demorest,R.A.
A1 - Halstead,M.E.
A1 - Koutures,C.G.
A1 - LaBella,C.R.
A1 - Labotz,M.
A1 - Loud,K.
A1 - Martin,S.M.
A1 - Weiss-Kelly,A.
A1 - Murray,R.
A1 - Devore,C.
A1 - Allison,M.
A1 - Barnett,S.
A1 - Gunther,R.
A1 - Holmes,B.W.
A1 - Lamont,J.
A1 - Minier,M.
A1 - Okamoto,J.
A1 - Wheeler,L.
Y1 - 2011///
N1 - 2011490224
English
Journal: Review
KW - EMBASE
KW - Adolescent
KW - Child
KW - Child Behavior
KW - child health care
KW - child safety
KW - Climate
KW - climatic heat stress/pc [Prevention]
KW - environmental temperature
KW - exercise intensity
KW - exercise tolerance
KW - Health Care Policy
KW - heat exhaustion/co [Complication]
KW - heat injury/pc [Prevention]
KW - high risk behavior

KW - Human
KW - hydration
KW - Physical Activity
KW - practice guideline
KW - priority journal
KW - Protective Clothing
KW - review
KW - sport
KW - Sports Medicine
KW - thermoregulation
KW - work environment
RP - NOT IN FILE
SP - e741
EP - e747
JF - Pediatrics
VL - 128
IS - 3
CY - United States
N2 - Results of new research indicate that, contrary to previous thinking, youth do not have less effective thermoregulatory ability, insufficient cardiovascular capacity, or lower physical exertion tolerance compared with adults during exercise in the heat when adequate hydration is maintained. Accordingly, besides poor hydration status, the primary determinants of reduced performance and exertional heat-illness risk in youth during sports and other physical activities in a hot environment include undue physical exertion, insufficient recovery between repeated exercise bouts or closely scheduled same-day training sessions or rounds of sports competition, and inappropriately wearing clothing, uniforms, and protective equipment that play a role in excessive heat retention. Because these known contributing risk factors are modifiable, exertional heat illness is usually preventable. With appropriate preparation, modifications, and monitoring, most healthy children and adolescents can safely participate in outdoor sports and other physical activities through a wide range of challenging warm to hot climatic conditions. Copyright 2011 by the American Academy of Pediatrics
SN - 0031-4005
ER -

TY - JOUR
ID - 3545
T1 - Ambient solar UV radiation and seasonal trends in potential sunburn risk among schoolchildren in South Africa
A1 - Wright,C.Y.
A1 - Coetzee,G.
A1 - Ncongwane,K.
Y1 - 2011///
N1 - 2011378647
English
Journal: Article
KW - EMBASE
KW - article
KW - Child
KW - controlled study
KW - environmental temperature
KW - Geography
KW - health hazard
KW - Human
KW - Public Health
KW - radiation exposure
KW - school child
KW - seasonal variation
KW - skin
KW - skin cancer/pc [Prevention]

KW - skin sensitivity
KW - skin type
KW - South Africa
KW - sunburn
KW - sunlight protection
KW - ultraviolet radiation
RP - NOT IN FILE
SP - 33
EP - 38
JF - SAJCH South African Journal of Child Health
VL - 5
IS - 2
CY - South Africa
N2 - Background. The detrimental effects of excess personal solar ultraviolet (UV) radiation exposure include sunburn, immunosuppression and skin cancer. In South Africa, individuals with minimum natural protection from melanin, including fair-skinned individuals and African albinos, and people spending extended unprotected periods outdoors are at risk of sunburn, a risk factor for skin cancer. Sunburn becomes increasingly likely during the high solar UV radiation hours around midday, and previous studies have shown that children are exposed to potentially high, sunburn-causing solar UV radiation levels while at school. Method. To estimate national potential child sunburn risk patterns, monitored ambient solar UV radiation levels at six sites in South Africa were converted into possible schoolchild solar UV radiation exposures by calculating the theoretical child exposure to 5% of the total daily ambient solar UV radiation as derived from personal child exposure studies. Results. Schoolgoing children with skin types I, II and III were identified as being at greatest risk of sunburn. There were 44 and 99 days in a year when schoolchildren with skin type III (moderately sensitive) living in Durban and De Aar, respectively, would be likely to experience sunburn. Schoolchildren with skin type I (extremely sensitive) were at risk of experiencing sunburn on 166 days in De Aar, and those with skin types I and II were at risk on at least 1 day per year at all six locations. Conclusion. Seasonal patterns show that schoolchildren with sensitive skin types may experience sunburn in spring, summer and autumn months. Differences in child sunburn risk were evident, mainly due to latitude and atmospheric aerosols. Additional factors affecting sunburn risk include schoolchildren's use of sun protection, sun-exposed activity, and timing and duration of exposure. Understanding risk patterns and obtaining locally relevant information will assist South African skin cancer prevention and sun protection awareness
SN - 1994-3032
AD - (Wright) Council for Scientific and Industrial Research, Pretoria, South Africa (Coetzee, Ncongwane) South African Weather Service, Pretoria, South Africa
ER -

TY - JOUR
ID - 3546
T1 - Food intake patterns and cardiovascular disease in different age cohorts: The relevance of food variety
A1 - Dzien,A.
A1 - Winner,H.
A1 - Theurl,E.
A1 - Dzien-Bischinger,C.
A1 - Lechleitner,M.
Y1 - 2011///
N1 - 2011298744
English
Journal: Article
KW - EMBASE
KW - Adolescent
KW - Adult
KW - Age Distribution
KW - article
KW - cardiovascular disease
KW - Child
KW - Cohort Analysis

KW - disease association
KW - drug use
KW - Feeding Behavior
KW - food frequency questionnaire
KW - Food Intake
KW - gender
KW - Human
KW - Infant
KW - major clinical study
KW - Physical Activity
KW - predictive value
KW - preschool child
KW - Probability
KW - quantitative analysis
KW - school child
KW - Smoking
KW - antidiabetic agent
KW - antihypertensive agent
KW - antilipemic agent
KW - antithrombocytic agent
KW - diuretic agent
RP - NOT IN FILE
SP - e116
EP - e120
JF - e-SPEN
VL - 6
IS - 3
CY - United Kingdom
N2 - Background: Dietary recommendations for healthy food intake are an essential part of preventive strategies, food variety might offer one additional approach. Methods: Between January 2005 and September 2009, a total of 2548 persons attended a medical outdoor centre for diagnostic and/or therapeutic interventions. To obtain information on their nutrition behaviour, patients were requested to complete a 51-item semi-quantitative food frequency questionnaire. Frequencies of consumption of food items were reported on a scale between 1 (seldom or never) and 6 (more than once per day). To investigate the impact of nutrition patterns on the probability of cardiovascular event and/or medication we estimated four alternative versions of logistic regressions. Results: Up to the age-decade 51-60 of years, the majority of the patients reported a moderate to high food consumption variety. A low variety was primarily found in the age decades older than 60 years. Within each age cohort, the predicted probability of cardiovascular events and/or medication was lower for patients with higher food variety. Our parameter estimates indicate that, on average, the consumption of one additional food item reduced the probability of a cardiovascular disease by about 0.5%. Adding overall daily intakes of energy or nutrient contents did not change this result. Conclusion: Our study shows that food diversity has a significant impact on the probability to stay healthy. 2011 European Society for Clinical Nutrition and Metabolism
SN - 1751-4991
AD - (Dzien, Dzien-Bischinger) Medical Center Hentschelhof, Buergerstrasse, A-6020 Innsbruck, Austria (Winner) Department of Economics and Social Sciences, University of Salzburg, Kapitelgasse 5-7, A-5010 Salzburg, Austria (Theurl) Department of Public Economics, University of Innsbruck, Universitatsstrasse 15/4, A-6020 Innsbruck, Austria (Lechleitner) Department of Internal and Geriatric Medicine, Hospital Hochzirl, Anna Dengel-Haus, A-6170 Zirl, Austria
ER -

TY - JOUR
ID - 3547
T1 - A low power wireless data acquisition device to monitor gait patterns for children with toe walking during daily activities
A1 - Lou,E.H.M.
A1 - Brunton,E.K.

A1 - Kamal,F.
A1 - Renggli,A.
A1 - Kemp,K.
A1 - Lewicke,J.
A1 - Sukhdeep,D.
A1 - Watt,J.M.
A1 - Andersen,J.
Y1 - 2011///
N1 - 2011296893
English
Journal: Article
KW - EMBASE
KW - Adult
KW - ambulatory monitoring
KW - article
KW - Case Report
KW - Child
KW - clinical assessment
KW - clinical trial
KW - daily life activity
KW - data analysis
KW - electric battery
KW - Female
KW - force
KW - gait
KW - Human
KW - information processing
KW - laboratory test
KW - Male
KW - school child
KW - toe
KW - Treatment Outcome
KW - walking difficulty
RP - NOT IN FILE
JF - Journal of Medical Devices, Transactions of the ASME
VL - 5
IS - 2
CY - United States
N2 - Clinical gait analysis is the accepted "gold standard" for evaluating an individual's walking pattern. However, in certain conditions such as idiopathic toe walking (ITW), the degree of voluntary control that a subject may elicit upon their walking pattern in a gait laboratory may not truly reflect their gait during daily activities. Therefore, a battery-powered, wireless data acquisition system was developed to record daily walking patterns to assist in the assessment of treatment outcomes in this patient population. The device was developed to be small (30x50x12 mm³), light-weight (15 g), easy to install, reliable, and consumed little power. It could be mounted across the laces of the shoe, while forces and walking activities were recorded to investigate the percentage of toe walking during the assessment. Laboratory tests were performed and preliminary clinical trials at a gait laboratory were done on six normal gait walkers. These volunteers also try to walk on their toes to simulate the toe walking at the gait laboratory. The system was able to track the gait pattern and determine the percentage of toe walking relative to normal gait. Three boys and one girl were diagnosed with ITW then participated into this study. A total of 4 sets thirty-three 10 min data sessions (5.5 h) were collected outside the laboratory. The results showed that the test subjects walked on their toes 70+4% of the total walking time, which was higher than that they performed 64+5% at the gait laboratory. This preliminary study shows promising results that the system should be able to use for clinical assessment and evaluation of children with ITW. 2011 American Society of Mechanical Engineers
SN - 1932-6181
AD - (Lou) Department of Electrical and Computer Engineering, University of Alberta, Edmonton, AB, T6G 2V4, Canada (Lou) Research and Technology Development, Alberta Health Services-Glenrose Rehabilitation

Hospital Site, 10230-111 Avenue, Edmonton, AB, T5G 0B7, Canada (Brunton) Research and Technology Development, Alberta Health Services-Glenrose Rehabilitation Hospital Site, 10230-111 Avenue, Edmonton, AB, T5G 0B7, Canada (Kamal) Department of Electrical and Computer Engineering, University of Alberta, Edmonton, AB, T6G 2V4, Canada (Renggli) Research and Technology Development, Alberta Health Services-Glenrose Rehabilitation Hospital Site, 10230-111 Avenue, Edmonton, AB, T5G 0B7, Canada (Kemp) Department of Surgery, University of Alberta, Edmonton, AB, T6G 2B7, Canada (Lewicke) Research and Technology Development, Alberta Health Services-Glenrose Rehabilitation Hospital Site, 10230-111 Avenue, Edmontonm, AB, T5G 0B7, Canada (Sukhdeep) Department of Surgery, University of Alberta, Edmonton, AB, T6G 2B7, Canada (Watt, Andersen) Department of Pediatrics, University of Alberta, Edmonton, AB, T6G 2B7, Canada
ER -

TY - JOUR
ID - 3548
T1 - Early antiretroviral therapy in HIV-1-infected infants, 1996-2008: Treatment response and duration of first-line regimens: The European Pregnancy and Paediatric HIV Cohort Collaboration (EPPICC) study group in EuroCoord
A1 - Judd,A.
Y1 - 2011///
N1 - 2011621654
English
Journal: Article
KW - EMBASE
KW - acquired immune deficiency syndrome
KW - article
KW - CD4 Lymphocyte Count
KW - Child
KW - cytomegalovirus infection
KW - drug substitution
KW - ethnicity
KW - Europe
KW - Female
KW - highly active antiretroviral therapy
KW - HIV associated dementia
KW - Human
KW - Human immunodeficiency virus 1
KW - Human immunodeficiency virus 1 infection/dt [Drug Therapy]
KW - Human immunodeficiency virus infected patient
KW - Infant
KW - major clinical study
KW - Male
KW - Pneumocystis jiroveci
KW - Pneumocystis pneumonia
KW - Prediction
KW - preschool child
KW - priority journal
KW - scoring system
KW - Sex
KW - treatment duration
KW - treatment response
KW - virus load
KW - abacavir/dt [Drug Therapy]
KW - abacavir plus lamivudine/dt [Drug Therapy]
KW - didanosine/dt [Drug Therapy]
KW - lamivudine/dt [Drug Therapy]
KW - lamivudine plus zidovudine/dt [Drug Therapy]
KW - nelfinavir/dt [Drug Therapy]

KW - nevirapine/dt [Drug Therapy]
KW - stavudine/dt [Drug Therapy]
KW - zidovudine/dt [Drug Therapy]
RP - NOT IN FILE
SP - 2279
EP - 2287
JF - AIDS
VL - 25
IS - 18
CY - United Kingdom
N2 - Objective: To investigate virological and immunological response to antiretroviral therapy (ART), and predictors of switching and interrupting treatment among infants starting ART across Europe. Design: Cohort study. Methods: Nine cohorts from 13 European countries contributed data on HIV-infected infants born 1996-2008 and starting ART before age 12 months. Logistic and linear regression, and competing risks methods were used to assess predictors of virological (viral load <400copies/ml) and immunological (change in CD4 Z-score) response, switching to second-line ART and treatment interruptions with viral load less than 400copies/ml. Results: A total of 437 infants were followed for median 5.9 (interquartile range 2.3-7.6) years after starting ART; 30% had an AIDS diagnosis prior to ART initiation. 53% had suppressed viral load <400copies/ml at 12 months in 1996-1999, increasing to 77% in 2004-2008. Virological and immunological responses at 12 months varied by initial ART type (P<0.001 and P=0.03, respectively), with four-drug nonnucleoside reverse transcriptase inhibitor (NNRTI)-based regimens being superior [virological response <400copies/ml adjusted odds ratio=3.00, 95% confidence interval (CI) 1.24-7.23; mean increase in CD4 Z-score coefficient=0.64, 95% CI 0.10-1.17] to both three-drug NNRTI-based (reference) and boosted protease inhibitor regimens which were similar. Rates of switching to second-line ART were lower among children starting four-drug NNRTI-based and boosted protease inhibitor-based regimens compared with three-drug NNRTI regimens (P=0.03). Sixty five percent of infants remained on first-line ART without treatment interruption after 5 years. Conclusion: Effective and prolonged responses to first-line ART can now be achieved in infants starting early ART outside trial settings. Superior responses to four-drug NNRTI compared with boosted protease inhibitor or three-drug NNRTI regimens need further evaluation, as does treatment interruption following early ART. 2011 Wolters Kluwer Health | Lippincott Williams & Wilkins
SN - 0269-9370
ER -

TY - JOUR
ID - 3549
T1 - Electromagnetic evidence of altered visual processing in autism
A1 - Neumann,N.
A1 - Dubischar-Krivec,A.M.
A1 - Poustka,F.
A1 - Birbaumer,N.
A1 - Bolte,S.
A1 - Braun,C.
Y1 - 2011///
N1 - 2011459181
English
Journal: Article
KW - EMBASE
KW - Adolescent
KW - Adult
KW - article
KW - Autism
KW - Child
KW - clinical article
KW - controlled study
KW - electromagnetic field
KW - event related potential
KW - Human

KW - Magnetoencephalography
KW - response time
KW - school child
KW - stimulus response
KW - striate cortex
KW - task performance
KW - visual disorder/di [Diagnosis]
KW - visual orientation
KW - visual stimulation
RP - NOT IN FILE
SP - 3011
EP - 3017
JF - Neuropsychologia
VL - 49
IS - 11
CY - United Kingdom
N2 - Individuals with autism spectrum disorder (ASD) demonstrate intact or superior local processing of visual-spatial tasks. We investigated the hypothesis that in a disembedding task, autistic individuals exhibit a more local processing style than controls, which is reflected by altered electromagnetic brain activity in response to embedded stimuli and enhanced activity of early visual areas. Ten autistic and ten matched control participants underwent 151-channel whole-head magnetoencephalography. Participants were presented with 400 embedded or isolated letters ('S' or 'H') and asked to indicate which of the two letters was shown. Performance was equal in both groups, but event-related magnetic fields differed between groups in an early (100-150. ms) and a later (350-400. ms) time window. In the early time window, autistic individuals differed from control participants in the embedded, but not in the isolated condition, reflecting reduced processing of the irrelevant context in autistic individuals. In the later time window, amplitude differences between the embedded and isolated conditions were measured in control participants only, suggesting that " disembedding" processes were not required in autistic individuals. Source localisation indicated that activity in individuals with ASD peaked in the primary visual cortex in both conditions and time windows indicating an effortless (automatic, bottom-up) local process, whereas activity in controls peaked outside the visual cortex. 2011 Elsevier Ltd
SN - 0028-3932
AD - (Neumann, Dubischar-Krivec, Birbaumer, Braun) Institute of Medical Psychology and Behavioral Neurobiology, University of Tubingen, Gartenstr. 29, 72074 Tubingen, Germany (Neumann) Institute of Psychology, Department of Physiological and Clinical Psychology and Psychotherapy, University of Greifswald, Franz-Mehring-Str. 47, 17487 Greifswald, Germany (Poustka) Department of Child and Adolescent Psychiatry, Johann Wolfgang Goethe-University, Deutschordenstr. 50, 60528 Frankfurt/M, Germany (Birbaumer) Ospedale San Camillo, Istituto di Ricovero e Cura a Caraterre Scientifico (IRCCS), 70, v. Alberoni, 30126 Venezia, Italy (Bolte) Department of Women's and Children's Health, Karolinska Institutet Center of Neurodevelopmental Disorders (KIND), Karolinska Institutet, 17177 Stockholm, Sweden
ER -

TY - JOUR
ID - 3550
T1 - Vitamin D supply in infancy, childhood, and adolescence: Abbreviated version of the statement issued by the Nutrition Committee of the German Society of Pediatrics and Adolescent Medicine
A1 - Wabitsch,M.
A1 - Koletzko,B.
A1 - Mo,A.
Y1 - 2011///
N1 - 2011576011
German
Journal: Article
KW - EMBASE
KW - Adolescent
KW - Age Distribution
KW - article
KW - Child

KW - Chronic Disease
KW - cost benefit analysis
KW - Germany
KW - high risk population
KW - Human
KW - Infant
KW - migration
KW - nutrient supply
KW - Nutritional Status
KW - Physical Activity
KW - sun exposure
KW - vegetarian diet
KW - vitamin intake
KW - vitamin supplementation
KW - 25 hydroxyvitamin D/ec [Endogenous Compound]
KW - vitamin D
RP - NOT IN FILE
SP - 766
EP - 774
JF - Monatsschrift fur Kinderheilkunde
VL - 159
IS - 8
CY - Germany
N2 - Current national and international publications describe a suboptimal vitamin D intake or an inadequate vitamin D status in all age groups. The aim of this statement is to summarize the available literature and evidence for reference values of vitamin D intake and vitamin D supply as well as for the current consumption. On this basis, recommendations for infants, children, and adolescents living in Germany were developed. Reasonable measures for achieving an improved vitamin D status are: protected sunlight exposure, intensive physical activity (at least 1 h daily) outside, and increase of vitamin D intake via supplements. Special attention is given to risk groups (children nourished on a vegetarian diet, migrants, persons with limited sunlight exposure, and chronically ill persons) for which regular control of serum 25-hydroxyvitamin D levels has to be considered. A systematic supervision of vitamin D supply in childhood and adolescence and a cost-benefit analysis of recommended or conducted measures should be performed. In the following an abbreviated version of the statement of the Nutrition Committee of the German Society of Pediatrics and Adolescent Medicine (DGKJ) is printed. The full version is published under http://www.dgkj.de. 2011 Springer-Verlag
SN - 0026-9298
AD - (Wabitsch, Koletzko, Mo) Ernahrungskommission, Deutsche Gesellschaft fur Kinder- und Jugendmedizin E.V., Chausseestrae 128/129, 10115 Berlin, Germany
ER -

TY - JOUR
ID - 3555
T1 - Coronary artery abnormalities in hyper-IgE syndrome
A1 - Freeman,A.F.
A1 - Avila,E.M.
A1 - Shaw,P.A.
A1 - Davis,J.
A1 - Hsu,A.P.
A1 - Welch,P.
A1 - Matta,J.R.
A1 - Hadigan,C.
A1 - Pettigrew,R.I.
A1 - Holland,S.M.
A1 - Gharib,A.M.
Y1 - 2011///

N1 - 2011430166
English
Journal: Article
KW - EMBASE
KW - Adolescent
KW - Adult
KW - Age Distribution
KW - aneurysm
KW - artery disease
KW - article
KW - atherosclerosis
KW - cardiovascular risk
KW - Child
KW - clinical article
KW - computer assisted tomography
KW - controlled study
KW - coronary artery dilatation
KW - coronary artery disease/co [Complication]
KW - coronary artery disease/ep [Epidemiology]
KW - coronary artery tortuosity
KW - disease association
KW - disease severity
KW - Female
KW - gene mutation
KW - Human
KW - hyper IgE syndrome
KW - Hypertension
KW - image analysis
KW - Male
KW - Morbidity
KW - nuclear magnetic resonance imaging
KW - population research
KW - priority journal
KW - protein determination
KW - protein expression
KW - protein function
KW - school child
KW - STAT3 protein/ec [Endogenous Compound]
RP - NOT IN FILE
SP - 338
EP - 345
JF - Journal of Clinical Immunology
VL - 31
IS - 3
CY - United States
N2 - Objective: Hyper-IgE syndrome (HIES) is a rare primary immunodeficiency caused by autosomal dominant STAT3 mutations resulting in recurrent infections and connective tissue abnormalities. Coronary artery abnormalities have been reported infrequently. We aimed to determine the frequency and characteristics of coronary artery abnormalities. Design: STAT3-mutated HIES patients (n = 38), ranging in age from 8 to 57 years, underwent coronary artery imaging by computed tomography or magnetic resonance imaging. Images were evaluated for tortuosity, dilation, and aneurysm. Charts were reviewed for cardiac risk factors. To allow blinded image interpretation, an age- and gender-matched non-HIES group was also evaluated (n = 33). Results: Coronary artery tortuosity or dilation occurred in 70% of HIES patients, with aneurysms present in 37%, incidences much higher than in the literature and in our non-HIES group, in which 21% had tortuosity or dilation and 3% had aneurysms. Hypertension was more common in the HIES group than in the general population and was associated with vessel abnormalities. Atherosclerosis was uncommon and mild. Conclusions: Coronary artery aneurysms and tortuosity are common in HIES, despite a paucity of

atherosclerosis, suggesting that STAT3 plays an integral role in human vascular remodeling and atherosclerosis. 2011 Springer Science+Business Media, LLC (outside the USA)
SN - 0271-9142
AD - (Freeman, Davis, Hsu, Holland) Immunopathogenesis Section, Laboratory of Clinical Infectious Diseases, National Institute of Allergy and Infectious Diseases (NIAID), 10 Center Drive, Bethesda, MD 20892-1899, United States (Avila) Clinical Research Training Program, School of Medicine, Duke University, Durham, NC, United States (Shaw) Biostatistics Research Branch, NIAID, NIH, Bethesda, MD, United States (Welch) SAIC-Frederick, National Cancer Institute (NCI) at Frederick, Frederick, MD, United States (Matta, Pettigrew, Gharib) National Institute of Diabetes and Digestive and Kidney Diseases, NIH, Bethesda, MD, United States (Hadigan) Laboratory of Immunoregulation, NIAID, NIH, Bethesda, MD, United States
ER -

TY - JOUR
ID - 3557
T1 - Teaching children with Autism Spectrum Disorder to mand "what is it?"
A1 - Marion,C.
A1 - Martin,G.L.
A1 - Yu,C.T.
A1 - Buhler,C.
Y1 - 2011///
N1 - 2011309153
English
Journal: Article
KW - EMBASE
KW - article
KW - Autism
KW - Child
KW - Environment
KW - Evaluation
KW - Human
KW - information
KW - Male
KW - Motivation
KW - preschool child
KW - priority journal
KW - school child
KW - Teaching
KW - vocalization
RP - NOT IN FILE
SP - 1584
EP - 1597
JF - Research in Autism Spectrum Disorders
VL - 5
IS - 4
CY - United Kingdom
N2 - In general terms a mand is a requesting response. Teaching children with autism to mand for information is often a difficult task given their language deficits. The present study examined a procedure consisting of contrived motivating operations, prompt fading, manded consequences, error correction, and a brief preference assessment for teaching the mand "What is it?" A modified multiple-baseline design across situations was used to evaluate the teaching procedure. Each of three children with Autism Spectrum Disorder learned the mands and generalized to situations, activities, scripts, the natural environment, and over time. 2011 Elsevier Ltd. All rights reserved
SN - 1750-9467
AD - (Marion, Martin, Yu) University of Manitoba, St. Paul's College, 129 Dysart Road, Winnipeg, MB R3T 2M6, Canada (Marion, Yu, Buhler) St. Amant Research Centre, 440 River Road, Winnipeg, MB R2M 3Z9, Canada
ER -

TY - JOUR
ID - 3558
T1 - Use of school recess time in the education and treatment of children with autism spectrum disorders: A systematic review
A1 - Lang,R.
A1 - Kuriakose,S.
A1 - Lyons,G.
A1 - Mulloy,A.
A1 - Boutot,A.
A1 - Britt,C.
A1 - Caruthers,S.
A1 - Ortega,L.
A1 - O'Reilly,M.
A1 - Lancioni,G.
Y1 - 2011///
N1 - 2011309143
English
Journal: Review
KW - EMBASE
KW - Academic Achievement
KW - Asperger syndrome
KW - Autism
KW - Child
KW - Evidence Based Practice
KW - Human
KW - preschool child
KW - priority journal
KW - Recreation
KW - review
KW - school child
KW - school health education
KW - Social Interaction
KW - student
KW - systematic review
KW - teacher
KW - Time Management
KW - Treatment Outcome
KW - Verbal Communication
RP - NOT IN FILE
SP - 1296
EP - 1305
JF - Research in Autism Spectrum Disorders
VL - 5
IS - 4
CY - United Kingdom
N2 - School recess is an opportunity to include students with autism spectrum disorders (ASD) with their typically developing peers and is a setting in which instruction can occur. However, the educational opportunities for children with ASD within recess are often overlooked and recess time is being reduced or eliminated in the United States. This review involved a systematic search and analysis of 15 studies that utilized recess to implement academic, social, or behavioral interventions for students with ASD. Each identified study that met pre-determined inclusion criteria was analyzed and summarized in terms of: (a) participant characteristics, (b) intervention procedures, (c) dependent variables, and (d) intervention outcomes. This review has three main aims: (a) to evaluate and synthesize the evidence-base, (b) to inform and guide teachers interested in utilizing recess time for educational purposes, and (c) to stimulate and guide future research in this area. Results demonstrate that recess time can indeed be used to teach target behaviors to students with ASD.
2011 Elsevier Ltd. All rights reserved

SN - 1750-9467
AD - (Lang, Boutot, Britt, Caruthers, Ortega) Texas State University-San Marcos, Clinic for Autism Research Evaluation and Support, United States (Kuriakose) University of California, Santa Barbara, United States (Lyons) University of Wisconsin-Madison, United States (Lang, Mulloy, O'Reilly) Meadows Center for the Prevention of Educational Risk, University of Texas, Austin, United States (Lancioni) University of Bari, Italy (Lang) Texas State University-San Marcos, Department of Curriculum and Instruction, 601 University Dr., San Marcos, TX 78666, United States
ER -

TY - JOUR
ID - 3559
T1 - Preschool outdoor play environment may combine promotion of children's physical activity and sun protection. Further evidence from Southern Sweden and North Carolina
A1 - Boldemann,C.
A1 - Dal,H.
A1 - Martensson,F.
A1 - Cosco,N.
A1 - Moore,R.
A1 - Bieber,B.
A1 - Blennow,M.
A1 - Pagels,P.
A1 - Raustorp,A.
A1 - Wester,U.
A1 - Soderstrom,M.
Y1 - 2011///
N1 - 2011282285
French
Journal: Article
KW - EMBASE
KW - Anthropometry
KW - article
KW - Child
KW - Environment
KW - Human
KW - outdoor environment
KW - Physical Activity
KW - play
KW - priority journal
KW - questionnaire
KW - radiation exposure
KW - radiation response
KW - risk factor
KW - school child
KW - scoring system
KW - sociometric status
KW - sun exposure
KW - sunburn
KW - ultraviolet radiation
RP - NOT IN FILE
SP - 72
EP - 82
JF - Science and Sports
VL - 26
IS - 2
CY - France
N2 - Objective: To study the impact of preschool outdoor environments on children's physical activity and solar ultraviolet (UV) exposure at different latitudes and countries.Methods. Outdoor environments of 11 preschools

(two in Raleigh NC, USA, Lat.36degreeN, nine in Malmo, Sweden, Lat.55degreeN) were scored (OPEC) regarding space, vegetation, hilly terrain and level of integration between open spaces, vegetation and play structures. Free sky (%) was determined by imaging. Physical activity was measured by pedometers and solar UV-exposure by polysulphone dosimeters during 5 days in spring 2009, and individual background data gathered by questionnaires and anthropometric measurements. Global UV radiation was measured and available individual UV radiation (%) computed. Results: In Malmo, mean step count/minute was 21.2 in preschools with high-scored environment vs. 17.6 in low-scored environments, and UV-exposure lower, 26% vs. 43% of available UV during outdoor stay. In Raleigh, step count/minute was 12.3 and UV-exposure of available UV 27% during outdoor stay. Conclusions: Step count/minute was lower in Raleigh than in Malmo, but in Malmo children at low-scored environments ran a higher risk of sunburn than in Raleigh. Trees and shrubbery integrated in children's playscape trigger both physical activity and sun-protective behaviour in Sweden, and previous measurements in Stockholm were confirmed. Such outdoor environment should be recommended, but the role of season and climate needs to be further explored. 2011 Elsevier Masson SAS
SN - 0765-1597
AD - (Boldemann, Dal) Karolinska Institutet, Department of Public Health Sciences, Stockholm, Sweden (Martensson) Department of Work Science, Business Economics and Environmental Psychology, Swedish University of Agricultural Sciences, Alnarp, Sweden (Cosco, Moore, Bieber) The Natural Learning Initiative, College of Design, North Carolina State University, Raleigh, NC, United States (Blennow) Karolinska Institutet, Dept. of Clinical Science and Education and Sachs' Children's Hospital, Soder Hospital, Stockholm, Sweden (Pagels, Raustorp) Linnaeus University, School of Sport Sciences, Kalmar, Sweden (Raustorp) Department of Neurobiology, Care Sciences and Society, Karolinska Institutet, Stockholm, Sweden (Wester) The Swedish Radiation Safety Authority, Stockholm, Sweden (Soderstrom) The Section of General Practice, Department of Public Health and The research Unit for General Practice, University of Copenhagen, Copenhagen, Denmark (Soderstrom) Department of Clinical Sciences in Lund, University of Lund, Lund, Sweden (Soderstrom) Department of Primary Health in Region Scania, Sweden
ER -

TY - JOUR
ID - 3560
T1 - Efficacy and safety of tacrolimus for induction therapy in patients with active lupus nephritis
A1 - Takahashi,S.
A1 - Hiromura,K.
A1 - Sakurai,N.
A1 - Matsumoto,T.
A1 - Ikeuchi,H.
A1 - Maeshima,A.
A1 - Kaneko,Y.
A1 - Kuroiwa,T.
A1 - Nojima,Y.
Y1 - 2011///
N1 - 2011377796
English
Journal: Article
KW - EMBASE
KW - abdominal skin ulcer/si [Side Effect]
KW - Adult
KW - article
KW - Child
KW - clinical article
KW - colitis/si [Side Effect]
KW - drug blood level
KW - drug efficacy
KW - drug safety
KW - drug withdrawal
KW - esophagus disease/si [Side Effect]
KW - Female
KW - glucose blood level

KW - Human
KW - hypertension/si [Side Effect]
KW - kidney biopsy
KW - low drug dose
KW - lung hemorrhage/si [Side Effect]
KW - lupus erythematosus nephritis/dt [Drug Therapy]
KW - Male
KW - mycosis/si [Side Effect]
KW - priority journal
KW - Remission
KW - retrospective study
KW - school child
KW - side effect/si [Side Effect]
KW - skin ulcer/si [Side Effect]
KW - systemic lupus erythematosus
KW - prednisone
KW - tacrolimus/ae [Adverse Drug Reaction]
KW - tacrolimus/cr [Drug Concentration]
KW - tacrolimus/do [Drug Dose]
KW - tacrolimus/dt [Drug Therapy]
RP - NOT IN FILE
SP - 282
EP - 289
JF - Modern Rheumatology
VL - 21
IS - 3
CY - Japan
N2 - After the completion of a double-blind placebo-controlled trial, tacrolimus (TAC) was approved for the treatment of lupus nephritis (LN) in Japan. However, the approved maximal dose, 3 mg/day, is almost half the dose used for induction therapy outside Japan. In this study, we retrospectively evaluated the efficacy and safety of low-dose TAC (<3 mg/day) for induction therapy in 13 adult patients (2 men and 11 women) with active LN. Eight patients were treated for LN flares. Twelve patients underwent renal biopsies: 8 with class IV, 2 with class III + V, 1 with class IV + V, and 1 with class V renal histology, according to the International Society of Nephrology/Renal Pathology Society (ISN/RPS) classification. The mean initial doses of prednisone and TAC were 34.6 + 14.5 and 2.7 + 0.6 mg/day, respectively. All the patients achieved a complete remission (CR) at 7.7 + 6.7 months (mean + SD) after the last administration of TAC, except for 2 patients who discontinued TAC treatment; 1 because of worsening systemic lupus erythematosus and 1 because of hypertension. Two patients experienced a flare-up after achieving CR. The mean blood TAC concentration 12 h after the last administration (C12) was significantly lower among the patients with flare-ups than among those with a sustained CR (1.5 + 1.5 vs. 5.1 + 1.9 ng/mL, P = 0.034). These data showed that low-dose TAC was effective for induction therapy in patients with active LN, although a lower TAC concentration may be associated with a poor outcome. 2010 Japan College of Rheumatology
SN - 1439-7595
AD - (Takahashi, Hiromura, Sakurai, Matsumoto, Ikeuchi, Maeshima, Kaneko, Kuroiwa, Nojima) Department of Medicine and Clinical Science, Gunma University, Graduate School of Medicine, 3-39-22 Showa, Maebashi, Gunma 371-8511, Japan
ER -

TY - JOUR
ID - 3561
T1 - Dorsal and ventral stream activation and object recognition performance in school-age children
A1 - Dekker,T.
A1 - Mareschal,D.
A1 - Sereno,M.I.
A1 - Johnson,M.H.
Y1 - 2011///

N1 - 2011358224
English
Journal: Article
KW - EMBASE
KW - Adult
KW - anatomical variation
KW - article
KW - BOLD signal
KW - brain cortex
KW - brain function
KW - Child
KW - Cognition
KW - controlled study
KW - electroencephalogram
KW - Female
KW - Functional Magnetic Resonance Imaging
KW - Human
KW - human experiment
KW - Male
KW - mental performance
KW - Neuroimaging
KW - normal human
KW - parietal cortex
KW - Pattern Recognition
KW - preschool child
KW - priority journal
KW - school child
KW - skill
KW - task performance
KW - temporal cortex
KW - visual cortex
KW - Visual Memory
RP - NOT IN FILE
SP - 659
EP - 670
JF - Neuroimage
VL - 57
IS - 3
CY - United States
N2 - We explored how developing neural artifact and animal representations in the dorsal and ventral stream play a role in children's increasingly more proficient interactions with objects. In thirty-three 6- to 10-year-old children and 11 adults, we used fMRI to track the development of (1) the cortical category preference for tools compared to animals and (2) the response to complex objects (as compared to scrambled objects) during a passive viewing task. In addition, we related a cognitive skill that improved substantially from age 6 to 10, namely the ability to recognize tools from unusual viewpoints, to the development of cortical object processing. In multiple complementary analyses we showed that those children who were better at recognizing tools from unusual viewpoints outside the scanner showed a reduced cortical response to tools and animals when viewed inside the scanner, bilaterally in intraparietal and inferotemporal cortex. In contrast, the cortical preference for tools in the dorsal and ventral visual stream did not predict object recognition performance, and was organized in an adult-like manner at six. While cortical tool preference did not change with age, the findings suggest that animal-preferring regions in the ventral visual stream may develop later, concordant with previous reports of a protracted development in similar regions for faces. We thus conclude that intraparietal and inferotemporal cortical networks that support aspects of object processing irrespective of tool or animal category, continue to develop during the school-age years and contribute to the development of object recognition skills during this period. 2010 Elsevier Inc
SN - 1053-8119

AD - (Dekker, Mareschal, Sereno, Johnson) Birkbeck College, University of London, Centre for Brain and Cognitive Development, Malet Street, London, United Kingdom (Sereno) University College London, Department of Psychology, 26 Bedford Way, London, United Kingdom
ER -

TY - JOUR
ID - 3562
T1 - Policy statement - Ultraviolet radiation: A hazard to children and adolescents
A1 - Balk,S.J.
A1 - Binns,H.J.
A1 - Brumberg,H.L.
A1 - Forman,J.A.
A1 - Karr,C.J.
A1 - Paulson,J.A.
A1 - Osterhoudt,K.C.
A1 - Seltzer,J.R.
A1 - Sandel,M.T.
A1 - Wright,R.O.
Y1 - 2011///
N1 - 2011124612
English
Journal: Review
KW - EMBASE
KW - Adolescent
KW - Awareness
KW - basal cell carcinoma
KW - Child
KW - erythema
KW - Health Care Policy
KW - Human
KW - melanoma/dt [Drug Therapy]
KW - pediatrician
KW - photosensitivity
KW - phototoxicity
KW - Physical Activity
KW - priority journal
KW - radiation hazard
KW - review
KW - skin cancer
KW - Skin Pigmentation
KW - squamous cell carcinoma
KW - sun exposure
KW - sunburn
KW - ultraviolet radiation
KW - nonsteroid antiinflammatory agent
KW - sunscreen/dt [Drug Therapy]
KW - tetracycline
KW - vitamin D/dt [Drug Therapy]
RP - NOT IN FILE
SP - 588
EP - 597
JF - Pediatrics
VL - 127
IS - 3
CY - United States
N2 - Ultraviolet radiation (UVR) causes the 3 major forms of skin cancer: basal cell carcinoma; squamous cell carcinoma; and cutaneous malignant melanoma. Public awareness of the risk is not optimal, overall compliance

with sun protection is inconsistent, and melanoma rates continue to rise. The risk of skin cancer increases when people overexpose themselves to sun and intentionally expose themselves to artificial sources of UVR. Yet, people continue to sunburn, and teenagers and adults alike remain frequent visitors to tanning parlors. Pediatricians should provide advice about UVR exposure during health-supervision visits and at other relevant times. Advice includes avoiding sunburning, wearing clothing and hats, timing activities (when possible) before or after periods of peak sun exposure, wearing protective sunglasses, and applying and reapplying sunscreen. Advice should be framed in the context of promoting outdoor physical activity. Adolescents should be strongly discouraged from visiting tanning parlors. Sun exposure and vitamin D status are intertwined. Cutaneous vitamin D production requires sunlight exposure, and many factors, such as skin pigmentation, season, and time of day, complicate efficiency of cutaneous vitamin D production that results from sun exposure. Adequate vitamin D is needed for bone health. Accumulating information suggests a beneficial influence of vitamin D on many health conditions. Although vitamin D is available through the diet, supplements, and incidental sun exposure, many children have low vitamin D concentrations. Ensuring vitamin D adequacy while promoting sun-protection strategies will require renewed attention to children's use of dietary and supplemental vitamin D. Copyright 2011 by the American Academy of Pediatrics
SN - 0031-4005
ER -

TY - JOUR
ID - 3563
T1 - A retrospective investigation of thiamin and energy intakes following an outbreak of beriberi in the Gambia
A1 - Thurnham,D.I.
A1 - Cathcart,A.E.
A1 - Livingstone,M.B.E.
Y1 - 2011///
N1 - 2011116581
English
Journal: Article
KW - EMBASE
KW - Adolescent
KW - Adult
KW - article
KW - beriberi/ep [Epidemiology]
KW - caloric intake
KW - Child
KW - Demography
KW - dietary intake
KW - epidemic
KW - Female
KW - Food Analysis
KW - food frequency questionnaire
KW - Food Intake
KW - Gambia
KW - harvest
KW - Human
KW - major clinical study
KW - Male
KW - preschool child
KW - retrospective study
KW - school child
KW - season
KW - Work
KW - thiamine
RP - NOT IN FILE
SP - 135
EP - 151

JF - Nutrients
VL - 3
IS - 1
CY - Switzerland
N2 - In the early part of the rainy season in 1988, an outbreak of beriberi occurred in free-living adults in a relatively small area in the North Bank region of The Gambia. In 1995 we selected two compounds in a village called Chilla situated within the affected district to retrospectively examine dietary factors potentially contributing to the outbreak. There had previously been cases of beriberi in one compound (BBC) but not in the other (NBC). We measured energy and thiamin intakes for four days on six occasions during the year. We calculated energy and thiamin intakes of people living in the two compounds and foods were collected for thiamin analysis through the year. Thiamin:Energy ratios only met international recommendations in the immediate post-harvest season when energy and thiamin intakes were highest and then fell through the year. In the rainy season when food was short and labour was heaviest, energy intakes were lower in the NBC but thiamin:energy ratios were lower in BBC. Records of rainfall in 1988 collected near the village indicated that the amount in August was twice the average. We suggest the heavy rainfall may have increased farm workload and reduced income from outside-village work activity. The lower energy intakes in the NBC may have forced adults to rest thus sparing thiamin demands and delaying onset of beriberi. In contrast, the higher energy intake of adults in the BBC may have enabled them to continue working, thus increasing demands for thiamin and inducing the earlier onset of beriberi. 2011 by the authors; licensee MDPI, Basel, Switzerland
AD - (Thurnham, Cathcart, Livingstone) Northern Ireland Centre for Food and Health, University of Ulster, Coleraine, BT52 1SA, United Kingdom
ER -

TY - JOUR
ID - 3565
T1 - Facilitating social initiations of preschoolers with autism spectrum disorders using video self-modeling
A1 - Buggey,T.
A1 - Hoomes,G.
A1 - Sherberger,M.E.
A1 - Williams,S.
Y1 - 2011///
N1 - 2011068866
English
Journal: Article
KW - EMBASE
KW - article
KW - autism/th [Therapy]
KW - Behavior
KW - Case Report
KW - Child
KW - communication skill
KW - Female
KW - Human
KW - imagery
KW - Male
KW - Motor Performance
KW - Nonverbal Communication
KW - play
KW - preschool child
KW - priority journal
KW - Social Interaction
KW - Socialization
KW - vide self modeling
KW - videorecording
KW - vocalization
RP - NOT IN FILE
SP - 25

EP - 36
JF - Focus on Autism and Other Developmental Disabilities
VL - 26
IS - 1
CY - United States
N2 - Video self-modeling (VSM) has accumulated a relatively impressive track record in the research literature across behaviors, ages, and types of disabilities. Using only positive imagery, VSM gives individuals the opportunity to view themselves performing a task just beyond their present functioning level via creative editing of videos using VCRs or video software. In this study, the authors used a single-subject multiple-baseline design across four preschool children on the autism spectrum to determine if self-modeling would facilitate their social initiations during playground time. The results were mostly positive, with two children exhibiting major treatment effects, one with questionable results, and one child being unaffected. Discussion of the results, the practicality of VSM, and suggestions for continued research are provided. 2011 Hammill Institute on Disabilities
SN - 1088-3576
AD - (Buggey, Hoomes, Sherberger, Williams) University of Tennessee at Chattanooga, United States
ER -

TY - JOUR
ID - 3566
T1 - Outdoor temperature, age, sex, body mass index, and diabetic status determine the prevalence, mass, and glucose-uptake activity of 18F-FDG-detected BAT in humans
A1 - Ouellet,V.
A1 - Routhier-Labadie,A.
A1 - Bellemare,W.
A1 - Lakhal-Chaieb,L.
A1 - Turcotte,E.
A1 - Carpentier,A.C.
A1 - Richard,D.
Y1 - 2011///
N1 - 2011014395
English
Journal: Article
KW - EMBASE
KW - Adolescent
KW - Adult
KW - Aged
KW - article
KW - body fat
KW - body mass
KW - brown adipose tissue
KW - Child
KW - computed tomography scanner
KW - computer assisted tomography
KW - Diabetes Mellitus/ep [Epidemiology]
KW - diabetic patient
KW - drug uptake
KW - environmental factor
KW - environmental temperature
KW - Female
KW - glucose blood level
KW - glucose transport
KW - Human
KW - lipid storage
KW - major clinical study
KW - Male
KW - mediastinum

KW - positron emission tomography
KW - preschool child
KW - Prevalence
KW - priority journal
KW - retrospective study
KW - risk factor
KW - school child
KW - sex difference
KW - winter
KW - fluorodeoxyglucose f 18/pk [Pharmacokinetics]
KW - glucose/ec [Endogenous Compound]
RP - NOT IN FILE
SP - 192
EP - 199
JF - Journal of Clinical Endocrinology and Metabolism
VL - 96
IS - 1
CY - United States
N2 - Context: In humans, the prevalence, mass, and glucose-uptake activity of ¹⁸F-fluorodeoxyglucose (¹⁸F-FDG)-detected brown adipose tissue (BAT), which are expectedly enhanced by a cold stimulus, also appear modulated by other factors that still have to be disentangled. Objective: The objective of the study was to investigate the factors determining the prevalence, mass, and glucose-uptake activity of ¹⁸F-FDG-detected BAT in humans. Research Design and Methods: We retrospectively analyzed all ¹⁸F-FDG positron emission tomography/computed tomography examinations performed between January 2007 and December 2008 at our institution for ¹⁸F-FDG uptake within the cervical/supraclavicular, mediastinal, paravertebral, and perirenal fat areas. The influence of outdoor temperature, sex, age, body mass index (BMI), plasma glucose level, diabetes diagnosis, day length, and cancer status on the prevalence, mass, and glucose-uptake activity of ¹⁸F-FDG-detected BAT depots was investigated. Results: Three hundred twenty-eight of the 4842 patients (6.8%) had ¹⁸F-FDG-detected BAT. The prevalence of ¹⁸F-FDG BAT was negatively associated with outdoor temperature (P<0.0001), age (P<0.0001), BMI (P<0.0001), and diabetes status (P=0.0003). Moreover, there was a significant age x sex interaction for the prevalence of ¹⁸F-FDG BAT (the younger the subjects, the greater the sex difference). The mass and glucose-uptake activity of ¹⁸F-FDG-detected BAT also decreased with increasing outdoor temperature (P < 0.0001), age (P < 0.0001), and BMI (P < 0.0001). They were lower in men than in women (P < 0.001) and lower in diabetic than in nondiabetic patients (P = 0.0002). Conclusions: The present study identifies outdoor temperature, age, sex, BMI, and diabetes status as determinants of the prevalence, mass, and glucose-uptake activity of ¹⁸F-FDG-detected BAT. Copyright 2011 by The Endocrine Society
SN - 0021-972X
AD - (Ouellet, Routhier-Labadie, Bellemare, Richard) Centre de Recherche de l'Institut Universitaire de Cardiologie et de Pneumologie de Quebec, Faculty of Medicine, Universite Laval, Quebec, QC G1V 4G5, Canada (Lakhal-Chaieb) Department of Mathematics and Statistics, Universite Laval, Quebec, QC G1V 0A6, Canada (Turcotte) Sherbrooke Molecular Imaging Center, Centre Hospitalier Universitaire de Sherbrooke, Universite de Sherbrooke, Sherbrooke, QC J1H 5N4, Canada (Carpentier) Department of Medicine, Centre Hospitalier Universitaire de Sherbrooke, Universite de Sherbrooke, Sherbrooke, QC J1H 5N4, Canada
ER -

TY - JOUR
ID - 3568
T1 - Respiratory effects of indoor particles in young children are size dependent
A1 - Franck,U.
A1 - Herbarth,O.
A1 - Roder,S.
A1 - Schlink,U.
A1 - Borte,M.
A1 - Diez,U.
A1 - Kramer,U.

A1 - Lehmann,I.
Y1 - 2011///
N1 - 2011113567
English
Journal: Article
KW - EMBASE
KW - article
KW - Child
KW - chronic obstructive lung disease
KW - cigarette smoking
KW - concentration (parameters)
KW - controlled study
KW - daily life activity
KW - Environmental Exposure
KW - Female
KW - health hazard
KW - Human
KW - indoor air pollution
KW - Infant
KW - major clinical study
KW - Male
KW - Newborn
KW - Particle Size
KW - Particulate Matter
KW - priority journal
KW - questionnaire
KW - respiratory tract disease
KW - Risk Assessment
KW - risk factor
KW - smoking habit
RP - NOT IN FILE
SP - 1621
EP - 1631
JF - Science of the Total Environment
JA - Sci Total Environ
VL - 409
IS - 9
CY - Netherlands
N2 - Background: Extensive epidemiological studies have provided evidence of an association between elevated outdoor particulate air pollution and adverse health effects. However, while people typically spend majority of time indoors, there is limited knowledge on airborne indoor particles and on the correlation between the concentrations of indoor particles and health effects. Even insights into the influence of differently sized indoor particles on human health are still rare. Objective: The association between differentially sized indoor air particles and the development of respiratory diseases was studied for three year aged children. Methods: Short-term measurements of particle mass and number concentrations were carried out in children's rooms. Information on possible particle sources (smoking habits, type of heating, and traffic) and respiratory outcomes were obtained from questionnaires. Measured indoor particle concentrations were correlated with possible sources of indoor particles and with respiratory health impacts. Results: Daily smoking, smoking more than 5 cigarettes per day at home and traffic density in front of the window of children's room were found to be related to indoor exposure by particles of different diameters. High indoor particle exposures were associated with an increased risk for the development of obstructive bronchitis and in some extent of non-obstructive bronchitis. The strongest impact was observed for the mass concentration of particles < 1 <m and the number concentration of particles > 0.5 mum. The risk increases still remain significant if tested for stability changing the number of adjustment variables or omitting randomly selected cases, respectively. Conclusion: Our results show significant associations between indoor particle concentrations and the risks for respiratory diseases in young children. The applied short-term measurements can help to assess the health risks of indoor particles with different sizes within epidemiological studies. 2010 Elsevier B.V

SN - 0048-9697
AD - (Franck, Roder, Schlink) Helmholtz Centre for Environmental Research, UFZ, Core Facility Studies, Permoserstrase 15, 04318 Leipzig, Germany (Herbarth) Environmental Medicine and Hygiene, Faculty of Medicine, University of Leipzig, Liebigstrasse 27, 04103 Leipzig, Germany (Borte, Diez) Children's Hospital, Municipal Hospital, Klinikum St. Georg gGmbH, Delitzscher Strase, 141 D-04129 Leipzig, Germany (Kramer) Institut fur Umweltmedizinische Forschung (IUF), Arbeitsbereich Epidemiologie, Auf'm Hennekamp 50, 40225, Dusseldorf, Germany (Lehmann) Helmholtz Centre for Environmental Research - UFZ, Department of Environmental Immunology, Permoserstrase 15, 04318 Leipzig, Germany
ER -

TY - JOUR
ID - 3569
T1 - Antibody levels and immune memory 23 years after primary plasma-derived hepatitis B vaccination: Results of a randomized placebo-controlled trial cohort from China where endemicity is high
A1 - Wu,Q.
A1 - Zhuang,G.-H.
A1 - Wang,X.-L.
A1 - Wang,L.-R.
A1 - Li,N.
A1 - Zhang,M.
Y1 - 2011///
N1 - 2011104707
English
Journal: Article
KW - EMBASE
KW - antibody blood level
KW - antibody production
KW - antibody titer
KW - article
KW - Child
KW - China
KW - controlled study
KW - endemic disease/dt [Drug Therapy]
KW - endemic disease/pc [Prevention]
KW - hepatitis B/dt [Drug Therapy]
KW - hepatitis B/pc [Prevention]
KW - Human
KW - immune response
KW - immunological memory
KW - major clinical study
KW - preschool child
KW - priority journal
KW - randomized controlled trial
KW - randomized controlled trial (topic)
KW - school child
KW - seroconversion
KW - Vaccination
KW - hepatitis B vaccine/dt [Drug Therapy]
KW - hepatitis B vaccine/im [Intramuscular Drug Administration]
KW - placebo
KW - recombinant vaccine
RP - NOT IN FILE
SP - 2302
EP - 2307
JF - Vaccine
VL - 29
IS - 12

CY - United Kingdom
N2 - The duration of protection of hepatitis B vaccine remains incompletely understood. To assess the long-term protection provided by a primary vaccine series, the current study again recruited all subjects of a previous randomized placebo-controlled trial cohort 23 years after vaccination. Two hundred and sixty-one healthy children aged 5-9 years living in a highly HBV-endemic country were enrolled in the primary trial and received three doses of plasma-derived vaccine or placebo. The primary placebo receivers who did not receive any immunization against hepatitis B were used as non-vaccinated controls in the current study. After eliminating the interference of an early booster dose and vaccines outside the study, 48.1% (39/81) vaccinees still maintained anti-HBs titers >10. mIU/mL at Year 23, higher than 34.7% (26/75) in non-vaccinated controls (P= 0.088). 75-100% of vaccinees with anti-HBs titer <10. mIU/mL at Year 23 in different sub-groups divided according to early immune backgrounds developed a rapid and robust antibody anamnestic response after a booster dose, highly significantly different from non-vaccinated controls who received the same dose of vaccine (7.5%, P< 0.01). No case of clinically significant HBV infection was found in the primary cohort during the whole 23 years, but 10 transient HBsAg seroconversions in the primary placebo group and one in the primary vaccine group were determined. Anti-HBc positive rate obviously tended to be lower in vaccinees compared with non-vaccinated controls at Year 23. These results suggest a persisting immune memory and certain protection for 23 years after primary vaccination in children living in highly HBV-endemic areas. Clinically insignificant infections, which cannot be avoided and may often occur in vaccinees, play a positive role in the maintaining of immunity to HBV. Booster doses should be unnecessary for more than 20 years after a full primary immunization in children (as catch-up vaccination) and, also likely, in newborns living in highly HBV-endemic areas. 2011 Elsevier Ltd
SN - 0264-410X
AD - (Wu, Zhuang, Wang, Wang, Li, Zhang) Department of Epidemiology and Biostatistics, Xi'an Jiaotong University College of Medicine, Xi'an, Shaanxi 710061, China
ER -

TY - JOUR
ID - 3570
T1 - Anti-allergic drug testing in an environmental challenge chamber is suitable both in and out of the relevant pollen season
A1 - Badorrek,P.
A1 - Dick,M.
A1 - Hecker,H.
A1 - Schaumann,F.
A1 - Sousa,A.R.
A1 - Murdoch,R.
A1 - Hohlfeld,J.M.
A1 - Krug,N.
Y1 - 2011///
N1 - 2011182303
English
Journal: Article
KW - EMBASE
KW - Adult
KW - allergy test
KW - article
KW - atopic dermatitis/si [Side Effect]
KW - Child
KW - clinical assessment tool
KW - controlled study
KW - double blind procedure
KW - drug efficacy
KW - drug eruption/si [Side Effect]
KW - drug induced headache/si [Side Effect]
KW - drug safety
KW - drug screening
KW - drug withdrawal

KW - environmental challenge chamber
KW - Female
KW - grass pollen
KW - hay fever/dt [Drug Therapy]
KW - Human
KW - Infant
KW - major clinical study
KW - Male
KW - pollen allergy/dt [Drug Therapy]
KW - priority journal
KW - questionnaire
KW - randomized controlled trial
KW - respiratory tract disease/si [Side Effect]
KW - respiratory tract infection/si [Side Effect]
KW - screening test
KW - treatment response
KW - antiallergic agent/dt [Drug Therapy]
KW - cetirizine/ae [Adverse Drug Reaction]
KW - cetirizine/ct [Clinical Trial]
KW - cetirizine/cb [Drug Combination]
KW - cetirizine/dt [Drug Therapy]
KW - placebo
KW - pseudoephedrine/ae [Adverse Drug Reaction]
KW - pseudoephedrine/ct [Clinical Trial]
KW - pseudoephedrine/cb [Drug Combination]
KW - pseudoephedrine/dt [Drug Therapy]
RP - NOT IN FILE
SP - 336
EP - 341
JF - Annals of Allergy, Asthma and Immunology
VL - 106
IS - 4
CY - United States
N2 - Background: An environmental challenge chamber (ECC) is a useful tool to expose allergic patients to relevant allergens in a controlled indoor setting and to test anti-allergic treatment. Hitherto, ECC studies with grass pollen are conducted primarily outside of the pollen season to avoid the influence of natural pollen exposure. Objective: To investigate whether an established anti-allergic treatment, a combination of cetirizine (CET) and pseudoephedrine (PSE), shows an equivalent treatment effect within and outside of the grass pollen season when tested in an ECC. Methods: In a randomized, placebo-controlled, double-blind, four-way crossover study, the effect of a combination of 10 mg CET and 120 mg PSE compared with placebo on nasal symptoms, nasal flow, and nasal secretion was investigated in 70 patients with seasonal allergic rhinitis. Subjects underwent four 6-hour pollen challenges in an ECC with administration of the drugs after 2 hours. Two challenges were conducted within the grass pollen season and two out of the grass pollen season. Results: The active treatment significantly improved nasal symptoms and nasal flow and significantly reduced the amount of nasal secretion compared with placebo both within and outside of the pollen season ($P < .0001$ each). The treatment effect was not different between the seasons ($P > .05$). Conclusion: Controlled allergen provocation in an ECC can be used to test anti-allergic treatment both within and outside of the grass pollen season. 2011 American College of Allergy, Asthma & Immunology
SN - 1081-1206
AD - (Badorrek, Dick, Schaumann, Hohlfeld, Krug) Fraunhofer Institute for Toxicology and Experimental Medicine, Nikolai-Fuchs-Str. 1A, 30625 Hannover, Germany (Hecker) Department of Biometrics, Hannover Medical School, Hannover, Germany (Sousa, Murdoch) GlaxoSmithKline, Stevenage, United Kingdom
ER -

TY - JOUR
ID - 3571

T1 - Accelerometer-determined physical activity among elementary school-aged children with autism spectrum disorders in Taiwan
A1 - Pan,C.-Y.
A1 - Tsai,C.-L.
A1 - Hsieh,K.-W.
A1 - Chu,C.-H.
A1 - Li,Y.-L.
A1 - Huang,S.-T.
Y1 - 2011///
N1 - 2011135096
English
Journal: Article
KW - EMBASE
KW - accelerometer
KW - Accelerometry
KW - age
KW - article
KW - Autism
KW - Child
KW - clinical article
KW - Human
KW - Male
KW - Physical Activity
KW - priority journal
KW - school child
KW - Taiwan
RP - NOT IN FILE
SP - 1042
EP - 1052
JF - Research in Autism Spectrum Disorders
VL - 5
IS - 3
CY - United Kingdom
N2 - To examine age-related physical activity (PA) patterns between- and within-day in elementary school-aged children with autism spectrum disorders (ASD). PA was recorded every 5-s by uniaxial accelerometry in 35 children (grades 1-2, n = 13; grades 3-4, n = 13; grades 5-6, n = 9) for up to five weekdays and two weekend days. Younger children were more active during weekend days compared with weekdays, while the opposite was observed in older children. Age variation also exists in children's PA levels within a weekday, with this effect being most evident during recess and after school. Weekend days and free time within school days seem appropriate targets when promoting PA in older children with ASD. 2010 Elsevier Ltd. All rights reserved
SN - 1750-9467
AD - (Pan, Chu, Li, Huang) Department of Physical Education, National Kaohsiung Normal University, No. 116, He-Ping First Road, Kaohsiung 802, Taiwan (Republic of China) (Tsai, Hsieh) Institute of Physical Education, Health and Leisure Studies, National Cheng Kung University, Tainan 701, Taiwan (Republic of China)
ER -

TY - JOUR
ID - 3572
T1 - Methodology for assessing exposure and impacts of air pollutants in school children: Data collection, analysis and health effects - A literature review
A1 - Mejia,J.F.
A1 - Choy,S.L.
A1 - Mengersen,K.
A1 - Morawska,L.
Y1 - 2011///

N1 - 2011019786
English
Journal: Review
KW - EMBASE
KW - air conditioning
KW - air pollutant
KW - Air Pollution
KW - air quality
KW - Child
KW - Comorbidity
KW - data analysis
KW - exhaust gas
KW - furniture
KW - Health Status
KW - Human
KW - information processing
KW - Methodology
KW - Morbidity
KW - Particle Size
KW - Physical Activity
KW - priority journal
KW - review
KW - school
KW - socioeconomics
KW - polycyclic aromatic hydrocarbon
RP - NOT IN FILE
SP - 813
EP - 823
JF - Atmospheric Environment
VL - 45
IS - 4
CY - United Kingdom
N2 - The aim of this review is to explore the methodologies employed to assess the exposure of children to air pollutants, in particular traffic emissions, at school, and how these methodologies influence the assessment of the impact of this exposure on the children's health. This involves four main steps: the measurement of air quality at school level, the association between measured air quality and children's exposure, the association between children's exposure and health; and source identification. The comparative advantages and disadvantages of the methods used at each of these steps are discussed.Air quality in schools can be measured at three scales: broad scale, across several city blocks using remote monitors; school-based scale, through ground-level monitors installed within the schools or their immediate surroundings (i.e. only a few metres outside the school); and personal exposure scale using portable monitors attached to a sample of children. Although studies have reported high exposure to PAHs (polycyclic aromatic hydrocarbons), submicrometre (<1.0 mum) and ultrafine particles (<100. nm) at school, no study has investigated the formation of new particles in school facilities and only a handful of studies have analysed children's exposure at school.Associating air quality measurements at the broad and medium scale with children's exposure is challenging: there is spatial and temporal heterogeneity in the distribution of air quality within a school, indoor measurements can often exceed outdoor measurements; and exposure in the classroom is affected by the penetration of outdoor pollutants, wall absorption, emissions from furniture and other materials, level and length of occupancy, and quality of ventilation. This is further exacerbated by the fact that children move around during their school day.Quantifying the contribution of school exposure with observed health symptoms presents further challenges. In addition to ascertaining the impact of non-school-based exposures and co-morbidities, the air pollutant dose intake is affected by daily patterns of physical and traffic activity during and outside school hours which make it difficult to compare the contribution of school-based and non-school-based exposures to the health effect under investigation. Finally, there is strong evidence that low socioeconomic level is highly correlated with the proximity of the school to pollution sources, yet this area of socioeconomic research has been largely unexplored in the assessment of traffic emission exposure. 2010 Elsevier Ltd
SN - 1352-2310

AD - (Mejia, Morawska) International Laboratory of Air Quality and Health (ILAQH), Queensland University of Technology, GPO Box 2434, Brisbane QLD 4001, Australia (Choy, Mengersen) School of Mathematical Sciences, Queensland University of Technology, Australia
ER -

TY - JOUR
ID - 3573
T1 - New cases and refinement of the critical region in the 1q41q42 microdeletion syndrome
A1 - Rosenfeld,J.A.
A1 - Lacassie,Y.
A1 - El-Khechen,D.
A1 - Escobar,L.F.
A1 - Reggin,J.
A1 - Heuer,C.
A1 - Chen,E.
A1 - Jenkins,L.S.
A1 - Collins,A.T.
A1 - Zinner,S.
A1 - Babcock,M.
A1 - Morrow,B.
A1 - Schultz,R.A.
A1 - Torchia,B.S.
A1 - Ballif,B.C.
A1 - Tsuchiya,K.D.
A1 - Shaffer,L.G.
Y1 - 2011///
N1 - 2011110817
English
Journal: Article
KW - EMBASE
KW - 1q41q42 microdeletion syndrome
KW - article
KW - behavior disorder
KW - body dysmorphic disorder
KW - Child
KW - chromosome 1q
KW - chromosome deletion
KW - cleft palate
KW - clinical article
KW - clubfoot
KW - comparative genomic hybridization
KW - congenital diaphragm hernia
KW - congenital heart malformation
KW - controlled study
KW - DISP1 gene
KW - Female
KW - fluorescence in situ hybridization
KW - Fryns syndrome
KW - gene
KW - genotype phenotype correlation
KW - Human
KW - hypertelorism
KW - hypospadias
KW - Infant
KW - inheritance
KW - Male
KW - mental deficiency

KW - microcephaly
KW - muscle hypotonia
KW - nystagmus
KW - Otitis Media
KW - preschool child
KW - real time polymerase chain reaction
KW - school child
KW - seizure
KW - Signal Transduction
KW - strabismus
KW - upper respiratory tract infection
KW - sonic hedgehog protein/ec [Endogenous Compound]
RP - NOT IN FILE
SP - 42
EP - 49
JF - European Journal of Medical Genetics
VL - 54
IS - 1
CY - France
N2 - Microdeletions of 1q41q42 have recently been classified as a syndrome. Features include significant developmental delay and characteristic dysmorphic features as well as cleft palate, clubfeet, seizures, and short stature in some individuals, with a clinical diagnosis of Fryns syndrome in two individuals with congenital diaphragmatic hernia at the severe end of the spectrum. The gene DISP1, which is involved in sonic hedgehog signaling, has been proposed as a candidate for the midline defects in this syndrome. We undertook a genotype-phenotype analysis of seven previously unreported individuals with deletions of 1q41q42 that range from 777 kb to 6.87 Mb. Three of the individuals in our cohort do not display the major features of the syndrome and have more proximal deletions that only overlap with the previously described 1q41q42 smallest region of overlap (SRO) at DISP1. One individual with several features of the syndrome has a more distal deletion that excludes DISP1. The three remaining individuals have larger deletions that include the entire SRO and demonstrate features of the microdeletion syndrome. Confounding genotype-phenotype correlations, one of the small deletions involving DISP1 was inherited from a phenotypically normal parent. DISP1 haploinsufficiency may not be solely responsible for the major features of 1q41q42 microdeletion syndrome, and other genes in the SRO likely play a role in the phenotype. Additionally, some features present in a minority of individuals, such as Pelger-Huet anomaly, may be attributed to deletions of genes outside of the SRO. 2010 Elsevier Masson SAS
SN - 1769-7212
AD - (Rosenfeld, Schultz, Torchia, Ballif, Shaffer) Signature Genomic Laboratories, 2820 N. Astor St., Spokane, WA 99207, United States (Lacassie) Division of Genetics, Department of Pediatrics, Louisiana State University Health Sciences Center and Children's Hospital, New Orleans, LA, United States (El-Khechen, Escobar) St. Vincent Children's Hospital, Indianapolis, IN, United States (Reggin) Sacred Heart Medical Center, Spokane, WA, United States (Heuer) MidSouth Maternal Fetal Medicine, Memphis, TN, United States (Chen) Department of Genetics, San Francisco Medical Center, San Francisco, CA, United States (Jenkins) Kaiser Permanente Regional Genetics Lab, San Jose, CA, United States (Collins) Seattle Children's Hospital, Seattle, WA, United States (Zinner) Department of Pediatrics, University of Washington School of Medicine, Seattle, WA, United States (Babcock, Morrow) Albert Einstein College of Medicine, Yeshiva University, Bronx, NY, United States (Tsuchiya) Department of Laboratories, Seattle Children's Hospital, Department of Laboratory Medicine, University of Washington School of Medicine, Seattle, WA, United States
ER -

TY - JOUR
ID - 3574
T1 - Non-human primate model of hemolytic uremic syndrome and therapeutic testing
A1 - Kurosawa,S.
A1 - Stearns-Kurosawa,D.J.
Y1 - 2011///
N1 - 70612541
English
Journal: Conference Abstract

KW - EMBASE
KW - Society
KW - Human
KW - model
KW - hemolytic uremic syndrome
KW - primate
KW - hemostasis
KW - thrombosis
KW - nonhuman
KW - baboon
KW - lethal dose
KW - acute kidney failure
KW - intestine infection
KW - Prevention
KW - disease course
KW - lethality
KW - Shiga toxin producing Escherichia coli
KW - patient
KW - Child
KW - toxemia
KW - urine volume
KW - thrombocytopenia
KW - cytokine response
KW - Anemia
KW - survivor
KW - plasma
KW - urine
KW - Survival Rate
KW - Therapy
KW - urea nitrogen blood level
KW - toxin
KW - peptide
KW - vasculotropin
KW - creatinine
RP - NOT IN FILE
SP - 56
JF - Journal of Thrombosis and Haemostasis
VL - Conference: 23rd Congress of the International Society on Thrombosis and Haemostasis 57th Annual SSC Meeting Kyoto Japan. Conference Start: 20110723 Conference End: 20110728. Conference Publication:
IS - var.pagings
N2 - Hemolytic-uremic syndrome (HUS) often follows intestinal infection with Shiga-toxin producing Escherichia coli (STEC). STEC affects 73,000 US patients annually and is the leading cause of acute renal failure in otherwise healthy children < 5 years. It is believed that developing therapeutic that acts intracellularly is the priority, because the toxin would have translocated from outside to inside the cells, by the time the disease develops. Objective: Evaluate efficacy of a novel cell-permeable tetravalent peptide (TVP) that binds to Stx2 beta subunit, in nonhuman primate models of Stx2 toxemia. Methods: Baboons were challenged with a lethal dose of Stx2 (50 ng/ kg); TVP was given i.v. at T (0) with toxin (prevention) or at 6 or 24 h after toxin with daily supplements up to day 4 (rescue). A comprehensive array of clinical and inflammatory indicators was monitored up to 28 days after challenge. Results: Peptide delivered with toxin (prevention) or after toxin (rescue) resulted in either complete absence of clinical signs of acute kidney injury and normal urine output, or delayed and reduced BUN and creatinine levels. Delayed peptide administration reduced thrombocytopenia and inflammatory cytokine responses, but surprisingly did not alter anemia even when monitored for 28 days in rescued survivors. Plasma VEGF levels were undetectable, whereas urine VEGF increased to 400 pg/mL after toxin, and were reduced to baseline by TVP. Conclusions: A cell-permeable peptide designed to counteract Stx2 activity within susceptible cells rescued baboons from an otherwise lethal dose of toxin. Even when delayed by 24 h, the survival rate was substantial and significant. Bedside measure of urinary VEGF levels may provide a non-invasive way to monitor treatment efficacy and clinical course. This is the first successful intracellular

therapeutic that counteracts Stx2 lethality in a nonhuman primate model which recapitulates many of the human responses to enteric infection
SN - 1538-7933
AD - (Kurosawa, Stearns-Kurosawa) Pathology and Laboratory Medicine, Boston University School of Medicine, United States
ER -

TY - JOUR
ID - 3575
T1 - The hypo-functional 7-repeat allele of DRD4 predicts both objective and reported fat intake in 4- to 6-year old girls
A1 - Levitan,R.
A1 - Silveira,P.
A1 - Portella,A.
A1 - Kennedy,J.
A1 - Gaudreau,H.
A1 - Davis,C.
A1 - Steiner,M.
A1 - Soares,C.
A1 - Matthews,S.
A1 - Dube,L.
A1 - Meaney,M.
Y1 - 2011///
N1 - 70607561
English
Journal: Conference Abstract
KW - EMBASE
KW - Human
KW - Female
KW - girl
KW - allele
KW - fat intake
KW - psychopharmacology
KW - college
KW - calorie
KW - Child
KW - date (fruit)
KW - Eating
KW - Food
KW - boy
KW - Male
KW - Obesity
KW - childhood
KW - laboratory
KW - Prevention
KW - Population
KW - Food Intake
KW - caloric intake
KW - gender
KW - Environment
KW - overnutrition
KW - Feeding Behavior
KW - questionnaire
KW - weight
KW - Genotype
KW - Adult
KW - validity

KW - Risk
KW - eating disorder
KW - human development
KW - gene
KW - receptor gene
KW - Imaging
KW - Reward
KW - Cohort Analysis
KW - nerve cell differentiation
KW - Canada
KW - mother
KW - Pregnancy
KW - food preference
KW - food frequency questionnaire
KW - dopamine
KW - dopamine 4 receptor
KW - dextropropoxyphene
RP - NOT IN FILE
SP - S261
JF - Neuropsychopharmacology
VL - Conference: 50th Annual Meeting of the American College of Neuropsychopharmacology, ACNP Waikoloa, HI United States. Conference Start: 20111204 Conference End: 20111208. Conference Publication:
IS - var.pagings
N2 - Background: The prevention and treatment of overeating and obesity continues to be a major challenge. While the ease of availability of highly palatable foods is a major factor in this regard, not all individuals overeat or become obese in this environment. The current project examines individual differences in palatable food intake at a critical time in human development. The current analysis studies the relationship between dopamine genes and eating behaviour in children from 4 to 6 years of age. A particular focus is the hypo-functional 7-repeat (7R) allele of the dopamine-4 receptor gene (DRD4), which associates with overeating and obesity in several female overeater populations (e.g. Levitan et al., 2004, 2006, 2010). Recent imaging work suggests that weaker activation of the brain0s reward circuitry may play a role in these associations (Stice et al., 2010). Methods: The current sample consists of children taking part in a longitudinal cohort study (Maternal Adversity, Vulnerability and Neurodevelopment) based in Canada. The mothers of the children, oversampled based on maternal depression and low SES, were recruited at 13-120 weeks of pregnancy, and the children have been followed intensively since birth. Key outcome measures include: 1. A laboratory-based snack test at 48 months of age which provides an objective measure of food preferences and overall caloric intake. 2. A food frequency questionnaire at 48 and 72 months, based on maternal report, which assesses naturalistic food intake and feeding behavior. 3. The childhood eating behaviour questionnaire (CEBQ; Wardle et al., 2001) which measures eating styles likely to promote over- or under- weight. Results: To date, 118 children have completed the 48 month snack test in the lab. Of these, 47 (39.8%) carry the 7R allele, while 71 (60.2%) do not. Controlling for total caloric intake, there is a significant gender X genotype interaction in predicting fat intake during the snack test (F=7.16, df=4, 113, p=.009). Girls who carry the 7R variant are consuming 31.2 % more fat during this snack than are non-carriers (mean=12.2 vs. 9.3 grams respectively) while in boys, 7R carriers are eating 19.2% less fat than are non-carriers (mean=11.3 vs. 13.5 grams respectively). This suggests that the 7R allele increases objective fat intake in girls but not boys by four years of age. Strikingly, when intake is measured using the 48 and 72 month food diaries, which were completed two years apart, the gender X 7R interaction is even more robust, accounting for 21 % of the variance in fat intake (F=11.3, df=3, 42; p=.002; N=46). Based on these diaries, girls who carry the 7R allele are consuming 39.8% more fat in their natural environments at 48 months than are non-7R carrier girls (820.5 vs. 587.0 calories/day respectively). Importantly, this difference remains robust at 72 months of age (881.5 calories as fat in 7R carriers vs. 609.2 calories as fat in non-carriers, a 44.7 % difference). In boys, 7R carriers are consuming 40.1 % less calories from fat than are non-carriers at 48 months, and 25.4 less calories from fat at 72 months based on these diaries. The consistency of these data across both laboratory based measures and food diaries, and the consistency of the food diary data over a two year time span, adds greatly to these findings. These findings are also highly consistent with earlier work in female adult overeater populations (see above). Discussion: These converging results show a highly robust association between the hypo-functional 7R allele of DRD4 and fat intake in girls, but not boys. The consistency of these findings across different outcome measures and across a two year age

span adds greatly to their validity and potential impact. Pending replication and detailed anthropomorphic outcomes later in childhood, these results may identify a significant subgroup of young girls at higher risk for childhood obesity and/or eating disorders. If so, this could inform novel prevention strategies implemented as early as birth in this subgroup
SN - 0893-133X
AD - (Levitan, Silveira, Portella, Kennedy, Gaudreau, Davis, Steiner, Soares, Matthews, Dube, Meaney) Centre for Addiction and Mental Health, Toronto, Canada
ER -

TY - JOUR
ID - 3576
T1 - Obesity risk factors in children: A case of Turkey
A1 - Bas,M.
A1 - Yilmaz,B.
A1 - Bilici,S.
Y1 - 2011///
N1 - 70604805
English
Journal: Conference Abstract
KW - EMBASE
KW - Human
KW - Child
KW - Obesity
KW - risk factor
KW - parent
KW - Eating
KW - questionnaire
KW - weight
KW - height
KW - mother
KW - Physical Activity
KW - Female
KW - student
KW - Lifestyle
KW - habit
KW - Education
KW - Waist Circumference
KW - fast food
KW - meal
KW - Responsibility
KW - childhood
KW - community
KW - Policy
KW - Child Development
RP - NOT IN FILE
SP - 117
JF - Obesity Reviews
JA - Obes Rev
VL - Conference: 18th European Congress on Obesity, ECO 2011 Istanbul Turkey. Conference Start: 20110525 Conference End: 20110528. Conference Publication:
IS - var.pagings
N2 - Introduction: This study was designed to determine the association of obesity with certain risk factors in children and to evaluate the associates of obesity and overweight in children. Methods: Data were collected from parents of 983 children by dietetic students. A questionnaire form consisting of the child's anthropometric measurements (weight, height, waist), physical activity patterns, eating and lifestyle habits were constructed by reviewing literature. There were also questions in the questionnaire form about the parents' anthropometric measurements (weight, height,waist) and level of education. Results: Mother's BMI were found as statistically

significant between obese and nonobese children (P < 0.05). In obese children, both values for parents were found grater than nonobese children (P < 0.05). In obese groups, mothers' waist circumference was found as different from nonobese group (P < 0.05). Number of fast food eatings was also a significant variable between groups (P < 0.05). Eating in view of TV, doing regularly breakfast everyday and performing physical activity were found as significant variables in the analysis (P < 0.05). Conclusions: The results of this study support previous work indicating that both fam,ly responsibility. Parents should be helped to recognise their child's overweight, and educational strategies should be targeted at both children and their parents. In the preventing and controlling childhood obesity will require multifaceted and community- wide programs and policies, with parents having a critical role to play. Successful intervention efforts, they argue, must involve and work directly with parents from the earliest stages of child development to support healthful practices both in and outside of the home
SN - 1467-7881
AD - (Bas, Yilmaz) Department of Nutrition and Dietetics, Health Sciences Faculty, Baskent University, Ankara, Turkey (Bilici) Department of Nutrition and Dietetics, Health Sciences Faculty, Gazi University, Ankara, Turkey
ER -

TY - JOUR
ID - 3577
T1 - Using observation to compare different accelerometer cut points for sedentary behavior in children
A1 - Yildirim,M.
A1 - Fischer,C.
A1 - Salmon,J.
A1 - Paw,C.A.M.
Y1 - 2011///
N1 - 70604511
English
Journal: Conference Abstract
KW - EMBASE
KW - accelerometer
KW - Child
KW - Human
KW - Obesity
KW - Physical Activity
KW - Television
KW - validity
KW - Accelerometry
KW - Television Viewing
KW - Monitoring
KW - Public Health
KW - childhood
KW - Population
KW - computer
RP - NOT IN FILE
SP - 24
EP - 25
JF - Obesity Reviews
JA - Obes Rev
VL - Conference: 18th European Congress on Obesity, ECO 2011 Istanbul Turkey. Conference Start: 20110525 Conference End: 20110528. Conference Publication:
IS - var.pagings
N2 - Introduction: Accurate objective methods for the assessment of sedentary behavior are crucial for population monitoring and evaluation of public health strategies for preventing childhood obesity. Actigraph accelerometers are a commonly used objective physical activity measurement tool. It is hypothesized that accelerometers may also provide a valid method for assessing children's sedentary time. However, there is considerable variation in published accelerometer cut-off points for measuring sedentary time in children. The objective of this study is therefore to compare different accelerometer sedentary cut points with observation of

children performing specific sedentary behaviors in free-living conditions. Methods: Direct observation and Actigraph uniaxial accelerometers were used to measure children's activity intensity while playing com- puter games, non-electronic sedentary games, watching television and playing outdoors. Direct observation was the criterion for assessing the validity of four different previously published sedentary cutpoints: i.e., 100, 300, 800, and 1100 counts per minute (cpm). Results: The median cpm were lowest for computer games (30 cpm), followed by television viewing (109 cpm) and non-electronic sedentary games (172 cpm) and highest for outdoor play (1452 cpm). The median counts during all sedentary behaviors were below the lowest cut-point of 100 cpm. The 75th percentile values for the sedentary behaviors were always below the cut-point of 300 cpm. Conclusion: Our results suggest that the Actigraph accelerometry cut-point of <100 cpm is the most appropriate, if not too high, for quantifying the time children spend sedentary
SN - 1467-7881
AD - (Yildirim, Paw) EMGO-Institute, Department of Public and Occupational Health, VU University Medical Center, Amsterdam, Netherlands (Fischer) Department of Public Health, University of Amsterdam, Academic Medical Center, Amsterdam, Netherlands (Salmon) Centre for Physical Activity and Nutrition Research, School of Exercise and Nutrition Sciences, Deakin University, Melbourne, VIC, Australia
ER -

TY - JOUR
ID - 3578
T1 - Physical activity interventions in children
A1 - Cardon,G.
Y1 - 2011///
N1 - 70604488
English
Journal: Conference Abstract
KW - EMBASE
KW - Physical Activity
KW - Human
KW - Child
KW - Obesity
KW - school
KW - sport
KW - childhood
KW - Prevention
KW - environmental factor
KW - Responsibility
KW - parent
KW - Environment
KW - Lifestyle
KW - intervention study
KW - environmental change
KW - Health Promotion
RP - NOT IN FILE
SP - 17
JF - Obesity Reviews
JA - Obes Rev
VL - Conference: 18th European Congress on Obesity, ECO 2011 Istanbul Turkey. Conference Start: 20110525 Conference End: 20110528. Conference Publication:
IS - var.pagings
N2 - Regular physical activity during childhood is associated with improvements in numerous physiological and psychological variables and is being promoted as an objective for obesity prevention. Although it is frequently assumed that physical activity is an integral part of growing up, numerous studies show that children are often physically inactive. All young people should participate in physical activity of at least moderate intensity for one hour per day. In most nations schools are the primary societal institution with responsibility for promoting physical activity in young people. PE classes, school-related physical activity outside PE and information to parents and other educational team members can contribute to the development of an active lifestyle. Moreover, through schoolcommunity partnerships schools can work together with local sport providers

to increase after school sports participation. Recent research focuses on environmental physical activity correlates. Also in children modifiable environmental factors were found to associate with physical activity, as well for neighborhoods (e.g. presence of sports fields, cycle track) as for school playgrounds (e.g. adequate space, diverse play opportunities, interaction with natural elements). However it is still unclear if changing the environment is enough to increase children's physical activity levels. Socio-ecological approaches to health promotion, consider children's PA levels as the result of transactions among multiple levels of influence and recommend to target intervention strategies at multiple settings. Moreover, the results of some intervention studies show that targeting both behavioural and environmental changes to promote PA is an effective strategy to target children's physical activity behaviour
SN - 1467-7881
AD - (Cardon) Ghent University, Ghent, Belgium
ER -

TY - JOUR
ID - 3579
T1 - "Floor the Pain" a novel approach for direct hospital admission and multidisciplinary team management of vasoocclusive pain crisis management in patients with sickle cell disease
A1 - Campbell,M.
A1 - Retort,C.
A1 - McLendon,P.
A1 - Krishnamurti,L.
Y1 - 2011///
N1 - 70601552
English
Journal: Conference Abstract
KW - EMBASE
KW - Human
KW - Pain
KW - patient
KW - sickle cell anemia
KW - hospital admission
KW - Writing
KW - hospital patient
KW - sickle cell
KW - emergency ward
KW - patient care
KW - Child
KW - nursing
KW - epidemic
KW - injury
KW - Hospitalization
KW - pharmacist
KW - patient controlled analgesia
KW - health care quality
KW - investment
KW - Plant
KW - model
KW - Quality of Life
KW - staff nurse
KW - emergency health service
KW - Hospital
KW - day hospital
KW - infusion
KW - nurse
RP - NOT IN FILE
SP - E51
EP - E52

JF - American Journal of Hematology
VL - Conference: 5th Annual Sickle Cell Disease Research and Educational Symposium and Grant Writing Institute and Annual National Sickle Cell Disease Scientific Meeting - Sickle Cell Disease: The Next Century Hollywood, FL United States. Conference Start: 20110221 Conference End: 20110225. Conference Publication:
IS - var.pagings
N2 - Purpose: "Floor the Pain" is an initiative to provide prompt pain management to children experiencing acute, uncomplicated episodes of sickle cell pain bypassing the Emergency Department (ED). Background: Pain crisis the hallmark of sickle cell disease. Frequently, systems of care in the emergency room and hospital are barriers to appropriate care. Dedicated day hospitals or infusion centers are an effective but potentially expensive option. While specialized, dedicated teams may be able to improve the care of these patients, such approaches typically leave out the regular nursing and resident staff, further limiting the spread of expertise in the care of patients with sickle cell disease. Emergency departments can improve care by following pain protocols and individualized pain plans. However, they are frequently overwhelmed by seasonal epidemics or trauma cases and are not able to consistently meet the needs of patients with SCD presenting with VOC. Further, in patients presenting with severe pain, sometimes it is evident that the pain is unlikely to be relieved by interventions offered in the ED and in-patient hospitalization is inevitable. In such cases the ED visit becomes an avoidable expense. Moreover, patients who receive some relief in the ED may have worsening of pain when there are delays in the instution of appropriate pain management in the transition to the in-patient floor. Aims: To develop an approach that eliminates avoidable ED visits, eliminates loss of pain control during transitions and involves a multidisciplinary in-patient team in order to improve the management of vasocclusive pain crises. Methods/Implementation Strategy: Collaborative of multi-disciplinary team has been developed to improve patient outcomes. The team includes floor nurses, residents, pharmacists, facilities management and sickle cell staff. The goal of this multidisciplinary team is to immediately asses and treat pain by immediately initiating pain management with intravenous opiods according to an individualized pain plan within 10 minutes of arrival on the floor and transition to a patient controlled analgesia, our standard of care within 30 minutes of arrival to floor. This service is available to clients triaged by the Sickle Cell Team and considered to be experiencing an uncomplicated vasoocclusive pain crisis. As soon as a patient is identified as a candidate for 'Floor the Pain, a group page alerts the multidisciplinary team. This initiative will improve pain management by assuring prompt, aggressive treatment for acute pain crises, comprehensive treatment delivery outside of the ED, Support and expertise of a highly knowledgeable staff with prior knowledge of the patient, Care in a quiet, comfortable setting on a patient care unit. All of this is achieved with no additional investment in physical plant or dedicated staff. Evaluation: This process began on June 21, 2010. Thus far we have applied this intervention to 18 patients. We have reached the goal of initiating intravenous opioids within 10 minutes of arrival on the floor and initiation of PCA within 30 minutes of arrival to the patient care unit in 15 of these patients. Three patients had intravenous opiods started within 30 minutes and PCA's started within 1 hour of arrival on the floor. Prior to initiation of this program these patients had received a PCA between 130min to 365 min after arrival on the floor. Implication for Practice: Floor the Pain promotes a new model for improved care which empowers members of interdisciplinary team to improve the quality of life of a child with SCD. This improvement actively involves staff nurses in pain management of complex patient's based on clear pathways as well as prior knowledge of the patients. Future plans are to continue this improvement 24 hours a day, 7 days a week, via phone triage by the clinical leader
SN - 0361-8609
AD - (Campbell, Retort, McLendon, Krishnamurti) Children's Hospital of Pittsburgh, UPMC, Pittsburgh, PA, United States
ER -

TY - JOUR
ID - 3580
T1 - A multi-center randomized controlled trial of pediatric constraint-induced movement therapy: 6-month follow-up
A1 - Case-Smith,J.
A1 - Deluca,S.
A1 - Stevenson,R.
A1 - Ramey,S.
Y1 - 2011///

N1 - 70594146
English
Journal: Conference Abstract
KW - EMBASE
KW - randomized controlled trial
KW - Cerebral Palsy
KW - follow up
KW - movement therapy
KW - Human
KW - Child
KW - Arm
KW - effect size
KW - hypothesis
KW - skill
KW - Environment
KW - parent
KW - Motor Activity
KW - systematic review (topic)
KW - occupational therapist
RP - NOT IN FILE
SP - 28
EP - 29
JF - Developmental Medicine and Child Neurology
VL - Conference: 65th Annual Meeting of the American Academy for Cerebral Palsy and Developmental Medicine Las Vegas, NV United States. Conference Start: 20111012 Conference End: 20111015. Conference Publication:
IS - var.pagings
N2 - Background and Objectives: Pediatric constraint-induced movement therapy (CIMT) is a promising intervention for children with unilateral cerebral palsy (CP). Recent systematic reviews have found moderate effects but intervention dose has varied from 6 to 126 total hours, without compelling evidence defining optimal CIMT dosage. This multisite randomized controlled trial (RCT) applied continuous casting of the less involved arm for 18 days of intervention with 3 additional days without constraint. We tested the hypothesis that 6 hours versus 3 hours/day for 21 days would produce greater maintenance of gains 6 months post-treatment. Design: Our study was a multisite randomized controlled trial. Participants and Setting: Three sites recruited 18 children (six per site) (mu=48.75 [SD=12.19] months) with unilateral CP. Children were randomly assigned to 3 or 6 hours/day of CIMT for 21 days, wearing a cast on the unaffected extremity the first 18 days. Occupational therapists applied a standardized pediatric CIMT protocol in the child's natural environment. Materials and Methods: Evaluators, blinded to condition, administered the Assisted Hand Assessment and the Quality of Upper Extremity Skills Test, and parents completed the Pediatric Motor Activity Log pre- and post-treatment (1wk, 1mo, and 6mo.) Results: Both CIMT dosage groups showed significant gains on all assessments with no significant group differences at 6 months post intervention. Effect sizes (partial n²) ranged from 0.34 to 0.63 for the Assisted Hand Assessment (AHA) and the Quality of Upper Extremity Skills Test, with continual improvement on the AHA at the three post intervention measurement times (post, 1mo and 6mo post). The parents rated their children's use of their affected arm as more frequent (F=20.7, p=0.001, effect size=0.78) and with higher quality (F=14.7, p<0.001, effect size 0.71) across intervention measurement times. Conclusions and Significance: This multisite RCT of pediatric CIMT using continuous casting and intervention in the child's natural environment affirms maintenance of positive effects at 6 months follow up, across multiple functional performance measures. The hypothesis that maintenance of effects would differ for children who received 6 versus 3 hours per day of CIMT (63 vs 126 total h) was not supported
SN - 0012-1622
AD - (Case-Smith) Occupational Therapy, Ohio State University, Columbus, Ohio, OH, United States (Deluca) Occupational Therapy, University of Alabama, Birmingham, AL, United States (Stevenson) Pediatrics, University of Virginia, Charlottesville, VA, United States (Ramey) Center on Health and Education, Georgetown University, Georgetown, DC, United States
ER -

TY - JOUR

ID - 3581
T1 - Clinical spectrum and predisposing factors in childhood obesity
A1 - Behera,B.
A1 - Thapa,B.
A1 - Rawal,P.
A1 - Singh,K.
Y1 - 2011///
N1 - 70590824
English
Journal: Conference Abstract
KW - EMBASE
KW - Obesity
KW - childhood
KW - Asian
KW - disease predisposition
KW - Human
KW - Child
KW - calorie
KW - Physical Activity
KW - Body Weight
KW - recall
KW - protein intake
KW - body mass
KW - liver function test
KW - kidney function test
KW - ultrasound
KW - Abdomen
KW - glucose blood level
KW - Prevalence
KW - Income
KW - fat intake
KW - caloric intake
KW - nutritional assessment
KW - lipid blood level
KW - sitting
KW - habit
KW - developing country
KW - malnutrition
KW - risk factor
KW - Anthropometry
KW - Physical Examination
KW - thyroid gland
KW - protein
KW - hemoglobin
KW - lipid
RP - NOT IN FILE
SP - 194
JF - Journal of Gastroenterology and Hepatology
VL - Conference: Asian Pacific Digestive Week 2011 Singapore Singapore. Conference Start: 20111001 Conference End: 20111004. Conference Publication:
IS - var.pagings
N2 - Background and objective Developing countries have double burden of malnutrition and obesity in children. The present study was conducted to determine the risk factors for childhood obesity in children with reference to their dietary pattern, physical activity and body mass index (BMI) levels. Subjects and methods 64 obese children and 40 healthy children were enrolled for the study. Subjects were evaluated for demographic details, anthropometry, general physical examination and clinical investigation (blood sugar, hemoglobin, liver function tests, renal function tests, ultrasound abdomen, lipid profile, thyroid profile). Dietary assessment was

done by one day recall method. Physical activity was assessed by one day activity recall. Results Prevalence of obesity was more among urban nuclear upper and lower middle income families. The mean calorie (2772.6 + 345.2 kcal/ day), protein (70.45 + 9.9 g/day) and fat (94.14 + 14.9 g/day) intake was higher among obese children compared to control, however their calorie and protein intake per kg of body weight was low. Mean calorie, protein, and fat consumption showed significant direct correlation with body mass index (BMI). However, an inverse correlation was found between BMI with calorie intake per kg body weight, serum lipids. 92% of obese children spent less than one hour per day on outdoor activities, more than 45% spent time 2 hours or more on sedentary activities. Conclusion Childhood obesity is associated with high calorie and low protein intake. Physical activity was grossly inadequate among 92% of obese children. Excess sedentary habits remained the most important determinant of childhood obesity
SN - 0815-9319
AD - (Rawal, Behera, Thapa, Rawal, Singh) Division of Pediatric Gastroenterology, Hepatology and Nutrition, Department of Superspeciality of Gastroenterology, Postgraduate Institute of Medical Education and Research (Pgimer), Chandigarh, India
ER -

TY - JOUR
ID - 3582
T1 - Follicular variant of papillary thyroid carcinoma presenting as a toxic nodule in an adolescent girl
A1 - Campenni,A.
A1 - Ruggeri,R.M.
A1 - Saraceno,G.
A1 - Carlotta,D.
A1 - Giovinazzo,S.
A1 - Nania,R.
A1 - Gangemi,V.
A1 - Cucinotta,M.
A1 - Baldari,S.
Y1 - 2011///
N1 - 70581078
English
Journal: Conference Abstract
KW - EMBASE
KW - Human
KW - nuclear medicine
KW - Adolescent
KW - Female
KW - girl
KW - thyroid carcinoma
KW - Mutation
KW - patient
KW - thyroid gland
KW - gene
KW - Codon
KW - thyroid nodule
KW - neck
KW - Therapy
KW - Child
KW - adenoma
KW - family history
KW - normal value
KW - thyroid scintiscanning
KW - Fatigue
KW - neoplasm
KW - heart palpitation
KW - Physical Examination
KW - skin

KW - tremor
KW - hyperreflexia
KW - weight
KW - pulse rate
KW - thyrotropin blood level
KW - mother
KW - serum
KW - blood flow
KW - weight reduction
KW - examination
KW - papillary carcinoma
KW - single nucleotide polymorphism
KW - exon
KW - thyroidectomy
KW - oral drug administration
KW - ablation therapy
KW - follow up
KW - nodular goiter
KW - thyrotropin
KW - iodine 131
KW - radioactive iodine
KW - thyrotropin receptor
KW - asparagine
KW - timonacic arginine
KW - isoleucine
KW - levothyroxine
RP - NOT IN FILE
SP - S398
JF - European Journal of Nuclear Medicine and Molecular Imaging
VL - Conference: Annual Congress of the European Association of Nuclear Medicine 2011 Birmingham United Kingdom. Conference Start: 20111015 Conference End: 20111019. Conference Publication:
IS - var.pagings
N2 - AIM: Discovery of a "hot" nodule is usually comforting due to the presumption that the nodule represents a benign toxic adenoma. Here, we present an adolescent with a hot nodule turned out to be a papillary cancer. PATIENT: A 15-year-old girl was referred to our Endocrine Unit, complaining of a painless mass in the right side of the neck that had appeared a few months earlier. Her family history was significant for benign nodular goiter (mother). RESULTS: Thyroid US showed a 25mm hypoechoic, non-homogenous nodule in the right lobe. Serum TSH was 0.26 mU/L (n.v. 0.27-4.2), with normal values of FT3 and FT4.¹³¹I thyroid scintigraphy was consistent with an autonomous thyroid nodule. Six months later, the girl returned complaining of fatigue, weight loss and palpitations of 1-month duration. Physical examination revealed warm, moist skin; tremors of the extremities; hyperreflexia. The patient's weight was 42.5 kg. Her pulse rate was 94 /min. At endocrine evaluation, TSH was 0.001 mU/L, and FT3 (7.68 pml/L; n.v. 3.0-6.7) and FT4 (26 pml/L; n.v. 12.0-22.0) were elevated. Thyroid US revealed an increase in the size of the nodule, that measured 35x30x21mm and displayed an abnormal intranodular blood flow. Treatment with MMI (20mg/day) was started, with normalization of FT3 and FT4 values. Subsequently, the patient underwent near-total thyroidectomy. Pathological examination revealed a papillary carcinoma, follicular variant. The excised nodule was examined for activating mutations of TSH receptor and Galphas genes by direct sequencing. No mutations were detected. Nevertheless, two combined non-functioning mutations were detected. The first mutation is a known single nucleotide polymorphism (SNP), in the transmembrane region of the TSHR gene, at codon 187 (AAC->AAT, both encoding asparagines). The second mutation is within exon 8 of Gsalpha gene, at codon 185, (ATC->ATT, both encoding isoleucine). Search for mutations in BRAF gene was negative. Few months later, patient underwent: Radioiodine thyroid uptake measurement (RTU=3%) after oral administration of ¹³¹I (1.85 MBq) and ¹³¹I ablation therapy (1850 MBq) (at this time: TSH: 45 mIU/L and Tg <1.6 ng/ml). ¹³¹I post-dose WBS did not reveal areas of radioiodine uptake outside the thyroid bed. At one year-follow-up, the patient is on levo-thyroxine therapy, with TSH values <0.2 mIU/ml and both basal and rhTSH-stimulated serum Tg <1.6 ng/mL, and neck US is negative. CONCLUSION: This case demonstrated that

the presence of hyperfunctioning thyroid nodule(s) does not preclude malignancy and warrants careful evaluation, especially in children and adolescents
SN - 1619-7070
AD - (Campenni, Ruggeri, Saraceno, Carlotta, Giovinazzo, Nania, Gangemi, Cucinotta, Baldari) Universita di Messina, Messina, Italy
ER -

TY - JOUR
ID - 3583
T1 - Systemic interaction in families engaging in physiological breastfeeding patterns: The contribution of family interchanges associated with breastfeeding to parental sensitivity and children's social emotional development
A1 - Epstein-Gilboa,K.
Y1 - 2011///
N1 - 70568607
English
Journal: Conference Abstract
KW - EMBASE
KW - Human
KW - Child
KW - nursing
KW - Interview
KW - Interpersonal Communication
KW - child parent relation
KW - videotape
KW - case study
KW - Infant
KW - risk factor
KW - Funding
KW - childhood
KW - pacifier
KW - feeding
KW - mother
KW - Female
KW - Systems Theory
KW - parent
RP - NOT IN FILE
SP - S9
EP - S10
JF - Breastfeeding Medicine
VL - Conference: 16th Annual International Meeting of the Academy of Breastfeeding Medicine Miami, FL United States. Conference Start: 20111103 Conference End: 20111106. Conference Publication:
IS - var.pagings
N2 - Background: Author's previous study on breastfeeding dyads demonstrates that nursing facilitates the development of intricate interactions, contributing to sensitive mothering styles and healthy psychological growth. Systems theory indicates that interchanges in dyads affect the family and vice versa. Objective: To broaden earlier research and investigate the reverberating impact of nursing on the family system. Methods: Observations, interviews, audio and videotape of families actively involved in physiological breastfeeding in natural settings over time and interviews with carefully selected key informants were organized into case studies and categories and analyzed. Results: Families engaging in physiological breastfeeding patterns display unique behaviors and interactions associated with evolving and reverberating themes related to breastfeeding. Themes facilitate interaction based on infant and child cues. Parents' attuned responses to cues for nursing and proximity evolve into an overall sensitive parenting and family style extending beyond nursing and early childhood. The circulation of themes associated with the development of sensitivity through nursing is influenced by complementary parenting task allocation, open communication and tutoring. Unresolved couple discord and closed communication interfere with these processes and is expressed through distancing, the use of artificial pacifiers, and naming nursing as feeding. Conclusions: The study concludes that interaction in the nursing sub-

system affects and is influenced by overall systemic interchanges, and facilitates the development of parental and family sensitivity extending beyond nursing. The conclusions have important implications for child outcome, including for children in families with multiple risk factors, due to the importance of parental sensitivity for healthy socio-emotional development. Funding Sources: None. The author has published the findings in a book and receives intermittent royalties
SN - 1556-8253
AD - (Epstein-Gilboa) Ryerson University, Toronto, ON, Canada
ER -

TY - JOUR
ID - 3584
T1 - Serum vitamin D (VitD) level in children with cystic fibrosis (CF) and its correlation with frequency of respiratory infections, respiratory severity score, and pulmonary function
A1 - Beattie,S.
A1 - Srivastava,T.
A1 - Geheb,J.
A1 - Black,P.
A1 - Carver,T.
Y1 - 2011///
N1 - 70555016
English
Journal: Conference Abstract
KW - EMBASE
KW - Human
KW - Child
KW - respiratory tract infection
KW - cystic fibrosis
KW - vitamin blood level
KW - lung function
KW - serum
KW - Risk
KW - mineral metabolism
KW - institutional review
KW - Forced Expiratory Volume
KW - statistical analysis
KW - computer program
KW - Kruskal Wallis test
KW - rank sum test
KW - patient
KW - Infection
KW - innate immunity
KW - immune system
KW - malabsorption
KW - Prevalence
KW - vitamin D
KW - antibiotic agent
KW - vitamin
KW - cathelicidin
KW - toll like receptor
KW - polypeptide antibiotic agent
RP - NOT IN FILE
SP - 261
EP - 262
JF - Pediatric Pulmonology
JA - Pediatr Pulmonol
VL - Conference: 25th Annual North American Cystic Fibrosis Conference Anaheim, CA United States. Conference Start: 20111103 Conference End: 20111105. Conference Publication:

IS - var.pagings
N2 - Background:Vitamin D has recently been shown to have a role outside of its well known effect on mineral metabolism. An immunological role for VitD in pediatric infections is being increasingly recognized. Toll like receptors (TLRs) are part of the innate immune system and function as sentinels of the immune system. Activation of TLRs is associated with production of antimicrobial peptides such as cathelicidin and others which are regulated by VitD. Serum from individuals with low VitD has poor cathelicidin induction which can be normalized by correction of serum VitD levels. Children with CF are vulnerable to VitD deficiency from malabsorption of fat soluble vitamins and are at risk for recurrent/chronic respiratory infections. We hypothesized that CF patients with low levels of VitD are at risk for increased respiratory infections, worse severity score, and lower pulmonary function. Methods: With Institutional Review Board (IRB) approval, data was collected over a one year period on 79 children with CF aged 6 to 17 years. We analyzed the prevalence of VitD deficiency (<15 ng/mL) or insufficien cy (15-30 ng/mL), and compared these vitamin levels to the frequency of antibiotic use, severity score per CF Pulmonary Guidelines, and forced expiratory volume in one second (FEV₁). We also analyzed the data using the new recommended VitD cut-off level of 20 ng/mL from Institute of Medicine (IOM). Statistical analysis was performed using SAS software. Results: There was significant correlation between serum VitD level and treatment with intravenous antibiotics (IVAbx) (p=0.036), worse severity scores (p=0.005) and lower FEV₁ measurements (p=0.02). In the 3 group comparison using Kruskal-Wallis test between deficient, insufficient and normal VitD levels, children with CF and VitD deficiency were more likely to receive IVAbx (p=0.016), have worse respiratory severity scores (p=0.006) and lower FEV₁ measurements (p=0.038). In 2 group analysis using Wilcoxan Rank Sum test with IOM cut-off of 20 ng/mL for VitD, children with CF and VitD deficiency had higher IVAbx use (p=0.009), worse respiratory severity scores (p=0.037) and lower FEV₁ measurements (p=0.05). Conclusions: The results suggest that VitD may have a role in infectious and pulmonary complications in children with CF. Additional studies are needed to confirm our preliminary observations from this retrospective analysis. Until further literature becomes available, we must strive to maintain normal serum VitD levels in children with CF
SN - 8755-6863
AD - (Beattie, Srivastava, Geheb, Black, Carver) Children's Mercy Hospitals and Clinics, Kansas City, MO, United States
ER -

TY - JOUR
ID - 3585
T1 - Developing physical activity targets
A1 - Cerny,F.J.
Y1 - 2011///
N1 - 70554818
English
Journal: Conference Abstract
KW - EMBASE
KW - Physical Activity
KW - cystic fibrosis
KW - Human
KW - Exercise
KW - Lifestyle
KW - Child
KW - prescription
KW - patient
KW - exercise intensity
KW - friend
KW - Walking
KW - bicycle
KW - leisure
KW - lung function
KW - energy cost
KW - Rehabilitation
KW - Adult

KW - Heart Rate
KW - car
KW - building
KW - pedometer
KW - Motivation
KW - Sedentary Lifestyle
KW - Conversation
KW - metabolic equivalent
KW - jogging
KW - hospital patient
KW - outpatient
KW - resistance training
KW - Aerobic Exercise
KW - Exercise Test
KW - sport
KW - sitting
KW - Reward
KW - lung clearance
KW - Diabetes Mellitus
KW - Health
KW - fatty acid desaturation
KW - lifestyle modification
KW - shopping
KW - habit
KW - dyspnea
KW - Snow
KW - marker
RP - NOT IN FILE
SP - 135
EP - 136
JF - Pediatric Pulmonology
JA - Pediatr Pulmonol
VL - Conference: 25th Annual North American Cystic Fibrosis Conference Anaheim, CA United States. Conference Start: 20111103 Conference End: 20111105. Conference Publication:
IS - var.pagings
N2 - It is clear that regular, intentional, physical activity (PA) should be part of a healthy lifestyle, including for patients with cystic fibrosis. There are both disease specific, e.g., improved airway clearance, improved lung function, better control of CF-related diabetes, etc., as well as more general, e.g., cardiovascular and musculoskeletal health, benefits of PA. It is equally clear that there is no "one-size-fits-all" target for PA. The following is intended to provide a general guide for PA recommendations followed by suggestions for children. PA recommendations of any kind should be based on knowledge of the clinical status, including pulmonary function, and of the exercise response, including whether desaturation occurs and at what level of exercise. PA targets can be attained using a combination of both formal, e.g., exercise prescription and leisure-time activity recommendations, and informal, but no less intentional, habitual or lifestyle PA recommendations. In any case the recommendation should be based on the patient's response to three critical questions: "What kinds of activities do you like to do?," "Who is going to be your partner?" and "What sorts of things keep you from being physically active?" Success in meeting PA targets will be improved if participants can select from a variety of favorite activities and participate in these activities on an "as desired" basis. Success is further enhanced by making a commitment with an activity partner. For children and young adults, promoting activity with best friends significantly increases time spent in moderate-to-vigorous activity while adults benefit from the accountability associated with a committed partner. Finally, PA is more easily integrated into a lifestyle with the identification of, and removal or minimization of barriers - excuses, in many cases. Making PA a regular part of one's daily routine is difficult for most people, particularly if they are currently sedentary. It is therefore best to start the exercise recommendation with advice and encouragement as to how to become a regular exerciser. For some the first step is to find ways to incorporate activity into one's daily routine. These lifestyle changes might include changing one's commute or shopping habits to include walking or riding a bicycle for all or part of the distance or simply parking the car at the far side of the parking lot and walking, taking the stairs instead of the

elevator, walking during work breaks, etc. An initial goal should be to accumulate 30 minutes of this kind of activity 5 days a week. The use of a pedometer can enhance motivation for lifestyle PA. Recognizing that less than 5,000 steps per day represents a sedentary lifestyle the initial goal should be to increase the daily step count to >10,000 per day over a period of time. As the individual approaches the goal of 30 minutes, 5 days a week or 10,000 steps the recommendation should include advice on increasing exercise intensity to moderate to vigorous levels. Moderate exercise intensity should allow the person to maintain a conversation without becoming breathless while vigorous intensity would involve a sense of breathlessness. Once a person has become accustomed to an increase in habitual, lifestyle PA it becomes easier to move to leisure-time PA or more formal exercise recommendations. The compendium of physical activities (1-3) provides a listing of hundreds of activities and their energy cost. Energy cost is listed in terms of METS (Metabolic Equivalents, with 1 MET = 3 mL O₂/kg/min) allowing one to recommend a variety of specific PA at moderate (3-6 METs) or vigorous (>6 METs) activity intensities. For instance, encouraging activities such as general carpentry or sweeping floors or bicycling with light effort would fulfill a recommendation for moderate activity while encouraging jogging at 6 mph, shoveling snow or higher intensity bicycling would fulfill a recommendation for vigorous activity. In this way a variety of activities can be combined to attain the goal of a minimum of 30 minutes of moderate-vigorous activity 5 days a week. Some patients will require a more formal exercise prescription to be carried out in an inpatient or outpatient rehabilitation setting; some may find that specific exercise prescriptions make it easier to attain the above-described goals outside of a rehabilitation setting. This more specific prescription approach also allows for safe recommendations for strength training activities. The prescription for aerobic exercise will use heart rate as the marker for exercise intensity and a target of >60% of Heart Rate Reserve calculated from a peak/maximal exercise test (HRR = (Peak HR - Rest HR)% intensity + Rest HR). The most effective way to involve children in regular activities that will meet the recommendation is to engage family and friends in an active lifestyle, including leisure activities and sports. Children can be effectively disengaged from sedentary activities by providing rewards for reducing those activities, allowing them to select their own alternatives. Similarly, connecting children with active friends is effective in increasing the amount of time spent in moderate to vigorous activity on a daily basis
SN - 8755-6863
AD - (Cerny) Children's Lung Center, Women and Children's Hospital of Buffalo, Buffalo, NY, United States
(Cerny) Pediatrics and Exercise, Nutrition Sciences, Univ. at Buffalo, Buffalo, NY, United States
ER -

TY - JOUR
ID - 3586
T1 - Maintaining paediatric anaesthetic skills in the district general hospital-the Portsmouth approach
A1 - Read,E.
A1 - Marsh,D.
Y1 - 2011///
N1 - 70547441
English
Journal: Conference Abstract
KW - EMBASE
KW - United Kingdom
KW - anesthesist
KW - skill
KW - general hospital
KW - Ireland
KW - Resuscitation
KW - Child
KW - Human
KW - protection
KW - feedback system
KW - Teaching
KW - college
KW - needle
KW - Hospital
KW - algorithm
KW - Learning

KW - Atmosphere
KW - Policy
KW - positive feedback
KW - teaching hospital
KW - anesthetic agent
RP - NOT IN FILE
SP - 29
JF - Anaesthesia
VL - Conference: Association of Anaesthetists of Great Britain and Ireland Annual Congress, AAGBI 2011 Edinburgh United Kingdom. Conference Start: 20110921 Conference End: 20110923. Conference Publication:
IS - var.pagings
N2 - Recent controversy regarding how anaesthetists outside major teaching hospitals can maintain their skills prompted us to develop an in-house half-day update [1]. Advanced Paediatric Life Support (APLS) and European Paediatric Life Support (EPLS) courses cover all aspects of paediatric resuscitation but are time consuming, expensive and not specifically designed for anaesthetists. The Association of Paediatric Anaesthetists (APA) suggest a list of core skills and knowledge, which they recommend are tailored to local requirements [2]. In addition, there is a national requirement for all anaesthetists who have contact with children to attend regular level 2 child protection training [3]. Methods We developed a scenario based update course designed specifically for the local anaesthetic department. The session was lead by anaesthetists who are trained APLS instructors and was based on recent APA recommendations, endorsed by the Royal College of Anaesthetists [2]. We ran five 25 minute sessions in small groups during a protected teaching half day. These sessions covered the following: a question and answer session on departmental guidelines and protocols, three scenarios incorporating all aspects of resuscitation training and intraosseous needle insertion practical session. Level 2 child protection training update was also included. We further supported clinicians by providing written information, designed as a small booklet, outlining local relevant and approved hospital guidelines and resuscitation algorithms. Feedback was actively promoted and has led to significant further changes to course design and structure. Results Feedback forms were received from 17 candidates, with 16/17 (94%) scoring all stations as excellent and 1/17 (6%) as adequate. The course was recommended by 100% of candidates. Other comments commended the effective learning atmosphere, and the useful forum to discuss local guidelines and policies. Estimated course costs were low and included those of protected departmental teaching time. Discussion This house paediatric anaesthetic update covered all aspects recommended by the APA. It was well received by local clinicians, and positive feedback confirmed suitability and effectiveness of the course format. Using local APLS expertise and resuscitation equipment kept costs to a minimum
SN - 0003-2409
AD - (Read, Marsh) Portsmouth Hospitals NHS Trust Debbie, United Kingdom
ER -

TY - JOUR
ID - 3587
T1 - Serum vitamin D levels in Czech overweight and obese children
A1 - Prusa,R.
A1 - Cepova,J.
A1 - Marinov,Z.
A1 - Pechova,M.
Y1 - 2011///
N1 - 70539889
English
Journal: Conference Abstract
KW - EMBASE
KW - Child
KW - Human
KW - Obesity
KW - vitamin blood level
KW - laboratory
KW - girl
KW - Female
KW - boy

KW - Male
KW - blood level
KW - serum
KW - Population
KW - Internet
KW - consensus
KW - Bone
KW - Health
KW - autumn
KW - bone metabolism
KW - Immunity
KW - exposure
KW - Sunlight
KW - Weather
KW - city
KW - Diagnosis
KW - vitamin D
KW - vitamin
KW - biochemical marker
RP - NOT IN FILE
SP - 541
JF - Clinical Biochemistry
VL - Conference: 12th International Congress of Pediatric Laboratory Medicine, ICPLM 2011 Berlin Germany. Conference Start: 20110513 Conference End: 20110515. Conference Publication:
IS - var.pagings
N2 - Background: Vitamin D is an important biochemical marker of bone metabolism and immunity. In recent years the exposure of children to sunlight decreased due to weather conditions and more time spent on the internet. Moreover they have also lower outdoor physical activity.We decided to study vitamin D status as serum levels of 25-OH vitamin D₃ in a group of obese children in the city of Prague. Methods: We measured serum 25-OH vitamin D₃ in the period between October and November 2010 in a group of overweight and obese children (BMI more than the 90th percentile, or 97th percentile respectively, of Czech population according the age and sex, http:// www.szu.cz/publikace/data/rustove-grafy), 53 boys aged 4-18 years and 60 girls aged 4-18 years. We used ECLIA method, cobas e411 (Roche Diagnostics). There is consensus opinion, that the minimal 25- OH vitamin D level for bone health is 50 nmol/L. Results: The median level of 25-OH vitaminD₃ in boys (n=53)was 25.9 nmol/L, 32 of them had levels below 30 nmol/L, and 20 boys had levels between 31 and 50 nmol/L. The median level of vitamin D₃ in girls (n=60)was 31.3 nmol/L, 26 of them had values below 30 nmol/L, 26 girls had levels between 31 and 50 nmol/L, and only 8 girls had values over 51 nmol/L. Conclusion: 98.1% of overweight and obese boys and 86.7% of overweight and obese girls had serum levels of 25-OH vitamin D₃ in autumn (October-November) less than recommended value, so less than 50 nmol/L
SN - 0009-9120
AD - (Prusa, Cepova, Pechova) Department of Clinical Biochemistry, 2nd Faculty of Medicine and Faculty Hospital Motol, Prague, Czech Republic (Marinov) Department of Paediatrics, 2nd Faculty of Medicine and Faculty Hospital Motol, Prague 15000, Czech Republic
ER -

TY - JOUR
ID - 3588
T1 - Critical values for the maternal-fetal unit, fetus, infant, child and adolescent: Bilirubin reporting practice in North American Children's Hospitals as a paradigm for critical value reporting assessment
A1 - Geaghan,S.M.
Y1 - 2011///
N1 - 70539803
English
Journal: Conference Abstract
KW - EMBASE
KW - Human

KW - pediatric hospital
KW - laboratory
KW - Infant
KW - fetus
KW - Child
KW - Adolescent
KW - kernicterus
KW - United States
KW - Canada
KW - jaundice
KW - Risk
KW - Newborn
KW - hyperbilirubinemia
KW - Hospital
KW - patient
KW - Prevention
KW - History
KW - physician
KW - groups by age
KW - college
KW - clinical laboratory
KW - hospital patient
KW - intensive care unit
KW - centralization
KW - standardization
KW - turnaround time
KW - patient safety
KW - Italy
KW - Safety
KW - Therapy
KW - university hospital
KW - landscape
KW - ontogeny
KW - patient care
KW - Pediatrics
KW - clinical decision making
KW - pathologist
KW - bilirubin
KW - glucose
KW - Sodium
KW - potassium
KW - Calcium
KW - magnesium
RP - NOT IN FILE
SP - 483
EP - 484
JF - Clinical Biochemistry
VL - Conference: 12th International Congress of Pediatric Laboratory Medicine, ICPLM 2011 Berlin Germany. Conference Start: 20110513 Conference End: 20110515. Conference Publication:
IS - var.pagings
N2 - Introduction Critical values are a concept introduced by Lundberg in 1972, defined as "pathophysiologic derangements of such variance with normal as to be life-threatening if therapy is not instituted immediately" [1]. Physician notification of critical values is currently a standard of practice in laboratory medicine. The definition of critical values must be a collaborative process between services and the clinical laboratory, which balances clinical needs and resource utilization. Periodic revision is recommended by the College of American Pathologists. The laboratory's critical value reporting practices should reflect activities critical to patient outcome [2]. The position of the American Academy of Pediatrics is that kernicterus is preventable. The AAP

focus is to reduce the incidence of severe hyperbilirubinemia and its sequelae of severe bilirubin encephalopathy, which begins with appropriate and timely evaluation of bilirubin levels. Specifically, a bilirubin level is recommended on every jaundiced infant in the first 24 h of life and if the jaundice appears excessive for the patient's age [3]. Bilirubin critical value reporting is a tool for the prevention of kernicterus and bilirubin-induced neurological damage. A bilirubin value elevated outside the range expected at a given age (in hours) has been demonstrated to represent high risk for kernicterus. In particular, values obtained during the hours of life, which represent the steepest slope of the curve for onset of physiologic jaundice [4], must be interpreted relative to age. Since bilirubin carries a uniquely pediatric impact as a critical value, and kernicterus is a devastating clinical outcome of global scope, an investigation of bilirubin critical value reporting practices in North American children's hospitals was undertaken, in the context of current theory and practice of critical value reporting. Methods A comprehensive review of current literature regarding critical value result reporting in the pediatric age group, including the fetus, neonate, child and adolescent, was performed. Evidence base for risks and benefits of analytes frequently chosen for critical value reporting was analyzed. Critical values for bilirubin were investigated in representative children's hospitals throughout the fifty United States, and two provinces of Canada. Bilirubin critical value reporting was surveyed in children's hospitals, if a children's hospital was available, in the fifty United States and three provinces of Canada. Performance characteristics (risks and benefits) for various critical value reporting strategies, as reported in the survey, were analyzed. Results The literature highlights that critical values are generally from inpatients, intensive care unit cases are overrepresented, and most are chemistries. Overall, they represent 0.25% of all tests and 0.75% of tests eligible to be critical values, based on broad-based survey [5]. Centralization of critical call notification is linked to standardization, fewer misses and a decrease in turnaround time [6]. The most recent comprehensive survey on pediatric critical values in fourteen Canadian laboratories reported only four analytes (sodium, glucose, potassium and calcium) eligible for critical value reporting in at least 90% of the laboratories. Considerable variability among these laboratories for thresholds-such as high critical glucose, high critical magnesium, and low critical O₂-was reported, and few laboratories could identify a source for their critical values [7]. Preliminary data and analysis of the frequency distribution of numerical values defined as critical for bilirubin in neonates from 93 children's hospitals in the United States and Canada is represented in Fig. 1 (including the number of children's hospitals that fail to define 1 critical value for bilirubin, and those institutions that offer age-related critical values for bilirubin). (Figure presented) Critical values for bilirubin that are age-related and based on the natural history of physiologic jaundice, as reported at Stanford University Hospitals and Clinics are presented (Table 1). Discussion The current landscape of pediatric critical values, as represented in the literature and this original data set, is characterized by a wide variety in analytes forwhich a critical value is chosen,whether or not the values are age-related, based on ontogeny of biochemical and hematological development, and in the numerical level chosen to alert the physician. Outcome-based studies are required to optimize this critically important laboratory activity: to define best practice in bilirubin reporting; insure relevance to patient care and clinical decision-making; and an appropriate balance of laboratory resource utilizationand patient safety. Variability in practices of notification and thresholds across countries (e.g. the United States and Italy) has been recently described, and this topic is of global interest and import [8]. For the first time we have defined the practice pattern for bilirubin critical value definition and reporting at representative children's hospitals over the United States and Canada These data demonstrate 1 high degree of variability in three variables: whether or not bilirubin is defined as a critical value; the numerical level at which the critical value, if existing, is defined; and whether the value is age-related in accordance with current knowledge of the natural history of physiologic jaundice in healthy newborns. Some hospitals do not define any value as a critical value for bilirubin, which may represent 1 missed opportunity in the course of prevention of severe hyperbilirubinemia and its sequelae. There are a small number of hospitals that offer bilirubin critical values that are age-related, which have the (Table presented) potential to offer greater safety and earlier manifestation of pathological elevation, and perhaps earlier intervention for neonates at risk for severe hyperbilirubinemia
SN - 0009-9120
AD - (Geaghan) Stanford Hospital, Clinics Clinical Laboratory, 300 Pasteur Drive, Palo Alto, CA, United States
ER -

TY - JOUR
ID - 3590
T1 - Evaluation and therapy of luts mental retarded children: A six years follow-up
A1 - Masnata,G.

A1 - Ponticelli,A.
A1 - Manca,V.
Y1 - 2011///
N1 - 70530446
English
Journal: Conference Abstract
KW - EMBASE
KW - Human
KW - Child
KW - urology
KW - urodynamics
KW - follow up
KW - pelvis floor
KW - continence
KW - Therapy
KW - Society
KW - patient
KW - lower urinary tract symptom
KW - habit
KW - Intelligence
KW - parent
KW - Interview
KW - health care quality
KW - health service
KW - mental deficiency
KW - intelligence quotient
KW - Hospital
KW - urinary tract
KW - psychologist
KW - Self Care
KW - Education
KW - micturition
KW - Self Esteem
KW - reassurance
KW - bladder
KW - disability
KW - skill
KW - Health
KW - health care utilization
RP - NOT IN FILE
SP - 49
JF - Neurourology and Urodynamics
VL - Conference: 35th Annual Congress of the Italian Urodynamics Society
IS - Continence, Neuro-Urology, Pelvic Floor
N2 - Introduction and Aim of the Study: Bladder re-training with behavioural approach is normally adopted in children affected by lower urinary tract symptoms (LUTS). For children with an intellectual disability, instructed method based on behavioural principles are likely to succeed in order to teach new skills and reduce dysfunctional habits, such us voiding behaviours. Childrenwith ID havemore frequent medical problems than age-matched peers without ID. Despite the higher rates of health problems, research has highlighted lower rates of health care use among children with ID. Less frequent approach to these patients may be related to environmental, behavioural and social barriers. Parents describe diverse barriers to access to care, in particular a lack of care in coordination services between providers. The possibility to improve the quality of health care services for children with ID is to organize individualized and specialized intervention programs, on the basis of the children specific needs. The aim of our study was to analyse the effect of a behavioural urotherapy in reducing LUTS in children with ID, compared with typical controls. Materials and Methods: This study included 10 patients (5 children with normal intelligence aged 6-12 years and 5 mildly mentally retarded children with IQ > 70 of the same age) with non-neurogenic voiding dysfunction , referred to our hospital

betweenMarch 2004 and April 2010. Of a total of 10 patients, 4 had recurrent urinary tract infecions (3 of the group with normal intelligence and 1 of the group of children with ID). In the course of the first tests the patients are checked by a psychologist and a paediatric urologist, to continue with the evaluation and collection of information and definition of objectives. The two groups were treated with a behavioural urotherapy program, that is a self-management programme on actual voiding behaviour using the Frequency-Volume Chart (FVC). Frequency-volume charts (FVCs) is a non-invasive tool that allows the assessment of LUTS outside the setting of a clinical interview but it's been used in this study also as a selfmanagement tool to re-educate the children to adopt a normal voiding behaviour. By using the behavioural approach it is possible to rectify bad urinary habits of their children. A careful history and general examinationwas performed on all patients. Therefore a programme of voiding re-education was proposed, with a systemof incentives. Patients are thus trained to use FVC frequently. In the first 15 days the FVC was without restriction therefore without influencing the patient's urinary behaviour. In the subsequent period, timed urination was advised, recommended children following FVC with their own cooperation. All children have been evaluated every 20 days in the first 6months of observation and later every 40 days. All the participants were monitored for voiding frequency for 12 months with monthly evaluations. After the end of the therapeutic program, all parents received a phone interview every six months for 5 years, to know urinary habits of the children. Results: Among the 10 children considered in this study, 9 (90%) had a improvement of symptoms during the application of the FVC. Of the 4 children with recurrent UTI, 3 had a complete lack of UTI in 1 year follow-up. The behavioural urotherapy programwas successful for both groups, meaning that the children reached a normal habit of voiding behaviour after 7 or 8months of treatment. The results were stable at 12 months and all patients got a normal voiding behaviour during the five years follow-up. Interpretation of Results: The use of an incentive system in mildlymentally retarded children and in children with normal intelligence, allows the strengthening of the children in a positiveway to avoid regression in the acquired behaviour and the FVC showed an improvement in voiding behaviour in both categories of patients. Analyzing in detailed the pattern of voiding behaviour at base-line, it seems that children with ID learn adaptive voiding behaviours at the same rate of typical children. This might be due to the fact that behavioural principles used in our urotherapy programare particularly suitable for them in order to learn new habits and new behaviours. Moreover, the collaboration of the parents have been critical for the program to succeed. Conclusions:Weshowed that even in mildlymentally retarded children with LUTS it is possible to obtain the same results in terms of quality and quantity as compared to other children with normal intelligence of the same age. The results obtained generate confidence and self-esteem as well as better quality relationships in the child, giving support and reassurance when difficulties arise during the educational course. Medical activity together with psychological advice which is applied the cognitive technical of behaviour supported of FVC, it reveals a efficacious, economical method, and non invasive in the treatment of LUTS in children
SN - 0733-2467
AD - (Masnata, Ponticelli, Manca) Paediatric Urodynamic, Brotzu Hospital, Cagliari, Italy
ER -

TY - JOUR
ID - 3591
T1 - Effect of anti TNF on Kawasaki disease
A1 - Boyapati,S.
A1 - Patra,K.P.
A1 - Gali,D.
A1 - Shahid,K.
A1 - Jackson,R.D.
A1 - Bass,R.P.
Y1 - 2011///
N1 - 70521825
English
Journal: Conference Abstract
KW - EMBASE
KW - mucocutaneous lymph node syndrome
KW - medical research
KW - Human
KW - patient
KW - T lymphocyte activation

KW - fever
KW - skin
KW - disease predisposition
KW - tongue
KW - Therapy
KW - Case Report
KW - Male
KW - Hospital
KW - rash
KW - Conjunctivitis
KW - coronary artery dilatation
KW - drug megadose
KW - thrombocyte
KW - heart transplantation
KW - Genetics
KW - genetic analysis
KW - Japanese
KW - Child
KW - pathogenesis
KW - clinical trial (topic)
KW - infliximab
KW - antibiotic agent
KW - methylprednisolone sodium succinate
KW - enoxaparin
KW - protein kinase C
KW - cyclic AMP dependent protein kinase
RP - NOT IN FILE
SP - 440
JF - Journal of Investigative Medicine
VL - Conference: American Federation for Medical Research Southern Regional Meeting, AFMR 2011 New Orleans, LA United States. Conference Start: 20110217 Conference End: 20110219. Conference Publication:
IS - var.pagings
N2 - Case Report: Case Presentation: A previously healthy 6 month old white male was transferred from outside hospital with uncontrolled fever and rash. Before being transferred, the patient was twice admitted and treated with IV antibiotics. Both the times, the septic workup including LP was negative. When transferred, the patient had a fever of 104 F and physical examiniation revealed a reddish tongue and peeling of the skin at the extremities. Detailed history revealed the progression of symptoms: fever followed by conjunctivitis, reddish discoloration of the tongue and finally peeling of the skin. Suspecting Kawasaki disease, a 2D echo was done which showed dilated coronaries. Patient immediately received Asprin and 2 doses IV IGG. However, symptoms persisted and repeat echo showed worsening of coronary dilatation. Patient was then started on high dose of solumedrol. After 3 doses, a repeat 2D showed further worsening of coronaries with RCA measuring 8 mm in diameter. The patient's platelets continued to increase from 300 K to 1200 K. At this time, anti-TNF (Infliximab) and Lovenox were started and patient was put on list for evaluation of cardiac transplantation. Post-Infliximab, echo showed improvement. Discussion: 'The contribution of genetics to disease predisposition is clear but the mechanisms involved in regulating the predisposition and the impact are not clearly understood. Two recent independent studies have identified regulation of T-cell activation as the critical factor in determining the susceptibility and severity of Kawasaki disease. In one study, genetic analysis of affected Japanese children identified ITPKC, 1,4,5-triphosphate 3-kinase C, a kinase involved in regulation of T-cell activation, to be significantly associated with Kawasaki disease. The other study also found that regulating T-cell activation is strongly correlated to the susceptibility and severity of the disease. The study demonstrated a central role for TNF-alpha in Kawasaki's pathogenesis. Controlled, randomized clinical trials are warranted to determine the role of anti-TNF-alpha therapy for treating this disease
SN - 1081-5589
AD - (Boyapati, Patra, Gali, Shahid, Jackson, Bass) LSUHSC, Shreveport, LA, United States
ER -

TY - JOUR

ID - 3592
T1 - Parental stress factors in a community paediatric case load
A1 - Rahman,F.R.
A1 - Adamson,E.A.
Y1 - 2011///
N1 - 70504184
English
Journal: Conference Abstract
KW - EMBASE
KW - parental stress
KW - community
KW - Pediatrics
KW - child health
KW - college
KW - Human
KW - Child
KW - child parent relation
KW - Adult
KW - physical disease
KW - mental disease
KW - Violence
KW - learning disorder
KW - childhood
KW - Child Abuse
KW - follow up
KW - abuse
KW - Mental Health
KW - case mix
KW - planning
KW - victim
KW - Funding
KW - physical disability
KW - mental health service
KW - Adolescent
KW - protection
KW - alcohol
RP - NOT IN FILE
SP - A67
JF - Archives of Disease in Childhood
JA - Arch Dis Child
VL - Conference: Annual Conference of the Royal College of Paediatrics and Child Health, RCPCH 2011 Warwick United Kingdom. Conference Start: 20110405 Conference End: 20110407. Conference Publication:
IS - var.pagings
N2 - Aims: To determine whether parental stress factors are present in a significant proportion of a community paediatric caseload. Methods: Analysis of routinely collected service activity data using parental factors codes defi ned in the national Child and Adolescent Mental Health Services dataset (CAMHS), that is, parental sensory impairment, physical illness, mental illness, drugs and alcohol problems, learning disability, history of being in care or the victim of abuse as a child, history of known violence or child abuse as an adult and other unspecified parenting difficulties. These data relates to cases seen generically, that is, outside dedicated child protection and LAC sessions. Results: Over the fi nancial year 2009/2010 the service had 2230 new and 4802 follow-up successful contacts. A total of 1519 separate instances of parental factors were identified in 436 new (19.55%) and 747(15.55%)of follow-up contacts. Specific factors (each contact may have more than one & up to four factors could be coded for each contact) were identified as follows: mental illness in 364, known history of violence in 208, learning disability in 159, physical illness in 157, problem drinking/ drugs in 132, known history of child abuse in 40,period of care in childhood in 30,childhood abuse in 24, sensory impairment in 18 and physical disability in 13.Unspecified parenting diffi-culties which could not be included in the previous categories were identified in another 374 instances. Conclusion: This study identifies for the fi rst time the

frequent presence of both specific and unspecified parenting difficulties, most often refl ecting adverse aspects of adult mental health, in this generic community paediatric caseload; this is another dimension of case mix complexity which has implications for training as well as planning and funding of services; it also illustrates the need for coordinated working across agencies as well as across services for vulnerable children and vulnerable adults & demonstrates the increasing overlap between CAMHS and community paediatrics. In our view the forthcoming child health dataset should therefore also include the CAMHS dataset parental stress factors codes
SN - 0003-9888
AD - (Rahman, Adamson) Specialist Community Children's Services, NHS Derby City PCT, Derby, United Kingdom
ER -

TY - JOUR
ID - 3593
T1 - A qualitative study investigating coping strategies in families with more than one autistic child
A1 - Waterson,I.M.
A1 - Stockl,A.
A1 - Langdon,P.
Y1 - 2011///
N1 - 70504122
English
Journal: Conference Abstract
KW - EMBASE
KW - infantile autism
KW - Pediatrics
KW - Coping Behavior
KW - college
KW - Qualitative Research
KW - child health
KW - Human
KW - Child
KW - emotion
KW - sibling
KW - Single Parent
KW - semi structured interview
KW - mother
KW - Female
KW - father
KW - Male
KW - grounded theory
KW - theoretical model
KW - Diagnosis
KW - grandparent
KW - bird
KW - Autism
KW - Praise
KW - Education
KW - social care
KW - health service
KW - parent
RP - NOT IN FILE
SP - A41
JF - Archives of Disease in Childhood
JA - Arch Dis Child
VL - Conference: Annual Conference of the Royal College of Paediatrics and Child Health, RCPCH 2011 Warwick United Kingdom. Conference Start: 20110405 Conference End: 20110407. Conference Publication:
IS - var.pagings

N2 - Aims: The purpose of this study was to investigate how families who have multiple autistic children function. Methods: 11 families were recruited and included in the study. The number of children per family varied between two and six, with up to four autistic children in a family. Married, single parent, and non married partnerships were sampled. 44 semi-structured interviews were untaken with mothers, fathers, children with autism and their siblings. These were transcribed and analysed using Grounded Theory. A selection of transcripts was read by another researcher and the fi ndings were sent to families for checking. Results: A variety of themes emerged from the data, including the use of practical strategies to reduce behavioural problems, the place of emotions in coping, the use of statutory and other services and the views of verbal autistic children and their siblings. A theoretical model encompassing the families' views of their children as normal was developed and highlighted that families dislike being pitied and pathologised by the non-autistic world. A surprising fi nding was that families with several autistic children in large families found coping easier, as they were more able to normalise the autistic children. Although several different types of emotions were expressed at the time of the diagnosis, there was also an overwhelming sense of relief. All of the families found there were several positive attributes to caring for autistic children. A small minority of families had very negative experiences and the reasons for these are discussed in my presentation. Conclusions Families rely mainly on their own internal family members for support, including grandparents, rather than on outside services. The Early Bird parent support scheme received high praise, with more criticism for education, social care and some health services. The main advice for newly diagnosed families was to treat your children as normal as much as possible, and to plan your day and activities ahead
SN - 0003-9888
AD - (Waterson, Stockl, Langdon) Department of Medicine, Health Policy and Practice, University of East Anglia, Norwich, United Kingdom
ER -

TY - JOUR
ID - 3594
T1 - Training executive attention and motor skills (TEAMS): A novel intervention for preschoolers with ADHD
A1 - Halperin,J.M.
A1 - Marks,D.J.
A1 - Chacko,A.
A1 - Curchack,J.
A1 - Yoon,C.
A1 - Bedard,A.
A1 - Healey,D.
Y1 - 2011///
N1 - 70495618
English
Journal: Conference Abstract
KW - EMBASE
KW - Human
KW - Attention Deficit Disorder
KW - Society
KW - preschool child
KW - manager
KW - Motor Performance
KW - Child
KW - parent
KW - teacher
KW - motor control
KW - Education
KW - model
KW - skill
KW - palatability
KW - follow up
KW - Satisfaction
KW - randomized controlled trial (topic)

KW - Prevention
KW - brain development
KW - working memory
RP - NOT IN FILE
SP - 25
JF - Brain Impairment
VL - Conference: 2011 International Neuropsychological Society Mid-Year Meeting/ASSBI 4th Pacific Rim Conference Auckland New Zealand. Conference Start: 20110706 Conference End: 20110709. Conference Publication:
IS - var.pagings
N2 - Objective: To assess the effectiveness of a novel neurocognitive prevention intervention for preschoolers with ADHD that was designed to enhance brain development and yield enduring reductions in ADHD symptom severity. Participants and Methods: 29 4 and 5 year-old children with ADHD and their parents participated in concurrently-run child (3-5 children/ group) and parent groups. Child groups focused on an array of games targeting distinct neurocognitive domains including inhibitory control, working memory, motor control, set-shifting and planning/organization. Parents received education about ADHD coupled with instruction, modeling, and a description of the cognitive constructs tapped by each of the children's game. Parents were directed to spend at least 30 min. per day playing these games with their children and were instructed in how to individualize (i.e., scaffold) the games to their child's level of proficiency and progressively increase the cognitive/behavioral load of each game as their child achieved mastery of the requisite skill. Daily diaries were collected to measure time engaged in activities outside of sessions. Treatment palatability was evaluated, and parent and teacher ratings on the ADHD-RS-IV were assessed pre-treatment, posttreatment and at 1- and 3-month follow-up. Data are reported for 7 child groups. Results: Only one child failed to complete the intervention, and session attendance was very high (92.7%). Mean daily playing at home was 35.3 min/day, and parent satisfaction ratings were quite high. Parent and teacher ratings on the ADHD-RS-IV significantly improved from pre- to post-treatment (both $p < .01$), and behavioral gains reported by parents ($p < .01$) and teachers ($p < .05$) persisted 1- and 3-months post-treatment. Conclusions: These data indicate that a structured play-based approach to neurocognitive training may be an effective treatment for preschoolers with ADHD. Double-blind, randomized controlled trials are beginning
SN - 1443-9646
AD - (Halperin) Dept. of Psychology, Queens College, 65-30 Kissena Blvd., Flushing, NY 11367, United States
ER -

TY - JOUR
ID - 3595
T1 - Obsessive-compulsive symptoms and physical activity in patients with anorexia nervosa: Possible relationships
A1 - Srebnicki,T.
A1 - Brynska,A.
A1 - Blachno,M.
A1 - Wolanczyk,T.
A1 - Wisniewski,A.
A1 - Tomaszewicz-Lubidzic,C.E.
Y1 - 2011///
N1 - 70489693
English
Journal: Conference Abstract
KW - EMBASE
KW - Human
KW - patient
KW - Physical Activity
KW - Child Psychiatry
KW - Child
KW - Society
KW - anorexia nervosa
KW - Risk

KW - school
KW - building
KW - hyperactivity
KW - weight
KW - psychiatric diagnosis
KW - Self Report
KW - habit
KW - Female
KW - high risk population
KW - parent
KW - winter
KW - computer
RP - NOT IN FILE
SP - S172
EP - S173
JF - European Child and Adolescent Psychiatry
VL - Conference: 14th International Congress of ESCAP European Society for Child and Adolescent Psychiatry Helsinki Finland. Conference Start: 20110611 Conference End: 20110615. Conference Publication:
IS - var.pagings
N2 - Objective: To find a possible relationship between presence of OCD symptoms thematically unrelated with weight or appearance and level and characteristics of physical activity AN. Method: 76 female patients, aged 14.76 + 1.78 years met the DSM-IV criteria for AN at the time of admission, with no comorbid psychiatric diagnosis, completed the Polish version of the 20-item self-report version of Leyton Obsessional Inventory-Child Version (LOI-CV). Two groups, high-risk (HR) and no high-risk (nHR), were defined according to the cut-off score of LOI-CV. Physical Acticity Index (KAF) was used to assess the intensity and type of physical activity. The information obtained allowed to measure the frequency and type physical activity as well as the change of behaviors and habits before and during anorectic episode and abscondence of AN symptoms (activities not controlled by parents). Results: 35 subjects (46%) were qualified to HR group and 41(54%) to nHR group. Compared to high-risk group no high risk subjects spent much less time outside in the winter (U = 5,760, p = 0.029), spent less time studying while standing (U = 10,371, p = 0.005), used elevators more often than stairs (U = 7,391, p = 0.015). Differences between patient-perceived Activity Index (U = 3,578, p = 0.061) and the total score on Activity Index (U = 3,334, p = 0.071) were on the tendency level. Patients in nHR group scored less on both indexes compared to HR patients. Negative correlation was found (t = -0,572; p = 0,026) between the number of YES answers in LOI-CV inventory and the time spent studying at school. Negative correlation on tendency level was found between the number of hours spent watching TV or playing computer and LOI-CV Interference Score (t = -0,458; p = 0.063). Positive correlation was found between the time spent on physical activities weekly and the number of YES answers. Conclusions: Results of the study showed that obsessiveness may be involved in the development of hyperactivity in AN patients
SN - 1018-8827
AD - (Srebnicki, Brynska, Blachno, Wolanczyk, Wisniewski, Tomaszewicz-Lubidzic) Warsaw Medical University, Poland
ER -

TY - JOUR
ID - 3596
T1 - Altered activation to working memory task in young subjects with autistic spectrum disorder: A functional MRI study
A1 - Rahko,J.
A1 - Vuontela,V.
A1 - Nikkinen,J.
A1 - Carlson,S.
A1 - Moilanen,I.
A1 - Kiviniemi,V.
Y1 - 2011///
N1 - 70489614
English
Journal: Conference Abstract

KW - EMBASE
KW - nuclear magnetic resonance imaging
KW - working memory
KW - Human
KW - Functional Magnetic Resonance Imaging
KW - Autism
KW - Child Psychiatry
KW - Society
KW - Child
KW - heart atrium septum defect
KW - cerebellum
KW - processing
KW - Memory
KW - Adolescent
KW - brain cortex
KW - brain
KW - postcentral gyrus
KW - gender
KW - temporal lobe
KW - insula
KW - supramarginal gyrus
KW - superior temporal gyrus
KW - Neuroimaging
RP - NOT IN FILE
SP - S147
EP - S148
JF - European Child and Adolescent Psychiatry
VL - Conference: 14th International Congress of ESCAP European Society for Child and Adolescent Psychiatry Helsinki Finland. Conference Start: 20110611 Conference End: 20110615. Conference Publication:
IS - var.pagings
N2 - Background: It has been shown that subjects with ASD have altered visuospatial processing and memory processing capabilities. The purpose of the present study was to examine both behaviorally and with neuroimaging methods the difference between adolescent ASD and TD controls during working memory task. Materials and methods: 30 adolescent ASD cases and 30 age and gender matched controls was assessed with multiple tests (DSM-IV). Resting and activation scans were imaged with GE 1.5 T, ASSET x 2, TR 1800 ms, TE 40 ms, flip angle 90, 64 x 64 matrix, FOV 25.6 cm, 28 4 mm slices. We investigated whether attentional processing (0-back) or more stronger task load (2-back) during working memory (WM) task is altered in ASD. Results: The memory load and task effects (2-back vs. 0-back) areas are in the right temporal regions S1 and insula. The power of deactivations, i.e. task <baseline activity, outside the default mode network during 0-back task was stronger in the ASD group and the activations were reduced compared to TD controls. There were deactivation during 0-back task in the right precentral and postcentral gyrus in ASD group. Activated areas were observed in the right Rolandic operculum, supramarginal gyrus and superior temporal gyrus. Conclusion: Our results show the differences between groups were largest in the cerebellum and they dominated compared to the differences in cerebral cortex. The cerebellum was the only area which had alterations in both 2-back and 0-back tasks in comparison to baseline. This was thought to be due to altered salience processing in the right insular/frontal region. In 0-back condition, the controls activated the cerebellum more strongly in the cerebellum lobules I, VII, VIIIb and in lobule IX. During 0-back task the brain is already de-activated and activated in excess and during the higher task load there is no reserve to take from and then the system overrides and gives un-controlled motor responses
SN - 1018-8827
AD - (Rahko) Institute of Clinical Medicine, University and University Hospital of Oulu, Finland (Rahko, Moilanen) Department of Child Psychiatry, Finland (Vuontela, Carlson) University of Helsinki, Helsinki, Finland (Vuontela, Carlson) Neuroscience Unit, Institute of Biomedicine/Physio, Finland (Nikkinen, Moilanen, Kiviniemi) University and University Hospital of Oulu, Finland (Nikkinen, Kiviniemi) Department of Diagnostic Radiology, Finland
ER -

TY - JOUR
ID - 3597
T1 - The price that children pay in the daily search for food in Soweto slums-Uganda
A1 - Muramizi,W.
A1 - Okoth,G.
Y1 - 2011///
N1 - 70489347
English
Journal: Conference Abstract
KW - EMBASE
KW - Human
KW - Child
KW - Society
KW - Food
KW - Uganda
KW - Child Psychiatry
KW - Risk
KW - Sexual Abuse
KW - Drug Abuse
KW - injury
KW - childhood
KW - Hunger
KW - community
KW - Diet
KW - growth,development and aging
KW - infancy
KW - non profit organization
KW - adulthood
KW - extended family
KW - Responsibility
KW - basic needs
KW - sampling
KW - questionnaire
KW - Adult
KW - study design
KW - Interview
KW - Water
RP - NOT IN FILE
SP - S62
JF - European Child and Adolescent Psychiatry
VL - Conference: 14th International Congress of ESCAP European Society for Child and Adolescent Psychiatry Helsinki Finland. Conference Start: 20110611 Conference End: 20110615. Conference Publication:
IS - var.pagings
N2 - A healthy diet is the key to normal growth and development in infancy and childhood and is the foundation to adulthood. Communities have absorbed these children in extended families with the desire to help them. The responsibility of raising these children is not easy and provision of food and other basic needs is a daily predicament in Soweto slums and most parts of Uganda at large. Purpose of study: This paper will discuss the risks that children encounter in their daily struggle and that of their families to provide food in Soweto slums in Kampala Uganda. Methods: Direct observation of the daily activities revolving around the children in Soweto slums were observed for fourteen consecutive days of the week. Cluster and simple random sampling methods were used to select 40 willing participants. Questionnaires were administered to adults and children. Study design: cross-sectional descriptive study. Results: Observation results showed that major activities in Soweto were driven by the dire need to provide at least a meal at the end of each day. In the daily struggle to provide food children were directly exposed to hunger, diseases, injuries, child labour, neglect, drug abuse, sexual abuse and child sacrifice. The residents used water from tunnels and Nakivubo channel for daily domestic use. The interview showed that hunger, injuries and diseases were the greatest risks. Neglect and child labour was the second greatest risk followed by drug abuse, sexual abuse and child sacrifice. Conclusion: Majority of children

are at a high risk of contracting diseases, injuries, child labour, neglect, drug abuse, sexual abuse, and child sacrifice. These risks are as a result of the struggle to earn daily living. Recommendations: Collaborative approach in provision of safe water, food and community safe playgrounds. Strengthen Advocacy for the rights of children, draw attention to the possibility of a safe childhood in Soweto slums
SN - 1018-8827
AD - (Muramizi, Okoth) Mulago Nursing School, Nursing, Uganda
ER -

TY - JOUR
ID - 3598
T1 - Dog-assisted activities in Finland
A1 - Haapasaari,M.
A1 - Ikaheimo,K.
Y1 - 2011///
N1 - 70489240
English
Journal: Conference Abstract
KW - EMBASE
KW - Child
KW - dog
KW - Human
KW - Finland
KW - Society
KW - Child Psychiatry
KW - Aged
KW - Adolescent
KW - Mental Health
KW - disabled person
KW - patient
KW - psychiatric department
KW - pet animal
KW - mental deficiency
KW - Day Care
KW - Pleasure
KW - puppy
KW - Child Welfare
KW - breed
KW - behavior disorder
KW - social adaptation
KW - Self Esteem
KW - Aggression
KW - Hostility
KW - love
RP - NOT IN FILE
SP - S29
JF - European Child and Adolescent Psychiatry
VL - Conference: 14th International Congress of ESCAP European Society for Child and Adolescent Psychiatry Helsinki Finland. Conference Start: 20110611 Conference End: 20110615. Conference Publication:
IS - var.pagings
N2 - Hali-Berni started in Finland in 1999. Who can be Hali-Berni dog? It does not depend on the breed but of the character of the dog. Dogs are trained pet dogs. The most important feature is that the dog likes people, comes actively to strangers, loves to be scratched and enjoys proximity. Dogs visit homes for elderly, mentally retarded, disabled people, mental health patients, children's homes, daycare centers. They give joy and pleasure just by their presence. They accept you as you are. Touching is very important. Hali-Berni holder must be committed to do this voluntary work, willing to give joy and good experiences to elderly, children and disabled and willing to share their own dog. Visits can be made from once a month to once a week. Amount of hugging portions (=one visit of a dog and a holder) has grown very rapidly from 72 in 2007 to 883 in 2010 in Turku and

to 1250 in Finland. Visits are free of charge. A dog and its holder are tested. The new holder must make some visits first without the dog and then with a dog with experienced Hali-Berni. Puppies are also accepted and they are welcome everywhere. In Finland Hali-Berni has taken part in many studies. In Turku area: how dogs can effectively be used in child welfare (Kallio and Rintamaa 2008). Newest is Luetaan koiralle-study. In Helsinki area there have been studies how they affect mental health patients (Tuunanen 2008) and elderly people (Lang in print). In Tampere area Hali-Berni dogs visit adolescent psychiatric department with very severe behavior disturbances (Reini in print). Studies have shown the same type of influence as other studies: positive results were related to behavior and affect, social skills, self-esteem, control and decreases in aggression and hostility especially in children and adolescents (Fine 2000; Kruger and Trachtenberg; Serpell 2004). You all are welcome to meet and feel our Haliberni dogs outside Finlandia Hall. They are waiting for you
SN - 1018-8827
AD - (Haapasaari) Hali-Bernit, Finland (Ikaheimo) Child Psychiatry, Turku, Finland
ER -

TY - JOUR
ID - 3599
T1 - The art of healing in health care-transforming lives through art
A1 - Devlin,B.
Y1 - 2011///
N1 - 70481792
English
Journal: Conference Abstract
KW - EMBASE
KW - Health Care
KW - healing
KW - brain
KW - neoplasm
KW - Human
KW - University
KW - Creativity
KW - Health
KW - non profit organization
KW - skill
KW - emotion
KW - Interpersonal Communication
KW - Photography
KW - United Kingdom
KW - general aspects of disease
KW - case study
KW - palliative therapy
KW - Consciousness
KW - ego development
KW - cognitive defect
KW - dysphasia
KW - Child
KW - Problem Solving
KW - neurobiology
KW - college
KW - brain function
KW - Self Concept
KW - Pleasure
KW - Language
KW - painting
KW - Quality of Life
KW - Philosophy
KW - patient
KW - Grief

KW - Death
KW - odor
KW - Environment
KW - Safety
KW - Health Promotion
KW - Teaching
KW - Ireland
KW - therapy effect
KW - Psychotherapy
KW - Art Therapy
KW - Society
RP - NOT IN FILE
SP - S206
EP - S207
JF - Radiotherapy and Oncology
VL - Conference: ESTRO Anniversary - GEC-ESTRO - EIOF - 11th Biennial London United Kingdom. Conference Start: 20110508 Conference End: 20110512. Conference Publication:
IS - var.pagings
N2 - Art work has been shown to be beneficial in health care.It can be used as a channel for communication. Art activity can bring to consciousness that which is unconscious. It is particularly helpful where individuals are unable to express feelings, due to cognitive impairment, dysphasia or because the person does not have the vocabulary to express their feelings. It addresses the existential dimension, in that it can also help in the search for meaning. This is especially important when one is faced with a health issue or life threatening condition. Art can improve one's sense of self-worth as creativity is enhanced. Examples of art work produced by those who have faced illness and its consequences clearly demonstrate some of the feelings and emotions they experienced.Art is very important in helping the brain reach its full potential.It introduces the brain to diverse cognitive skills that help us unravel intricate problems. It activates the creative part of our brain - the part that works without words and can only express itself non-verbally; the intuitive side of the brain. This is why art so important. It benefits the brain by training it to think outside the box. It helps children understand concepts more easily. It helps them to be greater achievers. In practice development of the artistic side of the brain helps in problem solving. It guides individuals to cerate solutions.It helps individuals to think beyond linear and logical thinking (Eckert accessed online 16-10-2010).Semir Zeki, a former professor of neurobiology at the University College, London detailed the relationship between the development of cognitive abilities and the creative process. He asserted that artistic expression is the key to comprehending ourselves. He also considered art and its expression as an expansion of brain function. In other words, art helps the brain in its search for knowledge.So what are the benefits of art on the brain? When individuals create art and reflect on it, the processes, increase self-awareness, initiate awareness of others and helps individuals cope with stressful, and traumatic experiences. Art enhances cognitive abilities and provides individuals with the ability to enjoy the life-affirming pleasures of making art (Zeki, 2001).In relation to cancer Fallowfield (2003) asserts that it is necessary to use modes of expression other than language to capture what cancer really means to those affected by itThe power of the arts as a channel for communication has been clearly demonstrated through Michele Angelo Petrone's (a professional artist) paintings of his cancer journey (Fallowfield, 2003;Richards, 2003).Health care must be holistic with emphasis on giving attention to the physical, social, emotional and spiritual domains of the individual. Art work helps address all domains. The emphasis on maintaining the quality of life for individuals in our current health care philosophy and mission statements is partly met through art work intervention.Qualitative findings through case studies of patients involved in art work demonstrate that clients regained a sense of control in their lives, after being given the opportunity to explore personal issues and emotions (Pratt, 1998).Art work may be completed alone or in groups. Completed alone it allows time for reflection; in groups it can be therapeutic for those with similar problems; for example a group who are experiencing grief through death or the thought of a loved one dying; or the losses individuals experience when faced with life threatening illness. Bonding can develop with individual group members helping, not only themselves but each other too as they identify and externalise common feelings.Facilitators of art work must be aware of the potential power of the art medium; just as any image, sound or smell can evoke a powerful reaction.It is important to highlight that it is the process of the art work that is important, not the artistic skill of the individual (Dalley, 1981). The environment must be psychologically safe. "Found images" may be used as a "safety net" that is, an image on a postcard or photograph which evokes some feeling for the individual. This is particularly helpful if art work seems threatening initially.It is important that a creative space is made in order to

assist with the process of the art work, that is a space that is physically comfortable and aesthetically appealing with the appropriate lighting.Breige Devlin MSc Health Promotion University of Ulster; Advanced Diploma in Teaching Studies University of Ulster RN N. Ireland SAA (The Society For All Artists) Member UKDalley, T(19981)Assessing the therapeutic effects of art: an illustrated case study - Art Psychotherapy 7 (1) 11-17Eckert, G http://www.self-help-healing-arts-journal.com/art-benefits- brain.html accessed 16-12-2010Fallowfield, L (2003) Forward. In: Petrone MA, ed. The Emotional Cancer Journey. MAP Foundation, Hove Pratt,M; Wood, M (1998)eds. Art Therapy in palliative care The creative response Routledge London Richards, M (2003) Preface. In: Petrone, MA, ed. The Emotional Cancer Journey. MAP Foundation, HoveZeki, S (2011) Artistic Creativity and the Brain - Science Magazine (293) 5527 51-52Photograph a helping hand image- sourced from :http://www.dreamstime.com/ "A Helping Hand"
SN - 0167-8140
AD - (Devlin) Marie Curie Cancer Care, Belfast, United Kingdom
ER -

TY - JOUR
ID - 3600
T1 - Healing journeys: Wellness retreats for aboriginal women living with HIV
A1 - Migwans,S.
A1 - Medjuck,M.
Y1 - 2011///
N1 - 70476032
English
Journal: Conference Abstract
KW - EMBASE
KW - Female
KW - Human
KW - Aborigine
KW - Human immunodeficiency virus
KW - wellbeing
KW - healing
KW - Health
KW - Canada
KW - traffic and transport
KW - Social Support
KW - health service
KW - Coping Behavior
KW - skill
KW - Child
KW - workshop
KW - catalyst
KW - gender
KW - Health Care
KW - Peer Group
KW - Quality of Life
KW - Human immunodeficiency virus infection
KW - school
KW - Poverty
KW - city
KW - genocide
KW - stereotypy
KW - Violence
KW - Fear
KW - Policy
KW - Social Isolation
KW - acquired immune deficiency syndrome
RP - NOT IN FILE
SP - 93B

JF - Canadian Journal of Infectious Diseases and Medical Microbiology
VL - Conference: 20th Annual Canadian Conference on HIV/AIDS Research: Honouring our History, Embracing our Diversity, CAHR 2011 Toronto, ON Canada. Conference Start: 20110414 Conference End: 20110417. Conference Publication:
IS - var.pagings
N2 - ISSUE: The staggering rates of HIV infection among Canadian Aboriginal women are connected to the legacy of colonization and the enduring effects of the residential school system, entrenched poverty and cultural genocide. Aboriginal women who are living with HIV (AWLWH) often contend with sexual stigma and stereotypes, dispossession of their rights, multiple family roles, gendered violence, unequal economic power, fear of rejection when seeking services, an unsympathetic medical system and invisibility in HIV/AIDS research and policy. Consequently, AWLWH frequently experience social isolation, poor health, and barriers to accessing HIV support services. DESCRIPTION: Since 1993, Positive Women's Network (PWN), a women-exclusive AIDS Service Organization in British Columbia, has organized weekend wellness retreats, allowing women living with HIV to come together safely. Given the number of Aboriginal women who are living with HIV and make up PWN membership, PWN has held an Aboriginal Women's Wellness Retreat every other year since 2004. Applicants who identify as having Aboriginal ancestry are prioritized. All costs, including transportation, are covered by PWN. Through workshops and activities facilitated by Aboriginal elders and diverse staff, there are opportunities to develop social support networks, discover personal strengths, health practices and coping skills, learn about HIV, and engage with Aboriginal history and culture. LESSONS LEARNED: To date, PWN has organized 4 Aboriginal Women's Wellness Retreats, 1 retreat included participants' children. On average, 16 women attend each retreat, 40% have not attended a retreat before, 35% are from outside city centre, and 95% have Aboriginal ancestry. During the evaluation phase, participants continually report the retreat serves as a catalyst to begin, maintain or enhance their healing journey. NExT STEPS: Our results indicate a retreat program focused on culturally sensitive gender specific healthcare strategies, traditional practices and peer support significantly improves quality of life for AWLWH
SN - 1712-9532
AD - (Migwans, Medjuck) VancouverBCCanada
ER -

TY - JOUR
ID - 3601
T1 - Medication adherence and caregiver's health locus of control in sickle cell disease
A1 - Viswanathan,K.
A1 - Swaminathan,N.
Y1 - 2011///
N1 - 70468306
English
Journal: Conference Abstract
KW - EMBASE
KW - sickle cell anemia
KW - oncology
KW - caregiver
KW - locus of control
KW - Health
KW - Patient Compliance
KW - hematology
KW - Society
KW - patient
KW - Child
KW - Multidimensional Health Locus of Control scale
KW - correlation coefficient
KW - health care personnel
KW - questionnaire
KW - screening
KW - Risk
KW - Health Behavior
KW - penicillin G

KW - hydroxyurea
RP - NOT IN FILE
SP - 938
JF - Pediatric Blood and Cancer
VL - Conference: 24th Annual Meeting of the American Society of Pediatric Hematology Oncology, ASPHO 2011 Baltimore, MD United States. Conference Start: 20110413 Conference End: 20110416. Conference Publication:
IS - var.pagings
N2 - Background: Poor compliance with prophylactic penicillin administration and good compliance with hydroxyurea has been reported in children with sickle cell disease. This difference could be because the effects of hydroxyurea are tangible whereas the effects of penicillin are not evident to the caregiver. The concept of "locus of control" (LOC) deals with the extent to which a person expects that the outcomes in life are under the control of one's own actions (internal LOC) in contrast to being controlled by outside factors such as chance (external LOC). The multidimensional health locus of control (MHLC) measure is a modification of this concept applicable to health behavior. A person's health locus of control has three categories: internal, chance and powerful others (outside influences such as health care professionals.) Objectives: We wanted to study the difference in the adherence rate between penicillin and hydroxyurea; and the relationship of LOC to medication adherence, to see if we can identify which caregivers are more likely to be non-adherent and thus might need more intervention. Design/Method: The Multidimensional Health Locus of Control Scale (MHLC), an 18-item questionnaire, a self-reported 8-item scale for medication adherence developed by Morisky, were administered to caregivers of patients. Results: 30 caregivers of children taking penicillin and 15 caregivers of children taking hydroxyurea. 1. Medication adherence was high in 45% of patients taking penicillin and 46% of patients taking HU. 2. The Pearson correlation coefficients of penicillin adherence with LOC subscales: internal LOC: 0.024143; chance: >0.5976208; powerful others: >0.46023241. 3. The Pearson correlation coefficients of HU adherence with LOC subscales: internal LOC: 0.147447; chance: >0.7444194; powerful others: >0.2308007. Conclusion: There was no difference between adherence to penicillin and adherence to hydroxyurea in our patients. There is a very strong (inverse) correlations between chance/powerful others and adherence. MHLC could be used as a screening instrument to identify a subgroup of people who believe that neither they nor the providers play a role in the health of a person and are at greater risk for non-adherence. We can design studies to see if interventions can be developed to improve adherence in this group
SN - 1545-5009
AD - (Viswanathan, Swaminathan) Brookdale Hospital Medical Center, Brooklyn, NY, United States
ER -

TY - JOUR
ID - 3602
T1 - Educational experience of patients with acute lymphoblastic leukemia at an urban medical center
A1 - Levine,J.
A1 - Orr,C.
Y1 - 2011///
N1 - 70468280
English
Journal: Conference Abstract
KW - EMBASE
KW - patient
KW - oncology
KW - hematology
KW - Society
KW - acute lymphoblastic leukemia
KW - school
KW - Therapy
KW - student
KW - parent
KW - African American
KW - Hispanic
KW - Education
KW - ethnicity

KW - Language
KW - Child
KW - Population
KW - survivor
KW - Leukemia
KW - sibling
KW - Colombia
KW - university hospital
KW - high school
KW - Male
KW - caregiver
KW - Caucasian
KW - Hospital
KW - Social Interaction
KW - neoplasm
RP - NOT IN FILE
SP - 931
JF - Pediatric Blood and Cancer
VL - Conference: 24th Annual Meeting of the American Society of Pediatric Hematology Oncology, ASPHO 2011 Baltimore, MD United States. Conference Start: 20110413 Conference End: 20110416. Conference Publication:
IS - var.pagings
N2 - Background: Survivors of leukemia are at an educational disadvantage compared to siblings as a direct result of both medical treatment and school days missed. For patients treated at the Columbia University Medical Center, this is of great concern as many attend NYC public schools where overall four year high school graduation rates are 63%, (51% for Hispanic students and 54% for African American students). Objectives: Define the educational experiences of patients with ALL focusing on minority patients compared with white patients. Design/Method: Educational experiences were assessed using a semi-structured IRB approved survey. Eighteen patients (11 female/7 male; mean age 12.7 years, range 7- 19) and/or their caregivers were interviewed by a single researcher. Thirteen patients were on therapy, five off. Twelve patients were Hispanic, two African American, and four Caucasian. Thirteen/fourteen (93%) of minority patients attended NYC public schools; whereas three/four (75%) of white patients attended schools outside of NYC. Results: Seventeen of eighteen (94%) patients received home schooling during therapy. All white patients had home schooling established by school while nine minority patients had home schooling set up by the hospital and four by a parent or school. All cited individual attention as a positive aspect of being home schooled and lack of social interaction as a negative aspect. All white patients reported home schooling to be effective in keeping them on pace in school while one-third of minority patients did not. All white patients reported successful re-entry into school while on treatment. Four minority patients have or will have received home schooling for the entire duration of medical therapy. White parents were more likely to know about 504 plans while minority parents were more likely to be aware of Individual Education Plans. Conclusion: Minority students had less support from the school system in setting up home schooling and establishing a re-entry plan than non-minority students. It is unclear from this sample what role race, ethnicity and language played in this discrepancy. Further studies will be conducted in a larger patient sample to assess the best way to enhance the educational experience of all children with cancer in our population
SN - 1545-5009
AD - (Levine, Orr) Columbia University Medical Center, New York, NY, United States
ER -

TY - JOUR
ID - 3603
T1 - From crawling to running: A provincial/territorial review of physical activity legislation in childcare centres across Canada
A1 - Vanderloo,L.
A1 - Tucker,T.
A1 - Ismail,A.
A1 - Van Zandvoort,M.M.
Y1 - 2011///

N1 - 70446299
English
Journal: Conference Abstract
KW - EMBASE
KW - Child Care
KW - law
KW - Physical Activity
KW - Canada
KW - Obesity
KW - epidemic
KW - preschool child
KW - systematic review
KW - childhood
RP - NOT IN FILE
SP - 199
JF - Canadian Journal of Diabetes
VL - Conference: 2nd National Obesity Summit Montreal, QC Canada. Conference Start: 20110428 Conference End: 20110501. Conference Publication:
IS - var.pagings
N2 - Introduction: Preschoolers spend a substantial portion of their day in child care; therefore, these facilities are an ideal venue to encourage healthy active behaviours. It is important that provinces'/territories' child care legislation encourages physical activity (PA) opportunities. The purpose of this study was to review Canadian provincial/territorial child care legislation regarding PA participation. Specifically, this review sought to: 1) appraise each provincial/territorial child care regulation for PA requirements; 2) compare such regulations with the NASPE PA guideline; and 3) appraise these regulations concerning required PA infrastructure. Methods: A systematic review of all provincial/territorial child care legislation was performed. Each document was reviewed separately by two researchers, and the PA regulations were coded and summarized. The specific provincial/territorial PA requirements (e.g., type/frequency of activity) were compared with the NASPE guideline. Results: PA legislation for Canadian child care centres varies greatly. Eight of the 13 provinces/territories provide PA recommendations; however, none provided specific time requirements for daily PA. All provinces/territories did require access to an outdoor play space. Conclusion: All Canadian provinces/territories lack specific PA guidelines for child care centres. The development and implementation of national PA legislation for child care centres may aid in tackling the childhood obesity epidemic and assist child care staff in facilitating PA participation
SN - 1499-2671
AD - (Vanderloo) Health and Rehabilitation Sciences, University of Western Ontario, London, ON, Canada (Tucker) School of Occupational Therapy, University of Western Ontario, London, ON, Canada (Ismail) School of Health Studies, University of Western Ontario, London, ON, Canada (Van Zandvoort) Middlesex-London Health Unit, London, ON, Canada
ER -

TY - JOUR
ID - 3605
T1 - What's your gut feeling? An unusual cause of intractable diarrhea
A1 - Xue,B.
A1 - Jiang,M.
Y1 - 2011///
N1 - 70423621
English
Journal: Conference Abstract
KW - EMBASE
KW - Diarrhea
KW - Hospital
KW - Intestine
KW - celiac disease
KW - lymphocytosis
KW - intestine villus atrophy

KW - enteropathy
KW - duodenum biopsy
KW - duodenitis
KW - colonoscopy
KW - erythema
KW - sigmoid
KW - descending colon
KW - ileum
KW - Biopsy
KW - colitis
KW - dry skin
KW - wheezing
KW - laboratory
KW - Leukocyte Count
KW - erythrocyte sedimentation rate
KW - serology
KW - mucosa
KW - duodenum
KW - enteritis
KW - Paneth cell
KW - outpatient
KW - Adult
KW - disease predisposition
KW - autoimmunity
KW - Child
KW - polyendocrinopathy
KW - Prevalence
KW - epidemiological data
KW - Diagnosis
KW - chronic diarrhea
KW - malabsorption
KW - goblet cell
KW - T lymphocyte
KW - immune dysregulation
KW - Medical Staff
KW - Differential Diagnosis
KW - Female
KW - medical history
KW - hypothyroidism
KW - Hypertension
KW - hypercholesterolemia
KW - deep vein thrombosis
KW - emergency ward
KW - dehydration
KW - vomiting
KW - intestine biopsy
KW - family history
KW - international normalized ratio
KW - IPEX syndrome
KW - colon cancer
KW - mother
KW - lung embolism
KW - daughter
KW - Abdomen
KW - pelvis
KW - esophagogastroduodenoscopy
KW - Smoking

KW - hiatus hernia
KW - gastritis
KW - abdominal cramp
KW - autoantibody
KW - azathioprine
KW - thyrotropin
KW - C reactive protein
KW - prednisone
KW - Antibody
KW - immunosuppressive agent
KW - cyclosporin
KW - mycophenolic acid
KW - infliximab
KW - immunoglobulin
KW - cyclophosphamide
KW - cell antibody
KW - warfarin
RP - NOT IN FILE
SP - S271
EP - S272
JF - Journal of Hospital Medicine
VL - Conference: Hospital Medicine 2011, HM 2011 Grapevine, TX United States. Conference Start: 20110510 Conference End: 20110513. Conference Publication:
IS - var.pagings
N2 - Case Presentation: A 63-year-old white woman with a medical history of hypothyroidism, hypertension, hypercholesterolemia, and recent deep vein thrombosis on Coumadin presented to the emergency department with dehydration because of intractable diarrhea and intermittent emesis. She had a 6-month history of daily greenish watery diarrhea with abdominal cramps. She also lost about 100 pounds. Her family history was remarkable for colon cancer in her mother and fatal pulmonary embolism in her daughter. She had a 30-pack-year smoking history. Her prior workup at an outside hospital included a CT of the abdomen and pelvis, which showed no definite inflammatory changes. Her esophagogastroduodenoscopy (EGD) showed a small hiatal hernia and gastritis. Her duodenal biopsy showed duodenitis with villous atrophy. She also underwent a colonoscopy, which showed some erythema in the sigmoid and descending colon with a normal terminal ileum. Random biopsies throughout the colon were negative for active chronic colitis. Physical exam revealed very dry skin with decreased turgor, occasional expiratory wheezes, and mild left lower quadrant tenderness. Laboratory was significant for white blood cell count of 15, INR of 1.8, normal thyroid-stimulating hormone, erythrocyte sedimentation rate, and C-reactive protein, negative stool infectious workup, and negative celiac disease serologies. Her repeated EGD showed diffuse mildly scalloped mucosa in the entire duodenum, which was suspicious of celiac disease. Yet duodenum biopsy revealed intense active chronic enteritis with loss of Goblet and Paneth cells, which was suggestive of autoimmune enteropathy (AE). Her symptoms improved significantly after prednisone was started. Subsequently she was transitioned to azathioprine as an outpatient. Discussion: AE is a rare cause of intractable diarrhea in adults associated with circulating gut autoantibodies and a predisposition to autoimmunity. In children it can be a part of IPEX (immunodysregulation, polyendocrinopathy, enteropathy, and X-linked) and APS-1 (autoimmune polyglandular syndrome 1). The prevalence and incidence is unknown because of limited epidemiological data. The diagnosis of this disorder is based on proposed Mayo criteria: chronic diarrhea (>6 weeks), malabsorption, exclusion of similar disorders, the presence of specific antibodies such as antienterocyte and anti-Goblet cell antibodies, and specific histological findings including partial/complete villous atrophy, increased crypt apoptotic bodies, deep crypt lymphocytosis, and minimal intraepithelial lymphocytosis. It is caused by dysregulation of CD4/ CD25 T cells, whereas the presence of antienterocyte autoantibodies seems to be secondary. Immunosuppressive agents such as corticosteroids, azathioprine, cyclosporine, tacrolimius, and mycophenolate have been used with apparent benefits. In refractory cases, infliximab, intravenous immunoglobulin, and cyclophosphamide can be used. Conclusions: Hospitalists should consider AE in the differential diagnosis of chronic intractable diarrhea. Because of its many similarities with celiac disease, small-bowel biopsy is a cornerstone of investigation
SN - 1553-5592
AD - (Xue, Jiang) University of Michigan, Ann Arbor, MI, United States
ER -

TY - JOUR
ID - 3606
T1 - Tap water enema and cryptosporidium infection in acute HIV infection
A1 - Francis,G.
A1 - Perlman,D.
A1 - Rizk,D.
Y1 - 2011///
N1 - 70423468
English
Journal: Conference Abstract
KW - EMBASE
KW - Human immunodeficiency virus
KW - cryptosporidiosis
KW - Hospital
KW - Human immunodeficiency virus infection
KW - patient
KW - Diarrhea
KW - general aspects of disease
KW - immunocompromised patient
KW - oocyte
KW - Cryptosporidium
KW - feces culture
KW - ingestion
KW - AIDS patient
KW - cell count
KW - Mortality
KW - risk factor
KW - Male
KW - syphilis
KW - emergency ward
KW - fever
KW - nausea
KW - vomiting
KW - abdominal pain
KW - Travel
KW - laboratory
KW - leukopenia
KW - hyponatremia
KW - Kidney Failure
KW - acquired immune deficiency syndrome
KW - Clostridium difficile
KW - assay
KW - parasite
KW - stain
KW - anus intercourse
KW - Polymerase Chain Reaction
KW - follow up
KW - Child
KW - Water Supply
KW - swallowing
KW - chlorination
KW - Therapy
KW - African American
KW - tap water
KW - enema
KW - Water

KW - CD4 antigen
KW - ciprofloxacin
KW - metronidazole
KW - RNA
KW - Human immunodeficiency virus antibody
KW - RNA polymerase
KW - well water
KW - nitazoxanide
RP - NOT IN FILE
SP - S180
EP - S181
JF - Journal of Hospital Medicine
VL - Conference: Hospital Medicine 2011, HM 2011 Grapevine, TX United States. Conference Start: 20110510 Conference End: 20110513. Conference Publication:
IS - var.pagings
N2 - Case Presentation: A 20-year-old African American man with a history of MSM (having sex with men) and previously treated for syphilis was admitted with 3 weeks of watery nonbloody diarrhea. Two weeks after symptoms started, the patient went to an outside hospital emergency room. At that time the patient was empirically started on ciprofloxacin and metronidazole and discharged home without stool cultures. The patient's symptoms did not improve, and subsequently he came to our institution. At that time the patient denied fever, nausea, vomiting, or abdominal pain. The patient also denied any international travel, water park activities, or consumption of well water. The patient's current partner was HIV+. Physical exam was unremarkable. Patient's laboratory analysis was significant for leukopenia (3.4 x 10³/muL), hyponatremia (131 mmol/ L), and renal insufficiency (1.62 mg/dL). Stool studies including stool cultures and Clostridium difficile assays were negative. Stool for ova and parasites revealed Cryptosporidium by Ziehl-Neelsen stain. The patient reported that he and his partner routinely use tap water enemas prior to unprotected anal intercourse. HIV antibody was negative. The HIV RNA polymerase chain reaction was >500,000 copies/mL, and CD4 count was 399 cells/mL. The patient was prescribed nitazoxanide 1 g twice daily for a total of 14 days. Patient's diarrheal symptoms improved from 20 episodes down to 3-4 episodes of diarrhea per day at the time of discharge. Patient was sent home with plans for follow-up in the hospital's HIV clinic. Discussion: Cryptosporidium infection is responsible for causing persistent watery diarrhea in children as well as in immunocompromised patients, including HIV+ patients, but also causes self-limited disease in healthy exposed persons. Patients are usually infected via ingestion of oocytes from a contaminated water source. Common sources of Cryptosporidium ingestion are usually attributed to unpurified water or from swallowing recreational water despite chlorination. The incidence of Cryptosporidium infection has fluctuated over the years because of various outbreaks; however, it generally presents at an average rate of 2-3 cases per 100,000 per year. These rates may be underreported, as the diarrhea is generally self-limited in immunocompetent individuals. The introduction of highly active retroviral therapy has seen a decrease in HIV-associated cryptosporidiosis. It should be noted that cryptosporidial disease in AIDS patients with low CD4 cell counts has a high mortality. The literature of a possible association between tap water enema and Cryptosporidium infection is limited. Conclusions: Providers should remember to evaluate stool for cryptosporidia in acute diarrheal illness, especially with known risk factors and in immunocompromised patients. Cryptosporidium infection may be associated with the use of tap water enema and may be the initial presenting illness in acute HIV infection
SN - 1553-5592
AD - (Francis, Perlman, Rizk) Beth Israel Medical Center, New York, NY, United States
ER -

TY - JOUR
ID - 3607
T1 - Auditory brainstem implant in four children with cochlear nerve aplasia
A1 - Pallares,N.
A1 - Diamante,V.
Y1 - 2011///
N1 - 70421730
English
Journal: Conference Abstract
KW - EMBASE

KW - implantation
KW - aplasia
KW - Child
KW - cochlear nerve
KW - auditory brain stem implant
KW - electrode
KW - patient
KW - Speech
KW - parent
KW - Speech Perception
KW - stimulation
KW - cochlear nucleus
KW - sigmoid
KW - postoperative complication
KW - sensation
RP - NOT IN FILE
SP - 55
JF - International Journal of Pediatric Otorhinolaryngology
JA - Int J Pediatr Otorhinolaryngol
VL - Conference: 10th European Symposium on Paediatric Cochlear Implantation Athens Greece. Conference Start: 20110512 Conference End: 20110515. Conference Publication:
IS - var.pagings
N2 - Aim: To verify the possibility of auditory habilitation and to present audiologial outcomes in four children with aplasia of the cochlear nerve, by direct stimulation of the cochlear nuclei (CN) using an Auditory Brainstem Implant (ABI). Material and Methods: Four children with cochlear nerve aplasia underwent retro sigmoid approach for placement of the ABI into the lateral recess. The correct positioning of the electrodes was monitored through the EABR. Results: No surgical or postoperative complications were observed. Auditory sensations were produced with the activation of the ABI. Patients have achieved speech detection and environmental sound awareness. They are showing auditory improvement in the MAIS Scale through the parents' opinion. All patients use the ABI in a permanent way. Active electrodes, audiological outcomes, free field and speech perception tests, will be reported separately. Conclusions: This study suggests that ABI in children with cochlear nerve aplasia is feasible, with variable results
SN - 0165-5876
AD - (Pallares, Diamante) Instituto Superior de ORL, Centro de Implantes Cocleares, Universidad del Salvador, Buenos Aires, Argentina
ER -

TY - JOUR
ID - 3608
T1 - Cochlear implantation in cases with congenital deformations and rare genetic syndromes: Goldenhar and Branchio-Oculo-Facial
A1 - Porowski,M.
A1 - Skarzynski,H.
A1 - Mrowka,M.
A1 - Mlotkowska-Klimek,P.
Y1 - 2011///
N1 - 70421525
English
Journal: Conference Abstract
KW - EMBASE
KW - implantation
KW - genetic disorder
KW - Hearing
KW - patient
KW - Child
KW - surgery
KW - Hospital

KW - Goldenhar syndrome
KW - cochlea prosthesis
KW - postoperative complication
KW - congenital malformation
KW - Society
KW - device
RP - NOT IN FILE
SP - 17
JF - International Journal of Pediatric Otorhinolaryngology
JA - Int J Pediatr Otorhinolaryngol
VL - Conference: 10th European Symposium on Paediatric Cochlear Implantation Athens Greece. Conference Start: 20110512 Conference End: 20110515. Conference Publication:
IS - var.pagings
N2 - Aim: The aim of the study was to specify the indications for cochlear implantation in patients with rare genetic syndromes: Goldenhar and BOF and assess the results obtained after surgical treatment. Material and Methods: Two children with Goldenhar syndrome and one with BOF syndrome were operated on in our Clinic. Various cochlear implant systems were used to each of these patients. The technique of cochlear implantation was in all cases facial recess. Results: In all cases we achieved satisfactory hearing level. No early or late surgical complications were observed. Conclusions: We point that even in cases with very complicated anatomy and congenital malformations there is possibility to improve hearing using implantable devices. It is of crucial importance to focus on hearing improvement to maintain appropriate hearing level and to give chance for those children to be active society members
SN - 0165-5876
AD - (Porowski, Skarzynski, Mrowka, Mlotkowska-Klimek) International Center of Hearing and Speech, Institute of Physiology and Pathology of Hearing, Warsaw, Poland
ER -

TY - JOUR
ID - 3609
T1 - How big should your PICU be?
A1 - Stickley,J.
A1 - Reynolds,F.
A1 - Pearson,G.
Y1 - 2011///
N1 - 70418483
English
Journal: Conference Abstract
KW - EMBASE
KW - intensive care
KW - Length of Stay
KW - catchment
KW - Monte Carlo Method
KW - Poisson Distribution
KW - patient
KW - computer program
KW - Hospital
KW - winter
KW - Child
KW - model
RP - NOT IN FILE
SP - A28
JF - Pediatric Critical Care Medicine
JA - Pediatr Crit Care Med
VL - Conference: 6th World Congress on Pediatric Critical Care: One World Sharing Knowledge Sydney, NSW Australia. Conference Start: 20110313 Conference End: 20110317. Conference Publication:
IS - var.pagings

N2 - Objectives: Our 20 bedded PICU attempts to provide supra-regional services as well as intensive care for the majority of a local catchment (1.3 million children). We sought to model demand because of high refusal rates. Methods: A conventional method based upon local catchment, using average length of stay and incidence and assuming a Poisson distribution, was used to find the number of beds required to cover 95% of the time. This was compared to the Monte Carlo method justified because: - supra regional referrals come from outside the catchment area - length of stay is a right skew distribution - refused admissions (exclusively referrals from other hospitals) would stay longer. Our data also shows marked increase in length of stay during winter. Using activity (admissions) and the refused admissions, we created 1,000 randomly sampled scenarios (with replacement) from 2 years data. Custom software established how many beds were occupied each minute. The maximum number of beds per calendar day under the worst scenario was compared to the percentage of time where this number of beds would accommodate all patients (i.e. percentage compliance). Results: Poisson suggested 27 beds covers 95% of the time. Monte Carlo showed 95% compliance with 35 beds. Conclusion: Methods using average length of stay for refused admissions underestimate required capacity compared to the Monte Carlo method. No additional capacity (required to meet the year on year increase in demand) was included
SN - 1529-7535
AD - (Stickley, Reynolds, Pearson) Birmingham Children's Hospital, United Kingdom
ER -

TY - JOUR
ID - 3610
T1 - Impact of a new combined neonatal and paediatric transport service on the yorkshire and humber region
A1 - Rajah,F.
A1 - Hancock,S.W.
A1 - Ganguly,S.
A1 - Harrison,C.
A1 - Harness,C.
A1 - Murch,L.
A1 - Perring,J.
Y1 - 2011///
N1 - 70418430
English
Journal: Conference Abstract
KW - EMBASE
KW - intensive care
KW - Child
KW - teaching hospital
KW - non profit organization
KW - data base
KW - Health
KW - Population
KW - pediatric hospital
KW - Hospital
KW - Pediatrics
KW - hospital personnel
KW - patient
RP - NOT IN FILE
SP - A16
JF - Pediatric Critical Care Medicine
JA - Pediatr Crit Care Med
VL - Conference: 6th World Congress on Pediatric Critical Care: One World Sharing Knowledge Sydney, NSW Australia. Conference Start: 20110313 Conference End: 20110317. Conference Publication:
IS - var.pagings
N2 - Aim: To examine the activity and impact of a new combined acute neonatal and paediatric transfer service, Embrace, on the Yorkshire and Humber region and to describe the evolution of a new service. Background: Embrace is the result of a 3-year effort between Sheffield Children's Hospital, Sheffield Teaching Hospitals NHS Foundation Trust, Leeds Teaching Hospital Trust and regional commissioners of children's

services to provide a combined neonatal and paediatric transport service, aid in finding delivery suite and neonatal cots for in utero transfers and act as bed bureau for children's critical care. Methods: Retrospective analysis of transport database from Dec 2009-June 2010 to determine service activity, levels of care required, resources use within and from outside the service. Results and Discussion: Yorkshire and Humber is the largest of nine English regions, population 5.1 million, covering 15,413 sq miles. Embrace supports 23 hospitals within the Strategic Health Authority (SHA). Embrace started in a phased manner with paediatrics retrievals going online first followed by neonatal retrievals in April 2010. Table 1 shows the activity breakdown since Embrace's start. (Table presented) 2000 retrievals are predicted for the first full year of operation. As the service is now fully staffed and operates 24 hours a day, there is a decreasing reliance on assistance from outside services except in the case of time critical transfers. Transport teams of the tertiary NICUs and PICUs within the Yorkshire and Humber SHA no longer formally exist thereby allowing hospital staff of these units to concentrate on in-patients and not be called away for retrievals
SN - 1529-7535
AD - (Rajah, Hancock, Ganguly, Harrison, Harness) Embrace Yorkshire and Humber Infant and Children's Transport Service Barnsley, United Kingdom (Murch, Perring) Sheffield Children's NHS Foundation Trust, Sheffield, United Kingdom
ER -

TY - JOUR
ID - 3611
T1 - One profession - Two worlds of employment - One aim
A1 - Gruber,I.
Y1 - 2011///
N1 - 70408496
English
Journal: Conference Abstract
KW - EMBASE
KW - Transplantation
KW - bone marrow
KW - occupation
KW - Employment
KW - Blood
KW - Child
KW - Rehabilitation
KW - nurse
KW - patient
KW - accident
KW - physician
KW - Hospital
KW - Motivation
KW - general aspects of disease
KW - teamwork
KW - Peer Group
KW - Aftercare
KW - Adolescent
KW - cancer therapy
KW - stem cell transplantation
KW - Hospitalization
KW - neoplasm
KW - groups by age
KW - psychologist
KW - psychotherapist
KW - leisure
KW - Therapy
KW - nursing
KW - pediatric hospital
KW - growth hormone

RP - NOT IN FILE
SP - S448
JF - Bone Marrow Transplantation
VL - Conference: European Group for Blood and Marrow Transplantation, EBMT 2011 Paris France. Conference Start: 20110403 Conference End: 20110406. Conference Publication:
IS - var.pagings
N2 - My tasks and experiences as a paediatric nurse caring for a rehabilitation camp for children and teenagers after cancer treatment Since 25 years I am qualified as paediatric nurse at the St. Anna Children Hospital stem cell transplantation-unit. An exciting but challenging profession. Everyone who works in it, has announced that after a hospital stay-of the daily life of patients is still far away from commonplace. Allthough we often expect that children return quickly to their normal life after successful treatment, the way back is quite stony for the whole familie. They are fare beyond their expectations and the burden of late effects is still present. The "national organization for children's cancer care" aims to assist patients and their families with reintegration. The "rehabilitation camps" play a centrale role and offer tailord programs for the needs of various age groups. The professional team of educators, psychologists, psychotherapist, and a paediatric nurse offer the children a platform of a well-prepared 10-day-game history to exchange experiences and disease specific. For this purpose I was trained as a leisure educator for one year to accompany these camps. My duties at the camp are medical treatment, supervizing and supporting the children with nursing measures. (e.c. subcutane application of growth hormones) Minor "accidents" are first aided from me, and I indicate a doctor's visit. The work outside the hospital is much joy and motivation for me. It's nice to see how they make their own progressions during the post-care-camp after a long and serious illness by making experiences to handle a change in living conditions. We trie to show them posibilities of ways back from the daily routine to self-determination. One of the majorgoals is not only but also teamwork within the peer group handling the daily routine. I want to point of the importance and effectiveness of aftercare camps, and aware of all concerned groups of professionals. For me, working in a rehabilitation camp is a very satisfactory enrichment and it influenced my personal attention in the stationary everyday anew
SN - 0268-3369
AD - (Gruber) St. Anna Children Hospital, Vienna, Austria
ER -

TY - JOUR
ID - 3612
T1 - KEEPING children connected-bringing the classroom into the bone marrow transplant room
A1 - Drew,D.
A1 - Punch,D.
A1 - Ellis,S.
A1 - Wakefield,C.E.
Y1 - 2011///
N1 - 70408375
English
Journal: Conference Abstract
KW - EMBASE
KW - Child
KW - Transplantation
KW - bone marrow
KW - Blood
KW - bone marrow transplantation
KW - patient
KW - school
KW - teacher
KW - parent
KW - Hospital
KW - Technology
KW - neoplasm
KW - videoconferencing
KW - friend
KW - pediatric hospital

KW - Adolescent
KW - student
RP - NOT IN FILE
SP - S401
JF - Bone Marrow Transplantation
VL - Conference: European Group for Blood and Marrow Transplantation, EBMT 2011 Paris France. Conference Start: 20110403 Conference End: 20110406. Conference Publication:
IS - var.pagings
N2 - Children and adolescents with cancer may be hospitalised for long periods of time, leading to isolation from their family, home, school and peers. The protracted nature of some treatments can impact negatively on educational outcomes, including progress in the classroom, socialisation and acceptance by peers. Treatments such as Bone Marrow Transplant (BMT) require the child to be isolated from weeks to months, thus imposing another layer of separation from the outside world. It is more important than ever, with available technology, that patients remain connected with their school for both school work and socialisation, yet there is a dearth of literature evaluating the impact of the use of interactive technology in this context. As part of a pilot program at Sydney Children's Hospital, a small cohort of patients "dialled in for lessons" at their home school and remained in contact with their school friends using Skype.The hospital school teacher was able to be present during the sessions, using either Skype or a video conferencing facility at the hospital school called 'Connected Classroom'. The aim of the program was to assist students to overcome the negative impact of prolonged absence and to assist their re-integration into the classroom post BMT. Teachers and parents both evaluated the program positively. Patients were able to join in lesson activities, teachers were able to successfully integrate the patient into the classroom from their bed and parents viewed the program as facilitating increased normality for their child. Classmates were able to see the physical changes in the patient as they occurred and being able to see the patient assisted with demystifying what was happening at the hospital. After discharge from the BMT unit but while still in isolation, patients continued with the program from their home. Data from a formal retrospective evaluation by patients, parents and teachers will be presented
SN - 0268-3369
AD - (Drew, Punch, Ellis) Sydney Children's Hospital, Randwick, Australia (Wakefield) University New South Wales, Randwick, Australia
ER -

TY - JOUR
ID - 3613
T1 - Vitamin D status of Belgian young children
A1 - Sioen,I.
A1 - Moratidou,T.
A1 - Kaufman,J.-M.
A1 - Bamman,K.
A1 - Michels,N.
A1 - Vanaelst,B.
A1 - Vyncke,K.
A1 - De,Henauw S.
Y1 - 2011///
N1 - 70394018
English
Journal: Conference Abstract
KW - EMBASE
KW - Child
KW - osteoarthritis
KW - osteoporosis
KW - sampling
KW - winter
KW - university hospital
KW - serum
KW - boy
KW - girl
KW - model

KW - Body Composition
KW - blood sampling
KW - Male
KW - Radioimmunoassay
KW - height
KW - weight
KW - Population
KW - Skinfold Thickness
KW - skinfold
KW - Blood
KW - vitamin D
RP - NOT IN FILE
SP - S272
JF - Osteoporosis International
JA - Osteoporos.Int
VL - Conference: European Congress on Osteoporosis and Osteoarthritis, ECCEO11-IOF Valencia Spain. Conference Start: 20110323 Conference End: 20110326 Sponsor: ROTTAPHARM, MADAUS, SERVIER, AMGEN, MSD, et al.. Conference Publication:
IS - var.pagings
N2 - Objectives: This study aims to describe vitamin D status of Belgian children assessed by blood samples taken during winter and spring 2010 and to examine the influence of non-nutritional factors on the outcome observed. Materials/Methods: Participating children (n=357; 51.5% male, 4-11 years) were drawn from the Belgian control region (Aalter) of the EU 6th FP IDEFICS cohort. Blood was sampled between February and June 2010. 25(OH)D was measured using radioimmunoassay I125 (INCSTAR 25 (OH)D-¹²⁵ IRIA kit, Stillwater, MN, USA). Vitamin D status was categorized as deficient (25(OH)D<25 nmol/l), insufficient (25-50 nmol/l), sufficient (50-75 nmol/l) and optimal (>75 nmol/l). Height and weight were measured with standard clinical stadiometer and balance. The BMI zscore was determined using the LMS method (with British reference population). Skinfold thickness (triceps and subscapular) was measured using Holtain Tanner/Whitehouse skinfold calipers. Ethical approval was granted by the Ethical Committee of the Ghent University Hospital. Results: Serum 25(OH)D ranged from 13.6 nmol/l to 123.5 nmol/l (mean 47.2+14.6 nmol/l). Within the group, 5% was deficient, 53.1% insufficient, 39.7% sufficient and 2.2% optimal. No significant difference in vitamin D status was found between boys and girls and no significant correlation with age. However, a significant difference in 25(OH)D concentration between the months of sampling was found, as well as a significant correlation with the number of h playing outside (r=0.14) and the sum of skinfolds (r=-0.11). Two different multivariate models (ANCOVA) indicated that 68% of the variability of the 25 (OH)D concentration was explained by the month of sampling, the number of h playing outside and the BMI z-score or sum of skinfolds, respectively. Conclusions: The majority of Belgian children have a suboptimal vitamin D status, with more than half having an insufficient status in winter and spring. Month of the year, number of h playing outside as well as body composition (BMI or sum of skinfolds) were identified as important determinants of vitamin D status in this group of children
SN - 0937-941X
AD - (Sioen, Michels, Vanaelst, Vyncke, De Henauw) Department of Public Health, Ghent University, Ghent, Belgium (Sioen, Vanaelst, Vyncke) Research Foundation Flanders (FWO), Brussels, Belgium (Moratidou) GENUD Research Group, University of Zaragoza, Zaragoza, Spain (Kaufman) Department of Endocrinology, Ghent University, Ghent, Belgium (Kaufman) Unit for Osteoporosis and Metabolic Bone Diseases, Ghent University Hospital, Ghent, Belgium (Bamman) Bremen Institute for Prevention Research and Social Medicine, University of Bremen, Bremen, Germany (De Henauw) Department of Health Sciences VESALIUS, Hogeschool Ghent, Ghent, Belgium
ER -

TY - JOUR
ID - 3614
T1 - Spreading awareness for prevention of obesity and diabetes in school age children [Project 'MARG': The Path]
A1 - Shah,P.
A1 - Misra,A.
Y1 - 2011///

N1 - 70391907
English
Journal: Conference Abstract
KW - EMBASE
KW - school
KW - Child
KW - child health
KW - Obesity
KW - Diabetes Mellitus
KW - workshop
KW - Prevention
KW - Europe
KW - India
KW - school child
KW - Prevalence
KW - city
KW - Food
KW - student
KW - parent
KW - dietary intake
KW - Sedentary Lifestyle
KW - Adolescent
KW - developing country
KW - Risk
KW - Morbidity
KW - overnutrition
KW - Mortality
KW - vascular disease
KW - childhood
KW - professional standard
KW - informed consent
KW - Eating
KW - calorie
KW - Health
KW - nutritional disorder
KW - Physical Activity
KW - Cooking
KW - cereal
KW - Fruit
KW - Adult
KW - fat intake
KW - Insulin Resistance
KW - diet restriction
KW - glucose blood level
KW - volunteer
KW - non profit organization
KW - saturated fatty acid
RP - NOT IN FILE
SP - S165
JF - International Journal of Obesity
JA - Int J Obes (Lond)
VL - Conference: 1st IDEFICS Symposium and Workshop Child Health in Europe - The IDEFICS Study: Towards a Better Understanding of Obesity Zaragoza Spain. Conference Start: 20101108 Conference End: 20101109. Conference Publication:
IS - var.pagings
N2 - Background: Obesity, a natural consequence of over-nutrition and sedentary lifestyle, is increasing rapidly in children and adolescents of developing countries including India. This is leading to an increased risk of

morbidity and mortality due to various metabolic problems such as diabetes, and cardio vascular diseases. Objectives: To assess the prevalence of childhood overweight/obesity among 9-18 year old school children and to assess impact of educative intervention on them. Materials and methods: Project MARG is a school based intervention program for school children aged 9-18 years initiated in 8 cities (Agra, Allahabad, Dehradun, Delhi, Jaipur, Lucknow, Mumbai, and Pantnagar of India. Each city enrolled 10 schools with a 50-50 split in the type of schools. The Ethics Committee of the Institution, Diabetes Foundation (India), approved the study. Informed consent from the principals of the schools, and parents of the students were procured before initiating the study. Students not willing to participate were excluded from the study. Results: A total of 57 225 school children participated in the study. Prevalence of overweight/obesity was 24.1 and 7.0 percent in private and public schools, respectively across 8 cities in India. This data increased to 32.9 and 9.2 percent in private and public schools, respectively when calculated for children between 14-18 years of age (n=25 738). Dietary intake pattern among schoolchildren revealed that 1 in every 3 eats out once or more in a week, with 22-50 percent of the children eating calorie dense foods at least once in a week, coupled with spending less time in outdoor activities (68%). Baseline knowledge and behaviours indicated better scores in private as compared to public schools. Educative interventions utilising locally appropriate innovative strategies showed improvements irrespective of the schools type. The percent increase in the score levels across all topics of assessment (health, nutrition, disease, physical activity, cooking practices) varied from 3.5 to 43 and 3.4 to 34.8 percent in private and public schools, respectively. Following intervention, more percent of children from public schools now identified chips as junk food (23.8%) and more than 80% of all children had improved knowledge in regards to cereals and fruits. Knowledge in regards to type-1 diabetes improved in 30 and 23.9 percent children from both schools, respectively. Dietary intake among children and young adults revealed a high total fat and saturated fat intake with a low intake of MUFAs and -3 PUFAs, predisposing them to an increased prevalence of obesity and insulin resistance. Biochemical parameters showed improvements as in decrease in fasting blood glucose levels and hs-CRP levels post educative intervention. Sustainability is being ensured by training volunteers for the initiative. Conclusion: Reports on educative interventions directed towards children and their parents show encouraging results, but large scale country wide programs need to be initiated
SN - 0307-0565
AD - (Shah, Misra) National Diabetes Obesity and Cholesterol Diseases Foundation (N-DOC), Safdarjung Development Area, New Delhi, India (Shah, Misra) Diabetes Foundation (India), Safdarjung Development Area, New Delhi, India (Misra) Department of Diabetes and Metabolic Diseases, Fortis Hospitals, Vasant Kunj, New Delhi, India
ER -

TY - JOUR
ID - 3615
T1 - Association between adiposity and urbanisation level in children: Results of the Italian cohort of the IDEFICS project
A1 - Donatiello,E.
A1 - Lauria,F.
A1 - Nappo,A.
A1 - Sparano,S.
A1 - Formisano,A.
A1 - Dello,Russo M.
A1 - Barba,G.
A1 - Russo,P.
A1 - Siani,A.
Y1 - 2011///
N1 - 70391870
English
Journal: Conference Abstract
KW - EMBASE
KW - Obesity
KW - Child
KW - child health
KW - workshop
KW - Europe
KW - Physical Activity

KW - Analysis of Variance
KW - Lifestyle
KW - parent
KW - Prevention
KW - school child
KW - boy
KW - girl
KW - biceps brachii muscle
KW - questionnaire
KW - Population Density
KW - kindergarten
KW - primary school
KW - Multivariate Analysis
KW - sexual education
KW - urban area
KW - rural area
KW - skinfold
RP - NOT IN FILE
SP - S154
JF - International Journal of Obesity
JA - Int J Obes (Lond)
VL - Conference: 1st IDEFICS Symposium and Workshop Child Health in Europe - The IDEFICS Study: Towards a Better Understanding of Obesity Zaragoza Spain. Conference Start: 20101108 Conference End: 20101109. Conference Publication:
IS - var.pagings
N2 - Objective: To investigate the association between adiposity and urbanisation level in a sample of schoolchildren of the Italian cohort of the IDEFICS Project. Materials and methods: The sample was composed of 2256 children (boys=1167; girls=1089; age=6.1+1.7 yrs).The degree of adiposity was estimated as the sum of the four measured skinfold (SST): biceps, triceps, suprailiac and subscapular. Lifestyles were investigated by a questionnaire filled in by parents. The sample was divided in three categories of urbanisation level, estimated according to the population density in each municipality: rural n=716; suburban n=600; urban n=940.The analysis was performed separately for kindergartens (KG) and primary schools (PS). Data are mean (95% CI). Results: A statistically significant -linear- increase in SST was observed going up across urbanisation categories (KG: rural=28.5 (23.5-33.5) mm; suburban=33.3 (28.8-37.9) mm; urban=37.1 (34.1-40.1) mm; PS: rural=46.1 (41.5-50.6) mm; suburban=48.6 (45.0-52.2) mm; urban=51.5 (48.6-54.2) mm; ANOVA P<0.0001).Children who lived in rural zone reported to spend significantly more time in outdoor activities (KG: rural=165 (149-181); suburban=96 (85-108); urban= 89 (80-97) minutes/day; PS: rural=160 (149-171); suburban=98 (89-107); urban=93 (84-100) minutes/day; ANOVA P<0.001) but on the contrary they practiced less structured physical activity (% of children regularly practicing structured physical activity; KG: rural=24%; suburban=24%; urban=52%; PS: rural=21%; suburban=30%; urban=49%; P<0.001). Multivariate analysis showed that sex, education level of parents and urbanisation level were independent determinants of the degree of adiposity in KG children (P<0.001). In PS children, age and time spent in outdoor activities were other statistically significant determinants of adiposity (P<0.01). Conclusions: Our data show that children living in an urban area had a higher degree of adiposity in comparison to the peers living in suburban and rural areas. Differences in lifestyle, in particular in physical activity patterns, could explain these results and may have implications for paediatric obesity prevention
SN - 0307-0565
AD - (Donatiello, Lauria, Nappo, Sparano, Formisano, Dello Russo, Barba, Russo, Siani) Epidemiology and Population Genetics, Institute of Food Sciences, CNR, Avellino, Italy
ER -

TY - JOUR
ID - 3616
T1 - Built environment, physical activity, and obesity
A1 - De,Bourdeaudhuij,I
A1 - Van,Dyck D.
A1 - De,Meester F.

A1 - Van,Cauwenberghe E.
A1 - Deforche,B.
A1 - Cardon,G.
Y1 - 2011///
N1 - 70391857
English
Journal: Conference Abstract
KW - EMBASE
KW - Physical Activity
KW - Obesity
KW - Environment
KW - child health
KW - workshop
KW - Europe
KW - Adult
KW - Adolescent
KW - Child
KW - pilot study
KW - boy
KW - accelerometer
KW - pedometer
KW - school
KW - Self Report
KW - active transport
KW - leisure
KW - Walking
KW - girl
KW - preschool child
KW - life cycle
KW - density
KW - land use
KW - juvenile
KW - geographic information system
RP - NOT IN FILE
SP - S149
JF - International Journal of Obesity
JA - Int J Obes (Lond)
VL - Conference: 1st IDEFICS Symposium and Workshop Child Health in Europe - The IDEFICS Study: Towards a Better Understanding of Obesity Zaragoza Spain. Conference Start: 20101108 Conference End: 20101109. Conference Publication:
IS - var.pagings
N2 - Objectives: A growing body of evidence is documenting the positive relationship between the built environment and physical activity. The aim of this presentation is to report on the evidence of this relationship and its impact upon obesity in children, adolescents, and adults, and to give a critical analysis on its similarities and differences. Material and methods: Several key studies from Europe are presented. For adults, the Belgian Environmental Physical Activity Study (BEPAS) will be presented. In this study 24 neighbourhoods in Ghent were selected based on GIS-based walkability and SES; 1166 adults (aged 20-65) participated in this study. In adult research, high-walkable neighbourhoods are defined by high street connectivity, residential density and land use mix. For adolescents, a small pilot study in 2 neighbourhoods and the Youth BEPAS will be presented in which 32 neighbourhoods were used based on walkability, and 509 adolescents (aged 13-15) were included. In children, the contribution of preschool playground factors in explaining children's physical activity was analysed in 39 randomly selected preschools (415 boys and 368 girls; 5.3+0.4 years old). Physical activity levels were assessed using accelerometers (7 days) or pedometers, supplemented with self-reports. Results: A clear association was found in adults between the built environment and physical activity. Adults living in a high-walkable neighbourhood reported more active transport and more leisure time walking than adults living in a low walkable neighbourhood. Physical activity mediated the associations of neighbourhood walkability with BMI. In adolescents, the relationship was less univocal. A first pilot study showed that, in contrast with results

in adults, lower walkability and larger distance to school were associated with more physical activity. However, the second study, which was larger, did show some positive relationships between the built environment and physical activity, but no relationship with overweight. In preschool children physical activity during outdoor play was associated with some modifiable playground factors, but not with others. Conclusion: The strength and the characteristics of the relationship between the built environment and physical activity are different across the life cycle
SN - 0307-0565
AD - (De Bourdeaudhuij, Van Dyck, De Meester, Van Cauwenberghe, Deforche, Cardon) Ghent University, Department of Movement and Sport Sciences, Ghent, Belgium (Van Dyck, Van Cauwenberghe, Deforche) Fund for Scientific Research Flanders (FWO), Belgium (Deforche) Vrije Universiteit Brussel, Department of Human Biometry and Biomechanics, Brussels, Belgium
ER -

TY - JOUR
ID - 3617
T1 - Extending the scope of pediatric palliative care
A1 - Davis,K.
Y1 - 2011///
N1 - 70371881
English
Journal: Conference Abstract
KW - EMBASE
KW - palliative therapy
KW - Child
KW - school
KW - Child Care
KW - Pediatrics
KW - Diagnosis
KW - health care personnel
KW - Training
KW - Health Status
KW - clergy
KW - community
KW - model
KW - hospital patient
KW - outpatient care
KW - World Health Organization
KW - hospice care
KW - Education
KW - United States
KW - Adult
KW - terminal care
KW - healing
KW - Hospital
KW - Environment
KW - University
RP - NOT IN FILE
SP - A4
EP - A5
JF - Journal of Palliative Medicine
VL - Conference: Center to Advance Palliative Care National Seminar Building New Paradigms in Palliative Care Phoenix, AZ United States. Conference Start: 20101021 Conference End: 20101023. Conference Publication:
IS - var.pagings
N2 - Topic: Expanded inpatient and outpatient services Description: The American Academy of Pediatrics (AAP) and the World Health Organization (WHO), in their respective definitions of pediatric palliative care (PPC), suggest that services should commence at the time of diagnosis of a child with a life limiting condition.

Despite these definitions, pediatric palliative care has traditionally been reserved for children nearing the end-of-life; those who are transitioning from curative treatment to hospice care. In the Department of Pediatrics at the University of Kansas Medical Center, a unique program is working to ensure that palliative supports begin at the time of diagnosis, and continues through cure, transition to adult care, or end-of-life care. The KU Kids Healing Place provides care for the physical, psychological, social, spiritual and educational needs of children with life limiting conditions, as well as traditional palliative care for youngsters nearing the end of life. The program initiates services within the hospital, and extends them into the natural environments of the youngster. As health care providers partner with schools, child care programs, extracurricular activities, athletics, places of worship and more, a system of support is developed that enables young people, regardless of health status, to continue to grow and develop normally. School professionals, peers, child care providers, coaches, clergy and other community members receive education and training which enables them to provide 'palliative care' in every facet of the youngster's world. This poster presentation will chart the development of this innovative model of pediatric palliative care, from inception to implementation
SN - 1096-6218
AD - (Davis) Department of Pediatrics, Medical Center, University of Kansas, 3901 Rainbow Blvd., Kansas City, KS 66160, United States
ER -

TY - JOUR
ID - 3618
T1 - Impact of exercise-related respiratory symptoms on school activities in children with asthma: Results from the EIB landmark survey
A1 - Ostrom,N.
A1 - Eid,N.
A1 - Craig,T.
A1 - Colice,G.
A1 - Hayden,M.
A1 - Parsons,J.
A1 - Stoloff,S.
Y1 - 2011///
N1 - 70359025
English
Journal: Conference Abstract
KW - EMBASE
KW - Child
KW - Exercise
KW - allergic asthma
KW - Asthma
KW - school
KW - immunology
KW - Physical Activity
KW - parent
KW - Adolescent
KW - Therapy
KW - Prevention
KW - Drug Therapy
KW - coughing
KW - dyspnea
KW - wheezing
KW - sport
KW - salbutamol
RP - NOT IN FILE
SP - AB78
JF - Journal of Allergy and Clinical Immunology
VL - Conference: 2011 American Academy of Allergy, Asthma and Immunology, AAAAI Annual Meeting San Francisco, CA United States. Conference Start: 20110318 Conference End: 20110322. Conference Publication:

IS - var.pagings
N2 - RATIONALE: This study evaluated the impact of exercise-related respiratory symptoms on physical activities of children with asthma. METHODS: A national survey was conducted with parents of 516 children (4-17 years) with asthma or taking medications for asthma in the prior year. Responses assessed whether up to 6 exercise-related respiratory symptoms influenced participation in exercise and school activities. Subgroup analyses (4-12 and 13-17 years) were also conducted. RESULTS: Majority (80%) of parents with children with asthma reported that their child experienced >1 and almost half (47.4%) experienced >4 exercise-related respiratory symptoms. Most commonly reported symptoms were coughing, shortness of breath, and wheezing. Older children were more likely to avoid activities because of exercise-related respiratory symptoms compared with younger children (31.9% vs 22.9%, respectively). Respondents felt that asthma limited their children's ability to participate either "a lot" or "some" in sports/recreation (30%), normal physical exertion (20.9%), and other outdoor activities (26.3%). A higher percentage of older children were reported to be limited "a lot" in sports participation compared with younger children (12.4% vs 6.4%, respectively). Only 22.2% of children with asthma took bronchodilators such as albuterol "always" or "most of the time" before exercise. CONCLUSIONS: Exercise-related respiratory symptoms substantially limit ability of children to participate normally and perform optimally in physical activities, with adolescents compromised to a greater extent than younger children. Many children are not using short-acting bronchodilators before exercise as recommended in national guidelines. Adherence to controller therapy and prevention with short-acting bronchodilators should be optimized per current treatment guidelines
SN - 0091-6749
AD - (Ostrom) Allergy and Asthma Medical Group and Research Center, San Diego, CA, United States (Eid) University of Louisville, School of Medicine, Louisville, KY, United States (Craig) Pennsylvania State University, Hershey, PA, United States (Colice) Washington Hospital Center, Silver Spring, MD, United States (Hayden) University of Virginia, Charlottesville, VA, United States (Parsons) Ohio State University, Asthma Center, Columbus, OH, United States (Stoloff) University of Nevada, School of Medicine, Reno, NV, United States
ER -

TY - JOUR
ID - 3619
T1 - Comprehensive evaluation of a sunscreen containing only inorganic UV filters
A1 - Appa,Y.
A1 - Ouyang,H.
Y1 - 2011///
N1 - 70331832
English
Journal: Conference Abstract
KW - EMBASE
KW - filter
KW - dermatology
KW - protection
KW - baby
KW - skin
KW - eye
KW - Child
KW - Infant
KW - consumer
KW - insect sting
KW - sunburn
KW - clinical study
KW - summer
KW - breathing
KW - tissue
KW - mother
KW - pediatric hospital
KW - sunscreen
KW - titanium dioxide

KW - zinc oxide
RP - NOT IN FILE
SP - AB138
JF - Journal of the American Academy of Dermatology
JA - J Am Acad Dermatol
VL - Conference: 69th Annual Meeting of the American Academy of Dermatology New Orleans, LA United States. Conference Start: 20110204 Conference End: 20110208. Conference Publication:
IS - var.pagings
N2 - Sunscreens formulated with exclusively inorganic UV filters are ideal for baby and sensitive skin. The combinations of titanium dioxide and zinc oxide can provide excellent and balanced UVA and UVB protection with a high skin tolerance. Until recently, high protection levels of inorganic sunscreens were typically accompanied by undesirable aesthetic properties, such as skin whitening and poor skin-feel. Formulation advances have overcome these aesthetic challenges as indicated by sensory testing and consumer in use testing. A comprehensive assessment of an SPF 60 sunscreen containing only titanium dioxide and zinc oxide was conducted, including in vivo UVA (UVAPF) assessment, critical wavelength measure to assess the breath of the protection, clinical and in use studies. A double-blind, crossover clinical in infants and young children at Rady Children's Hospital was conducted to compare the efficacy and tolerability of the inorganic sunscreen with a conventional organic sunscreen. A home use study involving about 200 babies and infants evaluated the aesthetic features and acceptability of the inorganic sunscreen. Finally, a double-blinded, randomized clinical eye-sting test was performed to assess the ocular irritation potential of a sunscreen product relative to the conventional organic filter product. The SPF 60 inorganic sunscreen had a PFA value of 20.8, higher than all other inorganic sunscreens tested, and a critical wavelength above 370 nm, indicating broad-spectrum protection. The SPF 60 sunscreen showed statistically better sunburn protection on the body and comparable sunburn protection on the face when compared to the organic sunscreen of SPF 50 in the baby clinical study with no adverse events. The majority of the mothers in the home usage study found the inorganic sunscreen nongreasy, nonwhitening, and easy to apply. The eye-sting evaluation showed that the inorganic sunscreen did not irritate the delicate tissue of the eyes or induce tearing in subjects after 30 minutes of outdoor activities during a hot summer day. The SPF 60 inorganic sunscreen demonstrates high protection and consumer acceptance, and has been clinically shown to be gentle and efficacious for use on babies and young children
SN - 0190-9622
AD - (Appa, Ouyang) Neutrogena Corp, Los Angeles, CA, United States
ER -

TY - JOUR
ID - 3620
T1 - Asthma progression and airway inflammation assessed by lung function in children with asthma
A1 - Baek,H.
A1 - Oh,J.
A1 - Lee,H.
Y1 - 2009///
N1 - 70020711
English
Journal: Conference Abstract
KW - EMBASE
KW - Asthma
KW - lung function
KW - allergy
KW - Child
KW - Human
KW - respiratory tract inflammation
KW - clinical immunology
KW - childhood
KW - risk factor
KW - wheezing
KW - body mass
KW - respiratory tract allergy
KW - Therapy

KW - laboratory test
KW - eczema
KW - Exercise
KW - Spirometry
KW - patient
KW - Inhalation
KW - serum
KW - parental smoking
KW - eosinophil
KW - atopy
KW - family history
KW - infancy
KW - prick test
KW - skin
KW - diseases
KW - atopic dermatitis
KW - corticosteroid
KW - methacholine
KW - immunoglobulin E
KW - allergen
RP - NOT IN FILE
SP - 303
JF - Allergy: European Journal of Allergy and Clinical Immunology
VL - Conference: 28th Congress of the European Academy of Allergy and Clinical Immunology Abstract Book Warszawa Poland. Conference Start: 20090606 Conference End: 20090610. Conference Publication:
IS - var.pagings
N2 - Background: The social and economic impact of asthma is substantial worldwide. Although current therapies targeting both airway inflammation and airway hyperresponsiveness (AHR) effectively relieve and prevent clinical symptoms in the majority of asthmatic children, some patients experience persistent symptoms and a progressive decline in lung function from early childhood. To date, there are many unanswered questions about the role of many factors in asthma progression and persistence. Purpose: This study was focused on finding possible risk factors for persistent asthma to allow better control of the illness from infancy to late childhood. Material and method: Sixty-eight children with persistent mild-to-moderate asthma: 23 early-onset (<2 years of age) and 45 late-onset (>2 years), according to GINA Guideline. Detailed information included personal or family history of atopy, breastfeeding, parental smoking, recent use of inhaled corticosteroid, and laboratory tests such as spirometry, body mass index, skin prick test, allergen-specific IgE, PB eosinophils and serum ECP. All the asthmatics were performed bronchial challenge by methacholine inhalation (BCM) and outdoor free running (ET). Results: Risk factors such as eczema and frequent wheezing were more common in early-onset asthmatics than in the lateonset (P < 0.05). On the other hand, there were no differences in overall incidence of AHR induced either by methacholine or by exercise as well as PC20 and postexercise decrease of FEV1 (P > 0.05) in both study groups. For the appropriate management, early-onset asthmatics required more frequent use of inhaled corticosteroid than the late-onset (P < 0.0001). Author also found a reciprocal relationship between FEV1/FVC and days of asthma in the study groups of persistent asthma (n = 68, r = -0.380, P = 0.004). Conclusion: One of the important risk factors for asthma persistence and lung function decline from early to late childhood was atopic dermatitis and frequent wheezing. In persistent asthmatics with AHR, early and proper treatment with inhaled corticosteroids is required for the support of better pulmonary function
SN - 0105-4538
AD - (Baek, Oh, Lee) Hanyang Univ. School of Medicine, Medical Center, Department of Pediatrics, Seoul, South Korea
ER -

TY - JOUR
ID - 3621
T1 - Sampling of fine particles in-door and out-door in two primary schools in Milan, Italy and evaluation of children's respiratory function and inflammation
A1 - Sala,M.

A1 - Riva,E.
A1 - Mandelli,M.
A1 - Cetta,F.
A1 - Bolzacchini,E.
A1 - Ferrini,B.
A1 - Giovannini,M.
Y1 - 2009///
N1 - 70020318
English
Journal: Conference Abstract
KW - EMBASE
KW - primary school
KW - Italy
KW - Child
KW - Human
KW - respiratory function
KW - Inflammation
KW - allergy
KW - clinical immunology
KW - sampling
KW - school
KW - clinical evaluation
KW - optics
KW - lung function
KW - air quality
KW - Pollution
KW - Spirometry
KW - Health
KW - chemical composition
KW - Nitric Oxide
RP - NOT IN FILE
SP - 159
EP - 160
JF - Allergy: European Journal of Allergy and Clinical Immunology
VL - Conference: 28th Congress of the European Academy of Allergy and Clinical Immunology Abstract Book Warszawa Poland. Conference Start: 20090606 Conference End: 20090610. Conference Publication:
IS - var.pagings
N2 - Background: Detrimental effects of pollution on children pulmonary function have been described, but few data relates the characteristics of air near and into schools. Methods: We selected two primary schools in Milan, one located near a large park (school 1) and the other downtown (school 2). For each school, from September 23th to October 3rd 2008, we sampled every 5 min in- and outdoor fine particles (PM1, PM2.5 and PM10, mug/m³) by Optical Particle Counters (OPC 1.107 environcheck and OPC 1.108 dustcheck). We compared the mean concentration during schooltime (7:30 am-18:30 pm) and during the rest of the hours (school empty). In the meantime we enrolled 232 children, mean age 8 years; 149 attending the first school, 83 the second school. All children underwent clinical evaluation, spirometry and exhaled fraction of nitric oxide (FeNO) dosage. Results: The mean concentrations of measured particles (SD) are summarized in the table. Fine particles are significantly higher in school 2 except PM10 indoor, during schooltime, which is higher in school 1. In both schools the concentration of indoor PM10 drastically increases during school activities. This increase is not observed for PM2.5 and PM1. Respiratory function measures were normal in all children. There are significant differences between the percentage values [mean (SD)] of FEV1, FVC and PEF in the two schools [106.1 (11.85) versus 102.1 (12.87) P = 0.007; 100.4 (10.77) versus 95.84 (11.45) P = 0.011; 92.8 (14.62) versus 88.6 (13.63) P = 0.31]. Mean (SD) concentration of FeNO (ppb) was normal in both school [14.5 (9.16) versus 17.1 (12.7) P = 0.456]. Conclusion: The higher value of PM10 in school 1 and its increase at the beginning of school activities show that the coarse fraction of particles depends on the location of the school but also on the presence/absence of children. PM10 could be not the most useful indicator of air quality and its effects on children pulmonary health. Further studies are needed to analyze the chemical composition of PM in schools

SN - 0105-4538
AD - (Sala, Riva, Mandelli, Ferrini, Giovannini) San Paolo Hospital, University of Milan, Department of Pediatrics, Milan, Italy (Cetta) University of Siena, Department of Surgery, Research Doctorate in Oncol, Siena, Italy (Bolzacchini) University of Milano, Bicocca, Department of Environmental Sciences, Milan, Italy
ER -

TY - JOUR
ID - 3623
T1 - Epidemiology and treatment of severe burns in the intensive care unit of Zagreb Children's Hospital from 2003 to 2008
A1 - Skaric,I.
A1 - Barcot,Z.
A1 - Jakobovic,J.
A1 - Kondza,K.
A1 - Mikecin,L.
A1 - Vrtar,Z.
Y1 - 2010///
N1 - 2011587668
Croatian
Journal: Article
KW - EMBASE
KW - Age Distribution
KW - article
KW - body regions
KW - body surface
KW - burn/ep [Epidemiology]
KW - burn/th [Therapy]
KW - Child
KW - Child Care
KW - child hospitalization
KW - Female
KW - Human
KW - intensive care unit
KW - Length of Stay
KW - Male
KW - pediatric hospital
KW - preschool child
KW - school child
KW - sex determination
KW - Treatment Outcome
RP - NOT IN FILE
SP - 139
EP - 142
JF - Paediatria Croatica
VL - 54
IS - 3
CY - Croatia
N2 - Zagreb Children's Hospital is the institution in which most children with burns are treated in Croatia. Analysis over a six-year period revealed that on average one child with a severe burn is treated per month in the intensive care unit (ICU). Here we present the distribution of patients according to age, sex, cause of burn, surface area and regions of the body affected, as well as some indicators of how demanding intensive care and surgical treatment of these patients is. Within the timeframe analysed, over 60% of children were in the age group up to 5 years old and were most frequently scalded by boiling water in their family home in the presence of adults, but lacking their supervision. In the group over 10 years of age, boys were more frequently burned than girls and it would occur while playing outdoors. Burns were equally distributed among all regions of the body in severely burned patients. Over 60% of children had burns covering 10%-30% of their body surface. There were 4 children (5.6%) with burns covering over 70% of their body surface. The average length of stay in

the ICU was 0.61 days per percent of affected body surface, while the overall average length of stay in the hospital was 2.05 days per percent of affected body surface. The purpose of this study is to warn about the relatively high number of children receiving severe burns in the earliest period of life and in the family home, and to discuss the complexities and demands of treating these patients in the ICU and on the ward
SN - 1330-1403
AD - (Skaric, Barcot, Jakobovic, Kondza, Mikecin, Vrtar) Klinika za Djecje Bolesti Zagreb, Klaiceva 16, 10000 Zagreb, Croatia
ER -

TY - JOUR
ID - 3624
T1 - Difficulties in prevention of overweight and obesity in children in general practice in Bulgaria
A1 - Asenova,R.
A1 - Forefa,G.
A1 - Madjova,V.
Y1 - 2010///
N1 - 2011125937
English
Journal: Article
KW - EMBASE
KW - article
KW - Bulgaria
KW - Child
KW - diet therapy
KW - Female
KW - Funding
KW - general practice
KW - general practitioner
KW - Human
KW - Interview
KW - Male
KW - medical documentation
KW - Obesity
KW - observational study
KW - Physical Activity
KW - prevention study
KW - preventive medicine
RP - NOT IN FILE
SP - 1
EP - 4
JF - Duzce Medical Journal
VL - 12
IS - 2
CY - Turkey
N2 - General practice is a suitable and natural environment for educating children on the issues of nutrition and physical activity. The aim of the study is to research the main difficulties in prevention of overweight and obesity encountered in general practice. 34 general practitioners are encompassed in the study. Interviews with general practitioners have been made as well as a direct open observation of the preventive measures organization has been applied. The insufficient funding, lack of materials in appropriate form on the obesity in children as well as the written documentation are the main barriers the general practitioners meet in the prevention of obesity in children. At this stage of the reform in Bulgaria there are substantial organizational difficulties with regard to the insufficient volume and quality of preventive measures on the problem with obesity in children. 2010 Duzce Medical Journal
SN - 1307-671X
AD - (Asenova, Forefa) Medical University - Plovdiv, Department of Health Management, Economics and General Medicine, Bulgaria (Madjova) Medical University - Varna, Department of General Medicine, Bulgaria
ER -

TY - JOUR
ID - 3625
T1 - Relationship between environmental exposures in children and adult lung disease: The case for outdoor exposures
A1 - Soto-Martinez,M.
A1 - Sly,P.D.
Y1 - 2010///
N1 - 2010457917
English
Journal: Review
KW - EMBASE
KW - Adult
KW - Air Pollution
KW - allergy
KW - antioxidant responsive element
KW - Asthma
KW - atopy
KW - behavior disorder
KW - bronchiectasis
KW - bronchus hyperreactivity
KW - cardiovascular disease
KW - Child
KW - chronic obstructive lung disease
KW - clinical assessment
KW - cognitive defect
KW - Diabetes Mellitus
KW - dietary intake
KW - disease association
KW - disease predisposition
KW - Educational Status
KW - Employment Status
KW - Environmental Exposure
KW - exercise tolerance
KW - fetus lung maturation
KW - Forced Expiratory Volume
KW - Fruit
KW - genetic association
KW - genetic polymorphism
KW - genetic risk
KW - genetic variability
KW - homozygosity
KW - Housing
KW - Human
KW - Hypertension
KW - immune response
KW - Income
KW - Inflammation
KW - Influenza virus
KW - intrauterine growth retardation
KW - Low Birth Weight
KW - lower respiratory tract infection
KW - lung development
KW - lung disease/et [Etiology]
KW - lung function
KW - lung parenchyma
KW - maternal smoking

KW - maximal mid expiratory flow
KW - Mediterranean diet
KW - Metapneumovirus
KW - Nutritional Status
KW - Oxidative Stress
KW - Parainfluenza virus
KW - Particulate Matter
KW - perinatal mortality
KW - placenta previa
KW - premature labor
KW - priority journal
KW - Respiratory syncytial pneumovirus
KW - respiratory tract infection
KW - review
KW - Rhinovirus
KW - risk factor
KW - skin defect
KW - social status
KW - sudden infant death syndrome
KW - teratogenicity
KW - tissue injury
KW - upper respiratory tract infection
KW - vegetable
KW - very low birth weight
KW - virus infection
KW - vitamin supplementation
KW - Water Pollution
KW - wheezing
KW - alpha tocopherol
KW - arsenic/to [Drug Toxicity]
KW - carbon monoxide/to [Drug Toxicity]
KW - chemokine/ec [Endogenous Compound]
KW - cytokine/ec [Endogenous Compound]
KW - fibroblast growth factor/ec [Endogenous Compound]
KW - glutathione transferase/ec [Endogenous Compound]
KW - glutathione transferase M2/ec [Endogenous Compound]
KW - glutathione transferase P1/ec [Endogenous Compound]
KW - house dust allergen
KW - nitric oxide/to [Drug Toxicity]
KW - nitrogen dioxide/to [Drug Toxicity]
KW - omega 3 fatty acid
KW - ozone/to [Drug Toxicity]
KW - reactive oxygen metabolite/ec [Endogenous Compound]
KW - sulfur dioxide/to [Drug Toxicity]
KW - tobacco smoke/to [Drug Toxicity]
KW - transforming growth factor alpha/ec [Endogenous Compound]
KW - transforming growth factor beta/ec [Endogenous Compound]
KW - tumor necrosis factor alpha/ec [Endogenous Compound]
KW - vasculotropin/ec [Endogenous Compound]
RP - NOT IN FILE
SP - 173
EP - 186
JF - Chronic Respiratory Disease
VL - 7
IS - 3
CY - United Kingdom

N2 - There is a growing understanding that chronic respiratory diseases in adults have their origins in early life. Adverse environmental exposures occurring in vulnerable periods during lung growth and development in the fetal period and in early childhood that alter lung structure and limit the growth in lung function may have lifelong consequences. Evidence is increasing that exposure to the ambient environment, including air pollutants, persistent toxic substances, water pollutants and respiratory viral infections, can inhibit lung function growth and predispose to chronic non-malignant lung diseases. These exposures generally interact with a genetic predisposition, and gene-environment interactions and epigenetic phenomena are attracting considerable study. An understanding of how ambient exposures impact on normal lung growth and development will aid in understanding of how chronic respiratory diseases of adults develop and may lead to new preventative strategies. 2010 The Author(s)
SN - 1479-9723
AD - (Soto-Martinez) Department of Respiratory Medicine, Royal Children's Hospital, Austria (Sly) Division of Clinical Sciences, Telethon Institute for Child Health Research, University of Western Australia, Australia (Sly) WHO Collaborating Centre for Research on Children's Environmental Health, Australia (Sly) School of Paediatrics and Child Health, University of Western Australia, Australia (Sly) School of Public Health, Curtin University of Technology, Australia
ER -

TY - JOUR
ID - 3628
T1 - Multiple sclerosis and solar exposure before the age of 15 years: Case-control study in Cuba, Martinique and Sicily
A1 - Dalmay,F.
A1 - Bhalla,D.
A1 - Nicoletti,A.
A1 - Cabrera-Gomez,J.A.
A1 - Cabre,P.
A1 - Ruiz,F.
A1 - Druet-Cabanac,M.
A1 - Dumas,M.
A1 - Preux,P.M.
Y1 - 2010///
N1 - 2010418086
English
Journal: Article
KW - EMBASE
KW - Adolescent
KW - Adult
KW - aquatic sport
KW - article
KW - autoimmune disease
KW - case control study
KW - Child
KW - controlled study
KW - Cuba
KW - disease course
KW - dose response
KW - ethnic group
KW - eye color
KW - family history
KW - Female
KW - hair color
KW - Human
KW - Italy
KW - leisure
KW - major clinical study
KW - Male

KW - Martinique
KW - multiple sclerosis/di [Diagnosis]
KW - Multiple Sclerosis/ep [Epidemiology]
KW - multiple sclerosis/pc [Prevention]
KW - Multivariate Analysis
KW - Protective Clothing
KW - radiation dose
KW - radiation exposure
KW - relapse
KW - Remission
KW - risk reduction
KW - skin color
KW - solar radiation
KW - sunburn/pc [Prevention]
RP - NOT IN FILE
SP - 899
EP - 908
JF - Multiple Sclerosis
VL - 16
IS - 8
CY - United Kingdom
N2 - Few studies report a protective role of childhood solar exposure to multiple sclerosis. Our objective was to confirm the protective role of childhood solar exposure in multiple sclerosis in Cuba, Martinique and Sicily. This was a matched case- control study, and cases met Poser criteria for clinically, laboratory (definite, probable) multiple sclerosis. Controls were resident population, without neurological disorder, living close to cases (within 100 km), matched for sex, age (+5 years), residence before age 15. We recruited 551 subjects during a 1-year period (193 cases, Cuba n = 95, Sicily n = 50, Martinique n = 48; 358 controls). Some (89%) met definite clinical multiple sclerosis criteria (relapsing remitting form (with and without sequel) (74%), secondary progressive (21%), primary progressive (5%)). Odds ratios in a uni-variate analysis were: family history of multiple sclerosis (5.1) and autoimmune disorder (4.0); wearing shirt (3.5), hat (2.7), pants (2.4); sun exposure causing sunburn (1.8); sun exposure duration (1 h more/day; weekends 0.91, weekdays 0.86); bare-chested (0.6); water sports (0.2). Independent factors in the multivariate analysis were family history of multiple sclerosis (4.8 (1.50-15.10)), wearing pants under sunlight (1.9 (1.10-3.20)), sun exposure duration (1 h more/ day, weekdays 0.90 (0.85-0.98), weekends 0.93 (0.87-0.99)), water sports (0.23 (0.13-0.40)). We conclude that outdoor leisure activities in addition to sun exposure reports are associated with a reduced multiple sclerosis risk, with evidence of dose response. 2010 The Author(s)
SN - 1352-4585
AD - (Dalmay, Bhalla, Druet-Cabanac, Dumas, Preux) Universite de Limoges, IFR 145 GEIST, Faculte de Medecine, Limoges, France (Nicoletti) Department of Neurosciences, University of Catania, Catania, Italy (Cabrera-Gomez) Multiple Sclerosis Society of Cuba, International Neurological Restoration Center, Havana City, Cuba (Cabre) Department of Neurology, CHU Fort de France, Fort de France, Martinique, France (Ruiz) Institute of Tropical Medicine, La Habana, Cuba
ER -

TY - JOUR
ID - 3629
T1 - Time-location patterns of a population living in an air pollution hotspot
A1 - Fan,Z.
A1 - Wu,X.
A1 - Ohman-Strickland,P.
Y1 - 2010///
N1 - 2010351779
English
Journal: Article
KW - EMBASE
KW - Adolescent
KW - Adult

KW - Aged
KW - Air Pollution
KW - ambient air
KW - article
KW - Child
KW - Cohort Analysis
KW - community
KW - controlled study
KW - Employment Status
KW - Environmental Exposure
KW - Female
KW - health hazard
KW - health survey
KW - high risk population
KW - Human
KW - Human Activities
KW - human experiment
KW - Male
KW - Poverty
KW - preschool child
KW - priority journal
KW - residential area
KW - school child
KW - seasonal variation
KW - socioeconomics
KW - time location pattern
KW - United States
RP - NOT IN FILE
JF - Journal of Environmental and Public Health
VL - 2010 , 2010. Article Number: 625461. Date of Publication: 2010.
CY - United States
N2 - This study characterized the time-location pattern of 107 residents living in air pollution hotspots, the Waterfront South and Copewood/Davis Streets communities in Camden, NJ. Most residents in the two communities are minority and impoverished individuals. Results showed that employment status played the fundamental role in determining time-location patterns of this study population, and the variations of time-location pattern by season and by day-type were partially attributed to employment status. Compared to the National Human Activity Pattern Survey, the Camden cohort spent significantly more time outdoors (3.8 hours versus 1.8 hours) and less time indoors (19.4 hours versus 20.9 hours) than the general US population, indicating a higher risk of exposure to ambient air pollution for the Camden cohort. The findings of the study are important for understanding exposure routes and sources for the socioeconomically disadvantaged subgroup and ultimately help develop effective strategies to reduce community exposure to ambient air pollution in hotspots. Copyright 2010 Xiangmei (May)Wu et al
AD - (Fan, Wu, Ohman-Strickland) Environmental and Occupational Health Sciences Institute (EOHSI), University of Medicine and Dentistry of New Jersey (UMDNJ), Rutgers University, Piscataway, NJ 08854, United States (Wu) Department of Public Health, University of California, Davis, CA 95616, United States (Fan) Environmental and Occupational Medicine, Robert Wood Johnson Medical School, UMDNJ, NJ 08854, United States (Ohman-Strickland) Department of Biostatistics, School of Public Health, UMDNJ, NJ 08854, United States
ER -

TY - JOUR
ID - 3630
T1 - Prevalence of overweight and obesity in six to ten year-old students from urban county schools
A1 - Mello,A.D.M.
A1 - Marcon,S.S.
A1 - Hulsmeyer,A.P.C.R.
A1 - Cattai,G.B.P.

A1 - Ayres,C.S.L.S.
A1 - Santana,R.G.
Y1 - 2010///
N1 - 2010307678
Portuguese
Journal: Article
KW - EMBASE
KW - article
KW - body mass
KW - Brazil
KW - Child
KW - Family Size
KW - Female
KW - Human
KW - Male
KW - Obesity
KW - Physical Activity
KW - Prevalence
KW - Urban Population
RP - NOT IN FILE
SP - 48
EP - 54
JF - Revista Paulista de Pediatria
VL - 28
IS - 1
CY - Brazil
N2 - Objective: To identify the prevalence of overweight and obesity and associated factors among six to ten-year-old students from public county schools in the urban area of Marialva in Parana, Brazil. Methods: Cross-sectional study that used a stratified random sample of 356 students. The following variables were evaluated: body mass index, gender, age, number of siblings, physical activity and sedentary activities. Descriptive statistics was used to verify the distribution of the sample in the strata. The chi-square test was used in order to verify the differences between the distribution of the sample for each category of variables. The association between variables was tested by univariate analysis. Results: Prevalence of overweight was 20% and obesity, 7%. Most students (72%) did not practice physical activity outside school and about half of them (53%) spent more than four hours a day in sedentary activities. The number of siblings was the only variable associated with excessive weight for those who had only one sibling (PR=1.64; 95%CI=1.10-2.45) and for those who had no siblings (PR=1.70; 95%CI=1.02-2.84), compared to those who had two or more siblings. Conclusions: There was a high prevalence of overweight and obesity in this sample. Further studies are needed in order to investigate the prevalence of this disease in children living in small Brazilian municipalities, as well as for research and actions to prevent and/or treat obesity
SN - 0103-0582
AD - (Marcon, Hulsmeyer, Cattai, Ayres, Santana) Programa de Pos-graduacao em Ciencias da Saude, Universidade Estadual de Maringa (UEM), Maringa, PR, Brazil (Mello) Nutricao Clinica, Universidade Federal do Parana (UFPR), Maringa, PR, Brazil (Marcon, Santana) Universidade Federal de Santa Catarina (UFSC), Brazil
ER -

TY - JOUR
ID - 3633
T1 - Pedometer-assessed physical activity of Singaporean youths
A1 - Chia,M.
Y1 - 2010///
N1 - 2010290416
English
Journal: Article
KW - EMBASE
KW - Adolescent

KW - adolescent health
KW - Adult
KW - article
KW - Child
KW - college student
KW - controlled study
KW - daily life activity
KW - elementary student
KW - Female
KW - functional assessment
KW - high school student
KW - Human
KW - Male
KW - pedometer
KW - Physical Activity
KW - priority journal
KW - school child
KW - school health service
KW - sex difference
KW - Singapore
KW - sitting
KW - validation process
RP - NOT IN FILE
SP - 262
EP - 264
JF - Preventive Medicine
JA - Prev Med
VL - 50
IS - 5-6
CY - United States
N2 - Objective: The aim of the study was to investigate the pedometer-assessed physical activity of Singaporean youths using an objective measurement of physical activity. Methods and results: Pedometer step count was monitored over the entire week in 877 participants aged 9-18. years in three schooling cohorts [primary (age, 9-12. years; n= 150 males; 156 females), secondary (age, 13-16. years; n= 137 males; 138 females) and junior college (age, 17-18. years; n= 140 males; 156 females)] in Singapore during July to September 2009. Analyses identified significant main effects for step count taken outside of school compared to within school (mean (SD): 5568 (4796) vs. 3881 (3149), p< 0.05). However, no significant difference was found for steps accumulated within or outside school in boys and girls across the schooling levels (steps x sex x level interaction, p> 0.05). Step counts were not significantly different between weekdays or weekends (9719 (6063) vs. 9483 (8056), p> 0.05), across schooling levels and between male and female participants (sex x level and steps x level x sex interactions, all p> 0.05). Conclusion: Step count decline is drastic for male adolescents after primary school but remains low across the schooling levels for female participants. Aggregated daily step count fell short by up to 35% of the 16,000 and 13,000 steps recommended respectively for male and female youths. 2010 Elsevier Inc
SN - 0091-7435
AD - (Chia) Physical Education and Sports Science, National Institute of Education, Nanyang Technological University, 1 Nanyang Walk, Singapore 637 616, Singapore
ER -

TY - JOUR
ID - 3635
T1 - Infectious morbidity in 18-month-old children with and without older siblings
A1 - Hedin,K.
A1 - Andre,M.
A1 - Hakansson,A.
A1 - Molstad,S.
A1 - Rodhe,N.

A1 - Petersson,C.
Y1 - 2010///
N1 - 2010518235
English
Journal: Article
KW - EMBASE
KW - article
KW - Child
KW - consultation
KW - Human
KW - Infection/dt [Drug Therapy]
KW - Infection/ep [Epidemiology]
KW - major clinical study
KW - preschool child
KW - prescription
KW - sibling
KW - socioeconomics
KW - antibiotic agent/dt [Drug Therapy]
RP - NOT IN FILE
SP - 507
EP - 512
JF - Family Practice
VL - 27
IS - 5
CY - United Kingdom
N2 - Background: Infections are the most commonly reported health problems in children. Younger age and day care outside the home are two factors of importance for infectious morbidity. The influence of siblings on infectious symptoms is not clear. Objectives: To compare families with one child and families with more than one child in terms of reported infectious symptoms, physician consultations and antibiotic prescriptions. Methods: A prospective population-based survey was performed. During 1 month, all infectious symptoms, physician consultations and antibiotic prescriptions for 18-month-old children were noted by the parents. The 789 families also answered questions about socio-economic factors, numbers of siblings in the family and type of day care. Results: No difference in number of symptom days was found between children with and without older siblings. Neither could we find any significance in terms of having older siblings in relation to physician consultations and antibiotic prescriptions. Conclusions: The results of our study indicate that having older siblings not was important in relation to number of symptoms days, physician consultations or antibiotic prescriptions for 18-month-old children in Sweden today. Changes in social activities and attitudes towards antibiotic prescription may explain our different findings as compared with previous Swedish studies and studies from other countries. The Author 2010. Published by Oxford University Press. All rights reserved
SN - 0263-2136
AD - (Hedin, Petersson) Unit of Research and Development, Kronoberg County Council, Vaxjo, Sweden (Hedin, Hakansson) Dept. of Clinical Sciences in Malmo-General practice/Family Medicine, Lund University, Malmo, Sweden (Andre, Rodhe) Centre for Clinical Research, Dalarna, Falun, Sweden (Molstad) Unit of Research and Development in Primary Health Care, Jonkoping, Sweden (Molstad) General Practice, Department of Health and Science, Faculty of Health Science, Linkoping, Sweden
ER -

TY - JOUR
ID - 3636
T1 - Bullying update: Are we making any progress?
A1 - Glew,G.M.
A1 - Frey,K.S.
A1 - Walker,W.O.
Y1 - 2010///
N1 - 2010493585
English
Journal: Article

KW - EMBASE
KW - Aggression
KW - Anger
KW - appetite
KW - article
KW - Asperger syndrome
KW - Behavior
KW - Bullying
KW - Case Report
KW - Child
KW - confidence interval
KW - denial
KW - developmental psychology
KW - emotional disorder
KW - Empathy
KW - Female
KW - habit
KW - Human
KW - Male
KW - metabolic rate
KW - Obesity
KW - Observation
KW - offender
KW - pediatrician
KW - Personality
KW - preschool child
KW - Prevalence
KW - primary school
KW - Research
KW - school child
KW - Self Concept
KW - Self Report
KW - social adaptation
KW - victim
RP - NOT IN FILE
SP - e68
EP - e74
JF - Pediatrics in Review
VL - 31
IS - 9
CY - United States
N2 - * Translation projects (implementation of evidence-based strategies) are desperately needed to get schools up to speed on what we already know about bullying. * School personnel can take numerous simple steps to make schools safer, such as increasing supervision in restrooms and on playgrounds. * Both students and teachers need to be taught the difference between tattling (telling an adult about bad behavior to get someone in trouble) and reporting (telling an adult about bad behavior to get help and stay safe). * Social-emotional education should be incorporated into the daily routine of all children both at home and at school. * Schools must be careful not to reward bad behavior by giving time off or special time with an adult to the bully, which inadvertently can reinforce bullying. * Some emotionally disturbed children are served best in special classrooms designed to meet their specific needs. * Adult indifference to bullying must end because it may be teaching young people to tolerate coercive and abusive behavior. * Parents, school personnel, and pediatricians can educate themselves by reading evidence-based literature on bullying. * Openness to feedback and discarding stereotypes about bullying may enable parents to respond constructively when their child has a behavior problem. * Parents can use community resources (eg, social skills and etiquette classes for children) to improve their children's social skills. * Pediatricians should advocate for parents with school personnel to make changes to stop bullying and should be open to supporting legal action if no change occurs
SN - 0191-9601

AD - (Glew) University of Washington School of Medicine, Department of Pediatrics, Seattle Children's Hospital, Seattle, WA, United States (Frey) Educational Psychology, University of Washington, Seattle, WA, United States (Walker) Department of Pediatrics, University of Washington School of Medicine, United States (Walker) Division of Developmental Medicine, United States (Walker) Developmental Behavioral Pediatrics Fellowship, Seattle Children's Hospital, Seattle, WA, United States
ER -

TY - JOUR
ID - 3637
T1 - Prenatal exposure to traffic-related air pollution and ultrasound measures of fetal growth in the INMA Sabadell cohort
A1 - Aguilera,I.
A1 - Garcia-Esteban,R.
A1 - Iniguez,C.
A1 - Nieuwenhuijsen,M.J.
A1 - Rodriguez,A.
A1 - Paez,M.
A1 - Ballester,F.
A1 - Sunyer,J.
Y1 - 2010///
N1 - 2010257179
English
Journal: Article
KW - EMBASE
KW - air pollutant
KW - anthropometric parameters
KW - article
KW - bone growth
KW - Child
KW - Cohort Analysis
KW - exhaust gas
KW - Female
KW - femur
KW - fetus
KW - fetus echography
KW - fetus growth
KW - fetus weight
KW - Gestational Age
KW - growth curve
KW - head circumference
KW - Human
KW - Male
KW - prenatal exposure
KW - priority journal
KW - aromatic hydrocarbon
KW - benzene
KW - ethylbenzene
KW - meta xylene
KW - nitrogen dioxide
KW - ortho xylene
KW - para xylene
KW - toluene
RP - NOT IN FILE
SP - 705
EP - 711
JF - Environmental Health Perspectives
JA - Environ Health Perspect

VL - 118
IS - 5
CY - United States
N2 - Background: Few studies have used longitudinal ultrasound measurements to assess the effect of traffic-related air pollution on fetal growth. Objective: We examined the relationship between exposure to nitrogen dioxide (NO₂) and aromatic hydrocarbons [benzene, toluene, ethylbenzene, m/p-xylene, and o-xylene (BTEX)] on fetal growth assessed by 1,692 ultrasound measurements among 562 pregnant women from the Sabadell cohort of the Spanish INMA (Environment and Childhood) study. Methods: We used temporally adjusted land-use regression models to estimate exposures to NO₂ and BTEX. We fitted mixed-effects models to estimate longitudinal growth curves for femur length (FL), head circumference (HC), abdominal circumference (AC), biparietal diameter (BPD), and estimated fetal weight (EFW). Unconditional and conditional SD scores were calculated at 12, 20, and 32 weeks of gestation. Sensitivity analyses were performed considering time-activity patterns during pregnancy. Results: Exposure to BTEX from early pregnancy was negatively associated with growth in BPD during weeks 20-32. None of the other fetal growth parameters were associated with exposure to air pollution during pregnancy. When considering only women who spent < 2 hr/day in nonresidential outdoor locations, effect estimates were stronger and statistically significant for the association between NO₂ and growth in HC during weeks 12-20 and growth in AC, BPD, and EFW during weeks 20-32. Conclusions: Our results lend some support to an effect of exposure to traffic-related air pollutants from early pregnancy on fetal growth during mid-pregnancy
SN - 0091-6765
AD - (Aguilera, Garcia-Esteban, Nieuwenhuijsen, Sunyer) Centre for Research in Environmental Epidemiology, Barcelona, Spain (Aguilera, Garcia-Esteban, Nieuwenhuijsen, Sunyer) Municipal Institute of Medical Research (IMIM-Hospital del Mar), Barcelona, Spain (Aguilera, Garcia-Esteban, Iniguez, Nieuwenhuijsen, Ballester, Sunyer) Centro de Investigacion Biomedica en Red de Epidemiologia y Salud Publica, Barcelona, Spain (Aguilera, Sunyer) Pompeu Fabra University, Barcelona, Spain (Iniguez, Ballester) Centre for Public Health Research, Conselleria de Sanitat, Generalitat Valenciana, Valencia, Spain (Rodriguez) Servei de Ginecologia i Obstetricia, Hospital Parc Tauli, Sabadell, Spain (Paez) Centre d'Atencio Primaria Sant Felix, Sabadell, Spain
ER -

TY - JOUR
ID - 3638
T1 - A Generalizability Study of the Instrumental Activities of Daily Living Profile
A1 - Bottari,C.
A1 - Dassa,C.
A1 - Rainville,C.
A1 - Dutil,E.
Y1 - 2010///
N1 - 2010241322
English
Journal: Article
KW - EMBASE
KW - Adolescent
KW - Adult
KW - Aged
KW - article
KW - Child
KW - clinical article
KW - community care
KW - daily life activity
KW - disease duration
KW - disease severity
KW - environmental factor
KW - Human
KW - human relation
KW - Observer Variation
KW - reliability

KW - Training
KW - traumatic brain injury
KW - workshop
RP - NOT IN FILE
SP - 734
EP - 742
JF - Archives of Physical Medicine and Rehabilitation
VL - 91
IS - 5
CY - United States
N2 - Bottari C, Dassa C, Rainville C, Dutil E. A generalizability study of the Instrumental Activities of Daily Living Profile. Objective: To establish generalizability estimates of the Instrumental Activities of Daily Living (IADL) Profile when administered to persons with a moderate or severe traumatic brain injury (TBI) within their home and community environment. Design: Generalizability theory was used to estimate generalizability and dependability coefficients as well as the relative contribution of identified sources of measurement error to total measurement error. Decision studies were used to enable the investigators to determine the optimal measurement design. Setting: The IADL Profile was administered in subjects' homes and community environments. Participants: Convenience sample of adults with a moderate or severe TBI (N=30, aged 16-65y). Interventions: Not applicable. Main Outcome Measures: IADL Profile scores include 6 factor scores (going to the grocery store/shopping for groceries, having a meal with guests/cleaning up, putting on outdoor clothing, obtaining information, making a budget, and preparing a hot meal) and 1 total score. Results: The greatest sources of measurement error were the subject-item interactions (3-random-facet design) and the subject-rater interactions (2-random-facet design). One hundred percent of generalizability coefficients of factor scores indicated acceptable to excellent reliability. Indices of dependability confirmed that 1 evaluator could reliably score the tool on a single occasion after having received a 3-day training workshop. Conclusions: The IADL Profile administered to persons with a moderate or severe TBI provides occupational therapists with a reliable set of measures of IADL independence capable of both capturing and analyzing the complex interactions between personal and environmental factors. 2010 American Congress of Rehabilitation Medicine
SN - 0003-9993
AD - (Bottari, Dutil) School of Rehabilitation, Universite de Montreal, Montreal, Que., Canada (Dassa) Department of Social and Preventative Medicine, Universite de Montreal, Montreal, Que., Canada (Rainville) Department of Psychology, Universite de Montreal, Montreal, Que., Canada (Bottari, Rainville, Dutil) Center for Interdisciplinary Research in Rehabilitation, Montreal, Que., Canada (Dassa) Interdisciplinary Research Group in Health, Montreal, Que., Canada
ER -

TY - JOUR
ID - 3639
T1 - Respiratory surveys of school children: Basis for communal health policy?
A1 - Moshammer,H.
Y1 - 2010///
N1 - 2010221779
German
Journal: Conference Paper
KW - EMBASE
KW - Air Pollution
KW - Breast Feeding
KW - Child
KW - child health care
KW - child nutrition
KW - clinical examination
KW - conference paper
KW - cost effectiveness analysis
KW - Decision Making
KW - environmental factor
KW - Health Care Policy
KW - Home

KW - household
KW - Human
KW - lung examination
KW - lung function
KW - major clinical study
KW - medical care
KW - preschool child
KW - Public Health
KW - Quality of Life
KW - questionnaire
KW - risk factor
KW - school child
KW - Smoking
KW - Spirometry
KW - sporting event
RP - NOT IN FILE
SP - 90
EP - 93
JF - Atemwegs- und Lungenkrankheiten
VL - 36
IS - 3
CY - Germany
N2 - In several campaigns we investigated the respiratory health of schoolchildren using questionnaires, clinical examinations and spirometry. The latter proved especially sensitive. The influence both of the outdoor air pollution and its improvement over time could be demonstrated. Among the indoor factors living in a smokers household as well as damp and mouldy homes were important as was the location of the home near a busy street. As protective factors we found breast feeding and sports activities of the children. 2010 Dustri-Verlag Dr. Karl Feistle
SN - 0341-3055
AD - (Moshammer) Institut fur Umwelthygiene, Medizinische Universitat Wien, Austria
ER -

TY - JOUR
ID - 3641
T1 - Genome-Wide Association Analysis in Primary Sclerosing Cholangitis
A1 - Karlsen,T.H.
A1 - Franke,A.
A1 - Melum,E.
A1 - Kaser,A.
A1 - Hov,J.R.
A1 - Balschun,T.
A1 - Lie,B.A.
A1 - Bergquist,A.
A1 - Schramm,C.
A1 - Weismuller,T.J.
A1 - Gotthardt,D.
A1 - Rust,C.
A1 - Philipp,E.E.R.
A1 - Fritz,T.
A1 - Henckaerts,L.
A1 - Weersma,R.K.
A1 - Stokkers,P.
A1 - Ponsioen,C.Y.
A1 - Wijmenga,C.
A1 - Sterneck,M.
A1 - Nothnagel,M.
A1 - Hampe,J.

A1 - Teufel,A.
A1 - Runz,H.
A1 - Rosenstiel,P.
A1 - Stiehl,A.
A1 - Vermeire,S.
A1 - Beuers,U.
A1 - Manns,M.P.
A1 - Schrumpf,E.
A1 - Boberg,K.M.
A1 - Schreiber,S.
Y1 - 2010///
N1 - 2010132069
English
Journal: Article
KW - EMBASE
KW - Adolescent
KW - Adult
KW - Aged
KW - article
KW - Child
KW - chromosome 13q
KW - chromosome 6p
KW - controlled study
KW - Female
KW - gene locus
KW - genetic susceptibility
KW - genome analysis
KW - Genotype
KW - HLA system
KW - Human
KW - major clinical study
KW - Male
KW - Phenotype
KW - primary sclerosing cholangitis/et [Etiology]
KW - priority journal
KW - school child
KW - single nucleotide polymorphism
KW - ulcerative colitis/et [Etiology]
KW - upregulation
KW - G protein coupled bile acid receptor 1/ec [Endogenous Compound]
KW - G protein coupled receptor/ec [Endogenous Compound]
KW - glypican/ec [Endogenous Compound]
KW - glypican 6/ec [Endogenous Compound]
KW - HLA B antigen/ec [Endogenous Compound]
KW - macrophage activating factor/ec [Endogenous Compound]
KW - unclassified drug
RP - NOT IN FILE
SP - 1102
EP - 1111
JF - Gastroenterology
VL - 138
IS - 3
CY - United States
N2 - Background & Aims: We aimed to characterize the genetic susceptibility to primary sclerosing cholangitis (PSC) by means of a genome-wide association analysis of single nucleotide polymorphism (SNP) markers. Methods: A total of 443,816 SNPs on the Affymetrix SNP Array 5.0 (Affymetrix, Santa Clara, CA) were genotyped in 285 Norwegian PSC patients and 298 healthy controls. Associations detected in this discovery

panel were re-examined in independent case-control panels from Scandinavia (137 PSC cases and 368 controls), Belgium/The Netherlands (229 PSC cases and 735 controls), and Germany (400 cases and 1832 controls). Results: The strongest associations were detected near HLA-B at chromosome 6p21 (rs3099844: odds ratio [OR], 4.8; 95% confidence interval [CI], 3.6-6.5; P = 2.6 x 10⁻²⁶; and rs2844559: OR, 4.7; 95% CI, 3.5-6.4; P = 4.2 x 10⁻²⁶ in the discovery panel). Outside the HLA complex, rs9524260 at chromosome 13q31 showed significant associations in 3 of 4 study panels. Lentiviral silencing of glypican 6, encoded at this locus, led to the up-regulation of proinflammatory markers in a cholangiocyte cell line. Of 15 established ulcerative colitis susceptibility loci, significant replication was obtained at chromosomes 2q35 and 3p21 (rs12612347: OR, 1.26; 95% CI, 1.06-1.50; and rs3197999: OR, 1.22; 95% CI, 1.02-1.47, respectively), with circumstantial evidence supporting the G-protein-coupled bile acid receptor 1 and macrophage-stimulating 1, respectively, as the likely disease genes. Conclusions: Strong HLA associations and a subset of genes involved in bile homeostasis and other inflammatory conditions constitute key components of the genetic architecture of PSC. 2010 AGA Institute
SN - 0016-5085
AD - (Karlsen, Melum, Hov, Schrumpf, Boberg) Medical Department, Rikshospitalet University Hospital, University of Oslo, Oslo, Norway (Franke, Balschun, Philipp, Rosenstiel, Schreiber) Institute of Clinical Molecular Biology, Christian-Albrechts-University, Kiel, Germany (Kaser, Fritz) Department of Gastroenterology and Hepatology, Innsbruck Medical University, Innsbruck, Austria (Lie) Institute of Immunology, Rikshospitalet University Hospital, Oslo, Norway (Bergquist) Department of Gastroenterology and Hepatology, Karolinska University Hospital Huddinge, Stockholm, Sweden (Schramm, Sterneck) 1st Department of Medicine, University Medical Center Hamburg-Eppendorf, Hamburg, Germany (Weismuller, Manns) Department of Gastroenterology, Hepatology and Endocrinology, Hannover Medical School, Hannover, Germany (Gotthardt, Stiehl) Department of Medicine, University Hospital of Heidelberg, Heidelberg, Germany (Rust) Department of Medicine 2, Grosshadern, University of Munich, Munich, Germany (Henckaerts, Vermeire) Department of Gastroenterology, University Hospital Gasthuisberg, Leuven, Belgium (Weersma) Department of Gastroenterology and Hepatology, University Medical Center Groningen, University of Groningen, Netherlands (Stokkers, Ponsioen, Beuers) Department of Gastroenterology and Hepatology, Academic Medical Center, University of Amsterdam, Amsterdam, Netherlands (Wijmenga) Department of Genetics, University Medical Center Groningen, University of Groningen, Groningen, Netherlands (Nothnagel) Institute of Medical Informatics and Statistics, Christian-Albrechts-University, Kiel, Germany (Hampe, Schreiber) Department for General Internal Medicine, Christian-Albrechts-University, Kiel, Germany (Teufel) 1st Department of Medicine, University of Mainz, Mainz, Germany (Runz) Department of Human Genetics, University Hospital of Heidelberg, Heidelberg, Germany
ER -

TY - JOUR
ID - 3642
T1 - Clinical report - Injuries in youth soccer
A1 - Koutures,C.G.
A1 - Gregory,A.J.M.
A1 - McCambridge,T.M.
A1 - Benjamin,H.J.
A1 - Brenner,J.S.
A1 - Cappetta,C.T.
A1 - Demorest,R.A.
A1 - Halstead,M.E.
A1 - LaBella,C.R.
A1 - Martin,S.S.
A1 - Rice,S.G.
Y1 - 2010///
N1 - 2010090882
English
Journal: Article
KW - EMBASE
KW - Adolescent
KW - article
KW - Child

KW - concussion
KW - cross-sectional study
KW - eye injury/pc [Prevention]
KW - eye protective device
KW - face injury
KW - Football
KW - head injury
KW - Human
KW - juvenile
KW - knee injury
KW - priority journal
KW - risk reduction
KW - sport injury
RP - NOT IN FILE
SP - 410
EP - 414
JF - Pediatrics
VL - 125
IS - 2
CY - United States
N2 - Injury rates in youth soccer, known as football outside the United States, are higher than in many other contact/collision sports and have greater relative numbers in younger, preadolescent players. With regard to musculoskeletal injuries, young females tend to suffer more knee injuries, and young males suffer more ankle injuries. Concussions are fairly prevalent in soccer as a result of contact/collision rather than purposeful attempts at heading the ball. Appropriate rule enforcement and emphasis on safe play can reduce the risk of soccer-related injuries. This report serves as a basis for encouraging safe participation in soccer for children and adolescents. Copyright 2010 by the American Academy of Pediatrics
SN - 0031-4005
ER -

TY - JOUR
ID - 3643
T1 - Effectiveness of planning and management interventions for improving age-appropriate immunization in rural India
A1 - Prinja,S.
A1 - Gupta,M.
A1 - Singh,A.
A1 - Kumar,R.
Y1 - 2010///
N1 - 2010078109
English
Journal: Article
KW - EMBASE
KW - article
KW - Attitude to Health
KW - Child
KW - clinical trial
KW - Cohort Analysis
KW - decentralization
KW - diphtheria/dt [Drug Therapy]
KW - diphtheria/pc [Prevention]
KW - Female
KW - Government
KW - health care personnel
KW - health care planning
KW - Hospitalization
KW - Human

KW - Immunization
KW - India
KW - Infant
KW - major clinical study
KW - Male
KW - patient attitude
KW - pertussis/dt [Drug Therapy]
KW - pertussis/pc [Prevention]
KW - preschool child
KW - preventive health service
KW - rural health care
KW - Rural Population
KW - tetanus/dt [Drug Therapy]
KW - tetanus/pc [Prevention]
KW - therapy delay
KW - diphtheria pertussis tetanus vaccine/dt [Drug Therapy]
RP - NOT IN FILE
SP - 97
EP - 103
JF - Bulletin of the World Health Organization
JA - Bull World Health Organ
VL - 88
IS - 2
CY - Switzerland
N2 - Objective: To study the effectiveness of planning and management interventions for ensuring children in India are immunized at the appropriate age. Methods: The study involved children aged less than 18 months recruited from Haryana, India, in 2005-2006: 4336 in a pre-intervention cohort and 5213 in a post-intervention cohort. In addition, immunization of 814 hospitalized children from outside the study area was also assessed. Operational barriers to age-appropriate immunization with diphtheria, pertussis and tetanus (DPT) vaccine were investigated by monitoring vaccination coverage, observing immunization sessions and interviewing parents and health-care providers. An intervention package was developed, with community volunteers playing a pivotal role. Its effectiveness was assessed by monitoring the ages at which the three DPT doses were administered. Findings: The main reasons for delayed immunization were staff shortages, non-adherence to plans and vaccine being out of stock. In the post-intervention cohort, 70% received a third DPT dose before the age of 6 months, significantly more than in the pre-intervention cohort (62%; P = 0.002). In addition, the mean age at which the first, second and third DPT doses were administered decreased by 17, 21 and 34 days, respectively, in the study area over a period of 18 months (P for trend < 0.0001). No change was observed in hospitalized children from outside the study area. Conclusion: An intervention package involving community volunteers significantly improved age-appropriate DPT immunization in India. The Indian Government's intention to recruit village-based volunteers as part of a health sector reform aimed at decentralizing administration could help increase timely immunization
SN - 0042-9686
AD - (Prinja, Gupta, Singh, Kumar) School of Public Health, Post Graduate Institute of Medical Education and Research (PGIMER), Chandigarh, India
ER -

TY - JOUR
ID - 3644
T1 - Trajectories of mobility and IADL function in older patients diagnosed with major depression
A1 - Hybels,C.F.
A1 - Pieper,C.F.
A1 - Blazer,D.G.
A1 - Fillenbaum,G.G.
A1 - Steffens,D.C.
Y1 - 2010///

N1 - 2010072036
English
Journal: Article
KW - EMBASE
KW - Aged
KW - article
KW - Child
KW - cleaning
KW - climbing
KW - controlled study
KW - daily life activity
KW - Female
KW - financial management
KW - functional status
KW - gardening
KW - gender
KW - geriatric patient
KW - Human
KW - major clinical study
KW - Major Depression
KW - Male
KW - patient mobility
KW - Self Concept
RP - NOT IN FILE
SP - 74
EP - 81
JF - International Journal of Geriatric Psychiatry
VL - 25
IS - 1
CY - United Kingdom
N2 - Objective: Research has shown an association between depression and functional limitations in older adults. Our aim was to explore the latent traits of trajectories of limitations in mobility and instrumental activities of daily living (IADL) tasks in a sample of older adults diagnosed with major depression. Methods: Participants were 248 patients enrolled in a naturalistic depression treatment study. Mobility/IADL tasks included walking one-fourth mile, going up/down stairs, getting around the neighborhood, shopping, handling money, taking care of children, cleaning house, preparing meals and doing yardwork/gardening. Latent class trajectory analysis was used to identify classes of mobility/IADL function over a 4-year period. Class membership was then used to predict functional status over time. Results: Using time as the only predictor, three latent class trajectories were identified: (1) Patients with few mobility/IADL limitations (42%), (2) Patients with considerable mobility/IADL limitations (37%) and (3) Patients with basically no limitations (21%). The classes differed primarily in their initial functional status, with some immediate improvement followed by no further change for patients in Classes 1 and 2 and a stable course for patients in Class 3. In a repeated measures mixed model controlling for potential confounders, class was a significant predictor of functional status. The effect of baseline depression score, cognitive status, self-perceived health and sex on mobility/IADL score differed by class. Conclusions: These findings show systematic variability in functional status over time among older patients with major depression, indicating that a single trajectory may not reflect the pattern for all patients. Copyright 2009 John Wiley & Sons, Ltd
SN - 0885-6230
AD - (Hybels, Blazer, Fillenbaum, Steffens) Department of Psychiatry and Behavioral Sciences, Center for the Study of Aging and Human Development, Duke University Medical Center, Box 3003, Durham, NC, United States (Pieper) Department of Biostatistics and Bioinformatics, Center for the Study of Aging and Human Development, Duke University Medical Center, Durham, NC, United States (Fillenbaum) Center for the Study of Aging and Human Development, Geriatrics, Education, and Clinical Center, Veterans Administration Medical Center, Durham, NC, United States
ER -

TY - JOUR

ID - 3648
T1 - Equine-assisted therapy and autism
A1 - Hameury,L.
A1 - Delavous,P.
A1 - Teste,B.
A1 - Leroy,C.
A1 - Gaboriau,J.-C.
A1 - Berthier,A.
Y1 - 2010///
N1 - 2010588322
French
Journal: Article
KW - EMBASE
KW - article
KW - autism/th [Therapy]
KW - Behavior Change
KW - Behavior Control
KW - Child
KW - Child Behavior
KW - Child Development
KW - clinical article
KW - communication skill
KW - Day Care
KW - emotionality
KW - hippotherapy
KW - horse
KW - Human
KW - imitation
KW - motor control
KW - outcome assessment
KW - Perception
KW - pet therapy
KW - social adaptation
KW - Social Interaction
KW - Treatment Planning
KW - treatment response
RP - NOT IN FILE
SP - 655
EP - 659
JF - Annales Medico-Psychologiques
VL - 168
IS - 9
CY - France
N2 - Equine-assisted therapy is used beside therapies realised at the Child Psychiatry Center and included into individualised therapeutic program. The goals are to adapt therapeutic principles outside hospital and with pony as mediator in order to exercise adjustment and control systems: Communication and relationships with others, cognitive regulation (attention, mental representation, understanding causality, anticipation), motor regulation (tonus, body consciousness, postural adjustment, balance, coordination), emotional regulation (self-esteem, relaxation, pleasure, coping emotionally with problems, expressing and sharing emotions), adjusting to change. Varied activities with tranquil and easy-going ponies were realised in a calm secure environment by three therapists with experience in horseback riding for a group of four children. An individual plan targeting specific goals was worked out for each child. Results were evaluated with standardized tools (Behavior Function Inventory and Behavior Summarized Evaluation Scale) for six children with autistic spectrum treated at the day-care hospital, which participated to equine-assisted therapy. Improvements are obtained since the first session and concerns all developmental functions, especially communication, imitation, perceptual, emotional and motor adjustment. With his/her pony the child is stable, calms down, adjusts his/her interactions, is more aware of the effects of his/her actions, and optimal sensory integration can take place. So, equine-assisted therapy is an

efficient remediation of the neurophysiologic functions involved in developing communication and social skills, and cognitivo-emotional regulation. 2010 Elsevier Masson SAS
SN - 0003-4487
AD - (Hameury, Delavous, Teste, Leroy, Gaboriau, Berthier) Centre universitaire de pedopsychiatrie, CHRU de Tours, 37044 Tours cedex 9, France
ER -

TY - JOUR
ID - 3649
T1 - PM2.5, soot and NO2 indoor-outdoor relationships at homes, pre-schools and schools in Stockholm, Sweden
A1 - Wichmann,J.
A1 - Lind,T.
A1 - Nilsson,M.A.-M.
A1 - Bellander,T.
Y1 - 2010///
N1 - 2010534443
English
Journal: Article
KW - EMBASE
KW - air monitoring
KW - Air Pollution
KW - ambient air
KW - article
KW - atmospheric dispersion
KW - Child
KW - clinical article
KW - combustion
KW - Female
KW - Human
KW - Male
KW - Measurement
KW - microenvironment
KW - Particulate Matter
KW - preschool child
KW - priority journal
KW - school child
KW - soot
KW - summer
KW - Sweden
KW - winter
KW - nitrogen dioxide
KW - tobacco smoke
RP - NOT IN FILE
SP - 4536
EP - 4544
JF - Atmospheric Environment
VL - 44
IS - 36
CY - United Kingdom
N2 - In developed nations people spend about 90% of their time indoors. The relationship between indoor and outdoor air pollution levels is important for the understanding of the health effects of outdoor air pollution. Although other studies describe both the outdoor and indoor atmospheric environment, few excluded a priori major indoor sources, measured the air exchange rate, included more than one micro-environment and included the presence of human activity. PM_{2.5}, soot, NO₂ and the air exchange rate were measured during winter and summer indoors and outdoors at 18 homes (mostly apartments) of 18 children (6-11-years-old) and also at the six schools and 10 pre-schools that the children attended. The three types of indoor

environments were free of environmental tobacco smoke and gas appliances, as the aim was to asses to what extent PM_{2.5}, soot and NO₂ infiltrate from outdoors to indoors. The median indoor and outdoor PM_{2.5} levels were 8.4 mug m⁻³ and 9.3 mug m⁻³, respectively. The median indoor levels for soot and NO₂ were 0.66 m⁻¹ x 10⁻⁵ and 10.0 mug m⁻³, respectively. The respective outdoor levels were 0.96 m⁻¹ x 10⁻⁵ and 12.4 mug m⁻³. The median indoor/outdoor (I/O) ratios were 0.93, 0.76 and 0.92 for PM_{2.5}, soot and NO₂, respectively. Their infiltration factors were influenced by the micro-environment, ventilation type and air exchange rate, with aggregated values of 0.25, 0.55 and 0.64, respectively. Indoor and outdoor NO₂ levels were strongly associated (R² = 0.71), followed by soot (R² = 0.50) and PM_{2.5} (R² = 0.16). In Stockholm, the three major indoor environments occupied by children offer little protection against combustion-related particles and gases in the outdoor air. Outdoor PM_{2.5} seems to infiltrate less, but indoor sources compensate. 2010 Elsevier Ltd
SN - 1352-2310
AD - (Wichmann, Lind, Nilsson, Bellander) Institute of Public Health, University of Copenhagen, Copenhagen, Denmark (Wichmann) Institute of Environmental Medicine, Karolinska Institutet, Stockholm, Sweden (Wichmann) School of Health Systems and Public Health, University of Pretoria, South Africa
ER -

TY - JOUR
ID - 3650
T1 - Examination of perceived neighborhood characteristics and transportation on changes in physical activity and sedentary behavior: The Trial of Activity in Adolescent Girls
A1 - Evenson,K.R.
A1 - Murray,D.M.
A1 - Birnbaum,A.S.
A1 - Cohen,D.A.
Y1 - 2010///
N1 - 2010424981
English
Journal: Article
KW - EMBASE
KW - article
KW - Child
KW - Child Behavior
KW - environmental factor
KW - Female
KW - Health Behavior
KW - Human
KW - human relation
KW - Physical Activity
KW - play
KW - priority journal
KW - school child
KW - Social Behavior
KW - Social Environment
KW - Social Interaction
KW - Walking
RP - NOT IN FILE
SP - 977
EP - 985
JF - Health and Place
VL - 16
IS - 5
CY - United Kingdom
N2 - We examined the association between perceived neighborhood characteristics and transport and 2-year changes in accelerometer-determined nonschool MET-weighted moderate-to-vigorous physical activity (MW-

MVPA) and sedentary behavior of adolescent girls. Reporting that children do not play outdoors in their neighborhood, that their neighborhood was well lit, and that there were trails in their neighborhood were each associated with significant decreases in nonschool MW-MVPA. None of the neighborhood or transportation measures was associated with changes in nonschool sedentary behavior. Further work is needed to understand the determinants of the decline in physical activity and the increase in sedentary behavior among adolescent girls. 2010 Elsevier Ltd
SN - 1353-8292
AD - (Evenson) University of North Carolina, Gillings School of Global Public Health, Department of Epidemiology, Bank of America Center, Suite 306, 137 East Franklin Street, Chapel Hill, NC 27514, United States (Murray) The Ohio State University, College of Public Health, Division of Epidemiology, Columbus, OH, United States (Birnbaum) Montclair State University, Department of Health and Nutrition Sciences, Montclair, NJ, United States (Cohen) RAND Corporation, Santa Monica, CA, United States
ER -

TY - JOUR
ID - 3651
T1 - Gender differences in personal, social and environmental influences on active travel to and from school for Australian adolescents
A1 - Leslie,E.
A1 - Kremer,P.
A1 - Toumbourou,J.W.
A1 - Williams,J.W.
Y1 - 2010///
N1 - 2010588453
English
Journal: Article
KW - EMBASE
KW - adolescence
KW - Adolescent
KW - article
KW - Australia
KW - bicycle
KW - Child
KW - community
KW - cycling
KW - environmental factor
KW - Family
KW - Female
KW - friend
KW - gender
KW - Home
KW - Human
KW - Male
KW - medical research
KW - normal human
KW - peer counseling
KW - Perception
KW - Physical Activity
KW - play
KW - Recreation
KW - school
KW - school child
KW - sex difference
KW - social aspect
KW - sport
KW - Traffic
KW - Travel

KW - Walking
RP - NOT IN FILE
SP - 597
EP - 601
JF - Journal of Science and Medicine in Sport
VL - 13
IS - 6
CY - United Kingdom
N2 - Active travel (walking or cycling for transport) is an important contributor to adolescents overall physical activity (PA). This study examines associations between personal, social and environmental variables and active travel to and from school using data from a large observational study to examine active travel in 2961 year 6 and 8 students (48.7% male), aged 10-14 years (M = 11.4, SD = 0.8. yrs) from 231 schools. Participants completed an on-line survey and all reported living within 2. km of school. Data collected included mode of travel to and from school, self-reported health, and PA variables. Social environmental variables included having playgrounds, parks or gyms close by, feeling safe to walk alone, barriers to walking in the neighbourhood (e.g. traffic, no footpaths), peer and family support for PA, existence of sports teams/scout groups, community disorder and perceived neighbourhood safety. Results showed that while more girls (44.3%) than boys (37.4%) walked to school, lower proportions rode bikes (8.3% vs 22.4%) and hence fewer were active travellers overall. Logistic regression models, adjusted for age, location and socio-economic status were conducted for active travel to/from school, separately for boys and girls. Predictors for boys and girls being 'active travellers' to/from school included recreational facilities close to home, higher perceived safety of the neighbourhood and higher community disorder. For boys, social support from friends, scout groups available and higher enjoyment of physical activity was also important. These findings suggest areas for future research and may be used to guide strategies to increase active travel to and from school. 2010 Sports Medicine Australia
SN - 1440-2440
AD - (Leslie, Kremer, Toumbourou) Deakin University, Australia (Toumbourou, Williams) Centre for Adolescent Health, Australia
ER -

TY - JOUR
ID - 3653
T1 - Playing with fire and getting burnt-A retrospective analysis of injuries presenting to the emergency department during 'firework season'
A1 - Ahmad,Z.
Y1 - 2010///
N1 - 2010431798
English
Journal: Article
KW - EMBASE
KW - abdominal injury
KW - Adolescent
KW - Adult
KW - Age Distribution
KW - Aged
KW - arm injury
KW - article
KW - burn
KW - burn unit
KW - Child
KW - clinical article
KW - conservative treatment
KW - emergency ward
KW - face injury
KW - Female
KW - foot injury
KW - hand burn
KW - Health Education

KW - hospital admission
KW - Human
KW - Male
KW - patient referral
KW - preschool child
KW - priority journal
KW - retrospective study
KW - school child
KW - thorax injury
RP - NOT IN FILE
SP - 199
EP - 201
JF - European Journal of Plastic Surgery
VL - 33
IS - 4
CY - Germany
N2 - During firework season in the UK (October-January), many patients present to emergency departments across the country with firework-related injuries. These seasonal injuries can vary from minor skin trauma to major burns, inhalation injuries and even death in some cases. Governments have tried to improve public awareness and safety with education using multi-million pound national safety campaigns as well as clamping down on fireworks both from within and outside of the EU which fail to meet safety standards. Despite these measures, firework-related injuries are increasing (2005 census Royal Society of Prevention of Accidents (RoSPA)). Reasons for this have been cited as new variations in firework design, antisocial behaviour and readily available cheap fireworks. The author conducted a retrospective study in the emergency department in Exeter from October 2006 to January 2007 highlighting 18 firework-related injuries. Of those, four patients needed referral to local burns centres and five needed admission for supportive treatment and specialty management. Prevention of firework injuries to the hand is of the utmost importance. It is hoped that an increase in public awareness will concentrate attention on precautionary measures to prevent these avoidable 'recreational' accidents. If and when accidents do occur, hand surgeons and therapists attempt to provide patients with the best possible return of hand function with a minimum disability and disfigurement. Further research together with national awareness is suggested to reduce these injuries, all for a tradition which can cost millions in healthcare and loss of earnings, but ultimately, it can cost peoples' lives. 2010 Springer-Verlag
SN - 0930-343X
AD - (Ahmad) Senior House Officer in Plastic and Reconstructive Surgery, Department of Plastic and Reconstructive Surgery, Royal Devon and Exeter NHS Foundation Trust, Exeter EX2 5DW, United Kingdom
ER -

TY - JOUR
ID - 3654
T1 - Association entre pollution particulaire et inflammation des bronches effet modulateur de l'asthme et de l'atopie
A1 - Hulin,M.
A1 - nnesi-Maesano,I.
A1 - Moreau,D.
A1 - Caillaud,D.
Y1 - 2010///
N1 - 2010677902
French
Journal: Article
KW - EMBASE
KW - Air Pollution
KW - airborne particle
KW - article
KW - Asthma/et [Etiology]
KW - atopy/et [Etiology]
KW - bronchus hyperreactivity/et [Etiology]
KW - Child

KW - controlled study
KW - Environmental Exposure
KW - Female
KW - Human
KW - major clinical study
KW - Male
KW - Particle Size
KW - respiratory tract allergy/et [Etiology]
KW - statistical significance
KW - biochemical marker/ec [Endogenous Compound]
KW - nitric oxide/ec [Endogenous Compound]
RP - NOT IN FILE
SP - 594
EP - 602
JF - Revue Francaise d'Allergologie
VL - 50
IS - 8
CY - France
N2 - The aim of the present study was to investigate, using exhaled nitric oxide (FeNO) as a marker of bronchial inflammation, the inflammatory effects of air-borne particles in children in the general population and to examine the role of asthma and atopy on any observed associations. FeNO was measured in 149 children at school in central Clemont-Ferrand participating in the Six Cities Study. Exposure to particles with a dimension of less than 2.5 micrometres (PM_{2.5}) was evaluated with a pump placed for one week in the classes and in the playground used by the children. Heavy exposure to PM_{2.5} was associated with a greater amount of exhaled FeNO in both asthmatic and non-asthmatic children. The non-asthmatic children who were heavily exposed in class exhaled 18.3ppb (95 % CI 14.5-23.2), against 11.3ppb (95 % CI 10.0-12.2) for those weakly exposed. In this same population, the difference in the FeNO between the non-atopic children weakly exposed and those heavily exposed (8.9ppb vs. 12.9ppb) was significantly less than it was between the weakly and the heavily exposed atopic children (11.1ppb vs. 27.4ppb). Our study confirms the inflammatory effect of air-borne particles on children with respiratory allergies, not only in asthmatic children but equally in those without pre-existing bronchial inflammation. The results obtained in non-asthmatic atopics confirm the existence of the known adjuvant effect of certain PM_{2.5} components on the intensity of the allergic inflammatory response. 2010 Elsevier Masson SAS
SN - 1877-0320
AD - (Hulin, Annesi-Maesano, Moreau) Epidemiologie des maladies allergiques et respiratoires (EPAR), UMR S 707, Inserm-UPMC, Universite Pierre-et-Marie-Curie Paris-6, 27, rue de Chaligny, 75571 Paris cedex 12, France (Caillaud) Service de pneumologie, Hopital Gabriel-Montpied, CHU de Clermont-Ferrand, 58, boulevard Montalembert, 63003 Clermont-Ferrand cedex 1, France
ER -

TY - JOUR
ID - 3655
T1 - Risk factors for Japanese encephalitis: A case-control study
A1 - Liu,W.
A1 - Gibbons,R.V.
A1 - Kari,K.
A1 - Clemens,J.D.
A1 - Nisalak,A.
A1 - Marks,F.
A1 - Xu,Z.Y.
Y1 - 2010///
N1 - 2010544276
English
Journal: Article
KW - EMBASE
KW - agricultural land
KW - article

KW - case control study
KW - Child
KW - clinical feature
KW - controlled study
KW - crop production
KW - Female
KW - health survey
KW - hospital admission
KW - Human
KW - Immunization
KW - Indonesia
KW - Infant
KW - infection prevention
KW - Japanese encephalitis/ep [Epidemiology]
KW - Japanese encephalitis/pc [Prevention]
KW - major clinical study
KW - Male
KW - night
KW - pig farming
KW - play
KW - preschool child
KW - residential area
KW - rice field
KW - Risk Assessment
KW - risk factor
KW - school child
KW - serology
KW - Sex Ratio
KW - insect repellent
RP - NOT IN FILE
SP - 1292
EP - 1297
JF - Epidemiology and Infection
VL - 138
IS - 9
CY - United Kingdom
N2 - Japanese encephalitis (JE) has been found to be endemic in Bali, Indonesia. A case-control study was conducted to identify factors associated with JE infection. All 94 serologically confirmed JE cases (cases) and 163 cases of encephalitis or aseptic meningitis without JE (controls) identified in Bali during 2001-2004 were included in the study. Potential risk factors were surveyed at hospital admission. Univariate analyses revealed the following factors to be associated with JE: older age, referral from sub-district health centre or private hospital, playing outdoors after dinner, use of mosquito repellent or spraying, proximity of the residence to rice fields, and pig ownership by the family or next-door neighbours. Multivariate analysis identified proximity to rice fields (OR 293, 95% CI 157-545), pig ownership (OR 224, 95% CI 117-426), and older age (OR 121, 95% CI 109-133) as being independently associated with the risk of JE. Because rice cultivation and pig rearing are essential to the economy of Bali, JE immunization is the best intervention for prevention of JE in Bali. Copyright 2010 Cambridge University Press
SN - 0950-2688
AD - (Liu, Clemens, Marks, Xu) International Vaccine Institute, San 4-8, Bongcheon-7 dong, Kwanak-ku, Seoul, South Korea (Gibbons, Nisalak) Department of Virology, Armed Forces Research Institute of Medical Sciences (AFRIMS), Bangkok, Thailand (Kari) Department of Paediatrics, Udayana University School of Medicine, Bali, Indonesia
ER -

TY - JOUR
ID - 3656
T1 - Balamuthia and Acanthamoeba-binding antibodies in West African human sera

A1 - Kiderlen,A.F.
A1 - Radam,E.
A1 - Schuster,F.L.
A1 - Adjogoua,E.V.
A1 - koua-Koffi,C.
A1 - Leendertz,F.H.
Y1 - 2010///
N1 - 2010396516
English
Journal: Article
KW - EMBASE
KW - Acanthamoeba
KW - Acanthamoeba castellanii
KW - Acanthamoeba lenticulata
KW - Adolescent
KW - Adult
KW - Africa
KW - Aged
KW - antibody affinity
KW - antibody blood level
KW - article
KW - Balamuthia
KW - Balamuthia mandrillaris
KW - blood analysis
KW - Child
KW - controlled study
KW - Environmental Exposure
KW - Female
KW - Flow Cytometry
KW - Human
KW - Male
KW - nonhuman
KW - parasitosis
KW - preschool child
KW - priority journal
KW - rain forest
KW - school child
KW - soil microflora
KW - species difference
KW - formaldehyde
KW - parasite antibody
RP - NOT IN FILE
SP - 28
EP - 32
JF - Experimental Parasitology
VL - 126
IS - 1
CY - United States
N2 - Little is known about the prevalence of Balamuthia mandrillaris amoebae and Balamuthia amoebic encephalitis in Africa. As an approach, relative concentrations of amoebae-binding serum antibodies (Ab) were assessed by flow cytometry using formaldehyde-fixed B. mandrillaris, Acanthamoeba lenticulata 72-2 and Acanthamoeba castellanii 1BU amoebae for specific Ab capture (B.m.-Ab, A.l.-Ab, A.c.-Ab). One hundred and ninety-two sera from West African (Cote d'Ivoire) donors aged 11-95. years (mean 38 a; 51% males), and living in villages surrounded by rainforest near the Liberian border, were tested and related to reference sera from Berlin. While B.m.-Ab tended to increase with donor age, A.l.-Ab and A.c.-Ab did not. Accordingly, B.m.-Ab correlated only weakly with A.l.-Ab or A.c.-Ab. Of the nine individuals with the highest B.m.-Ab

concentrations, most were elderly (mean 58 a), male (78%), and professed intensive outdoor activity (hunting, farming). Only three of these sera also showed elevated A.l.-Ab, and none elevated A.c.-Ab. 2009
SN - 0014-4894
AD - (Kiderlen, Radam) Robert Koch Institute, Cellular Immunology Unit P22, Berlin, Germany (Schuster) California Department of Health Services, Viral and Rickettsial Diseases Laboratory, Richmond, United States (Adjogoua, Akoua-Koffi) Department of Epidemic Viruses, Adiopodourme, 01 BP 490, Abidjan, Cote D'Ivoire (Leendertz) Robert Koch Institute, Emerging Zoonoses Unit NG2, Berlin, Germany
ER -

TY - JOUR
ID - 3657
T1 - Intervention to improve child care nutrition and activity environments
A1 - Wosje,K.S.
A1 - Sauers,H.
A1 - Norton,A.
A1 - Copeland,K.
Y1 - 2010///
N1 - 70546872
English
Journal: Conference Abstract
KW - EMBASE
KW - Environment
KW - Child Care
KW - Nutrition
KW - Human
KW - Child
KW - Muscle
KW - Physical Activity
KW - follow up
KW - preschool child
KW - United States
KW - Interview
KW - Self Evaluation
KW - milk
KW - Public Health
RP - NOT IN FILE
JF - FASEB Journal
VL - Conference: Experimental Biology 2010, EB Anaheim, CA United States. Conference Start: 20100424 Conference End: 20100428. Conference Publication:
IS - var.pagings
N2 - We determined the efficacy of a Southwest Ohio state-sponsored intervention to promote nutrition (Nu) and physical activity (PA) in preschool child care. We conducted baseline (7/08-4/09) and follow-up (9/09-12/09) phone interviews with child care center directors on Nu and PA environments of centers in an intervention (I, N=108 centers, Dayton area) and a demographically similar control (C, N=162 centers, Cincinnati area) county. A self-assessment was sent to the 108 I centers; 28 [active intervention (AI)] returned the application and received a Targeted Action Plan to address self identified deficiencies in Nu and PA environments. The 6mo (mean+SD) in global Nu (eg, meal environment, milk, juice, snacks, etc) and PA (eg, TV, outdoor play & muscle room features, etc) scores trended higher in AI than inactive intervention (II, N=80 centers) for Nu (0.86+1.71 v 0.10+2.74 p=0.21) and PA (0.96+3.74 v 0.00+3.35 p=0.18), but not C (Nu: 0.45+2.60, p=0.4; PA: 0.80+3.78, p=0.8). The trend in differences between AI and II remained after adjustment for race (25% and 27% primarily black in AI and II respectively) or % of children receiving meal subsidy (>75% of children for 39% of AI and 32% of II). These pilot data suggest the intervention could be effective as an annual state-wide public health initiative, but a larger study is needed
SN - 0892-6638
AD - (Wosje, Sauers, Copeland) General and Community Pediatrics, Cincinnati Children's Hospital Medical Center, Cincinnati, OH, United States (Norton) Ohio Department of Health, Columbus, OH, United States
ER -

TY - JOUR
ID - 3658
T1 - Polonium-210 in Diesel Particulate Matter (DPM)
A1 - Lykken,G.I.
A1 - Czapiewski,R.
A1 - Ward,T.
Y1 - 2010///
N1 - 70541566
English
Journal: Conference Abstract
KW - EMBASE
KW - Particulate Matter
KW - diesel engine
KW - Human
KW - Inhalation
KW - brain tissue
KW - brain tumor
KW - Child
KW - progeny
KW - brain
KW - combustion
KW - filter
KW - Atmosphere
KW - Risk
KW - polonium
KW - radon
KW - nitric acid
KW - silver
RP - NOT IN FILE
JF - FASEB Journal
VL - Conference: Experimental Biology 2010, EB Anaheim, CA United States. Conference Start: 20100424 Conference End: 20100428. Conference Publication:
IS - var.pagings
N2 - Inhalation of DPM has been reported to shorten the life of 70,000 Americans each year and to increase the risk of developing brain tumors in children. Environmental radon progeny (ERnP) have been measured in human brain tissues, but how they entered the brain remains uncertain. We hypothesize ERnP may be incorporated in DPM during combustion and enter the body through inhalation. Therefore, we captured DPM on 30 nm filters. The DPM, containing polonium-210 (Po-210) came from a variety of diesel engines operating in normal outdoor atmospheres with radon concentrations from 7 to 33 Bq/m3. Initially, the Po-210 alpha emissions were measured directly from the nanofilters. However, the activity dropped dramatically, presumably due to the volatility of the DPM particles. Therefore, immediately after loading the nanofilters were soaked for approximately 12 hours in nitric acid. Po-210 from this solution was deposited onto silver disks and counted. The observed Po-210 activities were dependent upon the type of diesel engine and the atmospheric ERn concentration
SN - 0892-6638
AD - (Lykken, Czapiewski) Department of Physics, University of North Dakota, Grand Forks, ND, United States (Ward) Techsource Inc., Germantown, MD, United States
ER -

TY - JOUR
ID - 3659
T1 - Acetaminophen use increases the risk of allergic respiratory diseases
A1 - Vlaski,E.
A1 - Stavric,K.
A1 - Seckova,L.
A1 - Kimovska,M.

A1 - Kirovski,I.
Y1 - 2010///
N1 - 70179107
English
Journal: Conference Abstract
KW - EMBASE
KW - clinical immunology
KW - allergy
KW - Risk
KW - respiratory tract disease
KW - hay fever
KW - Asthma
KW - wheezing
KW - Macedonia (republic)
KW - Rhinitis
KW - adolescence
KW - Biology
KW - physician
KW - allergic disease
KW - atopy
KW - childhood
KW - questionnaire
KW - Child
KW - city
KW - fruit vegetable
KW - cereal
KW - fish
KW - passive smoking
KW - Cooking
KW - heating
KW - exposure
KW - logistic regression analysis
KW - Speech
KW - Sleep
KW - night
KW - chest infection
KW - rhinoconjunctivitis
KW - coughing
KW - paracetamol
KW - antioxidant
KW - oxidizing agent
RP - NOT IN FILE
SP - 660
JF - Allergy: European Journal of Allergy and Clinical Immunology
VL - Conference: 29th Congress of the European Academy of Allergy and Clinical Immunology, EAACI London United Kingdom. Conference Start: 20100605 Conference End: 20100609. Conference Publication:
IS - var.pagings
N2 - Background: A positive association between acetaminophen use and allergic diseases is suggested with impaired respiratory oxidant/antioxidant balance and promotion of atopy as proposed underlying mechanisms. The aim of the study was to explore the impact of frequent acetaminophen use on asthma and hay fever. Methods: Self-reported data obtained through the International Study of Asthma and Allergies in Childhood (ISAAC) Phase Three written questionnaires of 5507 children aged 12/16 years from eight cities in The Republic of Macedonia were used for the analysis. The frequency of current acetaminophen use was correlated to current and ever-diagnosed asthma and hay fever. Current intake of fruit, vegetables, cereals and fish as dietary antioxidants and truck passage through the residential street, second-hand tobacco smoke, gas/wood cooking and wood/coal/oil heating at home as parameters of outdoor oxidant exposure were all employed as confounders for acetaminophen use adjustment. The data were statistically analyzed by odds ratios (OR, 95%

CI) in binary logistic regression. Results: Current acetaminophen use at least once monthly, compared to its use at least once yearly/never, was associated with an increased risk of current wheeze (OR: 1.53; 95% CI: 1.21-1.94; P = 0.000), current speech-limiting wheeze (OR: 2.06; 1.36-3.12; P = 0.001), current sleep-disturbing wheeze (OR: 1.69; 1.26-2.28; P = 0.001), current night dry cough apart from chest infection (OR: 1.82; 1.55-2.15; P = 0.000), current rhinoconjunctivitis symptoms (OR: 1.80; 1.46-2.23; P = 0.000), interference of current rhinitis symptoms with daily activities (OR: 1.66; 1.41-1.96; P = 0.000) and ever-diagnosed hay fever (OR: 1.55; 1.21-1.98; P = 0.000). Conclusion: The findings suggest an increased risk of asthma and hay fever associated with frequent acetaminophen use in early adolescence. Until further epi-demiological and biological research results come, acetaminophen should be used only if it is physician-recommended, within therapeutic doses and not frequently
SN - 0105-4538
AD - (Vlaski, Seckova, Kimovska, Kirovski) Department of Pulmonology and Allergology, University Children's Hospital, Skopje, Fyrom, Macedonia (Stavric) Department of Immunology, University Children's Hospital, Skopje, Fyrom, Macedonia
ER -

TY - JOUR
ID - 3660
T1 - The clinical development of specific immunotherapies: Specific methodological issues and clinical interpretation of results
A1 - Devillier,P.
A1 - Brehler,R.
A1 - Sastre,J.
A1 - Passalacqua,G.
A1 - Vervloet,D.
Y1 - 2010///
N1 - 70178854
English
Journal: Conference Abstract
KW - EMBASE
KW - Immunotherapy
KW - clinical immunology
KW - allergy
KW - Pollen
KW - season
KW - tablet
KW - disease severity
KW - Adult
KW - grass pollen
KW - patient
KW - Child
KW - allergic rhinitis
KW - rhinoconjunctivitis
KW - Population
KW - linear regression analysis
KW - exposure
KW - Safety
KW - meta analysis
KW - Patient Selection
KW - clinical trial
KW - placebo
RP - NOT IN FILE
SP - 567
JF - Allergy: European Journal of Allergy and Clinical Immunology
VL - Conference: 29th Congress of the European Academy of Allergy and Clinical Immunology, EAACI London United Kingdom. Conference Start: 20100605 Conference End: 20100609. Conference Publication:
IS - var.pagings

N2 - Background: Historically, the safety and efficacy of specific immunotherapy (SIT) has been demonstrated in (mainly small-scale) studies and confirmed in meta-analyses. However, the inherent sources of heterogeneity (patient selection outside the pollen season, pollen count variations, long evaluation periods) observed in recent, large-scale studies of sublingual immunotherapy (SLIT) tablets mean that highly susceptible patients may not exhibit uniformly a sufficient level of symptoms during the study period to assess the efficacy of SIT. Methods: We performed randomized, double-blind, placebo-controlled, multinational clinical trials of a 300IR pre- and co-seasonally administered 5-grass pollen SLIT tablet in adults and children with allergic rhinitis. The primary efficacy criterion was the mean average rhinoconjunctivitis total symptom score (ARTSS) over the pollen season, during which daily grass pollen counts were measured locally. To evaluate the potential relationship between disease severity and the treatment effect, investigating centres were divided in tiertiles according to their mean placebo group ARTSS. Results: Pre-seasonal inclusion of patients on the basis of retrospectively severe symptoms in the previous pollen season did not produce such a severely affected population in the study season. The apparent treatment effect of SLIT tablet was partially masked by the highly variable severity of symptoms during the whole pollen season (mean ARTSS in the adult placebo group: 4.93 + 3.229). For the adult trial, the mean active versus placebo ARTSSs and the relative difference versus placebo (%) were 15%, 26% and 37% for the low, moderate- and high-disease severity tiertiles, respectively. In the paediatric trial, these values were 10%, 33% and 34%, respectively. Linear regression of individual daily RTSS scores and pollen counts in active and placebo groups also showed that the more severe the disease, the greater the treatment effect of SLIT tablet. Conclusion: Inherent sources of heterogeneity must be taken into account when interpreting the results of natural-exposure immunotherapy trials but none obscured the statistically significant treatment effect of SLIT. In a subgroup analysis, SLIT tablets' beneficial effect increased with disease severity
SN - 0105-4538
AD - (Devillier) Foch Hospital, Suresnes, France (Brehler) UKM, Muenster, Germany (Sastre) Fundacion Jimenez Diaz, Servicio de Alergia, Madrid, Spain (Passalacqua) Allergy and Respiratory Diseases, Department of Internal Medicine, Genoa University, Italy (Vervloet) Universite de la Mediterranee, Marseille, France
ER -

TY - JOUR
ID - 3661
T1 - Importance of the IL-5 and eotaxin studies in children with the allergic diseases and eosinophilia
A1 - Vinogradova,T.
A1 - Potapova,O.
A1 - Smirnova,M.
A1 - Pampura,A.
A1 - Munblit,D.
Y1 - 2010///
N1 - 70178210
English
Journal: Conference Abstract
KW - EMBASE
KW - eosinophilia
KW - allergic disease
KW - Child
KW - clinical immunology
KW - allergy
KW - patient
KW - Pollen
KW - eosinophil
KW - season
KW - serum
KW - sensitization
KW - exposure
KW - atopic dermatitis
KW - allergic rhinitis
KW - Asthma
KW - immunoassay
KW - hay fever

KW - Dermatophagoides
KW - Austria
KW - eotaxin
KW - interleukin 5
KW - cytokine
KW - allergen
RP - NOT IN FILE
SP - 333
EP - 334
JF - Allergy: European Journal of Allergy and Clinical Immunology
VL - Conference: 29th Congress of the European Academy of Allergy and Clinical Immunology, EAACI London United Kingdom. Conference Start: 20100605 Conference End: 20100609. Conference Publication:
IS - var.pagings
N2 - Background and research objective: In the recent years the essential role of IL-5 and eotaxin in eosinophilia occurrence in patients with allergic diseases has been raised. The goal of this study was to evaluate the importance of IL-5 and eotaxin in blood serum in children with allergic diseases and coexisting eosinophilia. Materials and methods: We have studied a cohort of 27 patients from 3 to 17 years of age with allergic diseases (atopic dermatitis, allergic rhinitis and asthma) with a relative and absolute eosinophilia. IL-5 and eotaxin in blood serum has been detected using IFA method: Bender Medsy Stem (Austria) and Biosource immunoassay kit (USA) (sensitivity 0-1000 and 0-500 pg/ml accordingly). Results: Average value of relative quantity of eosinocytes was 7.65 + 0.1%, absolute - 0.65 + 0.08\10[*]9 In all patients we have deteced eotaxin, and IL-5 in 11 children. Average level of IL-5 was 5.9 + 3.1 pg/ml, eotaxin - 84.1 + 7.8 pg/ml. Cytokines level depended on eosinophilia degree, in patients with the absolute eosinophils level <0.4, IL-5 level was 1.3 + 1.06, and eotaxin level was 77.7 + 19.76, in those patients with absolute eosinophilia >0.4 (n = 17) it was detected as 8.9 + 4.96 and 84.7 + 6.6 accordingly, at higher eosinophilia >0.8 (n = 10) - 13.45 + 7.66 and 89.3 + 6.66 accordingly. We have found a positive correlation between eotaxin level and absolute quantity of eosinophils (r = 0.47, P > 0.05). In patients diagnosed with hay fever and coexisting eosinophilia IL-5 level was 2.07 + 1.05, and eotaxin - 82.1 + 8.6. Outside the pollen season (n = 8) IL-5 level was 1.92 + 1.06, and eotaxin - 67.6 + 8.8 while during the pollen season (n = 10) Il-5 level was 2.1 + 1.05, eotaxin - 88.9 + 13.8. Eotaxin level depended on a particular month: in February average eotaxin level was 82.9, while in May - 107.1. In patients sensitized to house dust mite and coexisting eosinophilia has been detected a higher level of IL-5 (10.8 + 7.02)compared to those patients who had a pollen sensitization, eotaxin level was 77.3 + 6.7. We haven't found any difference depending on the allergic disease. Conclusion: To summarise, Il-5 and eotaxin definitely play an important role in eosinophilia development in children with allergic diseases. Level of these cytokines depends on the type of sensitization and allergen exposure but it's requires further research
SN - 0105-4538
AD - (Vinogradova, Potapova, Smirnova, Pampura, Munblit) Russian Federation
ER -

TY - JOUR
ID - 3662
T1 - Prevalence of asthma among children of different nationalities in the Republic of Macedonia
A1 - Kirovski,I.
A1 - Vlaski,E.
A1 - Stavric,K.
A1 - Sazdovski,A.
A1 - Isjanovska,R.
A1 - Seckova,L.
A1 - Iseni,A.
Y1 - 2010///
N1 - 70177891
English
Journal: Conference Abstract
KW - EMBASE
KW - Macedonia (republic)
KW - Prevalence
KW - Asthma

KW - Child
KW - clinical immunology
KW - allergy
KW - Sleep
KW - fast food
KW - wheezing
KW - logistic regression analysis
KW - Exercise
KW - dog
KW - Fruit
KW - cereal
KW - Lifestyle
KW - Food Intake
KW - allergic disease
KW - questionnaire
KW - Adolescent
KW - city
KW - chi square test
KW - Risk
KW - paracetamol
KW - corticosteroid
RP - NOT IN FILE
SP - 221
JF - Allergy: European Journal of Allergy and Clinical Immunology
VL - Conference: 29th Congress of the European Academy of Allergy and Clinical Immunology, EAACI London United Kingdom. Conference Start: 20100605 Conference End: 20100609. Conference Publication:
IS - var.pagings
N2 - Background: Related to the suggested different prevalence rates of allergic diseases in different nationalities living in the same country, the study was aimed to explore the impact of the nationality on asthma in R. Macedonia. Methods: The self-reported data obtained through the ISAAC phase three questionnaire from 5507 adolescents aged 12/ 16 years from eight cities in R. Macedonia in 2006 were analysed. Chi-square test and odds ratios (OR, 95%CI) in logistic regression were performed for statistic analysis of the data. Results: In Macedonian in contrast to Albanian respondents significantly higher prevalence rates of current wheeze (W) and current sleep-disturbing W (P = 0.000 for both of them), nonsignificantly higher prevalence rates of current exercise-induced W and inhalant corticosteroids use (P > 0.05) and nonsignificantly lower prevalence of ever-diagnosed asthma (P > 0.05) were established. In Macedonian nationality a positive association of frequent fast food and paracetamol intake, dog at home, >3 hrs daily TV watching with current W (OR 1.49, 1.11-2.00 P = 0.009, OR 1.39, 1.03-1.87 P = 0.033, OR 1.38, 1.03-1.86 P = 0.031 and OR 1.56, 1.17-2.08 P = 0.002, respectively) and of frequent paracetamol intake, trucks passage through the residential street with current sleep-disturbing W (OR 1.72, 1.17-2.52 P = 0.005 and OR 1.52, 1.05-2.20 P = 0.025, respectively) were determined. In Albanian nationality a negative association only of fruit intake with current W and sleep-disturbing W (OR 0.26, 0.09-0.69 P = 0.007 and OR 0.16, 0.05-0.57 P = 0.004, respectively) and of cereal intake with current W (OR 0.37, 0.14-0.97 P = 0.043) were documented. Conclusion: It seems that the different lifestyle i.e. sedentary regimen, pets at home, frequent paracetamol and fast food intake and outdoor airpollution in Macedonian contrary to Albanian nationality may be responsible for frequent and more severe wheeze attacks
SN - 0105-4538
AD - (Kirovski, Vlaski, Sazdovski, Seckova) University Children's Hospital, Department of Pulmonology and Allergology, Skopje, Macedonia (Stavric) University Children's Hospital, Department of Immunology, Skopje, Macedonia (Isjanovska) Institute of Epidemiology, Biostatistics and Medical Informatics, Skopje, Macedonia (Iseni) Private Health Organisation, Department of Pediatrics, Kumanovo (Iseni) Private Health Organisation, Tetovo, Macedonia (Iseni) Private Health Organisation, Ohrid, Macedonia (Iseni) Private Health Organisation, Veles, Macedonia (Iseni) Private Health Organisation, Berovo, Macedonia (Iseni) Private Health Organisation, Delcevo, Macedonia (Iseni) Private Health Organisation, Pehcevo, Macedonia (Iseni) Private Health Organisation, Fyrom, Macedonia
ER -

TY - JOUR

ID - 3663
T1 - Particulate matter, allergic sensitisation and ISAAC questionnaire in schoolchildren of Milan, Italy
A1 - Sala,M.
A1 - Mandelli,M.
A1 - Rettani,G.
A1 - Palazzo,S.
A1 - Banderali,G.
A1 - Riva,E.
A1 - Giovannini,M.
Y1 - 2010///
N1 - 70177754
English
Journal: Conference Abstract
KW - EMBASE
KW - allergy
KW - Italy
KW - school child
KW - Particulate Matter
KW - questionnaire
KW - clinical immunology
KW - school
KW - Child
KW - Prevalence
KW - exposure
KW - girl
KW - boy
KW - sensitization
KW - skin
KW - prick test
KW - allergic rhinitis
KW - Asthma
KW - grass pollen
KW - Conjunctivitis
KW - respiratory function
KW - Rhinitis
KW - Pollution
KW - primary school
KW - respiratory system
KW - Air Pollution
KW - grass
KW - ragweed
KW - birch
KW - olive tree
KW - Pollen
KW - Dust
KW - mite
KW - dog
KW - cat
KW - dander
KW - Alternaria
KW - informed consent
KW - parent
KW - Spirometry
KW - Student t test
KW - Exercise
KW - allergen
KW - Nitric Oxide

RP - NOT IN FILE
SP - 169
JF - Allergy: European Journal of Allergy and Clinical Immunology
VL - Conference: 29th Congress of the European Academy of Allergy and Clinical Immunology, EAACI London United Kingdom. Conference Start: 20100605 Conference End: 20100609. Conference Publication:
IS - var.pagings
N2 - Background: Air pollution may exercise a detrimental effect on respiratory system, also influencing the allergic sensitization. Methods: We enrolled 228 children (127 boys, 101 girls; mean age 8, range 7-10 years) from two primary schools in different sites for pollution exposure in Milan: the first located downtown (74 children; 47 boys, 27 girls), the second in a large park (154 children; 80 boys, 74 girls). One hundred ninety three children (73 in downtown school and 120 in park school) agreed to undergo skin prick tests to common inhalant allergens: grass, ragweed, birch, olive tree pollens, dust mites, dog and cat dander and alternaria. Written informed consent was obtained from parents. Daily in- and outdoor levels of PM2.5 and PM1 (diameter <2.5 mum and 1 mum respectively) were measured by two detection units for a week during the study (October 2008). Children underwent spirometry, dosage of exhaled nitric oxide fraction (FeNO), skin prick tests and were administered the ISAAC questionnaire. Results: Mean (SD) values of indoor particulates (mug/m³) were significantly (P < 0.01) higher in the downtown than park school: PM1 48 (17.3) versus 21 (5.29), PM2.5 62.7 (20.5) versus 43.3 (17.9). The mean values of FEV1 (SD) and FVC (SD) were significantly lower in downtown school: 102.2 (12.8) versus 106.1 (11.8) (P = 0.02, t-test) and 95.8 (11.4) versus 100.4 (10.7) (P = 0.003). Mean (SD) concentration of FeNO (ppb) was 17 (12) in downtown school and 14.4 (9) in the park school (P = 0.456). Prevalence of sensitization to inhalant allergens did not differ between the two schools, both as a whole (downtown school 32.8% versus park school 27.5%; P = 0.427) and for any specific allergen. The prevalence of asthma exacerbations reported in the previous 12 months was higher in children from downtown school (8.4% in downtown school, 2.5% in park school; P = 0.05); in children allergic to grass pollen (16.4% in downtown school, 22.5% in park school), the prevalence of allergic rhinitis was higher in park school (30.8% in downtown school, 69.2% in park school P = 0.03) with more activity limitation due to rhinitis and associated conjunctivitis (0% in downtown school, 29.6% in park school; P = 0.03). Conclusion: Exposure to higher indoor levels of fine particulates could negatively affect respiratory function. The higher prevalence of allergic rhinitis and consequent activity limitation in the previous 12 months observed in park school might be related to allergen exposure
SN - 0105-4538
AD - (Sala, Mandelli, Rettani, Palazzo, Banderali, Riva, Giovannini) Pediatric Department, San Paolo Hospital, University of Milan, Milan, Italy
ER -

TY - JOUR
ID - 3664
T1 - The effect of neonatal binge ethanol exposure on the cingulate cortex of the rat
A1 - Shoemack,P.
A1 - Napper,R.M.A.
Y1 - 2010///
N1 - 70173237
English
Journal: Conference Abstract
KW - EMBASE
KW - Society
KW - cingulate gyrus
KW - rat
KW - exposure
KW - Alcoholism
KW - nerve cell
KW - Child
KW - Intubation
KW - brain
KW - Adolescent
KW - social adaptation
KW - control group

KW - apoptosis
KW - cell death
KW - adolescence
KW - Long Evans rat
KW - intragastric drug administration
KW - Death
KW - videorecording
KW - neuropathology
KW - alcohol
KW - thionine
KW - pentobarbital
KW - paraformaldehyde
RP - NOT IN FILE
SP - 97A
JF - Alcoholism: Clinical and Experimental Research
VL - Conference: 33rd Annual Scientific Meeting of the Research Society on Alcoholism, RSA San Antonio, TX United States. Conference Start: 20100626 Conference End: 20100630. Conference Publication:
IS - var.pagings
N2 - The cingulate cortex is a key region involved in the understanding of consequences and in the selection of appropriate behaviour. We have previously shown that a single binge exposure to ethanol during early neonatal life results in the acute death of neurons in the cingulate cortex via apoptosis. This study investigated the long-term consequences of ethanol-induced cell death on the structure of the mature cingulate cortex and on play fighting behaviour during adolescence. On postnatal day (PN) 6, Long-Evans rat pups were randomly assigned to either an alcohol exposure group (E); with daily doses of either 6.0, 5.25 or 4.5 g/kg ethanol via intragastric intubation as two feeds two hours apart; a sham intubation (IC) or a suckle control (SC) group. For one cohort, beginning on PN30, play fighting in pairs (an SC or dose matched E rat) was video recorded for 20 minutes a day on 3 consecutive days. Data was then manually scored. For a second cohort, animals were deeply anaesthetised (sodium pentobarbital 100 mg/kg i.p.) and perfused (4% paraformaldehyde) on PN365. Brains were removed, cryo-protected, frozen and sectioned in the coronal plane at 60 lm. A random systematic set of sections was stained with thionin and unbiased stereological methods used to determine the number of neurons in the anterior cingulate cortex (Acc). The 6.0 and 5.25 g/kg E groups showed a 39.6 and 30.8% ($p< 0.05$) mean decrease respectively in Acc cells relative to the IC group. The 6.0 g/kg E group also had significantly less Acc neurons than the 4.5 g/kg E group ($p < 0.05$). Preliminary data indicates that alcohol exposed animals differ in their rate of initiation of attack and in their response to attack compared to control animals. Alterations in play-fighting behaviour, that is characteristic of adolescent rats, may have implications for the playground behaviour of alcohol-affected children. For children that have been exposed intermittently to alcohol during fetal life, altered social skills that are used in typical playground interactions, may be the result of neuropathology in the cingulate cortex
SN - 0145-6008
AD - (Shoemack, Napper) University of Otago, School of Medical Sciences, Dunedin, Otago 9054, New Zealand (Napper) Anatomy and Structural Biology, School of Medical, Dunedin, Otago 9054, New Zealand
ER -

TY - JOUR
ID - 3665
T1 - Prenatal Alcohol Exposure (PAE) alters open field behavior in adult rats
A1 - Callaway,H.M.
A1 - Yang,Q.
A1 - Heck,D.H.
A1 - Waters,R.S.
Y1 - 2010///
N1 - 70173234
English
Journal: Conference Abstract
KW - EMBASE
KW - rat
KW - Adult

KW - open field behavior
KW - exposure
KW - Alcoholism
KW - Society
KW - velocity
KW - computer program
KW - hyperactivity
KW - Child
KW - Attention Deficit Disorder
KW - rodent
KW - animal model
KW - hypothesis
KW - Environment
KW - mare
KW - videorecording
KW - Acceleration
KW - Cognition
KW - motor control
KW - alcohol
RP - NOT IN FILE
SP - 96A
JF - Alcoholism: Clinical and Experimental Research
VL - Conference: 33rd Annual Scientific Meeting of the Research Society on Alcoholism, RSA San Antonio, TX United States. Conference Start: 20100626 Conference End: 20100630. Conference Publication:
IS - var.pagings
N2 - Objective: Prenatal Alcohol Exposure (PAE) often leads to Fetal Alcohol Spectrum Disorder (FASD). Children diagnosed with FASD frequently display symptoms of hyperactivity and deficits in attention span associated with Attention-Deficit Hyperactivity Disorder (ADHD). Rodent animal models have been used to study biological and behavioral consequences of PAE. In the present study, we tested the hypothesis that PAE rats would exhibit hyperactivity when placed in an open field environment. Methods: Pregnant Sprague-Dawley dams (Alc) were intragastrically intubated with ethanol (6 g/kg/day) on gestational days 1-20 and compared to chow-fed (CF) and pair-fed (PF) controls. At sixteen weeks of age, rats were individually placed in a cylindrical gray open field arena (8 ft in diameter and 3 ft high) surrounded on the outside by white sheet curtains. Video tracking software recorded the coordinates of the rat's movements within the arena. SEE (Software for Exploring Exploration) was used to analyze these coordinates. Results: Alc rats had significantly larger average (p>.05) (i) total distance traveled [26,631.221 cm] than PF [19,959.641 cm] and CF [20,674.931 cm] controls, (ii) Median Segment Acceleration to Max Speed (the median of a movement segment's maximum speed divided by its duration) [17.5198 cm/sec2] than PF [12.469 cm/sec2] and CF [13.274 cm/sec²] controls, (iii) Median of Move Segment Max Speed (the median of the maximum speed in progression segments taken over all progression segments) [48.831 cm/s] than PF [36.674 cm/s] and CF [41.708 cm/s] controls, and (iv) Quantile 95 of Move Segment Max Speed (the 95th quantile of the maximum speed in progression segments taken over all progression segments) [83.729 cm/s] than PF [58.877 cm/s] and CF [66.164 cm/s] controls. Alc rats also covered more distance in the arena center than the controls, which usually stayed near the arena's edge. Percent activity levels during lingering segments were significantly greater (p>.05) in CF [11.3% activity] and PF [12.0%] rats than Alc rats [7.9%]. Conclusion: These results suggest that Alc rats moved more rapidly, accelerated more quickly, covered a greater amount of distance, were less active in lingering segments, and were less confined to the arena's walls than the controls. We conclude that PAE greatly alters open field behavior in adult rats, and may reflect deficits in cognition and/or motor control
SN - 0145-6008
AD - (Callaway, Yang, Heck, Waters) Department of Anatomy and Neurobiology, University of Tennessee, Health Science Center, Memphis, TN 38163, United States
ER -

TY - JOUR
ID - 3666
T1 - Use of high-dose crotalidae polyvalent immune fab in a toddler with copperhead envenomation
A1 - Rose,S.R.

A1 - Murphy,C.M.
Y1 - 2010///
N1 - 70170036
English
Journal: Conference Abstract
KW - EMBASE
KW - snake
KW - envenomation
KW - drug megadose
KW - toddler
KW - patient
KW - Leg
KW - swelling
KW - edema
KW - Safety
KW - Case Report
KW - Female
KW - knee
KW - father
KW - university hospital
KW - Hospital
KW - ankle
KW - hip
KW - patient right
KW - thigh
KW - pulse rate
KW - capillary
KW - vital sign
KW - laboratory
KW - umbilicus
KW - perfusion
KW - Adult
KW - Child
KW - adverse drug reaction
KW - poison
KW - fibrinogen
RP - NOT IN FILE
SP - 300
JF - Clinical Toxicology
VL - Conference: 2010 International Congress of the European Association of Poisons Centres and Clinical Toxicologists Bordeaux France. Conference Start: 20100511 Conference End: 20100514. Conference Publication:
IS - var.pagings
N2 - Objective: The safety of Crotalidae polyvalent immune Fab (Crofab) for copperhead envenomations has not been studied in pediatric patients. This report documents the safety of high dose Crofab in a toddler bitten by a copperhead. Case report: A 28-month-old female was bitten on her right knee by a copperhead while playing in her backyard. Her father positively identified the snake, and the patient was transferred to an academic medical center (AMC) after brief evaluation in an outside hospital. On initial evaluation at the AMC (about 4.5 hours after the bite), the patient's right leg was noted to be ecchymotic and swollen from the ankle to the hip, with a right thigh circumference roughly three times greater than the left. Peripheral pulses were diminished and capillary refill was delayed. She was immediately treated with four vials of Crofab and the leg was elevated. Her vital signs and initial laboratory studies were normal, with exception of fibrinogen (149 mg/dL) and WBC (19,800 u/L). She was admitted to the ICU where swelling of her right lower extremity progressed to her umbilicus and both labia. The patient received an additional 18 vials over 24 hours to impede swelling and improve right lower extremity perfusion. Her edema significantly decreased by day three and she was discharged on day seven. No adverse reactions to Crofab were identified. Conclusion: Crofab is effective in treating copperhead envenomation in adults. Limited data is available on Crofab use in children. This case

demonstrates uncomplicated use of high dose Crofab in a pediatric patient with progressive edema following copperhead envenomation
SN - 1556-3650
AD - (Rose, Murphy) Department of Emergency Medicine, School of Medicine, Virginia Commonwealth University, Richmond, VA, United States (Rose) Division of Toxicology, School of Medicine, Virginia Commonwealth University, Richmond, VA, United States
ER -

TY - JOUR
ID - 3668
T1 - Psychosocial factors associated with failure to use contraception among adolescents with repeat pregnancies in Puerto Rico
A1 - Tulla,M.E.
A1 - Colon,M.
A1 - Laras,L.
Y1 - 2010///
N1 - 70141320
English
Journal: Conference Abstract
KW - EMBASE
KW - Puerto Rico
KW - Pregnancy
KW - Adolescent
KW - social psychology
KW - contraception
KW - gynecology
KW - Society
KW - Education
KW - birth rate
KW - Child
KW - Motivation
KW - Risk
KW - maternal morbidity
KW - Mortality
KW - Infant
KW - adolescent mother
KW - United States
KW - health care system
KW - Prevalence
KW - social aspect
KW - Hospital
KW - questionnaire
KW - social status
KW - Yates continuity correction
KW - school
KW - Government
KW - Morbidity
KW - contraceptive agent
RP - NOT IN FILE
SP - e81
JF - Journal of Pediatric and Adolescent Gynecology
VL - Conference: 24th Annual Clinical Meeting of the North American Society for Pediatric and Adolescent Gynecology, NASPAG Las Vegas, NV United States. Conference Start: 20100415 Conference End: 20100417. Conference Publication:
IS - var.pagings
N2 - Background: The birth rate for teenagers aged 15-19 years rose 3% in 2006, interrupting the long-term decline from 1991 through 2005. There are many risks associated with pregnancies among adolescents,

including increased maternal morbidity-mortality, infant morbidity, psychosocial and economical factors. In Puerto Rico, the birth rate to adolescent mothers 15 to 19 y/o, in 2000, (67 per 1,000) is higher than in the United States (49 per 1,000). The repeat births reported were 22% of a second child and 6% were of a third child. Adolescents that have repeat pregnancies have been through the health care system. The objectives of this research are to describe the profile of a group of Puerto Rican adolescents with a repeat pregnancy, identify the prevalence of psycho-social factors related to their repeat pregnancies and illustrate observed associations with psychosocial factors. Methods: An IRB approval, #9090109, was obtained for a cross sectional survey of adolescents younger than 21 years of age with a repeat pregnancy attending a prenatal clinic between November 2008 and February 2009. The questionnaire given included information related to: demographics, education, and socioeconomic status; support system; pregnancy history and reasons for not using contraception. The data was then gathered and analyzed using X² Yates Correction with a significance of p< 0.05. Results: The total participants recruited were seventy (70). The ages of the participants were from 12 to 19 (12, 1%; 16, 12%; 17, 11%; 18, 27%; 19, 46%). Their partner's ages ranged from 16 to 38, with a mode of 19 and 21, while 33% are between 21 and 23, 23% between 24 and 29, and 11% between 31 and 38. Their main activity at the time of the study was house work (36%), studying (26%), not working in or out of the house nor studying (23%) and working outside the home (16%). Among all of them, 63% had left school before becoming pregnant and 60% received government help. Only 3% of the participants live alone, while 56% lives with their partner and 41% lived with their family, some including their partners. Of all the participants, only 10% were legally married. The use of contraceptives was significantly (p<0.05) associated to older partners. Partners wanted the pregnancy in 49% and adolescent wanted the pregnancy in 48% of the cases. Other results will be presented. Conclusions: Psychosocial factors are associated with failure to use contraception among adolescents with repeat pregnancies in Puerto Rico. The factor that was found that influenced the most was a partner over 20 years of age. It is important to take into account the age of the partner, the level of education and the motivation in pregnancy postponement programs
SN - 1083-3188
AD - (Tulla, Colon, Laras) University of Puerto Rico, Medical Science Campus, San Juan, PR, United States
ER -

TY - JOUR
ID - 3669
T1 - Genetics of schizophrenia: Clinical and neurobiological implications
A1 - Boshes,R.A.
A1 - Manschreck,T.C.
A1 - Konigsberg,W.
Y1 - 2010///
N1 - 70132361
English
Journal: Conference Abstract
KW - EMBASE
KW - Schizophrenia
KW - Genetics
KW - Society
KW - gene
KW - patient
KW - Mutation
KW - gene pool
KW - heredity
KW - Human
KW - Child
KW - hypothesis
KW - preservation
KW - central nervous system
KW - human genome
KW - chromosome variant
KW - Genome
KW - fertilization
KW - childhood

KW - natural population
KW - fitness
KW - Population
KW - model
KW - organism
KW - Environment
KW - regulator gene
KW - nerve cell network
KW - organogenesis
KW - brain
KW - inheritance
KW - oncogene
KW - Prevalence
KW - Female
KW - Male
KW - lethal gene
KW - side effect
KW - clinical trial
KW - Autism
KW - Asperger syndrome
KW - nucleic acid hybridization
KW - Technology
KW - DNA
KW - neuroleptic agent
RP - NOT IN FILE
SP - 216
EP - 217
JF - Schizophrenia Research
VL - Conference: 2nd Schizophrenia International Research Society Conference, SIRS 2010 Florence Italy. Conference Start: 20100410 Conference End: 20100414. Conference Publication:
IS - var.pagings
N2 - Background: Schizophrenia (SZ) occurs throughout the world with a prevalence of 1%. As much as 80% can be attributed to genetic factors. Individuals with SZ reproduce at a lower rate: females produce 50% as many children as normal; males 25%. Genetic factors accounting for SZ should therefore behave like highly lethal genes. Laws of Hardy-Weinberg equilibrium predict that SZ should disappear from the human gene pool. How can we explain the persistence of SZ at a constant frequency of 1%? Methods: Clinicians appreciate that the natural course of SZ varies widely from patient to patient and cannot be predicted. The Clinical Antipsychotic Trial of Intervention Effectiveness (CATIE) concluded that the response of any individual with SZ to treatment with 2_{nd} generation antipsychotic agents cannot be predicted: either the effectiveness of the treatment or the myriad of possible side effects. Each clinical intervention is a clinical trial. More than 50 candidate genes have been identified within kindreds with high frequencies of SZ. There is no identified candidate gene common to all afflicted patients. The development of nucleic acid hybridization technology has revised our understanding of the structure and function of the human genome. Several recent publications have identified chromosomal rearrangements of large coding and non-coding regions including certain candidate or neurodevelopmental genes. Structural variations such as microdeletions and/or duplications range from 100 KB to 15 MB. Microduplications may include copy number variants Importantly, these chromosomal variants were not found in parental genomes. They are post fertilization or in vivo mutations. In vivo mutations are found in control DNA at a frequency of 5%, in SZ DNA at a frequency of 15%, and among children with the most severe childhood-onset SZ (COS) a rate of 22%. Results: Balanced polymorphism is the hypothesis explaining how non-adaptive genetic factors are maintained in natural populations in combination with other genes to confer increased fitness to the entire population, thus insuring their preservation in the gene pool. Diverse evidence suggests a neurodevelopmental model of SZ, the autism spectrum, Asperger's Syndrome, and others. A family of genes has been identified that are activated by diverse extracellular mechanisms. They are the interface between the organism and its environment, at the cellular and organismic level. An example of such a regulatory gene is Disrupted in Schizophrenia (DISC-1) Neurodevelopmental genes account for the organization of complex neuronal networks that are analogous to organogenesis In vivo mutations are a normal part of the development of the brain but occur more frequently in patients with SZ and most frequently among

patients with COS. In vivo mutations are not passed on to the next generation. Discussion: Genetic elements which are adaptive must exist to account for the persistence of SZ as well as its familial pattern of inheritance. A different class of neurodevelopmental genes in SZ, must exist to increase the rate of in vivo mutations within the CNS and must, in combination with other genetic elements, be adaptive to account for their preservation in the human gene pool. Such a gene, operating outside the CNS, would be expected to be highly lethal by generating oncogenes. These hypotheses, in conjunction with the findings of CATIE raise the possibility that each patient with SZ may be genetically unique, a phenocopy
SN - 0920-9964
AD - (Boshes, Manschreck) Harvard Medical School, Fall River, MA, United States (Konigsberg) Yale University, New Haven, CT, United States
ER -

TY - JOUR
ID - 3670
T1 - CTLA-4 haplotype associated with additional susceptibility factor in pediatric type-1 autoimmune hepatitis
A1 - Porta,G.
A1 - Oliveira,L.C.
A1 - Marin,M.L.
A1 - Kalil,J.
A1 - Goldberg,A.C.
Y1 - 2010///
N1 - 70131963
English
Journal: Conference Abstract
KW - EMBASE
KW - liver
KW - haplotype
KW - autoimmune hepatitis
KW - gene
KW - allele
KW - patient
KW - Child
KW - normal human
KW - Polymerase Chain Reaction
KW - restriction fragment
KW - Genotype
KW - statistical significance
KW - inflammatory disease
KW - Hepatitis
KW - lymphocytic infiltration
KW - hypergammaglobulinemia
KW - disease predisposition
KW - restriction fragment length polymorphism
KW - helper cell
KW - T lymphocyte activation
KW - autoimmune disease
KW - modulation
KW - T lymphocyte
KW - Inflammation
KW - cytotoxic T lymphocyte antigen 4
KW - protein
KW - autoantibody
KW - messenger RNA
KW - CD4 antigen
KW - Receptor
RP - NOT IN FILE
SP - S430

JF - Journal of Hepatology
VL - Conference: 45th Annual Meeting of the European Association for the Study of the Liver
IS - EASL
N2 - Background and Aims: Autoimmune Hepatitis (AIH) is a hepatocellular inflammatory disease characterized by interface hepatitis, portal lymphocyte infiltration, circulating autoantibodies, and hypergammaglobulinemia. In Brazilian patients HLA-DRB1[*]13 has been identified as the major susceptibility gene variant associated with the disease. Additional genes located outside of the MHC region may contribute to overall disease susceptibility. The cytotoxic T lymphocyte antigen-4 (CTLA-4) protein is an inhibitory receptor expressed on the surface of activated CD4 T helper cells playing a key role in the downregulation of T-cell activation, and has been associated with several different autoimmune diseases. At least three polymorphisms (SNPs) have been described for CTLA-4. The G allele called CT60 in the 3'UT portion of the CTLA4 gene is associated to lower mRNA CTLA4 levels, and consequently to decreased CTLA-4 protein levels, which would result in defective modulation of T cell-mediated inflammatory responses. This study looked for the association of CTLA4-318C/T, +49A/G and CT60A SNPs, and CTLA4 haplotypes, with AIH-1 in Brazilian children. Methods: SNPs were analyzed in 105 Type 1 AIH Brazilian children (AIH1) and in 228 healthy subjects (CTRL) by the polymerase chain reaction restriction-fragment length method (PCR-RFLP). Results: The G allele at CTLA4 CT60 and the corresponding GG genotype showed slightly higher frequencies in AIH1 compared to CTRL, with borderline p values (p = 0.0540 and p = 0.0508). The CT60 G allele is also part of the CTLA4-318C, +49A, CT60G haplotype, which exhibited a significant association with AIH1 compared with CTRL (23% vs 15%, p = 0.0166). The other SNPs had similar distribution in the two groups, suggesting that CT60 is the primary susceptibility variant marking this gene. Conclusions: The CT60G CTLA4 allele and the CTLA4-318C, +49A, CT60G haplotype are associated with AHI-1 in a pediatric sample of Brazilian patients. Supported by FAPESP
SN - 0168-8278
AD - (Porta) Department of Hepatology of Children, Institute of Clinics Hospital, Sao Paulo, Brazil (Oliveira) Laboratory of Pediatric Investigation (LIM-36), Clinics Hospital, Brazil (Marin) Laboratory of Immunology, Heart Institute (InCor), Brazil (Marin) Laboratory of Histocompatibility and Immunity (LIM19), Brazil (Kalil) Laboratory of Immunology, Heart Institute, University of Sao Paulo School of Medicine, Japan (Goldberg) Albert Einstein Institute for Education and Research, Japan (Goldberg) Institute of Investigation in Immunology, National Institute of Science and Technology - INCT, Sao Paulo, Brazil
ER -

TY - JOUR
ID - 3671
T1 - Predicting the effect of methylphenidate hydrochloride inchildren with ADHD using multi-channel NIRS
A1 - Takahashi,A.
A1 - Kawakubo,Y.
A1 - Nishimura,Y.
A1 - Kuwabara,H.
A1 - Takizawa,R.
A1 - Kasai,K.
Y1 - 2010///
N1 - 70129617
English
Journal: Conference Abstract
KW - EMBASE
KW - Attention Deficit Disorder
KW - psychiatry
KW - Society
KW - Child
KW - Neuroimaging
KW - Cognition
KW - tissue
KW - prefrontal cortex
KW - near infrared spectroscopy
KW - frontal lobe
KW - Methylphenidate

KW - hemoglobin
KW - central stimulant agent
RP - NOT IN FILE
SP - 42S
JF - Biological Psychiatry
JA - Biol Psychiatry
VL - Conference: 65th Annual Meeting of the Society of Biological Psychiatry New Orleans, LA United States. Conference Start: 20100520 Conference End: 20100522. Conference Publication:
IS - var.pagings
N2 - Background: The use of stimulant drugs for attention-deficit/hyperactivity disorder (ADHD) has raised questions about the possibility of overtreatment. It is necessary to develop biologically-based criteria to adjust treatment appropriately. Near-infrared spectroscopy (NIRS) is a noninvasive neuroimaging method that can measure relative changes in the concentration of oxygenated hemoglobin ([Oxy-Hb]) in cortical tissue in natural settings. The stop signal task is suitable for detecting the dysfunction of response inhibition in ADHD. The aims of our study were to examine the bilateral prefrontal activation associated with response inhibition by using multi-channel NIRS in children with ADHD, and to develop a neurophysiological index which predicts the effect of continuous administration of methylphenidate hydrochloride (MPH) on the cognitive function evaluated by Das-Naglieri Cognitive Assssment System (DN-CAS). Methods: Participants were 14 children with ADHD and 20 typically developing children. We defined the children with ADHD whose total score or subscale score of DN-CAS was improved after administration of MPH as responder (ADHD-R) and the children with ADHD whose score was not improved as non-responder (ADHD-NR). Results: ADHD-NR showed the lower [Oxy-Hb] increase than ADHD-R and typically developing children during the activation task in right dorsolateral prefrontal cortex (DLPFC) ($p<0.01$). Conclusions: The activation of [Oxy-Hb] in right DLPFC represented the difference of the frontal lobe function between responder and non-responder before administration of MPH. These results suggest that NIRS might be potentially useful as a neurophysiological index which predicts the effect of MPH in children with ADHD. Graph Presented
SN - 0006-3223
AD - (Takahashi, Kawakubo, Nishimura, Kuwabara, Takizawa, Kasai) NeuroPsychiatry, Tokyo University, Tokyo, Japan
ER -

TY - JOUR
ID - 3672
T1 - Household responses to school closures conducted in response to 2009 H1N1 pandemic influenza
A1 - Mak,D.
A1 - Giele,C.
A1 - Carcione,D.
A1 - Effler,P.
Y1 - 2010///
N1 - 70126633
English
Journal: Conference Abstract
KW - EMBASE
KW - school
KW - pandemic influenza
KW - physician
KW - Internal Medicine
KW - household
KW - student
KW - Child
KW - parent
KW - control strategy
KW - Child Care
KW - disease control
KW - general aspects of disease
RP - NOT IN FILE
SP - 33

JF - Internal Medicine Journal
VL - Conference: World Congress of Internal Medicine, WCIM 2010 in Conjunction with Physicians Week Melbourne, VIC Australia. Conference Start: 20100320 Conference End: 20100325. Conference Publication:
IS - var.pagings
N2 - Introduction: School closure is often recommended for mitigating the impact of an influenza pandemic. Little is known, however, about the impact of school closures on students and families. Aim: To describe the behaviour of students excluded from school due to school closures and parental opinions of school closure as a pandemic influenza control strategy. Methods: Parents/guardians of all students at the three schools excluded from school attendance due to school closure were surveyed seven days after the closure ended to ascertain their child's age and illness status, childcare arrangements, whether the child went out of the home during the school closure, and parental opinions of school closure. Results: Surveys were returned for 233 (58%) of the 402 students (range 5- 13 years, median = 11) affected by school closure. Twelve (5%) of the 233 responses were from confirmed cases, 143 (61%) from contacts and 78 (34%) from student peers (neither a case nor contact). Fourteen contacts (10%) and five peers (6%) developed respiratory symptoms during the closure. A total of 172 (74%) students went outside the home on >1 occasion during the week of closure, including 42% and 66% of cases and contacts. The mean number of outings was 3.7 (range: 0-24). Less than half the parents (47%) thought that school closure was appropriate. Cases and contacts, and children of parents who thought school closure was appropriate were less likely to report activities outside the home. Forty-five percent of parents of asymptomatic students reported taking time off work (range: 1-5 days; median = 3) to care for their child and 71 (35%) had to make additional special childcare arrangements. Conclusion: Nearly three-quarters of students undertook out-of-home activities during school closure. These findings can be used to predict more accurately the effectiveness of school closure as a disease control strategy
SN - 1444-0903
AD - (Mak, Giele, Carcione, Effler) Department of Health Western Australia, Perth, Australia
ER -

TY - JOUR
ID - 3673
T1 - Explanation of temporary effects during cryo catheter ablation
A1 - Haemmerich,D.
A1 - Morley,G.E.
A1 - Bernstein,S.
A1 - Saul,P.J.
A1 - Laura,K.
Y1 - 2010///
N1 - 70120731
English
Journal: Conference Abstract
KW - EMBASE
KW - heart rhythm
KW - catheter ablation
KW - Society
KW - cryoablation
KW - electric potential
KW - velocity
KW - Temperature
KW - tissue
KW - camera
KW - Swine
KW - heart arrhythmia
KW - radiofrequency ablation
KW - heart muscle refractory period
KW - Child
KW - heart
KW - optics
KW - Light
KW - endocardium

KW - action potential
KW - repolarization
KW - epicardium
KW - body temperature
KW - infrared radiation
KW - warming
KW - heart conduction
KW - dye
RP - NOT IN FILE
SP - S2
JF - Heart Rhythm
VL - Conference: 31st Annual Scientific Sessions of the Heart Rhythm Society, Heart Rhythm 2010 Denver, CO United States. Conference Start: 20100512 Conference End: 20100515 Sponsor: St Jude Medical. Conference Publication:
IS - var.pagings
N2 - Introduction: Cryoablation is an alternative to radiofrequency ablation of cardiac arrhythmias. In some circumstances it may be the treatment of choice, e.g in small children. Temporary effects during cryoablation such as increases in effective refractory period and AV nodal block have been observed clinically but have so far not been explained. Methods: Hearts from healthy Yorkshire pigs (40-60 kg) pigs were isolated, Langendorff perfused and optically mapped with a voltage sensitive dye (di-4-ANNEPS). Cryoablation was performed with a 5 mm tip at -120degreeC at three endocardial ventricular locations for 8 min. Optical voltage mapping data was obtained using two high-speed CCD cameras and a custom 520nm light source. Epicardial temperature profile was imaged via an infrared camera at 1 frame/s. Both voltage and thermal mapping were performed during and for 15 min following cryoablation. Maps of activation time, signal magnitude, APD90, and conduction velocity were generated. Correlation between epicardial temperature and signal parameters was performed. Loss of tissue function was defined as regions where voltage magnitude was below 10% of baseline level. Results: Regions outside the visible ice ball experienced temporary conduction changes such as reduced action potential magnitude, reduced conduction velocity, slower de- and repolarization, and an increase in APD90. As a result, late activation of epicardium was observed located proximal of the permanent lesion in relation to the pacing site, and disappeared after tissue warmed back to body temperature. APD90 increased from 262.1+/-12.2 ms at 36 degreeC to 295.0+/-7.1 ms at 22 degreeC, with no further increase at lower temperatures. Conduction velocity slowed by a factor of ~2.4 for every 10degreeC, and regions of slow conduction correlated with locations of reduced epicardial surface tissue temperature. These changes were completely reversible and disappeared upon rewarming, approximately 4 min after the end of cryoablation. Conclusions: Temporary changes in cardiac conduction were observed surrounding the permanent cryo lesion and may explain temporary effects observed during clinical cryoablation procedures
SN - 1547-5271
ER -

TY - JOUR
ID - 3674
T1 - The effect of 6-weeks active video game on body mass index and physical activity during school recess
A1 - Duncan,M.J.
A1 - Staples,V.
Y1 - 2010///
N1 - 70091696
English
Journal: Conference Abstract
KW - EMBASE
KW - childhood
KW - Physical Activity
KW - school
KW - Recreation
KW - Obesity
KW - Health
KW - body mass
KW - Child

KW - videorecording
KW - control group
KW - personal experience
KW - Group Dynamics
KW - stimulus
KW - Heart Rate
KW - boy
KW - girl
KW - height
KW - pedometer
KW - information processing
KW - Interview
KW - Analysis of Variance
KW - nitrogen 15
RP - NOT IN FILE
SP - 55
JF - International Journal of Pediatric Obesity
JA - Int J Pediatr Obes
VL - Conference: 19th European Childhood Obesity Group Meeting 'Moving Towards Health' Dublin Ireland. Conference Start: 20090917 Conference End: 20090919. Conference Publication:
IS - var.pagings
N2 - Objective. To compare a 6 week, recess-based, active video game intervention with traditional recess activity. Methods. Thirty, 10-11 year old children (12 boys, 18 girls, 16% overweight; 6% obese) were randomly allocated to intervention (N =15) or control (N =15) groups. The intervention group undertook twice weekly sessions of active video gaming during school lunch breaks. The control group took part in traditional' recess activity. Height and body mass (from which BMI was derived) were assessed pre and post the intervention period. Heart rate and pedometer data, assessed during the first, third and sixth weeks, were used to quantify physical activity over time and between groups. The children also participated in focus group interviews pre and post the intervention to determine their lived experiences of active video gaming as a physical activity tool. Results. Repeated measures ANOVA indicated a significant steps by group interaction (F_{2, 56} =3.88, P =0.02) with number of steps/min being greater for the intervention group on the first week of the intervention period but lower than the control group at the mid and end points of the 6-week period. The percentage of time spent in moderate and vigorous physical activity (F_{1, 28} =15.6, P =.0001) was significantly lower in the intervention group across the intervention period. BMI was not significantly different across time or group (P > 0.05). Conclusions. These findings suggest active video gaming may not be a sufficient stimulus to enhance children's recessbased physical activity in the long term
SN - 1747-7166
AD - (Duncan, Staples) School of Science, University of Derby, Derby, United Kingdom
ER -

TY - JOUR
ID - 3675
T1 - Periscope project: Physical activity and play in kindergarten age children
A1 - Malecka-Tendera,E.
A1 - Caroli,M.
A1 - Epifani,S.
A1 - Losacco,G.
A1 - Larsen,S.
A1 - Matusik,P.
A1 - Mikkelsen,B.E.
Y1 - 2010///
N1 - 70091695
English
Journal: Conference Abstract
KW - EMBASE
KW - Child
KW - Physical Activity

KW - kindergarten
KW - Obesity
KW - childhood
KW - Health
KW - parent
KW - Italy
KW - Denmark
KW - urban area
KW - Traffic
KW - city
KW - Risk
KW - Public Health
KW - health program
KW - habit
KW - questionnaire
KW - Poland
KW - statistical analysis
RP - NOT IN FILE
SP - 54
EP - 55
JF - International Journal of Pediatric Obesity
JA - Int J Pediatr Obes
VL - Conference: 19th European Childhood Obesity Group Meeting 'Moving Towards Health' Dublin Ireland. Conference Start: 20090917 Conference End: 20090919. Conference Publication:
IS - var.pagings
N2 - Objective. Assessment of physical activity habits and factors that can promote or inhibit activity in kindergarten age children. Methods. Self-administered questionnaires were filled in by the parents of 1063 children attending kindergarten in Italy, Poland and Denmark. Statistical analysis: X² test. Results. 50% of Italian parents as compared with 99% of Danish parents and 96% of Polish parents stated that, in the area where they lived, children could play outside their home. The most frequently reported obstacles were heavy traffic (52%), and the absence of a park/house yard (24.7%). On weekdays, only 19.8% of Italian children and 19.5% of Danish children spent more than one hour playing outside per day as compared with 35% of Polish children. 11% of Italian children never played outside (p > 0.0001). On weekends, 91% of Polish children and 86.7% of Danish children, but only 50% of Italian children played outside for more than one hour. 8.4% of Italian children never play outside (p > 0.0001). Conclusions. The urban environment in Italy, even in small towns, is not built according to children's needs or Italian parents perception of risk is higher than in other countries. For 3-6 years old children the most natural way to be active and to develop healthy and positive attitudes to physical activity is to play freely. Low levels of PA in Italian children can partially explain their higher rate of being overweight. Grants: Periscope (AN 2006341) EU Public Health Programme framework
SN - 1747-7166
AD - (Malecka-Tendera, Matusik) Medical University of Silesia, Katowice, Poland (Caroli, Epifani, Losacco) Nutrition Unit, Department of Prevention, ASL Brindisi, Italy (Larsen, Mikkelsen) Aalborg University, Ballerup, Denmark
ER -

TY - JOUR
ID - 3676
T1 - Roundtable discussion: Thinking outside the box to facilitate change
A1 - Lobstein,T.
A1 - Flynn,M.
A1 - Malina,R.
A1 - Poskitt,E.
A1 - Faenza,C.
A1 - Waligora,J.
A1 - Mullen,N.
A1 - Dunphy,M.L.
A1 - Roche,E.

A1 - Caroli,M.
Y1 - 2010///
N1 - 70091689
English
Journal: Conference Abstract
KW - EMBASE
KW - Obesity
KW - Health
KW - childhood
KW - Child
KW - Physical Activity
KW - school
KW - parent
KW - sport
KW - law
KW - Industry
KW - Policy
KW - Ireland
KW - baby
KW - Food
KW - Marketing
KW - machine
KW - Environment
KW - Prevention
KW - cacao
KW - consumer
KW - model
KW - body mass
KW - girl
KW - weight
KW - Public Health
KW - fitness
KW - Primary Prevention
KW - Health Care
KW - Health Care Policy
KW - Day Care
KW - juvenile
KW - Statistics
KW - blood sampling
KW - Infant
KW - Internet
KW - Recreation
KW - adolescence
KW - Exercise
KW - mother
KW - environmental change
KW - bicycle
KW - workshop
KW - Voice
KW - eating habit
KW - Sweden
KW - France
KW - high school
KW - primary school
KW - freedom
KW - money
KW - Behavior Change

KW - Competition
KW - Home Environment
KW - Poverty
KW - developing country
KW - commercial phenomena
KW - planning
KW - advertizing
KW - market
KW - Responsibility
KW - Quality Control
KW - prescription
KW - budget
KW - social psychology
KW - fiber
KW - consensus
KW - Early Intervention
KW - Weight Gain
KW - Weaning
KW - Pregnancy
KW - Education
KW - sugar
KW - carbohydrate
KW - Sodium
RP - NOT IN FILE
SP - 50
EP - 52
JF - International Journal of Pediatric Obesity
JA - Int J Pediatr Obes
VL - Conference: 19th European Childhood Obesity Group Meeting 'Moving Towards Health' Dublin Ireland. Conference Start: 20090917 Conference End: 20090919. Conference Publication:
IS - var.pagings
N2 - Early Intervention: E. Poskitt: When one looks at policy and legislation it's relevant to make note that obesity is well established by the time the child gets to school. Research has indicated some of the things that happen in pregnancy or in the first weeks of life (rapid weight gain and fatness) are the things that contribute to the obesity later on. It is physiologically normal for a baby to be fat. In the past (30 years ago) 80-90% of fat babies slimmed down to normal five-year old children. We have to get the environment at weaning and beyond right so that we can go back to this natural slimming down before they start school at age five. Audience-T. Wilkin: It is important to remember that once children get to five they are set on a trajectory. Therefore the opportunity for intervention by implication lies before age five. M. Flynn: Recent work by our group found that in Ireland, by the time an infant is six-months they have already tasted chips, salty bacon, crisps and chocolate pudding. The fact that chocolate pudding exists for babies at 4-6 months raises questions regarding the type of message this gives parents. E. Roche: We need to intervene with education and advice on primary prevention before children are born and then at every healthcare meeting thereafter. M. Lordon Dunphy: In Ireland we have a national nutrition policy that specifically targets those less than five years. On the parental side, our national campaign looks at parents as role models for their children. We have seen a shift in parents' ability to identify their important role as role models to their children. E. Poskitt: Children need to be taught that "no" means "no" and that though the child is important in the family, he/she does not rule the family. Another important aspect is having a regular bedtime, which may be very important in the development of obesity. Finally, we must not send the message that parents of children who are obese are bad parents. R. Malina: We need to monitor day-care and pre-school more closely. Some centres want the child to sit and be quiet throughout the day. As such, we need to educate those people who take care of the child and this will require major changes. Activity: R. Malina: Physical activity peaks at ten-eleven years and then declines. If we look at the participation of children in youth sport, it increases in general from six to eleven years and then declines. Kids define physical activity as sport. As such, one strategy is to get local organisation to modify sport to meet the needs of our children. It might contribute to increased physical activity levels and may contribute to the prevention of obesity. Regarding research in the area of physical activity, papers focus on group means and the cult of significance. We need to be aware of individual differences, as some youngsters may gain weight and some lose weight. We need to do

our statistics carefully and not overlook variability as not all will respond to conditions in the same manner. Audience-T. Wilkin: There are two different aspects to physical activity. One is that physical activity is effective at improving cardiorespiratory and metabolic fitness. The other is whether we can indeed use physical activity to improve body mass. If we can, does it have a meaningful impact? These distinctions are important and at the moment they are being confused. If we use body mass as an outcome measure of physical activity intervention we may not see any change. We may need to shift our focus away from body mass and instead move toward the use of fitness testing or blood sampling to test the effect of physical activity interventions. Audience-ML. Frelut: When asking why people escape moving and choose sedentary pursuits we must remember that children live now in a world of major permanent constraint at school and elsewhere. Sedentary games at home are their space for freedom. Also, many children are lonely and competition is only another way for them to be lonely, while using the internet, watching TV and playing video games are ways to be with the others. Audience: It's vital to link these children with activities that they enjoy. M. Lordan Dunphy: Ireland's Participation Strategy aims to engage children at all levels with their local sports partnerships and there is a special focus on teenage girls. E. Roche: Girls are falling out of exercise early and these are the future mothers of future generations. Sport is very oriented toward team games and the Irish sports council has made the attempt to broaden this definition. Policy: T. Lobstein: If we are to think outside the box we need to think on a public health level rather than the individual level. Once the environment is altered then we can work on individual behaviour. Some environmental changes are very simple like providing healthy school meals and bicycle lanes. We need to make the opportunity to be active more secure and safe and also food policy and taxing need to be discussed. M. Flynn: Indeed laws on marketing exist as does good legislation from the EU on the regulation of health claims. However, having been involved in the legislation workshops since 2006, I'm dismayed to have seen this legislation picked apart. I do not know where the consumer group-voice is. It appears we are tiny and that the industry lobby are winning. Audience-A. Vazeou: It is clear that if we improve the eating habits of the masses then we will have better results on the individual level. Audience-H. Thibault: In France, there is a law since 2004 that vending machines must be banned in middle and high schools. It has been effective. At the same time morning snacks in primary school are not recommended. We are for individual liberty but sometime we need to recognise that law can be useful. Audience: Regarding that point, the schools are not happy that vending machines are forbidden as they usually yield a lot of money for the schools. Audience: We know that structural prevention by imposing rules works, and that influencing the individual's behaviour change is difficult as we are talking to archaic feelings within us. For example in the STOP programme in Sweden, bringing candy and unhealthy foods to school was banned. It is evident now that children are bringing this message home to their parents who in turn can modify the home environment with less resistance. E. Poskitt: We have to think hard about how we get people to do healthy things as imposing rules may not be successful Sometimes coming sideways at these issues by removing poverty and deprivation might be effective though this method is slow. In addition, similar to the case in developing countries, non-governmental organisations work in parallel rather than together. This leads to duplication of materials and wasted resources. M. lordon Dunphy: I think that joint business planning across the various governmental departments is imperative as there is a lot of duplication of information, which can confuse the public and uses up resources. Industry: M. Caroli: Ted Kennedy once said "who is responsible for the control of advertising, marketing and promotional activities which market products in spite of public health consideration ". If we are honest that responsibility lies with everybody. C. Faenza: I work in the product's quality control department of COOP, the most important Italian retailer, and I can say that formulating recipes with fats and sugars is easier and cheaper from a technical point of view. Coop's actions are the concrete demonstration that it is really possible to find innovative solutions in terms of healthier product- formulation and still keeping in mind the need to achieve a positive budget at the end of the year. COOP is an ethical company and believes in social values; thus, with the aim to facilitate our consumer in making healthy choices, COOP has reformulated its children's products. We have developed a new specific range of healthy products for children. These have an increased fibre content, a lower content of sugar, sodium, and fats and use only natural flavourings. Secondly for all our existing products which cannot be feasibly re-formulated we have decided to discontinue their production altogether. This policy is very different from one which only adds a healthier line of products to an existing unhealthy one. J. Waligora: Regarding health regulation, the final legislation that we have for industry is not perfect. We have to realise that so many differences exist between each member state of the EU and what each would like to achieve. As such, it's difficult to find consensus. Secondly, we need to have dialogue with both the member states and industry if we want to achieve our aims

SN - 1747-7166

AD - (Lobstein) Food Commission, United Kingdom (Flynn) Food Safety Authority of Ireland, Ireland (Malina) University of Texas at Austin, United States (Poskitt) United Kingdom (Faenza) Italian Cooperative,

Italy (Waligora) European Commission, Luxembourg (Mullen) Irish Medical Times, Ireland (Dunphy) Irish Obesity Task Force, Ireland (Roche) Ireland (Caroli) ECOG, Italy
ER -

TY - JOUR
ID - 3677
T1 - Self regulation or legislation? That is the queston!
A1 - Lobstein,T.
Y1 - 2010///
N1 - 70091688
English
Journal: Conference Abstract
KW - EMBASE
KW - Health
KW - law
KW - Obesity
KW - childhood
KW - autoregulation
KW - Child
KW - Marketing
KW - Industry
KW - Food
KW - advertizing
KW - Persuasive Communication
KW - Internet
KW - Norway
KW - model
KW - Government
KW - World Health Organization
KW - nutrient
KW - computer
KW - market
KW - United Kingdom
KW - protection
KW - Writing
KW - telecommunication
KW - budget
KW - economic aspect
KW - Climate
KW - packaging
KW - Color
KW - flavor
KW - Europe
KW - audiovisual equipment
KW - consumer
KW - consultation
KW - solid
KW - Ireland
KW - public figure
KW - Sweden
KW - France
KW - nutritional value
KW - primary school
KW - Punishment
RP - NOT IN FILE
SP - 49
EP - 50

JF - International Journal of Pediatric Obesity
JA - Int J Pediatr Obes
VL - Conference: 19th European Childhood Obesity Group Meeting 'Moving Towards Health' Dublin Ireland. Conference Start: 20090917 Conference End: 20090919. Conference Publication:
IS - var.pagings
N2 - In this presentation I shall focus on the issue of protecting children from commercially-led persuasions to purchase and consume unhealthy foods. This immediately raises a question - what foods are unhealthy? I shall return to that issue shortly. Before discussing how to protect children, we need to consider what is currently happening - what is the nature and extent of commercial persuasion. When we have an understanding of this, we can discuss what measures would be appropriate to protect children. The forms of persuasion are many: I can only show a few in this presentation. hey range from the familiar, such as TV commercials and advertising in cinemas, through to more unusual techniques, including internet sites, branded toys, packaging and the compositions of the foods themselves. Text messages to phones, viral marketing messages on Facebook, product placements in computer games, plus for younger children, product placement in story books and counting books. Free gifts with products, games and puzzles on product labels, and I would argue that the addition of colours and artificial flavours are really marketing methods, to make an object more appealing to children. The extent of marketing can be considered in terms of the amount of spending by industry on promoting their products. This is not easy to identify, especially as the available figures do not distinguish child-oriented and non-child-oriented commercial promotions. Further, the figures available do not include all the activities used by companies, such as internet sites, product labelling, and product composition. We can also get some feel for the problem by asking how many advertisements children are exposed to on TV, for example. The figures vary across countries and interestingly the variation matches to some extent the variation in obesity levels among children in those countries (Lobstein and Dibb, 2005). Advocacy organisations have been arguing for many years that children should not be exposed to junk food marketing messages. The Sydney Principles launched at the ICO in 2006 made a clear statement of the need to protect children. In Europe, the WHO organised a meeting on the issue in 2006 and launched a Network of governments sympathetic to the protection of children from marketing in 2007, chaired by Norway. The World Health Assembly in Geneva in 2007 called for its officials to prepare a draft set of recommendations on the issue, to be presented in 2010. IASO, with Consumers International, launched a set of recommendations in the form of an International Code in 2008 (see www.iotf.org/documents/Consumers- InternationalMarketingCode.pdf) and has contributed to the WHO consultation on the issue. The Network chaired by Norway has also drafted an international code (in press), with some very similar recommendations to the IASO-CI code. The European Commission has made some bold statements about the need to control marketing to children, but has been slow to take any solid action, preferring a voluntary model. Meanwhile there have been moves at Member State government level to exert some control over the types of ads and the numbers of ads seen by children. Ireland has banned specifictechniques - such as the use of celebrities - and is considering further controls. Both Sweden and Norway have general restrictions on marketing of any products to children under age 12. France requires health messages to be included on any food product advertisement on TV or in print. The UK has perhaps gone the furthest, by banning ads in children's programmes or directed to children under age 16, and the UK has specified what it means by junk food: using a nutrient profile' model based on positive and negative aspects of the nutritional quality of a product. It is now possible to say what is healthy' and what is not, and to get industry to comply with this distinction, even if they dislike it. I believe this could not have happened if we had relied on a voluntary approach. I do accept that some food companies have taken steps towards recognising the need to restrict marketing to children, and have offered some new thinking in just the last year or two, including some nutrient profiling' of their own (but this differs between different companies). Companies have also offered a higher age threshold, increasing it from 6 years to 12 years and in some examples 14 years. Companies have promised not to market to children in primary school settings. They have also promised not to advertise in programmes where children are over 50% of the audience (and some companies have strengthened this to 35% and even 30% of the audience). But big questions remain: what about the companies that do not join in the agreement? What happens to voluntary agreements after a few years when we have stopped making such a fuss? What penalties are there for a company if it breaks the rules it made? And what about all the loopholes in the voluntary agreements that allow a wide range of techniques to continue? The problems with a statutory approach are that legislation would have to be very detailed to cover all the possible methods for marketing to children. TV controls are relatively straightforward, but writing the same sort of legislation for all the different possible media would be extremely difficult. The other issue is the cross-border problem, where marketing is coming in from outside the jurisdiction of a country, thorough internet, satellite, and imported media such as Olympic Games broadcasting, computer games and so on. A compromise approach being tried in some countries is co-regulation' which can take various

forms, but could include a tough set of standards set by government to which industry signs up. There has to be some persuasion to get industry agreement, such as the threat of tough legislation. Then the tandards are monitored and enforced by a body including industry and non-industry stakeholders. There are problems with this model, and there will be loopholes that industry will exploit. But it may represent the best available compromise when large multinational corporations have advertising budgets greater than the economies of several countries, and in a political climate that is strongly pro-free market
SN - 1747-7166
AD - (Lobstein) Policy and Programmes, International Association for the Study of Obesity, International Obesity TaskForce, London, United Kingdom
ER -

TY - JOUR
ID - 3678
T1 - What's really causing the chidhood obesity epidemic? What kids say
A1 - Pretlow,R.
Y1 - 2010///
N1 - 70091674
English
Journal: Conference Abstract
KW - EMBASE
KW - Obesity
KW - childhood
KW - epidemic
KW - Health
KW - Food
KW - Child
KW - weight
KW - withdrawal syndrome
KW - Eating
KW - Loneliness
KW - friend
KW - parent
KW - Boredom
KW - Prevention
KW - Policy
KW - Coping Behavior
KW - Anxiety
KW - health practitioner
KW - Industry
KW - Genetics
KW - gene
KW - Metabolism
KW - Lifestyle
KW - computer
KW - school
KW - search engine
KW - Europe
KW - United States
KW - honesty
KW - shame
KW - physician
KW - Grief
KW - Happiness
KW - Weight Gain
KW - habit
KW - community
KW - Diet

KW - Exercise
KW - juvenile
KW - food industry
KW - substance abuse
KW - Weaning
KW - portion size
KW - skill
KW - relapse
KW - exposure
KW - weight reduction
KW - Self Esteem
KW - videorecording
KW - clip
KW - Learning
KW - Etiology
KW - sodium carbonate
RP - NOT IN FILE
SP - 28
JF - International Journal of Pediatric Obesity
JA - Int J Pediatr Obes
VL - Conference: 19th European Childhood Obesity Group Meeting 'Moving Towards Health' Dublin Ireland. Conference Start: 20090917 Conference End: 20090919. Conference Publication:
IS - var.pagings
N2 - Several etiologies have been postulated for the childhood obesity epidemic, including genetics (e.g. "thrifty gene" theory), the "weight set point", and "low metabolism." Sedentary lifestyle (e.g. playing on the computer instead of the playground), less nutritious food at school, "supersized" portions, and consumption of soda and junk food' all contribute. However, none of these theories or factors adequately explains the meteoric rise in childhood obesity or why treatment is only marginally successful, with dismal long-term results. What does explain it? Kids do! This presentation will reveal what tens of thousands of overweight children say in nearly 130,000 bulletin board posts on an open access website for overweight teens and preteens over a nine-year period. Children come to the site mainly on their own via search engines, links on other sites, and links in books. Many children are from Europe, as well as the U.S. With stunning honesty, these children share their difficult lives, their shame and secrecy, and their struggles and successes. Most say they're too embarrassed to talk to their friends, parents, or even their doctor. Numerous messages express a striking struggle to lose weight. Many explicitly describe using the pleasure of food and the displacement activity of eating to cope with depression, stress, anger, loneliness, anxiety, and boredom. Even grief is described from giving up "my best friend", i.e. food. The majority of messages ask, "How do I lose weight?" If a child posts any degree of success, multiple replies immediately ask, "How'd you do it?" Success stories typically describe distractions from food, improvement in the happiness level of the child as a predecessor for losing weight, and tolerating withdrawal-type symptoms. These children use food to cope with life. They become hooked on this behavior and are unable to stop. They struggle to resist cravings for pleasurable food, particularly junk food,' in spite of full awareness of the negative impact that overweight has on their lives and the dreadful effects of further weight gain. Many say that their eating is "out of control. " This is suggestive of an addictive quality, or psychological food dependence, which is further supported by the many explicit descriptions of using food as: 1) a stress coping mechanism (displacement activity similar to nail biting); 2) selfmedication for depression, loneliness, and anxiety (comfort eating); and 3) entertainment (to combat boredom). Some children explicitly state that they use food "like a drug." The professional community generally offers healthy diet and exercise as the solution to the overweight problem, however these children say that information is often unhelpful. A new paradigm will be explored in regard to what is really causing the childhood obesity epidemic and what should be done about it from a treatment, prevention, and policy standpoint. The perfect storm' of increasing youth stress levels combined with the rise of the Comfort Food' industry will be examined. Perhaps overweight and obesity in childhood should be treated similarly to forms of substance dependence, by: 1) sequential weaning from each abused food or excessive portion size; 2) management of resulting withdrawal' symptoms; 3) coping skill training; and 4) long-term support to prevent relapse. Public policy would control exposure of children to highly abused foods. Proposed weight loss techniques to combat psychological food dependence will be presented, e.g. getting through withdrawal -type cravings, substituting non-food ways to cope, combating stress, and generating healthy self-esteem and joy in living. The session also will include multiple, compelling video and audio clips.

Learning Objectives: a) Appreciate what the lives of overweight children are like and why they struggle to lose weight. b) Learn what overweight kids say about their parents and health professionals. c) Understand why the current theories on the causes of the childhood obesity epidemic do not fit. d) Realize the role of the "comfort food" industry in the childhood obesity epidemic. e) Comprehend what may be a little understood but substantial component of the childhood obesity epidemic, including fresh ideas on treatment and prevention
SN - 1747-7166
AD - (Pretlow) Weigh Rock.com - Online Weight Loss for Teens and Tweens, Seattle, WA, United States
ER -

TY - JOUR
ID - 3679
T1 - Are childbearing motivations getting in the way of STI prevention in young women?
A1 - Trent,M.
A1 - Chung,S.
A1 - Vyas,A.
A1 - Jennings,J.
A1 - Ellen,J.
Y1 - 2010///
N1 - 70079395
English
Journal: Conference Abstract
KW - EMBASE
KW - Pregnancy
KW - Society
KW - Female
KW - Prevention
KW - Motivation
KW - Adolescent
KW - Medicine
KW - condom
KW - hormonal contraception
KW - Child
KW - child parent relation
KW - United States
KW - unplanned pregnancy
KW - contraception
KW - parenthood
KW - infancy
KW - childbirth
KW - African American
KW - family planning
KW - Risk
KW - health disparity
KW - adolescence
KW - questionnaire
KW - Urban Population
KW - sexually transmitted disease
KW - Prevalence
KW - household
KW - Interview
KW - reproductive history
KW - juvenile
KW - Adaptation
KW - Satisfaction
KW - Regression Analysis
RP - NOT IN FILE
SP - S6

JF - Journal of Adolescent Health
JA - J Adolesc Health
VL - Conference: Society for Adolescent Medicine Annual Meeting - Adolescent Clinical Care: Integrating Art and Science Toronto, ON Canada. Conference Start: 20100407 Conference End: 20100410. Conference Publication:
IS - var.pagings
N2 - Purpose: Urban communities with high sexually transmitted infection (STI) prevalence often have disparities in unplanned pregnancies among young women. Research using single measures of pregnancy desire and/or intention has not yielded guidance on dual prevention strategies. Exploring childbearing motivation (CBM), the first step in the psychological sequence to childbearing behavior, may enable us to better understand reproductive decisions. The objective of this study is to examine the relationship between CBM and use of hormonal contraception, condoms, and dual contraception. Methods: A cross-sectional household study was conducted from 2004-2007. Data was collected from 297 sexually active females 15-25 years in age residing in high STI- prevalent neighborhoods in Baltimore, Maryland. Participants completed audio-computerized assisted self- interviews eliciting demographic, reproductive history and behaviors, and CBM. CBM was measured using a youth-specific adaptation of Miller's childbearing questionnaire. Positive (P)CBM subscales included measures of satisfaction from raising a child, feeling needed/connected, value of children/ parenthood, and positive aspects of pregnancy, birth, and infancy (alpha = .94). Negative (N) CBM subscales included discomforts of pregnancy and childbirth, fears/worries/stressors of parenthood, and negatives of caring for child (alpha = .85). The highest and lowest quartiles from each subscale were combined to create overall positive (high PCBM/low NCBM) and negative (low PCBM/high NCBM). Data outside of these groups was characterized as intermediate (I). Data were evaluated using multivariable regression analyses. Results: Participants were 90% African American with a mean age of 19.4 (SD 2.8). More than half (55%) had a history of pregnancy and 42% had children. 35% had a history of STIs. Using the quar-tile-based groups (21%; n = 64) were in the positive CBM group, 39.3 were in the negative group, and 39.7% (n = 118) were in the ICBM group. Those in the ICBM group were less likely to use condoms at last sex with main partner compared to the NCBM group (AOR: .54; 95% CI: .32-.93), controlling for STI history, pregnancy history, and age. Those in the PCBM group were 2.8 times more likely to use hormonal contraception (AOR: 2.75; 95% CI: 1.35, 5.62), and 3.3 times more likely to use dual contraception (consistent condom use with hormonal contraception) with their main partner (AOR: 3.31; 95% CI: 1.46, 7.50) compared with those in the NCBM group, controlling for STI history, pregnancy history, and age. Conclusions: Young women with overall positive attitudes about parenting are more likely to engage in simultaneous family planning and STI/HIV prevention behaviors than those with NCBM. Those in the intermediate group who appear to be ambivalent or indifferent about parenting are at highest risk for STIs due to inconsistent condom use compared with those in NCBM group. Given the health disparities associated with early childbearing and STIs, additional research exploring how CBM evolves over adolescence and the potential role of CBM in the prevention of STIs and unplanned pregnancy is warranted
SN - 1054-139X
AD - (Vyas) George Washington University, School of Public Health, United States (Trent, Ellen) Johns Hopkins School of Medicine, United States (Chung, Jennings) Johns Hopkins School of Medicine, Bayview Pediatric Unit, United States
ER -

TY - JOUR
ID - 3680
T1 - The contribution of psychosomatic medicine in IBD research
A1 - Paluzzi,C.
A1 - Di,Blasio C.
A1 - D'Amario,C.
A1 - Illiceto,M.T.
A1 - Filippone,M.
A1 - Lombardi,G.
Y1 - 2010///
N1 - 70460004
English
Journal: Conference Abstract
KW - EMBASE
KW - psychosomatics

KW - patient
KW - Psychotherapy
KW - Child
KW - relapse
KW - Interview
KW - Intestine
KW - household
KW - brain
KW - central nervous system
KW - Therapy
KW - medical specialist
KW - psychologist
KW - Crohn disease
KW - Diagnosis
KW - ulcerative colitis
KW - rigidity
KW - triangulation
KW - hypothesis
KW - model
KW - Family Conflict
KW - homeostasis
KW - enteritis
KW - mental stress
KW - medical literature
KW - Adult
KW - immunology
KW - biogenesis
KW - gastrointestinal tract
KW - Food
KW - nerve cell
KW - human dignity
KW - autonomic nervous system
KW - antigen antibody complex
KW - emotional disorder
KW - earthquake
KW - Italy
KW - dynamics
KW - Family Therapy
KW - physician
KW - Reinforcement
KW - peptide
RP - NOT IN FILE
SP - S340
JF - Digestive and Liver Disease
VL - Conference: 17th National Congress SIGENP Pescara Italy. Conference Start: 20101007 Conference End: 20101009. Conference Publication:
IS - var.pagings
N2 - Aim: Previous studies have shown a relationship between Inflammatory Bowel Disease (IBD) and psychological stress (PS); there is increasing evidence that PS can adversely affect the course of IBD in children and young adults. Emerging trials tend to demonstrate that psychological intervention is necessary as well as conventional therapy. Medical literature is not so rich about the role of psyche in biogenesis and maintenance of IBD in pediatric patients. Studies of Psycho-Neuro-Endocrine-Immunology (PNEI) have pointed out how stress can become disease, giving a scientific dignity to assumptions that in the past were only scientific speculation. PNEI studies have shown in gastrointestinal tract the presence of about 100,000 neurons secreting peptides, independent of Central Nervous System (CNS), that are however linked to CNS through the Autonomic Nervous System, today better known as Gut-Brain. Thus, our gut must be considered as a potent neuro-endocrine-immune complex, autonomous and in continuous contact with the outside (food) and the internal

(brain, emotion, disorders, diseases) world. Objectives: Our observation started from the relief of a high number of relapses in IBD patients already receiving medical therapy, after the devastating earthquake interesting Abruzzo (a region of Central Italy) in April 2009. Our study started with the objective of capturing the correlation between stress (triggering event) and relapse or first manifestation of the disease. We are also in charge of studying the relational dynamics of families with a child affected by IBD, to highlight any common relational mode. Another important objective was to motivate willing families to a path of individual or family psychotherapy and therefore to assess the evolution of the disease after a certain period. Material and methods: 70 families of children with IBD and treated in our Centre have been called for a psycho-diagnostic interview lasting 60-120 minutes (in any case all the necessary time to collect relevant information). The interviews were conducted by a doctor specialist in psychotherapy and a psychologist. Results: 80% of cases showed distrust and skepticism toward the initiative, a 10% refused the interview. There were no significance differences in the relationship of families of patients with Crohn Disease (CD) or Ulcerative Colitis (UC). It was always revealed a relationship between perceived stressful event and the onset of symptoms. The following transactional characteristics were common to 90% of households: 1. enmeshment; 2. hyper-protectiveness; 3. rigidity; 4. lack of conflict resolution. Particularly, three patterns of involvement have emerged, giving the impression to prevent the emotional disabling: a. triangulation; b. coalition; c. deviation. We were able to motivate 50% of households to undertake psychotherapy. Preliminary data are suggestive of hypothesis that relapses can be reduced by psychotherapy. Conclusion: It seems clear that in those families with a child affected by CD or UC, the family system is characterized by rigid and poor transactions and that the patient plays a major role in interactive models of avoidance of family conflict and of maintenance of family homeostasis. It seems also evident that this role is a source of reinforcement for his own symptoms. The study is still ongoing, rather we are just beginning: we intend to review the evolution of the disease after an appropriate psychotherapy path
SN - 1590-8658
AD - (Paluzzi, Di Blasio) Pediatric Gastroenterology Unit, Hospital of Pescara, Psychodiagnostic Service, Italy (D'Amario) Clinical Pathology, Hospital of Atri, Teramo, Italy (Illiceto, Filippone, Lombardi) Pediatric Gastroenterology Unit, Hospital of Pescara, Italy
ER -

TY - JOUR
ID - 3681
T1 - Development and validation of a short 31P cardiac magnetic resonance spectroscopy protocol
A1 - Dass,S.
A1 - Cochlin,L.E.
A1 - Holloway,C.J.
A1 - Suttie,J.J.
A1 - Johnson,A.W.
A1 - Tyler,D.J.
A1 - Watkins,H.
A1 - Robson,M.D.
A1 - Clarke,K.
A1 - Neubauer,S.
Y1 - 2010///
N1 - 70456095
English
Journal: Conference Abstract
KW - EMBASE
KW - nuclear magnetic resonance spectroscopy
KW - cardiovascular magnetic resonance
KW - phosphorus nuclear magnetic resonance
KW - heart disease
KW - contamination
KW - patient
KW - validity
KW - thorax wall
KW - Muscle
KW - liver
KW - information processing

KW - reproducibility
KW - telecommunication
KW - pulse rate
KW - normal human
KW - Male
KW - Female
KW - Child
KW - clinical assessment
KW - high energy phosphate
KW - Spectroscopy
KW - nuclear Overhauser effect
KW - phosphate metabolism
KW - Diagnosis
KW - pathology
KW - Heart Rate
KW - signal noise ratio
KW - nuclear magnetic resonance
KW - phosphorus 31
KW - creatine phosphate
KW - adenosine triphosphate
RP - NOT IN FILE
SP - 186
EP - 187
JF - Journal of Cardiovascular Magnetic Resonance
VL - Conference: 13th Annual SCMR Scientific Sessions - 2010 Phoenix, AZ United States. Conference Start: 20100121 Conference End: 20100124. Conference Publication:
IS - var.pagings
N2 - Introduction: Cardiac 31P-MRS is the only non-invasive in vivo technique for the determination of cardiac high energy phosphate metabolism. Changes in cardiac phosphocreatine to adenosine triphosphate ratios (PCr/ATP) occur in common cardiac pathologies and have diagnostic, prognostic and therapeutic utility. However, long acquisition times (20 minutes or more, depending on heart rate) required to achieve sufficient signal to noise ratios for reliable interpretation have limited the clinical utility of 31P-MRS studies in patients with severe cardiac disease. We have developed an 8 minute 31P-MRS protocol and demonstrate the validity of this 'short' acquisition by comparison with a 'long' (at least 20 minutes) method of published reproducibility (Tyler, NMR Biomed:2008). Purpose: To design a robust, 'short' cardiac 31P-magnetic resonance spectroscopy (31P-MRS) protocol which facilitates acquisition within a clinically acceptable timeframe. Methods: Protocol development This 'short' protocol essentially incorporates a larger voxel (93 mls compared to 39 mls for 'long' protocol) but eliminates extra myocardial contamination by: * active suppression of chest wall muscle and liver signals, * raw data acquisition weighted to reduce contamination arising from outside the nominal voxel. The accuracy of the data and its interpretation is improved by: * optimised radio frequency () pulse, * flip angle calibration (at voxel of interest) used during postprocessing to calculate and correct for subject variation to coil loading. * calibrated signal enhancement (Nuclear Overhauser Enhancement (NOE)), * rapid repetition time (with calibrated saturation correction). Validity: 22 healthy volunteers (age 42 + 16.5; 13 males, 9 females), were scanned (3 T Siemens Trio) with both the 'long' and 'short' acquisitions. Acquisition parameters for both protocols are summarised and compared in Table 1. (Table Presented) Results: There was no difference of derived PCr/ATP ratios for both methods ('short' 1.83 + 0.32; 'long' 1.78 + 0.27). Bland-Altman analysis demonstrates excellent agreement between the two methods (Figure 1) confirming equivalence for clinical purposes. Figure 2 shows an example of spectra acquired from one subject, using both methods. Conclusion: We have developed a novel 'short' cardiac 31P-MRS protocol of high data quality. This protocol allows cardiac spectroscopy to be measured in patients who are often intolerant of long acquisition times, such as those with severe cardiac disease and children. Hence this work provides a useful tool for the routine clinical assessment of cardiac 31P-MRS. (Figure Presented) (Figure Presented)
SN - 1097-6647
AD - (Dass, Suttie, Robson, Neubauer) OCMR, Department of Cardiovascular Medicine, University of Oxford, United Kingdom (Cochlin, Holloway, Johnson, Tyler, Clarke) Department of Physiology, University of Oxford, United Kingdom (Watkins) Department of Cardiovascular Medicine, University of Oxford, United Kingdom
ER -

TY - JOUR
ID - 3682
T1 - Asthma frequency among swimmers
A1 - Siegel,M.J.
A1 - Siegel,C.J.
A1 - Stekoll,L.H.
Y1 - 2010///
N1 - 70452715
English
Journal: Conference Abstract
KW - EMBASE
KW - Swimming
KW - Asthma
KW - immunology
KW - allergic asthma
KW - college
KW - Female
KW - United States
KW - city
KW - Male
KW - exposure
KW - Child
KW - Hospital
KW - parent
KW - health care personnel
KW - gender
KW - sexually transmitted disease
KW - groups by age
KW - Risk
KW - sport
KW - Chlorine
RP - NOT IN FILE
SP - A6
JF - Annals of Allergy, Asthma and Immunology
VL - Conference: 2010 Annual Meeting of the American College of Allergy, Asthma and Immunology Phoenix, Arizona United States. Conference Start: 20101111 Conference End: 20101116. Conference Publication:
IS - var.pagings
N2 - Background: Studies have questioned swimming, especially total hours swam in a life time (THS), and swimming in indoor chlorinated pools (ICP), to the development or worsening of asthma (A), based on chlorine chemical exposure. This study explored the frequency of active asthma (AA) among swimmers(S)based on:(1) THS;(2)and the frequency of AA versus THS of S using ICP compared to chlorinated outdoor (OCP) pool facilities. Methods: IRB approval was obtained from Children's Mercy Hospital in Kansas City. 1500 surveys were distributed by coaches to the parents of S ages 5-18y/o participating in a variety of Kansas City competitive swimming programs. Responses were collected by the study investigators. Each survey queried THS in ICP and OCP. Estimations of swimming hours spent in swim venues were given. THS were categorized as follows. 0-99(A);100-249(B);250-499(C);500-999(D);1000-1499(E);>1500(F) for each type of facility. Queried was whether S have health care provider diagnosed A and if A meds were used in the last year (criteria for AA). S gender and age were also noted. S were age bracketed by std USA swimming divisions. Results: An independent samplest test was used. For AA, there was a marginal difference overall between S in groups (grp) A-F t=1.73 (301), p=.085, and S in ICP in groups A-F t=1.68(209), p=.094. There was a sig differences between S in OCP grp A-F t=2.48(287), p=.014. There was no difference of AA between male S in ICP, OPC or overall in grp A-F. In female S, AA was significant in hours spend in OCP t=2.45(139), p=.016 and ICP t=2.15(104), p=.034. AA frequency by age bracket had a significant correlation between 9-10 y/o S in OCP t=2.039(69), p=.045 (primarily driven by females at this age), and 11-12 y/o in OPC t=2.55(60), p=.013. No differences in AA noted between age groups for A-F overall and ICP. Conclusion: Our study supports the notion that

increased hours of swimming may contribute to the persistence of AA in both ICP and OCP and female S may be more at risk for persistence of AA than males as THS increases. S ages 9-12y/o appeared to have an increased frequency of AA, particularly in females. Although the results are interesting, as swimming is often recommended as a sport for AA, further results are needed to validate the above conclusions. This study is ongoing. The specific role of inhaled chlorine products being responsible for the persistence of AA in S is yet to be determined
SN - 1081-1206
AD - (Siegel, Siegel, Stekoll) Kansas CityMOUnited States
ER -

TY - JOUR
ID - 3683
T1 - 24-h heart rate patterns in children with ADHD: A naturalistic approach
A1 - Imeraj,L.
A1 - Antrop,I.
A1 - Deboutte,D.
A1 - Roeyers,H.
Y1 - 2010///
N1 - 70451646
English
Journal: Conference Abstract
KW - EMBASE
KW - Attention Deficit Disorder
KW - Child
KW - Heart Rate
KW - clinical practice
KW - night
KW - Arousal
KW - Sleep
KW - Circadian Rhythm
KW - Physical Activity
KW - marker
RP - NOT IN FILE
SP - S35
JF - European Child and Adolescent Psychiatry
VL - Conference: Eunethydis 1st International ADHD Conference: From Data to Best Clinical Practice Amsterdam Netherlands. Conference Start: 20100526 Conference End: 20100528. Conference Publication:
IS - var.pagings
N2 - Introduction and objective: Fluctuations in ADHD symptoms are theoretically explained by variations in arousal, a factor influenced by circadian rhythms. This study investigates whether 24-h patterns of heart rate (HR), an arousal-related marker, support differential circadian processes in children with ADHD. Mediating effects of physical activity, chronotypology and sleep-wake problems are explored. Methods: 30 children with ADHD and 30 sex-matched normal classmates, aged 6-12, were examined in their naturalistic setting during 5 days: HR and activity (24/24 h), chronotypology and sleep-wake problems. Results: Both activity and HR analyses showed significant group differences, with higher levels in the ADHD group, and significant day-night differences. Only for activity an interaction effect was detected, with no group differences during night but marked differences in the afternoon. Subjective evaluation revealed a later time-ofday preference in the ADHD group. Discussion: Raised HR in ADHD during night could not be explained by nighttime activity levels as these were only higher during day. Daytime HR levels did not follow daytime activity peaks in ADHD. Conclusion: Results revealed differential autonomic functioning in children with ADHD compared to controls
SN - 1018-8827
AD - (Imeraj, Antrop, Deboutte) Ghent University, Department of Psychiatry and Medical Psychology, Ghent, Belgium (Antrop) University Hospital Ghent, Child and Adolescent Psychiatry, Ghent, Belgium (Deboutte) Antwerp University, Collaborate Antwerp Psychiatry Research, Antwerp, Belgium (Roeyers) Ghent University, Department of Experimental-Clinical and Health Psychology, Ghent, Belgium
ER -

TY - JOUR
ID - 3684
T1 - Patient safety and reporting of spontaneous adverse drug reactions in paediatric inpatients
A1 - Kakosova,V.
Y1 - 2010///
N1 - 70449561
English
Journal: Conference Abstract
KW - EMBASE
KW - workshop
KW - adverse drug reaction
KW - patient safety
KW - clinical pharmacy
KW - hospital pharmacy
KW - pharmacy
KW - Society
KW - hospital patient
KW - physician
KW - pharmacist
KW - ward
KW - Monitoring
KW - patient
KW - Child
KW - Pediatrics
KW - Hospitalization
KW - polypharmacy
KW - questionnaire
KW - medical record review
KW - university hospital
KW - drug administration
KW - drug industry
KW - health care personnel
KW - nurse
KW - licence
KW - side effect
KW - drug control
KW - Death
KW - growth hormone
RP - NOT IN FILE
SP - 685
JF - Pharmacy World and Science
VL - Conference: European Society of Clinical Pharmacy International Workshop on Patient Safety and Pharmacy Uppsala Sweden. Conference Start: 20100510 Conference End: 20100511. Conference Publication:
IS - var.pagings
N2 - Background and objective Adverse drug reactions (ADRs) occur frequently in paediatric patients. Information about ADRs in children are often lacking or are insufficient. Frequently, they are not supplied by drug administration authorities or pharmaceutical industry. Many drugs used are unlicensed for the treatment of children or are prescribed outside the terms of the product license (off-label use). Occurrence especially of the preventable ADRs can by reduced by a monitoring performed by health care professionals (physicians, pharmacist and nurses). The ADRs are recorded and analysed by a physician and a clinical pharmacist. Design Spontaneous adverse reactions (ADRs) reporting in paediatric patients. Setting Paediatric Wards, Department of Pharmacy, Paediatric University Hospital. Main outcome measures Side effect monitoring and reporting. Case reporting to The State Institute for Drug Control. Results A 4 years survey of spontaneous ADRs is given. Among them one very serious-resulting in death (amphotericine B) and one world's first published adverse event (growth hormone somatothropine). Conclusions Number of adverse drug reactions reported by our paediatrics wards was influenced by several factors. First, usually only severe or rare events were spontaneously reported by physicians. Second, the prolonged hospital stay, the pharmaco-therapeutic groups of drugs, and the

polypharmacy also play a significant role. More ever, the reports are provided spontaneously. To increase detection of adverse reactions or events questionnaires and chart reviews should by implemented
SN - 0928-1231
AD - (Kakosova) Paediatric University Hospital, Bratislava, Slovakia
ER -

TY - JOUR
ID - 3685
T1 - Age-related improvement in social functioning and motor simulation within the mirror neuron system in autism
A1 - Thioux,M.
A1 - Bastiaansen,J.A.C.J.
A1 - Keysers,C.
Y1 - 2010///
N1 - 70444068
English
Journal: Conference Abstract
KW - EMBASE
KW - health care organization
KW - Autism
KW - Social Interaction
KW - Simulation
KW - nerve cell
KW - psychiatry
KW - inferior frontal gyrus
KW - Facial Expression
KW - Child
KW - Adult
KW - gaze
KW - taste
KW - social adaptation
KW - Diagnosis
KW - Remission
KW - adolescence
KW - adulthood
KW - laboratory
KW - patient
KW - Male
KW - gender
KW - intelligence quotient
KW - audiovisual equipment
RP - NOT IN FILE
SP - S7
JF - European Archives of Psychiatry and Clinical Neuroscience
VL - Conference: 3rd Meeting of West European Societies of Biological Psychiatry Berlin Germany. Conference Start: 20100602 Conference End: 20100604. Conference Publication:
IS - var.pagings
N2 - Introduction and Aims: Several studies have described an hypoactivity in the inferior frontal gyrus in children with autism during the observation of facial expressions1,2,3. This hypoactivation has been interpreted as evidence for a deficit of the mirror neuron system. The deficit is thought to have serious consequences on the ability to simulate and understand the feelings of others4. We examined the outcome of the motor simulation deficits in a large sample of adults with autism. In addition, we investigated the relationship between inferior frontal gyrus activity and social functioning outside the laboratory. Patients and Methods: Twenty-one adult males with autism spectrum disorders and 21 typically developing subjects matched for age, gender, and IQ were scanned in three conditions: observing short movies showing emotional facial expressions, performing a facial movement, and experiencing a disgusting taste. Symptom severity and level of social adjustment were measured with the Autism Diagnostic Observation Schedule and the Social Functioning Scale. Results: Activity

triggered in the inferior frontal gyrus by the observation of facial expressions increased with age in autism, but not in controls. This age-related increase of activity was associated with changes in gaze behaviour, and improvements in social functioning, but not with the remission of autistic symptoms. Conclusions: The results suggest that activity in the mirror neuron system normalizes with age in autism, and this normalization is accompanied by changes in gaze behaviour and improved social functioning. Better simulation of facial expressions in the inferior frontal gyrus may contribute to the amelioration in social functioning commonly observed throughout adolescence and adulthood. It is the first demonstration of an age-related neurocognitive improvement in autism. This finding should encourage the development of new therapeutic interventions aimed at augmenting motor simulation of emotional facial expressions in children with autism
SN - 0940-1334
AD - (Thioux, Bastiaansen, Keysers) University Medical Centre Groningen, Dept. of Neuroscience, NeuroImaging Centre, Groningen, Netherlands (Bastiaansen) Autism Team North Netherlands, Groningen, Netherlands
ER -

TY - JOUR
ID - 3686
T1 - Effects of plasmodium falciparum dihydropteroate synthase mutations on parasite fitness
A1 - Ochong,E.O.
A1 - Rosenthal,P.J.
Y1 - 2010///
N1 - 70443285
English
Journal: Conference Abstract
KW - EMBASE
KW - fitness
KW - Mutation
KW - parasite
KW - Hygiene
KW - Plasmodium falciparum
KW - tropical medicine
KW - Society
KW - wild type
KW - Competition
KW - Africa
KW - biosynthesis
KW - Point Mutation
KW - assay
KW - mutant
KW - type strain
KW - prophylaxis
KW - pregnant woman
KW - Child
KW - opportunistic infection
KW - Human immunodeficiency virus infection
KW - Human immunodeficiency virus
KW - dihydropteroate synthase
KW - enzyme
KW - folic acid
KW - pyrimethamine plus sulfadoxine
KW - cotrimoxazole
KW - dihydrofolate reductase
RP - NOT IN FILE
SP - 380
JF - American Journal of Tropical Medicine and Hygiene

VL - Conference: 59th Annual Meeting of the American Society of Tropical Medicine and Hygiene, ASTMH 2010 Atlanta, GA United States. Conference Start: 20101103 Conference End: 20101107. Conference Publication:
IS - var.pagings
N2 - The antifolates sulfadoxine-pyrimethamine (SP) and trimethoprimsulfamethoxazole (TMP/SMX) have potent activity against wild type Plasmodium falciparum, but activity is decreased due to resistancemediating mutations in many areas. However, SP remains the drug of choice for intermittent preventive therapy in pregnant women and children, and TMP/SMX is widely used to prevent opportunistic infections in those with HIV infection. Resistance to antifolates is mediated by a series of mutations in the target enzymes dihydrofolate reductase (DHFR) and dihydropteroate synthase (DHPS), key enzymes in the folic acid biosynthetic pathway. In East Africa, parasites commonly harbor 3 DHFR mutations (S108N, N51I and C59R) and 2 DHPS mutations (A437G and K540E) that mediate an intermediate level of resistance. Additional point mutations, seen more commonly outside Africa, mediate higher-level resistance. We are studying the impact of various resistancemediating mutations in DHFR and DHPS on the relative fitness of P. falciparum. To this aim, we are exploring the growth of parasites under different conditions and in competition assays. We are studying D10-strain parasites engineered to express DHPS with 1-3 mutations. In initial competition experiments, D10 wild-type and single mutant (A437G) strains outcompeted parasites with 2 (A437G + A581G or S436A + A437G) or 3 (S436A + A437G + K540E) mutations over 2 months of culture, as assessed by strain-specific PCR. Experiments to more stringently characterize relative growth of these strains utilizing folate-limiting culture conditions and quantitative PCR and to evaluate parasites with mutations in DHFR are underway. Our preliminary results suggest that resistancemediating mutations in DHPS engender a loss of fitness compared to that of wild type strains of P. falciparum
SN - 0002-9637
AD - (Ochong, Rosenthal) University of California at San Francisco, San Francisco, CA, United States
ER -

TY - JOUR
ID - 3687
T1 - HFOs (80-450Hz) recorded with standard intracranial electrodes reflect epileptogenicity in children with focal cortical dysplasia
A1 - Jacobs,J.
A1 - Kerber,K.
A1 - Dumpelmann,M.
A1 - LeVan,P.
A1 - Schulze-Bonhage,A.
A1 - Korinthenberg,R.
Y1 - 2010///
N1 - 70441923
English
Journal: Conference Abstract
KW - EMBASE
KW - Child
KW - electrode
KW - cortical dysplasia
KW - Society
KW - electric potential
KW - patient
KW - Classification
KW - electroencephalogram
KW - microelectrode
KW - high frequency oscillation
KW - Epilepsy
KW - pathology
KW - slow wave sleep
KW - filter
KW - seizure
KW - Analysis of Variance

RP - NOT IN FILE
JF - Neuropediatrics
VL - Conference: 36th Annual Meeting of the Society of Neuropediatrics Mannheim Germany. Conference Start: 20100922 Conference End: 20100925. Conference Publication:
IS - var.pagings
N2 - Introduction: High frequency oscillations (HFO) ranging between 80 and 500Hz recorded with intracranial electrodes have been linked to epileptogenic regions in patients with epilepsy. The removal of HFO-generating areas correlates with a good postsurgical outcome. Most studies have been performed with microelectrodes or custom made macroelectrodes. Data on children and different pathologies is limited. We here investigate HFOs in children with Focal Cortical Dysplasia (FCD) during a routine investigation with commercially available grid electrodes. Methods: Patients under the age of 18 recorded in our centre between 2005 and 2009 and with pathologically confirmed FCD were included in this study. EEG was filtered at 450Hz and sampled at 1024Hz. HFOs (Ripples 80-200Hz, Fast Ripples 200-450Hz) were visually identified on bipolar montage in a five-minute segment of slow wave sleep using high-pass filters at 80Hz and 200Hz. Rates of HFOs in areas inside and outside the seizure onset zone (SOZ) were calculated for each channel and compared using ANOVA (p<0.05). Results: Six children were included (mean age 13.6 years), four had FCD type 2a and two type 1b (Palmini classification). Rates of ripples (19.1/min) and fast ripples (2.5/min) were similar to those described before. They were significantly higher inside the SOZ (R: 29.1/min; FR: 4.7/min) than outside (R: 15.8/min, FR: 1.8/min, p<0.001). Two patterns of HFO occurrence were seen: in pattern 1, channels had a flat baseline without high frequency activity and intermittent very high voltage HFOs. Pattern 2 was defined as channels with a continuously oscillating baseline with intermittently occurring higher voltage HFOs. Of the SOZ channels 71.1% showed pattern 1 and 17.1% showed pattern 2. Of non-SOZ channels, 20.5% showed pattern 1 and 17.5% pattern 2. The remaining channels showed few or no HFO activity; nevertheless HFOs were generated in a large number of channels. Conclusion: Children with FCDs showed a large extent of areas generating HFOs. Rates of HFOs could distinguish between channels inside and outside the SOZ. Therefore, HFOs recorded during only 5 minutes in a routine clinical setting in children may help to further delineate epileptogenic areas. Two patterns of HFOs were distinguished, of which pattern 1 consisting of single high voltage HFOs may show a closer link to the SOZ. Whether these neocortical HFO patterns can be linked to FCD or its subtypes will have to be evaluated in larger studies
SN - 0174-304X
AD - (Jacobs, Kerber, Korinthenberg) Neuropediatric Department, Freiburg, Germany (Dumpelmann, Schulze-Bonhage) Section for Epileptology, Freiburg, Germany (LeVan) Radiology, Freiburg, Germany
ER -

TY - JOUR
ID - 3688
T1 - The European pediatric immediate life support (EPILS) course: Experience in an Italian hospital to implement quality in primary care setting
A1 - Garani,G.
A1 - Garani,M.C.
A1 - Bertacca,L.
A1 - Falorni,S.
A1 - Barlocco,E.
Y1 - 2010///
N1 - 70431012
English
Journal: Conference Abstract
KW - EMBASE
KW - Resuscitation
KW - Hospital
KW - primary medical care
KW - physician
KW - nurse
KW - Child
KW - feedback system
KW - emergency ward
KW - United Kingdom

KW - critically ill patient
KW - rapid response team
KW - respiratory arrest
KW - Teaching
KW - course evaluation
RP - NOT IN FILE
SP - S102
JF - Resuscitation
VL - Conference: 10th Scientific Congress of the European Resuscitation Council Porto Portugal. Conference Start: 20101202 Conference End: 20101204. Conference Publication:
IS - var.pagings
N2 - Purpose of the study: The EPILS is a one day course and is aimed at the training attendee to recognise and manage critically ill children and children in cardio-respiratory arrest whilst awaiting the arrival of the Medical Emergency Team (MET) (1-5 min). The course also trained the candidate to work as a member of the Resuscitation team. In this paper we describe our experience of teaching EPILS to nurses and physicians of a Paediatric Unit. We have also measured the impact that EPILS has on the resuscitation knowledge. Methods: The EPILS course materials were translated by Italian EPLS instructors. From April to June 2010 nurses and physicians attended the Italian EPILS course. Candidate feedback was collected using an evaluation form. The change in knowledge of candidates was measured using a pre- and post-course test. Variables associated with candidate performance on course papers were investigated. Results: A total of 49 participants attended five EPILS courses. All candidates completed the course successfully and gave high evaluation scores. EPILS produced a significant increase from pre- to post-course test score (87.8+5.8 to 95+4.2,p<0.001). Only 8.1% of the participants obtain a post-test score under 90% (in contrast with 67.5% in pre-test). The pre-course score was higher for nurses and physicians working in Paediatric Emergency Unit compared with those coming from Paediatric unit, but this difference disappeared in the post-course evaluation. A detailed analysis of question evidenced the difficulty to understand when activate MET (limited availability in Italian actuality). Conclusions: This local Italian experience shows that the EPILS course format can be applied successfully outside the UK, and adapted to local needs. Better attention must be focalized to MET alert. The course improves knowledge scores and abolishes knowledge differences due to different working backgrounds of candidates
SN - 0300-9572
AD - (Garani, Garani, Bertacca, Falorni, Barlocco) NICU and Neonatology, Azienda Ospedaliero-Universitaria S.Anna, Ferrara, Italy (Garani, Garani, Bertacca, Falorni, Barlocco) Pediatric Unit, Misericordia Hospital, Grosseto, Italy
ER -

TY - JOUR
ID - 3689
T1 - Two modalities of delivery following TVT-O and TVT-Secur
A1 - Carmen,F.
A1 - Maddalena,S.M.
A1 - Fabiana,S.
Y1 - 2010///
N1 - 70428634
English
Journal: Conference Abstract
KW - EMBASE
KW - patient
KW - Pregnancy
KW - vaginal delivery
KW - Cesarean Section
KW - device
KW - Female
KW - follow up
KW - incision
KW - continence
KW - incontinence
KW - clinical evaluation

KW - tension free vaginal tape
KW - suburethral sling
KW - Exercise Test
KW - urethra
KW - echography
KW - gold standard
KW - surgery
KW - stress incontinence
KW - bladder
KW - intestine perforation
KW - inguinal region
KW - Pain
KW - Case Report
KW - pelvic floor muscle training
KW - urine retention
KW - unplanned pregnancy
KW - Diagnosis
KW - pregnancy test
KW - ward
KW - spine
KW - informed consent
KW - Newborn
KW - episiotomy
KW - Child
KW - Birth Weight
KW - questionnaire
KW - Hospital
KW - polypropylene
RP - NOT IN FILE
SP - 88
EP - 89
JF - Neurourology and Urodynamics
VL - Conference: 34th Congresso Nazionale SIUD, 8th Congresso SIUD - Fisioterapisti Infermieri Ostetriche Verona Italy. Conference Start: 20100617 Conference End: 20100619. Conference Publication:
IS - var.pagings
N2 - Introduction and Aim of the Study: Retropubic tension-free vaginal tape (TVT) has become the gold standard in the surgical treatment of female stress urinary incontinence (SUI), with good clinical results and limited complications. Nevertheless, some severe complications have been reported, among which bladder and bowel perforation and vessel damages. For this reason, a novel route for the introduction of polypropylene sling has been proposed via the obturator foramen, both from the outside to the inside and from the inside to the outside, with similar cure rates.1 Finally, to further reduce invasiveness and complications (such as groin pain and urinary retention), single incision devices were produced. This continuous reduction of invasiveness leads to an increase of younger women suffering from SUI requesting surgical resolution of incontinence. Hence, a number of case reports on pregnancies after suburethral sling positioning. We here report on two cases of pregnancy after suburethral sling for SUI, one after TVT-O and one after the single incision TVT-Secur device - with different management of delivery. Materials andMethods:A34-year oldwoman (gravid 2, para 2) was referred on January 2007 to our Urogynecology Clinic with symptoms of SUI, appeared after her second delivery. After unsuccessful pelvic floor training, on May 2007she underwent VT-O procedure as described by de Leval.2 After the procedure, the patient was continent, as well as at the sixth month follow-up visit. Soon after that follow-up visit, a third unplanned pregnancy occurred. After a thorough discussion of the mode of delivery, considering the pros and cons of vaginal delivery as opposed to cesarean section, the patient decided for the latter. A 41-year old woman (gravida 3, para 3), suffering of SUI symptoms after her second vaginal delivery, on February 2008.underwent TVT-Secur after confirmation of SUI diagnosis with clinical evaluation and urodynamic testing. After the procedure she was continent, as well as at the three-month follow-up visit. Eight months after the procedure, the patient returned with a positive pregnancy test. Since no data on pregnancy after TVT-Secur positioning, and considering the presence of absorbable fixation tips instead of the tape passing out fromthe obturator foramen, the patient was advised to undergo a cesarean section. Ten days

before the planned cesarean section, the patient was admitted to our Obstetric Ward with strong contractions, a 5 cm cervical opening, a complete effacement of the cervix and fetal index at the ischiatic spine level. Considering the favourable obstetric conditions, after receiving an informed consent by the patients, it was decided to let her complete her labour. Thus, a spontaneous vaginal delivery of a newborn weighing 3,250 g followed one hour later, without episiotomy. Results: During pregnancy, the first patientwas continent and delivered a child with a birthweight of 3,560 g at 38 1 week gestation bymeans of cesarean section.One year after delivery, the patient is still continent as shown both by QoL questionnaires, clinical evaluation and urodynamic testing. After three months, the second patient did not show any incontinence symptoms. Clinical stress test, post-voidal residue and urodynamic findings were negative. Interpretation of Results: Reports of pregnancies following retropubic and transobtuator TVT show successful outcomes both in case of cesarean section and vaginal delivery.3 The first reports are on deliveriesmanaged with cesarean section due to the lack of information on the correct modality of delivery following TVT. Nevertheless, reports of vaginal delivery after TVT indicate that, at least in the short term, continence is not altered, as well as the tape position.3 We here report on two women, treated with two different types of slings, delivering with two different modalities. Both women were continent before, during and after delivery. In the case of TVT-O we have a follow-up at one year with no incontinence symptoms, both objectively and subjectively. In that case, the choice of the type of delivery was left to the patients, after having discussed all pros and cons. Our patient treated with TVT-Secur is, to our knowledge, the first case of a pregnancy and a delivery following single incision device positioning. For this patient, vaginal delivery was not planned, since too few information is available on the novel single incision device. The fixation system of this device is different from both retropubic and transobturator TVTs, thus the safest advice would have been to deliver by cesarean section. Nevertheless, she delivered vaginally with no recurrence of SUI. Follow-up of this patient is quite short, so that no conclusions can be drawn from this case, even though it seems that no immediate harm to the urethra has been caused by vaginal delivery and, apparently, function of the tape was preserved. Unfortunately, we did not manage to perform ultrasonography to confirm tape positioning, but we can hypothesize that, since continence was preserved, tape positioning should have been preserved as well. Conclusions: In conclusion, primary treatment for SUI in younger patients should be conservative, butwomen suffering from persistent SUI and desiring further pregnancies should be offered TVT procedures, since pregnancy seems no to alter TVTinduced continence. Themode of delivery should be thoroughly discussed with the patients, and should be customized for each of them, not precluding vaginal delivery, at least for retropubic and transobturator TVTs. More data on themode of delivery in case of the presence of a TVT-Secur device are needed to better counsel patients and thus cesarean section should be advised, at least for the moment
SN - 0733-2467
AD - (Carmen, Maddalena, Fabiana) Tommaselli Giovanni Antonio, Di Carlo Costantino, Nappi Carmine University Federico II, Ginecology Department, Naples, Italy
ER -

TY - JOUR
ID - 3690
T1 - Circulating cytokines influence the fetal growth in pregnant women with rheumatoid arthritis
A1 - De Steenwinkel,F.D.O.
A1 - Hazes,J.M.W.
A1 - De Man,Y.A.
A1 - Hokken,A.C.S.
A1 - De Rijke,Y.B.
A1 - Dolhain,R.J.E.M.
Y1 - 2010///
N1 - 70380523
English
Journal: Conference Abstract
KW - EMBASE
KW - pregnant woman
KW - fetus growth
KW - rheumatoid arthritis
KW - college
KW - health practitioner
KW - rheumatology

KW - disease activity
KW - Birth Weight
KW - third trimester pregnancy
KW - patient
KW - Pregnancy
KW - first trimester pregnancy
KW - prospective study
KW - Gestational Age
KW - Child
KW - cardiovascular disease
KW - Parity
KW - metabolic syndrome X
KW - stratification
KW - maternal disease
KW - cytokine
KW - interleukin 6
KW - interleukin 10
KW - prednisone
RP - NOT IN FILE
SP - 1073
JF - Arthritis and Rheumatism
VL - Conference: American College of Rheumatology/Association of Rheumatology Health Professionals Annual Scientific Meeting, ACR/ARHP 10 Philadelphia, PA United States. Conference Start: 20091016 Conference End: 20091021. Conference Publication:
IS - var.pagings
N2 - Background: High RA disease activity during pregnancy is associated with lower birth weight. Lower birth weight, even within the normal range, has been linked with cardiovascular diseases and metabolic syndromes later in life. Outside pregnancy, active RA is associated with high levels of circulating cytokines. Purpose: To asses the levels of IL-10, IL-6 and TNFa in pregnant women with RA and to evaluate correlations between these levels, disease activity, and birth weight. Methods: Current study is embedded in the PARA-study, a prospective study on RA and pregnancy. 134 pregnant RA patients are enrolled in first and 168 in third trimester. 33 healthy pregnant women served as control. We analyse data using birth weight standard deviation scores (bwsds), a measure for birth weight adjusting for gestational age and sex of the child. Maternal disease activity is based on DAS28. IL-10, IL-6 and TNFa are determined by Immulite 1000. Results: Levels of IL-10, IL-6 and TNFa decrease during pregnancy. Strong correlations are found between these cytokines in first and third trimester and between DAS28 and IL-10, IL-6. First trimester patients with detectable IL-10 (n=12) show a higher disease activity than the IL-10 negative patients, a mean DAS28 4.4 (SD 1.2) and 3.6 (SD 1.1) respectively. To diminish the influence of disease activity on birth weight we match the IL-10 positive to an IL-10 negative group. Matching is on disease activity, parity and prednisone use. Mean bwsds is significantly higher (p=0.02) in the IL-10 positive group 0.92 (SD 0.7), than in the IL-10 negative match, 0.15 (SD 0.7). No such association is found in third trimester. To determine the additional effect of IL-6 to disease activity on bwsds stratification is done. In first trimester we stratify IL-6 and DAS28 in high and low group based on their median, resulting in 4 groups. In the two high DAS28 groups (defined median over 3.8), bwsds is significantly lower in high IL-6 than in low IL-6 (p<0.05). In the high IL-6 group bwsds is -0.19 (SD 1.12) in the low IL-6 group bwsds is 0.36 (SD 0.93). No such association is found in third trimester. TNFa is stratified the same way. In third trimester, only in patients with low disease activity, bwsds is lower in the low TNFa group than in the high TNFa group, bwsds of 0.05 (SD 0.97) and 0.52 (SD 0.96) respectively (p<0.05). Same trend is seen in the healthy cohort group, although not significant. No such association is found in first trimester or in patients with high RA disease activity. Conclusion: Circulating cytokines influence fetal growth in pregnant women with RA. In first trimester elevated IL-10 seems to protect against the negative influence of RA disease activity on birth weight, IL-6 seems to amplify this negative influence. Both cytokines create a bwsds deviation of more than 0.50 which is considered clinically relevant. In third trimester there is no influence suggesting an early critical window. Finally, our data might indicate a physiological role of TNFa in fetal growth during third trimester in healthy controls and patients with low disease activity. Underscoring that TNF-blockers should be used with caution during pregnancy
SN - 0004-3591

AD - (Hazes, De Man, Dolhain) Erasmus Medical Center, Rotterdam, Netherlands (De Steenwinkel, Hokken, De Rijke) Erasmus Medical Center, Sophia Children's Hospital, Rotterdam, Netherlands
ER -

TY - JOUR
ID - 3691
T1 - Cerebellar fMRI lateralization of language in children after pilocytic astrocytoma resection: Comparison with healty children
A1 - Ghielmetti,F.
A1 - Erbetta,A.
A1 - Bulgheroni,S.
A1 - Vitali,P.
A1 - Riva,D.
A1 - Bruzzone,M.G.
Y1 - 2010///
N1 - 70363689
English
Journal: Conference Abstract
KW - EMBASE
KW - Child
KW - Language
KW - Functional Magnetic Resonance Imaging
KW - surgery
KW - astrocytoma
KW - neuroradiology
KW - Diagnosis
KW - hemisphere
KW - normal human
KW - right hemisphere
KW - nuclear magnetic resonance imaging
KW - patient
KW - brain
KW - left hemisphere
KW - Imaging
KW - statistical analysis
KW - cerebellum
KW - computer program
KW - volunteer
KW - processing
KW - cognitive defect
KW - frontal lobe
KW - Cognition
RP - NOT IN FILE
SP - 289
EP - 290
JF - Neuroradiology Journal
VL - Conference: 19th Symposium Neuroradiologicum - The World Congress of Diagnostic and Therapeutic Neuroradiology Bologna Italy. Conference Start: 20101004 Conference End: 20101009. Conference Publication:
IS - var.pagings
N2 - Introduction: Cerebellum is not only involved in motor and articulatoy control but also in higher cognitive functions. Studies with functional MRI in right-handed healthy volunteers show that cerebellar activation during language tasks is mainly localized in the right hemisphere, contralateral to the dominant cerebral hemisphere. Our purpose was to evaluate cerebellar lateralization of language by functional MRI in healthy children and to study the functional reorganization in children after cerebellar pilocytic astrocytoma resection. Subjects and methods: We performed fMRI studies in 12 healthy right handers children (mean 11.3, range 8-16) and 6 patients (mean 11.6, range 9-17), 5 right handers, 1 left, with surgical resection of vermal (3) and right

cerebellar hemisphere (3). The tasks chosen for the healthy subjects were: overt verbal fluency (OVF), silent verbal fluency (SVF) and silent verb generation (SVG). Patients underwent OVF and SVG tasks. All subjects were trained outside the scanner before the MRI exam. During the fMRI sessions also morpholocical T1 and T2 imaging was done for each subject. All acquisitions were performed using a 1.5T Siemens Avanto scanner. Imaging processing and statistical analysis were conducted using the Brain Voyager (Brain Innovation) and SPM (Statistical Parametric Mapping) software. Results: Both in OVF and in SVF 11 volunteers presented fronto-parietal activations lateralized in the left cerebral hemisphere, while the cerebellar activations were lateralized in the right hemisphere. In one healthy subject where the activations were right lateralized, the cerebellar activations were localized in the left hemisphere. In SVG, all subjects presented left fronto-parietal and left temporal activations, while the cerebellar activations were lateralized in the right hemisphere. Also a II level study group has been performed on the healthy subjects for all tasks. The random effect analysis gave the localization of the tasks in the right cerebellar emisphere. The 6 patients presented slightly more bilateral cerebral activations if compared with healthy subjects. Among these, 3 presented bilateral cerebellar activation, 3 more localized activations in the right cerebellar hemisphere. Discussion: Assessment of cerebellar fMRI lateralization of language is feasible in children, even with mild cognitive deficits. According to literature, both VF and VG are associated with significant activation in the cerebellar hemisphere, contralateral to the dominant cerebral hemisphere. The calculated cerebellar Asymetry Indexes seem to anti-correlate with the frontal lobe Asymmetry Indexes and activations are significantly lateralized for silent tasks, probably due to the bilateral contribution of the mouth movements in overt tasks. Children with right cerebellar resections, especially of the lateral part and for wide lesions, present the most significant language deficits and bilateral cerebellar activations. This activation pattern is likely to be an attempt of compensatory reorganization. However the reorganization takes place also in the right damaged cerebellar hemisphere. Future analysis of correlation between cerebellar lateralization and language performance will disclose if this reorganization is successful to compensate the language deficit. The random effect analysis (study group) conducted on the healthy subjects gave the topology of the cerebellar activations, showing the differences between VF and VG
SN - 1971-4009
AD - (Ghielmetti, Erbetta, Bulgheroni, Vitali, Riva, Bruzzone) Fondazione IRCCS, Istituto Nazionale Neurologico C. Besta, Milano, Italy
ER -

TY - JOUR
ID - 3692
T1 - Energy intake, dietary habits, and physical activity of obese children's mothers: Obesogenic family environment
A1 - Chavez-Palencia,C.
A1 - Larrosa-Haro,A.
A1 - Vasquez-Garibay,E.M.
A1 - Romero-Velarde,E.
A1 - Salazar-Preciado,L.
A1 - Troyo-Sanroman,R.
A1 - Rodriguez-Anguiano,A.K.
A1 - Camara-Lopez,M.E.
A1 - Sepulveda-Vazquez,H.E.
Y1 - 2010///
N1 - 70326659
English
Journal: Conference Abstract
KW - EMBASE
KW - Child
KW - mother
KW - gastroenterology
KW - caloric intake
KW - Physical Activity
KW - Nutrition
KW - habit
KW - Society
KW - Environment

KW - Obesity
KW - questionnaire
KW - weight
KW - logistic regression analysis
KW - school
KW - sitting
KW - model
KW - protein intake
KW - Demography
KW - father
KW - Work
KW - Lifestyle
KW - childhood
KW - primary school
KW - Mexico
KW - recall
KW - traffic and transport
KW - protein
RP - NOT IN FILE
SP - E23
JF - Journal of Pediatric Gastroenterology and Nutrition
VL - Conference: North American Society for Pediatric Gastroenterology, Hepatology, and Nutrition Annual Meeting New Orleans, LA United States. Conference Start: 20101021 Conference End: 20101023. Conference Publication:
IS - var.pagings
N2 - Background: The aim was to compare the energy intake (EI), dietary habits (DH) and physical activity (PA) of mothers of children with obesity with mothers of children with healthy weight. Methods: Design: Case-control (cases: mothers of obese children, n=63; controls: mothers of healthy weight children, n=62). Setting: An elementary school at Guadalajara Mexico, 2009. EI was evaluated by a 24-hour recall questionnaire; HA and PA were evaluated with an ad hoc questionnaire. Analyses: OR, CI95%, logistic regression. Results: Overweight and obesity were significantly more frequent in the mothers of the obese children. They ingested a higher amount of energy and protein and with a significant higher frequency had lunch or dinner at work and watched TV at mealtimes. Their PA differed from controls mothers PA in outdoors or indoors moderate PA, less time of transportation to school and work and longer periods of sedentary activity. The regression models included the variables of the energy equation (energy and protein intake, active plus sedentary PA) plus specific DH and some socio-demographic characteristics (as families with one son and fathers job as independent professionals). Conclusions: The obese children's mothers' lifestyle seems to be obesogenic in variables related to energy intake and consumption -both as a part of the energy equation- as well as in dietary habits and the socio-demographic condition, underlining the multifactorial condition of childhood obesity
SN - 0277-2116
AD - (Larrosa-Haro) Gastroenterology and Nutrition, UMAE Hospital de Pediatra CMNO IMSS, Guadalajara, Mexico (Chavez-Palencia, Larrosa-Haro, Vasquez-Garibay, Romero-Velarde, Troyo-Sanroman) Instituto de Nutricion Humana, Universidad de Guadalajara, Guadalajara, Mexico (Salazar-Preciado, Rodriguez-Anguiano, Camara-Lopez, Sepulveda-Vazquez) Unidad de Investigacion en Epidemiologia Clinica UMAE HE CMNO, Instituto Mexicano del Seguro Social, Guadalajara, Mexico
ER -

TY - JOUR
ID - 3693
T1 - How body mass index and body fat mass correlate with physical activity in urban Belarusian school children
A1 - Vainilovich,Y.
A1 - Danilova,L.
A1 - Lushchyk,M.
A1 - Sretenskaya,Z.
Y1 - 2010///

N1 - 70325537
English
Journal: Conference Abstract
KW - EMBASE
KW - Society
KW - school child
KW - body mass
KW - Physical Activity
KW - body fat
KW - Diabetes Mellitus
KW - Adolescent
KW - Child
KW - Male
KW - Female
KW - school
KW - Obesity
KW - questionnaire
KW - computer
KW - sitting
KW - Body Weight
KW - cardiopulmonary hemodynamics
KW - urban area
KW - analyzer
KW - girl
RP - NOT IN FILE
SP - 57
JF - Pediatric Diabetes
JA - Pediatr Diabetes
VL - Conference: 36th Annual Meeting of the International Society for Pediatric and Adolescent Diabetes, ISPAD Buenos Aires Argentina. Conference Start: 20101027 Conference End: 20101030. Conference Publication:
IS - var.pagings
N2 - Objectives: Regular physical activity (PA) decreases adiposity in children and benefit cardiopulmonary system. Aim of this study: To estimate frequency, duration and intensity of PA and the sedentary time outside school in Belarusian school children from urban area and to analyze their correlation with body mass index (BMI) and percent of body fat mass (%FM). Methods: The study was conducted in two randomly selected schools. Body weight and %FM were measured using foot-to-foot bioimpedance analyser Tanita. The level of PA and sedentary time outside school was assessed by a selfadministered questionnaire. General sedentary time was estimated as screen time (spent at TV, computer) and time for home work and other sedentary activities. Results: Three hundred and eighty-five children and adolescents aged 10-17 years, M/F 165/220 with completed anthropometric data and questionnaires were included in analysis. Mean age (13.1 + 1.85 years) and mean BMI (19.89 + 3.34 kg/m²) were not statistically different between males and females, males had significantly lower %FM (P = 0.02). Th13% of children reported undertaking PA every day, males more often than females (17% vs. 10%). 25% of children exercised more than 60 minutes per section (38% males vs. 16% females). Totally 6% of children reported recommended level of PA: every day and more than 60 minutes per section (11% males vs. 2% females). 33% of children had screen time more than 3 hours per day (35% males vs. 31% females) and 63% reported general sedentary time more than 4 hours per day. BMI and %FM showed weak positive correlation with screen time (r = 0.151, P = 0.003 and r = 0.104, P = 0.041, respectively), %FM in addition demonstrated weak negative correlation with frequency (r =)0.112, P = 0.028) and duration (r =)0.144, P = 0.005) of PA. Conclusion: Our study showed that level of PA did not influence BMI, however higher frequency and duration of PA decreased adiposity (%FM) in children. Generally Belarusian school children, especially girls, reported low levels of PA
SN - 1399-543X
AD - (Vainilovich, Danilova, Lushchyk, Sretenskaya) Belarusian Medical Academy for Postgraduate Education, Endocrinology Department, Minsk, Belarus
ER -

TY - JOUR
ID - 3694
T1 - Motor performance of children with developmental coordination disorder is supported by different neural networks than in typical children
A1 - Zwicker,J.G.
A1 - Missiuna,C.
A1 - Harris,S.R.
A1 - Boyd,L.A.
Y1 - 2010///
N1 - 70324807
English
Journal: Conference Abstract
KW - EMBASE
KW - Child
KW - Motor Performance
KW - developmental coordination disorder
KW - Cerebral Palsy
KW - Functional Magnetic Resonance Imaging
KW - electroencephalogram
KW - Learning
KW - brain
KW - cerebellum
KW - nuclear magnetic resonance imaging
KW - pilot study
KW - volunteer
KW - Parietal Lobe
KW - laboratory
KW - nuclear magnetic resonance scanner
KW - oxygen blood level
KW - brain region
KW - prefrontal cortex
KW - thalamus
KW - putamen
KW - Neuroimaging
KW - learning disorder
KW - model
RP - NOT IN FILE
SP - 9
EP - 10
JF - Developmental Medicine and Child Neurology
VL - Conference: 64th Annual Meeting of the American Academy for Cerebral Palsy and Developmental Medicine Washington, DC United States. Conference Start: 20100922 Conference End: 20100925. Conference Publication:
IS - var.pagings
N2 - Background/Objectives: Children with developmental coordination disorder (DCD) struggle to learn new motor skills and demonstrate more variable motor performance than their typically-developing (TD) peers. Because no neuroimaging studies have been conducted to examine the mechanisms underlying motor learning deficits in children with DCD, the objective of this pilot study was to determine if patterns of brain activity differed between children with and without DCD while learning a motor task. We expected that children with DCD would demonstrate more accurate tracing of our trail-tracing task from early practice to retention, but that they would be significantly less accurate on the task as compared to TD children. We predicted that children with DCD would under-activate the cerebellum when tracing, and that they would show a compensatory pattern of brain activity in the prefrontal and posterior parietal cortices when compared with typical peers. Design: Behavioral and functional magnetic resonance imaging (fMRI) blocked design. Participants and Setting: A volunteer sample of seven children with DCD (ages 8-12 y) and seven age-matched TD children in the Brain Behavioral Laboratory and UBC MRI Research Centre. Materials/Methods: Using fMRI, we mapped brain activity associated with learning of a trail-tracing task in children with and without DCD. We indexed motor

learning as a reduction of tracing error from early practice to retention on our experimental task. We assessed changes in brain activation across two fMRI sessions. Early task practice (Day 1) and a retention test (Day 5) took place inside a 3-Tesla Phillips MRI scanner. Three days (Days 2-4) of behavioral practice (tracing outside the fMRI) occurred in between the scanning sessions. Brain activity was represented by using fMRI as a summed-weighted threshold of percent signal change. Results: Consistent with their clinical presentation, children with DCD did not show an improvement in motor accuracy with equivalent practice to TD children. They also showed less blood oxygen level dependent response than TD children in a broad network of brain regions associated with motor learning and performance, including the right inferior parietal lobule, bilateral dorsolateral prefrontal cortices, right middle occipitotemporal area, lobule VI of the cerebellum, bilateral posterior cingulate, and left thalamus. In contrast, significantly greater activation of the caudate and putamen was apparent in children with DCD as compared to control children. Conclusions/Significance: Our results indicate that differences inmotor learningmay be related to differences in brain activation. Compared to TD peers, the DCD group demonstrated under-activation in the cerebellumand in brain areas associated with the cerebello-thalamo-cortical network. Findings suggest that children withDCDmay have a deficit in updating internal models of movement and use the cortico- striatal network to support their motor performance
SN - 0012-1622
AD - (Zwicker) Rehabilitation Sciences, University of British Columbia, Vancouver, BC, Canada (Missiuna) School of Rehabilitation Science, McMaster University, Hamilton, ON, Canada (Missiuna) CanChild Centre for Childhood Disability Research, McMaster University, Hamilton, ON, Canada (Harris, Boyd) Department of Physical Therapy, University of British Columbia, Vancouver, BC, Canada (Boyd) Brain Research Centre, University of British Columbia, Vancouver, BC, Canada
ER -

TY - JOUR
ID - 3695
T1 - Lifestyle factors in childhood obesity
A1 - Patankar,N.
A1 - Prabhu,S.
A1 - Venkatesh,S.
A1 - Chhabria,D.
Y1 - 2010///
N1 - 70323745
English
Journal: Conference Abstract
KW - EMBASE
KW - Obesity
KW - Lifestyle
KW - Society
KW - childhood
KW - India
KW - Child
KW - statistical significance
KW - heart left ventricle mass
KW - diastolic blood pressure
KW - Reading
KW - onset age
KW - Physical Activity
KW - Diet
KW - epidemic
KW - Physical Examination
KW - body mass
KW - Classification
KW - patient
KW - Nuclear Family
KW - Motivation
KW - protein intake
KW - Food

KW - metabolic parameters
KW - saturated fatty acid
KW - unsaturated fatty acid
KW - carbohydrate
KW - protein
RP - NOT IN FILE
SP - 224
EP - December
JF - Annals of Pediatric Cardiology
VL - Conference: 12th Annual Conference of Pediatric Cardiac Society of India Mumbai India. Conference Start: 20101008 Conference End: 20101010. Conference Publication:
IS - var.pagings
N2 - Obesity is the new age epidemic and a slow killer with its roots predominantly in our lifestyle and behaviour. MATERIAL AND METHODS: Thirty overweight children who met inclusion criteria were evaluated over a period of 1 year. A detailed history physical examination was done in all. Body Mass Index Percentile (BMI % tile) for their age and sex as per the NCHS/CDC charts was used for classification. Relevant investigations to assess metabolic parameters were done. RESULTS: Thirteen of 30 patients had onset of obesity before one year of age, with 10 being obese and 3 overweight (P=0.036). No correlation was found between parental obesity and BMI of children. Fourteen of the obese children belonged to nuclear families (66.7%) as compared to 9 (100%) who stayed in a joint family (P=0.048). Poor level of motivation was observed in 22/30 children and average level in 8/30 (P=0.037). Excess carbohydrates and fats consumed and the decrease in protein intake by the children correlated significantly with the BMI (P values of 0.001, 0.002, and 0.003 respectively). Amount of protein consumed by those children who were overweight was significantly more than those who were obese (P value 0.001). 93.3% consumed food prepared using saturated fat as compared to 2 (6.7%) who used unsaturated fat (P=0.008). Sedentary hours spent correlated significantly with the BMI (P=0.01), with statistically significant hours spent outdoors by overweight group than in the obese group (P=0.007). Children with high BMI had increased Left ventricular mass index (LVMI). LV Mass showed a positive correlation with systolic and diastolic blood pressure readings. Total Chol/HDL ratio was found to be statistically significant in relation to BMI (P=0.032). CONCLUSION: Grade of obesity statistically correlates with age of onset, type of family, degree of physical activity, diet and raised total Cholesterol/HDL ratio and motivational level. Childhood obesity definitely has a significant correlation with the lifestyle factors
SN - 0974-2069
AD - (Patankar, Prabhu, Venkatesh, Chhabria) Division of Pediatric Cardiology, Department of Pediatric Medicine, B. J. Wadia Hospital for Children, Mumbai, India
ER -

TY - JOUR
ID - 3696
T1 - Effect of the cellular irradiation on neuro-behavior of mice after acute exposure
A1 - Kumar,N.
A1 - Chaturvedi,R.K.
A1 - Khan,R.A.
Y1 - 2010///
N1 - 70320507
English
Journal: Conference Abstract
KW - EMBASE
KW - mouse
KW - exposure
KW - molecular imaging
KW - irradiation
KW - mobile phone
KW - brain
KW - Locomotion
KW - laboratory
KW - headache
KW - central nervous system

KW - Child
KW - Technology
KW - weight
KW - Environment
KW - device
KW - radius
KW - electromagnetic radiation
RP - NOT IN FILE
SP - S1594
JF - Molecular Imaging and Biology
VL - Conference: 2010 World Molecular Imaging Congress, WMIC 2010 Kyoto Japan. Conference Start: 20100908 Conference End: 20100911. Conference Publication:
IS - var.pagings
N2 - Extension of the mobile phone technology raises concern about the implications of electromagnetic radiation (EMR) on the central nervous system (CNS). Children are more sensitive to EMR due to developing phase of their brain. Generally Mobile phone radiates an average power of 0.2-0.6 watts when hand-held and operated close to the head, background levels are sharply distorted, with likely 40- 50 percent of radiated phone energy absorbed into the brain. Our previous study has concluded that the extensive users, they hold cell phone for a period above 3 years, were more attentive for 'headache' symptom in comparison to normal users. In this study we assessed the spontaneous locomotors activity (SLA) of the mice after the acute exposure of cellular telephone (CDMA). 10 mice of the 30 days old (average weight 30gm) were grouped as control and EMR and they were habitualised for one week prior to start the experiment in well designed circular cellphone exposure (CCPE) cage. CCPE cage was specially designed for EMR exposure through the cellular phone over the mice in the natural environment and it was placed inside the exposure room where no electric appliances were exist. In the cage, cellular phone was placed in the center and mice were allowed to move around the device within 10 cm. radius. 3 hours/day exposure was applied to the mice of EMR group by the cellular phone in CCPE cage. Locomotor activity was assessed using computerized Optovarimax animal activity monitor, following the method Ali et. al., (1990). In this study Distance traveled, Time resting, Time moving and Stereotypic Time was measured with the help of the activity monitor. No significant changes were found in the EMR exposed mice in comparison of control mice. More laboratory research is essential to clarify the contradictions
SN - 1536-1632
AD - (Kumar, Khan) SIST-DIT, Babasaheb Bhimrao Ambedkar University, Lucknow, India (Kumar, Chaturvedi) DTD, Indian Institute of Toxicology Research, Lucknow, India
ER -

TY - JOUR
ID - 3697
T1 - Power-up: A collaborative after school and family pilot study to prevent obesity
A1 - Choudhry,S.
A1 - Selvaraj,K.
A1 - Gliessen,K.
A1 - Myers,A.
A1 - Darukhanavala,A.
A1 - Lipton,R.
A1 - Davis,D.
A1 - Burnet,D.
A1 - Solomon,M.
A1 - Salahuddin,R.
A1 - Embil,K.
A1 - Love,L.
A1 - McClinton,L.
Y1 - 2010///
N1 - 70315107
English
Journal: Conference Abstract
KW - EMBASE
KW - school

KW - Obesity
KW - Internal Medicine
KW - Society
KW - pilot study
KW - Child
KW - parent
KW - Physical Activity
KW - teacher
KW - juvenile
KW - Nutrition
KW - United States
KW - eating habit
KW - African American
KW - Prevention
KW - Environment
KW - Risk
KW - Population
KW - participatory research
KW - skill
KW - Blood Pressure
KW - dietary intake
KW - boy
KW - girl
KW - Female
KW - Personnel
KW - Health
KW - community
RP - NOT IN FILE
SP - S355
JF - Journal of General Internal Medicine
VL - Conference: 33rd Annual Meeting of the Society of General Internal Medicine Minneapolis, MN United States. Conference Start: 20100428 Conference End: 20100501. Conference Publication:
IS - var.pagings
N2 - BACKGROUND: Obesity has risen dramatically in young people over the past two decades; this trend is especially prominent among minority populations, including African American (AA) youth. Schools represent a key venue in which to address the problem of obesity, through creative combination of curricular and environmental interventions. Given parental work demands and schedules and the need for safe and structured after-school opportunities for youth, Chicago Public Schools (CPS) has developed an extensive after-school program that provides an ideal potential setting for a healthy nutrition and physical activity intervention for children and families. METHODS: Using Community Based Participatory Research (CBPR) principles, we developed a partnership with Woodlawn Community School (WCS) on Chicago's South Side to implement and assess the feasibility of integrating into the after-school environment a behavioral intervention designed to decrease overweight and risk of obesity among AA youth and their parents. The Power-Up intervention, which comprises 14 interactive weekly sessions for children on various topics concerning nutrition and physical activity, was led by CPS teachers in the after school setting. Children were grouped by age, with approximately 15 children per group. Weekly parent discussion sessions held at pick-up time addressed similar topics in a discussion format, and families practiced Power-Up skills at home throughout the week by completing Power-Up 'homework' assignments. Data for participating families were collected pre- and post-intervention. Parent and child measures included BMI (BMI z-scores for children), blood pressure, self reported dietary intake and levels of physical activity, and health knowledge, beliefs and attitudes. RESULTS: 40 AA children (16 boys and 24 girls) and their 28 parents/guardians (all women) consented to participate in the Power-Up study. Children's ages ranged from 5 - 12 years (mean= 8). At baseline, over half of the children were either overweight (26%, 85th-95th %ile) or obese (28%, > 95th %ile). Post-intervention, children significantly reduced their mean BMI z-score from 1.05 to 0.81 (p<0.0001). Changes were more pronounced in the overweight children than in the obese category. CONCLUSIONS: Children, parents, teachers and school personnel enjoyed the Power-Up program, which was effectively integrated into school activities and culture through support of the Principal and other school leaders, presentations at Parent-Teacher events, and

participation in the annual All School Festival at the end of the school year. The after-school setting proved to be a feasible venue with promising pilot results for this obesity prevention program for children and families. Using CBPR principles in the development and implementation of the program was crucial to the success of this pilot project, and has led to long-term, sustainable impact in the environmental context at WCS; the school is working to install a green house to encourage healthier eating habits and new playground equipment to promote physical activity. Plans are underway for a larger, randomized trial to continue the Power-Up study in additional schools
SN - 0884-8734
AD - (Choudhry, Darukhanavala, Lipton, Davis, Burnet, Solomon) University of Chicago, Chicago, IL, United States (Selvaraj, Gliessen) Pritzker School of Medicine, Chicago, IL, United States (Myers) Rush University Medical Center, Chicago, IL, United States (Salahuddin, Embil, Love, McClinton) Woodlawn Community School, Chicago, IL, United States
ER -

TY - JOUR
ID - 3698
T1 - Predictors of Vitamin D status in children: A great Britain population based study
A1 - Absoud,M.
A1 - Cummins,C.
A1 - Lim,M.
A1 - Wassmer,E.
A1 - Shaw,N.
Y1 - 2010///
N1 - 70313141
English
Journal: Conference Abstract
KW - EMBASE
KW - Pediatrics
KW - United Kingdom
KW - Population
KW - Child
KW - childhood
KW - Risk
KW - Physical Activity
KW - Lifestyle
KW - blood sampling
KW - vitamin D deficiency
KW - dietary intake
KW - logistic regression analysis
KW - model
KW - Prevalence
KW - Adolescent
KW - Income
KW - risk factor
KW - Sunlight
KW - exposure
KW - supplementation
KW - autoimmune disease
KW - Diabetes Mellitus
KW - secondary analysis
KW - Diet
KW - Nutrition
KW - Male
KW - household
KW - field work
KW - Interview
KW - habit

KW - Multiple Sclerosis
KW - vitamin D
RP - NOT IN FILE
SP - 118
JF - Acta Paediatrica, International Journal of Paediatrics
VL - Conference: 2nd Excellence in Paediatrics Conference London United Kingdom. Conference Start: 20101202 Conference End: 20101204. Conference Publication:
IS - var.pagings
N2 - Introduction: Vitamin D insufficiency (VID) may result in autoimmune diseases such as diabetes and multiple sclerosis. There is a lack of data on the relative importance of childhood VID predictors. Purpose: To evaluate predictors of childhood VID by undertaking a secondary analysis of the Great Britain National Diet and Nutrition Survey (NDNS) of young people (YP) dataset. Material: One thousand one hundred and two YP aged 4-18 years (999 white, 570 male). Methods: Design: nationally representative survey of YP living in private households in Great Britain. Fieldwork covered 12 months (January 1997-1998). Interventions: interview and record providing information about; dietary habits; physical activity; lifestyle and socio-demographics; and blood sample. Outcomes: Vitamin D deficiency (<25 nmol/L) and VID (<50 nmol/L). Results: Vitamin D levels (mean = 62.1 nmol/L, 95% CI 60.4-63.7) showed deficiency = 7.4% and insufficiency = 35.1%. Levels were higher for those living between 53 and 59degree latitude (compared with 50-53, P = 0.045). Dietary intake had no effect on vitamin D status. A logistic regression model showed the following significant (P < 0.05) associations for VID: (Table presented) Conclusion: We report the high prevalence of childhood VID and a previously under-recognised risk of VID in adolescents. The marked higher odds for VID in non-white children and those on income support suggest these groups should be targeted in preventative strategies. Marked seasonal differences and the association of higher risk of VID amongst children who exercised less outdoors and watched more TV highlights potentially modifiable risk factors. Clearer guidelines for safe sunlight exposure and an increased awareness are needed as there are no recommendations for supplementation in YP
SN - 0803-5253
AD - (Absoud, Cummins, Wassmer, Shaw) Institute of Child Health, Birmingham Children's Hospital NHS Foundation Trust, Birmingham, United Kingdom (Lim) Department of Paediatric Neurology, Evelina Children's Hospital at St Thomas' Hospital, Westminster Bridge Road, London, United Kingdom
ER -

TY - JOUR
ID - 3699
T1 - The fitness benefits of pushing a baby stroller outdoors
A1 - Hanadel,K.
A1 - Greany,J.F.
A1 - Greany,K.A.
Y1 - 2010///
N1 - 70312897
English
Journal: Conference Abstract
KW - EMBASE
KW - fitness
KW - baby
KW - pulmonary rehabilitation
KW - velocity
KW - Walking
KW - Energy Expenditure
KW - weight
KW - Oxygen Consumption
KW - Exercise
KW - walking speed
KW - post hoc analysis
KW - college
KW - volunteer
KW - treadmill
KW - gas

KW - analyzer
KW - Sports Medicine
KW - mother
KW - Health
KW - Female
KW - Child
KW - diaper
KW - Heart Rate
KW - Analysis of Variance
KW - steady state
KW - aerobic capacity
RP - NOT IN FILE
SP - 271
EP - August
JF - Journal of Cardiopulmonary Rehabilitation and Prevention
VL - Conference: 25th Annual Meeting of the American Association of Cardiovascular and Pulmonary Rehabilitation Milwaukee, WI United States. Conference Start: 20101006 Conference End: 20101009. Conference Publication:
IS - var.pagings
N2 - Introduction: New mothers are often seeking professional advice about restarting or beginning a fitness program. Purpose: The aim of this study was to compare cardiorespiratory responses and energy expenditure of various walking speeds while pushing a baby stroller outdoors. Design: Descriptive (Observational) study. Methods: Eleven female volunteers (mean age 24 yrs; range 22 - 41) completed a treadmill VO2 max test to determine their aerobic capacity. Approximately one week later a portable gas analyzer was used to measure oxygen consumption while subjects performed 3 exercise sessions in random order pushing a baby stroller at 3.0, 4.0 mph and a selfselected speed to simulate the speed they considered walking for exercise. The stroller included an additional 35 pounds of weight to simulate the weight of a one-year old child plus a diaper bag. Oxygen consumption (V02), heart rate (HR) and walking speed (GPS watch) were measured. Data were analyzed by one-way analysis of variance (ANOVA) for each factor and pairwise comparisons were performed. Results: At steady state there was a difference (p < .001) between V02 (ml - kg⁻¹ . min⁻¹) for walk speed. Post hoc comparisons revealed differences between 3.0 mph (19.3 + 4.4 ml - kg⁻¹ . min⁻¹; 43% of max) and both 4.0 mph (25.1 + 2.4 ml - kg⁻¹ min⁻¹; 55% of max) and self-select (26.6 + 1.6 ml . kg⁻¹ - min⁻¹; 58% of max). Similar differences were observed for HR (p < .002) between 3.0 mph (103.2 + 18.1 bpm) and 4.0 mph (121.6 + 12.6 bpm) and self-select (128.7 + 16.2 bpm). Total caloric expenditure was also significantly greater during the 4.0 mph (7.5 + 0.6 kcal/min) and self-select (8.4 + 1.0 kcal/min) conditions compared to the slower walk (5.7 + 1.0 kcal/min). Pushing a weighted stroller at 4.0 mph and at a self-selected speed (mean speed 4.3 mph) was sufficient to meet the American College of Sports Medicine's threshold for improving cardiorespiratory fitness. Conclusions: These data suggest that walking at least 4.0 mph outdoors while pushing a baby stroller can be an effective way to obtain health and fitness benefits
SN - 1932-7501
AD - (Hanadel, Greany, Greany) University of Wisconsin - La Crosse, United States
ER -

TY - JOUR
ID - 3700
T1 - CF can't stop me, I'm becoming a woman
A1 - Dettenmeier,P.A.
Y1 - 2010///
N1 - 70304452
English
Journal: Conference Abstract
KW - EMBASE
KW - Female
KW - cystic fibrosis
KW - adolescence
KW - Adolescent

KW - parent
KW - lung function
KW - sexual health
KW - coughing
KW - Sexual Behavior
KW - Health
KW - Environment
KW - Adult
KW - venous thromboembolism
KW - social evolution
KW - general aspects of disease
KW - social status
KW - sport
KW - daughter
KW - Weather
KW - Smoke
KW - lung infection
KW - Risk
KW - breathing
KW - Intimacy
KW - underweight
KW - moon face
KW - breast
KW - hair
KW - gastrointestinal symptom
KW - flatulence
KW - hazard
KW - Health Care
KW - Child Care
KW - girl
KW - health care personnel
KW - Child
KW - Body Image
KW - Chronic Disease
KW - Sexual Development
KW - Nutritional Status
KW - Male
KW - Clothing
KW - patient
KW - sexually transmitted disease
KW - Menarche
KW - menstruation
KW - Puberty
KW - sexual intercourse
KW - uterine cervix mucus
KW - unplanned pregnancy
KW - Therapy
KW - Population
KW - contraception
KW - health program
KW - sex difference
KW - Bone Density
KW - oral contraceptive agent
KW - antibiotic agent
RP - NOT IN FILE
SP - 209
EP - 210

JF - Pediatric Pulmonology
JA - Pediatr Pulmonol
VL - Conference: 24th Annual North American Cystic Fibrosis Conference Baltimore, MD United States. Conference Start: 20101021 Conference End: 20101023. Conference Publication:
IS - var.pagings
N2 - Adolescence is defined as a period of biological, psychological, and social transition from a dependent child into an independent autonomous adult (1). It commonly occurs in the teenage years, but may extend well into the early twenties. During adolescence additional challenges emerge as the body and mind undergo rapid changes and care is transitioned from parents to the adolescent. The wishes of the young adult begin to surface and must be addressed. While adolescence can be difficult for healthy teens, the trials associated with having cystic fibrosis (CF) can magnify the struggles. Chronic health issues influence perception of body image and this is no less important in young women with CF. Few studies have addressed the impact of chronic diseases on sexual health in women with cystic fibrosis. What studies are available suggest that social and sexual development is delayed. The delay of puberty may be 2 years or more depending on nutritional status and results in a female (or male) of shorter statue with decreased bone density (2,3). Zeitlin suggests that the onset of menarche in CF women may predispose them to impaired lung function as a result of the monthly menses; this may account for some of the gender differences in morbidity/mortality (4). Roberts and Green report that 60% of CF adolescents want to know about sexual health before the age of 16 years (5). CF adolescents engage in sexual activity at the same age as their unaffected peers. Lung function impairment may require alternative positions for sexual activity. Coughing during sexual intercourse can be particularly disturbing for teens. Even though the cervical mucus is thicker, sexually transmitted diseases (STD) and unplanned pregnancy can occur. The incidence of STD and the response to therapy are unknown in this population. Contraception as part of a comprehensive sexual health program should be addressed early in CF affected women as many believe that they cannot get pregnant, but there are no guidelines to suggest an optimal method for all CF women. In addition, CF women who choose hormonal birth control pills must be cautioned about the interaction of antibiotics and oral contraceptives and the risk of venous thromboembolism which can further affect lung function. On another front is the social development of the young CF woman. Illnesses may restrict attendance at common social functions like sports events, homecoming and prom. Sometimes parents are overprotective and limit involvement of CF daughters. Exacerbations of coughing may occur at outdoor events in cooler weather or in smoke filled rooms. Recurrent sinus or lung infections causing coughing or foul breath may affect social intimacy like kissing and result in rejection by boyfriends. Changes in the physical appearance of CF women (such as clubbing, underweight, barrel chested, moon faces, small breasts) cause feelings of inadequacy and result in adjusting hair or clothing styles to hide inadequacies (6). Gastrointestinal disturbances, especially flatus, are particularly embarrassing for CF women, but may be a source of bravado for CF men. Adolescence is also a time of choosing a career path. Adolescents must be guided to choose appropriately. CF women should be steered toward careers in clean environments that pose minimal or no hazard to them or to the people they are around. Healthcare and child care may not be appropriate choices for many reasons (7,8). The treatment of adolescent girls with CF poses specific challenges for promoting health and treating CF related diseases. At this point the relationship between the health care provider and the patient, not the parents, becomes more important.A safe environment separate from the parents to discuss confidential issues of importance to teens is required and can result in improved adherence at a difficult time
SN - 8755-6863
AD - (Dettenmeier) Internal Medicine, St. Louis University, St. Louis, MO, United States
ER -

TY - JOUR
ID - 3701
T1 - Neurobehavioral effects in mice through the acute exposure of cellular irradiation
A1 - Kumar,N.
A1 - Khan,R.A.
A1 - Sharma,V.P.
Y1 - 2010///
N1 - 70279201
English
Journal: Conference Abstract
KW - EMBASE
KW - mouse

KW - exposure
KW - Society
KW - Asia
KW - irradiation
KW - neurochemistry
KW - mobile phone
KW - brain
KW - Environment
KW - device
KW - radius
KW - Locomotion
KW - laboratory
KW - headache
KW - Child
KW - weight
RP - NOT IN FILE
SP - 74
EP - 75
JF - Journal of Neurochemistry
VL - Conference: 10th Biennial Meeting of the Asia-Pacific Society of Neurochemistry Phuket Thailand. Conference Start: 20101018 Conference End: 20101020. Conference Publication:
IS - var.pagings
N2 - Neurological abnormalities are complained by the cellular users from the corners of the globe in last few years. Our previous study has concluded that the extensive users, they hold cell phone for a period above 3 years, were more attentive for 'headache' symptom in comparison to normal users. Children are more sensitive to EMR due to developing phase of their brain. Generally cellular phone radiates an average power of 0.2-0.6 watts when hand-held and operated close to the head, background levels are sharply distorted, with likely 40-50% of radiated phone energy absorbed into the brain. In this study we assessed the spontaneous locomotors activity (SLA) of the mice after the acute exposure of cellular telephone (CDMA). 10 mice of the 30 days old (average weight 30 gm) were grouped as control and EMR and they were habitualised for 1 week prior to start the experiment in well designed circular cellphone exposure (CCPE) cage. CCPE cage was specially designed for EMR exposure through the cellular phone over the mice in the natural environment and it placed inside the exposure room where other electric appliances were not exist. In the cage, cellular phone was placed in the center and mice were allowed to move around the device within 10 cm. radius. 3 h/day exposure was applied to the mice of EMR group by the cellular phone in CCPE cage. Locomotor activity was assessed using computerized Optovarimax animal activity monitor, following the method Ali et al., (1990). In this study sub parameters such as Distance traveled, Time resting, Time moving and Stereotypic time was measured with the help of the activity monitor. No significant changes were found in the EMR exposed mice in comparison of control mice. More laboratory research is essential to clarify the contradictions
SN - 0022-3042
AD - (Kumar, Khan) School for Information Sciences, Department of Information Technology, Babasaheb Bhimrao Ambedkar University, Lucknow, UP, India (Sharma) Developmental Toxicology Division, Indian Institute of Toxicology Research, Lucknow, UP, India
ER -

TY - JOUR
ID - 3702
T1 - Suspected mass sociogenic illness reported as carbon monoxide poisoning
A1 - Nordt,S.P.
A1 - Minns,A.
A1 - Carstairs,S.
A1 - Kreshak,A.
A1 - Tomaszweski,C.
A1 - Campbell,C.
A1 - Joshua,A.
A1 - Hayden,S.
A1 - Clark,R.

A1 - Ly,B.
Y1 - 2010///
N1 - 70273135
English
Journal: Conference Abstract
KW - EMBASE
KW - general aspects of disease
KW - toxicology
KW - carbon monoxide intoxication
KW - Child
KW - sitting
KW - Religion
KW - ambulance
KW - oximeter
KW - Etiology
KW - laboratory
KW - fire
KW - patient
KW - Adult
KW - cyanosis
KW - tertiary health care
KW - Male
KW - dizziness
KW - headache
KW - paresthesia
KW - Consciousness
KW - nausea
KW - Blood
KW - Diagnosis
KW - association
KW - hyperventilation
KW - syncope
KW - Physical Examination
KW - case study
KW - seizure
KW - Environment
KW - toxic gas
RP - NOT IN FILE
SP - 625
JF - Clinical Toxicology
VL - Conference: 2010 North American Congress of Clinical Toxicology, NACCT Annual Meeting Denver, CO United States. Conference Start: 20101007 Conference End: 20101012. Conference Publication:
IS - var.pagings
N2 - Background: Suspected mass sociogenic illness (MSI) initially attributed to carbon monoxide (CO) poisoning. Case series: Over 100 people at church on hot day when child fainted followed by another. No seizure activity and awoke normal and removed. Others then had various complaints and building evacuated. The fire department evaluated patients including: COHb and O2 sat with Masimo oximeter, several COHb reported up to 19%. Paramedics and Haz Mat to scene. After ambulances more children and two adults became symptomatic outside. No cyanosis at any time. Seventeen of twentytwo taken to tertiary hospital with HBO chamber and other five to local ED. All other attendees asymptomatic. All asymptomatic in ED after 15 min transport time with normal exams. Majority not treated with O2 en route. Mean age 13-year-old (7-50), seven males, complaints at scene: nine dizziness, six headache, three paresthesia, three loss of consciousness, seven nausea. All treated in ED 100% O2 at 15 L/min. VBG COHb immediately sent and different Masimo oximeter repeated. Mean blood COHb 0.6 (0.2-1.2), O2 sat 99.8 (99-100), Masimo COHb 0.2 (0-3), MetHb 1.5 (0-0.4). Discussion: MSI challenging diagnosis. Primarily seen in children, rapid onset and resolution, visual cues trigger, no illness in other persons sharing environment, no clinical or laboratory evidence of illness, hyperventilation and syncope common. None had elevated COHb. Haz Mat did not measure CO or other toxic

gases. No heater or air conditioner on at time. Haz Mat reproduced scenario, including running heater, over several hours and no toxic gases. Heater evaluated and operating normally. Other potential etiology includes simple asphyxiant. Unlikely as affected persons sitting in various areas and majority unaffected sitting near affected persons. Furthermore, several became symptomatic after leaving church. Escalation and increased persons affected with increasing ambulance presence is common in MSI. We suspect field COHb aberrant from inexperienced operator error. Conclusion: Clinicians should consider MSI following mass outbreaks of illness, particularly with the rapid onset and rapid resolution of symptoms coupled with normal physical examination and laboratory analysis. However, MSI should only be entertained after potential toxicologic etiologies have been excluded
SN - 1556-3650
AD - (Nordt) University of Southern California, Los Angeles, CA, United States (Minns, Kreshak, Tomaszweski, Campbell, Joshua, Hayden, Clark, Ly) University of California, San Diego, CA, United States (Carstairs) Naval Medical Center, San Diego, CA, United States
ER -

TY - JOUR
ID - 3703
T1 - A cross-sectional survey to assess the prevalence and symptoms associated with laxity of the vaginal introitus
A1 - Millheiser,L.
A1 - Kingsberg,S.
A1 - Pauls,R.
Y1 - 2010///
N1 - 70272938
English
Journal: Conference Abstract
KW - EMBASE
KW - Prevalence
KW - Society
KW - continence
KW - Female
KW - vaginal delivery
KW - information processing
KW - Body Image
KW - Income
KW - sensation
KW - childbirth
KW - sexuality
KW - physician
KW - Child
KW - household
KW - Graduate
KW - orgasm
KW - sexual satisfaction
KW - data base
KW - patient
KW - sexual health
KW - pelvic floor muscle training
KW - hypothesis
KW - Evidence Based Practice
KW - sexual intercourse
KW - study design
KW - city
KW - health care personnel
KW - friend
KW - ethnicity
KW - Education

KW - Employment Status
KW - Marriage
KW - sexual function
KW - Intimacy
KW - questionnaire
KW - Self Concept
KW - groups by age
KW - Satisfaction
KW - Sexual Behavior
KW - vagina
RP - NOT IN FILE
SP - 1102
EP - 1103
JF - Neurourology and Urodynamics
VL - Conference: Joint Annual Meeting of the International Continence Society, ICS and International Urogynecological Association, IUGA Toronto, ON Canada. Conference Start: 20100823 Conference End: 20100827. Conference Publication:
IS - var.pagings
N2 - Hypothesis / aims of study Laxity of the vaginal introitus may occur following vaginal delivery. However, this condition is infrequently discussed between patients and physicians, likely due to a paucity of evidence-based treatment options. We hypothesized that introital laxity leads to loss of physical sensation and the development of sexual dissatisfaction during vaginal intercourse. Therefore, a survey was conducted with two primary aims: 1) to better quantify the prevalence of vaginal laxity among parous women ages 25-55 years of age and 2) to explore the association between vaginal laxity and changes in physical sensation or sexual dissatisfaction. Study design, materials and methods A cross-sectional survey was conducted utilizing focus groups and a national database of on-line registrants for women ages 25-55 who had experienced at least one vaginal delivery. This survey was conducted in two phases; a qualitative assessment survey utilizing focus groups from which directional information was collected followed by the second phase, a quantitative survey. For the qualitative phase, nine focus groups were conducted each comprised of six to eight women per group. Three groups per city were organized in Portland, Cleveland and Atlanta. Discussions were lead by a female moderator using a standardized script to explore perceptions and concerns of women presenting with vaginal laxity after vaginal childbirth and their attitudes toward resolution of this medical condition. Key areas for discussion included physical changes experienced since vaginal childbirth, confidence and relationship with partner, physical and psychological consequences specifically related to vaginal laxity, and barriers to discussion of vaginal laxity with health care providers, friends and family. Each focus group lasted approximately two hours; participants received $100 for attending. The second phase of the survey utilized a national database of on-line registrants of women aged 25-55 who had experienced at least one vaginal delivery. The survey consisted of five parts, namely (i) demographic data, e.g., age, ethnicity, education, employment status, household income, marital status and number of vaginal births, plans for future children, (ii) interaction with her obstetrician/gynecologist (OB/GYN) regarding sexual function, (iii) 16 questions on body image and sexuality following childbirth, (iv) body image procedures contemplated or had, and (v) potential benefits to improving vaginal laxity. Results A total of 50 women participated in the focus groups. In the pre-selected age range of 25 to 45, slightly more than half were 25- 35 years of age, predominantly college graduates, and evenly distributed amongst household incomes. Participants included a mix of women working outside the home or homemakers. They candidly described body image changes following childbirth and the complexity of physical and emotional consequences due to vaginal laxity (e.g., reduced vaginal sensation, difficulty reaching orgasm). Vaginal changes were equated with loss of sexuality, burdensome sexual performance, and a yearning for intimacy and sexual confidence experienced before having children. The participants acknowledged the impediments to discussing sexual concerns with their physicians. In the second, quantitative phase of the survey, 421 (69.8%) of the 635 women who received on-line questionnaires completed the survey. Surveys were collected from women in every state in the continental US (14% of all responses were from CA, 5-6% from NY, MI, TX and FL). The respondents were predominately white (81%), college graduates (55%), employed full or part time (59%), suburban living (57%), income $70K or greater (71%), and married (90%), and a median of two childbirths. Some of the survey responses related to concerns about self-image, sexuality and sexual satisfaction are presented in Table 1. (Table presented). Interpretation of results Approximately half of the women in all age groups expressed a diminished interest in sex after having children, initiated sex less frequently and felt like it was more of an effort. However, half of the respondents also indicated satisfaction

with their sex life. Laxity of the vaginal introitus was expressed as a concern ("some" or "a little" or "a lot") by 48% (201 of 421) of respondents. Eighty percent of respondents (335 of 421) had not discussed the topic with their OB/GYN. Eighty-five percent (357 of 421) of women knew how to perform the Kegel exercises, 76% (318) had done them, 48% (204) thought they were effective in tightening the vagina, 15% (65) indicated they were not effective and 36% (152) did not know if the Kegel exercises were effective in improving their vaginal tone. Approximately 50% or more of women felt that improving the tightness of their vaginal introitus would have a positive effect on the relationship with their partner, both their sexual confidence and overall confidence, the orgasmic experience of both them and their partner, and sexual satisfaction. Concluding message The results from this cross-sectional survey on sexual health after vaginal birth provide a new focus on laxity of the vaginal introitus as an important medical concern after vaginal childbirth, one that is infrequently discussed between patients and physicians, and under reported in the literature. The surveys are unique in assessing women's attitudes about their body image, sexual health, and sexual relationships. Among the respondents who represented a 30-year age range from 25 to 55, approximately half expressed some degree of concern for "looseness" of the vaginal introitus. Potential treatment to tighten the vaginal introitus was considered to be positive for personal renewal, to improve orgasms and benefit the sexual relationship for both partners. (Table presented)
SN - 0733-2467
AD - (Millheiser) Stanford University, School of Medicine, United States (Kingsberg) Case Western Reserve University, School of Medicine, United States (Pauls) Good Samaritan Hospital, United States
ER -

TY - JOUR
ID - 3705
T1 - HIV/AIDS epidemic features and trends in setif city (Algeria) from 1986 to 2009
A1 - Ouyahia,A.
A1 - Rais,M.
A1 - Gasmi,A.
A1 - Guenifi,W.
A1 - Mechakra,S.
A1 - Lacheheb,A.
Y1 - 2010///
N1 - 70261820
English
Journal: Conference Abstract
KW - EMBASE
KW - Human immunodeficiency virus
KW - Algeria
KW - Infection
KW - epidemic
KW - city
KW - acquired immune deficiency syndrome
KW - Blood
KW - patient
KW - Travel
KW - Prevalence
KW - Human immunodeficiency virus infection
KW - heterosexuality
KW - Death
KW - Safety
KW - gender
KW - highly active antiretroviral therapy
KW - Female
KW - enzyme linked immunosorbent assay
KW - Prognosis
KW - Diagnosis
KW - married man
KW - Marriage

KW - married woman
KW - mother
KW - Child
KW - Africa
KW - sexual transmission
KW - Male
KW - tourism
KW - Risk
KW - exposure
KW - sexually transmitted disease
KW - Adult
KW - Blood Transfusion
KW - Policy
KW - risk factor
RP - NOT IN FILE
SP - 99
JF - Retrovirology
VL - Conference: 16th International Symposium on HIV and Emerging Infectious Diseases Marseille France. Conference Start: 20100324 Conference End: 20100326. Conference Publication:
IS - var.pagings
N2 - Background To identify the epidemiologic characteristics of HIV and AIDS in SETIF during the period between 1986, and 2009. Methods This report presents a descriptive analysis of HIV and AIDS surveillance data. The subjects of this study were all notified HIV and AIDS cases in SETIF diagnosed by ELISA and confirmatoryWestern blot, we worked with epi info 3.5.2. Results The HIV epidemic started in SETIF in 1986 with the first diagnosed AIDS cases. The number of cases slowly but steadily increased, to reach, by 2009, 152 cases. The median age of infected persons was 39,77 years. 40.8% were WOMEN; The most frequent mode of transmission is sexual (76,9% of all cases; with mainly heterosexual transmission 77.9 percnt; with a strong correlation to travel and migration 37.20%). the proportion of persons diagnosed late (within 12 months before AIDS diagnosis) was 82,3%; 91.4% of married men (36) have sexual relationships outside marriage as compared to 0.0% of married women(27). We report also 13.60% discordant couples among married patients, Among documented HIV-positive mothers (36), their children were screend in 61.10%, and 95.5% of them were seronegatif, 55,9% of all cases were treated with ARV. HIV/ AIDS deaths 36,2% decreased markedly from 2002, associated with the advent of HAART. Discussion Gender distribution and distribution by mode of transmission among HIV-infected revealed an equal gender distribution of infection wich contrary to sub-Saharan Africa, where women are more affected. a higher female prevalence of HIV infection might be expected in algeria since heterosexuality is the prevailing mode of sexual transmission. Non-marital sex is the main risk factor, as it is worldwide for several reasons: Our data might be explained by u the large number of males who travel to other countries, especially to areas with a high prevalence of HIV infection. Travel and tourism enhance the probability of having casual sex, a fact that increases the risk of exposure to sexually transmitted infections u The ever-decreasing religious values Adults 23- to 45-years old were found to constitute 65,8% of all cases, a fact that points to the serious social and economical impact of the disease The trend of mode of transmission by blood transfusion shows that most of the cases infected through contaminated blood were registered before 1995. These patients most probably received blood unscreened for HIV in the 1980s before blood safety measures were undertaken. However, the incidence of this type of transmission significantly declined after implementation of blood safety policies. 32,90% of infected had died, This high percentage can be explained by the fact that: most infections are detected among patients, who already have a bad prognosis 66,40% 68,6% of death occurs before 1996 it means before the introduction of highly active antiretroviral therapy
SN - 1742-4690
AD - (Ouyahia, Rais, Gasmi, Guenifi, Mechakra, Lacheheb) Faculte De Medecine Centre Hospitalo Universitaire, Setif, Algeria
ER -

TY - JOUR
ID - 3706
T1 - The influence of the use of multiple devices of asthma inhalers on the adequacy of inhalation technique in asthmatic children

A1 - Alotaibi,S.
A1 - Alenezi,H.
A1 - Spier,S.
Y1 - 2010///
N1 - 70236159
English
Journal: Conference Abstract
KW - EMBASE
KW - Child
KW - Asthma
KW - Inhalation
KW - inhaler
KW - device
KW - pulmonology
KW - patient
KW - Hospital
KW - non profit organization
KW - Lung
KW - Male
KW - Teaching
KW - Therapy
KW - physician
KW - Checklist
RP - NOT IN FILE
SP - S84
JF - Paediatric Respiratory Reviews
VL - Conference: 9th International Congress on Pediatric Pulmonology Vienna Austria. Conference Start: 20100619 Conference End: 20100621. Conference Publication:
IS - var.pagings
N2 - The purpose of the current study was to examine whether asthmatic children using multiple types of inhalers demonstrate poor inhalation technique scores in comparison to those patients using only one type of inhaler. The DPI inhalers investigated were Turbuhaler (TH; AstraZeneca), Diskus (DK; GlaxoWellcome), and Diskhaler. Subjects used one of these DPI inhalers alone or in combination with MDIS. The inhalation technique was evaluated during the clinic visit and scored using a modified inhaler-specific checklist adapted from the Dutch Asthma Foundation technique score. The total score was obtained by multiplication of all the scores then multiplying the total by 100 to get a percent score. This system incorporates the relative importance of each step in the inhalation technique. For each inhaler, items essential to the delivery of active drug into the lungs were identified. These key items involve preparing or loading the device prior to inhalation, which is performed differently for MDI and DPI, and the inspiration maneuver. In 97 asthmatic children (mean age 11.59+SD 3.9 years) inhalation technique was assessed. Of these, 59 (60.8%) were males and 76 (78.4%) received instruction in the asthma clinic. Those who received teaching from outside the clinic (21 children) had asthma for a mean of 4.5 years. Twenty-eight patients (25 from within the asthma clinic and 3 from outside) used the Turbuhaler, with 12 of these 28 patients using it in combination with MDIS. Meanwhile, 40 children used the Diskus inhaler (35 from within the asthma clinic and 5 from outside), with 26 of these users combining the Diskus device with MDIS. Overall, there were 30 patients (30.9%) in the DPI treatment group and 38 patients (39.2%) using both DPI and MDI inhalers. The DPI alone group exhibited a significantly higher inhalation technique score (85.7% vs. 61.9%; $p < 0.05$). The inhalation technique scores associated with both the Diskus and Turbuhaler were significantly reduced when used in combination with MDIS. The mean of the Turbuhaler technique score was 87% when used alone, which decreased to 61.3% when used in combination with MDIS ($p < 0.05$). Similarly, the mean of the Diskus technique score was also significantly reduced (83.4% to 62%) under these same conditions ($p < 0.05$). We conclude that the use of DPI inhalers in conjunction with MDIS inhalers confuses patients and reduces the likelihood of proper inhalation technique. When prescribing asthma therapy for patients, we advise physicians to consider similar devices to reduce inhalation technique errors, which may improve the drug deposition and lead to better asthma control
SN - 1526-0542
AD - (Alotaibi, Alenezi) Farwanyah Hospital, Kuwait Ministry of Health, Ardeyah City, Kuwait (Spier) Alberta Children Hospital, Calgary University, Calgary, Canada

ER -

TY - JOUR
ID - 3707
T1 - Protracted bacterial bronchitis (PBB)
A1 - Chang,A.B.
Y1 - 2010///
N1 - 70236139
English
Journal: Conference Abstract
KW - EMBASE
KW - bronchitis
KW - pulmonology
KW - Child
KW - coughing
KW - airway
KW - bacterial infection
KW - Inflammation
KW - lung disease
KW - bronchiectasis
KW - pathogenesis
KW - lower respiratory tract
KW - Morbidity
KW - physician
KW - antibiotic therapy
KW - esophagitis
KW - parent
KW - microbiology
KW - neutrophilia
KW - Methodology
KW - Diagnosis
KW - neutrophil
KW - Society
KW - respiratory tract infection
KW - follow up
KW - innate immunity
KW - Asthma
KW - Risk
KW - prospective study
KW - Etiology
KW - Drug Therapy
KW - Infection
KW - Haemophilus influenzae
KW - Bronchoscopy
KW - clinical practice
KW - consultation
KW - New Zealand
KW - Australia
KW - sinusitis
KW - ear disease
KW - wheezing
KW - relapse
KW - community acquired pneumonia
KW - respiratory tract disease
KW - adaptive immunity
KW - gastroscopy
KW - stomach juice

KW - thorax radiography
KW - gene
KW - immune response
KW - Gene Expression
KW - cystic fibrosis
KW - control group
KW - United Kingdom
KW - general aspects of disease
KW - health care cost
KW - antigen recognition
KW - antibiotic agent
KW - toll like receptor 2
KW - gelatinase B
KW - substance P
KW - chemokine receptor
KW - interleukin 8
KW - Receptor
KW - messenger RNA
KW - toll like receptor 4
KW - preprotachykinin
KW - defensin
KW - chemokine
RP - NOT IN FILE
SP - S71
EP - S73
JF - Paediatric Respiratory Reviews
VL - Conference: 9th International Congress on Pediatric Pulmonology Vienna Austria. Conference Start: 20100619 Conference End: 20100621. Conference Publication:
IS - var.pagings
N2 - What is PBB? PBB, long been recognized by pediatric pulmonologists worldwide, was only adequately characterized (clinically and BAL) recently [1-3]. PBB's original description was derived from a prospective study that evaluated the aetiology of chronic cough in children using a research protocol. The study [1] used a priori defined criteria and validated cough diaries to assess response with a clear time period relating to when medications were given. In the 108 young children with chronic cough in the study, bacterial infection of the airways (defined as growth of single pathogens of >10⁵ cfu/ml) was the most common (40% of children) finding [1]. Airway neutrophilia was also present and the respiratory pathogens found in the endobronchial infection were H. influenzae, S. pneumoniae and M. catarrhalis [1,3]. The criteria for PBB in the research study were; (1) history of chronic moist cough, (2) positive BAL culture, and (3) response to antibiotic treatment with resolution of the cough within two weeks. Outside of research protocols, bronchoscopies cannot be routinely undertaken for uncomplicated chronic wet cough in clinical practice. Thus PBB, sometimes truncated to protracted bronchitis (PB) is clinically defined as (a) the presence of isolated chronic (>4 weeks) wet/moist cough, (b) resolution of cough with antibiotic treatment and (c) absence of pointers suggestive of an alternative specific cause of cough [2]. Hence while the diagnosis of PB can be suspected at the time of consultation, it can only be made down the track on review. PB has been officially recognized by the Thoracic Society of New Zealand and Australia and the British Thoracic Society [2]. The clinical profile of children with PB: Children with PB are typically young (<5 years of age, median age - 3 years [1,3]), do not have any other systemic symptoms including the absence of clinical sinusitis and ear disease. Some parents may report a 'wheeze' but it usually represents a misinterpretation of the sound [3]. Many of these children have been misdiagnosed as having asthma [1,3]. Their chest x-rays usually show peribronchiolar changes or may be reported as 'normal' [1,3]. Like children with chronic cough, children with PB have significant morbidity; parents typically have seen multiple medical practitioners for their child's chronic cough in the last 12 months. In PB the child's cough resolves only after a prolonged course of appropriate antibiotics (12-14 days). When a typical course (5 days) of antibiotics is used, the cough either relapses within 2-3 days, or slightly subsides but does not resolve completely. This is in contrast to the short course of antibiotics (5-7 days) required to treat community acquired pneumonia in otherwise well children. Children with PB also have higher Canadian Acute Respiratory Infection Scale (CARIFS) scores in subsequent respiratory illness. When compared with children with acute asthma and normal controls, children with PB, there was no difference between groups on day-1.

However days 7, 10 and 14 later, children with PB had significantly ($p < 0.0001$ for all) higher CARIFS scores [2]. Pathogenesis of PBB: PBB is associated with persistent bacterial infection in the airways [1,3] and it is widely accepted that persistent bacteria infection (with accompanying inflammation) is harmful to the airways. PBB is likely non-homogenous with neutrophilic airway inflammation developing by a variety of mechanisms. It is likely that an innate immune dysfunction or immature adaptive immunity is present, at least in a subgroup of these children. A group of children with bacterial colonisation, airway neutrophilia and protracted cough that was unrelated to the presence or absence of oesophagitis, was identified from 150 children undergoing gastroscopy [4]. In children without lung disease, cough was more likely to result from airway bacterial infection and not to esophagitis [4]. The abnormal microbiology of the airways in children with cough, was not reflected in the microbiology of gastric juices. In a subset of these children (n = 69), bacterial colonisation of the lower airways was associated with neutrophilic inflammation and reduced expression of both the toll-like receptor (TLR) -4 and the preprotachykinin gene, TAC1, that encodes substance P [5]. Substance P has a defensin-like function which may explain the association between reduced TAC1 and persistent bacterial infection. In order to provide additional evidence for a dysfunctional host response to bacterial infection, chemokine receptor expression was assessed. Elevated gene expression for neutrophil chemo-attractant chemokine IL-8 cellular receptor, CXCR1, was detected, while the chemokine receptors (CCR3, CCR5) were similar between groups. In another cohort of children with established chronic cough who presented to respiratory physicians and had PBB, intense neutrophilic airway inflammation with marked inflammatory mediator response (IL-8, active matrix metalloproteinase 9 [MMP-9]) was present, compared to controls [6]. The median levels of IL-8 (0.69, IQR 0.30, 1.72 ng/ml) in the BAL was similar to those found in other studies that examined chronic airway inflammation in cystic fibrosis and other forms of chronic suppurative lung disease [6]. In contrast to above data, TLR-2 and TLR-4mRNA expression were significantly elevated in this cohort when compared to the control group [6]. However when corrected for neutrophil numbers (it remains unclear if values should be corrected), TLR-2 and -4 were reduced [6]. Furthermore when followed up those with recurrent PBB has significantly reduced TLR-2 and 4mRNA expression (adjusted values) compared to those who had did not have recurrent PBB in the 12-mo follow-up period (unpublished). Nevertheless there were other differences between these cohorts that include age (innate immunity is age-dependent), duration of cough and key presentation history. Thus the nature and duration of innate immune dysfunction remain undefined, nor is it clear whether the dysfunction is specific to the lower airways or is more generalised (ie systemic). Only a long term study will elucidate the pathogenesis and evolution of PB in children. Treatment and follow-up of children with PB: Both the Australian and UK groups have described the response of the wet cough to a prolonged (at least 2 weeks) course of antibiotics [1-3]. In a Cochrane review (albeit consisting of 2 limited studies with total number of 140 children), chronic wet cough in children responded to antibiotics with a number needed to treat of 3 (95% CI 2, 4) and the progression of illness, defined by requirement for further antibiotics, was significantly lower in the antibiotic group with a number needed to treat for benefit of 4 (95% CI 3, 5) [2]. However until a double blinded randomized control trial that specifically addresses this question is performed, it remains indefinite which children with wet cough should receive antibiotics. Children with PB may have recurrent episodes. In the Brisbane cohort's preliminary study, approximately 35% of children have recurrent (>2 episodes a year) PB. Also whether PB is antecedent to chronic suppurative lung disease and bronchiectasis in some children is unknown and important to evaluate. It is likely that, at least in a small but significant number of children, PBB is an early spectrum of the same process leading to chronic suppurative lung disease and bronchiectasis [2]. Children with PB do not have established bronchiectasis as those with established bronchiectasis usually have a different clinical profile and are unlikely to recover after 12-14 days of oral antibiotics. It is suggested that children with recurrent PB (>2 episodes per year) are evaluated like a child with bronchiectasis. Summary: In summary, chronic wet cough in young children is common and causes considerable morbidity and health care costs. A clinical diagnostic entity called PBB is the most common cause of chronic wet cough on young children. Even though many children with PBB get better with 2 weeks of antibiotics, the long term consequences in young children whose pulmonary system are still developing, remains undefined. In published preliminary studies, reduced levels of receptors involved in innate immune recognition in the BAL cells of children with PBB and airway infection were described. It is likely that, at least some if not many, children with recurrent PBB have innate immune dysfunction and are at risk of chronic suppurative lung disease, which has been lately recognised to be not as rare as previously thought. Studies elucidating the determinants of chronic airway inflammation and bacterial colonisation in children, in particular the role of innate immunity dysfunction in children with recurrent PBB and their clinical outcomes are required

SN - 1526-0542

AD - (Chang) Menzies School of Health Research, Child Health Division, Darwin, Australia (Chang) Queensland Children's Respiratory Centre, Royal Children's Hospital, Brisbane, Herston, QLD 4029, Australia

ER -

TY - JOUR
ID - 3708
T1 - The Forgotten Majority - Late preterm infants and their vulnerability to lung disease
A1 - Colin,A.A.
Y1 - 2010///
N1 - 70236126
English
Journal: Conference Abstract
KW - EMBASE
KW - prematurity
KW - airway
KW - Morbidity
KW - pulmonology
KW - functional residual capacity
KW - lung disease
KW - lung dysplasia
KW - airway conductance
KW - Infant
KW - Gestational Age
KW - Risk
KW - lung function
KW - Lung
KW - lung volume
KW - thorax wall
KW - Hospitalization
KW - lung development
KW - gas exchange
KW - Child
KW - Infection
KW - respiratory system
KW - Mortality
KW - Hospital
KW - bronchiolitis
KW - Death
KW - growth,development and aging
KW - expiratory flow
KW - hospital readmission
KW - Population
KW - respiratory tract disease
KW - follow up
KW - lung alveolus wall
KW - longitudinal study
KW - adulthood
KW - Adult
KW - lifespan
KW - sacculus
KW - chronic obstructive lung disease
KW - live birth
KW - hospital discharge
KW - newborn morbidity
KW - risk factor
KW - Infant Mortality
KW - pathogenesis
KW - Respiratory syncytial pneumovirus
KW - outpatient

KW - Newborn
KW - United States
KW - Human
KW - continuous process
KW - maturation
KW - gas
KW - capillary
KW - breathing
KW - lung compliance
KW - fatty acid desaturation
KW - bronchus
KW - Airway Resistance
KW - total lung capacity
KW - Pregnancy
KW - maturity
KW - hyperoxia
KW - uterus
KW - Environment
KW - hypothesis
KW - premature labor
KW - adverse drug reaction
KW - compression
KW - forced vital capacity
KW - muscle contraction
RP - NOT IN FILE
SP - S51
EP - S53
JF - Paediatric Respiratory Reviews
VL - Conference: 9th International Congress on Pediatric Pulmonology Vienna Austria. Conference Start: 20100619 Conference End: 20100621. Conference Publication:
IS - var.pagings
N2 - The frequency of late preterm births has generally increased in the United States over the past 2 decades [1]. Infants 34-36 weeks gestational age (wGA) comprise approximately 71% of all preterm births (in 2007 approximately 390,000 live births) [1]. Infants of this gestational age termed "late preterm" are often considered to be as physiologically and metabolically mature as term infants, and therefore at low risk for morbidity and mortality. The resulting early discharge from hospital may be a factor in an increased overall risk of re-hospitalization [2]. A growing body of literature has documented the broad range of complications that late preterm infants may experience [3]. They have a higher risk of developing a large array of medical complications, and mortality rate for late preterm infants are 3-5 fold higher than that for term infants. Each weekly reduction in estimated gestational age at birth was found to be associated with increased morbidity before hospital discharge and higher rates of hospital readmission in the first months of life [2]. A study involving 377,638 term and 26,170 late preterm (34-36 wGA) infants showed that 22.2% of the late preterm vs. 3.0% of term infants had newborn morbidity during their birth hospitalization. Preterm infants born at 34 wGA had 20-fold (relative risk [RR]: 20.6) and at 35wGA had 10-fold (RR: 10.2) higher risk of morbidity compared with infants born at 40wGA. Relative morbidity increased approximately 2-fold with every week of decreasing gestational age earlier than 38wGA [4]. A recent review on the respiratory consequences of preterm birth suggests that many adverse respiratory consequences of preterm birth are likely the result of persistent pulmonary structural abnormalities [5]. Retrospective analyses of records of infants born to US residents showed higher infant mortality rates in 34-36wGA infants compared with term infants [6]. All forms of respiratory morbidity affect late preterm infants at a higher rate than infants of more advanced gestational age, but in particular preterm infants (33-36wGA) who are outside of the typical bronchopulmonary dysplasia (BPD) age range are highly susceptible to infection by such pathogens as respiratory syncytial virus (RSV). Boyce et al. [7] showed that infants born at 33-36wGA had similar rates of admission to the hospital for RSV infection as infants <33wGA, and that there were substantially higher rates of admission in these 2 populations compared with term infants. In other studies infants 33-35wGA had hospital outcomes that were similar to or worse than those of infants <32wGA, whether RSV infection was confirmed or they were hospitalized for nonspecific bronchiolitis. After RSV-hospitalization infants 32-36wGA experienced rehospitalization rates twice as high,

hospital stays 3 times as long, and outpatient visits twice as frequent as infants of similar gestational age who were not hospitalized for RSV [8]. Prematurity was also associated with an increased risk of bronchiolitis-associated death. The odds ratio for death related to RSV in 32-35wGA compared with full-term infants was approximately 5 [9]. The mechanisms explaining the morbidity in infants 34-36wGA are at least in part related to physiologic deficiencies related to their stage of lung development. Stages of lung development: Early growth and development of the human lung is a continuous process that is highly variable between individuals and has traditionally been divided into 5 stages. Premature infants are predominantly born in the saccular stage (28-36wGA) that precedes the final alveolar stage [10]. The saccular period, is a transitional phase before full maturation of alveoli occurs characterized by an increased number of primitive alveoli (saccules) that become gradually more effective as gas exchangers and may be sufficient in number and quality to sustain life in the preterm infant. The alveolar walls are more compact and thick than the final thin walls of alveoli and include an immature capillary system that is capable of carrying out the function of gas exchange and also fully matures in the alveolar phase. Mature alveoli are not uniformly present until 36wGA [10]. These structural changes not only affect gas exchange but have profound effects on the mechanical properties of the lung and hence the respiratory system as a whole. Impaired maintenance of FRC due to chest wall compliance, deficient airway tethering, and small lung volumes: The physiologic consequences of this early birth are that maintenance of a stable and adequate functional residual capacity (FRC) that secures stable gas exchange is impaired. FRC is determined by the balance between the opposing forces of the chest wall and lung and is thus a direct function of their respective mechanical properties. In early life, a compliant chest wall offers little outward recoil to the respiratory system and thus the elastic characteristics of the respiratory system approximate those of the lung with a tendency to decrease to lower lung volumes. Only within the first 2 years of life is this vulnerability corrected as the chest wall stiffens. To circumvent this limitation, infants actively elevate their FRC by modulating expiratory flow using laryngeal braking during tidal expiration and by maintaining inspiratory muscle activity into the expiratory phase. In addition they initiate inspiration early within the expiratory phase. Thus it is reasonable to assume that breathing with an overly compliant chest wall, high lung compliance, and reduced number of air-containing units is a challenge for infants delivered before term. The challenge of maintaining an FRC that permits stable gas exchange is likely compounded in the premature infant by apneic events, which have been shown to drive the system to critically low lung volumes and result in rapid desaturation. An additional crucial mechanism that secures airway patency and thus adequate maintenance of FRC is airway tethering. Tethering depends on a complex mesh-like elastic network within the alveolar walls that transmits tension from the pleural surface and exerts a circumferential pull on individual bronchi. Thus, tethering couples lung volume changes with airway caliber. Tethering of airways is less effective in infants born prematurely because alveolarization and the associated development of the parenchymal elastic network are still in the saccular stage of development at 32-36 wGA. The effect of reduced tethering is decreased airway stability, increased tendency to closure, increased airway resistance, and, ultimately, a tendency to collapse alveolar units in the lung periphery. Total lung volume undergoes rapid changes during the last trimester of gestation. At 30wGA, the lung volume is only 34% of the ultimate volume at mature birth, and at 34 weeks only reaches 47% of the final volume at maturity. This change parallels a marked thinning of air space walls associated with a dramatic increases in air space surface area from 1.0-2.0m² at 30-32wGA, to 3.0-4.0m² at term. These volume changes likely have direct mechanical implications in reducing the vulnerability caused by a low and unstable FRC. Altered lung development and function in association with preterm (30-36 wGA) birth: It is now recognized that postnatal hyperoxia plays a key role in the development of BPD. Premature birth interrupts normal in utero lung development and results in an early transition from the hypoxic intrauterine environment to a comparatively hyperoxic atmospheric environment. There is increasing evidence to support the hypothesis that preterm delivery, even in the absence of any neonatal respiratory disease, may have adverse effects on subsequent lung growth and development, and that these alterations may persist during the first 5 years of life and possibly beyond. Using pulmonary function testing a number of studies have shown a direct association between premature birth and altered pulmonary function. These studies demonstrated reduced expiratory flows in premature infants of varying gestational ages, but born without clinical lung disease. Most convincing was a recent study using the raised volume rapid thoracic compression technique [11]. In this study healthy preterm infants (mean, 33.4wGA) were studied at 8 weeks and had reduced airway flows in the presence of normal forced vital capacity compared with term infants. In a follow-up analysis, the reduced flows did not normalize in these children by 16 months of age, thus demonstrating a lack of "catch-up" growth in airway function [12]. The long-term significance of reduced airway function early in life has recently been emphasized in a longitudinal study involving a large group of non-selected infants who had participated in the Tucson Children's Respiratory study [13]. In this study Stern et al. showed that infants whose pulmonary function was in the lowest quartile also had pulmonary function in the lowest quartile through

the years of follow-up until early adulthood. These findings in a normal unselected population, suggest that the level of pulmonary function in early life tracks and changes little with growth. Weiss and Ware [14] suggest that deficits in lung function during early life, especially if associated with lower respiratory illnesses, increase the risk of chronic obstructive pulmonary disease (COPD) in late adult life. Of particular importance in this context may be the role played by RSV, which affects most children during their first year of life.Conclusions: Long term prospective data are needed to elucidate the lifetime impact of birth in the late preterm stage and the consequences of the morbidities associated with such birth
SN - 1526-0542
AD - (Colin) Division of Pediatric Pulmonology, Miller School of Medicine, University of Miami, 1580 NW 10th Avenue, Miami, FL 33136, United States
ER -

TY - JOUR
ID - 3709
T1 - Improving our understanding of childhood obesity and the need for early intervention: Nutrition and health behaviours of primary and secondary Australian school students
A1 - Bolton,K.
A1 - Kremer,P.
A1 - Swinburn,B.
A1 - Waters,E.
A1 - Gibbs,L.
A1 - Meloni,L.
A1 - de Silva-Sanigorski,A.
Y1 - 2010///
N1 - 70227927
English
Journal: Conference Abstract
KW - EMBASE
KW - student
KW - Obesity
KW - school
KW - Nutrition
KW - Early Intervention
KW - Health Behavior
KW - childhood
KW - high school
KW - Physical Activity
KW - Child
KW - Television
KW - computer
KW - meal
KW - Health
KW - Environment
KW - adulthood
KW - Australia
KW - questionnaire
KW - primary school
KW - Fruit
RP - NOT IN FILE
SP - 467
EP - 468
JF - Obesity Reviews
JA - Obes Rev
VL - Conference: 11th International Congress on Obesity, ICO 2010 Stockholm Sweden. Conference Start: 20100711 Conference End: 20100715. Conference Publication:
IS - var.pagings

N2 - Introduction: Recent evidence suggests that the nutrition and physical activity behaviours learnt early in life are subsequently carried into adulthood. The aim of this study was to examine the nutrition and physical activity behaviours of elementary (primary) and secondary school students in schools across Victoria, Australia. Methods: Students from 27 primary and 24 secondary schools (n = 1213 and n = 1413 students, respectively) completed a questionnaire regarding nutrition- and physical activity-related behaviours. Results: Compared to primary school students, on average in one day, secondary school students consumed less serves of fruit (mean + SE; 1.68 + 0.03 vs. 2.00 + 0.03; P < 0.001); more soft drink (249.30 mL + 12.71 mL vs. 203.46 ml + 13.47 mL; P = 0.014), more cordial/fruit juice/fruit drinks/sports drinks (385.07 mL + 12.63 mL vs. 310.84 ml + 13.38 mL; P < 0.001), watched more television (1.78 hour + 0.03 hour vs. 1.47 hour + 0.04 hour; P < 0.001) and spent more time playing electronic games or the computer (not for homework; 1.08 hour + 0.03 hour vs. 0.85 hour + 0.03 hour; P < 0.001). The amount of outside play after school was similar (2.09 hour + 0.04 hour vs. 2.19 hour + 0.04 hour). Additionally, there was an increased proportion of secondary school students whom missed breakfast 'yesterday' (15.1% vs. 6.3%; P < 0.001) and reported having a tv in their bedroom (42.5% vs. 38.5%; P = 0.034). Conclusion: Obesogenic behaviours are frequent in both primary and secondary students however they appear to be more prevalent in the older students. Creating consistent health promoting environments for children as they move from preschool, through primary and then into secondary school is an important strategy for reducing obesity both in childhood and in later life
SN - 1467-7881
AD - (Bolton, Kremer, Swinburn, Meloni, De Silva-Sanigorski) Deakin University, Geelong, Australia (Waters, Gibbs, De Silva-Sanigorski) University of Melbourne, Carlton, Australia
ER -

TY - JOUR
ID - 3710
T1 - The impact of the Kids - 'Go for your life' award program on the nutrition and physical activity environments of Australian preschools
A1 - Carpenter,L.
A1 - de Silva-Sanigorski,A.
A1 - Prosser,L.
A1 - Gibbs,L.
A1 - Swinburn,B.
A1 - Waters,E.
Y1 - 2010///
N1 - 70227881
English
Journal: Conference Abstract
KW - EMBASE
KW - Obesity
KW - Environment
KW - Physical Activity
KW - awards and prizes
KW - Nutrition
KW - Policy
KW - Child
KW - Health Care Policy
KW - school
KW - Health Promotion
KW - teacher
KW - Australia
KW - Health
KW - parent
KW - Fruit
KW - vegetable
KW - Weather
KW - Risk
KW - childhood
KW - Eating

KW - questionnaire
KW - Water
RP - NOT IN FILE
SP - 454
JF - Obesity Reviews
JA - Obes Rev
VL - Conference: 11th International Congress on Obesity, ICO 2010 Stockholm Sweden. Conference Start: 20100711 Conference End: 20100715. Conference Publication:
IS - var.pagings
N2 - Introduction: The Kid's - 'Go for your life' (KGFYL) intervention program aims to reduce the risk of childhood obesity by improving children's environments and promoting healthy eating and physical activity related behaviours. The program involves an award scheme whereby elementary and pre-schools join as 'members' and then implement health promotion initiatives to meet the KGFYL criteria and become 'awarded'. The aim of this study is to examine differences in the environment between member and awarded preschools. Methods: Teachers from 36 KGFYL preschools in Victoria, Australia (RR: 65%) completed a 244-item questionnaire examining the nutrition and physical activity environment (socio-cultural, policy and physical) of their preschool. Results: Compared to member preschools, a greater proportion of awarded preschools had written physical activity policies (7% vs. 76%; P < 0.001); provided parents with a copy of their nutrition policy (64% vs. 95%; P < 0.05); had nutrition policies restricting sweet drinks and packaged snacks (73%, vs. 100%; 64% vs. 100%; P < 0.05) and promoting fruit and vegetables (64% vs. 100%; P < 0.05). A significantly smaller proportion of awarded preschools reported lack of equipment (53% vs. 19%; P < 0.05) and lack of alternatives when weather prevents outdoor play (60% vs. 29%; P < 0.01) as barriers to physical activity. There was also a higher proportion of children who brought water for snack time (P < 0.05). Conclusion: There is strong evidence that major differences exist between the nutrition and physical activity environments of KGFYL member and awarded preschools. KGFYL appears to be a positive, enabling and motivating program for making the preschool setting more health promoting
SN - 1467-7881
AD - (Carpenter, De Silva-Sanigorski, Prosser, Gibbs, Waters) University of Melbourne, Melbourne, Australia (De Silva-Sanigorski, Swinburn) Deakin University, Melbourne, Australia
ER -

TY - JOUR
ID - 3711
T1 - Obesity related to free time activities in Portuguese children
A1 - Mourao-Carvalhal,I.
A1 - Coelho,E.
A1 - Fonseca,S.
Y1 - 2010///
N1 - 70227789
English
Journal: Conference Abstract
KW - EMBASE
KW - Obesity
KW - Child
KW - Body Image
KW - Risk
KW - risk factor
KW - swimming pool
KW - logistic regression analysis
KW - questionnaire
KW - boy
KW - high school
KW - parent
KW - Internet
KW - Physical Activity
KW - girl
RP - NOT IN FILE

SP - 424
JF - Obesity Reviews
JA - Obes Rev
VL - Conference: 11th International Congress on Obesity, ICO 2010 Stockholm Sweden. Conference Start: 20100711 Conference End: 20100715. Conference Publication:
IS - var.pagings
N2 - Introduction: The purpose of this study was to assess the association among free time activities, body image perception and obesity. Methods: The sample included 588 children (269 girls and 319 boys) from elementary and secondary school. Obesity was estimated by BMI and the cut off points of Cole et al. A questionnaire was completed by parents and children to provide information about age, hours spent per week and weekend in screen activities (TV, electronic games, internet), playing, studying, doing physical activity, the use of public spaces (playground, gymnasium, swimming pool, gardens) and body image perception (Stunkard's silhouettes). Results: The results from binary logistic regression were only significant for age (OR=0.807; 95% CI 0.714-0.911), playing at weekend (OR=0.678; 95% CI 0.461-0.997) and body image perception (OR=11.219; 95% CI 6.988-18.010). The odds ratio of being obese were 0.678 times less for children that play more at weekend, and 0.807 times less for older children. In other hand body image is a risk factor for obesity and the odds ratio were 11,219 times more for children that have the perception of a more obese image. Conclusions: Age and playing at weekend are preventive factors; meanwhile, the perception of body image is a risk factor for obesity. These results are very important to take into account in programs designed to combat obesity
SN - 1467-7881
AD - (Mourao-Carvalhal, Coelho, Fonseca) University of Tras-os-Montes e Alto Douro, CIDESD, Vila Real, Portugal
ER -

TY - JOUR
ID - 3712
T1 - Risk factors of obesity in Algerian school children
A1 - Oulamara,H.
A1 - Agli,A.N.
A1 - Frelut,M.L.
Y1 - 2010///
N1 - 70227518
English
Journal: Conference Abstract
KW - EMBASE
KW - Obesity
KW - school child
KW - risk factor
KW - Child
KW - Risk
KW - Birth Weight
KW - feeding
KW - eating habit
KW - Statistics
KW - computer program
KW - statistical significance
KW - caloric intake
KW - Physical Activity
KW - meal
KW - Food
KW - Lifestyle
KW - Algeria
KW - childhood
KW - retrospective study
KW - girl
KW - urban area

KW - boy
KW - school
KW - city
KW - questionnaire
KW - random sample
RP - NOT IN FILE
SP - 341
JF - Obesity Reviews
JA - Obes Rev
VL - Conference: 11th International Congress on Obesity, ICO 2010 Stockholm Sweden. Conference Start: 20100711 Conference End: 20100715. Conference Publication:
IS - var.pagings
N2 - Objective: To identify at risk social and behaviours factors linked to obesity in Algerian school children living in an urban environment. Methods: A retrospective study was conducted in a random sample of 810 children (girls = 421, boys = 389) aged 6 to 12 years old, attending 9 schools of the city of Constantine, between April and November 2006. Data included anthropometric measurements, questionnaires about socio economic factors (SES), birth weight (BW), early feeding practices, eating habits, physical activity. IOTF criteria were used to assess overweight including obesity (OB). Statistics were performed using Statview software with statistical significance set at P = 0.05. The study was approved by the local ethical committee. Results: RR for OB is increased by parental OB (RR: 2.5, 95% CI[1.66-3.76]). Obese children have higher BW (3.51 + 0.59 vs 3.31 + 0.55 kg, P = 0.0008), are less breastfed (16 vs 20%, P = 0.0004). BMI is negatively correlated to time spent to outdoor activities (P = 0.002) and positively to energy intakes (P = 0.0003). Less OB children eat breakfast (69 vs 83%, P = 0.0004). Junk foods snacking is more common in OB children than in non OB ones (65% vs 56 %, P < 0.05). SES status does not differ among OB and non OB children. Conclusion: Recent changes in urban lifestyle in Algeria, but not SES, favour at risk behaviours leading to OB, starting from early childhood onward. Urgent preventive actions need to be undertaken
SN - 1467-7881
AD - (Oulamara, Agli) Nutrition Research Centre, INATAA, University of Constantine, Algeria (Frelut) APHP, Hospital St Vincent de Paul, Paris, France
ER -

TY - JOUR
ID - 3713
T1 - Romp & Chomp: Improving young children's eating and activity behaviours through an environmental intervention in family day care
A1 - de Silva-Sanigorski,A.
A1 - Kremer,P.
A1 - Elea,D.
A1 - Boak,R.
A1 - Swinburn,B.
Y1 - 2010///
N1 - 70226636
English
Journal: Conference Abstract
KW - EMBASE
KW - Child
KW - Obesity
KW - Eating
KW - Day Care
KW - Food
KW - Child Care
KW - questionnaire
KW - follow up
KW - Nutrition
KW - Policy
KW - Environment
KW - Television

KW - childhood
KW - parent
KW - Australia
KW - community
KW - Capacity Building
KW - Government
KW - Physical Activity
RP - NOT IN FILE
SP - 59
JF - Obesity Reviews
JA - Obes Rev
VL - Conference: 11th International Congress on Obesity, ICO 2010 Stockholm Sweden. Conference Start: 20100711 Conference End: 20100715. Conference Publication:
IS - var.pagings
N2 - Background: Romp & Chomp (R&C) was an intervention conducted in Geelong, Australia (2004-2008) which aimed to prevent childhood obesity and promote healthy eating and active play in 0-5-year old children through community capacity-building and environmental (policy, socio-cultural, physical) interventions in earlychildhood settings. This paper presents the intervention impacts in family day care (FDC); a government funded and accredited homebased childcare service. Methods: Evaluation of R&C was cross-sectional and quasi-experimental with a comparison sample. An environmental questionnaire was completed by FDC providers at follow-up in intervention (n = 28) and comparison (n = 347) samples. The 45-item survey captured information about the nutrition and physical activity environment. Results: Post intervention, children in the intervention sample spent significantly less time watching television (P < 0.001) and using computer/ electronic games (P < 0.05). Intervention children spent significantly less time in organised active play (P < 0.001), and free inside play (P < 0.001), but were taken more often for active play at another location (P < 0.001) and minimum time in outside play was higher (P < 0.05) compared to the comparison sample. Related to healthy eating, FDC providers in the intervention area had significantly higher odds of: written guidelines related to appropriate foods/drinks for parents to send to care (OR:2.49, 95%CI:1.61-3.86), rules related to healthy eating (OR:1.46, 1.14-1.88) and, taking action if foods provided do not meet guidelines (OR:3.63, 2.63-4.99). Conclusions: The improvements made in the FDC service through the R&C intervention have created more opportunities for children to be physically active and eat more nutritious foods during their time in care
SN - 1467-7881
AD - (De Silva-Sanigorski, Boak, Swinburn) WHO Collaborating Centre for Obesity Prevention, Deakin University, Geelong 3217, Australia (Kremer) School of Psychology, Deakin University, Geelong 3217, Australia (Elea) City of Greater Geelong, Geelong 3220, Australia
ER -

TY - JOUR
ID - 3714
T1 - Single dose rFVIIa for severe haemophilia A (HA) with inhibitors: Report of the experience in Argentina
A1 - Bianco,R.P.
A1 - Candela,M.
A1 - Neme,D.
Y1 - 2010///
N1 - 70225129
English
Journal: Conference Abstract
KW - EMBASE
KW - single drug dose
KW - hemophilia
KW - hemophilia A
KW - Argentina
KW - patient
KW - bleeding
KW - clinical trial
KW - Safety
KW - thromboembolism

KW - Child
KW - blood clotting factor 7
RP - NOT IN FILE
SP - 70
JF - Haemophilia
VL - Conference: 29th International Congress of the World Federation of Hemophilia Buenos Aires Argentina. Conference Start: 20100710 Conference End: 20100714. Conference Publication:
IS - var.pagings
N2 - Introduction: Recombinant activated coagulation factor VII (rFVIIa) is currently a firstline treatment of bleeding episodes in patients with haemophilia A and B with inhibitors. Several studies have been published showing a new dosing schedule of a single dose of 270 ug/kg to be as effective and safe as the standard 90 ug/kg every 2-3 hours. Objective: To evaluate, outside the setting of a clinical trial, the efficacy and safety of single dose rFVIIa treatment in mild/moderate bleeding episodes. Methods: We reviewed 24 mild/moderate bleeds from 10 severe HA patients with inhibitors from our institution. Mean age was 9 years old (4-24), mean inhibitor title was 50.6 BU/mL (5-450). All patients received treatment with single dose rFVIIa and response was evaluated every three hours. If bleeding persisted, further doses were administered. Efficacy was evaluated according to the need of additional doses and re-bleeding within five days of the original episode. Results: The mean dose administered was 217.77 ug/kg (177-309). 20/24 patients (84%) responded to the initial dose, with only 4 (16%) requiring additional doses. Only 1 patient required a new treatment with rFVIIa 3 days after the original episode. No thromboembolic events were registered. Conclusions: Single dose rFVIIa proved to be an efficacious treatment for mild/moderate bleeding episodes in inhibitor patients. The efficacy showed in our patients (84%) was similar to that reported in the literature (81-92%). This treatment provided rapid bleeding control and could be a convenient option, especially for younger children and those with poor venous access
SN - 1351-8216
AD - (Bianco, Neme) Fundacion Nacional de la Hemofilia, Buenos Aires, Argentina (Bianco, Candela) Institute de Investigaciones Hematologicas Mariano R. Castex, Academia Nacional de Medicina, Buenos Aires, Argentina
ER -

TY - JOUR
ID - 3715
T1 - Reliability of cycle ergometer tests and the LifeShirt in children with CF
A1 - Kent,L.
A1 - O'Neill,B.
A1 - Murray,J.
A1 - Reid,A.
A1 - Davison,G.
A1 - Nevill,A.
A1 - Elborn,J.S.
A1 - Bradley,J.
Y1 - 2010///
N1 - 70211767
English
Journal: Conference Abstract
KW - EMBASE
KW - Child
KW - reliability
KW - cystic fibrosis
KW - bicycle ergometer
KW - Ergometry
KW - Heart Rate
KW - breathing rate
KW - clinical laboratory
KW - air conditioning
KW - breathing
KW - Exercise Test
KW - lung disease

KW - Muscle Strength
KW - fitness
KW - questionnaire
KW - work capacity
KW - tidal volume
RP - NOT IN FILE
SP - S67
JF - Journal of Cystic Fibrosis
JA - J Cyst.Fibros.
VL - Conference: 33rd European Cystic Fibrosis Conference Valencia Spain. Conference Start: 20100616 Conference End: 20100619. Conference Publication:
IS - var.pagings
N2 - Introduction: There is limited research on the use of exercise tests outside the clinical laboratory setting in children with CF. Aims: To assess reliability and acceptability of (a) cycle ergometry tests, and (b) using the LifeShirt to record cardiorespiratory responses during cycle ergometry tests, in children with CF. Methods: Children with CF and stable lung disease wore the LifeShirt during 2 cycle ergometry tests (intermittent sprint test (IST) measuring muscle power; continuous incremental test (CIT) measuring cardiorespiratory fitness) on 2 occasions 7-8 days apart. Acceptability data were collected by questionnaire. Results: 10 children with CF completed the study: (5M:5F), 9(2) y, FEV₁: 83(14)%. Peak power (IST) and peak work capacity (CIT) were reliable (bias p>0.05, CV < 10%). Tidal volume, ventilation, respiratory rate, fractional inspiratory time, heart rate and SpO₂ recorded by the LifeShirt demonstrated no significant bias (p>0.05). Respiratory rate, fractional inspiratory time, heart rate and SpO₂ demonstrated acceptable CVs (<10%). Acceptability questions indicated that cycle ergometer tests and the LifeShirt were mainly acceptable to the children. Conclusion: This is the first study to report reliability of cycle ergometry in children with CF. The LifeShirt may be useful for simultaneous measurement of respiratory rate, fractional inspiratory time, heart rate and SpO₂ outside the normal clinical laboratory setting, however, there is concern over volume components of breathing in children with CF
SN - 1569-1993
AD - (Kent, O'Neill, Davison, Bradley) University of Ulster, Jordanstown, United Kingdom (Murray, Reid) Royal Belfast Hospital for Sick Children, Belfast, United Kingdom (Nevill) University of Wolverhampton, Wolverhampton, United Kingdom (Elborn) Queen's University Belfast, Belfast, United Kingdom (Elborn, Bradley) Belfast City Hospital, Belfast, United Kingdom
ER -

TY - JOUR
ID - 3716
T1 - Nutrition & physical education in the summer migrant classroom (MEP)
A1 - Kilanowski,J.F.
Y1 - 2010///
N1 - 70206113
English
Journal: Conference Abstract
KW - EMBASE
KW - summer
KW - Education
KW - Nutrition
KW - Physical Education
KW - Child
KW - student
KW - Curriculum
KW - weight
KW - pilot study
KW - Hispanic
KW - Eating
KW - Cooking
KW - bivariate analysis
KW - body mass

RP - NOT IN FILE
SP - S30
EP - S31
JF - Clinical and Translational Science
VL - Conference: 2010 Clinical and Translational Research and Education Meeting: ACRT/SCTS Joint Annual Meeting Washington, DC United States. Conference Start: 20100405 Conference End: 20100407. Conference Publication:
IS - var.pagings
N2 - OBJECTIVES: The aims of this pilot study were to test effectiveness of a supplemental curriculum intervention in nutrition and physical education in a summer Midwest MEP with the children of migrant farmworkers (MFWs), and to evaluate the study feasibility. Outcomes were: changes in body mass index (BMI), BMI percentile, weight, and knowledge. We hypothesize that students assigned to the intervention group will show greater improvement in outcomes in contrast to the comparison site. METHODS AND POPULATION: Two summer MEPs that support MFW children in remedial education were study sites: intervention group n = 138, comparison group n = 33. Students had 4 interventions a week (2 of each) and structured recess. Curriculum was ethnically-tailored with subject oriented, Latino themed children's trade books, cooking classes and a field day. RESULTS: Bivariate analysis showed that more MEP days attended, the larger change in wt (r = .24, p = .03, n = 78), and larger change in BMI (r = .27, p = .01, n = 78); the means of change in BMI and change in BMI % were negative(n = 110). The intervention site had marginal significance with change in BMI (Kendall Tau's, r = - 0.16, p = 0.07, n = 110). In the nonparametric Wilcoxon 2 sample test, the intervention site was significant for change in wt (p = .05) with the comparison group being heavier in wt. When looking at change in BMI, the intervention group had a greater than 4-fold decrease in change in BMI (-.14 vs. -.03) with marginal significant (Wilcoxon 2 sample test, p = .06, n = 110). Increases in age-appropriate knowledge scores from baseline to intervention conclusion were seen in 2 assessments by 70% (n = 47) and 63% (n = 46) of the students. SIGNIFICANCE OF STUDY: Summer MEPs can provide opportunities to instruct MFW children on healthy eating and activity with positive results
SN - 1752-8054
AD - (Kilanowski) Case Western Reserve University, Cleveland, OH, United States
ER -

TY - JOUR
ID - 3717
T1 - Impact of transition to high school on leisure participation of adolescents with cerebral palsy
A1 - Imms,C.
A1 - Reilly,S.
A1 - Carlin,J.
A1 - Dodd,K.
Y1 - 2010///
N1 - 70202644
English
Journal: Conference Abstract
KW - EMBASE
KW - high school
KW - Cerebral Palsy
KW - Adolescent
KW - leisure
KW - Child
KW - school
KW - social adaptation
KW - boy
KW - book
KW - girl
KW - Classification
KW - Gross Motor Function Classification System
KW - Student t test
KW - computer
KW - Population

KW - Australia
KW - Social Behavior
RP - NOT IN FILE
SP - 22
JF - Developmental Medicine and Child Neurology
VL - Conference: 5th Biennial Conference of the Australasian Academy of Cerebral Palsy and Developmental Medicine Christchurch New Zealand. Conference Start: 20100303 Conference End: 20100306. Conference Publication:
IS - var.pagings
N2 - Objective: This study investigated the impact of transition to high school on the range, frequency, enjoyment of, and preferences for participation in activities outside school. We hypothesised that activity preferences would be stable across the period that included transition to high school but that the range of activities adolescents with cerebral palsy engaged in would be reduced. Design: A component of a longitudinal (3 waves, 12mo apart), population-based study of children born in Victoria Australia in 1994 or 1995 who had cerebral palsy. Method: Participants were 26 boys (53%) and 23 girls (47%), classified at Manual Ability Classification System levels I (22%), II (45%), III (19%), IV (8%) and V (6%) and Gross Motor Function Classification System levels I (35%), II (41%), III (6%), IV (10%) and V (8%). All 49 participants contributed wave-1 data in 2006 [mean age 11y 10mo (SD: 6mo)], and wave-2 data in 2007 when n=19 of the 49 had transitioned to high school [mean age 12y10mo (SD: 6mo)]. 30 participants contributed wave-3 data after transitioning to high school in 2008 [mean age 13y9mo (SD: 6mo)]. Participation in recreational, active-physical, social, skill-based and self-improvement activities outside of school was measured using the Children's Assessment of Participation and Enjoyment (CAPE) and the Preferences for Activity of Children (PAC).¹ Changes in diversity (number of activities) intensity (relative frequency), enjoyment and preference were assessed using paired t-tests between assessments conducted 12 months apart (before and after transition). Results: There was some evidence that participation in recreational activities (e.g. crafts, puzzles, pet play, computer games) was reduced after transition to high school in diversity (t₄₈=-2.47, P=0.017; average reduction of one activity), intensity (t₄₈=-1.97, P=0.055), and preference (t₄₈=-3.06, P=0.004). There was no evidence that diversity or intensity of participation in active-physical, social, skill-based or self-improvement activities were different following transition (all P>0.05). Apart from changes in recreational preferences, preferences and enjoyment for all other activity types were stable (all P>0.05). Conclusion: In this study, participation in activities outside school was predominantly stable across the transition to high school. The slight reduction in participation in recreational activities more suited to younger children was similar to that shown previously,² but was not balanced by an increase in participation in social activities as is expected in typically developing adolescents.³
SN - 0012-1622
AD - (Imms, Dodd) La Trobe University, Melbourne, VIC, Australia (Imms) Royal Children's Hospital, Melbourne, VIC, Australia (Imms, Reilly, Carlin) Murdoch Childrens Research Institute, VIC, Australia (Reilly, Carlin) University of Melbourne, VIC, Australia
ER -

TY - JOUR
ID - 3718
T1 - Playground sand as an environmental source of a localized community salmonella outbreak
A1 - Staff,M.
A1 - Musto,J.
A1 - Hogg,G.
Y1 - 2010///
N1 - 70195766
English
Journal: Conference Abstract
KW - EMBASE
KW - sand
KW - community
KW - Salmonella
KW - Human
KW - bacteriophage typing
KW - case control study

KW - Government
KW - hypothesis
KW - Population
KW - tandem repeat
KW - gastrointestinal symptom
KW - bloody diarrhea
KW - organism
KW - exposure
KW - Risk
KW - Interview
KW - Salmonella paratyphi B
KW - Public Health
KW - Australia
KW - seashore
KW - law
KW - Infection
KW - health service
KW - Child
KW - hazard
KW - biotype
RP - NOT IN FILE
SP - S320
EP - S321
JF - Clinical Microbiology and Infection
VL - Conference: 20th ECCMID Vienna Austria. Conference Start: 20100410 Conference End: 20100413. Conference Publication:
IS - var.pagings
N2 - Objectives: To identify the source of a protracted Salmonella Paratyphi B biovar Java (S. Java) outbreak localized to a single local government area within metropolitan Sydney, Australia. Methods: From 2007 to 2009 S. Java cases notified under public health legislation to the Northern Sydney Central Coast Area Health Service were interviewed to collect clinical information and to identify likely sources of infection. Human isolates were classified by phage typing and Multi-locus Variable number tandem repeat Analysis (MLVA). Environmental samples were collected from various sources and isolates classified using both phage typing and MLVA. Contaminated sand from one playground was tested second monthly to assess environmental persistence of the bacterium. An age-matched case control study was conducted for outbreak cases identified in the first half of 2008 to test the hypothesis that close contact with playground sand was the source of outbreak. Results: A total of 69 cases of S. Java were notified among residents in a single local government area (population 57,000) between 2007 and 2009 with 41% aged less than 2yrs. Most cases had significant gastrointestinal symptoms (including bloody diarrhoea) lasting a median of 7 days. Of the 65 human isolates for which phage typing was available 72% were reported as Dundee with remainder typed as reacts does not conform (RDNC). MLVA typing identified a specific outbreak strain with no variation at the first and fifth loci and only minor variation at the second locus. Case interviews implicated playground sand as a possible source and subsequent environmental testing isolated S. Java with the outbreak MLVA pattern from 5 of13 playgrounds tested. Second monthly testing of sand from one playground left undisturbed and closed to public access demonstrated that the organism could survive for at least 6 months in sand. The case control study confirmed playground sand exposure as the source of the outbreak with cases being significantly more likely to have played with sand in known contaminated playgrounds (odds ratio 3.7, 95% ci 1.1 to 12.1). Conclusion: This research demonstrates that S. Java can survive in sand for a prolonged period and lead to a sustained community outbreak of human disease. The use of sand in playgrounds can pose a microbiological hazard especially to young children
SN - 1198-743X
AD - (Staff, Musto, Hogg) Sydney, MelbourneAustralia
ER -

TY - JOUR
ID - 3719
T1 - Functional neuroanatomy of working memory in children with ADHD: Dysfunction of neural circuits involving cerebro-cerebellar patterns

A1 - Massat,I.
A1 - Mary,A.
A1 - Slama,H.
A1 - Kavec,M.
A1 - Metens,T.
A1 - Baleriaux,D.
A1 - Peigneux,P.
Y1 - 2010///
N1 - 70188542
English
Journal: Conference Abstract
KW - EMBASE
KW - Attention Deficit Disorder
KW - disability
KW - Child
KW - childhood
KW - working memory
KW - neuroanatomy
KW - cerebellum
KW - Comorbidity
KW - Impulsiveness
KW - pathophysiology
KW - Adolescent
KW - Functional Magnetic Resonance Imaging
KW - Prevalence
KW - Drug Therapy
KW - control group
KW - brain region
KW - patient
KW - hyperactivity
KW - manager
RP - NOT IN FILE
SP - 13
JF - Developmental Medicine and Child Neurology
VL - Conference: 22nd Annual Meeting of the European Academy of Childhood Disability, EACD Brussels Belgium. Conference Start: 20100526 Conference End: 20100529 Sponsor: Medtronic. Conference Publication:
IS - var.pagings
N2 - Attention deficit/hyperactivity disorder (ADHD) is one of the most common childhood disorders characterized by three clusters of age inappropriate symptoms: inattention, hyperactivity and impulsivity, with a high worldwide prevalence (around 5%). Several authors have hypothesized that deficits in executive function, behavioral inhibition, and working memory (WM) are considered as one of the key neuropsychological features in children and adolescents with ADHD. Althought there is a strong evidence suggesting that frontostriatal dysfunctions is central to the pathophysiology of ADHD, there is now growing evidence in the most recent studies, of dysfunctions outside the frontostriatal circuity, including the cerebellum and the temporoparietal lobes. The purpose of the present study was to investigate in fMRI, the cerebro-cerebellar networks to verbal WM (n-back task) in prepubertal 8-12-years old, right-handed and medication naIve children with ADHD combined type (n=18) in comparison with a control group (n=17). Because ADHD is associated to high rates of comorbidity, we decided to only include ADHD participants without comorbidity, to avoid their own impact on different brain regions and functions. In conclusion, functional data indicate significant different activation patterns in WM task between healthy controls and patients with ADHD suggesting less activity in ADHD in left occipital region (area 17/18), and in crus1 region in right cerebellum. These results will be discussed in light of recent literature in this field, and specially the cognitive contribution of the cerebellum in working memory
SN - 0012-1622
AD - (Massat) Laboratoire de Neurologie Experimentale, Faculty of Medicine, Universite Libre de Bruxelles, Brussels, Belgium (Mary, Slama, Kavec) Unite de Recherches en Neuropsychologie et Neuroimagerie Fonctionnelle, Faculty of Psychological and Educational Sciences, Universite Libre de Bruxelles, Brussels, Belgium (Metens, Baleriaux, Peigneux) Department of Neurology, Hopital Erasme, ULB, Brussels, Belgium

ER -

TY - JOUR
ID - 3720
T1 - Community-based occupational therapy services for children: A Quebec survey on service delivery
A1 - Cotellesso,A.
A1 - Mazer,B.
A1 - Majnemer,A.
Y1 - 2009///
N1 - 19916826
English
Journal: Article
KW - EMBASE
KW - article
KW - Canada
KW - Child
KW - community care
KW - Human
KW - Occupational Therapy
KW - Safety
KW - Statistics
RP - NOT IN FILE
SP - 426
EP - 444
JF - Physical and Occupational Therapy in Pediatrics
VL - 29
IS - 4
CY - United States
N2 - Community-based occupational therapy (OT) services are intended to promote social integration and minimize disability. The objective of this study was to describe community-based OT services for children in the province of Quebec, Canada. Specific aims included (a) to determine the proportion of Centres Locaux de Services Communautaires (CLSCs) that provide OT services to children, (b) to describe the nature of the services offered, and (c) to identify factors that are associated with the availability of these services. Surveys were sent to all CLSCs where at least one OT was employed. The response rate was 80. Only 12 of CLSCs offered OT services to children on a regular basis (≥ 1 day/week). Home adaptations were the most frequent service offered, whereas health promotion was rarely addressed. Perceived obstacles to servicing children included prioritization of services to the elderly, limited funding, and shortage of OTs. CLSCs in rural areas and those with a psychologist had significantly more dedicated OT time spent for services to children. The findings suggest that community-based OT services for children in Quebec are poorly developed, which may negatively impact upon timely access to specialized rehabilitation services and may increase activity limitations and family burden. © 2009 by Informa Healthcare USA, Inc. All rights reserved
SN - 0194-2638
AD - (Cotellesso) Centres Locaux de Services Communautaires, Cote-des-Neiges, Montreal, QC, Canada (Mazer) School of Physical and Occupational Therapy, McGill University (Mazer) Jewish Rehabilitation Hospital, Centre de Recherche Interdisciplinaire en Radaptation du Montral Mtropolitain (CRIR), Montreal, QC, Canada (Majnemer) School of Physical and Occupational Therapy (Majnemer) Departments of Pediatrics and Neurology and Neurosurgery, McGill University (Majnemer) Montreal Children's Hospital, 2300 Tupper St., Montreal, QC H3H 1P3, Canada
ER -

TY - JOUR
ID - 3721
T1 - Does BMI impact activity level?
A1 - Rosehill,K.H.K.
Y1 - 2009///

N1 - 2011536833
English
Journal: Article
KW - EMBASE
KW - article
KW - Body Height
KW - body mass
KW - Body Weight
KW - Child
KW - childhood obesity
KW - controlled study
KW - Female
KW - Human
KW - major clinical study
KW - Male
KW - Obesity
KW - Physical Activity
KW - Physical Education
KW - questionnaire
KW - United States
RP - NOT IN FILE
SP - S319
EP - S320
JF - Ethnicity and Disease
VL - 19
IS - 2 SUPPL.3
CY - United States
N2 - Research has found that obese and overweight boys and girls are prone to less physical activity. in this study, we reinvestigate the relationship between physical activity and obesity among children. We examined the differences in physical activity between overweight and normal weight children. One-hundred and twenty six Hawaii school-system children participated in this study. The children were measured for height and weight, and answered questions about their activity level. Activity level was assessed by participation in physical education, active play during recess and lunch, and other activities during the past week. Children were divided into obese, overweight and normal weight based on their age and body mass index (EMI). Age-adapted EMI was used as overweight and obesity indicator. The hypothesis for this project is that physical activity will be greater with normal weight boys and overweight girls than it is with overweight boys and normal weight girls
SN - 1049-510X
ER -

TY - JOUR
ID - 3722
T1 - Prospective study in schoolchildren of Milan of health effects (respiratory damage and airway inflammation) from traffic related air pollution
A1 - Cetta,F.
A1 - Sala,M.
A1 - Argiro,S.
A1 - Ballista,P.
A1 - Perrone,G.
A1 - Zangari,R.
A1 - Giovannini,M.
Y1 - 2009///
N1 - 2011299124
English
Journal: Review
KW - EMBASE
KW - Air Pollution
KW - air quality

KW - allergic rhinitis
KW - Asthma
KW - breath analysis
KW - bronchitis
KW - Child
KW - clinical trial
KW - Conjunctivitis
KW - controlled study
KW - disease exacerbation
KW - Environmental Exposure
KW - exhaust gas
KW - Female
KW - Forced Expiratory Volume
KW - grass pollen
KW - health hazard
KW - Human
KW - Italy
KW - lung function test
KW - Male
KW - maximal mid expiratory flow
KW - Particulate Matter
KW - pollution monitoring
KW - Prevalence
KW - prick test
KW - review
KW - school child
KW - sensitization
KW - Spirometry
KW - Nitric Oxide
RP - NOT IN FILE
SP - 447
EP - 455
JF - GIMT - Giornale Italiano delle Malattie del Torace
VL - 63
IS - 6
CY - Italy
N2 - Two-hundreds and twenty-eight children (127 males and 101 females), mean age 8 years, were enrolled from 2 primary schools, which were located in different sites, for studying pollution related respiratory symptoms and/or diseases in different places of Milan with a different traffic related exposure. The former (School 1) was located near a large park, the latter was located downtown, close to main crossroads (School 2). Daily levels of PM10 and PM 2,5 (diameter < 10 mum and 2,5 mum, respectively) were measured using a mobile detection unit, which was placed either outside the schools (in the school garden) and within common places (corridors), for 7 consecutive days during 2 different campaigns (winter and spring-summer). Children underwent skin prick testing for inhaled allergens, analysis of exhaled nitric oxide and spirometry. The mean values of respiratory function measured by spirometry was within the normal limits in both schools. The mean value of Tiffeneau index (SD) was significantly lower in downtown school: 93.2 (5.5) vs 96.5 (4.1) (p=0.02). Mean (SD) concentration of FeNO (ppb) was higher in downtown school: 13.6 (14.6) vs 12.5 (14.9), and the distribution of FeNo concentration significatively differs between the two schools (p=0.02). 193 subjects (73 in downtown school and 120 in park school) agreed to undergo to skin prick tests. Prevalence of sensitization to inhaled allergens did not differ significatively between the two schools (downtown school, 32.8% vs park school, 27.5%; p=n.s.). Based on ISAAC questionnaire the percentage of asthma exacerbations in the previous 12 months was higher in children from school downtown (p=0.05); the prevalence of persistent allergic rhinitis in children allergic to grass pollen was higher in park school (p=0.03) with more activity limitation due to rhinitis and associated conjunctivitis (p=0.03). Present findings show that different degrees of respiratory function and bronchial inflammation were found in the 2 groups of children. Even if great caution is required when relating data from air quality monitoring to clinical outcomes in humans, it can be inferred that: 1) the different air quality in the 2 schools could have a role in determining observed differences; 2) traffic could be, at

least in part, responsible for the different air quality; 3) reduction of traffic related pollution should be a logical and suitable objective of policy makers
SN - 1127-0810
AD - (Cetta, Zangari) Department of Surgery, University of Siena, Siena, Italy (Sala, Argiro, Ballista, Perrone, Giovannini) Department of Pediatrics, San Paolo Hospital, University of Milan, Milan, Italy
ER -

TY - JOUR
ID - 3723
T1 - The prevalence of exercise-induced asthma among school children
A1 - Mtshali,B.F.
A1 - Mokwena,K.E.
Y1 - 2009///
N1 - 2010075740
English
Journal: Article
KW - EMBASE
KW - Adolescent
KW - allergic rhinitis
KW - article
KW - Asthma
KW - Child
KW - exercise induced asthma/ep [Epidemiology]
KW - Female
KW - Heart Rate
KW - Human
KW - major clinical study
KW - Male
KW - peak expiratory flow
KW - Prevalence
KW - respiratory tract infection
KW - school child
KW - screening test
KW - South Africa
RP - NOT IN FILE
SP - 489
EP - 491
JF - South African Family Practice
VL - 51
IS - 6
CY - South Africa
N2 - Exercise-induced asthma (EIA) is one of the major factors that affect optimal performance in sport. The prevalence of EIA is reported to be on the increase among school children worldwide. The aim of this study was to indicate EIA prevalence among primary-school children in South Africa. A field study determined the prevalence of EIA. A convenience sample of 127 children aged 8 to 16 years was selected. A health- screening questionnaire was used. The criteria for selection were that the subjects should be non- asthmatics and should not have any other illness during the screening that could interfere with the results. Peak expiratory flow rate (PEFR) was measured pre- and post-exercise using an ASSESS peak flow meter. The subjects did a six-minute run on a school playground. The post-exercise PEFR was measured within 10 minutes of exercise. The determinant of EIA was > 10% decrease in PEFR after exercise. A total of 112 (88%) subjects participated in the study. Fifteen subjects were excluded (eight had asthma, five had respiratory tract infection and two had other illnesses). The average exercise heart rate was + 80% of predicted maximal heart rate. The prevalence of EIA was 23% (n = 26). The incidence of a history of allergic rhinitis was statistically significantly increased among those with positive tests (p = 0.027). This study highlights the need to screen all children so that EIA can be determined and addressed appropriately. SAAFP
SN - 1726-426X

AD - (Mtshali) Department of Physiotherapy, Medunsa Campus, University of Limpopo, South Africa
(Mokwena) Department of Social and Behavioural Health Sciences, School of Public Health, University of Limpopo, South Africa
ER -

TY - JOUR
ID - 3724
T1 - Chlorinated pool attendance and risk of asthma during childhood
A1 - Chladkova,J.
A1 - Chladek,J.
Y1 - 2009///
N1 - 2010055428
Czech
Journal: Article
KW - EMBASE
KW - article
KW - Asthma/dt [Drug Therapy]
KW - Asthma/et [Etiology]
KW - asthma/pc [Prevention]
KW - Atmosphere
KW - bronchus hyperreactivity
KW - Child
KW - childhood
KW - chlorination
KW - developed country
KW - Environmental Exposure
KW - Human
KW - Inhalation
KW - leisure
KW - odor
KW - Prevalence
KW - respiratory tract inflammation
KW - risk factor
KW - Swimming
KW - swimming pool
KW - beclometasone dipropionate/dt [Drug Therapy]
KW - budesonide/cb [Drug Combination]
KW - budesonide/dt [Drug Therapy]
KW - chlorine/to [Drug Toxicity]
KW - fluticasone/dt [Drug Therapy]
KW - fluticasone/na [Intranasal Drug Administration]
KW - formoterol/cb [Drug Combination]
KW - formoterol/dt [Drug Therapy]
KW - immunoglobulin E/ec [Endogenous Compound]
RP - NOT IN FILE
SP - 298
EP - 303
JF - Alergie
VL - 11
IS - 4
CY - Czech Republic
N2 - Exposure of human population in developed countries to chlorination products has considerably increased during the 20th century especially after the 1960s with the development of public swimming pools. Swimmers, however, are mainly exposed when they actively inhale aerolised chlorination products in the air just above pool surface. Studies on elite swimmers has shown that the chlorine-laden atmosphere of indoor pools could be detrimental to the lungs, by increasing the risk of asthma, bronchial hyperreactivity and airways inflammation. Recent studies have suggested these adverse effects of pool chlorine on the airways and increased

risk of asthma even in children with recreational swimming, especially with atopy who regularly attend indoor pools before the age of 5 years. Prospective follow-up studies are needed whether or not a cause-effect relationship really exists between recreational swimming and asthma development. Trichloramine, the gas that builds up un the air of indoor pools, giving them their distinctive chlorine smell, has been identified as a cause of asthma and respiratory problems in pool workers. Even frequent attendance of outdoor chlorinated swimming pool is associated with higher risks of asthma and airways inflammation
SN - 1212-3536
AD - (Chladkova) Detska Klinika, LF a FN, Sokolska 581, 500 05 Hradec Kralove, Czech Republic (Chladek) Ustav Farmakologie Lekarska Fakulta, Hradec Kralove, Czech Republic
ER -

TY - JOUR
ID - 3731
T1 - ADHD and problem-solving in play
A1 - Borg,S.
Y1 - 2009///
N1 - 2009613019
English
Journal: Article
KW - EMBASE
KW - article
KW - Attention
KW - attention deficit disorder/di [Diagnosis]
KW - attention deficit disorder/th [Therapy]
KW - Child
KW - Child Behavior
KW - clinical article
KW - Cognition
KW - controlled study
KW - Diagnostic and Statistical Manual of Mental Disorders
KW - emotion
KW - Human
KW - human relation
KW - Male
KW - maturation
KW - Peer Group
KW - Play Therapy
KW - priority journal
KW - Problem Solving
KW - school child
KW - Self Control
KW - social adaptation
KW - Social Environment
KW - Social Interaction
KW - social status
KW - placebo
RP - NOT IN FILE
SP - 325
EP - 336
JF - Emotional and Behavioural Difficulties
VL - 14
IS - 4
CY - United States
N2 - This paper reports a small-scale study to determine whether there is a difference in problem-solving abilities, from a play perspective, between individuals who are diagnosed as ADHD and are on medication and those not on medication. Ten children, five of whom where on medication and five not, diagnosed as ADHD predominantly inattentive type, were observed in school playgrounds in Malta during recess to verify any

differences in the ways real-life problem-solving situations are tackled. Results indicated that children on medication tend to avoid group situations and discussion more often than their non-medicated peers. Additionally, non-medicated individuals tend to jump more quickly from one emotional state to the other and resort to physical aggression more often compared with medicated individuals. Such findings suggest that medication has a negative effect on social interaction processes, while it helps to maintain self-control, at least from a physical stand point. 2009 SEBDA
SN - 1363-2752
AD - (Borg) Nursery Assistant, Birmingham, United Kingdom
ER -

TY - JOUR
ID - 3732
T1 - Virtual environments for powered wheelchair learner drivers: Case studies
A1 - Adelola,I.A.
A1 - Cox,S.L.
A1 - Rahman,A.
Y1 - 2009///
N1 - 2009610720
English
Journal: Article
KW - EMBASE
KW - Adolescent
KW - article
KW - assistive technology
KW - Case Report
KW - Cerebral Palsy/rh [Rehabilitation]
KW - Child
KW - computer interface
KW - daily life activity
KW - Early Intervention
KW - Educational Technology
KW - Female
KW - Human
KW - Human Computer Interaction
KW - Learning
KW - learning disorder
KW - Male
KW - motor dysfunction/rh [Rehabilitation]
KW - outcome assessment
KW - patient education
KW - powered wheelchair
KW - preschool child
KW - skill retention
KW - task performance
KW - Virtual Reality
RP - NOT IN FILE
SP - 97
EP - 106
JF - Technology and Disability
VL - 21
IS - 3
CY - Netherlands
N2 - Early intervention through the use of a powered wheelchair can meliorate the developmental experience of children with mobility impairment. It is therefore important to design an effective training and assessment strategy to facilitate the potentials of each child in readiness to drive the device. The use of virtual reality (VR) technology for wheelchair training purposes is therefore considered. The skills acquired during training in a virtual reality system should be observable in the functional activities of the learner. It is necessary to evaluate

the degree and the permanence of the skills learnt from the virtual environment to other activities outside the training environment. The mode of evaluation applied during conventional wheelchair training could provide the basis upon which permanence of skills is determined after training in virtual reality. Thus, an induction factor is proposed as a measure of the transfer of powered wheelchair control skills from virtual reality to the functional activities of daily living by the learner. The outcomes show that virtual reality technology could offer an appropriate means of providing powered wheelchair training that can be tailored to the needs of the learner.
2009 IOS Press. All rights reserved
SN - 1055-4181
AD - (Adelola, Rahman) Department of Electronic and Computer Engineering, University of Limerick, Limerick, Ireland (Cox) Department of Manufacturing and Operations Engineering, University of Limerick, Limerick, Ireland
ER -

TY - JOUR
ID - 3733
T1 - ENT physicians' efforts to treat allergic diseases at schools
A1 - Shimada,K.
Y1 - 2009///
N1 - 2009551675
English
Journal: Review
KW - EMBASE
KW - Air Pollution
KW - allergic disease
KW - allergic rhinitis/ep [Epidemiology]
KW - Child
KW - disease classification
KW - Human
KW - Hygiene
KW - medical information
KW - physician
KW - pollen allergy
KW - Prevalence
KW - review
KW - school
KW - Swimming
RP - NOT IN FILE
SP - 178
EP - 183
JF - Japan Medical Association Journal
VL - 52
IS - 3
CY - Japan
N2 - Recently Sinusitis is declining in severity, while allergic rhinitis is increasing in prevalence. Although improved nutrition and the spread of hygiene information have caused a decline of sinusitis, air pollution has caused an increase in rhinitis. Behind these changes, the first factor to consider is air pollution. While areas with no air pollution have more schoolchildren with infectious rhinitis, sinusitis and allergic rhinitis are both notably more prevalent among schoolchildren in air-polluted areas. In a nationwide survey, the prevalence of cedar and cypress pollen allergies was the highest at 26.9% in Yamanashi Prefecture, followed by Nagano, Kochi, and Shizuoka Prefectures. Although it is said that pollen allergies have come to affect children at younger ages, teenagers and younger children are also more likely to have perennial allergic rhinitis. Analysis of the association between allergic rhinitis and sports showed that the prevalence rate was higher among children playing indoor sports and lower among children playing outdoor sports or no sports. The prevalence rate was lower among children who received treatment, health management guidance, and hygiene management guidance. With respect to the association between allergic rhinitis and learning, healthy children had better academic performance records, while placebo-treated children performed worse, and those who received

medication showed varying results, with some children performing well depending on what drugs were administered
SN - 1346-8650
AD - (Shimada) Yamanashi Medical Association, Kofu, Japan
ER -

TY - JOUR
ID - 3734
T1 - Levetiracetam for managing neurologic and psychiatric disorders
A1 - Farooq,M.U.
A1 - Bhatt,A.
A1 - Majid,A.
A1 - Gupta,R.
A1 - Khasnis,A.
A1 - Kassab,M.Y.
Y1 - 2009///
N1 - 2009518967
English
Journal: Review
KW - EMBASE
KW - accidental injury/si [Side Effect]
KW - add on therapy
KW - Aggression
KW - agitation
KW - Alzheimer disease
KW - anorexia/si [Side Effect]
KW - anticonvulsant activity
KW - Anxiety
KW - anxiety disorder/dt [Drug Therapy]
KW - asthenia/si [Side Effect]
KW - ataxia/si [Side Effect]
KW - autism/dt [Drug Therapy]
KW - automutilation/si [Side Effect]
KW - backache/si [Side Effect]
KW - behavior disorder/si [Side Effect]
KW - bipolar disorder/dt [Drug Therapy]
KW - Child
KW - clinical trial
KW - cognitive defect
KW - complex partial seizure/dt [Drug Therapy]
KW - confusion/si [Side Effect]
KW - convulsion/si [Side Effect]
KW - depersonalization/si [Side Effect]
KW - depression/si [Side Effect]
KW - distress syndrome/dt [Drug Therapy]
KW - dizziness/si [Side Effect]
KW - dose kidney function relation
KW - drowsiness/si [Side Effect]
KW - drug clearance
KW - drug distribution
KW - drug dose comparison
KW - drug elimination
KW - drug half life
KW - drug indication
KW - drug metabolism
KW - drug overdose/th [Therapy]
KW - drug protein binding

KW - dyskinesia/dt [Drug Therapy]
KW - Dystonia/dt [Drug Therapy]
KW - epilepsy/dm [Disease Management]
KW - epilepsy/dr [Drug Resistance]
KW - Epilepsy/dt [Drug Therapy]
KW - epileptic state/dt [Drug Therapy]
KW - fatigue/si [Side Effect]
KW - flu like syndrome/si [Side Effect]
KW - focal epilepsy/dt [Drug Therapy]
KW - Gilles de la Tourette syndrome/dt [Drug Therapy]
KW - grand mal epilepsy/dt [Drug Therapy]
KW - hallucination/si [Side Effect]
KW - headache/dt [Drug Therapy]
KW - headache/si [Side Effect]
KW - Hostility
KW - Human
KW - Huntington chorea/dt [Drug Therapy]
KW - hyperactivity/si [Side Effect]
KW - Impulsiveness
KW - infection/si [Side Effect]
KW - insomnia/si [Side Effect]
KW - irritability
KW - mania/dt [Drug Therapy]
KW - mental disease
KW - mental instability/si [Side Effect]
KW - monotherapy
KW - mood disorder/dt [Drug Therapy]
KW - multiple sclerosis/dt [Drug Therapy]
KW - myoclonus epilepsy/dt [Drug Therapy]
KW - nausea/si [Side Effect]
KW - neurologic disease
KW - neuromodulation
KW - neuropathic pain/dt [Drug Therapy]
KW - panic/dt [Drug Therapy]
KW - Parkinson disease/dt [Drug Therapy]
KW - posttraumatic stress disorder/dt [Drug Therapy]
KW - priority journal
KW - psychosis/dt [Drug Therapy]
KW - Quality of Life
KW - review
KW - rhinitis/si [Side Effect]
KW - sedation
KW - side effect/si [Side Effect]
KW - social phobia/dt [Drug Therapy]
KW - somnolence/si [Side Effect]
KW - spasticity/dt [Drug Therapy]
KW - suicidal ideation/dt [Drug Therapy]
KW - suicidal ideation/si [Side Effect]
KW - tardive dyskinesia/dt [Drug Therapy]
KW - tremor/dt [Drug Therapy]
KW - urinary tract infection/si [Side Effect]
KW - urine incontinence/si [Side Effect]
KW - Weight Gain
KW - weight reduction
KW - 4 aminobutyric acid receptor/ec [Endogenous Compound]
KW - AMPA receptor/ec [Endogenous Compound]
KW - antidepressant agent/ae [Adverse Drug Reaction]

KW - antidepressant agent/it [Drug Interaction]
KW - antihistaminic agent/ae [Adverse Drug Reaction]
KW - antihistaminic agent/it [Drug Interaction]
KW - benzodiazepine receptor/ec [Endogenous Compound]
KW - carbamazepine/ae [Adverse Drug Reaction]
KW - carbamazepine/cm [Drug Comparison]
KW - carbamazepine/dt [Drug Therapy]
KW - cytochrome P450 isoenzyme/ec [Endogenous Compound]
KW - digoxin/it [Drug Interaction]
KW - etiracetam/ae [Adverse Drug Reaction]
KW - etiracetam/ct [Clinical Trial]
KW - etiracetam/cm [Drug Comparison]
KW - etiracetam/do [Drug Dose]
KW - etiracetam/it [Drug Interaction]
KW - etiracetam/dt [Drug Therapy]
KW - etiracetam/to [Drug Toxicity]
KW - etiracetam/iv [Intravenous Drug Administration]
KW - etiracetam/po [Oral Drug Administration]
KW - etiracetam/pk [Pharmacokinetics]
KW - etiracetam/pd [Pharmacology]
KW - glycine receptor/ec [Endogenous Compound]
KW - n methyl dextro aspartic acid receptor/ec [Endogenous Compound]
KW - oral contraceptive agent/it [Drug Interaction]
KW - phenytoin/it [Drug Interaction]
KW - placebo
KW - sedative agent/ae [Adverse Drug Reaction]
KW - sedative agent/it [Drug Interaction]
KW - valproic acid/it [Drug Interaction]
KW - warfarin/it [Drug Interaction]
RP - NOT IN FILE
SP - 541
EP - 561
JF - American Journal of Health-System Pharmacy
VL - 66
IS - 6
CY - United States
N2 - Purpose. The role of levetiracetam in different epileptic, nonepileptic, neurologic, and psychiatric disorders is discussed. Summary. Levetiracetam, an antiepileptic drug (AED), was first approved as an adjunctive therapy for the treatment of partial epilepsy in adults. It is currently being used in the treatment of multiple seizure disorders, including generalized tonic-clonic; absence; myoclonic, especially juvenile myoclonic; Lennox-Gastaut syndrome; and refractory epilepsy in children and adults. Data are emerging on possible uses of levetiracetam outside the realm of epilepsy because of its unique mechanisms of action. There is preliminary evidence about the efficacy of levetiracetam in the treatment of different psychiatric disorders, including anxiety, panic, stress, mood and bipolar, autism, and Tourette's syndrome. The most serious adverse effects associated with levetiracetam use are behavioral in nature and might be more common in patients with a history of psychiatric and neurobehavioral problems. Conclusion. Levetiracetam is an effective AED with potential benefits in other neurologic and psychiatric disorders. The benefit-risk ratio in an individual patient with a specific condition should be used to determine its optimal use. Levetiracetam's use in nonepileptic conditions is not recommended until more data become available from larger trials. Copyright 2009, American Society of Health-System Pharmacists, Inc. All rights reserved
SN - 1079-2082
AD - (Farooq, Bhatt, Majid) Division of Cerebrovascular Disorders, Michigan State University, East Lansing, MI, United States (Gupta) Department of Neurology and Ophthalmology, Michigan State University, East Lansing, MI, United States (Kassab) Department of Neurology and Ophthalmology, Division of Cerebrovascular Disorders, Michigan State University (Farooq) Department of Neurology and Ophthalmology, Michigan State University, A 217, 138 Service Road, East Lansing, MI 48824, United States
ER -

TY - JOUR
ID - 3736
T1 - The Ponseti method of treatment of congenital clubfoot - First experiences
A1 - Chomiak,J.
A1 - Frydrychova,M.
A1 - Ostadal,M.
A1 - Matejicek,M.
Y1 - 2009///
N1 - 2009369325
Czech
Journal: Article
KW - EMBASE
KW - article
KW - brace
KW - Child
KW - clubfoot/th [Therapy]
KW - controlled study
KW - family attitude
KW - Female
KW - Human
KW - Infant
KW - major clinical study
KW - Male
KW - orthopedic shoe
KW - Patient Compliance
KW - plaster cast
KW - ponseti method
KW - preschool child
KW - splint
KW - technique
KW - therapy delay
KW - tibialis anterior muscle
KW - Treatment Outcome
RP - NOT IN FILE
SP - 194
EP - 201
JF - Acta Chirurgiae Orthopaedicae et Traumatologiae Cechoslovaca
JA - Acta Chir Orthop Traumatol Cech.
VL - 76
IS - 3
CY - Czech Republic
N2 - PURPOSE OF THE STUDY: To provide a detailed description of the Ponseti method and report the first results of its use, including factors that played a role. MATERIAL AND METHODS: In the 2005-2007 period, 91 patients with idiopathic rigid clubfoot (133 feet) were treated by the Ponseti method. The group comprised 62 boys and 29 girls. In most patients the Ponseti method was used as primary treatment, or by 3 months of age when previous treatment failed. In five children this treatment was started between the 3rd and 8th months of age. The result were evaluated by the criteria described by Richards et al., who distinguished four groups. The result was regarded as good when a permanent plantigrade foot was achieved (group 1). Plantigrade feet likely to require posterior release later were considered indeterminate rusults (group 2). Feet that needed posterior release, anterior tibial muscle transfer or lateral column shortening fell in the fair result group (3). Feet requiring complete subtalar release were classified as poor results (group 4). The results achieved in each year were statistically evaluated using Fisher's test ($p<0.05$). RESULTS: The overall evaluation for 3 years showed good results in 70 %, indeterminate in 7.5 %, fair in 6.76 % and poor in 15.8 % of the treated feet. A detailed analysis for each year revealed that, in 2005, good results (50 %) were recorded in a significantly lower number of feet than in 2006 (72.2 %; $p=0.032$) and 2007 (93 %; $p<0.001$). On comparison of the years 2006 and 2007, good results in 2007 were found in a significantly higher number of feet than in 2006 ($p=0.019$). The poor results

were due to 1) very rigid feet (6 %); 2) initial problems with availability of Denis-Brown splints (19 feet; 14.5 %); 3) problems with shoes not made to custom and not fitting patient's little feet (20 feet; 15 %) 4) faulty techniques of correcting the deformity (4 feet); 5) poor family cooperation in compliance with the bracing protocol (15 feet; 11.2 %). Some of the factors were combined. A delayed beginning of the treatment had no significant effect on the results. DISCUSSION: Our 3-year results of clubfoot treatment, by which plantigrade foot position was acheved on average in 77.5 % of the patients, are in agreement with those achieved outside Ponseti centres. However, there were clear differences, with the worst results in 2005. The results comparable with those of Ponseti and his co-workers were achieved by us only in 2007. In accordance with the findings of Richards et al. we suggest that the percentage of short-term good results can change insignificantly within 4 years because of increased recurrence of deformities. CONCLUSIONS: Although our initial results were worse than reported in the literature, it can be concluded that the Ponseti method of treating idiopathic clubfoot is more efficient that the methods used previously and can be recommended as an efficient, safe and economical technique. Good compliance with the protocol improves the therapeutic results
SN - 0001-5415
AD - (Chomiak, Frydrychova, Ostadal, Matejicek) Ortopedicka Klinika IPVZ, 1. LF UK, FN Na Bulovce, Budinova 2, 180 81 Praha 8
ER -

TY - JOUR
ID - 3737
T1 - Epidemiology of drowning in Isfahan province, center of Iran
A1 - Sheikhazadi,A.
A1 - Ghadyani,M.H.
Y1 - 2009///
N1 - 2009333166
English
Journal: Article
KW - EMBASE
KW - accidental death
KW - Adolescent
KW - Adult
KW - age
KW - Aged
KW - alcohol consumption
KW - article
KW - Child
KW - controlled study
KW - Demography
KW - drowning
KW - Educational Status
KW - Employment Status
KW - falling
KW - Female
KW - Health Care Policy
KW - health program
KW - Health Status
KW - Homicide
KW - Human
KW - Incidence
KW - Iran
KW - lake
KW - major clinical study
KW - Male
KW - Morbidity
KW - Mortality
KW - pond
KW - preschool child

KW - river
KW - school child
KW - seasonal variation
KW - Sex Ratio
KW - Suicide
KW - Swimming
RP - NOT IN FILE
SP - 79
EP - 87
JF - Journal of Research in Medical Sciences
VL - 14
IS - 2
CY - Iran, Islamic Republic of
N2 - Background: Drowning is a preventable cause of morbidity and mortality. We studied drowning deaths in Isfahan province, focusing on the activity victims were involved in and the setting of the incident. Methods: Retrospective analysis was done on all drowning deaths referred to Legal Medicine Organization of Isfahan province over a period of 5 years from 1 January 2002 to 31 December 2006. Results: During these 5 years, 355 persons suffered fatal drowning in Isfahan province. The annual incidence rate was 1.6 per 100000 population (2.6 & 0.4 for males and females, respectively). Mean age was 21.99 + 14.86 and M/F ratio was 6.5:1. Most victims were 15-24 and 5-14 years of age (172 and 62 decedents, 3.0 and 1.8 per 100000 population/year, respectively). Most incidents occurred in natural settings, of which rivers, canals, lakes and ponds were the most common sites, respectively. The most frequently connected activity was swimming and wading, followed by accidental fall into water. The manner of death was found as following: 85.1% accidental, 1.7% suicidal, 0.3% homicidal and 12.9% undetermined. Conclusions: Although the drowning related mortality in Isfahan province is comparable to developed countries, effective prevention of drowning with better programming and policies might reduce its rate
SN - 1735-1995
AD - (Sheikhazadi) Department of Forensic Medicine, School of Medicine, Tehran University of Medical Sciences, Tehran, Iran, Islamic Republic of (Ghadyani) Legal Medicine Organization of Iran, Tehran, Iran, Islamic Republic of
ER -

TY - JOUR
ID - 3738
T1 - Sevoflurane inside and outside the operating room
A1 - Michel,F.
A1 - Constantin,J.-M.
Y1 - 2009///
N1 - 2009322568
English
Journal: Review
KW - EMBASE
KW - Adult
KW - agitation
KW - amnesia/si [Side Effect]
KW - bradycardia/si [Side Effect]
KW - brain ischemia/dt [Drug Therapy]
KW - cardiovascular disease/si [Side Effect]
KW - Child
KW - coughing/si [Side Effect]
KW - delirium/dt [Drug Therapy]
KW - delirium/pc [Prevention]
KW - drug activity
KW - drug distribution
KW - drug elimination
KW - drug excretion
KW - drug half life

KW - drug mechanism
KW - drug metabolism
KW - drug receptor binding
KW - drug structure
KW - drug tolerability
KW - Epilepsy/dt [Drug Therapy]
KW - general anesthesia
KW - heart arrhythmia/si [Side Effect]
KW - heart muscle contractility
KW - Hemodynamics
KW - hepatitis/si [Side Effect]
KW - Human
KW - hypnosis
KW - hypotension/si [Side Effect]
KW - Immobilization
KW - Inhalation
KW - intensive care unit
KW - larynx spasm/si [Side Effect]
KW - low drug dose
KW - malignant hyperthermia/dt [Drug Therapy]
KW - malignant hyperthermia/si [Side Effect]
KW - myocardial disease/si [Side Effect]
KW - nephrotoxicity/si [Side Effect]
KW - nonhuman
KW - nuclear magnetic resonance imaging
KW - operating room
KW - Pain
KW - Pediatrics
KW - physical chemistry
KW - postoperative nausea and vomiting/si [Side Effect]
KW - postoperative pain/dt [Drug Therapy]
KW - respiration depression/si [Side Effect]
KW - respiratory tract disease/si [Side Effect]
KW - review
KW - sedation
KW - side effect/si [Side Effect]
KW - tachycardia/si [Side Effect]
KW - 4 aminobutyric acid receptor/ec [Endogenous Compound]
KW - chloral hydrate/dt [Drug Therapy]
KW - clonidine/dt [Drug Therapy]
KW - dantrolene/dt [Drug Therapy]
KW - dantrolene/iv [Intravenous Drug Administration]
KW - desflurane/ae [Adverse Drug Reaction]
KW - desflurane/cm [Drug Comparison]
KW - desflurane/dt [Drug Therapy]
KW - desflurane/ih [Inhalational Drug Administration]
KW - dexmedetomidine/dt [Drug Therapy]
KW - enflurane/ae [Adverse Drug Reaction]
KW - enflurane/dt [Drug Therapy]
KW - fluoride ion/ae [Adverse Drug Reaction]
KW - fluoride ion/dt [Drug Therapy]
KW - fluoride ion/pk [Pharmacokinetics]
KW - halothane/ae [Adverse Drug Reaction]
KW - halothane/cm [Drug Comparison]
KW - halothane/dt [Drug Therapy]
KW - isoflurane/ae [Adverse Drug Reaction]
KW - isoflurane/cm [Drug Comparison]

KW - isoflurane/dt [Drug Therapy]
KW - isoflurane/ih [Inhalational Drug Administration]
KW - ketamine/do [Drug Dose]
KW - ketamine/dt [Drug Therapy]
KW - methoxyflurane/dt [Drug Therapy]
KW - methoxyflurane/pk [Pharmacokinetics]
KW - midazolam/cm [Drug Comparison]
KW - midazolam/dt [Drug Therapy]
KW - neurotransmitter/ec [Endogenous Compound]
KW - nitrous oxide/dt [Drug Therapy]
KW - nitrous oxide/ih [Inhalational Drug Administration]
KW - propofol/ae [Adverse Drug Reaction]
KW - propofol/cm [Drug Comparison]
KW - propofol/dt [Drug Therapy]
KW - propofol/iv [Intravenous Drug Administration]
KW - sevoflurane/ae [Adverse Drug Reaction]
KW - sevoflurane/an [Drug Analysis]
KW - sevoflurane/cm [Drug Comparison]
KW - sevoflurane/dt [Drug Therapy]
KW - sevoflurane/ih [Inhalational Drug Administration]
KW - sevoflurane/pk [Pharmacokinetics]
KW - sevoflurane/pd [Pharmacology]
KW - suxamethonium/ae [Adverse Drug Reaction]
KW - suxamethonium/dt [Drug Therapy]
RP - NOT IN FILE
SP - 861
EP - 873
JF - Expert Opinion on Pharmacotherapy
VL - 10
IS - 5
CY - United Kingdom
N2 - Background: Sevoflurane is often presented as a near-perfect anaesthetic. After 10 years in the operating room, new uses are emerging outside. Objective: To remind readers of the principal characteristics of sevoflurane, to affirm its usefulness for day-case anaesthesia and to consider the recent new uses. Methods: The discussion of the physical properties, pharmacokinetics, metabolism, mechanisms of action and clinical effects is based on classic, essential papers. Recent literature concerning emerging utilizations of sevoflurane was analysed. Results: Sevoflurane presents many benefits with minimum inconvenience. It allows rapid inhalation induction, maintenance and rapid recovery. It has little toxicity and its haemodynamic and respiratory depressive effects are moderate and well tolerated. It is already widely use for sedation for magnetic resonance imaging in children. Its use in paediatric or adult intensive care could improve the management of pain and sedation. 2009 Informa UK Ltd. All rights reserved
SN - 1465-6566
AD - (Michel) University of the Mediterranean, Faculty of Medicine, North University Hospital, 13915 Marseille Cedex 20, France (Constantin) CHU Clermont-Ferrand Hopital Hotel-Dieu, Service de Reanimation Adultes et Unite de Transplantation, Pole Anesthesie-Reanimation HD, Clermont-Ferrand, France
ER -

TY - JOUR
ID - 3741
T1 - Increased activity of coxsackievirus B1 strains associated with severe disease among young infants in the United States, 2007-2008
A1 - Wikswo,M.E.
A1 - Khetsuriani,N.
A1 - Fowlkes,A.L.
A1 - Zheng,X.
A1 - Penaranda,S.
A1 - Verma,N.

A1 - Shulman,S.T.
A1 - Sircar,K.
A1 - Robinson,C.C.
A1 - Schmidt,T.
A1 - Schnurr,D.
A1 - Steven,Oberste M.
Y1 - 2009///
N1 - 2009483014
English
Journal: Article
KW - EMBASE
KW - Adolescent
KW - article
KW - Child
KW - Coxsackie virus B
KW - coxsackie virus b1
KW - disease surveillance
KW - Enterovirus infection/ep [Epidemiology]
KW - Enterovirus infection/et [Etiology]
KW - Human
KW - major clinical study
KW - molecular typing
KW - Newborn
KW - newborn death
KW - newborn disease/ep [Epidemiology]
KW - newborn disease/et [Etiology]
KW - nucleotide sequence
KW - Phylogeny
KW - priority journal
KW - school child
KW - unindexed sequence
KW - United States
KW - virus characterization
KW - virus isolation
KW - virus strain
RP - NOT IN FILE
SP - e44
EP - e51
JF - Clinical Infectious Diseases
JA - Clin Infect Dis
VL - 49
IS - 5
CY - United States
N2 - Background. Enterovirus infections are very common and typically cause mild illness, although neonates arc at higher risk for severe illness. In 2007, the Centers for Disease Control and Prevention (CDC) received multiple reports of severe neonatal illness and death associated with coxsackievirus B1 (CVBl), a less common enterovirus serotype not previously associated with death in surveillance reports to the CDC. Methods. This report includes clinical, epidemiologic, and virologie data from cases of severe neonatal illness associated with CVB1 reported during the period from 2007 through 2008 to the National Enterovirus Surveillance System (NESS), a voluntary, passive surveillance system. Also included are data on additional cases reported to the CDC outside of the NESS. Virus isolates or original specimens obtained from patients from 25 states were referred to the CDC Picornavirus laboratory for molecular typing or characterization. Results. During 2007-2008, the NESS received 1079 reports of enterovirus infection. CVB1 accounted for 176 (23%) of 775 reported cases with known serotype, making it the most commonly reported serotype for the first time ever in the NESS. Six neonatal deaths due to CVB1 infection were also reported to the CDC during that time. Phylogenctic analysis of the 2007 and 2008 CVB1 strains indicated that the increase in cases resulted from widespread circulation of a single genetic lineage that had been present in the United States since at least 2001. Conclusions.

Healthcare providers and public health departments should be vigilant to the possibility of continuing CVB1-associated neonatal illness, and testing and continued reporting of enterovirus infections should be encouraged. 2009 by the Infectious Diseases Society of America. All rights reserved
SN - 1058-4838
AD - (Wikswo, Khetsuriani, Fowlkes, Penaranda, Steven Oberste) Div. of Viral Diseases, National Center for Immunization and Respiratory Diseases, Centers for Disease Control and Prevention, 1600 Clifton Rd., NE, Mailstop A-34, Atlanta, GA 30333, United States (Sircar) Epidemiologic Intelligence Service, Centers for Disease Control and Prevention, Atlanta GA, United States (Zheng) Departments of Pathology, Laboratory Medicine (Verma, Shulman) Pediatrics, Children's Memorial Hospital (Zheng, Verma, Shulman) Northwestern University, Feinberg School of Medicine, Chicago IL, United States (Sircar) Los Angeles County Department of Public Health, Los Angeles, United States (Schnurr) California Department of Public Health, Richmond CA, United States (Robinson) Department of Pathology, Laboratory Medicine, The Children's Hospital, Aurora CO, United States (Schmidt) Alaska State Public Health Virology Laboratory, Fairbanks AK, United States
ER -

TY - JOUR
ID - 3743
T1 - Childhood injuries seen at Emergency Department DHQ Hospital Rawalpindi in 2007
A1 - Hanif,M.
A1 - Hamid,A.
A1 - Rashid,M.A.
A1 - nwaar Alvina Uz,Zaman F.
Y1 - 2009///
N1 - 2009196785
English
Journal: Article
KW - EMBASE
KW - Adolescent
KW - Age Distribution
KW - arm injury
KW - article
KW - Child
KW - childhood injury
KW - controlled study
KW - emergency ward
KW - falling
KW - Female
KW - head injury
KW - Human
KW - Infant
KW - major clinical study
KW - Male
KW - Pakistan
KW - retrospective study
RP - NOT IN FILE
SP - 32
EP - 35
JF - Medical Forum Monthly
VL - 20
IS - 1
CY - Pakistan
N2 - Objective:- To determine the frequency & pattern of injuries in children less than 15 years of age. Material & Methods:- 500 oases are studied in children less than 15 years of age at Emergency Department, DHQ Hospital Rawalpindi. Place of Study: Emergency Department DHQ Hospital Rawalpindi. Duration of Study: Retrospective study to determine the relative frequency and pattern of injuries in children less than 15 yrs of age. Results:- A total number of 500 children were seen in ED and ages ranged from 1 month to 15 years. There were 321 males & 179 Females representing about 61.2% & 35.8% female respectively with ratio of about 2: 1.

A total no of 210 children were injured by fall. In 0-5 year age group fall is either from stair furniture, beds or walker. In 5-15 years age group the playground injuries like fall from owings, slide & sea saws. The injuries were mostly on head and upper limbs
SN - 1029-385X
AD - (Hanif, Anwaar) FLJMC, Rawalpindi, Pakistan (Hamid, Alvina) FMC, Abbottabad, Pakistan (Rashid) PGMI, Lahore, Pakistan (Uz Zaman) GMC, Dera Ismail Khan, Pakistan
ER -

TY - JOUR
ID - 3745
T1 - Dietary habits and physical activity after one year of health intervention in the school: A pilot study
A1 - Espejo Garcia,M.P.
A1 - Vazquez Garcia,M.D.
A1 - Benedi,Curiel,V
A1 - Lopez Lacoma,J.C.
Y1 - 2009///
N1 - 2009122240
Spanish
Journal: Article
KW - EMBASE
KW - Adolescent
KW - article
KW - Child
KW - controlled study
KW - eating habit
KW - health program
KW - health survey
KW - Human
KW - nutritional assessment
KW - Physical Activity
KW - preschool child
KW - school
KW - school child
KW - sitting
RP - NOT IN FILE
SP - 21
EP - 25
JF - Acta Pediatrica Espanola
VL - 67
IS - 1
CY - Spain
N2 - Introduction: Unsuitable dietary habits and sedentary life are perhaps the main causes of the alarming increase in childhood obesity, and they are closely linked to eating disorders. The school is an ideal place to foster healthier lifestyles. Materials and methods: This is a community-based, interventional, longitudinal, prospective study for the purpose of measuring the improvement in eating habits and physical activity after a year of health education in two schools during the school year 2003-2004. Five hundred one school children ranging in age from 3 to 16 years took part in the project. Surveys were designed and were completed before and after the program. The intervention was designed for children, parents and teachers. Results: We must highlight the low intake of legumes, fruits, vegetables and fish. Of the children who took part in the project, 5.1 % attended class without having had breakfast. The improvements achieved after the program were: at breakfast, a decrease in the intake of industrial bakery and pastry products (p <0.000); at lunch time, a decrease in the consumption of snacks (p <0.000) and, in mid-afternoon, the intake of industrial baked goods, soft drinks (juices not included) and snacks also decreased. In all, 40% of the children involved do not practice any physical activity outside of school and a large number of them watch TV for more than two hours. Despite this fact, we have observed a decrease in the time spent watching TV (p <0.005). Conclusions: After a year of health education in the school, we find little improvement regarding eating habits. It is necessary to promote physical

activity at an early age and reduce the time spent in front of the TV, video games and computers. The school is the best place to develop these programs, but getting parents involved in this task is difficult
SN - 0001-6640
AD - (Espejo Garcia) Servicio de Pediatra, Centro de Salud de Barrio Peral, Cartagena (Murcia), Spain (Vazquez Garcia, Benedi Curiel) Servicio de Medico de Familia, Centro de Salud de Barrio Peral, Cartagena (Murcia), Spain (Lopez Lacoma) Servicio de Enfermero, Centro de Salud de Barrio Peral, Cartagena (Murcia), Spain (Espejo Garcia) Ulloa, s/n, 30300 Cartagena (Murcia), Spain
ER -

TY - JOUR
ID - 3746
T1 - Six-year dialysis freedom in end-stage renal disease
A1 - Al Mosawi,A.J.
Y1 - 2009///
N1 - 2009525512
English
Journal: Article
KW - EMBASE
KW - Adolescent
KW - Anemia
KW - article
KW - Body Composition
KW - Case Report
KW - Child
KW - Female
KW - Human
KW - kidney failure/dt [Drug Therapy]
KW - Male
KW - Peritoneal Dialysis
KW - renal osteodystrophy
KW - school child
KW - uremia
KW - gum arabic/dt [Drug Therapy]
KW - Urea
RP - NOT IN FILE
SP - 494
EP - 500
JF - Clinical and Experimental Nephrology
VL - 13
IS - 5
CY - Japan
N2 - Background: Patients with end-stage renal disease (ESRD) cannot sustain life in the absence of renal replacement therapy (RRT). However, a 4-year dialysis freedom with improved well-being has been reported in ESRD using a new therapeutic approach combining conservative measures and acacia gum (AG) supplementation. The aim of this paper is to report the achievement of 6-year dialysis freedom. Patients and methods: During December 2001, six patients with ESRD and significant uremia that required at least one dialysis session to maintain life were enrolled in a clinical trial investigating the use of a new therapeutic approach combining conservative measures and AG supplementation aiming at improving well-being and providing patients with ESRD dialysis freedom. Three patients were treated with this approach. One patient complied with protocol for only 10 days and died after 6 months peritoneal dialysis. Two patients completed 1 year on this therapeutic regimen. Both patients reported improved well-being. Neither became acidotic nor uremic, and neither required dialysis during the 1 year of the study period. Both patients maintained serum creatinine and urea levels not previously achieved without dialysis. The other three patients were managed with intermittent peritoneal dialysis (IPD). All died within less than 6 months. Of the two surviving patients on AG supplementation, one patient stopped AG supplementation after 1 year and died within 1 month despite IPD. The other patient continued to be treated with this novel approach and continued to experience improved well-being and dialysis freedom. The aim of this paper is to report the achievement of 6-year dialysis freedom in this

patient. Results: During 6 years of therapy the girl continued in experiencing improved well-being and good participation in outdoor activities. Mild uremic symptoms occurred only during periods of noncompliance. Periods of decreased compliance with pharmacologic therapies were associated with anemia and renal osteodystrophy and some degree genue vulgum has resulted. Conclusion: It was possible to address ESRD in this particular patient as a disease process that can be treated by totally different medical treatment approaches without the use of either chronic dialysis or transplant. 2009 Japanese Society of Nephrology
SN - 1342-1751
AD - (Al Mosawi) Department of Pediatrics, University Hospital in Al Kadhimiyia, PO Box 70025, Baghdad, Iraq
ER -

TY - JOUR
ID - 3747
T1 - Removal of inhaled foreign bodies-Middle of the night or the next morning?
A1 - Mani,N.
A1 - Soma,M.
A1 - Massey,S.
A1 - Albert,D.
A1 - Bailey,C.M.
Y1 - 2009///
N1 - 2009309605
English
Journal: Article
KW - EMBASE
KW - Adolescent
KW - airway obstruction
KW - article
KW - breathing exercise
KW - Bronchoscopy
KW - bronchus
KW - Child
KW - clinical feature
KW - conservative treatment
KW - coughing
KW - cyanosis
KW - epiglottis
KW - Female
KW - foreign body aspiration/di [Diagnosis]
KW - foreign body aspiration/dt [Drug Therapy]
KW - foreign body aspiration/th [Therapy]
KW - hoarseness
KW - Human
KW - Infant
KW - larynx
KW - larynx surgery
KW - lower respiratory tract infection/co [Complication]
KW - lower respiratory tract infection/dt [Drug Therapy]
KW - lung collapse/co [Complication]
KW - major clinical study
KW - Male
KW - Morbidity
KW - Preoperative Care
KW - priority journal
KW - stridor
KW - surgical technique
KW - thorax
KW - Time

KW - trachea
KW - wheezing
KW - antibiotic agent/dt [Drug Therapy]
KW - steroid/dt [Drug Therapy]
RP - NOT IN FILE
SP - 1085
EP - 1089
JF - International Journal of Pediatric Otorhinolaryngology
JA - Int J Pediatr Otorhinolaryngol
VL - 73
IS - 8
CY - Ireland
N2 - Objective: Foreign body inhalation is a potentially life-threatening emergency and is the commonest cause of accidental death in children under one year old. There is varying opinion regarding the urgency for removal of inhaled foreign bodies; most centres in the United Kingdom will take the patient to theatre as soon as can be arranged, regardless of the time of day. At Great Ormond Street Hospital for children it has been standard practice to perform rigid bronchoscopy and removal of an inhaled foreign body on the next available daytime operating list, providing the patient is clinically stable, even if this incurs a delay until the following day. We aimed to identify if any additional morbidity resulted from delaying removal of the foreign body. Methods: Retrospective case note review of all cases of foreign body inhalation seen at our institution over an 11-year period between July 1996 and July 2007. Results: 165 patients were referred to our institution with a suspected inhaled foreign body in the study period. 14 patients were managed conservatively due to low clinical suspicion and 57 patients underwent a negative bronchoscopy: these groups were excluded from our analysis. Of the remaining 94 patients, only 7 were taken to theatre outside a scheduled daytime operating list. All of these patients had signs of severe respiratory distress and were operated upon within 4 h of their arrival. Of the remaining patients, 41 were taken to theatre on the day of presentation and 46 on the day after presentation; all within normal daytime working hours. No additional post-operative morbidity was identified as a result of our policy to delay bronchoscopy until the next available daytime operating list. Conclusions: It is our perception that delaying removal of suspected inhaled foreign bodies to allow optimal circumstances for manipulation of the paediatric airway is a safe practice at our institution. We have not identified any adverse outcomes related to delaying bronchoscopy to the next available daytime operating list in the clinically stable patient. This remains our preferred method of practice. 2009 Elsevier Ireland Ltd. All rights reserved
SN - 0165-5876
AD - (Mani, Soma, Massey, Albert, Bailey) Great Ormond Street Hospital for Children NHS Trust, Great Ormond Street, London, WC1N 3JH, United Kingdom
ER -

TY - JOUR
ID - 3748
T1 - Increasing play and decreasing the challenging behavior of children with autism during recess with activity schedules and task correspondence training
A1 - Machalicek,W.
A1 - Shogren,K.
A1 - Lang,R.
A1 - Rispoli,M.
A1 - O'Reilly,M.F.
A1 - Franco,J.H.
A1 - Sigafoos,J.
Y1 - 2009///
N1 - 2009092390
English
Journal: Article
KW - EMBASE
KW - article
KW - autism/th [Therapy]
KW - behavior disorder
KW - Behavior Therapy

KW - Case Report
KW - Child
KW - Child Behavior
KW - education program
KW - Human
KW - intervention study
KW - learning style
KW - leisure
KW - Male
KW - play
KW - preschool child
KW - priority journal
KW - school child
KW - Special Education
KW - Teaching
RP - NOT IN FILE
SP - 547
EP - 555
JF - Research in Autism Spectrum Disorders
VL - 3
IS - 2
CY - United Kingdom
N2 - We examined the effects of a teacher implemented playground intervention consisting of activity schedules and task correspondence training on the challenging behaviors and play of three school age children with moderate to severe autism. A multiple baseline design across participants was used to evaluate the intervention effects. Results indicated that each of the participants learned to use an activity schedule to follow a schedule of play activities and demonstrated improved play. Moreover, challenging behavior decreased for two participants during the intervention and remained at low levels for the third participant. The findings from this study provide support for the use of activity schedules and task correspondence training to reinforce appropriate play and decrease challenging behaviors during recess. 2008 Elsevier Ltd. All rights reserved
SN - 1750-9467
AD - (Machalicek) Department of Special Education, GSE, Portland State University, PO Box 751, Portland, OR 97207, United States (Shogren, Lang, Rispoli, O'Reilly, Franco) The Meadows Center for Preventing Educational Risk, The University of Texas at Austin, United States (Sigafoos) Victoria University, New Zealand
ER -

TY - JOUR
ID - 3749
T1 - Personal exposure of children to air pollution
A1 - Ashmore,M.R.
A1 - Dimitroulopoulou,C.
Y1 - 2009///
N1 - 2008550972
English
Journal: Article
KW - EMBASE
KW - air pollutant
KW - Air Pollution
KW - air quality
KW - ambient air
KW - Architecture
KW - article
KW - building
KW - Child
KW - child health
KW - energy consumption

KW - Environmental Exposure
KW - Epidemiology
KW - Home Environment
KW - Human
KW - indoor air pollution
KW - microenvironment
KW - North America
KW - Particulate Matter
KW - Physical Activity
KW - pollution control
KW - pollution monitoring
KW - priority journal
KW - process model
KW - room ventilation
KW - school
KW - smog
KW - Social Isolation
KW - socioeconomics
KW - traffic and transport
KW - Travel
KW - Urbanization
KW - Western Europe
KW - benzene
KW - carbon monoxide
KW - nitrogen dioxide
KW - sulfur dioxide
KW - volatile organic compound
RP - NOT IN FILE
SP - 128
EP - 141
JF - Atmospheric Environment
VL - 43
IS - 1
CY - United Kingdom
N2 - Changes over recent decades in outdoor concentrations of air pollutants are well documented. However, the impacts of air pollution on an individual's health actually relate not to these outdoor concentrations but to their personal exposure in the different locations in which they spend time. Assessing how personal exposures differ from outdoor concentrations, and how they have changed over recent decades, is challenging. This review focuses on the exposure of children, since they are a particularly sensitive group. Much of children's time is spent indoors, and childhood exposure is closely related to concentrations in the home, at school, and in transport. For this reason, children's personal exposures to air pollutants differ significantly from both those of adults and from outdoor concentrations. They depend on a range of factors, including urbanisation, energy use, building design, travel patterns, and activity profiles; analysis of these factors can identify a wider range of policy measures to reduce children's exposure than direct emission control. There is a very large variation in personal exposure between individual children, caused by differences in building design, indoor and outdoor sources, and activity patterns. Identifying groups of children with high personal exposure, and their underlying causes, is particularly important in regions of the world where emissions are increasing, but there are limited resources for environmental and health protection. Although the science of personal exposure assessment, with the associated measurement and modelling techniques, has developed to maturity in North America and western Europe over the last 50 years, there is an urgent need to apply this science in other parts of the world where the effects of air pollution are now much more serious. 2008 Elsevier Ltd. All rights reserved
SN - 1352-2310
AD - (Ashmore) Environment Department, University of York, York, YO10 5DD, United Kingdom
(Dimitroulopoulou) BRE Environment, Building Research Establishment Ltd, United Kingdom
ER -

TY - JOUR

ID - 3750
T1 - Access to medicines in primary care for paediatric patients: Involvement of hospital clinical nurse specialists and advanced nurse practitioners
A1 - Terry,D.R.P.
A1 - Sinclair,A.G.
A1 - Marriott,J.F.
A1 - Wilson,K.A.
A1 - Daniels,A.
Y1 - 2009///
N1 - 70139951
English
Journal: Conference Abstract
KW - EMBASE
KW - patient
KW - Hospital
KW - primary medical care
KW - clinical nurse specialist
KW - nurse practitioner
KW - nurse
KW - central nervous system
KW - Drug Therapy
KW - prescription
KW - Work
KW - Responsibility
KW - consultation
KW - Child
KW - community
KW - pharmacy
KW - Pediatrics
KW - general practitioner
KW - e-mail
KW - questionnaire
KW - Risk
KW - pharmacist
KW - health care cost
KW - pediatric hospital
RP - NOT IN FILE
SP - B40
JF - International Journal of Pharmacy Practice
VL - Conference: British Pharmaceutical Conference 2009 Manchester United Kingdom. Conference Start: 20090906 Conference End: 20090909. Conference Publication:
IS - var.pagings
N2 - Introduction and Objectives: Children and their carers may experience difficulties in obtaining continuing prescribed medicines whilst in primary care. Issues include who will prescribe continuing medication (general practitioner (GP) or consultant) and problems in obtaining appropriate medication from community pharmacies. These issues may be enhanced where the required medicines are unlicensed or used 'off-label', which is frequent in paediatrics.^[1] This study describes the role senior hospital nurses undertake in response to medication issues for patients residing in primary care. Methods An electronic survey tool was developed and created using LimeSurvey version 1.71+(5498). The development included discussion with clinical nurse specialist/advanced nurse practitioners (CNS/ANPs) and was piloted before use. The survey was tokenised to ensure that one invite elicited one response only. The invitation to participate was sent via e-mail with a link to the study to the CNS and ANP of Birmingham Children's Hospital commencing September 2008. Two reminders were sent if necessary at weekly intervals. Responses were entered into MS Excel 2003 for analysis. Results and Discussion A 78% response rate was obtained from study cohort of 100 nurses. Nurses from 23 specialties completed the questionnaire with 49 nurses (63%) working more than 30.1 h per week (0.8 whole time equivalent), nine as prescribers. A majority (n = 57, 73%) reported that in the 3 months preceding the study, they were called upon to organise repeat prescriptions at least once, with 28 (36%) doing so on 6 or more

occasions, and 55 (70%) of respondents organised repeat prescriptions for at least 514 patients over the 3-month period. Fifty nurses (64%) described the risk to patients if they did not assist patients in accessing medicines as significant or highly significant, Only 10 nurses (12.8%) considered that this activity was included in their job descriptions. The respondents indicated that the most common reason for such problems was prescriber issue(s). The respondents gave a range of responses when asked in what ways services can be better organised including require GPs to prescribe all continuing medicines (59% frequency) and require hospitals to prescribe and dispense unlicensed/uncommon medicines (46% frequency). Conclusions Hospital CNS and ANP report frequent interventions to assist patients outside hospital in obtaining the medicines they need. Prescriber-related issues were the most common reason cited for these problems. Further work will be required to identify the rational for this opinion. Hospital CNS and ANP provide an important service to patients in primary care although this may not be reflected in their job description. Pharmacists in all healthcare sectors will benefit from being aware of this activity. The findings of this study suggest that responsibility for prescribing between hospitals and GPs described in 1991^[2] may benefit from review
SN - 0961-7671
AD - (Terry, Sinclair) Department of Pharmacy, Birmingham Children's Hospital, Birmingham, United Kingdom (Marriott, Wilson) School of Pharmacy, Aston University, Birmingham, United Kingdom (Daniels) Department of Cardiology, Birmingham Children's Hospital, Birmingham, United Kingdom
ER -

TY - JOUR
ID - 3751
T1 - In vitro toxicity and molecular epidemiology of indoor Aspergillus versicolor
A1 - Majorosova,M.
A1 - Pieckova,E.
A1 - Pangallo,D.
Y1 - 2009///
N1 - 70114785
English
Journal: Conference Abstract
KW - EMBASE
KW - in vitro study
KW - toxicity
KW - Aspergillus
KW - molecular epidemiology
KW - health care organization
KW - toxicology
KW - metabolite
KW - organ culture
KW - fungus
KW - trachea mucosa
KW - species
KW - Environment
KW - Child
KW - Slovakia
KW - airway
KW - bioassay
KW - chick
KW - vulnerable population
KW - Clothing
KW - micromycete
KW - upper respiratory tract
KW - toxin
KW - chloroform
RP - NOT IN FILE
SP - S80
JF - Toxicology Letters
JA - Toxicol Lett

VL - Conference: 46th Congress of the European Societies of Toxicology, EUROTOX 2009 Dresden Germany. Conference Start: 20090913 Conference End: 20090916 Sponsor: Deutsche Forschungsgemeinschaft
IS - DFG
N2 - In vitro toxicity of complex chloroform-extractable endo- and exo-metabolites of 10 indoor, and related outdoor, A. versicolor isolates from a heavily mouldy kids' fashion store in Slovakia with complaints of occupants on irritation of their airways - has been evaluated by a bioassay with 1-day-old chicks' tracheal organ cultures (20 mug of toxicants per mL of cultivation medium). Searching for their possible source, molecular epidemiological study of the isolates was performed using RAMP PCR. Strains colonizing indoor walls of the shop were the highest correlated to the outdoor airborne ones (Pearson correlation 97%). While indoor airborne isolates correlated to the strains growing on retailed clothes at the levels of 90% or 86% according to Pearson. All micromycetes produced secondary metabolites that ceased ciliary beating in tracheal epithelium in the organ cultures already in 24 h of the activity, i.e. in the sense of the method used, they belong to strong toxicants. Two of the isolates tested so far produced also extrolites without toxic effects detectable by the method. It has been proven that toxin production in fungi depends not only onto the species but may vary between every single isolates as well. The most important outcome of the study is that microscopic filamentous fungi present in the dwelling indoor environment under Slovak (Central European) building/housing conditions might produce compounds even with a strong potential to damage upper airways of occupants, while children remain the most vulnerable population
SN - 0378-4274
AD - (Majorosova, Pieckova) Slovak Medical University, Mycology, Bratislava, Slovakia (Pangallo) Institute of Molecular Biology, Slovak Academy of Sciences, Bratislava, Slovakia
ER -

TY - JOUR
ID - 3752
T1 - Toxic and inflammatory effects of particulate matter PM10 sampled during teaching hours in elementary school classrooms
A1 - Buters,J.
A1 - Oeder,S.
A1 - Weichenmeier,I.
A1 - Schober,W.
A1 - Dietrich,S.
A1 - Fromme,H.
A1 - Behrendt,H.
Y1 - 2009///
N1 - 70114654
English
Journal: Conference Abstract
KW - EMBASE
KW - health care organization
KW - primary school
KW - Particulate Matter
KW - toxicology
KW - Teaching
KW - toxicity
KW - Human
KW - metabolic activation
KW - gene
KW - keratinocyte
KW - epithelium cell
KW - B lymphocyte
KW - cytotoxicity
KW - exposure
KW - school child
KW - Health
KW - ambient air
KW - school

KW - Lung
KW - Chinese hamster
KW - lung fibroblast
KW - Genome
KW - Gene Expression
KW - cytokine
KW - cytochrome P450
KW - xenobiotic agent
KW - cytochrome
KW - interleukin 1
KW - interleukin 6
KW - interleukin 8
RP - NOT IN FILE
SP - S35
JF - Toxicology Letters
JA - Toxicol Lett
VL - Conference: 46th Congress of the European Societies of Toxicology, EUROTOX 2009 Dresden Germany. Conference Start: 20090913 Conference End: 20090916 Sponsor: Deutsche Forschungsgemeinschaft
IS - DFG
N2 - Background: Because most individuals spend about 85% of their time indoors, we investigated the health effects of indoor air PM₁₀. Methods: PM₁₀ was collected in five schools in Munich during teaching hours. Toxicity was assayed in human primary keratinocytes, human lung epithelial A549 cells and Chinese hamster V79 lung fibroblasts at concentrations from 3 ng/ml to 10 mug/ml. Toxicity after metabolic activation was assayed in cells expressing human cytochrome P450 1A1, 1A2, 1B1, 2A6, 2B6, 2C9, 2D6, 2E1, 3A4, or 3A5. Additional toxic effects were analyzed with affimetrix genome wide gene expression analysis in BEAS-2B bronchial epithelial cells at 10 mug/ml PM₁₀. Results: In A549 and V79 cells no toxicity was observed. In human primary keratinocytes PM10 evoked a slight, but significant decrease in vitality. PM₁₀ was also toxic after metabolic activation by CYP1A1 or CYP2C9. PM₁₀ induced in BEAS-2B cells the expression of xenobiotic metabolizing genes (CYP1A1, CYP1B1) and inflammatory cytokines (IL-1A, IL-1B, IL-6, IL-8). Indoor PM₁₀ induced xenobiotic metabolizing genes less but inflammatory cytokines up to 6-fold more than outdoor PM₁₀. Conclusion: Direct cytotoxicity and toxicity after metabolic activation by cytochrome P450 1A1 and 2C9 were significant at a PM₁₀ concentration of 10 mug/ml (about 100l indoor air/ml), which is more than 10,000 times higher than exposure encountered in classrooms. We therefore expect no toxic effects of these particles in school children. The induction of inflammatory cytokines in indoor PM₁₀ treated cells suggests that classroom particles have a higher inflammatory potential than outdoor air particles
SN - 0378-4274
AD - (Buters, Oeder, Weichenmeier, Schober, Behrendt) Division of Environmental Dermatology and Allergy, Helmholtz Zentrum Munchen/TUM, ZAUM- Center for Allergy and Environment, Munich, Germany (Dietrich, Fromme) Bavarian Health and Food Safety Authority, Department of Environmental Health, Oberschleisheim, Germany
ER -

TY - JOUR
ID - 3753
T1 - Establishing a tertiary level child and Adolescent weight management service: Lessons learned
A1 - Baur,L.A.
Y1 - 2009///
N1 - 70110304
English
Journal: Conference Abstract
KW - EMBASE
KW - Obesity
KW - Adolescent
KW - Child
KW - Prevention

KW - Pediatrics
KW - weight
KW - childhood
KW - workshop
KW - patient
KW - Morbidity
KW - Exercise
KW - Health Care
KW - Chronic Disease
KW - Disease Management
KW - bariatrics
KW - psychiatry
KW - child parent relation
KW - Social Work
KW - protection
KW - social psychology
KW - Health
KW - general practitioner
KW - community
KW - Case Management
KW - planning
KW - pediatric hospital
KW - teamwork
KW - skill
KW - cultural competence
KW - nurse
KW - conductor
KW - physician
KW - dietitian
KW - Clinical Psychology
KW - social worker
KW - physiotherapist
KW - Music
KW - Hospital
KW - disabled person
KW - blood pressure cuff
KW - data base
KW - Medical Audit
KW - health practitioner
KW - vocational education
KW - Sleep
KW - endocrinology
KW - dermatology
KW - general practice
KW - gynecology
KW - orthopedics
KW - Medicine
KW - model
RP - NOT IN FILE
SP - 13
JF - International Journal of Pediatric Obesity
JA - Int J Pediatr Obes
VL - Conference: 1st International Conference and Workshops on Childhood and Adolescent Obesity: Research, Prevention and Intervention Riyadh Saudi Arabia. Conference Start: 20090330 Conference End: 20090401. Conference Publication:
IS - var.pagings

N2 - Despite the increasing public recognition of the problem of obesity in many countries, the planning for effective treatment services, especially for children, has lagged behind. Following are some personal reflections on the elements found useful on developing a bariatric medicine service within a large pediatric hospital. Effective staffing is vital. Staff members should be flexible and willing to move outside traditional professional "silos" of knowledge; they should have good teamwork skills, cultural competence and a respectful attitude towards obese people. A multidisciplinary clinical team is required, and ideally should include senior nurse (often the "conductor of the orchestra"), pediatric physician, dietitian, clinical psychologist, social worker, and exercise professional (ideally a dual-trained exercise scientist/ physiotherapist). The clinic facility should have disabled access and wide corridors and doorways, with chairs that are sturdy and can seat large people, weight scales that allow sensitive measurement of very large people (e.g., 250⁺ kg) and large blood pressure cuffs. The service should be supported by a database that can also allow audit and research studies to be undertaken. In establishing such a service, it is important to make the issue of obesity the problem of the institution, and not just of a few enthusiastic clinicians, and hence senior management and clinicians need to be engaged with the issue early. Ongoing health professional training of many levels of staff is required, from undergraduate level through to continuing postgraduate professional education, and for many types of clinician. Coordination with services that manage co-morbidities (e.g., Sleep Unit, Hepatology, Endocrinology, Dermatology, Psychiatry, General Medicine, Gynaecology, Orthopaedics, and Adolescent Medicine) is vital - shared protocols for assessment and management may need to be developed and key staff in the other services may require additional training regarding obesity. Families and young people with severe obesity may have significant family dysfunction, psychiatric co-morbidities and/ or parenting concerns; hence the importance of effective links with Psychiatry, Social Work and even Child Protection Services, and the need for obesity clinical staff to have a good understanding of the psychosocial aspects of pediatric medicine. Finally, a tertiary pediatric bariatric medicine service should only be one part of a much larger coordinated system of delivering health care within a region or country to obese children and adolescents. The Kaiser Permanente Chronic Disease Management model recognises that three different levels of chronic disease care should be offered: Level 1 care, suitable for the vast majority of those affected, emphasises patients' (or patients' families') central role in managing their health, supported by family doctors and community and other health system resources. Level 2 is care of highrisk patients by multidisciplinary disease management protocols and Level 3 is active case management of highly complex patients
SN - 1747-7166
AD - (Baur) Discipline of Pediatrics and Child Health, University of Sydney, Children's Hospital at Westmead, Sydney, Australia (Baur) Advisory of King Saud University, Obesity Department, Saudi Arabia
ER -

TY - JOUR
ID - 3754
T1 - Obesity in Saudi children in the Eastern Province of Saudi Arabia
A1 - Al-Dossary,S.S.
A1 - Sarkis,P.E.
A1 - Hassan,A.
A1 - El Regal,M.E.
A1 - Fouda,A.E.
Y1 - 2009///
N1 - 70110294
English
Journal: Conference Abstract
KW - EMBASE
KW - Obesity
KW - Child
KW - Prevention
KW - Pediatrics
KW - Adolescent
KW - Saudi Arabia
KW - workshop
KW - childhood
KW - Prevalence
KW - school

KW - weight
KW - Adult
KW - consultation
KW - Breast Feeding
KW - screening
KW - Population
KW - Male
KW - Health
KW - follow up
KW - adolescence
KW - Female
KW - Weight Control
KW - community
KW - body mass
KW - environmental factor
KW - adolescent health
KW - health program
KW - Curriculum
KW - Lifestyle
KW - Health Care
KW - wellbeing
KW - education program
KW - weight reduction
KW - Diet
KW - habit
KW - Etiology
KW - Diagnosis
KW - medical specialist
KW - Hospital
KW - eating habit
KW - Nutrition
KW - Television Viewing
KW - computer
KW - Recreation
KW - gender
KW - school child
KW - heredity
RP - NOT IN FILE
SP - 7
EP - 8
JF - International Journal of Pediatric Obesity
JA - Int J Pediatr Obes
VL - Conference: 1st International Conference and Workshops on Childhood and Adolescent Obesity: Research, Prevention and Intervention Riyadh Saudi Arabia. Conference Start: 20090330 Conference End: 20090401. Conference Publication:
IS - var.pagings
N2 - During the end of the last century, obesity was identified as a worldwide health care problem that is affecting the well-being of the population, previously known in the adult population, but in reality, studies are reporting an increasing problem in children too (1). The Gulf region is not exempt. Many surveys have reported the prevalence of overweight and obesity in Saudi children. All areas and provinces of the country have been affected through all ages (2,3). Governmental and local authorities have implemented education programs to help weight reduction or prevention. The abnormal weight in children is still considered by experts as an imbalance between diet and habit, although hormonal etiology in children is a diagnosis that should be ruled out first. In order to add valid information about the status of weight imbalance and child weight status in our region, this study has been conducted in the Eastern Province of Saudi Arabia with the following objectives: 1. To determine the prevalence of overweight and obesity in children from the Eastern Province of Saudi Arabia and study its basic demographic distribution, and to compare it with the Gulf Countries and other areas of the

world. 2. To compare the prevalence with Non-Saudi children living in Saudi Arabia. 3. To propose a prevention and treatment plan for overweight and obesity. This study collected first 10 509 files, including 9 249 consultations done in OPD in Saad Specialist Hospital, from January to June 2006. After excluding follow-up consultations, repetitions and non-completed file data, a total of 7 497 files were enrolled in the study. According to our study findings, 42% of Saudi children in the present Eastern Province sample have a BMI greater than the 85th percentile. It was evident that Saudi children start developing overweight when they are 5-9 years of age, and continue to increase up to their precious adolescent years. Twenty-one percent of children at 5-9 years are overweight and 21% are obese. This could be attributed to the fact that children start going to school at that age, and hence, there is poor control of eating habits and nutrition at this stage. Moreover, children have been less active; few, or none, of them walk to school, spending more time in sedentary entertainment activities, including television viewing, and playing computer and video games. On average, a child spends 6 hours per day in front of screens (4). Our results agree with a previously published study in the country that obesity in both genders is lowest in pre-school children (31%), and highest among adolescents (50-76%) (2,5,6). In our sample, the peak of obesity starts at 10-13 years of age (28%) and keeps the same high prevalence at age 14-18 years. Studies have shown that 80% of obese adolescents become obese adults (7). Adolescence has been addressed as the "critical period for the development for adult obesity" (8). Hence, if intervention before this stage is unsuccessful, intervention at this age becomes vital for both future health and ability to sustain long term weight control. We developed, in our institution, a plan of action for obesity prevention and detection (7). It is oriented towards the community in general and towards children particularly. In our opinion, prevention should start at birth by putting more emphasis on exclusively breast feeding for the first 6 months of age, and continue throughout school age with a special interest starting at the age of 10 years. Regarding screening for early detection and management of these cases, we propose a plan that is based on regular follow-up and supervision. Fifty percent of Saudi children from the Eastern Province in our sample have a body mass index (BMI) greater than the 85th percentile, being overweight or obese. Our study showed that obesity starts early in life (10-14 years), and continues all through the ages of adolescence. Prevalence of obesity is higher among males compared with females, who show a higher prevalence of overweight rather than obesity. Environmental factors seem to play more of an influence on the prevalence of obesity than do genetic factors, because there was no difference in obesity among Saudi and Non-Saudi children, as well as similar weight distribution between males and females in children below one year of age. It is difficult to reduce excessive weight once it becomes established. Children, therefore, should start prevention of obesity from birth by putting more emphasis on exclusively breast feeding for the first 6 months of life. It is becoming a priority to establish natural settings for pre-school, school and adolescent health programs with the emphasis on increasing physical educational hours, and incorporating health messages into school curricula. Integrated preventative measures, screening for early detection/management and educational programs towards a healthier lifestyle are required at the national level
SN - 1747-7166
AD - (Al-Dossary, Sarkis) Saad Specialist Hospital, Al Khobar, Saudi Arabia (Hassan) Department of Bone Marrow Transplant/Immunology, London WC1N, United Kingdom (El Regal, Fouda) Faculty of Medicine, Mansoura University, Al-Mansoura, Egypt
ER -

TY - JOUR
ID - 3755
T1 - An outdoor activity weekend for patients with a metabolic disorder - A novel approach to dietetic management of adult patients
A1 - Adam,S.A.
A1 - Wildgoose,J.
A1 - Ferguson,C.
Y1 - 2009///
N1 - 70101505
English
Journal: Conference Abstract
KW - EMBASE
KW - patient
KW - Adult
KW - Metabolism
KW - metabolic disorder

KW - Diet
KW - Cooking
KW - skill
KW - leisure
KW - Child
KW - United Kingdom
KW - Food
KW - phenylketonuria
KW - Female
KW - lake
KW - protein intake
KW - homocystinuria
KW - teamwork
KW - Competition
KW - Learning
KW - diet therapy
KW - Self Esteem
KW - Friendship
KW - follow up
KW - Education
KW - Male
KW - financial management
KW - protein
RP - NOT IN FILE
SP - 16
EP - October
JF - Molecular Genetics and Metabolism
VL - Conference: 11th International Congress of Inborn Errors of Metabolism San Diego, CA United States. Conference Start: 20090829 Conference End: 20090902. Conference Publication:
IS - var.pagings
N2 - The outdoor activity weekend is a popular method for supporting children with Phenylketonuria (PKU) but equivalent opportunities for adults do not exist in the United Kingdom. Given its success with children, we felt a similar activity for adults would prove valuable in their dietetic management. The weekend away was planned as an exciting opportunity to promote and support the continuation of the diet and was designed to give opportunities for patients to meet other people with the same condition, talk about their experiences in managing their diet, and to share ideas and support. The activity weekend was held over 3 days at an outdoor centre in the Lake District, England. Eleven adults with PKU and one adult with Homocystinuria from Glasgow, Newcastle, and West Yorkshire participated. They were aged between 17 and 37 years with an even mix of males and females. The weekend was split into discussion groups, cookery and leisure activities. For the discussion groups, we facilitated sharing of ideas and experiences on protein substitutes, their importance and the varieties available. They were informed of new low protein foods and how they could be incorporated into their diets. For the cooking activity, the patients were divided into teams for a low-protein cookery competition (there was a wide range of cooking skills in each team, some people's skills were very limited but all could obtain advice from ourselves and the chefs from the centre). The leisure activities such as the high ropes challenges and gorge sliding were designed to build teamwork, self-esteem, and confidence. At the end of the weekend the patients were asked to complete evaluation forms. The results of the evaluation and our own experiences during the weekend demonstrated that it was a success. The patients reported learning the importance of continuing on their diet. Many had the opportunity to try new foods, learn new cookery skills, and exchange practical tips and ideas about their diets with their peers. The cookery teams worked well together and produced some delicious food which inspired confidence amongst the patients. The discussion groups provided an ideal forum for participants to share ideas and discuss the practical aspects of dietary treatment. The leisure activities promoted friendship and sharing across the group. We intend to follow-up the patients in due course to see if they have made any changes in their diet. If the weekend did indeed lead to long-term changes in the patients' dietary choices then it demonstrates that such an event could prove to be a valuable treatment method for metabolic patients and for educating, engaging, and maintaining links with them. We thank the staff of the Derwent Hill Outdoor Education and Training Centre for their support through the weekend. Financial support was provided by the patients, Yorkhill Metabolic Fund, Vitaflo, and SHS International

SN - 1096-7192
AD - (Adam) Dietetics and Nutrition, Royal Hospital for Sick Children, Glasgow, Lanarkshire, United Kingdom (Wildgoose) Dietetics and Nutrition, St Lukes Hospital, Bradford, West Yorkshire, United Kingdom (Ferguson) Dietetics and Nutrition, Newcastle General Hospital, Newcastle, Newcastle Upon Tyne, United Kingdom
ER -

TY - JOUR
ID - 3756
T1 - Human genome variability and host-pathogen interactions
A1 - Quintana-Murci,L.
Y1 - 2009///
N1 - 70091836
English
Journal: Conference Abstract
KW - EMBASE
KW - virus infection
KW - human genome
KW - Child
KW - host pathogen interaction
KW - mother
KW - gene
KW - Human
KW - natural selection
KW - Infection
KW - population differentiation
KW - Population
KW - Immunity
KW - fitness
KW - organism
KW - Genome
KW - immune response
KW - Health
KW - survival
KW - pathogenesis
KW - genetic variability
KW - toll like receptor
KW - antibiotic agent
KW - vaccine
RP - NOT IN FILE
JF - Retrovirology
VL - Conference: 5th Dominique Dormont International Conference. Mother-to-child transmitted viral diseases: From transmission to children care Paris France. Conference Start: 20090326 Conference End: 20090328. Conference Publication:
IS - var.pagings
N2 - Natural selection is a major force behind the shaping of patterns of human genome variability. Inferences concerning the action of selection in the human genome provide a powerful tool for predicting regions of the genome of major functional importance. Genetic variants influencing human susceptibility to disease are likely to affect the fitness of the organism, unless the disease concerned begins late in the life. There is therefore an intimate relationship between disease and selection that can be exploited for the identification of candidate disease loci. To date, some of the strongest evidence for selection in the human genome has been obtained for genes involved in the immune response or host-pathogen interactions. Indeed, before the advent of antibiotics and vaccines, infectious diseases have been paramount among the threats to health and survival for most of human evolutionary history. I will review our most recent studies searching for the footprints of natural selection in the human genome. These studies, which go from global genomewide scans to more fine-tuned analyses in specific genes, highlight how the identification of selected loci or variants may provide insight into host genes or pathways playing an important role in pathogen resistance. For example, we have shown that

natural selection has significantly driven the processes of population differentiation in modern human populations. Specifically, we have identified a number of genes under strong geographicallyrestricted positive selection, some of them involved in host-pathogen interactions. In addition, I will present our most recent data on the Toll-like receptor (TLR) signalling pathway. Our evolutionary data indicate that the different members of TLR family differ in their ecological relevance and increase our understanding of how variation in these genes results in different contributions to the outcome of infectious diseases. More generally, I will show how the identification of the extent and type of selection acting upon human genes involved in host-pathogen interactions make it possible to define the redundant and nonredundant functions of individual immunity-related genes in the natural setting
SN - 1742-4690
AD - (Quintana-Murci) Institut Pasteur, CNRS, Paris, France
ER -

TY - JOUR
ID - 3757
T1 - Treating insomnia in comorbid disorders
A1 - Lader,M.
Y1 - 2009///
N1 - 70090973
English
Journal: Conference Abstract
KW - EMBASE
KW - insomnia
KW - anxiety disorder
KW - mood
KW - Sleep
KW - patient
KW - relapse
KW - night
KW - mental disease
KW - panic
KW - Drug Therapy
KW - Therapy
KW - Arousal
KW - Population
KW - compliance (physical)
KW - Anxiety
KW - substance abuse
KW - dementia
KW - depression
KW - Schizophrenia
KW - dream
KW - Sleep Deprivation
KW - detoxification
KW - hypersomnia
KW - Etiology
KW - suicide attempt
KW - dyspnea
KW - Pain
KW - Early Intervention
KW - Needs Assessment
KW - enantiomer
KW - psychiatrist
KW - physician
KW - Morality
KW - bipolar disorder
KW - mental patient

KW - agonist
KW - Posttraumatic Stress Disorder
KW - parasomnia
KW - Circadian Rhythm
KW - model
KW - Child
KW - intensive care
KW - Major Depression
KW - accident
KW - clinical feature
KW - antidepressant agent
KW - melatonin
KW - mirtazapine
KW - hypnotic agent
KW - sedative agent
KW - trazodone
KW - psychotropic agent
KW - tricyclic antidepressant agent
KW - trimipramine
KW - antihistaminic agent
KW - eszopiclone
KW - serotonin antagonist
KW - agomelatine
KW - ramelteon
KW - alcohol
KW - opiate
RP - NOT IN FILE
SP - 20
EP - 22
JF - International Journal of Psychiatry in Clinical Practice
VL - Conference: 9th International Forum on Mood and Anxiety Disorders Monte Carlo Monaco. Conference Start: 20091111 Conference End: 20091113. Conference Publication:
IS - var.pagings
N2 - Insomnia has long provided a series of challenges to the various classes of physicians responsible for care of those with this complaint. I shall concentrate on the assessment and treatment of insomnia by psychiatrists. One of the challenges is whether the insomnia is a primary symptom in itself, secondary to medical or psychiatric disorders, or a comorbid disorder, existing in conjunction with the primary psychiatric problem. The assessment of this factor is important as it has a major influence on the conceptual approach, management and type of therapy. Primary, secondary and comorbid insomnia: Primary insomnia is a specific disorder defined in the DSM-IV as a condition of at least one month's duration, not caused by a medical or psychiatric disorder. The insomnia can be a disturbed sleep initiation, problem maintaining sleep or early morning awakening; one or more or these must be associated with complaints of daytime dysfunction. Many psychiatric patients have chronic insomnia but in the general population primary insomnia occurs in about 10% of the population. Secondary insomnia is found in a whole series of conditions. The most common is in association with physical disorders such as pain and breathing difficulties. An important recent State-of-the-Science conference focused on the role that comorbid conditions can have in association with insomnia. It was noted that insomnia usually appears in the presence of at least one other disorder. Particularly common psychiatric comorbidities are major depression, generalised anxiety, substance abuse, attention deficithyperactivity in children, and dementia. This concept of comorbid as opposed to secondary insomnia is becoming accepted as a valid model for understanding the relationships between insomnia and other concomitant disorders. If the insomnia is "secondary" to a psychiatric condition, treatment of the primary disorder would be expected to help the insomnia. However, if the disorder is comorbid, for example, with depression, successful treatment of the depression with antidepressants will not necessarily alleviate the insomnia complaint. Also, people with severe sleep complaints that occurs comorbidly, are less likely to respond than those with milder complaints and may need quite intensive therapy. The most important of these disorders is depression. Sleep difficulties are common symptoms in depressed patients. Insomnia is often the reason that causes depressed patients to seek help. Severe insomnia may lead to exhaustion, poor functioning during the day and accidents. About three quarters of

depressed patients complain of difficulties with sleep, in particular fragmented sleep with disturbing dreams. They wake feeling unrefreshed in the morning and feel tired during the day. Paradoxically, total sleep deprivation can help as a temporary expedient in depression but this lies outside my remit. In many cases the insomnia and depression can be conceptualised as comorbidities. One clue can be adduced from the fact that in depressive disorder a proportion of patients show comorbid hypersomnia not insomnia. The relationship between sleep and mood are closely related. Insomnia can significantly worsen the disorder of depression and can increase the likelihood of a suicide attempt. Insomnia is often a prodromal warning sign of depression that will manifest itself in the next two years. After a depressive episode it is a predictive sign of an incipient relapse and can warn the prescriber not to withdraw the antidepressants while insomnia persists. Early intervention to improve sleep may help abort a possible relapse. Antidepressant treatment can help sleep in depressed patients. Many of the treatments used in depression are sedative. The classic examples are the tricyclic antidepressants, such as trimipramine, with antihistaminic actions. More recently, compounds such as trazodone and mirtazapine have been used to treat depression associated with insomnia. The use of such medication may result in an early amelioration of sleep problems and this can improve the morale of the insomniac depressive and also help improve compliance. An interesting compound is the antidepressant agomelatine but the relationship between its melatoninergic properties, antidepressant efficacy and effects on sleep are still being worked out. Other conditions in which the insomnia appears comorbid include bipolar disorder where the manic phase is usually preceded by a shortened duration of sleep. This pattern can often be quite stereotyped. Generalised anxiety is also accompanied by sleep onset insomnia and increased night time awakenings. The sufferers may describe a sense of insufficient and unsatisfying sleep. Other anxiety disorders include post traumatic stress disorders in which there is not only insomnia but parasomnias. Panic disorder may be associated with night time panic attacks that awaken the patient. Patients with schizophrenia can suffer from insomnia at the time of acute symptoms. A circadian rhythm disorder with gradual delay of the sleep period relative to the day may ensue. Carers of schizophrenic patients may recognise this as the onset of a relapse. Dementia is also associated with comorbid insomnia with often fragmentation of the 24-hour sleep wake pattern or even reversal with more sleeping during the day and less at night. Such behaviour may be very difficult to manage and actually be a factor leading the carers to insist that the patient be admitted to full time care. Substance abuse includes alcohol problems, opiate detoxification and the use of sedative hypnotic drugs themselves. Various patterns are seen. One particular source of severe insomnia is withdrawal from psychotropic drugs, in particular sedatives/hypnotics and some antidepressants. Assessment and management: The complaint of insomnia needs assessment and treatment in its own right as the therapeutic approach will vary according to the comorbid psychiatric disorder, its severity and pattern, and the characteristics of the insomnia itself. The treatment of insomnia can be roughly divided into 2 major divisions - the use of medication and behavioural techniques. The latter are becoming more available with evidence that they can be effective when judiciously chosen and applied. The usual hypnotics used are essentially general depressants and have no specific effects on sleep itself. They lie along the sedative/hypnotic/deep sedation/anaesthetic continuum. More recently a series of new approaches have been applied. One of these is a variant of the depressant approach and is eszopiclone. This active enantiomer appears to have long-term efficacy without problems during withdrawal. 5-HT blockers have also been studied for some time and such blockade is part of the profile of action of drugs such as trazodone and mirtazapine. However, newer compounds are being studied. Melatonin agonists have been developed and one, ramelteon, is licensed in some countries. Melatonin itself is too shortacting for much efficacy but long-acting formulations (such as Circadin) have recently been licensed. Another set of compounds involved in sleep regulation are the orexins, and their antagonists are in development. In conclusion, a whole range of compounds are available to treat insomnia. These treatments can be applied symptomatically to primary, secondary and comorbid insomnia. As research progresses into both basic mechanisms and clinical features, distinctions between these aetiologies will become more apparent, and will lead to more focused therapies
SN - 1365-1501
AD - (Lader) King's College London, London, United Kingdom
ER -

TY - JOUR
ID - 3758
T1 - Long term use of implanted subcutaneous ports for photopheresis
A1 - Schiller,D.
A1 - Simon,G.
A1 - Adams,K.
A1 - Wiltbank,T.B.

Y1 - 2009///
N1 - 70090212
English
Journal: Conference Abstract
KW - EMBASE
KW - PUVA
KW - apheresis
KW - Society
KW - patient
KW - sepsis
KW - blood flow
KW - Quality of Life
KW - vascular access
KW - catheter
KW - device
KW - Liquid
KW - Infection
KW - Therapy
KW - allograft
KW - cutaneous T cell lymphoma
KW - solid
KW - peripheral vein
KW - Risk
KW - arteriovenous fistula
KW - subcutaneous drug administration
KW - bacterium
KW - needle
KW - central venous catheter
KW - erythrocyte
KW - catheter infection
KW - Child
KW - Transplantation
KW - heparin
KW - anticoagulant agent
KW - tissue plasminogen activator
RP - NOT IN FILE
SP - 63
EP - 64
JF - Journal of Clinical Apheresis
VL - Conference: American Society for Apheresis 30th Annual Meeting San Diego, CA United States. Conference Start: 20090520 Conference End: 20090523. Conference Publication:
IS - var.pagings
N2 - Background: Vascular access for long term extracorporeal photopheresis (ECP) therapy in post allograft or cutaneous T-cell lymphoma patients is a critical problem. Most patients, who have had solid organ transplants, have had their peripheral veins lost during the peri-operative period and thus require some form of central vein catheter. While the primary risk, namely sepsis, associated with percutaneous central line catheters (PCC) has improved over the decade, there are alternatives: either arteriovenous fistulas or subcutaneous infusion ports (SIP). In these alternatives there is no constant residual percutaneous entry way for bacteria, since each access occurs utilizing a needle that is removed at the procedure's conclusion. The use of a specific Vortex SIP has been previously described for long term use in children undergoing erythrocyte exchange. Methods: The records of patients requiring ECP during the past two years were reviewed to compare those requiring a PCC versus SIP. Specific procedures were followed for access and discontinuation with attention given to the volume and concentration of heparin dwelling in the catheter during non-use periods. We recorded the number of catheter associated infections, the number of occasions access occurred through the central line by individuals outside the treatment facility and the frequency with which blood flow problems occurred requiring the use of tissue plasminogen activator (TPA). Patients were also asked quality of life questions relevant to the central line device and their ability to enjoy certain activities. Results: Ten patients had a tangent conical SIP implanted with

244 (range 4-38) procedures performed and 10 patients had standard PCC lines with 246 (range 3-42) procedures performed. The current longest period for evaluation is 24 months for either access. During this time 3 SIP patients had admissions to other facilities, which used the SIP for fluid administration, and did not adhere to the procedures for post SIP use heparin dwell. These 3 patients had 8/11 (73%) of the TPA treatments for SIP blood flow problems occurring after such an admission. No SIP patient had a bacterial line infection. In contrast, there were 20 episodes of blood flow difficulty requiring TPA in 6 of the PCC patients. Two PCC patients had 3 separate line sepsis episodes requiring an exchange of the line each time for control of the sepsis. All SIP patients were able to shower/bathe as they desired, while PCC patients followed a restricted bath procedure. Conclusion: The use of an implanted subcutaneous port with a tangential design appears functional for vascular access, appears to have a reduced incidence in bacterial sepsis related to the time in place, has a reduced frequency for TPA antithrombotic treatment when specific procedures are routinely followed and permits an improved mobility and quality of life for patients
SN - 0733-2459
AD - (Schiller, Simon, Adams, Wiltbank) United Blood Services, Scottsdale, AZ, United States
ER -

TY - JOUR
ID - 3759
T1 - Air pollution and asthma in childhood
A1 - Rahimi-Rad,M.H.
A1 - Hamzezadeh,A.
Y1 - 2009///
N1 - 70072896
English
Journal: Conference Abstract
KW - EMBASE
KW - Asthma
KW - Air Pollution
KW - childhood
KW - wheezing
KW - Exercise
KW - school child
KW - city
KW - Speech
KW - parent
KW - night
KW - Risk
KW - coughing
KW - exposure
KW - passive smoking
KW - heating
KW - logistic regression analysis
KW - Sleep
KW - groups by age
KW - car
KW - Smoking
KW - air pollutant
KW - Iran
KW - Child
KW - long term exposure
KW - tube
KW - allergy
KW - questionnaire
KW - Cooking
RP - NOT IN FILE
SP - A222
JF - Respirology

VL - Conference: 14th Congress of the APSR and 3rd Joint Congress of the APSR/ACCP Seoul South Korea. Conference Start: 20091114 Conference End: 20091118. Conference Publication:
IS - var.pagings
N2 - Background/Purpose: To examine the influence of indoor and outdoors air pollution on asthma in schoolchildren in Urmia, Iran. Although Urmia is not an industrialized city but similar to other Iranian cities popular use of worn out cars with high tailed-pipe emission leads to air pollution in this city. Methods: Parents of 3000 schoolchildren aged 6-7 and 3000 schoolchildren aged 13-14 years themselves filled of International Study of Asthma and Allergies in Childhood (ISAAC) questionnaires. The influence of trucks passage, exposure to passive smoking, electricity/gas/wood cooking and electricity/ wood/central heating at home on wheezing ever, current wheezing, number of attacks, disturbed sleep, limited speech, exercise induced wheeze, dry cough at night and asthma ever was determined. Odds ratios in logistic regression for statistic analysis of the data were used. Results: Among 13-14-year-old group, always or frequent trucks passage through the residential street, compared to seldom one, significantly increased the risk of wheezing ever OR 1.19 (95%CI 1.08-1.31); current wheezing OR 1.32 (1.18-1.47); speech limiting wheezing: OR 1.46 (1.26-1.70); wheezing during exercise OR 1.42 (1.30-1.55) and dry night cough OR 1.21 (1.10-1.34). In 6-7 years old group only exercise induced wheezing was significantly associated with busy roads OR 1.27 (95%CI 1.02-1.59). In both age group parent smoking was strongly associated with asthma symptoms. A significant influence of other investigated air pollutants on other asthma symptoms was not found. Conclusions: Our results suggest that outdoors air pollution significantly associated with asthma symptoms in older children but not younger group. This might be explained by progressive effect of long term exposure to air pollution
SN - 1323-7799
AD - (Rahimi-Rad, Hamzezadeh) Medicine, Urmia University of Medical Sciences
ER -

TY - JOUR
ID - 3760
T1 - Radiographic findings of PULMONARY TB and NTM lung disease
A1 - Ok,C.K.
Y1 - 2009///
N1 - 70072545
English
Journal: Conference Abstract
KW - EMBASE
KW - lung disease
KW - Infection
KW - tuberculoma
KW - Calcification
KW - Lung
KW - delayed hypersensitivity
KW - attenuation
KW - pathogenesis
KW - Human
KW - necrosis
KW - bronchiectasis
KW - lymph vessel
KW - airway
KW - tree
KW - bronchus stenosis
KW - Child
KW - effusion
KW - lymphadenitis
KW - Hypersensitivity
KW - empyema
KW - Aged
KW - pneumonia
KW - macrophage
KW - patient

KW - bacterium
KW - lung tuberculosis
KW - pleura
KW - Adolescent
KW - Adult
KW - Mosaicism
KW - lobar pneumonia
KW - blood vessel
KW - mediastinum lymph node
KW - traction therapy
KW - healing
KW - radiologist
KW - Drug Resistance
KW - immunocompromised patient
KW - epidemic
KW - Immunity
KW - Bacilli
KW - lung lobe
KW - Human immunodeficiency virus
KW - fibrosis
KW - bronchiolitis obliterans
KW - lymphadenopathy
KW - bronchiolitis
KW - Mycobacterium
KW - Environmental Exposure
KW - bronchus
KW - bronchiole
KW - sputum culture
KW - Radiography
KW - antigen presentation
KW - emphysema
KW - giant cell
KW - lymphocyte
KW - fibroblast
KW - down regulation
KW - immune status
KW - Diagnosis
KW - exposure
KW - life cycle
KW - cytoplasm
KW - granulomatosis
KW - Male
KW - implantation
KW - sputum
KW - Female
KW - Mycobacterium tuberculosis
KW - glass
KW - tuberculin
RP - NOT IN FILE
SP - A121
EP - A122
JF - Respirology
VL - Conference: 14th Congress of the APSR and 3rd Joint Congress of the APSR/ACCP Seoul South Korea. Conference Start: 20091114 Conference End: 20091118. Conference Publication:
IS - var.pagings
N2 - PULMONARY TUBERCULOSIS Pulmonary TB caused by Mycobacterium tuberculosis has traditionally been classified into primary (non-immune) and secondary (delayed hypersensitivity) reactivation. Delayed

hypersensitivity manifests 4-10 weeks after initial infection. Caseation necrosis consequent to development of delayed hypersensitivity is pathologic hallmark of pulmonary TB. Radiologic manifestations of pulmonary tuberculosis (TB) can vary according to several host factors, including prior exposure to TB and underlying immune status. M.TB has capacity to survive and replicate in human macrophages, by down-regulation of the host's antigen processing (latent infection). Continuous lifelong interaction between the individuals and the mycobacteria results in considerable variety of morphologic features. It is a matter of chance when the radiologist first sees the patient. Recently multi-drug resistance TB and increase in immune-compromised patients, including HIV epidemics, make the control of the pulmonary TB more difficult. Atypical manifestation of clinical and radiographic features is common in patients with decreased host immunity, and CT can play a role in this situation. PRIMARY PULMONARY TB No prior contact with bacilli in this form. Most infections are air-borne, so it manifests as a parenchymal consolidation in any pulmonary lobe or segment. The bacteria spread via lymphatics from parenchymal lesion to ipsilateral hilar and/or mediastinal lymph nodes. Combination of lobar pneumonia with lymphadenopathy is called the primary complex. REACTIVATION PULMONARY TB Reactivation in majority is reactivation of the dormant lesion at the apical portion of bilateral upper and lower lobes. Caseation necrosis is the pathologic hallmark in this type. It manifests as a heterogeneous, often cavitary nodules or consolidation. Then the bacteria spread via airway to the other healthy lungs. It typically manifests as tree-in-bud appearance, corresponding to the caseous material involving small bronchi and bronchioles. Radiographic stability for 6 months and negative sputum cultures is the best indicator of inactive disease. Activity may not be determined by plain radiography and CT may be more accurate when it can detect cavitation and tree-in-bud appearance, and differentiation of cavity from cystic bronchiectasis or paracicatricial emphysema. COMMON TO THE PRIMARY AND REACTIVATION PULMONARY TB TUBERCULOMA It most often originates from healed primary TB. Pathologically, its centre contains caseous necrosis and viable AFB in most healed lesion, so it has potential to reactivate sometime. The Inner wall consists of epithelioid and Langerhan's giant cells and the Outer consists of lymphocytes and fibroblasts. Stable tuberculoma The majority remains stable. Its typical findings are the size usually <2 cm in size, smooth or fine speculated outer margin or calcification. When it shows central, laminated, homogeneous Calcification, then the diagnosis is almost certain. CT is helpful in detection of calcification. The contrast enhance of tuberculoma is <20 HU in CT. Reactivated tuberculoma Any tuberculoma has potential to reactivate. When it reactivates, the nodule Enlarges and calcification disappears. Low attenuation area, thin enhanced capsule and cavitation, Bronchogenic dissemination to the lung adjacent to nodule, so called satellite nodules, can be and in CT. Cavitation means Seminal event in the life cycle of M.TB; aerobic Bacillary proliferation outside the macrophage's cytoplasm. Cavitation implies a high bacillary burden, high infectivity, and is associated with numerous complications including endobronchial spread. ENDOBRONCHIAL TUBERCULOSIS TB granulomatous change in the bronchial wall is defined as endobronchial TB. The Pathogenesis is usually Erosion from lymphadenitis in primary TB, but usually Lymphogenous spread through submucosal lymphatics or Implantation from infected sputum in reactivation TB. Plain film sometimes detects Narrowing or irregularity of the major airway and more frequently detects the secondary signs, such as Collapse of a lobe or segment or Blocked or tension cavity. CT on hyperplastic stage show Uniform thickening of bronchial wall, Concentric and long bronchostenosis and Lymphadenitis (56%). While CT in fibrostenotic phase, concentric and long bronchostenosis is main finding, and thinner bronchial wall. PLEURAL INVOLVEMENT A TB infection of the pleura is more common in teenagers and adults than in children. The pathogenesis of the effusion is the result of hypersensitivity to tuberculin in the majority. It usually resolves spontaneously, but it needs treatment to prevent pulmonary TB and fibrothorax. Rarely the effusion is empyema, with complication such as bronchopleural fistua or empyema necessitates. MILIARY TUBERCULOSIS This disease is more common in small children <2 years old, the elderly, and immunocompromised. Miliary spread may evolve from lung or mediastinal infection eroding into a blood vessel. Typical finding are diffuse, random, subpleural small nodules, but in the early stage it may be subclinical, and seen as a diffuse GGO. BRONCHIECTASIS, CONSTRICTIVE BRONCHIOLITIS, FIBROSIS Bronchiectasia and post-TB bronchiolitis obliterans are common as sequel of primary or reactivation pulmonary TB, these are secondary to traction by parenchymal fibrosis, distal obstructive pneumonia following endobronchial-TB, or healing of bronchiolitis after bronchogenic dissemination of caseous material. NONTUBERCULOUS MYCOBACTERIAL (NTM) INFECTION NTM organisms are ubiquitous and infection arises from environmental exposure, not from human to human contact. M. kansasii, M avium-intracellulare, M. xenop are commonest pathogen. This infection can be classified in to two groups. M. avium complex can complex can cause both types, but M. Kansasii & xenopii usually manifests as TB-like pattern. "Classic" pattern The one group is affection mainly of males > 50 years old with pre-existing lung disease. The constitutional symptoms and the radiographic findings are very similar to that of pulmonary TB. It is characterized by upper lobe cavitary lesion and bronchogenic dissemination. Cavities tend to be multiple,

smaller and thin-walled. More pleural reaction and less parenchymal infiltration "Non-classical" pattern The other group is affected by mainly elderly women without pre-existing lung disease, and the radiographic findings consist of bronchiectasis, multiple small nodules and branching centrilobular nodules, involving right middle lobe and lingular segment. "Hypersensitivity pneumonia" pattern The radiographic features are similar to the HP of other origin, such as diffuse ground glass attenuation, micro centrilobular nodules, and mosaic attenuation
SN - 1323-7799
AD - (Ok) Radiology, Yonsei University, Severance Hospital, South Korea
ER -

TY - JOUR
ID - 3761
T1 - Association of serum soluble CD26 and CD30 levels with asthma, lung function and bronchial hyperresponsiveness at school age
A1 - Remes,S.T.
A1 - Delezuch,W.
A1 - Pulkki,K.
A1 - Matinlauri,I.
Y1 - 2009///
N1 - 70072362
English
Journal: Conference Abstract
KW - EMBASE
KW - serum
KW - Asthma
KW - school
KW - lung function
KW - allergy
KW - Child
KW - Inhalation
KW - Spirometry
KW - Lymphocyte Activation
KW - plasma
KW - childhood
KW - provocation test
KW - Infection
KW - expiratory flow
KW - Diagnosis
KW - marker
KW - methacholine
RP - NOT IN FILE
SP - 24
JF - Pediatric Allergy and Immunology
VL - Conference: EAACI Pediatric Allergy and Asthma Meeting Venice Italy. Conference Start: 20091112 Conference End: 20091114. Conference Publication:
IS - var.pagings
N2 - Aim: Lymphocyte activation markers CD26 and CD30 are also present as soluble forms in circulating plasma. Serum soluble CD30 (sCD30) has been suggested to be associated with childhood asthma. We examined the association of serum soluble CD26 (sCD26) and sCD30 concentrations with asthma at school age. Methods: Serum sCD26 and sCD30 were measured in34 children with clinical asthma (positive history and pathological lung function or challenge test finding), in 31 children with possible asthma and in 147 controls. In addition, the association of flow-volume spirometry, methacholine inhalation challenge and free running test results with serum sCD26 or sCD30 were analysed. The tests were taken at a non-acute phase (outside an acute infection). Results: The levels of serum sCD30 were significantly higher in children with asthma (mean 91.5 IU/ml, SD 23,0) than in children with possible asthma (85.0 IU/ml, 28.5) or the control children (78.8 IU/ml, 25.9) (P = 0.042). However, there was a considerable overlap between the groups in serum sCD30 levels. No significant differences were found in serum sCD26 levels between the groups. In flow-volume spirometry, there

was a significant negative correlation of mean mid expiratory flow values with serum sCD26 (r = -0.22, P = 0.0018) and of borderline significance with serum sCD30 (r = -0.14, P = 0.054). Neither methacholine inhalation challenge nor free running test results were associated with serum sCD26 or sCD30 levels. Conclusions: Serum sCD30 levels were higher in children with clinical asthma. However, a marked overlap in serum sCD30 levels between asthmatic and healthy children may limit the usefulness of serum sCD30 as a diagnostic marker of asthma. The finding that neither serum sCD26 nor sCD30 was associated with bronchial hyperresponsiveness supports this notion
SN - 0905-6157
AD - (Remes, Delezuch, Pulkki) Kuopio University Hospital, Kuopio, Finland (Matinlauri) Turku University Hospital, Turku, Finland
ER -

TY - JOUR
ID - 3762
T1 - Dietary habits, media usage, and physical activity in preschoolers born to overweight mothers
A1 - Bowman,A.E.
A1 - Lovelady,C.
A1 - Ostbye,T.
Y1 - -32676///
N1 - 70059089
English
Journal: Conference Abstract
KW - EMBASE
KW - preschool child
KW - mother
KW - Physical Activity
KW - Obesity
KW - habit
KW - beverage
KW - computer
KW - fast food
KW - fruit juice
KW - videorecording
KW - Food
KW - vegetable
KW - African American
KW - bivariate analysis
KW - milk
KW - Food Intake
KW - Fruit
KW - sodium carbonate
KW - yoghurt
RP - NOT IN FILE
JF - The FASEB Journal
VL - Conference: Experimental Biology 2009
IS - EB 2009
N2 - The purpose of this study was to describe dietary habits, media use, and physical activity in 2 to 5-year-olds born to overweight (BMI = 25) mothers. Data were collected on 174 preschoolers, of whom 72.8% were white, 23.1% African-American, and 4.1% other races. Mothers reported the time their preschooler spent watching TV and videos and using the computer, the number of servings of certain food groups eaten, and the duration of outdoor play. Preschoolers spent 3.4 + 1.0 hours watching TV and videos and 1.4 + 0.7 hours using the computer each day. They spent 4.4 + 3.1 hours each week playing outdoors. In bivariate analyses, there was a positive relationship between hours of TV watched and servings of soda, sweetened beverages, fruit juice, fast food, and French fries; and a negative relationship between hours of TV and milk servings. Time spent using the computer was positively related to sweetened beverage and fast food intake. Outdoor play was positively related to fruit and sweetened beverage intake, but not to soda, fruit juice, fast food, or French fries. Media use and physical activity were not related to yogurt, dessert, or vegetable consumption. Results of this exploratory

analysis suggest that there is a relationship between increased media use by preschoolers and a higher intake of less healthy foods. Higher levels of physical activity are related to increased sweetened beverage intake
SN - 0892-6638
AD - (Bowman, Lovelady) Nutrition, University of North Carolina - Greensboro, Greensboro, NC, United States (Ostbye) Community and Family Medicine, Duke University, Durham, NC, United States
ER -

TY - JOUR
ID - 3763
T1 - The relationship of dietary habits with TV watching and internet usage in part of Gwangju and Jeonnam elementary school children
A1 - Khil,J.
A1 - Cho,A.R.
Y1 - -32676///
N1 - 70054753
English
Journal: Conference Abstract
KW - EMBASE
KW - habit
KW - Internet
KW - school child
KW - primary school
KW - Child
KW - eating habit
KW - questionnaire
KW - Program Development
RP - NOT IN FILE
JF - The FASEB Journal
VL - Conference: Experimental Biology 2009
IS - EB 2009
N2 - The purpose of this study was to examine the dietary habits according to TV watching and internet usage and to establish the desirable extracurricular activity and eating habits in elementary school children. A questionnaire was filled out by children in 4th, 5th and 6th grades in 6 elementary schools within Gwangju and Jeonnam region and 712 cases were analyzed by SPSS 12.0. TV watching time was related with dietary habit score in subjects. The children who watch TV "=1 hour/day" in weekday had a better dietary habit score than the children who watch TV "=1 hour (p<0.0001). The children who watch TV"=2 hours/day in weekend had a better dietary habit than the children who watch TV "=2 hours" (p<0.0001). The internet usage was related with dietary habit score in children. The children who use internet "=1 hour/day" in weekdays had a better dietary habit score than the children who use internet "=2 hours/day" (p<0.0001). The children who use internet "=2 hours/day" in weekend had a better dietary habit than the children who use internet "=2 hours/day" (p<0.0001). These results indicate that the children who have more appropriate daily activity pattern showed better dietary habit score and TV watching and internet usage time occupy much of the elementary school children's extracurricular activities. Therefore, it is essential that the program development of both educational and social levels for active and outdoor extracurricular activity of school children
SN - 0892-6638
AD - (Khil) Dept. of Culinary Science, Honam University, Gwangju, South Korea (Cho) Dept. of Nutrition Education, Honam University, Graduate School of Education, Gwangju, South Korea
ER -

TY - JOUR
ID - 3764
T1 - Childhood-onset idiopathic generalized epilepsy, not otherwise specified (IGE NOS) is medically benign but socially depressing: A population-based cohort with >20 year follow up
A1 - Camfield,C.S.
A1 - Camfield,P.R.
Y1 - 2009///

N1 - 70039108
English
Journal: Conference Abstract
KW - EMBASE
KW - follow up
KW - Epilepsy
KW - Population
KW - generalized epilepsy
KW - Society
KW - childhood
KW - seizure
KW - Child
KW - patient
KW - Pregnancy
KW - Female
KW - Remission
KW - Drug Therapy
KW - learning disorder
KW - high school
KW - convulsion
KW - tonic clonic seizure
KW - juvenile
KW - electroencephalogram
KW - Death
KW - school
KW - University
KW - Mental Health
KW - Government
KW - Friendship
KW - Social Behavior
KW - psychiatric diagnosis
KW - unemployment
KW - injury
KW - spike wave
KW - Prognosis
KW - Canada
KW - benign childhood epilepsy
KW - onset age
KW - epileptic state
KW - intractable epilepsy
KW - myoclonus epilepsy
KW - immunoglobulin E
KW - marker
KW - valproic acid
KW - lamotrigine
KW - topiramate
RP - NOT IN FILE
SP - 207
JF - Epilepsia
VL - Conference: 2009 Annual Meeting of the American Epilepsy Society Boston, MA United States. Conference Start: 20091204 Conference End: 20091208. Conference Publication:
IS - var.pagings
N2 - Rationale: Children with generalized epilepsy may have a non-specific IGE syndrome (IGE NOS). They are intellectually and neurologically normal with generalized tonic-clonic seizures (GTC) and an interictal EEG showing generalized spike-wave without a known cause. The prognosis is not well described. Methods: Children with IGE NOS were selected from the Nova Scotia Childhood Epilepsy Study - a population-based cohort including children with >1 unprovoked seizure with onset between 1977-1985. Follow-up was in 2007-

2008 by personal contact. Results: Of 692 in the cohort, 39(5%) had IGE NOS. 5 had <5 years follow up and were omitted. Age at onset averaged 6.3 + 4 years. Follow-up averaged 22.5 + 7.5 years, at age 29.3 + 8.5 years. There were 18 females. 26 (76%) had a terminal epilepsy remission (seizure-free and off medication) for 16.7 + 8.3 years (range 1-31). Only 3 had an episode of status epilepticus but no one developed intractable epilepsy. Even though 41% had >10 convulsions, there was only one injury-related seizure. All patients had at least 4 years seizure-free during the follow up period (average 17.5 years, range 4-33). During the last year of follow-up, only 3 had any seizures (2 GTC). Of the 8 patients with persistent AED treatment at the end of follow-up, 4 had never received valproic acid, lamotrigine or topiramate suggesting suboptimal treatment. Only one developed Juvenile Myoclonic Epilepsy (JME) while no other patient changed epilepsy syndrome over time. There were no deaths. (For comparison 3.5% of the overall cohort had JME.) At the end of follow-up, of those >18 years of age (N=29), 22 (76%) had significant learning problems as evidenced by >1 school grade repeated (16), resource help (12) or special class (6). Only 17 (59%) completed high school with 4 attending university. 11 (38%) had mental health visits with 21% having depression sufficient for medication treatment. 17(59%) lived with a "life partner" and 7(25%) alone. 9 women had >1 pregnancy and 7 men fathered a child. Only 4 pregnancies were planned and 11 were outside a stable relationship. 35% were unemployed and 14% received government assistance. 60-65% were satisfied with their lives, friendships and social activities. We defined markers of poor social outcome as: a psychiatric diagnosis, no high school graduation, pregnancy outside of a stable relationship, living alone, unemployment. 7(24%) had no poor social outcome markers, 8 had 1, 7 had 2, 5 had 3, 1 had 4 and 1 had all 5. Conclusions: IGE NOS is a recognizable syndrome within the spectrum of IGE. The seizures are nearly exclusively GTC and are transiently problematic for most. Eventually 75% have complete epilepsy remission. 76% have learning difficulties and overall 75% have at least one major indicator of unsatisfactory social outcome
SN - 0013-9580
AD - (Camfield, Camfield) Pediatrics, IWK Health Centre, Dalhousie University, Halifax, NS, Canada
ER -

TY - JOUR
ID - 3765
T1 - Participation and quality of life among youth with SCI
A1 - Kelly,E.H.
A1 - Anderson,C.J.
A1 - Klaas,S.J.
A1 - Russell,H.F.
A1 - Daharsh,E.
A1 - Vogel,L.C.
Y1 - 2009///
N1 - 70036434
English
Journal: Conference Abstract
KW - EMBASE
KW - juvenile
KW - Quality of Life
KW - Rehabilitation
KW - spinal cord
KW - injury
KW - school
KW - Child
KW - Anxiety
KW - exposure
KW - social network
KW - spinal cord injury
KW - personality test
KW - caregiver
KW - questionnaire
KW - medical record
KW - linear regression analysis
KW - Male

KW - Caucasian
KW - paraplegia
KW - Hospital
RP - NOT IN FILE
SP - 462
JF - Journal of Spinal Cord Medicine
VL - Conference: Congress on Spinal Cord Medicine and Rehabilitation Dallas, TX United States. Conference Start: 20090923 Conference End: 20090926. Conference Publication:
IS - var.pagings
N2 - Objective: To determine the relationship between aspects of participation and health-related quality of life (QOL) among youth with spinal cord injury (SCI). Design: Survey research. Participants/Methods: Youth ages 7-18 years who had been injured at least one year and were receiving care within 3 specialty hospitals completed the Children's Assessment of Participation and Enjoyment, Pediatric Quality of Life Inventory, and Revised Children's Manifest Anxiety Scale. Caregivers completed a demographics questionnaire. Injury-related information was gathered from medical records. Analyses included hierarchical linear regression. Results: 175 youth were enrolled: mean age was 12.77 years (SD=3.12), 54% were male, 63% were Caucasian, 73% had paraplegia, 56% had complete injuries, and mean age at injury was 6.90 years (SD=5.30). Participating in more activities was related to increased school QOL (p=.028). As youth participated with others outside of family or participated further from home they experienced higher emotional (p=.023, p=.024, respectively), social (p=.046, p=.020, respectively), and overall psychosocial (p=.045, p=.016, respectively) QOL. After controlling for current age and youth anxiety, participating in more activities significantly predicted higher school QOL (p=.021), and participating with a broader group of people significantly predicted higher emotional QOL(p=.011). Conclusion: There are relationships between diversity of activities and with whom youth participate and aspects of QOL. Results suggest that exposure to more types of activities and increased social networks can increase QOL among youth with SCI
SN - 1079-0268
AD - (Kelly, Anderson, Klaas, Vogel) Shriners Hospitals for Children, Chicago, IL, United States (Russell) Shriners Hospitals for Children, Philadelphia, PA, United States (Daharsh) Shriners Hospitals for Children, Northern California, CA, United States (Vogel) Rush Medical College, Chicago, IL, United States
ER -

TY - JOUR
ID - 3766
T1 - Assessment of quality of life of patient members of a web-based hydroa vacciniforme support group
A1 - Huggins,R.H.
A1 - Leithauser,L.A.
A1 - Eide,M.J.
A1 - Hexsel,C.L.
A1 - Jacobsen,G.
A1 - Lim,H.W.
Y1 - 2009///
N1 - 70386007
English
Journal: Conference Abstract
KW - EMBASE
KW - patient
KW - Society
KW - Quality of Life
KW - support group
KW - hydroa vacciniforme
KW - dermatology
KW - Adult
KW - Child
KW - childhood
KW - skin disease
KW - scar formation
KW - questionnaire

KW - Female
KW - onset age
KW - Caucasian
KW - psoriasis
KW - eczema
KW - atopic dermatitis
KW - Clothing
KW - skin
KW - photodermatosis
KW - blister
KW - Male
RP - NOT IN FILE
SP - S62
JF - Journal of Investigative Dermatology
VL - Conference: 69th Annual Society for Investigative Dermatology Meeting Montreal, QC Canada. Conference Start: 20090506 Conference End: 20090509. Conference Publication:
IS - var.pagings
N2 - Hydroa vacciniforme (HV) is a rare pediatric photodermatosis characterized by pruritic blisters which heal with scarring. The purpose of this study was to investigate the effect of HV on quality of life (QoL) and to compare our results with life quality indices reported for other dermatoses. A disease-specific questionnaire was developed, and participants recruited from a web-based, international HV support group. Impact of HV on QoL was measured using the Dermatology Life Quality Index (DLQI) or the Children's DLQI. Fifteen HV patients participated: 9 (60%) males and 6 (40%) females. Median age at onset was 7 years, and 11/15 (73%) were younger than 18 years. Most patients self-identified as Caucasian (93%). The mean CLDLQI and DLQI scores for HV were 12.1 and 8.5, respectively, which suggests equal or greater impact on patient QoL compared to previously reported indices for childhood psoriasis (9.2) and generalized eczema (9.1), and atopic dermatitis in adults (4.5). Children cited QoL as being negatively impacted most by an inability to play outdoors while adults noted QoL influences due to limitations on choice of clothing. Embarrassment associated with their skin condition was also noted by children and adults surveyed, but personal relationships were not reported as being significantly impacted by either group. Patients with HV have significant impairment of QoL. When compared to other common childhood dermatoses, HV appears to have an equal or greater impact on patients' QoL. Dermatologists should be aware of the substantial psychosociologic impact of this disease
SN - 0022-202X
AD - (Huggins, Eide, Hexsel, Lim) Dermatology, Henry Ford Hospital, Detroit, MI, United States (Eide, Jacobsen) Biostatistics and Research Epidemiology, Henry Ford Hospital, Detroit, MI, United States (Leithauser) School of Medicine, Wayne State University, Detroit, MI, United States
ER -

TY - JOUR
ID - 3767
T1 - Death cap may thrive in playgrounds in urban areas - A case report
A1 - Thorsen,J.
A1 - Bang,J.
A1 - Byrsting,A.M.
A1 - Hjelt,K.
Y1 - 2009///
N1 - 70024808
English
Journal: Conference Abstract
KW - EMBASE
KW - Death
KW - Case Report
KW - urban area
KW - Child
KW - mushroom
KW - Amanita phalloides
KW - intoxication

KW - ingestion
KW - toxicity
KW - Adult
KW - Waste Management
KW - Female
KW - Nursery
KW - forest
KW - Hospital
KW - intensive care unit
KW - gastrointestinal symptom
KW - liver
KW - beech
KW - Risk
KW - fungus
KW - swallowing
KW - poison
KW - activated carbon
KW - antidote
KW - silibinin
RP - NOT IN FILE
SP - 474
JF - Clinical Toxicology
VL - Conference: 29th International Congress of the European Association of Poison Centres and Clinical Toxicologists Stockholm Sweden. Conference Start: 20090512 Conference End: 20090515. Conference Publication:
IS - var.pagings
N2 - Objective: Amanita phalloides, known as the death cap, is a poisonous fungus and when ingested it can have fatal consequences of poisoning. It has high toxic potential, so that even a small intake can cause serious toxicity. Children are considered more sensitive than adults. Besides early gastrointestinal decontamination, there are only a few effective treatment regimens, and a child should therefore always receive treatment if ingestion is suspected.¹ Case report: A 14-month old female was found by the staff at a nursery playground with a mushroom in the mouth. The location was suburban and distant from a forest. Albeit the unusual place of growth, the preliminary identification by the poison centre arouse suspicion of an Amanita phalloides mushroom. Although there was no observation of the child swallowing the mushroom, which was broken in several pieces, she had been under no observation for minutes and could theoretically have ingested a toxic dose. She was immediately brought to the ER where she was aspirated and treated with activated charcoal. Simultaneously, the mushroom was identified by an expert as Amanita phalloides. Treatment with the antidote silibinin was started within 3 hours of ingestion and the child was moved to an intensive care unit for continued treatment. During the following day the child did not show clinical signs of poisoning including gastrointestinal symptoms and the liver parameters remained normal. Conclusion: The deadly Amanita phalloides may thrive in unexpected surroundings as in the presented case where it grew in the playground in an urban area. It grew together with several similar mushrooms close to a beech hedge. Thus the case represents an unanticipated and in some ways unpreventable risk for serious poisoning in a setting with expected high security standards. The child in our case underwent immediate and relevant treatment in collaboration with the Danish Poison Centre and showed no clinical signs of poisoning or rise in biochemistry. The child was discharged from the hospital on the second day of admission without sequelae
SN - 1556-3650
AD - (Thorsen) Department of Clinical Pharmacology, Bispebjerg Hospital, Copenhagen, Denmark (Bang) The Danish Poison Centre, Bispebjerg Hospital, Copenhagen, Denmark (Byrsting) Department of Anaesthesiology and Anaesthesia, Bispebjerg Hospital, Copenhagen, Denmark (Hjelt) Department of Paediatrics, Glostrup Hospital, Copenhagen, Denmark
ER -

TY - JOUR
ID - 3768
T1 - A middle aged woman with back pain
A1 - Boursoulian,L.J.

A1 - Rudnicki,S.A.
Y1 - 2009///
N1 - 70023485
English
Journal: Conference Abstract
KW - EMBASE
KW - Female
KW - Middle Aged
KW - backache
KW - Leg
KW - Muscle
KW - weakness
KW - gene
KW - examination
KW - lordosis
KW - gait
KW - father
KW - hypertrophy
KW - cranial nerve
KW - nerve conduction
KW - action potential
KW - Hoffmann reflex
KW - needle
KW - motor unit
KW - Diagnosis
KW - hip pain
KW - electromyogram
KW - ankle
KW - lumbar spine
KW - family history
KW - neuromuscular disease
KW - mother
KW - arm weakness
KW - Child
KW - Arm
KW - nuclear magnetic resonance imaging
RP - NOT IN FILE
SP - 152
JF - Journal of Clinical Neuromuscular Disease
VL - Conference: 31st Annual Carrell-Krusen Symposium Dallas, TX United States. Conference Start: 20090219 Conference End: 20090220. Conference Publication:
IS - var.pagings
N2 - A 50-year-old woman presented with a 10 year history of progressive back and right hip pain radiating to the ankle. After an MRI of the lumbar spine revealed fatty infiltration of her paraspinal muscles, she was referred to an outside neurologist. She reported spells of weakness, once or twice a year for the past 10 years, where her right leg would give out and she would fall. With further questioning, she admitted to mild leg and arm weakness causing difficulty in exercising at the gym in recent years. Family history was negative for neuromuscular disorders on the mother's side, father'side was unknown. Three half sibs and 2 grown children are healthy. Examination revealed relatively symmetric proximal > distal weakness of the legs > arms. She had an exaggerated lordosis with a waddling gait. There was equivocal calf hypertrophy. She was diffusely areflexic except for preserved triceps jerks. Sensory exam and cranial nerve exam was unremarkable. CK level was 288 IU/L (ULN 235). Nerve conduction study showed mild reduction in amplitude of the right peroneal compound action potential and bilateral absence of H reflexes, otherwise NCS were normal. Needle examination revealed occasional fibs and positive sharp waves and prolonged duration, polyphasic motor units some with satellite potentials in proximal leg and paraspinal muscles. Interference pattern was reduced. Given the neurogenic nature of her EMG and her clinical exam, genetic testing for SMA was done. The diagnosis of SMA was confirmed genetically with zero copies of SMN1 gene and more than four copies of SMN2 gene

SN - 1522-0443
AD - (Boursoulian, Rudnicki) Little RockARUnited States
ER -

TY - JOUR
ID - 3769
T1 - Implementation of EPLS European Pediatric Life Support courses improves survival and quality of life of pediatric sepsis patients
A1 - Wermter,B.
A1 - Wessel,A.
A1 - Ganzer,C.
A1 - Sasse,M.
Y1 - 2009///
N1 - 70371763
English
Journal: Conference Abstract
KW - EMBASE
KW - patient
KW - sepsis
KW - survival
KW - Quality of Life
KW - septic shock
KW - Tertiary (period)
KW - Hospital
KW - Child
KW - necrosis
KW - consultation
KW - Resuscitation
KW - epidemic meningitis
KW - Simulation
KW - emergency
KW - Diagnosis
KW - Human
KW - workshop
KW - physician
KW - traffic and transport
KW - pediatric hospital
KW - ambulance
KW - helicopter
KW - fulminating purpura
KW - skin
KW - lower leg
KW - adverse drug reaction
KW - Survival Rate
KW - Therapy
KW - Mortality
KW - Morbidity
KW - intensive care
KW - Liquid
KW - International Cooperation
KW - protein C
RP - NOT IN FILE
SP - 68
JF - Infection
VL - Conference: 4th International Congress "Sepsis and Multiorgan Dysfunction" Weimar Germany. Conference Start: 20090909 Conference End: 20090912. Conference Publication:
IS - var.pagings

N2 - Introduction: Since 2000/2001 clear progress in the treatment of severe sepsis: immediate & aggressive therapy; mortality & morbidity decrease significantly in our unit, but non in our region. Objectives: to improve treatment of paediatric septic shock in children. Methods: Regional paediatric intensive care network (PIN) reference PICU & 25 children's hospitals in Lower Saxony, Saxony-Anhalt & Nordrhine-Westphaly, (2003).: 3.000.000 inhabitants. Standardised treatment/training of septic shock including standard resuscitation courses EPLS with intraosseous access, fluid-/drug-management, recognition of critical ill child, septic shock scenarios & post resuscitation care. Highly advanced Simulation Training since 2008. Implementation of emergency training, diagnosis & treatment of Waterhouse-Friedrichsen syndrome with human protein C concentrate (PC) by symposia, workshops & 6-months training periods for physicians of the network in reference PICU. 24-h-Service for transport & consultation provided by central PICU. Results: Implementation of EPLS in own PICU & network with international cooperation: 24 courses, 2 instructor courses, 450 participants & 30 instructors inside the network. 8 courses outside the network. Treatment of 26 children with septic shock (herein 17 patients with Waterhouse-Friedrichsen syndrome WFS) within the network, 1 patient of non network hospital. Announcement-time: 15 min.-1 h (not network patient: 8 h.). Consultation was requested by hospitals via phone; treatment was induced immediately. Transportation-Team of tertiary centre went to patients by ambulance or helicopter. Continuation of treatment on site & after transfer, in the tertiary centre. All patients showed typical signs of WFS with purpura fulminans & severe multi organ failure. None of the patient died. One patient had necrosis of skin, already present at admission. The others had restitution ad integrum. The patient from outside presented several areas of necrosis, needed ECMO, 1 lower leg was amputated. No adverse effects submistrating PC concentrate. Patients were retransferred to network hospitals as soon as possible. Conclusions: EPLS is a significant factor for pediatric survival leading to active networking with high acceptance. EPLS it is an important tool for standard treatment and training. Survival rate & outcome after implementation of new treatments & trainings were excellent
SN - 0300-8126
AD - (Wermter, Wessel, Ganzer, Sasse) Paediatric Intensive Care Unit, Department of Paediatric Cardiology and Intensive Care, MHH Medical School Hannover, Germany (Sasse) Paediatric/Neonatal Section, German Sepsis Society, Germany (Wermter) Educational Department, White Cross Medical Emergency Association Southtyrol, Germany (Wermter) Paediatric Working Group, European Resuscitation Council, Germany
ER -

TY - JOUR
ID - 3770
T1 - Adherence to the Mediterranean diet and albuminuria levels in adolescents: Emerging data from the Lyceum Leontio Albuminuria (3L) study
A1 - Mazaraki,A.
A1 - Tsioufis,C.
A1 - Dimitriadis,K.
A1 - Syrseloudis,D.
A1 - Thomopoulos,C.
A1 - Tolis,P.
A1 - Selima,M.
A1 - Stefanadi,E.
A1 - Kallikazaros,I.
A1 - Stefanadis,C.
Y1 - 2009///
N1 - 70354031
English
Journal: Conference Abstract
KW - EMBASE
KW - albuminuria
KW - Adolescent
KW - cardiology
KW - Mediterranean diet
KW - Society
KW - Male
KW - body mass
KW - habit

KW - Heart Rate
KW - Waist Circumference
KW - sport
KW - school
KW - Food
KW - Television
KW - leisure
KW - pulse pressure
KW - Risk
KW - Population
KW - Obesity
KW - Cardiovascular System
KW - Blood Pressure
KW - urine
KW - questionnaire
KW - food frequency questionnaire
KW - Lifestyle
KW - Child
KW - atherosclerosis
KW - excretion
KW - albumin
KW - creatinine
KW - marker
RP - NOT IN FILE
SP - 236
JF - European Heart Journal
VL - Conference: European Society of Cardiology, ESC Congress 2009 Barcelona Spain. Conference Start: 20090829 Conference End: 20090902. Conference Publication:
IS - var.pagings
N2 - Purpose: Mounting evidence supports that Mediterranean diet has favorable effects on the cardiovascular system, while albuminuria is associated with atherosclerosis progression and increased risk. The aim of the study was to investigate the relationship of dietary habits with urinary albumin excretion, expressed as the albumin to creatinine ratio (ACR), in a cohort of adolescents. Methods: A total of 365 adolescents 12-17 years of age [212 males, aged 13.9 years, office blood pressure (BP)=115/67 mmHg] that were included in the Lyceum Leontio Albuminuria (3L) study were considered for analysis. In all participants ACR values were determined in a morning spot urine and for each adolescent a questionnaire was completed in order to retrieve information on dietary habits (through a semi-quantitative Food Frequency Questionnaire), lifestyle and socio-economic characteristics. Moreover, the Mediterranean Diet Quality Index for children and adolescents (KIDMED) was estimated and accordingly subjects were divided into those with optimal (>7), average (4-7) and low (<4) score. Results: Only 6.8% of the participants had optimal KIDMED score, whereas 51.2% had an average and 42% had a low score. Participants with at least average KIDMED score (n=187) compared to those with low KIDMED score (n=153) were more frequently males (64.5 vs 53.4%, p=0.029), had higher body mass index (22.2 vs 21.4 kg/m², p=0.043) and waist circumference (77.6 vs 75.4 cm, p=0.044), spent more frequently time for sports activities outside school (75.2% vs 58%, p=0.001), reported less consumption of foods outside home (3% vs 14%, p<0.001), less hours of watching television (1.75 vs 2.05 hours, p=0.013) and more hours of leisure time (5.4 vs 4.4 hours, p=0.026). Moreover, those with at least average compared to those with low KIDMED score exhibited higher systolic BP (117 vs 114 mmHg, p=0.039) and pulse pressure (49 vs 47 mmHg, p=0.01), whereas had lower heart rate (84 vs 87 bpm, p=0.014) and ACR levels (12.6 vs 20.5 mg/g, p=0.015). In the total population, ACR was associated with age (r=-0.11, p=0.044), male sex (r=0.160, p=0.003), body mass index (r=- 0.131, p=0.016), systolic BP (r=-0.144, p=0.008), heart rate (r=0.141, p=0.011) and KIDMED score (r=-0.111, p=0.041). Conclusions: In adolescents there is an inverse relation of KIDMED score with albuminuria and those who adhere to the Mediterranean diet exhibit lower levels of ACR. However, the paradoxical associations of both ACR and KIDMED score with obesity markers and BP levels suggest distinct mechanisms of albuminuria development in adolescents
SN - 0195-668X
AD - (Mazaraki, Tsioufis, Dimitriadis, Syrseloudis, Thomopoulos, Tolis, Selima, Stefanadi, Kallikazaros, Stefanadis) First Cardiology Clinic, University of Athens, Hippokration Hospital, Athens, Greece

ER -

TY - JOUR
ID - 3771
T1 - Multipolar radiofrequency ablation for the treatment of HCC using no touch technique: Initial experience
A1 - Seror,O.
A1 - N'Kontchou,G.
A1 - Ajavon,Y.
A1 - Amerane,H.
A1 - Coderc,E.
A1 - Trinchet,J.
A1 - Beaugrand,M.
A1 - Sellier,N.
Y1 - 2009///
N1 - 70339809
English
Journal: Conference Abstract
KW - EMBASE
KW - radiofrequency ablation
KW - Society
KW - Europe
KW - patient
KW - Tumor
KW - electrode
KW - tumor ablation
KW - Death
KW - pneumonia
KW - hospital patient
KW - ascites
KW - jaundice
KW - pleura effusion
KW - follow up
KW - tumor growth
KW - cancer control
KW - liver cell carcinoma
KW - Liver Cirrhosis
KW - Child
KW - Safety
KW - treatment response
KW - computer assisted tomography
KW - liver
RP - NOT IN FILE
SP - 410
JF - CardioVascular and Interventional Radiology
VL - Conference: Cardiovascular and Interventional Radiological Society of Europe, CIRSE 2009 Lisbon Portugal. Conference Start: 20090919 Conference End: 20090923. Conference Publication:
IS - var.pagings
N2 - Purpose: to assess feasibility and effectiveness of extranodular no touch multipolar radiofrequency ablation of hepatocellular carcinoma (HCC). Materials/methods: 26 patients (56-82 years, mean: 68+7 years) with cirrhosis (Child-Pugh A/B: 21/5) and one or two (22/4) HCC (1.5-5.5 cm, mean: 3+0.9 cm) were treated with no touch multipolar radiofrequency ablation consisting of simultaneous activation of two to four bipolar coaxial electrodes inserted just outside the tumors. Safety and effectiveness of the no touch technique were assessed. Treatment response was evaluated with CT scan. Results: to achieve complete ablation of all tumors (100%) one to two sessions (mean: 1+0.3) including one to two applications (mean: 1+0.2) of 16 min to 45 minutes were required. The mean number of electrodes used per tumor ablation was 3.1+0.6. 40 to 220 kJ were delivered per session into the tumors (mean: 119+43 kJ). Two deaths occurred within three months after the procedure: one at 82 days in a patient who experienced uncontrolled pneumonia and the other at 54 days in

patient who had infected ascites. Two other patient experienced minor complications: transient jaundice in one and pleural effusion in other. After a mean follow up of 6.6+5 months, no local tumor progression was detected. Distant multinodular recurrence occurred in one patient 2 months after the treatment. Conclusion: multipolar radiofrequency ablation of HCC using no touch technique seems to be a very effective method in term of tumor control. However, owing to the large volume of non tumorous liver tissue ablated, a careful selection of patients is mandatory
SN - 0174-1551
AD - (Seror, Ajavon, Amerane, Coderc, Beaugrand, Sellier) Radiology, Hopital Jean Verdier, AP-HP, Bondy, France (N'Kontchou, Trinchet) Hepatology, Hopital Jean Verdier, AP-HP, Bondy, France
ER -

TY - JOUR
ID - 3772
T1 - Bland embolization
A1 - Orsi,F.
A1 - Bonomo,G.
A1 - Monfardini,L.
A1 - Della,Vigna P.
Y1 - 2009///
N1 - 70339108
English
Journal: Conference Abstract
KW - EMBASE
KW - artificial embolism
KW - Europe
KW - Society
KW - patient
KW - Tumor
KW - Hypoxia
KW - survival
KW - multidetector computed tomography
KW - necrosis
KW - feeding
KW - Therapy
KW - ischemia
KW - hepatic vein
KW - injection
KW - catheter
KW - surgery
KW - Inflammation
KW - palliative therapy
KW - liver tumor
KW - shunting
KW - occlusion
KW - cancer cell
KW - liver
KW - microbubble
KW - follow up
KW - Chemotherapy
KW - Lung
KW - tumor necrosis
KW - tissue
KW - Abdomen
KW - Prognosis
KW - cancer staging
KW - liver function
KW - conservative treatment

KW - promoter region
KW - mitosis
KW - cell survival
KW - protein expression
KW - angiogenesis
KW - blood level
KW - stimulation
KW - blood vessel
KW - systemic circulation
KW - Cell Adhesion
KW - endothelium
KW - consensus
KW - Particle Size
KW - Diagnosis
KW - Biopsy
KW - liver disease
KW - Child
KW - gastrointestinal hemorrhage
KW - brain disease
KW - ascites
KW - artery
KW - angiography
KW - celiac artery
KW - superior mesenteric artery
KW - Catheterization
KW - meta analysis
KW - thrombosis
KW - ultrasound
KW - intraarterial drug administration
KW - scintigraphy
KW - hepatic artery
KW - artery blood flow
KW - analgesia
KW - minimally invasive procedure
KW - chemoembolization
KW - Fluoroscopy
KW - blood flow
KW - arterial blood
KW - relapse
KW - Imaging
KW - contrast enhancement
KW - liver scintiscanning
KW - pancreatitis
KW - breast cancer
KW - heart infarction
KW - liver surgery
KW - Autopsy
KW - embolism
KW - pulmonary artery
KW - liver blood vessel
KW - antiinflammatory activity
KW - pathologist
KW - Rupture
KW - liver cell carcinoma
KW - apoptosis
KW - microsphere
KW - survivin

KW - antineoplastic agent
KW - gelatin
KW - protein
KW - vasculotropin
KW - polyvinyl alcohol
KW - resin
KW - glue
KW - fetoprotein
KW - levobupivacaine
RP - NOT IN FILE
SP - 140
EP - 142
JF - CardioVascular and Interventional Radiology
VL - Conference: Cardiovascular and Interventional Radiological Society of Europe, CIRSE 2009 Lisbon Portugal. Conference Start: 20090919 Conference End: 20090923. Conference Publication:
IS - var.pagings
N2 - Introduction: Only 20 to 30% of patients with hepatocellular carcinoma (HCC) are candidates for curative treatments and most of them are suitable only for locoregional therapies or palliative care. Intra-arterial treatment as transarterial chemoembolization (TACE) and transarterial embolization (TAE) are considered as palliative therapy for multifocal HCC in advanced stage. Several alternative minimally invasive treatments have been proposed but recently only TACE has been shown to improve survival compared with best supportive care in meta-analyses of randomized trials. However, it is not clear whether TAE alone gives the same survival advantage nor whether specific patient characteristics affect outcome or any particular technique in performing transarterial therapy is better than any other. Some authors have reported experience in treating liver tumor by mean TAE by using different materials and techniques: not selective, selective and superselective injection of fibrine sponge, glue, particles and so on. The main purpose of this study is to assess feasibility and local response in patients affected by unresectable HCC, treated with TAE using microparticles sized 40 and 100 microns. Rationale for using well calibrated small sized microparticles for treating liver tumors is mainly based on the evidence that all hepatic malignancies are mainly or exclusively fed by arterial blood flow. Vessels within the tumor have been reported to have a size ranged between 20 to 120 microns and it should be, in our opinion, the target for achieving a deep ischemia and necrosis by using TAE. Bigger particles will embolize bigger vessels outside the target nodule: the more bigger the particles used, the more far from the tumor will be the embolization. Methods: Between October 2007 and April 2008, 22 patients underwent TAE. Diagnosis of HCC was based on radiological findings (MDCT scan), alfa-fetoprotein level and biopsy according to the Barcellona criteria. Inclusion criteria were HCC nodules not suitable for surgical resection or percutaneous therapies. Exclusion criteria were patients older than 80 years, advanced liver disease (Child Pugh class-C), active gastrointestinal bleeding, encephalopathy, refractory ascites, portal branch occlusion and documented arterovenous hepatic shunts. All patients underwent upper-abdomen multi-phase MDCT before treatment. MDCT scans were also used for defining vascular anatomy supplying the liver and for assessing tumor feeding vessels. A total of 32 target lesions were embolised using 40 and/or 100 i micro-spheres (Embozene /Celonova); average treated nodule diameter was 5.7 cm. Angiography of celiac trunk and superior mesenteric artery were always obtained before every treatment, in order to assess arterial anatomy and possible variants and for detecting the feeding vessels to the target lesions. Superselective catheterization of tumor feeding arterial branches was obtained by using micro-catheters (Renegade High Flow/Boston Scientific). When it was not possible to detect the feeding vessels to the lesion, CO₂-microbubbles-enhanced liver ultrasound has been performed; it consists into the intra-arterial injection of CO₂ microbubbles through the micro-catheter, by changing the positions of the tip within different arterial branches feeding the tumour area. Right vessel to be used for TAE was detected when the target lesion enhanced during micro-bubbles injection. Hepatic perfusional scintigraphy with intrarterial injection via the main hepatic artery of macro-aggregates of albumine (TC99-MAA) was performed just before TAE, in order to exclude pulmonary shunting. Just before the embolization, 5 mg of levobupivacaine was injected via the micro-catheter in order to obtain local parenchymal analgesia; then, microspheres were gently injected under fluoroscopy, until blood flow was stopped. Upper abdomen MDCT was performed 24 hours after treatment, for assessing early local result and for detecting any possible complication. MDCT scan was then repeated at 30 days, three and every six months. Local efficacy of the treatment was defined according to RECIST criteria on CT follow-up imaging: complete response was defined as the disappearance of treated target lesion. Moreover, post TAE amount of tumour devascularisation (i.e. necrosis) has been also evaluated: complete response (=devascularisation) was defined as

the absence of any contrast enhancement during arterial phases in MDCT, massive response as the necrosis of the huge portion of the lesion and partial response as the necrosis of a portion of the lesion. We considered a complication due to the treatment if it occurred within 3 weeks from TAE. Results: Patients received a total of 29 sessions of TAE. Technical success was achieved in all TAE procedures. Only one pt within the study had a 6% pulmonary shunting detected at MAA liver scintigraphy. Minor complications were observed in 2 patients: 2 pancreatitis restored in few days. Major complication occurred in one patient who died within 24 hr after TAE. This 74-y.o. patient had a previous history of breast cancer, CRC and heart infarction due to chemotherapy. She had even liver surgery for HCC two years before (S2-S3 resection), with early recurrence within S4, just between the right and the median hepatic vein. Post-mortem examination showed the presence of a large necrotic portion of the lesion and the presence of necrotic emboli in the right pulmonary artery. Microspheres were observed in arterial and portal liver vessels, but also into both lungs. Pathologists supposed that a massive tumor necrosis and wall rupture of the tumor involved hepatic vein occurred few hours after TAE with passage of necrotic tissue and microspheres to the lungs via the median hepatic vein. Complete necrosis (hypodensity) was observed in 30 nodules at 24 h CT, while 2 nodules showed poor response. All patients included have at least one month FU (average: 10 months) and evaluation based on RECIST criteria shown 2 progressive disease (PD), 13 stable disease (SD), 6 partial response (PR= ranging between 33 and 78% of diameter reduction) and 1 complete response (CR). Discussion: There is no standard therapy for patients with HCC unsuitable for surgical treatment. Cirrhotic patients with HCC have a poor prognosis, mainly influenced by hepatic reserve function and tumor staging. In these cases, TAE and TACE are the most used treatments with proven improvement on survival in selected patients with well preserved liver function. Actually, there is no absolute evidence that TACE is better than TAE. Six randomised trials of arterial embolizations, with or without chemotherapy, have shown a strong antitumoral effect, but none detected survival benefits in comparison with conservative management or suboptimum treatments. Some authors describe an important rule represented by survivin, a member of family of inhibitors of apoptosis protein and promoter of mitosis and therefore cancer cell survival and growth. Theoretically, TACE, combining the effect of drug with hypoxia, should be more effective than TAE. Survivin expression, increase under anticancer drug and further increase over, after the administration of a combination of hypoxia and anticancer drug. No survivin protein expression is observed in the hypoxia condition without drug. There is no evidence of an additive or synergistic antitumoral effect of TACE vs TAE alone. Three different randomized trial studies have failed in demonstrating a significant difference in survival between the two different treatments. Probably, ischemia resulting from embolization might be the main factor inducing reduction in tumour size after TACE. Conversely, hypoxia and inflammation, as they are powerful stimulators of angiogenesis, might inadvertently promote tumour growth. Kobayashi et al. showed an increasing of serum concentration of vascular endothelial growth factor in patients who underwent TACE, suggesting a direct link between the degree of embolization, tumour hypoxia, and the stimulation of new blood vessel growth. Moreover, it has been suggested that TACE may facilitate the hematogenous dissemination of malignant cells in the systemic circulation by disrupting cell-cell adhesion, and by damaging the endothelium. In the major part of the studies concerning HCC treatment with TAE, the embolizing agent is gelatine sponge that induces hypoxia only temporarily. In some studies, polyvinyl alcohol (PVA) particles were used to cause a permanent or semi-permanent vessel occlusion. Nowadays, there are few studies on the new embolizing agents, such as resin or gelatine microspheres. There is no consensus about which is the most effective embolizing agent. Theoretically, an embolizing agent that has a standardized particle size and that can be delivered into smaller arteries and cause permanent thrombosis should be more effective than temporary or eterogeneous sized embolizing agents. Moreover, smaller particles will effect within the tumor vessels, avoiding any possible arterial blood relapse as it can occur if more distal vessels are occluded. In this scenario, some other new vessels from outside the nodule will develop and feed the tumor. The new microparticles (Embozenes) we used for TAE have some distinctive features, which may lead some other advantages in obtaining tumor necrosis than other materials. Precise and well calibrated small size and antiinflammatory effect (due to the coating film with Polyzene-F) are the most important characteristics, which may allow for deep ischemia without inflammatory reaction of surrounding tissue. According to the RECIST criteria, we had one patient with complete response (tumor disappearance) at 14 months follow up

SN - 0174-1551

AD - (Orsi, Bonomo) Unit of Interventional Radiology, European Institute of Oncology, Milan, Italy (Monfardini, Della Vigna) Radiology, Unit of Interventional Radiology, European Institute of Oncology, Milan, Italy

ER -

TY - JOUR

ID - 3773
T1 - Previous exposure with cephalosporins and macrolides but not cotrimoxazole as a risk factor for colonization with MRSA in HIV-infected children
A1 - Kalavska,A.
A1 - Krcmery,V.
A1 - Liskova,A.
Y1 - 2009///
N1 - 70336953
English
Journal: Conference Abstract
KW - EMBASE
KW - methicillin resistant Staphylococcus aureus
KW - Child
KW - risk factor
KW - tropical medicine
KW - Human immunodeficiency virus
KW - exposure
KW - Hygiene
KW - Society
KW - Infection
KW - methicillin resistant Staphylococcus aureus infection
KW - patient
KW - Cambodia
KW - Hospital
KW - Risk
KW - Asia
KW - prophylaxis
KW - high risk population
KW - meta analysis
KW - community
KW - drug self administration
KW - orphanage
KW - University
KW - Prevalence
KW - antibiotic therapy
KW - highly active antiretroviral therapy
KW - World Health Organization
KW - cotrimoxazole
KW - cephalosporin derivative
KW - macrolide
KW - antibiotic agent
KW - quinolone derivative
KW - glycopeptide
KW - lactam
RP - NOT IN FILE
SP - 136
JF - American Journal of Tropical Medicine and Hygiene
VL - Conference: 58th Annual Meeting of the American Society of Tropical Medicine and Hygiene, ASTMH Washington, DE United States. Conference Start: 20091118 Conference End: 20091122. Conference Publication:
IS - var.pagings
N2 - MRSA infections in community accounted for 3-11% of S. aureus infections in resource-limited regions of Asia especially due to availability of over-the-counter antibiotics, their frequent self-administration for inappropriate indications and taken for irregular durations, low cost and substandard quality. We would like to expand those data by a small cohort of 102 HIV-infected children from Cambodia treated with HAART for 4 to 5 years. Of 102 HIV-infected children in the orphanage House of Family run by St. Elizabeth University, 62 were colonized within last 58 months with S. aureus, which caused 149 episodes of infection, 118 MRSA and

31 MSSA. Proportion of MRSA colonization was very high at the baseline (75-100%) without any previous known hospital contact as well as during first 24 months, then dropped to 57,1% and 43% after 27 respectively 30 months but then rose again to the level at baseline of 76% after 51 months. Meta-analysis which analyzed exposure of antibiotics as a risk factor for MRSA infection showed that risk of acquiring MRSA was increased by 1.8-fold in patients who had taken antibiotics. Quinolones increased risk 3-fold, glycopeptides 2.9-fold, cephalosporins 2.2-fold and other beta-lactams 1.9-fold. When looking for previous exposure with antibiotics one month before infection, children infected with MRSA were significantly more frequently receiving oral 2nd and 3rd generation cephalosporins (67% vs. 23%, P<0.01) and macrolides (47% vs. 23%, P<0.05). However aminopenicillins, quinolones and cotrimoxazole were not significantly related with MRSA in HIV-infected children. Quinolones were less associated with MRSA in our group of children because of less frequent use in children. The prevalence of MRSA remained high but did not increase despite frequent antibiotic treatment of S. aureus infections during entire 58 months assessed period. Use of cephalosporins and macrolides were significantly related to the increased of MRSA infection but surprisingly despite children were receiving cotrimoxazole during the entire period of 4 to 5 years for prophylaxis as recommended by WHO, use of cotrimoxazole was not related to resistance selection. Emergence of MRSA in a resource-limited countries in Asia fuelled by uncontrolled antibiotic use outside hospital settings may pose a significant threat for risk group of patients such as our group of HIV-infected children from Cambodia
SN - 0002-9637
AD - (Kalavska, Krcmery, Liskova) St. Elizabeth University College of Health and Social Sciences, Bratislava, Slovakia
ER -

TY - JOUR
ID - 3774
T1 - A living donor liver transplantation for uncorrectable intra- and extrahepatic biloma after transcatheter arterial chemoembolization
A1 - Kim,Y.J.
A1 - Chang,S.-H.
A1 - Yun,I.J.
A1 - Lee,H.W.
Y1 - 2009///
N1 - 70321684
English
Journal: Conference Abstract
KW - EMBASE
KW - biloma
KW - Liver Transplantation
KW - chemoembolization
KW - Society
KW - living donor
KW - liver
KW - Infection
KW - bile duct obstruction
KW - abscess
KW - Cholangitis
KW - peritoneal cavity
KW - outpatient department
KW - percutaneous drainage
KW - liver cell carcinoma
KW - patient
KW - biliary tract drainage
KW - Child
KW - Klebsiella pneumoniae
KW - adhesion
KW - recurrent infection
KW - Inflammation
KW - pus

KW - abdominal wall
KW - peritoneum
KW - recipient
KW - transfusion
KW - Biopsy
KW - bile duct stone
KW - erythrocyte
KW - hepatitis B surface antigen
KW - antibiotic agent
RP - NOT IN FILE
SP - S266
JF - Liver Transplantation
VL - Conference: International Liver Transplantation Society 15th Annual International Congress New York, NY United States. Conference Start: 20090708 Conference End: 20090711. Conference Publication:
IS - var.pagings
N2 - We experienced a living liver transplantation of an unusual indication of uncorrectable intra- and extrahepatic biloma after transcatheter arterial chemoembolization (TACE) which could be a relative contraindication so that we are reporting it. A 45 year old patient was a carrier of HBsAg for more than 25 years. He was diagnosed to be hepatocellular carcinoma (HCC) in 2004. He then underwent TACE 6 times and had a complication of biliary stricture in Nov. 2007. At that time the problem was solved with an interventional procedure. In Apr. 2008, he underwent another TACE for HCC, and after that a long segment of biliary stricture was developed. With recurrent cholangitis, there developed two huge biloma, one in the liver and one outside the liver. Percuatenous transhepatic biliary drainage for the intrahepatic biloma and percutaneous drainage for the extrahepatic biloma with long standing antibiotics administration were done, but with all effort there was no improvement. The Child-Turcote-Pugh score was 6 and Klebsiella pneumoniae was detected in culture for the drain discharge. The CT shows two large biloma; one in the liver and one outside the liver. There was massive adhesion due to recurrent infection and inflammation in the abdominal cavity. Small amount of pus was inevitably spilled into the abdominal cavity. The extrahepatic biloma was found to be abdominal wall abscess which was covered with thick peritoneum. The total recipient operative time was 12 hours and 25 minutes and required transfusion was 8 units of packed RBC and 4 units of FFP. The biopsy showed HCC of 3.0 x 1.5 x 1.2 cm in the caudate lobe. Hepatolithiasis with chronic and active cholangitis and multiple abscess formation was also seen. He is now visiting the outpatient clinic without a problem. Although usually active and uncontrolled infection outside of the hepatobliliary system is thought to be an absolute contraindication to liver transplantation and chronic and refractory infection is thought to be a relative contraindication. In the present case, however, the localized, controlled infection and biloma did not cause the posttransplant septic complications. Therefore, a liver transplantation could be considered for uncorrectable, infected biloma if other treatement modalities failed
SN - 1527-6465
AD - (Kim, Chang, Yun, Lee) Organ Transplant Center, Konkuk University Hospital, Seoul, South Korea (Chang, Yun, Lee) Department of Surgery, Konkuk University, School of Medicine, Seoul, South Korea
ER -

TY - JOUR
ID - 3775
T1 - Two year old male with foreign body ingestion
A1 - Kamboj,S.
A1 - McGeady,S.J.
A1 - Yousef,E.
Y1 - 2009///
N1 - 70255054
English
Journal: Conference Abstract
KW - EMBASE
KW - allergic asthma
KW - ingestion
KW - Male
KW - college

KW - foreign body
KW - immunology
KW - esophagus
KW - Biopsy
KW - Child
KW - allergy
KW - patient
KW - gastrointestinal symptom
KW - wheat
KW - peanut
KW - rice
KW - beef
KW - potato
KW - oat
KW - disease course
KW - eosinophilic esophagitis
KW - Drug Therapy
KW - elemental diet
KW - esophagus biopsy
KW - pathology
KW - endoscopy
KW - parent
KW - witness
KW - dysphagia
KW - abdominal pain
KW - vital sign
KW - hypersalivation
KW - telecommunication
KW - gastroenterology
KW - esophagogastroduodenoscopy
KW - forceps
KW - eosinophil
KW - hyperplasia
KW - squamous epithelium
KW - skin
KW - egg
KW - Food
KW - milk
KW - thorax radiography
KW - proton pump inhibitor
KW - allergen
RP - NOT IN FILE
SP - A94
JF - Annals of Allergy, Asthma and Immunology
VL - Conference: 2009 Annual Scientific Meeting of the American College of Allergy, Asthma and Immunology, ACAAI Miami, FL United States. Conference Start: 20091105 Conference End: 20091110. Conference Publication:
IS - var.pagings
N2 - Introduction: A 2 year old male presented to the ER after refusal to eat for the past 6 hours, gesturing to his mouth and saying "coin." Parents did not witness ingestion of a foreign body (FB) and denied any previous complaints of dysphagia or abdominal pain. ER vital signs were stable and physical exam was unremarkable, revealing a playful, interactive child with no drooling or distress. Review of symptoms did not identify any previous gastrointestinal(GI) complaints in this child. Initial evaluation: Chest X ray (CXR) revealed a 2.0 cm radio-opaque FB in upper esophagus at the T3 level. Subsequent CXR taken 18 hours later showed no change in the FB location. Gastroenterology (GI) team performed an esophagogastroduodenoscopy (EGD) and removed a coin from the upper esophagus by using forceps. Evidence of furrowing was found in the middle and lower thirds of the esophagus, of which biopsies were collected. Results: Pathologic evaluation of the biopsies

revealed more than 70 eosinophils(EOS) per high-power field (HPF) in both the middle and lower thirds of the esophagus. Epithelial reactive changes, spongiosis, and hyperplasia of the squamous epithelium were also appreciated. The patient was referred to GI and Allergy for further evaluation. Allergy evaluation revealed positive skin prick testing (SPT) to multiple indoor, outdoor allergens, and egg. Patch testing revealed numerous positives to multiple foods including: milk, soy, wheat, peanut, rice, beef, potato, turkey, and oat. Clinical Course: The patient was diagnosed with eosinophilic esophagitis (EE) by the GI team. He was started on a daily proton pump inhibitor medication in addition to an elemental diet. Subsequent EGD and biopsy 6 months later showed interval improvement in the mid and distal esophageal biopsies revealing 30 and 9 EOS/HPF, respectively. Conclusion: This case illustrates an unusual presentation for EE in a healthy child with no previous GI complaints. This case also highlights that failure of a FB to pass through the esophagus may reflect underlying pathology. Therefore, endoscopy to remove such a FB should include evaluation of the overall condition of the esophagus
SN - 1081-1206
AD - (Kamboj, McGeady) PhiladelphiaPAUnited States (Yousef) WilmingtonDEUnited States
ER -

TY - JOUR
ID - 3777
T1 - Pet therapy: Study of effectiveness on childhood headache
A1 - Moscato,D.
A1 - Calabrese,B.
A1 - Moscato,F.R.
Y1 - 2009///
N1 - 70216790
English
Journal: Conference Abstract
KW - EMBASE
KW - headache
KW - pet therapy
KW - Society
KW - childhood
KW - patient
KW - migraine
KW - Child
KW - Anxiety
KW - pathology
KW - Environment
KW - processing
KW - nerve cell plasticity
KW - longitudinal study
KW - experimental model
KW - Statistics
KW - Therapy
KW - marker
RP - NOT IN FILE
SP - 105
EP - 106
JF - Cephalalgia
VL - Conference: 14th Congress of the International Headache Society Philadelphia, PA United States. Conference Start: 20090910 Conference End: 20090913. Conference Publication:
IS - var.pagings
N2 - Objectives: The goal of this work is to evaluate the salient features of headache patients, to whom the Pet Therapy had long-lasting benign effect. Background: Pet Therapy effectiveness studies are rarely found in international publications, although in the common practice, also outside clinical environment, there exist very high statistics of well responding patients. Our group has been using for several years the Pet Therapy as child's headache first choice Therapy. In several works we authored, we noticed a reduction of headache-defining parameters, as well as of associated psychological markers. Methods: All patients which underwent Pet Therapy

in 2006 have been contacted and questioned. Out of the 74 patients, 65 patients have been reached; out of the group of questioned 65, 55 patients declared to not show anymore headache symptoms. Data from 2006 clinical charts of the responding 55 patients have been reanalyzed (35MWoutA, 7 MwA, 13FETTH,)36f, 19m, mean age 10,4+/-3,4 range 6/15). Results: At birth, neuro-behavioral and developmental aspects in the first years of life there is no noticeable difference between the two groups. Regarding headache features outside TTH details and migraine, it has been found a precocious insurgency of migraine 3 years versus 1 year for TTH, as the presence of a traumatic psychological event in the pathology genesis in 75% of the migraine patients, versus 30% TTH. Differences have been spotted in the psychological features of the patients: 95% of migraine patients suffering from anxiety, out of which in 25% of those associated with depression; while in the TTH anxiety was present 69% of the times. Main difference was found in the duration of the Pet Therapy treatment: 22 + 3 sessions for TTH, against 37 + 12 in migraine patients. Conclusions: In the Pet Therapy, we found no specific characteristics in the two diseases, in addition to a significant difference in processing time. This once again suggests that the child's headache could be a continum from one form to another; so that the Pet Therapy seems to elicit adaptive psychological structures that activate the unrealized potential of neuronal plasticity. This longitudinal study confirms the effectiveness of the stabilization of the results of Pet Therapy, paradoxically, without fail, even if we have not an experimental model explanatory
SN - 0333-1024
AD - (Moscato, Calabrese, Moscato) Childhood Headache Centre, Saint Charles IDI Hospital, Roma, Italy
ER -

TY - JOUR
ID - 3778
T1 - Don't get cold feet-winter fun is for everyone! (2009 Fred P. Sage Award Winner)
A1 - Anthony,B.
A1 - Hahn,T.
Y1 - 2009///
N1 - 70212280
English
Journal: Conference Abstract
KW - EMBASE
KW - winter
KW - awards and prizes
KW - Cerebral Palsy
KW - Child
KW - disability
KW - Recreation
KW - videorecording
KW - wheelchair
KW - community
KW - consultation
KW - Risk
KW - Obesity
KW - Health
KW - skill
KW - volunteer
KW - juvenile
KW - Language
KW - health care personnel
RP - NOT IN FILE
SP - 85
JF - Developmental Medicine and Child Neurology
VL - Conference: 63rd Annual Meeting of the American Academy for Cerebral Palsy and Developmental Medicine Scottsdale, AZ United States. Conference Start: 20090923 Conference End: 20090926. Conference Publication:
IS - var.pagings
N2 - Participating in recreation is widely recognized by healthcare professionals as beneficial fro all children. Meaningful activity contributes to a child's physical, social, emotional, spiritual and intellectual growth. This is

even more important for children with disabilities because recreation is by its very nature, nurturing and restorative. Many studies, however, show that children with disabilities are often less involved than their peers. They have been found to be less active and more at risk of obesity and impaired health due to lack of activity (Steeles et al., 2000). Bloorview Kids Rehab's Therapeutic Recreation Life Skills program undertook a series of consultations with our families, clients, staff, volunteers and service providers to look at some of the reasons for diminished participation. One of the key findings was the need for engaging, easy-to-access information (e.g., appealing to youth; realistic; easy-to-understand for those whose first language is not English; available for viewing on our website and/or while waiting for appointments at Bloorview) about winter activities, especially for children with higher needs. As a result, we developed a 12-minute, open-captioned video inviting people with disabilities to get outside in the winter. Shot at WindReach, a wheelchair-accessible farm, the film demonstrates a variety of adapted winter equipment, tips and activities to promote healthy, active living in the community
SN - 0012-1622
AD - (Anthony, Hahn) Therapeutic Recreation and Life Skills, Life Skills and Wellness Institute, Bloorview Kids Rehab, Toronto, ON, Canada
ER -

TY - JOUR
ID - 3779
T1 - Accuracy and precision of the StepWatch in stride counting and oxygen consumption
A1 - Bjornson,K.
A1 - Yung,D.
A1 - Burr,Msee R.
A1 - Jacques,K.
A1 - Christakis,D.
Y1 - 2009///
N1 - 70212179
English
Journal: Conference Abstract
KW - EMBASE
KW - Oxygen Consumption
KW - accuracy
KW - Cerebral Palsy
KW - Prediction
KW - Walking
KW - book
KW - Child
KW - walking speed
KW - juvenile
KW - Physical Activity
KW - Energy Expenditure
KW - treadmill
KW - velocity
KW - gender
KW - leg length
KW - Exercise Test
KW - community
KW - ankle
KW - linear regression analysis
KW - accelerometer
KW - Adult
KW - gold standard
KW - case study
KW - volunteer
KW - boy
KW - girl
KW - weight

KW - employee
KW - tertiary health care
KW - gait
KW - computer program
KW - pediatric hospital
KW - Oxygen
RP - NOT IN FILE
SP - 32
EP - 33
JF - Developmental Medicine and Child Neurology
VL - Conference: 63rd Annual Meeting of the American Academy for Cerebral Palsy and Developmental Medicine Scottsdale, AZ United States. Conference Start: 20090923 Conference End: 20090926. Conference Publication:
IS - var.pagings
N2 - Background/Objectives: Studies testing methods to promote physical activity require accurate and precise measurements of both physical activity and energy expenditure in children. StepWatch (SW) accelerometer data has been found to accurately count strides across speeds and predict oxygen costs of walking in lean and obese adults. This project aims to test the accuracy and precision of the SW across walking speeds against the gold standard of manual stride counting and to pilot the prediction of oxygen consumption modeled from SW counts in youth developing typically (YDT). Design: Instrument development and validation with a clinical case series. Participants and Setting: A volunteer sample of ten YDT (5 boys, 5 girls), average age of 14.1 (SD 2.2) years, average leg length 86.2 (SD 7.5) cm, weight 50.2 (SD 11.2) kg and BMI 18.3 (SD 2.2) participated with recruitment through (children of) employees of a tertiary care children's hospital. Materials/Methods: Two SW monitors were calibrated to individual walking patterns using SW software and leg length. A self-selected walking (100+ stride) sample with the SW was compared to manual count with accuracy of 99% (SD 0.47). Participants underwent treadmill cardiopulmonary exercise testing (CPET) wearing two SW monitors (outside right, inside left ankles) at 1, 2, 3 and 4 mph for 3 minutes. Absolute disagreement, with a ratio and interclass coefficients (ICC) to manual counts were employed with hierarchical linear regression to develop a prediction equation. Results: Average sign-corrected disagreement for strides counted between the two SW monitors was 0.4 (0.51), 0.2 (0.42), 0.1 (0.32) and 0.1 (0.32) for 1, 2, 3 and 4 mph respectively. SW stride counts to manual counts (accuracy ratio) averaged 100.03 (0.10) with ICC=0.995 (precision) cross the four walking speeds. The derived prediction equation for oxygen consumption was: VO₂=-1.2* gender +-0.58* age. (years) + 0.31* SW strides + 8.35 with r2=0.84. Conclusions/Significance: The SW data demonstrates excellent accuracy and precision for treadmill walking across speeds in YDT.With SW data corrected for gender and age, acceptable prediction of walking oxygen consumption in youth 10-16 years of age appears possible within the context of day to day life versus the current lab based measures. Future work will explore prediction of oxygen consumption with the SW during selfselected walking speeds, with varying terrain and community based activity. Such information will allow testing of interventions to improve walking efficiency, physical activity and/or energy expenditure in children and youth
SN - 0012-1622
AD - (Bjornson) Developmental Medicine, Seattle Children's Research Institute, University of Washington, Seattle, WA, United States (Yung, Jacques) Pediatric Cardiology, Seattle Children's, University of Washington, Seattle, WA, United States (Burr Msee) School of Nursing, University of Washington, Seattle, WA, United States (Christakis) Pediatrics, Seattle Children's Research Institute, University of Washington, Seattle, WA, United States
ER -

TY - JOUR
ID - 3780
T1 - Ambulatory activity patterns in cerebral palsy with the StepWatch
A1 - Bjornson,K.F.
A1 - Song,K.
A1 - Zhou,C.
A1 - Coleman,K.
Y1 - 2009///

N1 - 70212178
English
Journal: Conference Abstract
KW - EMBASE
KW - Cerebral Palsy
KW - juvenile
KW - Walking
KW - Gross Motor Function Classification System
KW - ankle
KW - Child
KW - Wakefulness
KW - Accelerometry
KW - community
KW - Hospital
KW - clinical practice
RP - NOT IN FILE
SP - 32
JF - Developmental Medicine and Child Neurology
VL - Conference: 63rd Annual Meeting of the American Academy for Cerebral Palsy and Developmental Medicine Scottsdale, AZ United States. Conference Start: 20090923 Conference End: 20090926. Conference Publication:
IS - var.pagings
N2 - Background/Objectives: Current StepWatch (SW) accelerometry data describes walking activity with the numeric variables of strides/day, % time active and peak strides/durations of time. Ambulatory activity curves allow interpretation of these variables in a combined format. The purpose of this study is to characterize community based daily ambulatory activity patterns in cerebral palsy as compared to non-impaired youth. Design: Cross-sectional comparison cohort. Participants and Setting: Eighty-one children with CP, gross motor function classification system [GMFCS] levels I [n=31], II [n=30] and III [n=20]; seen for care at three regional pediatric specialty care hospitals and 204 youth developing typically (YDT), all ages 10-13 years. Materials/Methods: Participants wore the SW on their lateral ankle for 7 days during waking hours. Five days of data were plotted for average minutes per day spend at each stride rate from one stride per minute to the highest rate per participant. Time spent at each intensity level (strides/ min) was represented in log-scale with a Lowest curve fit for visualization of the youth with CP by GMFCS level and YDT. Y axis is the natural log and average strides taken with X axis the # of strides/minute. Results: Youth with CP at level I on average were as active, have similar stride/min rates between <20 and >60 strides and are capable of peak stride rates (85 strides/min) similar to YDT. Their primarily limitation appears in stride/min rates between 20 and 60 strides [Fig H1]. Participants at Level II were less active on a daily basis demonstrating rates consistently lower than the YDT peaking at 75 strides/min as documented by a initially steeper curve that remains flat. Youth at level III demonstrate a low level of daily activity with the average stride rates outside the confidence band for YDT between10 and 50 with a peak stride/min of 70. Conclusions/Significance: Day to day walking activity of youth with CP demonstrates characteristic patterns of lower levels of walking activity, intensity levels across stride rates and peak stride rates than YDT as expected by GMFCS level. Youth with CP are more heterogeneous across stride rates than YDT. Ambulatory activity curves with the StepWatch have the potential to capture levels of daily ambulatory function temporally as well as in terms of intensity. This 'snapshot' analysis of average daily walking activity patterns may have application for clinical practice and/or outcome research (Graph presented)
SN - 0012-1622
AD - (Bjornson) Developmental Medicine, Seattle Children's Research Institute, University of Washington, Seattle, WA, United States (Song) Orthopedics, Seattle Childrens, University of Washington, Seattle, WA, United States (Zhou) Biostatistics, Seattle Childrens, University of Washington, Seattle, WA, United States (Coleman) Clinical Research, OrthoCare Innovations, Seattle, WA, United States
ER -

TY - JOUR
ID - 3781
T1 - Evaluation of balance in youth with an acquired brain injury: Reliability of the community balance and mobility scale

A1 - Wright,V.
A1 - Brewer,K.
Y1 - 2009///
N1 - 70212169
English
Journal: Conference Abstract
KW - EMBASE
KW - Cerebral Palsy
KW - juvenile
KW - community
KW - reliability
KW - brain injury
KW - Gross Motor Function Measure
KW - Adult
KW - test retest reliability
KW - patient
KW - Child
KW - physiotherapist
KW - follow up
KW - Rehabilitation
KW - balance impairment
KW - Pediatrics
KW - Female
KW - Male
KW - pediatric rehabilitation
KW - interrater reliability
KW - confidence interval
KW - sport
KW - skill
RP - NOT IN FILE
SP - 25
EP - 26
JF - Developmental Medicine and Child Neurology
VL - Conference: 63rd Annual Meeting of the American Academy for Cerebral Palsy and Developmental Medicine Scottsdale, AZ United States. Conference Start: 20090923 Conference End: 20090926. Conference Publication:
IS - var.pagings
N2 - Background/Objectives: Balance impairments are common following acquired brain injury (ABI). Children with ABI often have goals related to participation in sports and recess activities that require high-level balance skills. The community balance and mobility (CB&M) scale (Howe et al, 2006) is used by physiotherapists (PTs) at our pediatric centre to measure balance, and may be better at detecting changes in this area than the Gross Motor Function Measure (GMFM). While the CB&M's psychometric strength has been shown with adults, it has not been tested in pediatrics. The purpose of this study was to determine inter-rater and test-retest reliability of the CB&Min ambulatory youth who have an ABI. Design: Prospective, repeated measures design. Participants and Setting: Twenty-four ambulatory youth (13 females and 11 males) with an ABI, ages 8 to 17 years (mean age=14 year 1 month, SD=2.1) who were in-patients or day-patients at a pediatric rehabilitation centre. Materials/Methods: Six PTs were trained as assessors. Each passed the study's CB&M criterion test. The PTs were assigned in pairs to do the CB&M assessment. At baseline, one PT assessor administered and scored the CB&M, while a second PT observed and independently scored it (inter-rater reliability). Children were reassessed 3-10 days later by the PT assessor who administered the baseline CB&M (test-retest reliability). Each PT had equal opportunity to serve as assessor or observer over the course of the study. Results: The participants' GMFM mean score at baseline was 93.4% (SD=7.8). CB&M baseline mean scores were 63.5% (SD=18.4) and 64.7% (SD=18.5) for PT assessors and observers respectively. The interrater reliability ICC was 0.93 (95% confidence interval [CI]=0.85-0.97), and a Bland-Altman plot revealed an indication of smaller inter-rater differences for lower CB&M scores (i.e., slightly greater agreement on lower scores). The assessor retest mean score was 68.7% (SD=17.0). The testretest ICC was 0.92 (95% CI=0.84-0.96). No systematic bias was indicated in the test-retest Bland-Altman plot. The minimum detectable change

(MDC90) estimate was 12.6% points. Previous clinical CB&M follow-up at our centre of 17 youth with ABI with similar baseline scores to our reliability sample showed mean change score of 23.6% points [SD=13.5] from baseline to discharge assessment, suggesting that this study's MDC estimate is clinically realistic. Conclusions/Significance: The CB&M showed excellent inter-rater and test-retest reliability in youth with an ABI with estimates similar to those of the published reliability work by Howe et al (2006) with adults post-ABI. In our sample, there was little room for gain on the GMFM, while the CB&M showed scoring scope to detect improvement. Future work should focus on formal evaluation of the CB&M's responsiveness to change in rehabilitation centre and community intervention contexts
SN - 0012-1622
AD - (Wright) Research, Bloorview Research Institute, Toronto, ON, Canada (Brewer) Brain Injury Rehab Team, Bloorview Kids Rehab, Toronto, ON, Canada
ER -

TY - JOUR
ID - 3782
T1 - Factor and item level analysis of the activity scale for kids - Performance (ASKp)
A1 - Bagley,A.
A1 - Gorton,G.
A1 - Bjornson,K.
A1 - Tucker,C.
Y1 - 2009///
N1 - 70212163
English
Journal: Conference Abstract
KW - EMBASE
KW - Cerebral Palsy
KW - model
KW - Statistics
KW - factorial analysis
KW - Physical Activity
KW - Child
KW - sport
KW - Rasch analysis
KW - Eating
KW - Motivation
KW - Hospital
KW - clinical research
KW - planning
KW - Self Report
KW - physical disease
KW - quartimax rotation
KW - daily life activity
KW - Regression Analysis
KW - Drinking
KW - Locomotion
KW - skill
KW - friend
KW - musculoskeletal disease
RP - NOT IN FILE
SP - 22
EP - 23
JF - Developmental Medicine and Child Neurology
VL - Conference: 63rd Annual Meeting of the American Academy for Cerebral Palsy and Developmental Medicine Scottsdale, AZ United States. Conference Start: 20090923 Conference End: 20090926. Conference Publication:
IS - var.pagings

N2 - Background/Objectives: The ASKp is a self-report measure of common physical activity for children 5-15 years of age with limitations due to musculoskeletal disorders. The ASKp measures how often a child performed specific activities over the past week using a five point Likert scale. Since the original psychometric properties were published, the ASKp has appeared in 30 and 38 item versions. The objective of this study was to perform exploratory and confirmatory factor analyses of the 38 item ASKp and determine model and item fit statistics. Design: Instrument Development and Validation. Participants and Setting: The ASKp38 was administered to 200 children (mean age 12.2 + 2.3 years) with a variety of physical impairments. Administration of the ASKp occurred during IRB approved home or center based research visits. Materials/Methods: Confirmatory (CFA) and exploratory (EFA) factor analyses were done in MPlus. Item fit statistics were calculated using the Rasch partial credit model in Winsteps. Results: EFA with unweighted least squares and Quartimax rotation was done. Five factors with eigenvalues >1 were identified, but only two factors explained >5% variance. Comparison of one and two factor models indicated better fit for the two factor model based on higher communalities and higher factor loadings. There was good separation between the two factors with three cross-loaded items (Figure 1). CFA fit statistics for a two factor model were acceptable (CFI=0.974, TLI=0.991, RMSEA=0.049). Fit statistics for higher factor models were better but exhibited significant item cross-loading. The first factor consisted of "physical activities of daily living" (ADLs): all Personal Care, Dressing, Eating and Drinking, Miscellaneous, Locomotion, Standing Skills, Stairs, and Transfers items, with the exception of #19 "kept up with friends outside" , and #28 "sat on the floor" . The second factor consisted of the one Play item, the eight sports-focused Additional items, and items 19 and 28. Rasch analysis created a hierarchy based on the frequency of successful performance of items. "ADLs" showed a range from-0.67 (least frequently performed: stood still for 10 minutes) to 0.85 logits (most frequently performed: got around inside my home). For the "sports" activities, the range was from-1.19 (least frequently performed: ran in a race) to 0.63 logits (most frequently performed: sat on the floor). Infit (range 0.42-1.56 logits) and outfit statistics (range 0.13-1.59 logits) were acceptable. Conclusions/Significance: Factor analysis reveals a two-factor model underlying the ASKp38 instrument indicating two different constructs. One factor consists of 27 "ADLs" and the other factor consists of 11 sports-related physical activities. Clinicians should consider other factors such as motivation, opportunity, inability, and lack of need when interpreting this performance measure. Acknowledgement: Research Supported by NIH Grant 5U01AR052171 (D. Amtmann, PI), NINDS F31-NS048740, Staheli Endowment Fund, Seattle Childrens' Hospital and 2008 AACPDM Clinical Research Planning Grant. (Graph presented)
SN - 0012-1622
AD - (Bagley) Motion Analysis Laboratory, Shriners Hospitals for Children Northern California, Sacramento, CA, United States (Gorton) Clinical Outcomes Assessment, Shriners Hospitals for Children, Springfield, MA, United States (Tucker) College of Health Professions, Temple University, Philadelphia, PA, United States (Bjornson) Pediatrics, Seattle Children's Research Institute, Seattle, WA, United States
ER -

TY - JOUR
ID - 3783
T1 - Ambulation, activity, and participation of young adults with cerebral palsy
A1 - Gannotti,M.E.
A1 - Gorton,G.E.
A1 - Nahorniak,M.T.
A1 - Masso,P.D.
Y1 - 2009///
N1 - 70212140
English
Journal: Conference Abstract
KW - EMBASE
KW - Cerebral Palsy
KW - Adult
KW - mobilization
KW - Walking
KW - Gross Motor Function Classification System
KW - adulthood
KW - Education
KW - Employment
KW - adolescence

KW - parent
KW - spasticity
KW - tertiary health care
KW - Hospital
KW - Health
KW - gait
KW - health survey
KW - high school
KW - student
KW - unemployment
KW - traffic and transport
KW - Socialization
KW - Child
KW - follow up
KW - outcomes research
KW - Society
RP - NOT IN FILE
SP - 9
JF - Developmental Medicine and Child Neurology
VL - Conference: 63rd Annual Meeting of the American Academy for Cerebral Palsy and Developmental Medicine Scottsdale, AZ United States. Conference Start: 20090923 Conference End: 20090926. Conference Publication:
IS - var.pagings
N2 - Background/Objectives: To accurately assess the long term impact of orthopedic interventions, more information is needed about changes in the walking abilities of people with cerebral palsy (CP) that occur from adolescence to young adulthood. Additional information about current levels of activity and participation can document the interrelationships among walking abilities and other aspects of health. The purpose of this study was to document changes in walking abilities from adolescence to young adulthood in people with CP, and current level of activity and participation. Design: Outcomes research. Participants and Setting: Thirty-three (50/191 located, 17 cancelled or refused) people with spastic CP who had a previous motion analysis evaluation (3DGA) at a tertiary care hospital (initial gross motor functional classification system [GMFCS] levels I [n=1], II [n=14], III [n=14], IV[n=4]; ages: 20-36 years; time between evaluations: 5-19 years). Materials/Methods: Follow-up evaluation included GMFCS level, functional abilities (FIM), Gillette Gait Index (GGI), living arrangements, employment, level of education, canadian occupational performance measure (COPM), short form health survey (SF-36), and 5 year goals. Results: Thirty out of 33 maintained or improved GMFCS Level since initial evaluation; 22/26 maintained or improved GGI. Sixteen out of 33 were living with parents, 15/33 apart from parents, and two in skilled care facilities. Twenty-two out of 33 had education or specialized training past high school. Nineteen worked outside the home for pay, eight were students, six were not employed. Family values, medical issues, and lack of personal care attendants were reasons for unemployment. Lack of transportation, employment, socialization, and personal care attendants were the most frequently cited perceived problems. No relationships were found among changes in GMFCS levels or GGI with FIM scores, SF-36 sub-domain scores, COPM barriers, level of education, employment, or living arrangements. The two subjects with the greatest declines in walking abilities were either working full time or married, with children. Subgroup analysis of subjects who did not decline demonstrated an association of orthopedic interventions with maintained or improved walking abilities (R^2=0.82, F=35.0, P=0.000). Conclusions/Significance: The majority of the subjects maintained walking abilities into young adulthood, and the relationship between changes in walking abilities, orthopedic interventions, personal and environmental characteristics, and participation in society requires additional investigation
SN - 0012-1622
AD - (Gannotti) Physical Therapy Department, University of Hartford, West Hartford, CT, United States (Gorton, Masso) Clinical Outcomes Assessment Laboratory, Shriners Hospital for Children, Springfield, MA, United States (Nahorniak) Physical Therapy Department, Shriners Hospital for Children, Springfield, MA, United States
ER -

TY - JOUR
ID - 3784

T1 - Assessing changes in tumour metabolism using magnetic resonance spectroscopy
A1 - Peet,A.
Y1 - 2009///
N1 - 70209607
English
Journal: Conference Abstract
KW - EMBASE
KW - Tumor
KW - nuclear magnetic resonance spectroscopy
KW - Metabolism
KW - metabolite
KW - proton nuclear magnetic resonance
KW - brain
KW - Imaging
KW - nuclear magnetic resonance imaging
KW - nuclear magnetic resonance
KW - Child
KW - prostate
KW - Biopsy
KW - prostate biopsy
KW - Radiotherapy
KW - planning
KW - phosphorus nuclear magnetic resonance
KW - medulloblastoma
KW - parent
KW - clinical study
KW - apoptosis
KW - tissue
KW - breast
KW - Diagnosis
KW - Pattern Recognition
KW - prostate cancer
KW - prospective study
KW - diagnostic accuracy
KW - glioblastoma
KW - metastasis
KW - Aggressiveness
KW - glioma
KW - Cell Line
KW - cell death
KW - macromolecule
KW - lipid
KW - marker
KW - choline
KW - phosphorylcholine
KW - glycerophosphorylcholine
KW - tracer
KW - pyruvic acid
KW - inositol
RP - NOT IN FILE
SP - 55
JF - European Journal of Cancer, Supplement
VL - Conference: Joint ECCO 15 - 34th ESMO Multidisciplinary Congress Berlin Germany. Conference Start: 20090920 Conference End: 20090924. Conference Publication:
IS - var.pagings
N2 - Magnetic resonance spectroscopy (MRS) is a technique which can be readily combined with conventional magnetic resonance imaging to measure the levels of various metabolites, lipids and macromolecules in a

specified volume of tissue. The most clinically available form is 1H MRS which provides a broad metabolite profile with approximately 15 metabolites being quantitated in brain tumours. Due to the comparative ease of performing 1H MRS in the brain, brain tumours have been the most widely studied tumour group, however, there have also been many studies of prostate cancer and breast tumours. It is well established that 1H MRS metabolite profiles are a powerful characteristic of brain tumours and the use of 1H MRS as a non-invasive diagnostic tool is well investigated. The most impressive results have been obtained by coupling 1H MRS with pattern recognition techniques and large multicentre prospective studies have shown a high level of diagnostic accuracy for many tumour types but a lower accuracy when trying to distinguish between glioblastoma and metastases. Fewer studies have been published in children but small studies indicate that the accuracy is similar. Total choline and mobile lipids have been noted as indicators of tumour aggressiveness and a decrease in choline has been used as an indicator of tumour response to drugs. Similarly, myoinositol correlates with lower grade in gliomas and is a good prognostic marker in pilocytic astrocytomas in children. Animal and cell line studies show particular promise for mobile lipids as early indicators of cell death, however these findings are yet to be verified clinically. MR spectroscopic imaging allows MRS to be collected from several locations at the same time with a spatial resolution down to 1 cubic cm in the brain using a 1.5T clinical scanner and much higher resolution in the prostate using endorectal coils. Using choline as a marker of active tumour, often as a ratio to another metabolite, has allowed accurate targeting of biopsies and is used commonly in some centres for improving the accuracy of prostate biopsies. MR spectroscopic imaging is also useful for identifying tumour invasion and has detected diffuse tumour outside the regions delineated by conventional MRI. This shows promise in radiotherapy planning. 31P MRS can detect phosphorous containing metabolites and phospho-lipids. It is technically more demanding than 1H MRS but is useful in certain circumstances. Phosphocholine and glycerophosphocholine can be quantitated individually rather than the combined value usually provided by 1H MRS. The ratio of these two metabolites is a powerful discriminant of some tumour types such as medulloblastoma and of response to treatment with the phosphocholine/glycerophosphocholine ratio decreasing in responding tumours. Dynamic Nuclear Polarisation (DNP) is an exciting new technique for the production of tracer metabolites which can be detected by MRS and has the particular advantage that the parent molecule and its metabolites can be detected separately. The first clinical studies of DNP using pyruvate as a marker of apoptosis are about to start
SN - 1359-6349
AD - (Peet) University of Birmingham, Cancer Sciences, Birmingham, United Kingdom
ER -

TY - JOUR
ID - 3785
T1 - The effect of seasons on sleep in preschool children and their mothers
A1 - Okamoto-Mizuno,K.
A1 - Mizuno,K.
A1 - Yamamoto,M.
A1 - Shirakawa,S.
Y1 - 2009///
N1 - 70209165
English
Journal: Conference Abstract
KW - EMBASE
KW - Sleep
KW - mother
KW - season
KW - preschool child
KW - Asian
KW - Society
KW - summer
KW - winter
KW - Humidity
KW - Temperature
KW - illumination
KW - actimetry
KW - night

KW - sleep time
KW - Wrist
RP - NOT IN FILE
SP - A6
JF - Sleep and Biological Rhythms
VL - Conference: 6th Congress of Asian Sleep Research Society, ASRS Osaka Japan. Conference Start: 20091024 Conference End: 20091028. Conference Publication:
IS - var.pagings
N2 - The objective of this study was to investigate the effect of seasons on sleep in 22 pairs of healthy preschool children and their mothers. They were informed of the study protocol and provided their written consent. The study was performed at the subjects' homes during spring, summer, fall, and winter. Wrist actigraphy and temperature and relative humidity (RH) outside and within the bedroom were measured for 7 consecutive days. Bedroom illumination and subjective sleep evaluation were determined on 2 nights. The bedroom temperature significantly increased in summer and decreased in winter as compared to fall or spring, while no significant difference was observed in the humidity. Bedroom illumination was significantly higher in the morning in summer and spring than in fall or winter. Among the mothers, the sleep efficiency index increased and the wake after sleep onset decreased in spring as compared to the other seasons. The mean activity during sleep increased and the sleep time decreased in summer as compared to the other seasons in the preschool children. Among the mothers, the subjective sleep evaluation was improved in spring as compared to the other seasons. The subjective sleep onset was delayed in summer in preschool children. These results indicate that effect of season on sleep may differ between preschool children and their mothers. Furthermore, care should be taken to prevent disturbed sleep in preschool children in summer
SN - 1446-9235
AD - (Okamoto-Mizuno) Tohoku Fukushi University, Center for Welfare and Health Improvement, Miyagi, Japan (Mizuno) Faculty of Child and Family Studies, Tohoku Fukushi University, Miyagi, Japan (Yamamoto) Tohoku Fukushi University, Kansei Fukushi Research Center, Miyagi, Japan (Shirakawa) Department of Psychogeriatrics, National Institute of Mental Health, NCNP, Tokyo, Japan
ER -

TY - JOUR
ID - 3786
T1 - Cytotoxic and inflammatory effects of PM10 in classrooms
A1 - Oeder,S.
A1 - Weichenmeier,I.
A1 - Schober,W.
A1 - Dietrich,S.
A1 - Fromme,H.
A1 - Behrendt,H.
A1 - Buters,J.
A1 - Poblete-Gutierrez,P.
Y1 - 2009///
N1 - 70203097
English
Journal: Conference Abstract
KW - EMBASE
KW - Human
KW - cytotoxicity
KW - metabolic activation
KW - toxicity
KW - gene
KW - Genome
KW - keratinocyte
KW - ambient air
KW - Health
KW - Particulate Matter
KW - school
KW - Child

KW - Lung
KW - Chinese hamster
KW - Teaching
KW - lung fibroblast
KW - epithelium cell
KW - exposure
KW - school child
KW - cytokine
KW - xenobiotic agent
KW - cytochrome
KW - cytochrome P450
KW - adenosine triphosphate
KW - RNA
RP - NOT IN FILE
SP - 324
JF - Experimental Dermatology
VL - Conference: 36th Annual Meeting of the Arbeitsgemeinschaft Dermatologische Forschung, ADF Heidelberg Germany. Conference Start: 20090305 Conference End: 20090307. Conference Publication:
IS - var.pagings
N2 - Outdoor particulate matter (PM10) is associated with a wide range of health effects and a European threshold limit of 50 lg/m3 was established in 2005. However, most individuals spend at least 85% of their time indoors where particle concentrations are mostly higher than outdoors. Since children represent a vulnerable group, we investigated the health effects of indoor air PM10 collected in classrooms compared to outdoor air PM10. PM10 was collected in five schools in Munich during teaching hours. Cytotoxicity was assayed as a decline of cellular ATP concentration in human primary keratinocytes, human lung epithelial A549 cells and Chinese hamster V79 lung fibroblasts at concentrations up to 10 mug/ml. In addition, toxicity after metabolic activation was assayed in V79 cells expressing human cytochrome P450 1A1, 1A2, 1B1, 2A6, 2B6, 2C9, 2D6, 2E1, 3A4 or 3A5. For a genome wide expression analysis BEAS-2B bronchial epithelial cells were incubated with 10 mug/ml PM10. RNA was isolated and analyzed on Affymetrix HG U133A 2.0 expression arrays. While in A549 and V79 cells no toxicity was observed, in human primary keratinocytes PM10 at a concentration of 10 mug/ml caused a slight decrease in vitality. This cytotoxic effect was also found in V79 cells after metabolic activation by CYP1A1 or CYP2C9. Genome wide analysis of PM10 from outdoor and indoor air showed the overexpression of xenobiotic metabolizing genes (CYP1A1, CYP1B1) and of inflammatory cytokines (IL1A, IL1B, IL6, IL8). Indoor PM10 caused a lower induction of xenobiotic metabolizing genes but a up to six fold higher induction of inflammatory cytokines compared to outdoor PM10. Direct cytotoxicity and metabolic activation by cytochrome P450 isoforms 1A1 and 2C9 were statistically significant at a PM10 concentration of 10 mug/ml, which is about 10 000 times higher than exposure encountered in classrooms. We therefore expect no toxic effects of these particles in school children. The reduced induction of xenobiotic metabolizing genes but increased induction of inflammatory cytokines in indoor PM10 treated cells suggests that classroom PM10 is less toxic but has a higher inflammatory potential than outdoor PM10
SN - 0906-6705
AD - (Oeder, Weichenmeier, Schober, Behrendt, Buters) Division of Environmental Dermatology and Allergy Helmholtz ZentrumMunchen/TUM, ZAUM-Center for Allergy and Environment, Technische Universitat Munchen, Munich 80802, Germany (Dietrich, Fromme) Bavarian Health and Food Safety Authority, Department of Environmental Health, Oberschleibetaheim 85764, Germany
ER -

TY - JOUR
ID - 3787
T1 - Gross motor function through the lifespan of individuals with cerebral palsy
A1 - Palisano,R.J.
Y1 - 2009///
N1 - 70199002
English
Journal: Conference Abstract
KW - EMBASE
KW - Cerebral Palsy

KW - Motor Performance
KW - lifespan
KW - Child
KW - juvenile
KW - Walking
KW - Environment
KW - Prognosis
KW - environmental factor
KW - Accommodation
KW - assistive technology
KW - adulthood
KW - body posture
KW - brain
KW - parent
KW - longitudinal study
KW - Canada
KW - adolescence
KW - Gross Motor Function Measure
KW - Exercise
KW - childhood
KW - disability
KW - Motor Development
KW - Gross Motor Function Classification System
KW - school
KW - Quality of Life
RP - NOT IN FILE
SP - 86
JF - Developmental Medicine and Child Neurology
VL - Conference: 3rd International Cerebral Palsy Conference Sydney, NSW Australia. Conference Start: 20090218 Conference End: 20090221 Sponsor: Allergan Australia. Conference Publication:
IS - var.pagings
N2 - Cerebral palsy (CP) describes a group of disorders of posture and movement that occur early in life. Although the disturbance in the developing brain is non-progressive, motor function may change over time. From the outset, parents want to know whether their child will walk. Although walking is valued by individuals ofall ages, youth with CP may be more concerned with mobility; getting from place to place in a safe and efficient manner to do things with other people. The objective of this presentation is to provide a lifespan perspective of gross motor function of individuals with CP. The perspective is informed by two longitudinal studies, the Ontario Motor Growth Study and the Adolescence Study of Quality of Life, Mobility and Exercise, completed at the CanChild Centre for Childhood Disability Research. The rate of gross motor development among children with CP is highest at younger ages. Children approach the upper limit of their motor capacity at approximately 3-6 years of age depending on Gross Motor Function Classification System (GMFCS) level. Children and youth in Levels I and II maintain motor capacity; while children in Levels III, IV, and V demonstrate peak motor capacity at 7-9 years of age before declining an average of 5-8 points on the GMFM-66. By 3 years of age, almost all children in Level I walk at home, school, and outdoors. By age nine, the probability of walking is high in all settings for children and youth in Level II. Children and youth in Level III demonstrate variability in usual method of mobility in all settings. For children in Level IV, the probability of using powered mobility increases with age. Only a small percentage of children and youth in Level V mobilize (powered mobility) without physical assistance of a person. Youth spoke of constantly adapting to situations that often reflected a lack of control over the environment and having to plan ahead to go places and do things. The findings provide evidence of prognosis for gross motor function and have implications for decisions on goals, outcomes, services, and supports. Personal and environmental factors are important when making decisions on mobility including attitudes and beliefs regarding walking and wheeled mobility. The preferred method in one setting may not optimize participation in another setting. Task accommodations, environmental modifications, and assistive technology are interventions that have the potential to optimize activity and participation. Implications for adulthood are discussed
SN - 0012-1622
AD - (Palisano) Drexel University, Philadelphia, PA, United States

ER -

TY - JOUR
ID - 3788
T1 - Does self-report measure performance? A study of the construct validity of the Functional Mobility Scale
A1 - Harvey,A.
A1 - Graham,K.
A1 - Morris,M.
A1 - Baker,R.
Y1 - 2009///
N1 - 70198969
English
Journal: Conference Abstract
KW - EMBASE
KW - Self Report
KW - Cerebral Palsy
KW - construct validity
KW - Child
KW - school
KW - physiotherapist
KW - Gross Motor Function Classification System
KW - tertiary health care
KW - kappa statistics
KW - Male
KW - Environment
KW - prospective study
KW - Female
KW - community
KW - instrument validation
RP - NOT IN FILE
SP - 61
JF - Developmental Medicine and Child Neurology
VL - Conference: 3rd International Cerebral Palsy Conference Sydney, NSW Australia. Conference Start: 20090218 Conference End: 20090221 Sponsor: Allergan Australia. Conference Publication:
IS - var.pagings
N2 - Background: There are various instruments designed to measure activity limitation in children with cerebral palsy (CP) that rely on self-report to measure performance. Performance can be described as what a child 'does do' and differs from capability which describes what a child 'can do'. Performance instruments are often more relevant to the child and family because they assess function in everyday life, however they are used with the assumption that what is reported accurately reflects what is really happening. Objective: The aim of this study was to determine if a self- report instrument, the Functional Mobility Scale (FMS), truly measures performance by correlating it with direct observation of mobility of a group of children with CP. Design: A prospective study of instrument validation. Participants/setting: Included were children with CP aged 417 years, classified as GMFCS levels I-IV. They were recruited from a special school and were clients of a tertiary care facility. Materials/Methods: Participants were rated on the FMS by an independent experienced physiotherapist. The same children were observed directly at home and at school by a different physiotherapist and their various mobility methods were recorded. These observations were used to generate an 'observed' FMS score which was correlated with the usual self-report FMS score using unweighted kappa statistics. Results: 18 children (11 males, 7 females) of 27 eligible to enterwereincluded. Theage rangewas 8-17 years(mean 12.7 years). Direct observation showed that many children used more than one method of mobility, particularly those classified as GMFCS III. Seven children used more than one method at home and 13 used more than one method at school. Correlations between 'observed' and self-report FMS ratings were fair to moderate with unweighted kappas of 0.27 for 5 m (home), 0.45 for 50 m (school) and 0.29 for 500 m (community). Conclusions/clinical implications: The fair to moderate correlation of self-report and direct observation using the FMS was lower than expected. This is potentially the result of the multiplicity of mobility methods children with CP are observed to use. Because it is not always practical for clinicians to measure children in their natural environments, self-report measures remain the most convenient method of assessing performance. Further investigation of this clinically

important issue with other self-report measures is required to ensure that it is a valid and clinically relevant method of reporting
SN - 0012-1622
AD - (Harvey, Baker) Murdoch Children's Research Institute, Melbourne, VIC, Australia (Graham) Royal Children's Hospital Melbourne, Melbourne, VIC, Australia (Morris) University of Melbourne, Melbourne, VIC, Australia
ER -

TY - JOUR
ID - 3789
T1 - Factors influencing participation of children with cerebral palsy in Australia
A1 - Imms,C.
A1 - Reilly,S.
A1 - Carlin,J.
A1 - Dodd,K.
Y1 - 2009///
N1 - 70198920
English
Journal: Conference Abstract
KW - EMBASE
KW - Child
KW - Cerebral Palsy
KW - Australia
KW - school
KW - Environment
KW - book
KW - Motor Performance
KW - Classification
KW - gender
KW - Population
KW - model
KW - multiple regression
KW - childhood
KW - Gross Motor Function Classification System
KW - child health care
KW - Policy
KW - United States
KW - independent variable
KW - handicapped child
KW - skill
KW - Interpersonal Communication
KW - Temperament
KW - parent
KW - linear regression analysis
KW - girl
KW - Family Size
KW - Physical Activity
KW - valerian
RP - NOT IN FILE
SP - 23
EP - 24
JF - Developmental Medicine and Child Neurology
VL - Conference: 3rd International Cerebral Palsy Conference Sydney, NSW Australia. Conference Start: 20090218 Conference End: 20090221 Sponsor: Allergan Australia. Conference Publication:
IS - var.pagings
N2 - Background: Active and pleasurable involvement of disabled children in activities outside school is a key measure ofsuccessful participation. Participation is influenced by many factors including the skills and interests

of the child and social and environmental supports (Hammal et al. Dev Med Child Neurol 2004; 46: 292-298; King et al. Child Health Care 2006; 35(3): 209-234; Forsyth et al. Dev Med Child Neurol 2007; 49(5): 345-349). Policies and social expectations in different countries vary and these may also influence participation. Objective: This study investigated the extent to which selected individual, family and environmental variables were associated with participation of Australian children with cerebral palsy in formal and informal activities outside school. Design: Prospective population-based survey. Participants: Allchildrenbornin 1994 or 1995 in Victoria and diagnosed with cerebral palsy were eligible for inclusion. Complete data were available for 108 of 114 participating children (49.3% of the known living population) in Gross Motor Function Classification (GMFCS) levels I:23%; 11:37%; IV:9%; V:20%. Methods: Participation was measured using the Children's Assessment of Participation and Enjoyment (CAPE) (King et al. Children's Assessment of Participation and Enjoyment and Preferences for Activities of Kids. San Antonio, Texas: PsychCorp 2004). Independent variables included gender, child's activity preference, motor function measured by the GMFCS and the Manual Ability Classification Systems (MACS), child's temperament and communication ability. Family variables included family type (single or two-parent family), background (Australian-born or not) and socio-economic advantage/disadvantage. Environment variables included living and school environments. Linear regression analysis was used to assess associations between these variables and diversity of participation in informal and formal activities. Results: Other than where children lived, all variables had univariate associations with informal participation. The variables with the strongest multivariate associations with informal participation were manual ability (MACS level independently accounted for 22.6% of the variance), a preference for informal activities (accounted for 7% of the variance and gender (2.6% of variance - girls participated more). Only two variables were important in the multivariate model for participation in formal activities: preference for formal activities (2.9% of the variance) and attendance at mainstream school (7.1% of the variance). Discussion/Conclusion: Child characteristics were more strongly associated with informal participation than environment or family characteristics. The explanatory power of the multiple regression model for formal participation was very limited but attendance at mainstream school was important. It is likely that MACS level is relatively stable, although improved manual ability may influence participation. Preference for activity in childhood may be more amenable to change. Being exposed to a range of activities within supportive environments may provide the opportunity to develop preferences, especially in activities where children with cerebral palsy have reduced participation, such as in physical activities (Maher et al. Dev Med Child Neurol 2007; 49(6): 45 0-457)
SN - 0012-1622
AD - (Imms, Dodd) La Trobe University, Melbourne, VIC, Australia (Imms, Reilly, Carlin) Murdoch Childrens Research Institute, Melbourne, VIC, Australia (Reilly, Carlin) University of Melbourne, Melbourne, VIC, Australia
ER -

TY - JOUR
ID - 3790
T1 - Preschool nurseries: Are they sunsmart?
A1 - Shalders,K.
A1 - Zaki,I.
Y1 - 2009///
N1 - 70018972
English
Journal: Conference Abstract
KW - EMBASE
KW - Nursery
KW - Policy
KW - protection
KW - Child
KW - parent
KW - questionnaire
KW - cancer research
KW - United Kingdom
KW - shade
KW - Child Care
KW - childhood

KW - organization and management
KW - exposure
KW - Sunlight
KW - Weather
KW - Risk
KW - Public Health
KW - skin cancer
KW - sunscreen
RP - NOT IN FILE
SP - 122
JF - British Journal of Dermatology
JA - Br J Dermatol
VL - Conference: British Association of Dermatologists 89th Annual Meeting Glasgow United Kingdom. Conference Start: 20090707 Conference End: 20090710. Conference Publication:
IS - var.pagings
N2 - Excessive sunlight exposure in early childhood is associated with an increased risk of developing cutaneous malignancies (Masso M. Policy and practice in preventing skin cancer in children. Public Health Nurs 2006; 23: 361-5). In 2004, Cancer Research UK's SunSmart campaign produced guidelines on developing sun protection policies for day nurseries and preschools. We devised and distributed a postal questionnaire to 137 local day nurseries to assess awareness of the SunSmart campaign and evaluate its impact on sun protection policy development. Of the 137 surveys sent, 68 were received back (50%). Of all the nurseries that returned their surveys, 53 (78%) were aware of the SunSmart campaign. Fifty-five nurseries (81%) had a sun protection policy in place and only eight of these (14%) stated that their policy was in place before 2004. Comparing policies with the SunSmart guidelines, 93% of nurseries stated that they asked parents to provide sun hats for their children and 38% asked parents to provide long-sleeved tops. Parents were asked to provide suncream for their children by 84% of nurseries and a further 10% of nurseries provided their own suncream. Of these, 55% suggested a minimal SPF of at least 15. No nursery stated that staff were not permitted to apply suncream to children. Usually, written consent was obtained (63%) before staff would undertake this activity. Outside activities during sunny weather were restricted by 82% of nurseries. Almost two-thirds (61%) of these were in line with the SunSmart guidelines of staying in the shade between 11am and 3pm and 48 (71%) had a designated shady area for activities. Of the nurseries surveyed 90% aimed to educate their children about sun protection and 72% of nurseries hoped to inform parents about the importance of sun protection. A nursery without a sun protection policy may be less likely to return their questionnaire, which would obviously introduce bias in our results. From these data however, it is apparent that nurseries who are aware of the SunSmart campaign have often taken steps towards introducing sun protection policies in line with the guidance provided and the campaign therefore has had a positive effect. The number of policies and the timing of their development would suggest that other sources of information must also be involved in influencing childcare providers into introducing sun protection policies. Cancer Research UK continues to redevelop its guidelines for this public sector. More active targeted campaigns may be beneficial in raising awareness and influencing policy in the remaining nurseries
SN - 0007-0963
AD - (Shalders, Zaki) Solihull Hospital, Solihull, West Midlands, United Kingdom
ER -

TY - JOUR
ID - 3791
T1 - Consecuences of a social suport program in the inmunnosupressant treatment of patiens with liver transplant
A1 - Vinoly,M.
A1 - Gonzalez,S.
A1 - Rando,K.
A1 - Xavier,A.
A1 - Lagomarsino,G.
A1 - Lopez,C.
A1 - Texeira,J.
A1 - Scalone,P.
A1 - Gerona,S.

N1 - 70183070
English
Journal: Conference Abstract
KW - EMBASE
KW - liver graft
KW - Society
KW - Liver Transplantation
KW - patient
KW - liver
KW - Policy
KW - drug administration
KW - Biopsy
KW - Social Work
KW - adhesion
KW - Uruguay
KW - graft rejection
KW - prescription
KW - social problem
KW - retrospective study
KW - Child
KW - Adult
KW - Transplantation
KW - pediatrician
KW - Telephone
KW - Food
KW - city
KW - night
KW - social worker
KW - salary
KW - medical care
KW - immunosuppressive agent
RP - NOT IN FILE
SP - S231
EP - S232
JF - Liver Transplantation
VL - Conference: 16th Annual International Congress of the International Liver Transplantation Society Hong Kong China. Conference Start: 20100616 Conference End: 20100619. Conference Publication:
IS - var.pagings
N2 - Many patients with liver transplant in poor countries have difficulties to follow medical prescriptions due to economical and social problems; that may have some infl uence in rejection rates. Our objective was to evaluate the impact of a new social policy that gives educational and economical support to liver transplanted patients in the Uruguayan Liver Transplant Program. Methods: We conducted a descriptive and retrospective study including 40 liver transplanted patients (24 children and 16 adults. All of them have post-transplant medical assistance in the Uruguayan Liver Transplant Unit. Data on two periods of time were compared: from January to June 2008 (6 months) and to July 2008 to June 2009 (12 months). During the first period, there was not a specific social assistance program for transplanted patients. During the second period, y of social and educational support was conducted by the Social Assistant and the Pediatricians of our team. The policy included: regular telephone supervision, educational activities, economical help (for bus tickets and food) and hosting (for patients living outside the city of Montevideo). The total cost of the social program (for the 40 patients) was 100 dollars per month, 36 hosting nights per year and the 400 dollars per month (Social Worker salary). We evaluate the rate of assistance to the scheduled postoperative medical controls, the incidence of rejection and the correct (Graphe Presented) immunosuppressant drug administration. Results: In the second period, the patients followed the immunosuppressant treatment much better than in the first period ($p<0.0001$); they also had a tendency to go to scheduled postoperative medical controls than in the first period. In the first 6 month of the study, 5 patient had rejection (confirmed by biopsy); while in the following 12 months, only 3. Conclusions: social work was an inexpensive and very effective policy to improve immunosuppressant treatment adhesion in liver transplanted patients in Uruguay. This might help to reduce the rate of graft rejection

SN - 1527-6465
AD - (Vinoly, Gonzalez, Rando, Xavier, Lagomarsino, Lopez, Texeira, Scalone, Gerona) Liver Transplant Unit, Montevideo, Uruguay (Vinoly, Gonzalez, Rando, Scalone, Gerona) Unidad Docente Asistencial de Higado, Military Hospital, Montevideo, Uruguay
ER -

TY - JOUR
ID - 3794
T1 - Crime rates and sedentary behavior among 4th grade Texas school children
A1 - Shelton III,H.S.
A1 - Perez,A.
A1 - Mirchandani,G.G.
A1 - Hoelscher,D.M.
A1 - Kelder,S.H.
Y1 - 2008///
N1 - 2008279178
English
Journal: Article
KW - EMBASE
KW - air conditioning
KW - article
KW - assault
KW - boy
KW - Child Behavior
KW - childhood
KW - community
KW - Crime
KW - data analysis
KW - dependent variable
KW - epidemic
KW - Female
KW - girl
KW - Homicide
KW - Human
KW - Internet
KW - Male
KW - Obesity
KW - parent
KW - play
KW - Probability
KW - rape
KW - Recreation
KW - Regression Analysis
KW - sample (statistics)
KW - school child
KW - sexual crime
KW - sitting
KW - Technology
KW - Television
KW - theft
KW - United States
RP - NOT IN FILE
JF - International Journal of Behavioral Nutrition and Physical Activity
VL - 5 , 2008. Article Number: 28. Date of Publication: 14 May 2008.
CY - United Kingdom
N2 - Introduction: Although per capita crime has generally fallen over the period which coincides with the obesity epidemic, it has not fallen uniformly across communities. It also has not fallen enough to allay fears on

the part of parents. Over the past 30 years, technological changes have made the indoor alternatives to playing outside, where children are more vulnerable to criminal activity, more enjoyable (cable TV, video games, and the internet) and comfortable (the spread of air conditioning to low income neighborhoods). We determined whether indoor sedentary behavior patterns are associated with community crime statistics. 4th graders in the U.S. are typically 9 or 10 years old. Methods: We used data from the 2004-2005 Texas School Physical Activity and Nutrition (SPAN) survey linked with U.S. Department of Justice, Office of Justice Programs, Bureau of Justice Statistics data for the years 2000 through 2005 and Texas State data on sexual offenders. The probability-based sample included a total of 7,907 children in grade four. Multistage probability sampling weights were used. The dependent variables included were hours of TV watching, video game playing, computer use and total indoor sedentary behavior after school. Incremental Relative Rates were computed for community crime rates including robberies, all violent crimes, murders, assaults, property crimes, rapes, burglaries, larcenies and motor vehicle thefts as well as for sexual offenders living in the neighborhood. The neighborhood refers to the areas where the students at each school live. In the case of sexual offenders, sexual offenders per capita are estimated using the per capita rate in the zip code of the school attended; all other crime statistics are estimated by the crimes per capita in the police department jurisdiction covering the school attended. After controlling for sex, age, and African-American and Hispanic, cross-sectional associations were determined using multivariate Poisson regression. Results: 4th grade boys were more likely to play video games in communities with increased per 100 population rates of larceny and burglary as well as in communities with increased per capita sexual offenders; 4th grade girls were more likely to watch television in communities with increased per capita sexual offenders. While 4th grade girls were more likely to watch TV in communities with increased per capita sex offenders, they were less likely to use computers. Per capita sexual offenders were negatively related to computer use amongst 4th grade girls. Conclusion: By combining community crime and cross-sectional individual level data on indoor sedentary behavior, we found that there is an association between community crimes/sex offender rates and certain types of indoor sedentary behavior. The development of technologies in recent decades which makes supervising children easier indoors, where children are much less vulnerable to crime, may be contributing to the epidemic of childhood obesity. 2008 Brown et al; licensee BioMed Central Ltd
AD - (Shelton III) Michael and Susan Dell Center for Advancement of Healthy Living, Division of Management, Policy and Community Health, University of Texas School of Public Health, 313 E. 12th Street, Austin, TX 78701, United States (Perez) Department of Bioinformatics and Biostatistics, School of Public Health and Information Sciences, University of Louisville, 555 S. Floyd Street, Louisville, KY 40292, United States (Mirchandani) Family Health Research and Program Development, Texas Department of State Health Services, Austin, TX 78756, United States (Hoelscher) Michael and Susan Dell Center for Advancement of Healthy Living, Division of Behavioral Science, University of Texas School of Public Health, 313 E. 12th Street, Austin, TX 78701, United States (Kelder) Michael and Susan Dell Center for Advancement of Healthy Living, Division of Epidemiology, University of Texas School of Public Health, 313 E. 12th Street, Austin, TX 78701, United States
ER -

TY - JOUR
ID - 3797
T1 - Value of a clinical test for assessing physical activity in children
A1 - Claude,Godard M.
A1 - Maria Del Pilar,Rodriguez N.
A1 - Diaz,N.
A1 - Lydia,Lera M.
A1 - Gabriela,Salazar R.
A1 - Raquel,Burrows A.
Y1 - 2008///
N1 - 19030660
Spanish
Journal: Article
KW - EMBASE
KW - Acceleration
KW - Adolescent
KW - Anthropometry
KW - article

KW - Child
KW - comparative study
KW - Evaluation
KW - Female
KW - fitness
KW - Human
KW - leisure
KW - Lifestyle
KW - Male
KW - Motor Activity
KW - Obesity
KW - pathophysiology
KW - physiology
KW - questionnaire
KW - reproducibility
KW - Sensitivity and Specificity
KW - standard
RP - NOT IN FILE
SP - 1155
EP - 1162
JF - Revista Medica de Chile
JA - Rev Med Chil.
VL - 136
IS - 9
CY - Chile
N2 - Background: An appropriate measurement of physical activity (PA) in children is useful, since inactivity is associated to obesity, cardiovascular and metabolic risk. Aim: To assess me reliability of the INTA questionnaire of PA, to compare the derived PA score with accelerometry and to assess its ability to identify excessively inactive children. Material and methods: One hundred eighty children aged 8 to 13 years answered an interviewer-administered questionnaire about their usual PA, consisting in 5 items (recumbent, seated, walking, playing outdoor, sports). The answers were converted to a PA score with a 0-10 points scale. Reliability was tested in 87 children by test/retest conducted 3-5 days apart. The PA score was compared with 3-day accelerometry in 77 of 93 children (35 obese and 42 non obese). Receiver operating characteristic (ROC) curves were used to determine the optimal cutpoint for identify an excessively sedentary child. Results: The test/retest reliability of the questionnaire was 0.69 to 0.93 (Lin coefficient). Accelerometry was dgnificantly associated with PA score (RHO: 0.60, p =0.008), outdoor plays (RHO: 0.37, p =0.0009) and practicing of sports (RHO: 0.33, p =0.003). Obese children were less active than non obese children, according both to PA score and to accelerometry. The optimal cut-point for classifying a child as too sedentary was a score of 5 (sensitivity =0.89). Conclusions: The INTA-test is a valuable instrument for measuring usual PA in clinical practice and is easy to administer
SN - 0034-9887
AD - (Claude Godard, Maria Del Pilar Rodriguez, Diaz, Lydia Lera, Gabriela Salazar, Raquel Burrows) Proyecto Financiado Por Nestle Foundation, Suiza Instituto de Nutricion y Tecnologia de Los Alimentos, Universidad de Chile, Santiago, Chile (Claude Godard) El Libano 5524, Macul, Chile
ER -

TY - JOUR
ID - 3798
T1 - Patterns of antiretroviral drug prescripcion in 997 Colombian patients
A1 - Machado,J.E.
A1 - Alzate,J.A.
Y1 - 2008///
N1 - 18645663
Spanish
Journal: Article
KW - EMBASE
KW - Adult

KW - Aged
KW - article
KW - Child
KW - clinical practice
KW - Colombia
KW - drug combination
KW - Female
KW - Human
KW - Human immunodeficiency virus infection/dt [Drug Therapy]
KW - Infant
KW - Male
KW - Middle Aged
KW - Newborn
KW - preschool child
KW - prescription
KW - Statistics
KW - antiretrovirus agent/dt [Drug Therapy]
RP - NOT IN FILE
SP - 78
EP - 86
JF - Biomedica
JA - Biomedica (Bogota)
VL - 28
IS - 1
CY - Colombia
N2 - Introduction. Antiretroviral therapy for treatment of human immunodeficiency virus type 1 (HIV-1) infection has improved steadily since the advent of combination therapy in 1996. Objective. The pharmacological therapies of the infection by HIV/AIDS were documented in order to determine if effective treatment regimes were prescribed. Materials and methods. Pharmacological therapies of the human immunodeficiency virus infection were compared in 997 patients affiliated with the health security system, of both sexes and all ages. All had been in treatment for more than three months (July-September 2006) and were distributed in 15 Colombian cities. The data were retrieved from medication consumption files which were maintained by the institutions that had distributed medications to the selected patients. Results. The average age of patients was 37.7+13.2 and 82.6% were men. All the patients received between three to five antiretrovirals. The medications most commonly prescribed were nucleoside reverse transcriptase inhibitors (96.4%), nonnucleoside reverse transcriptase inhibitors (54.9%), protease inhibitors (39.8%) and others (0.4%). All of the doses were at recommended levels. The most common combinations were lamivudine-zidovudine-efavirenz (35%), lamivudine-zidovudine+lopinavir/ritonavir (8.4%), abacavir+lamivudine-zidovudine (5.5%), lamivudine-zidovudine+nevirapine (5.2%) and others (45.9%), consisting of 65 different combinations. Conclusions. All agents were used at internationally recommended doses and rational prescription patterns prevailed in the initial therapy. However, the use of 69 different associations suggested that after the beginning of treatment, clinicians do not have adequate criteria to prescribe in accordance with international guidelines of antiretroviral therapy; they adopt a variety of options that may be outside of established recommendations
SN - 0120-4157
AD - (Machado, Alzate) Grupo de Investigacion de Farmacoepidemiologia y Farmacovigilancia, Departamento de Ciencias Basicas, Universidad Tecnologica de Pereira, Pereira, Colombia (Machado) Departamento de Farmacoepidemiologia, Audifarma S.A., Pereira, Colombia (Machado) Calle 105 No 14-140, Risaralda, Pereira, Colombia
ER -

TY - JOUR
ID - 3799
T1 - When a child says 'no': experiences of nurses working with children having invasive procedures
A1 - Lloyd,M.
A1 - Urquhart,G.
A1 - Heard,A.
A1 - Kroese,B.

Y1 - 2008///
N1 - 18547007
English
Journal: Article
KW - EMBASE
KW - article
KW - Child
KW - emotion
KW - Exercise
KW - Human
KW - nurse patient relationship
KW - surgery
RP - NOT IN FILE
SP - 29
EP - 34
JF - Paediatric nursing
VL - 20
IS - 4
CY - United Kingdom
N2 - Experiencing invasive medical procedures can be a devastating experience for some children and their parents. The potential impact on staff who perform the procedure and who may have to restrain the child who is unwilling to have an essential procedure is a neglected area of research. Children's distress and their coping are affected by those around them so it is important to understand how nurses react in these situations. AIM: To explore the experiences of nursing staff involved in facilitating invasive procedures for children who do not want them. METHOD: Participants were selected at random from staff lists of one hospital in the West Midlands. Data collection was undertaken using unstructured qualitative interviews with ten paediatric nurses and in two focus groups. Theories generated from each interview were tested and validated with participants in subsequent interviews and then in the focus groups. FINDINGS: The most common experiences reported by the participants were 'getting upset' and 'getting stressed' by some aspect of the medical procedure, either because the child or parents became upset or the procedure had gone wrong in some way. Procedural protocols that exist to protect children, for example, by limiting the number of unsuccessful attempts to undertake the procedure, also protect staff by providing a framework to manage emotions during the procedure. Being able to explain the process and need for the procedure to the child and parents, obtaining consent where possible for the use of certain techniques, such as restraint, and having the time to adequately prepare a child for a procedure, all helped minimise the likelihood of an unsuccessful procedure, thereby reducing the risk of the nurse being emotionally affected by a distressed child. CONCLUSION: Nurses working with children who are unwilling to undergo invasive procedures experience negative emotions but these are short lived due to a combination of protective factors and coping strategies. Further research is needed to understand the experiences of medical staff and of nurses working outside paediatric environments who may not experience the same support and protection as those in paediatric settings
SN - 0962-9513
AD - (Lloyd, Urquhart, Heard, Kroese) Blurton Child and Adolescent Mental Health Team, Stoke-on-Trent
ER -

TY - JOUR
ID - 3800
T1 - Bullying among school children in postwar Bosnia and Herzegovina: Cross-sectional study
A1 - Obrdalj,E.C.
A1 - Rumboldt,M.
Y1 - 2008///
N1 - 2008574460
English
Journal: Article
KW - EMBASE
KW - Adolescent
KW - article
KW - battle injury

KW - Bosnia and Herzegovina
KW - Bullying
KW - controlled study
KW - Female
KW - Human
KW - major clinical study
KW - Male
KW - questionnaire
KW - risk factor
KW - school child
RP - NOT IN FILE
SP - 528
EP - 535
JF - Croatian Medical Journal
JA - Croat.Med J
VL - 49
IS - 4
CY - Croatia
N2 - Aim: To compare the prevalence and characteristics of bullying between two towns in Bosnia and Herzegovina - Stolac, which was exposed to firearm conflict during the 1992-1995 war in Bosnia and Herzegovina, and Posusje, which was outside of the active combat zone. Methods: In this cross-sectional study, we included 484 primary school pupils attending 4th-8th grade of elementary school, 217 (44.8%) of them from Stolac and 267 (55.2%) from Posusje. The pupils were interviewed using a standardized questionnaire on the experience of bullying. Results: Every sixth pupil (16.4%) experienced at least one form of bullying almost every day, while 34 (7.0%) pupils constantly bullied other children. Sixth-eighth graders were more often bullies than 4th-5th graders (P = 0.044). Girls were most often victims of bullying, while boys were most often bullies (P = 0.036). The expected difference in bullying between the two towns was not observed, except for older pupils in Posusje, who were more violent than their peers in Stolac (P = 0.044). Among the analyzed variables of sex, age, town, and school achievement, only male sex was significant predictor of bullying (P = 0.010), increasing the relative risk by 3.005 times. Conclusion: Bullying among primary school pupils did not differ between areas that experienced war activities in 1992-1995. Our results could be useful in the introduction of specific prevention measures against bullying in postwar situation
SN - 0353-9504
AD - (Obrdalj, Rumboldt) Department of Family Medicine, Mostar University School of Medicine, Mostar, Bosnia and Herzegovina (Obrdalj) Department of Family Medicine, Mostar University School of Medicine, Bijeli Brijeg bb, 88000 Mostar, Bosnia and Herzegovina
ER -

TY - JOUR
ID - 3801
T1 - Arterial thrombosis in children
A1 - Price,V.E.
A1 - Chan,A.K.C.
Y1 - 2008///
N1 - 2008515013
English
Journal: Review
KW - EMBASE
KW - angiography
KW - anticoagulant therapy
KW - aorta arch syndrome/di [Diagnosis]
KW - aorta arch syndrome/dt [Drug Therapy]
KW - aorta arch syndrome/su [Surgery]
KW - artery blood flow
KW - artery catheterization
KW - artery thrombosis/co [Complication]
KW - artery thrombosis/di [Diagnosis]

KW - artery thrombosis/dt [Drug Therapy]
KW - artery thrombosis/et [Etiology]
KW - artery thrombosis/pc [Prevention]
KW - artery thrombosis/su [Surgery]
KW - balloon dilatation
KW - Blalock Taussig shunt
KW - bleeding/si [Side Effect]
KW - bleeding/th [Therapy]
KW - Blood Transfusion
KW - catheter thrombosis/co [Complication]
KW - catheter thrombosis/pc [Prevention]
KW - Child
KW - childhood mortality
KW - clinical feature
KW - clinical trial
KW - computed tomographic angiography
KW - congenital heart disease/cn [Congenital Disorder]
KW - continuous infusion
KW - contrast enhancement
KW - disease association
KW - disease course
KW - Doppler echography
KW - drug contraindication
KW - drug dose increase
KW - drug efficacy
KW - drug hypersensitivity/si [Side Effect]
KW - drug response
KW - drug safety
KW - drug substitution
KW - drug withdrawal
KW - gene mutation
KW - heart catheterization
KW - heparin induced thrombocytopenia/di [Diagnosis]
KW - heparin induced thrombocytopenia/si [Side Effect]
KW - hepatic artery
KW - Human
KW - hyperhomocysteinemia/cn [Congenital Disorder]
KW - hyperhomocysteinemia/dt [Drug Therapy]
KW - hyperhomocysteinemia/et [Etiology]
KW - hyperlipidemia
KW - Incidence
KW - international normalized ratio
KW - loading drug dose
KW - low drug dose
KW - magnetic resonance angiography
KW - maintenance drug dose
KW - mucocutaneous lymph node syndrome/dt [Drug Therapy]
KW - optimal drug dose
KW - pathophysiology
KW - plethysmography
KW - recommended drug dose
KW - renal artery thrombosis/co [Complication]
KW - renal artery thrombosis/dt [Drug Therapy]
KW - renal artery thrombosis/pc [Prevention]
KW - renal artery thrombosis/su [Surgery]
KW - review
KW - Risk Assessment

KW - risk factor
KW - single drug dose
KW - surgical thrombectomy
KW - symptom
KW - thromboembolism/co [Complication]
KW - thromboembolism/di [Diagnosis]
KW - thromboembolism/dt [Drug Therapy]
KW - Thromboembolism/et [Etiology]
KW - thromboembolism/pc [Prevention]
KW - thromboembolism/su [Surgery]
KW - thrombogenesis
KW - thrombophilia/cn [Congenital Disorder]
KW - thrombophilia/et [Etiology]
KW - umbilical artery catheterization
KW - vascular surgery
KW - acetylsalicylic acid/ct [Clinical Trial]
KW - acetylsalicylic acid/cb [Drug Combination]
KW - acetylsalicylic acid/do [Drug Dose]
KW - acetylsalicylic acid/dt [Drug Therapy]
KW - anticoagulant agent/ct [Clinical Trial]
KW - anticoagulant agent/cb [Drug Combination]
KW - anticoagulant agent/dt [Drug Therapy]
KW - anticoagulant agent/po [Oral Drug Administration]
KW - argatroban/do [Drug Dose]
KW - argatroban/dt [Drug Therapy]
KW - blood clotting factor 5 Leiden/ec [Endogenous Compound]
KW - corticosteroid/dt [Drug Therapy]
KW - cytokine antibody/dt [Drug Therapy]
KW - danaparoid/do [Drug Dose]
KW - danaparoid/dt [Drug Therapy]
KW - fibrinogen receptor antagonist/dt [Drug Therapy]
KW - folic acid/dt [Drug Therapy]
KW - heparin/ae [Adverse Drug Reaction]
KW - heparin/do [Drug Dose]
KW - heparin/dt [Drug Therapy]
KW - hirulog/do [Drug Dose]
KW - hirulog/dt [Drug Therapy]
KW - immunoglobulin/cb [Drug Combination]
KW - immunoglobulin/do [Drug Dose]
KW - immunoglobulin/dt [Drug Therapy]
KW - immunoglobulin/iv [Intravenous Drug Administration]
KW - immunosuppressive agent/dt [Drug Therapy]
KW - interleukin 2 antibody/dt [Drug Therapy]
KW - lepirudin/do [Drug Dose]
KW - lepirudin/dt [Drug Therapy]
KW - low molecular weight heparin/ae [Adverse Drug Reaction]
KW - low molecular weight heparin/dt [Drug Therapy]
KW - methylprednisolone/ct [Clinical Trial]
KW - methylprednisolone/cb [Drug Combination]
KW - methylprednisolone/do [Drug Dose]
KW - methylprednisolone/dt [Drug Therapy]
KW - methylprednisolone/iv [Intravenous Drug Administration]
KW - placebo
KW - prothrombin/ec [Endogenous Compound]
KW - streptokinase/ae [Adverse Drug Reaction]
KW - streptokinase/dt [Drug Therapy]
KW - tissue plasminogen activator/do [Drug Dose]

KW - tissue plasminogen activator/dt [Drug Therapy]
KW - unclassified drug
KW - urokinase/dt [Drug Therapy]
KW - warfarin/do [Drug Dose]
KW - warfarin/dt [Drug Therapy]
RP - NOT IN FILE
SP - 419
EP - 428
JF - Expert Review of Cardiovascular Therapy
VL - 6
IS - 3
CY - United Kingdom
N2 - Arterial thromboembolism is rare in children. The frequency of arterial thromboembolisms has paralleled the advances in pediatric tertiary care with increasing numbers of children surviving life-threatening primary illnesses and, paradoxically, developing previously rare complications. The pathophysiology of thrombosis in children differs from that of adults, underscoring the need for a unique diagnostic and therapeutic approach in pediatrics. This review is a summary of the available data on the etiology, diagnosis and management of arterial thrombosis, occurring outside of the CNS, in children. 2008 Future Drugs Ltd
SN - 1477-9072
AD - (Price) IWK Health Centre, Division of Hematology/Oncology, Dalhousie University, 5850/5890 University Ave., Halifax, NS B3K 6R8, Canada (Chan) Division of Pediatric Hematology/Oncology, Department of Pediatrics, McMaster University, Hamilton, ON, Canada
ER -

TY - JOUR
ID - 3802
T1 - Outbreak of Cryptosporidium parvum among children after a school excursion to an adventure farm, South West England
A1 - Hoek,M.R.
A1 - Oliver,I.
A1 - Barlow,M.
A1 - Heard,L.
A1 - Chalmers,R.
A1 - Paynter,S.
Y1 - 2008///
N1 - 2008469740
English
Journal: Article
KW - EMBASE
KW - article
KW - clinical article
KW - Cohort Analysis
KW - controlled study
KW - Cryptosporidiosis/ep [Epidemiology]
KW - cryptosporidiosis/et [Etiology]
KW - Cryptosporidium parvum
KW - disinfection
KW - effluent
KW - Environmental Exposure
KW - epidemic
KW - feces analysis
KW - Filtration
KW - fluid intake
KW - Human
KW - infection risk
KW - infectious diarrhea
KW - livestock

KW - microbial contamination
KW - parasite transmission
KW - school
KW - school child
KW - United Kingdom
KW - vomiting
KW - water contamination
KW - Water Supply
KW - Rain
KW - surface water
KW - well water
RP - NOT IN FILE
SP - 333
EP - 338
JF - Journal of Water and Health
VL - 6
IS - 3
CY - United Kingdom
N2 - We present the results of an outbreak investigation on a Cryptosporidium parvum outbreak among 35 people, (27 students and 8 teachers), who participated in a school excursion to an outdoor adventure farm in South West England, from 22 to 26 May 2006. A cohort study was implemented to investigate possible sources of infection during the farm visit. The most likely transmission route was contact with faecally contaminated surface water following heavy rainfall, or consumption of water from the private well. Disinfection of the water reservoir was by chlorination, to which cryptosporidium is resistant. Supplemental disinfection or filtration methods of private water supplies on livestock farms may be needed. This study highlights the fact that epidemiological investigations of outbreaks as a result of environmental exposures are complex but important to inform the public and health professionals of the risks posed by private water supplies and outdoor activities. This is particularly so after heavy rainfall, as this may result in an increased effluent from faecally contaminated land, causing a wide variety of pathogens to wash into surface water and potentially, private wells. This poses risks for public health. IWA Publishing 2008
SN - 1477-8920
AD - (Hoek, Oliver) Health Protection Agency, The Wheelhouse, Bond's Mill, Stonehouse, Glos GL10 3RF, United Kingdom (Barlow, Heard, Paynter) Health Protection Agency, Sedgemoor Centre, Priory Road, St. Austell PL25 5AS, United Kingdom (Chalmers) Singleton Hospital, Sketty, Swansea SA2 8QA, United Kingdom
ER -

TY - JOUR
ID - 3803
T1 - An investigation of non-participation in health promotion interventions and its impact on population level outcome
A1 - Davies,G.M.
A1 - Boothman,N.J.
A1 - Duxbury,J.T.
A1 - Davies,R.M.
A1 - Blinkhorn,A.S.
Y1 - 2008///
N1 - 2008453515
English
Journal: Article
KW - EMBASE
KW - article
KW - Attitude to Health
KW - Child
KW - child health care
KW - community assessment
KW - health care access

KW - Health Education
KW - health program
KW - Health Promotion
KW - Human
KW - human relation
KW - Immunization
KW - medical examination
KW - patient attitude
KW - Patient Participation
KW - population movement pattern
KW - public health service
KW - United Kingdom
KW - measles mumps rubella vaccine
RP - NOT IN FILE
SP - 107
EP - 112
JF - International Journal of Health Promotion and Education
VL - 46
IS - 3
CY - United Kingdom
N2 - Background: Many health promotion interventions rely on active participation, if uptake is low the impact of the intervention is diminished at population level. This study aimed to identify the main reasons for non-participation in a modelled health promotion programme. Methods: A classification for eligibility and for participation in a modelled health promotion intervention was applied to a database of all 3-5 year olds in 3 PCTs in Manchester, UK. This identified which children would have been participants and which non-participants. Results: Of the 29,220 children in the cohort, 90% would have been eligible to have received the model intervention as they were resident in Manchester before they became 24 months old. Of these, 75% would have participated by having either of the required contacts with health care personnel in the Manchester PCT area. Of these only 43% were attending Manchester LEA schools or pre-schools at the age of 3-5 years when the health benefits would be evaluated. Population churn, uptake of health services outside of the intervention area and non-uptake all accounted for a high level of 'non-participation'. Conclusions: The success of public health interventions are seriously threatened by high levels of non-participation due to population transience in some areas
SN - 1463-5240
AD - (Davies, Boothman, Duxbury) Dept Dental Public Health, Manchester Primary Care Trust, Mauldeth Road West, Manchester M21 7RL, United Kingdom (Davies) Dental Health Unit, University of Manchester, Lloyd Street North, Manchester M15 6SH, United Kingdom (Blinkhorn) Population Oral Health, Faculty of Dentistry, University of Sydney, 1 Mons Road, Westmead, NSW 2145, Australia
ER -

TY - JOUR
ID - 3805
T1 - Natural and traumatic sports-related fatalities: A 10-year retrospective study
A1 - Turk,E.E.
A1 - Riedel,A.
A1 - Pueschel,K.
Y1 - 2008///
N1 - 2008342053
English
Journal: Article
KW - EMBASE
KW - Adolescent
KW - Adult
KW - Aged
KW - article
KW - Autopsy
KW - Cause of Death

KW - controlled study
KW - fatality
KW - Female
KW - Football
KW - Germany
KW - Human
KW - major clinical study
KW - Male
KW - medical examination
KW - outcome assessment
KW - preschool child
KW - retrospective study
KW - risk factor
KW - Running
KW - sport injury
RP - NOT IN FILE
SP - 604
EP - 608
JF - British Journal of Sports Medicine
JA - BJSM online
VL - 42
IS - 7
CY - United Kingdom
N2 - Objective: To identify the most common causes of death during sports activity in the population of Hamburg, Germany, and to identify which groups of sportspeople are particularly in danger of dying during sports and in which types of sport most fatalities occur. Design and setting: We performed a 10-year autopsybased retrospective study of all 48 335 fatalities in Hamburg and the surrounding areas that were subjected to police investigations between 1997 and 2006 and screened for sports-related deaths. The main outcome measure was cause of death depending on form of sport, age and risk factors. Results: Most of the fatalities were male. In natural deaths, cardiac causes were the most frequent causes found, with running and football being the most frequent forms of sport in which death had occurred. In some of the cases, sports medical examinations had been performed before death, certifying eligibility for the respective activity. Traumatic deaths were found in all age groups, with younger age groups more likely to have traumatic than natural deaths, and as expected, occurred more commonly in "risky" outdoor activities. Conclusions: Although exercise can have beneficial effects on health, fatalities related to sports activity occur. Cardiac disease is the main cause of sudden death from natural causes. In patients with pre-existing coronary heart disease, left ventricular hypertrophy constitutes a risk factor for exercise-related sudden death. Traumatic deaths often happen on holiday outside the person's country of dwelling, and are most commonly attributable to drowning and blunt trauma. Preparticipation medical screening cannot always prevent fatal incidents during sports activity. Postmortem macroscopic and histological examination can clarify the cause of death and legal issues
SN - 0306-3674
AD - (Turk, Riedel, Pueschel) Institute of Legal Medicine, University Medical, Centre Hamburg-Eppendorf, Hamburg, Germany
ER -

TY - JOUR
ID - 3807
T1 - The human-canine environment: A risk factor for non-play bites?
A1 - Messam,L.L.M.
A1 - Kass,P.H.
A1 - Chomel,B.B.
A1 - Hart,L.A.
Y1 - 2008///
N1 - 2008273732
English
Journal: Article
KW - EMBASE

KW - Adolescent
KW - Adult
KW - article
KW - Child
KW - Cohort Analysis
KW - controlled study
KW - cultural factor
KW - data analysis
KW - dog bite
KW - Environmental Exposure
KW - environmental factor
KW - Female
KW - Human
KW - Jamaica
KW - major clinical study
KW - Male
KW - nonhuman
KW - object relation
KW - Poisson Distribution
KW - Regression Analysis
KW - retrospective study
KW - risk factor
KW - United States
RP - NOT IN FILE
SP - 205
EP - 215
JF - Veterinary Journal
VL - 177
IS - 2
CY - United Kingdom
N2 - Few dog bite risk factor studies have been conducted. This veterinary clinic-based retrospective cohort study was aimed at identifying human-canine environmental risk factors for non-play bites in Kingston, Jamaica (660) and San Francisco (SF), USA (452). Data were analysed using modified Poisson regression with confounders selected using directed acyclic graphs (DAGs) and the change-in-estimate procedure. Dogs acquired for companionship were more likely (RR = 1.66; 95% CI 1.02-2.70) to bite than those acquired for protection. Routinely allowing a dog into the presence of visitors was also positively associated with it biting. A dog sleeping in a family member's bedroom was a risk factor for biting in Kingston (RR = 2.54; 95% CI 1.43-4.54) but not in SF, while being able to leave the yard unaccompanied was a risk factor for biting in SF (RR = 3.40; 95% CI 1.98-5.85) but not in Kingston. Overall, dogs which were less restricted in their interactions with humans were at elevated risk for biting. An observed association with dog bites in one cultural setting might not exist in another. 2007 Elsevier Ltd. All rights reserved
SN - 1090-0233
AD - (Messam, Kass, Chomel, Hart) School of Veterinary Medicine, Department of Population Health and Reproduction, University of California Davis, 1114 Tupper Hall, Davis, CA 95616, United States
ER -

TY - JOUR
ID - 3808
T1 - Outdoor and indoor air pollution and COPD-related diseases in high- and low-income countries
A1 - Liu,Y.
A1 - Lee,K.
A1 - Perez-Padilla,R.
A1 - Hudson,N.L.
A1 - Mannino,D.M.
Y1 - 2008///

N1 - 2008068426
English
Journal: Review
KW - EMBASE
KW - Adolescent
KW - Adult
KW - Aged
KW - Air Pollution
KW - Child
KW - chronic obstructive lung disease/ep [Epidemiology]
KW - cigarette smoking
KW - Environmental Exposure
KW - Human
KW - Infant
KW - lowest income group
KW - major clinical study
KW - population research
KW - Prevalence
KW - priority journal
KW - review
KW - risk factor
RP - NOT IN FILE
SP - 115
EP - 127
JF - International Journal of Tuberculosis and Lung Disease
VL - 12
IS - 2
CY - France
N2 - Chronic obstructive pulmonary disease (COPD) is an important cause of morbidity and mortality in both high- and low-income countries. While active cigarette smoking is the most important preventable risk factor globally, outdoor and indoor air pollutants can cause or exacerbate COPD. In high-income countries, historic air pollution events provide clear evidence that exposure to high levels of outdoor air pollutants is associated with increased mortality and morbidity due to COPD and related cardiorespiratory diseases. Studies in the last 20 years continue to show increased risk associated mainly with particulate matters, even at much lower levels. Populations in low-income countries are largely exposed to indoor air pollutants from the combustion of solid fuels, which contributes significantly to the burden of COPD-related diseases, particularly in non-smoking women. Effective preventive strategies for COPD may vary between countries, and include continued improvements in air cleaning technology, air quality legislation and dissemination of improved cooking stoves. A joint effort from both society and governments is needed for these endeavors. 2008 The Union
SN - 1027-3719
AD - (Liu, Lee, Hudson, Mannino) Department of Preventive Medicine and Environmental Health, University of Kentucky College of Public Health, Lexington, KY, United States (Lee) School of Public Health, Seoul National University, South Korea (Perez-Padilla) National Institute of Respiratory Diseases, Mexico City, Mexico (Liu) Department of Preventive Medicine and Environmental Health, University of Kentucky, College of Public Health, 121 Washington Ave, Lexington, KY 40536, United States
ER -

TY - JOUR
ID - 3809
T1 - Educational stay for asthmatic children in Ardeche: An innovative program
A1 - Fourot-Bauzon,M.
A1 - Morisson,M.-D.
A1 - Galle,M.
Y1 - 2008///
N1 - 2008507613
French
Journal: Article

KW - EMBASE
KW - Altitude
KW - article
KW - breathing exercise
KW - Child
KW - clinical article
KW - clinical practice
KW - consultation
KW - controlled study
KW - disease association
KW - disease severity
KW - exercise induced asthma/th [Therapy]
KW - Female
KW - health care personnel
KW - health program
KW - Hospitalization
KW - Human
KW - Male
KW - medical education
KW - Motivation
KW - Muscle Strength
KW - Patient Satisfaction
KW - Patient Selection
KW - Physical Activity
KW - questionnaire
KW - Rhinitis
KW - sport
RP - NOT IN FILE
SP - 490
EP - 494
JF - Revue Francaise d'Allergologie et d'Immunologie Clinique
VL - 48
IS - 7
CY - France
N2 - Asthma is children's foremost chronic disease. If the medicinal progress is unambiguous, therapeutic education as well as rehabilitation efforts are integral parts of asthmatic children's global care. Initiated and proposed by the Association asthme et allergies, a protocol that defines place, dates, purposes and means, received the agreement of the French ministry for Youth and Sports. Thirteen children (eight boys, five girls, with an average age of eleven) who suffer from persistent asthma, from moderate to severe and/or exercise-induced asthma, were selected by two Parisian hospitals. The children spent fourteen days on vacation in Ardeche (altitude, 1400 m) during the month of July and were supervised by a medical and an animation team. Motivating children to engage in sports activities and strengthening therapeutic education trained in asthma's schools (ecoles de l'asthme) were the dual purpose of the stay. An educational diagnosis and an evaluation of the knowledge of each child were realized at the beginning and at the end of the stay. A flexible program was set up and combined outdoor games, role-playing, educational workshops on breathing, techniques of inhalation and measurement of breath... associated with tests and coupled with physical or sportive activities practiced individually or collectively. Climatic conditions did not provoke any asthma attack or intercurrent affection. The questionnaire dispensed at the end of the stay reveals the children's global satisfaction. In terms of health, preliminary results are promising, particularly regarding their aerobic capacity and physical performances. A medium-term evaluation is in progress, but consequences of this experience - shared by children coming from different social milieus and in a less polluted environment - seem to confirm the interest for promoting educational and sportive stays supervised by health professionals specialized in therapeutic education and spent in specific places which correspond to the standards of allergens' eviction. 2008
SN - 0335-7457
AD - (Fourot-Bauzon) Ecole de l'asthme de La-Bourboule, 150, rue Vercingetorix, 63150 La-Bourboule, France (Morisson) Reseau asthme et allergies 44, 1, place Beaumanoir, 44100 Nantes, France (Galle) Service de pediatrie, centre hospitalier de Beziers, 2, rue Valentin-Hauy, 34525 Beziers cedex, France

ER -

TY - JOUR
ID - 3810
T1 - Metabolic syndrome in children and adolescents in Crete, Greece, and association with diet quality and physical fitness
A1 - Linardakis,M.
A1 - Bertsias,G.
A1 - Sarri,K.
A1 - Papadaki,A.
A1 - Kafatos,A.
Y1 - 2008///
N1 - 2008508726
English
Journal: Article
KW - EMBASE
KW - abdominal obesity/ep [Epidemiology]
KW - Adolescent
KW - article
KW - body mass
KW - Child
KW - cholesterol blood level
KW - controlled study
KW - dietary intake
KW - Female
KW - fitness
KW - food quality
KW - Greece
KW - Human
KW - human cell
KW - Hypertension
KW - hypertriglyceridemia/ep [Epidemiology]
KW - impaired glucose tolerance
KW - Male
KW - metabolic syndrome X/ep [Epidemiology]
KW - outcome assessment
KW - Prevalence
KW - risk factor
KW - systolic blood pressure
KW - high density lipoprotein cholesterol/ec [Endogenous Compound]
RP - NOT IN FILE
SP - 421
EP - 428
JF - Journal of Public Health
VL - 16
IS - 6
CY - Germany
N2 - Aim: To determine the prevalence of metabolic syndrome in children and adolescents in Crete (Greece) and examine associations with diet quality and physical fitness. Subjects and Methods: A representative sample of 1,209 Cretan children and adolescents aged 3-17 1/2 years participated in this cross-sectional survey. The study took place in kindergartens, primary and secondary schools in the cities of Heraklion and Chania and kindergartens and schools in semi-urban and rural regions outside Heraklion. The metabolic syndrome was defined as the presence of >3 of: impaired fasting blood glucose, hypertension, abdominal obesity, hypertriglyceridaemia, and low HDL-cholesterol. Diet quality was assessed by the Healthy Eating Index score (USDA) and physical fitness by the 20-m shuttle run test. Results: Approximately 4% of Cretan children and adolescents assessed (4.2% of boys, 3.6% of girls) had three or more metabolic syndrome factors. Over 25% of the subjects had a "poor" diet, defined as Healthy Eating Index <51, and higher diet quality scores correlated

with decreased mean body mass index, waist/height ratio, systolic blood pressure, total cholesterol, and number of metabolic syndrome risk factors present. Clustering of metabolic syndrome risk factors was also associated with decreased physical fitness, as well as increased body mass index, waist/height ratio and total cholesterol/HDL-cholesterol ratio. Conclusions: Presence of three or more factors related to the metabolic syndrome was strongly associated with poor diet quality and low physical fitness, as well as overweight, in Cretan children and adolescents. Health promotion strategies in childhood should encourage weight control, the establishment of healthier dietary patterns, and adequate physical activity, as a means of preventing the increase in the prevalence of this public health issue. 2008 Springer-Verlag
SN - 0943-1853
AD - (Linardakis, Bertsias, Sarri, Papadaki, Kafatos) Preventive Medicine and Nutrition Clinic, University of Crete School of Medicine, Heraklion, Crete, Greece (Papadaki) Department of Social Medicine, Faculty of Medicine, University of Crete, P.O. Box 2208, Heraklion, Crete 71003, Greece
ER -

TY - JOUR
ID - 3812
T1 - Current status of rickets among children under 6 years of age in Shanghai
A1 - Gong,Q.
A1 - Yu,S.-M.
A1 - Zhou,X.-J.
A1 - He,L.
A1 - Zhang,J.F.
A1 - Hong,Q.
A1 - Lu,G.F.
A1 - Zhou,W.L.
A1 - Huang,J.Y.
Y1 - 2007///
N1 - 2008036204
Chinese
Journal: Article
KW - EMBASE
KW - anamnesis
KW - article
KW - bottle feeding
KW - Breast Feeding
KW - calcium deficiency
KW - Child
KW - China
KW - controlled study
KW - Female
KW - Human
KW - Infant
KW - major clinical study
KW - Male
KW - Morbidity
KW - Nutrition
KW - Pregnancy
KW - Prevalence
KW - questionnaire
KW - respiratory tract infection
KW - Rickets/ep [Epidemiology]
KW - rickets/et [Etiology]
KW - risk factor
KW - sampling
KW - urban area
KW - Calcium
KW - cod liver oil

KW - vitamin D
RP - NOT IN FILE
SP - 10461
EP - 10463
JF - Journal of Clinical Rehabilitative Tissue Engineering Research
VL - 11
IS - 52
CY - China
N2 - Aim: To assess the current prevalence and influencing factors of rickets in children of Shanghai city. Methods: In Spring of 2005, the cluster and stratified random sampling study was used to examine 821 children under 6 years of age living in Shanghai to evaluate the living environment, feeding patterns, outdoor activity, nutrition, past history of disease, and pregnancy of mother by questionnaires. Rickets was defined as the guideline of "Prevention and Treatment of Rickets in Nurseling" by the State Ministry of Health in 1996. Results: Totally 769 cases were included in the analysis, consisting of 396 boys and 373 girls; 456 collective residences and 313 scattered livers. 1 The morbidity rate of rickets aged from 0-6 years in Shanghai was 17.3% (133/769), 17.4% in boys and 17.2% in girls. 2 The morbidity rate of rickets in children was associated with feeding patterns (breast feeding, mix feeding and bottle feeding were 13.0%, 17.5%, 25.2%), supplement of cod liver oil (regular supplement, occasional or no supplement of vitamin D was 13.5% versus 32.5%), living environment (living in urban, suburb was 23.6% versus 10.56%; collective residences, scattered liver were 13.8% versus 22.4%), time of outdoor activity (do out door activity often, occasionally was 12.9% versus 31.8%), repeatedly respiratory tract infection (with or without infection was 26.9% versus 12.9%), and the deficiency of calcium in the duration of mother's pregnancy (the morbidity rate of rickets in their children was 33.2% versus 12.0% who with or without the deficiency of calcium ($P < 0.01$). Conclusion: The morbidity rate of children rickets in Shanghai increases recently, and is associated with multiple factors, such as pollution of city, reduction of breast feeding and insufficient knowledge of child care by Parents
SN - 1673-8225
AD - (Gong, He, Zhang) Shanghai Child Health Care Institute, Shanghai Children's Hospital, Shanghai Jiao Tong University, Shanghai 200040, China (Yu, Zhou, Hong) Department of Pediatdcal Internal Medicine, First People's Hospital, Shanghai Jiao Tong University, Shanghai 200080, China (Lu) Institute of Maternal and Child Health, Qingpu District, Shanghai 201700, China (Zhou) Institute of Maternal and Child Health, Changning Dictrict, Shanghai 200052, China (Huang) Institute of Maternal and Child Health, Luwan Dictrict, Shanghai 200023, China
ER -

TY - JOUR
ID - 3813
T1 - Pulmonary function in adolescents living in an outdoor environmental air polluted area and in a mountain area
A1 - Longhini,E.
A1 - Fanari,P.
A1 - Fontana,M.
A1 - Giacomotti,E.
A1 - Codecasa,F.
A1 - Pacetti,M.
A1 - Cherubini,P.
A1 - Barlera,S.
A1 - Tognoni,G.
A1 - Salvadori,A.
Y1 - 2007///
N1 - 2007590017
English
Journal: Article
KW - EMBASE
KW - Adolescent
KW - Air Pollution
KW - article
KW - atopy

KW - Child
KW - controlled study
KW - Environmental Monitoring
KW - Female
KW - Forced Expiratory Volume
KW - Human
KW - immunoglobulin blood level
KW - industrialization
KW - lung function test
KW - Male
KW - prick test
KW - questionnaire
KW - respiratory tract disease
KW - skin test
KW - urban area
KW - immunoglobulin E/ec [Endogenous Compound]
RP - NOT IN FILE
SP - 157
EP - 166
JF - Minerva Pneumologica
VL - 46
IS - 3
CY - Italy
N2 - Aim. Human activity generates mixtures of pollutants and outdoor air pollution has been known to cause clinically significant adverse effects. Methods. We studied the possibility of associations among long-term exposure to environmental air pollution and respiratory symptoms, and pulmonary function and atopy in a cross-sectional study. The target population was a sample of 744 adolescents living in an industrialised town and a sample of 287 adolescents living in mountain valleys in the Alps. Notes on respiratory symptoms were obtained from a questionnaire; all the subjects performed a lung function test, skin tests and plasma levels IgE determinations (PRIST). Results. Significant lower values of forced vital capacity (FVC) and even lower values of forced expiratory volume in 1 s (FEV₁), of FEV₁/FVC and of maximal expiratory flow at 25% of FVC (MEF 25%) were detected in the metropolitan population. The tests resulted pathological in a significantly higher number of clinical silent subjects of this group. About atopy, high plasma IgE levels were significantly more frequent in the metropolitan group, while PRICK tests more frequent in the mountain group. Conclusion. This study seems to provide evidence that long-term exposure to air pollution in adolescents may be associated with lower lung volumes, and especially expiratory flows. The lack of a concordance between anamnestic respiratory symptoms from questionnaires and pulmonary functions may point out the suitability for periodical controls of them in young subjects of air polluted areas
SN - 0026-4954
AD - (Longhini, Fanari, Fontana, Giacomotti, Codecasa, Salvadori) Laboratory of Respiratory Pathophysiology, Istituto Auxologico Italiano, Ospedale San Giuseppe, Verbania, Italy (Pacetti, Cherubini) D. e G. Campari Medical Division, Ospedale Citta Sesto San Giovanni, Sesto San Giovanni, Italy (Barlera, Tognoni) Istituto Ricerche Farmacologiche Mario Negri, Milan, Italy (Fanari) Laboratorio di Fisiopatologia Respiratoria, Ospedale S. Giuseppe, Istituto Auxologico Italiano, P.O. Box 1, Verbania 28921, Italy
ER -

TY - JOUR
ID - 3814
T1 - Interdependence of domestic malaria prevention measures and mosquito-human interactions in urban Dar es Salaam, Tanzania
A1 - Geissbuhler,Y.
A1 - Chaki,P.
A1 - Emidi,B.
A1 - Govella,N.J.
A1 - Shirima,R.
A1 - Mayagaya,V.
A1 - Mtasiwa,D.

A1 - Mshinda,H.
A1 - Fillinger,U.
A1 - Lindsay,S.W.
A1 - Kannady,K.
A1 - De Castro,M.C.
A1 - Tanner,M.
A1 - Killeen,G.F.
Y1 - 2007///
N1 - 2007517967
English
Journal: Article
KW - EMBASE
KW - Adolescent
KW - Adult
KW - Anopheles gambiae
KW - article
KW - Child
KW - disease carrier
KW - Human
KW - insect bite
KW - insecticide treated net
KW - Interview
KW - major clinical study
KW - Malaria/ep [Epidemiology]
KW - malaria/pc [Prevention]
KW - mosquito
KW - night
KW - protective equipment
KW - Sleep
KW - Tanzania
KW - urban area
KW - Wakefulness
RP - NOT IN FILE
JF - Malaria Journal
JA - Malar.J
VL - 6 , 2007. Article Number: 126. Date of Publication: 2007.
CY - United Kingdom
N2 - Background. Successful malaria vector control depends on understanding behavioural interactions between mosquitoes and humans, which are highly setting-specific and may have characteristic features in urban environments. Here mosquito biting patterns in Dar es Salaam, Tanzania are examined and the protection against exposure to malaria transmission that is afforded to residents by using an insecticide-treated net (ITN) is estimated. Methods. Mosquito biting activity over the course of the night was estimated by human landing catch in 216 houses and 1,064 residents were interviewed to determine usage of protection measures and the proportion of each hour of the night spent sleeping indoors, awake indoors, and outdoors. Results. Hourly variations in biting activity by members of the Anopheles gambiae complex were consistent with classical reports but the proportion of these vectors caught outdoors in Dar es Salaam was almost double that of rural Tanzania. Overall, ITNs confer less protection against exophagic vectors in Dar es Salaam than in rural southern Tanzania (59% versus 70%). More alarmingly, a biting activity maximum that precedes 10 pm and much lower levels of ITN protection against exposure (38%) were observed for Anopheles arabiensis, a vector of modest importance locally, but which predominates transmission in large parts of Africa. Conclusion. In a situation of changing mosquito and human behaviour, ITNs may confer lower, but still useful, levels of personal protection which can be complemented by communal transmission suppression at high coverage. Mosquito-proofing houses appeared to be the intervention of choice amongst residents and further options for preventing outdoor transmission include larviciding and environmental management. 2007 Geissbuhler et al; licensee BioMed Central Ltd
AD - (Geissbuhler, Tanner, Killeen) Swiss Tropical Institute, Department of Public Health and Epidemiology, Socinstrasse 57, 4002 Basel, Switzerland (Geissbuhler, Chaki, Govella, Mayagaya, Mshinda, Killeen) Ifakara

Health Research and Development Centre, Co-ordination Office, Kiko Avenue, Dar es Salaam, Tanzania (Chaki, Emidi, Govella, Shirima, Mtasiwa, Kannady) Dar Es Salaam City Council, Dar es Salaam, Tanzania (Emidi) University of Dar Es Salaam, Dar es Salaam, Tanzania (Chaki, Govella, Fillinger, Lindsay, Killeen) School of Biological and Biomedical Sciences, South Road, Durham DH1 3LE, United Kingdom (De Castro) Department of Population and International Health, Harvard School of Public Health, 655 Huntington Avenue, Boston, MA 02115, United States
ER -

TY - JOUR
ID - 3815
T1 - The psychological sequelae of thermal injury on children and adolescents: Part 1
A1 - Rivlin,E.
A1 - Faragher,E.B.
Y1 - 2007///
N1 - 2007496160
English
Journal: Article
KW - EMBASE
KW - Adolescent
KW - Age Distribution
KW - antisocial behavior/di [Diagnosis]
KW - anxiety neurosis/di [Diagnosis]
KW - article
KW - Child Psychiatry
KW - controlled study
KW - Depression/di [Diagnosis]
KW - distress syndrome
KW - Female
KW - Human
KW - Interview
KW - major clinical study
KW - Male
KW - priority journal
KW - psychologic assessment
KW - Psychotherapy
KW - questionnaire
KW - Recreation
KW - Sample Size
KW - school child
KW - Sex Ratio
KW - social aspect
KW - thermal injury
RP - NOT IN FILE
SP - 161
EP - 172
JF - Developmental neurorehabilitation
JA - Dev Neurorehabil
VL - 10
IS - 2
CY - United Kingdom
N2 - The psychological effects of thermal injury and children and their mothers were investigated in a three-part study; Part 1 is concerned with group comparisons regarding the psychological effects of thermal injury on children; Part 2 with aspects of the thermally injured group and Part 3 with psychological effects on their mothers. A total of 44 thermally injured (aged 11-16 years) injured 3-14 years previously, were matched according to age, sex, burn percentage and site of injury. In-depth interviewing and questionnaire responses on measures of psychological disturbance indicated that thermally injured children were differentiated in terms of psychopathology from matched Fracture Controls and Normal Controls. Such differences embraced many

aspects of social and recreational functioning, and group differences emphasised depression, anxiety (particularly situational anxiety) and anti-social disorder as being particularly prominent in the thermally injured group. Therapeutic approaches are briefly discussed. 2007 Informa UK Ltd
SN - 1751-8423
AD - (Rivlin) Central Manchester and Manchester Children's University Hospitals NHS Trust, Department of Child/Adolescent Clinical Psychology, United Kingdom (Rivlin) Postgraduate Medical Institute, University of Hull, Department of Clinical Psychology, United Kingdom (Faragher) Clinical Research Group, Liverpool School of Tropical Medicine, Pembroke Place, Liverpool, L3 5QA, United Kingdom (Rivlin) Central Manchester and Manchester Children's University Hospitals NHS Trust, Booth Hall Children's Hospital, Department of Clinical Psychology, Charlestown Road, Manchester, M9 2AA, United Kingdom
ER -

TY - JOUR
ID - 3816
T1 - Group A antibody persistence five years after meningococcal polysaccharide vaccination in the Sudan
A1 - Flitter,B.A.
A1 - Ismail,A.
A1 - Vu,D.
A1 - Granoff,D.M.
Y1 - 2007///
N1 - 2007479878
English
Journal: Article
KW - EMBASE
KW - Adolescent
KW - Adult
KW - antibody titer
KW - article
KW - clinical article
KW - controlled study
KW - Human
KW - immunogenicity
KW - meningococcosis/dt [Drug Therapy]
KW - meningococcosis/pc [Prevention]
KW - Neisseria meningitidis
KW - North America
KW - school child
KW - Sudan
KW - Vaccination
KW - Meningococcus vaccine/dt [Drug Therapy]
RP - NOT IN FILE
SP - 135
EP - 138
JF - Human Vaccines
JA - Hum
VL - 3
IS - 4
CY - United States
N2 - Large meningococcal group A epidemics occur periodically in the Sudan, a country within the "meningitis belt" of Sub-Saharan Africa. Immunization with meningococcal polysaccharide vaccine induces protective serum bactericidal titers but little information is available on the duration of protection. Serum samples were obtained from 20 subjects, aged 11-47 years, who resided in the Sudan, and who had participated in a meningococcal polysaccharide immunogenicity study five years earlier. Persistence of serum group A bactericidal titers (measured with human complement) was compared to that of 12 immunized adults in North America with no known exposure to group A organisms. One month after vaccination, there were no significant differences in the serum bactericidal titers of the two groups. By five years the respective reciprocal geometric mean bactericidal titers had declined in both groups (82 to 34 in Sudanese, and 69-11 in North Americans, p <

0.03). However, the proportion of sera with protective bactericidal titers (>1:4) at five years was higher in the Sudanese than North Americans (80% vs. 42%, p < 0.05). Recommendations for periodic meningococcal polysaccharide vaccination every 3-5 years to maintain group A immunity may be more appropriate for persons living outside of endemic areas than for persons residing in endemic regions since immunity in endemic areas can be maintained by periodic exposure to group A organisms, even during periods between epidemics. 2007 Landes Bioscience
SN - 1554-8600
AD - (Flitter, Vu, Granoff) Center for Immunobiology and Vaccine Development, Children's Hospital Oakland Research Institute, Oakland, CA, United States (Ismail) National Health Laboratory, Ministry of Health, Sudan (Granoff) Children's Hospital Oakland Research Institute, 5700 Martin Luther King Jr. Way, Oakland, CA 94609, United States
ER -

TY - JOUR
ID - 3817
T1 - Participant and parent experiences in the parenteral insulin arm of the diabetes prevention trial for type 1 diabetes
A1 - Johnson,S.B.
A1 - Baughcum,A.E.
A1 - Hood,K.
A1 - Rafkin-Mervis,L.E.
A1 - Schatz,D.A.
Y1 - 2007///
N1 - 2007434258
English
Journal: Article
KW - EMBASE
KW - Adolescent
KW - alternative medicine
KW - article
KW - blood sampling
KW - controlled study
KW - Decision Making
KW - Diet
KW - distress syndrome
KW - Exercise
KW - Female
KW - glucose blood level
KW - health hazard
KW - Human
KW - hypoglycemia
KW - insulin dependent diabetes mellitus/dt [Drug Therapy]
KW - insulin dependent diabetes mellitus/pc [Prevention]
KW - low drug dose
KW - major clinical study
KW - Male
KW - Patient Participation
KW - school child
KW - glucose/ec [Endogenous Compound]
KW - insulin/do [Drug Dose]
KW - insulin/dt [Drug Therapy]
KW - insulin/pa [Parenteral Drug Administration]
KW - vitamin
RP - NOT IN FILE
SP - 2193
EP - 2198
JF - Diabetes Care

VL - 30
IS - 9
CY - United States
N2 - OBJECTIVE - To assess participant and parent experiences in the parenteral insulin arm of the Diabetes Prevention Trial for Type 1 Diabetes (DPT-1). RESEARCH DESIGN AND METHODS - Before trial results were publicized, surveys were completed by 82 intervention participants (the intervention group) (who received annual 4-day insulin infusions and daily insulin injections), 81 closely monitored control subjects (the closely monitored group), and 135 parents of children in the trial. RESULTS - Survey results suggest that participant perspective (adult, child, parent, and sex), study procedures, and group assignment have important implications when planning clinical trials. Parents rated the trial more favorably but worried about hypoglycemia and diabetes onset. Children had the least favorable reaction to the study. Parents preferred assignment to the intervention group; child/adult participants preferred assignment to the closely monitored group. The intervention group rated the annual 4-day insulin infusions more negatively than all other study procedures. Intervention group participants/parents reported poorer insulin injection adherence over the course of the study. Intervention group participants, parents, and female subjects expressed an interest in additional psychosocial support during the trial. Random assignment was viewed negatively by both study groups. Close observation for diabetes onset was viewed as the most favorable aspect of the study. Behaviors outside of the study protocol to prevent or delay diabetes onset were common and should be monitored in future prevention studies. CONCLUSIONS - Overall, most participants were positive about the trial, and many expressed optimism about the intervention's potential for success. These results have implications for study design, recruitment, and retention procedures in future prevention trials. 2007 by the American Diabetes Association
SN - 0149-5992
AD - (Johnson, Baughcum) Department of Medical Humanities and Social Sciences, Florida State College of Medicine, Tallahassee, FL, United States (Hood) Joslin Diabetes Center, Harvard Medical School, Boston, MA, United States (Rafkin-Mervis) Diabetes Research Institute, University of Miami Medical School, Miami, FL, United States (Schatz) Department of Pediatrics, College of Medicine, University of Florida, Gainesville, FL, United States (Johnson) Department of Medical Humanities and Social Sciences, 1115 West Call St., Tallahassee, FL 32306-4300, United States
ER -

TY - JOUR
ID - 3818
T1 - Childhood obesity: Contributing factors and consequences in Indian children
A1 - Singh,S.K.
A1 - Kapoor,D.
A1 - Goyal,R.
A1 - Rastogi,A.
A1 - Kumar,S.
A1 - Mishra,O.P.
Y1 - 2007///
N1 - 2007375816
English
Journal: Article
KW - EMBASE
KW - article
KW - Body Height
KW - body mass
KW - Body Weight
KW - caloric intake
KW - Child
KW - child nutrition
KW - cholesterol blood level
KW - clinical article
KW - controlled study
KW - diet restriction
KW - environmental factor
KW - enzyme blood level

KW - fat intake
KW - fatty acid blood level
KW - Female
KW - glucose blood level
KW - Human
KW - hypercholesterolemia/co [Complication]
KW - hypercholesterolemia/et [Etiology]
KW - hyperglycemia
KW - hyperinsulinemia/co [Complication]
KW - hyperinsulinemia/et [Etiology]
KW - Hypertension
KW - Indian
KW - insulin blood level
KW - insulin resistance/co [Complication]
KW - insulin resistance/et [Etiology]
KW - Male
KW - Obesity/et [Etiology]
KW - Physical Activity
KW - priority journal
KW - risk factor
KW - statistical significance
KW - triacylglycerol blood level
KW - fatty acid/ec [Endogenous Compound]
KW - glucose/ec [Endogenous Compound]
KW - high density lipoprotein cholesterol/ec [Endogenous Compound]
KW - insulin/ec [Endogenous Compound]
KW - liver enzyme/ec [Endogenous Compound]
KW - low density lipoprotein cholesterol/ec [Endogenous Compound]
KW - triacylglycerol/ec [Endogenous Compound]
RP - NOT IN FILE
SP - 167
EP - 172
JF - Diabetes and Metabolic Syndrome: Clinical Research and Reviews
VL - 1
IS - 3
CY - United Kingdom
N2 - Introduction: Childhood obesity is a risk factor for adult morbidity and mortality, independent of body mass index (BMI) in adulthood, family history of cardiovascular disease or cancer, and smoking. The present study was designed with the objectives to study contributing factors of childhood obesity and its metabolic consequences. Material and methods: Forty-nine obese and 13 normal children in the age range of 5-12 years were recruited. Subjects with hypothyroidism, Cushing's syndrome and any other disease contributing to endogenous obesity were excluded. Weight and height were recorded and BMI was calculated (weight in kg divided by square of height in meters). Children with weight above 90th percentile for age and sex and BMI > 28.5 or >95th percentile for that age were defined as obese. After an overnight fast (10-12 h) blood was drawn for the determination of plasma glucose, serum insulin, lipid profile and free fatty acids. Observations: The calorie intake in obese children was significantly higher than controls and fatty meal contributed significantly to high calorie intake. Obese children indulged less in outdoor activities than controls. Liver enzymes were significantly higher in the study group. The levels of hepatic enzymes were raised above normal in 26 (53.06%) children. Total cholesterol and LDL cholesterol were higher in the study group, but only the LDL values reached statistical significance. Fasting plasma glucose in the study and control groups were 75.25 + 13.57 and 66.07 + 9.52 mg/dL respectively and it was significantly higher in the study group (p = 0.009). Fasting insulin and fasting free fatty acid levels in study group were 31.66 + 17.58 muU/mL and 379.35 + 148.00 mumol/L and in control group were 8.6 + 4.98 muU/mL and 263.84 + 71.00 mumol/L respectively hence both are significantly higher in the obese children. The mean insulin glucose ratio in the study group was 0.41 + 11.20. Sixteen (32.65%) children had values of more than 0.4, suggesting insulin resistance but none of the control group had insulin glucose ratio above this level. Insulin free fatty acid ratio was also higher in the study group 8.58 + 6.34 than control group 3.10 + 1.19. The mean HOMA-R in the study group and control group were 6.08

+ 3.83 and 1.37 + 0.73 respectively indicating significant insulin resistance in the obese children. Conclusion: The environment influence like increased calorie intake and decreased physical activity are major determinants of childhood obesity. The biochemical consequences of childhood obesity are hyperinsulinemia, increased LDL, FFA levels and increased hepatic transaminases. The triglycerides, HDL, fasting hyperglycemia and hypertension do not seem to have association with metabolic syndrome in children as seen in adults. Therefore it is proposed that the diagnostic parameters of metabolic syndrome should be redefined in children with population-based values of these new biochemical parameters. 2007 Diabetes India
SN - 1871-4021
AD - (Singh, Kapoor, Goyal, Rastogi, Kumar) Department of Endocrinology and Metabolism, Institute of Medical Sciences, Banaras Hindu University, Varanasi, 221005, India (Mishra) Department of Pediatrics, Institute of Medical Sciences, Banaras Hindu University, Varanasi, 221005, India
ER -

TY - JOUR
ID - 3819
T1 - An assessment of the applicability of a test to measure physical activity in Indian school children
A1 - Srivastva,A.K.
A1 - Albadri,S.
Y1 - 2007///
N1 - 2007312694
English
Journal: Article
KW - EMBASE
KW - article
KW - Child Care
KW - Exercise Test
KW - Human
KW - Indian
KW - Jumping
KW - Physical Activity
KW - physical capacity
KW - psychological well being
KW - Running
KW - school
KW - school child
RP - NOT IN FILE
SP - 67
EP - 68
JF - International Journal of Health Promotion and Education
VL - 45
IS - 2
CY - United Kingdom
N2 - Objectives: To assess the applicability of a test to measure the physical activity of Indian school children in the school environment. Subjects and Method: 2,008 school children aged between 9 and 12 years were included. The test used to measure physical efficiency included four items: standing long jump, cricket ball throw, 60 yard run (54.86 metres) and chin-ups. All children completed the four components of the test. Results: The majority of children in the three age groups studied managed to reach the standard norm values for the standing long jump (77%), 60 yard run (90%) and the chin-up tests (86%). However, only just over half (51%) of the children managed to qualify in the cricket ball throw test. Conclusion: The majority of the school children achieved the standard norms of the physical activity test which was practical to use in the school environment
SN - 1463-5240
AD - (Srivastva) Delhi College of Engineering, Bawana Road, Delhi 110042, India (Albadri) Department of Child Dental Health, University Dental Hospital of Manchester, Manchester, United Kingdom
ER -

TY - JOUR
ID - 3820

T1 - Comparison of global positioning system (GPS) tracking and parent-report diaries to characterize children's time-location patterns
A1 - Elgethun,K.
A1 - Yost,M.G.
A1 - Fitzpatrick,C.T.E.
A1 - Nyerges,T.L.
A1 - Fenske,R.A.
Y1 - 2007///
N1 - 2007164485
English
Journal: Article
KW - EMBASE
KW - article
KW - comparative study
KW - controlled study
KW - Employment
KW - Environmental Exposure
KW - Female
KW - global positioning system
KW - Home Environment
KW - household
KW - Human
KW - Male
KW - parent
KW - preschool child
RP - NOT IN FILE
SP - 196
EP - 206
JF - Journal of Exposure Science and Environmental Epidemiology
VL - 17
IS - 2
CY - United States
N2 - Respondent error, low resolution, and study participant burden are known limitations of diary timelines used in exposure studies such as the National Human Exposure Assessment Survey (NHEXAS). Recent advances in global positioning system (GPS) technology have produced tracking devices sufficiently portable, functional and affordable to utilize in exposure assessment science. In this study, a differentially corrected GPS (dGPS) tracking device was compared to the NHEXAS diary timeline. The study also explored how GPS can be used to evaluate and improve such diary timelines by determining which location categories and which respondents are least likely to record "correct" time-location responses. A total of 31 children ages 3-5 years old wore a dGPS device for all waking hours on a weekend day while their parents completed the NHEXAS diary timeline to document the child's time-location pattern. Parents misclassified child time-location approximately 48% of the time using the NHEXAS timeline in comparison to dGPS. Overall concordance between methods was marginal (=0.33-0.35). The dGPS device found that on average, children spent 76% of the 24-h study period in the home. The diary underestimated time the child spent in the home by 17%, while overestimating time spent inside other locations, outside at home, outside in other locations, and time spent in transit. Diary data for time spent outside at home and time in transit had the lowest response concordance with dGPS. The diaries of stay-at-home mothers and mothers working unskilled labor jobs had lower concordance with dGPS than did those of the other participants. The ability of dGPS tracking to collect continuous rather than categorical (ordinal) data was also demonstrated. It is concluded that automated GPS tracking measurements can improve the quality and collection efficiency of time-location data in exposure assessment studies, albeit for small cohorts. 2007 Nature Publishing Group All rights reserved
SN - 1559-0631
AD - (Elgethun) Department of Geography, School of Rural Public Health, Texas A and M University, College Station, TX, United States (Yost, Fitzpatrick, Fenske) Department of Environmental and Occupational Health Sciences, School of Public Health and Community Medicine, University of Washington, Seattle, WA, United States (Fitzpatrick, Nyerges) Department of Geography, University of Washington, Seattle, WA, United States

(Elgethun) Department of Geography, School of Rural Public Health, Texas A and M University, College Station, TX 77843-3147, United States
ER -

TY - JOUR
ID - 3821
T1 - Long-term personal exposure to PM2.5, soot and NOx in children attending schools located near busy roads, a validation study
A1 - van,Roosbroeck S.
A1 - Jacobs,J.
A1 - Janssen,N.A.H.
A1 - Oldenwening,M.
A1 - Hoek,G.
A1 - Brunekreef,B.
Y1 - 2007///
N1 - 2007163580
English
Journal: Article
KW - EMBASE
KW - air pollutant
KW - airborne particle
KW - article
KW - child health
KW - Environmental Exposure
KW - exhaust gas
KW - Human
KW - normal human
KW - Particulate Matter
KW - priority journal
KW - school
KW - school child
KW - soot
KW - Traffic
KW - validation study
RP - NOT IN FILE
SP - 3381
EP - 3394
JF - Atmospheric Environment
VL - 41
IS - 16
CY - United Kingdom
N2 - Several studies have investigated the health of children attending schools located near busy roads. In this study, we have measured personal exposure to traffic-related pollutants in children to validate exposure classification based on school location. Personal exposure to PM_{2.5}, soot, NO_x and NO₂ was measured during four 48-h periods. The study involved 54 children attending four different schools, two of which were located within 100 m of a major road (one ring road and one freeway) and the other two were located at a background location in the city of Utrecht, The Netherlands. Outdoor monitoring was conducted at all school sites, during the personal measurements. A questionnaire was administered on time activity patterns and indoor sources at home. The outdoor concentration of soot was 74% higher at the freeway school compared to its matched background school. Personal exposure to soot was 30% higher. For NO_x the outdoor concentration was 52% higher at the freeway school compared to its background school. The personal concentration of NO_x was 37% higher for children attending the freeway school. Differences were smaller and insignificant for PM_{2.5} and NO₂. No elevated personal exposure to air pollutants was found for the children attending the school near the ring road. We conclude that the school's proximity to a freeway can be used as a valid estimate of exposure in epidemiological studies on the effects of the traffic-related air pollutants soot and NO_x in children. 2007 Elsevier Ltd. All rights reserved

SN - 1352-2310
AD - (Van Roosbroeck, Jacobs, Oldenwening, Hoek, Brunekreef) Institute for Risk Assessment Sciences, Utrecht University, P.O. Box 80178, 3508 TD Utrecht, Netherlands (Janssen) Center for Environmental Health Research, National Institute for Public Health and the Environment (RIVM), P.O. Box 1, 3720 BA Bilthoven, Netherlands (Brunekreef) Julius Center for Health Sciences and Primary Care, University Medical Center Utrecht, Netherlands
ER -

TY - JOUR
ID - 3822
T1 - The relationship between fundamental motor skills and outside-school physical activity of elementary school children
A1 - Raudsepp,L.
A1 - Pall,P.
Y1 - 2006///
N1 - 2007367628
English
Journal: Article
KW - EMBASE
KW - accelerometer
KW - article
KW - calculation
KW - Child
KW - controlled study
KW - Female
KW - Human
KW - Jumping
KW - Male
KW - Motor Development
KW - Motor Performance
KW - normal human
KW - observational method
KW - Physical Activity
KW - Physical Development
KW - primary school
KW - scoring system
KW - statistical analysis
RP - NOT IN FILE
SP - 426
EP - 435
JF - Pediatric Exercise Science
JA - Pediatr Exerc Sci
VL - 18
IS - 4
CY - United States
N2 - The purpose of the present study was to examine the association between fundamental motor skill development and various types of outside-school physical activity. Outside-school physical activity of 133 elementary schoolchildren was measured using a modified observational method validated by O'Hara et al. (18) and Caltrac accelerometers (Hemokinetics, Madison, Wisconsin). Developmental level of overhand throwing and jumping was assessed using total-body developmental sequences. The results revealed that developmental levels of both overhand throwing and jumping were significantly correlated with the skill-specific physical activity (r = .44 and .55 for overhand throwing and jumping, respectively). Caltrac score was not significantly related to jumping and overhand throwing skills. Skill-specific physical activities accounted for 20% of the variance (adjusted R²) in overhand throwing and 17% of the variance in jumping performance. Findings supported the hypothesis that developmental level of fundamental motor skills would be related with skill-specific outside-school physical activity but not with general level of physical activity of elementary schoolchildren. 2006 Human Kinetics, Inc

SN - 0899-8493
AD - (Raudsepp, Pall) Institute of Sport Pedagogy and Coaching Science, University of Tartu, Tartu, Jakobi 5, 51014, Estonia
ER -

TY - JOUR
ID - 3823
T1 - 'You can't wrap them up in cotton wool!' Constructing risk in young people's access to outdoor play
A1 - Jenkins,N.E.
Y1 - 2006///
N1 - 2006627267
English
Journal: Article
KW - EMBASE
KW - Accident Prevention
KW - Adolescent
KW - Adult
KW - article
KW - Child Behavior
KW - child parent relation
KW - child safety
KW - controlled study
KW - Decision Making
KW - expectation
KW - falling
KW - family study
KW - Female
KW - hazard assessment
KW - health care organization
KW - Health Care Policy
KW - Human
KW - leisure
KW - Male
KW - normal human
KW - paranoia
KW - parental behavior
KW - play
KW - Responsibility
KW - Risk Assessment
KW - Risk Management
KW - risk reduction
KW - school child
KW - semi structured interview
KW - Social Behavior
KW - sport injury
KW - United Kingdom
RP - NOT IN FILE
SP - 379
EP - 393
JF - Health, Risk and Society
VL - 8
IS - 4
CY - United Kingdom
N2 - Policy approaches towards risk in relation to young people's leisure activities are characterized by uncertainty. While some organizations have sought to emphasize the need to reduce or even eliminate the risk of 'unintentional injuries' in outdoor play, others highlight the dangers in excessively restricting young people's access to the outside world. Drawing on semi-structured interviews with families in the South Wales area, this

paper argues that while parents are not in the grips of 'paranoia' (Furedi 2001) they are wrestling with competing sets of social expectations, as well as their own rational and emotive judgements. Young people tended to respond to their parents' concerns by demonstrating their street-wisdom, claiming sanctuary behind the notion of 'accident' and asserting their moral right to access the outside world
SN - 1369-8575
AD - (Jenkins) Glasgow University, Glasgow, United Kingdom
ER -

TY - JOUR
ID - 3824
T1 - Mercury exposures in informal gold miners and relatives in southern Peru
A1 - Hurtado,J.
A1 - Gonzales,G.F.
A1 - Steenland,K.
Y1 - 2006///
N1 - 2006599907
English
Journal: Article
KW - EMBASE
KW - Adolescent
KW - Adult
KW - article
KW - Child
KW - controlled study
KW - Environmental Exposure
KW - Female
KW - Human
KW - Male
KW - Mining
KW - Peru
KW - priority journal
KW - relative
KW - smelter
KW - urine level
KW - amalgam
KW - gold
RP - NOT IN FILE
SP - 340
EP - 345
JF - International Journal of Occupational and Environmental Health
VL - 12
IS - 4
CY - United States
N2 - Subjects working in or living near informal gold mining and processing in southern Peru were studied to determine mercury exposures from two tasks: amalgamation and amalgam smelting. The authors collected 17 airborne and 41 urinary mercury levels. The mean urinary levels were 728 (range: 321-1,662) and 113 (45-197) mug/L for working in smelters and living near smelters, respectively. A third group working in amalgamation had a mean 18 mug/L (range 8-37). People living in the mining town but with no mining activities had 8 mug/L (5-10), while a control group outside the town had 4 mug/L (2-6). Mean airborne mercury exposure was 2,423 mug/m³ (range 530-4,430) during smelting, 30.5 mug/m³ (12-55) during amalgamation, and 12 mug/m³ (3-23) in the mining town. Smelters are highly contaminated with mercury, as are the people living around smelters
SN - 1077-3525
AD - (Hurtado) Department of Microbiology, Faculty of Sciences and Philosophy, Universidad Peruana Cayetano Heredia, Lima, Peru (Gonzales) Department of Biological and Physiological Sciences, Instituto de Investigaciones de la Altura, Faculty of Sciences and Philosophy (Steenland) Department of Environmental and Occupational Health, Rollins School of Public Health, Emory University, Atlanta, GA, United States (Hurtado)

Department of Microbiology, Faculty of Sciences and Philosophy, Universidad Peruana Cayetano Heredia, Av. Honorio Delgado No430 Lima 31, Lima 100, Peru
ER -

TY - JOUR
ID - 3825
T1 - Early summer meningoencephalitis in children and adolescents. A prospective study conducted over 10 years in Baden-Wurttemberg and South Hessen
A1 - Kaiser,R.
Y1 - 2006///
N1 - 2006553978
German
Journal: Article
KW - EMBASE
KW - Adolescent
KW - article
KW - ataxia
KW - Child
KW - clinical feature
KW - consciousness disorder
KW - cranial nerve paralysis
KW - disease course
KW - Germany
KW - Human
KW - laboratory test
KW - leukocytosis
KW - major clinical study
KW - medical record
KW - neurologic disease
KW - paresis
KW - Prognosis
KW - questionnaire
KW - sedimentation rate
KW - summer
KW - symptomatology
KW - tick bite
KW - tick borne encephalitis
KW - C reactive protein/ec [Endogenous Compound]
RP - NOT IN FILE
SP - 1111
EP - 1116
JF - Monatsschrift fur Kinderheilkunde
VL - 154
IS - 11
CY - Germany
N2 - Background. The purpose of this study was to investigate the frequency and course of tick-borne encephalitis (TBE) in children and young adults (ages 1-15 years) in southwestern Germany. Patients and methods. Clinical and epidemiological data from children were recorded by means of a questionnaire by Departments of Paediatrics in Baden-Wurttemberg and Southern Hessen. Results. Between 1994 and 2003 a total of 124 children with TBE were recorded. Seventy-seven children (62%) had noticed a tick bite, and the first symptoms occurred an average of 7 days later. The disease had a biphasic course in 89 (72%) of the 124 children. TBE presented as meningitis in 80 children (64%), as meningoencephalitis in 43 (35%), and as encephalomyelitis in 1 child (1%). The most frequent neurological symptoms of meningoencephalitis were impaired consciousness (35%), ataxia (4%), and paresis of the extremities (2.4%) and cranial nerves (2.4%). Laboratory investigations revealed leukocytosis in the peripheral blood in 102 of the 124 patients (82%), elevation of the sedimentation rate in 102 (82%), and increased C-reactive protein in 92 (74%). Conclusion. Even though the course of TBE is less dramatic, and its prognosis better, in children than in adults, there is just

as much reason to protect children against TBE by active immunisation as there s to immunise adults in any area where any cases of TBE are known. The beginning of a vaccination course should be linked to the frequency of exposure through outdoor activities in areas where there is a risk of TBE. 2006 Springer Medizin Verlag
SN - 0026-9298
AD - (Kaiser) Neurologische Klinik, Klinikum Pforzheim GmbH (Kaiser) Neurologische Klinik, Klinikum Pforzheim GmbH, Kanzlerstrase 2-6, 75175 Pforzheim, Germany
ER -

TY - JOUR
ID - 3827
T1 - Skin cancer primary prevention programme for schoolchildren. Analysis of behavioural practices
A1 - Correia,O.
A1 - Barros,A.M.
A1 - Rocha,N.
A1 - Quirino,P.
A1 - Fernandes,J.C.
A1 - Tavares,C.
A1 - Robalo,Cordeiro M.
A1 - Rebelo,C.
A1 - Baptista,Rodrigues J.
A1 - Pereira,A.
Y1 - 2006///
N1 - 2006416277
English
Journal: Article
KW - EMBASE
KW - article
KW - cancer incidence
KW - cancer prevention
KW - Child Behavior
KW - controlled study
KW - Human
KW - medical education
KW - medical practice
KW - Physical Education
KW - questionnaire
KW - risk factor
KW - school
KW - school child
KW - skin cancer
KW - skin sensitization
KW - sun exposure
KW - sunburn
KW - sunscreen
RP - NOT IN FILE
SP - 67
EP - 76
JF - Skin Cancer
VL - 21
IS - 2
CY - Portugal
N2 - The incidence of skin cancer has been increasing steadily, and a direct correlation with sun exposure has long been recognised. Primary prevention actions, mainly directed at children, are important to promote behavioural changes regarding sun exposure. A questionnaire-based enquiry, followed by a sensitisation action, with distribution of didactic material, was carried out in several private and public schools, in June 2003. A significant number of children reported the existence of only a few trees at their schools' playground and the practice of outdoor gymnastics during risky hours. Although they admitted to usually applying sunscreens when

going to the beach, this was not a normal practice when going to school on sunny days. A history of sunburn was reported by 53% of the children. We found some changes in behaviour after the summer holidays following the sensitisation action, which emphasizes the importance of this type of campaigns
SN - 0871-2549
AD - (Correia, Barros, Rocha, Quirino, Fernandes, Robalo Cordeiro, Rebelo, Baptista Rodrigues) Photobiology Group of the Portuguese Society of Dermatology and Venereology, Faculty of Medicine, Oporto, Portugal (Tavares, Pereira) Department of Biostatistics, Faculty of Medicine, Oporto, Portugal (Correia) Centre de Dermatologia Epidermis, Rua Piedade, 43, 4050-481 Porto, Portugal
ER -

TY - JOUR
ID - 3829
T1 - Activity Levels Among Adolescents with Migraine
A1 - Dooley,J.M.
A1 - Gordon,K.E.
A1 - Wood,E.P.
A1 - Brna,P.M.
Y1 - 2006///
N1 - 2006349303
English
Journal: Article
KW - EMBASE
KW - Adolescent
KW - Adult
KW - Age Distribution
KW - article
KW - Canada
KW - controlled study
KW - daily life activity
KW - Exercise
KW - Female
KW - fishing
KW - health survey
KW - Human
KW - jogging
KW - Male
KW - migraine
KW - Physical Activity
KW - priority journal
KW - Running
KW - school child
KW - Sex Ratio
KW - sport
KW - Walking
RP - NOT IN FILE
SP - 119
EP - 121
JF - Pediatric Neurology
JA - Pediatr Neurol
VL - 35
IS - 2
CY - United States
N2 - Data was analyzed from the nationally representative Canadian Community Health Survey. A total of 17,549 adolescents reported whether they had "migraine headaches" (response rate 99.9%) and in what exercise activities they participated. Those with migraine reported more daily activity than migraine-free peers when corrected for age and sex. They were as likely to play contact sports but were more involved in other noncompetitive activities, such as walking (P < 0.001), gardening and yard work (P = 0.008), jogging and

running (P = 0.002), and fishing (P = 0.03). Canadian adolescents with migraine are therefore overall more active than their peers without migraine. 2006 Elsevier Inc. All rights reserved
SN - 0887-8994
AD - (Dooley, Gordon, Wood, Brna) Pediatric Neurology Division, Dalhousie University, the IWK Health Centre, Halifax, NS, Canada
ER -

TY - JOUR
ID - 3830
T1 - Management of procedure-related pain in children and adolescents
Y1 - 2006///
N1 - 2006313160
English
Journal: Review
KW - EMBASE
KW - Adolescent
KW - airway obstruction/si [Side Effect]
KW - allergic reaction/si [Side Effect]
KW - Anxiety
KW - artery catheterization
KW - artery puncture
KW - Australia
KW - Behavior Therapy
KW - bone marrow biopsy
KW - burn dressing
KW - Child
KW - clinical practice
KW - Cognitive Therapy
KW - computer assisted tomography
KW - Coping Behavior
KW - diagnostic procedure
KW - distress syndrome/co [Complication]
KW - distress syndrome/dt [Drug Therapy]
KW - distress syndrome/pc [Prevention]
KW - dizziness/si [Side Effect]
KW - dysphoria/si [Side Effect]
KW - endotracheal intubation
KW - euphoria
KW - feedback system
KW - foreign body/th [Therapy]
KW - fracture/th [Therapy]
KW - general anesthesia
KW - heart arrest/si [Side Effect]
KW - heart arrhythmia/si [Side Effect]
KW - Human
KW - hypnosis
KW - hypotension/si [Side Effect]
KW - hypoxia/si [Side Effect]
KW - Immunization
KW - informed consent
KW - injection
KW - intracranial hypertension/si [Side Effect]
KW - intraocular hypertension/si [Side Effect]
KW - intravenous regional anesthesia
KW - kidney biopsy
KW - laceration/th [Therapy]
KW - larynx spasm/si [Side Effect]

KW - lumbar puncture
KW - medical examination
KW - medical research
KW - MEDLINE
KW - methemoglobinemia/si [Side Effect]
KW - nasogastric tube
KW - nausea/si [Side Effect]
KW - nerve block
KW - neurotoxicity/si [Side Effect]
KW - New Zealand
KW - nuclear magnetic resonance imaging
KW - nystagmus/si [Side Effect]
KW - pain/co [Complication]
KW - Pain/di [Diagnosis]
KW - pain/dt [Drug Therapy]
KW - pain/pc [Prevention]
KW - pain assessment
KW - parent
KW - Pediatrics
KW - practice guideline
KW - priority journal
KW - procedural pain/co [Complication]
KW - procedural pain/di [Diagnosis]
KW - procedural pain/dt [Drug Therapy]
KW - procedural pain/pc [Prevention]
KW - PsycInfo
KW - rash/si [Side Effect]
KW - relaxation training
KW - review
KW - salivation disorder/si [Side Effect]
KW - sedation
KW - side effect/si [Side Effect]
KW - therapy effect
KW - topical anesthesia
KW - urine catheter
KW - vein catheterization
KW - vein puncture
KW - vomiting/si [Side Effect]
KW - adrenalin/cb [Drug Combination]
KW - adrenalin/dt [Drug Therapy]
KW - adrenalin/tp [Topical Drug Administration]
KW - analgesic agent/do [Drug Dose]
KW - analgesic agent/dt [Drug Therapy]
KW - anesthetic agent/ae [Adverse Drug Reaction]
KW - anesthetic agent/dt [Drug Therapy]
KW - benzodiazepine derivative/dt [Drug Therapy]
KW - bupivacaine/dt [Drug Therapy]
KW - cocaine/cb [Drug Combination]
KW - cocaine/dt [Drug Therapy]
KW - cocaine/tp [Topical Drug Administration]
KW - codeine/cb [Drug Combination]
KW - codeine/dt [Drug Therapy]
KW - codeine/po [Oral Drug Administration]
KW - EMLA/dt [Drug Therapy]
KW - EMLA/tp [Topical Drug Administration]
KW - flumazenil
KW - ketamine/ae [Adverse Drug Reaction]

KW - ketamine/dt [Drug Therapy]
KW - ketamine/iv [Intravenous Drug Administration]
KW - ketamine/po [Oral Drug Administration]
KW - levobupivacaine/dt [Drug Therapy]
KW - lidocaine/cb [Drug Combination]
KW - lidocaine/dt [Drug Therapy]
KW - lidocaine/tp [Topical Drug Administration]
KW - local anesthetic agent/ae [Adverse Drug Reaction]
KW - local anesthetic agent/dt [Drug Therapy]
KW - local anesthetic agent/tp [Topical Drug Administration]
KW - midazolam/ae [Adverse Drug Reaction]
KW - midazolam/dt [Drug Therapy]
KW - midazolam/na [Intranasal Drug Administration]
KW - midazolam/iv [Intravenous Drug Administration]
KW - midazolam/po [Oral Drug Administration]
KW - morphine/dt [Drug Therapy]
KW - morphine/po [Oral Drug Administration]
KW - naloxone
KW - nitrous oxide/ae [Adverse Drug Reaction]
KW - nitrous oxide/dt [Drug Therapy]
KW - nitrous oxide/ih [Inhalational Drug Administration]
KW - opiate/dt [Drug Therapy]
KW - opiate/po [Oral Drug Administration]
KW - paracetamol/cb [Drug Combination]
KW - paracetamol/dt [Drug Therapy]
KW - paracetamol/po [Oral Drug Administration]
KW - prilocaine/dt [Drug Therapy]
KW - ropivacaine/dt [Drug Therapy]
KW - sedative agent/dt [Drug Therapy]
KW - tetracaine/cb [Drug Combination]
KW - tetracaine/dt [Drug Therapy]
KW - tetracaine/tp [Topical Drug Administration]
RP - NOT IN FILE
SP - S1
EP - S29
JF - Journal of Paediatrics and Child Health
VL - 42
IS - SUPPL. 1
CY - Australia
N2 - In the last decade, several bodies (American Academy of Pediatrics 1,2, American College of Emergency Physicians 3, United States Department of Health and Human Services 4, Royal College of Paediatrics and Child Health 5,6) have published guidelines for management of acute pain, including procedural pain. 3 In 2003, the Royal Australasian College of Physicians recognized the need for a local document to:* Raise awareness that the treatment of pain is essential and should be a priority. * Invite paediatricians and other child health professionals to reflect on their own attitudes, beliefs and practices. * Make recommendations relevant to clinical settings in Australia and New Zealand which can then be a resource for individuals and institutions developing local protocols. * Bridge the gap between research and clinical practice. Significant advances have been made in the field of pain management in recent years. The essential question is no longer whether children feel pain but how best to treat and prevent it.⁷ However, despite the established efficacy of pain management techniques, multiple studies and reviews show that pain and distress is poorly managed and that children, in particular, continue to suffer unnecessarily.^{8,10} The values and attitudes of society, family members and health care professionals, together with health care practices within an organizational system, all present obstacles to optimal pain control.^{8,11-13} Preventing pain is not only humane, it can also reduce the risk of subsequent morbidity¹⁴ including delayed healing.¹⁵ One of the consequences of untreated pain is that children of all ages become sensitized to pain.^{16,17} This is because pain may activate the physical, biochemical and cellular processes that change the future response to pain. This central plasticity or sensitization may cause previously non-painful stimuli to be painful

or may enhance the response to painful stimuli.¹⁸ As health care providers, we have a clinical, moral and ethical obligation to minimize pain and suffering in children. There are a number of ways to achieve this, with regard to procedu res: * Adopt a child-centred approach (listening to the needs of the child and family) rather than a procedure-focused 'get-it-over-with' approach.¹⁹ * Make the child and their family active participants and members of the team, rather than passive recipients.¹⁹ * Use parents for positive assistance, not negative restraint.²⁰ * Ensure that all procedures undertaken are necessary that is, the benefit outweighs any negative impact caused by the procedure. * Ensure that all procedures are carried out in order to maximize safety for the child. * Perform procedures in a child friendly environment, away from the bed.²¹ * Use pain assessment routinely.⁵ * Use the least invasive equipment where possible. * Ensure that the person performing the procedure has appropriate technical expertise, or is closely supervised by someone who does.¹ * Use appropriate combinations of non-pharmacological and pharmacological interventions to manage pain and anxiety.²² Sedation alone does not provide pain relief. * Optimize waiting time: too little time increases distress but too much time increases anticipatory anxiety. Time required for preparation is child-specific.^{1,19} * Ensure that the development of anticipatory anxiety is prevented as far as possible by maximizing the intervention to alleviate pain and distress for the first procedure (e.g., general anaesthetic for bone marrow aspirate).²¹ The guideline statement associated with this executive summary is an evidence-based approach to procedural pain management, outside of the operating theatre and neonatal intensive care unit environments, for all children. The document integrates both non-pharmacological and pharmacological approaches to common childhood procedures. In addition, specific information is available on pain assessment, pre-procedure preparation, resources required for procedural pain management, special situations unique to the care of children, a consumer's perspective, and details of common techniques, including non-pharmacological techniques. 2006 Blackwell Publishing Ltd
SN - 1034-4810
ER -

TY - JOUR
ID - 3832
T1 - Towards responsible management of prenatal diagnosis: Arguments for and against
A1 - Viafora,C.
Y1 - 2006///
N1 - 2006139950
English
Journal: Article
KW - EMBASE
KW - Abortion
KW - article
KW - Child
KW - congenital malformation/cn [Congenital Disorder]
KW - congenital malformation/di [Diagnosis]
KW - Decision Making
KW - fetal rights
KW - fetus
KW - genetic disorder/di [Diagnosis]
KW - genetic screening
KW - handicapped child
KW - Human
KW - medical ethics
KW - parental attitude
KW - physician attitude
KW - Pregnancy
KW - Prenatal Diagnosis
KW - Responsibility
KW - Social Behavior
RP - NOT IN FILE
SP - 5
EP - 12

JF - Rivista di Neuroradiologia
VL - 19
IS - 1
CY - Italy
N2 - These bioethical reflections review the comparative arguments surrounding specific responsibilities raised by prenatal diagnosis: i) in the face of a risk pregnancy, attested by precise medical documentation, should recourse to prenatal diagnosis figure as a moral obligation or, conversely, is unconditional acceptance of the child incompatible with all prior "quality control"? (parental responsibility) ii) if so, does the final decision rest with the parents, and is a completely neutral attitude by the doctor justified even when the foetus has a complaint for which adequate forms of treatment are available? (medical and professional responsibility) iii) is it right to promote health campaigns introducing compulsory screening for risk categories with the prospect of automatically selecting out foetuses bearing genetic anomalies? (social responsibility). After analysis and discussion of the conflicting arguments over responsible management of prenatal diagnosis, there is a review of the stages through which the diagnostic process passes when a couple go for prenatal diagnosis under the shadow of a risk pregnancy. The aim is to assess the role that may be played by specific ethically-oriented support within the counselling process. For such support to be properly framed, the following suggestions are made: i) Helping towards an aware decision: the consultant should help the couple take a sufficient distance from the outside world and face up to the situation in personal terms. They must be able to decide by criteria they see as their own, worked out as a couple and not absorbed from outside (which mostly means ratified by hearsay). As part of helping the couple to an authentic decision, one notes the concern not to reduce prenatal diagnosis to an automatic stage in a medical routine. Being referred for diagnosis by one's G.P. does not dispense with the need for the parents involved in the first person to size up the position for themselves, ii) Helping to pinpoint the responsibilities that can in all conscience be shouldered: amid the ambivalence that most couples experience, it is important that some outsider should let them know the difficulties that await them, but also the resources they can draw on. Though it is up to the couple to decide whether to terminate or go on with the pregnancy, the process of talking, confiding, reflecting and judging nonetheless gives its own weight of humanity to the decision that will come of it. At the same time it creates the right space in which to identify the real responsibility that one can in all conscience take upon oneself; iii) Creating the conditions for authentic ethical discernment: prenatal diagnosis puts the couple in a paradoxical situation precisely because every pregnancy lives in the hope of a child as its achievement and outcome; the handicap that one learns of through prenatal diagnosis just when the investment is at its ideal height may prove to be something truly intolerable. There are two particular ways of imaging the "blow" of a positive outcome which strongly condition the ethical discernment or even make it impossible: seeing it as an "error"; seeing it as a "horror". The real alternative to these lies in configuring limitation and impairment as an integral part of our human condition, through which discerning attitude one can come to identify with the handicapped child and recognise the right it has to lead "its own" life
SN - 1120-9976
AD - (Viafora) Department of Philosophy, University of Padua, Padua, Italy (Viafora) Department of Philosophy, University of Padua, P.zza Capitaniato, 7, 35139 Padua, Italy
ER -

TY - JOUR
ID - 3833
T1 - Impact of accessible sexual and reproductive health care on poor and underserved adolescents in Managua, Nicaragua: A quasi-experimental intervention study
A1 - Meuwissen,L.E.
A1 - Gorter,A.C.
A1 - Knottnerus,A.J.A.
Y1 - 2006///
N1 - 2006005946
English
Journal: Article
KW - EMBASE
KW - Adolescent
KW - adolescent health
KW - Adult
KW - article

KW - condom
KW - confidence interval
KW - contraception
KW - Evaluation
KW - Female
KW - Health Care
KW - health care access
KW - health center
KW - health program
KW - Human
KW - knowledge
KW - major clinical study
KW - Male
KW - market
KW - Nicaragua
KW - priority journal
KW - quasi experimental study
KW - questionnaire
KW - reproductive health
KW - school
KW - school child
KW - sexual health
KW - sexual intercourse
KW - sexually transmitted disease/pc [Prevention]
KW - contraceptive agent
RP - NOT IN FILE
SP - 56
EP - 56
JF - Journal of Adolescent Health
JA - J Adolesc Health
VL - 38
IS - 1
CY - United States
N2 - Background: The objective of this study was to evaluate a competitive voucher program intended to make sexual and reproductive health care (SRHC) accessible to adolescents from disadvantaged areas of Managua. Methods: A quasi-experimental intervention study was performed in which 28,711 vouchers that gave free access to SRHC in 20 health centers, were distributed to adolescents. To evaluate the impact, community sampling took place in markets, neighborhoods, and outside schools where self-administered questionnaires were distributed. The study comprised a random sample of 3,009 female adolescents, ages 12 to 20 years old, 904 voucher receivers and 2,105 nonreceivers. Their use of SRHC, and knowledge and use of contraceptives and condoms were measured. Results: Voucher receivers had a significantly higher use of SRHC compared with nonreceivers, 34% versus 19% (adjusted odds ratio, 3.1; 95% confidence interval, 2.5-3.8). The highest influence was seen among respondents at schools, where use was 24% relative to 6% in nonreceivers (adjusted odds ratio, 5.9; 95% confidence interval, 3.7-9.5). Voucher receivers answered significantly more questions correctly that were related to knowledge of contraceptives and sexually transmitted infections than nonreceivers. At schools, sexually active voucher receivers had a significantly higher use of modern contraceptives than nonreceivers, 48% versus 33% (adjusted odds ratio, 2.3; 95% confidence interval, 1.2-4.4); and in neighborhoods, condom use during last sexual contact was significantly greater among voucher receivers than nonreceivers (adjusted odds ratio, 2.5; 95% confidence interval, 1.4-4.5). Conclusion: The voucher program succeeded in increasing access to SRHC for poor and underserved girls. The needs of adolescents were met with a relatively simple intervention through existing health facilities. Many adolescents appeared willing to protect themselves against the risks of sexual intercourse. This suggest that access to SRHC can play an important role in changing youth behavior and increase the use of contraceptives and condoms. 2006 Society for Adolescent Medicine. All rights reserved
SN - 1054-139X
AD - (Meuwissen) Instituto CentroAmericano de la Salud, Managua, Nicaragua (Gorter) London School of Hygiene and Tropical Medicine, London, United Kingdom (Knottnerus) University of Maastricht, Maastricht,

Netherlands (Knottnerus) Health Council of the Netherlands, The Hague, Netherlands (Meuwissen)
Ruysdaelplein 10, 3723 CD Bilthoven, Netherlands
ER -

TY - JOUR
ID - 3836
T1 - Effects of outward-bound training on children's self-consciousness
A1 - Fang,P.-S.
A1 - Luan,Y.-M.
Y1 - 2005///
N1 - 2006052462
Chinese
Journal: Article
KW - EMBASE
KW - Adolescent
KW - Age Distribution
KW - article
KW - Camping
KW - Child Behavior
KW - China
KW - clinical trial
KW - controlled clinical trial
KW - controlled study
KW - groups by age
KW - Human
KW - major clinical study
KW - population distribution
KW - randomized controlled trial
KW - school child
KW - scoring system
KW - Self Concept
KW - Self Esteem
KW - statistical analysis
KW - statistical significance
RP - NOT IN FILE
SP - 28
EP - 29
JF - Chinese Journal of Clinical Rehabilitation
VL - 9
IS - 36
CY - China
